# A Markovian Entropy Measure for the Analysis of Calcium Activity Time Series

**DOI:** 10.1371/journal.pone.0168342

**Published:** 2016-12-15

**Authors:** John P. Marken, Andrew D. Halleran, Atiqur Rahman, Laura Odorizzi, Michael C. LeFew, Caroline A. Golino, Peter Kemper, Margaret S. Saha

**Affiliations:** 1 Department of Mathematics, College of William and Mary, Williamsburg, Virginia, United States of America; 2 Department of Biology, College of William and Mary, Williamsburg, Virginia, United States of America; 3 Department of Computer Science, College of William and Mary, Williamsburg, Virginia, United States of America; Universitatsklinikum Wurzburg, GERMANY

## Abstract

Methods to analyze the dynamics of calcium activity often rely on visually distinguishable features in time series data such as spikes, waves, or oscillations. However, systems such as the developing nervous system display a complex, irregular type of calcium activity which makes the use of such methods less appropriate. Instead, for such systems there exists a class of methods (including information theoretic, power spectral, and fractal analysis approaches) which use more fundamental properties of the time series to analyze the observed calcium dynamics. We present a new analysis method in this class, the Markovian Entropy measure, which is an easily implementable calcium time series analysis method which represents the observed calcium activity as a realization of a Markov Process and describes its dynamics in terms of the level of predictability underlying the transitions between the states of the process. We applied our and other commonly used calcium analysis methods on a dataset from *Xenopus laevis* neural progenitors which displays irregular calcium activity and a dataset from murine synaptic neurons which displays activity time series that are well-described by visually-distinguishable features. We find that the Markovian Entropy measure is able to distinguish between biologically distinct populations in both datasets, and that it can separate biologically distinct populations to a greater extent than other methods in the dataset exhibiting irregular calcium activity. These results support the benefit of using the Markovian Entropy measure to analyze calcium dynamics, particularly for studies using time series data which do not exhibit easily distinguishable features.

## Introduction

Calcium ions are essential messenger molecules that perform diverse functional roles across all phyla. These functions include the regulation of nearly all cellular processes, including differentiation, synaptic signaling, apoptosis, and gene expression [1, 2, 3, 4]. Calcium’s myriad roles are associated with a host of activity patterns which are categorized as distinct activity features based on the shape of their waveforms along a temporal or spatial scale [5]. Such features include spikes, which are transient fluxes of a significant amplitude, waves, which are spatially-propagating fluxes of calcium, and oscillations [6].

These features have been characterized and defined based on observations in biological systems where they are easily identified. A time series recording of the calcium activity in a mature, synaptic neuron tends to exhibit clearly-defined action potentials that are well-represented as spikes [7]. Calcium imaging of *Xenopus* oocytes reveals clearly delineated wave behavior [8]. These systems have motivated the development of a suite of feature-based analysis methods for calcium activity data. Methods such as wavelet analysis, temporal deconvolution, and supervised learning approaches perform extremely well when they are applied to datasets from such well-characterized systems, where the calcium activity both strongly exhibits and is predominantly characterized by these canonical types of visual features [9, 10, 11]. Continued development of these methods to both better analyze such time series by integrating techniques such as image recognition or noise deconvolution and to improve their computational efficiency is a current and active field of research, exemplified by recent developments [12, 13, 14]. However, at their core these methods still characterize and analyze calcium activity with respect to these visual features.

**Fig 1:**
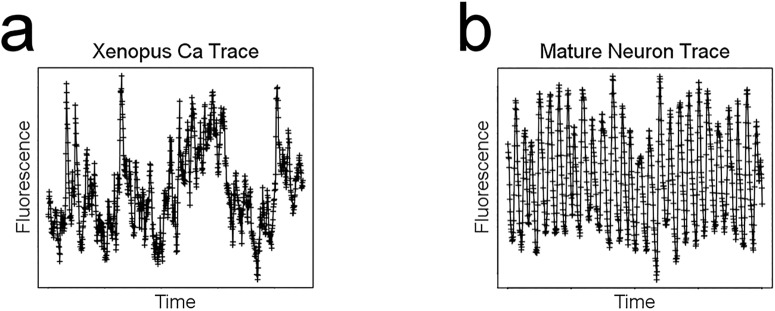
Measured calcium time series exhibit different levels of distinguishability for visual features. Representative single-cell calcium activity time series from (a) a progenitor neuron from embryonic *Xenopus laevis* or (b) a mature, synaptic neuron from embryonic mouse. Crosses represent individual time points from (a) our own dataset or (b) data received from [15]. While both time series exhibit calcium spikes, they are more easily identified in the cell in (b) than in the cell in (a). Furthermore, the calcium dynamics in (b) are governed almost entirely by spikes whereas in (a), one can see activity patterns that are more complex than the simple spiking behavior. The *Xenopus* neural progenitor time series consists of 900 data points measured at 0.25 Hz, while the murine synaptic neuron time series consists of 800 data points measured at 10 Hz.

This limitation is particularly relevant for scientists who investigate systems whose calcium dynamics do not manifest themselves as easily-distinguishable forms of features like spikes or waves, but rather exhibit an irregular type of calcium activity. An example of such a system is a neural progenitor cell in the developing nervous system (Fig 1a), where in marked contrast to the cleanly distinguishable spiking of a mature, synaptic neuron (Fig 1b), one can observe dynamic activity in the time series which is not easily classifiable as a particular ‘type’ of visual feature.

In order to analyze such time-series data, one would want to use a method that examines the entirety of the activity patterns without focusing on one or two visually discernible features. A wide class of such methods exist, including power spectral analysis, which measures properties of intrinsic oscillations in the signal, fractal analysis, which quantifies predictability via self-similarity in a given signal, and information-theoretic approaches that describe the predictability or information content in a signal. Though each of these types of methods analyze time series data in a broader fashion than the specificity of feature-focused methods, they do so by focusing on different properties of these measured activity patterns. This makes these methods ideal to use in complement with each other, as a more holistic sense of the system under study can be gained through aggregating insights from detailed analyses of different properties of the system’s behavior. This is particularly advantageous for exploratory studies of poorly-characterized systems which, like the developing nervous system, display an irregular type of calcium activity that makes it difficult to justify the use of an analysis method that emphasizes the importance of a specific visual feature.

The information-theoretic approaches to calcium analysis, in particular, focus on the idea of Shannon Entropy, which can be used to quantify notions of both predictability and information storage capacity in a system’s dynamics [16]. The latter interpretation of Shannon Entropy has been employed in calcium analysis methods that view calcium activity explicitly as a signal in the communication sense, propagating the flow of information between or within cells [17, 18, 19]. However, to our knowledge, there is currently no information-theoretic calcium analysis method in the literature which explicitly interprets its entropy measure in terms of the level of predictability in the system’s dynamics, specifically with respect to the transitions between levels of observed activity. We therefore developed the Markovian Entropy method, which calculates the information entropy associated with the state-transition probabilities of a Markov Process which is generated from a calcium activity time series obtained from a cell. Our Markovian Entropy method returns a single numerical value, in units of Bits, for a given time series to distinguish cells’ calcium activity by their level of predictability.

## Materials and Methods

### Calcium Imaging

Because of their ease of manipulation and ability to grow in a serum-free defined media consisting of a buffered salt solution, embryos from *Xenopus laevis*, a classic and widely used system for studying early neural development, were employed for analysis of calcium activity [20]. Embryos were obtained following standard procedures [21] and all staging was performed according to the standard staging table published by Nieuwkoop and Faber [22]. Briefly, adult *Xenopus laevis* pairs were injected with human chorionic gonadotropin (1000U/mL, Chorulon) to induce natural matings with females receiving 600 U and males receiving 400 U. Embryos were collected and dejellied by gently washing with a 2% cysteine solution (pH 8.0) for 2–4 minutes and rinsing three times in 0.1X Mark’s Modified Ringer’s solution (MMR) composed of 100 mM NaCl, 2 mM KCl, 1 mM MgSO_4_, 2 mM CaCl_2_, and 5 mM HEPES. Embryos were transferred to 100mm Petri dishes containing 0.1X MMR with gentamycin (50μg/mL) and raised until either stage 14 (neural plate stage), stage 18 (neural tube stage), or stage 22 (late neurula/early tailbud stage).

In order to obtain cells for calcium imaging, the presumptive neural plates of five morphologically normal embryos at one of the stages listed above were dissected from each embryo and transferred to a 35 mm Petri dish containing calcium-magnesium-free medium (CMF; 116 mM NaCl, 0.67 mM KCl, 4.6 mM Tris, 0.4 mM EDTA) [23, 24, 25]. The five pieces of presumptive neural tissue were incubated for 60 minutes to allow complete dissociation into individual cells. The cells were then evenly spread onto a Nunclon dish containing 116 mM NaCl, 0.67 mM KCl, 10 mM CaCl_2_, 1.31 mM MgSO_4_, 4.6 mM Tris, and 5 μM Fluo-4 AM/0.01% pluronic acid and incubated for 60 min to allow for cells to load the Fluo-4 and adhere to the plate. After this incubation, the plate was washed three times to remove excess Fluo-4. Remaining embryos (at least two per image) were transferred to a 35 mm dish containing 0.1X MMR to serve as sibling controls. The stages in the paper refer to the time at which the neural plates were dissected and dissociated. Imaging was performed on a Zeiss LSM 510 confocal microscope using a Pan-Neofluor 20X/0.3 objective and an argon laser at 4% power with 488 nm excitation and 510–530 nm emission settings. Cells were imaged for 1 hr (0.25 Hz) resulting in a 900 frame time series.

Following imaging, it was necessary to assign each cell a unique Region Of Interest (ROI). However, given that cells frequently shifted positions during the imaging period, the cell tracking program in Nikon Elements was used to ensure that measured fluorescence units were accurate at each time point. To do so the calcium image.lsm files were converted to.nd2 files and opened in Nikon Elements for further analysis. Using the “Binary” menu and the “spot detection” tool under the FITC fluorescence channel all cells within a particular image were circled. Diameter, circularity, and contrast settings were adjusted to ensure that cells are appropriately captured as individual ROIs. The Nikon Elements cell tracking program was then employed to track individual cells across the entire image. After the program completed, the image was viewed with ROI overlay to ensure that cells are accurately tracked. Any ROIs that did not accurately correspond to an individual cell across all frames of the image were manually deleted. Once each cell was identified throughout the 900 frames of the image, fluorescence intensity data were collected for each individual cell and exported as a csv file for further analysis.

All protocols were conducted in accordance with the Institutional Animal Care and Use Committee (IUCAC) guidelines at the College of William and Mary. The protocols were approved by the William and Mary IUCAC committee (IACUC-2010-10-31-6991-mssaha and IACUC-2013-11-21-9110-mssaha).

### Time Series Trend Removal

In this manuscript, in addition to analyzing calcium activity datasets using our Markovian Entropy method, we also compute each time series’ Average Power, Hurst Exponent, or number of spikes. The use of these methods are often preceded by a detrending algorithm as the underlying trend can influence the efficacy of the analysis methods. To assess the extent of this influence we analyzed our *Xenopus laevis* calcium activity time series data using these methods, with and without detrending via the Baseline Correction algorithm developed by Eilers and Boelen [26]. This algorithm was chosen because it was able to consistently remove the diverse types of trends present in our time series while still preserving the visually discernible dynamics in the time series (S1, S3, S4 and S6 Files). We found for our *Xenopus* dataset that the qualitative results of the analyses by our Markovian Entropy method, the Average Power, and the Hurst Exponent of each time series, as assessed by the sign of Cohen’s d statistic, were insensitive to the presence of long-term trends in the time-series but that this was not the case for analyses performed via spike counting (S1 Fig, S1 Table). In order to provide an accurate comparison of these various methods, we performed all analyses in the main text on baseline-corrected data. Time series from [15] were provided to us in normalized, detrended form according to the ΔF/F technique outlined in [15] (S7 and S8 Files).

### The Markovian Entropy Measure

#### Outline of the analysis method

The underlying concept of our method is that it represents a calcium activity time series as an empirically observed realization of a Markov Process. A Markov Process is a mathematical object that satisfies two properties: (1) it consists of a set number of discrete states, which it can transition between with defined probabilities, and (2) the system is memoryless, in that the probability of a transition from a state *i* to another (possibly the same) state *j* is determined only by the fact that the system is currently in state *i*, and is not affected by the past history of the system. By representing the activity of the time series in this way, our method is able to explicitly analyze the observed calcium dynamics in terms of the transitions between activity levels.

**Fig 2:**
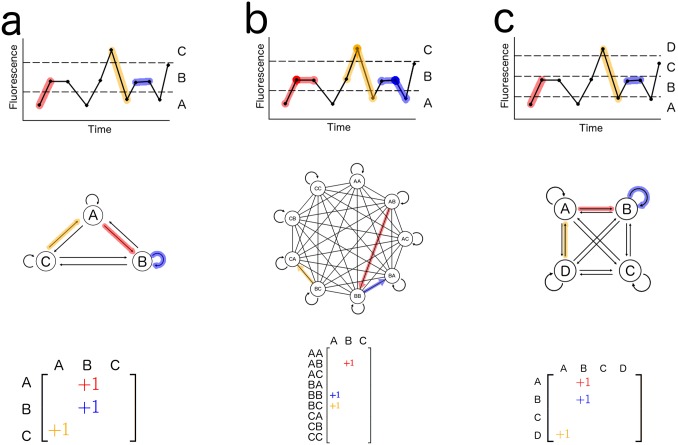
Illustrations of the Markov Processes which represent the observed calcium activity time series. Processes are defined with (a) *n* = 3 and *k* = 1, (b) *n* = 3 and *k* = 2, and (c) *n* = 4 and *k* = 1. Colored transitions in the observed schematic time series are correspondingly colored as state transitions in the Markov Process below the time series, and also designated in the state transition matrices. Note that the time series are identical between (a), (b), and (c). For clarity, each line between states in the central state-transition graph of (b) is condensed to represent both a forward and a reverse state transition.

**Fig 3:**
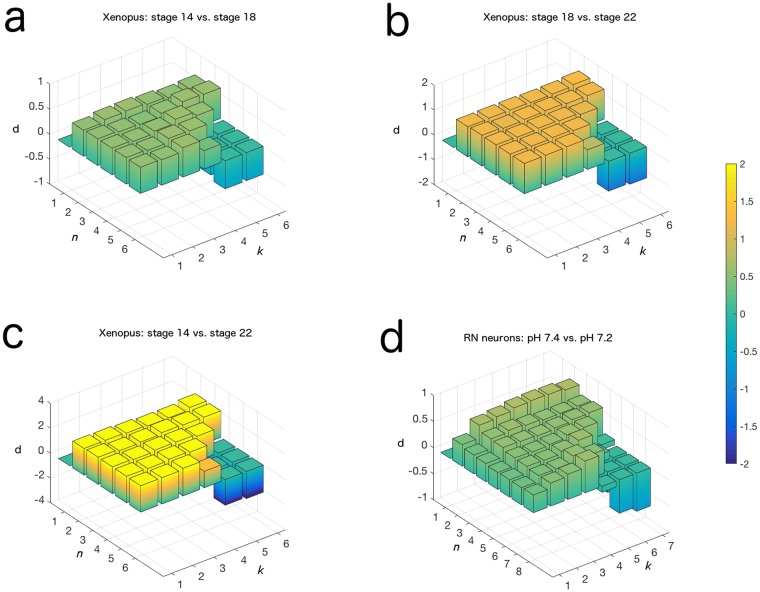
Parameter choices do not qualitatively change the biological interpretation of our information entropy measure. Cohen’s d statistic [29] comparing distributions of entropy values for cellular calcium activity of (a) stage 14 and stage 18 *Xenopus laevis* embryos, (b) stage 18 and stage 22 *Xenopus laevis* embryos, (c) stage 14 and stage 22 *Xenopus laevis* embryos, and (d) mature retrotrapezoid nucleus neurons from embryonic mice in pH 7.4 solution vs. pH 7.2 solution (data in (d) obtained from [15]). At large values of *n* and *k*, a sign change in d value occurs which is a technical artifact arising from there being more entries in the transition matrix than can be filled by data from our time series. The numerical values of d which generated this figure can be found in S2 Table.

In order to generate and analyze the Markov Process which best approximates the empirically observed calcium time series, our method follows a simple three-step pipeline that consists of time series discretization, transition probability matrix construction, and calculation of information entropy. The calcium fluorescence values are first discretized into a character sequence by splitting the observed fluorescence values of the time series into *n* quantiles and converting each value into a character representing its appropriate quantile (Fig 2). This process discretizes the continuous values of calcium activity levels into the *n* distinct states of the Markov Process. In our datasets, we observed the best results at small values of *n*, in particular *n =* 2 (Fig 3).

The discrete signal is then used to empirically calculate an *n*^*k*^-by-*n* transition probability matrix (Fig 2), where *k* is an integer value which represents the number of observed time steps which make up a state in the Markov Process. In our datasets, we observed the best results at small values of *k*, in particular *k* = 1 or 2.

The method then calculates the Shannon Entropy of the *n*^*k*^ discrete probability distributions contained within the matrix, then sums and normalizes the results to obtain a single, scalar value which ranges from 0 to 1 to represent the level of predictability in the transitions between levels of calcium activity in the observed dynamics.

We implemented our method using a series of custom Python scripts that convert a csv file of single-cell calcium activity time series into a csv file of Markovian Entropy values. These are uploaded to Github along with our entire *Xenopus laevis* dataset in csv format [27].

#### Time series discretization and transition matrix generation

Our method represents the observed calcium time series as a *k*-th order *n-*state Markov Process. For a given calcium activity time series, we calculated its *n* quantiles using the Python package numPy and assigned a symbol to each quantile. Any fluorescence value within a given quantile was converted from its numeric value to the quantile’s representative symbol, transforming the continuous fluorescence values of the measured time series into a discrete symbol sequence with the same length as the original time series. The use of quantile-based binning was chosen in order to avoid forcing the experimenter to set thresholds to explicitly define the levels of calcium activity in their system. Furthermore, the quantiles ensure that each state of the Markov process is represented by an equal number of data points, which prevents data sparseness effects from disproportionately affecting some states in the Markov Process over others.

We then generated an empty *n*^*k*^*-*by-*n* transition matrix whose rows enumerate all possible *k*-grams of the *n* symbols, and whose columns enumerate the symbols themselves. We iterated through the symbol sequence and recorded every *k*-gram-to-symbol transition in the appropriate entry in the matrix (Fig 2). After the iteration, the entries in the matrix were divided by their row sum to convert each row into a discrete probability distribution representing the probability that a Markov Process will transition into one of the *n* symbols, given that its current and past *k*-1 states are given by the row’s *k*-gram. This matrix is the transition probability matrix which defines the *k*-th order *n*-state Markov Process which is most likely to produce the observed calcium activity dynamics [28].

#### Guidelines for the choice of *n* and *k* parameters

There are two parameters in our Markovian Entropy method: the number of states *n* in the Markov Process, and the length *k* of each of these states. Together, these parameters serve to represent the dynamics of the calcium activity as a *k*-th order *n*-state Markov Process. Because of this dependence, the size of the transition probability matrix is explicitly dependent on the size of the values of *n* and *k*. Since the probabilities in each entry of the matrix are estimated from the empirically observed frequencies of these transitions in the given time series, one should ensure that the matrix is not so large that the time series’ data points are unable to densely populate the entries of the matrix and sufficiently support the estimates of its transition probabilities. Thus as transition matrix size increases, there is a trade-off between obtaining a higher resolution of activity states and losing confidence in the accuracy of the generated Markov Process through sparseness effects in the sampling.

The benefit of obtaining a particular number of states should be seriously considered if the design of the experiment lends itself to focusing on a specified set of distinct levels of calcium activity, such as “low” vs. “high” or “low” vs. “medium” vs. “high”. In this case the parameter *n* should be specified to correspond to the appropriate number of such levels, and if the boundaries of these states are known, then the method can be modified to define the states accordingly instead of using the default quantile-based binning method. Otherwise, avoiding sparseness concerns becomes the more important priority, and the experimenter should aim to keep *n* at a low level. We found that across the datasets studied in this paper, *n = 2* was sufficient to capture the calcium activity dynamics for time series of length 800 or 900 (Fig 3, S2 Table).

The *k* parameter defines the order of the Markov Process and is used to increase the ability of the Process to capture short-term autocorrelations in the observed dynamics. Assuming that *k = 1* is not the ideal value (which it may be), as *k* increases from 1 the fit in using the Markov Process to approximate the dynamics will become better, and analyses will perform better at tasks such as separating distinct populations. At a certain level of *k*, however, further increases in the parameter will not contribute significant additional information to the process. In order to conserve the size of the transition matrix, the experimenter should set *k* equal to the minimal value at which this stabilization in information gain occurs, which we call k*. We determined k* as the value of *k* past which the separation of two distinct populations does not become stronger, and found that in our *Xenopus* dataset that k* = 1, and that in the retrotrapezoid nucleus neuron dataset that k* = 2 (Fig 3, S2 Table).

Finally, we note that the length of the measured calcium activity time series can influence the flexibility one has with the choice of *n* and *k*. Large values of *n* or *k* lead to a large transition matrix with a correspondingly larger number of possible state transitions in the Markov Process. This decreases the number of observed state transitions in the time series which support a given transition probability value in the Markov Process, decreasing the confidence one can have in the validity of the estimate. Furthermore, such a matrix can lead to technical artifacts in the results of the analysis, where rows in the matrix become maximized in entropy and cause the effect size between distributions to change in sign (Fig 3; S2 Fig).

#### Calculation of information entropy

For each row in the transition probability matrix, we calculated the information entropy *E* using Shannon’s formula [30]

$$E=-\sum_{i=1}^{n}p_{i}{\text{log}}_{2}\left(p_{i}\right)$$

where each *p*_*i*_ is the value of the entry in that column. We then summed the information entropies from each row and divided the result by *n*^*k*^ log_2_(*n*), which is the maximum entropy value which can be obtained this way from a transition probability matrix. Thus we obtained a Markovian Entropy value that ranges from 0 to 1 representing the entropic content of the observed calcium activity. This allows comparisons to be made between Markovian Entropy values from calcium dynamics in disparate systems or across different experiments in a standardized way, provided that the *n* and *k* values which define their corresponding Markov Processes are also reported.

The information entropy of a probability distribution takes units of Bits and is a measure of uncertainty in the distribution. A high Bits value suggests that the probability distribution describes a system with less predictable dynamics, and a low Bits value suggests a system with more predictable dynamics [16]. Because we compute the information entropy associated with the state transition probabilities, the final value returned by our Markovian Entropy measure is an aggregate measure of the predictability of the state transition dynamics between the *n* relative levels of calcium activity observed in the time series for a given cell.

### Calculation of Spike Counts

In order to accurately and consistently determine the number of calcium spikes in our datasets, we developed an algorithm that followed the guidelines described in [31, 32] and defines the onset of a spike to be a time point with a fluorescence value that is at least 150% of the fluorescence value at the previous time point. The spike ends once the time series reaches a fluorescence value that is below the value attained at the spike’s onset. Spikes must last for at least two time points to be counted. We implemented this algorithm using a custom Python script.

### Calculation of Average Power

In our datasets, we compared the performance of our Markovian Entropy measure to a method from power spectral analysis, which investigates the properties of the intrinsic oscillations in a signal. We calculated the average power *P* for a given calcium activity time series *X* using Parseval’s Theorem for discrete-time signals [33]

$$P=\frac{1}{T}\sum_{i=1}^{T}X_{i}{}^{2}$$

where the length of the time series *T* is 900 for the *Xenopus* progenitor neuron dataset and 800 for the murine synaptic neuron dataset from [15]. We implemented the calculation using a custom MATLAB script.

### Calculation of Hurst Exponent

We also compared the performance of our Markovian information entropy measure to that of the Hurst Exponent of the time series, which is a type of fractal analysis which investigates self-similarity and predictability in a time series via long-term correlations in the dynamics [34]. The value of the Hurst Exponent *H* ranges between 0 and 1, and 0.5 < *H* < 1 indicates persistent dynamics in the time series (if the value is increasing at a given time point, it is more likely that the value will continue to increase in the following time points, and similarly for a decreasing trend) whereas 0 < *H* < 0.5 indicates antipersistent dynamics (if the value is increasing at a given time point, it is more likely that the value will decrease in the following time points, and vice versa) and *H* = 0.5 indicates a purely random time series [34].

We estimated the Hurst Exponent of each time series in our datasets by computing their rescaled ranges using the procedure described in [34]. To summarize, we selected a 512-timepoint window randomly from within each cell’s time series, and computed the rescaled range of the window. We then computed the average of the rescaled ranges of the two 256-timepoint windows within the first window, then the average of the rescaled ranges of the four 128-timepoint windows within the original window, and so on until the window size was 8 timepoints. The rescaled range of a section of a time series *X* was determined by first computing the mean-adjusted series

$$Y_{i}=X_{i}-E\left[X\right],$$

where *E[X]* denotes the mean of *X*. We then calculated the cumulative deviate series

$$Z_{i}=\sum_{k=1}^{i}Y_{k}$$

and used it to determine the range series

$$R_{i}=\underset{}{\text{max}}\left\{Z_{1},Z_{2},\ldots ,Z_{i}\right\}-\underset{}{\text{min}}\left\{Z_{1},Z_{2},\ldots ,Z_{i}\right\}.$$

We then calculated the standard deviation series

$$S_{i}=\sqrt{\frac{1}{i}\sum_{k=1}^{i}{\left(X_{k}-u_{k}\right)}^{2}},$$

where

$$u_{k}=E[\left\{X_{1},X_{2},\ldots ,X_{k}\right\}],$$

and used it to compute the rescaled range series *(R/S)*_*i*_ *= R*_*i*_ */ S*_*i*_. The average value of this series is the rescaled range of the section *X*. We executed this algorithm using a custom Python script, then used MATLAB to perform a linear regression between the logarithm (base 2) of the window lengths and the averaged rescaled range values obtained at each window length, as described in [33]. We took the slope of this regression line to be the estimate for the Hurst Exponent. Due to the imperfect nature of the approximation, <0.5% of the cells from each stage in our detrended *Xenopus* dataset yielded an *H > 1*, which we discarded from our analyses.

### Statistical Analysis

In assessing the performance of the data analysis methods in this paper, we used a two-sample Kolmogorov-Smirnov Test on the resulting distributions of analysis results to determine the statistical significance of the difference between two populations as determined by the analysis method. In addition to this significance measure, we also computed an effect size measure to assess the magnitude of the difference between the distributions. To do this we used Cohen’s d statistic [29], defined for two distributions *A* and *B* as

$$d=\frac{E\left[A\right]-E\left[B\right]}{s}$$

where *E[]* denotes the mean and

$$s=\sqrt{\frac{\left(\left|A\right|-1\right)s_{A}^{2}+\left(\left|B\right|-1\right)s_{B}^{2}}{\left|A\right|+\left|B\right|-2}},$$

where *| |* represents the number of points in the distribution and *s*^*2*^ represents the sample variance of a distribution.

Cohen’s d provides a signed measure of the difference between two population means with respect to their pooled standard deviation. Cohen determined guideline values of d to qualitatively describe the magnitude of separation between two distributions, labeling 0.20 < |d| <0.50 as ‘small’, 0.50 < |d| < 0.80 as ‘medium’, and |d| > 0.80 as ‘large’ [29]. In our datasets we observed a wide range of d values well above |d| > 0.80, so we chose two additional guideline values of |d| = 1.00 and |d| = 2.00 to aid in visually distinguishing the differences in population separation between analysis methods.

## Results

To test our Markovian Entropy method in a system which displays dynamics which are not governed by an easily classifiable set of visual features, we analyzed the dynamics of calcium activity in neural progenitor cells from *Xenopus laevis* embryos. Progenitor neurons exhibit a diverse range of calcium signals whose waveforms blur the distinctions between the canonical shapes of features like spikes, waves, or oscillations. We measured *in vitro* calcium activity from developing neuronal tissue from *Xenopus laevis* embryos at stages 14 (neural plate stage), 18 (neural tube stage) and 22 (early tailbud stage).

**Fig 4:**
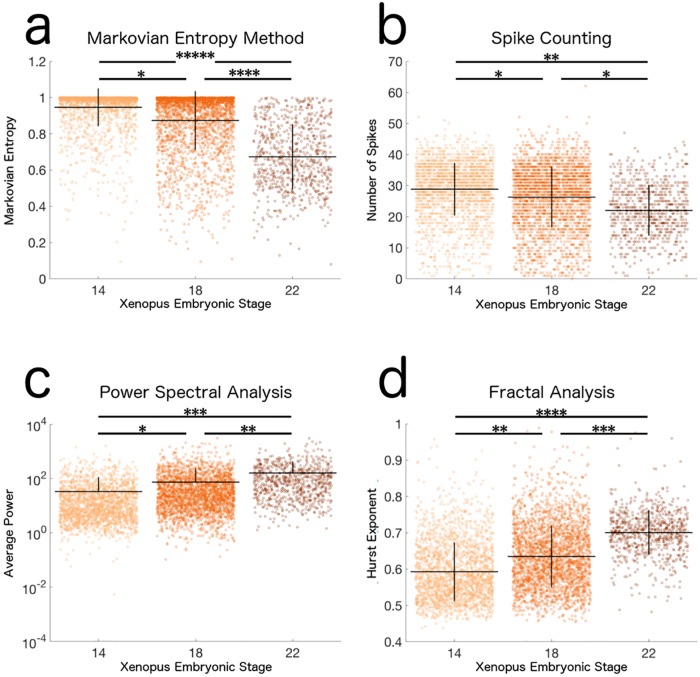
Distributions of Markovian Entropy and other analysis measures of calcium activity from Xenopus laevis neural progenitors. Univariate scatterplots represent the (a) Markovian Entropy, (b) Number of Spikes, (c) Average Power, and (d) Hurst Exponent of Xenopus laevis neural progenitor cells’ calcium activity at embryonic stages 14, 18, and 22. Lines represent mean ± SD of 2,176, 2,664, and 757 cells, respectively. All comparisons between distribution were statistically significant according to a Bonferroni-corrected two-sample Kolmogorov-Smirnov Test (p < 0.01). Hence stars are used to represent the effect size, rather than the significance of difference, between distributions via Cohen’s d statistic (*: |d| ≥ 0.20, **: |d| ≥ 0.50, ***: |d| ≥ 0.80, ****: |d| ≥ 1.00, *****: |d| ≥ 2.00) [29]. Markovian Entropy is calculated with *n* = 2 and *k* = 1.

By applying our method to this data, we found a prominent decrease in the Markovian Entropy of cells’ calcium activity as they progress through development (Cohen’s d: 14 vs. 18, 0.47; 18 vs. 22, 1.18; 14 vs. 22, 2.04) (Fig 4a, Table 1). These results confirm that our method can effectively distinguish between populations which are known to be biologically distinct.

**Table 1:** Separation of Biologically Distinct Populations by Markovian Information Entropy, Average Power, and Hurst Exponent for Xenopus Data and Synaptic Neuron data.

	*Xenopus* dataset						Synaptic Neuron Dataset	
	Stage 14 vs Stage 18		Stage 18 vs Stage 22		Stage 14 vs Stage 22		pH 7.2 vs pH 7.4	
	p	d	p	d	p	d	p	d
Markovian Entropy	2.50E-64	**0.4738**	3.99E-145	**1.1837**	9.33E-240	**2.0429**	8.14E-15	**-0.6598**
Spike Counting	7.72E-20	**0.2895**	9.02E-44	**0.4337**	5.83E-89	**0.7965**	5.12E-132	**2.4956**
Average Power	7.17E-55	**-0.2827**	1.51E-58	**-0.5625**	9.06E-151	**-0.8558**	4.36E-22	**0.6462**
Hurst Exponent	4.45E-57	**-0.5064**	8.92E-95	**-0.8154**	4.42E-182	**-1.4174**	6.06E-07	**-0.3458**

The numerical p values (from a two-sample Komogorov-Smirnov Test) and Cohen’s d values which correspond to the comparisons presented in this paper. Markovian Entropy is calculated using *n* = 2 and *k* = 1 in the Xenopus dataset, and using *n* = 2 and *k* = 2 in the synaptic neuron dataset.

We then assessed the performance of our Markovian Entropy method on this dataset compared to that of other standard data analysis techniques in the calcium analysis field. We computed the number of spikes, the average power, and the Hurst Exponent of each time series from the *Xenopus* dataset (Fig 4; Table 1). All methods separated the populations significantly, and the results from all four analyses suggested an increase in the predictability of the calcium dynamics as the neural progenitors progress through development. However, the extent to which each analysis separated the distinct populations differed with the chosen method. These differences highlight the fact that each method is investigating distinct aspects of the observed calcium dynamics, and hence should be used in complement with each other for a full analysis of the system. In particular, we found that the Markovian Entropy of the calcium activity generally separated the biologically distinct populations to a greater extent than the other three methods, although the Hurst Exponent provided a larger separation between the stage 14 cells and the stage 18 cells. This suggests that the differences between the calcium dynamics in the neural progenitor cells at these different developmental stages of *Xenopus laevis* can be described meaningfully in terms of the level of predictability in the transitions between levels of calcium activity, and perhaps even more meaningfully than a description of increases or decreases in signal power, long-term self-similarity, or spike frequency. We also found that the spike counting method separated the distinct populations to a lesser extent than the other three methods, which suggests that the dynamics in our neural progenitor cells’ calcium activity can be better explained by analysis methods which consider a wider range of time series properties instead of focusing exclusively on spikes.

**Fig 5:**
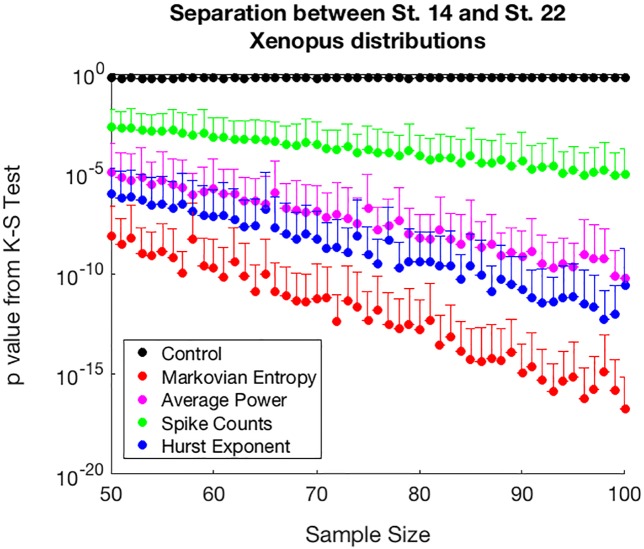
Separation between calcium activity distributions from two biologically distinct populations as a function of sample size. The p-value obtained from a two-sample Kolmogorov-Smirnov test between distributions of calcium activity traces processed by a given analysis method from stage 14 Xenopus neural progenitors and stage 22 Xenopus neural progenitors is used as a measure of separation between the two biologically distinct populations. A smaller p-value indicates a more confident separation between the distributions. Each point represents mean + SD of 5,000 comparisons between samples of a given size taken with replacement from the two distributions. Markovian Entropy is calculated with *n* = 2 and *k =* 1. A randomized control is included that compares two samples which both come from the stage 14 Xenopus population. The Cohen’s d values associated with this data can be found in S3 Fig.

Given that the two-sample Kolmogorov-Smirnov test is a highly sensitive test which tends to return low p values when comparing distributions with large sample sizes, we also assessed the performance of our method in analyses of distributions with small sample size. We computed the p value from a two-sample Kolmogorov-Smirnov test comparing the calcium activity from a subsample of stage 14 *Xenopus* neural progenitor cells to that from a subsample of stage 22 *Xenopus* neural progenitor cells. We found that Markovian Entropy still separates the biologically distinct populations to the greatest extent, and also find that its ability to do so increases with sample size at a faster rate than the other three methods (Fig 5). The strong performance of the methods at separating the data at low sample sizes, and the fact that all four methods return a larger separation between the distributions as sample size increases, both support the notion that these populations do indeed exhibit distinct calcium activity dynamics, and that the Markovian Entropy method’s strong performance at separating the distinct populations in the *Xenopus* dataset is not contingent upon the large sample sizes present in the data.

**Fig 6:**
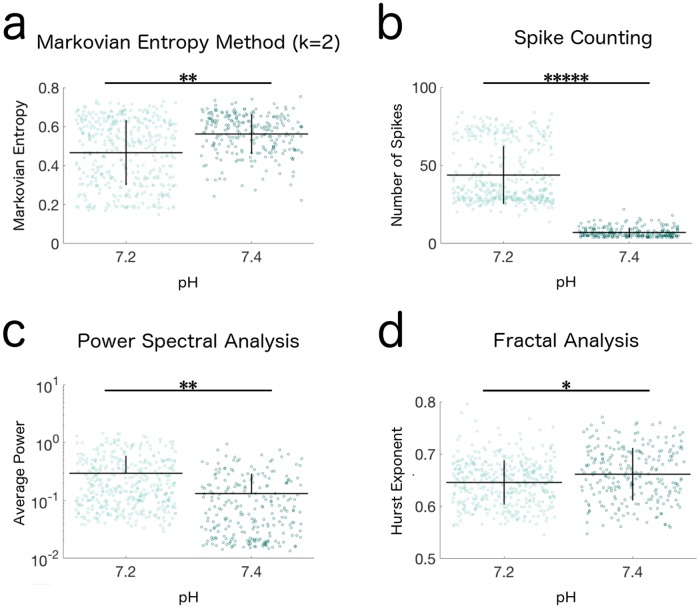
Distributions of Markovian Entropy and other analysis measures of calcium activity from synaptic neurons. Univariate scatterplots represent the (a) Markovian Entropy, (b) Number of Spikes, (c) Average Power, and (d) Hurst Exponent of murine retrotrapezoid nucleus neurons’ calcium activity in solution with pH 7.2 or 7.4. Data received from [15]. Lines represent mean ± SD of 397 and 244 cells, respectively. All comparisons between distributions were statistically significant according to a two-sample Kolmogorov-Smirnov Test (p < 0.01). Hence stars are used to represent the effect size, rather than the significance of difference, between distributions via Cohen’s d statistic (*: |d| ≥ 0.20, **: |d| ≥ 0.50, ***: |d| ≥ 0.80, ****: |d| ≥ 1.00, *****: |d| ≥ 2.00) [29]. Markovian Entropy is calculated with *n* = 2 and *k* = 2.

In addition to establishing the effectiveness of our method on calcium activity data with the complex, irregular dynamics often associated with neural progenitor cells, we also wanted to evaluate the generality of its use by assessing its performance on the analysis of calcium activity which consists predominantly of easily-discernible visual features. While such data would be best analyzed by feature-specific analysis methods, any valid analysis method should still be able to separate distinct populations of feature-dominated calcium activity. We obtained calcium activity data from mature mouse neurons used by Ruffault and colleagues, who found that the activity patterns of retrotrapezoid nucleus neurons positive for two marker genes, Atoh1 and Phox2b, were dependent on the pH of their surrounding solution [15]. As expected for such feature-dominated signals (Fig 1b), spike counting provided the best separation between the measurements from the two pH conditions (Fig 6; Table 1). However, by applying all four analysis methods to these data, we both confirm the authors’ original findings and demonstrate that even in feature-dominated signals, the Markovian Entropy method can still confidently distinguish the biologically distinct populations, and can even do so to a slightly greater extent than the average power or the Hurst Exponent (Fig 6; Table 1).

**Fig 7:**
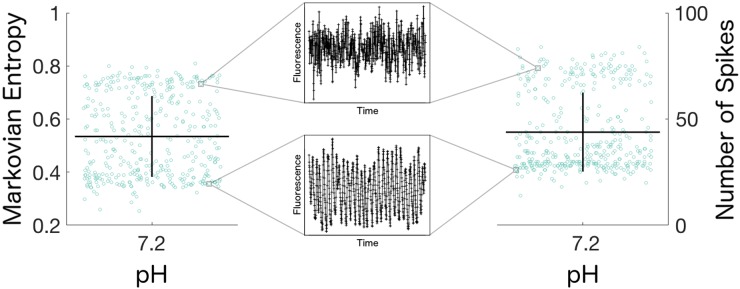
Markovian entropy and spike counting detect two modes of calcium activity. Representative calcium traces from selected cells in the high-entropy / high-spiking cluster and the low-entropy / low-spiking cluster of retrotrapezoid nucleus neurons from embryonic mice in a pH 7.2 solution reveal that both methods detect two distinct modes of calcium activity dynamics in these cells. Lines represent mean ± SD of 397 cells. Markovian Entropy is calculated with *n* = 2 and *k* = 1.

Additionally, we noted that the distribution of spike counts from the cells in the pH 7.2 condition exhibited a bimodality which was not reported in the authors’ original paper. By examining the two subpopulations of cells, we found that these clusters corresponded to two distinct modes of calcium activity (Fig 7). Interestingly, when we evaluated the Markovian Entropy of the calcium activity of the cells in the pH 7.2 condition using an *n* of 2 and a *k* of 1, we found that the measure was also able to observe the presence of these two subpopulations in the data and that the cells in a low- or high-entropy cluster were also contained in the low- or high-spike frequency clusters (Fig 7, S4 Fig). Together, the results from the analyses on the synaptic neuron dataset support the validity of applying the Markovian Entropy measure to calcium activity time series which are dominated by visually-distinguishable features, as well as the potential versatility of the Markovian Entropy measure in detecting population-wide effects that other analysis methods may not be able to discern.

## Discussion

Through the analysis of the *Xenopus laevis* neural progenitor dataset and the murine synaptic neuron dataset, we have shown that holistic data analysis methods such as power spectral analysis, fractal analysis, and our Markovian Entropy method are able to distinguish biologically distinct populations and elucidate insights into the systems of study, regardless of whether their dynamics are predominantly characterized by a set of distinct visual features or if they are more irregular (Figs 4 and 6; Table 1). Our analysis also supports the case for using the Markovian Entropy measure in combination with these other methods to obtain the most insight when investigating the calcium dynamics of a system. Although our Markovian Entropy measure was able to provide the overall greatest degree of separation between the biologically distinct populations amongst the methods tested on the *Xenopus* dataset, for each analysis method that we tested the differences in the extent of this separation and the way that these separations were influenced by the nature of the calcium dynamics were distinct (Figs 4 and 6; Table 1). Together, these results highlight the fact that each method analyzes a distinct aspect of the observed calcium activity, and that the aspect analyzed by the Markovian Entropy measure, the level of predictability in the state transitions of a Markov Process which approximates the observed activity, is potentially a more effective way to describe the underlying calcium dynamics in time series like those from our *Xenopus* dataset which display an irregular type of calcium activity.

Furthermore, our analysis of the calcium activity from murine synaptic neurons in pH 7.2 solution reveals that the distinctions between the analysis methods are not restricted to quantitative differences in the extent of separation, but can also be differences in the qualitative insights obtained regarding the nature of the measured populations, where the Markovian Entropy measure and spike counting were able to reveal the presence of distinct subpopulations of calcium activity that we did not observe using the average power or the Hurst Exponent (Fig 7). Though these analyses do not explicitly point to potential mechanistic causes for such phenomena in these datasets, and although the Markovian Entropy method in particular does not reveal which specific aspects of the time series’ dynamics contributed in what ways to the eventual entropy value, our results demonstrate that the insights obtained from the use of the Markovian Entropy method in concert with other analysis methods can open new avenues for exploratory investigations in poorly-characterized systems.

The presence of free parameters in an analysis method can often induce uncertainties into its results and interpretations, particularly when choices for the values of these parameters are not supported by methodological or physiological justifications. This is the case for spike counting methods, where the choice of amplitude threshold for defining a spike can qualitatively change the results for the analysis (S5 Fig). However, our Markovian Entropy method provides simple guidelines for the methodical selection of parameter values, and we have found that the choices for *n* and *k* in the analyses on disparate datasets do not qualitatively affect the interpretations of the results provided they are within these guidelines (Fig 3). Thus the presence of these guidelines alleviates the introduction of parameter-based uncertainties into the results, allowing for easier implementation and adoption of this method in future analyses. This allows the parameterization in our method to instead confer a versatility in its analyses, as demonstrated by the fact that by altering *k* from 2 to 1 in the synaptic neuron dataset, the Markovian Entropy method was able to reveal the presence of distinct subpopulations of cells distinguished by their calcium activity in the pH 7.2 condition (Fig 7).

Given these promising initial results on the effectiveness of the Markovian Entropy measure, future work would expand the scope of the experimental pipeline which the analysis method covers in order to further improve its accessibility and ease of implementation. Recently developed highly sophisticated feature-based methods can take fluorescent image files of populations of cells as their inputs, and return statistical analyses that incorporate both spatial and temporal information from the experimental measurements into their assessments of calcium dynamics [12, 35, 36]. Our Markovian Information Entropy method is currently restricted to the subset of the analysis process which converts a calcium activity time series into a single numerical value for downstream analysis, and so any upstream image processing or downstream statistical analysis must be conceived and performed by the experimenter. Recent progress in the field of calcium activity analysis has, however, led to the development of holistic analysis methods which explicitly integrate upstream and downstream aspects. A particularly influential example is the method developed by Mukamel and coauthors [37] which extracts calcium activity information from image files and performs an independent component analysis to relatively cluster the cells in an experimental condition with respect to each others’ activity patterns before proceeding to a feature identification algorithm. As more comprehensive and sophisticated methods for calcium activity analysis emerge, we anticipate that they will integrate the results from several complementary analysis methods, such as our Markovian Entropy measure, to yield deeper insights in the study of such a ubiquitous phenomenon.

## Supporting Information

S1 Fig Distributions of Markovian Entropy and other analysis measures of calcium activity from raw, non-detrended time series from Xenopus laevis neural progenitors. Univariate scatterplots represent the (a) Markovian Entropy, (b) Number of Spikes, (c) Average Power, and (d) Hurst Exponent of Xenopus laevis neural progenitor cells’ calcium activity at embryonic stages 14, 18, and 22. Lines represent mean ± SD of 2,176, 2,664, and 757 cells, respectively. All comparisons between distribution were statistically significant according to a Bonferroni-corrected two-sample Kolmogorov-Smirnov Test (p < 0.01). Hence stars are used to represent the effect size, rather than the significance of difference, between distributions via Cohen’s d statistic (*: |d| ≥ 0.20, **: |d| ≥ 0.50, ***: |d| ≥ 0.80, ****: |d| ≥ 1.00, *****: |d| ≥ 2.00) [29]. Markovian Entropy is calculated with *n* = 2 and *k* = 1.
(TIF)

S2 Fig Parameter choices do not qualitatively change the biological interpretation of our markovian entropy measure. Cohen’s d values [29] comparing the effect size between distributions of markovian entropy values for time series of cellular calcium activity between stage 14 and stage 22 Xenopus laevis embryos, using (a) the full 900-point time series, (b) only the first 400 timepoints of each time series, (c) only the first 300 timepoints of each time series, or (d) only the first 100 timepoints of each time series. The sign change in d value occurs at lower values of n and k when the Xenopus time series are truncated to shorter lengths, which supports the conclusion that the sign-change phenomenon is an artifact arising from an insufficient time series length for a given matrix size. Panel (a) is replicated from panel (c) in Fig 3 of the main text.
(TIF)

S3 Fig Separation between calcium activity distributions from two biologically distinct populations as a function of sample size. The Cohen’s d values obtained from the comparisons, presented in Fig 5 of the main text, between distributions of calcium activity traces processed by a given analysis method from stage 14 Xenopus neural progenitors and stage 22 Xenopus neural progenitors. Each point represents mean + SD of 5,000 comparisons between samples of a given size taken with replacement from the two distributions. A randomized control is included that compares two samples which both come from the stage 14 Xenopus population.
(TIF)

S4 Fig The relationship between spike counts and Markovian Entropy in cells in the pH 7.2 condition of the synaptic neuron dataset. Cells from the pH 7.2 condition of the dataset from [15] with low or high spike frequency correspond to having low or high Markovian Entropy, calculated with *n* = 2 and *k* = 1.
(TIF)

S5 Fig The choice of amplitude threshold for spike counting algorithms can influence the qualitative interpretation of the analysis results. Varying the choice of amplitude threshold which defines a spike in the spike counting algorithm used in the main text causes a change in the sign of the effect size (measured by Cohen’s d value) in the comparison between calcium activity from neuronal progenitors in stage 14 and stage 22 Xenopus laevis embryos. Without thorough guidelines or physiological justifications for the choice of amplitude threshold, this introduces uncertainty into the results and interpretations of analyses conducted with such methods.
(TIF)

S1 Table Separation of Biologically Distinct Populations by Markovian Information Entropy, Average Power, and Hurst Exponent for raw, non-detrended Xenopus laevis time series. (XLSX)

S2 Table Parameter choices do not qualitatively change the biological interpretation of our markovian entropy measure. Cohen’s d values [29] corresponding to Fig 3 of the main text, comparing the effect size between distributions of markovian entropy values for cellular calcium activity of stage 14 and stage 18 Xenopus laevis embryos, stage 18 and stage 22 Xenopus laevis embryos, stage 14 and stage 22 Xenopus laevis embryos, and mature retrotrapezoid nucleus neurons from embryonic mice in pH 7.4 solution vs. pH 7.2 solution (retrotrapezoid nucleus neuron data obtained from [15]). At large values of n and k, a sign change in d value occurs which is a technical artifact arising from there being more entries in the transition matrix than can be filled by data from our time series.
(XLSX)

S1 File Traces of every time series from Xenopus stage 14 dataset, baseline corrected. (PDF)

S2 File Traces of every time series from Xenopus stage 18 dataset, baseline corrected. (PDF)

S3 File Traces of every time series from Xenopus stage 22 dataset, baseline corrected. (PDF)

S4 File Traces of every time series from Xenopus stage 14 dataset. (PDF)

S5 File Traces of every time series from Xenopus stage 18 dataset. (PDF)

S6 File Traces of every time series from Xenopus stage 22 dataset. (PDF)

S7 File Traces of every time series from the Ruffault et al. dataset in the pH 7.2 condition. (PDF)

S8 File Traces of every time series from the Ruffault et al. dataset in the pH 7.4 condition. (PDF)

## References

[1] Hennings H, Michael D, Cheng C, Steinert P, Holbrook K, Yuspa SH. Calcium regulation of growth and differentiation of mouse epidermal cells in culture. Cell. 1980 1 31;19(1):245–54. 6153576 10.1016/0092-8674(80)90406-7

[2] Mintz IM, Sabatini BL, Regehr WG. Calcium control of transmitter release at a cerebellar synapse. Neuron. 1995 9 30;15(3):675–88. 7546746 10.1016/0896-6273(95)90155-8

[3] Smaili SS, Pereira GJ, Costa MM, Rocha KK, Rodrigues L, do Carmo LG, et al The role of calcium stores in apoptosis and autophagy. Current molecular medicine. 2013 2 1;13(2):252–65. 23228221 10.2174/156652413804810772

[4] Michod D, Bartesaghi S, Khelifi A, Bellodi C, Berliocchi L, Nicotera P, et al Calcium-dependent dephosphorylation of the histone chaperone DAXX regulates H3. 3 loading and transcription upon neuronal activation. Neuron. 2012 4 12;74(1):122–35. 10.1016/j.neuron.2012.02.021 22500635 PMC3657165

[5] Berridge MJ, Bootman MD, Roderick HL. Calcium signalling: dynamics, homeostasis and remodelling. Nature reviews Molecular cell biology. 2003 7 1;4(7):517–29. 10.1038/nrm1155 12838335

[6] Spitzer NC, Olson E, Gu X. Spontaneous calcium transients regulate neuronal plasticity in developing neurons. Developmental Neurobiology. 1995, 3; 26(3):316–324. 10.1002/neu.480260304 7775965

[7] Khodagholy D, Gelinas JN, Thesen T, Doyle W, Devinsky O, Malliaras GG, et al NeuroGrid: recording action potentials from the surface of the brain. Nature neuroscience. 2015 2 1;18(2):310–5. 10.1038/nn.3905 25531570 PMC4308485

[8] Lechleiter J, Girard S. Spiral calcium wave propagation and annihilation in Xenopus laevis oocytes. Science. 1991 4 5;252(5002):123 2011747 10.1126/science.2011747

[9] Quiroga RQ, Nadasdy Z, Ben-Shaul Y. Unsupervised spike detection and sorting with wavelets and superparamagnetic clustering. Neural computation. 2004 8;16(8):1661–87. 10.1162/089976604774201631 15228749

[10] Yaksi E, Friedrich RW. Reconstruction of firing rate changes across neuronal populations by temporally deconvolved Ca2+ imaging. Nature Methods. 2006 5 1;3(5):377–83. 10.1038/nmeth874 16628208

[11] Theis L, Berens P, Froudarakis E, Reimer J, Rosón MR, Baden T, et al. Supervised learning sets benchmark for robust spike detection from calcium imaging signals. arXiv preprint arXiv:1503.00135. 2015 Feb 28.

[12] Pnevmatikakis EA, Soudry D, Gao Y, Machado TA, Merel J, Pfau D, et al Simultaneous denoising, deconvolution, and demixing of calcium imaging data. Neuron. 2016 1 20;89(2):285–99. 10.1016/j.neuron.2015.11.037 26774160 PMC4881387

[13] Oñativia J, Schultz SR, Dragotti PL. A finite rate of innovation algorithm for fast and accurate spike detection from two-photon calcium imaging. Journal of neural engineering. 2013 7 17;10(4):046017 10.1088/1741-2560/10/4/046017 23860257 PMC4038919

[14] Freeman J, Vladimirov N, Kawashima T, Mu Y, Sofroniew NJ, Bennett DV, et al Mapping brain activity at scale with cluster computing. Nature methods. 2014 9 1;11(9):941–50. 10.1038/nmeth.3041 25068736

[15] Ruffault PL, D'Autréaux F, Hayes JA, Nomaksteinsky M, Autran S, Fujiyama T, et al The retrotrapezoid nucleus neurons expressing Atoh1 and Phox2b are essential for the respiratory response to CO2. Elife. 2015 4 13;4:e07051. 10.7554/eLife.07051 PMC4429526 25866925

[16] Cover TM, Thomas JA. Elements of information theory. John Wiley & Sons; 2012 11 28.

[17] Nakano T, Liu JQ. Information transfer through calcium signaling. In International Conference on Nano-Networks 2009 Oct 18 (pp. 29–33). Springer Berlin Heidelberg.

[18] Prank K, Gabbiani F, Brabant G. Coding efficiency and information rates in transmembrane signaling. Biosystems. 2000 2 29;55(1):15–22. 10745104 10.1016/s0303-2647(99)00078-7

[19] Pahle J, Green AK, Dixon CJ, Kummer U. Information transfer in signaling pathways: a study using coupled simulated and experimental data. BMC bioinformatics. 2008 3 4;9(1):1 18318909 10.1186/1471-2105-9-139 PMC2323387

[20] Cline HT, Kelley D eds. Special Issue: Xenopus as an Experimental System for Developmental Neuroscience. Developmental Neurobiology. 2012 4 1; 72(4):463–675. 10.1002/dneu.22012 22328291

[21] Sive HL, Grainger RM, Harland RM. Early Development of *Xenopus laevis*: A Laboratory Manual. Cold Spring Harbor, NY: Cold Spring Harbor Press, 2000.

[22] Nieuwkoop PD Faber J, (1994) Normal Table of *Xenopus laevis* (Daudin). New York: Garland Publishing Inc., 1994.

[23] Gu X, Spitzer NC. Distinct aspects of neuronal differentiation encoded by frequency of spontaneous Ca2+ transients. Nature. 1995 6 29;375(6534):784–7. 10.1038/375784a0 7596410

[24] Root CM, Velázquez-Ulloa NA, Monsalve GC, Minakova E, Spitzer NC. Embryonically expressed GABA and glutamate drive electrical activity regulating neurotransmitter specification. J Neurosci. 2008 4 30;28(18):4777–84. 10.1523/JNEUROSCI.4873-07.2008 18448654 PMC3318922

[25] Rosenberg SS, Spitzer NC. Calcium signaling in neuronal development. Cold Spring Harbor perspectives in biology. 2011 10 1;3(10):a004259 10.1101/cshperspect.a004259 21730044 PMC3179332

[26] Eilers PH, Boelens HF. Baseline correction with asymmetric least squares smoothing. Leiden University Medical Centre Report. 2005 Oct 21;1:1.

[27] Marken J, Halleran A. A Markovian Entropy Measure for Calcium Activity. https://github.com/jpmarken/markovian-entropy-calcium 10.1371/journal.pone.0168342 PMC5158058 27977764

[28] Bickenbach F, Bode E. Evaluating the Markov property in studies of economic convergence. International Regional Science Review. 2003 7 1;26(3):363–92.

[29] Cohen J. Statistical power analysis for the behavioral sciences. 2nd ed Hillsdale, NJ: Lawrence Erlbaum Associates; 1988.

[30] Shannon CE. A mathematical theory of communication. ACM SIGMOBILE Mobile Computing and Communications Review. 2001 1 1;5(1):3–55.

[31] Gorbunova YV, Spitzer NC. Dynamic interactions of cyclic AMP transients and spontaneous Ca2&plus; spikes. Nature. 2002 7 4;418(6893):93–6. 10.1038/nature00835 12097913

[32] Gu X, Olson EC, Spitzer NC. Spontaneous neuronal calcium spikes and waves during early differentiation. The Journal of neuroscience. 1994 11 1;14(11):6325–35. 7965039 10.1523/JNEUROSCI.14-11-06325.1994 PMC6577261

[33] Jenkins GM, Watts D. Spectral analysis and its applications. 1st ed San Francisco: Holden-Day; 1968.

[34] Rasheed K, Qian B. Hurst exponent and financial market predictability. In IASTED conference on Financial Engineering and Applications (FEA 2004) 2004 (pp. 203–209).

[35] Vogelstein JT, Packer AM, Machado TA, Sippy T, Babadi B, Yuste R, et al Fast nonnegative deconvolution for spike train inference from population calcium imaging. Journal of neurophysiology. 2010 12 1;104(6):3691–704. 10.1152/jn.01073.2009 20554834 PMC3007657

[36] Salles A, Billaudeau C, Sergé A, Bernard AM, Phélipot MC, Bertaux N, et al Barcoding T cell calcium response diversity with methods for automated and accurate analysis of cell signals (MAAACS). PLoS Comput Biol. 2013 9 26;9(9):e1003245 10.1371/journal.pcbi.1003245 24086124 PMC3784497

[37] Mukamel EA, Nimmerjahn A, Schnitzer MJ. Automated analysis of cellular signals from large-scale calcium imaging data. Neuron. 2009 9 24;63(6):747–60. 10.1016/j.neuron.2009.08.009 19778505 PMC3282191

