# Supplementary figures and images for: A Markovian Entropy Measure for the Analysis of Calcium Activity Time Series
Source: PLoS One. 2016 Dec 15;11(12):e0168342. doi: 10.1371/journal.pone.0168342 (PMC5158058; doi:10.1371/journal.pone.0168342)

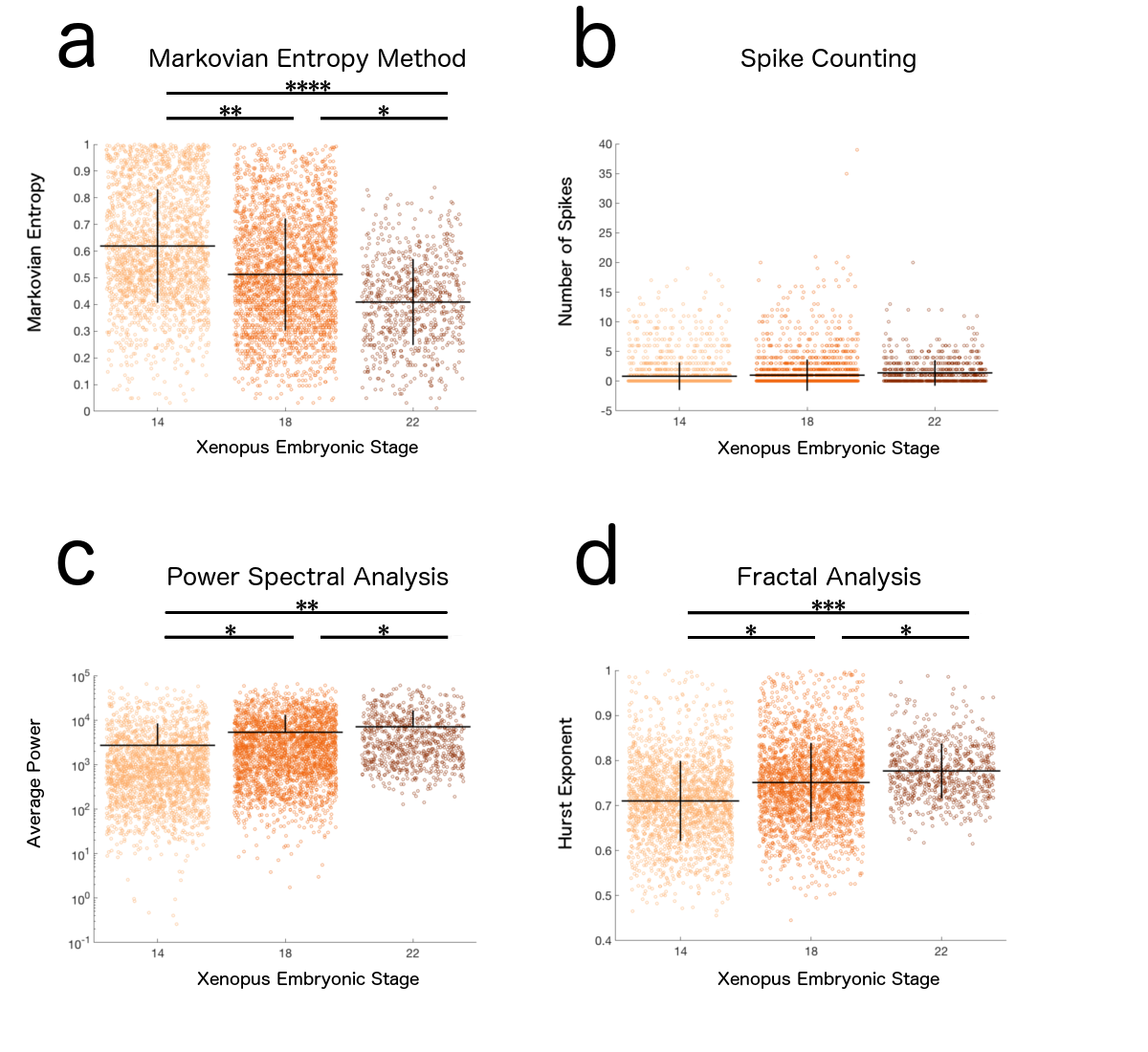

Supplement: S1 Fig — Univariate scatterplots represent the (a) Markovian Entropy, (b) Number of Spikes, (c) Average Power, and (d) Hurst Exponent of Xenopus laevis neural progenitor cells’ calcium activity at embryonic stages 14, 18, and 22. Lines represent mean ± SD of 2,176, 2,664, and 757 cells, respectively. All comparisons between distribution were statistically significant according to a Bonferroni-corrected two-sample Kolmogorov-Smirnov Test (p < 0.01). Hence stars are used to represent the effect size, rather than the significance of difference, between distributions via Cohen’s d statistic (*: |d| ≥ 0.20, **: |d| ≥ 0.50, ***: |d| ≥ 0.80, ****: |d| ≥ 1.00, *****: |d| ≥ 2.00) [29]. Markovian Entropy is calculated with n = 2 and k = 1. (TIF) [file pone.0168342.s001.tif]

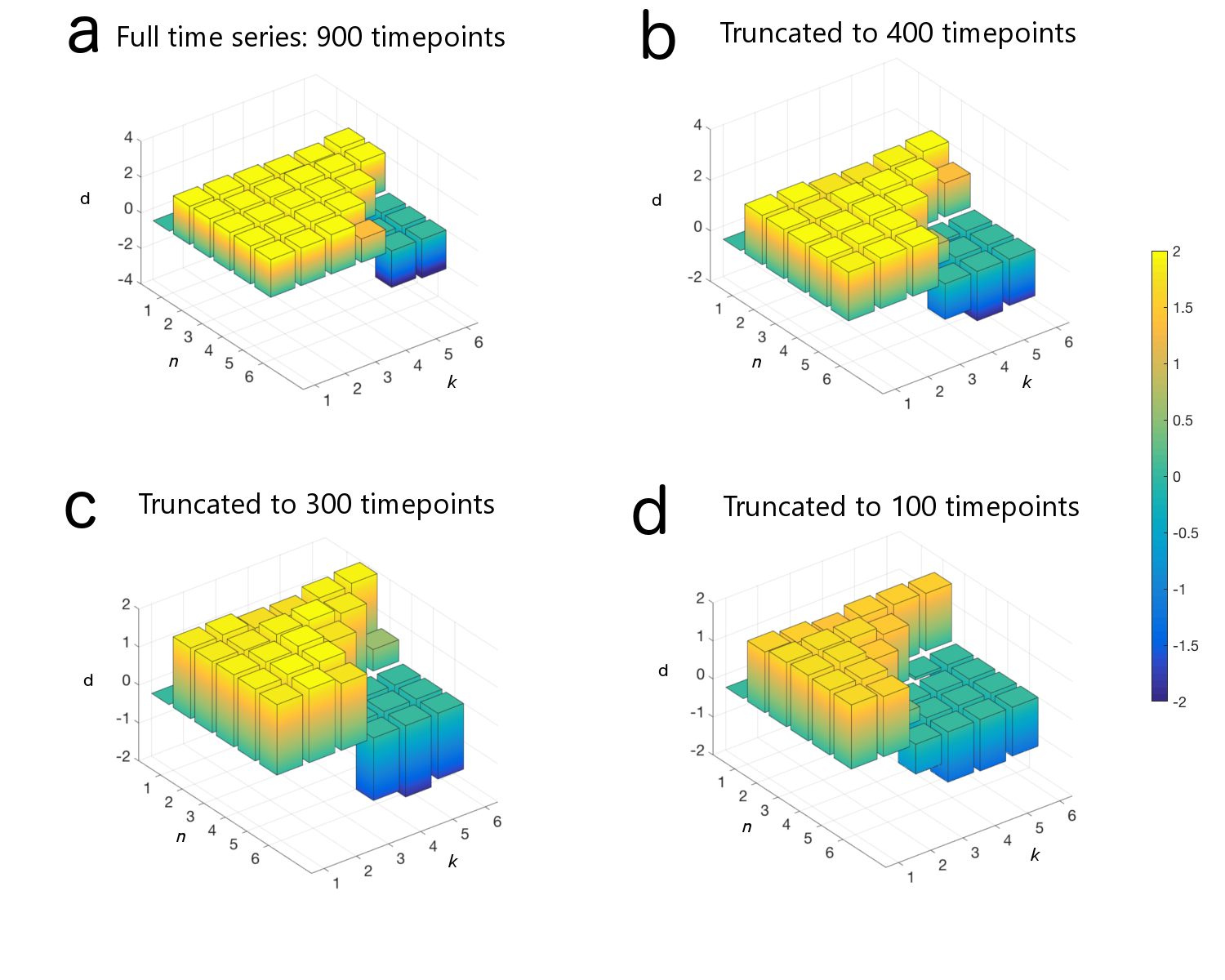

Supplement: S2 Fig — Cohen’s d values [29] comparing the effect size between distributions of markovian entropy values for time series of cellular calcium activity between stage 14 and stage 22 Xenopus laevis embryos, using (a) the full 900-point time series, (b) only the first 400 timepoints of each time series, (c) only the first 300 timepoints of each time series, or (d) only the first 100 timepoints of each time series. The sign change in d value occurs at lower values of n and k when the Xenopus time series are truncated to shorter lengths, which supports the conclusion that the sign-change phenomenon is an artifact arising from an insufficient time series length for a given matrix size. Panel (a) is replicated from panel (c) in Fig 3 of the main text. (TIF) [file pone.0168342.s002.tif]

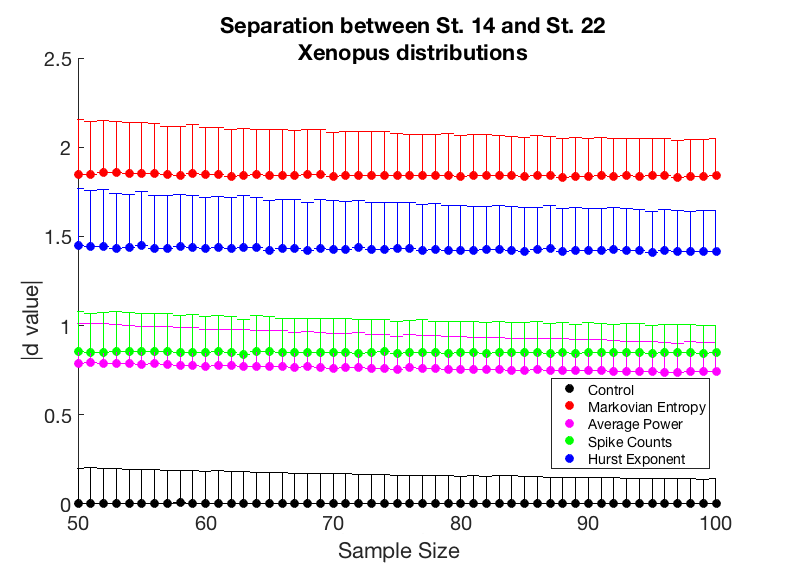

Supplement: S3 Fig — The Cohen’s d values obtained from the comparisons, presented in Fig 5 of the main text, between distributions of calcium activity traces processed by a given analysis method from stage 14 Xenopus neural progenitors and stage 22 Xenopus neural progenitors. Each point represents mean + SD of 5,000 comparisons between samples of a given size taken with replacement from the two distributions. A randomized control is included that compares two samples which both come from the stage 14 Xenopus population. (TIF) [file pone.0168342.s003.tif]

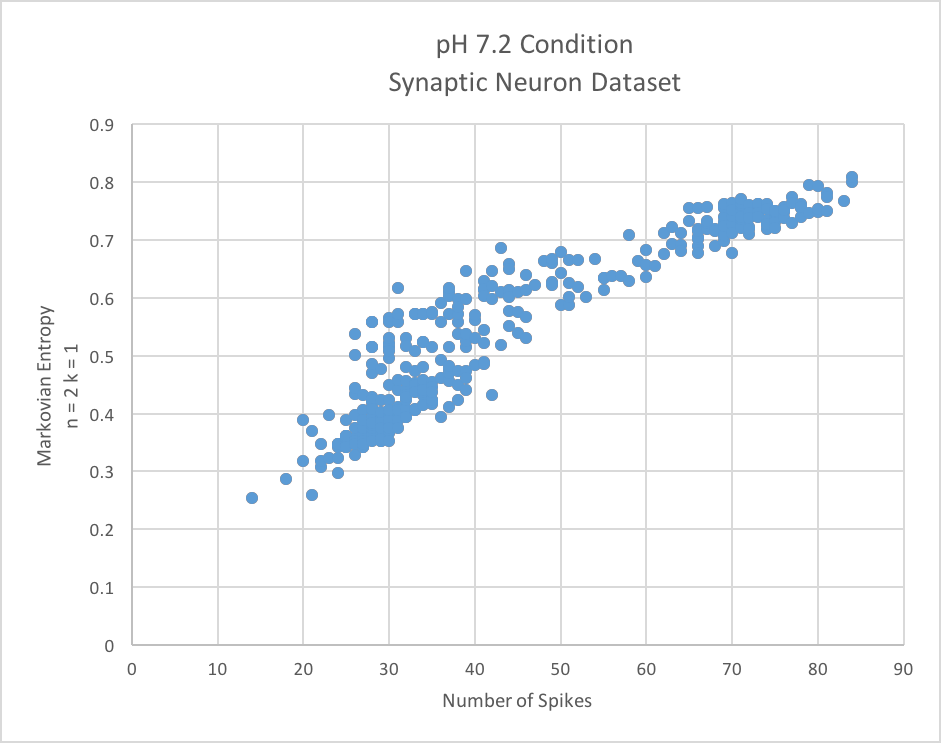

Supplement: S4 Fig — Cells from the pH 7.2 condition of the dataset from [15] with low or high spike frequency correspond to having low or high Markovian Entropy, calculated with n = 2 and k = 1. (TIF) [file pone.0168342.s004.tif]

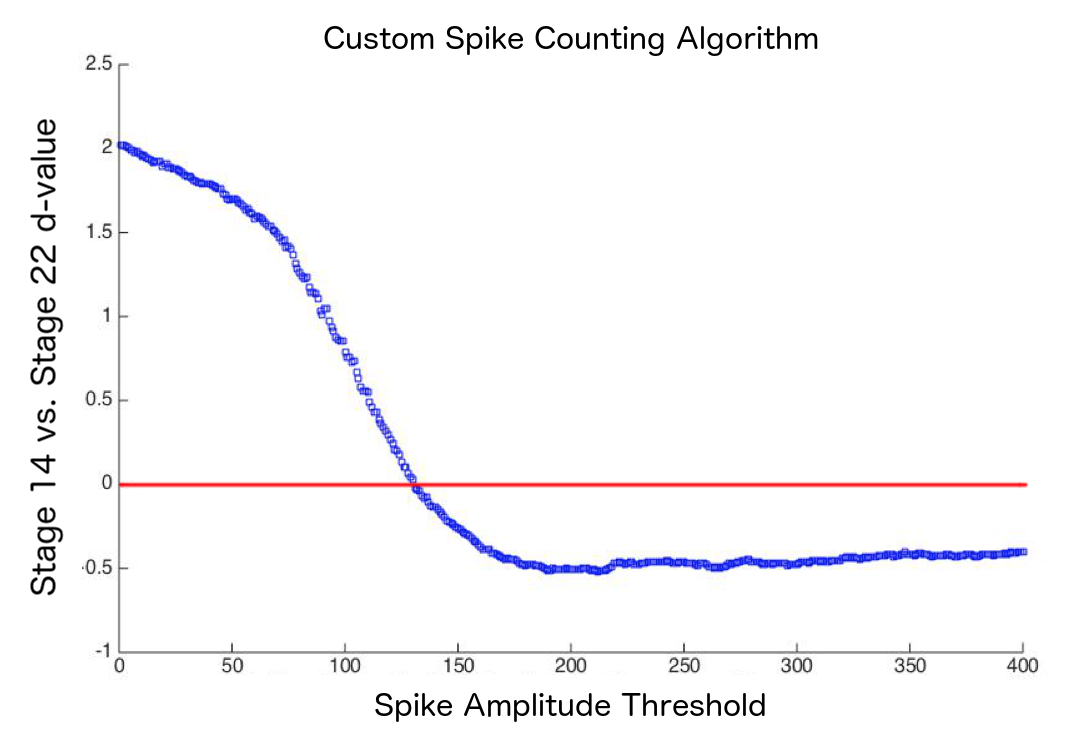

Supplement: S5 Fig — Varying the choice of amplitude threshold which defines a spike in the spike counting algorithm used in the main text causes a change in the sign of the effect size (measured by Cohen’s d value) in the comparison between calcium activity from neuronal progenitors in stage 14 and stage 22 Xenopus laevis embryos. Without thorough guidelines or physiological justifications for the choice of amplitude threshold, this introduces uncertainty into the results and interpretations of analyses conducted with such methods. (TIF) [file pone.0168342.s005.tif]
