# Supplementary material for: A Markovian Entropy Measure for the Analysis of Calcium Activity Time Series
Source: PLoS One. 2016 Dec 15;11(12):e0168342. doi: 10.1371/journal.pone.0168342 (PMC5158058; doi:10.1371/journal.pone.0168342)
Supplement: S3 File — (PDF) [file pone.0168342.s010.pdf]

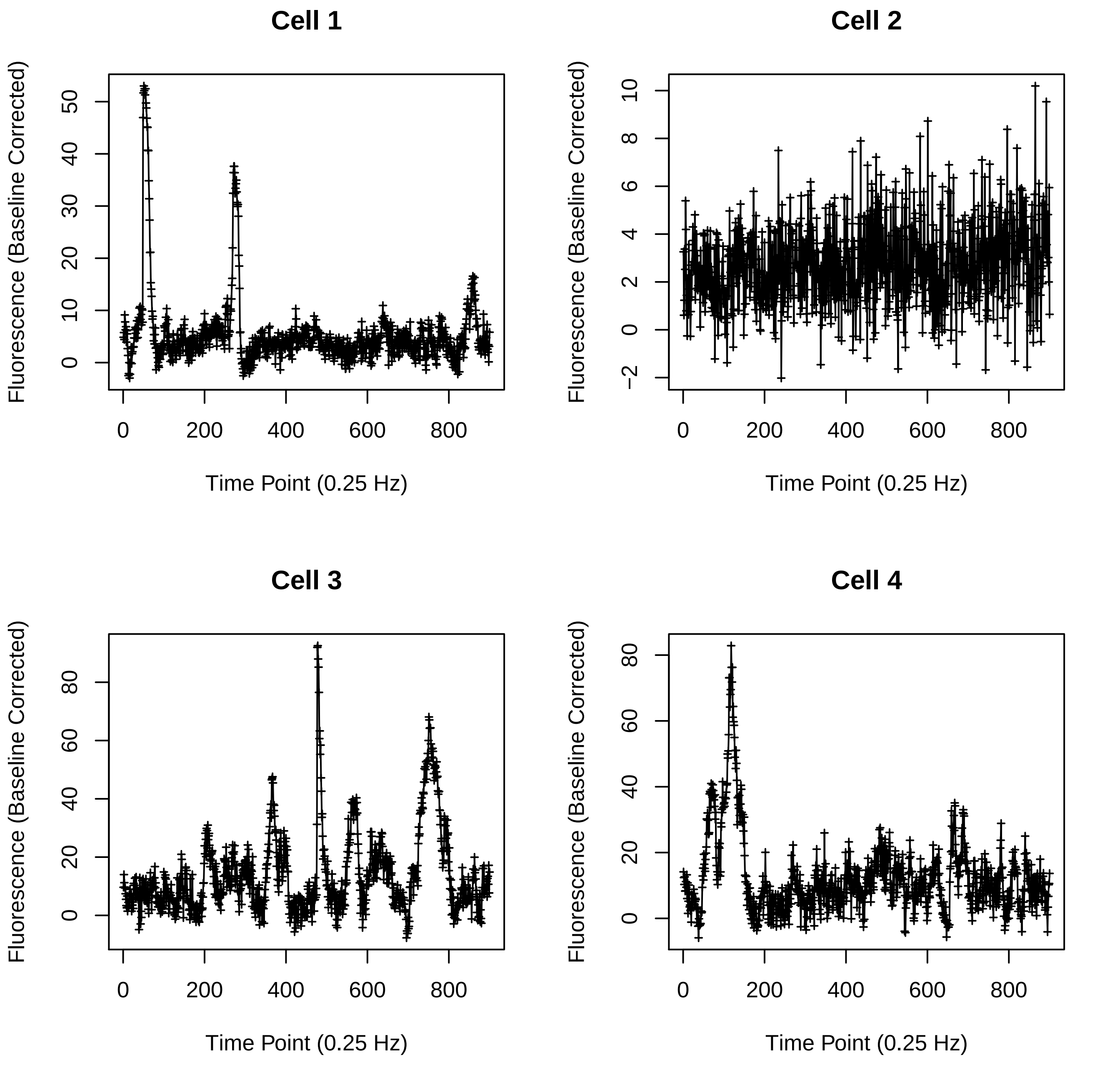
Cell 1
Cell 2
Cell 3
Cell 4
Fluorescence (Baseline Corrected)
Time Point (0.25 Hz)
0
200
400
600
800
10
20
30
40
50
−2
2
4
6
8
60
80

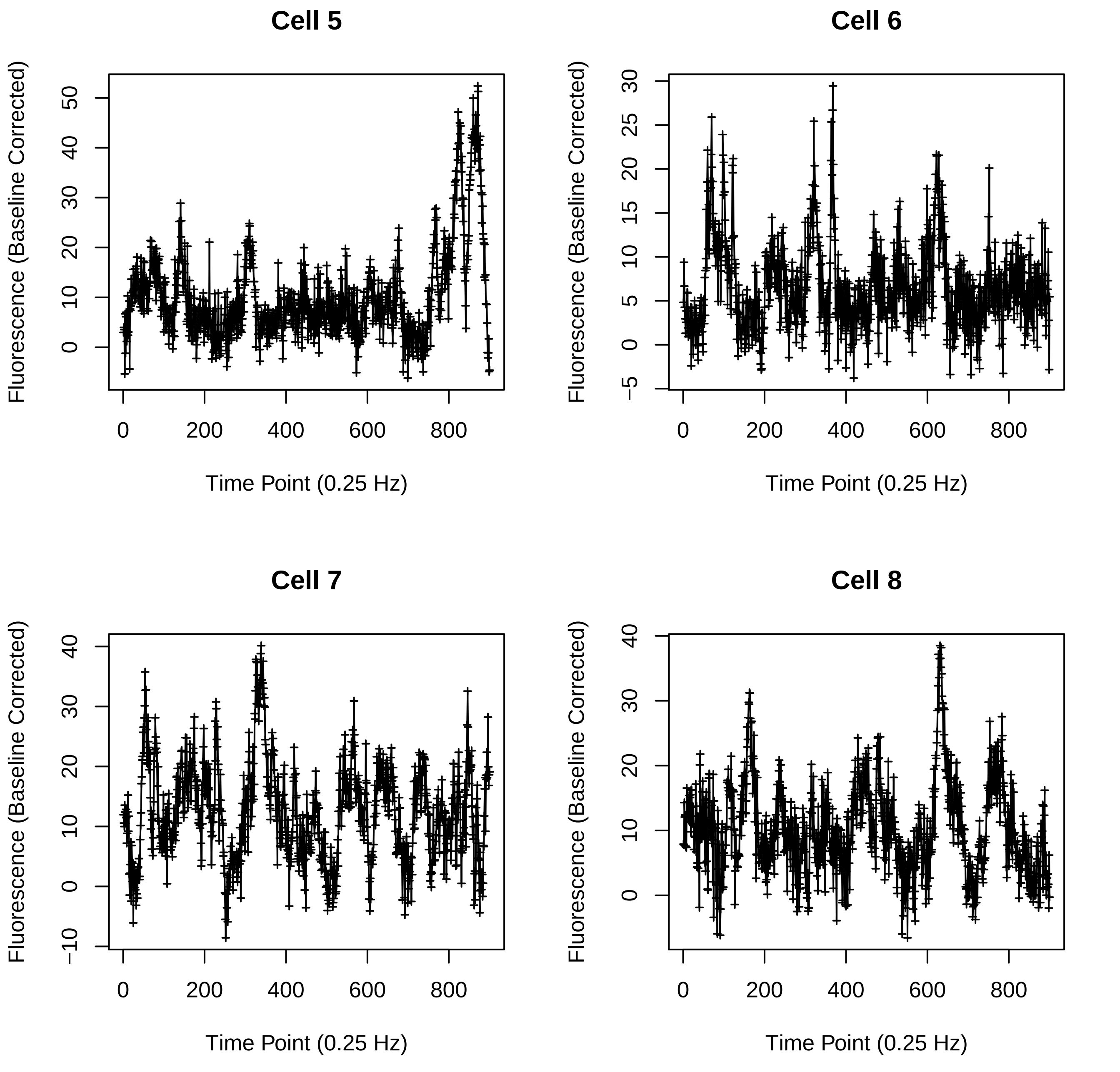
Cell 5
Fluorescence (Baseline Corrected)
0
10
20
30
40
50
0
200
400
600
800
Time Point (0.25 Hz)
Cell 6
Fluorescence (Baseline Corrected)
−5
0
5
10
15
20
25
30
0
200
400
600
800
Time Point (0.25 Hz)
Cell 7
Fluorescence (Baseline Corrected)
−10
0
10
20
30
40
0
200
400
600
800
Time Point (0.25 Hz)
Cell 8
Fluorescence (Baseline Corrected)
0
10
20
30
40
0
200
400
600
800
Time Point (0.25 Hz)

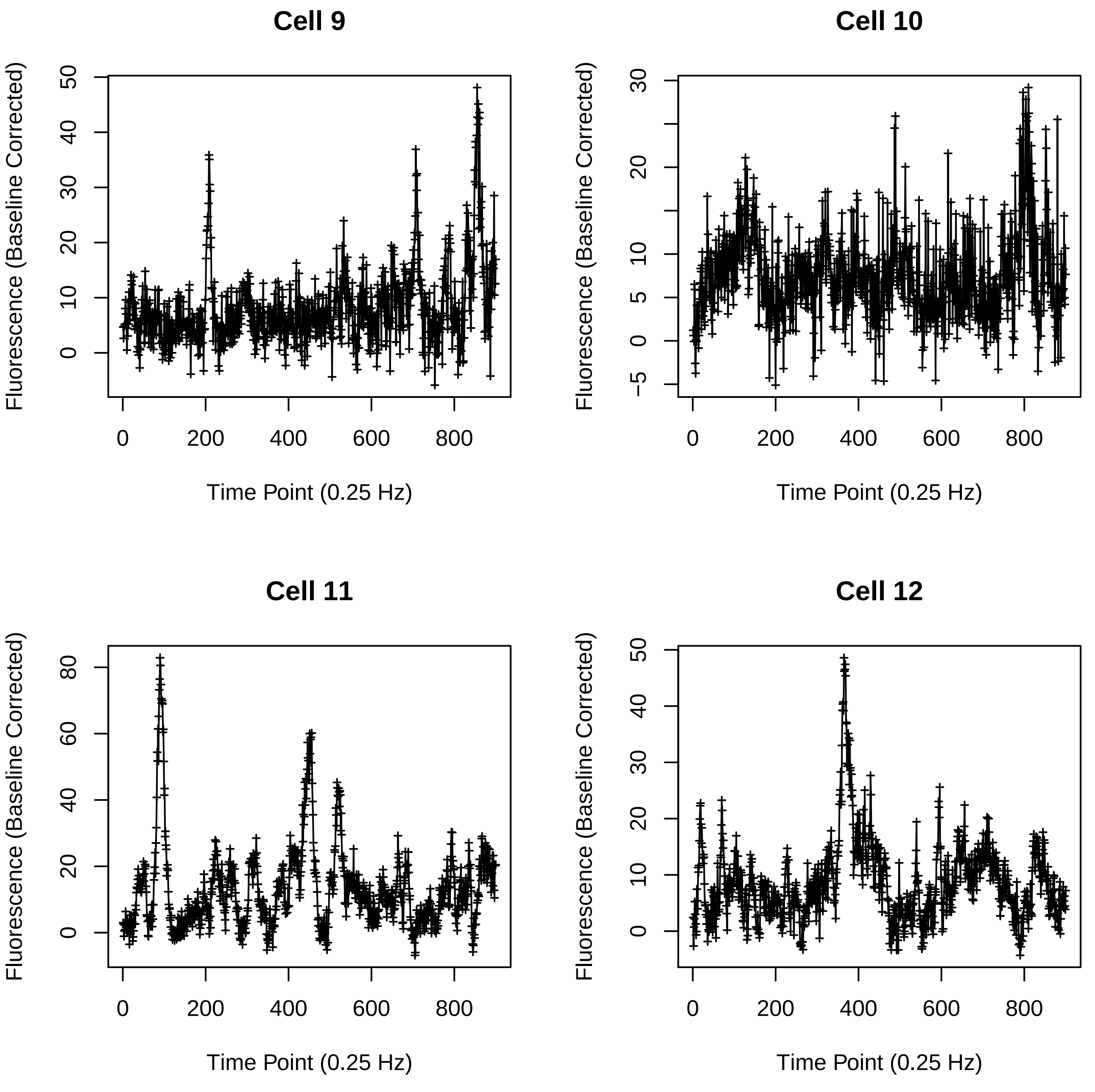
Cell 9
Cell 10
Cell 11
Cell 12
Fluorescence (Baseline Corrected)
Time Point (0.25 Hz)
0
200
400
600
800
0
10
20
30
40
50
−5
5
15
25
60
80

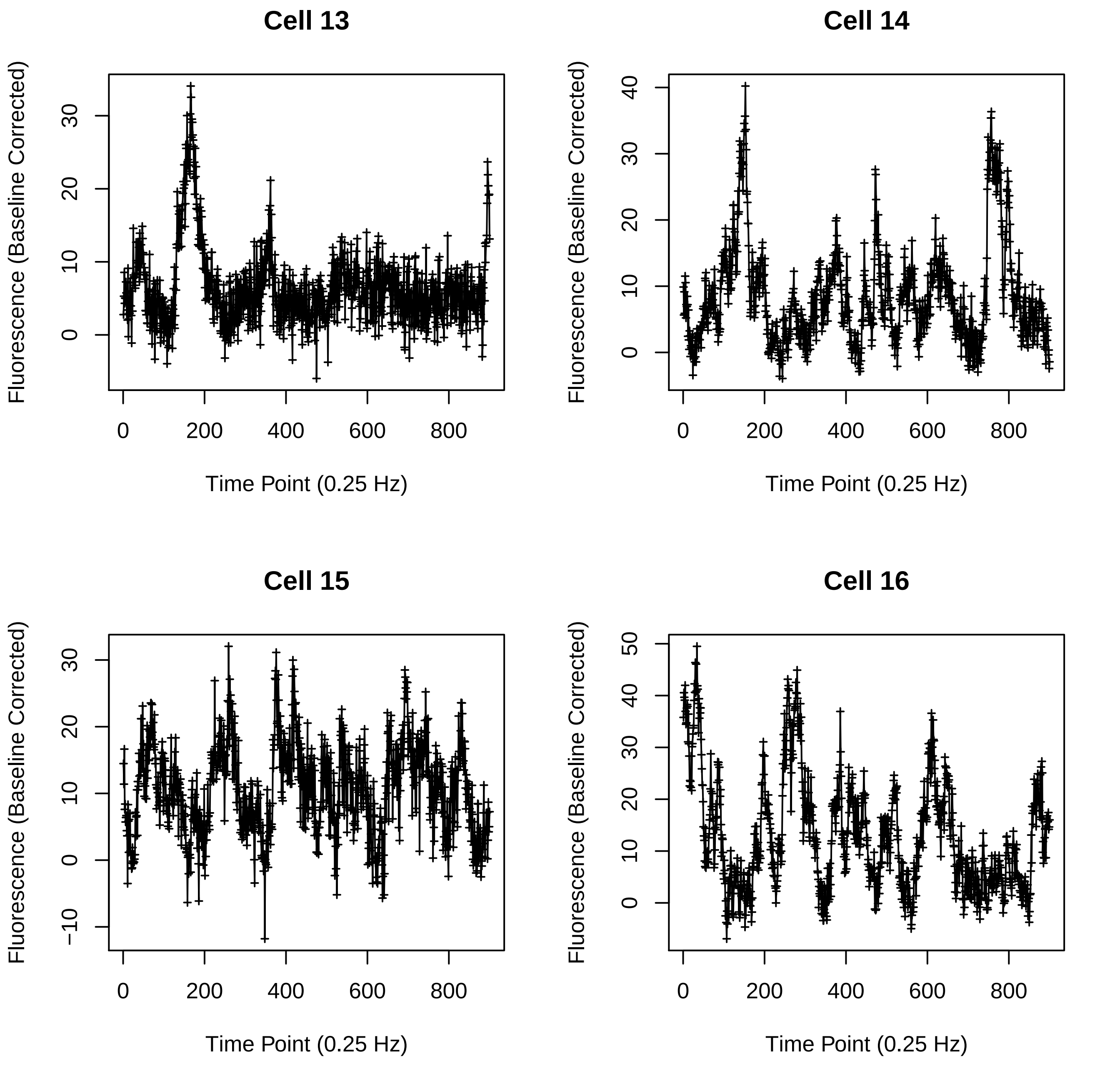

Cell 13
Cell 14
Cell 15
Cell 16
Fluorescence (Baseline Corrected)
Time Point (0.25 Hz)
0
200
400
600
800
-10
10
20
30
40
50

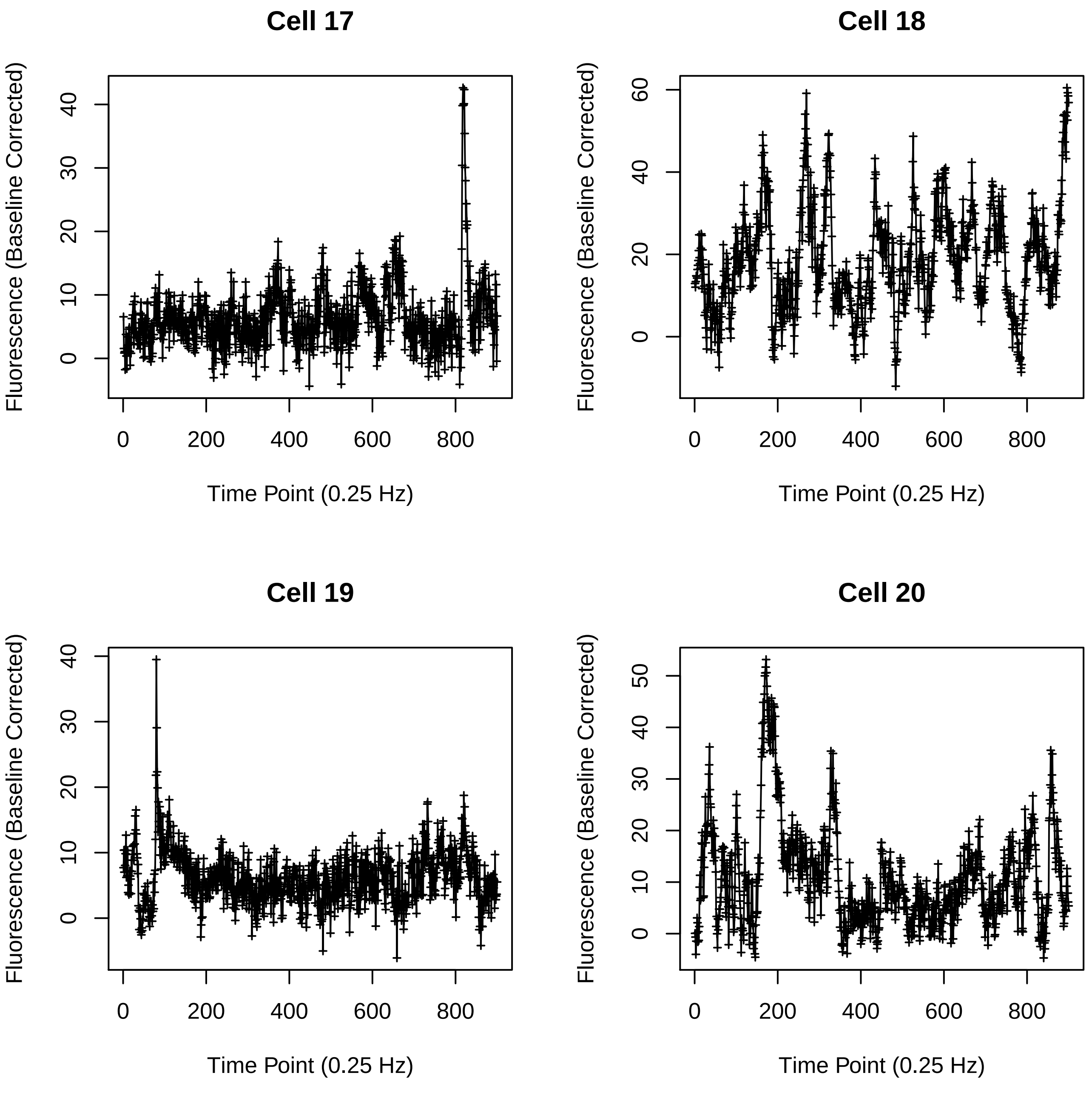
Cell 17
Fluorescence (Baseline Corrected)
0
10
20
30
40
0
200
400
600
800
Time Point (0.25 Hz)
Cell 18
Fluorescence (Baseline Corrected)
0
20
40
60
0
200
400
600
800
Time Point (0.25 Hz)
Cell 19
Fluorescence (Baseline Corrected)
0
10
20
30
40
0
200
400
600
800
Time Point (0.25 Hz)
Cell 20
Fluorescence (Baseline Corrected)
0
10
20
30
40
50
0
200
400
600
800
Time Point (0.25 Hz)

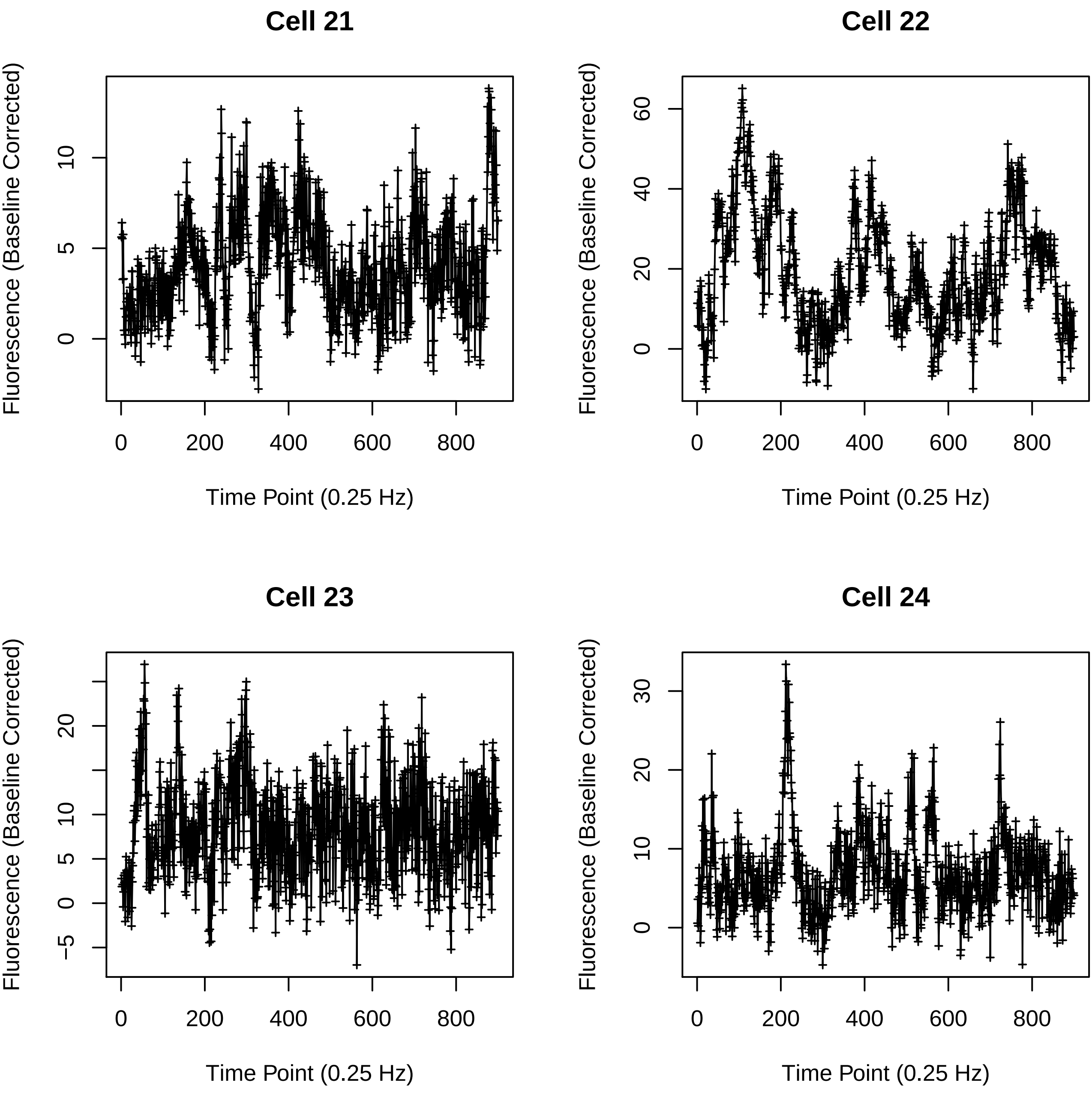
Cell 21
Cell 22
Cell 23
Cell 24
Fluorescence (Baseline Corrected)
Time Point (0.25 Hz)
0
200
400
600
800
0
5
10
20
40
60
−5
15
20
30

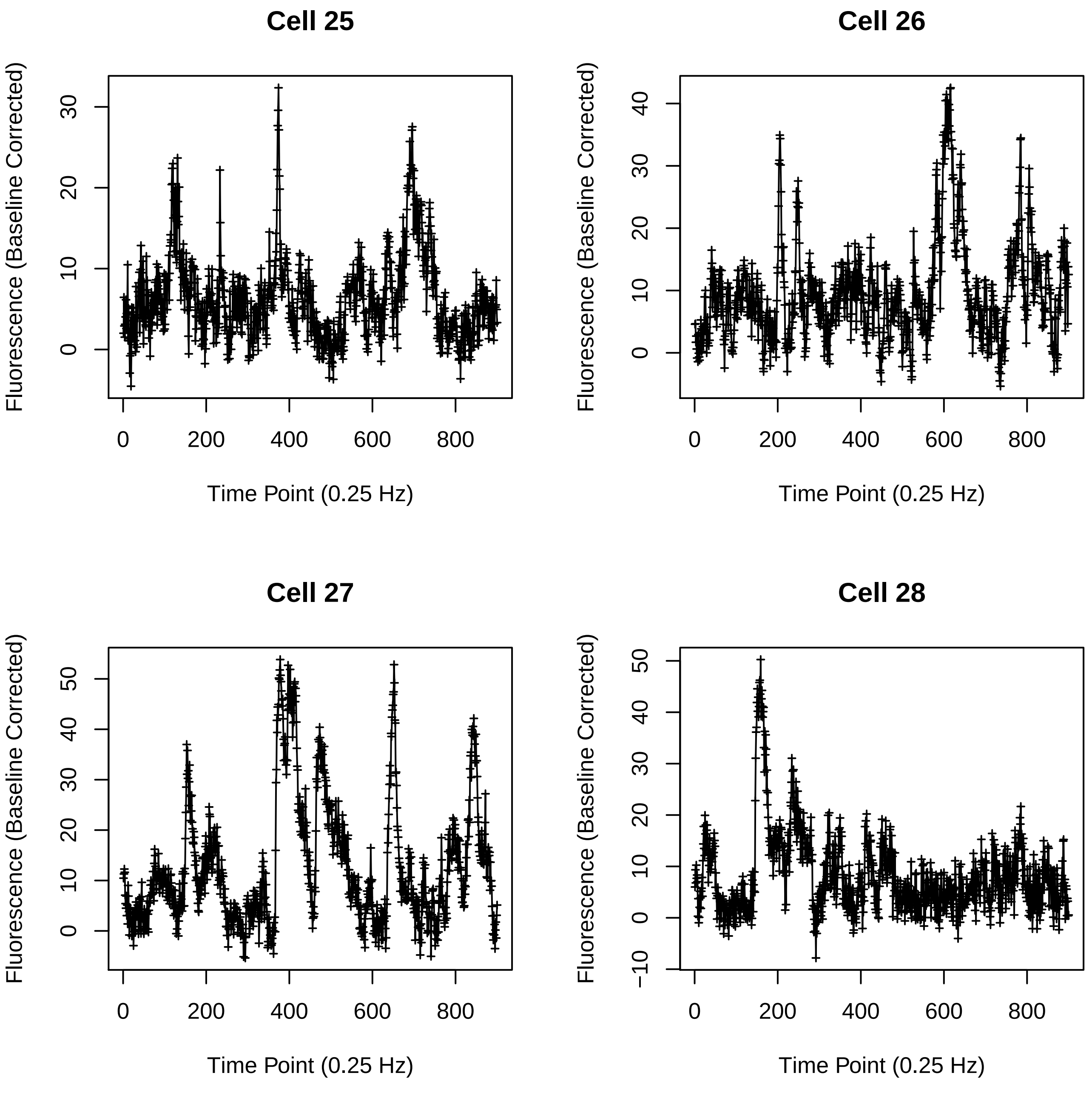
Cell 25
Fluorescence (Baseline Corrected)
0
10
20
30
0
200
400
600
800
Time Point (0.25 Hz)
Cell 26
Fluorescence (Baseline Corrected)
0
10
20
30
40
0
200
400
600
800
Time Point (0.25 Hz)
Cell 27
Fluorescence (Baseline Corrected)
0
10
20
30
40
50
0
200
400
600
800
Time Point (0.25 Hz)
Cell 28
Fluorescence (Baseline Corrected)
−10
0
10
20
30
40
50
0
200
400
600
800
Time Point (0.25 Hz)

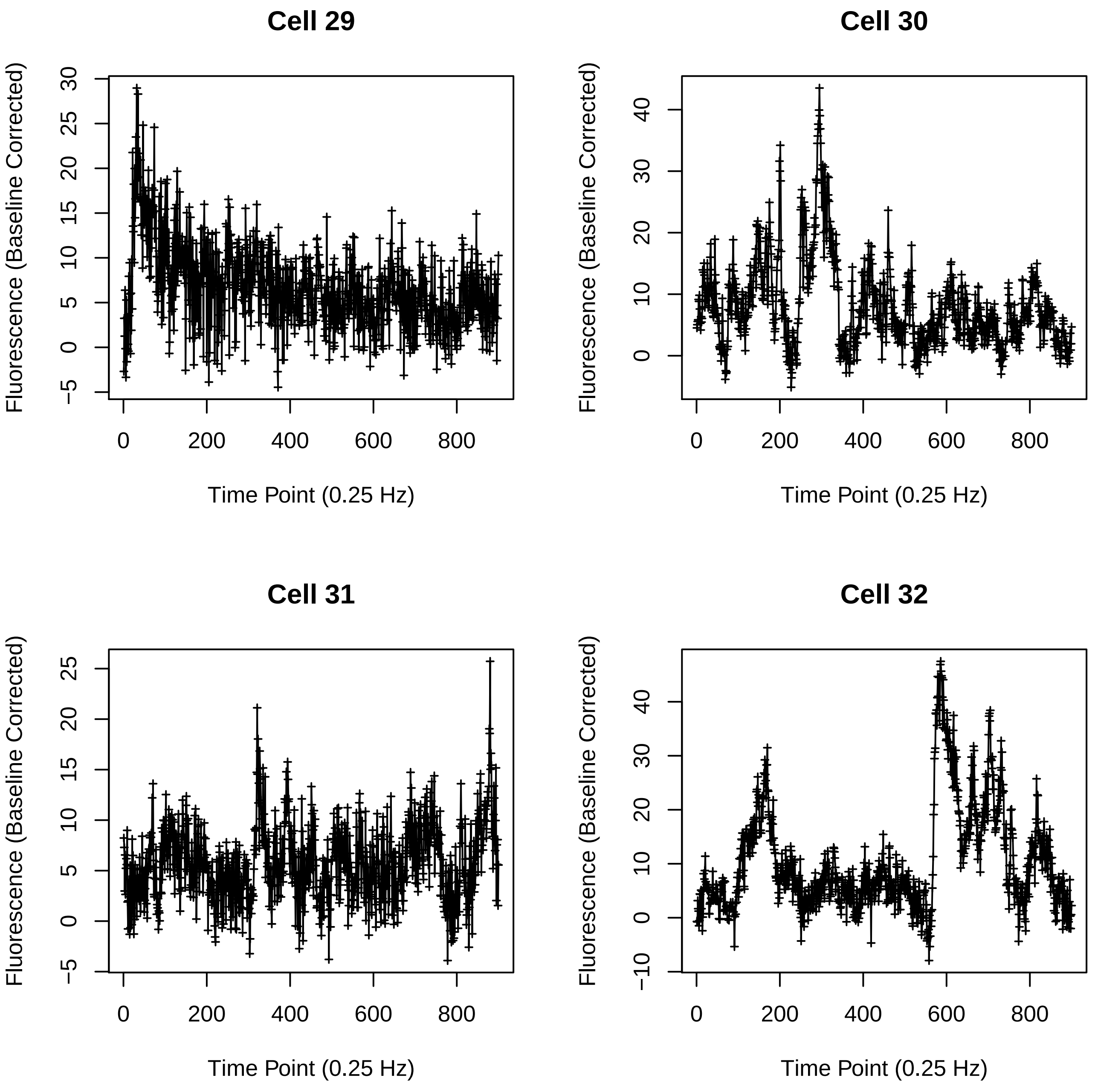
Cell 29
Fluorescence (Baseline Corrected)
−5
0
5
10
15
20
25
30
0
200
400
600
800
Time Point (0.25 Hz)
Cell 30
Fluorescence (Baseline Corrected)
0
10
20
30
40
0
200
400
600
800
Time Point (0.25 Hz)
Cell 31
Fluorescence (Baseline Corrected)
−5
0
5
10
15
20
25
0
200
400
600
800
Time Point (0.25 Hz)
Cell 32
Fluorescence (Baseline Corrected)
−10
0
10
20
30
40
0
200
400
600
800
Time Point (0.25 Hz)

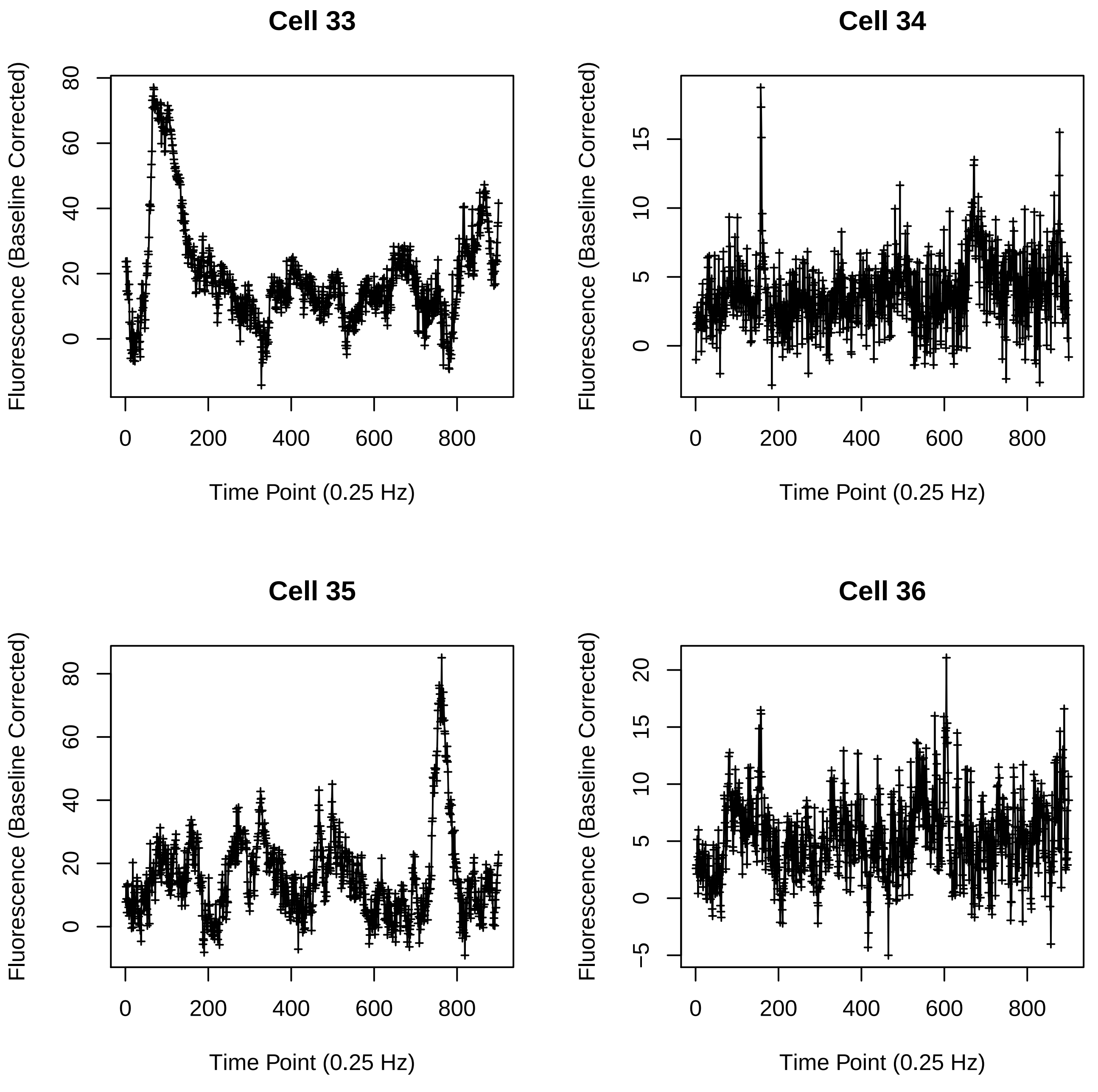
Cell 33
Cell 34
Cell 35
Cell 36
Fluorescence (Baseline Corrected)
Time Point (0.25 Hz)
0
200
400
600
800
80
60
40
20
15
10
5
−5

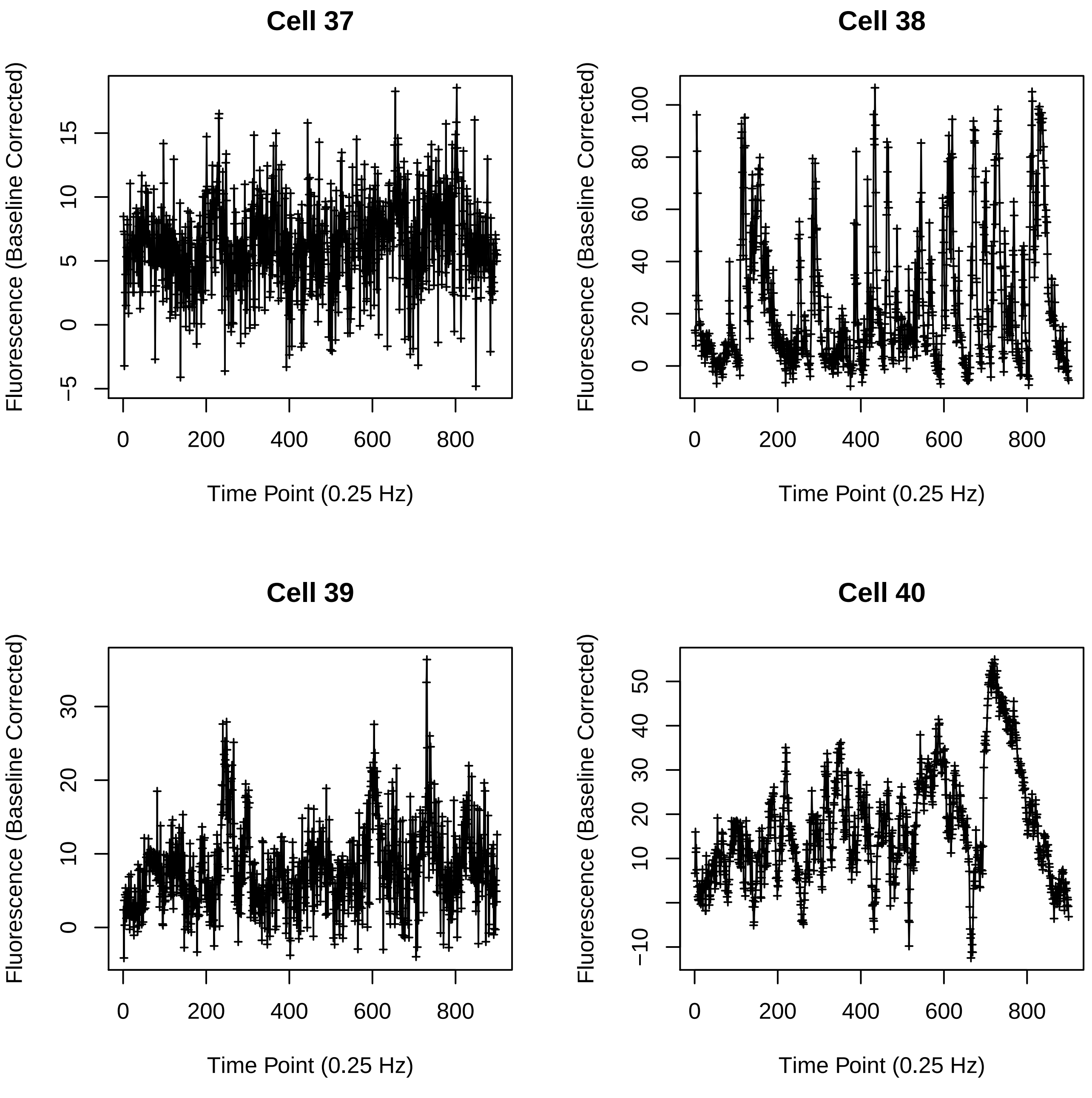

Cell 37
Cell 38
Cell 39
Cell 40
Fluorescence (Baseline Corrected)
Time Point (0.25 Hz)

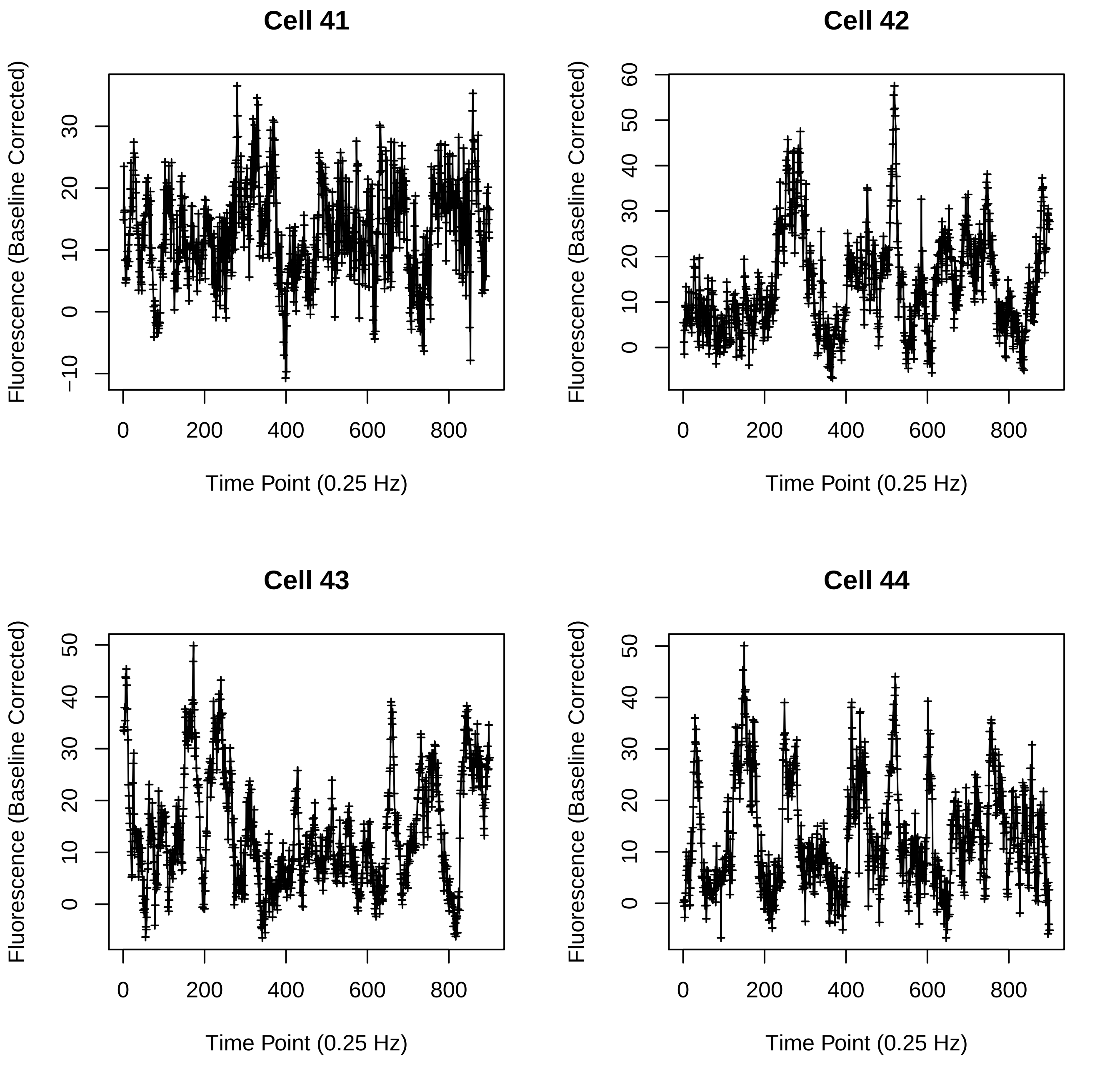
Cell 41
Cell 42
Cell 43
Cell 44
Fluorescence (Baseline Corrected)
Time Point (0.25 Hz)
−10
0
10
20
30
40
50
60
0
200
400
600
800

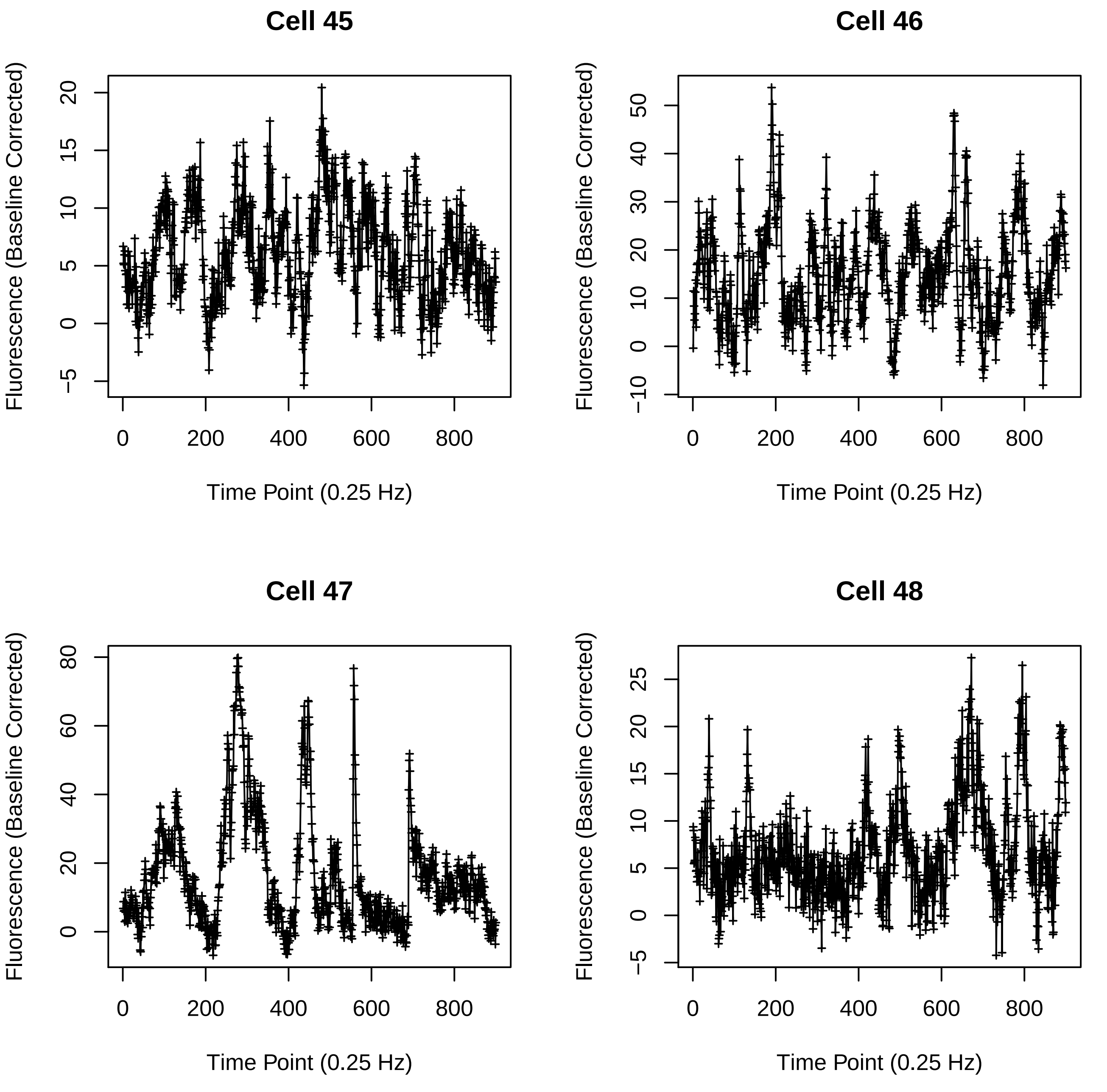
Cell 45
Cell 46
Cell 47
Cell 48
Fluorescence (Baseline Corrected)
Time Point (0.25 Hz)
−5
0
5
10
15
20
25
30
40
50
60
80
−10
200
400
600
800

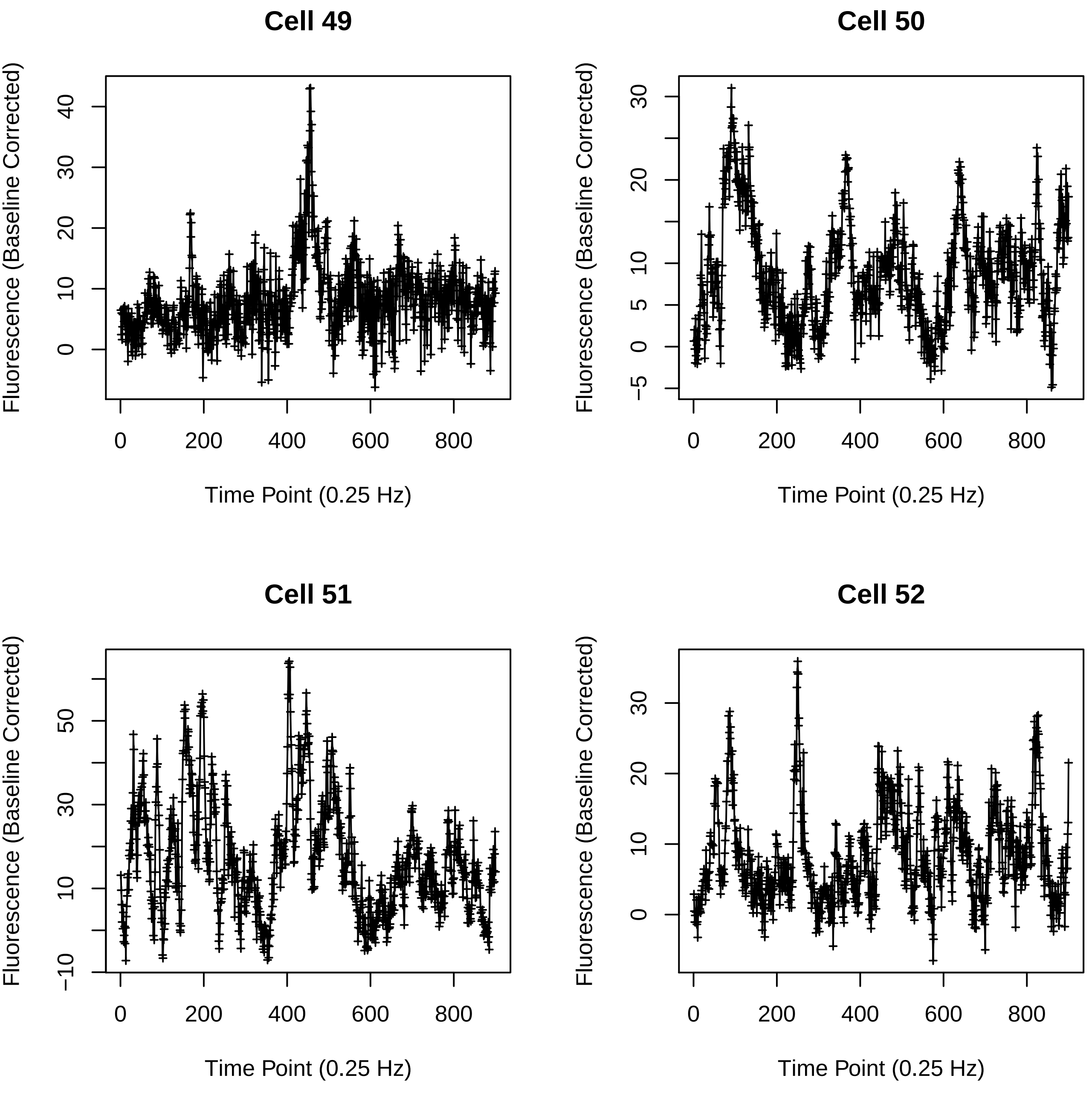
Cell 49
Cell 50
Cell 51
Cell 52
Fluorescence (Baseline Corrected)
Time Point (0.25 Hz)
0
200
400
600
800
0
10
20
30
40
−5
5
15
25
−10
50

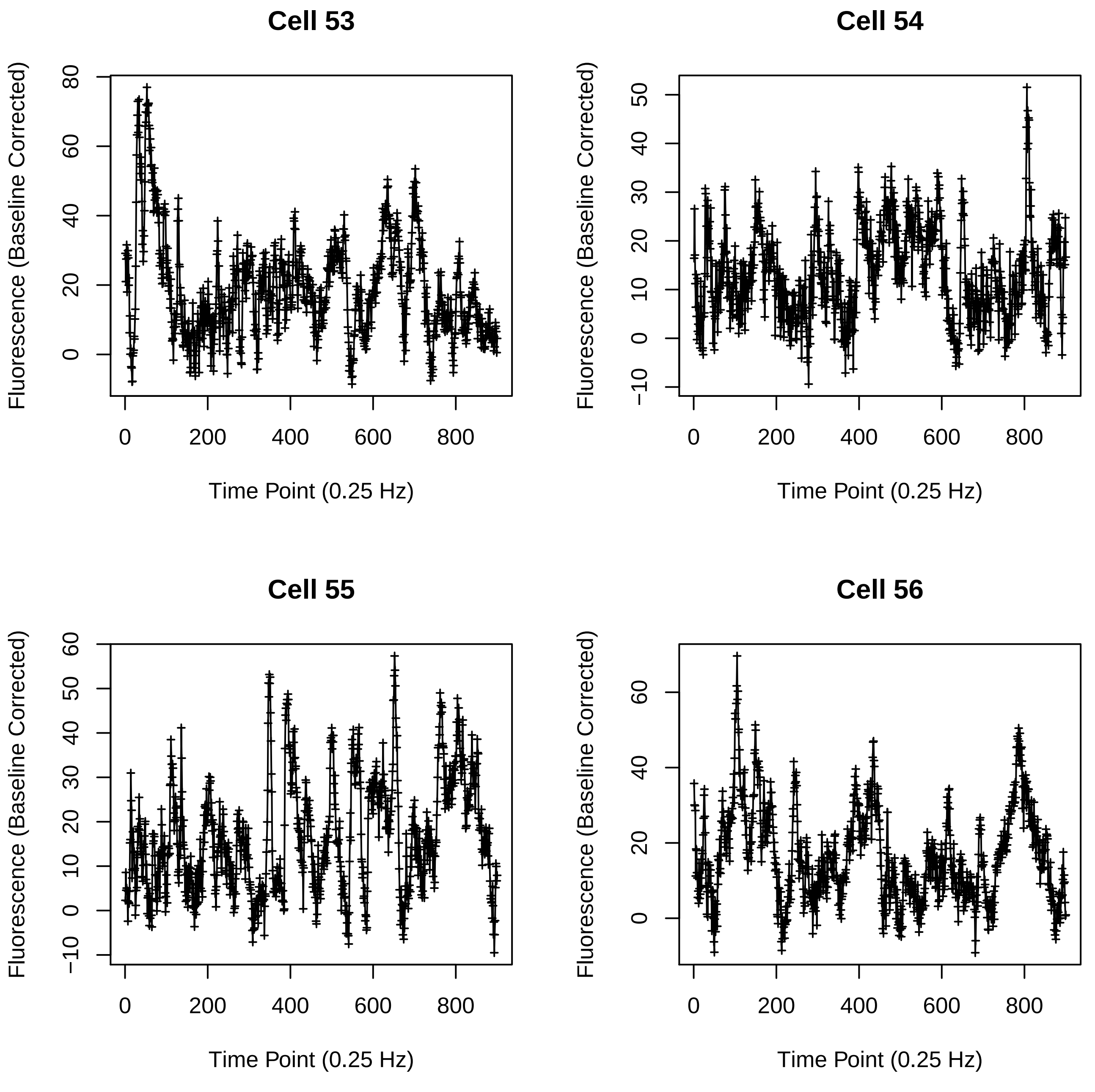
Cell 53
Cell 54
Cell 55
Cell 56
Fluorescence (Baseline Corrected)
Time Point (0.25 Hz)
0
200
400
600
800

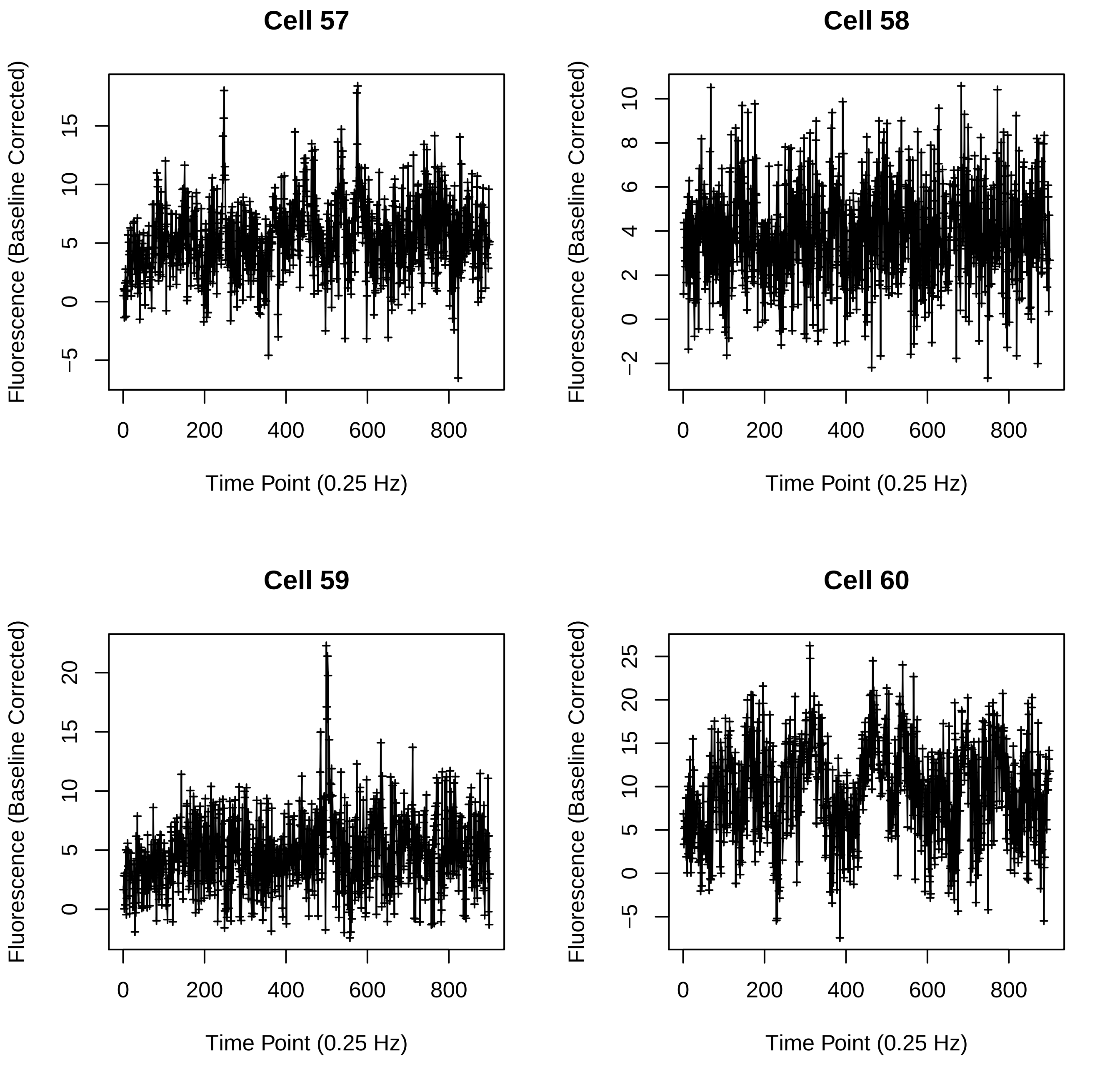
Cell 57
Cell 58
Cell 59
Cell 60
Fluorescence (Baseline Corrected)
Time Point (0.25 Hz)
0
200
400
600
800
−5
0
5
10
15
−2
2
4
6
8
20
25

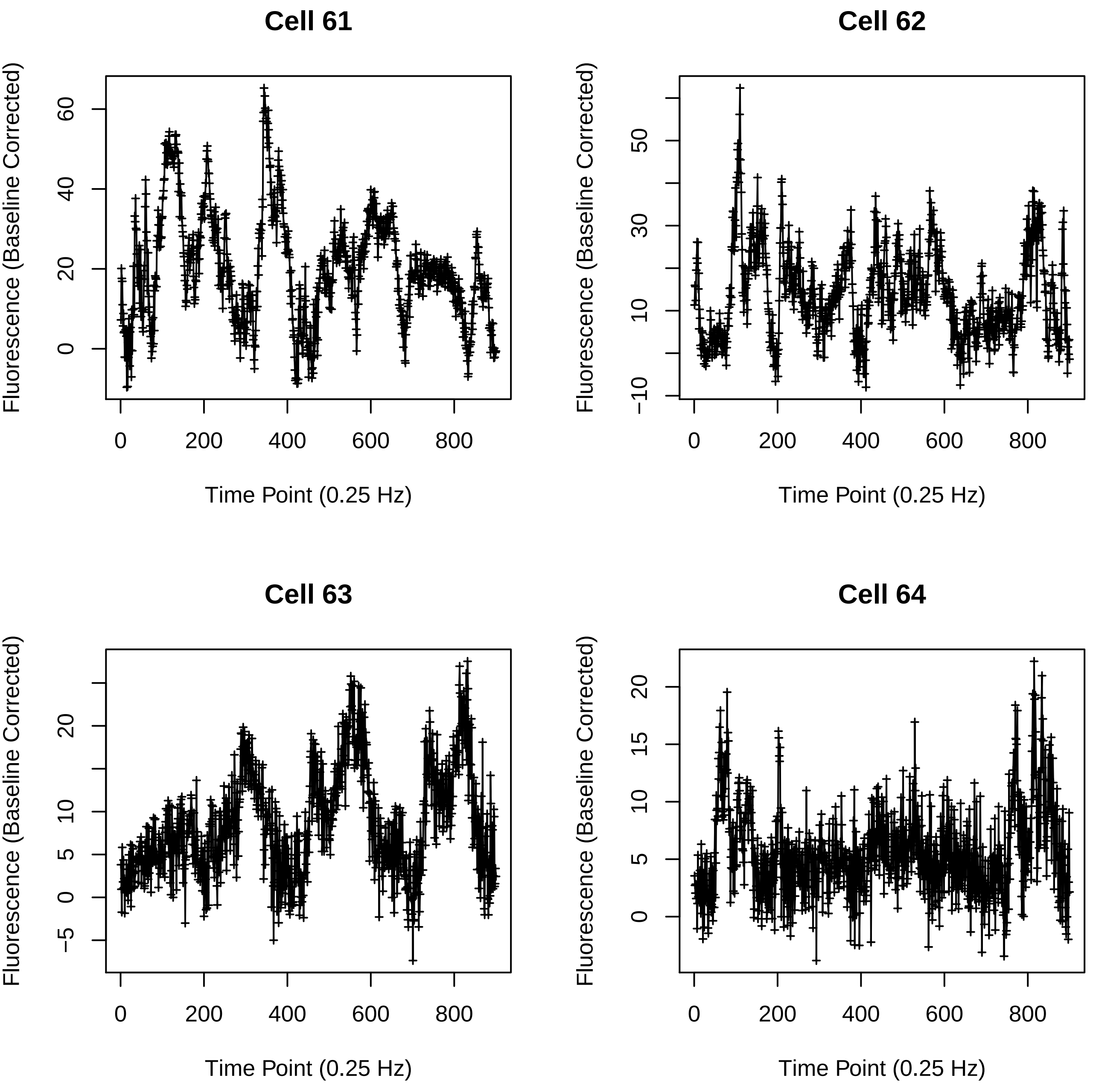
Cell 61
Cell 62
Cell 63
Cell 64
Fluorescence (Baseline Corrected)
Time Point (0.25 Hz)
0
200
400
600
800
60
40
20
0
50
30
10
−10
20
10
5
0
−5
15

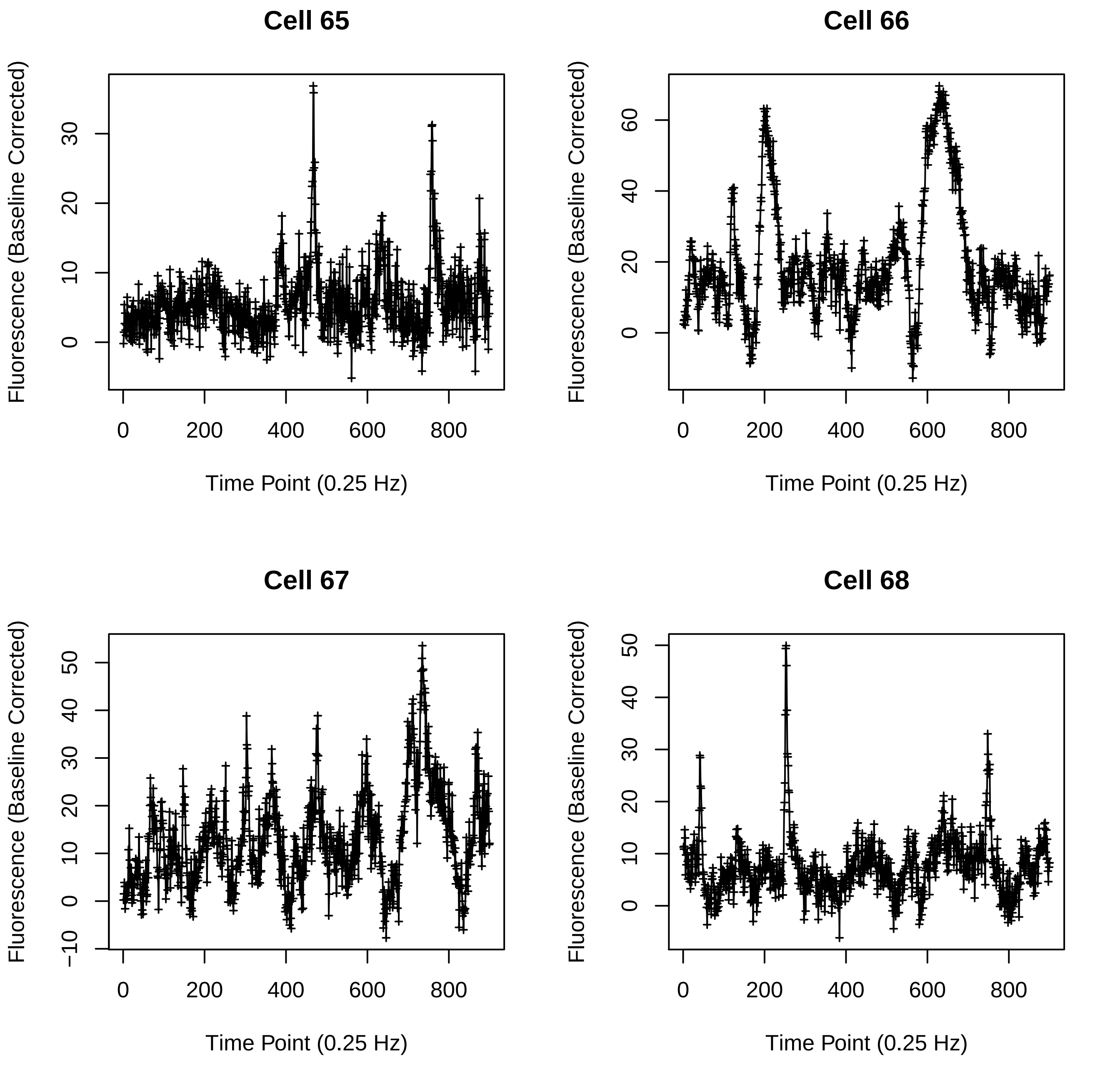

Cell 65
Cell 66
Cell 67
Cell 68
Fluorescence (Baseline Corrected)
Time Point (0.25 Hz)
0
200
400
600
800
0
10
20
30
40
50
60
−10

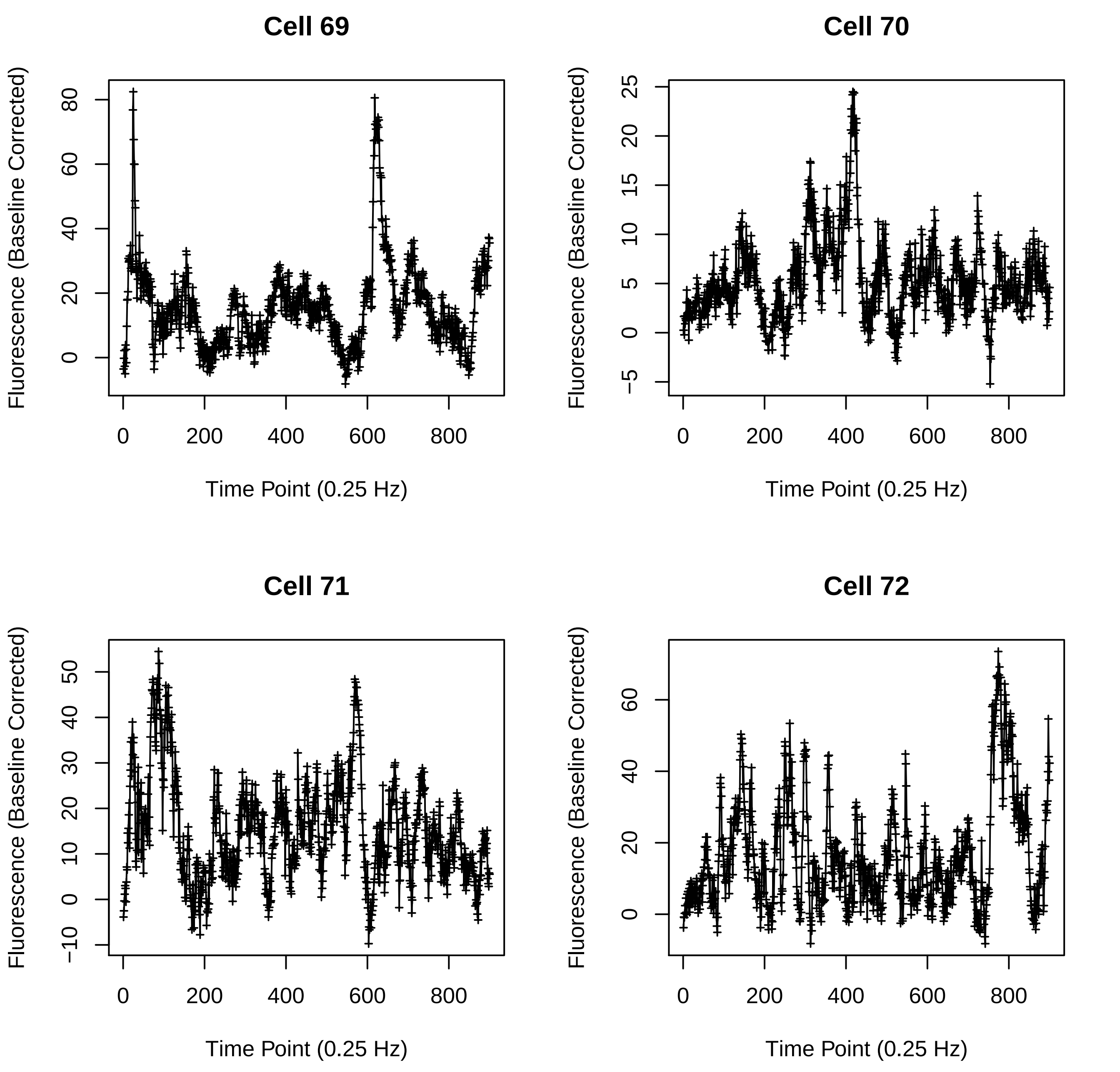
Cell 69
Cell 70
Cell 71
Cell 72
Fluorescence (Baseline Corrected)
Time Point (0.25 Hz)
0
200
400
600
800
−10
0
10
20
30
40
50
60
80
−5
5
15
25

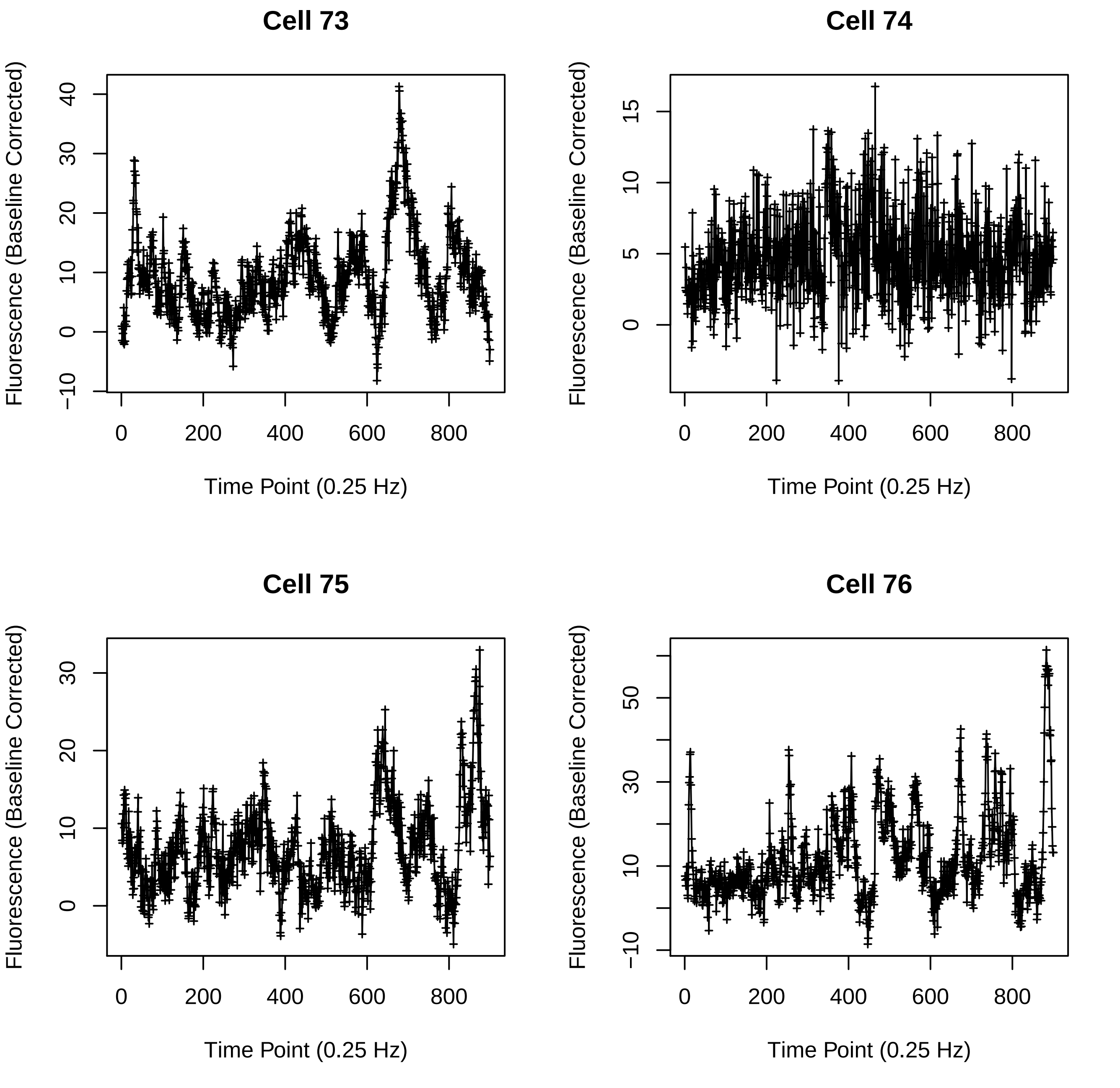

Cell 73
Cell 74
Cell 75
Cell 76
Fluorescence (Baseline Corrected)
Time Point (0.25 Hz)

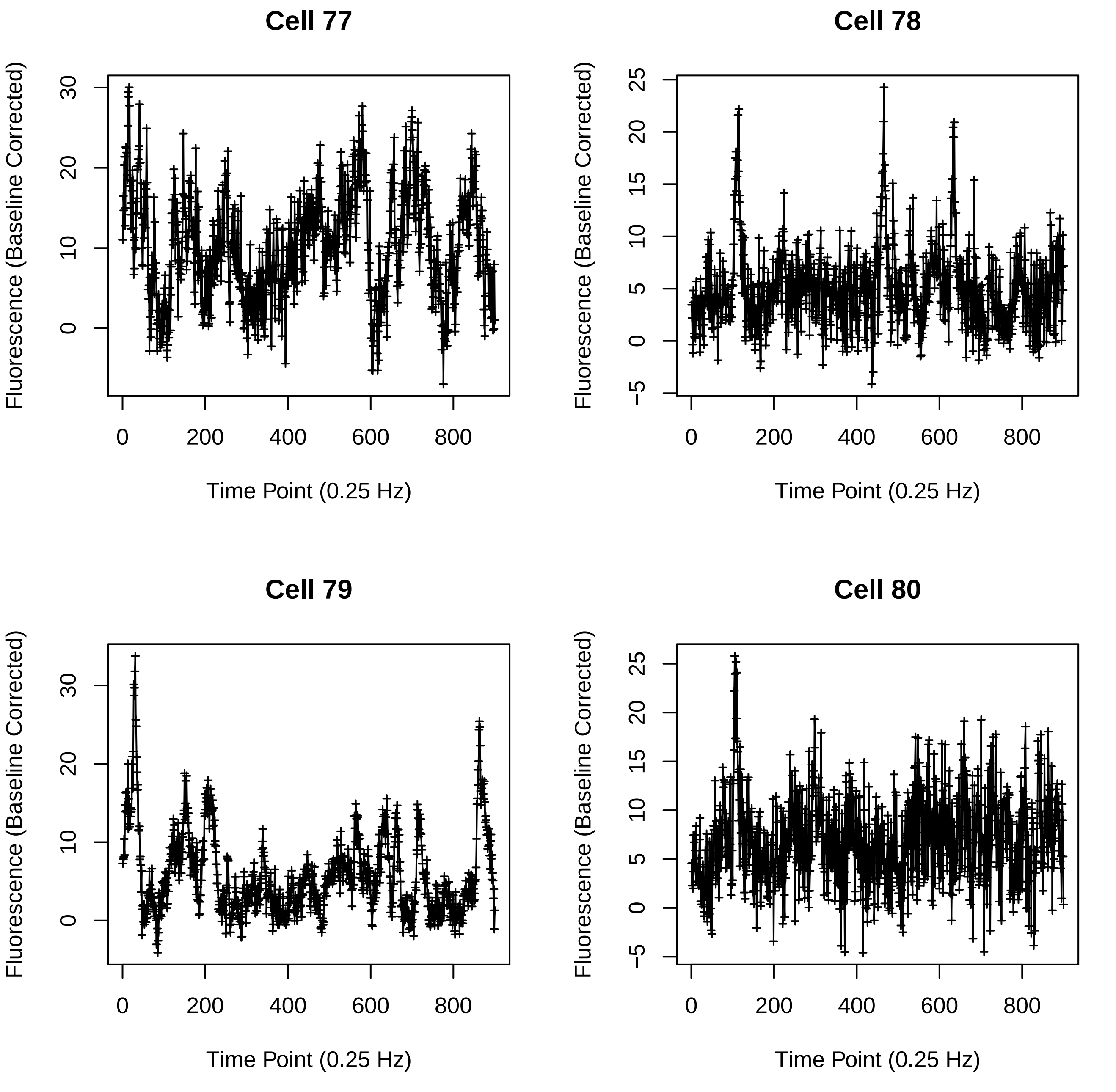

Cell 77
Cell 78
Cell 79
Cell 80
Fluorescence (Baseline Corrected)
Time Point (0.25 Hz)
0
200
400
600
800
-5
5
10
15
20
25
30

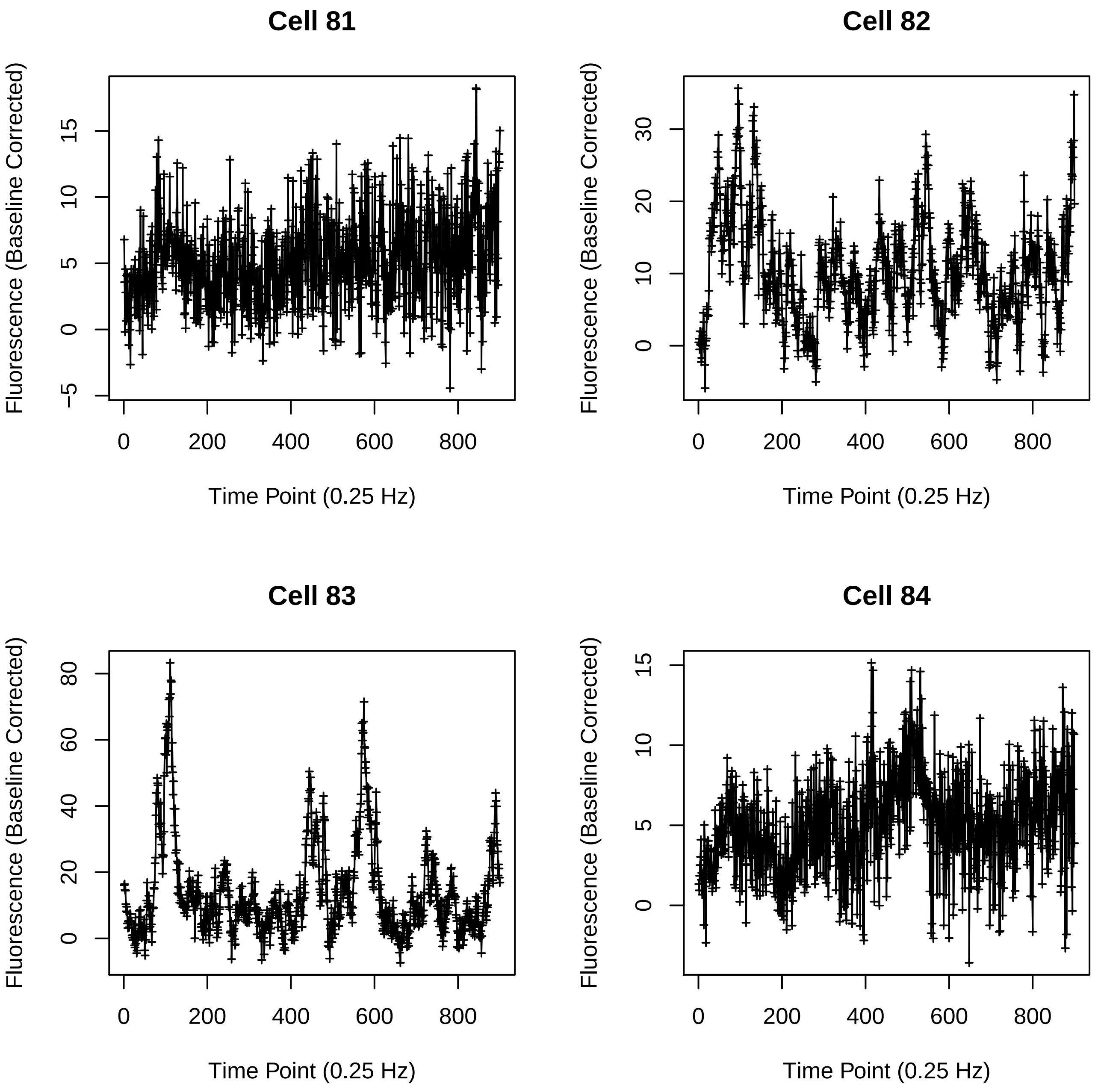
Cell 81
Cell 82
Cell 83
Cell 84
Fluorescence (Baseline Corrected)
Time Point (0.25 Hz)
0
200
400
600
800
−5
0
5
10
15
20
30
40
60
80

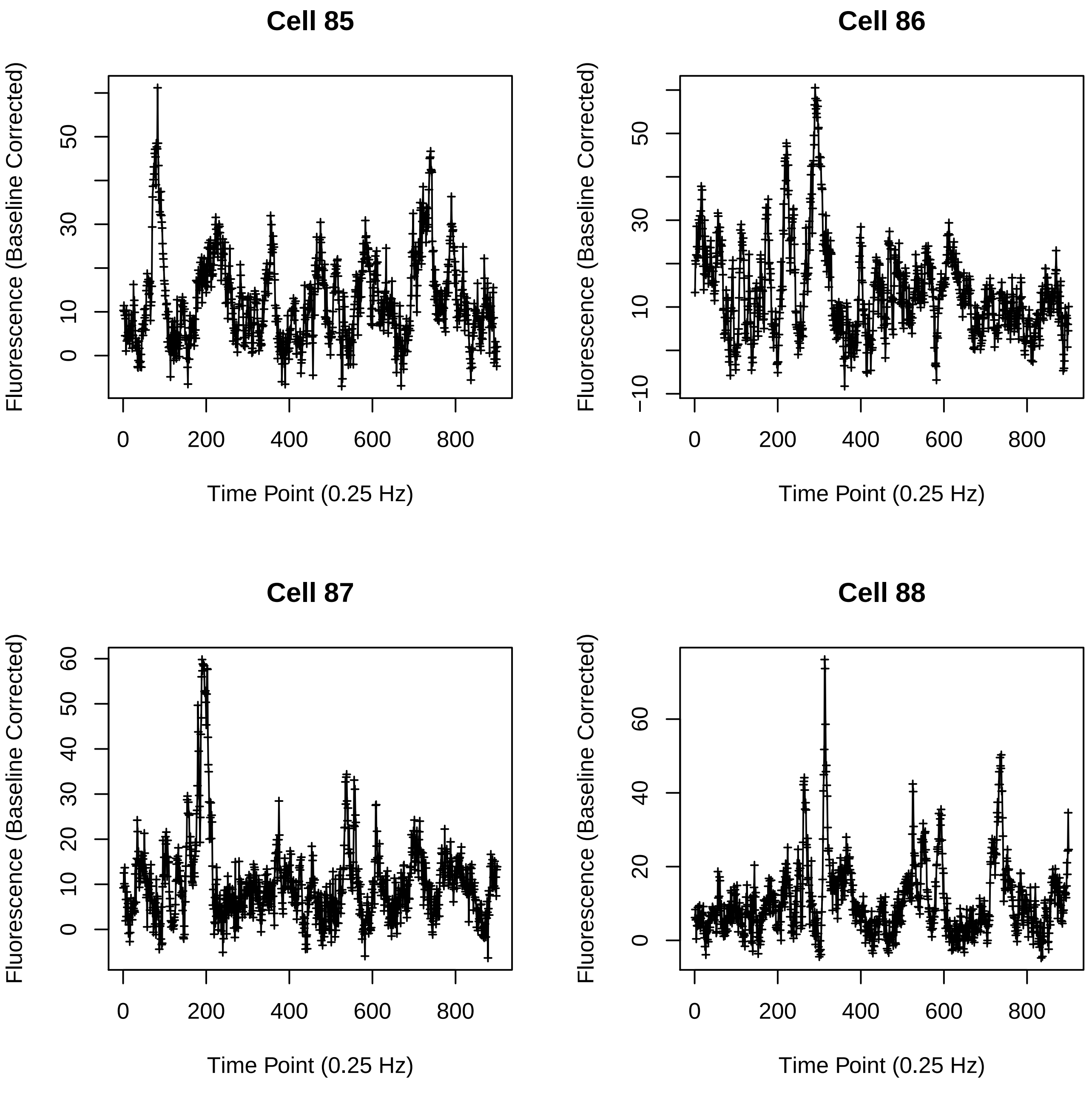

Cell 85
Cell 86
Cell 87
Cell 88
Fluorescence (Baseline Corrected)
Time Point (0.25 Hz)
0
200
400
600
800
-10
0
10
20
30
40
50
60

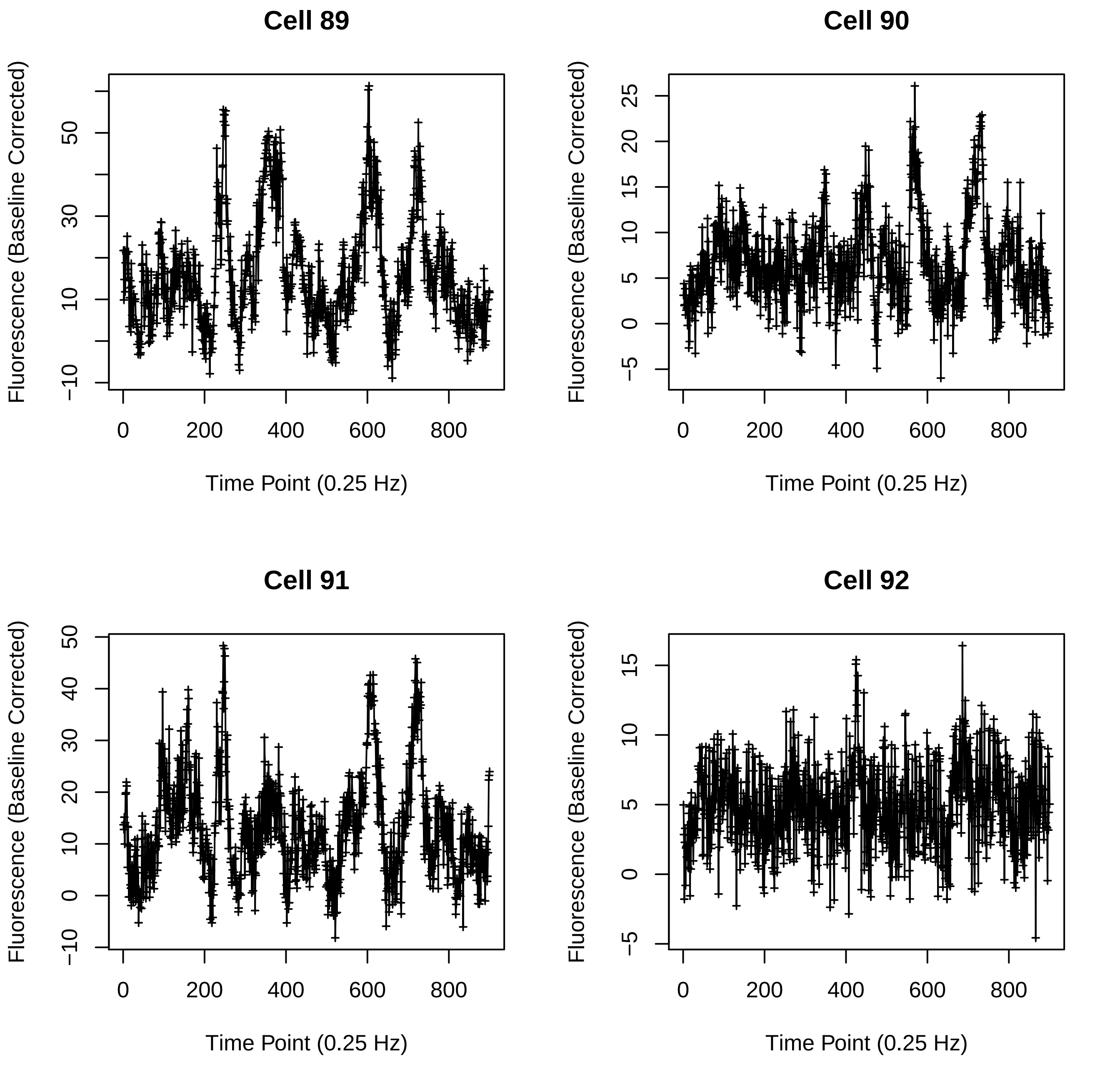

Cell 89
Cell 90
Cell 91
Cell 92
Fluorescence (Baseline Corrected)
Time Point (0.25 Hz)

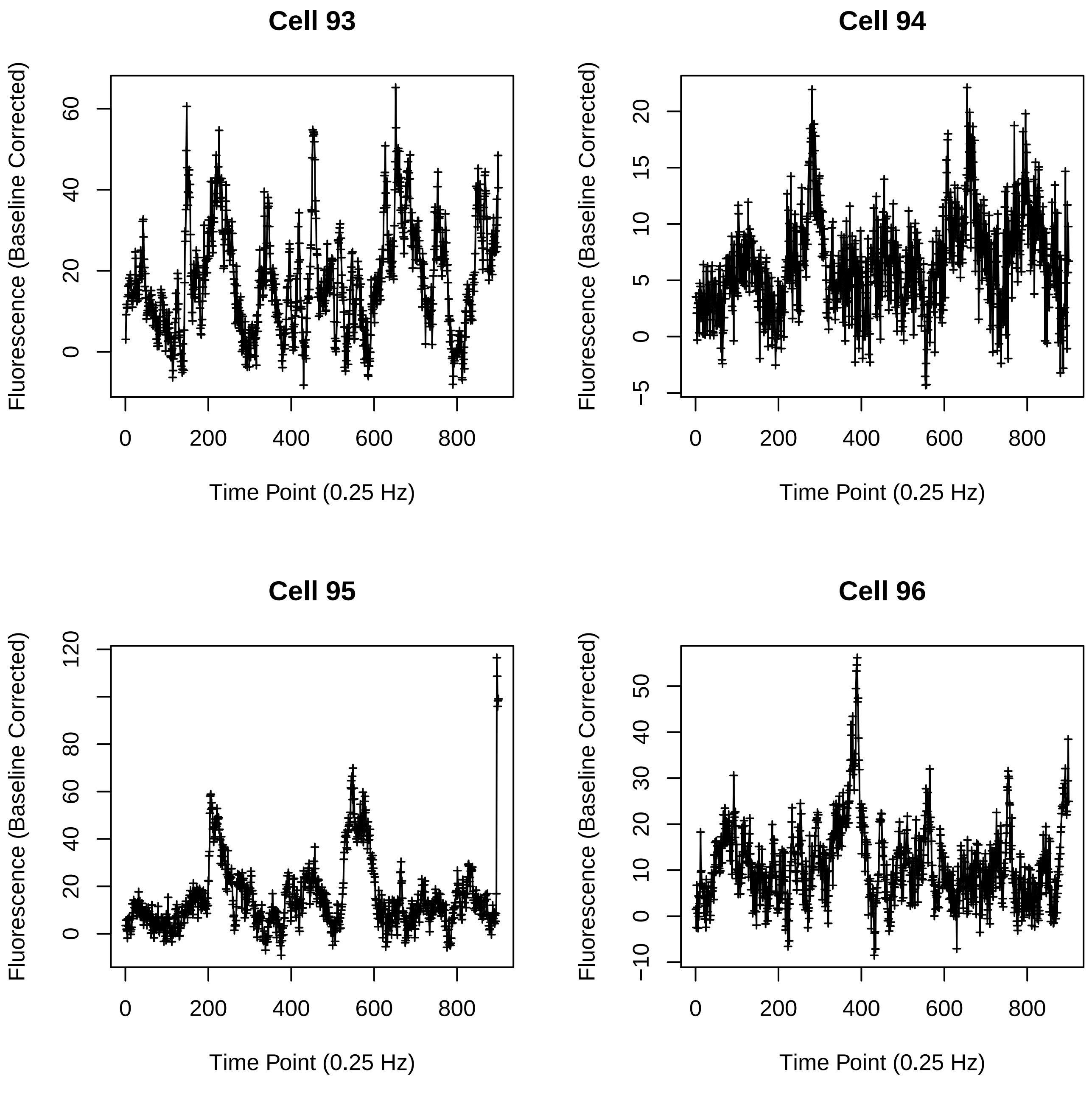

Cell 93
Fluorescence (Baseline Corrected)
Time Point (0.25 Hz)
0
20
40
60
0
200
400
600
800
Cell 94
Fluorescence (Baseline Corrected)
Time Point (0.25 Hz)
−5
0
5
10
15
20
0
200
400
600
800
Cell 95
Fluorescence (Baseline Corrected)
Time Point (0.25 Hz)
0
20
40
60
80
120
0
200
400
600
800
Cell 96
Fluorescence (Baseline Corrected)
Time Point (0.25 Hz)
−10
0
10
20
30
40
50
0
200
400
600
800

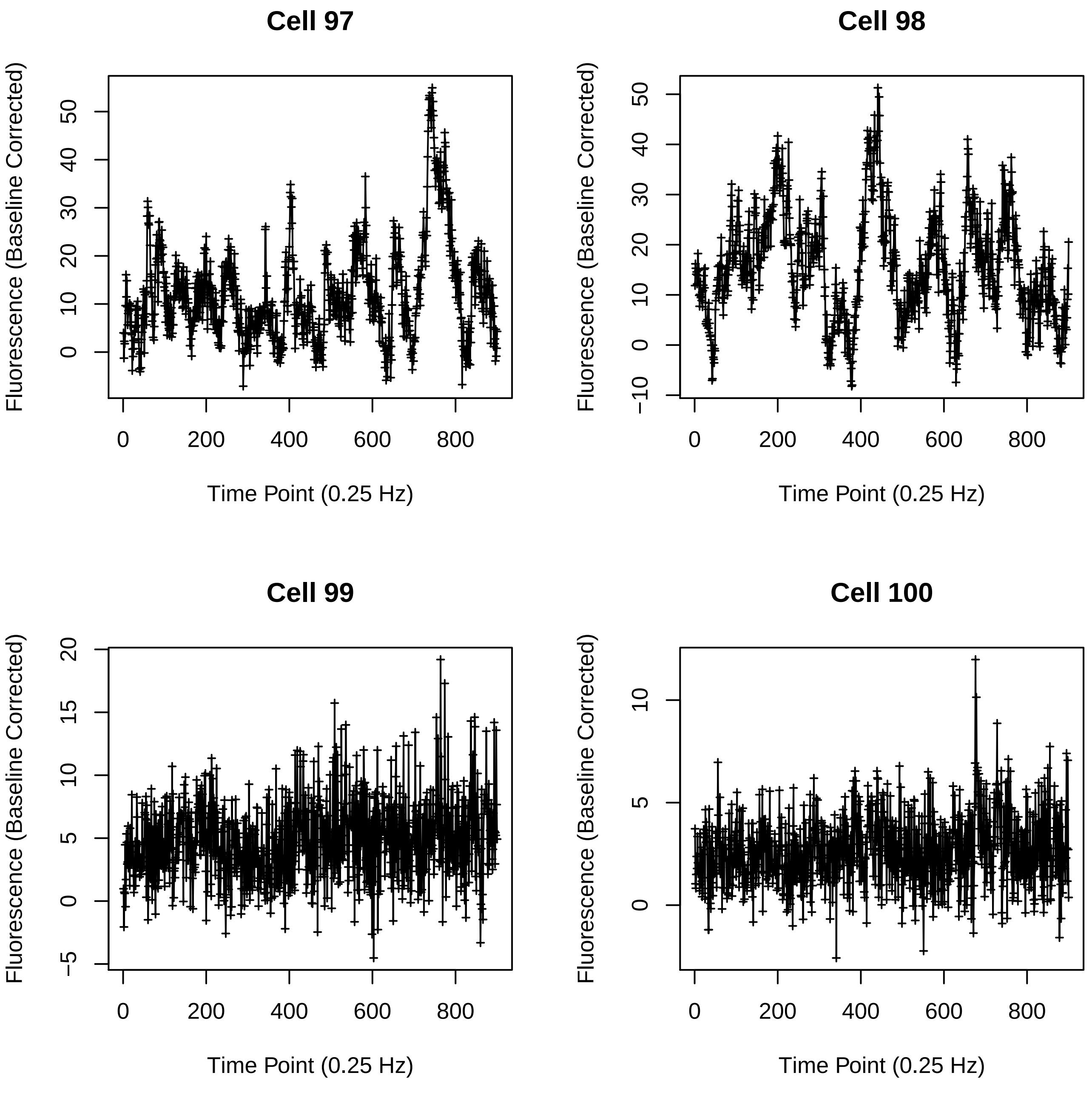

Cell 97
Cell 98
Cell 99
Cell 100
Fluorescence (Baseline Corrected)
Time Point (0.25 Hz)
0
10
20
30
40
50
−10
−5
5
15
200
400
600
800

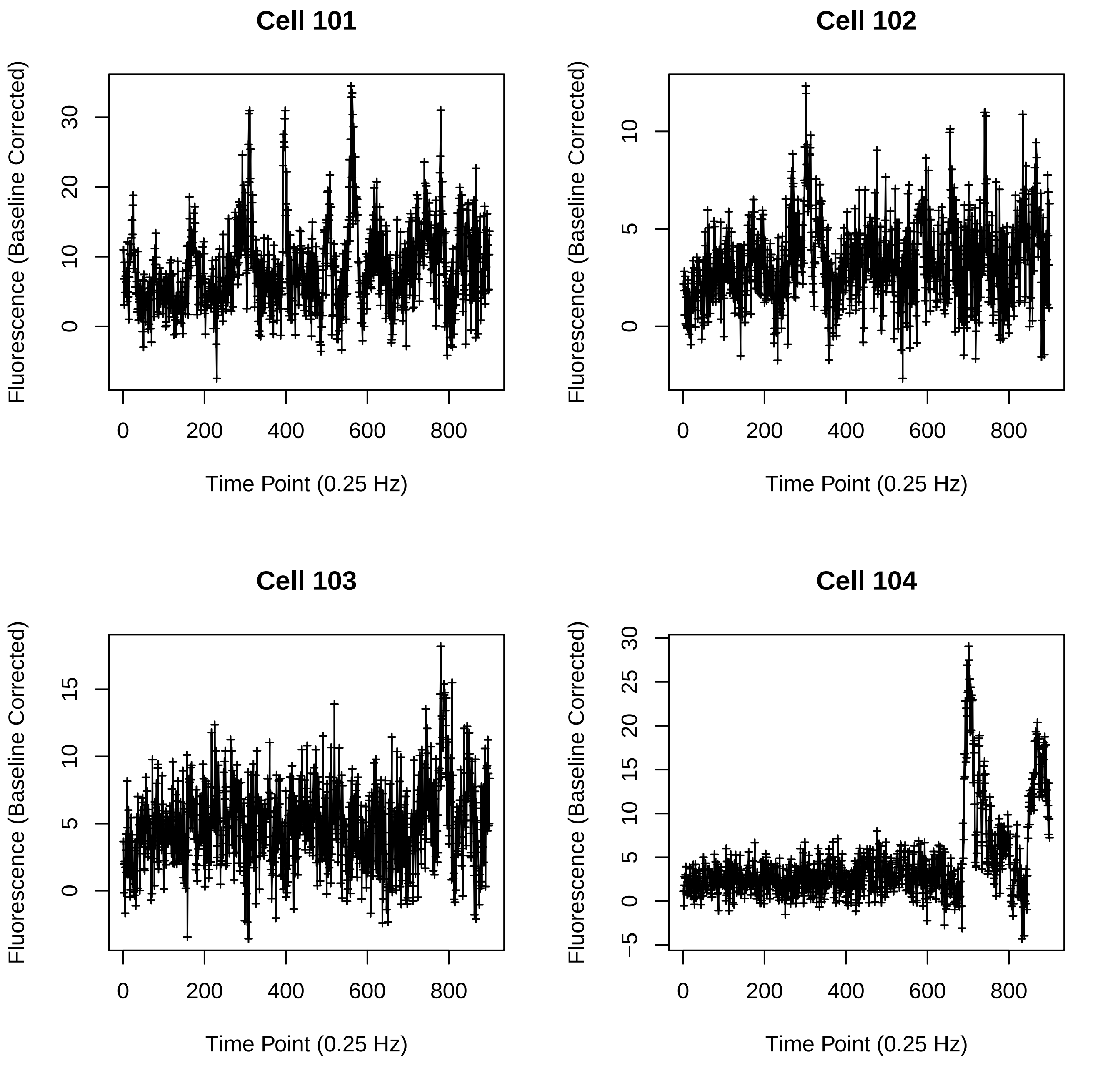
Cell 101
Cell 102
Cell 103
Cell 104
Fluorescence (Baseline Corrected)
Time Point (0.25 Hz)
0
200
400
600
800
0
10
20
30
5
15
25
−5

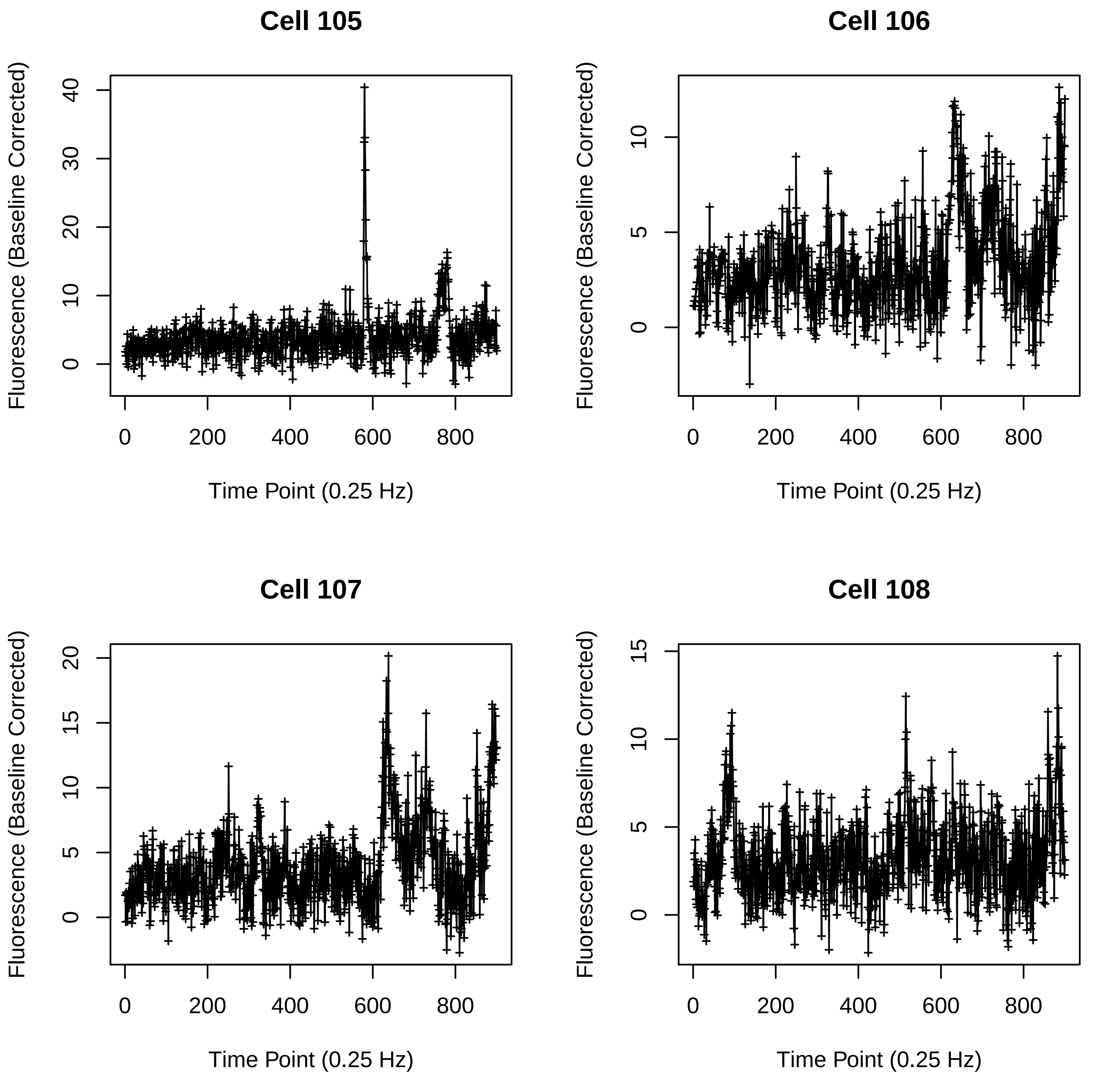
Cell 105
Cell 106
Cell 107
Cell 108
Fluorescence (Baseline Corrected)
Time Point (0.25 Hz)
0
200
400
600
800
0
10
20
30
40
5
15

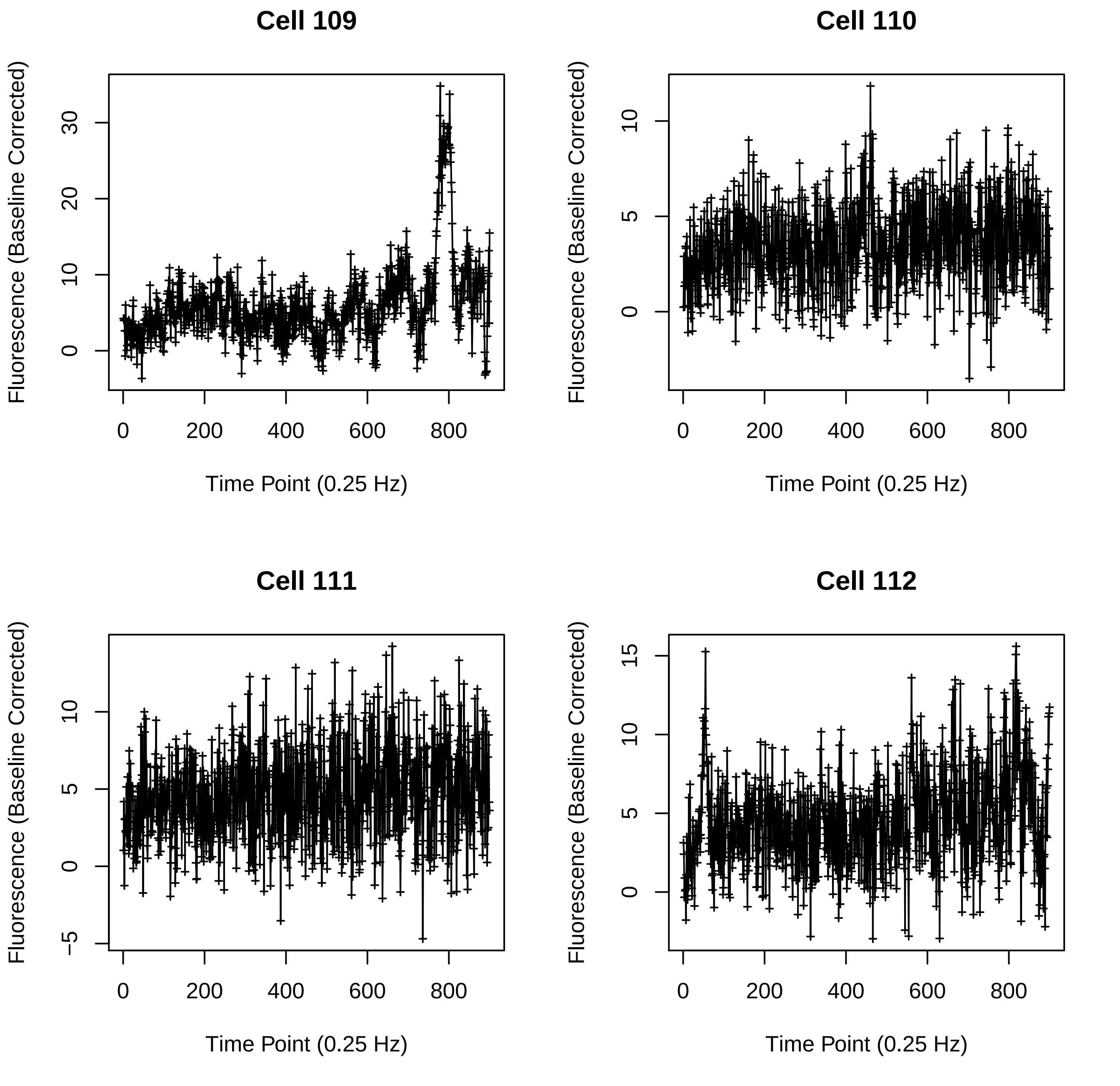
Cell 109
Cell 110
Cell 111
Cell 112
Fluorescence (Baseline Corrected)
Time Point (0.25 Hz)
0
200
400
600
800
-5
5
10
15
20
30

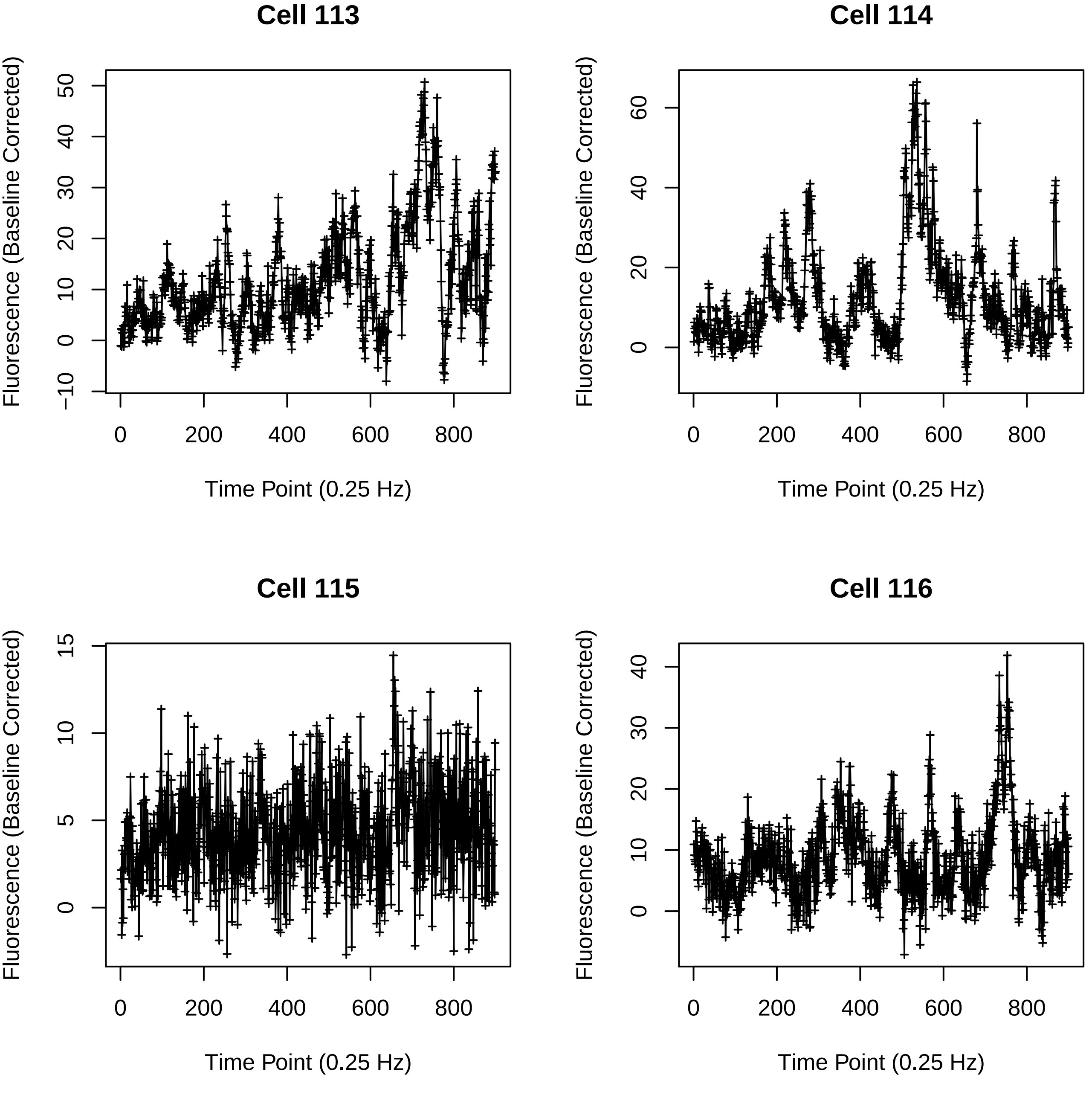
Cell 113
Cell 114
Cell 115
Cell 116
Fluorescence (Baseline Corrected)
Time Point (0.25 Hz)
0
200
400
600
800
−10
10
20
30
40
50
60
5
15

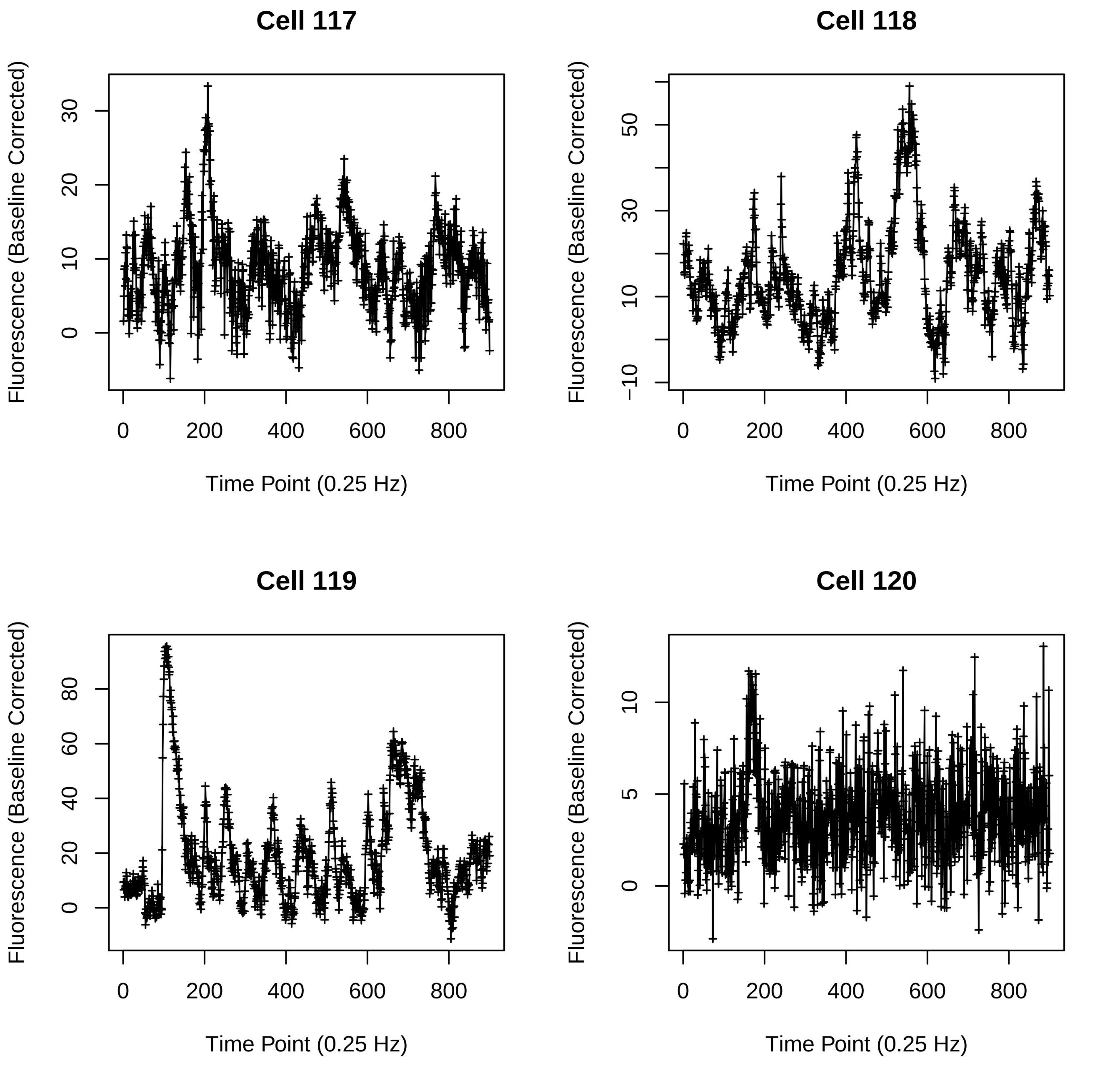
Cell 117
Cell 118
Cell 119
Cell 120
Fluorescence (Baseline Corrected)
Time Point (0.25 Hz)
0
200
400
600
800
30
20
10
50
−10
80
60
40
5

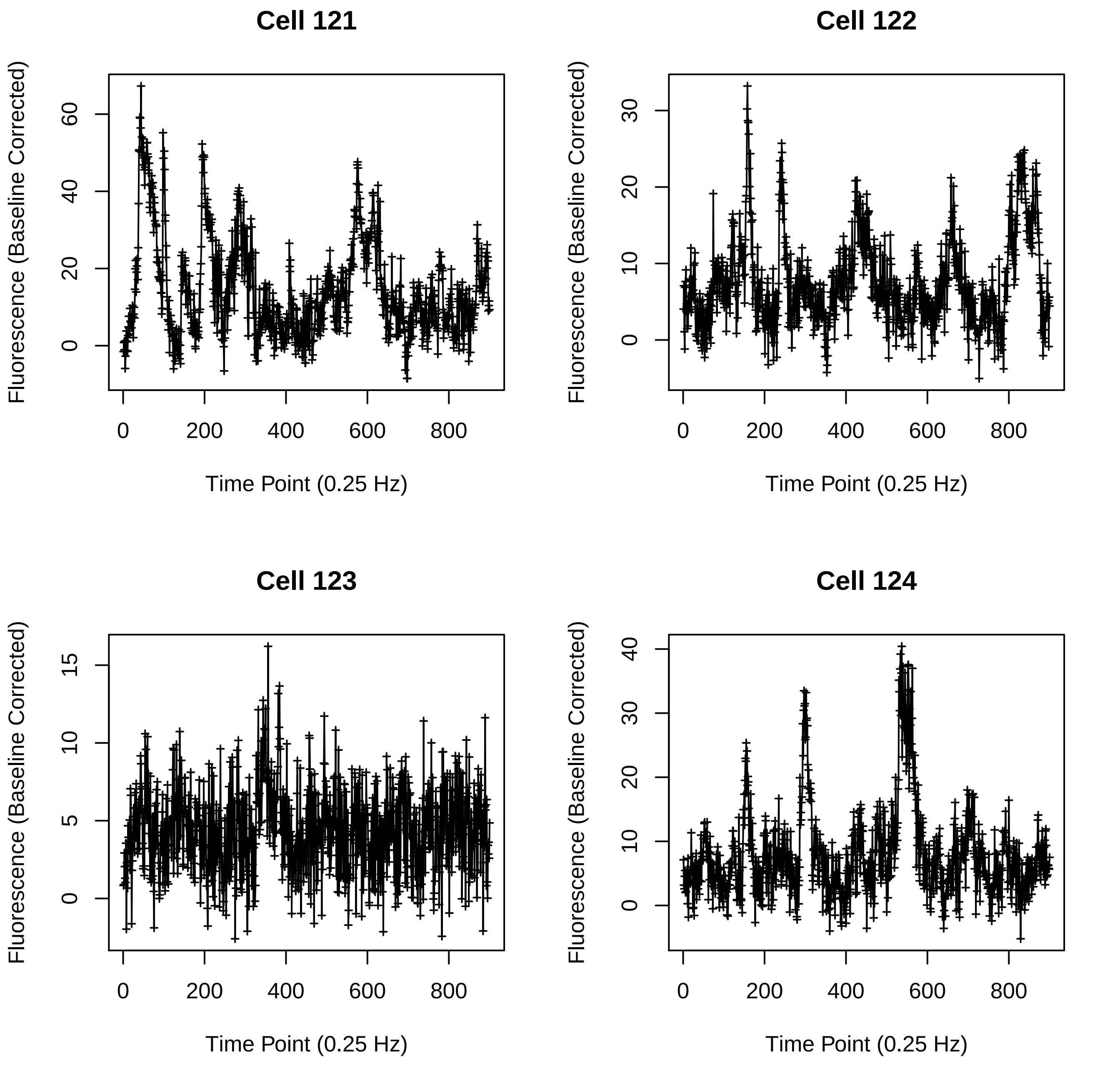

Cell 121
Cell 122
Cell 123
Cell 124
Fluorescence (Baseline Corrected)
Time Point (0.25 Hz)
0
200
400
600
800
0
20
40
60
0
10
20
30
0
5
10
15
0
10
20
30
40

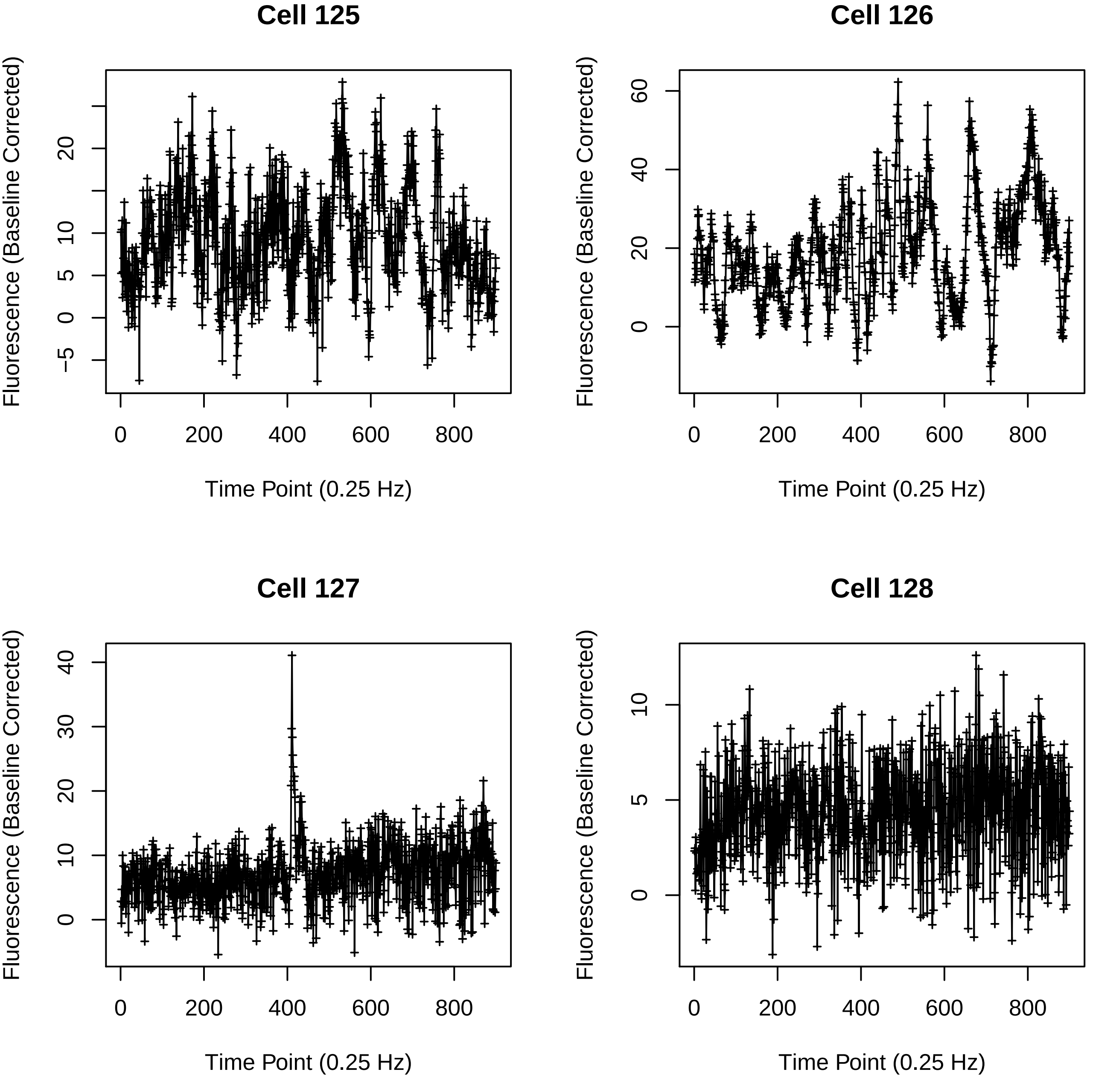
Cell 125
Cell 126
Cell 127
Cell 128
Fluorescence (Baseline Corrected)
Time Point (0.25 Hz)
0
200
400
600
800
−5
0
5
10
20
30
40
60

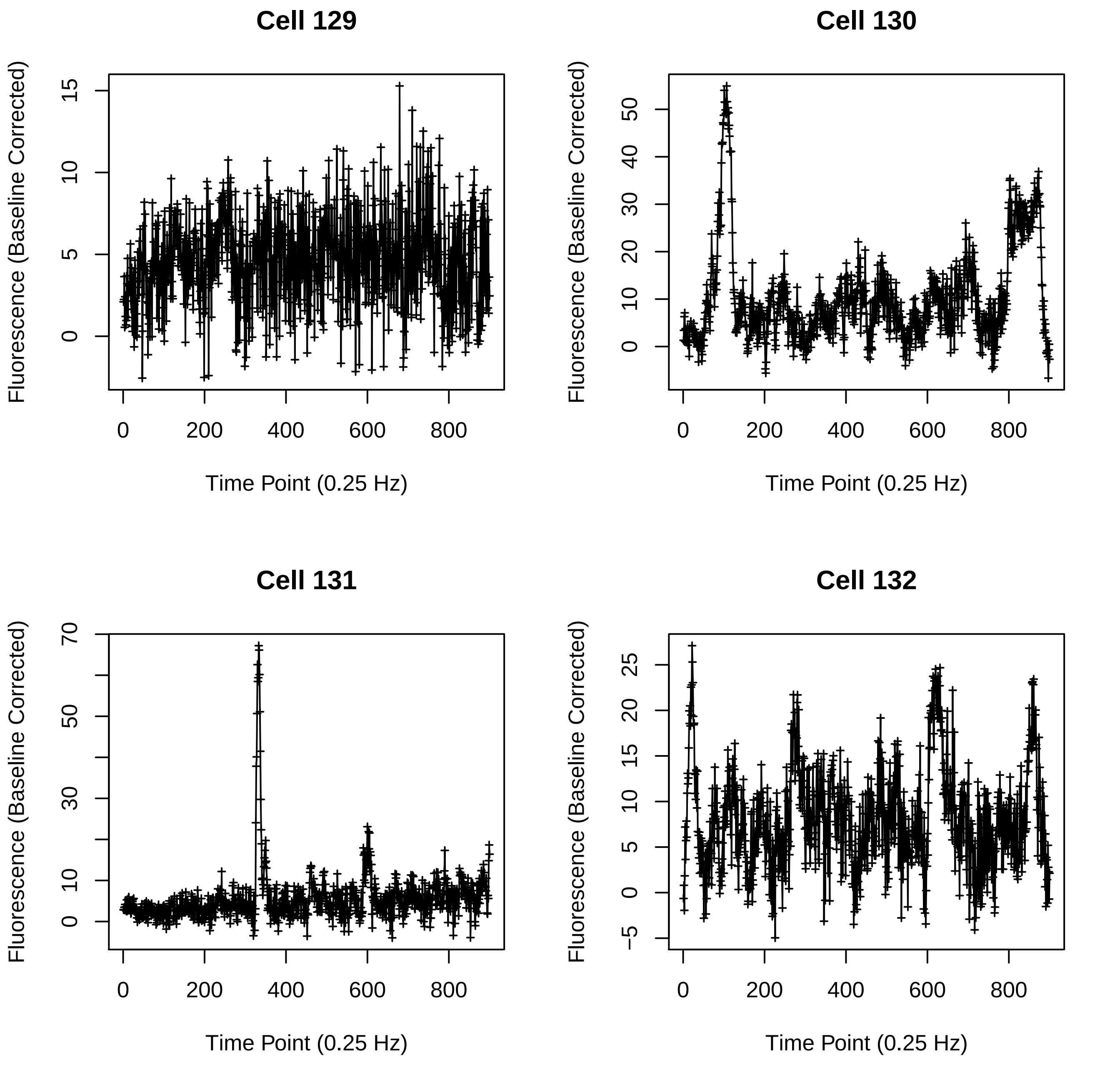
Cell 129
Cell 130
Cell 131
Cell 132
Fluorescence (Baseline Corrected)
Time Point (0.25 Hz)
0
200
400
600
800
-5
0
5
10
15
20
25
30
40
50
70

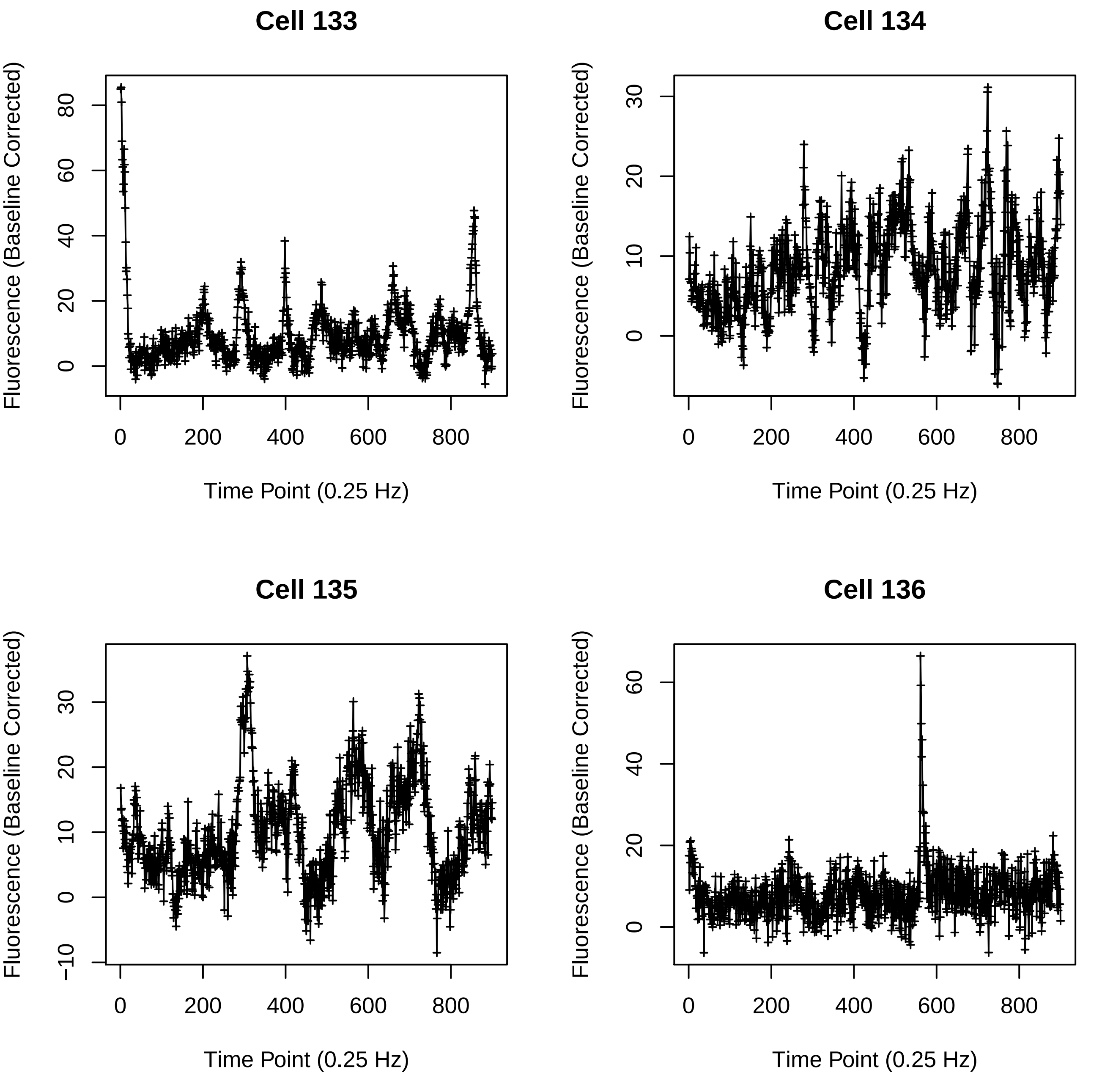
Cell 133
Cell 134
Cell 135
Cell 136
Fluorescence (Baseline Corrected)
Time Point (0.25 Hz)
0
200
400
600
800
−10
10
20
30
40
60
80

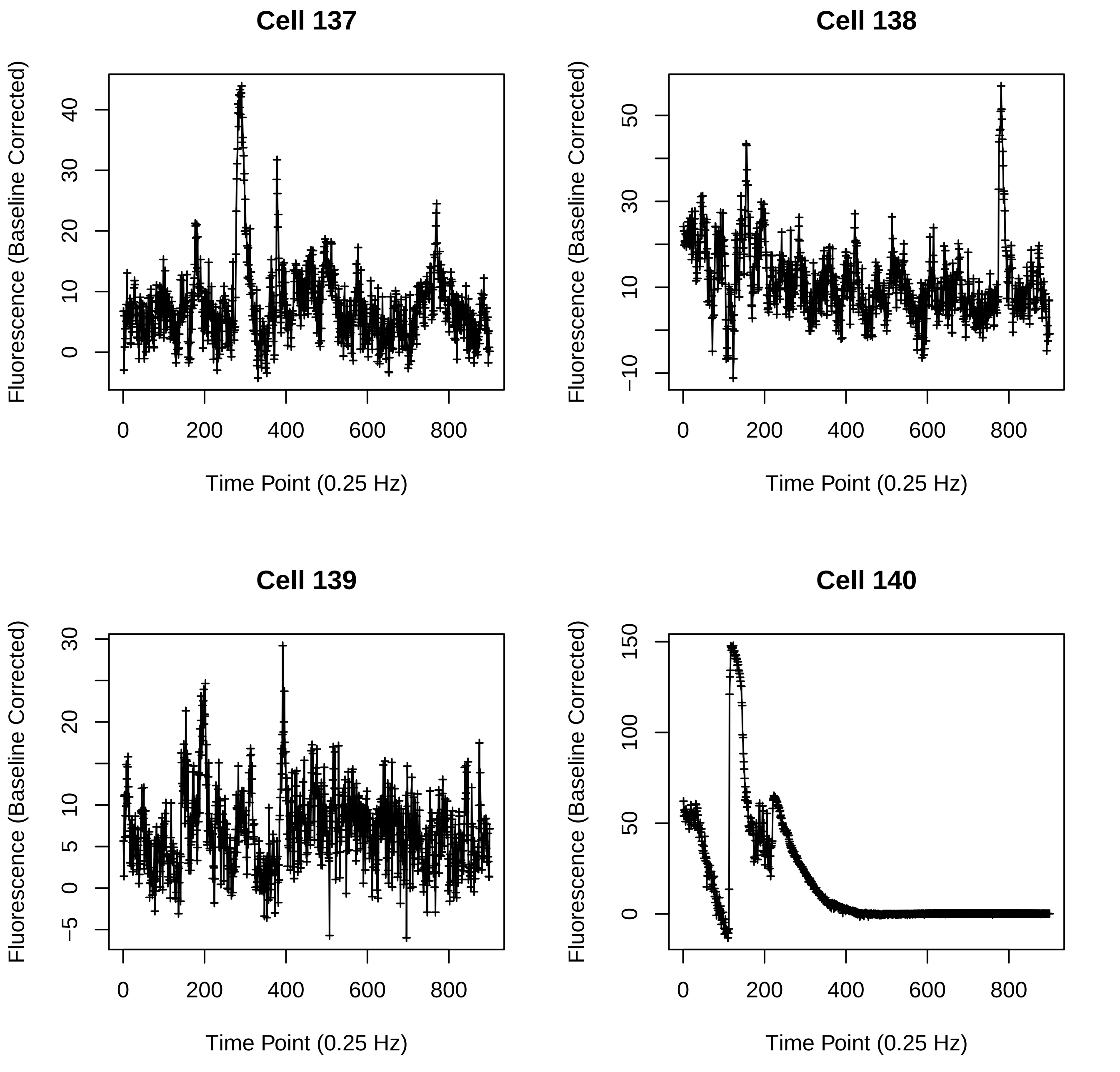
Cell 137
Cell 138
Cell 139
Cell 140
Fluorescence (Baseline Corrected)
Time Point (0.25 Hz)
0
10
20
30
40
−10
50
−5
5
15
25
100
150
200
400
600
800

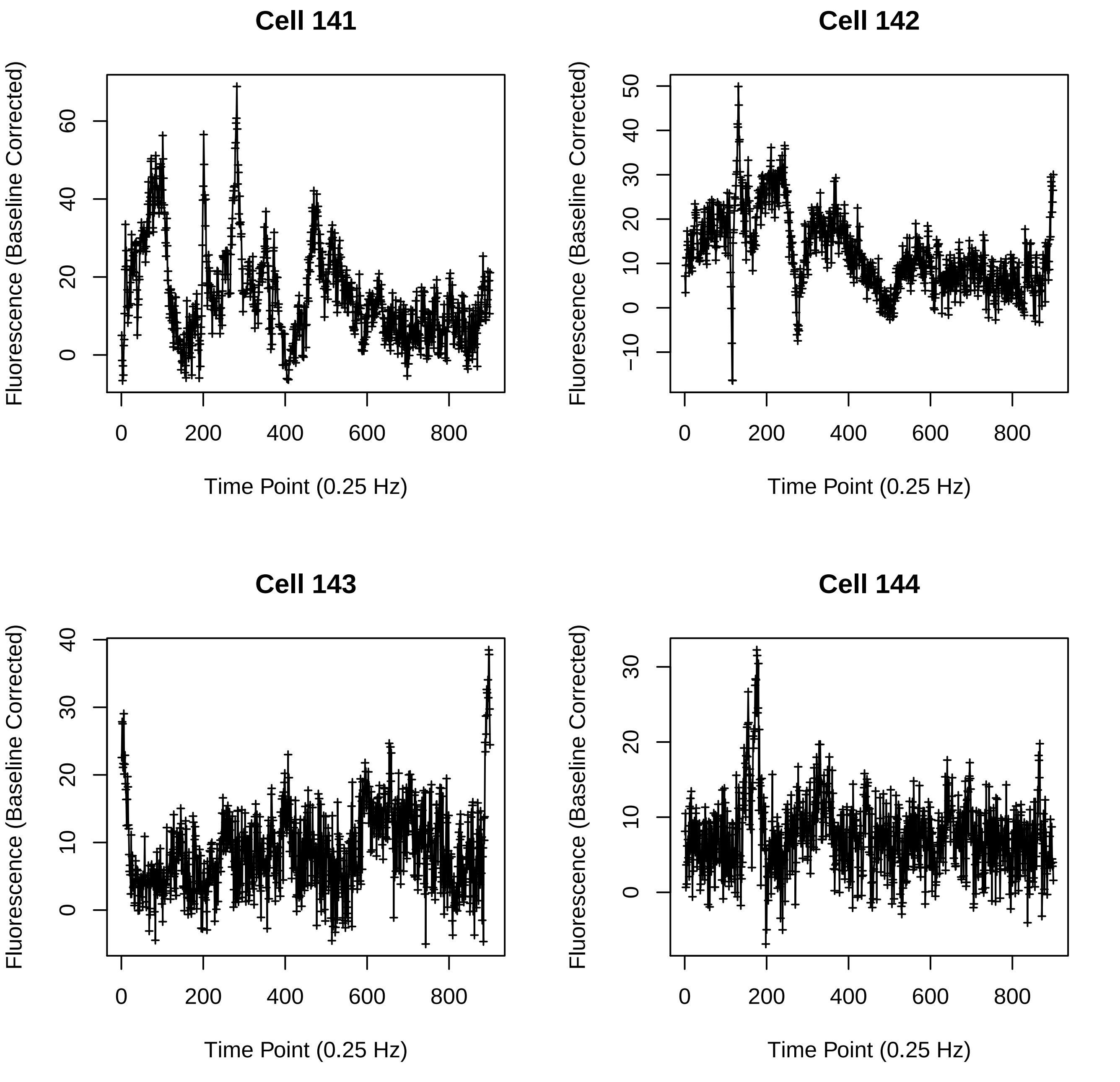
Cell 141
Cell 142
Cell 143
Cell 144
Fluorescence (Baseline Corrected)
Time Point (0.25 Hz)
0
20
40
60
−10
0
10
20
30
40
50
0
200
400
600
800

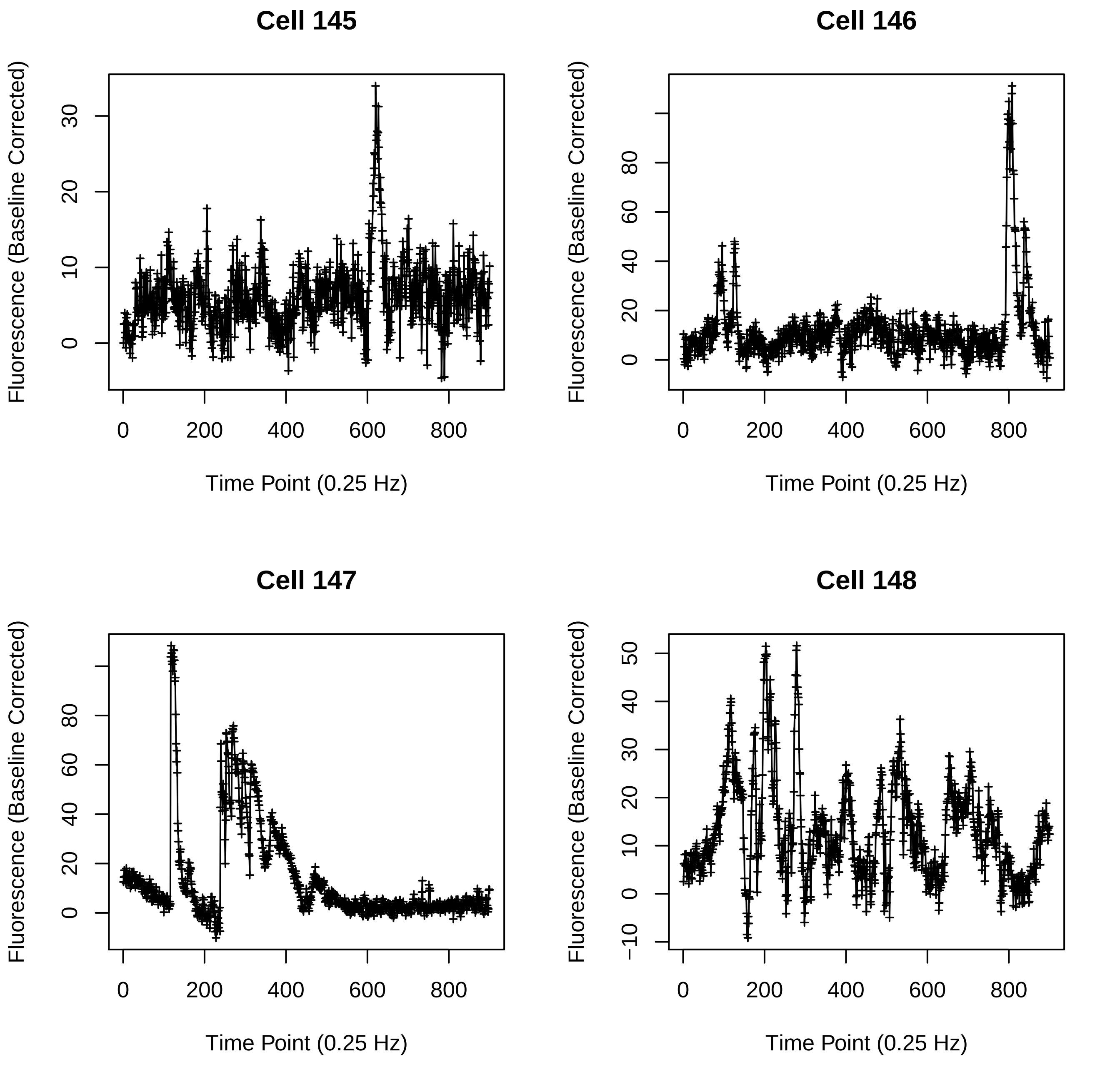

Cell 145
Cell 146
Cell 147
Cell 148
Fluorescence (Baseline Corrected)
Time Point (0.25 Hz)
0
200
400
600
800
-10
0
10
20
30
40
50
60
80

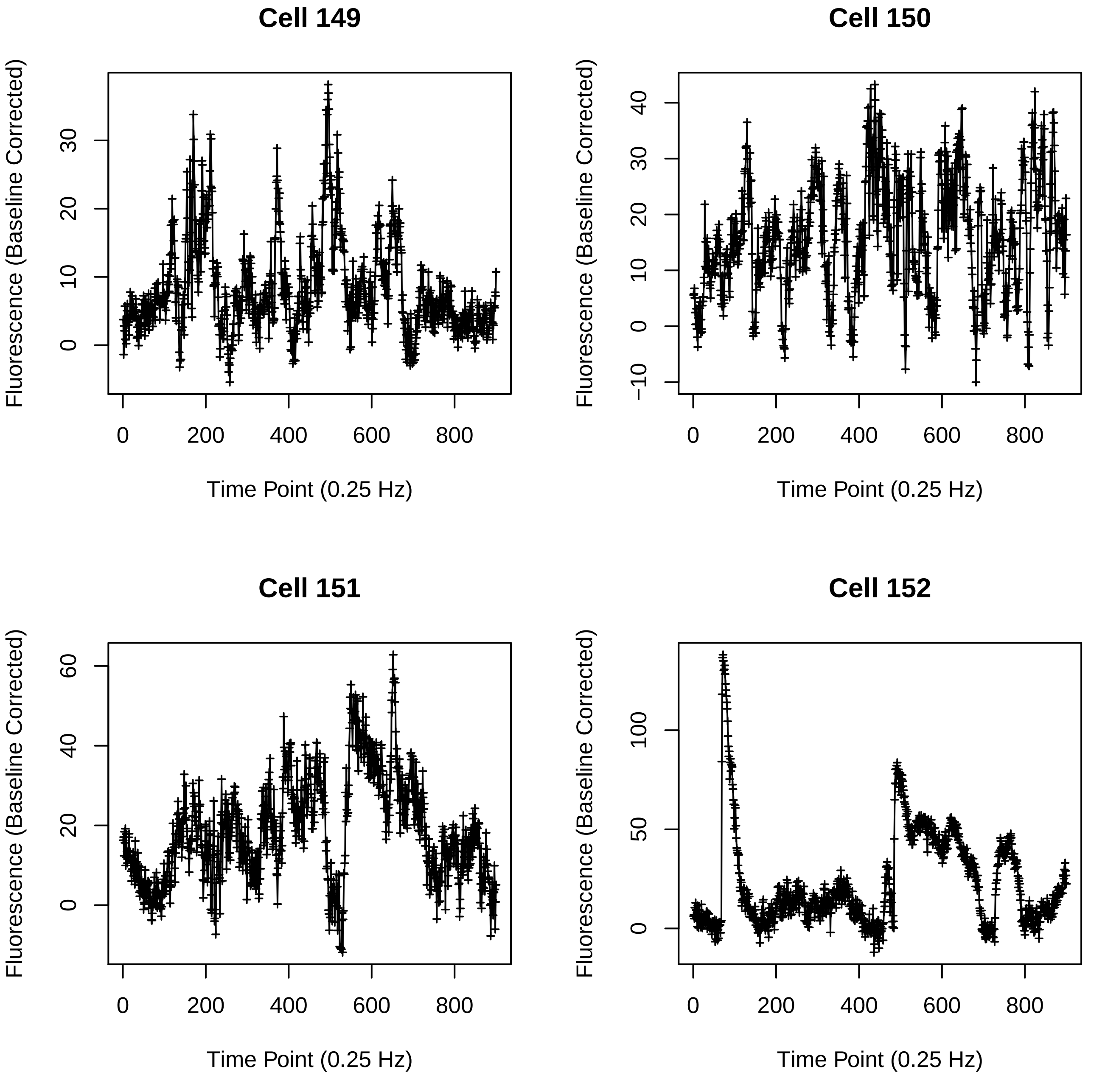
Cell 149
Cell 150
Cell 151
Cell 152
Fluorescence (Baseline Corrected)
Time Point (0.25 Hz)
0
200
400
600
800
0
10
20
30
−10
40
60
50
100

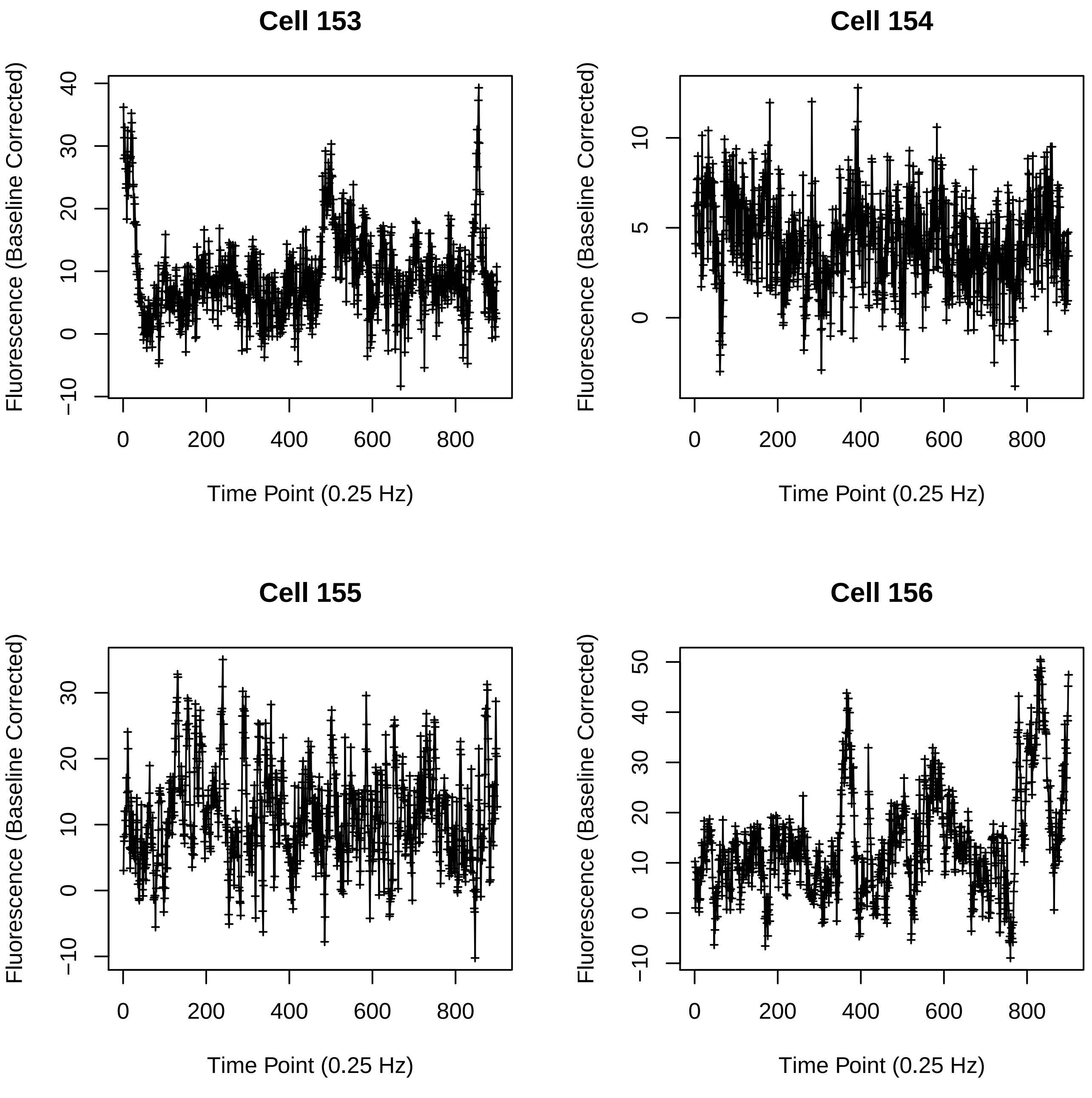
Cell 153
Cell 154
Cell 155
Cell 156
Fluorescence (Baseline Corrected)
Time Point (0.25 Hz)
0
200
400
600
800
−10
0
10
20
30
40
50
5

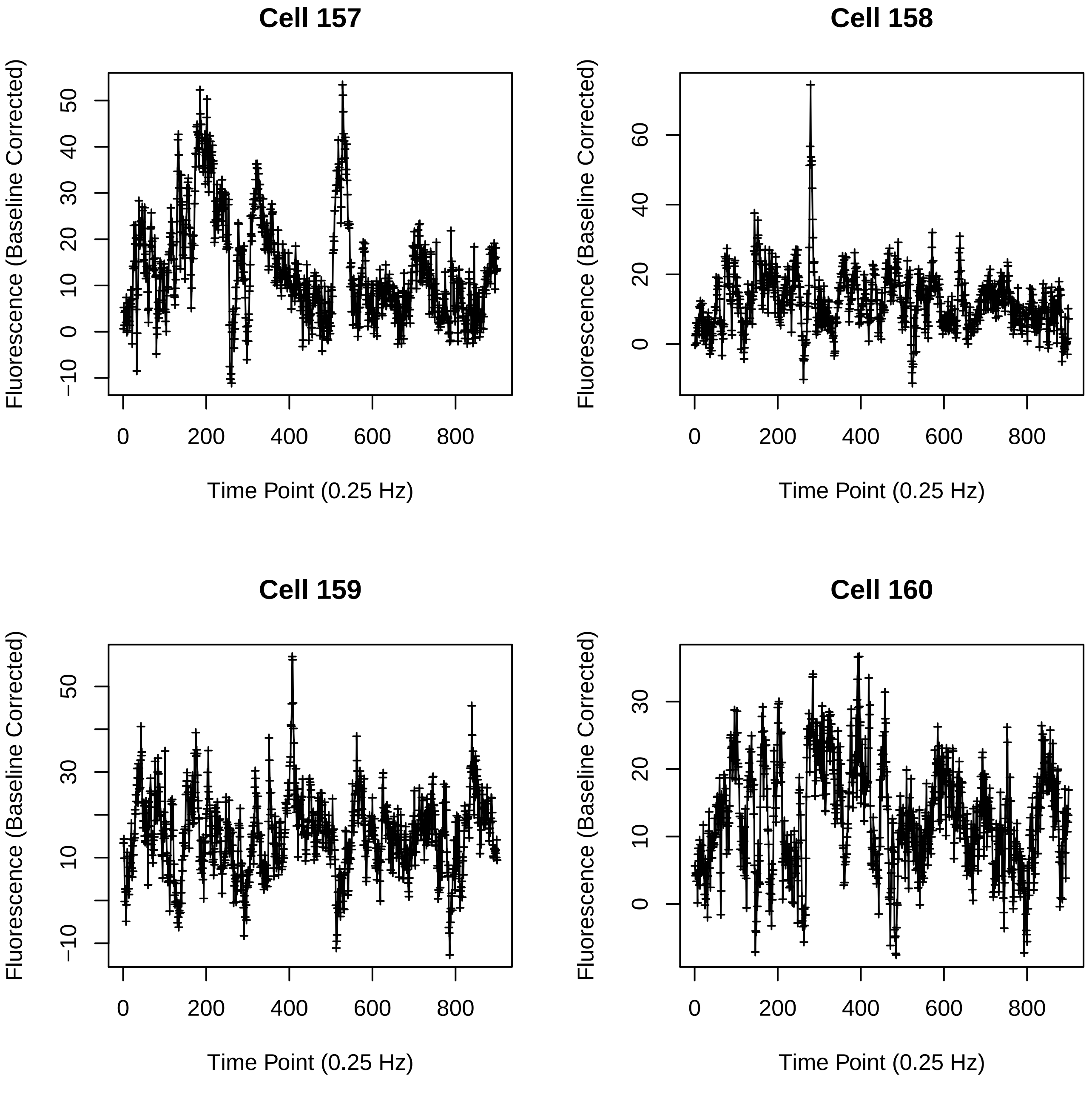
Cell 157
Cell 158
Cell 159
Cell 160
Fluorescence (Baseline Corrected)
Time Point (0.25 Hz)
0
200
400
600
800
−10
10
20
30
40
50
60

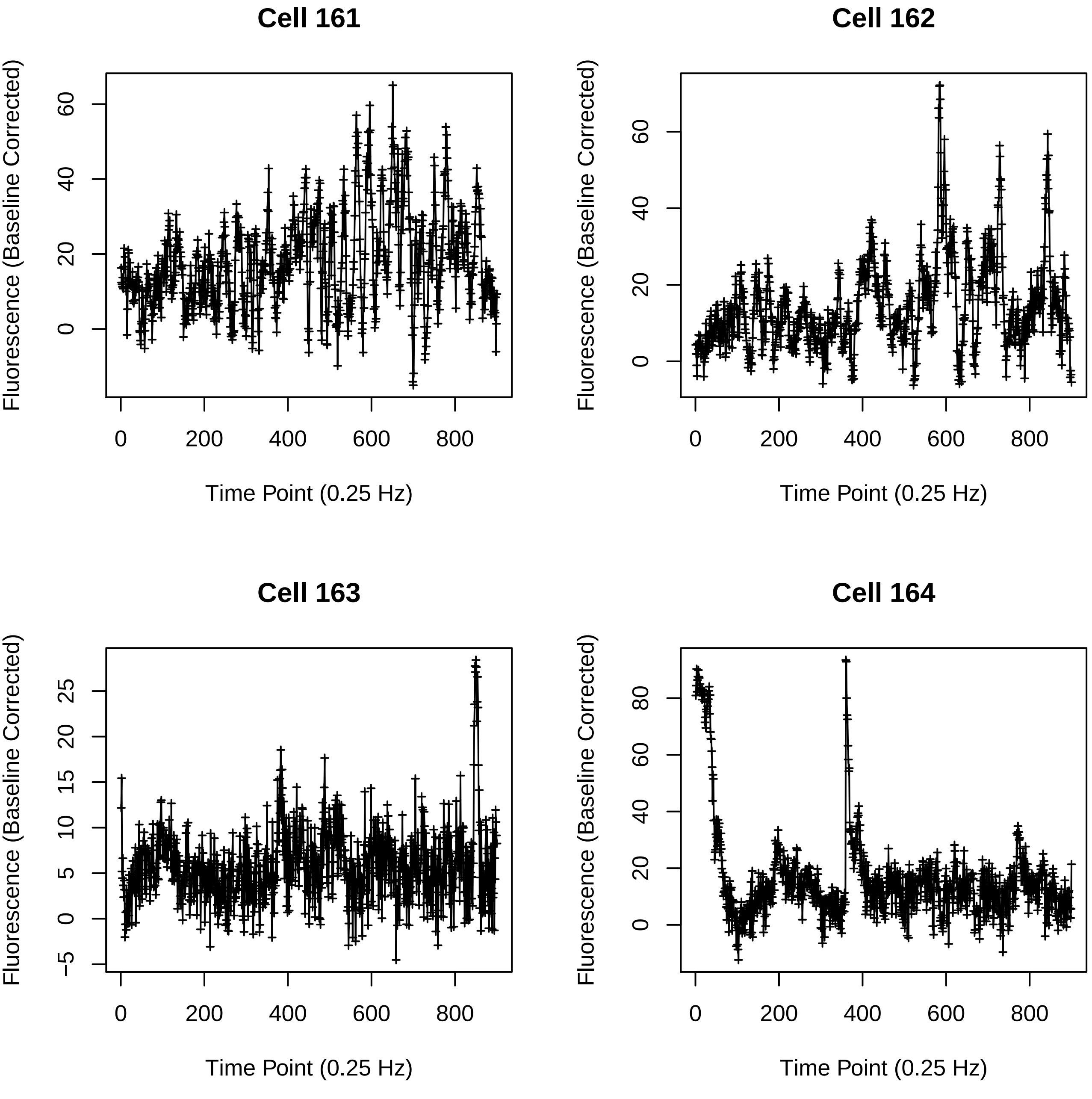
Cell 161
Cell 162
Cell 163
Cell 164
Fluorescence (Baseline Corrected)
Time Point (0.25 Hz)
0
200
400
600
800
-5
5
10
15
20
25
40
60
80

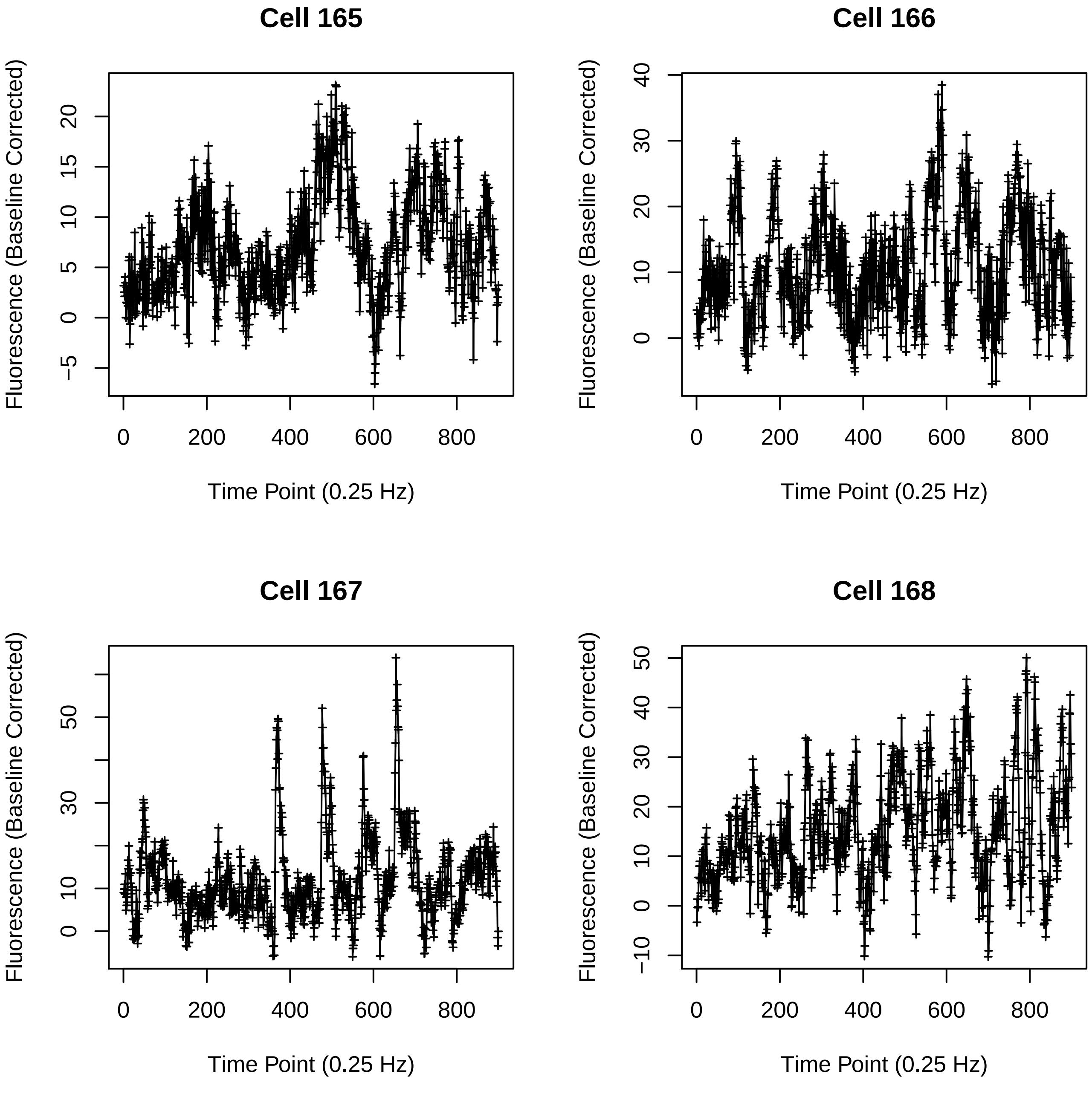

Cell 165
Cell 166
Cell 167
Cell 168
Fluorescence (Baseline Corrected)
Time Point (0.25 Hz)
−5
0
5
10
15
20
0
10
20
30
40
50
−10
0
200
400
600
800

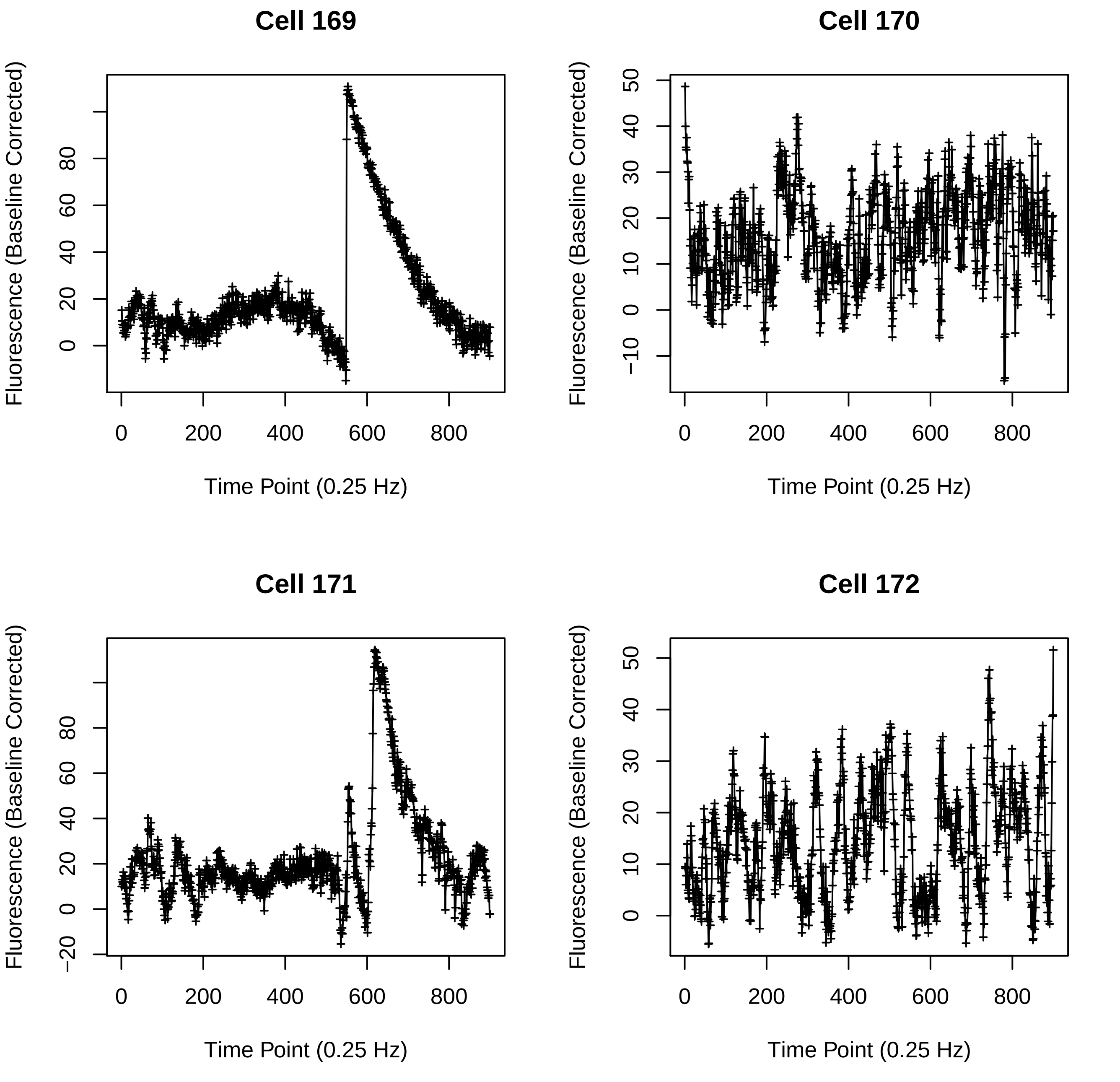

Cell 169
Cell 170
Cell 171
Cell 172
Fluorescence (Baseline Corrected)
Time Point (0.25 Hz)
0
20
40
60
80
−10
10
30
50
−20
200
400
600
800

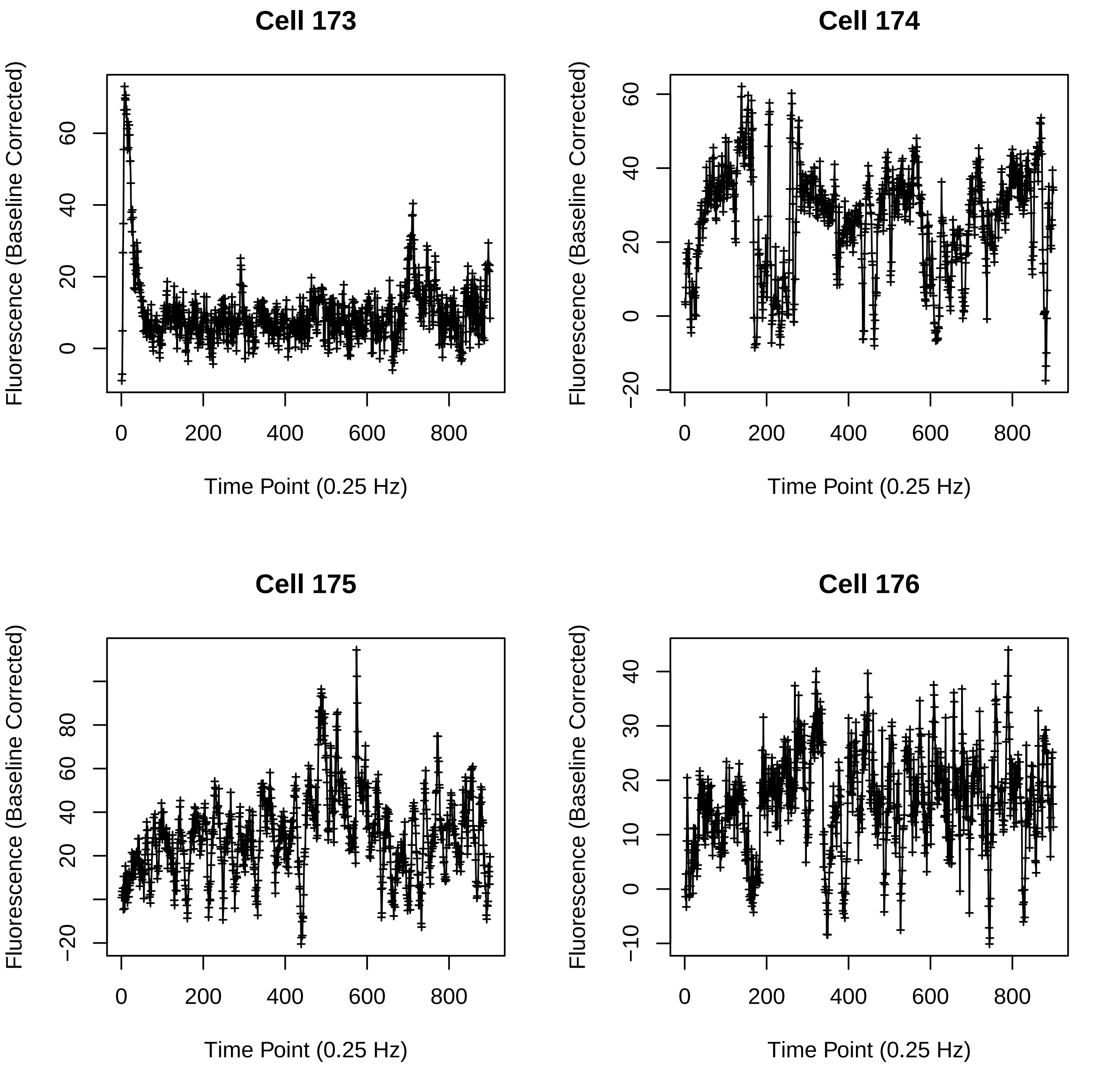

Cell 173
Cell 174
Cell 175
Cell 176
Fluorescence (Baseline Corrected)
Time Point (0.25 Hz)
0
20
40
60
80
−20
−10
10
30
200
400
600
800

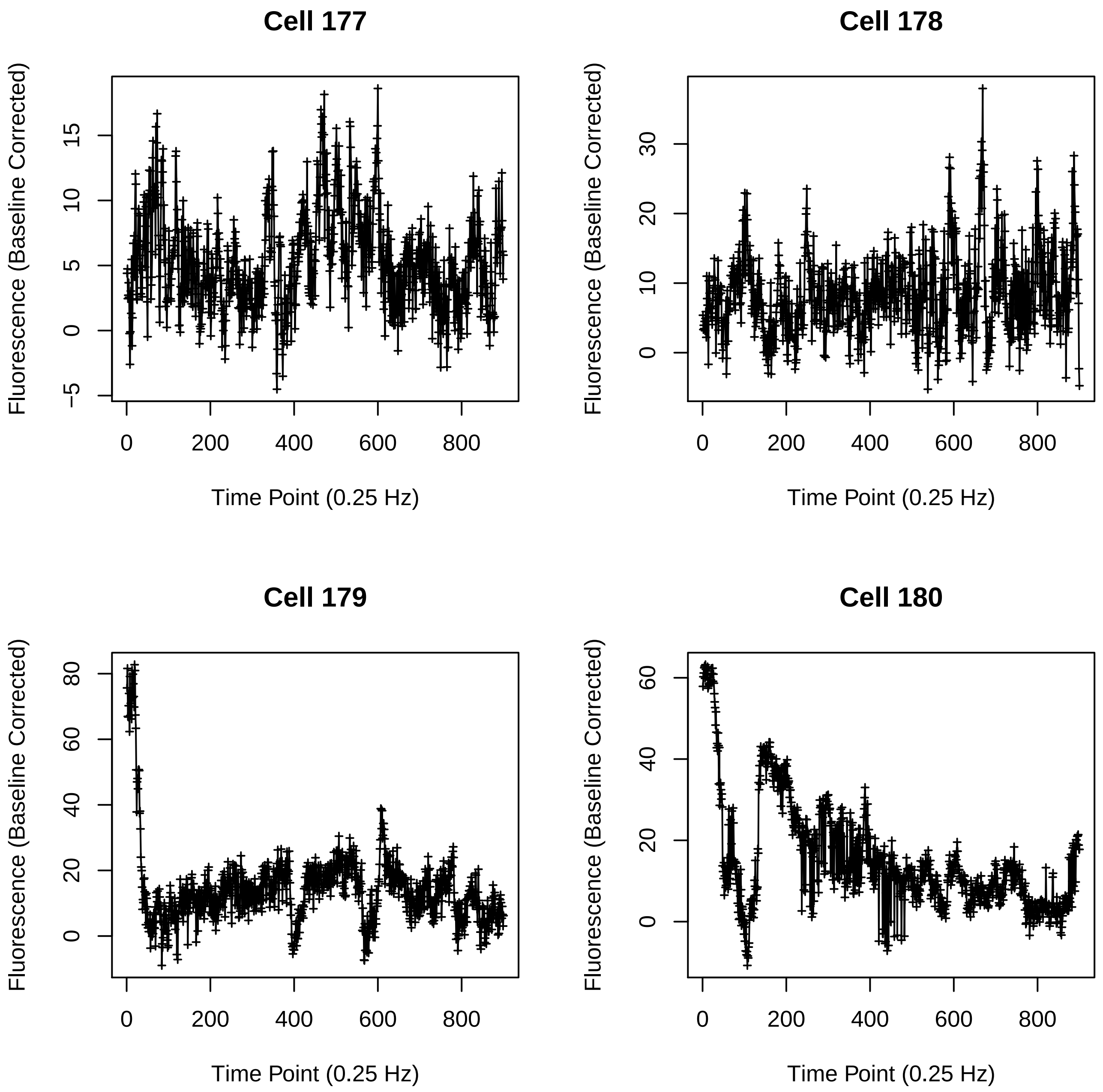
Cell 177
Cell 178
Cell 179
Cell 180
Fluorescence (Baseline Corrected)
Time Point (0.25 Hz)
-5
0
5
10
15
20
30
40
60
80
0
200
400
600
800

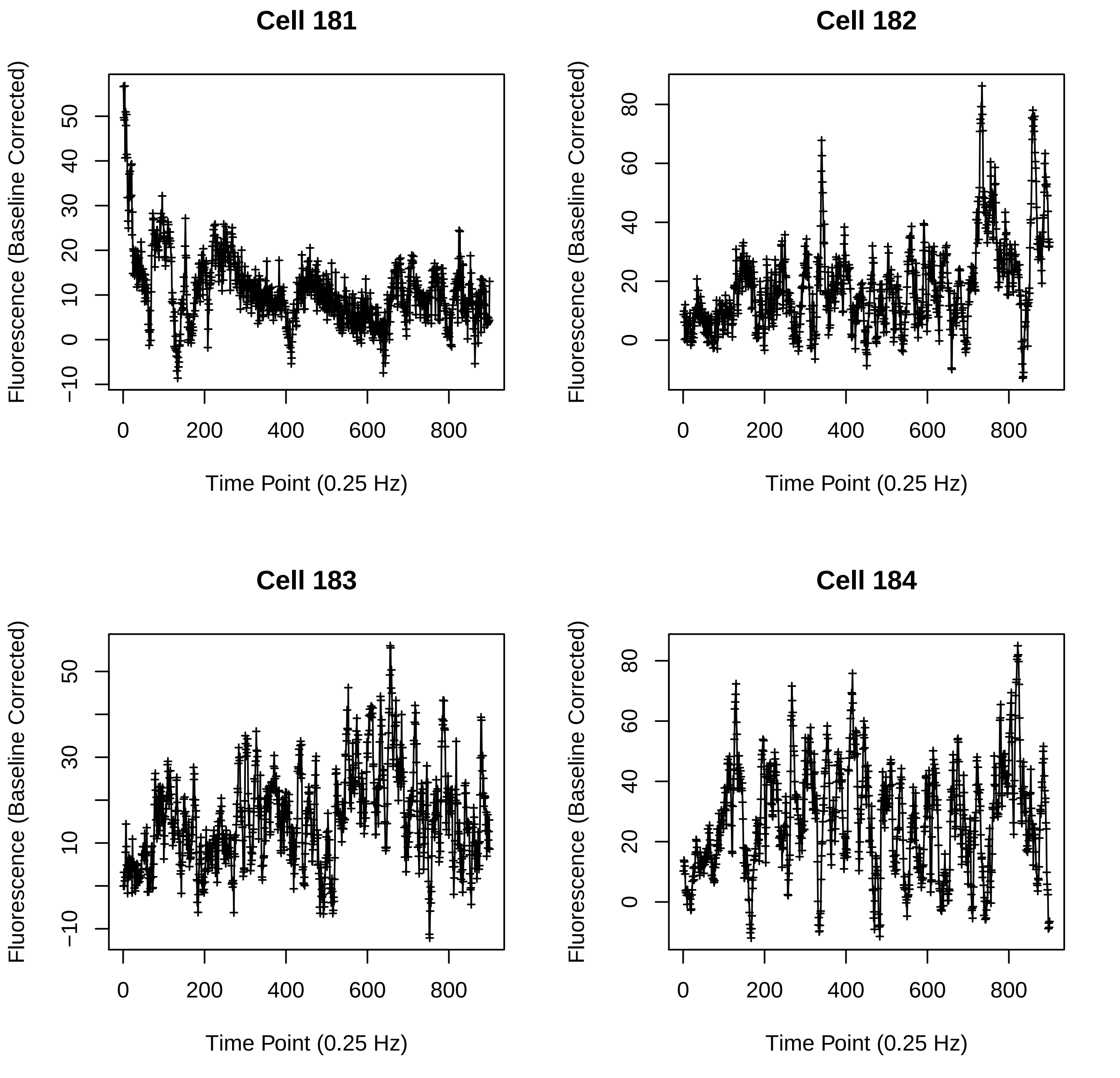
Cell 181
Cell 182
Cell 183
Cell 184
Fluorescence (Baseline Corrected)
Time Point (0.25 Hz)
0
200
400
600
800
−10
0
10
20
30
40
50
60
80

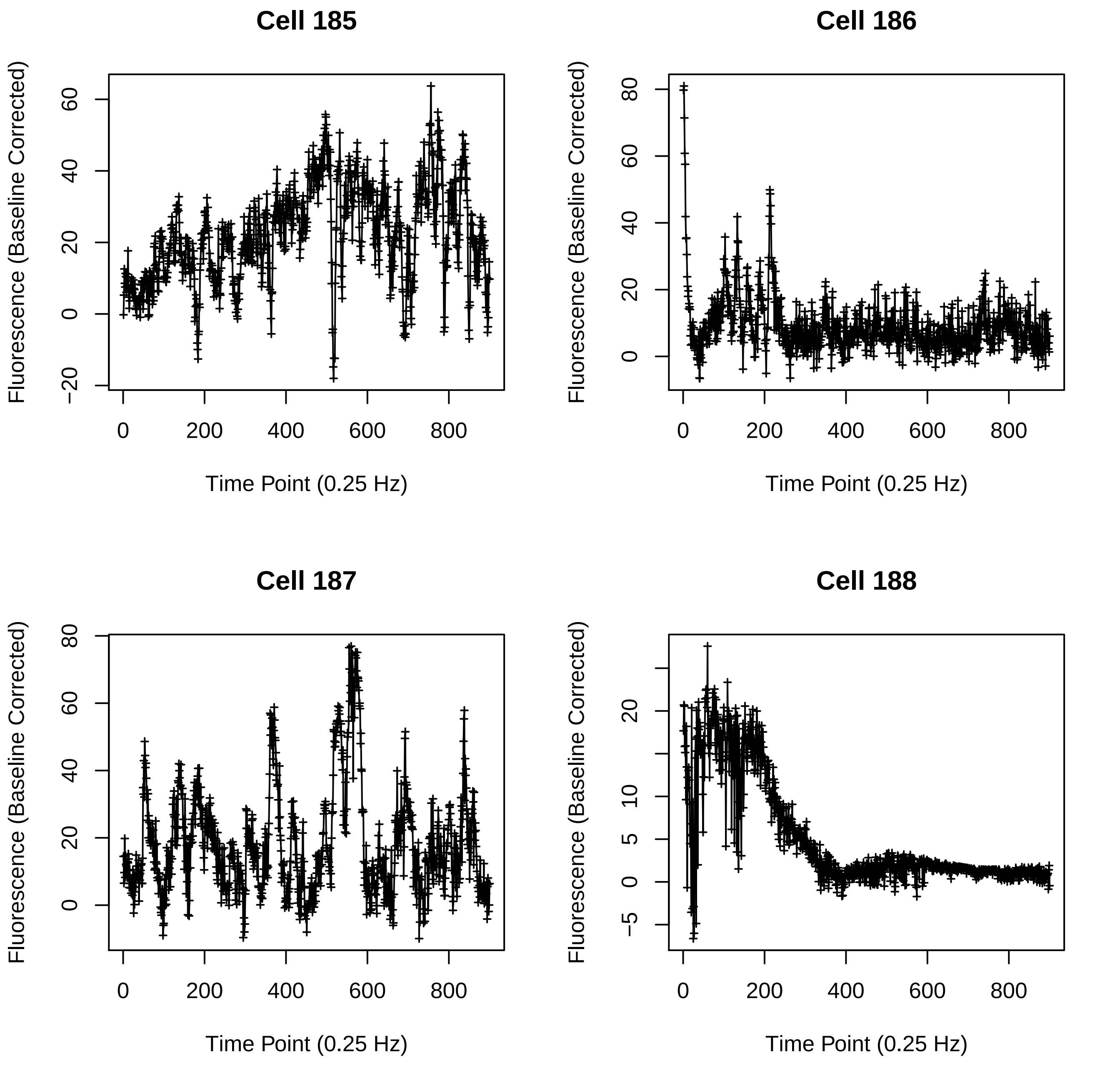
Cell 185
Fluorescence (Baseline Corrected)
−20
0
20
40
60
0
200
400
600
800
Time Point (0.25 Hz)
Cell 186
Fluorescence (Baseline Corrected)
0
20
40
60
80
0
200
400
600
800
Time Point (0.25 Hz)
Cell 187
Fluorescence (Baseline Corrected)
0
20
40
60
80
0
200
400
600
800
Time Point (0.25 Hz)
Cell 188
Fluorescence (Baseline Corrected)
−5
0
5
10
20
0
200
400
600
800
Time Point (0.25 Hz)

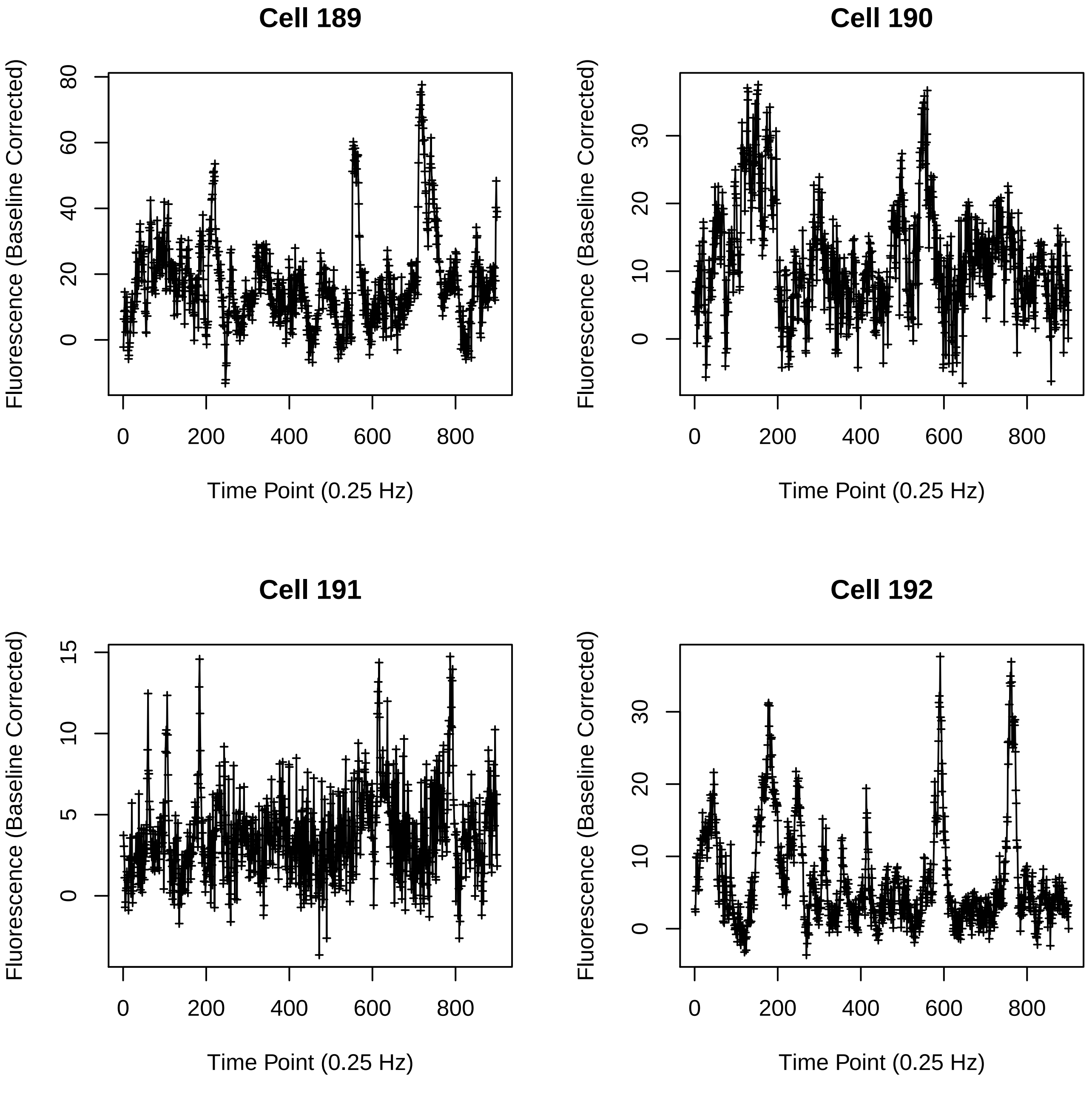
Cell 189
Cell 190
Cell 191
Cell 192
Fluorescence (Baseline Corrected)
Time Point (0.25 Hz)
0
200
400
600
800

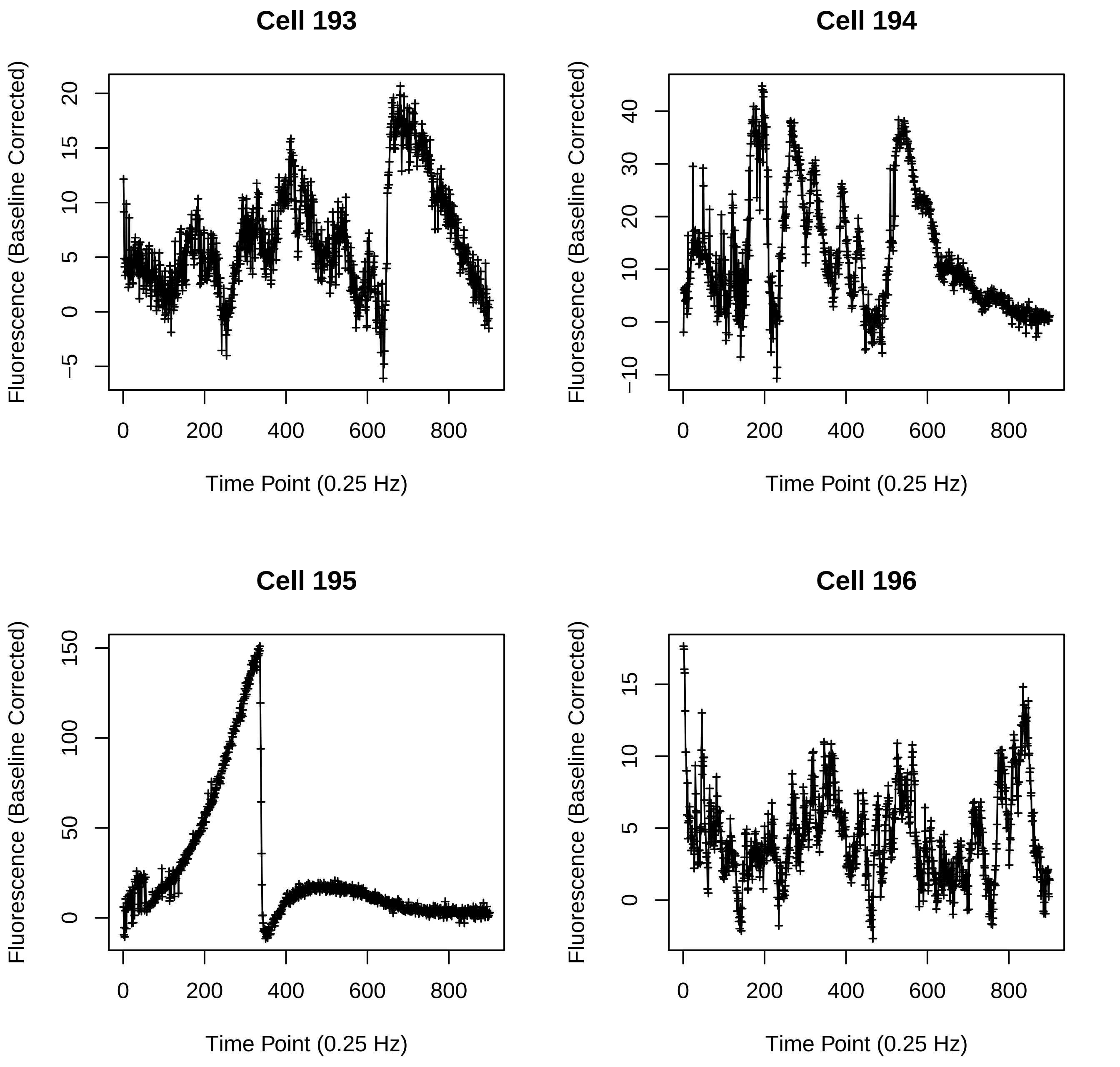
Cell 193
Cell 194
Cell 195
Cell 196
Fluorescence (Baseline Corrected)
Time Point (0.25 Hz)
0
200
400
600
800
−5
0
5
10
15
20
−10
30
40
50
100
150

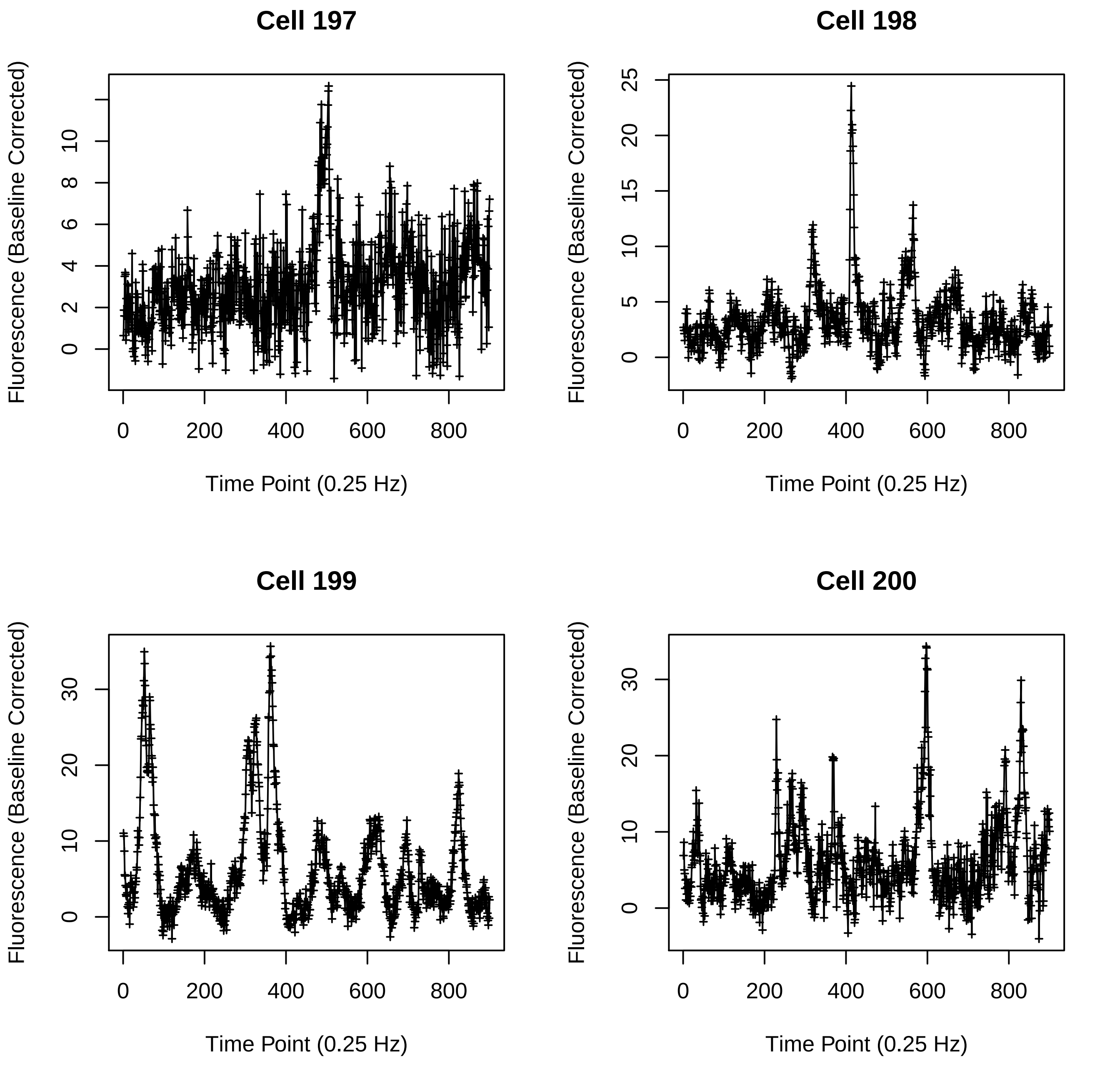
Cell 197
Cell 198
Cell 199
Cell 200
Fluorescence (Baseline Corrected)
Time Point (0.25 Hz)
0
200
400
600
800
2
4
6
8
10
5
15
20
25
30

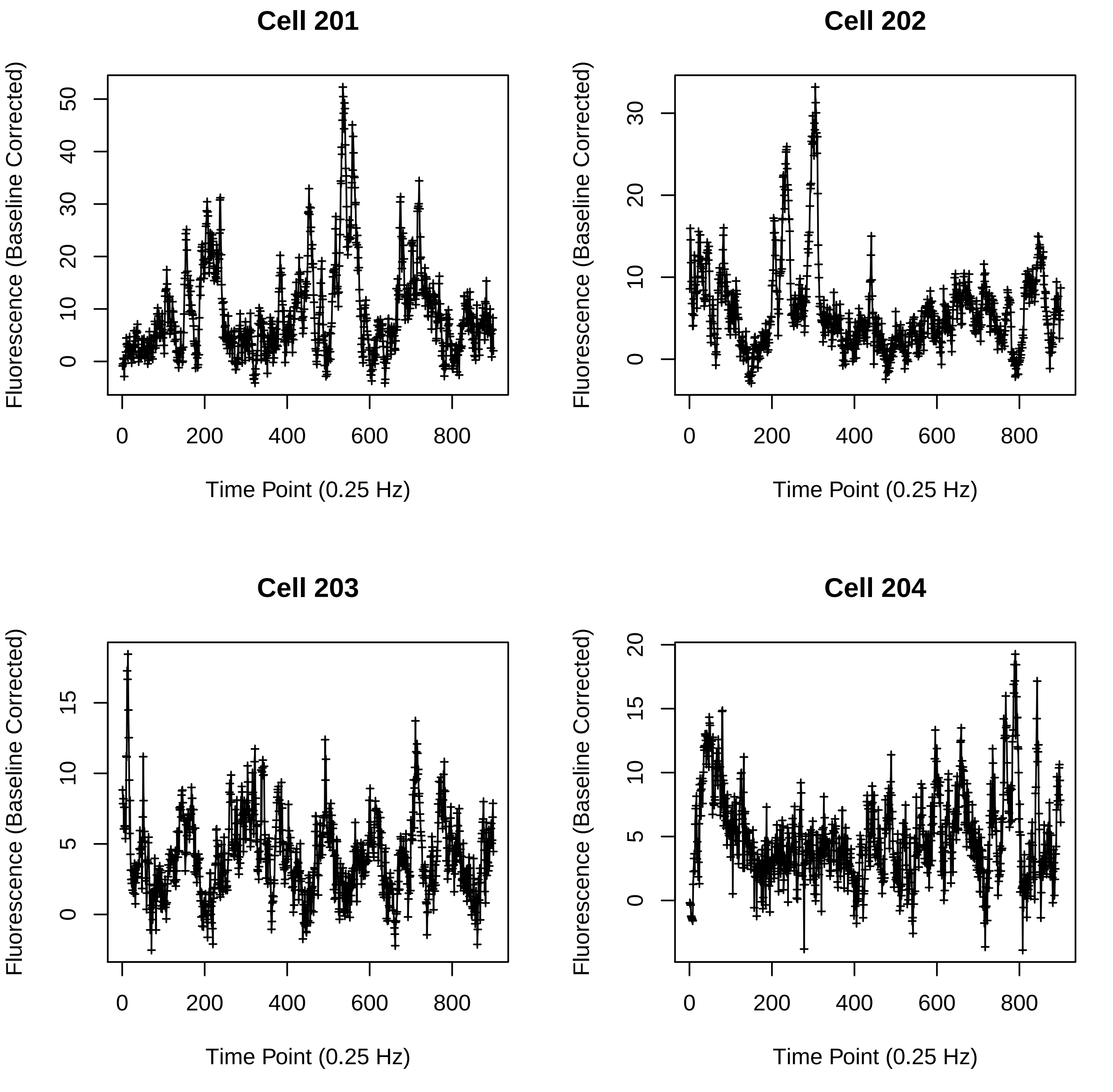
Cell 201
Cell 202
Cell 203
Cell 204
Fluorescence (Baseline Corrected)
Time Point (0.25 Hz)
0
200
400
600
800
0
10
20
30
40
50
5
15

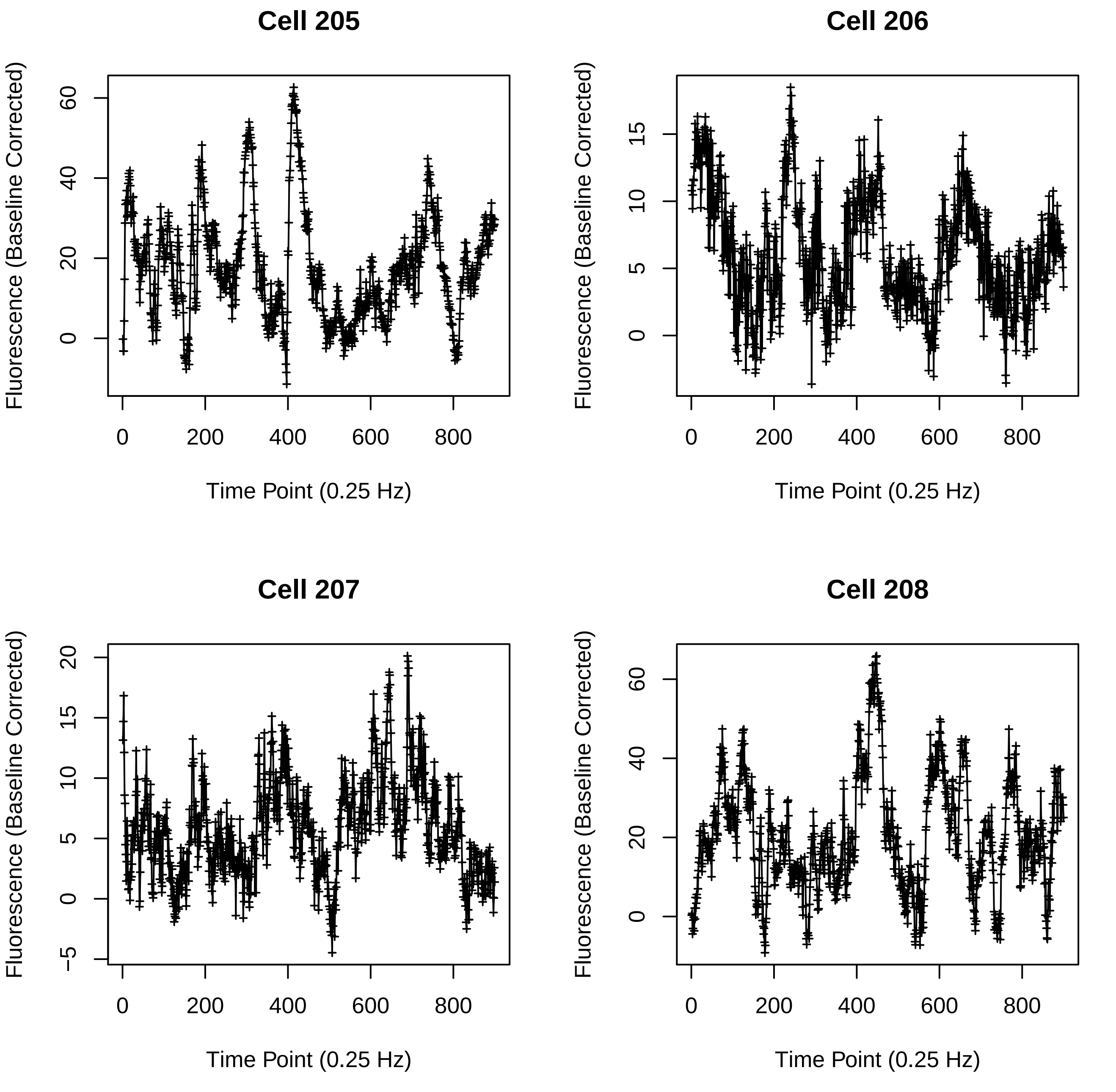
Cell 205
Cell 206
Cell 207
Cell 208
Fluorescence (Baseline Corrected)
Time Point (0.25 Hz)
0
200
400
600
800
-5
0
5
10
15
20
40
60

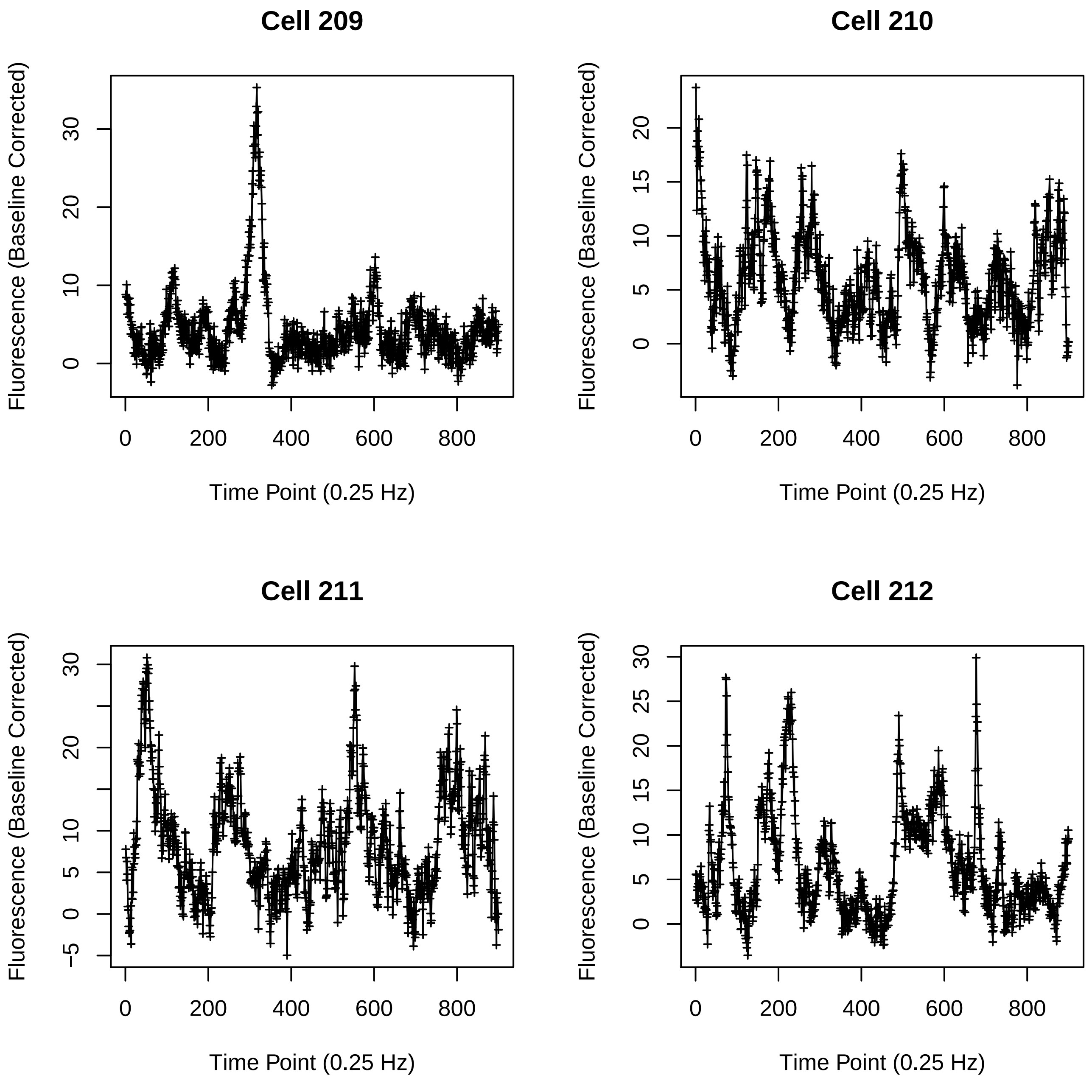
Cell 209
Fluorescence (Baseline Corrected)
0
10
20
30
0
200
400
600
800
Time Point (0.25 Hz)
Cell 210
Fluorescence (Baseline Corrected)
0
5
10
15
20
0
200
400
600
800
Time Point (0.25 Hz)
Cell 211
Fluorescence (Baseline Corrected)
−5
0
5
10
20
30
0
200
400
600
800
Time Point (0.25 Hz)
Cell 212
Fluorescence (Baseline Corrected)
0
5
10
15
20
25
30
0
200
400
600
800
Time Point (0.25 Hz)

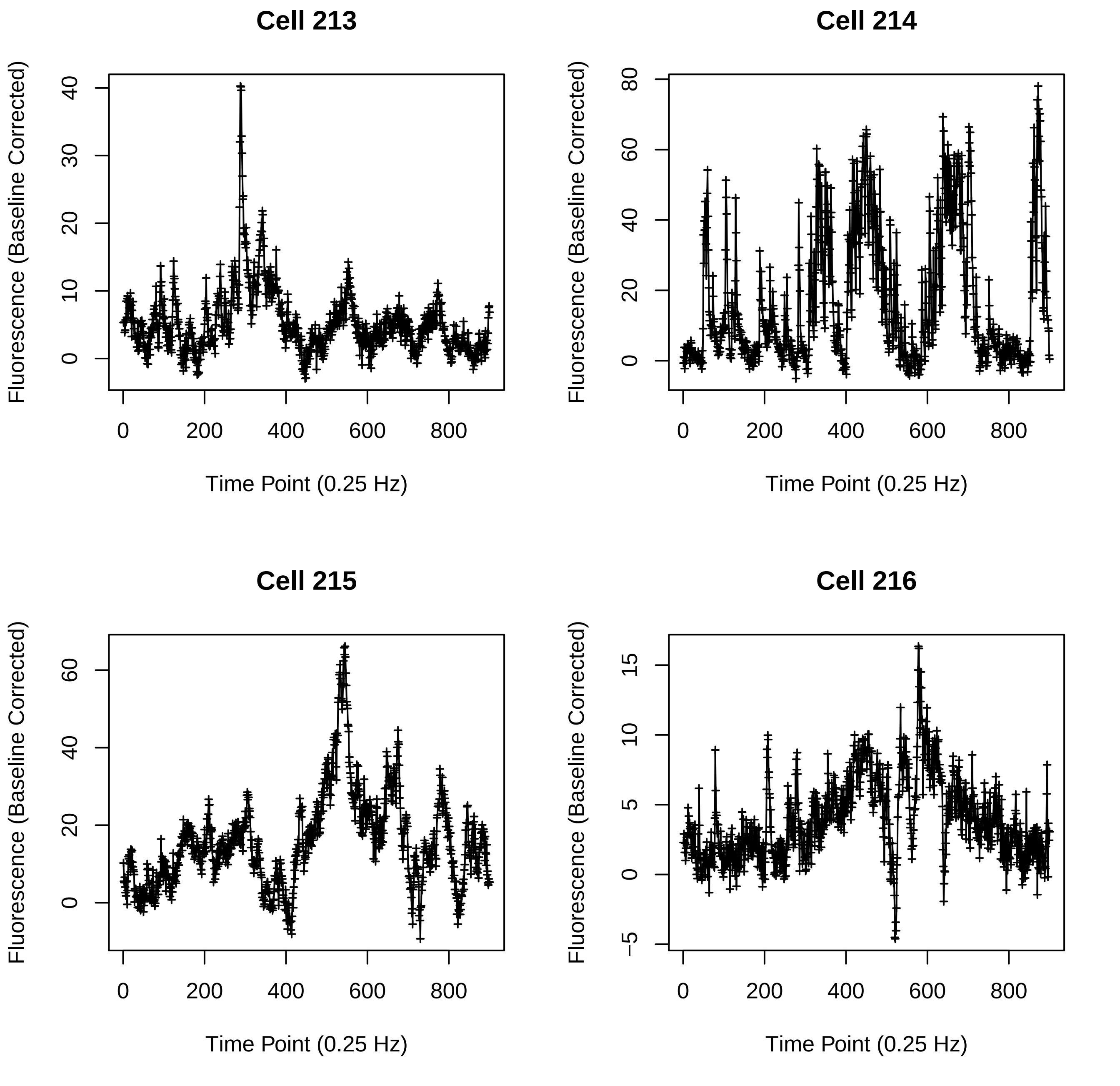

Cell 213
Cell 214
Cell 215
Cell 216
Fluorescence (Baseline Corrected)
Time Point (0.25 Hz)
0
200
400
600
800
0
10
20
30
40
60
80
-5
5
15

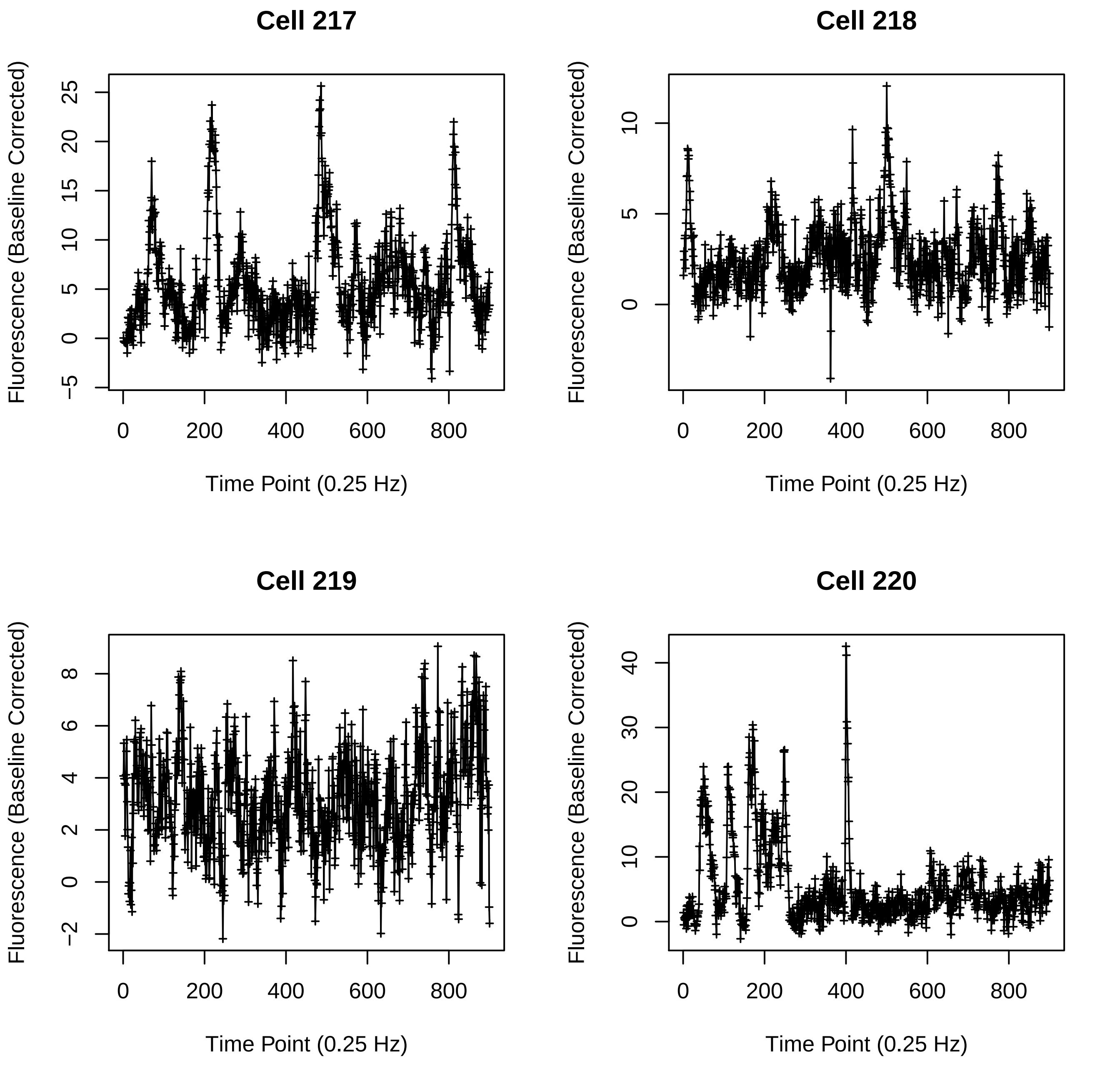

Cell 217
Cell 218
Cell 219
Cell 220
Fluorescence (Baseline Corrected)
Time Point (0.25 Hz)

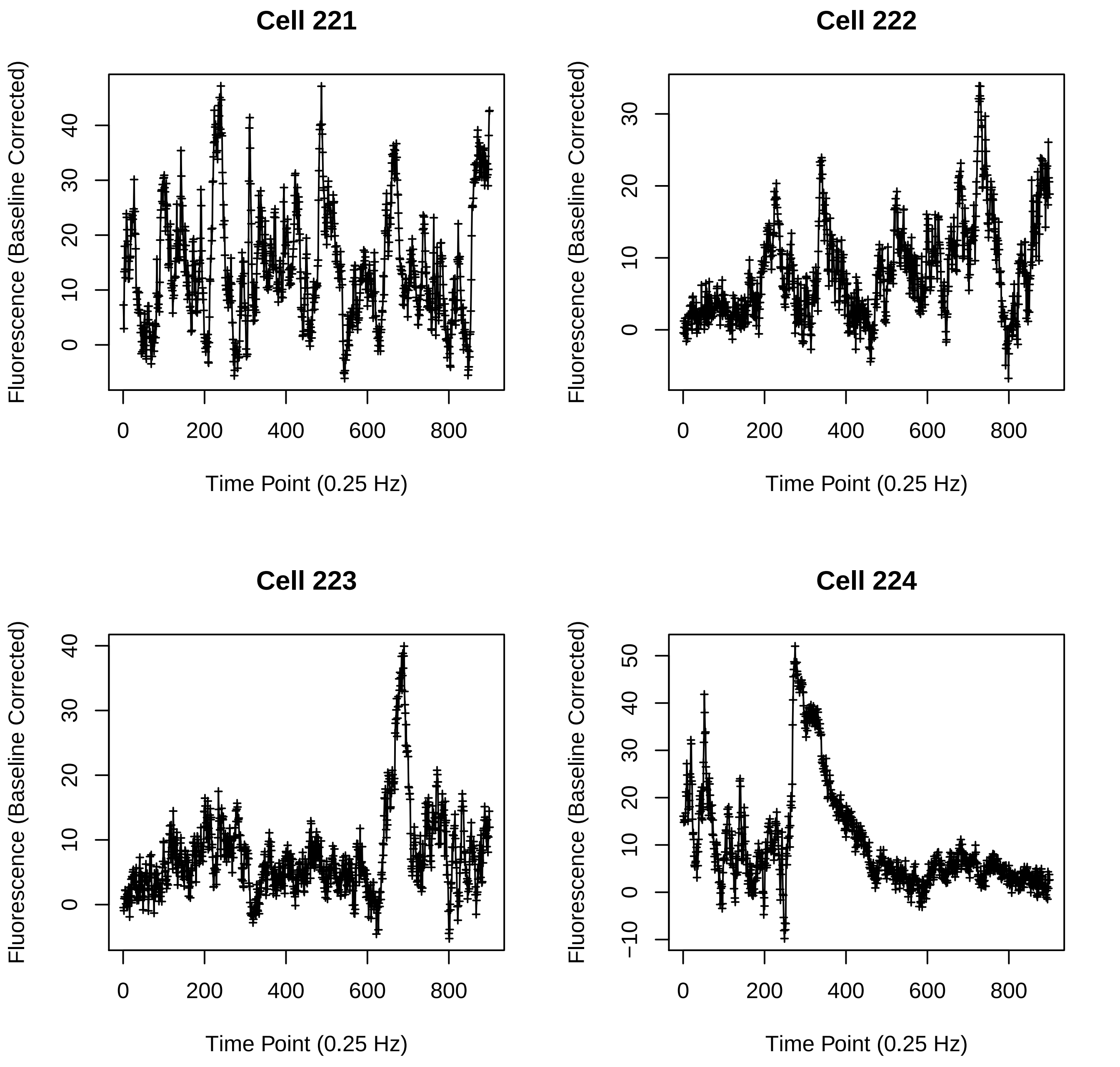
Cell 221
Cell 222
Cell 223
Cell 224
Fluorescence (Baseline Corrected)
Time Point (0.25 Hz)
0
200
400
600
800
10
20
30
40
50
−10

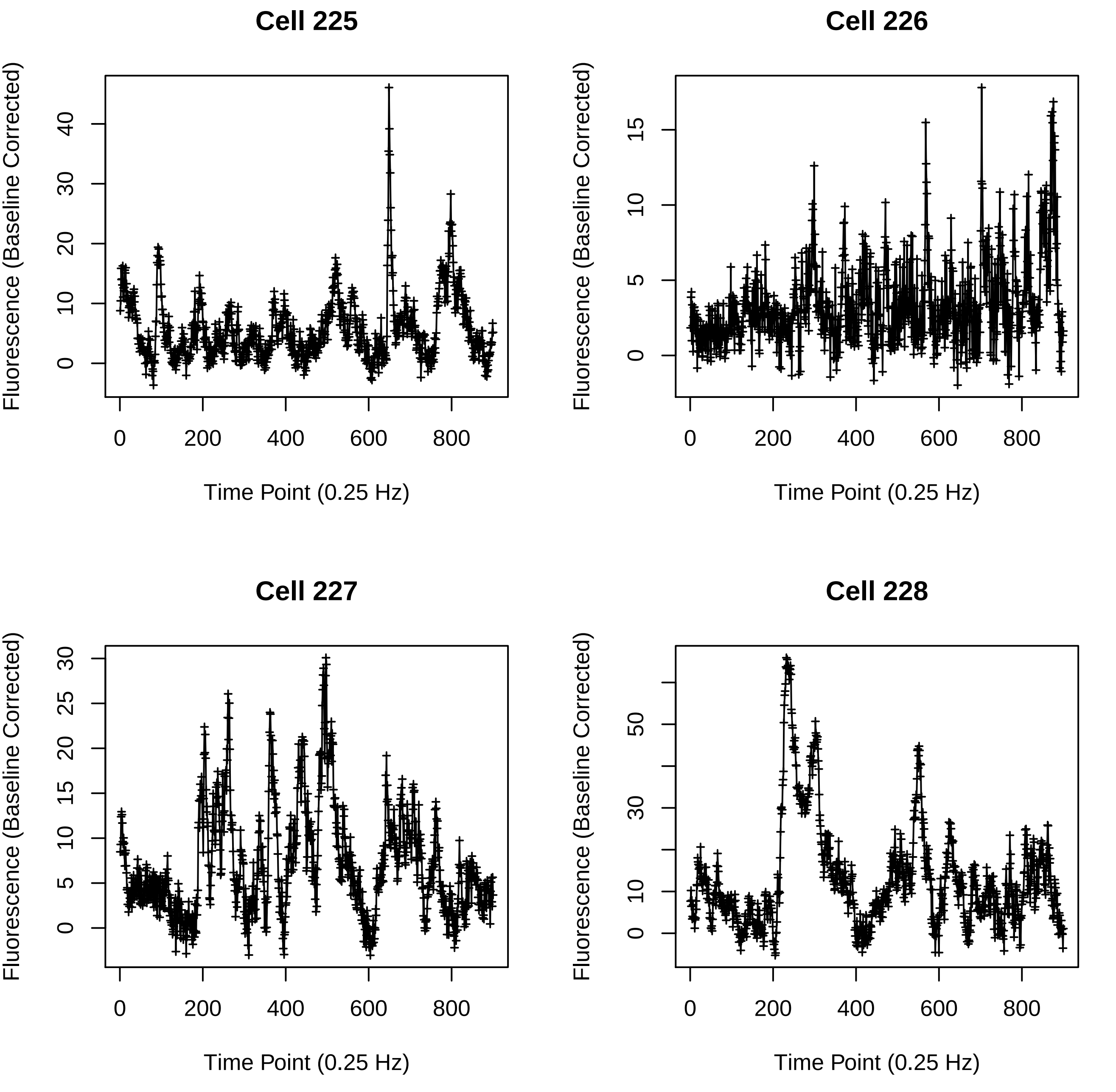

Cell 225
Cell 226
Cell 227
Cell 228
Fluorescence (Baseline Corrected)
Time Point (0.25 Hz)
0
200
400
600
800
0
10
20
30
40
0
5
10
15
0
5
10
15
20
25
30
0
10
30
50

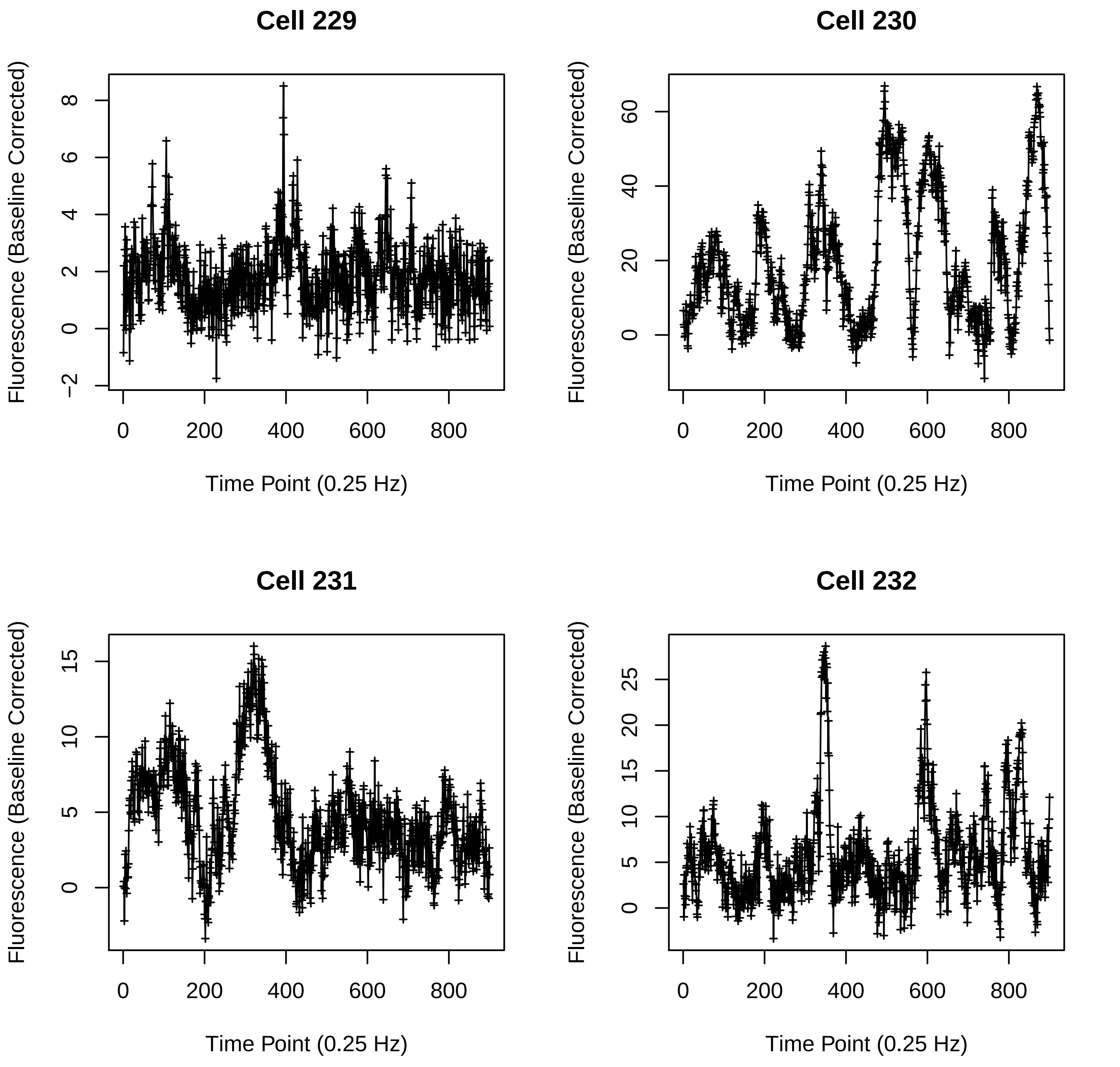
Cell 229
Cell 230
Cell 231
Cell 232
Fluorescence (Baseline Corrected)
Time Point (0.25 Hz)
0
200
400
600
800
−2
2
4
6
8
20
40
60
5
10
15
25

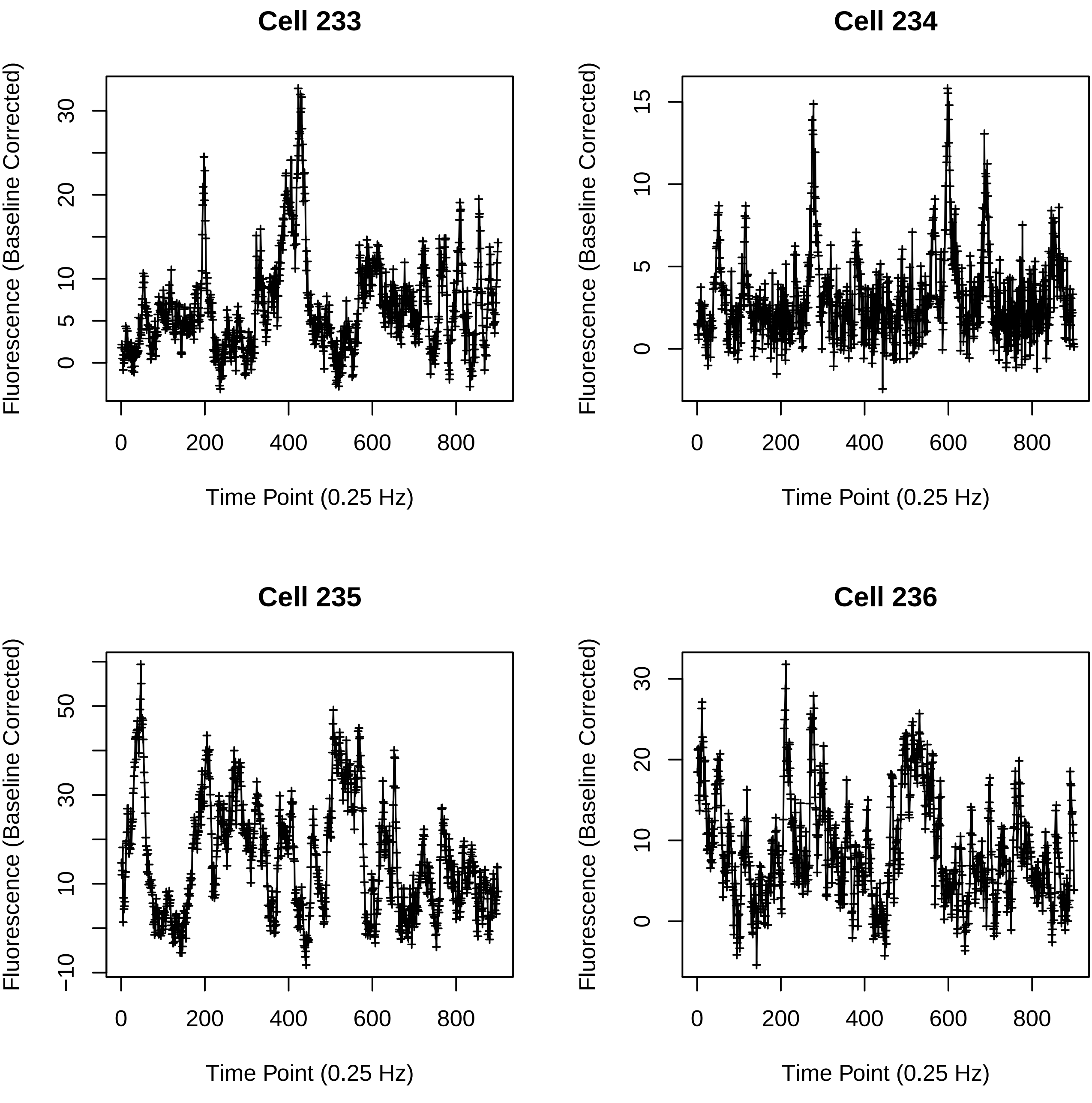
Cell 233
Cell 234
Cell 235
Cell 236
Fluorescence (Baseline Corrected)
Time Point (0.25 Hz)
0
200
400
600
800
0
5
10
15
20
30
50
−10

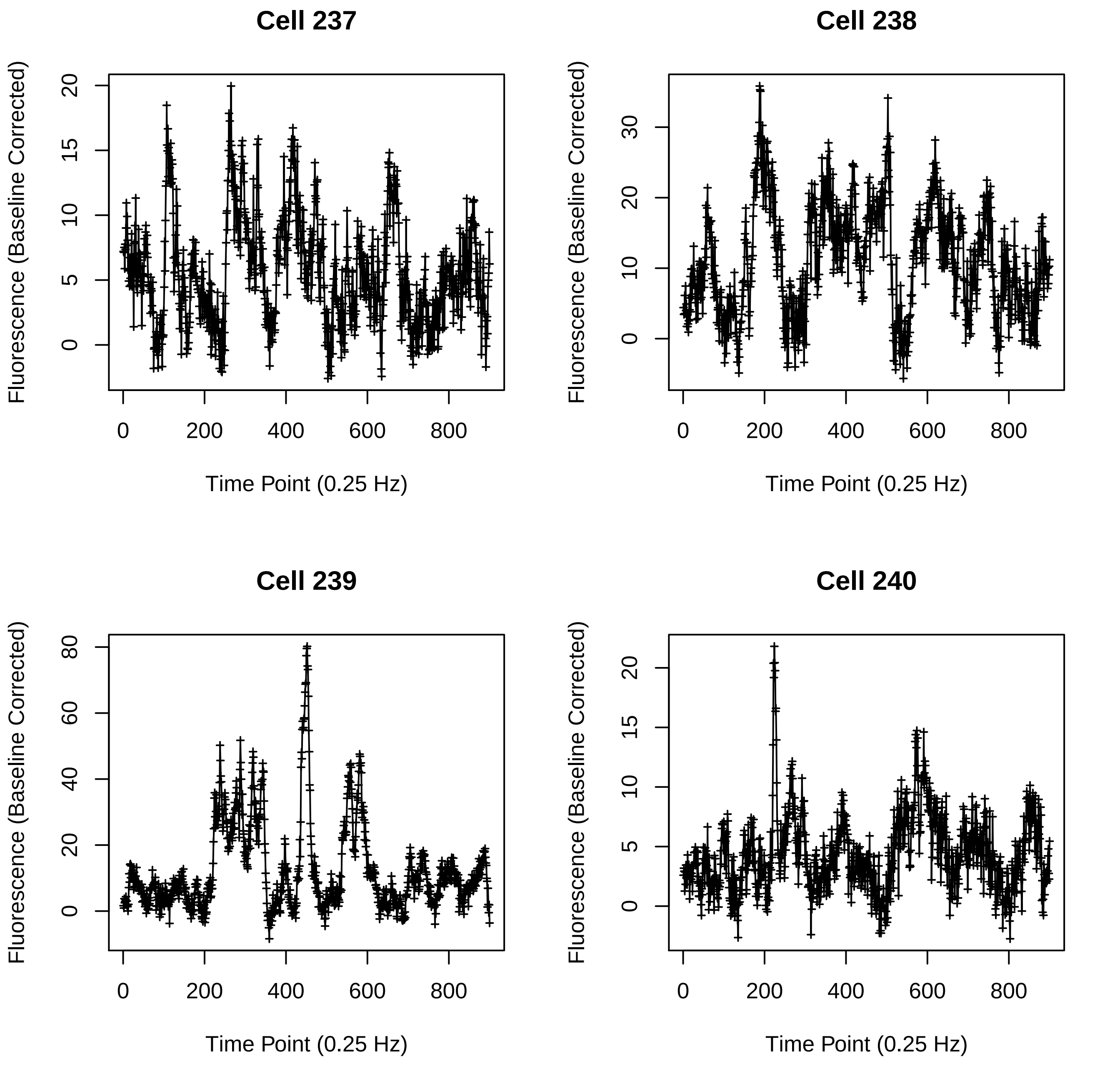
Cell 237
Cell 238
Cell 239
Cell 240
Fluorescence (Baseline Corrected)
Time Point (0.25 Hz)
0
200
400
600
800
0
5
10
15
20
30
40
60
80

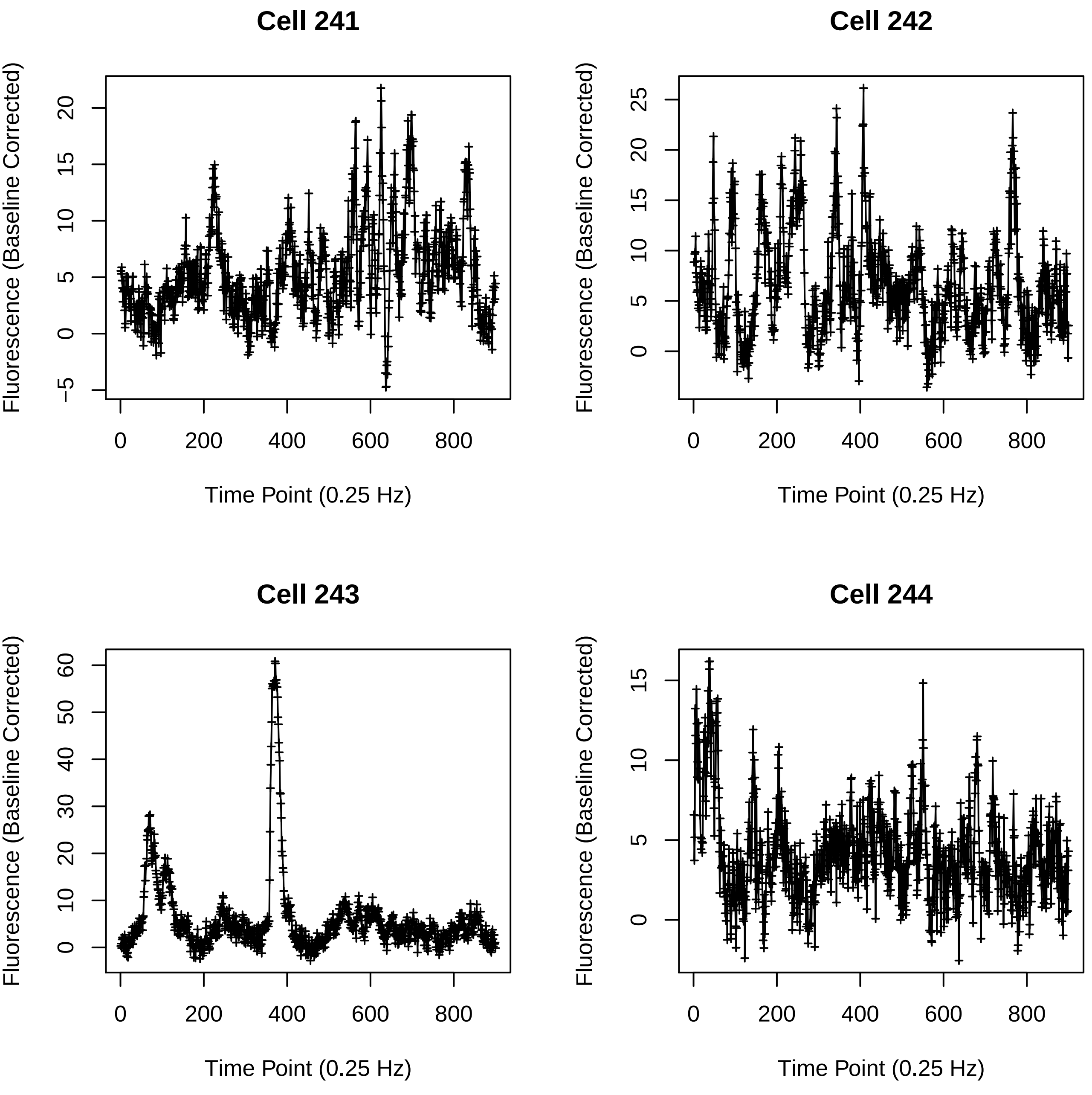
Cell 241
Cell 242
Cell 243
Cell 244
Fluorescence (Baseline Corrected)
Time Point (0.25 Hz)
0
200
400
600
800
−5
0
5
10
15
20
25
30
40
50
60

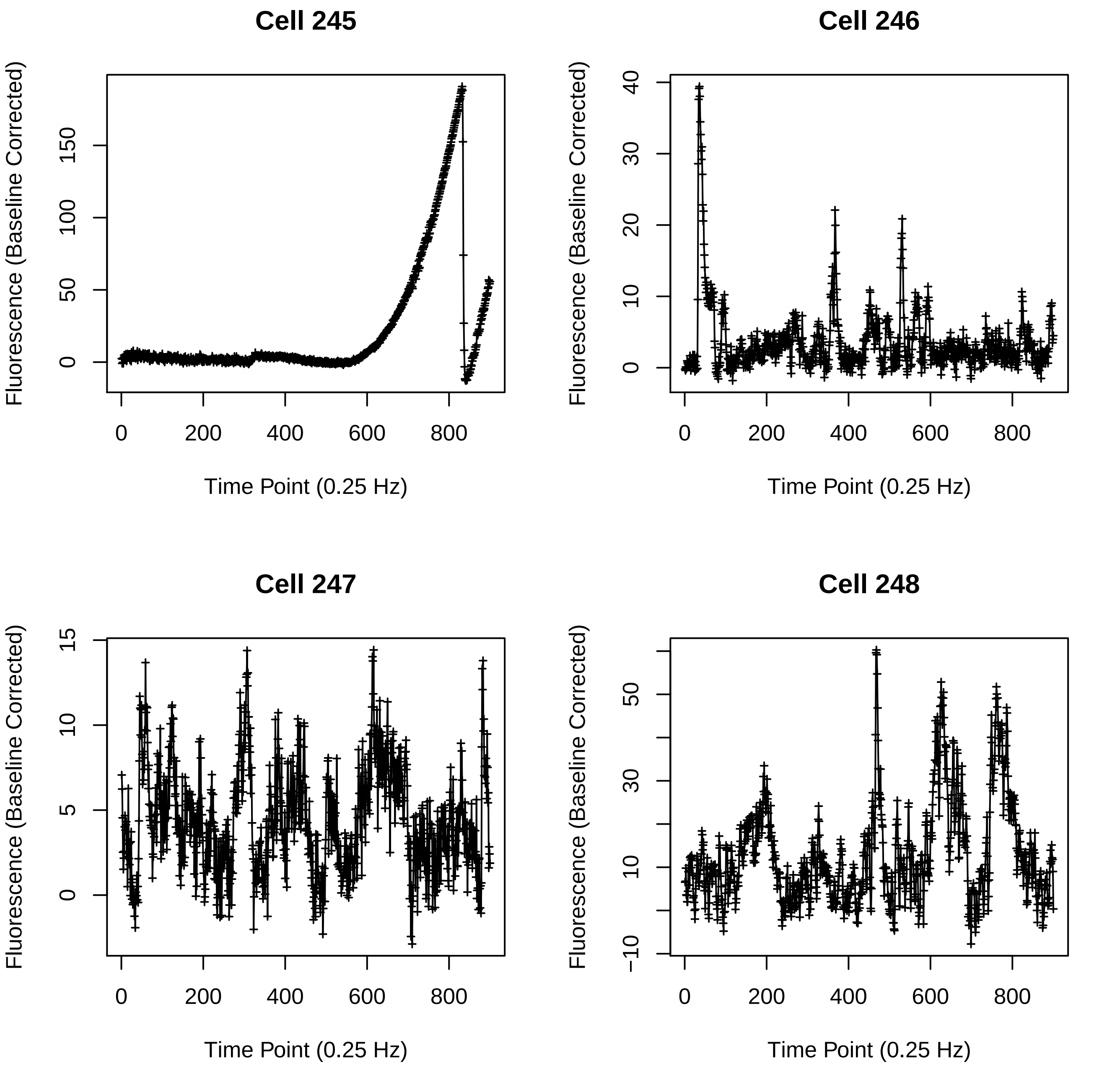
Cell 245
Cell 246
Cell 247
Cell 248
Fluorescence (Baseline Corrected)
Time Point (0.25 Hz)
0
50
100
150
0
10
20
30
40
0
5
10
15
−10
10
30
50
0
200
400
600
800

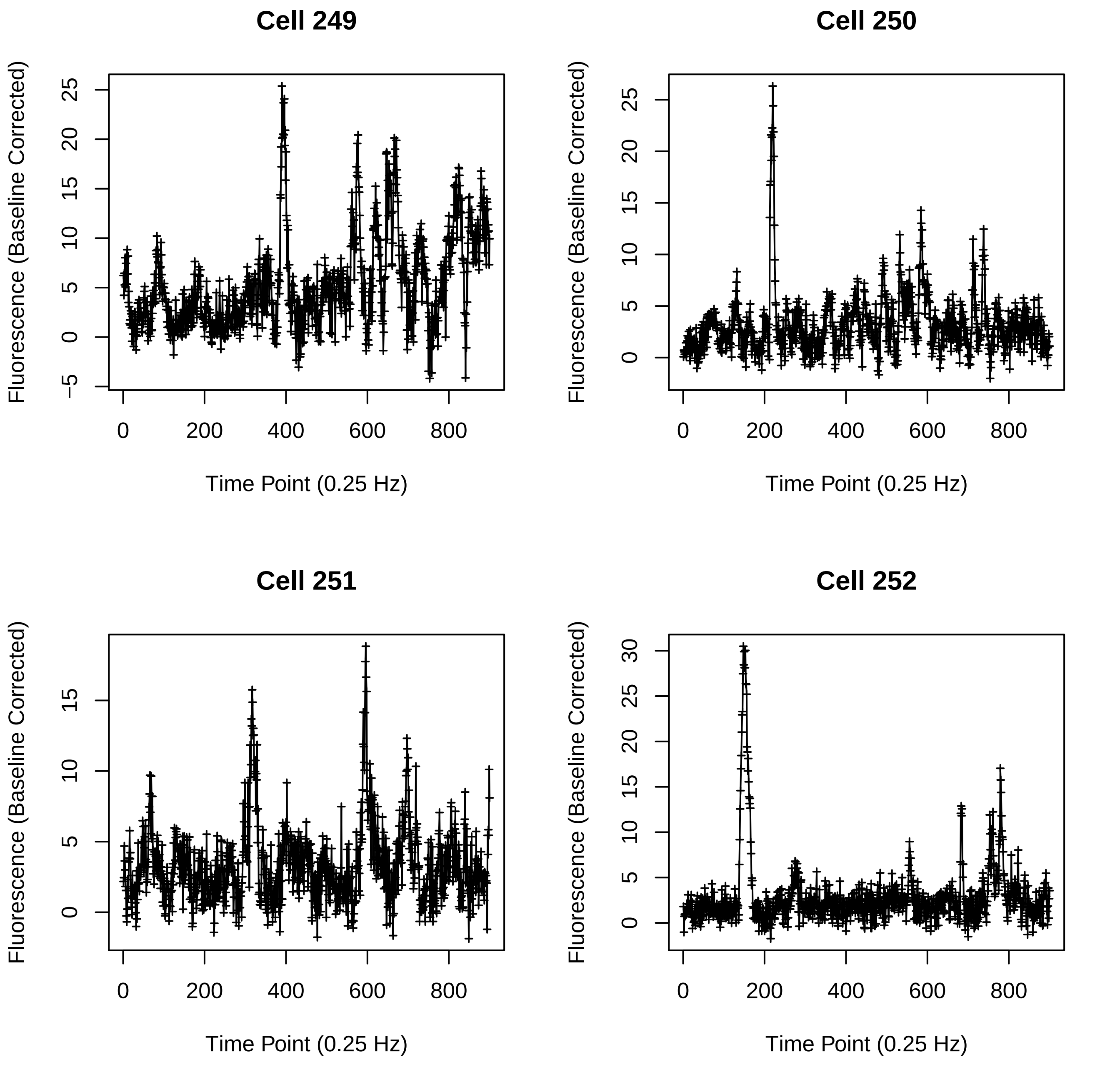
Cell 249
Cell 250
Cell 251
Cell 252
Fluorescence (Baseline Corrected)
Time Point (0.25 Hz)
0
200
400
600
800
−5
5
10
15
20
25
30

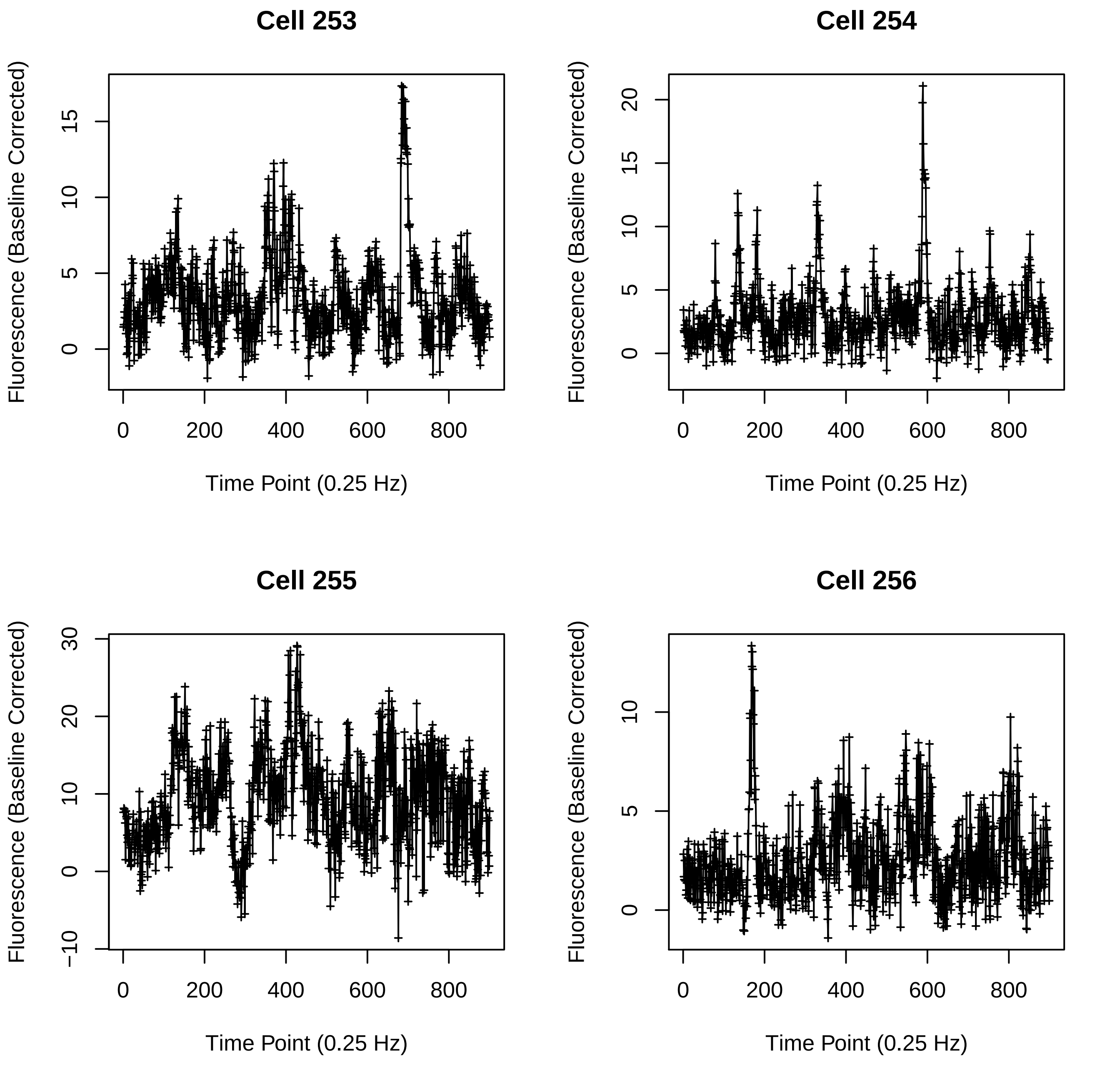
Cell 253
Fluorescence (Baseline Corrected)
0
5
10
15
0
200
400
600
800
Time Point (0.25 Hz)
Cell 254
Fluorescence (Baseline Corrected)
0
5
10
15
20
0
200
400
600
800
Time Point (0.25 Hz)
Cell 255
Fluorescence (Baseline Corrected)
−10
0
10
20
30
0
200
400
600
800
Time Point (0.25 Hz)
Cell 256
Fluorescence (Baseline Corrected)
0
5
10
0
200
400
600
800
Time Point (0.25 Hz)

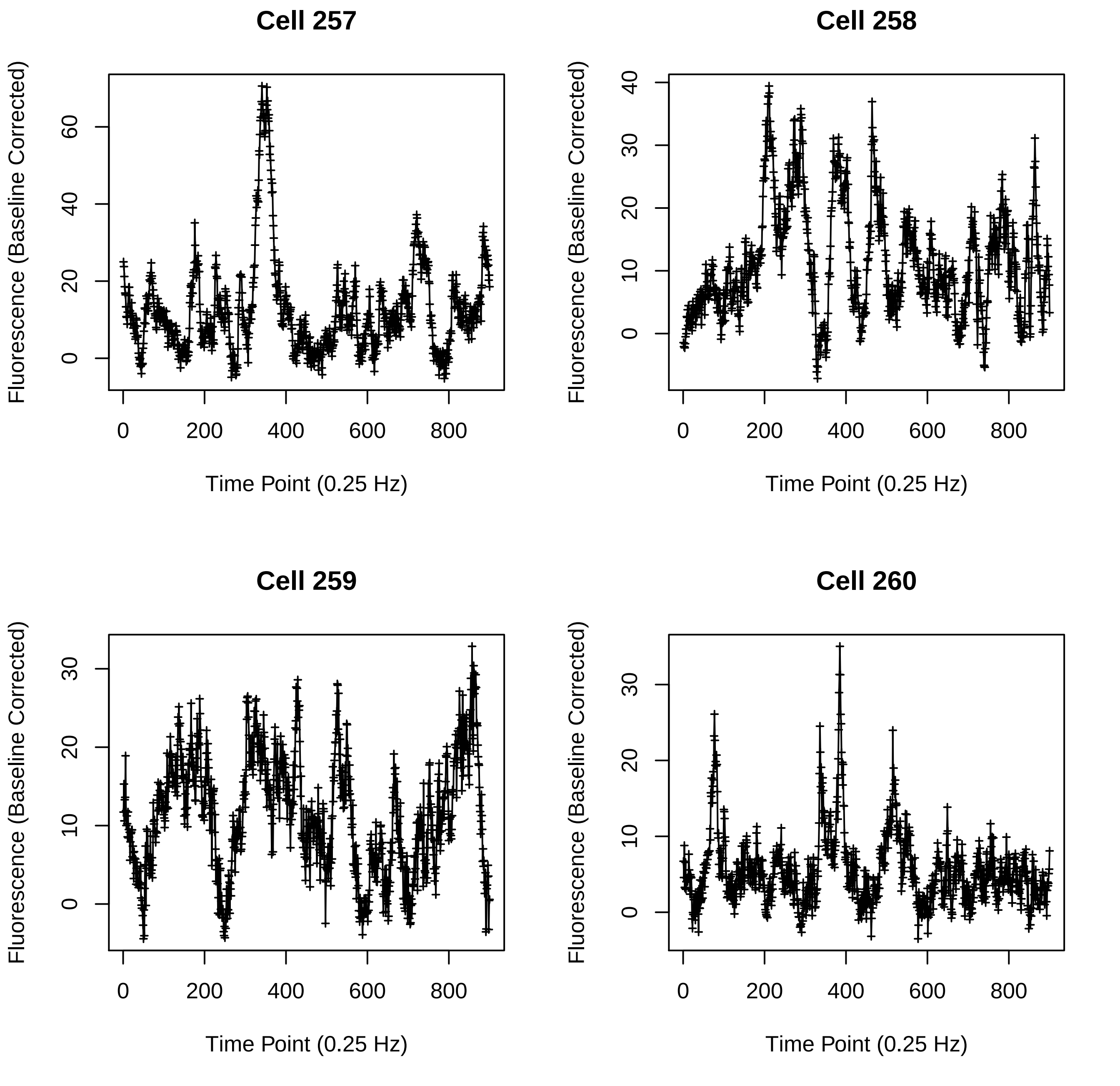
Cell 257
Cell 258
Cell 259
Cell 260
Fluorescence (Baseline Corrected)
Time Point (0.25 Hz)
0
200
400
600
800
0
20
40
60
10
30

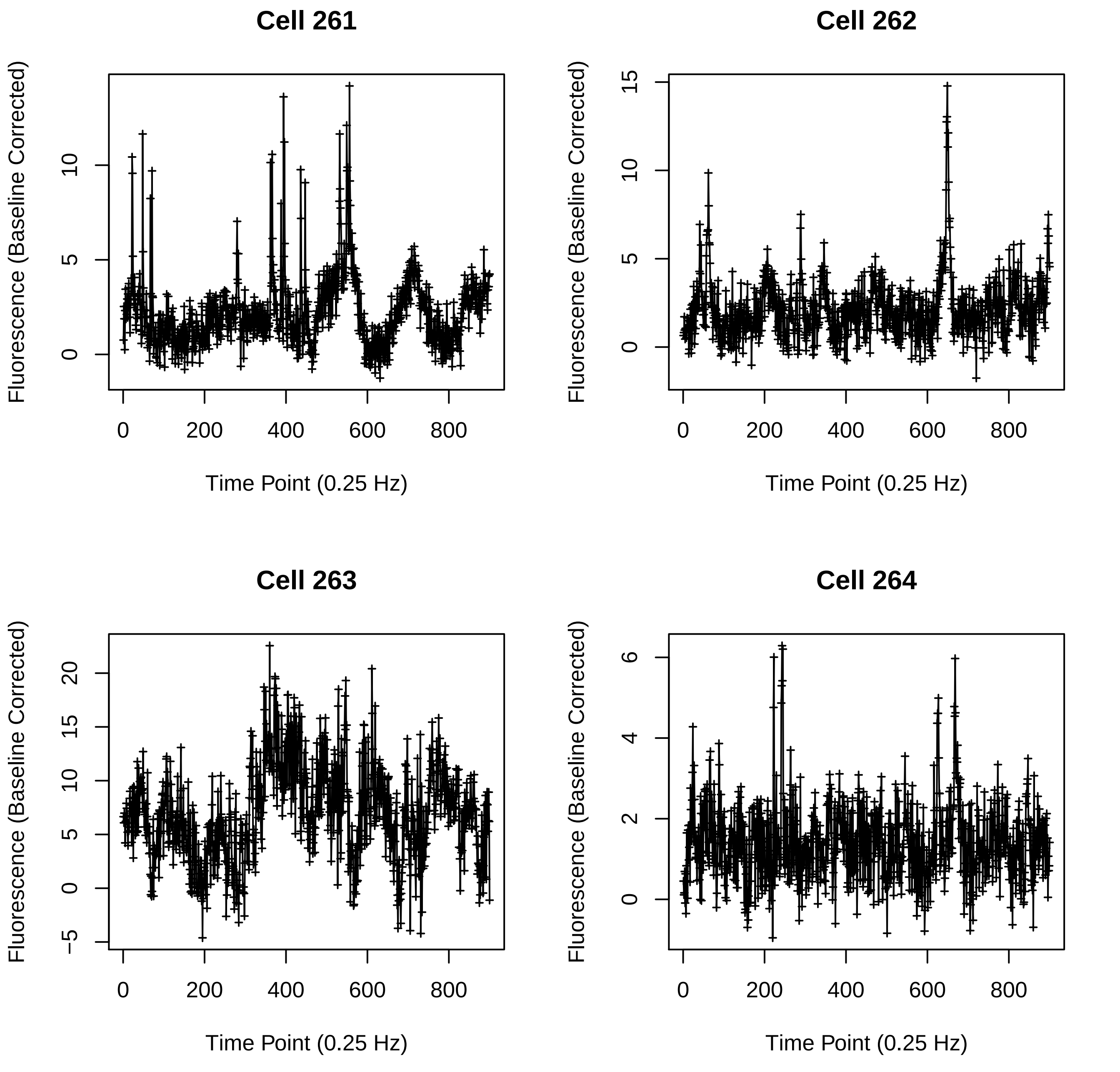
Cell 261
Fluorescence (Baseline Corrected)
0
5
10
0
200
400
600
800
Time Point (0.25 Hz)
Cell 262
Fluorescence (Baseline Corrected)
0
5
10
15
0
200
400
600
800
Time Point (0.25 Hz)
Cell 263
Fluorescence (Baseline Corrected)
−5
0
5
10
15
20
0
200
400
600
800
Time Point (0.25 Hz)
Cell 264
Fluorescence (Baseline Corrected)
0
2
4
6
0
200
400
600
800
Time Point (0.25 Hz)

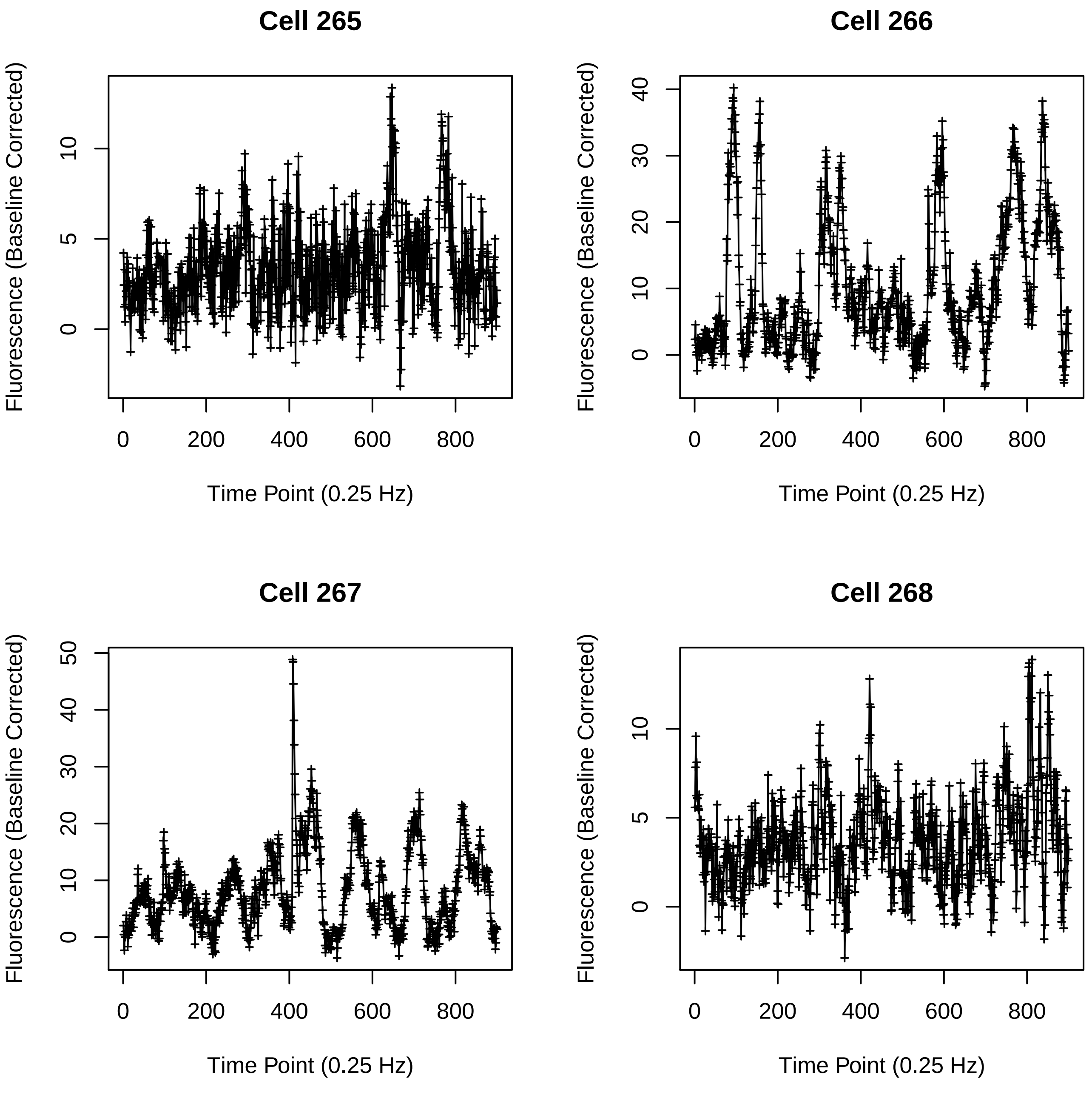
Cell 265
Fluorescence (Baseline Corrected)
Time Point (0.25 Hz)
Cell 266
Fluorescence (Baseline Corrected)
Time Point (0.25 Hz)
Cell 267
Fluorescence (Baseline Corrected)
Time Point (0.25 Hz)
Cell 268
Fluorescence (Baseline Corrected)
Time Point (0.25 Hz)

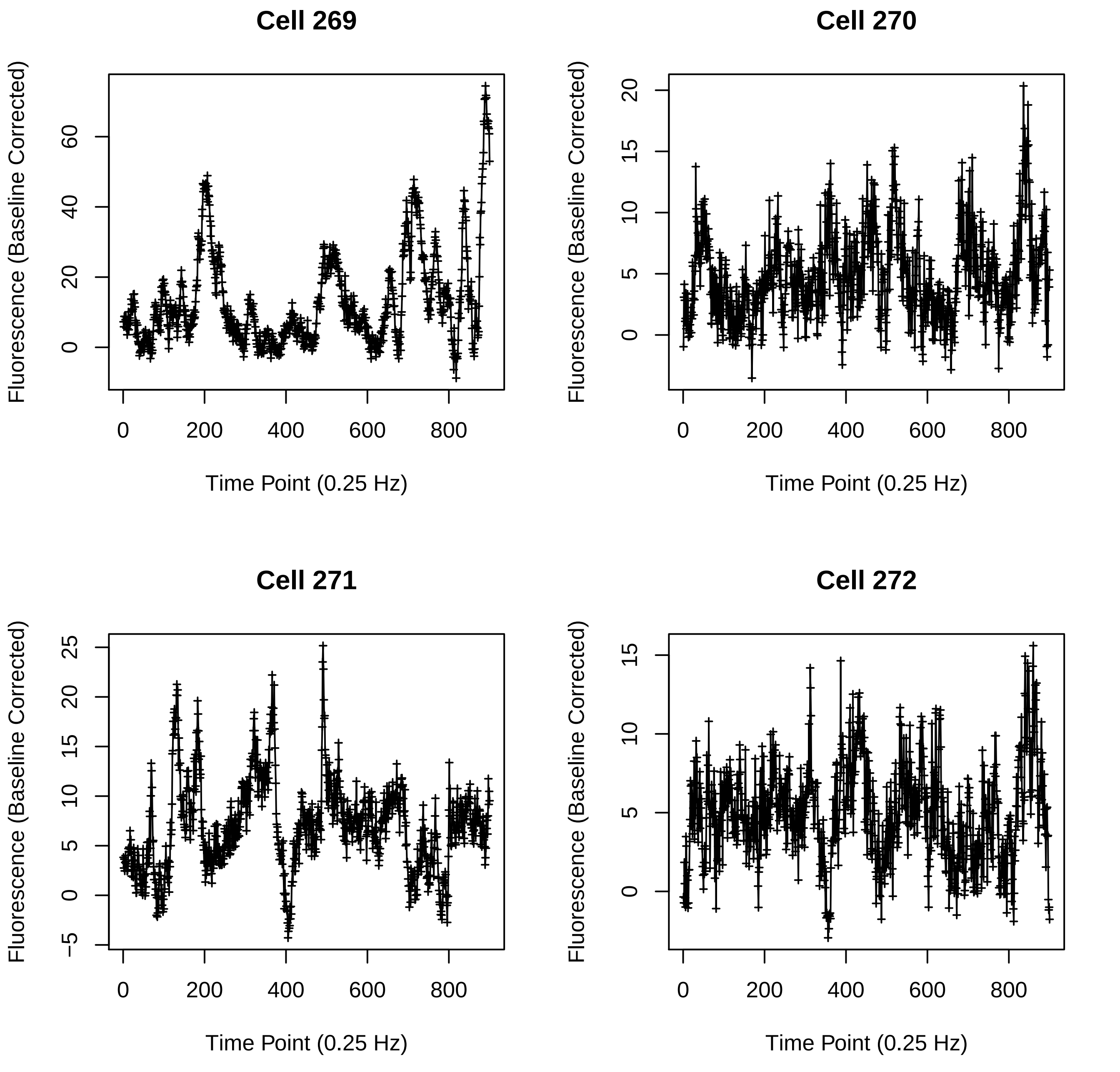

Cell 269
Fluorescence (Baseline Corrected)
0
20
40
60
0
200
400
600
800
Time Point (0.25 Hz)
Cell 270
Fluorescence (Baseline Corrected)
0
5
10
15
20
0
200
400
600
800
Time Point (0.25 Hz)
Cell 271
Fluorescence (Baseline Corrected)
−5
0
5
10
15
20
25
0
200
400
600
800
Time Point (0.25 Hz)
Cell 272
Fluorescence (Baseline Corrected)
0
5
10
15
0
200
400
600
800
Time Point (0.25 Hz)

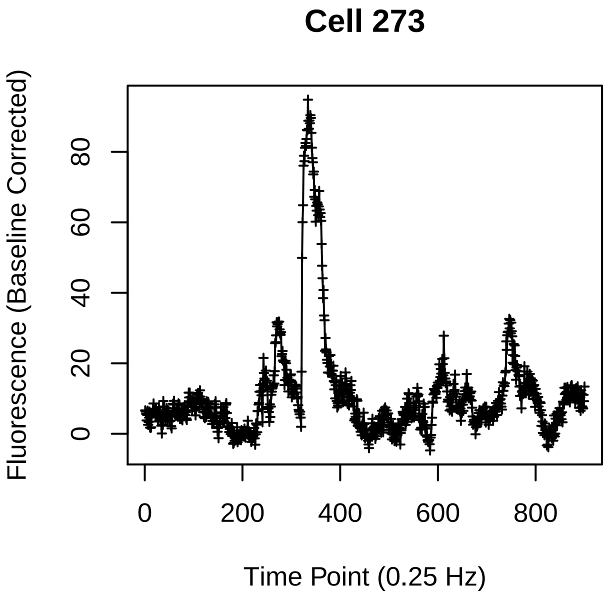
Cell 273
Fluorescence (Baseline Corrected)
0
20
40
60
80
0
200
400
600
800
Time Point (0.25 Hz)
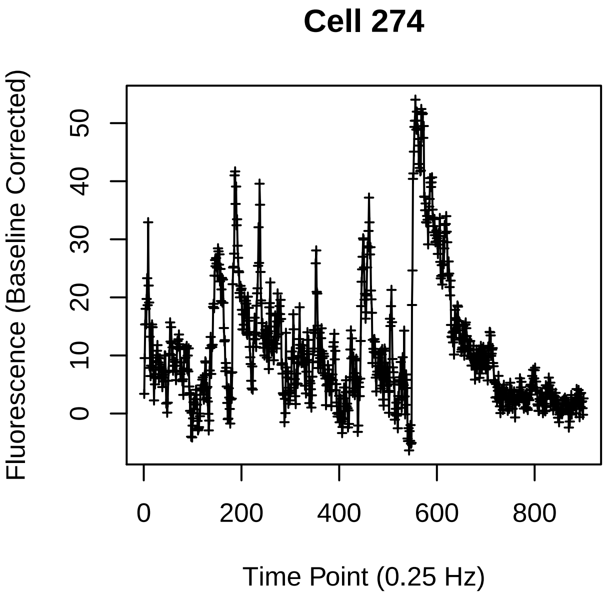
Cell 274
Fluorescence (Baseline Corrected)
0
10
20
30
40
50
0
200
400
600
800
Time Point (0.25 Hz)
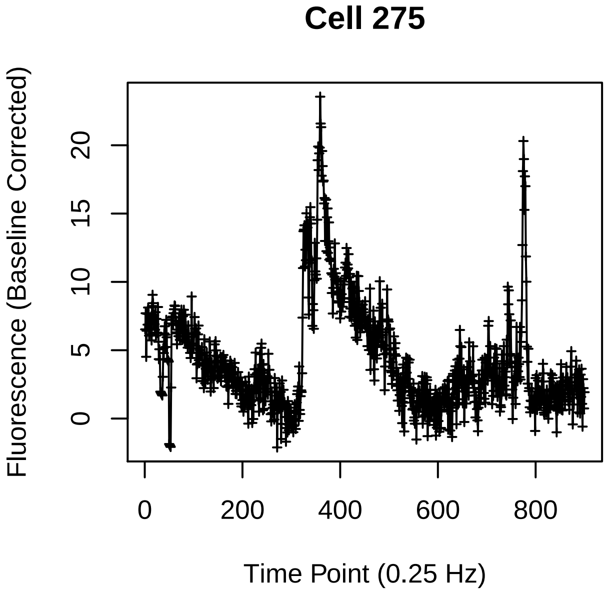
Cell 275
Fluorescence (Baseline Corrected)
0
5
10
15
20
0
200
400
600
800
Time Point (0.25 Hz)
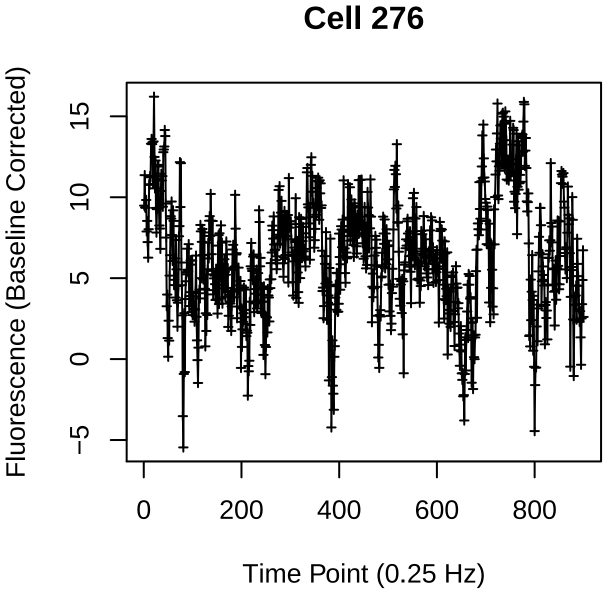
Cell 276
Fluorescence (Baseline Corrected)
−5
0
5
10
15
0
200
400
600
800
Time Point (0.25 Hz)

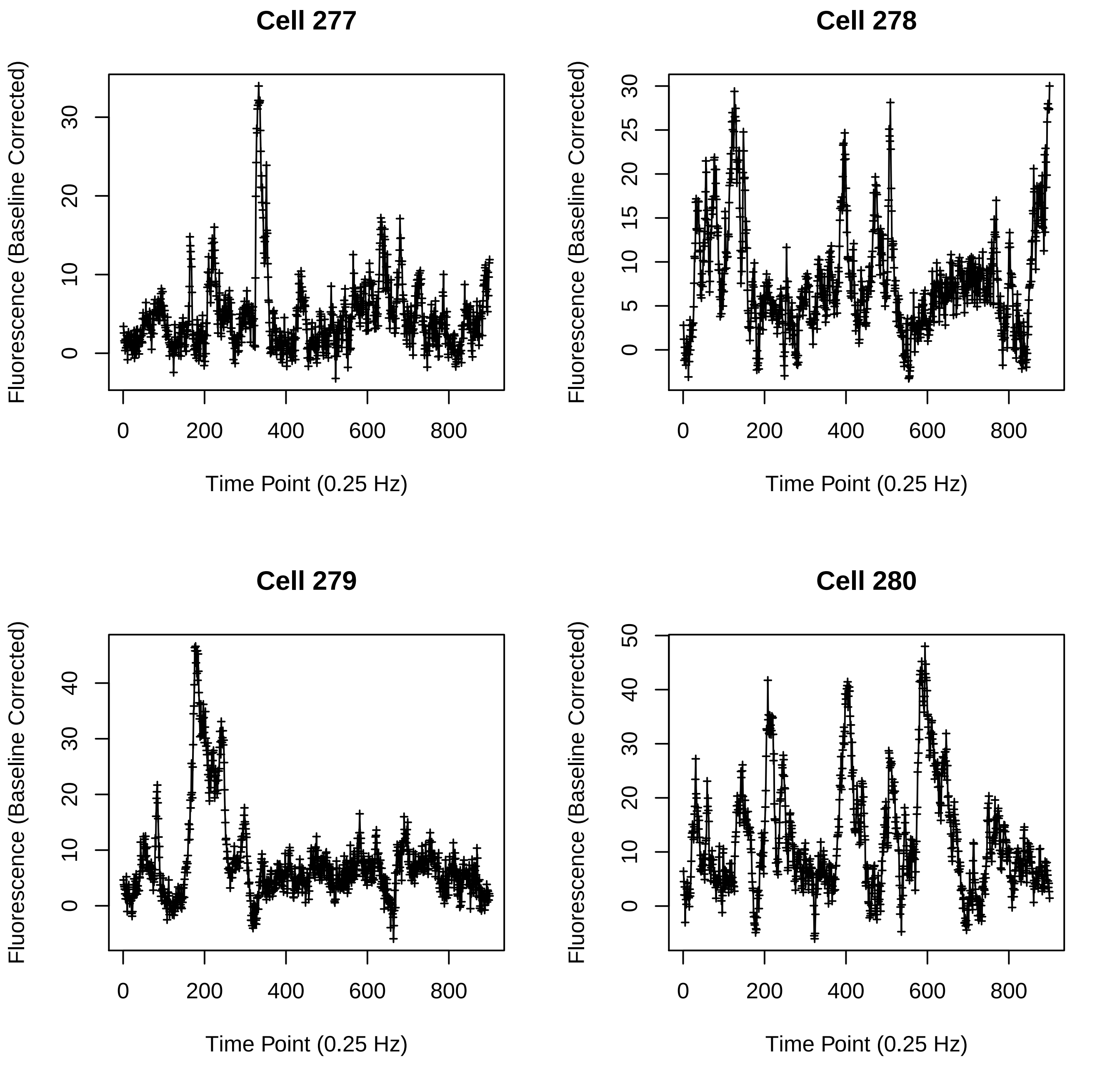

Cell 277
Cell 278
Cell 279
Cell 280
Fluorescence (Baseline Corrected)
Time Point (0.25 Hz)

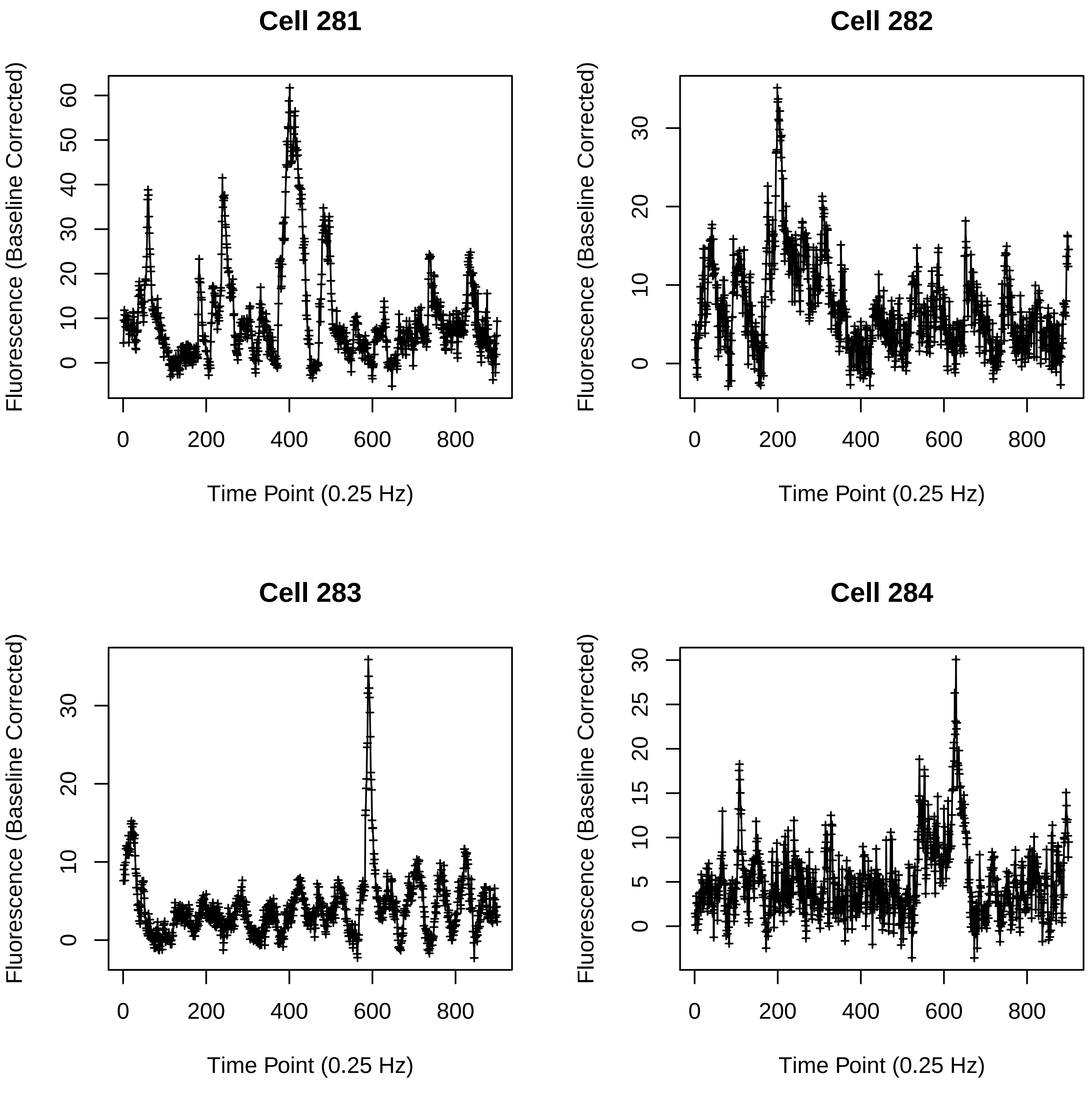

Cell 281
Cell 282
Cell 283
Cell 284
Fluorescence (Baseline Corrected)
Time Point (0.25 Hz)
0
200
400
600
800
10
20
30
40
50
60
5
15
25

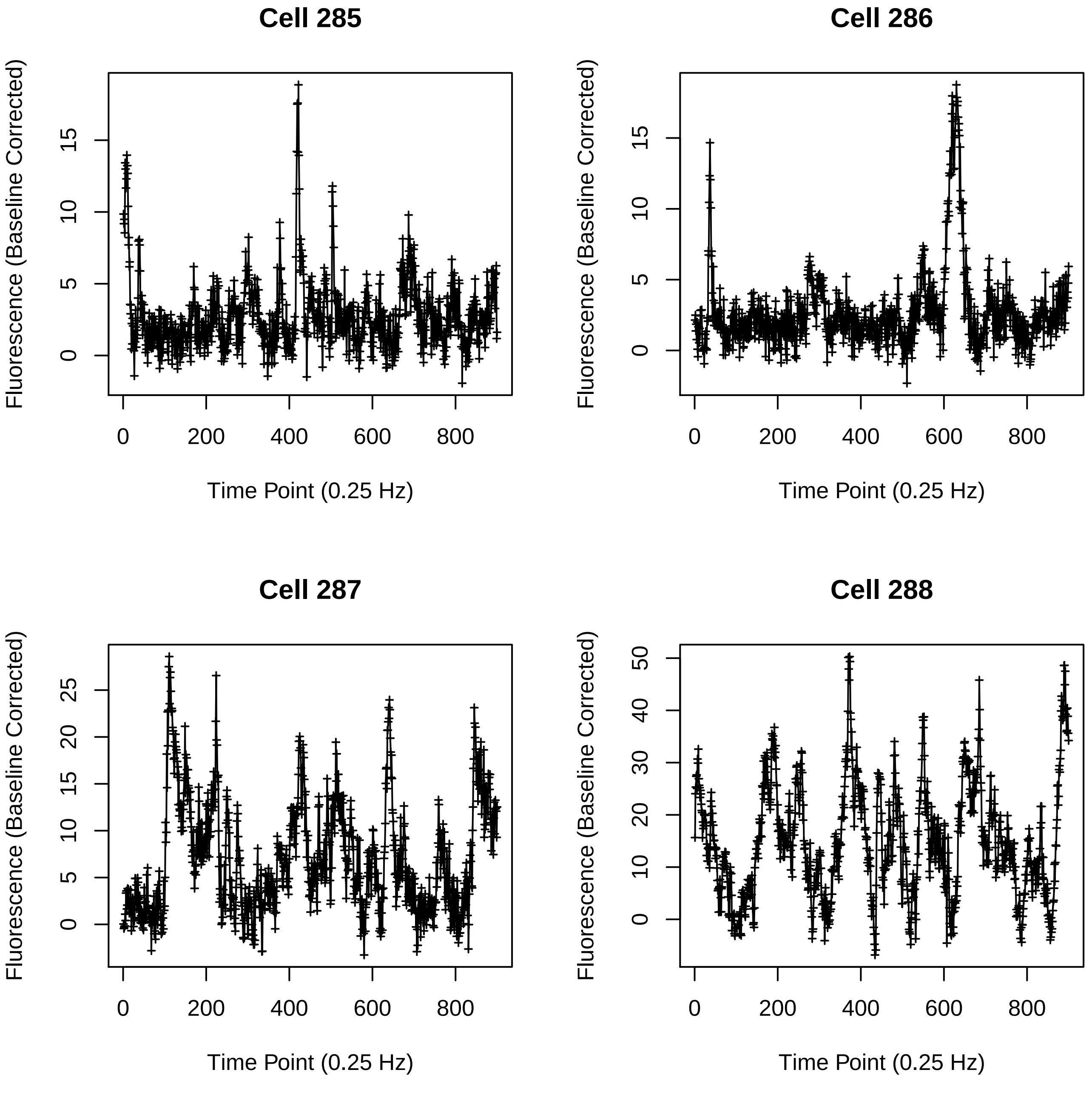
Cell 285
Cell 286
Cell 287
Cell 288
Fluorescence (Baseline Corrected)
Time Point (0.25 Hz)
0
200
400
600
800
0
5
10
15
20
25
30
40
50

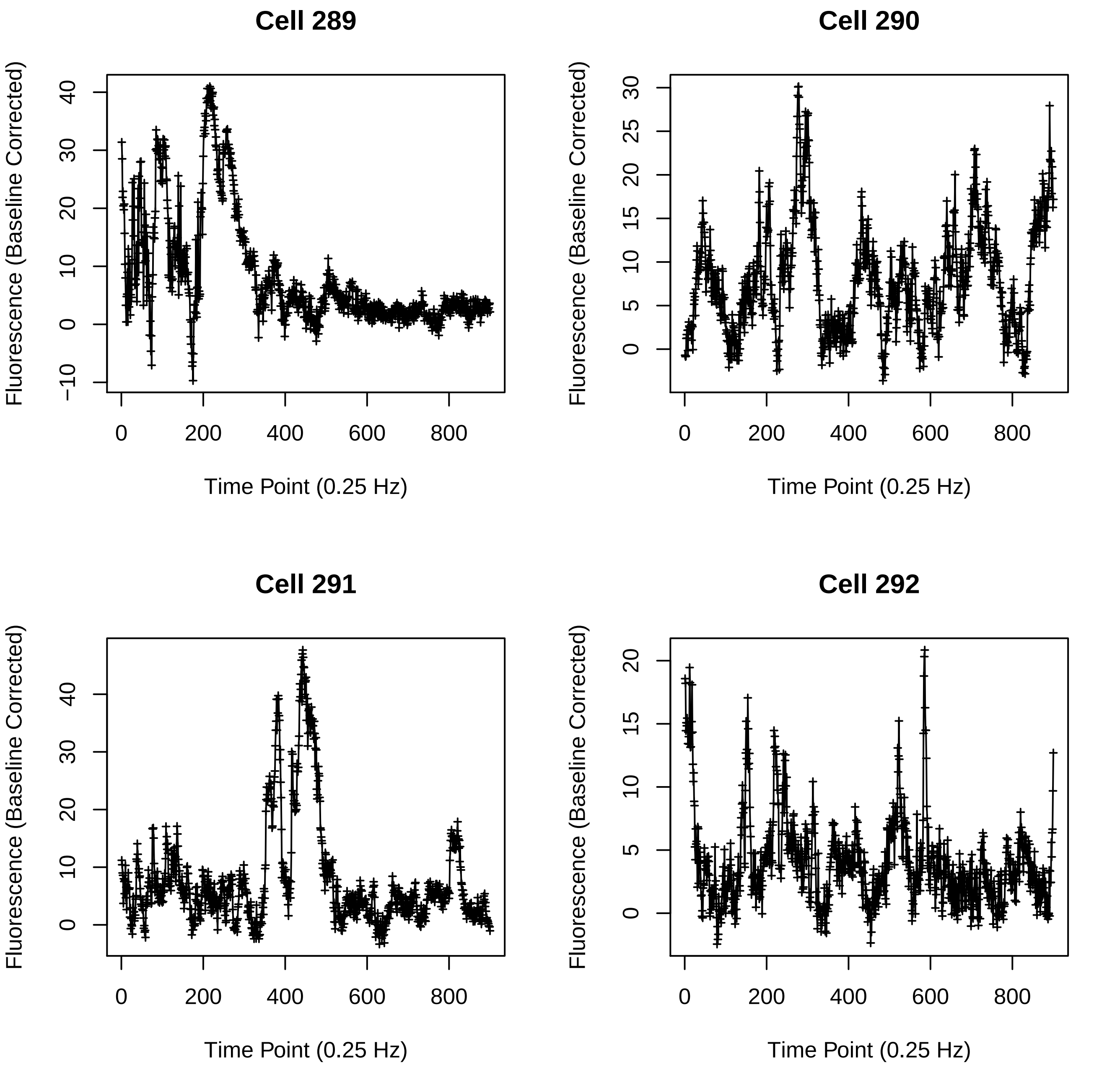
Cell 289
Fluorescence (Baseline Corrected)
Time Point (0.25 Hz)
Cell 290
Fluorescence (Baseline Corrected)
Time Point (0.25 Hz)
Cell 291
Fluorescence (Baseline Corrected)
Time Point (0.25 Hz)
Cell 292
Fluorescence (Baseline Corrected)
Time Point (0.25 Hz)

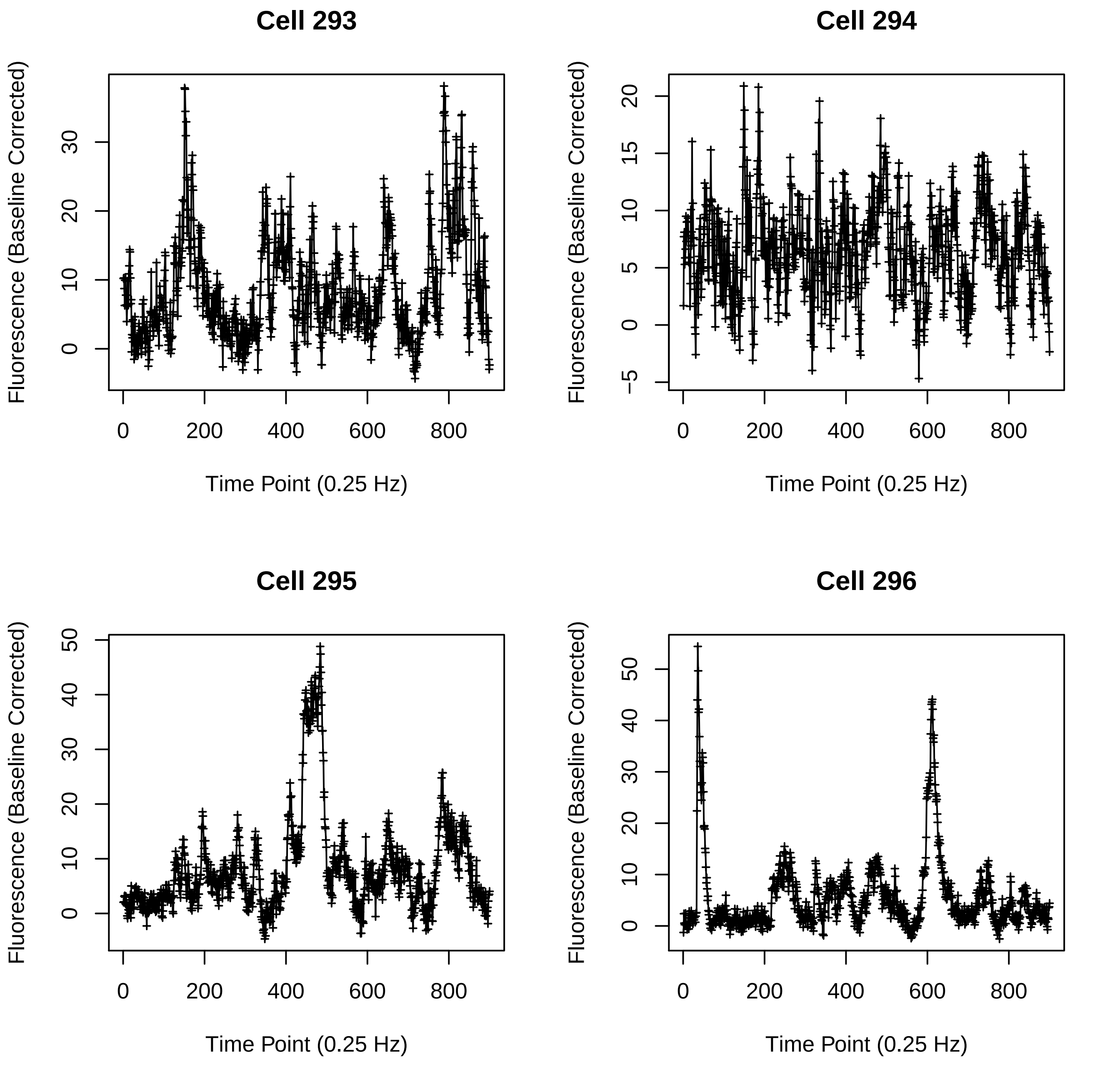
Cell 293
Cell 294
Cell 295
Cell 296
Fluorescence (Baseline Corrected)
Time Point (0.25 Hz)
0
200
400
600
800
-5
5
10
15
20
30
40
50

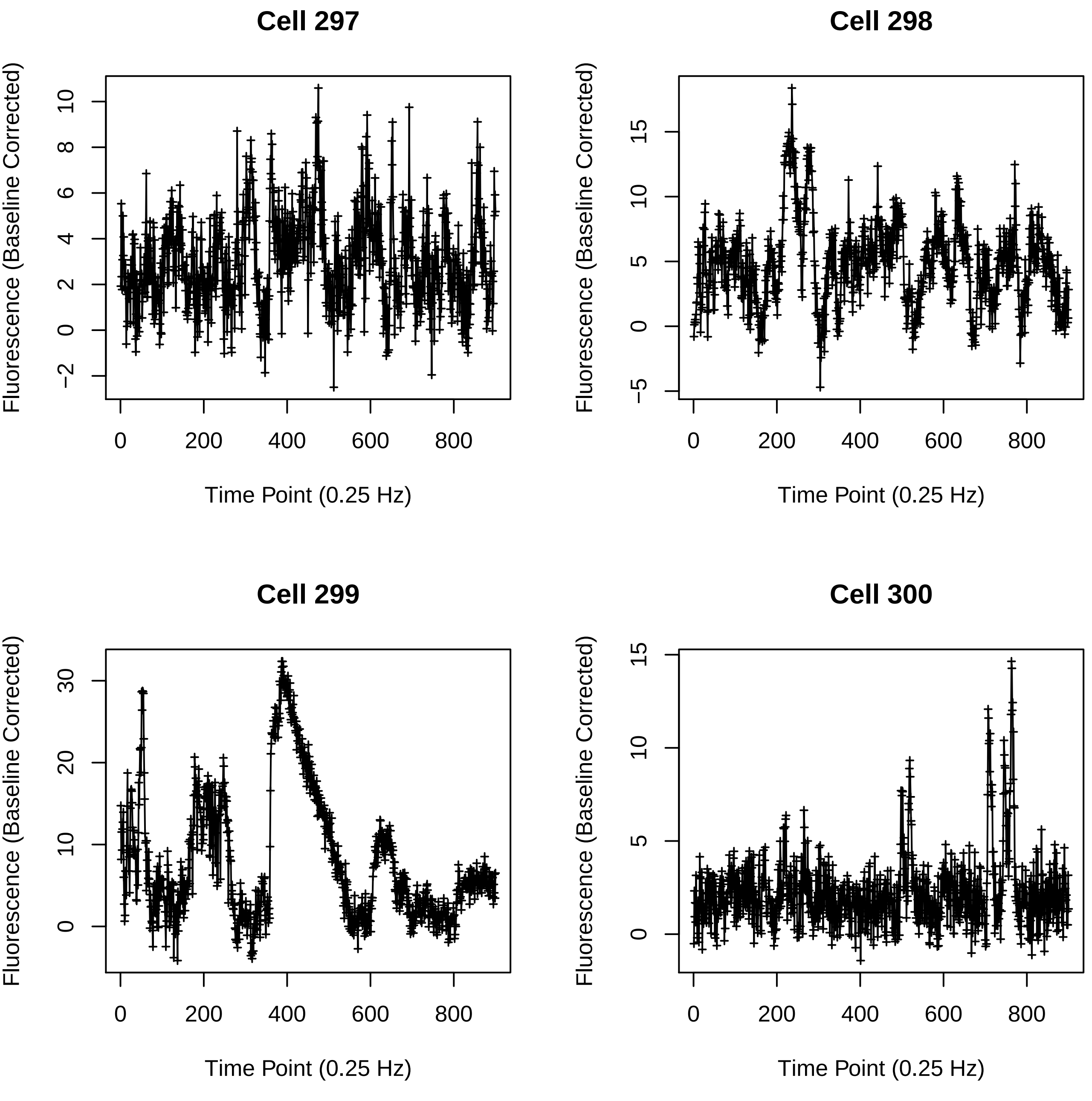
Cell 297
Cell 298
Cell 299
Cell 300
Fluorescence (Baseline Corrected)
Time Point (0.25 Hz)
0
200
400
600
800
−2
2
4
6
8
10
−5
5
15
20
30

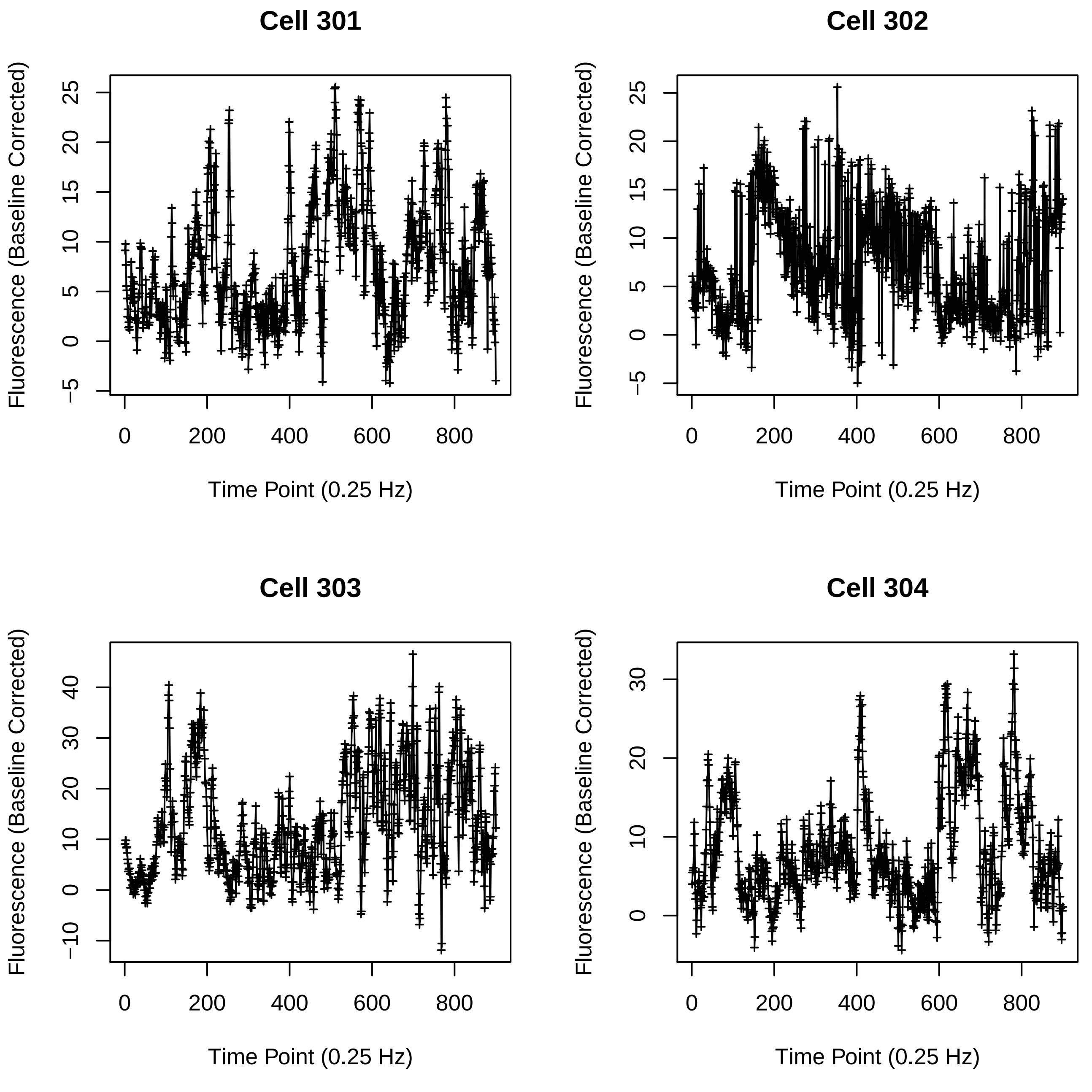
Cell 301
Cell 302
Cell 303
Cell 304
Fluorescence (Baseline Corrected)
Time Point (0.25 Hz)
-5
0
5
10
15
20
25
30
40
-10
0
200
400
600
800

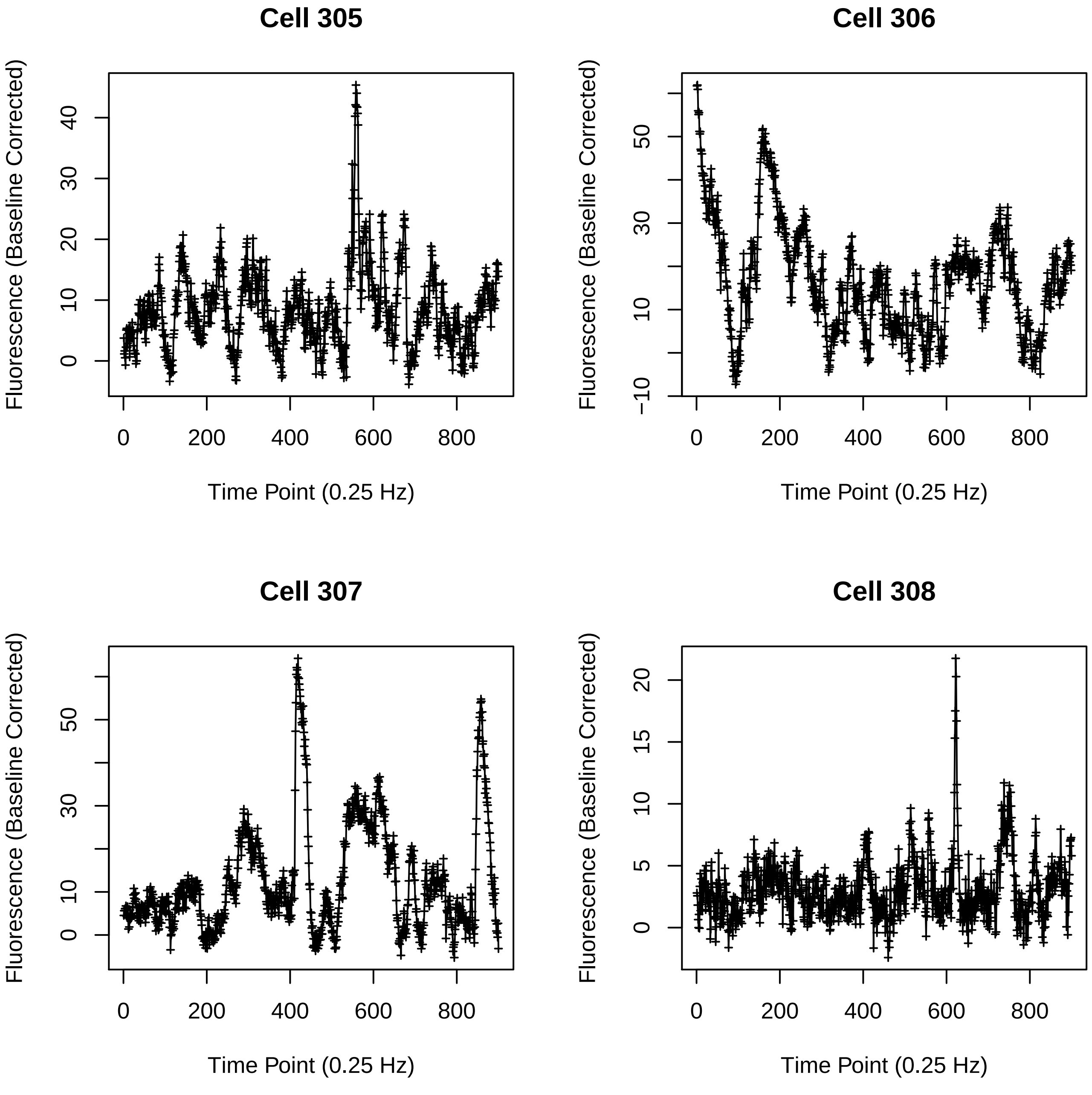
Cell 305
Cell 306
Cell 307
Cell 308
Fluorescence (Baseline Corrected)
Time Point (0.25 Hz)
0
10
20
30
40
50
−10
15
5
200
400
600
800

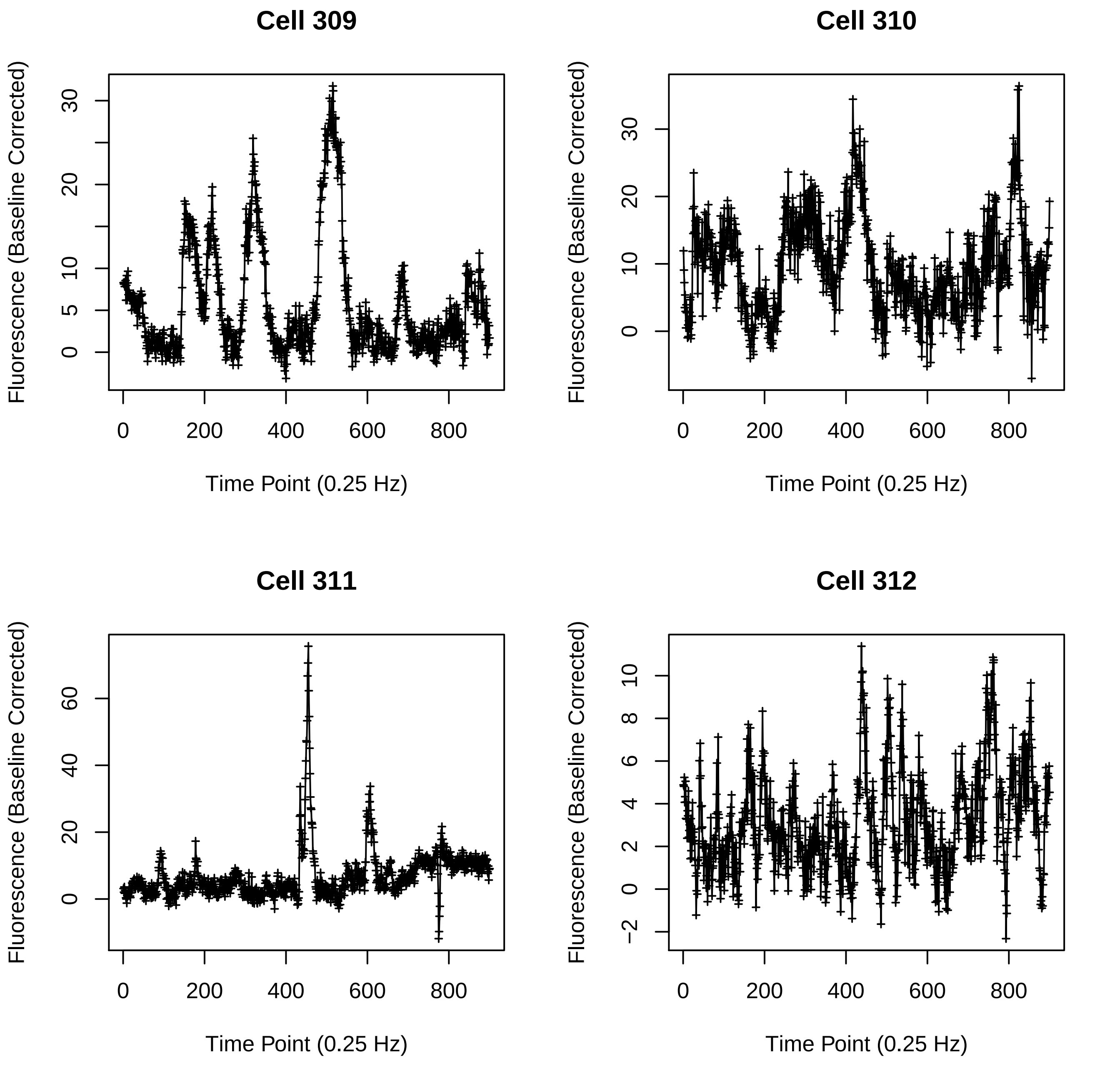
Cell 309
Cell 310
Cell 311
Cell 312
Fluorescence (Baseline Corrected)
Time Point (0.25 Hz)
0
5
10
20
30
200
400
600
800
40
60
8
6
4
2
−2

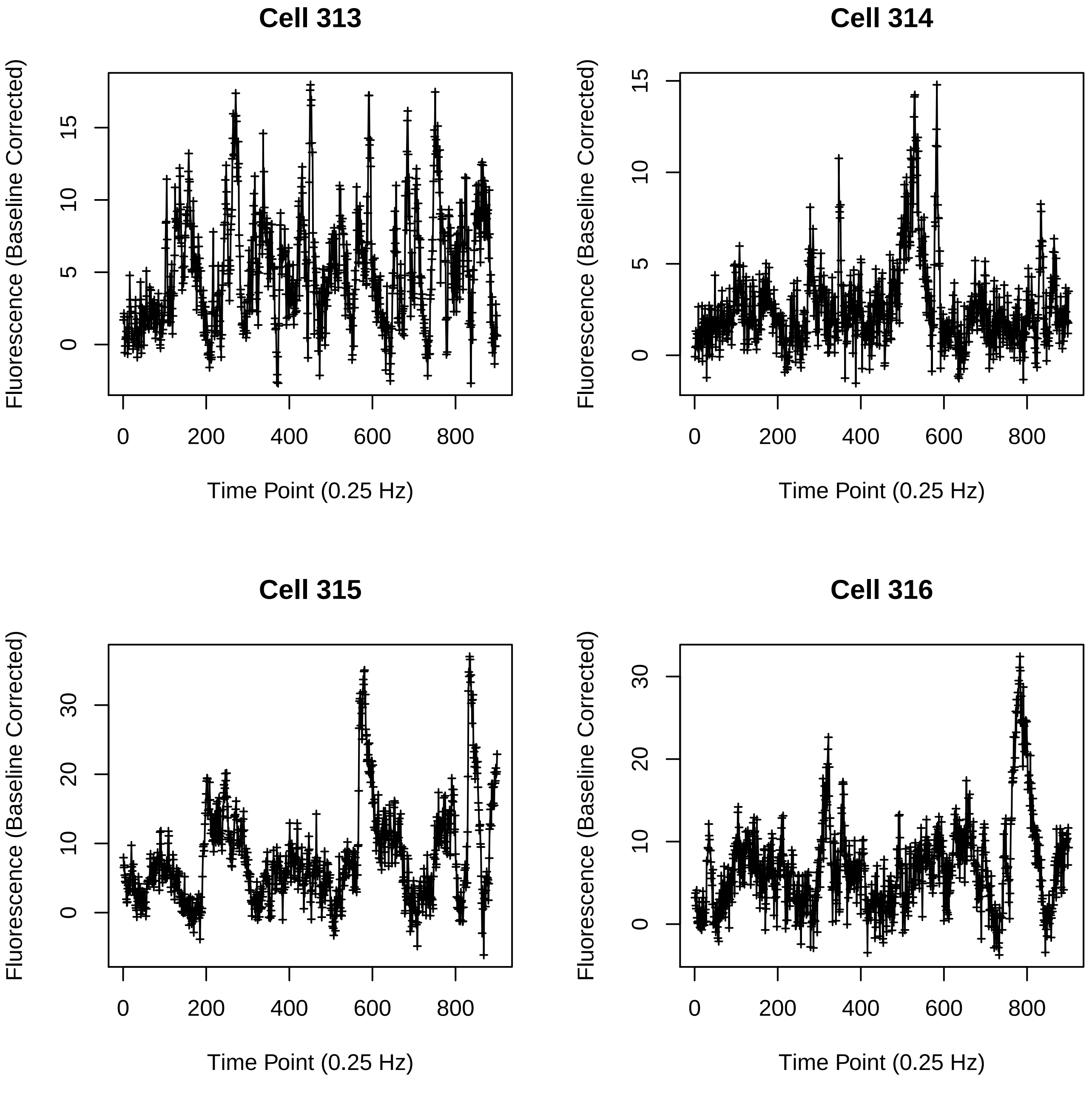

Cell 313
Cell 314
Cell 315
Cell 316
Fluorescence (Baseline Corrected)
Time Point (0.25 Hz)
0
200
400
600
800
0
5
10
15
20
30

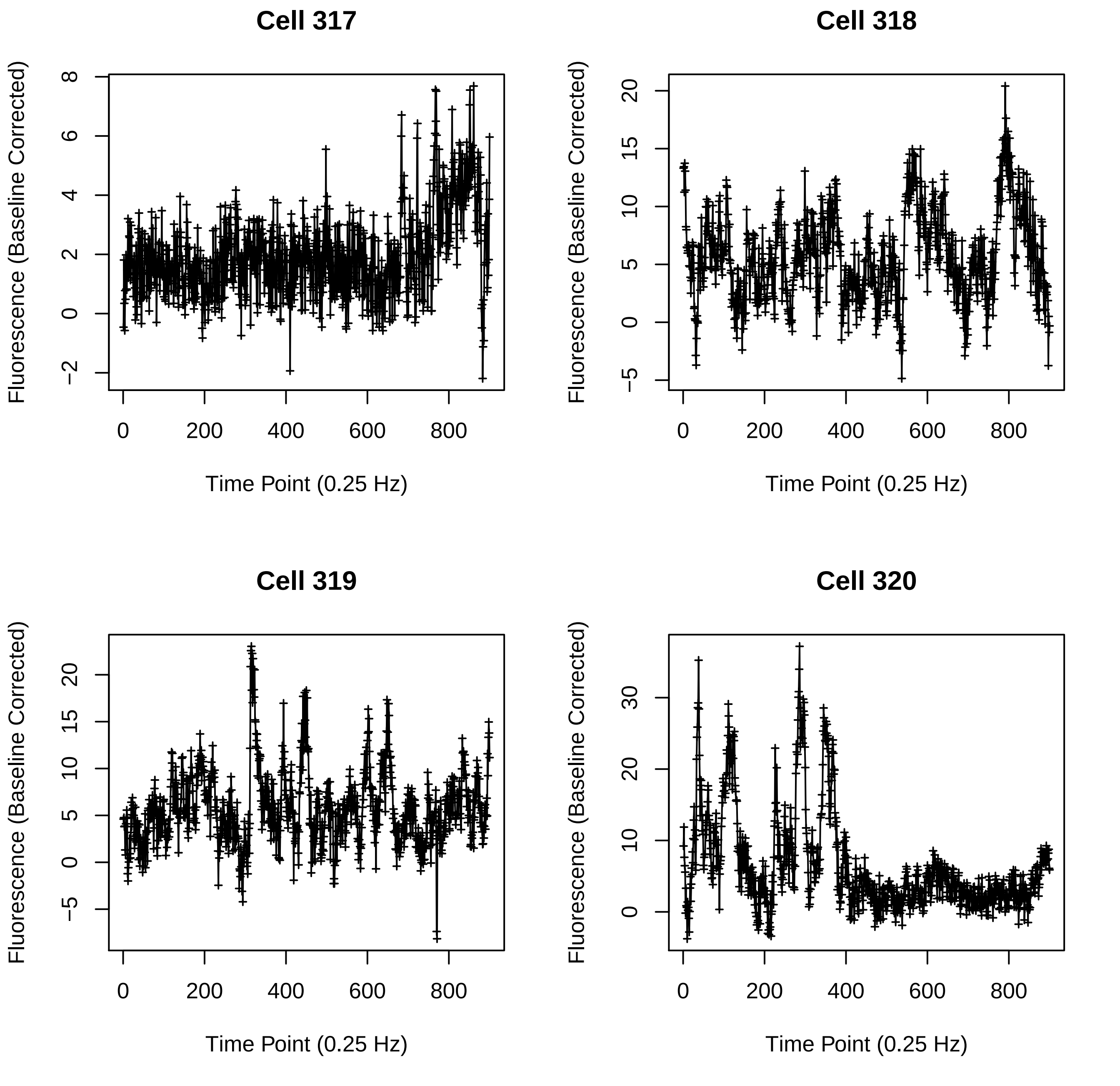

Cell 317
Cell 318
Cell 319
Cell 320
Fluorescence (Baseline Corrected)
Time Point (0.25 Hz)
0
200
400
600
800

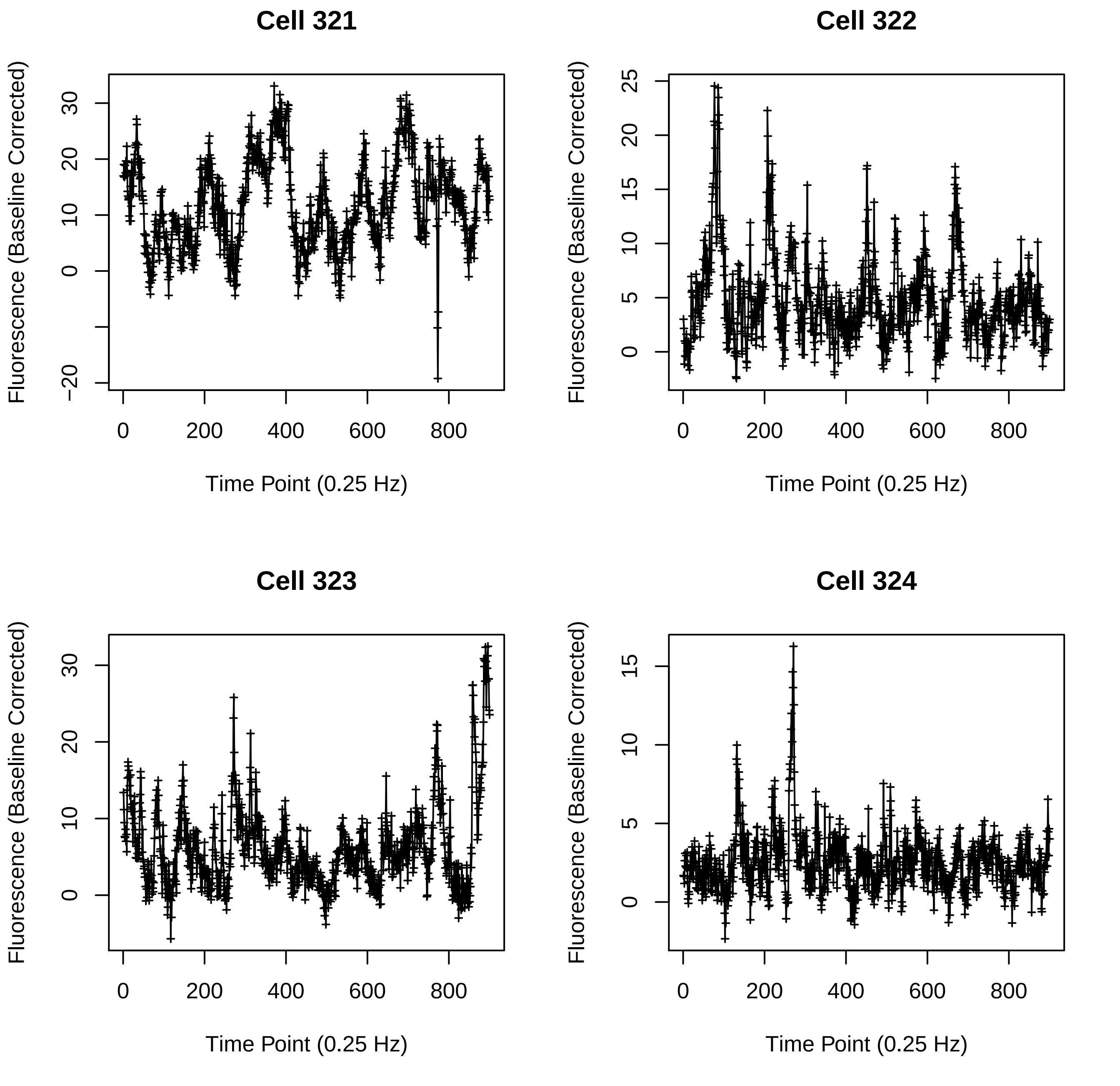

Cell 321
Cell 322
Cell 323
Cell 324
Fluorescence (Baseline Corrected)
Time Point (0.25 Hz)
0
200
400
600
800

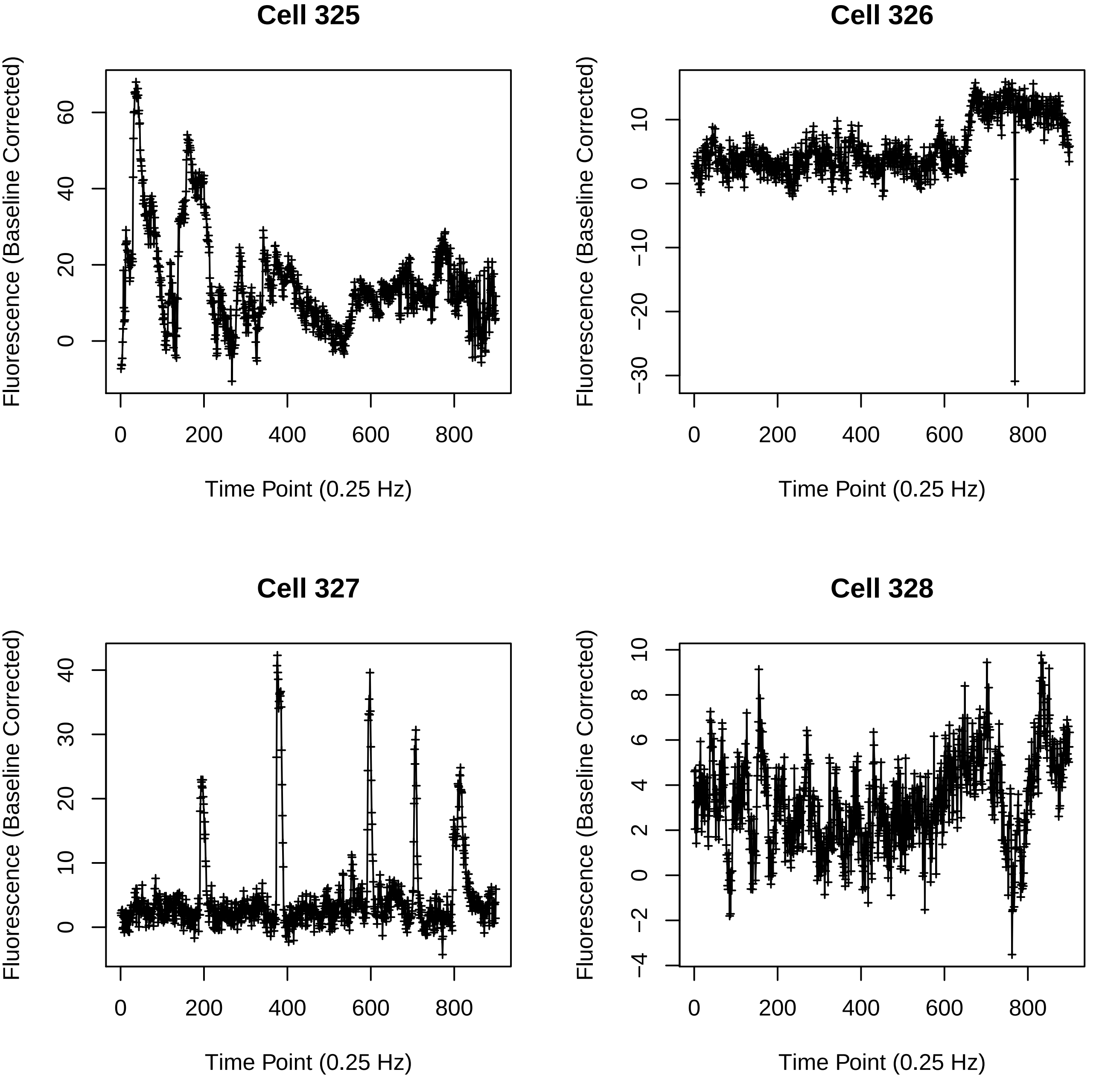
Cell 325
Fluorescence (Baseline Corrected)
Time Point (0.25 Hz)
Cell 326
Fluorescence (Baseline Corrected)
Time Point (0.25 Hz)
Cell 327
Fluorescence (Baseline Corrected)
Time Point (0.25 Hz)
Cell 328
Fluorescence (Baseline Corrected)
Time Point (0.25 Hz)

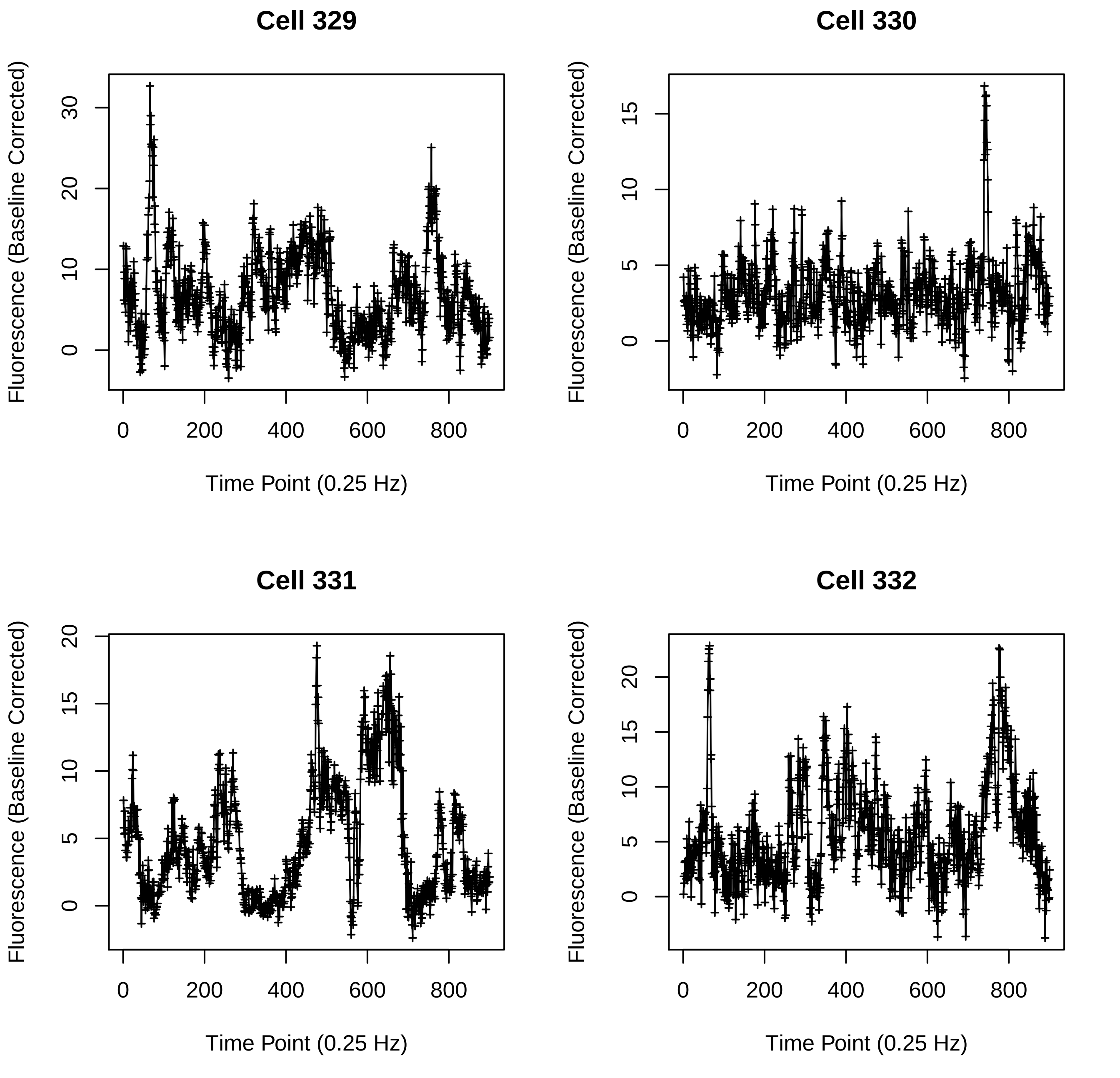
Cell 329
Cell 330
Cell 331
Cell 332
Fluorescence (Baseline Corrected)
Time Point (0.25 Hz)
0
200
400
600
800
-5
0
5
10
15
20
30

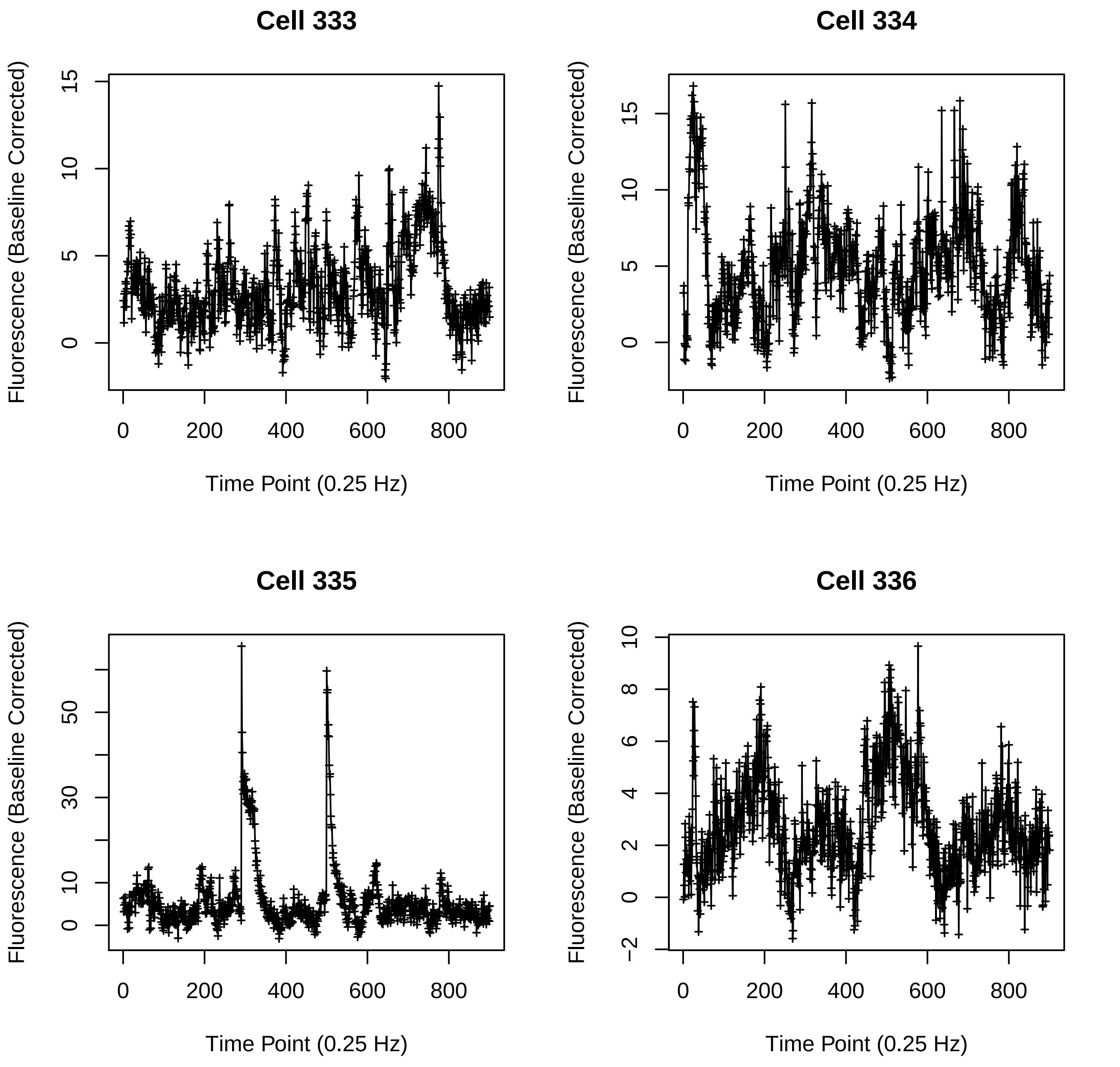
Cell 333
Cell 334
Cell 335
Cell 336
Fluorescence (Baseline Corrected)
Time Point (0.25 Hz)
0
200
400
600
800
0
5
10
15
30
50
−2
2
4
6
8

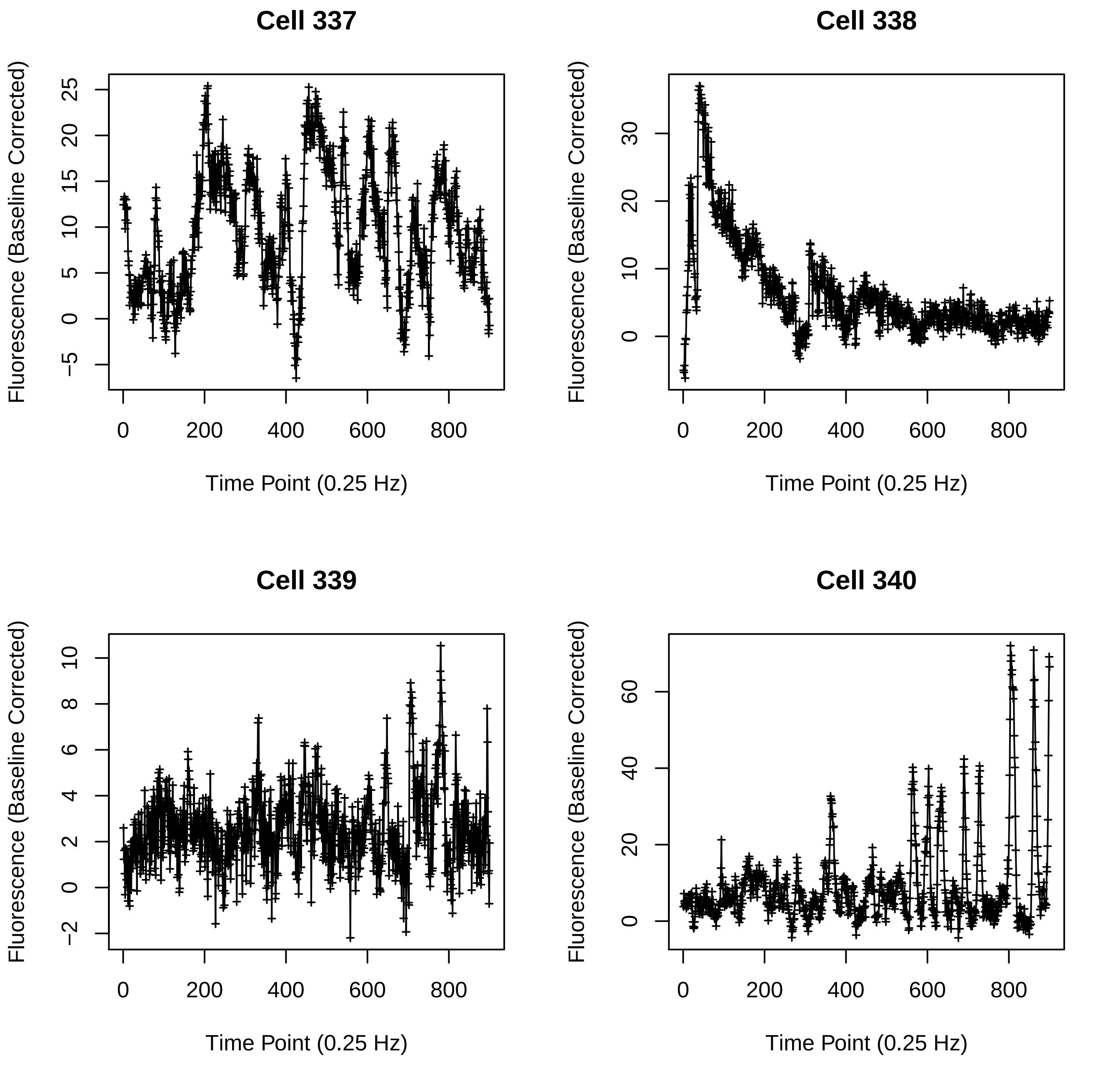
Cell 337
Cell 338
Cell 339
Cell 340
Fluorescence (Baseline Corrected)
Time Point (0.25 Hz)
0
200
400
600
800
−5
5
10
15
20
25
30
−2
2
4
6
8
40
60

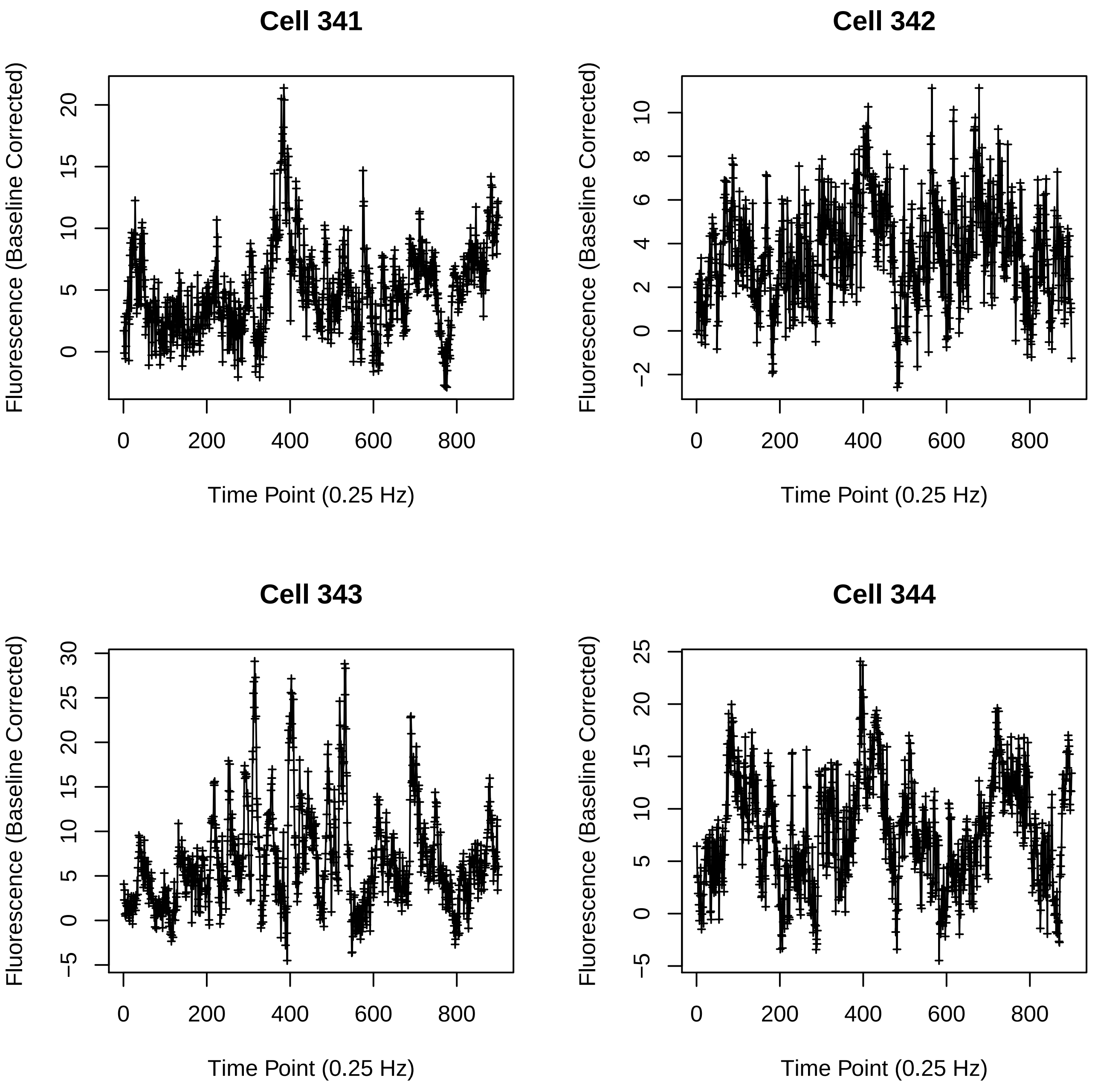
Cell 341
Fluorescence (Baseline Corrected)
0
5
10
15
20
Time Point (0.25 Hz)
0
200
400
600
800
Cell 342
Fluorescence (Baseline Corrected)
−2
0
2
4
6
8
10
Time Point (0.25 Hz)
0
200
400
600
800
Cell 343
Fluorescence (Baseline Corrected)
−5
0
5
10
15
20
25
30
Time Point (0.25 Hz)
0
200
400
600
800
Cell 344
Fluorescence (Baseline Corrected)
−5
0
5
10
15
20
25
Time Point (0.25 Hz)
0
200
400
600
800

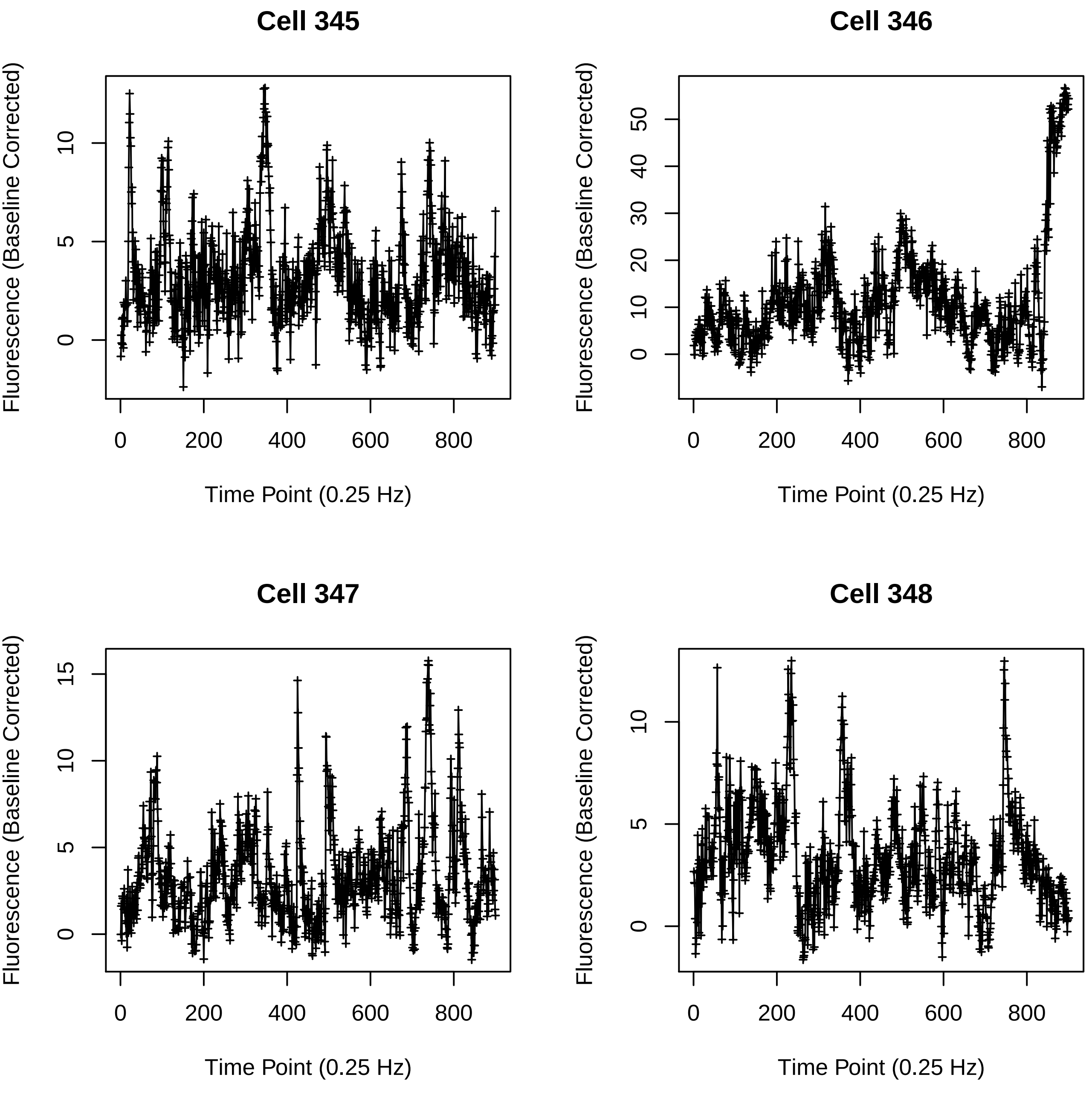
Cell 345
Cell 346
Cell 347
Cell 348
Fluorescence (Baseline Corrected)
Time Point (0.25 Hz)
0
200
400
600
800
-0
5
10
15
20
30
40
50

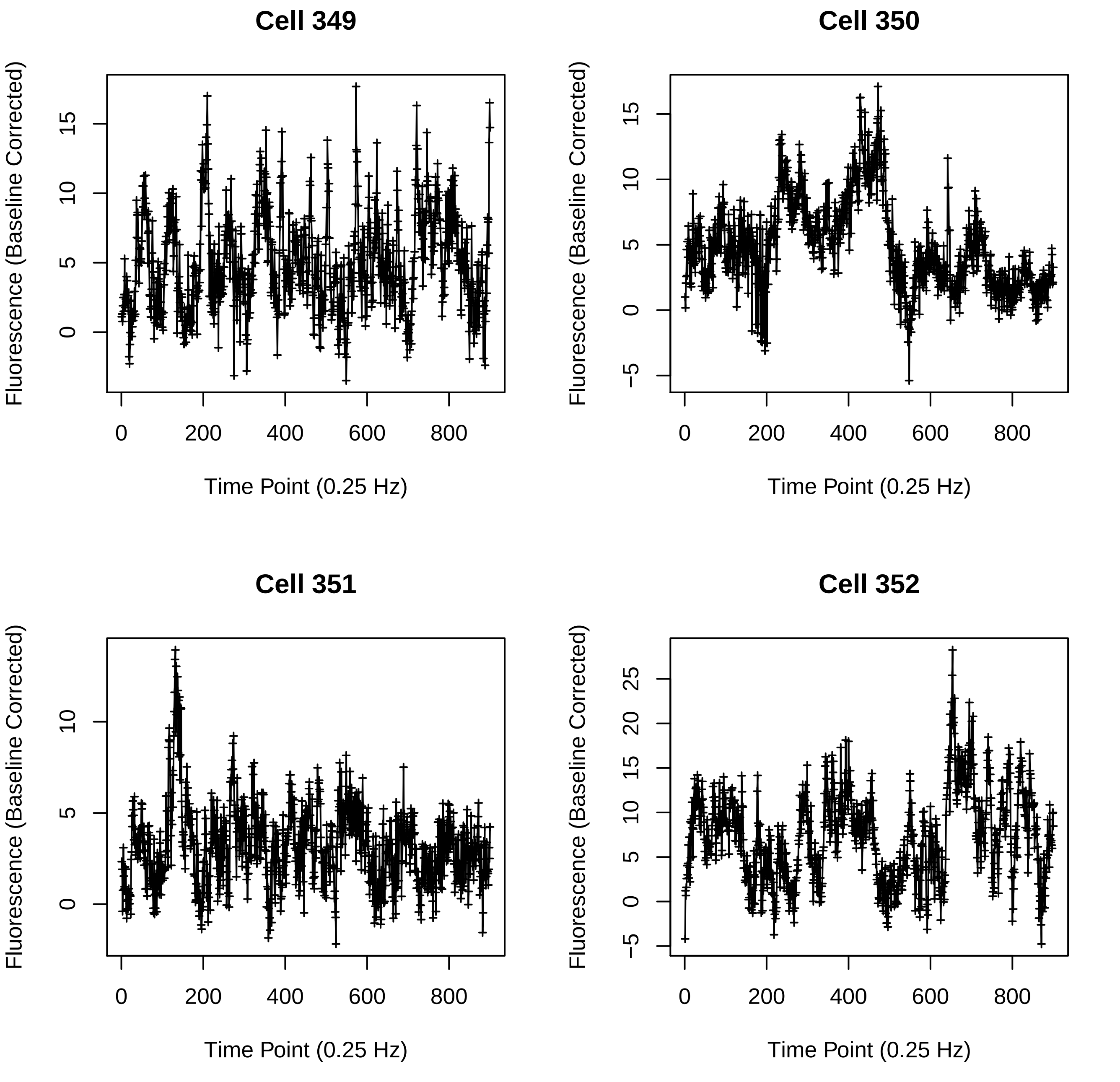

Cell 349
Cell 350
Cell 351
Cell 352
Fluorescence (Baseline Corrected)
Time Point (0.25 Hz)
0
5
10
15
20
25
−5
200
400
600
800

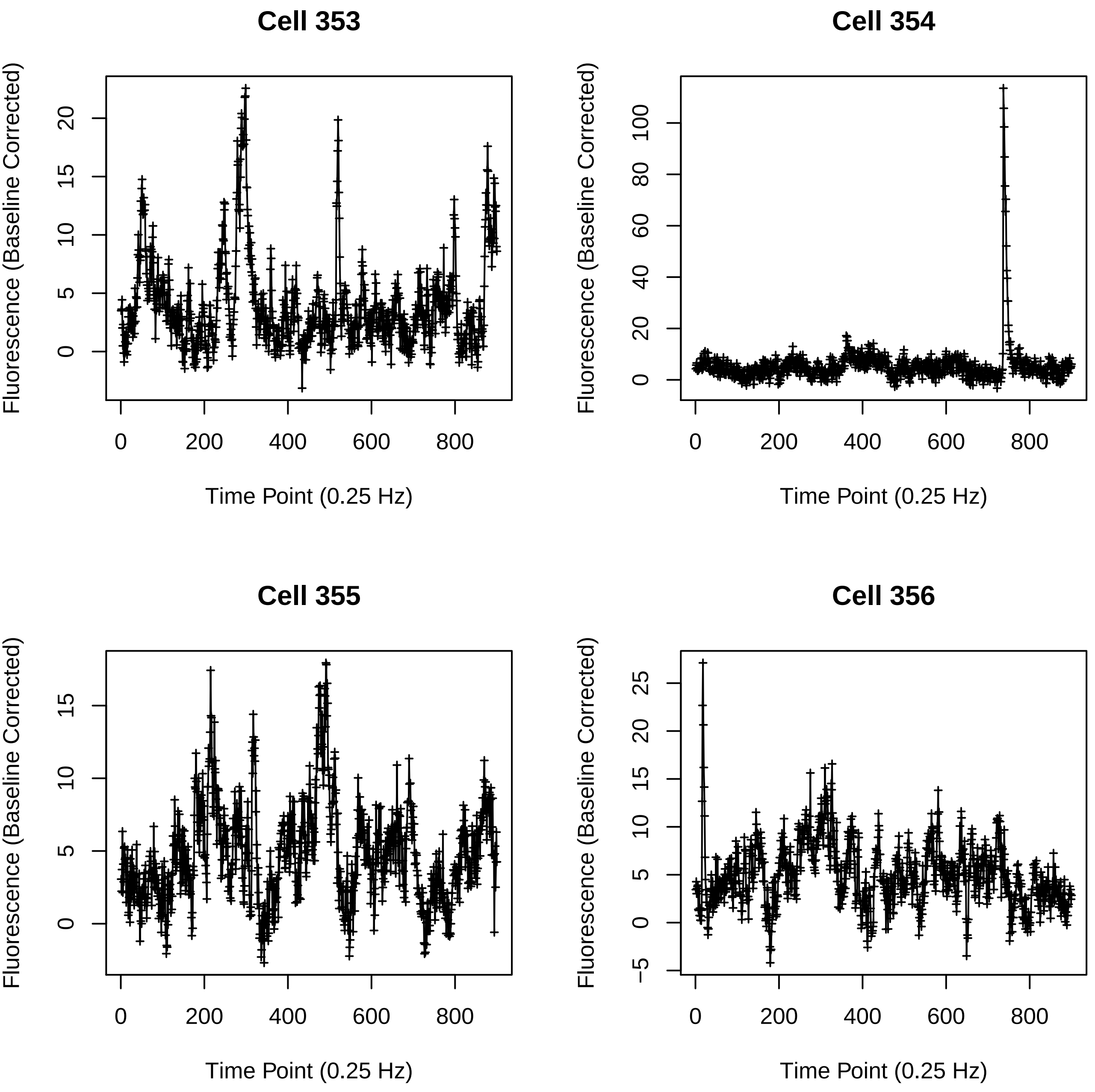
Cell 353
Cell 354
Cell 355
Cell 356
Fluorescence (Baseline Corrected)
Time Point (0.25 Hz)
0
200
400
600
800
0
5
10
15
20
0
20
40
60
80
100
−5
25

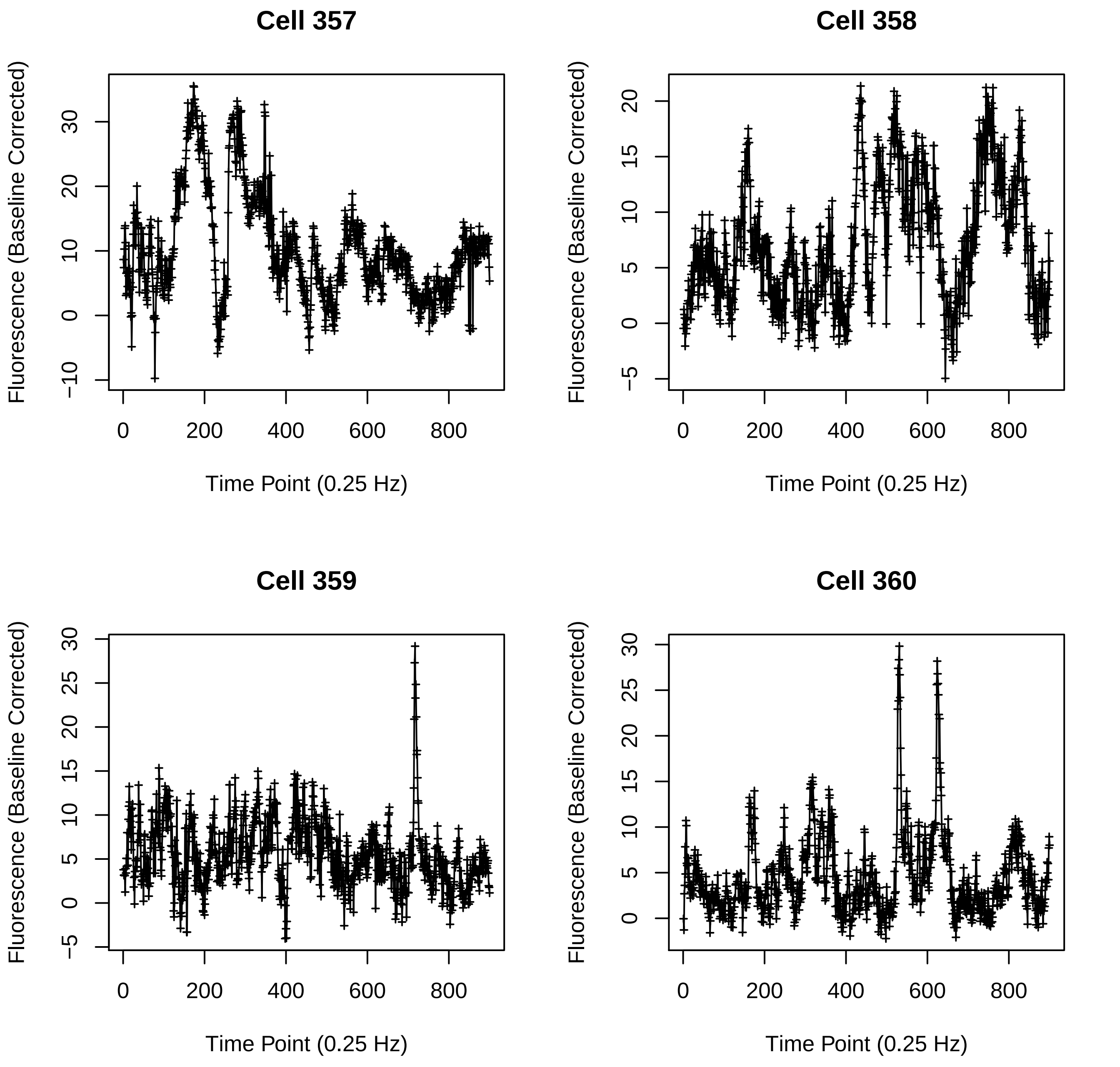

Cell 357
Cell 358
Cell 359
Cell 360
Fluorescence (Baseline Corrected)
Time Point (0.25 Hz)

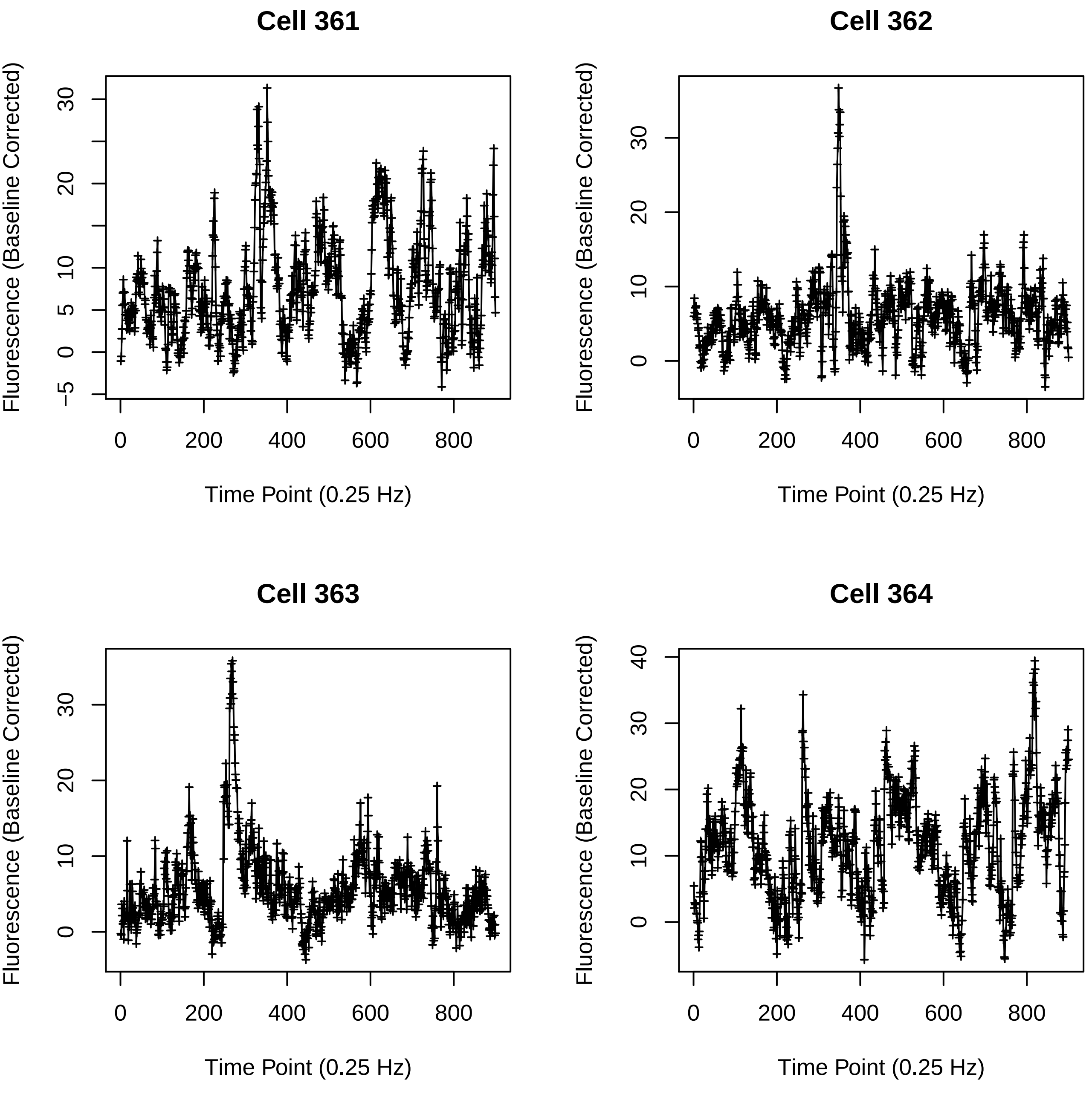
Cell 361
Cell 362
Cell 363
Cell 364
Fluorescence (Baseline Corrected)
Time Point (0.25 Hz)
0
200
400
600
800
−5
5
10
20
30
40

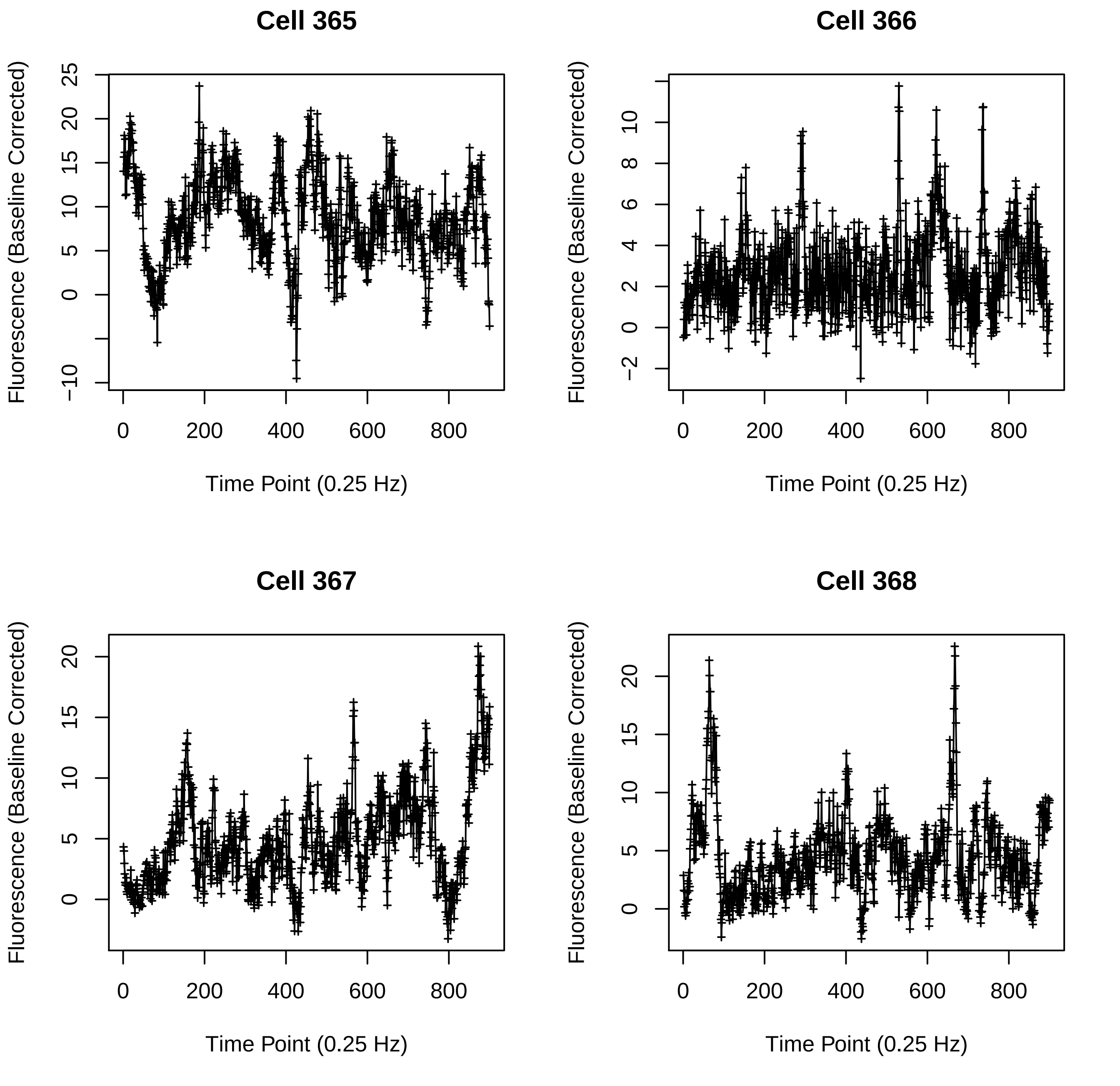
Cell 365
Cell 366
Cell 367
Cell 368
Fluorescence (Baseline Corrected)
Time Point (0.25 Hz)
0
200
400
600
800
−10
0
5
10
15
20
25
−2
2
4
6
8

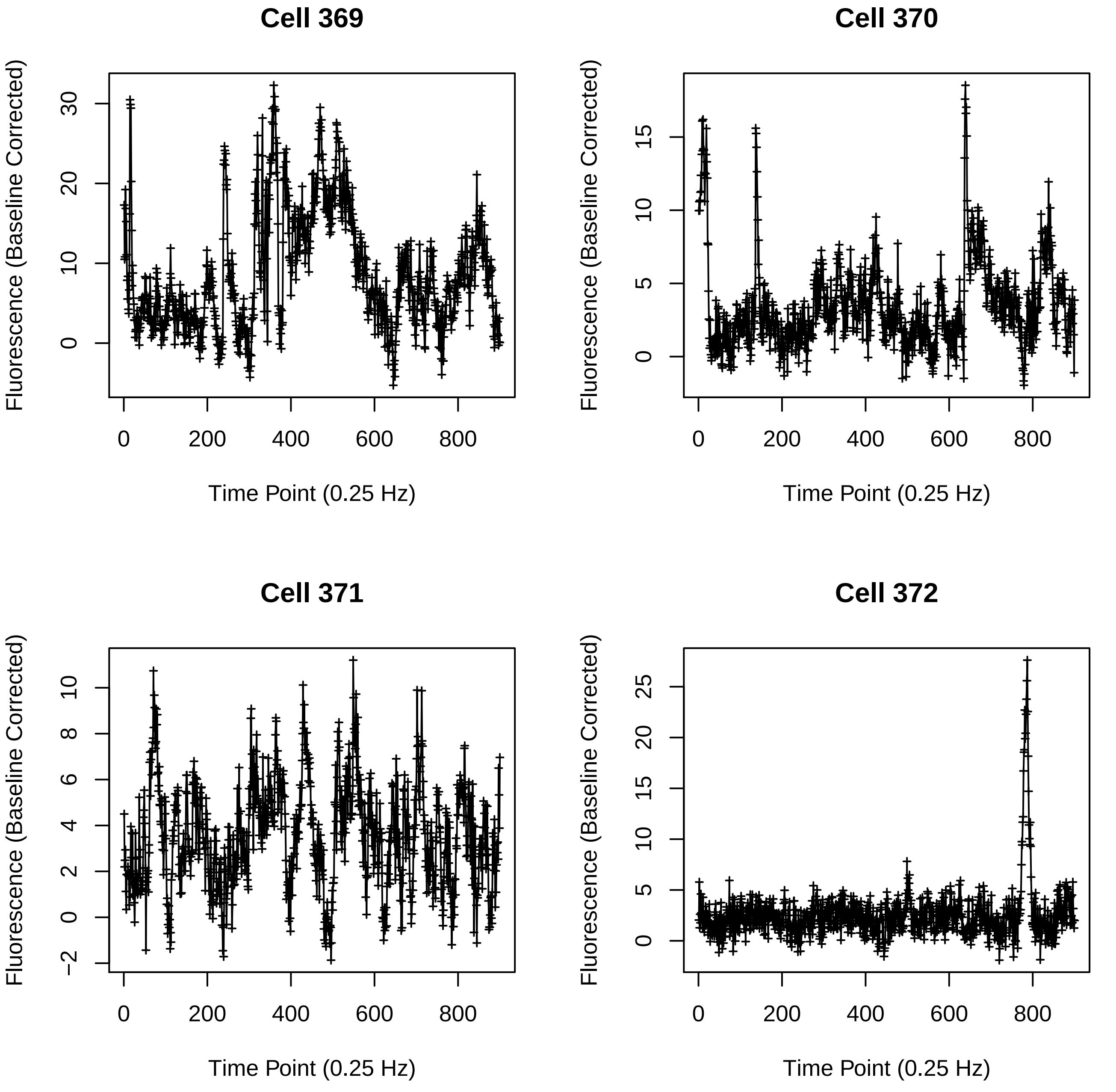
Cell 369
Fluorescence (Baseline Corrected)
Time Point (0.25 Hz)
Cell 370
Fluorescence (Baseline Corrected)
Time Point (0.25 Hz)
Cell 371
Fluorescence (Baseline Corrected)
Time Point (0.25 Hz)
Cell 372
Fluorescence (Baseline Corrected)
Time Point (0.25 Hz)

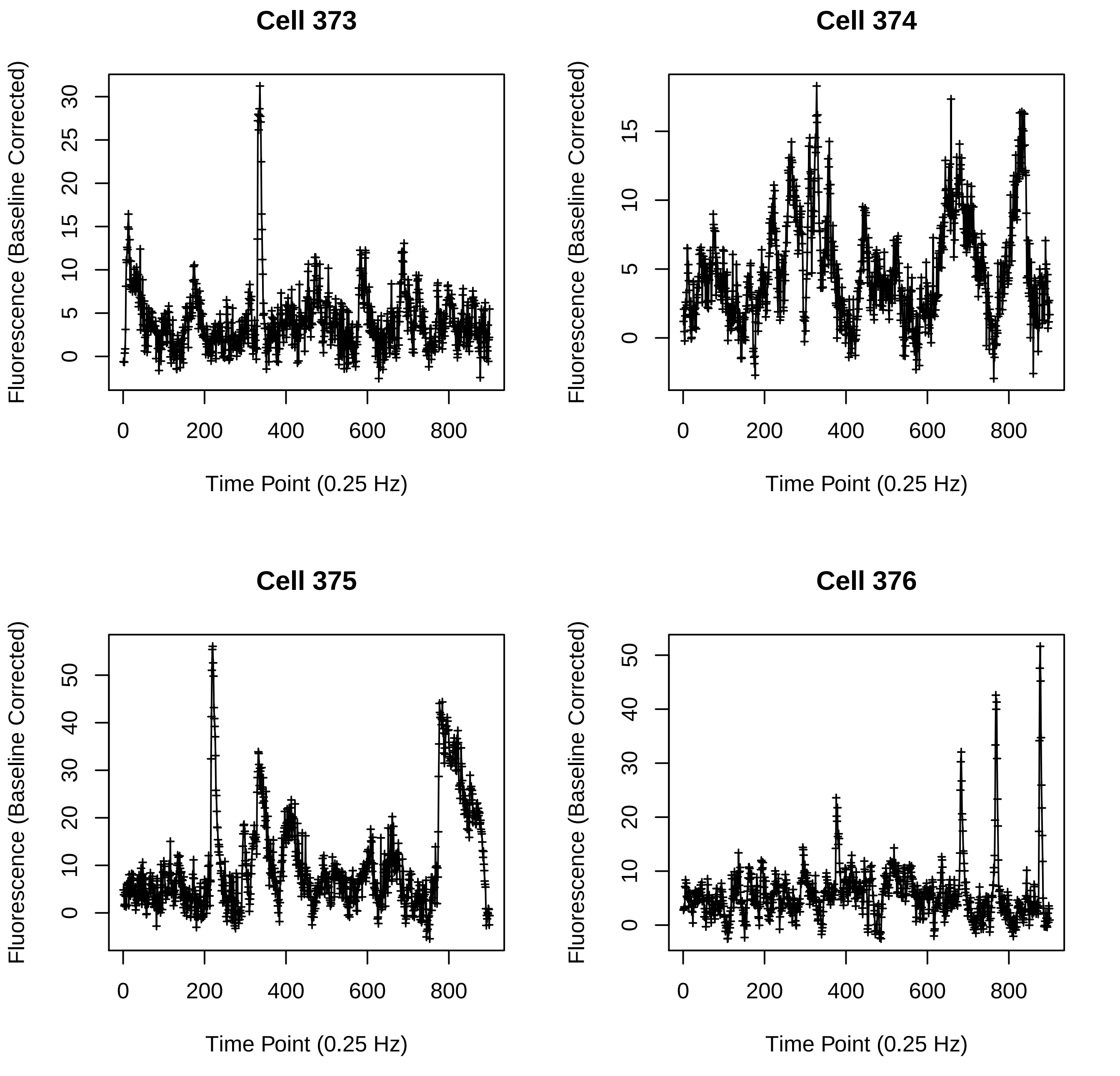
Cell 373
Cell 374
Cell 375
Cell 376
Fluorescence (Baseline Corrected)
Time Point (0.25 Hz)
0
200
400
600
800
0
5
10
15
20
25
30
40
50

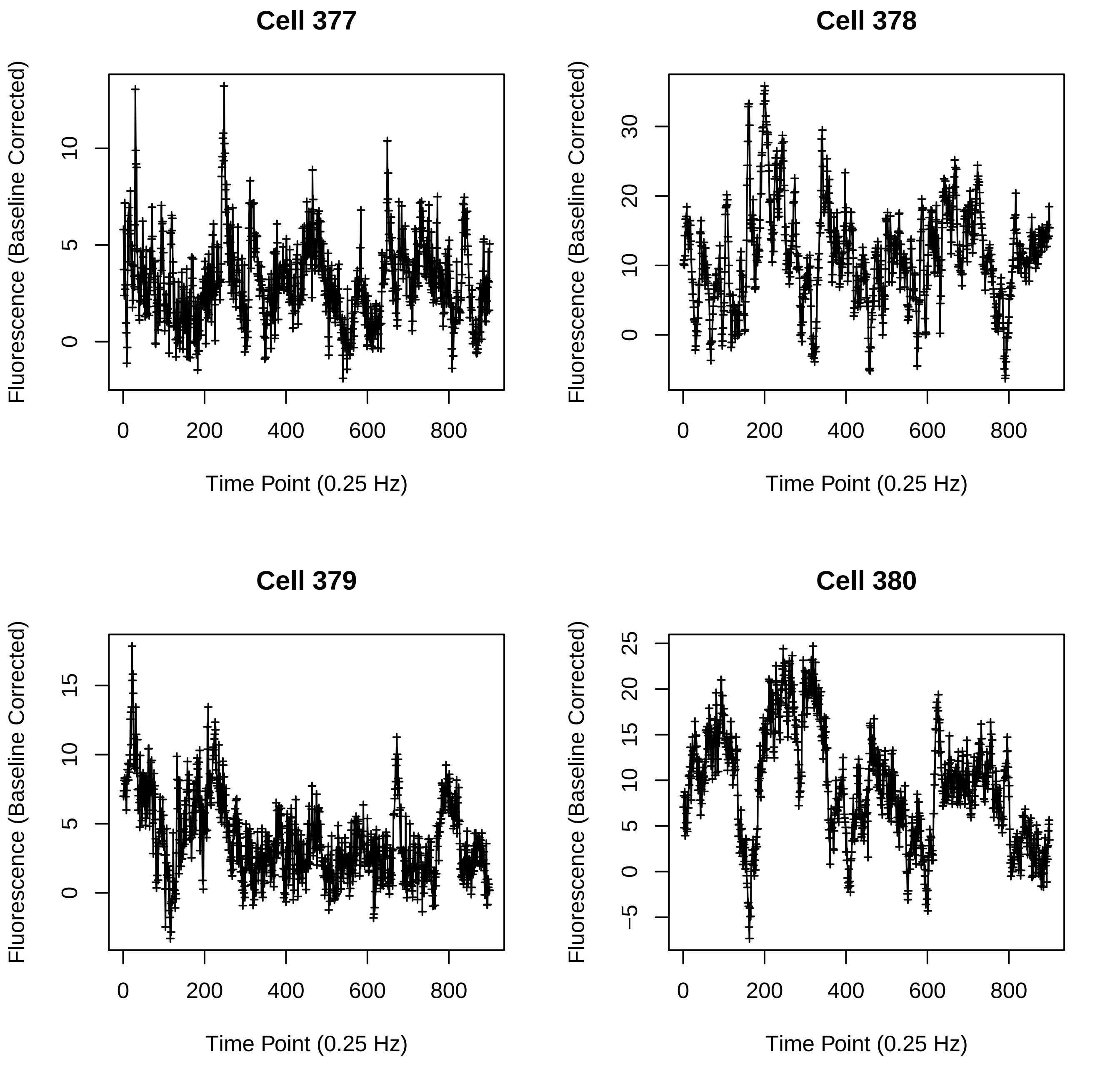
Cell 377
Cell 378
Cell 379
Cell 380
Fluorescence (Baseline Corrected)
Time Point (0.25 Hz)
0
200
400
600
800
0
5
10
15
20
25
30
−5

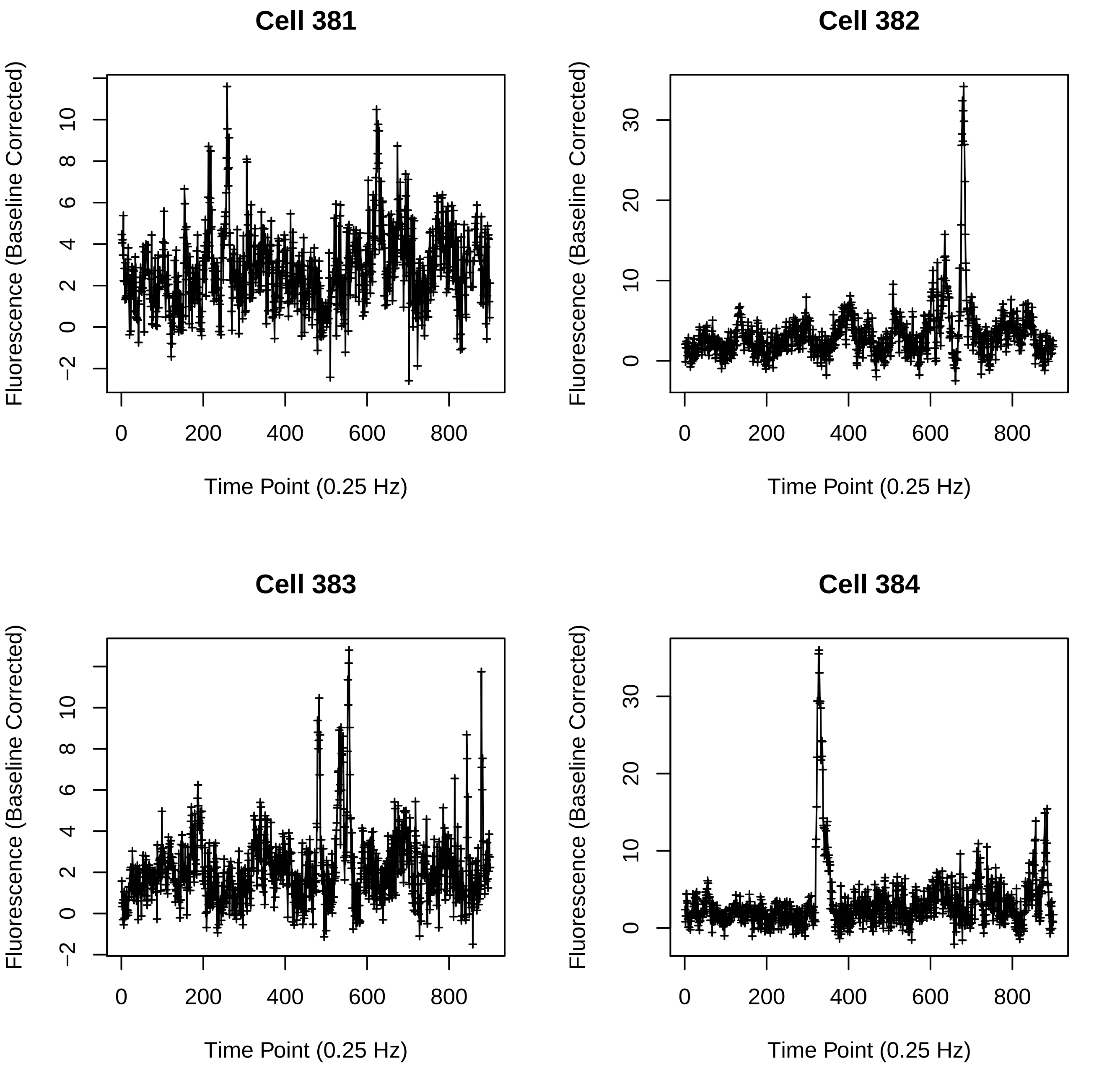
Cell 381
Cell 382
Cell 383
Cell 384
Fluorescence (Baseline Corrected)
Time Point (0.25 Hz)
0
200
400
600
800
−2
0
2
4
6
8
10
20
30

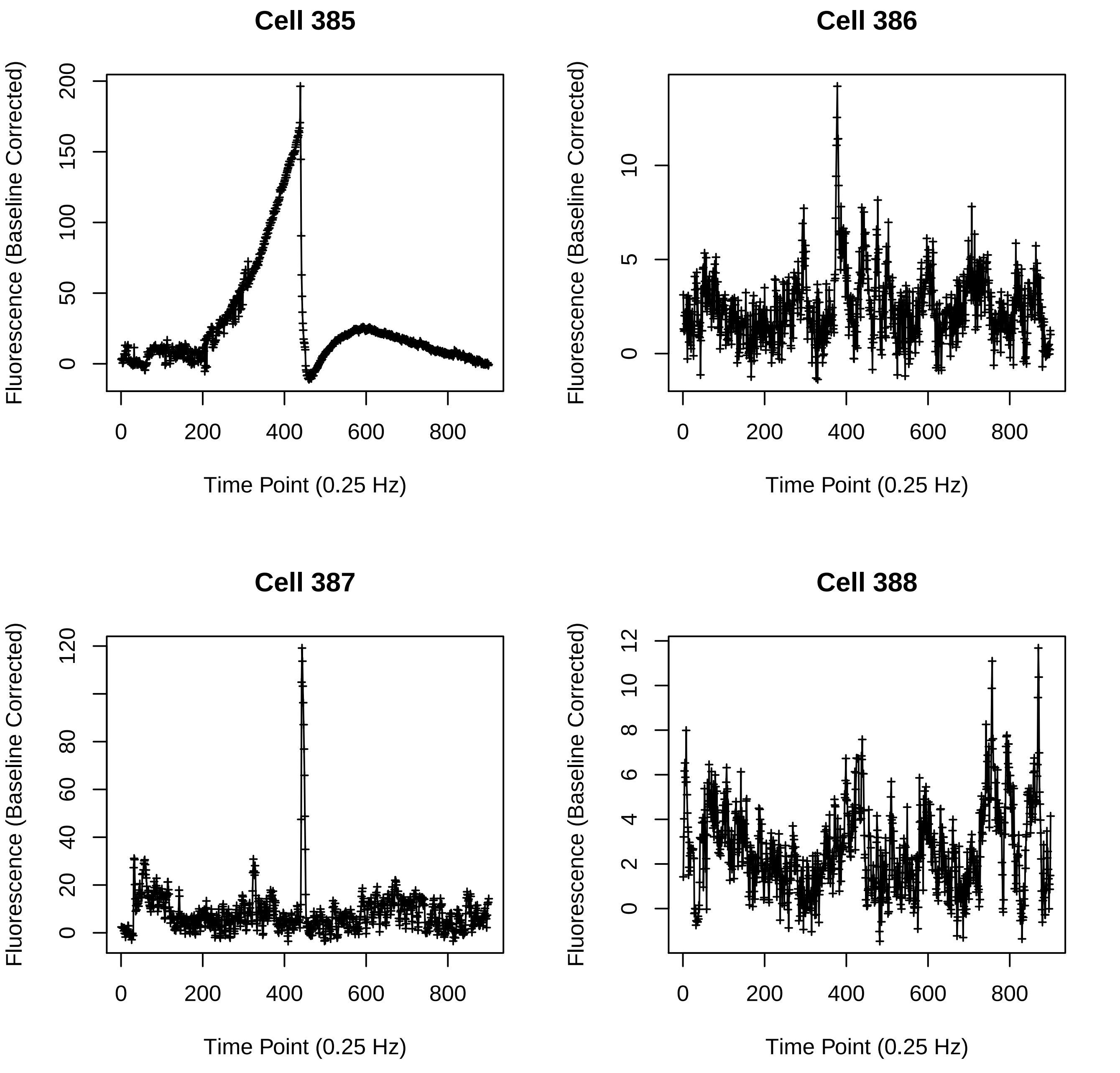
Cell 385
Cell 386
Cell 387
Cell 388
Fluorescence (Baseline Corrected)
Time Point (0.25 Hz)
0
50
100
150
200
0
5
10
0
20
40
60
80
120
0
2
4
6
8
10
12
0
200
400
600
800

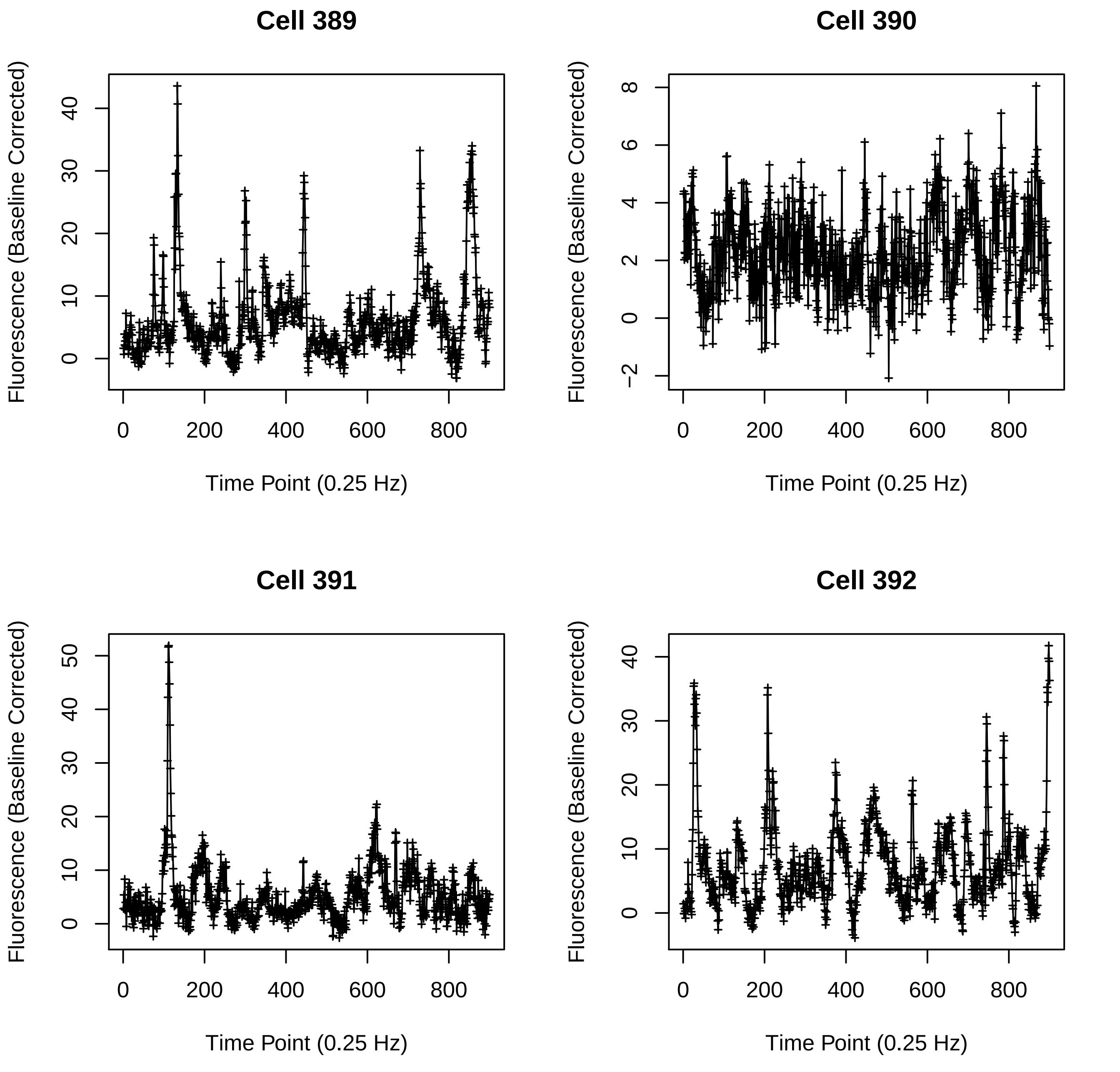

Cell 389
Cell 390
Cell 391
Cell 392
Fluorescence (Baseline Corrected)
Time Point (0.25 Hz)
0
200
400
600
800
10
20
30
40
50
−2
2
4
6
8

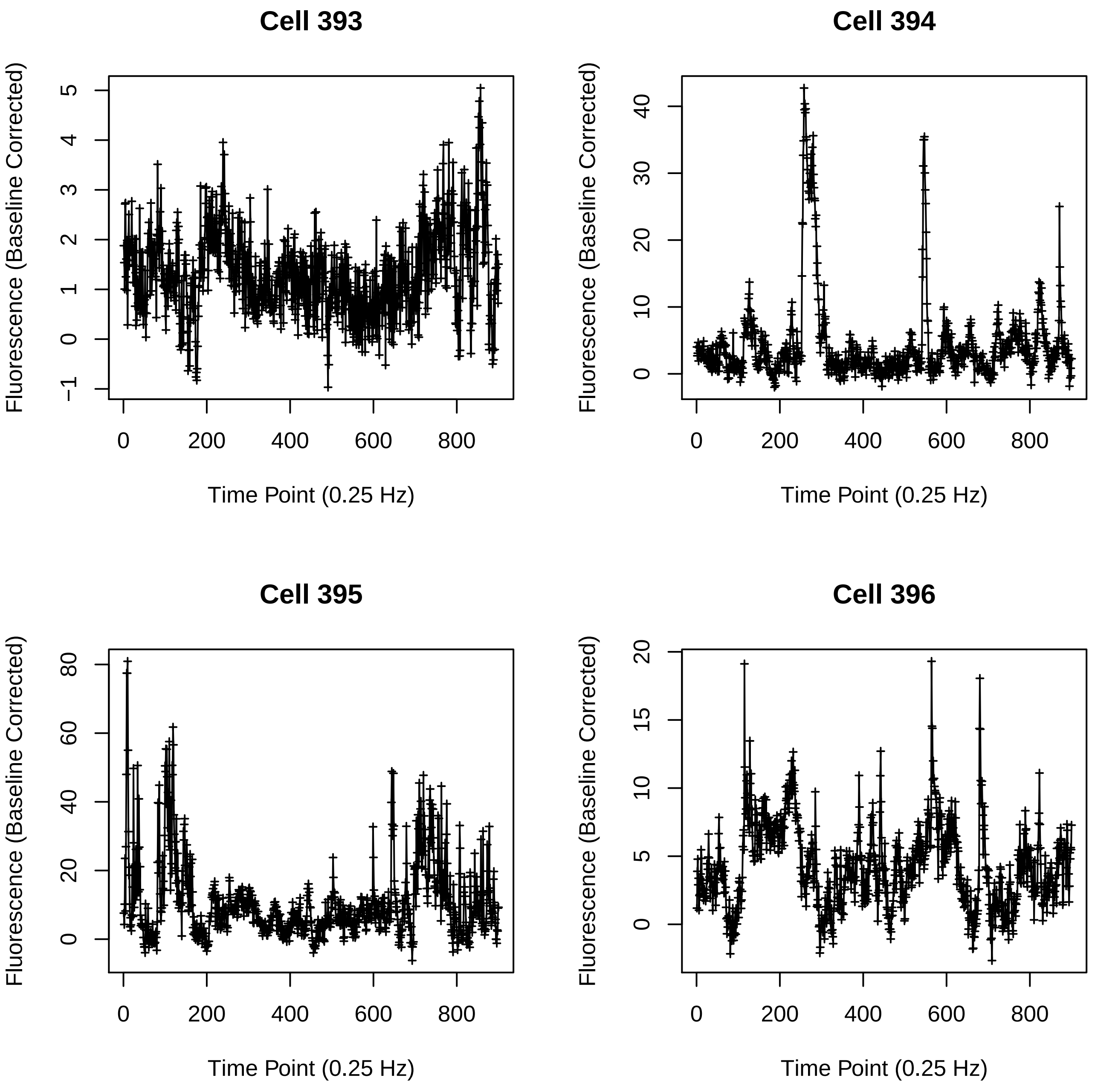
Cell 393
Fluorescence (Baseline Corrected)
−1
0
1
2
3
4
5
Time Point (0.25 Hz)
0
200
400
600
800
Cell 394
Fluorescence (Baseline Corrected)
0
10
20
30
40
Time Point (0.25 Hz)
0
200
400
600
800
Cell 395
Fluorescence (Baseline Corrected)
0
20
40
60
80
Time Point (0.25 Hz)
0
200
400
600
800
Cell 396
Fluorescence (Baseline Corrected)
0
5
10
15
20
Time Point (0.25 Hz)
0
200
400
600
800

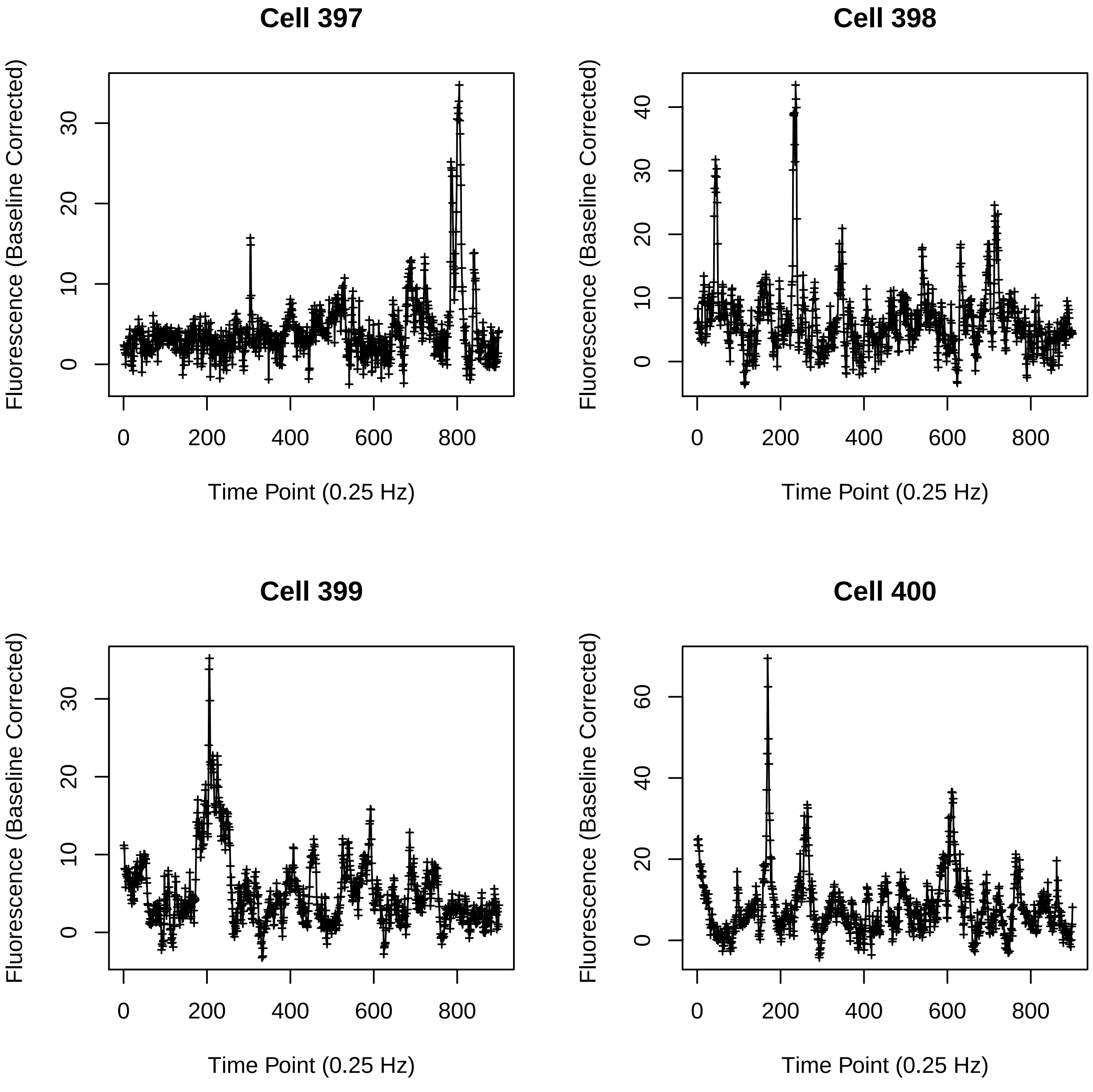
Cell 397
Cell 398
Cell 399
Cell 400
Fluorescence (Baseline Corrected)
Time Point (0.25 Hz)
0
10
20
30
40
60
0
200
400
600
800

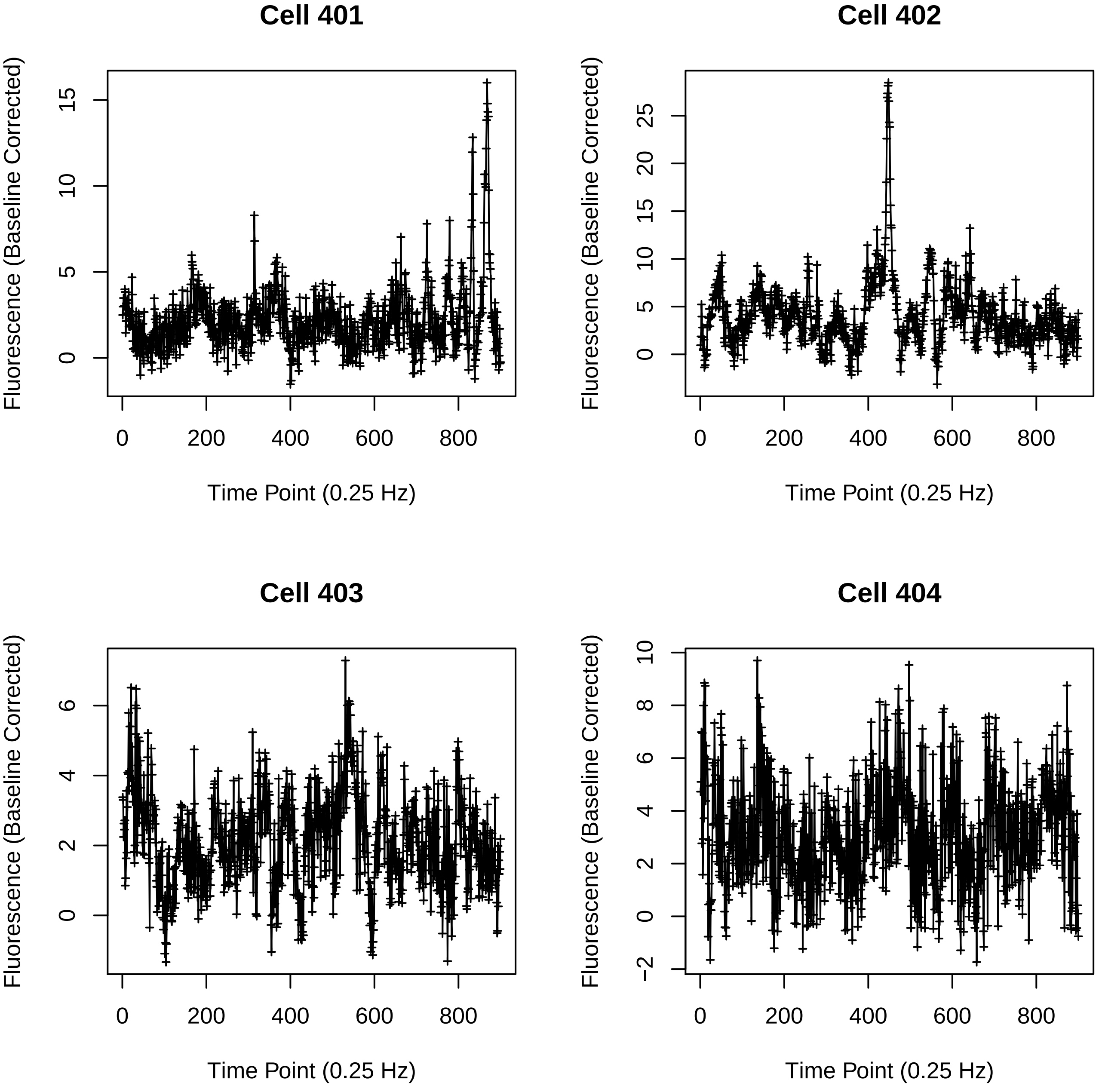

Cell 401
Fluorescence (Baseline Corrected)
0
5
10
15
0
200
400
600
800
Time Point (0.25 Hz)
Cell 402
Fluorescence (Baseline Corrected)
0
5
10
15
20
25
0
200
400
600
800
Time Point (0.25 Hz)
Cell 403
Fluorescence (Baseline Corrected)
0
2
4
6
0
200
400
600
800
Time Point (0.25 Hz)
Cell 404
Fluorescence (Baseline Corrected)
−2
0
2
4
6
8
10
0
200
400
600
800
Time Point (0.25 Hz)

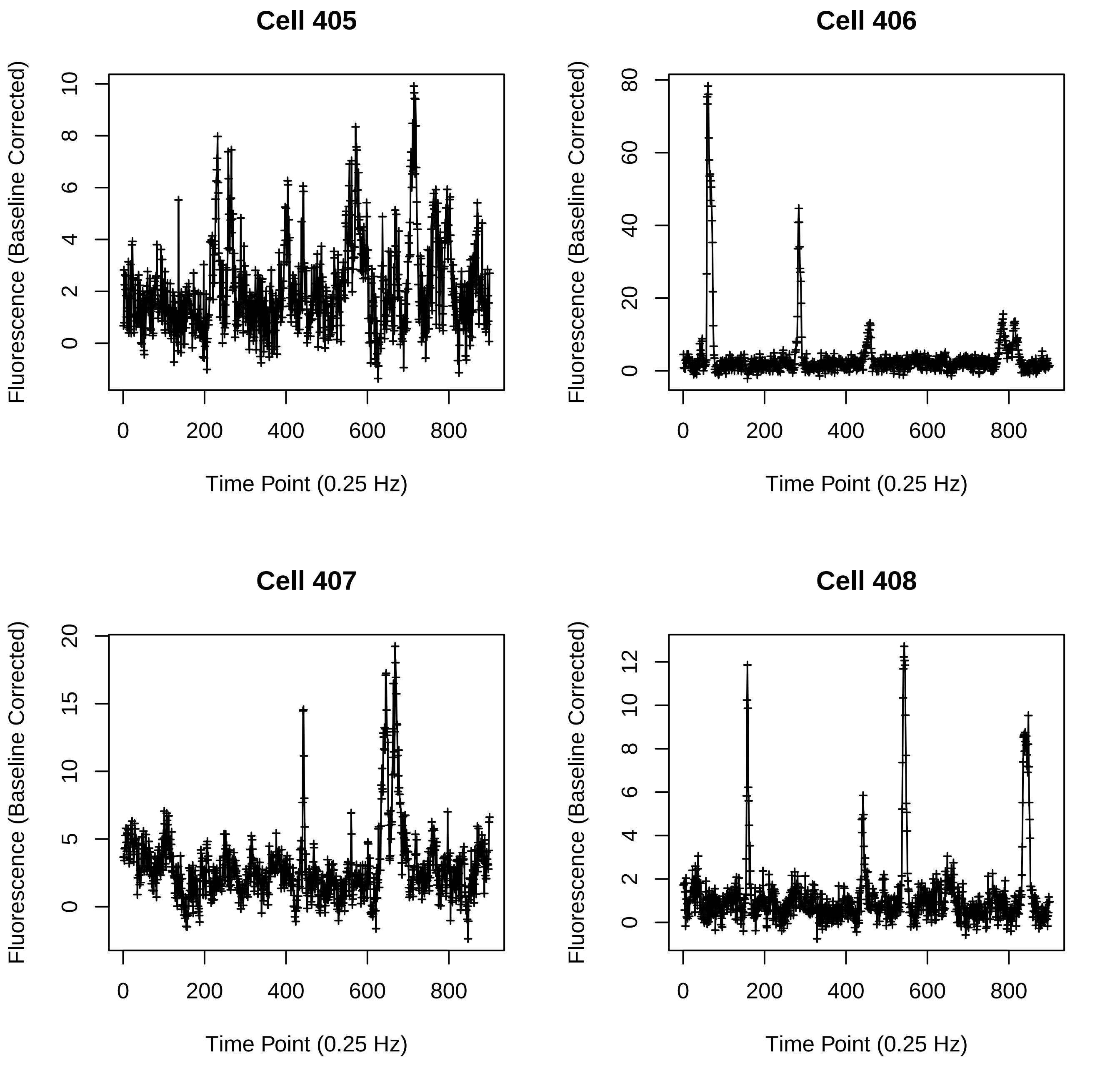
Cell 405
Cell 406
Cell 407
Cell 408
Fluorescence (Baseline Corrected)
Time Point (0.25 Hz)
0
200
400
600
800

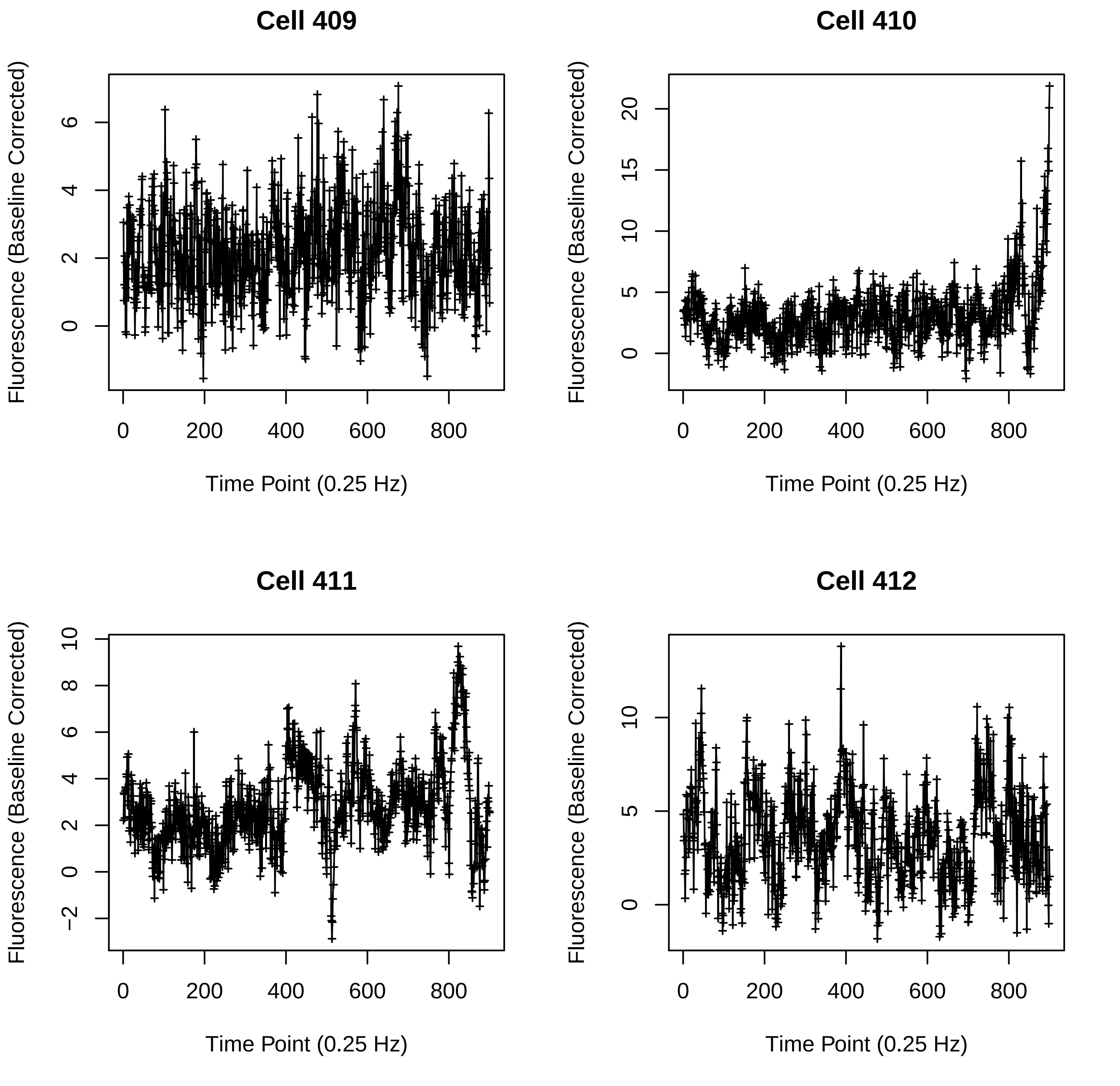

Cell 409
Cell 410
Cell 411
Cell 412
Fluorescence (Baseline Corrected)
Time Point (0.25 Hz)
0
200
400
600
800

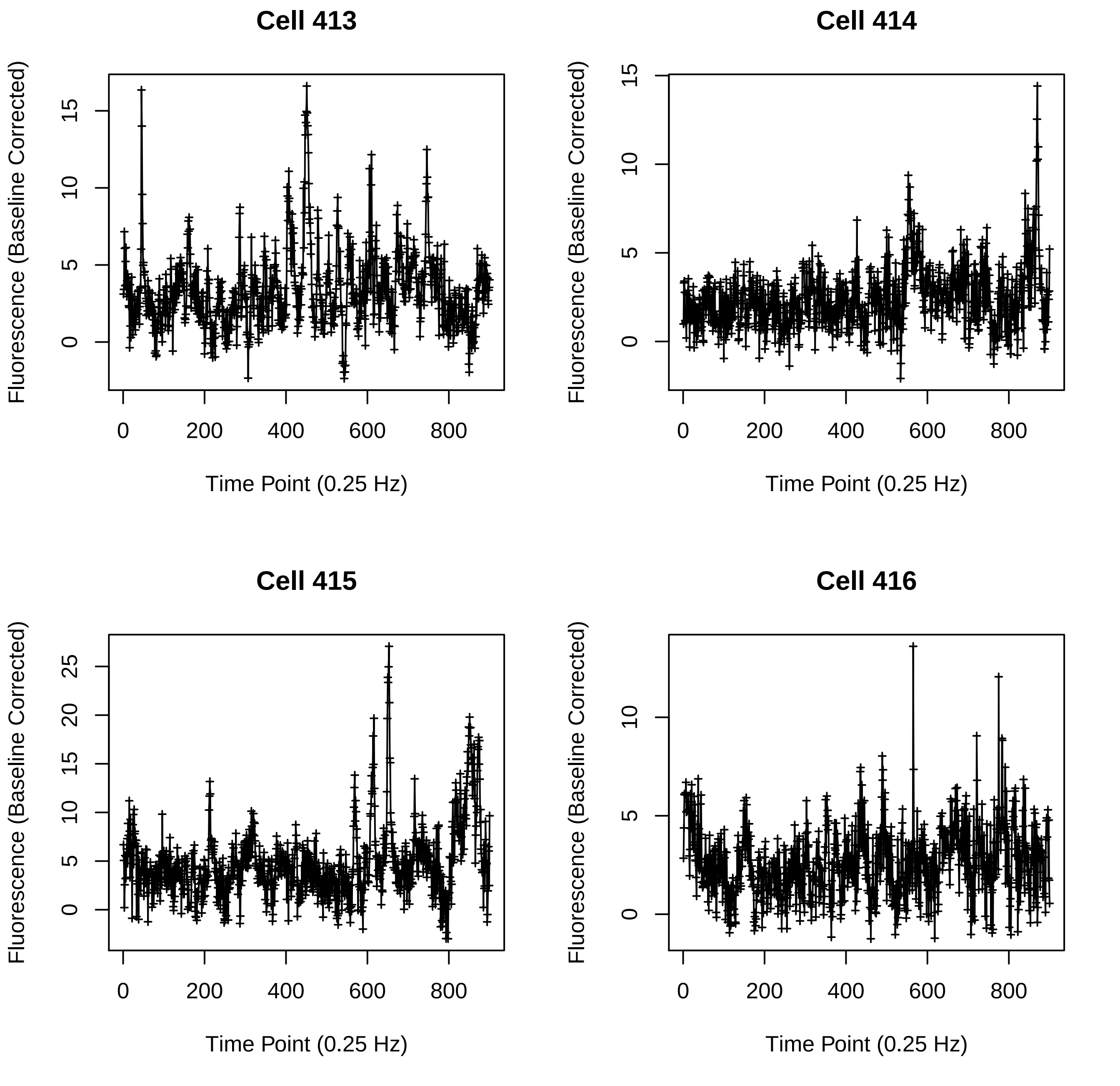
Cell 413
Cell 414
Cell 415
Cell 416
Fluorescence (Baseline Corrected)
Time Point (0.25 Hz)
0
200
400
600
800
0
5
10
15
20
25

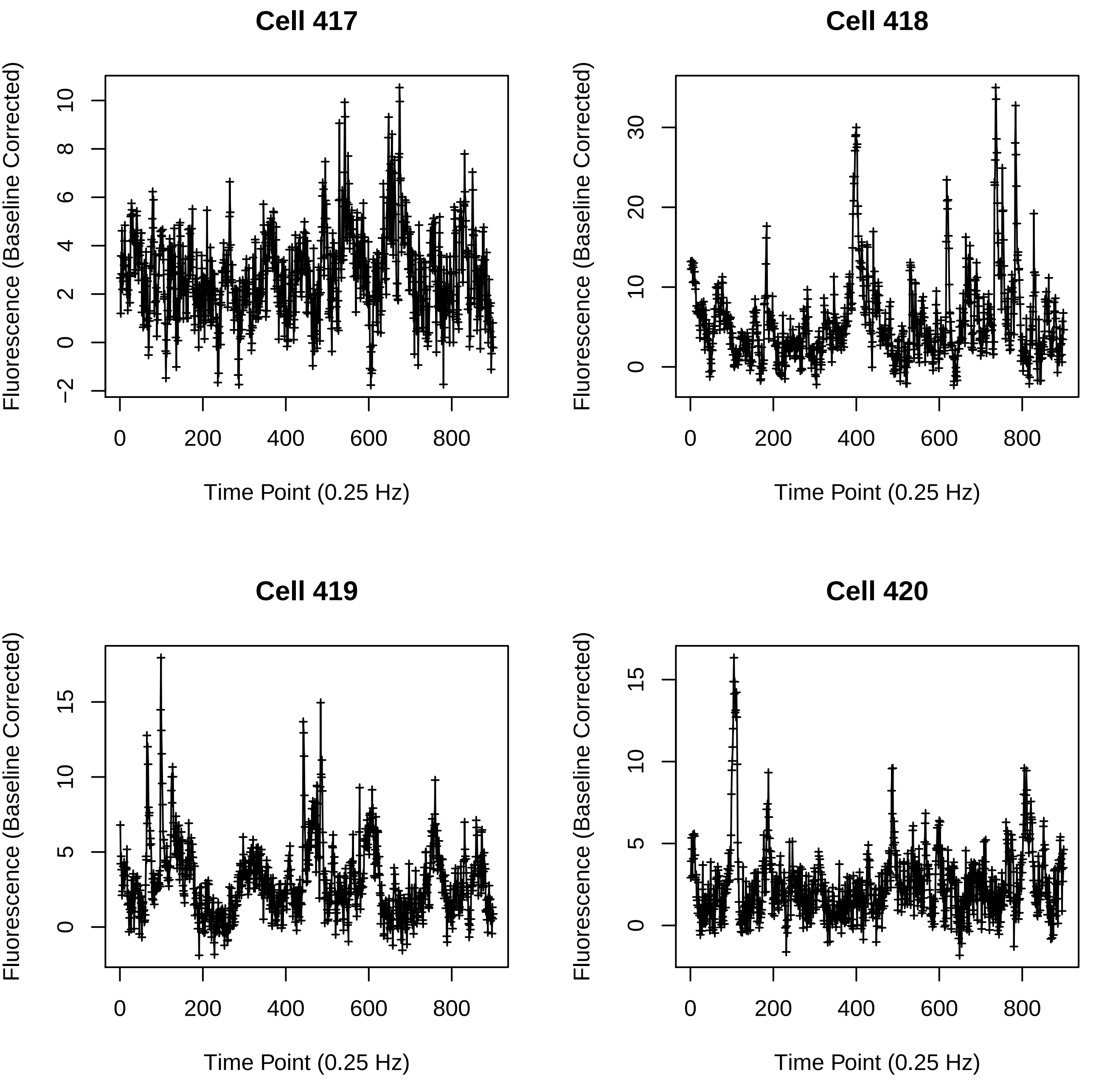
Cell 417
Cell 418
Cell 419
Cell 420
Fluorescence (Baseline Corrected)
Time Point (0.25 Hz)
0
200
400
600
800
−2
2
4
6
8
10
20
30
5
15

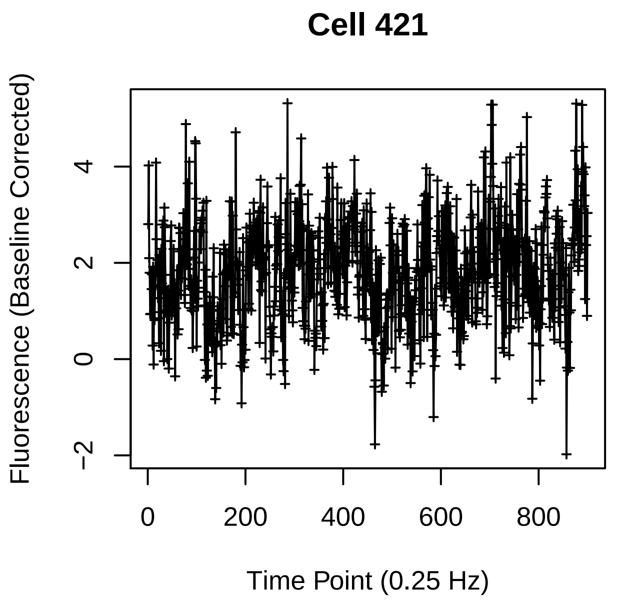
Cell 421
Fluorescence (Baseline Corrected)
−2
0
2
4
0
200
400
600
800
Time Point (0.25 Hz)

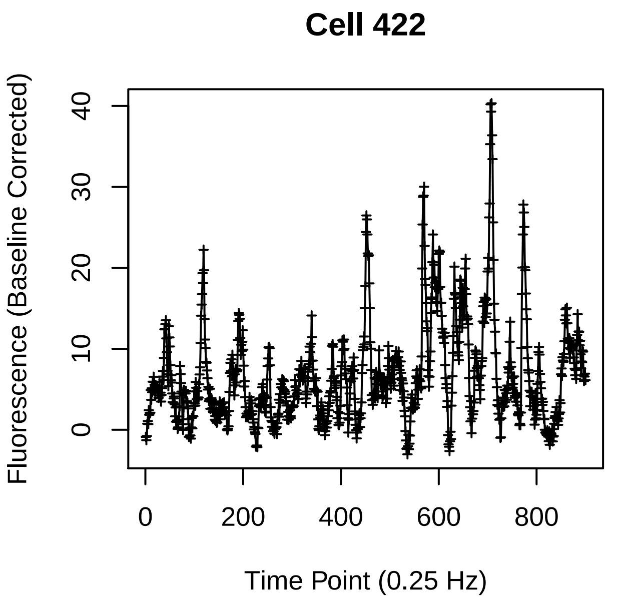
Cell 422
Fluorescence (Baseline Corrected)
0
10
20
30
40
0
200
400
600
800
Time Point (0.25 Hz)

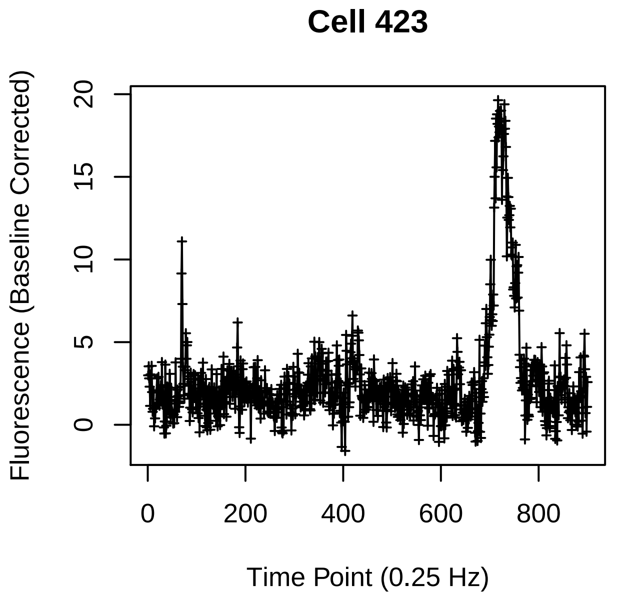
Cell 423
Fluorescence (Baseline Corrected)
0
5
10
15
20
0
200
400
600
800
Time Point (0.25 Hz)

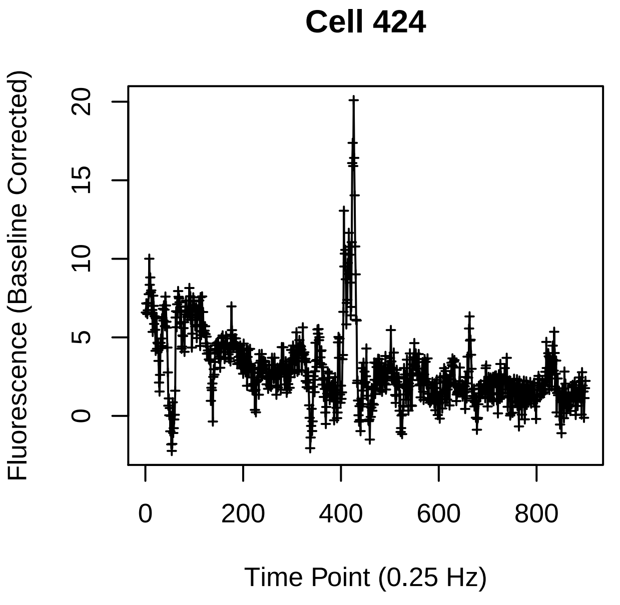
Cell 424
Fluorescence (Baseline Corrected)
0
5
10
15
20
0
200
400
600
800
Time Point (0.25 Hz)

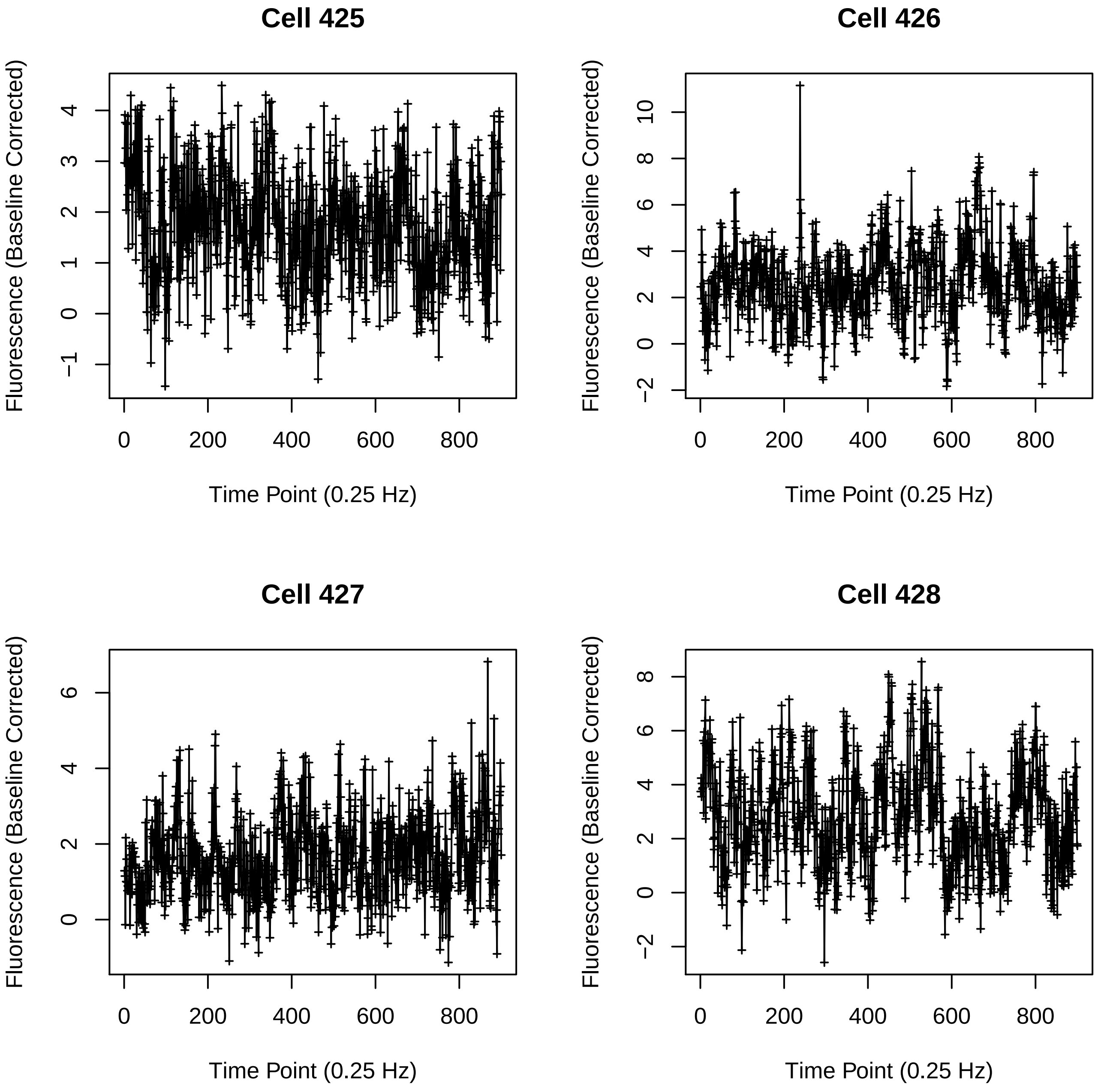
Cell 425
Cell 426
Cell 427
Cell 428
Fluorescence (Baseline Corrected)
Time Point (0.25 Hz)
0
200
400
600
800
−1
0
1
2
3
4
−2
0
2
4
6
8
10
0
2
4
6
−2
0
2
4
6
8

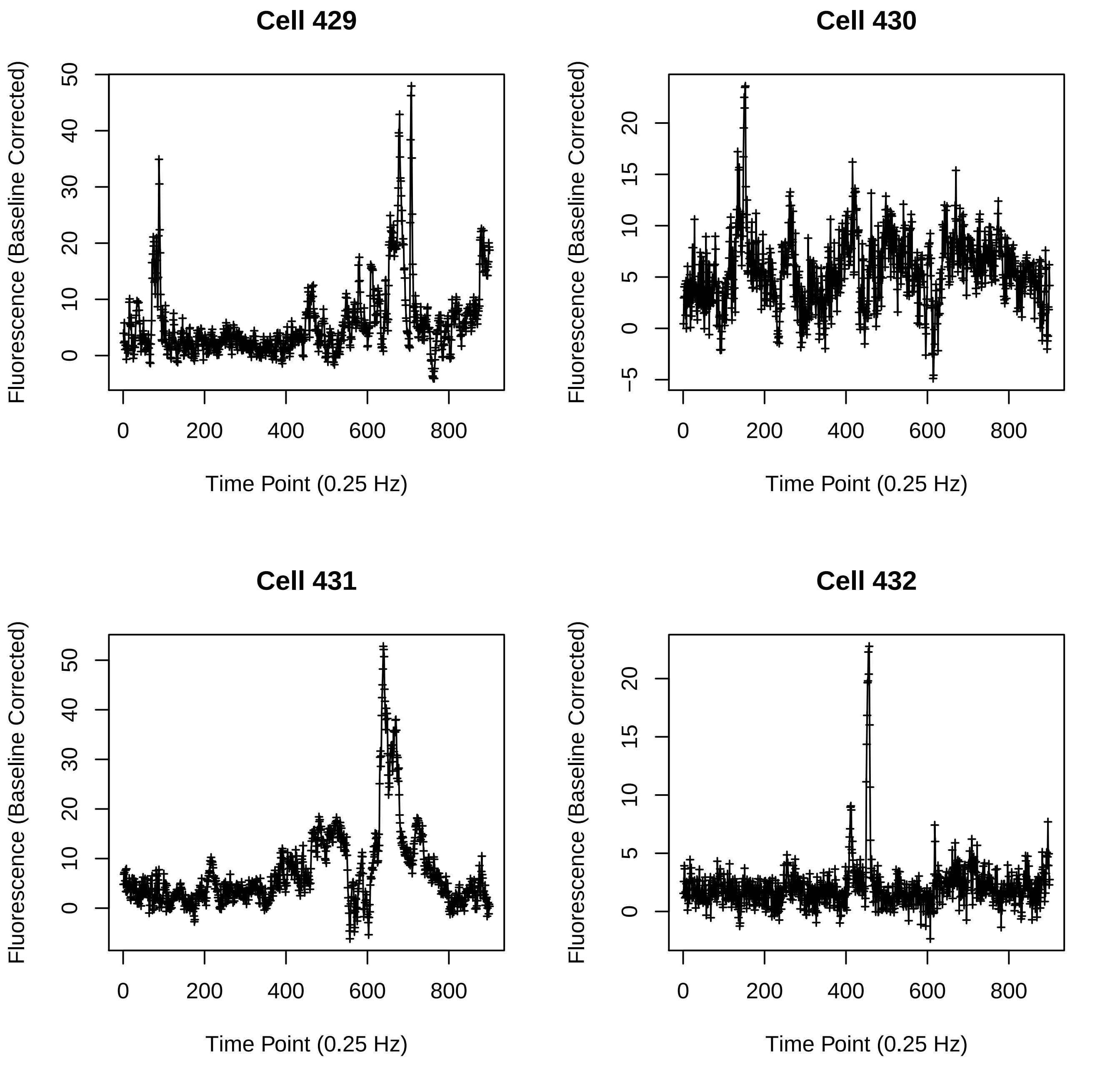

Cell 429
Cell 430
Cell 431
Cell 432
Fluorescence (Baseline Corrected)
Time Point (0.25 Hz)
0
200
400
600
800
−5
5
10
15
20
30
40
50

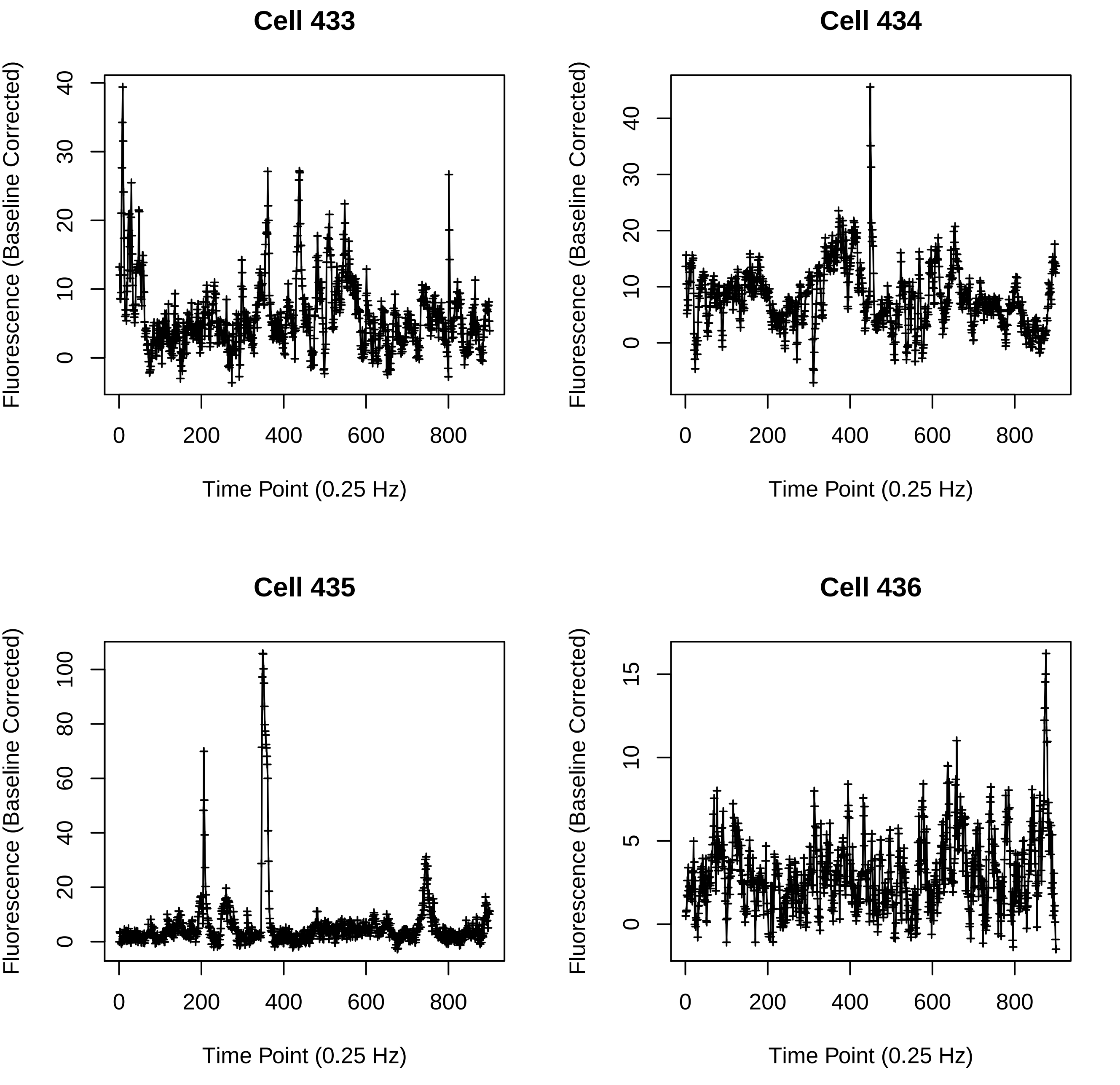
Cell 433
Cell 434
Cell 435
Cell 436
Fluorescence (Baseline Corrected)
Time Point (0.25 Hz)
0
10
20
30
40
200
400
600
800
60
80
100
5
15

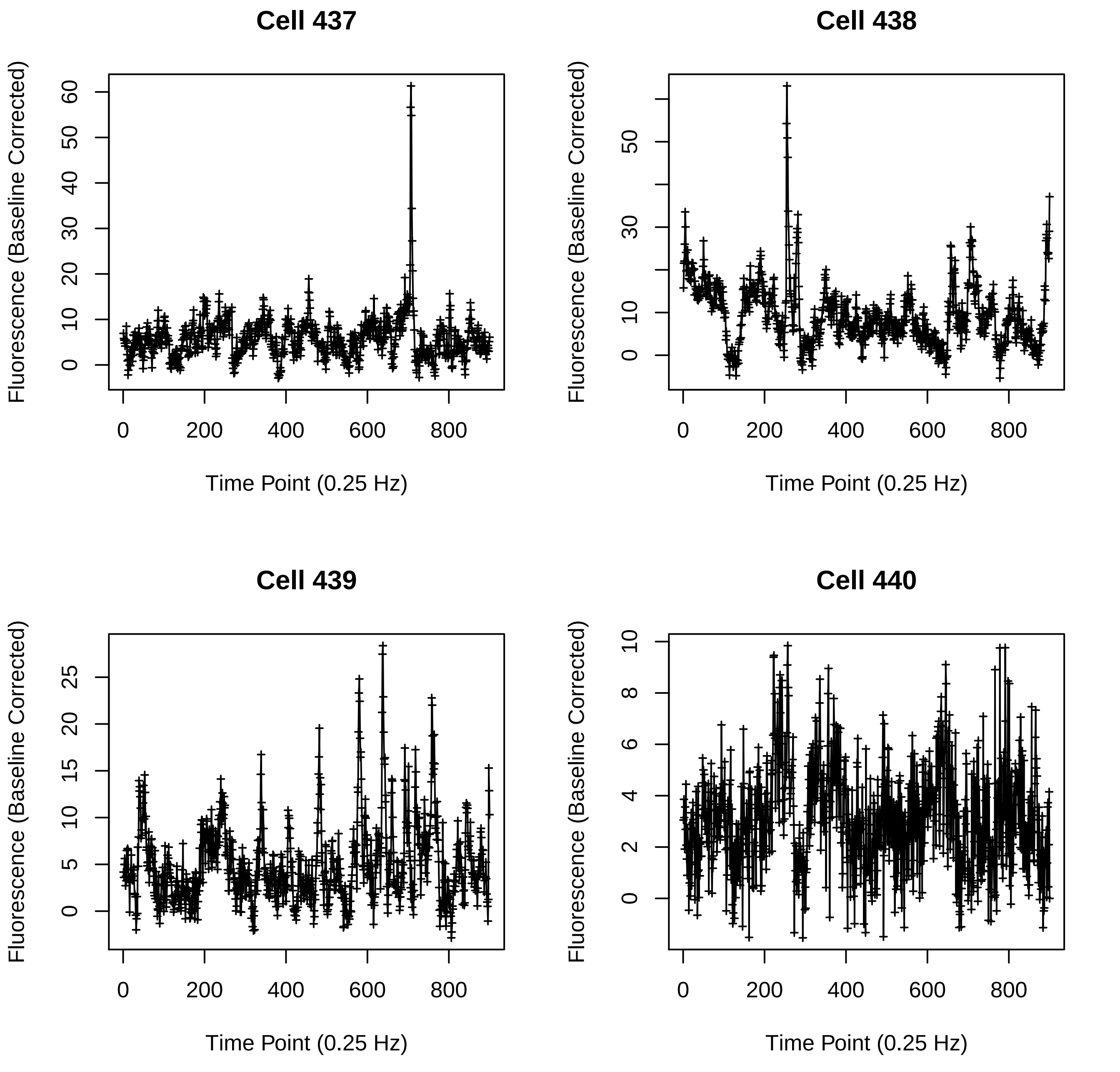
Cell 437
Cell 438
Cell 439
Cell 440
Fluorescence (Baseline Corrected)
Time Point (0.25 Hz)
0
200
400
600
800
10
20
30
40
50
60
5
15
25

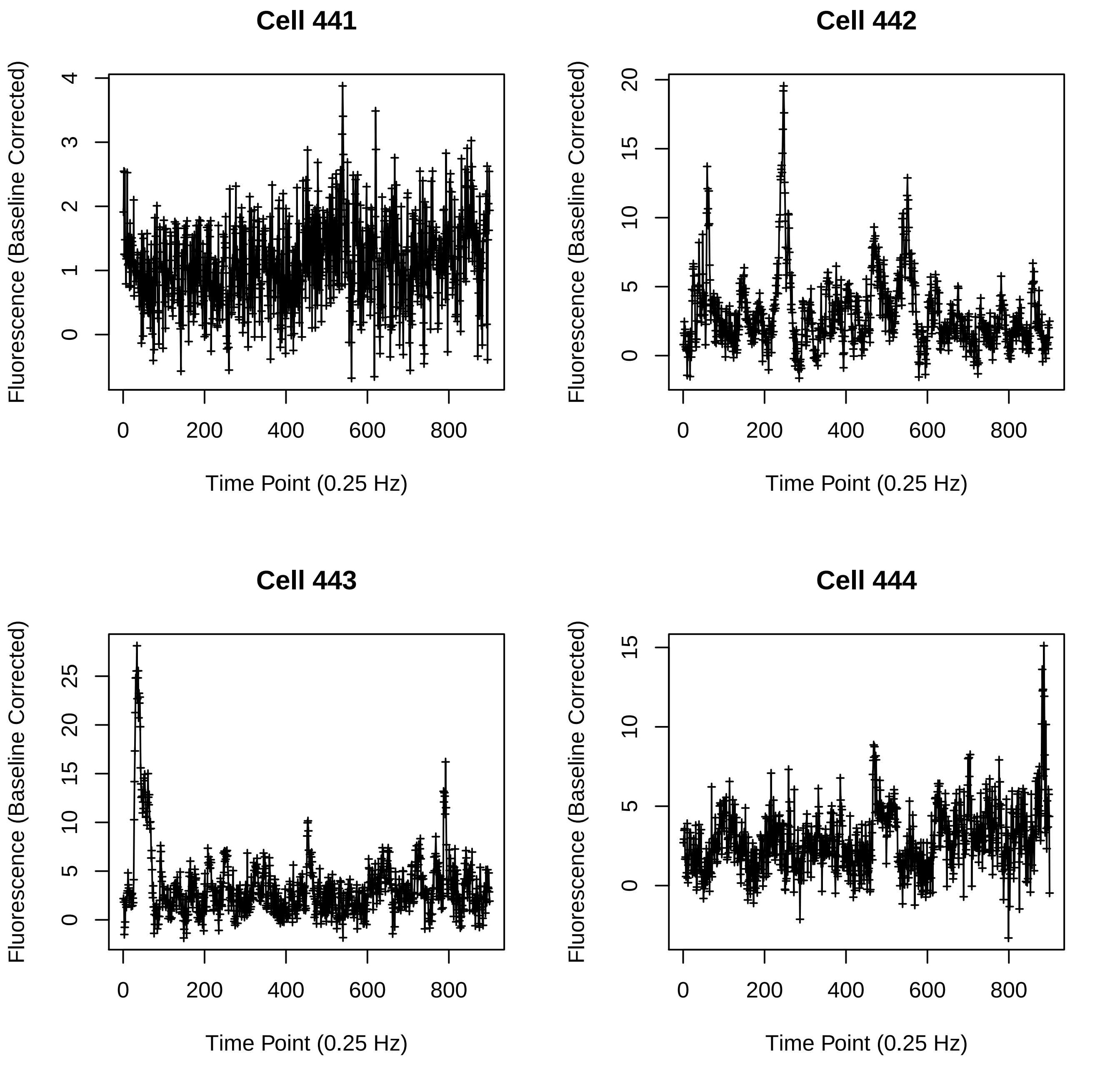
Cell 441
Cell 442
Cell 443
Cell 444
Fluorescence (Baseline Corrected)
Time Point (0.25 Hz)
0
200
400
600
800

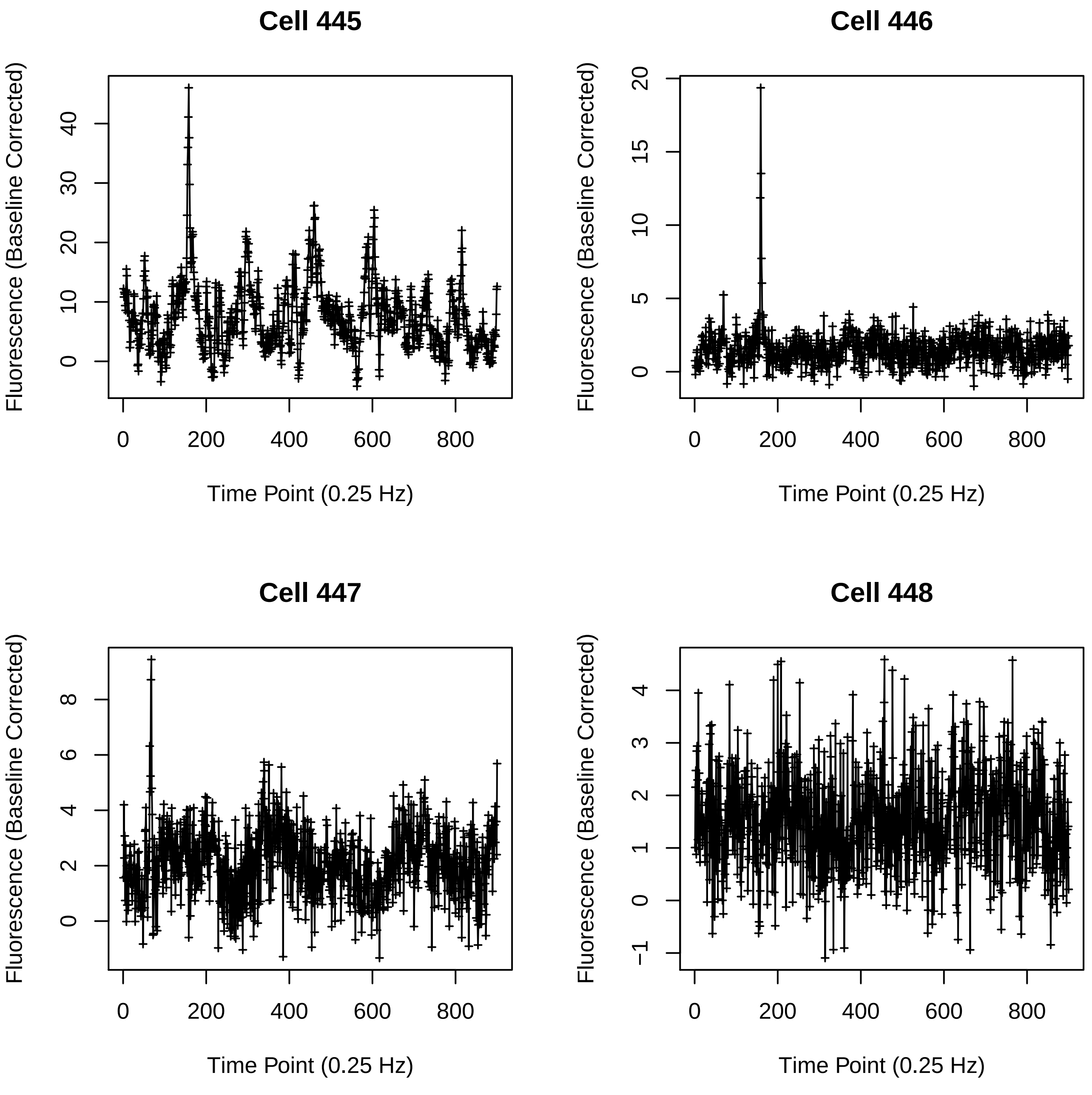

Cell 445
Cell 446
Cell 447
Cell 448
Fluorescence (Baseline Corrected)
Time Point (0.25 Hz)
0
10
20
30
40
5
15
2
4
6
8
−1
1
3
200
400
600
800

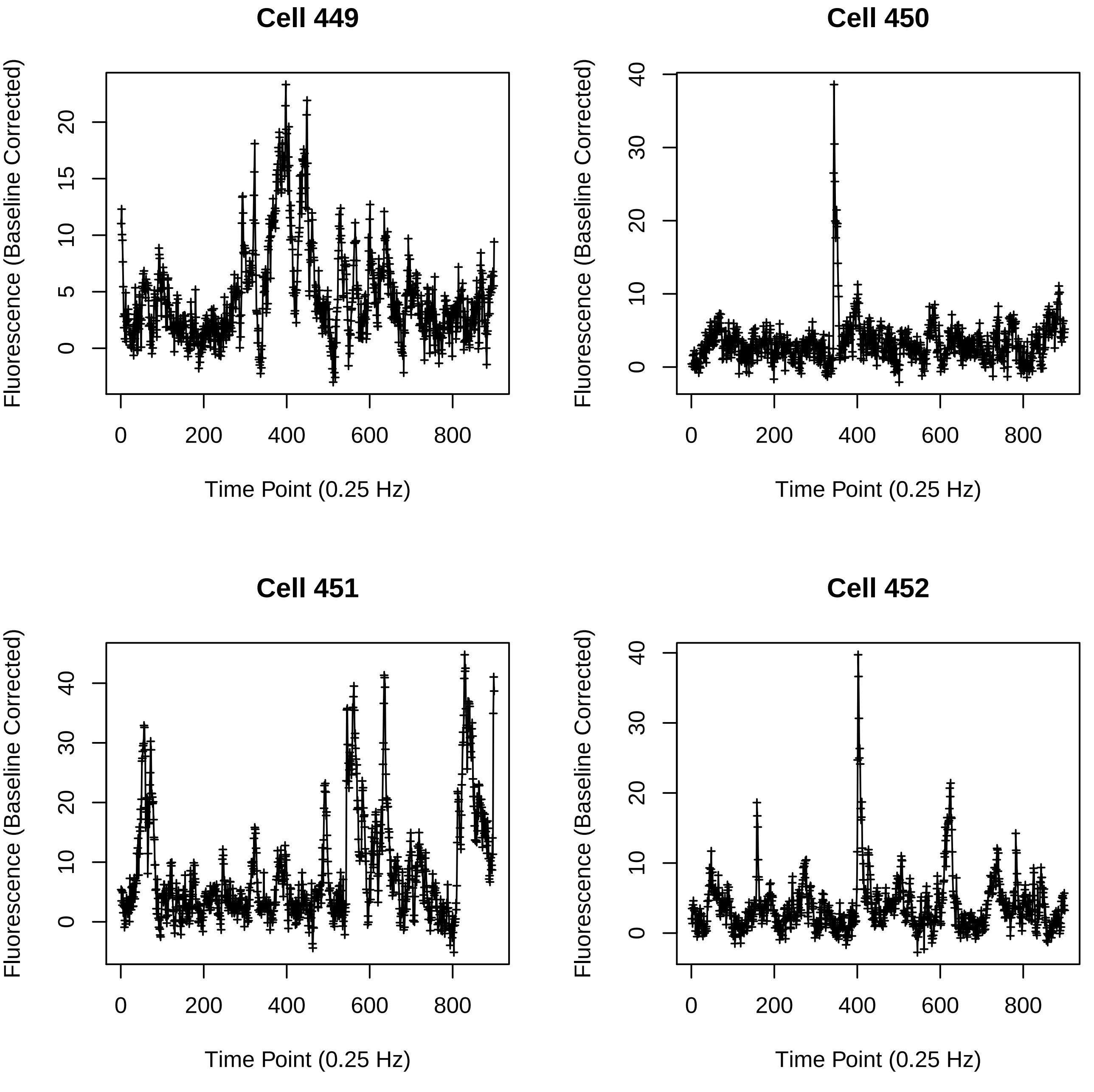
Cell 449
Cell 450
Cell 451
Cell 452
Fluorescence (Baseline Corrected)
Time Point (0.25 Hz)
0
5
10
15
20
30
40
200
400
600
800

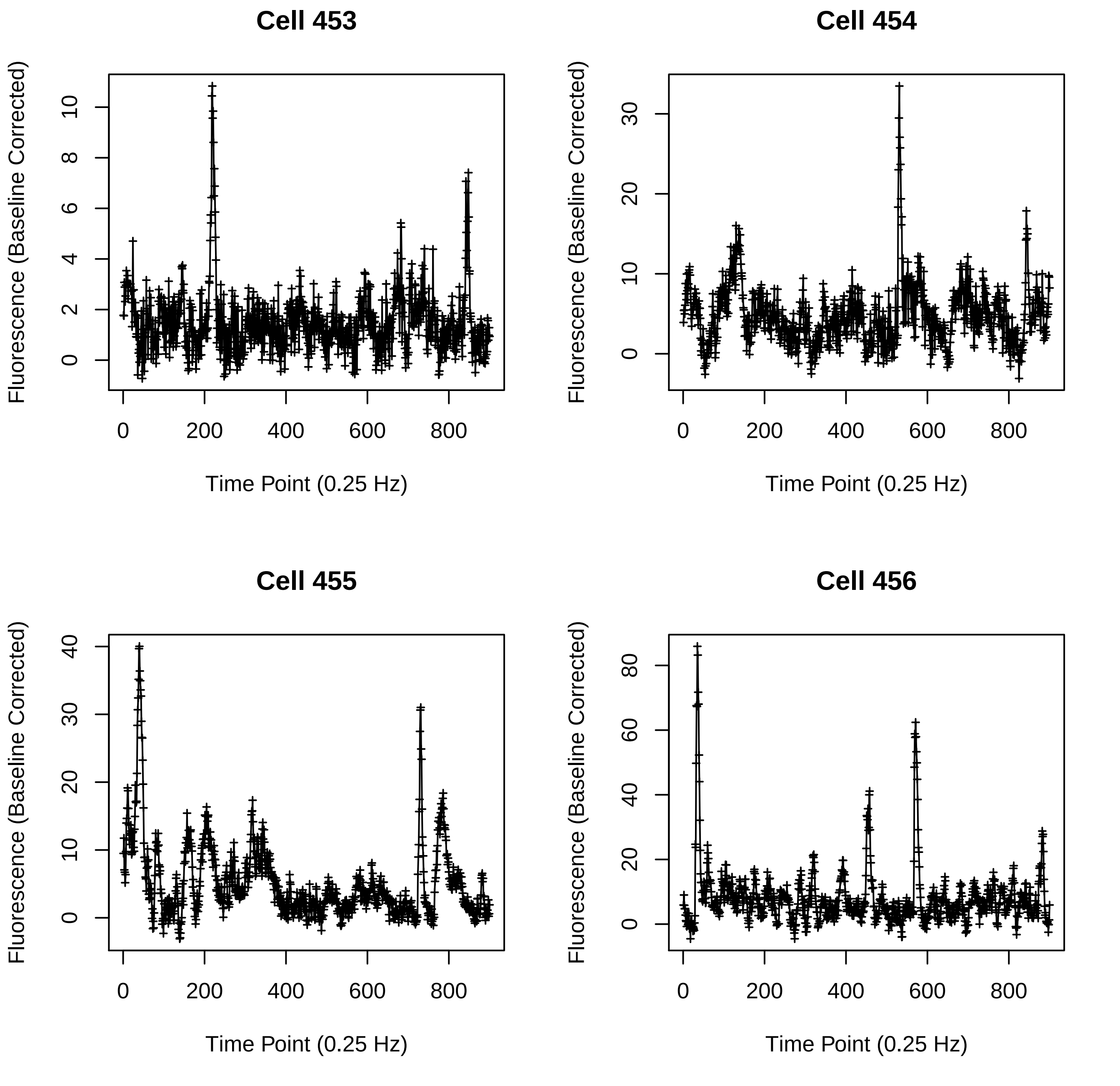
Cell 453
Cell 454
Cell 455
Cell 456
Fluorescence (Baseline Corrected)
Time Point (0.25 Hz)
0
200
400
600
800
0
2
4
6
8
10
0
10
20
30
40
60
80

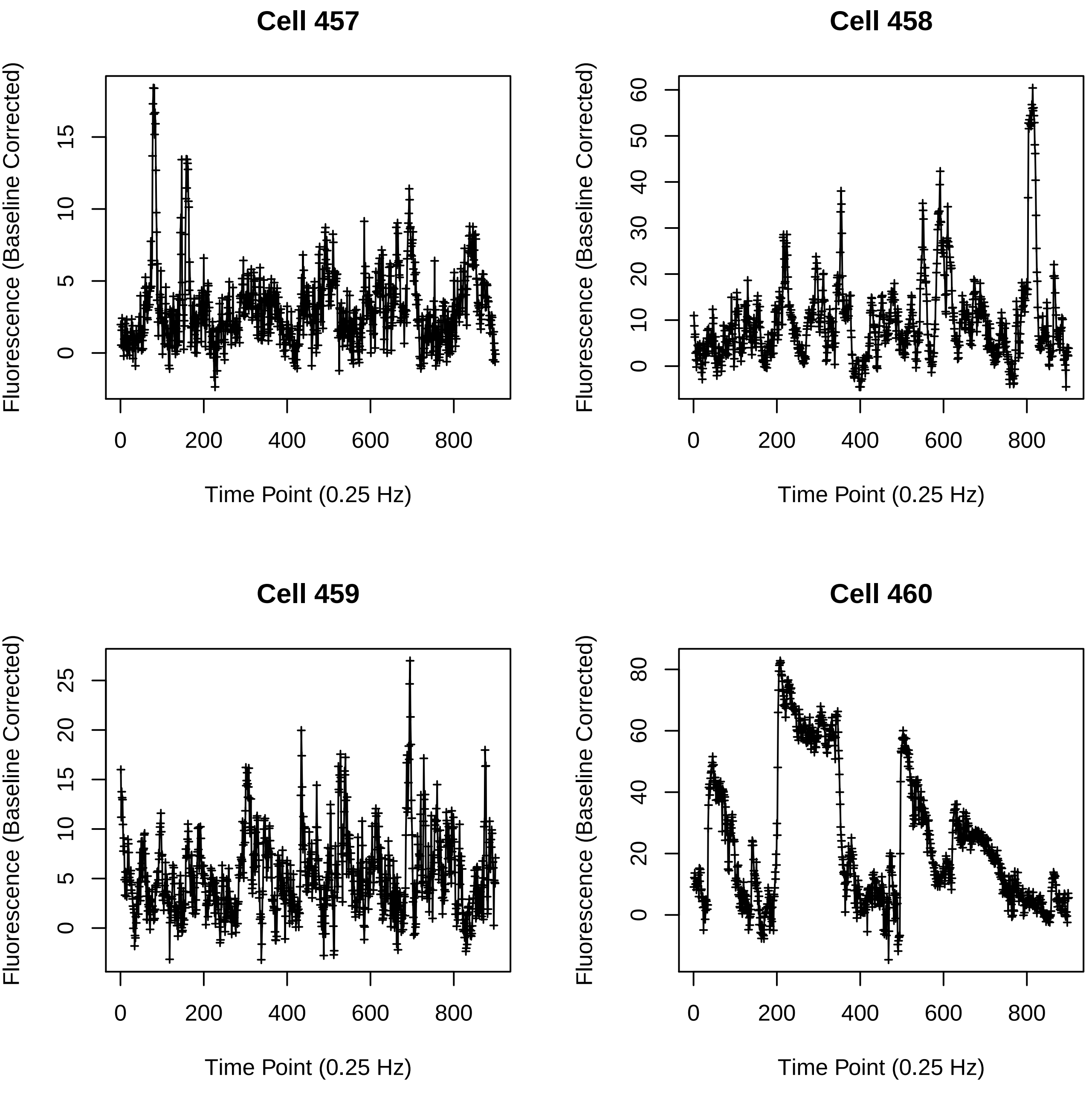
Cell 457
Cell 458
Cell 459
Cell 460
Fluorescence (Baseline Corrected)
Time Point (0.25 Hz)
0
200
400
600
800
5
10
15
20
25
30
40
50
60
80

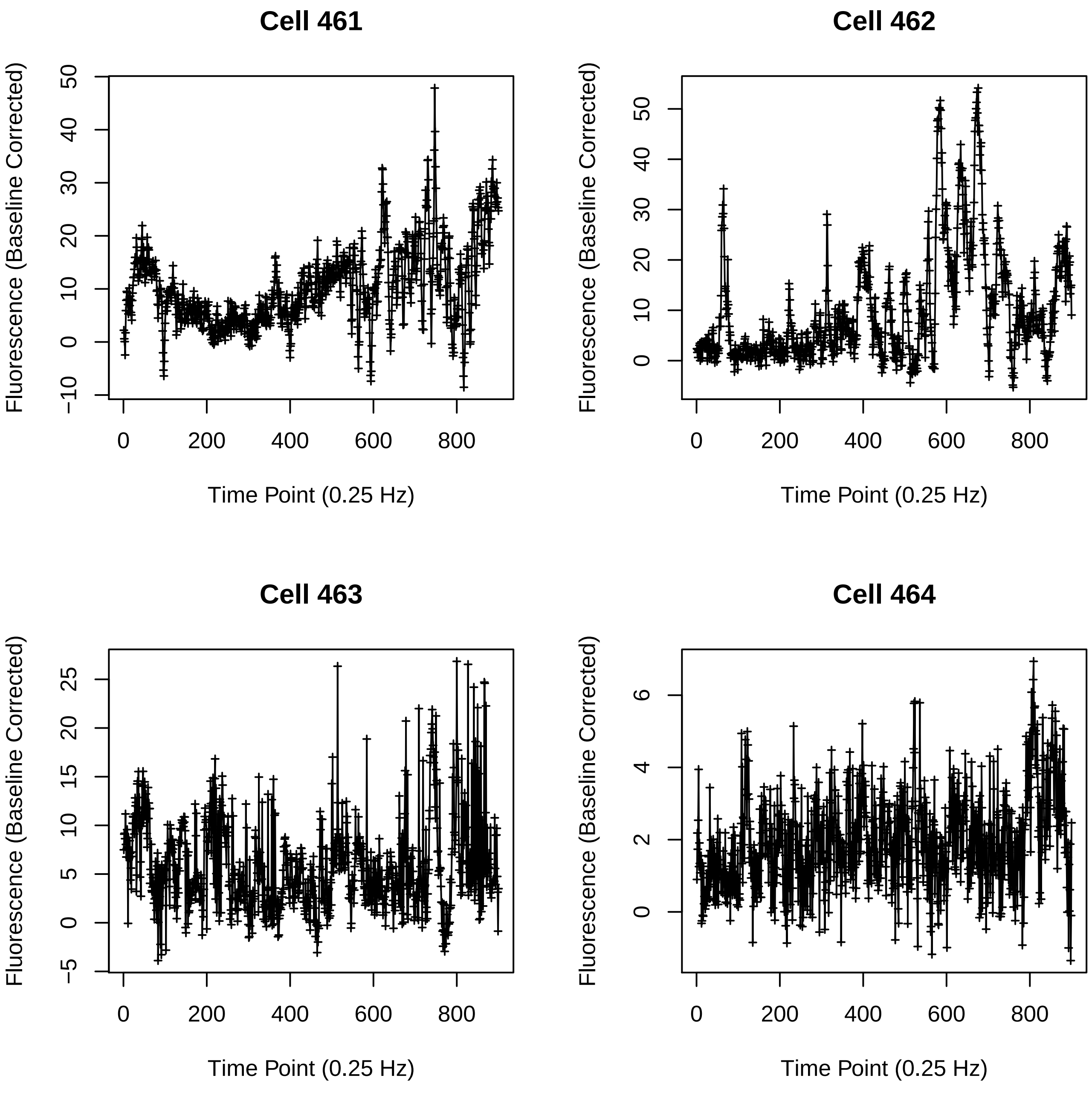
Cell 461
Cell 462
Cell 463
Cell 464
Fluorescence (Baseline Corrected)
Time Point (0.25 Hz)
−10
0
10
20
30
40
50
−5
5
15
25
2
4
6
200
400
600
800

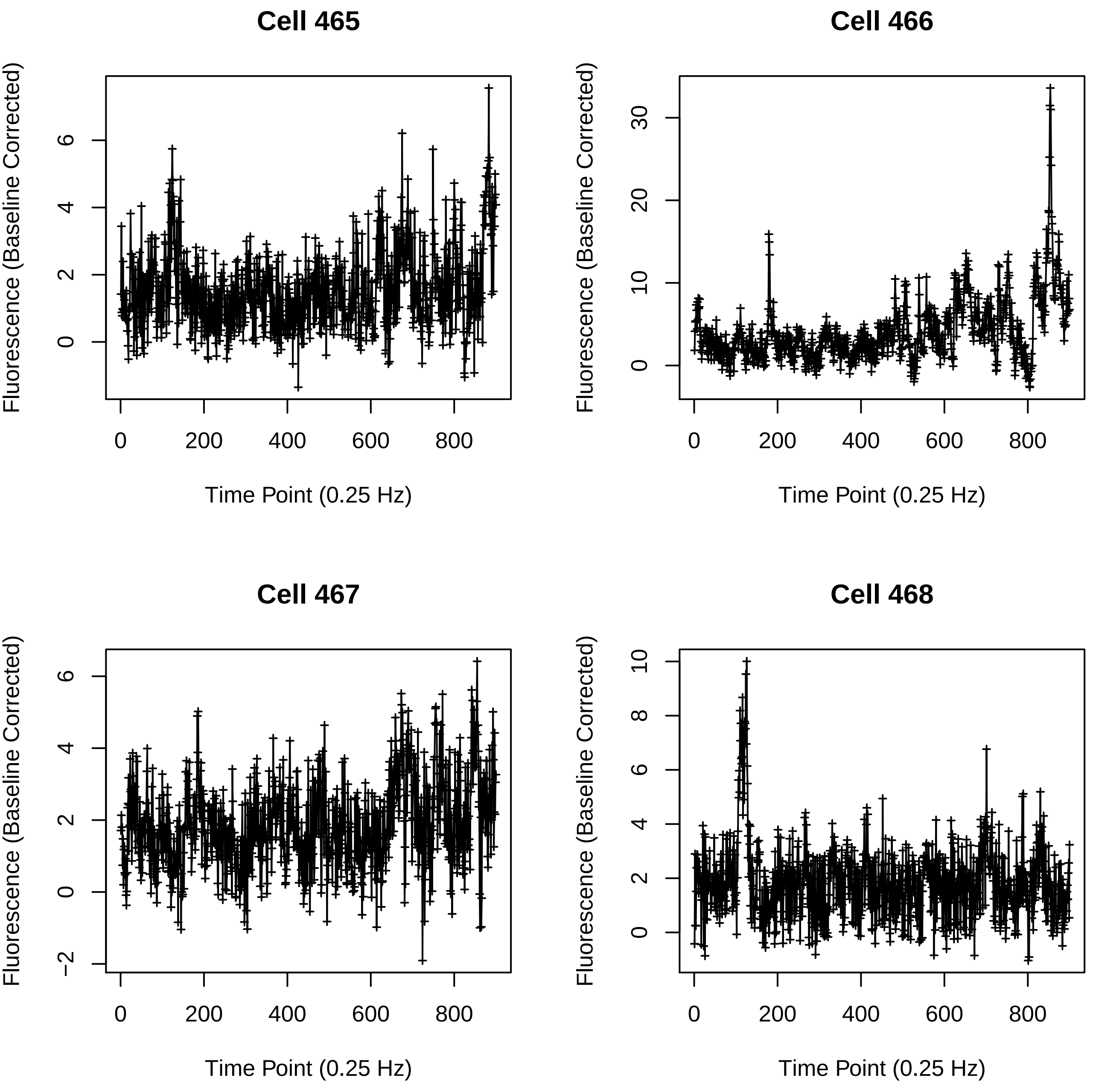
Cell 465
Cell 466
Cell 467
Cell 468
Fluorescence (Baseline Corrected)
Time Point (0.25 Hz)
0
200
400
600
800
−2
2
4
6
10
20
30
8

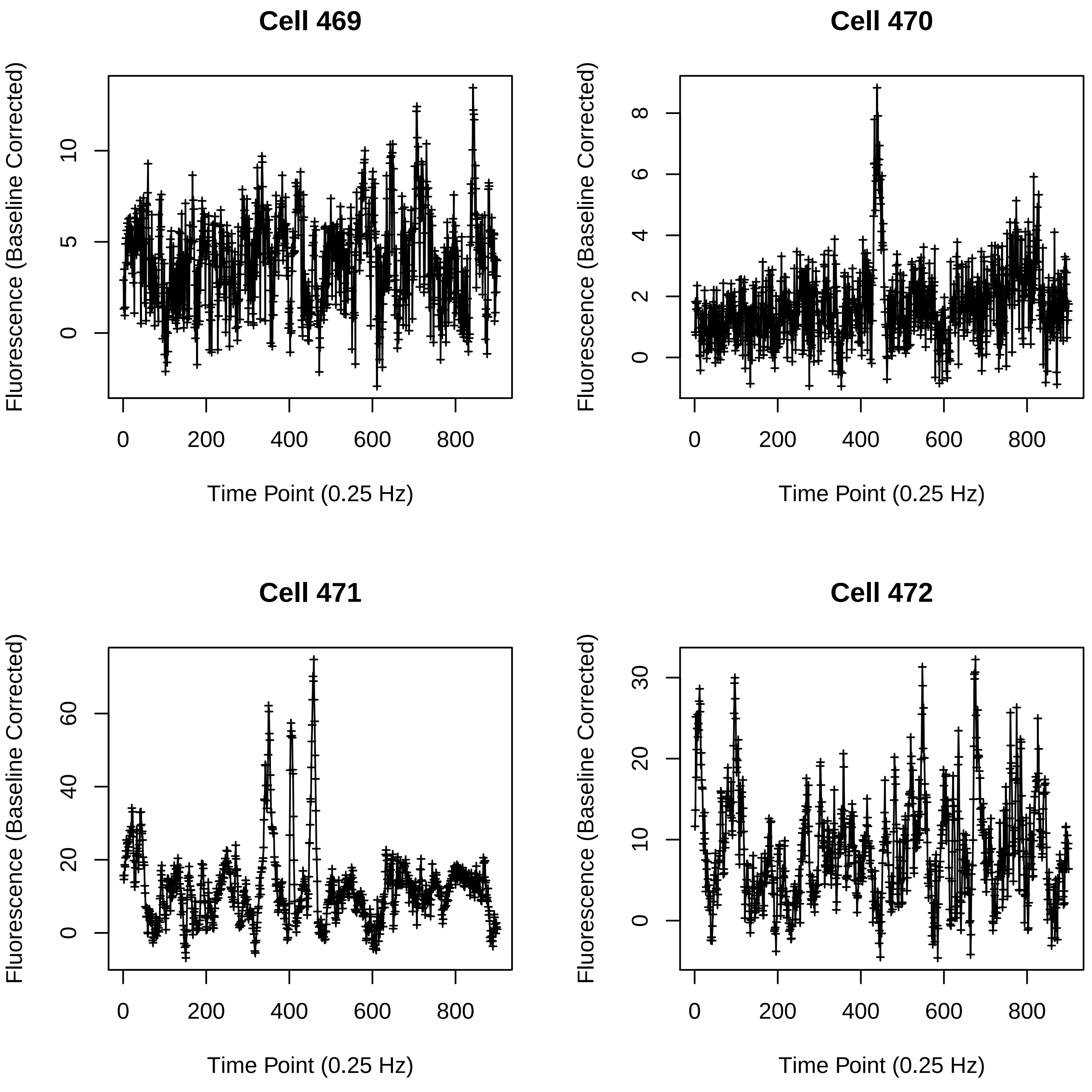
Cell 469
Cell 470
Cell 471
Cell 472
Fluorescence (Baseline Corrected)
Time Point (0.25 Hz)
0
200
400
600
800
0
5
10
2
4
6
8
20
40
60
30

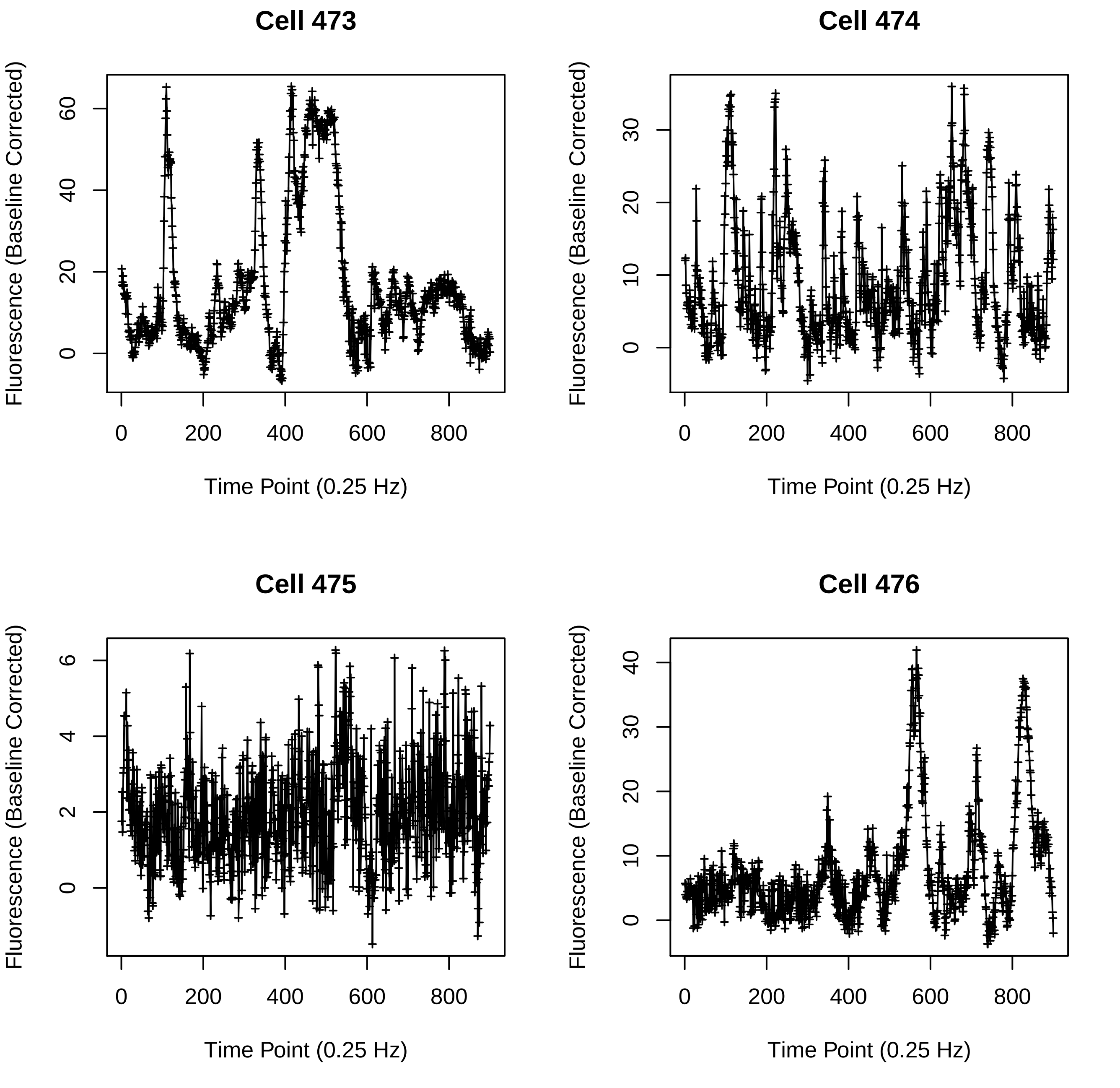
Cell 473
Cell 474
Cell 475
Cell 476
Fluorescence (Baseline Corrected)
Time Point (0.25 Hz)
0
200
400
600
800
-
0
20
40
60
0
10
20
30
0
2
4
6
0
10
20
30
40

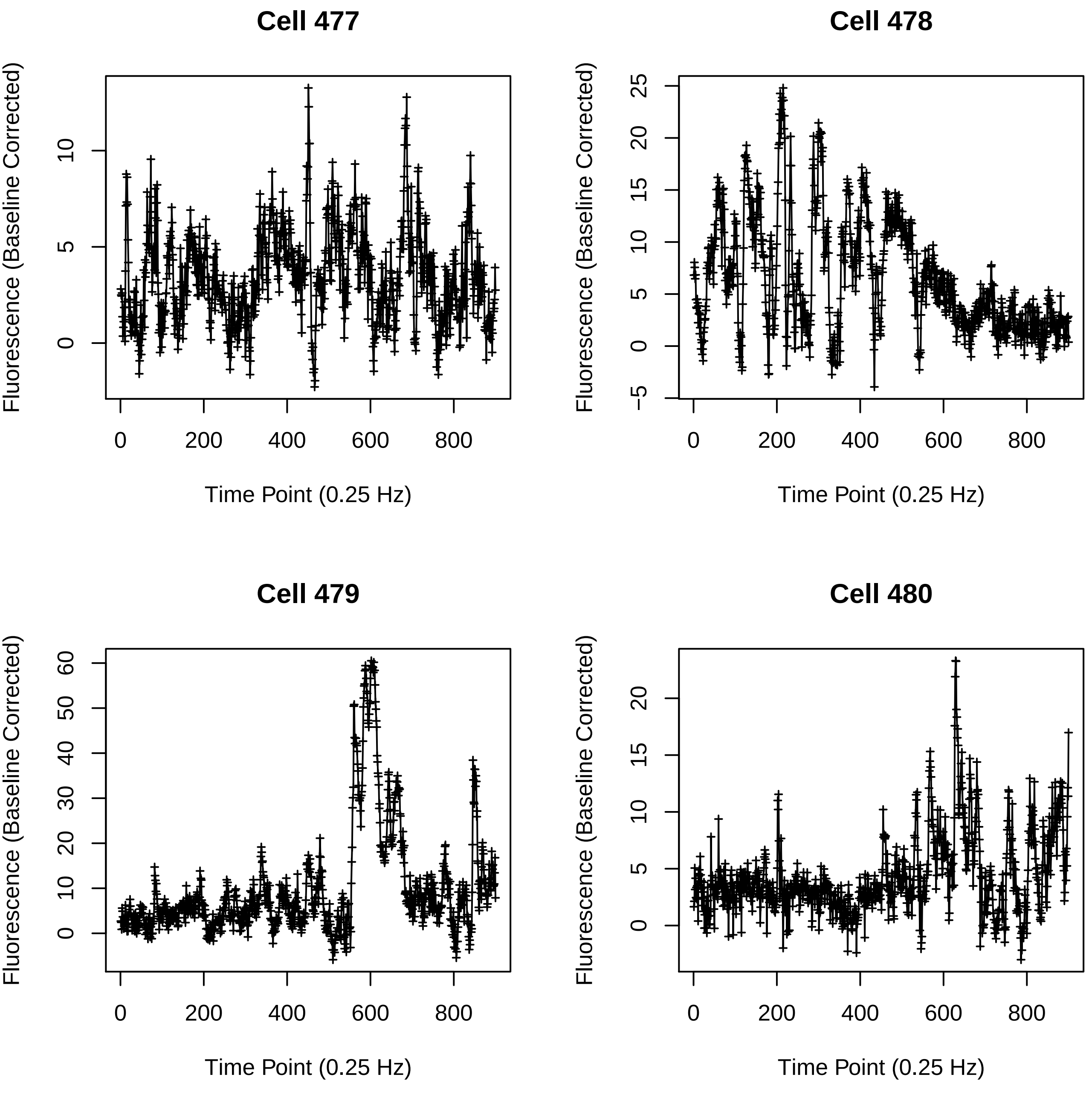
Cell 477
Cell 478
Cell 479
Cell 480
Fluorescence (Baseline Corrected)
Time Point (0.25 Hz)
0
200
400
600
800
−5
5
10
15
20
25
30
40
50
60

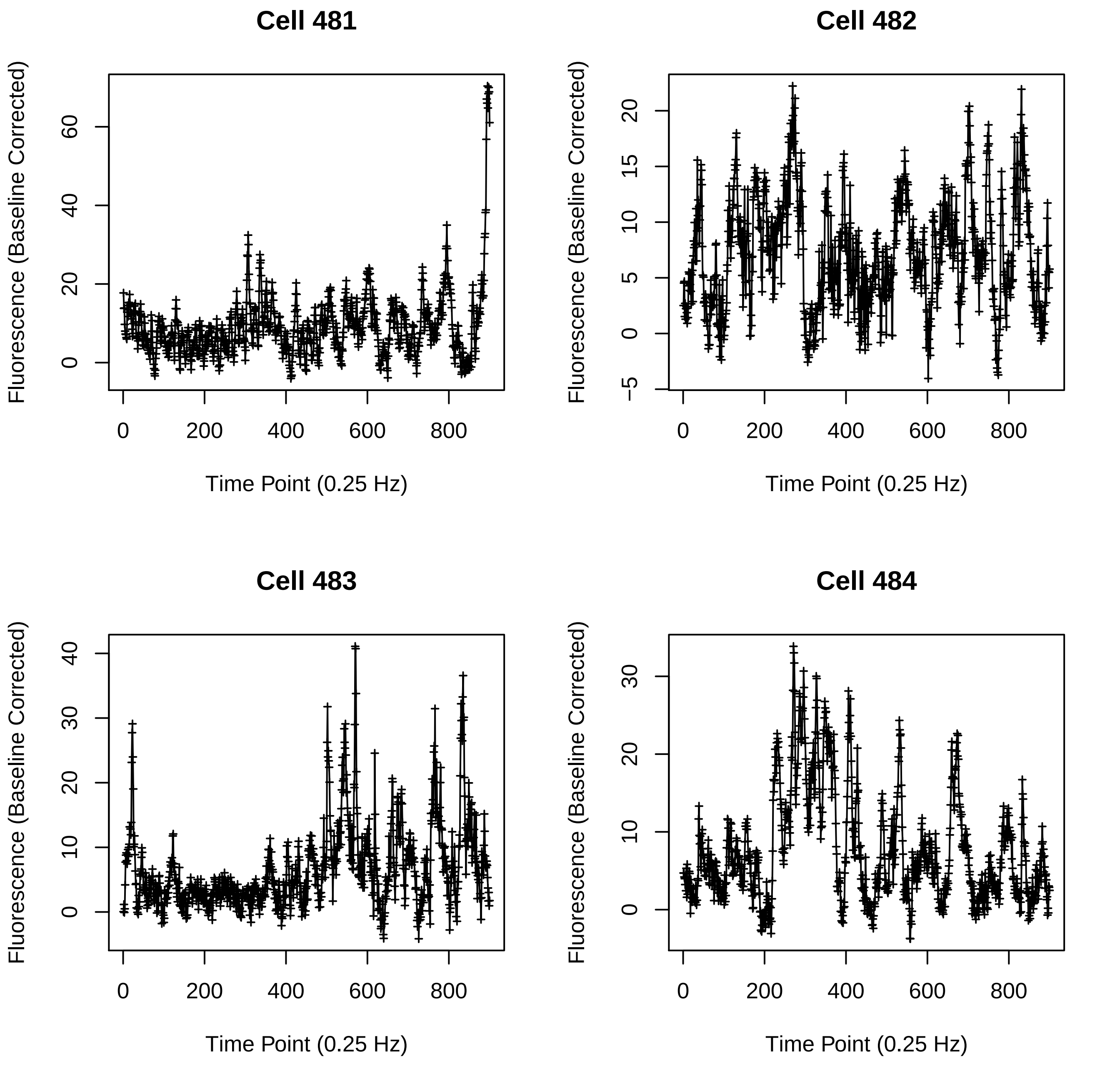
Cell 481
Cell 482
Cell 483
Cell 484
Fluorescence (Baseline Corrected)
Time Point (0.25 Hz)
0
200
400
600
800
20
40
60
-5
5
10
15
30

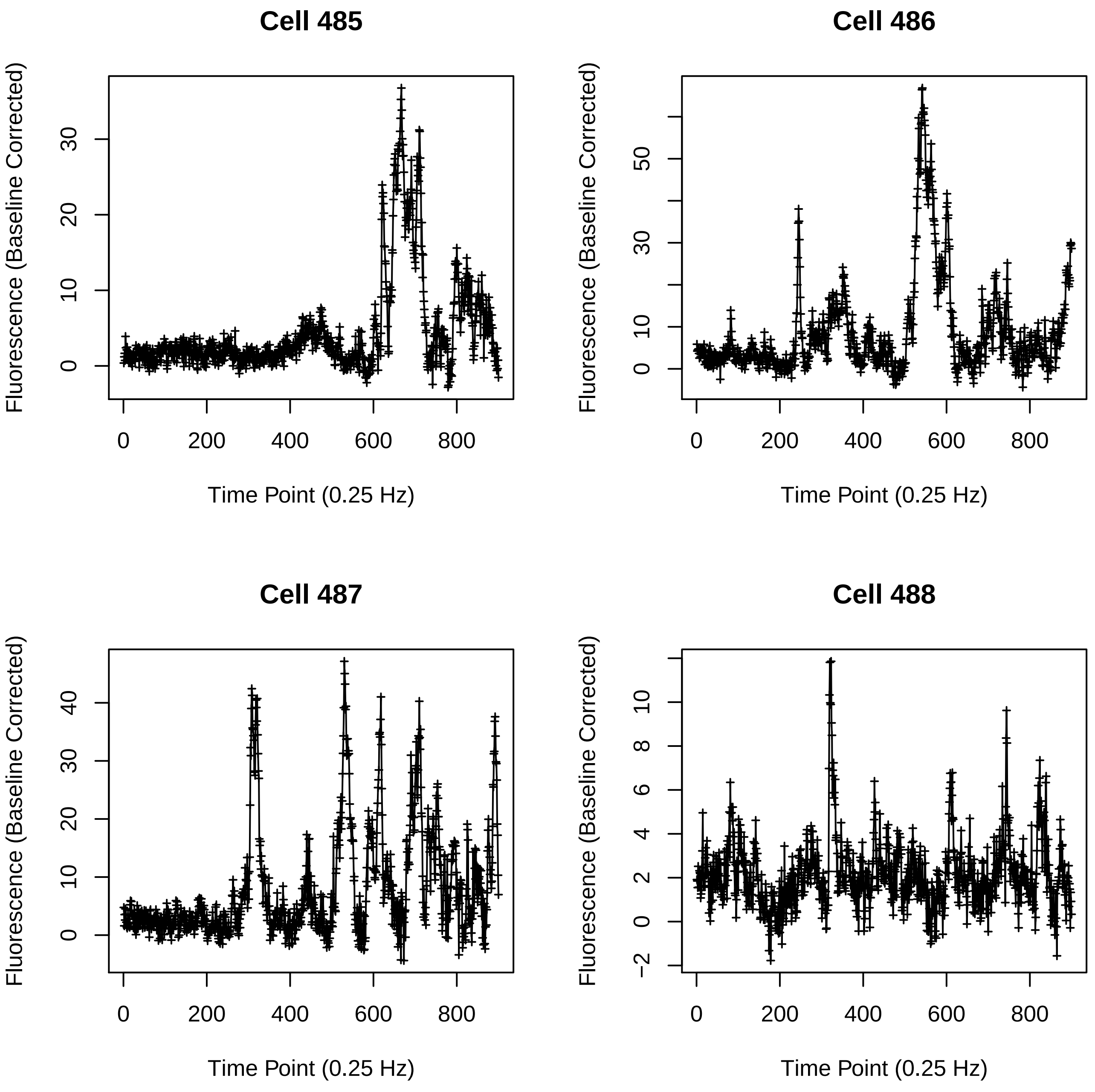
Cell 485
Cell 486
Cell 487
Cell 488
Fluorescence (Baseline Corrected)
Time Point (0.25 Hz)
0
200
400
600
800
0
10
20
30
40
50
-2
2
4
6
8

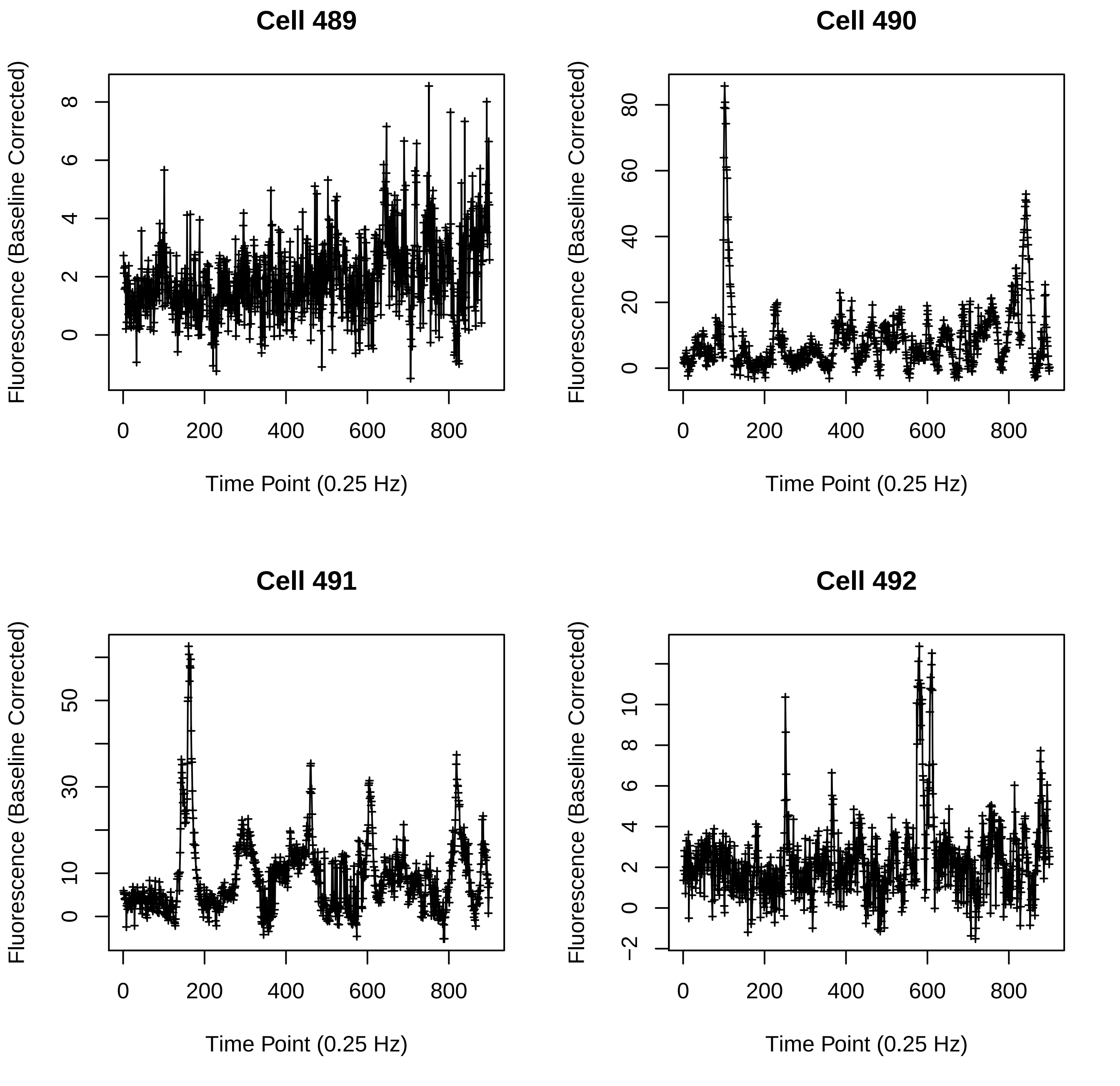

Cell 489
Cell 490
Cell 491
Cell 492
Fluorescence (Baseline Corrected)
Time Point (0.25 Hz)
0
200
400
600
800

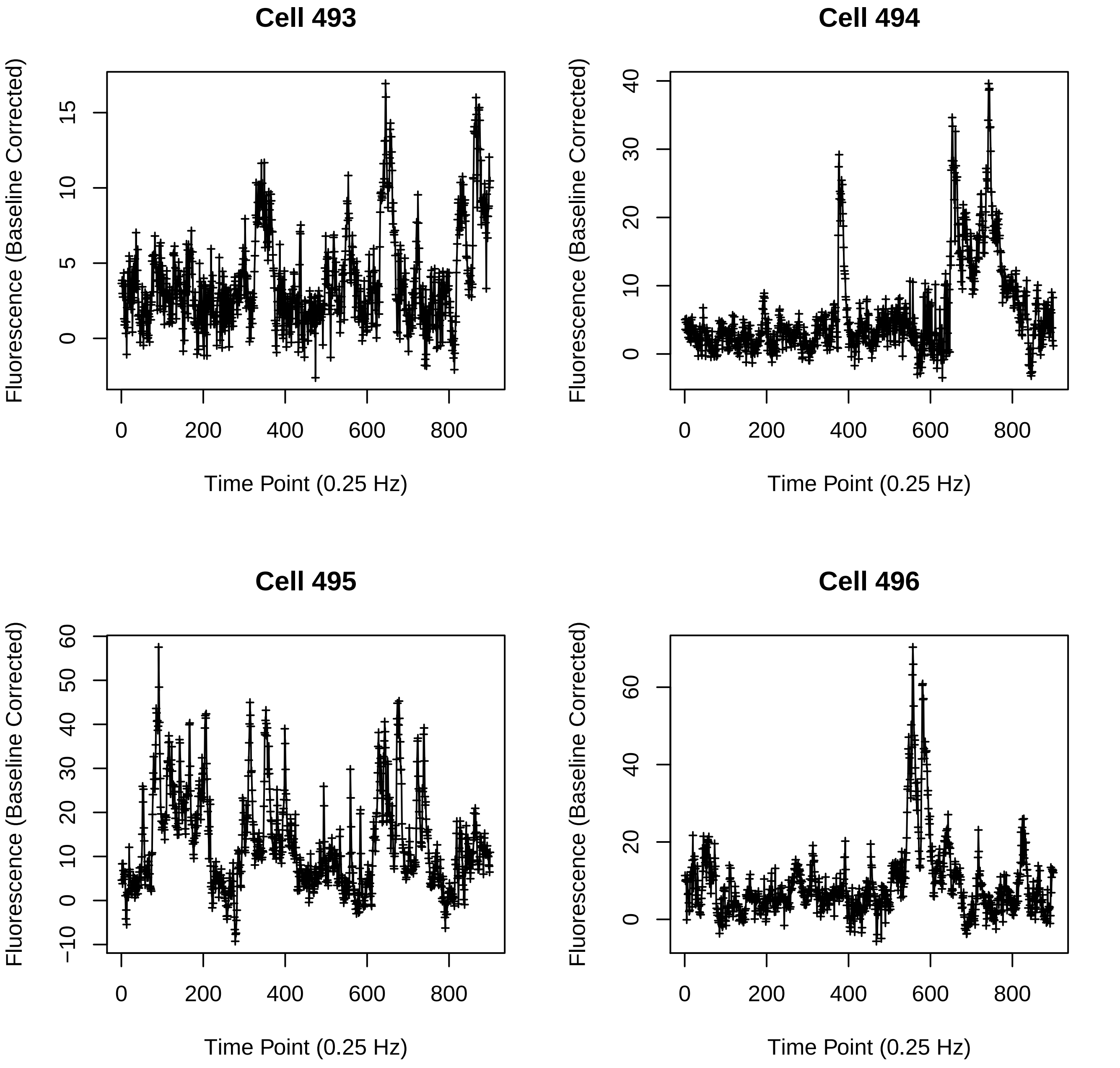
Cell 493
Fluorescence (Baseline Corrected)
Time Point (0.25 Hz)
0
5
10
15
0
200
400
600
800
Cell 494
Fluorescence (Baseline Corrected)
Time Point (0.25 Hz)
0
10
20
30
40
0
200
400
600
800
Cell 495
Fluorescence (Baseline Corrected)
Time Point (0.25 Hz)
−10
0
10
20
30
40
50
60
0
200
400
600
800
Cell 496
Fluorescence (Baseline Corrected)
Time Point (0.25 Hz)
0
20
40
60
0
200
400
600
800

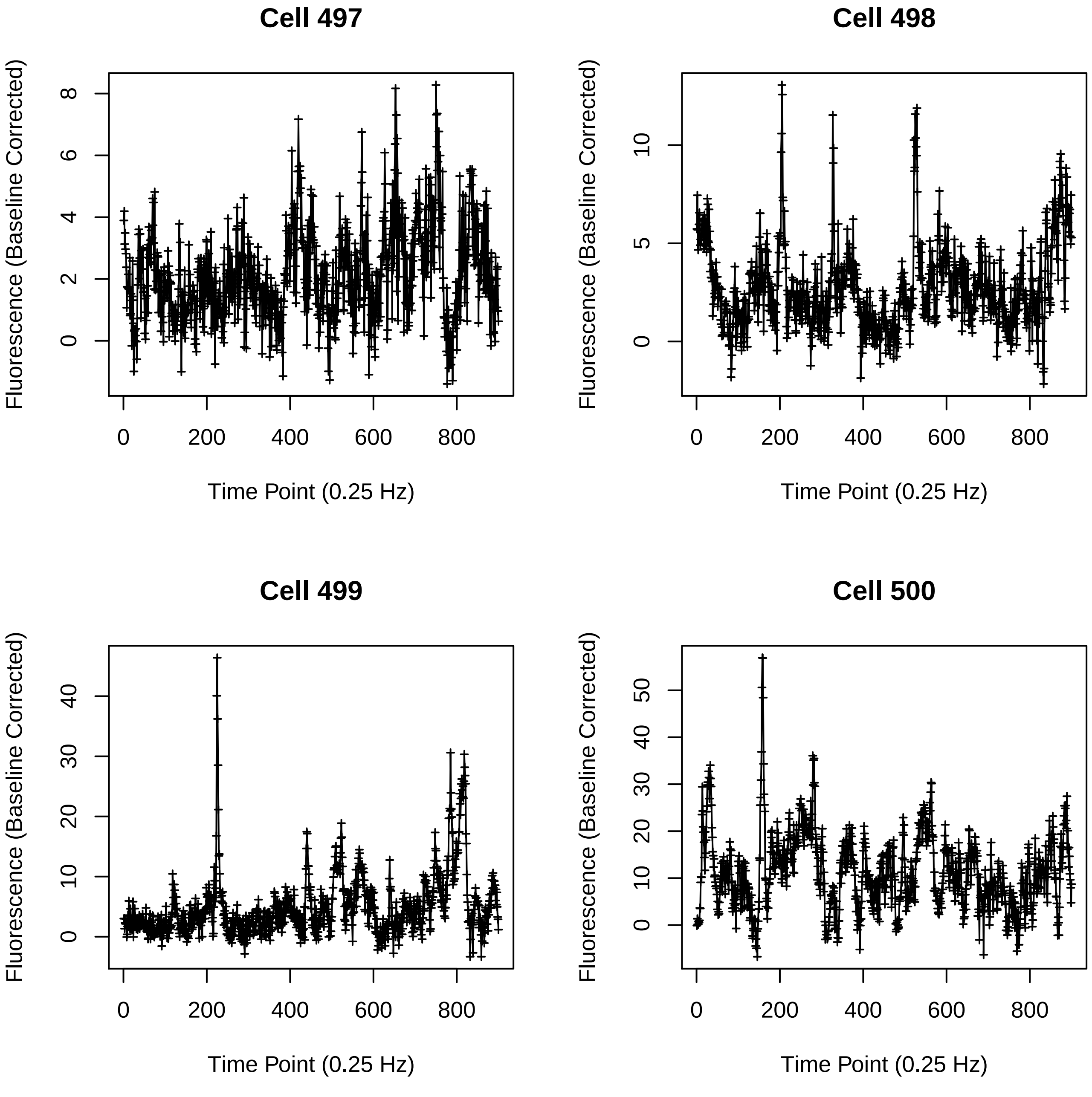
Cell 497
Cell 498
Cell 499
Cell 500
Fluorescence (Baseline Corrected)
Time Point (0.25 Hz)
0
200
400
600
800
2
4
6
8
5
10
20
30
40
50

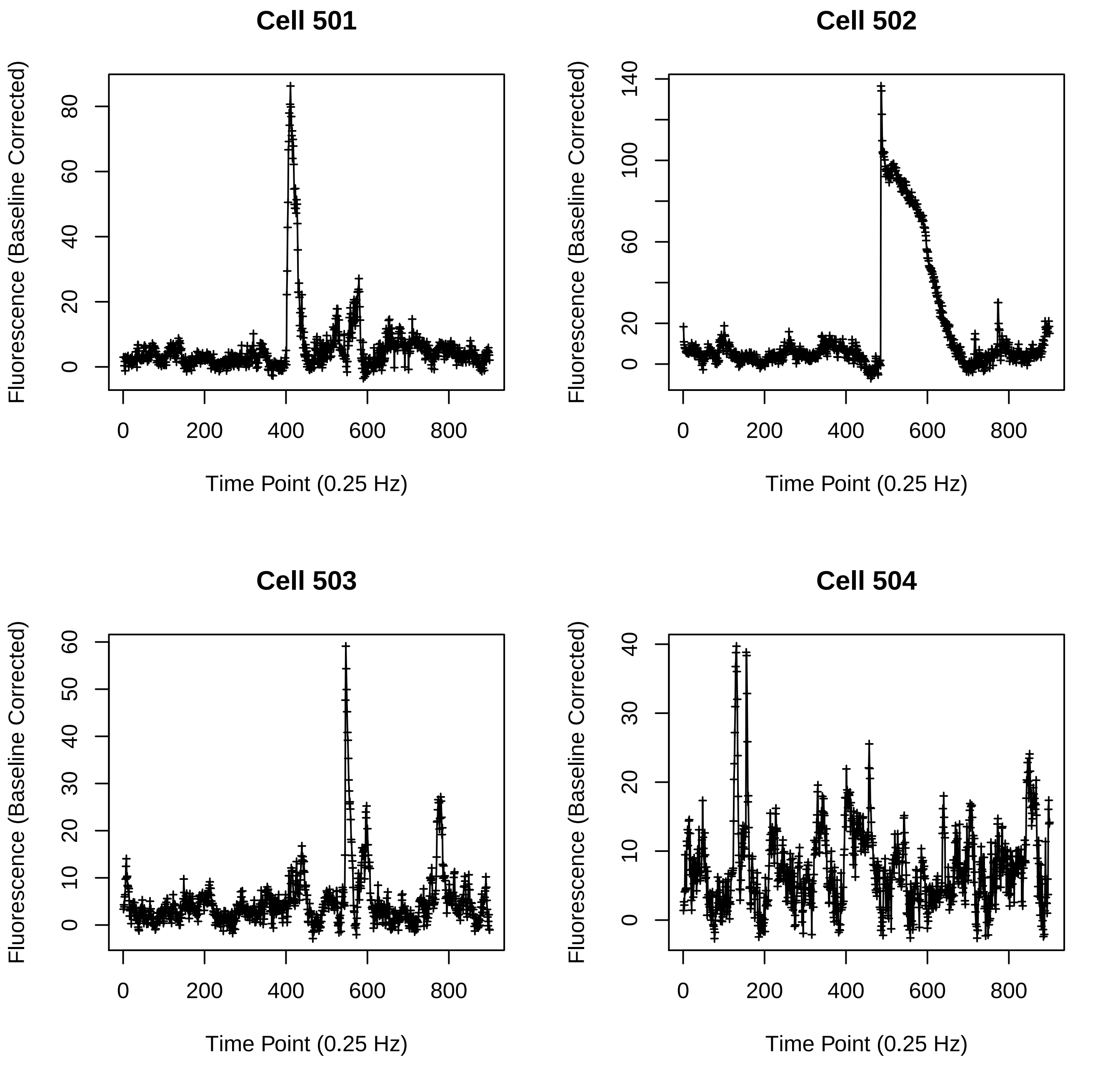
Cell 501
Cell 502
Cell 503
Cell 504
Fluorescence (Baseline Corrected)
Time Point (0.25 Hz)
0
20
40
60
80
100
140
0
10
20
30
40
50
60
200
400
600
800

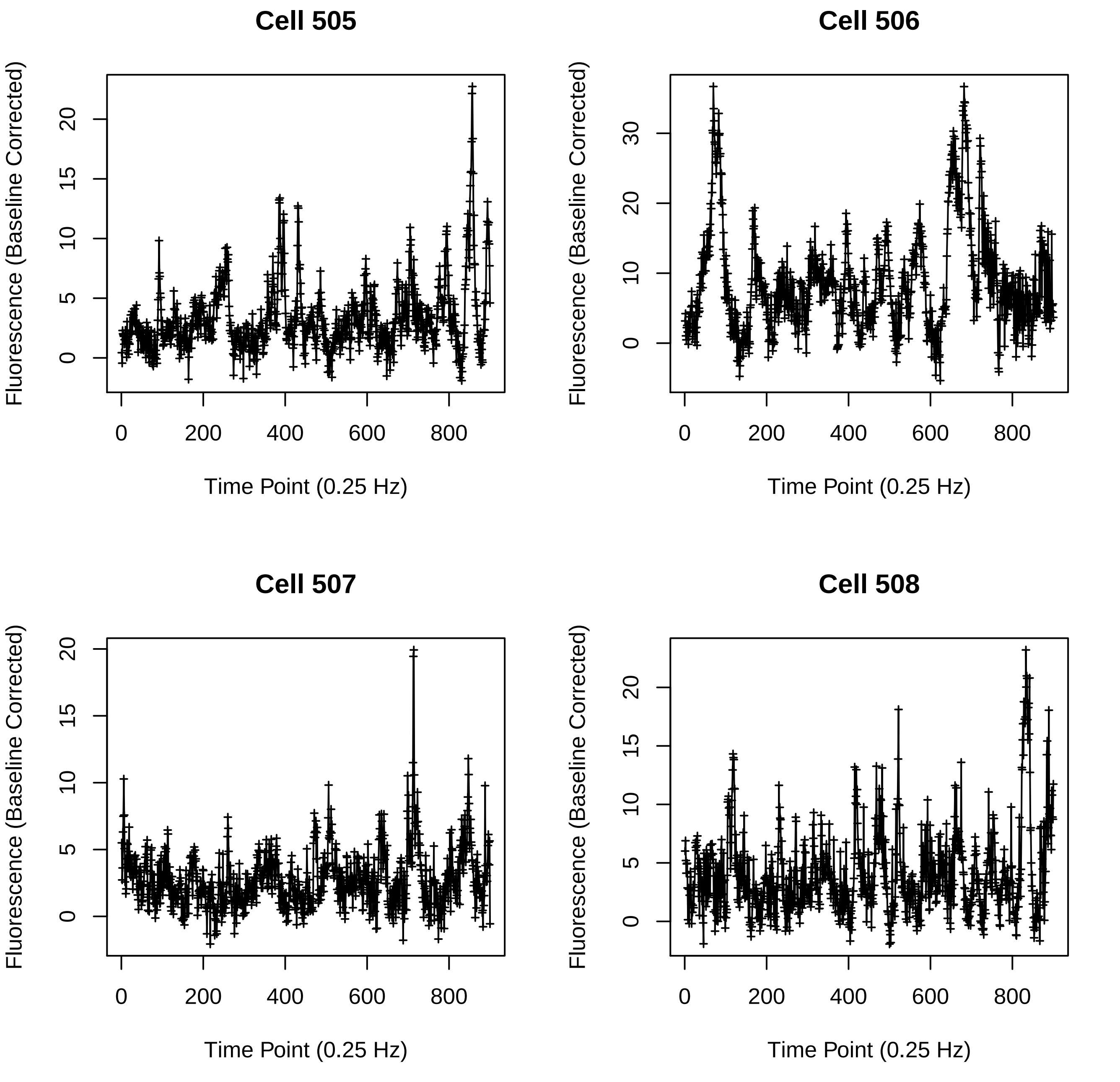
Cell 505
Fluorescence (Baseline Corrected)
0
5
10
15
20
0
200
400
600
800
Time Point (0.25 Hz)
Cell 506
Fluorescence (Baseline Corrected)
0
10
20
30
0
200
400
600
800
Time Point (0.25 Hz)
Cell 507
Fluorescence (Baseline Corrected)
0
5
10
15
20
0
200
400
600
800
Time Point (0.25 Hz)
Cell 508
Fluorescence (Baseline Corrected)
0
5
10
15
20
0
200
400
600
800
Time Point (0.25 Hz)

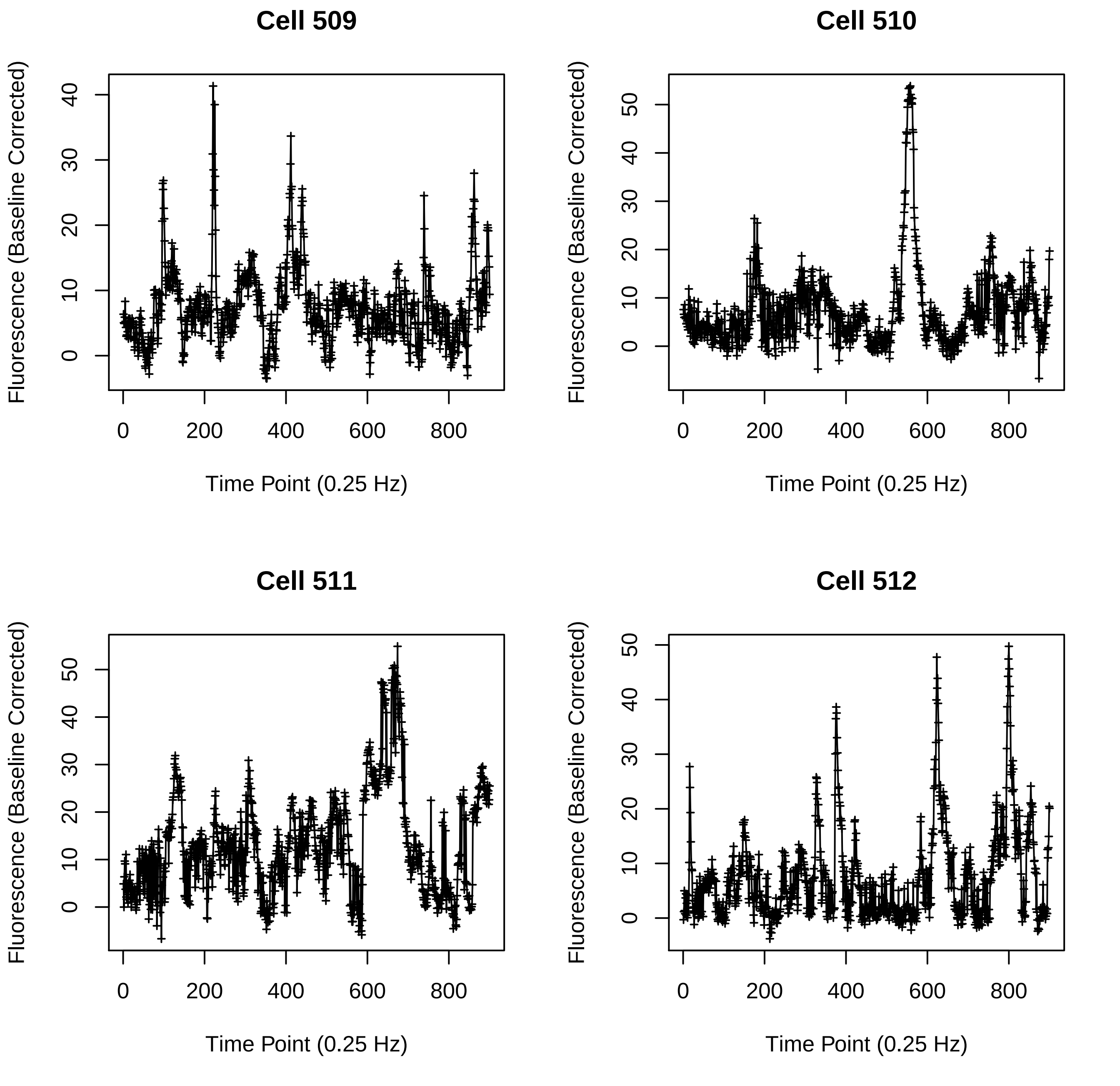
Cell 509
Cell 510
Cell 511
Cell 512
Fluorescence (Baseline Corrected)
Time Point (0.25 Hz)
0
10
20
30
40
50
0
200
400
600
800

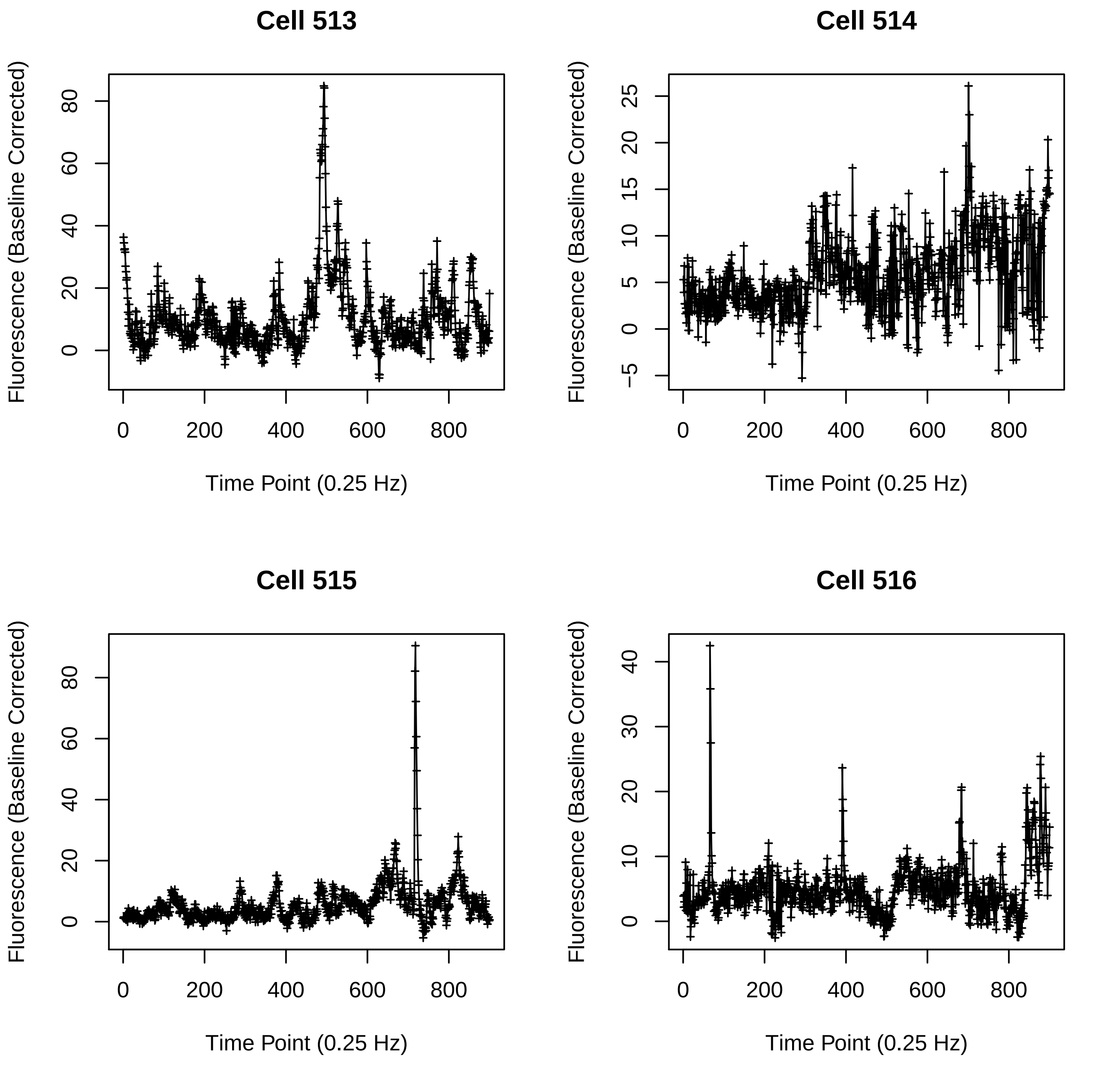
Cell 513
Cell 514
Cell 515
Cell 516
Fluorescence (Baseline Corrected)
Time Point (0.25 Hz)
0
200
400
600
800
20
40
60
80
-5
5
10
15
25
30

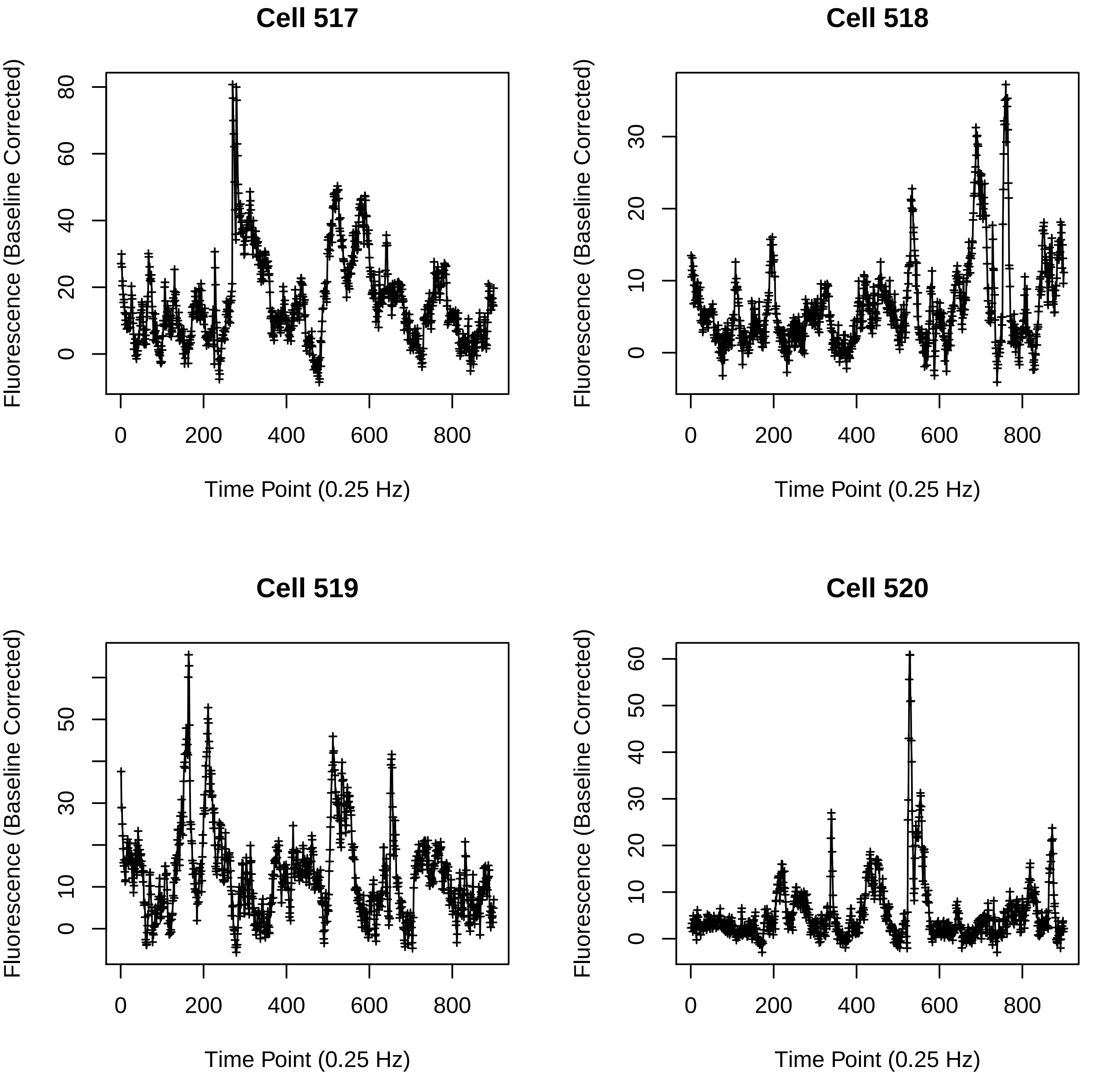
Cell 517
Cell 518
Cell 519
Cell 520
Fluorescence (Baseline Corrected)
Time Point (0.25 Hz)
0
200
400
600
800
20
40
60
80
10
30
50

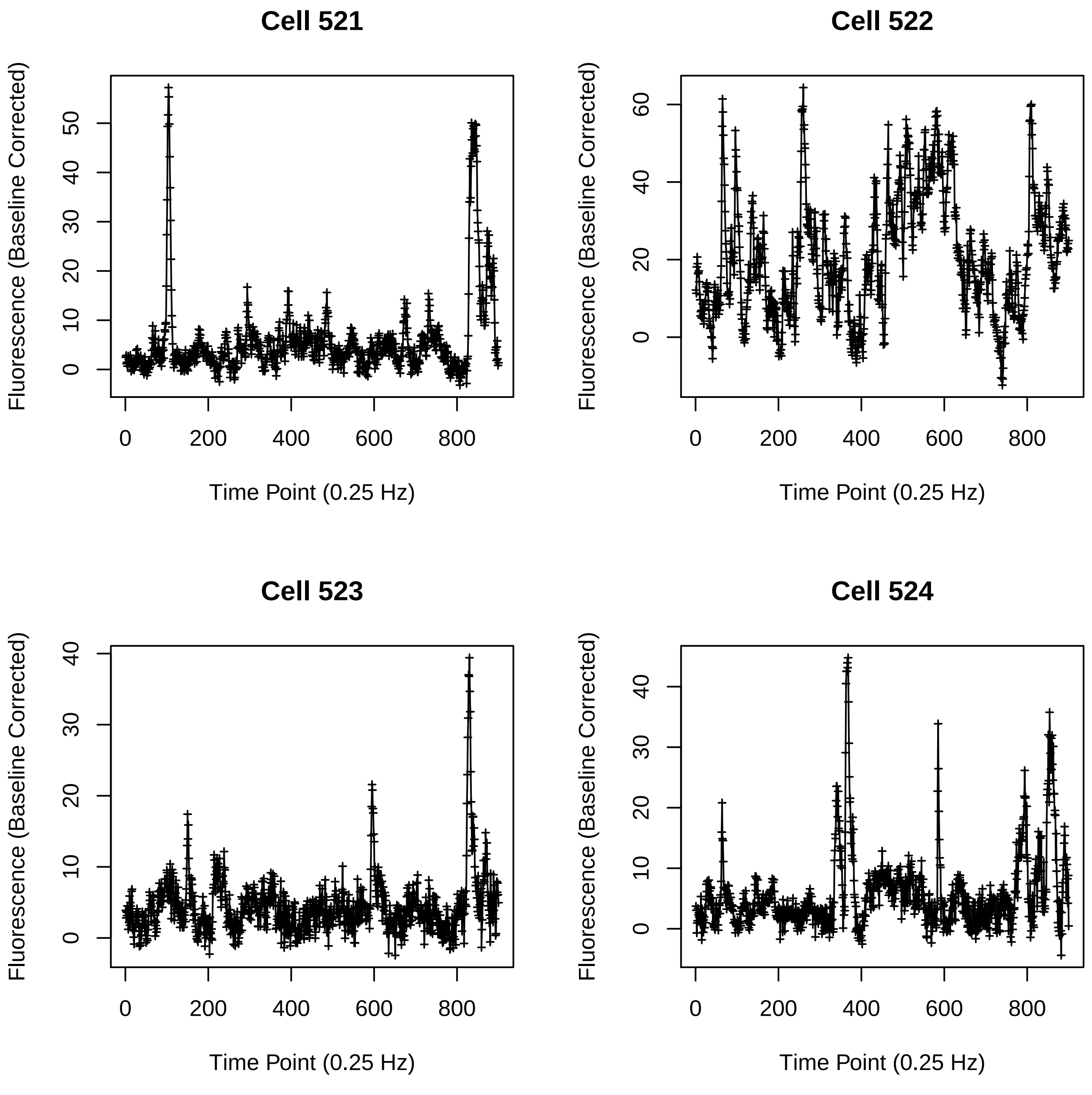

Cell 521
Cell 522
Cell 523
Cell 524
Fluorescence (Baseline Corrected)
Time Point (0.25 Hz)
0
200
400
600
800
-10
10
20
30
40
50
60

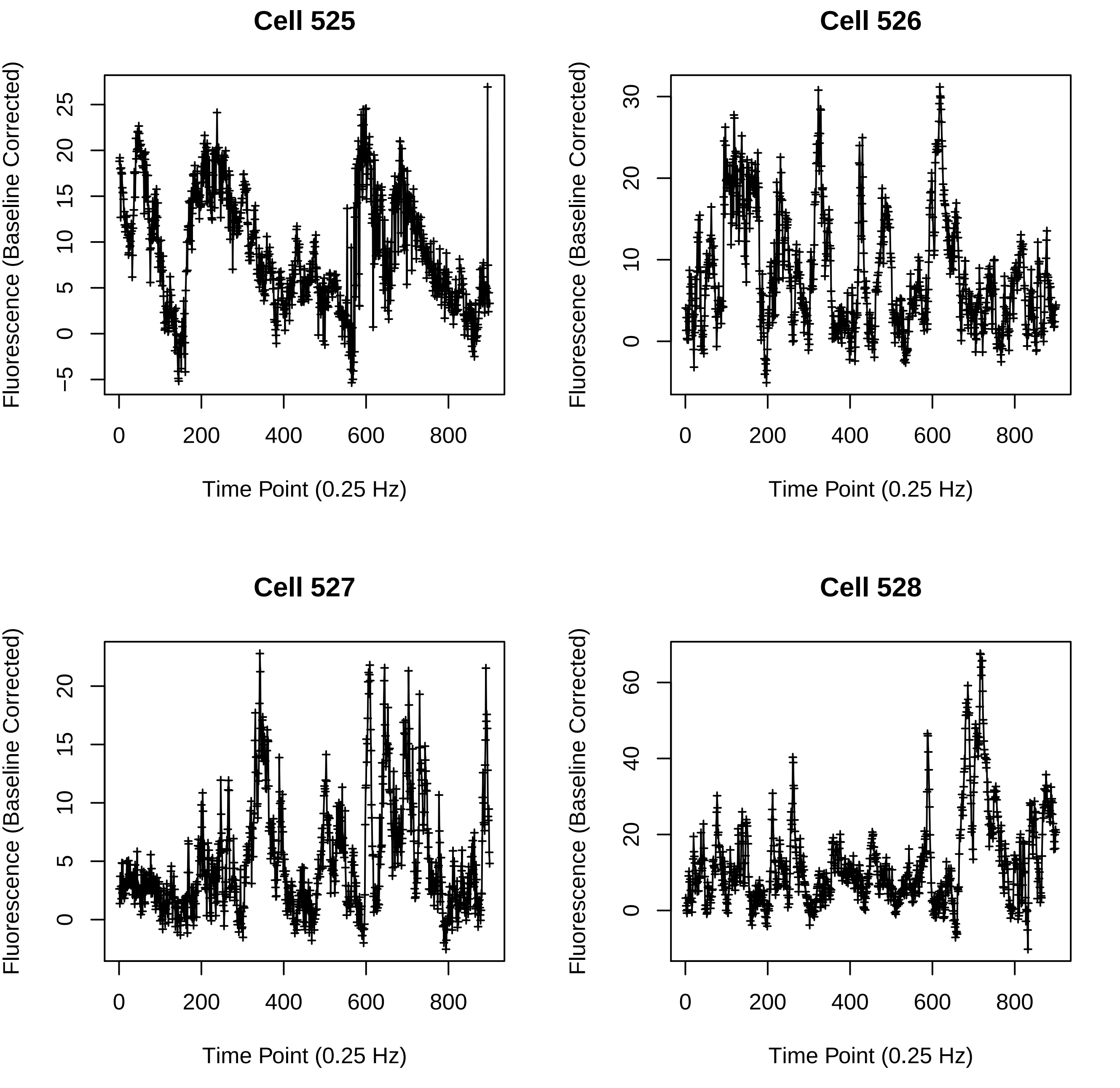
Cell 525
Fluorescence (Baseline Corrected)
−5
0
5
10
15
20
25
0
200
400
600
800
Time Point (0.25 Hz)
Cell 526
Fluorescence (Baseline Corrected)
0
10
20
30
0
200
400
600
800
Time Point (0.25 Hz)
Cell 527
Fluorescence (Baseline Corrected)
0
5
10
15
20
0
200
400
600
800
Time Point (0.25 Hz)
Cell 528
Fluorescence (Baseline Corrected)
0
20
40
60
0
200
400
600
800
Time Point (0.25 Hz)

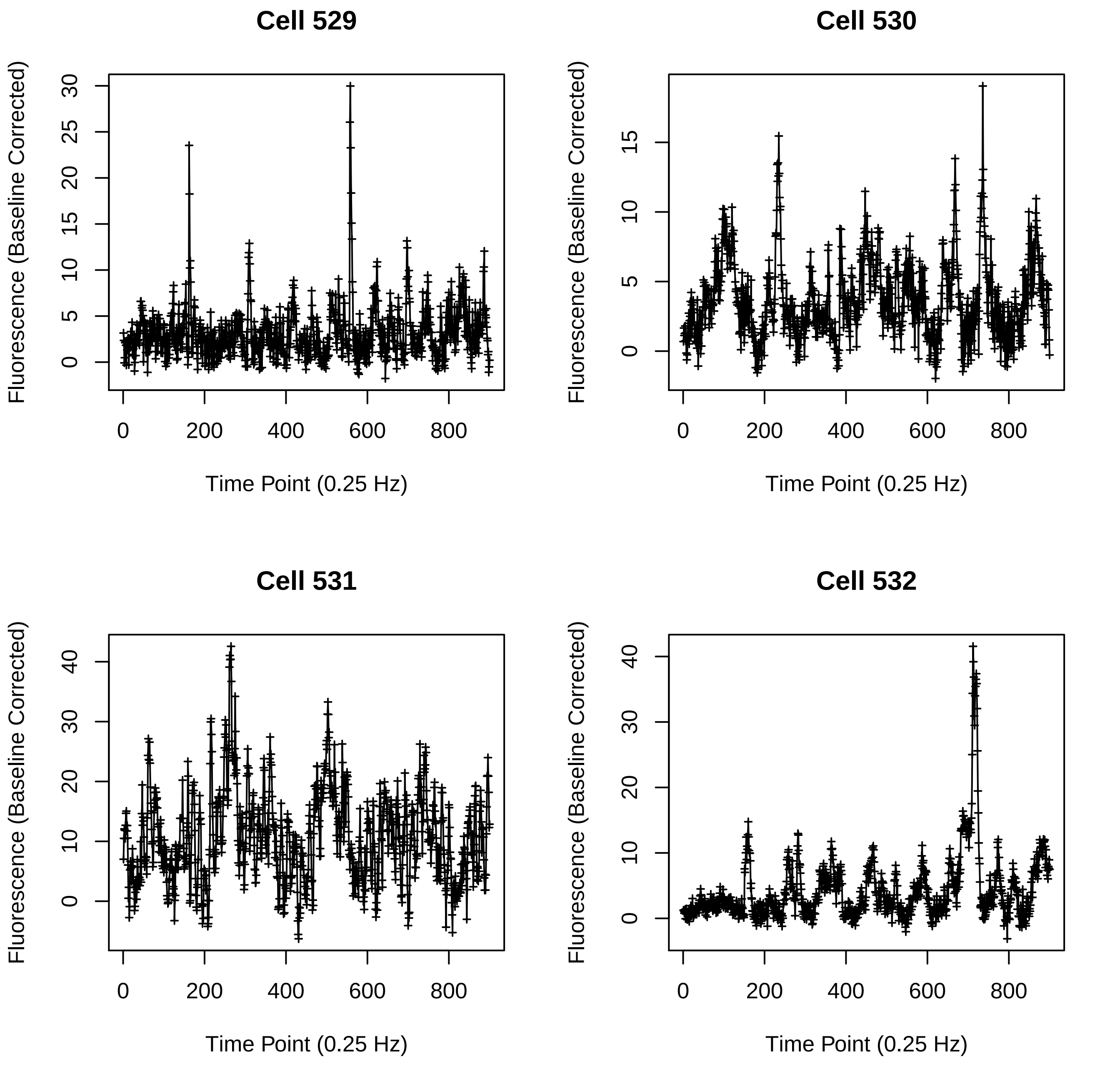
Cell 529
Cell 530
Cell 531
Cell 532
Fluorescence (Baseline Corrected)
Time Point (0.25 Hz)
0
200
400
600
800
0
5
10
15
20
25
30
40

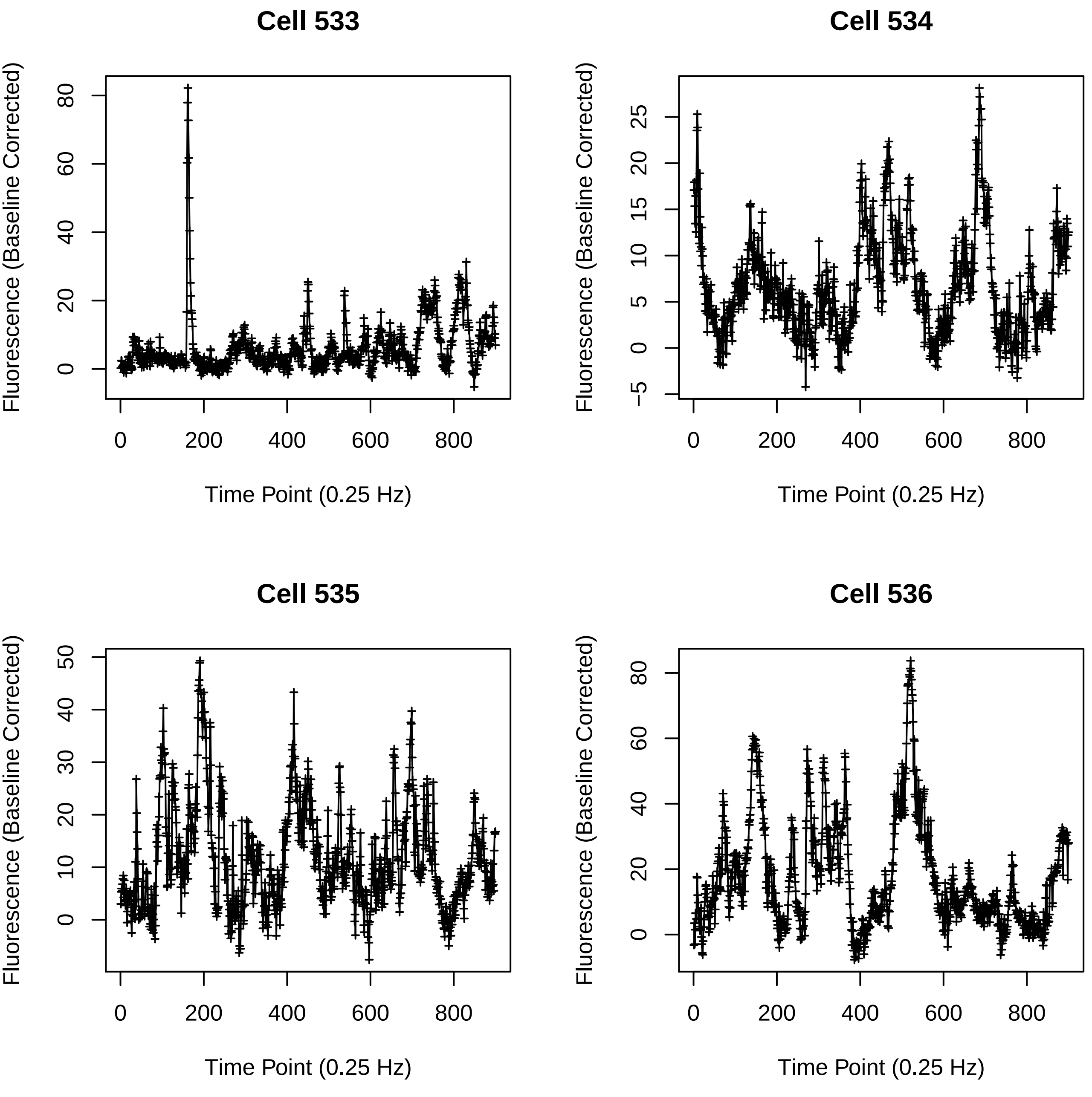
Cell 533
Cell 534
Cell 535
Cell 536
Fluorescence (Baseline Corrected)
Time Point (0.25 Hz)
0
200
400
600
800
20
40
60
80
-5
5
10
15
25
30
50

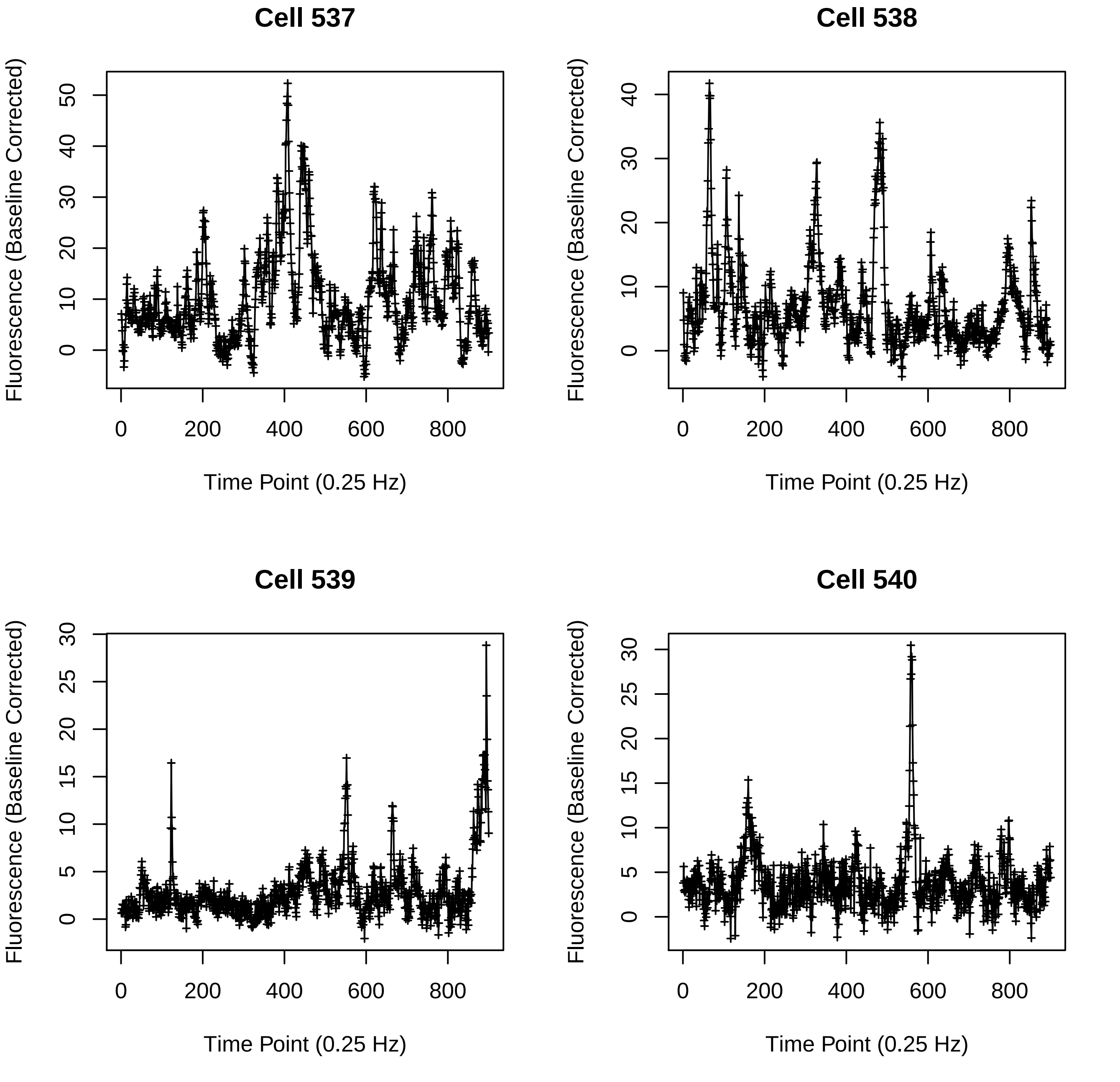
Cell 537
Cell 538
Cell 539
Cell 540
Fluorescence (Baseline Corrected)
Time Point (0.25 Hz)
0
200
400
600
800
0
10
20
30
40
50
5
15
25

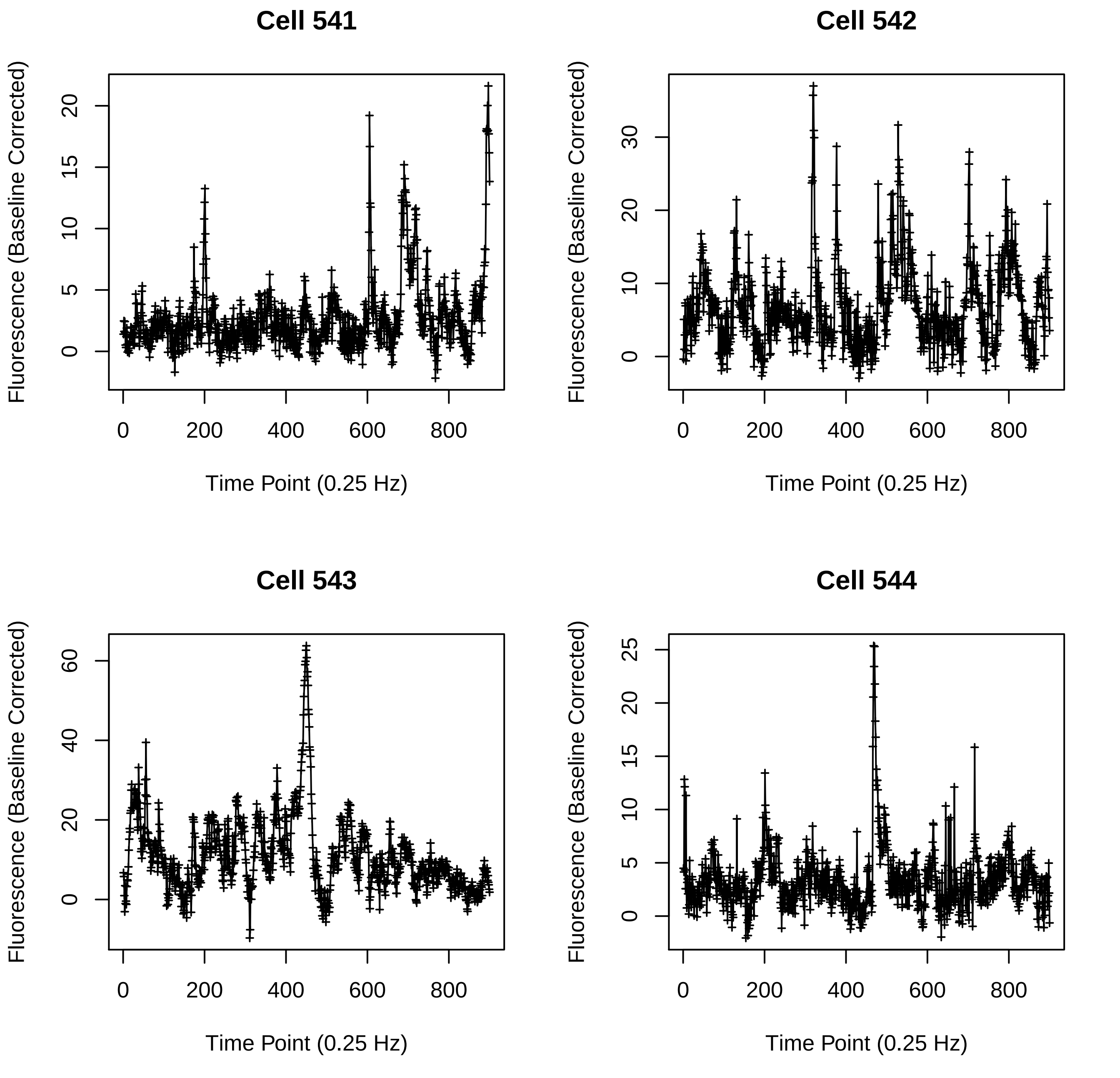
Cell 541
Cell 542
Cell 543
Cell 544
Fluorescence (Baseline Corrected)
Time Point (0.25 Hz)
0
200
400
600
800

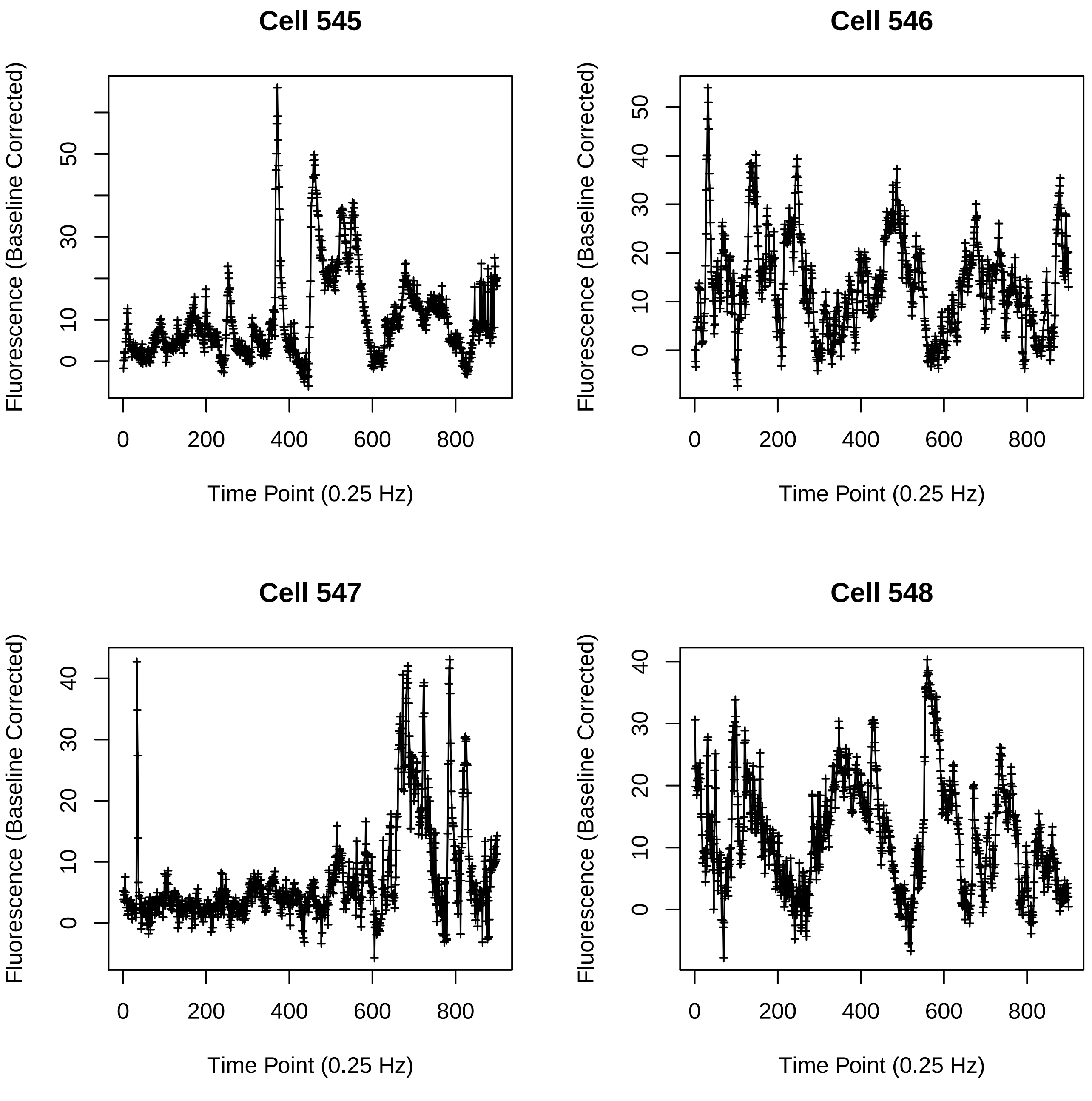

Cell 545
Cell 546
Cell 547
Cell 548
Fluorescence (Baseline Corrected)
Time Point (0.25 Hz)
0
200
400
600
800
10
30
50
20
40

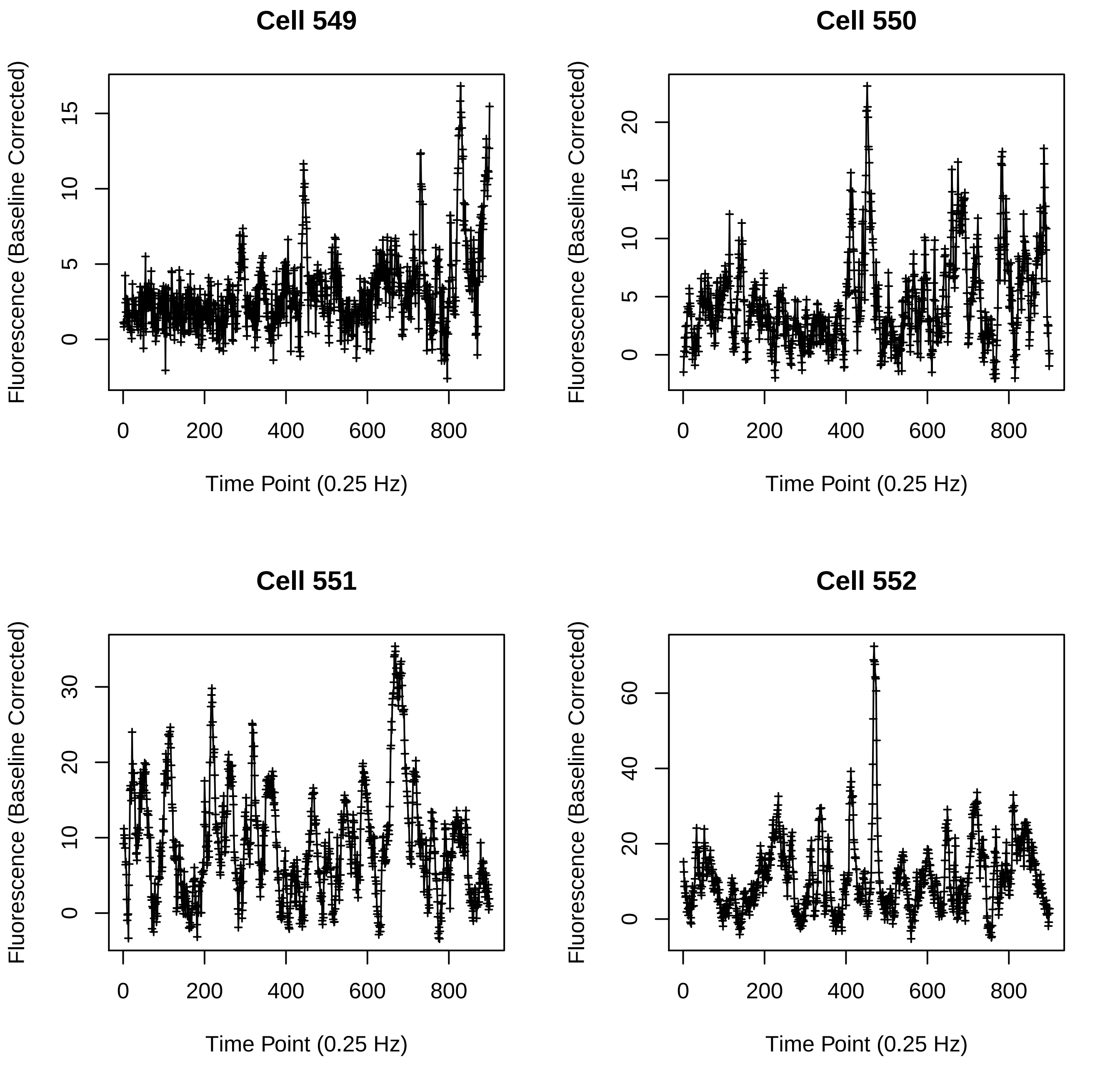

Cell 549
Cell 550
Cell 551
Cell 552
Fluorescence (Baseline Corrected)
Time Point (0.25 Hz)
0
200
400
600
800
0
5
10
15
20
30
40
60

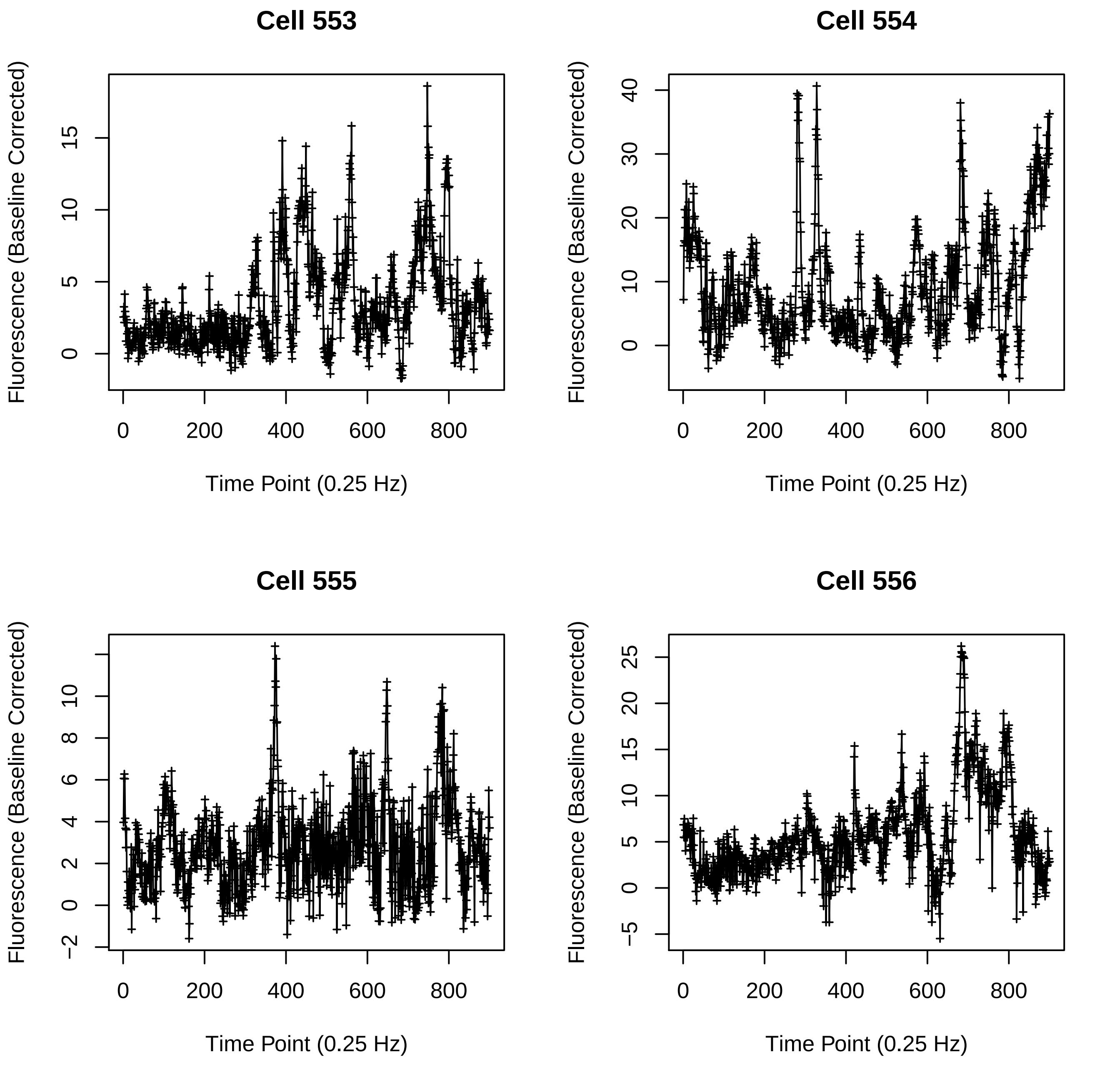
Cell 553
Fluorescence (Baseline Corrected)
0
5
10
15
0
200
400
600
800
Time Point (0.25 Hz)
Cell 554
Fluorescence (Baseline Corrected)
0
10
20
30
40
0
200
400
600
800
Time Point (0.25 Hz)
Cell 555
Fluorescence (Baseline Corrected)
−2
0
2
4
6
8
10
0
200
400
600
800
Time Point (0.25 Hz)
Cell 556
Fluorescence (Baseline Corrected)
−5
0
5
10
15
20
25
0
200
400
600
800
Time Point (0.25 Hz)

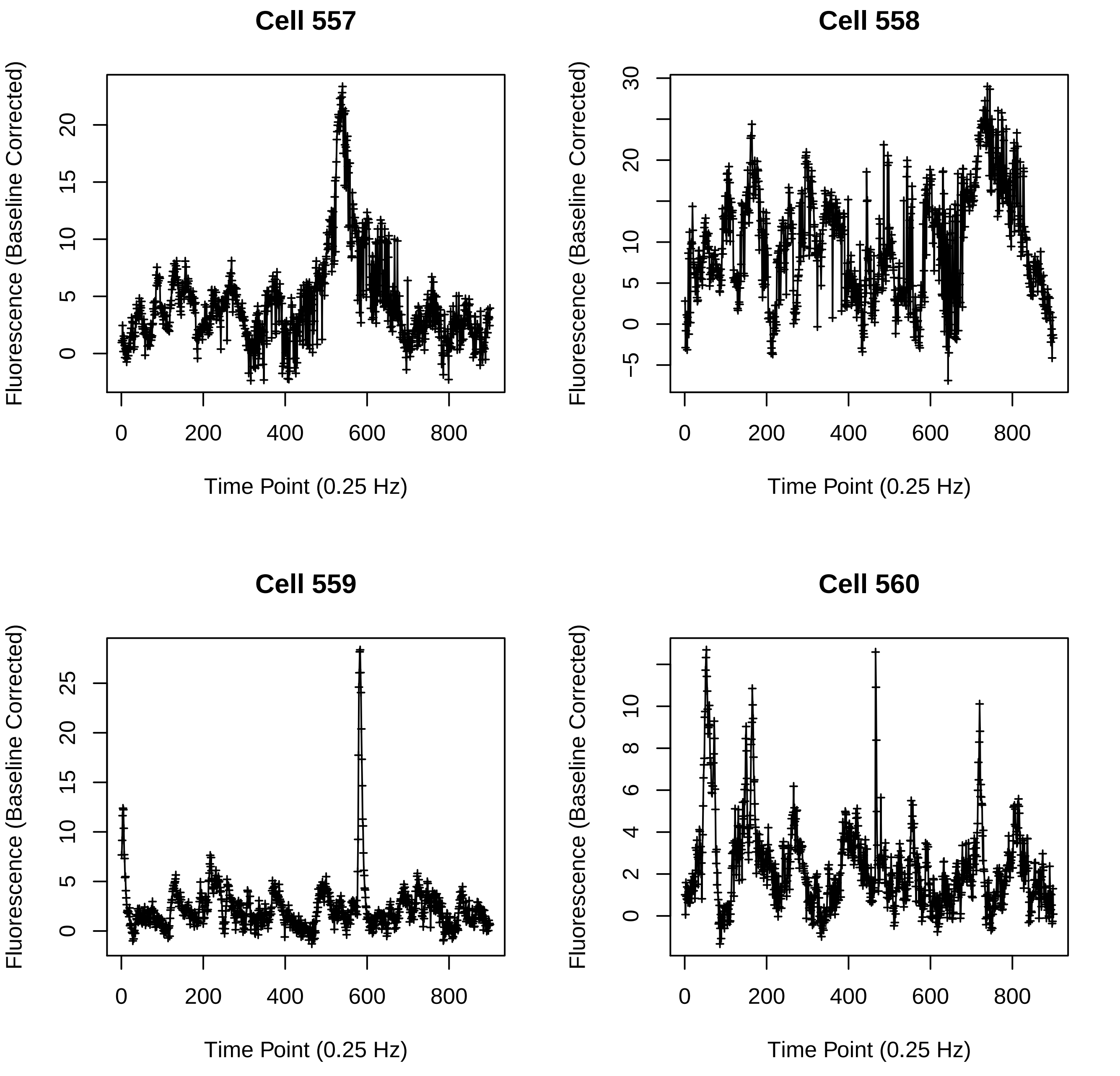
Cell 557
Fluorescence (Baseline Corrected)
Time Point (0.25 Hz)
Cell 558
Fluorescence (Baseline Corrected)
Time Point (0.25 Hz)
Cell 559
Fluorescence (Baseline Corrected)
Time Point (0.25 Hz)
Cell 560
Fluorescence (Baseline Corrected)
Time Point (0.25 Hz)

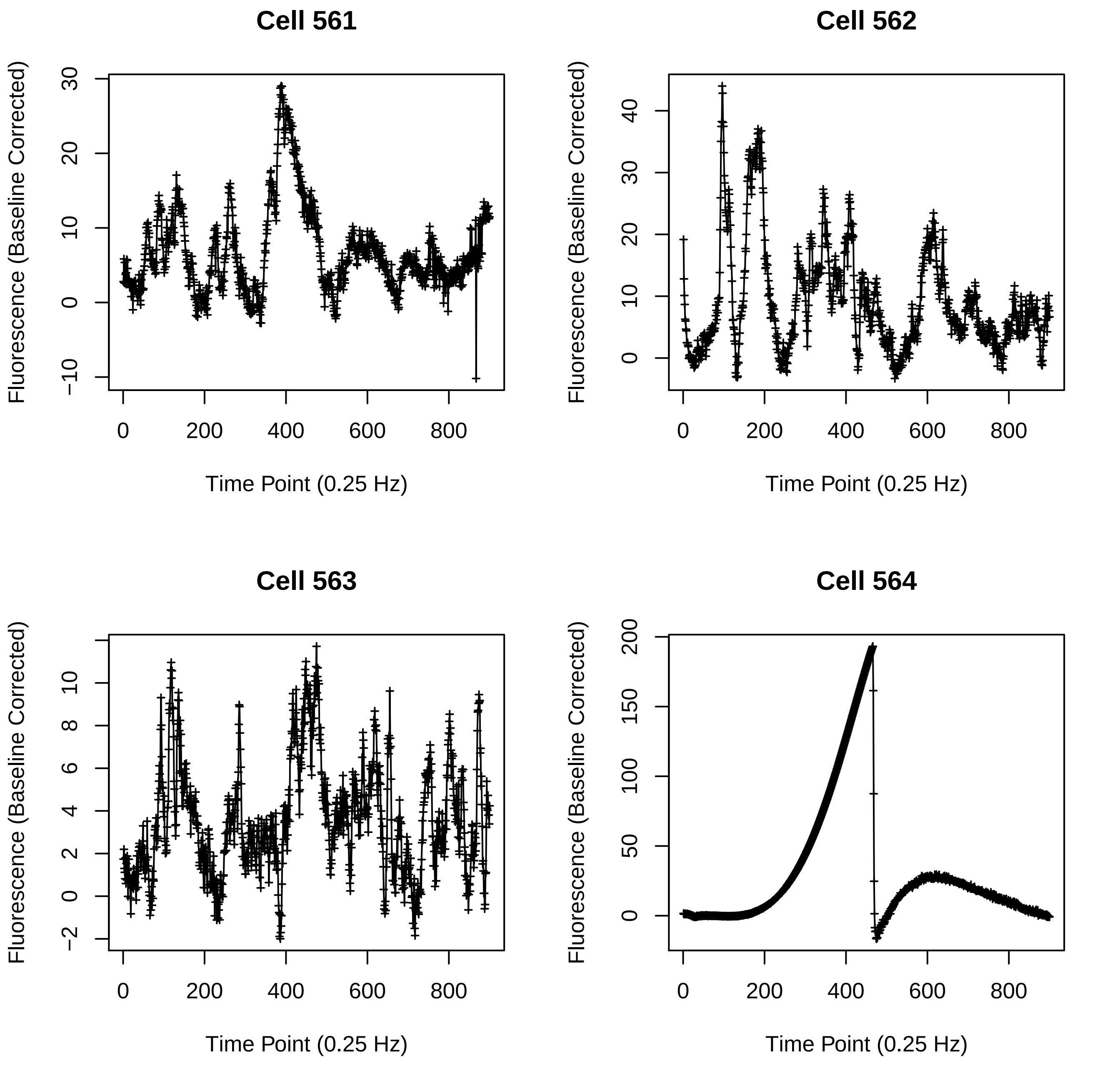
Cell 561
Cell 562
Cell 563
Cell 564
Fluorescence (Baseline Corrected)
Time Point (0.25 Hz)
−10
0
10
20
30
40
−2
2
4
6
8
50
100
150
200
0
200
400
600
800

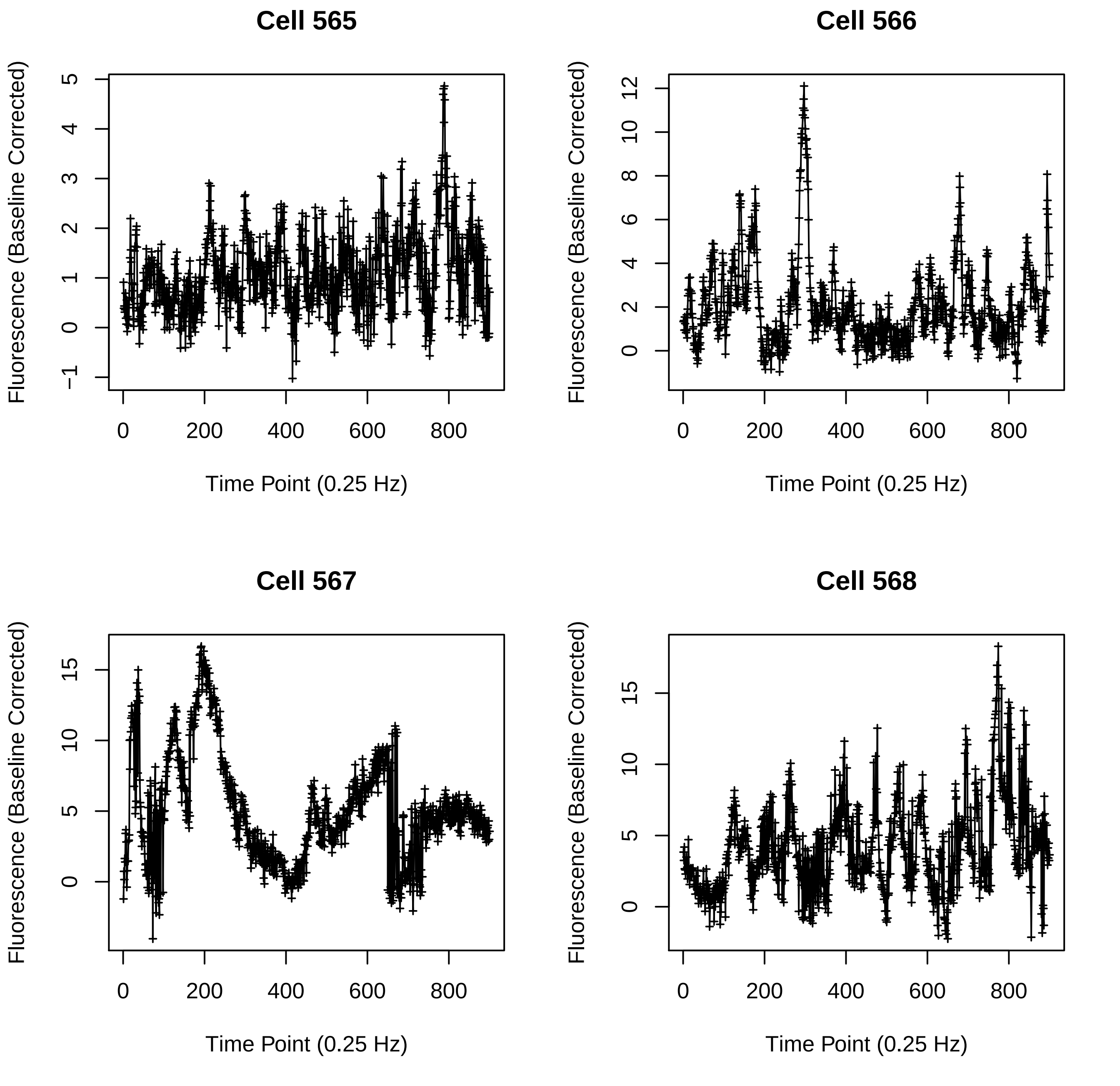
Cell 565
Cell 566
Cell 567
Cell 568
Fluorescence (Baseline Corrected)
Time Point (0.25 Hz)
−1
0
1
2
3
4
5
6
8
10
12
15
200
400
600
800

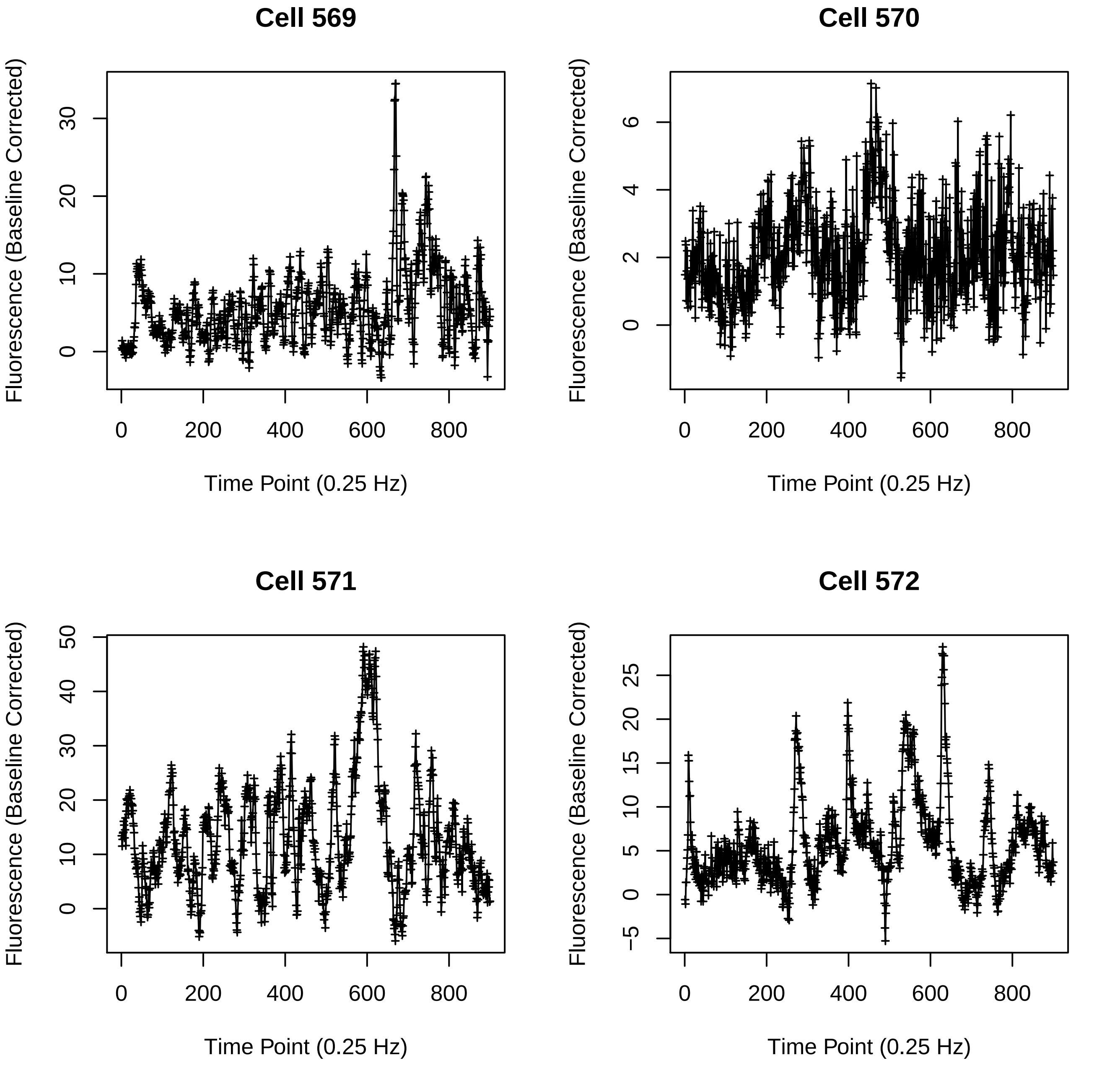
Cell 569
Cell 570
Cell 571
Cell 572
Fluorescence (Baseline Corrected)
Time Point (0.25 Hz)
0
200
400
600
800
0
10
20
30
0
2
4
6
0
10
20
30
40
50
−5
0
5
10
15
20
25

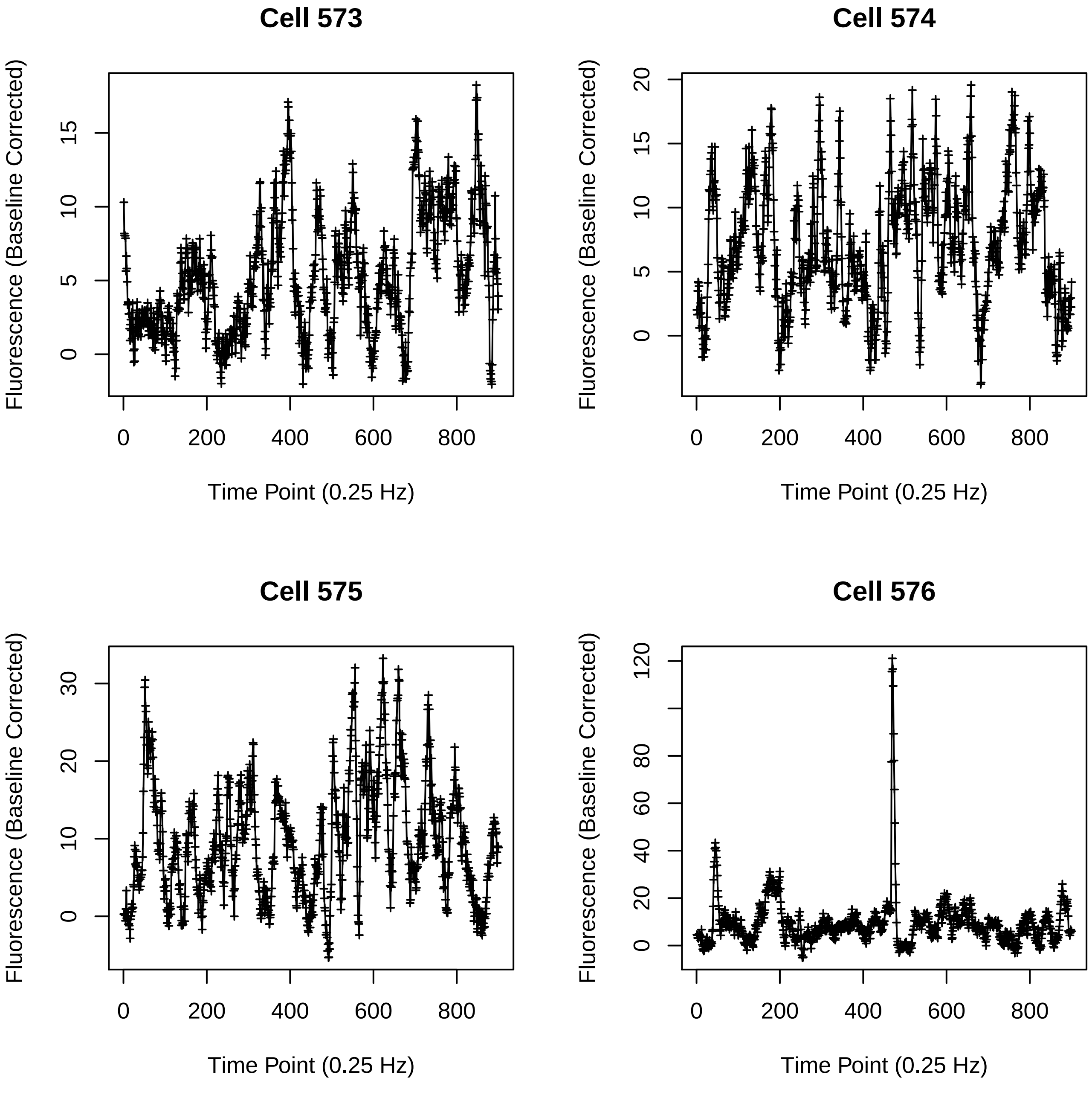
Cell 573
Cell 574
Cell 575
Cell 576
Fluorescence (Baseline Corrected)
Time Point (0.25 Hz)
0
200
400
600
800
0
5
10
15
20
30
40
60
80
120

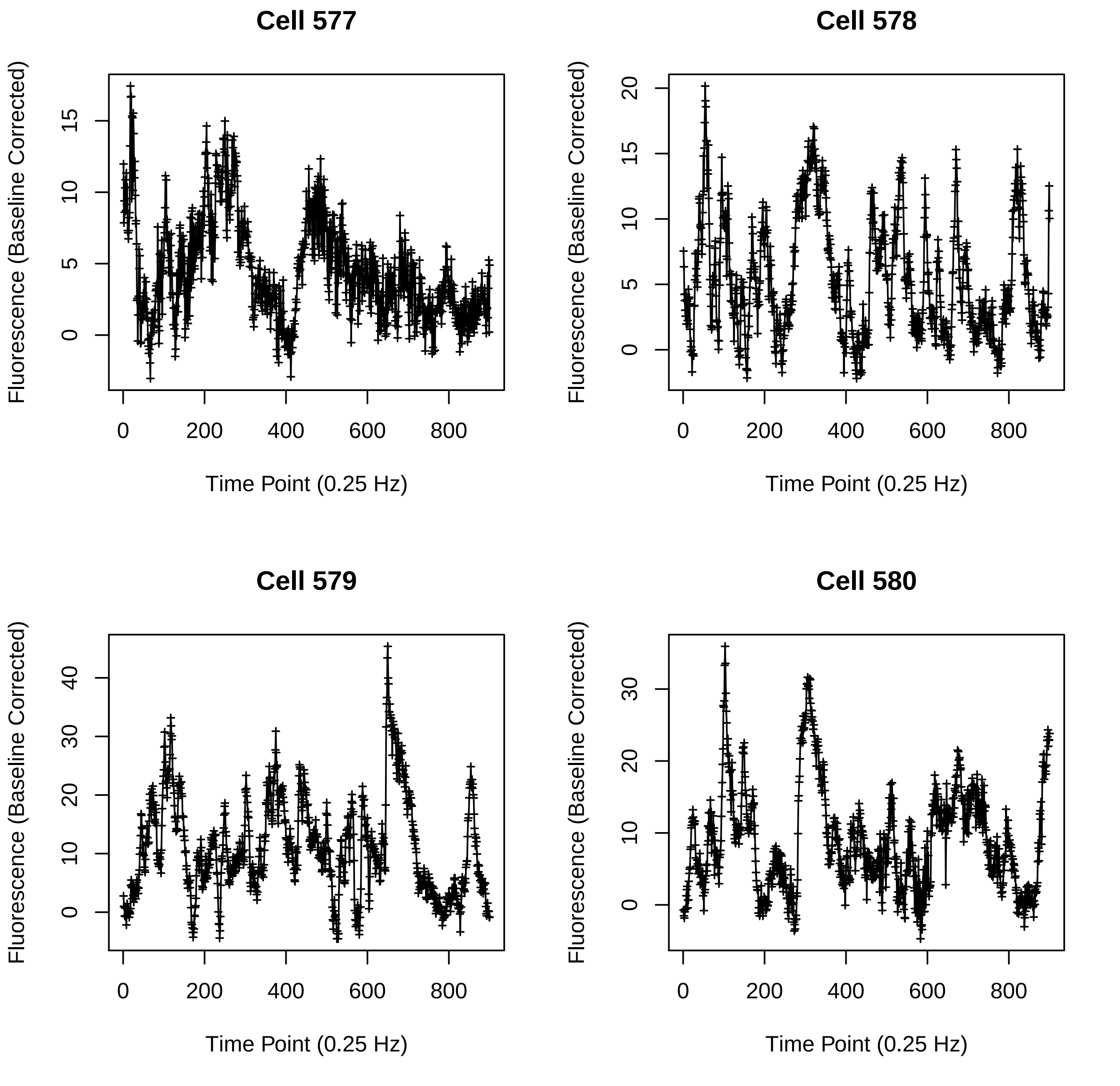

Cell 577
Cell 578
Cell 579
Cell 580
Fluorescence (Baseline Corrected)
Time Point (0.25 Hz)

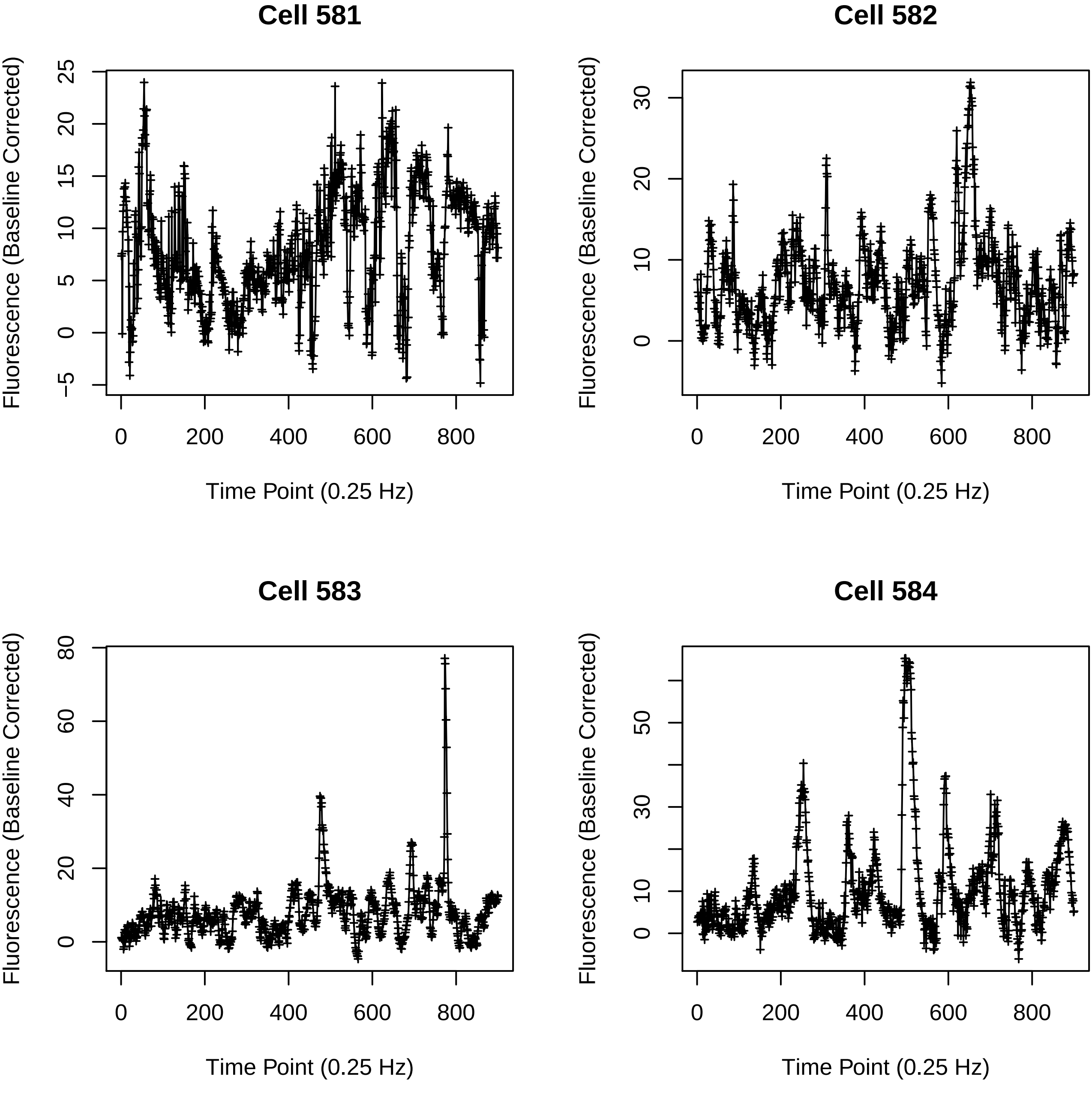
Cell 581
Cell 582
Cell 583
Cell 584
Fluorescence (Baseline Corrected)
Time Point (0.25 Hz)
−5
0
5
10
15
20
25
30
40
50
60
80
0
200
400
600
800

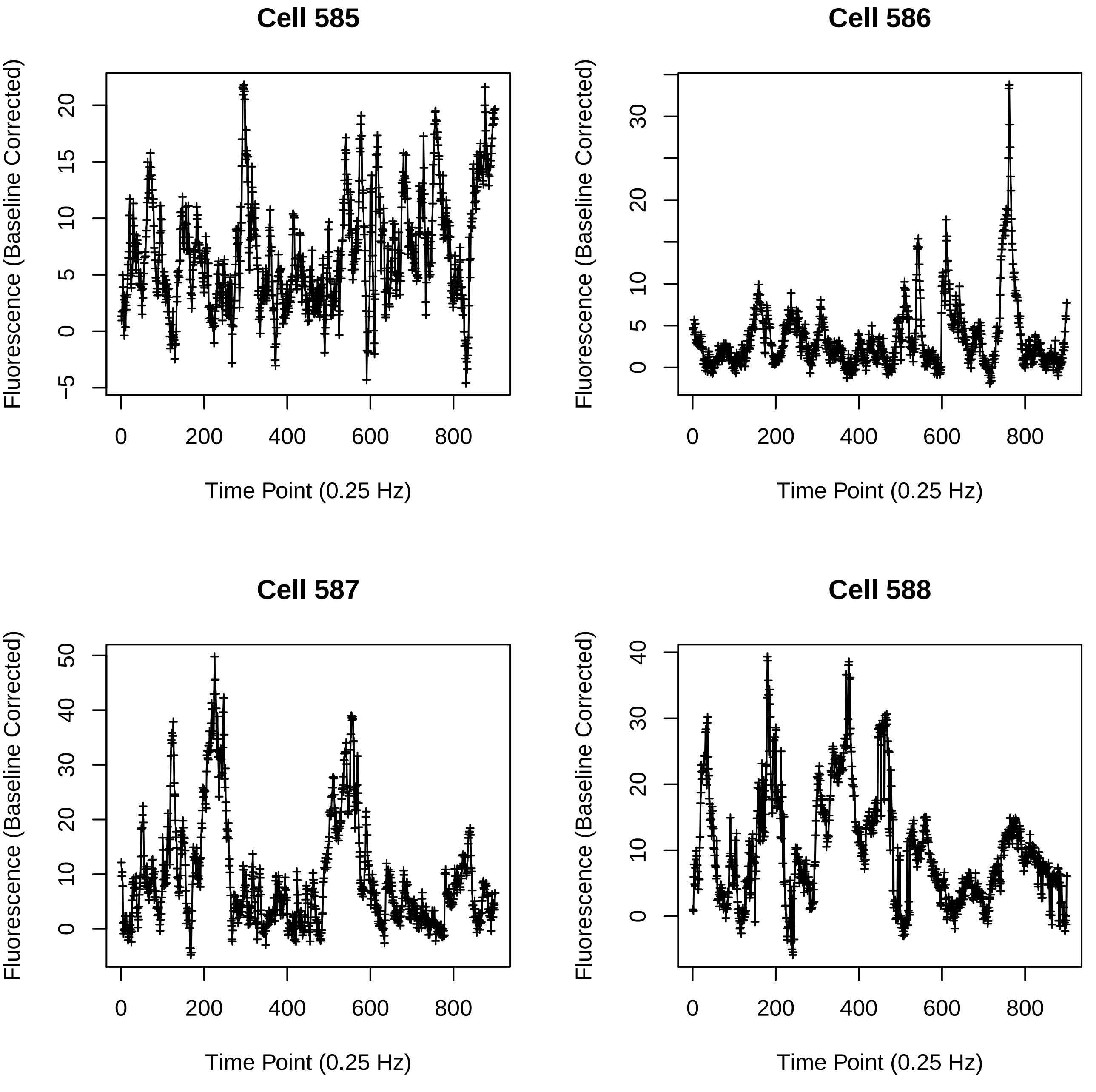
Cell 585
Cell 586
Cell 587
Cell 588
Fluorescence (Baseline Corrected)
Time Point (0.25 Hz)
0
200
400
600
800
−5
5
10
15
20
25
30
40
50

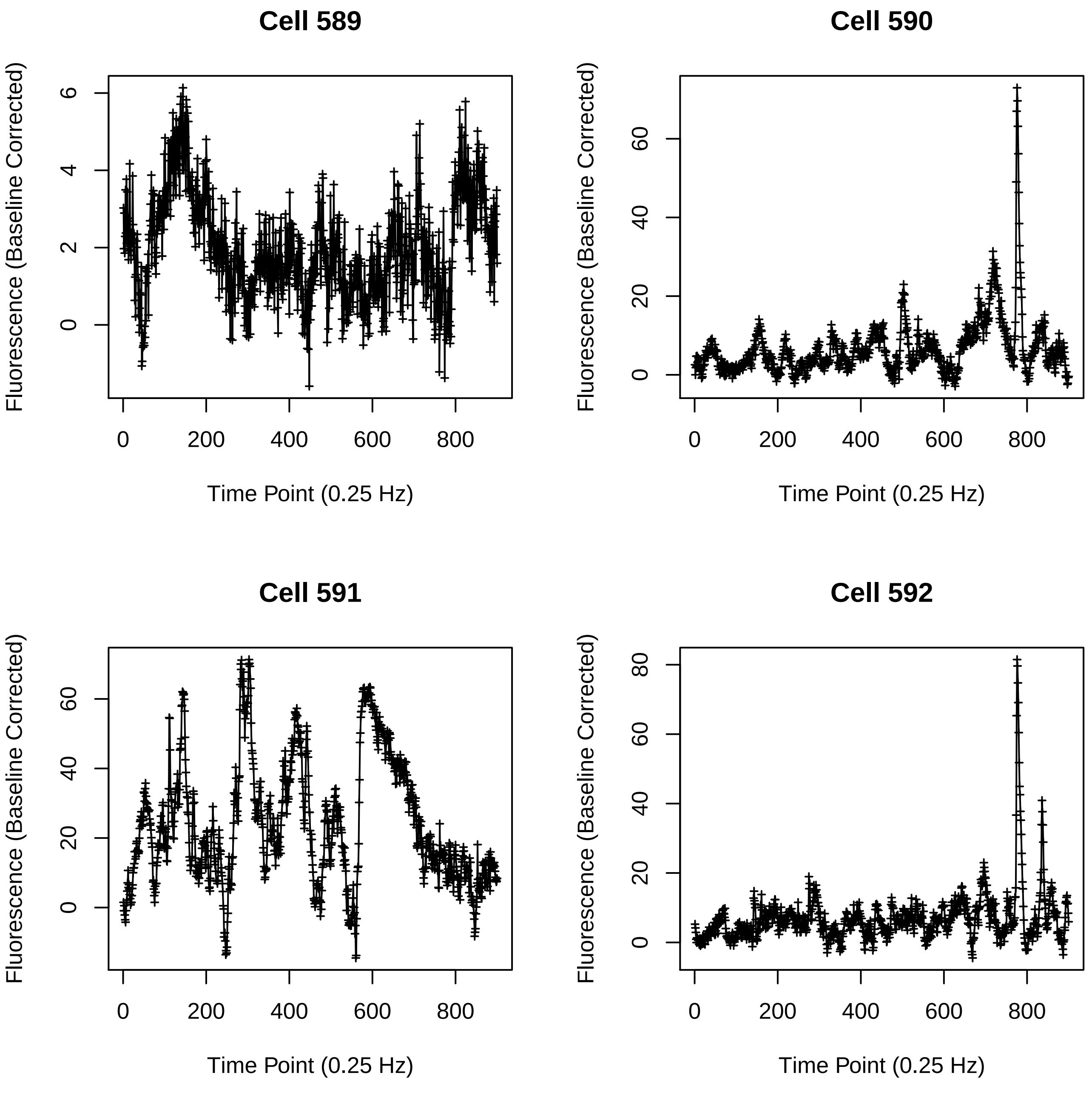
Cell 589
Cell 590
Cell 591
Cell 592
Fluorescence (Baseline Corrected)
Time Point (0.25 Hz)
0
200
400
600
800
2
4
6
20
40
60
80

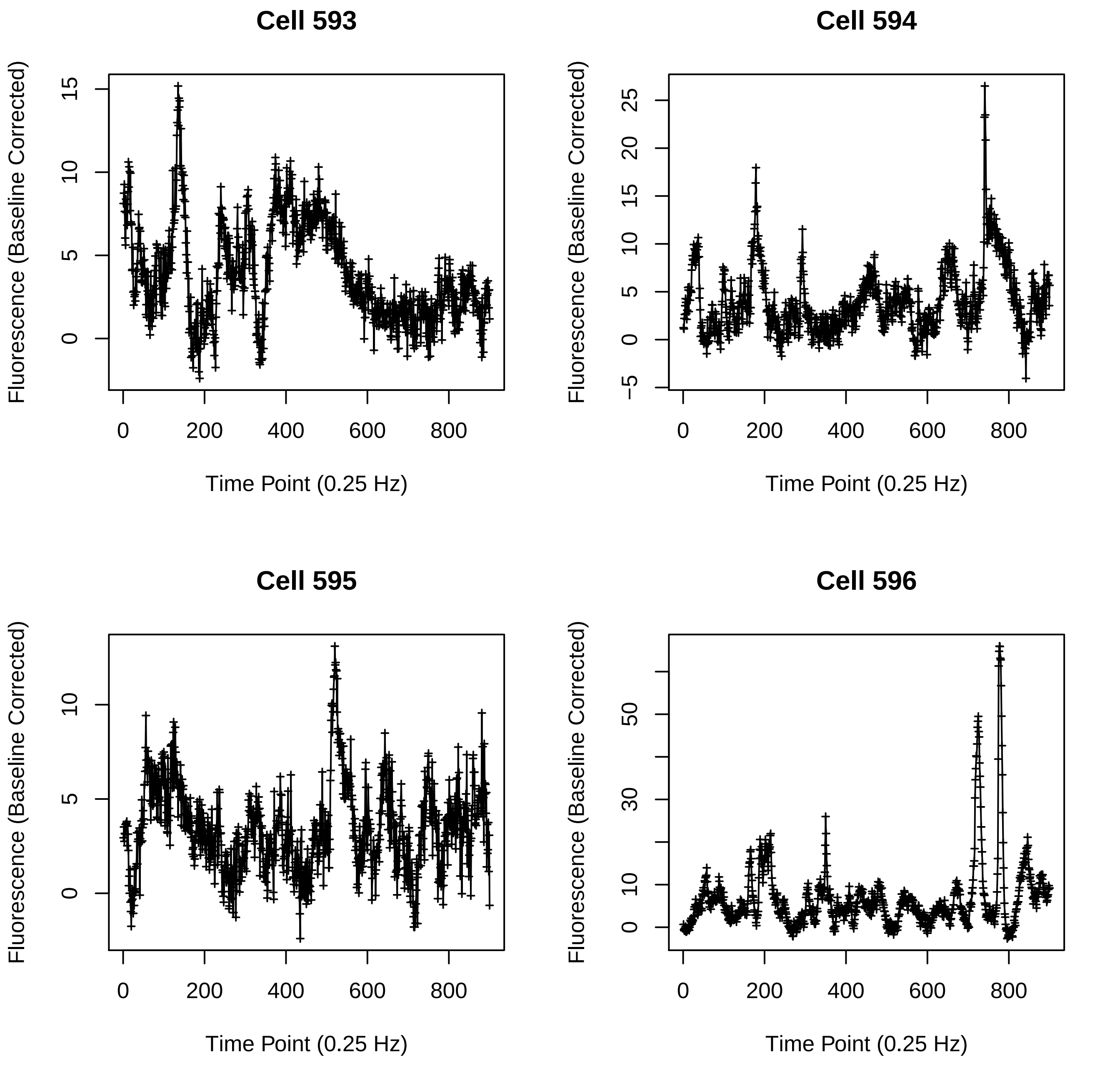
Cell 593
Fluorescence (Baseline Corrected)
0
5
10
15
0
200
400
600
800
Time Point (0.25 Hz)
Cell 594
Fluorescence (Baseline Corrected)
−5
0
5
10
15
20
25
0
200
400
600
800
Time Point (0.25 Hz)
Cell 595
Fluorescence (Baseline Corrected)
0
5
10
0
200
400
600
800
Time Point (0.25 Hz)
Cell 596
Fluorescence (Baseline Corrected)
0
10
30
50
0
200
400
600
800
Time Point (0.25 Hz)

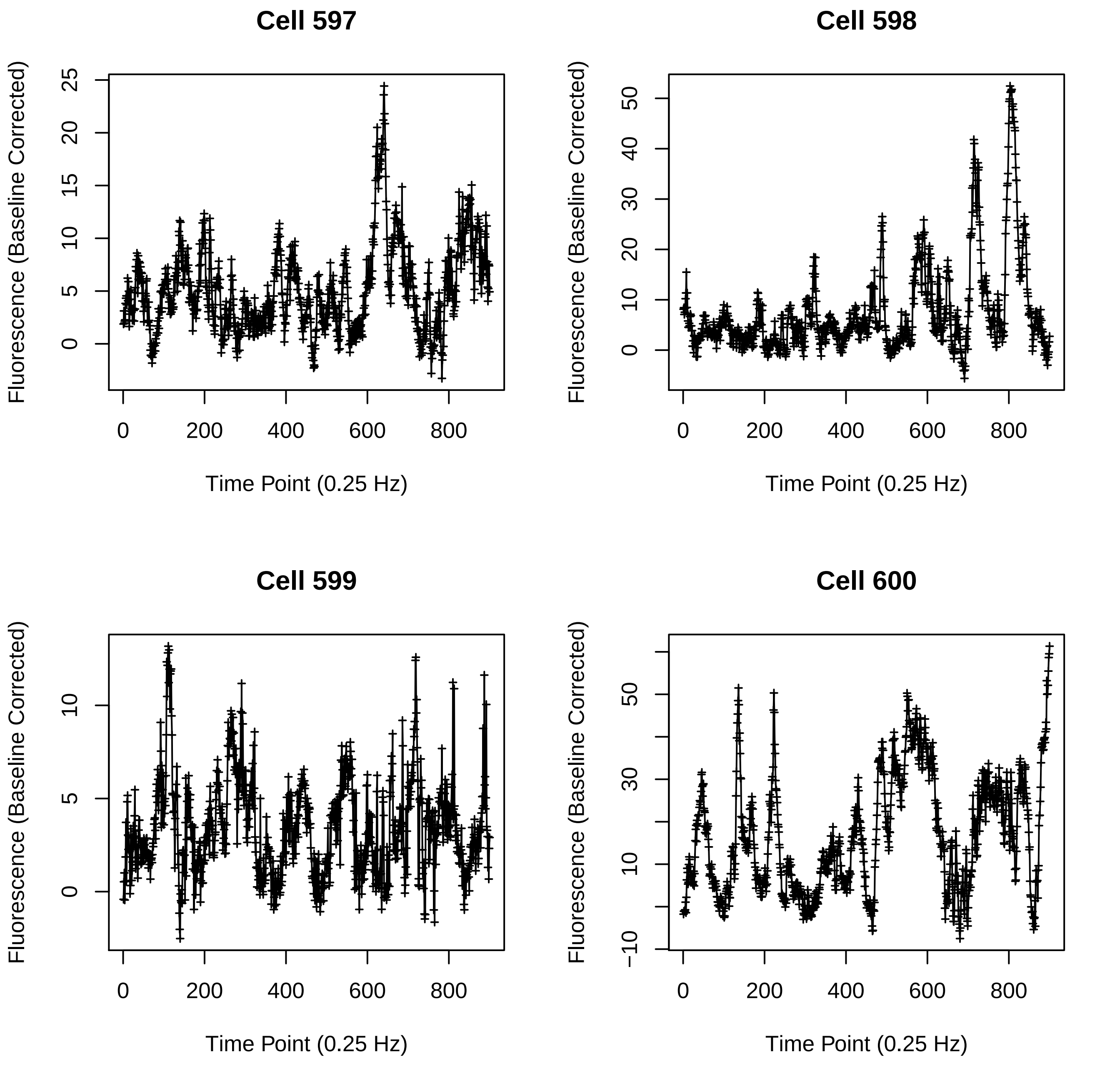

Cell 597
Cell 598
Cell 599
Cell 600
Fluorescence (Baseline Corrected)
Time Point (0.25 Hz)
0
200
400
600
800
5
10
15
20
25
30
40
50
−10

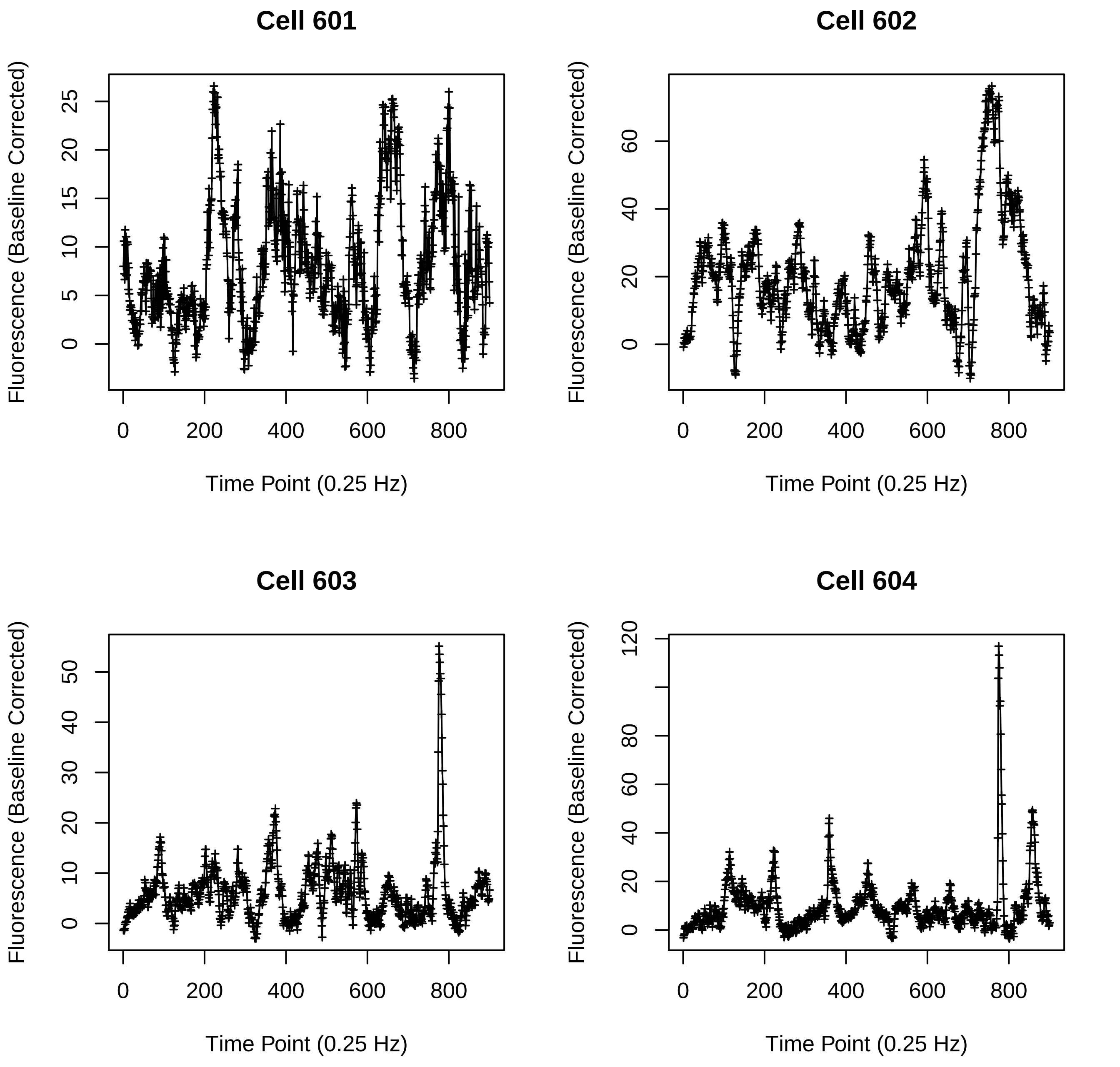
Cell 601
Cell 602
Cell 603
Cell 604
Fluorescence (Baseline Corrected)
Time Point (0.25 Hz)
0
5
10
15
20
25
20
40
60
30
50
80
100
120
200
400
600
800

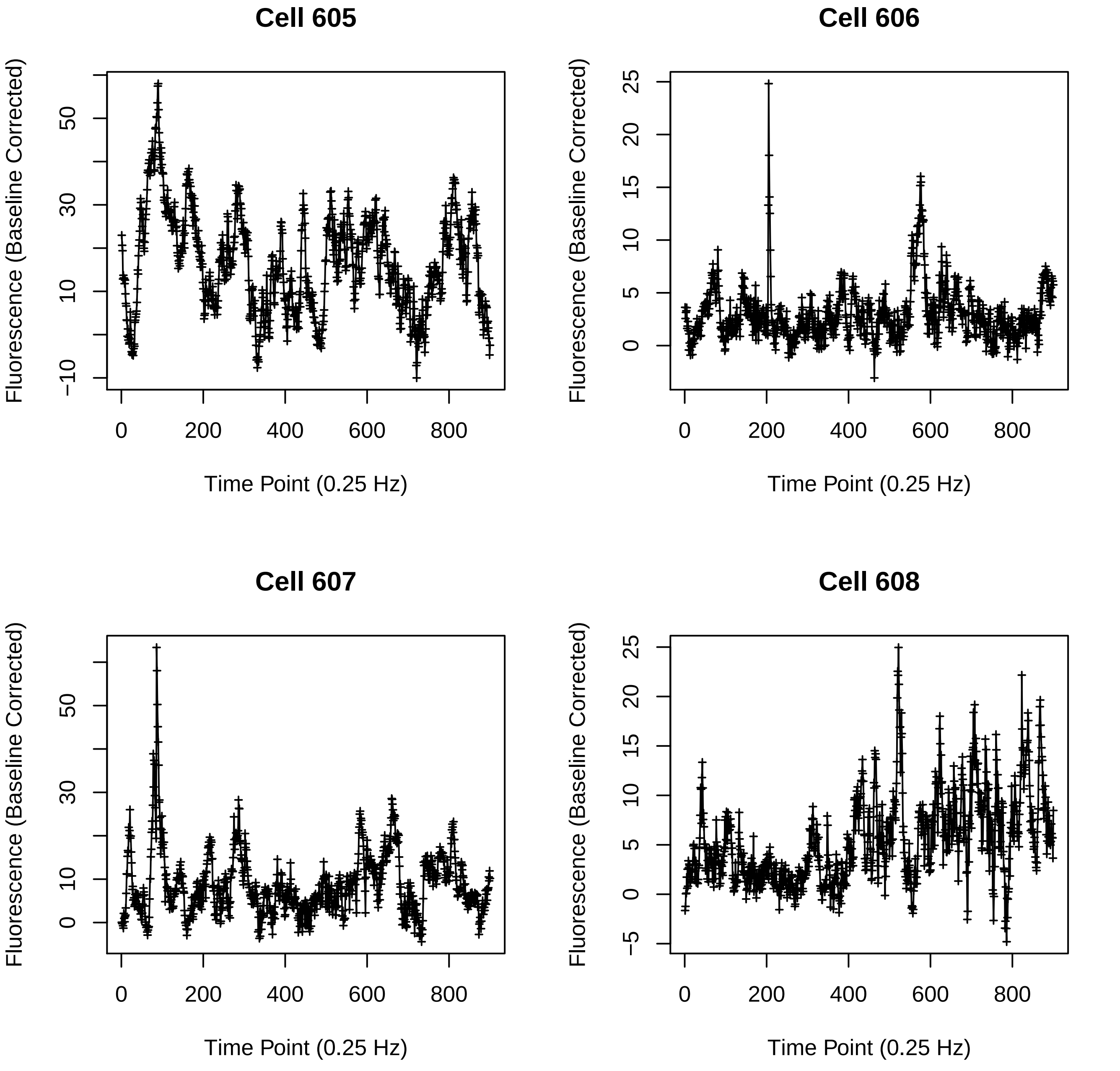

Cell 605
Fluorescence (Baseline Corrected)
−10
10
30
50
0
200
400
600
800
Time Point (0.25 Hz)
Cell 606
Fluorescence (Baseline Corrected)
0
5
10
15
20
25
0
200
400
600
800
Time Point (0.25 Hz)
Cell 607
Fluorescence (Baseline Corrected)
0
10
30
50
0
200
400
600
800
Time Point (0.25 Hz)
Cell 608
Fluorescence (Baseline Corrected)
−5
0
5
10
15
20
25
0
200
400
600
800
Time Point (0.25 Hz)

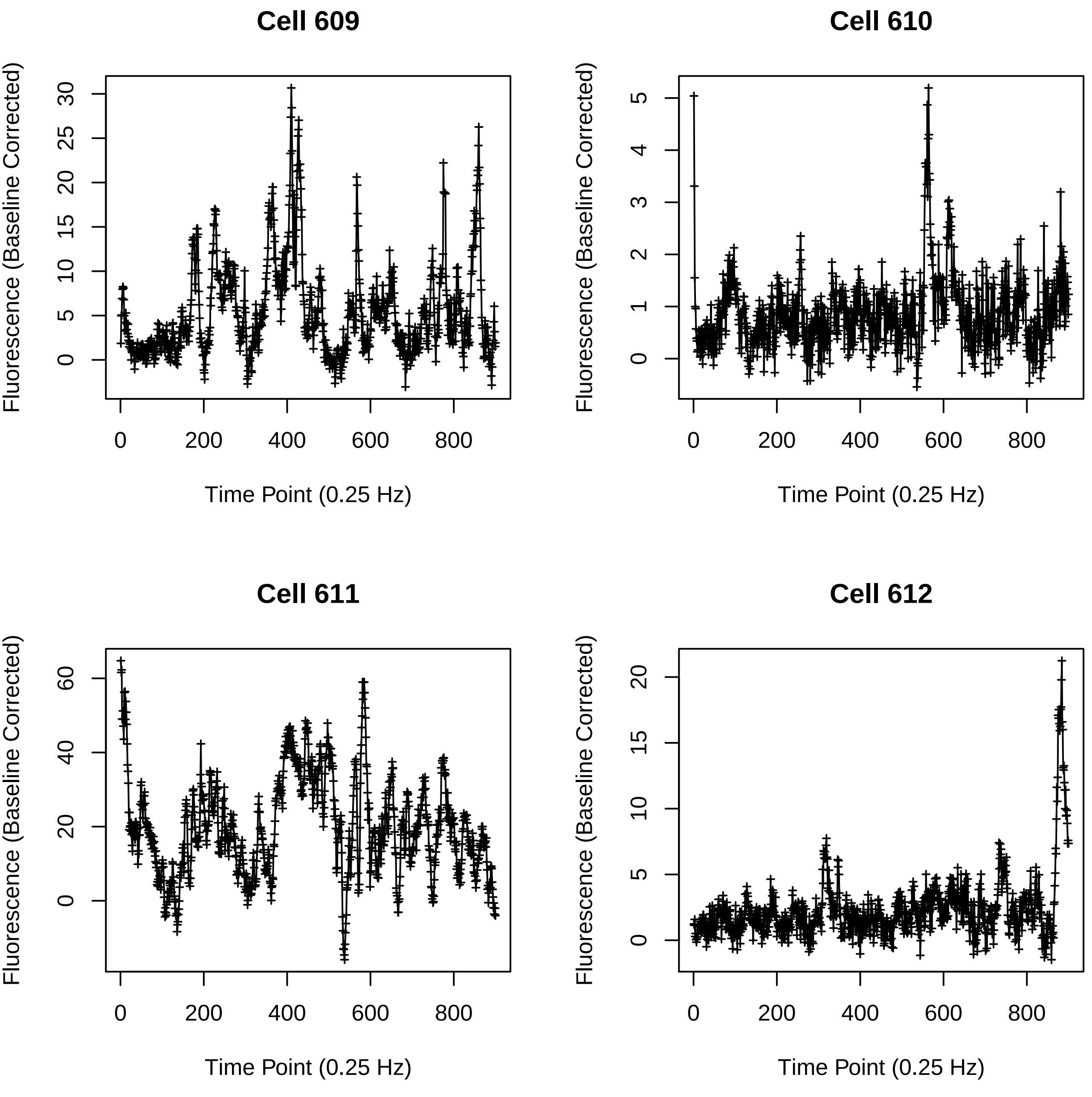

Cell 609
Cell 610
Cell 611
Cell 612
Fluorescence (Baseline Corrected)
Time Point (0.25 Hz)
0
200
400
600
800
5
10
15
20
25
30
1
2
3
4
40
60

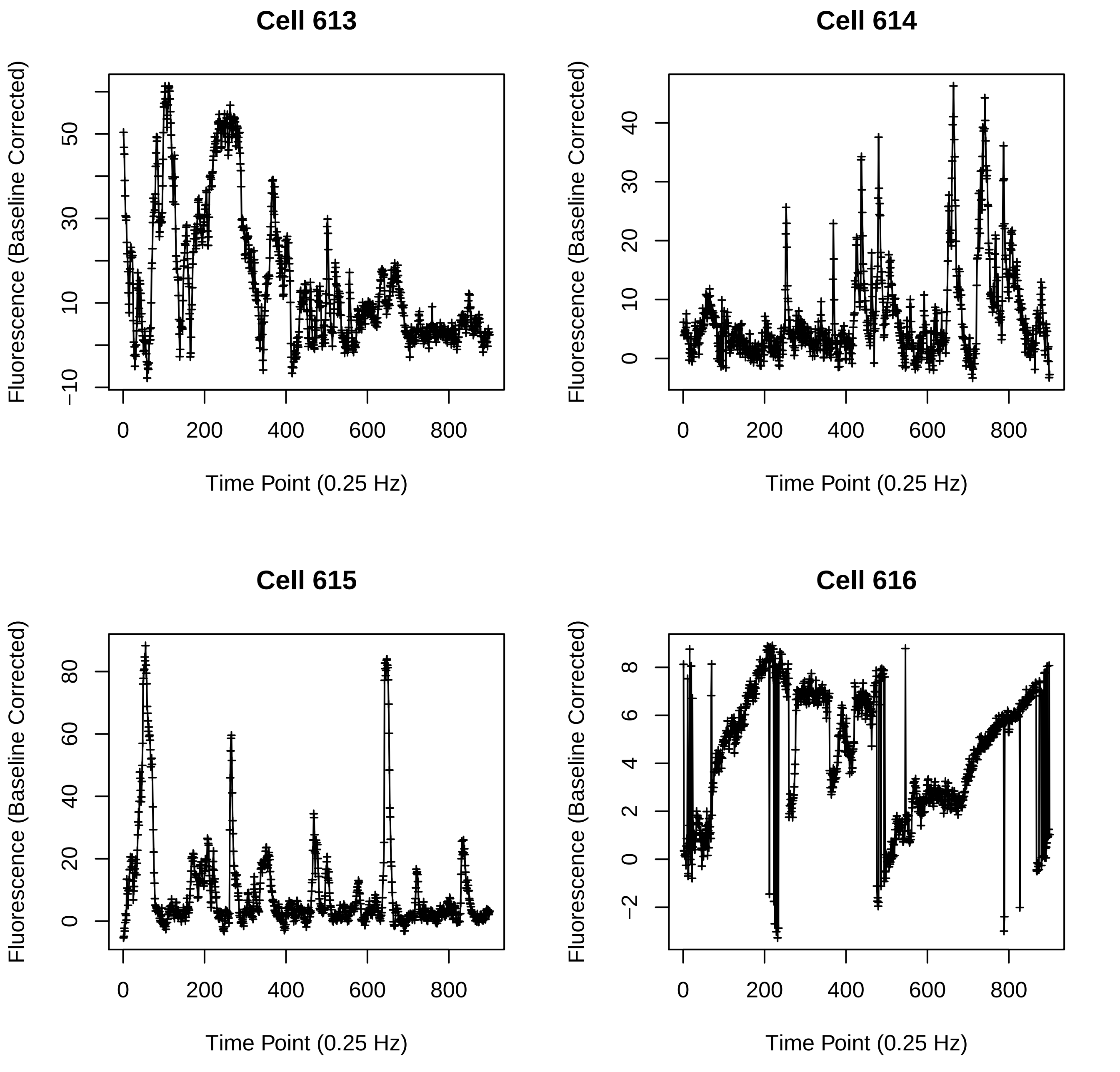

Cell 613
Cell 614
Cell 615
Cell 616
Fluorescence (Baseline Corrected)
Time Point (0.25 Hz)

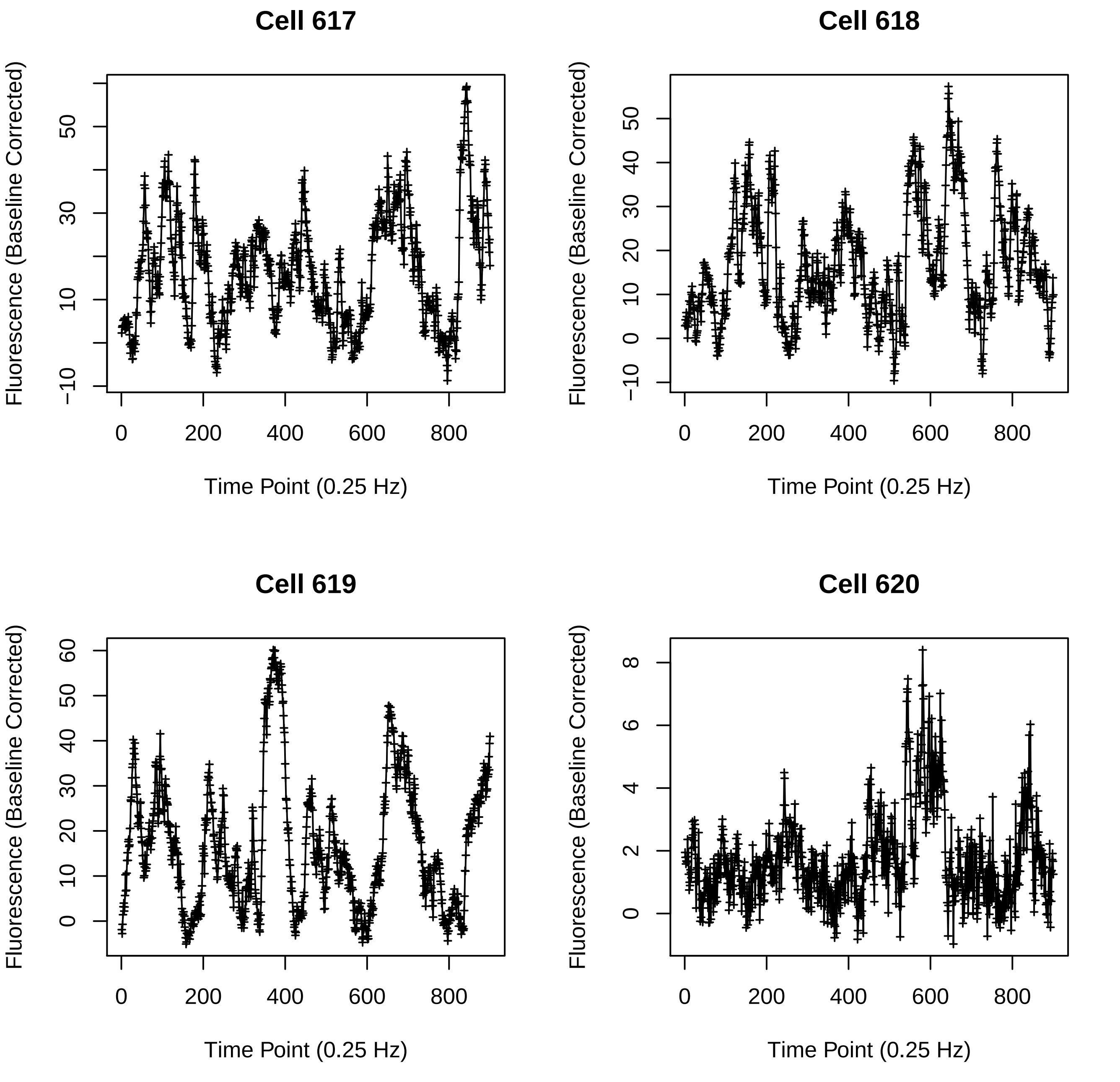
Cell 617
Fluorescence (Baseline Corrected)
Time Point (0.25 Hz)
−10
10
30
50
0
200
400
600
800
Cell 618
Fluorescence (Baseline Corrected)
Time Point (0.25 Hz)
−10
0
10
20
30
40
50
0
200
400
600
800
Cell 619
Fluorescence (Baseline Corrected)
Time Point (0.25 Hz)
0
10
20
30
40
50
60
0
200
400
600
800
Cell 620
Fluorescence (Baseline Corrected)
Time Point (0.25 Hz)
0
2
4
6
8
0
200
400
600
800

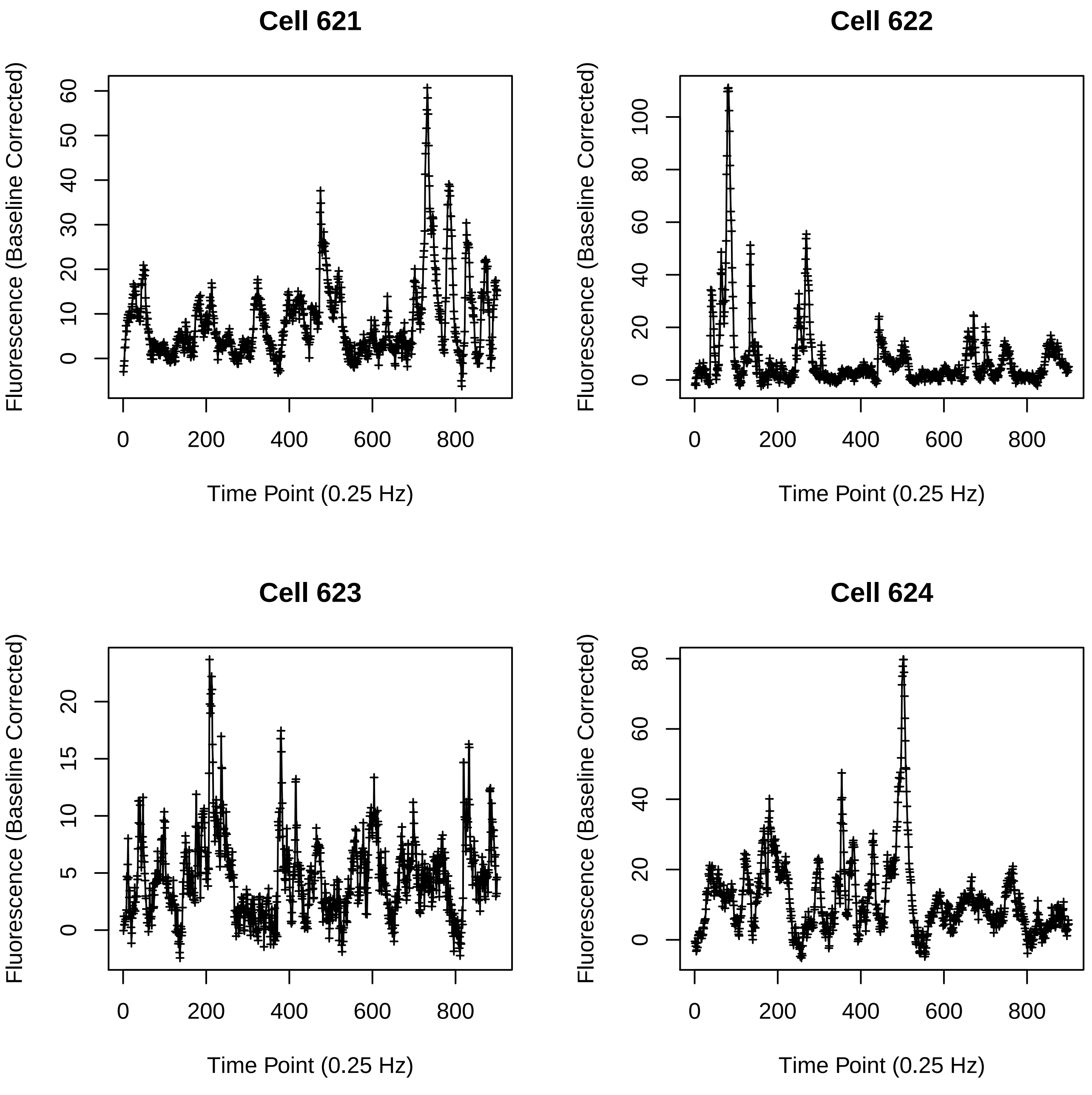
Cell 621
Cell 622
Cell 623
Cell 624
Fluorescence (Baseline Corrected)
Time Point (0.25 Hz)
0
10
20
30
40
50
60
80
100
5
15
200
400
600
800

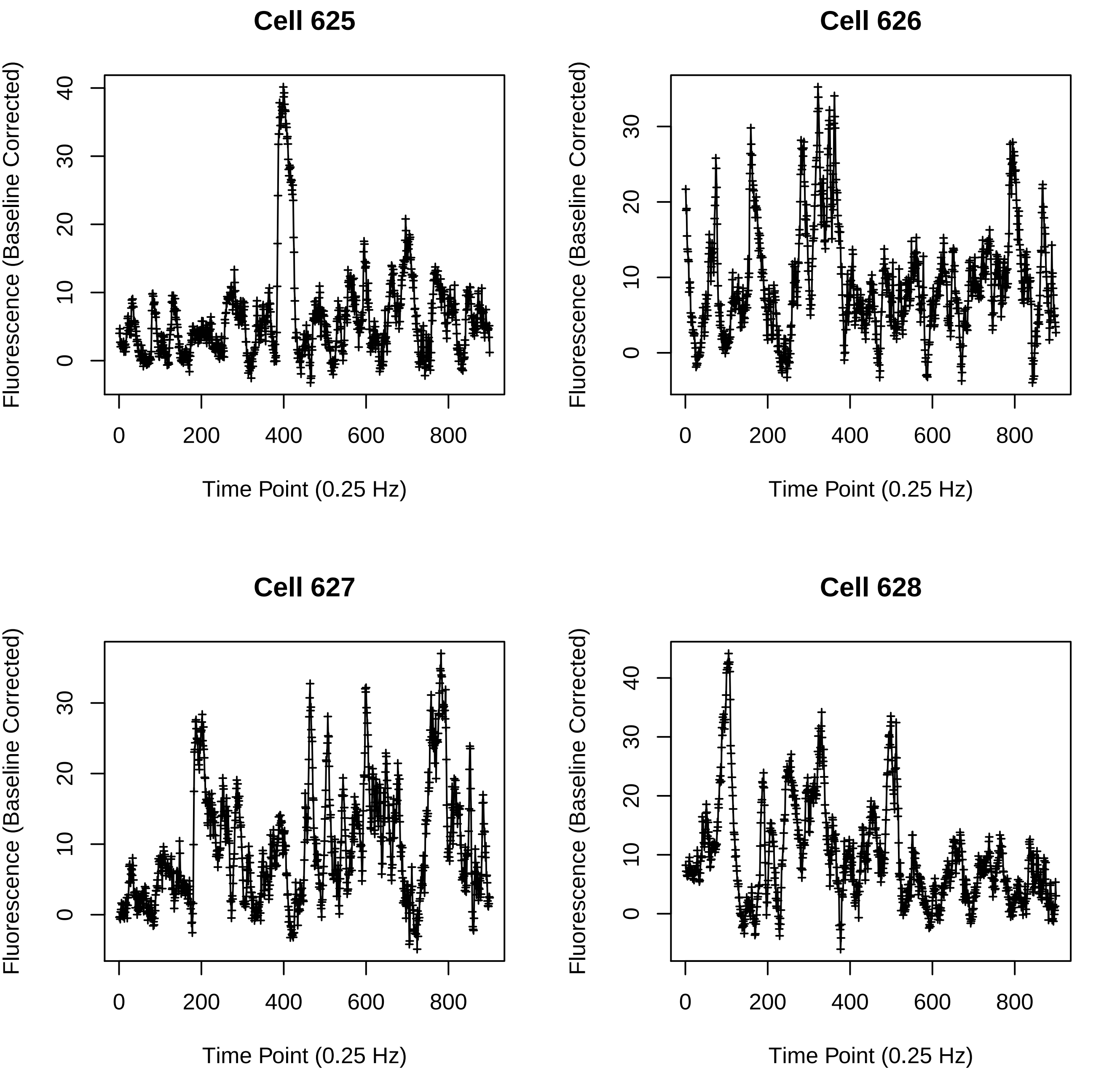
Cell 625
Cell 626
Cell 627
Cell 628
Fluorescence (Baseline Corrected)
Time Point (0.25 Hz)
0
200
400
600
800
0
10
20
30
40

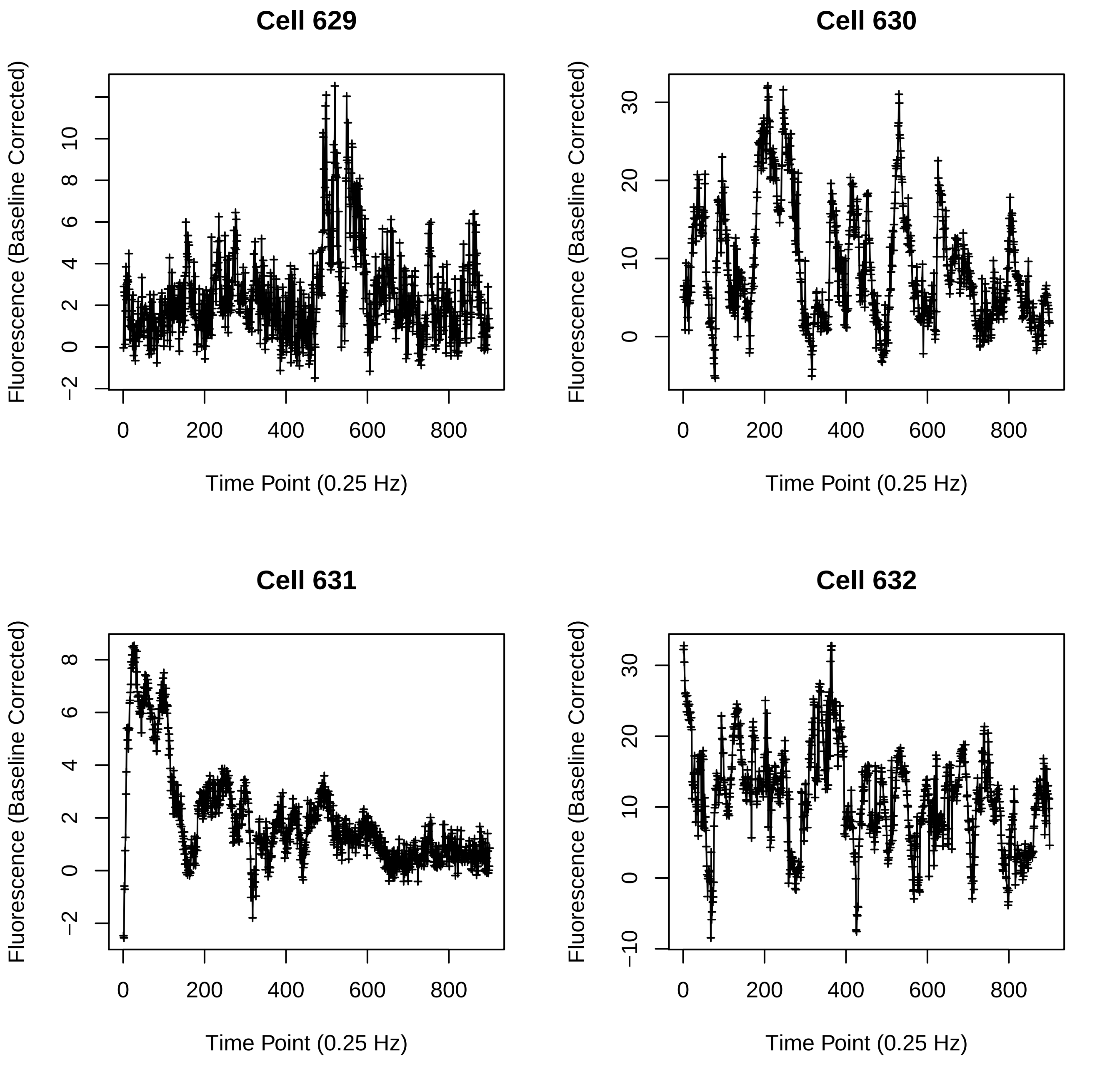
Cell 629
Cell 630
Cell 631
Cell 632
Fluorescence (Baseline Corrected)
Time Point (0.25 Hz)
−2
0
2
4
6
8
10
−10
0
10
20
30
0
200
400
600
800

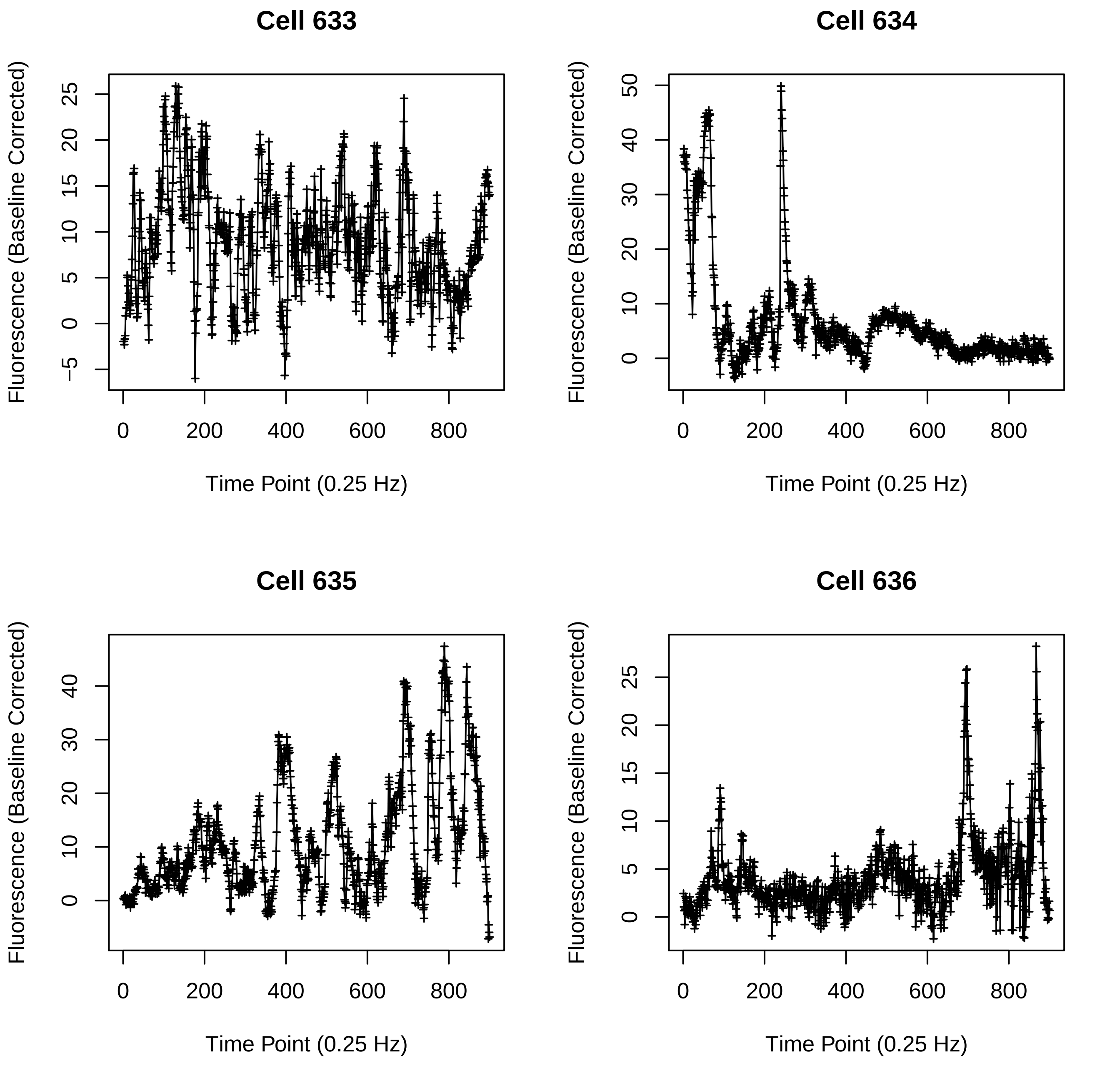

Cell 633
Cell 634
Cell 635
Cell 636
Fluorescence (Baseline Corrected)
Time Point (0.25 Hz)

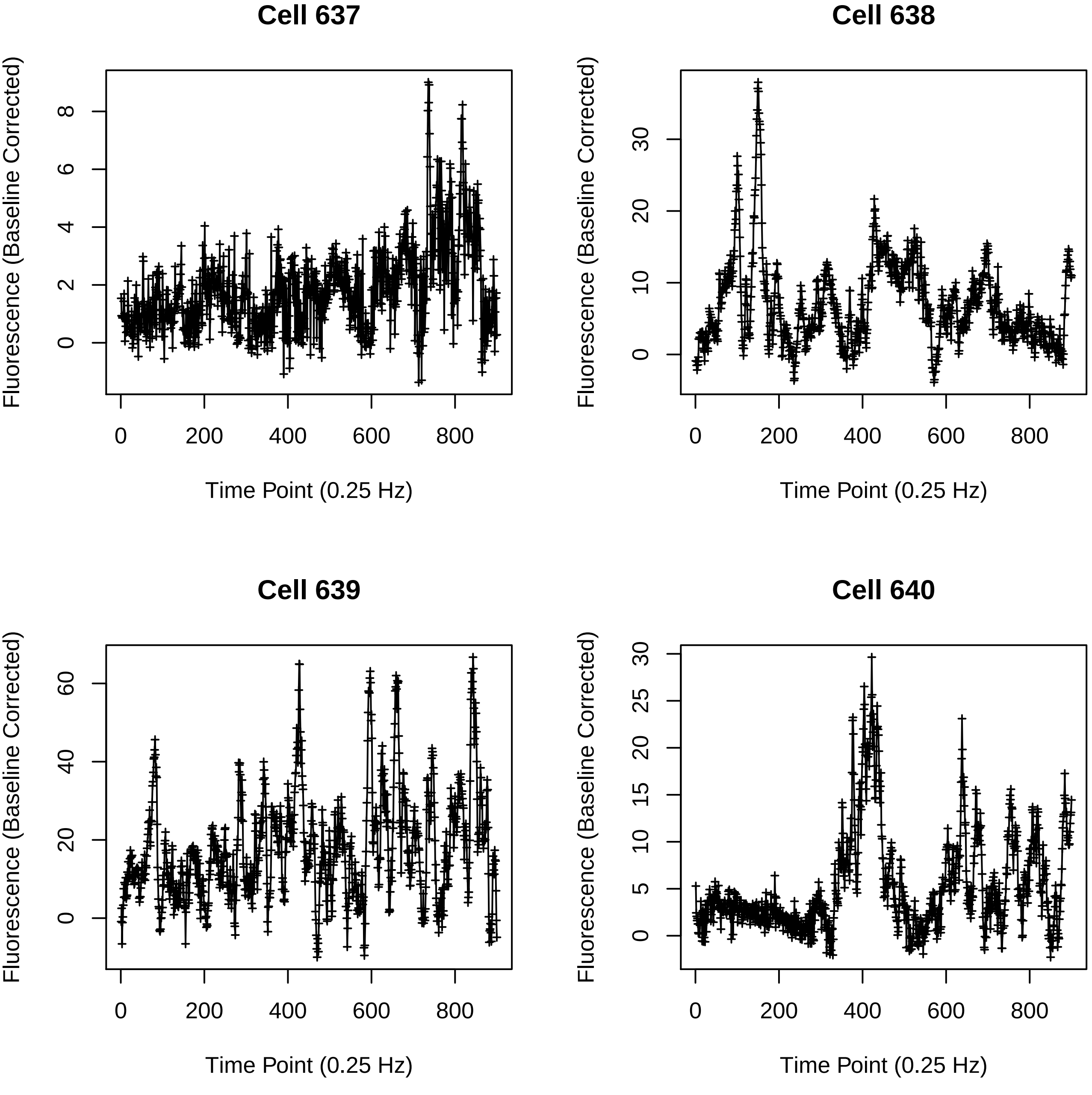
Cell 637
Fluorescence (Baseline Corrected)
Time Point (0.25 Hz)
0
2
4
6
8
200
400
600
800
Cell 638
Fluorescence (Baseline Corrected)
Time Point (0.25 Hz)
0
10
20
30
200
400
600
800
Cell 639
Fluorescence (Baseline Corrected)
Time Point (0.25 Hz)
0
20
40
60
200
400
600
800
Cell 640
Fluorescence (Baseline Corrected)
Time Point (0.25 Hz)
0
5
10
15
20
25
30
200
400
600
800

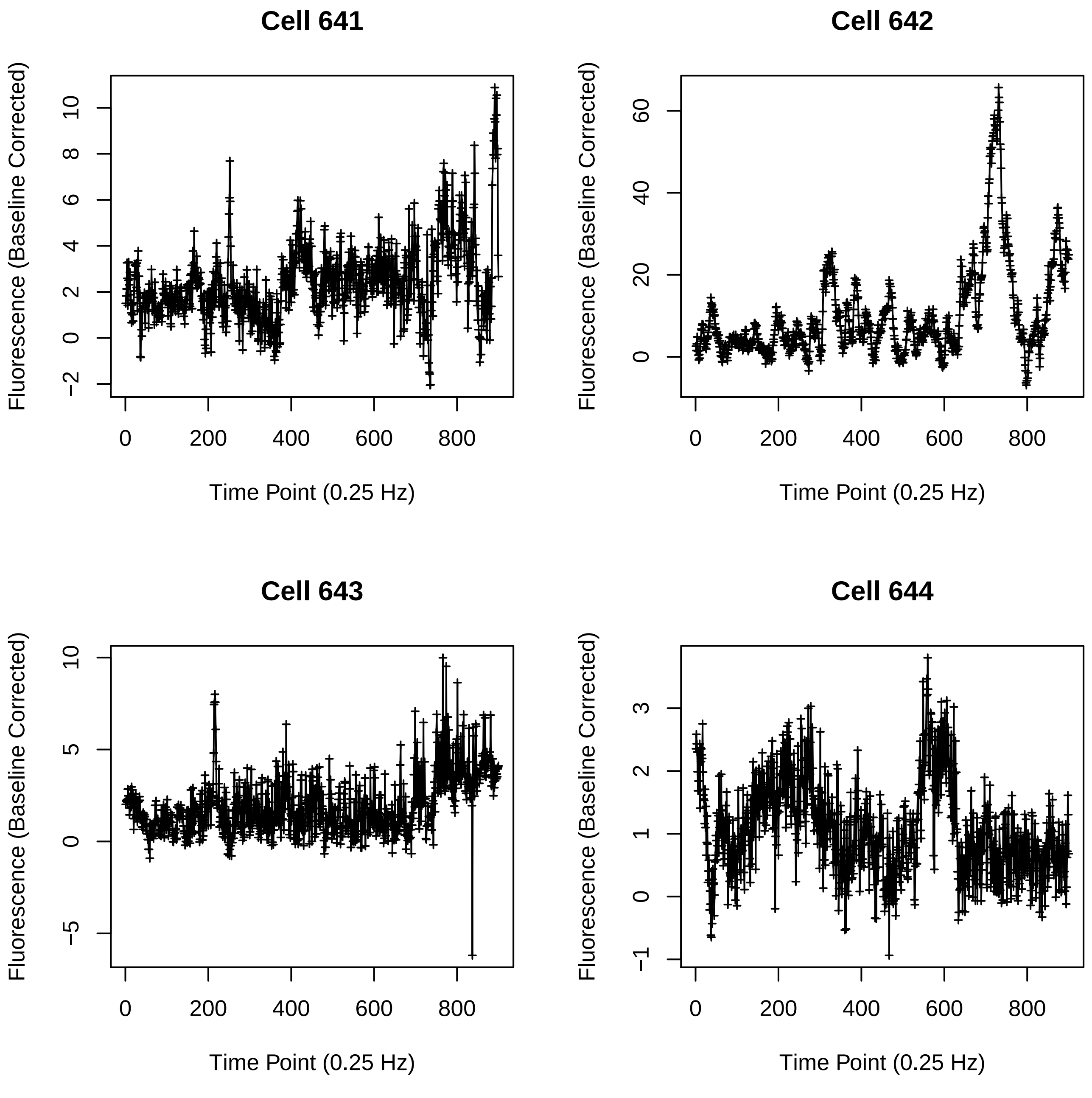
Cell 641
Fluorescence (Baseline Corrected)
Time Point (0.25 Hz)
−2
0
2
4
6
8
10
0
200
400
600
800
Cell 642
Fluorescence (Baseline Corrected)
Time Point (0.25 Hz)
0
20
40
60
0
200
400
600
800
Cell 643
Fluorescence (Baseline Corrected)
Time Point (0.25 Hz)
−5
0
5
10
0
200
400
600
800
Cell 644
Fluorescence (Baseline Corrected)
Time Point (0.25 Hz)
−1
0
1
2
3
0
200
400
600
800

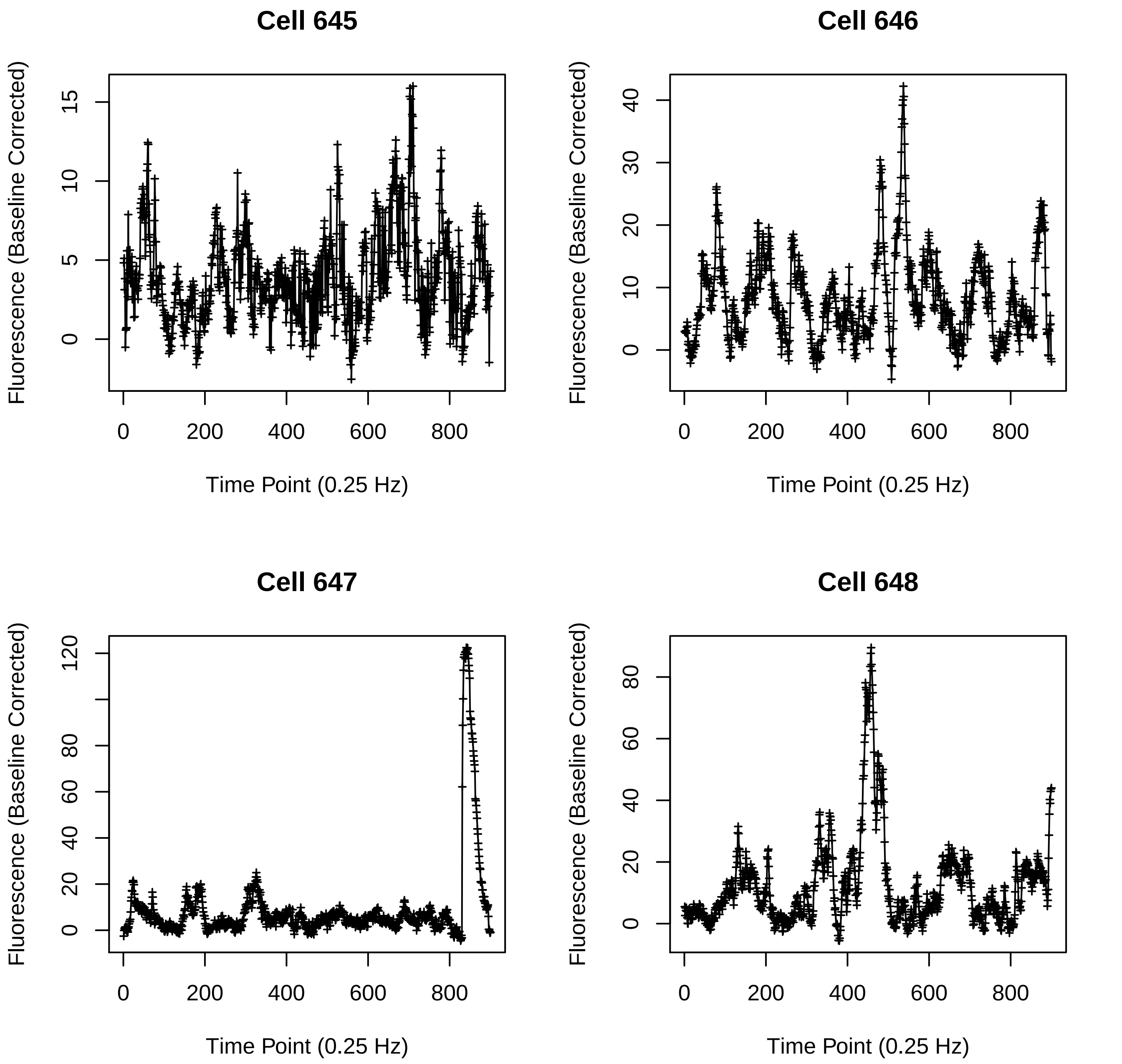

Cell 645
Cell 646
Cell 647
Cell 648
Fluorescence (Baseline Corrected)
Time Point (0.25 Hz)
0
200
400
600
800

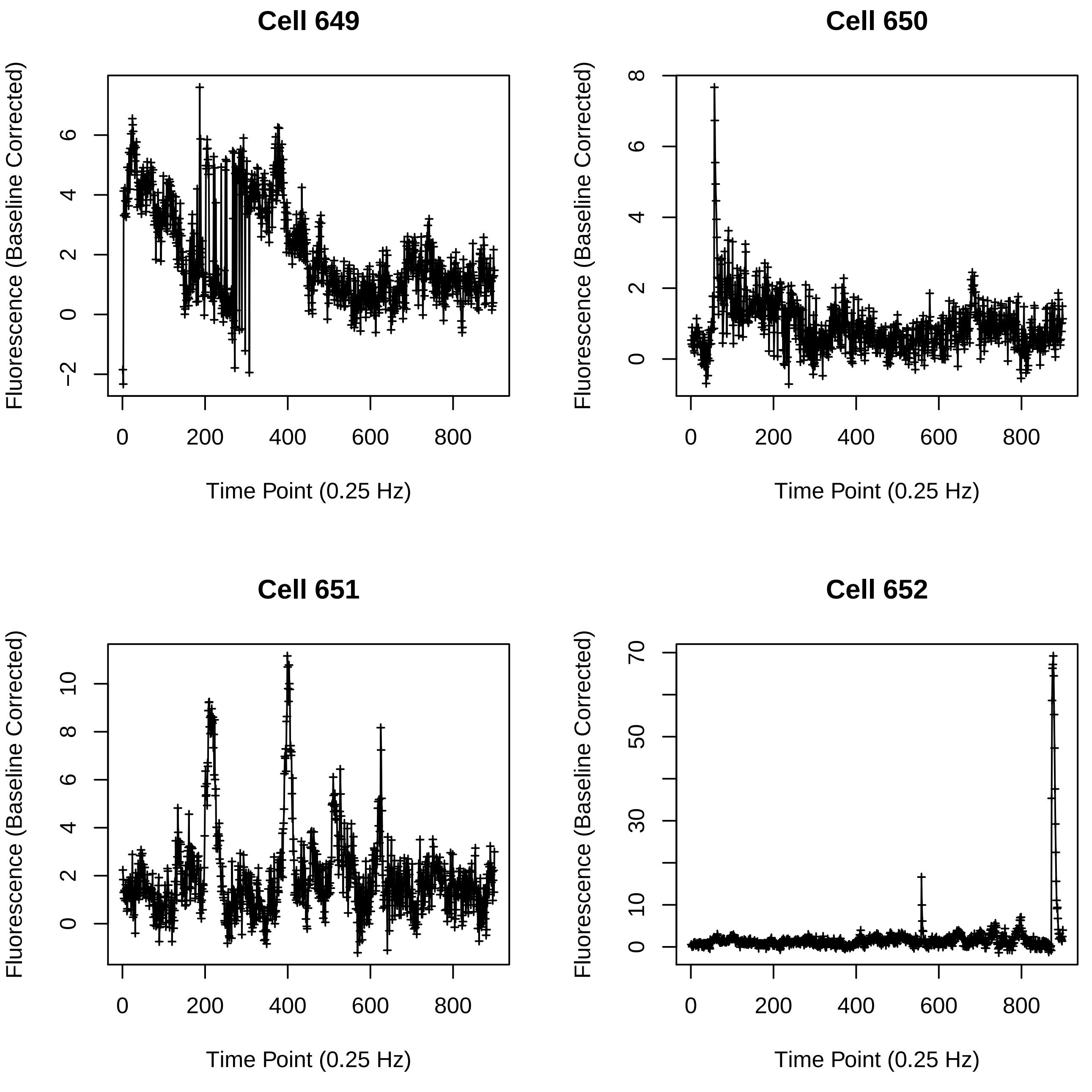
Cell 649
Cell 650
Cell 651
Cell 652
Fluorescence (Baseline Corrected)
Time Point (0.25 Hz)
−2
0
2
4
6
8
10
30
50
70
0
200
400
600
800

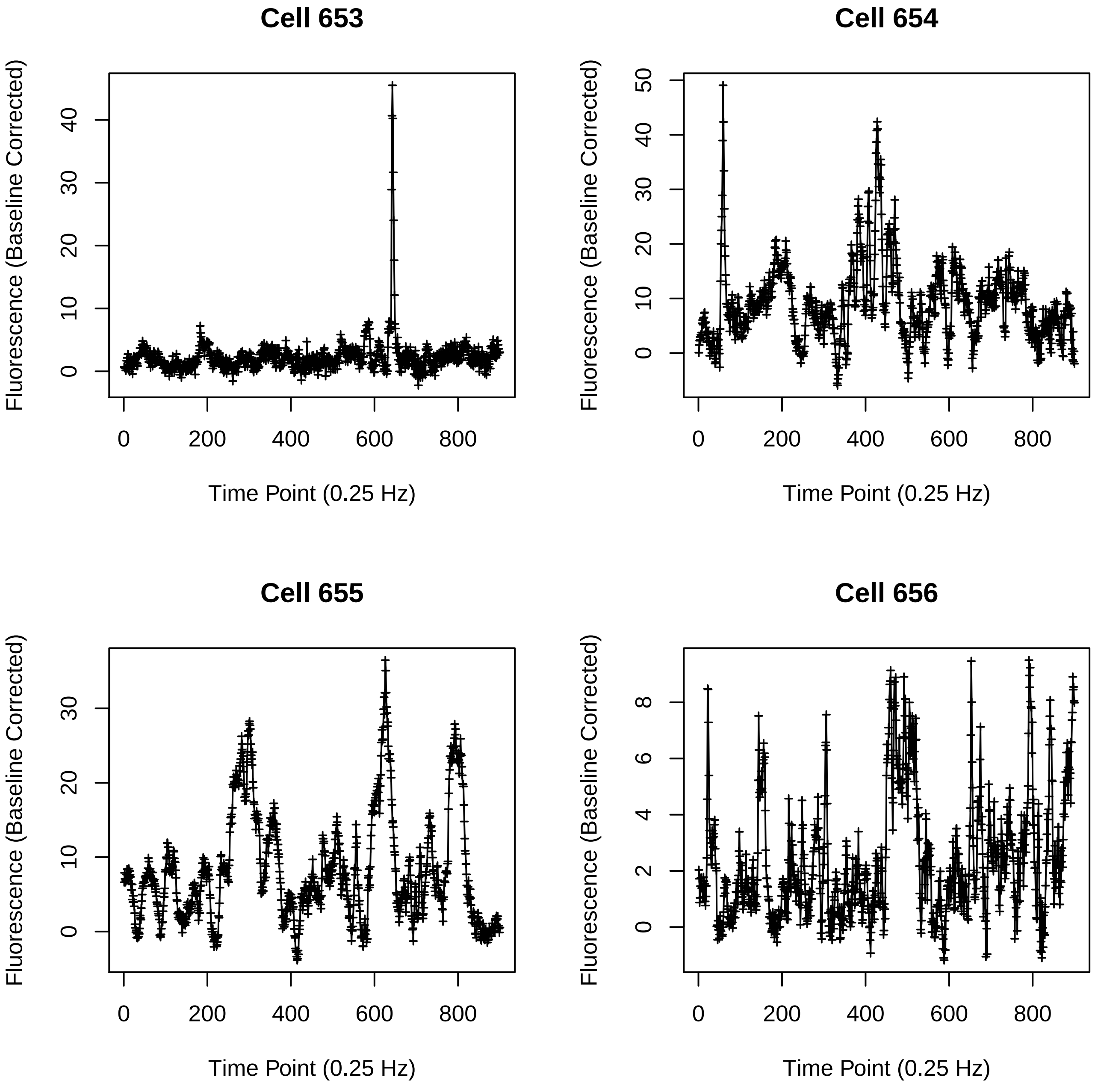
Cell 653
Cell 654
Cell 655
Cell 656
Fluorescence (Baseline Corrected)
Time Point (0.25 Hz)
0
200
400
600
800
10
20
30
40
50
2
4
6
8

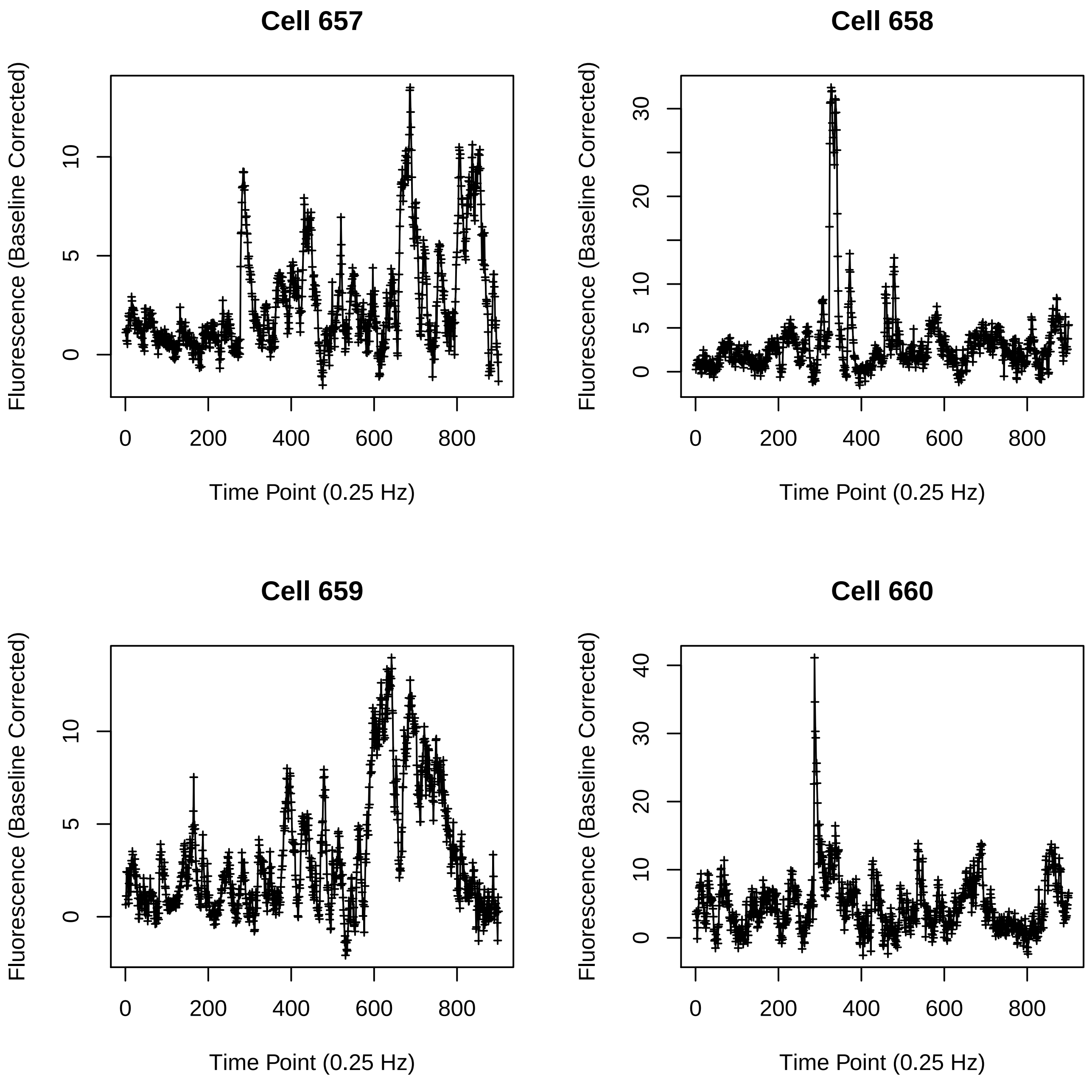
Cell 657
Cell 658
Cell 659
Cell 660
Fluorescence (Baseline Corrected)
Time Point (0.25 Hz)
0
5
10
10
20
30
40
200
400
600
800

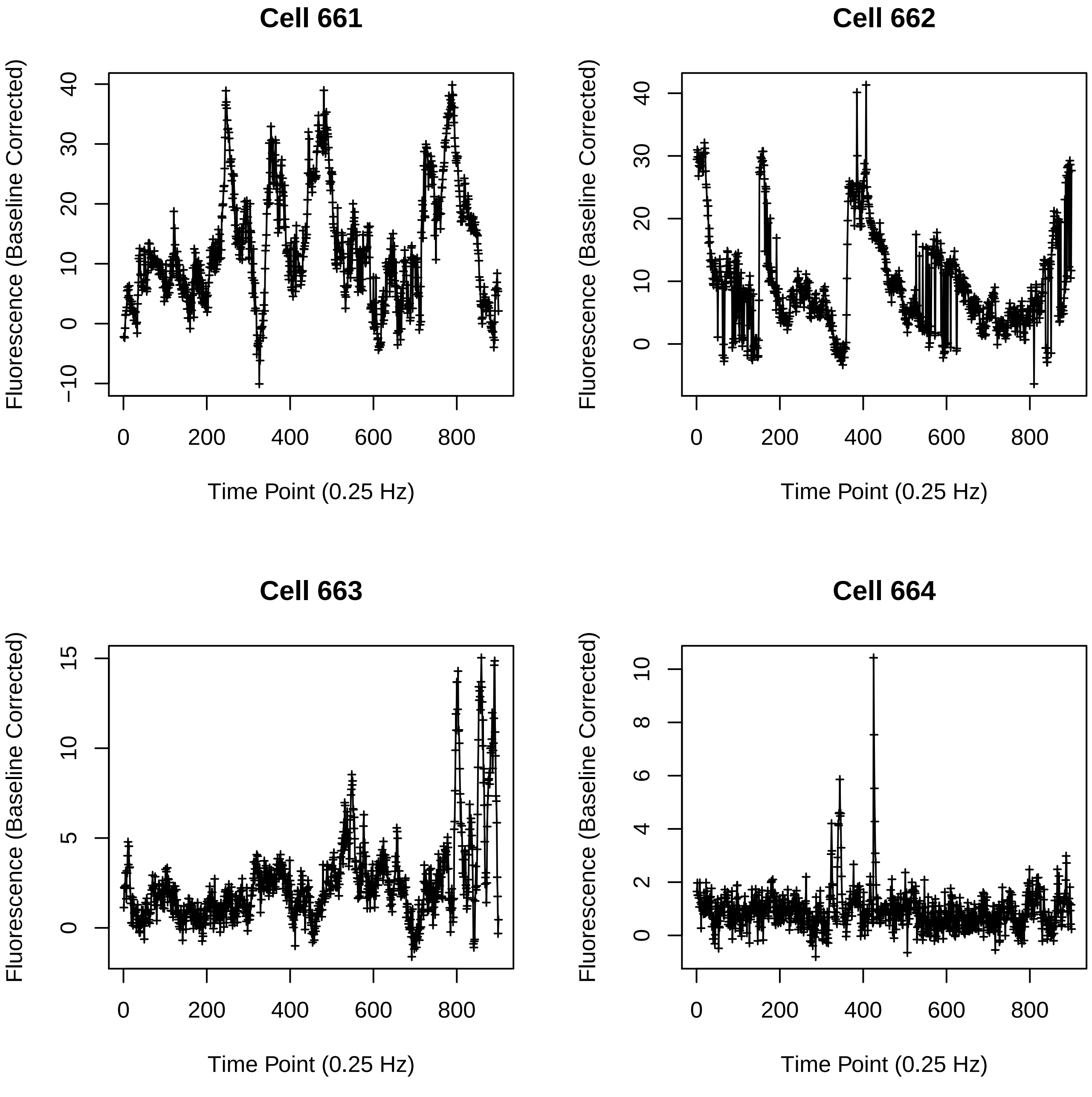
Cell 661
Cell 662
Cell 663
Cell 664
Fluorescence (Baseline Corrected)
Time Point (0.25 Hz)
-10
0
10
20
30
40
0
2
4
5
6
8
15
200
400
600
800

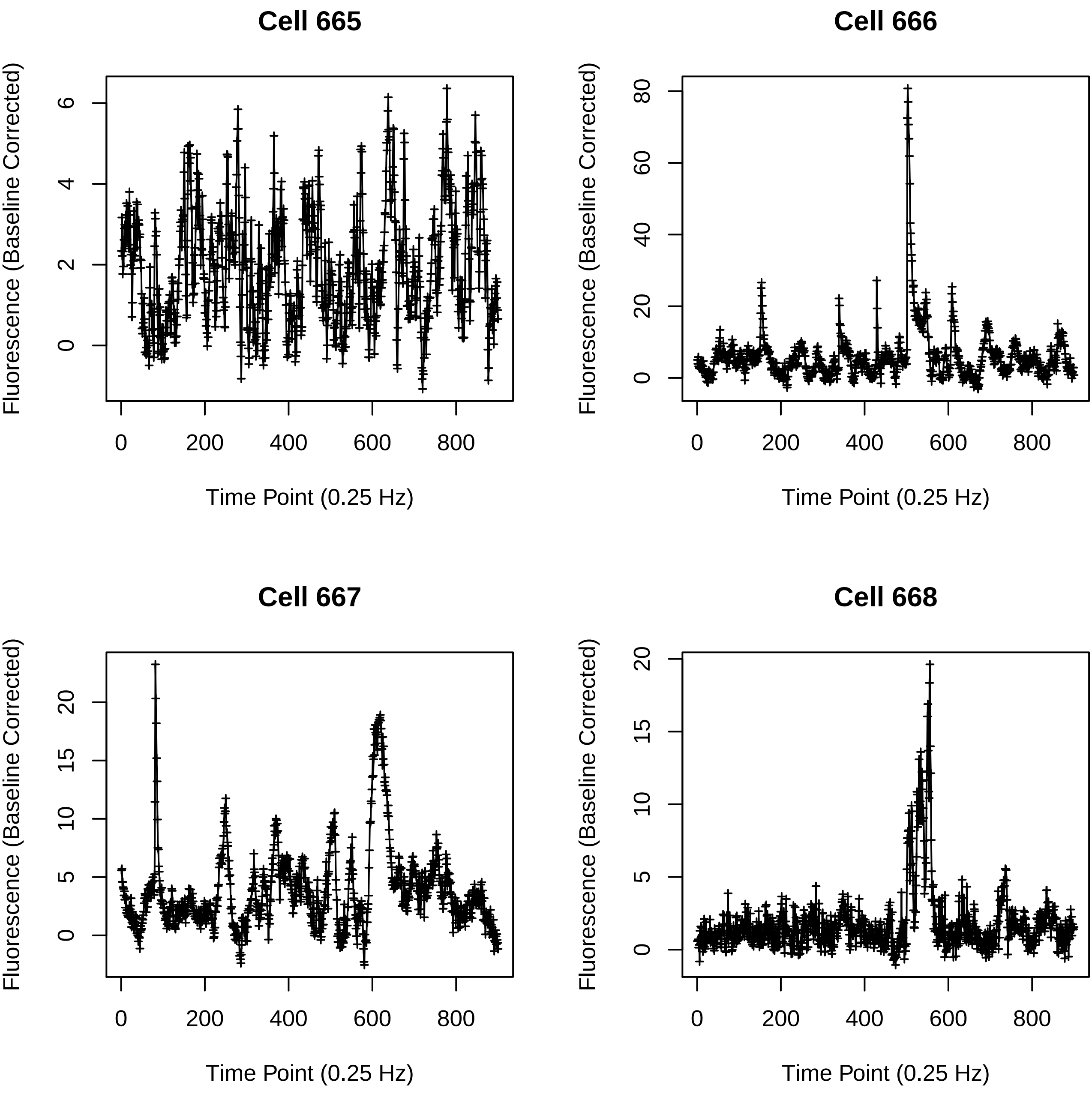
Cell 665
Cell 666
Cell 667
Cell 668
Fluorescence (Baseline Corrected)
Time Point (0.25 Hz)
0
200
400
600
800
2
4
6
20
40
60
80
5
10
15

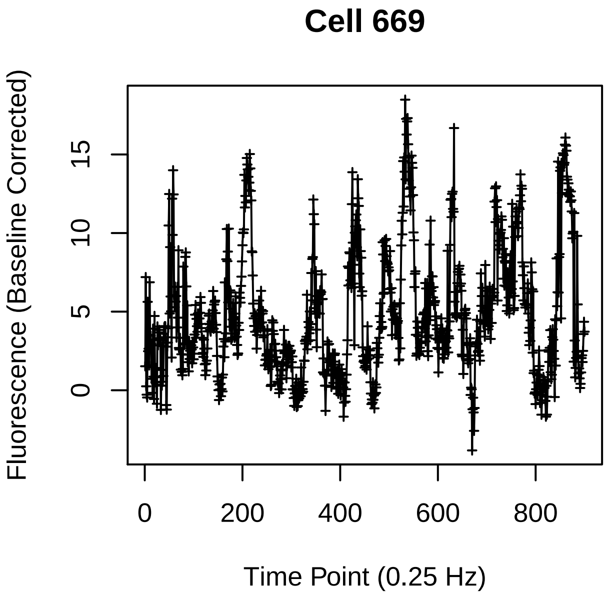
Cell 669
Fluorescence (Baseline Corrected)
0
5
10
15
0
200
400
600
800
Time Point (0.25 Hz)

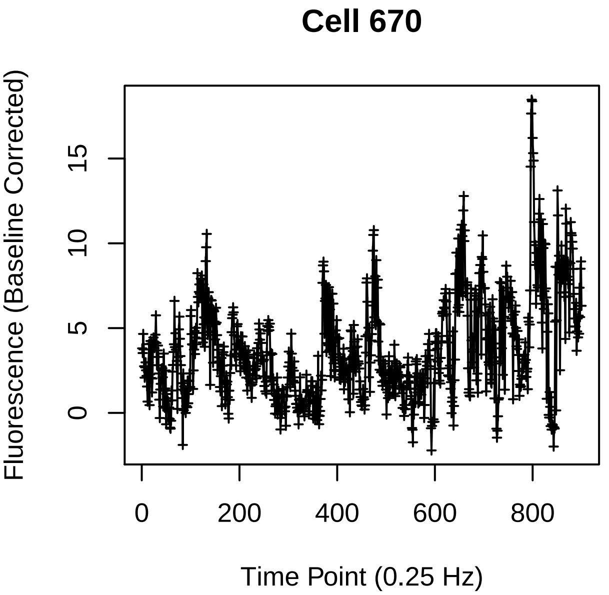
Cell 670
Fluorescence (Baseline Corrected)
0
5
10
15
0
200
400
600
800
Time Point (0.25 Hz)

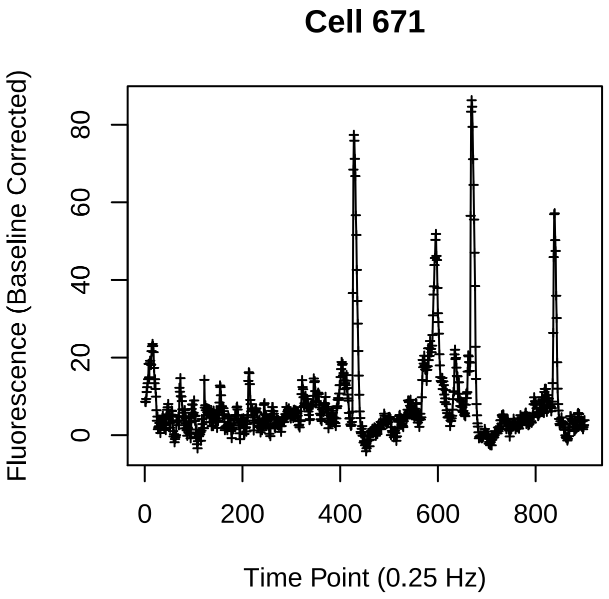
Cell 671
Fluorescence (Baseline Corrected)
0
20
40
60
80
0
200
400
600
800
Time Point (0.25 Hz)

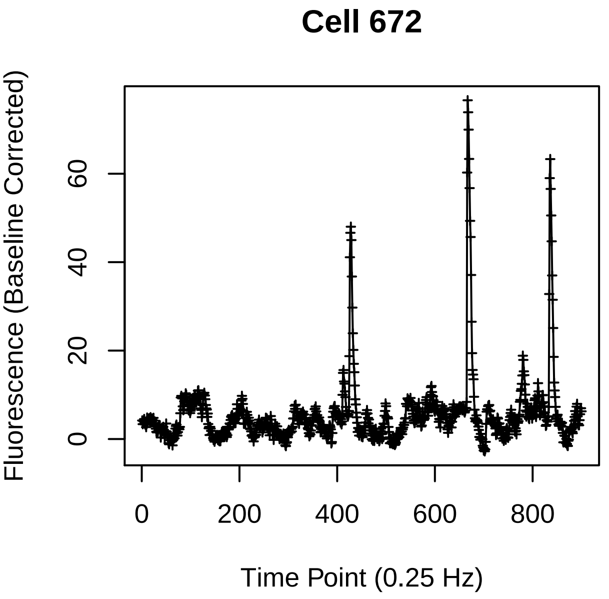
Cell 672
Fluorescence (Baseline Corrected)
0
20
40
60
0
200
400
600
800
Time Point (0.25 Hz)

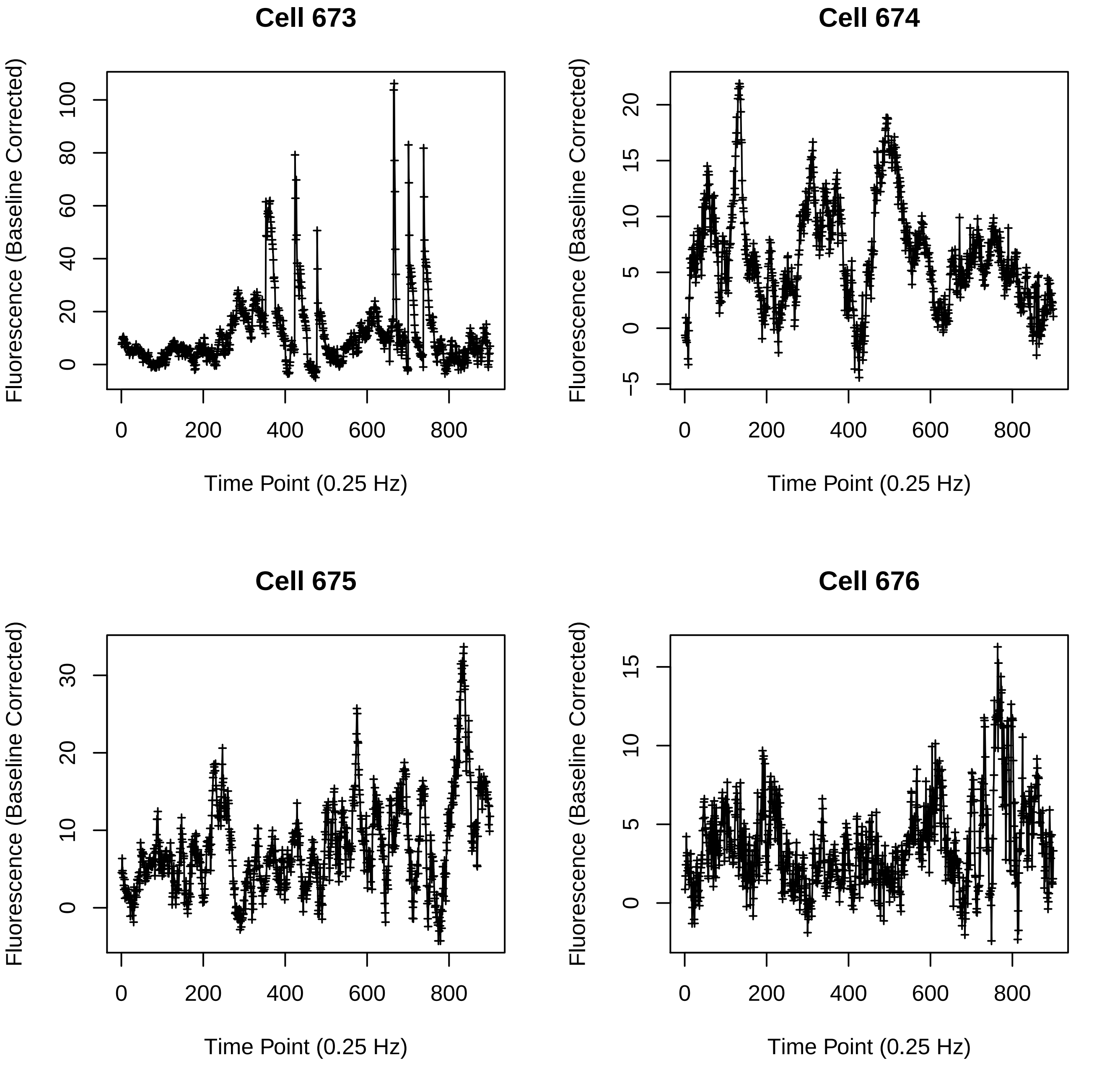
Cell 673
Cell 674
Cell 675
Cell 676
Fluorescence (Baseline Corrected)
Time Point (0.25 Hz)
0
200
400
600
800
20
40
60
80
100
-5
5
10
15
30

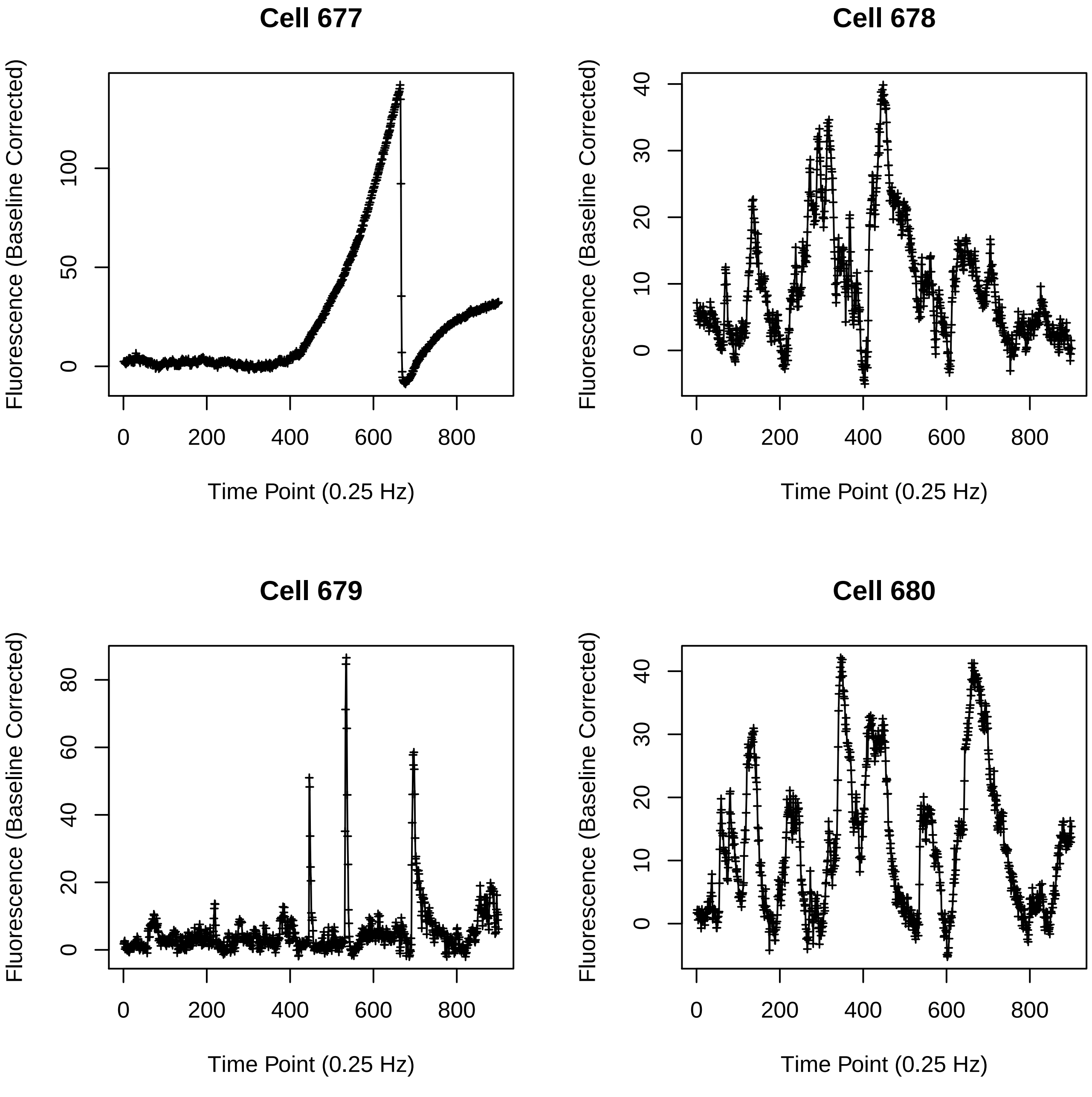
Cell 677
Cell 678
Cell 679
Cell 680
Fluorescence (Baseline Corrected)
Time Point (0.25 Hz)
0
50
100
10
20
30
40
60
80
-0
200
400
600
800

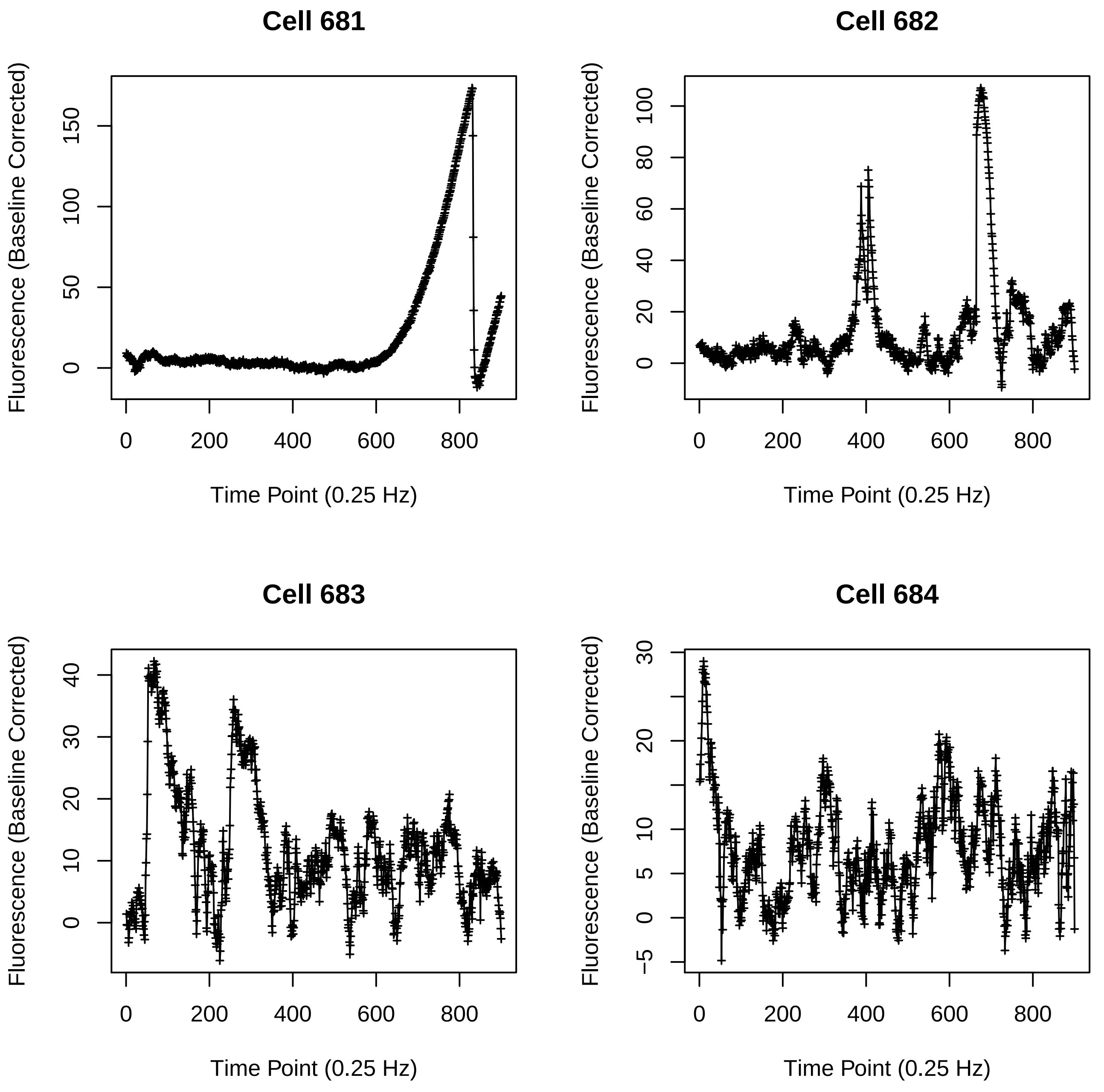
Cell 681
Cell 682
Cell 683
Cell 684
Fluorescence (Baseline Corrected)
Time Point (0.25 Hz)
0
50
100
150
200
400
600
800
20
40
60
80
10
30
−5
5
15
25

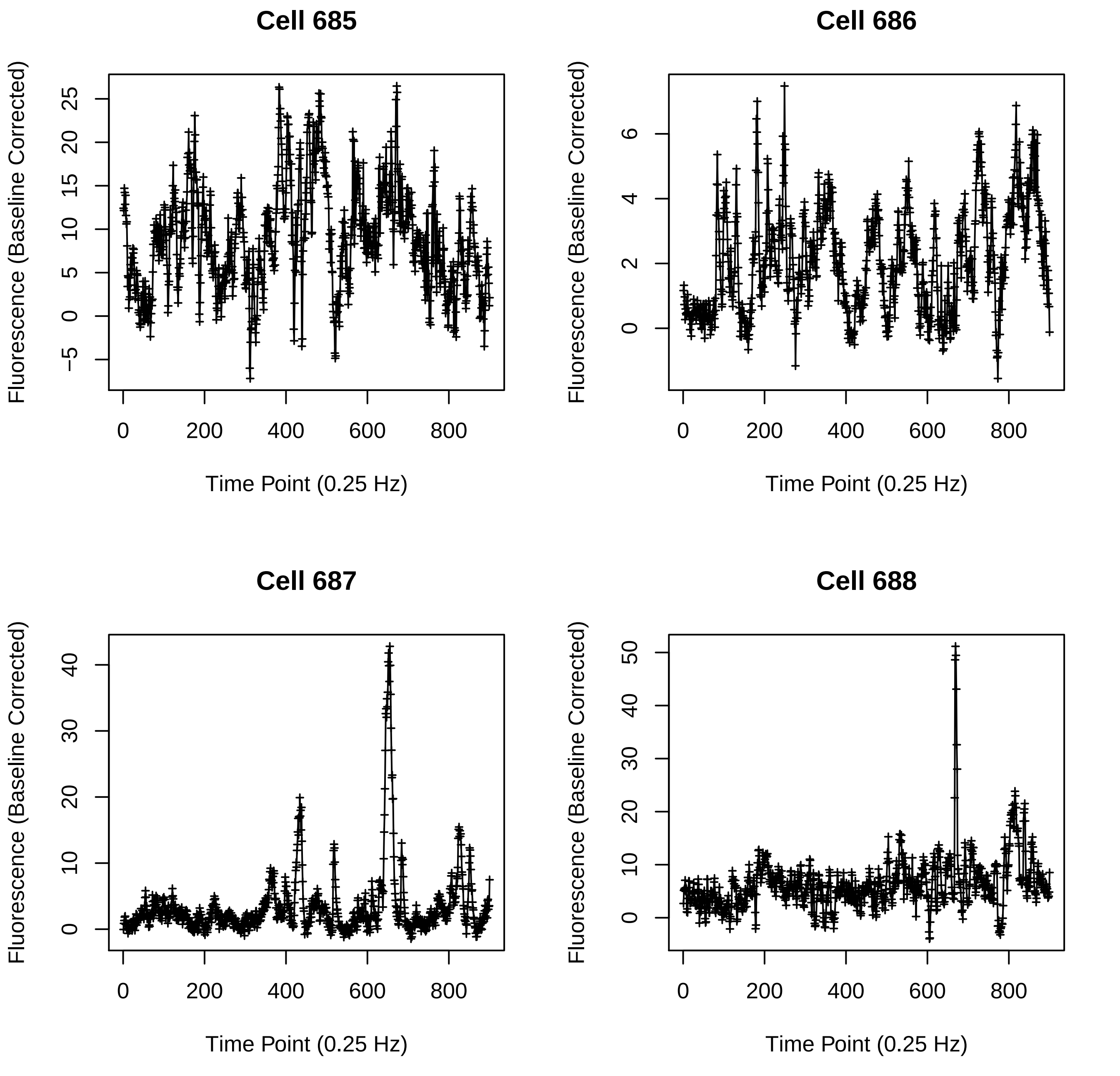
Cell 685
Cell 686
Cell 687
Cell 688
Fluorescence (Baseline Corrected)
Time Point (0.25 Hz)
0
200
400
600
800
−5
0
5
10
15
20
25
2
4
6
30
40
50

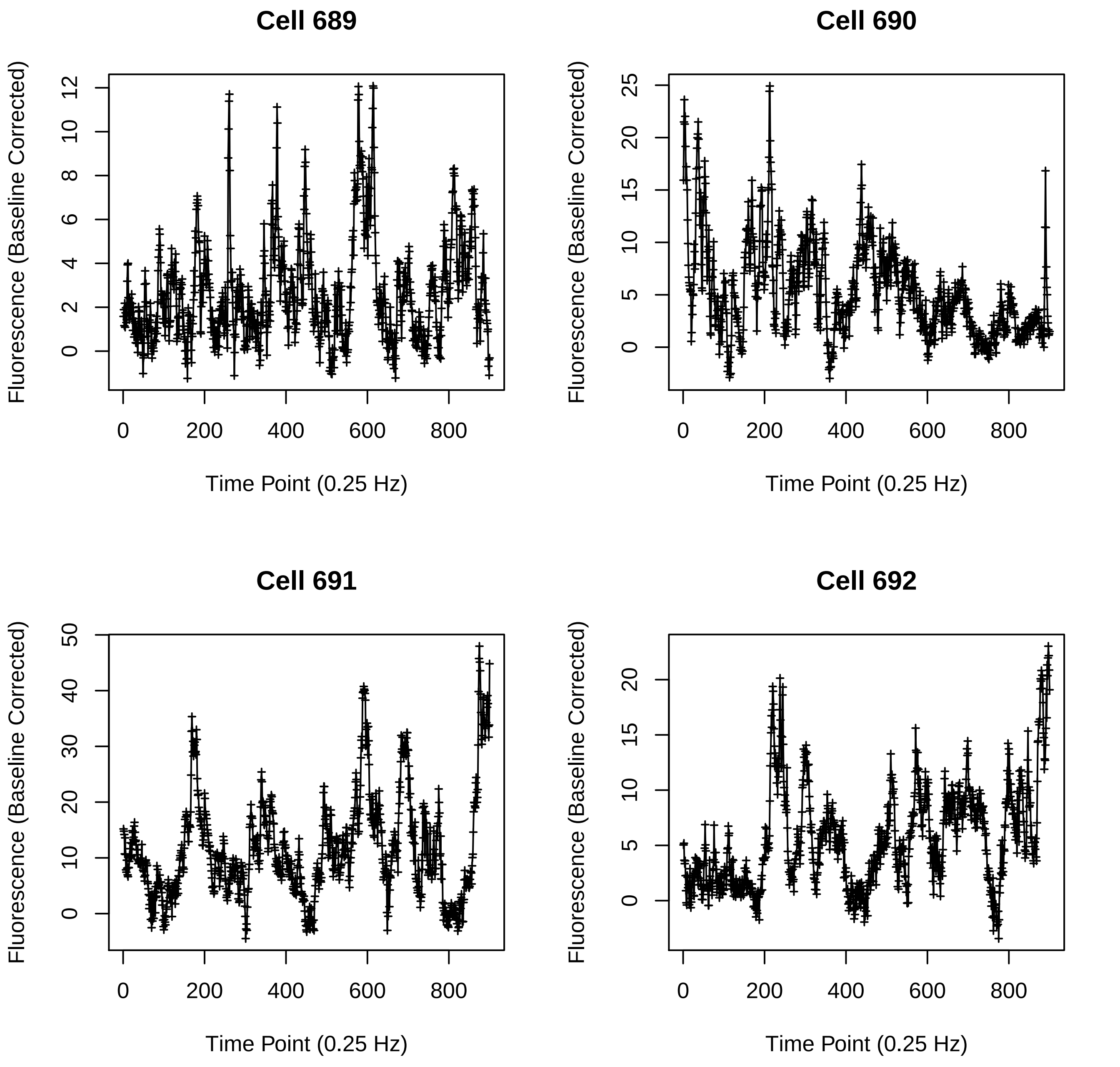
Cell 689
Cell 690
Cell 691
Cell 692
Fluorescence (Baseline Corrected)
Time Point (0.25 Hz)
0
200
400
600
800
-2
0
2
4
6
8
10
12
5
15
20
25
30
40
50

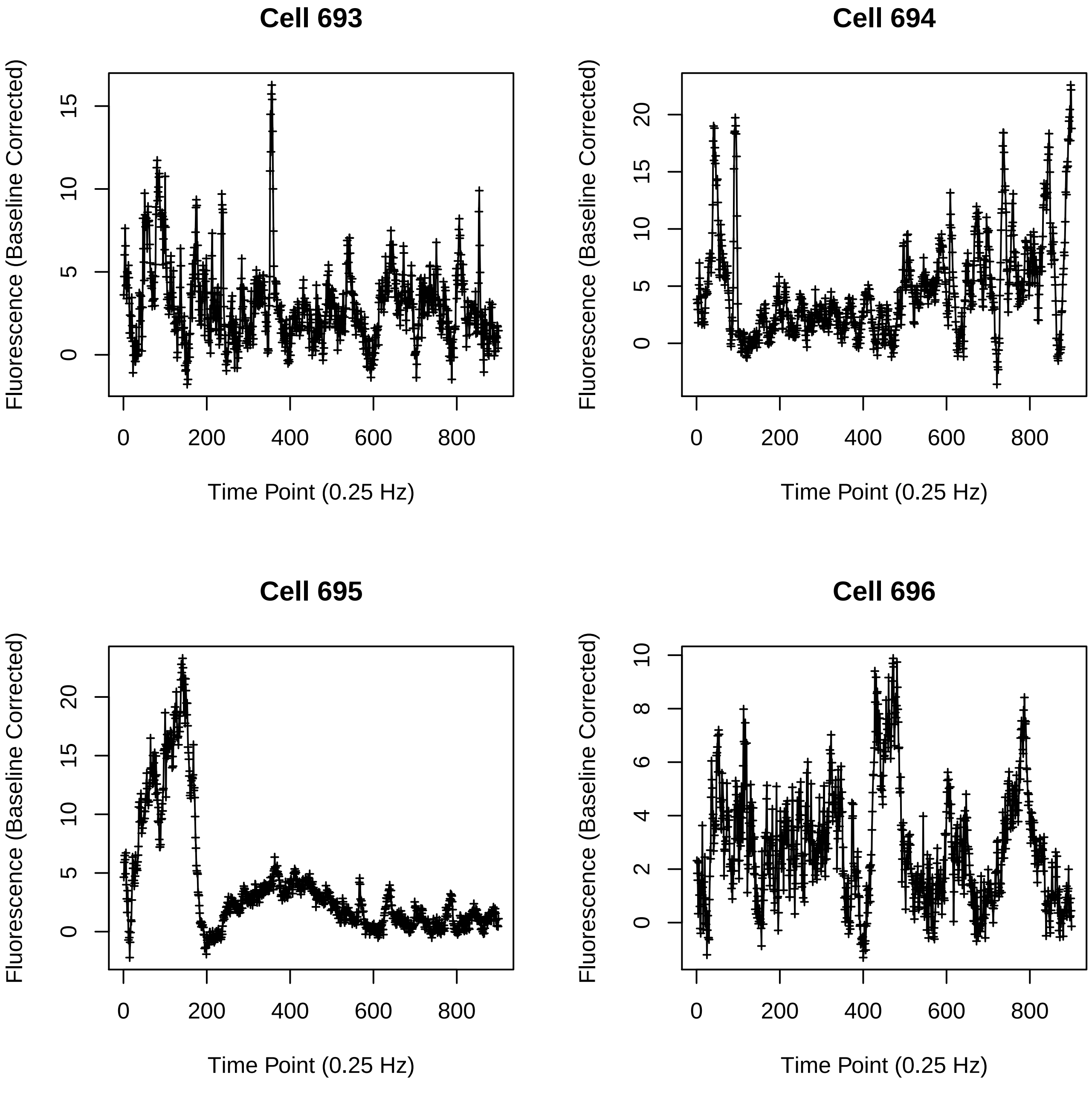

Cell 693
Cell 694
Cell 695
Cell 696
Fluorescence (Baseline Corrected)
Time Point (0.25 Hz)
0
200
400
600
800
5
10
15
20
2
4
6
8

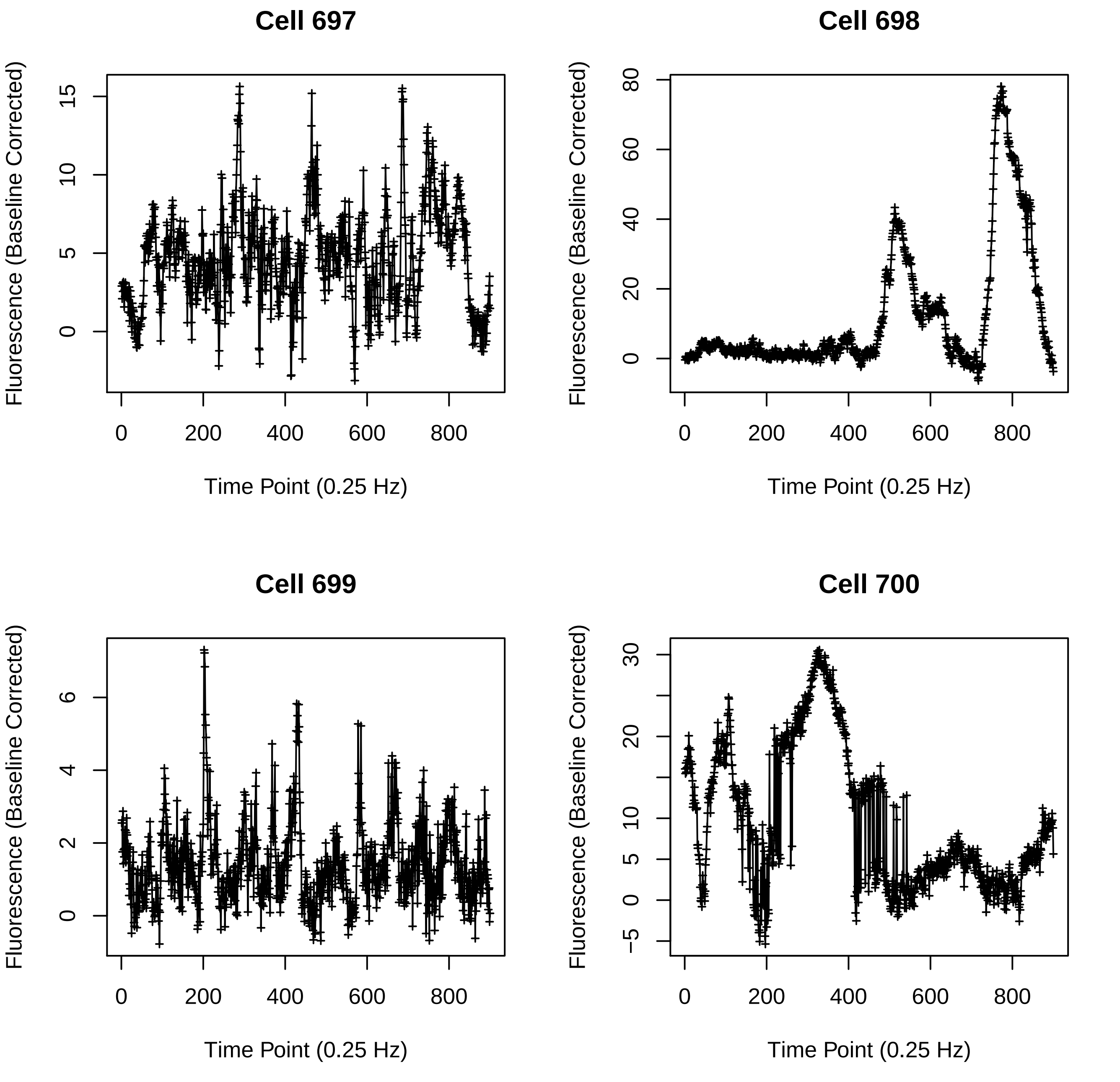
Cell 697
Cell 698
Cell 699
Cell 700
Fluorescence (Baseline Corrected)
Time Point (0.25 Hz)

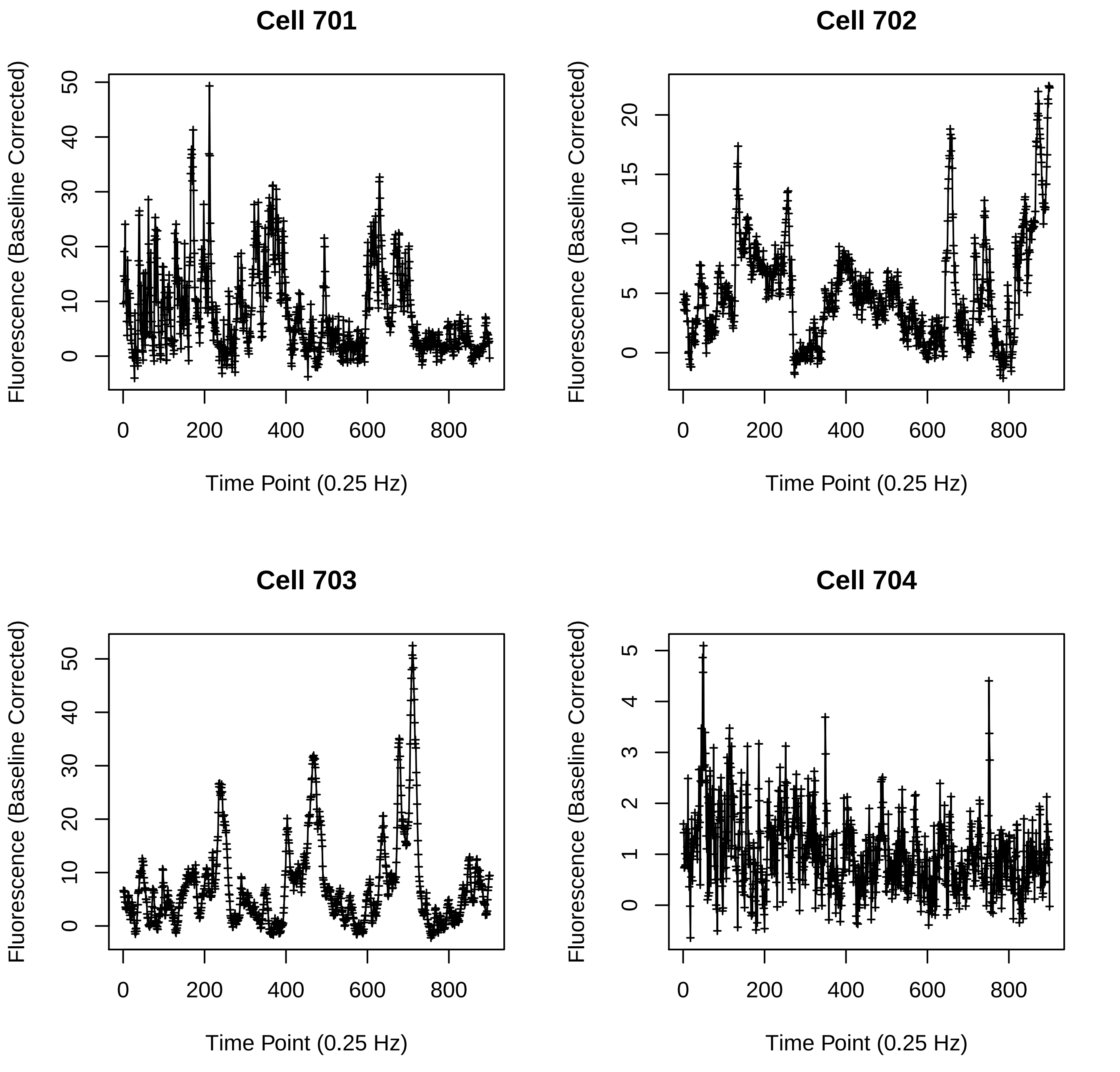

Cell 701
Cell 702
Cell 703
Cell 704
Fluorescence (Baseline Corrected)
Time Point (0.25 Hz)
0
200
400
600
800
-0
10
20
30
40
50
5
15
1
2
3
4

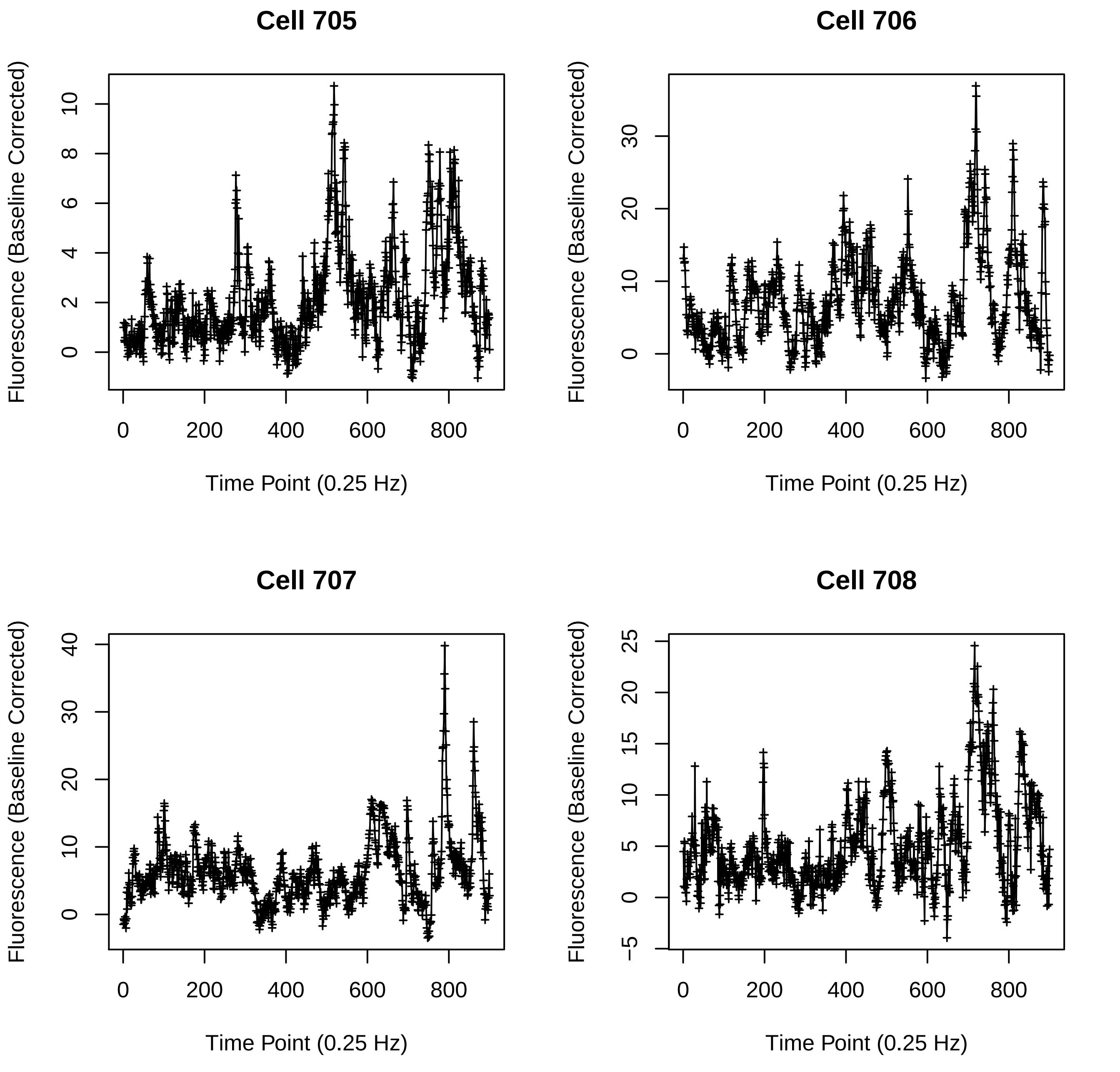
Cell 705
Fluorescence (Baseline Corrected)
0
2
4
6
8
10
0
200
400
600
800
Time Point (0.25 Hz)
Cell 706
Fluorescence (Baseline Corrected)
0
10
20
30
0
200
400
600
800
Time Point (0.25 Hz)
Cell 707
Fluorescence (Baseline Corrected)
0
10
20
30
40
0
200
400
600
800
Time Point (0.25 Hz)
Cell 708
Fluorescence (Baseline Corrected)
−5
0
5
10
15
20
25
0
200
400
600
800
Time Point (0.25 Hz)

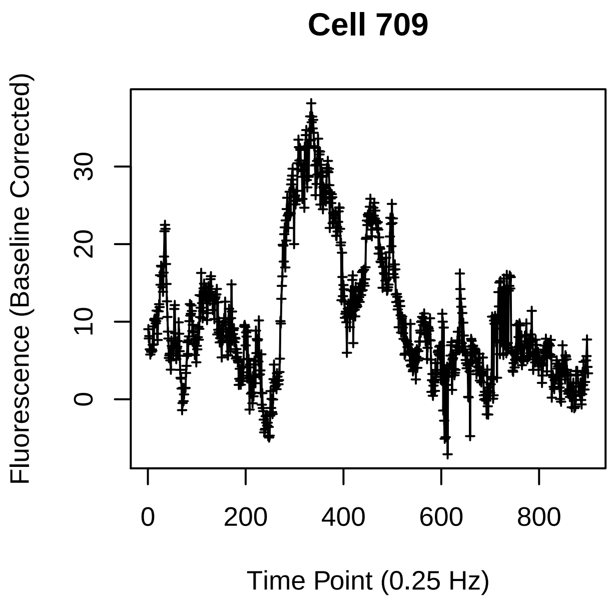
Cell 709
Fluorescence (Baseline Corrected)
0
10
20
30
0
200
400
600
800
Time Point (0.25 Hz)

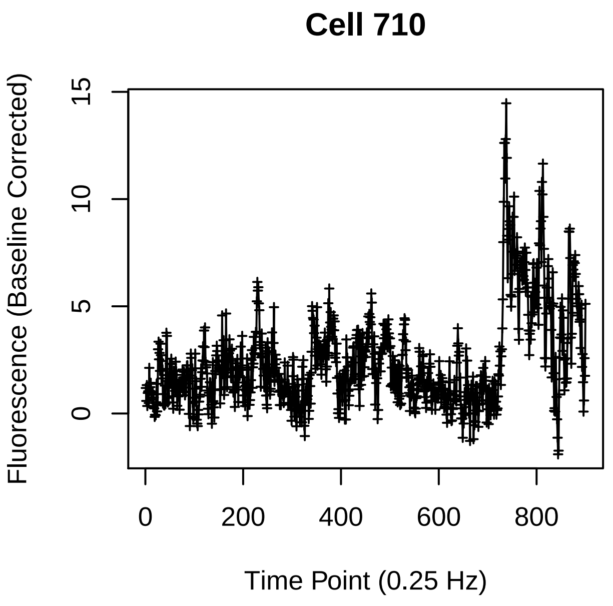
Cell 710
Fluorescence (Baseline Corrected)
0
5
10
15
0
200
400
600
800
Time Point (0.25 Hz)

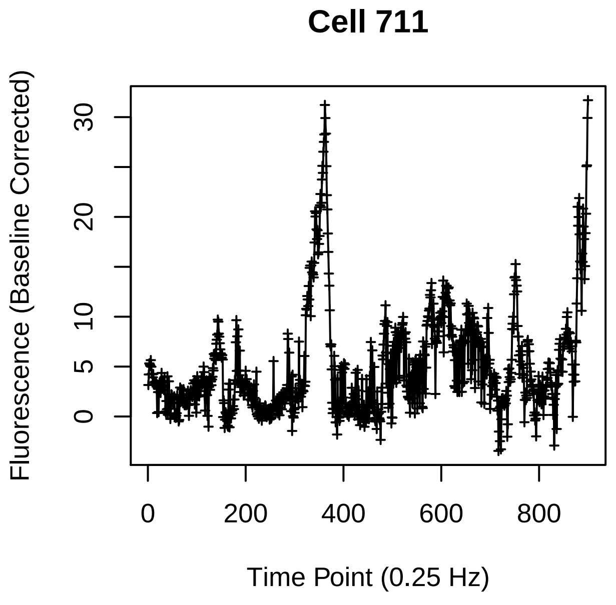
Cell 711
Fluorescence (Baseline Corrected)
0
5
10
20
30
0
200
400
600
800
Time Point (0.25 Hz)

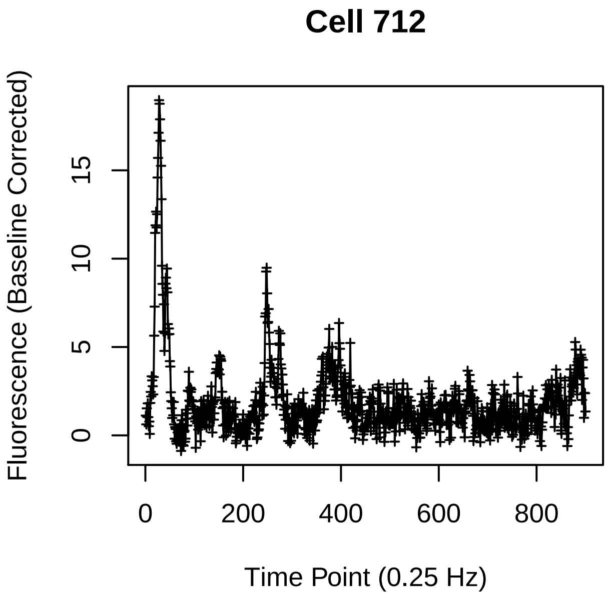
Cell 712
Fluorescence (Baseline Corrected)
0
5
10
15
0
200
400
600
800
Time Point (0.25 Hz)

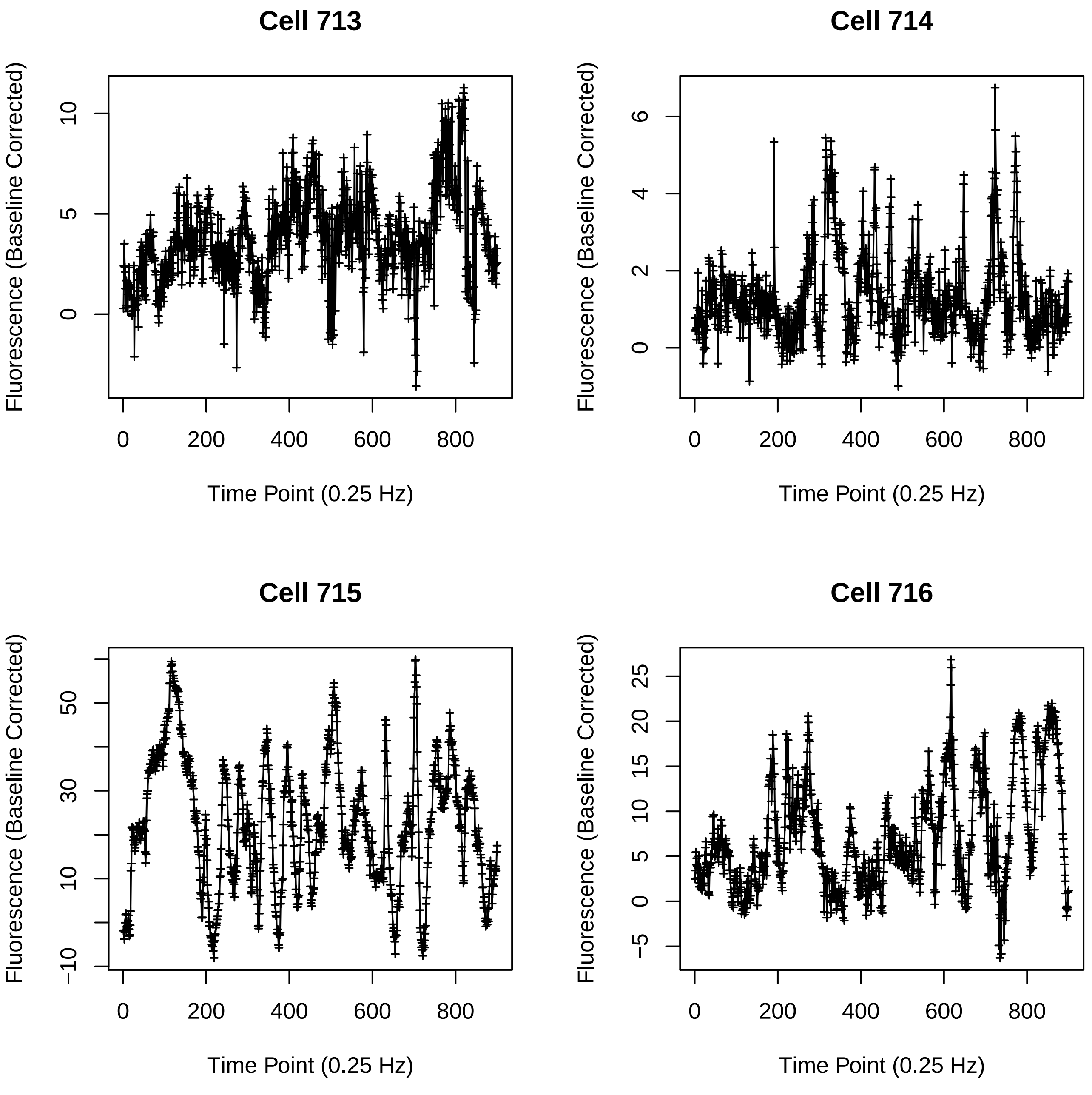
Cell 713
Cell 714
Cell 715
Cell 716
Fluorescence (Baseline Corrected)
Time Point (0.25 Hz)
0
200
400
600
800
10
5
0
6
4
2
50
30
10
−10
25
20
15
−5

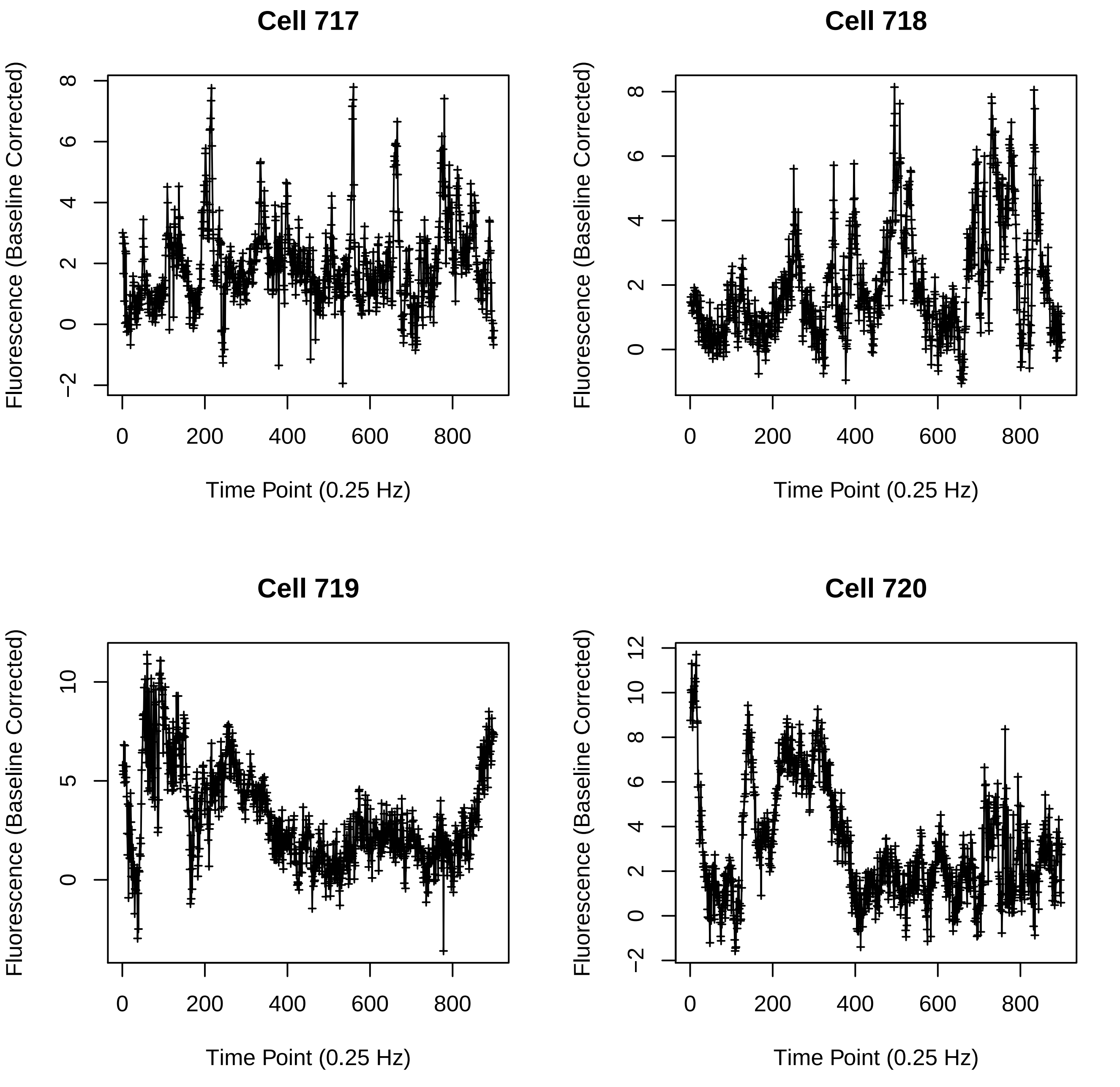
Cell 717
Cell 718
Cell 719
Cell 720
Fluorescence (Baseline Corrected)
Time Point (0.25 Hz)
0
200
400
600
800
−2
2
4
6
8
5
10
12

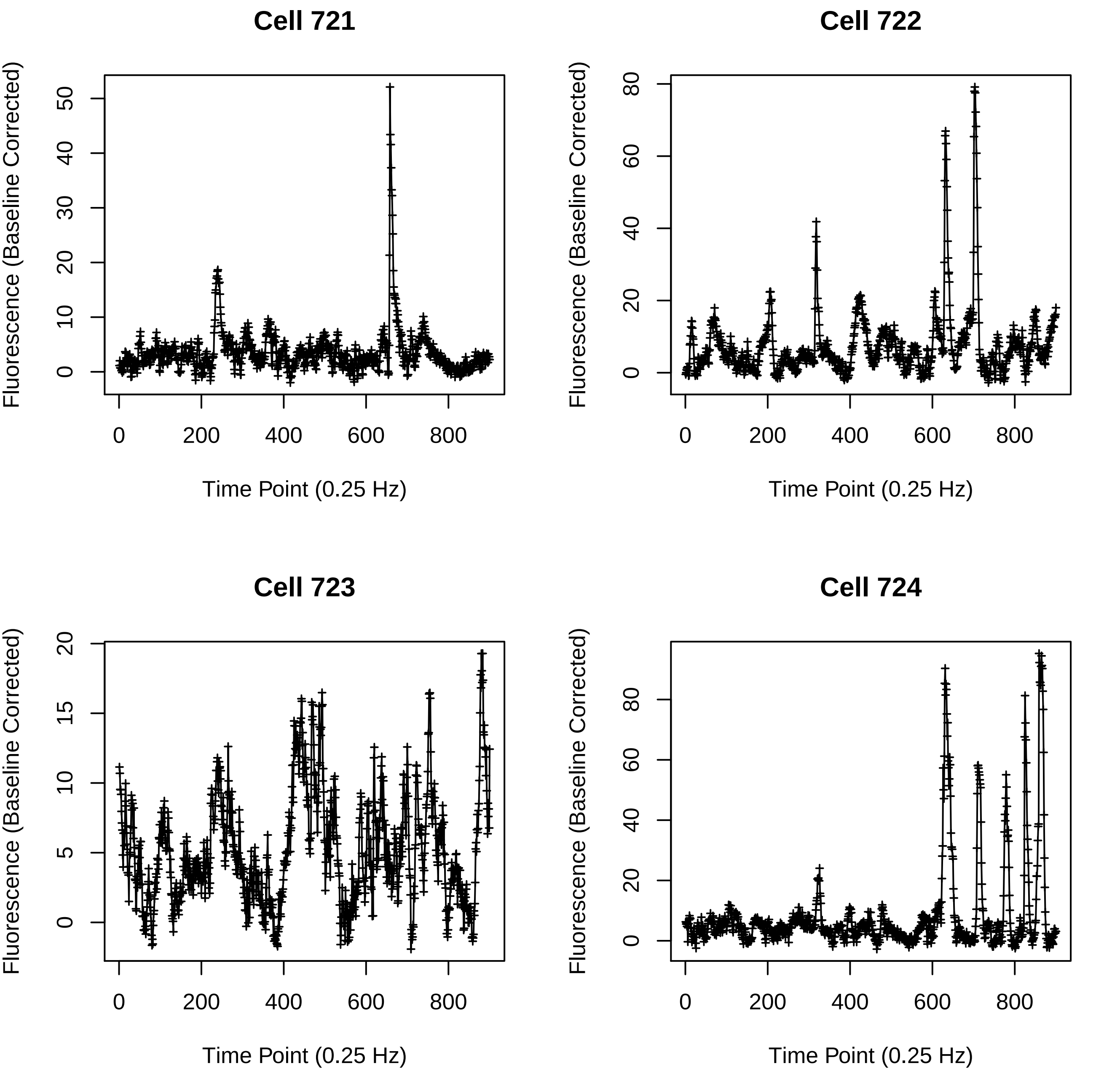

Cell 721
Fluorescence (Baseline Corrected)
0
10
20
30
40
50
0
200
400
600
800
Time Point (0.25 Hz)
Cell 722
Fluorescence (Baseline Corrected)
0
20
40
60
80
0
200
400
600
800
Time Point (0.25 Hz)
Cell 723
Fluorescence (Baseline Corrected)
0
5
10
15
20
0
200
400
600
800
Time Point (0.25 Hz)
Cell 724
Fluorescence (Baseline Corrected)
0
20
40
60
80
0
200
400
600
800
Time Point (0.25 Hz)

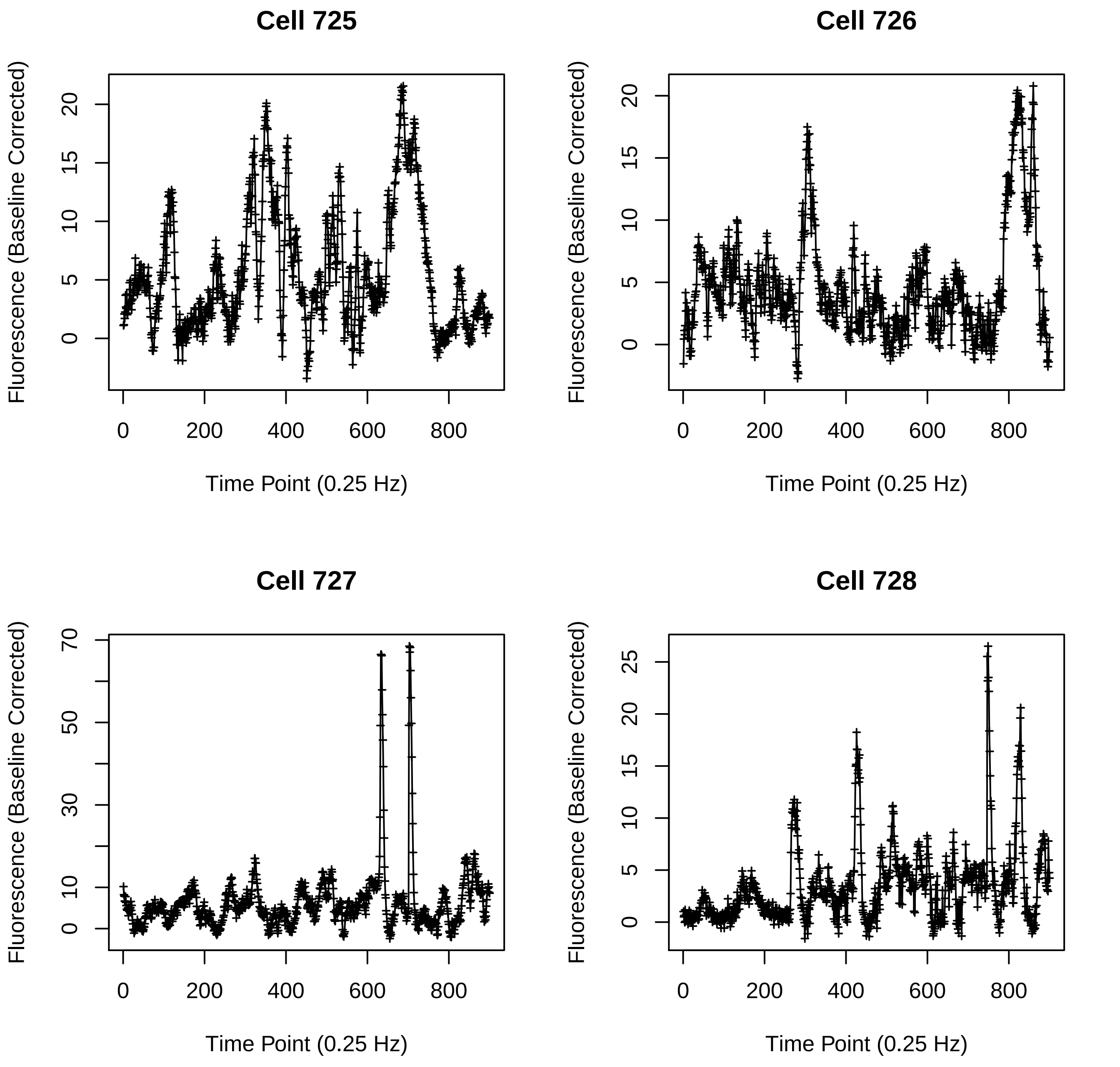
Cell 725
Cell 726
Cell 727
Cell 728
Fluorescence (Baseline Corrected)
Time Point (0.25 Hz)
0
5
10
15
20
25
30
50
70
200
400
600
800

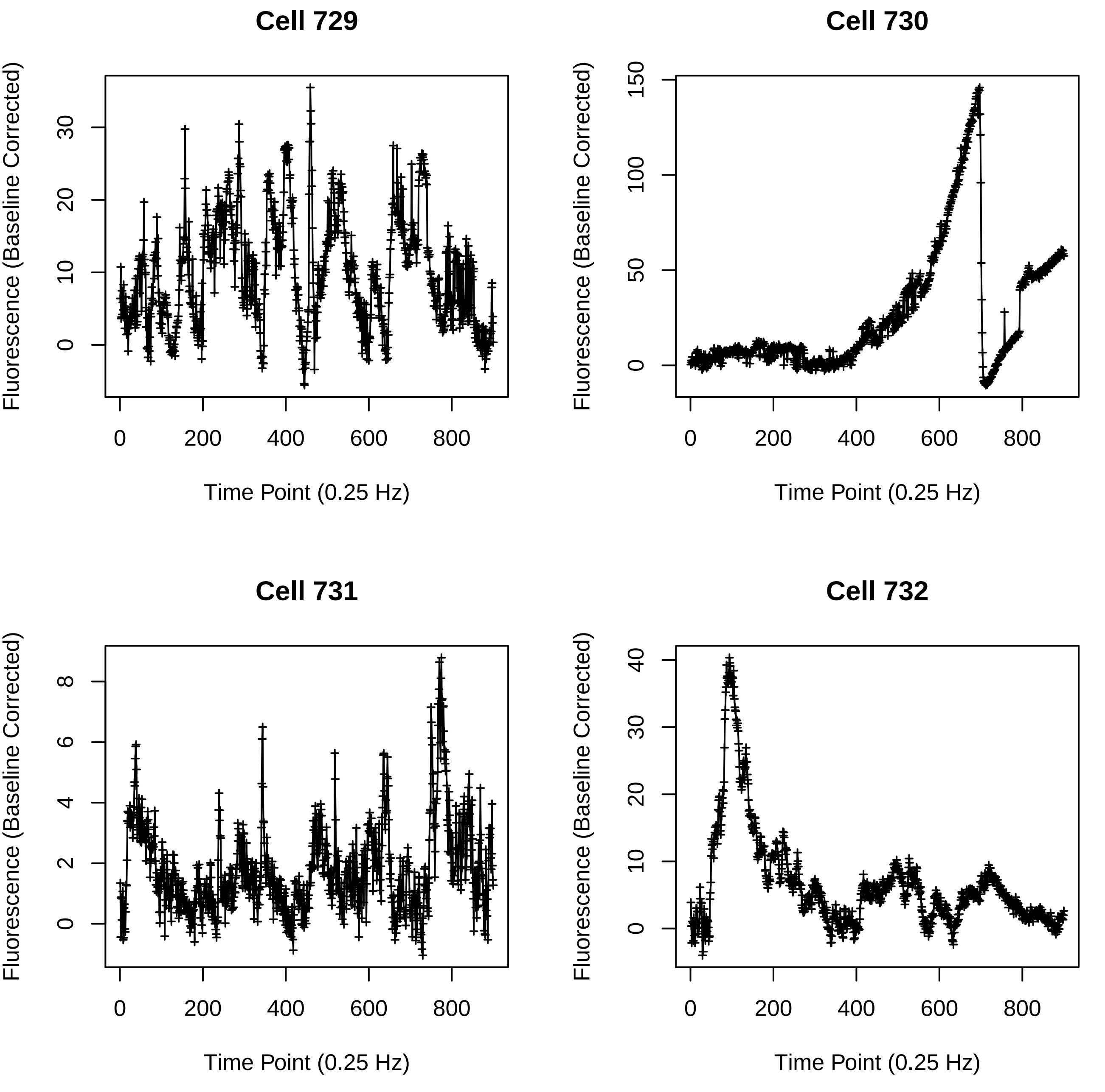

Cell 729
Cell 730
Cell 731
Cell 732
Fluorescence (Baseline Corrected)
Time Point (0.25 Hz)

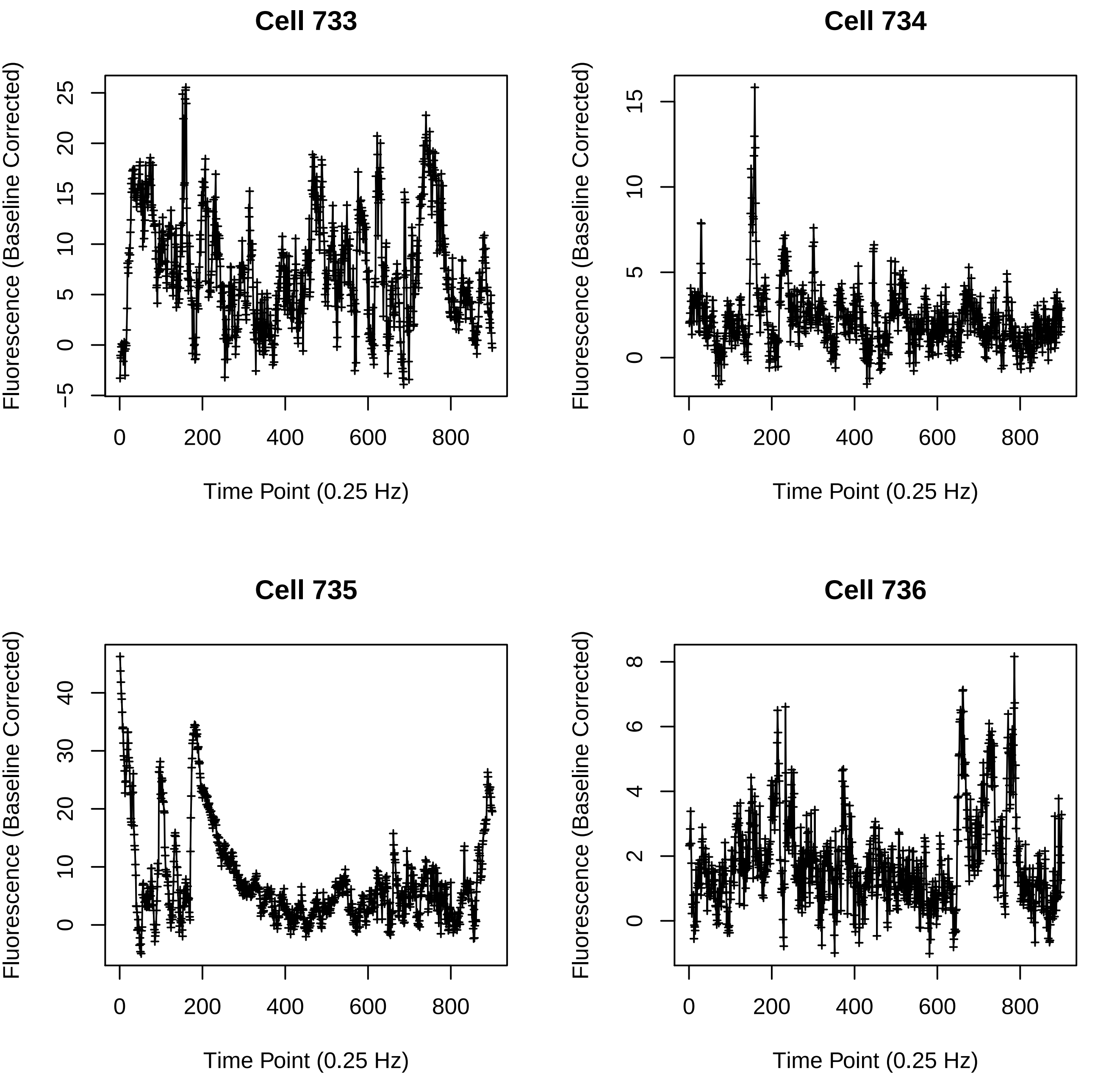
Cell 733
Fluorescence (Baseline Corrected)
−5
0
5
10
15
20
25
0
200
400
600
800
Time Point (0.25 Hz)
Cell 734
Fluorescence (Baseline Corrected)
0
5
10
15
0
200
400
600
800
Time Point (0.25 Hz)
Cell 735
Fluorescence (Baseline Corrected)
0
10
20
30
40
0
200
400
600
800
Time Point (0.25 Hz)
Cell 736
Fluorescence (Baseline Corrected)
0
2
4
6
8
0
200
400
600
800
Time Point (0.25 Hz)

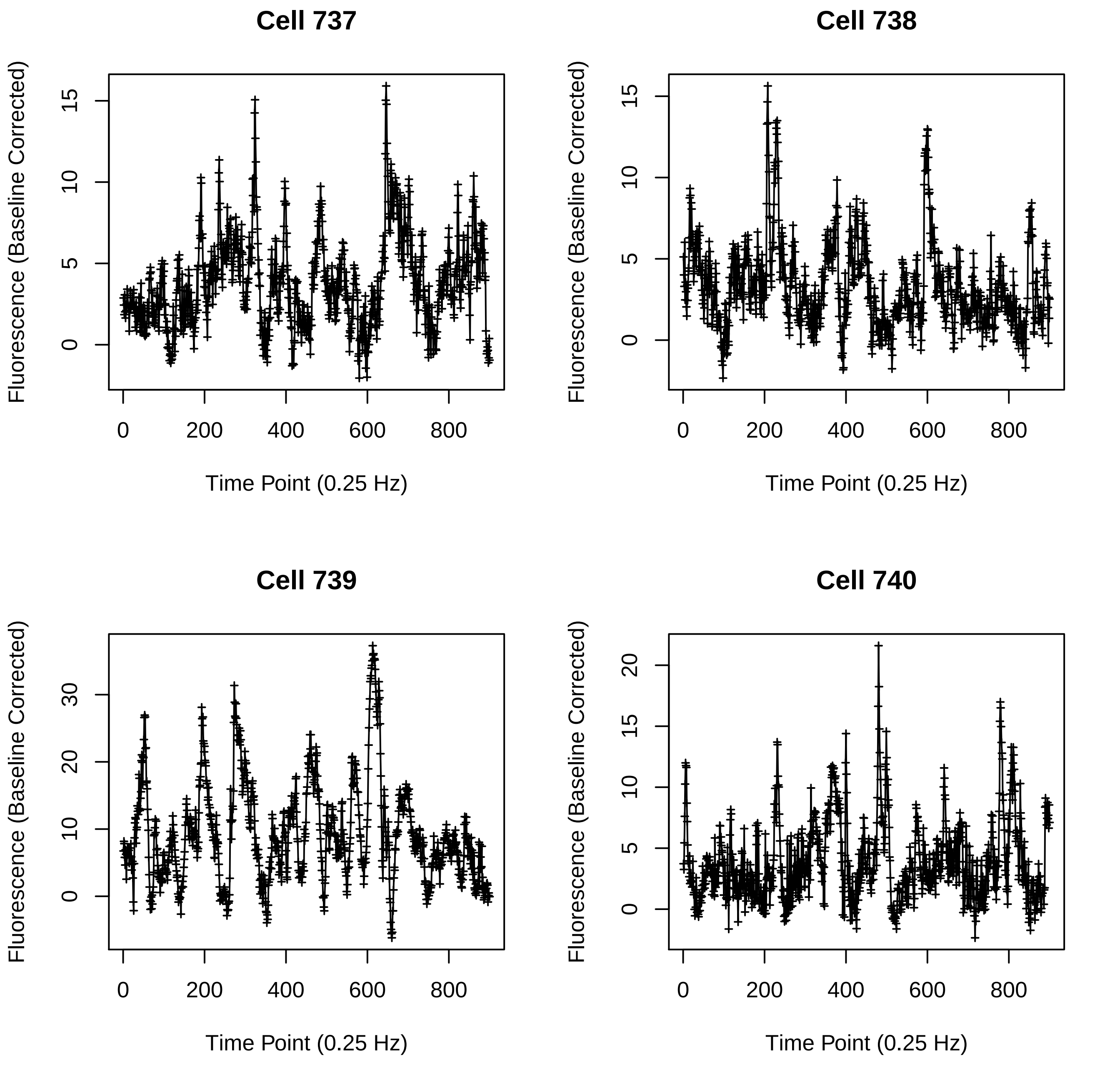
Cell 737
Fluorescence (Baseline Corrected)
Time Point (0.25 Hz)
Cell 738
Fluorescence (Baseline Corrected)
Time Point (0.25 Hz)
Cell 739
Fluorescence (Baseline Corrected)
Time Point (0.25 Hz)
Cell 740
Fluorescence (Baseline Corrected)
Time Point (0.25 Hz)

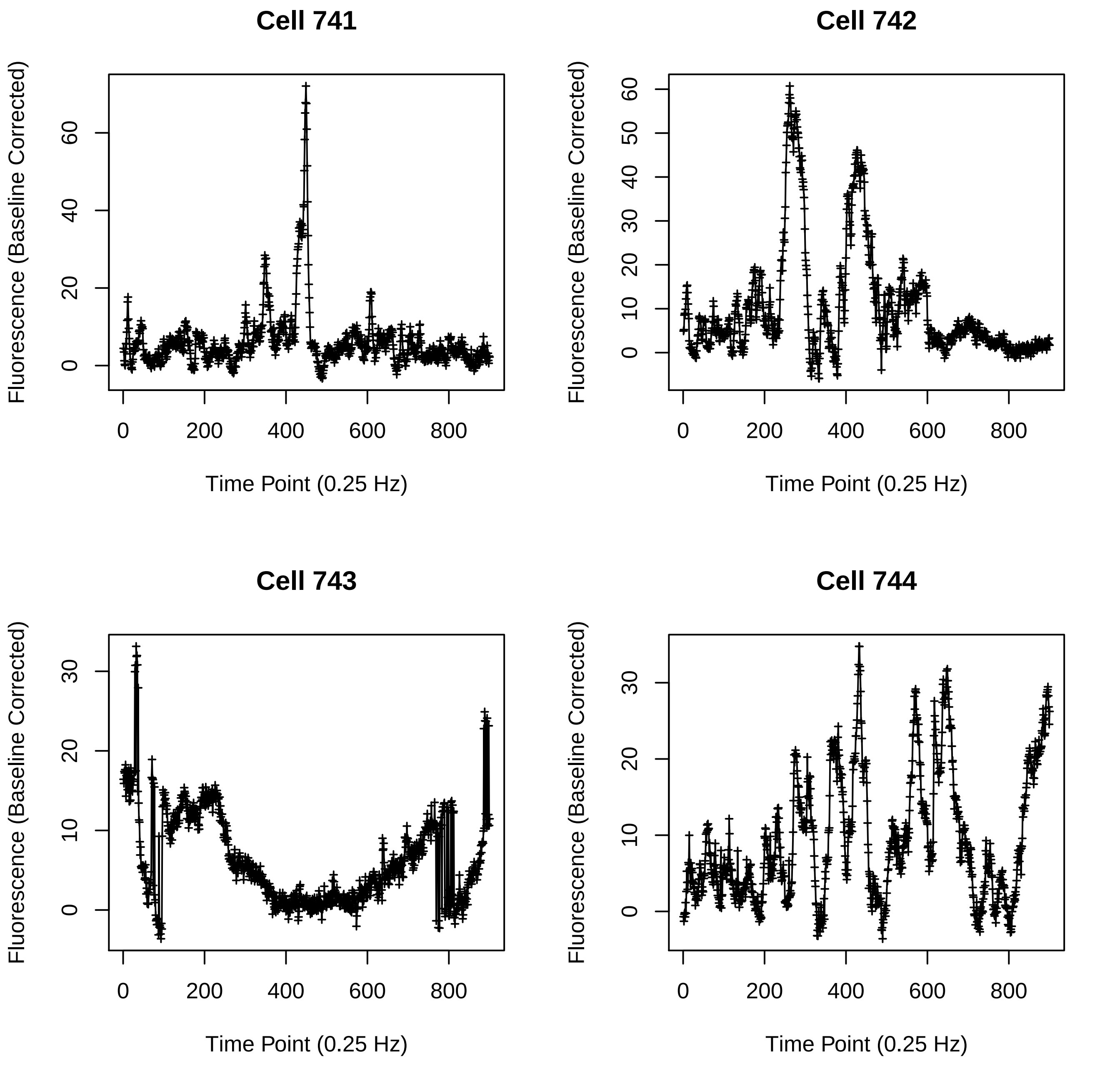

Cell 741
Cell 742
Cell 743
Cell 744
Fluorescence (Baseline Corrected)
Time Point (0.25 Hz)
0
200
400
600
800
0
20
40
60
10
30
50

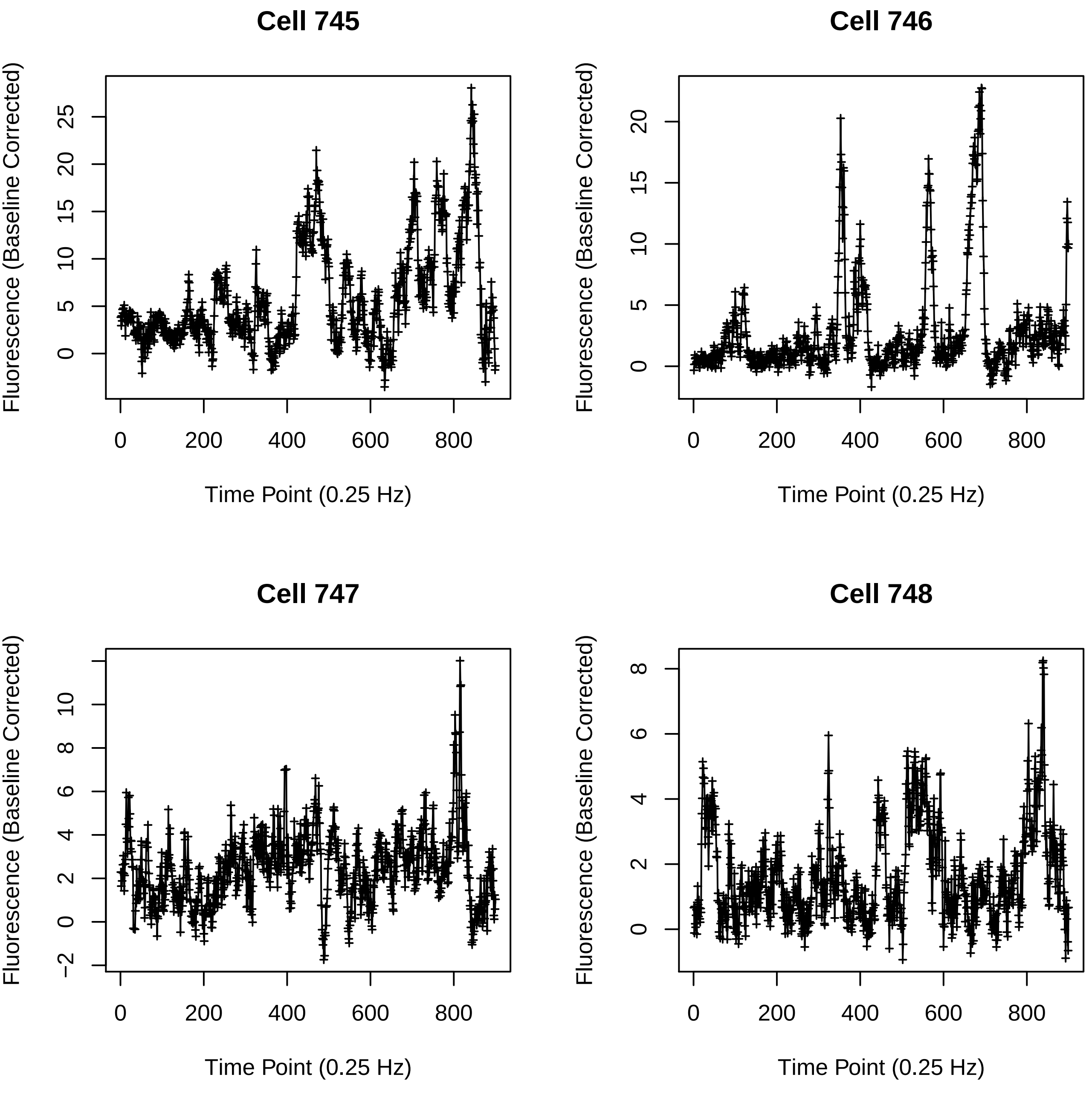
Cell 745
Fluorescence (Baseline Corrected)
Time Point (0.25 Hz)
Cell 746
Fluorescence (Baseline Corrected)
Time Point (0.25 Hz)
Cell 747
Fluorescence (Baseline Corrected)
Time Point (0.25 Hz)
Cell 748
Fluorescence (Baseline Corrected)
Time Point (0.25 Hz)

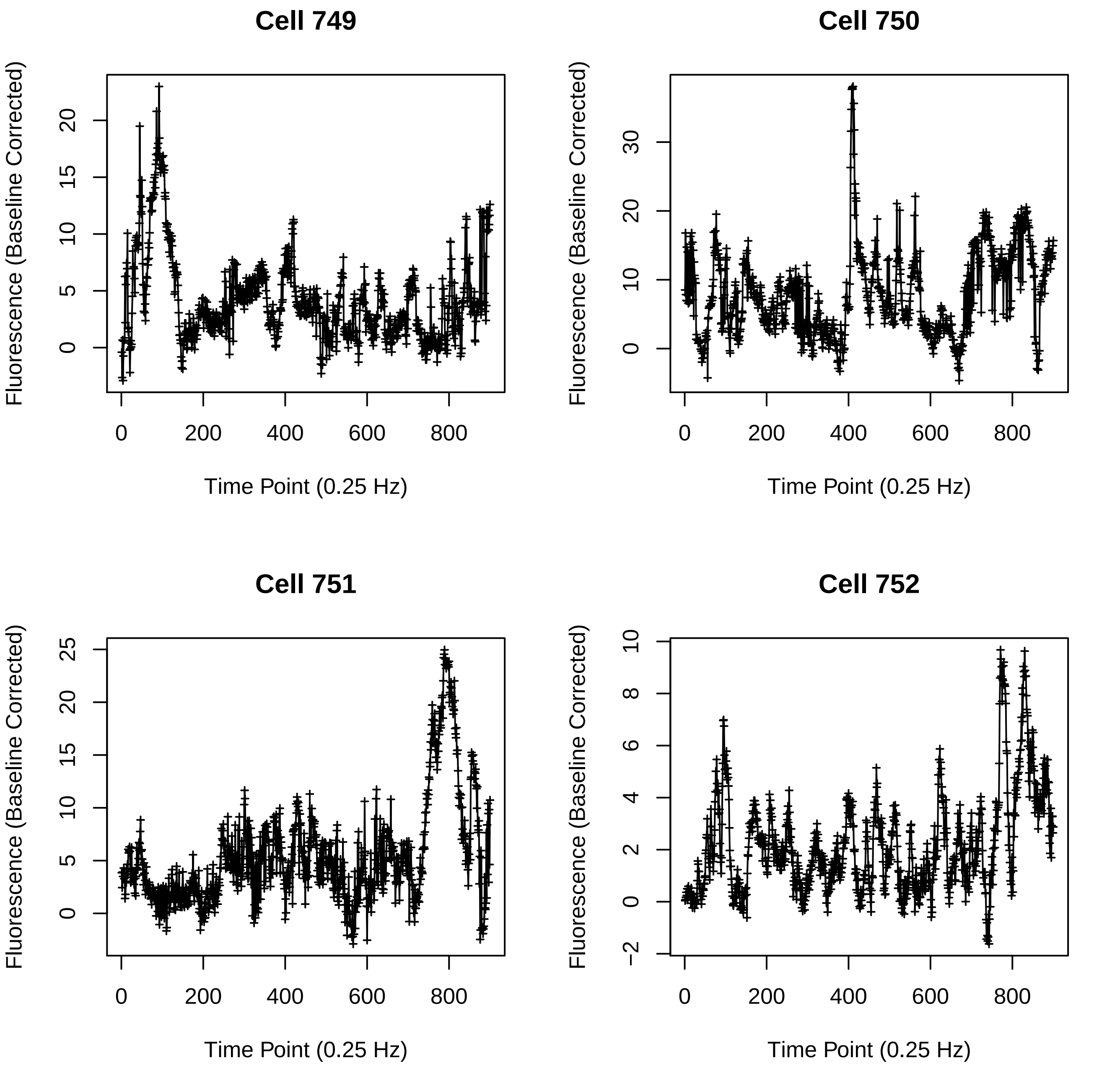
Cell 749
Fluorescence (Baseline Corrected)
0
5
10
15
20
0
200
400
600
800
Time Point (0.25 Hz)
Cell 750
Fluorescence (Baseline Corrected)
0
10
20
30
0
200
400
600
800
Time Point (0.25 Hz)
Cell 751
Fluorescence (Baseline Corrected)
0
5
10
15
20
25
0
200
400
600
800
Time Point (0.25 Hz)
Cell 752
Fluorescence (Baseline Corrected)
−2
0
2
4
6
8
10
0
200
400
600
800
Time Point (0.25 Hz)

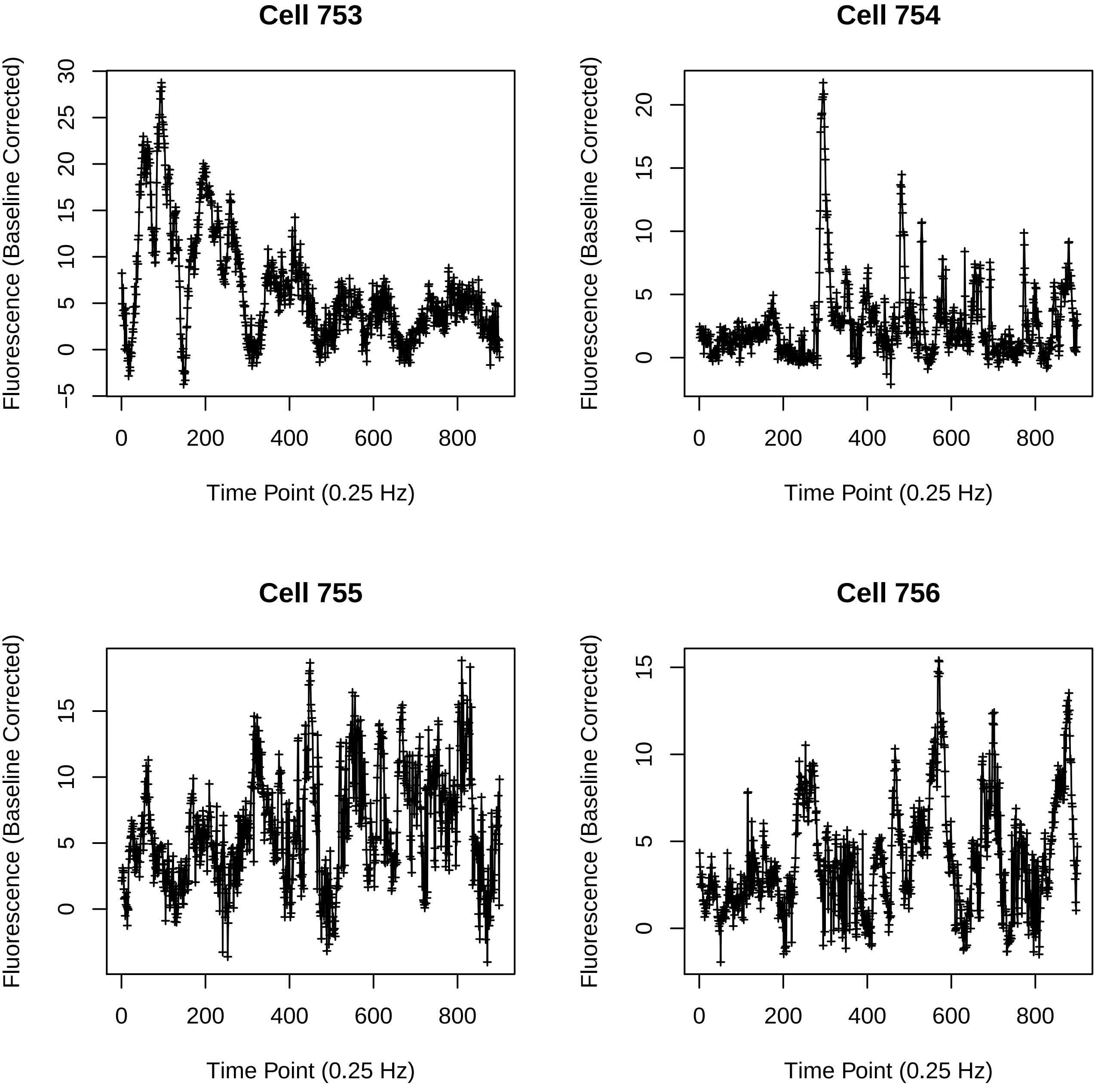

Cell 753
Cell 754
Cell 755
Cell 756
Fluorescence (Baseline Corrected)
Time Point (0.25 Hz)
−5
0
5
10
15
20
25
30
0
200
400
600
800

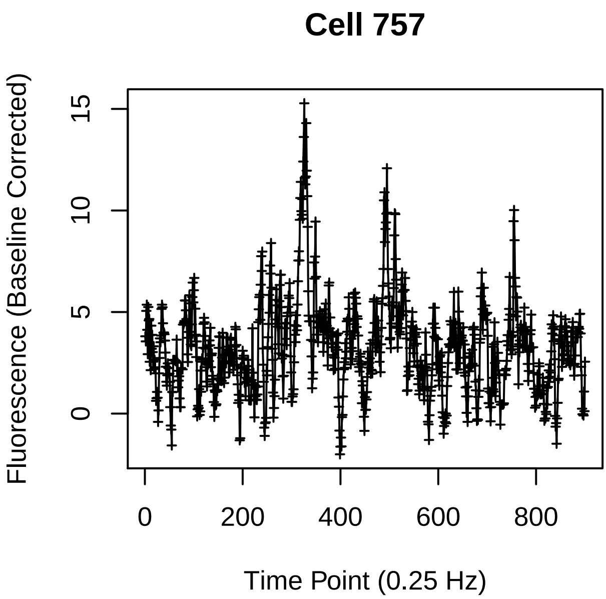
Cell 757
Fluorescence (Baseline Corrected)
0
5
10
15
0
200
400
600
800
Time Point (0.25 Hz)
