# Supplementary material for: A Markovian Entropy Measure for the Analysis of Calcium Activity Time Series
Source: PLoS One. 2016 Dec 15;11(12):e0168342. doi: 10.1371/journal.pone.0168342 (PMC5158058; doi:10.1371/journal.pone.0168342)

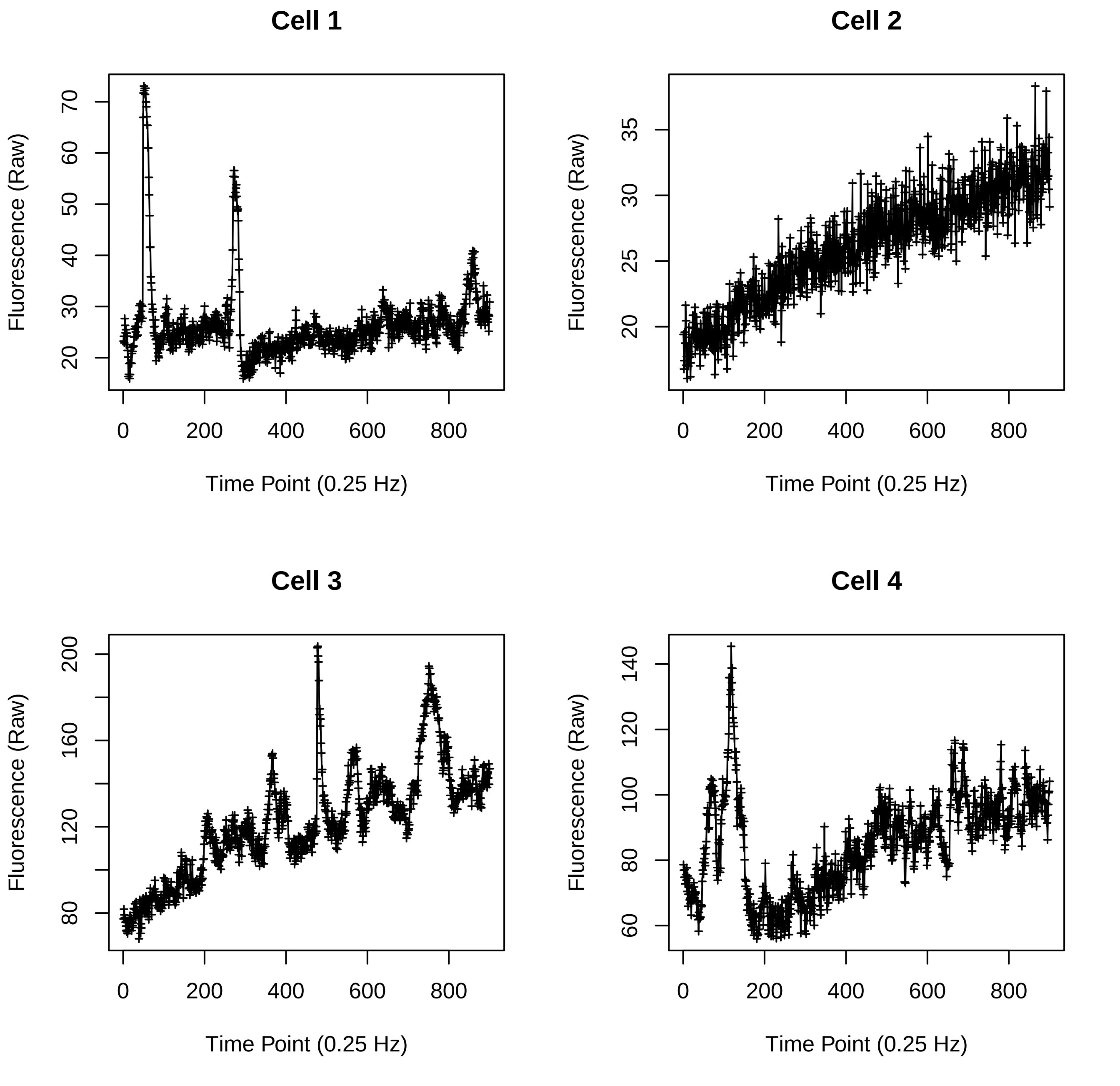

Cell 1
Cell 2
Cell 3
Cell 4
Fluorescence (Raw)
Time Point (0.25 Hz)
20
30
40
50
60
70
25
35
80
120
160
200
60
100
140
0
200
400
600
800

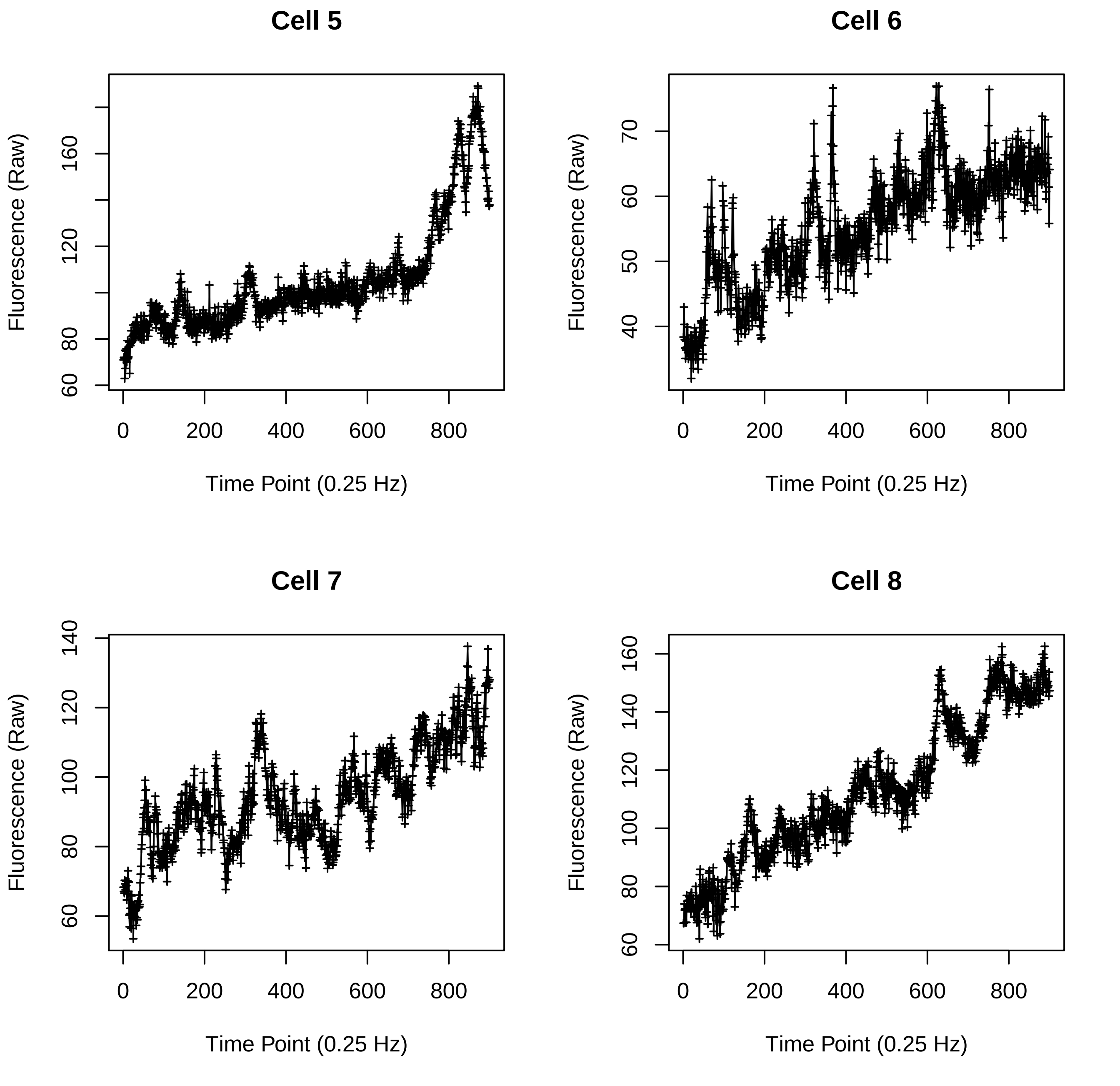
Cell 5
Fluorescence (Raw)
60
80
120
160
0
200
400
600
800
Time Point (0.25 Hz)
Cell 6
Fluorescence (Raw)
40
50
60
70
0
200
400
600
800
Time Point (0.25 Hz)
Cell 7
Fluorescence (Raw)
60
80
100
120
140
0
200
400
600
800
Time Point (0.25 Hz)
Cell 8
Fluorescence (Raw)
60
80
100
120
140
160
0
200
400
600
800
Time Point (0.25 Hz)

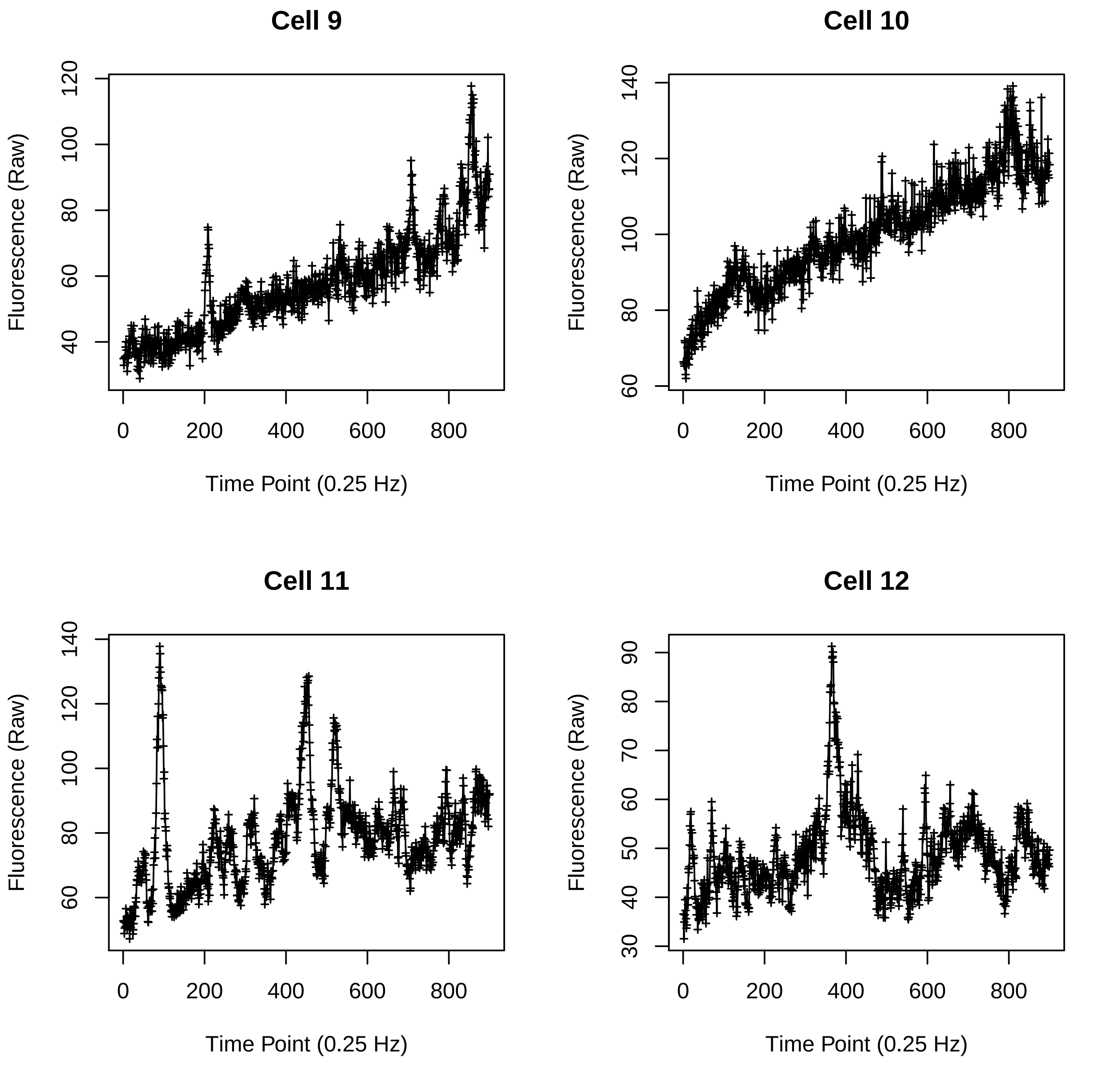

Cell 9
Cell 10
Cell 11
Cell 12
Fluorescence (Raw)
Time Point (0.25 Hz)
0
200
400
600
800

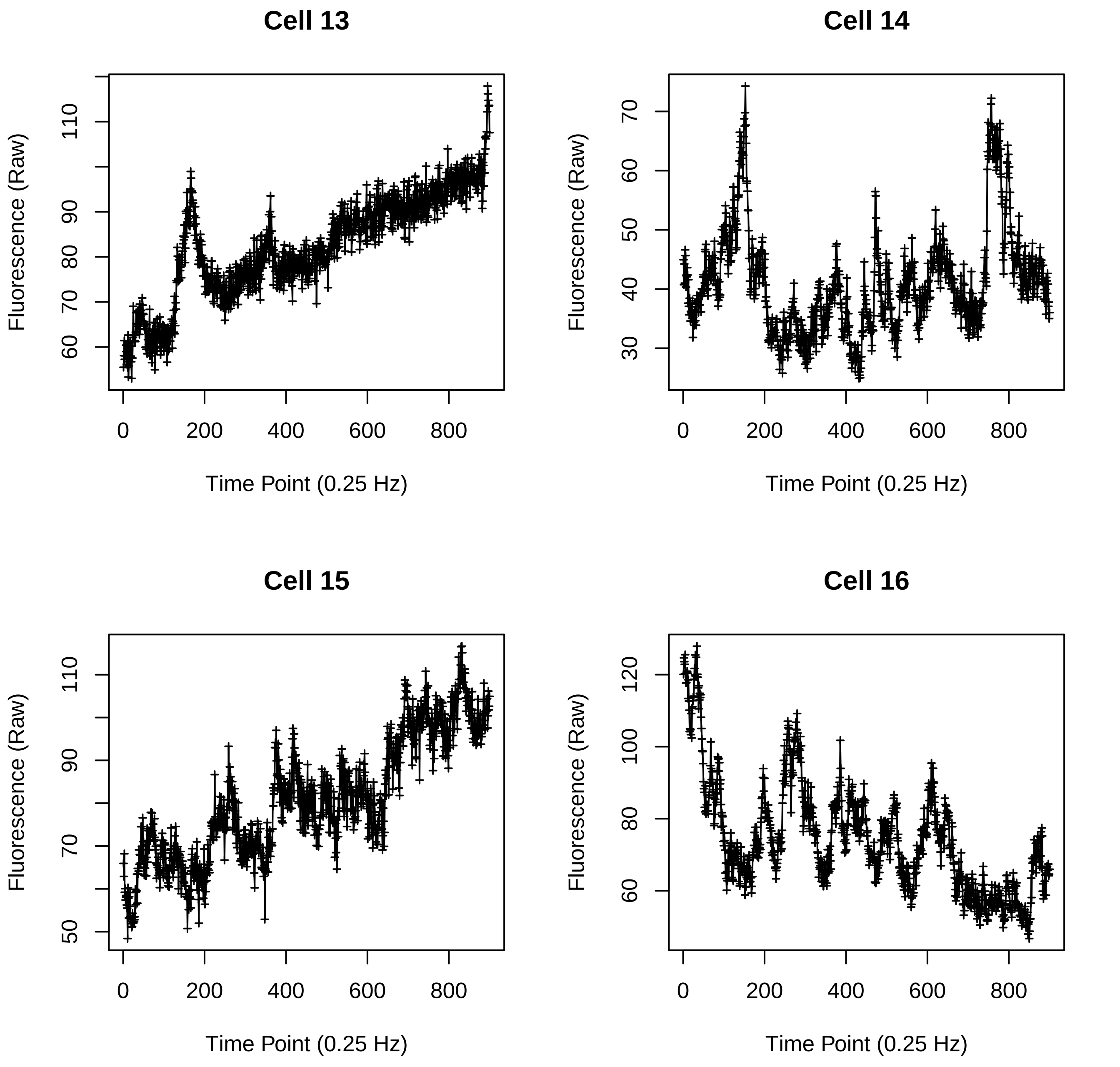
Cell 13
Cell 14
Cell 15
Cell 16
Fluorescence (Raw)
Time Point (0.25 Hz)
0
200
400
600
800
60
70
80
90
110
30
40
50
60
70
50
70
90
110
60
80
100
120

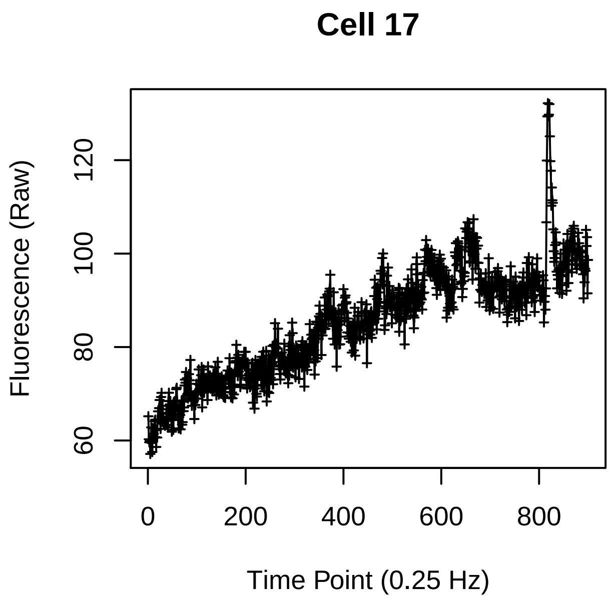
Cell 17
Fluorescence (Raw)
60
80
100
120
0
200
400
600
800
Time Point (0.25 Hz)

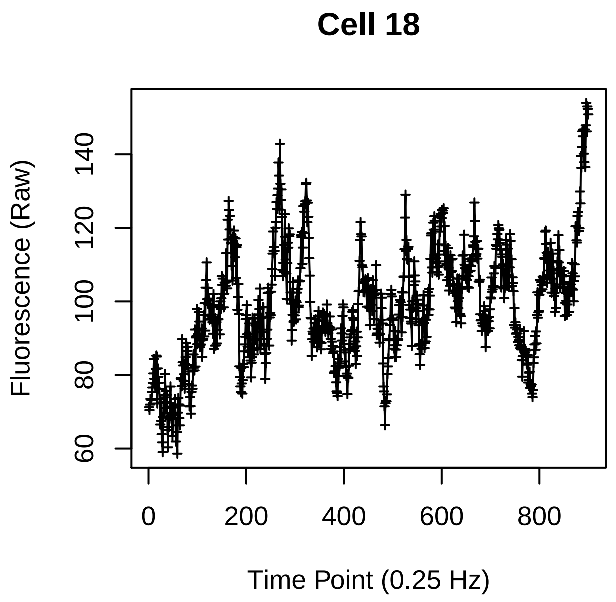
Cell 18
Fluorescence (Raw)
60
80
100
120
140
0
200
400
600
800
Time Point (0.25 Hz)

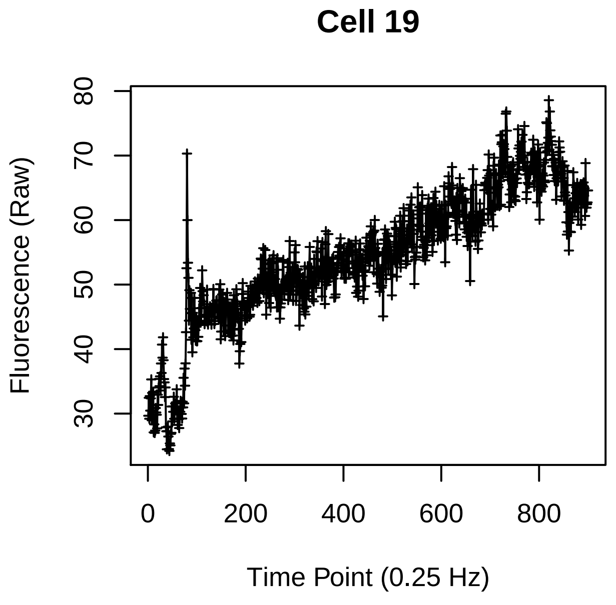
Cell 19
Fluorescence (Raw)
30
40
50
60
70
80
0
200
400
600
800
Time Point (0.25 Hz)

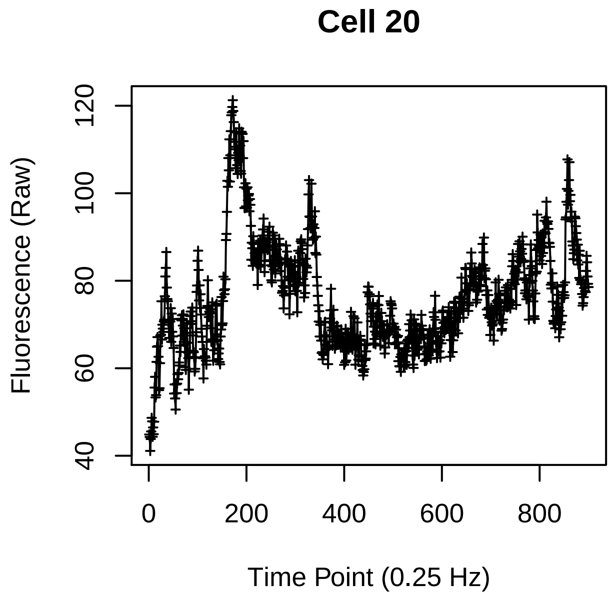
Cell 20
Fluorescence (Raw)
40
60
80
100
120
0
200
400
600
800
Time Point (0.25 Hz)

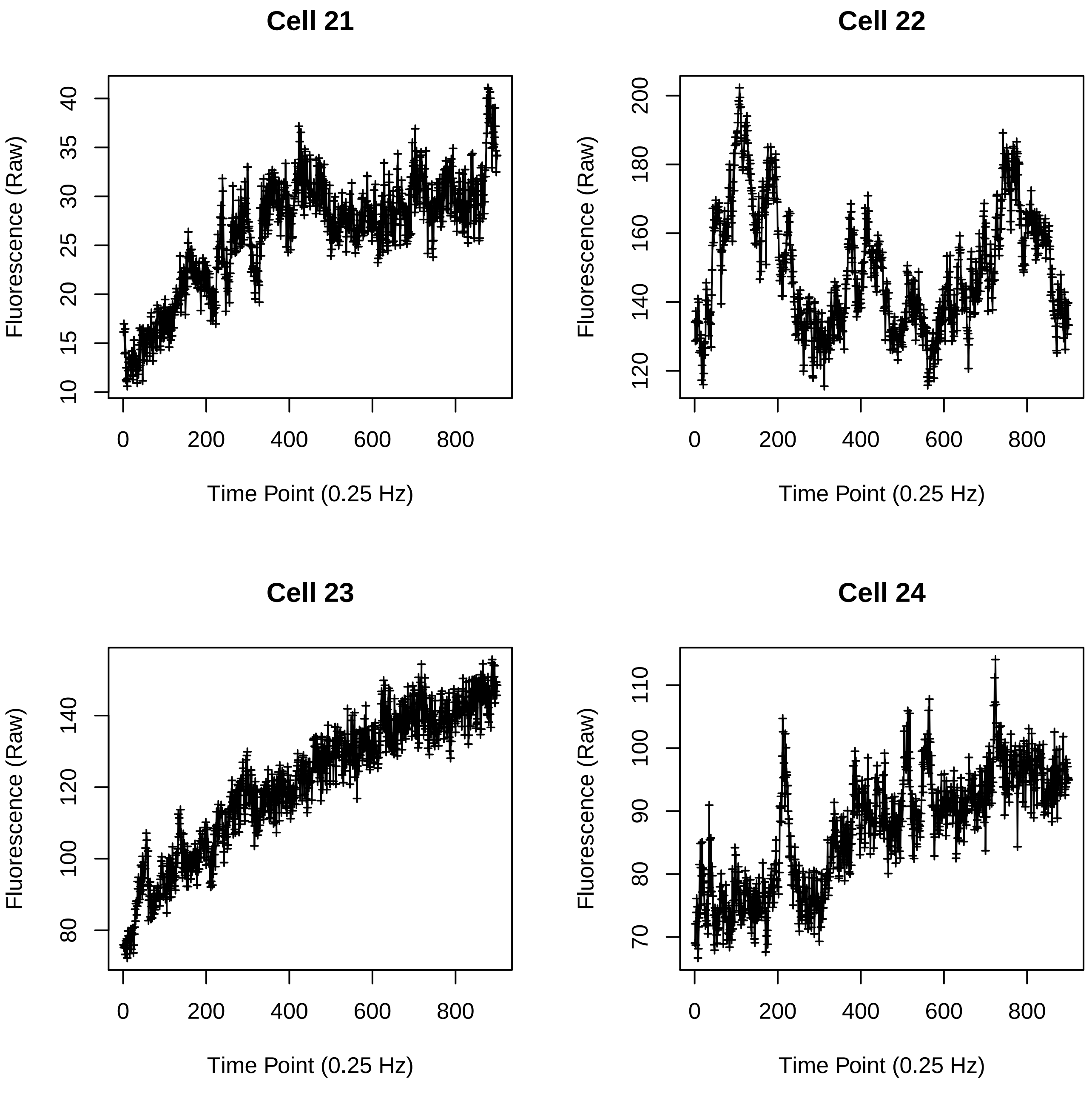

Cell 21
Fluorescence (Raw)
10
15
20
25
30
35
40
0
200
400
600
800
Time Point (0.25 Hz)
Cell 22
Fluorescence (Raw)
120
140
160
180
200
0
200
400
600
800
Time Point (0.25 Hz)
Cell 23
Fluorescence (Raw)
80
100
120
140
0
200
400
600
800
Time Point (0.25 Hz)
Cell 24
Fluorescence (Raw)
70
80
90
100
110
0
200
400
600
800
Time Point (0.25 Hz)

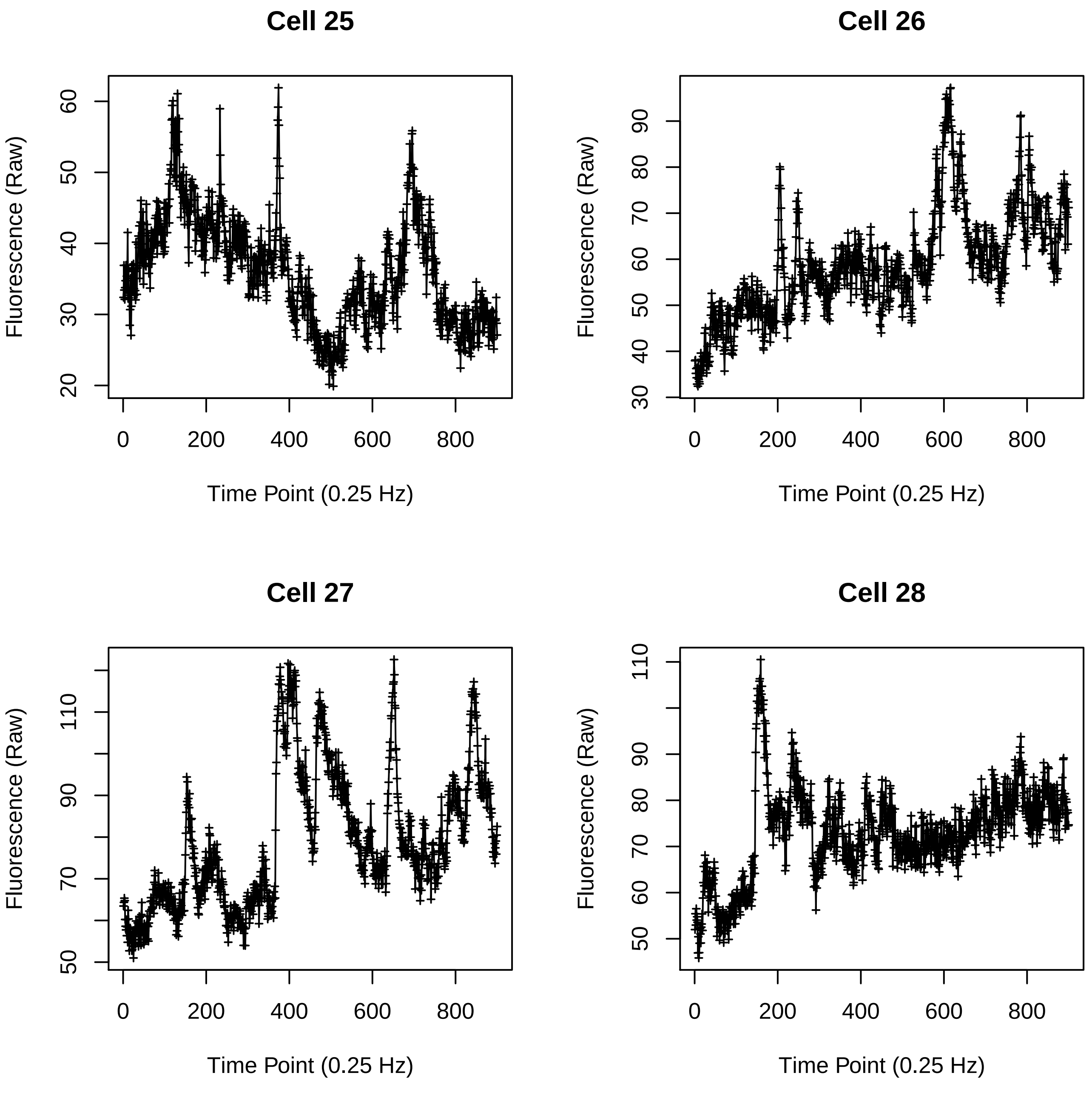
Cell 25
Fluorescence (Raw)
20 30 40 50 60
0 200 400 600 800
Time Point (0.25 Hz)
Cell 26
Fluorescence (Raw)
30 40 50 60 70 80 90
0 200 400 600 800
Time Point (0.25 Hz)
Cell 27
Fluorescence (Raw)
50 70 90 110
0 200 400 600 800
Time Point (0.25 Hz)
Cell 28
Fluorescence (Raw)
50 60 70 80 90 110
0 200 400 600 800
Time Point (0.25 Hz)

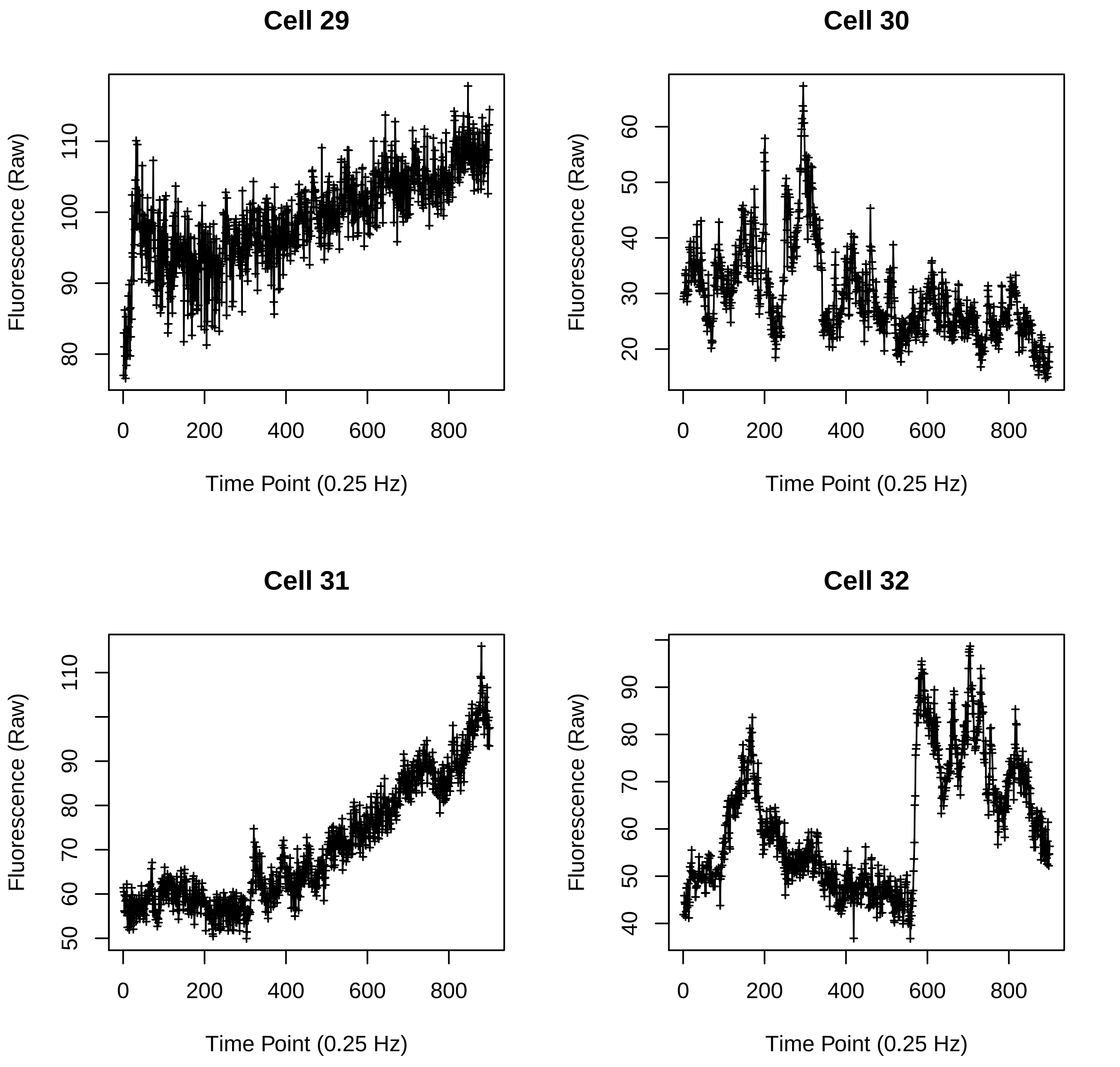

Cell 29
Cell 30
Cell 31
Cell 32
Fluorescence (Raw)
Time Point (0.25 Hz)
0
200
400
600
800
80
90
100
110
20
30
40
50
60
70

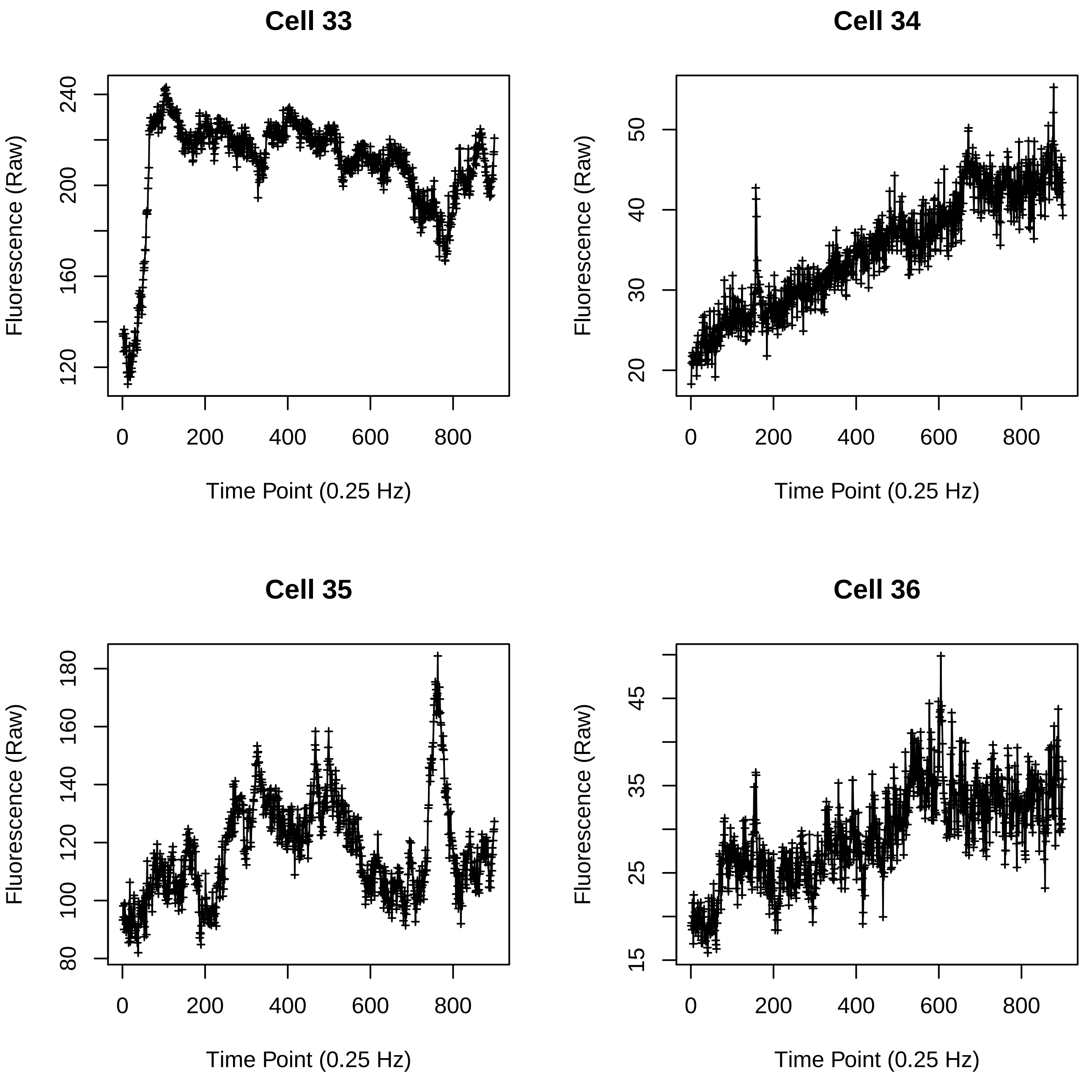
Cell 33
Fluorescence (Raw)
120
160
200
240
0
200
400
600
800
Time Point (0.25 Hz)
Cell 34
Fluorescence (Raw)
20
30
40
50
0
200
400
600
800
Time Point (0.25 Hz)
Cell 35
Fluorescence (Raw)
80
100
120
140
160
180
0
200
400
600
800
Time Point (0.25 Hz)
Cell 36
Fluorescence (Raw)
15
25
35
45
0
200
400
600
800
Time Point (0.25 Hz)

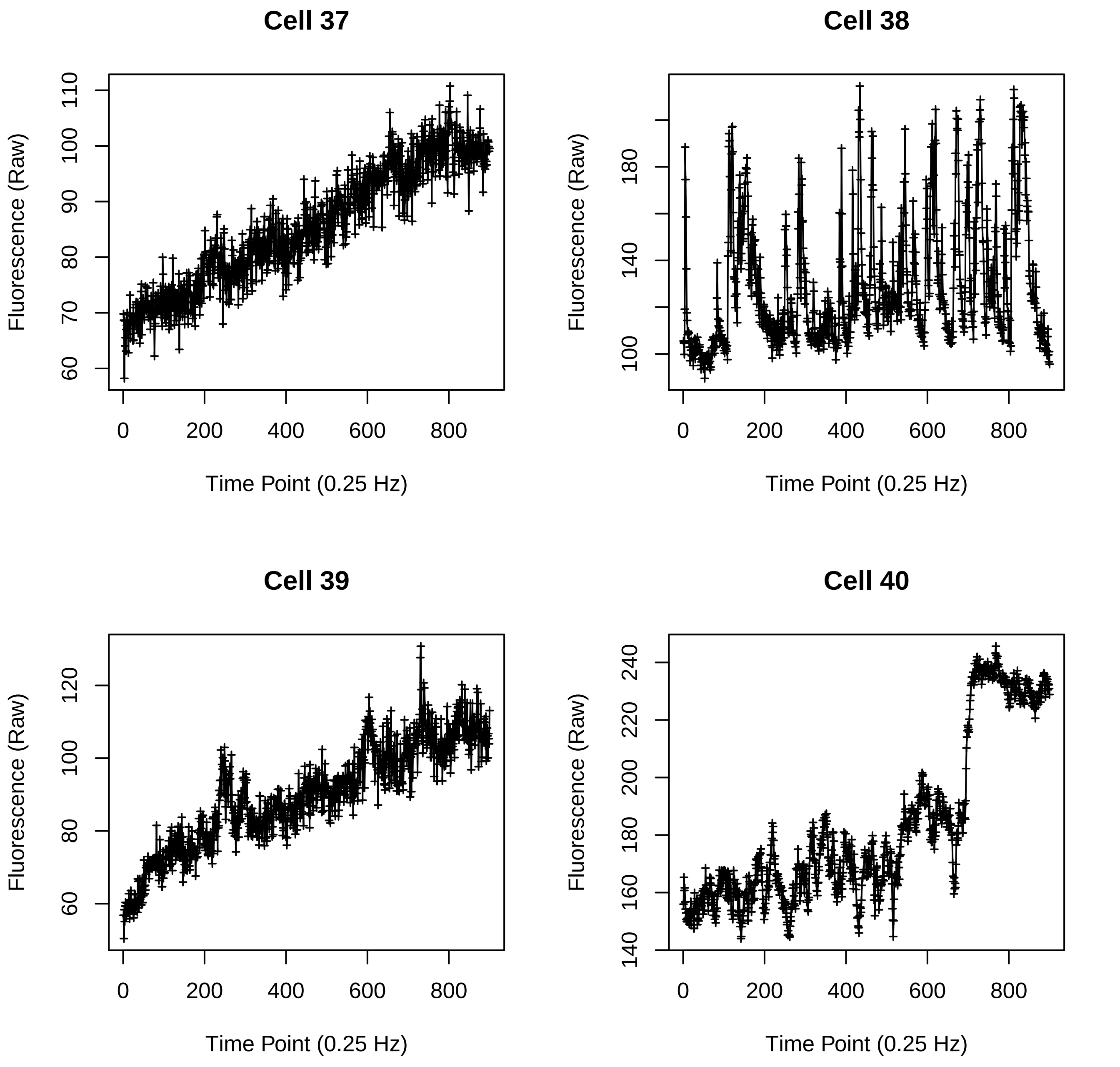
Cell 37
Cell 38
Cell 39
Cell 40
Fluorescence (Raw)
Time Point (0.25 Hz)
60
70
80
90
100
110
120
140
160
180
200
220
240
0
200
400
600
800

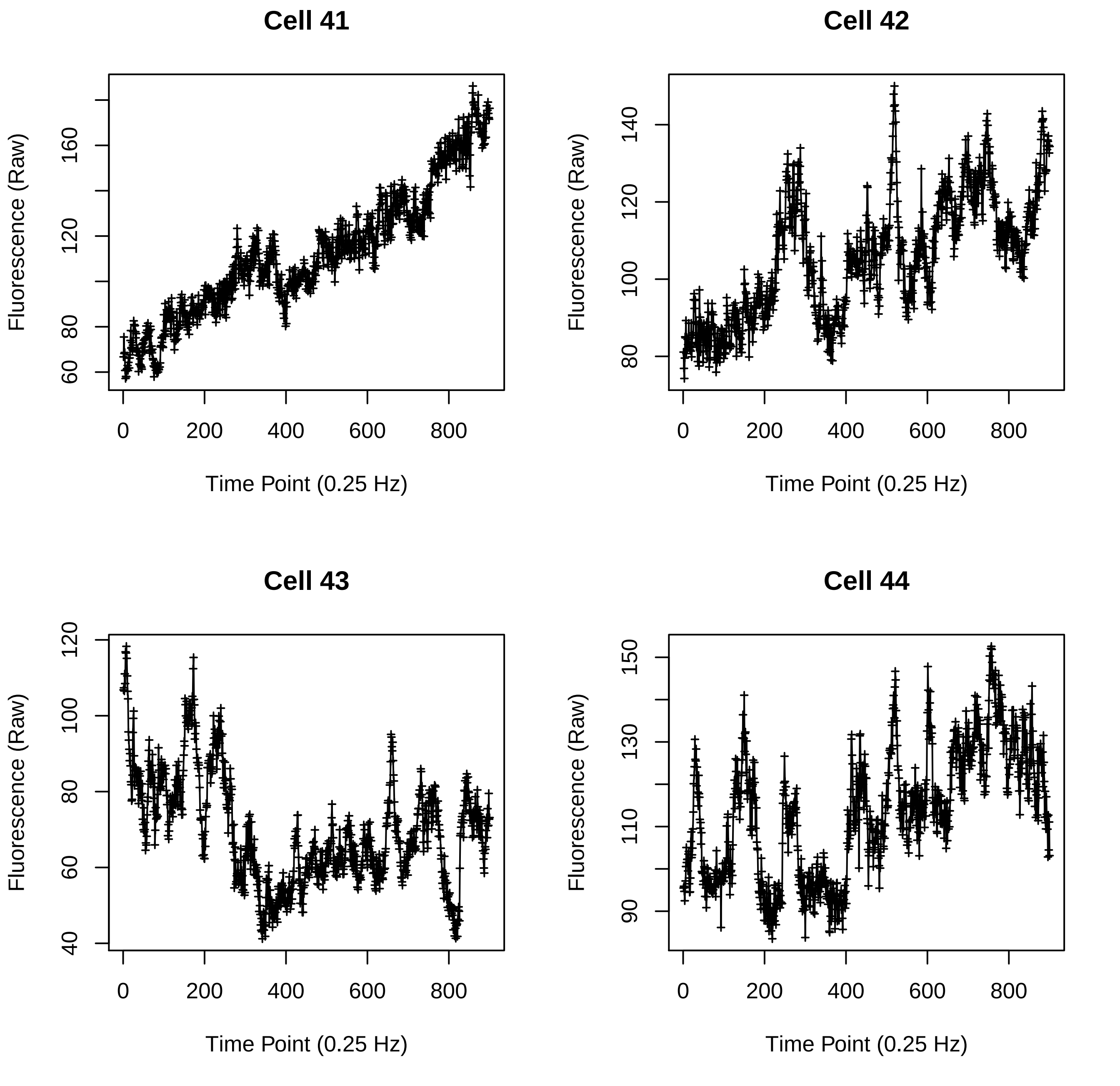
Cell 41
Cell 42
Cell 43
Cell 44
Fluorescence (Raw)
Time Point (0.25 Hz)
0
200
400
600
800
60
80
120
160
100
140
40
90
110
130
150

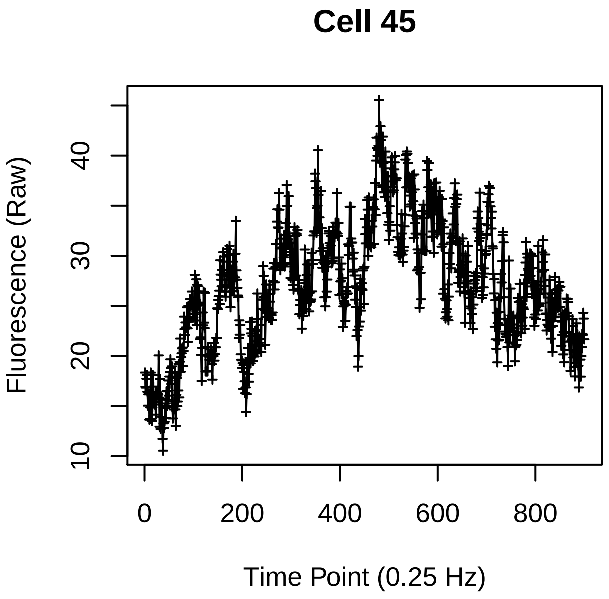
Cell 45
Fluorescence (Raw)
10
20
30
40
0
200
400
600
800
Time Point (0.25 Hz)

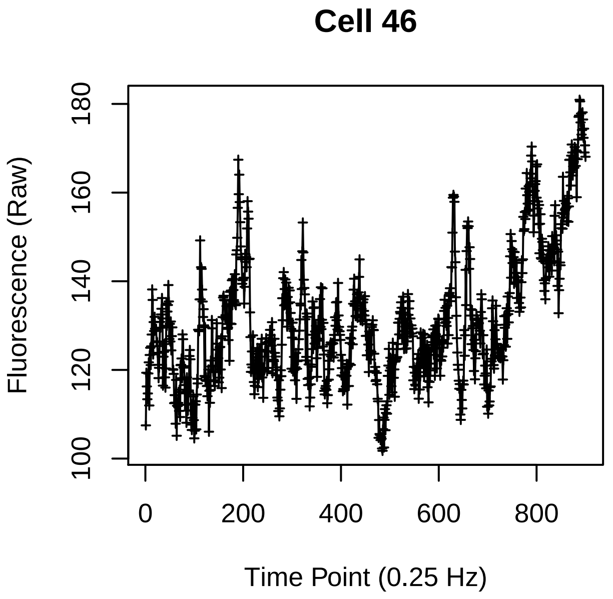
Cell 46
Fluorescence (Raw)
100
120
140
160
180
0
200
400
600
800
Time Point (0.25 Hz)

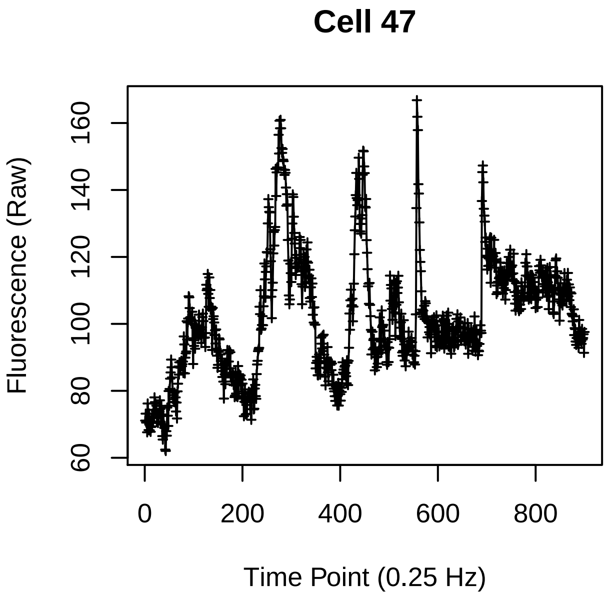
Cell 47
Fluorescence (Raw)
60
80
100
120
140
160
0
200
400
600
800
Time Point (0.25 Hz)

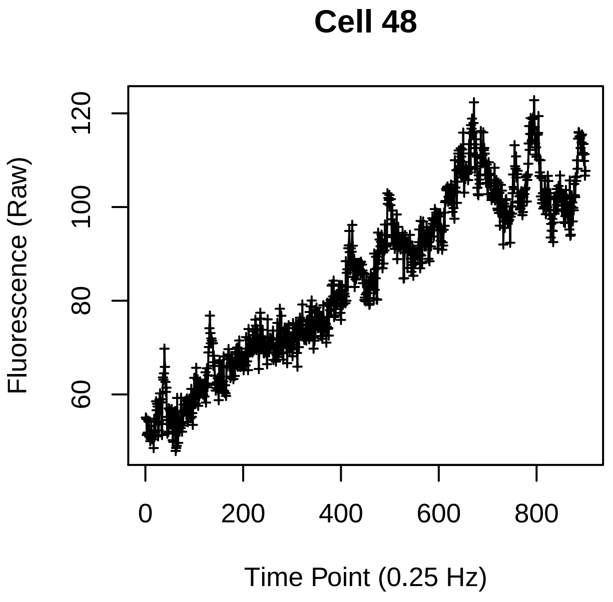
Cell 48
Fluorescence (Raw)
60
80
100
120
0
200
400
600
800
Time Point (0.25 Hz)

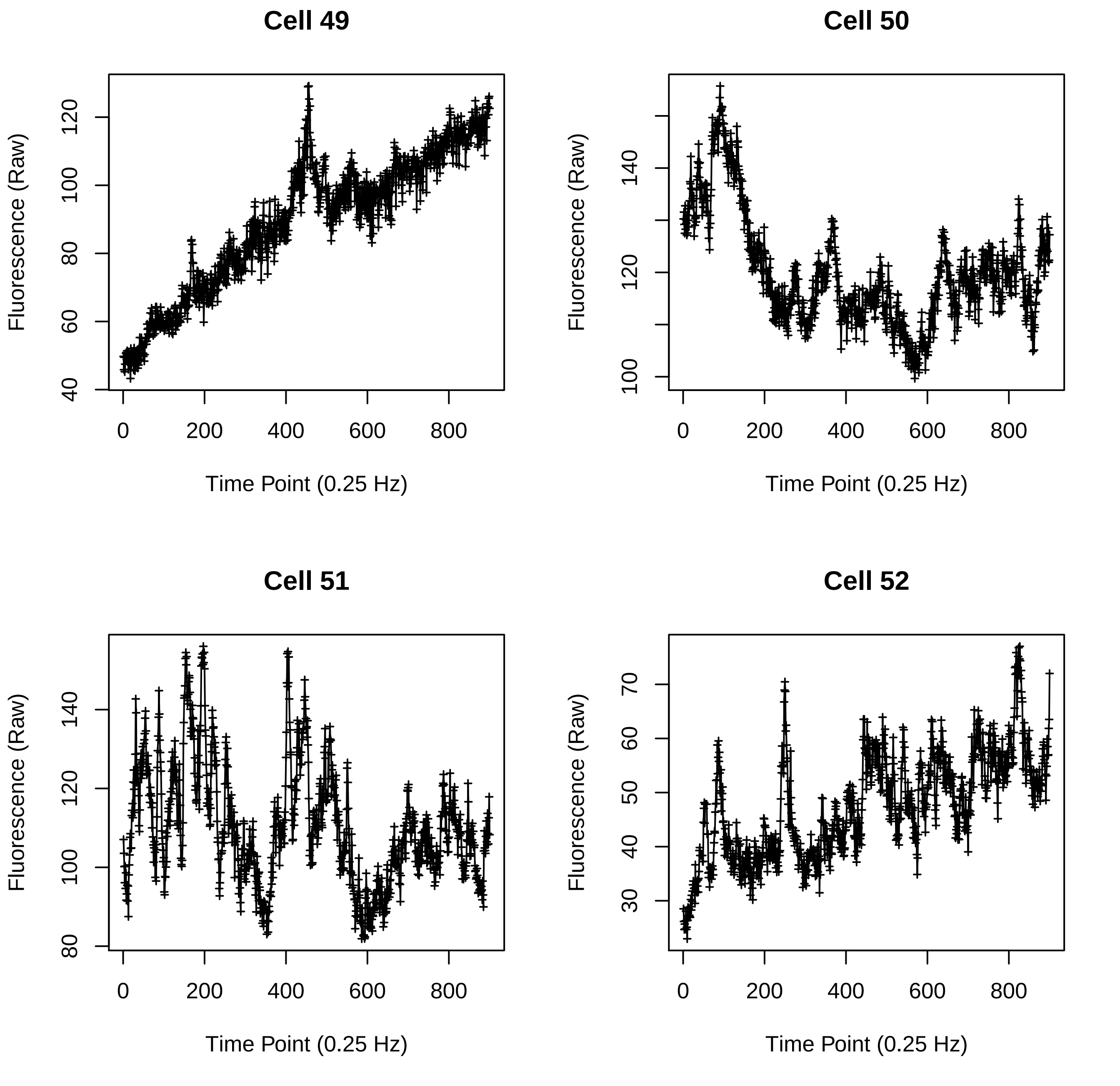
Cell 49
Cell 50
Cell 51
Cell 52
Fluorescence (Raw)
Time Point (0.25 Hz)
0
200
400
600
800
40
60
80
100
120
140
30
50
70

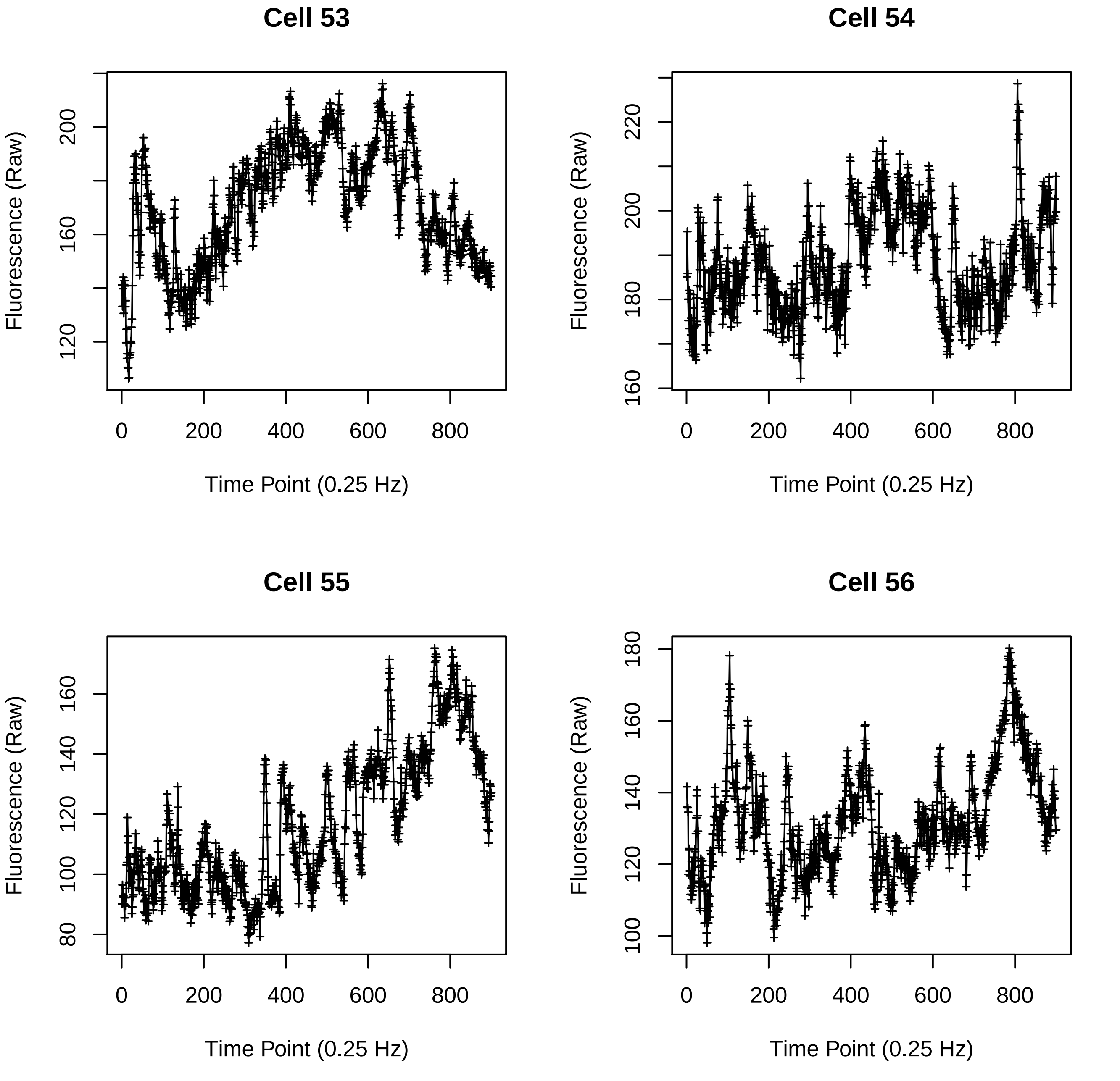
Cell 53
Fluorescence (Raw)
120
160
200
0
200
400
600
800
Time Point (0.25 Hz)
Cell 54
Fluorescence (Raw)
160
180
200
220
0
200
400
600
800
Time Point (0.25 Hz)
Cell 55
Fluorescence (Raw)
80
100
120
140
160
0
200
400
600
800
Time Point (0.25 Hz)
Cell 56
Fluorescence (Raw)
100
120
140
160
180
0
200
400
600
800
Time Point (0.25 Hz)

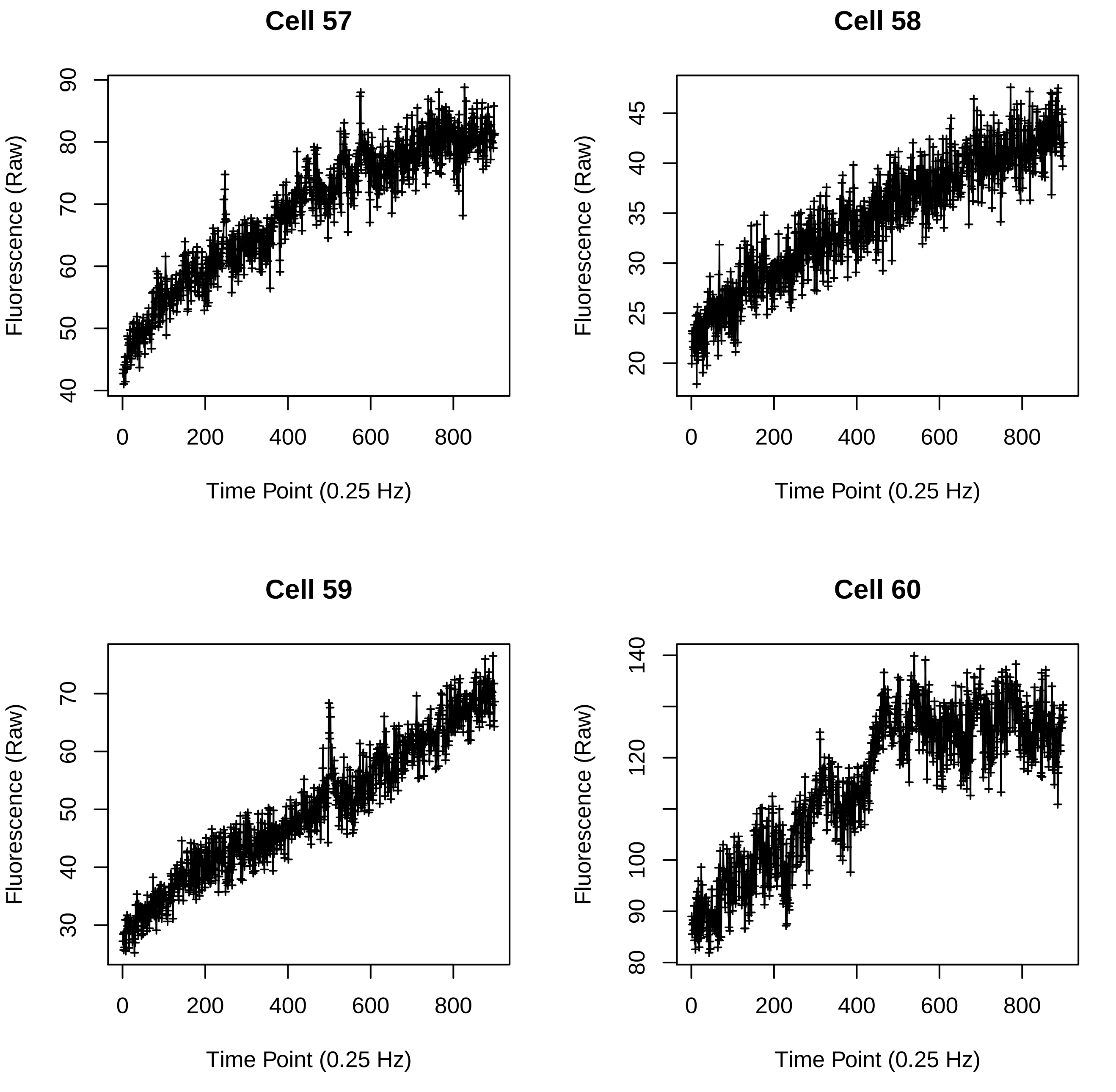
Cell 57
Fluorescence (Raw)
Time Point (0.25 Hz)
Cell 58
Fluorescence (Raw)
Time Point (0.25 Hz)
Cell 59
Fluorescence (Raw)
Time Point (0.25 Hz)
Cell 60
Fluorescence (Raw)
Time Point (0.25 Hz)

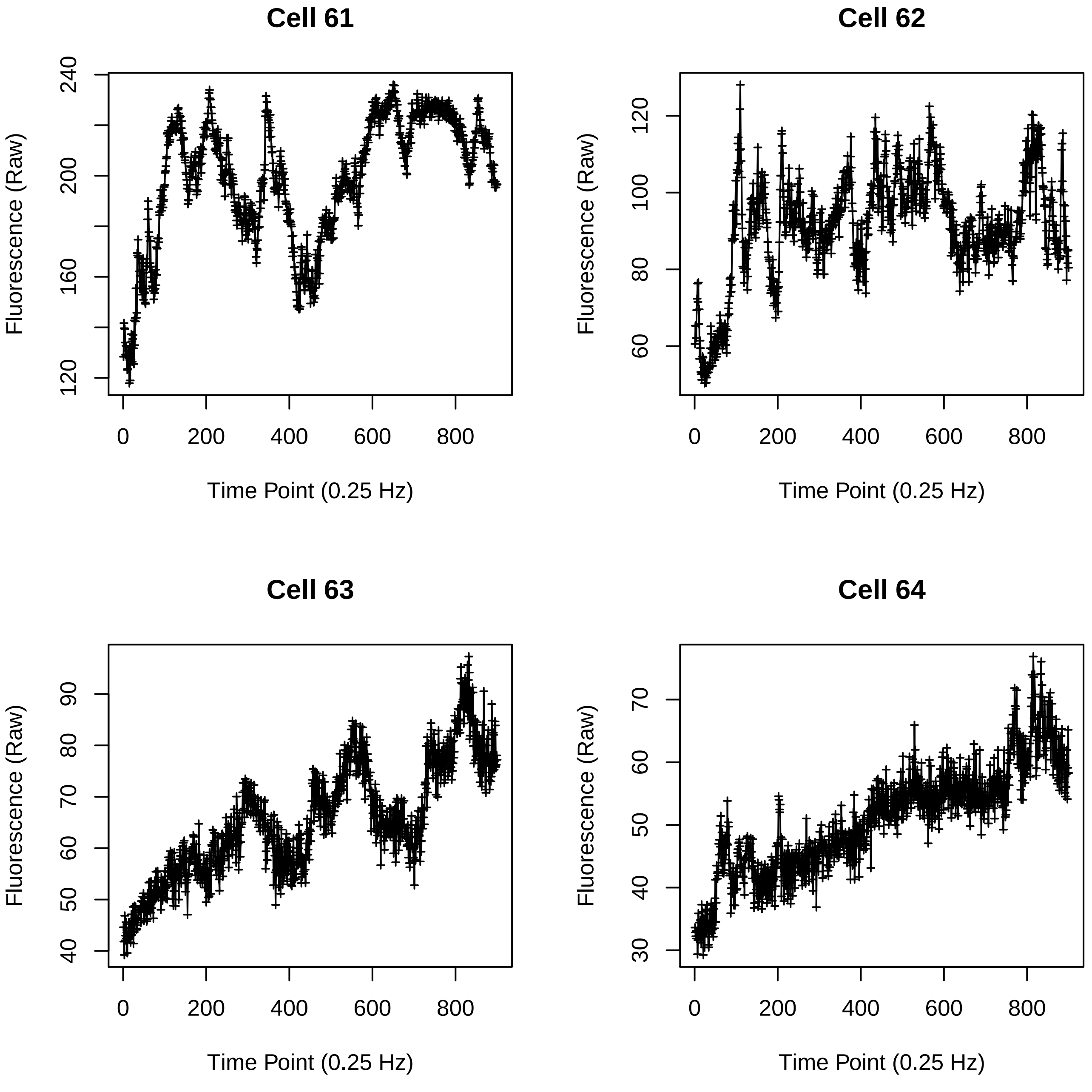
Cell 61
Cell 62
Cell 63
Cell 64
Fluorescence (Raw)
Time Point (0.25 Hz)
0
200
400
600
800
120
160
200
240
60
80
100
120
40
50
60
70
80
90
30
40
50
60
70

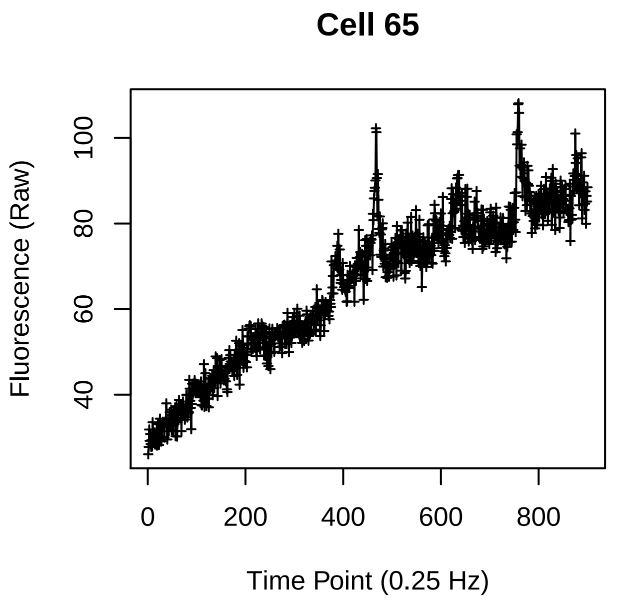
Cell 65
Fluorescence (Raw)
40
60
80
100
0
200
400
600
800
Time Point (0.25 Hz)

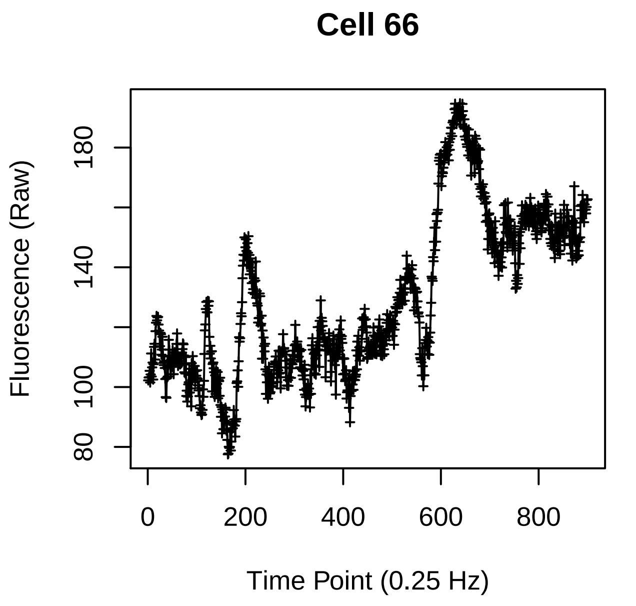
Cell 66
Fluorescence (Raw)
80
100
140
180
0
200
400
600
800
Time Point (0.25 Hz)

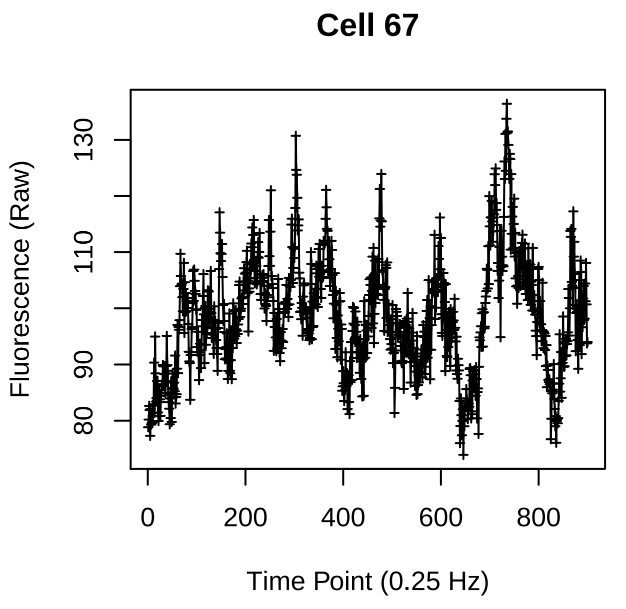
Cell 67
Fluorescence (Raw)
80
90
110
130
0
200
400
600
800
Time Point (0.25 Hz)

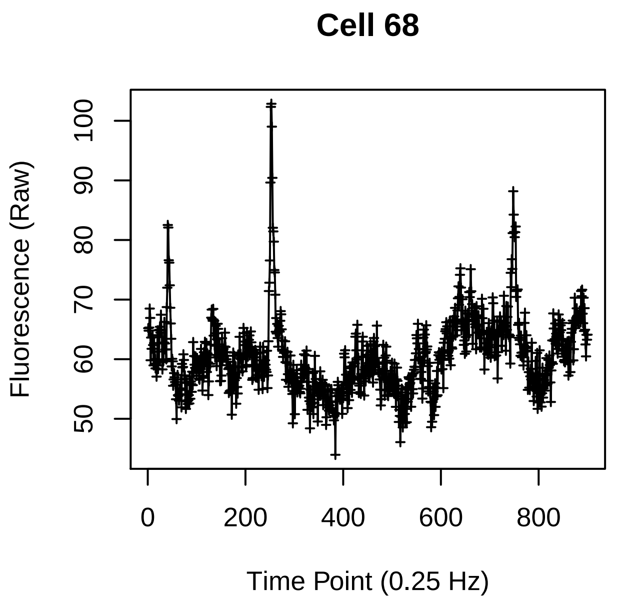
Cell 68
Fluorescence (Raw)
50
60
70
80
90
100
0
200
400
600
800
Time Point (0.25 Hz)

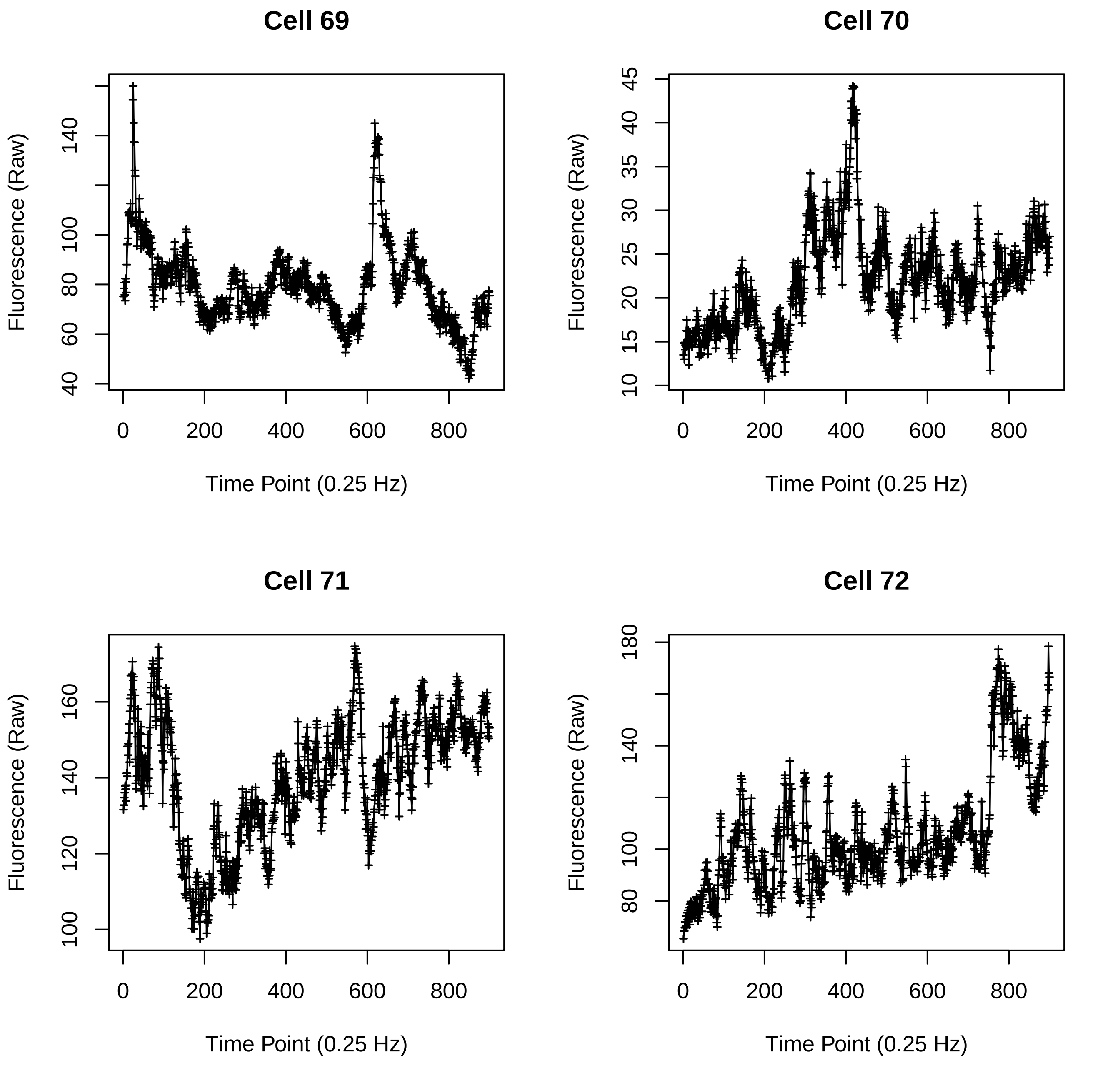

Cell 69
Cell 70
Cell 71
Cell 72
Fluorescence (Raw)
Time Point (0.25 Hz)
0
200
400
600
800
40
60
80
100
140
10
15
20
25
30
35
40
45
100
120
140
160
80
100
140
180

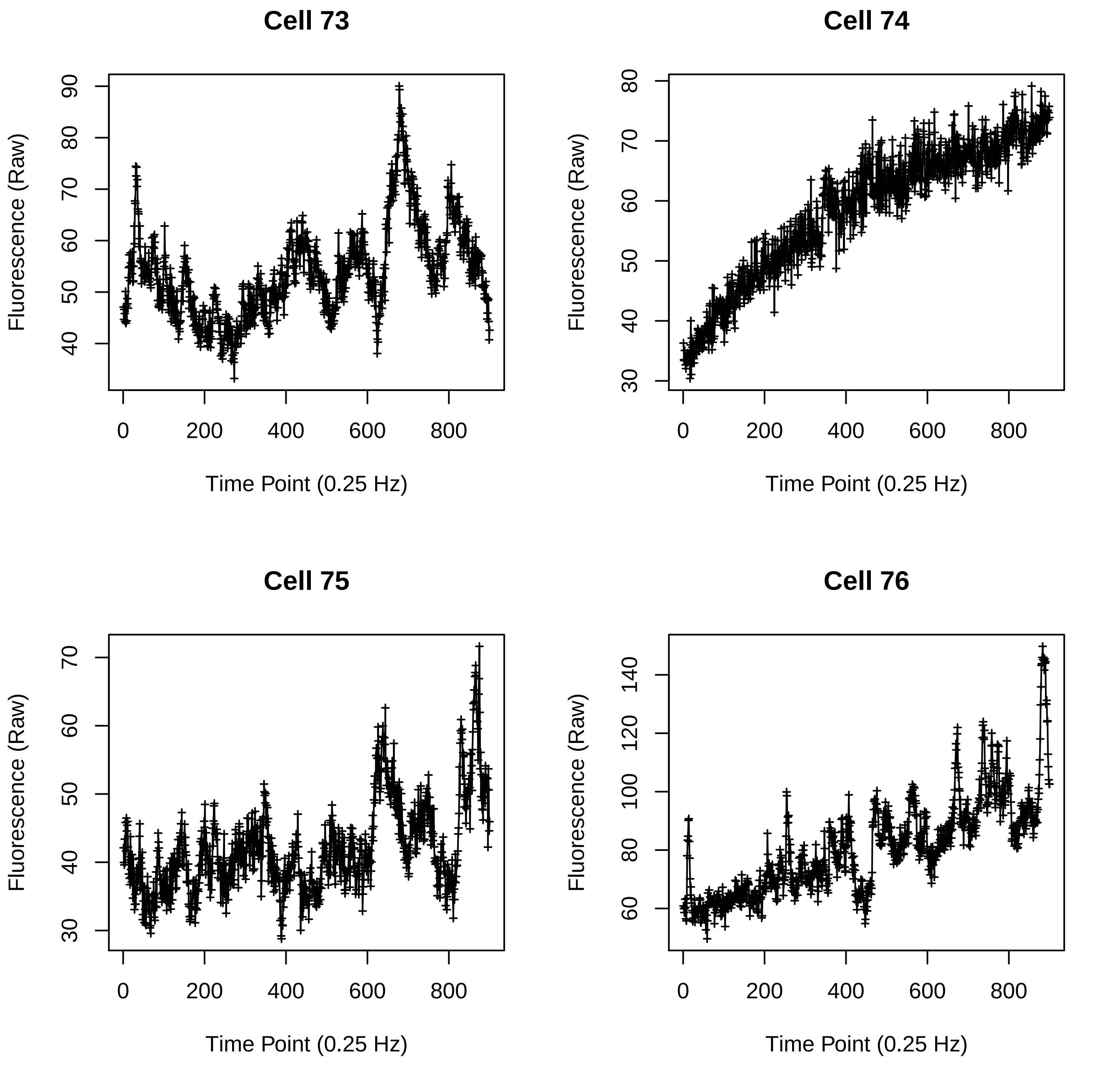

Cell 73
Fluorescence (Raw)
40
50
60
70
80
90
0
200
400
600
800
Time Point (0.25 Hz)
Cell 74
Fluorescence (Raw)
30
40
50
60
70
80
0
200
400
600
800
Time Point (0.25 Hz)
Cell 75
Fluorescence (Raw)
30
40
50
60
70
0
200
400
600
800
Time Point (0.25 Hz)
Cell 76
Fluorescence (Raw)
60
80
100
120
140
0
200
400
600
800
Time Point (0.25 Hz)

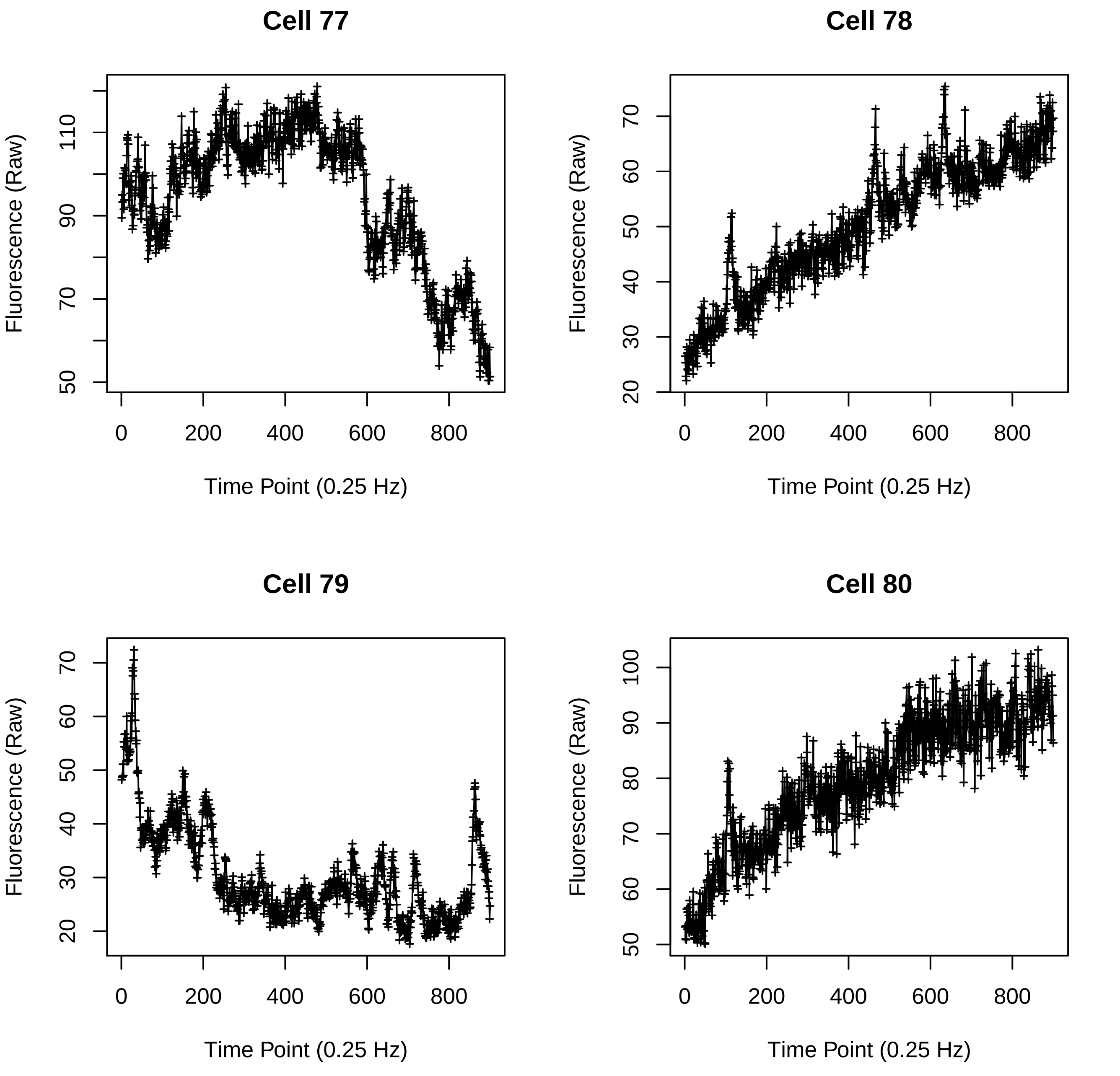
Cell 77
Cell 78
Cell 79
Cell 80
Fluorescence (Raw)
Time Point (0.25 Hz)
0
200
400
600
800
50
70
90
110
20
30
40
50
60
70
50
60
70
80
90
100

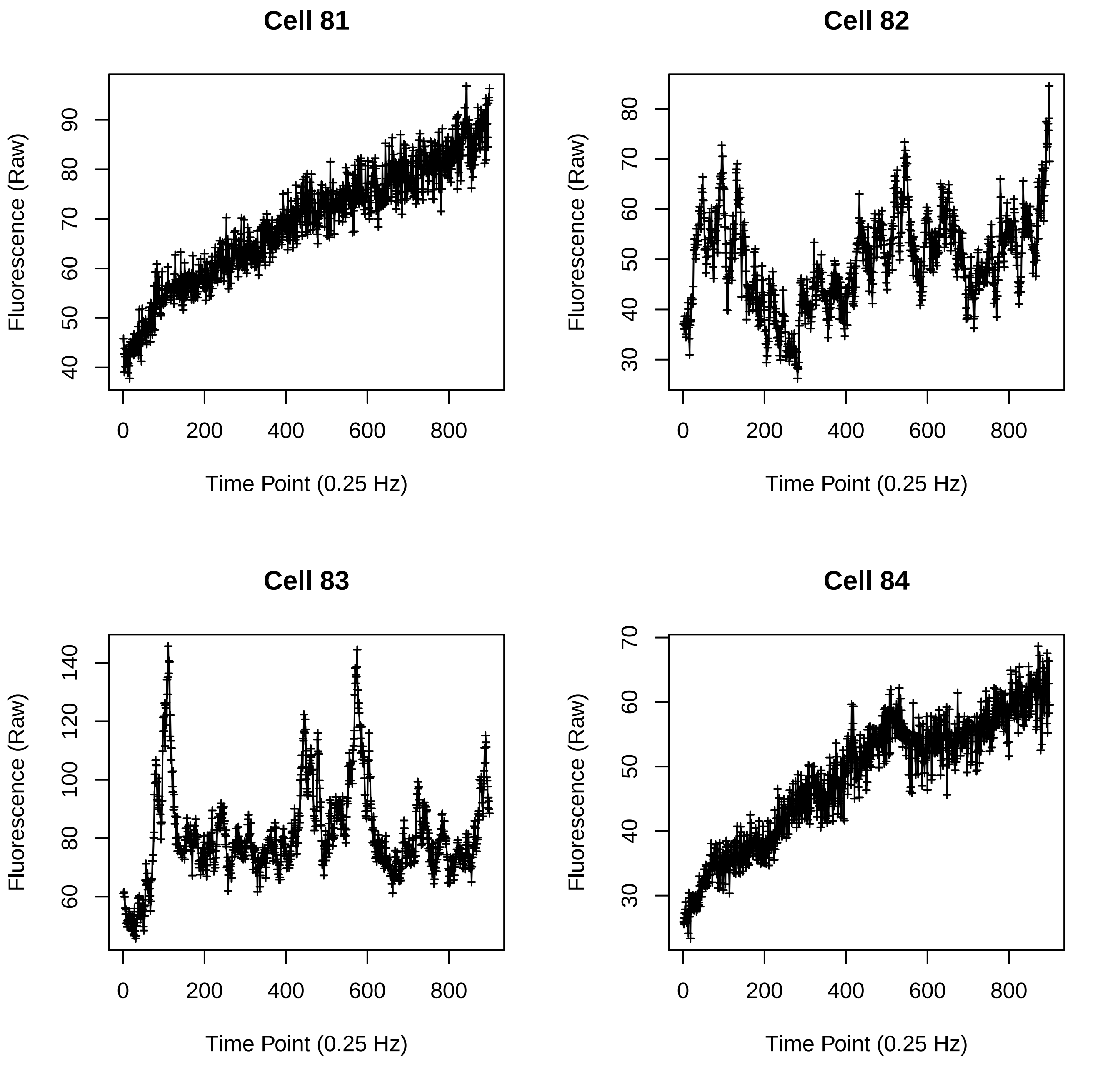
Cell 81
Cell 82
Cell 83
Cell 84
Fluorescence (Raw)
Time Point (0.25 Hz)
0
200
400
600
800
40
50
60
70
80
90
30
140
120
100

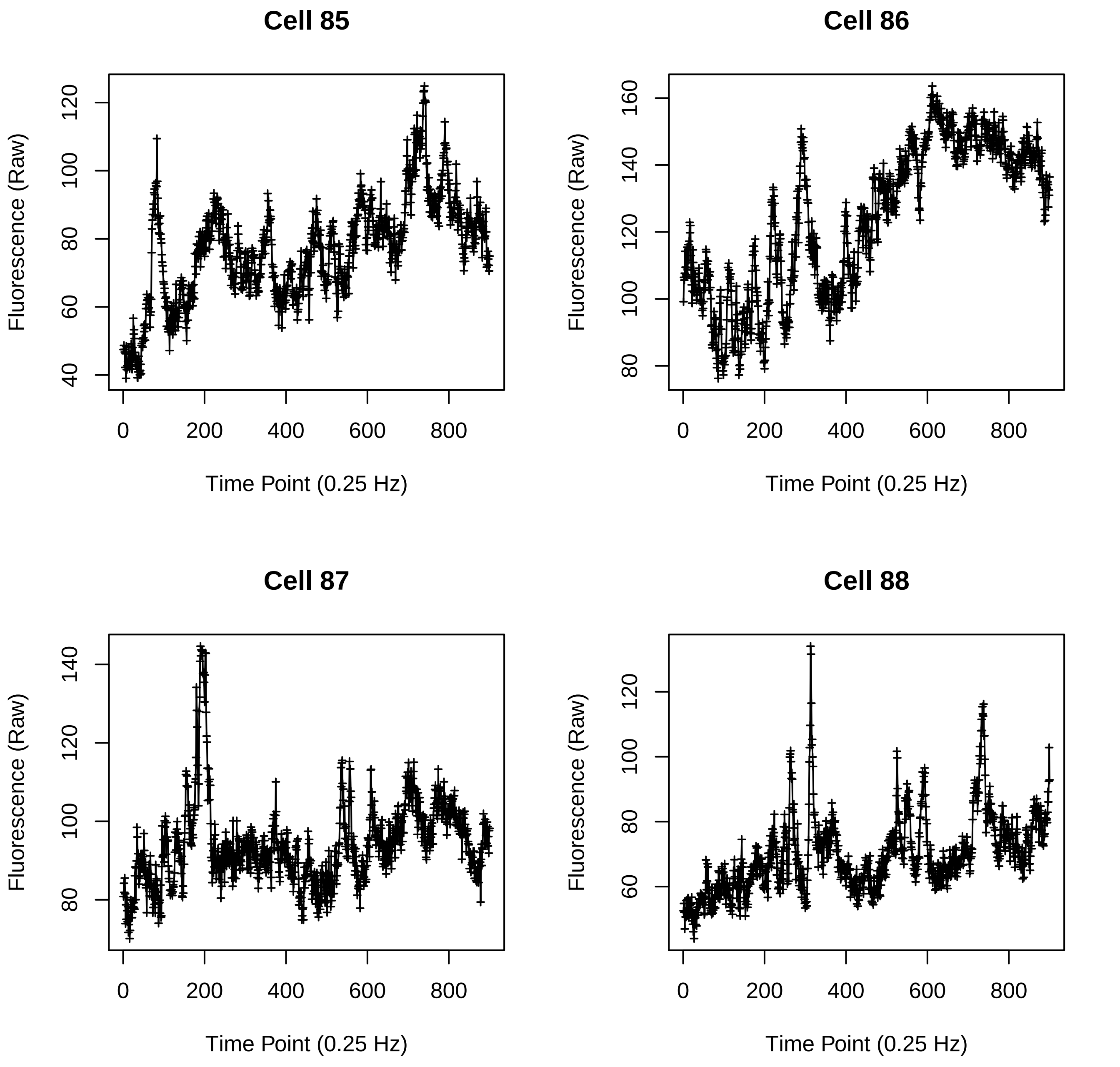
Cell 85
Fluorescence (Raw)
40
60
80
100
120
0
200
400
600
800
Time Point (0.25 Hz)
Cell 86
Fluorescence (Raw)
80
100
120
140
160
0
200
400
600
800
Time Point (0.25 Hz)
Cell 87
Fluorescence (Raw)
80
100
120
140
0
200
400
600
800
Time Point (0.25 Hz)
Cell 88
Fluorescence (Raw)
60
80
100
120
0
200
400
600
800
Time Point (0.25 Hz)

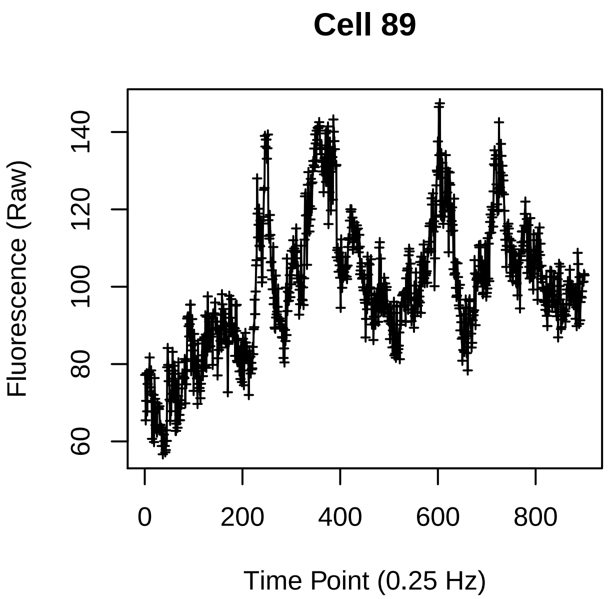
Cell 89
Fluorescence (Raw)
60
80
100
120
140
0
200
400
600
800
Time Point (0.25 Hz)

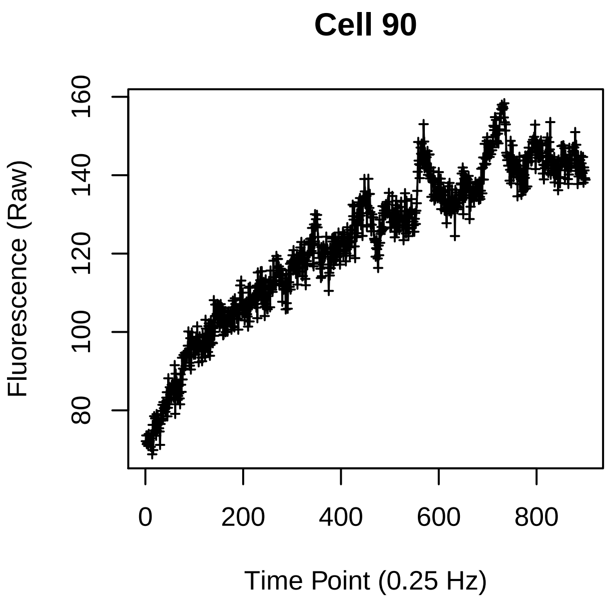
Cell 90
Fluorescence (Raw)
80
100
120
140
160
0
200
400
600
800
Time Point (0.25 Hz)

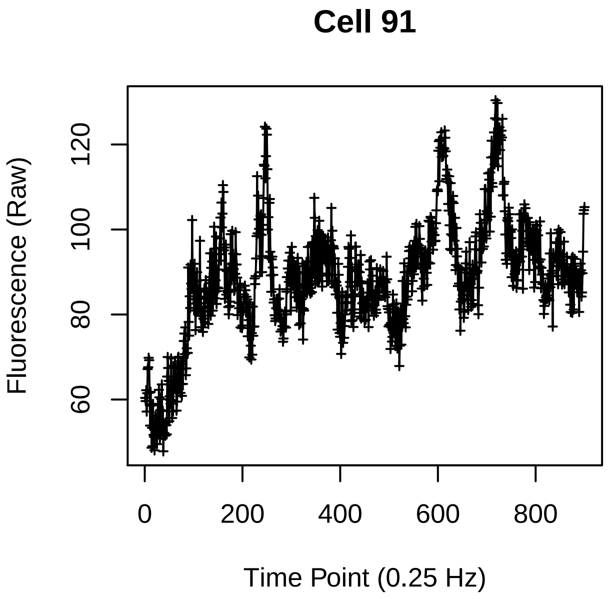
Cell 91
Fluorescence (Raw)
60
80
100
120
0
200
400
600
800
Time Point (0.25 Hz)

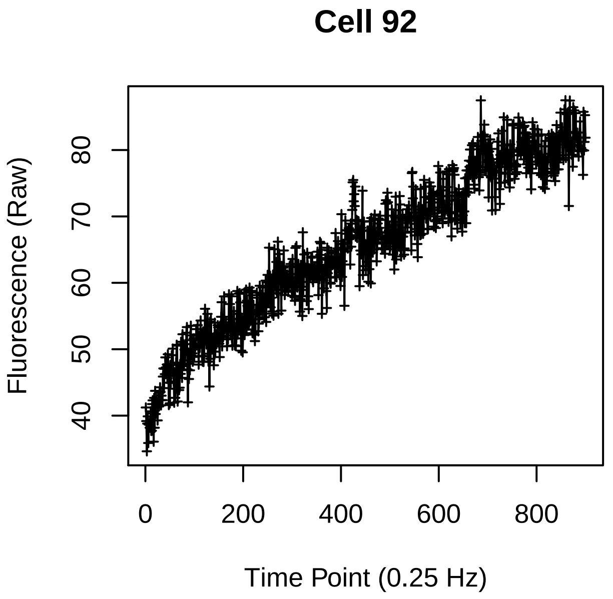
Cell 92
Fluorescence (Raw)
40
50
60
70
80
0
200
400
600
800
Time Point (0.25 Hz)

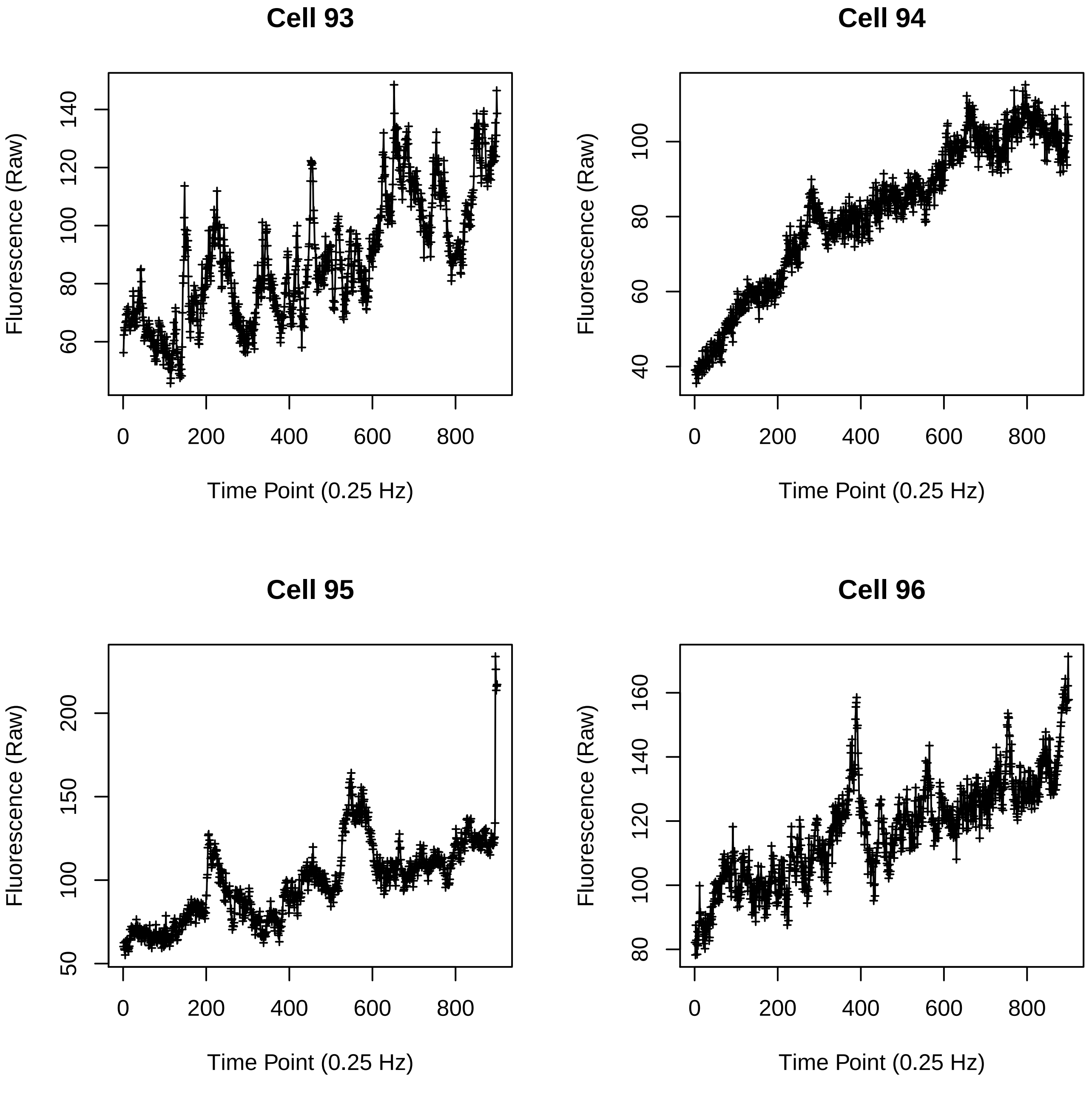

Cell 93
Fluorescence (Raw)
60
80
100
120
140
0
200
400
600
800
Time Point (0.25 Hz)
Cell 94
Fluorescence (Raw)
40
60
80
100
0
200
400
600
800
Time Point (0.25 Hz)
Cell 95
Fluorescence (Raw)
50
100
150
200
0
200
400
600
800
Time Point (0.25 Hz)
Cell 96
Fluorescence (Raw)
80
100
120
140
160
0
200
400
600
800
Time Point (0.25 Hz)

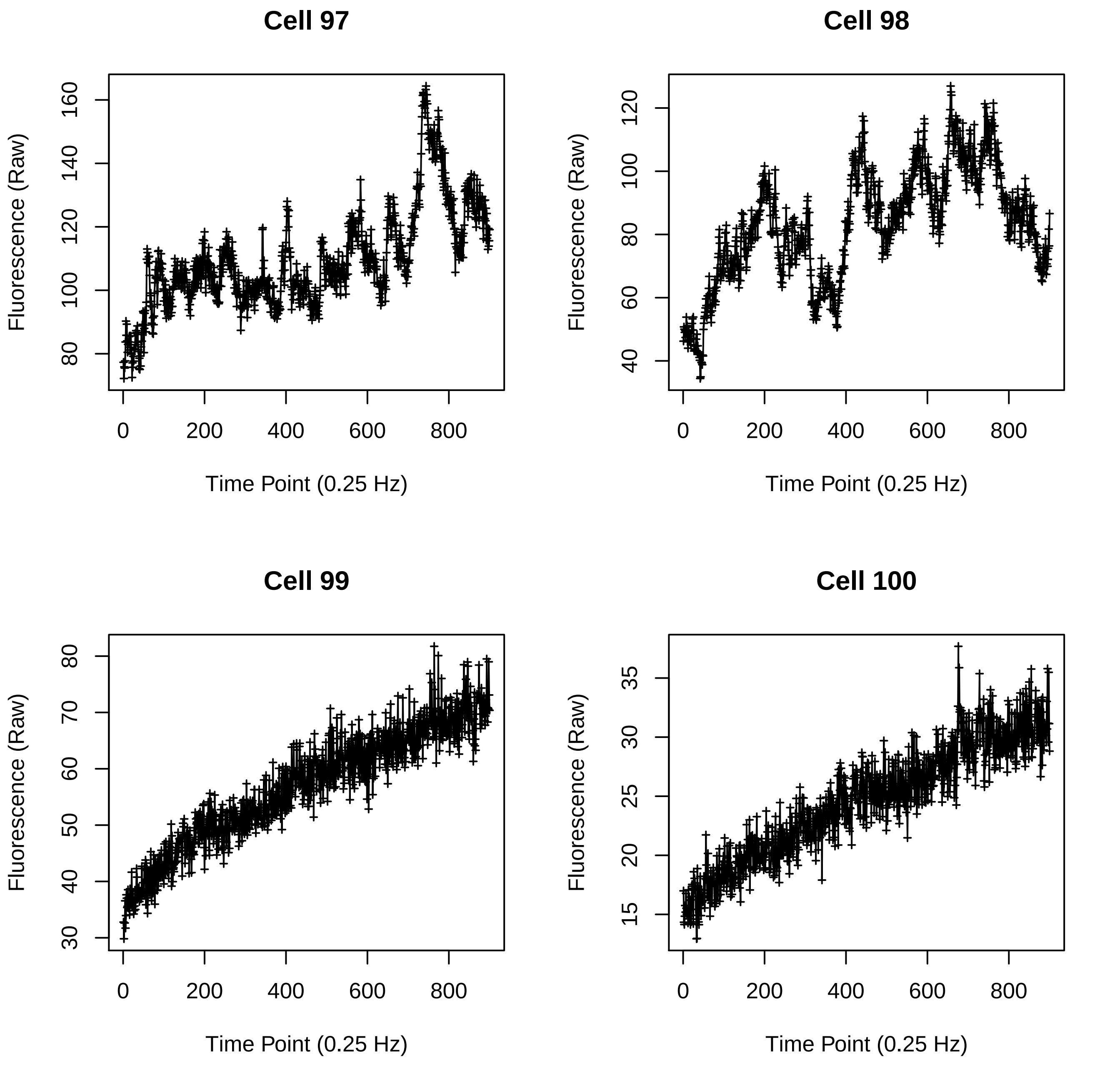

Cell 97
Cell 98
Cell 99
Cell 100
Fluorescence (Raw)
Time Point (0.25 Hz)
0
200
400
600
800
80
100
120
140
160
40
60
30
50
70
15
20
25
30
35

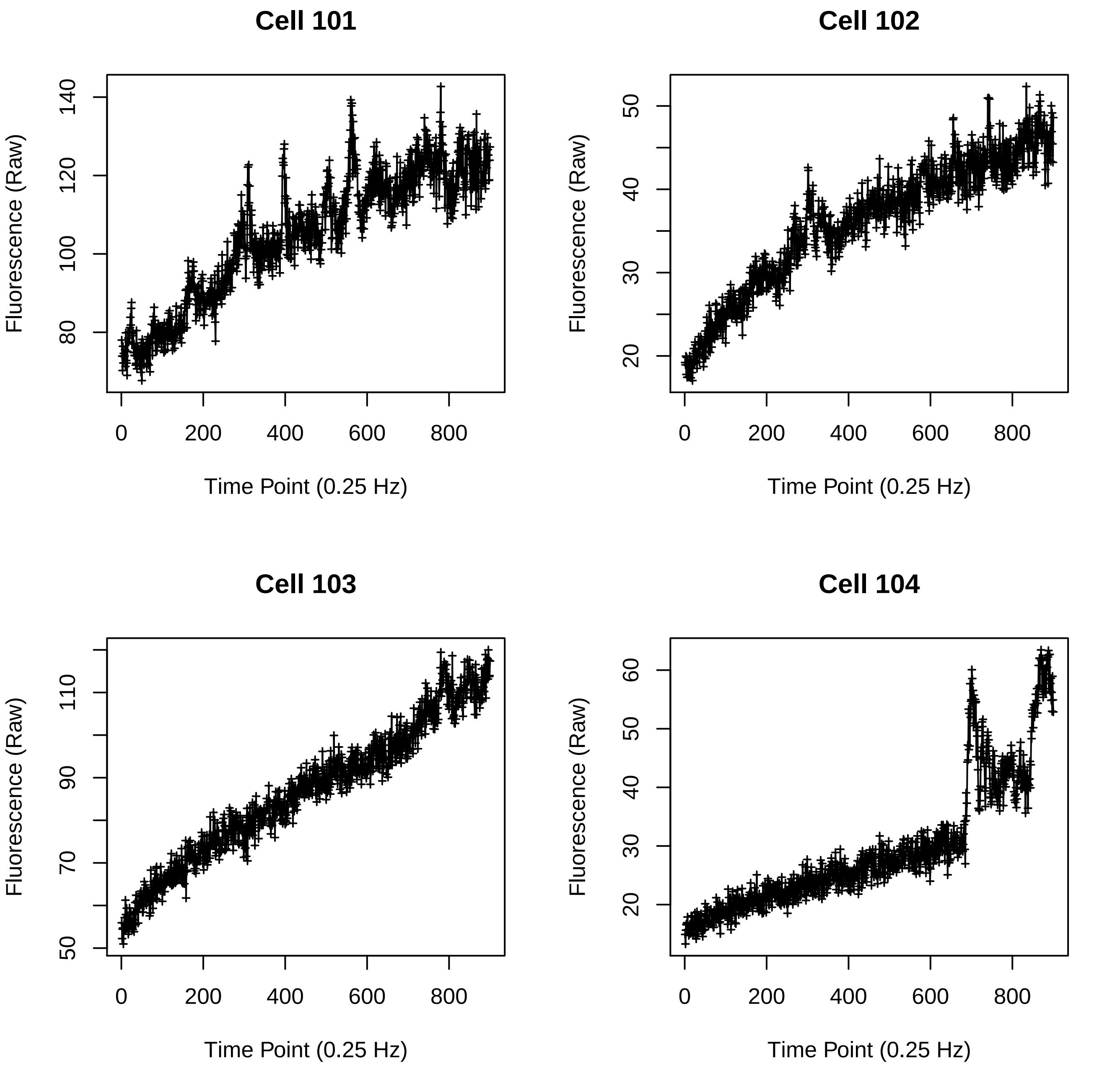
Cell 101
Fluorescence (Raw)
80
100
120
140
0
200
400
600
800
Time Point (0.25 Hz)
Cell 102
Fluorescence (Raw)
20
30
40
50
0
200
400
600
800
Time Point (0.25 Hz)
Cell 103
Fluorescence (Raw)
50
70
90
110
0
200
400
600
800
Time Point (0.25 Hz)
Cell 104
Fluorescence (Raw)
20
30
40
50
60
0
200
400
600
800
Time Point (0.25 Hz)

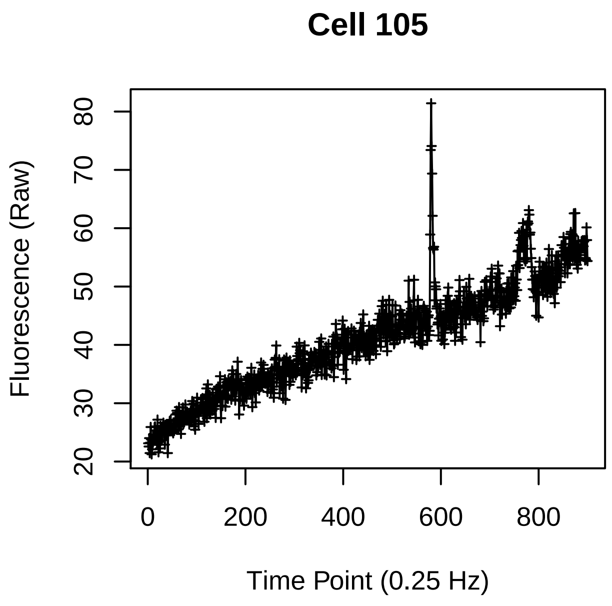
Cell 105
Fluorescence (Raw)
Time Point (0.25 Hz)
20
30
40
50
60
70
80
0
200
400
600
800
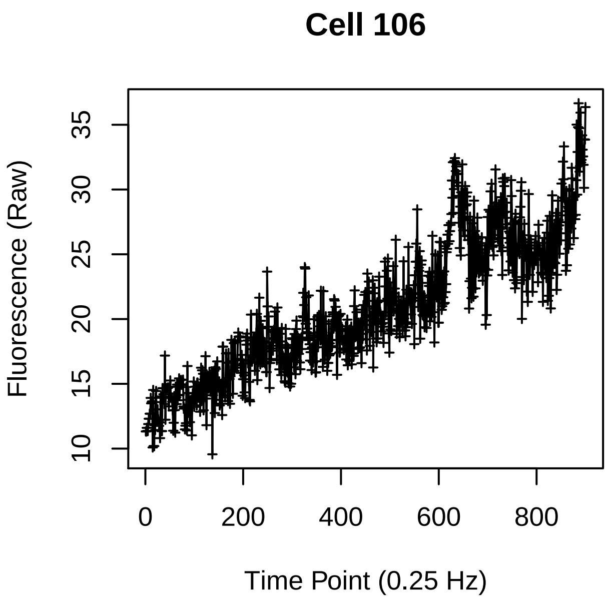
Cell 106
Fluorescence (Raw)
Time Point (0.25 Hz)
10
15
20
25
30
35
0
200
400
600
800
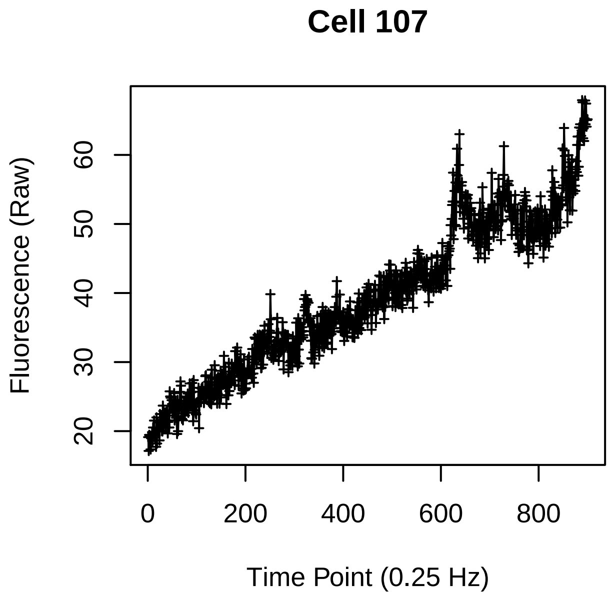
Cell 107
Fluorescence (Raw)
Time Point (0.25 Hz)
20
30
40
50
60
0
200
400
600
800
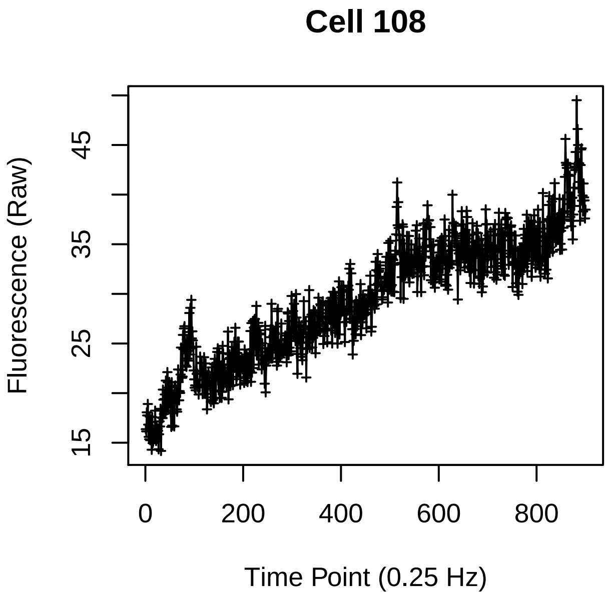
Cell 108
Fluorescence (Raw)
Time Point (0.25 Hz)
15
25
35
45
0
200
400
600
800

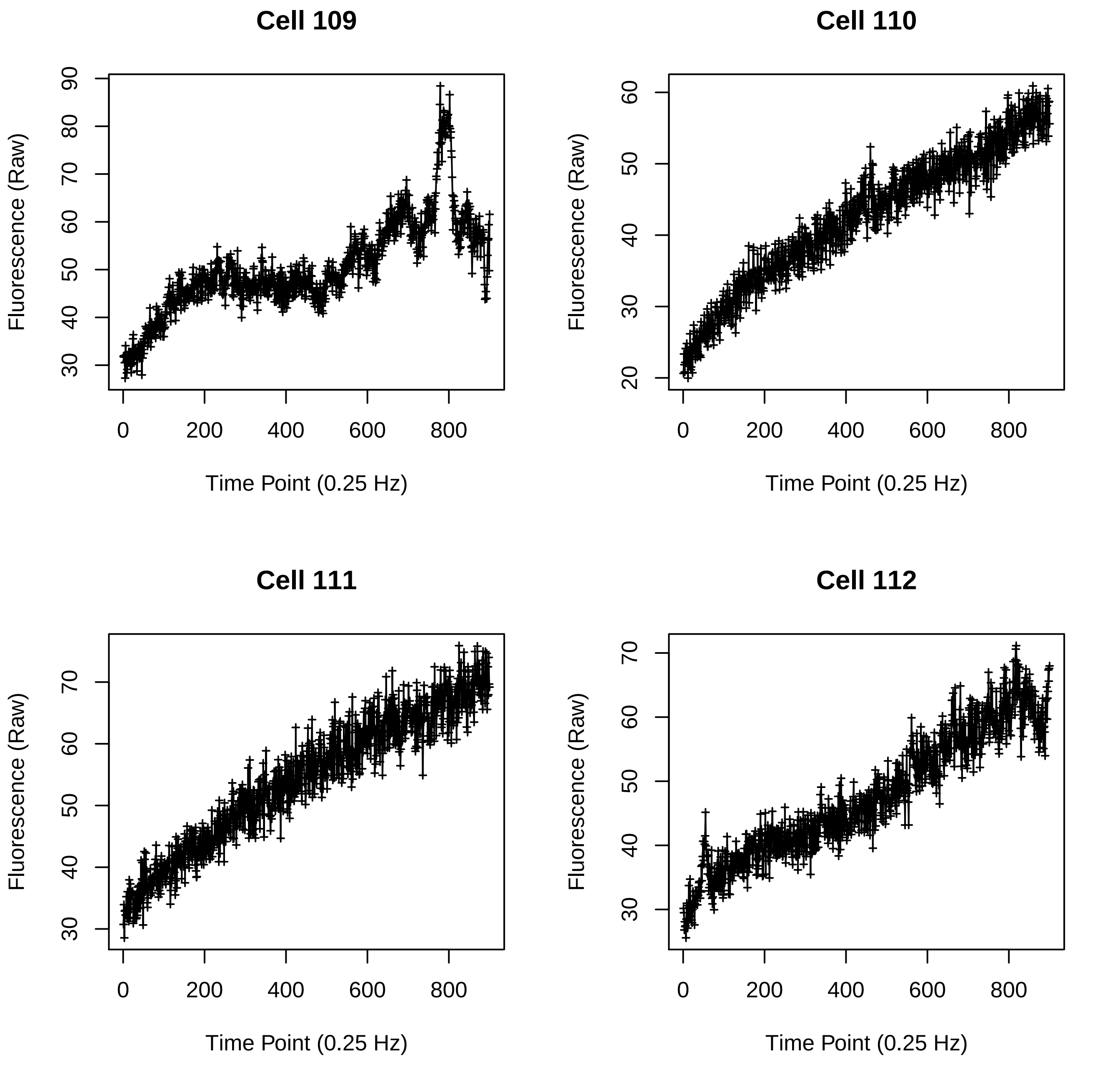

Cell 109
Fluorescence (Raw)
30
40
50
60
70
80
90
0
200
400
600
800
Time Point (0.25 Hz)
Cell 110
Fluorescence (Raw)
20
30
40
50
60
0
200
400
600
800
Time Point (0.25 Hz)
Cell 111
Fluorescence (Raw)
30
40
50
60
70
0
200
400
600
800
Time Point (0.25 Hz)
Cell 112
Fluorescence (Raw)
30
40
50
60
70
0
200
400
600
800
Time Point (0.25 Hz)

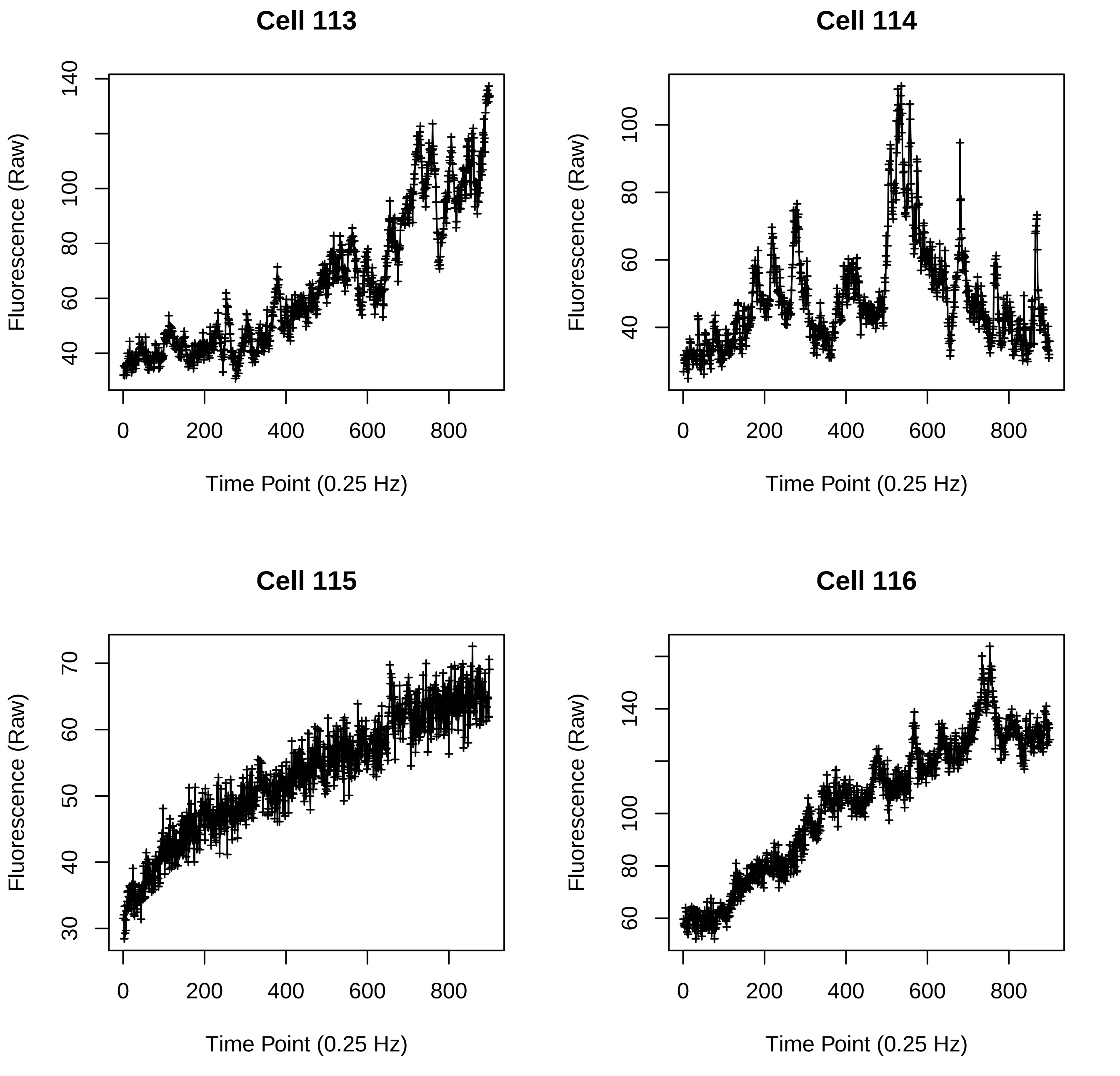

Cell 113
Fluorescence (Raw)
40
60
80
100
140
0
200
400
600
800
Time Point (0.25 Hz)
Cell 114
Fluorescence (Raw)
40
60
80
100
0
200
400
600
800
Time Point (0.25 Hz)
Cell 115
Fluorescence (Raw)
30
40
50
60
70
0
200
400
600
800
Time Point (0.25 Hz)
Cell 116
Fluorescence (Raw)
60
80
100
140
0
200
400
600
800
Time Point (0.25 Hz)

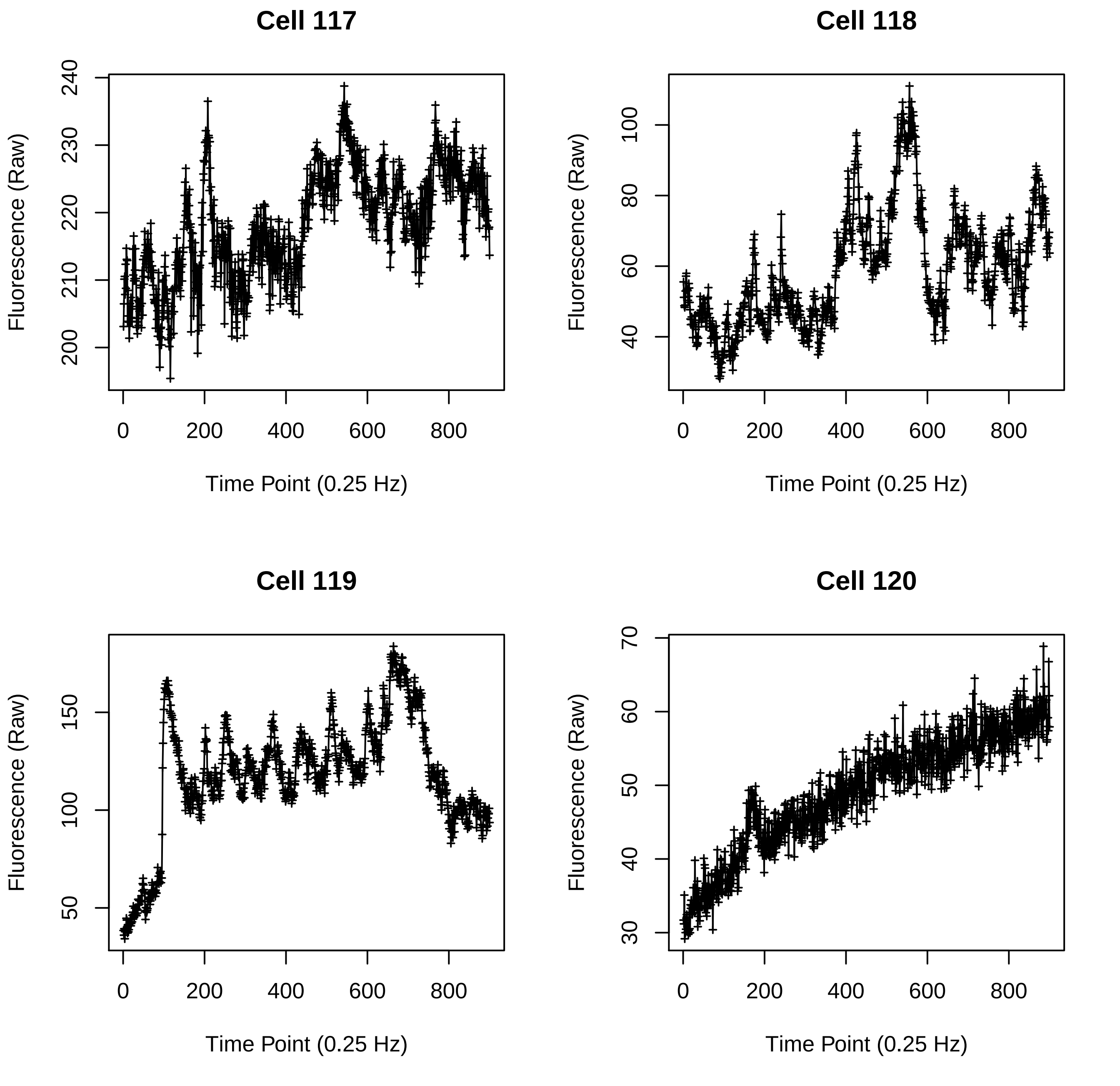
Cell 117
Cell 118
Cell 119
Cell 120
Fluorescence (Raw)
Time Point (0.25 Hz)
0
200
400
600
800
200
210
220
230
240
40
60
80
100
50
100
150
30
40
50
60
70

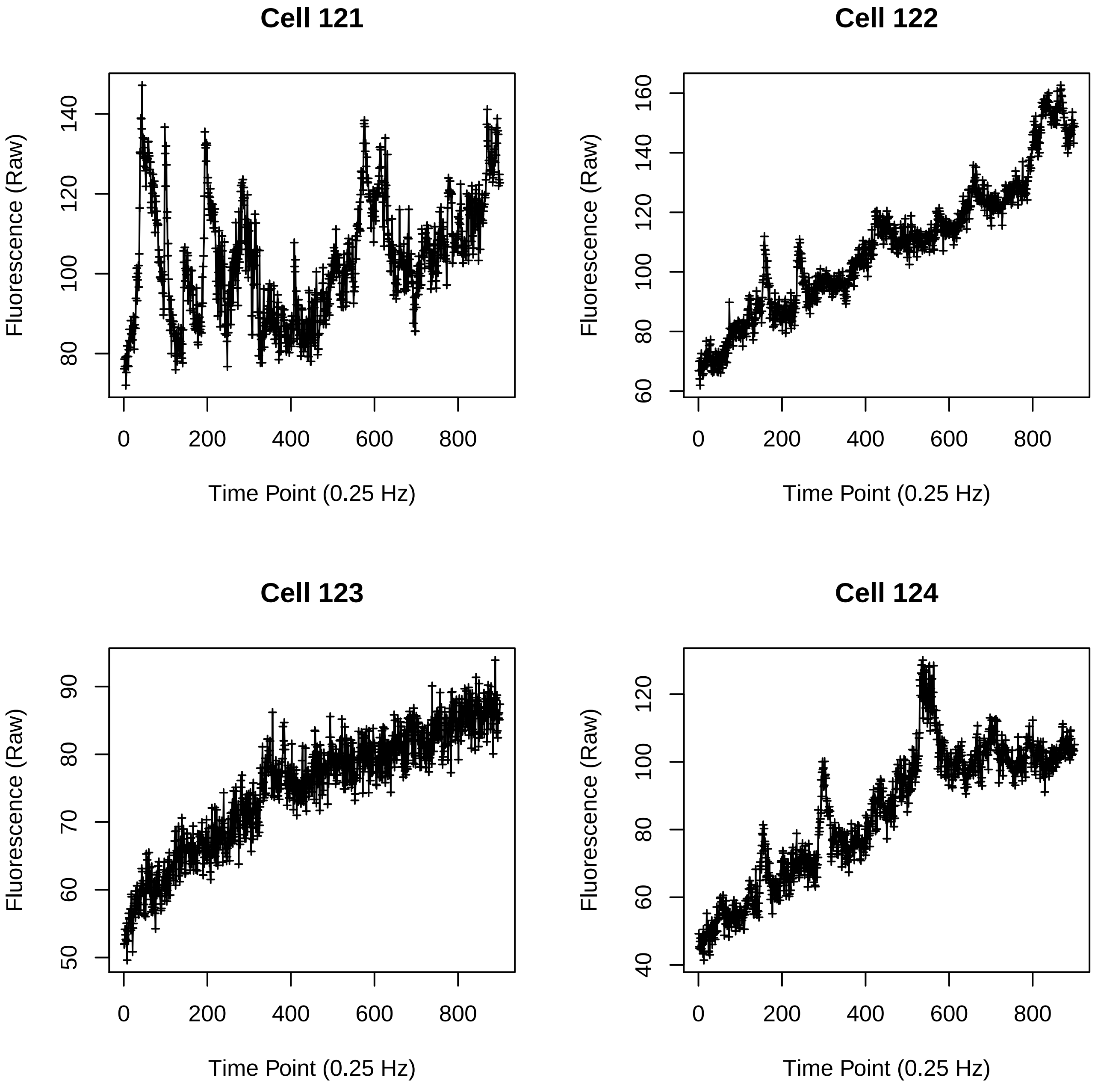

Cell 121
Cell 122
Cell 123
Cell 124
Fluorescence (Raw)
Time Point (0.25 Hz)
0
200
400
600
800
80
100
120
140
60
160
50
70
90
40

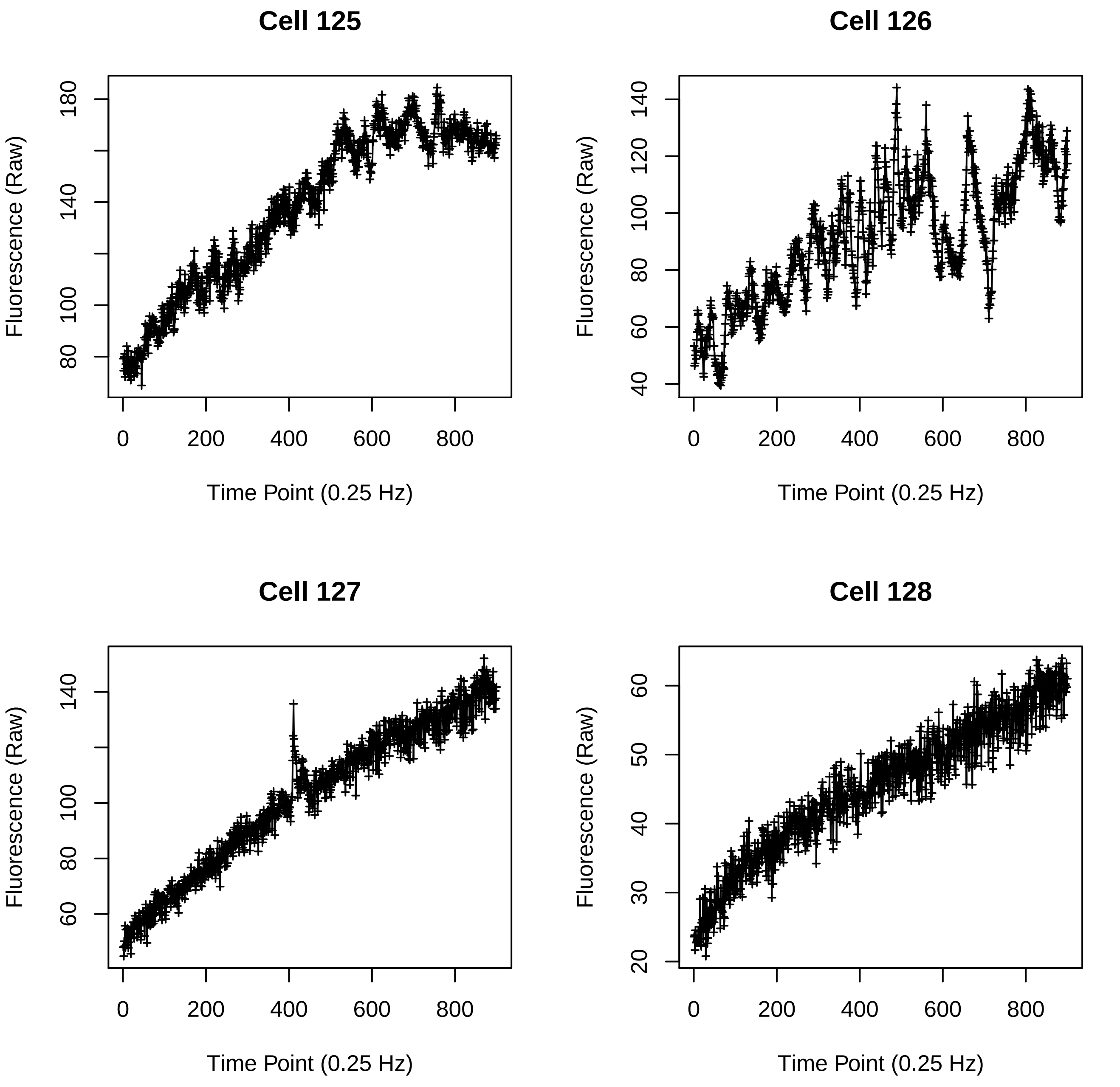
Cell 125
Fluorescence (Raw)
80
100
140
180
0
200
400
600
800
Time Point (0.25 Hz)
Cell 126
Fluorescence (Raw)
40
60
80
100
120
140
0
200
400
600
800
Time Point (0.25 Hz)
Cell 127
Fluorescence (Raw)
60
80
100
140
0
200
400
600
800
Time Point (0.25 Hz)
Cell 128
Fluorescence (Raw)
20
30
40
50
60
0
200
400
600
800
Time Point (0.25 Hz)

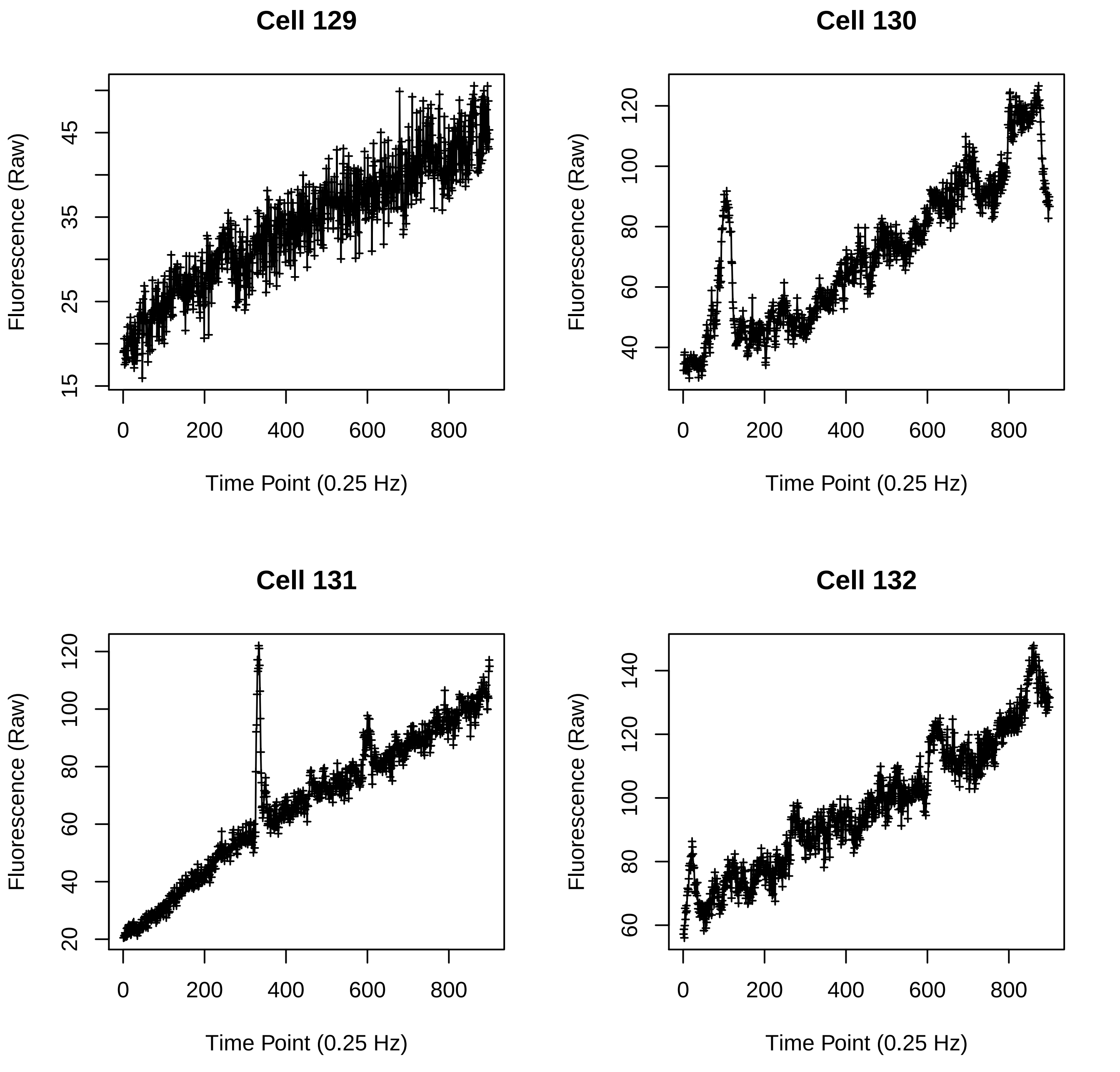

Cell 129
Fluorescence (Raw)
15
25
35
45
0
200
400
600
800
Time Point (0.25 Hz)
Cell 130
Fluorescence (Raw)
40
60
80
100
120
0
200
400
600
800
Time Point (0.25 Hz)
Cell 131
Fluorescence (Raw)
20
40
60
80
100
120
0
200
400
600
800
Time Point (0.25 Hz)
Cell 132
Fluorescence (Raw)
60
80
100
120
140
0
200
400
600
800
Time Point (0.25 Hz)

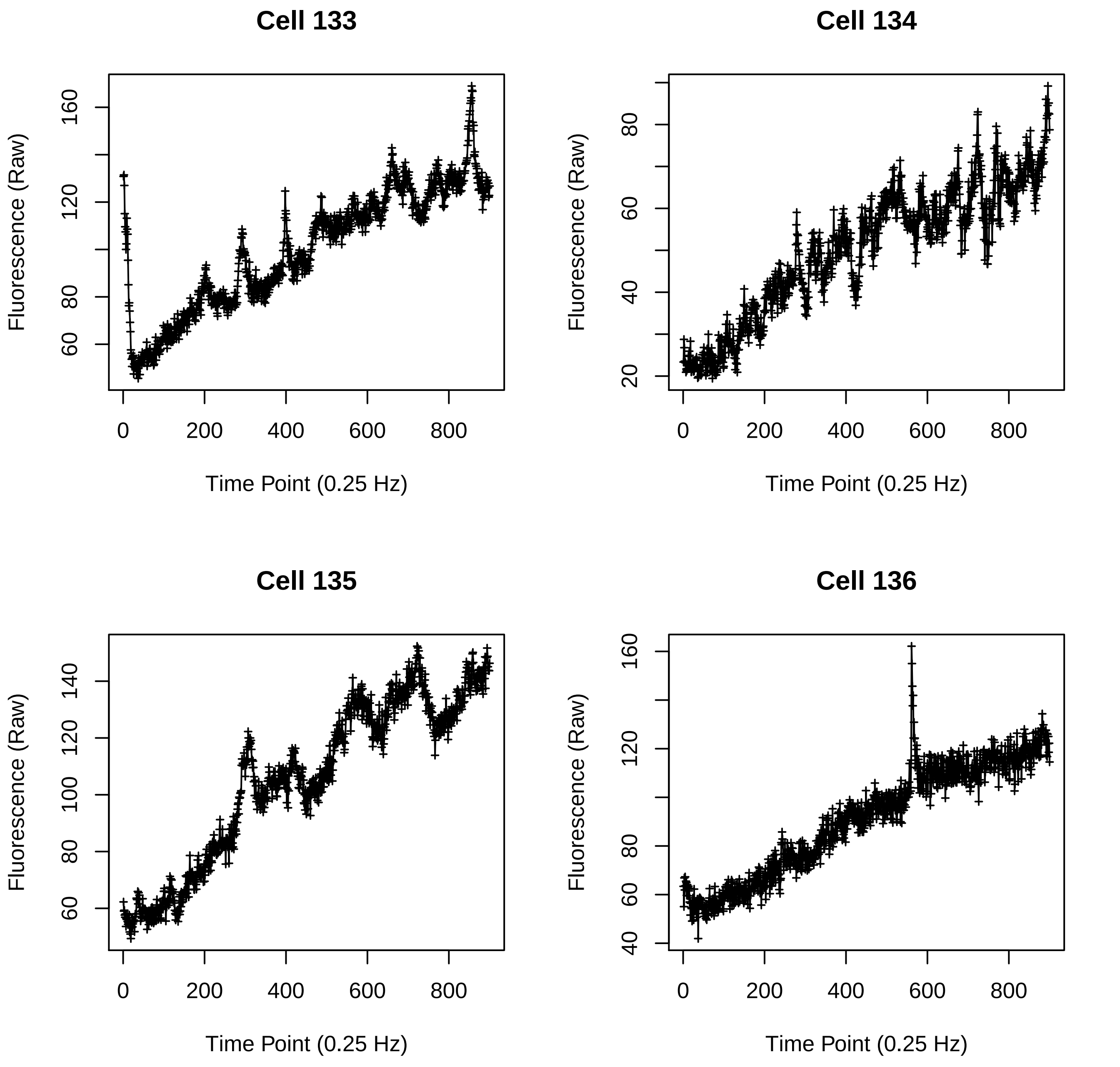
Cell 133
Cell 134
Cell 135
Cell 136
Fluorescence (Raw)
Time Point (0.25 Hz)
0
200
400
600
800
20
40
60
80
100
120
140
160

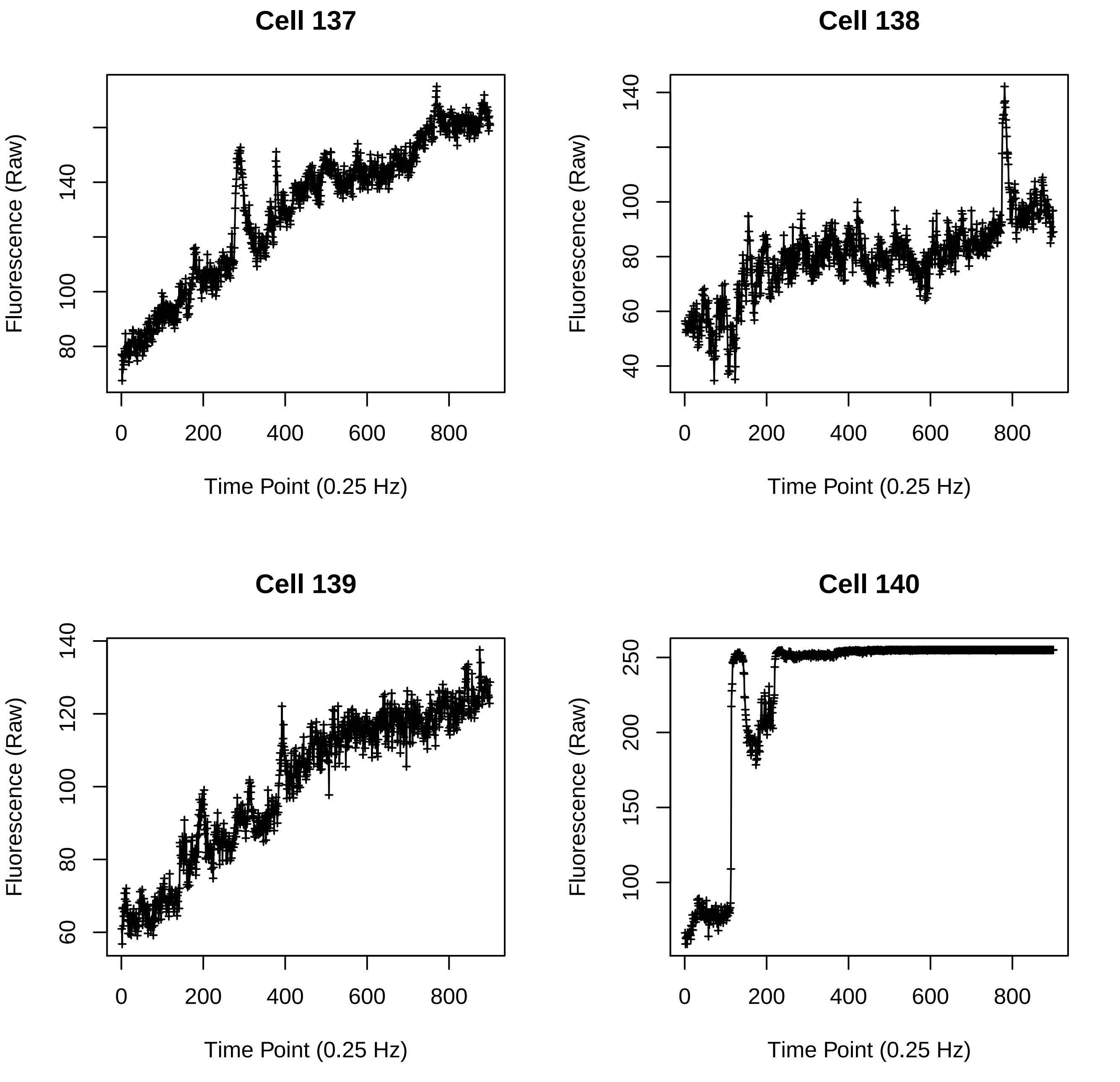
Cell 137
Cell 138
Cell 139
Cell 140
Fluorescence (Raw)
Time Point (0.25 Hz)
0
200
400
600
800
80
100
140
40
60
120
250
200
150

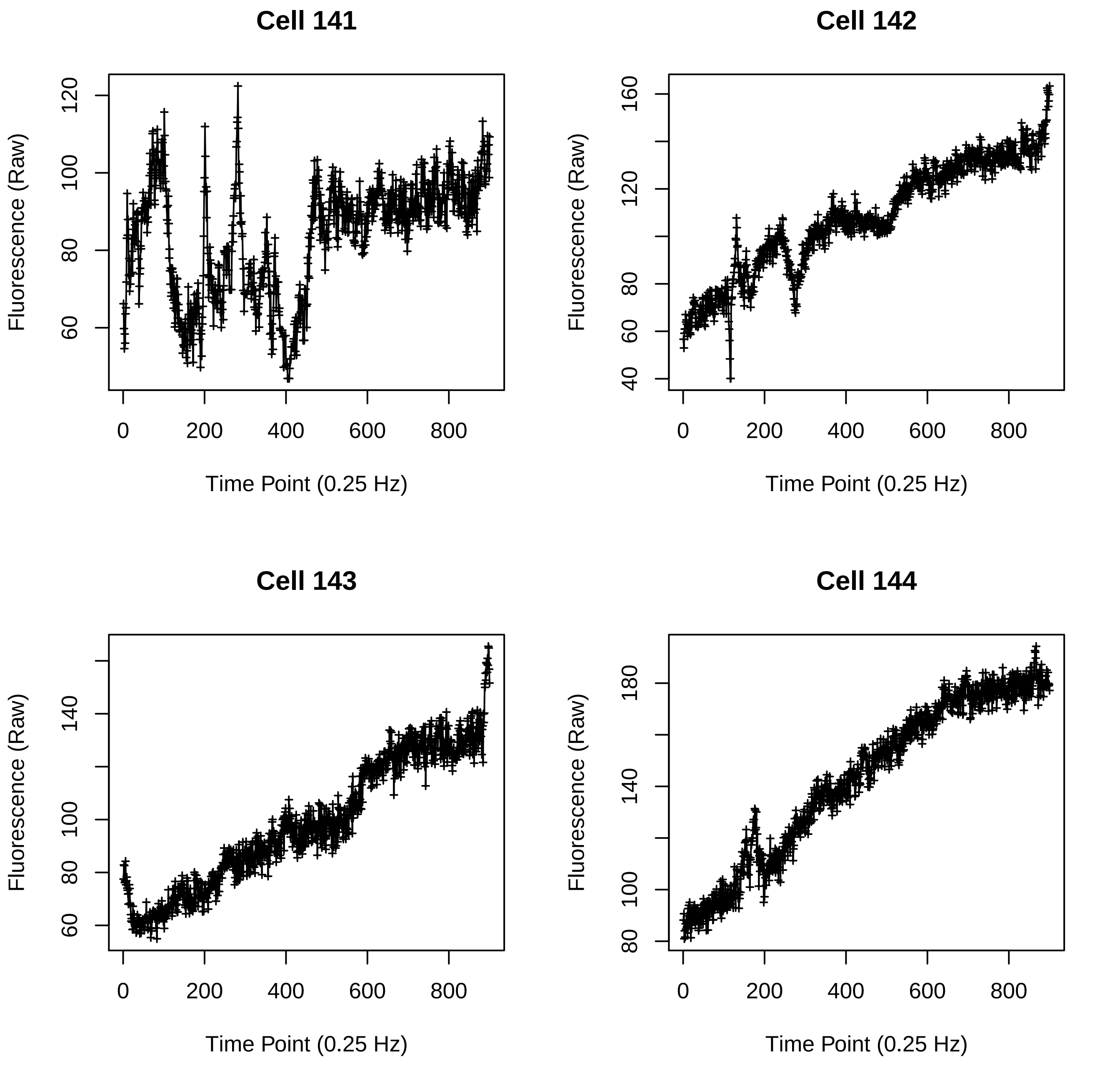

Cell 141
Cell 142
Cell 143
Cell 144
Fluorescence (Raw)
Time Point (0.25 Hz)
0
200
400
600
800
60
80
100
120
40
140
160
180

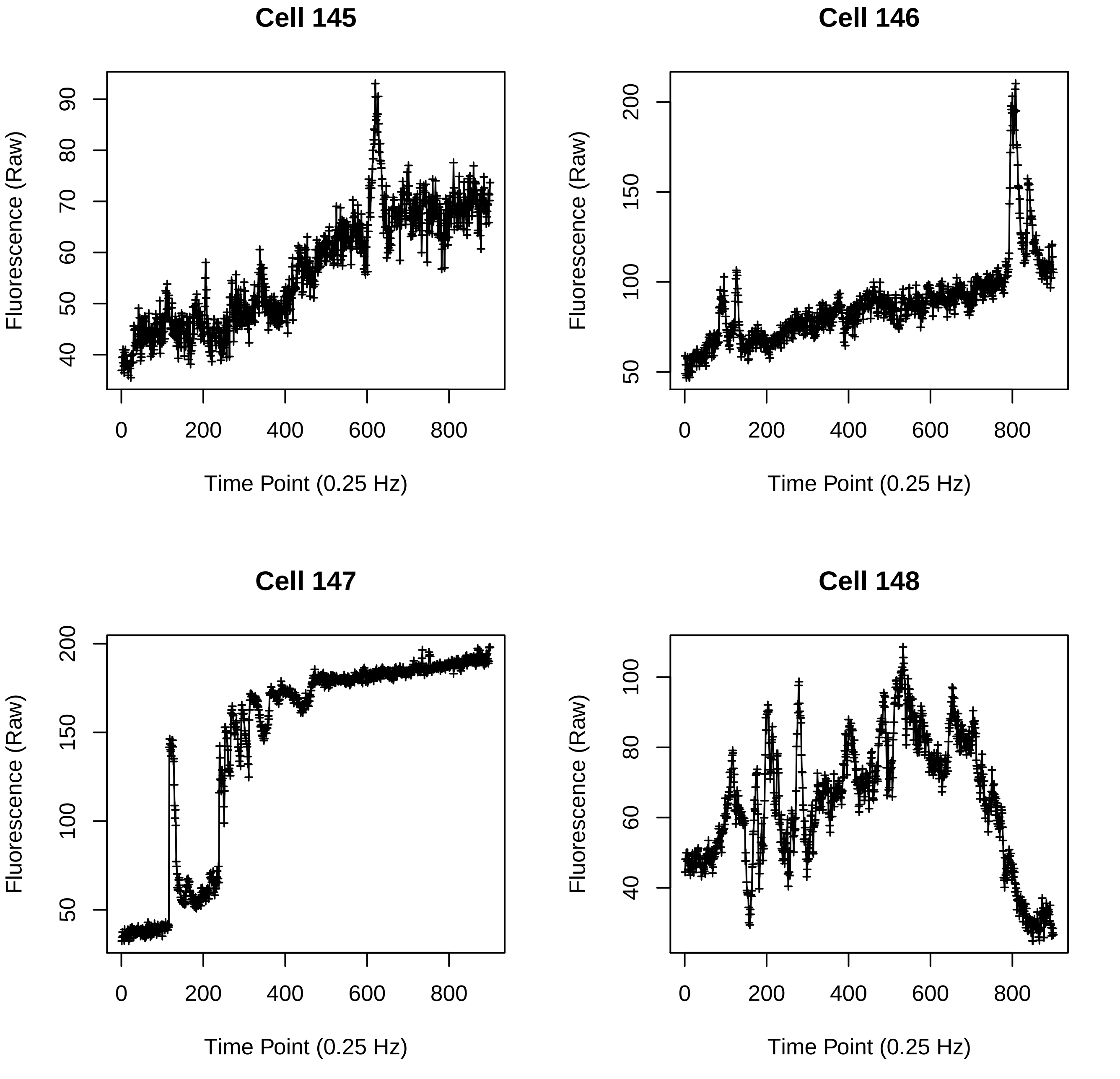
Cell 145
Fluorescence (Raw)
40
50
60
70
80
90
0
200
400
600
800
Time Point (0.25 Hz)
Cell 146
Fluorescence (Raw)
50
100
150
200
0
200
400
600
800
Time Point (0.25 Hz)
Cell 147
Fluorescence (Raw)
50
100
150
200
0
200
400
600
800
Time Point (0.25 Hz)
Cell 148
Fluorescence (Raw)
40
60
80
100
0
200
400
600
800
Time Point (0.25 Hz)

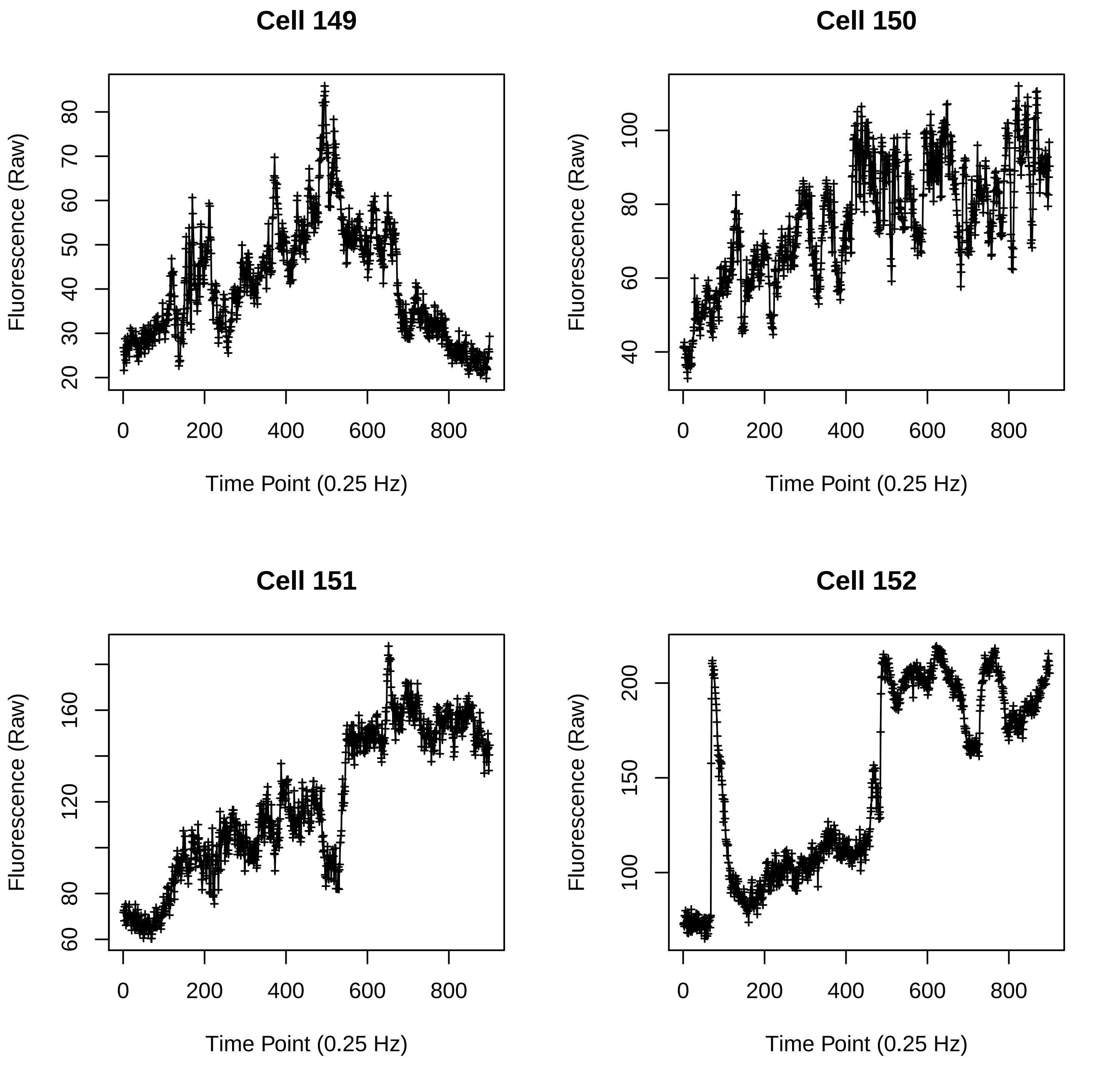
Cell 149
Cell 150
Cell 151
Cell 152
Fluorescence (Raw)
Time Point (0.25 Hz)
0
200
400
600
800
20
30
40
50
60
70
80
100
60
120
160
150
200

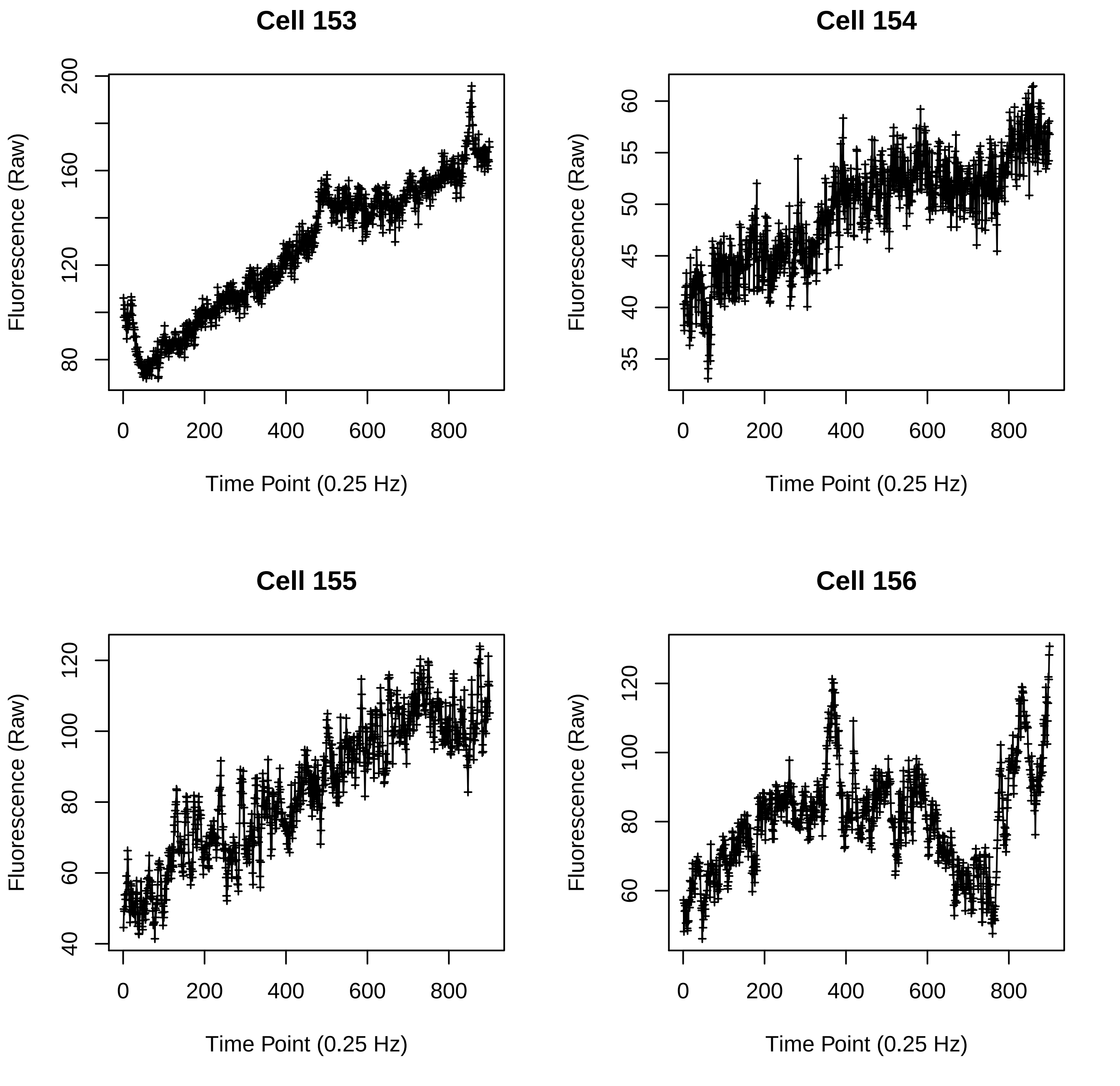

Cell 153
Cell 154
Cell 155
Cell 156
Fluorescence (Raw)
Time Point (0.25 Hz)
0
200
400
600
800
80
120
160
200
35
40
45
50
55
60
40
60
80
100
120

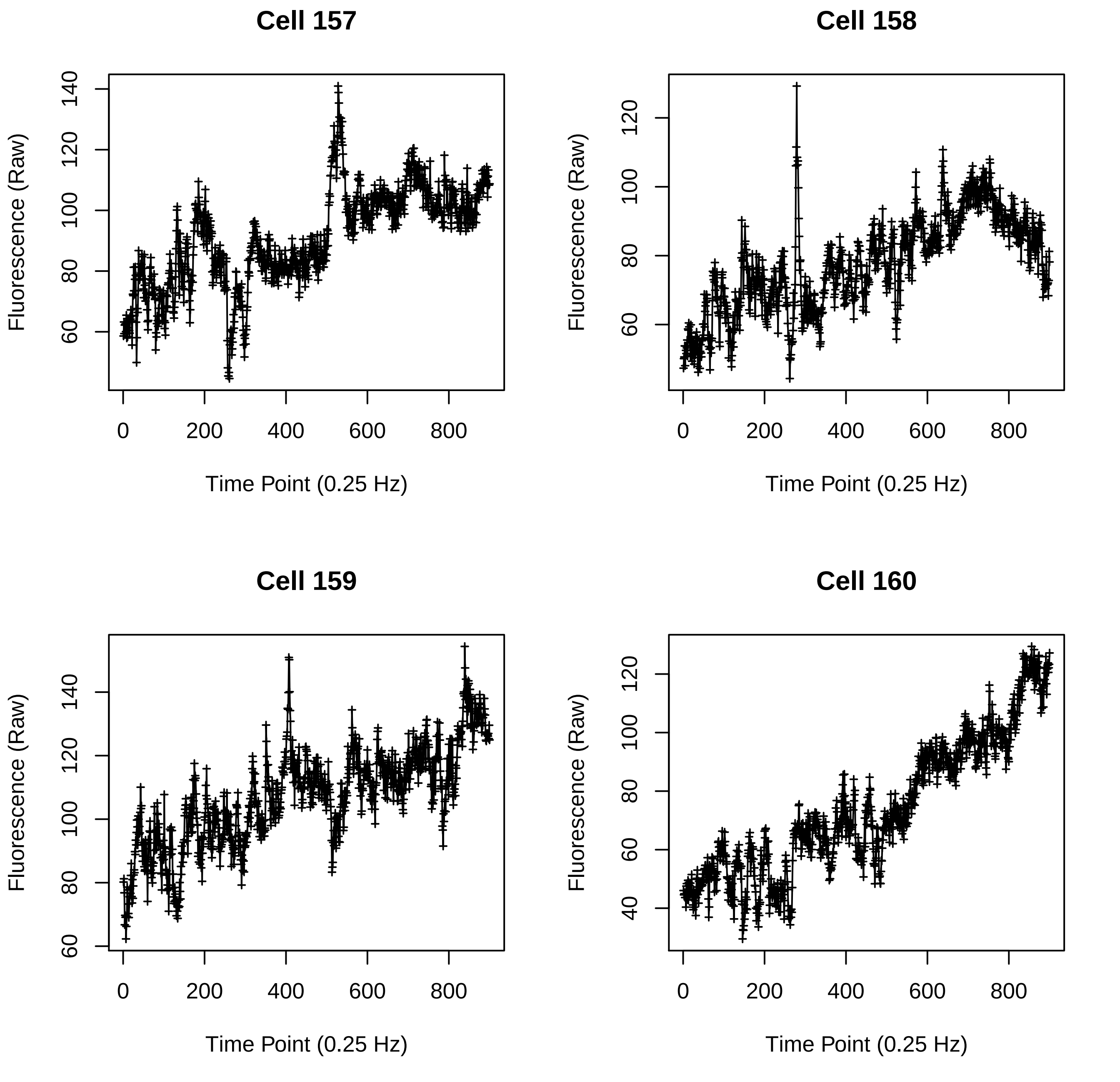
Cell 157
Fluorescence (Raw)
60
80
100
120
140
0
200
400
600
800
Time Point (0.25 Hz)
Cell 158
Fluorescence (Raw)
60
80
100
120
0
200
400
600
800
Time Point (0.25 Hz)
Cell 159
Fluorescence (Raw)
60
80
100
120
140
0
200
400
600
800
Time Point (0.25 Hz)
Cell 160
Fluorescence (Raw)
40
60
80
100
120
0
200
400
600
800
Time Point (0.25 Hz)

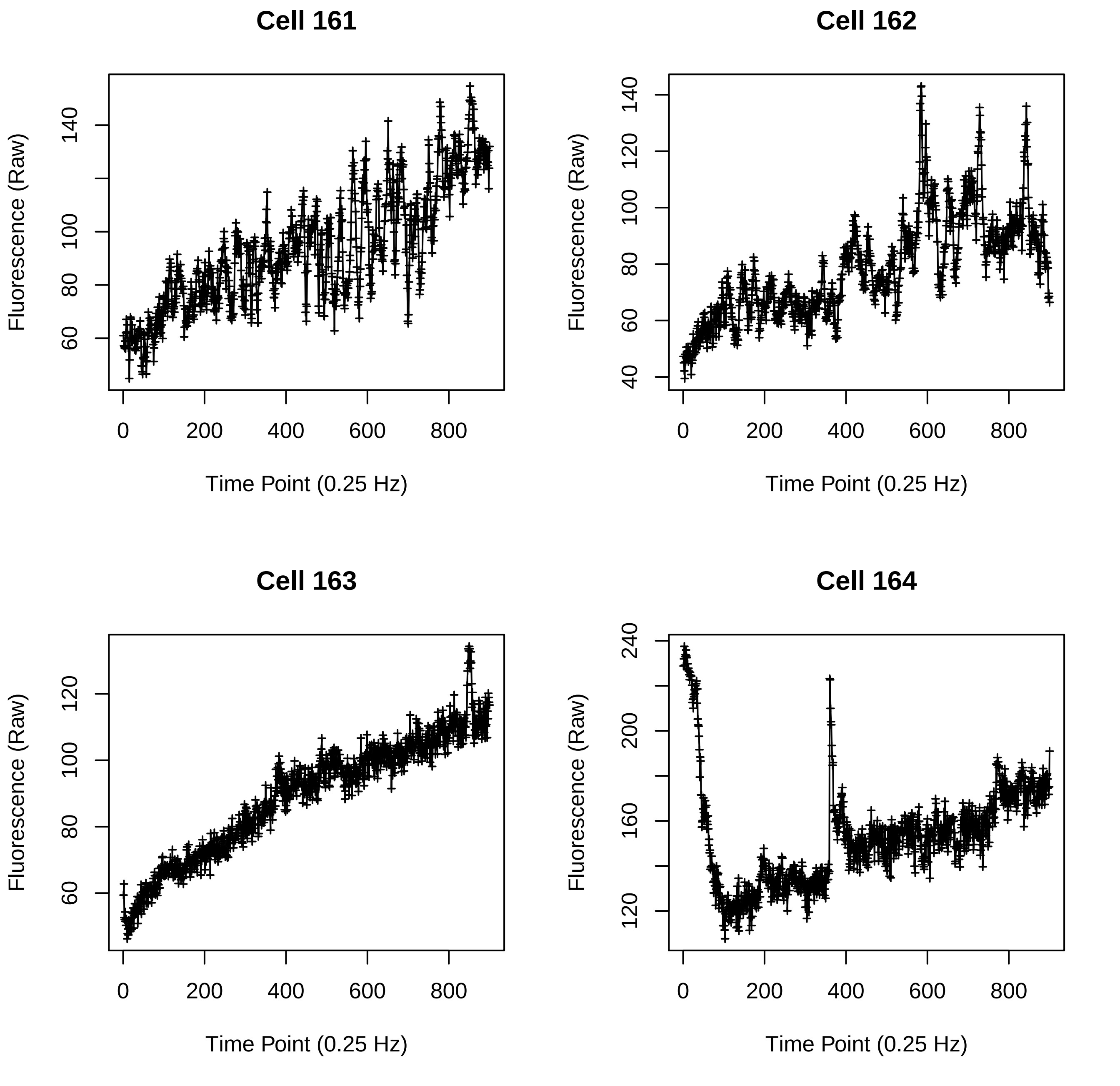
Cell 161
Cell 162
Cell 163
Cell 164
Fluorescence (Raw)
Time Point (0.25 Hz)
0
200
400
600
800
60
80
100
140
40
120
160
200
240

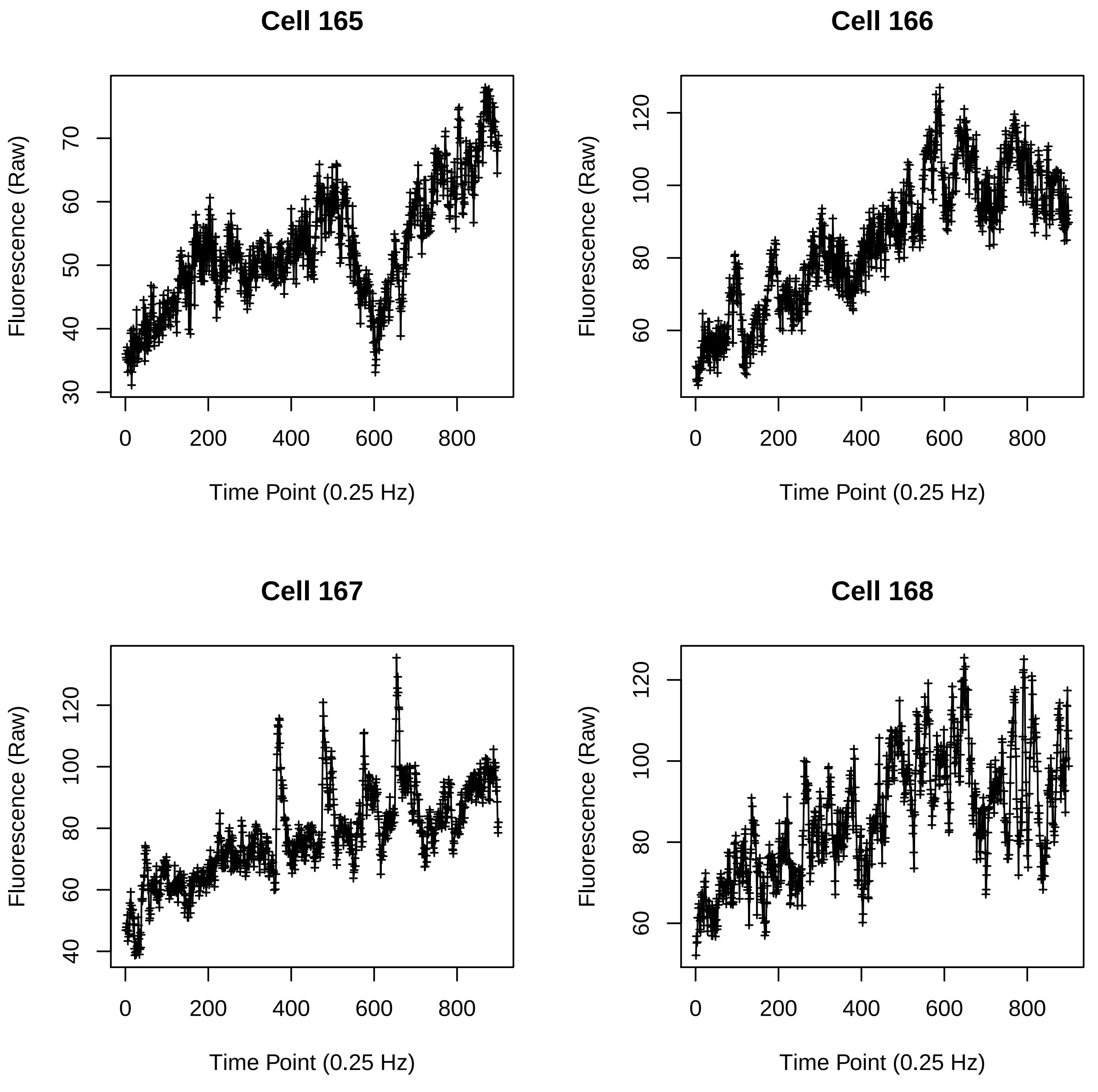
Cell 165
Cell 166
Cell 167
Cell 168
Fluorescence (Raw)
Time Point (0.25 Hz)
30
40
50
60
70
60
80
100
120
40
60
80
100
120
0
200
400
600
800

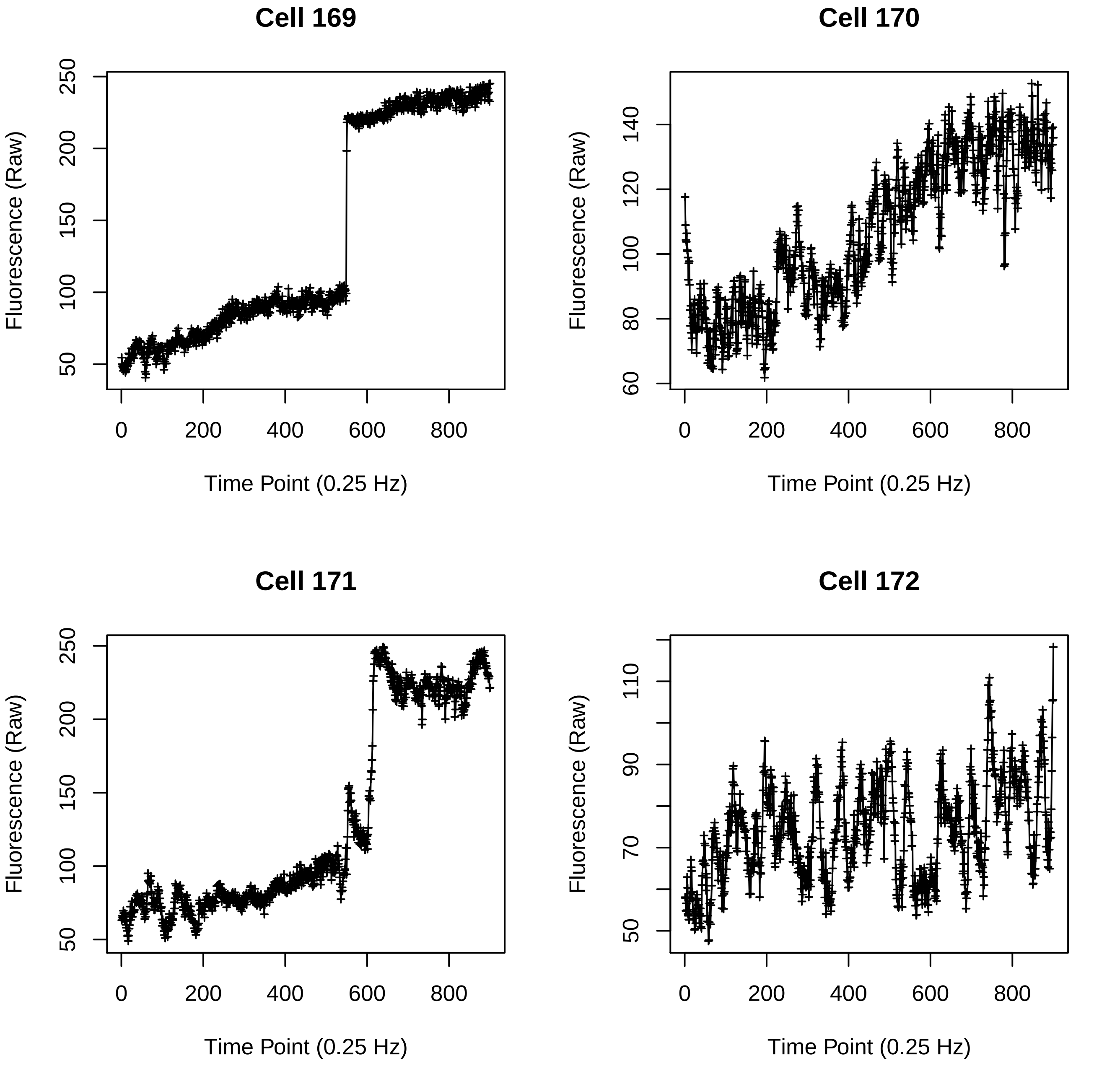

Cell 169
Cell 170
Cell 171
Cell 172
Fluorescence (Raw)
Time Point (0.25 Hz)
0
200
400
600
800
50
100
150
200
250
60
80
100
120
140
70
90
110

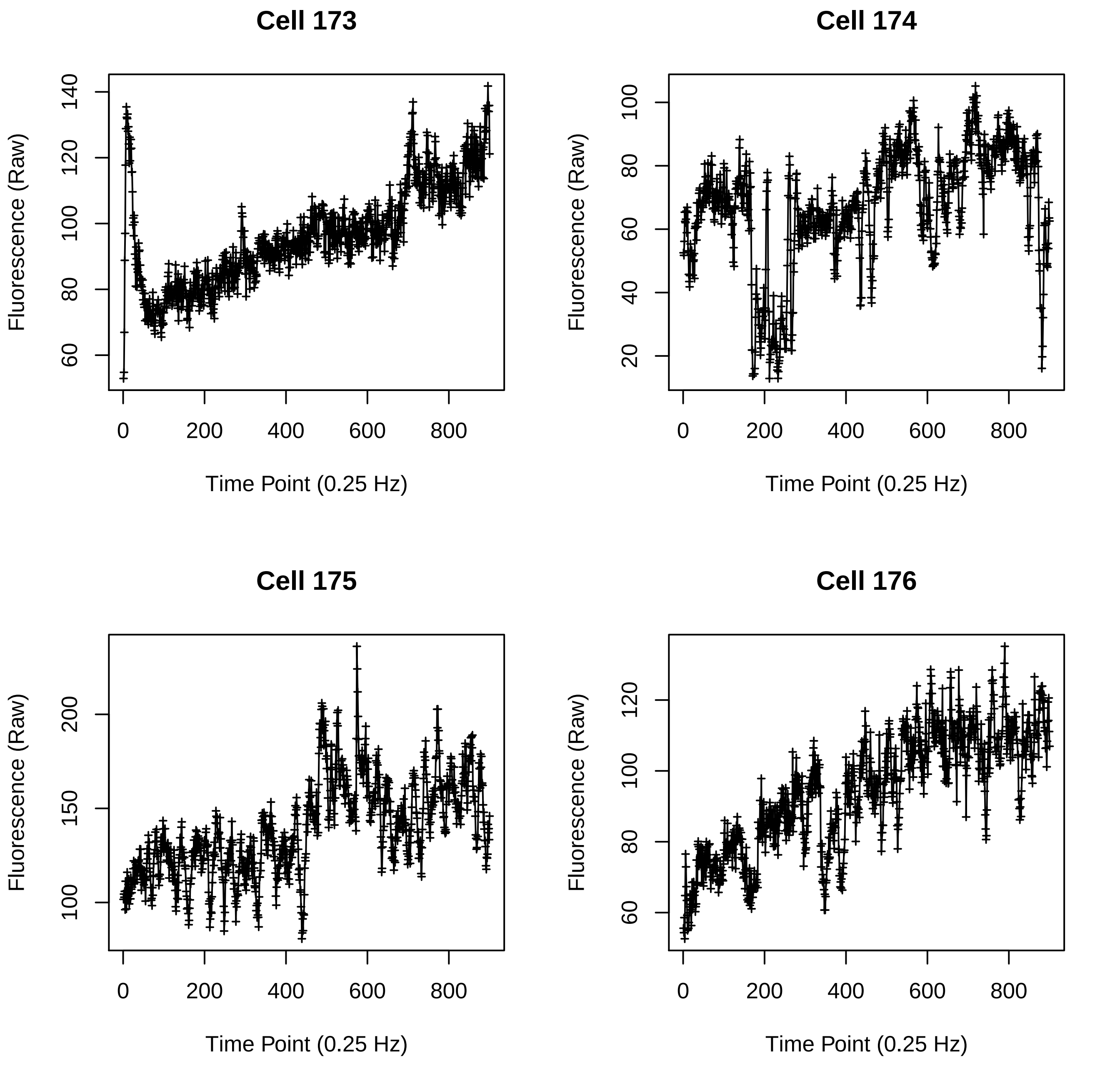
Cell 173
Cell 174
Cell 175
Cell 176
Fluorescence (Raw)
Time Point (0.25 Hz)
0
200
400
600
800
60
80
100
120
140
20
40
150
200

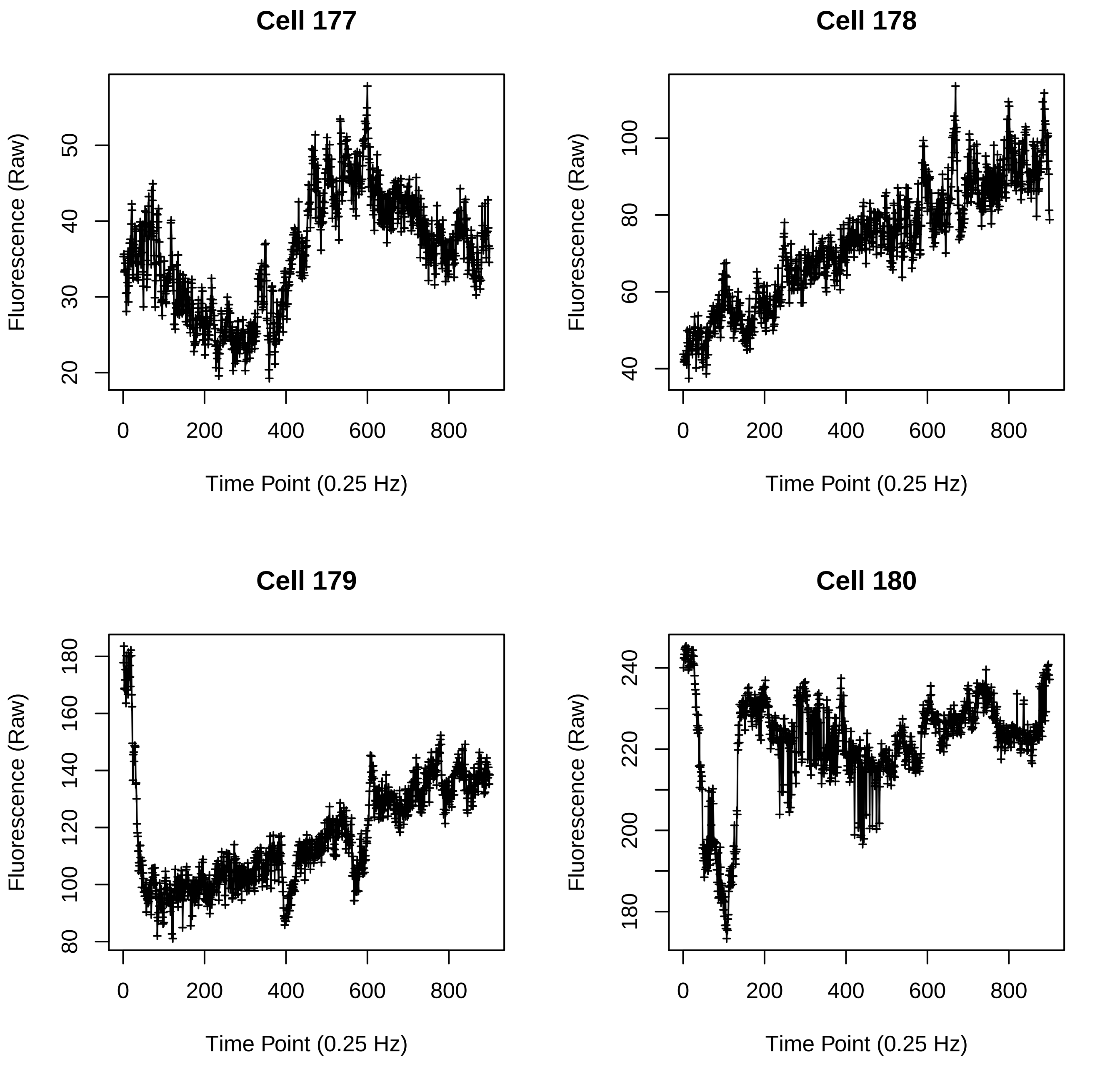
Cell 177
Cell 178
Cell 179
Cell 180
Fluorescence (Raw)
Time Point (0.25 Hz)
0
200
400
600
800
20
30
40
50
60
80
100
120
140
160
180
200
220
240

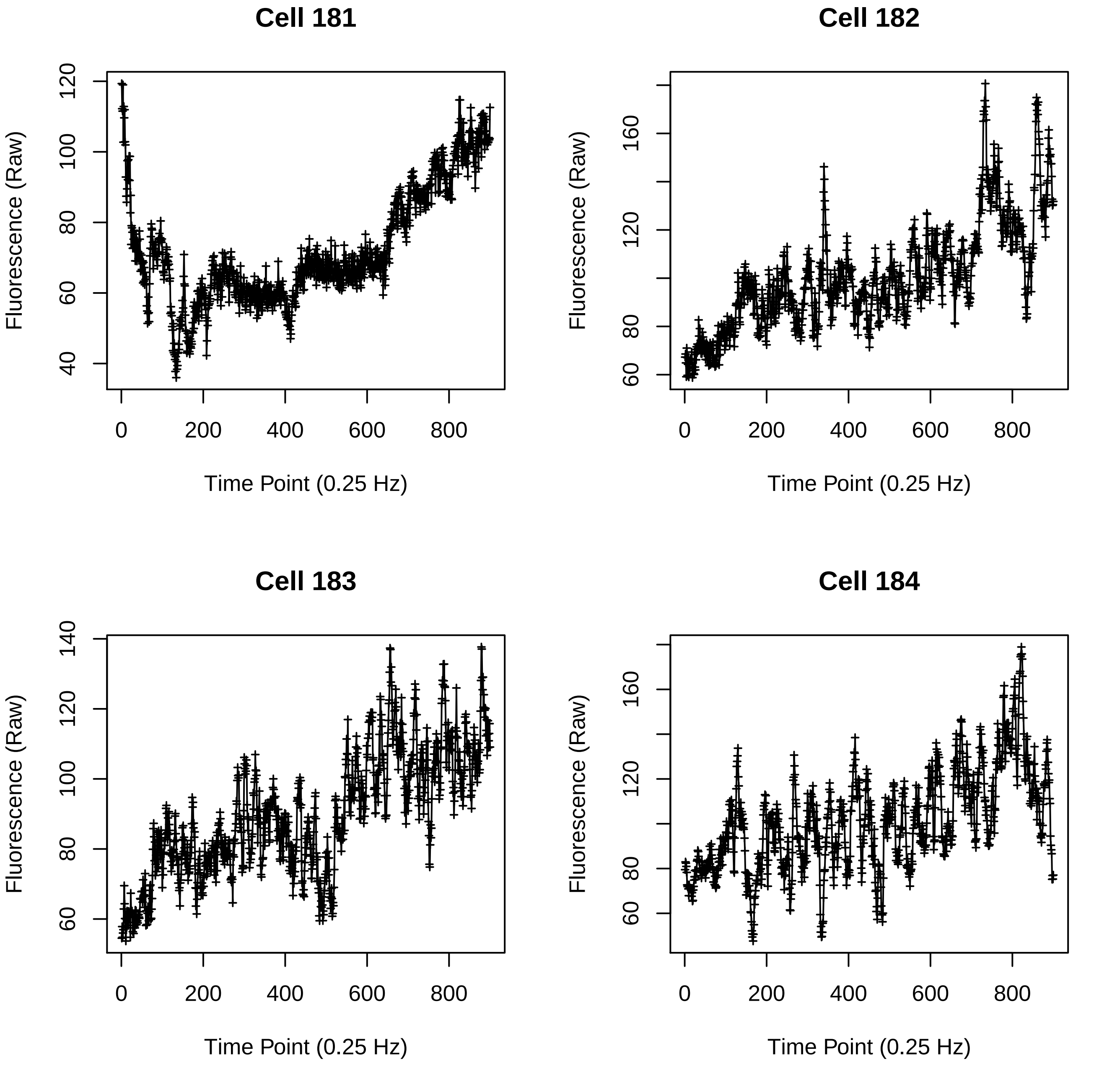
Cell 181
Cell 182
Cell 183
Cell 184
Fluorescence (Raw)
Time Point (0.25 Hz)
0
200
400
600
800
40
60
80
100
120
140
160

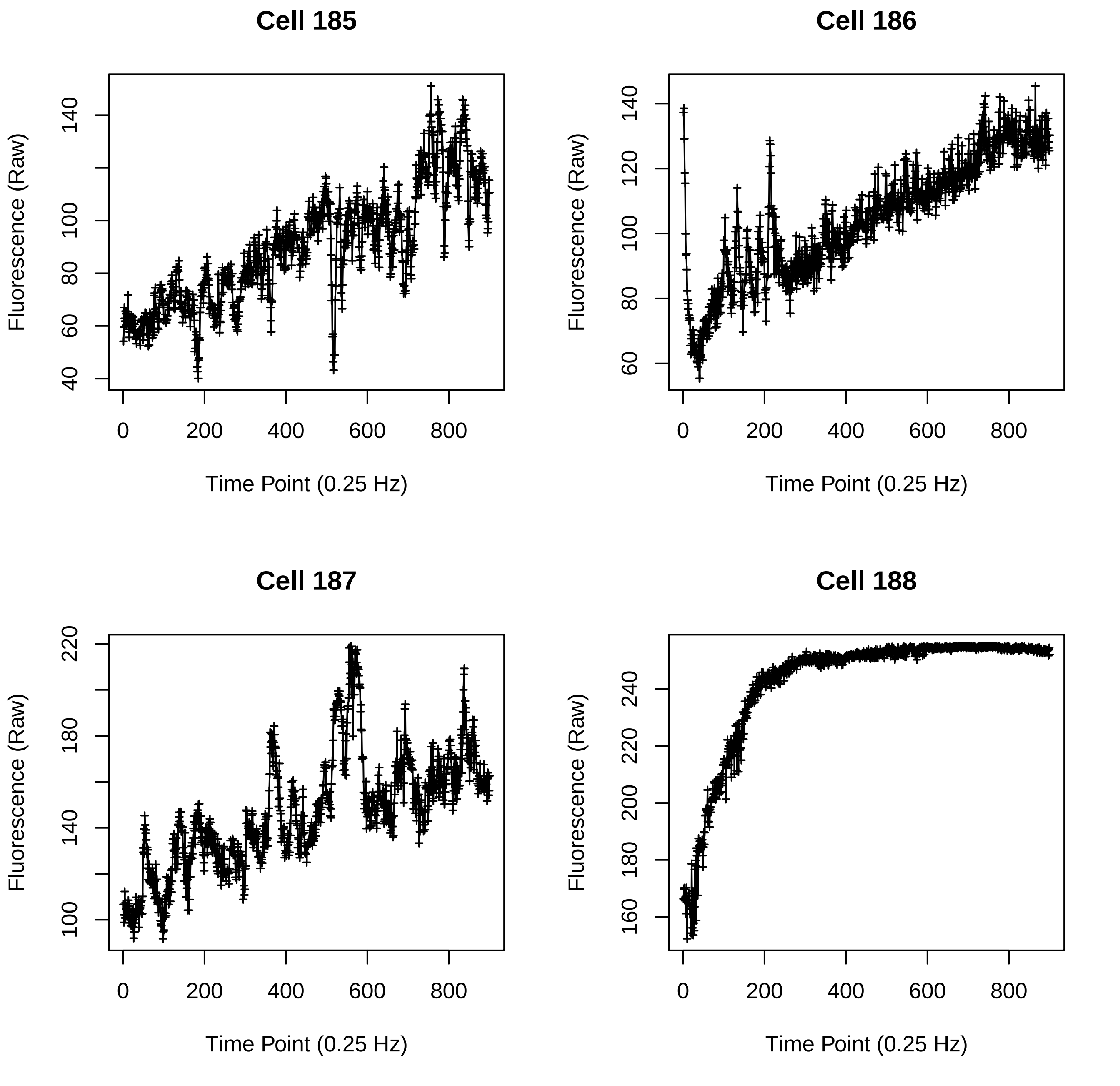

Cell 185
Fluorescence (Raw)
40
60
80
100
140
0
200
400
600
800
Time Point (0.25 Hz)
Cell 186
Fluorescence (Raw)
60
80
100
120
140
0
200
400
600
800
Time Point (0.25 Hz)
Cell 187
Fluorescence (Raw)
100
140
180
220
0
200
400
600
800
Time Point (0.25 Hz)
Cell 188
Fluorescence (Raw)
160
180
200
220
240
0
200
400
600
800
Time Point (0.25 Hz)

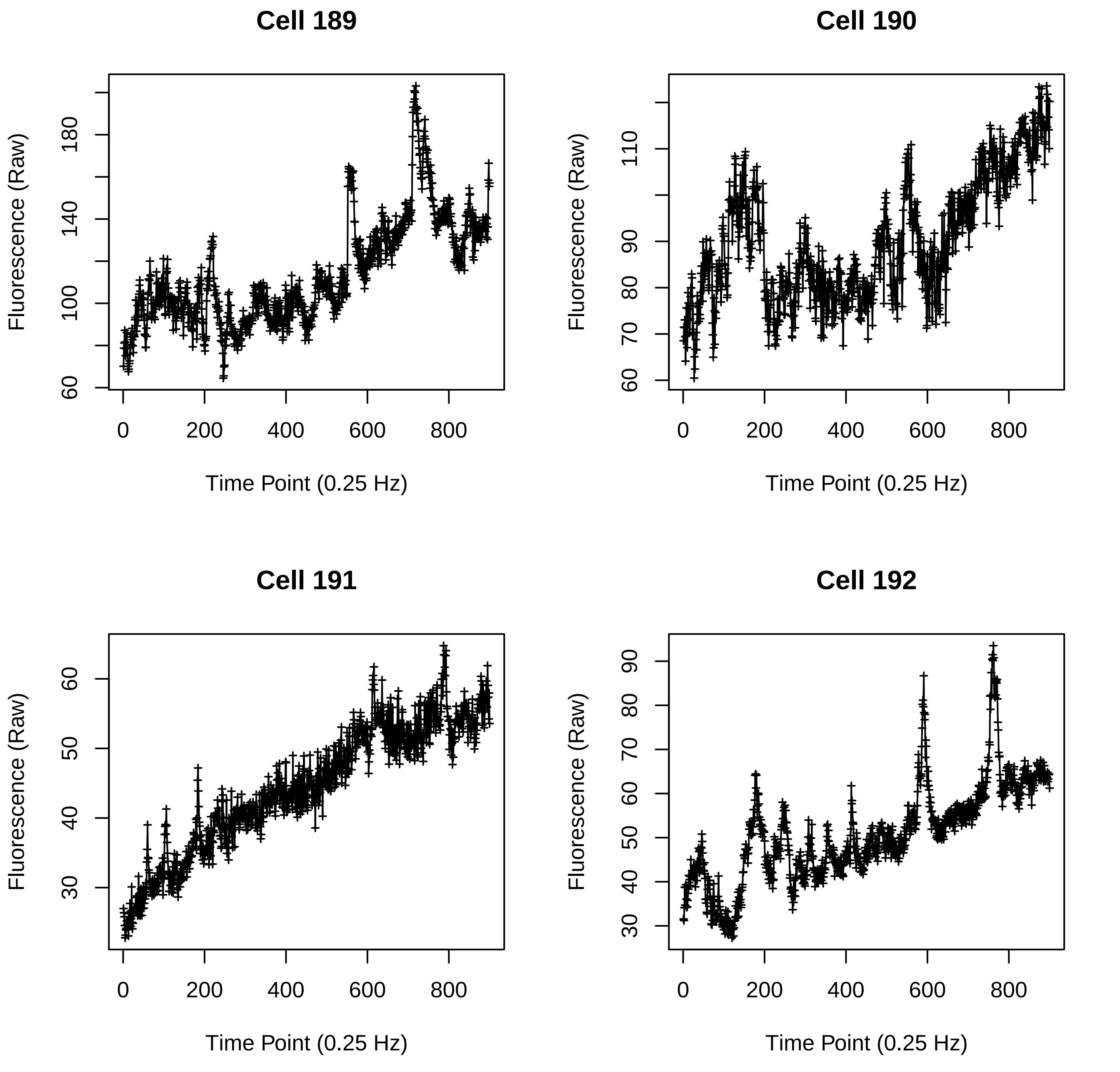

Cell 189
Fluorescence (Raw)
60
100
140
180
0
200
400
600
800
Time Point (0.25 Hz)
Cell 190
Fluorescence (Raw)
60
70
80
90
110
0
200
400
600
800
Time Point (0.25 Hz)
Cell 191
Fluorescence (Raw)
30
40
50
60
0
200
400
600
800
Time Point (0.25 Hz)
Cell 192
Fluorescence (Raw)
30
40
50
60
70
80
90
0
200
400
600
800
Time Point (0.25 Hz)

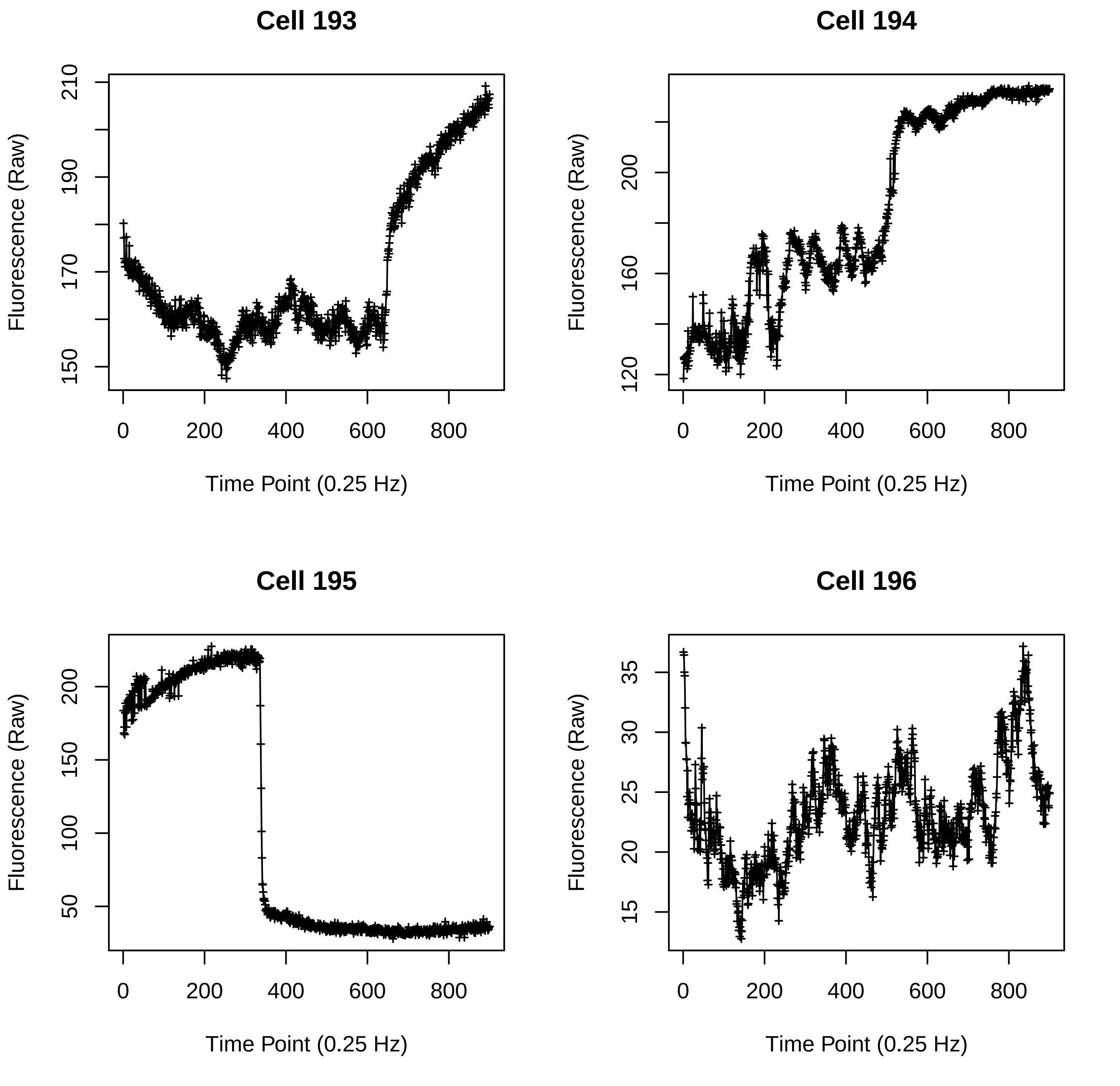
Cell 193
Fluorescence (Raw)
150
170
190
210
0
200
400
600
800
Time Point (0.25 Hz)
Cell 194
Fluorescence (Raw)
120
160
200
0
200
400
600
800
Time Point (0.25 Hz)
Cell 195
Fluorescence (Raw)
50
100
150
200
0
200
400
600
800
Time Point (0.25 Hz)
Cell 196
Fluorescence (Raw)
15
20
25
30
35
0
200
400
600
800
Time Point (0.25 Hz)

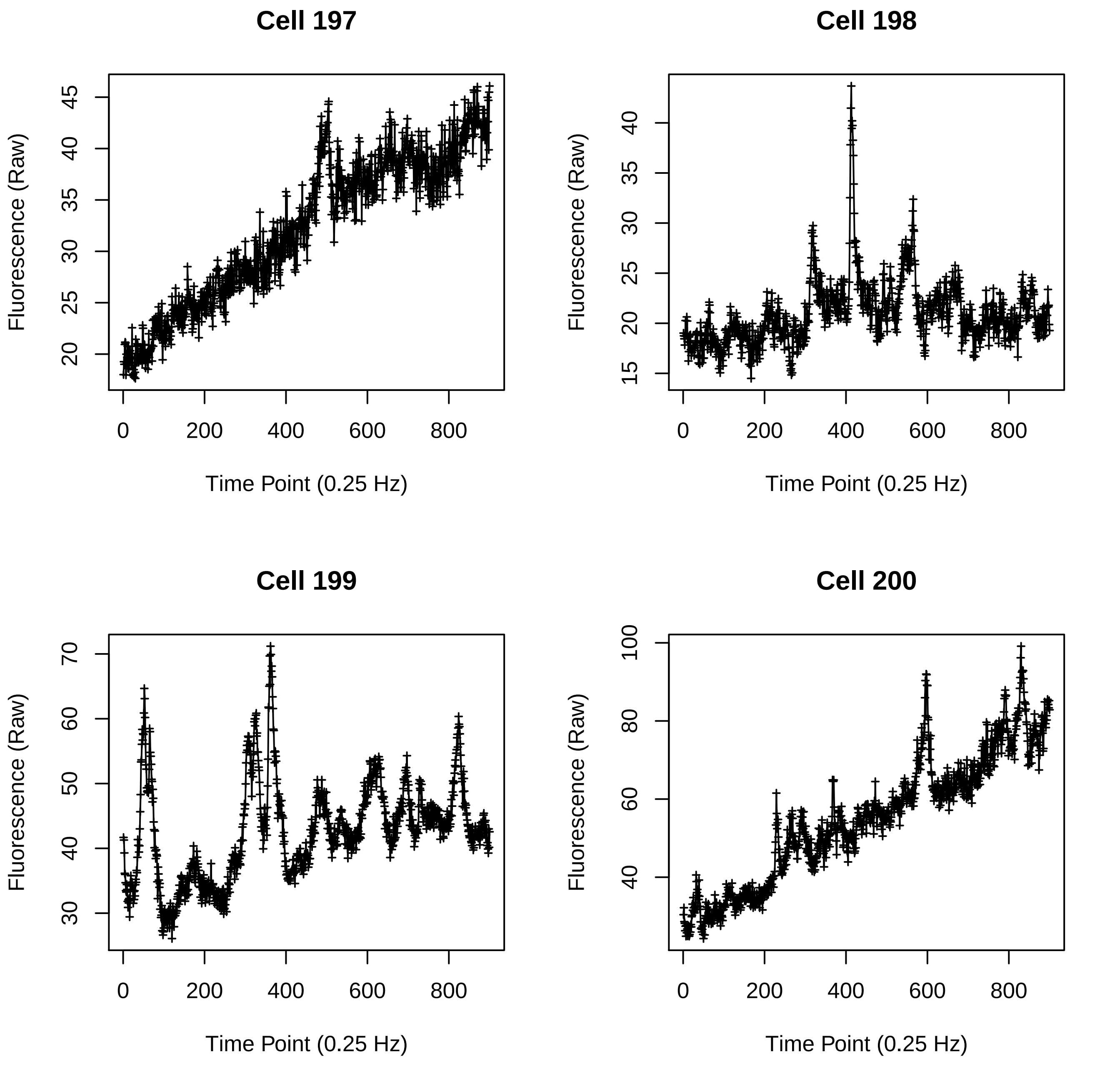

Cell 197
Cell 198
Cell 199
Cell 200
Fluorescence (Raw)
Time Point (0.25 Hz)
0
200
400
600
800
20
25
30
35
40
45
15
50
60
70
80
100

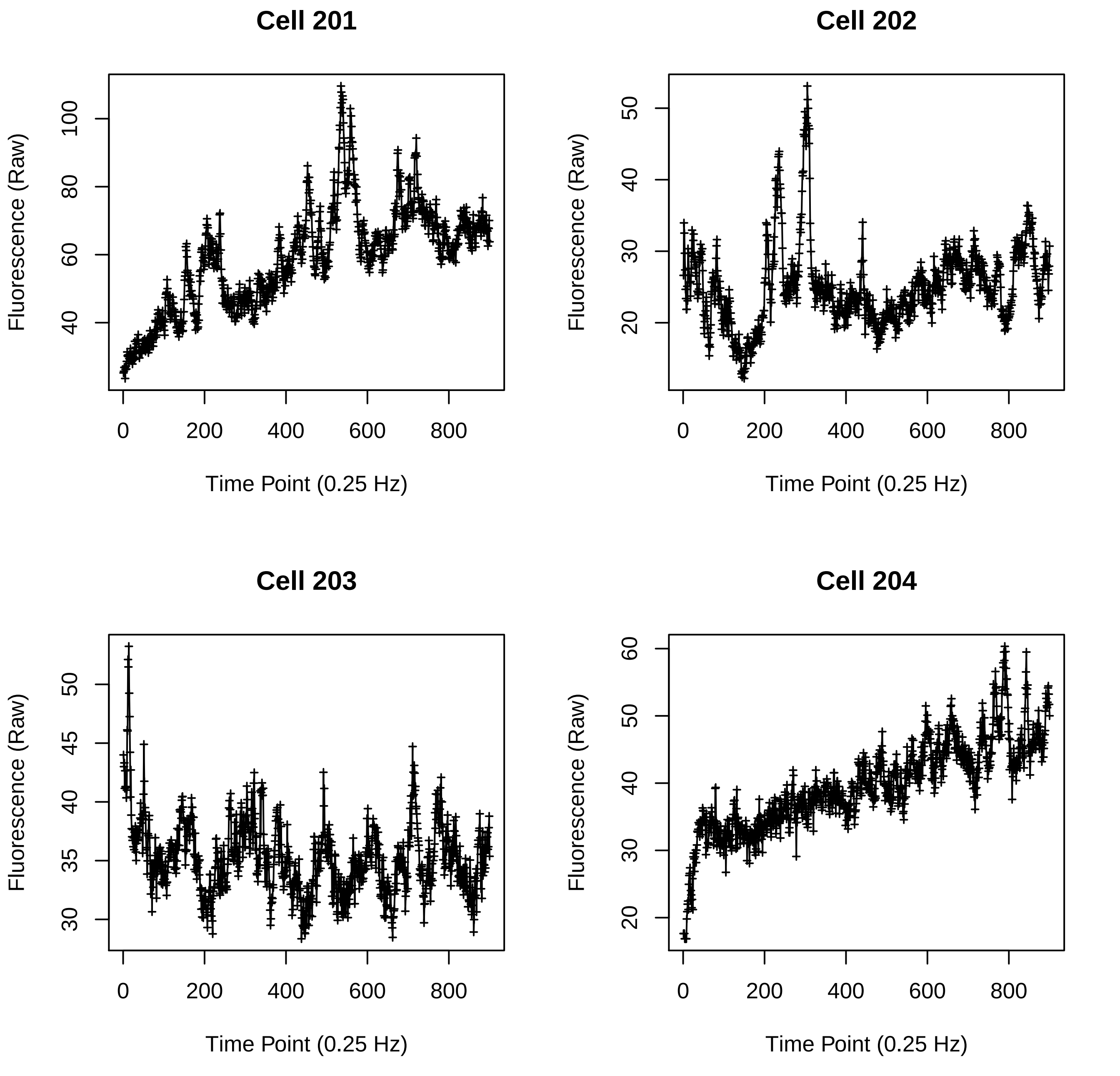
Cell 201
Cell 202
Cell 203
Cell 204
Fluorescence (Raw)
Time Point (0.25 Hz)
0
200
400
600
800
40
60
80
100
20
30
40
50
30
35
40
45
50
20
30
40
50
60

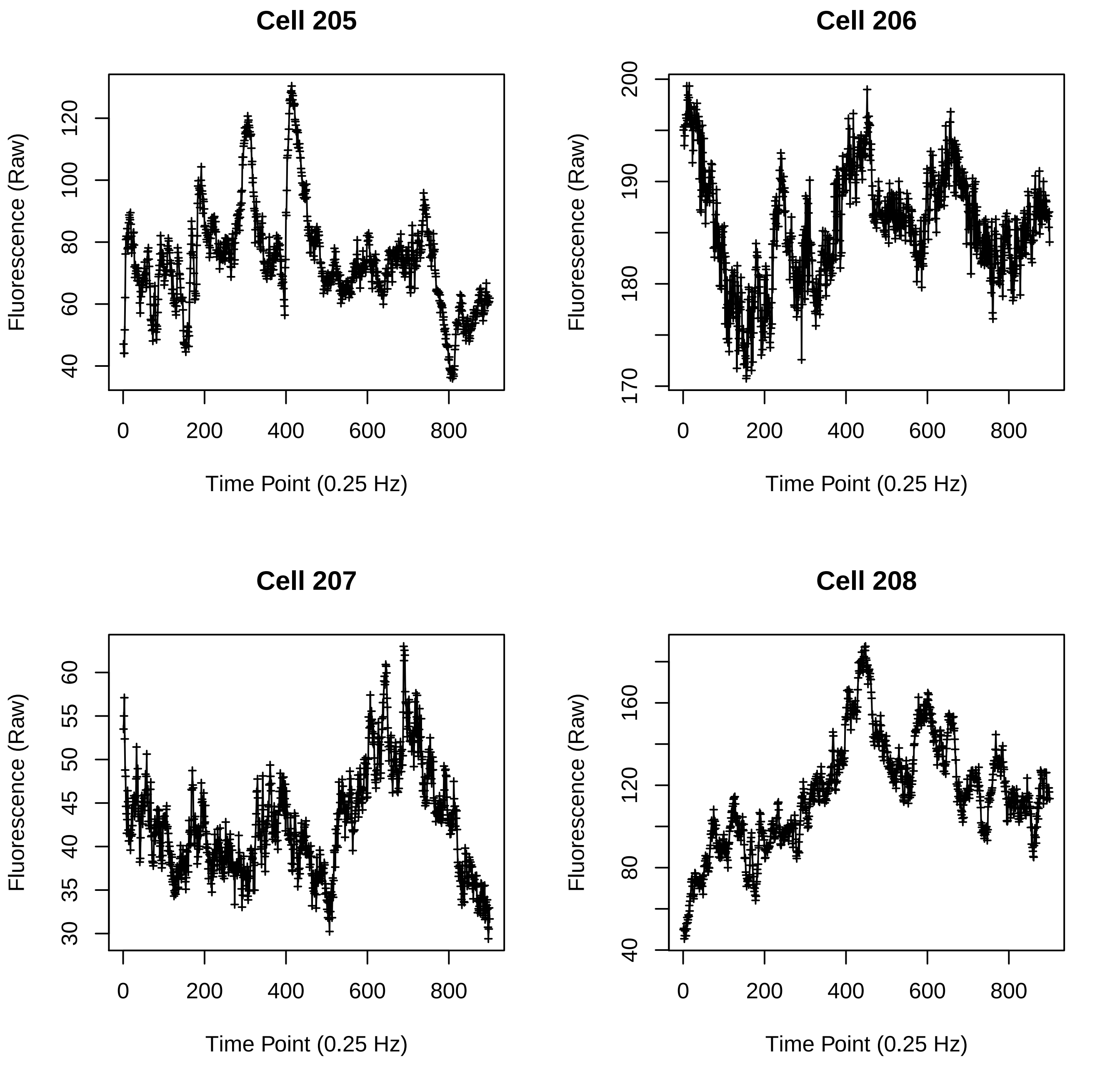
Cell 205
Fluorescence (Raw)
Time Point (0.25 Hz)
Cell 206
Fluorescence (Raw)
Time Point (0.25 Hz)
Cell 207
Fluorescence (Raw)
Time Point (0.25 Hz)
Cell 208
Fluorescence (Raw)
Time Point (0.25 Hz)

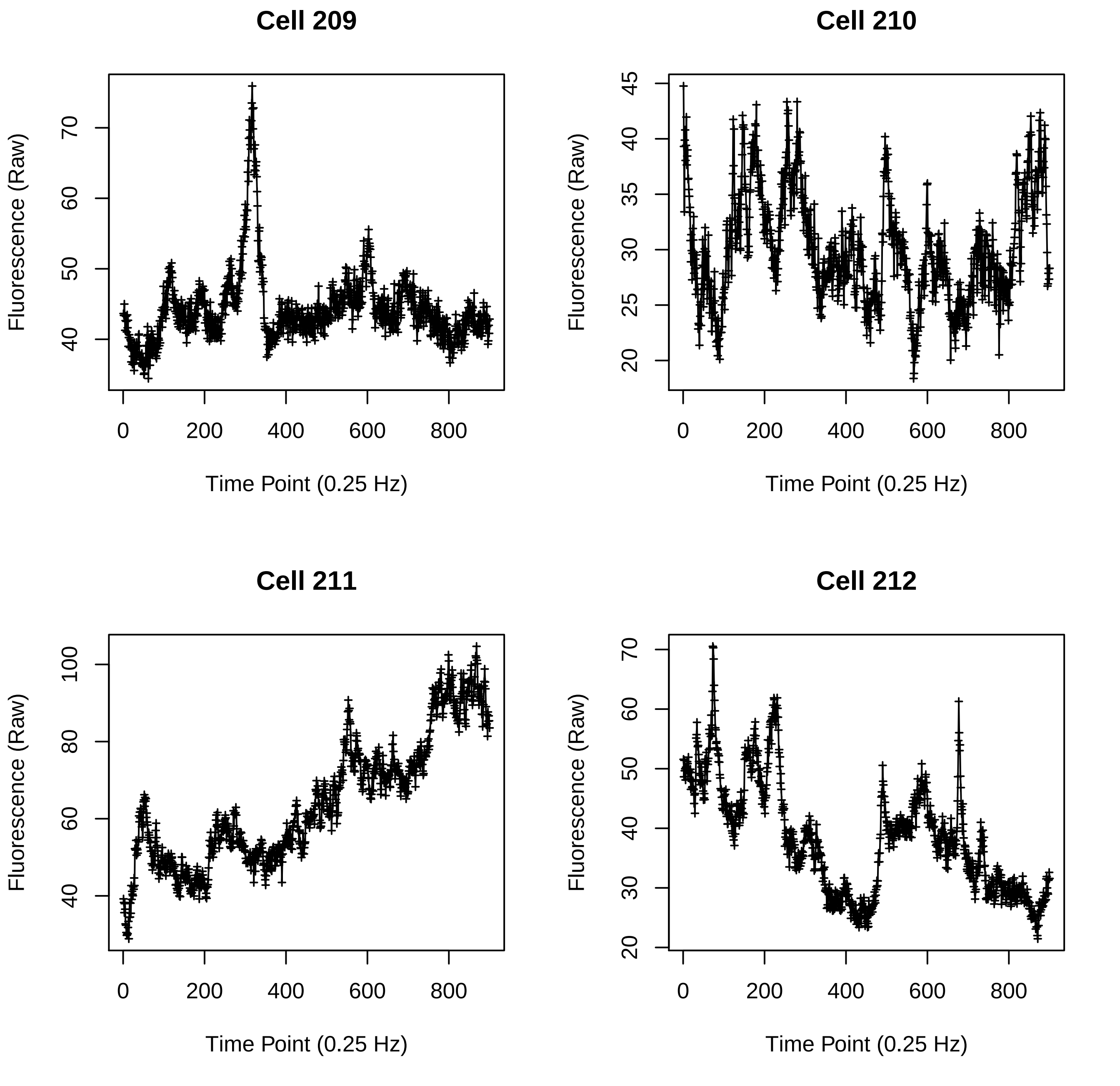

Cell 209
Fluorescence (Raw)
Time Point (0.25 Hz)
Cell 210
Fluorescence (Raw)
Time Point (0.25 Hz)
Cell 211
Fluorescence (Raw)
Time Point (0.25 Hz)
Cell 212
Fluorescence (Raw)
Time Point (0.25 Hz)

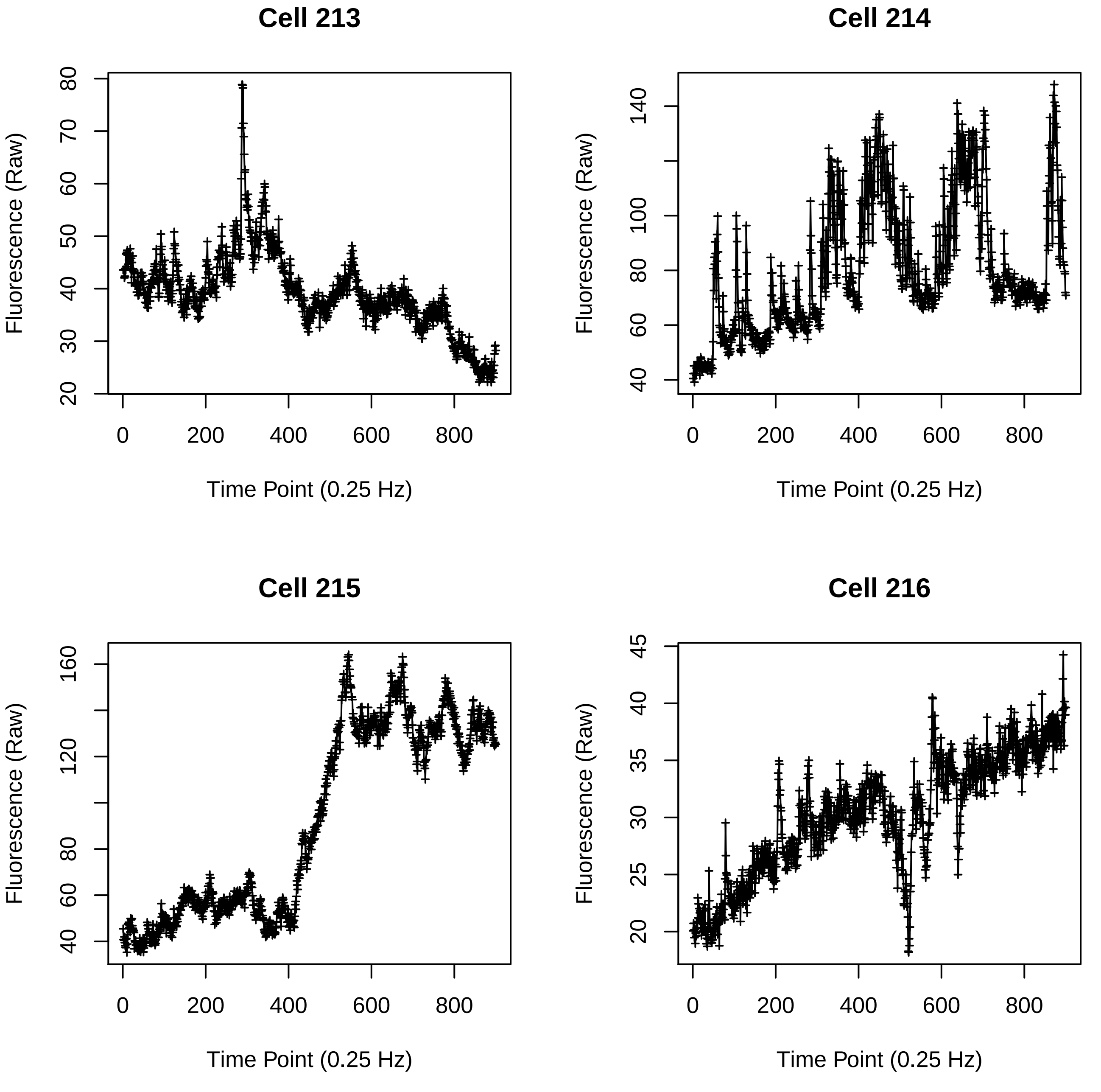

Cell 213
Fluorescence (Raw)
20
30
40
50
60
70
80
0
200
400
600
800
Time Point (0.25 Hz)
Cell 214
Fluorescence (Raw)
40
60
80
100
140
0
200
400
600
800
Time Point (0.25 Hz)
Cell 215
Fluorescence (Raw)
40
60
80
120
160
0
200
400
600
800
Time Point (0.25 Hz)
Cell 216
Fluorescence (Raw)
20
25
30
35
40
45
0
200
400
600
800
Time Point (0.25 Hz)

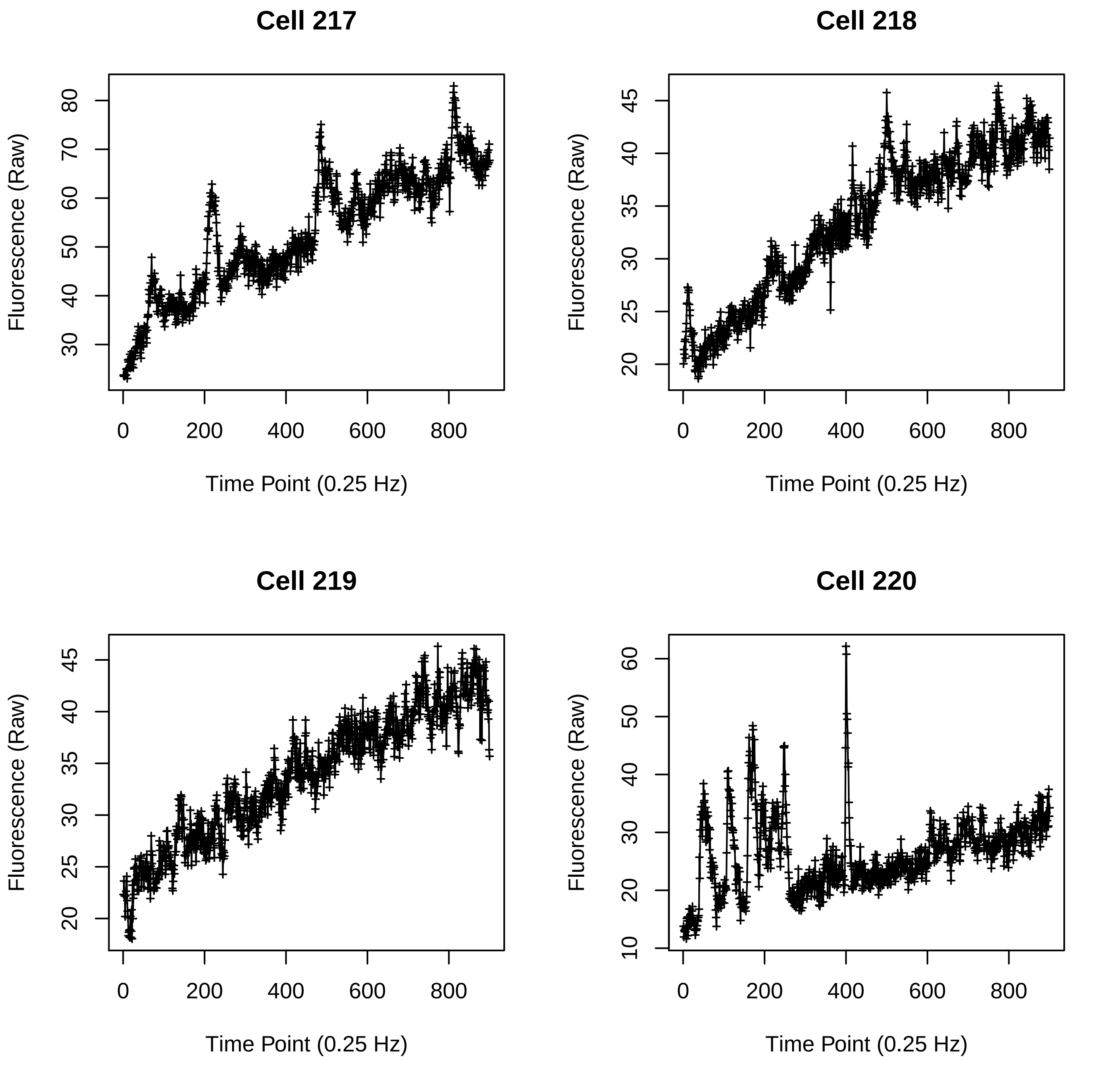
Cell 217
Fluorescence (Raw)
30
40
50
60
70
80
0
200
400
600
800
Time Point (0.25 Hz)
Cell 218
Fluorescence (Raw)
20
25
30
35
40
45
0
200
400
600
800
Time Point (0.25 Hz)
Cell 219
Fluorescence (Raw)
20
25
30
35
40
45
0
200
400
600
800
Time Point (0.25 Hz)
Cell 220
Fluorescence (Raw)
10
20
30
40
50
60
0
200
400
600
800
Time Point (0.25 Hz)

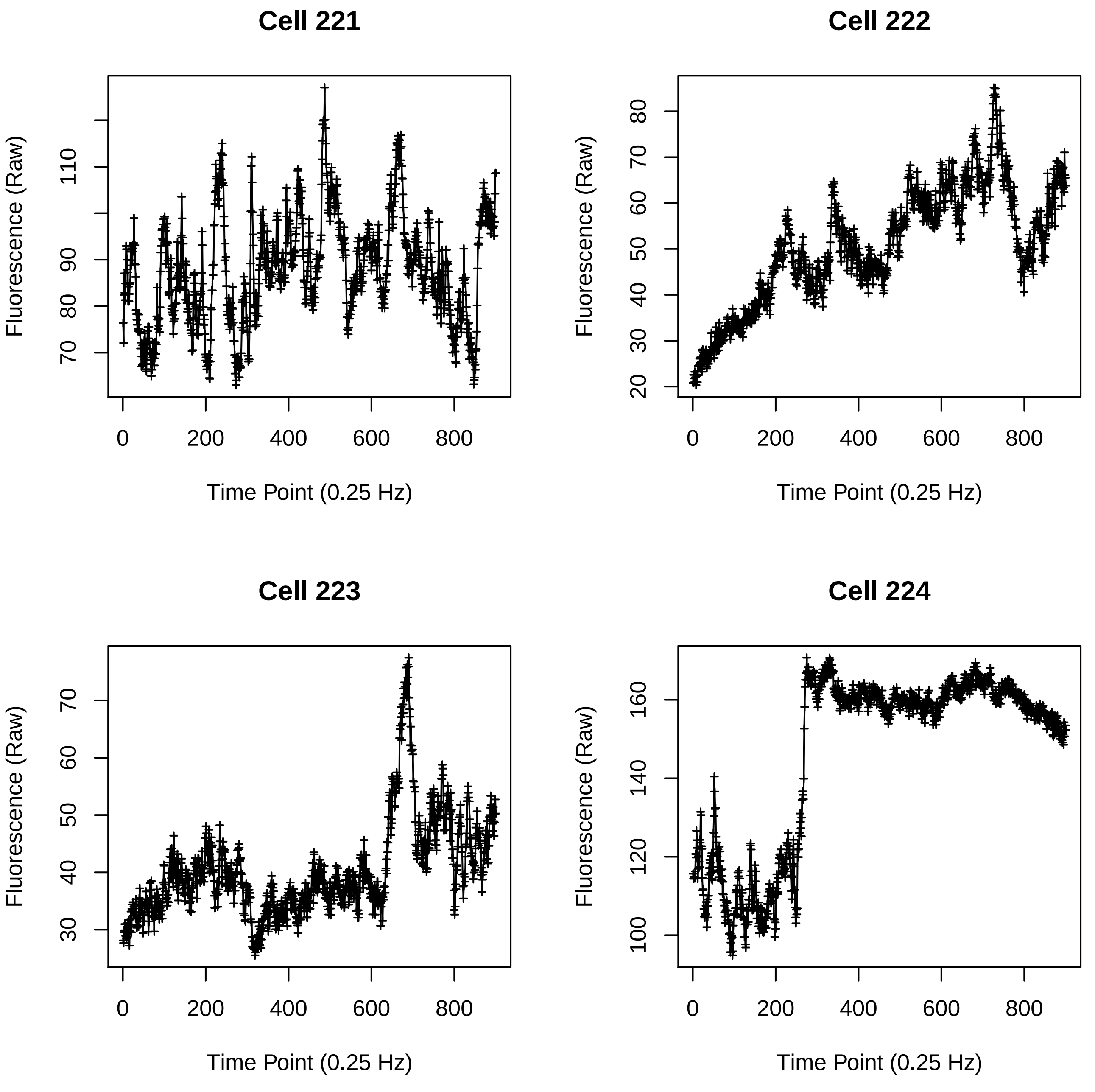
Cell 221
Fluorescence (Raw)
70
80
90
110
0
200
400
600
800
Time Point (0.25 Hz)
Cell 222
Fluorescence (Raw)
20
30
40
50
60
70
80
0
200
400
600
800
Time Point (0.25 Hz)
Cell 223
Fluorescence (Raw)
30
40
50
60
70
0
200
400
600
800
Time Point (0.25 Hz)
Cell 224
Fluorescence (Raw)
100
120
140
160
0
200
400
600
800
Time Point (0.25 Hz)

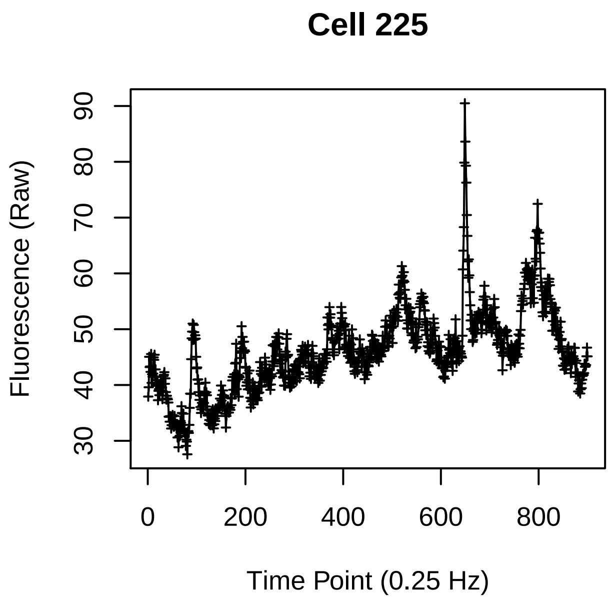

Cell 225
Fluorescence (Raw)
30
40
50
60
70
80
90
0
200
400
600
800
Time Point (0.25 Hz)

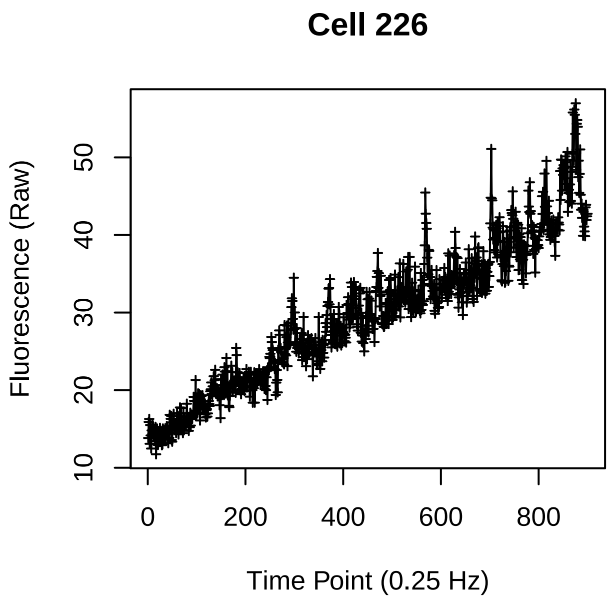

Cell 226
Fluorescence (Raw)
10
20
30
40
50
0
200
400
600
800
Time Point (0.25 Hz)

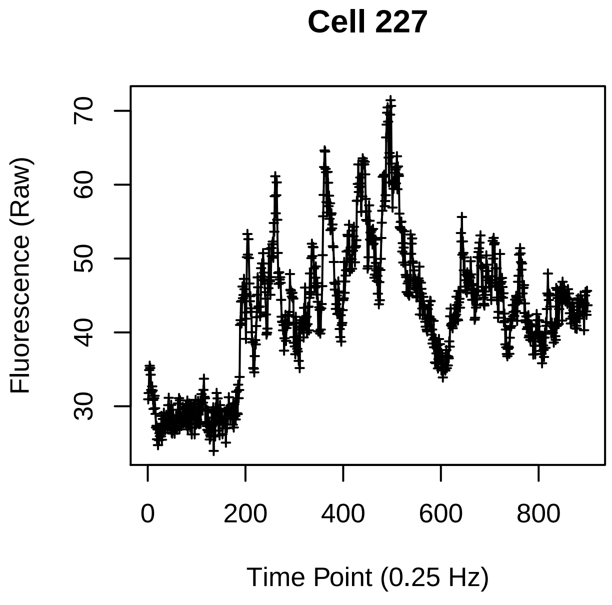

Cell 227
Fluorescence (Raw)
30
40
50
60
70
0
200
400
600
800
Time Point (0.25 Hz)

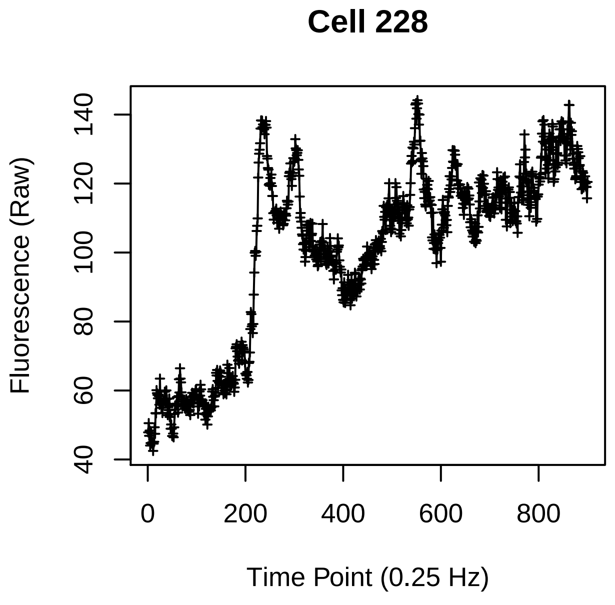

Cell 228
Fluorescence (Raw)
40
60
80
100
120
140
0
200
400
600
800
Time Point (0.25 Hz)

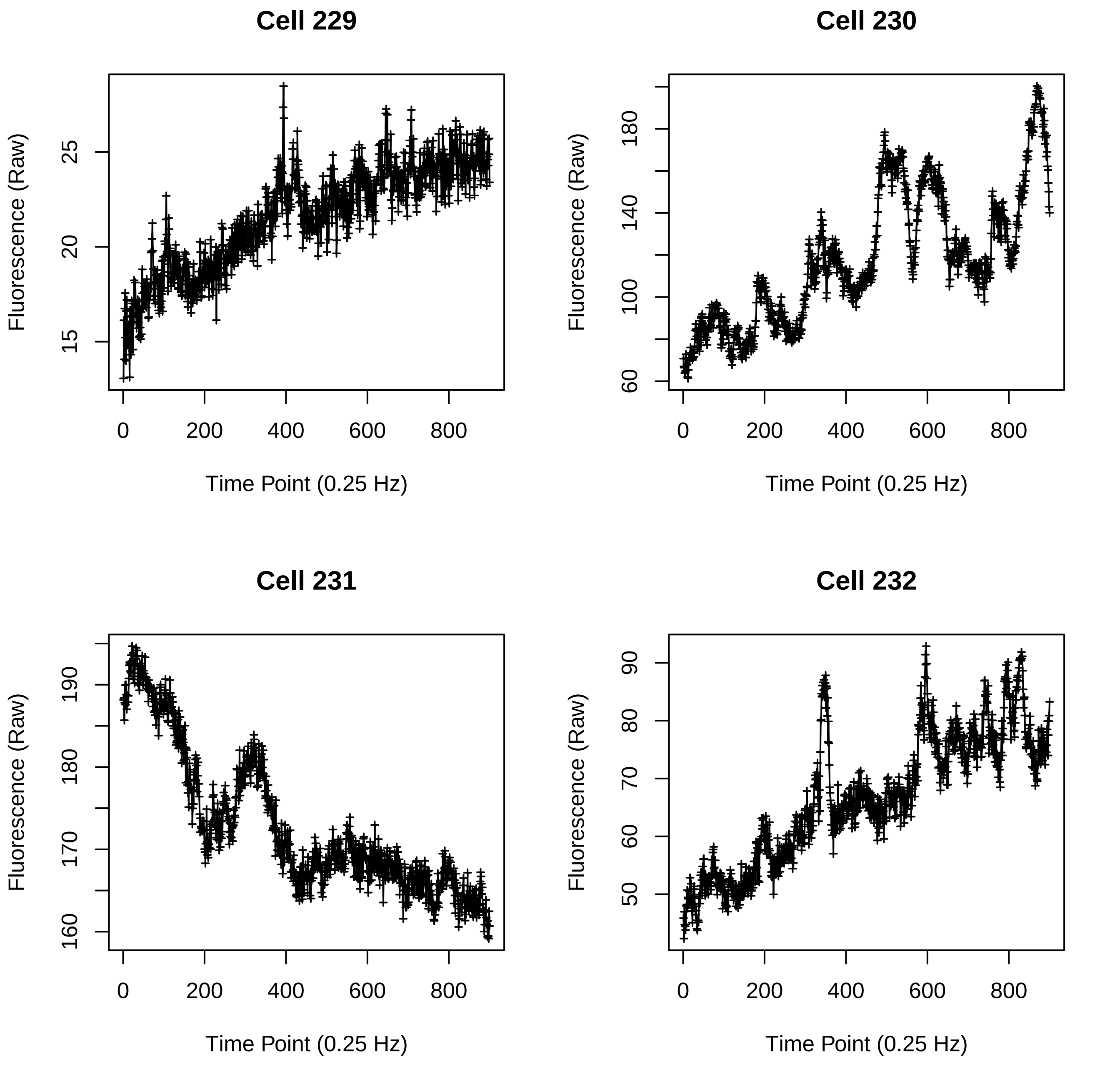
Cell 229
Cell 230
Cell 231
Cell 232
Fluorescence (Raw)
Time Point (0.25 Hz)
0
200
400
600
800
15
20
25
60
100
140
180
160
170
180
190
50
60
70
80
90

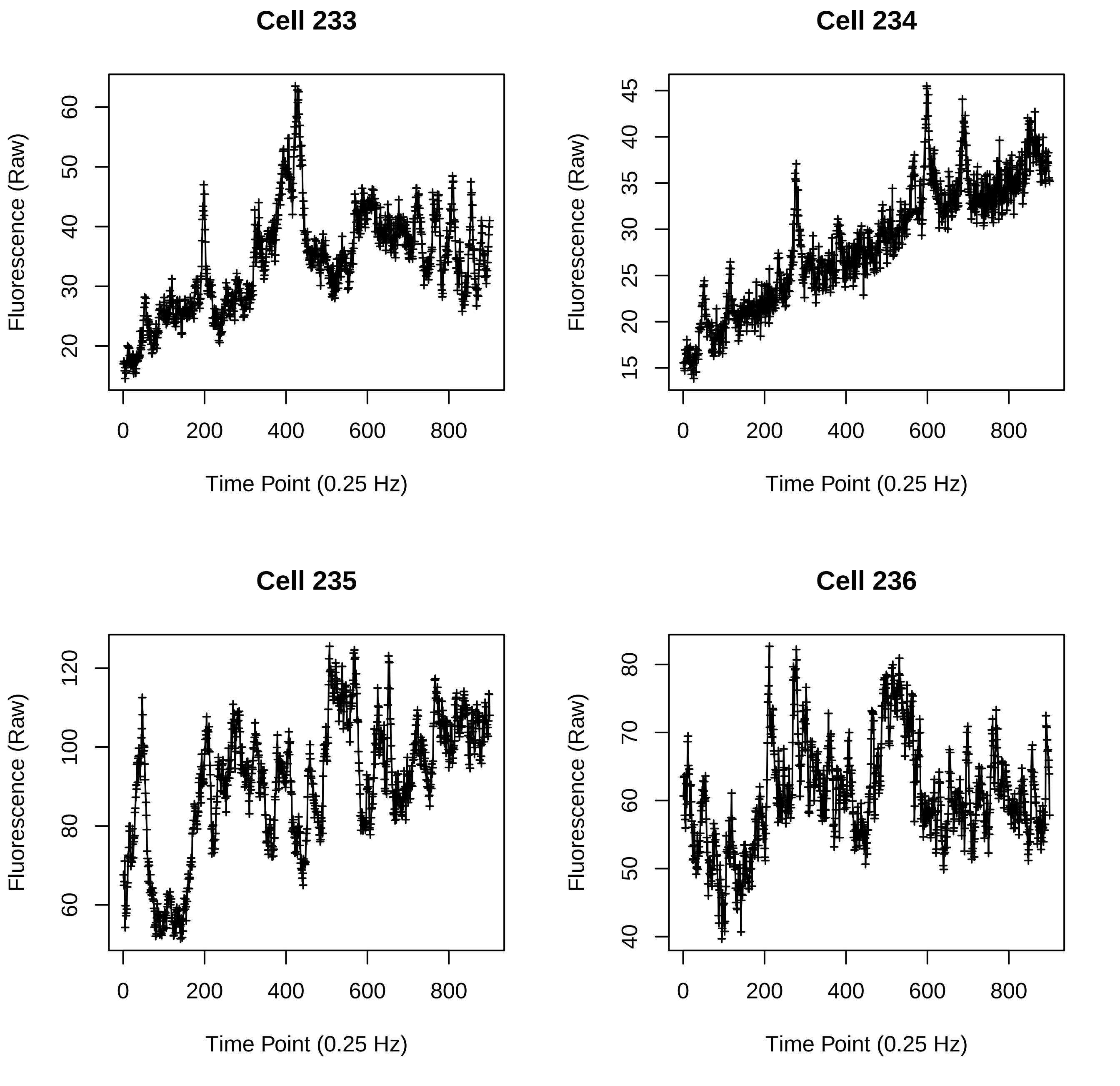

Cell 233
Cell 234
Cell 235
Cell 236
Fluorescence (Raw)
Time Point (0.25 Hz)
0
200
400
600
800

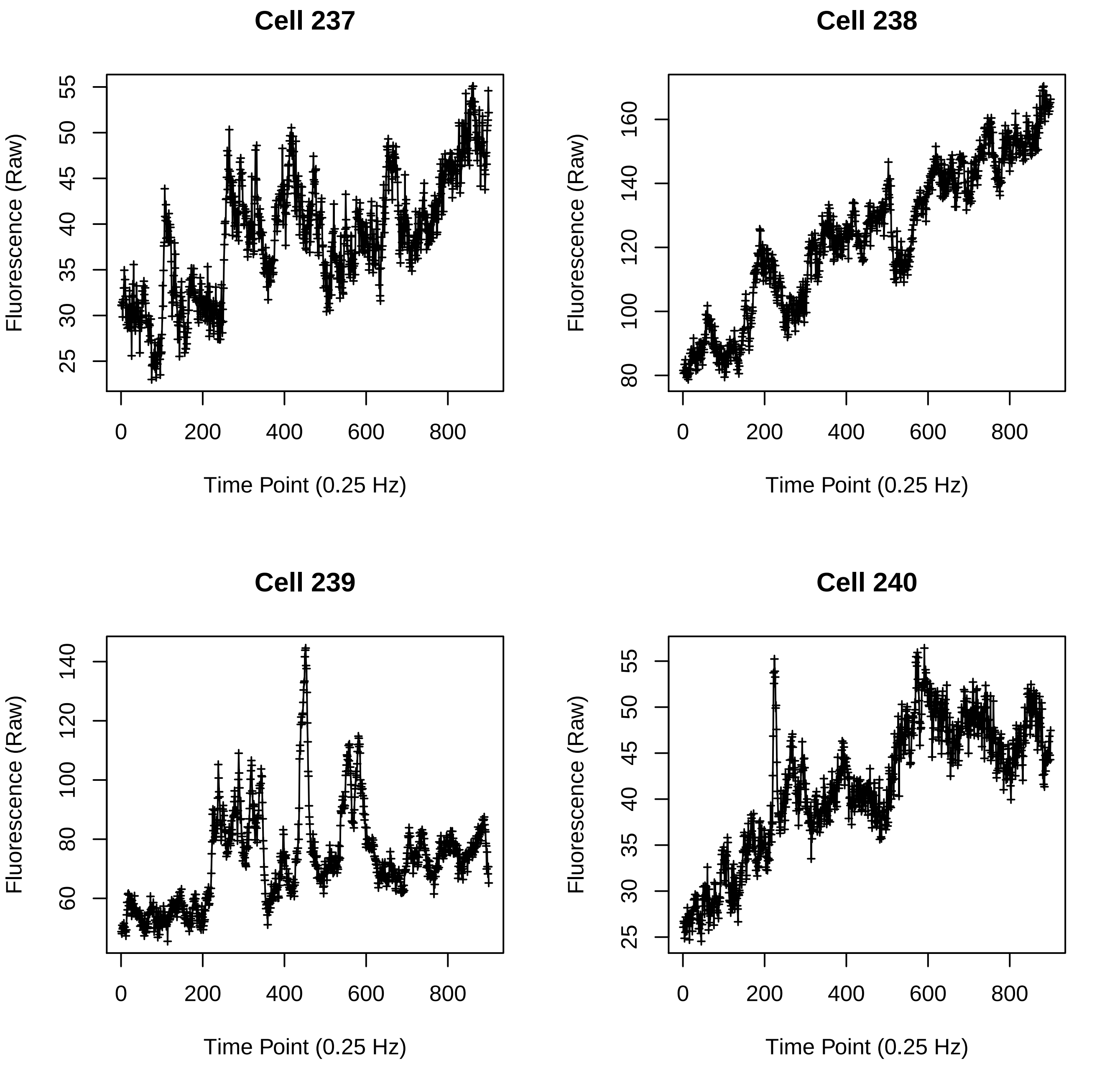
Cell 237
Cell 238
Cell 239
Cell 240
Fluorescence (Raw)
Time Point (0.25 Hz)
0
200
400
600
800
25
30
35
40
45
50
55
80
100
120
140
160
60

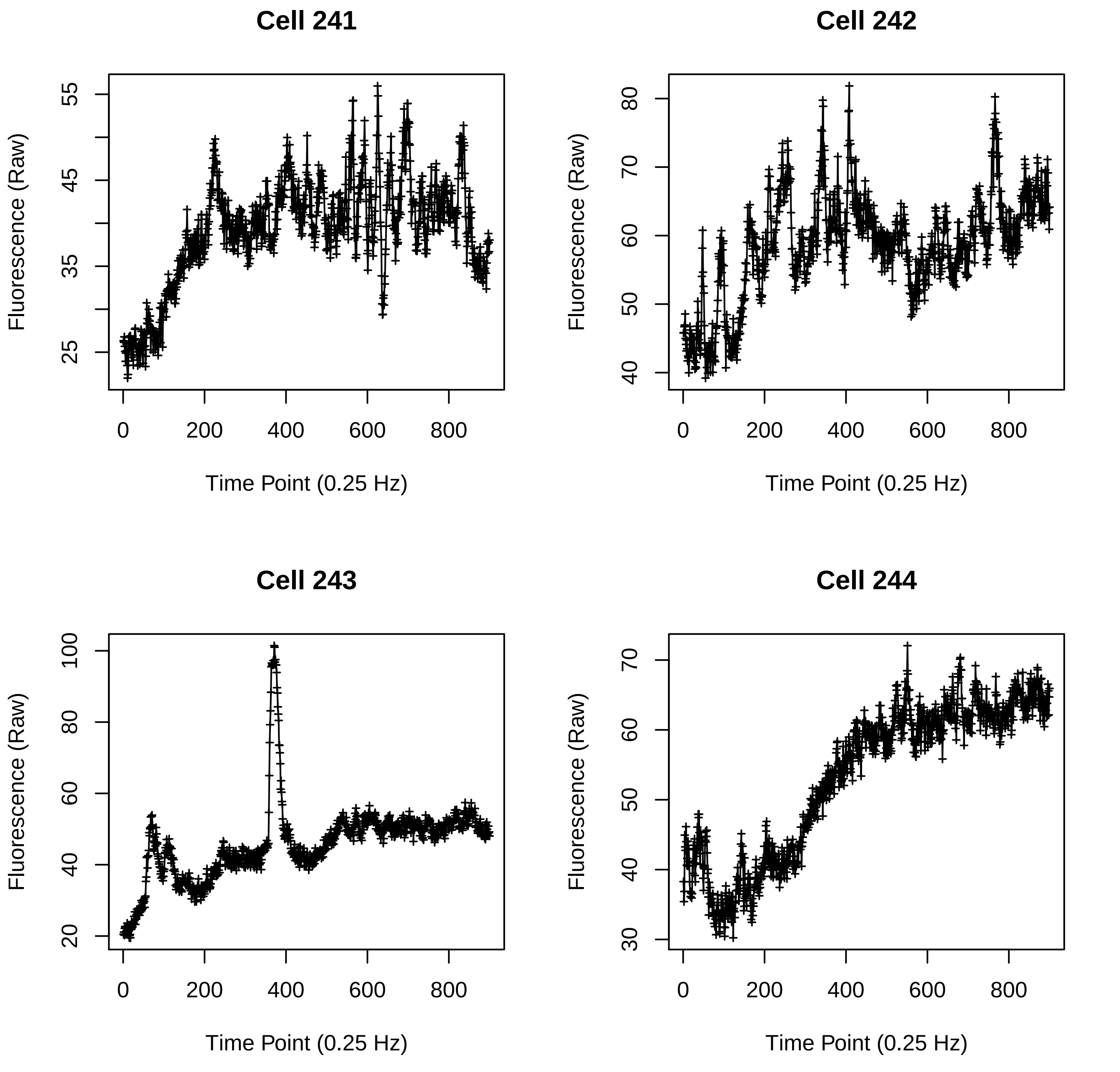
Cell 241
Fluorescence (Raw)
25
35
45
55
0
200
400
600
800
Time Point (0.25 Hz)
Cell 242
Fluorescence (Raw)
40
50
60
70
80
0
200
400
600
800
Time Point (0.25 Hz)
Cell 243
Fluorescence (Raw)
20
40
60
80
100
0
200
400
600
800
Time Point (0.25 Hz)
Cell 244
Fluorescence (Raw)
30
40
50
60
70
0
200
400
600
800
Time Point (0.25 Hz)

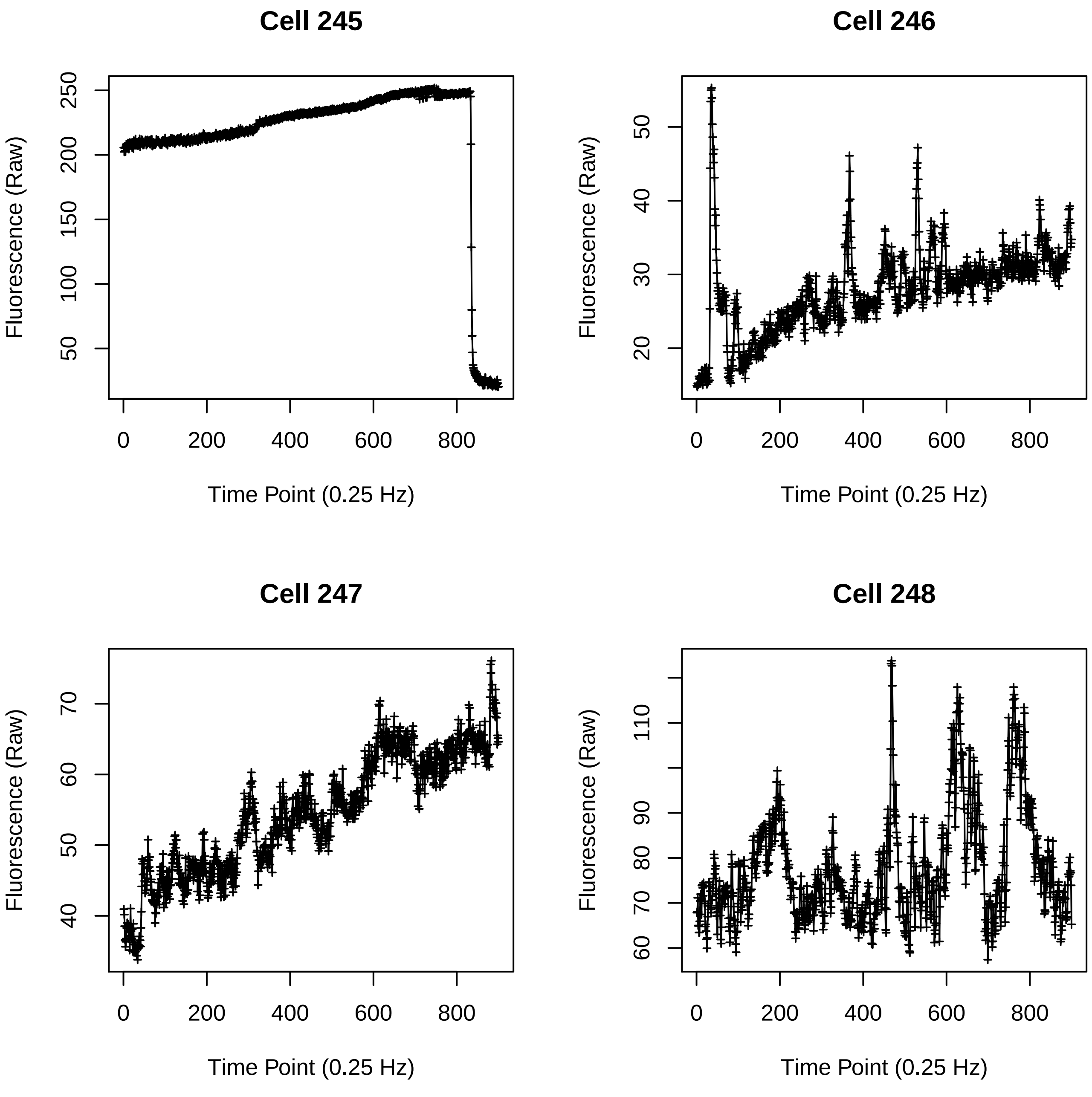
Cell 245
Cell 246
Cell 247
Cell 248
Fluorescence (Raw)
Time Point (0.25 Hz)
0
200
400
600
800
50
100
150
200
250
20
30
40
50
40
50
60
70
60
70
80
90
110

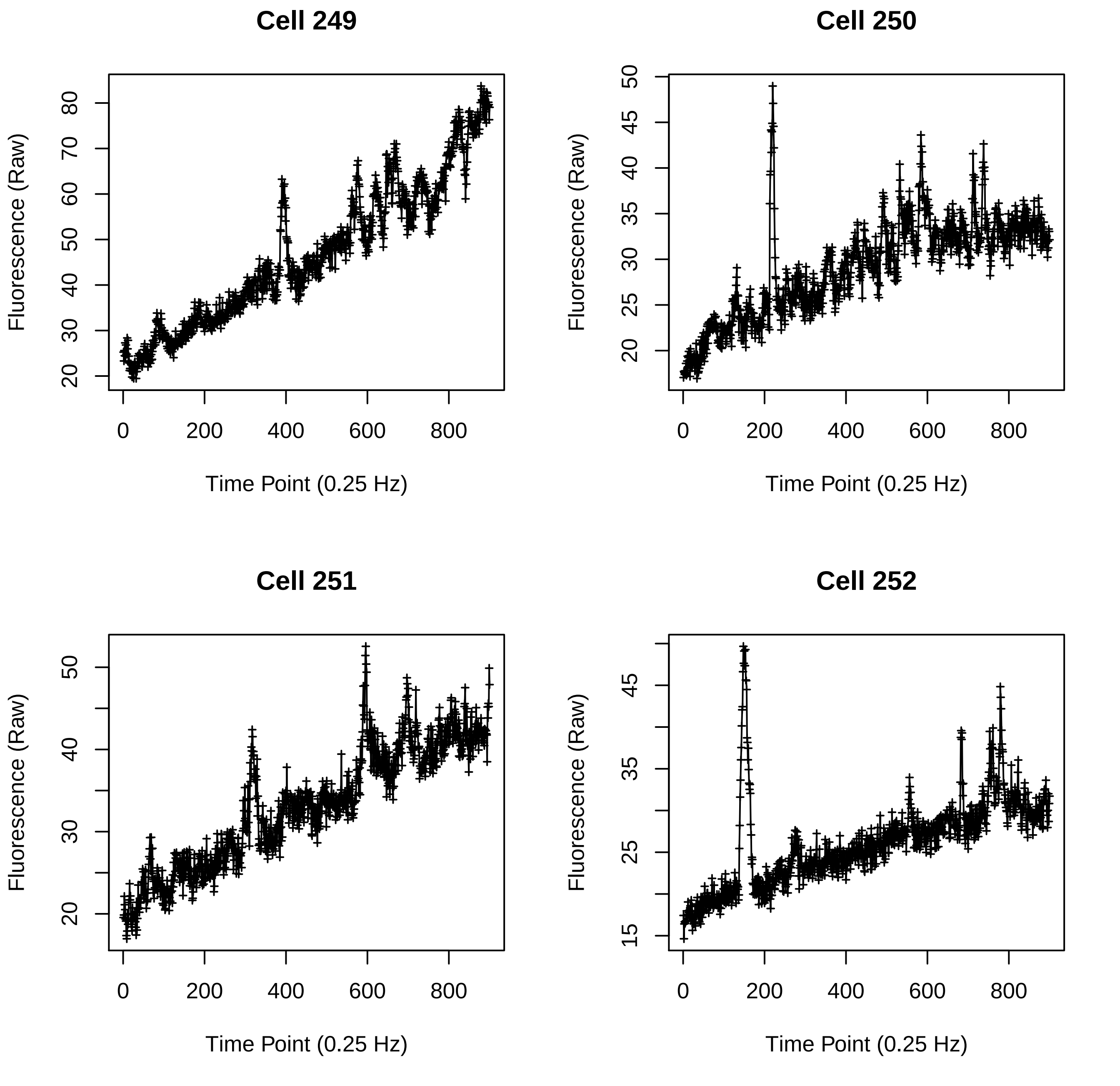
Cell 249
Fluorescence (Raw)
20
30
40
50
60
70
80
0
200
400
600
800
Time Point (0.25 Hz)
Cell 250
Fluorescence (Raw)
20
25
30
35
40
45
50
0
200
400
600
800
Time Point (0.25 Hz)
Cell 251
Fluorescence (Raw)
20
30
40
50
0
200
400
600
800
Time Point (0.25 Hz)
Cell 252
Fluorescence (Raw)
15
25
35
45
0
200
400
600
800
Time Point (0.25 Hz)

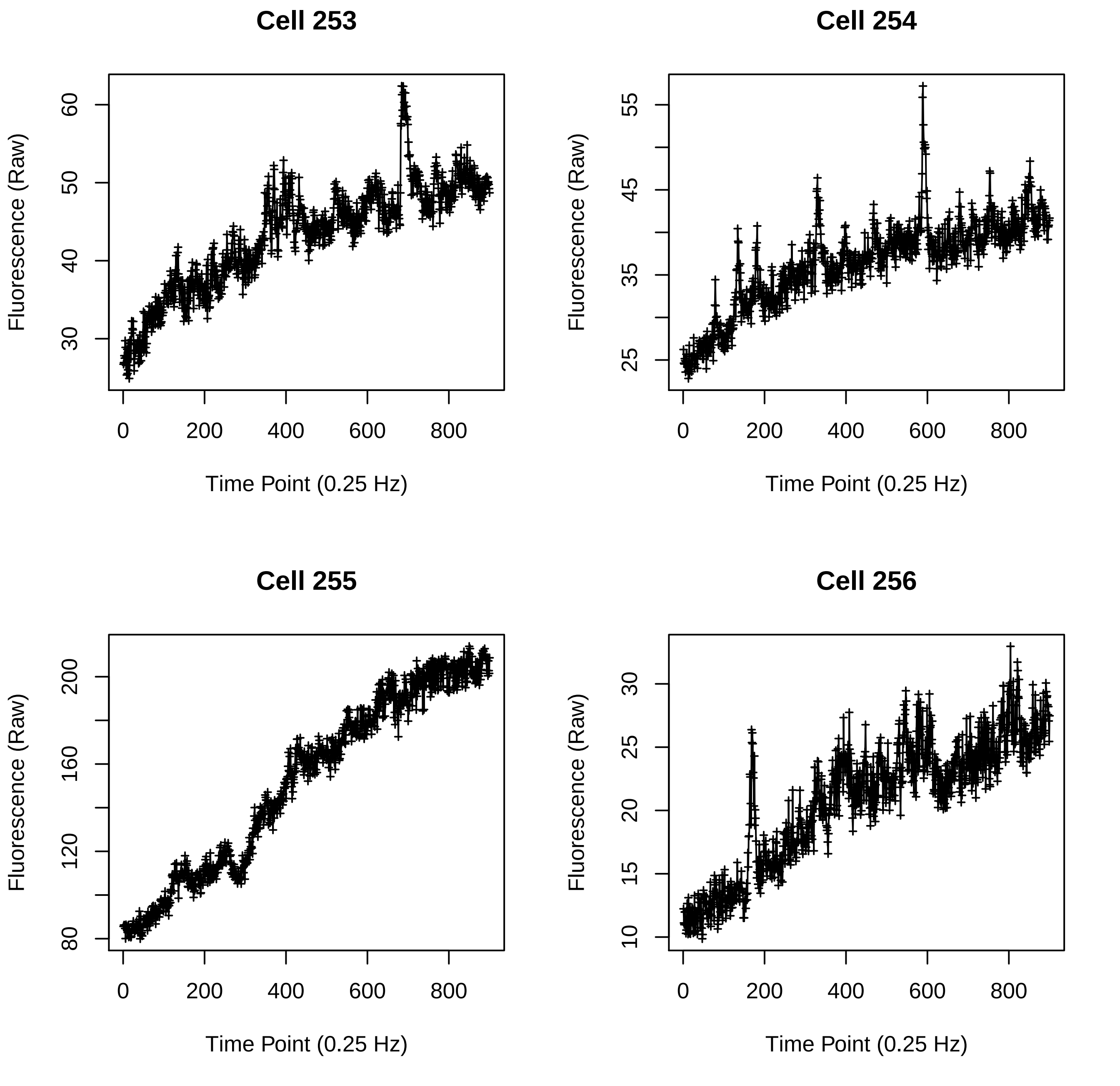
Cell 253
Cell 254
Cell 255
Cell 256
Fluorescence (Raw)
Time Point (0.25 Hz)
0
200
400
600
800
30
40
50
60
25
35
45
55
80
120
160
200
10
15
20
25
30

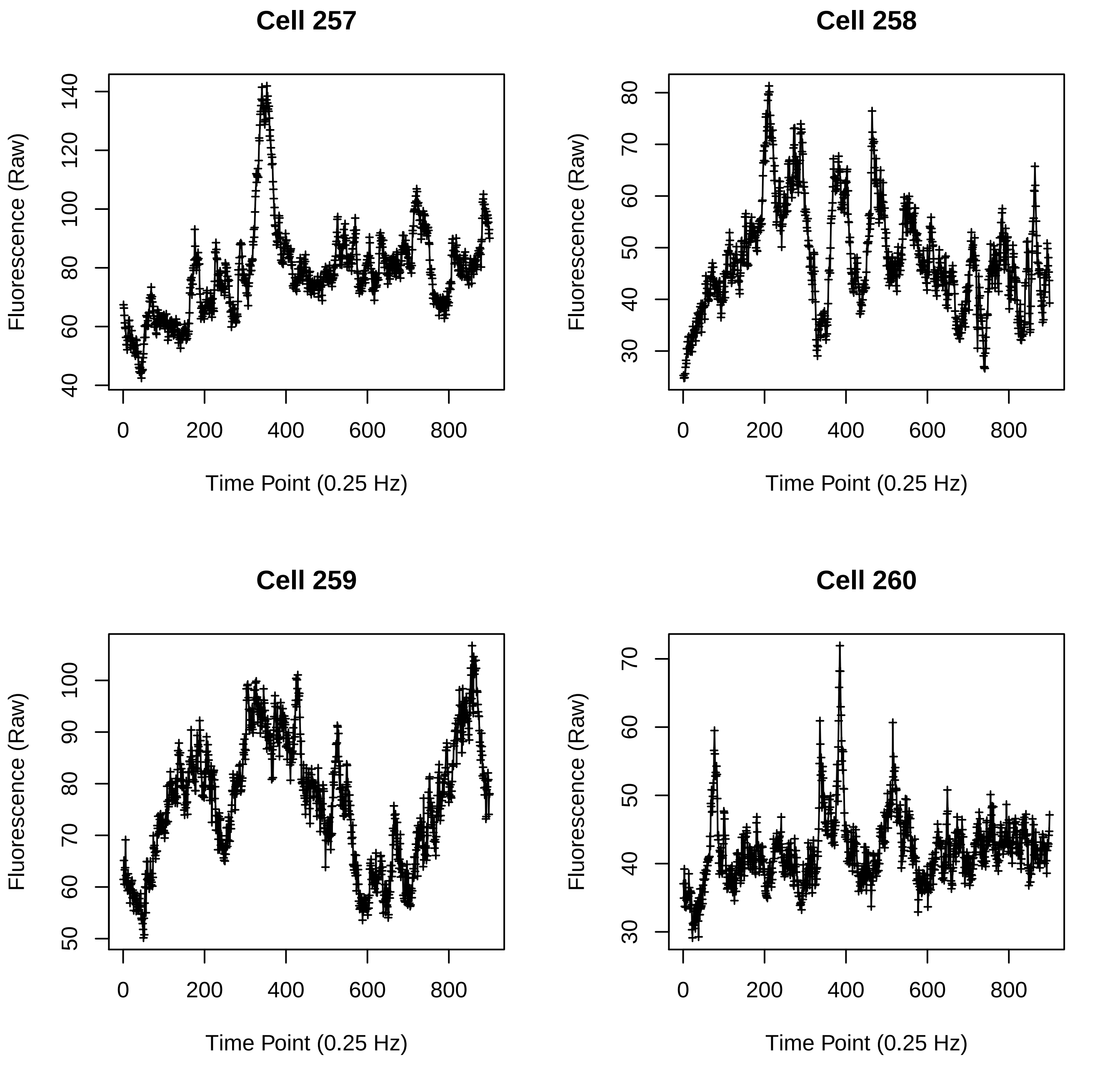
Cell 257
Cell 258
Cell 259
Cell 260
Fluorescence (Raw)
Time Point (0.25 Hz)
0
200
400
600
800
40
60
80
100
120
140
30
50
70
90

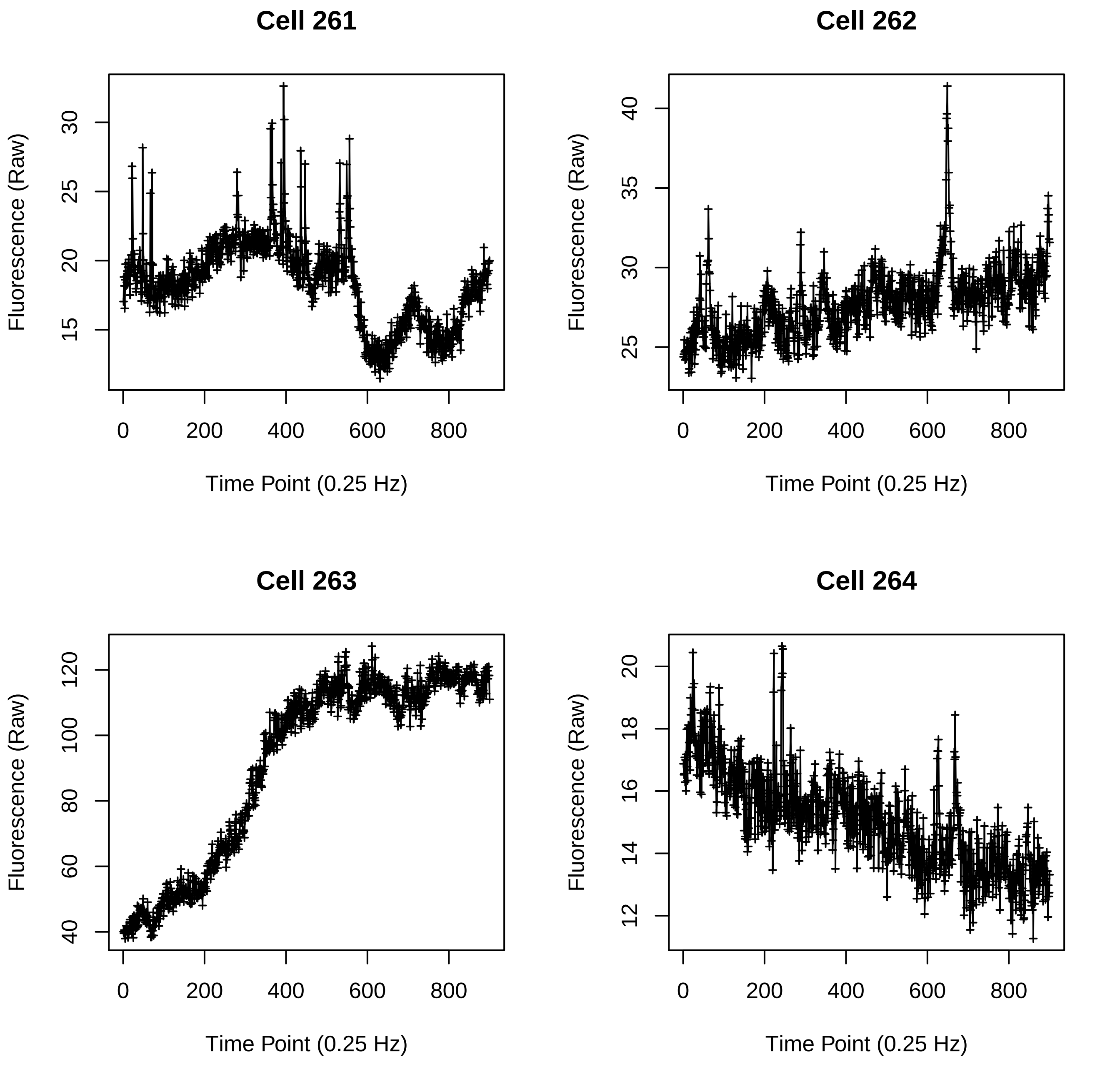

Cell 261
Cell 262
Cell 263
Cell 264
Fluorescence (Raw)
Time Point (0.25 Hz)
0
200
400
600
800
15
20
25
30
35
40
12
14
16
18
60
80
100
120

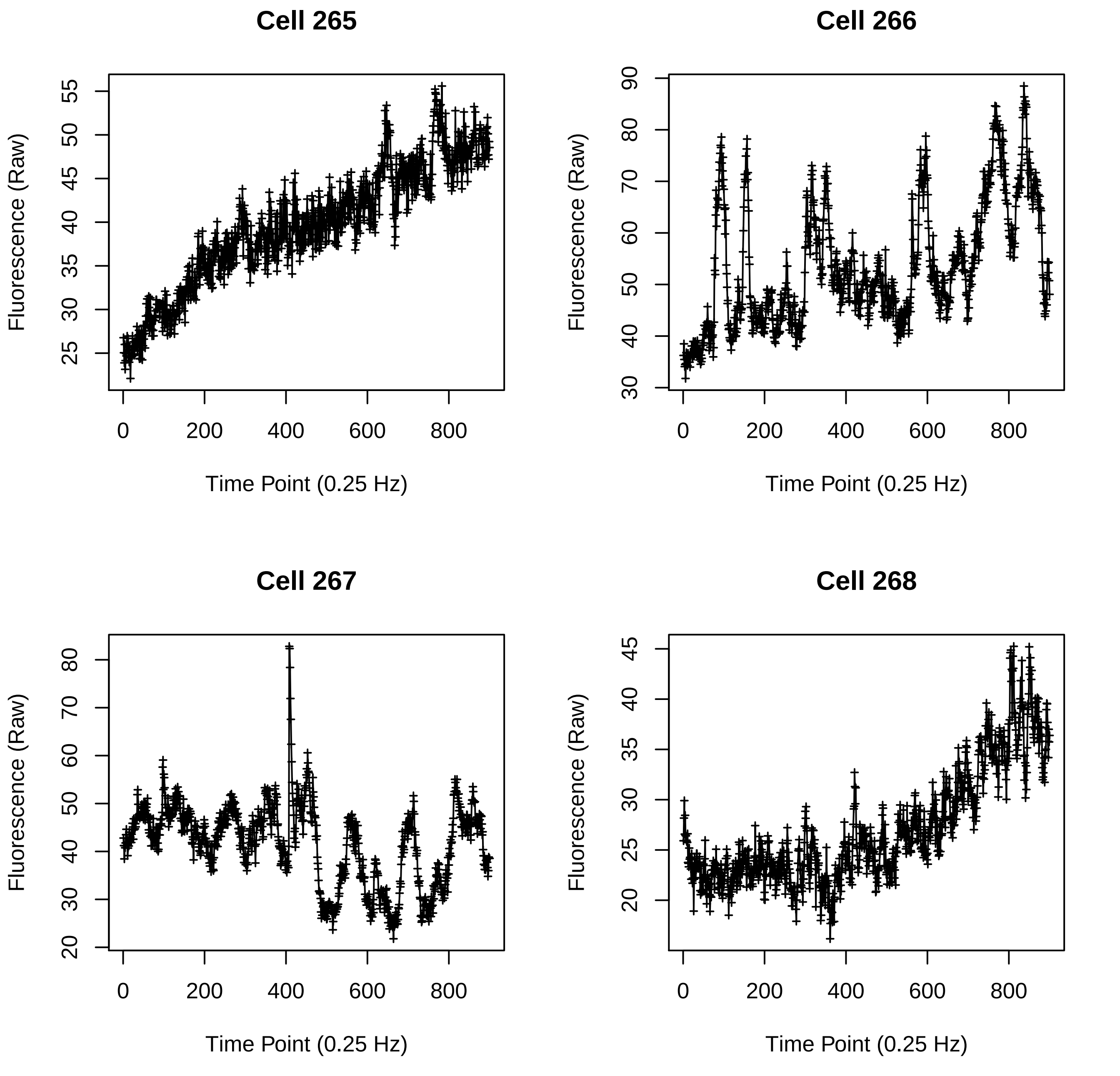

Cell 265
Cell 266
Cell 267
Cell 268
Fluorescence (Raw)
Time Point (0.25 Hz)
0
200
400
600
800
25
30
35
40
45
50
55
60
70
80
90
20

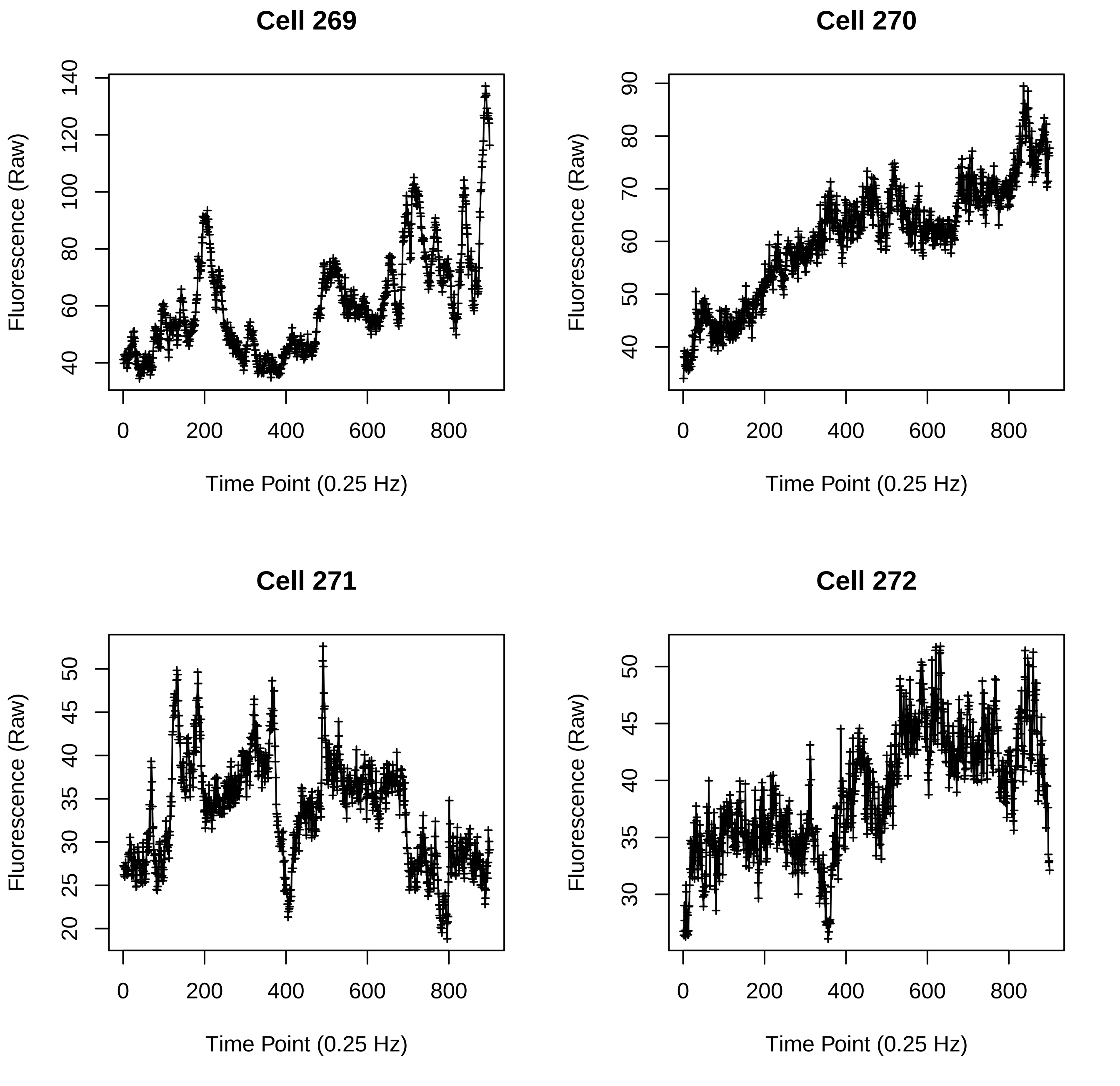

Cell 269
Fluorescence (Raw)
Time Point (0.25 Hz)
Cell 270
Fluorescence (Raw)
Time Point (0.25 Hz)
Cell 271
Fluorescence (Raw)
Time Point (0.25 Hz)
Cell 272
Fluorescence (Raw)
Time Point (0.25 Hz)

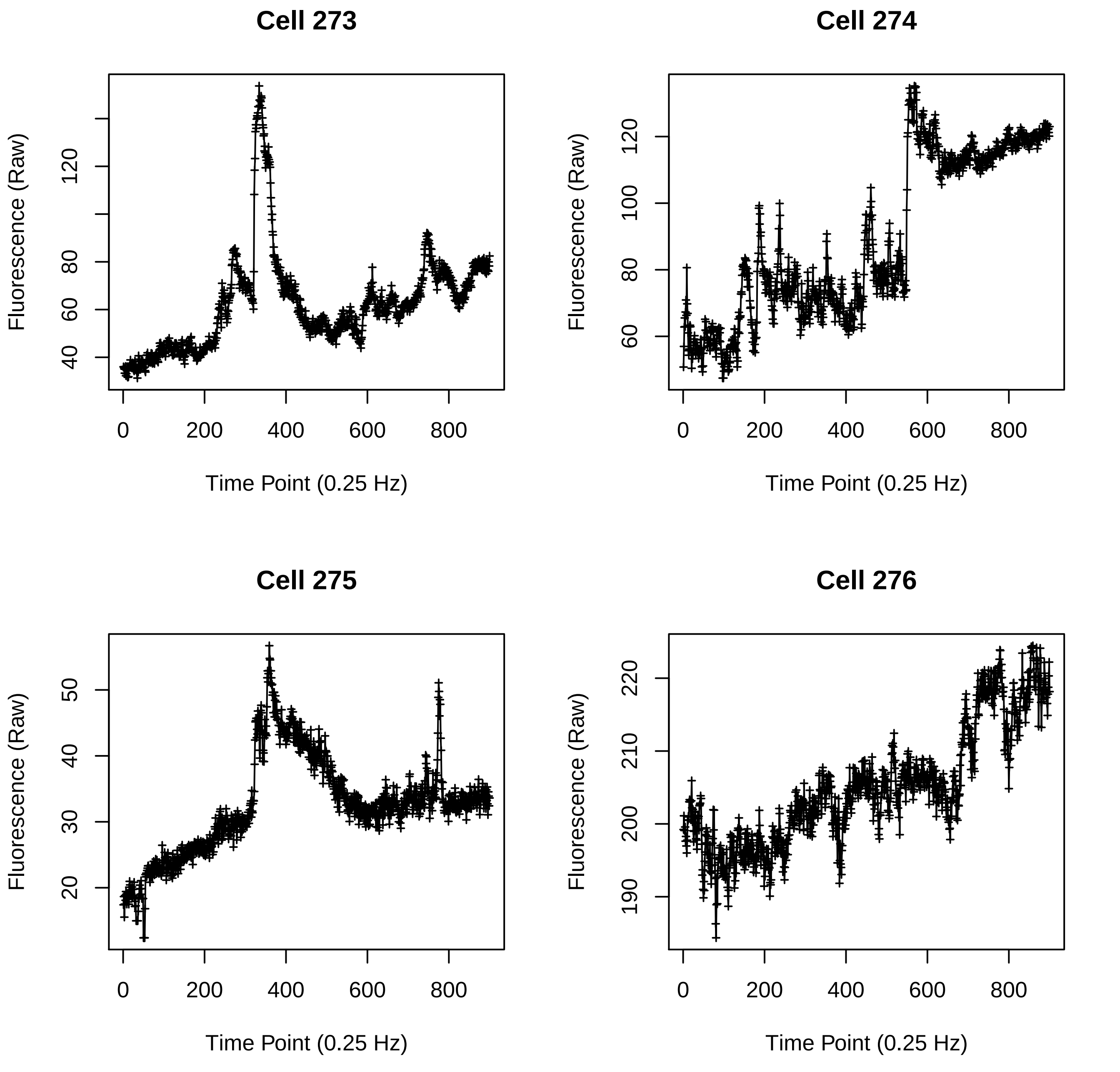

Cell 273
Cell 274
Cell 275
Cell 276
Fluorescence (Raw)
Time Point (0.25 Hz)
0
200
400
600
800
40
60
80
120
100
20
30
50
190
210
220

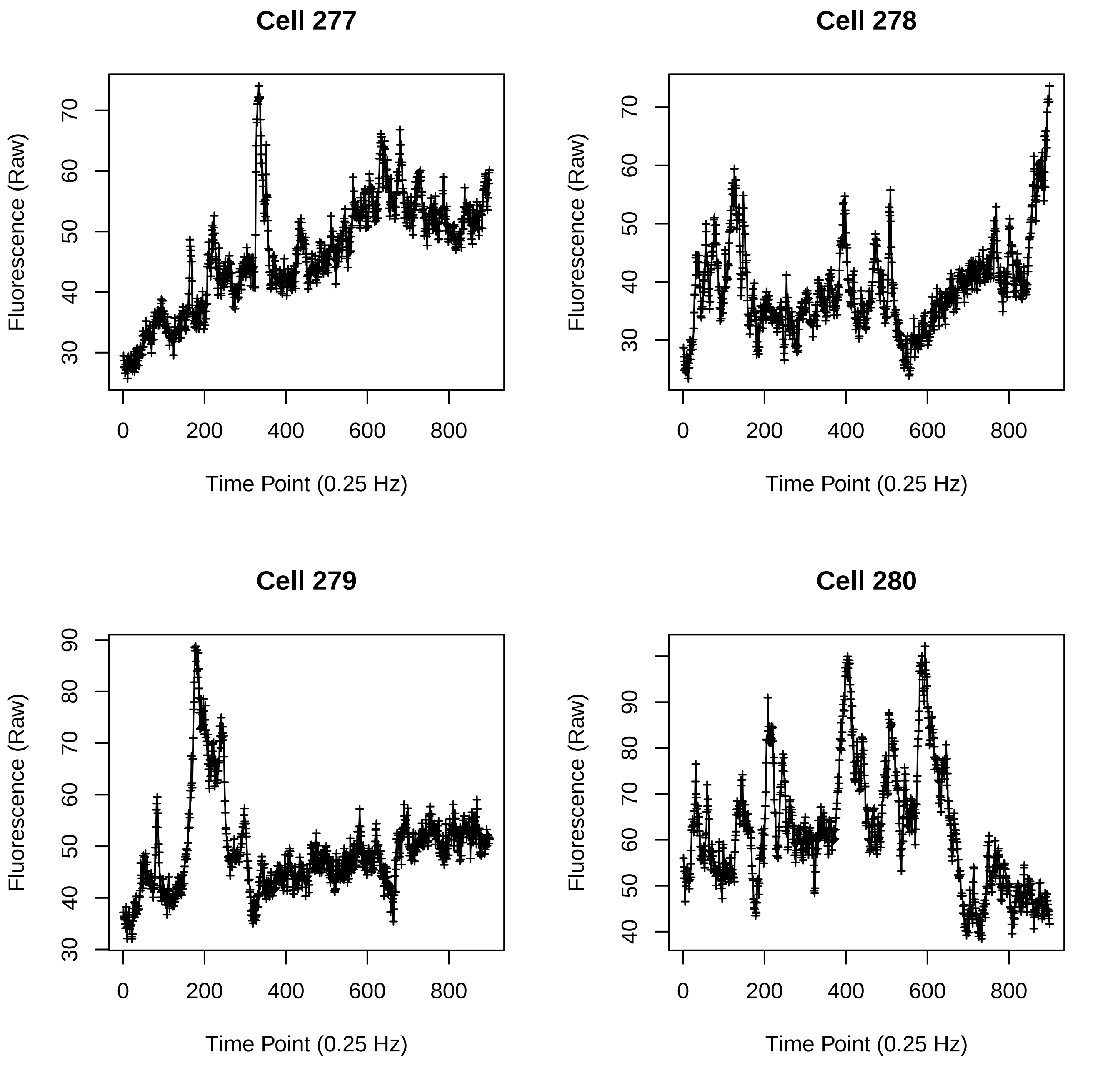

Cell 277
Cell 278
Cell 279
Cell 280
Fluorescence (Raw)
Time Point (0.25 Hz)
0
200
400
600
800
30
40
50
60
70
80
90

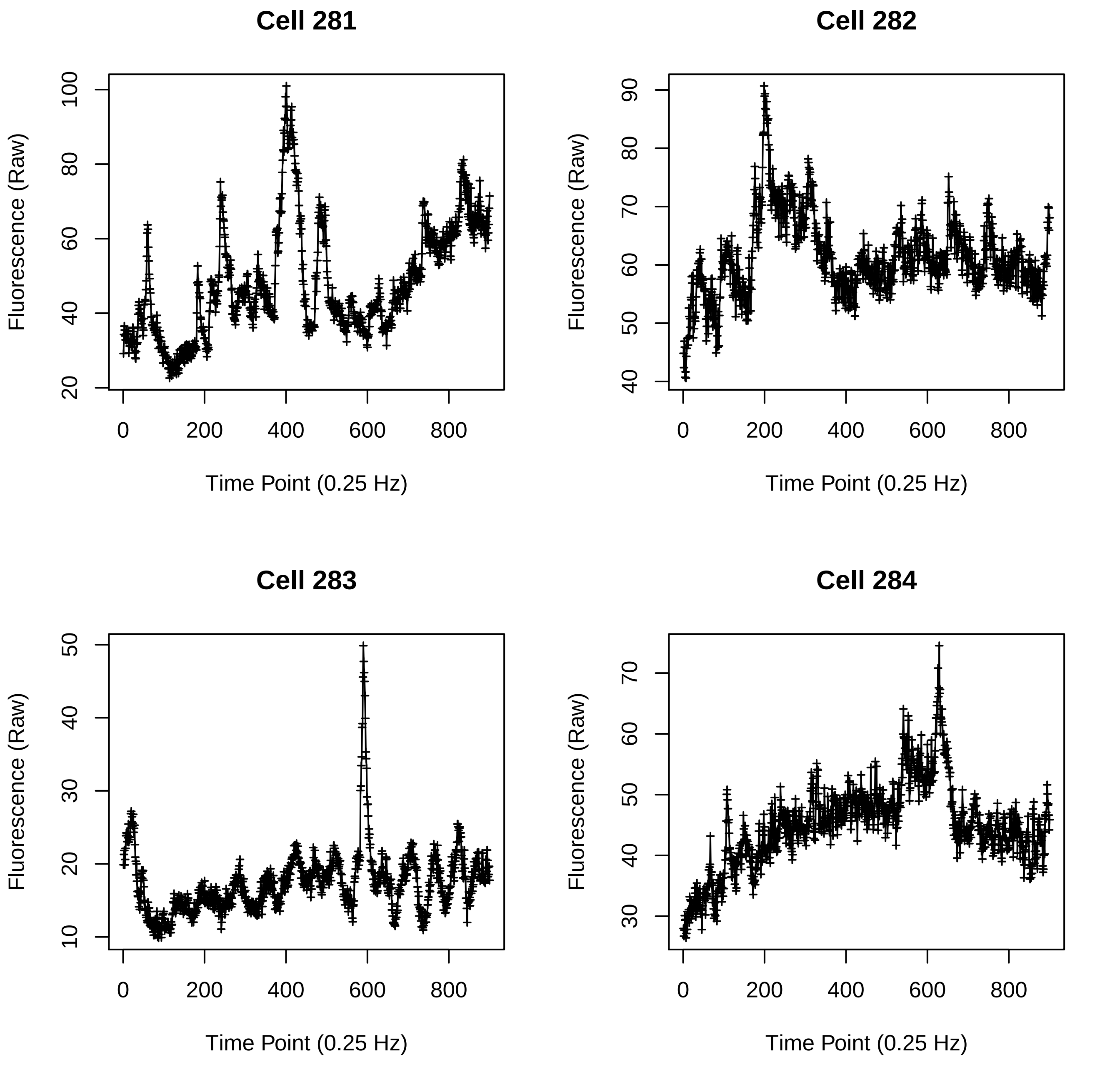
Cell 281
Fluorescence (Raw)
Time Point (0.25 Hz)
20
40
60
80
100
0
200
400
600
800
Cell 282
Fluorescence (Raw)
Time Point (0.25 Hz)
40
50
60
70
80
90
0
200
400
600
800
Cell 283
Fluorescence (Raw)
Time Point (0.25 Hz)
10
20
30
40
50
0
200
400
600
800
Cell 284
Fluorescence (Raw)
Time Point (0.25 Hz)
30
40
50
60
70
0
200
400
600
800

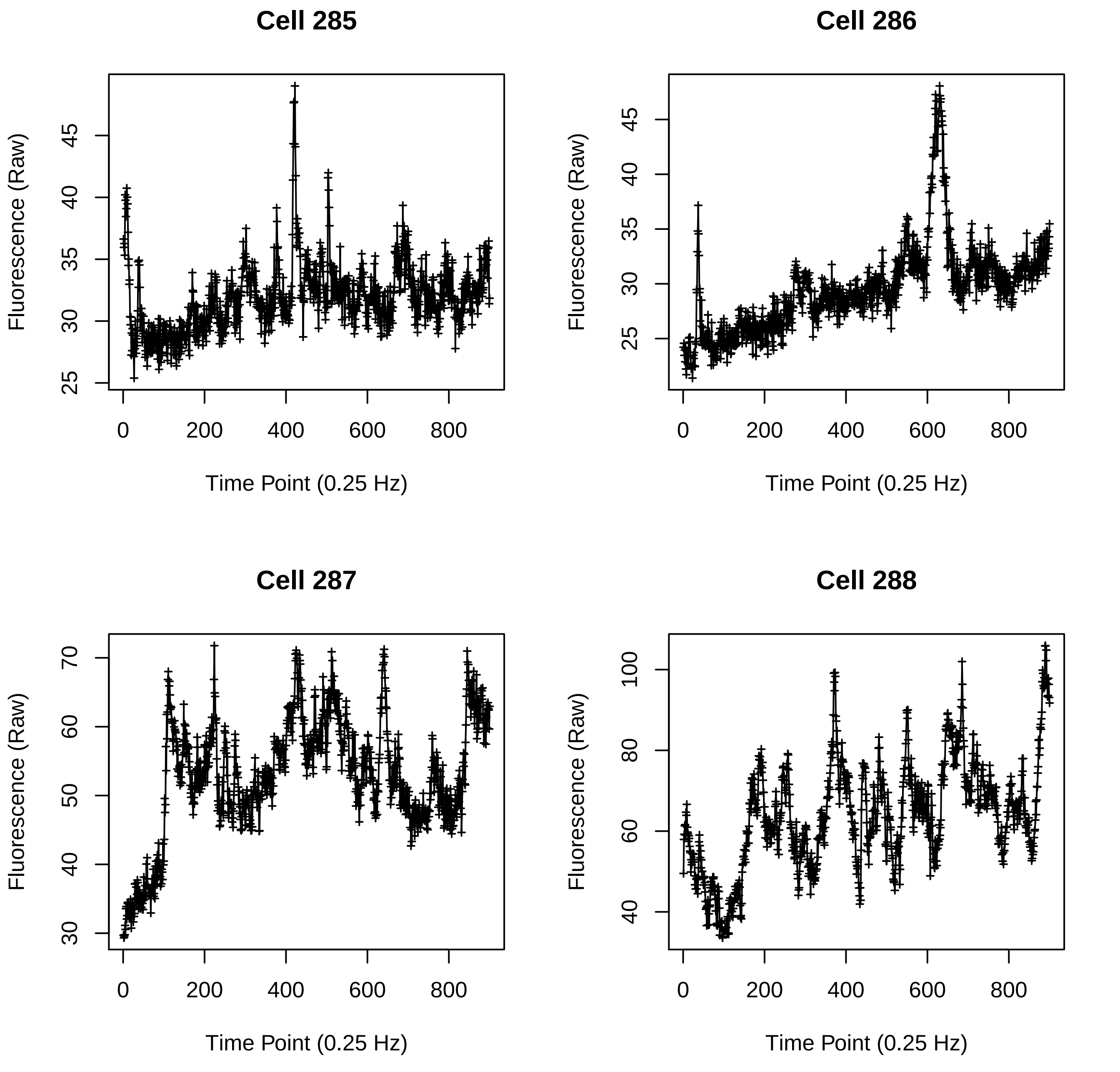
Cell 285
Fluorescence (Raw)
25
30
35
40
45
0
200
400
600
800
Time Point (0.25 Hz)
Cell 286
Fluorescence (Raw)
25
30
35
40
45
0
200
400
600
800
Time Point (0.25 Hz)
Cell 287
Fluorescence (Raw)
30
40
50
60
70
0
200
400
600
800
Time Point (0.25 Hz)
Cell 288
Fluorescence (Raw)
40
60
80
100
0
200
400
600
800
Time Point (0.25 Hz)

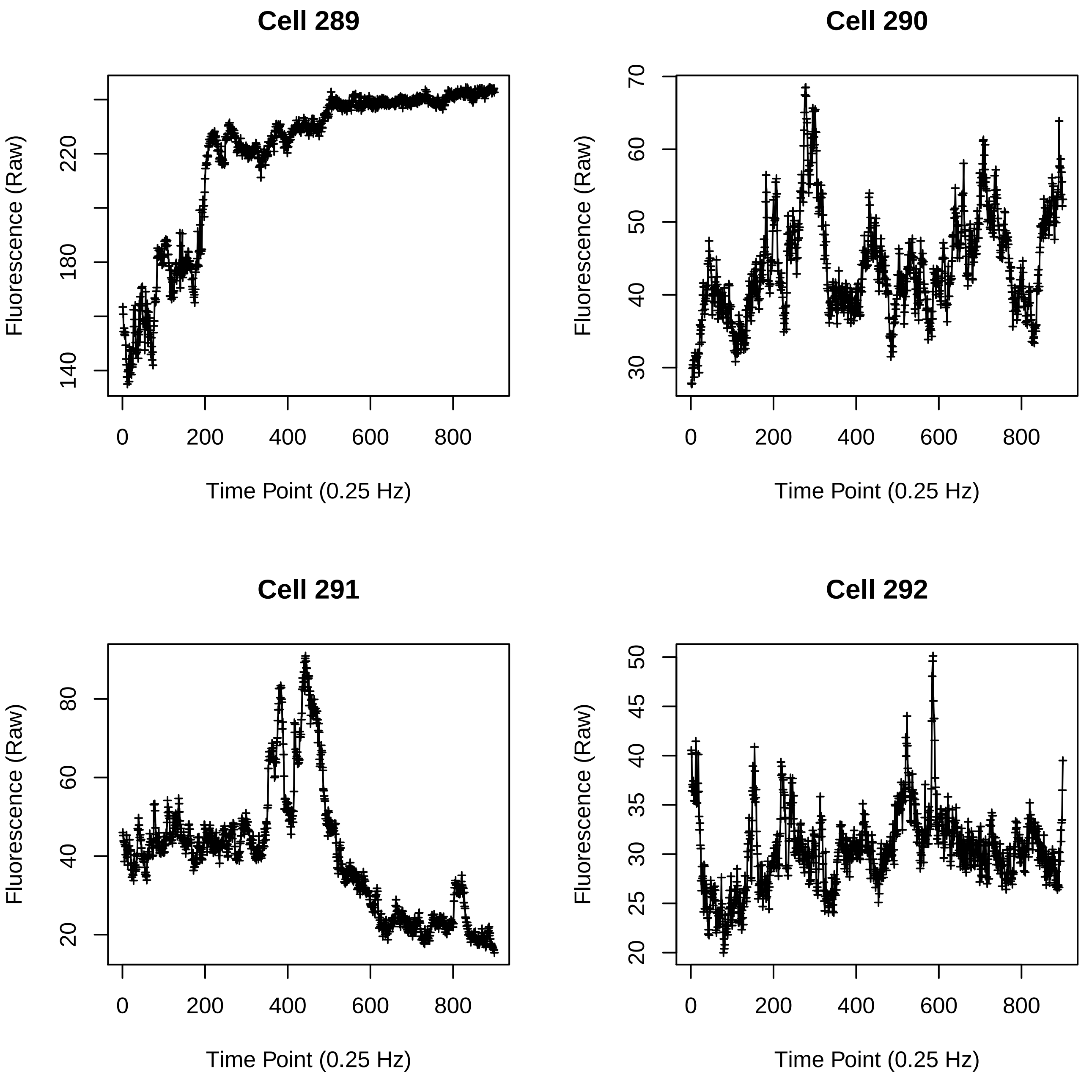

Cell 289
Fluorescence (Raw)
140
180
220
0
200
400
600
800
Time Point (0.25 Hz)
Cell 290
Fluorescence (Raw)
30
40
50
60
70
0
200
400
600
800
Time Point (0.25 Hz)
Cell 291
Fluorescence (Raw)
20
40
60
80
0
200
400
600
800
Time Point (0.25 Hz)
Cell 292
Fluorescence (Raw)
20
25
30
35
40
45
50
0
200
400
600
800
Time Point (0.25 Hz)

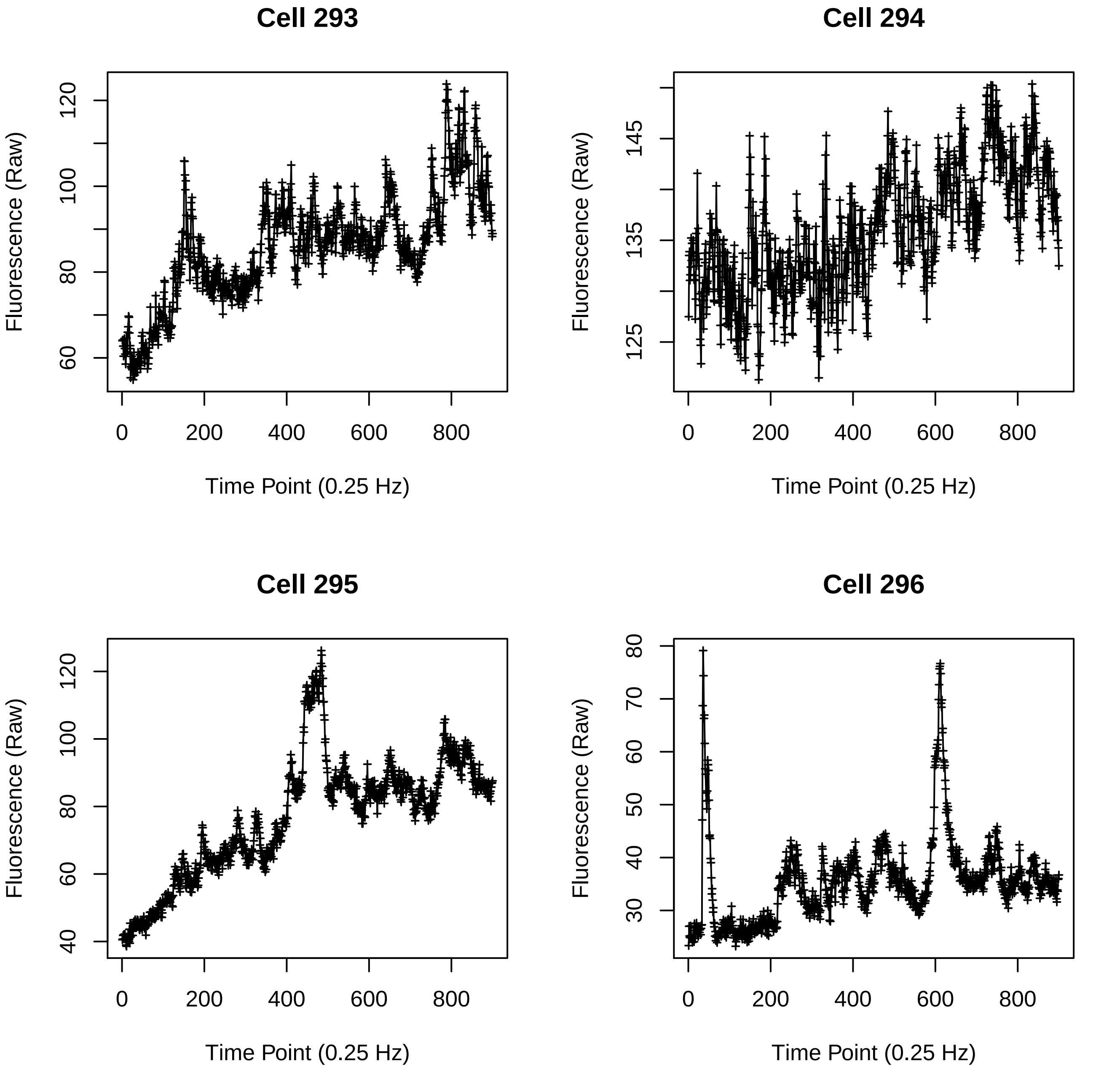
Cell 293
Cell 294
Cell 295
Cell 296
Fluorescence (Raw)
Time Point (0.25 Hz)
0
200
400
600
800
60
80
100
120
125
135
145
40
30
50
70

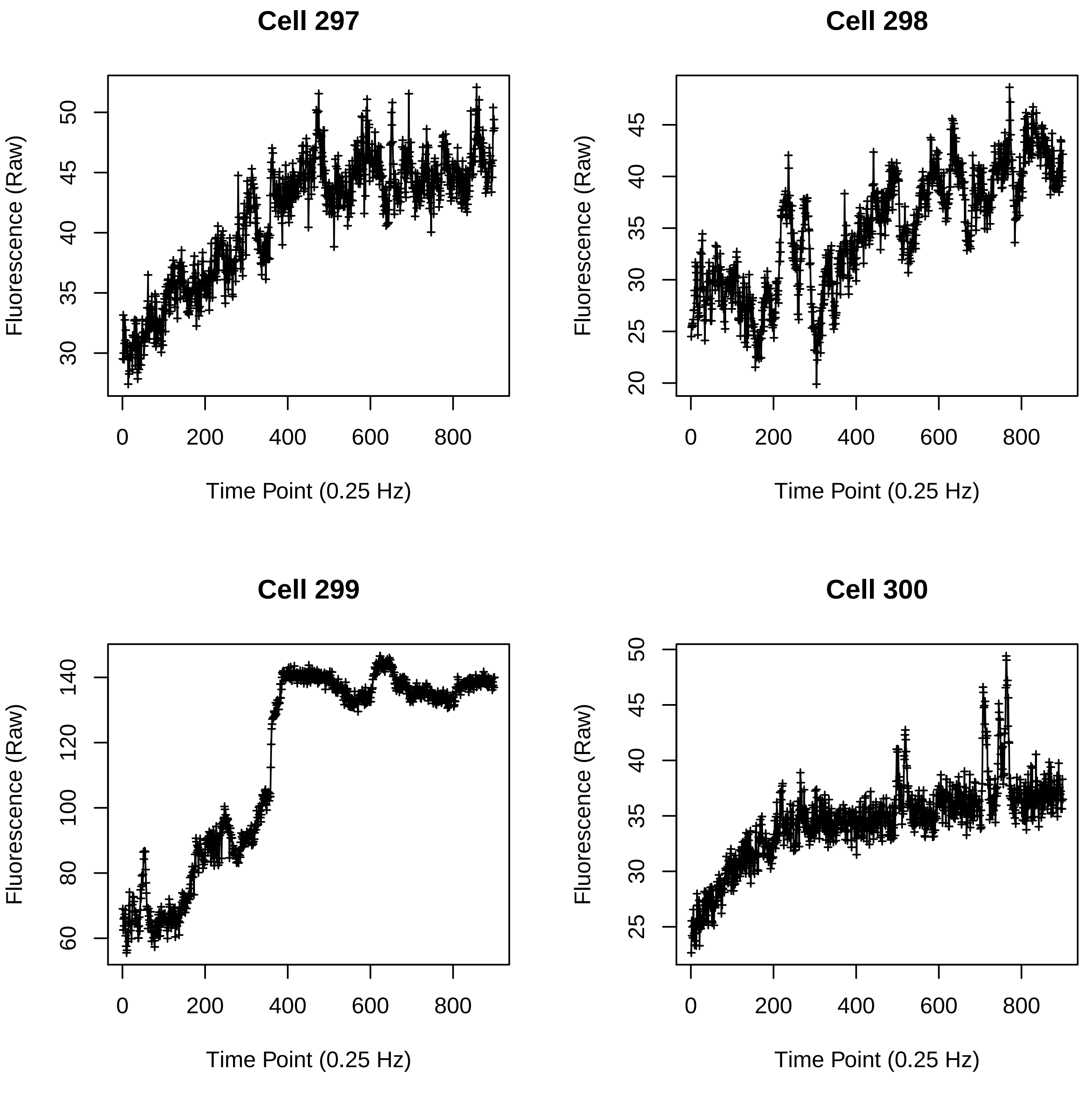

Cell 297
Fluorescence (Raw)
30
35
40
45
50
0
200
400
600
800
Time Point (0.25 Hz)
Cell 298
Fluorescence (Raw)
20
25
30
35
40
45
0
200
400
600
800
Time Point (0.25 Hz)
Cell 299
Fluorescence (Raw)
60
80
100
120
140
0
200
400
600
800
Time Point (0.25 Hz)
Cell 300
Fluorescence (Raw)
25
30
35
40
45
50
0
200
400
600
800
Time Point (0.25 Hz)

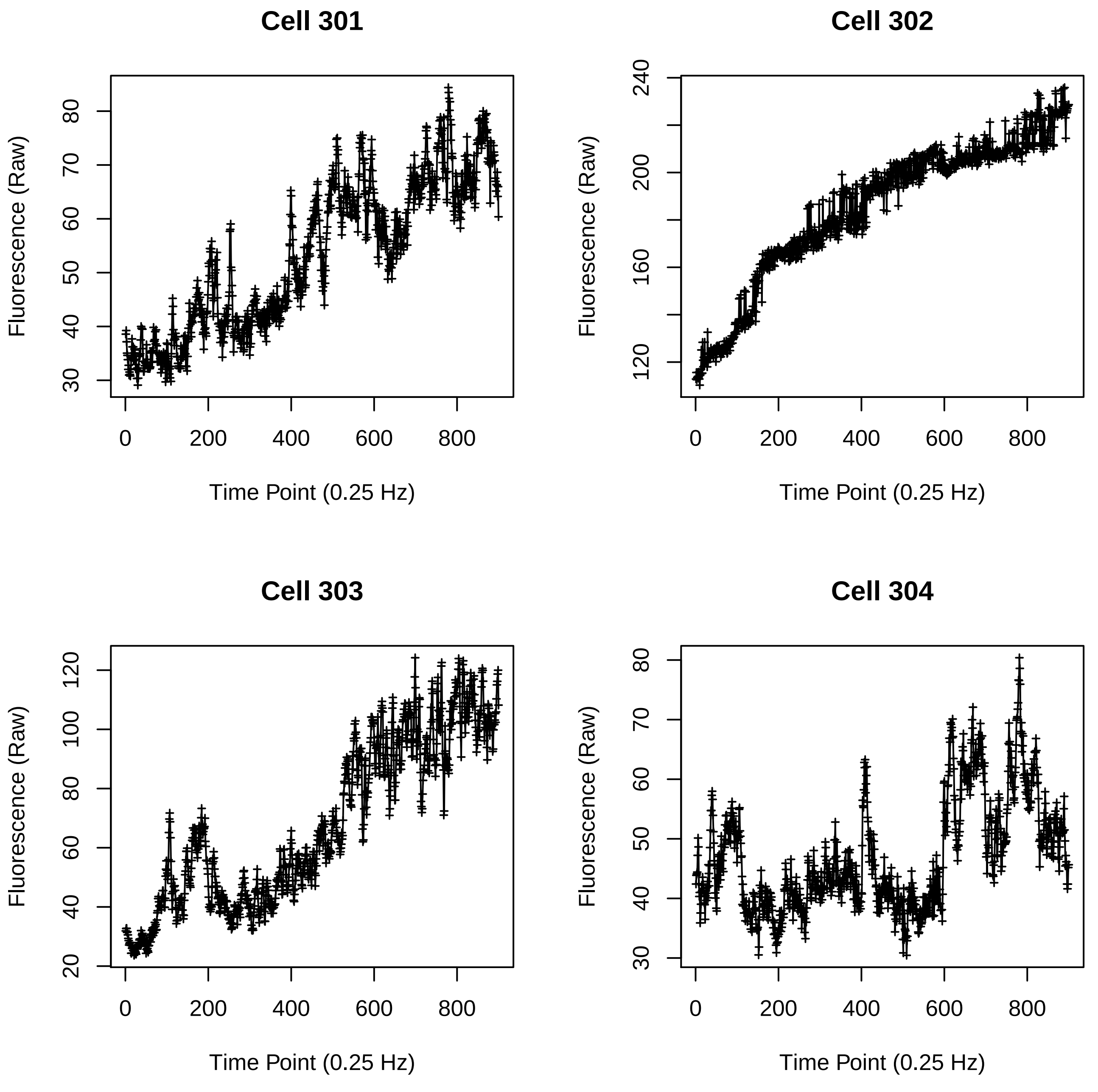
Cell 301
Fluorescence (Raw)
30
40
50
60
70
80
0
200
400
600
800
Time Point (0.25 Hz)
Cell 302
Fluorescence (Raw)
120
160
200
240
0
200
400
600
800
Time Point (0.25 Hz)
Cell 303
Fluorescence (Raw)
20
40
60
80
100
120
0
200
400
600
800
Time Point (0.25 Hz)
Cell 304
Fluorescence (Raw)
30
40
50
60
70
80
0
200
400
600
800
Time Point (0.25 Hz)

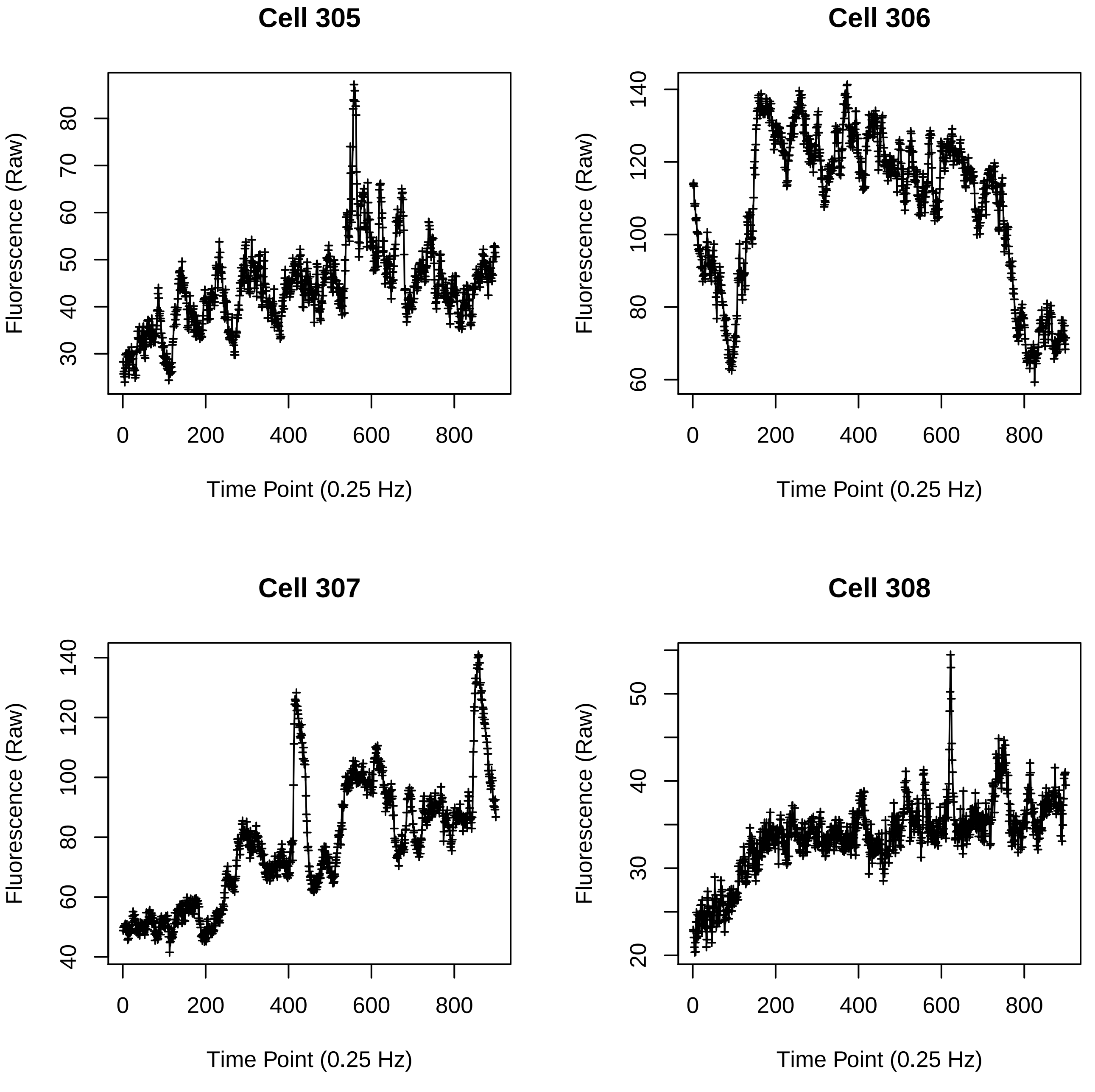

Cell 305
Cell 306
Cell 307
Cell 308
Fluorescence (Raw)
Time Point (0.25 Hz)

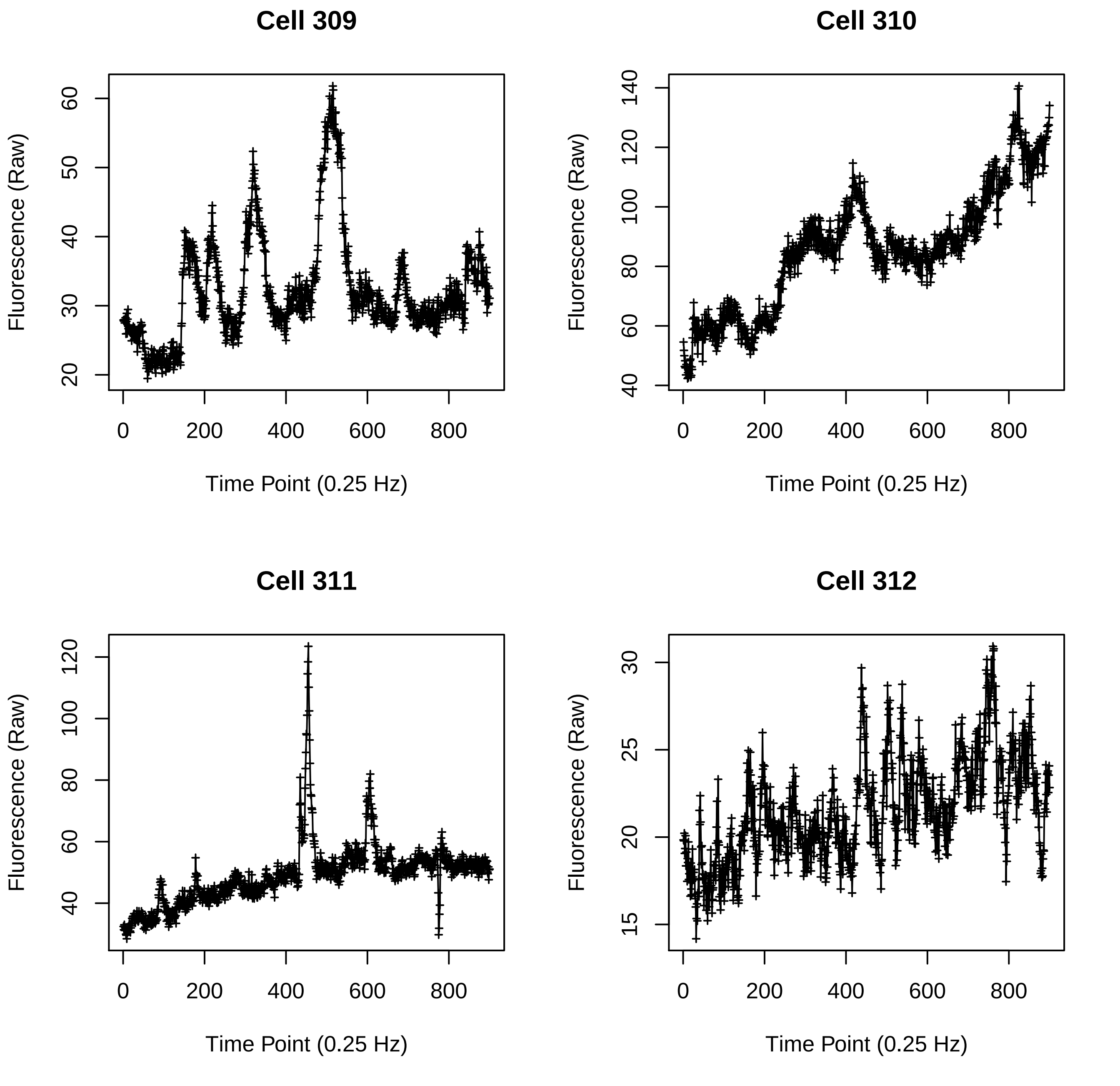

Cell 309
Fluorescence (Raw)
Time Point (0.25 Hz)
Cell 310
Fluorescence (Raw)
Time Point (0.25 Hz)
Cell 311
Fluorescence (Raw)
Time Point (0.25 Hz)
Cell 312
Fluorescence (Raw)
Time Point (0.25 Hz)

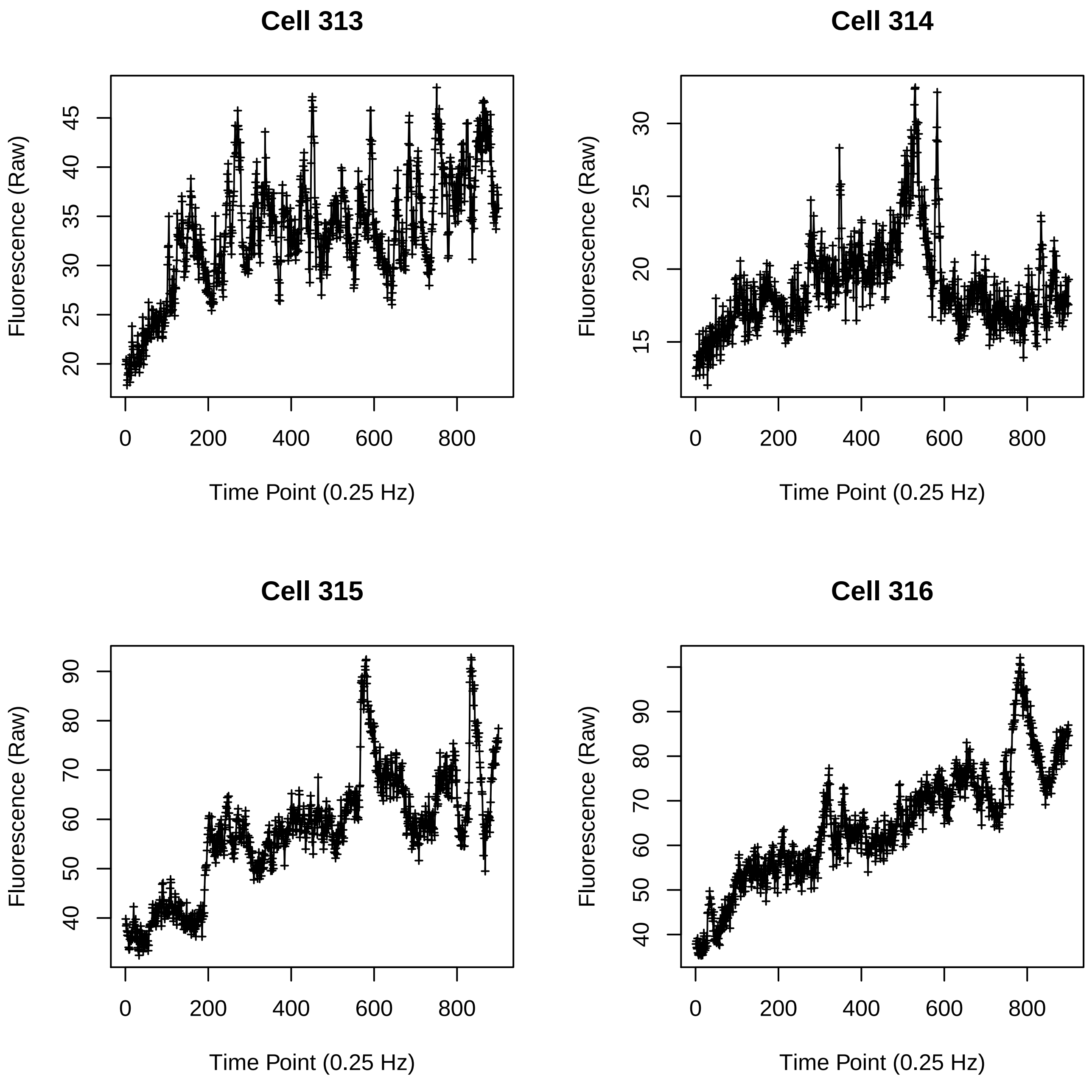
Cell 313
Cell 314
Cell 315
Cell 316
Fluorescence (Raw)
Time Point (0.25 Hz)
0
200
400
600
800
20
25
30
35
40
45
15
50
60
70
80
90

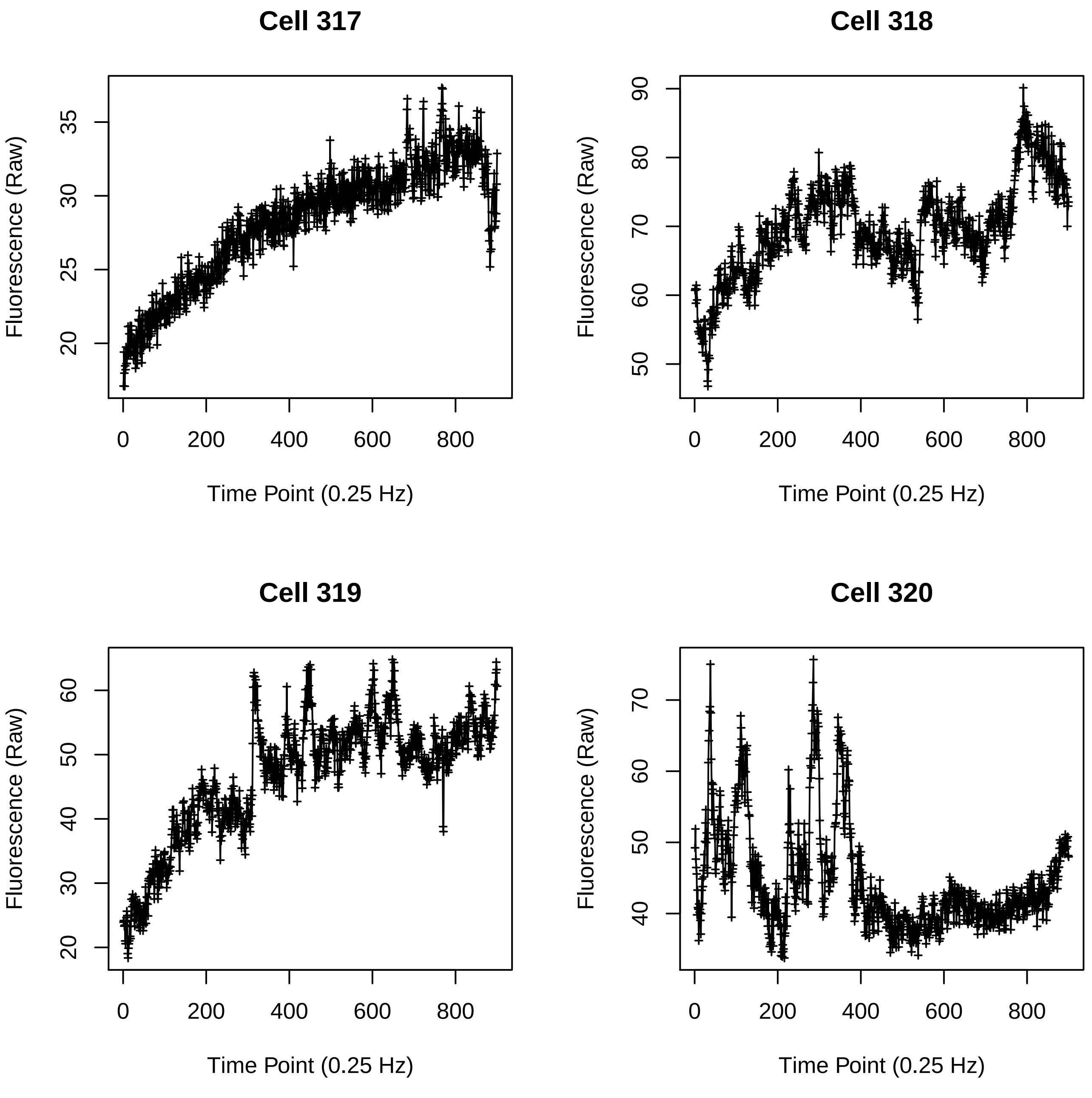
Cell 317
Cell 318
Cell 319
Cell 320
Fluorescence (Raw)
Time Point (0.25 Hz)
20
25
30
35
50
60
70
80
90
40
0
200
400
600
800

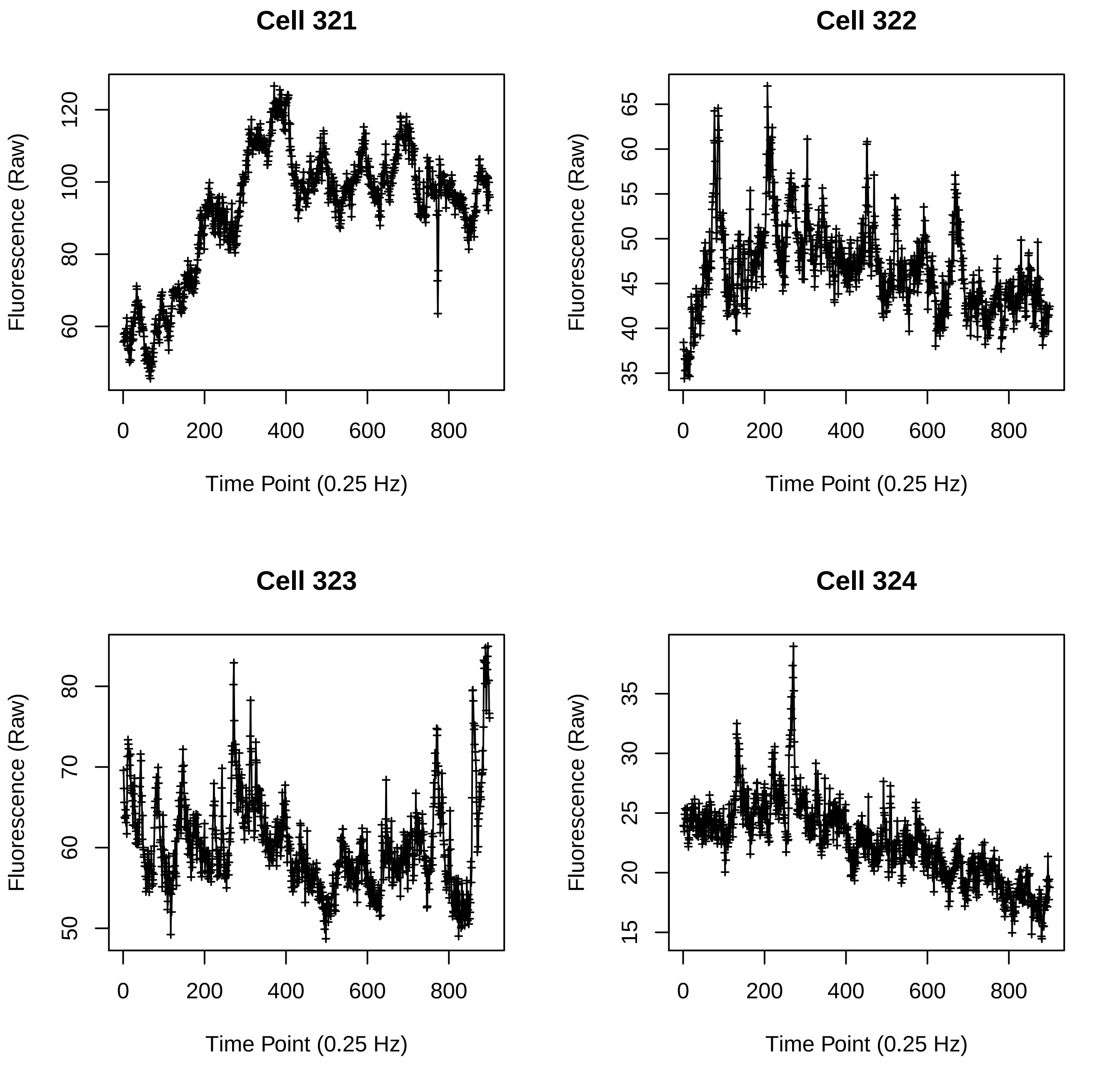
Cell 321
Fluorescence (Raw)
60
80
100
120
0
200
400
600
800
Time Point (0.25 Hz)
Cell 322
Fluorescence (Raw)
35
40
45
50
55
60
65
0
200
400
600
800
Time Point (0.25 Hz)
Cell 323
Fluorescence (Raw)
50
60
70
80
0
200
400
600
800
Time Point (0.25 Hz)
Cell 324
Fluorescence (Raw)
15
20
25
30
35
0
200
400
600
800
Time Point (0.25 Hz)

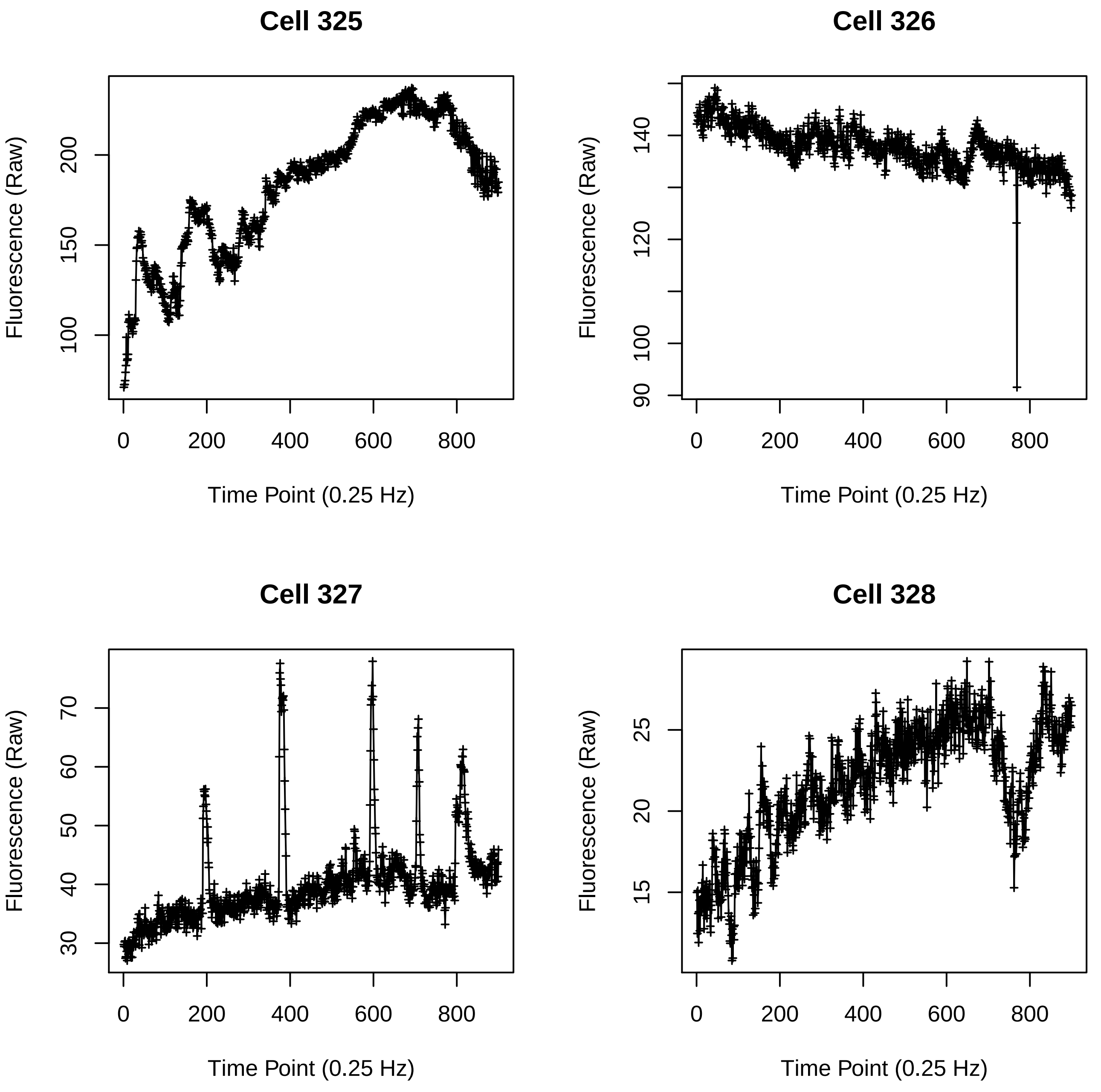
Cell 325
Fluorescence (Raw)
100
150
200
0
200
400
600
800
Time Point (0.25 Hz)
Cell 326
Fluorescence (Raw)
90
100
120
140
0
200
400
600
800
Time Point (0.25 Hz)
Cell 327
Fluorescence (Raw)
30
40
50
60
70
0
200
400
600
800
Time Point (0.25 Hz)
Cell 328
Fluorescence (Raw)
15
20
25
0
200
400
600
800
Time Point (0.25 Hz)

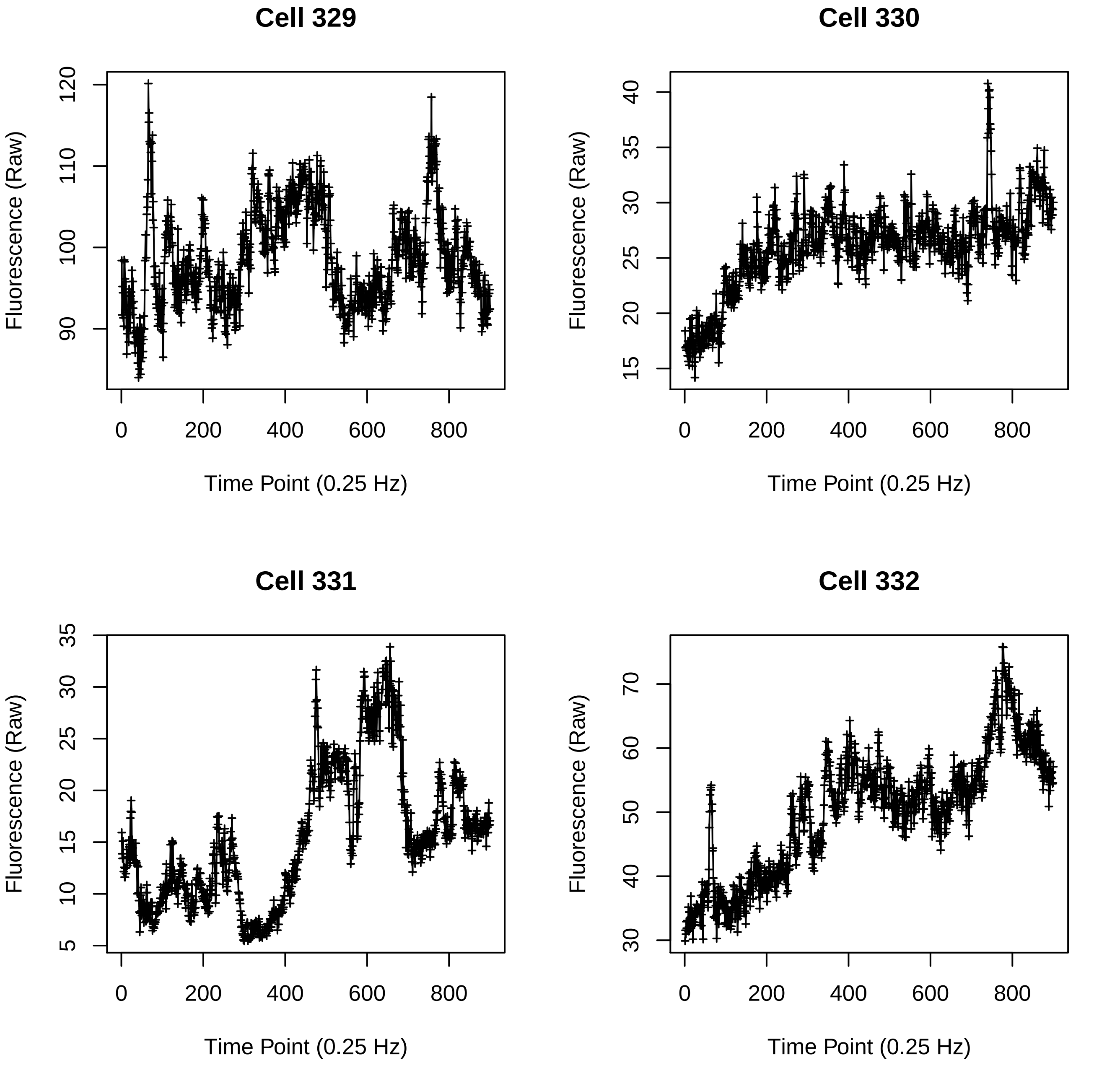
Cell 329
Fluorescence (Raw)
90
100
110
120
0
200
400
600
800
Time Point (0.25 Hz)
Cell 330
Fluorescence (Raw)
15
20
25
30
35
40
0
200
400
600
800
Time Point (0.25 Hz)
Cell 331
Fluorescence (Raw)
5
10
15
20
25
30
35
0
200
400
600
800
Time Point (0.25 Hz)
Cell 332
Fluorescence (Raw)
30
40
50
60
70
0
200
400
600
800
Time Point (0.25 Hz)

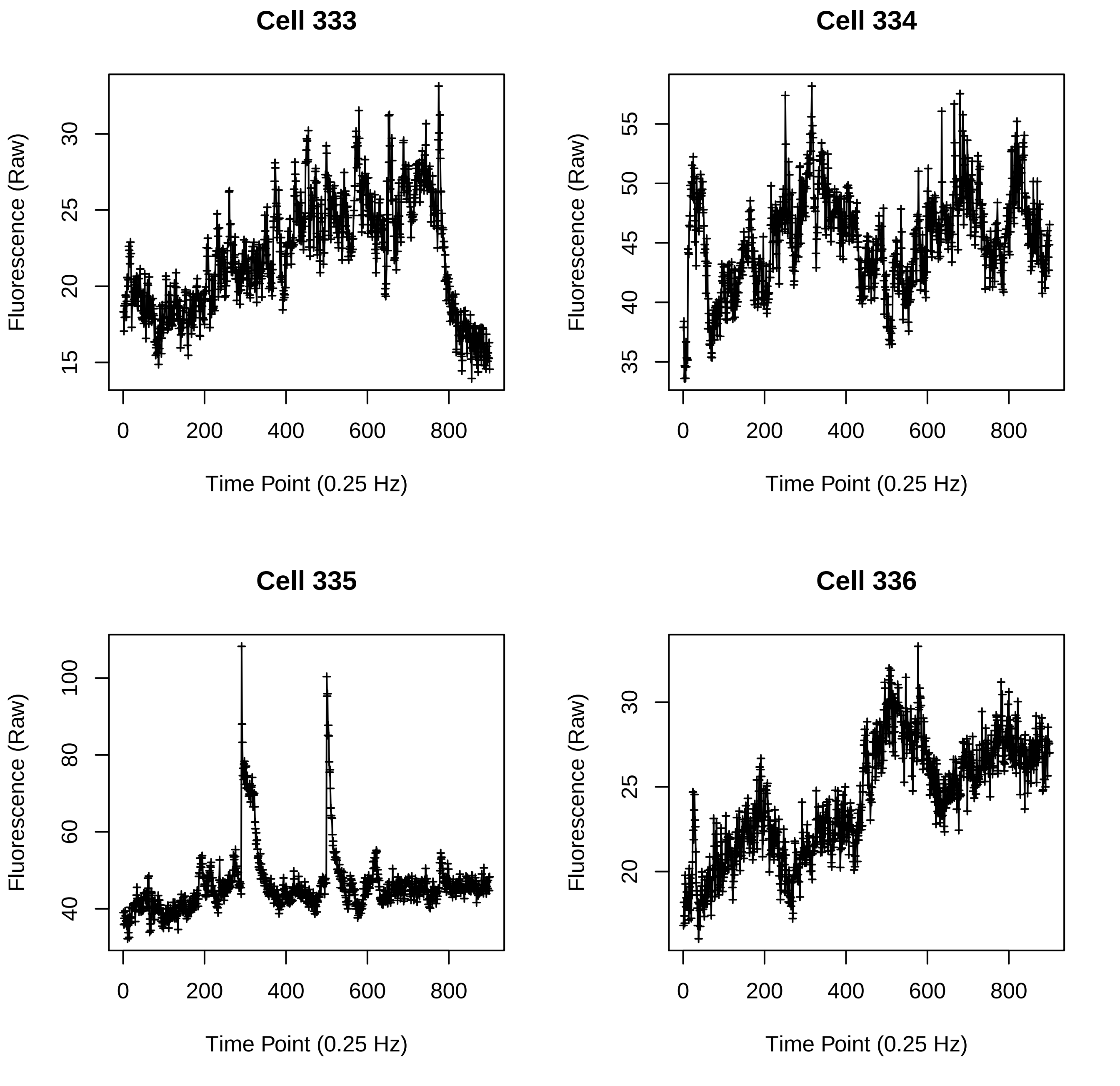

Cell 333
Cell 334
Cell 335
Cell 336
Fluorescence (Raw)
Time Point (0.25 Hz)
0
200
400
600
800
15
20
25
30
35
40
45
50
55
60
80
100

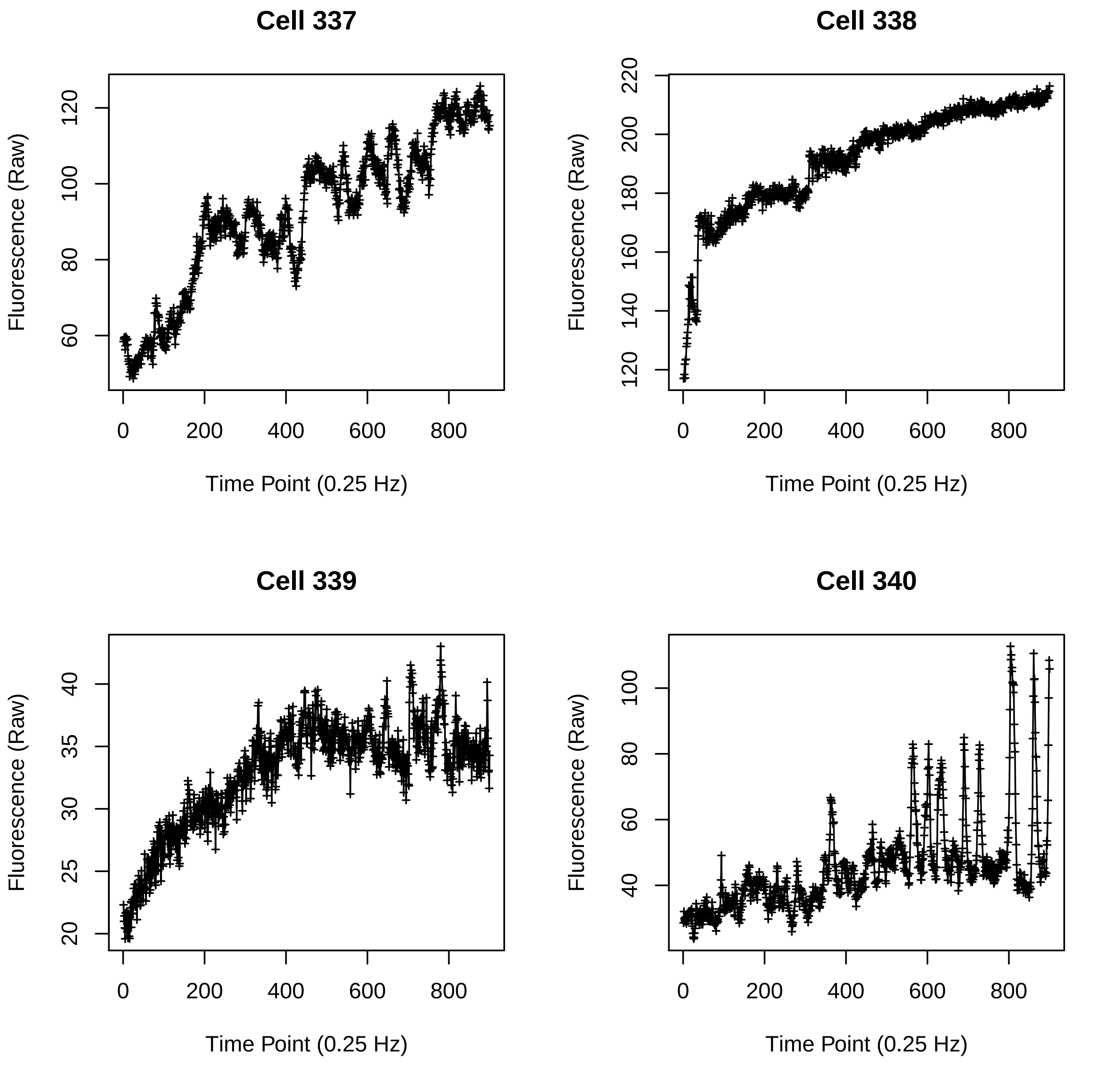

Cell 337
Fluorescence (Raw)
Time Point (0.25 Hz)
Cell 338
Fluorescence (Raw)
Time Point (0.25 Hz)
Cell 339
Fluorescence (Raw)
Time Point (0.25 Hz)
Cell 340
Fluorescence (Raw)
Time Point (0.25 Hz)

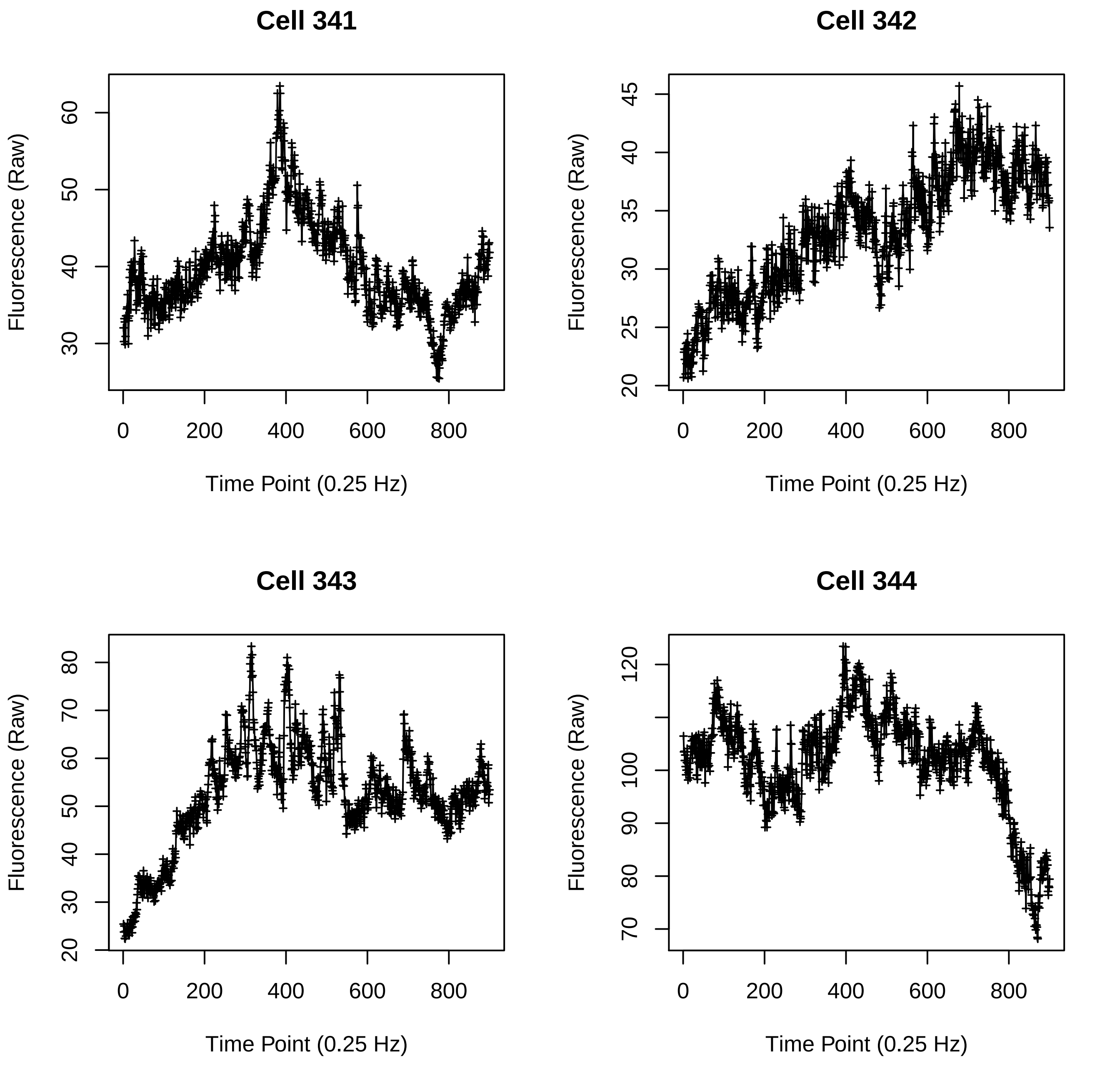

Cell 341
Fluorescence (Raw)
Time Point (0.25 Hz)
Cell 342
Fluorescence (Raw)
Time Point (0.25 Hz)
Cell 343
Fluorescence (Raw)
Time Point (0.25 Hz)
Cell 344
Fluorescence (Raw)
Time Point (0.25 Hz)

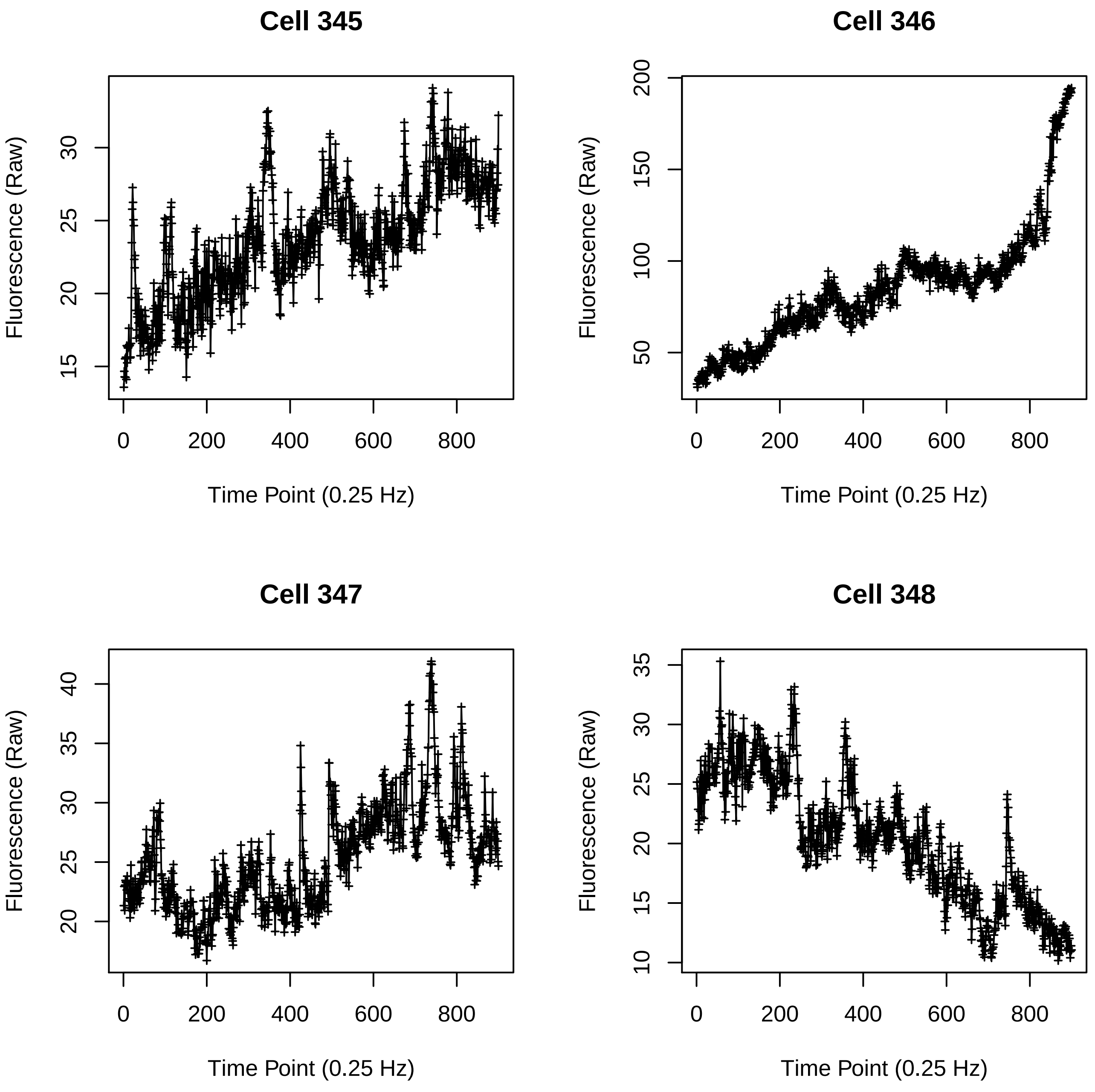
Cell 345
Fluorescence (Raw)
15
20
25
30
0
200
400
600
800
Time Point (0.25 Hz)
Cell 346
Fluorescence (Raw)
50
100
150
200
0
200
400
600
800
Time Point (0.25 Hz)
Cell 347
Fluorescence (Raw)
20
25
30
35
40
0
200
400
600
800
Time Point (0.25 Hz)
Cell 348
Fluorescence (Raw)
10
15
20
25
30
35
0
200
400
600
800
Time Point (0.25 Hz)

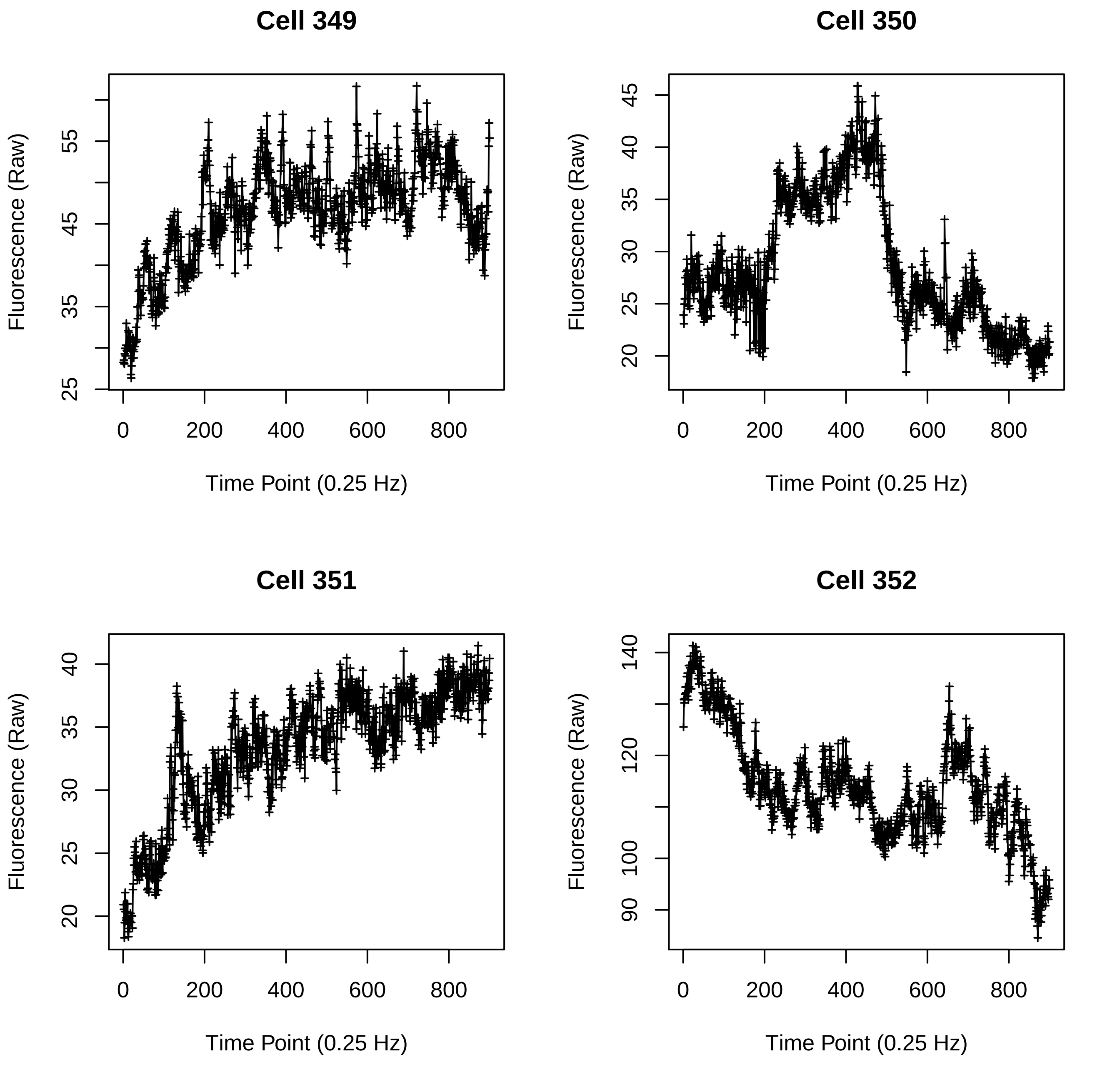
Cell 349
Cell 350
Cell 351
Cell 352
Fluorescence (Raw)
Time Point (0.25 Hz)
25
35
45
55
20
25
30
35
40
45
90
100
120
140
0
200
400
600
800

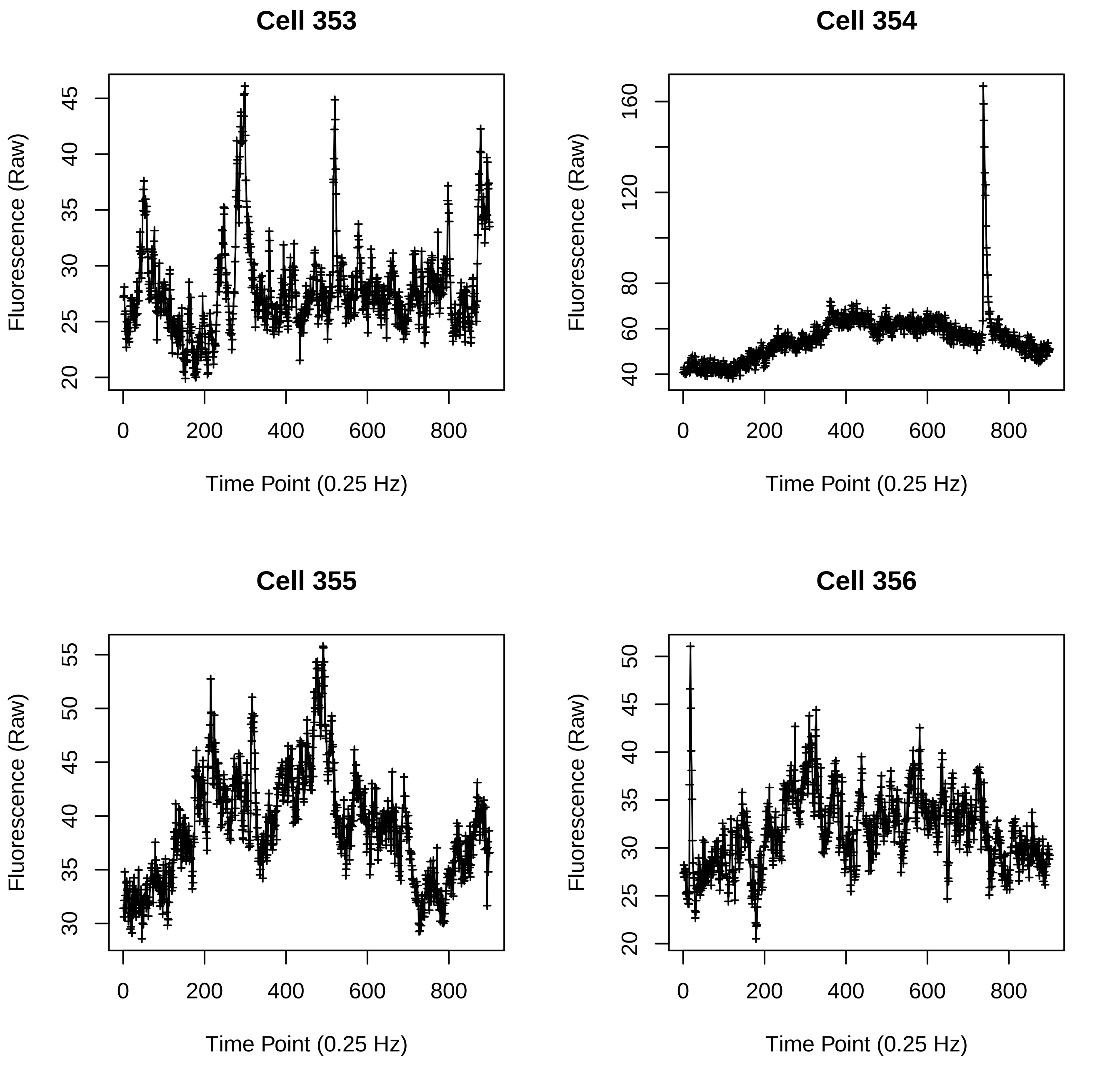

Cell 353
Cell 354
Cell 355
Cell 356
Fluorescence (Raw)
Time Point (0.25 Hz)

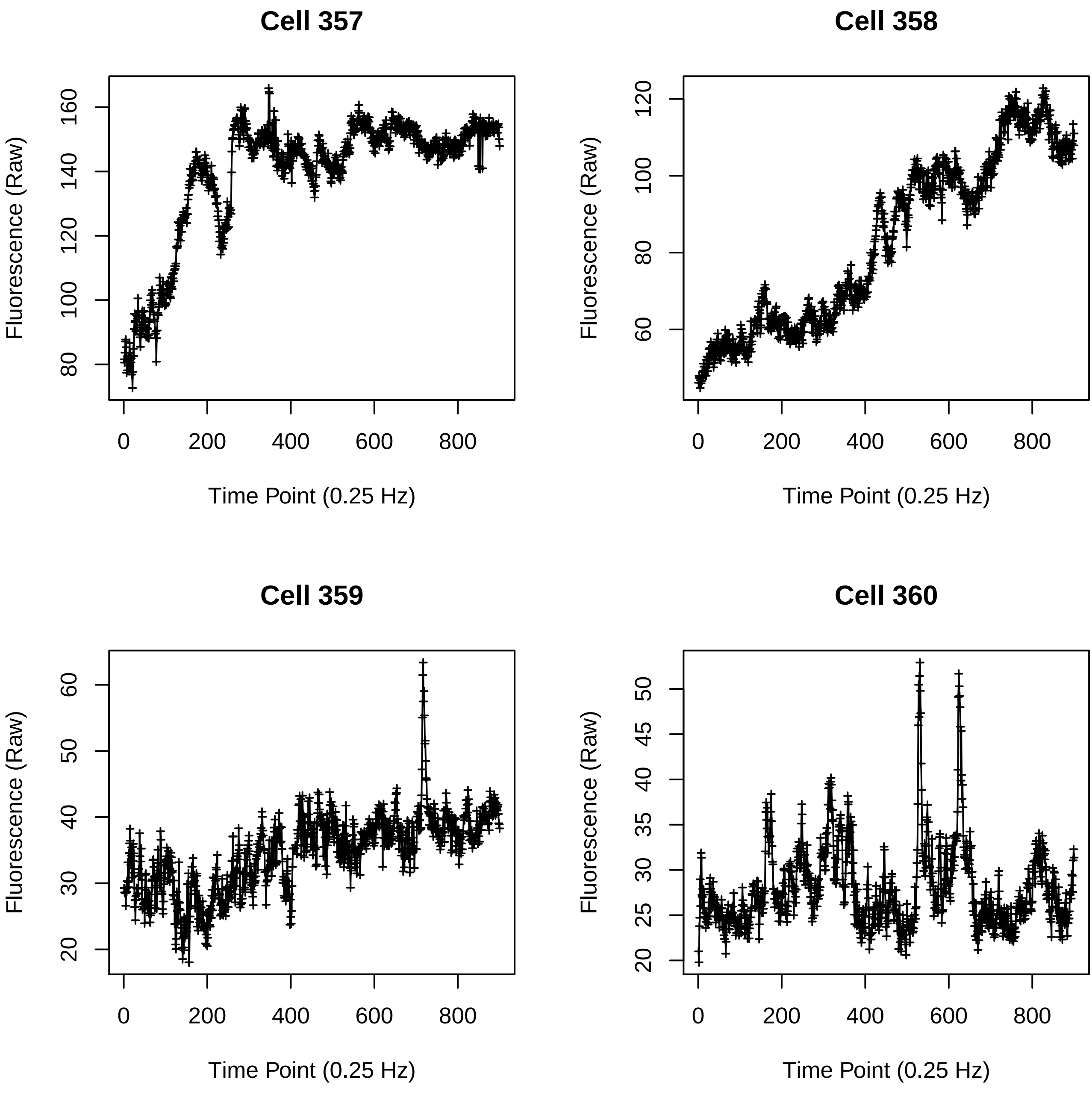
Cell 357
Fluorescence (Raw)
80
100
120
140
160
0
200
400
600
800
Time Point (0.25 Hz)
Cell 358
Fluorescence (Raw)
60
80
100
120
0
200
400
600
800
Time Point (0.25 Hz)
Cell 359
Fluorescence (Raw)
20
30
40
50
60
0
200
400
600
800
Time Point (0.25 Hz)
Cell 360
Fluorescence (Raw)
20
25
30
35
40
45
50
0
200
400
600
800
Time Point (0.25 Hz)

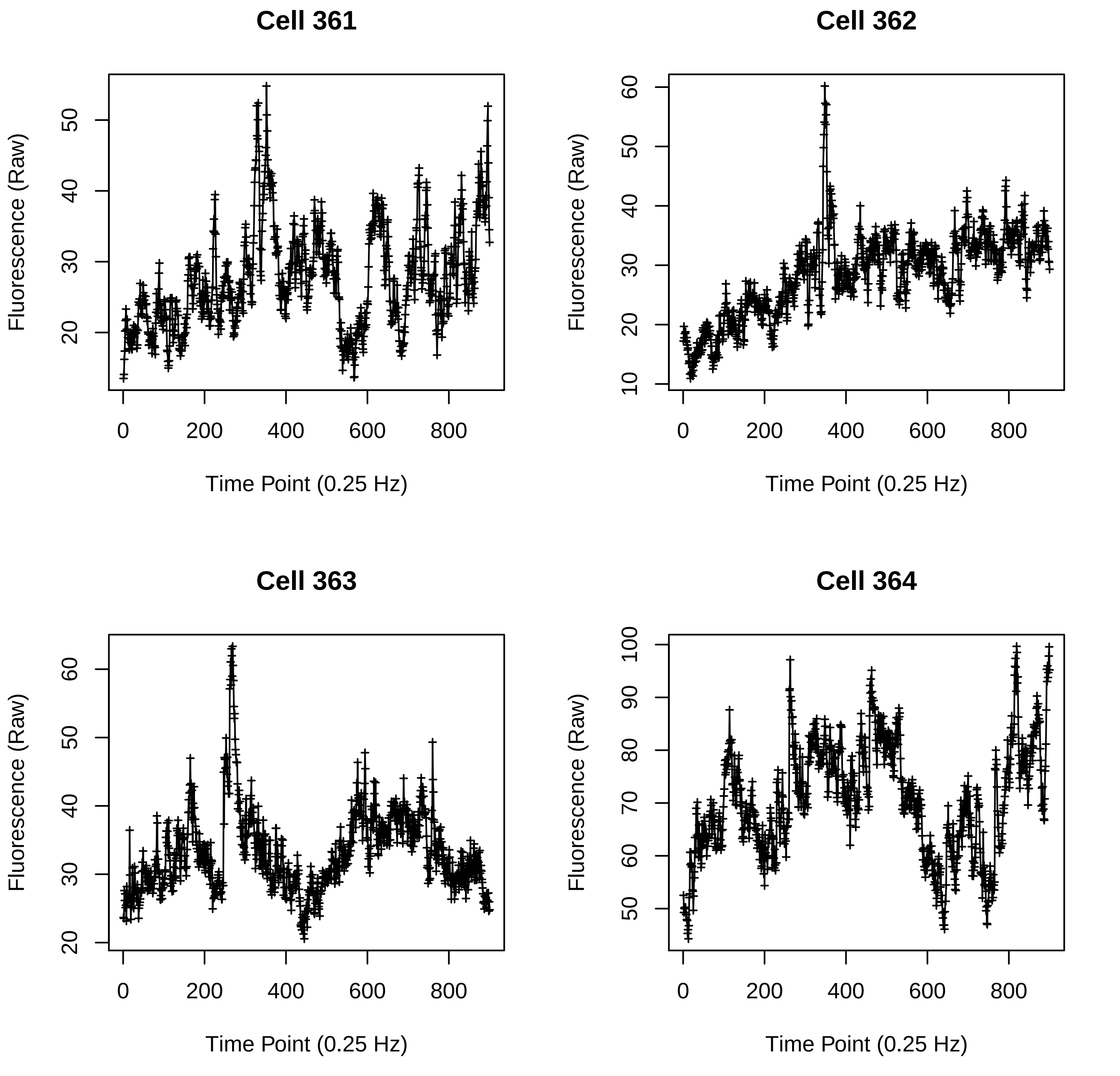
Cell 361
Cell 362
Cell 363
Cell 364
Fluorescence (Raw)
Time Point (0.25 Hz)
0
200
400
600
800
20
30
40
50
10
60
70
80
90
100

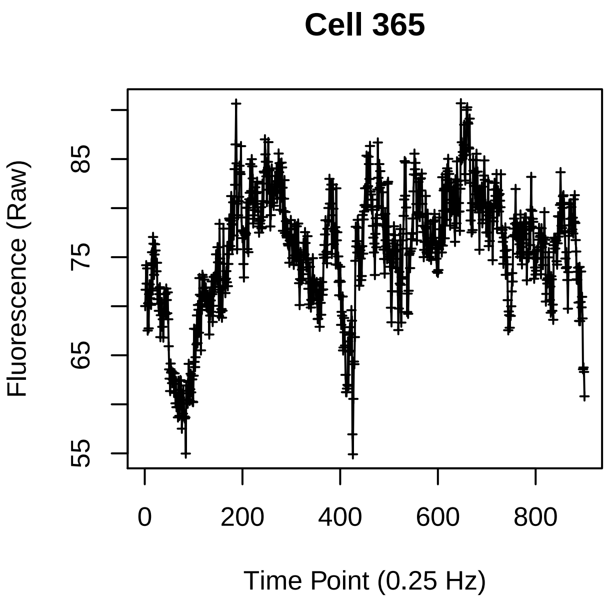
Cell 365
Fluorescence (Raw)
55
65
75
85
0
200
400
600
800
Time Point (0.25 Hz)

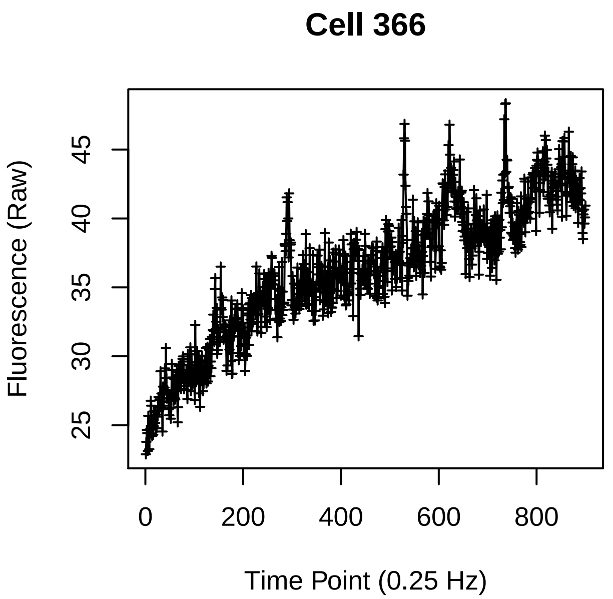
Cell 366
Fluorescence (Raw)
25
30
35
40
45
0
200
400
600
800
Time Point (0.25 Hz)

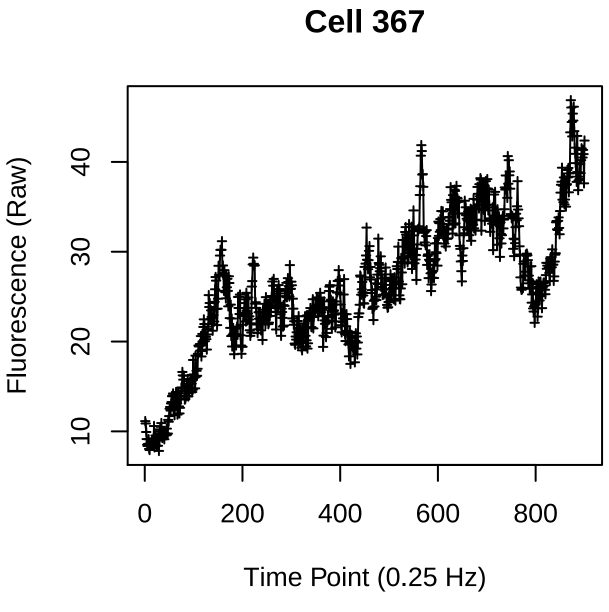
Cell 367
Fluorescence (Raw)
10
20
30
40
0
200
400
600
800
Time Point (0.25 Hz)

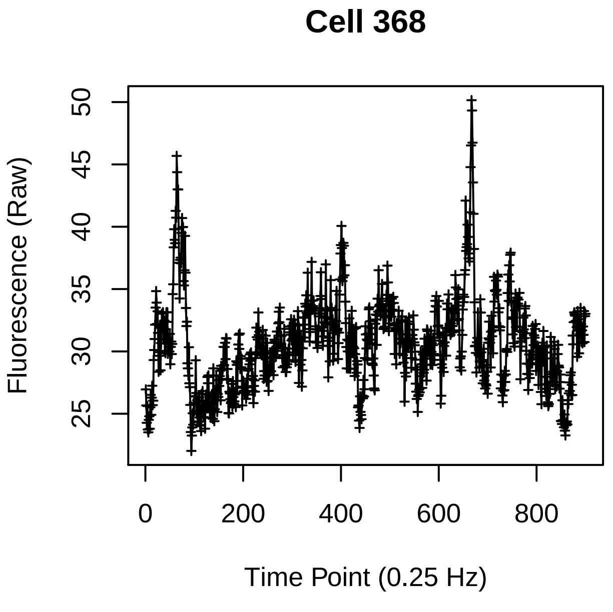
Cell 368
Fluorescence (Raw)
25
30
35
40
45
50
0
200
400
600
800
Time Point (0.25 Hz)

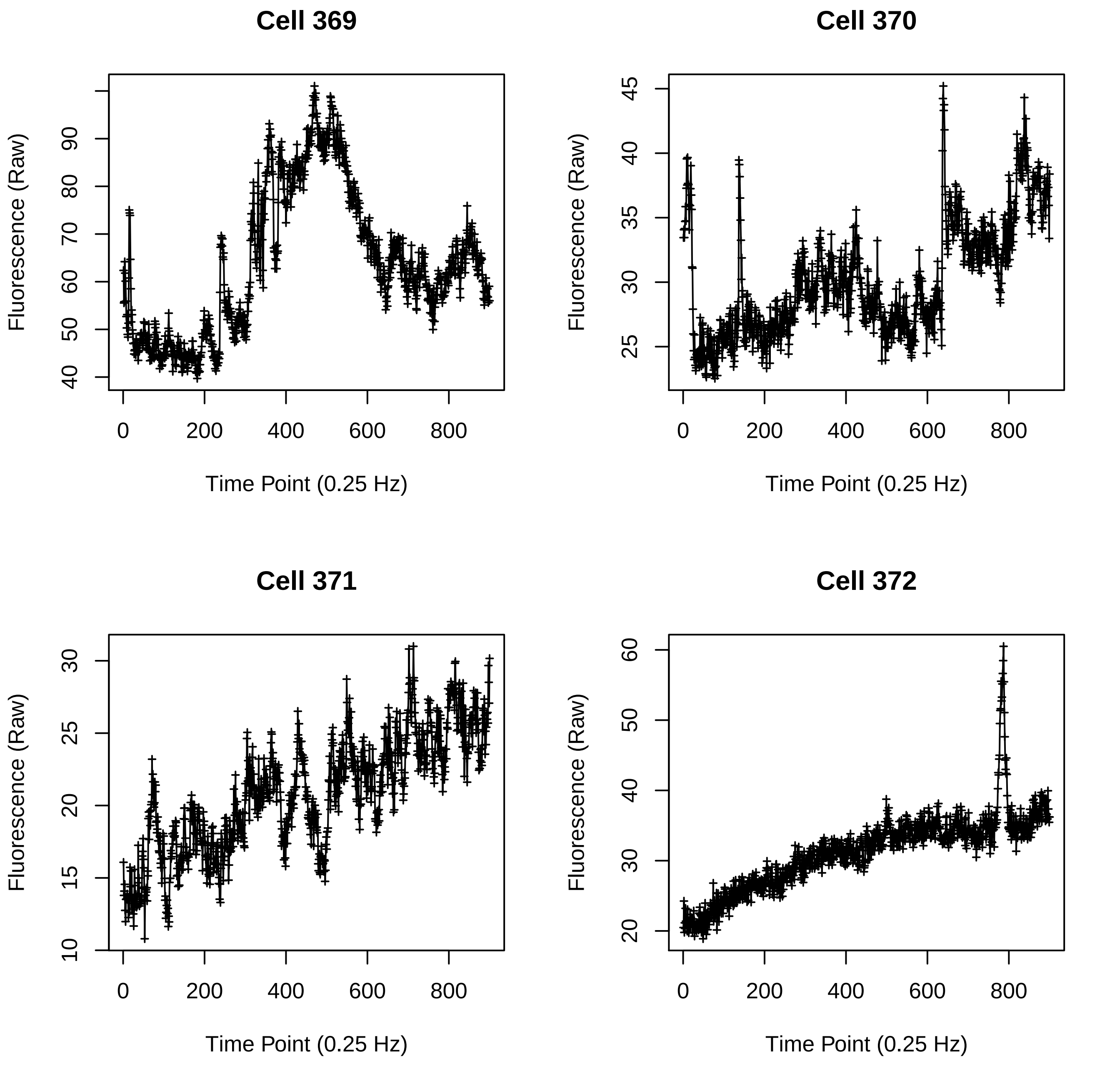
Cell 369
Fluorescence (Raw)
40
50
60
70
80
90
0
200
400
600
800
Time Point (0.25 Hz)
Cell 370
Fluorescence (Raw)
25
30
35
40
45
0
200
400
600
800
Time Point (0.25 Hz)
Cell 371
Fluorescence (Raw)
10
15
20
25
30
0
200
400
600
800
Time Point (0.25 Hz)
Cell 372
Fluorescence (Raw)
20
30
40
50
60
0
200
400
600
800
Time Point (0.25 Hz)

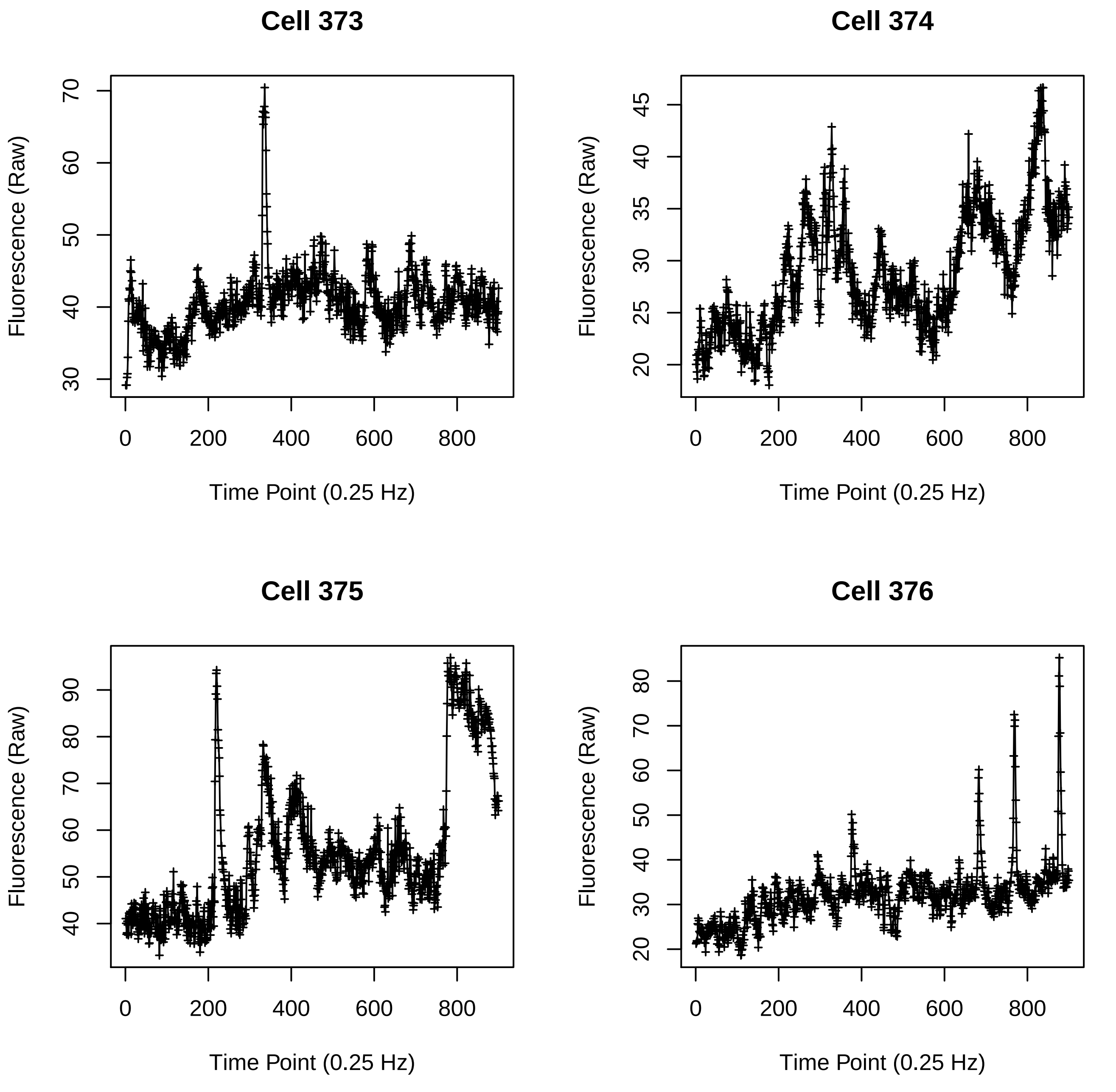
Cell 373
Fluorescence (Raw)
30
40
50
60
70
0
200
400
600
800
Time Point (0.25 Hz)
Cell 374
Fluorescence (Raw)
20
25
30
35
40
45
0
200
400
600
800
Time Point (0.25 Hz)
Cell 375
Fluorescence (Raw)
40
50
60
70
80
90
0
200
400
600
800
Time Point (0.25 Hz)
Cell 376
Fluorescence (Raw)
20
30
40
50
60
70
80
0
200
400
600
800
Time Point (0.25 Hz)

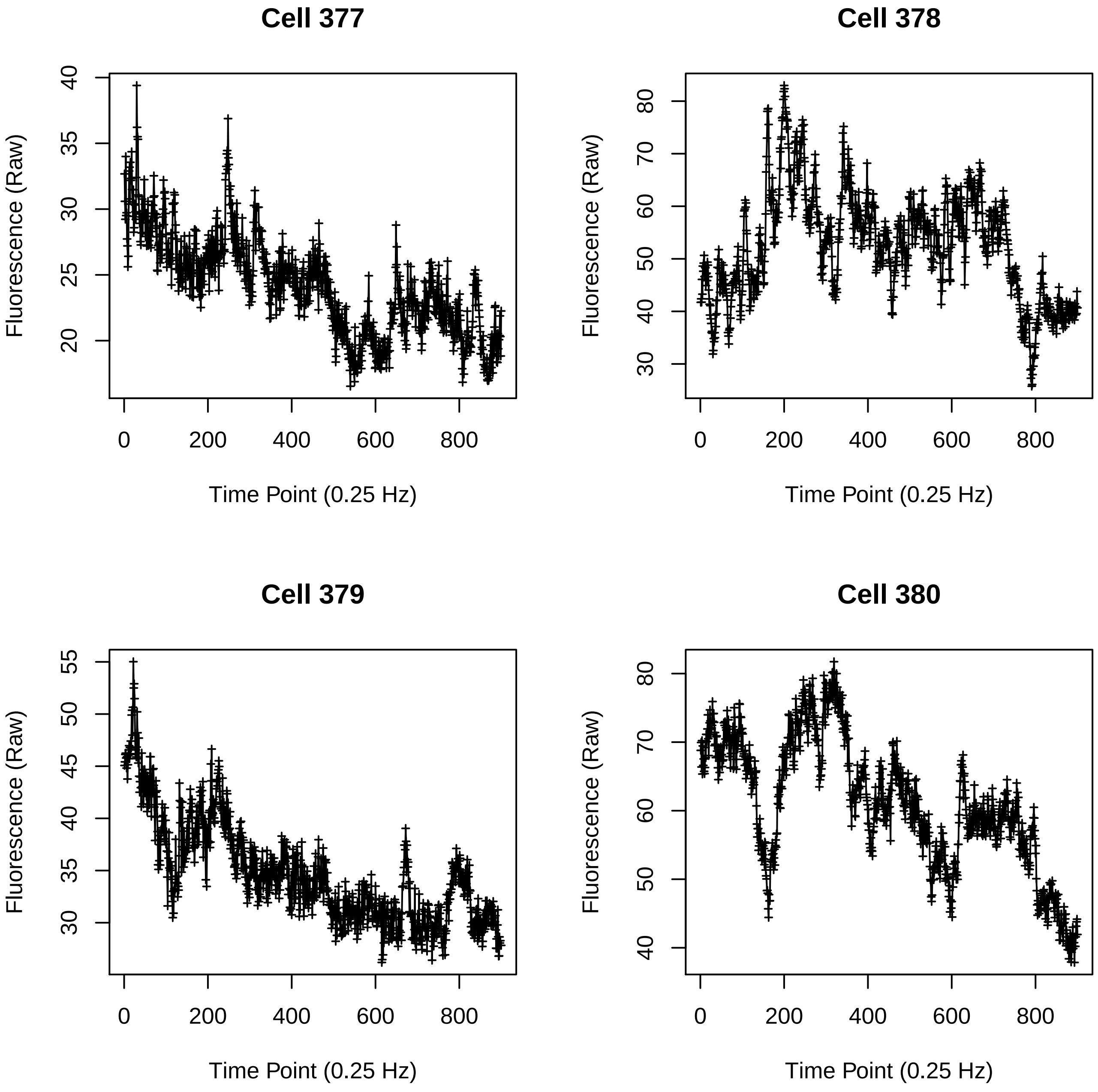
Cell 377
Cell 378
Cell 379
Cell 380
Fluorescence (Raw)
Time Point (0.25 Hz)
0
200
400
600
800
20
25
30
35
40
30
40
50
60
70
80
45
50
55

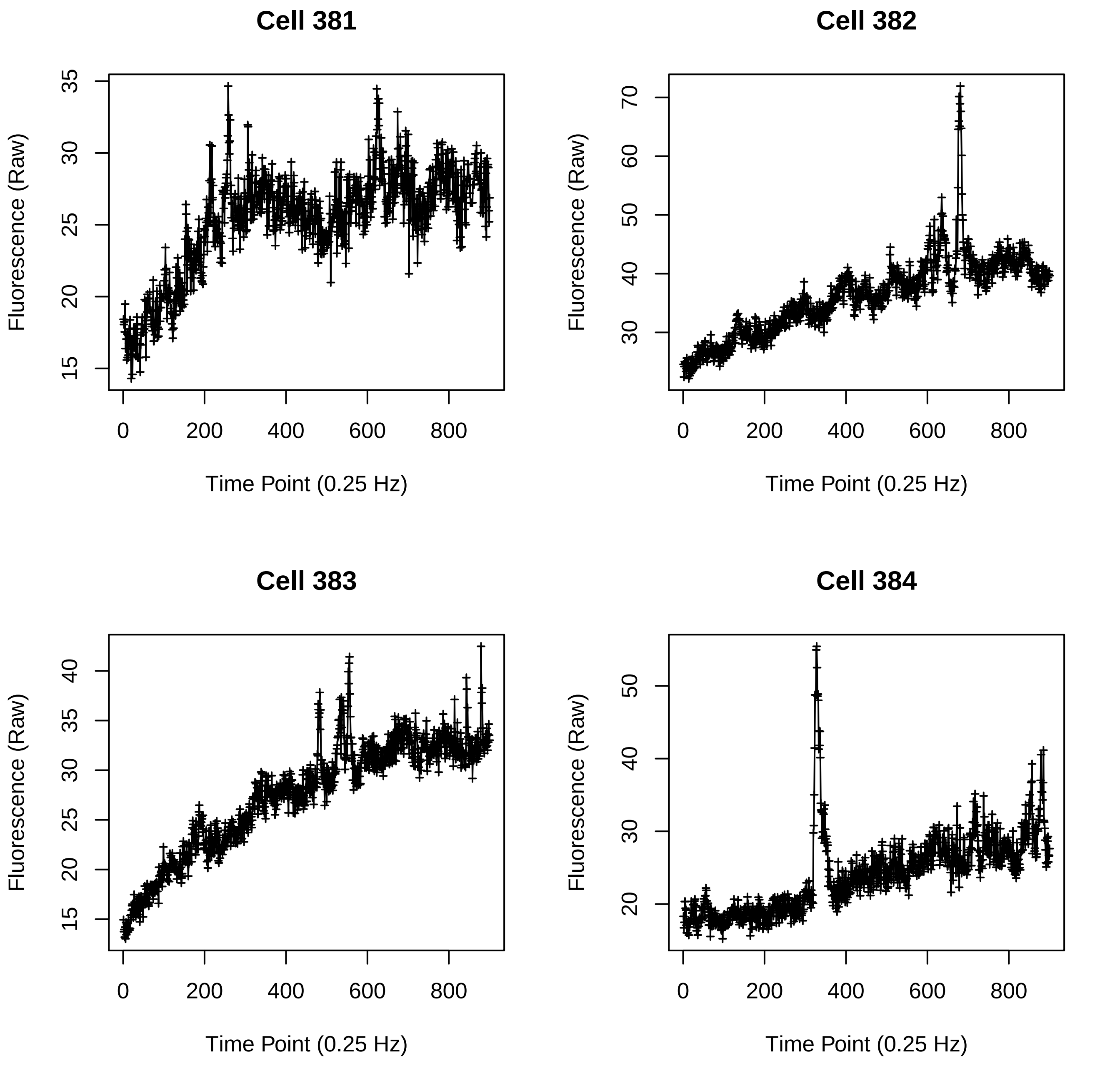
Cell 381
Fluorescence (Raw)
15
20
25
30
35
0
200
400
600
800
Time Point (0.25 Hz)
Cell 382
Fluorescence (Raw)
30
40
50
60
70
0
200
400
600
800
Time Point (0.25 Hz)
Cell 383
Fluorescence (Raw)
15
20
25
30
35
40
0
200
400
600
800
Time Point (0.25 Hz)
Cell 384
Fluorescence (Raw)
20
30
40
50
0
200
400
600
800
Time Point (0.25 Hz)

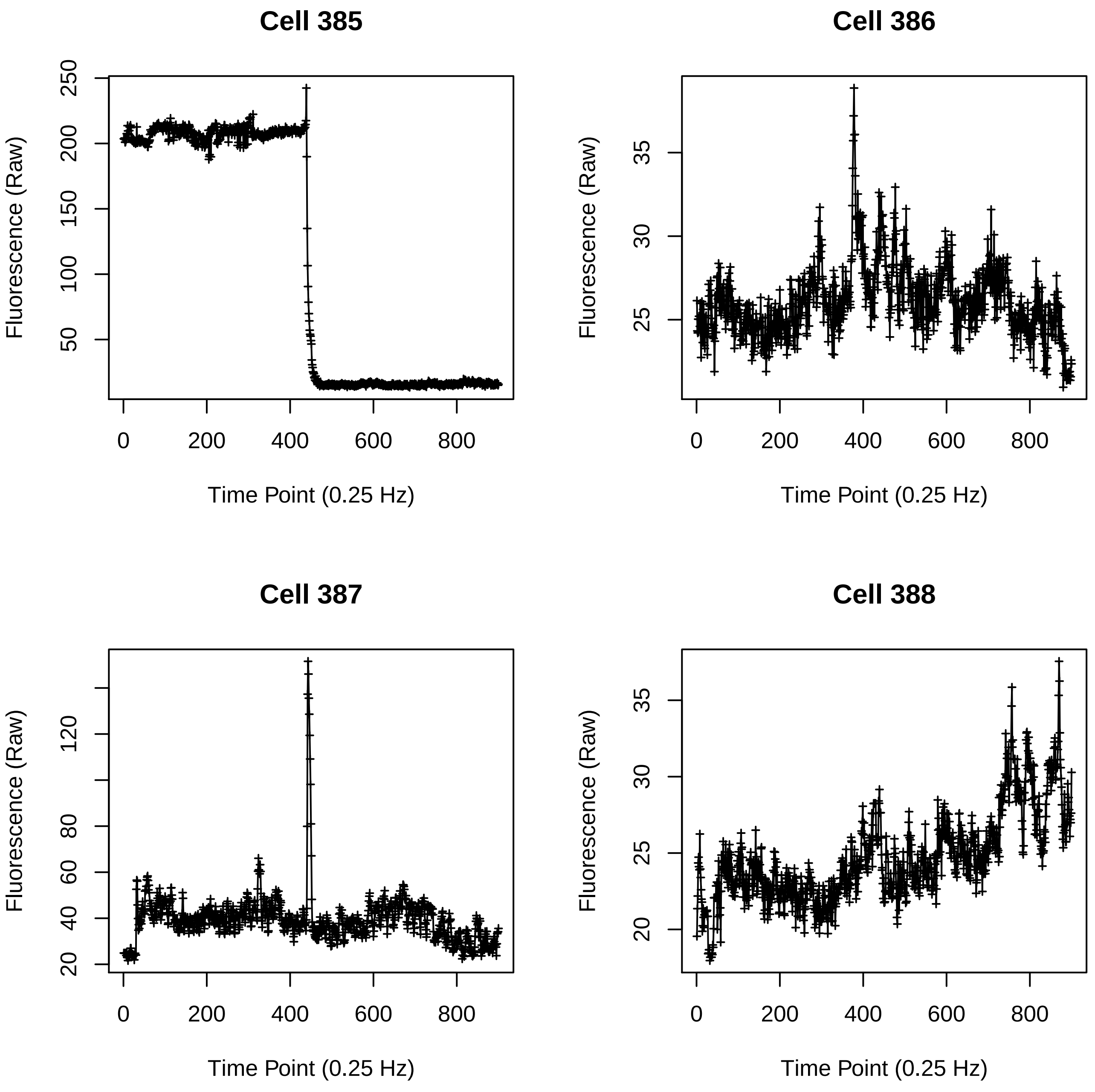
Cell 385
Fluorescence (Raw)
50
100
150
200
250
0
200
400
600
800
Time Point (0.25 Hz)
Cell 386
Fluorescence (Raw)
25
30
35
0
200
400
600
800
Time Point (0.25 Hz)
Cell 387
Fluorescence (Raw)
20
40
60
80
120
0
200
400
600
800
Time Point (0.25 Hz)
Cell 388
Fluorescence (Raw)
20
25
30
35
0
200
400
600
800
Time Point (0.25 Hz)

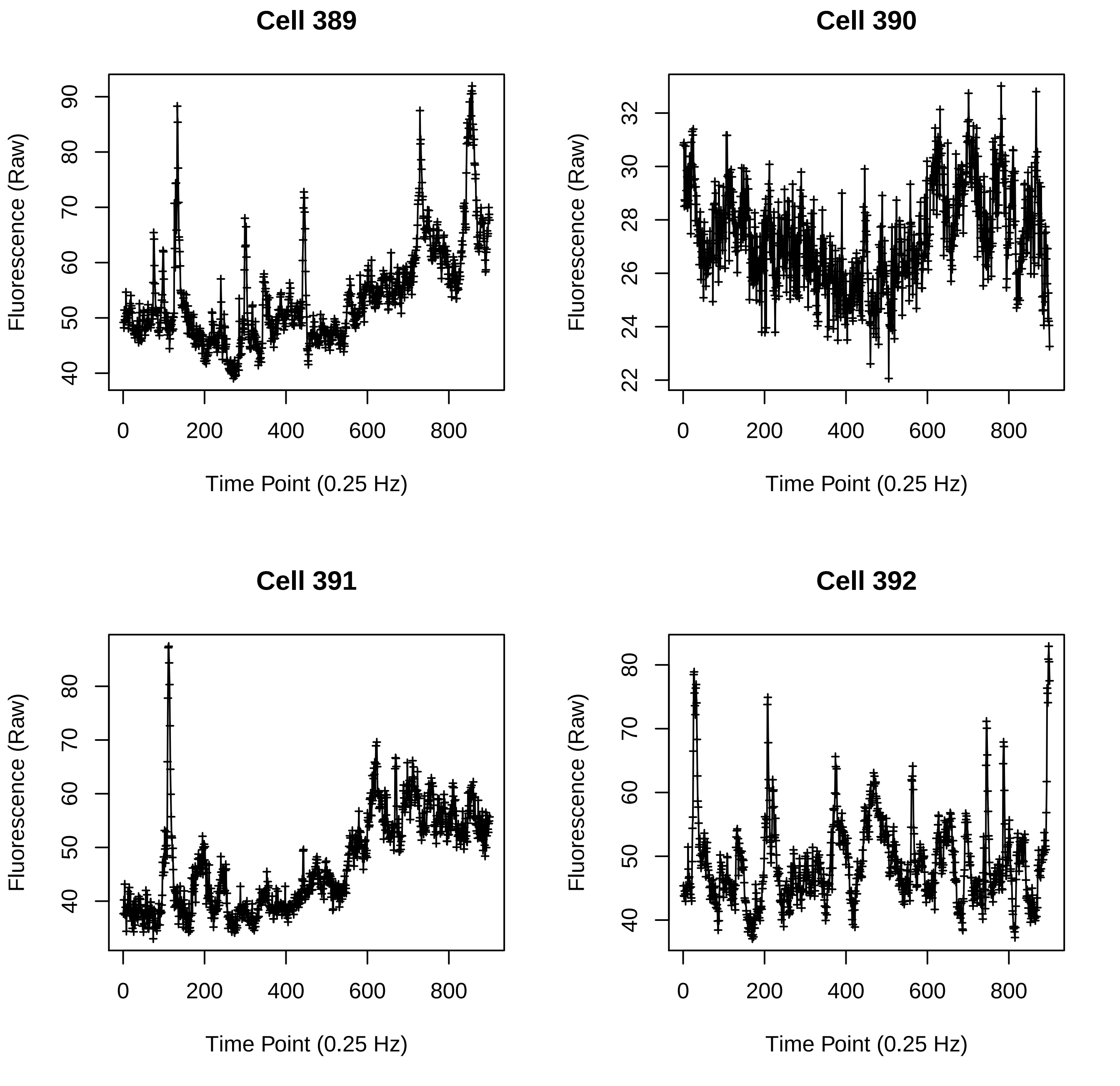
Cell 389
Cell 390
Cell 391
Cell 392
Fluorescence (Raw)
Time Point (0.25 Hz)
0
200
400
600
800
40
50
60
70
80
90
22
24
26
28
30
32

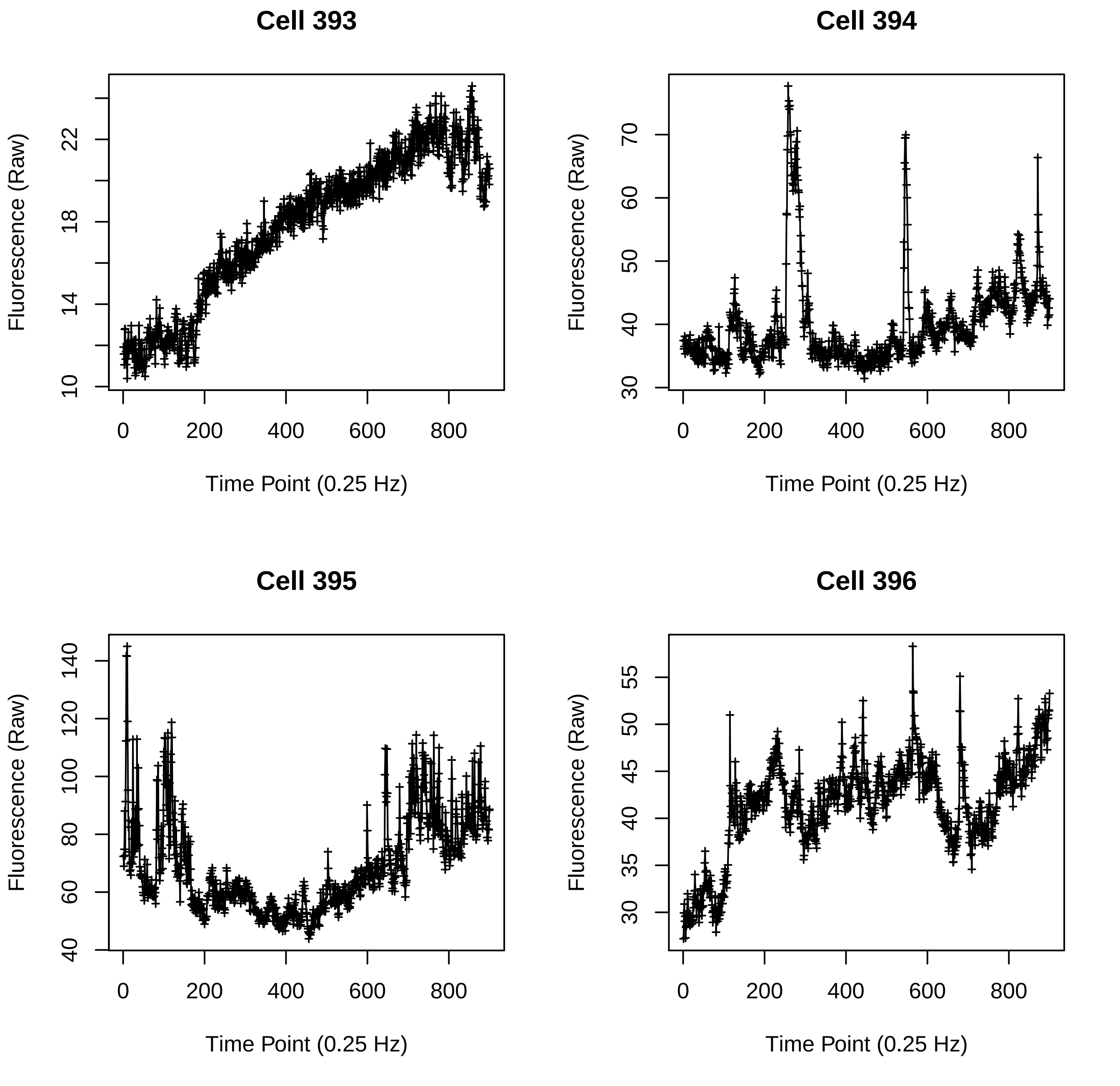
Cell 393
Fluorescence (Raw)
10
14
18
22
0
200
400
600
800
Time Point (0.25 Hz)
Cell 394
Fluorescence (Raw)
30
40
50
60
70
0
200
400
600
800
Time Point (0.25 Hz)
Cell 395
Fluorescence (Raw)
40
60
80
100
120
140
0
200
400
600
800
Time Point (0.25 Hz)
Cell 396
Fluorescence (Raw)
30
35
40
45
50
55
0
200
400
600
800
Time Point (0.25 Hz)

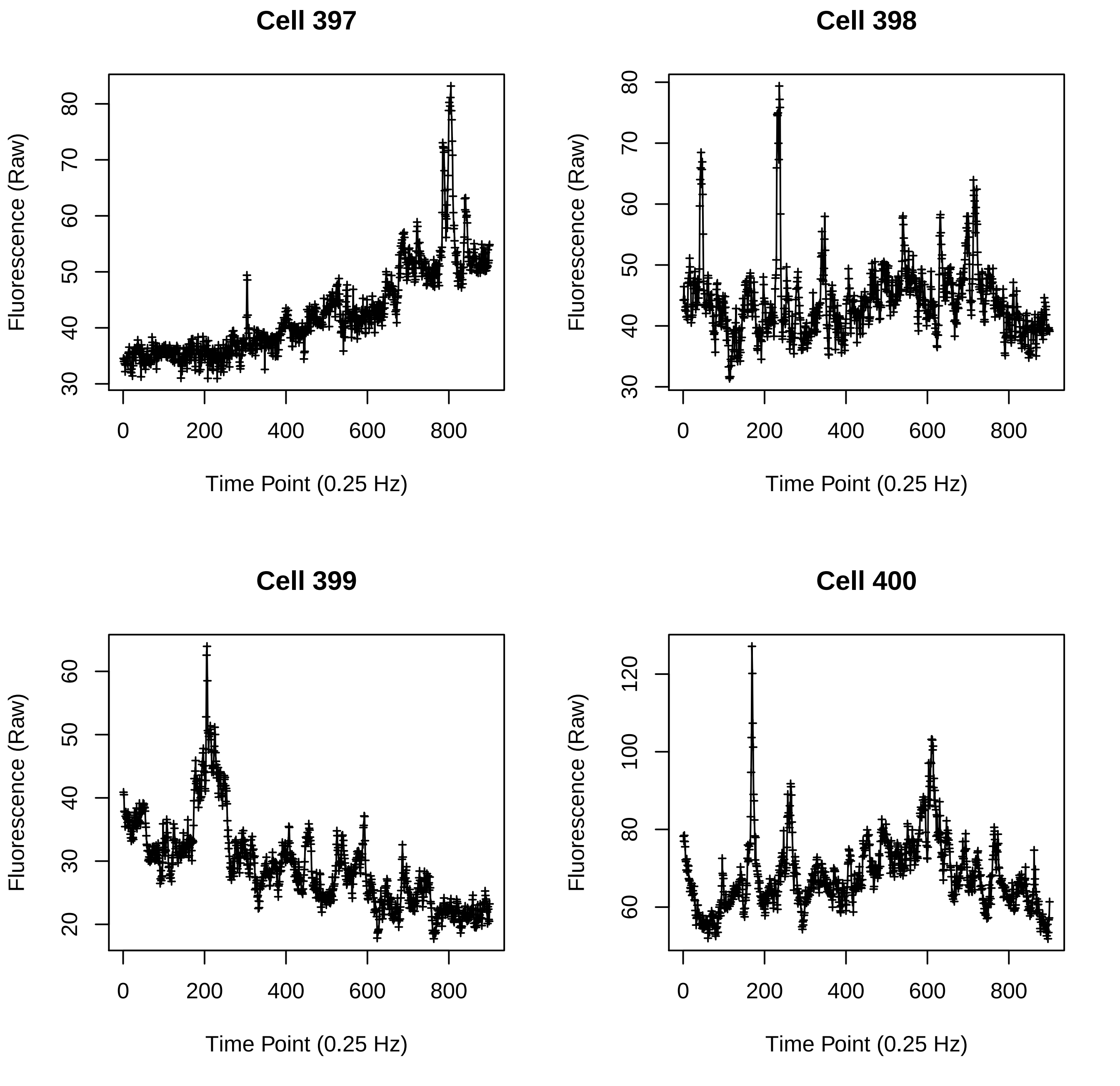

Cell 397
Cell 398
Cell 399
Cell 400
Fluorescence (Raw)
Time Point (0.25 Hz)
0
200
400
600
800
30
40
50
60
70
80
20
100
120

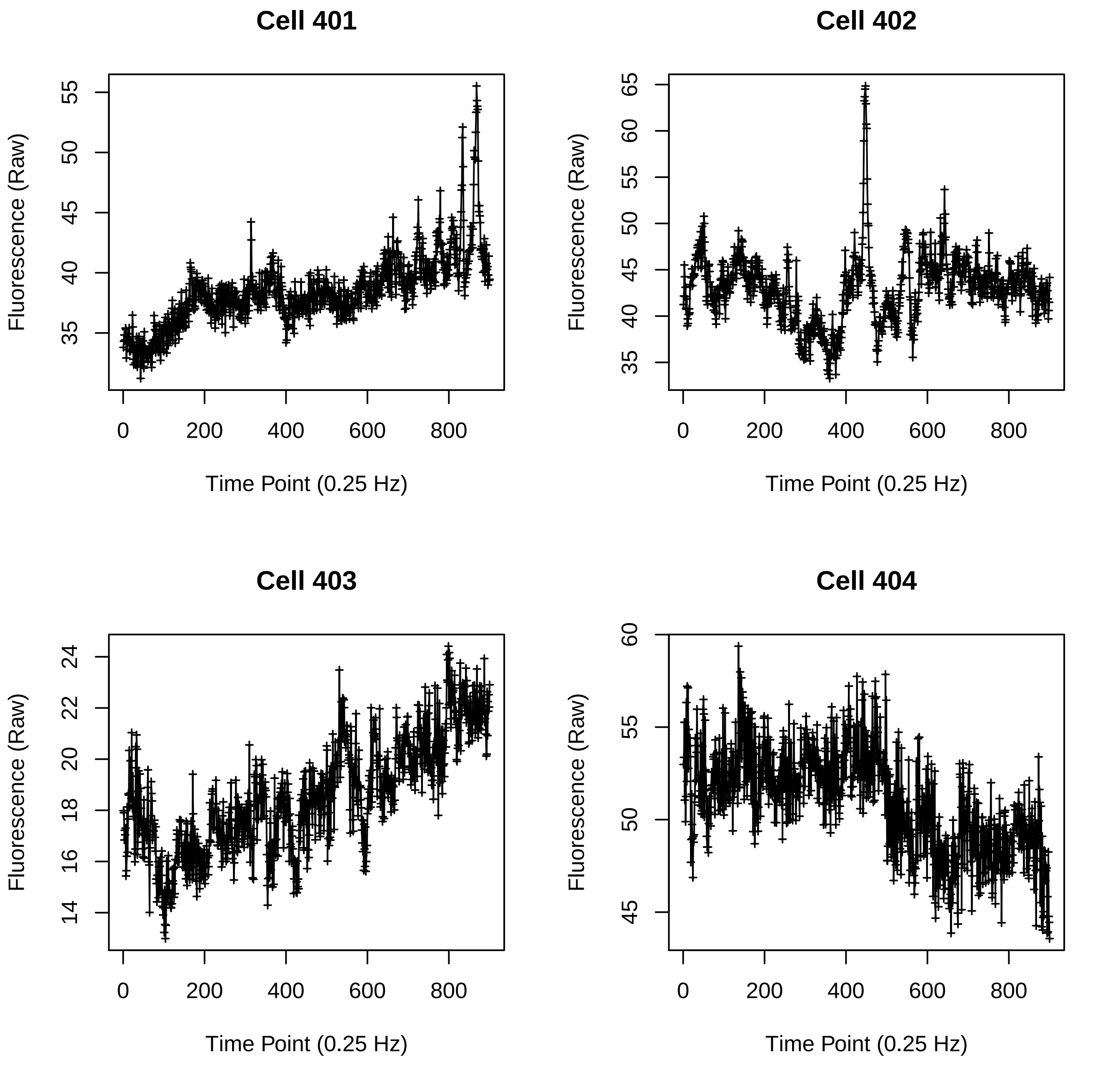
Cell 401
Cell 402
Cell 403
Cell 404
Fluorescence (Raw)
Time Point (0.25 Hz)
0
200
400
600
800
35
40
45
50
55
60
65
14
16
18
20
22
24

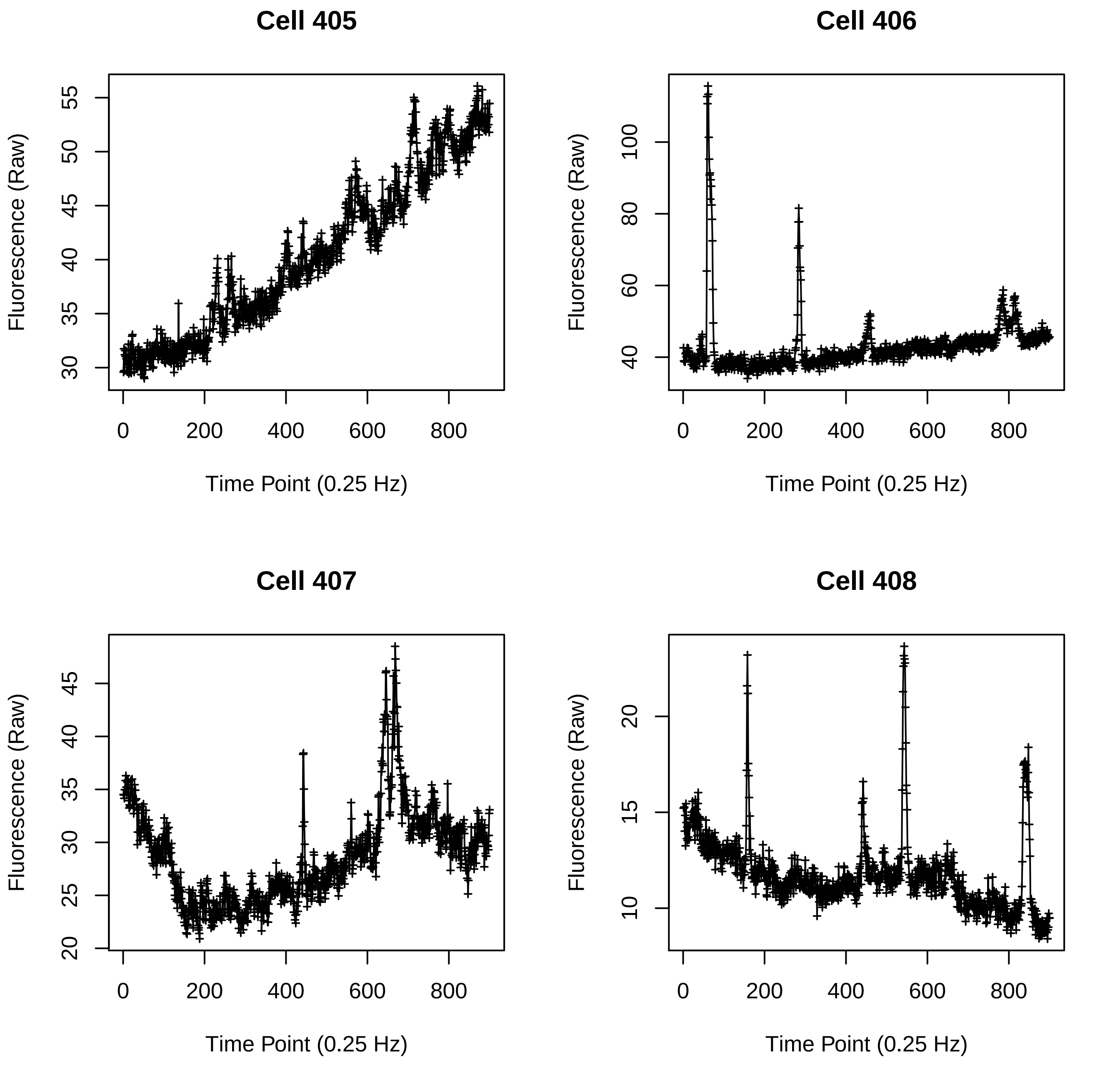
Cell 405
Fluorescence (Raw)
Time Point (0.25 Hz)
Cell 406
Fluorescence (Raw)
Time Point (0.25 Hz)
Cell 407
Fluorescence (Raw)
Time Point (0.25 Hz)
Cell 408
Fluorescence (Raw)
Time Point (0.25 Hz)

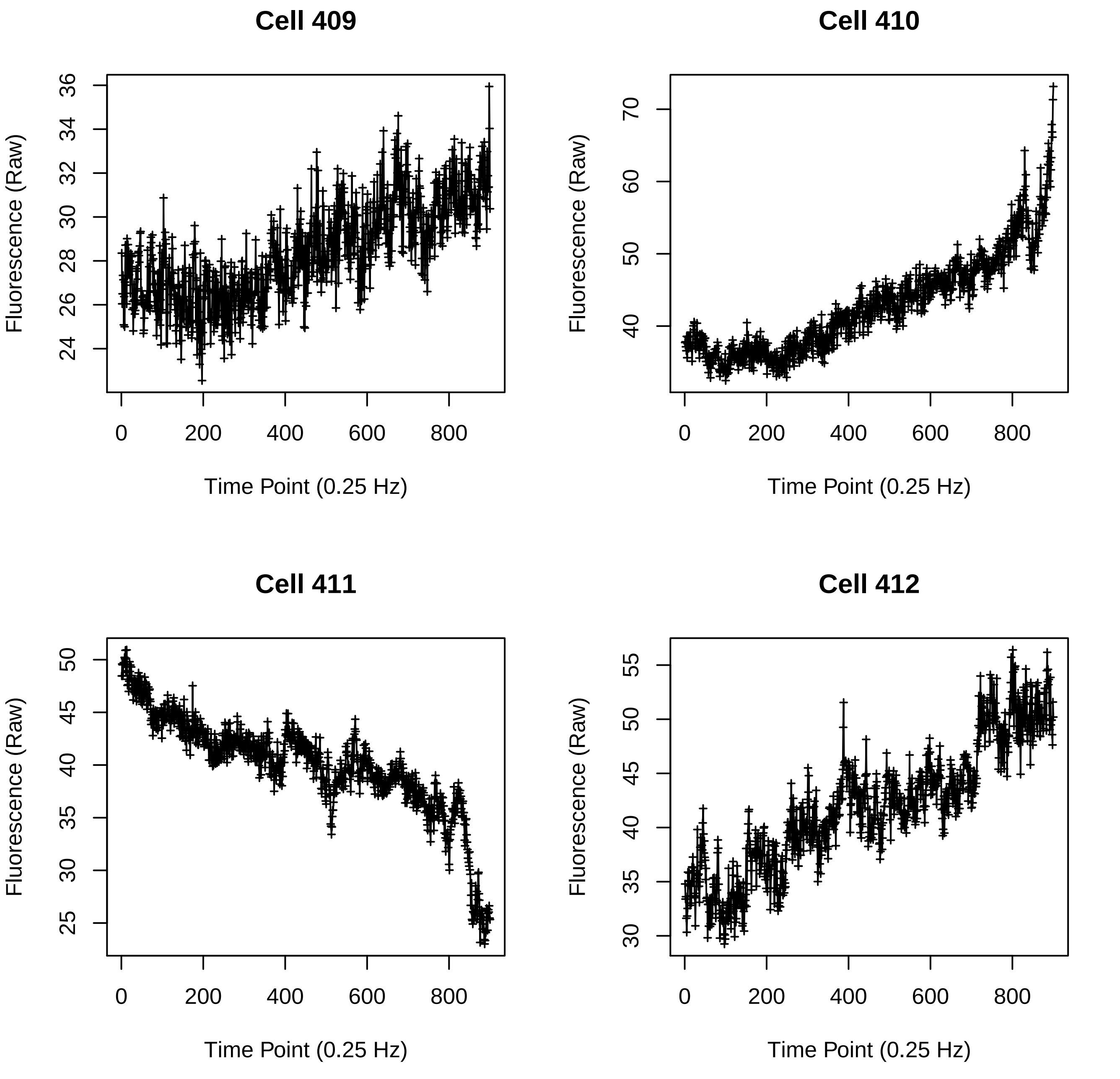
Cell 409
Fluorescence (Raw)
24
26
28
30
32
34
36
0
200
400
600
800
Time Point (0.25 Hz)
Cell 410
Fluorescence (Raw)
40
50
60
70
0
200
400
600
800
Time Point (0.25 Hz)
Cell 411
Fluorescence (Raw)
25
30
35
40
45
50
0
200
400
600
800
Time Point (0.25 Hz)
Cell 412
Fluorescence (Raw)
30
35
40
45
50
55
0
200
400
600
800
Time Point (0.25 Hz)

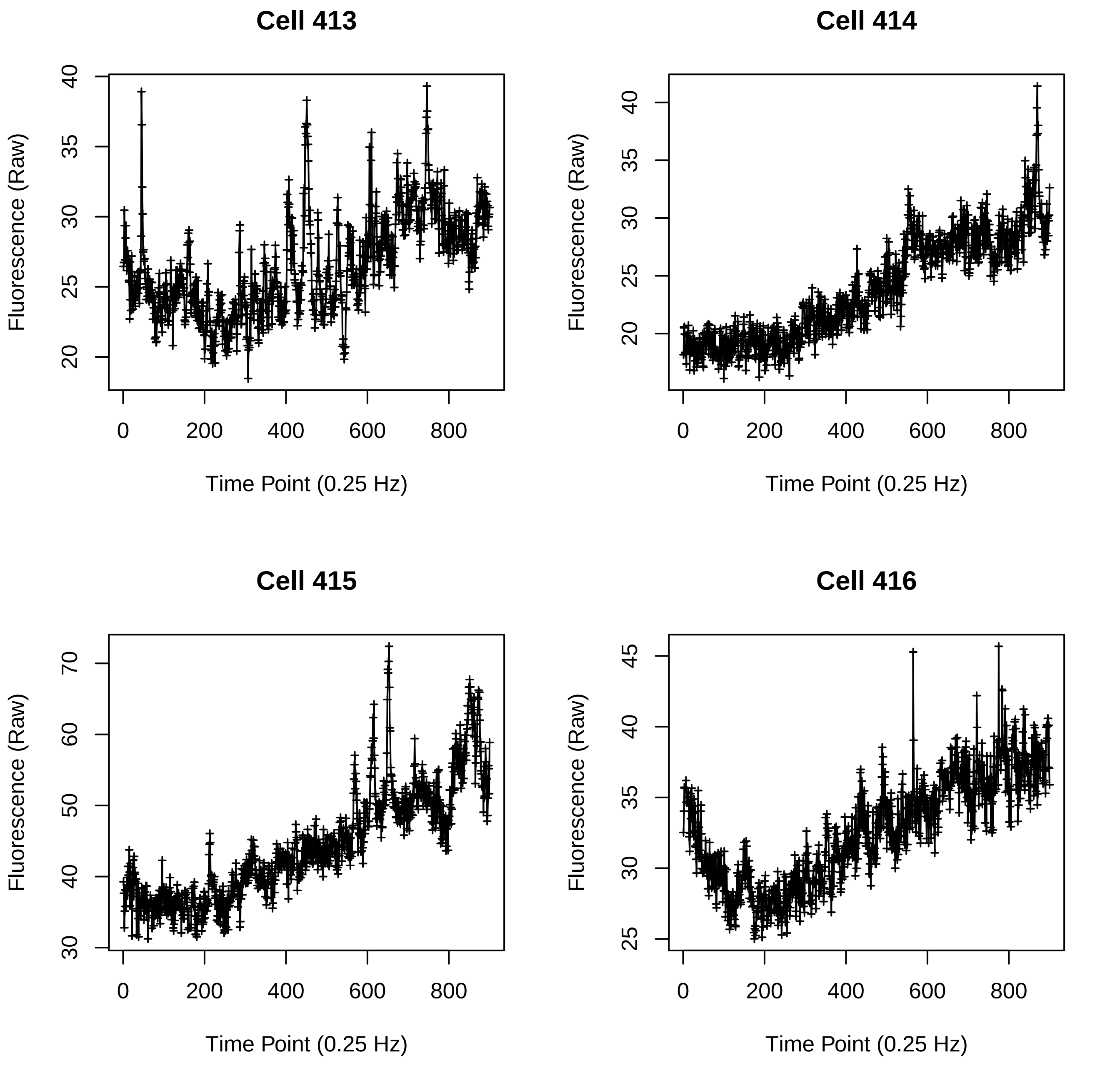

Cell 413
Cell 414
Cell 415
Cell 416
Fluorescence (Raw)
Time Point (0.25 Hz)
0
200
400
600
800
20
25
30
35
40
45
50
60
70

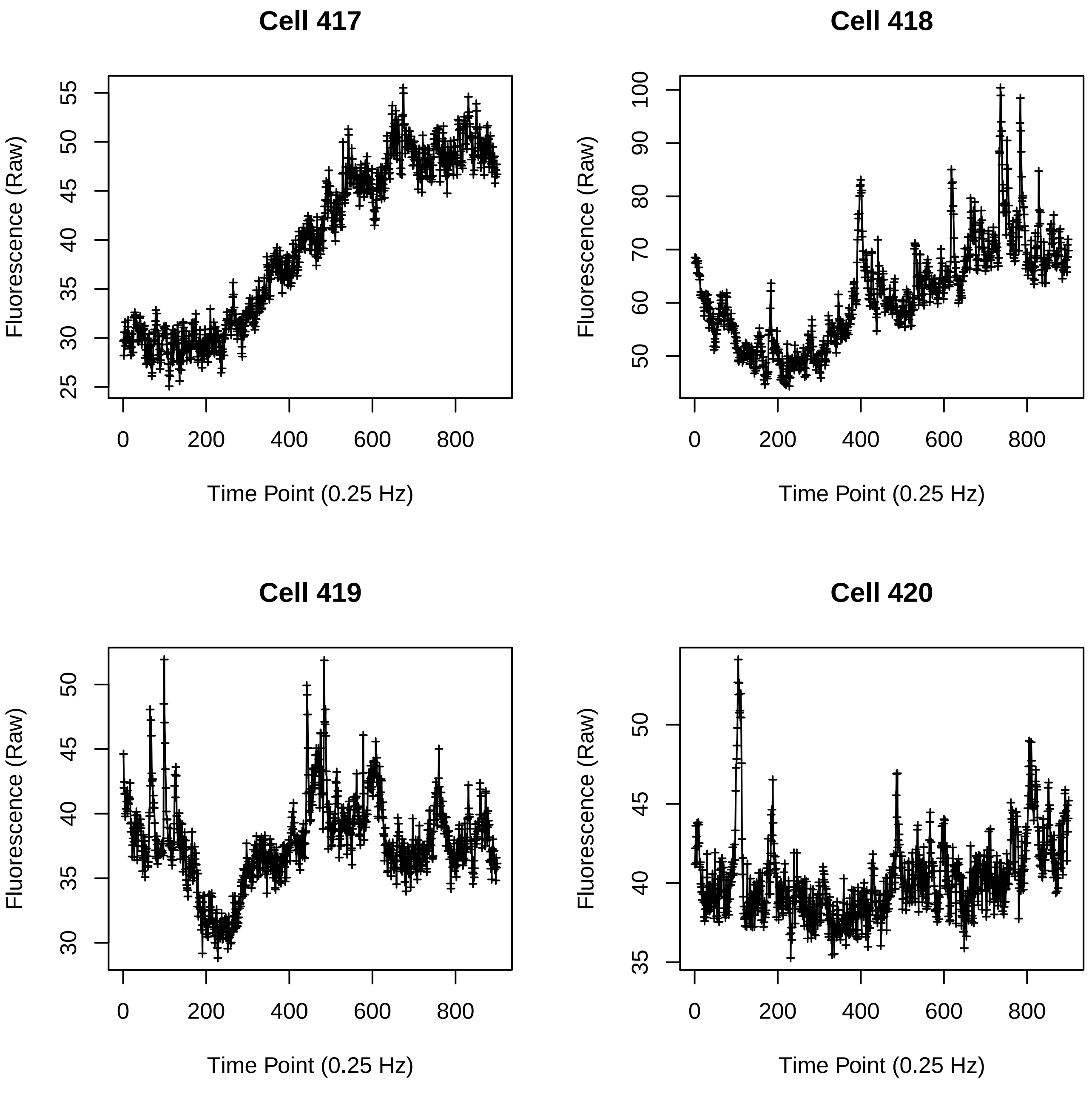
Cell 417
Fluorescence (Raw)
25
30
35
40
45
50
55
0
200
400
600
800
Time Point (0.25 Hz)
Cell 418
Fluorescence (Raw)
50
60
70
80
90
100
0
200
400
600
800
Time Point (0.25 Hz)
Cell 419
Fluorescence (Raw)
30
35
40
45
50
0
200
400
600
800
Time Point (0.25 Hz)
Cell 420
Fluorescence (Raw)
35
40
45
50
0
200
400
600
800
Time Point (0.25 Hz)

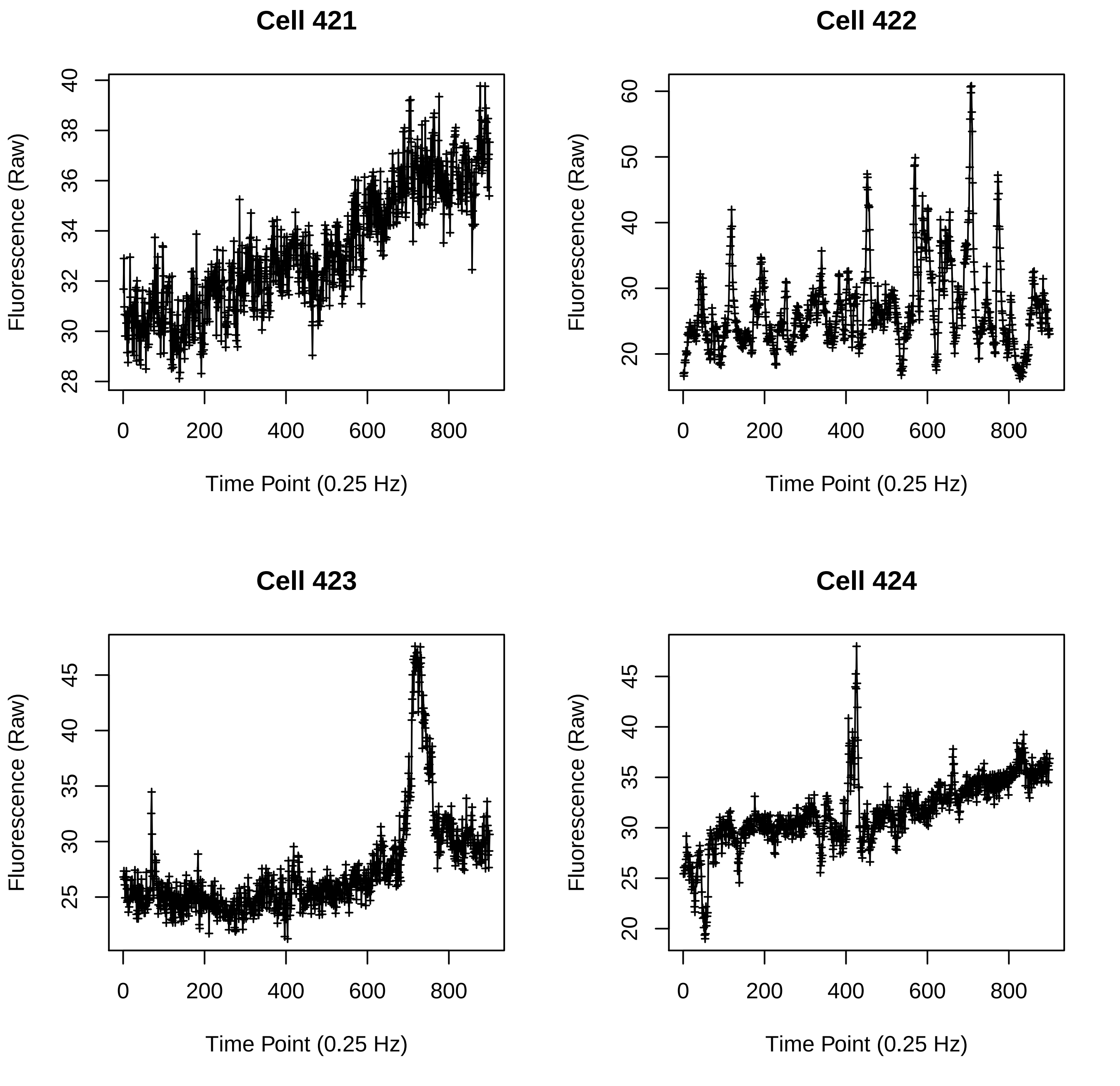
Cell 421
Cell 422
Cell 423
Cell 424
Fluorescence (Raw)
Time Point (0.25 Hz)
0
200
400
600
800
28
30
32
34
36
38
40
20
30
40
50
60
25
35
45

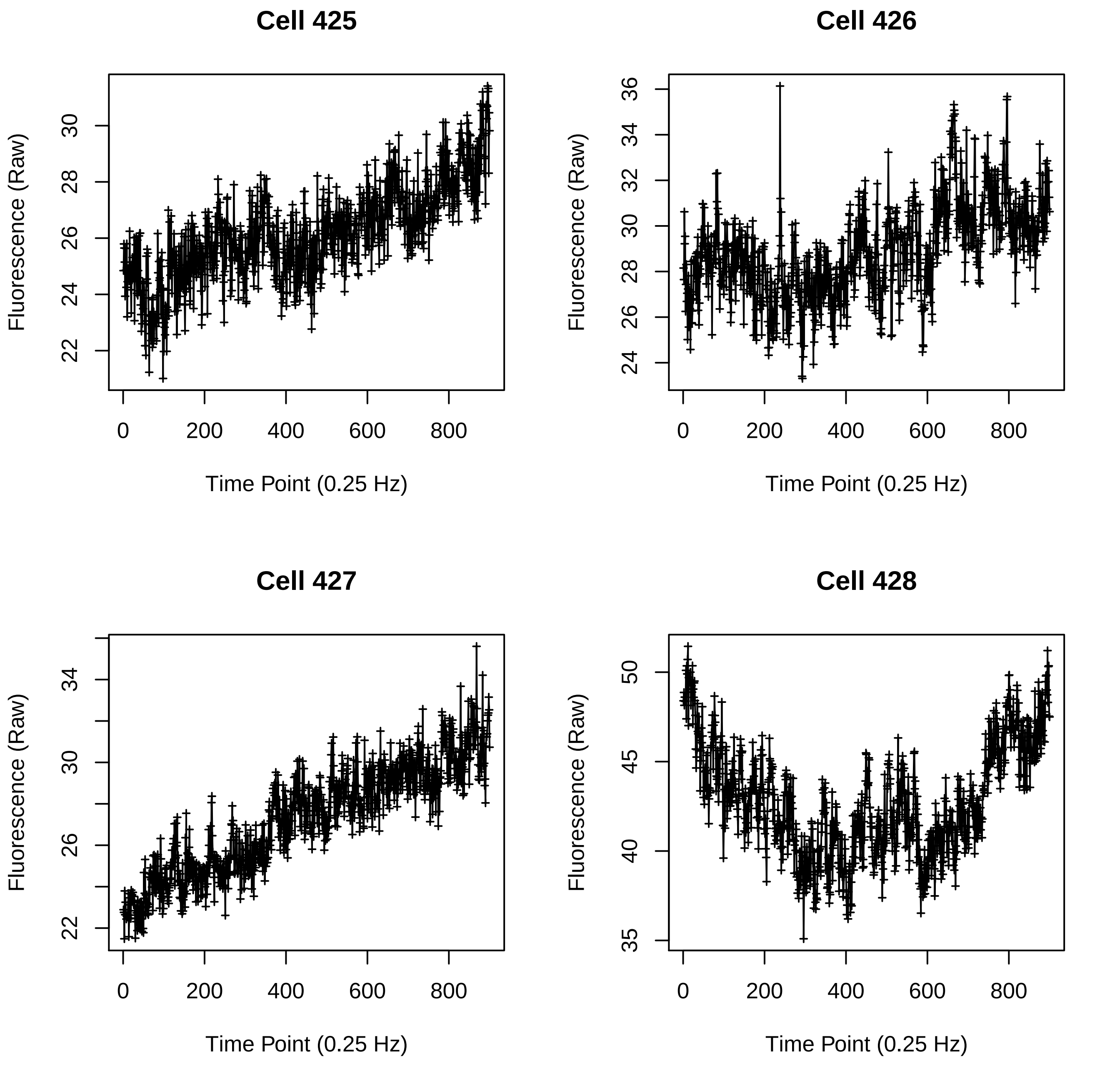
Cell 425
Cell 426
Cell 427
Cell 428
Fluorescence (Raw)
Time Point (0.25 Hz)
0
200
400
600
800
22
24
26
28
30
32
34
36
35
40
45
50

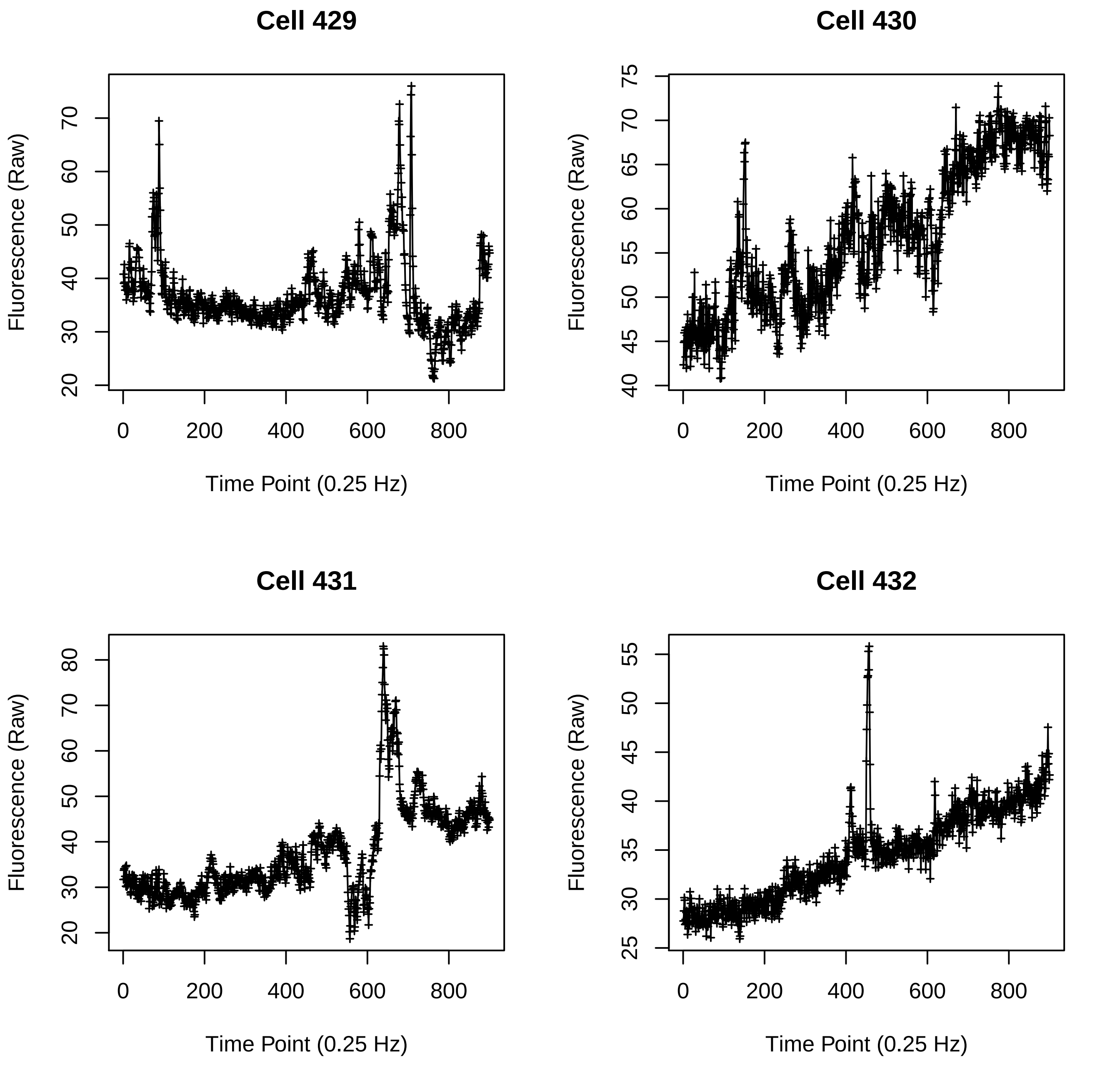
Cell 429
Cell 430
Cell 431
Cell 432
Fluorescence (Raw)
Time Point (0.25 Hz)
0
200
400
600
800
20
30
40
50
60
70
75
25
35
45
55
65
80

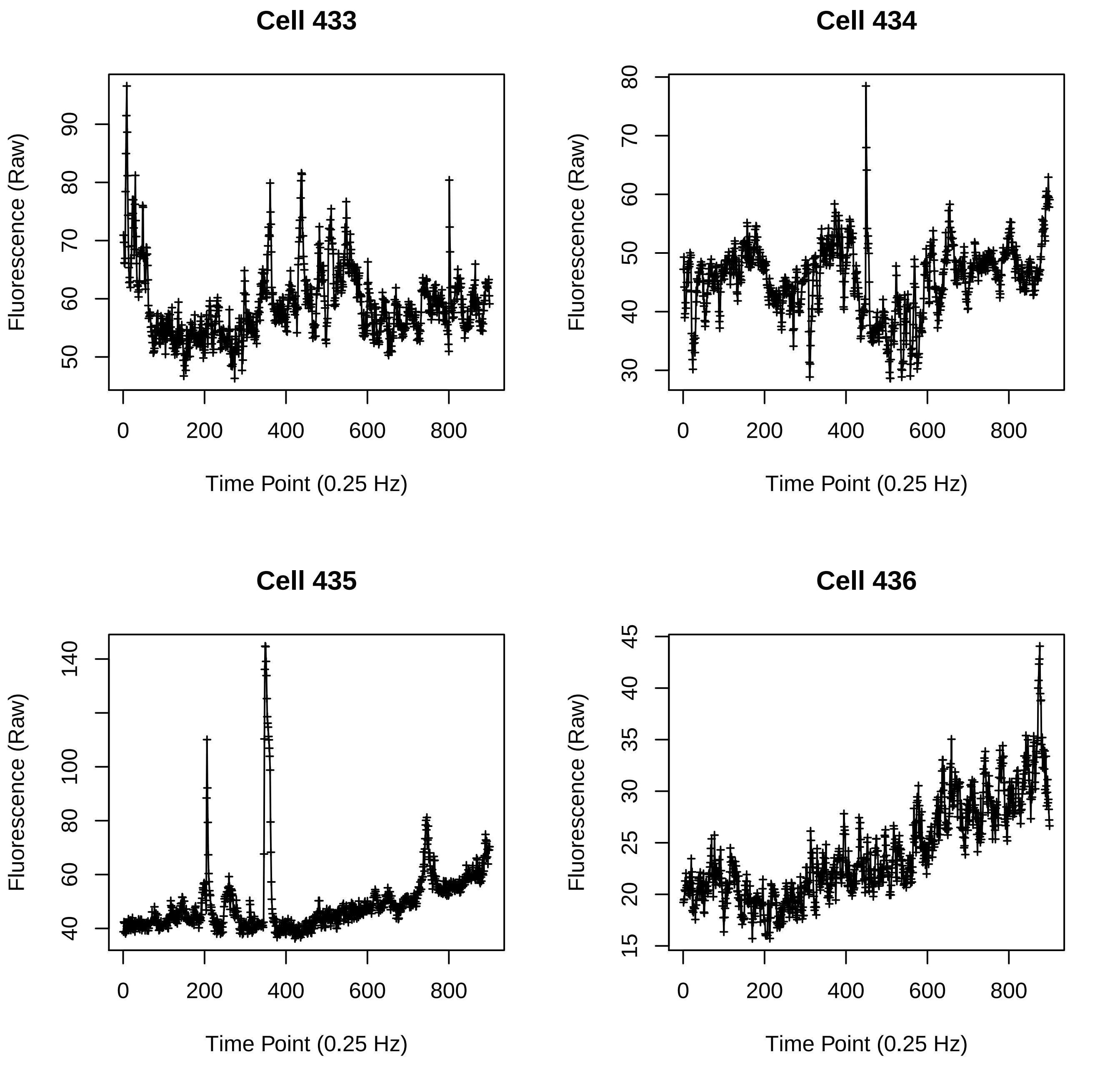
Cell 433
Fluorescence (Raw)
50
60
70
80
90
0
200
400
600
800
Time Point (0.25 Hz)
Cell 434
Fluorescence (Raw)
30
40
50
60
70
80
0
200
400
600
800
Time Point (0.25 Hz)
Cell 435
Fluorescence (Raw)
40
60
80
100
140
0
200
400
600
800
Time Point (0.25 Hz)
Cell 436
Fluorescence (Raw)
15
20
25
30
35
40
45
0
200
400
600
800
Time Point (0.25 Hz)

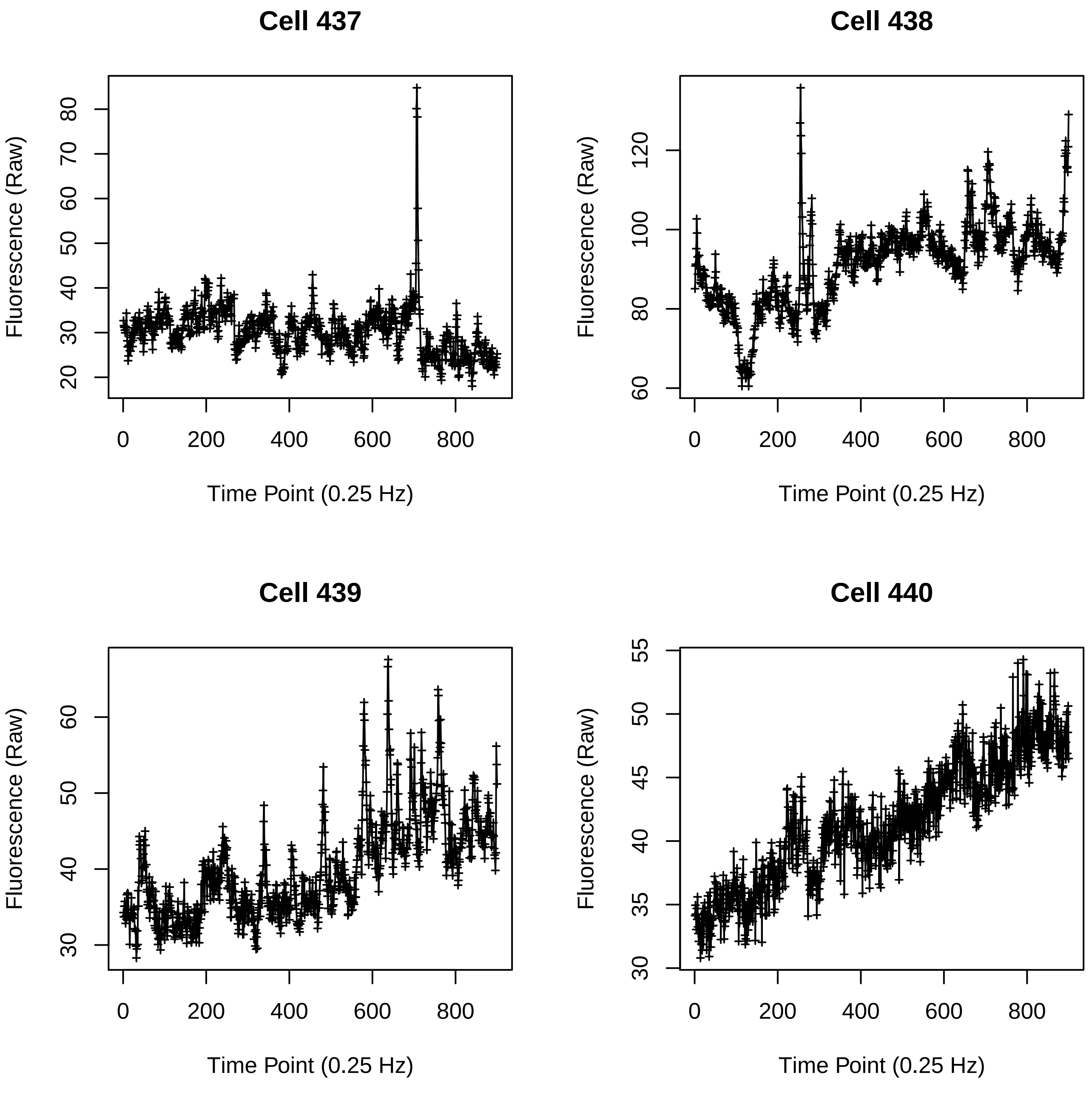
Cell 437
Cell 438
Cell 439
Cell 440
Fluorescence (Raw)
Time Point (0.25 Hz)
0
200
400
600
800
20
30
40
50
60
70
80
100
120
35
45
55

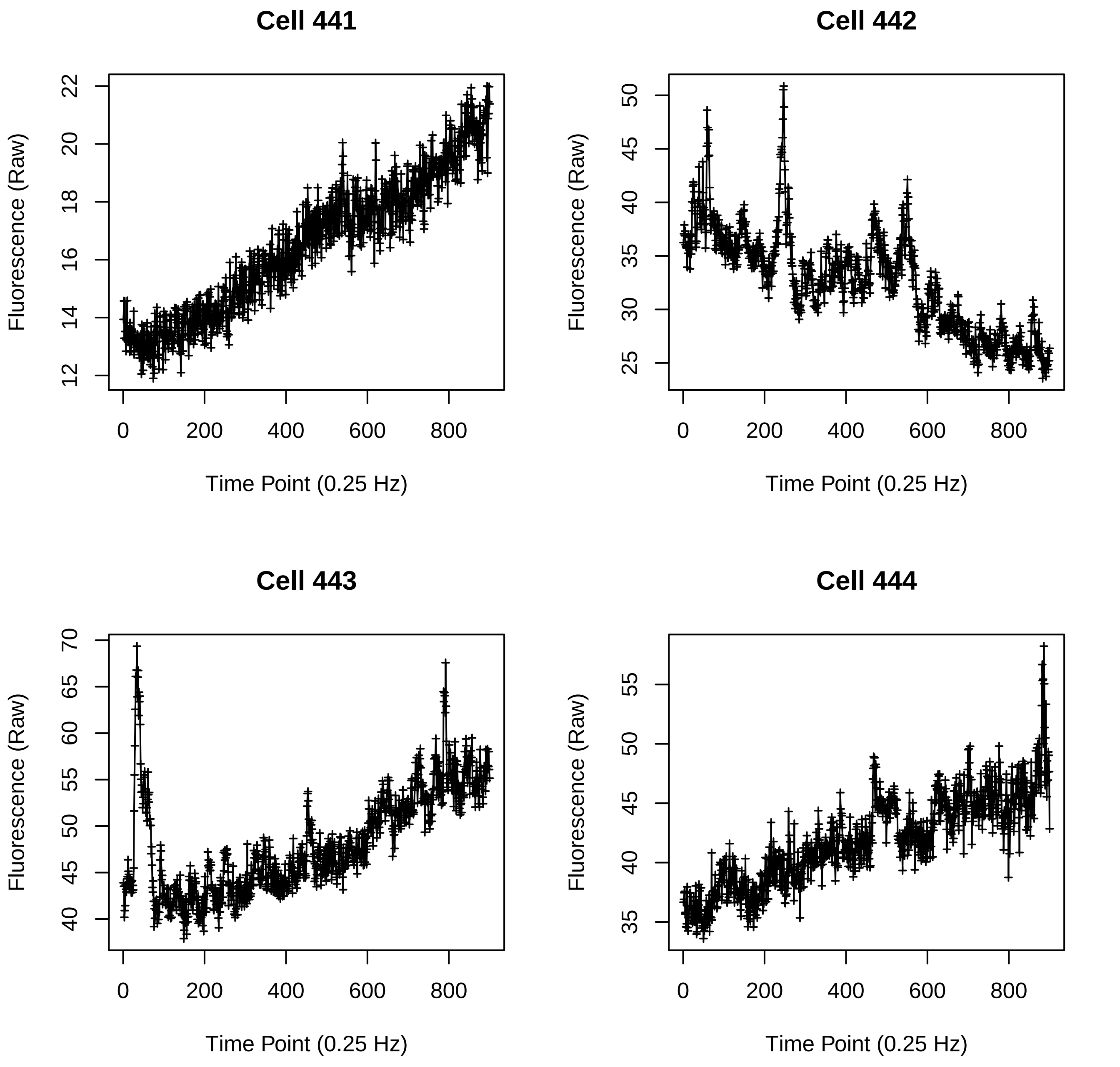
Cell 441
Cell 442
Cell 443
Cell 444
Fluorescence (Raw)
Time Point (0.25 Hz)
0
200
400
600
800
12
14
16
18
20
22
25
30
35
40
45
50
55
60
65
70

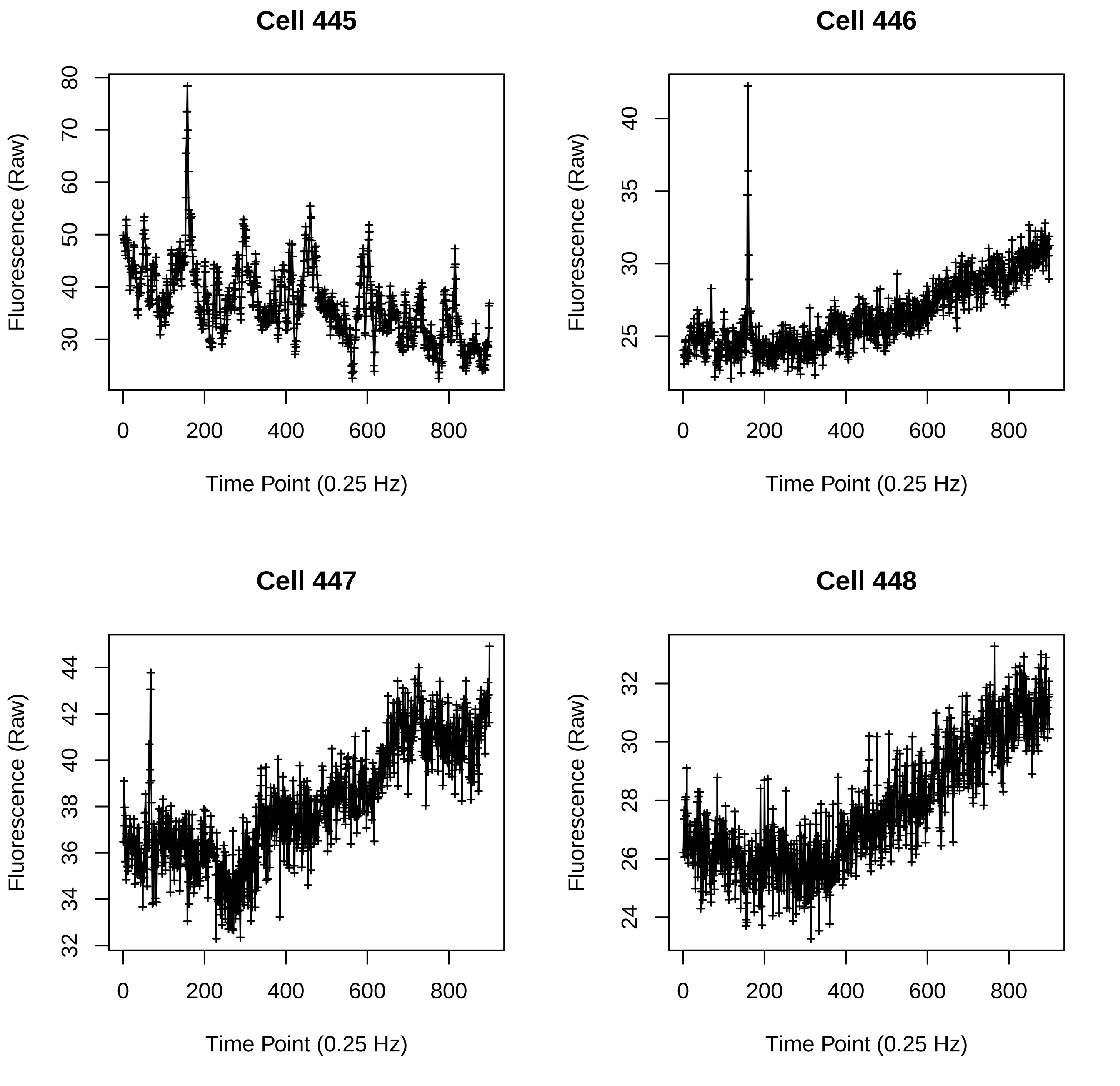

Cell 445
Cell 446
Cell 447
Cell 448
Fluorescence (Raw)
Time Point (0.25 Hz)
0
200
400
600
800
30
40
50
60
70
80
25
35
32
34
36
38
42
44
24
26
28

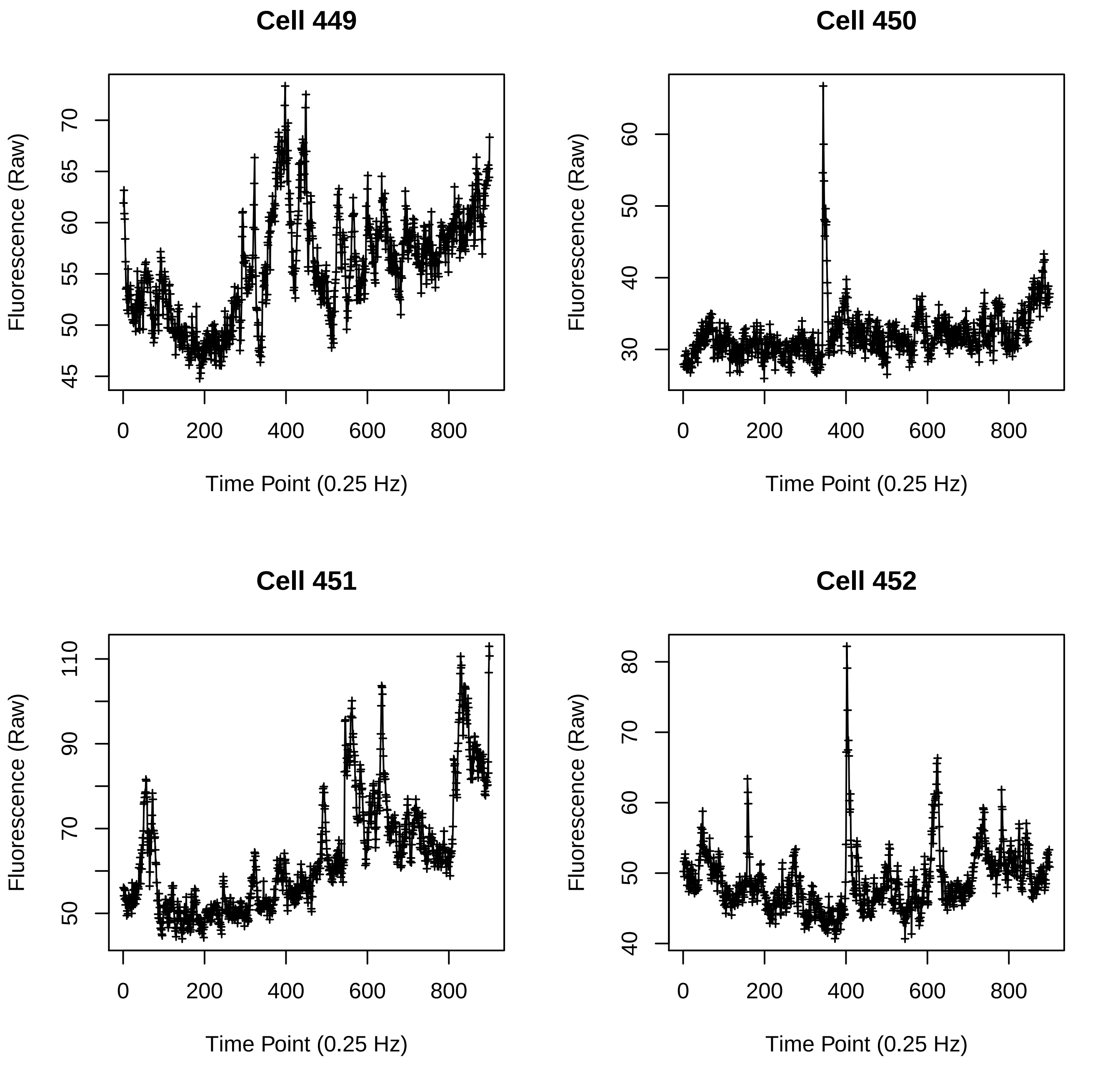
Cell 449
Cell 450
Cell 451
Cell 452
Fluorescence (Raw)
Time Point (0.25 Hz)
0
200
400
600
800
45
50
55
60
65
70
30
40
50
60
90
110
80

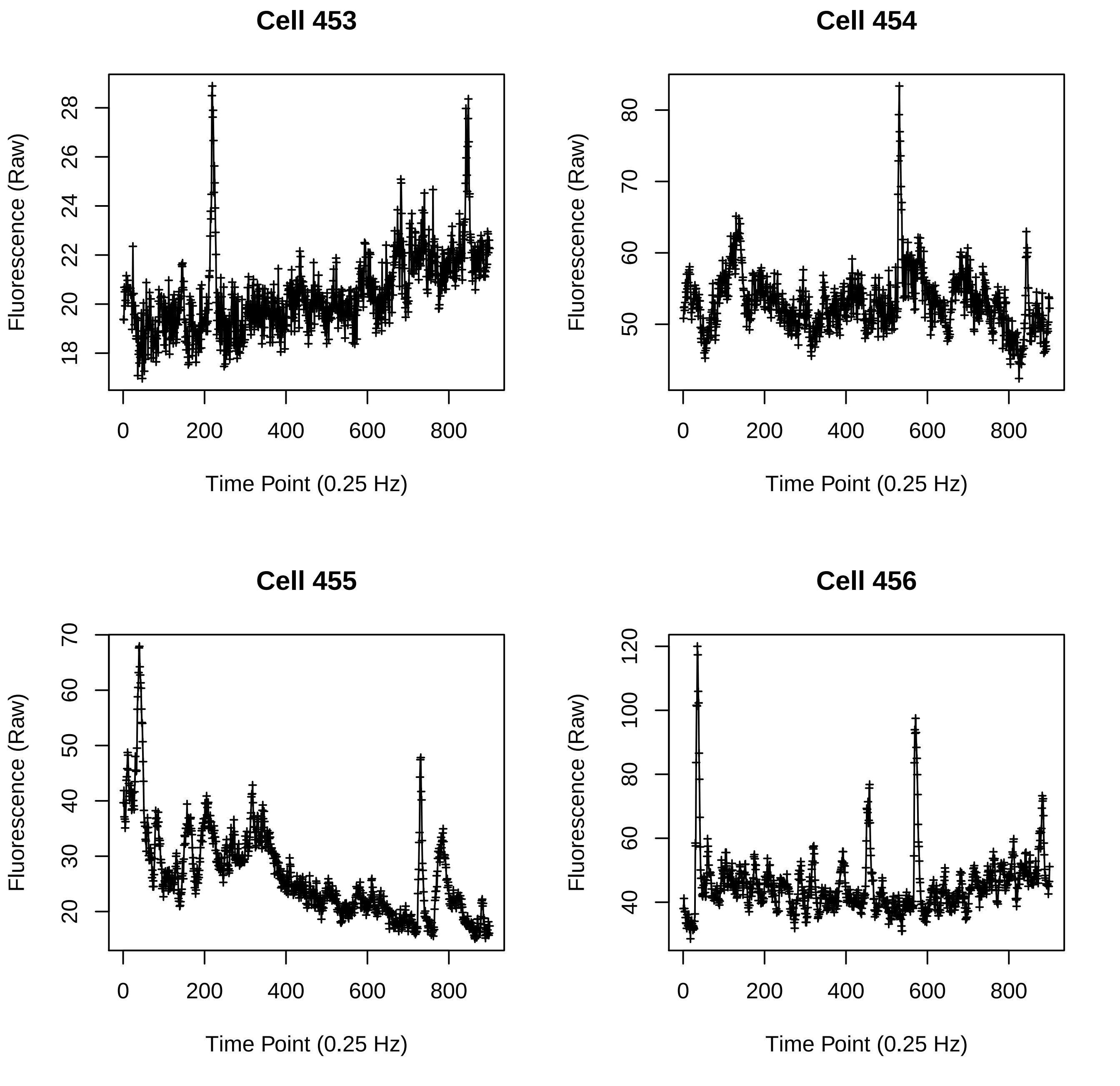
Cell 453
Cell 454
Cell 455
Cell 456
Fluorescence (Raw)
Time Point (0.25 Hz)
0
200
400
600
800
18
20
22
24
26
28
50
60
70
80
20
30
40
50
60
70
40
60
80
100
120

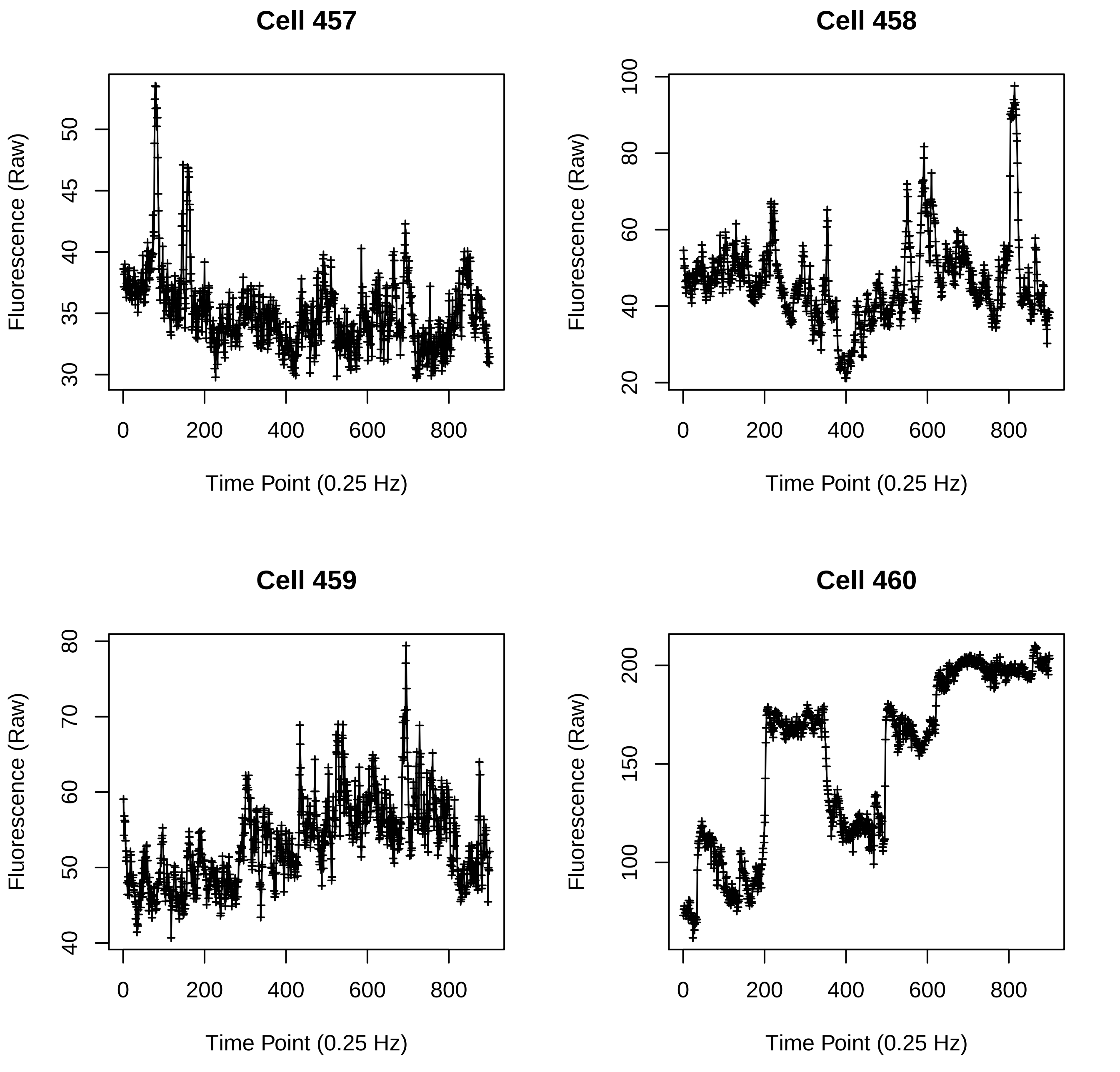
Cell 457
Fluorescence (Raw)
30
35
40
45
50
0
200
400
600
800
Time Point (0.25 Hz)
Cell 458
Fluorescence (Raw)
20
40
60
80
100
0
200
400
600
800
Time Point (0.25 Hz)
Cell 459
Fluorescence (Raw)
40
50
60
70
80
0
200
400
600
800
Time Point (0.25 Hz)
Cell 460
Fluorescence (Raw)
100
150
200
0
200
400
600
800
Time Point (0.25 Hz)

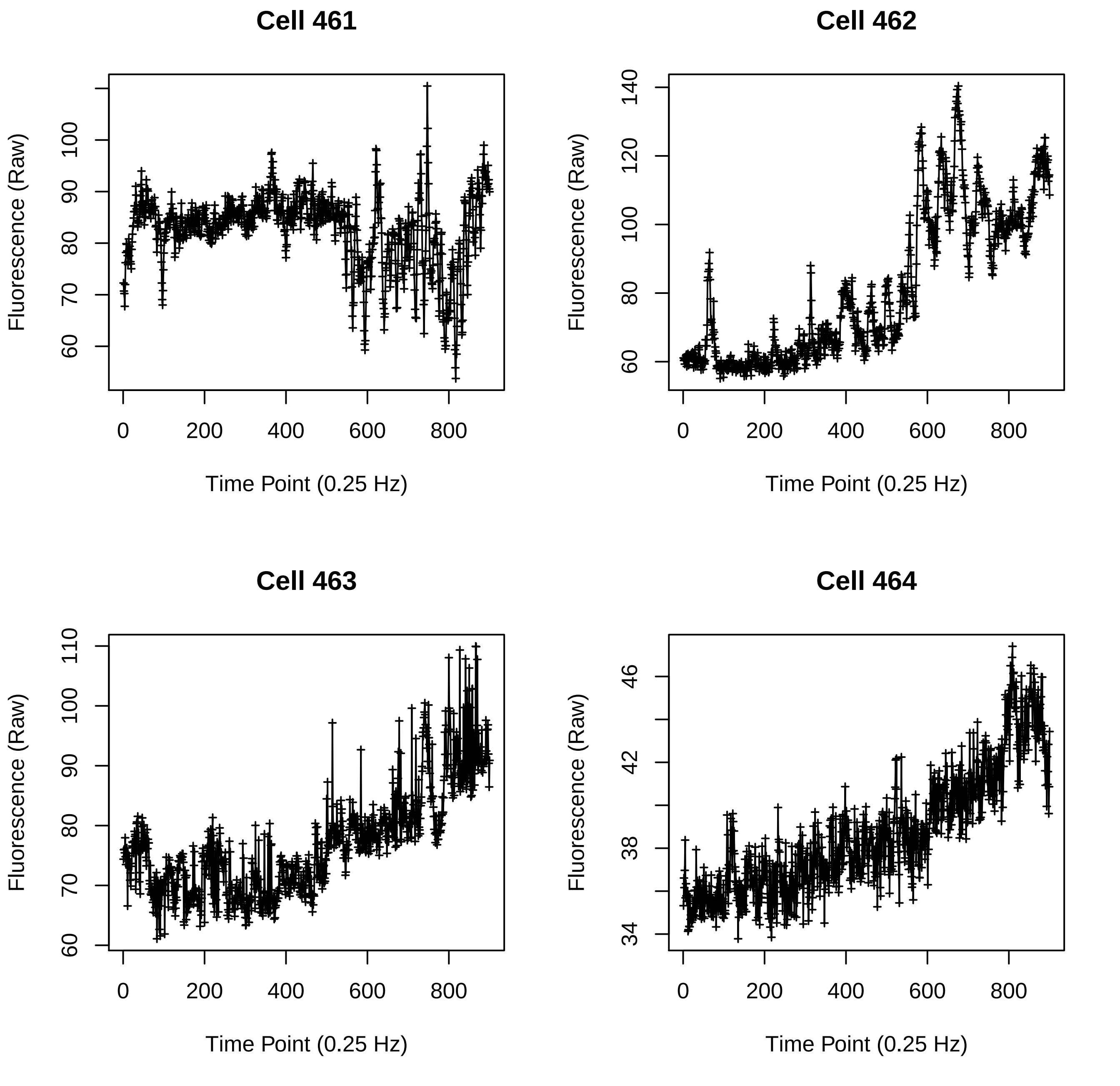

Cell 461
Cell 462
Cell 463
Cell 464
Fluorescence (Raw)
Time Point (0.25 Hz)
0
200
400
600
800
60
70
80
90
100
120
140
110
34
38
42
46

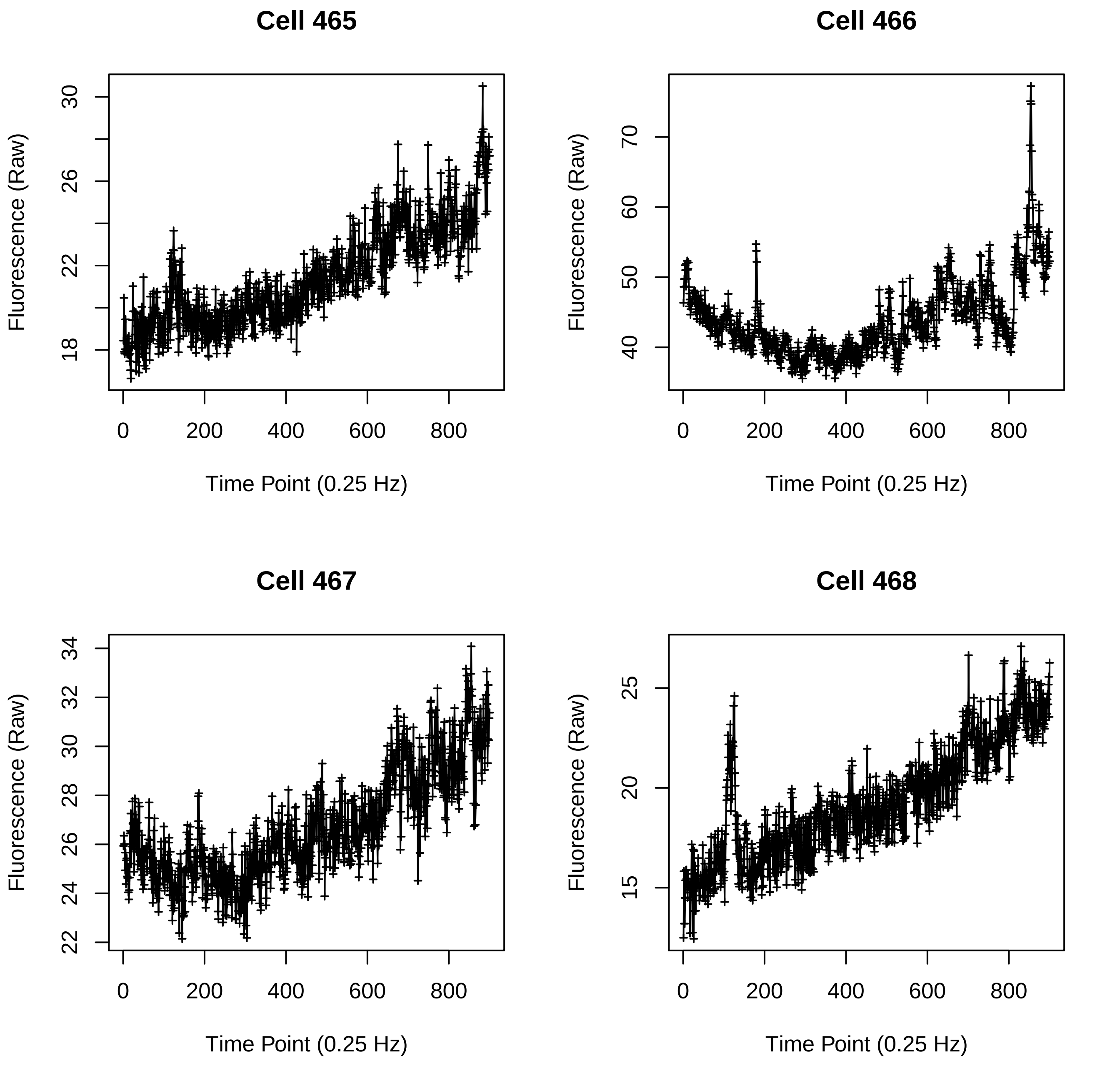
Cell 465
Cell 466
Cell 467
Cell 468
Fluorescence (Raw)
Time Point (0.25 Hz)
0
200
400
600
800
18
22
26
30
40
50
60
70
22
24
26
28
30
32
34
15
20
25

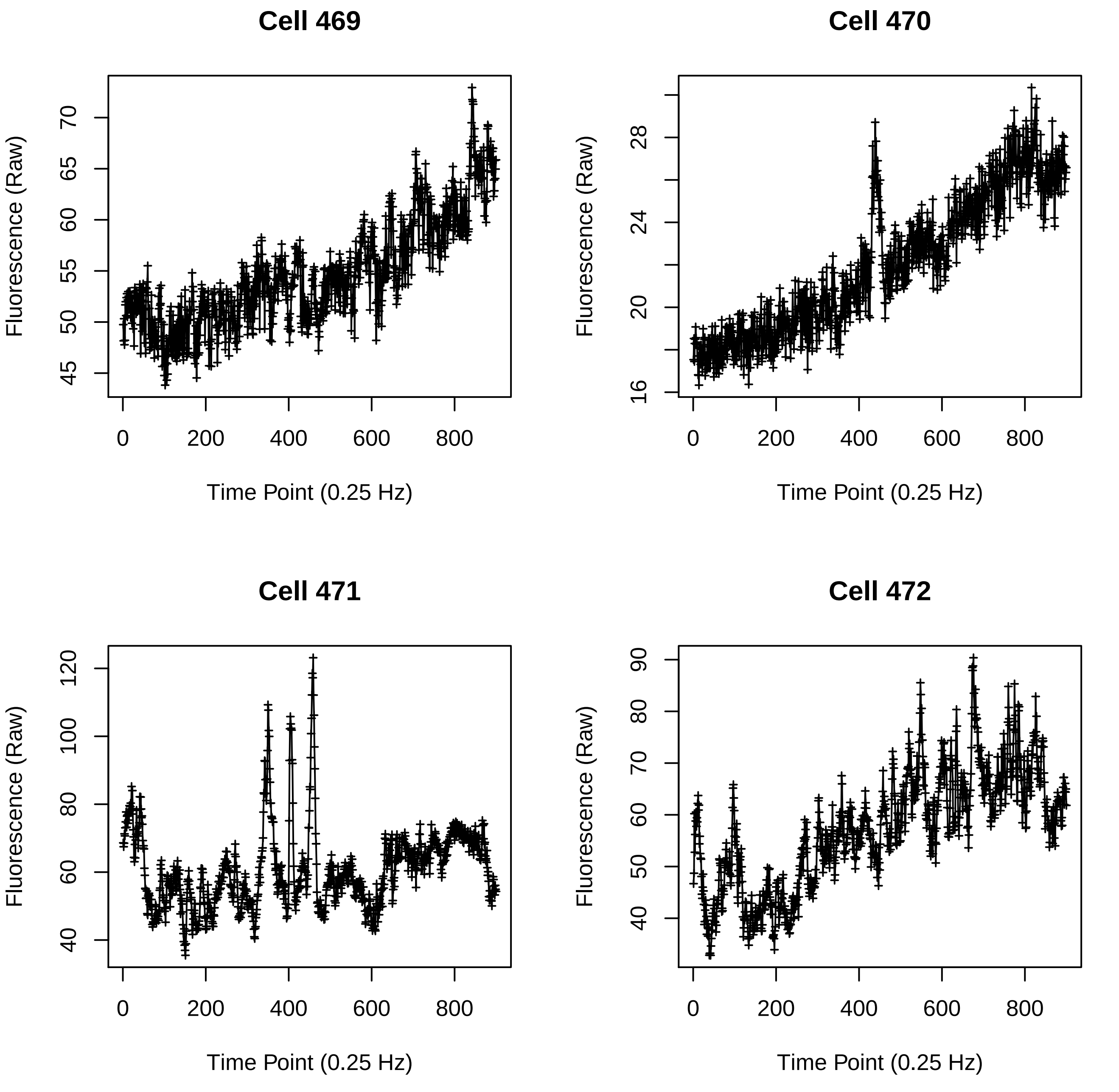
Cell 469
Fluorescence (Raw)
45
50
55
60
65
70
0
200
400
600
800
Time Point (0.25 Hz)
Cell 470
Fluorescence (Raw)
16
20
24
28
0
200
400
600
800
Time Point (0.25 Hz)
Cell 471
Fluorescence (Raw)
40
60
80
100
120
0
200
400
600
800
Time Point (0.25 Hz)
Cell 472
Fluorescence (Raw)
40
50
60
70
80
90
0
200
400
600
800
Time Point (0.25 Hz)

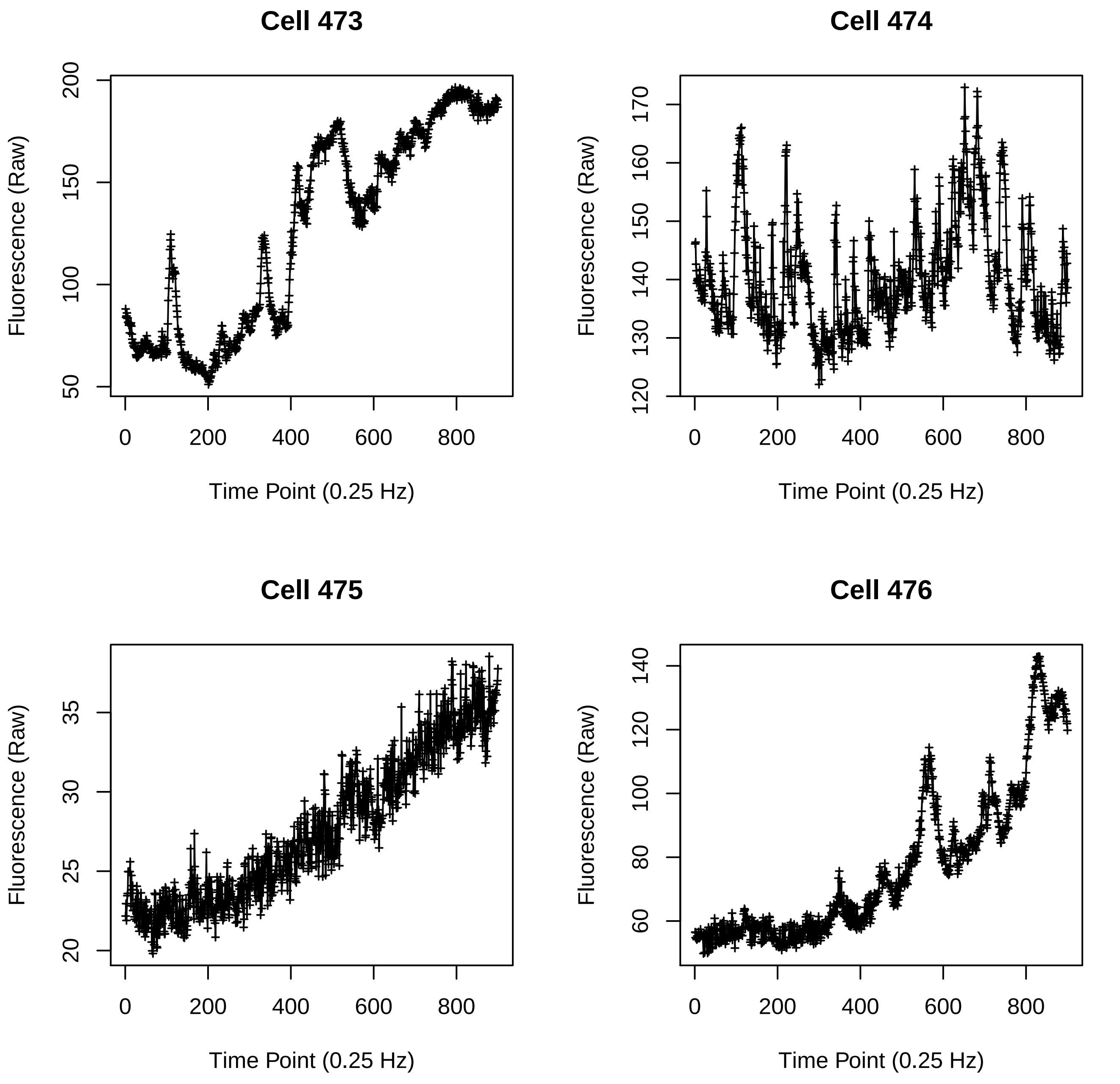
Cell 473
Cell 474
Cell 475
Cell 476
Fluorescence (Raw)
Time Point (0.25 Hz)
0
200
400
600
800
50
100
150
200
120
130
140
150
160
170
20
25
30
35
60
80
100
120
140

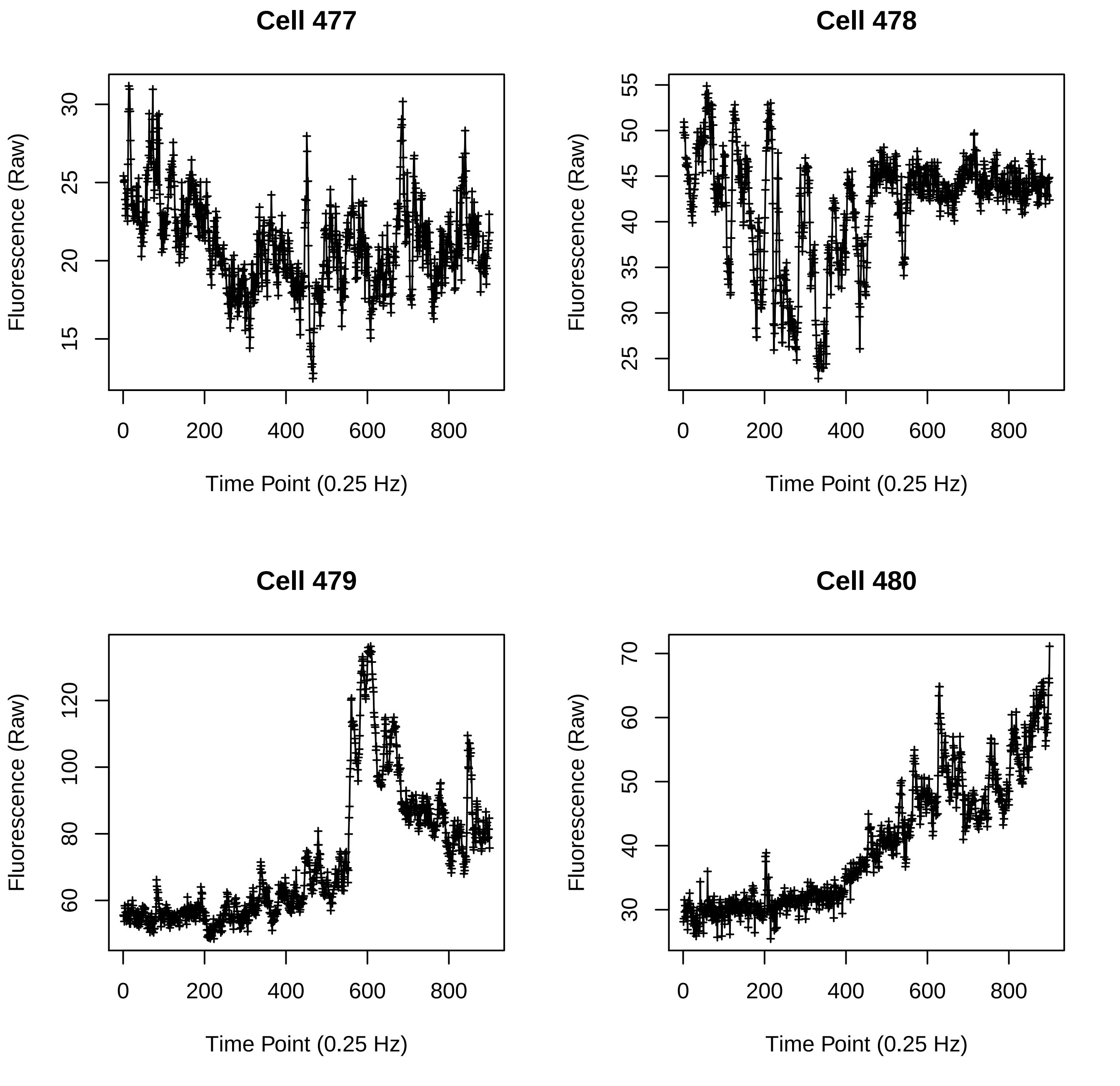

Cell 477
Cell 478
Cell 479
Cell 480
Fluorescence (Raw)
Time Point (0.25 Hz)
0
200
400
600
800
15
20
25
30
35
40
45
50
55
60
70
80
100
120

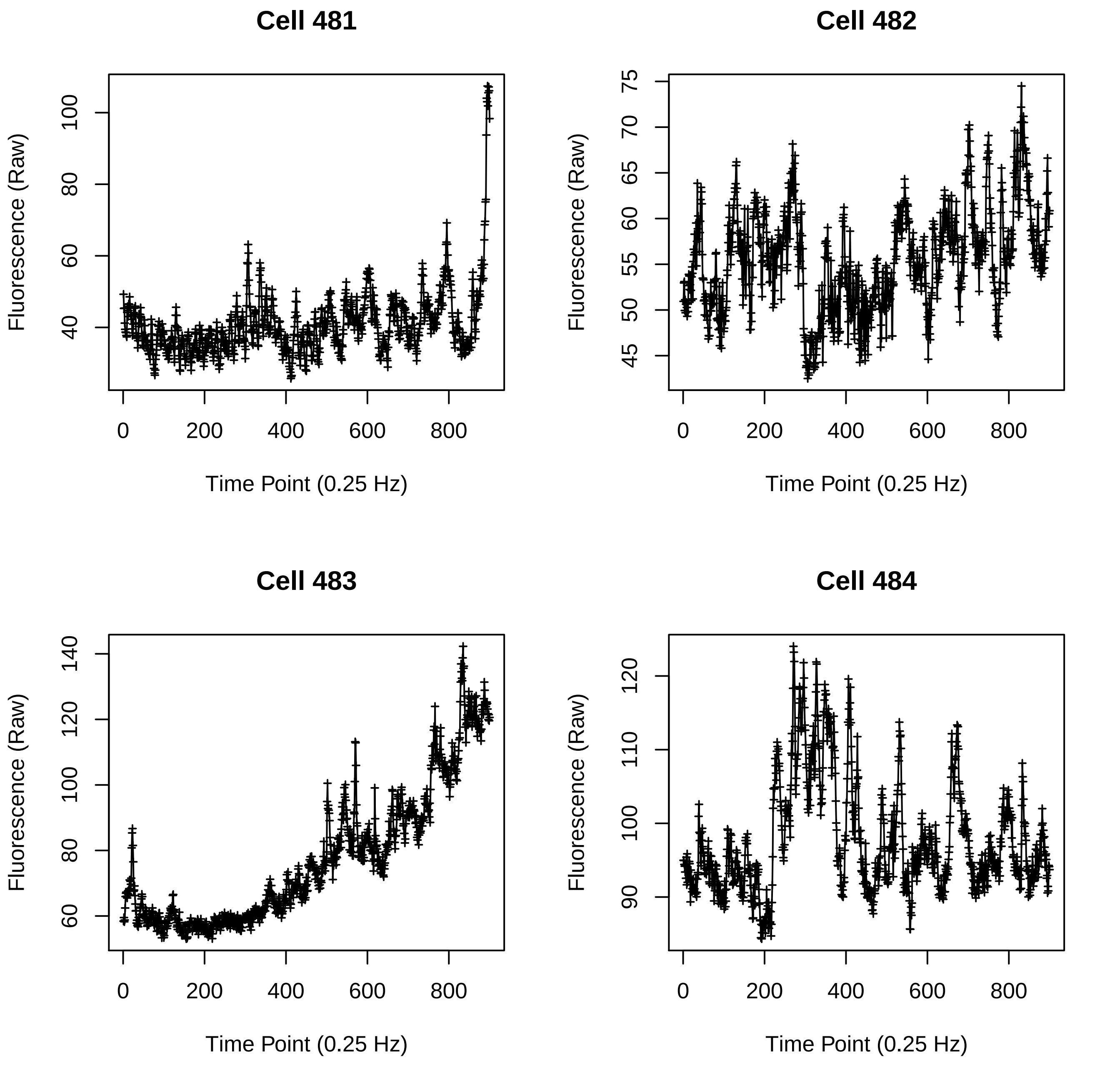
Cell 481
Cell 482
Cell 483
Cell 484
Fluorescence (Raw)
Time Point (0.25 Hz)
0
200
400
600
800
40
60
80
100
45
50
55
60
65
70
75
60
80
100
120
140
90
100
110
120

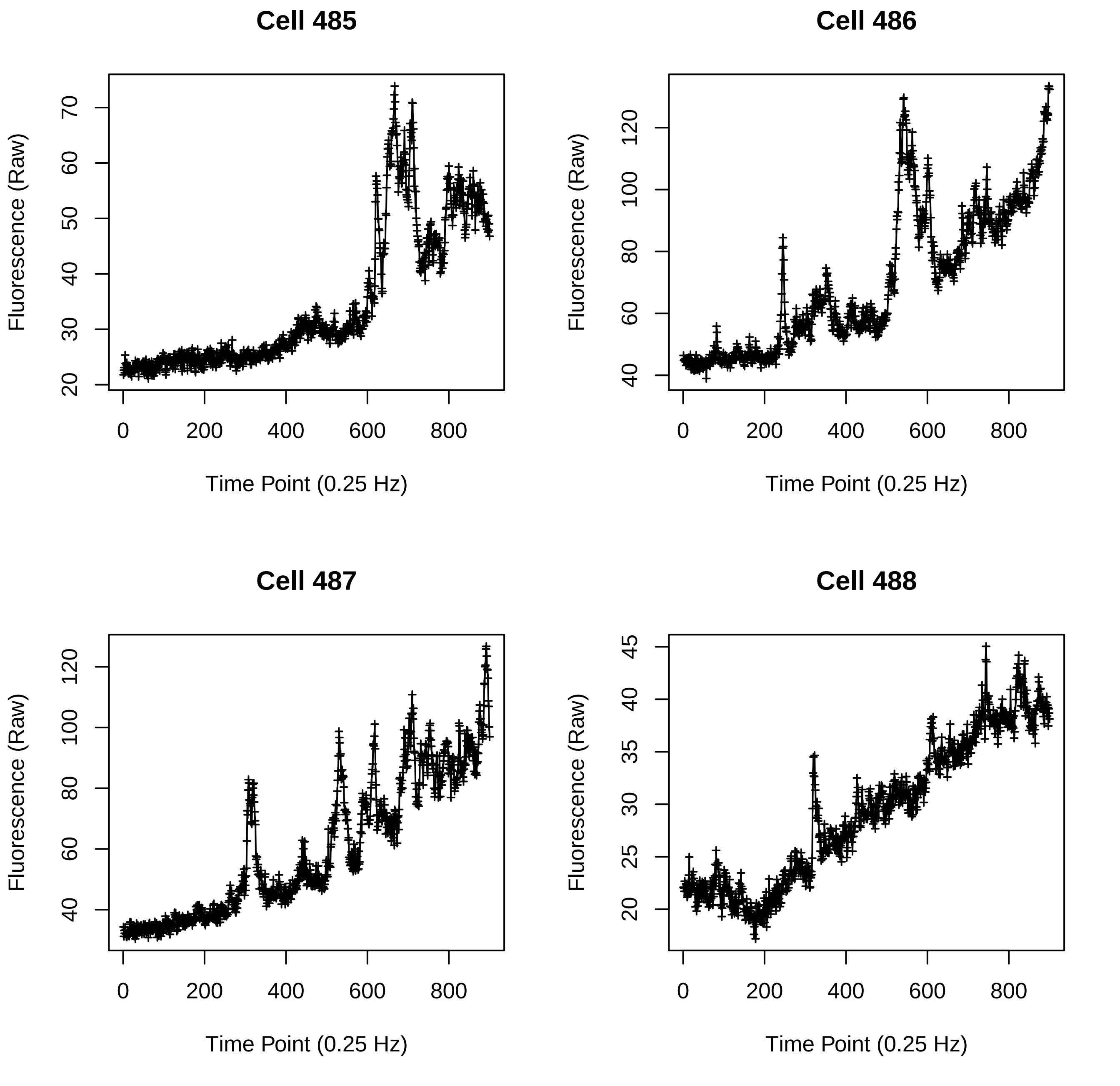
Cell 485
Cell 486
Cell 487
Cell 488
Fluorescence (Raw)
Time Point (0.25 Hz)
0
200
400
600
800
20
30
40
50
60
70
40
60
80
100
120
20
25
30
35
40
45

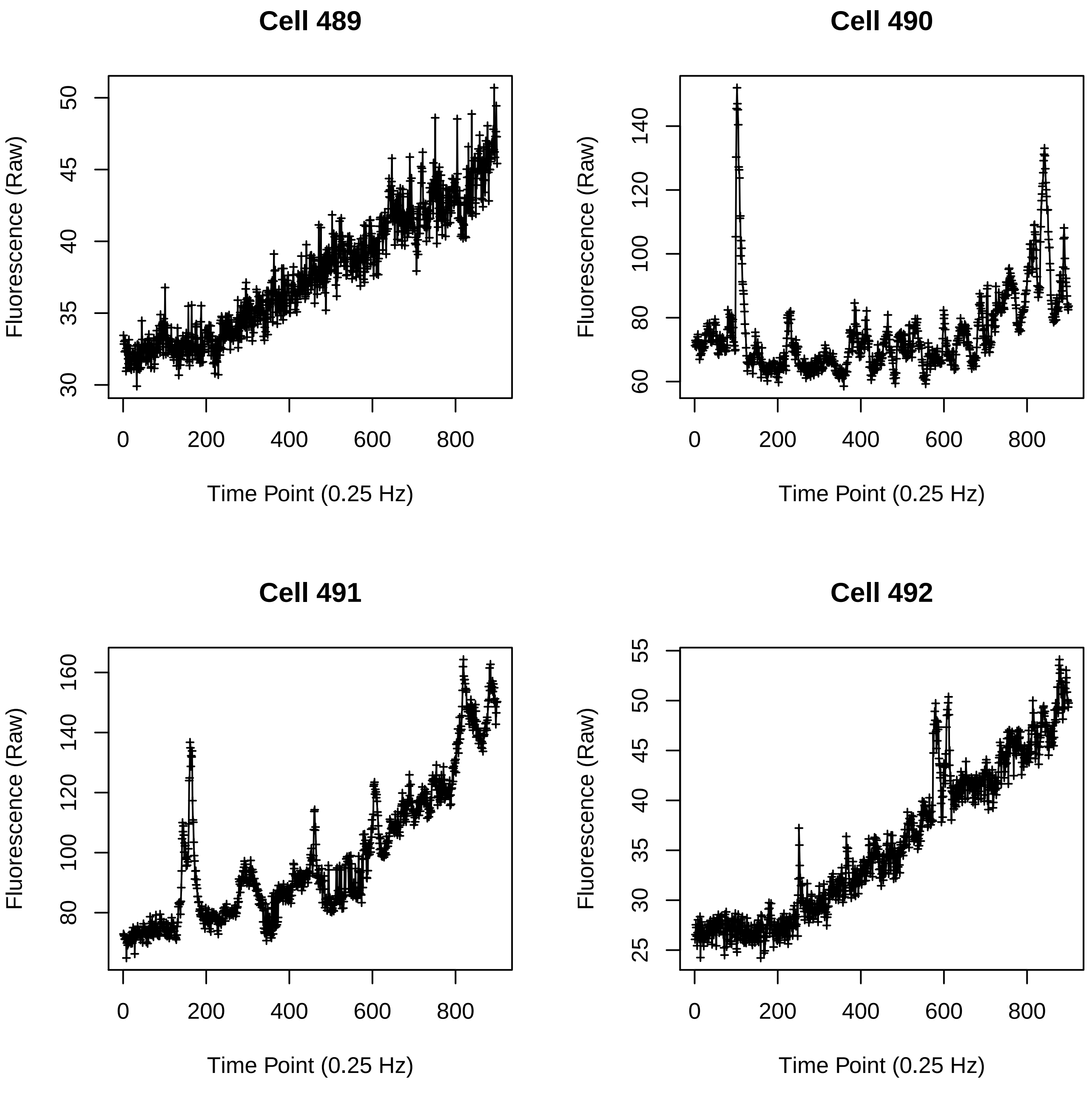
Cell 489
Fluorescence (Raw)
30
35
40
45
50
0
200
400
600
800
Time Point (0.25 Hz)
Cell 490
Fluorescence (Raw)
60
80
100
120
140
0
200
400
600
800
Time Point (0.25 Hz)
Cell 491
Fluorescence (Raw)
80
100
120
140
160
0
200
400
600
800
Time Point (0.25 Hz)
Cell 492
Fluorescence (Raw)
25
30
35
40
45
50
55
0
200
400
600
800
Time Point (0.25 Hz)

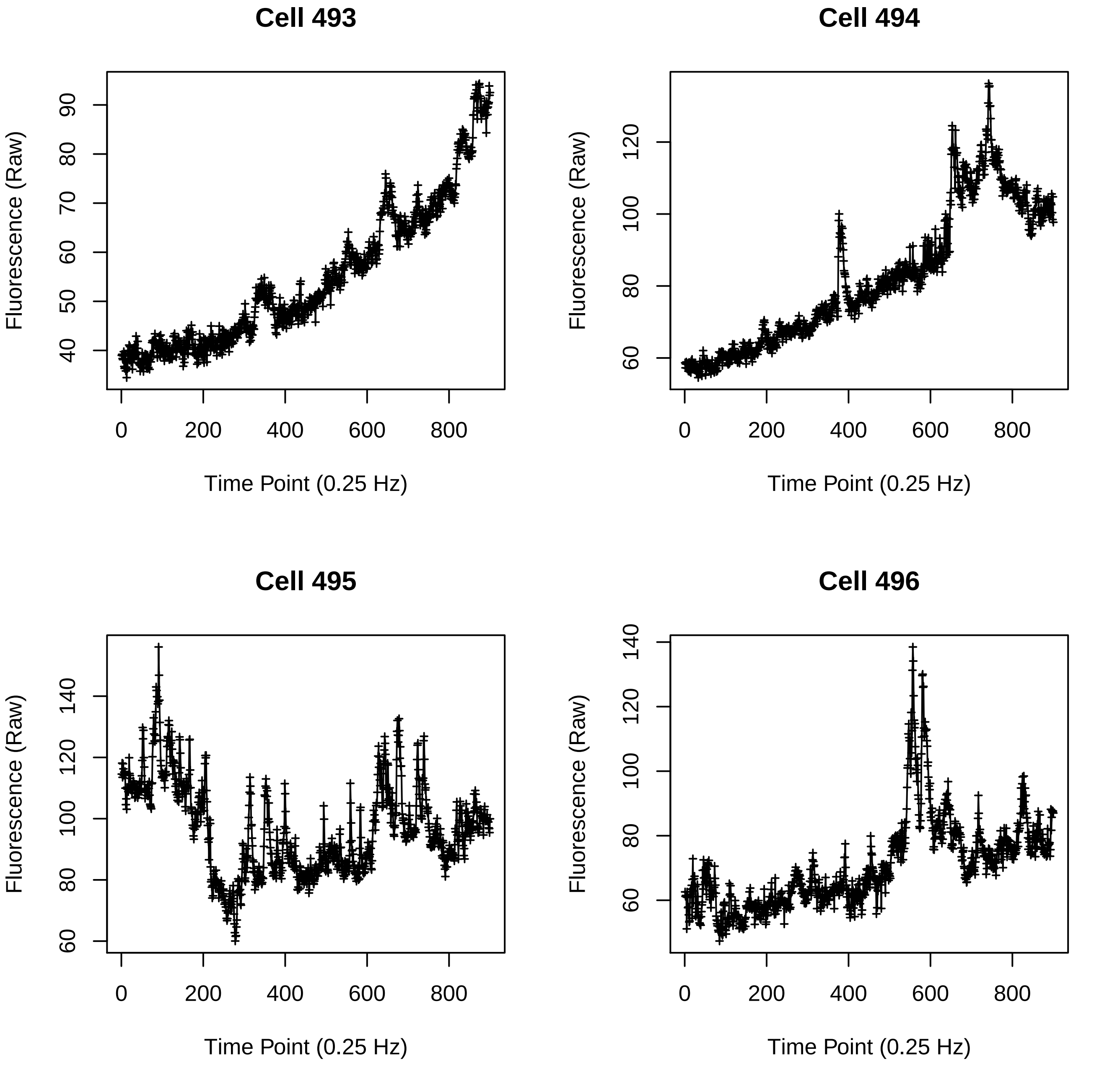
Cell 493
Fluorescence (Raw)
Time Point (0.25 Hz)
40
50
60
70
80
90
0
200
400
600
800
Cell 494
Fluorescence (Raw)
Time Point (0.25 Hz)
60
80
100
120
0
200
400
600
800
Cell 495
Fluorescence (Raw)
Time Point (0.25 Hz)
60
80
100
120
140
0
200
400
600
800
Cell 496
Fluorescence (Raw)
Time Point (0.25 Hz)
60
80
100
120
140
0
200
400
600
800

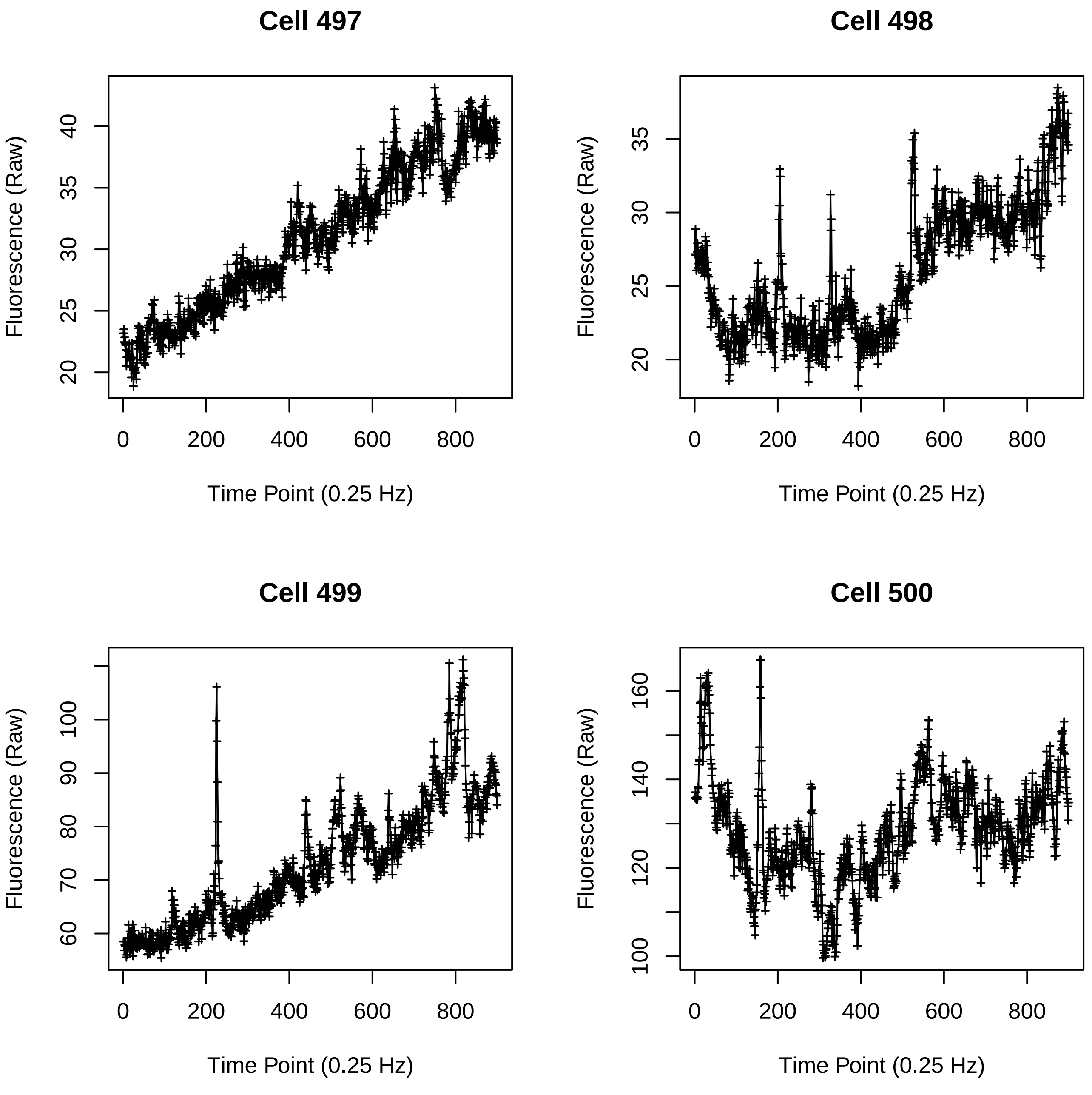

Cell 497
Cell 498
Cell 499
Cell 500
Fluorescence (Raw)
Time Point (0.25 Hz)
0
200
400
600
800
20
25
30
35
40
60
70
80
90
100
120
140
160

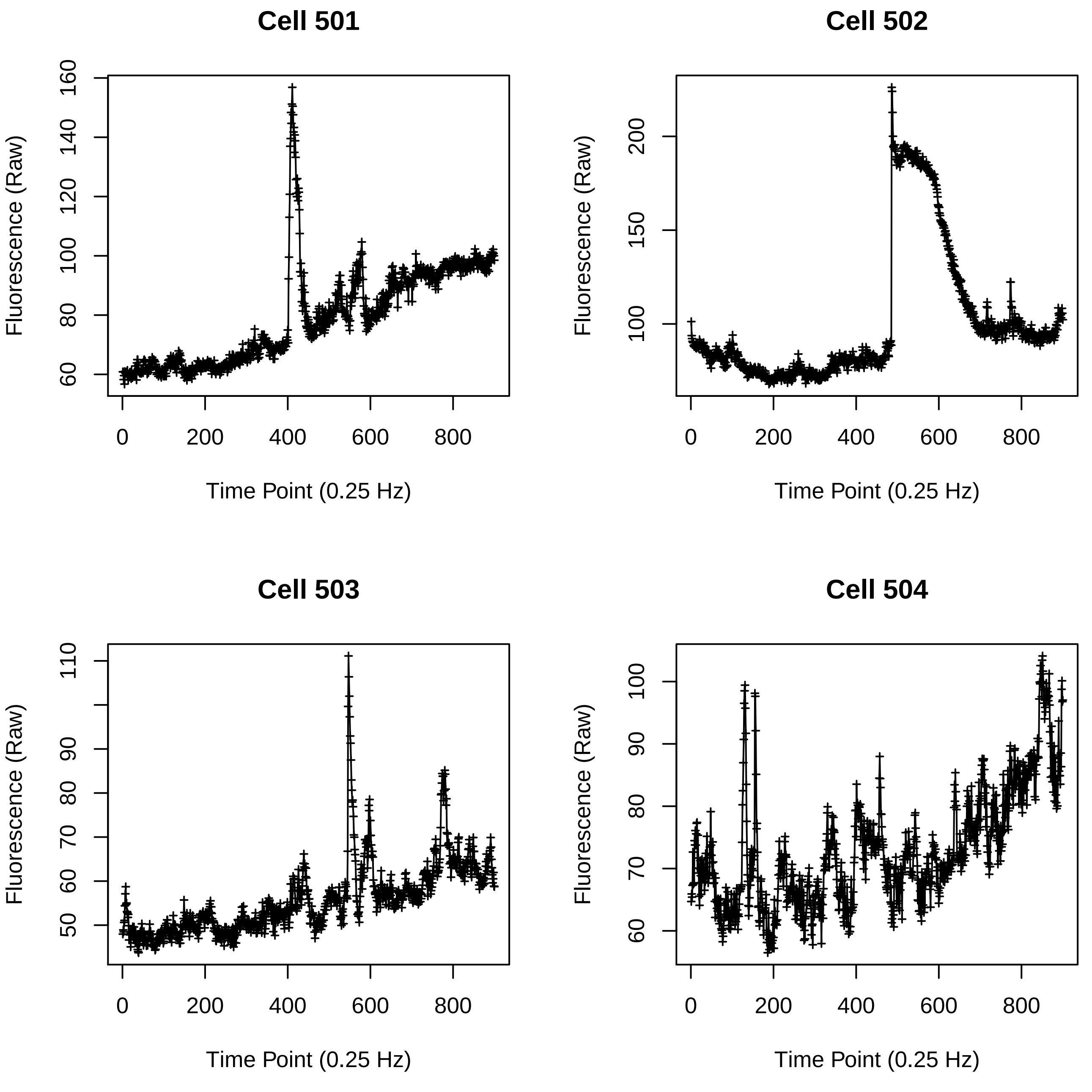
Cell 501
Fluorescence (Raw)
60
80
100
120
140
160
0
200
400
600
800
Time Point (0.25 Hz)
Cell 502
Fluorescence (Raw)
100
150
200
0
200
400
600
800
Time Point (0.25 Hz)
Cell 503
Fluorescence (Raw)
50
60
70
80
90
110
0
200
400
600
800
Time Point (0.25 Hz)
Cell 504
Fluorescence (Raw)
60
70
80
90
100
0
200
400
600
800
Time Point (0.25 Hz)

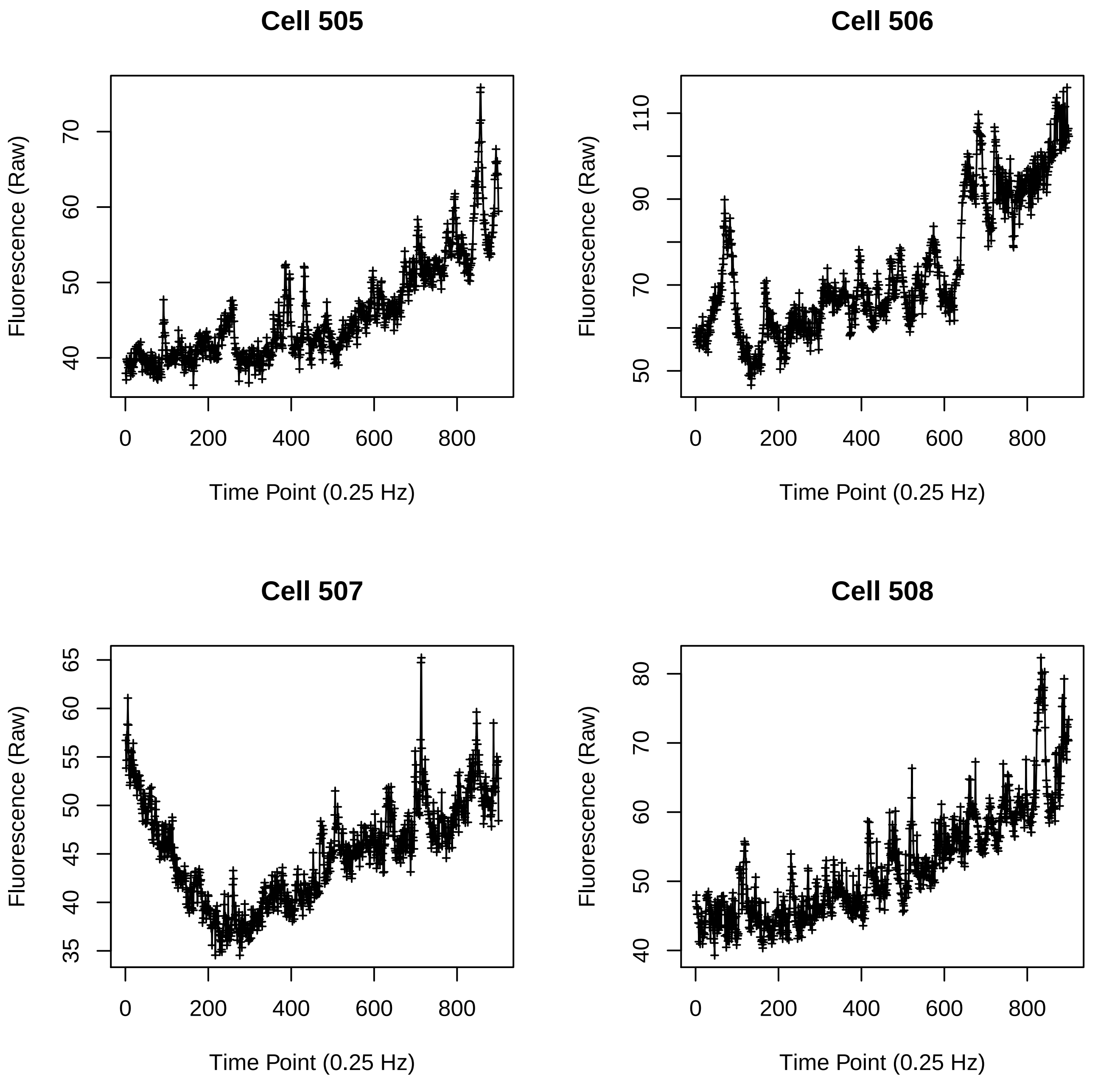
Cell 505
Cell 506
Cell 507
Cell 508
Fluorescence (Raw)
Time Point (0.25 Hz)
0
200
400
600
800
40
50
60
70
90
110
35
45
55
65
80

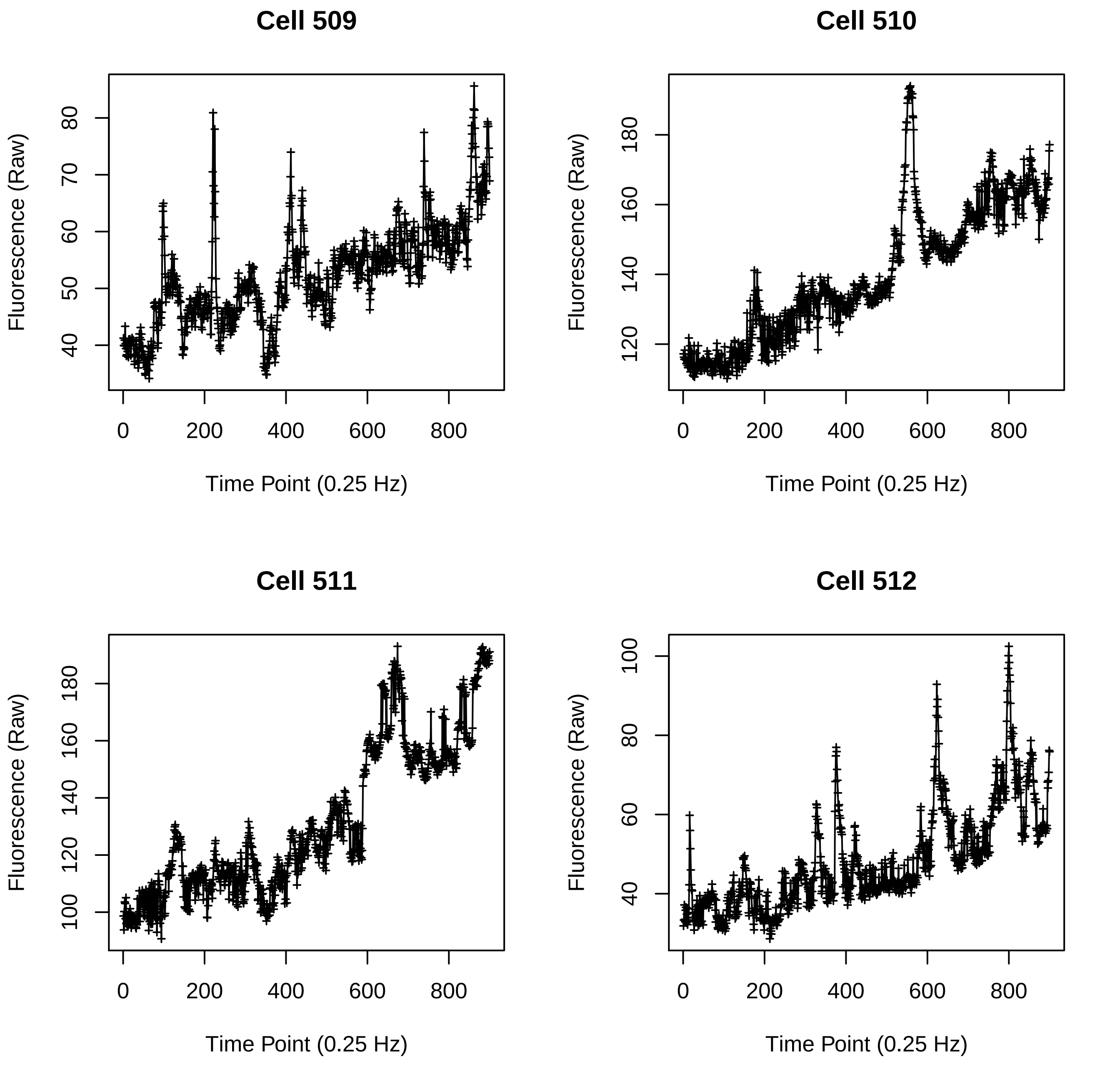

Cell 509
Cell 510
Cell 511
Cell 512
Fluorescence (Raw)
Time Point (0.25 Hz)
0
200
400
600
800
40
50
60
70
80
120
140
160
180
100
20
40
60
80
100

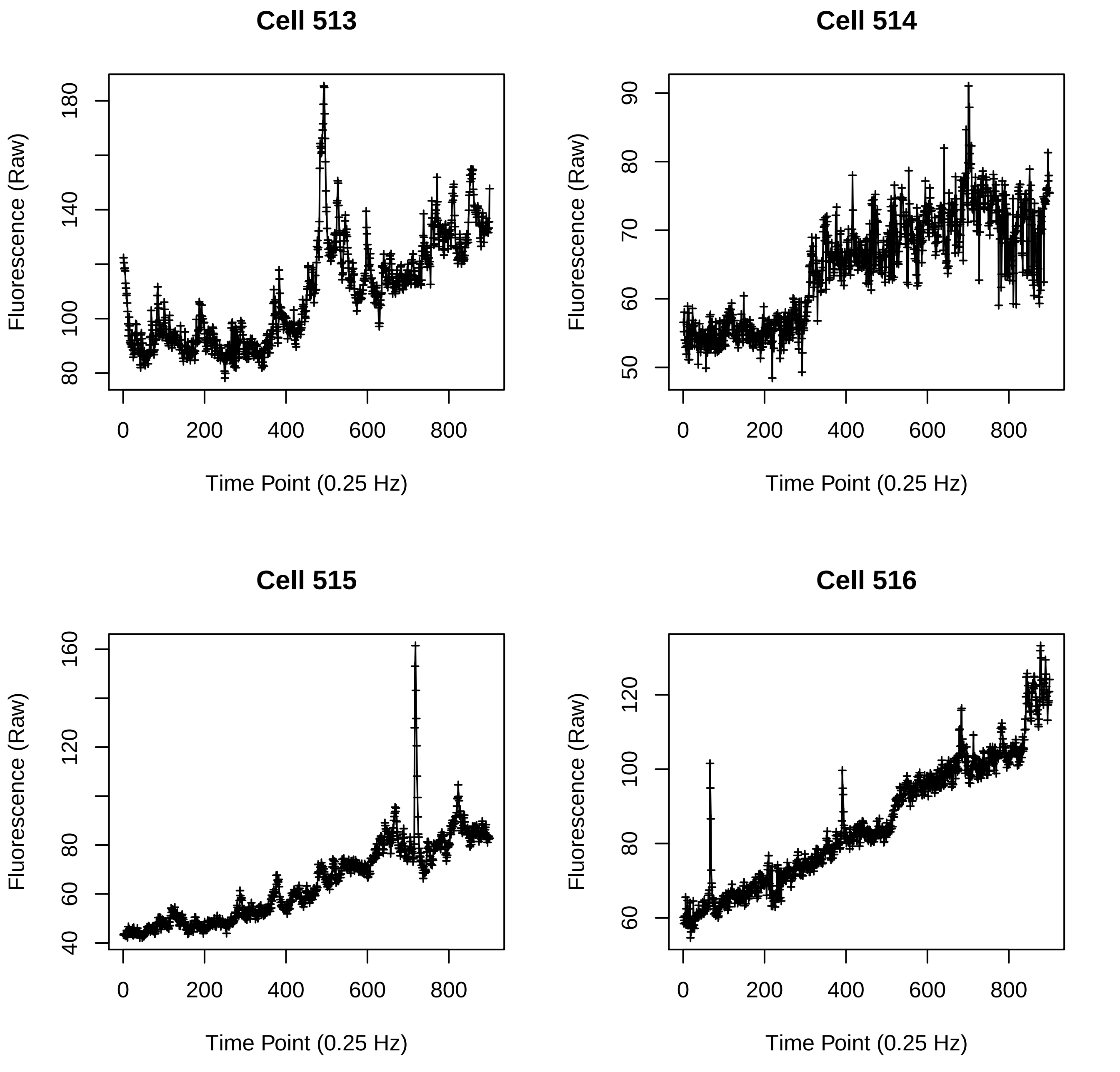

Cell 513
Fluorescence (Raw)
80
100
140
180
0
200
400
600
800
Time Point (0.25 Hz)
Cell 514
Fluorescence (Raw)
50
60
70
80
90
0
200
400
600
800
Time Point (0.25 Hz)
Cell 515
Fluorescence (Raw)
40
60
80
120
160
0
200
400
600
800
Time Point (0.25 Hz)
Cell 516
Fluorescence (Raw)
60
80
100
120
0
200
400
600
800
Time Point (0.25 Hz)

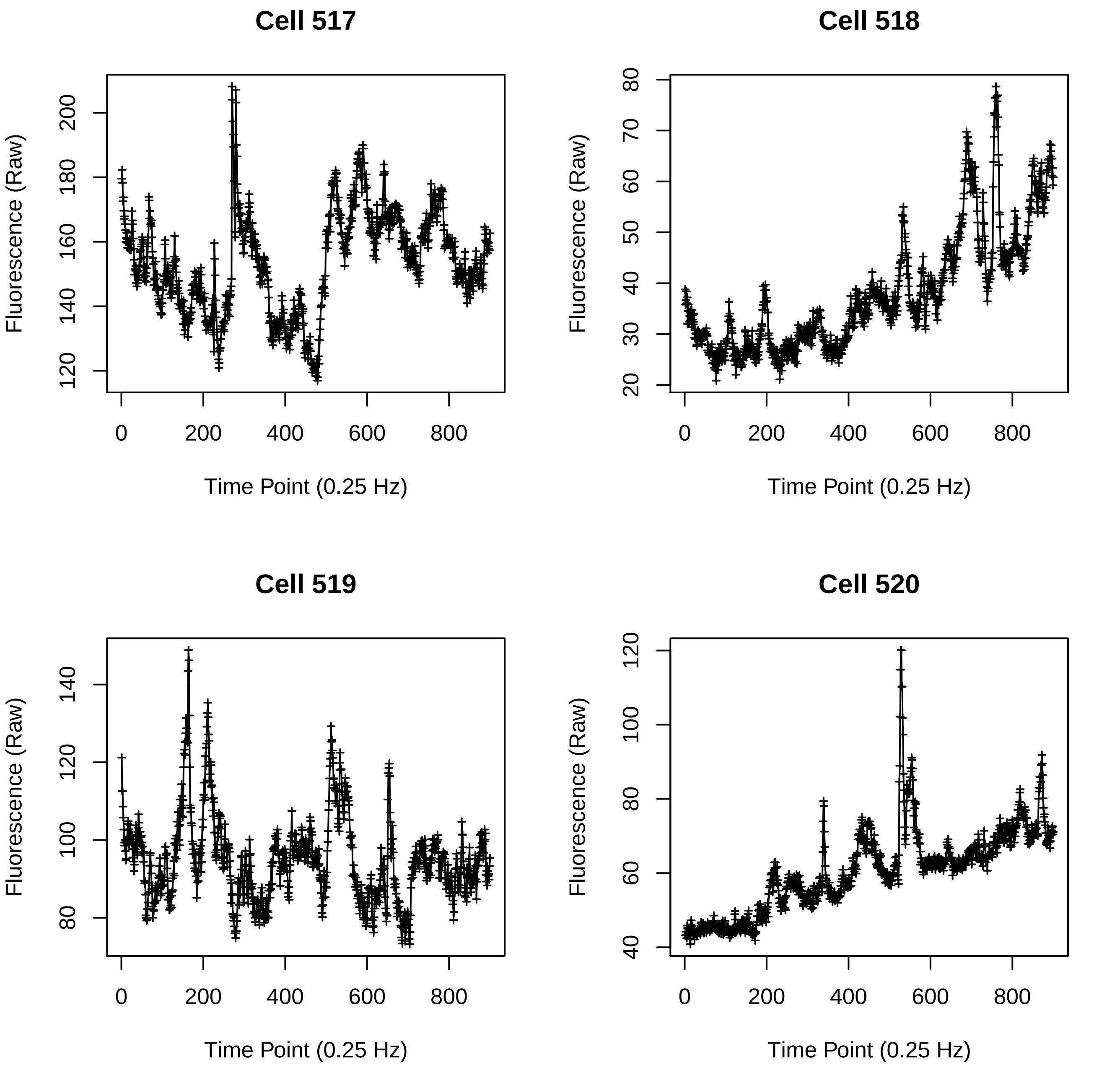
Cell 517
Cell 518
Cell 519
Cell 520
Fluorescence (Raw)
Time Point (0.25 Hz)
120
140
160
180
200
20
30
40
50
60
70
80
100
0
200
400
600
800

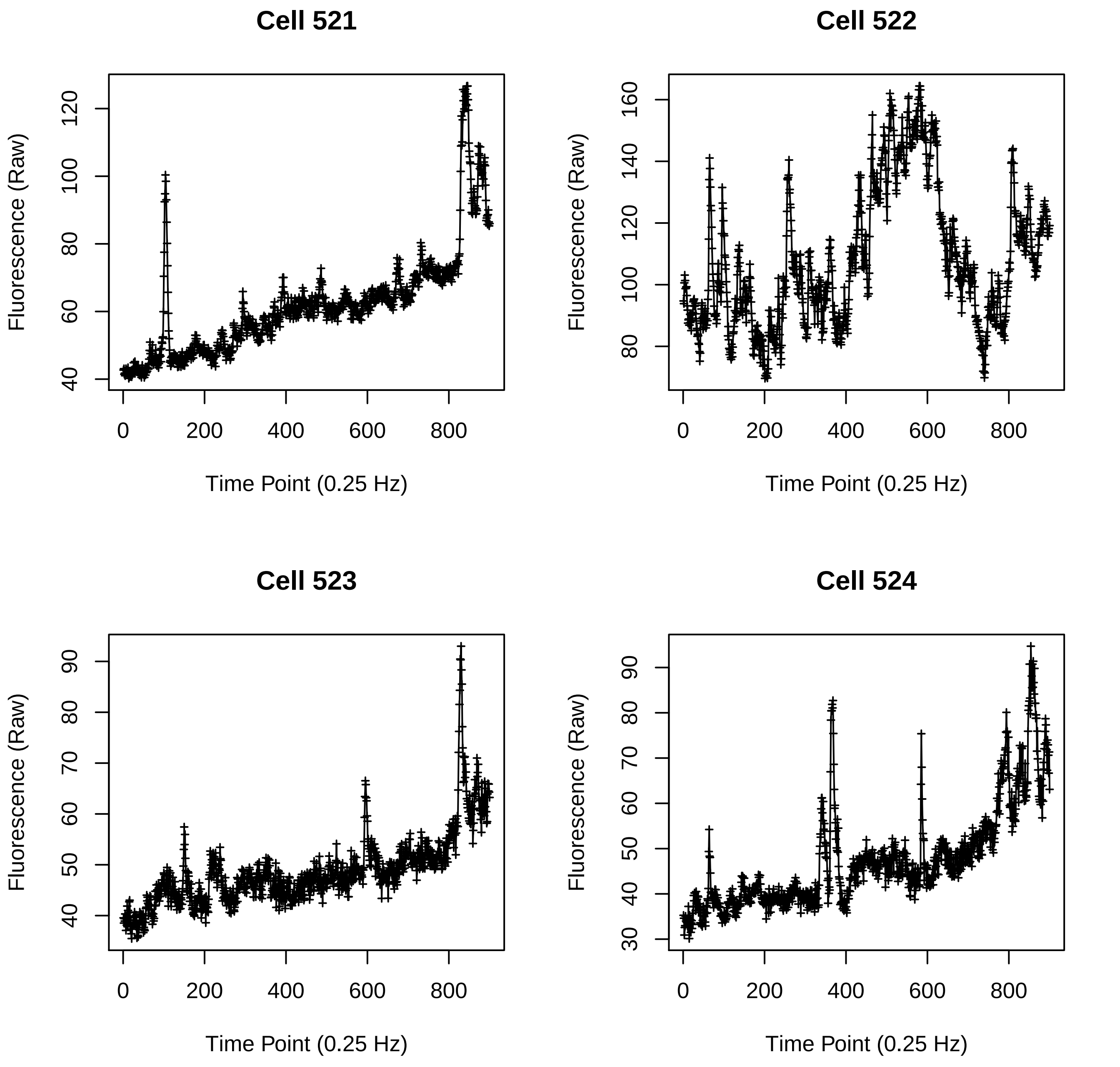
Cell 521
Fluorescence (Raw)
40
60
80
100
120
0
200
400
600
800
Time Point (0.25 Hz)
Cell 522
Fluorescence (Raw)
80
100
120
140
160
0
200
400
600
800
Time Point (0.25 Hz)
Cell 523
Fluorescence (Raw)
40
50
60
70
80
90
0
200
400
600
800
Time Point (0.25 Hz)
Cell 524
Fluorescence (Raw)
30
40
50
60
70
80
90
0
200
400
600
800
Time Point (0.25 Hz)

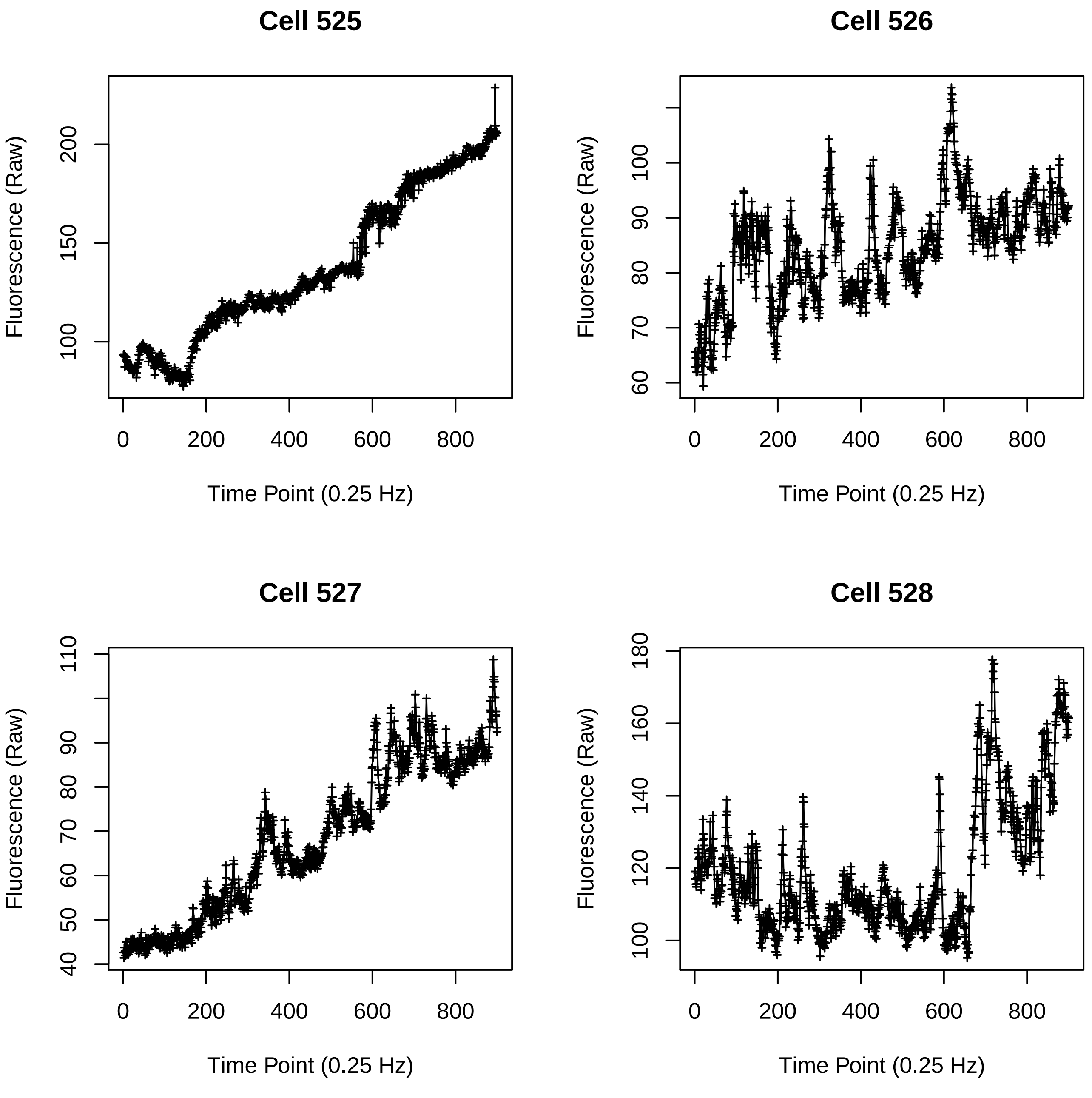
Cell 525
Cell 526
Cell 527
Cell 528
Fluorescence (Raw)
Time Point (0.25 Hz)
0
200
400
600
800
100
150
200
60
70
80
90
100
40
50
60
70
80
90
110
120
140
160
180

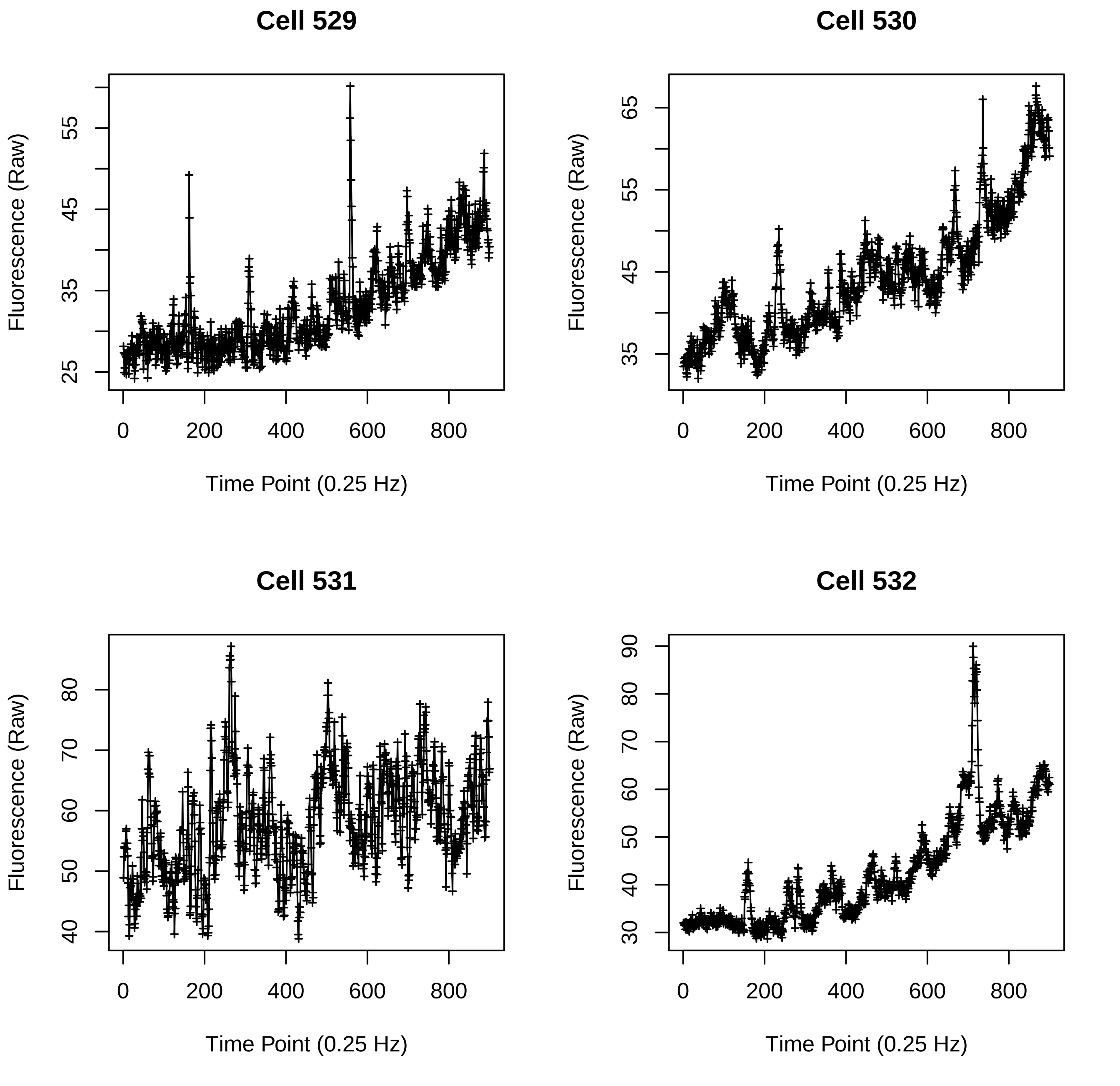

Cell 529
Fluorescence (Raw)
25
35
45
55
0
200
400
600
800
Time Point (0.25 Hz)
Cell 530
Fluorescence (Raw)
35
45
55
65
0
200
400
600
800
Time Point (0.25 Hz)
Cell 531
Fluorescence (Raw)
40
50
60
70
80
0
200
400
600
800
Time Point (0.25 Hz)
Cell 532
Fluorescence (Raw)
30
40
50
60
70
80
90
0
200
400
600
800
Time Point (0.25 Hz)

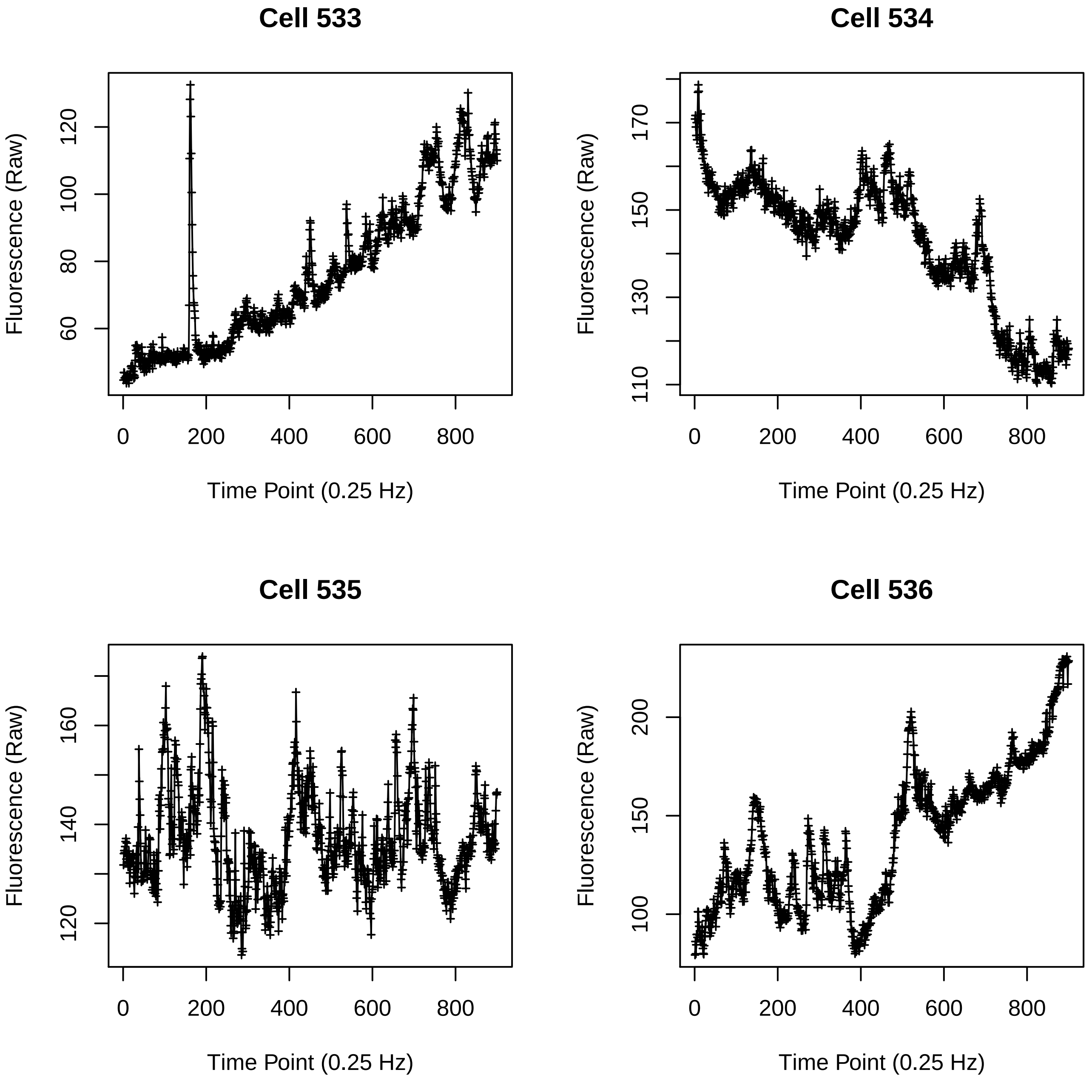
Cell 533
Cell 534
Cell 535
Cell 536
Fluorescence (Raw)
Time Point (0.25 Hz)
0
200
400
600
800
60
80
100
120
110
130
150
170
120
140
160
100
150
200

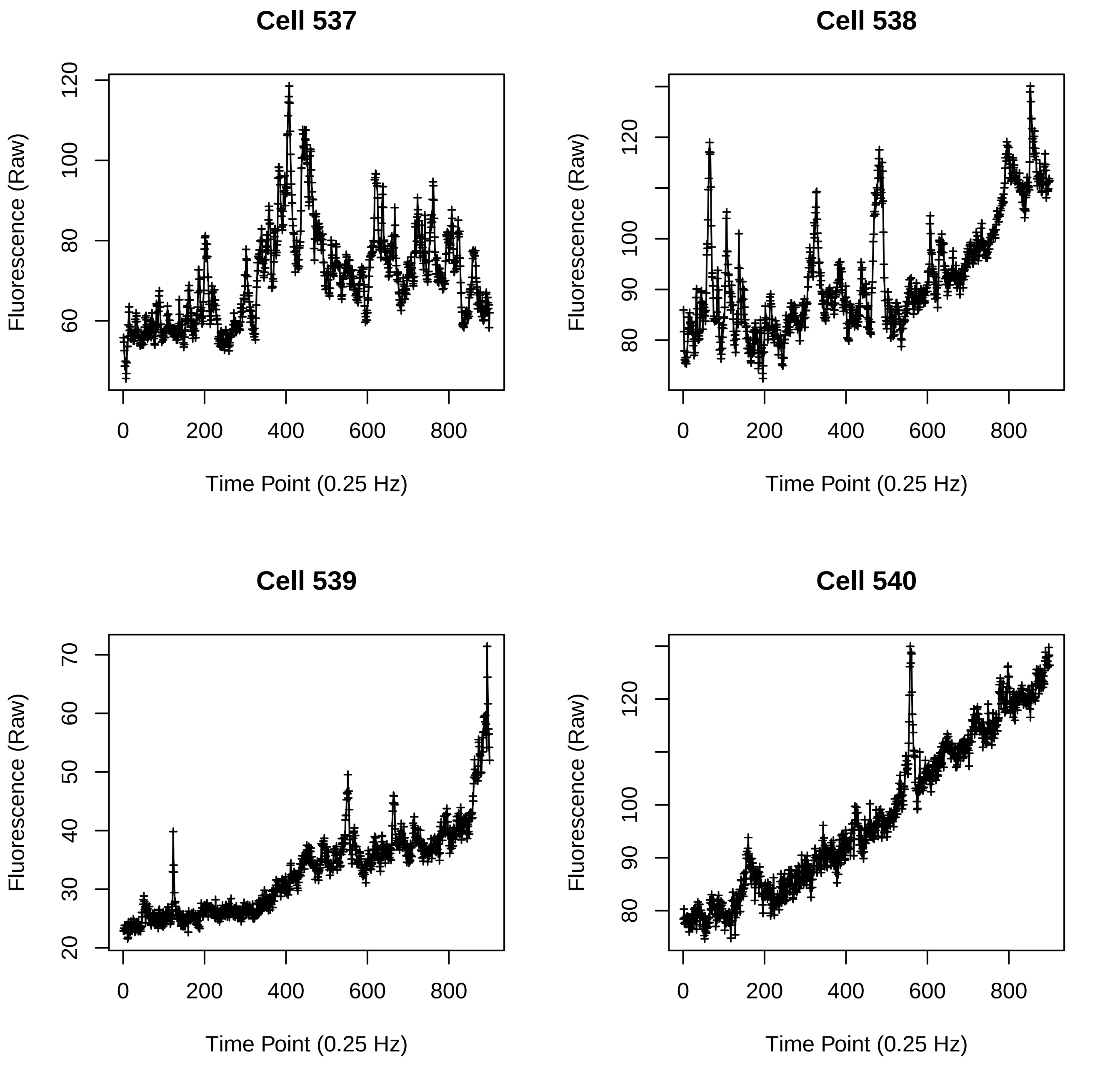
Cell 537
Cell 538
Cell 539
Cell 540
Fluorescence (Raw)
Time Point (0.25 Hz)
0
200
400
600
800
60
80
100
120
90
70
50
40
30
20

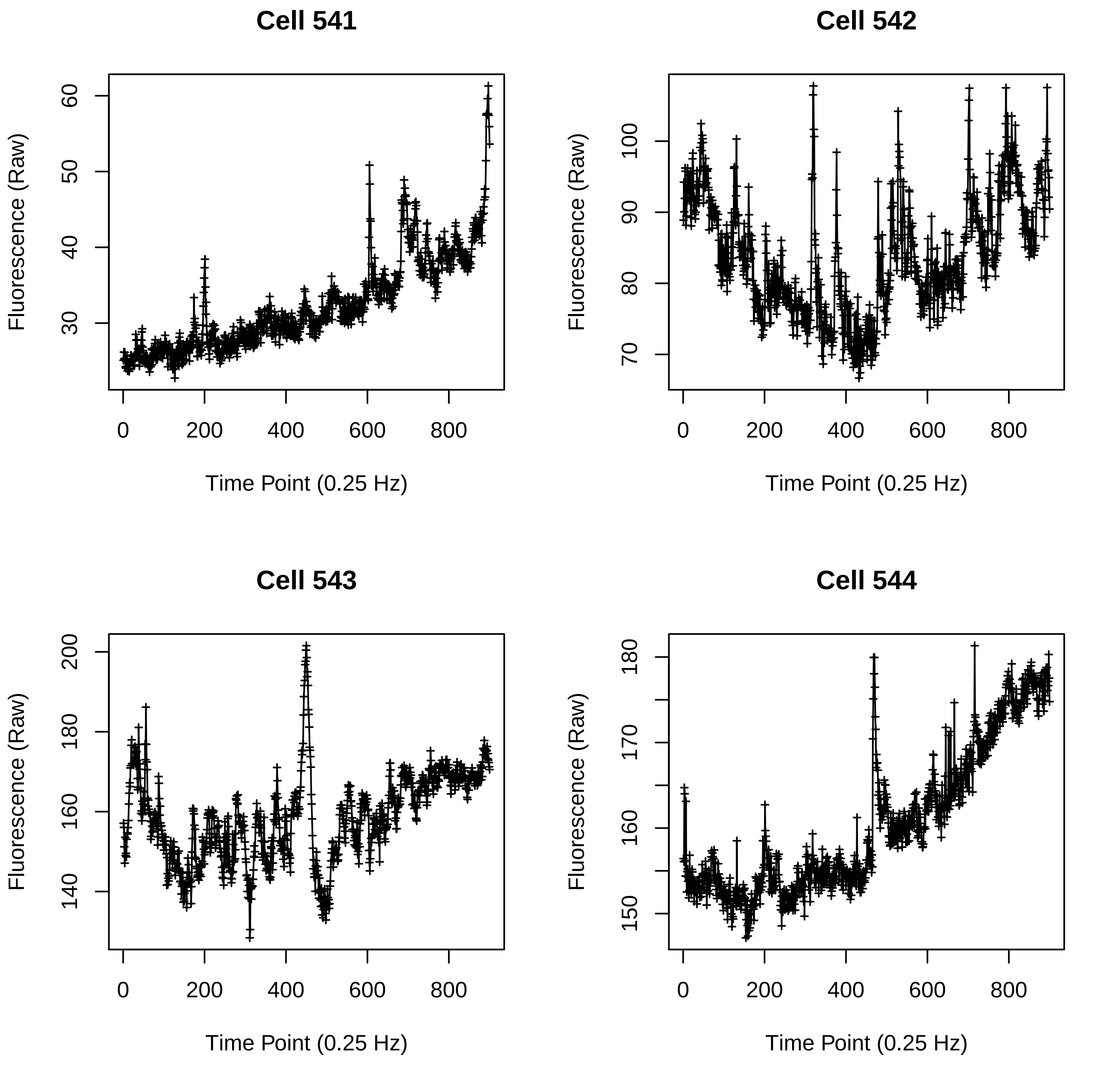

Cell 541
Cell 542
Cell 543
Cell 544
Fluorescence (Raw)
Time Point (0.25 Hz)
0
200
400
600
800

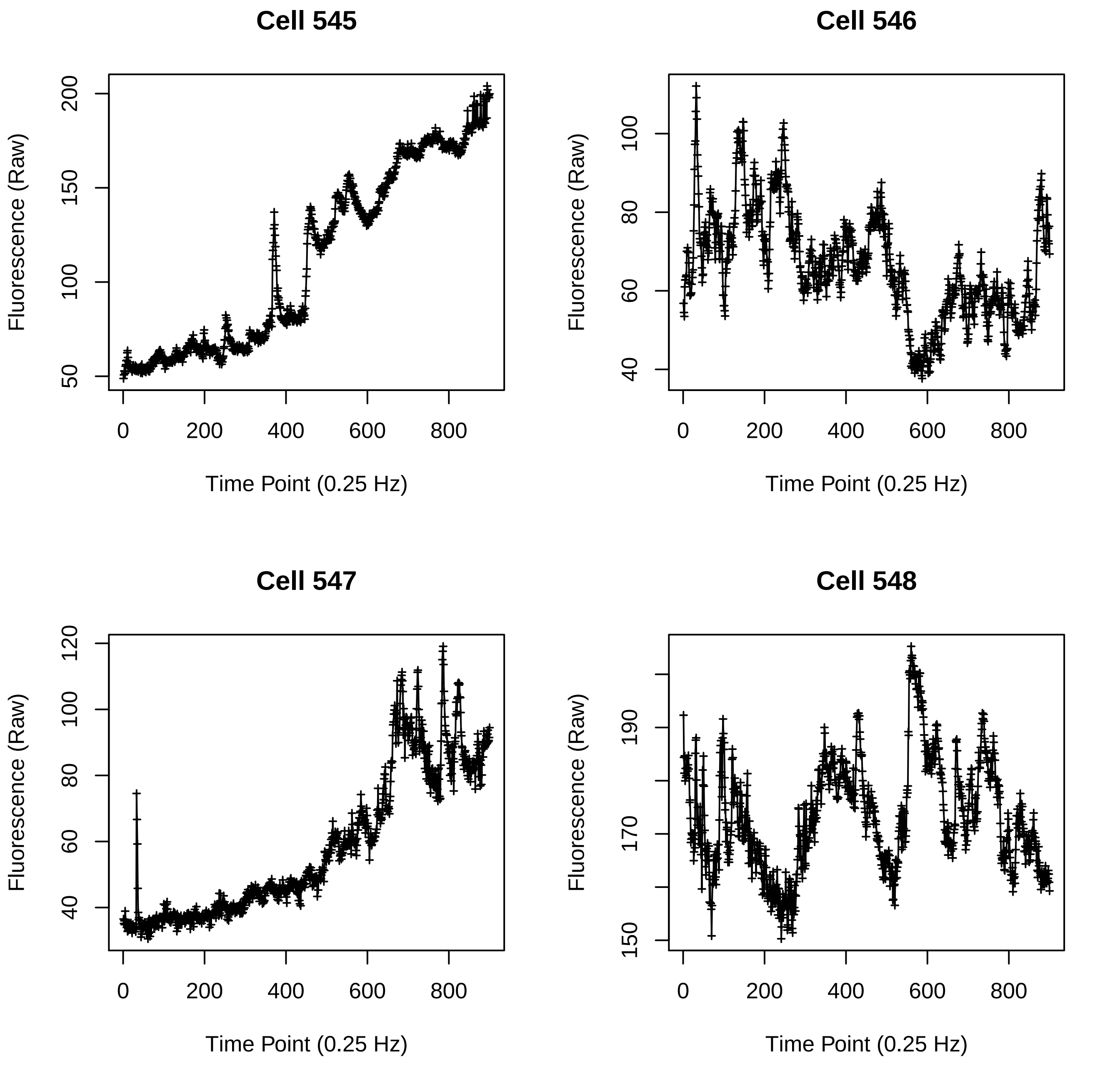
Cell 545
Fluorescence (Raw)
50
100
150
200
0
200
400
600
800
Time Point (0.25 Hz)
Cell 546
Fluorescence (Raw)
40
60
80
100
0
200
400
600
800
Time Point (0.25 Hz)
Cell 547
Fluorescence (Raw)
40
60
80
100
120
0
200
400
600
800
Time Point (0.25 Hz)
Cell 548
Fluorescence (Raw)
150
170
190
0
200
400
600
800
Time Point (0.25 Hz)

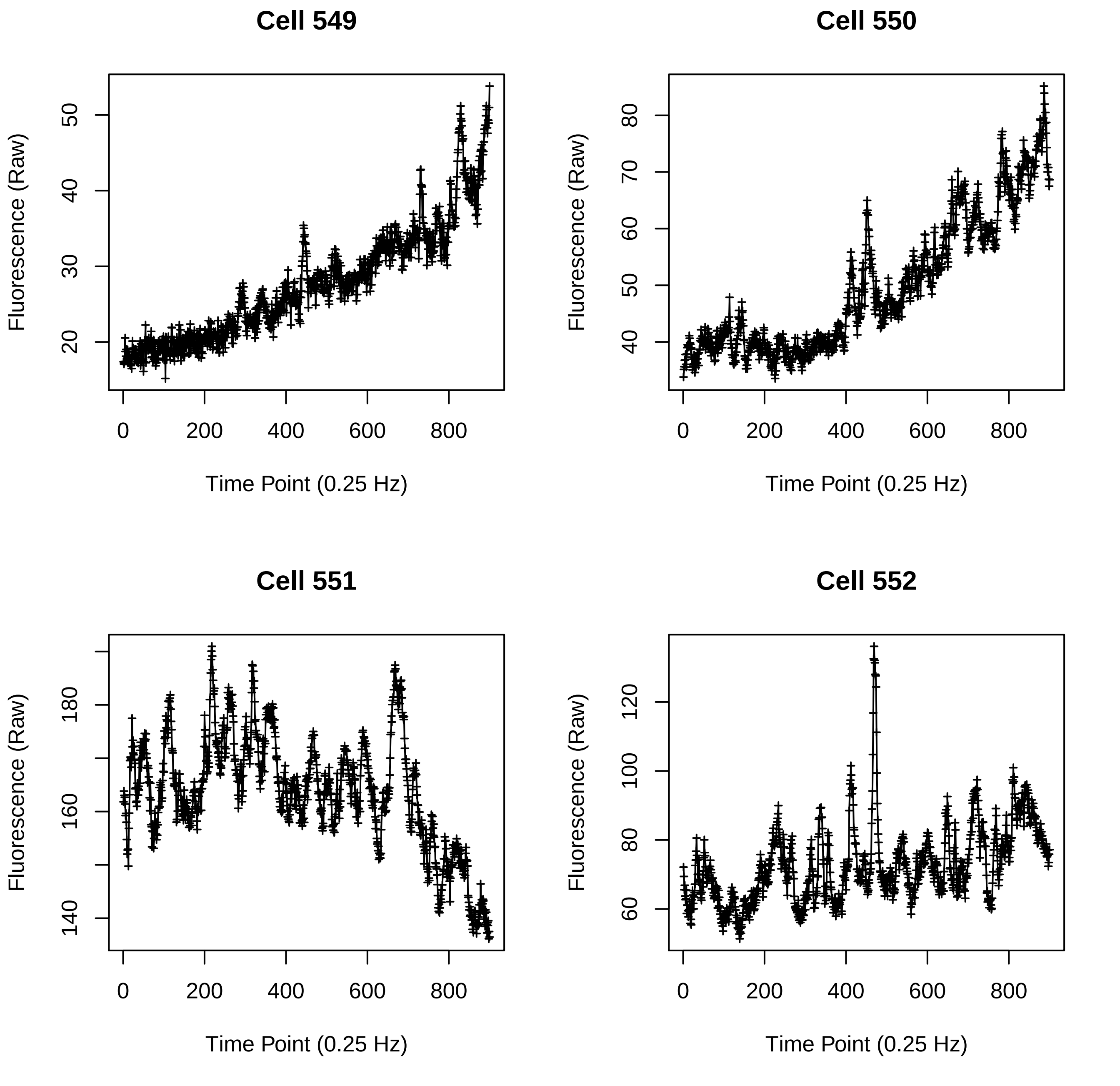
Cell 549
Fluorescence (Raw)
Time Point (0.25 Hz)
Cell 550
Fluorescence (Raw)
Time Point (0.25 Hz)
Cell 551
Fluorescence (Raw)
Time Point (0.25 Hz)
Cell 552
Fluorescence (Raw)
Time Point (0.25 Hz)

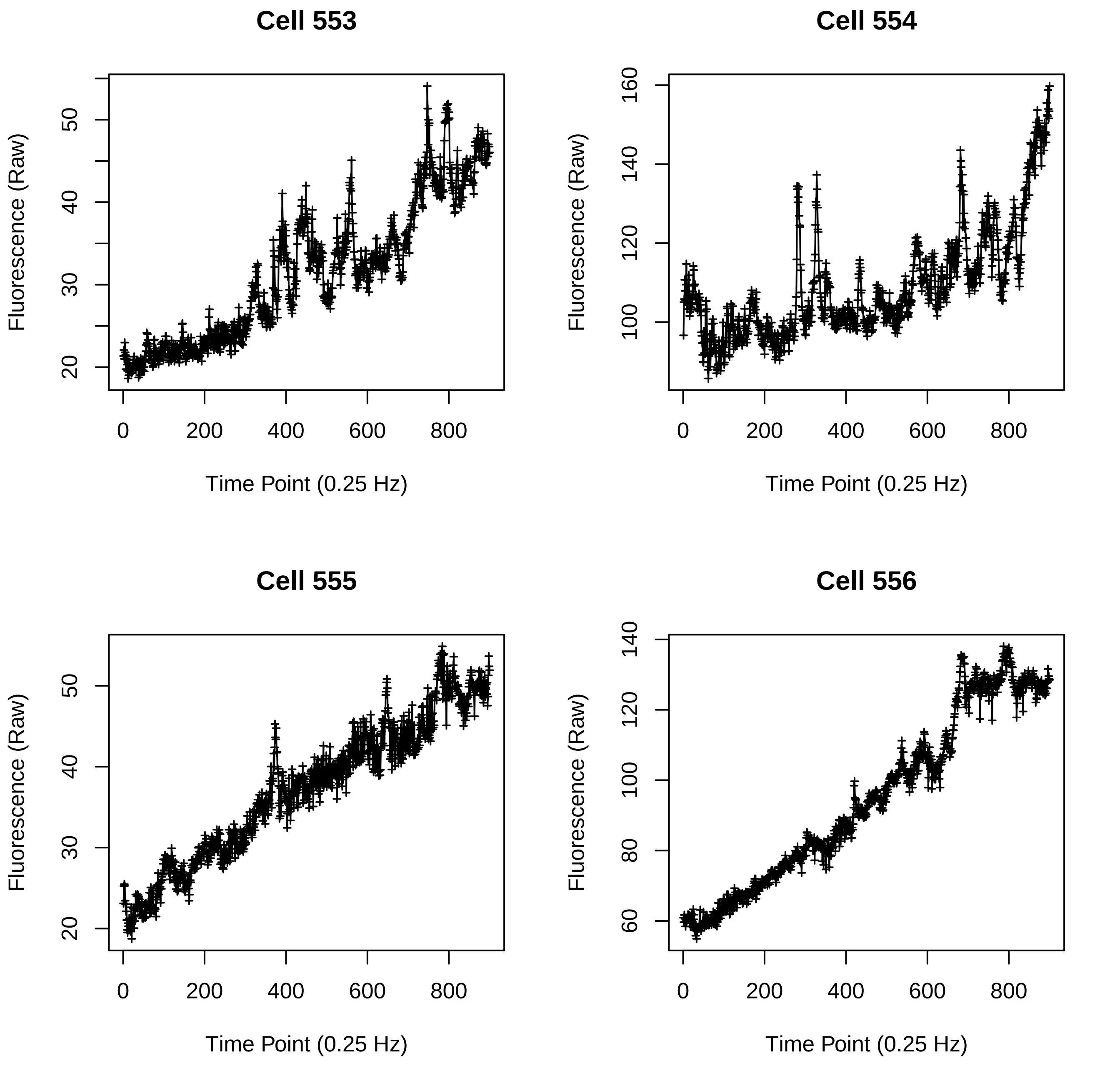
Cell 553
Cell 554
Cell 555
Cell 556
Fluorescence (Raw)
Time Point (0.25 Hz)
0
200
400
600
800
20
30
40
50
100
120
140
160
60
80

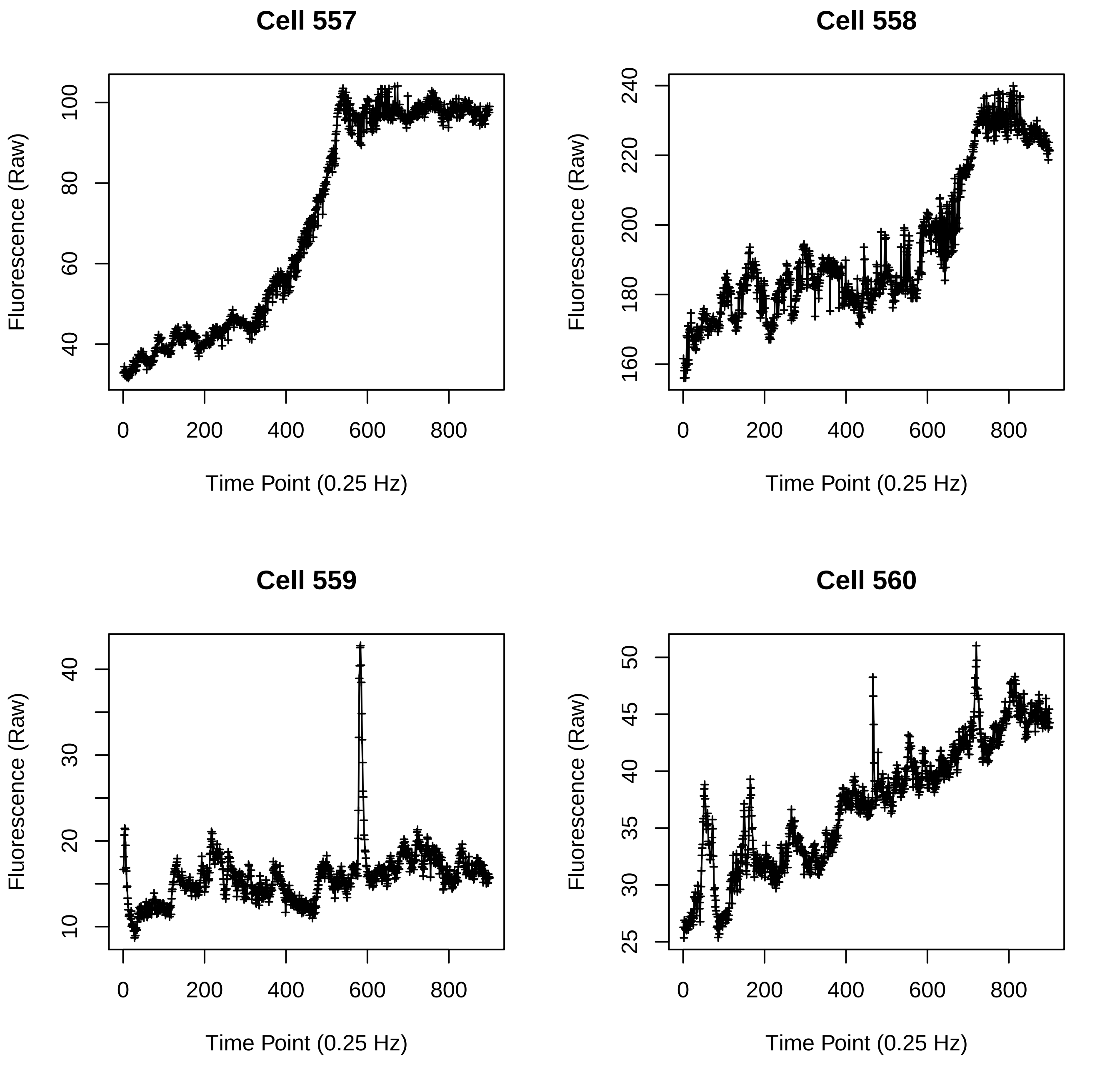

Cell 557
Fluorescence (Raw)
Time Point (0.25 Hz)
Cell 558
Fluorescence (Raw)
Time Point (0.25 Hz)
Cell 559
Fluorescence (Raw)
Time Point (0.25 Hz)
Cell 560
Fluorescence (Raw)
Time Point (0.25 Hz)

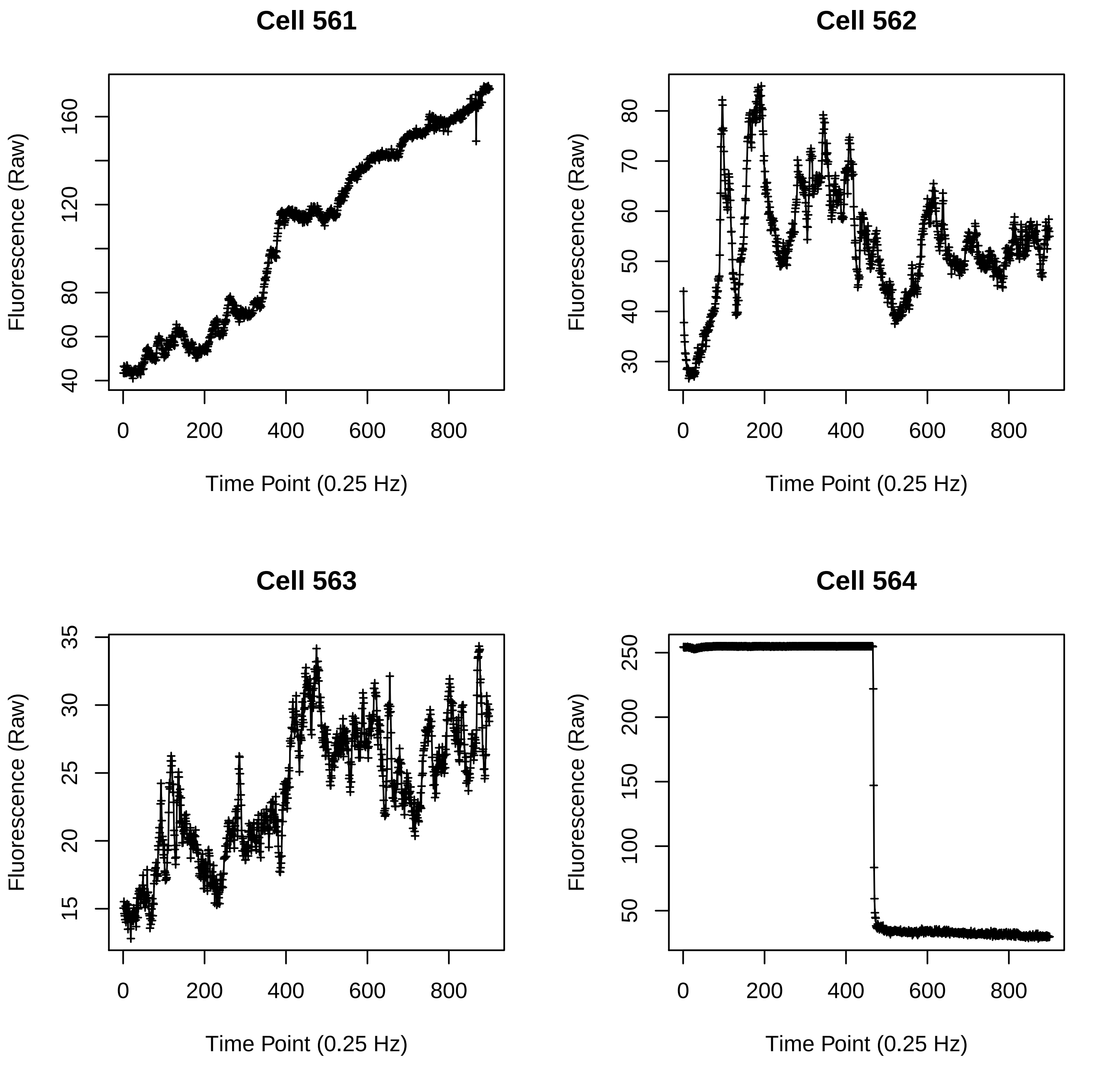
Cell 561
Cell 562
Cell 563
Cell 564
Fluorescence (Raw)
Time Point (0.25 Hz)
0
200
400
600
800
40
60
80
120
160
30
40
50
60
70
80
15
20
25
30
35
50
100
150
200
250

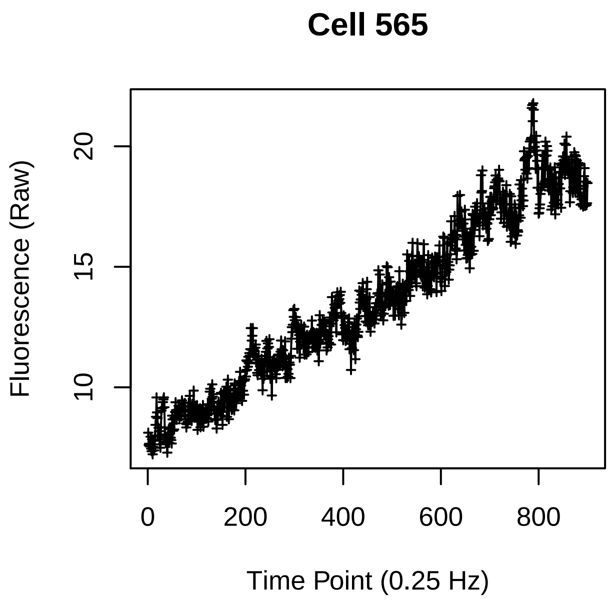
Cell 565
Fluorescence (Raw)
10
15
20
0
200
400
600
800
Time Point (0.25 Hz)

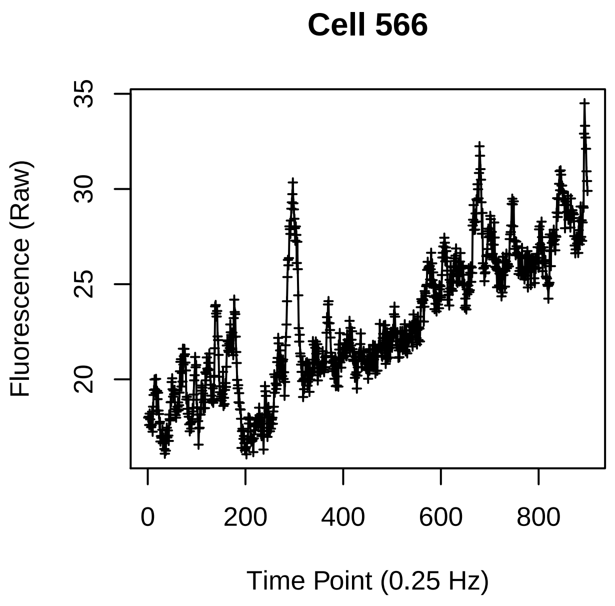
Cell 566
Fluorescence (Raw)
20
25
30
35
0
200
400
600
800
Time Point (0.25 Hz)

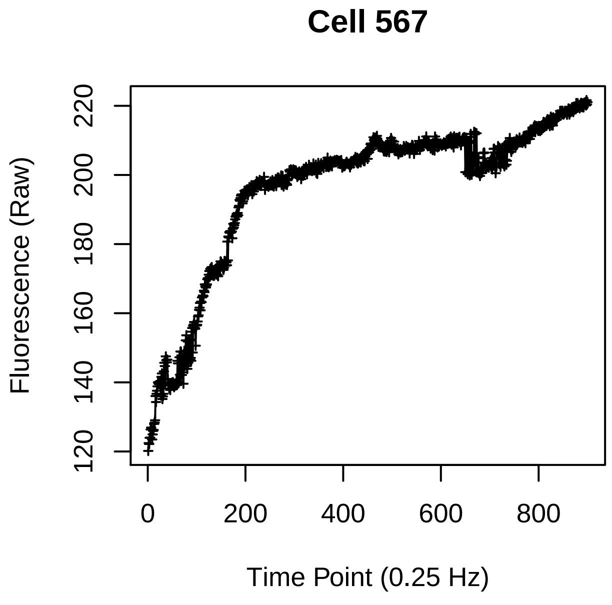
Cell 567
Fluorescence (Raw)
120
140
160
180
200
220
0
200
400
600
800
Time Point (0.25 Hz)

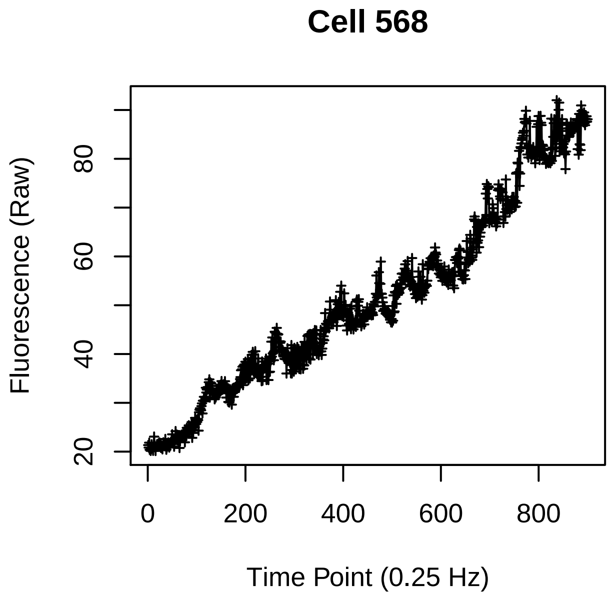
Cell 568
Fluorescence (Raw)
20
40
60
80
0
200
400
600
800
Time Point (0.25 Hz)

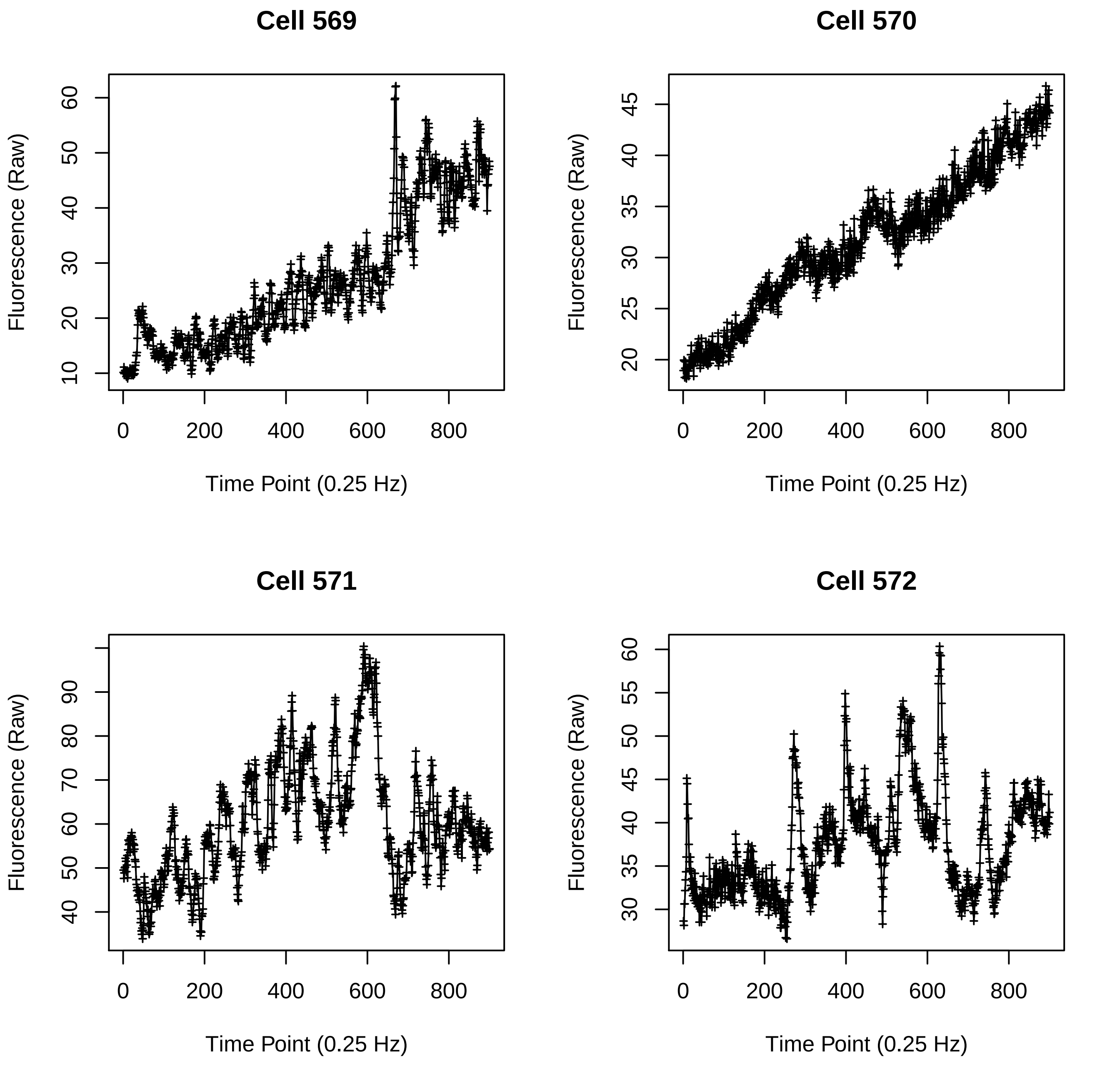
Cell 569
Fluorescence (Raw)
Time Point (0.25 Hz)
Cell 570
Fluorescence (Raw)
Time Point (0.25 Hz)
Cell 571
Fluorescence (Raw)
Time Point (0.25 Hz)
Cell 572
Fluorescence (Raw)
Time Point (0.25 Hz)

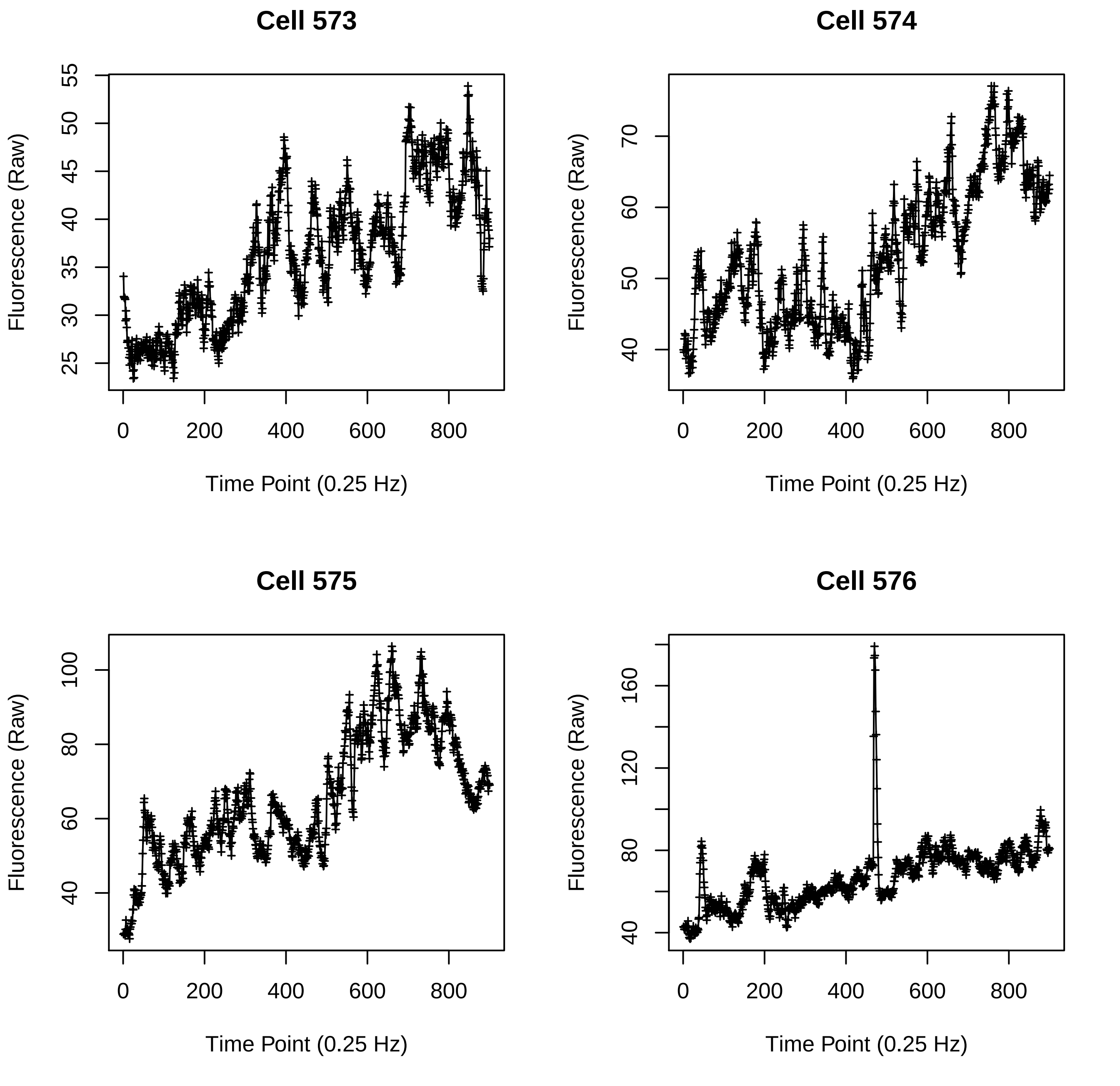
Cell 573
Fluorescence (Raw)
Time Point (0.25 Hz)
Cell 574
Fluorescence (Raw)
Time Point (0.25 Hz)
Cell 575
Fluorescence (Raw)
Time Point (0.25 Hz)
Cell 576
Fluorescence (Raw)
Time Point (0.25 Hz)

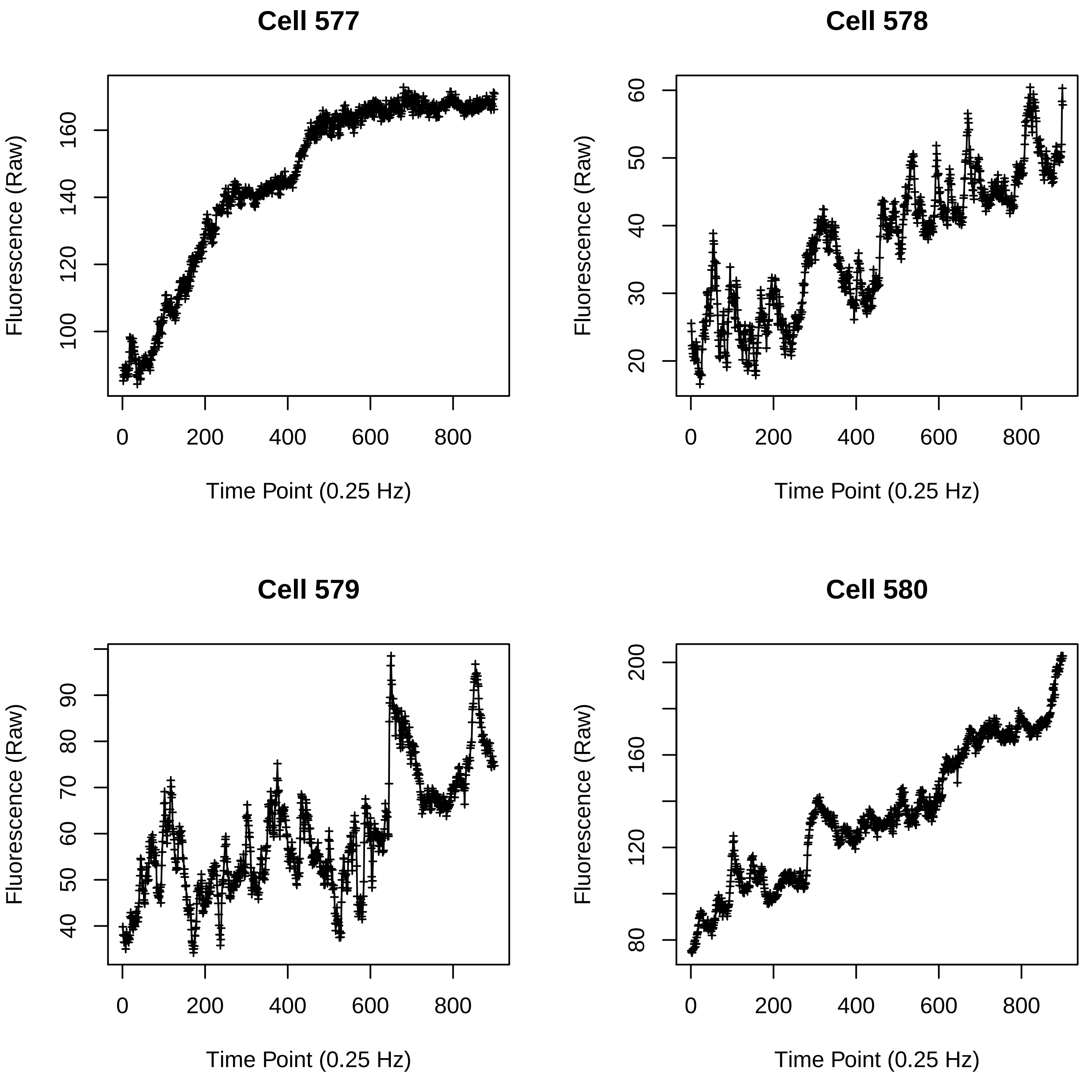
Cell 577
Fluorescence (Raw)
100
120
140
160
0
200
400
600
800
Time Point (0.25 Hz)
Cell 578
Fluorescence (Raw)
20
30
40
50
60
0
200
400
600
800
Time Point (0.25 Hz)
Cell 579
Fluorescence (Raw)
40
50
60
70
80
90
0
200
400
600
800
Time Point (0.25 Hz)
Cell 580
Fluorescence (Raw)
80
120
160
200
0
200
400
600
800
Time Point (0.25 Hz)

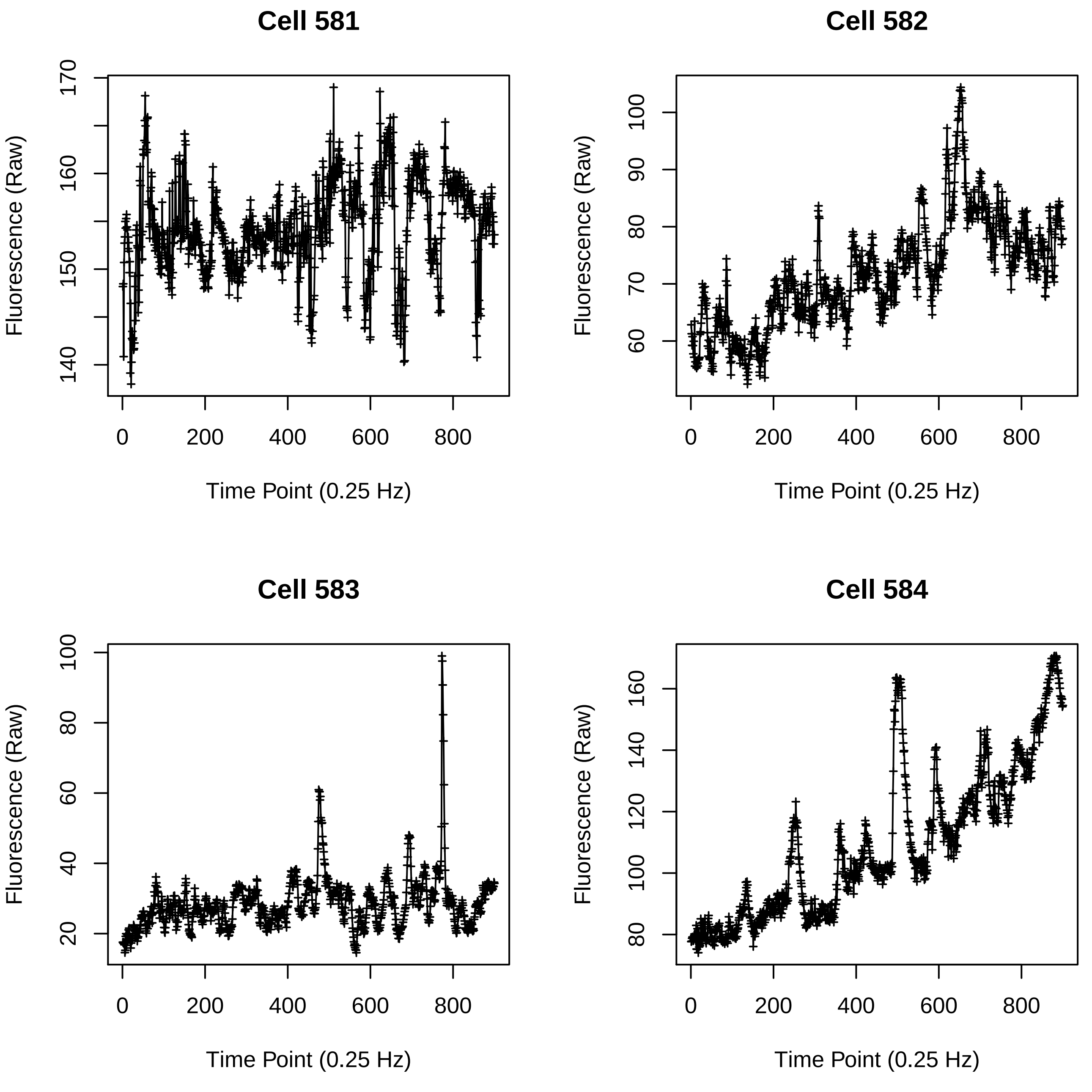
Cell 581
Fluorescence (Raw)
140
150
160
170
Time Point (0.25 Hz)
0
200
400
600
800
Cell 582
Fluorescence (Raw)
60
70
80
90
100
Time Point (0.25 Hz)
0
200
400
600
800
Cell 583
Fluorescence (Raw)
20
40
60
80
100
Time Point (0.25 Hz)
0
200
400
600
800
Cell 584
Fluorescence (Raw)
80
100
120
140
160
Time Point (0.25 Hz)
0
200
400
600
800

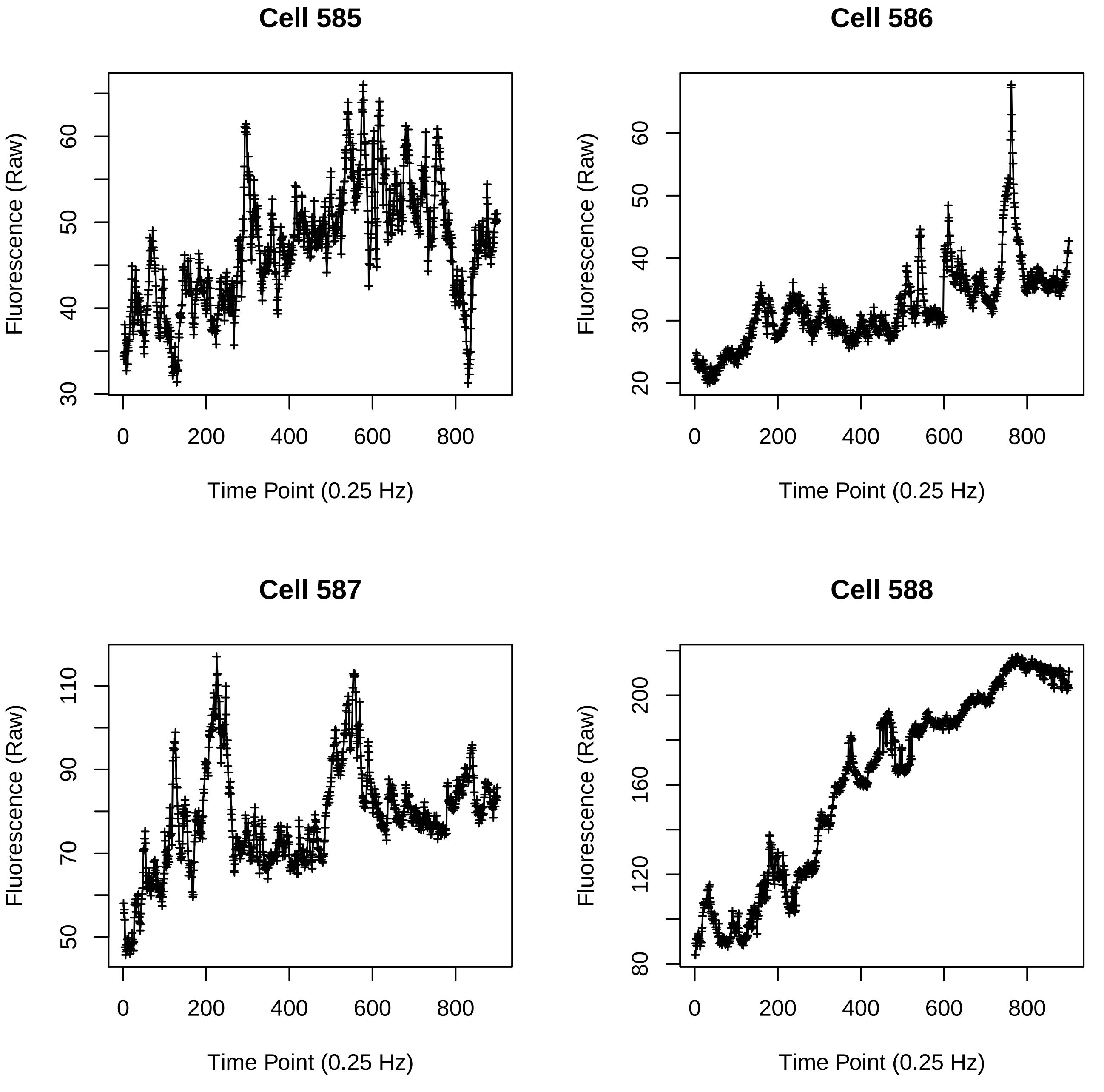

Cell 585
Fluorescence (Raw)
30
40
50
60
0
200
400
600
800
Time Point (0.25 Hz)
Cell 586
Fluorescence (Raw)
20
30
40
50
60
0
200
400
600
800
Time Point (0.25 Hz)
Cell 587
Fluorescence (Raw)
50
70
90
110
0
200
400
600
800
Time Point (0.25 Hz)
Cell 588
Fluorescence (Raw)
80
120
160
200
0
200
400
600
800
Time Point (0.25 Hz)

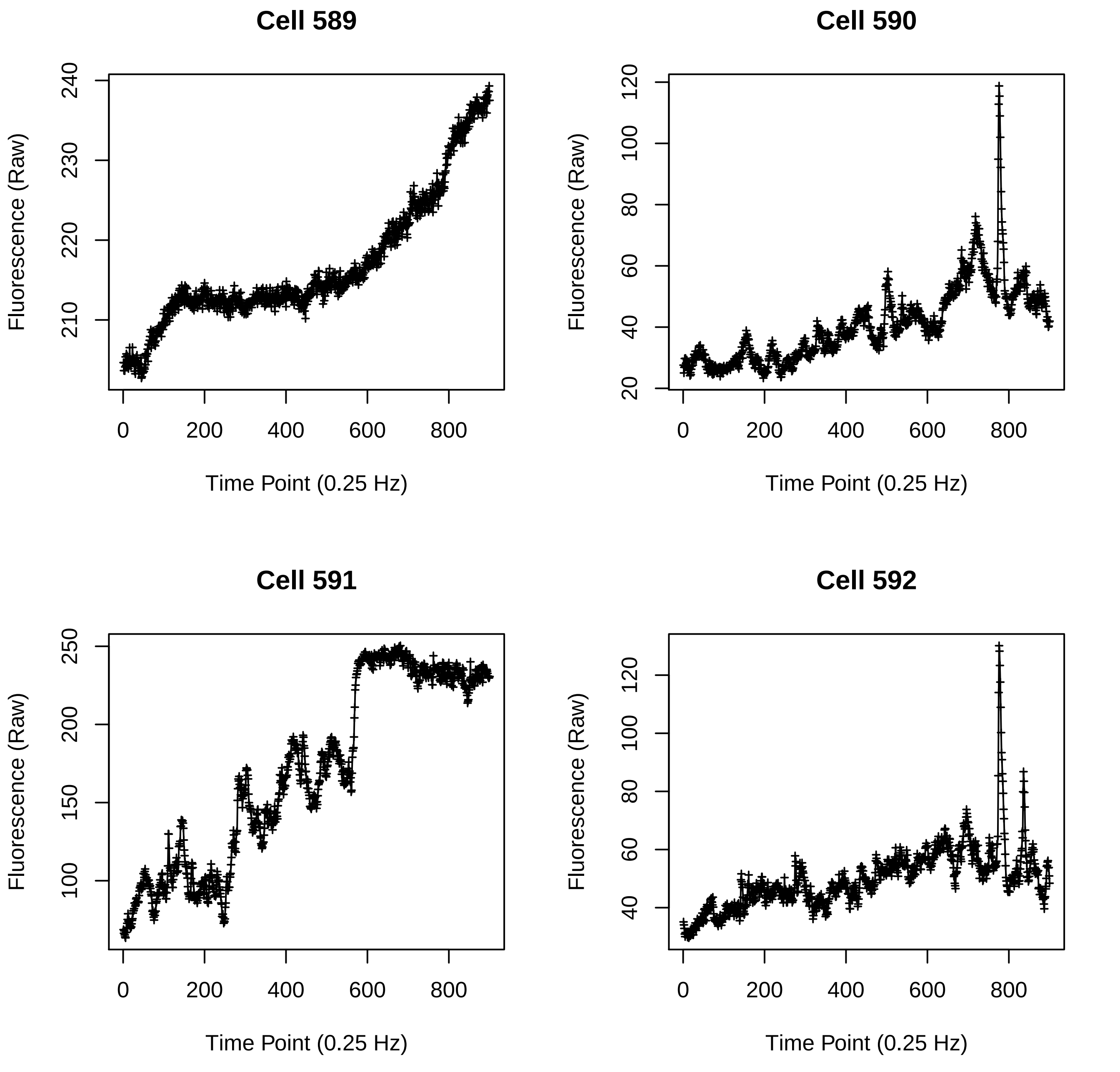
Cell 589
Fluorescence (Raw)
210
220
230
240
0
200
400
600
800
Time Point (0.25 Hz)
Cell 590
Fluorescence (Raw)
20
40
60
80
100
120
0
200
400
600
800
Time Point (0.25 Hz)
Cell 591
Fluorescence (Raw)
100
150
200
250
0
200
400
600
800
Time Point (0.25 Hz)
Cell 592
Fluorescence (Raw)
40
60
80
100
120
0
200
400
600
800
Time Point (0.25 Hz)

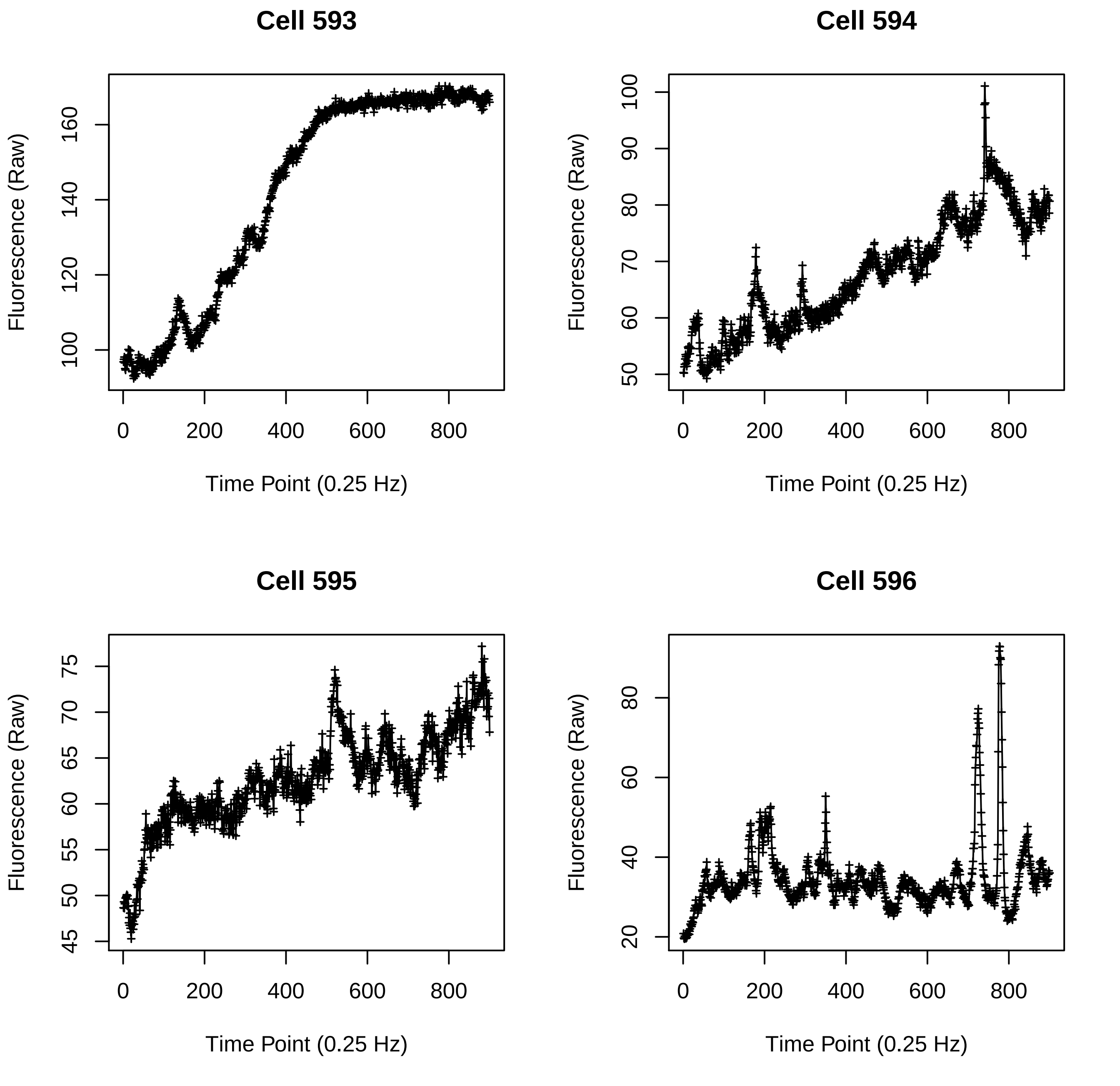
Cell 593
Cell 594
Cell 595
Cell 596
Fluorescence (Raw)
Time Point (0.25 Hz)
0
200
400
600
800
100
120
140
160
50
60
70
80
90
100
45
50
55
60
65
70
75
20
40
60
80

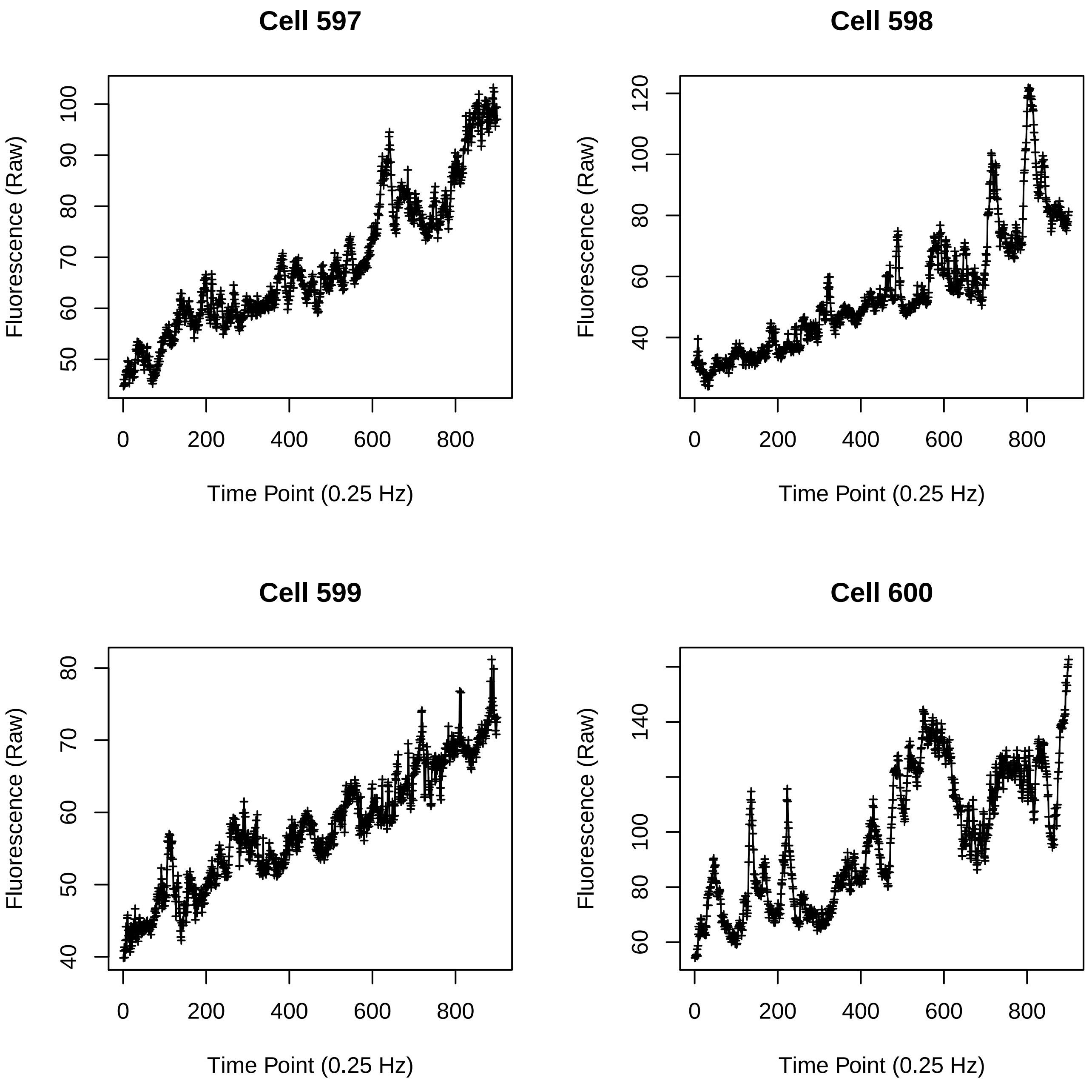
Cell 597
Cell 598
Cell 599
Cell 600
Fluorescence (Raw)
Time Point (0.25 Hz)
0
200
400
600
800
50
60
70
80
90
100
40
60
80
100
120
40
50
60
70
80
60
80
100
140

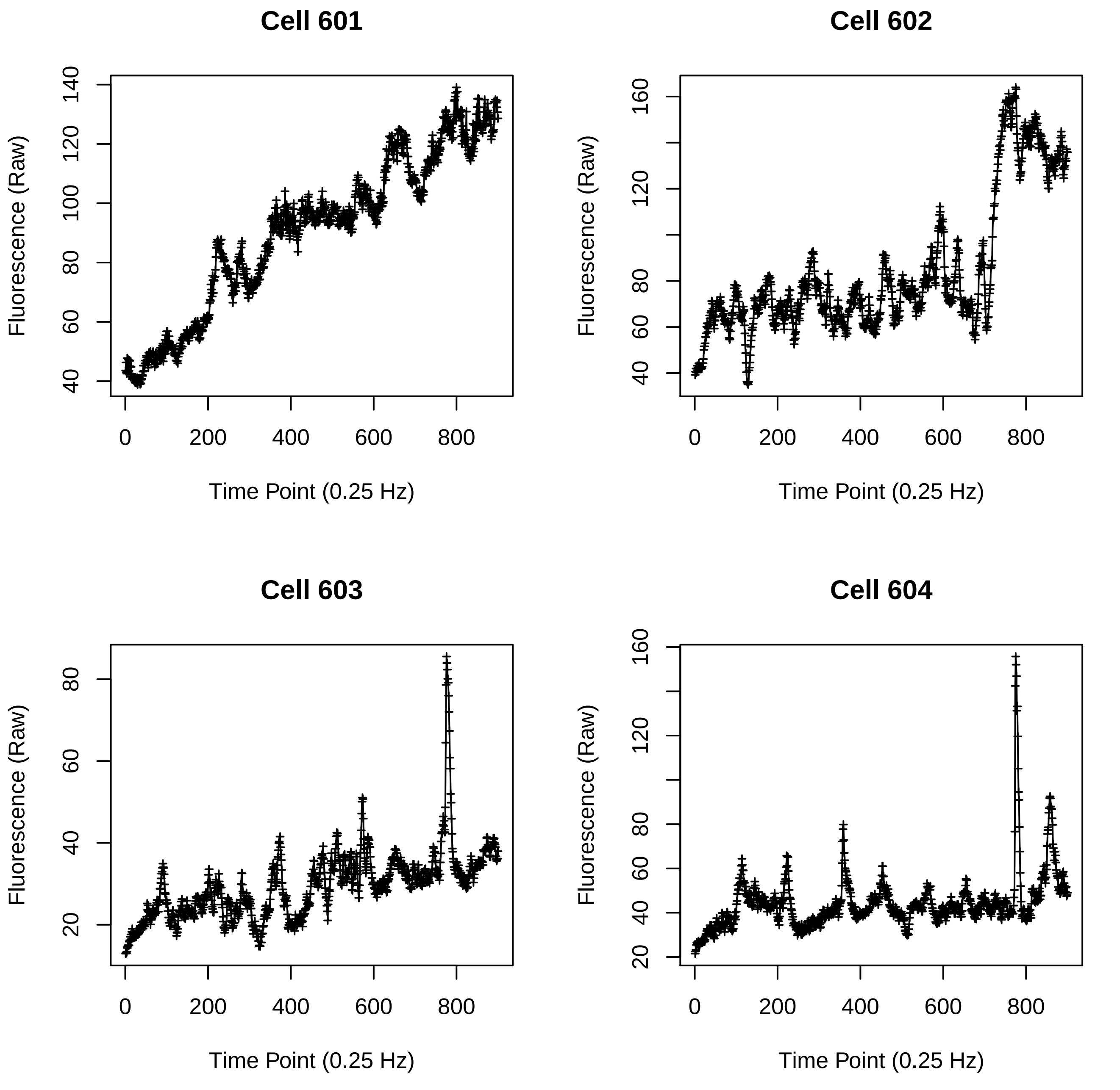
Cell 601
Cell 602
Cell 603
Cell 604
Fluorescence (Raw)
Time Point (0.25 Hz)
0
200
400
600
800
20
40
60
80
100
120
140
160

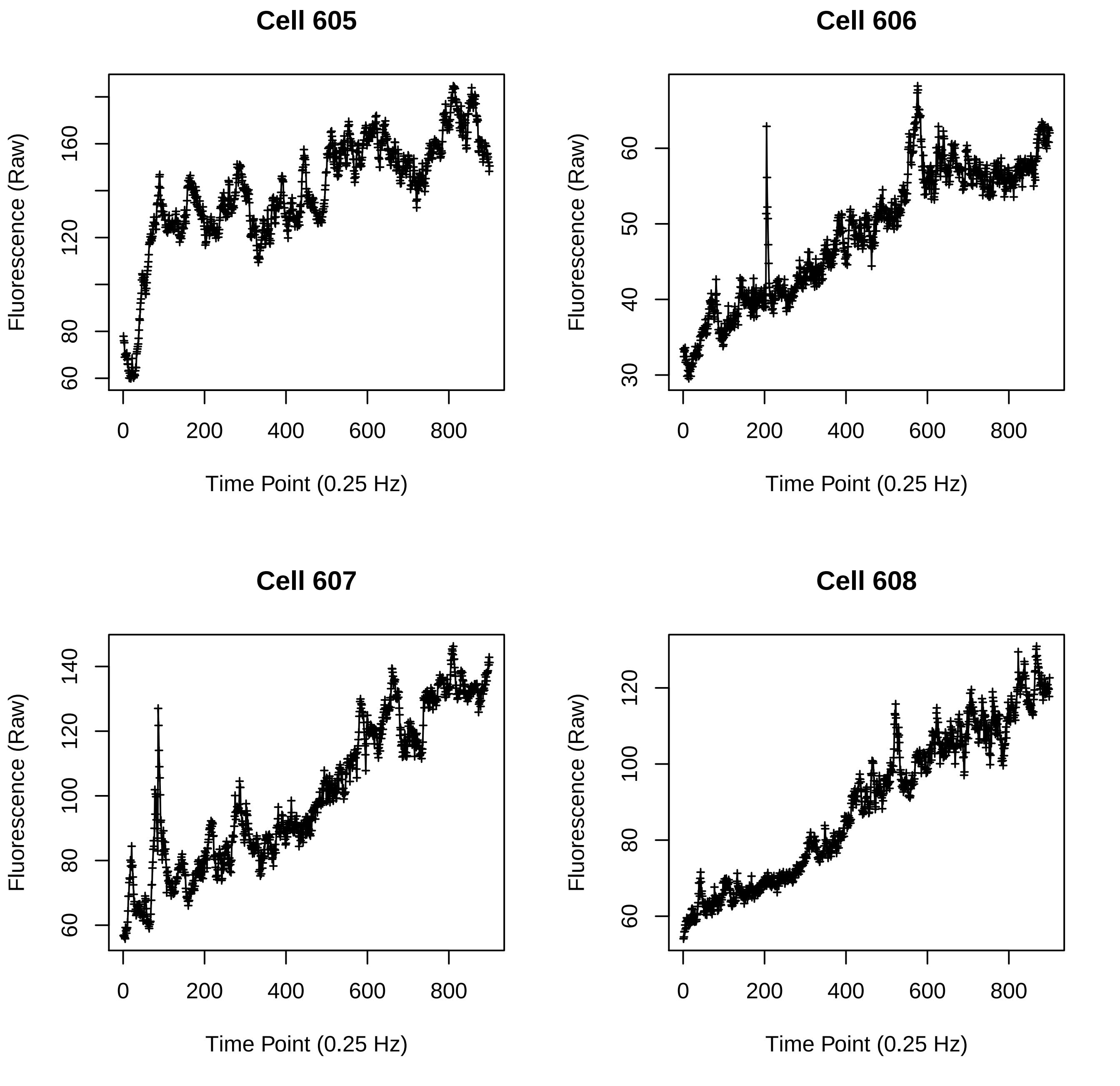
Cell 605
Fluorescence (Raw)
60
80
120
160
0
200
400
600
800
Time Point (0.25 Hz)
Cell 606
Fluorescence (Raw)
30
40
50
60
0
200
400
600
800
Time Point (0.25 Hz)
Cell 607
Fluorescence (Raw)
60
80
100
120
140
0
200
400
600
800
Time Point (0.25 Hz)
Cell 608
Fluorescence (Raw)
60
80
100
120
0
200
400
600
800
Time Point (0.25 Hz)

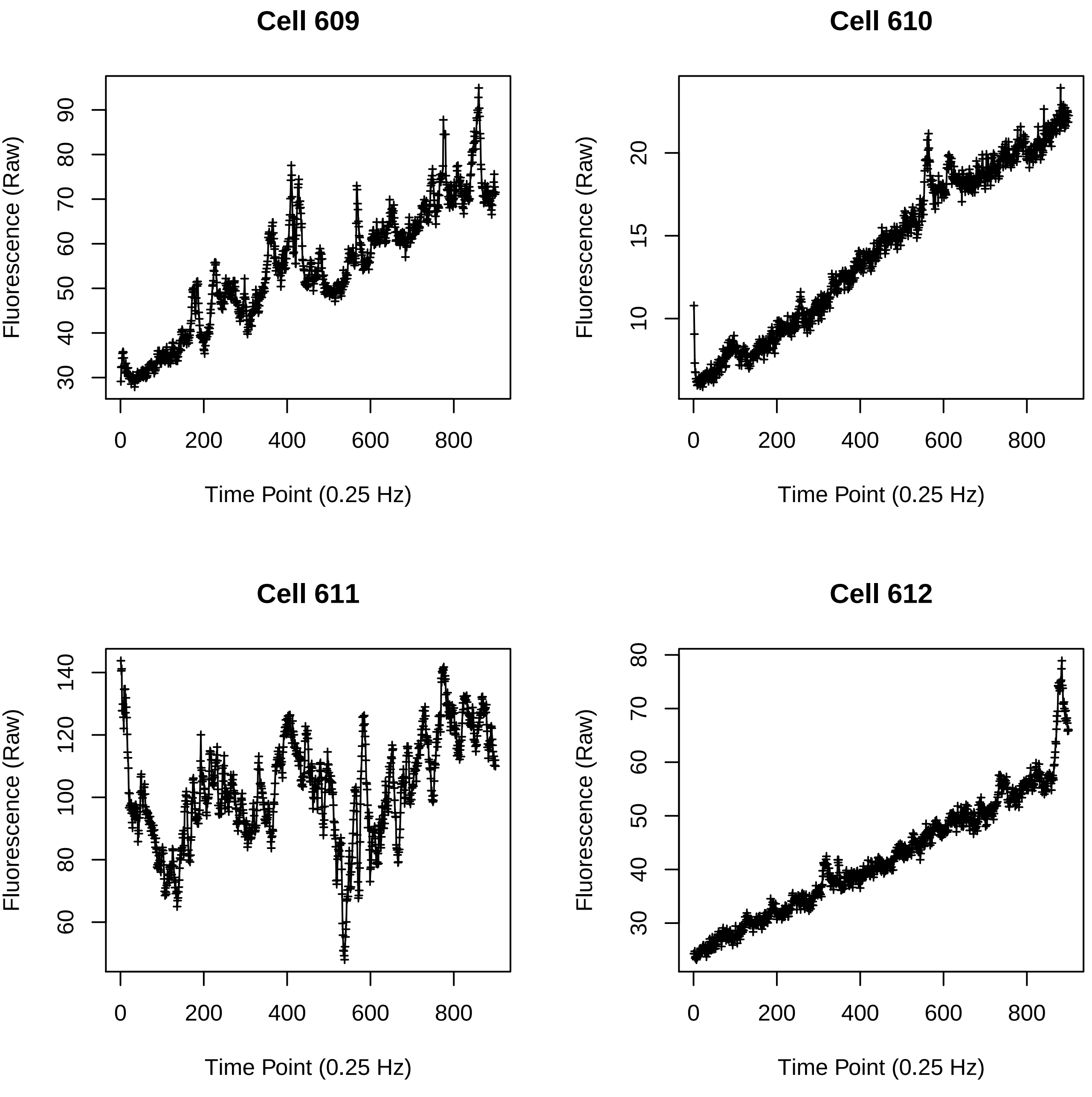

Cell 609
Fluorescence (Raw)
30
40
50
60
70
80
90
0
200
400
600
800
Time Point (0.25 Hz)
Cell 610
Fluorescence (Raw)
10
15
20
0
200
400
600
800
Time Point (0.25 Hz)
Cell 611
Fluorescence (Raw)
60
80
100
120
140
0
200
400
600
800
Time Point (0.25 Hz)
Cell 612
Fluorescence (Raw)
30
40
50
60
70
80
0
200
400
600
800
Time Point (0.25 Hz)

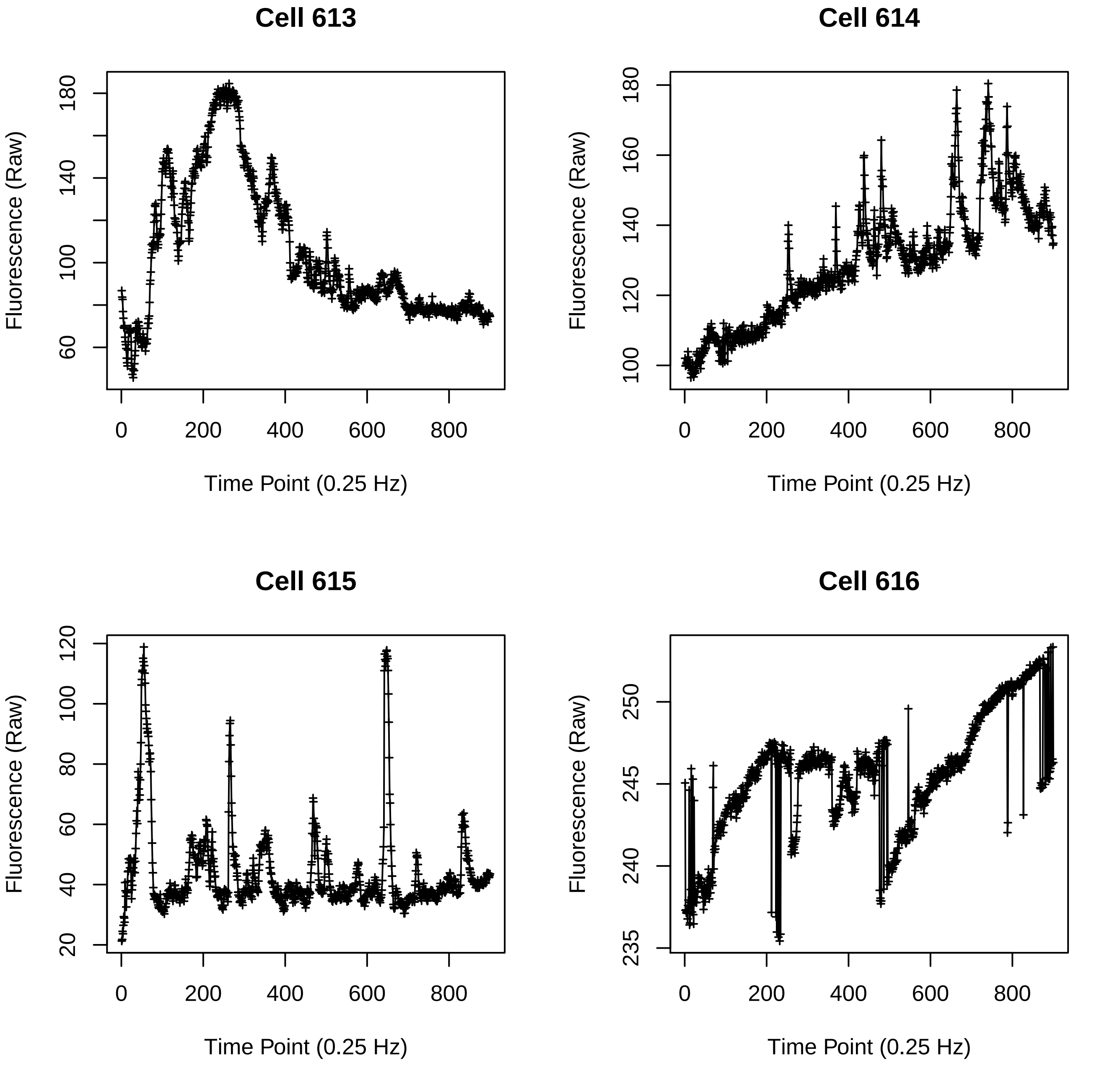

Cell 613
Fluorescence (Raw)
60
100
140
180
0
200
400
600
800
Time Point (0.25 Hz)
Cell 614
Fluorescence (Raw)
100
120
140
160
180
0
200
400
600
800
Time Point (0.25 Hz)
Cell 615
Fluorescence (Raw)
20
40
60
80
100
120
0
200
400
600
800
Time Point (0.25 Hz)
Cell 616
Fluorescence (Raw)
235
240
245
250
0
200
400
600
800
Time Point (0.25 Hz)

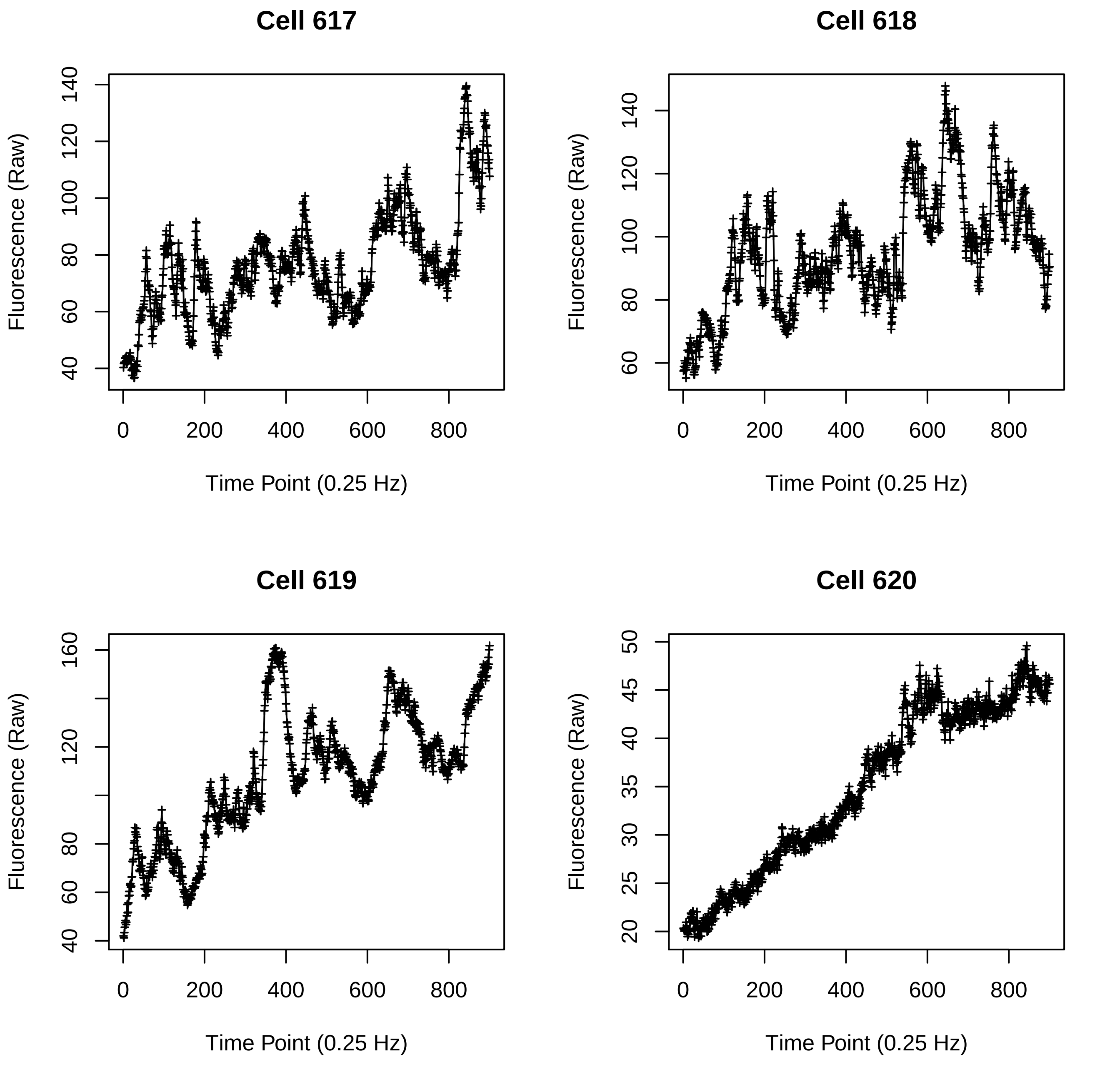
Cell 617
Cell 618
Cell 619
Cell 620
Fluorescence (Raw)
Time Point (0.25 Hz)
0
200
400
600
800
40
60
80
100
120
140
160
20
25
30
35
45
50

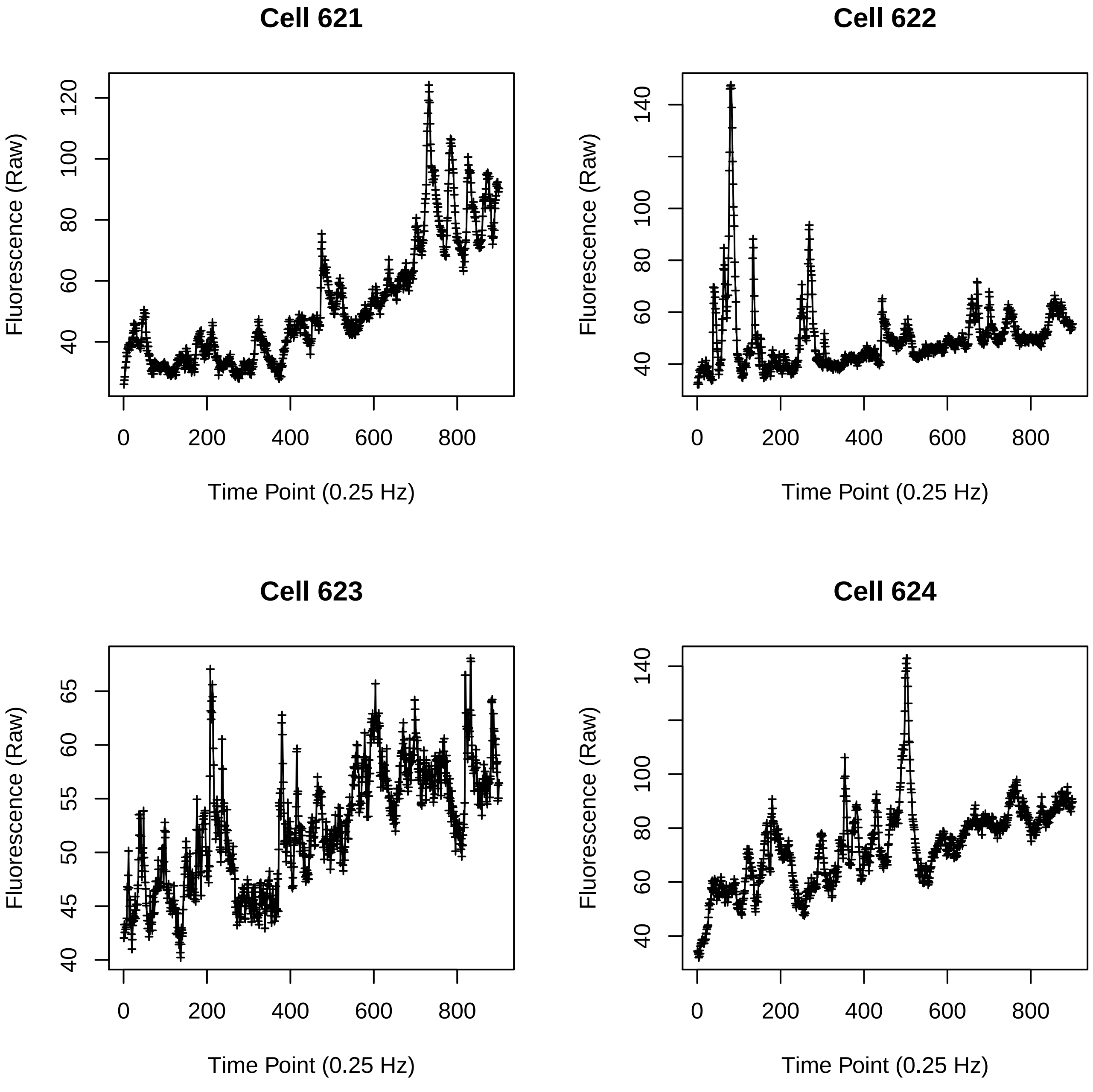
Cell 621
Fluorescence (Raw)
40
60
80
100
120
0
200
400
600
800
Time Point (0.25 Hz)
Cell 622
Fluorescence (Raw)
40
60
80
100
140
0
200
400
600
800
Time Point (0.25 Hz)
Cell 623
Fluorescence (Raw)
40
45
50
55
60
65
0
200
400
600
800
Time Point (0.25 Hz)
Cell 624
Fluorescence (Raw)
40
60
80
100
140
0
200
400
600
800
Time Point (0.25 Hz)

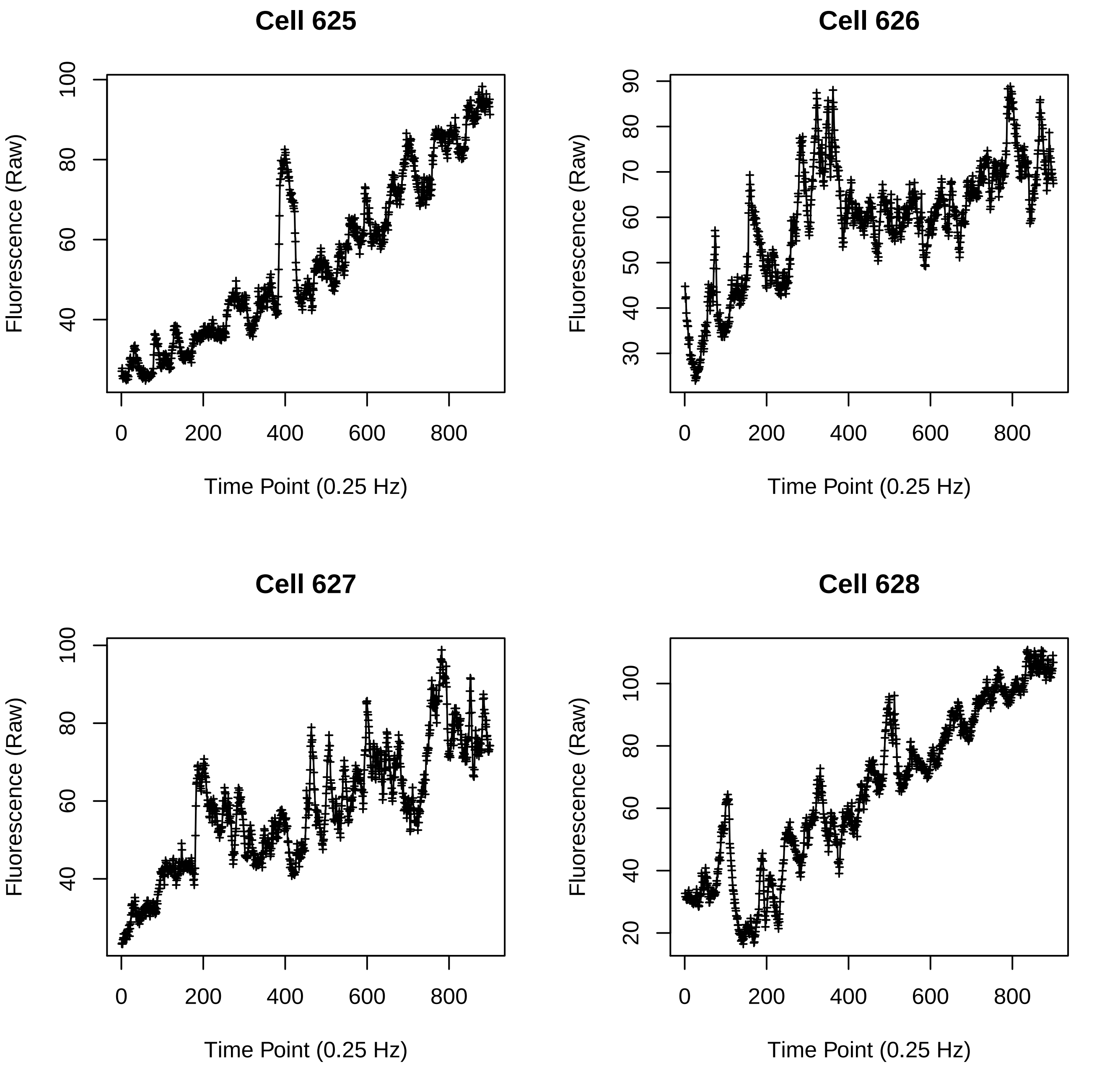
Cell 625
Fluorescence (Raw)
40
60
80
100
0
200
400
600
800
Time Point (0.25 Hz)
Cell 626
Fluorescence (Raw)
30
40
50
60
70
80
90
0
200
400
600
800
Time Point (0.25 Hz)
Cell 627
Fluorescence (Raw)
40
60
80
100
0
200
400
600
800
Time Point (0.25 Hz)
Cell 628
Fluorescence (Raw)
20
40
60
80
100
0
200
400
600
800
Time Point (0.25 Hz)

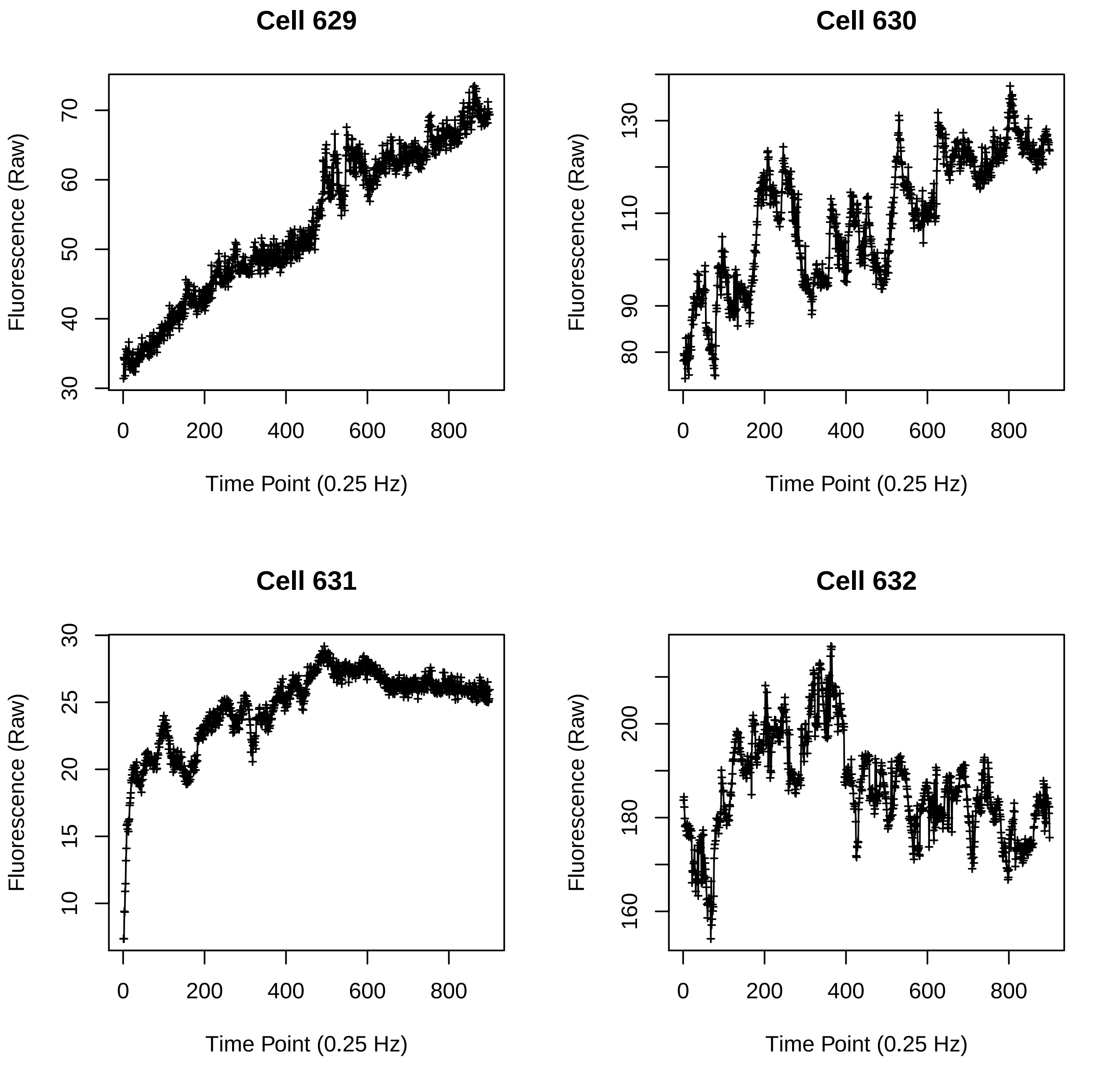

Cell 629
Fluorescence (Raw)
30
40
50
60
70
0
200
400
600
800
Time Point (0.25 Hz)
Cell 630
Fluorescence (Raw)
80
90
110
130
0
200
400
600
800
Time Point (0.25 Hz)
Cell 631
Fluorescence (Raw)
10
15
20
25
30
0
200
400
600
800
Time Point (0.25 Hz)
Cell 632
Fluorescence (Raw)
160
180
200
0
200
400
600
800
Time Point (0.25 Hz)

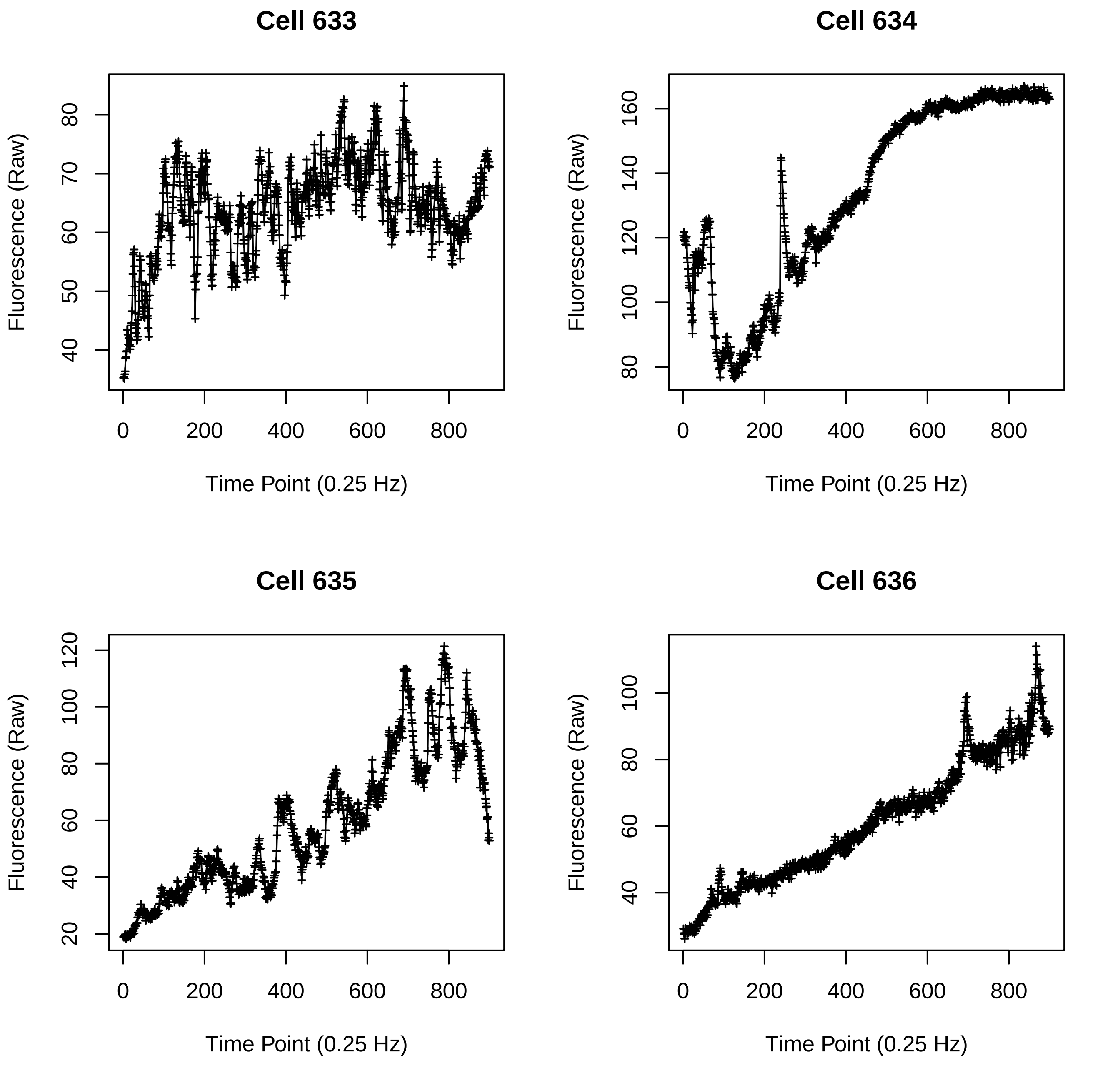

Cell 633
Cell 634
Cell 635
Cell 636
Fluorescence (Raw)
Time Point (0.25 Hz)
0
200
400
600
800
40
50
60
70
80
100
120
140
160
20

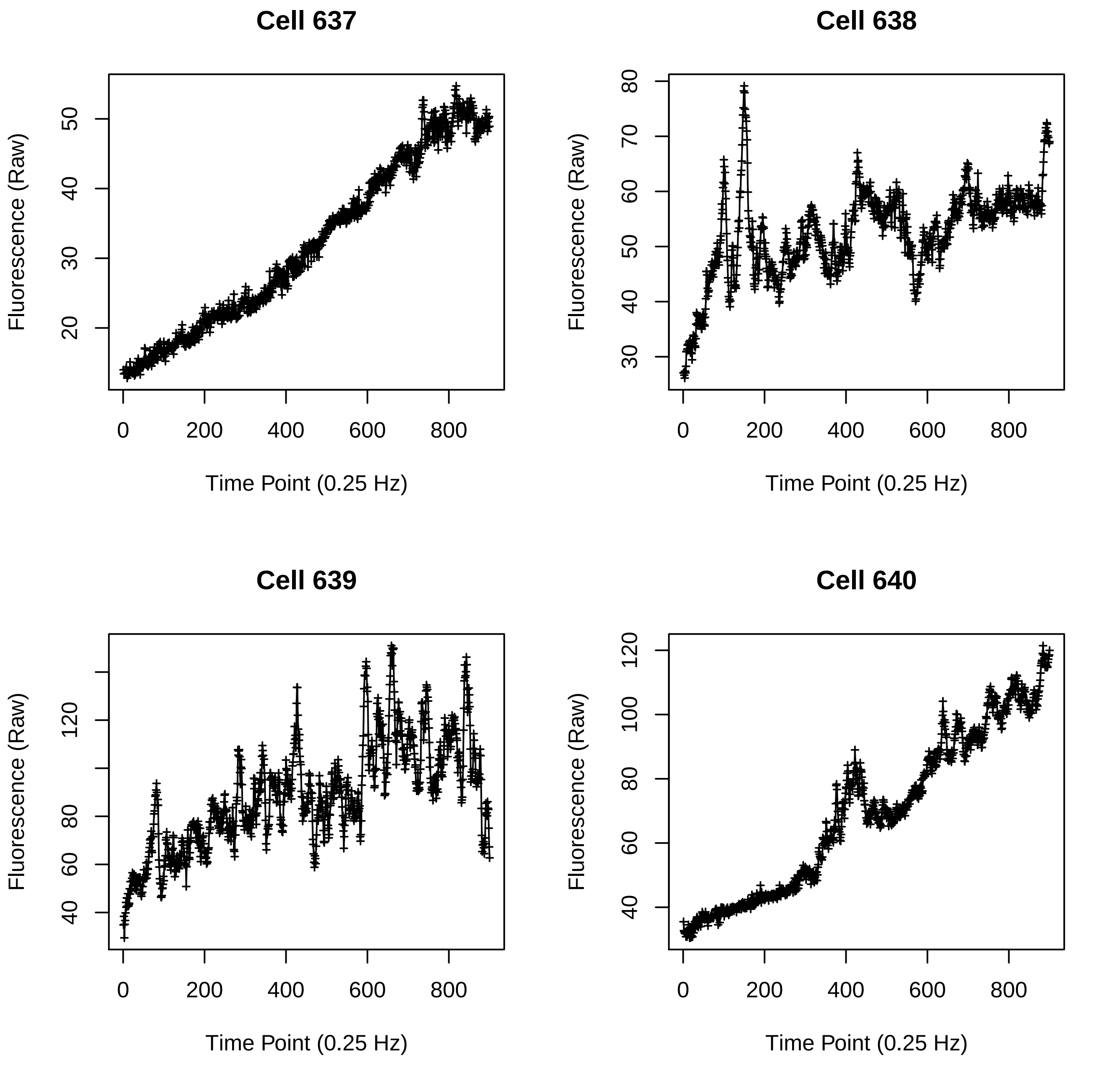
Cell 637
Fluorescence (Raw)
20
30
40
50
Time Point (0.25 Hz)
0
200
400
600
800
Cell 638
Fluorescence (Raw)
30
40
50
60
70
80
Time Point (0.25 Hz)
0
200
400
600
800
Cell 639
Fluorescence (Raw)
40
60
80
120
Time Point (0.25 Hz)
0
200
400
600
800
Cell 640
Fluorescence (Raw)
40
60
80
100
120
Time Point (0.25 Hz)
0
200
400
600
800

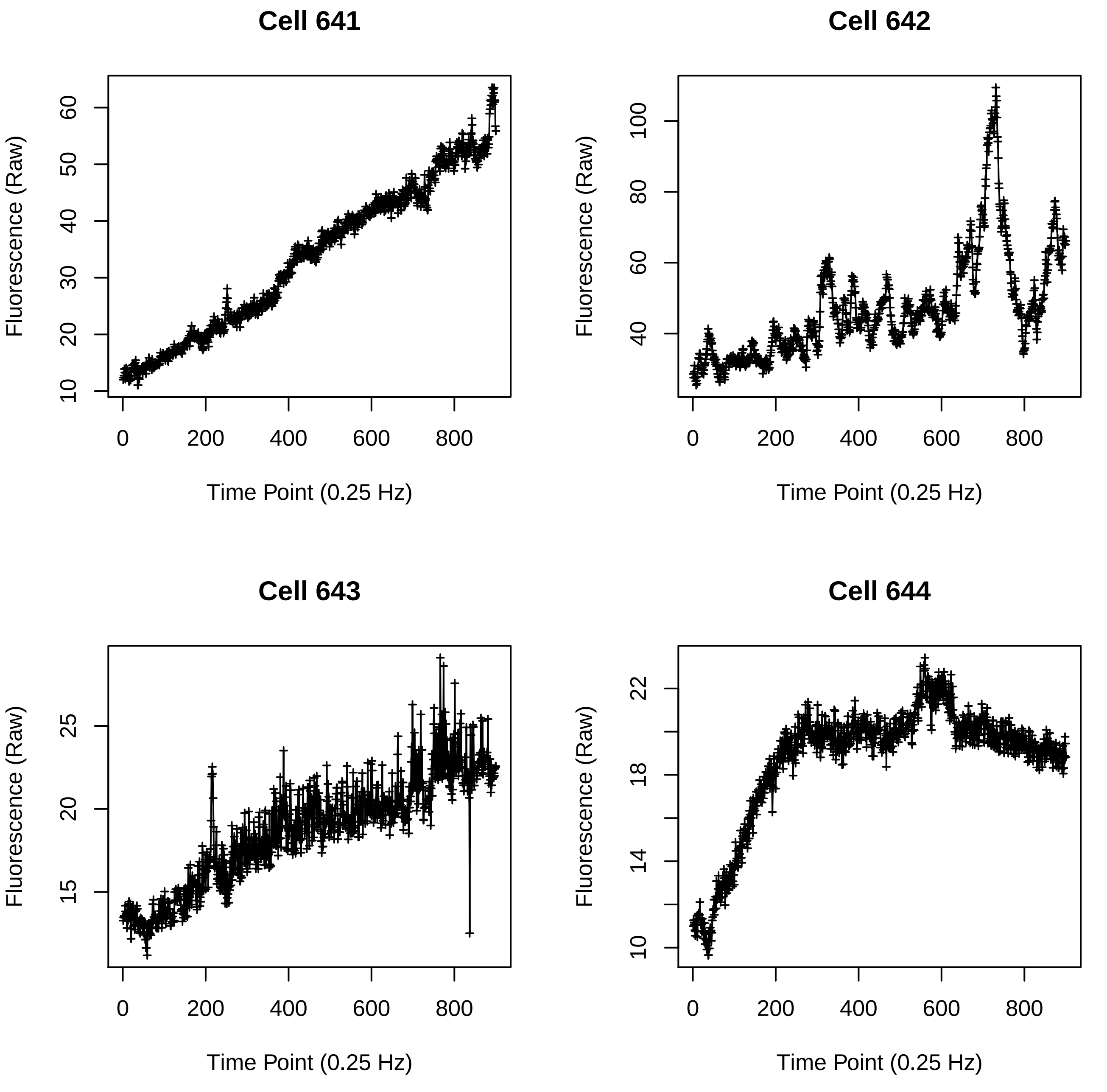
Cell 641
Fluorescence (Raw)
10
20
30
40
50
60
0
200
400
600
800
Time Point (0.25 Hz)
Cell 642
Fluorescence (Raw)
40
60
80
100
0
200
400
600
800
Time Point (0.25 Hz)
Cell 643
Fluorescence (Raw)
15
20
25
0
200
400
600
800
Time Point (0.25 Hz)
Cell 644
Fluorescence (Raw)
10
14
18
22
0
200
400
600
800
Time Point (0.25 Hz)

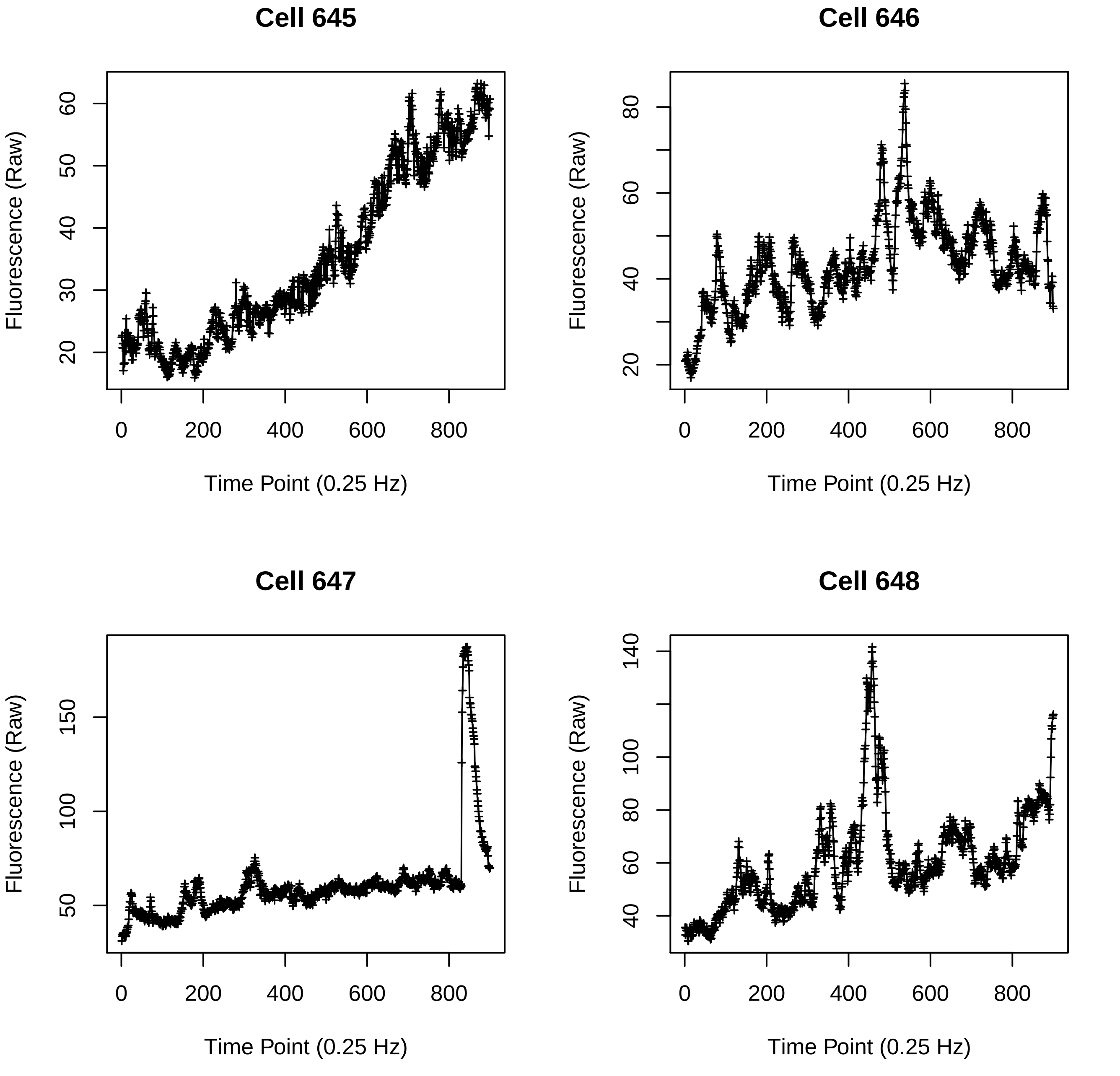
Cell 645
Fluorescence (Raw)
20
30
40
50
60
0
200
400
600
800
Time Point (0.25 Hz)
Cell 646
Fluorescence (Raw)
20
40
60
80
0
200
400
600
800
Time Point (0.25 Hz)
Cell 647
Fluorescence (Raw)
50
100
150
0
200
400
600
800
Time Point (0.25 Hz)
Cell 648
Fluorescence (Raw)
40
60
80
100
140
0
200
400
600
800
Time Point (0.25 Hz)

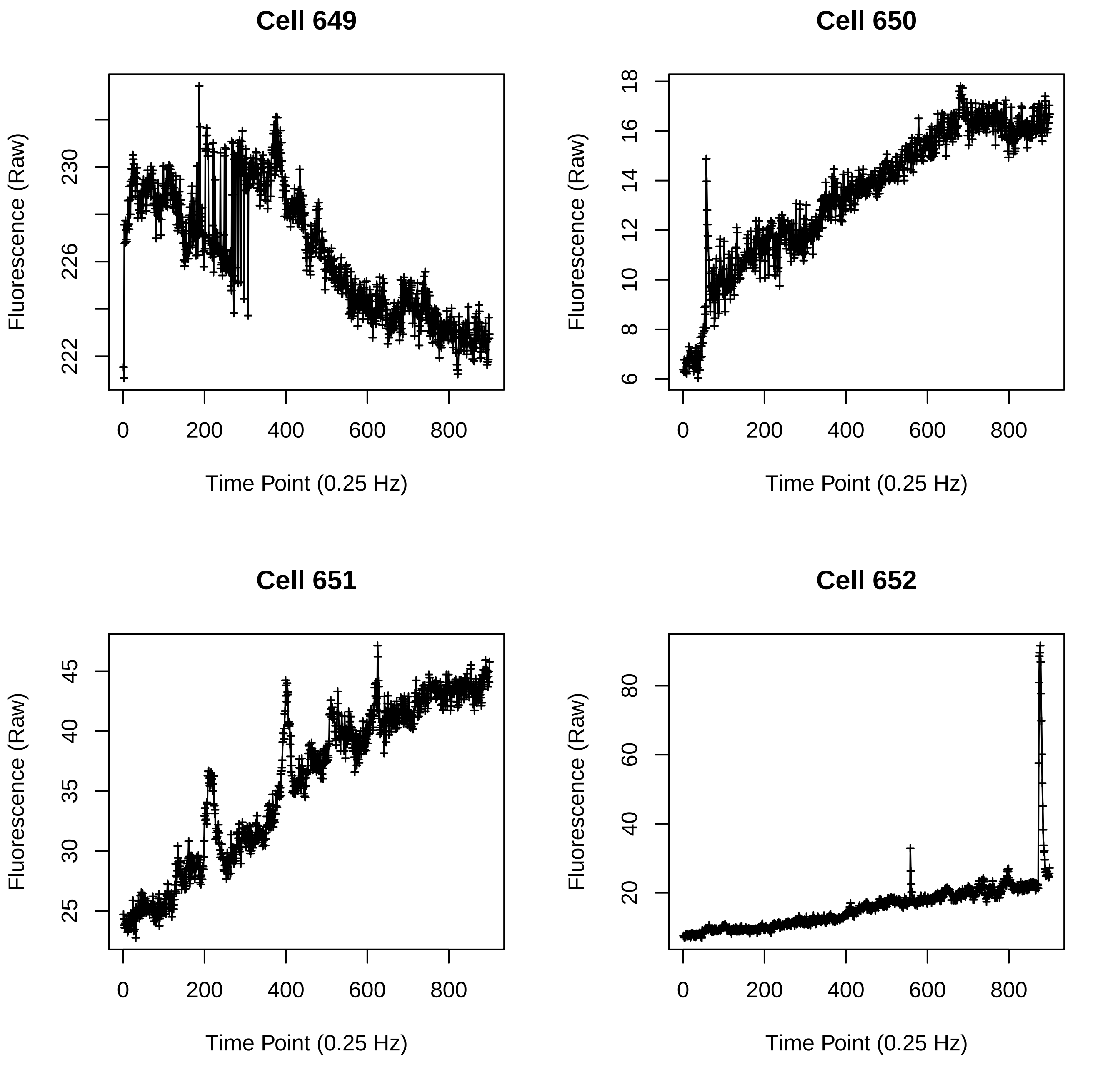

Cell 649
Fluorescence (Raw)
222
226
230
0
200
400
600
800
Time Point (0.25 Hz)
Cell 650
Fluorescence (Raw)
6
8
10
12
14
16
18
0
200
400
600
800
Time Point (0.25 Hz)
Cell 651
Fluorescence (Raw)
25
30
35
40
45
0
200
400
600
800
Time Point (0.25 Hz)
Cell 652
Fluorescence (Raw)
20
40
60
80
0
200
400
600
800
Time Point (0.25 Hz)

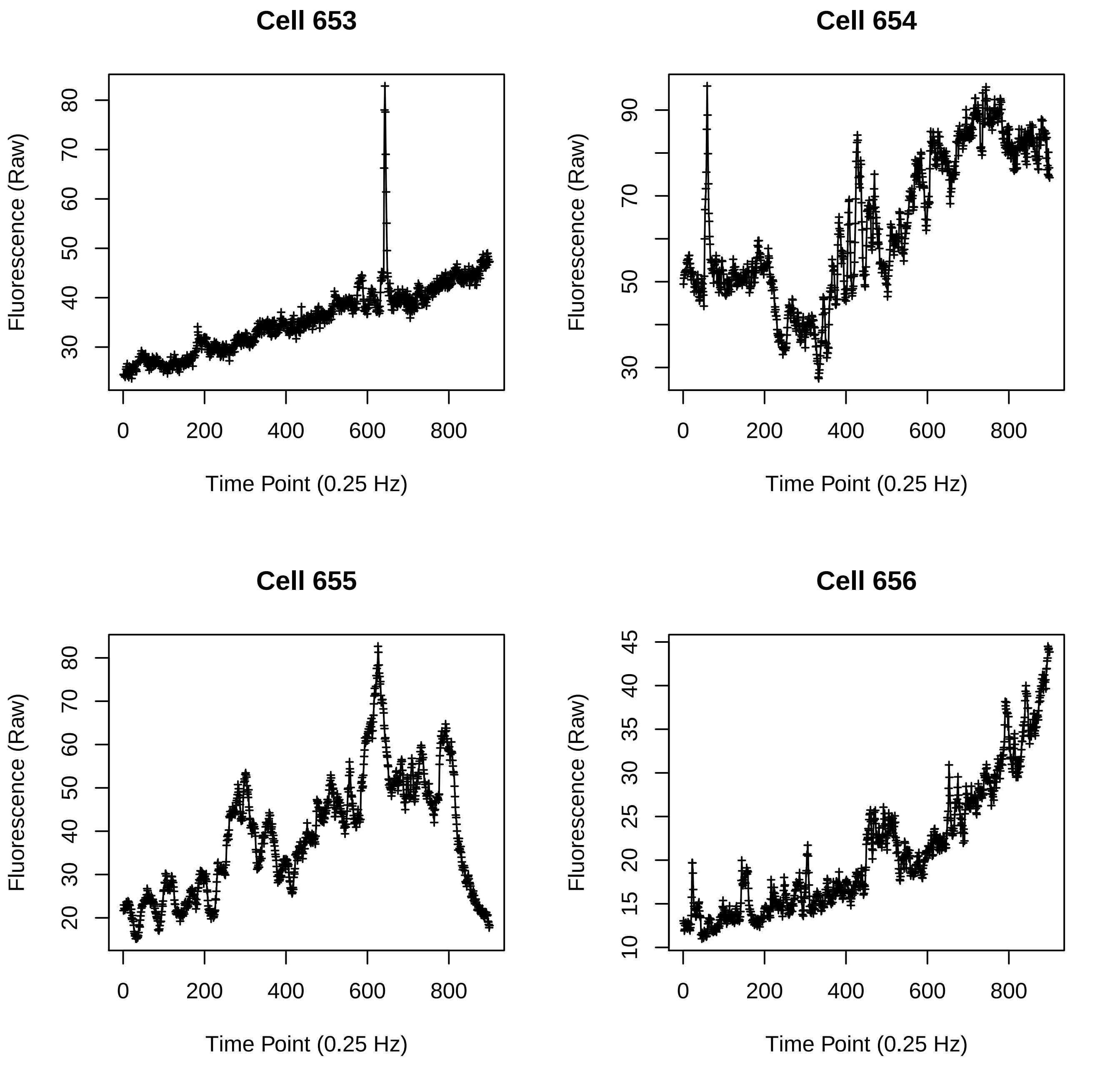
Cell 653
Cell 654
Cell 655
Cell 656
Fluorescence (Raw)
Time Point (0.25 Hz)
0
200
400
600
800
10
15
20
25
30
35
40
45
50
60
70
80
90

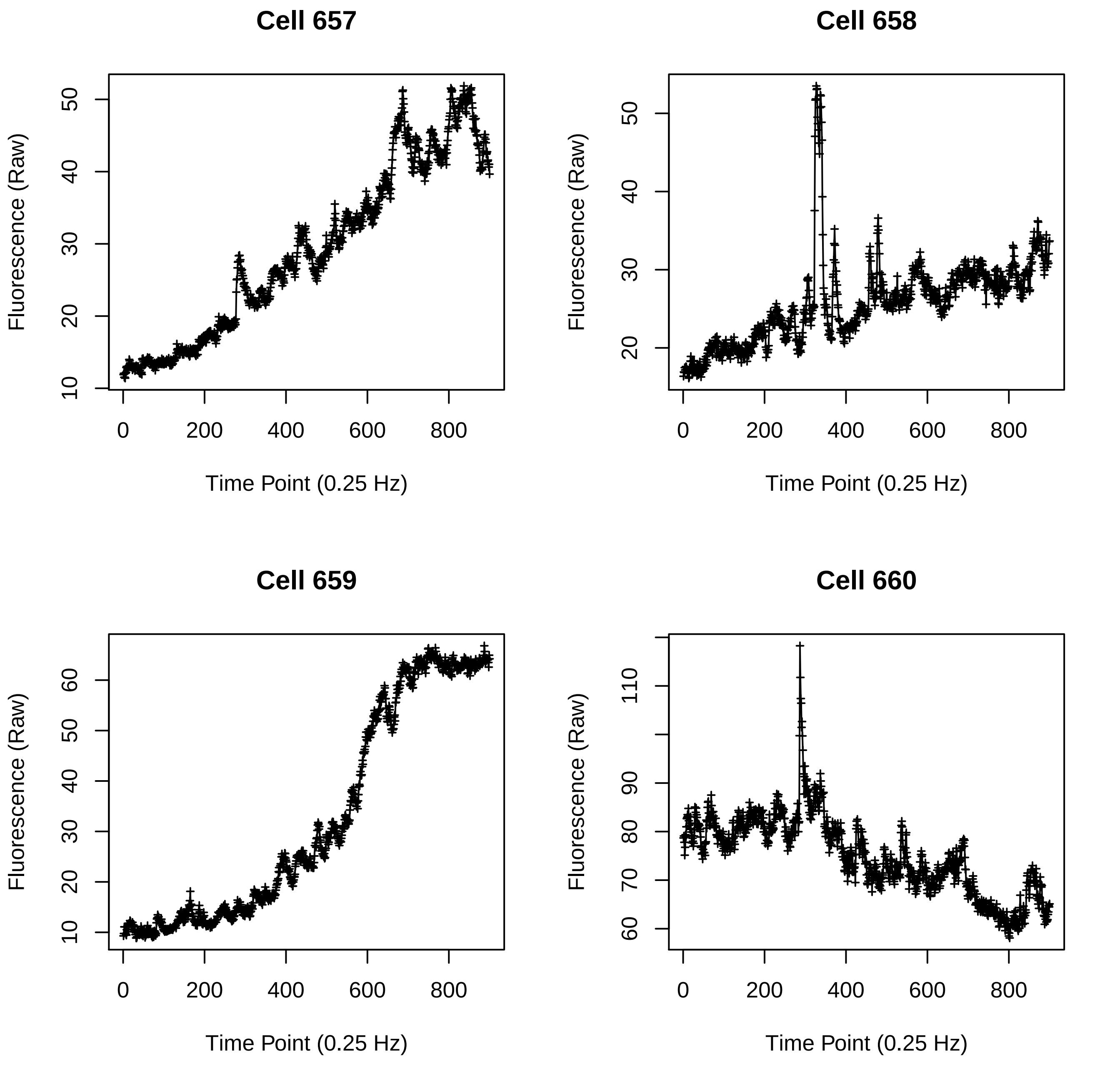

Cell 657
Cell 658
Cell 659
Cell 660
Fluorescence (Raw)
Time Point (0.25 Hz)
0
200
400
600
800
10
20
30
40
50
60
70
80
90
110

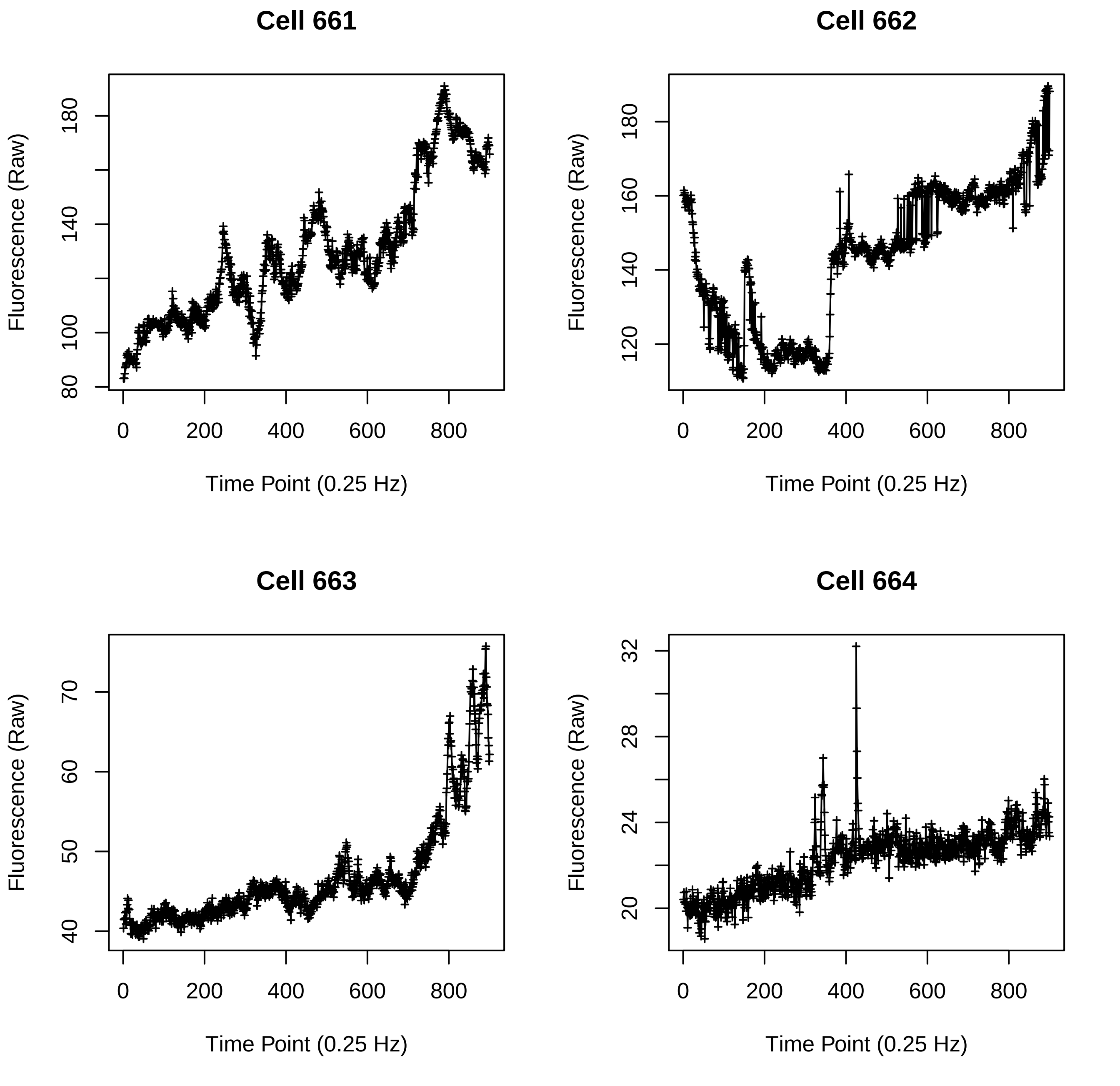
Cell 661
Cell 662
Cell 663
Cell 664
Fluorescence (Raw)
Time Point (0.25 Hz)
0
200
400
600
800
80
100
140
180
120
140
160
180
40
50
60
70
20
24
28
32

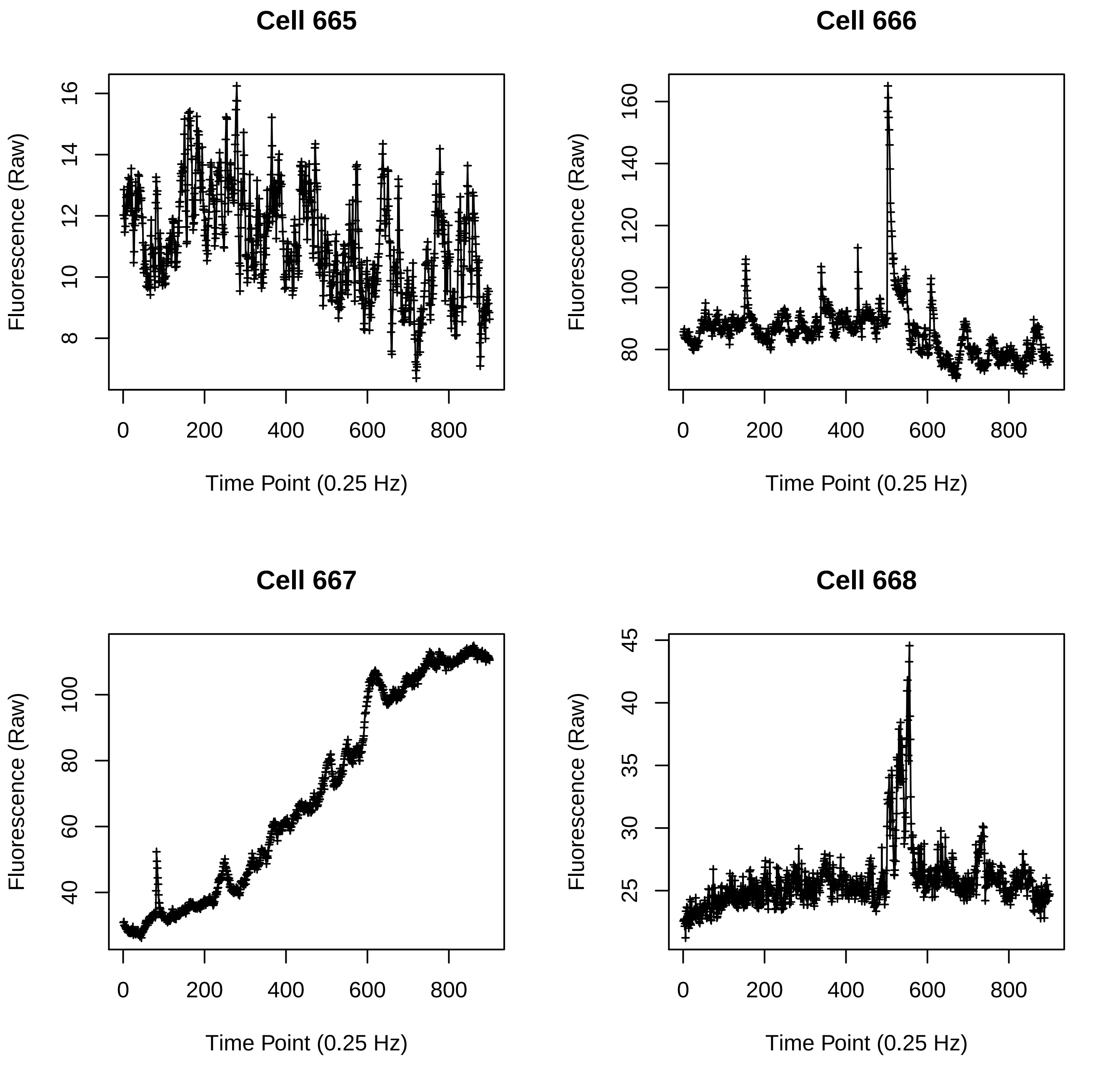

Cell 665
Cell 666
Cell 667
Cell 668
Fluorescence (Raw)
Time Point (0.25 Hz)
0
200
400
600
800
8
10
12
14
16
80
100
120
140
160
40
60
80
100
25
30
35
40
45

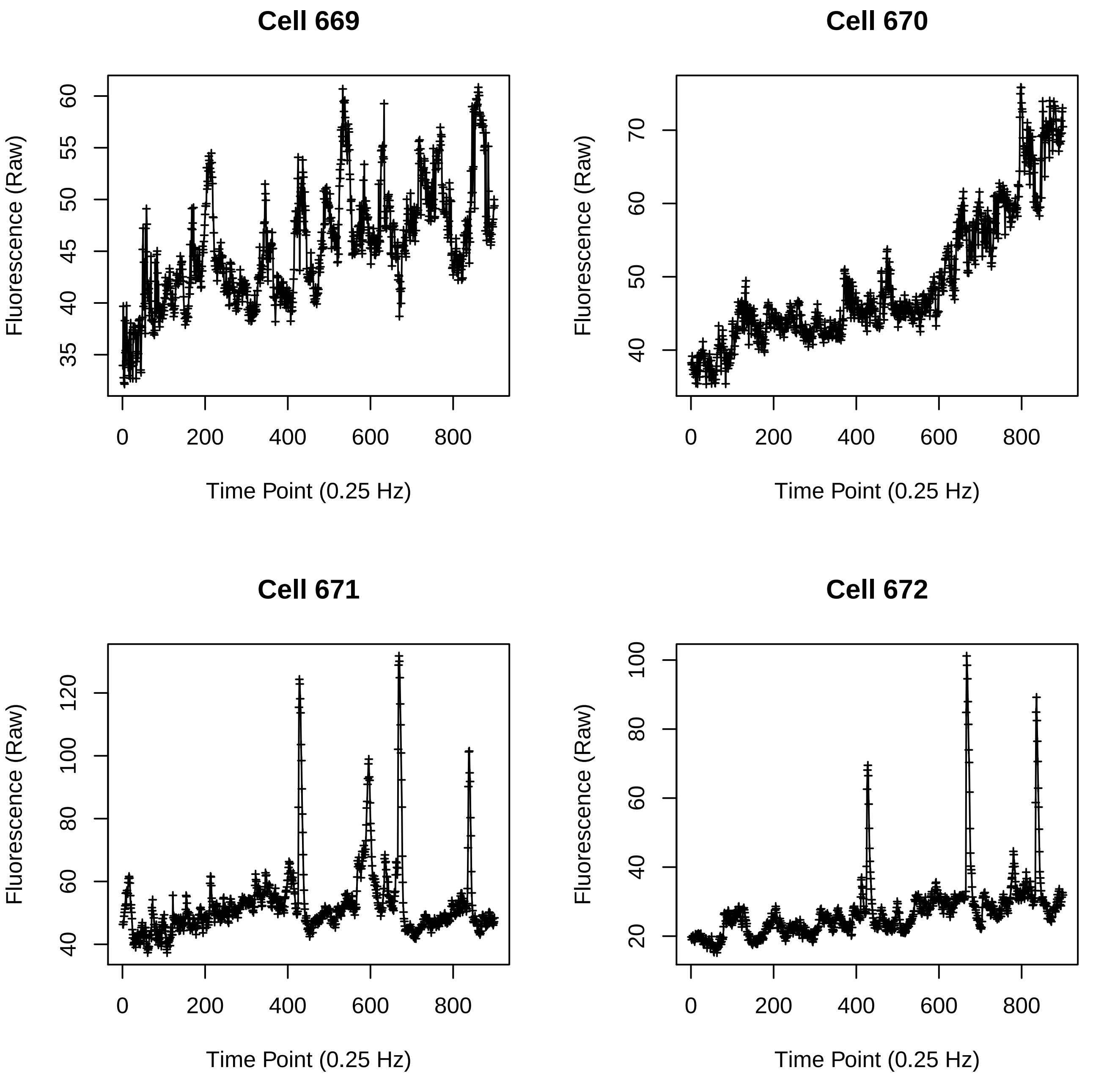
Cell 669
Cell 670
Cell 671
Cell 672
Fluorescence (Raw)
Time Point (0.25 Hz)
0
200
400
600
800
35
40
45
50
55
60
70
20
80
100
120

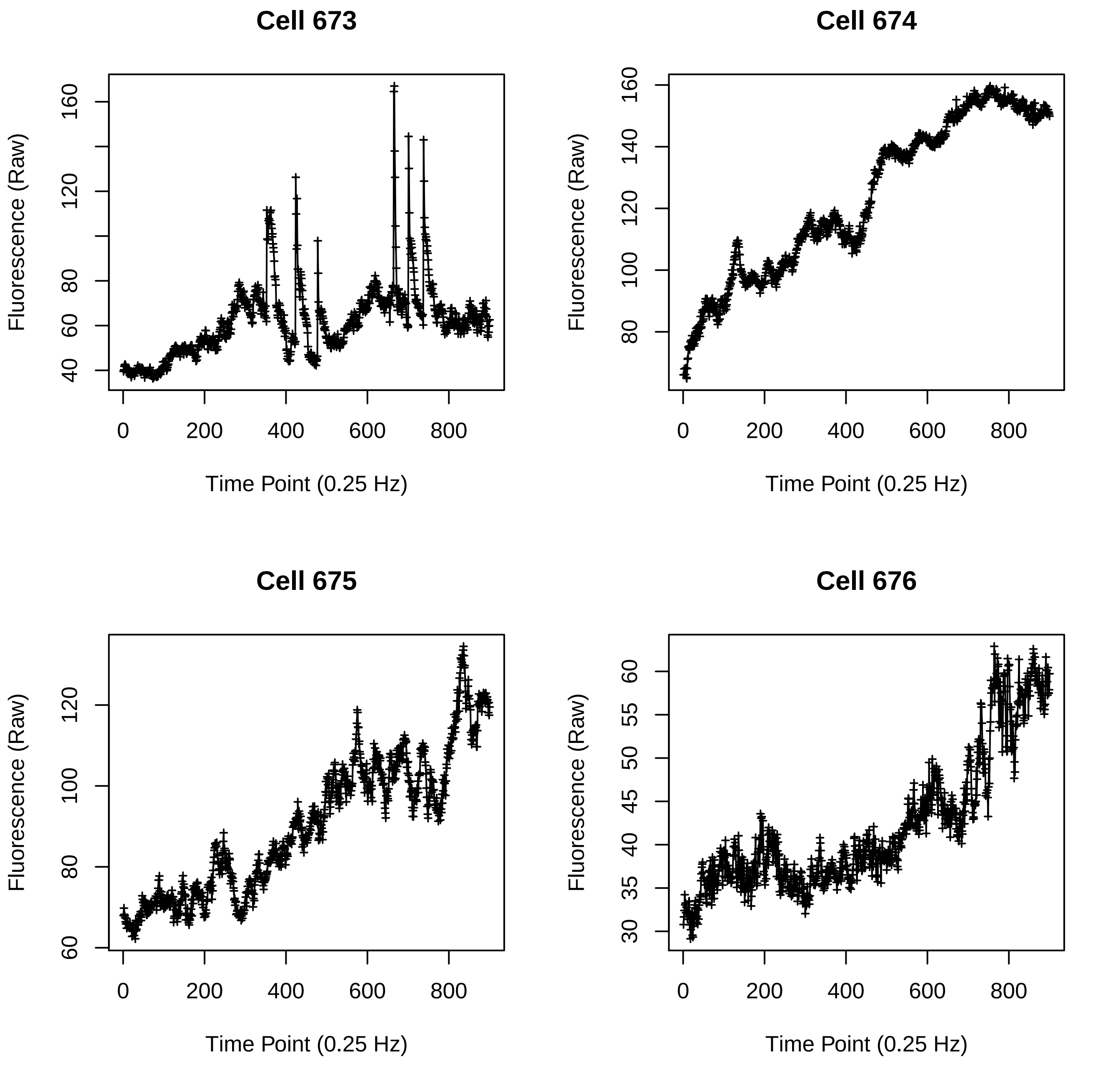
Cell 673
Cell 674
Cell 675
Cell 676
Fluorescence (Raw)
Time Point (0.25 Hz)
0
200
400
600
800
40
60
80
120
160
100
140
30
35
45
50
55

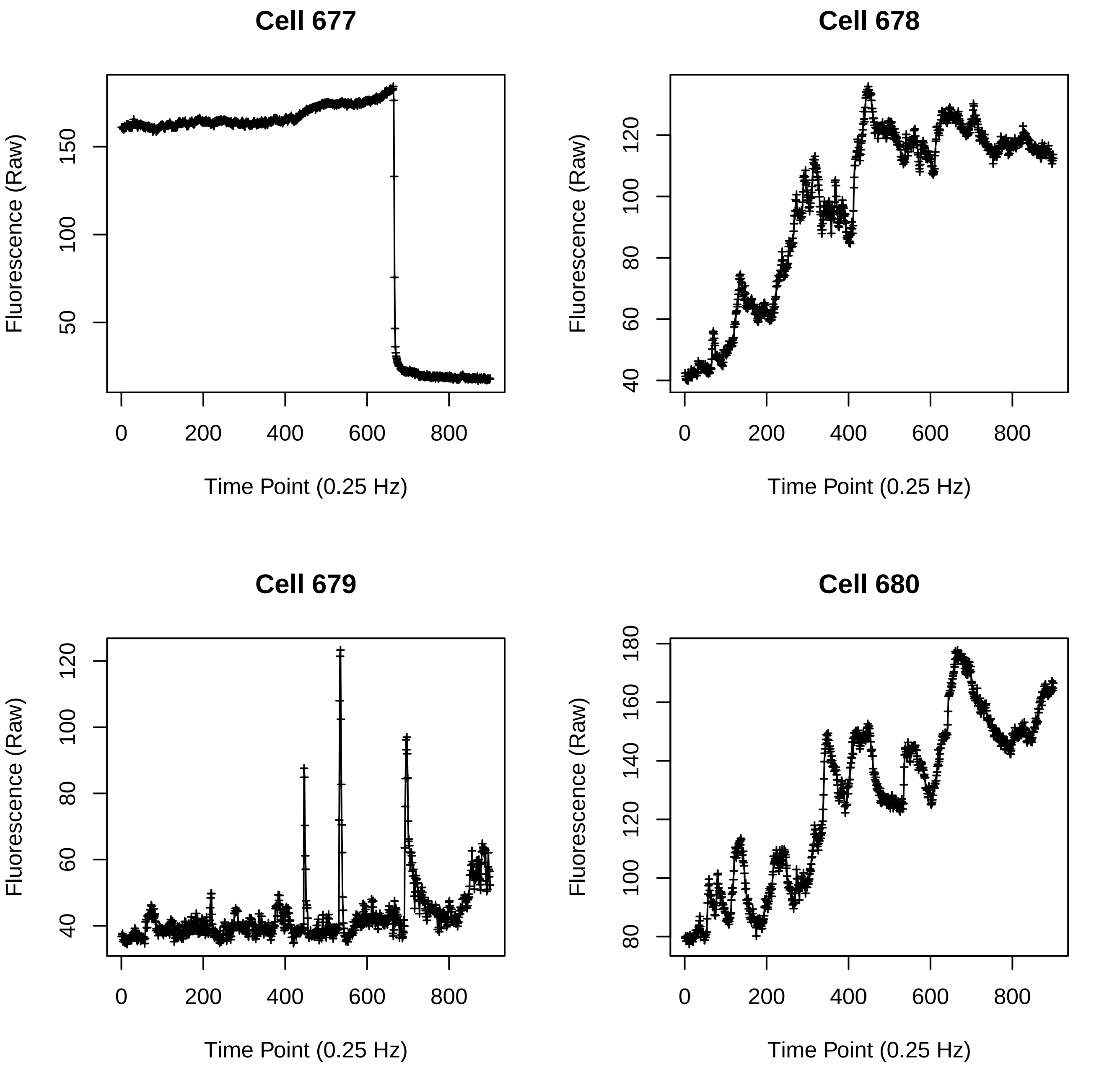

Cell 677
Fluorescence (Raw)
50
100
150
0
200
400
600
800
Time Point (0.25 Hz)
Cell 678
Fluorescence (Raw)
40
60
80
100
120
0
200
400
600
800
Time Point (0.25 Hz)
Cell 679
Fluorescence (Raw)
40
60
80
100
120
0
200
400
600
800
Time Point (0.25 Hz)
Cell 680
Fluorescence (Raw)
80
100
120
140
160
180
0
200
400
600
800
Time Point (0.25 Hz)

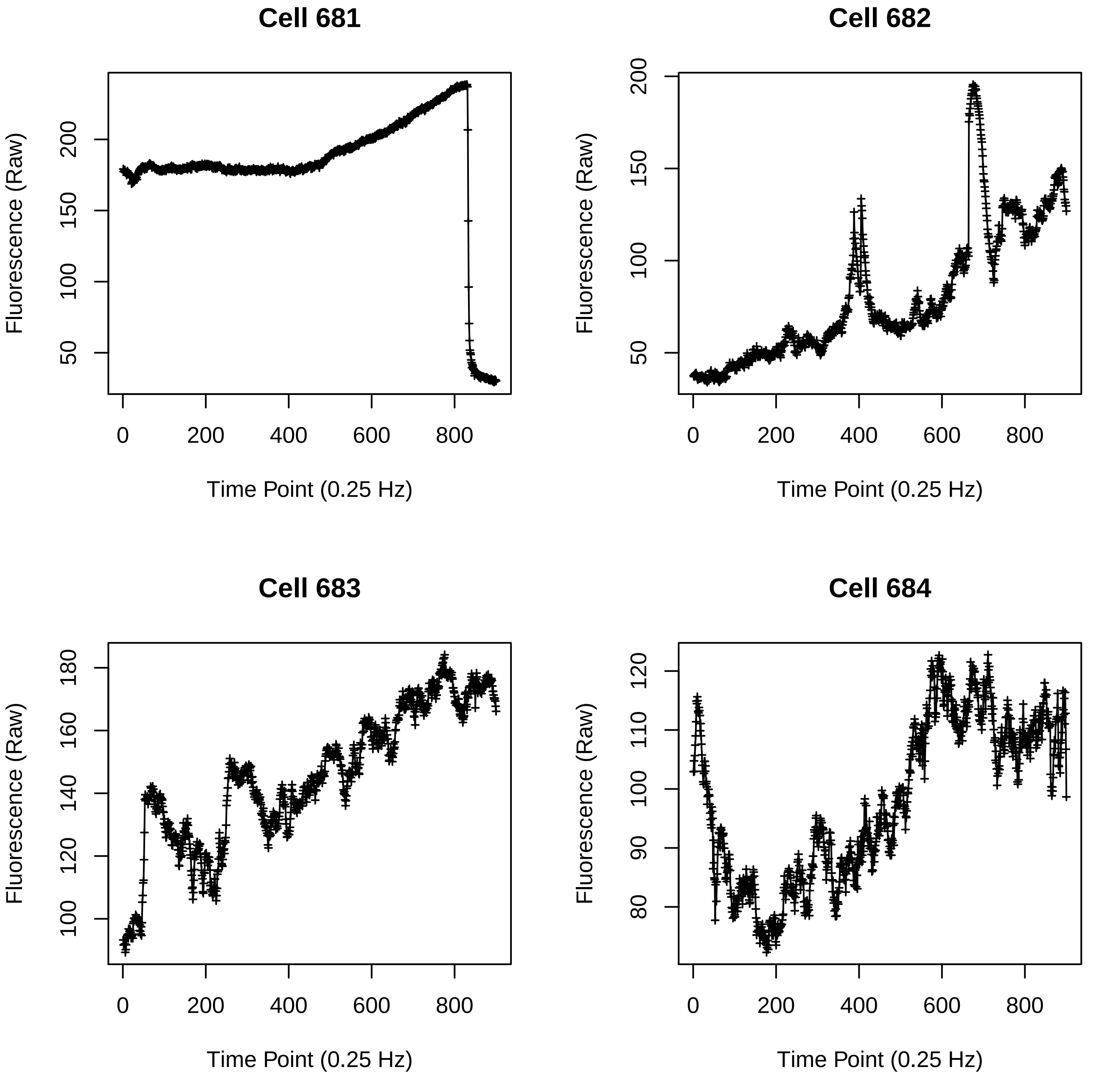
Cell 681
Cell 682
Cell 683
Cell 684
Fluorescence (Raw)
Time Point (0.25 Hz)
0
200
400
600
800
50
100
150
200
80
90
100
110
120
140
160
180

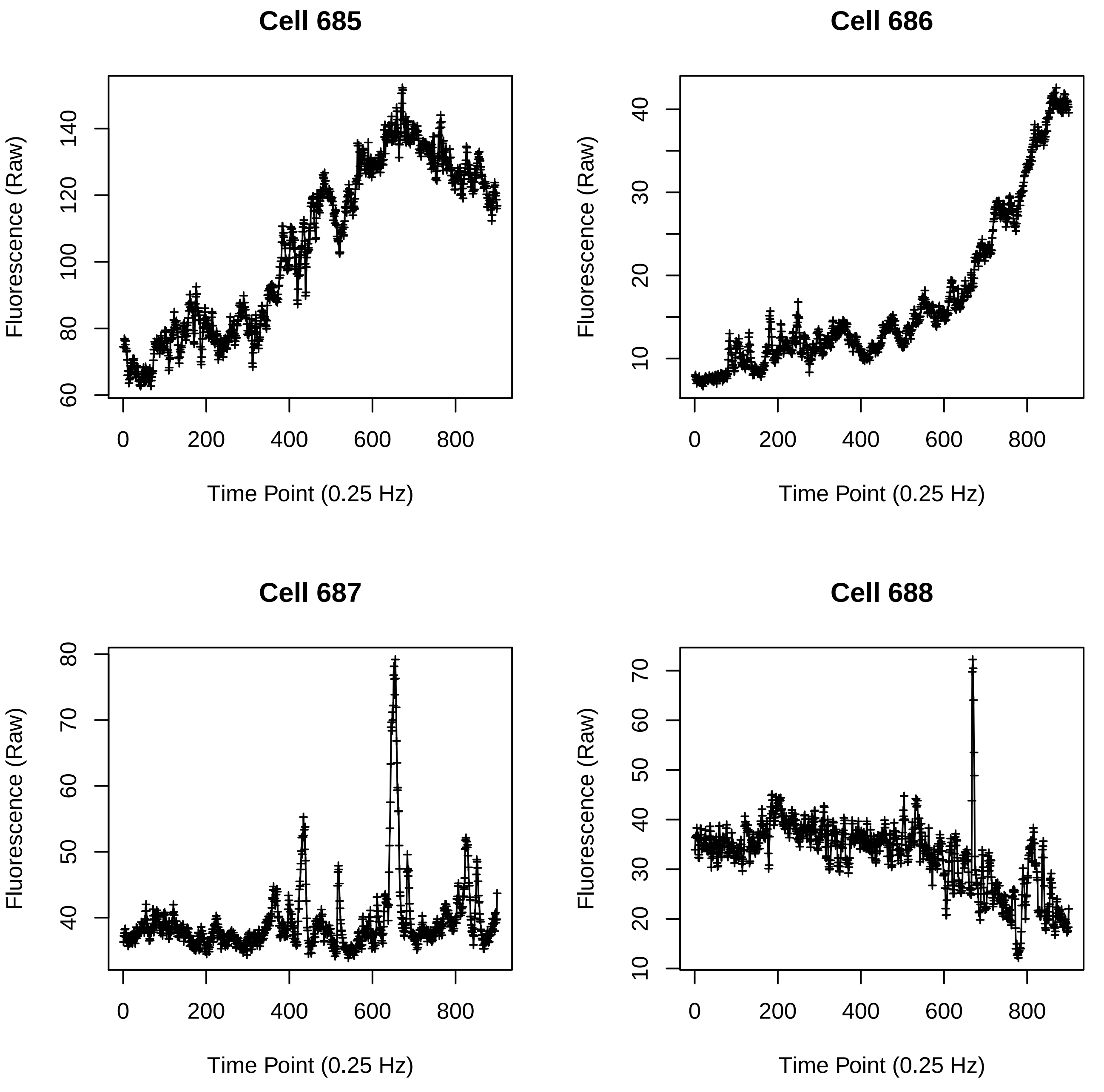

Cell 685
Cell 686
Cell 687
Cell 688
Fluorescence (Raw)
Time Point (0.25 Hz)
0
200
400
600
800
60
80
100
120
140
10
20
30
40
50
70

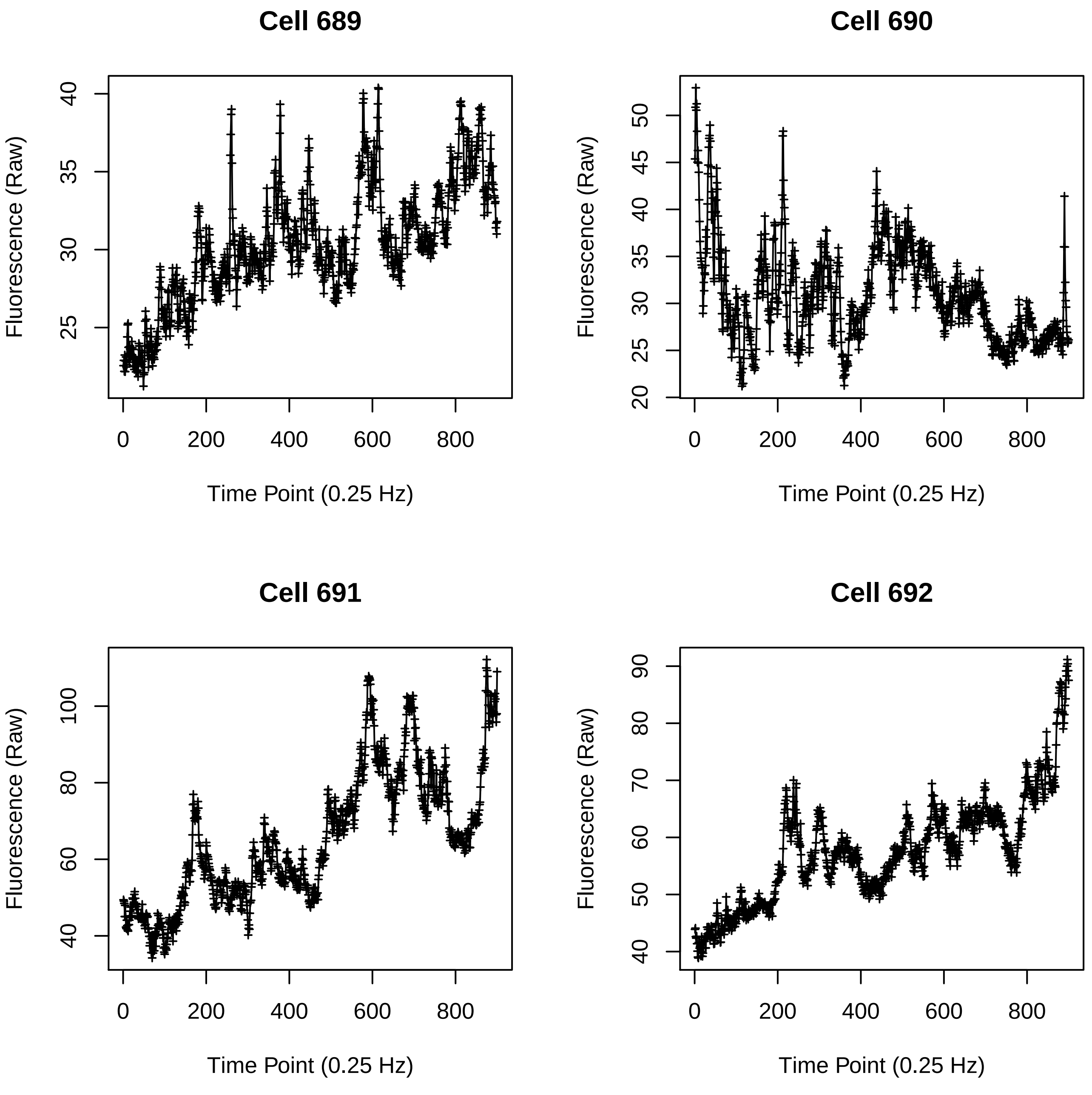
Cell 689
Fluorescence (Raw)
Time Point (0.25 Hz)
25
30
35
40
0
200
400
600
800
Cell 690
Fluorescence (Raw)
Time Point (0.25 Hz)
20
25
30
35
40
45
50
0
200
400
600
800
Cell 691
Fluorescence (Raw)
Time Point (0.25 Hz)
40
60
80
100
0
200
400
600
800
Cell 692
Fluorescence (Raw)
Time Point (0.25 Hz)
40
50
60
70
80
90
0
200
400
600
800

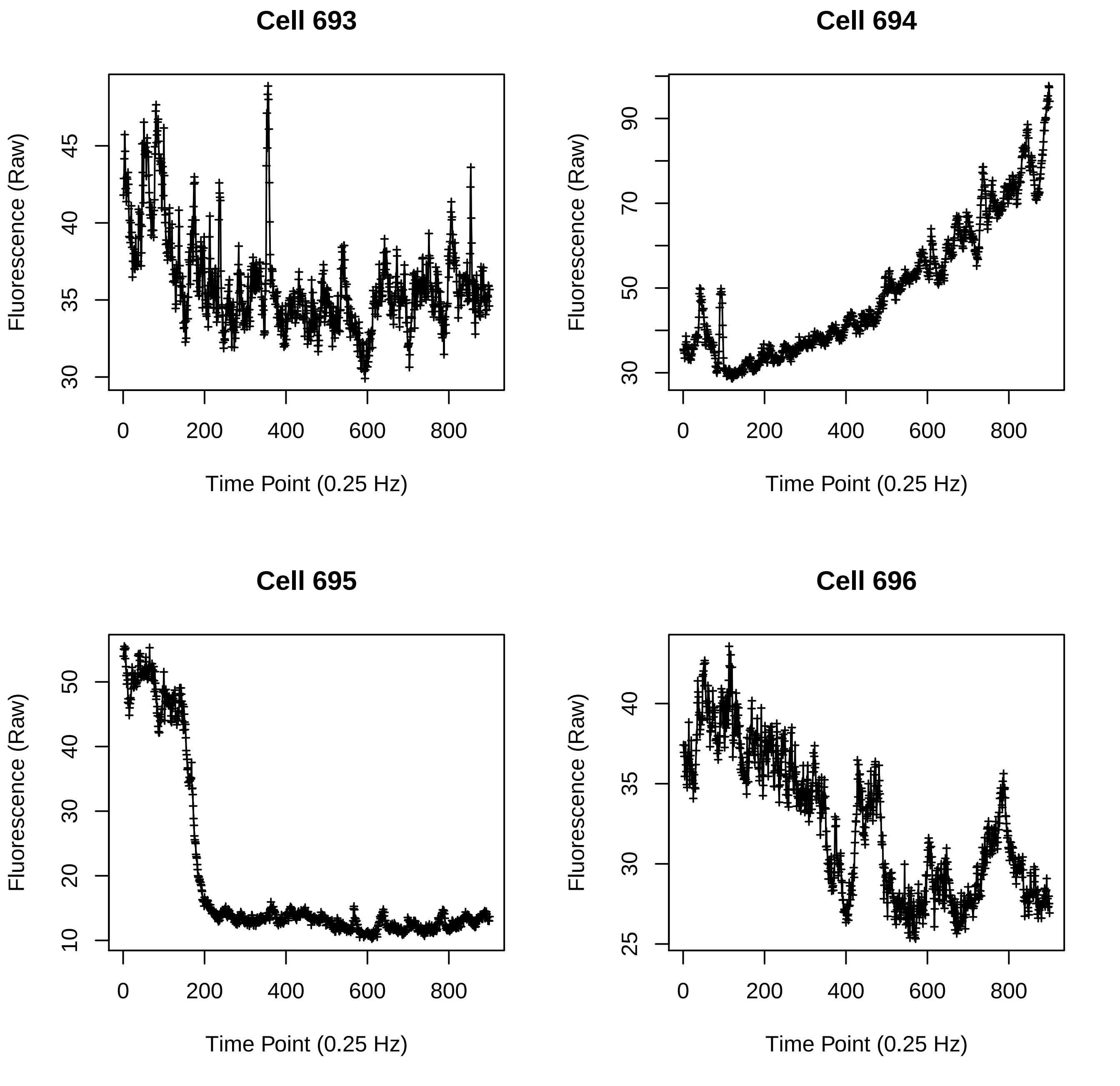
Cell 693
Fluorescence (Raw)
Time Point (0.25 Hz)
Cell 694
Fluorescence (Raw)
Time Point (0.25 Hz)
Cell 695
Fluorescence (Raw)
Time Point (0.25 Hz)
Cell 696
Fluorescence (Raw)
Time Point (0.25 Hz)

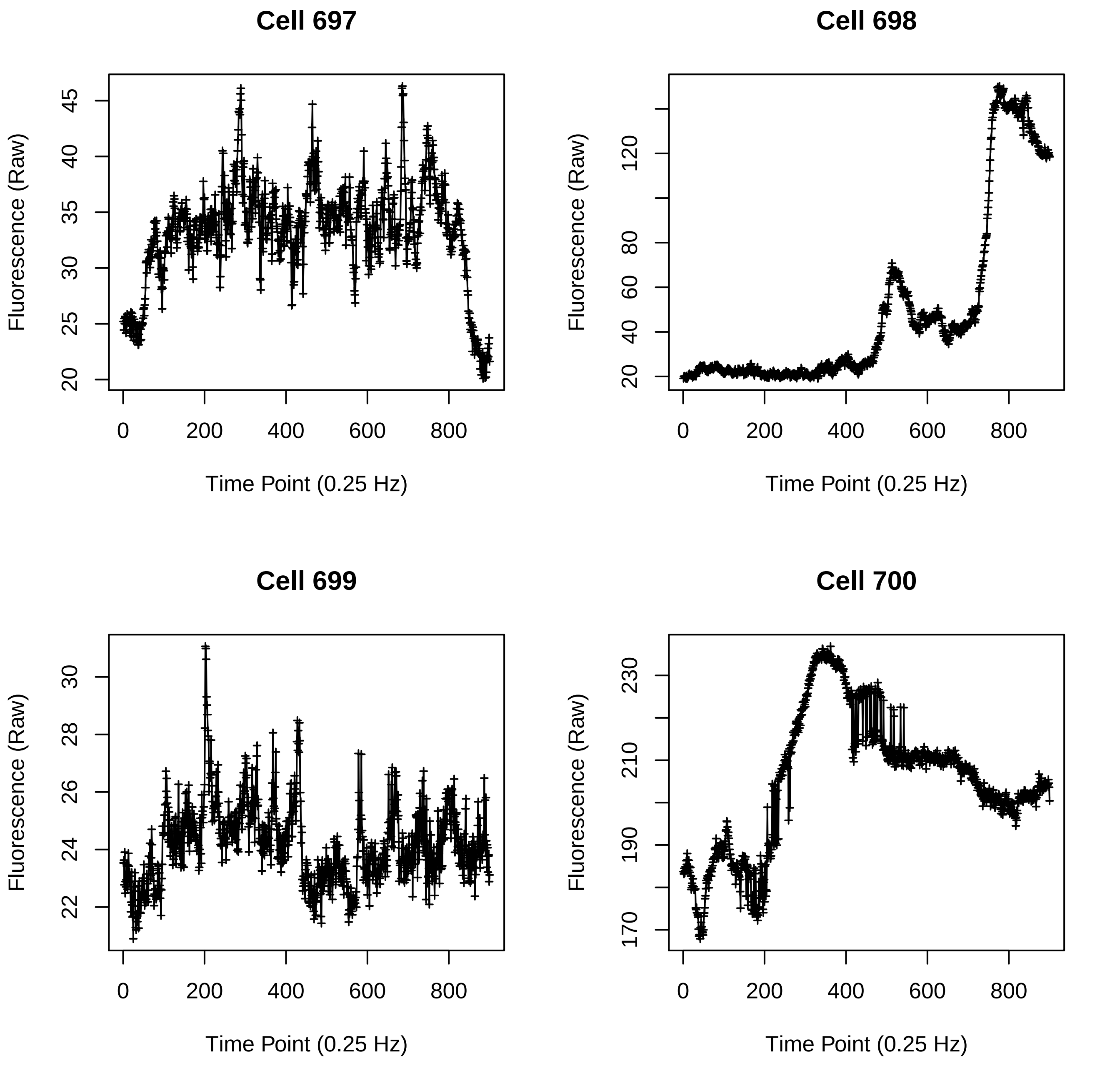
Cell 697
Cell 698
Cell 699
Cell 700
Fluorescence (Raw)
Time Point (0.25 Hz)
0
200
400
600
800
20
25
30
35
40
45
60
80
120
22
24
26
28
170
190
210
230

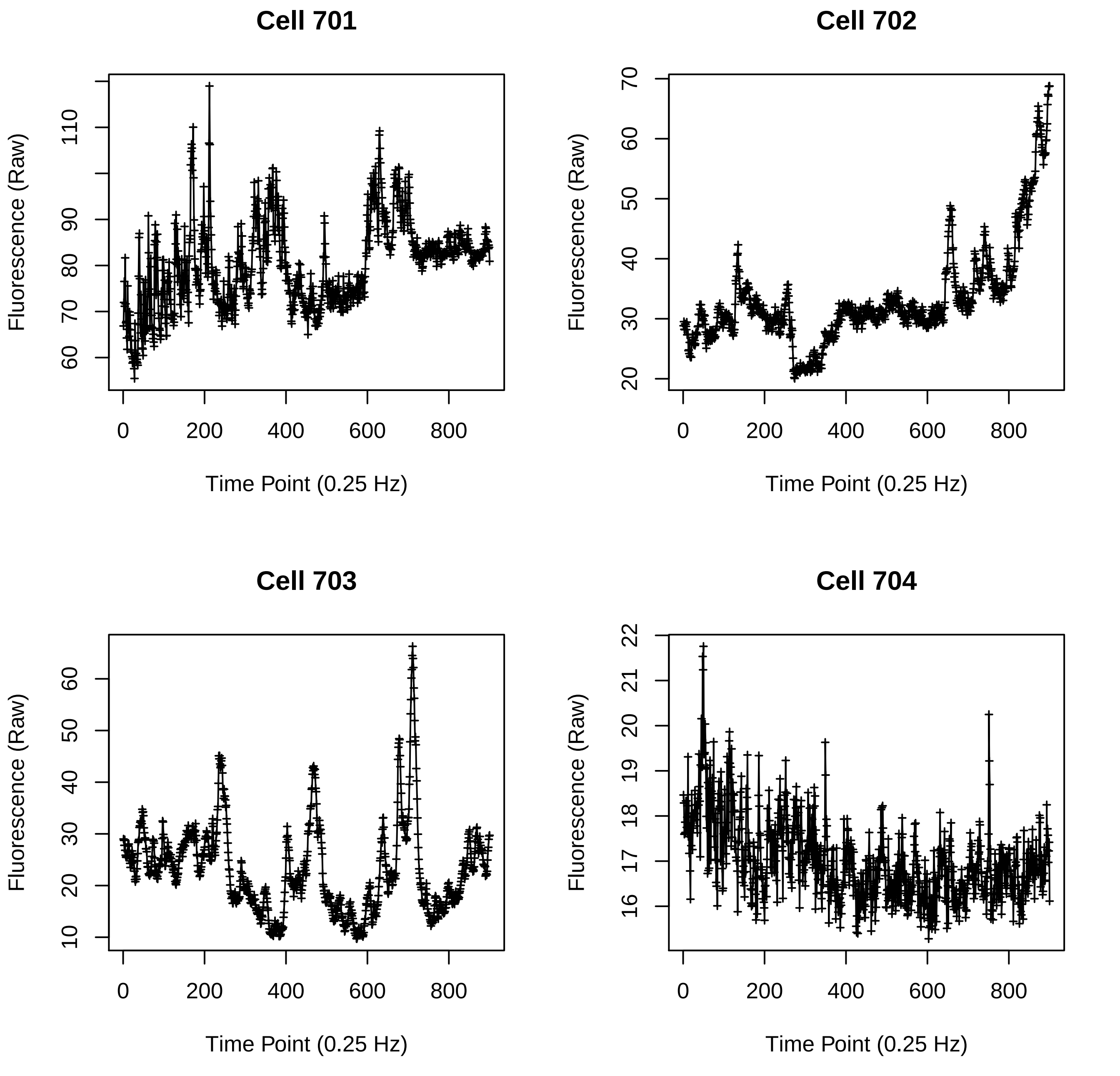
Cell 701
Cell 702
Cell 703
Cell 704
Fluorescence (Raw)
Time Point (0.25 Hz)
0
200
400
600
800
60
70
80
90
110
20
30
40
50
60
70
10
20
30
40
50
60
16
17
18
19
20
21
22

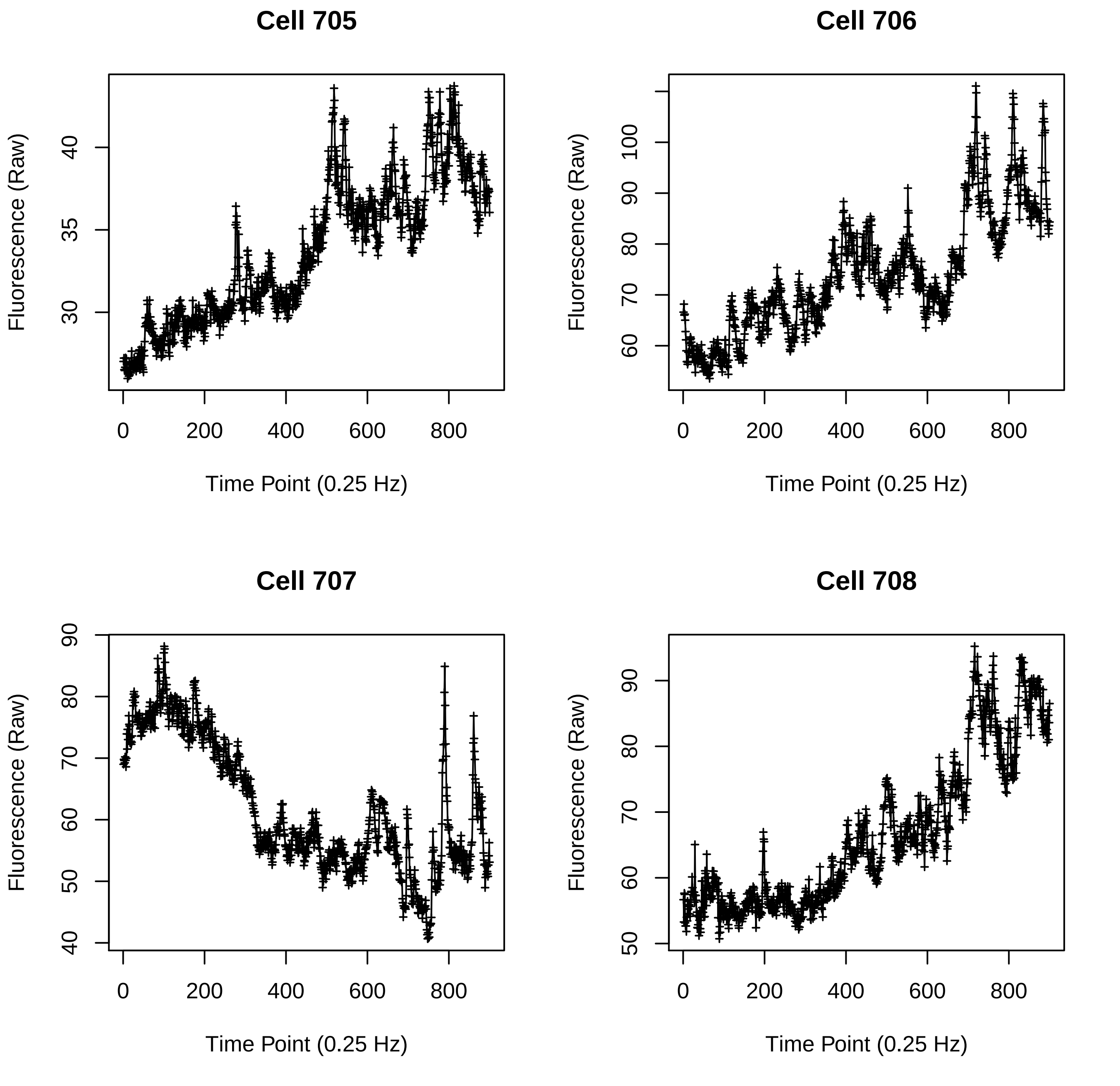
Cell 705
Fluorescence (Raw)
30
35
40
0
200
400
600
800
Time Point (0.25 Hz)
Cell 706
Fluorescence (Raw)
60
70
80
90
100
0
200
400
600
800
Time Point (0.25 Hz)
Cell 707
Fluorescence (Raw)
40
50
60
70
80
90
0
200
400
600
800
Time Point (0.25 Hz)
Cell 708
Fluorescence (Raw)
50
60
70
80
90
0
200
400
600
800
Time Point (0.25 Hz)

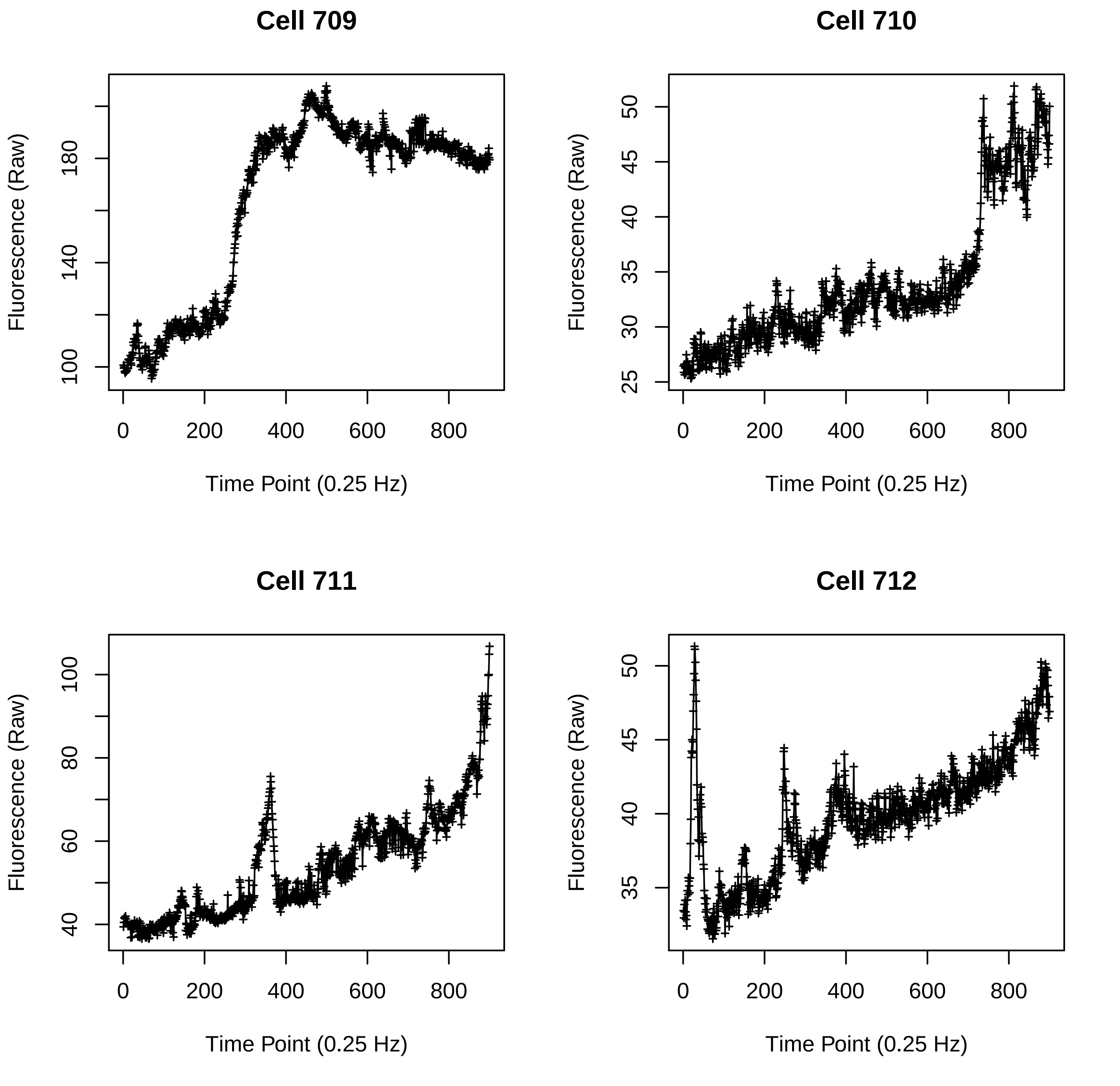
Cell 709
Fluorescence (Raw)
Time Point (0.25 Hz)
Cell 710
Fluorescence (Raw)
Time Point (0.25 Hz)
Cell 711
Fluorescence (Raw)
Time Point (0.25 Hz)
Cell 712
Fluorescence (Raw)
Time Point (0.25 Hz)

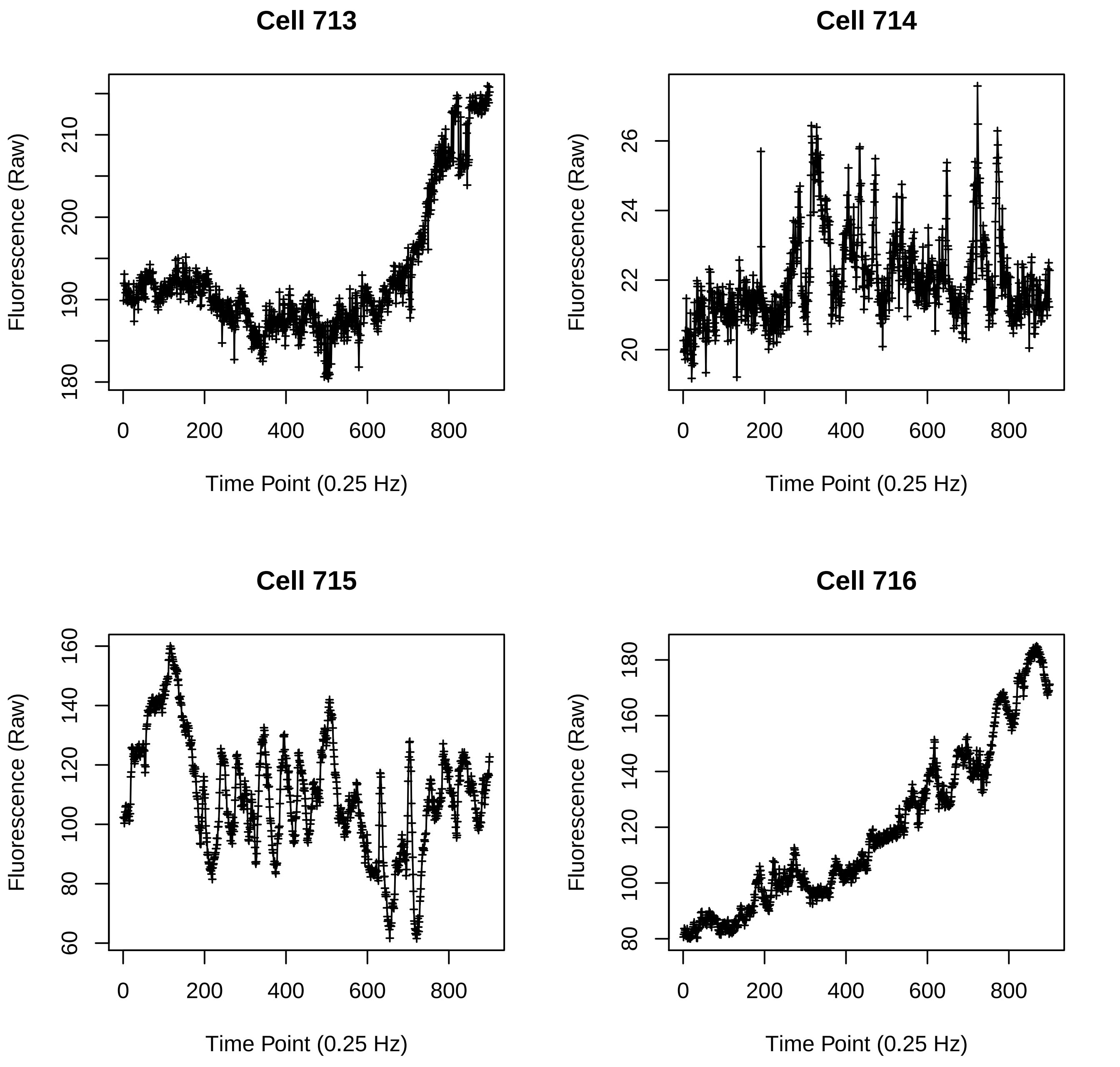
Cell 713
Fluorescence (Raw)
180
190
200
210
0
200
400
600
800
Time Point (0.25 Hz)
Cell 714
Fluorescence (Raw)
20
22
24
26
0
200
400
600
800
Time Point (0.25 Hz)
Cell 715
Fluorescence (Raw)
60
80
100
120
140
160
0
200
400
600
800
Time Point (0.25 Hz)
Cell 716
Fluorescence (Raw)
80
100
120
140
160
180
0
200
400
600
800
Time Point (0.25 Hz)

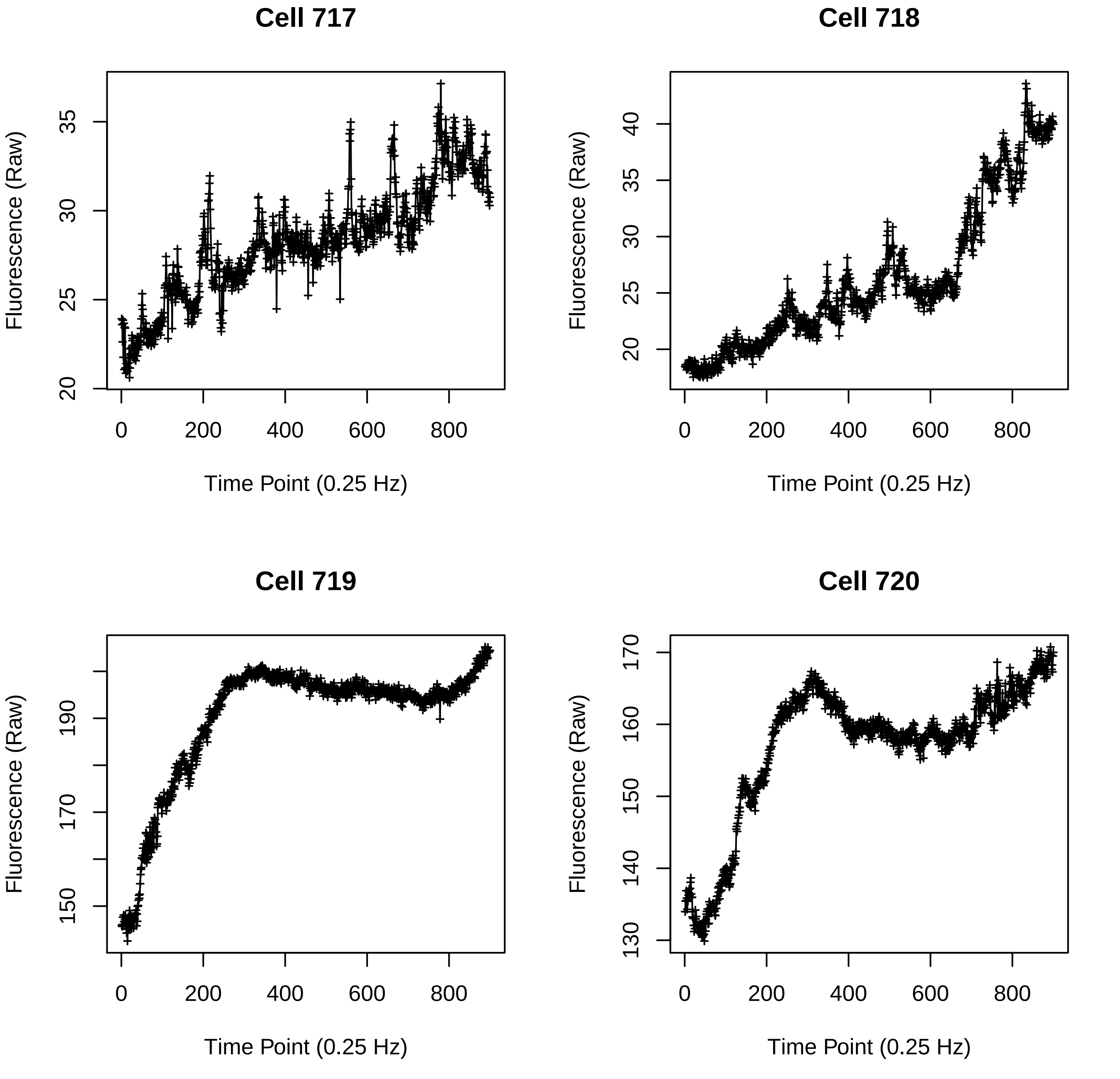
Cell 717
Fluorescence (Raw)
Time Point (0.25 Hz)
20
25
30
35
0
200
400
600
800
Cell 718
Fluorescence (Raw)
Time Point (0.25 Hz)
20
25
30
35
40
0
200
400
600
800
Cell 719
Fluorescence (Raw)
Time Point (0.25 Hz)
150
170
190
0
200
400
600
800
Cell 720
Fluorescence (Raw)
Time Point (0.25 Hz)
130
140
150
160
170
0
200
400
600
800

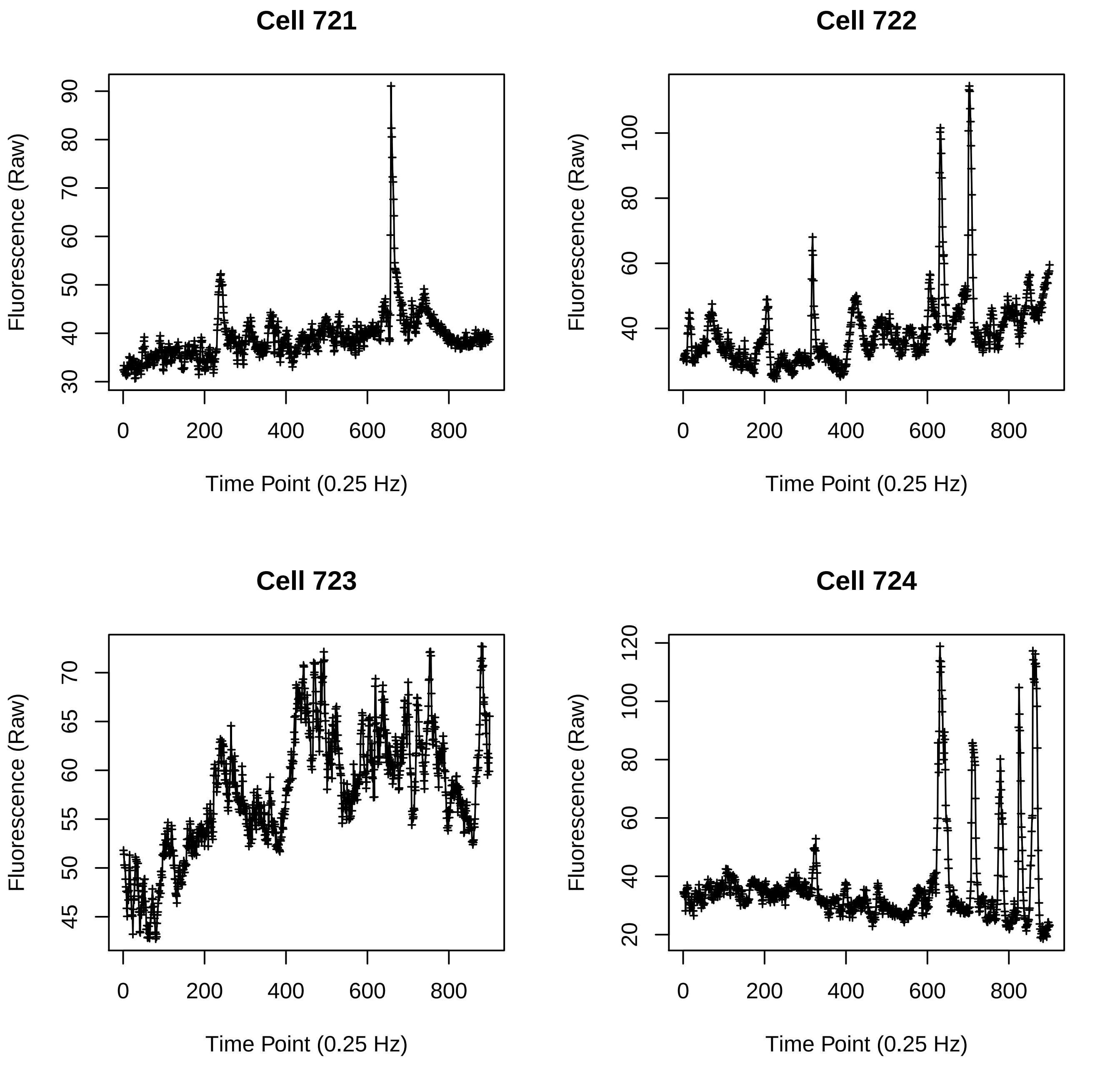
Cell 721
Cell 722
Cell 723
Cell 724
Fluorescence (Raw)
Time Point (0.25 Hz)
30 40 50 60 70 80 90
40 60 80 100
45 50 55 60 65 70
20 40 60 80 100 120
0 200 400 600 800

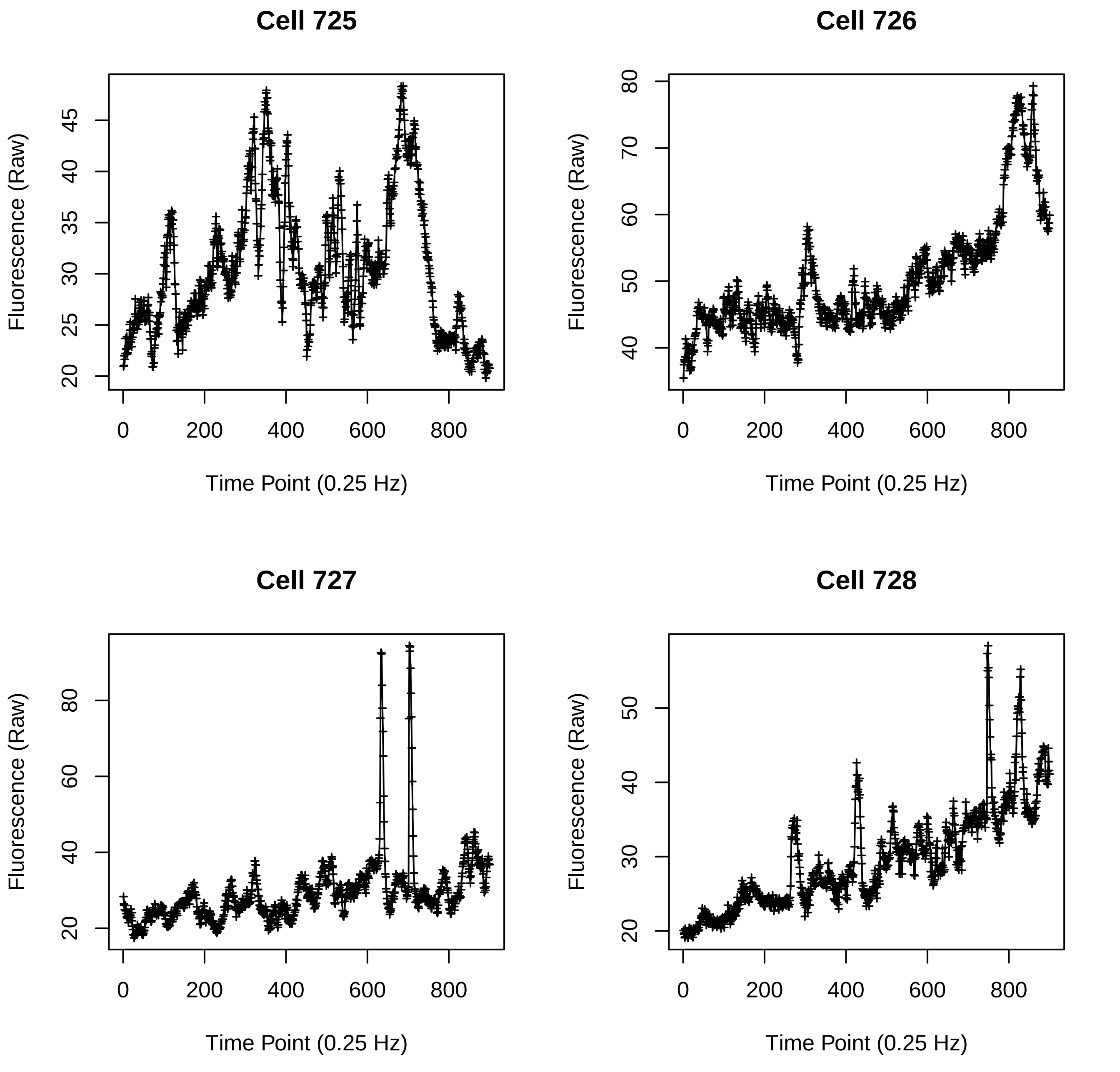
Cell 725
Fluorescence (Raw)
20
25
30
35
40
45
0
200
400
600
800
Time Point (0.25 Hz)
Cell 726
Fluorescence (Raw)
40
50
60
70
80
0
200
400
600
800
Time Point (0.25 Hz)
Cell 727
Fluorescence (Raw)
20
40
60
80
0
200
400
600
800
Time Point (0.25 Hz)
Cell 728
Fluorescence (Raw)
20
30
40
50
0
200
400
600
800
Time Point (0.25 Hz)

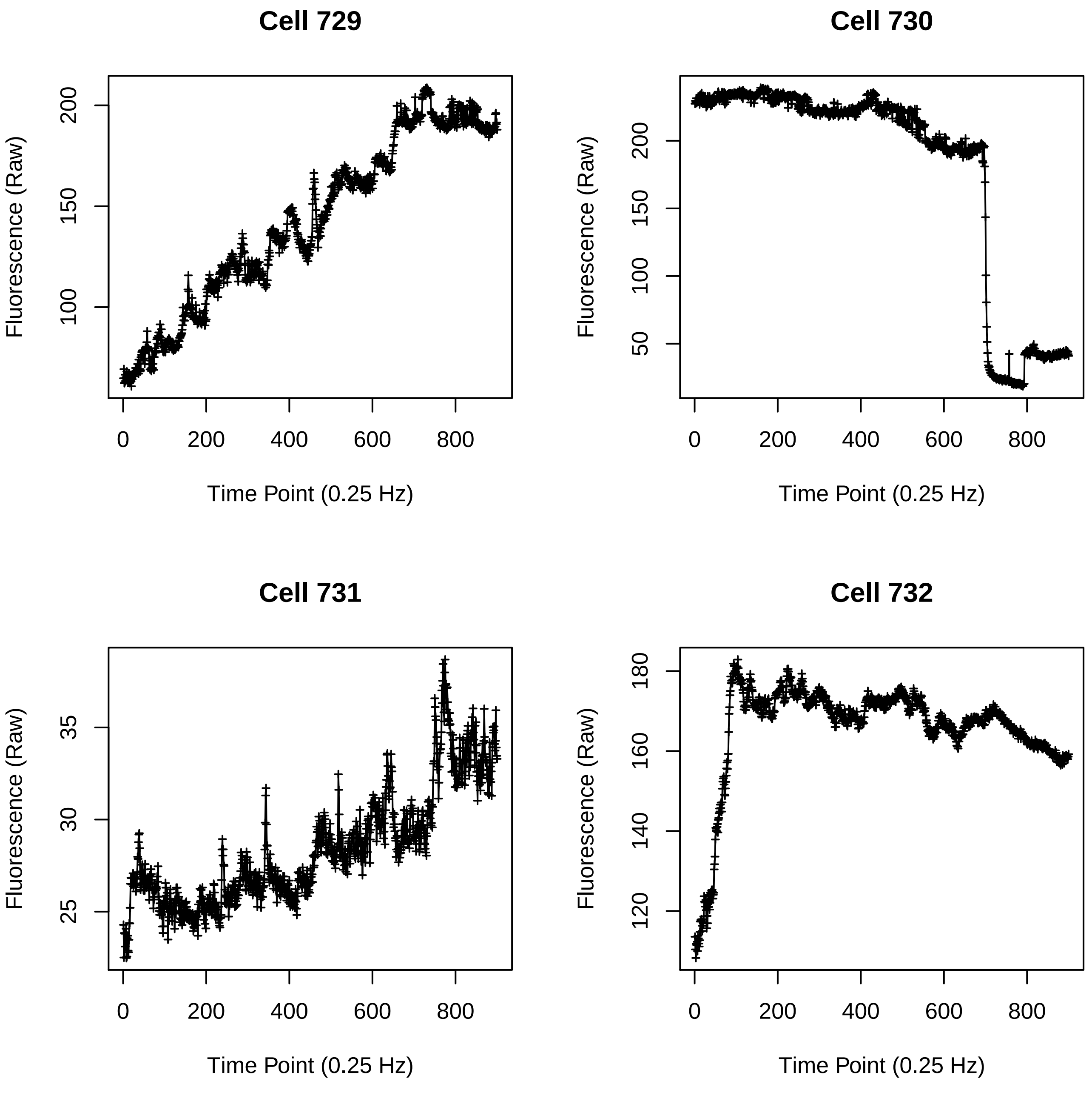

Cell 729
Fluorescence (Raw)
100
150
200
0
200
400
600
800
Time Point (0.25 Hz)
Cell 730
Fluorescence (Raw)
50
100
150
200
0
200
400
600
800
Time Point (0.25 Hz)
Cell 731
Fluorescence (Raw)
25
30
35
0
200
400
600
800
Time Point (0.25 Hz)
Cell 732
Fluorescence (Raw)
120
140
160
180
0
200
400
600
800
Time Point (0.25 Hz)

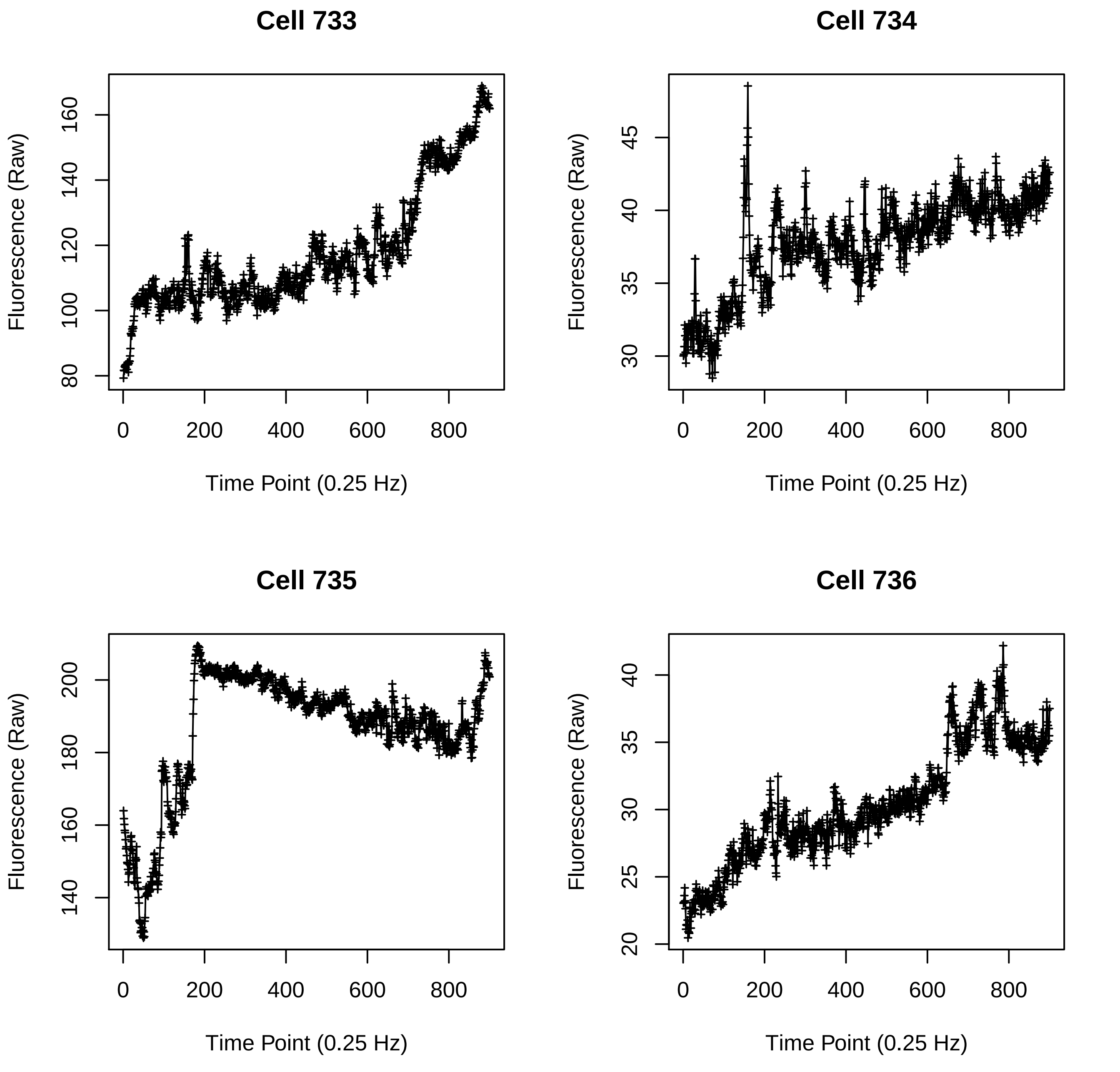

Cell 733
Fluorescence (Raw)
80
100
120
140
160
0
200
400
600
800
Time Point (0.25 Hz)
Cell 734
Fluorescence (Raw)
30
35
40
45
0
200
400
600
800
Time Point (0.25 Hz)
Cell 735
Fluorescence (Raw)
140
160
180
200
0
200
400
600
800
Time Point (0.25 Hz)
Cell 736
Fluorescence (Raw)
20
25
30
35
40
0
200
400
600
800
Time Point (0.25 Hz)

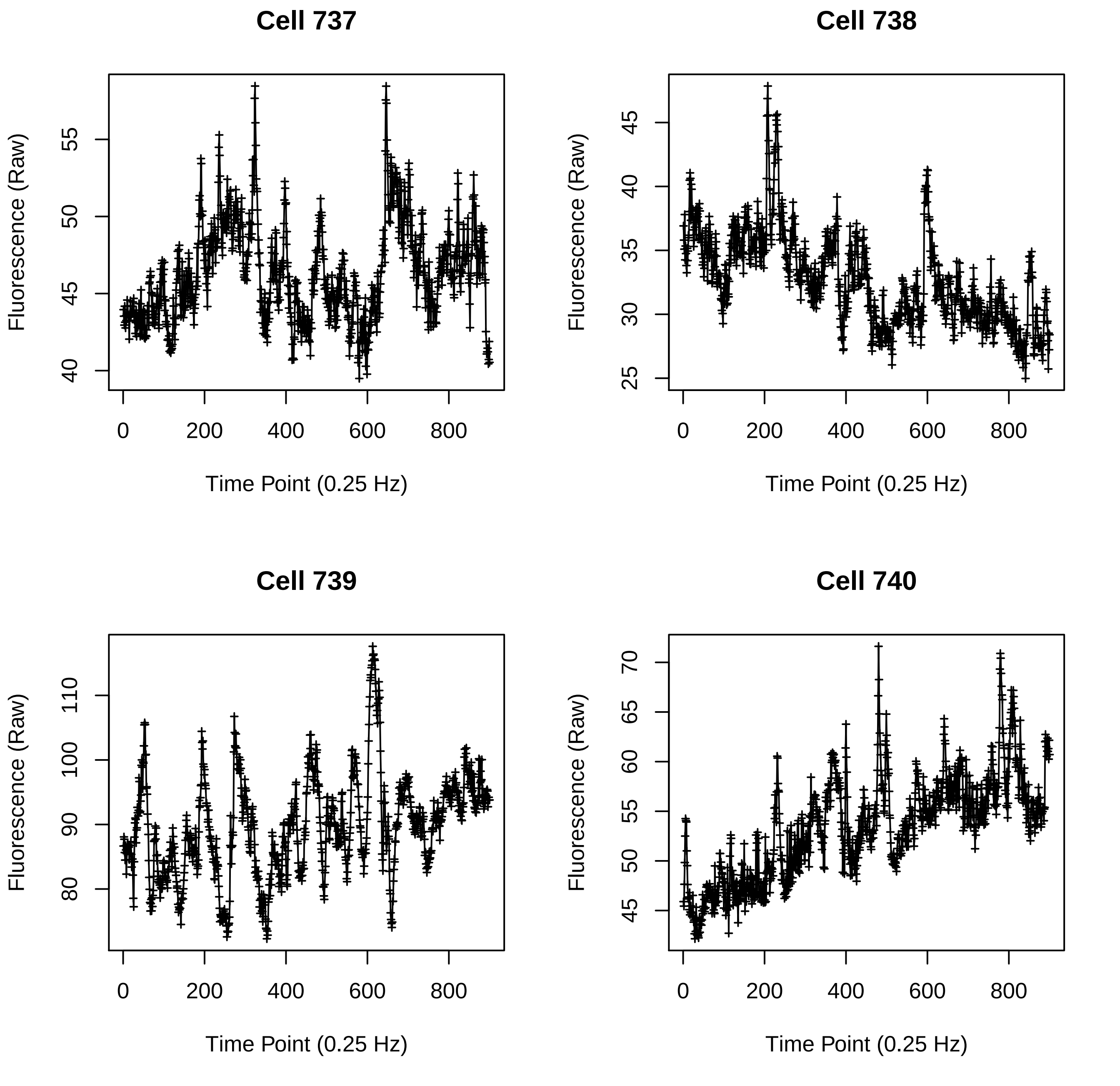

Cell 737
Cell 738
Cell 739
Cell 740
Fluorescence (Raw)
Time Point (0.25 Hz)
0
200
400
600
800
40
45
50
55
25
30
35
40
45
80
90
100
110
45
50
55
60
65
70

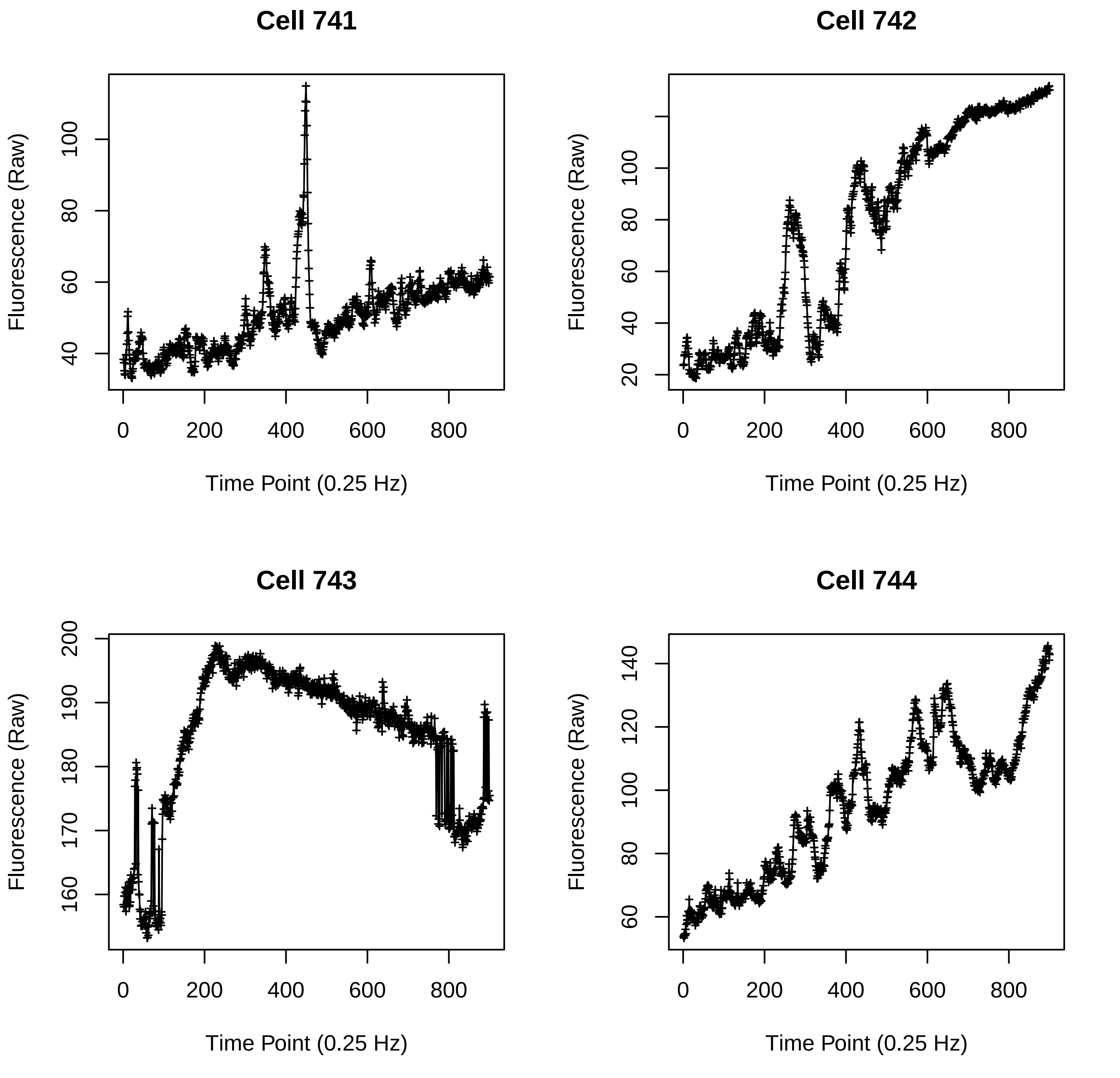
Cell 741
Fluorescence (Raw)
40
60
80
100
0
200
400
600
800
Time Point (0.25 Hz)
Cell 742
Fluorescence (Raw)
20
40
60
80
100
0
200
400
600
800
Time Point (0.25 Hz)
Cell 743
Fluorescence (Raw)
160
170
180
190
200
0
200
400
600
800
Time Point (0.25 Hz)
Cell 744
Fluorescence (Raw)
60
80
100
120
140
0
200
400
600
800
Time Point (0.25 Hz)

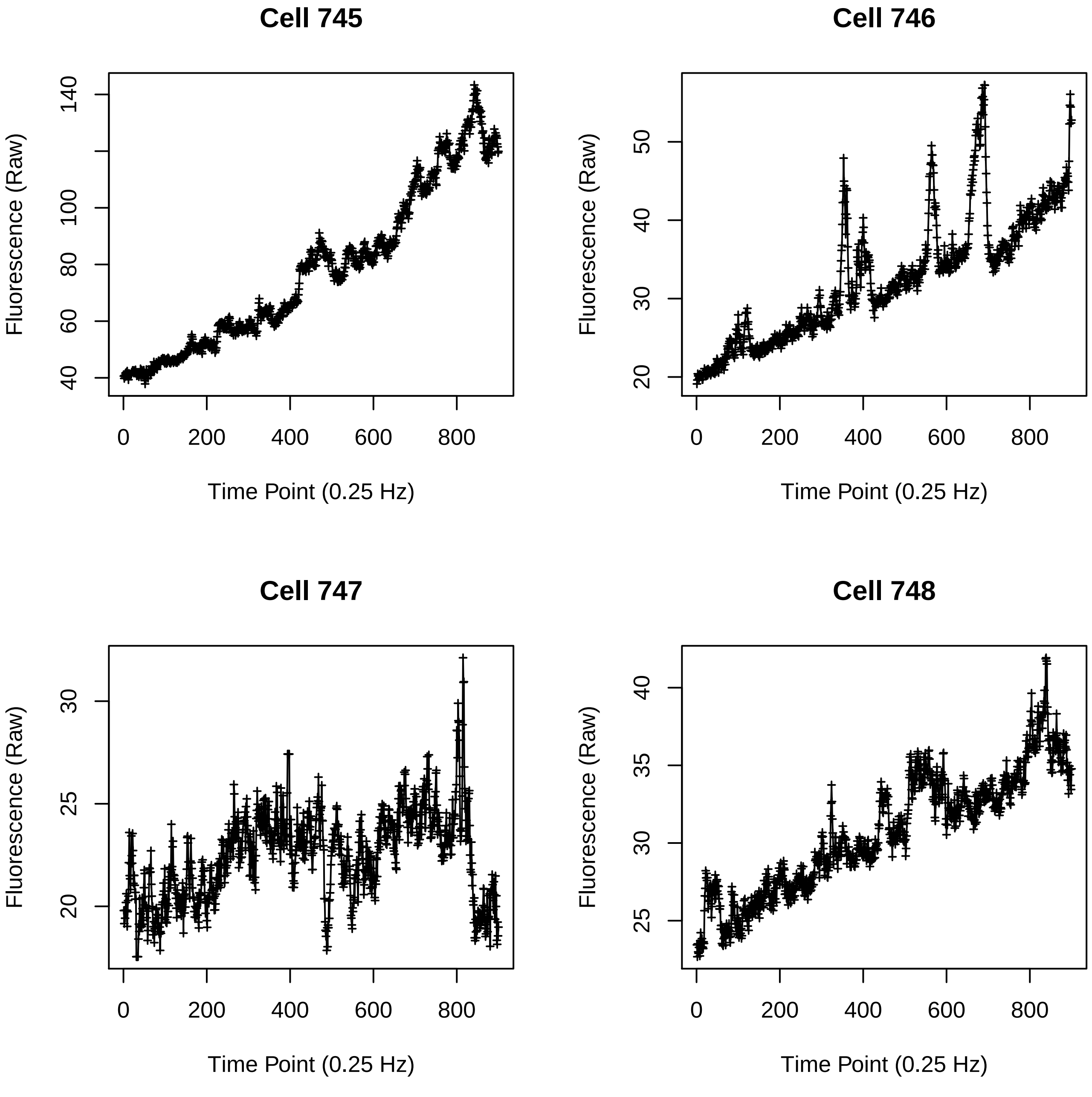
Cell 745
Fluorescence (Raw)
40
60
80
100
140
0
200
400
600
800
Time Point (0.25 Hz)
Cell 746
Fluorescence (Raw)
20
30
40
50
0
200
400
600
800
Time Point (0.25 Hz)
Cell 747
Fluorescence (Raw)
20
25
30
0
200
400
600
800
Time Point (0.25 Hz)
Cell 748
Fluorescence (Raw)
25
30
35
40
0
200
400
600
800
Time Point (0.25 Hz)

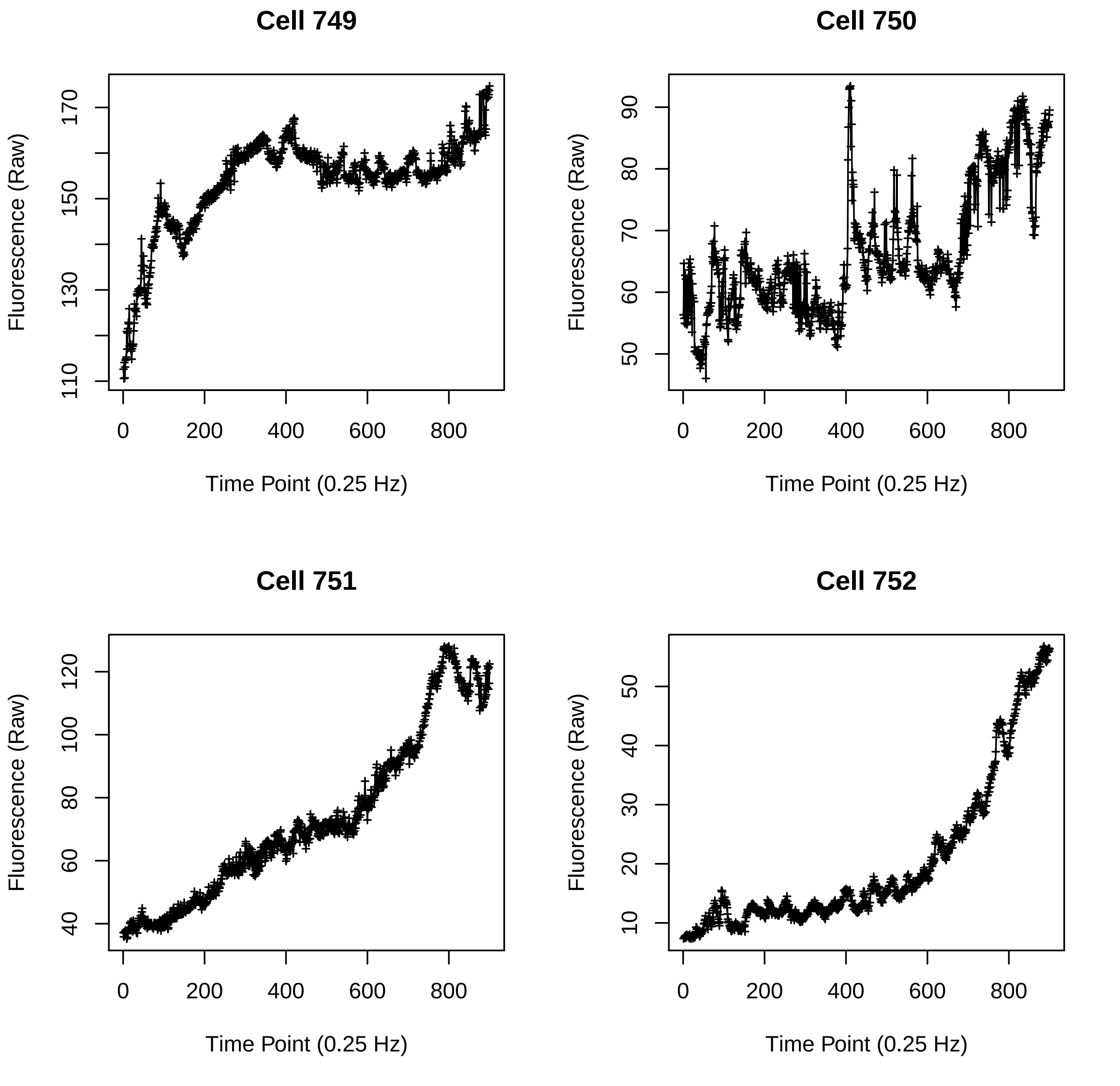
Cell 749
Fluorescence (Raw)
110
130
150
170
0
200
400
600
800
Time Point (0.25 Hz)
Cell 750
Fluorescence (Raw)
50
60
70
80
90
0
200
400
600
800
Time Point (0.25 Hz)
Cell 751
Fluorescence (Raw)
40
60
80
100
120
0
200
400
600
800
Time Point (0.25 Hz)
Cell 752
Fluorescence (Raw)
10
20
30
40
50
0
200
400
600
800
Time Point (0.25 Hz)

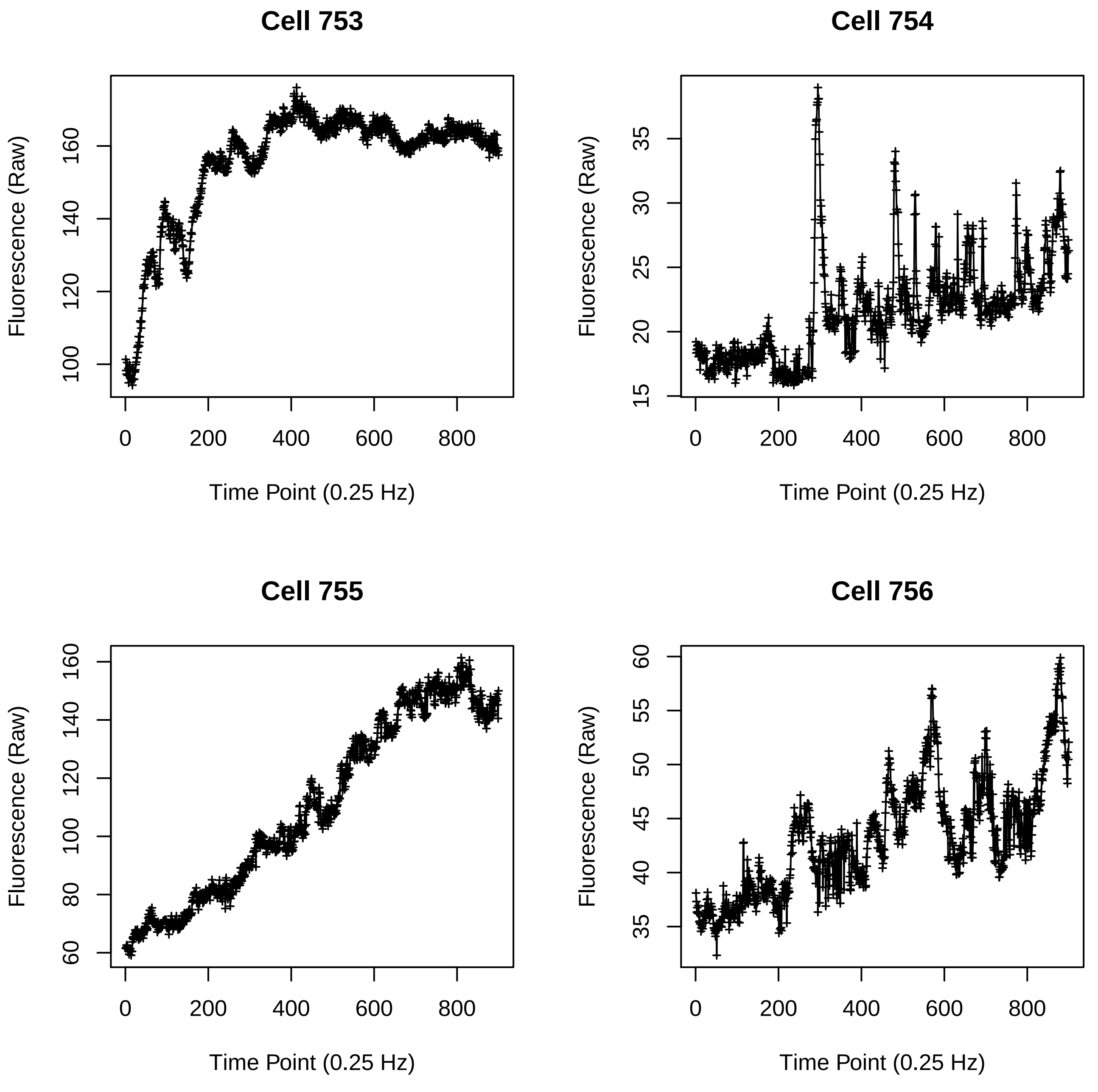

Cell 753
Cell 754
Cell 755
Cell 756
Fluorescence (Raw)
Time Point (0.25 Hz)
0
200
400
600
800
100
120
140
160
15
20
25
30
35
60
80
40
45
50
55

Cell 757

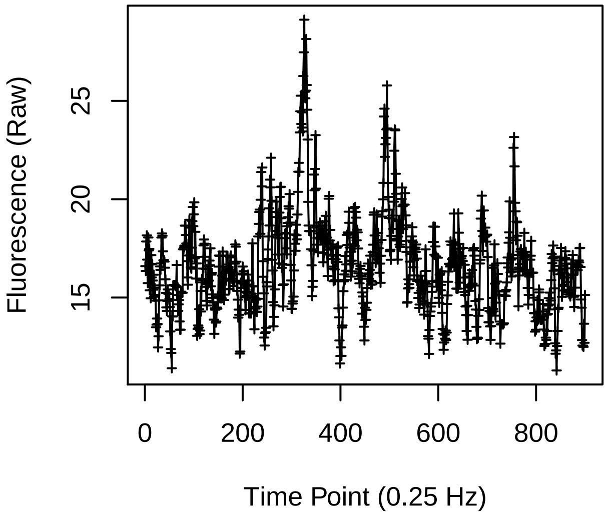

Supplement: S6 File — (PDF) [file pone.0168342.s013.pdf]
