# Supplementary material for: A Markovian Entropy Measure for the Analysis of Calcium Activity Time Series
Source: PLoS One. 2016 Dec 15;11(12):e0168342. doi: 10.1371/journal.pone.0168342 (PMC5158058; doi:10.1371/journal.pone.0168342)
Supplement: S7 File — (PDF) [file pone.0168342.s014.pdf]

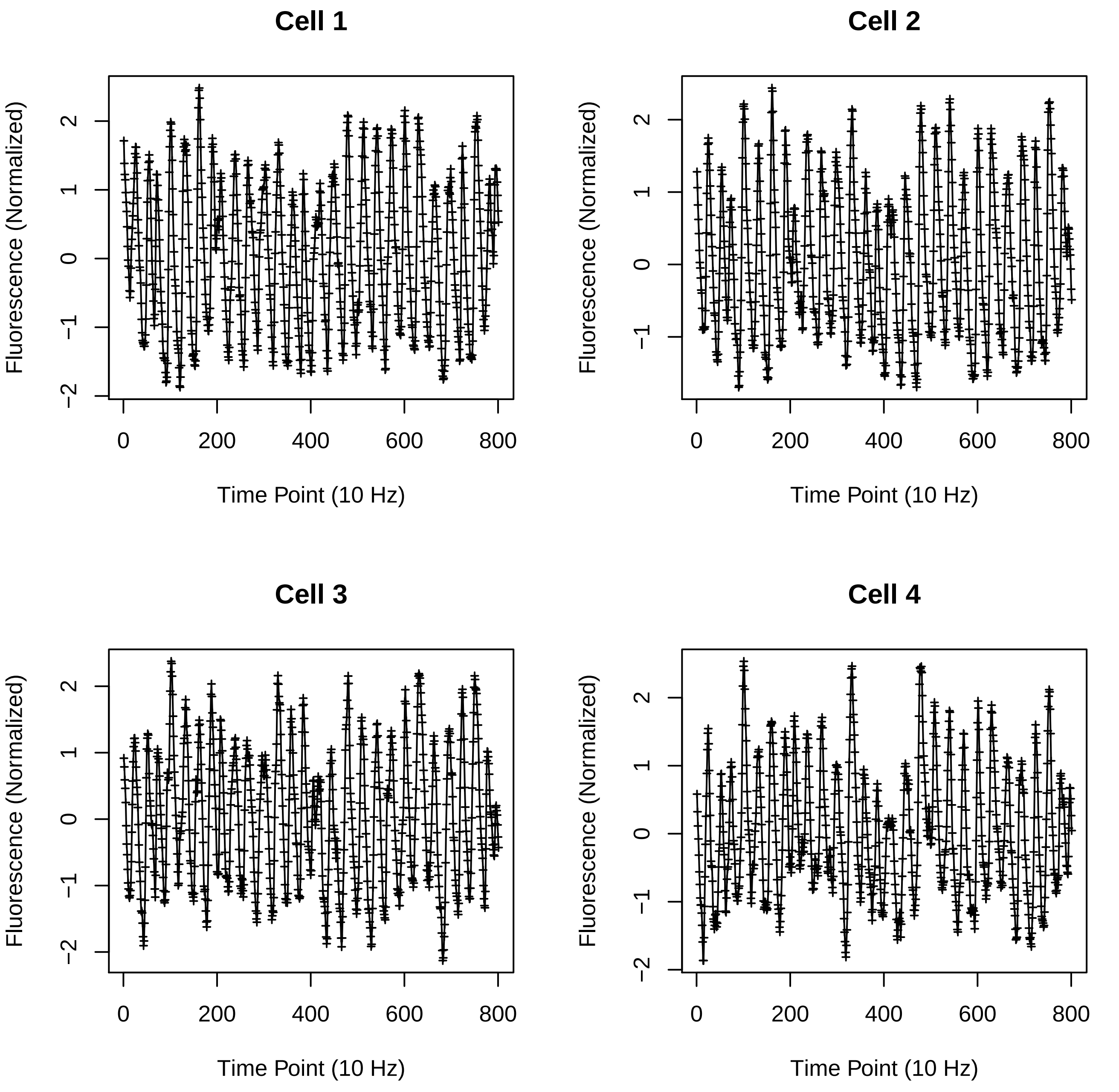
Cell 1
Cell 2
Cell 3
Cell 4
Fluorescence (Normalized)
Time Point (10 Hz)
−2
−1
0
1
2
0
200
400
600
800

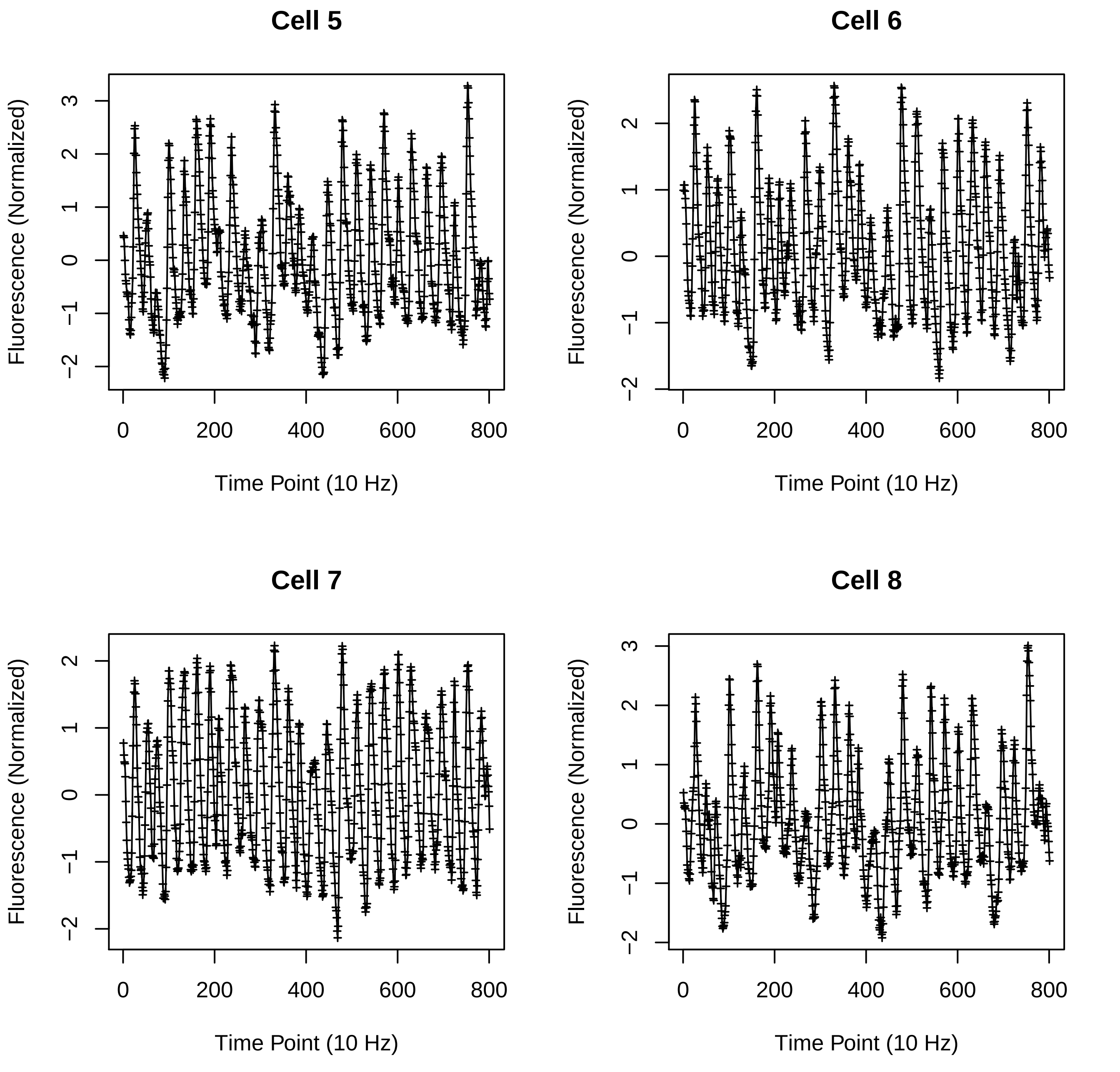
Cell 5
Cell 6
Cell 7
Cell 8
Fluorescence (Normalized)
Time Point (10 Hz)
0
200
400
600
800
−2
−1
0
1
2
3

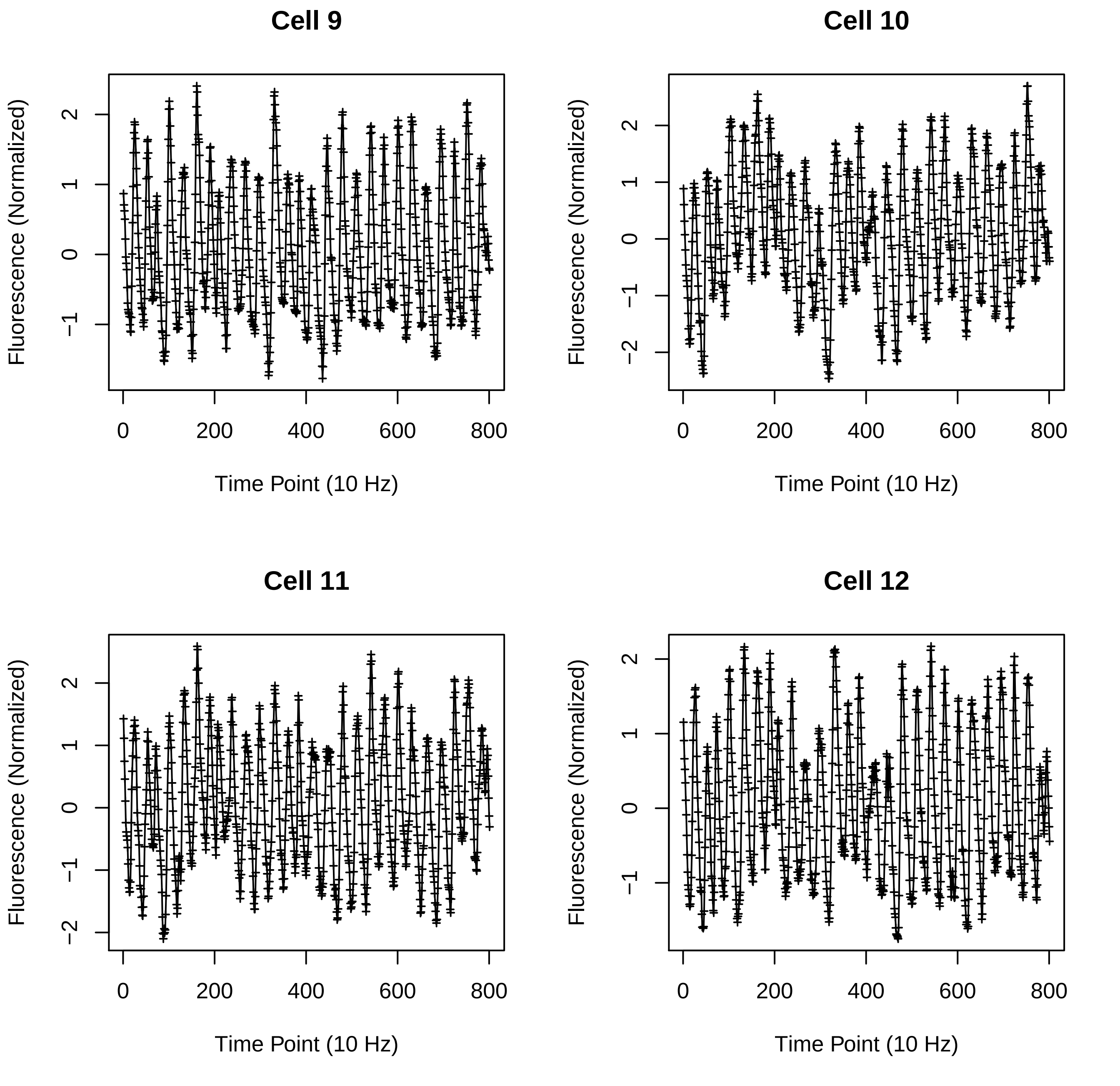

Cell 9
Cell 10
Cell 11
Cell 12
Fluorescence (Normalized)
Time Point (10 Hz)
0
200
400
600
800
−2
−1
0
1
2

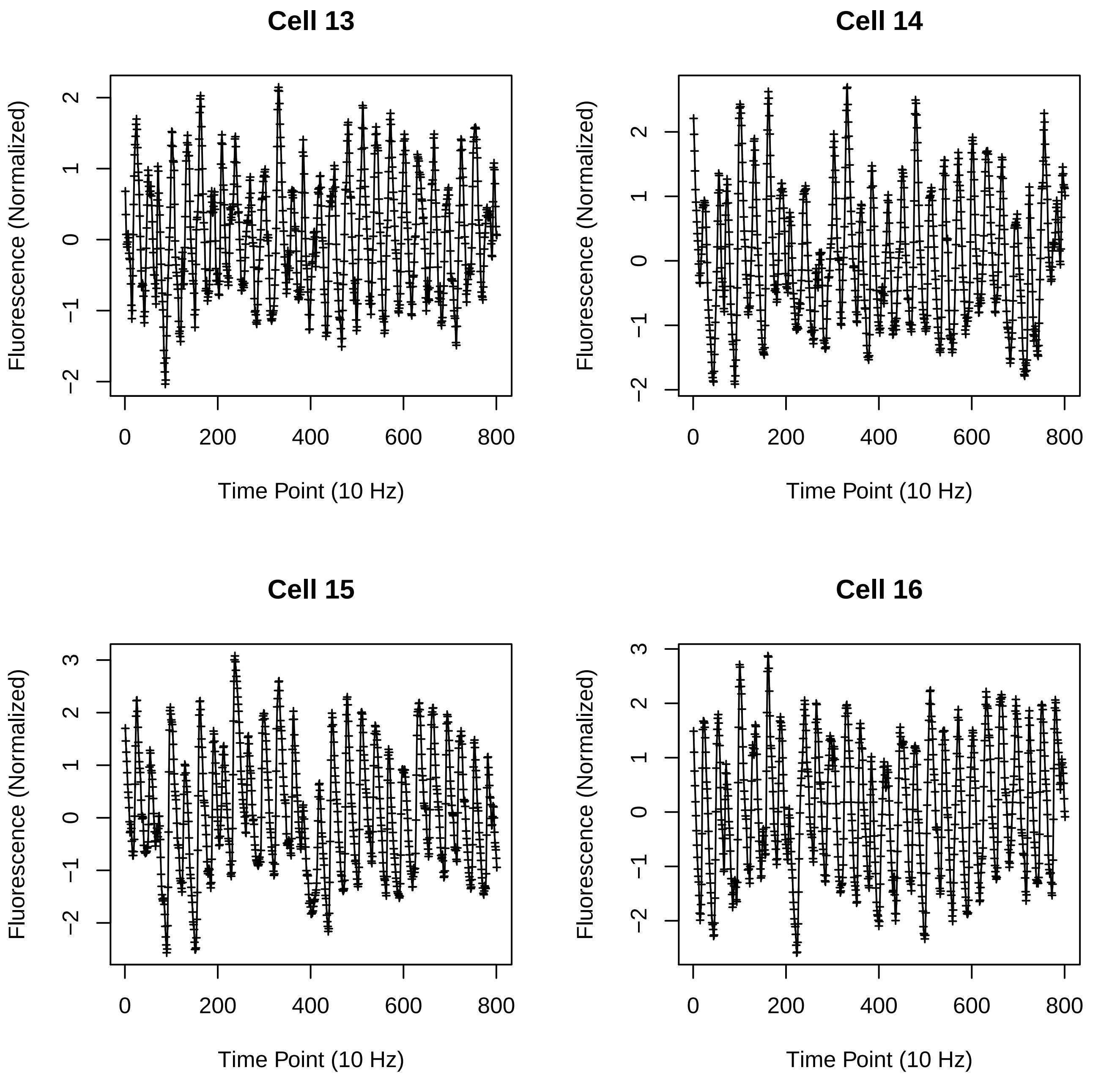

Cell 13
Cell 14
Cell 15
Cell 16
Fluorescence (Normalized)
Time Point (10 Hz)
0
200
400
600
800
−2
−1
0
1
2
3

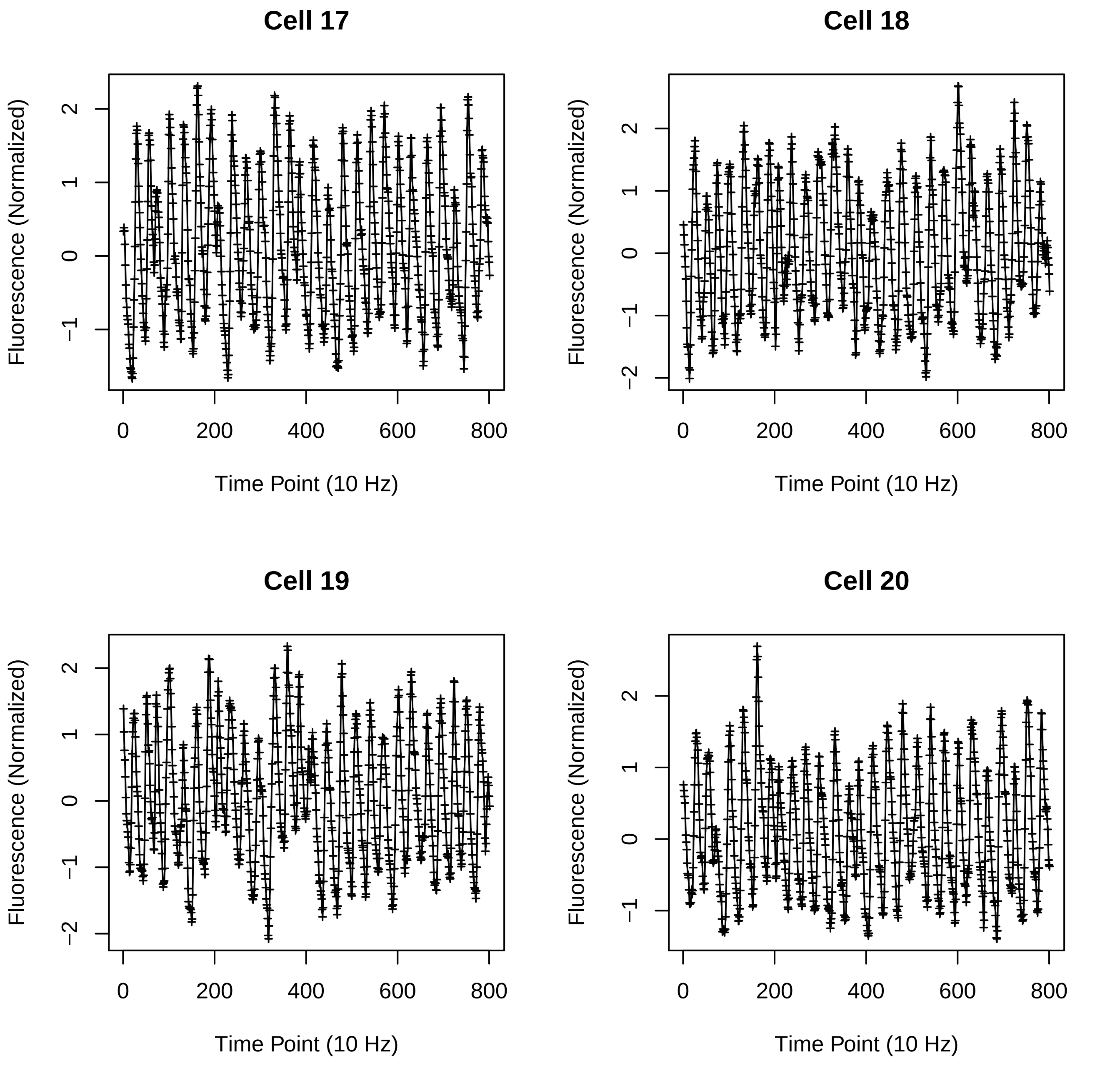
Cell 17
Cell 18
Cell 19
Cell 20
Fluorescence (Normalized)
Time Point (10 Hz)
0
200
400
600
800
−2
−1
0
1
2

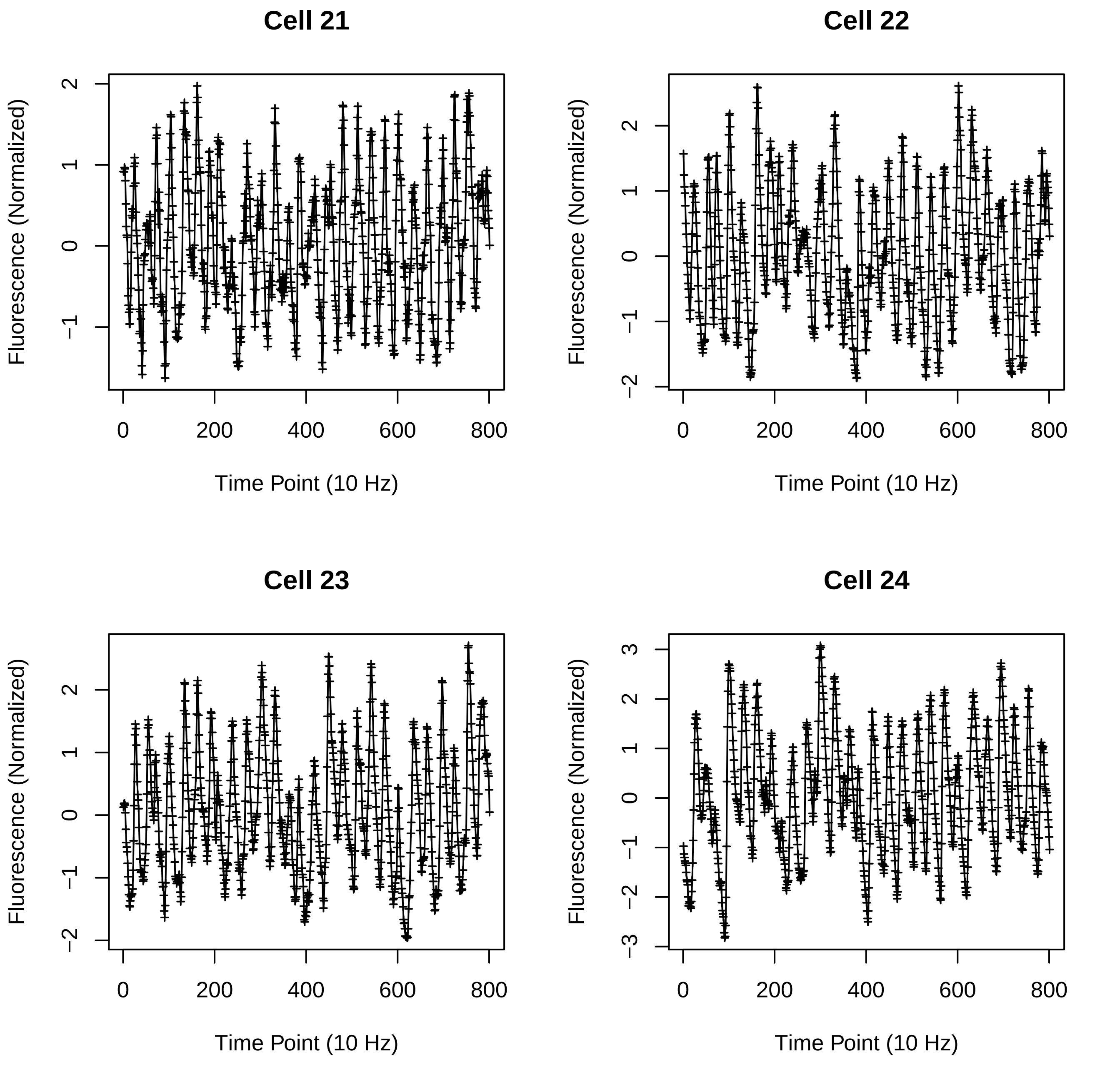

Cell 21
Cell 22
Cell 23
Cell 24
Fluorescence (Normalized)
Time Point (10 Hz)
0
200
400
600
800

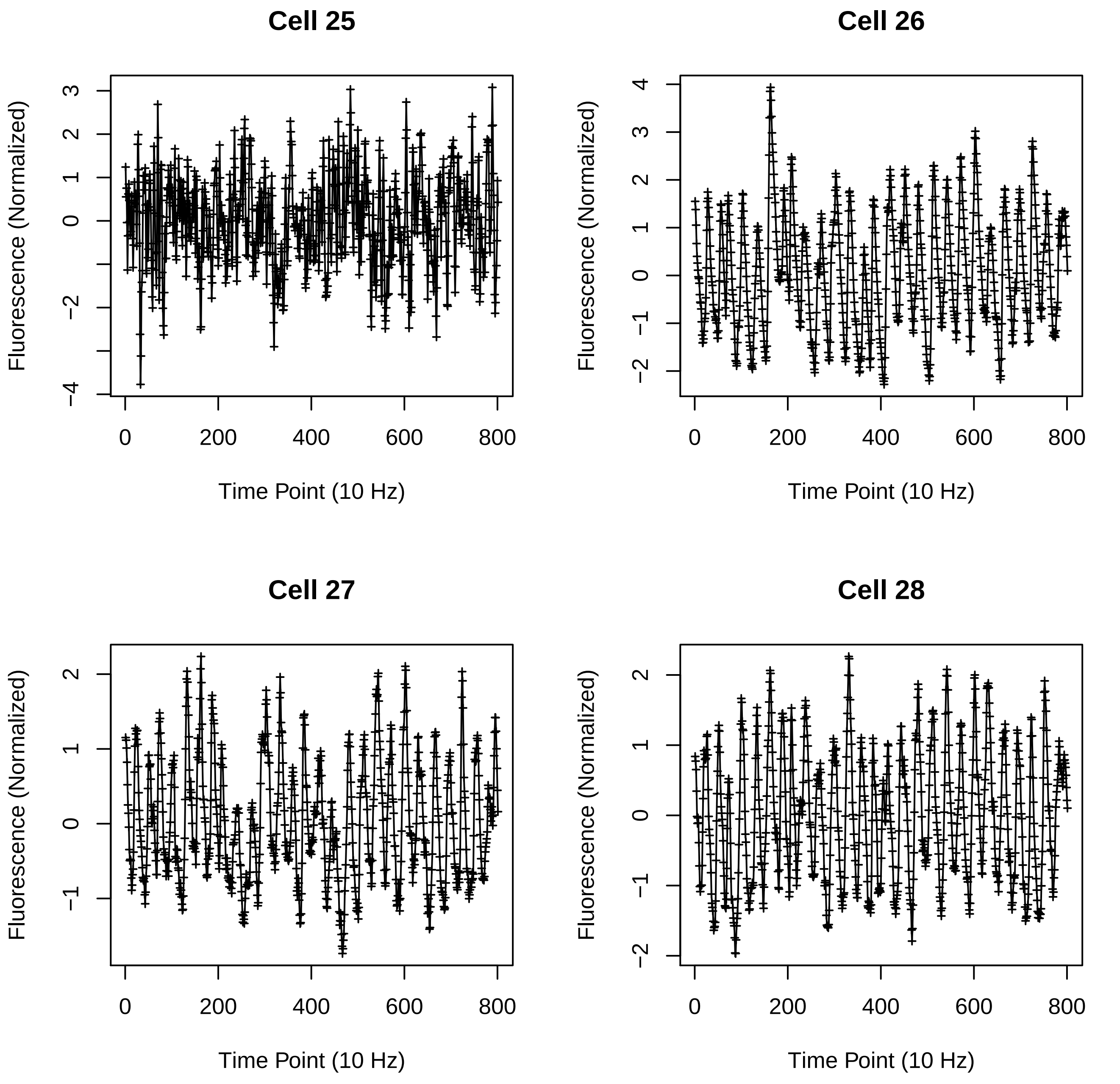
Cell 25
Cell 26
Cell 27
Cell 28
Fluorescence (Normalized)
Time Point (10 Hz)
0
200
400
600
800
-4
-2
0
1
2
3
4

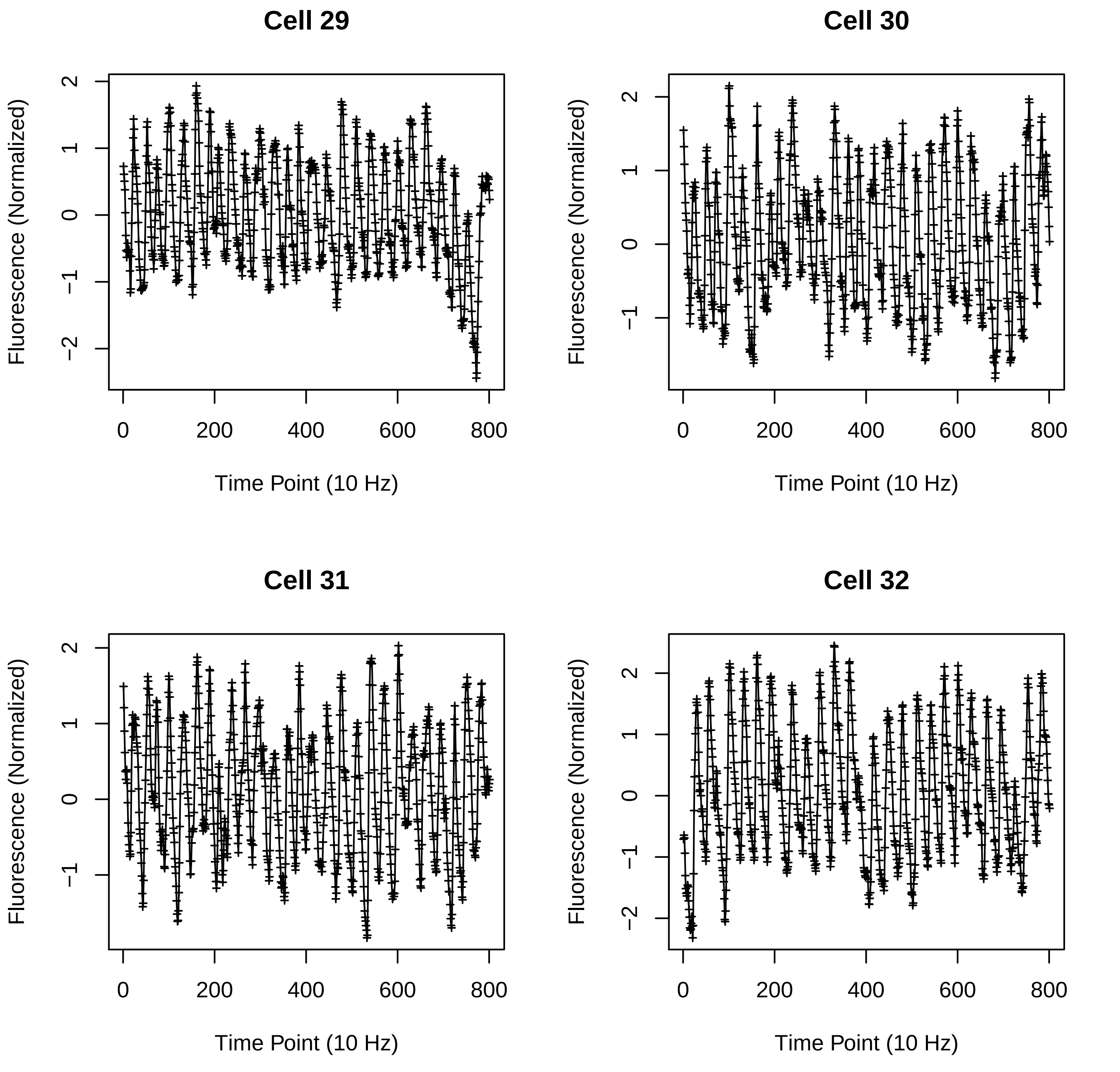
Cell 29
Cell 30
Cell 31
Cell 32
Fluorescence (Normalized)
Time Point (10 Hz)
0
200
400
600
800
−2
−1
0
1
2

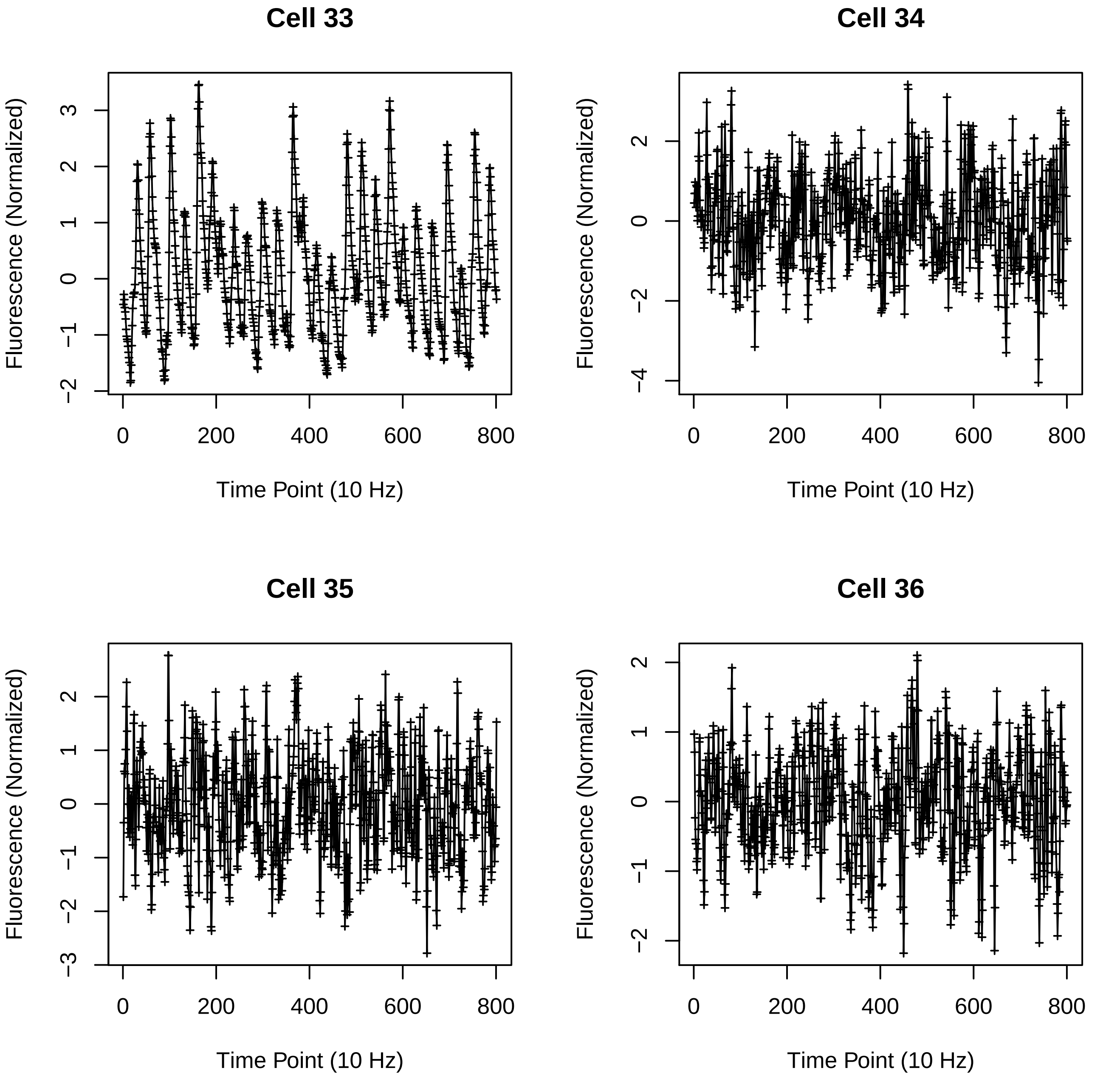

Cell 33
Cell 34
Cell 35
Cell 36
Fluorescence (Normalized)
Time Point (10 Hz)
0
200
400
600
800
−4
−3
−2
−1
0
1
2
3

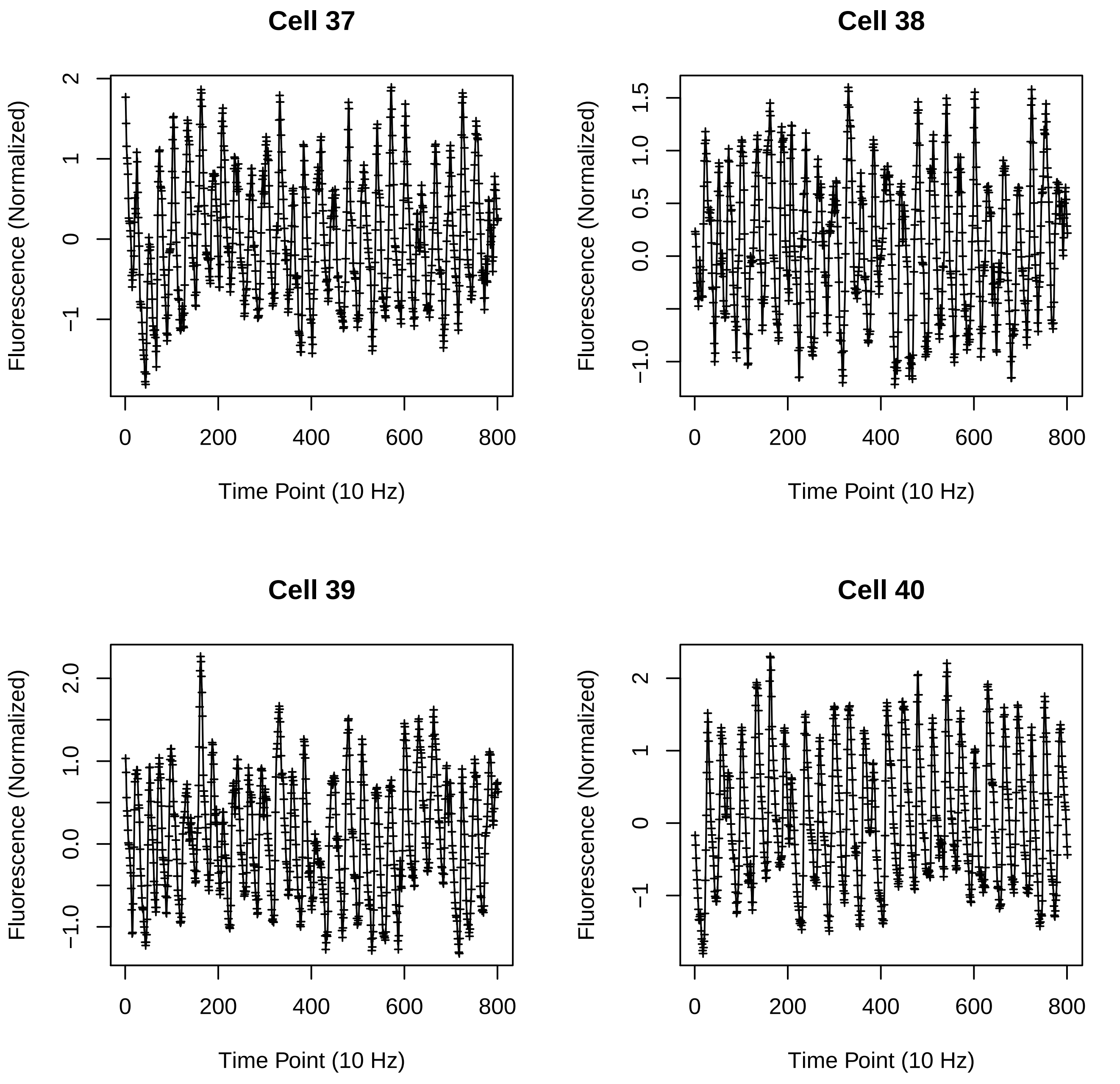
Cell 37
Cell 38
Cell 39
Cell 40
Fluorescence (Normalized)
Time Point (10 Hz)
0
200
400
600
800
2
1
0
−1
1.5
1.0
0.5
0.0
−1.0
2.0
1.0
0.0
−1.0
2
1
0
−1

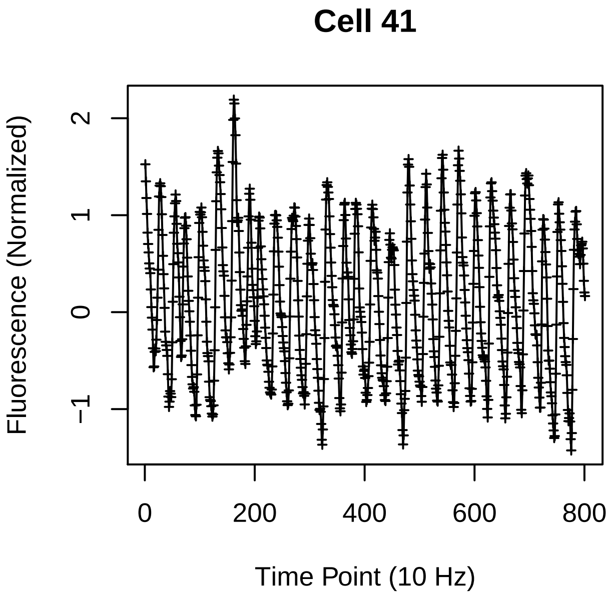
Cell 41
Fluorescence (Normalized)
Time Point (10 Hz)
−1
0
1
2
0
200
400
600
800

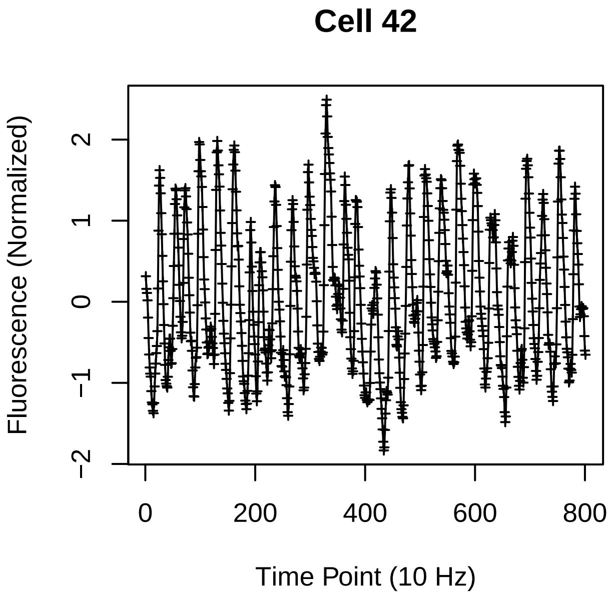
Cell 42
Fluorescence (Normalized)
Time Point (10 Hz)
−2
−1
0
1
2
0
200
400
600
800

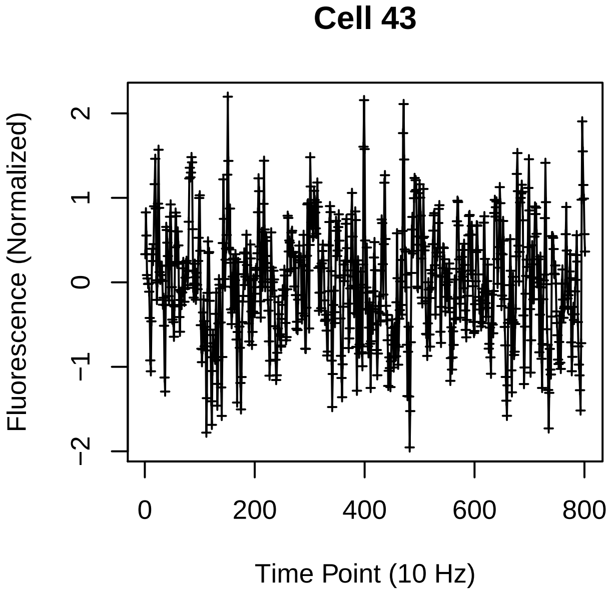
Cell 43
Fluorescence (Normalized)
Time Point (10 Hz)
−2
−1
0
1
2
0
200
400
600
800

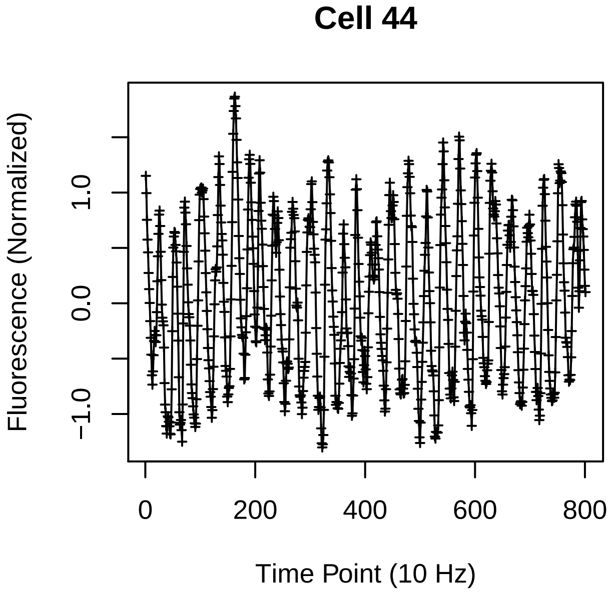
Cell 44
Fluorescence (Normalized)
Time Point (10 Hz)
−1.0
0.0
1.0
0
200
400
600
800

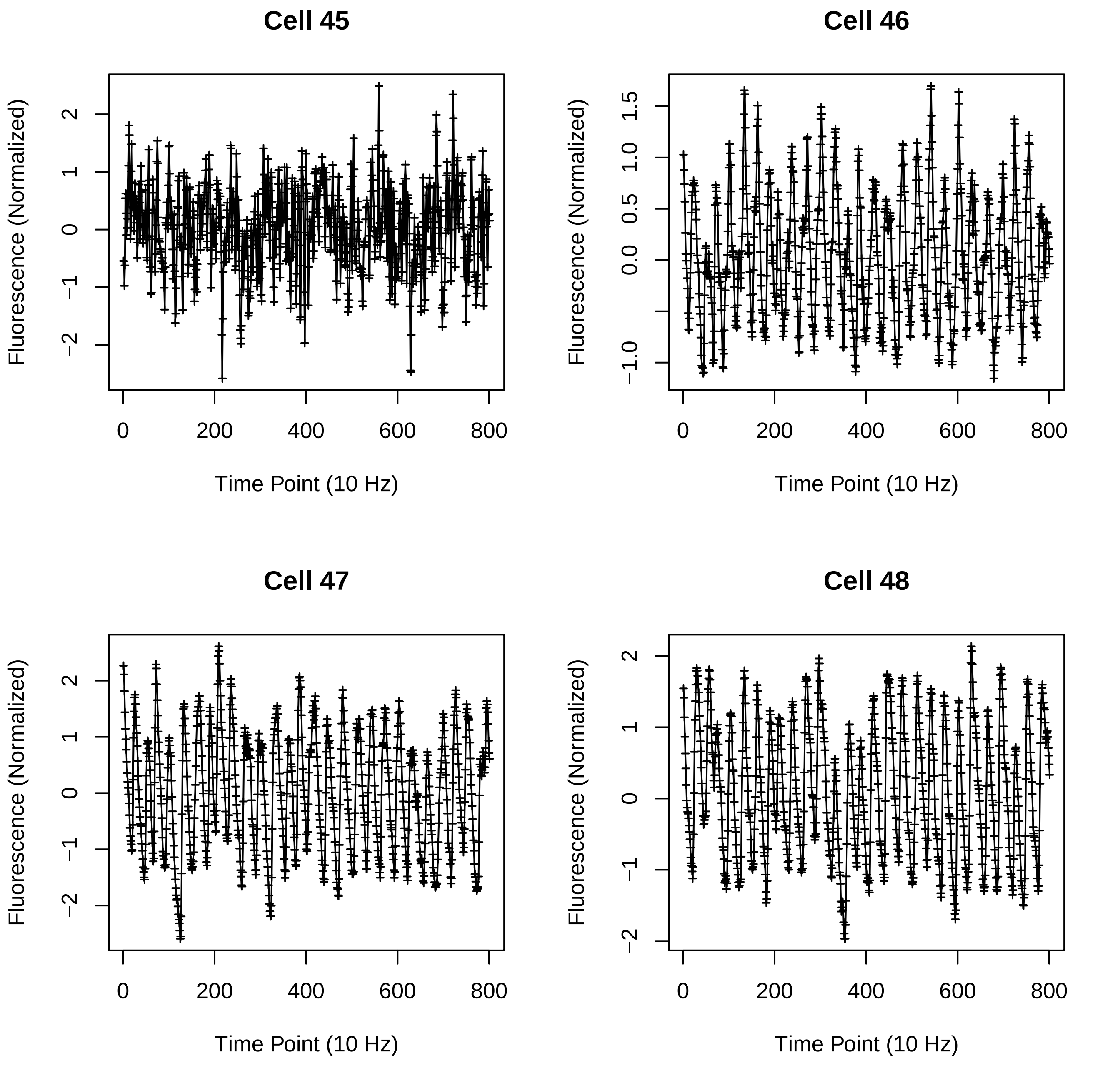
Cell 45
Cell 46
Cell 47
Cell 48
Fluorescence (Normalized)
Time Point (10 Hz)
0
200
400
600
800
−2
−1
0
1
2
−1.0
0.0
0.5
1.0
1.5

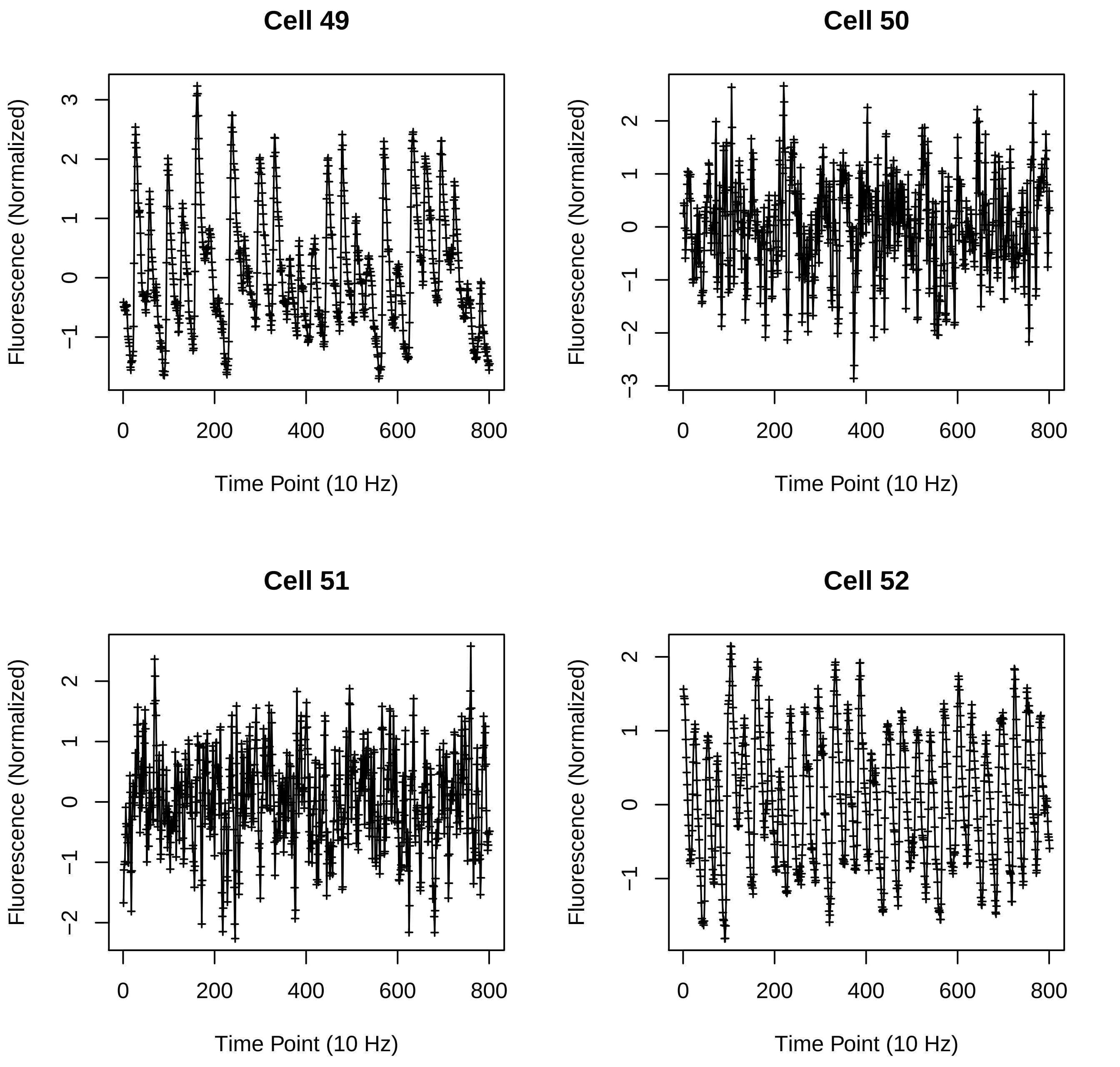
Cell 49
Cell 50
Cell 51
Cell 52
Fluorescence (Normalized)
Time Point (10 Hz)
0
200
400
600
800
−3
−2
−1
0
1
2
3

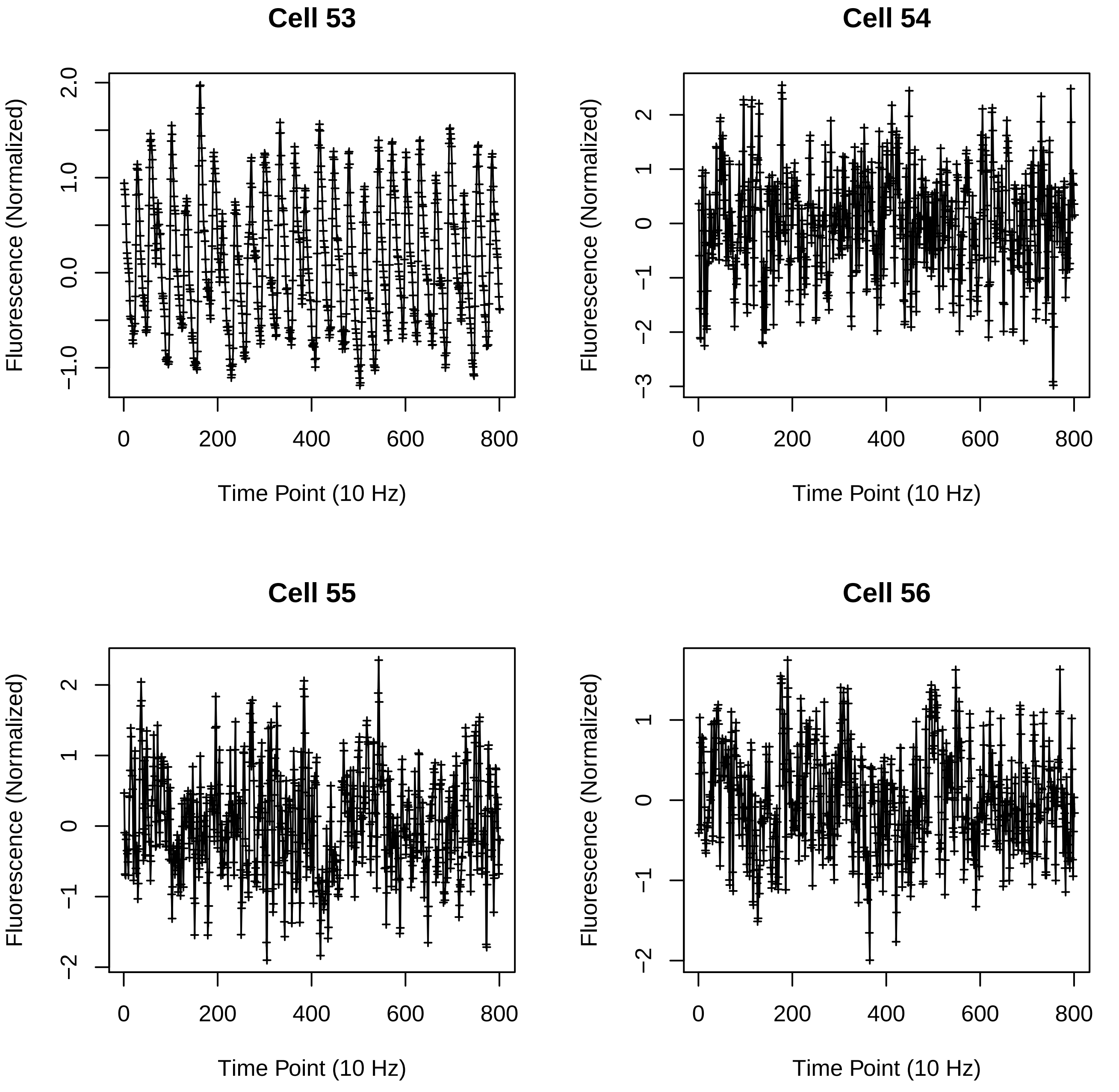
Cell 53
Cell 54
Cell 55
Cell 56
Fluorescence (Normalized)
Time Point (10 Hz)
2.0
1.0
0.0
−1.0
2
1
0
−1
−2
−3
0
200
400
600
800

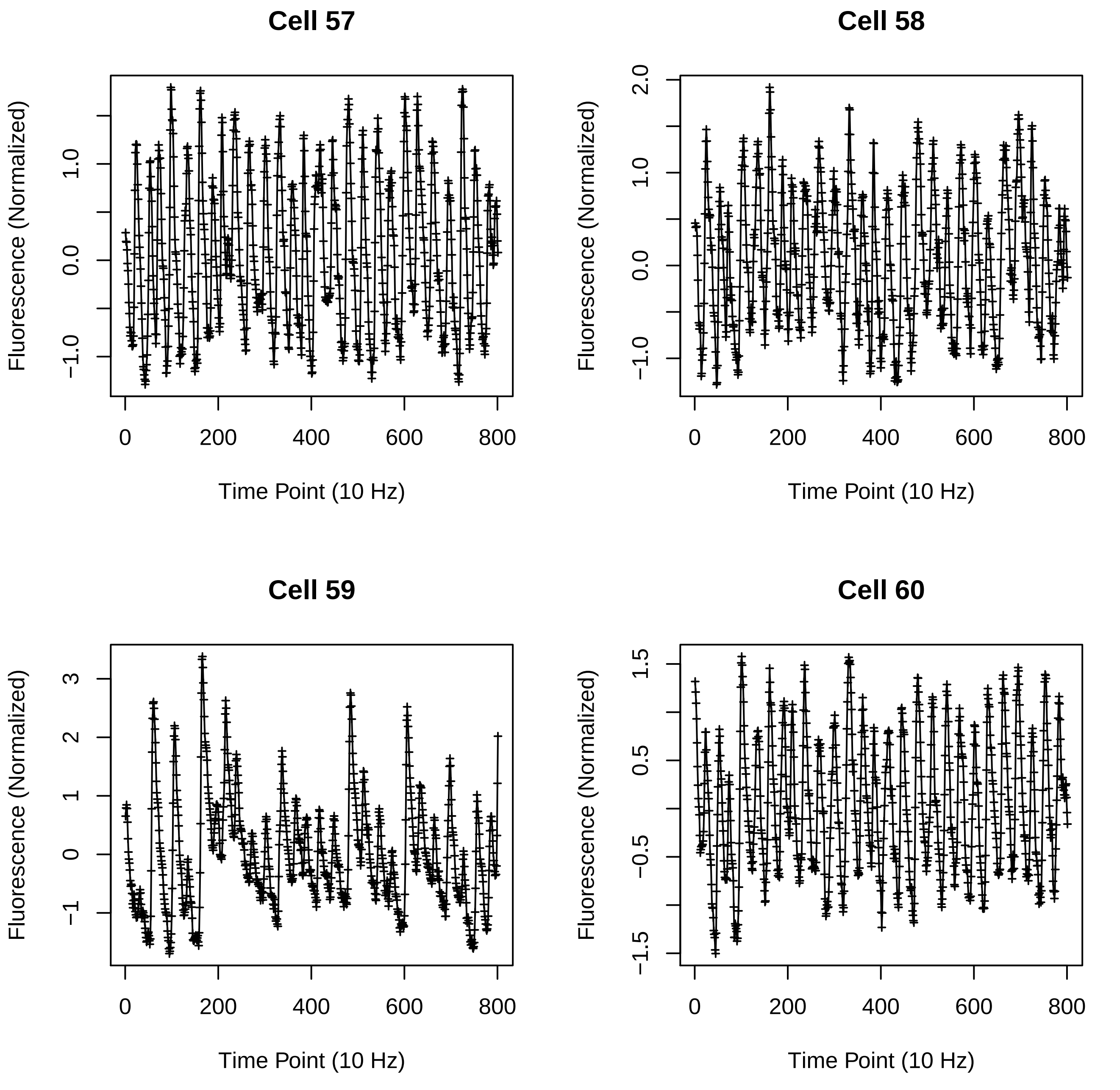
Cell 57
Cell 58
Cell 59
Cell 60
Fluorescence (Normalized)
Time Point (10 Hz)
0
200
400
600
800
−1.0
0.0
1.0
2.0
−1
1
2
3
−1.5
−0.5
0.5
1.5

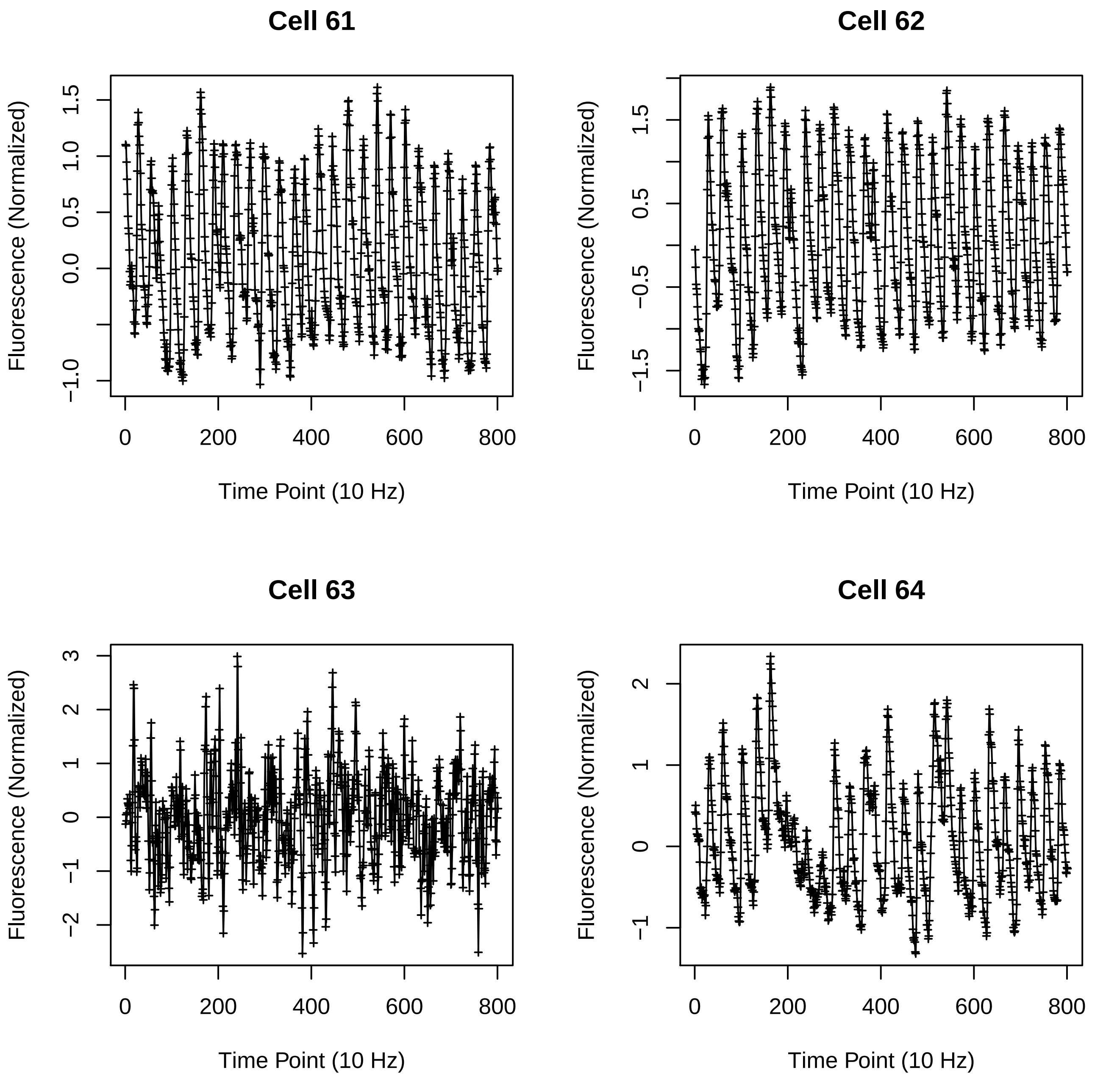
Cell 61
Cell 62
Cell 63
Cell 64
Fluorescence (Normalized)
Time Point (10 Hz)
0
200
400
600
800

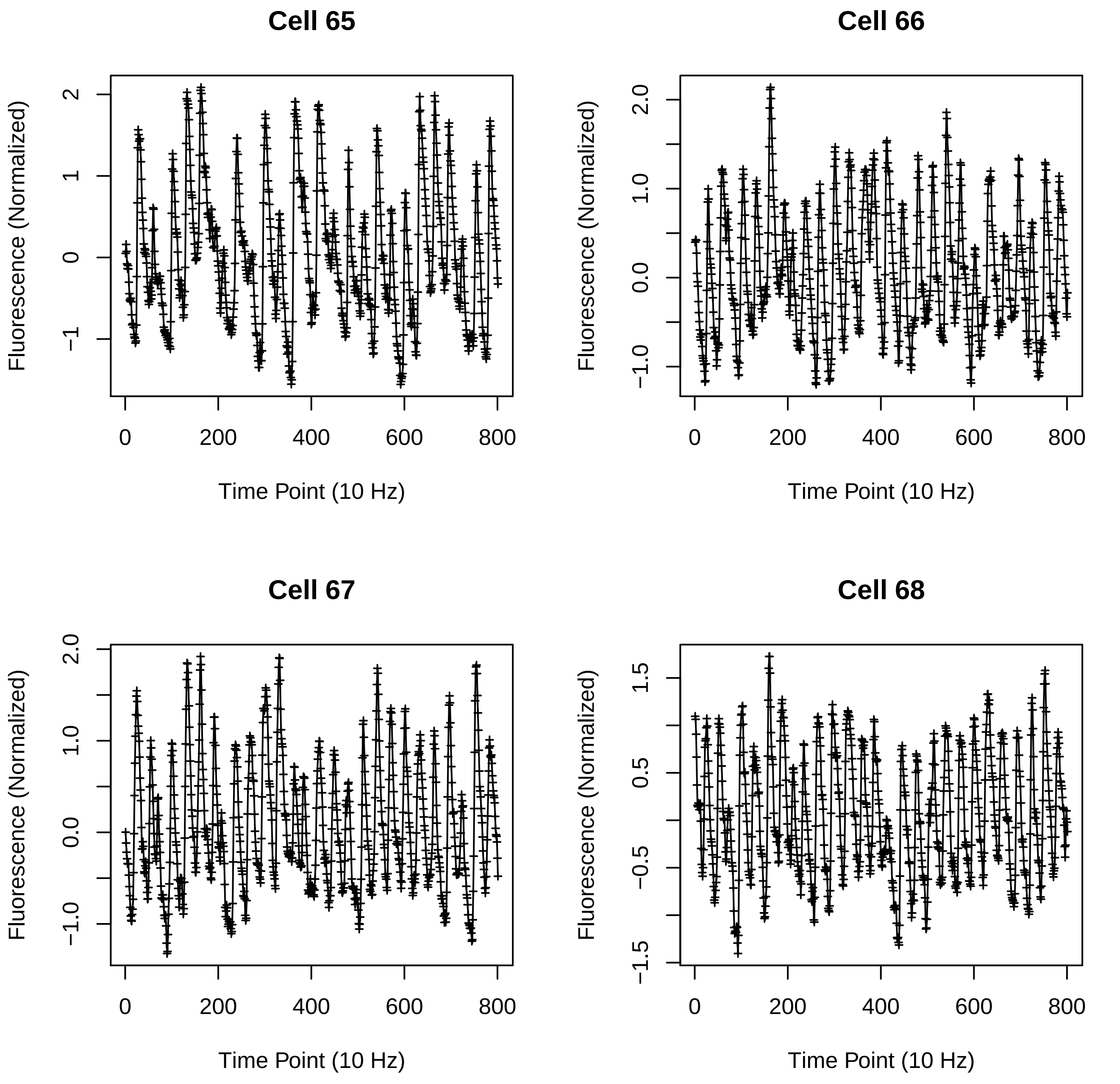

Cell 65
Cell 66
Cell 67
Cell 68
Fluorescence (Normalized)
Time Point (10 Hz)
0
200
400
600
800
−1
0
1
2
−1.0
0.0
1.0
2.0
−1.5
−0.5
0.5
1.5

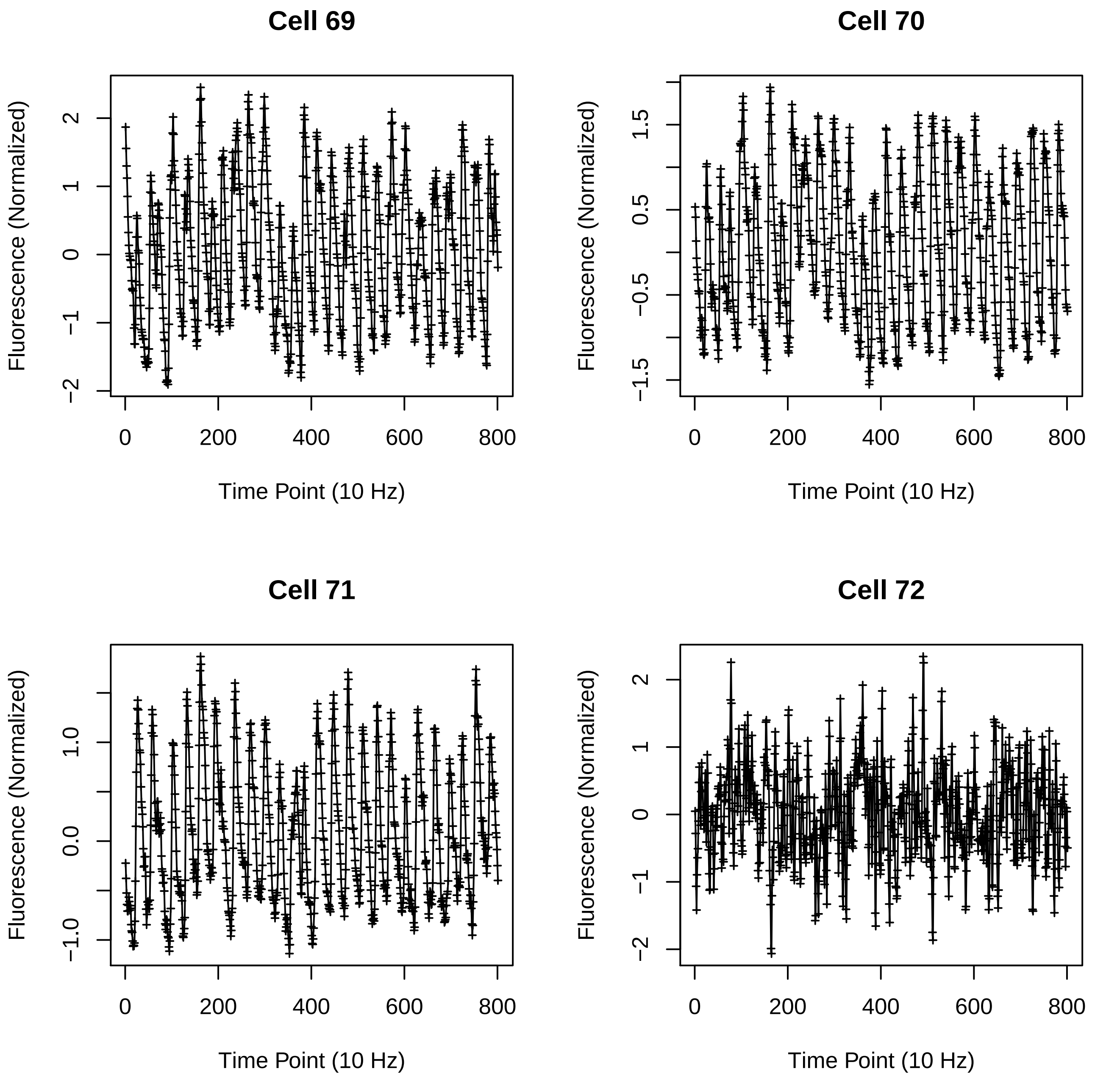
Cell 69
Cell 70
Cell 71
Cell 72
Fluorescence (Normalized)
Time Point (10 Hz)
0
200
400
600
800
−2
−1
0
1
2
−1.5
−0.5
0.5
1.5
−1.0
0.0
1.0

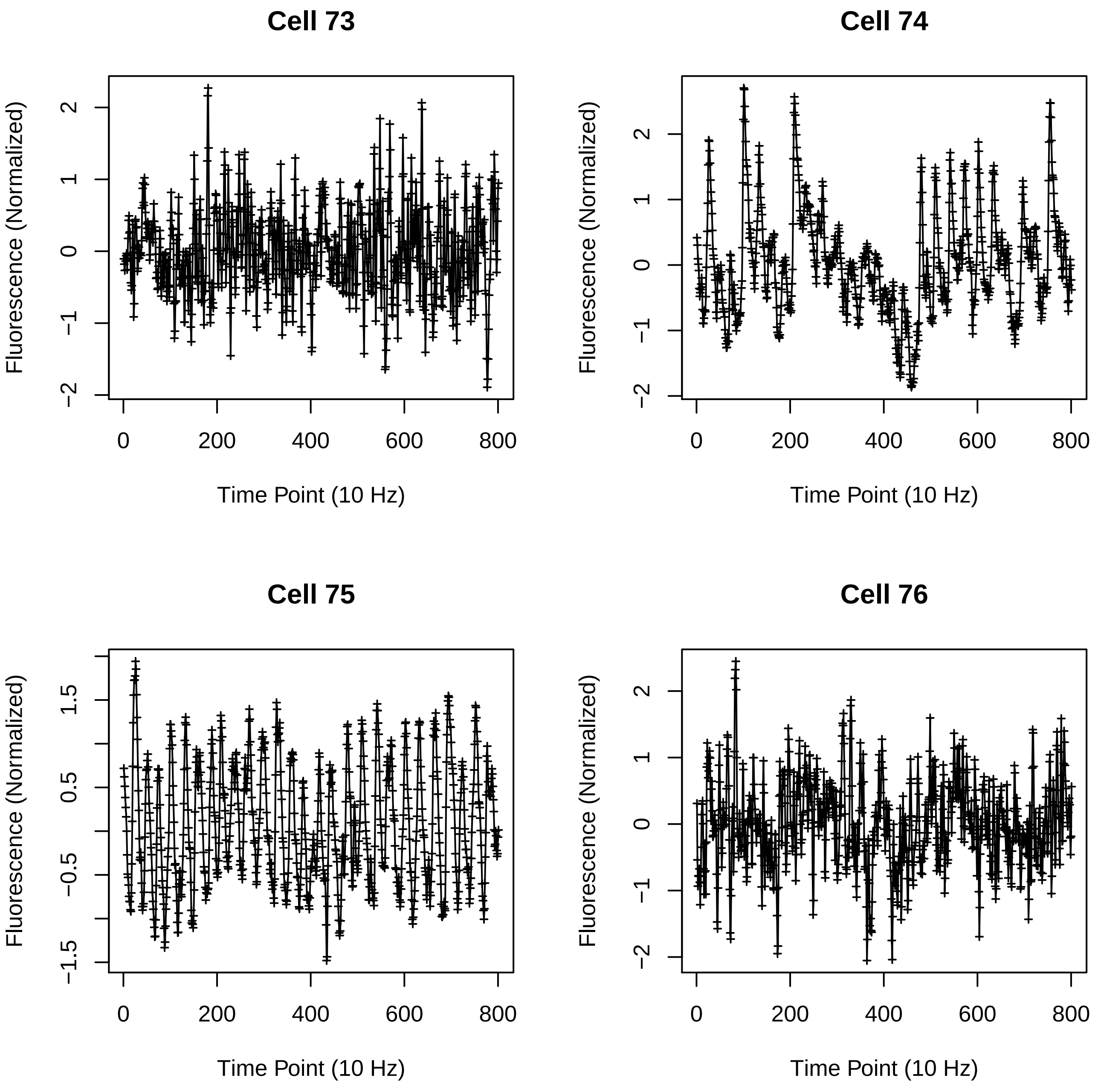
Cell 73
Cell 74
Cell 75
Cell 76
Fluorescence (Normalized)
Time Point (10 Hz)
0
200
400
600
800
−2
−1
0
1
2
−1.5
−0.5
0.5
1.5

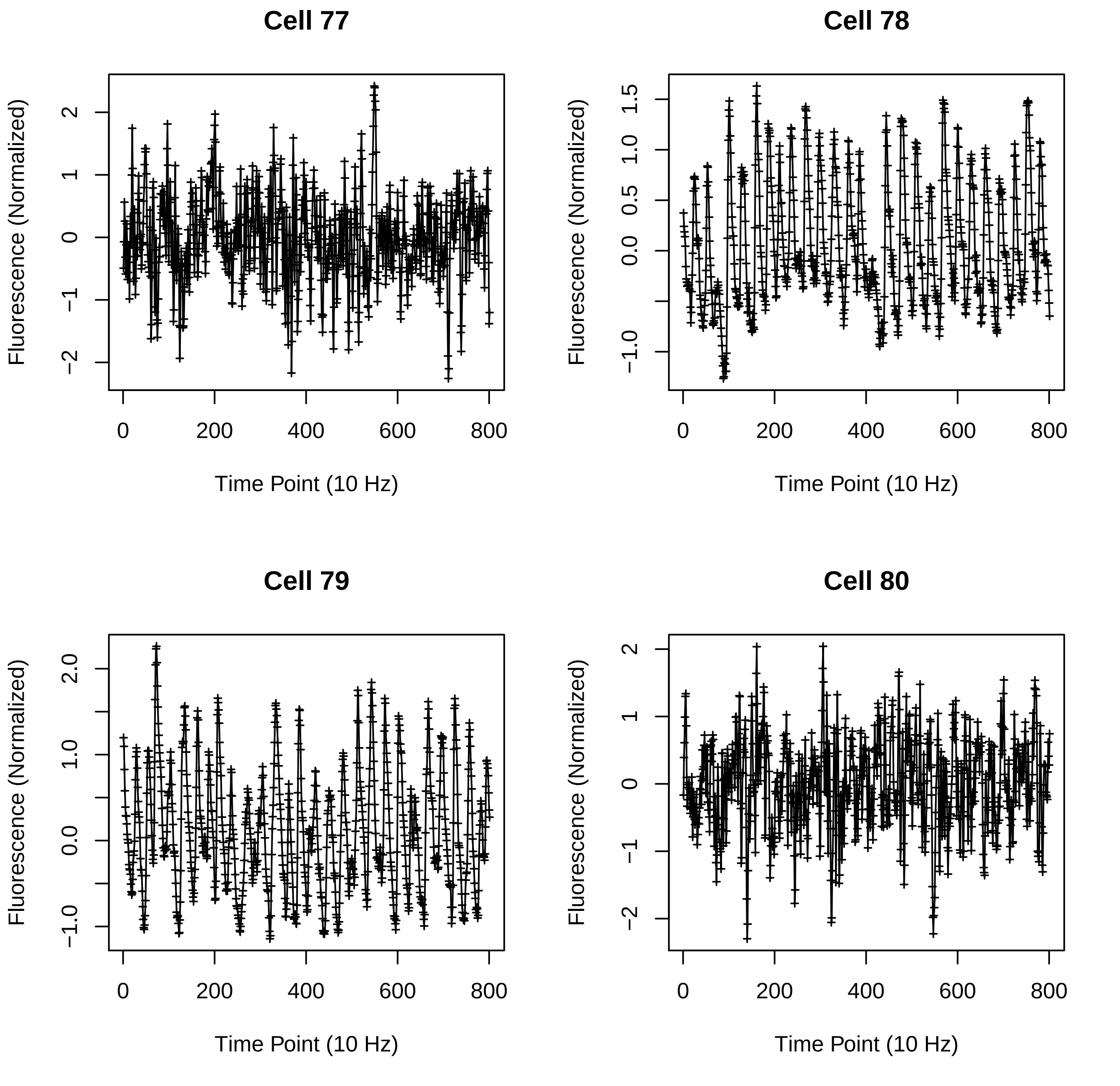

Cell 77
Cell 78
Cell 79
Cell 80
Fluorescence (Normalized)
Time Point (10 Hz)
0
200
400
600
800
−2
−1
0
1
2
−1.0
−0.5
0.0
0.5
1.0
1.5
2.0

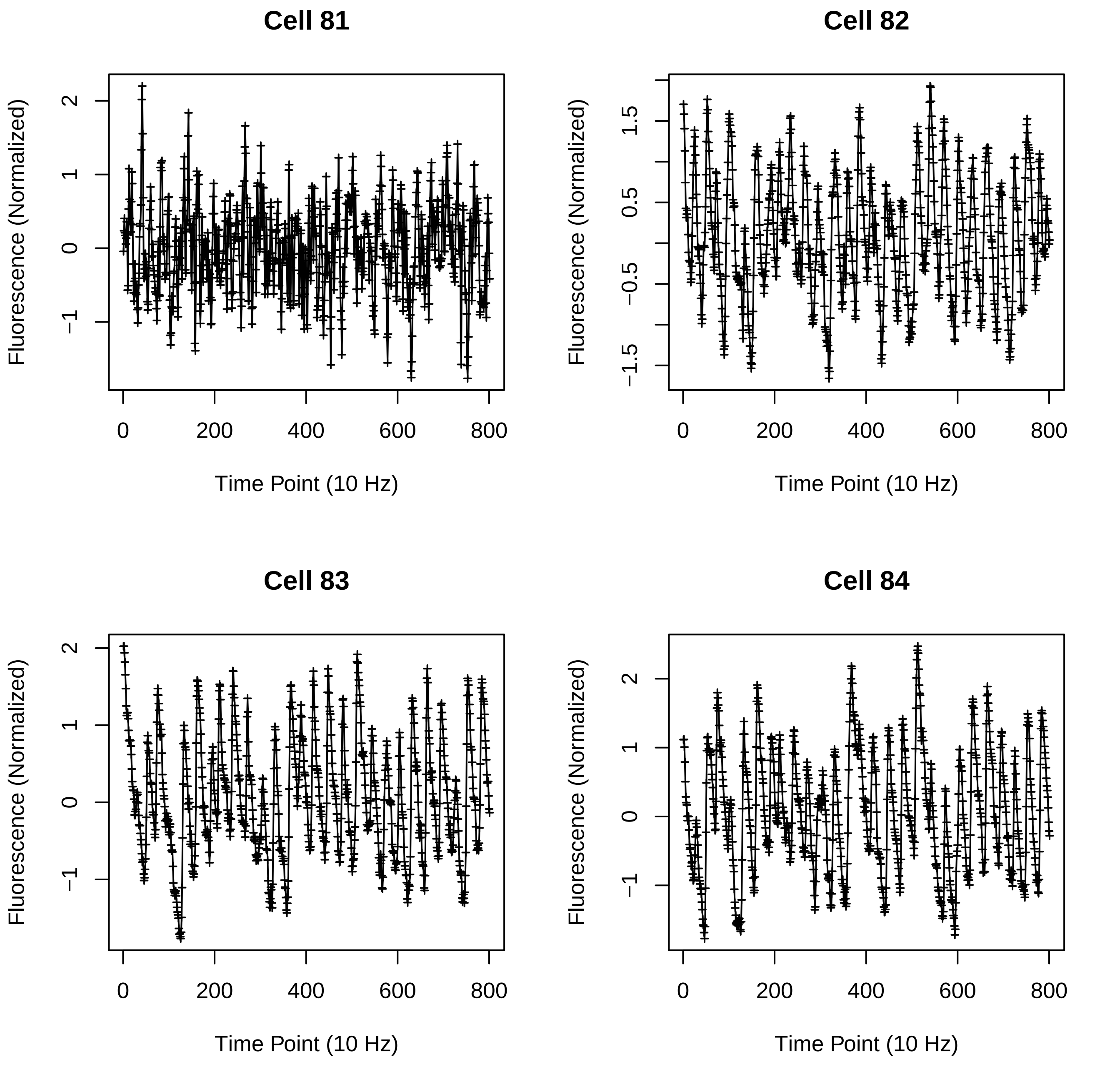
Cell 81
Cell 82
Cell 83
Cell 84
Fluorescence (Normalized)
Time Point (10 Hz)
0
200
400
600
800
−1
0
1
2
−1.5
−0.5
0.5
1.5

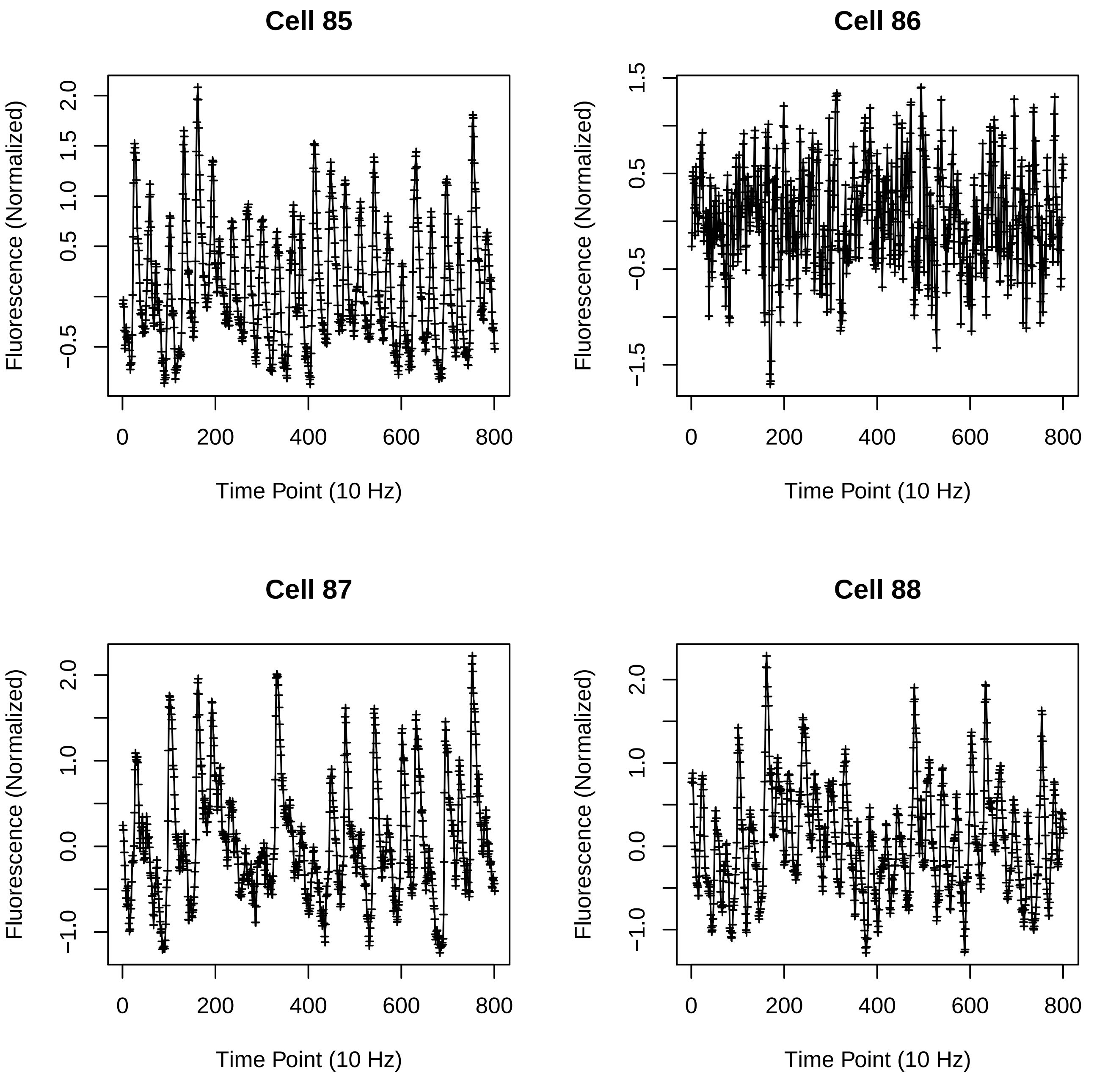
Cell 85
Cell 86
Cell 87
Cell 88
Fluorescence (Normalized)
Time Point (10 Hz)
0
200
400
600
800
−0.5
0.5
1.0
1.5
2.0
−1.5
−0.5
0.5
1.5
−1.0
0.0
1.0
2.0

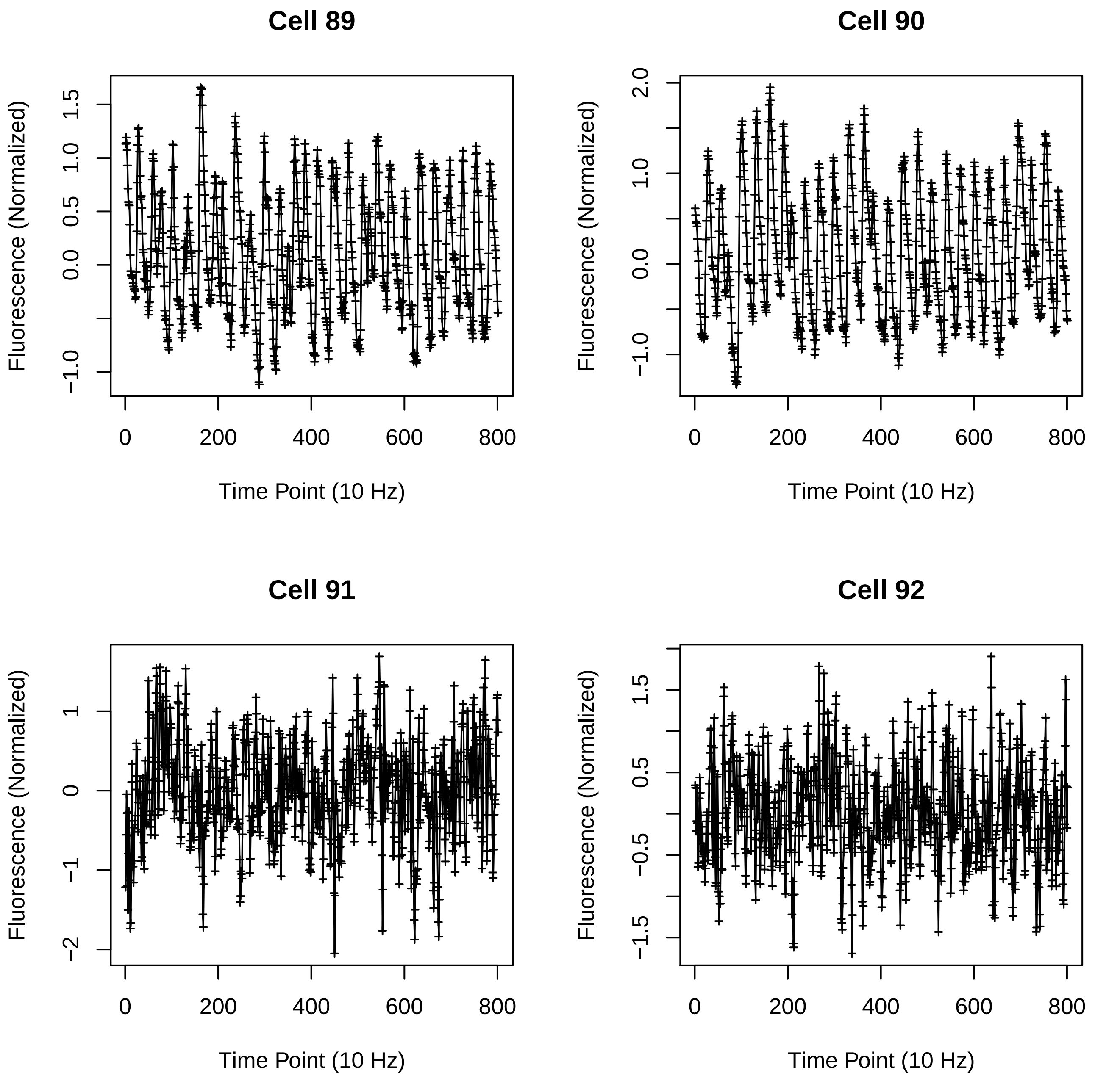
Cell 89
Fluorescence (Normalized)
−1.0
0.0
0.5
1.0
1.5
0
200
400
600
800
Time Point (10 Hz)
Cell 90
Fluorescence (Normalized)
−1.0
0.0
1.0
2.0
0
200
400
600
800
Time Point (10 Hz)
Cell 91
Fluorescence (Normalized)
−2
−1
0
1
0
200
400
600
800
Time Point (10 Hz)
Cell 92
Fluorescence (Normalized)
−1.5
−0.5
0.5
1.5
0
200
400
600
800
Time Point (10 Hz)

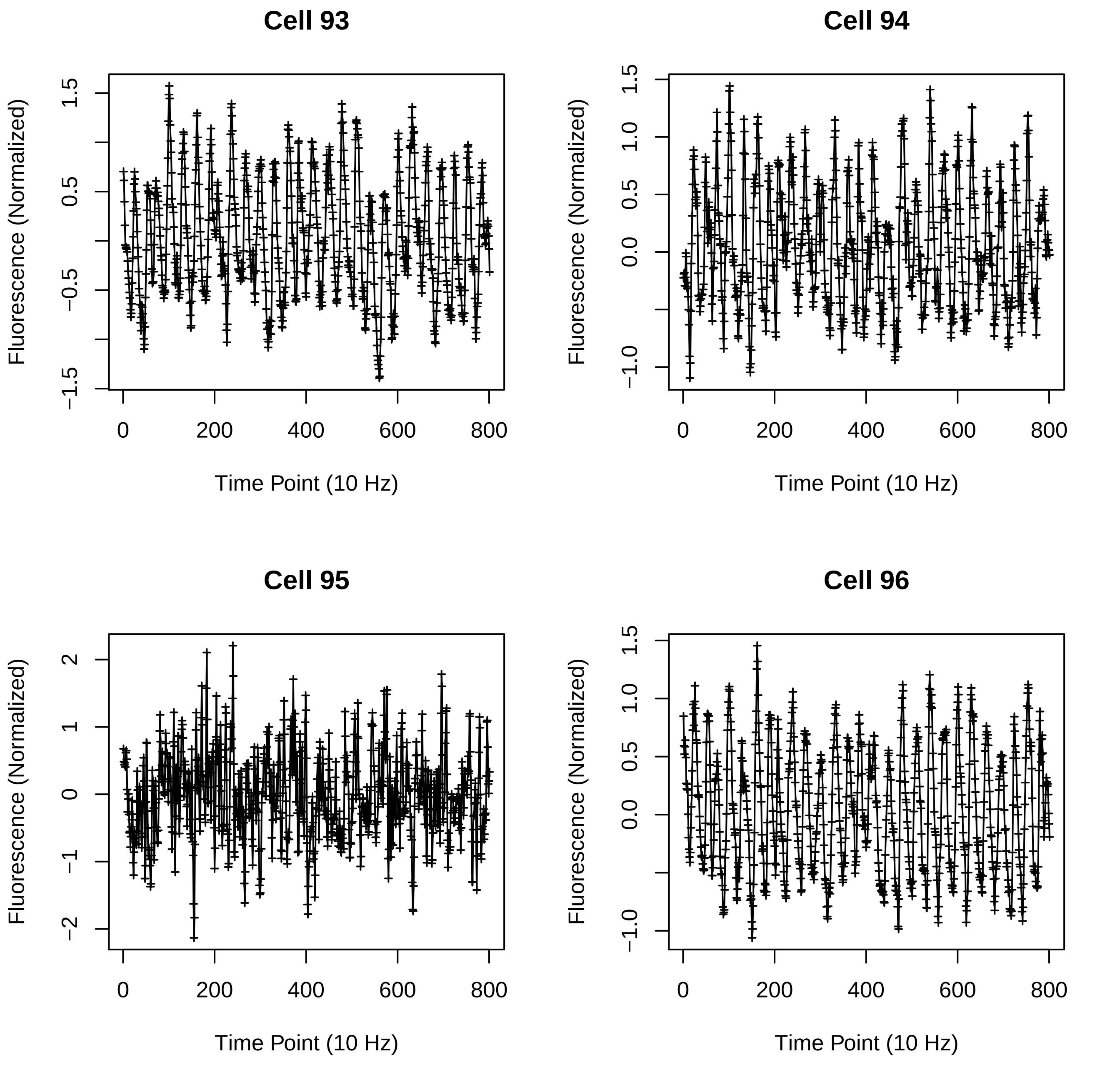
Cell 93
Cell 94
Cell 95
Cell 96
Fluorescence (Normalized)
Time Point (10 Hz)
0
200
400
600
800
1.5
0.5
−0.5
−1.5
1.0
0.0
−1.0
2
1
0
−1
−2

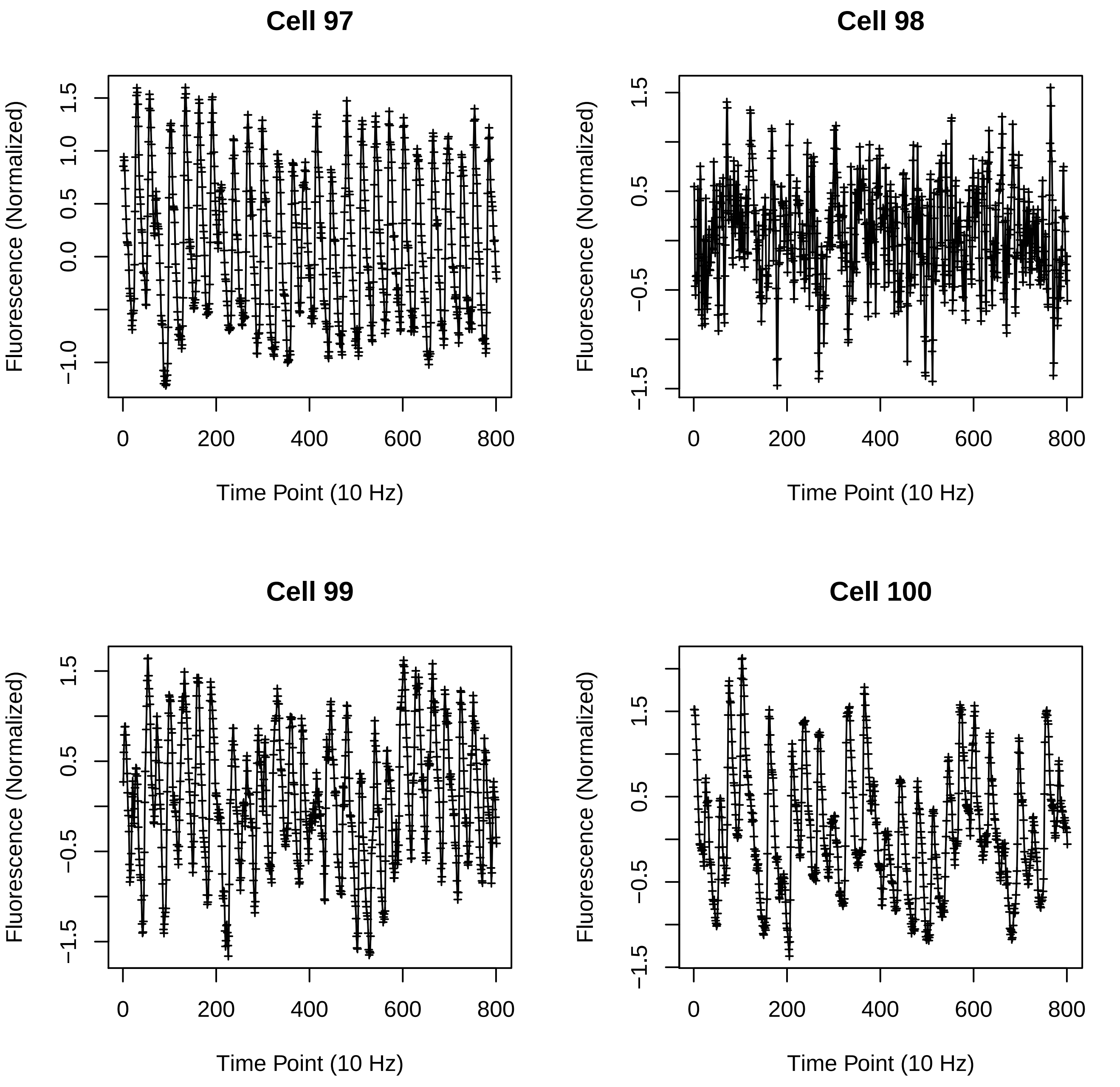
Cell 97
Cell 98
Cell 99
Cell 100
Fluorescence (Normalized)
Time Point (10 Hz)
−1.0
0.0
0.5
1.0
1.5
−1.5
−0.5
0.5
1.5
0
200
400
600
800

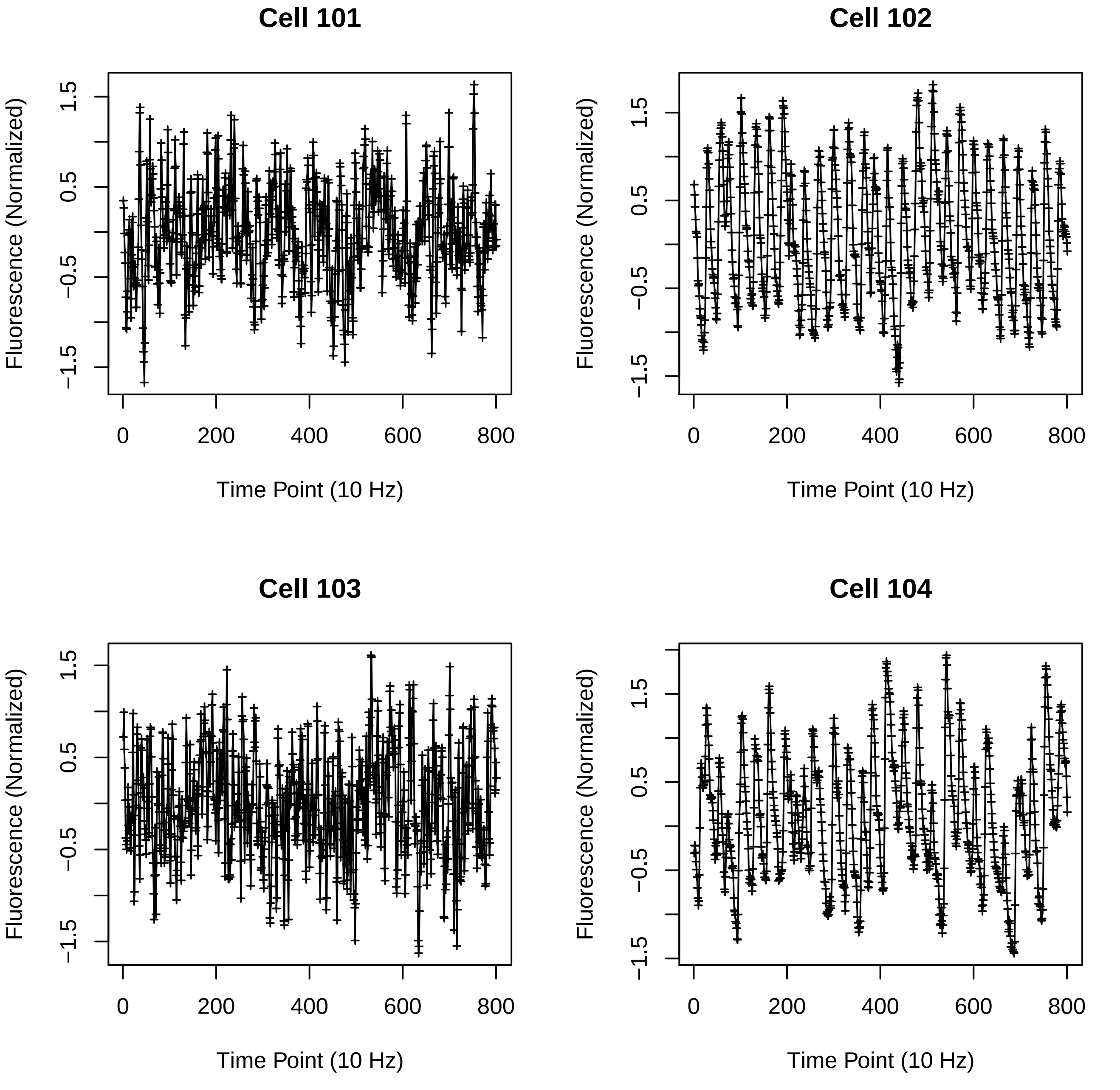
Cell 101
Cell 102
Cell 103
Cell 104
Fluorescence (Normalized)
Time Point (10 Hz)
1.5
0.5
−0.5
−1.5
0
200
400
600
800

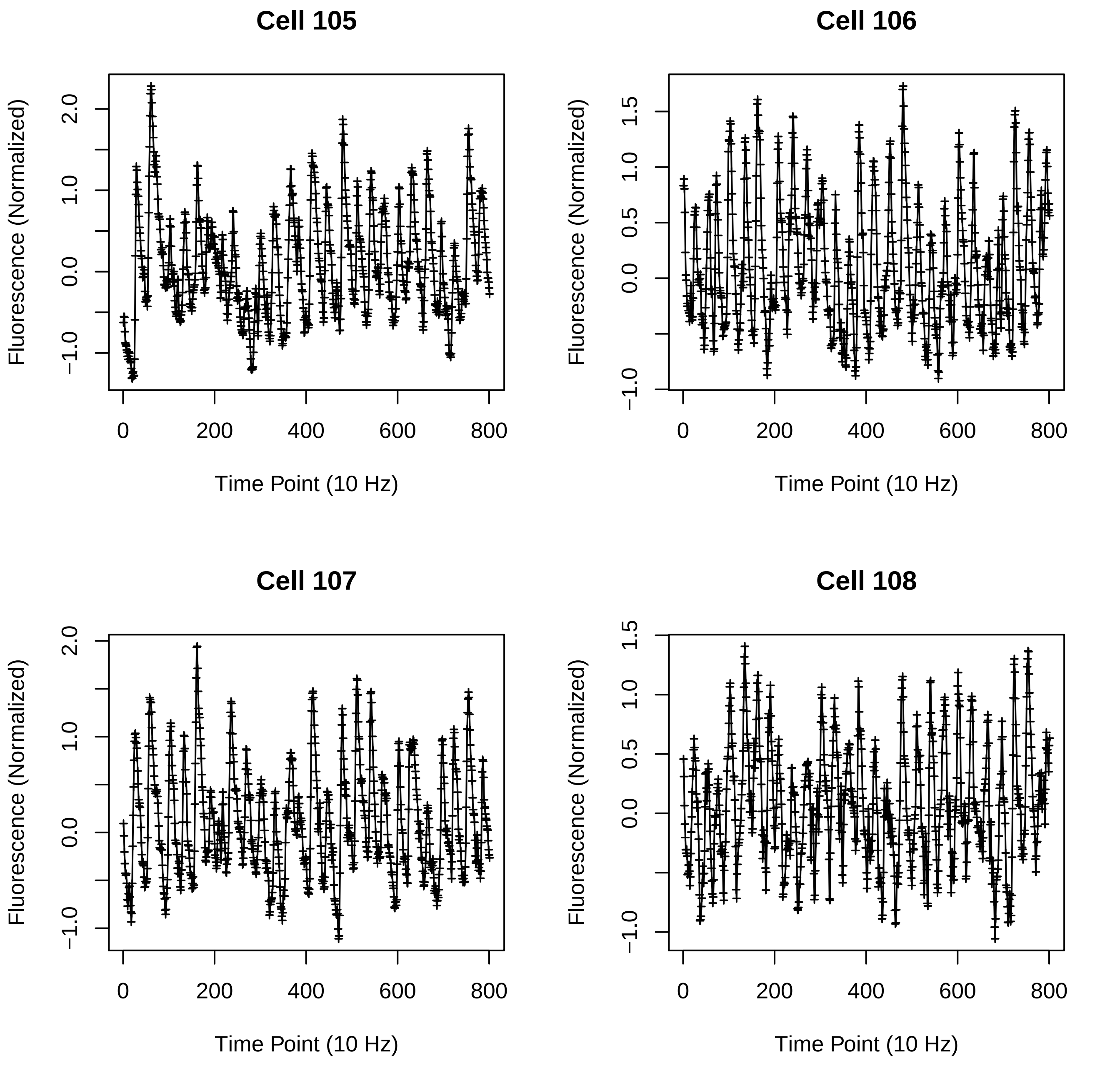
Cell 105
Cell 106
Cell 107
Cell 108
Fluorescence (Normalized)
Time Point (10 Hz)
0
200
400
600
800
−1.0
0.0
1.0
2.0
−1.0
0.0
0.5
1.0
1.5

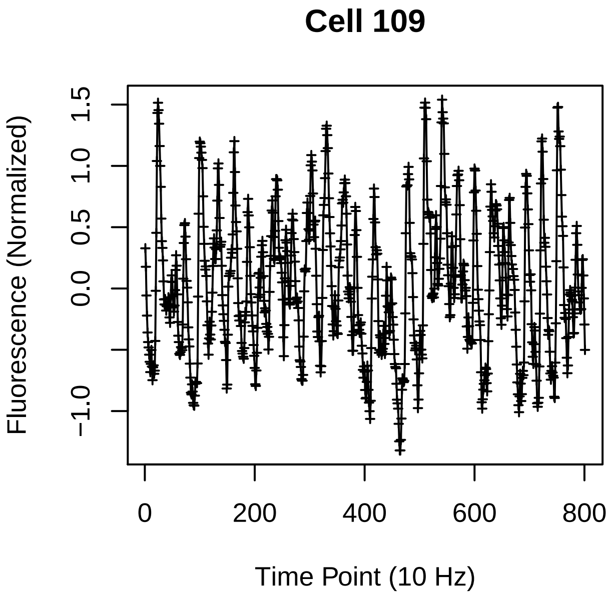

Cell 109
Fluorescence (Normalized)
Time Point (10 Hz)

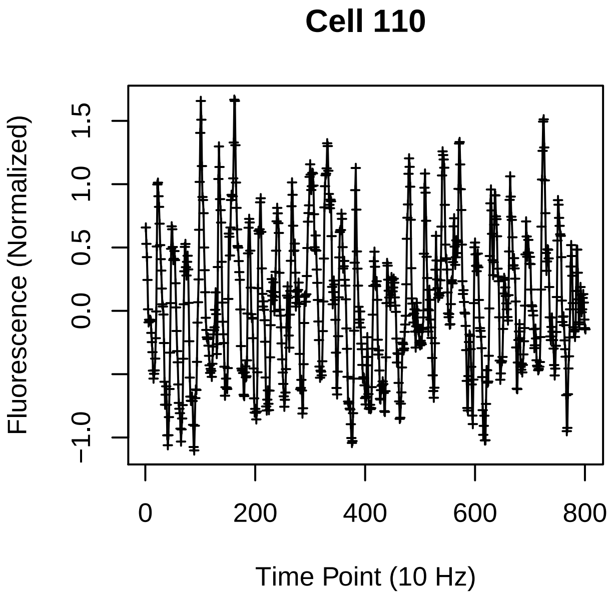

Cell 110
Fluorescence (Normalized)
Time Point (10 Hz)

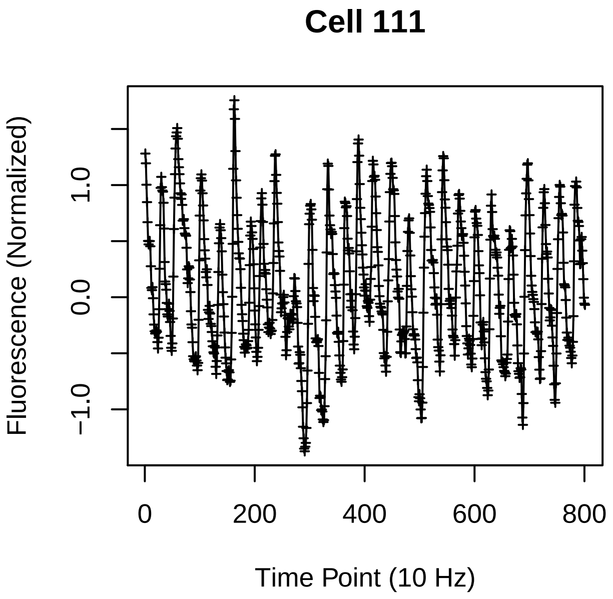

Cell 111
Fluorescence (Normalized)
Time Point (10 Hz)

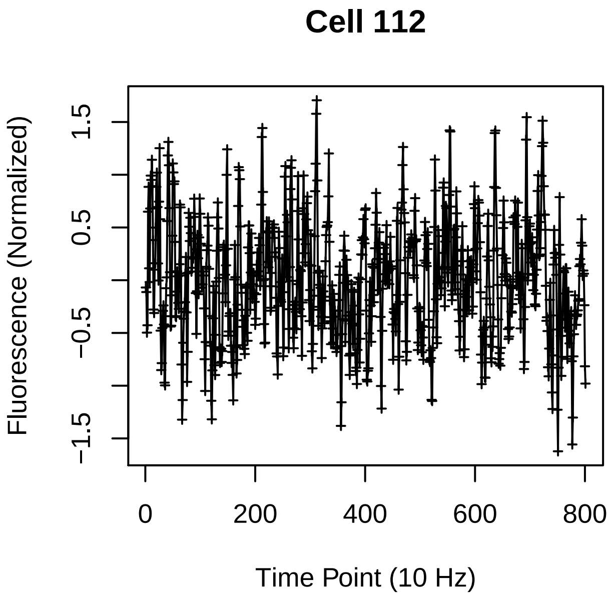

Cell 112
Fluorescence (Normalized)
Time Point (10 Hz)

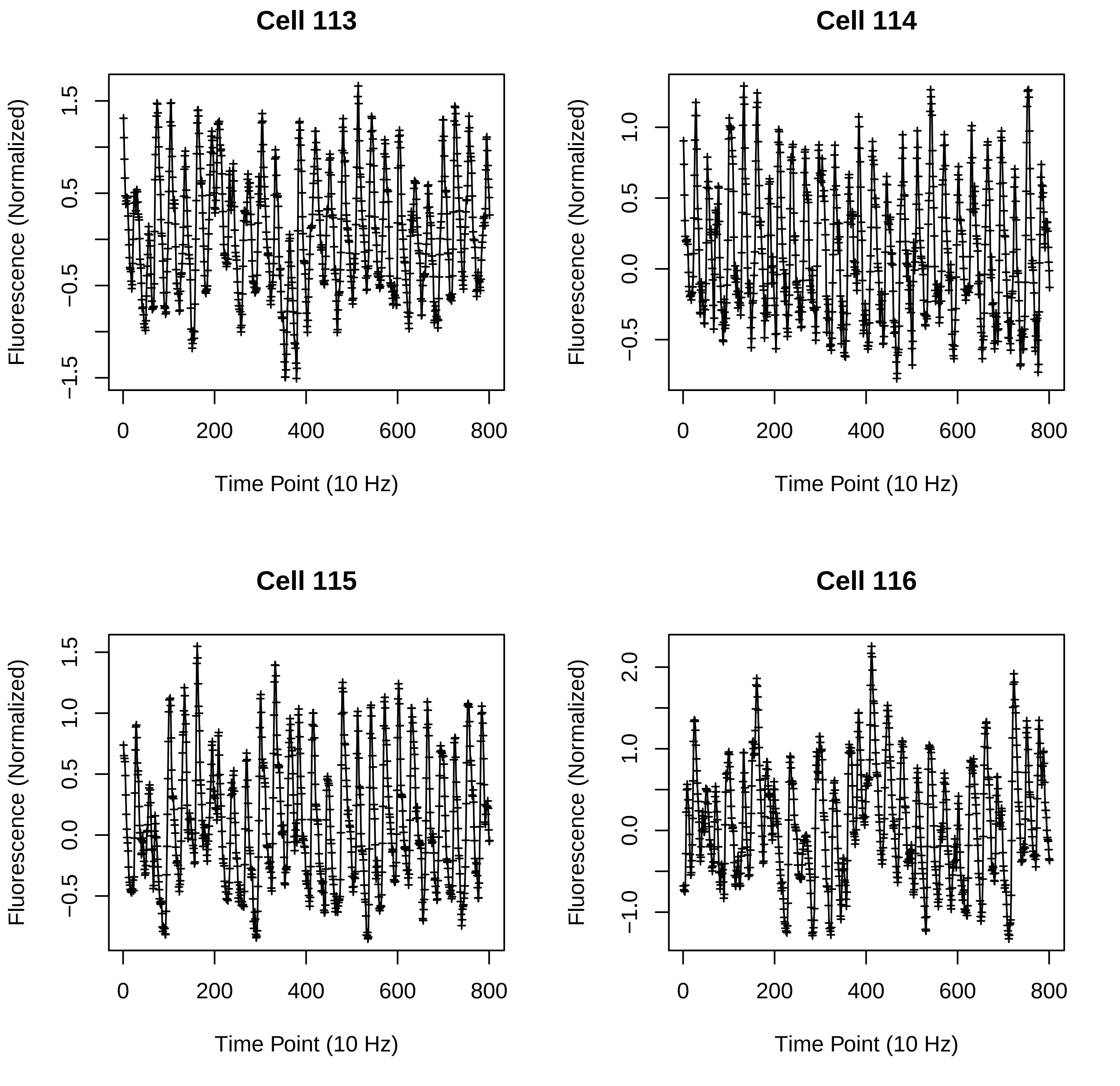
Cell 113
Cell 114
Cell 115
Cell 116
Fluorescence (Normalized)
Time Point (10 Hz)
0
200
400
600
800
−1.5
−0.5
0.5
1.5
0.0
1.0
2.0
−1.0

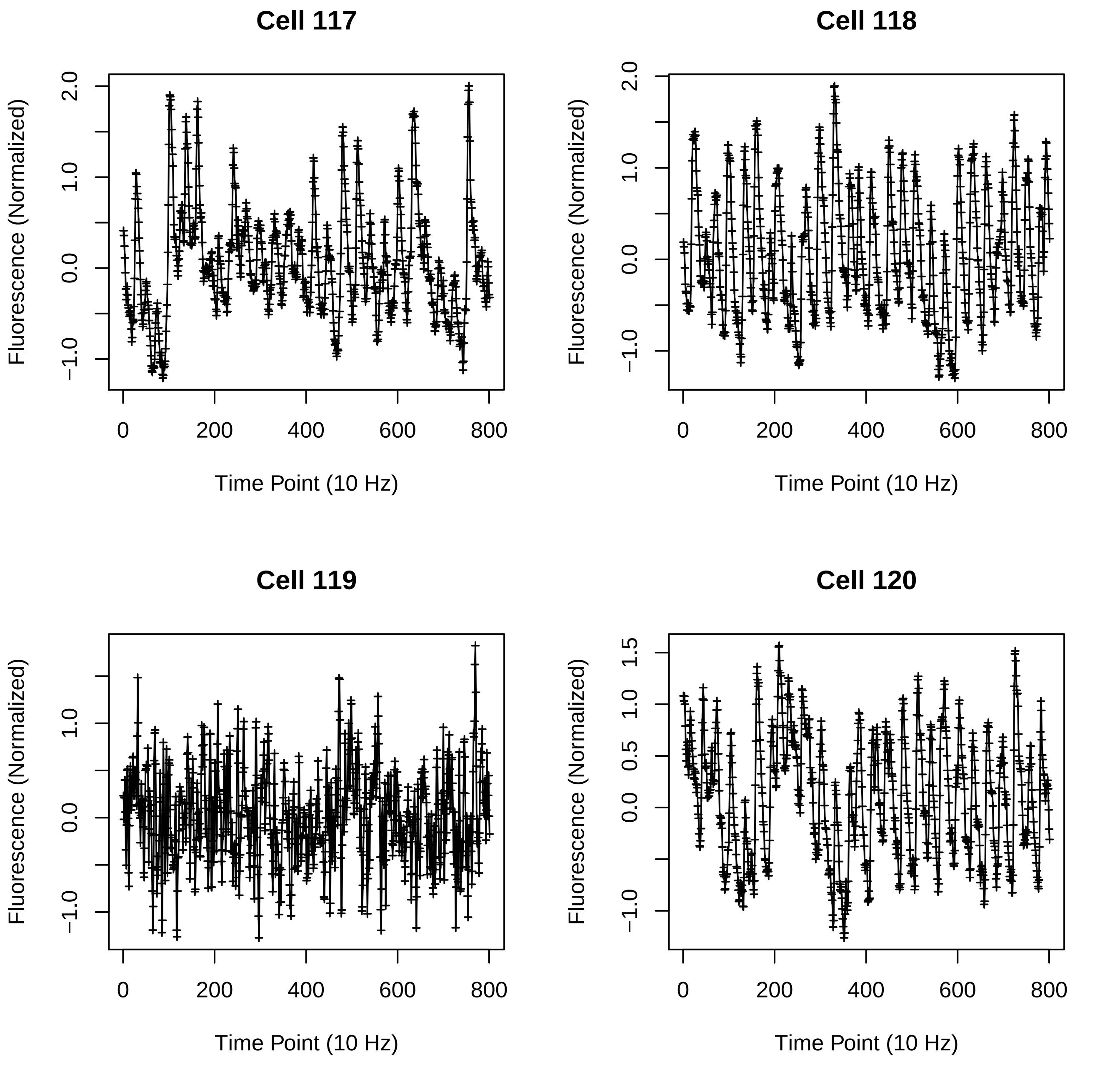
Cell 117
Cell 118
Cell 119
Cell 120
Fluorescence (Normalized)
Time Point (10 Hz)
0
200
400
600
800
2.0
1.0
0.0
−1.0
1.5
0.5

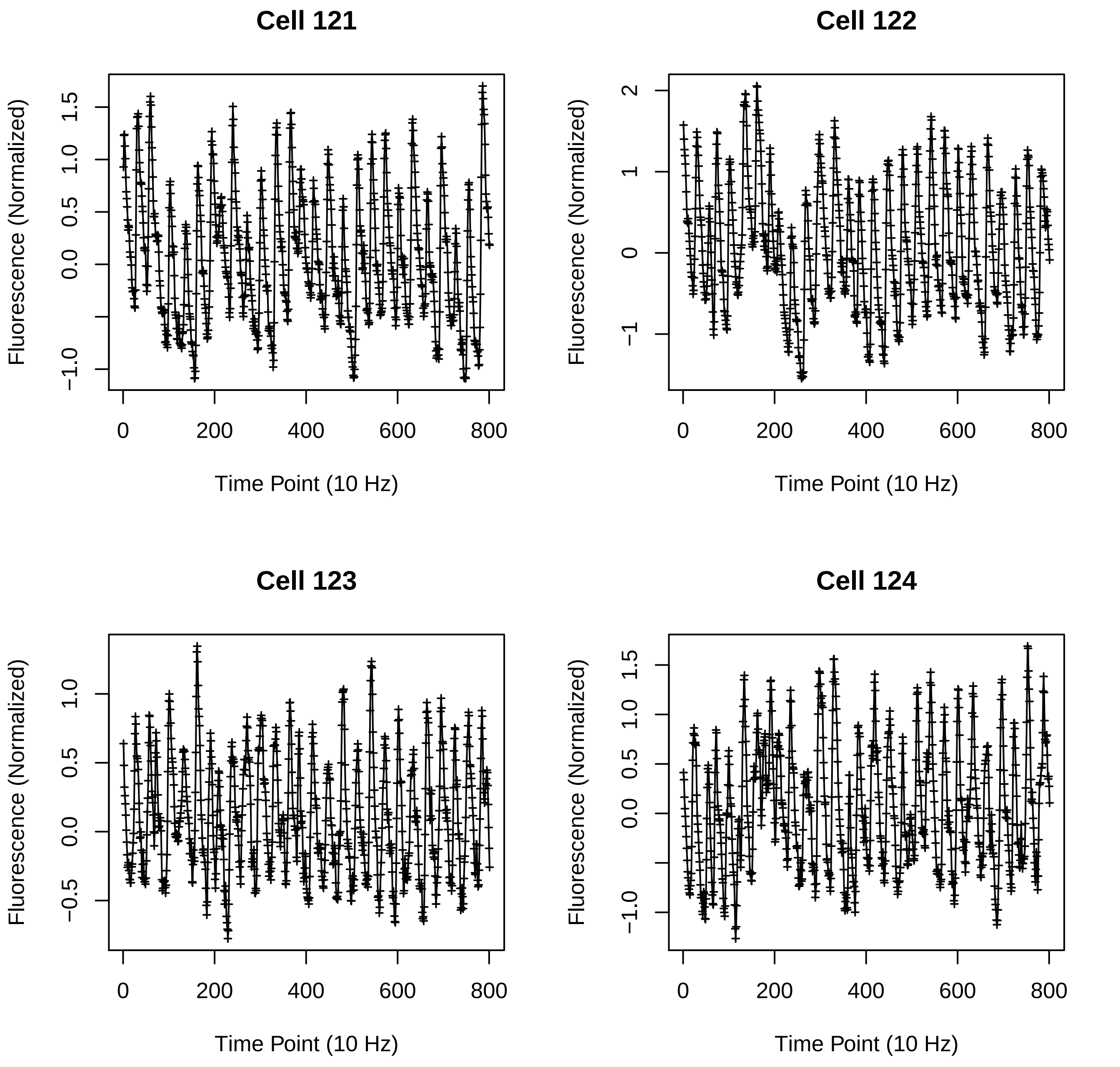
Cell 121
Cell 122
Cell 123
Cell 124
Fluorescence (Normalized)
Time Point (10 Hz)

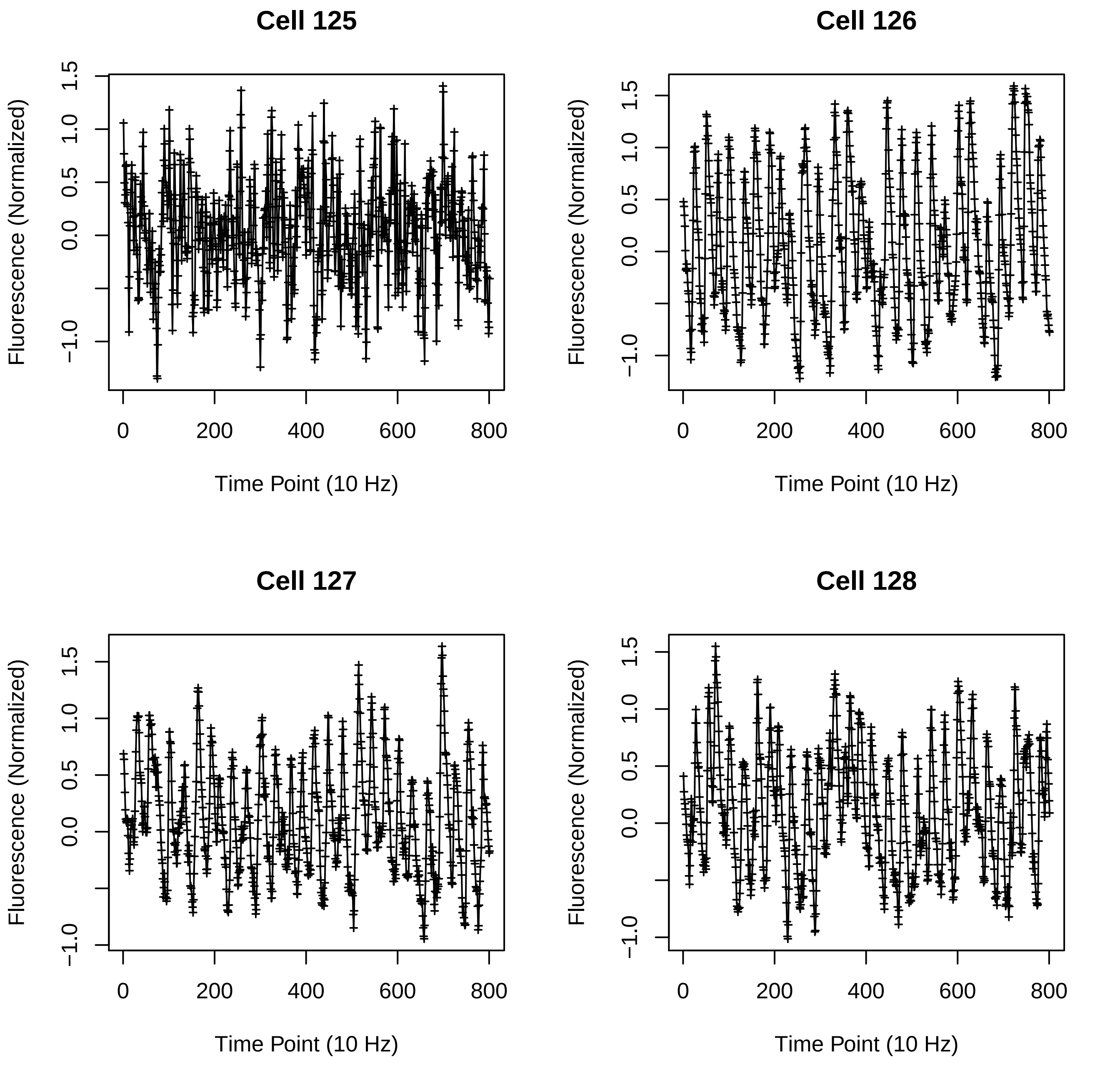

Cell 125
Cell 126
Cell 127
Cell 128
Fluorescence (Normalized)
Time Point (10 Hz)
0
200
400
600
800
−1.0
0.0
0.5
1.0
1.5

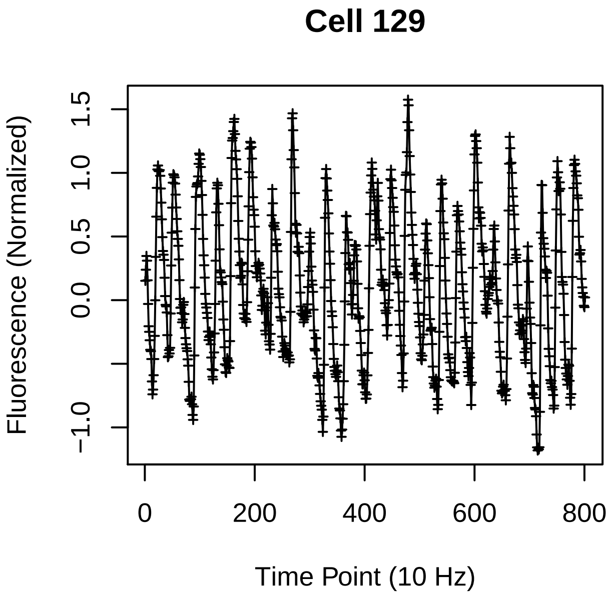
Cell 129
Fluorescence (Normalized)
-1.0
0.0
0.5
1.0
1.5
0
200
400
600
800
Time Point (10 Hz)

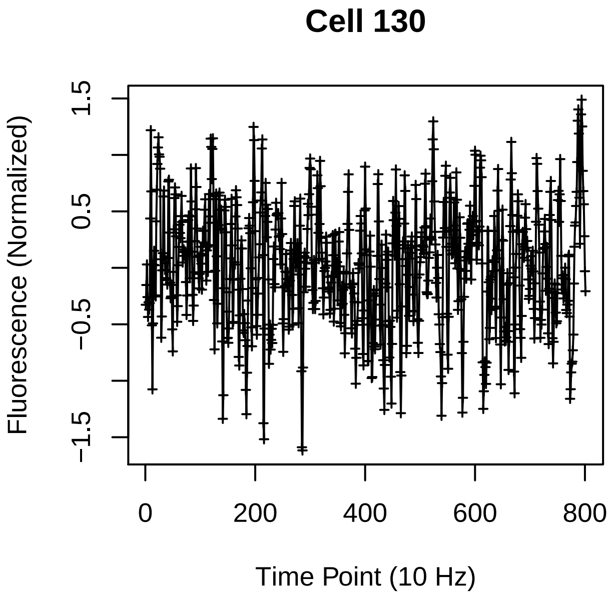
Cell 130
Fluorescence (Normalized)
-1.5
-0.5
0.5
1.5
0
200
400
600
800
Time Point (10 Hz)

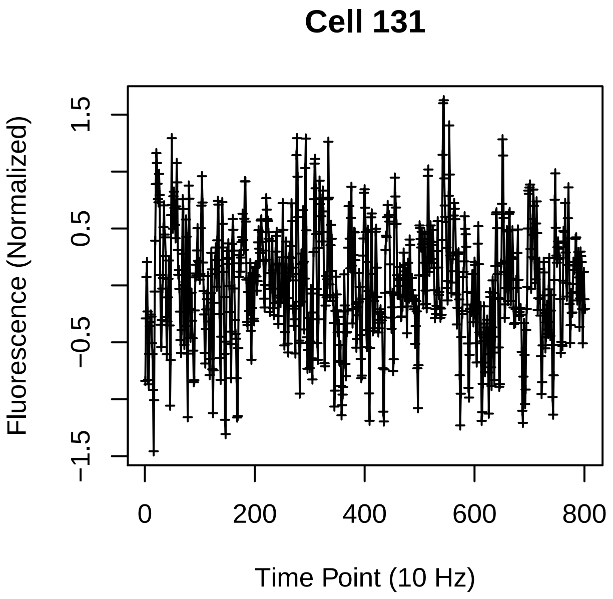
Cell 131
Fluorescence (Normalized)
-1.5
-0.5
0.5
1.5
0
200
400
600
800
Time Point (10 Hz)

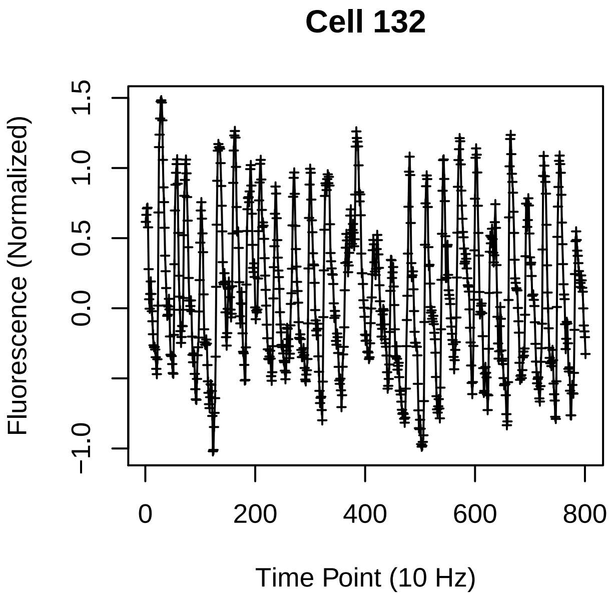
Cell 132
Fluorescence (Normalized)
-1.0
0.0
0.5
1.0
1.5
0
200
400
600
800
Time Point (10 Hz)

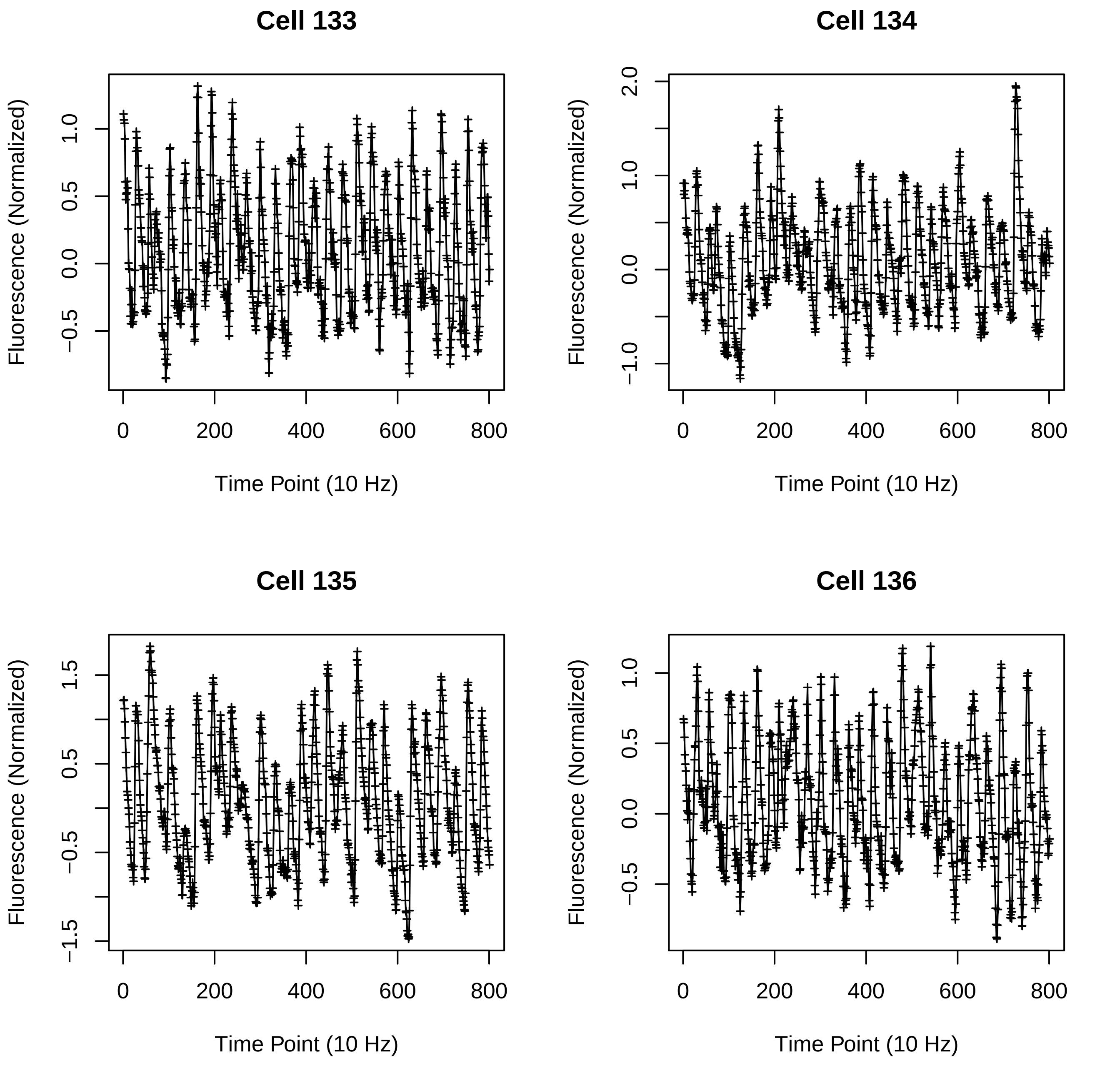
Cell 133
Cell 134
Cell 135
Cell 136
Fluorescence (Normalized)
Time Point (10 Hz)
0
200
400
600
800
−0.5
0.0
0.5
1.0
−1.0
2.0
−1.5
1.5

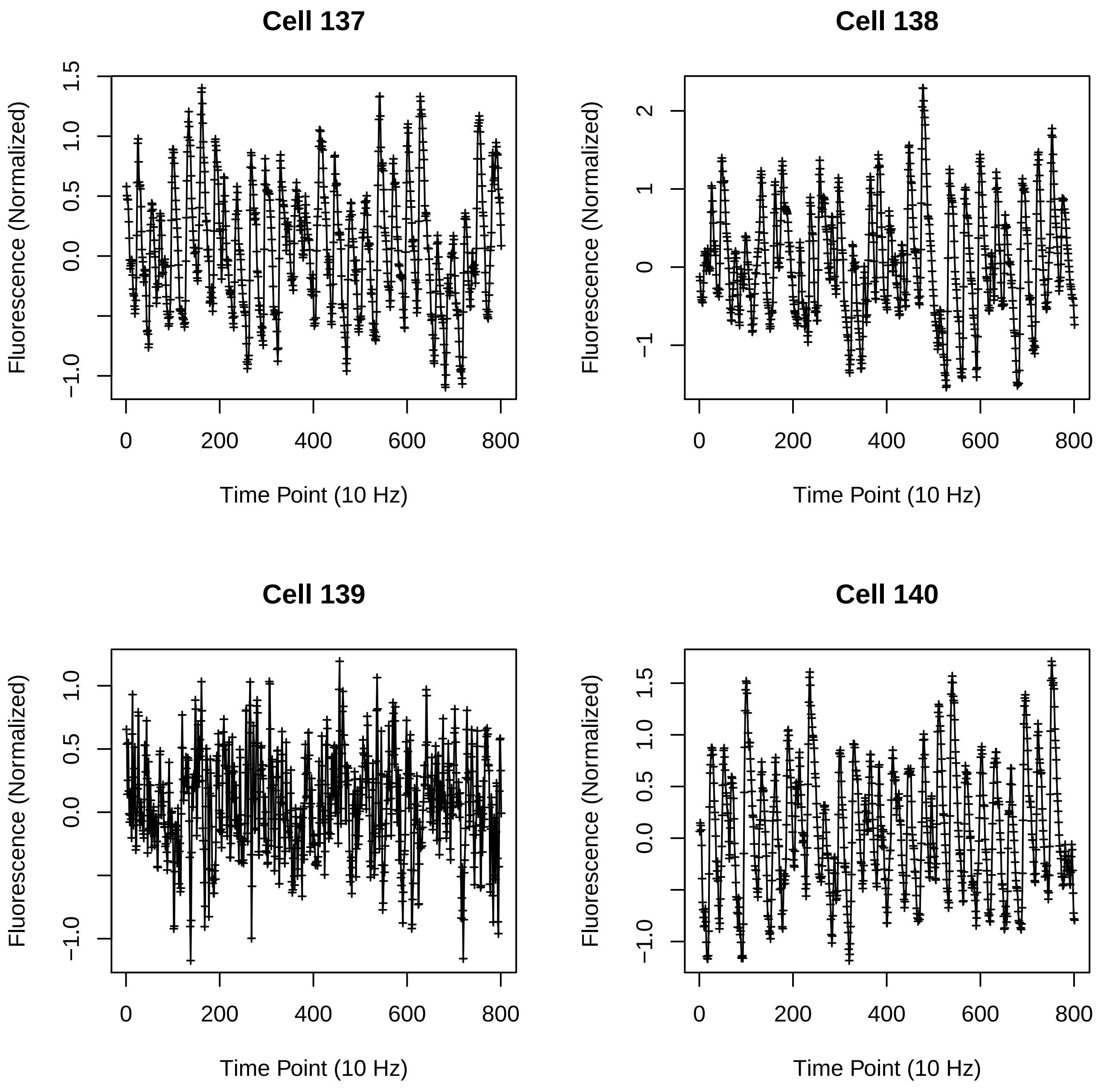
Cell 137
Cell 138
Cell 139
Cell 140
Fluorescence (Normalized)
Time Point (10 Hz)
0
200
400
600
800
−1.0
0.0
0.5
1.0
1.5
−1
0
1
2

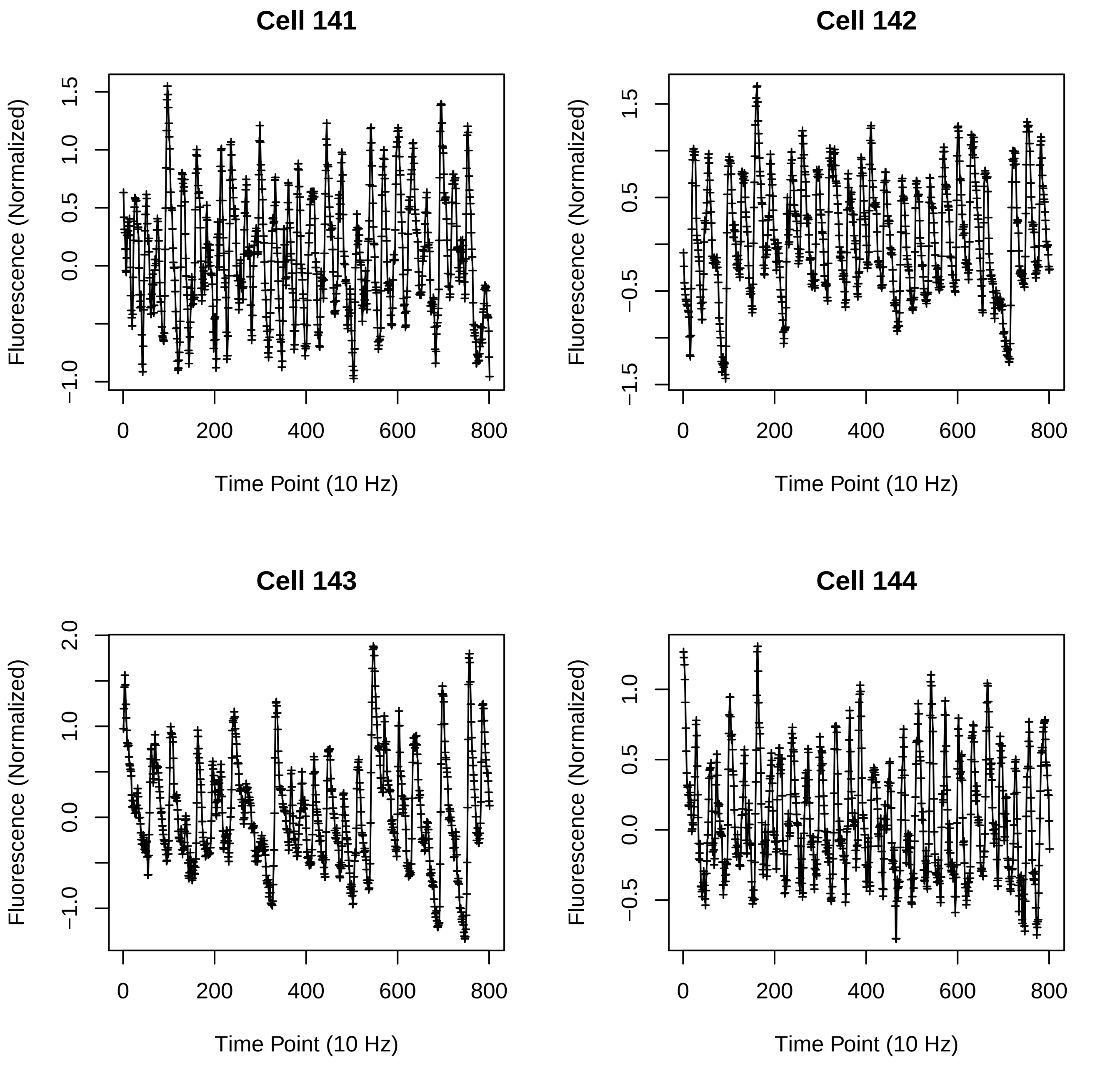
Cell 141
Cell 142
Cell 143
Cell 144
Fluorescence (Normalized)
Time Point (10 Hz)
0
200
400
600
800
−1.0
0.0
0.5
1.0
1.5
−1.5
−0.5
2.0

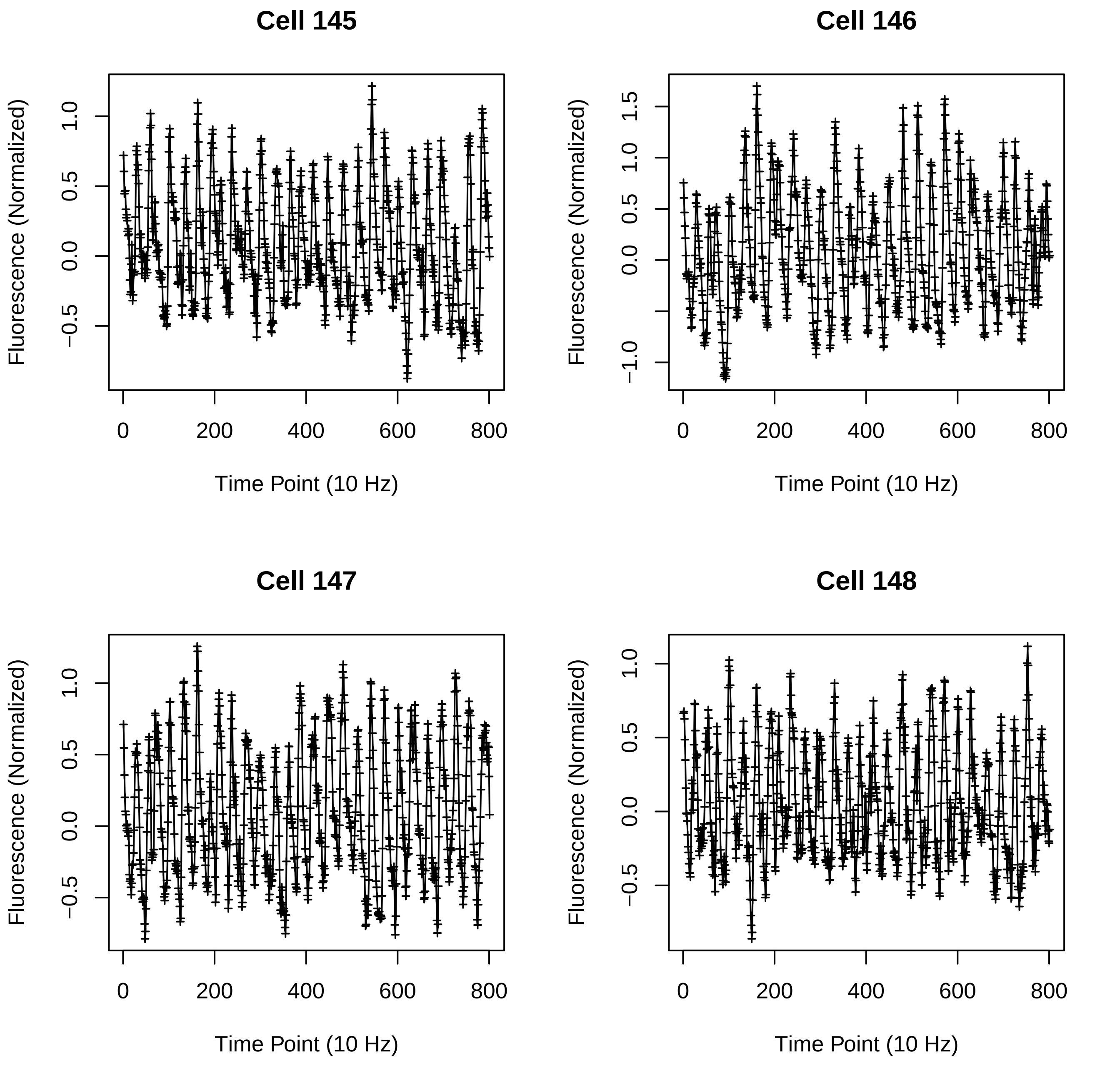
Cell 145
Cell 146
Cell 147
Cell 148
Fluorescence (Normalized)
Time Point (10 Hz)
0
200
400
600
800
−0.5
0.0
0.5
1.0
1.5
−1.0

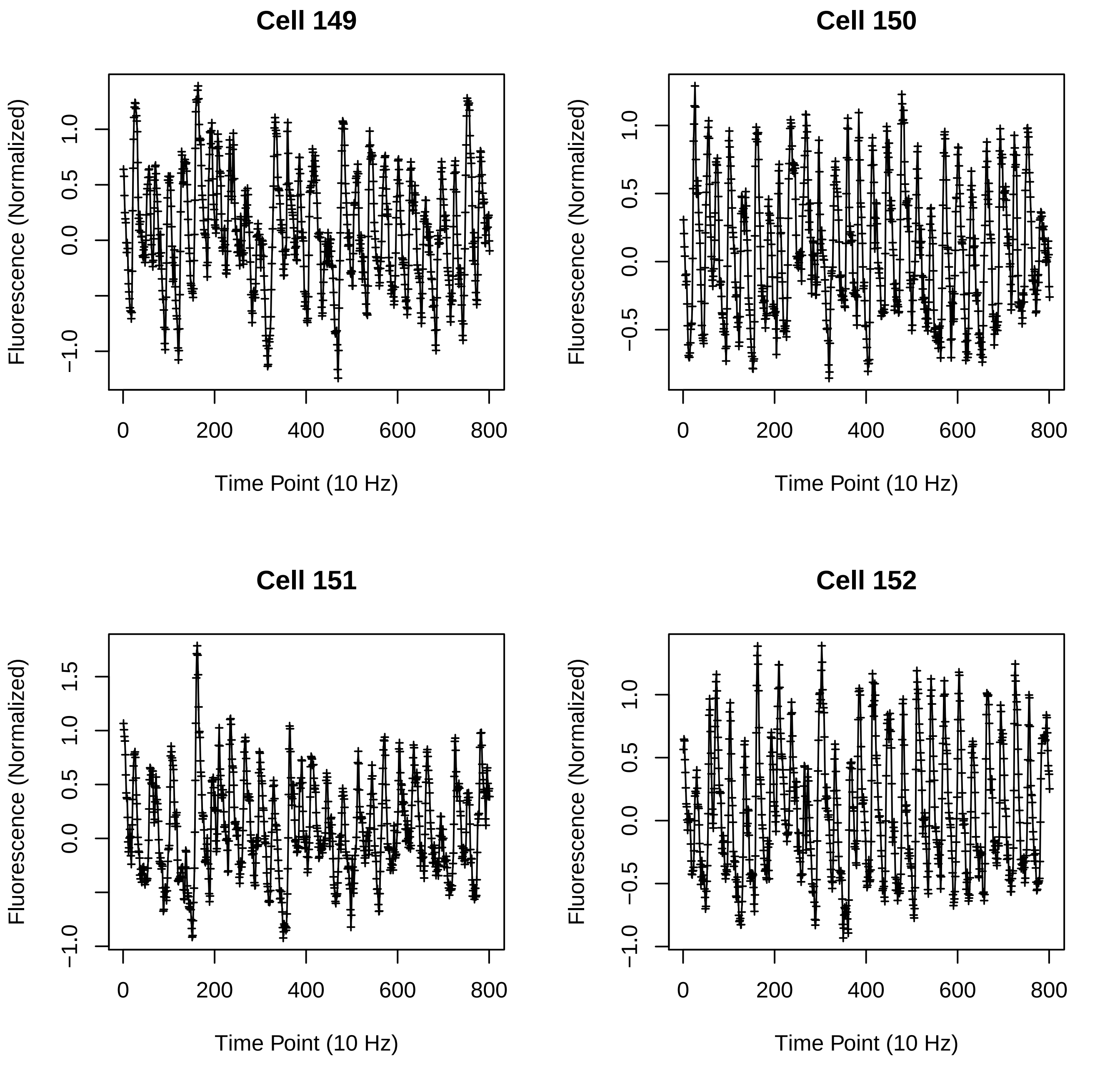
Cell 149
Cell 150
Cell 151
Cell 152
Fluorescence (Normalized)
Time Point (10 Hz)
0
200
400
600
800
−1.0
−0.5
0.0
0.5
1.0
1.5

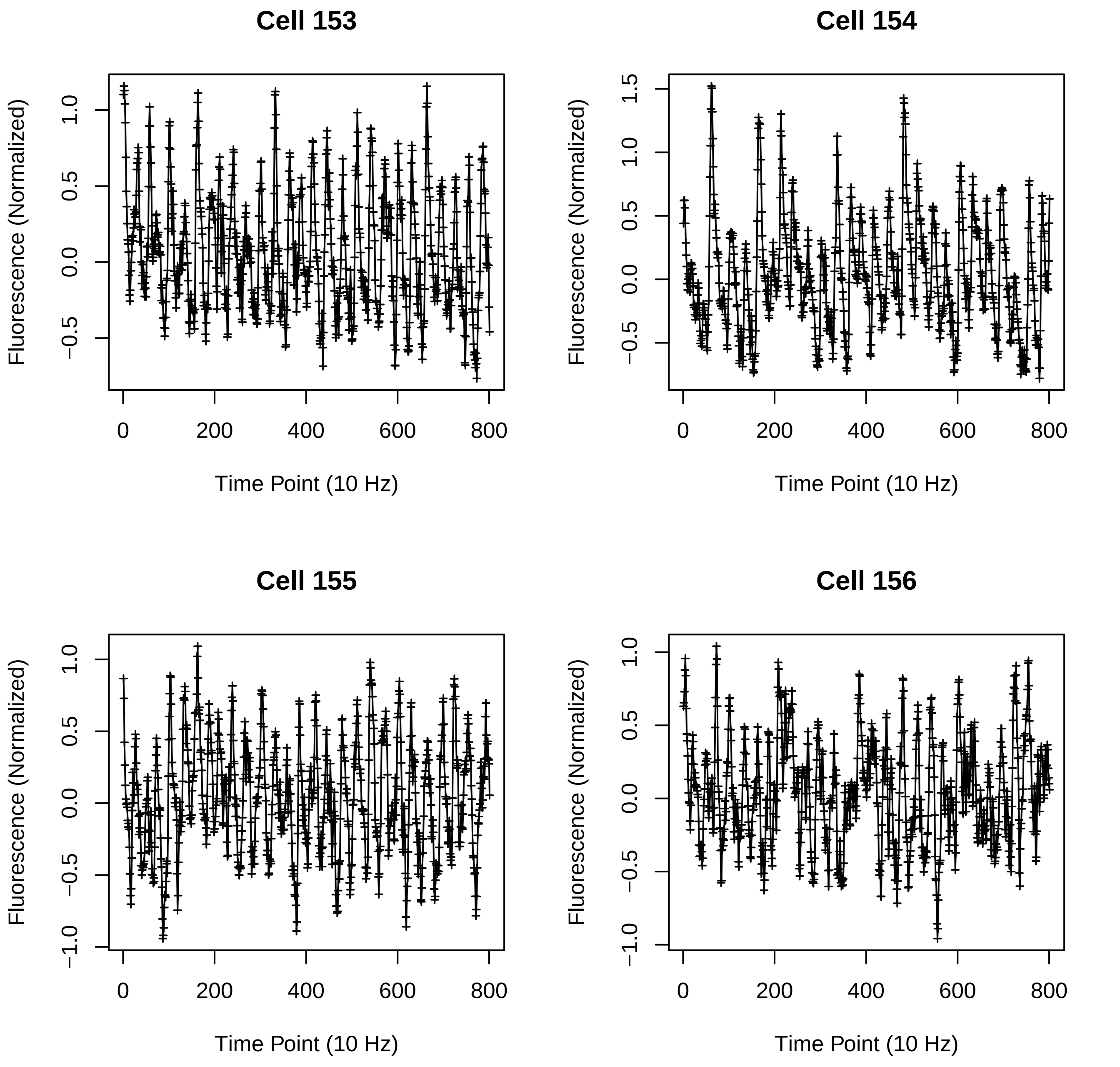
Cell 153
Cell 154
Cell 155
Cell 156
Fluorescence (Normalized)
Time Point (10 Hz)
0
200
400
600
800
−1.0
−0.5
0.0
0.5
1.0
1.5

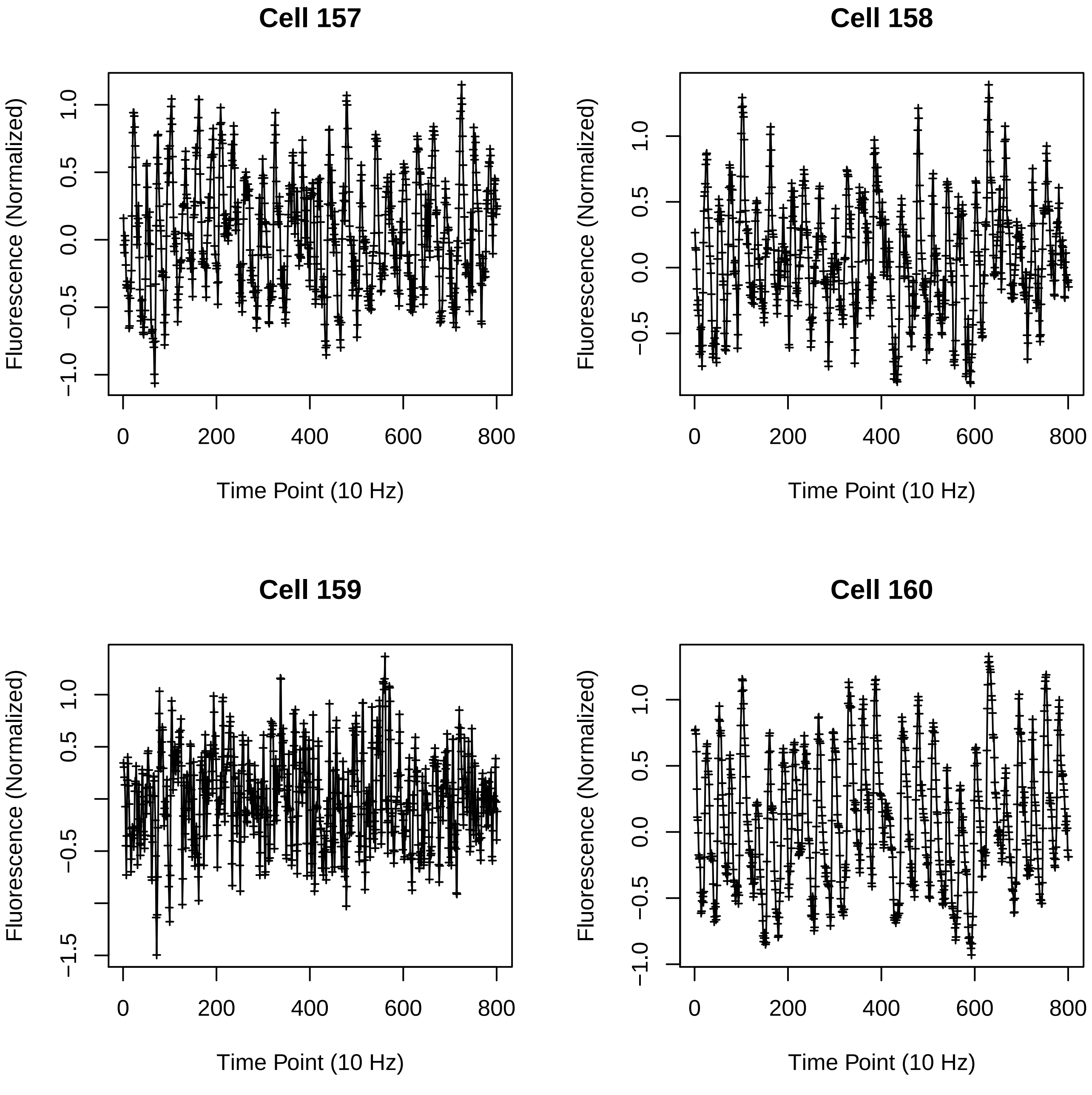
Cell 157
Cell 158
Cell 159
Cell 160
Fluorescence (Normalized)
Time Point (10 Hz)
0
200
400
600
800
−1.5
−1.0
−0.5
0.0
0.5
1.0

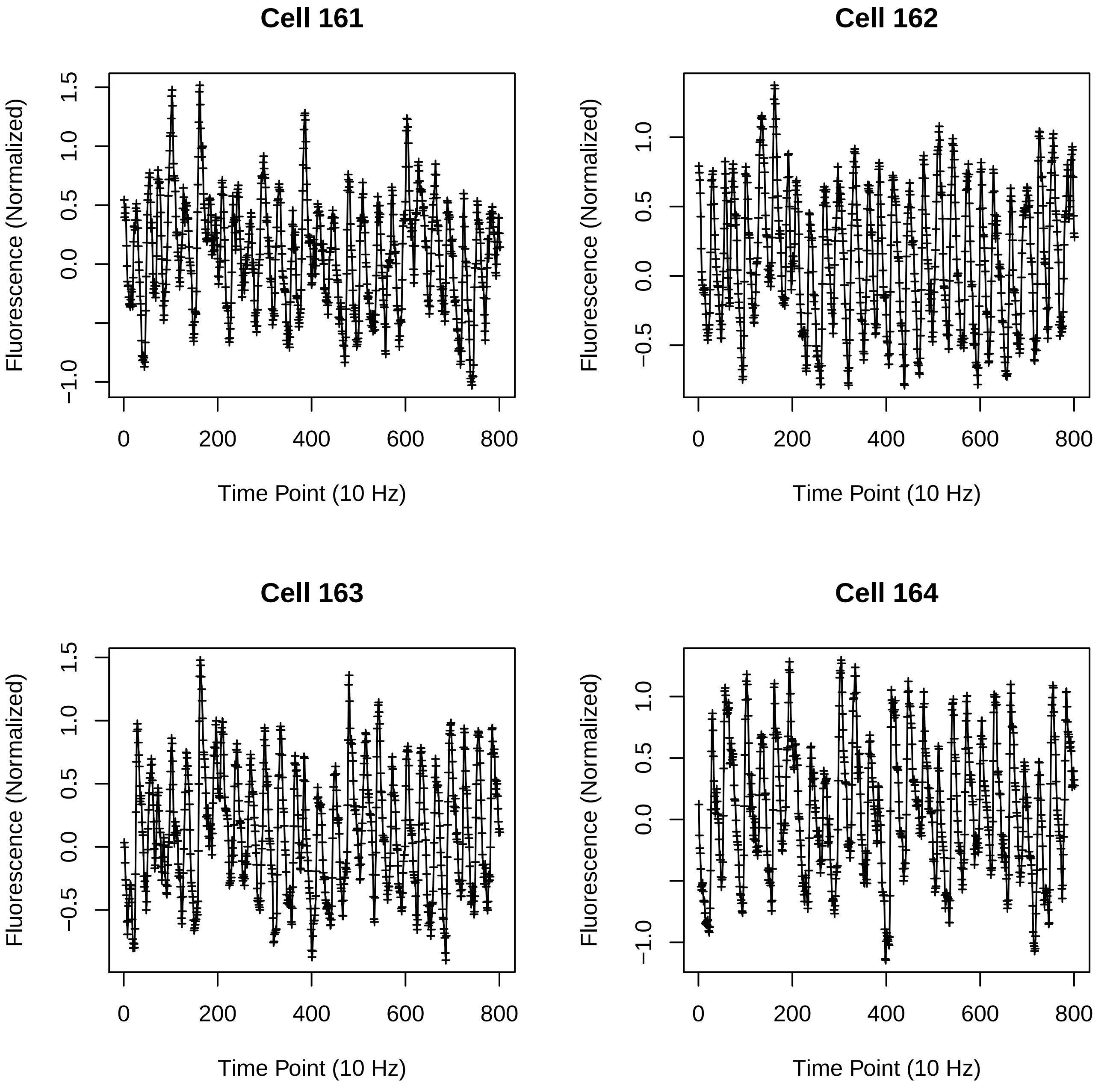
Cell 161
Cell 162
Cell 163
Cell 164
Fluorescence (Normalized)
Time Point (10 Hz)
0
200
400
600
800
−1.0
−0.5
0.0
0.5
1.0
1.5

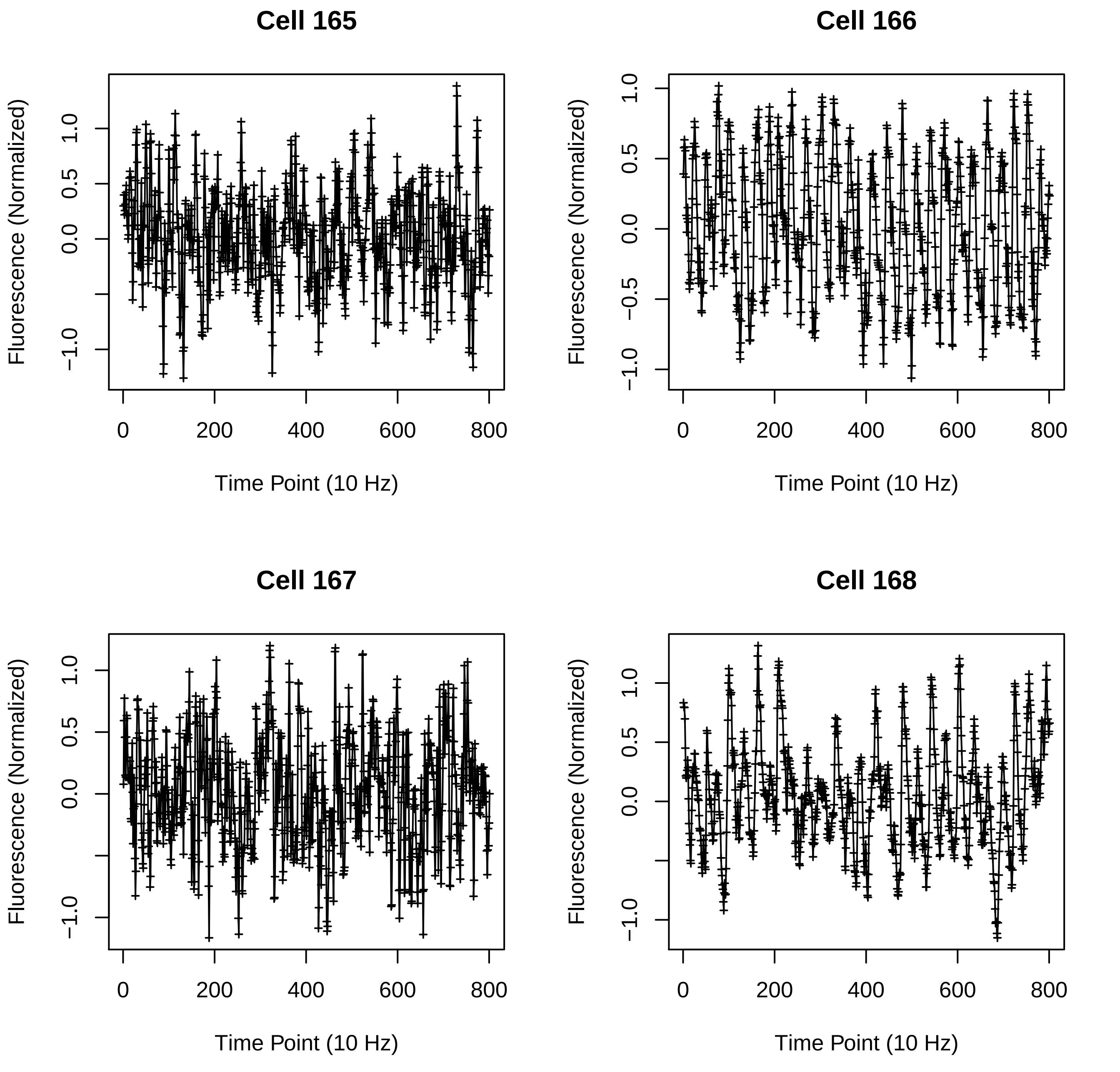
Cell 165
Cell 166
Cell 167
Cell 168
Fluorescence (Normalized)
Time Point (10 Hz)
−1.0
−0.5
0.0
0.5
1.0
0
200
400
600
800

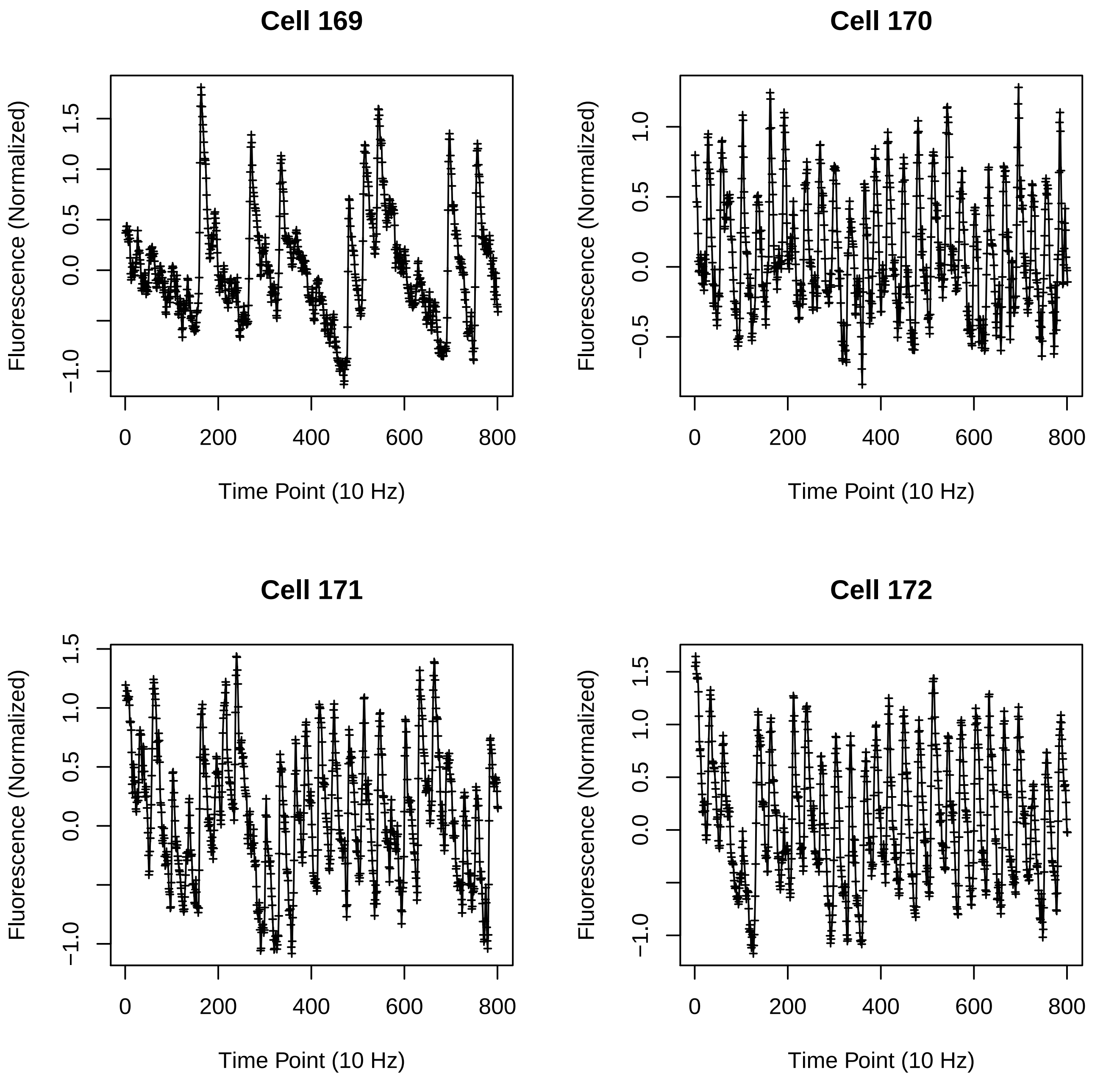

Cell 169
Cell 170
Cell 171
Cell 172
Fluorescence (Normalized)
Time Point (10 Hz)

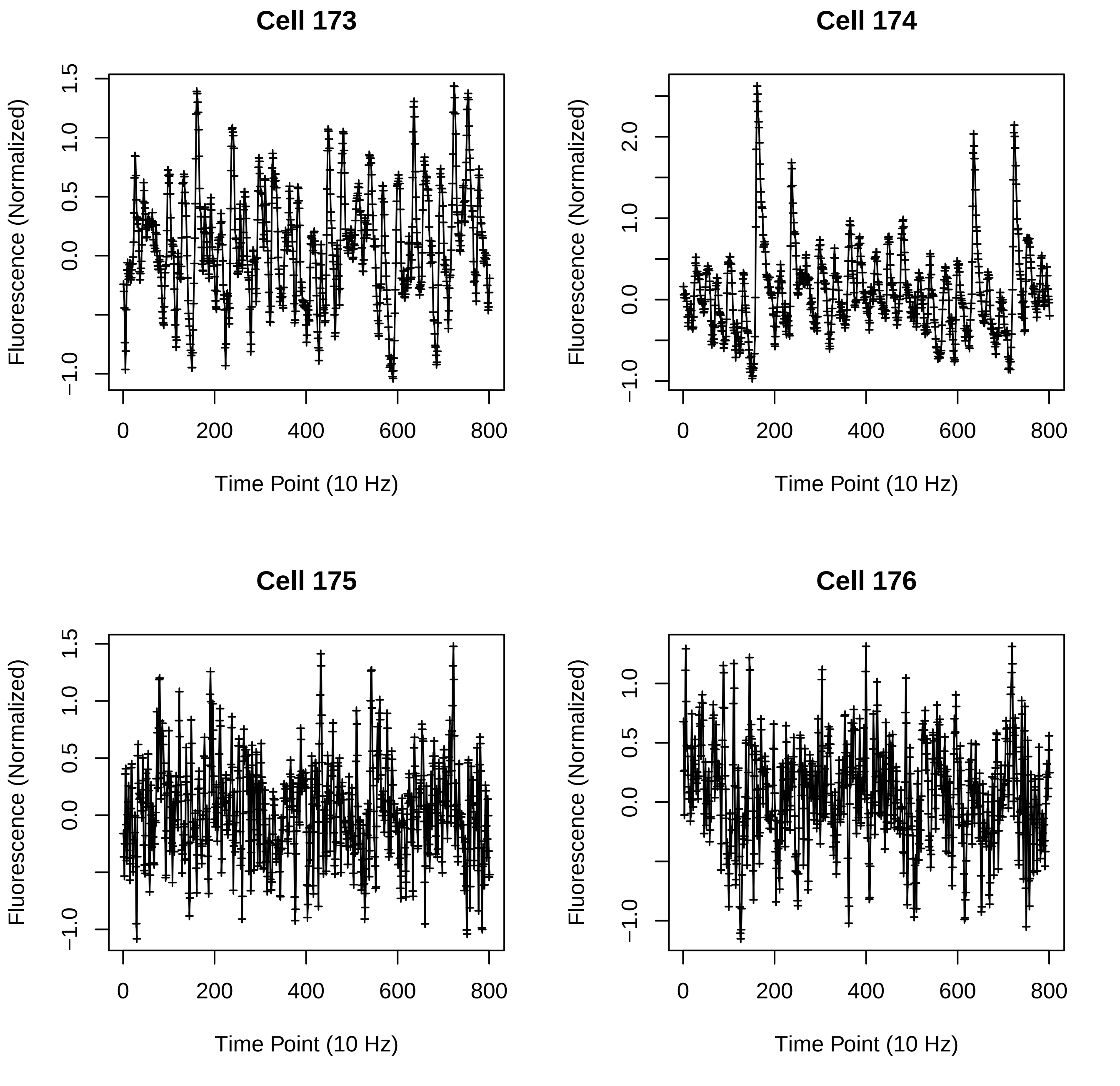

Cell 173
Cell 174
Cell 175
Cell 176
Fluorescence (Normalized)
Time Point (10 Hz)
0
200
400
600
800
−1.0
0.0
0.5
1.0
1.5
2.0

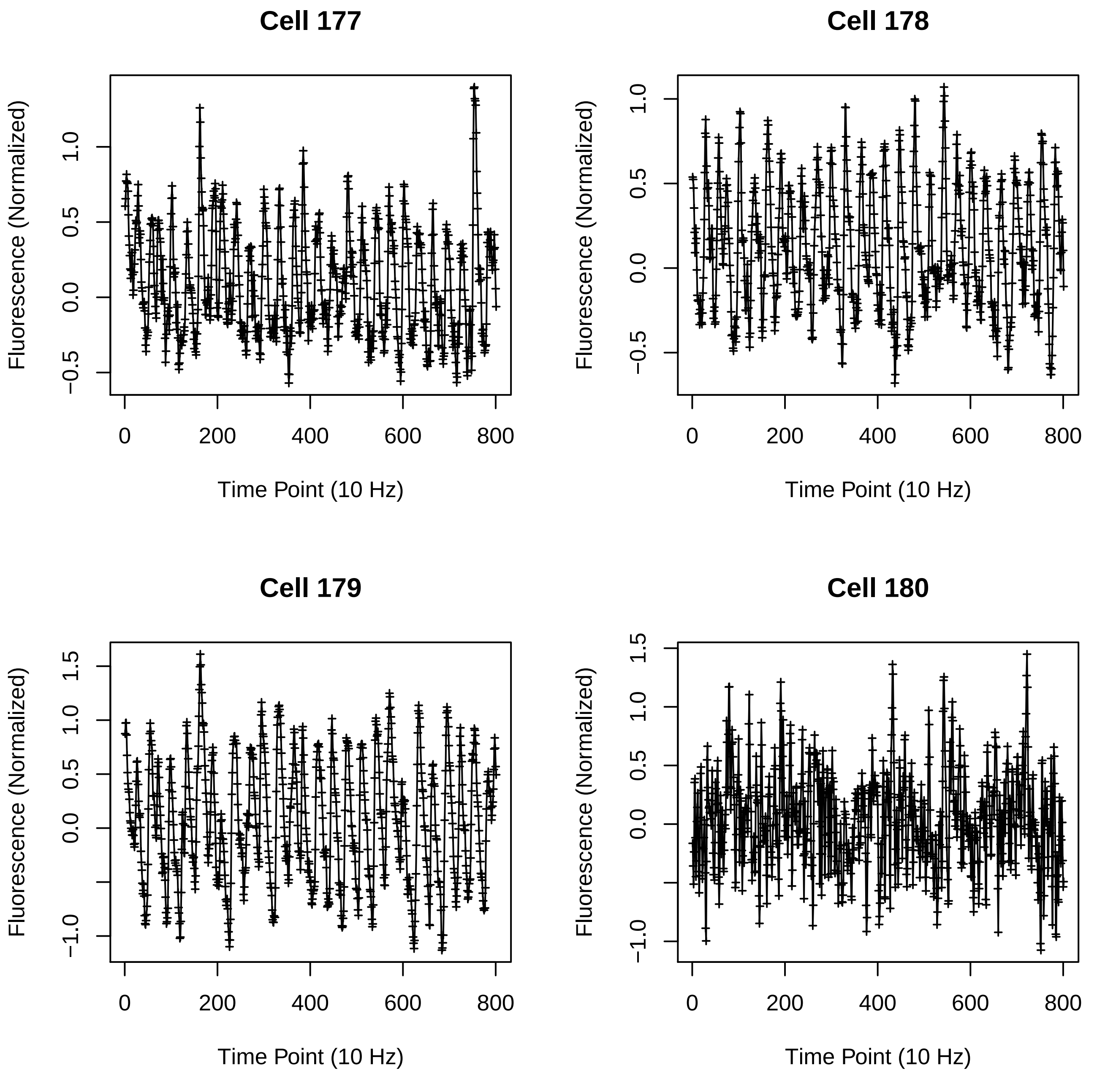
Cell 177
Cell 178
Cell 179
Cell 180
Fluorescence (Normalized)
Time Point (10 Hz)
0
200
400
600
800
−0.5
0.0
0.5
1.0
1.5
−1.0

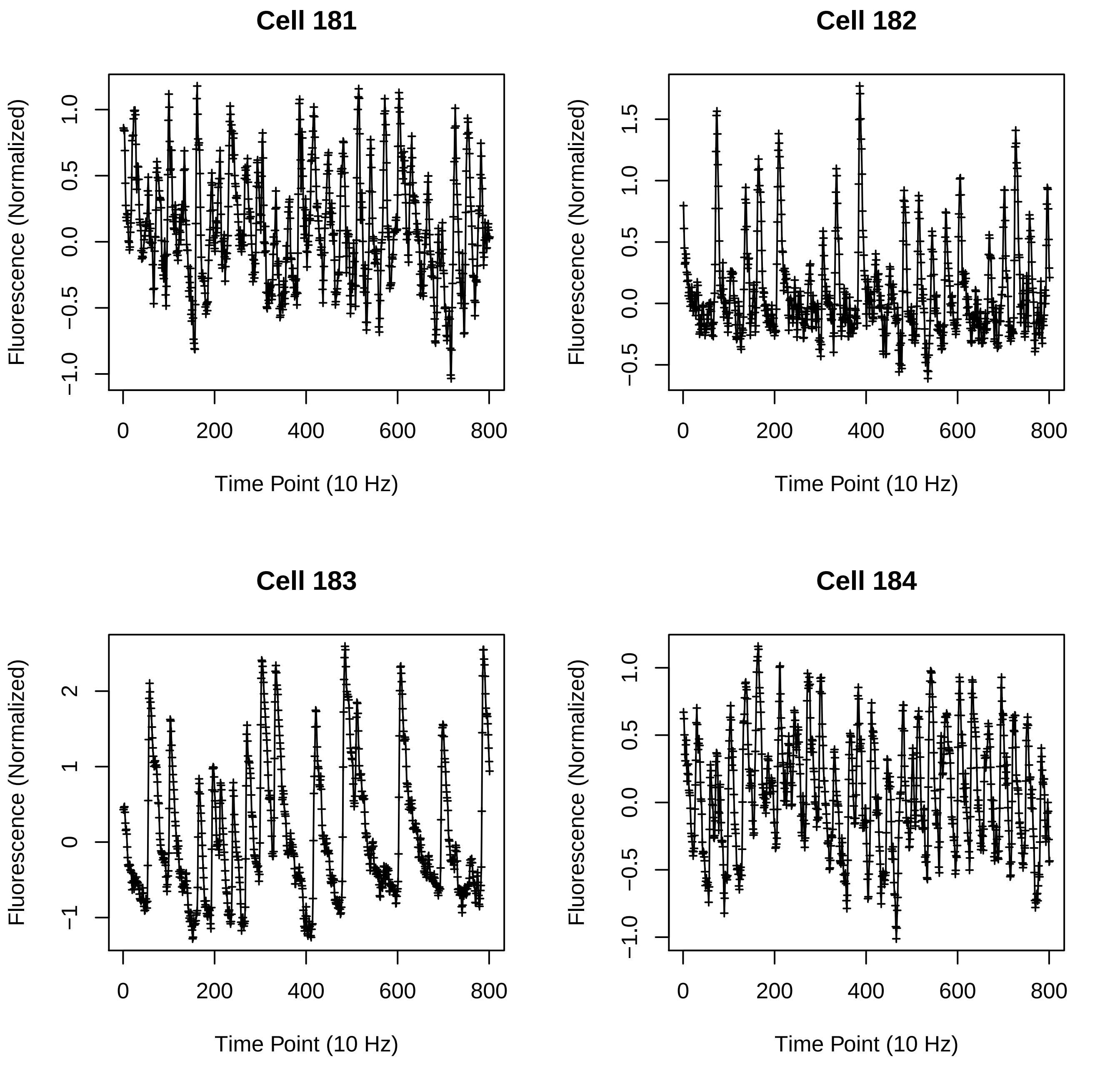

Cell 181
Cell 182
Cell 183
Cell 184
Fluorescence (Normalized)
Time Point (10 Hz)
0
200
400
600
800
−1.0
−0.5
0.0
0.5
1.0
1.5
−1
0
1
2

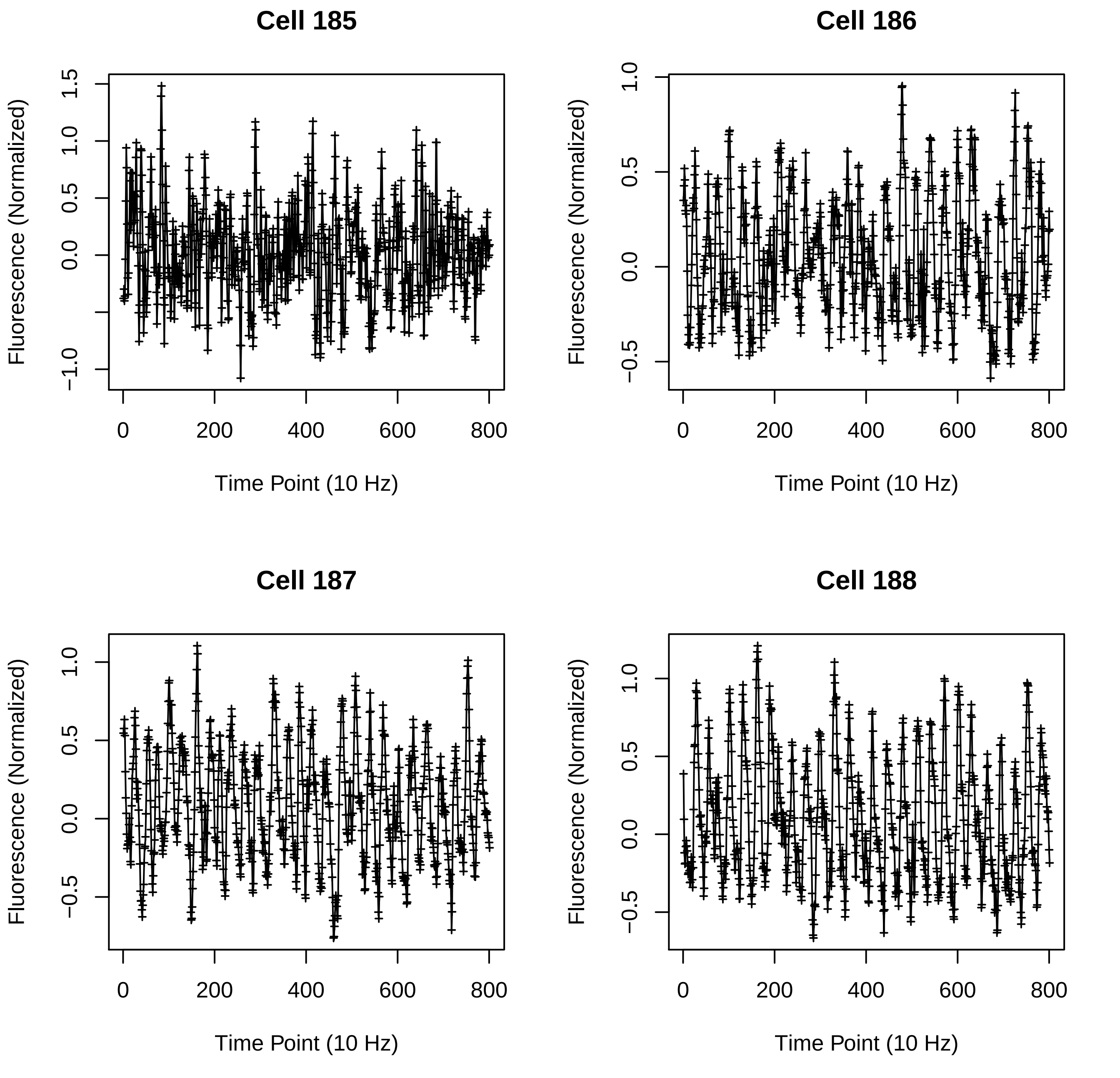
Cell 185
Cell 186
Cell 187
Cell 188
Fluorescence (Normalized)
Time Point (10 Hz)
1.5
1.0
0.5
0.0
−1.0
−0.5
0
200
400
600
800

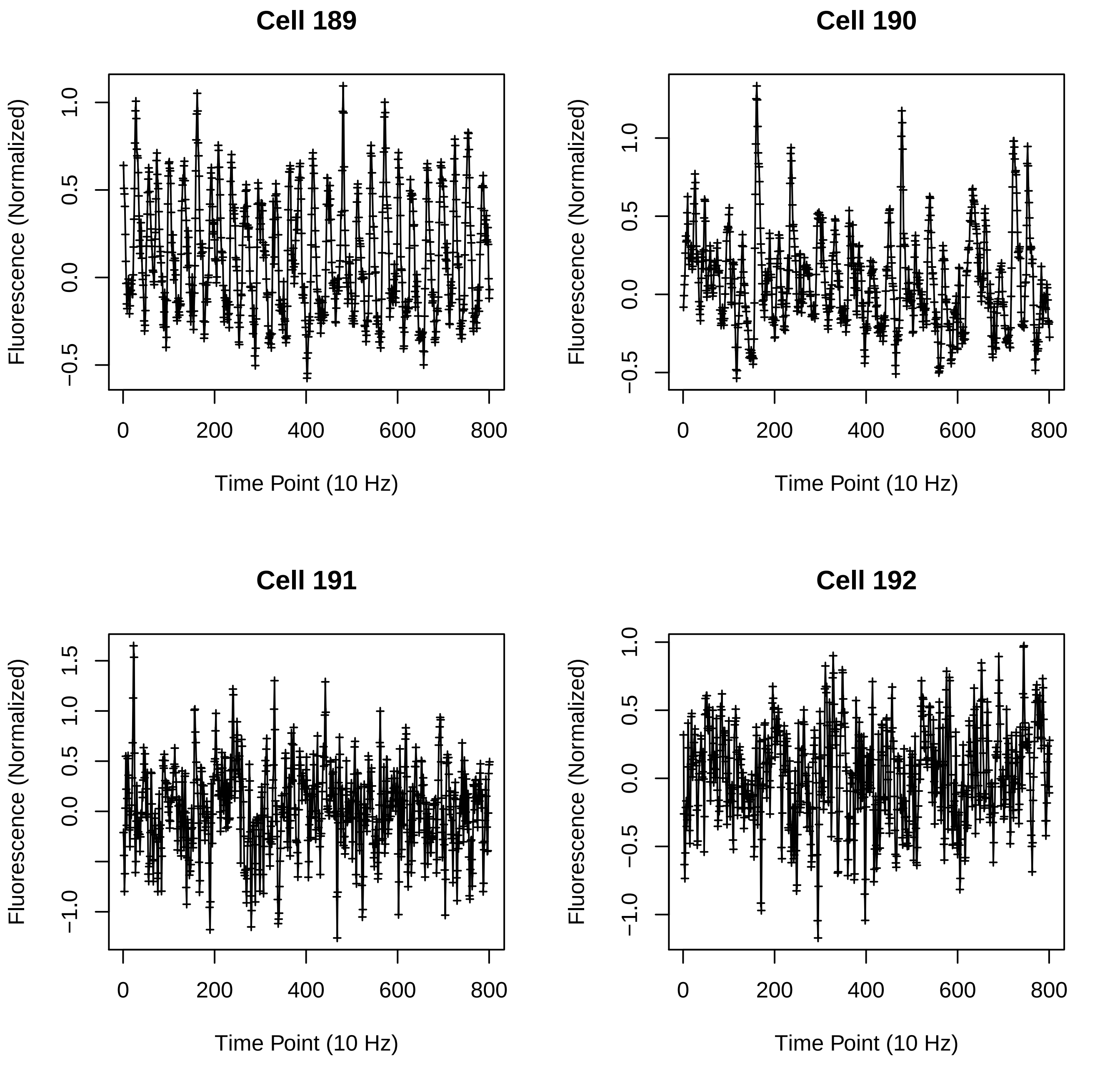
Cell 189
Cell 190
Cell 191
Cell 192
Fluorescence (Normalized)
Time Point (10 Hz)
0
200
400
600
800
−0.5
0.0
0.5
1.0
1.5
−1.0

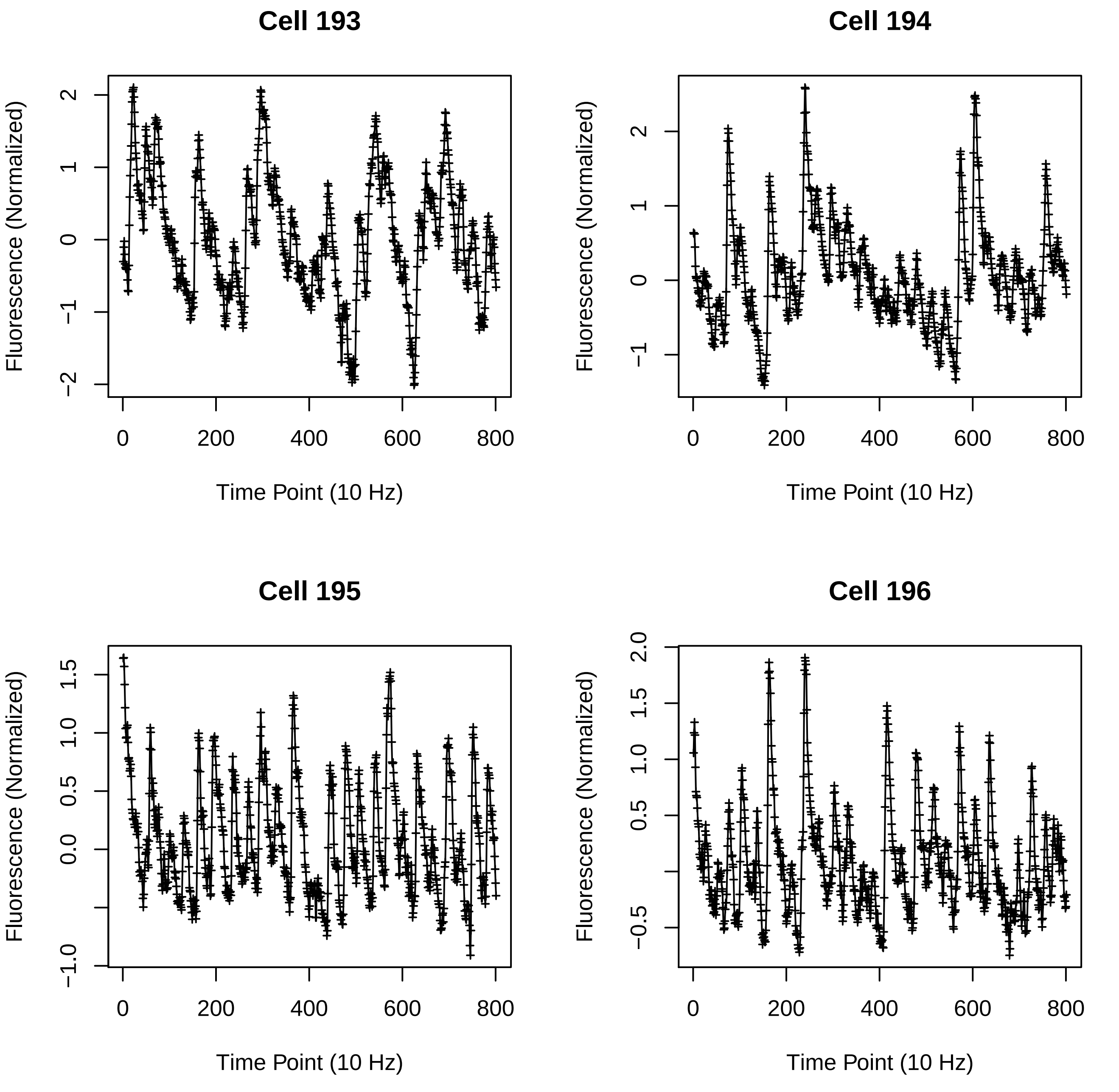
Cell 193
Cell 194
Cell 195
Cell 196
Fluorescence (Normalized)
Time Point (10 Hz)
0
200
400
600
800
−2
−1
0
1
2
−1.0
0.0
0.5
1.0
1.5
−0.5
2.0

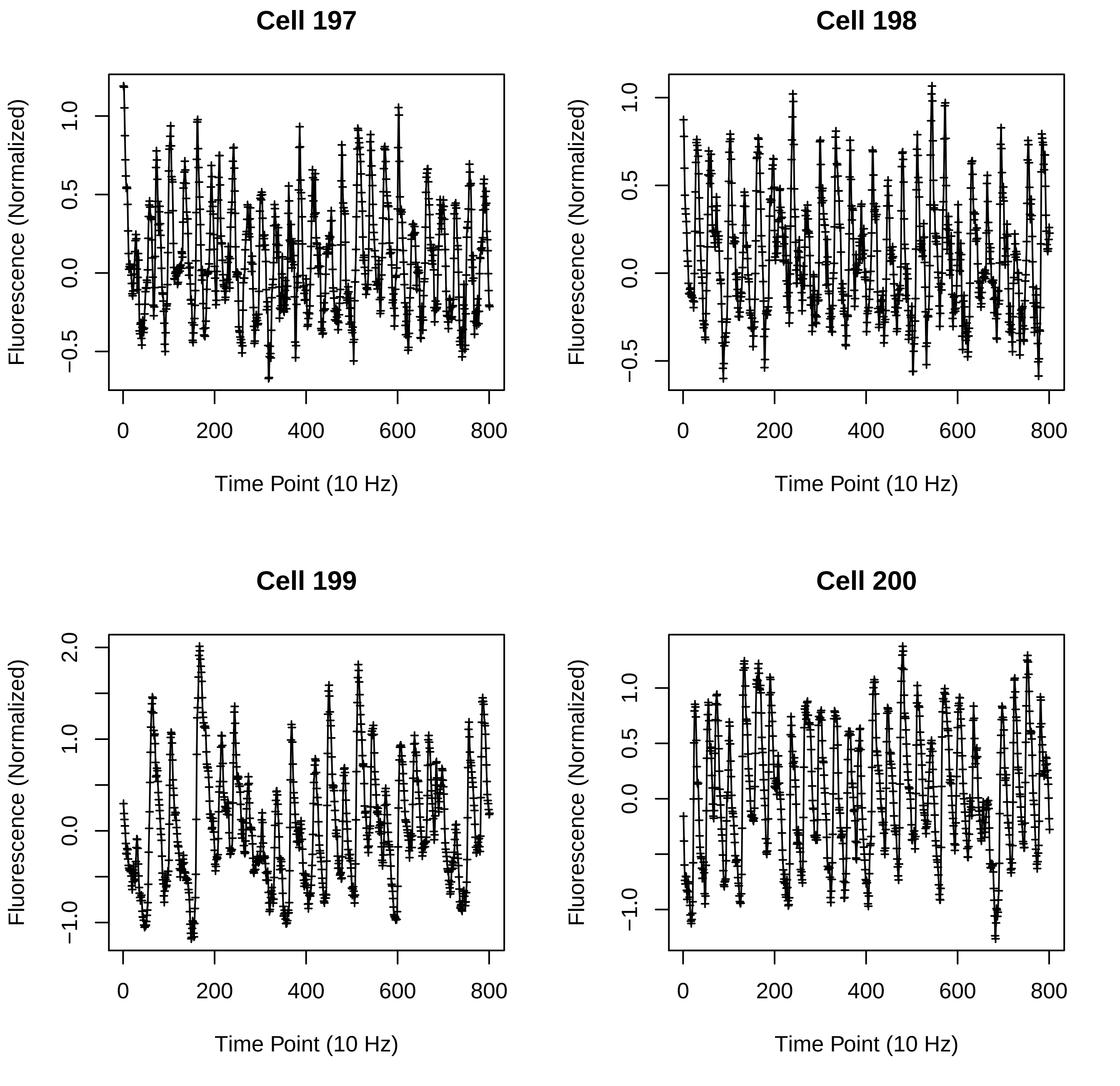
Cell 197
Cell 198
Cell 199
Cell 200
Fluorescence (Normalized)
Time Point (10 Hz)
0
200
400
600
800
−0.5
0.0
0.5
1.0
−1.0
2.0

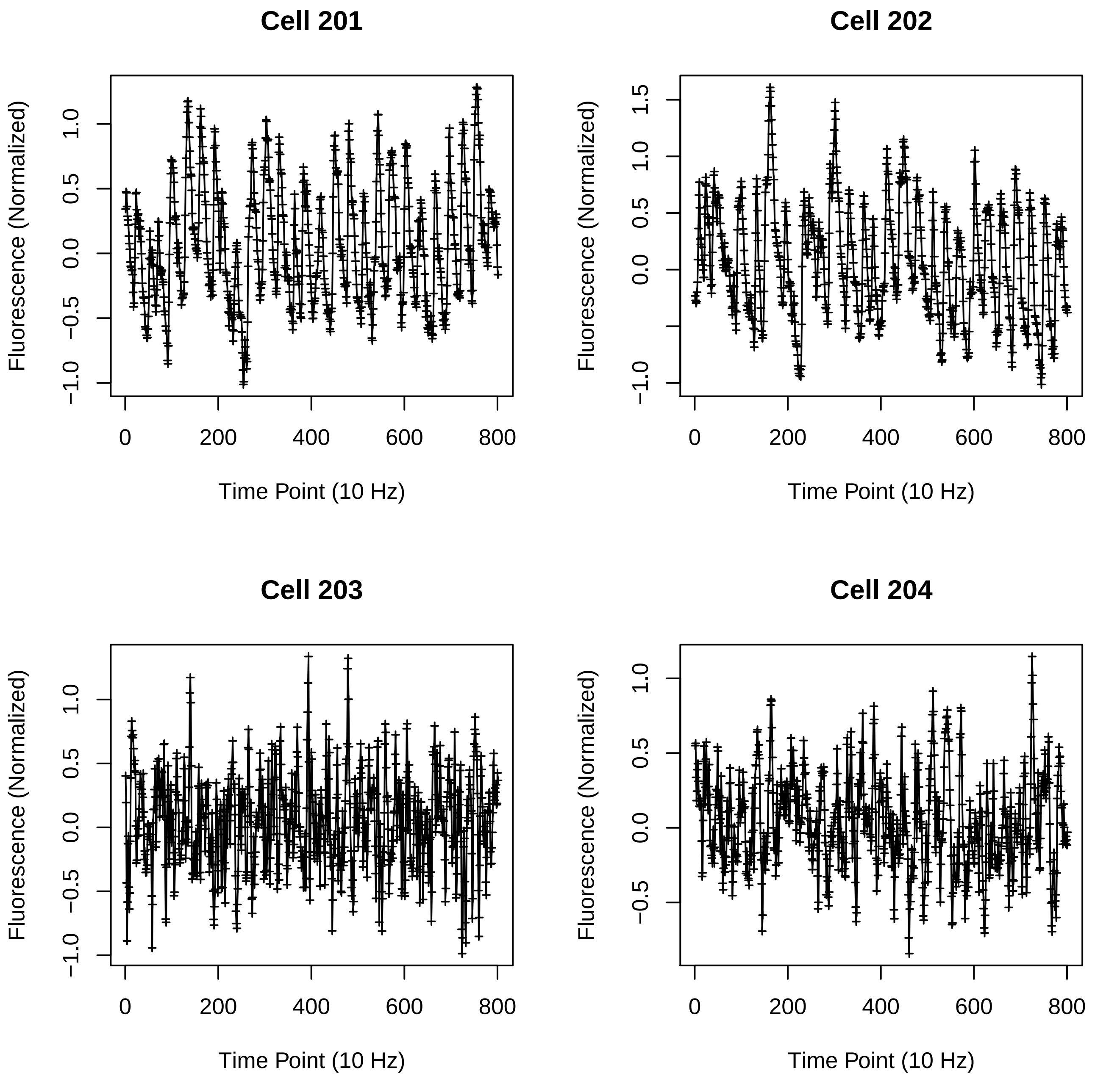
Cell 201
Cell 202
Cell 203
Cell 204
Fluorescence (Normalized)
Time Point (10 Hz)
0
200
400
600
800
−1.0
−0.5
0.0
0.5
1.0
1.5

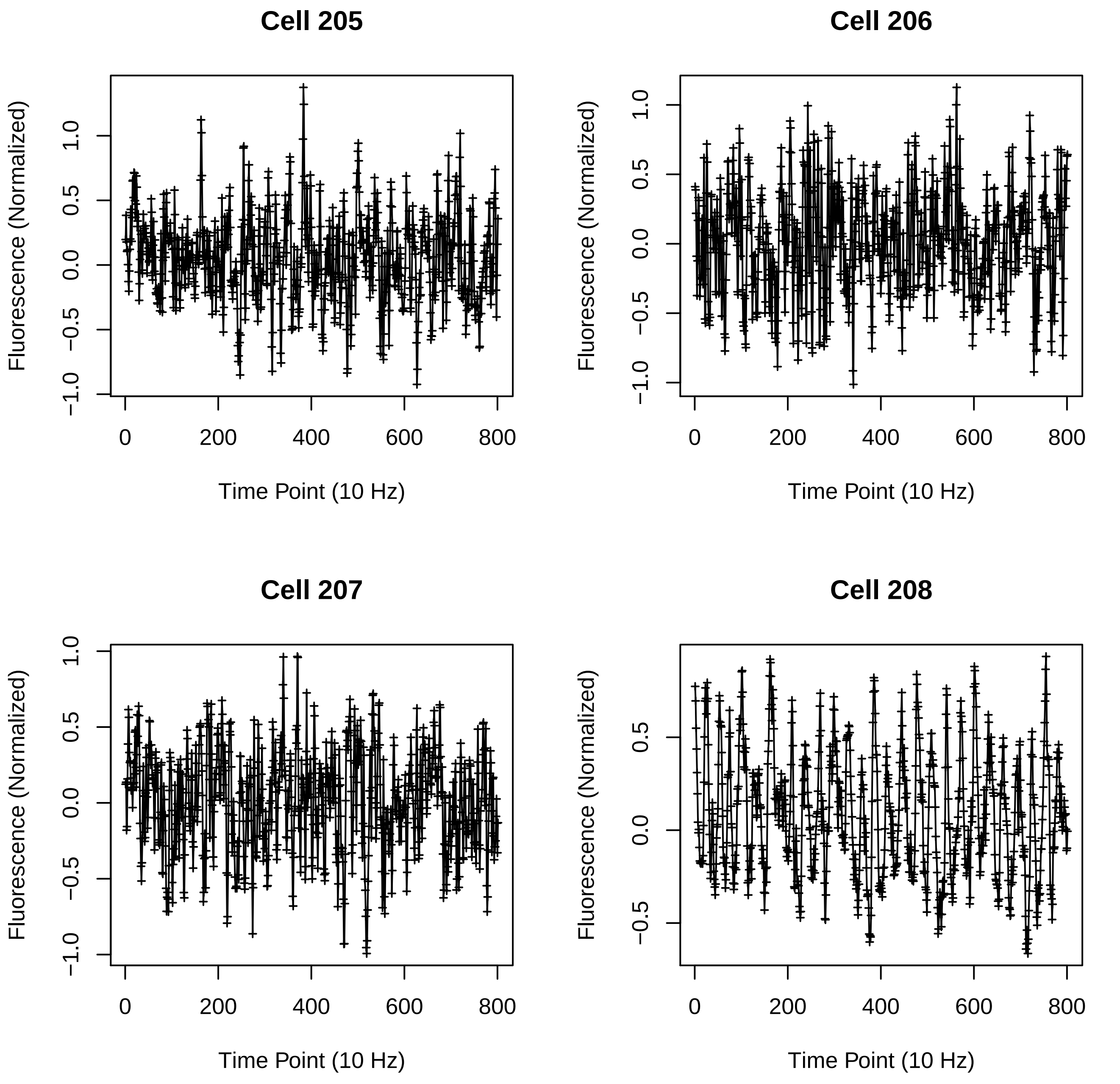
Cell 205
Cell 206
Cell 207
Cell 208
Fluorescence (Normalized)
Time Point (10 Hz)
−1.0
−0.5
0.0
0.5
1.0
0
200
400
600
800

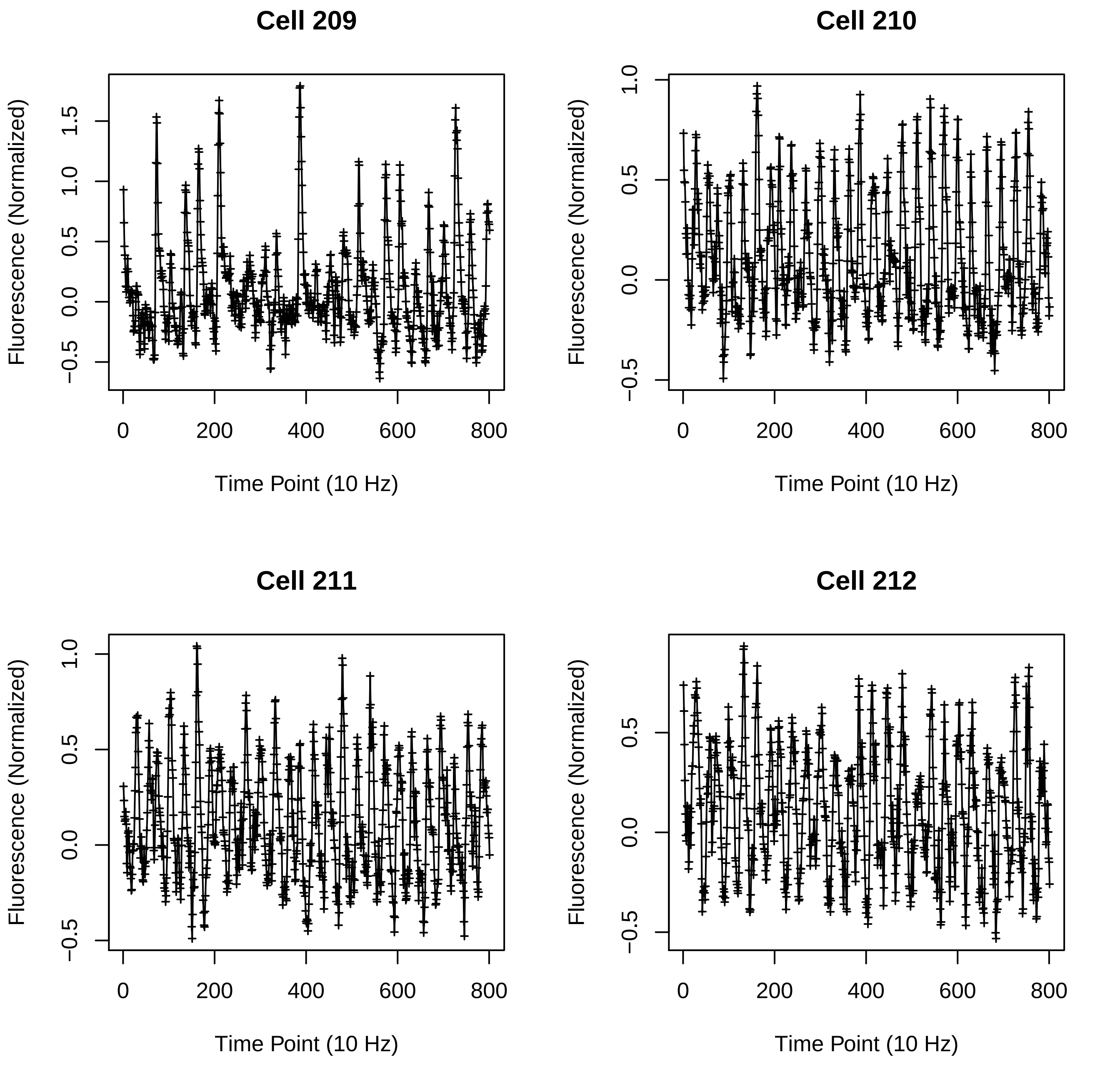

Cell 209
Cell 210
Cell 211
Cell 212
Fluorescence (Normalized)
Time Point (10 Hz)
0
200
400
600
800
−0.5
0.0
0.5
1.0
1.5

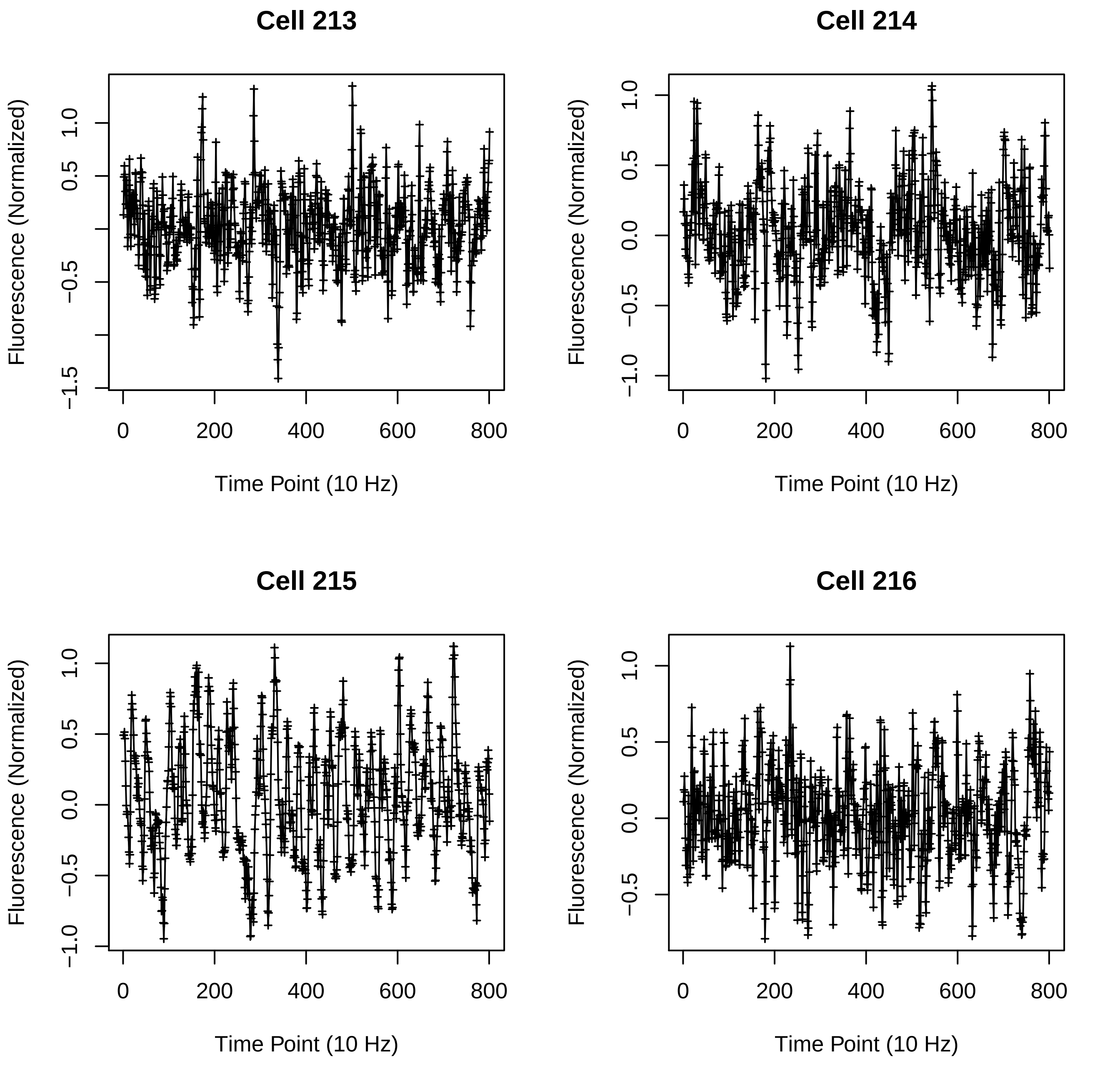
Cell 213
Cell 214
Cell 215
Cell 216
Fluorescence (Normalized)
Time Point (10 Hz)
0
200
400
600
800
1.0
0.5
0.0
−0.5
−1.0
−1.5

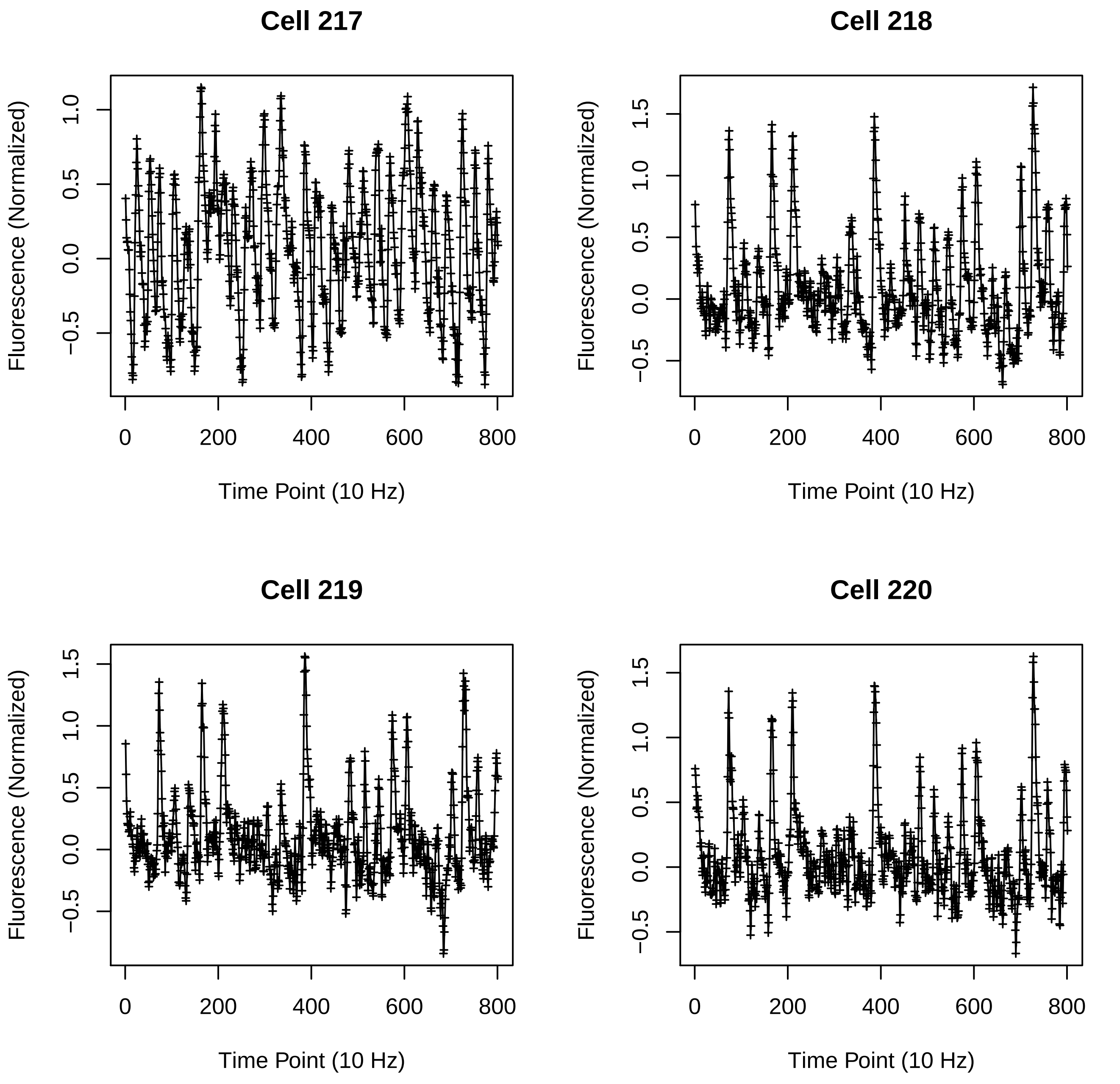

Cell 217
Cell 218
Cell 219
Cell 220
Fluorescence (Normalized)
Time Point (10 Hz)
0
200
400
600
800
−0.5
0.0
0.5
1.0
1.5

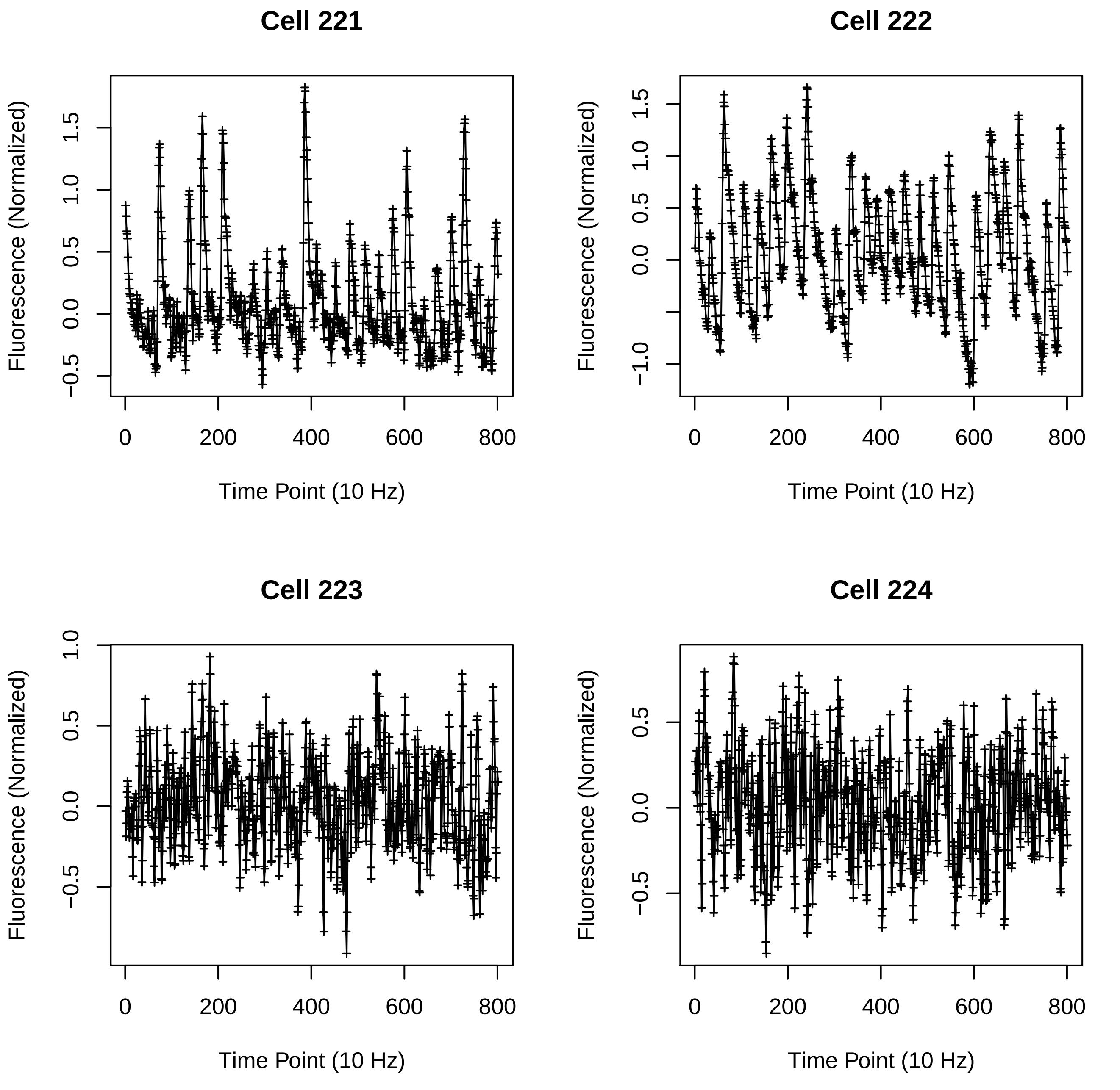
Cell 221
Fluorescence (Normalized)
−0.5 0.0 0.5 1.0 1.5
0 200 400 600 800
Time Point (10 Hz)
Cell 222
Fluorescence (Normalized)
−1.0 −0.5 0.0 0.5 1.0 1.5
0 200 400 600 800
Time Point (10 Hz)
Cell 223
Fluorescence (Normalized)
−0.5 0.0 0.5 1.0
0 200 400 600 800
Time Point (10 Hz)
Cell 224
Fluorescence (Normalized)
−0.5 0.0 0.5
0 200 400 600 800
Time Point (10 Hz)

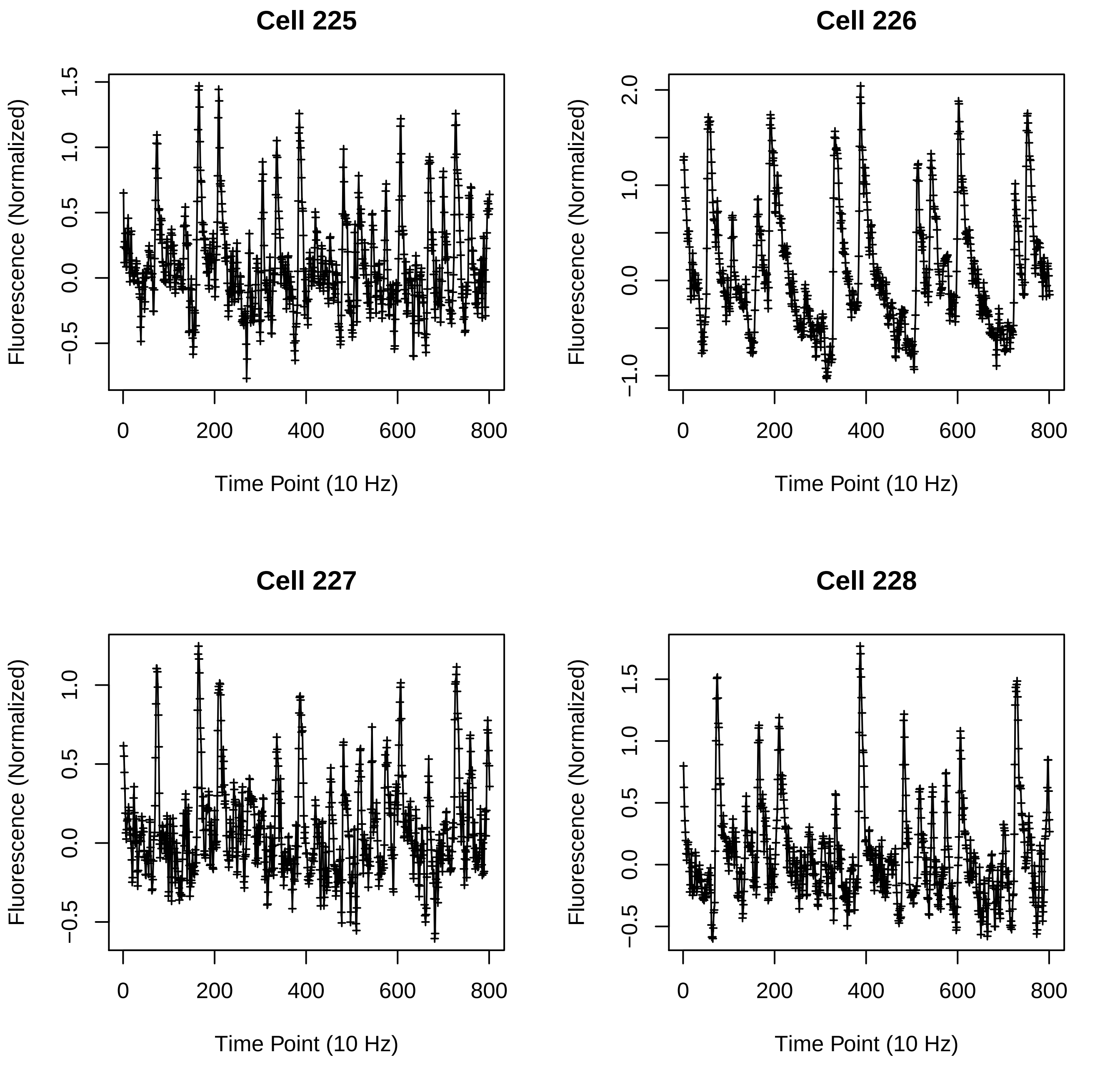
Cell 225
Cell 226
Cell 227
Cell 228
Fluorescence (Normalized)
Time Point (10 Hz)
0
200
400
600
800
−0.5
0.0
0.5
1.0
1.5
2.0

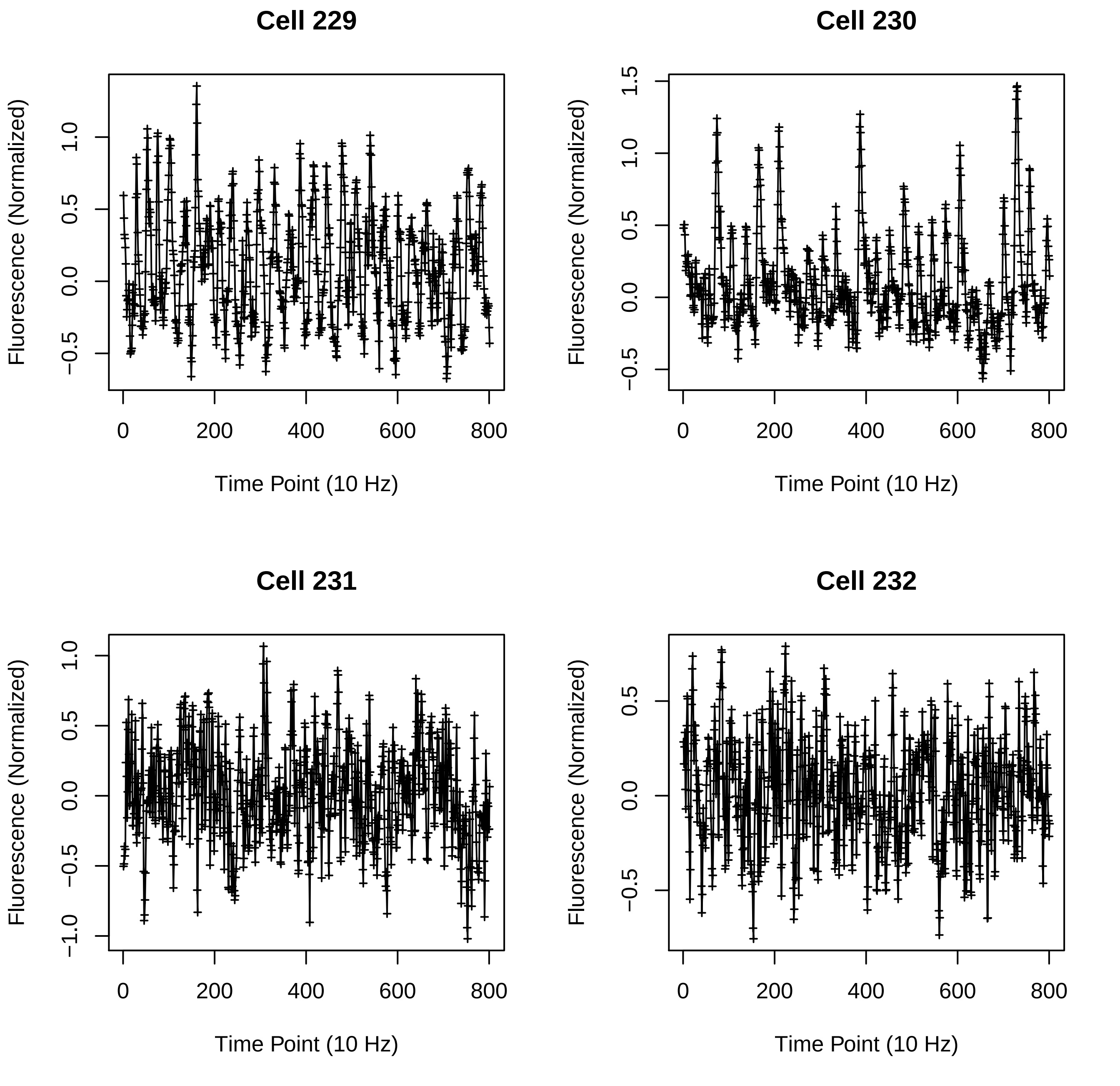
Cell 229
Cell 230
Cell 231
Cell 232
Fluorescence (Normalized)
Time Point (10 Hz)
0
200
400
600
800
-0.5
0.0
0.5
1.0
1.5
-1.0

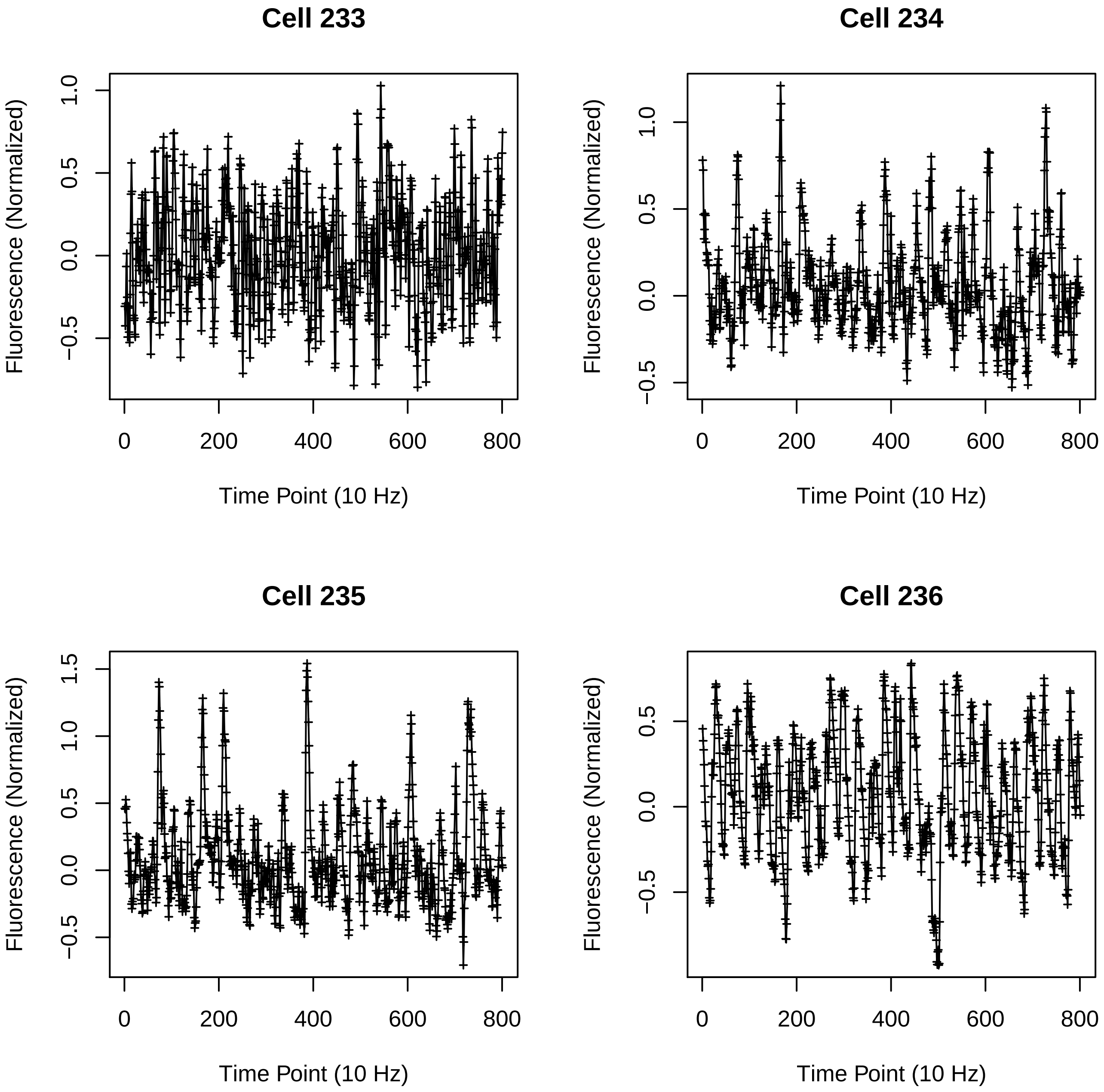
Cell 233
Cell 234
Cell 235
Cell 236
Fluorescence (Normalized)
Time Point (10 Hz)
0
200
400
600
800
−0.5
0.0
0.5
1.0
1.5

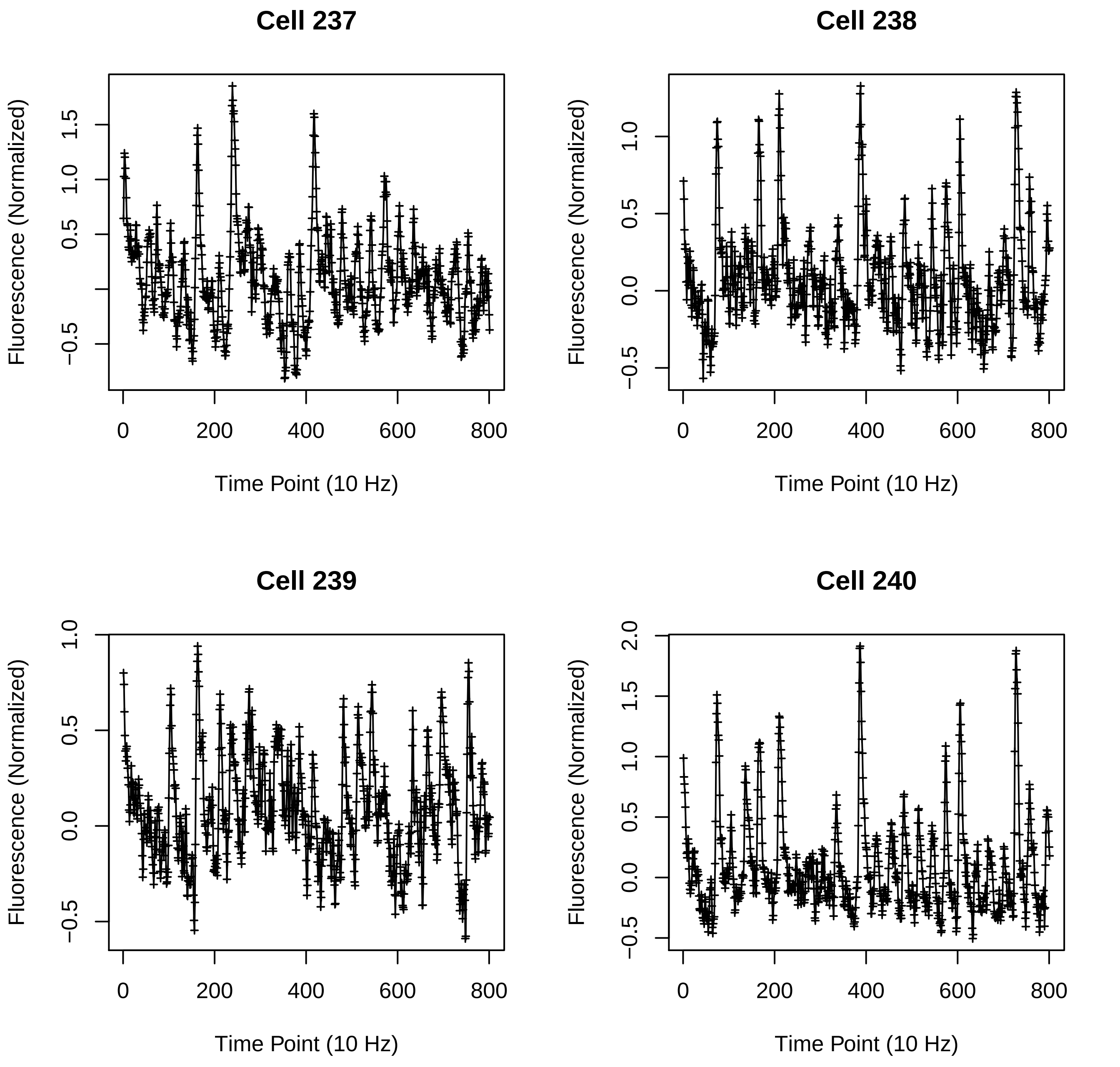
Cell 237
Cell 238
Cell 239
Cell 240
Fluorescence (Normalized)
Time Point (10 Hz)
0
200
400
600
800
−0.5
0.0
0.5
1.0
1.5
2.0

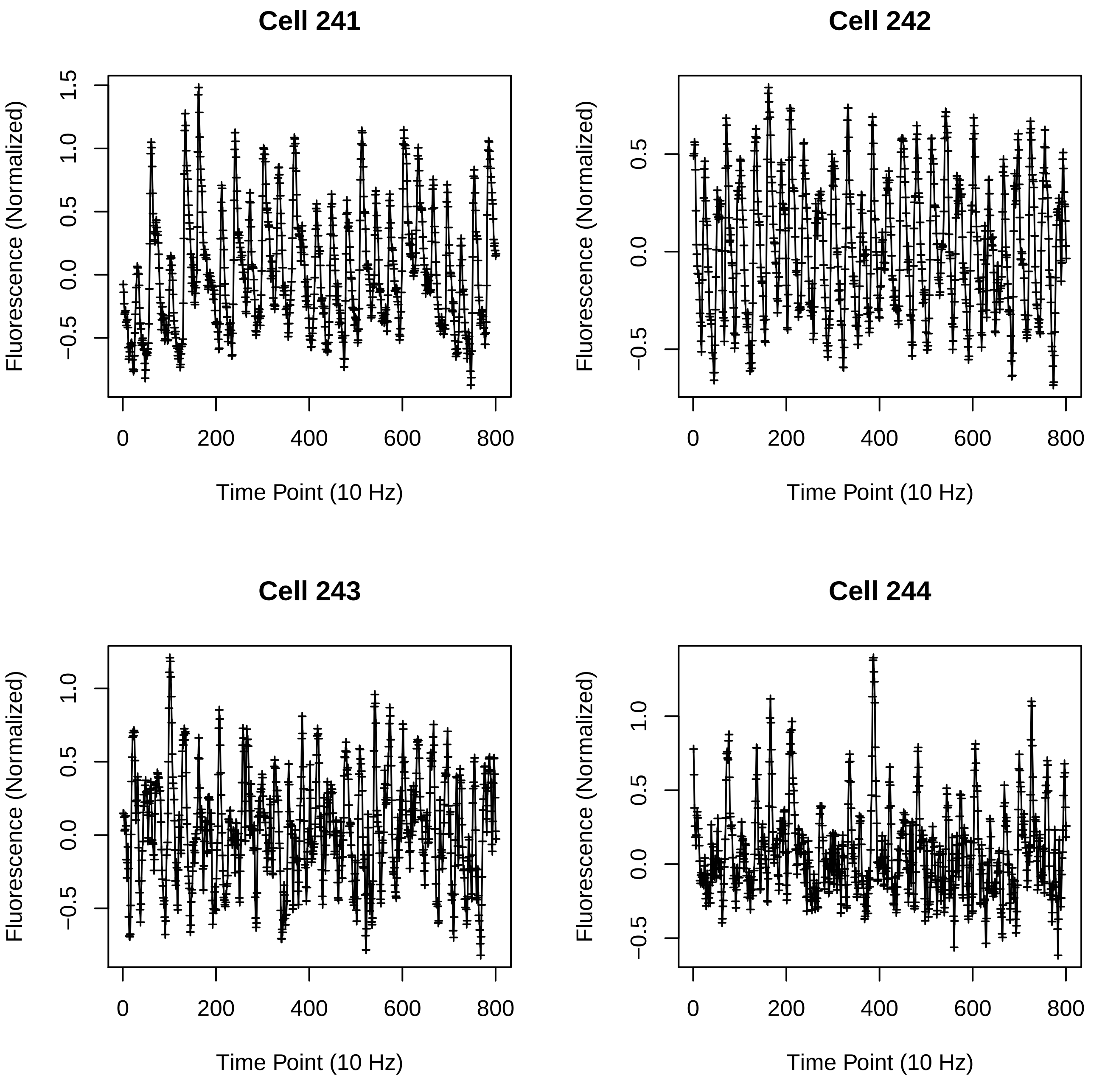
Cell 241
Cell 242
Cell 243
Cell 244
Fluorescence (Normalized)
Time Point (10 Hz)
0
200
400
600
800
−0.5
0.0
0.5
1.0
1.5

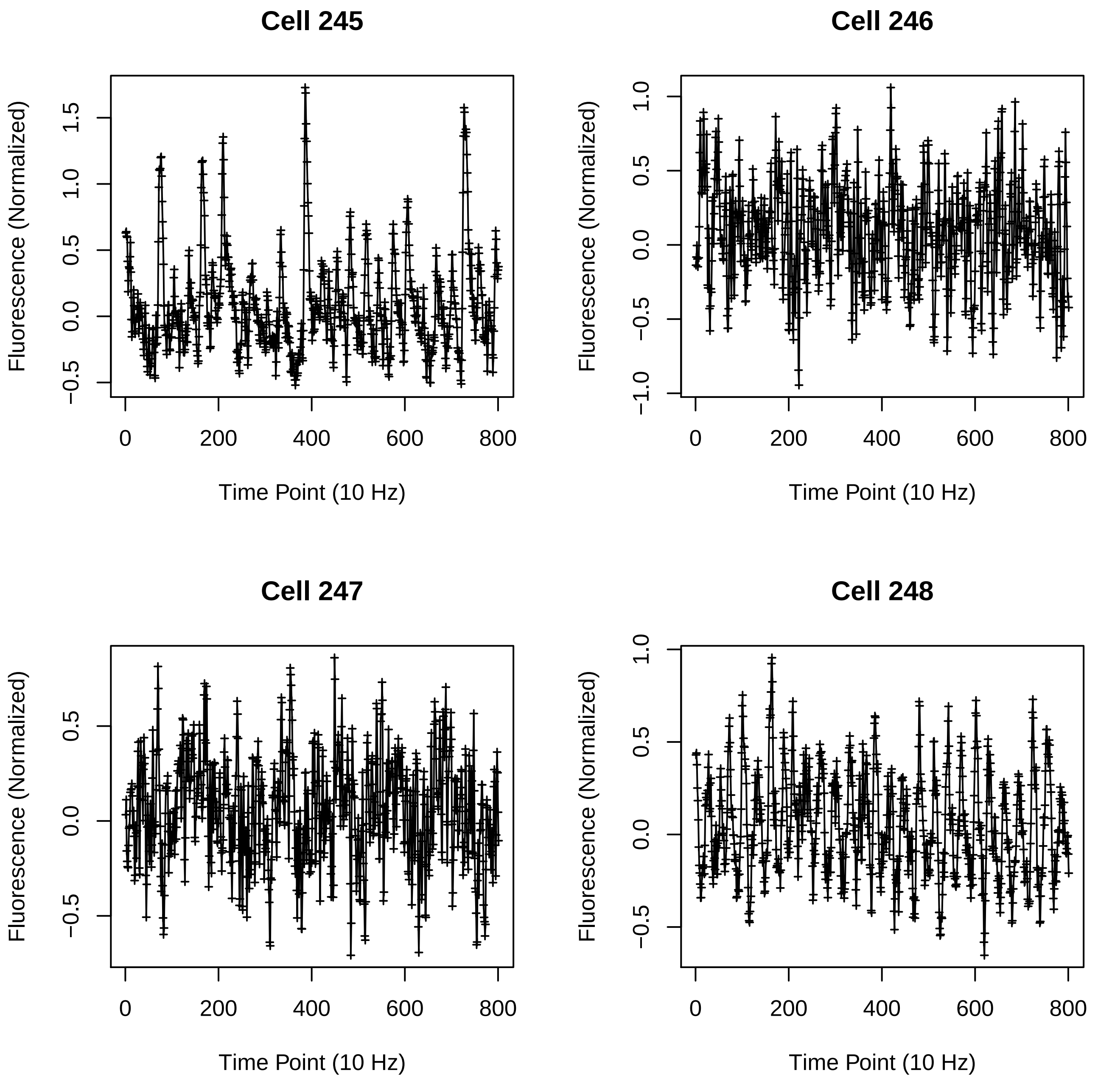

Cell 245
Cell 246
Cell 247
Cell 248
Fluorescence (Normalized)
Time Point (10 Hz)
0
200
400
600
800
−1.0
−0.5
0.0
0.5
1.0
1.5

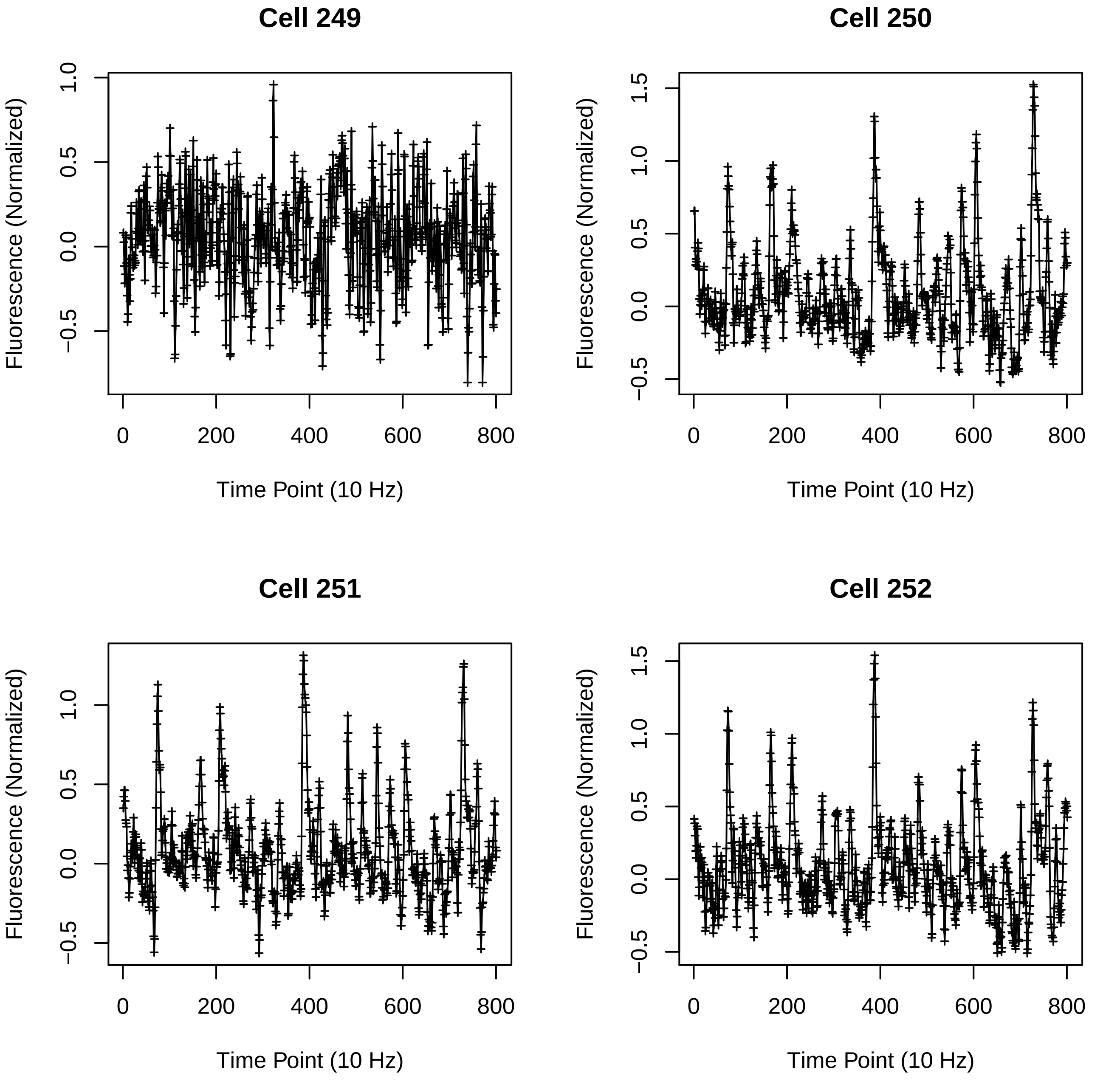
Cell 249
Cell 250
Cell 251
Cell 252
Fluorescence (Normalized)
Time Point (10 Hz)
0
200
400
600
800
−0.5
0.0
0.5
1.0
1.5

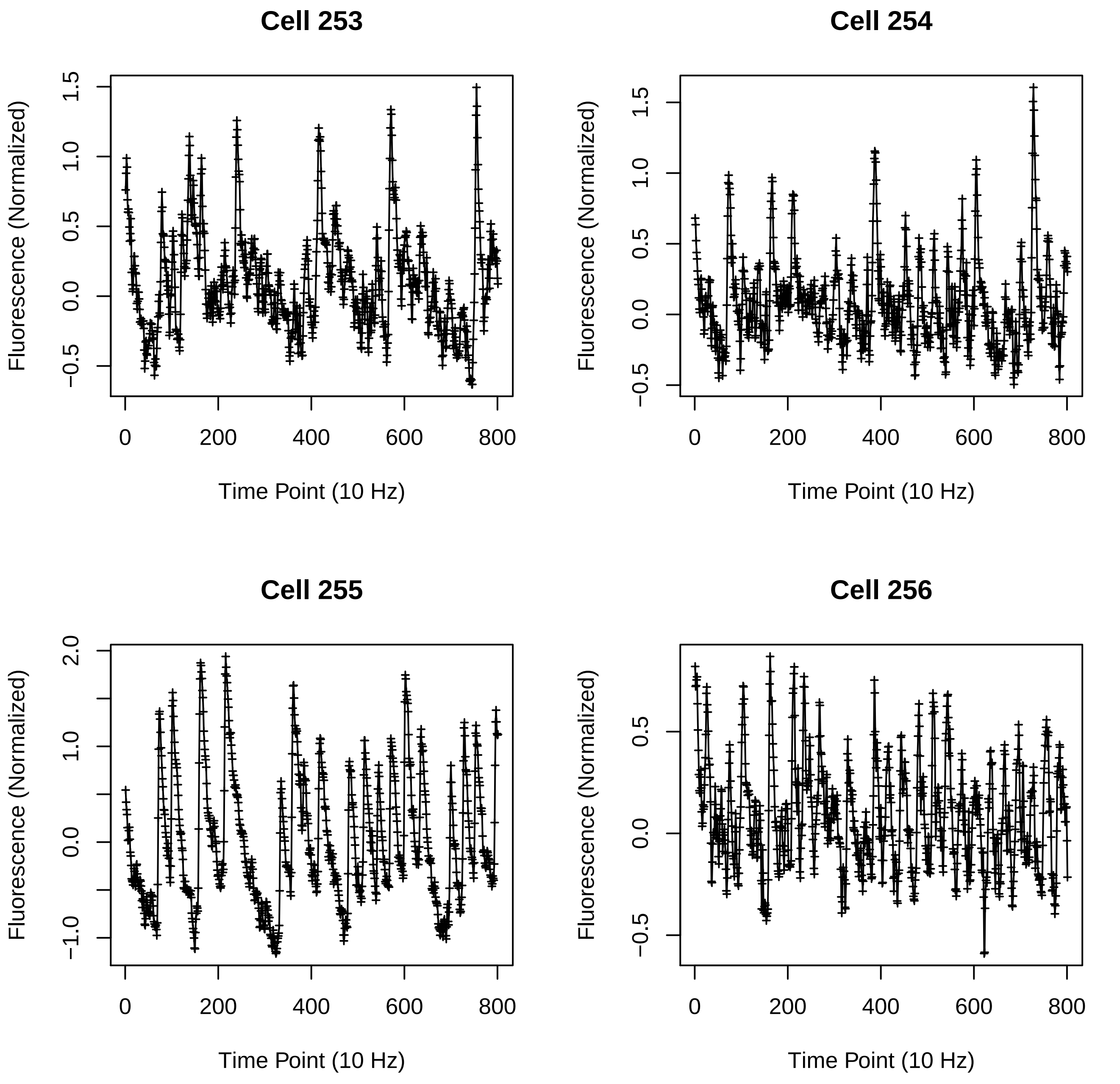

Cell 253
Cell 254
Cell 255
Cell 256
Fluorescence (Normalized)
Time Point (10 Hz)
0
200
400
600
800
−0.5
0.0
0.5
1.0
1.5
2.0
−1.0

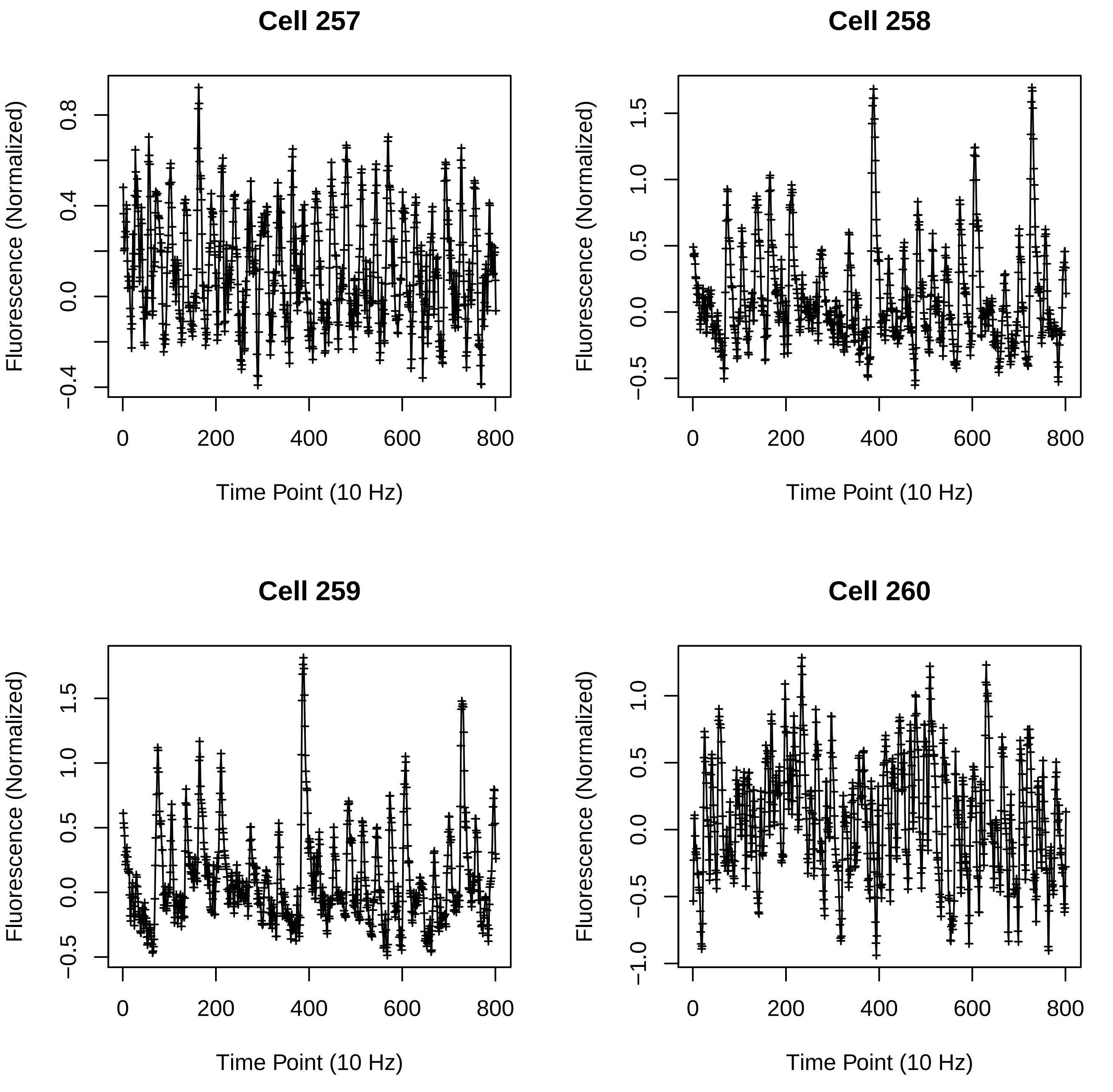
Cell 257
Cell 258
Cell 259
Cell 260
Fluorescence (Normalized)
Time Point (10 Hz)
0
200
400
600
800
−0.4
0.0
0.4
0.8
−0.5
0.0
0.5
1.0
1.5
−1.0

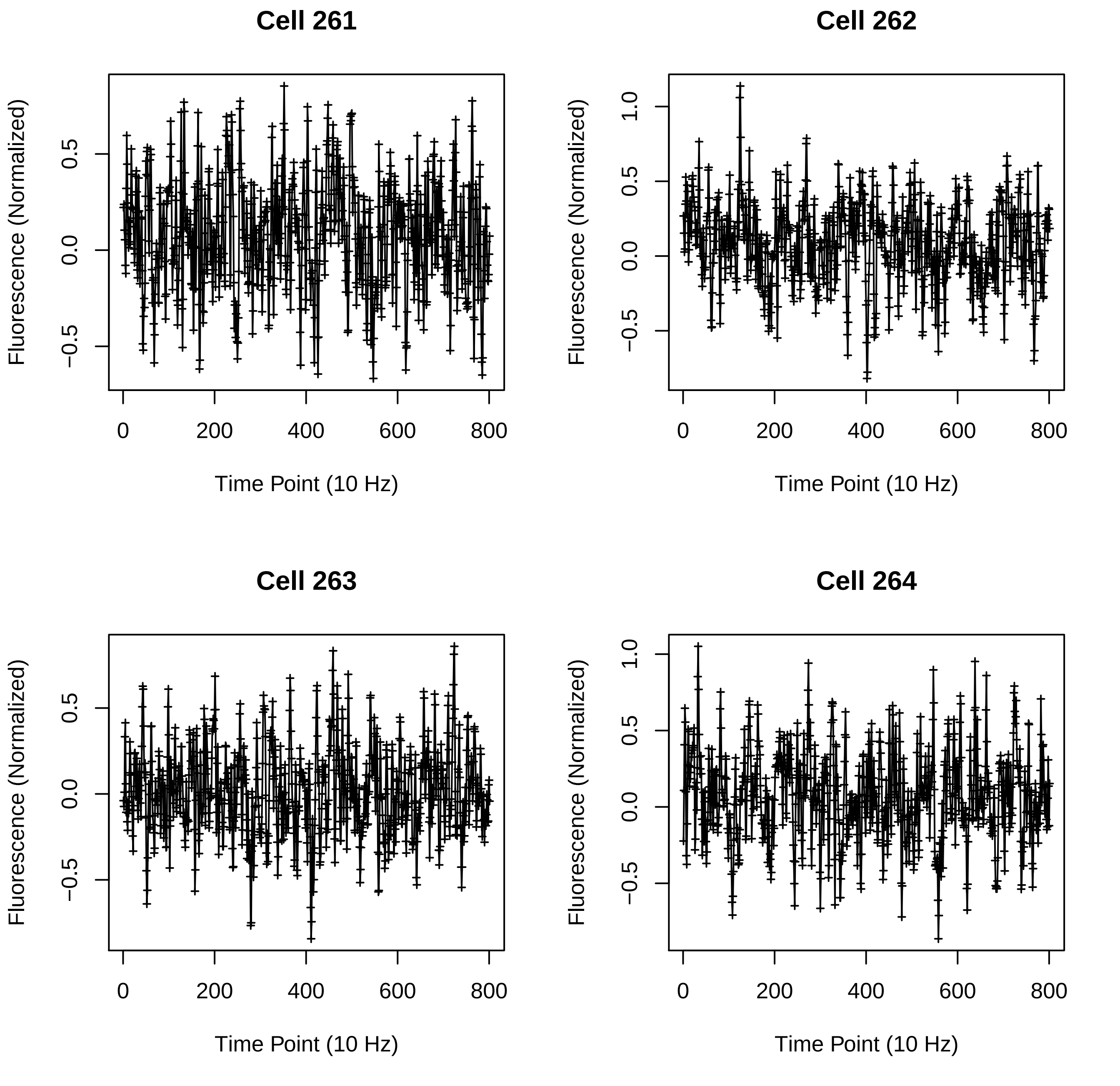
Cell 261
Cell 262
Cell 263
Cell 264
Fluorescence (Normalized)
Time Point (10 Hz)
−0.5
0.0
0.5
1.0
0
200
400
600
800

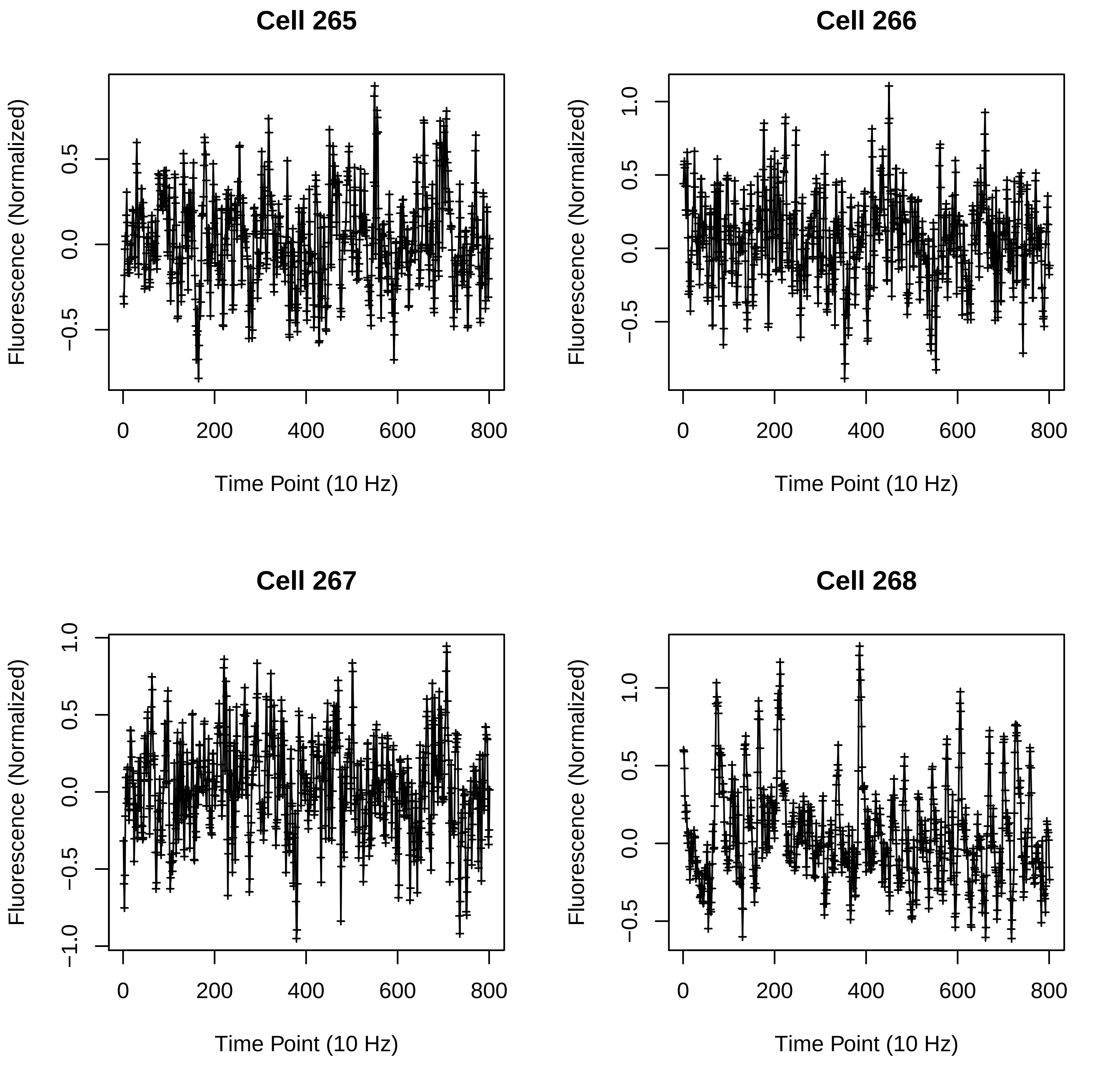

Cell 265
Cell 266
Cell 267
Cell 268
Fluorescence (Normalized)
Time Point (10 Hz)
0
200
400
600
800
−1.0
−0.5
0.0
0.5
1.0

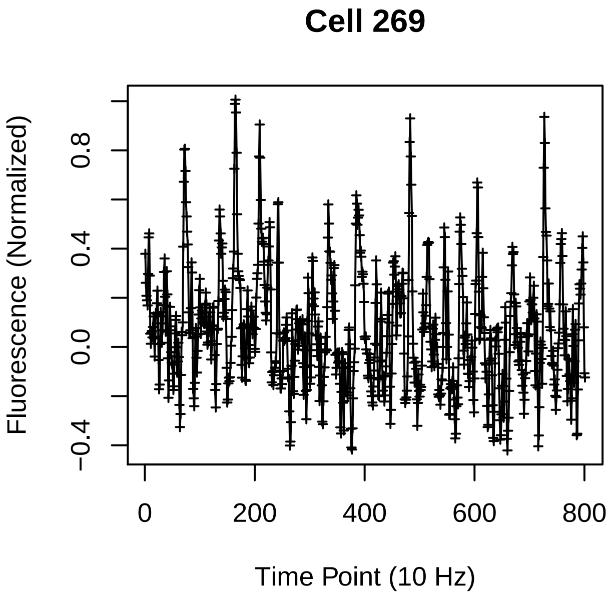
Cell 269
Fluorescence (Normalized)
−0.4
0.0
0.4
0.8
0
200
400
600
800
Time Point (10 Hz)

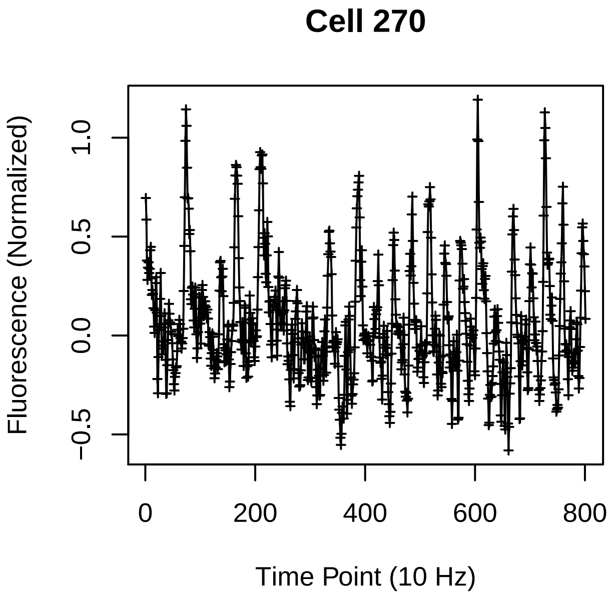
Cell 270
Fluorescence (Normalized)
−0.5
0.0
0.5
1.0
0
200
400
600
800
Time Point (10 Hz)

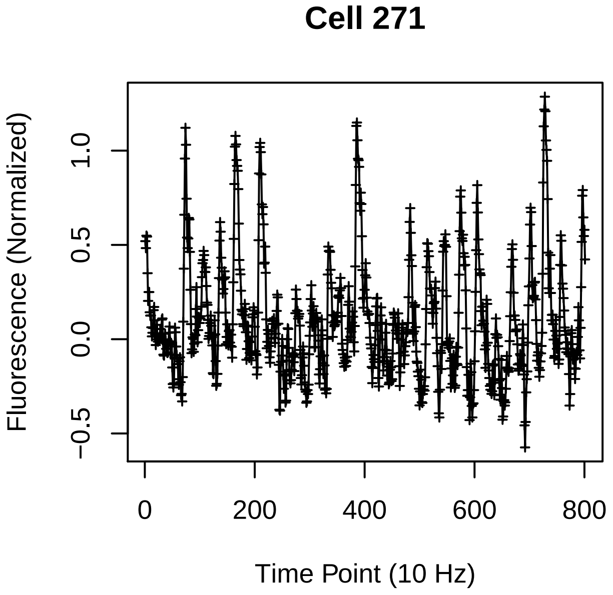
Cell 271
Fluorescence (Normalized)
−0.5
0.0
0.5
1.0
0
200
400
600
800
Time Point (10 Hz)

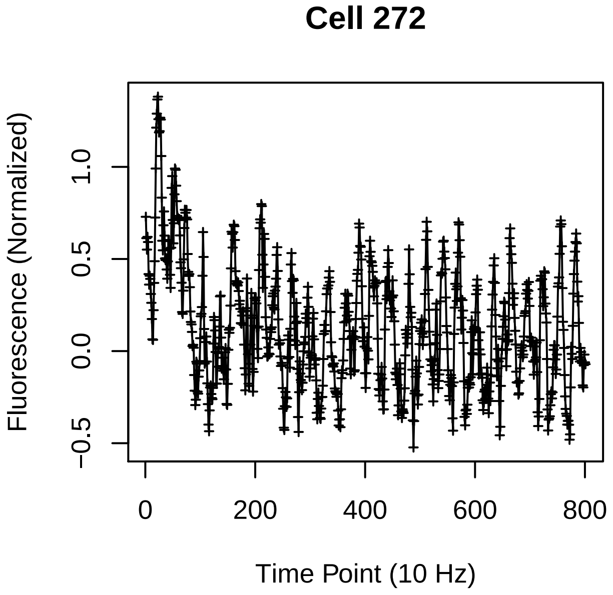
Cell 272
Fluorescence (Normalized)
−0.5
0.0
0.5
1.0
0
200
400
600
800
Time Point (10 Hz)

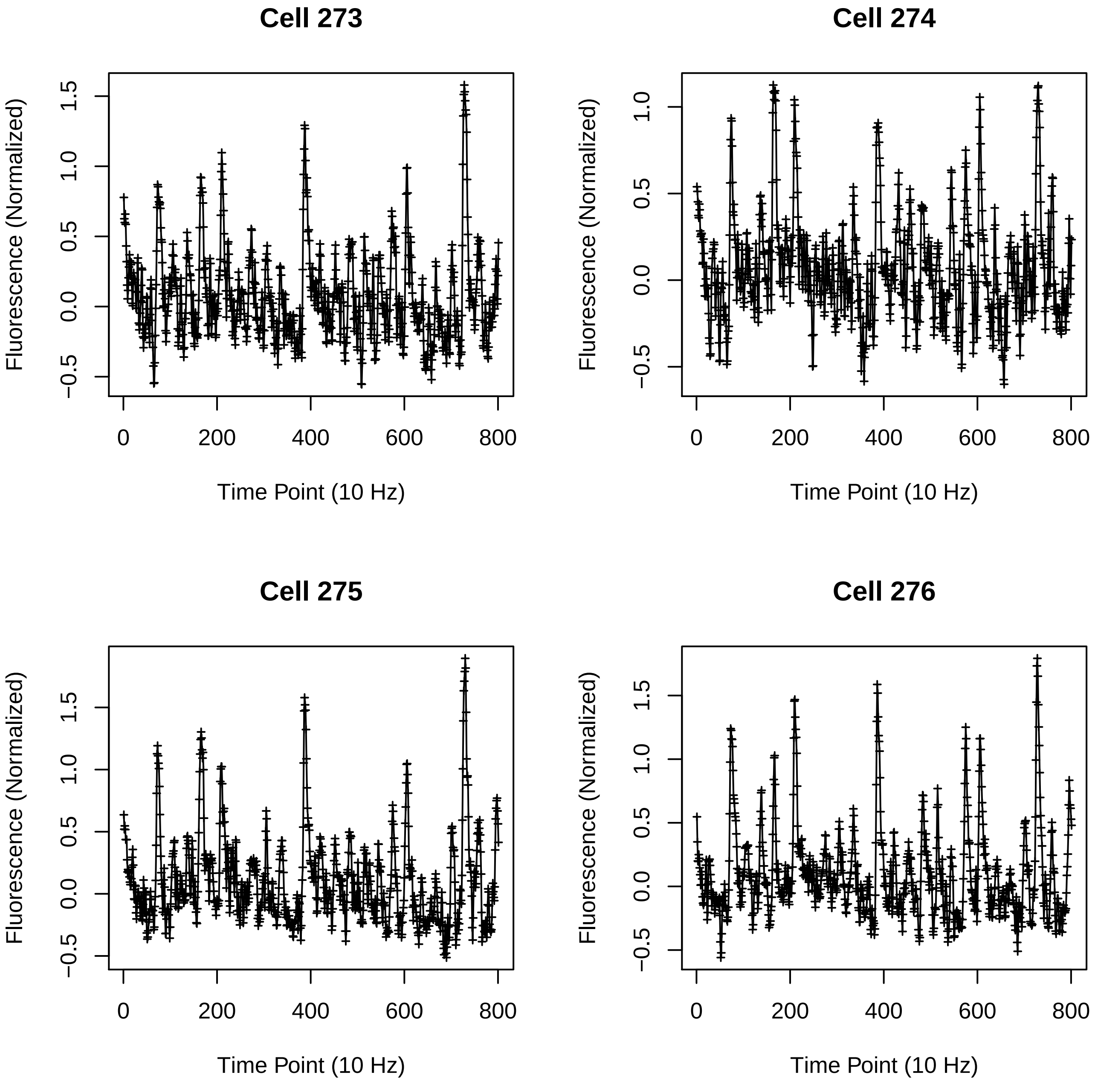
Cell 273
Cell 274
Cell 275
Cell 276
Fluorescence (Normalized)
Time Point (10 Hz)
−0.5
0.0
0.5
1.0
1.5
0
200
400
600
800

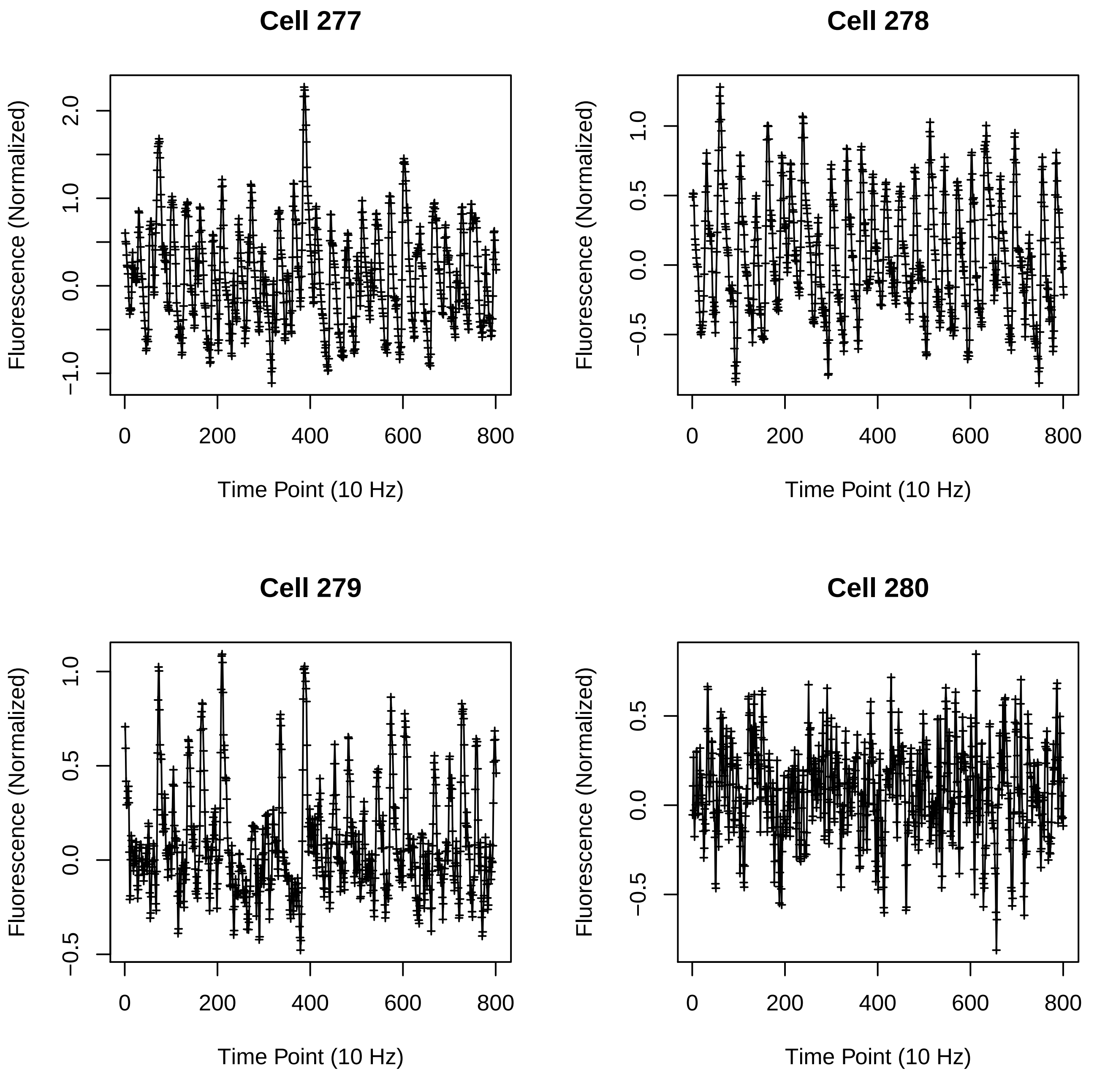

Cell 277
Cell 278
Cell 279
Cell 280
Fluorescence (Normalized)
Time Point (10 Hz)
0
200
400
600
800
−1.0
0.0
1.0
2.0
−0.5
0.5

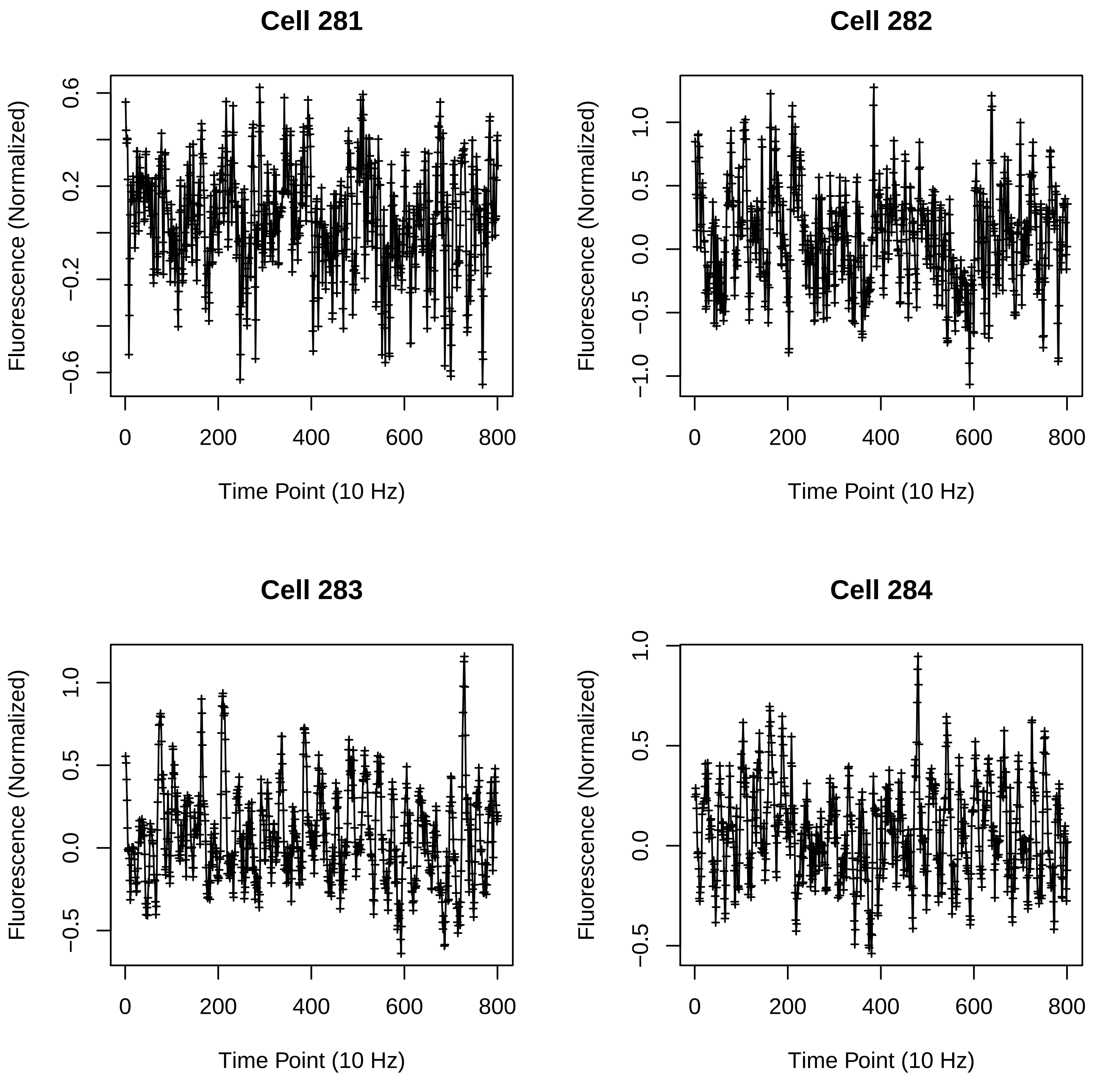
Cell 281
Cell 282
Cell 283
Cell 284
Fluorescence (Normalized)
Time Point (10 Hz)
0.6
0.2
−0.2
−0.6
1.0
0.5
0.0
−0.5
−1.0
0
200
400
600
800

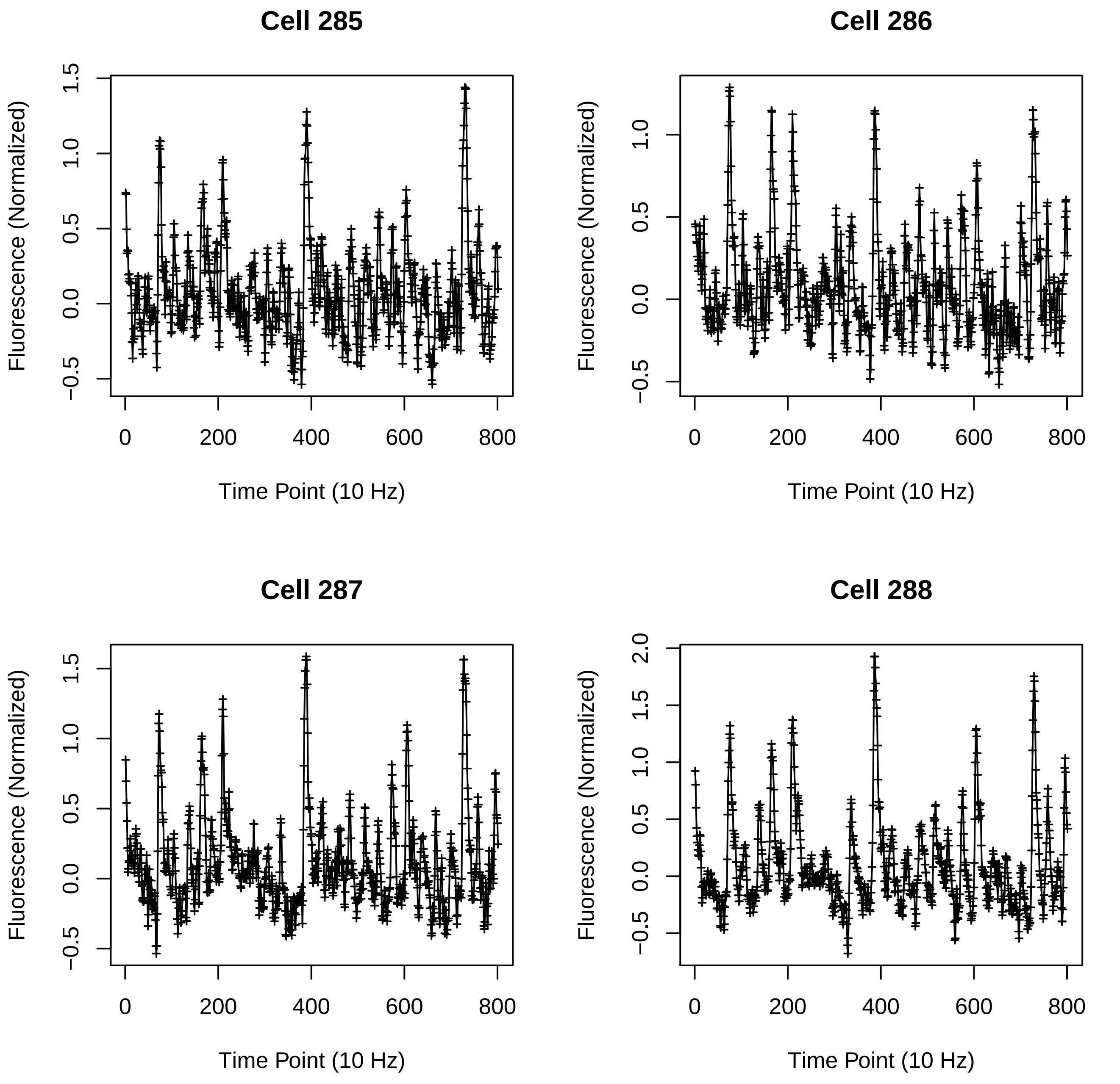
Cell 285
Cell 286
Cell 287
Cell 288
Fluorescence (Normalized)
Time Point (10 Hz)
0
200
400
600
800
−0.5
0.0
0.5
1.0
1.5
2.0

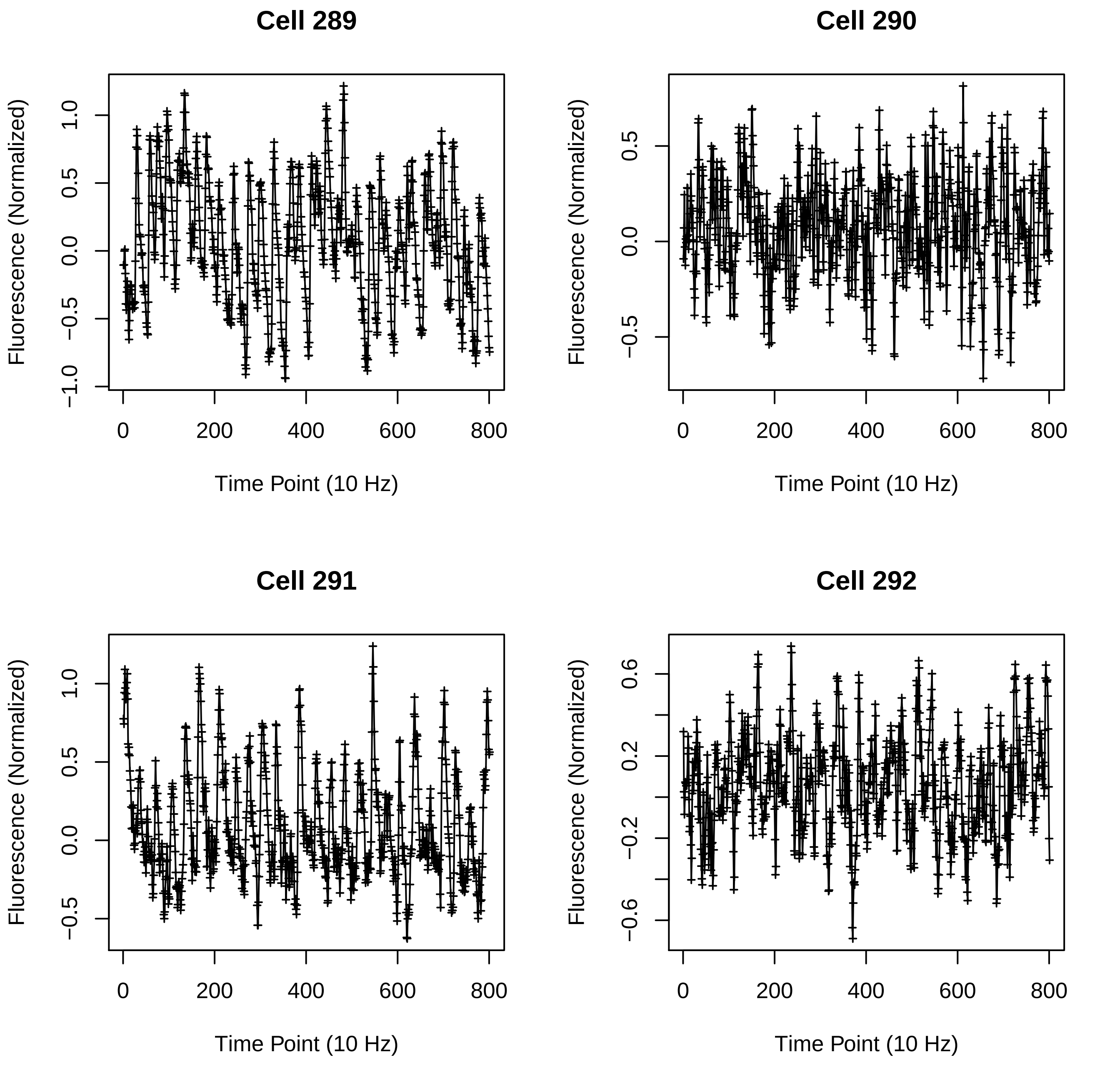
Cell 289
Cell 290
Cell 291
Cell 292
Fluorescence (Normalized)
Time Point (10 Hz)
0
200
400
600
800
−1.0
−0.5
0.0
0.5
1.0
−0.6
−0.2
0.2
0.6

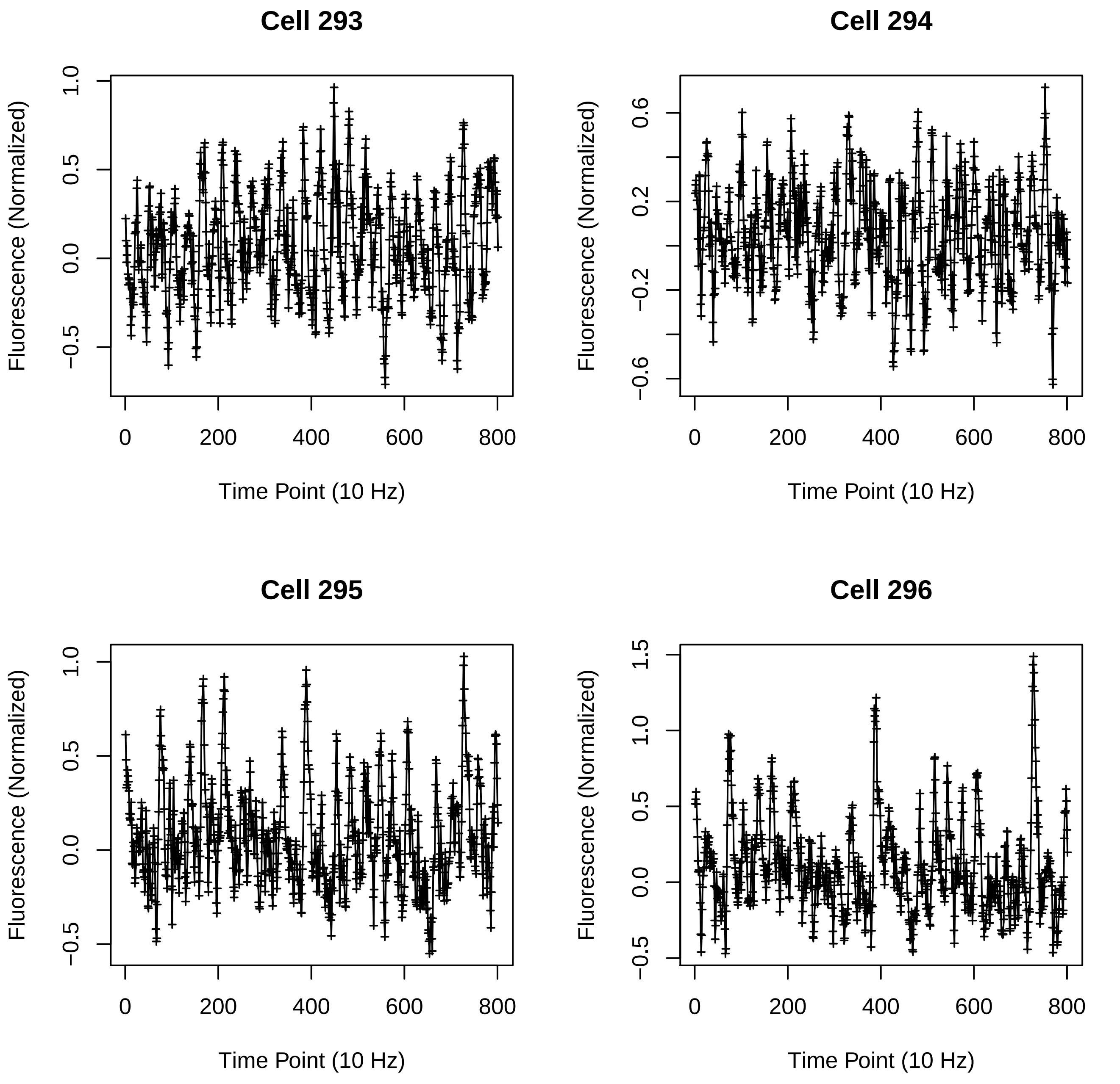

Cell 293
Cell 294
Cell 295
Cell 296
Fluorescence (Normalized)
Time Point (10 Hz)
0
200
400
600
800
1.0
0.5
0.0
−0.5
0.6
0.2
−0.2
−0.6
1.5

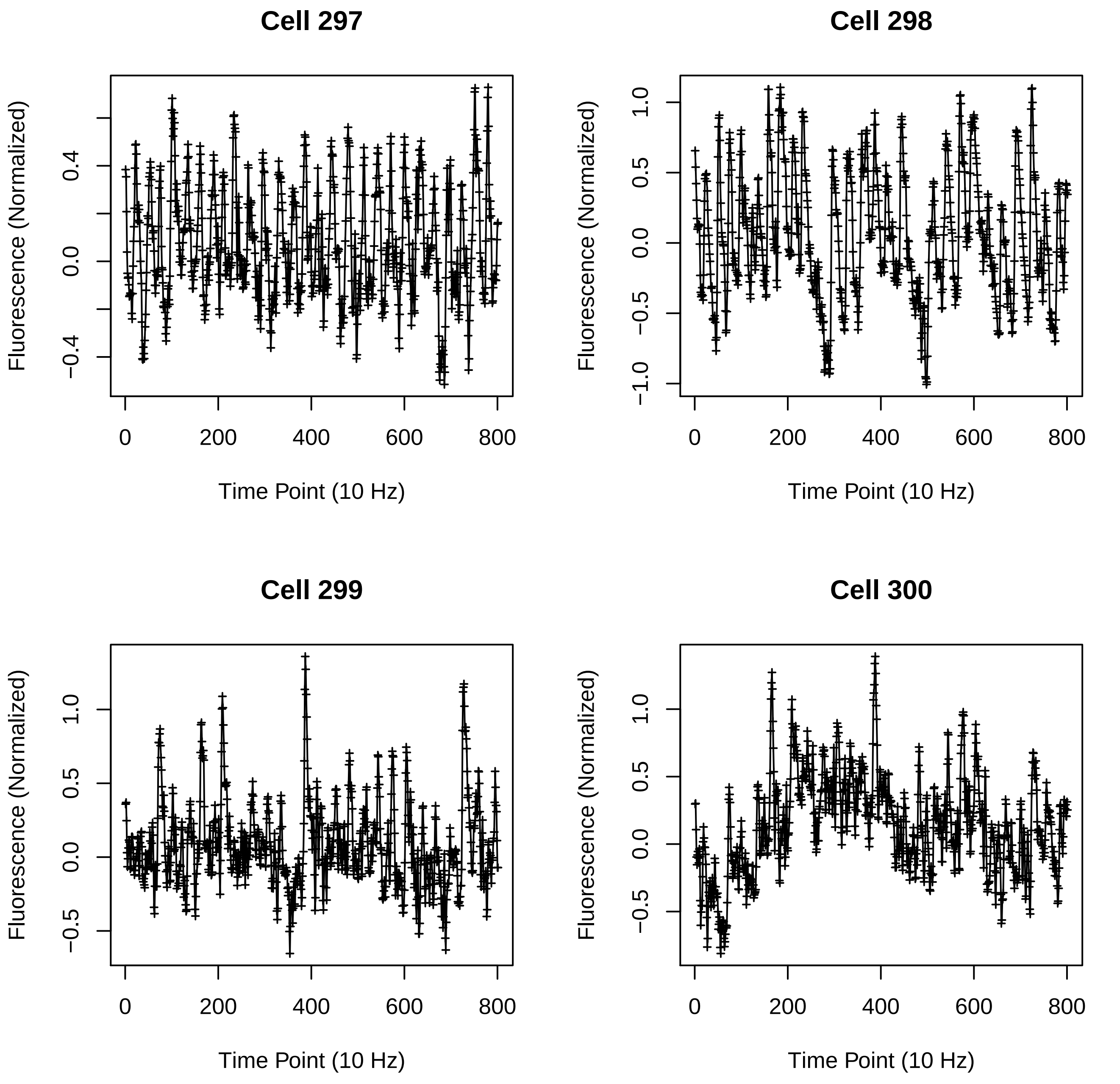

Cell 297
Fluorescence (Normalized)
0.4
0.0
−0.4
0
200
400
600
800
Time Point (10 Hz)
Cell 298
Fluorescence (Normalized)
1.0
0.5
0.0
−0.5
−1.0
0
200
400
600
800
Time Point (10 Hz)
Cell 299
Fluorescence (Normalized)
1.0
0.5
0.0
−0.5
0
200
400
600
800
Time Point (10 Hz)
Cell 300
Fluorescence (Normalized)
1.0
0.5
0.0
−0.5
0
200
400
600
800
Time Point (10 Hz)

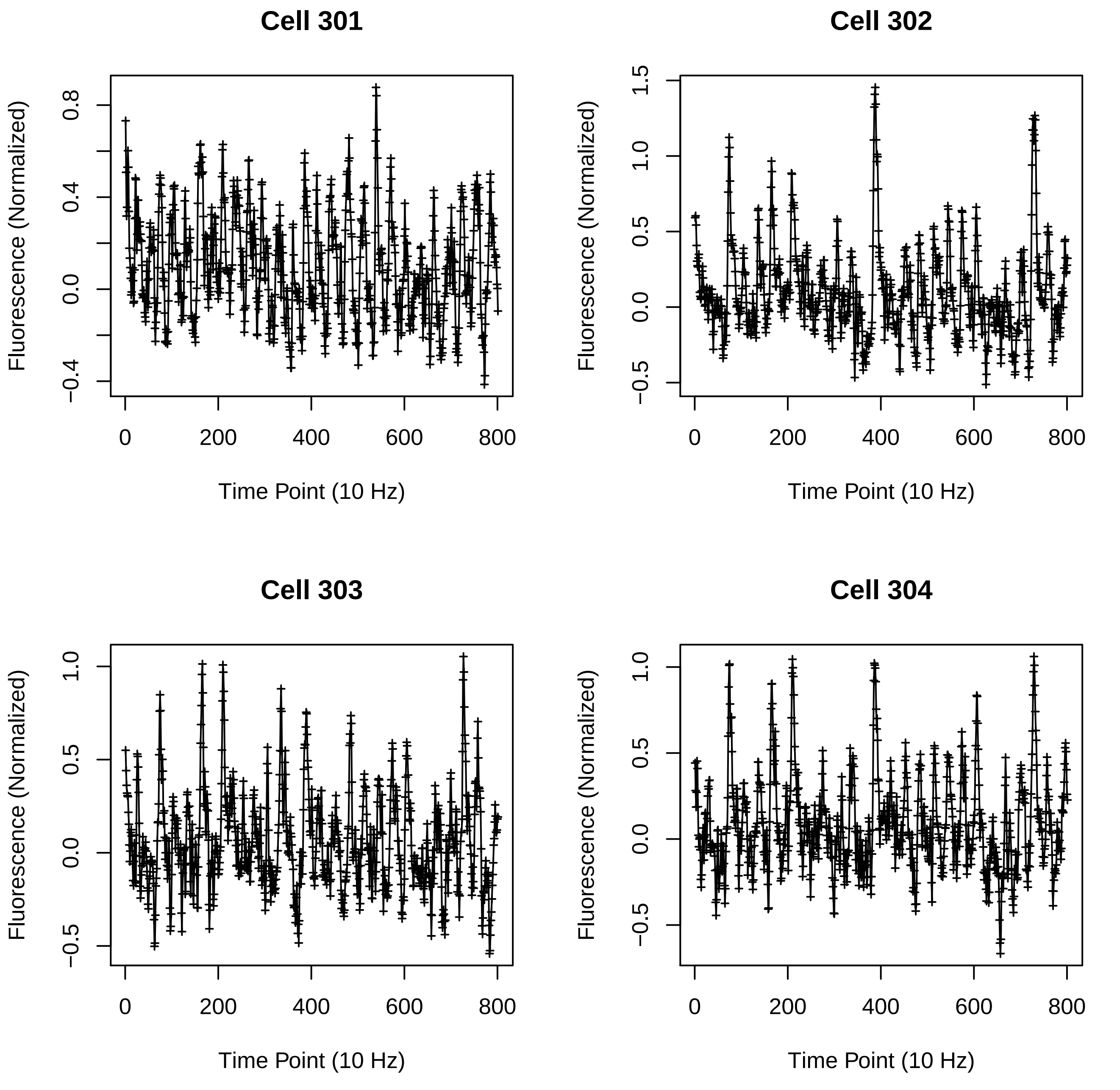
Cell 301
Cell 302
Cell 303
Cell 304
Fluorescence (Normalized)
Time Point (10 Hz)
-0.4
0.0
0.4
0.8
-0.5
0.0
0.5
1.0
1.5
0
200
400
600
800

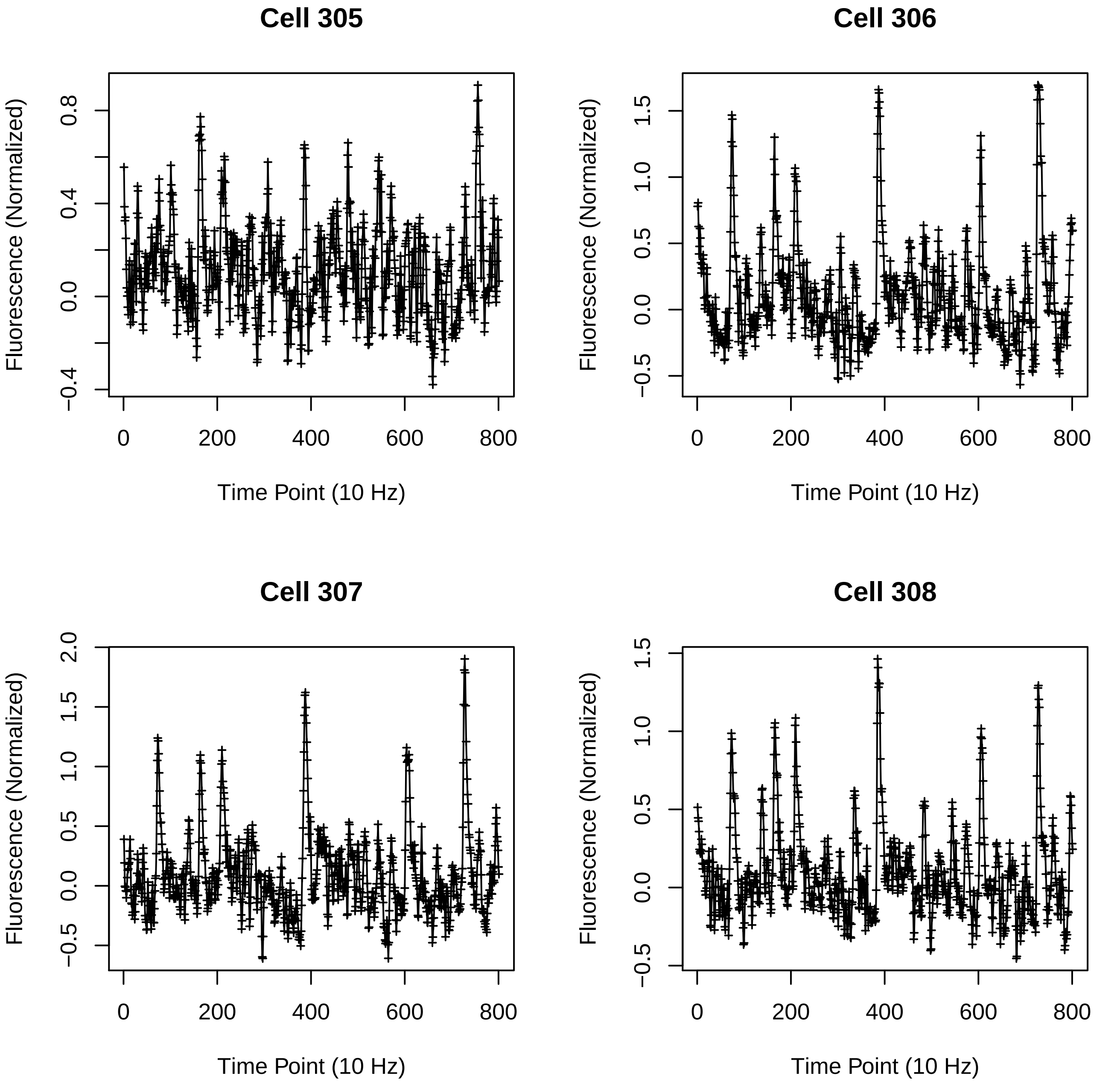
Cell 305
Cell 306
Cell 307
Cell 308
Fluorescence (Normalized)
Time Point (10 Hz)
0
200
400
600
800
−0.4
0.0
0.4
0.8
−0.5
0.0
0.5
1.0
1.5
2.0

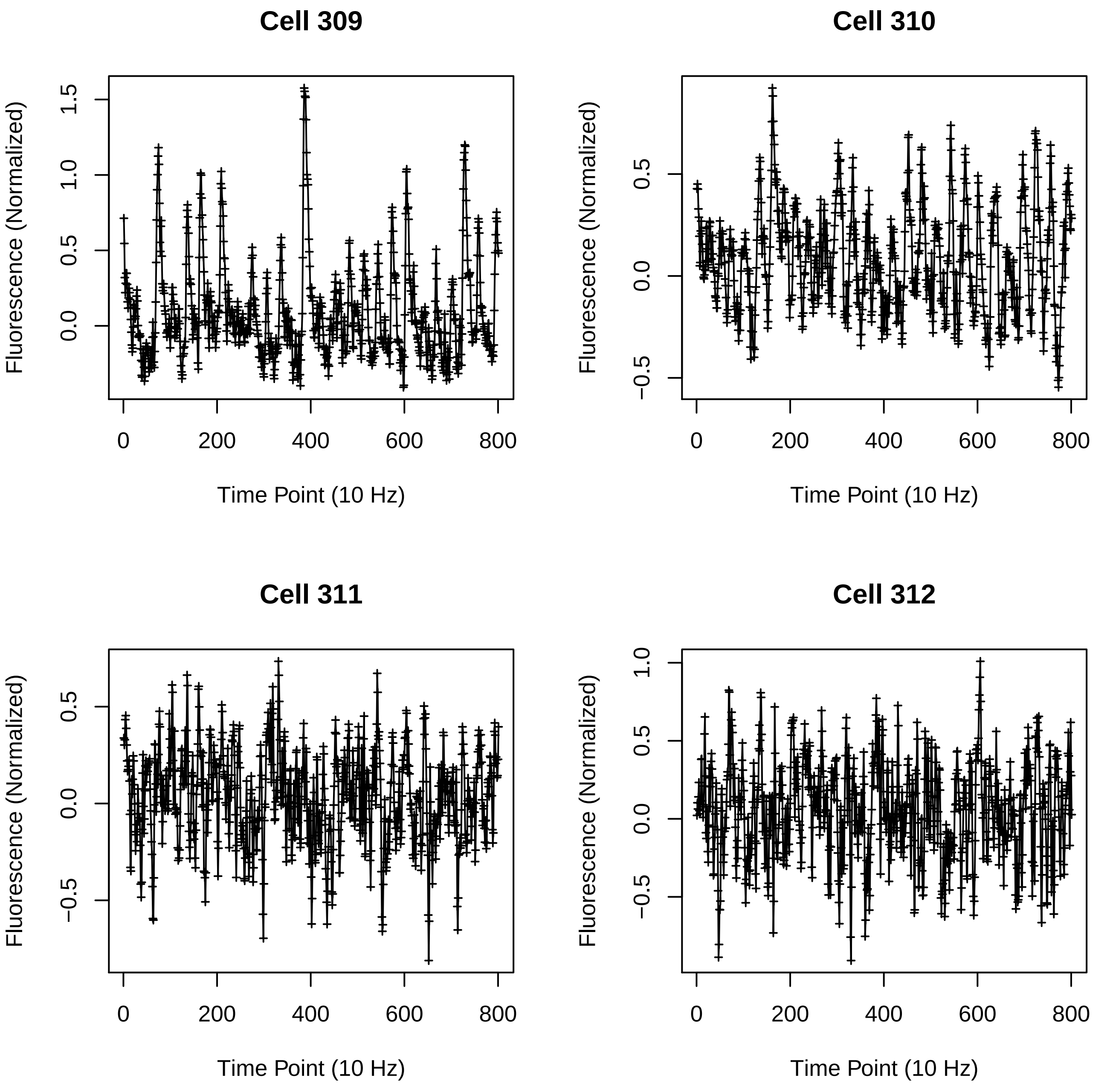
Cell 309
Cell 310
Cell 311
Cell 312
Fluorescence (Normalized)
Time Point (10 Hz)
0
200
400
600
800
−0.5
0.0
0.5
1.0
1.5

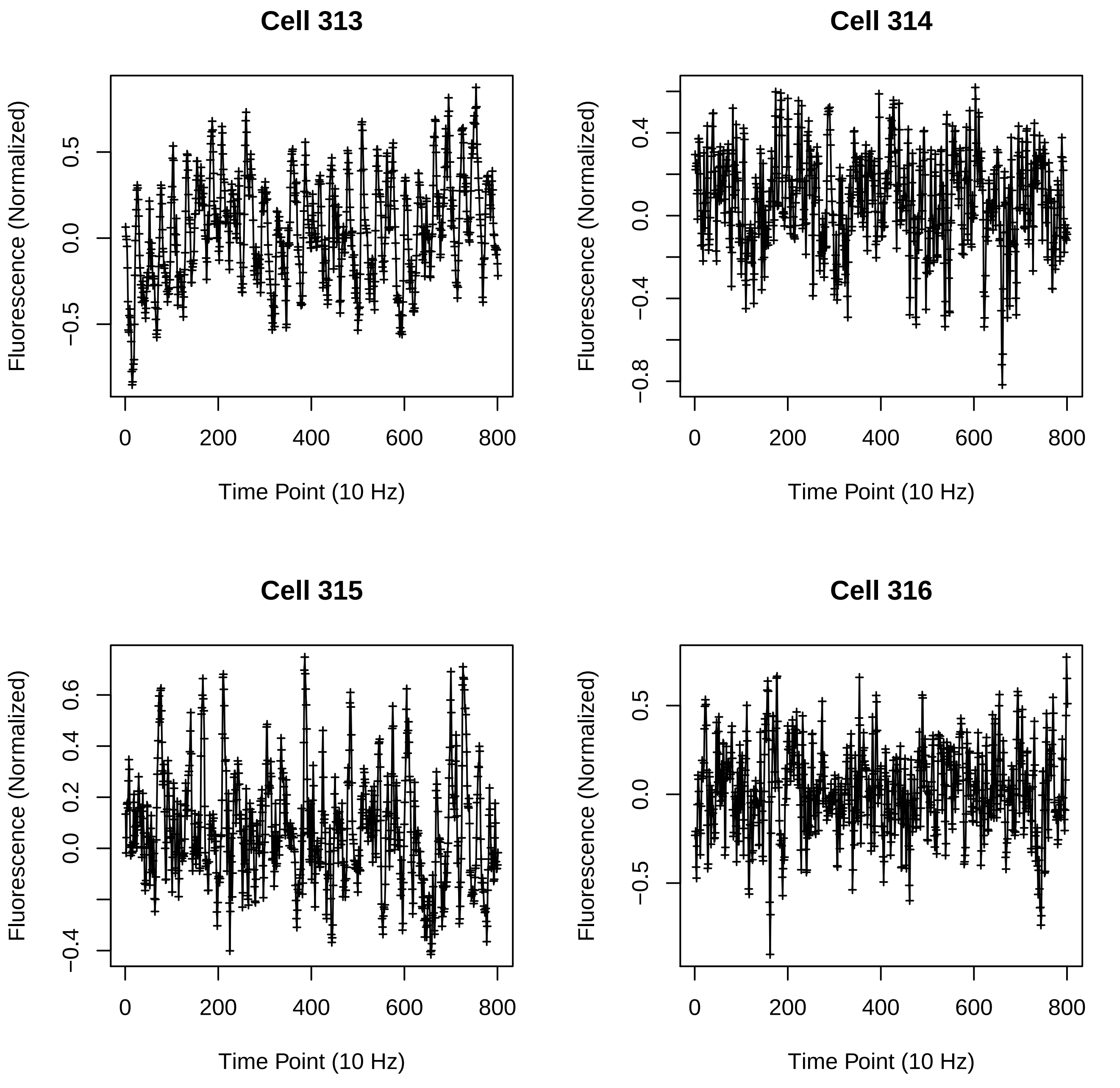
Cell 313
Cell 314
Cell 315
Cell 316
Fluorescence (Normalized)
Time Point (10 Hz)
0
200
400
600
800
0.5
0.0
−0.5
0.4
−0.4
−0.8
0.6
0.2

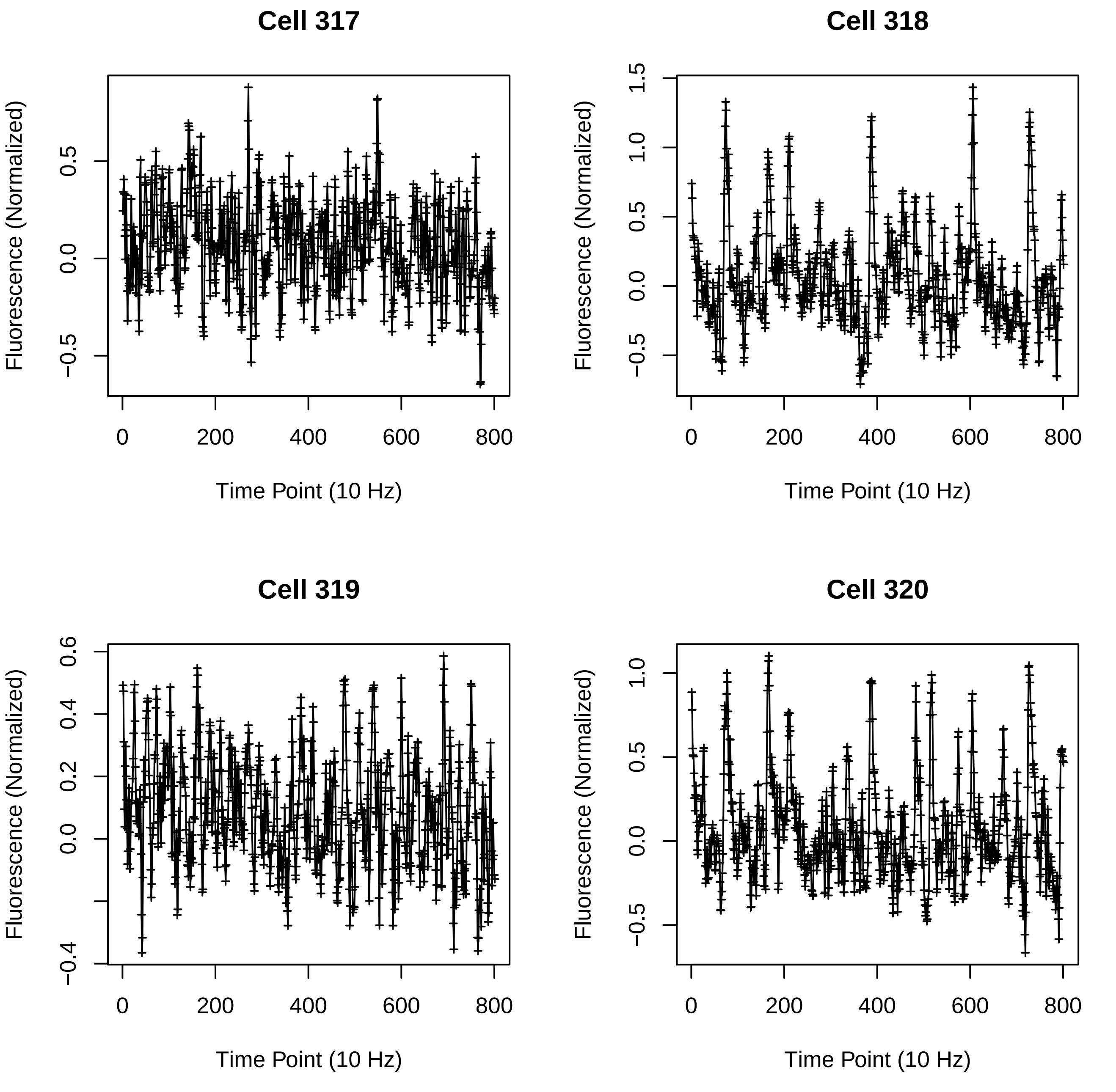

Cell 317
Fluorescence (Normalized)
−0.5
0.0
0.5
0
200
400
600
800
Time Point (10 Hz)
Cell 318
Fluorescence (Normalized)
−0.5
0.0
0.5
1.0
1.5
0
200
400
600
800
Time Point (10 Hz)
Cell 319
Fluorescence (Normalized)
−0.4
0.0
0.2
0.4
0.6
0
200
400
600
800
Time Point (10 Hz)
Cell 320
Fluorescence (Normalized)
−0.5
0.0
0.5
1.0
0
200
400
600
800
Time Point (10 Hz)

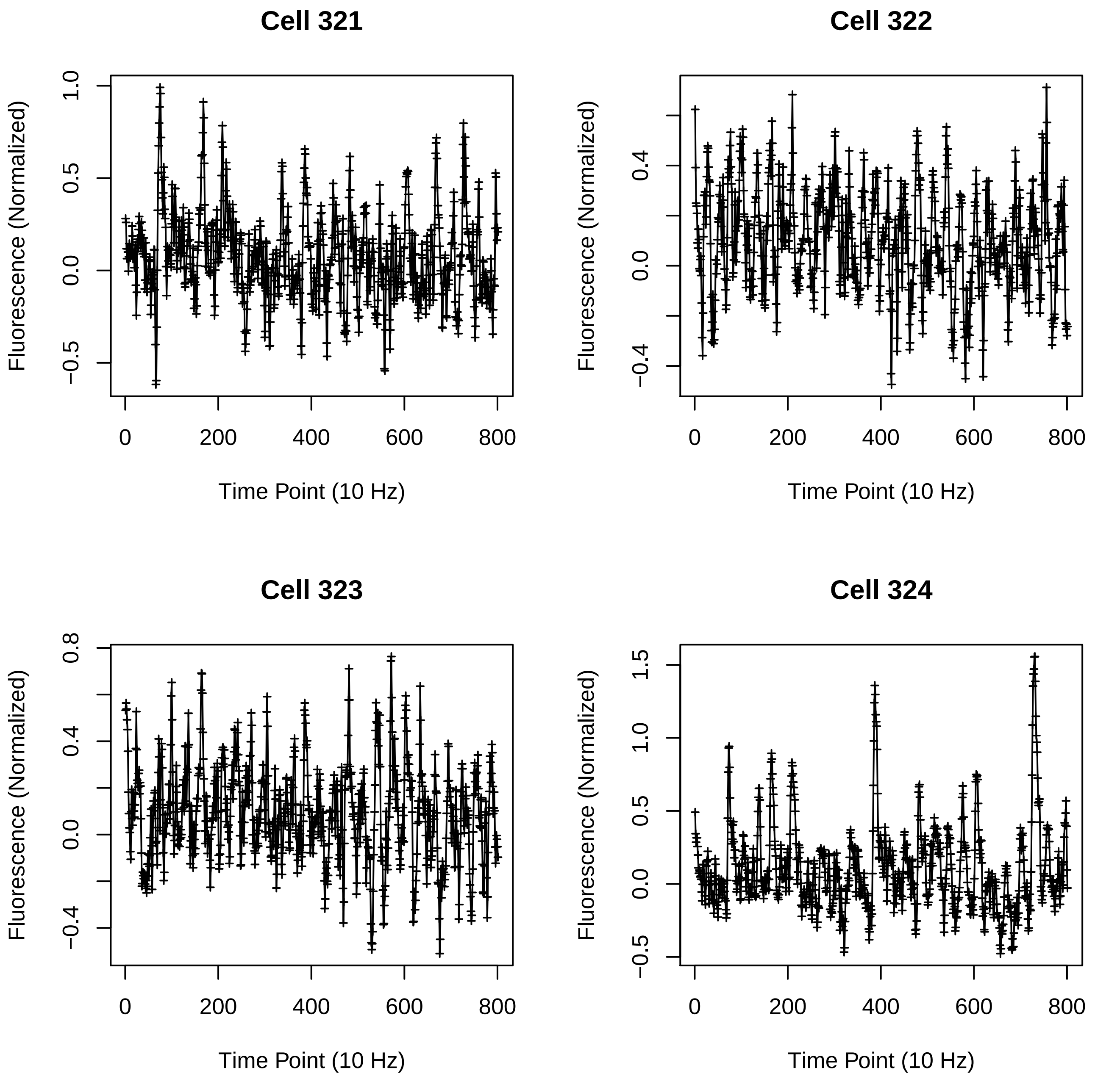

Cell 321
Cell 322
Cell 323
Cell 324
Fluorescence (Normalized)
Time Point (10 Hz)
0
200
400
600
800
1.0
0.5
0.0
−0.5
0.4
0.0
−0.4
0.8
1.5

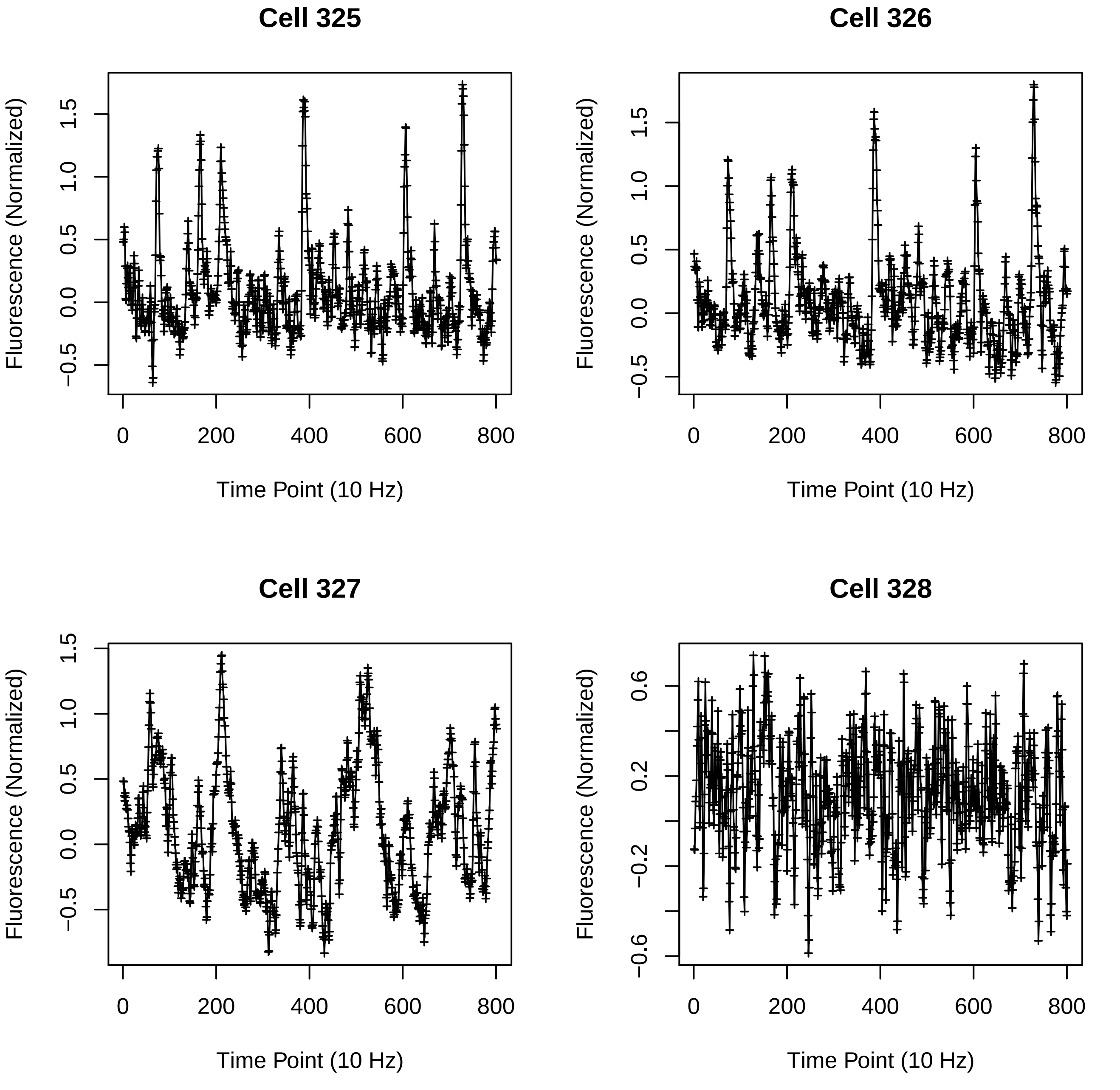
Cell 325
Cell 326
Cell 327
Cell 328
Fluorescence (Normalized)
Time Point (10 Hz)
−0.5
0.0
0.5
1.0
1.5
−0.6
−0.2
0.2
0.6
0
200
400
600
800

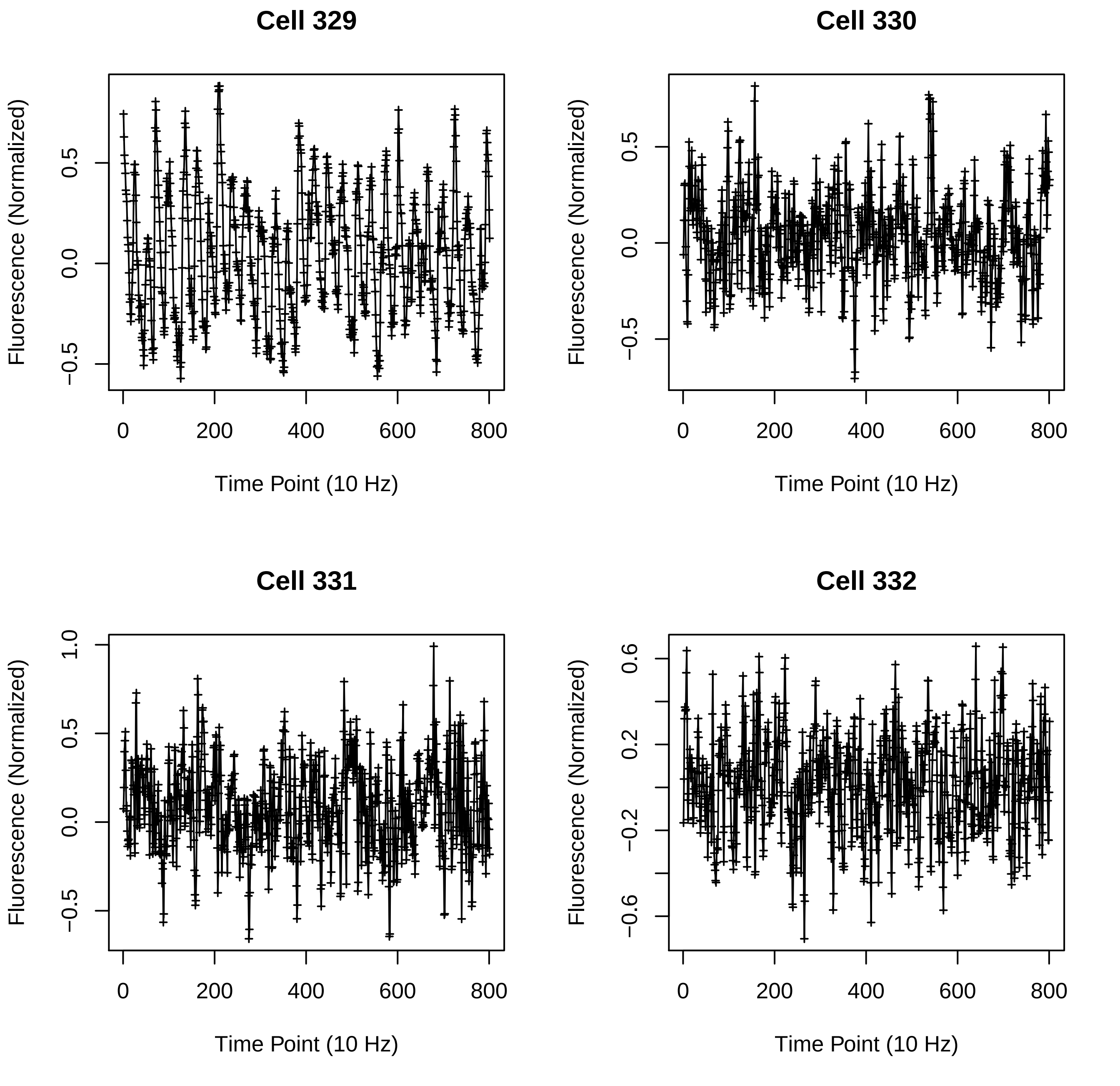
Cell 329
Cell 330
Cell 331
Cell 332
Fluorescence (Normalized)
Time Point (10 Hz)
0
200
400
600
800
−0.5
0.0
0.5
1.0
−0.6
−0.2
0.2
0.6

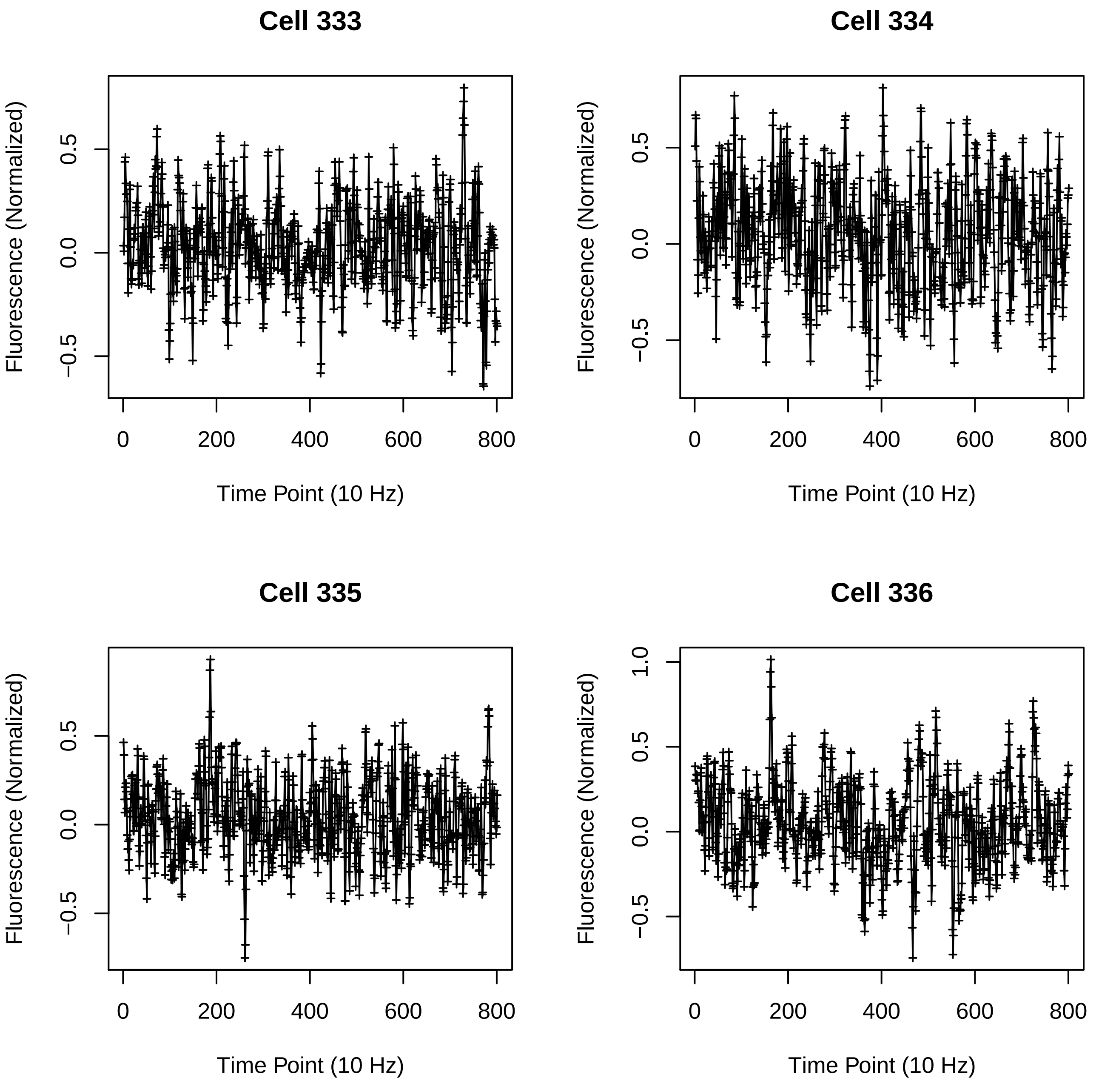
Cell 333
Cell 334
Cell 335
Cell 336
Fluorescence (Normalized)
Time Point (10 Hz)
0
200
400
600
800
−0.5
0.0
0.5
1.0

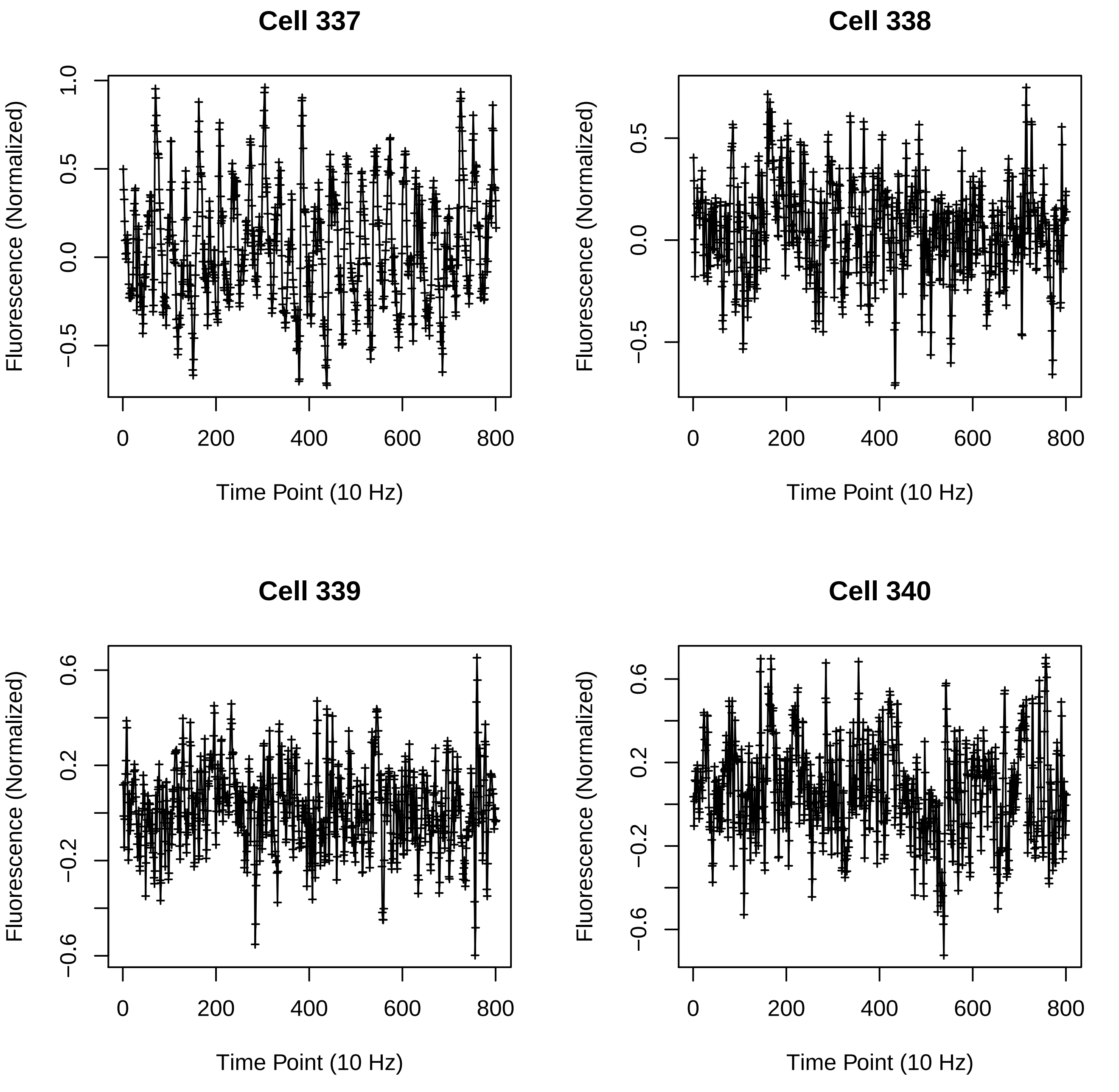
Cell 337
Cell 338
Cell 339
Cell 340
Fluorescence (Normalized)
Time Point (10 Hz)
1.0
0.5
0.0
−0.5
0.6
0.2
−0.2
−0.6
0
200
400
600
800

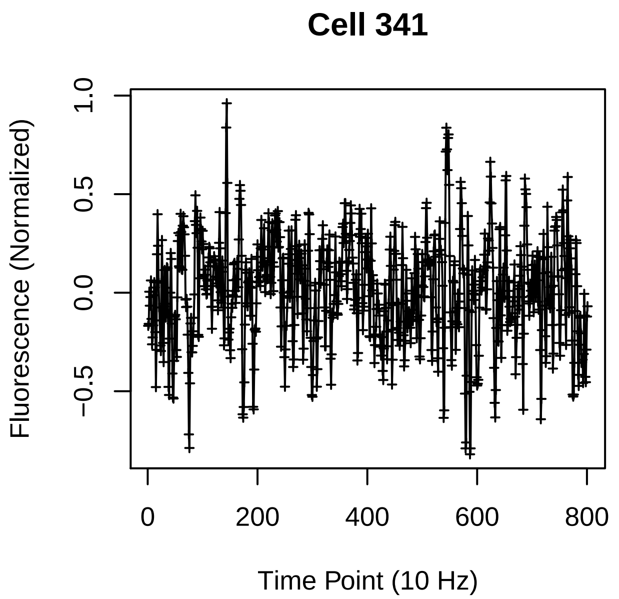
Cell 341
Fluorescence (Normalized)
1.0
0.5
0.0
−0.5
0
200
400
600
800
Time Point (10 Hz)

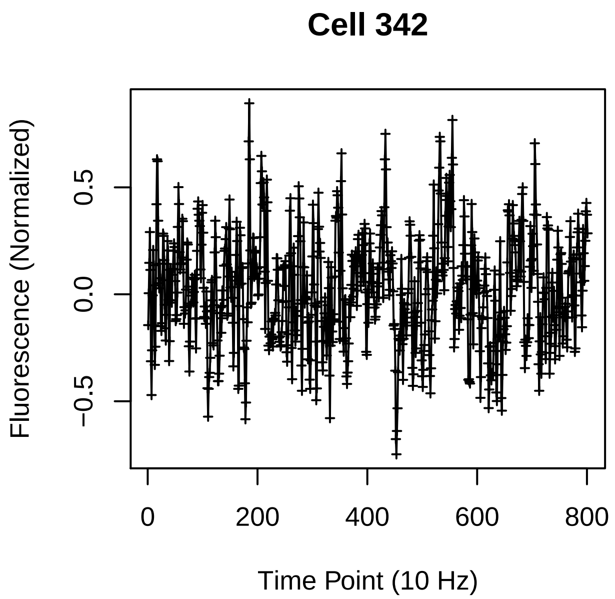
Cell 342
Fluorescence (Normalized)
0.5
0.0
−0.5
0
200
400
600
800
Time Point (10 Hz)

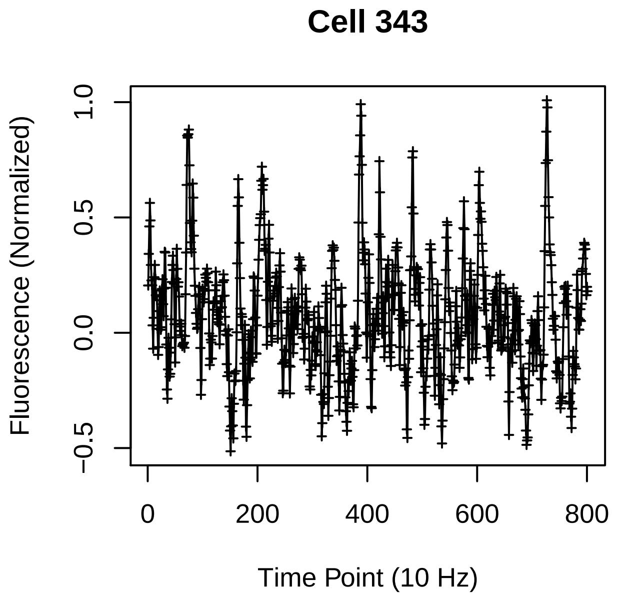
Cell 343
Fluorescence (Normalized)
1.0
0.5
0.0
−0.5
0
200
400
600
800
Time Point (10 Hz)

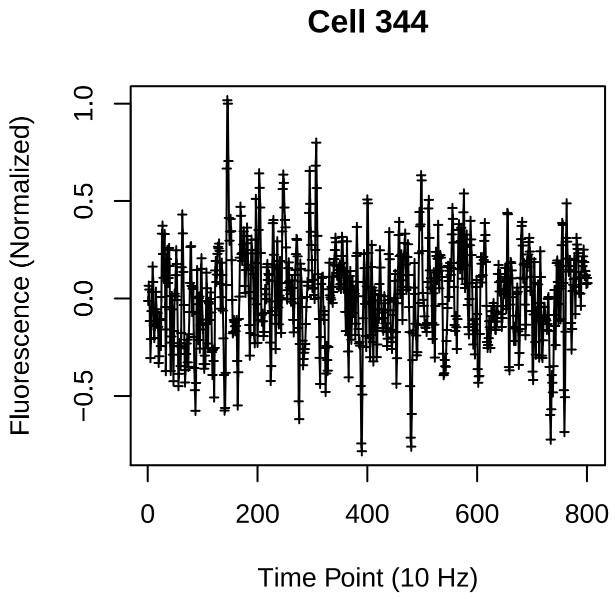
Cell 344
Fluorescence (Normalized)
1.0
0.5
0.0
−0.5
0
200
400
600
800
Time Point (10 Hz)

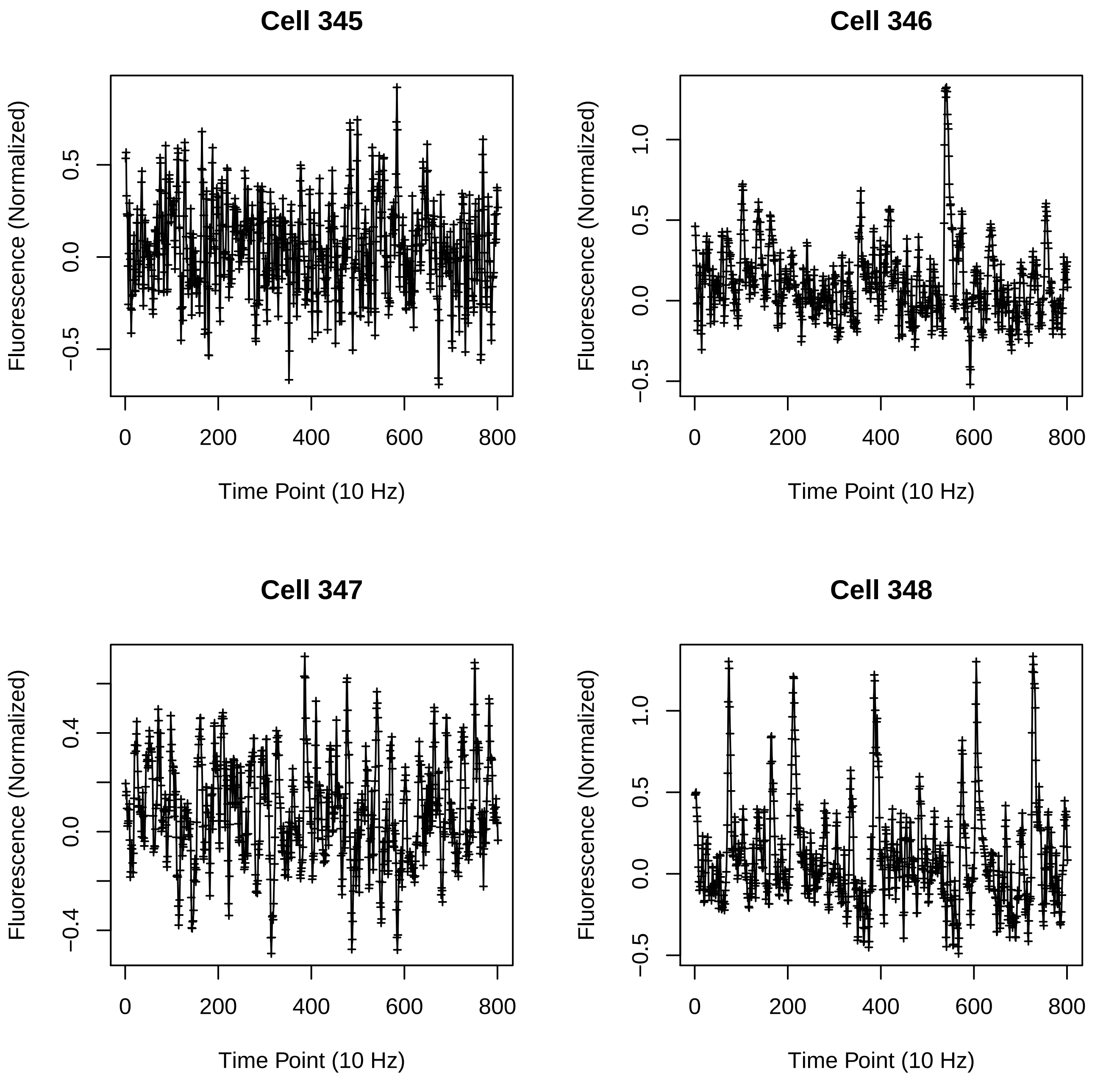
Cell 345
Cell 346
Cell 347
Cell 348
Fluorescence (Normalized)
Time Point (10 Hz)
0
200
400
600
800
−0.5
0.0
0.5
1.0
−0.4
0.4

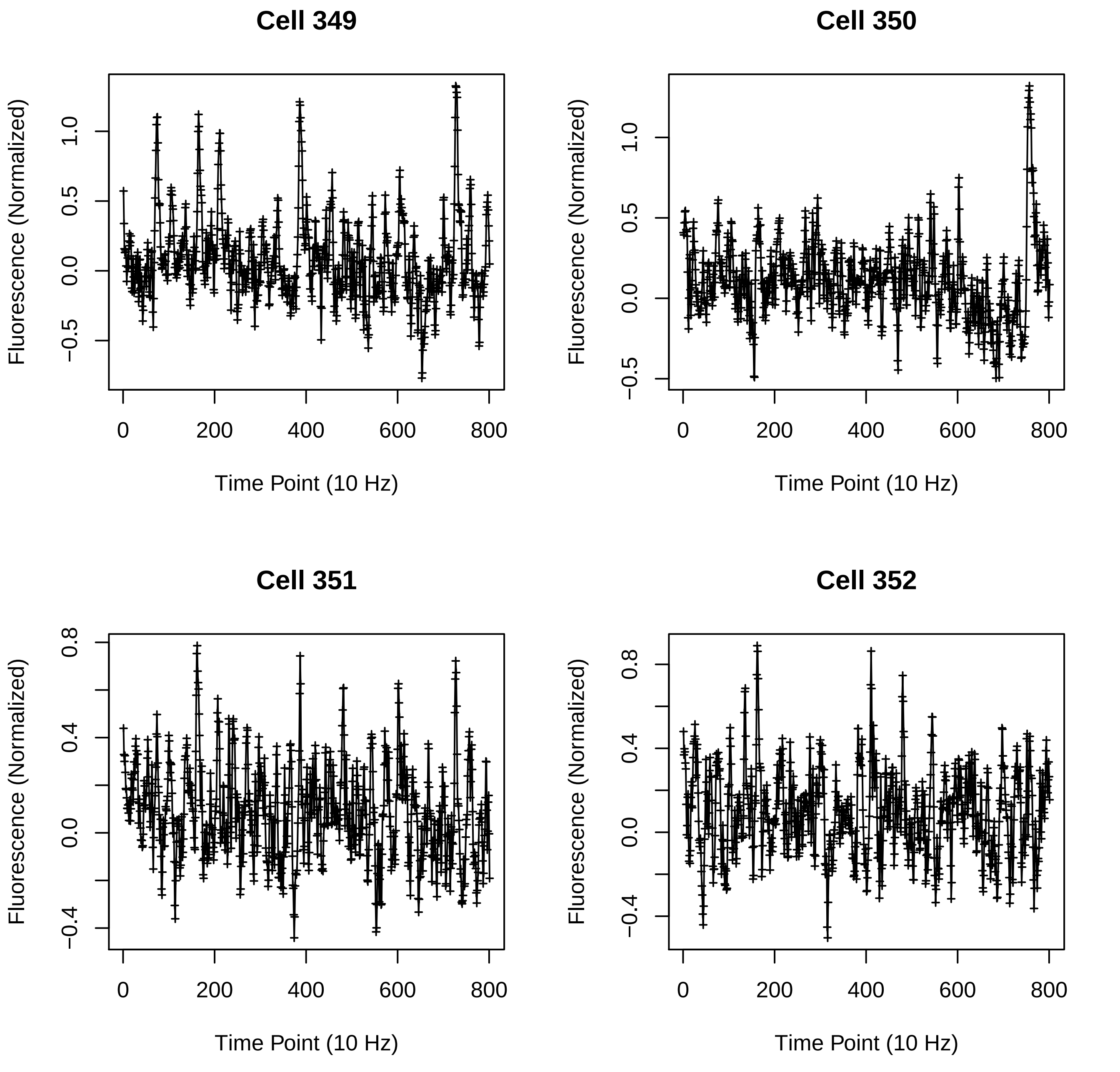
Cell 349
Cell 350
Cell 351
Cell 352
Fluorescence (Normalized)
Time Point (10 Hz)
1.0
0.5
0.0
−0.5
0.8
0.4
−0.4
0
200
400
600
800

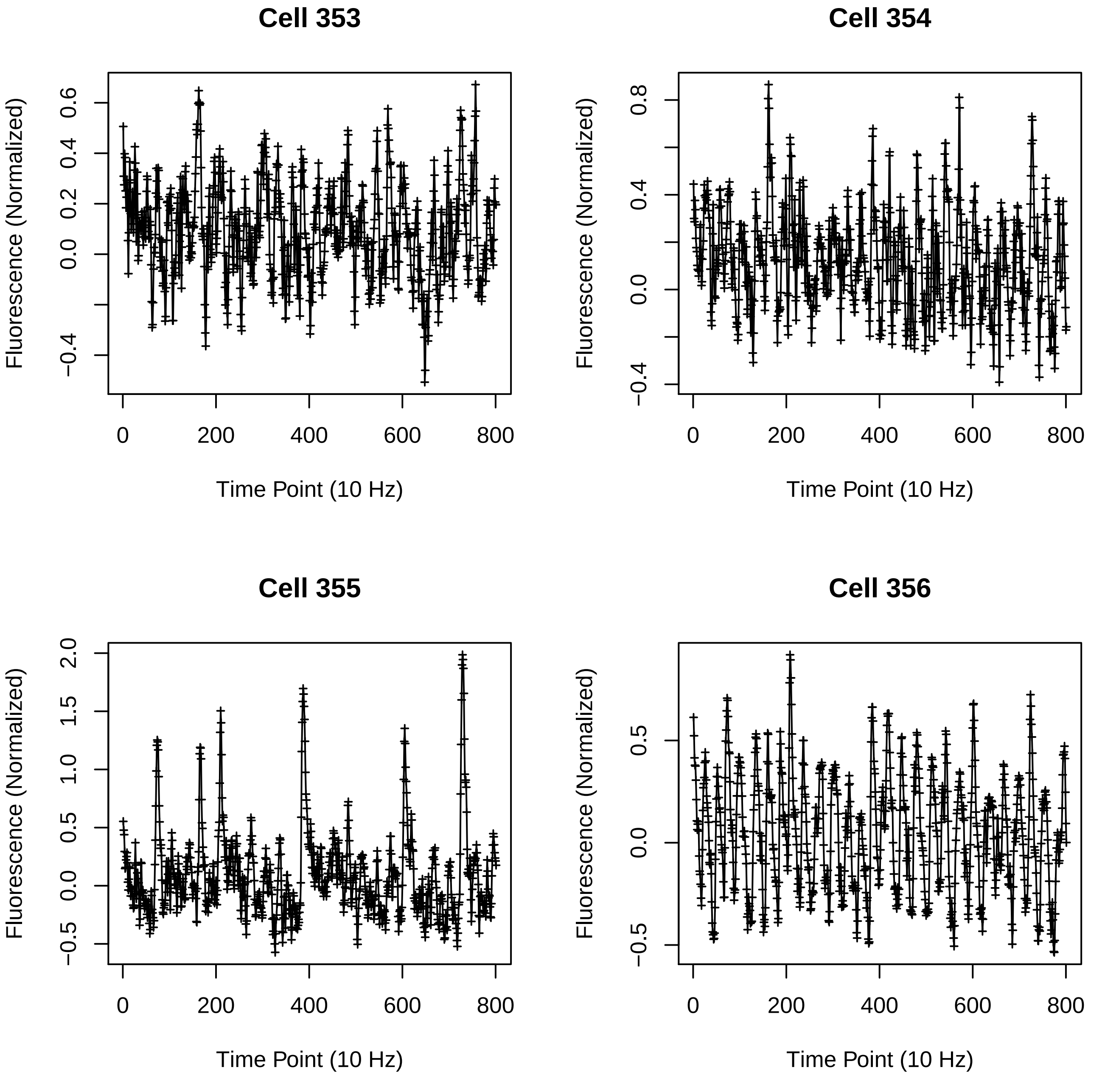
Cell 353
Cell 354
Cell 355
Cell 356
Fluorescence (Normalized)
Time Point (10 Hz)
0
200
400
600
800
−0.4
0.0
0.2
0.4
0.6
0.8
−0.5
0.5
1.0
1.5
2.0

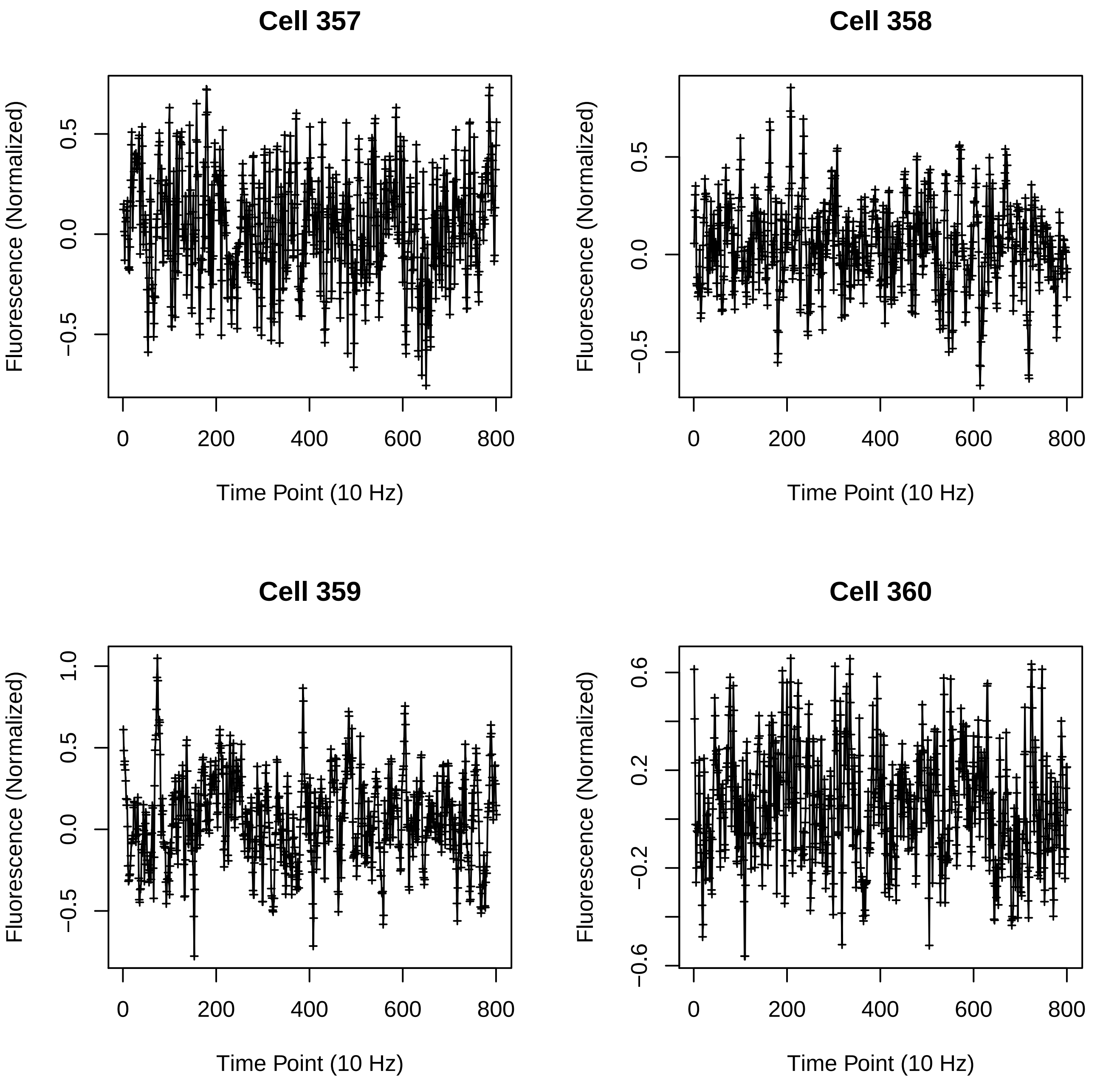

Cell 357
Cell 358
Cell 359
Cell 360
Fluorescence (Normalized)
Time Point (10 Hz)
0
200
400
600
800
−0.5
0.0
0.5
1.0
−0.6
−0.2
0.2
0.6

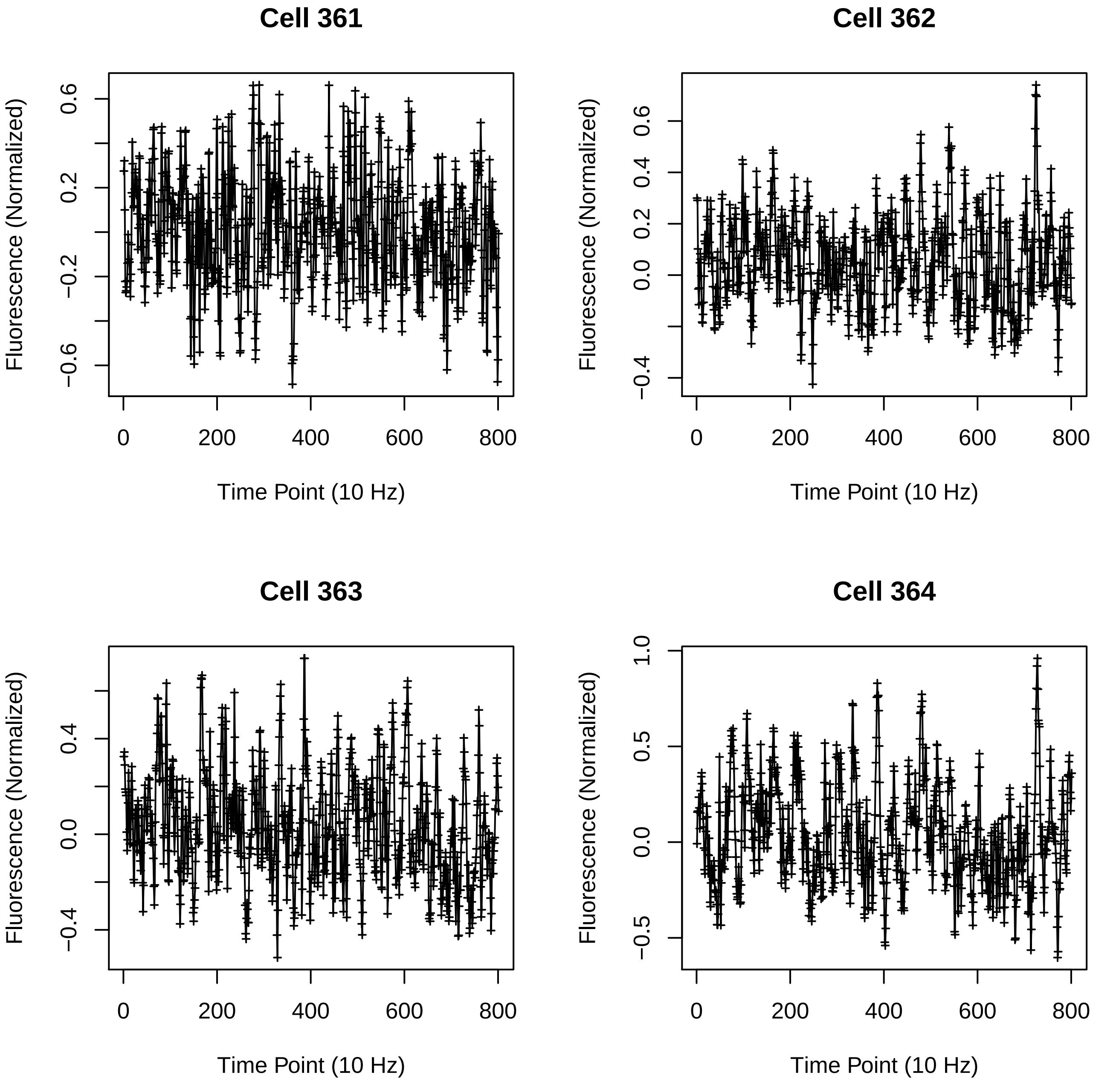
Cell 361
Cell 362
Cell 363
Cell 364
Fluorescence (Normalized)
Time Point (10 Hz)
0
200
400
600
800
−0.6
−0.2
0.2
0.6
−0.4
0.0
0.2
0.4
0.6
−0.4
0.0
0.4
−0.5
0.0
0.5
1.0

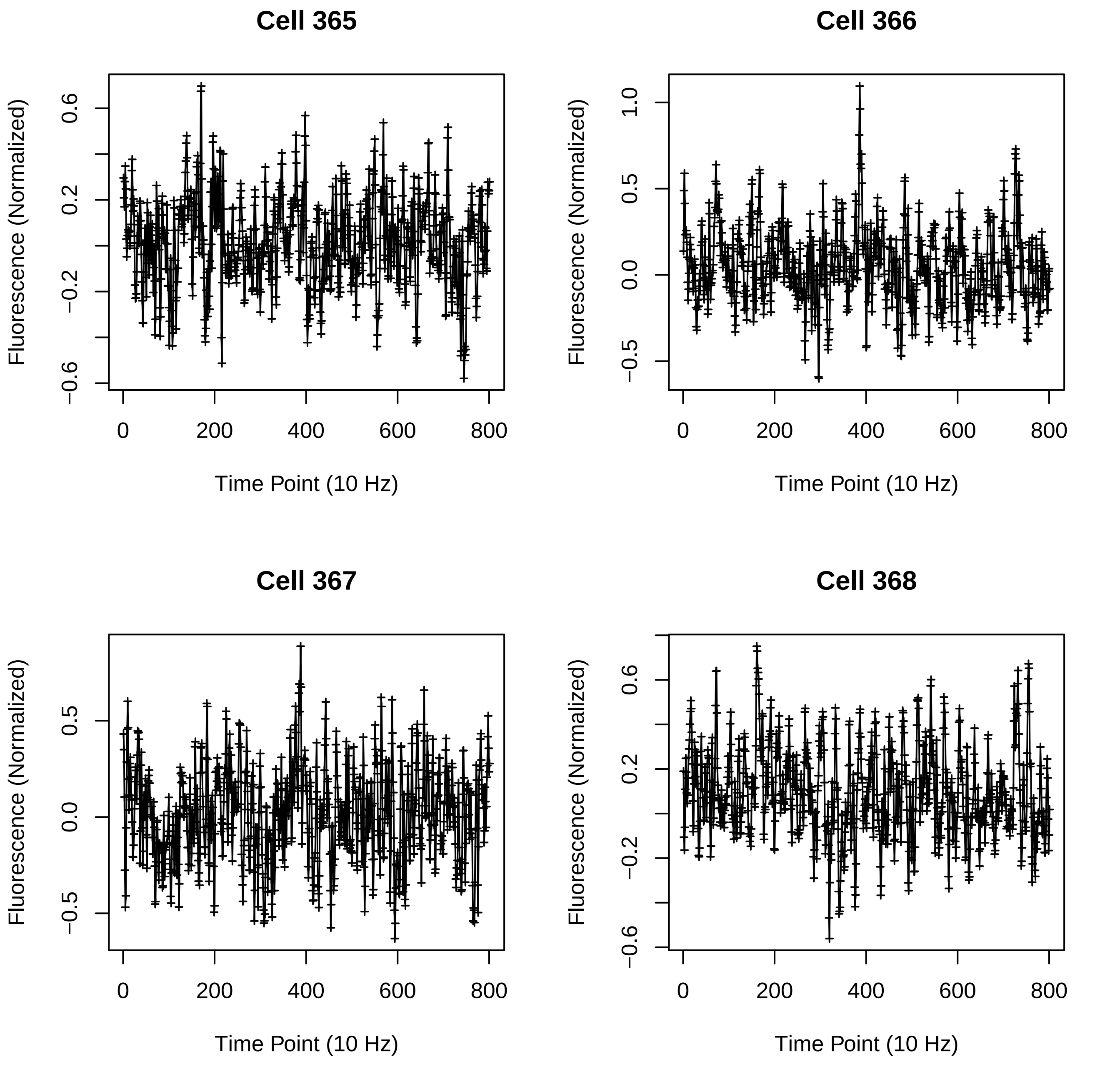

Cell 365
Cell 366
Cell 367
Cell 368
Fluorescence (Normalized)
Time Point (10 Hz)
0
200
400
600
800
−0.6
−0.2
0.2
0.6
−0.5
0.0
0.5
1.0

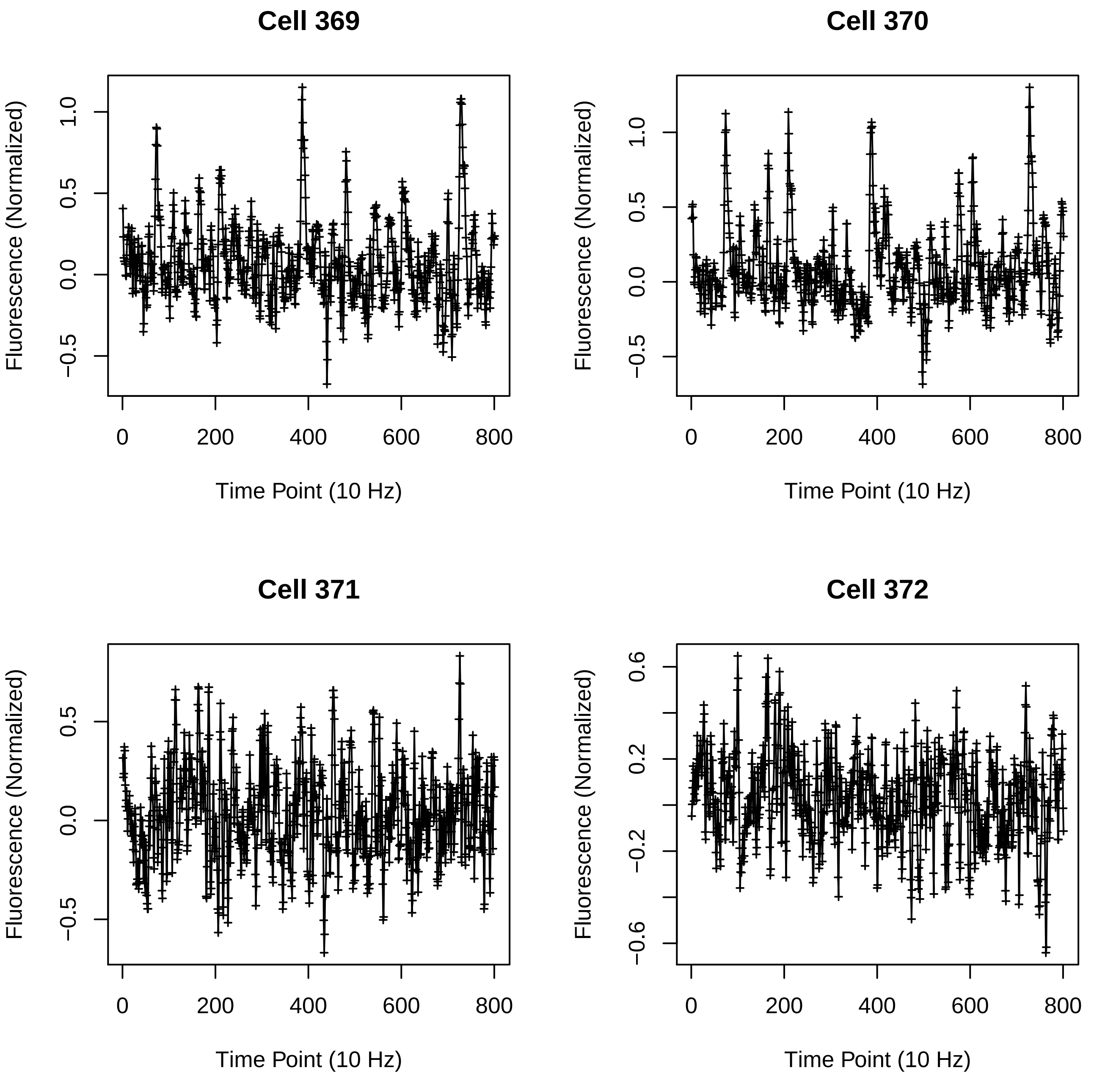
Cell 369
Cell 370
Cell 371
Cell 372
Fluorescence (Normalized)
Time Point (10 Hz)
−0.5
0.0
0.5
1.0
−0.6
−0.2
0.2
0.6
0
200
400
600
800

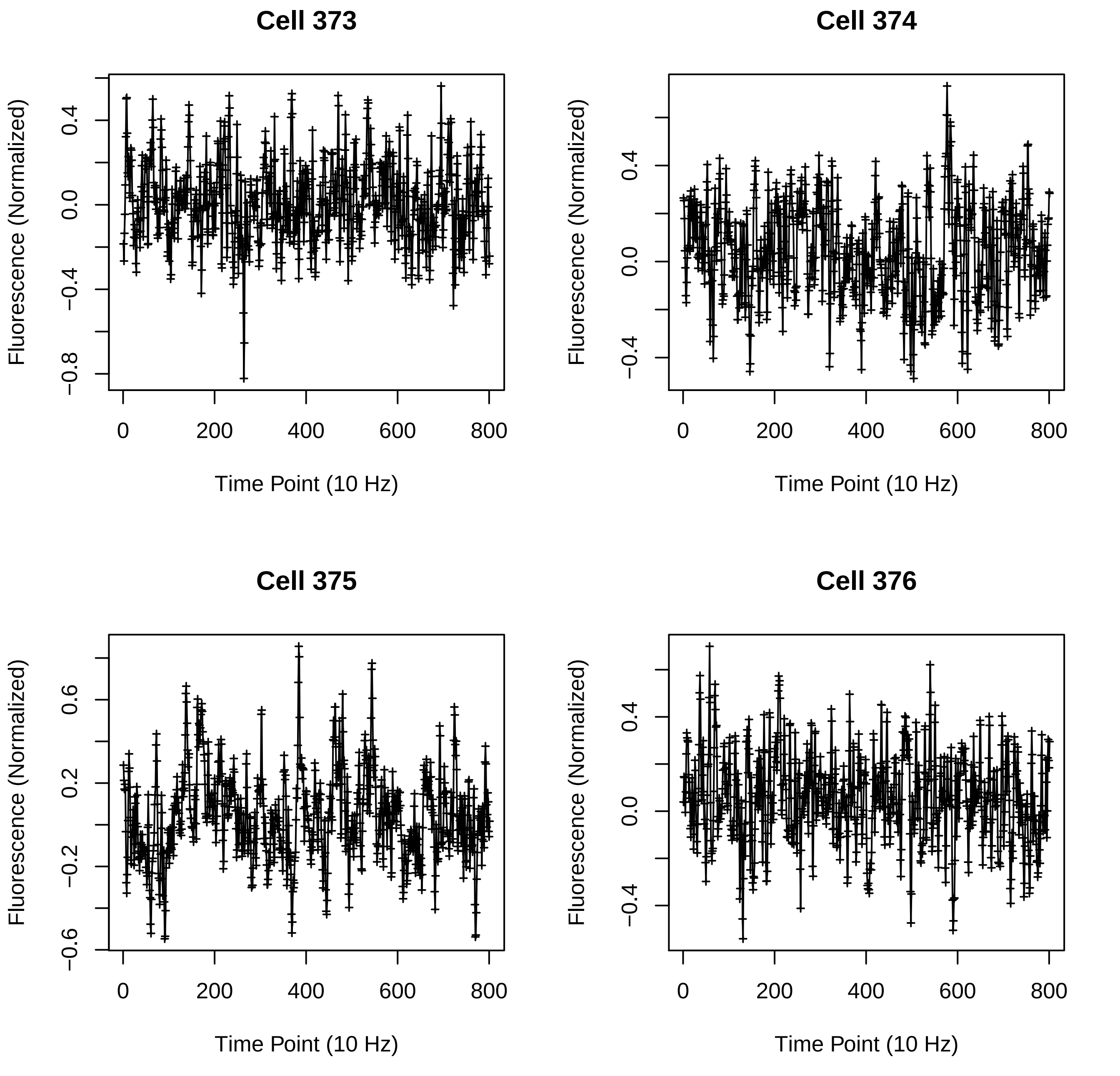
Cell 373
Cell 374
Cell 375
Cell 376
Fluorescence (Normalized)
Time Point (10 Hz)
0
200
400
600
800
0.4
0.0
−0.4
−0.8
0.6
0.2
−0.2
−0.6

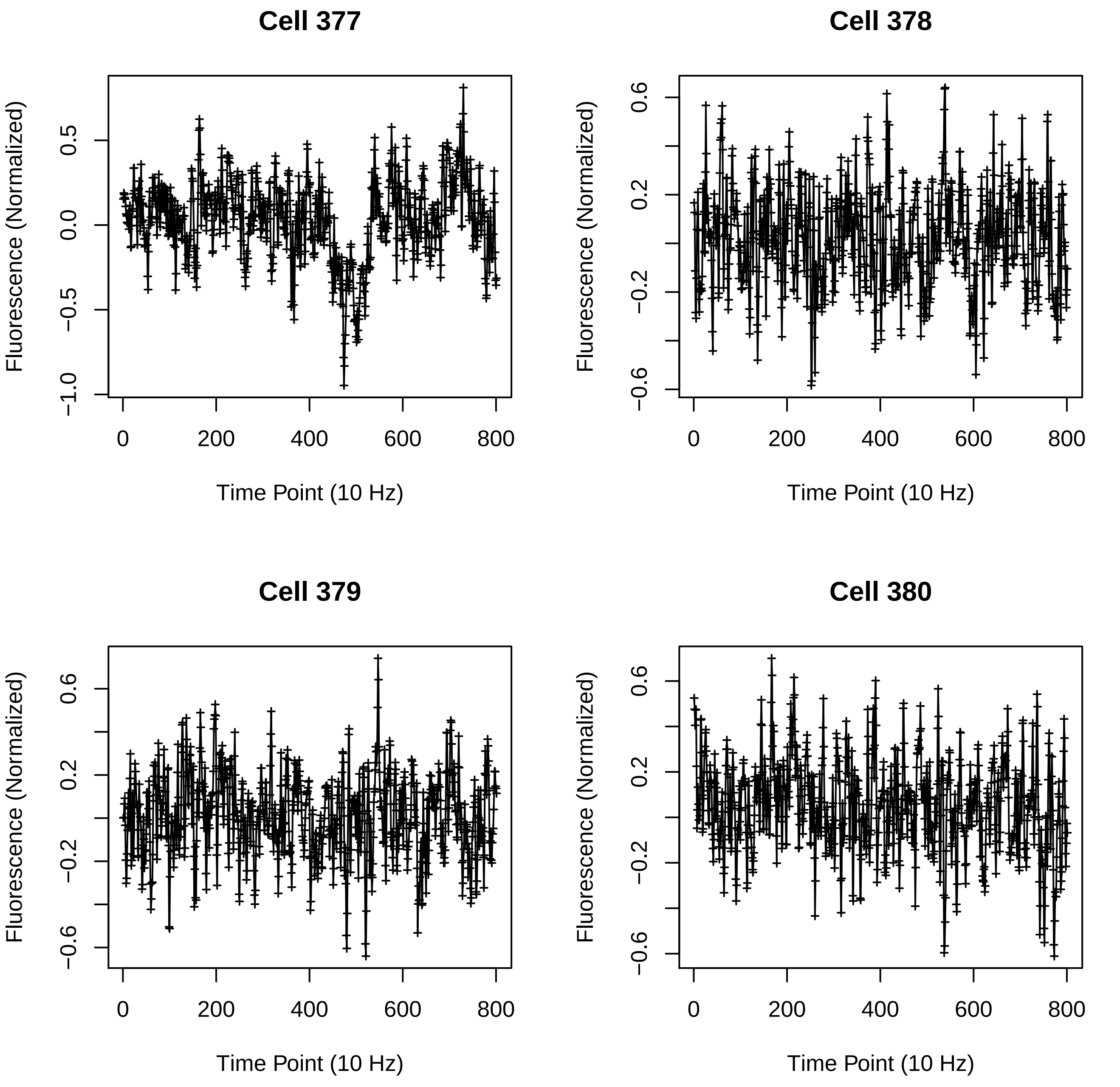

Cell 377
Cell 378
Cell 379
Cell 380
Fluorescence (Normalized)
Time Point (10 Hz)
0
200
400
600
800
0.5
0.0
−0.5
−1.0
0.6
0.2
−0.2
−0.6

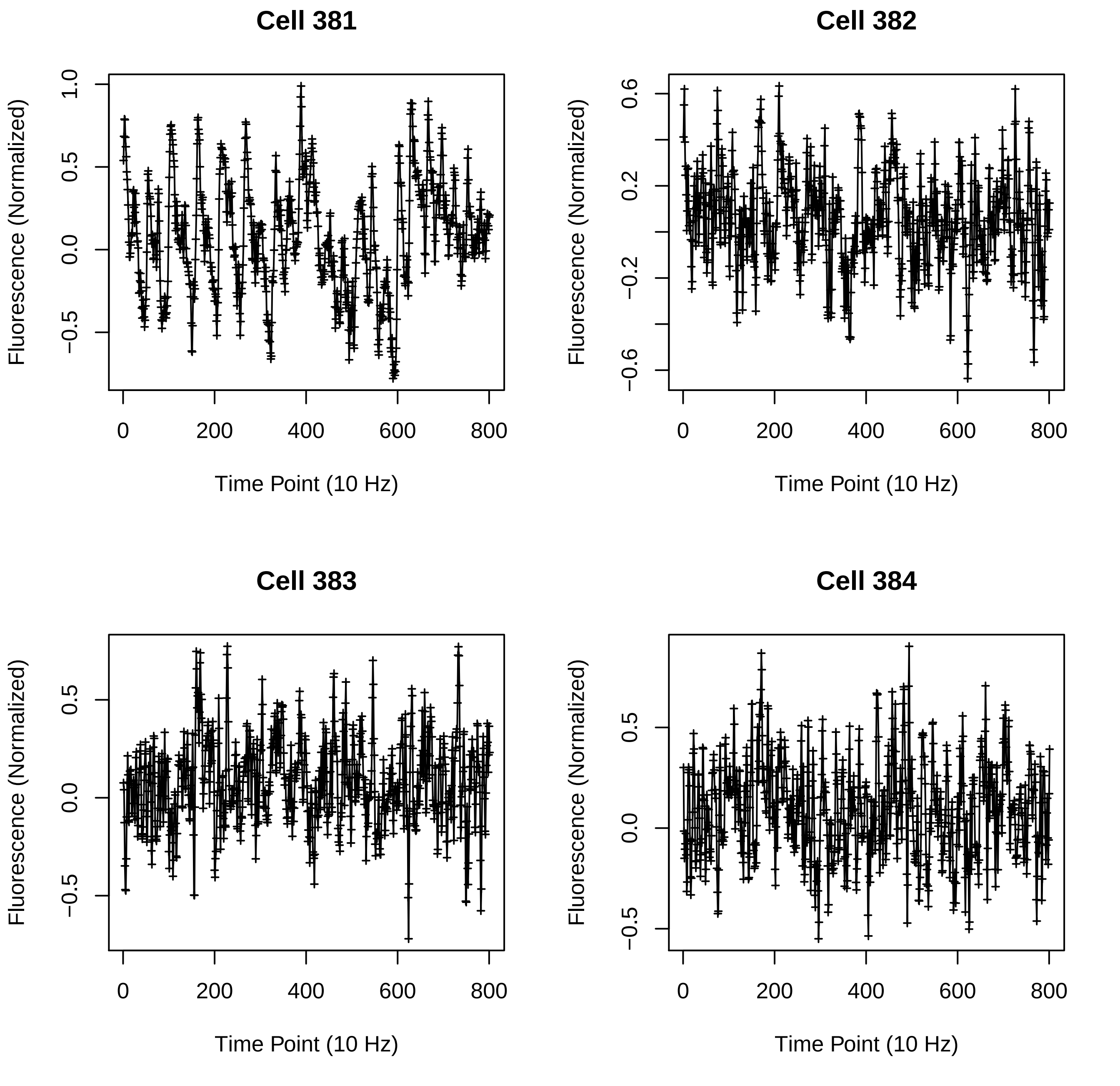
Cell 381
Cell 382
Cell 383
Cell 384
Fluorescence (Normalized)
Time Point (10 Hz)
0
200
400
600
800
1.0
0.5
0.0
−0.5
0.6
0.2
−0.2
−0.6

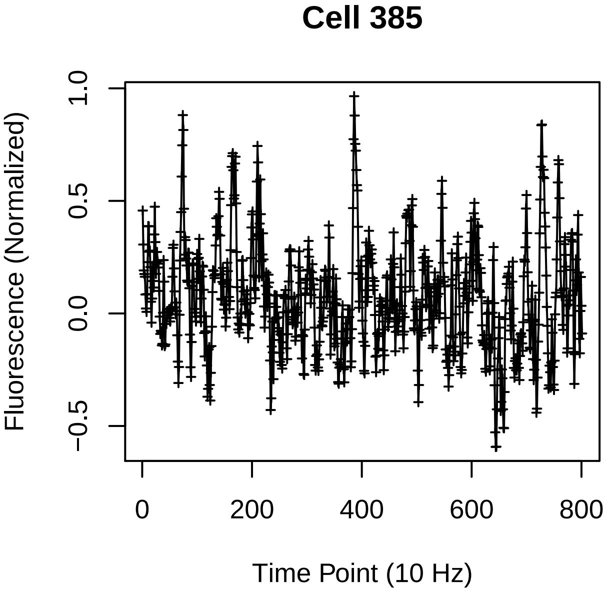
Cell 385
Fluorescence (Normalized)
−0.5
0.0
0.5
1.0
0
200
400
600
800
Time Point (10 Hz)

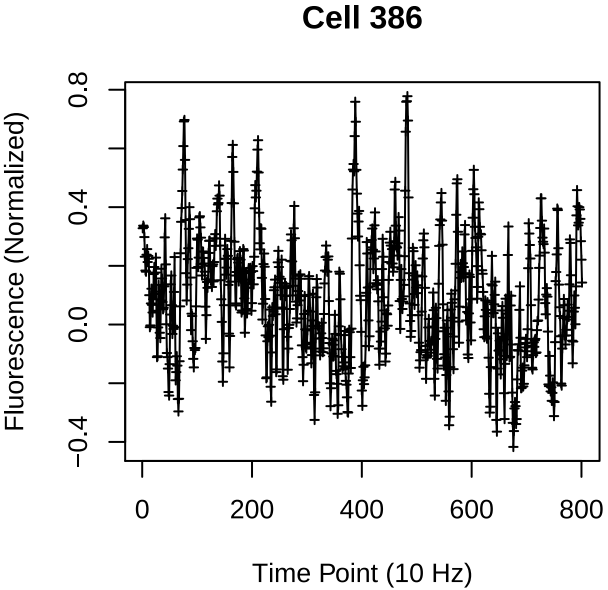
Cell 386
Fluorescence (Normalized)
−0.4
0.0
0.4
0.8
0
200
400
600
800
Time Point (10 Hz)

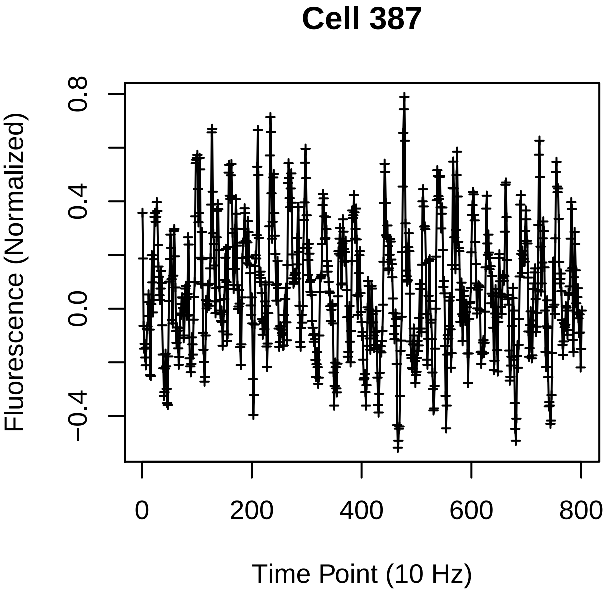
Cell 387
Fluorescence (Normalized)
−0.4
0.0
0.4
0.8
0
200
400
600
800
Time Point (10 Hz)

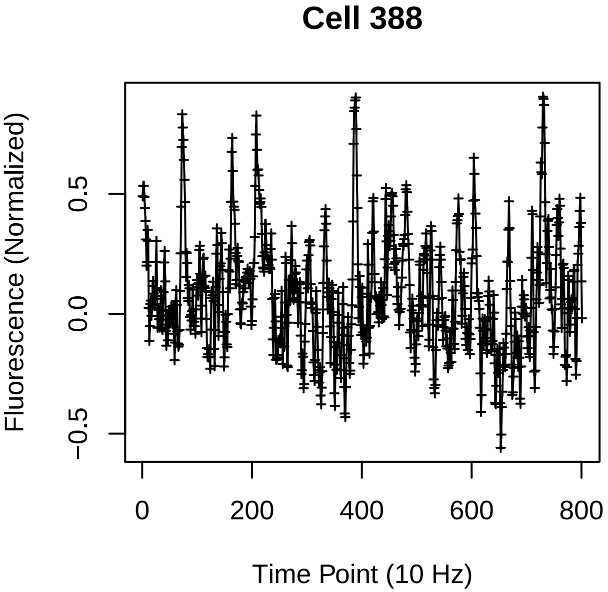
Cell 388
Fluorescence (Normalized)
−0.5
0.0
0.5
0
200
400
600
800
Time Point (10 Hz)

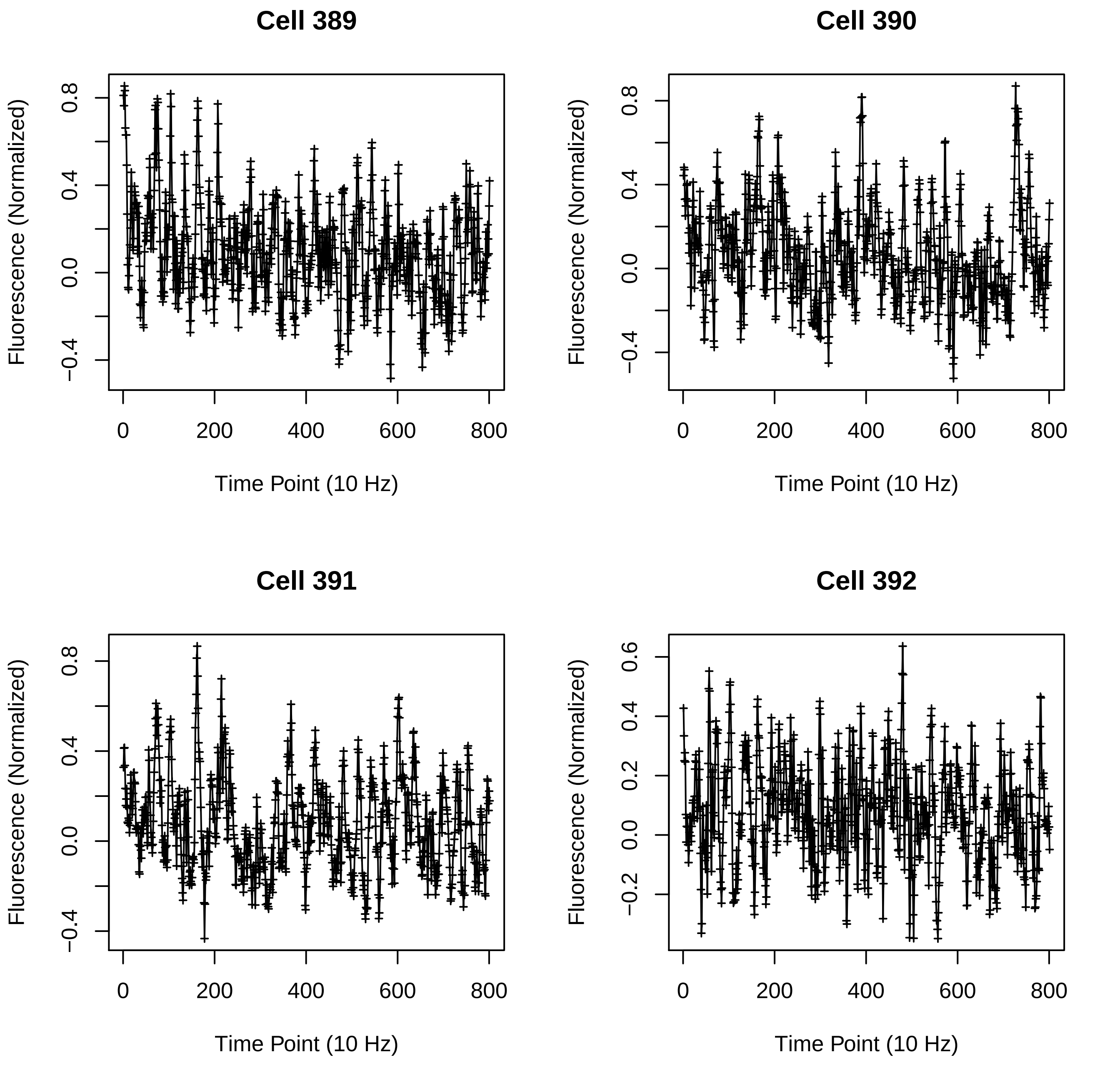

Cell 389
Cell 390
Cell 391
Cell 392
Fluorescence (Normalized)
Time Point (10 Hz)
0
200
400
600
800
−0.4
0.0
0.4
0.8
−0.2
0.2
0.6

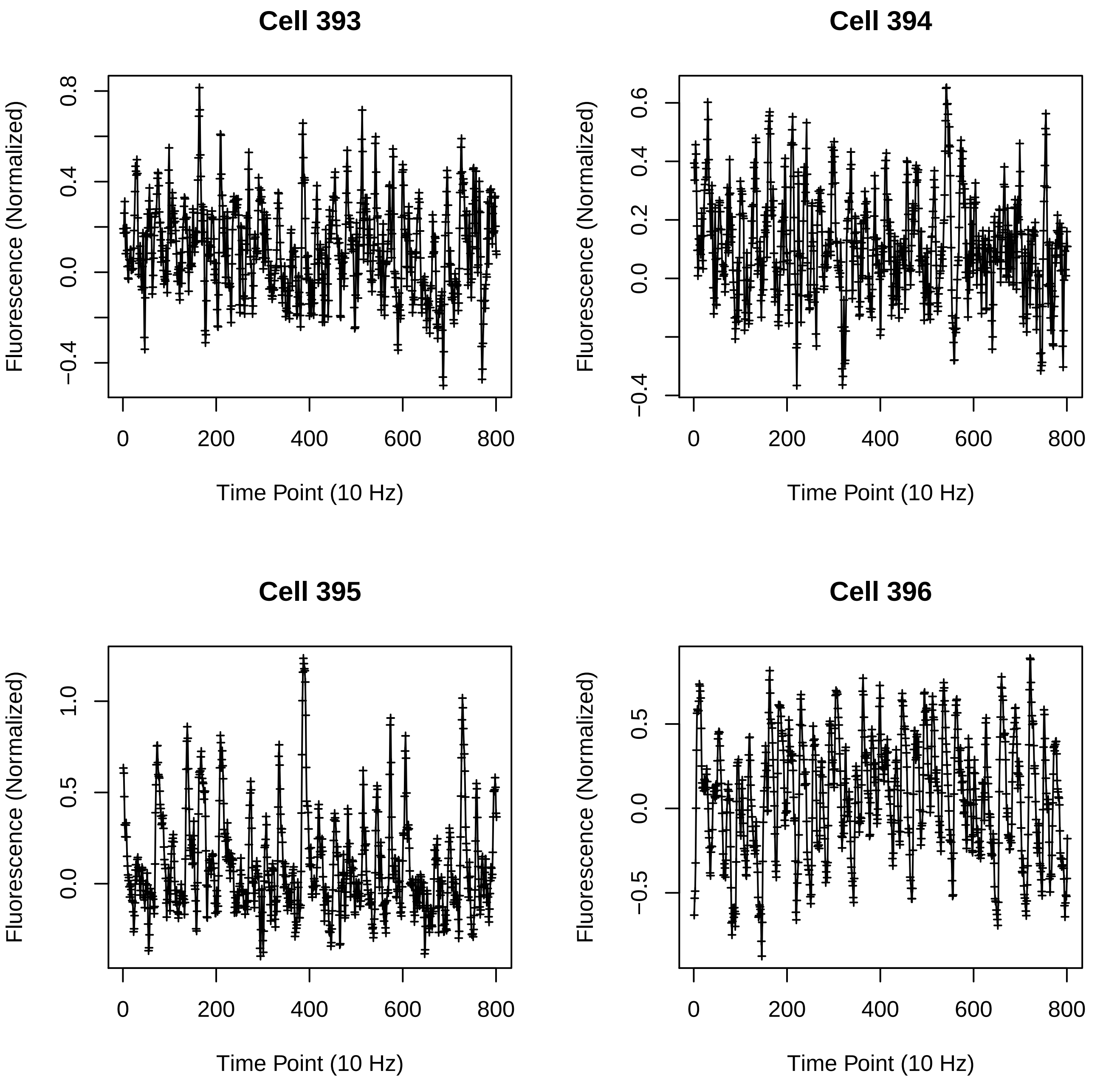
Cell 393
Cell 394
Cell 395
Cell 396
Fluorescence (Normalized)
Time Point (10 Hz)
0
200
400
600
800
−0.4
0.0
0.4
0.8
0.2
0.6
0.5
1.0
−0.5

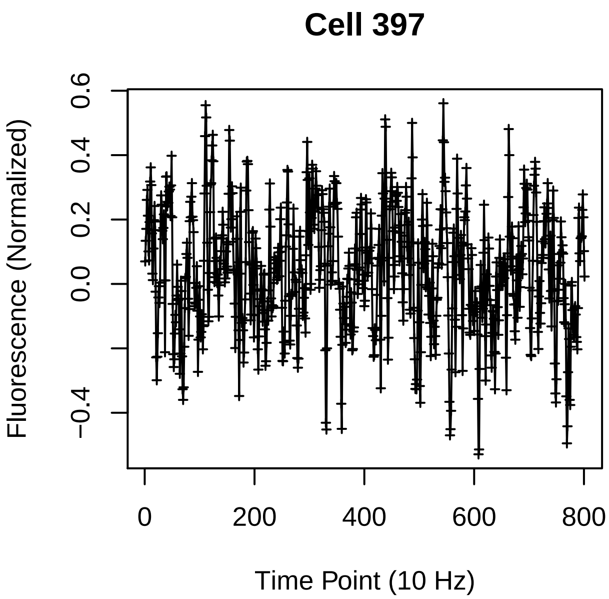

Cell 397
Fluorescence (Normalized)
0.6
0.4
0.2
0.0
−0.4
0
200
400
600
800
Time Point (10 Hz)
