# Supplementary material for: A Markovian Entropy Measure for the Analysis of Calcium Activity Time Series
Source: PLoS One. 2016 Dec 15;11(12):e0168342. doi: 10.1371/journal.pone.0168342 (PMC5158058; doi:10.1371/journal.pone.0168342)
Supplement: S8 File — (PDF) [file pone.0168342.s015.pdf]

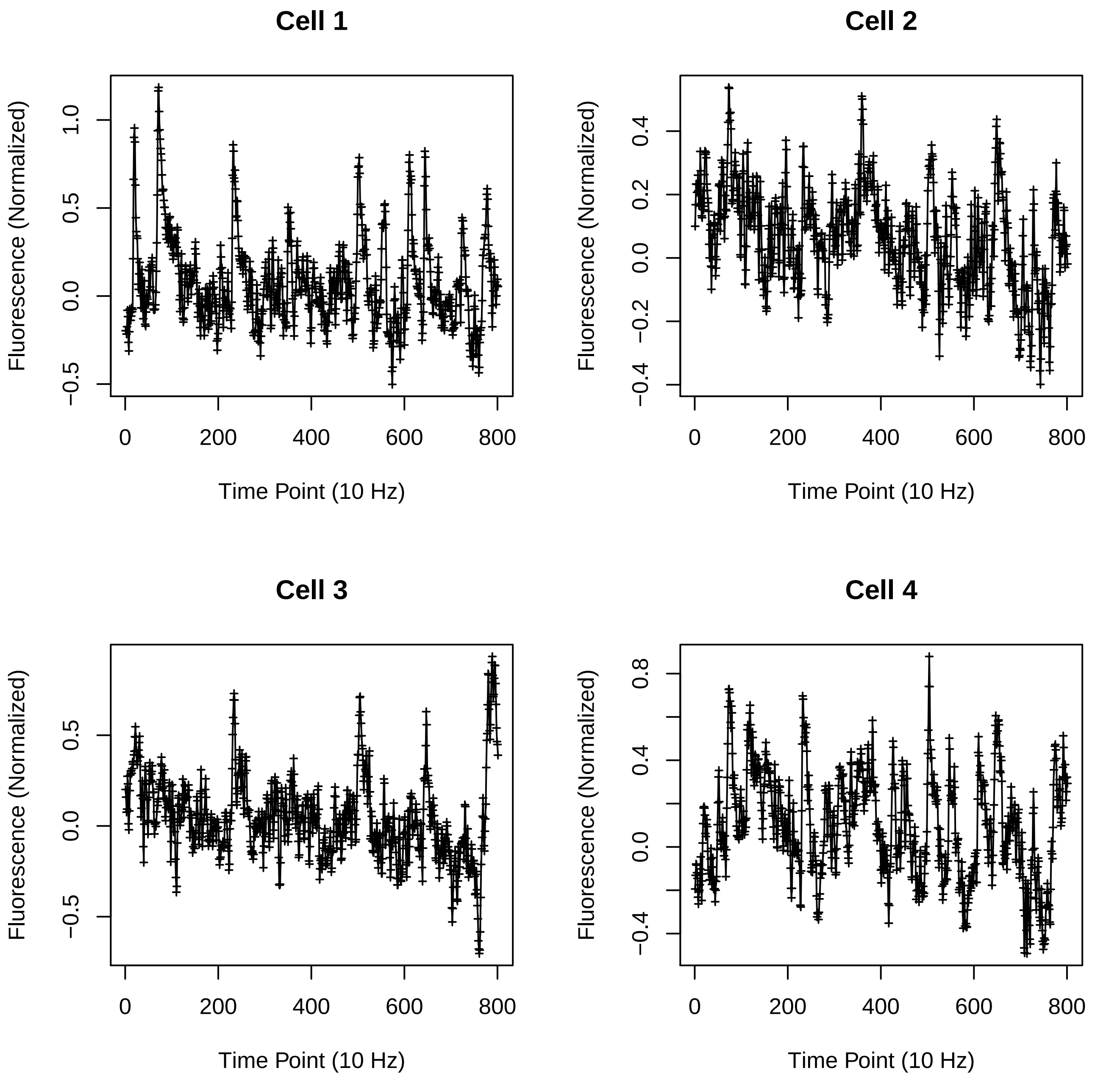

Cell 1
Cell 2
Cell 3
Cell 4
Fluorescence (Normalized)
Time Point (10 Hz)
0
200
400
600
800
−0.5
0.0
0.5
1.0
−0.4
−0.2
0.0
0.2
0.4
0.8

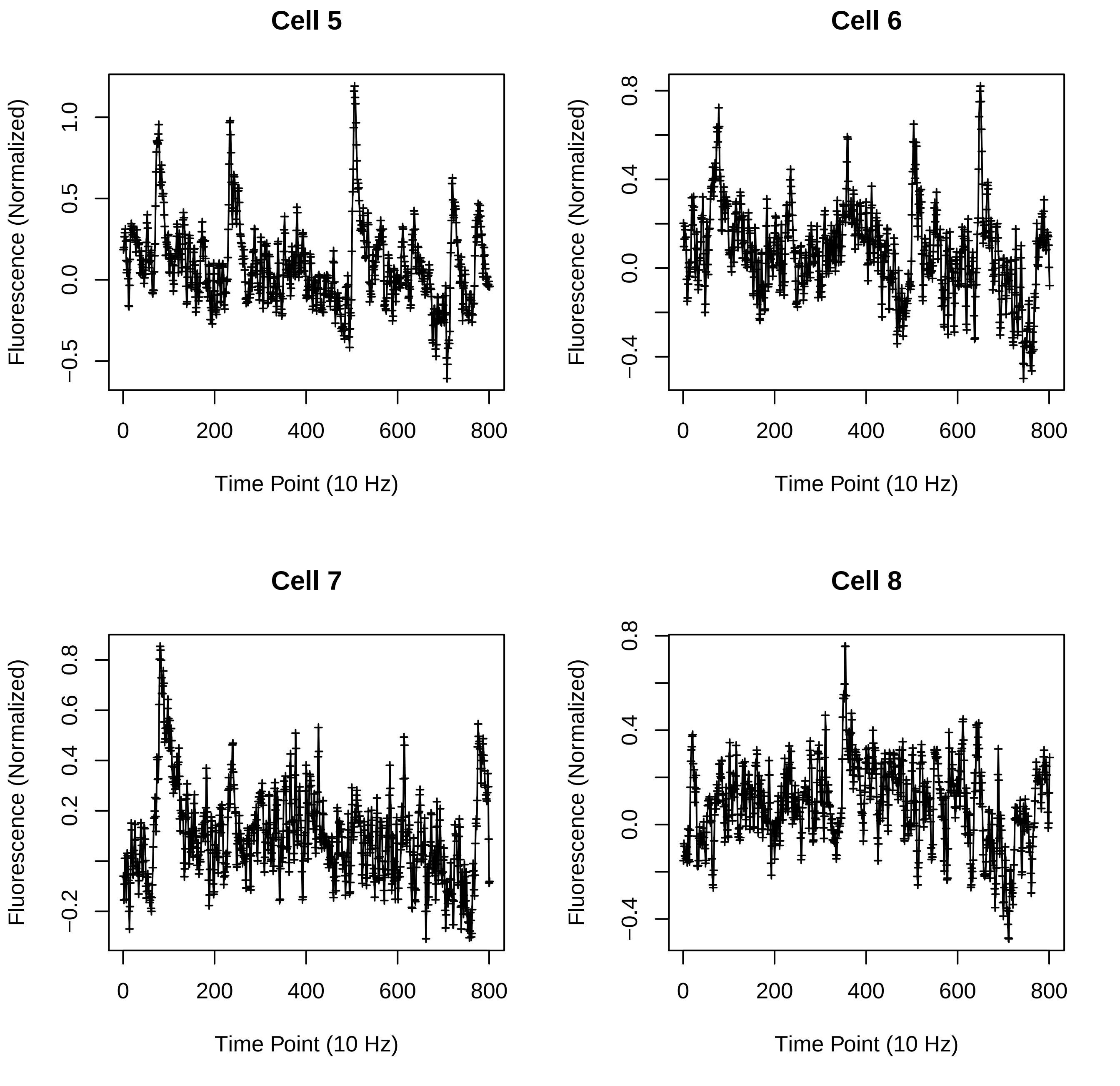
Cell 5
Cell 6
Cell 7
Cell 8
Fluorescence (Normalized)
Time Point (10 Hz)
0
200
400
600
800
1.0
0.5
0.0
−0.5
0.8
0.4
−0.4
0.6
0.2
−0.2

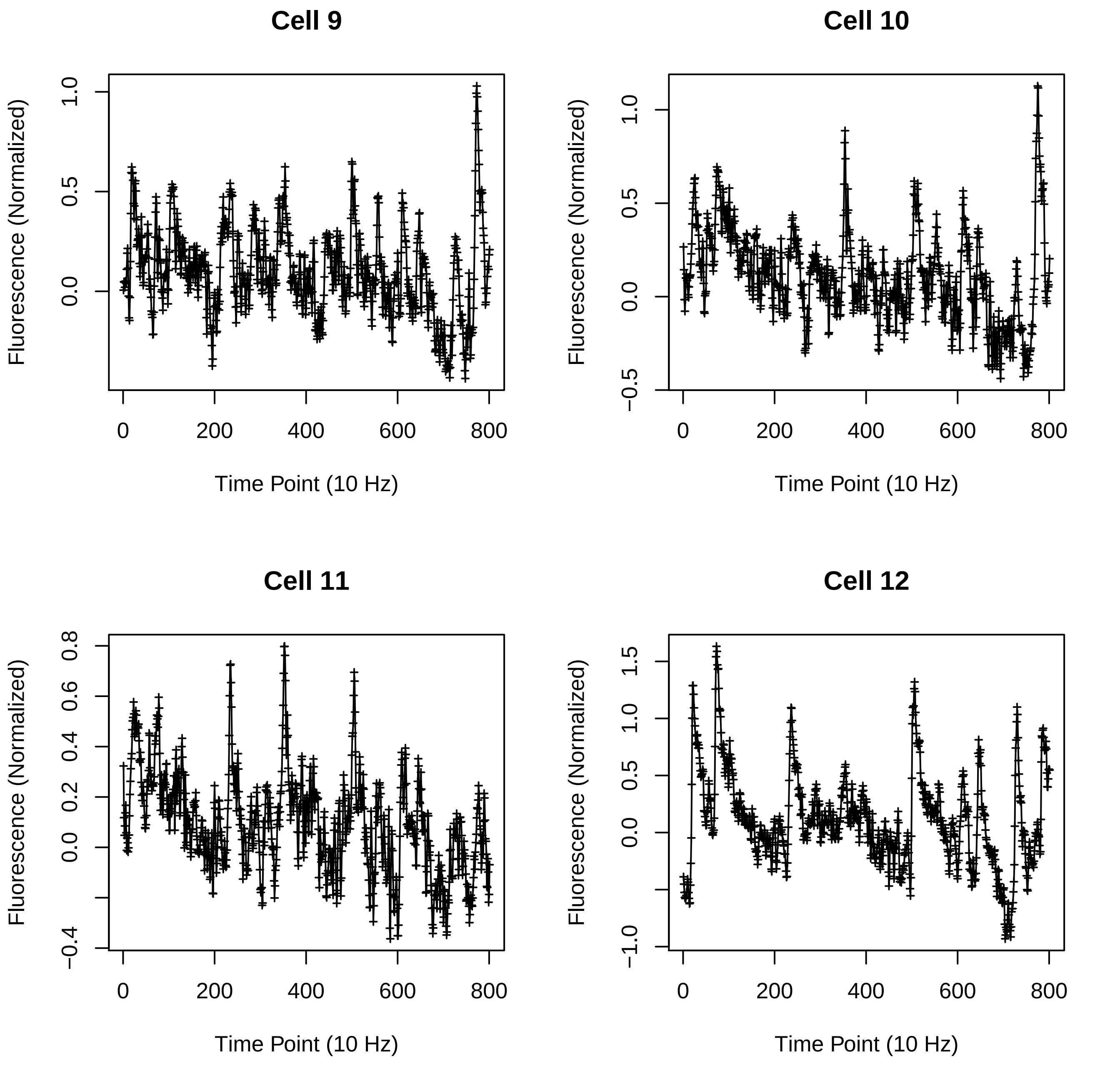
Cell 9
Cell 10
Cell 11
Cell 12
Fluorescence (Normalized)
Time Point (10 Hz)
0
200
400
600
800
1.0
0.5
0.0
−0.5
0.8
0.6
0.4
0.2
0.0
−0.4
1.5
1.0
0.5
0.0
−1.0

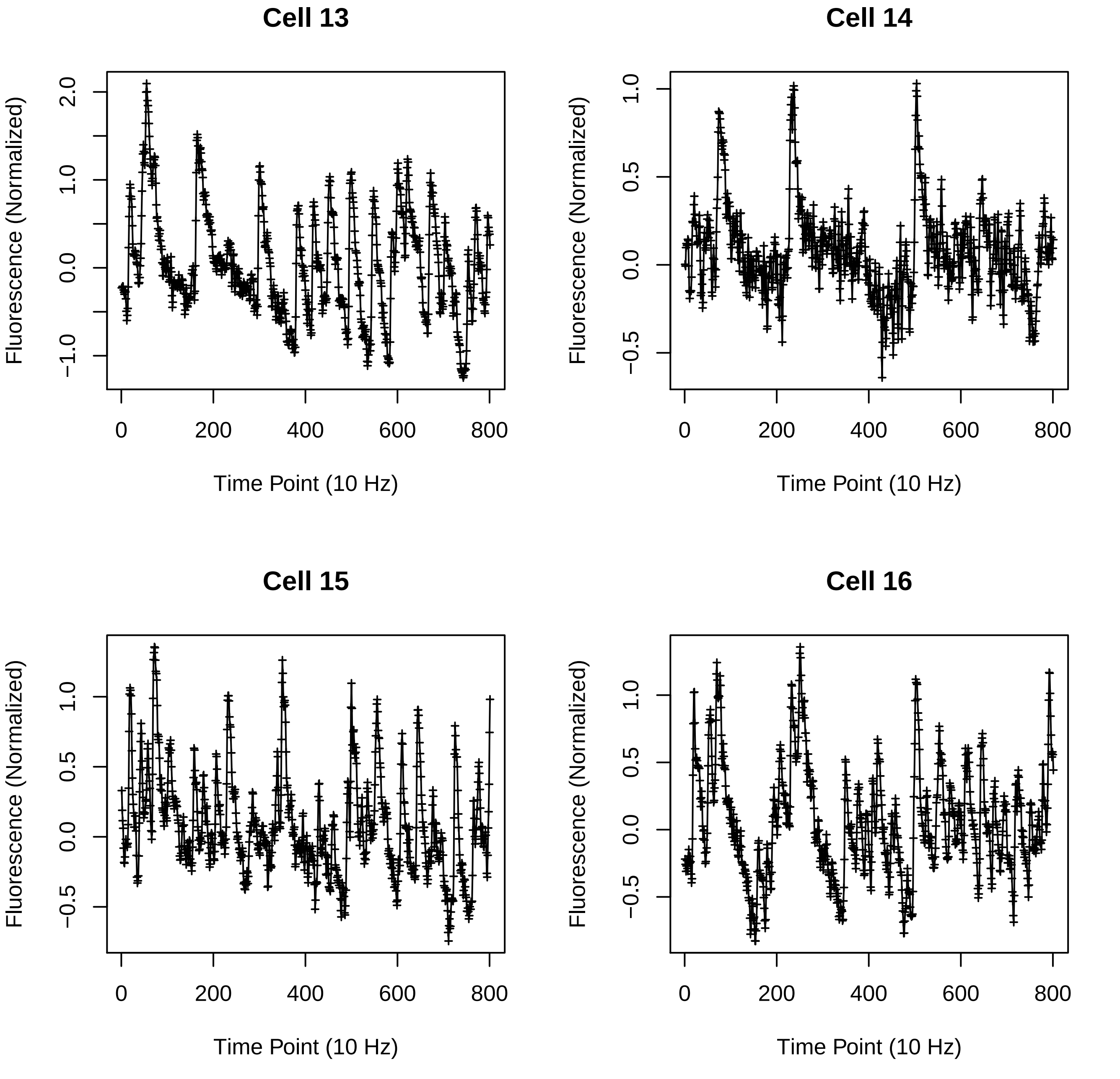
Cell 13
Cell 14
Cell 15
Cell 16
Fluorescence (Normalized)
Time Point (10 Hz)
-1.0
0.0
1.0
2.0
-0.5
0.0
0.5
1.0
0
200
400
600
800

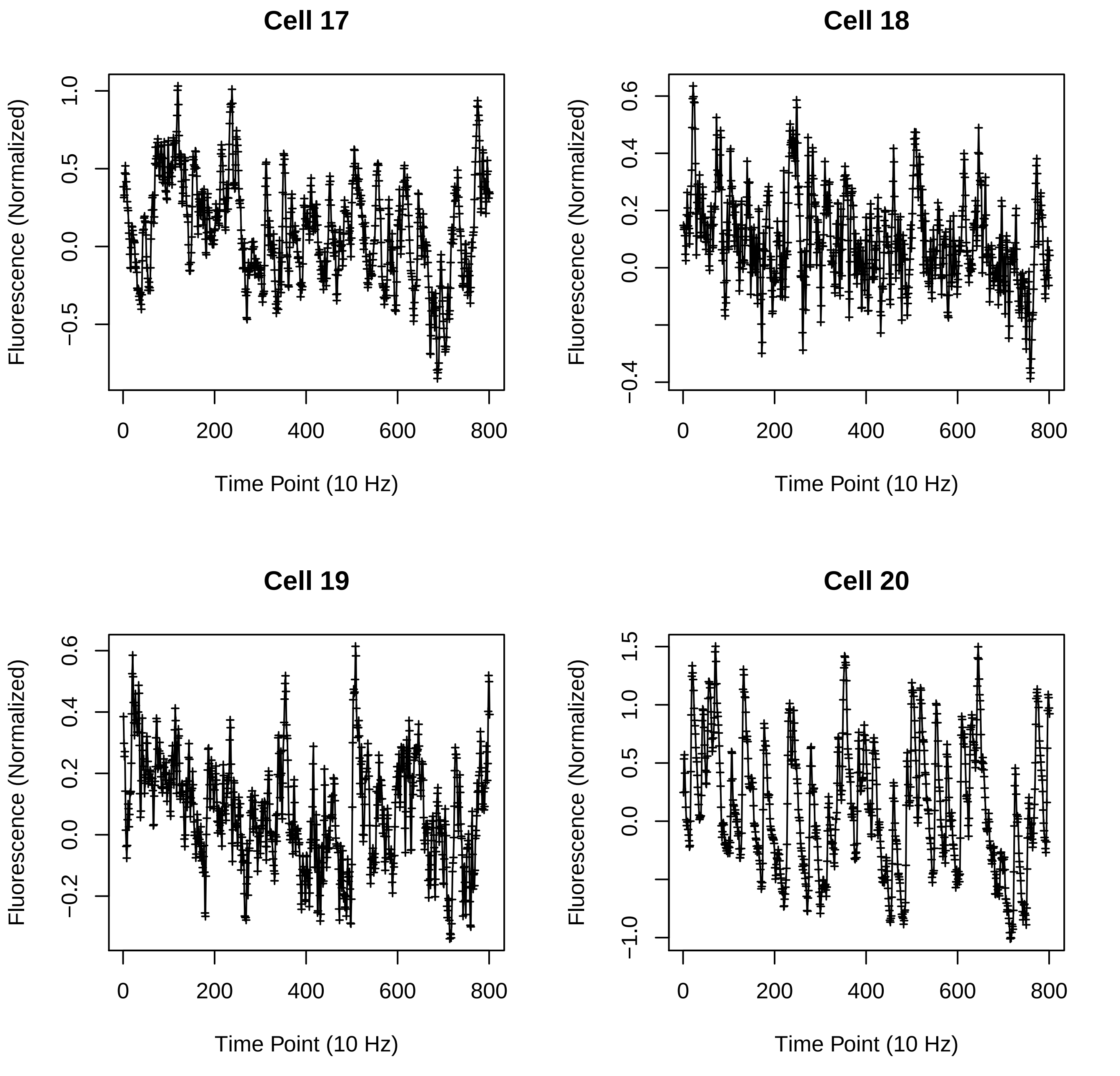
Cell 17
Cell 18
Cell 19
Cell 20
Fluorescence (Normalized)
Time Point (10 Hz)
0
200
400
600
800
1.0
0.5
0.0
−0.5
0.6
0.4
0.2
−0.2
−0.4
1.5
−1.0

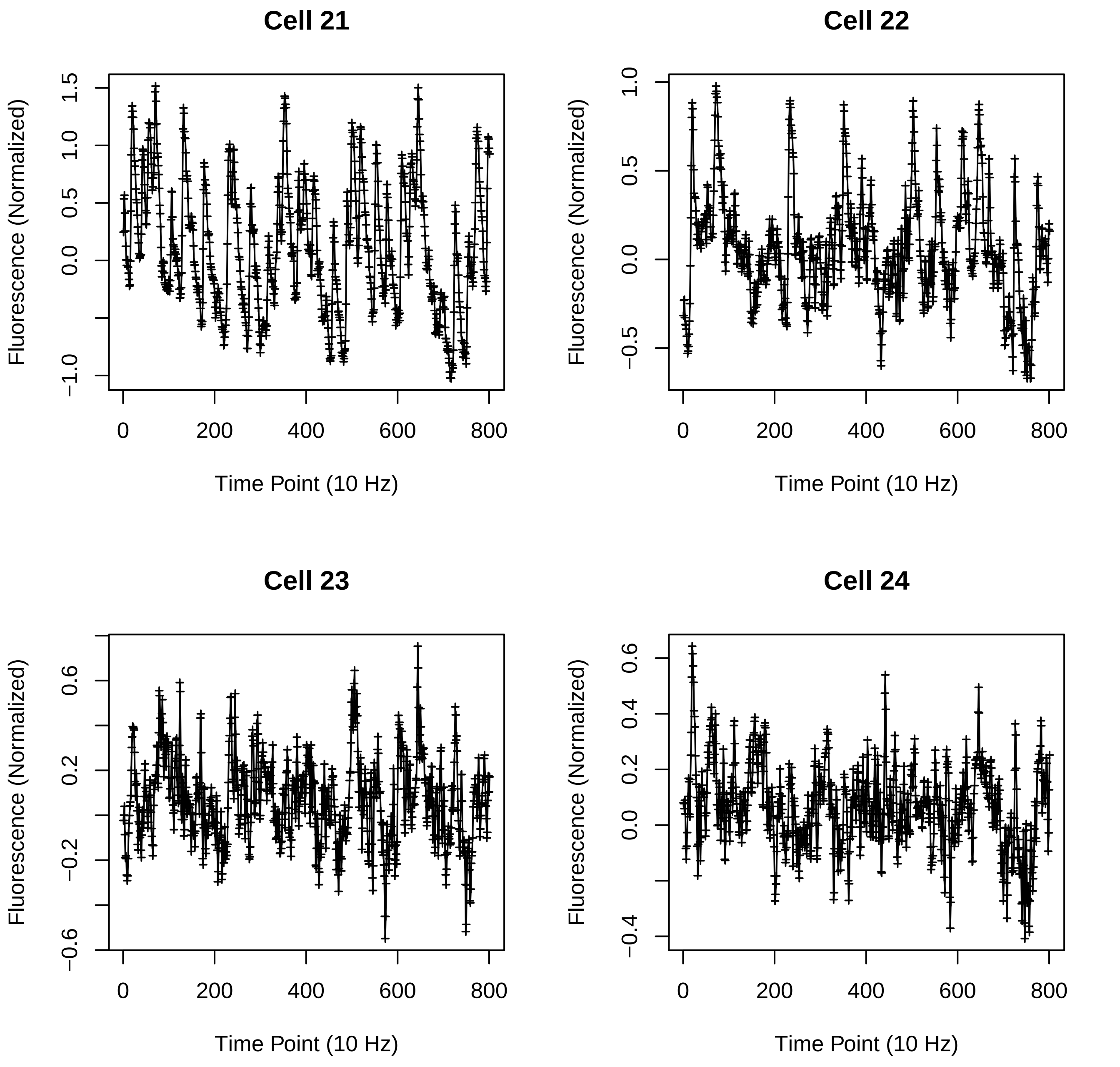
Cell 21
Cell 22
Cell 23
Cell 24
Fluorescence (Normalized)
Time Point (10 Hz)
0
200
400
600
800
1.5
1.0
0.5
0.0
−1.0
−0.5
0.6
0.2
−0.2
−0.6
0.4
−0.4

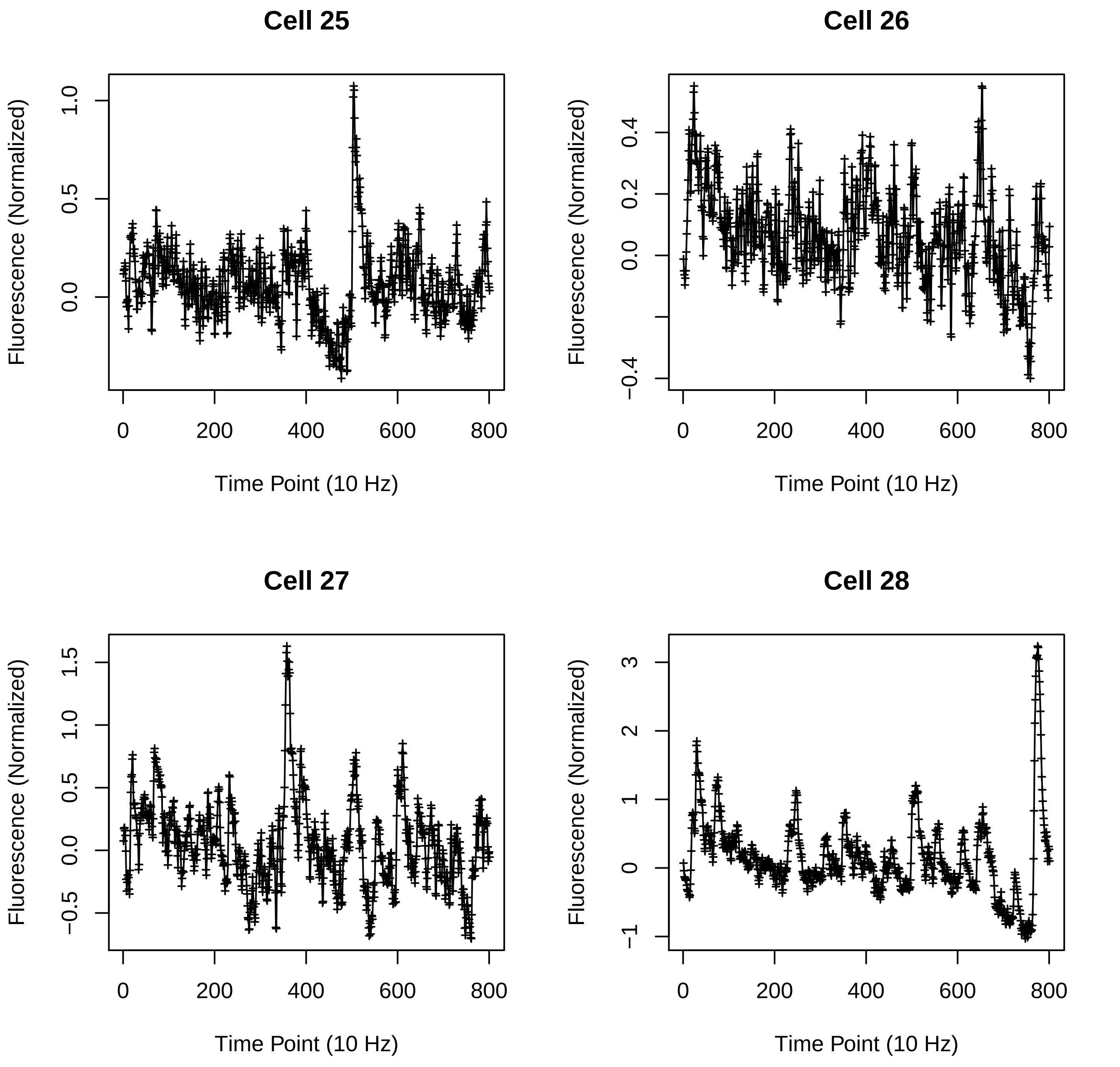

Cell 25
Fluorescence (Normalized)
Time Point (10 Hz)
Cell 26
Fluorescence (Normalized)
Time Point (10 Hz)
Cell 27
Fluorescence (Normalized)
Time Point (10 Hz)
Cell 28
Fluorescence (Normalized)
Time Point (10 Hz)

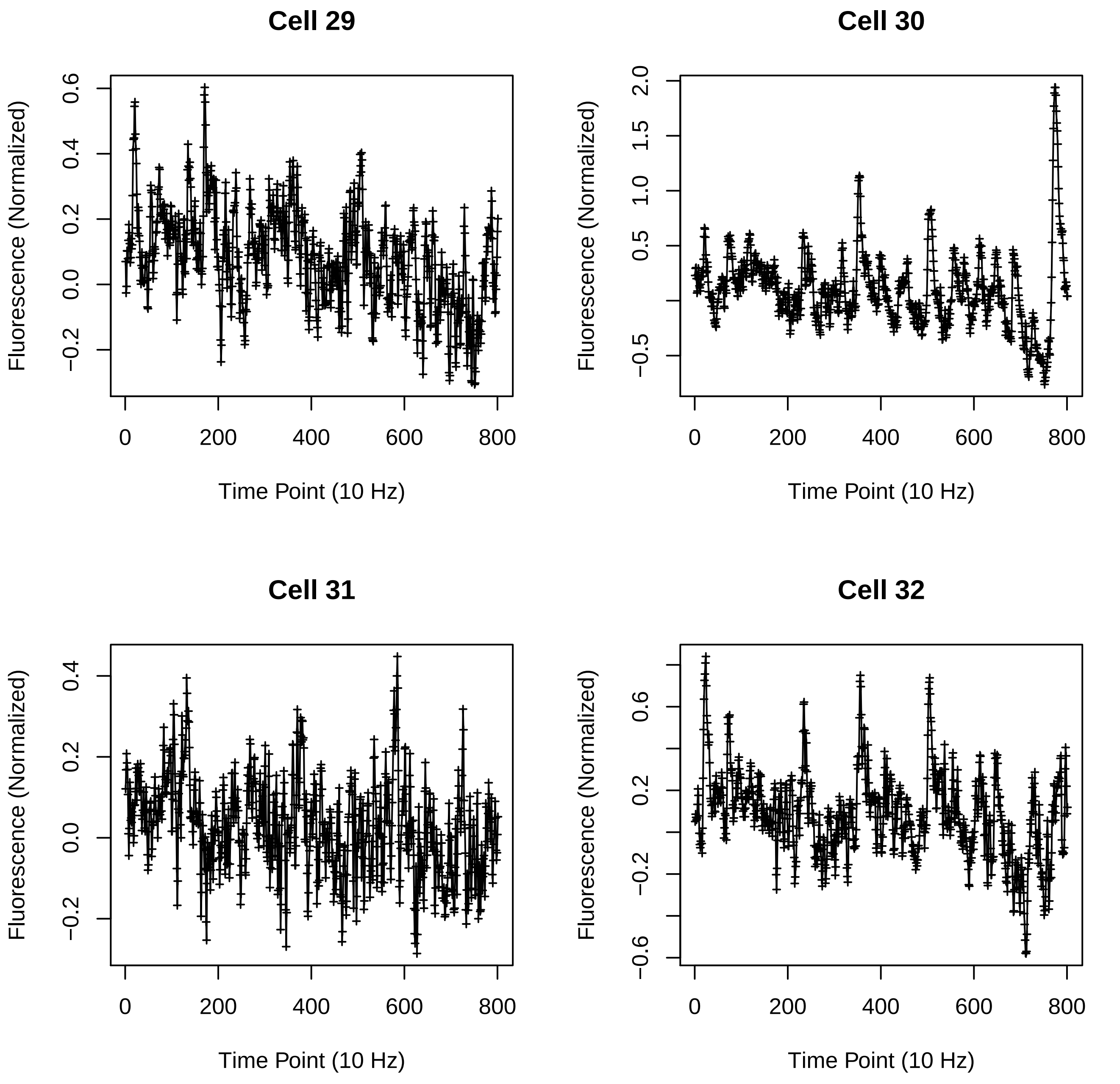
Cell 29
Cell 30
Cell 31
Cell 32
Fluorescence (Normalized)
Time Point (10 Hz)
0
200
400
600
800
−0.2
0.0
0.2
0.4
0.6
−0.5
0.5
1.0
1.5
2.0
−0.6
−0.2
0.2
0.6

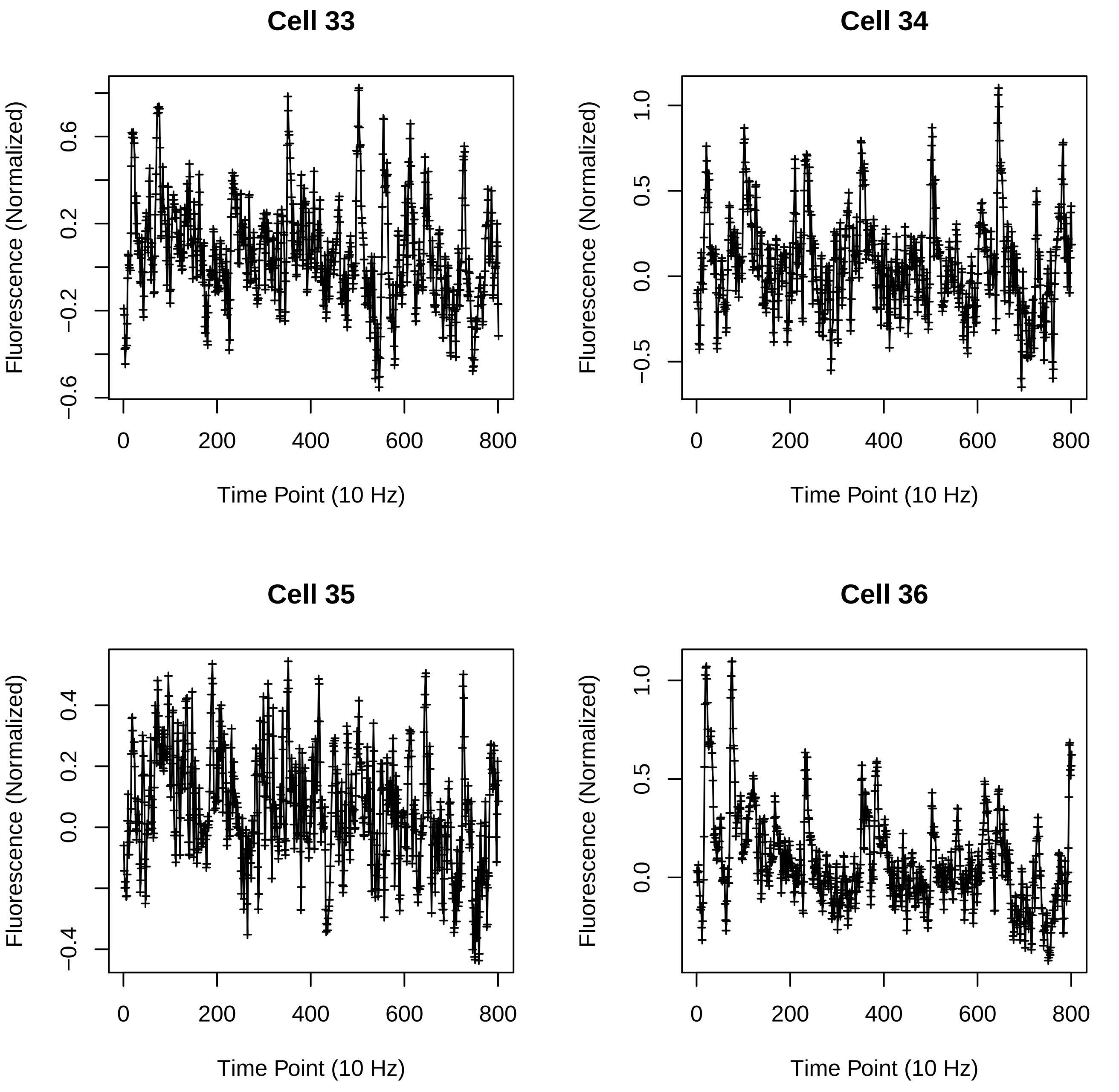
Cell 33
Cell 34
Cell 35
Cell 36
Fluorescence (Normalized)
Time Point (10 Hz)
0
200
400
600
800
−0.6
−0.2
0.2
0.6
−0.5
0.0
0.5
1.0
−0.4
0.0
0.2
0.4

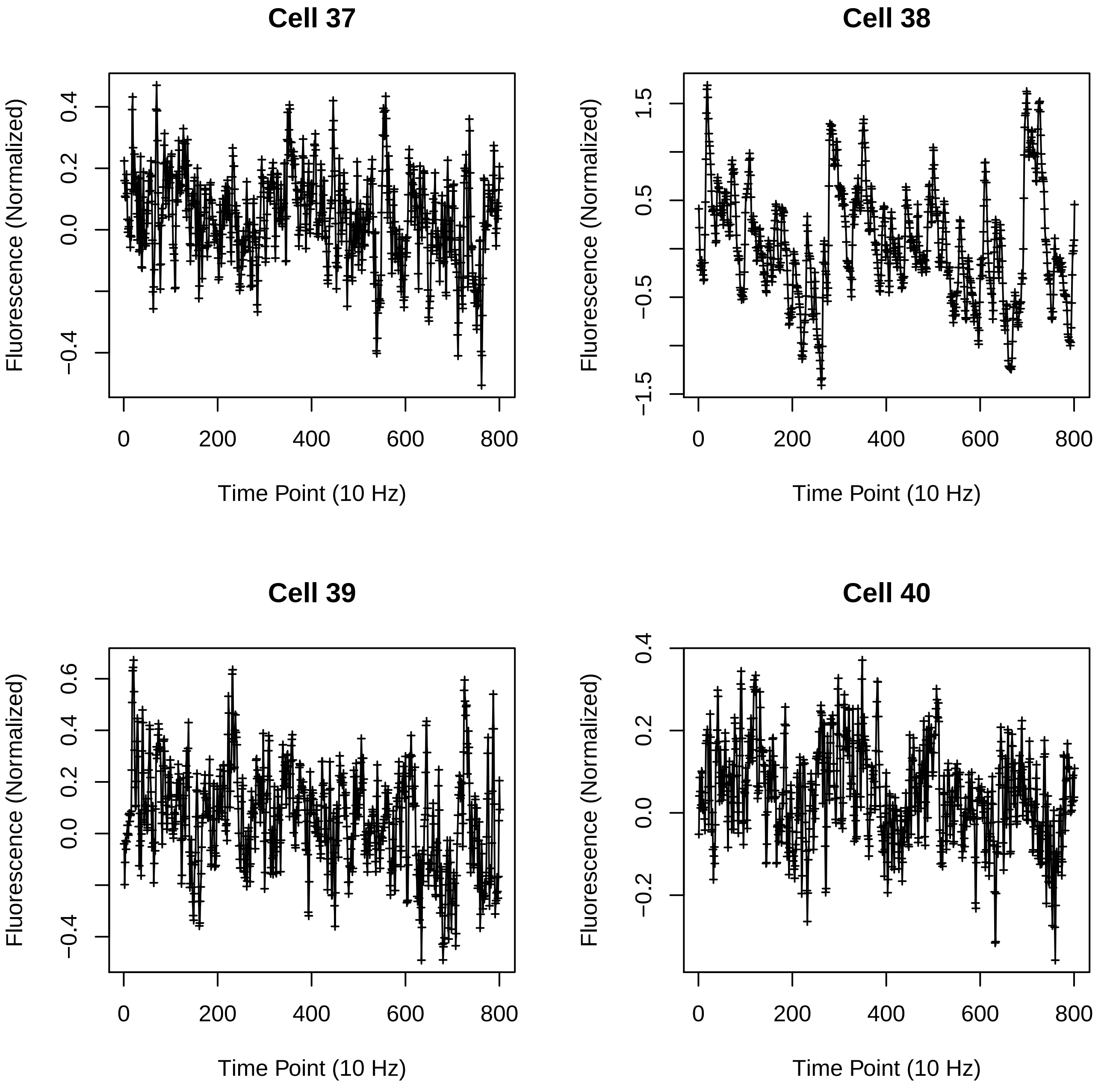
Cell 37
Cell 38
Cell 39
Cell 40
Fluorescence (Normalized)
Time Point (10 Hz)
0
200
400
600
800
−0.4
0.0
0.2
0.4
−1.5
−0.5
0.5
1.5
0.6
−0.2

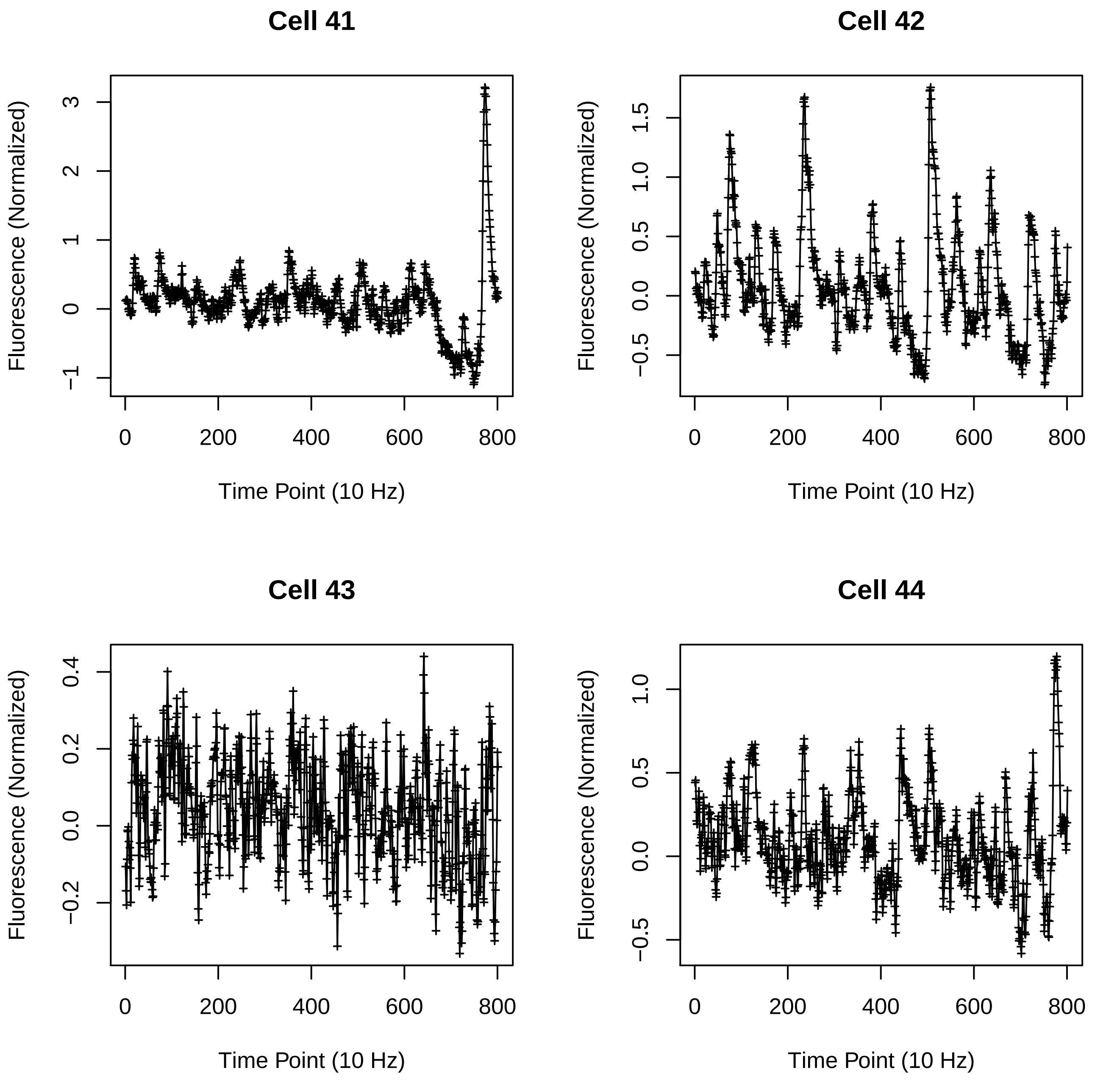

Cell 41
Cell 42
Cell 43
Cell 44
Fluorescence (Normalized)
Time Point (10 Hz)

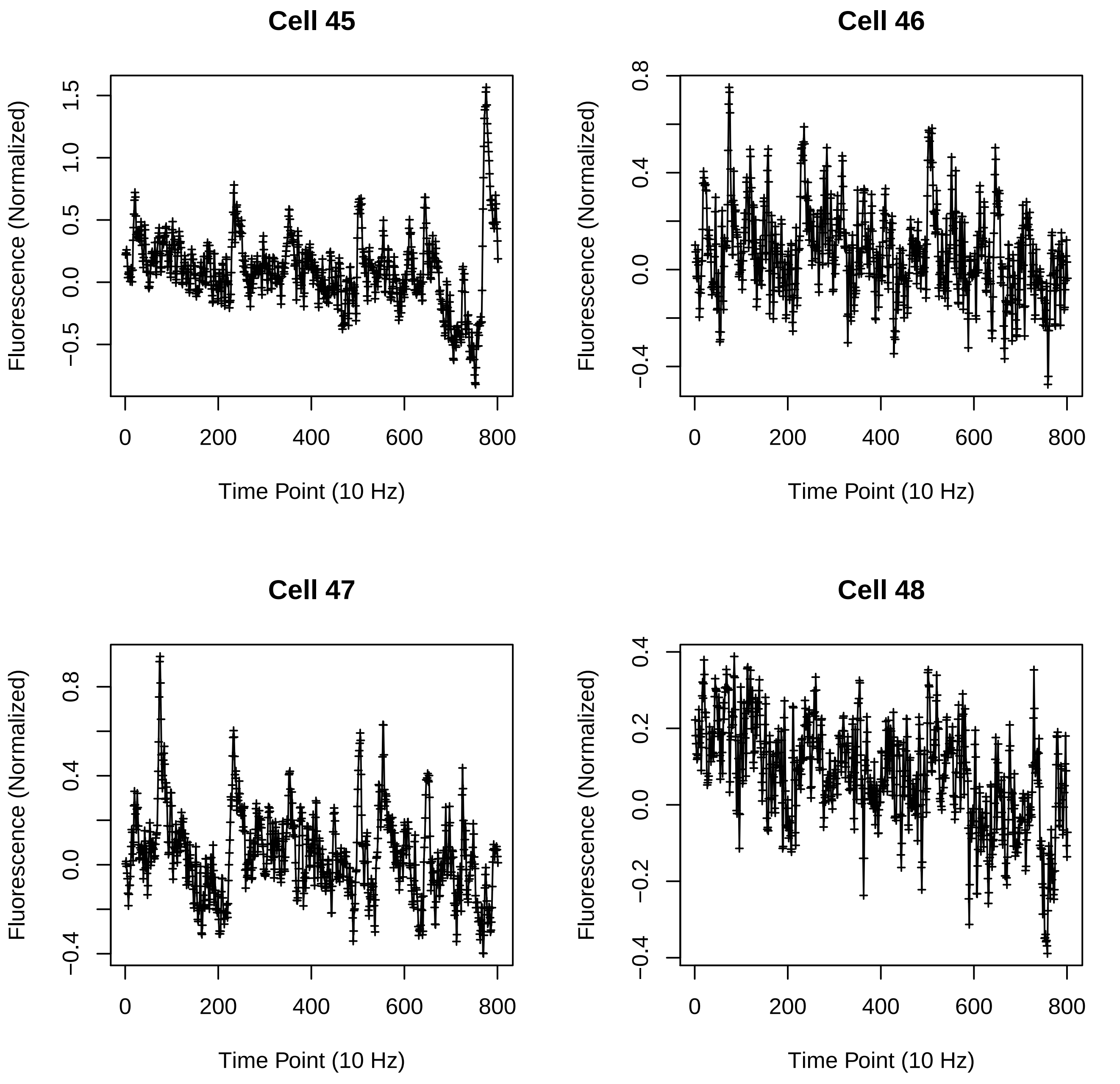

Cell 45
Cell 46
Cell 47
Cell 48
Fluorescence (Normalized)
Time Point (10 Hz)
0
200
400
600
800
−0.5
0.0
0.5
1.0
1.5
−0.4
0.0
0.4
0.8
−0.2
0.2

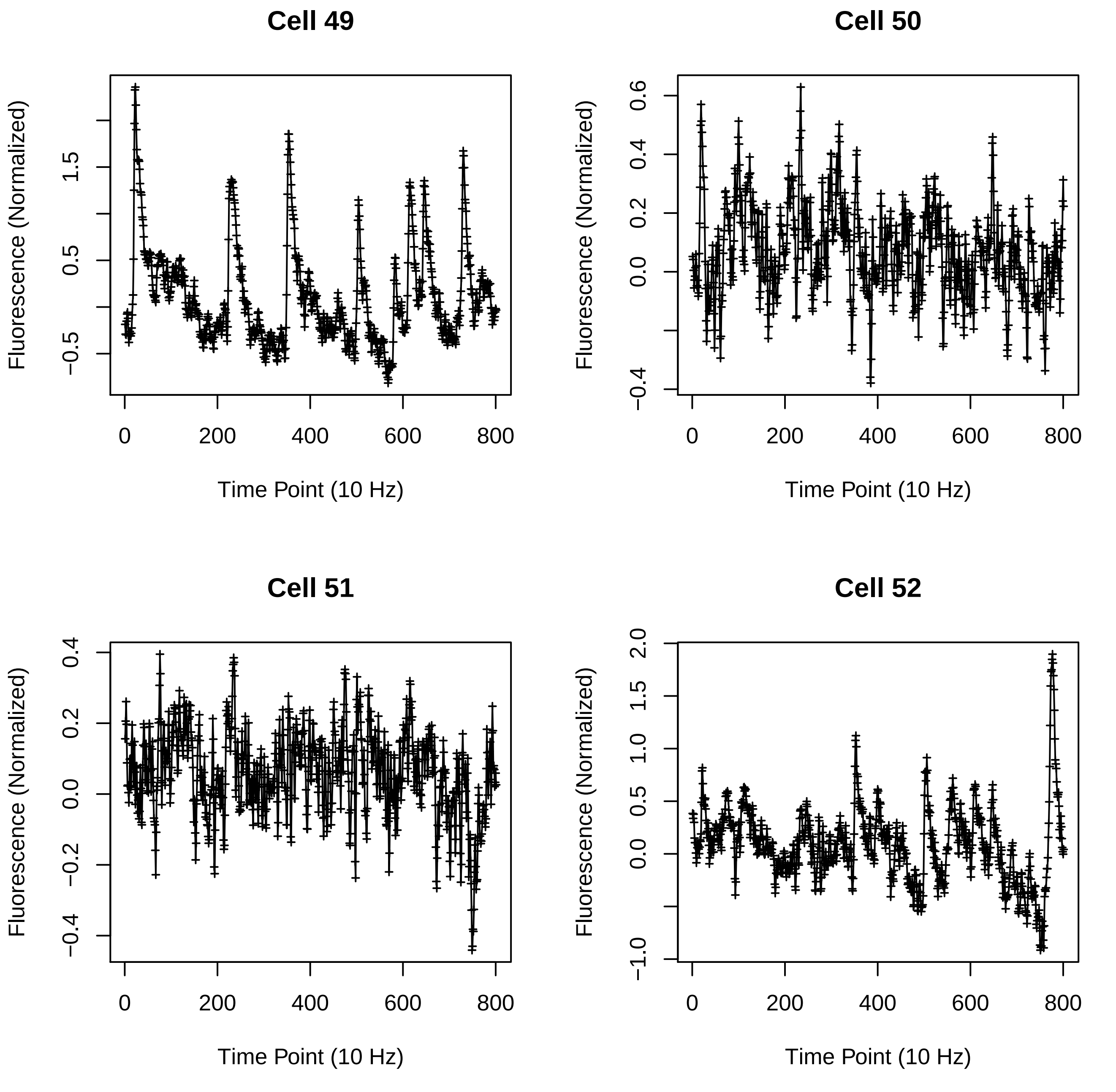

Cell 49
Cell 50
Cell 51
Cell 52
Fluorescence (Normalized)
Time Point (10 Hz)
0
200
400
600
800
−0.5
0.5
1.5
−0.4
0.0
0.2
0.4
0.6
−0.2
−1.0
1.0
2.0

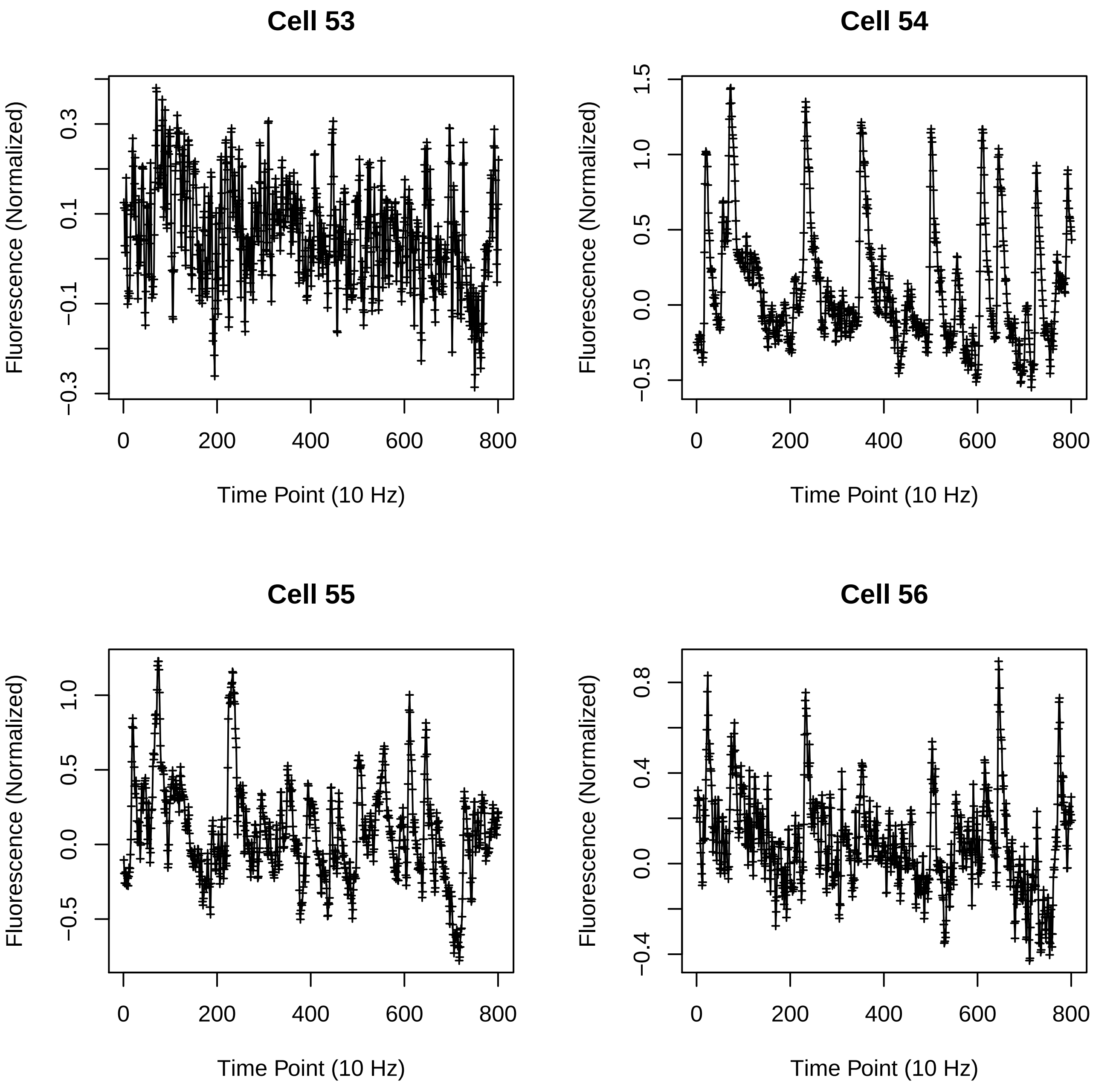
Cell 53
Cell 54
Cell 55
Cell 56
Fluorescence (Normalized)
Time Point (10 Hz)
0
200
400
600
800
0.3
0.1
−0.1
−0.3
1.5
1.0
0.5
0.0
−0.5
0.8
0.4
−0.4

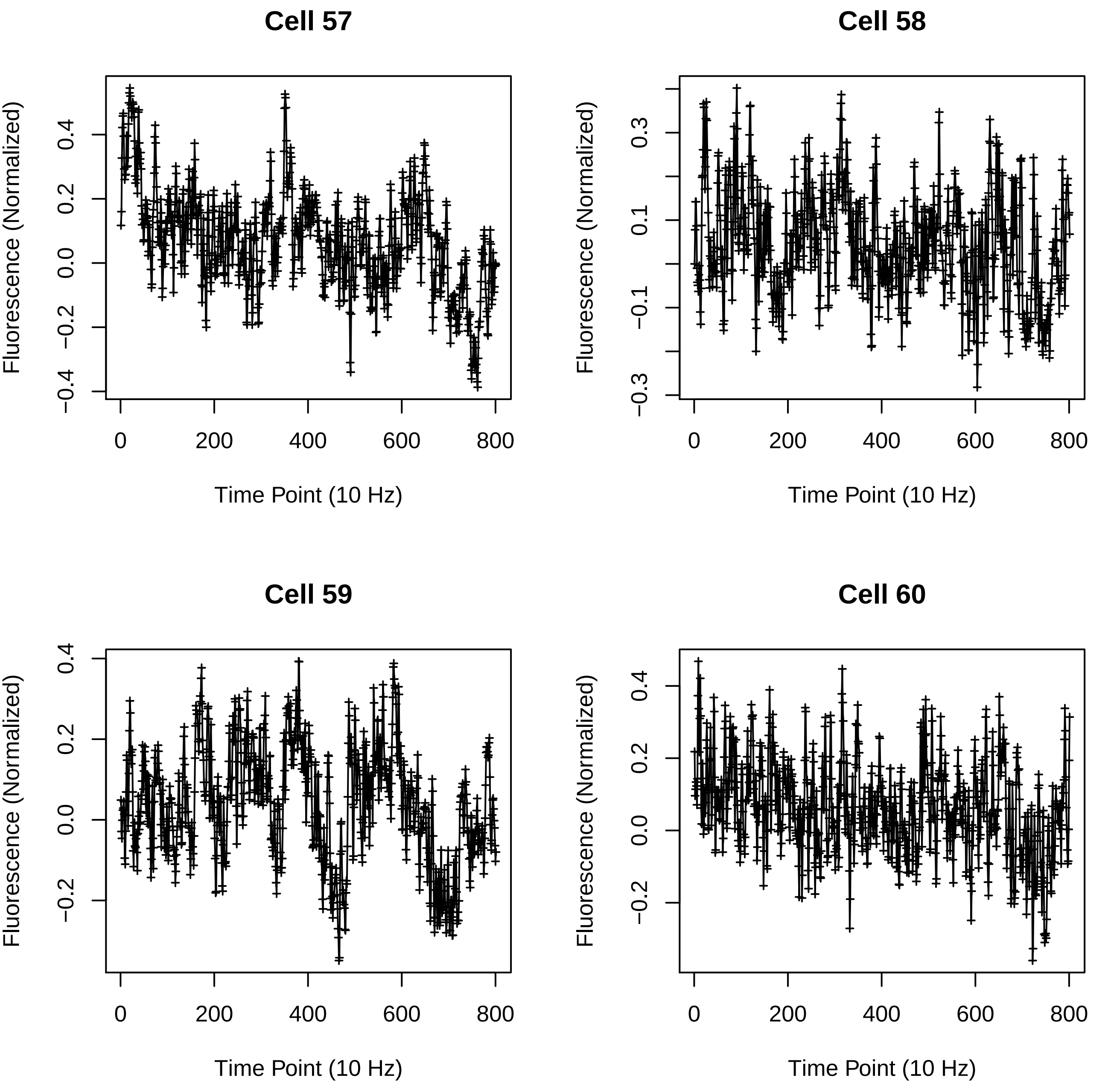
Cell 57
Cell 58
Cell 59
Cell 60
Fluorescence (Normalized)
Time Point (10 Hz)
0
200
400
600
800
−0.4
−0.2
0.0
0.2
0.4
−0.3
−0.1
0.1
0.3

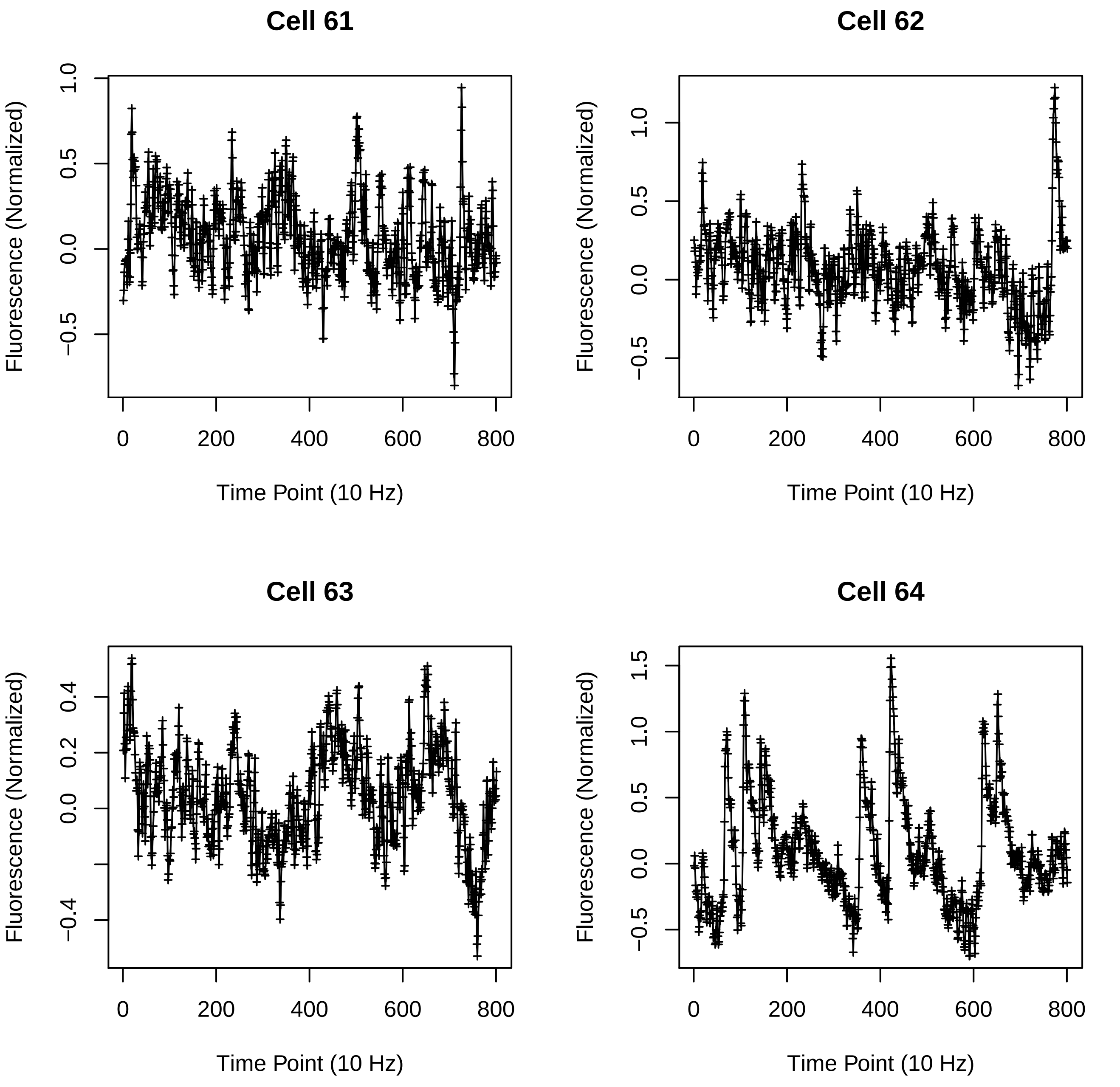
Cell 61
Cell 62
Cell 63
Cell 64
Fluorescence (Normalized)
Time Point (10 Hz)
0
200
400
600
800
1.0
0.5
0.0
−0.5
0.4
0.2
0.0
−0.4
1.5

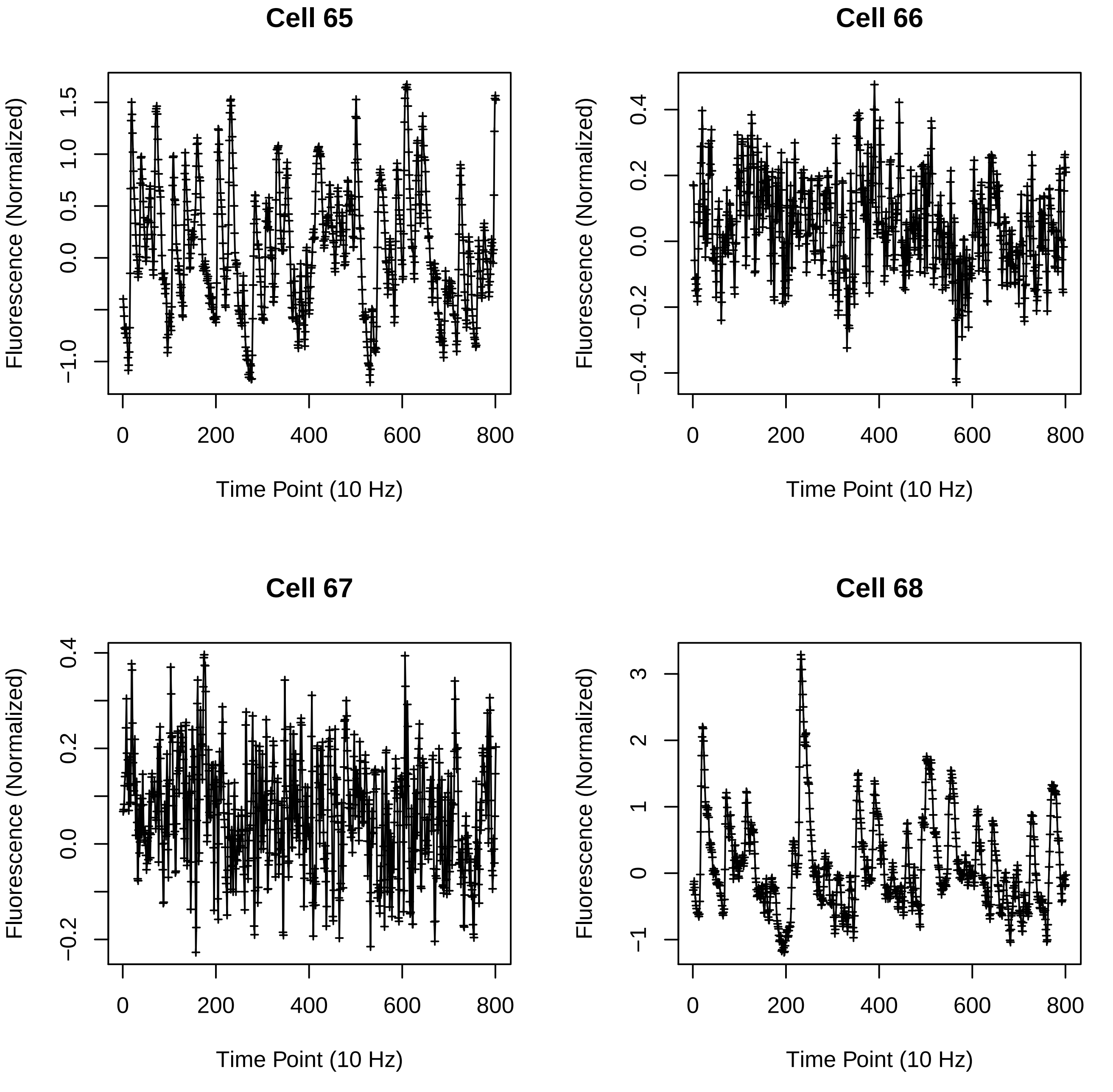
Cell 65
Cell 66
Cell 67
Cell 68
Fluorescence (Normalized)
Time Point (10 Hz)
0
200
400
600
800
−1.0
0.0
0.5
1.0
1.5
−0.4
−0.2
0.0
0.2
0.4
−0.2
0.0
0.2
0.4
−1
0
1
2
3

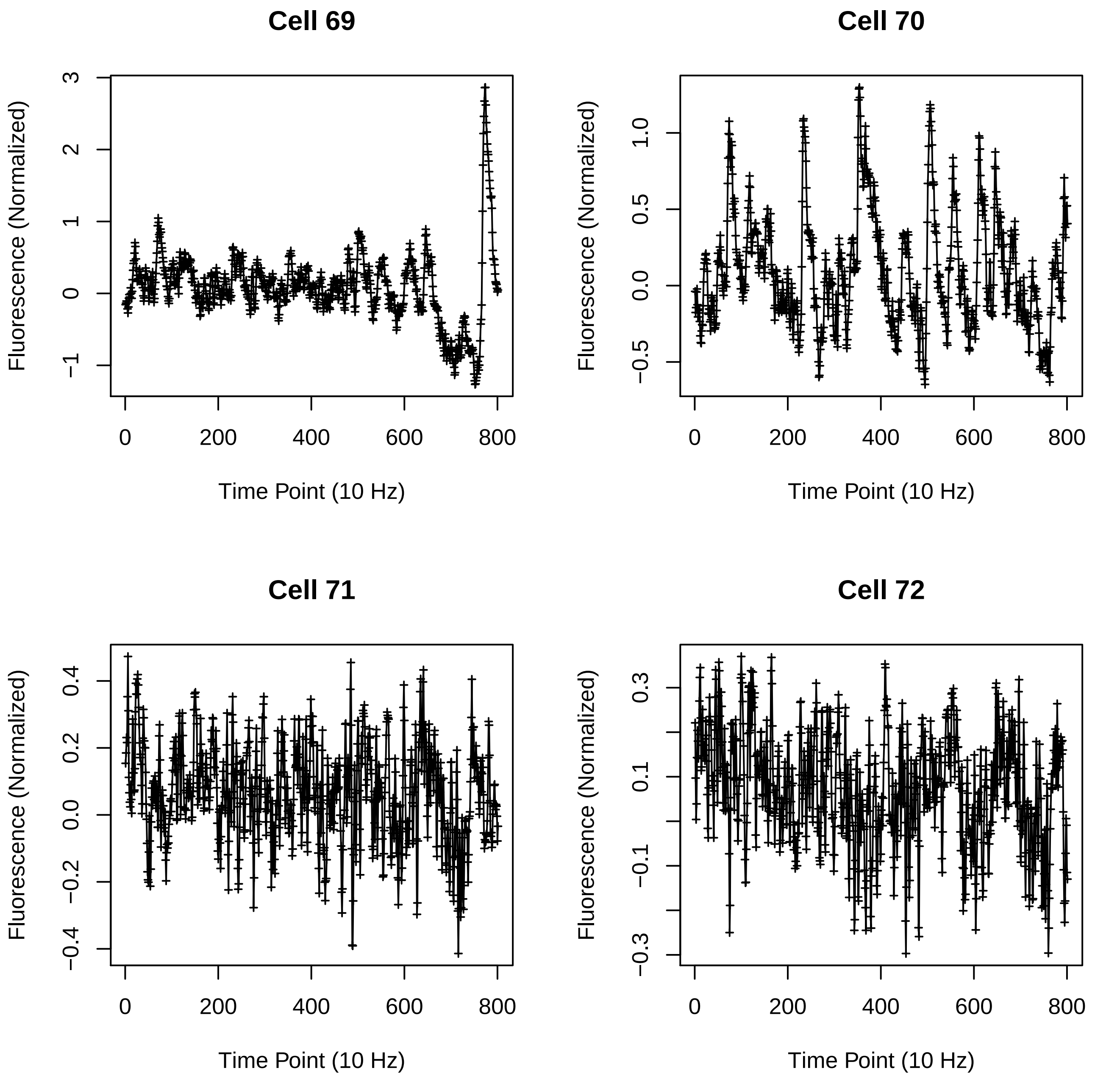

Cell 69
Cell 70
Cell 71
Cell 72
Fluorescence (Normalized)
Time Point (10 Hz)
0
200
400
600
800
3
2
1
0
−1
1.0
0.5
0.0
−0.5
0.4
0.2
0.0
−0.2
−0.4
0.3
0.1
−0.1
−0.3

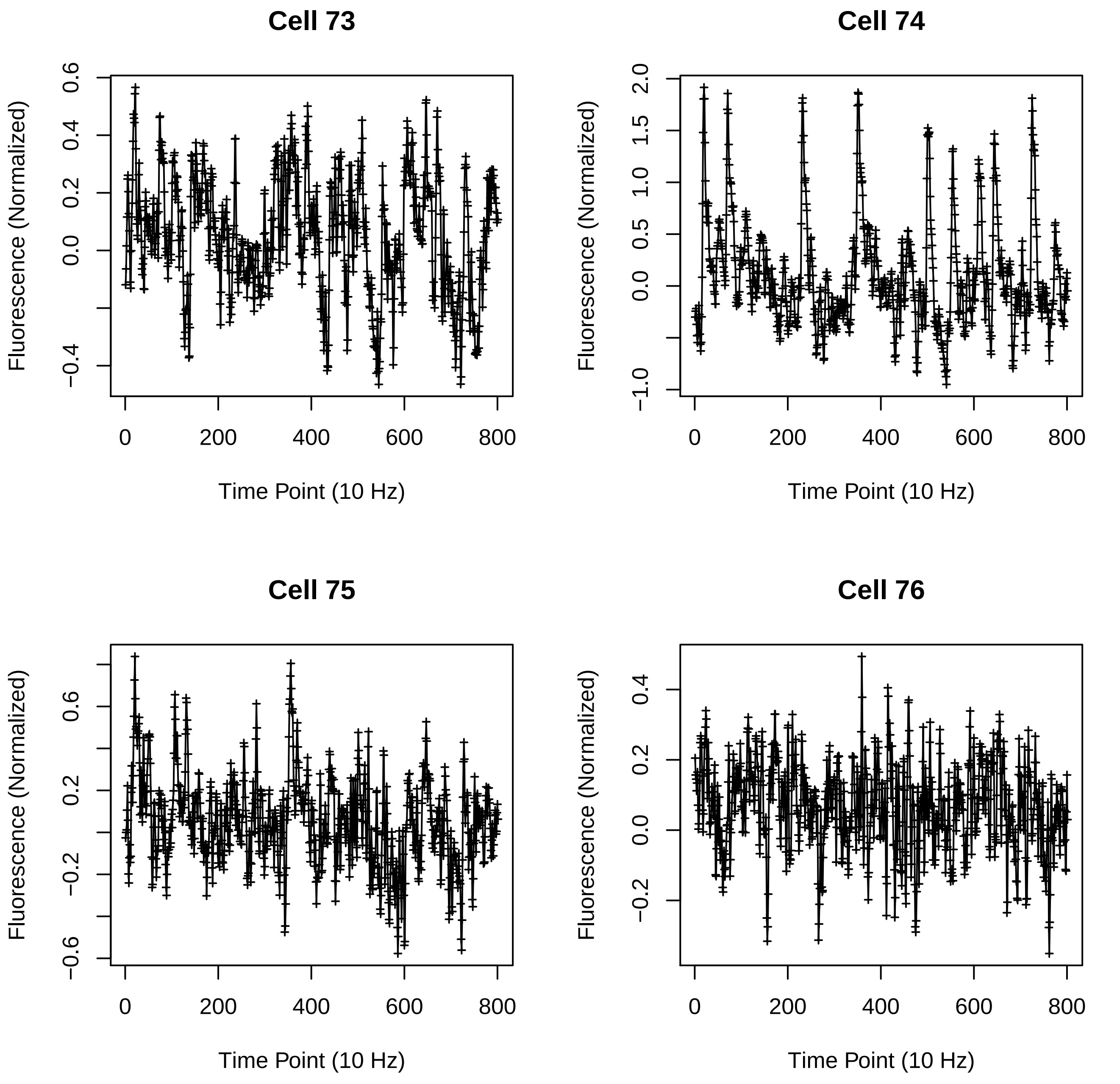
Cell 73
Cell 74
Cell 75
Cell 76
Fluorescence (Normalized)
Time Point (10 Hz)
0
200
400
600
800
0.6
0.4
0.2
0.0
−0.4
2.0
1.5
1.0
0.5
0.0
−1.0
0.6
0.2
−0.2
−0.6
0.4
0.2
0.0
−0.2

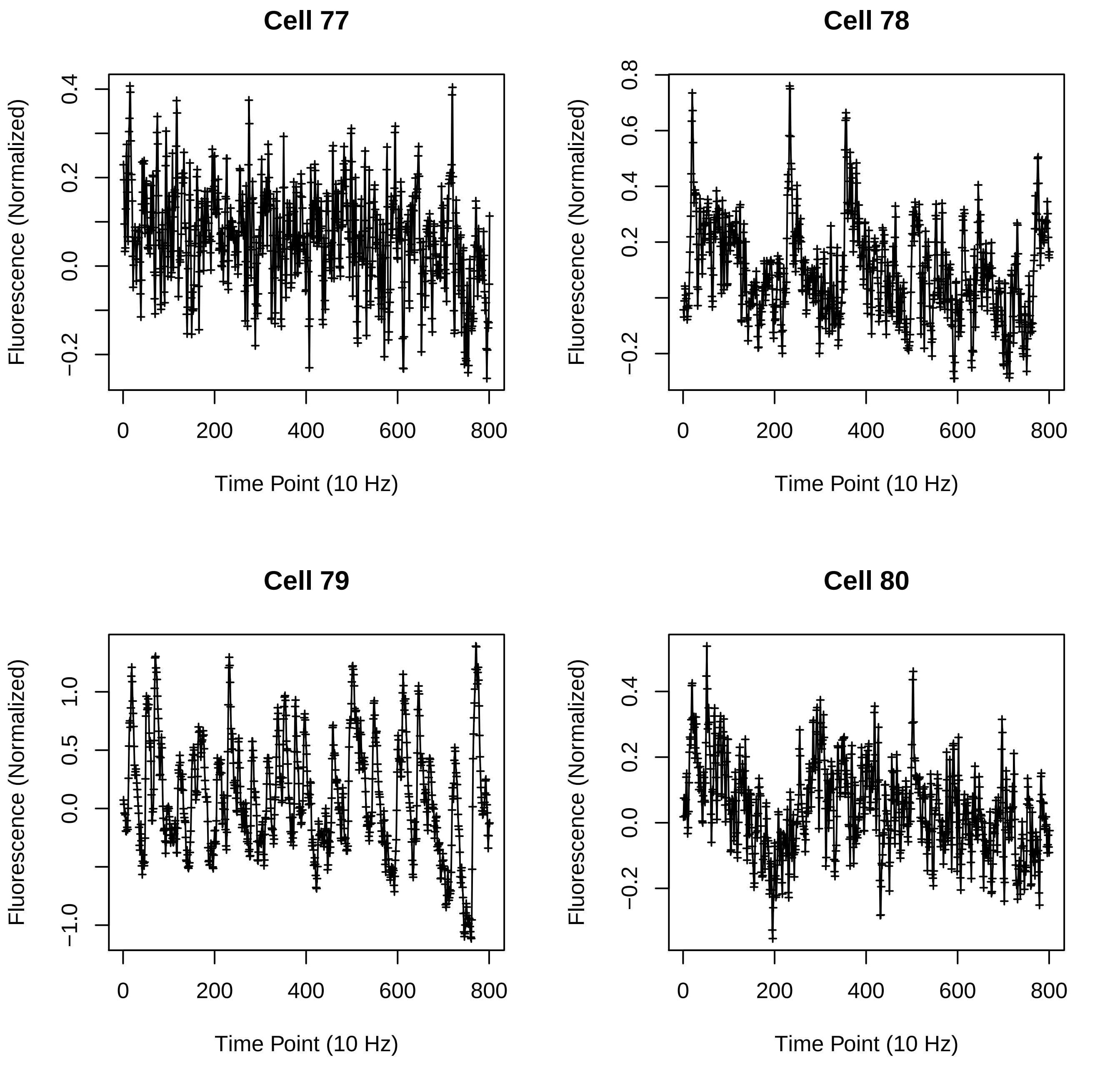

Cell 77
Cell 78
Cell 79
Cell 80
Fluorescence (Normalized)
Time Point (10 Hz)
0
200
400
600
800
−0.2
0.0
0.2
0.4
0.6
0.8
−1.0
0.5
1.0

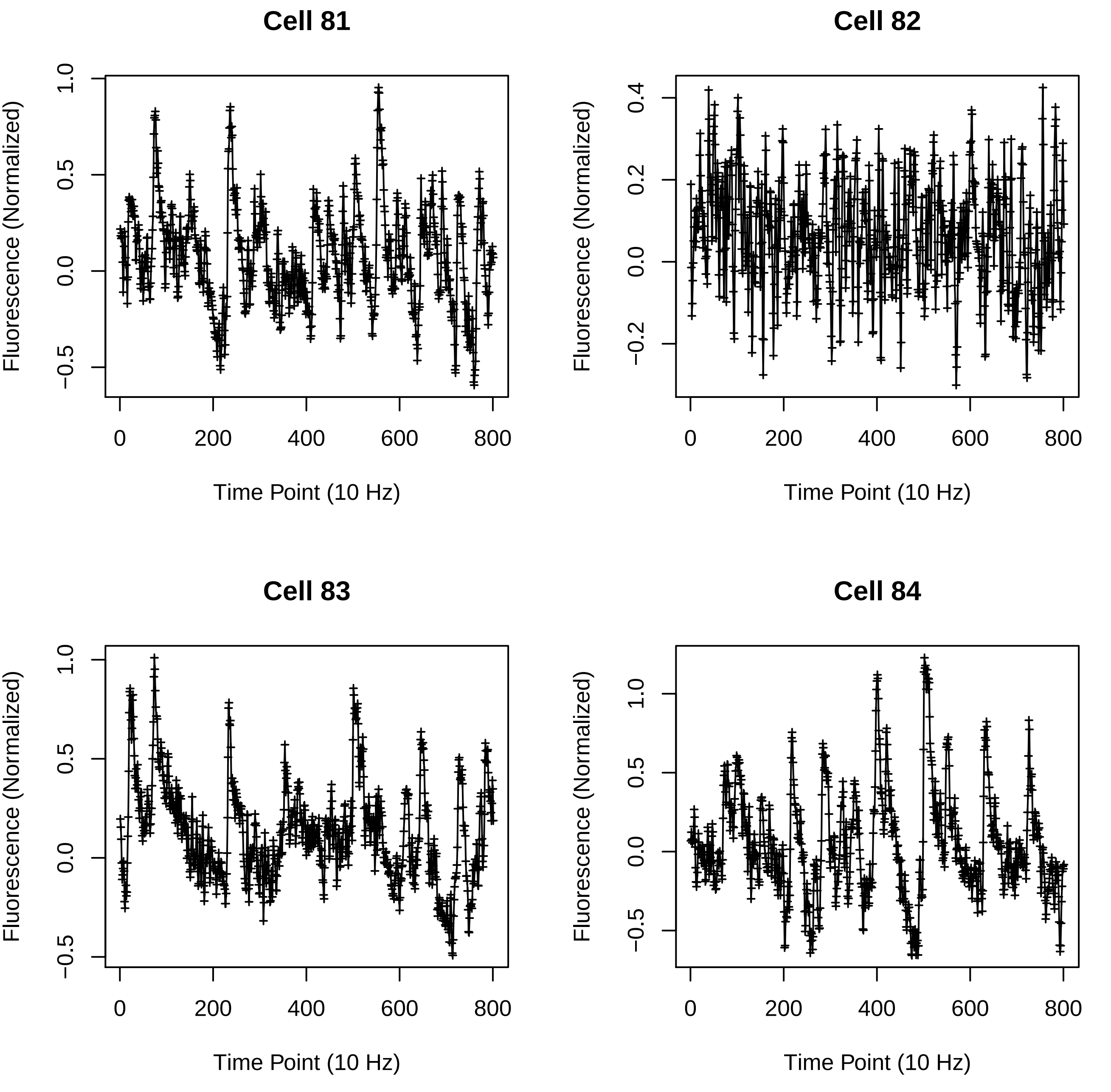
Cell 81
Cell 82
Cell 83
Cell 84
Fluorescence (Normalized)
Time Point (10 Hz)
0
200
400
600
800
−0.5
0.0
0.5
1.0
−0.2
0.2
0.4

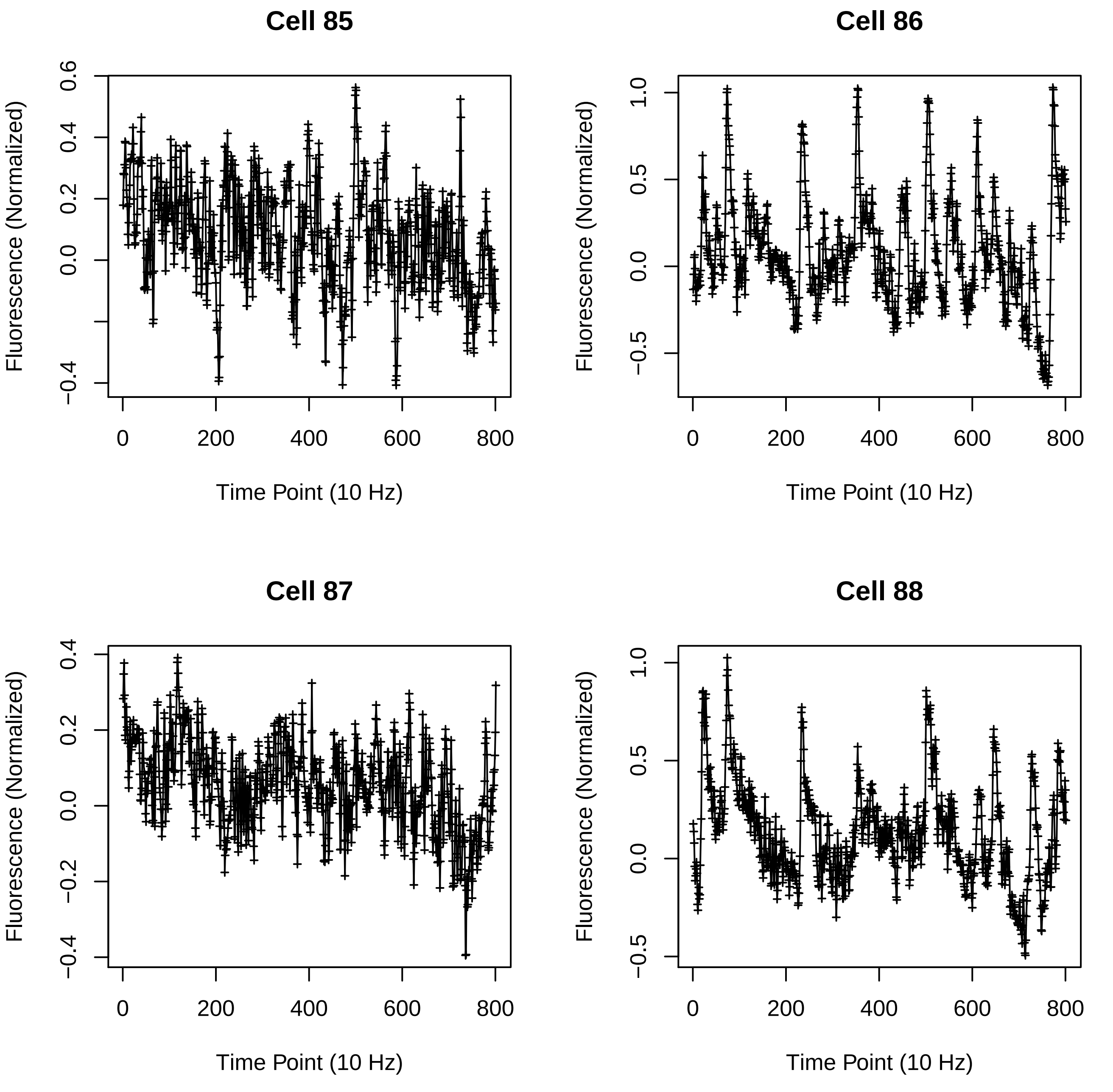
Cell 85
Cell 86
Cell 87
Cell 88
Fluorescence (Normalized)
Time Point (10 Hz)
0
200
400
600
800
0.6
0.4
0.2
0.0
−0.4
1.0
0.5
0.0
−0.5
0.4
0.2
0.0
−0.2
−0.4

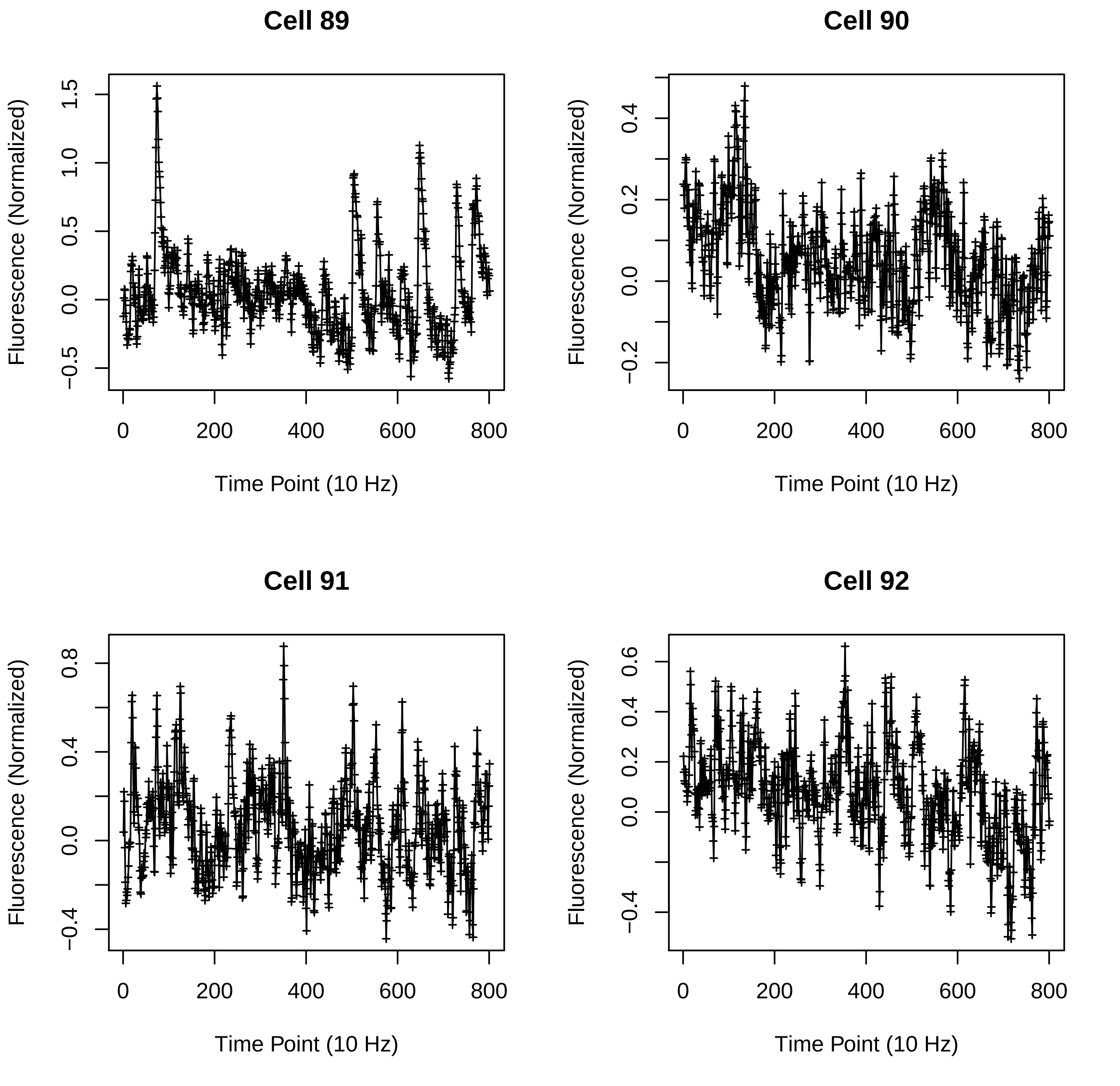
Cell 89
Fluorescence (Normalized)
Time Point (10 Hz)
Cell 90
Fluorescence (Normalized)
Time Point (10 Hz)
Cell 91
Fluorescence (Normalized)
Time Point (10 Hz)
Cell 92
Fluorescence (Normalized)
Time Point (10 Hz)

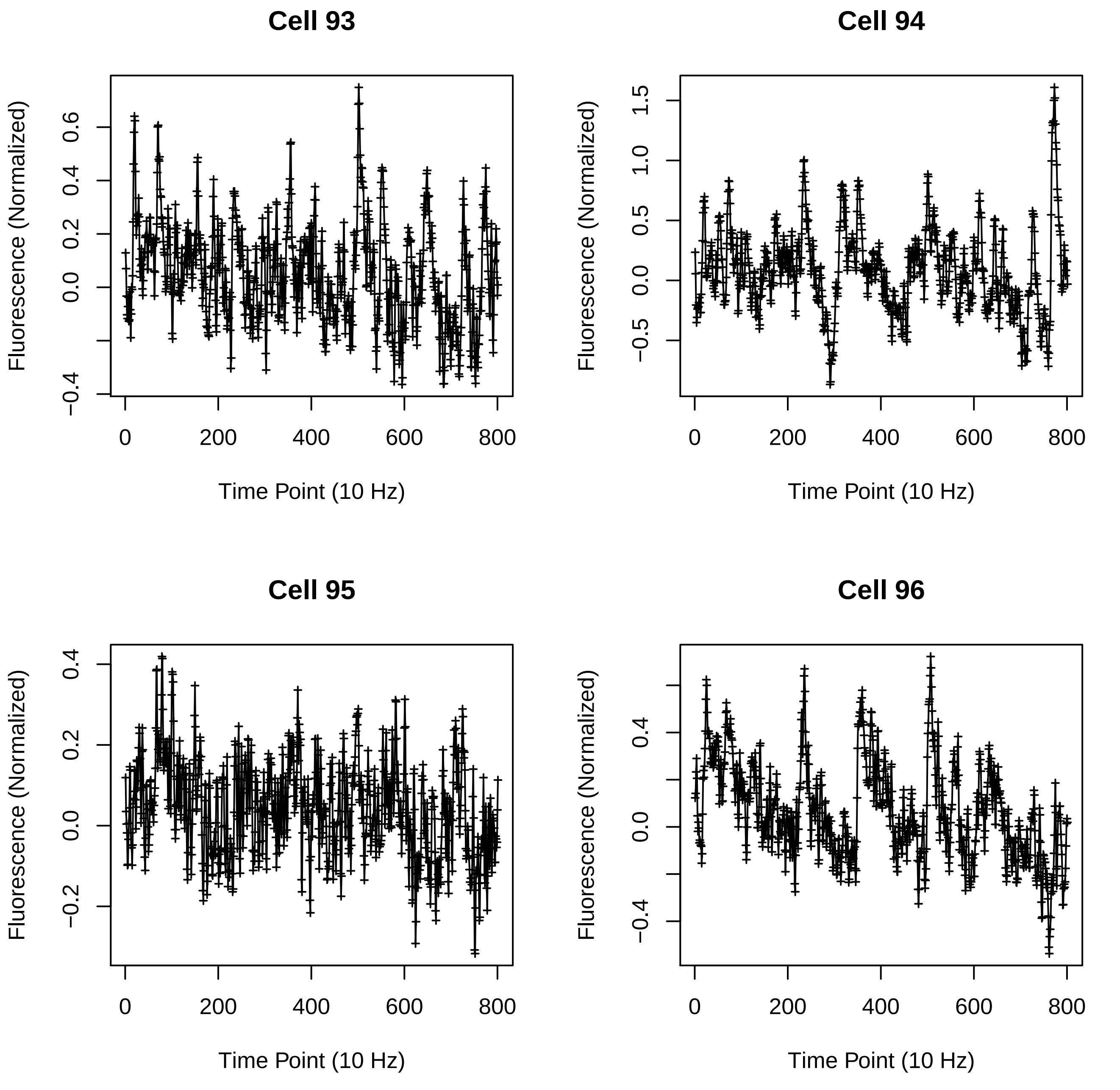

Cell 93
Fluorescence (Normalized)
Time Point (10 Hz)
Cell 94
Fluorescence (Normalized)
Time Point (10 Hz)
Cell 95
Fluorescence (Normalized)
Time Point (10 Hz)
Cell 96
Fluorescence (Normalized)
Time Point (10 Hz)

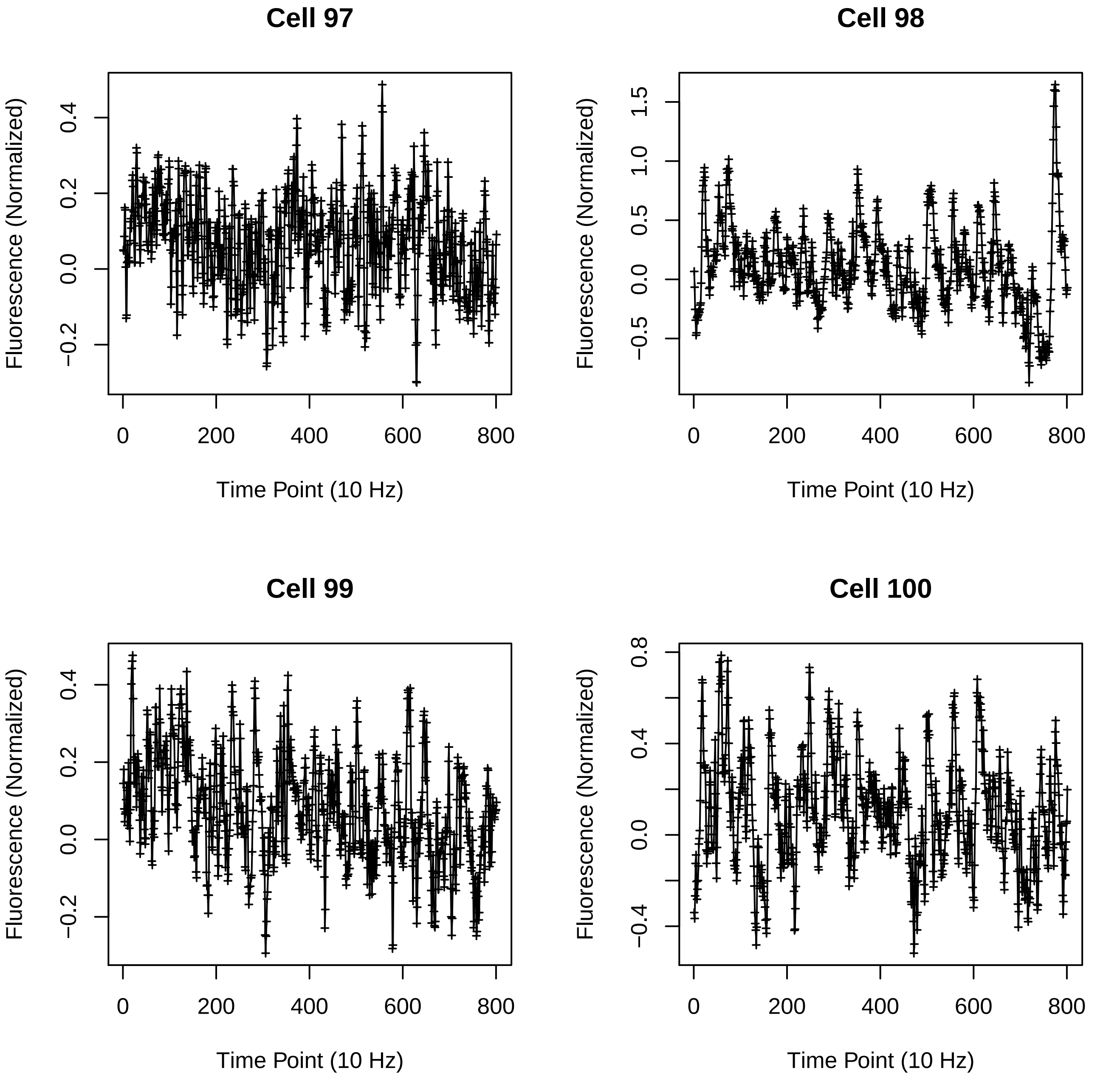

Cell 97
Cell 98
Cell 99
Cell 100
Fluorescence (Normalized)
Time Point (10 Hz)
0
200
400
600
800
−0.2
0.0
0.2
0.4
−0.5
0.5
1.0
1.5
−0.4
0.8

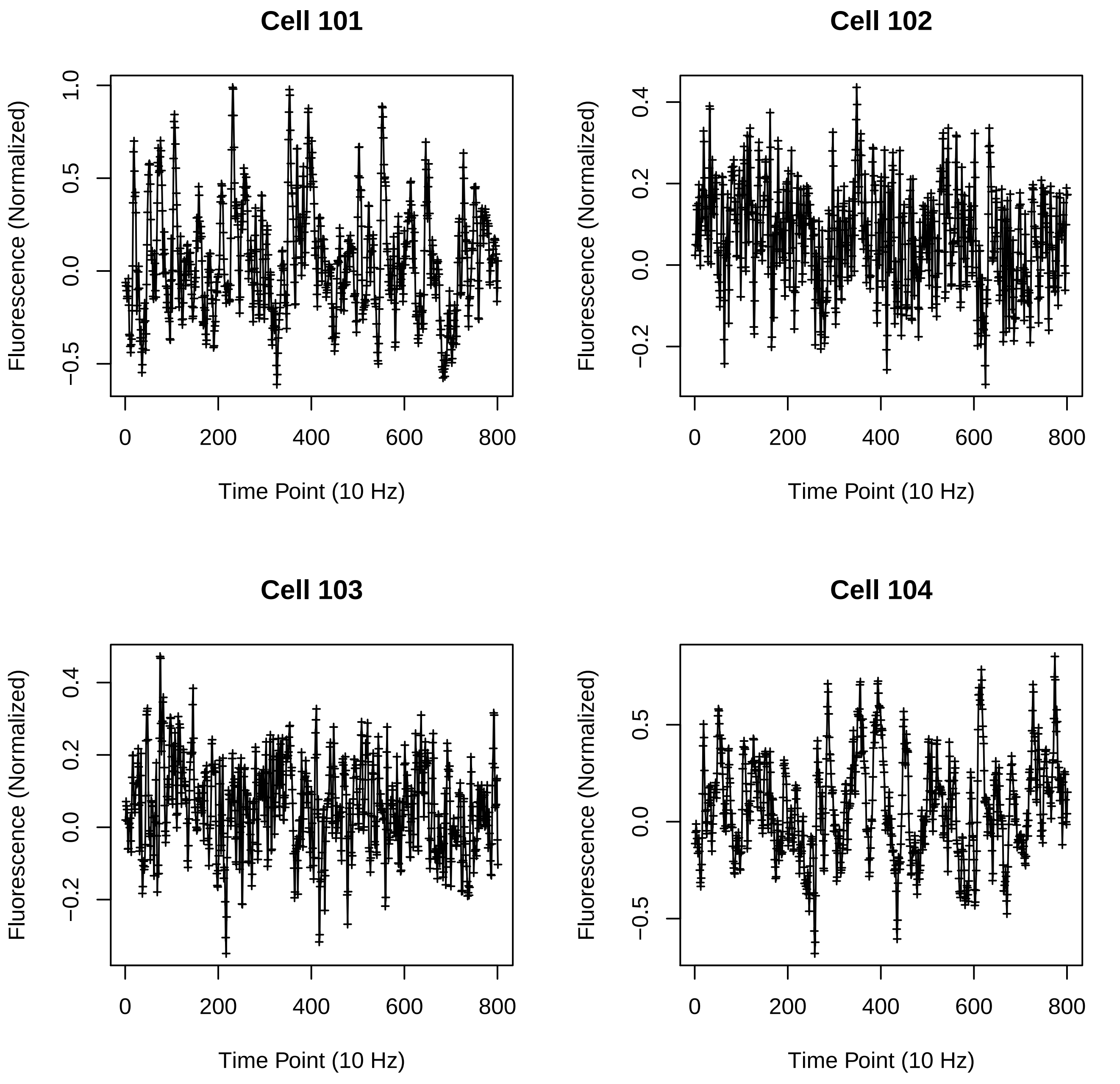
Cell 101
Cell 102
Cell 103
Cell 104
Fluorescence (Normalized)
Time Point (10 Hz)
0
200
400
600
800
1.0
0.5
0.0
−0.5
0.4
0.2
0.0
−0.2

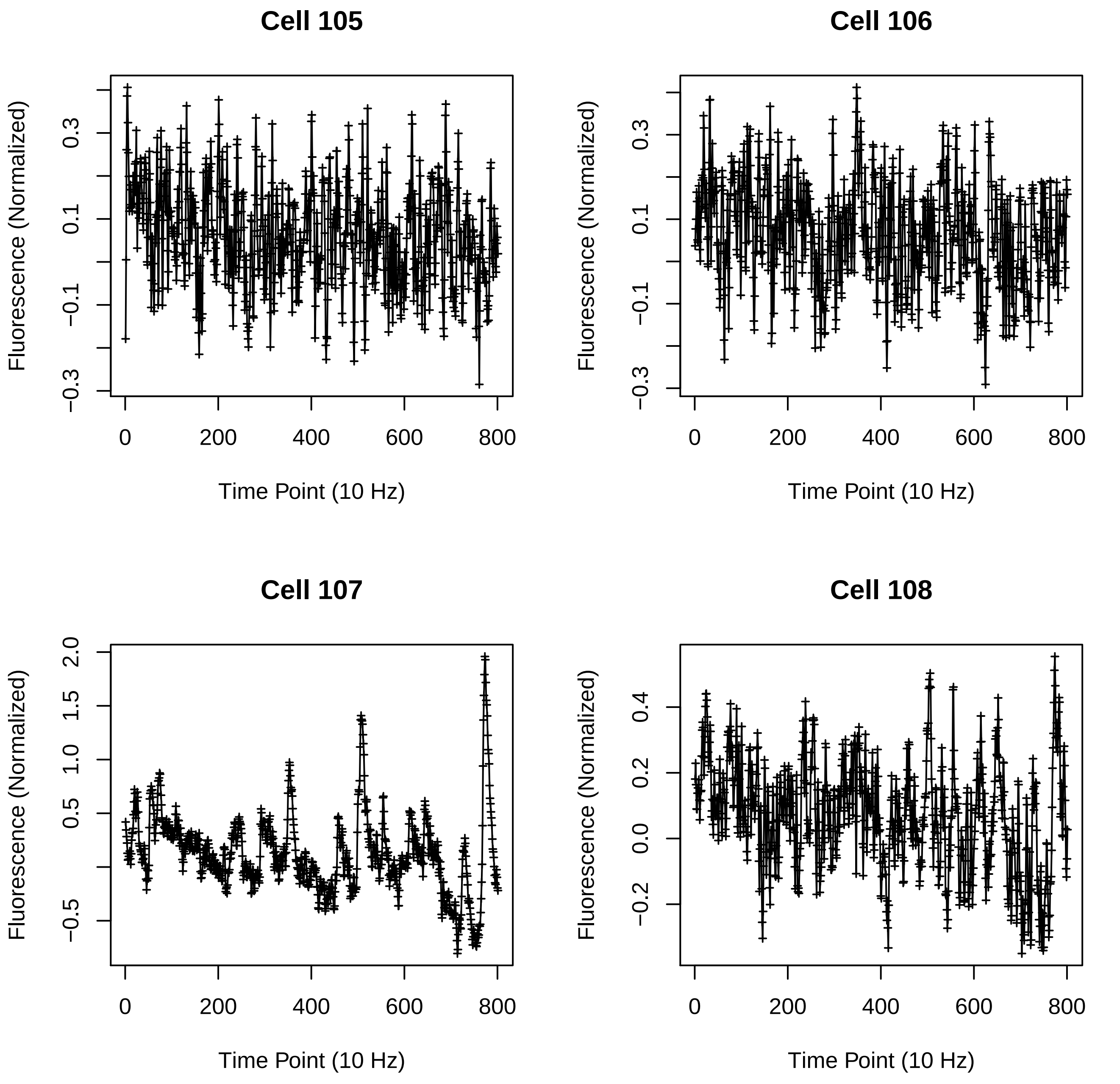
Cell 105
Fluorescence (Normalized)
Time Point (10 Hz)
Cell 106
Fluorescence (Normalized)
Time Point (10 Hz)
Cell 107
Fluorescence (Normalized)
Time Point (10 Hz)
Cell 108
Fluorescence (Normalized)
Time Point (10 Hz)

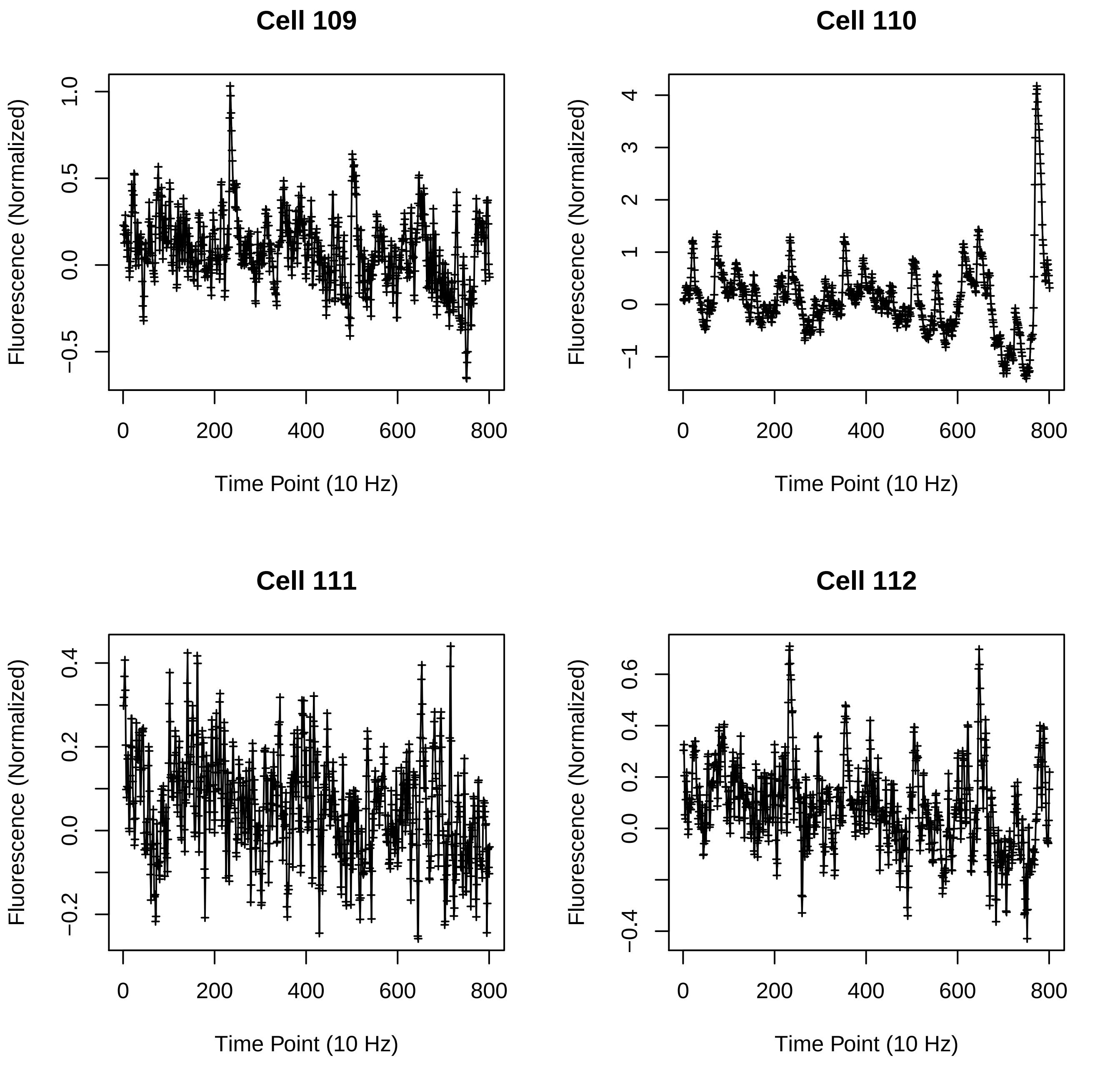
Cell 109
Fluorescence (Normalized)
Time Point (10 Hz)
Cell 110
Fluorescence (Normalized)
Time Point (10 Hz)
Cell 111
Fluorescence (Normalized)
Time Point (10 Hz)
Cell 112
Fluorescence (Normalized)
Time Point (10 Hz)

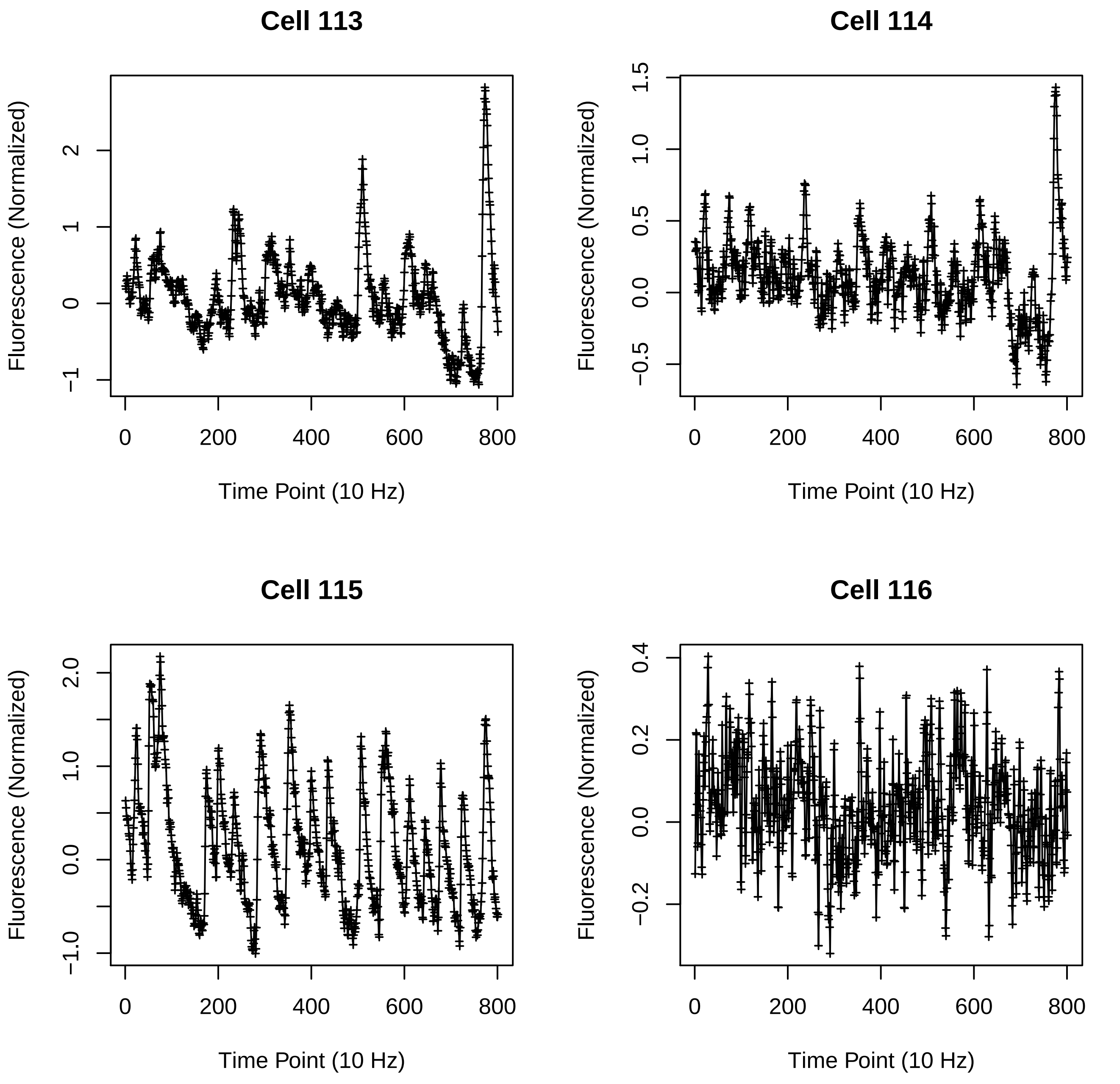
Cell 113
Fluorescence (Normalized)
−1
0
1
2
0
200
400
600
800
Time Point (10 Hz)
Cell 114
Fluorescence (Normalized)
−0.5
0.0
0.5
1.0
1.5
0
200
400
600
800
Time Point (10 Hz)
Cell 115
Fluorescence (Normalized)
−1.0
0.0
1.0
2.0
0
200
400
600
800
Time Point (10 Hz)
Cell 116
Fluorescence (Normalized)
−0.2
0.0
0.2
0.4
0
200
400
600
800
Time Point (10 Hz)

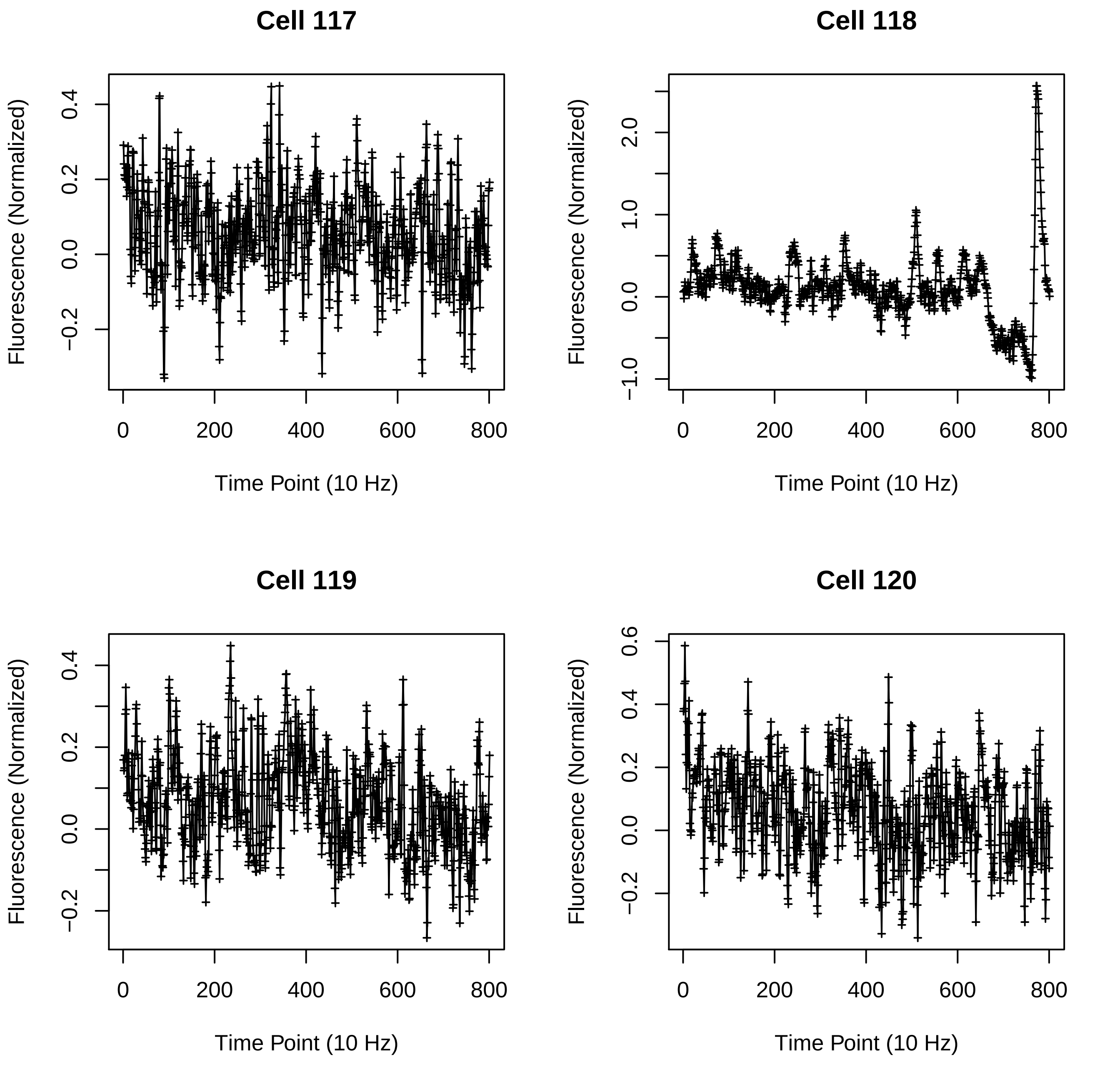
Cell 117
Cell 118
Cell 119
Cell 120
Fluorescence (Normalized)
Time Point (10 Hz)
0
200
400
600
800
−0.2
0.0
0.2
0.4
−1.0
0.0
1.0
2.0
0.6

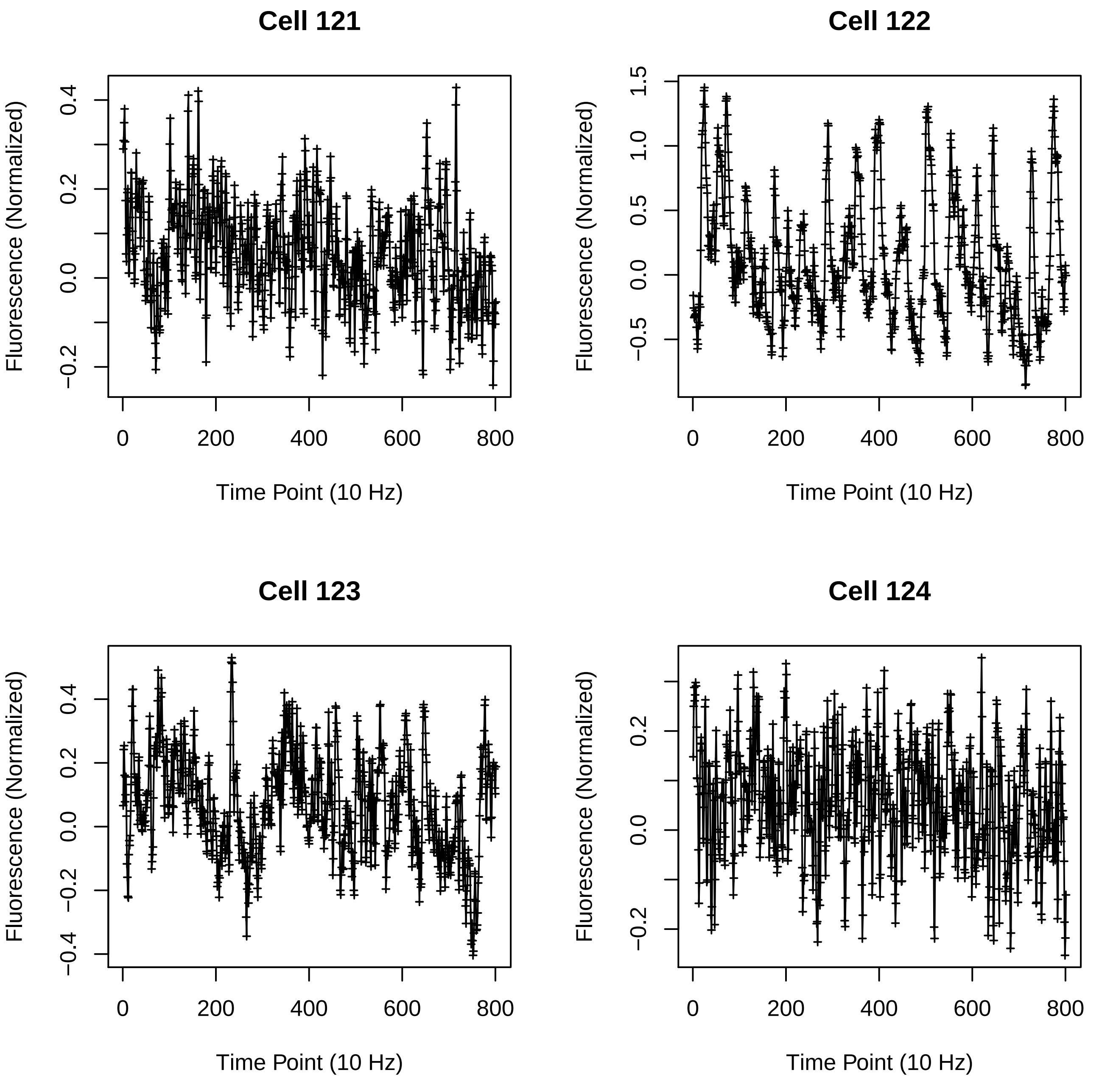
Cell 121
Fluorescence (Normalized)
−0.2
0.0
0.2
0.4
0
200
400
600
800
Time Point (10 Hz)
Cell 122
Fluorescence (Normalized)
−0.5
0.0
0.5
1.0
1.5
0
200
400
600
800
Time Point (10 Hz)
Cell 123
Fluorescence (Normalized)
−0.4
−0.2
0.0
0.2
0.4
0
200
400
600
800
Time Point (10 Hz)
Cell 124
Fluorescence (Normalized)
−0.2
0.0
0.2
0
200
400
600
800
Time Point (10 Hz)

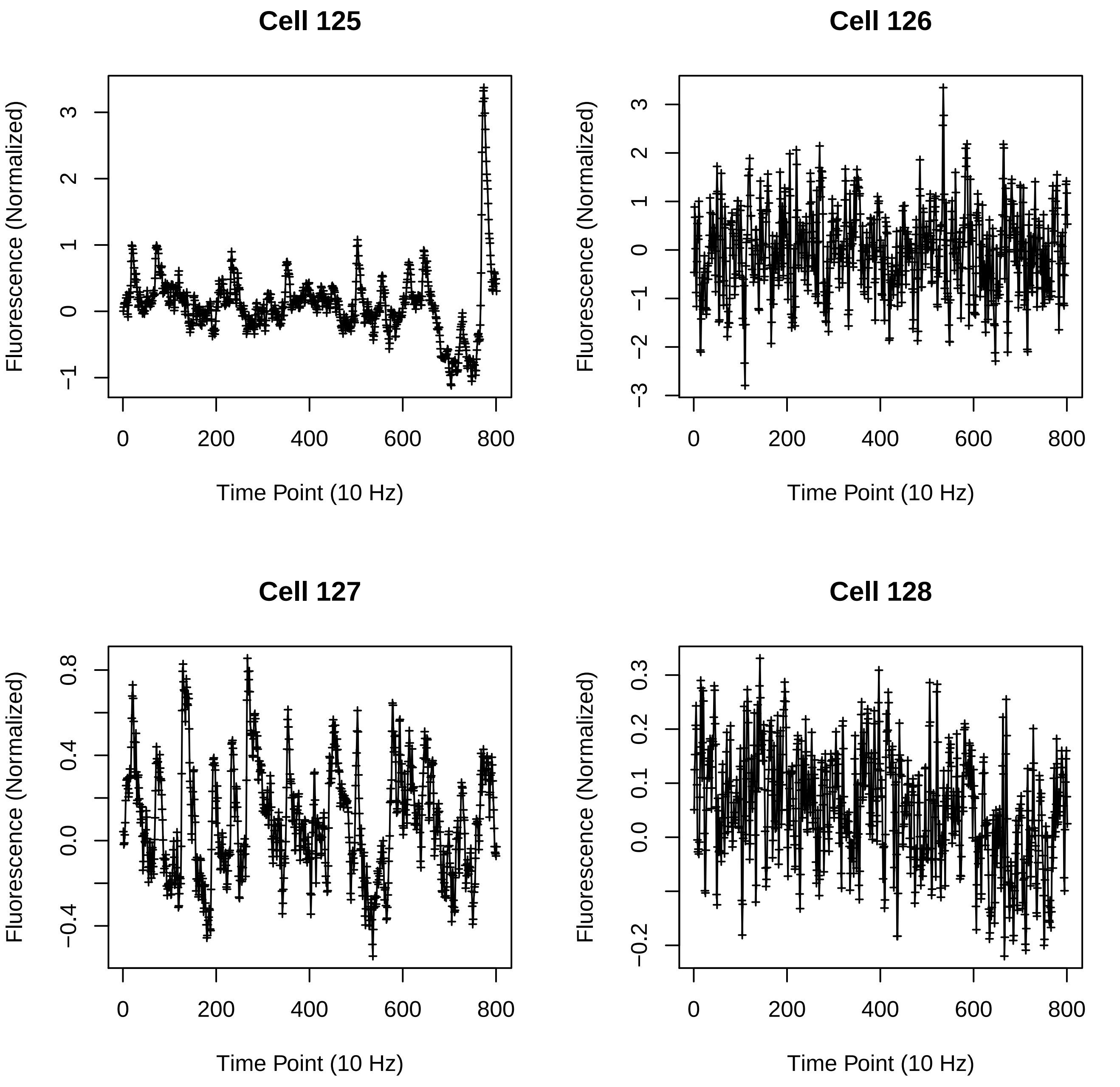
Cell 125
Cell 126
Cell 127
Cell 128
Fluorescence (Normalized)
Time Point (10 Hz)
0
200
400
600
800
−1
0
1
2
3
−3
−2
−0.4
0.0
0.4
0.8
−0.2
0.1
0.2
0.3

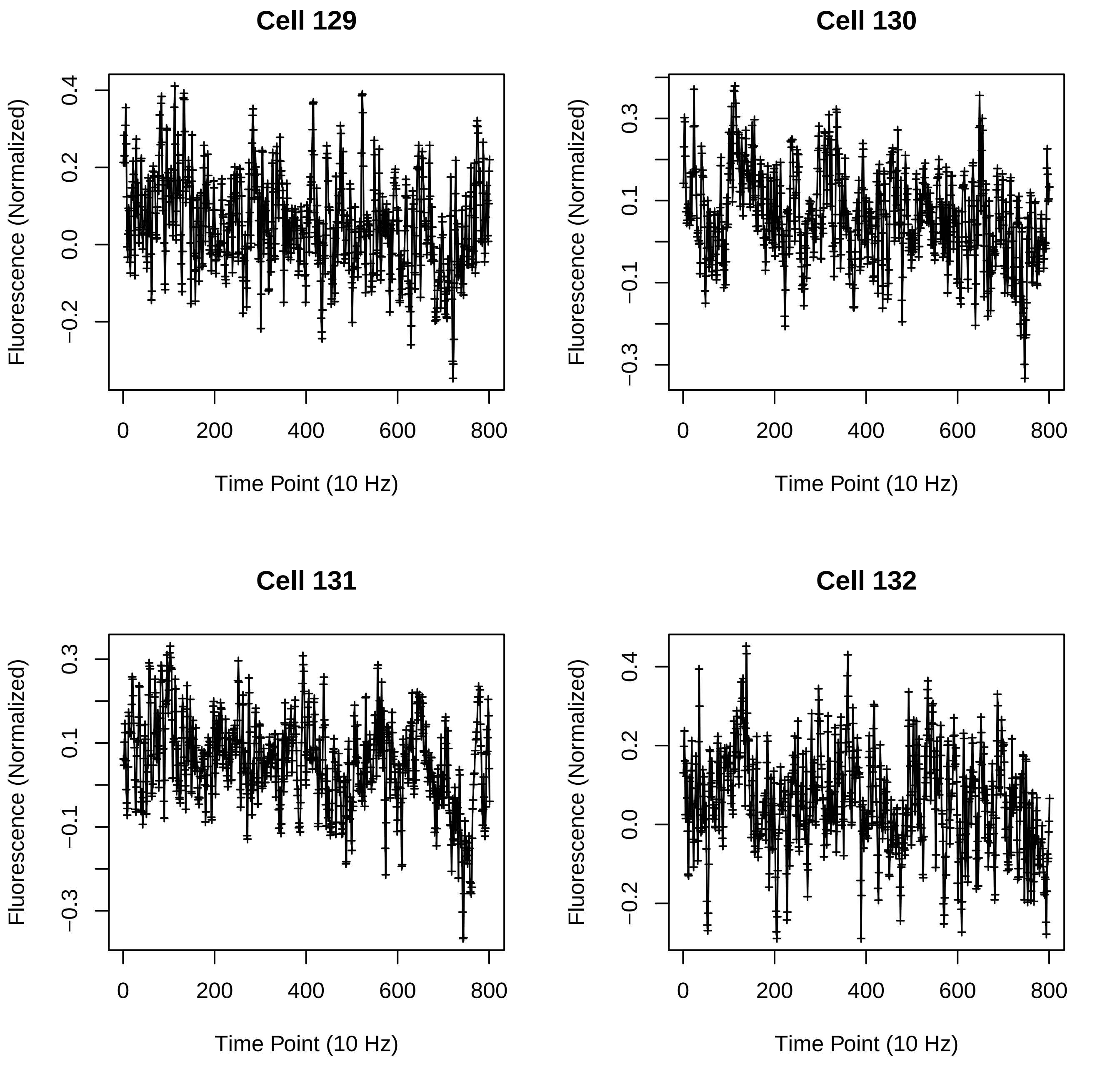

Cell 129
Cell 130
Cell 131
Cell 132
Fluorescence (Normalized)
Time Point (10 Hz)
0
200
400
600
800
0.4
0.2
0.0
−0.2
0.3
0.1
−0.1
−0.3

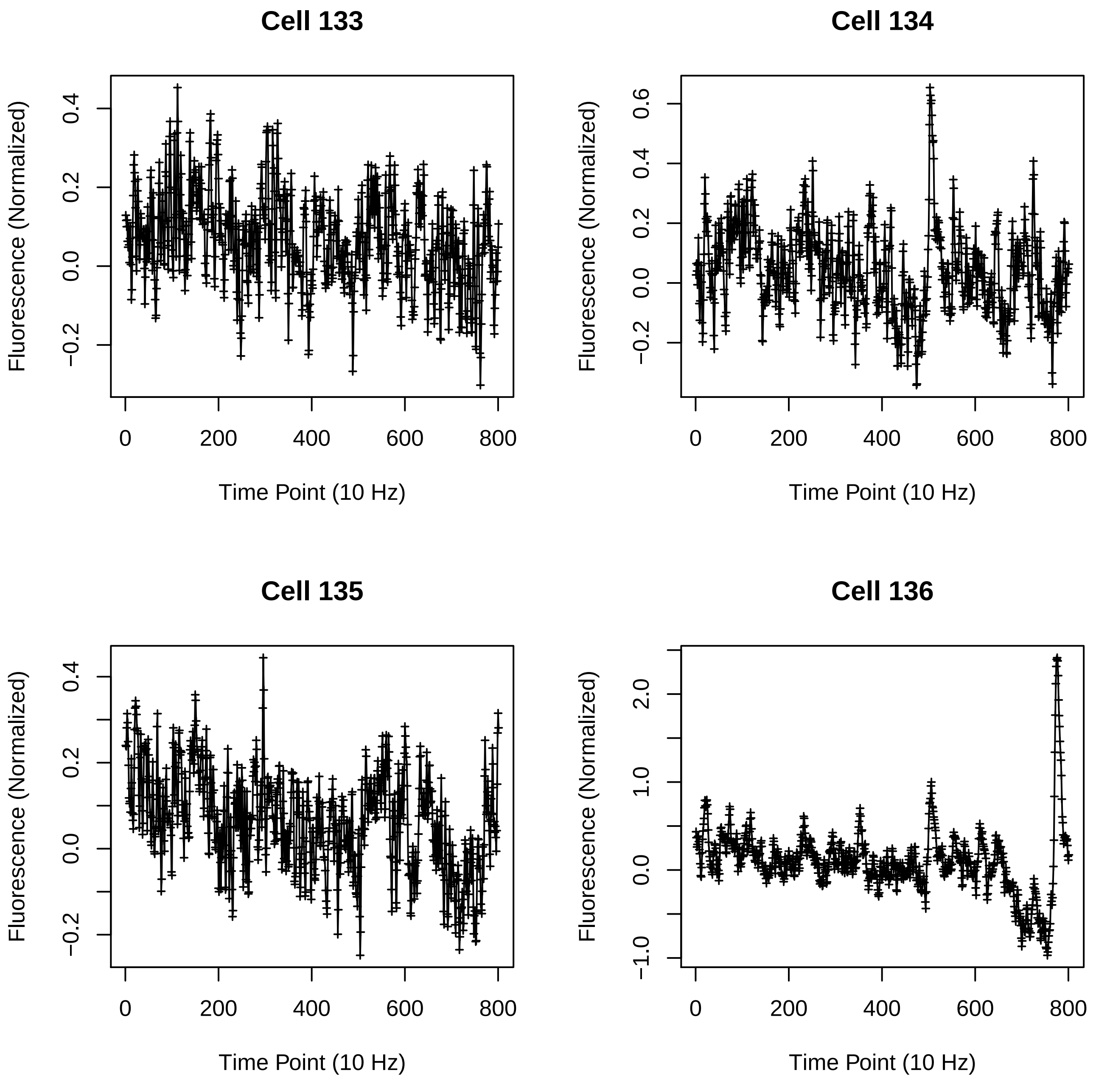

Cell 133
Cell 134
Cell 135
Cell 136
Fluorescence (Normalized)
Time Point (10 Hz)
0
200
400
600
800
−0.2
0.0
0.2
0.4
0.6
−1.0
1.0
2.0

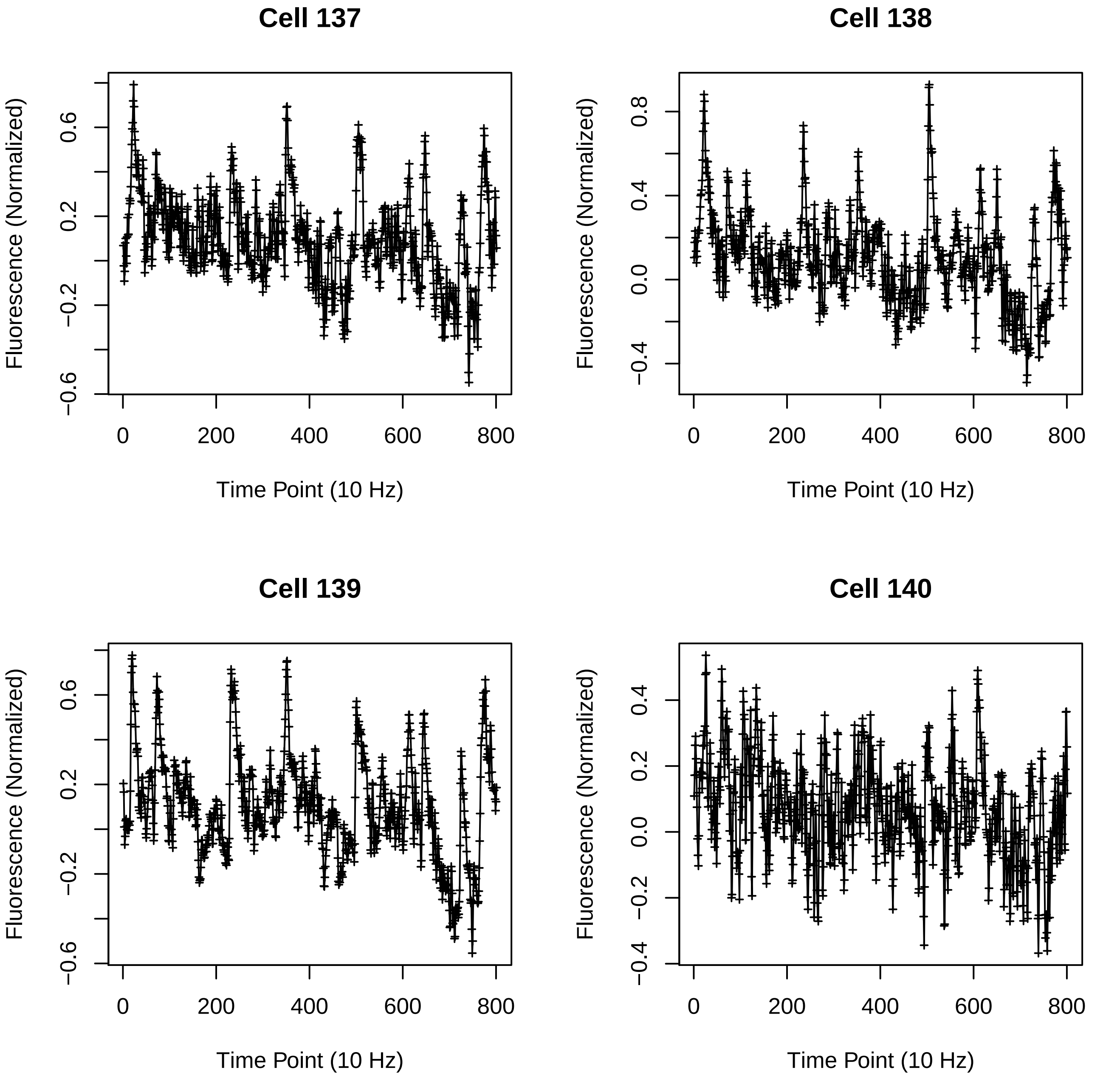
Cell 137
Cell 138
Cell 139
Cell 140
Fluorescence (Normalized)
Time Point (10 Hz)
0
200
400
600
800
−0.6
−0.2
0.2
0.6
−0.4
0.0
0.4
0.8

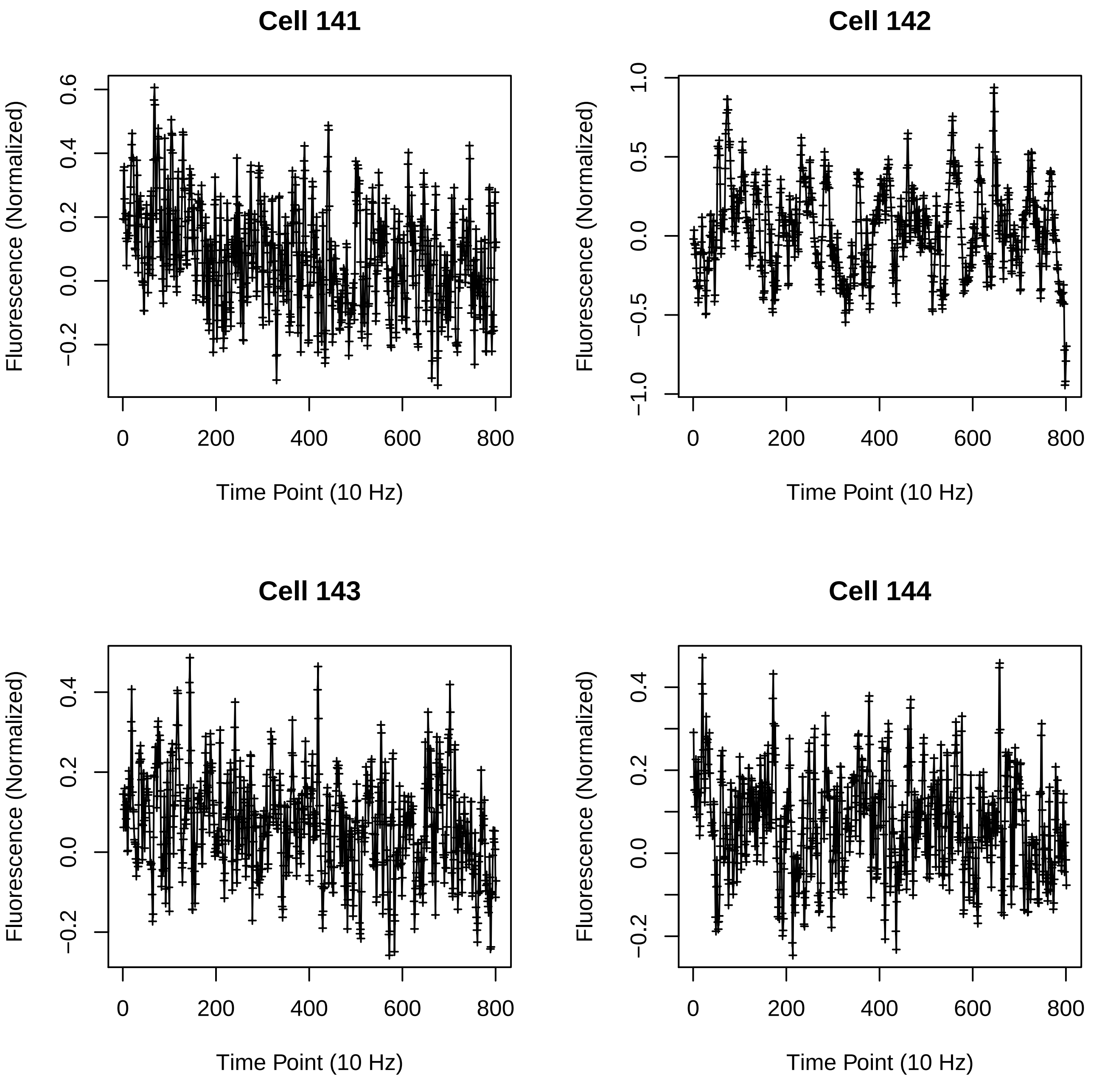
Cell 141
Cell 142
Cell 143
Cell 144
Fluorescence (Normalized)
Time Point (10 Hz)
0
200
400
600
800
0.6
0.4
0.2
0.0
−0.2
1.0
0.5
0.0
−0.5
−1.0

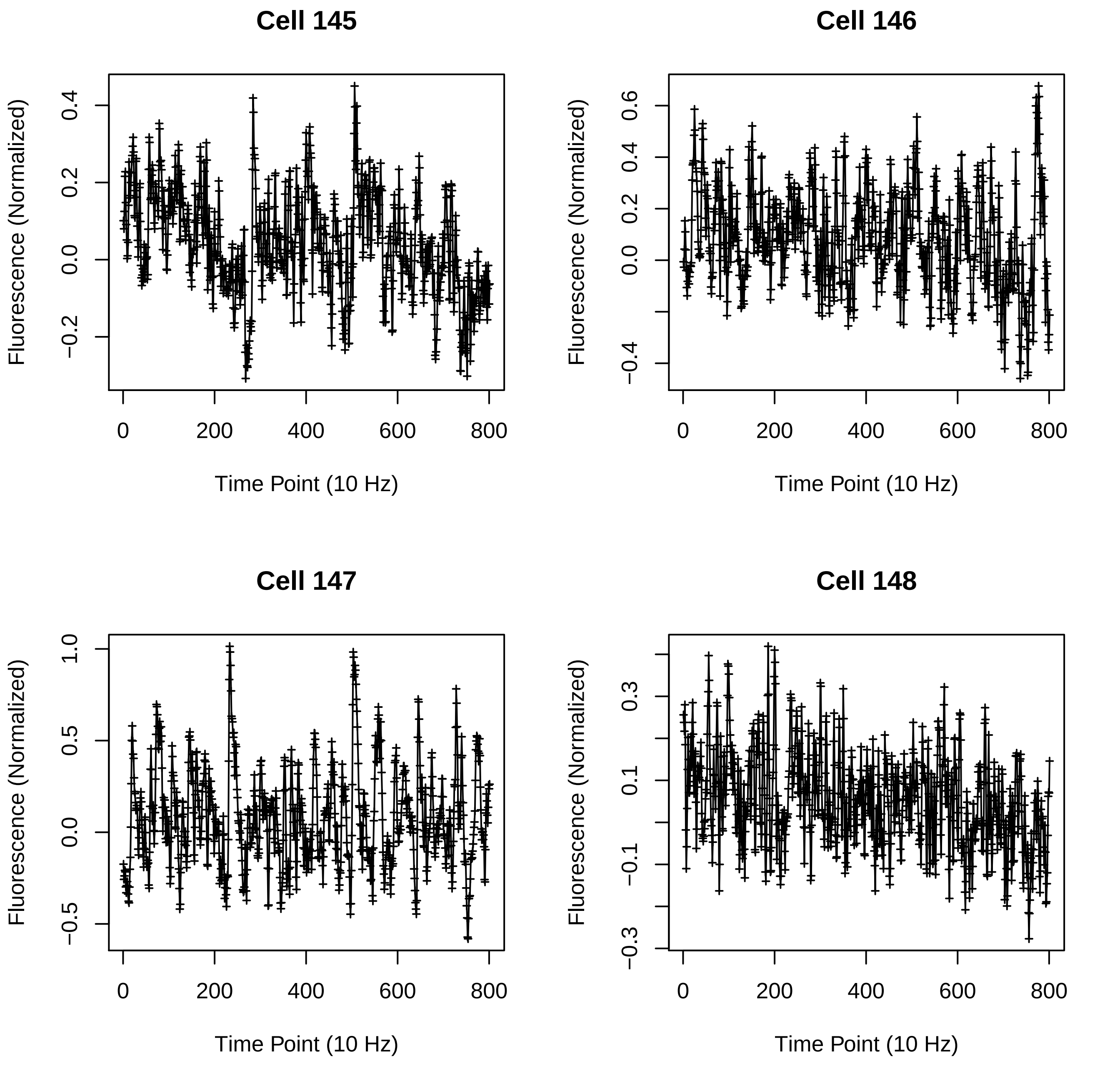
Cell 145
Fluorescence (Normalized)
Time Point (10 Hz)
−0.2
0.0
0.2
0.4
0
200
400
600
800
Cell 146
Fluorescence (Normalized)
Time Point (10 Hz)
−0.4
0.0
0.2
0.4
0.6
0
200
400
600
800
Cell 147
Fluorescence (Normalized)
Time Point (10 Hz)
−0.5
0.0
0.5
1.0
0
200
400
600
800
Cell 148
Fluorescence (Normalized)
Time Point (10 Hz)
−0.3
−0.1
0.1
0.3
0
200
400
600
800

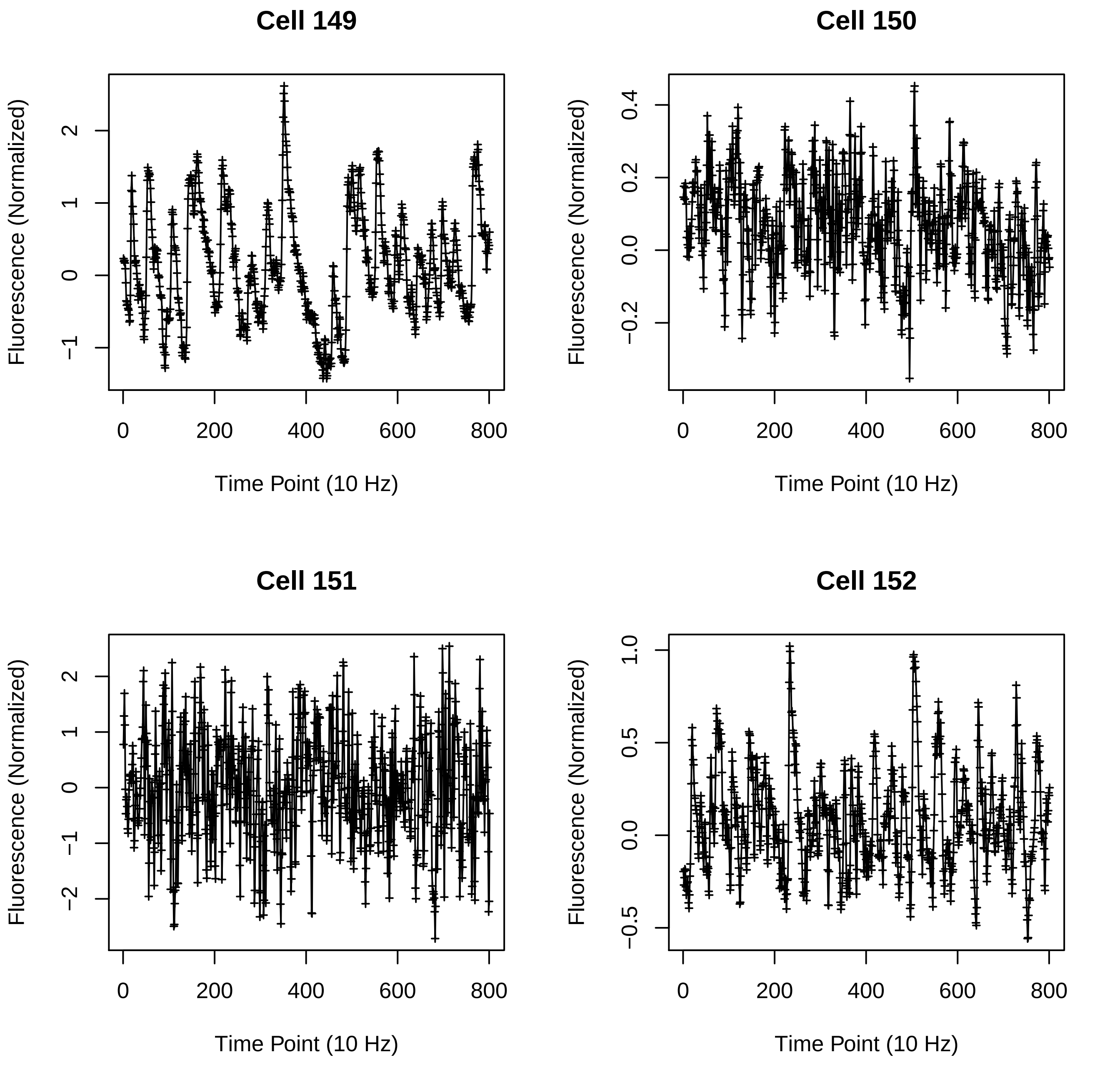
Cell 149
Cell 150
Cell 151
Cell 152
Fluorescence (Normalized)
Time Point (10 Hz)
0
200
400
600
800
−1
0
1
2
−0.2
0.0
0.2
0.4
−2
−1
0
1
2
−0.5
0.0
0.5
1.0

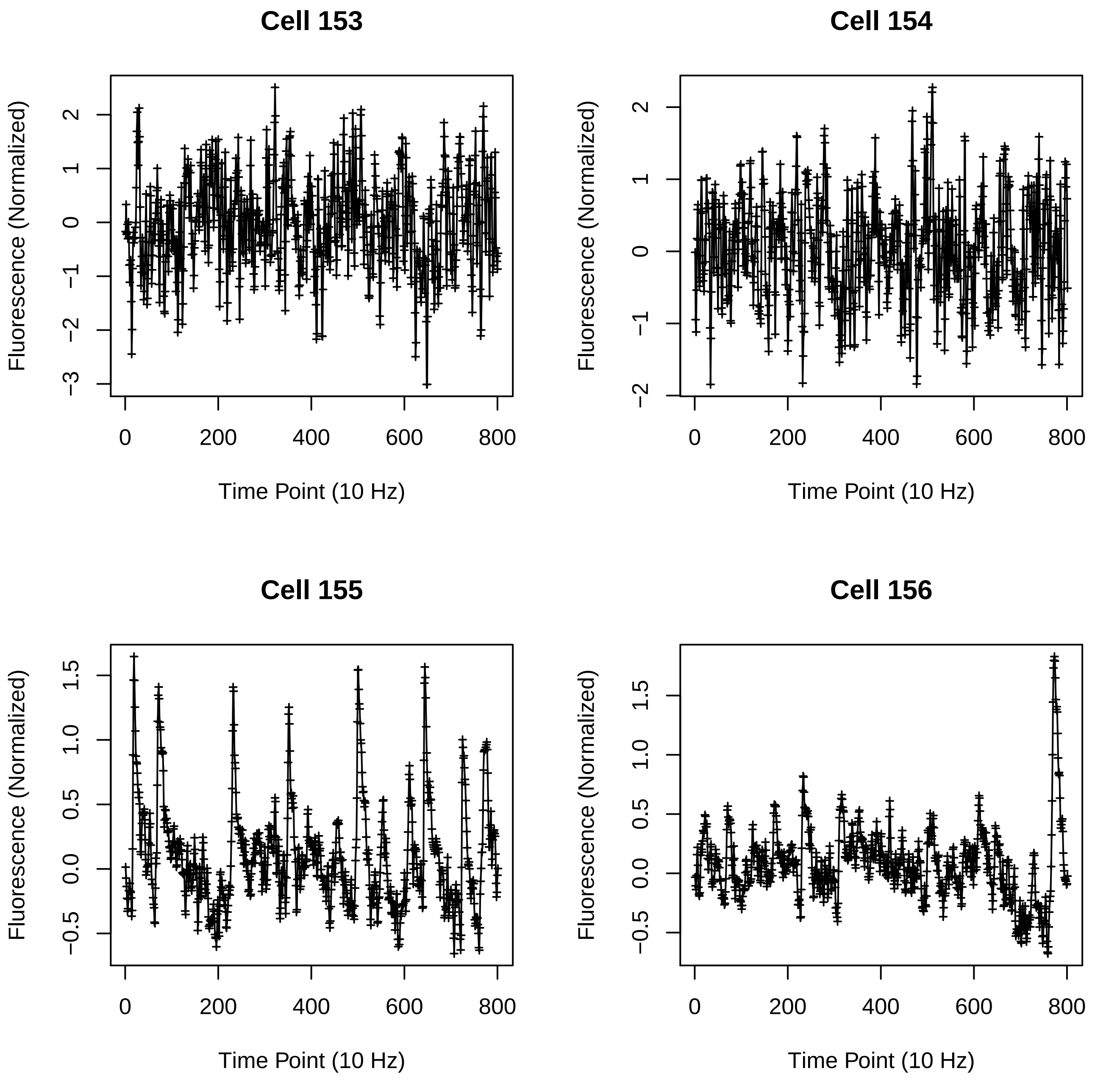

Cell 153
Cell 154
Cell 155
Cell 156
Fluorescence (Normalized)
Time Point (10 Hz)
0
200
400
600
800

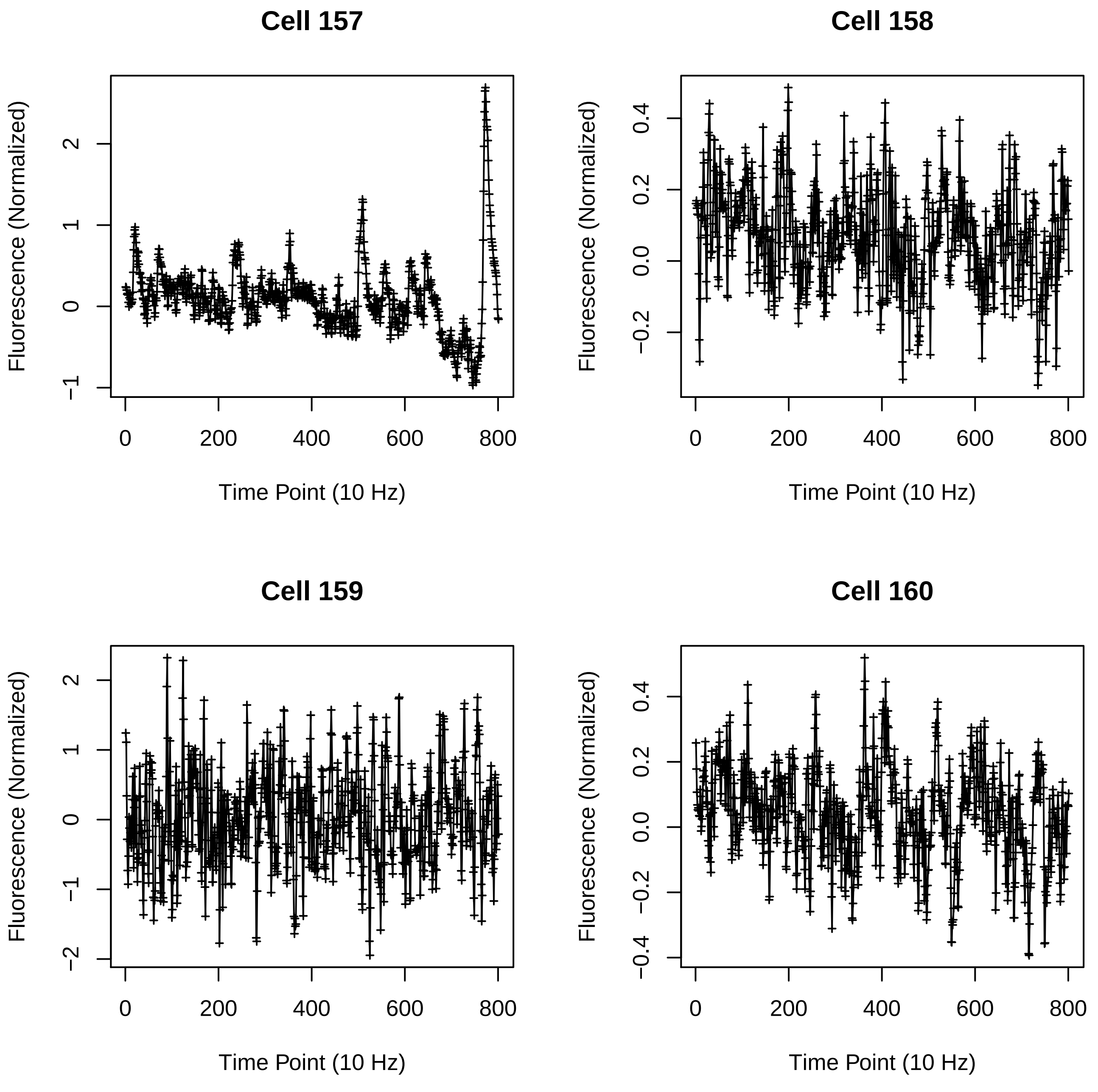
Cell 157
Cell 158
Cell 159
Cell 160
Fluorescence (Normalized)
Time Point (10 Hz)
0
200
400
600
800
−1
1
2
−0.2
0.0
0.2
0.4
−2
−0.4

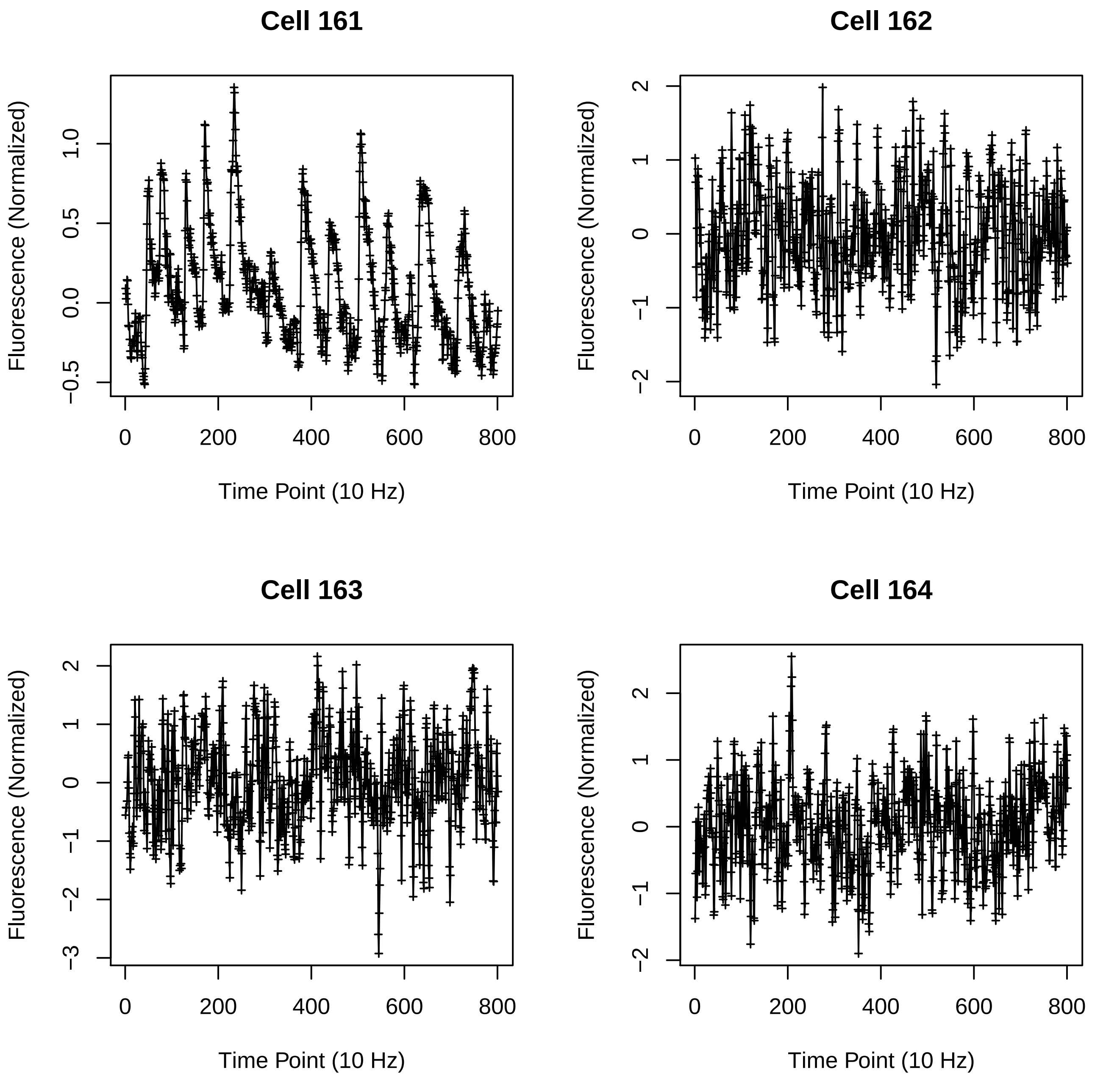
Cell 161
Cell 162
Cell 163
Cell 164
Fluorescence (Normalized)
Time Point (10 Hz)
0
200
400
600
800
−0.5
0.0
0.5
1.0
−3
−2
−1
0
1
2

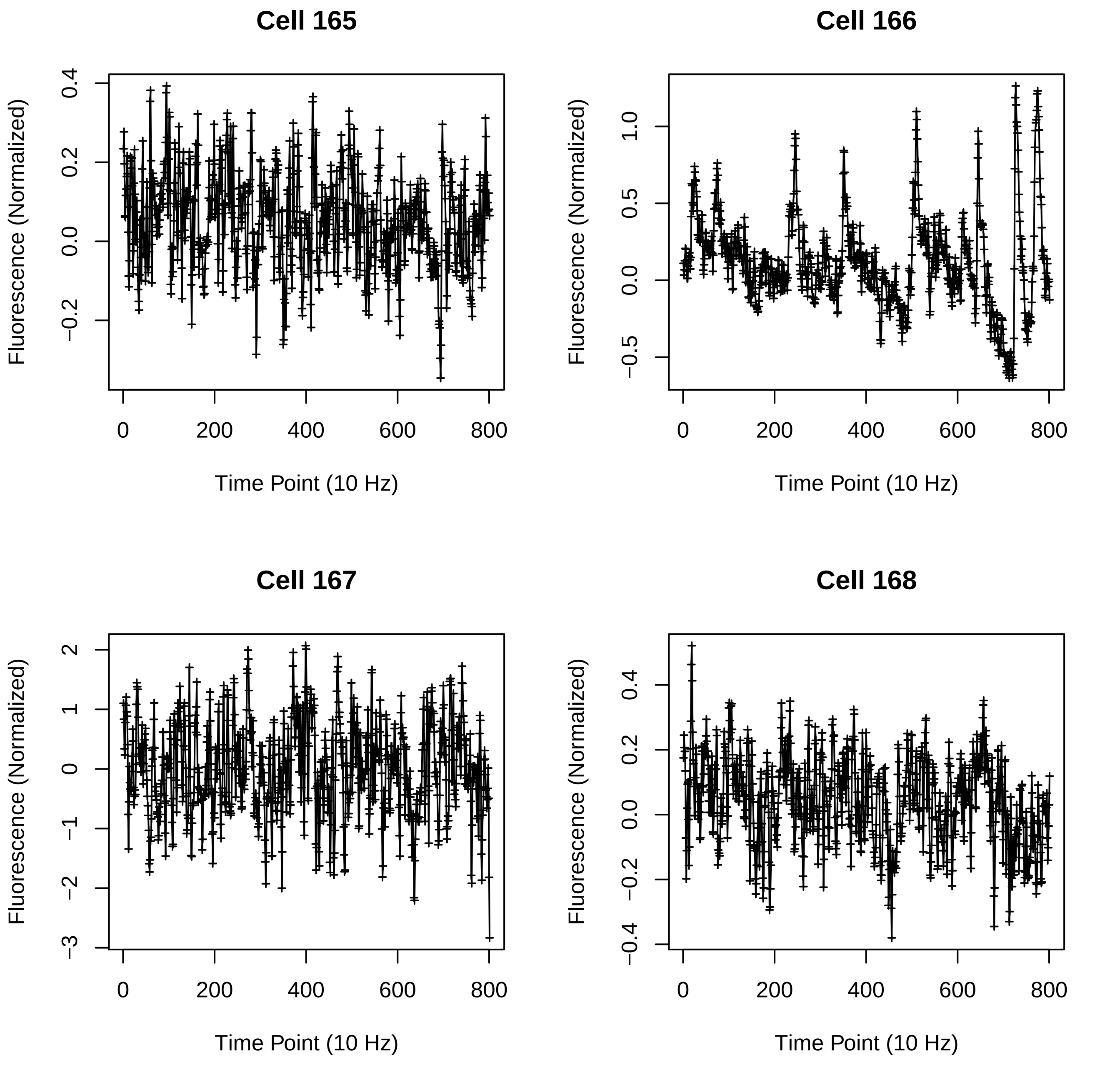
Cell 165
Cell 166
Cell 167
Cell 168
Fluorescence (Normalized)
Time Point (10 Hz)
0
200
400
600
800
0.4
0.2
0.0
−0.2
1.0
0.5
0.0
−0.5
2
1
0
−1
−2
−3
0.4
0.2
0.0
−0.2
−0.4

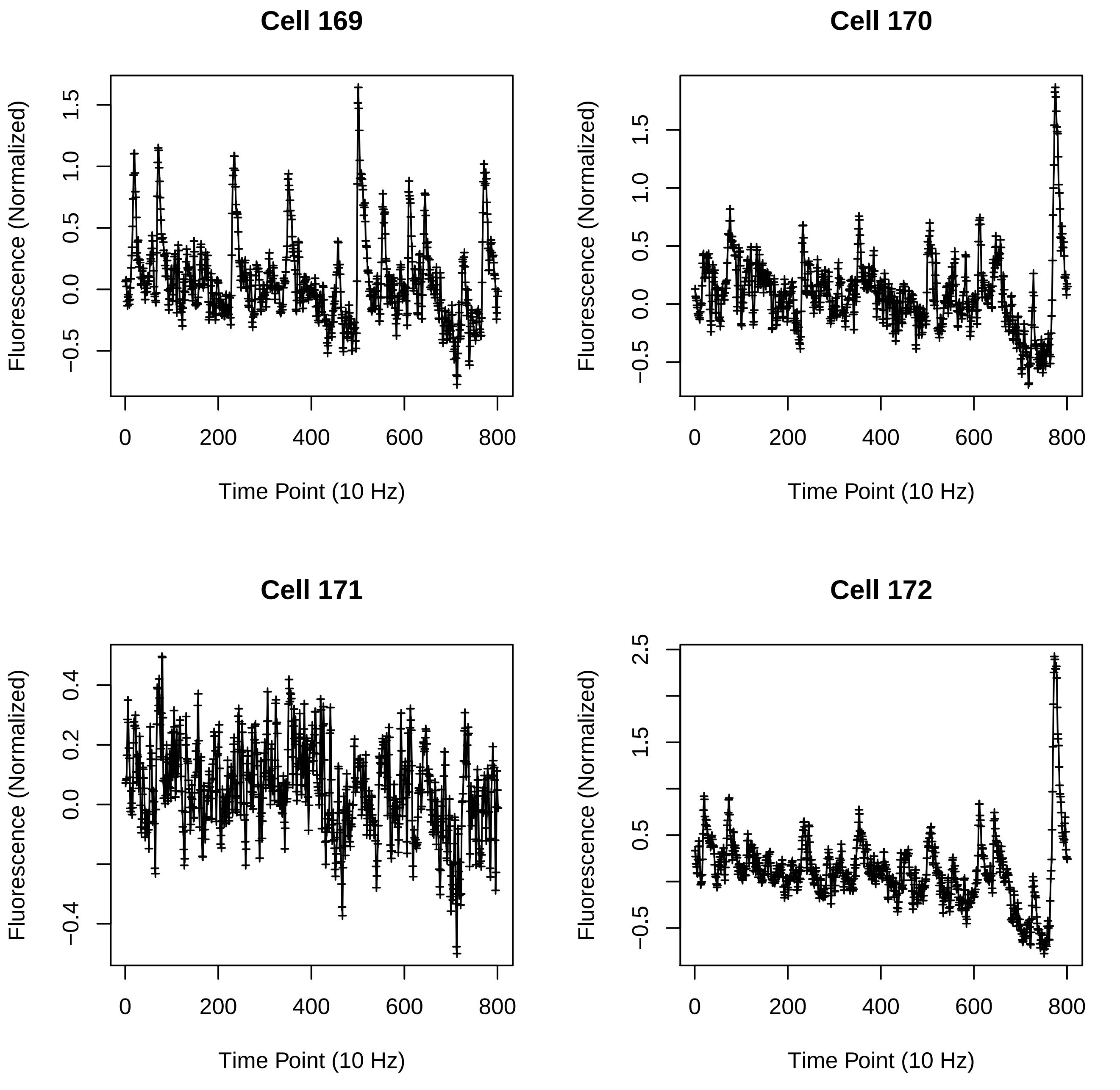
Cell 169
Cell 170
Cell 171
Cell 172
Fluorescence (Normalized)
Time Point (10 Hz)
0
200
400
600
800
−0.5
0.0
0.5
1.0
1.5
2.5
−0.4
0.2
0.4

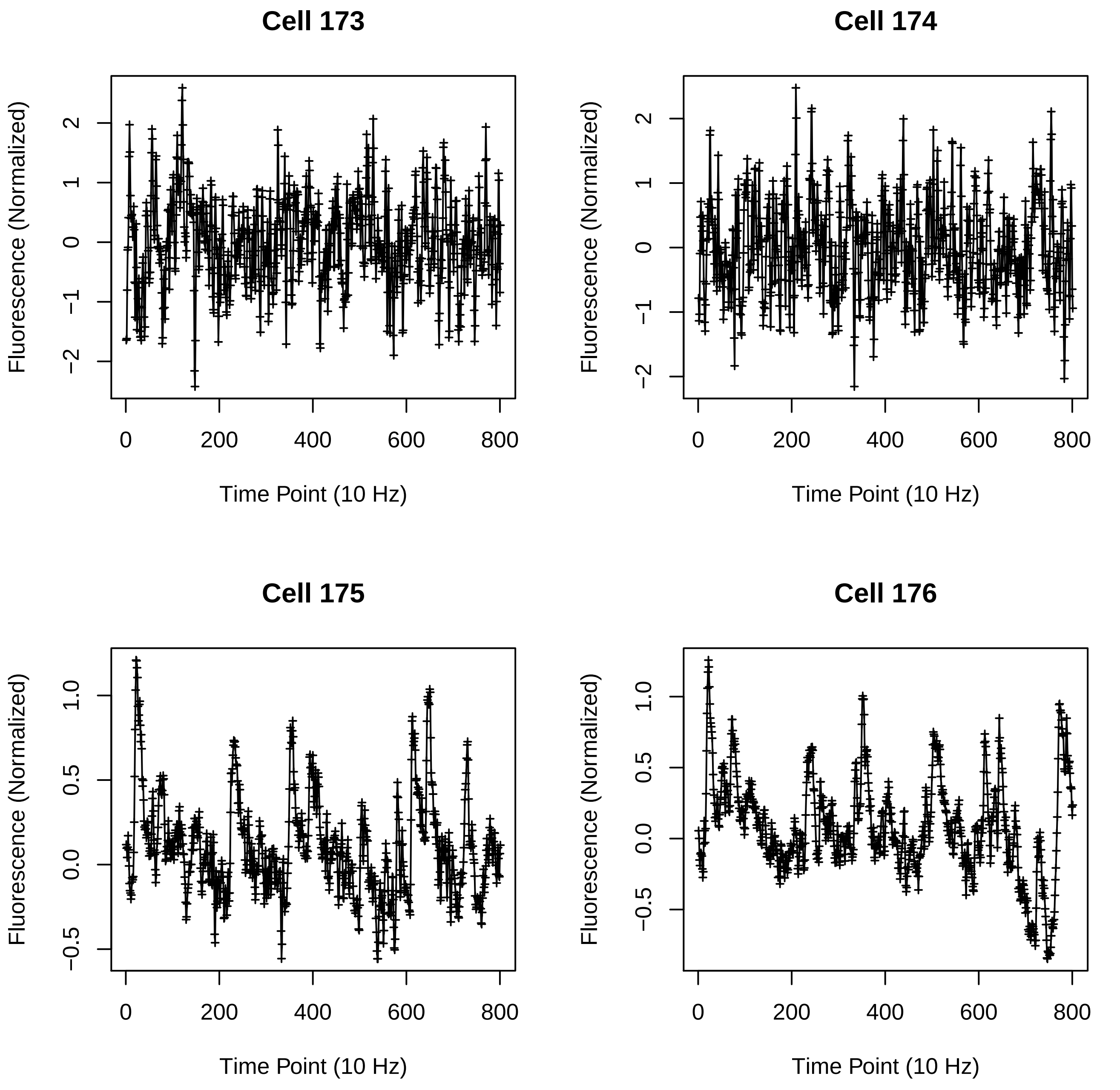
Cell 173
Cell 174
Cell 175
Cell 176
Fluorescence (Normalized)
Time Point (10 Hz)
0
200
400
600
800
−2
−1
0
1
2
−0.5
0.0
0.5
1.0

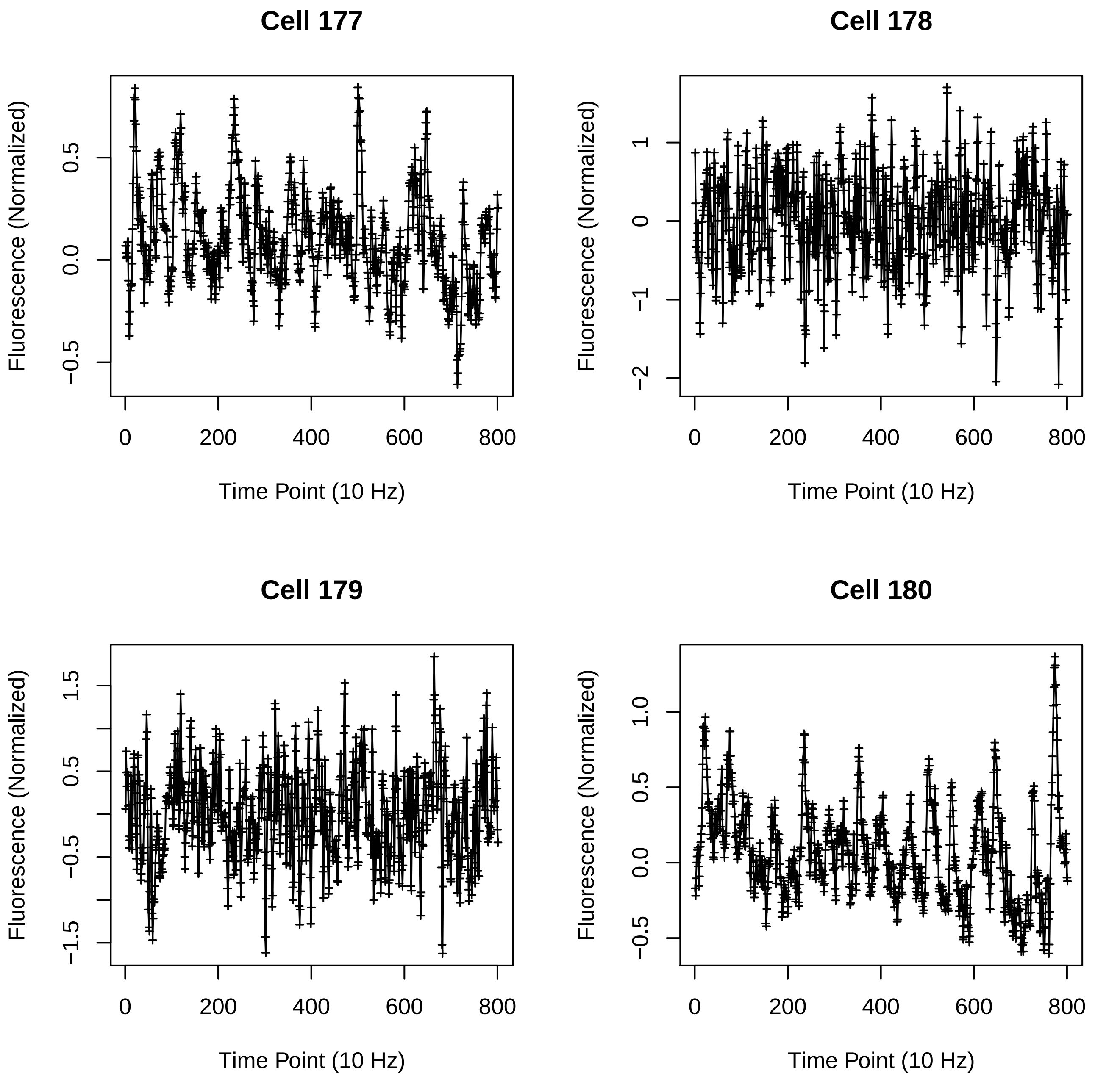

Cell 177
Fluorescence (Normalized)
Time Point (10 Hz)
Cell 178
Fluorescence (Normalized)
Time Point (10 Hz)
Cell 179
Fluorescence (Normalized)
Time Point (10 Hz)
Cell 180
Fluorescence (Normalized)
Time Point (10 Hz)

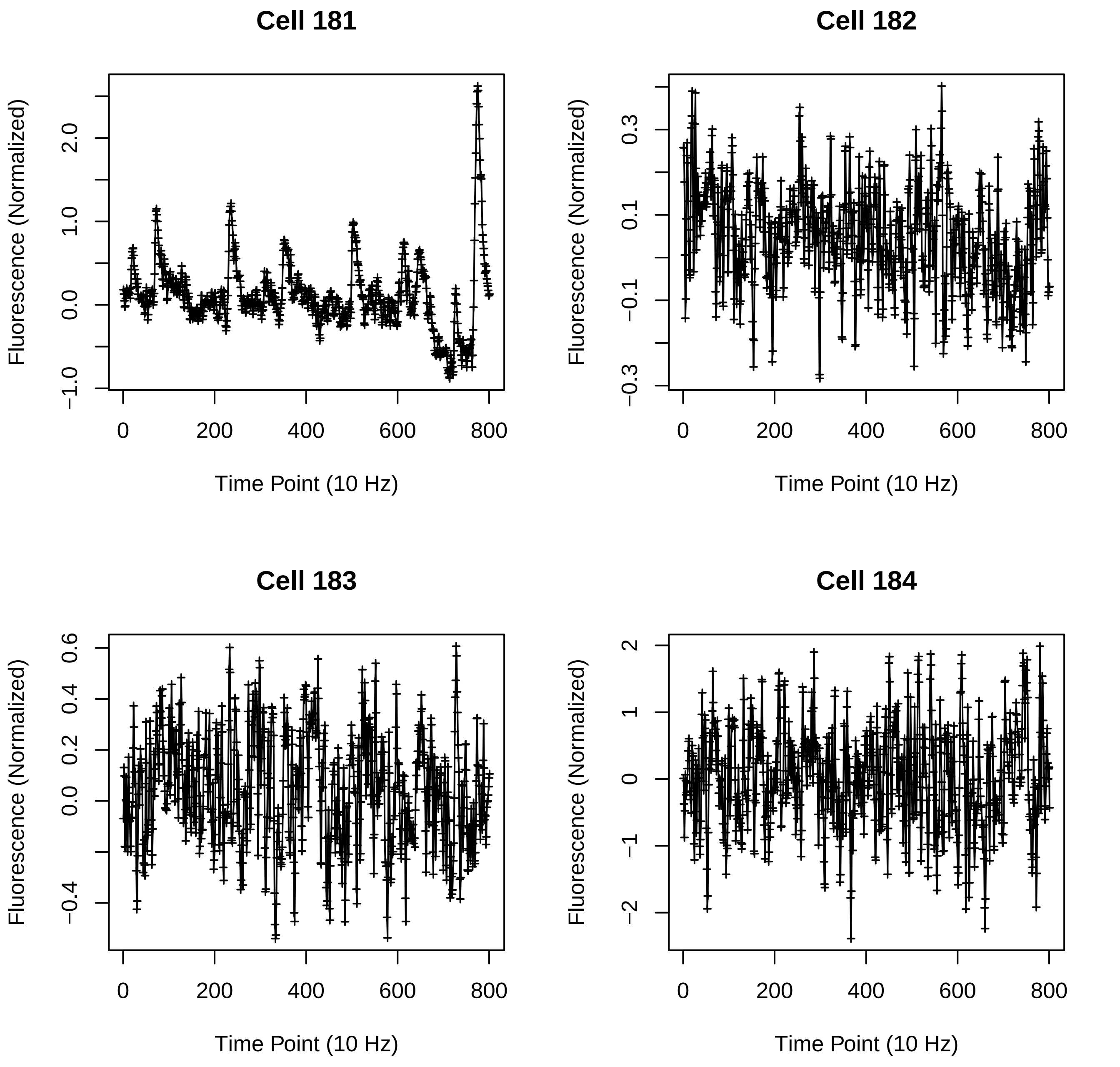
Cell 181
Cell 182
Cell 183
Cell 184
Fluorescence (Normalized)
Time Point (10 Hz)
0
200
400
600
800
−1.0
0.0
1.0
2.0
−0.3
−0.1
0.1
0.3
−0.4
0.0
0.2
0.4
0.6
−2
−1
0
1
2

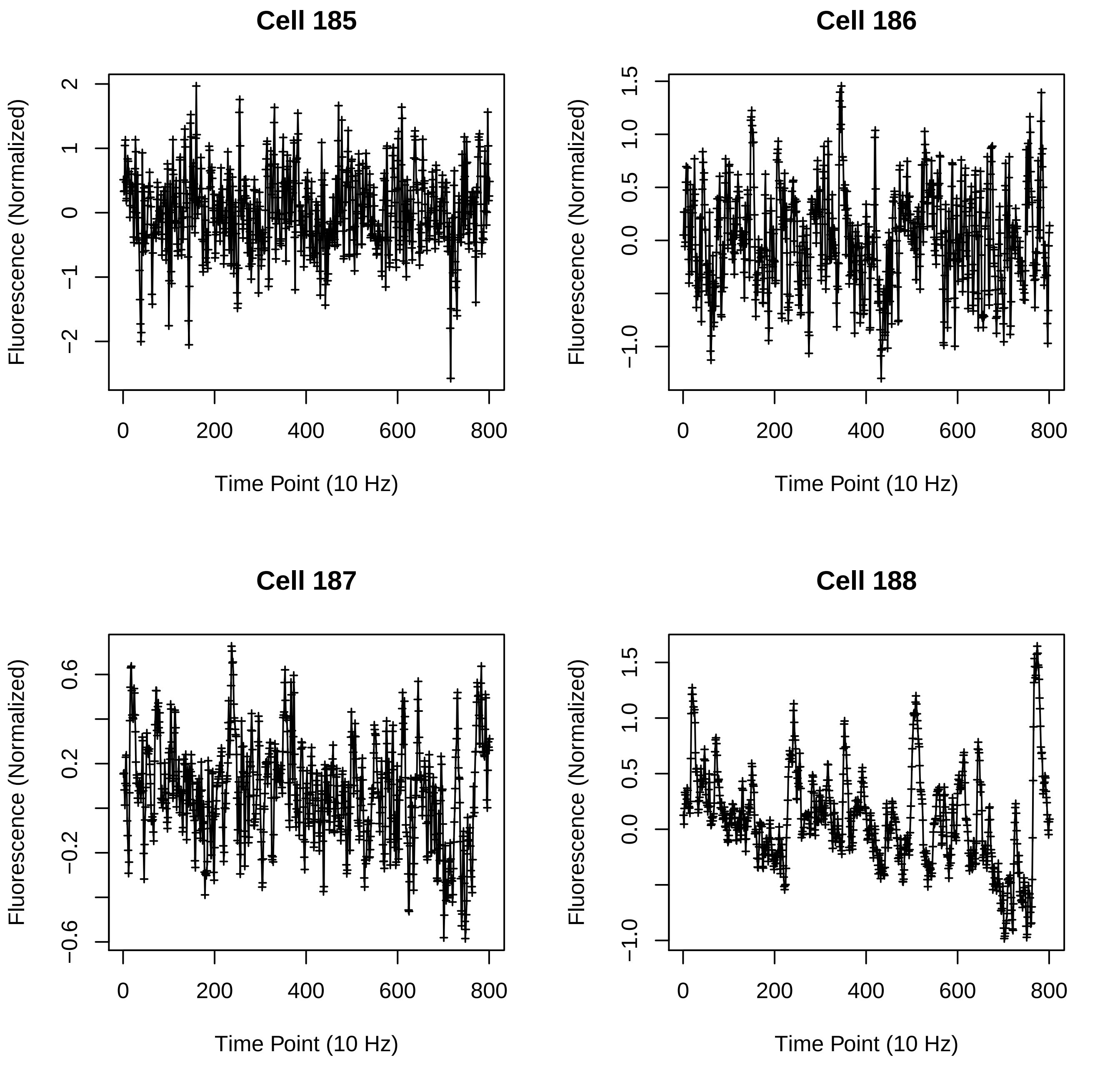

Cell 185
Cell 186
Cell 187
Cell 188
Fluorescence (Normalized)
Time Point (10 Hz)
0
200
400
600
800
2
1
0
−1
−2
1.5
1.0
0.5
0.0
−1.0
0.6
0.2
−0.2
−0.6

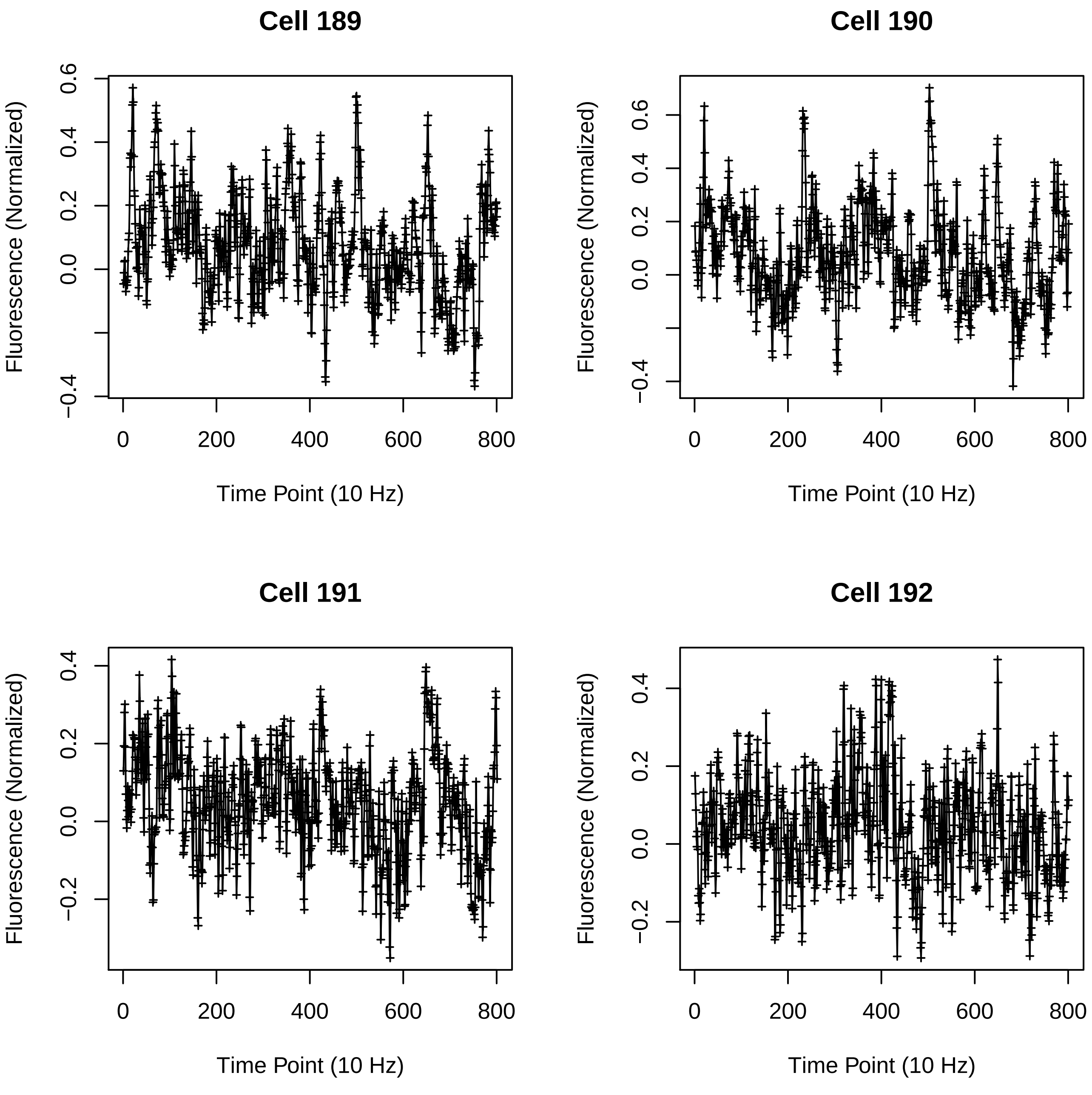
Cell 189
Cell 190
Cell 191
Cell 192
Fluorescence (Normalized)
Time Point (10 Hz)
0
200
400
600
800
−0.4
0.0
0.2
0.4
0.6
−0.2

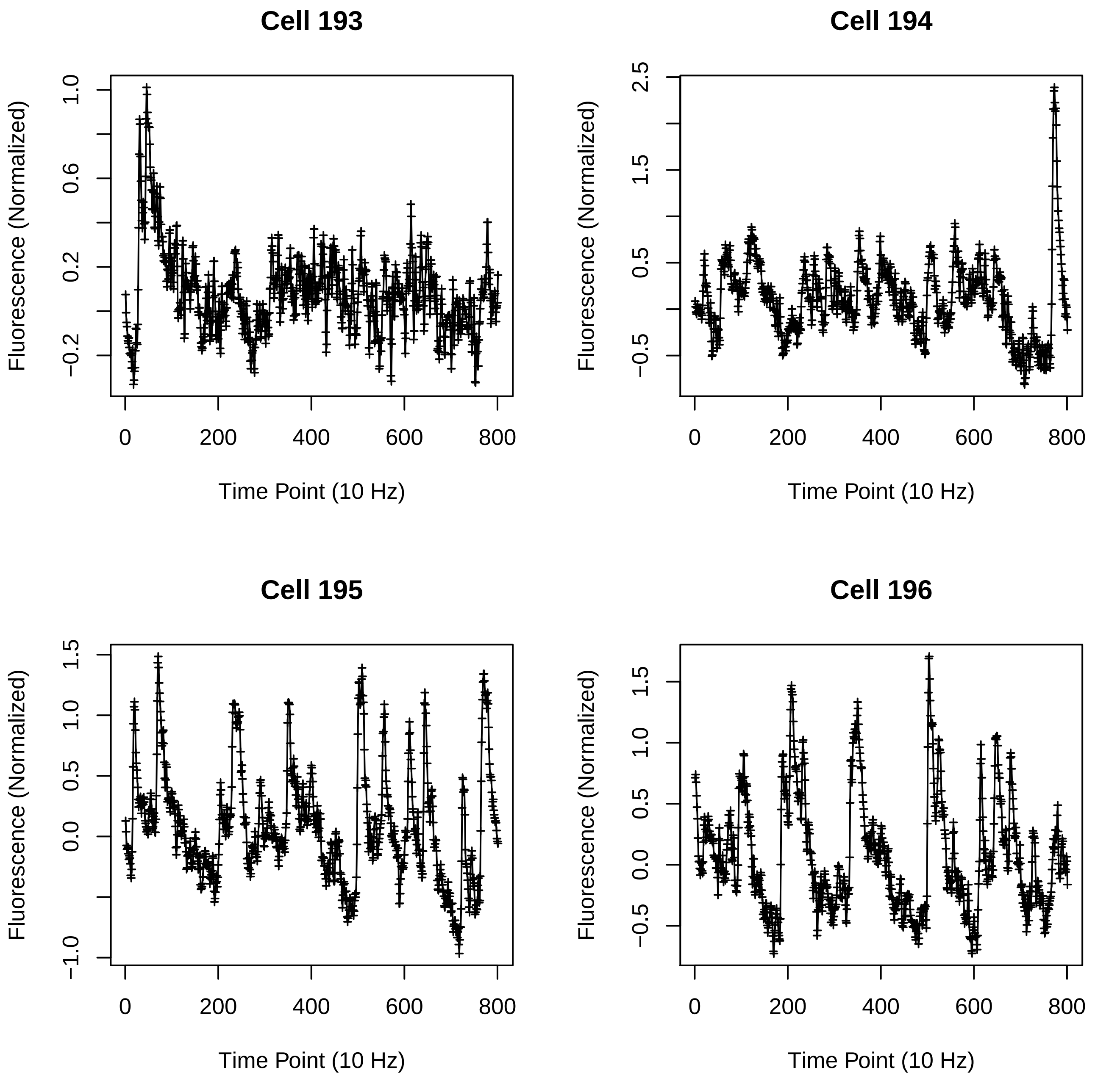

Cell 193
Cell 194
Cell 195
Cell 196
Fluorescence (Normalized)
Time Point (10 Hz)
0
200
400
600
800
−0.2
0.2
0.6
1.0
−0.5
0.5
1.5
2.5
−1.0
0.0
0.5
1.0
1.5

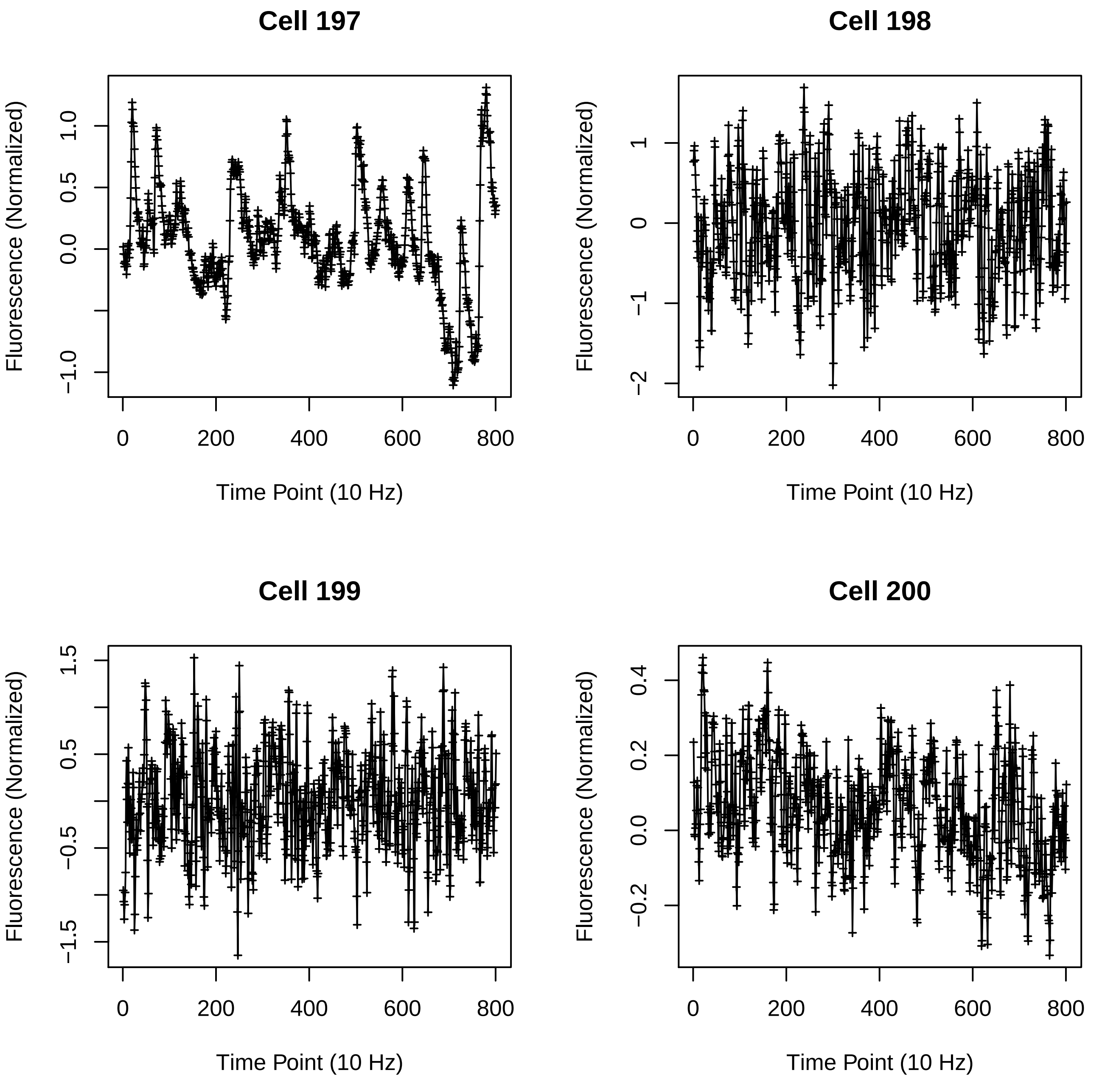
Cell 197
Cell 198
Cell 199
Cell 200
Fluorescence (Normalized)
Time Point (10 Hz)
0
200
400
600
800
−1.0
0.0
0.5
1.0
−2
−1
0
1
−1.5
−0.5
0.5
1.5
−0.2
0.0
0.2
0.4

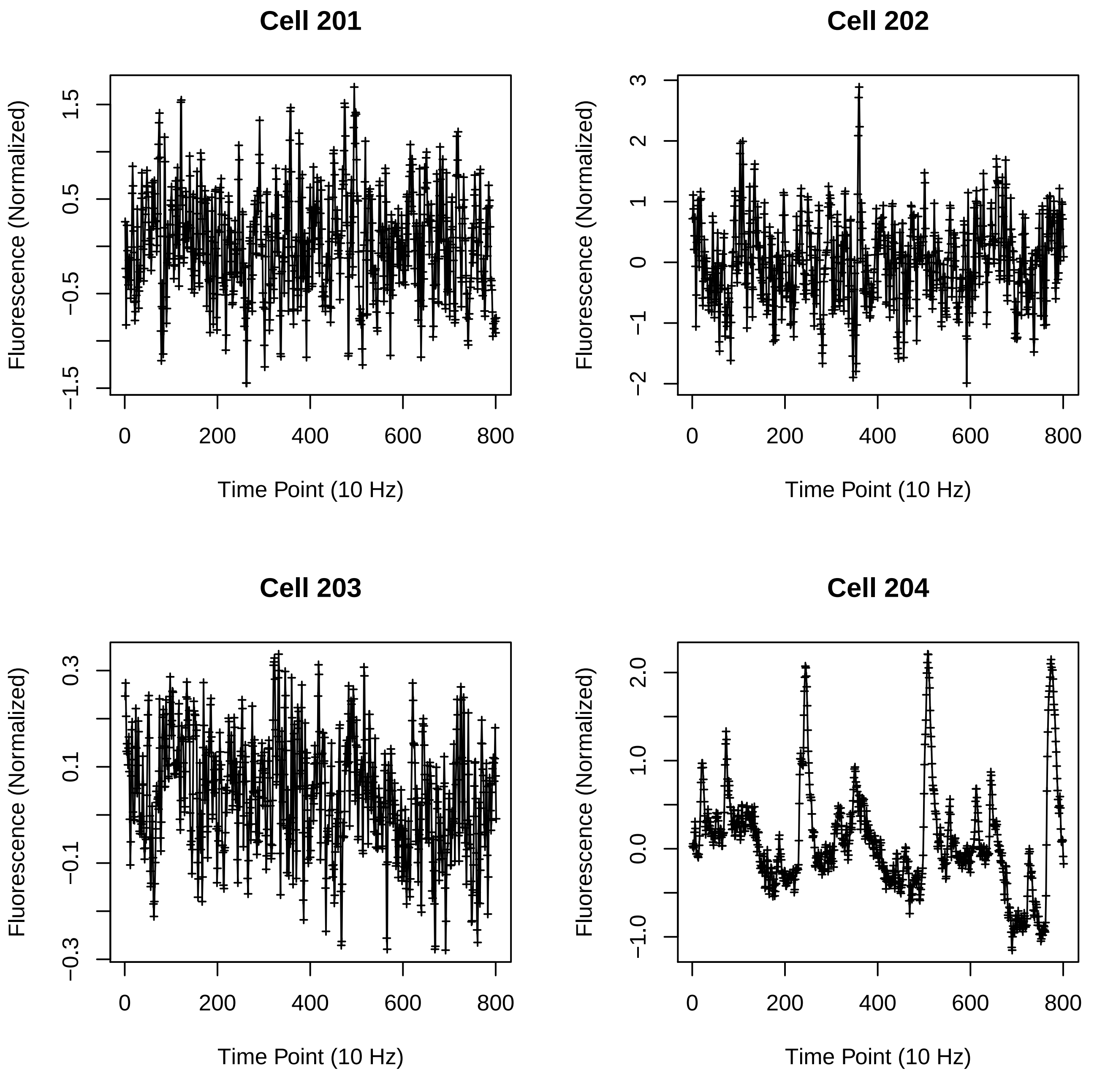
Cell 201
Cell 202
Cell 203
Cell 204
Fluorescence (Normalized)
Time Point (10 Hz)
0
200
400
600
800
1.5
0.5
−0.5
−1.5
3
2
1
0
−1
−2
0.3
0.1
−0.1
−0.3
2.0
1.0
0.0
−1.0

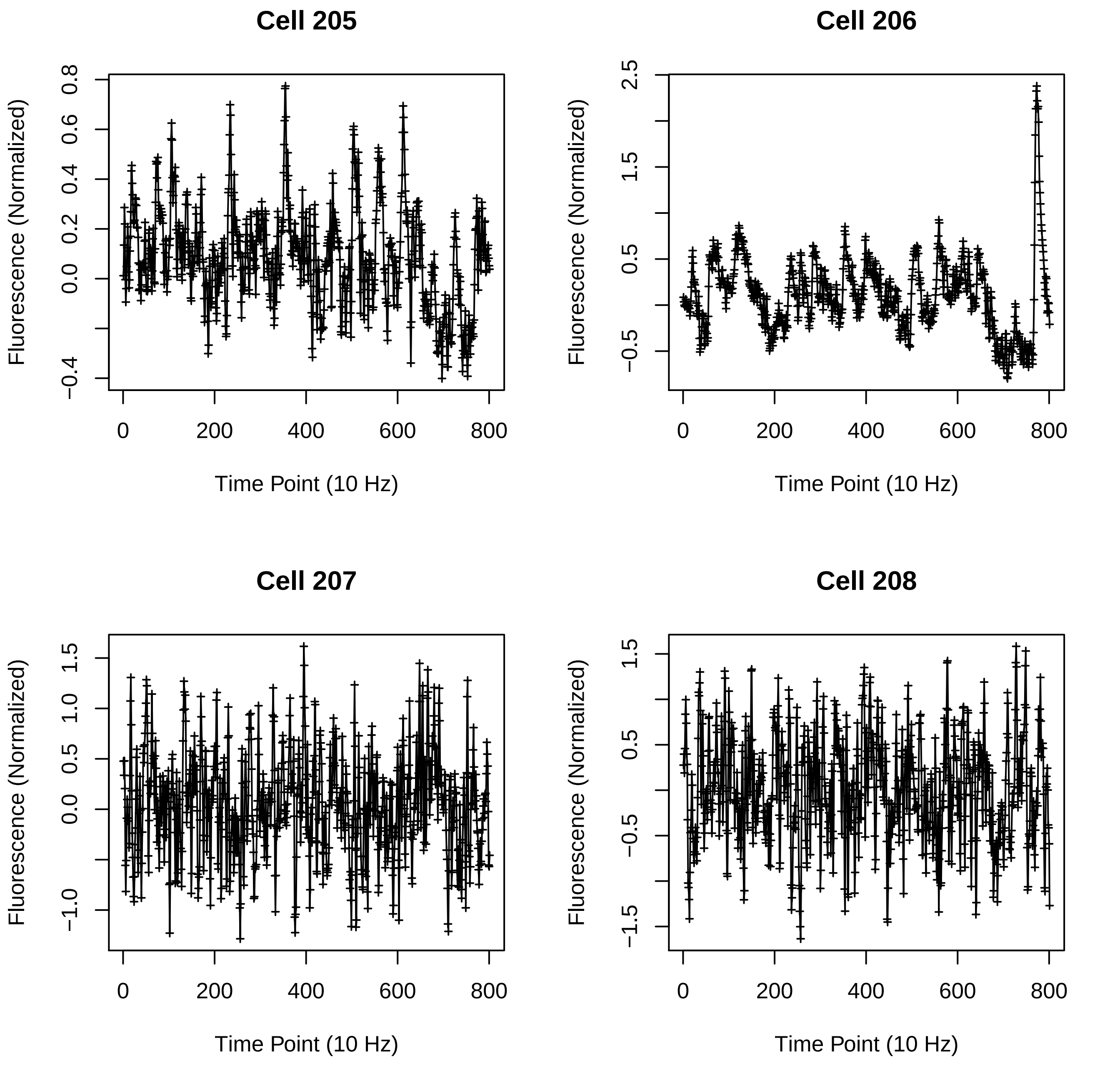
Cell 205
Cell 206
Cell 207
Cell 208
Fluorescence (Normalized)
Time Point (10 Hz)
0
200
400
600
800
−0.4
0.0
0.2
0.4
0.6
0.8
−0.5
0.5
1.5
2.5
−1.0
0.0
0.5
1.0
1.5
−1.5
−0.5
0.5
1.5

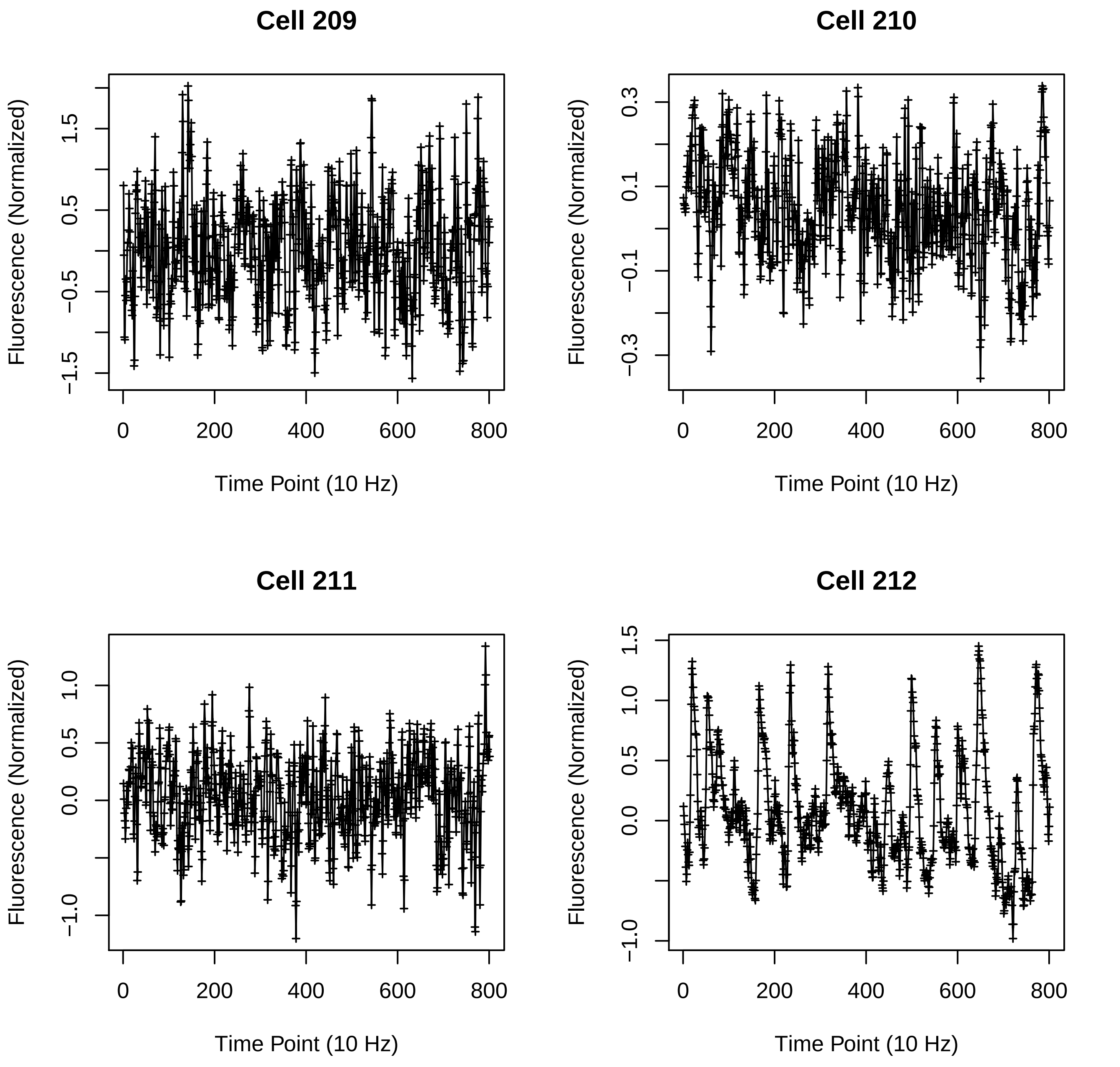
Cell 209
Cell 210
Cell 211
Cell 212
Fluorescence (Normalized)
Time Point (10 Hz)
0
200
400
600
800
1.5
0.5
−0.5
−1.5
0.3
0.1
−0.1
−0.3
1.0
0.5
0.0
−1.0

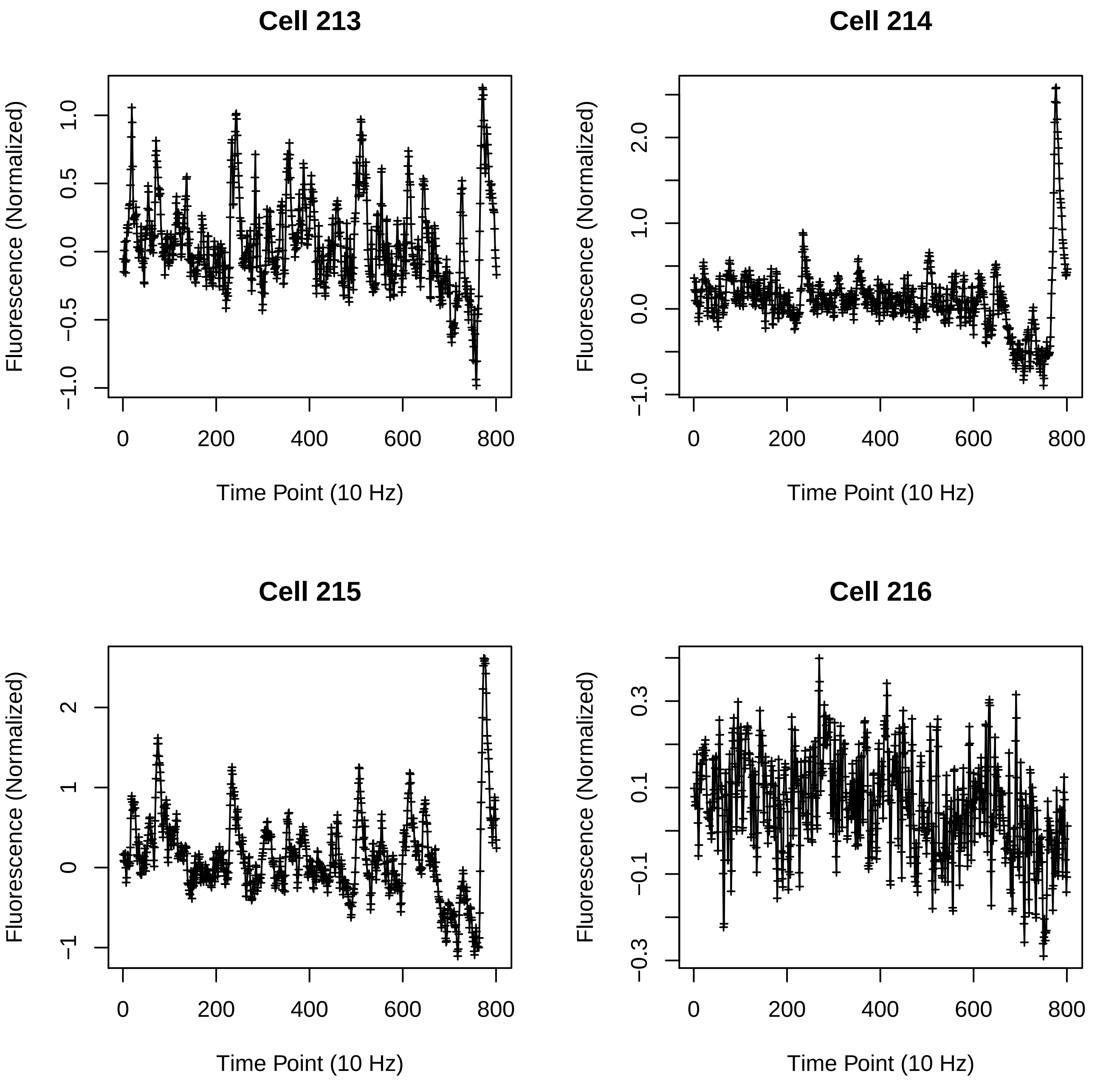
Cell 213
Cell 214
Cell 215
Cell 216
Fluorescence (Normalized)
Time Point (10 Hz)
−1.0
−0.5
0.0
0.5
1.0
2.0
−1
0
1
2
−0.3
−0.1
0.1
0.3
0
200
400
600
800

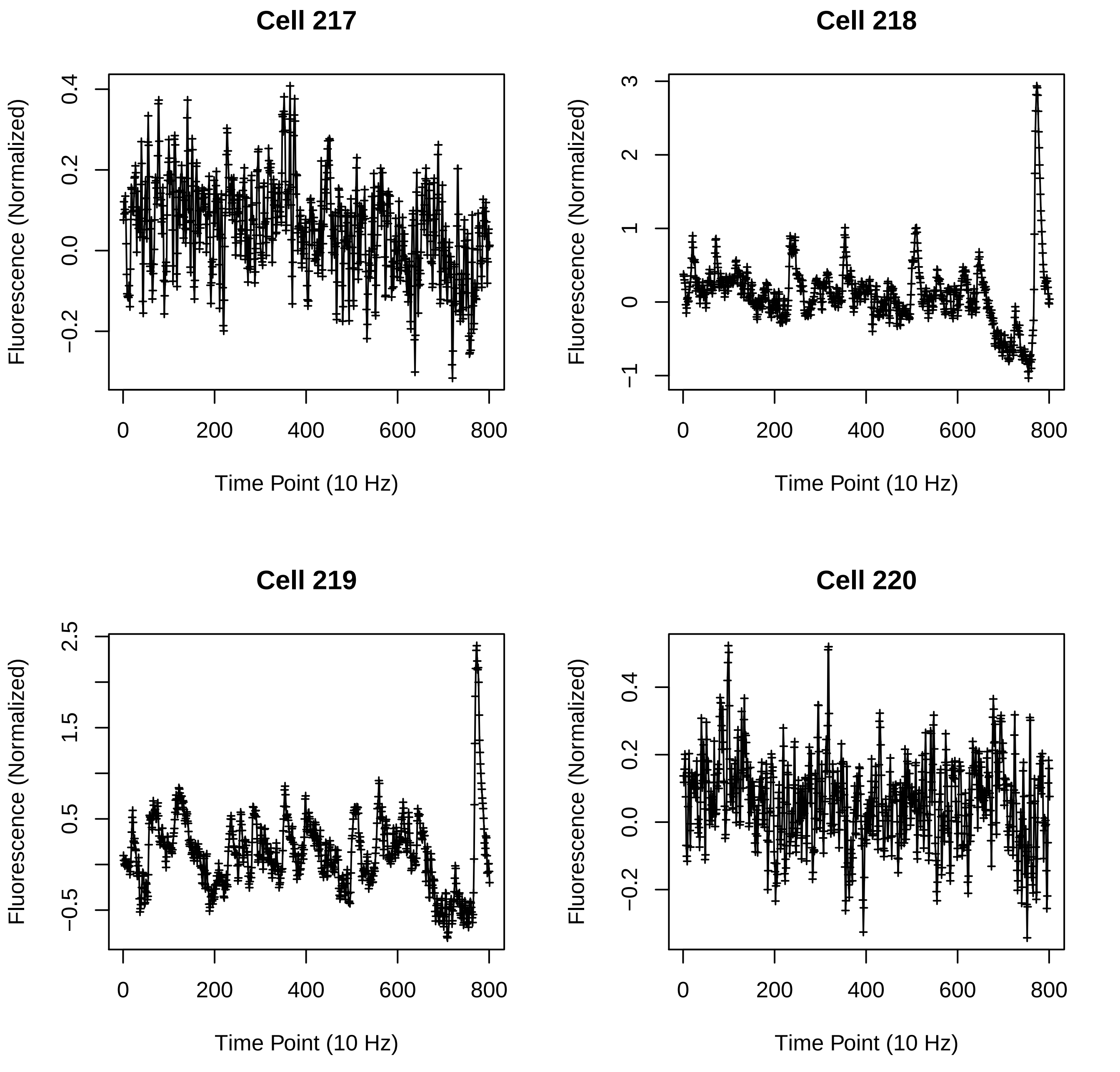

Cell 217
Fluorescence (Normalized)
−0.2
0.0
0.2
0.4
0
200
400
600
800
Time Point (10 Hz)
Cell 218
Fluorescence (Normalized)
−1
0
1
2
3
0
200
400
600
800
Time Point (10 Hz)
Cell 219
Fluorescence (Normalized)
−0.5
0.5
1.5
2.5
0
200
400
600
800
Time Point (10 Hz)
Cell 220
Fluorescence (Normalized)
−0.2
0.0
0.2
0.4
0
200
400
600
800
Time Point (10 Hz)

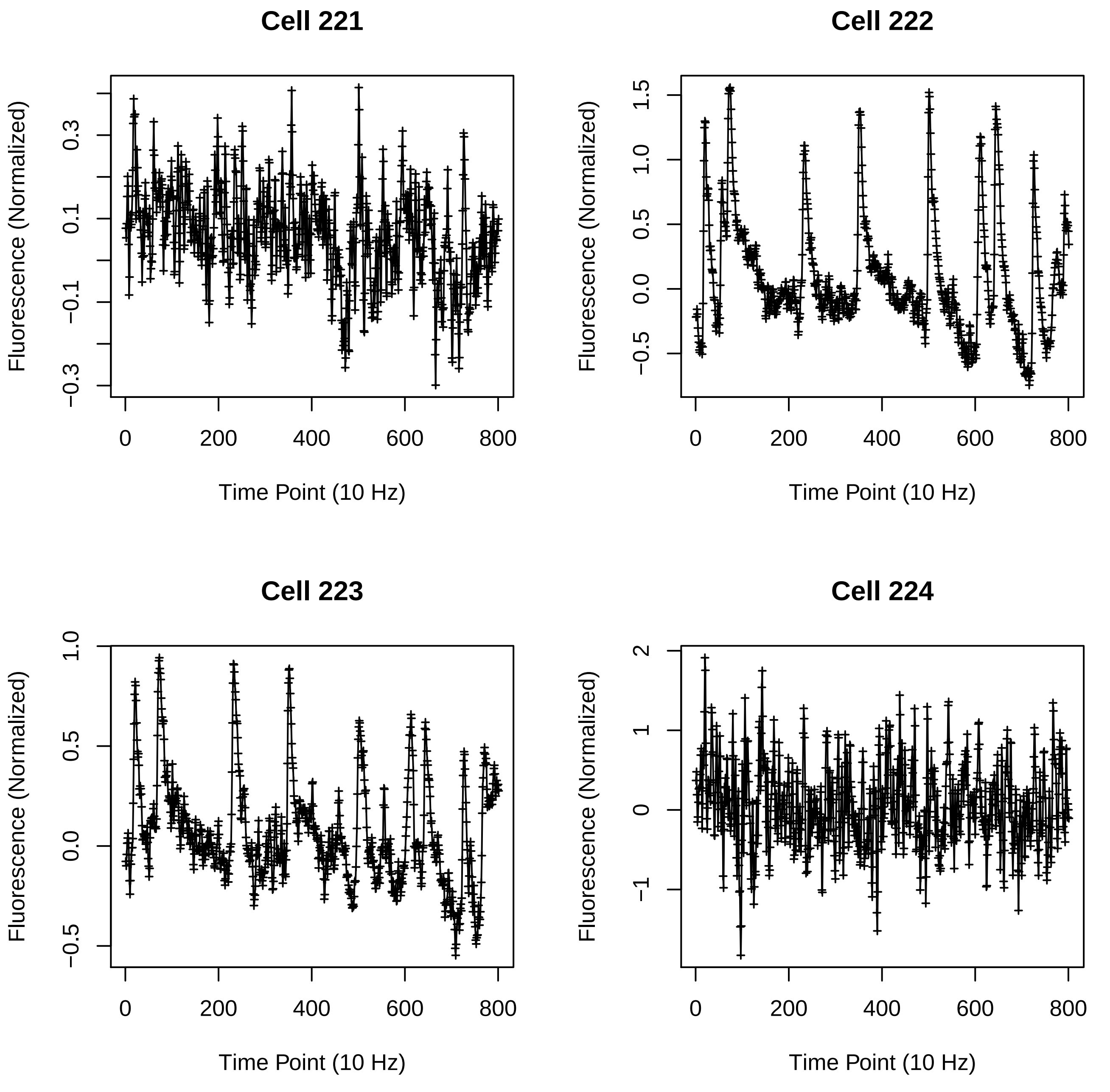
Cell 221
Cell 222
Cell 223
Cell 224
Fluorescence (Normalized)
Time Point (10 Hz)
0
200
400
600
800
−0.3
−0.1
0.1
0.3
−0.5
0.0
0.5
1.0
1.5
−1
0
1
2

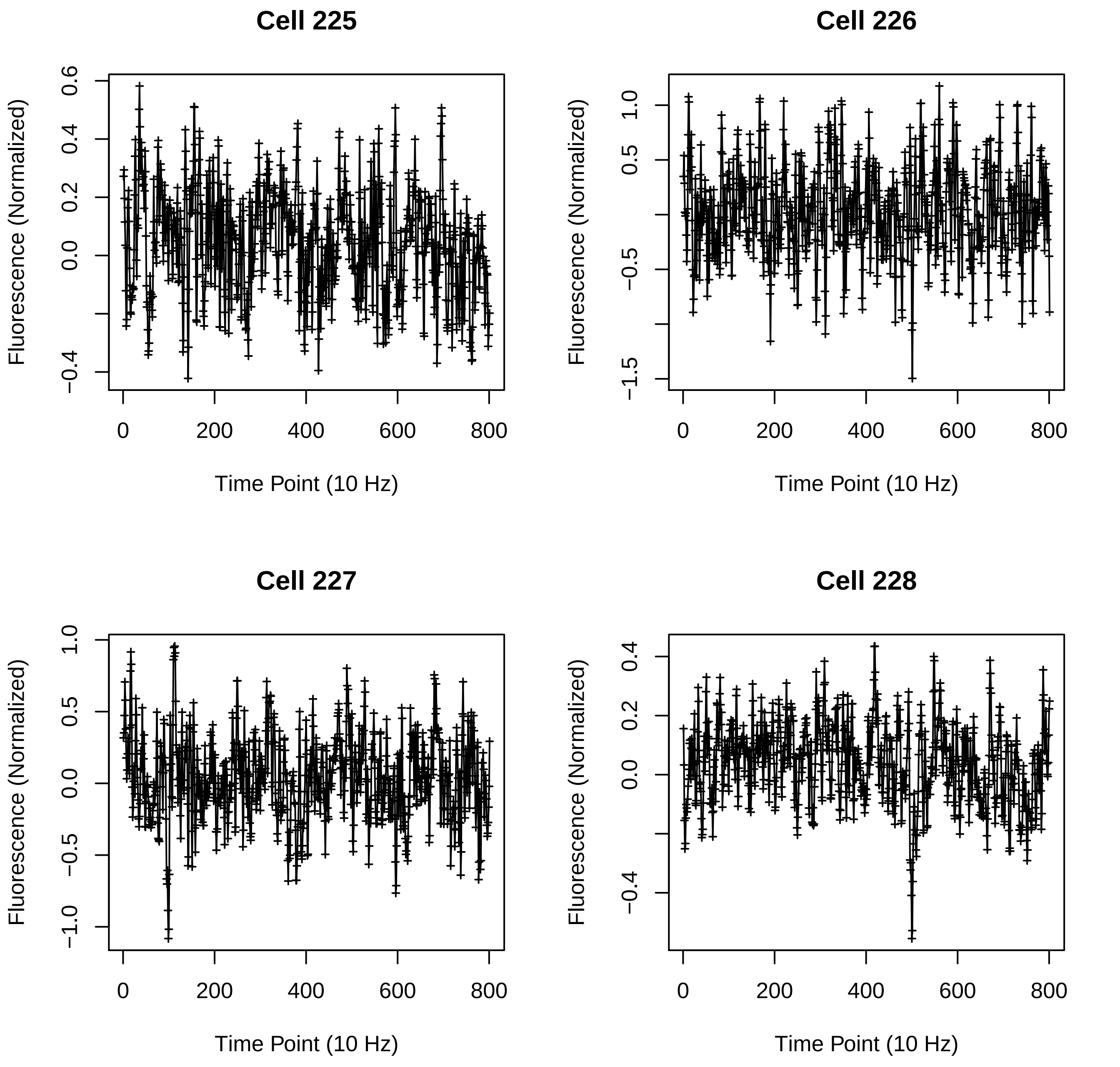
Cell 225
Cell 226
Cell 227
Cell 228
Fluorescence (Normalized)
Time Point (10 Hz)
0
200
400
600
800
0.6
0.4
0.2
0.0
−0.4
1.0
0.5
−0.5
−1.5
−1.0

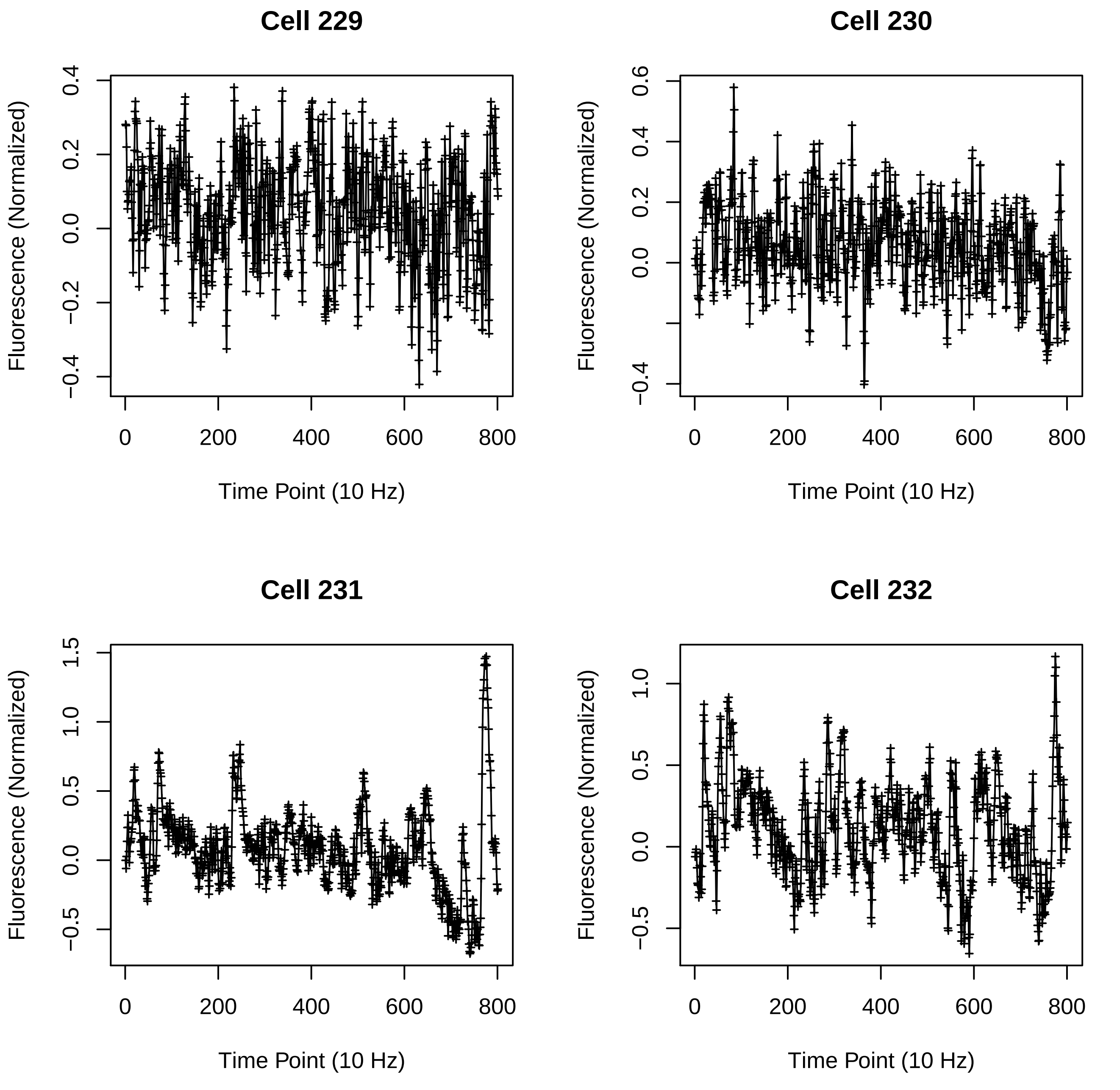
Cell 229
Cell 230
Cell 231
Cell 232
Fluorescence (Normalized)
Time Point (10 Hz)
0
200
400
600
800
−0.4
−0.2
0.0
0.2
0.4
0.6
−0.5
0.5
1.0
1.5

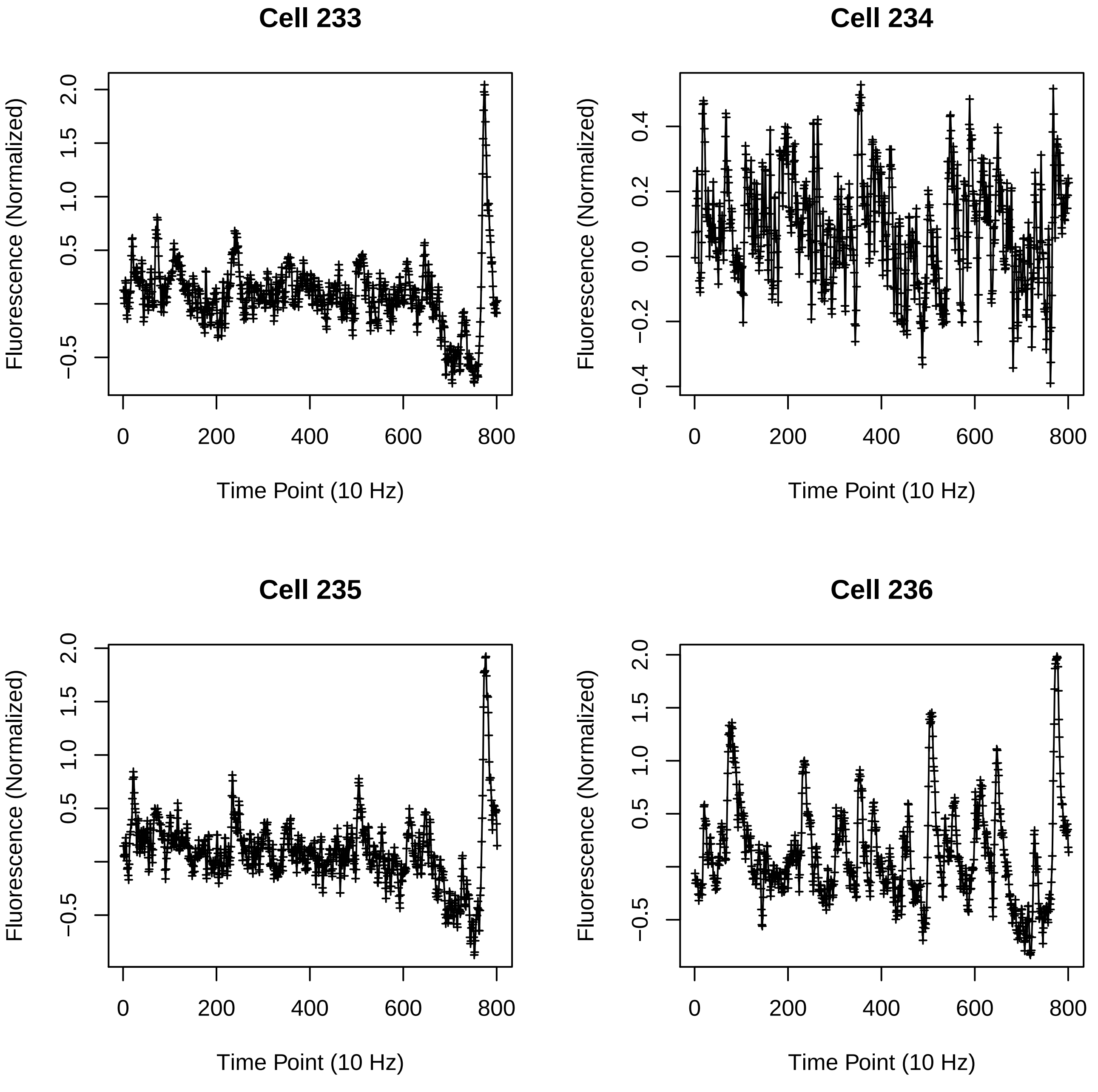
Cell 233
Cell 234
Cell 235
Cell 236
Fluorescence (Normalized)
Time Point (10 Hz)
0
200
400
600
800
−0.5
0.5
1.0
1.5
2.0
−0.4
−0.2
0.0
0.2
0.4

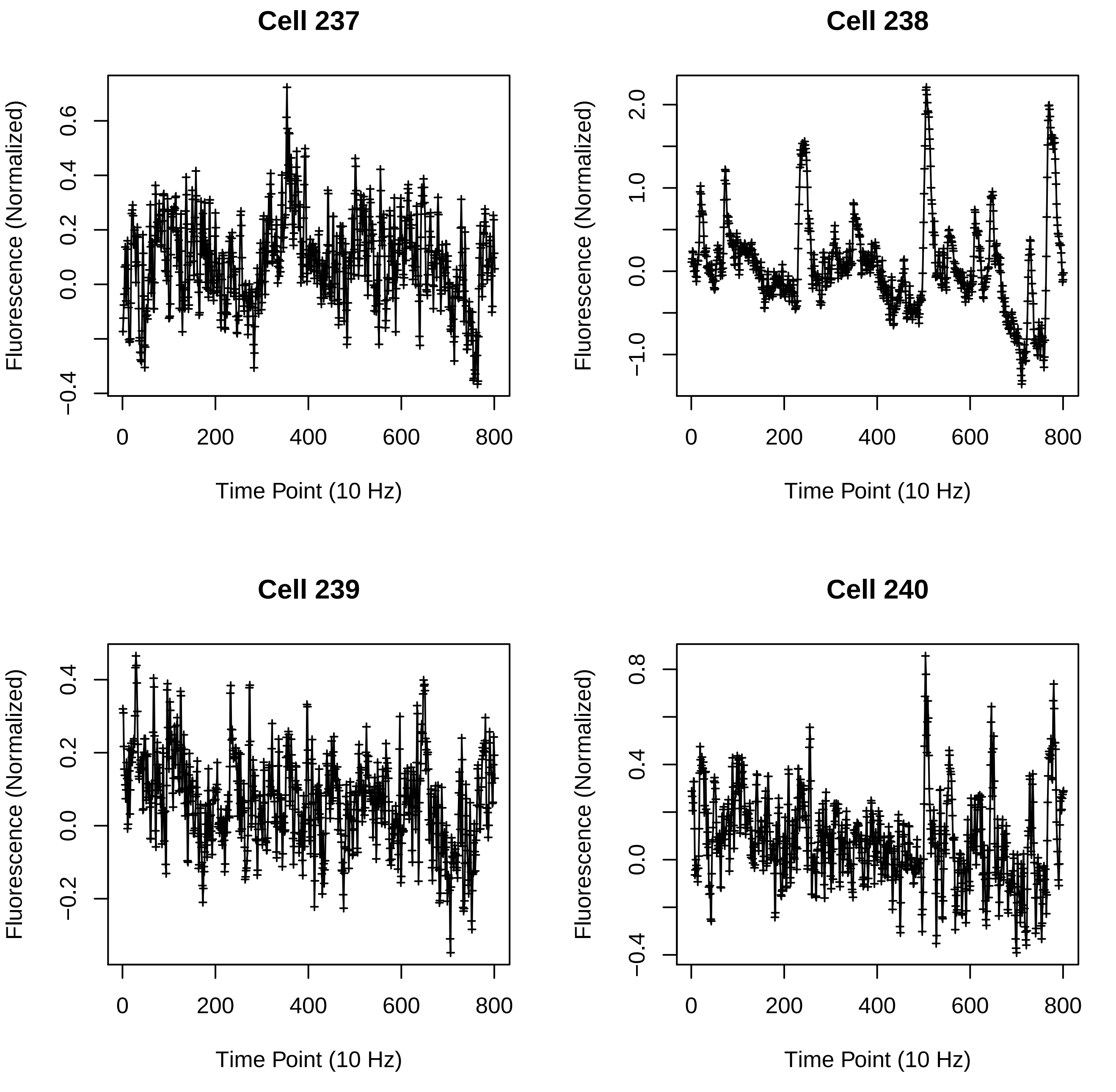
Cell 237
Cell 238
Cell 239
Cell 240
Fluorescence (Normalized)
Time Point (10 Hz)
0
200
400
600
800
−0.4
0.0
0.2
0.4
0.6
−1.0
1.0
2.0
−0.2
0.8

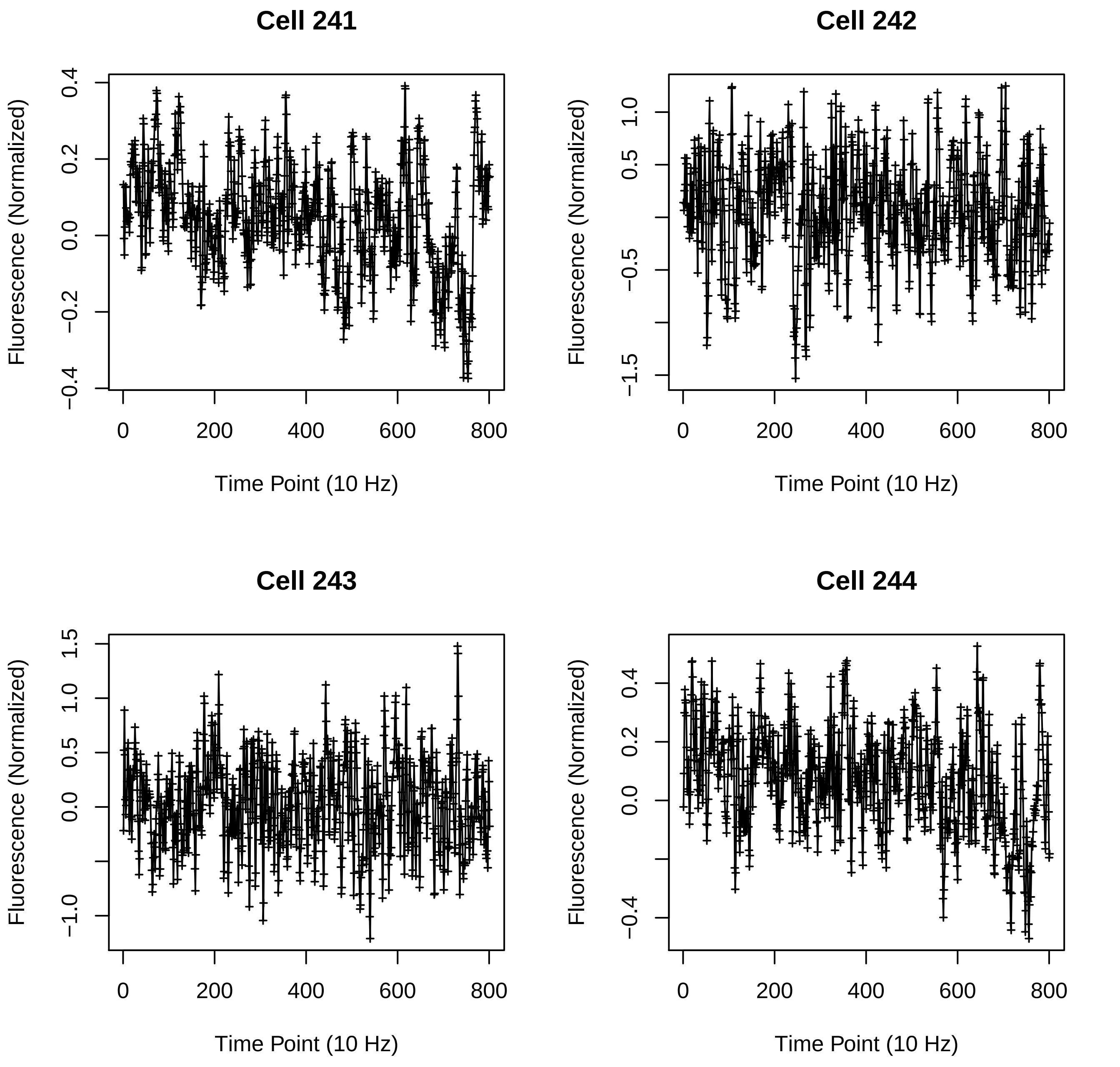
Cell 241
Cell 242
Cell 243
Cell 244
Fluorescence (Normalized)
Time Point (10 Hz)
0
200
400
600
800
0.4
0.2
0.0
−0.2
−0.4
1.0
0.5
−0.5
−1.5
1.5
−1.0
