# Supplementary material for: A Markovian Entropy Measure for the Analysis of Calcium Activity Time Series
Source: PLoS One. 2016 Dec 15;11(12):e0168342. doi: 10.1371/journal.pone.0168342 (PMC5158058; doi:10.1371/journal.pone.0168342)
Supplement: S1 File — (PDF) [file pone.0168342.s008.pdf]

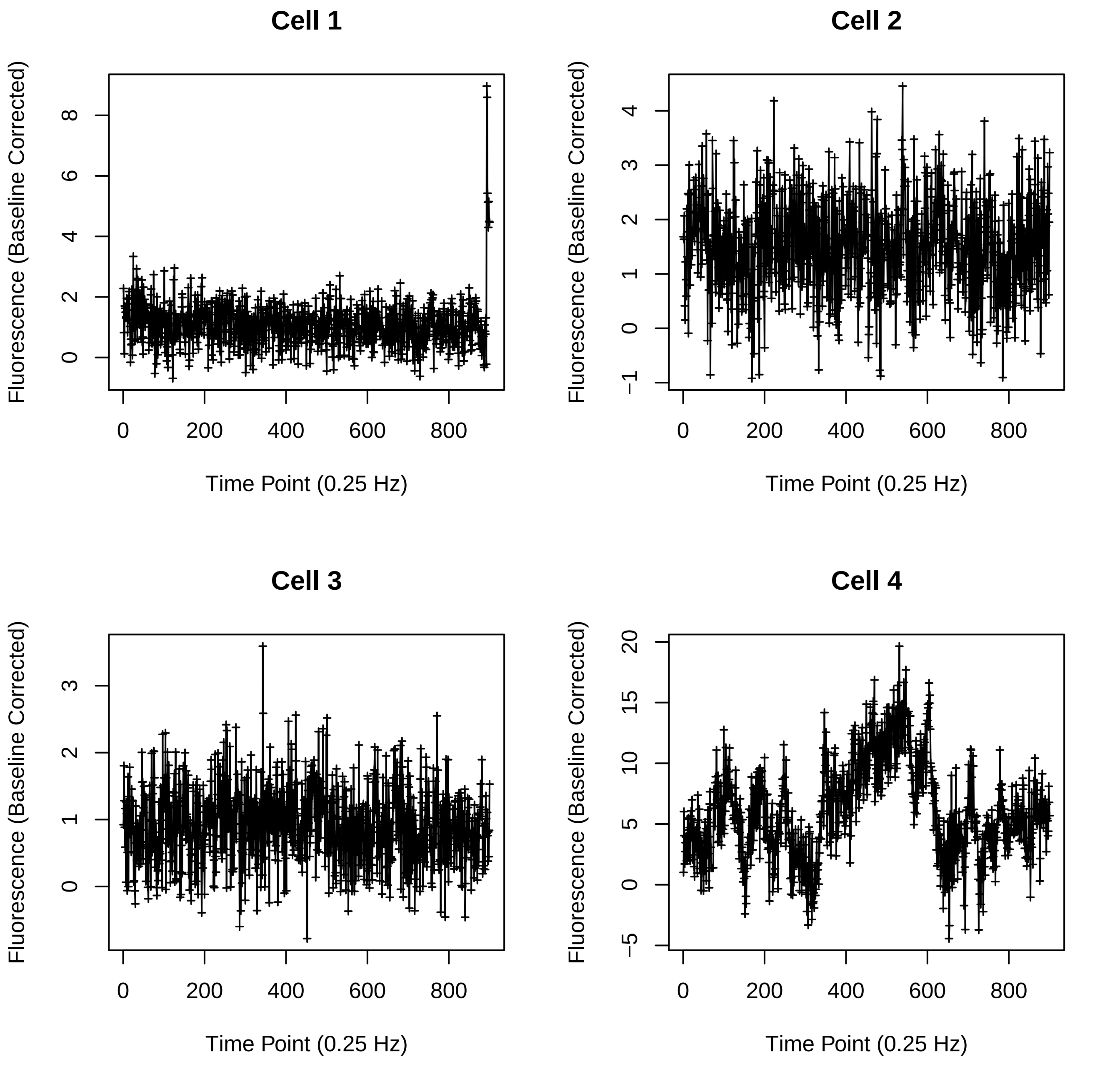
Cell 1
Cell 2
Cell 3
Cell 4
Fluorescence (Baseline Corrected)
Time Point (0.25 Hz)
0
200
400
600
800
-1
2
4
6
8
3
-5
5
10
15
20

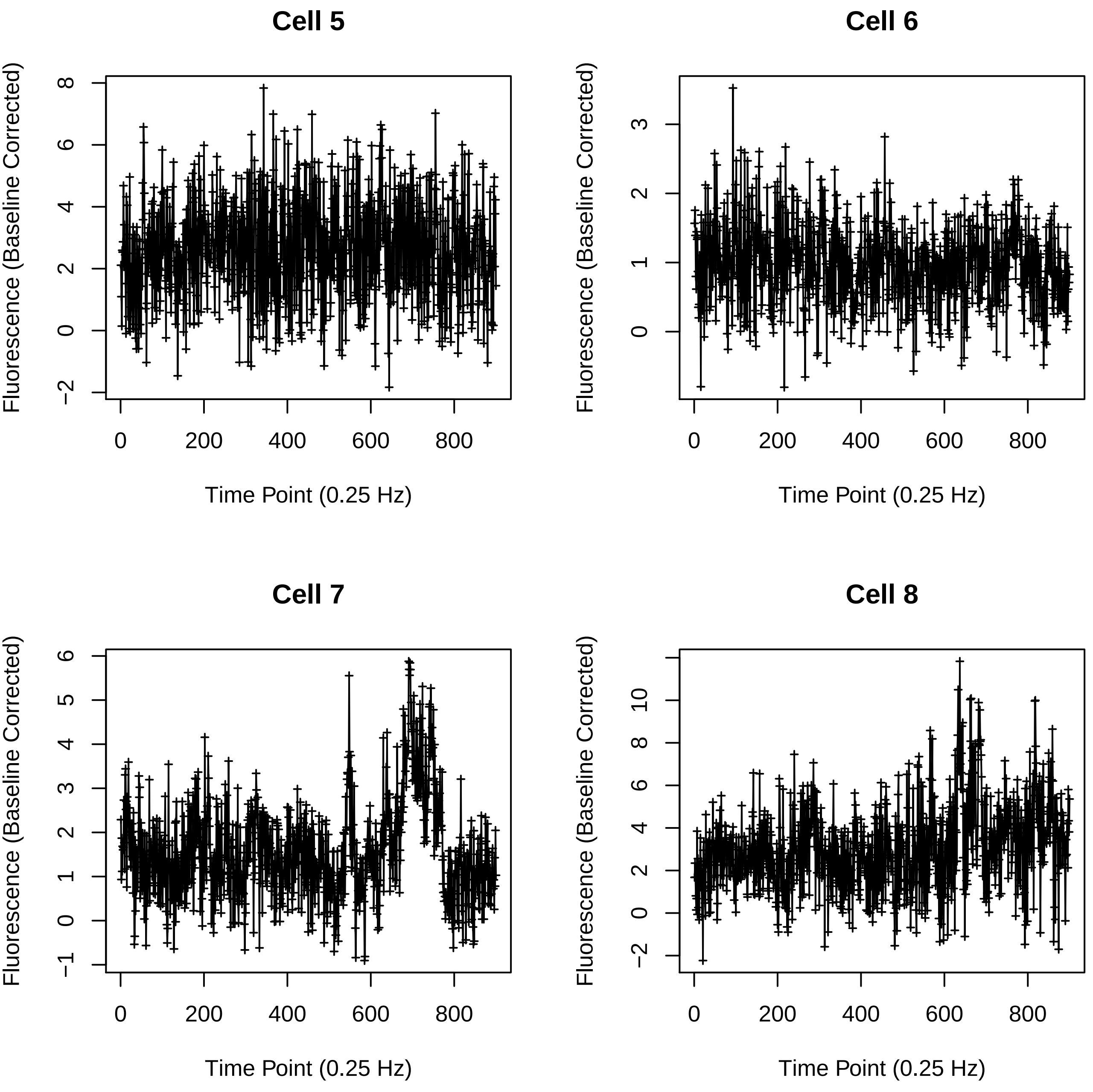
Cell 5
Cell 6
Cell 7
Cell 8
Fluorescence (Baseline Corrected)
Time Point (0.25 Hz)
−2
0
2
4
6
8
0
1
2
3
−1
5
10
0
200
400
600
800

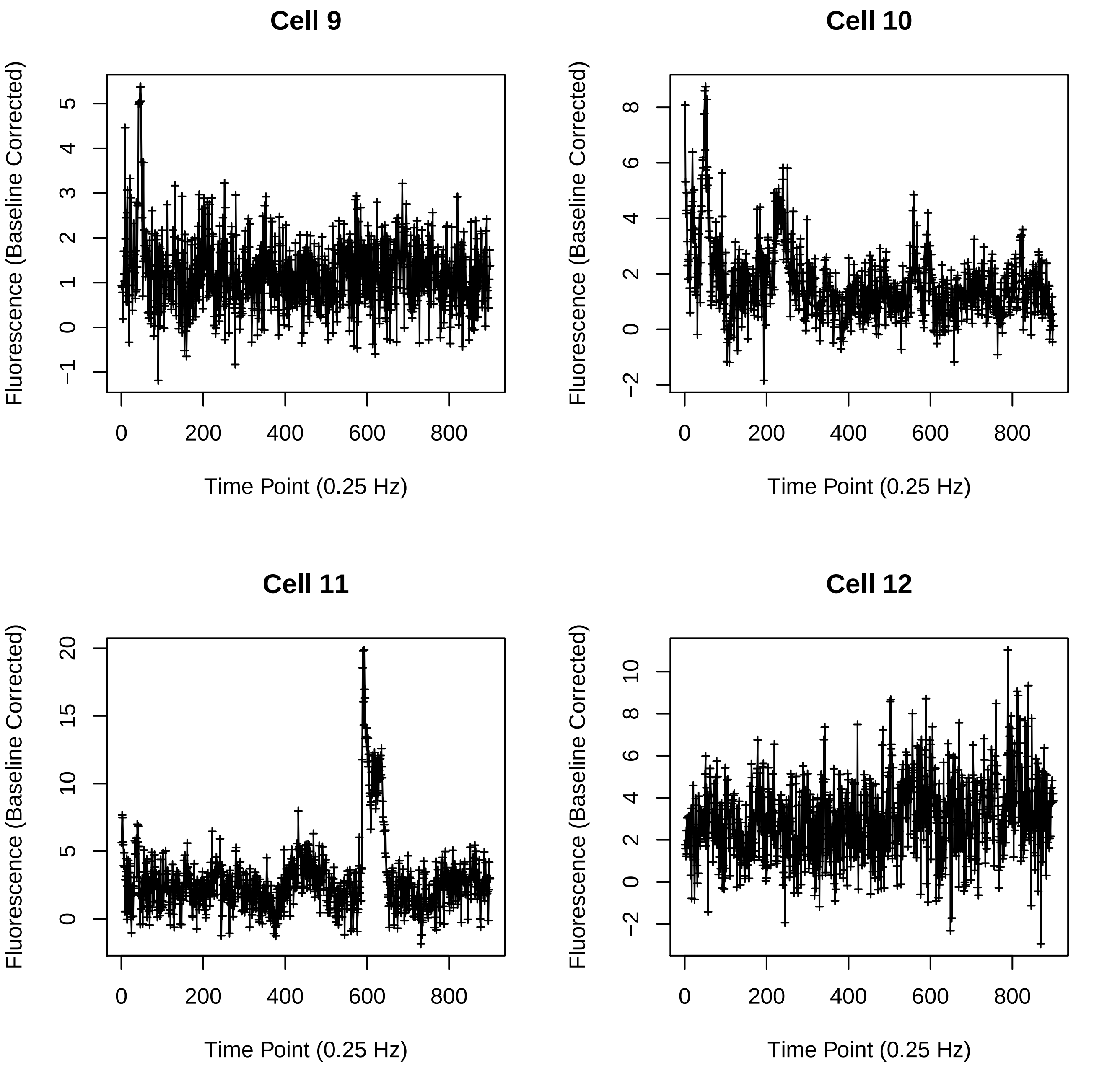
Cell 9
Cell 10
Cell 11
Cell 12
Fluorescence (Baseline Corrected)
Time Point (0.25 Hz)
0
200
400
600
800
−1
1
2
3
4
5
−2
6
8
10
15
20

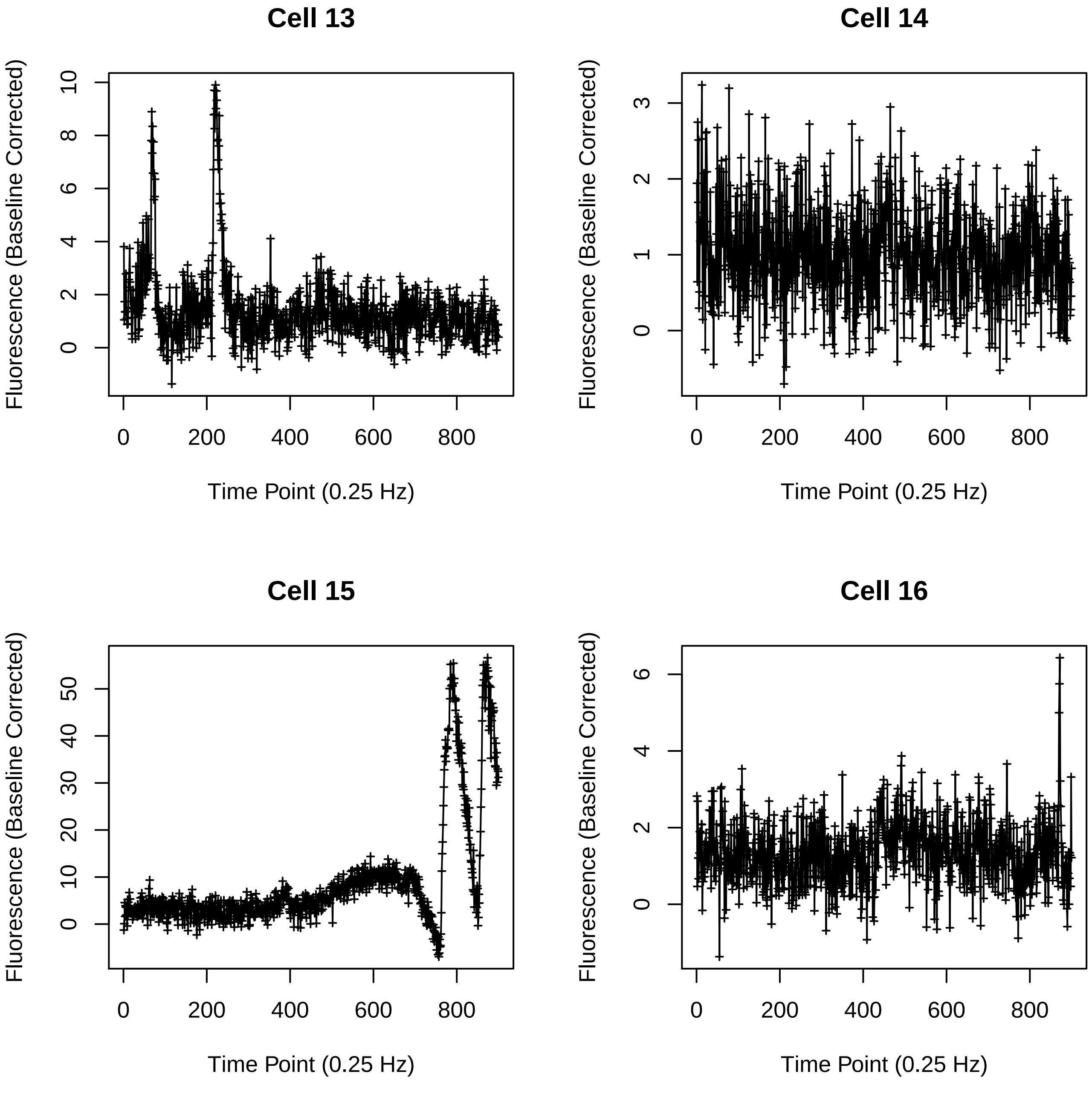
Cell 13
Cell 14
Cell 15
Cell 16
Fluorescence (Baseline Corrected)
Time Point (0.25 Hz)
0
200
400
600
800
2
4
6
8
10
1
3
20
30
40
50

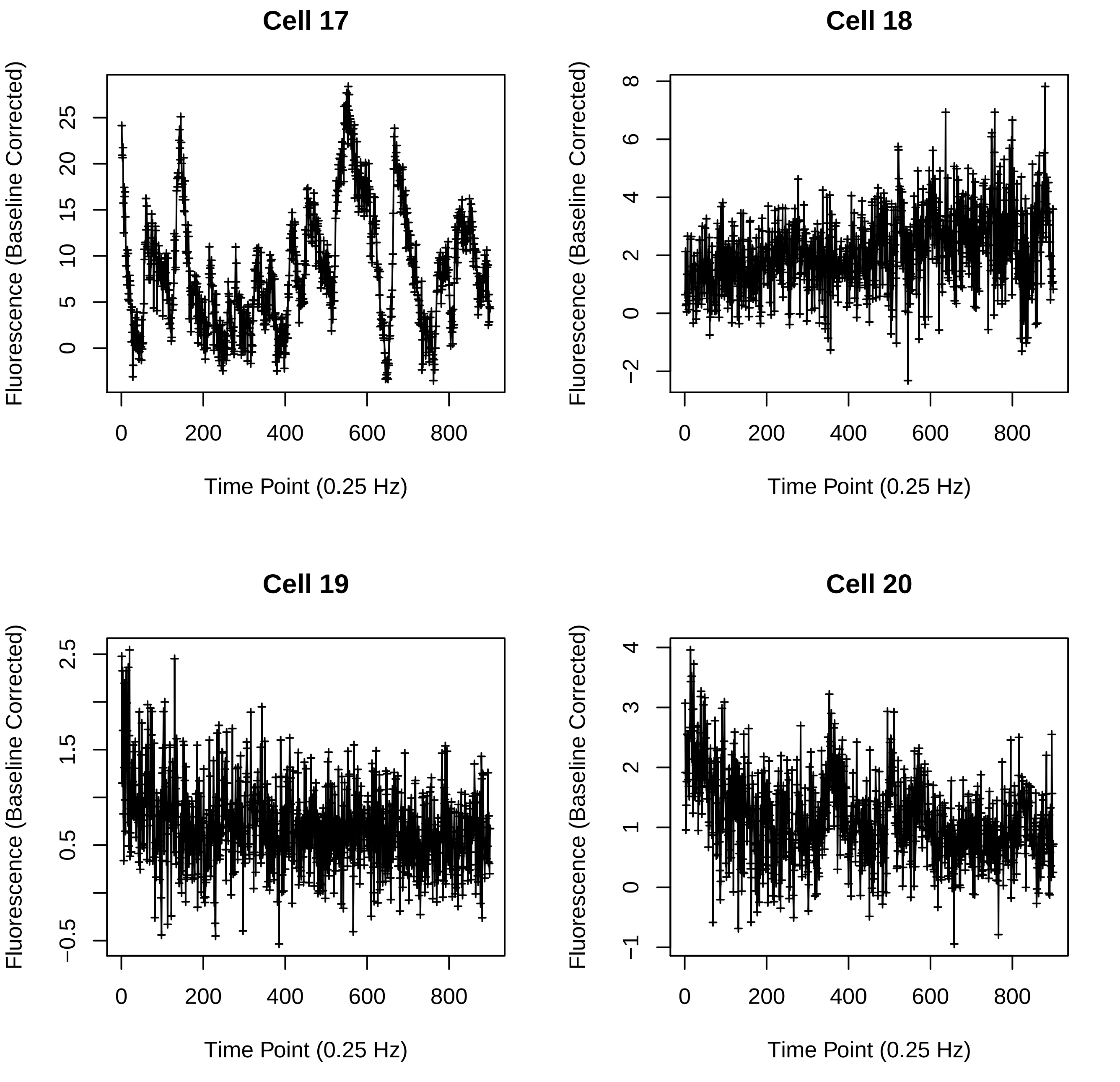
Cell 17
Cell 18
Cell 19
Cell 20
Fluorescence (Baseline Corrected)
Time Point (0.25 Hz)
0
5
10
15
20
25
200
400
600
800
8
6
4
2
−2
2.5
1.5
0.5
−0.5
3
1
−1

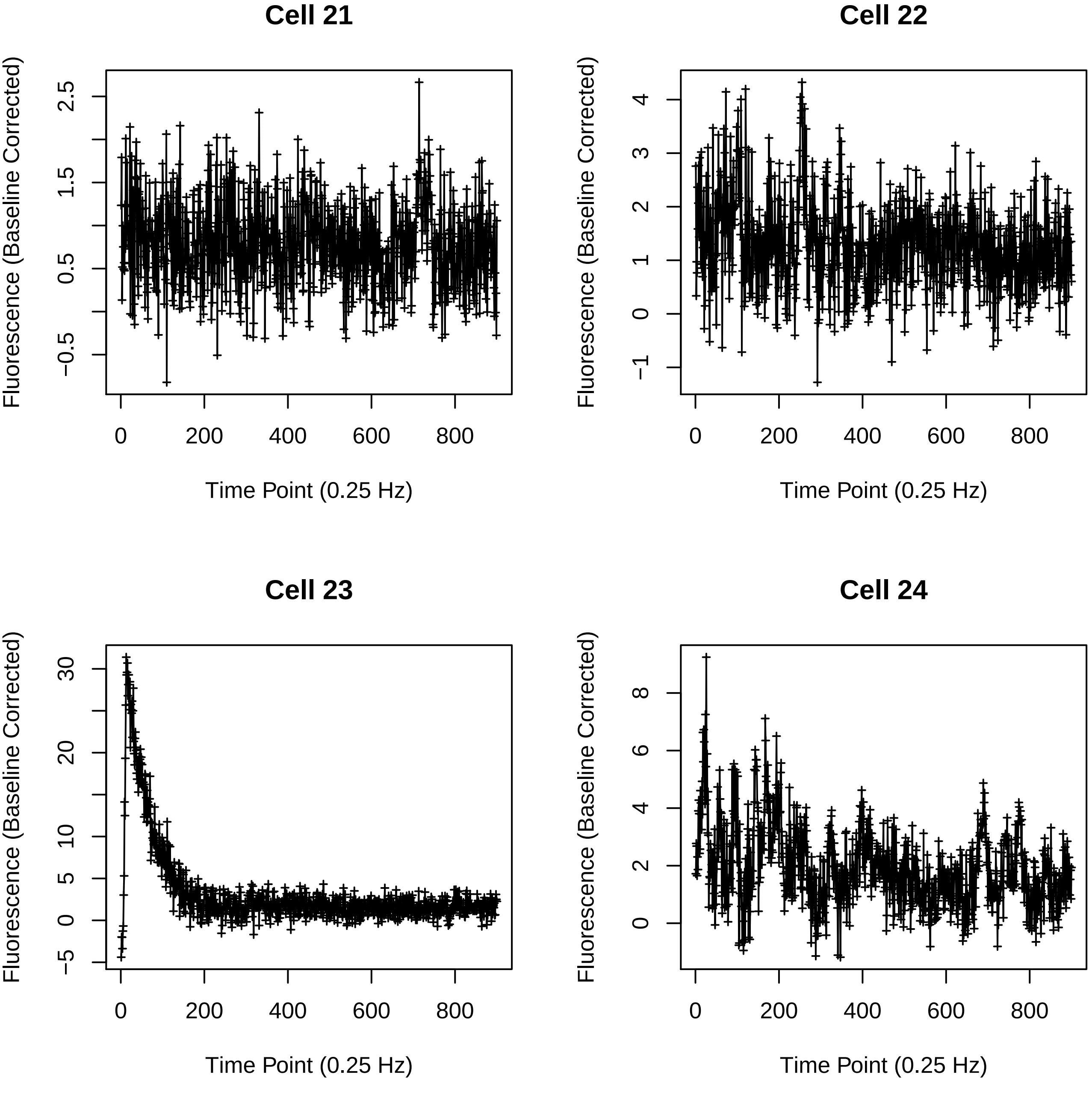
Cell 21
Cell 22
Cell 23
Cell 24
Fluorescence (Baseline Corrected)
Time Point (0.25 Hz)
−0.5
0.5
1.5
2.5
−1
0
1
2
3
4
−5
0
5
10
20
30
0
2
4
6
8
0
200
400
600
800

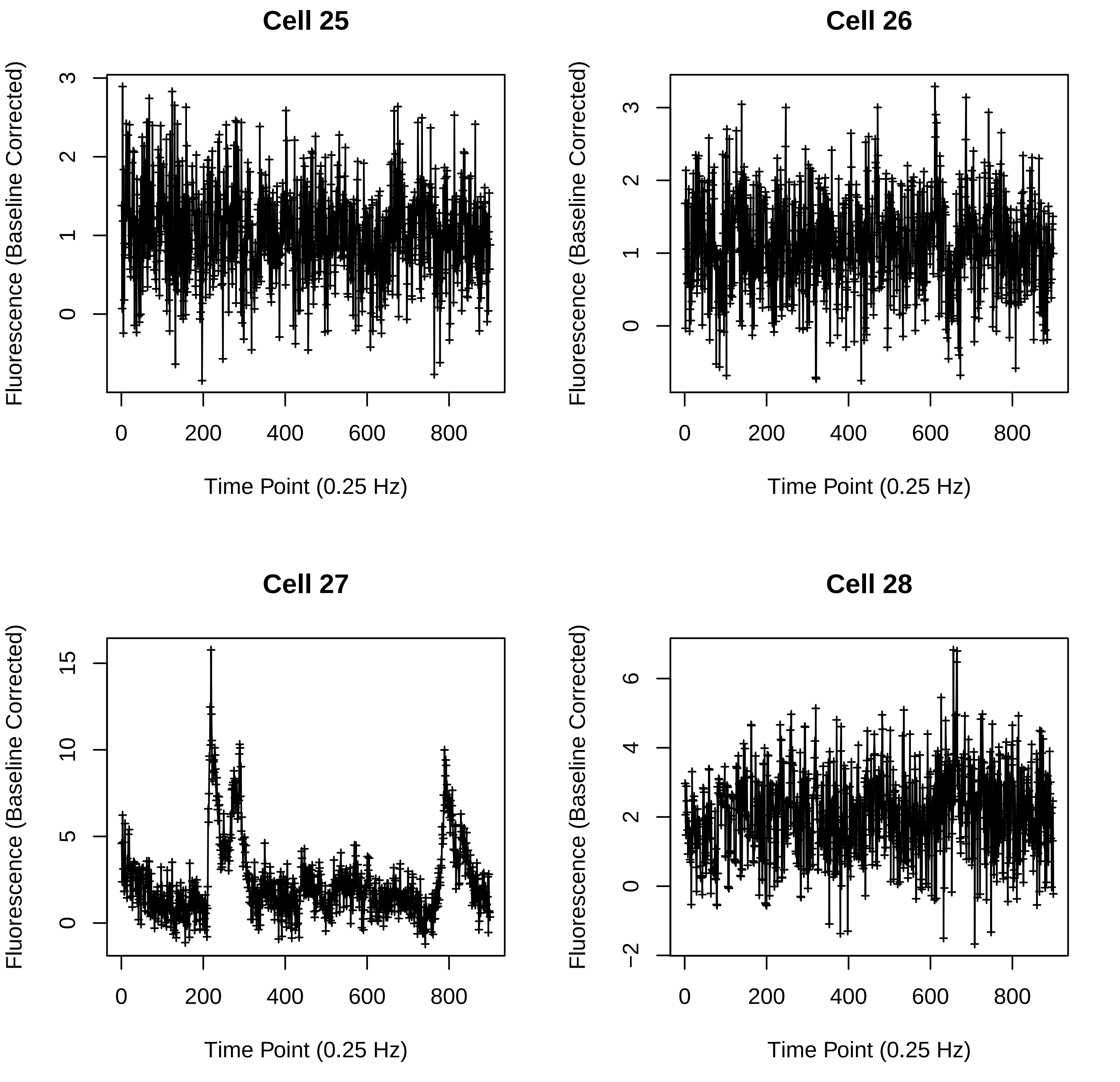
Cell 25
Cell 26
Cell 27
Cell 28
Fluorescence (Baseline Corrected)
Time Point (0.25 Hz)
0
1
2
3
−2
4
5
6
10
15
200
400
600
800

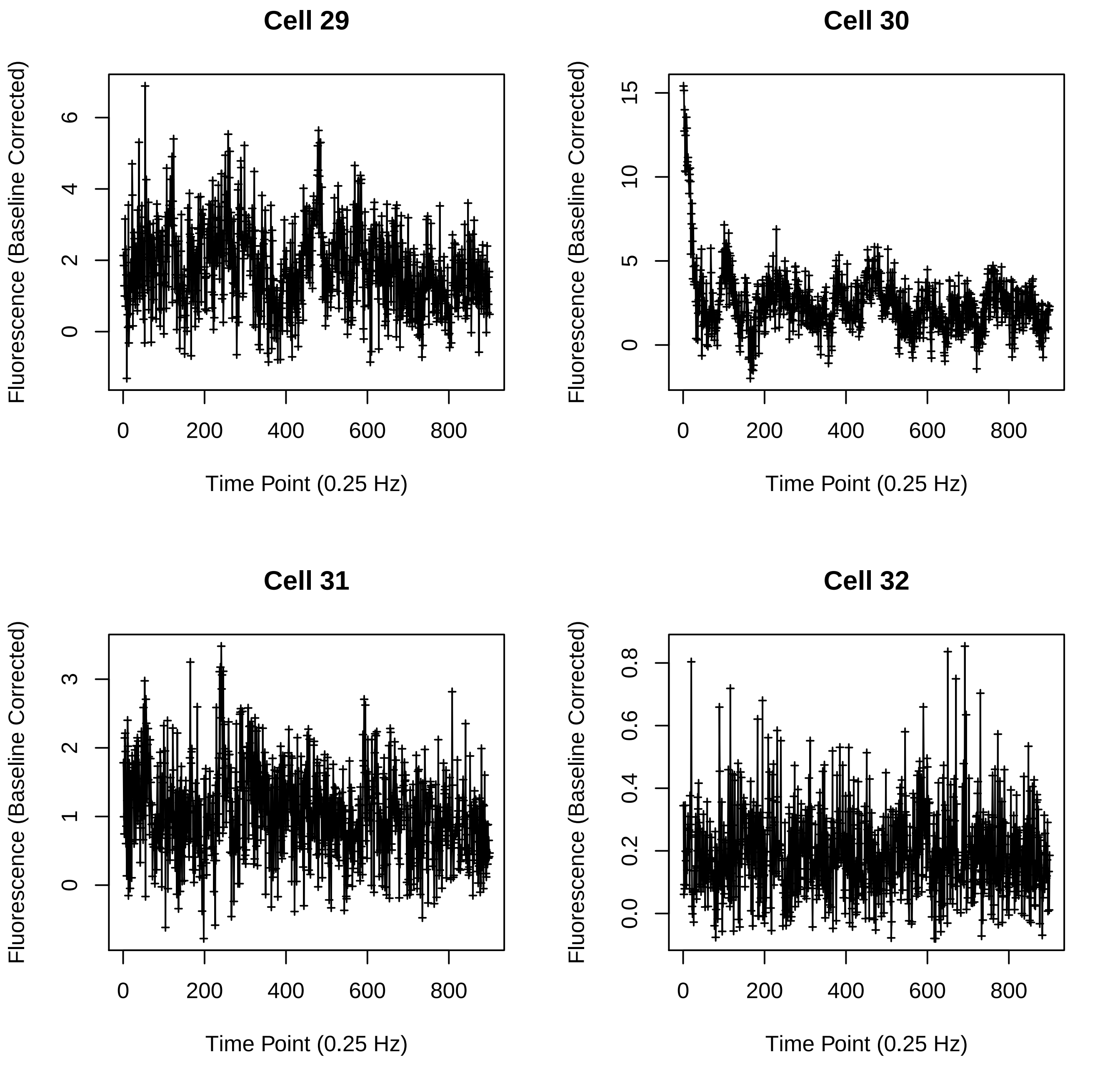
Cell 29
Cell 30
Cell 31
Cell 32
Fluorescence (Baseline Corrected)
Time Point (0.25 Hz)
0
200
400
600
800
0
2
4
6
5
10
15
1
3
0.0
0.2
0.4
0.6
0.8

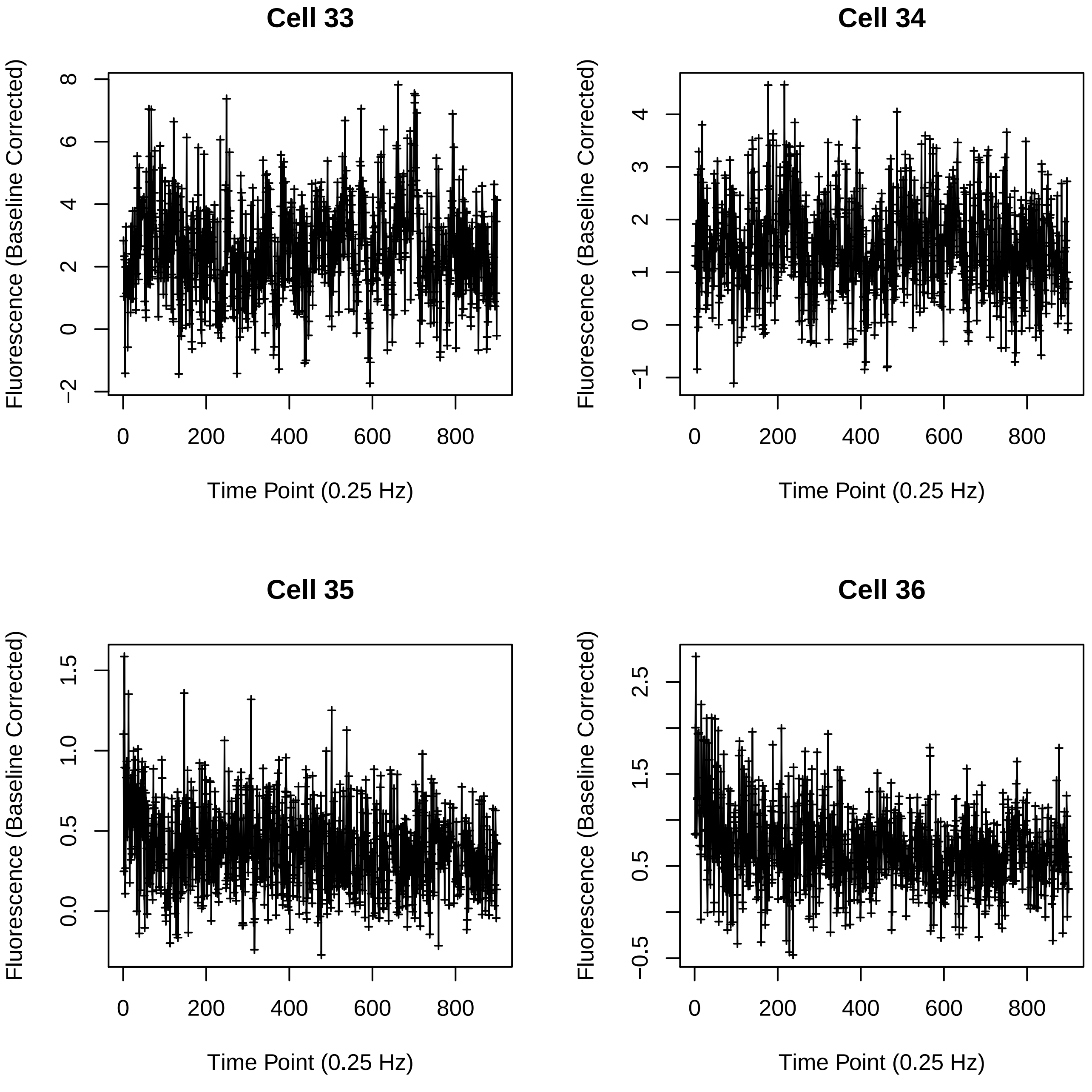
Cell 33
Fluorescence (Baseline Corrected)
−2
0
2
4
6
8
0
200
400
600
800
Time Point (0.25 Hz)
Cell 34
Fluorescence (Baseline Corrected)
−1
0
1
2
3
4
0
200
400
600
800
Time Point (0.25 Hz)
Cell 35
Fluorescence (Baseline Corrected)
0.0
0.5
1.0
1.5
0
200
400
600
800
Time Point (0.25 Hz)
Cell 36
Fluorescence (Baseline Corrected)
−0.5
0.5
1.5
2.5
0
200
400
600
800
Time Point (0.25 Hz)

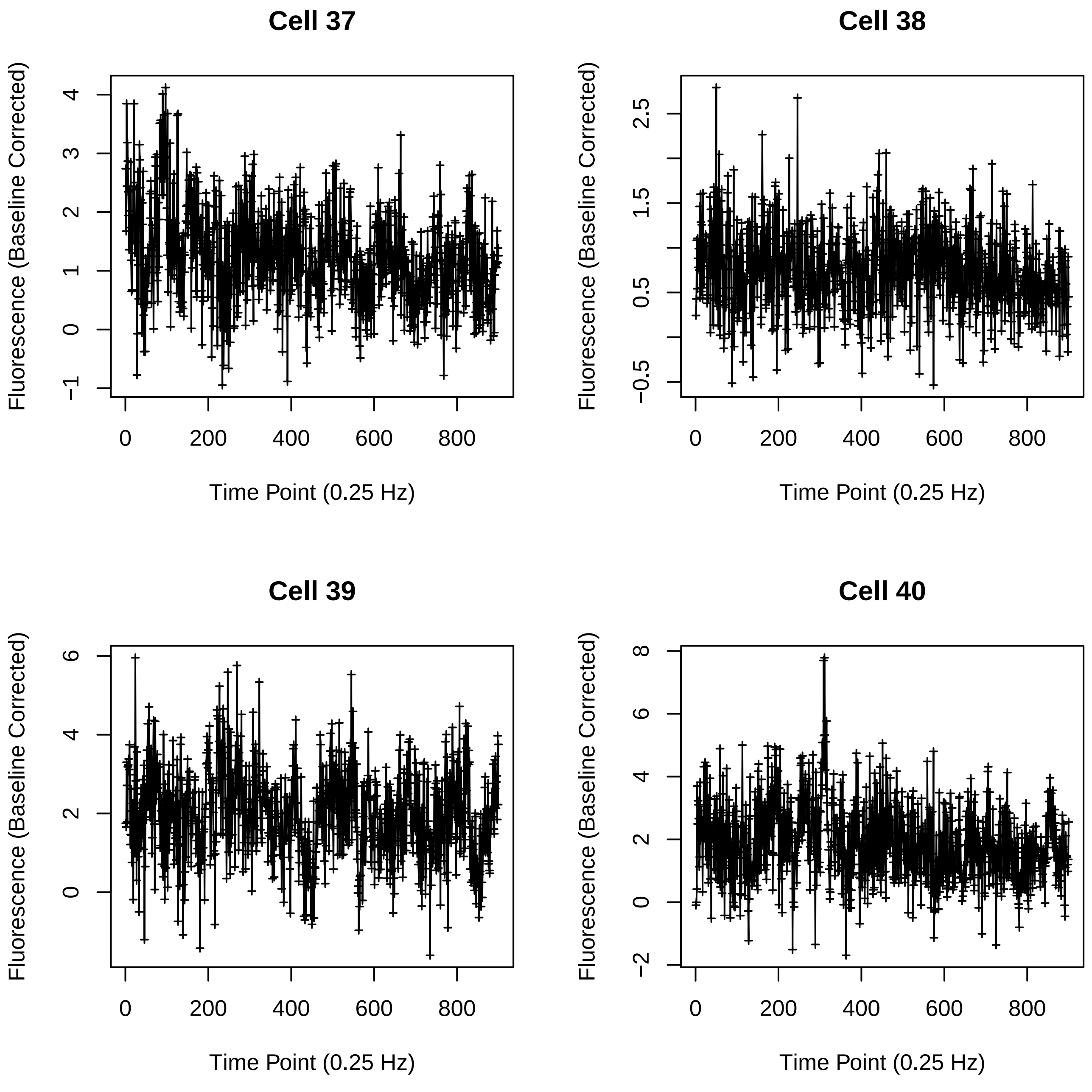
Cell 37
Cell 38
Cell 39
Cell 40
Fluorescence (Baseline Corrected)
Time Point (0.25 Hz)
0
200
400
600
800
−1
0
1
2
3
4
−0.5
0.5
1.5
2.5
6
−2
8

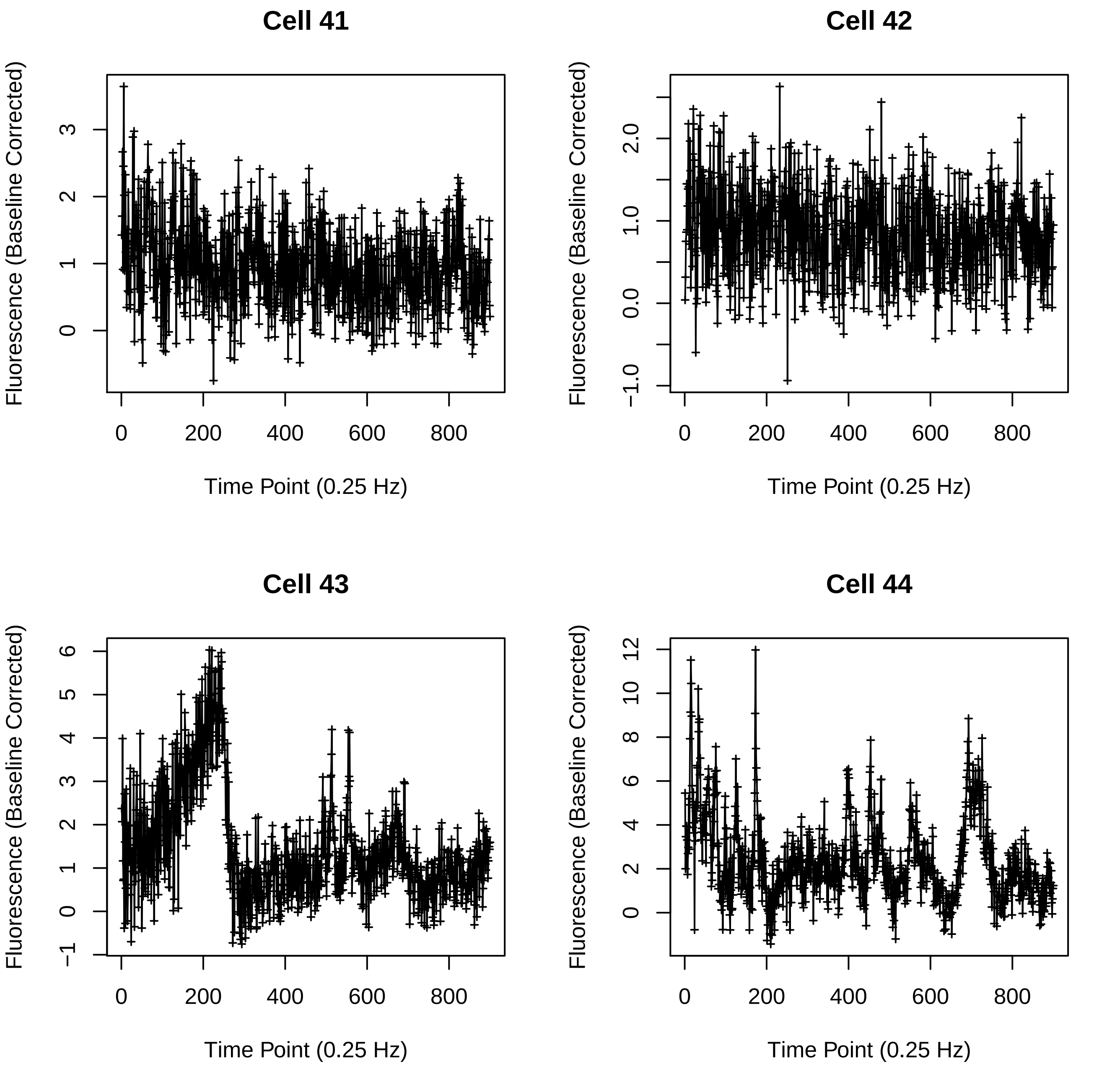

Cell 41
Cell 42
Cell 43
Cell 44
Fluorescence (Baseline Corrected)
Time Point (0.25 Hz)
0
200
400
600
800
-1
0
1
2
3
4
5
6
-1.0
0.0
1.0
2.0
8
10
12

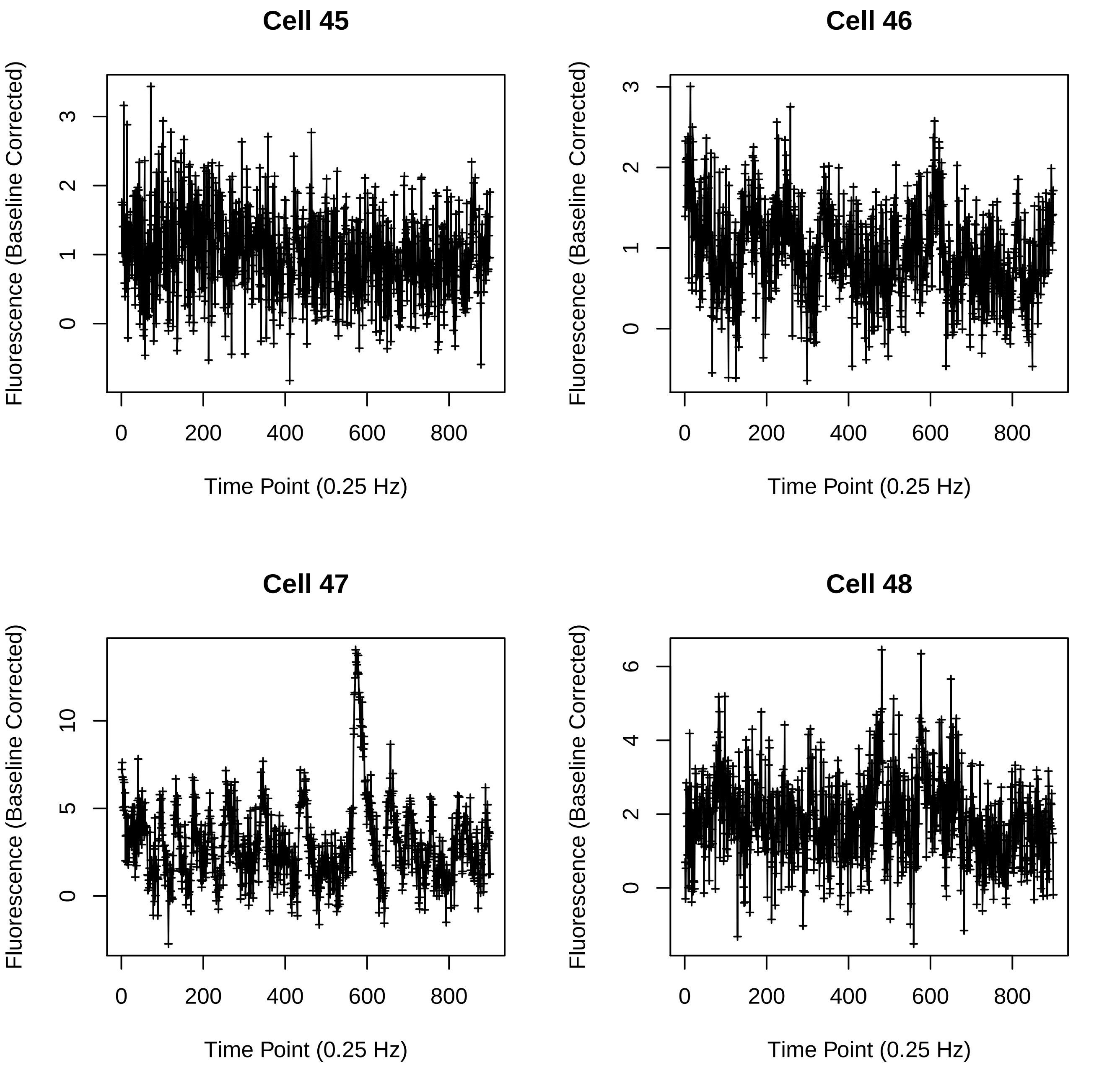
Cell 45
Fluorescence (Baseline Corrected)
0
1
2
3
0
200
400
600
800
Time Point (0.25 Hz)
Cell 46
Fluorescence (Baseline Corrected)
0
1
2
3
0
200
400
600
800
Time Point (0.25 Hz)
Cell 47
Fluorescence (Baseline Corrected)
0
5
10
0
200
400
600
800
Time Point (0.25 Hz)
Cell 48
Fluorescence (Baseline Corrected)
0
2
4
6
0
200
400
600
800
Time Point (0.25 Hz)

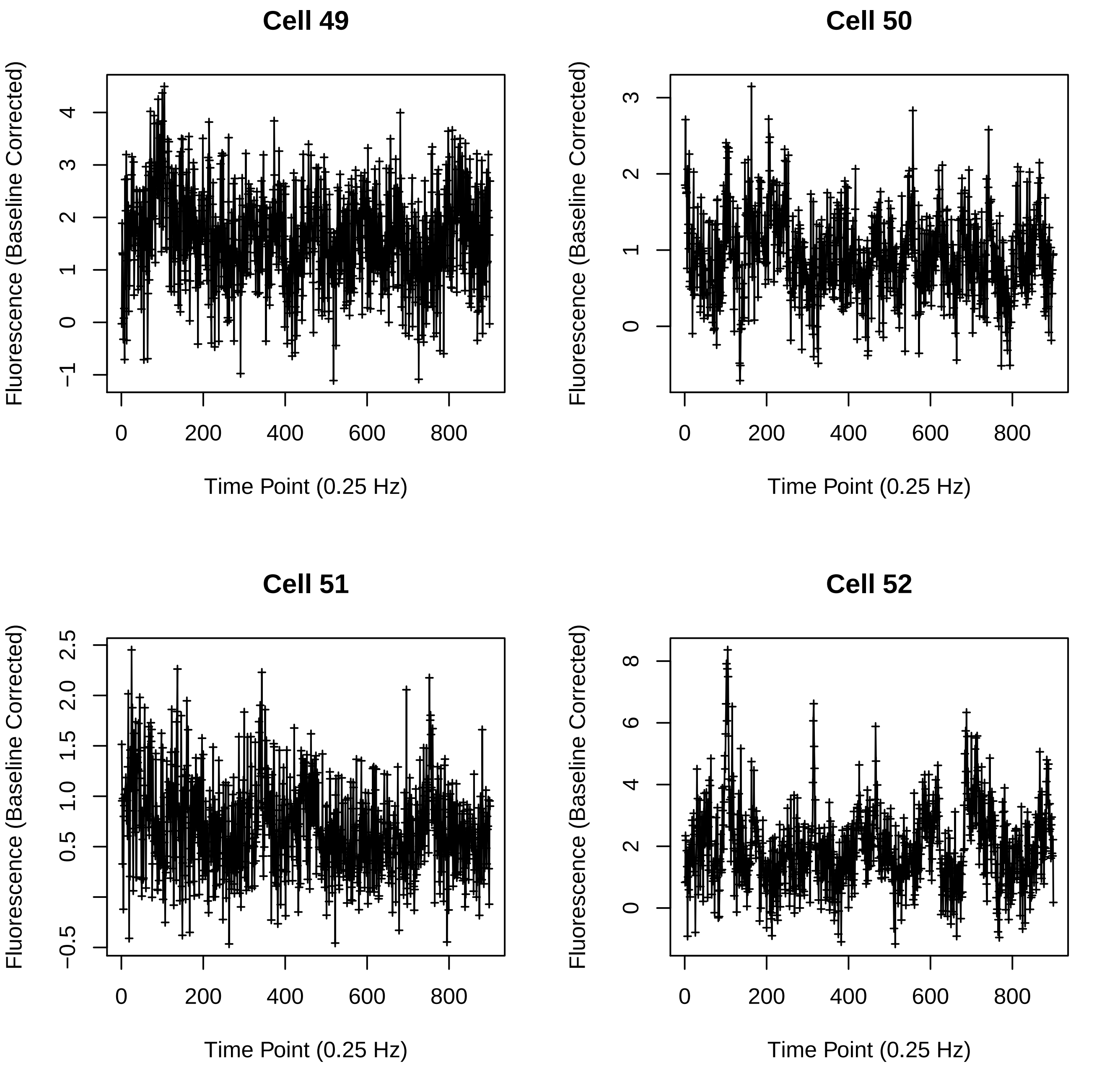
Cell 49
Cell 50
Cell 51
Cell 52
Fluorescence (Baseline Corrected)
Time Point (0.25 Hz)
0
200
400
600
800

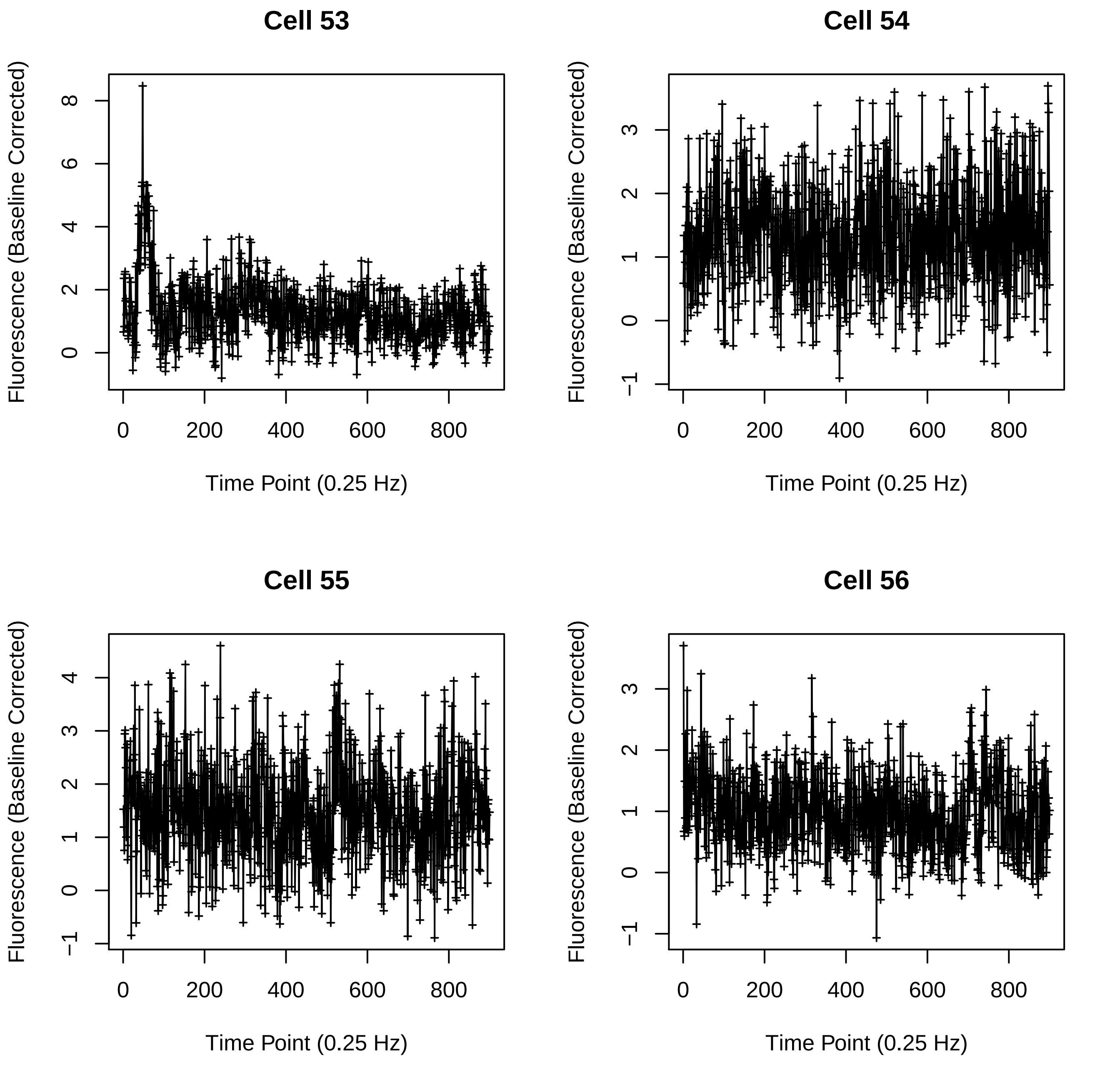

Cell 53
Cell 54
Cell 55
Cell 56
Fluorescence (Baseline Corrected)
Time Point (0.25 Hz)
0
200
400
600
800
−1
1
2
3
4
6
8

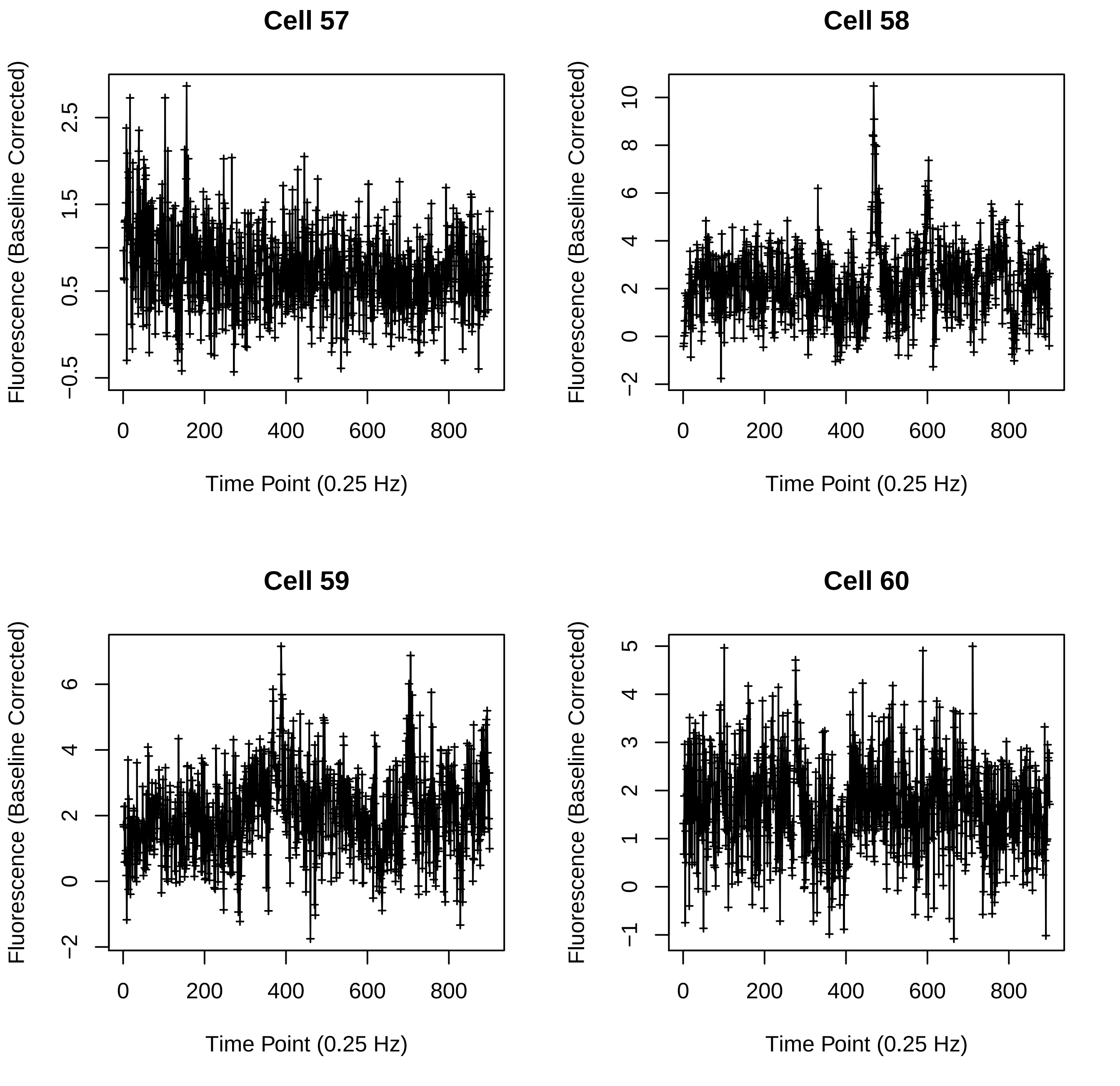

Cell 57
Cell 58
Cell 59
Cell 60
Fluorescence (Baseline Corrected)
Time Point (0.25 Hz)
−0.5
0.5
1.5
2.5
−2
0
2
4
6
8
10
−1
1
3
5
200
400
600
800

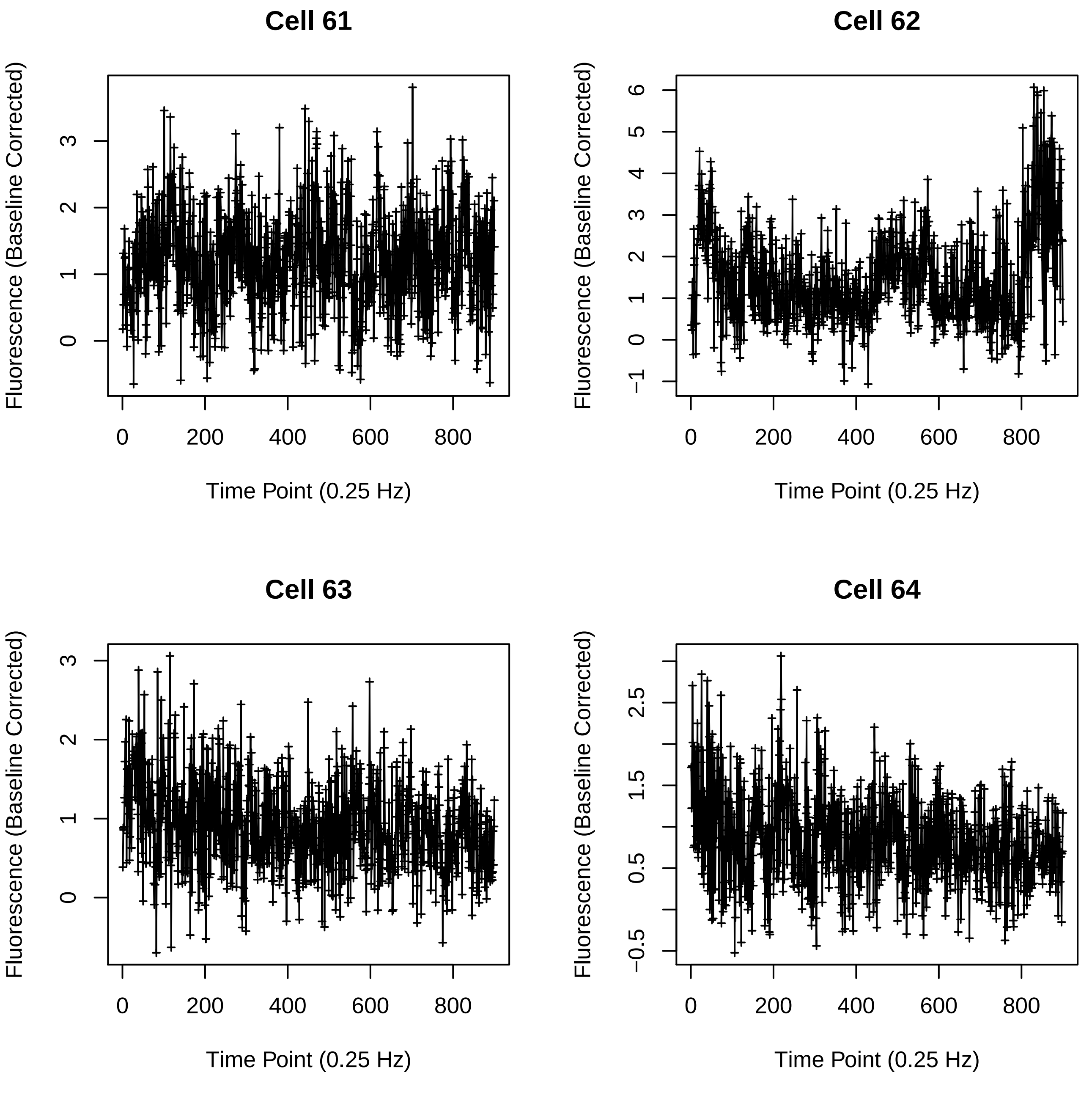

Cell 61
Cell 62
Cell 63
Cell 64
Fluorescence (Baseline Corrected)
Time Point (0.25 Hz)

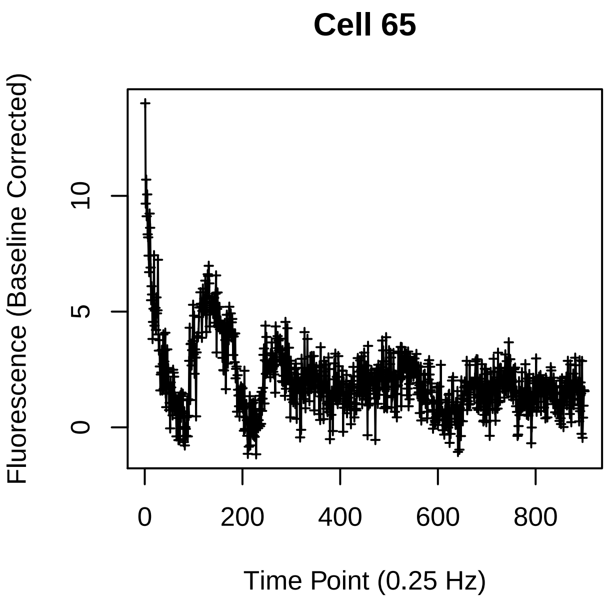
Cell 65
Fluorescence (Baseline Corrected)
0
5
10
0
200
400
600
800
Time Point (0.25 Hz)

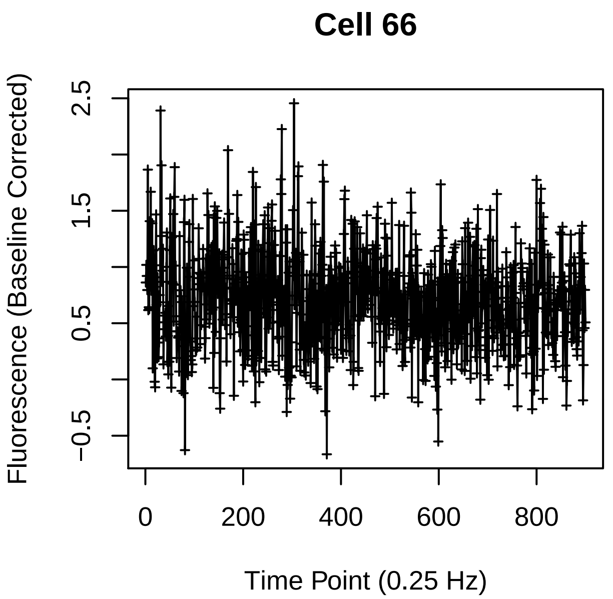
Cell 66
Fluorescence (Baseline Corrected)
−0.5
0.5
1.5
2.5
0
200
400
600
800
Time Point (0.25 Hz)

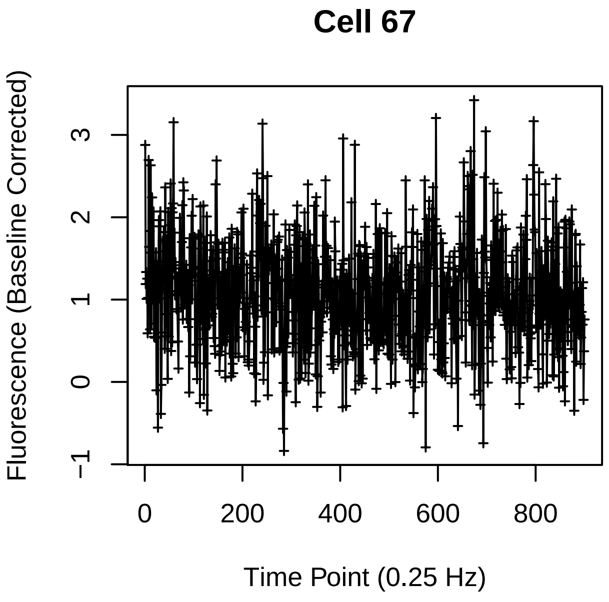
Cell 67
Fluorescence (Baseline Corrected)
−1
0
1
2
3
0
200
400
600
800
Time Point (0.25 Hz)

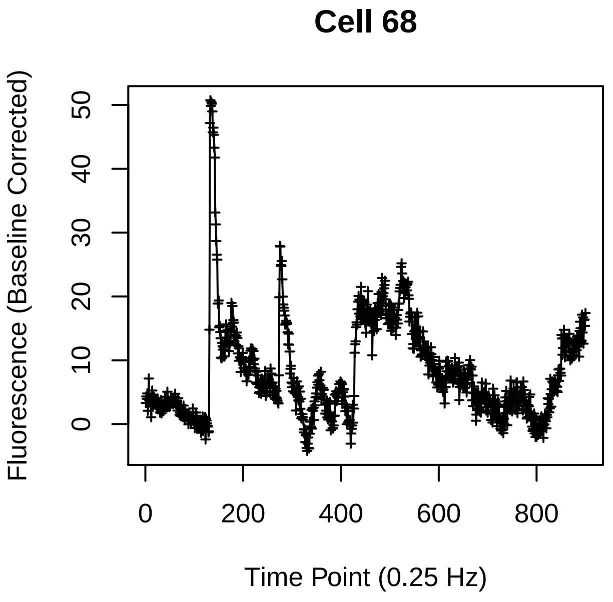
Cell 68
Fluorescence (Baseline Corrected)
0
10
20
30
40
50
0
200
400
600
800
Time Point (0.25 Hz)

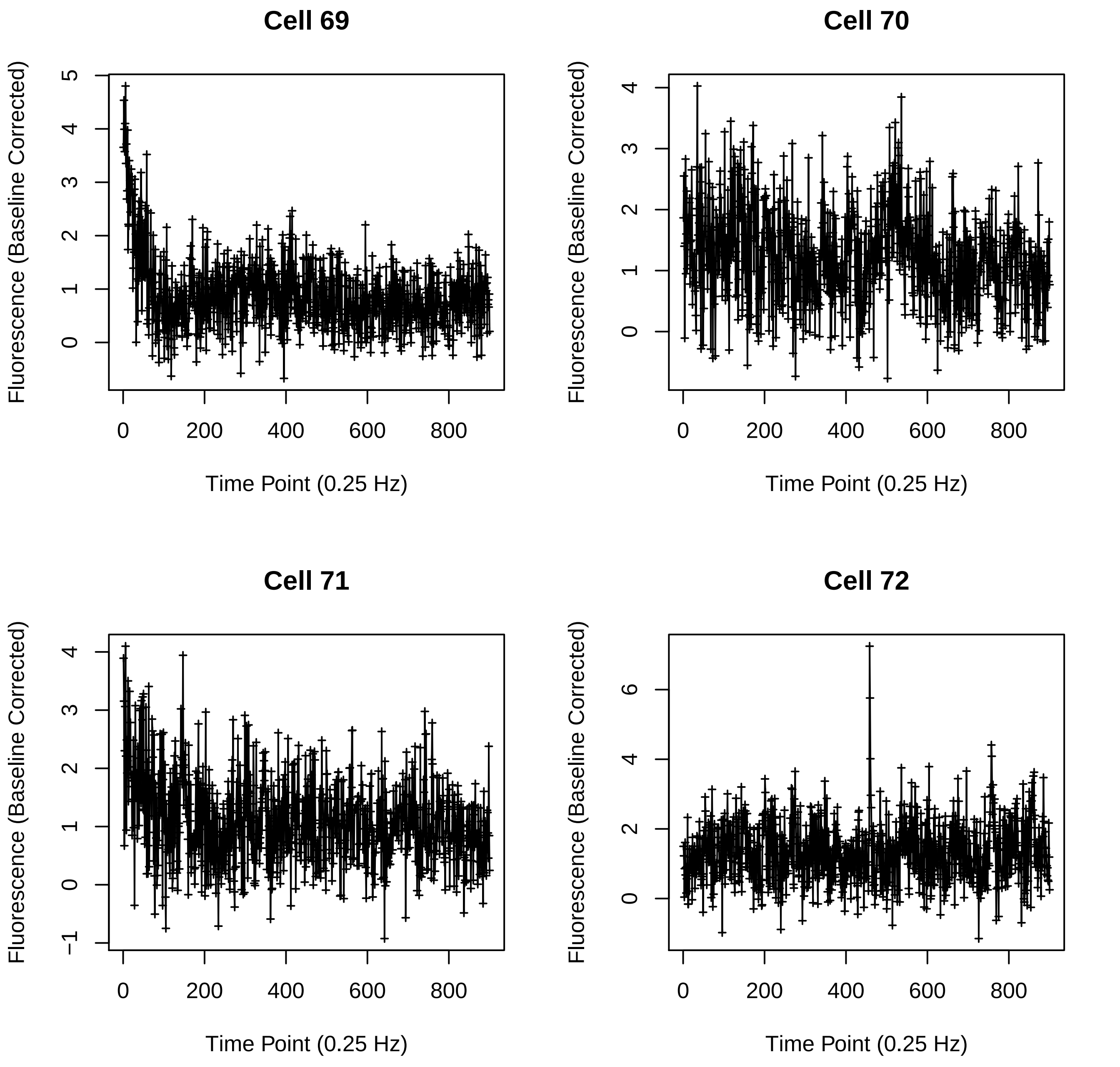

Cell 69
Cell 70
Cell 71
Cell 72
Fluorescence (Baseline Corrected)
Time Point (0.25 Hz)

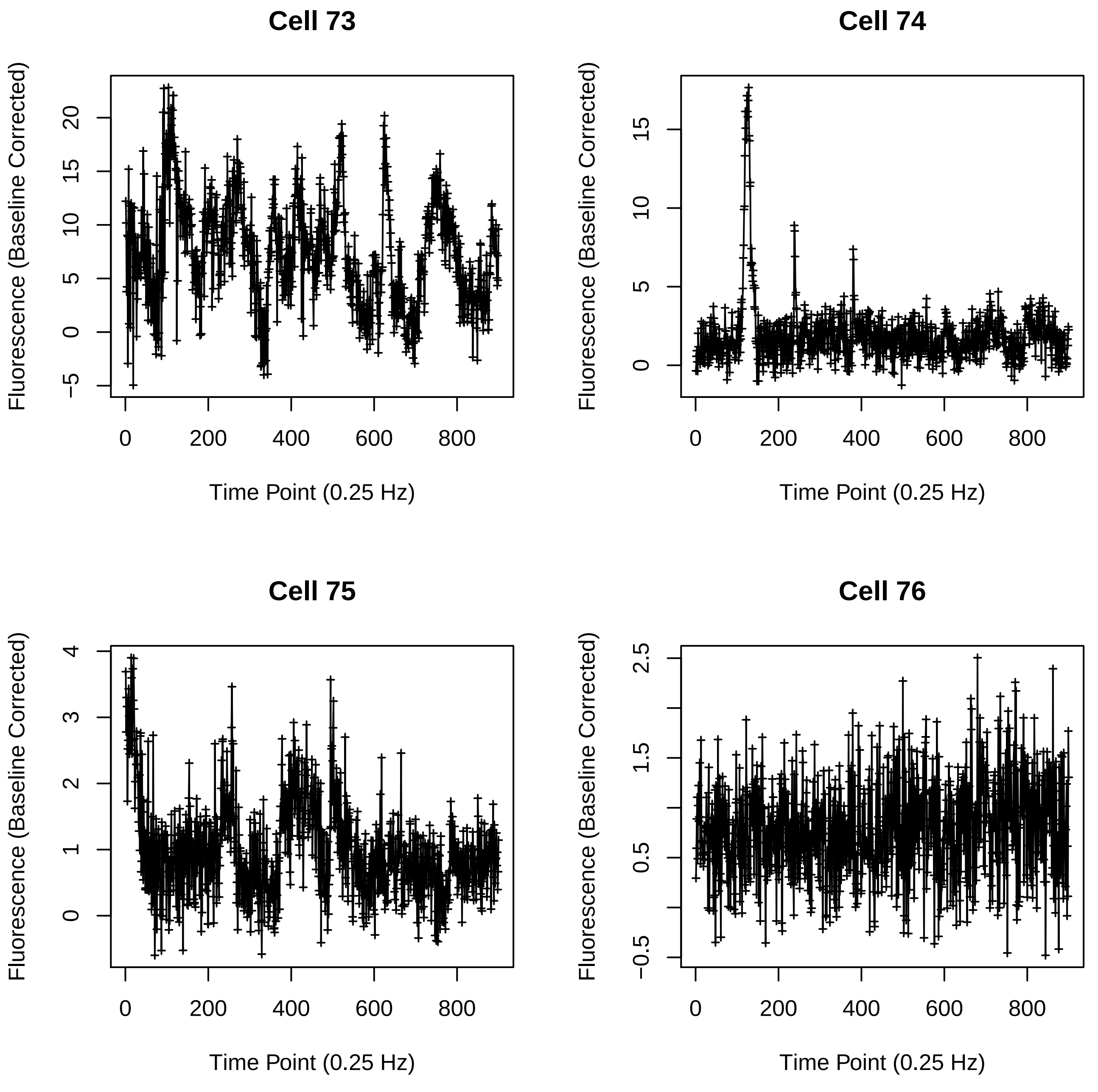
Cell 73
Cell 74
Cell 75
Cell 76
Fluorescence (Baseline Corrected)
Time Point (0.25 Hz)
0
200
400
600
800
−5
0
5
10
15
20
0
5
10
15
0
1
2
3
4
−0.5
0.5
1.5
2.5

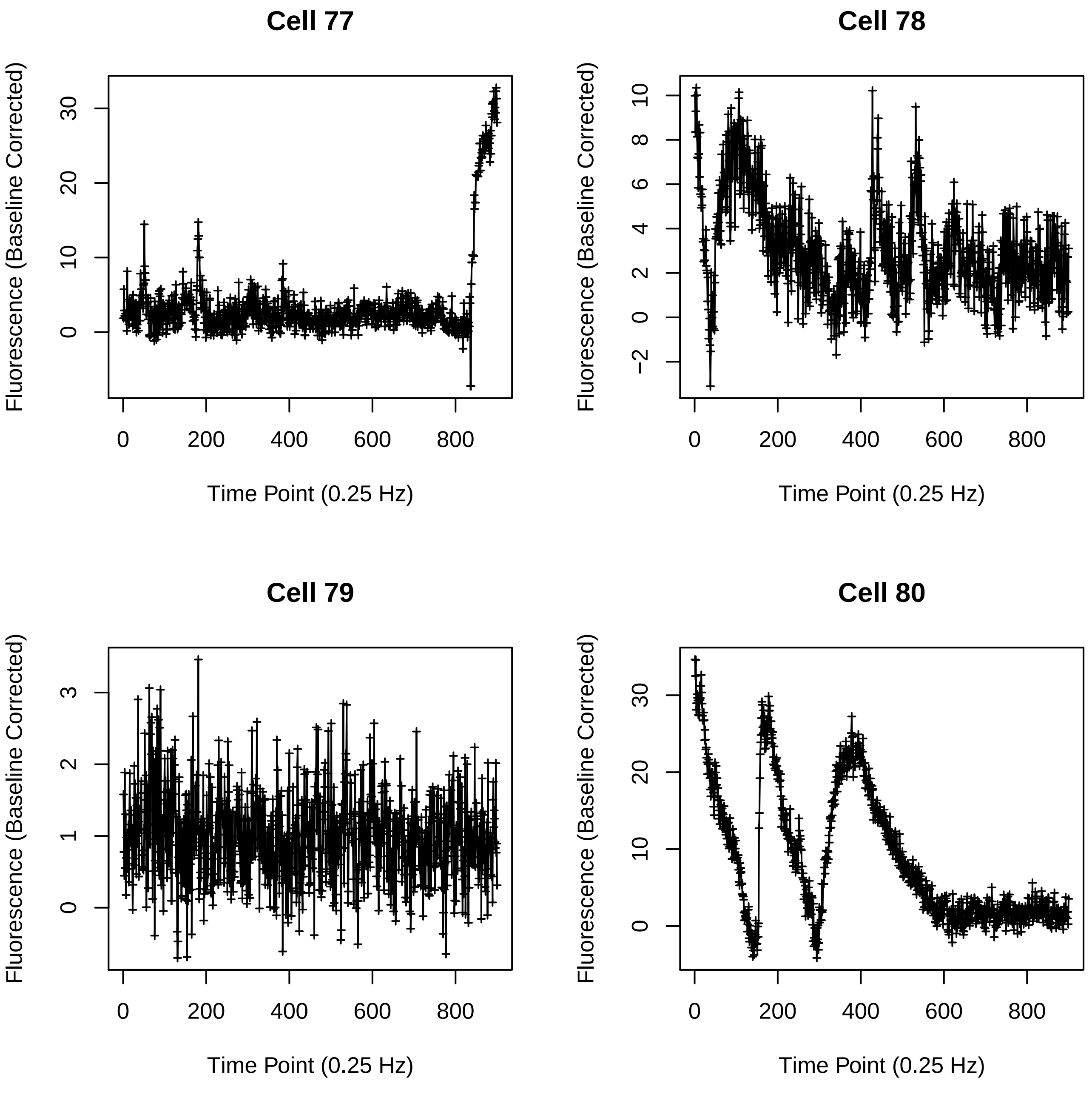
Cell 77
Cell 78
Cell 79
Cell 80
Fluorescence (Baseline Corrected)
Time Point (0.25 Hz)
0
200
400
600
800
-2
2
4
6
8
10
20
30
1
3

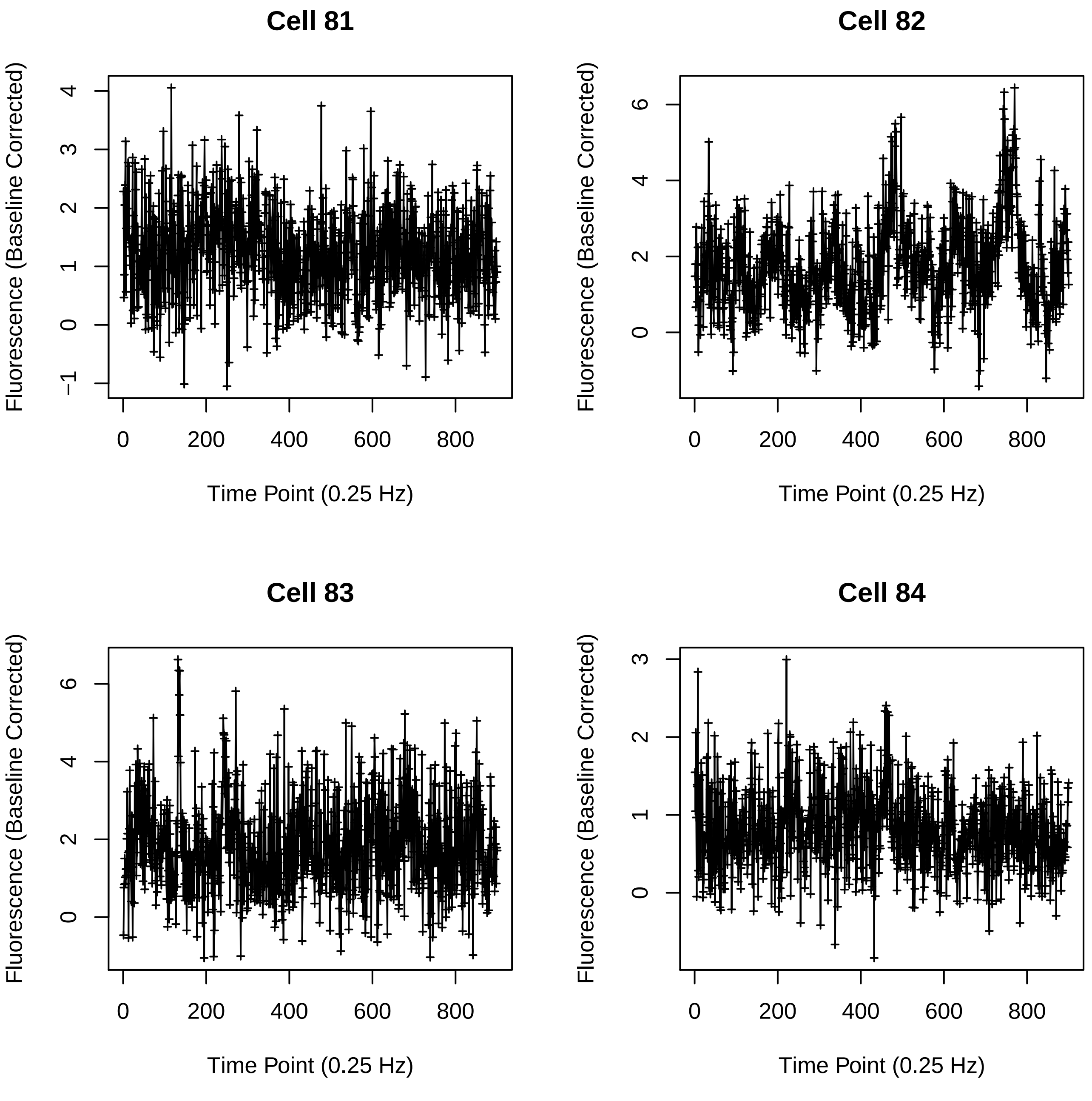

Cell 81
Cell 82
Cell 83
Cell 84
Fluorescence (Baseline Corrected)
Time Point (0.25 Hz)
0
200
400
600
800
−1
1
2
3
4
6

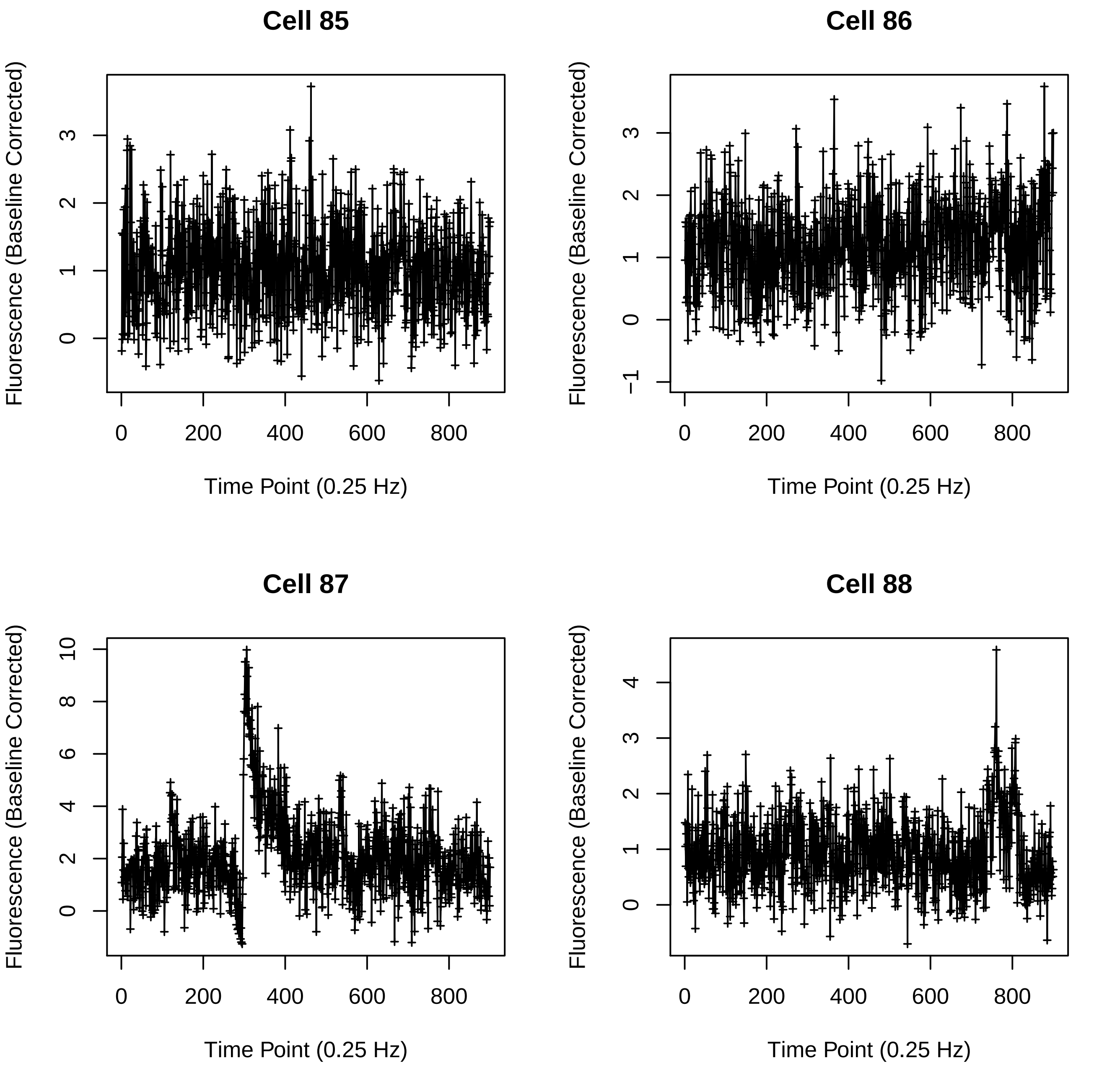
Cell 85
Cell 86
Cell 87
Cell 88
Fluorescence (Baseline Corrected)
Time Point (0.25 Hz)
0
200
400
600
800
−1
1
2
3
4
6
8
10

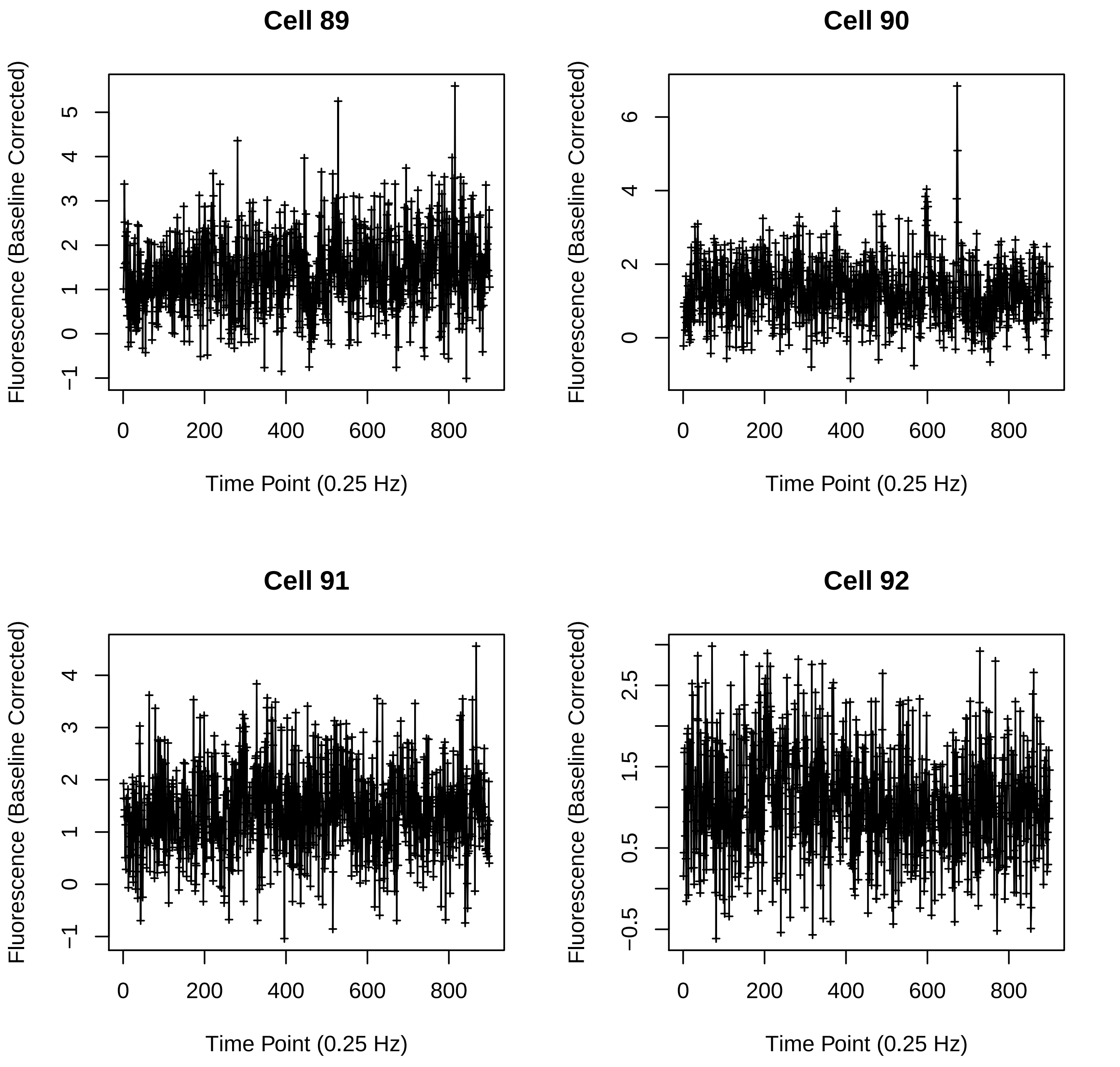
Cell 89
Cell 90
Cell 91
Cell 92
Fluorescence (Baseline Corrected)
Time Point (0.25 Hz)
0
200
400
600
800
−1
0
1
2
3
4
5
6
−0.5
0.5
1.5
2.5

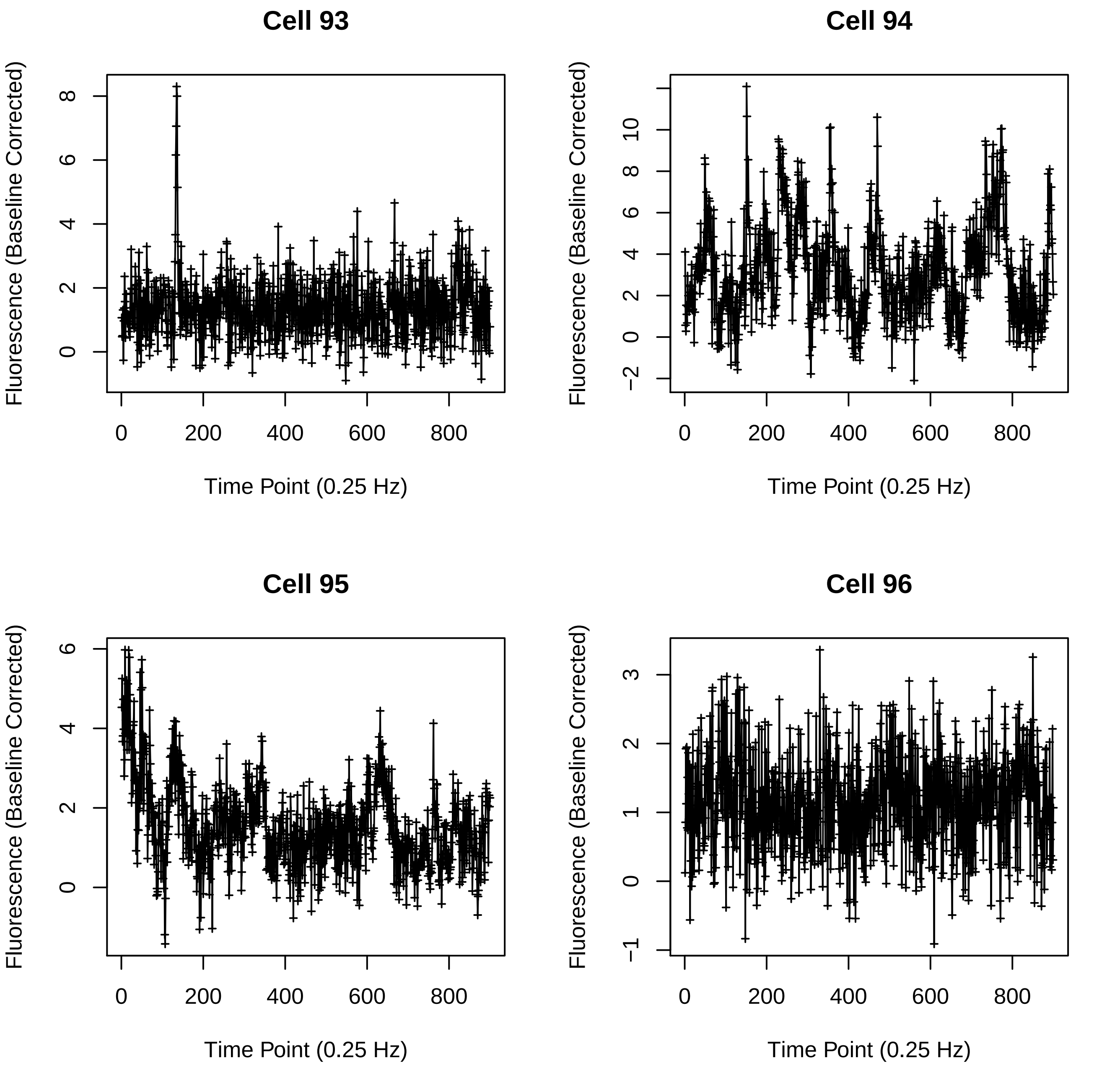
Cell 93
Fluorescence (Baseline Corrected)
Time Point (0.25 Hz)
0
2
4
6
8
0
200
400
600
800
Cell 94
Fluorescence (Baseline Corrected)
Time Point (0.25 Hz)
−2
0
2
4
6
8
10
0
200
400
600
800
Cell 95
Fluorescence (Baseline Corrected)
Time Point (0.25 Hz)
0
2
4
6
0
200
400
600
800
Cell 96
Fluorescence (Baseline Corrected)
Time Point (0.25 Hz)
−1
0
1
2
3
0
200
400
600
800

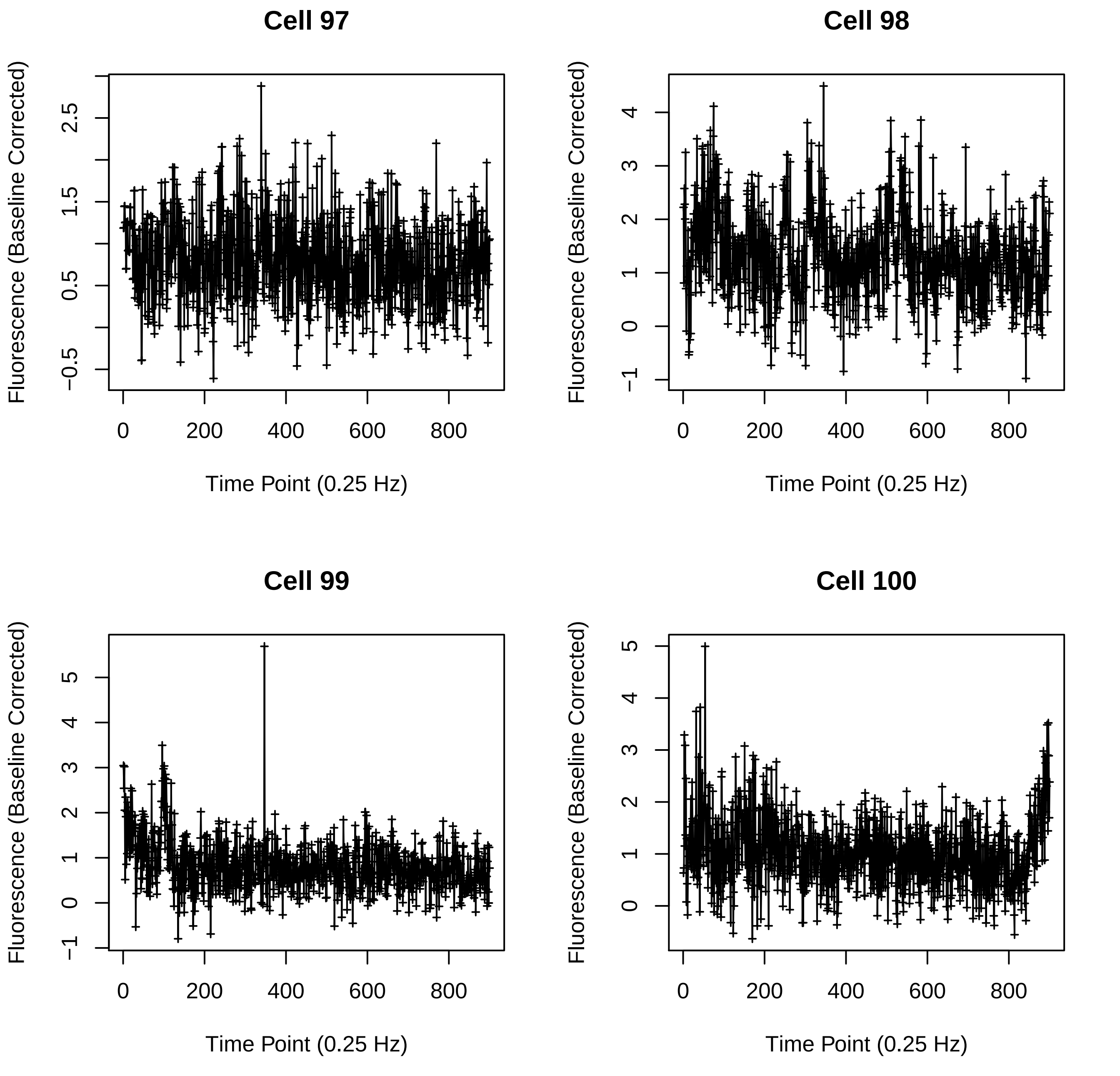
Cell 97
Fluorescence (Baseline Corrected)
-0.5
0.5
1.5
2.5
0
200
400
600
800
Time Point (0.25 Hz)
Cell 98
Fluorescence (Baseline Corrected)
-1
0
1
2
3
4
0
200
400
600
800
Time Point (0.25 Hz)
Cell 99
Fluorescence (Baseline Corrected)
-1
0
1
2
3
4
5
0
200
400
600
800
Time Point (0.25 Hz)
Cell 100
Fluorescence (Baseline Corrected)
0
1
2
3
4
5
0
200
400
600
800
Time Point (0.25 Hz)

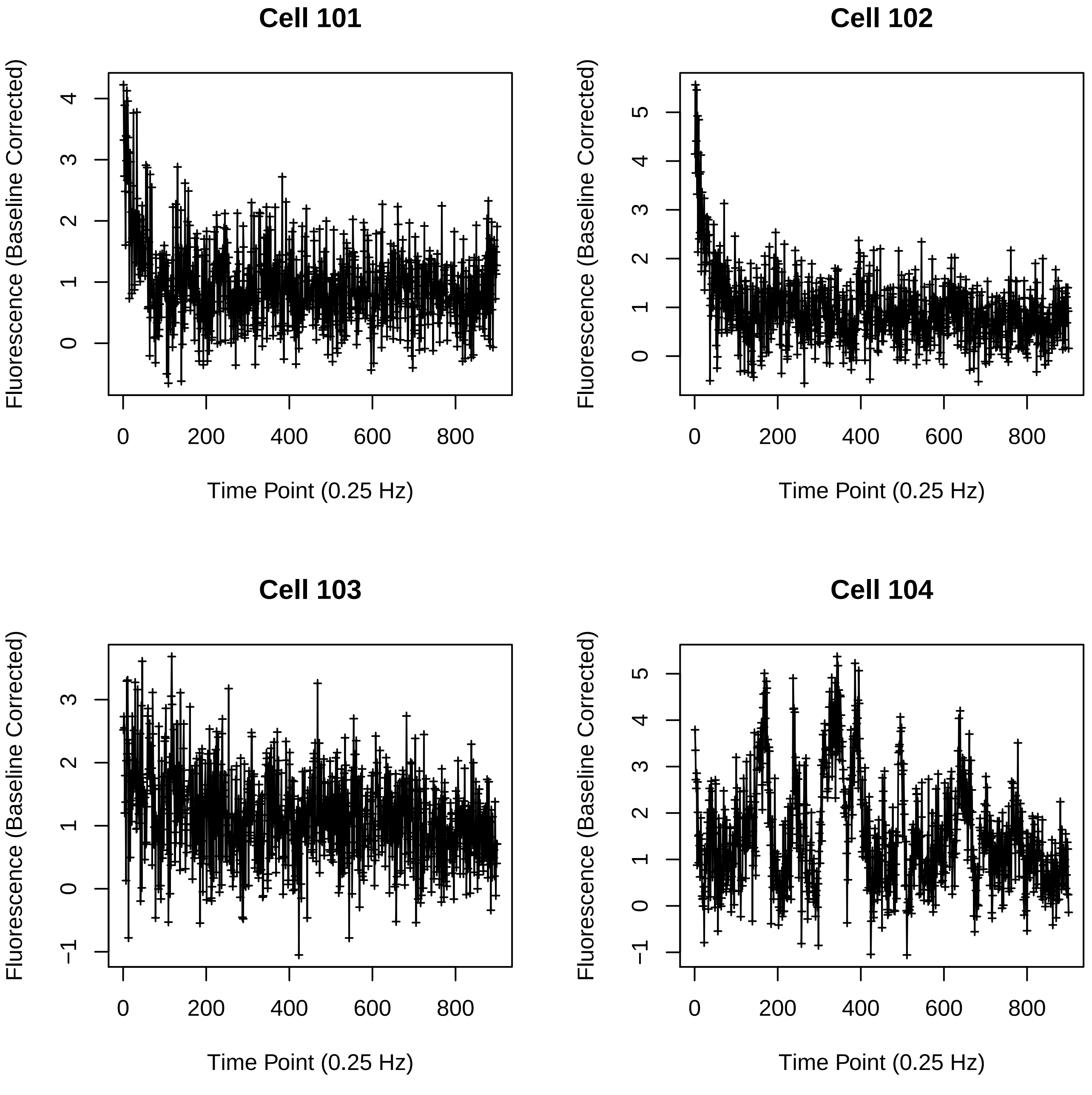
Cell 101
Cell 102
Cell 103
Cell 104
Fluorescence (Baseline Corrected)
Time Point (0.25 Hz)
0
200
400
600
800
−1
1
2
3
4
5

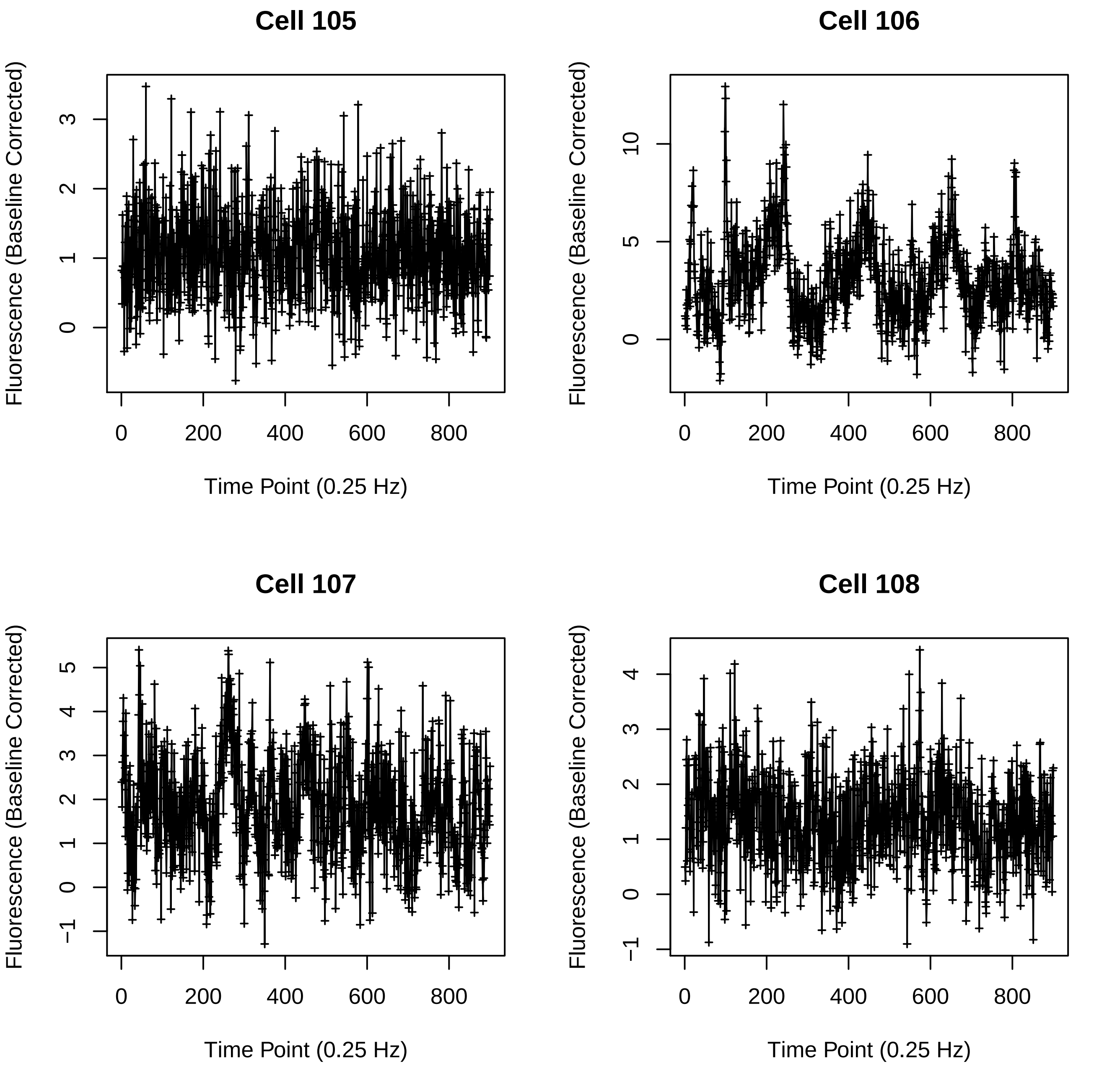
Cell 105
Fluorescence (Baseline Corrected)
Time Point (0.25 Hz)
0
1
2
3
0
200
400
600
800
Cell 106
Fluorescence (Baseline Corrected)
Time Point (0.25 Hz)
0
5
10
0
200
400
600
800
Cell 107
Fluorescence (Baseline Corrected)
Time Point (0.25 Hz)
−1
0
1
2
3
4
5
0
200
400
600
800
Cell 108
Fluorescence (Baseline Corrected)
Time Point (0.25 Hz)
−1
0
1
2
3
4
0
200
400
600
800

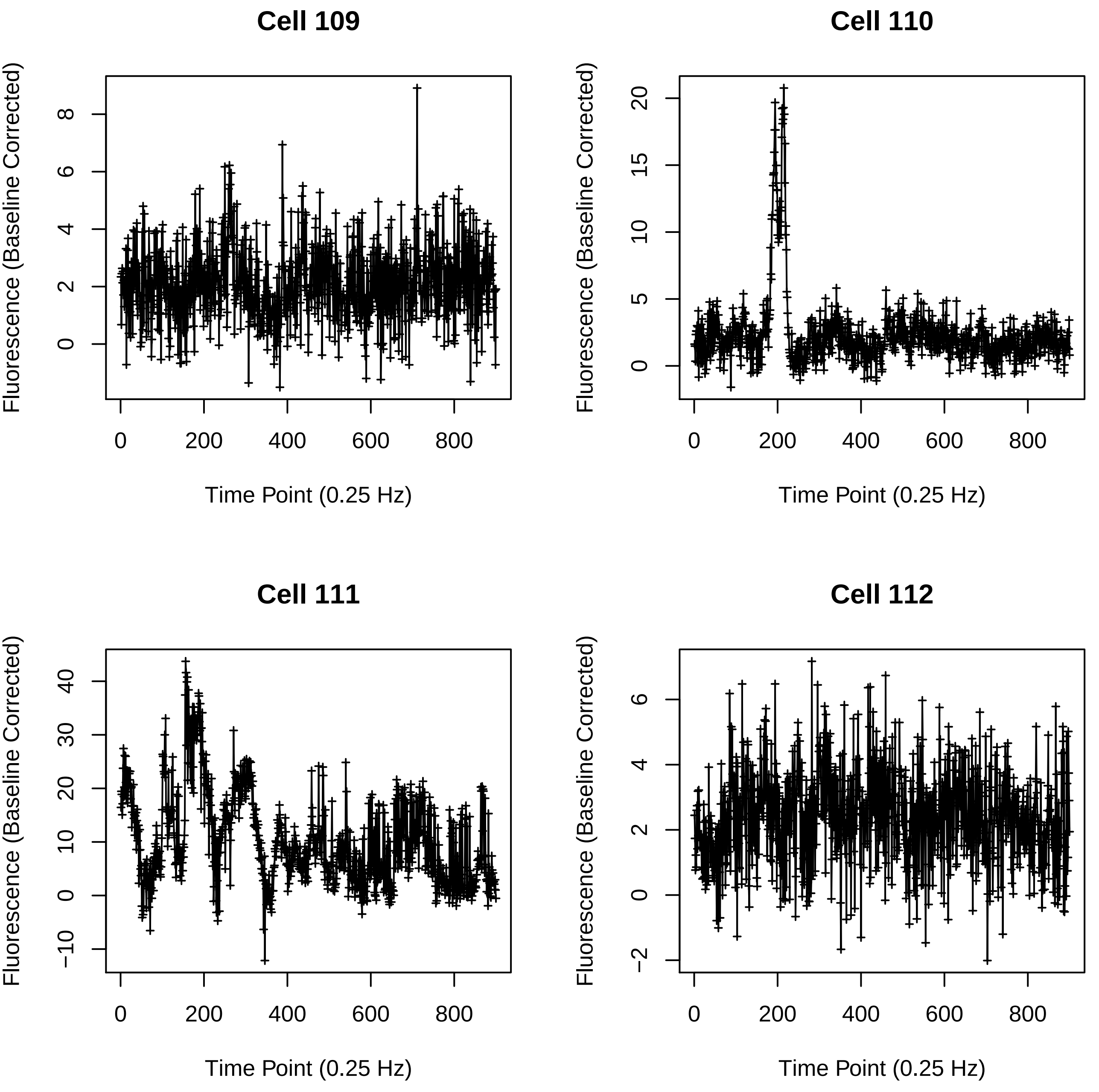
Cell 109
Cell 110
Cell 111
Cell 112
Fluorescence (Baseline Corrected)
Time Point (0.25 Hz)
0
200
400
600
800
0
2
4
6
8
5
10
15
20
−10
30
40
−2

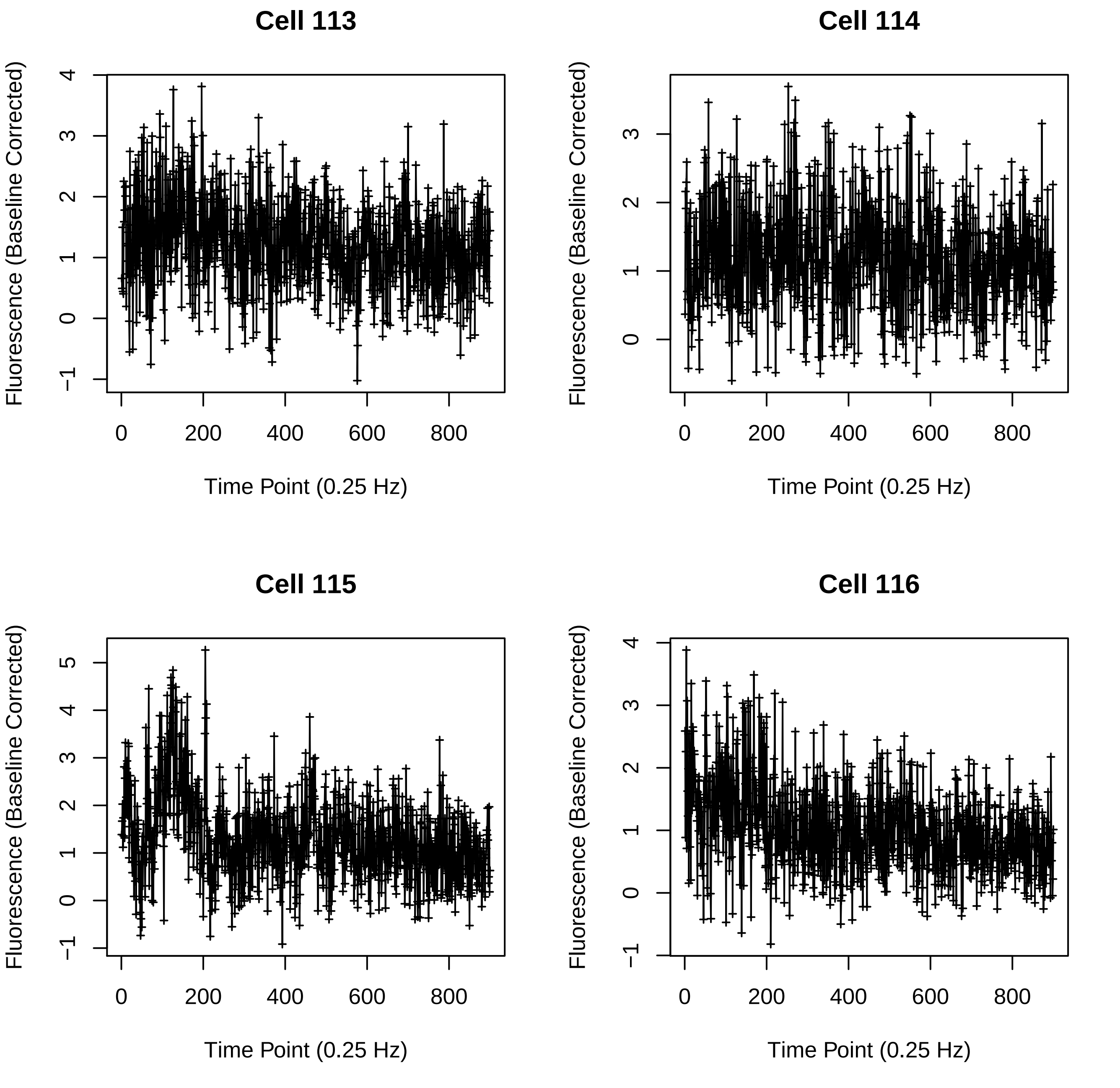
Cell 113
Cell 114
Cell 115
Cell 116
Fluorescence (Baseline Corrected)
Time Point (0.25 Hz)
0
200
400
600
800
−1
0
1
2
3
4
5

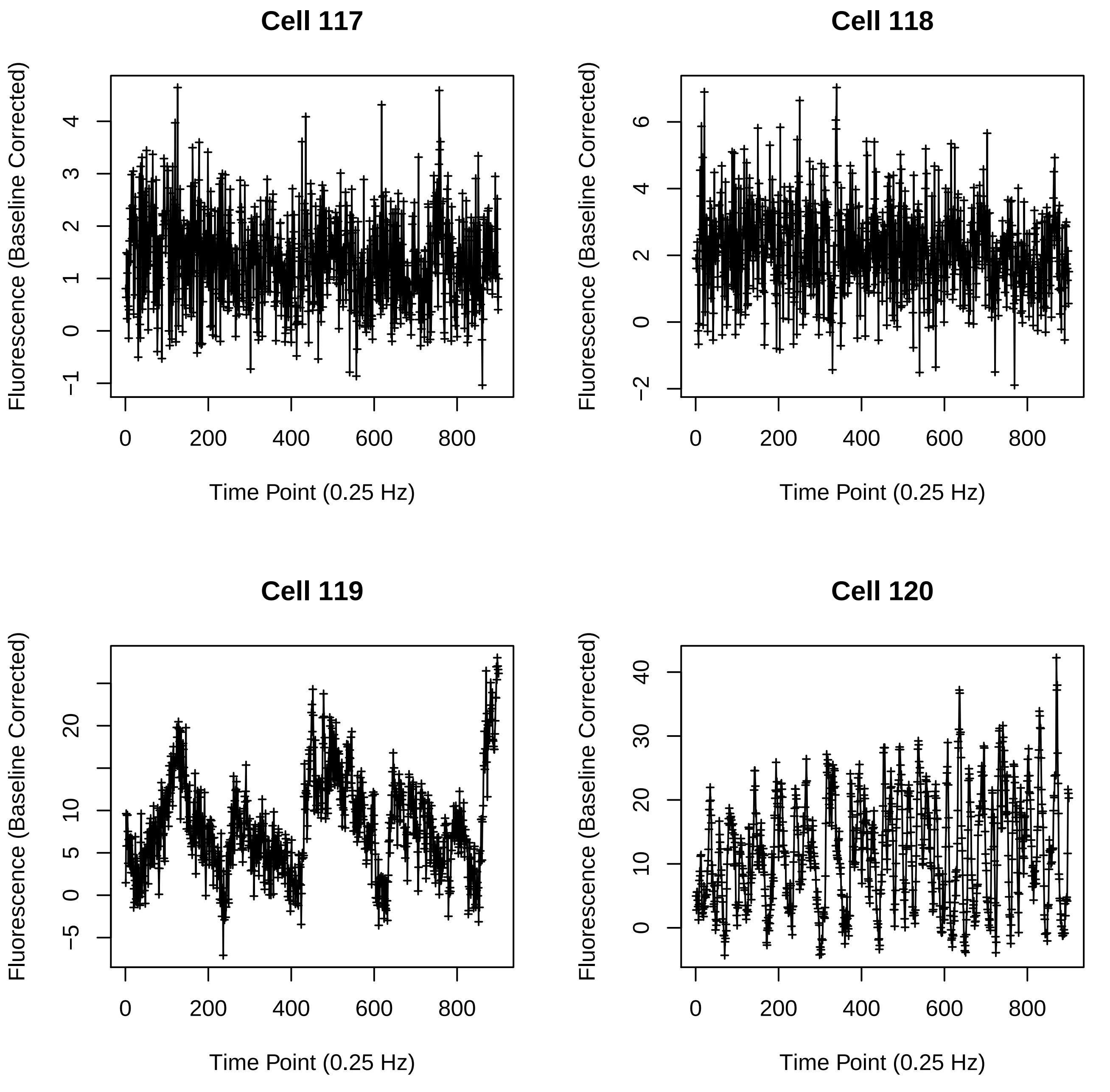
Cell 117
Cell 118
Cell 119
Cell 120
Fluorescence (Baseline Corrected)
Time Point (0.25 Hz)
−1
0
1
2
3
4
−2
6
−5
5
10
20
30
40
0
200
400
600
800

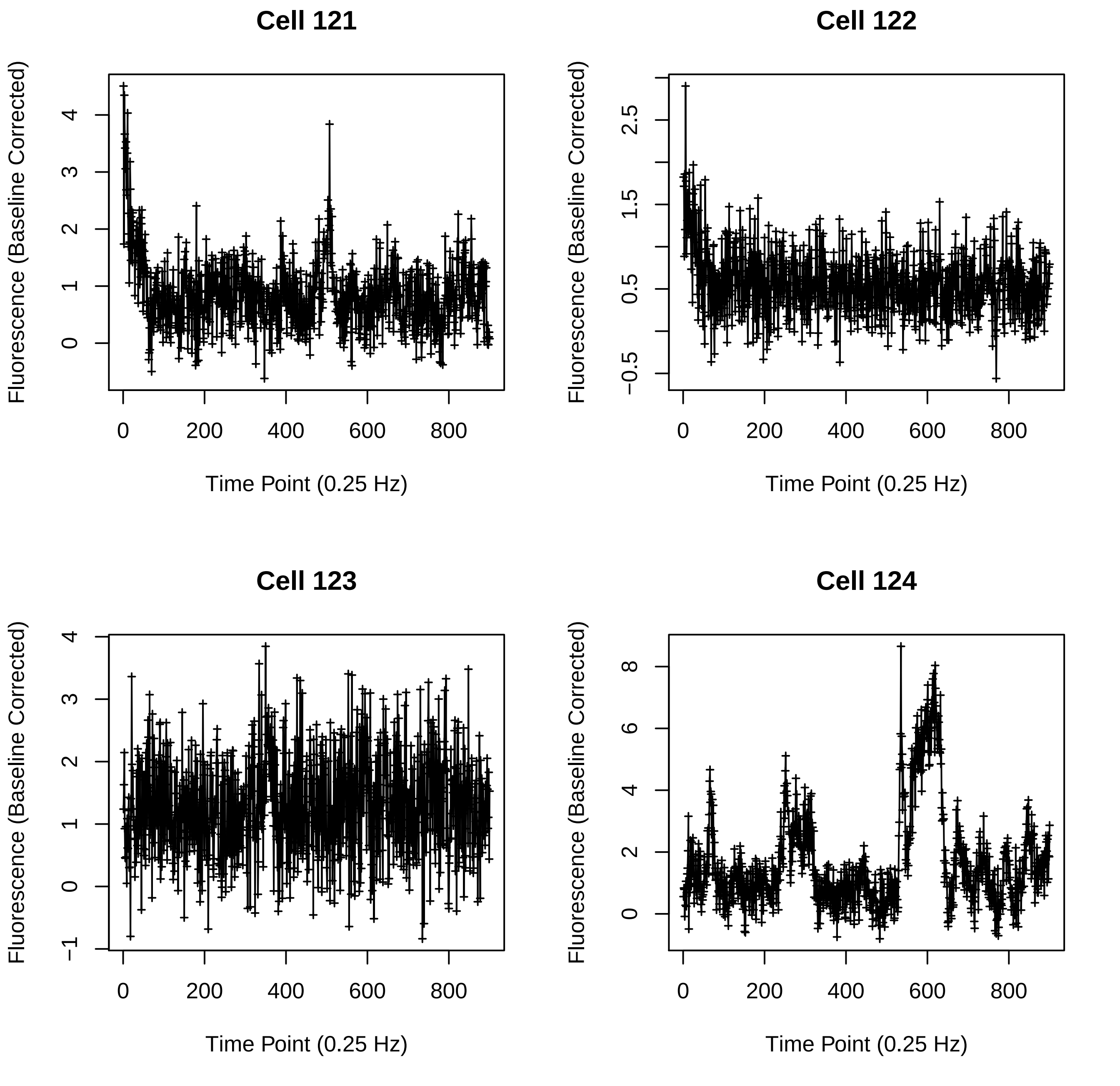

Cell 121
Cell 122
Cell 123
Cell 124
Fluorescence (Baseline Corrected)
Time Point (0.25 Hz)
0
200
400
600
800
−0.5
0.5
1.5
2.5
−1
1
2
3
4
6
8

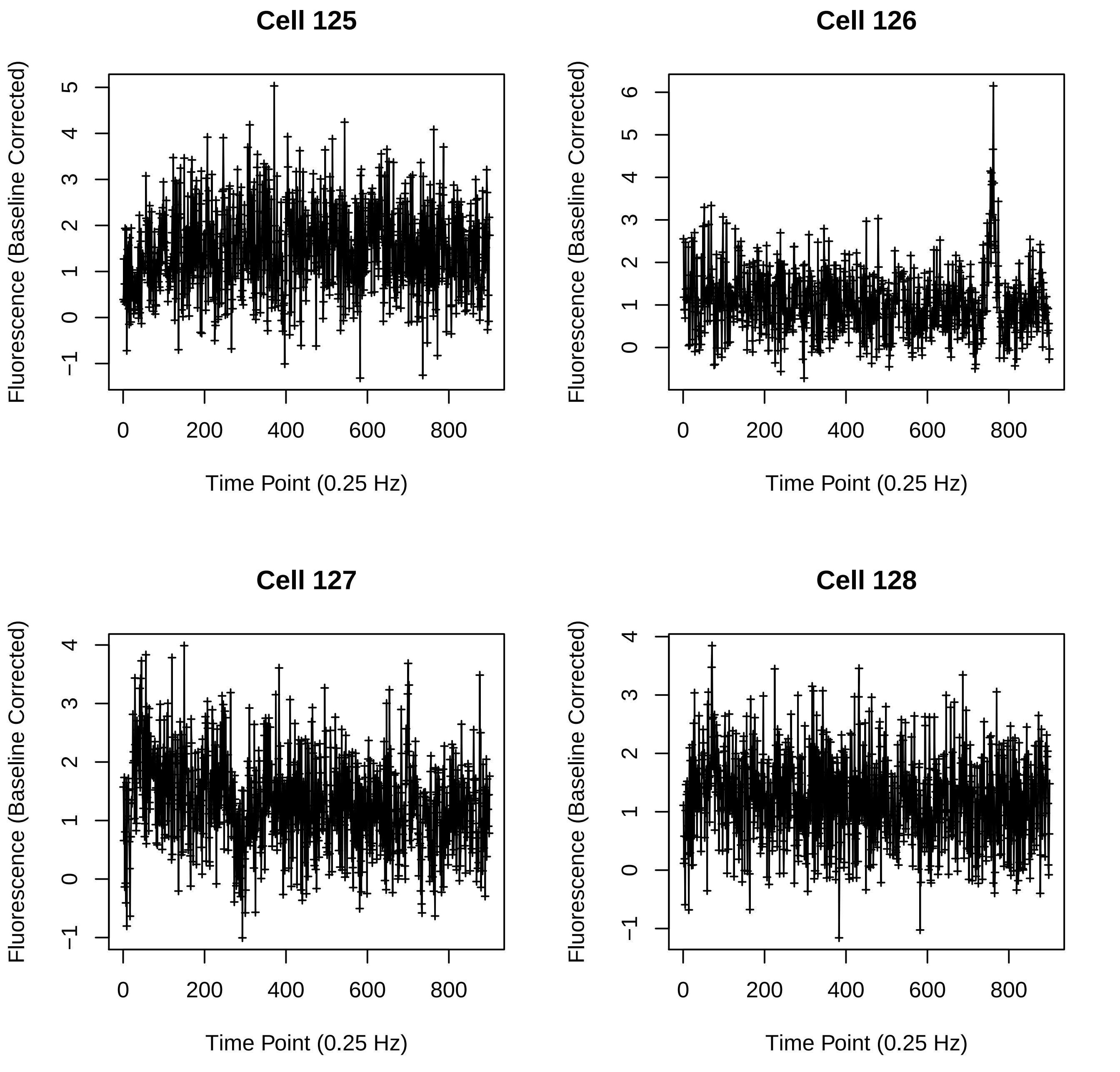
Cell 125
Cell 126
Cell 127
Cell 128
Fluorescence (Baseline Corrected)
Time Point (0.25 Hz)
0
200
400
600
800
−1
1
2
3
4
5
6

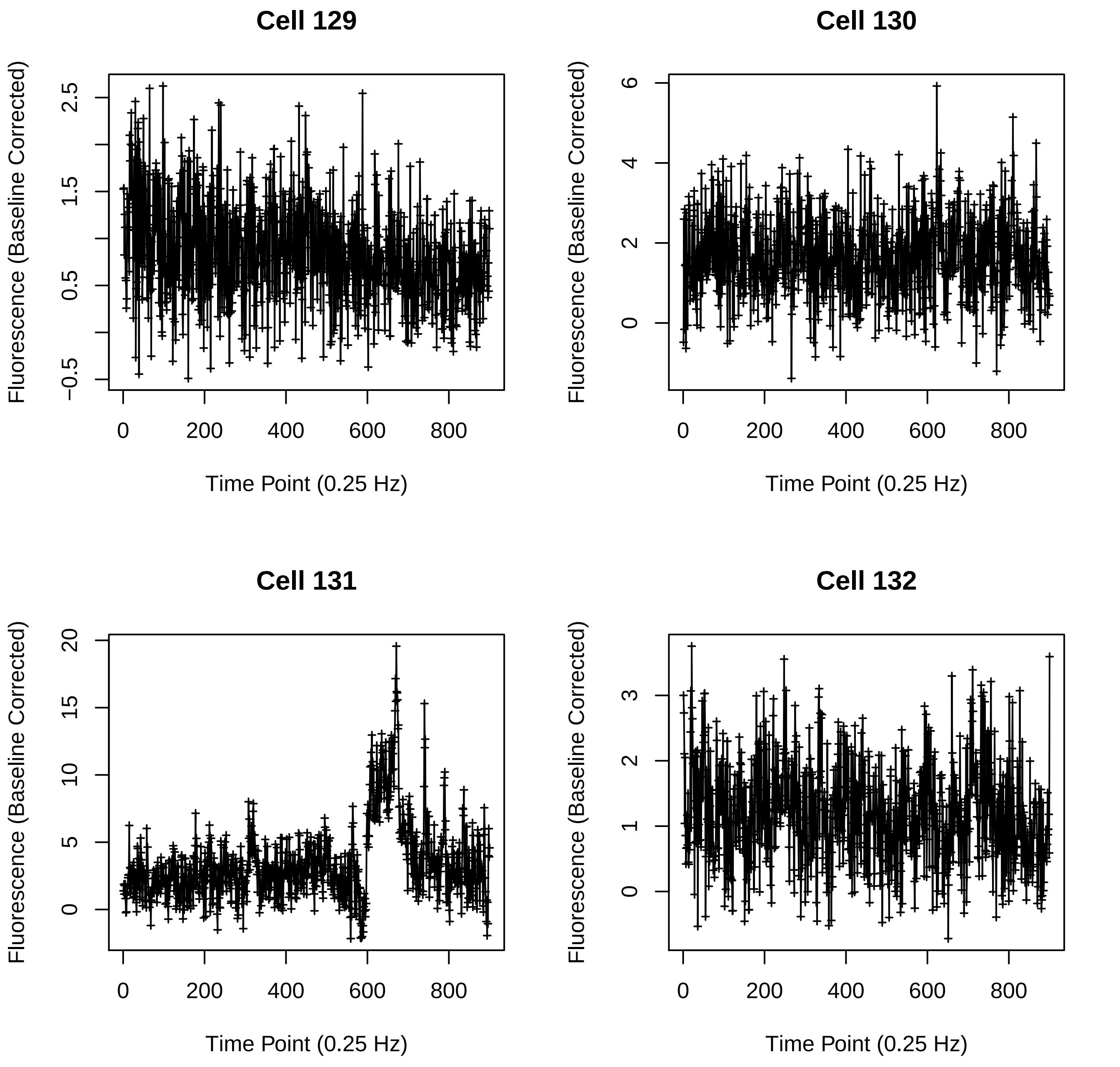
Cell 129
Cell 130
Cell 131
Cell 132
Fluorescence (Baseline Corrected)
Time Point (0.25 Hz)
−0.5
0.5
1.5
2.5
0
2
4
6
0
5
10
15
20
0
1
2
3
0
200
400
600
800

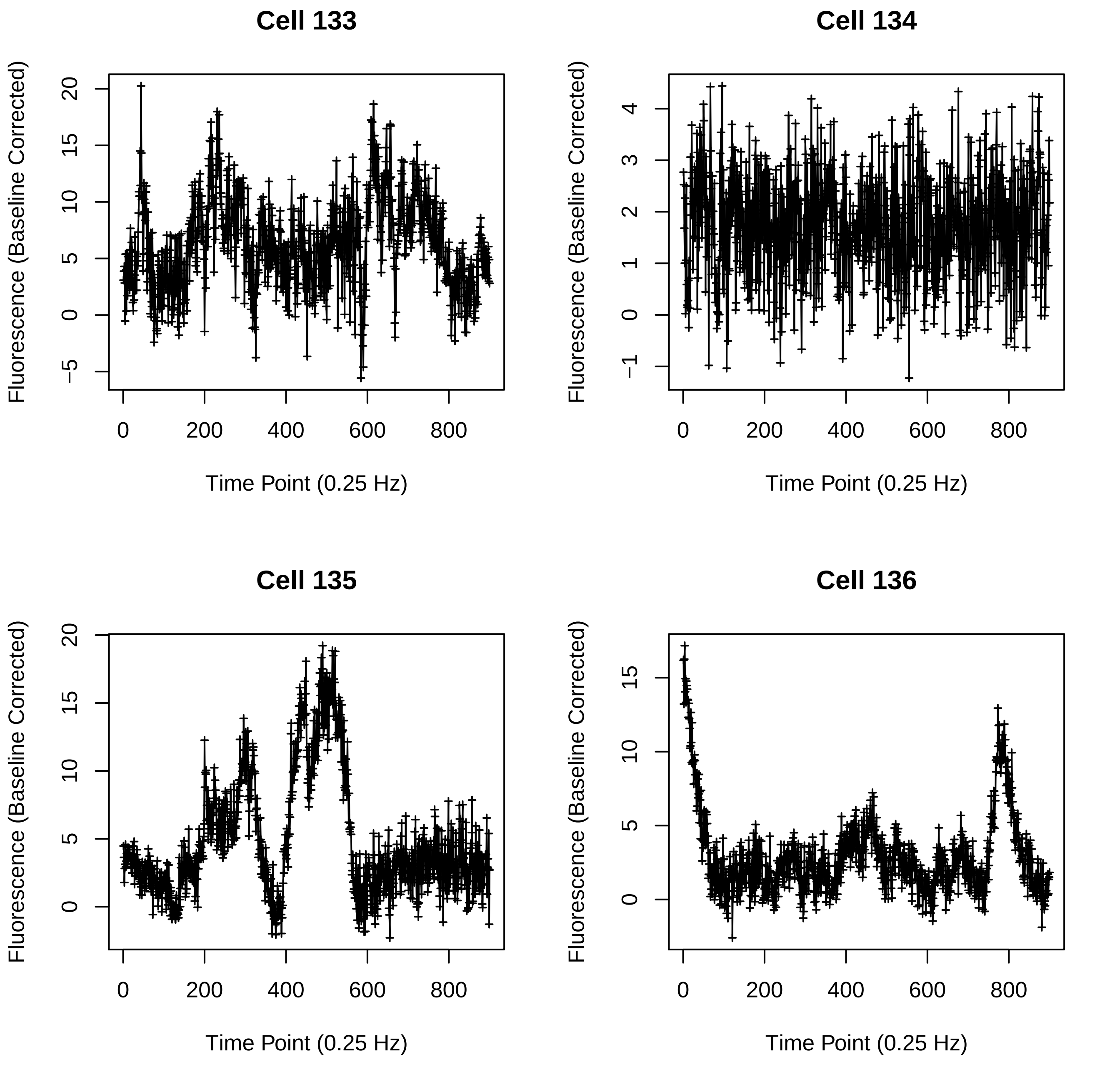
Cell 133
Cell 134
Cell 135
Cell 136
Fluorescence (Baseline Corrected)
Time Point (0.25 Hz)
0
200
400
600
800
−5
5
10
15
20
−1
1
2
3
4

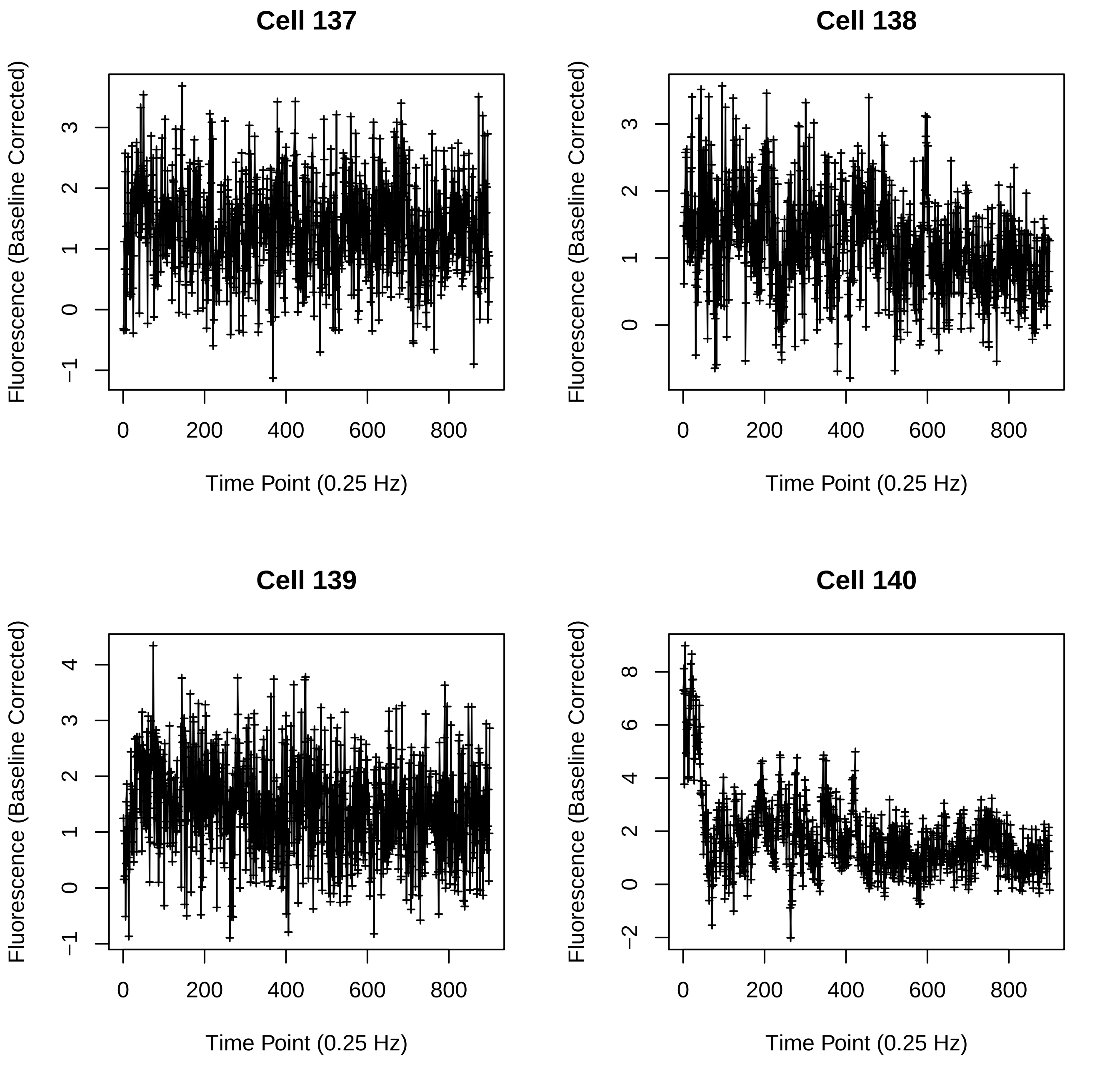

Cell 137
Cell 138
Cell 139
Cell 140
Fluorescence (Baseline Corrected)
Time Point (0.25 Hz)

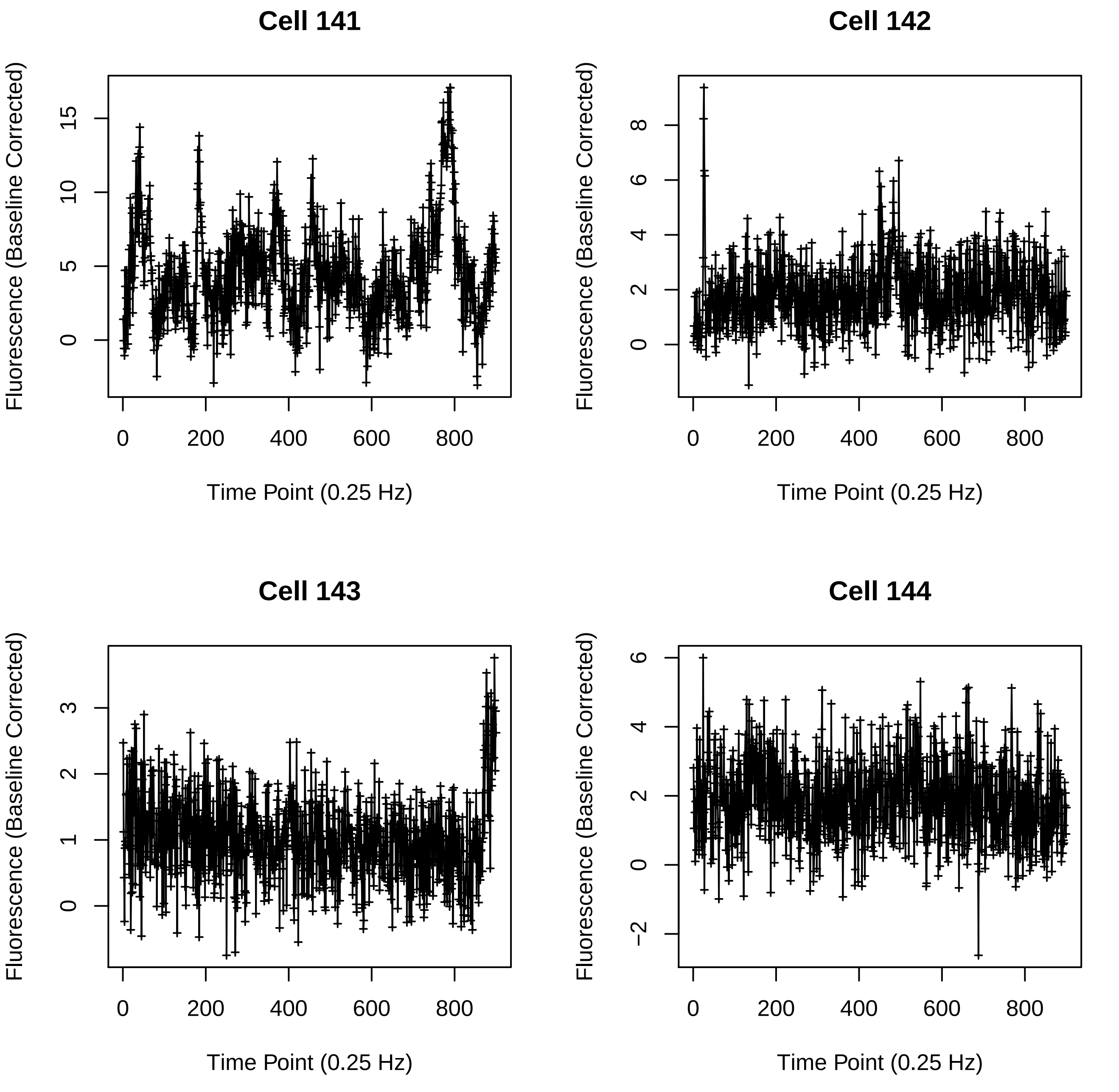
Cell 141
Fluorescence (Baseline Corrected)
0
5
10
15
0
200
400
600
800
Time Point (0.25 Hz)
Cell 142
Fluorescence (Baseline Corrected)
0
2
4
6
8
0
200
400
600
800
Time Point (0.25 Hz)
Cell 143
Fluorescence (Baseline Corrected)
0
1
2
3
0
200
400
600
800
Time Point (0.25 Hz)
Cell 144
Fluorescence (Baseline Corrected)
−2
0
2
4
6
0
200
400
600
800
Time Point (0.25 Hz)

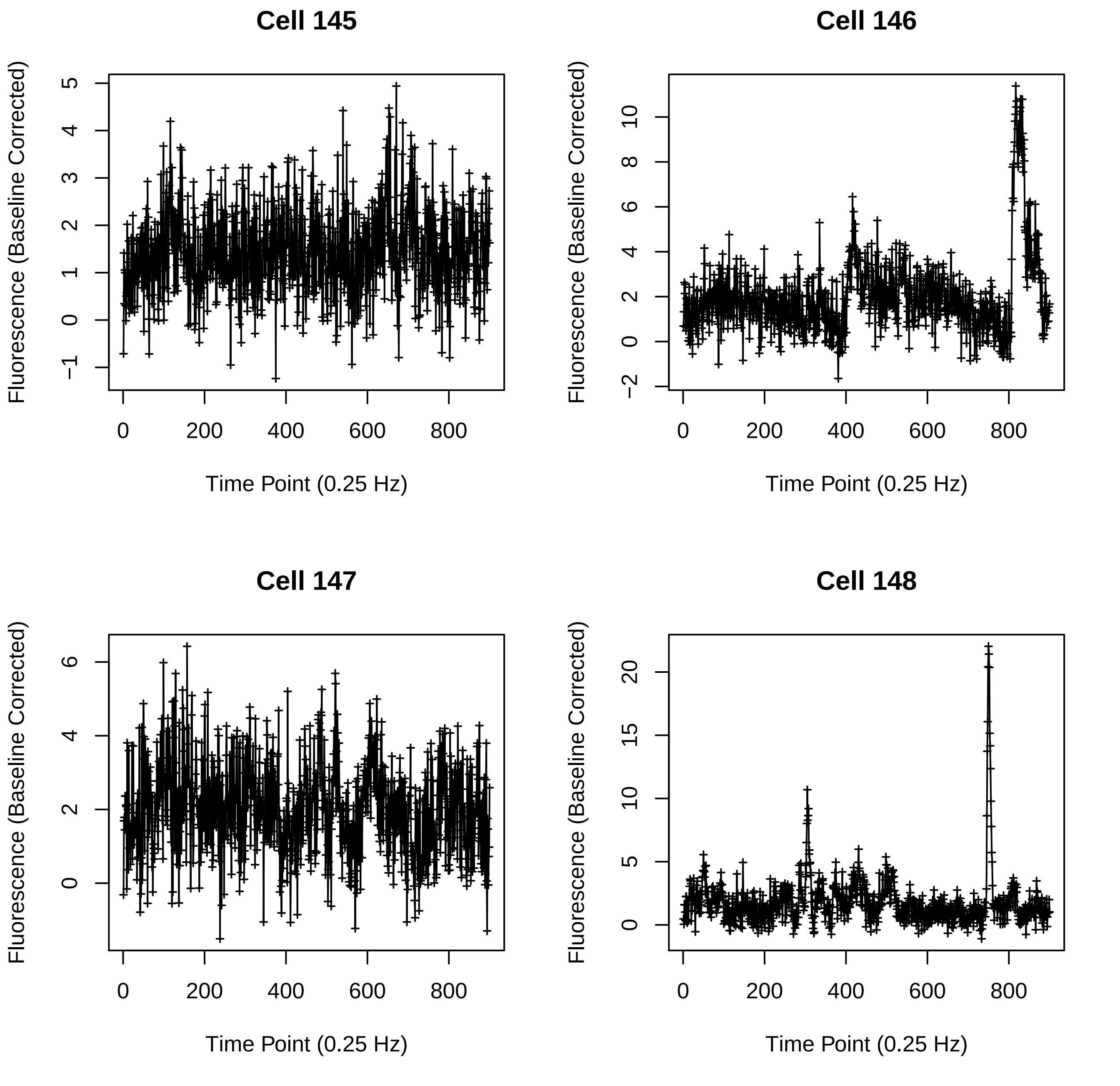
Cell 145
Cell 146
Cell 147
Cell 148
Fluorescence (Baseline Corrected)
Time Point (0.25 Hz)
0
200
400
600
800
−1
1
2
3
4
5
−2
6
8
10
15
20

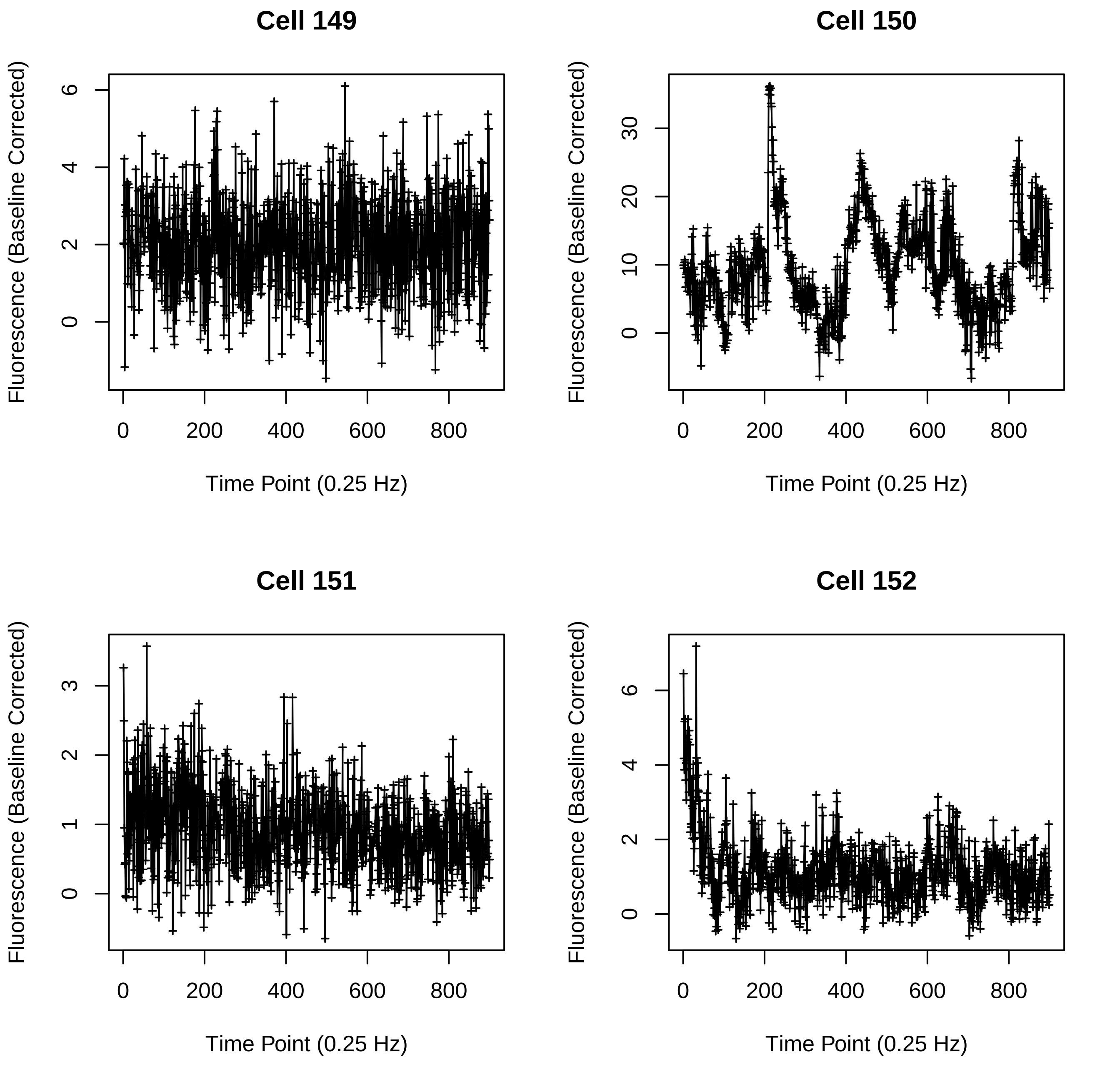
Cell 149
Cell 150
Cell 151
Cell 152
Fluorescence (Baseline Corrected)
Time Point (0.25 Hz)
0
200
400
600
800
6
4
2
0
30
20
10
3
1

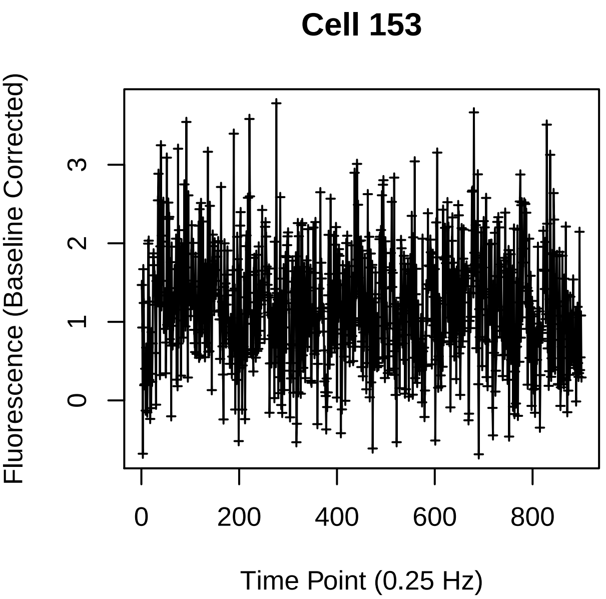
Cell 153
Fluorescence (Baseline Corrected)
0
1
2
3
0
200
400
600
800
Time Point (0.25 Hz)

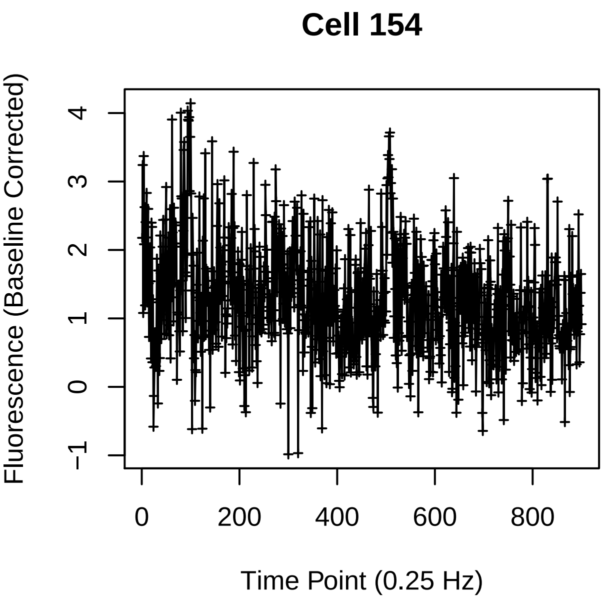
Cell 154
Fluorescence (Baseline Corrected)
−1
0
1
2
3
4
0
200
400
600
800
Time Point (0.25 Hz)

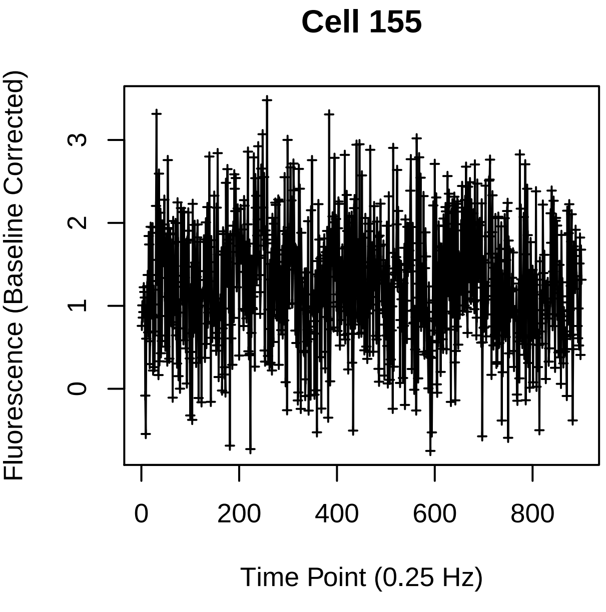
Cell 155
Fluorescence (Baseline Corrected)
0
1
2
3
0
200
400
600
800
Time Point (0.25 Hz)

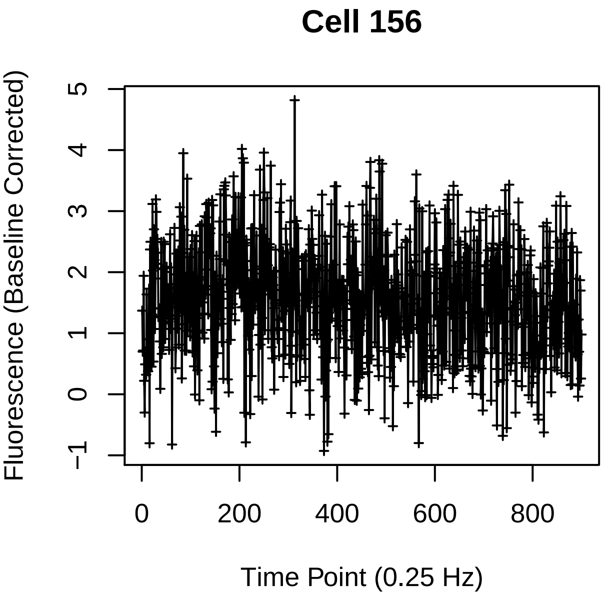
Cell 156
Fluorescence (Baseline Corrected)
−1
0
1
2
3
4
5
0
200
400
600
800
Time Point (0.25 Hz)

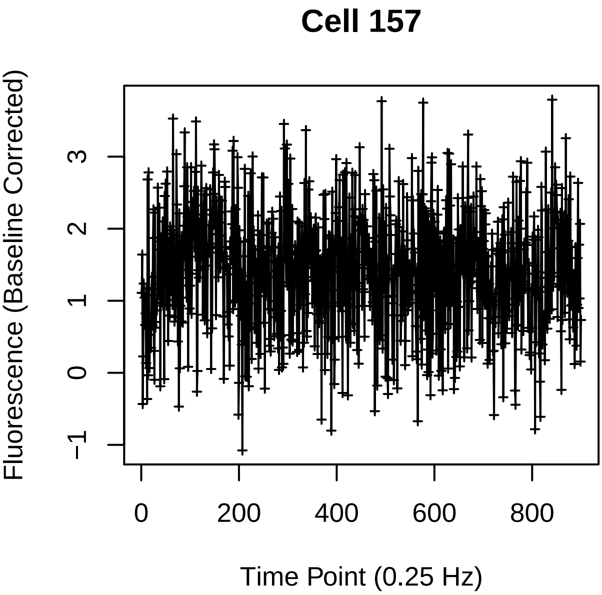

Cell 157
Fluorescence (Baseline Corrected)
Time Point (0.25 Hz)
−1
0
1
2
3
0
200
400
600
800

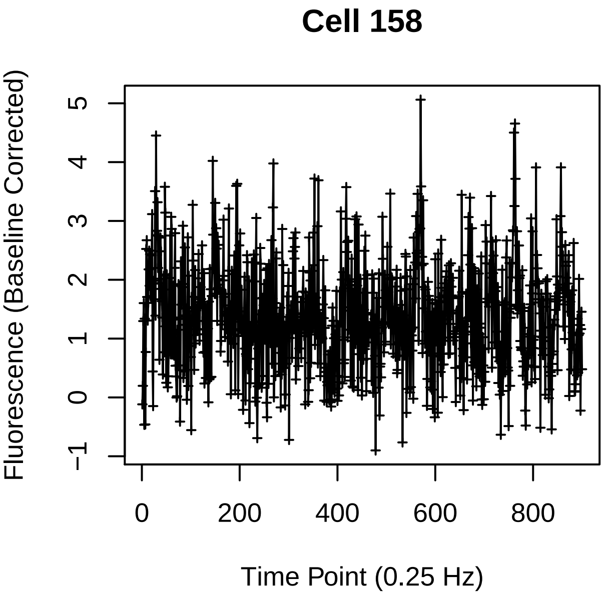

Cell 158
Fluorescence (Baseline Corrected)
Time Point (0.25 Hz)
−1
0
1
2
3
4
5
0
200
400
600
800

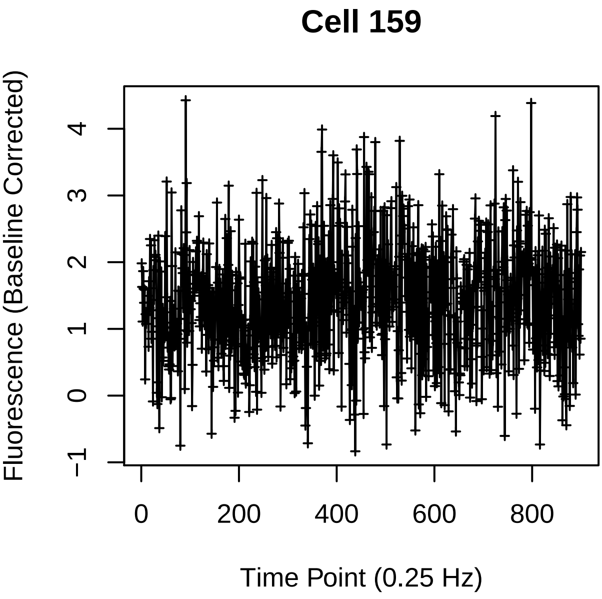

Cell 159
Fluorescence (Baseline Corrected)
Time Point (0.25 Hz)
−1
0
1
2
3
4
0
200
400
600
800

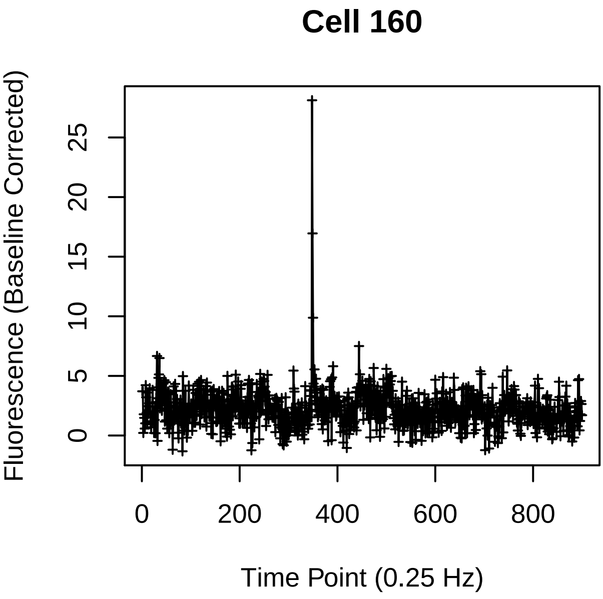

Cell 160
Fluorescence (Baseline Corrected)
Time Point (0.25 Hz)
0
5
10
15
20
25
0
200
400
600
800

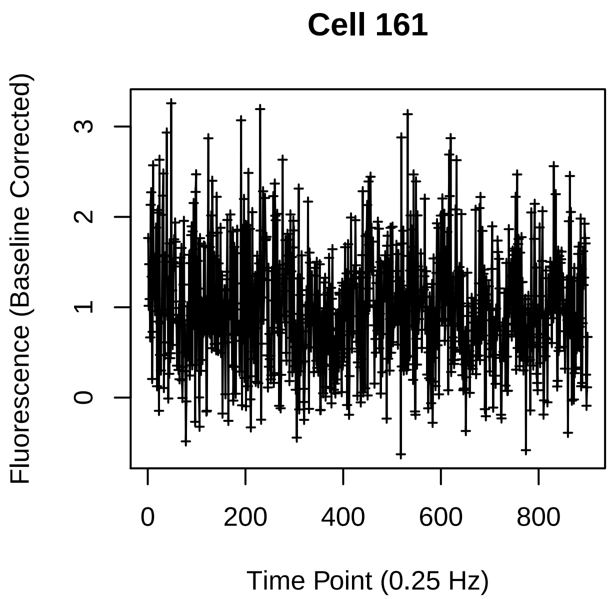
Cell 161
Fluorescence (Baseline Corrected)
Time Point (0.25 Hz)
0
1
2
3
0
200
400
600
800

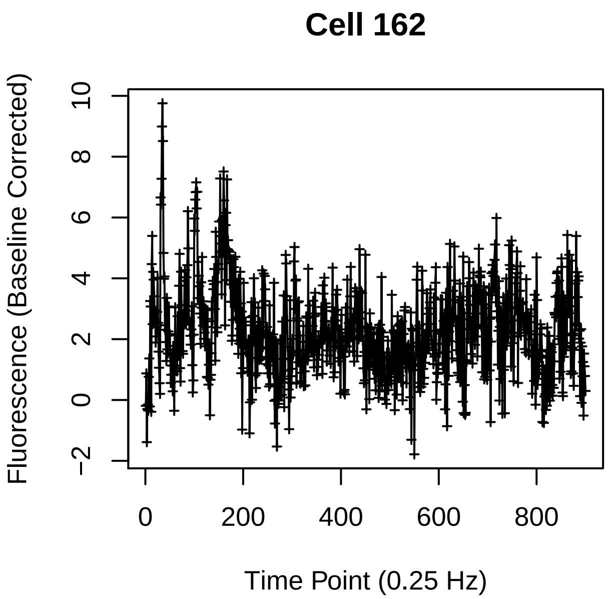
Cell 162
Fluorescence (Baseline Corrected)
Time Point (0.25 Hz)
−2
0
2
4
6
8
10
0
200
400
600
800

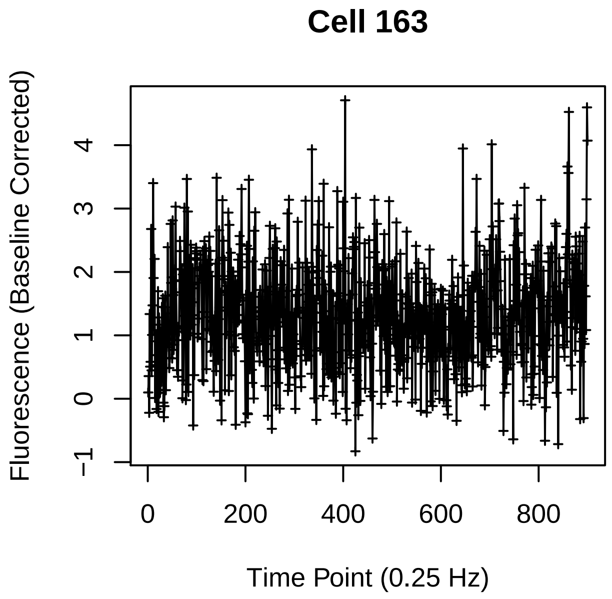
Cell 163
Fluorescence (Baseline Corrected)
Time Point (0.25 Hz)
−1
0
1
2
3
4
0
200
400
600
800

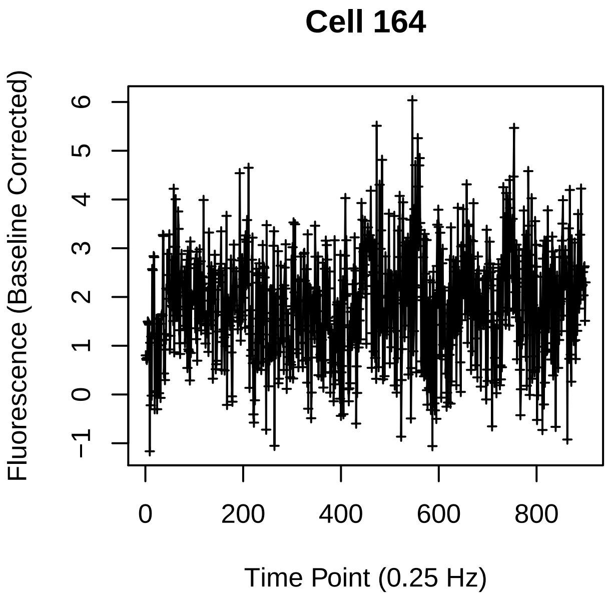
Cell 164
Fluorescence (Baseline Corrected)
Time Point (0.25 Hz)
−1
0
1
2
3
4
5
6
0
200
400
600
800

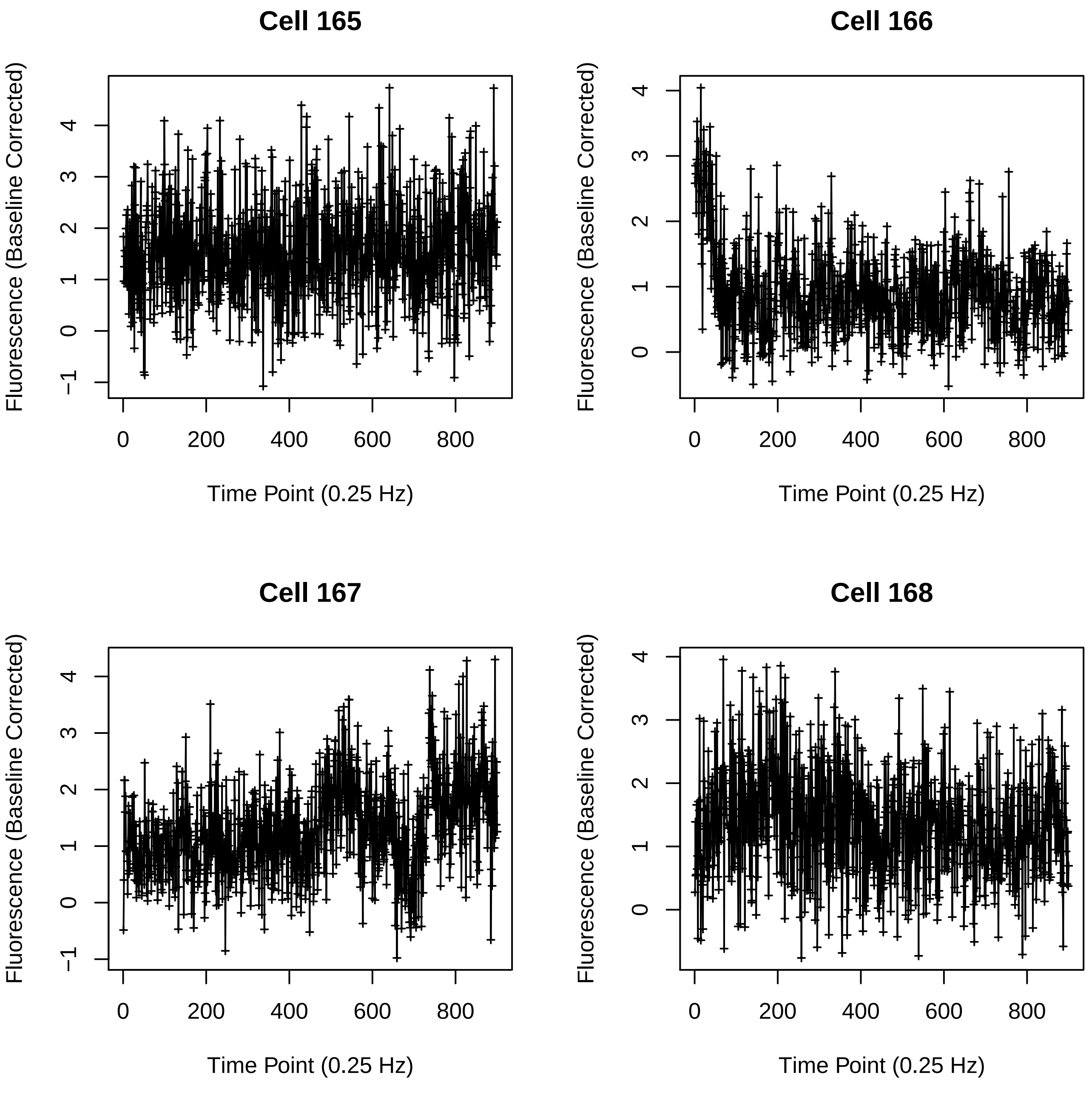
Cell 165
Cell 166
Cell 167
Cell 168
Fluorescence (Baseline Corrected)
Time Point (0.25 Hz)
−1
0
1
2
3
4
200
400
600
800

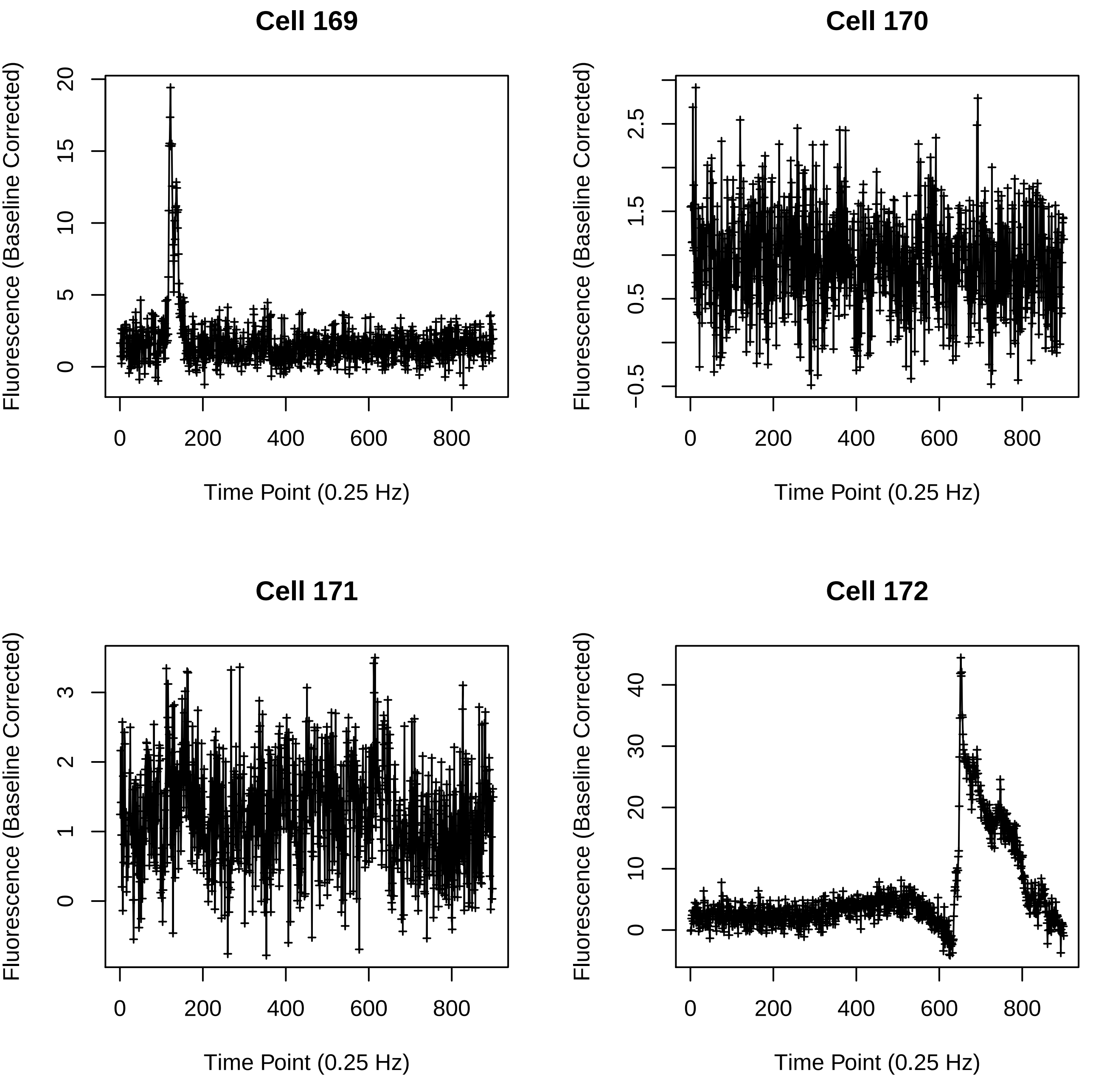
Cell 169
Fluorescence (Baseline Corrected)
Time Point (0.25 Hz)
Cell 170
Fluorescence (Baseline Corrected)
Time Point (0.25 Hz)
Cell 171
Fluorescence (Baseline Corrected)
Time Point (0.25 Hz)
Cell 172
Fluorescence (Baseline Corrected)
Time Point (0.25 Hz)

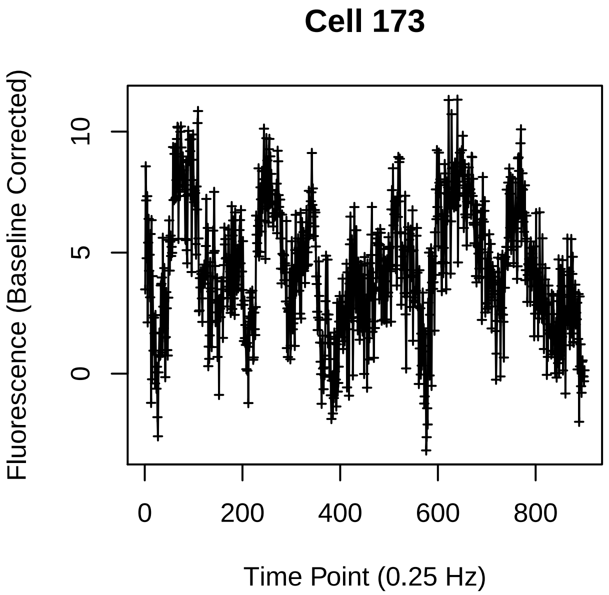
Cell 173
Fluorescence (Baseline Corrected)
Time Point (0.25 Hz)
0
5
10
0
200
400
600
800

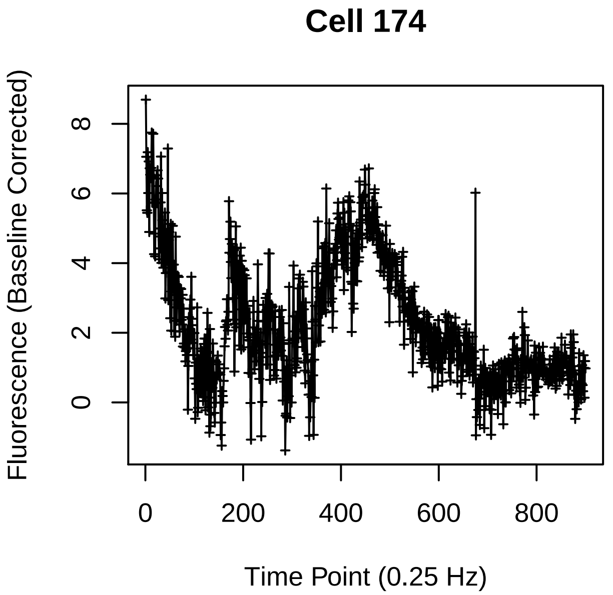
Cell 174
Fluorescence (Baseline Corrected)
Time Point (0.25 Hz)
0
2
4
6
8
0
200
400
600
800

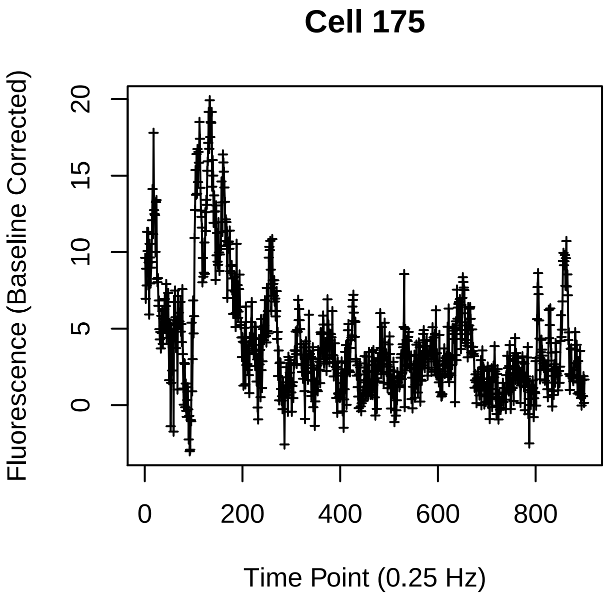
Cell 175
Fluorescence (Baseline Corrected)
Time Point (0.25 Hz)
0
5
10
15
20
0
200
400
600
800

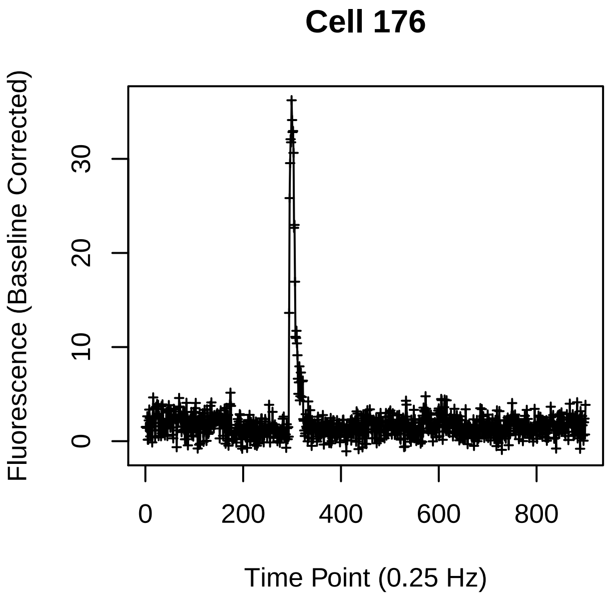
Cell 176
Fluorescence (Baseline Corrected)
Time Point (0.25 Hz)
0
10
20
30
0
200
400
600
800

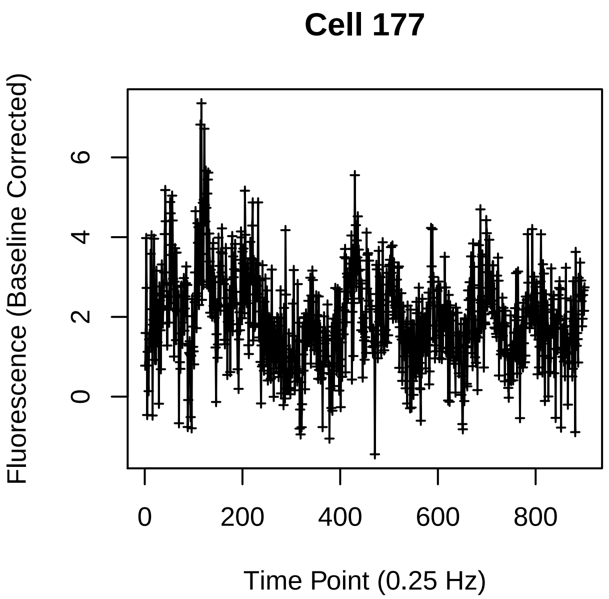
Cell 177
Fluorescence (Baseline Corrected)
Time Point (0.25 Hz)
0
2
4
6
0
200
400
600
800

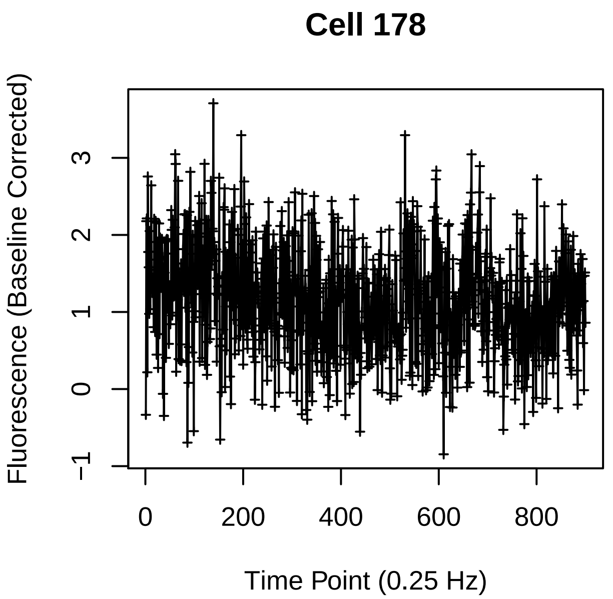
Cell 178
Fluorescence (Baseline Corrected)
Time Point (0.25 Hz)
−1
0
1
2
3
0
200
400
600
800

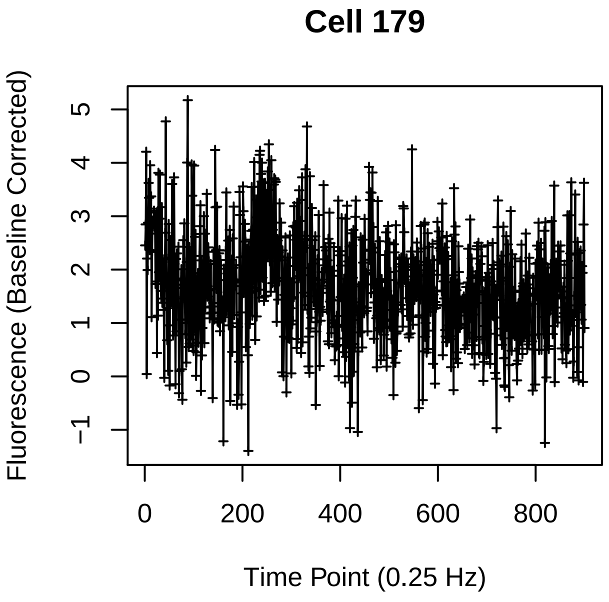
Cell 179
Fluorescence (Baseline Corrected)
Time Point (0.25 Hz)
−1
0
1
2
3
4
5
0
200
400
600
800

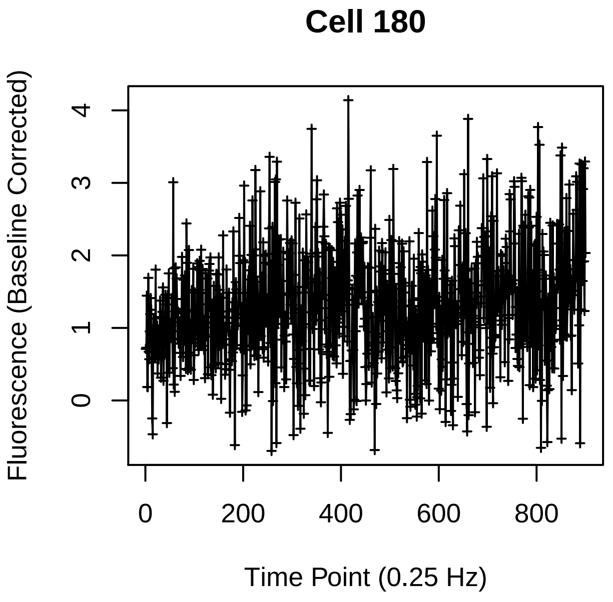
Cell 180
Fluorescence (Baseline Corrected)
Time Point (0.25 Hz)
0
1
2
3
4
0
200
400
600
800

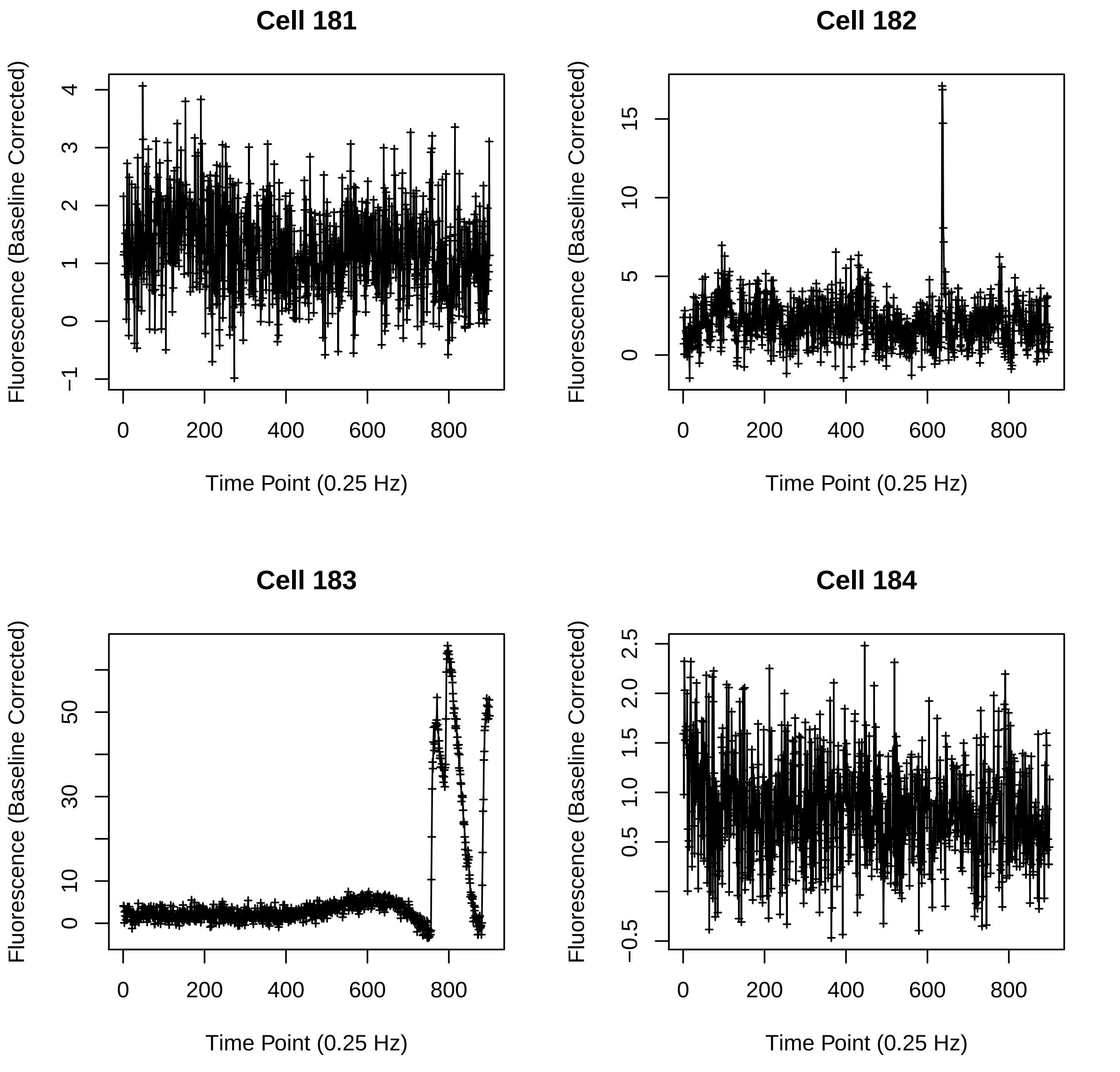
Cell 181
Cell 182
Cell 183
Cell 184
Fluorescence (Baseline Corrected)
Time Point (0.25 Hz)
−1
0
1
2
3
4
0
5
10
15
0
10
30
50
−0.5
0.5
1.0
1.5
2.0
2.5
0
200
400
600
800

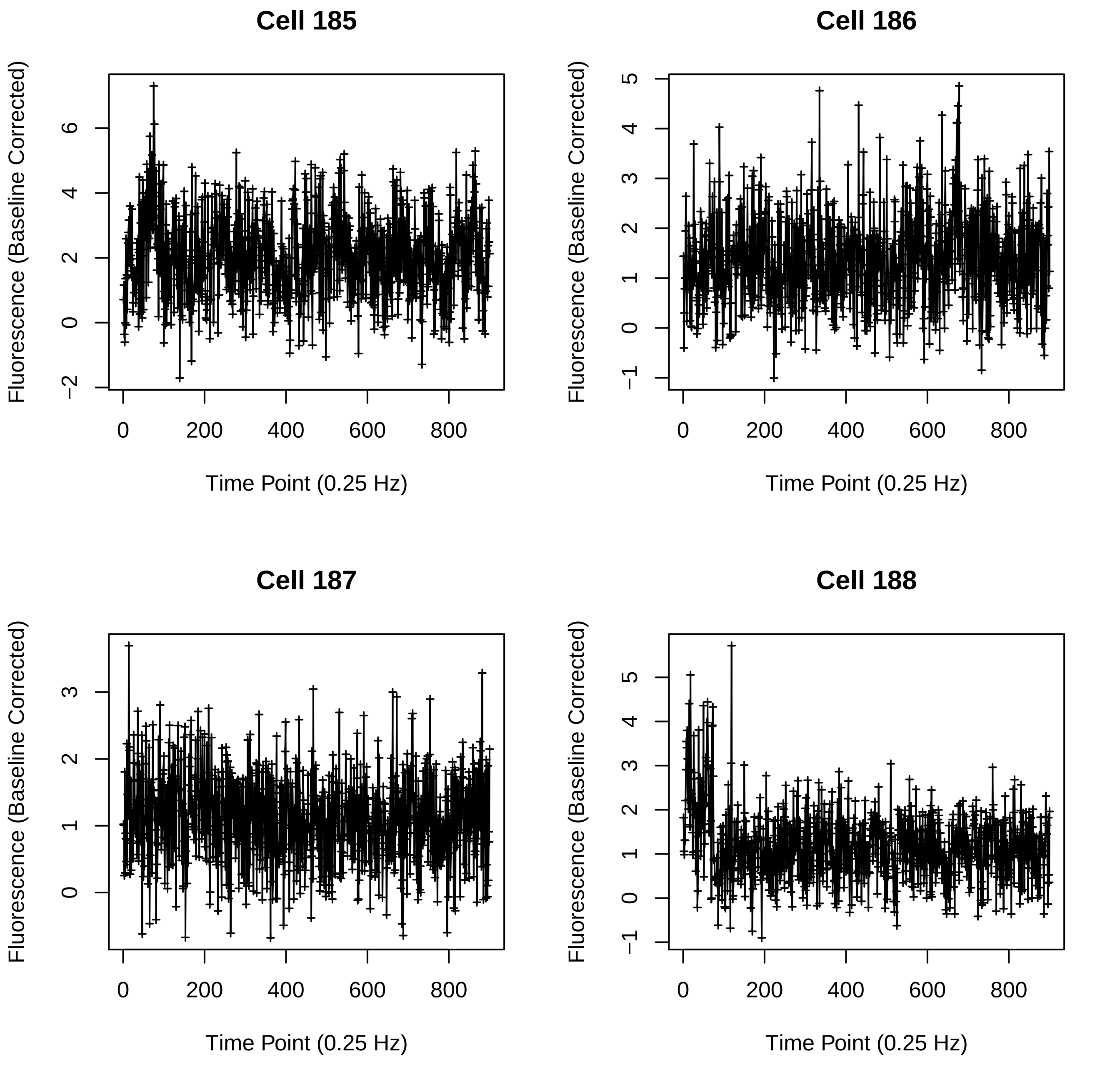
Cell 185
Fluorescence (Baseline Corrected)
−2
0
2
4
6
0
200
400
600
800
Time Point (0.25 Hz)
Cell 186
Fluorescence (Baseline Corrected)
−1
0
1
2
3
4
5
0
200
400
600
800
Time Point (0.25 Hz)
Cell 187
Fluorescence (Baseline Corrected)
0
1
2
3
0
200
400
600
800
Time Point (0.25 Hz)
Cell 188
Fluorescence (Baseline Corrected)
−1
0
1
2
3
4
5
0
200
400
600
800
Time Point (0.25 Hz)

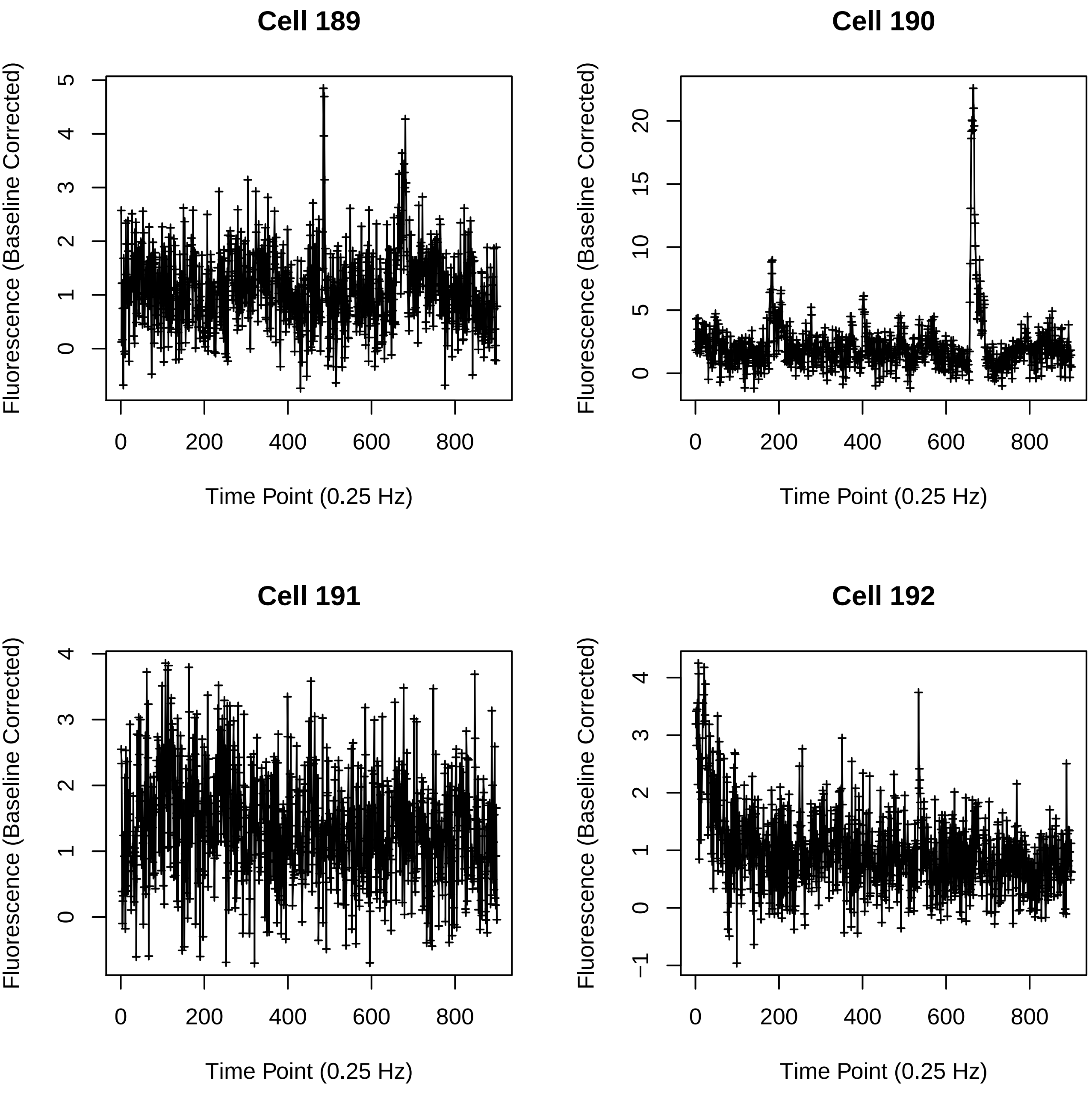
Cell 189
Cell 190
Cell 191
Cell 192
Fluorescence (Baseline Corrected)
Time Point (0.25 Hz)
0
200
400
600
800
0
1
2
3
4
5
10
15
20
−1

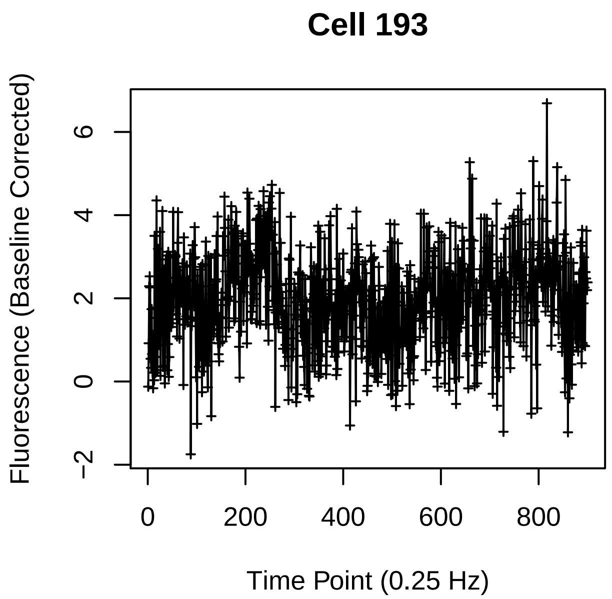
Cell 193
Fluorescence (Baseline Corrected)
−2
0
2
4
6
0
200
400
600
800
Time Point (0.25 Hz)

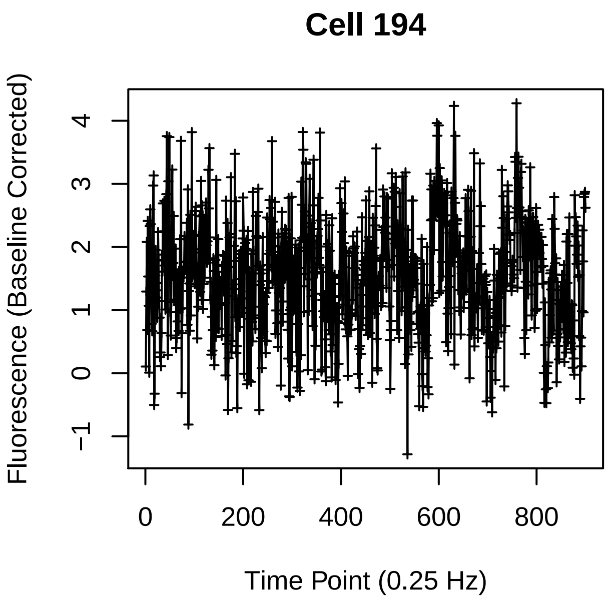
Cell 194
Fluorescence (Baseline Corrected)
−1
0
1
2
3
4
0
200
400
600
800
Time Point (0.25 Hz)

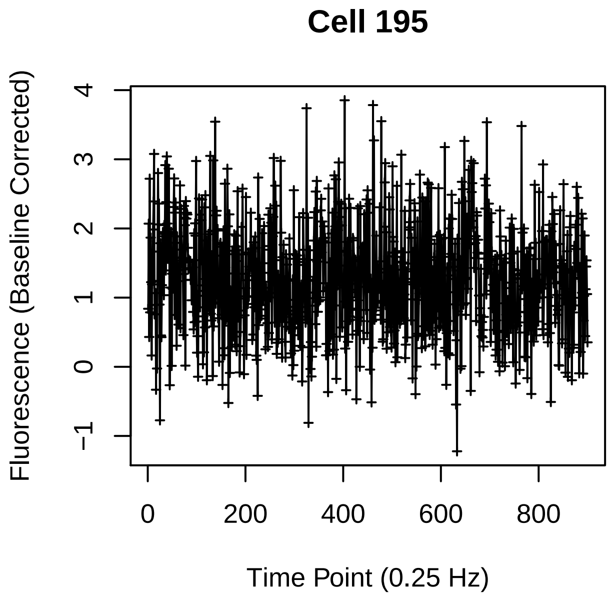
Cell 195
Fluorescence (Baseline Corrected)
−1
0
1
2
3
4
0
200
400
600
800
Time Point (0.25 Hz)

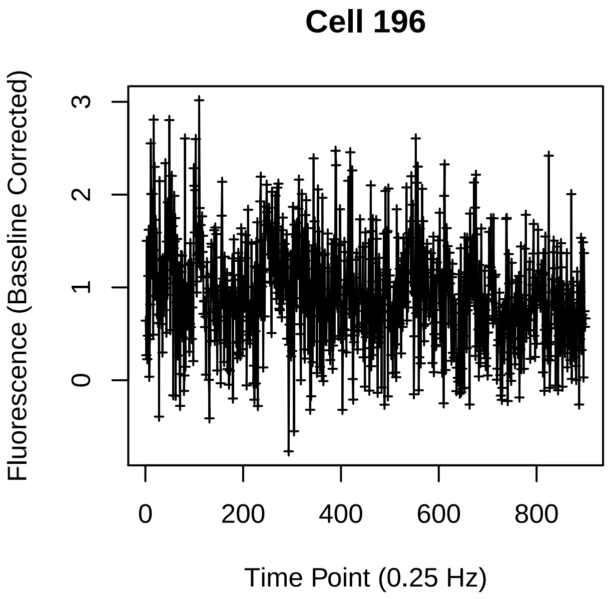
Cell 196
Fluorescence (Baseline Corrected)
0
1
2
3
0
200
400
600
800
Time Point (0.25 Hz)

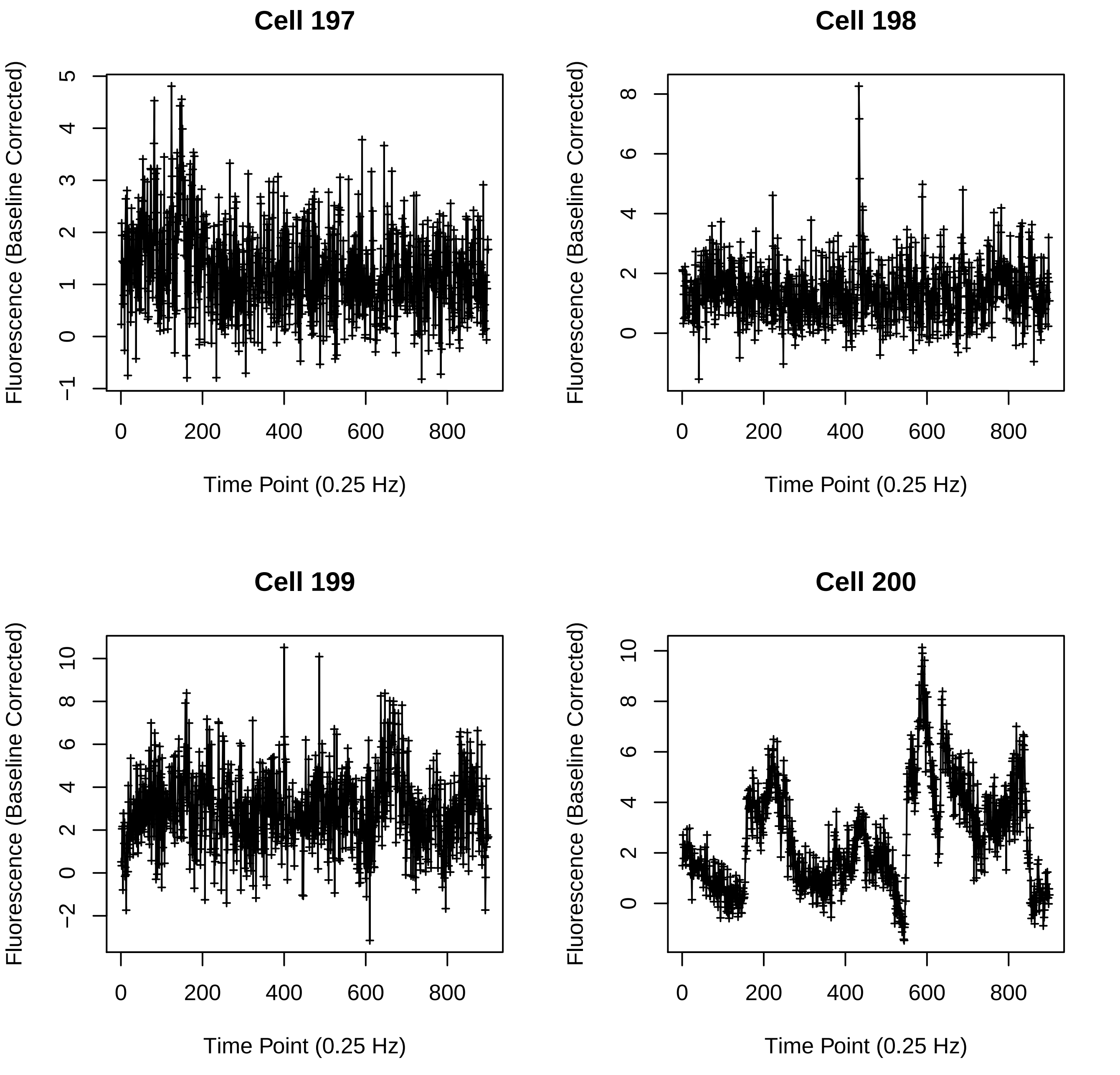
Cell 197
Cell 198
Cell 199
Cell 200
Fluorescence (Baseline Corrected)
Time Point (0.25 Hz)
−1
0
1
2
3
4
5
6
8
10
−2
0
200
400
600
800

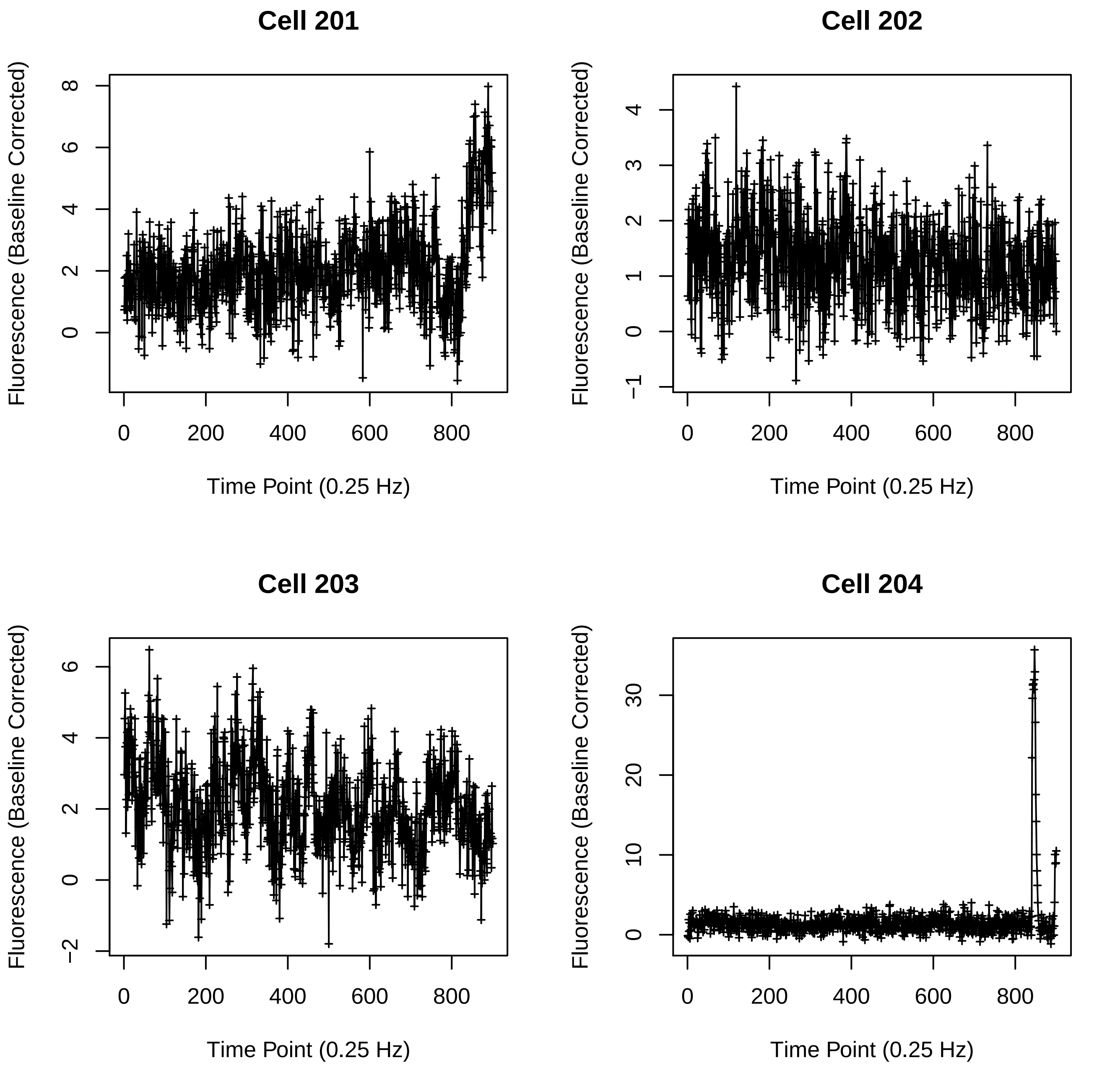
Cell 201
Fluorescence (Baseline Corrected)
0
2
4
6
8
0
200
400
600
800
Time Point (0.25 Hz)
Cell 202
Fluorescence (Baseline Corrected)
−1
0
1
2
3
4
0
200
400
600
800
Time Point (0.25 Hz)
Cell 203
Fluorescence (Baseline Corrected)
−2
0
2
4
6
0
200
400
600
800
Time Point (0.25 Hz)
Cell 204
Fluorescence (Baseline Corrected)
0
10
20
30
0
200
400
600
800
Time Point (0.25 Hz)

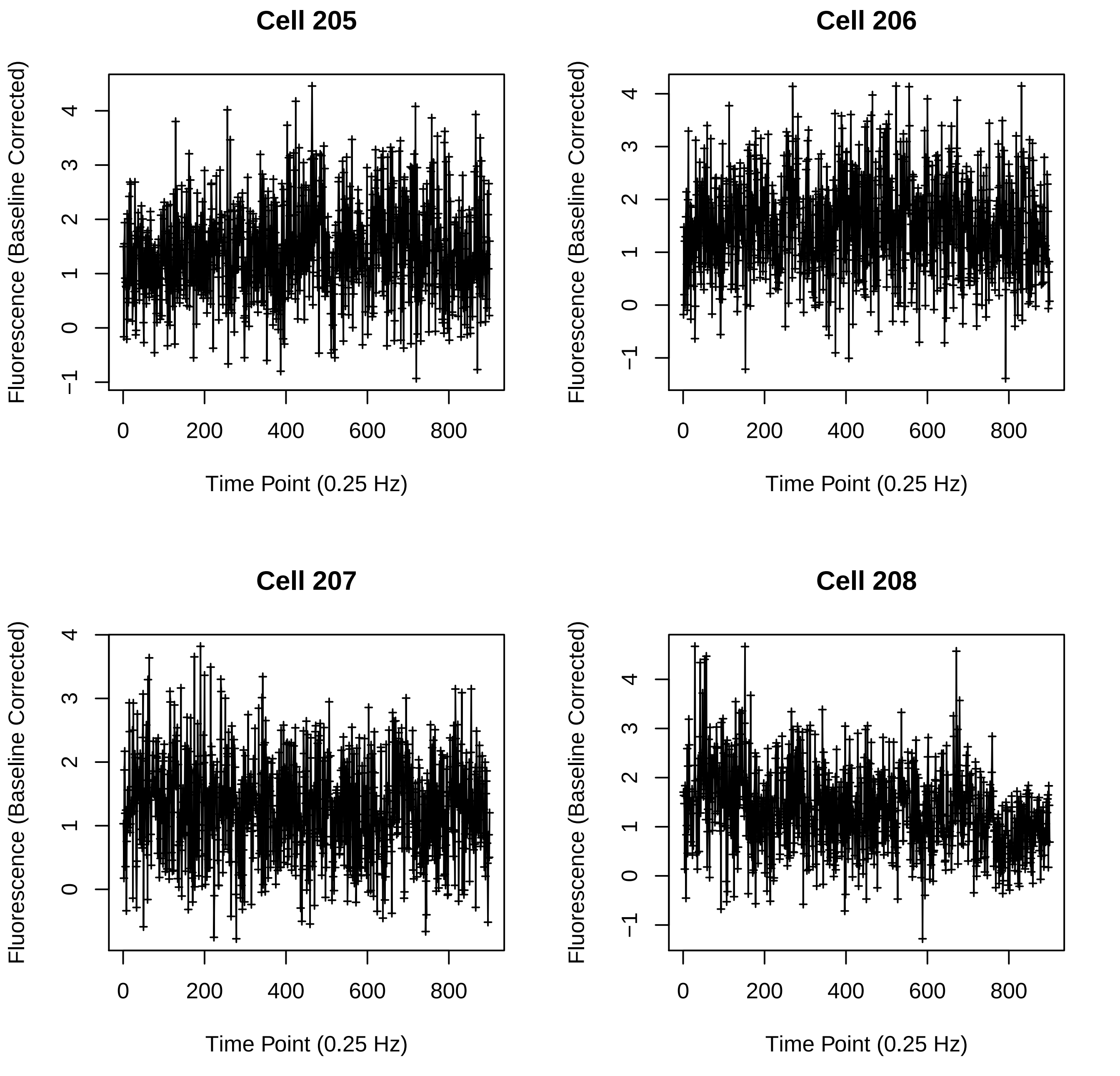
Cell 205
Cell 206
Cell 207
Cell 208
Fluorescence (Baseline Corrected)
Time Point (0.25 Hz)
0
200
400
600
800
−1
0
1
2
3
4

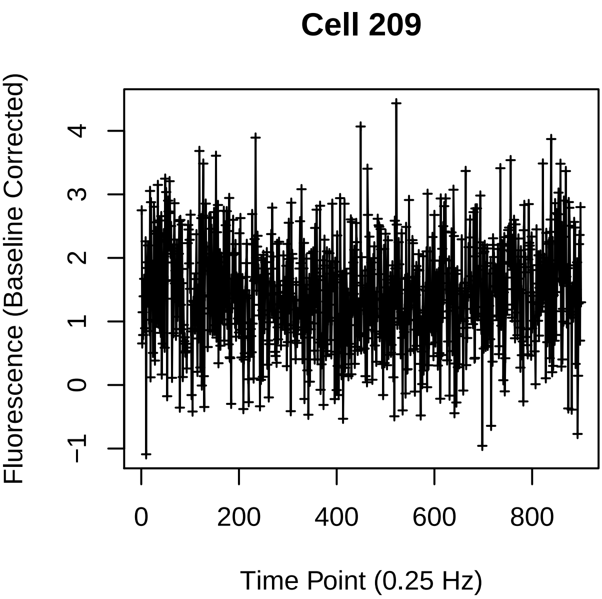
Cell 209
Fluorescence (Baseline Corrected)
Time Point (0.25 Hz)
−1
0
1
2
3
4
0
200
400
600
800

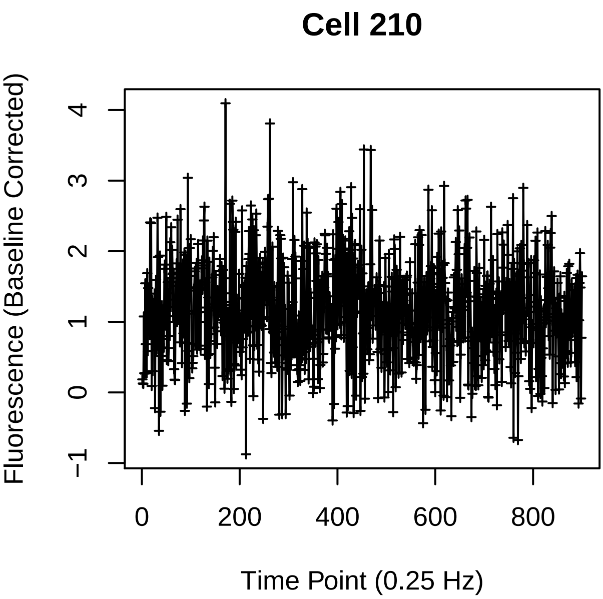
Cell 210
Fluorescence (Baseline Corrected)
Time Point (0.25 Hz)
−1
0
1
2
3
4
0
200
400
600
800

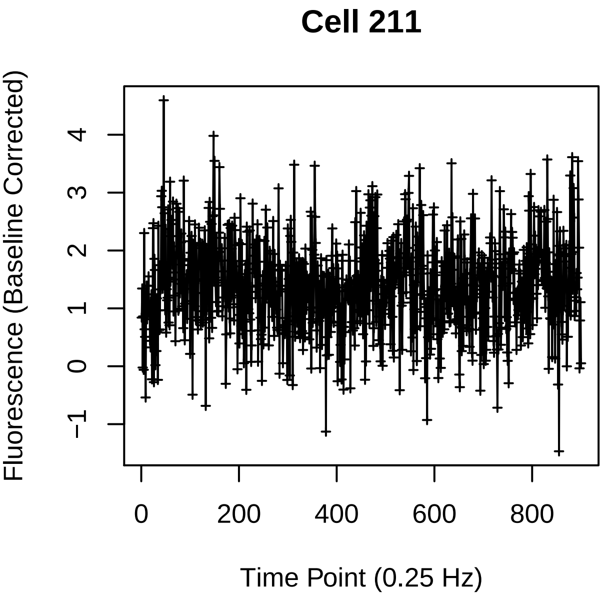
Cell 211
Fluorescence (Baseline Corrected)
Time Point (0.25 Hz)
−1
0
1
2
3
4
0
200
400
600
800

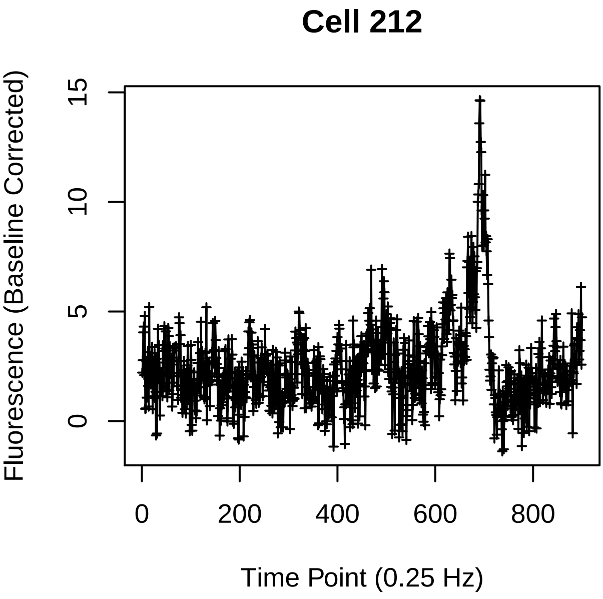
Cell 212
Fluorescence (Baseline Corrected)
Time Point (0.25 Hz)
0
5
10
15
0
200
400
600
800

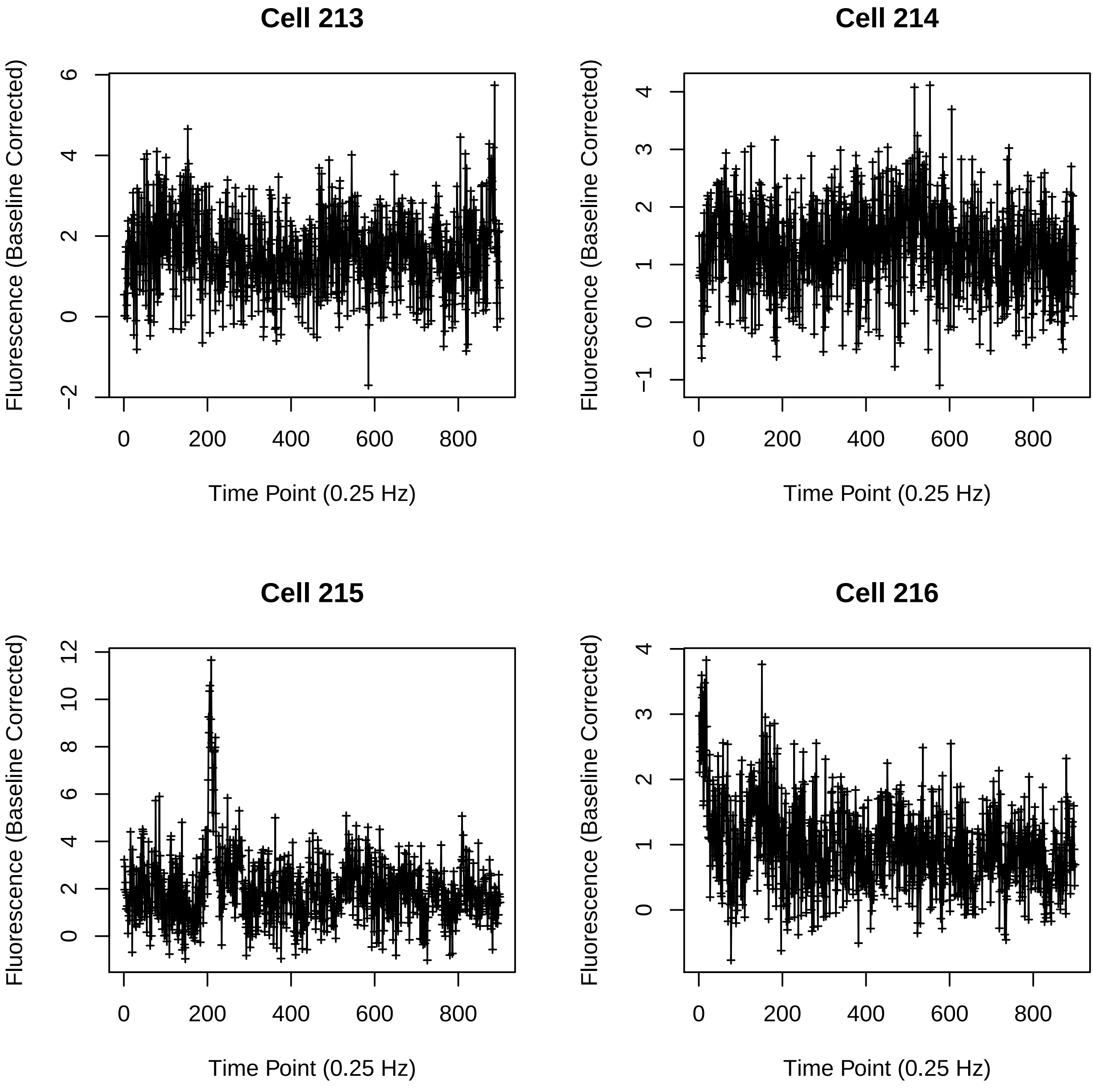

Cell 213
Cell 214
Cell 215
Cell 216
Fluorescence (Baseline Corrected)
Time Point (0.25 Hz)
-2
0
2
4
6
-1
0
1
2
3
4
0
2
4
6
8
10
12
0
1
2
3
4
0
200
400
600
800

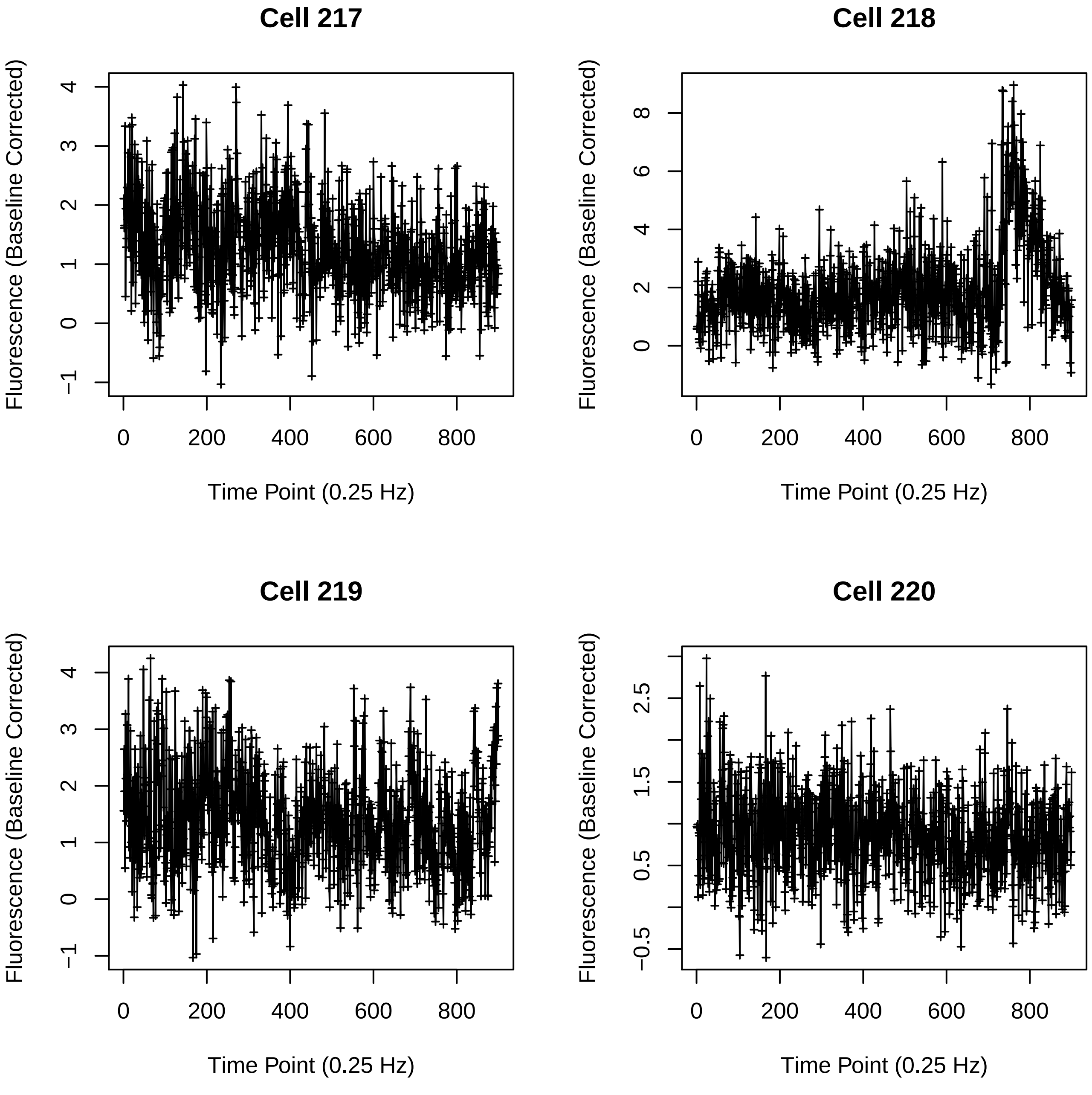
Cell 217
Cell 218
Cell 219
Cell 220
Fluorescence (Baseline Corrected)
Time Point (0.25 Hz)
0
200
400
600
800
−1
1
2
3
4
6
8
−0.5
0.5
1.5
2.5

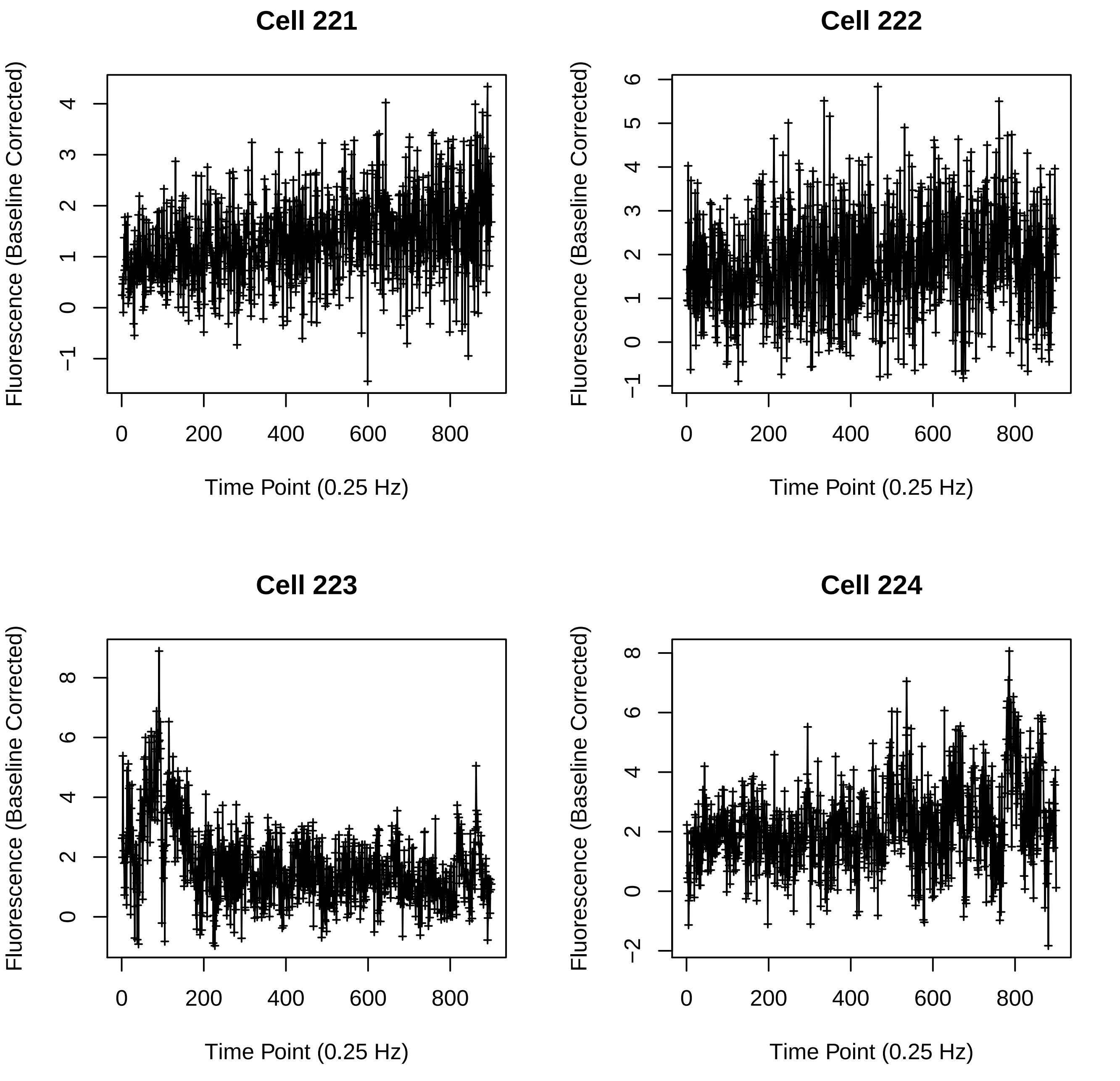
Cell 221
Cell 222
Cell 223
Cell 224
Fluorescence (Baseline Corrected)
Time Point (0.25 Hz)
0
200
400
600
800

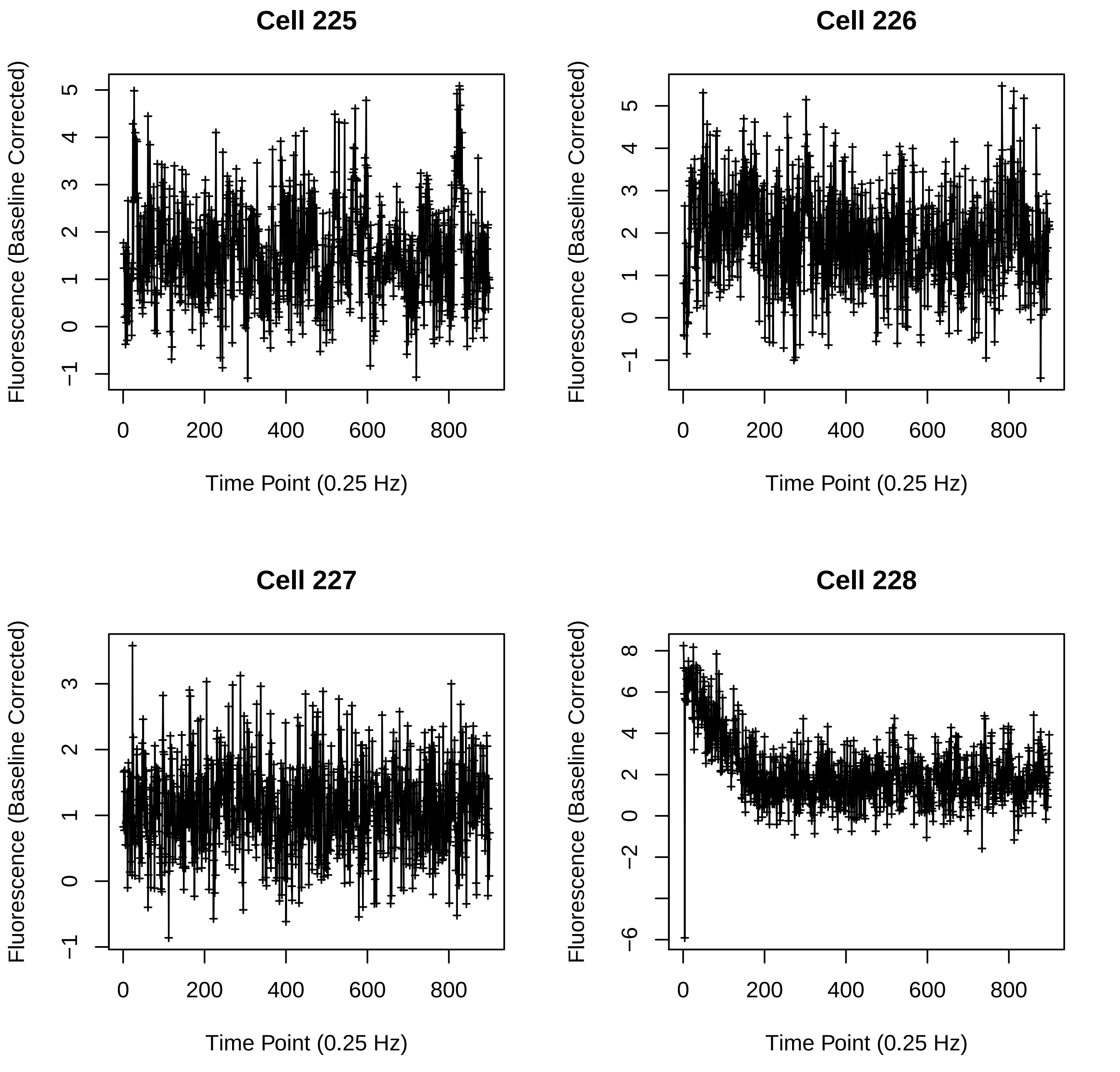
Cell 225
Cell 226
Cell 227
Cell 228
Fluorescence (Baseline Corrected)
Time Point (0.25 Hz)
0
200
400
600
800
−1
0
1
2
3
4
5
8
6
−2
−6

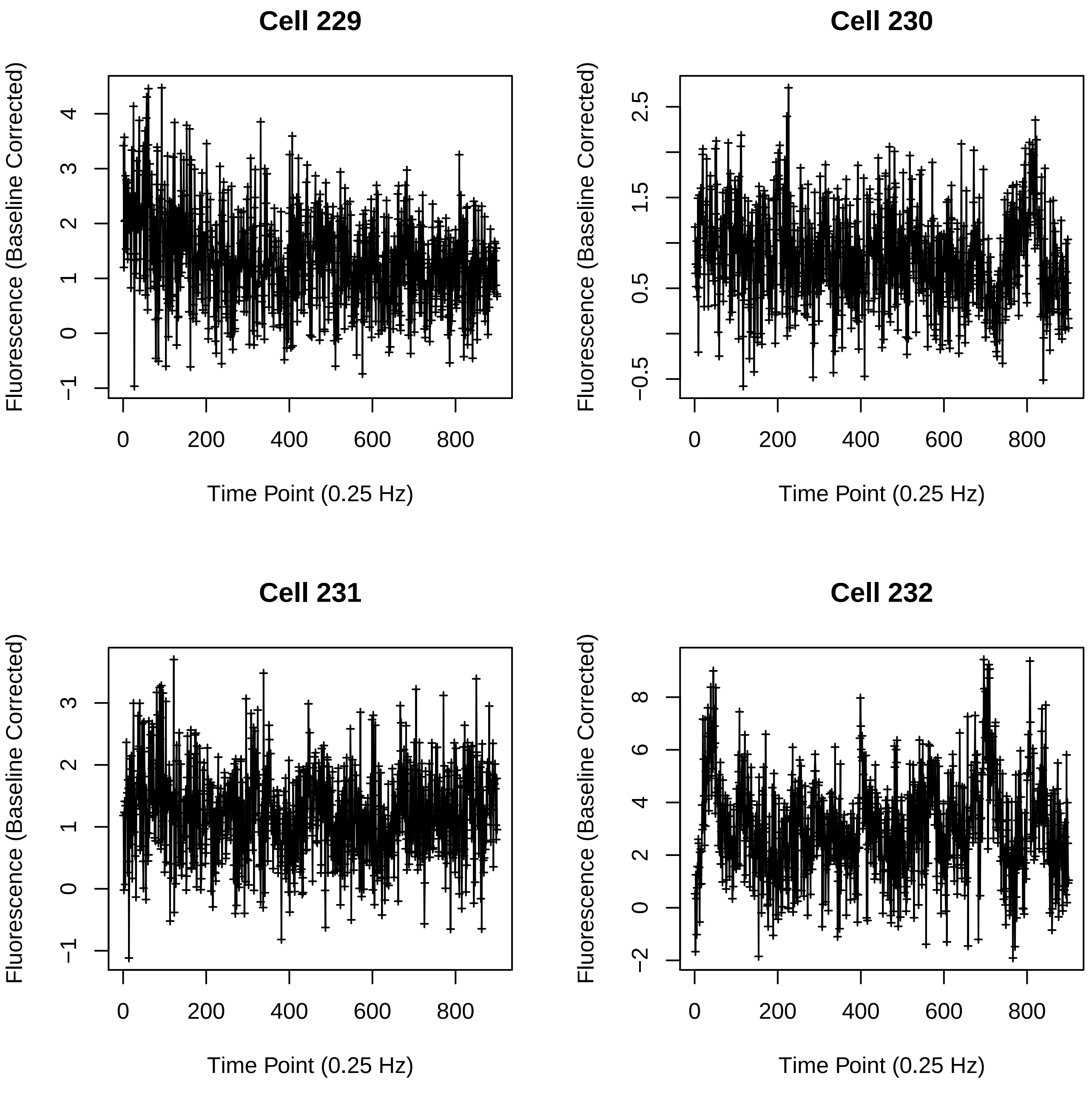
Cell 229
Cell 230
Cell 231
Cell 232
Fluorescence (Baseline Corrected)
Time Point (0.25 Hz)
0
200
400
600
800
−1
1
2
3
4
−0.5
0.5
1.5
2.5
−2
6
8

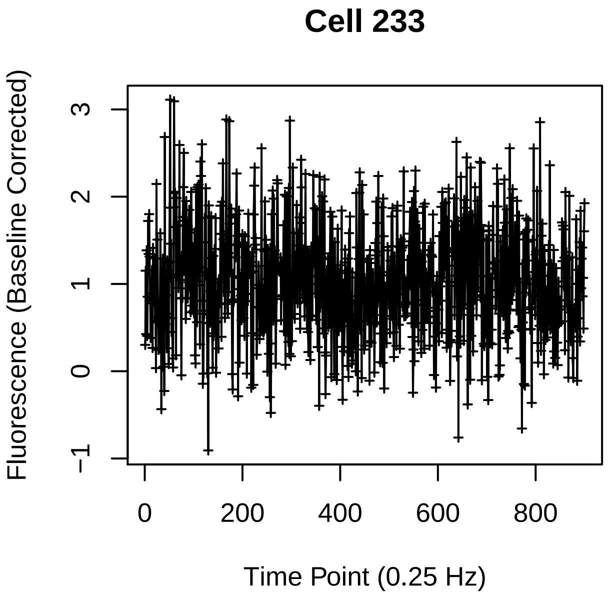
Cell 233
Fluorescence (Baseline Corrected)
Time Point (0.25 Hz)
−1 0 1 2 3
0 200 400 600 800

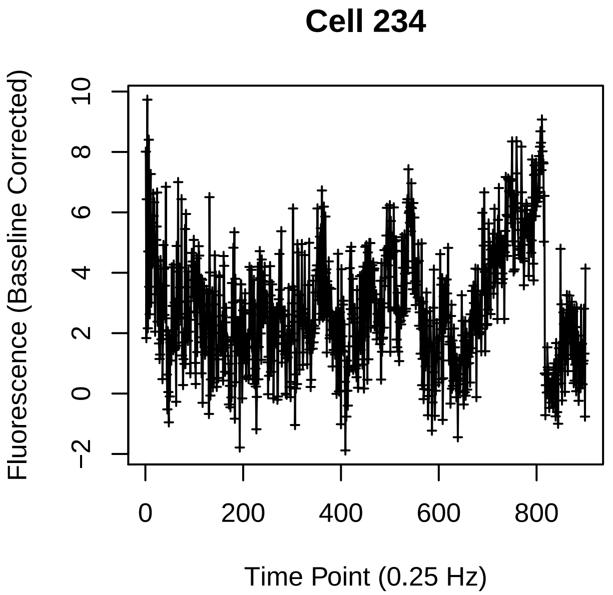
Cell 234
Fluorescence (Baseline Corrected)
Time Point (0.25 Hz)
−2 0 2 4 6 8 10
0 200 400 600 800

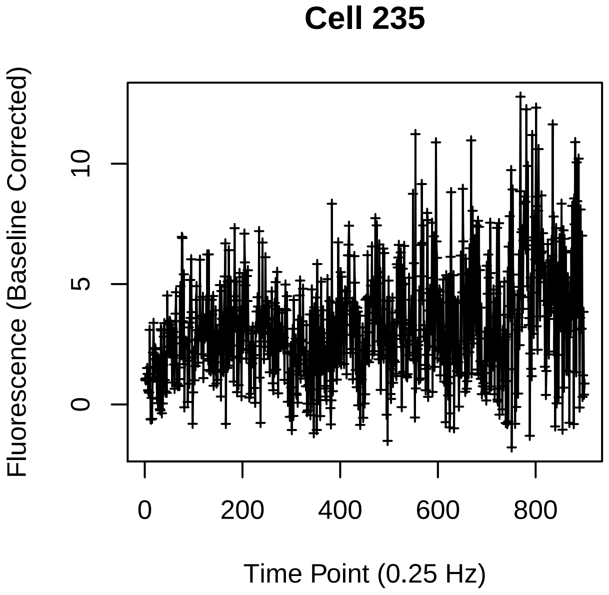
Cell 235
Fluorescence (Baseline Corrected)
Time Point (0.25 Hz)
0 5 10
0 200 400 600 800

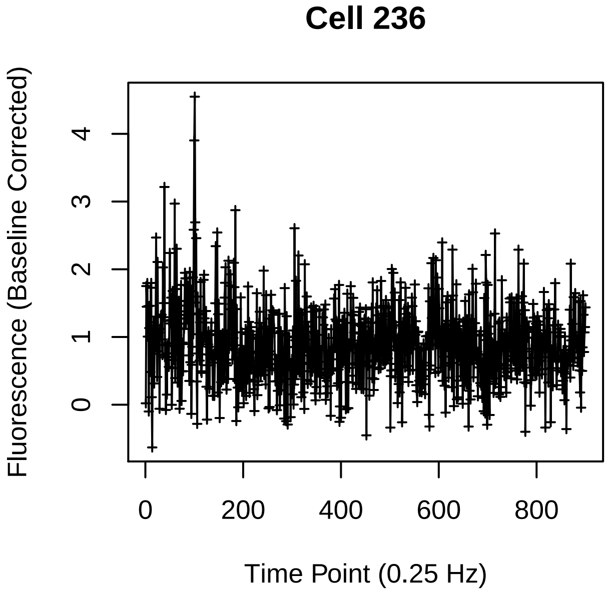
Cell 236
Fluorescence (Baseline Corrected)
Time Point (0.25 Hz)
0 1 2 3 4
0 200 400 600 800

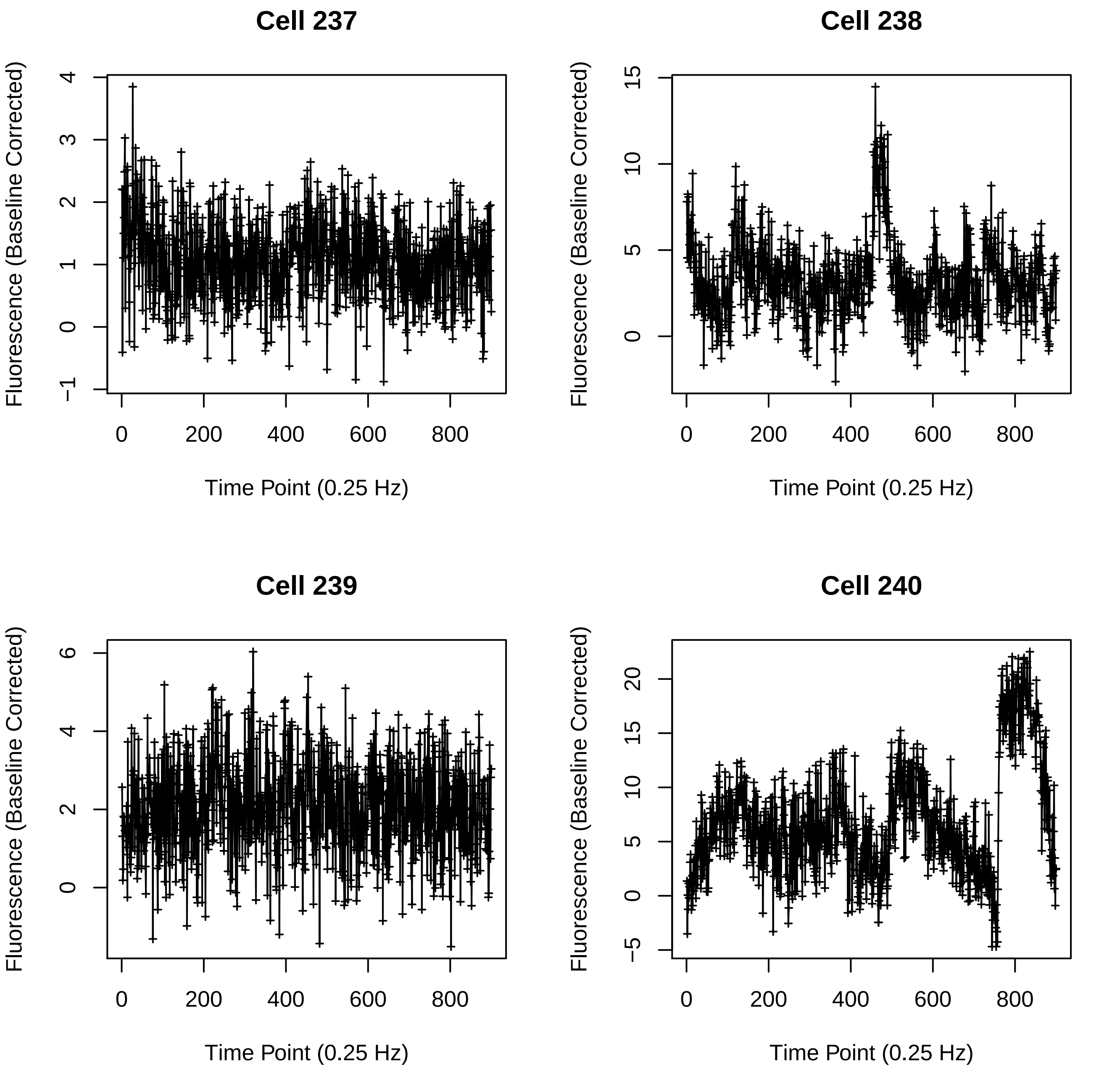

Cell 237
Cell 238
Cell 239
Cell 240
Fluorescence (Baseline Corrected)
Time Point (0.25 Hz)

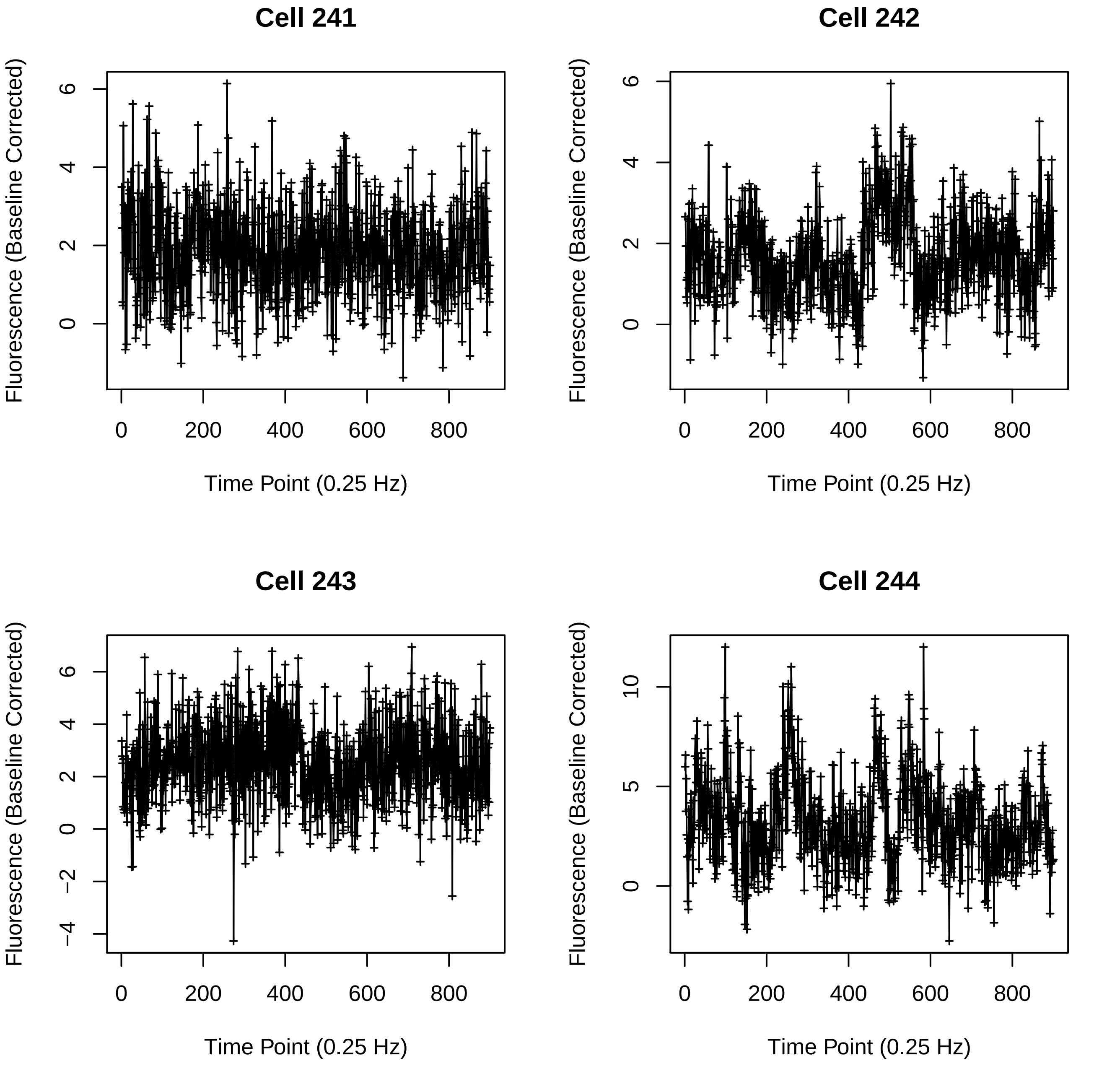
Cell 241
Cell 242
Cell 243
Cell 244
Fluorescence (Baseline Corrected)
Time Point (0.25 Hz)
0
200
400
600
800
−4
−2
0
2
4
6
5
10

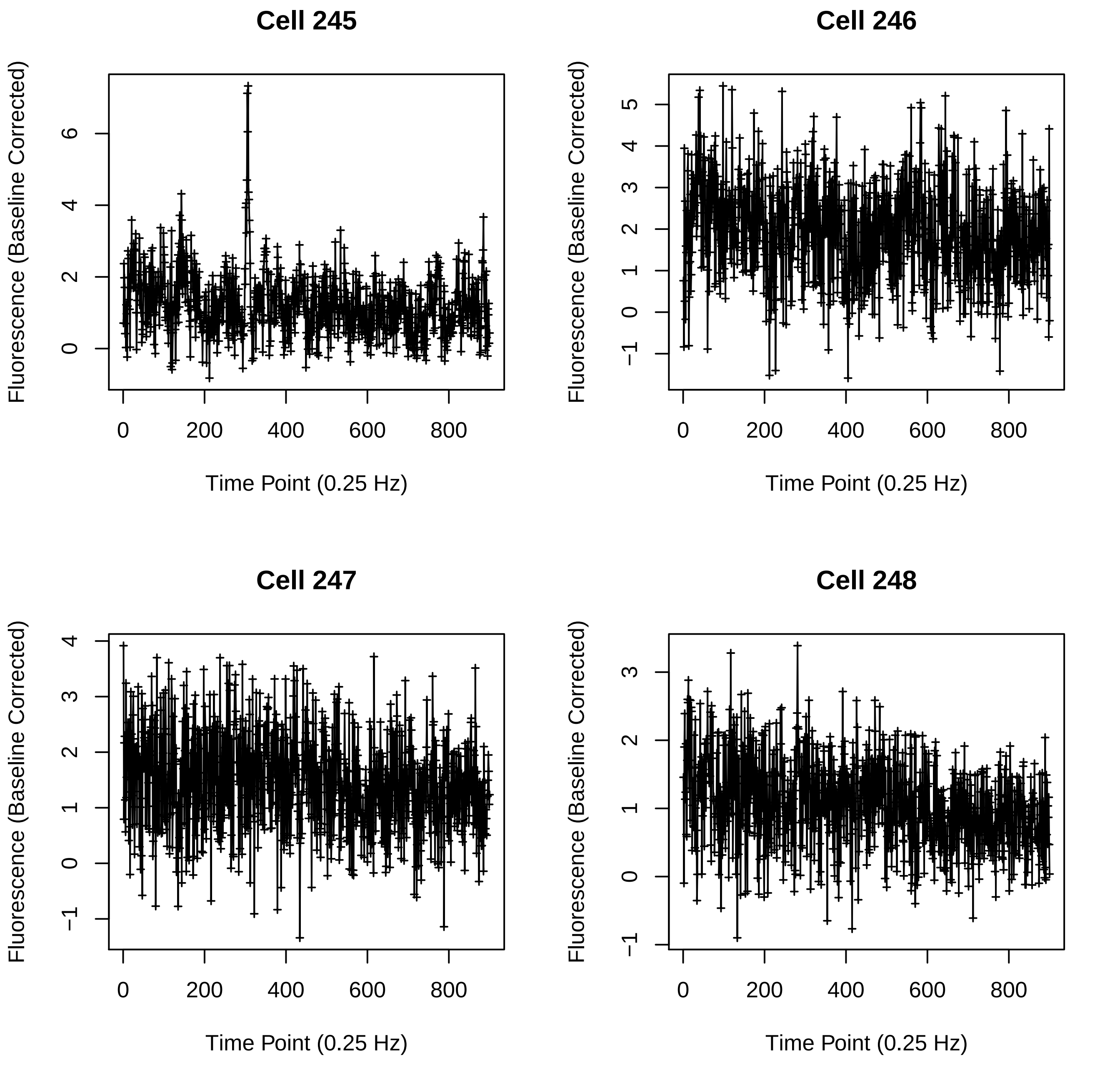

Cell 245
Cell 246
Cell 247
Cell 248
Fluorescence (Baseline Corrected)
Time Point (0.25 Hz)

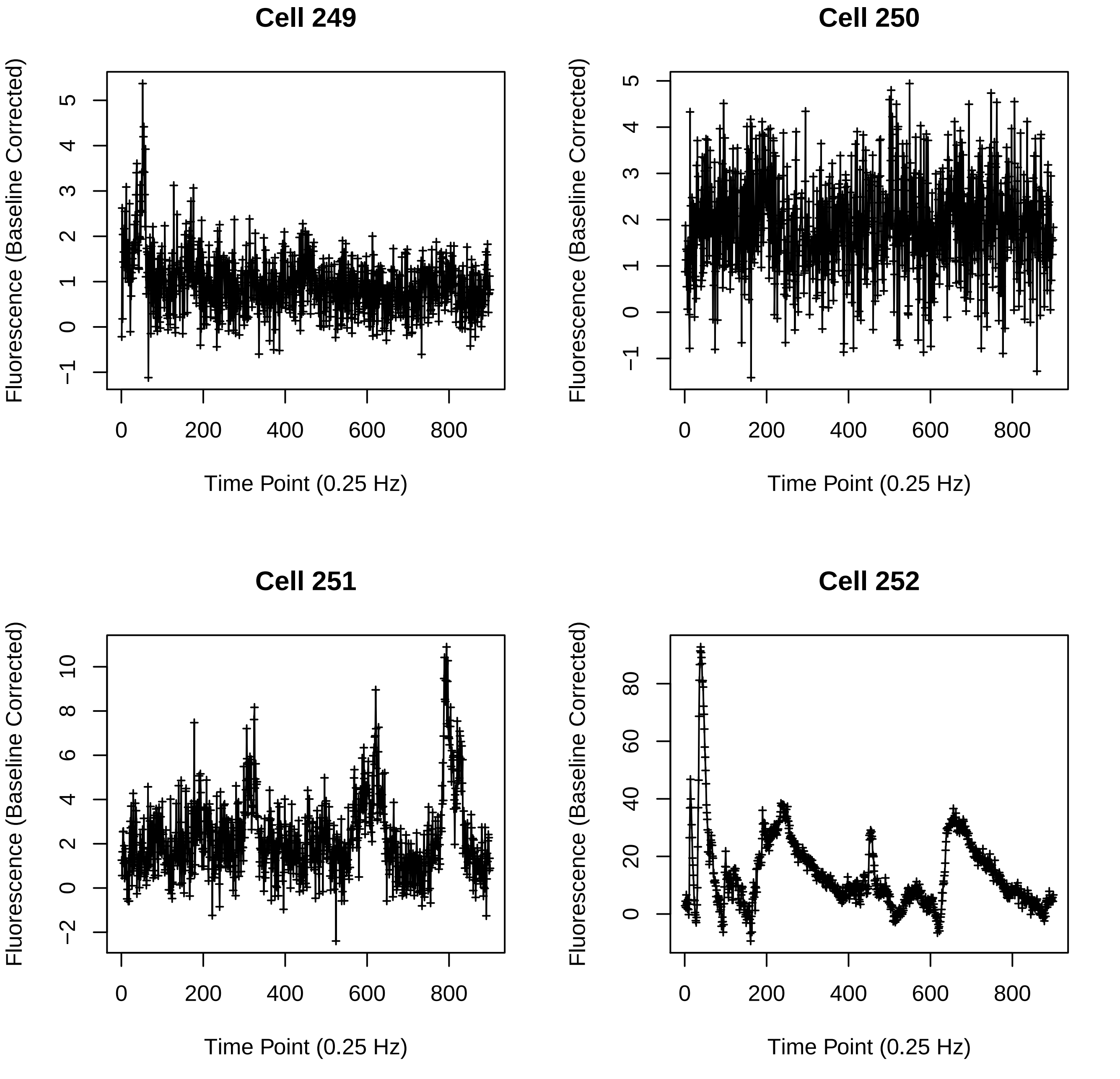
Cell 249
Fluorescence (Baseline Corrected)
−1 0 1 2 3 4 5
0 200 400 600 800
Time Point (0.25 Hz)
Cell 250
Fluorescence (Baseline Corrected)
−1 0 1 2 3 4 5
0 200 400 600 800
Time Point (0.25 Hz)
Cell 251
Fluorescence (Baseline Corrected)
−2 0 2 4 6 8 10
0 200 400 600 800
Time Point (0.25 Hz)
Cell 252
Fluorescence (Baseline Corrected)
0 20 40 60 80
0 200 400 600 800
Time Point (0.25 Hz)

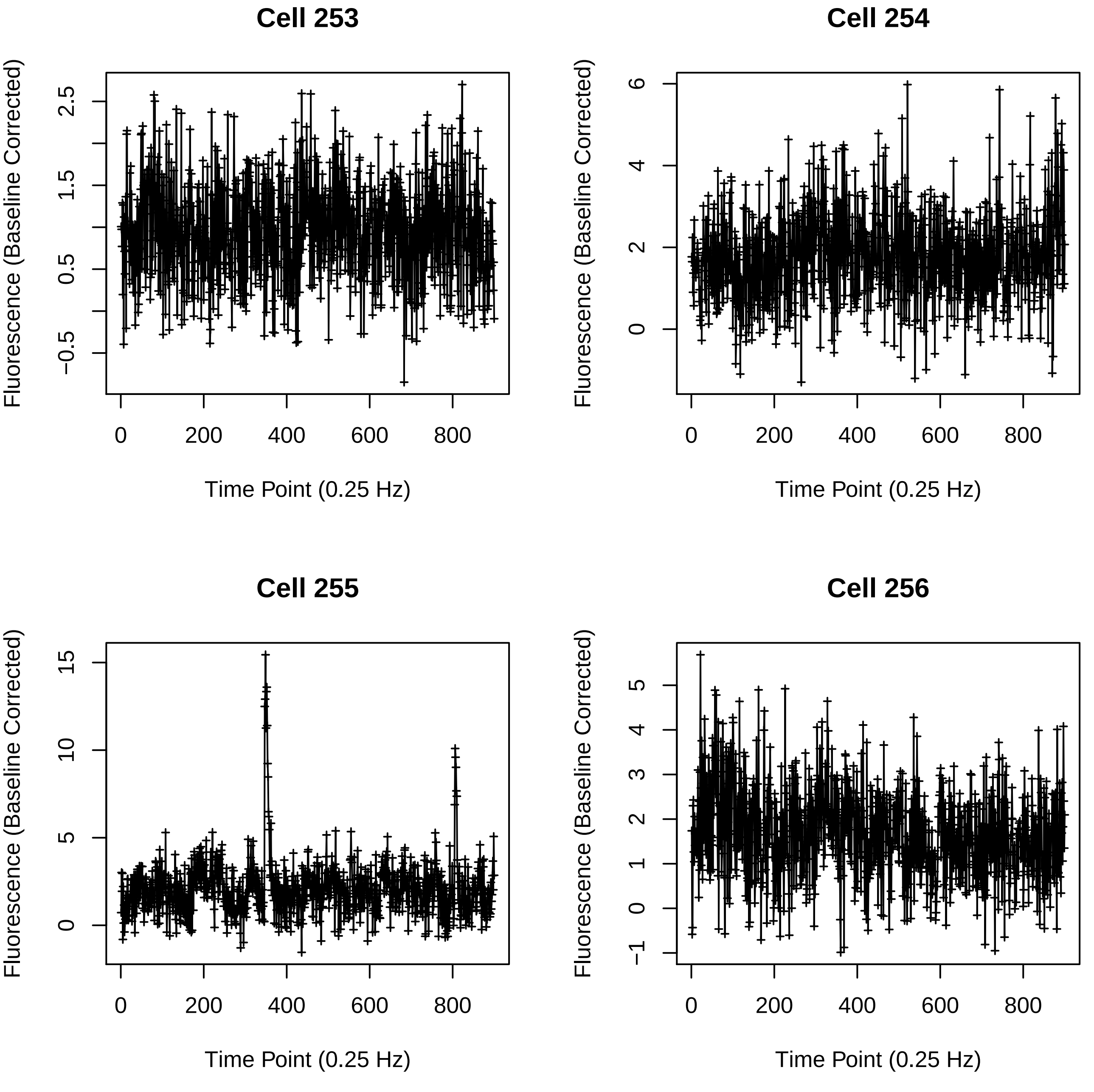
Cell 253
Fluorescence (Baseline Corrected)
−0.5
0.5
1.5
2.5
0
200
400
600
800
Time Point (0.25 Hz)
Cell 254
Fluorescence (Baseline Corrected)
0
2
4
6
0
200
400
600
800
Time Point (0.25 Hz)
Cell 255
Fluorescence (Baseline Corrected)
0
5
10
15
0
200
400
600
800
Time Point (0.25 Hz)
Cell 256
Fluorescence (Baseline Corrected)
−1
0
1
2
3
4
5
0
200
400
600
800
Time Point (0.25 Hz)

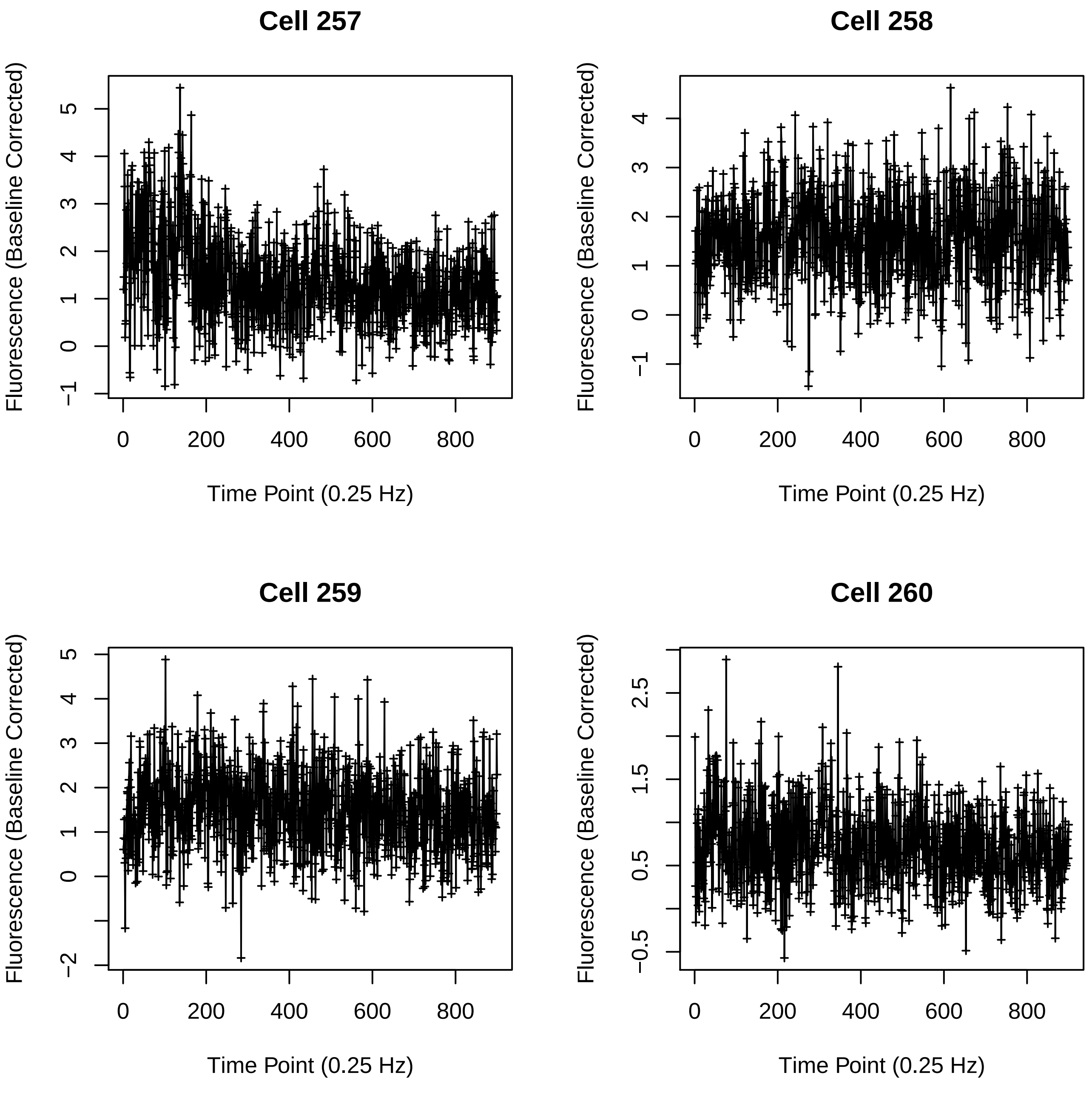
Cell 257
Cell 258
Cell 259
Cell 260
Fluorescence (Baseline Corrected)
Time Point (0.25 Hz)
0
200
400
600
800
−1
0
1
2
3
4
5
−2
−0.5
0.5
1.5
2.5

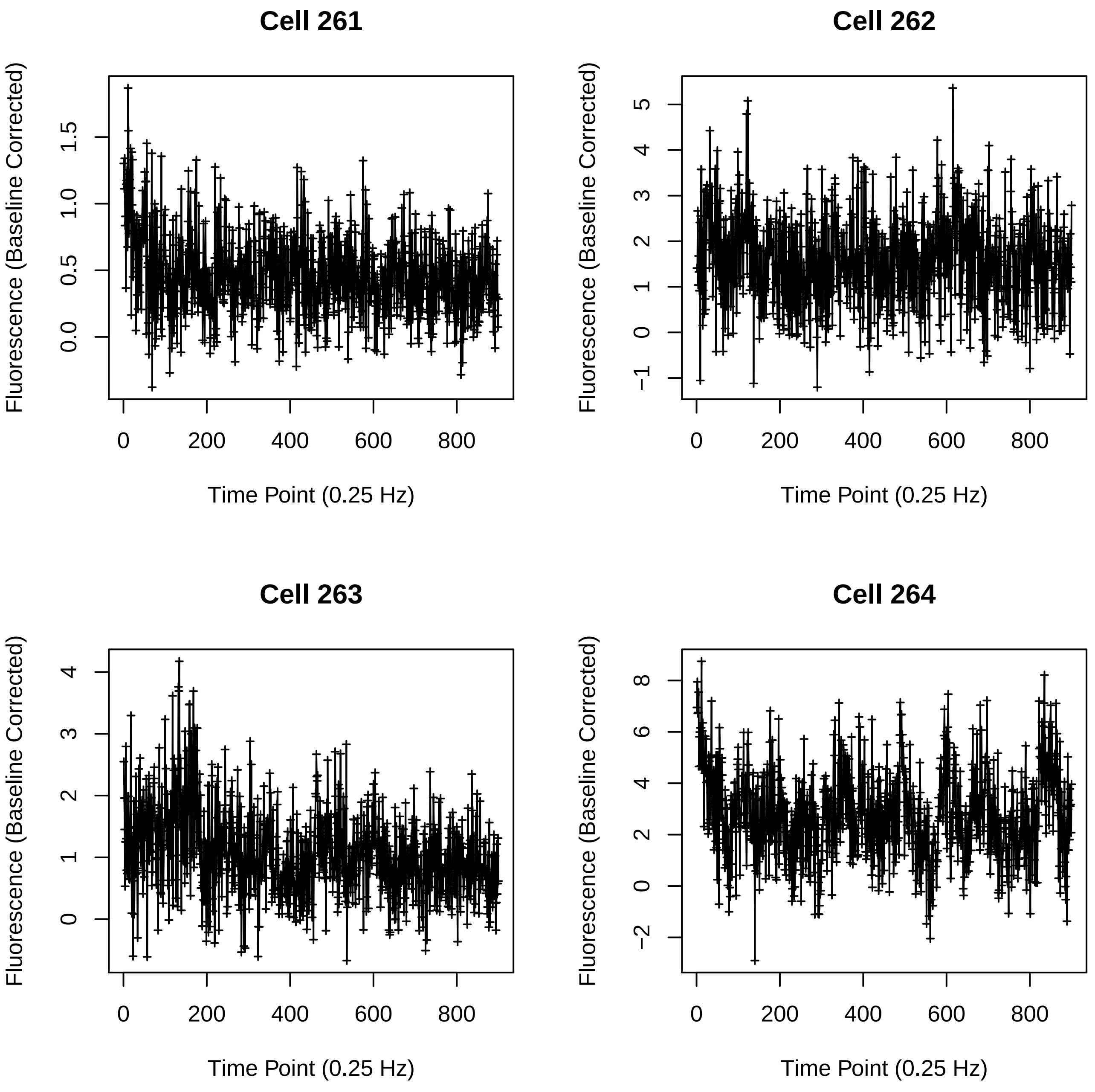
Cell 261
Cell 262
Cell 263
Cell 264
Fluorescence (Baseline Corrected)
Time Point (0.25 Hz)
0.0
0.5
1.0
1.5
−1
0
1
2
3
4
5
6
8
−2
0
200
400
600
800

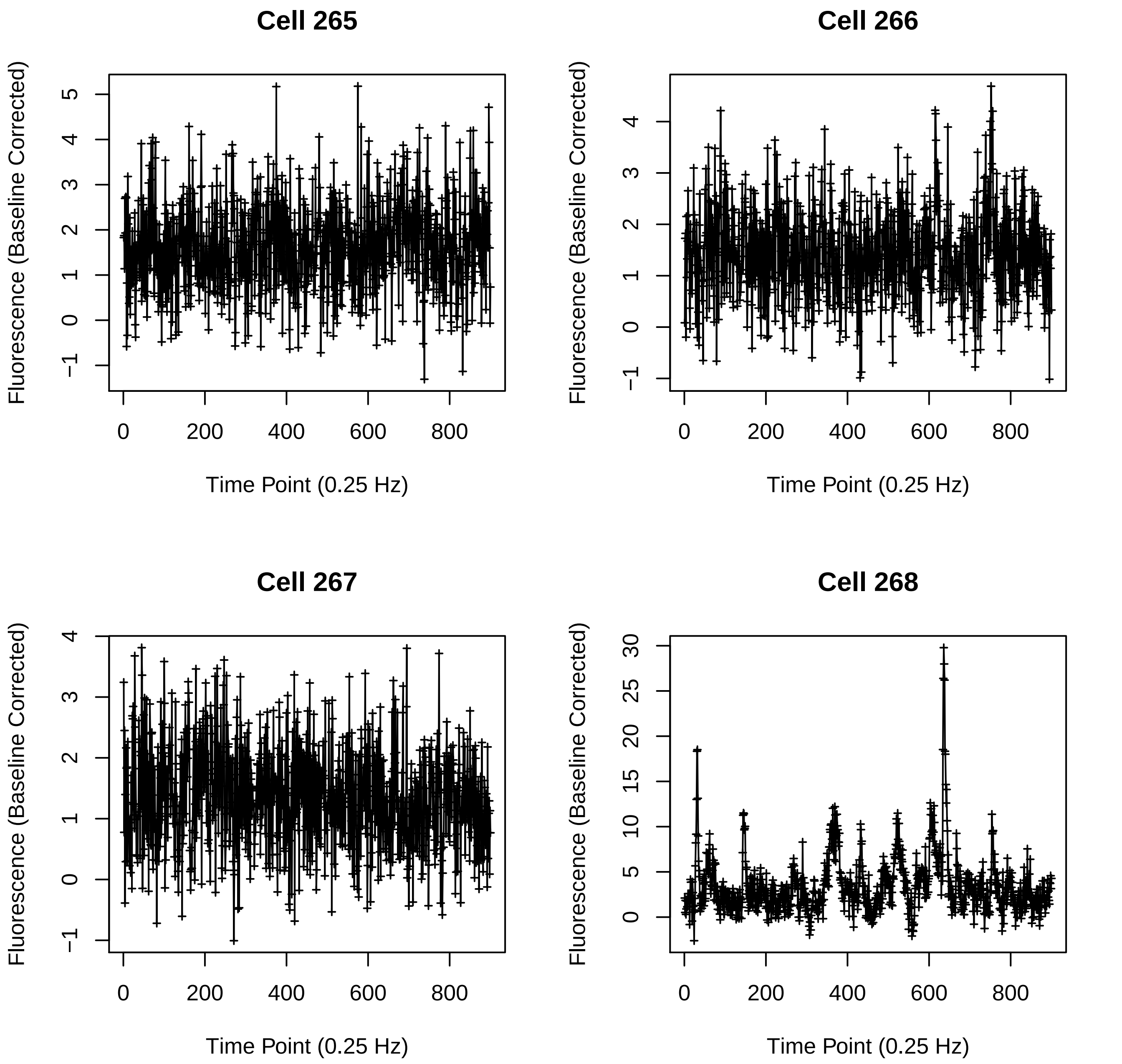

Cell 265
Cell 266
Cell 267
Cell 268
Fluorescence (Baseline Corrected)
Time Point (0.25 Hz)

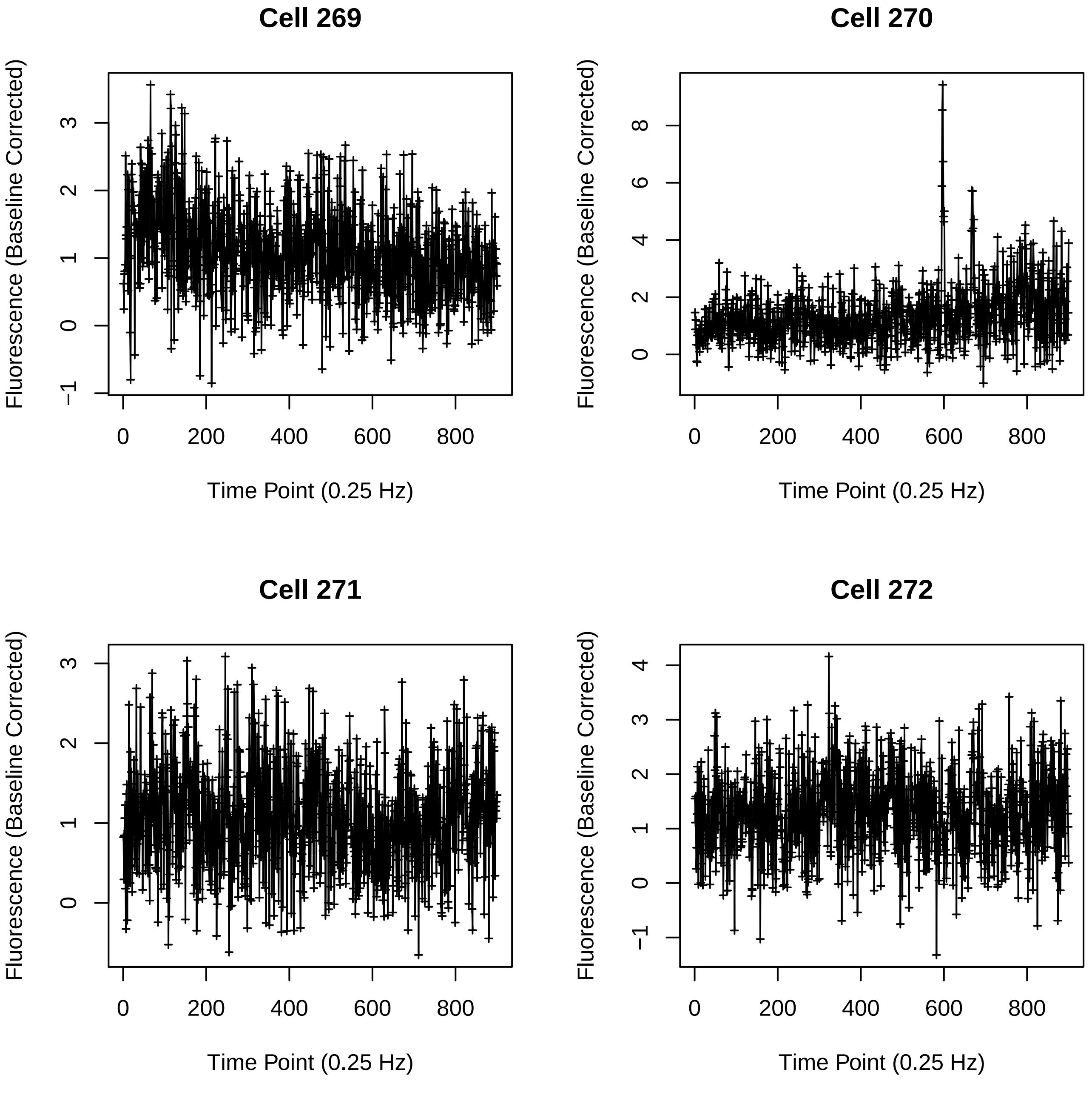

Cell 269
Cell 270
Cell 271
Cell 272
Fluorescence (Baseline Corrected)
Time Point (0.25 Hz)

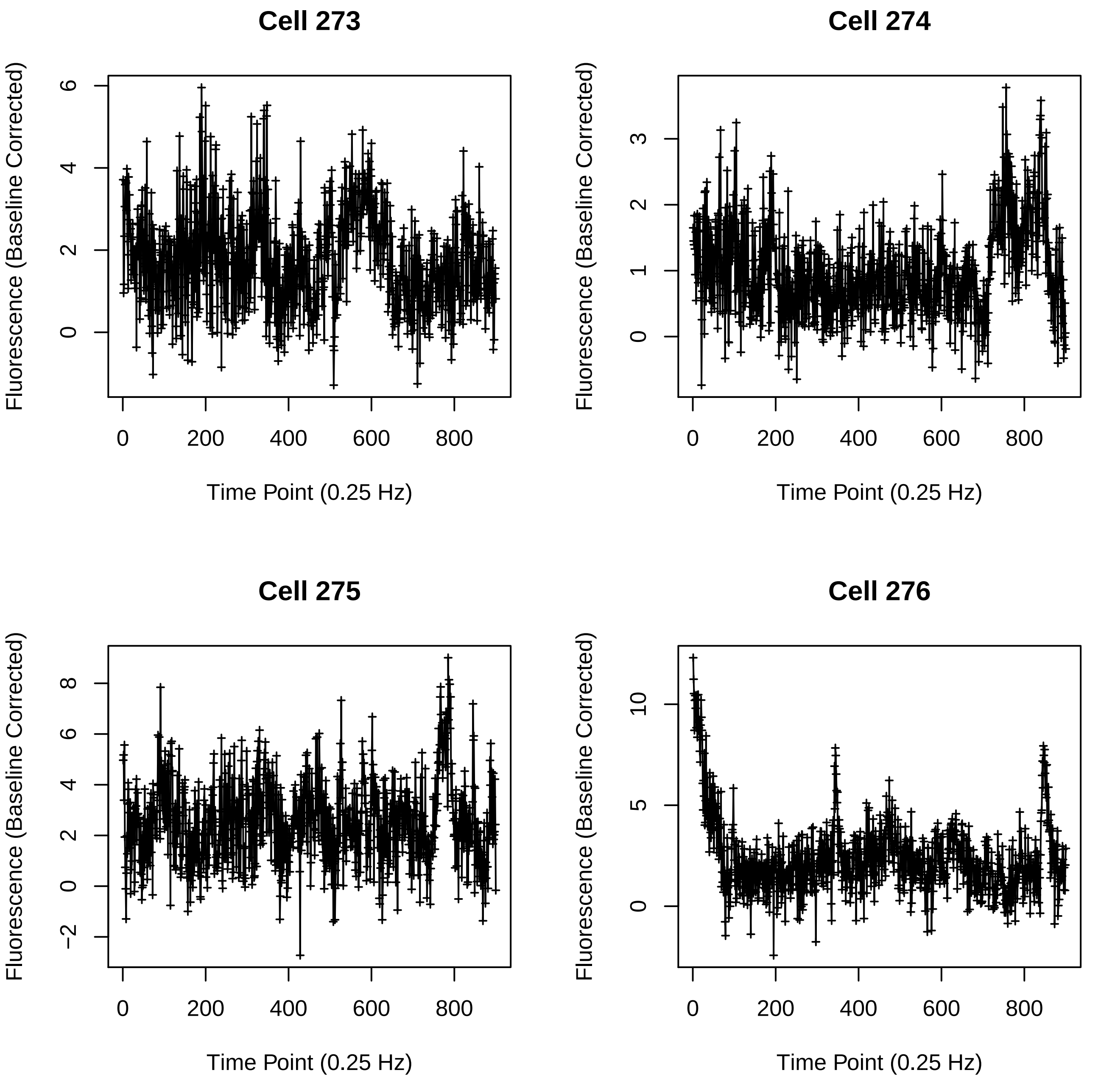
Cell 273
Cell 274
Cell 275
Cell 276
Fluorescence (Baseline Corrected)
Time Point (0.25 Hz)
0
200
400
600
800
−2
2
4
6
8
10
5
1
3

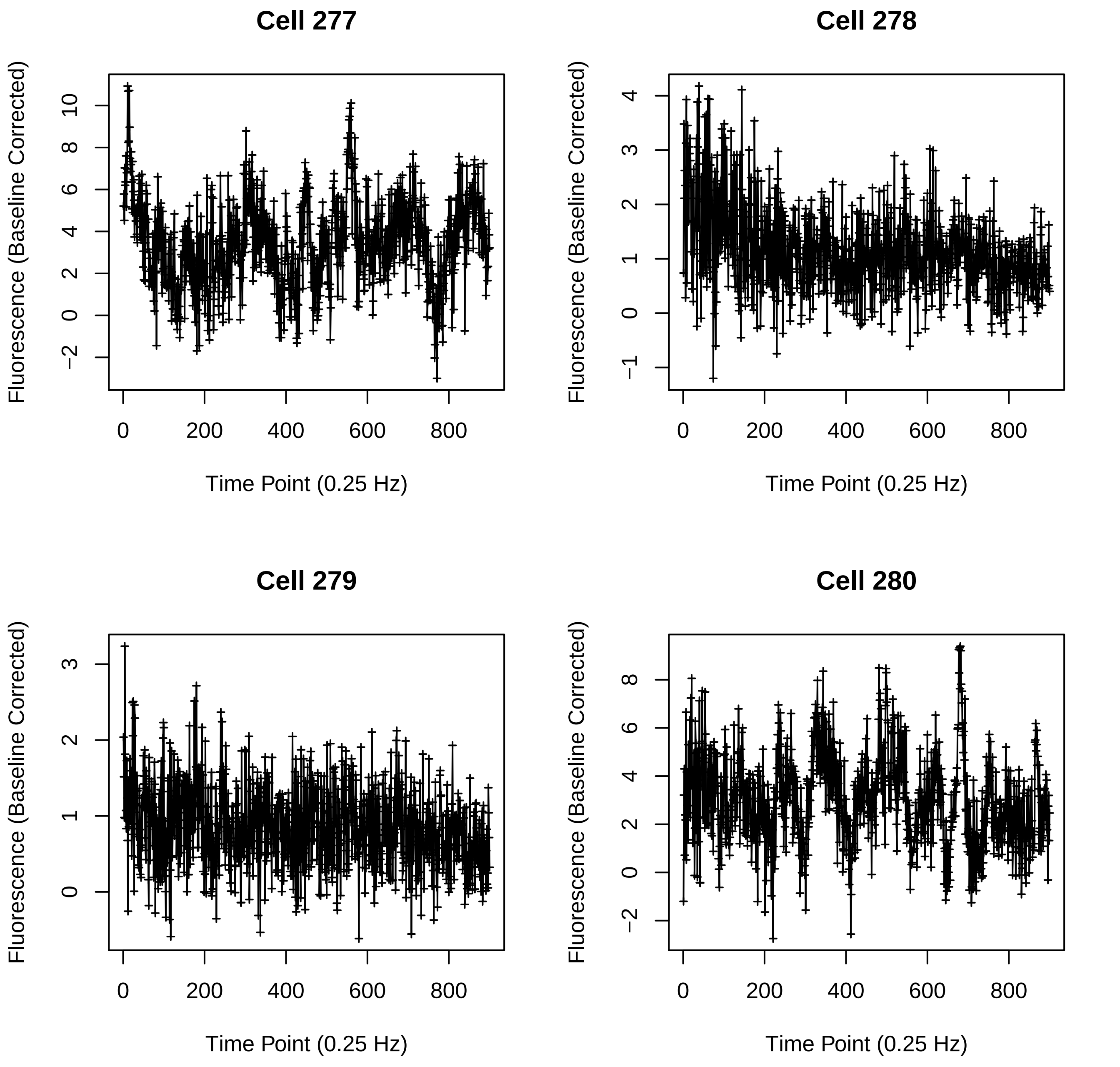
Cell 277
Cell 278
Cell 279
Cell 280
Fluorescence (Baseline Corrected)
Time Point (0.25 Hz)
0
200
400
600
800

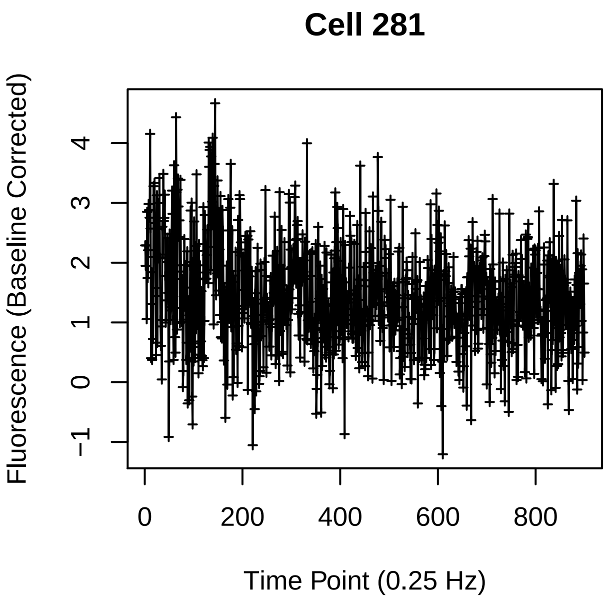
Cell 281
Fluorescence (Baseline Corrected)
−1
0
1
2
3
4
0
200
400
600
800
Time Point (0.25 Hz)

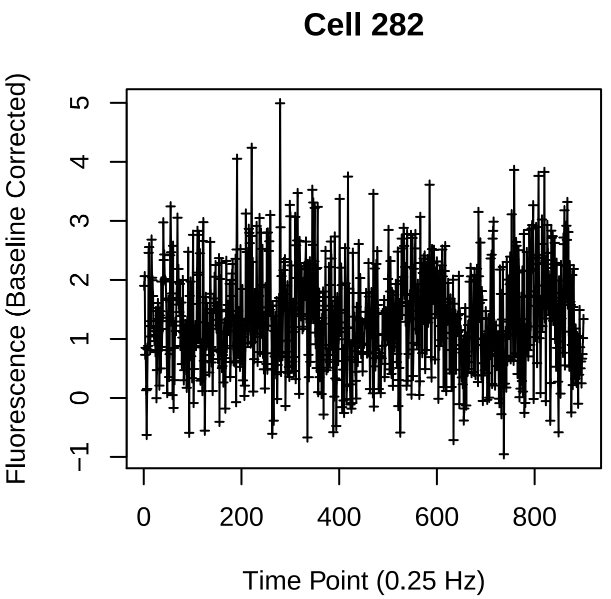
Cell 282
Fluorescence (Baseline Corrected)
−1
0
1
2
3
4
5
0
200
400
600
800
Time Point (0.25 Hz)

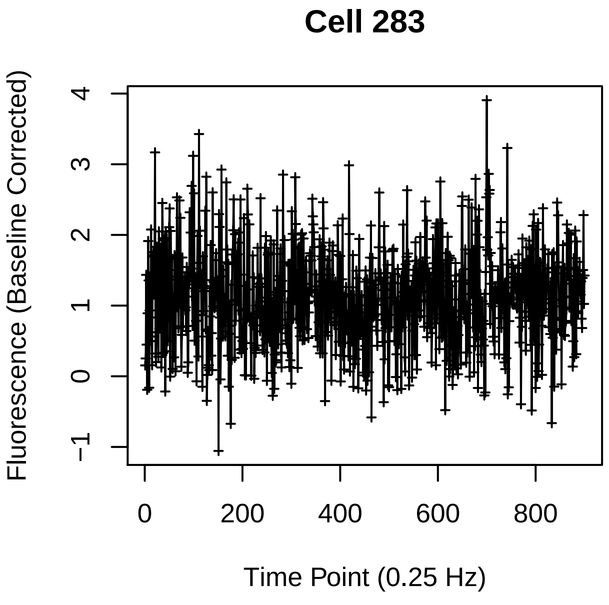
Cell 283
Fluorescence (Baseline Corrected)
−1
0
1
2
3
4
0
200
400
600
800
Time Point (0.25 Hz)

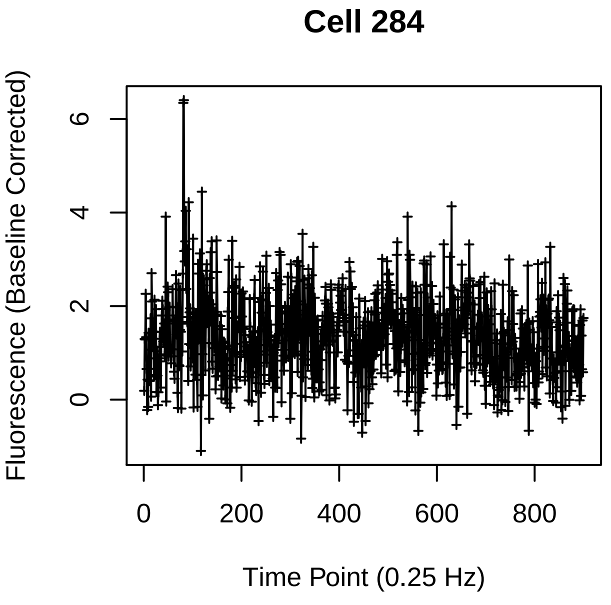
Cell 284
Fluorescence (Baseline Corrected)
0
2
4
6
0
200
400
600
800
Time Point (0.25 Hz)

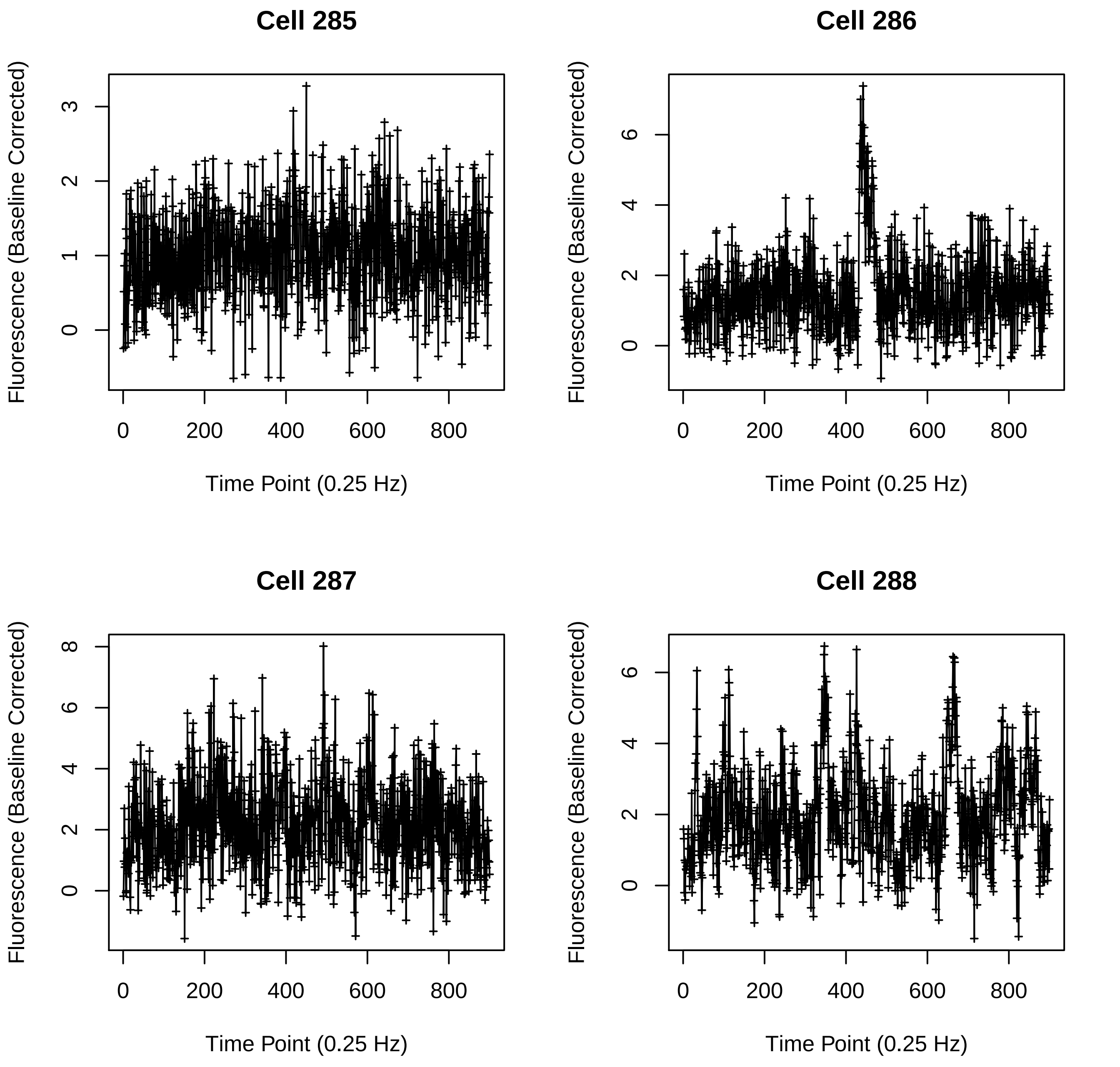
Cell 285
Fluorescence (Baseline Corrected)
Time Point (0.25 Hz)
0
1
2
3
0
200
400
600
800
Cell 286
Fluorescence (Baseline Corrected)
Time Point (0.25 Hz)
0
2
4
6
0
200
400
600
800
Cell 287
Fluorescence (Baseline Corrected)
Time Point (0.25 Hz)
0
2
4
6
8
0
200
400
600
800
Cell 288
Fluorescence (Baseline Corrected)
Time Point (0.25 Hz)
0
2
4
6
0
200
400
600
800

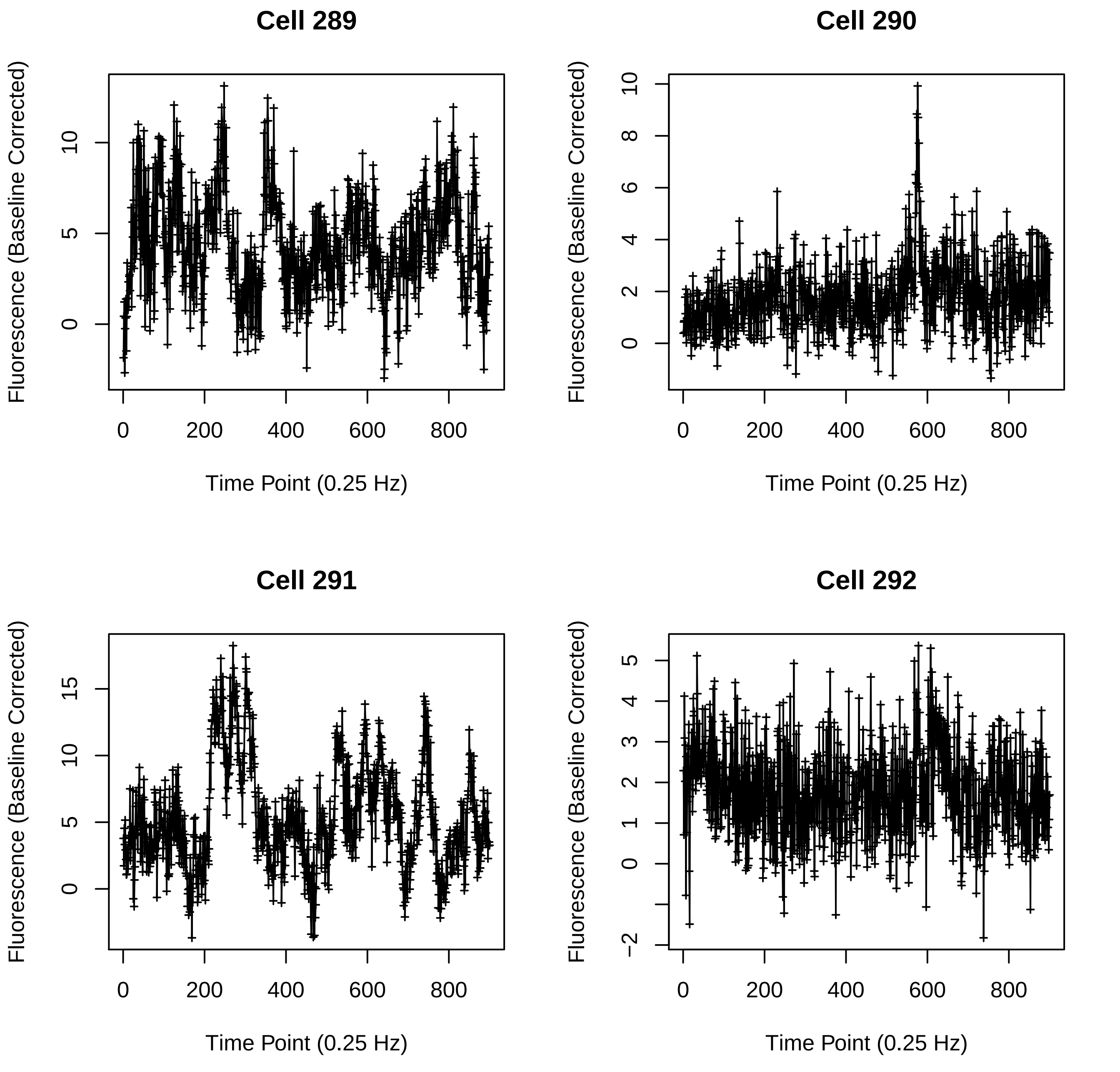
Cell 289
Cell 290
Cell 291
Cell 292
Fluorescence (Baseline Corrected)
Time Point (0.25 Hz)
0
200
400
600
800
-2
0
1
2
3
4
5
6
8
10
15

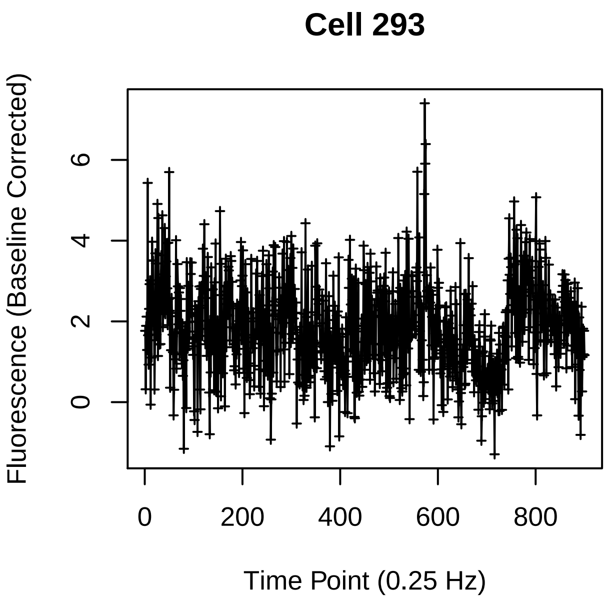

Cell 293
Fluorescence (Baseline Corrected)
0
2
4
6
0
200
400
600
800
Time Point (0.25 Hz)

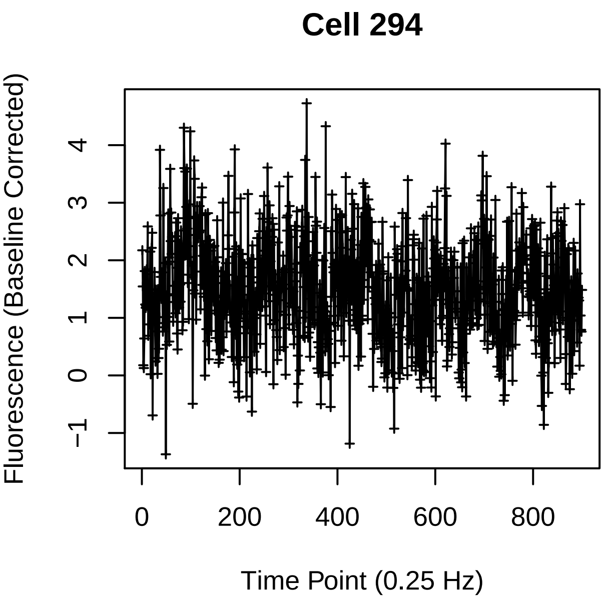

Cell 294
Fluorescence (Baseline Corrected)
−1
0
1
2
3
4
0
200
400
600
800
Time Point (0.25 Hz)

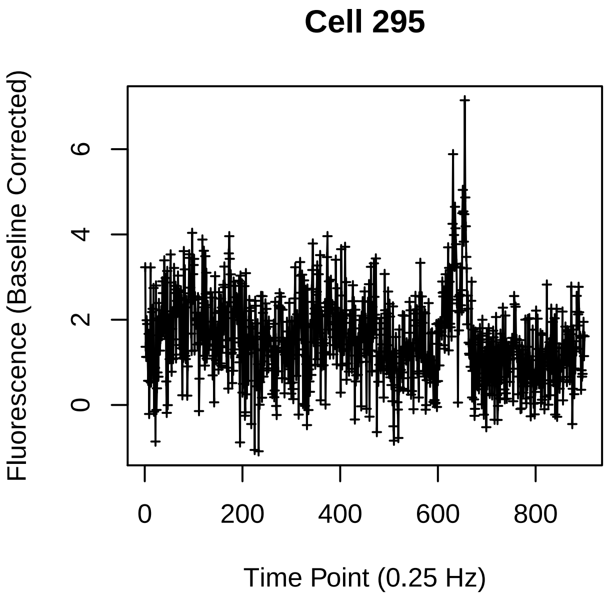

Cell 295
Fluorescence (Baseline Corrected)
0
2
4
6
0
200
400
600
800
Time Point (0.25 Hz)

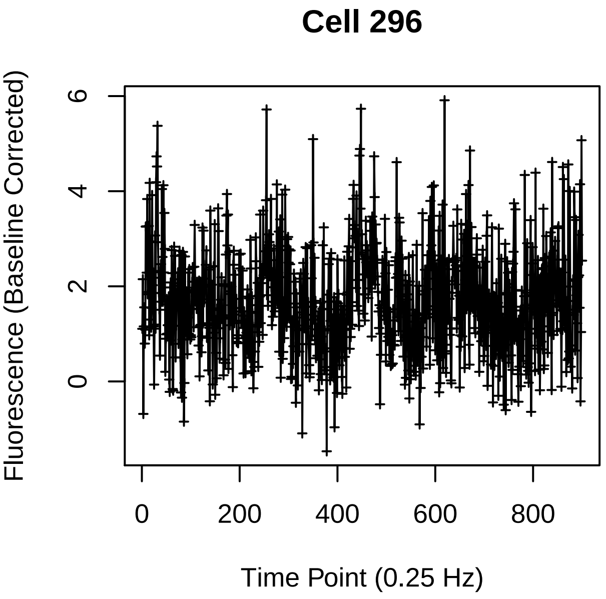

Cell 296
Fluorescence (Baseline Corrected)
0
2
4
6
0
200
400
600
800
Time Point (0.25 Hz)

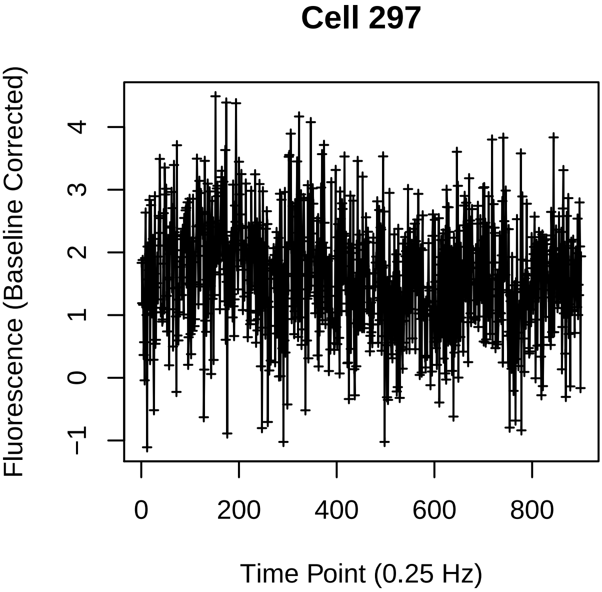
Cell 297
Fluorescence (Baseline Corrected)
−1
0
1
2
3
4
0
200
400
600
800
Time Point (0.25 Hz)

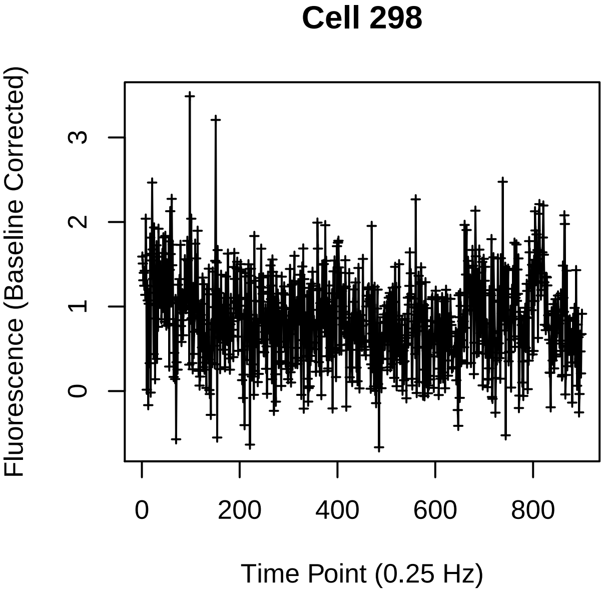
Cell 298
Fluorescence (Baseline Corrected)
0
1
2
3
0
200
400
600
800
Time Point (0.25 Hz)

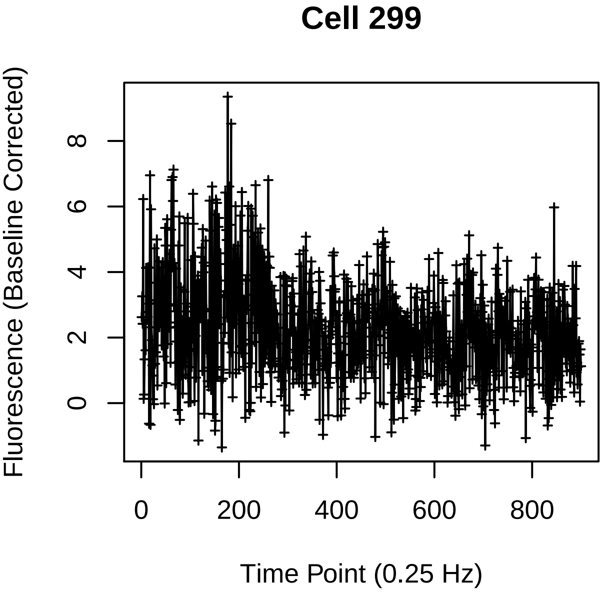
Cell 299
Fluorescence (Baseline Corrected)
0
2
4
6
8
0
200
400
600
800
Time Point (0.25 Hz)

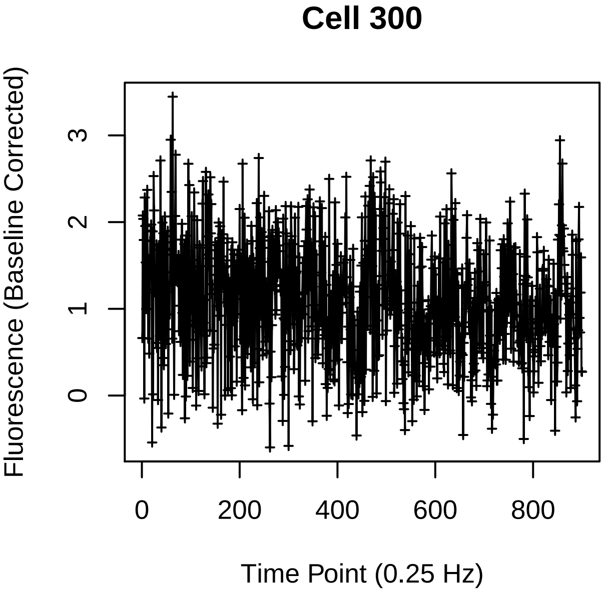
Cell 300
Fluorescence (Baseline Corrected)
0
1
2
3
0
200
400
600
800
Time Point (0.25 Hz)

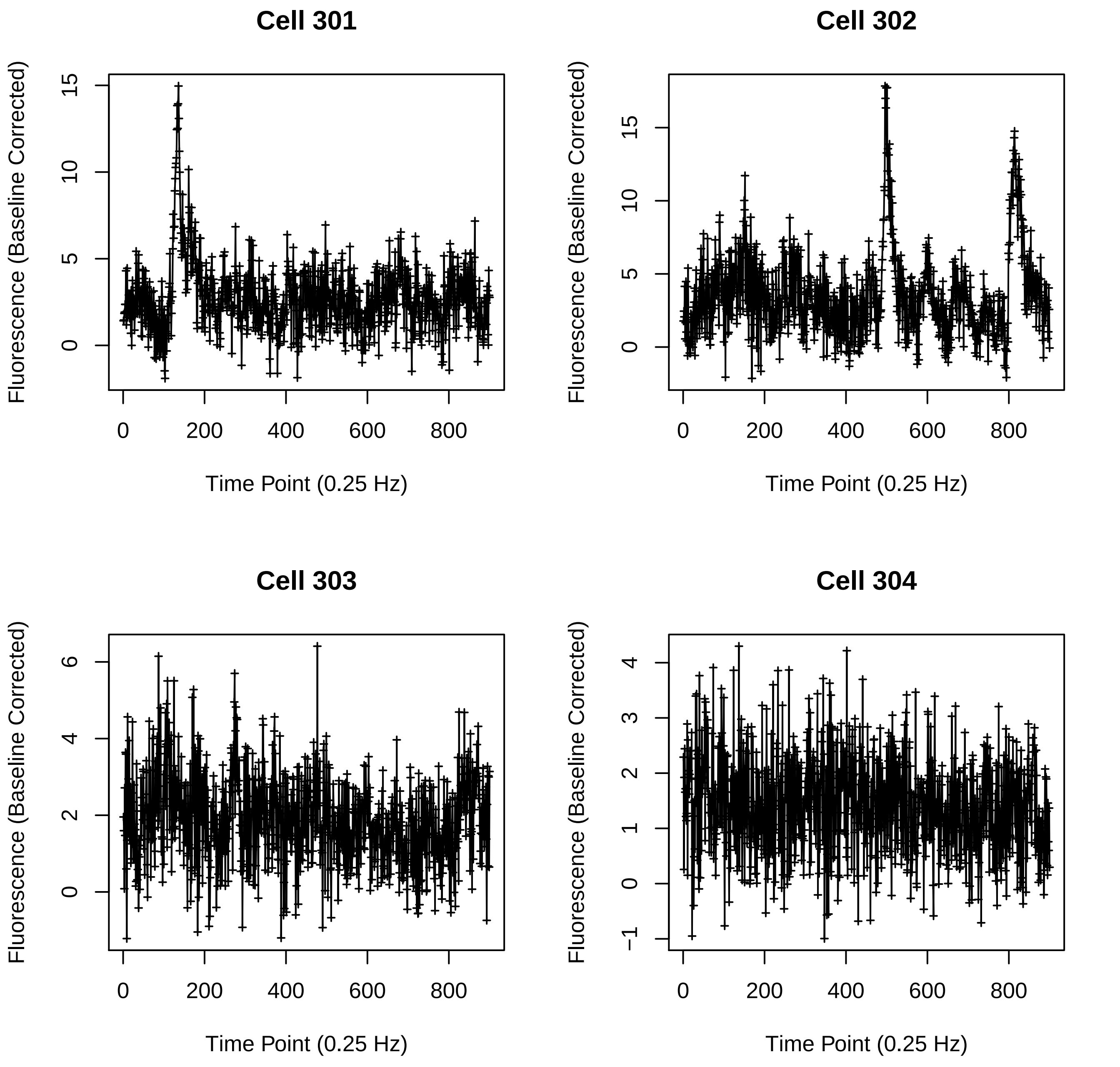
Cell 301
Cell 302
Cell 303
Cell 304
Fluorescence (Baseline Corrected)
Time Point (0.25 Hz)
0
5
10
15
200
400
600
800
2
4
6
3
−1

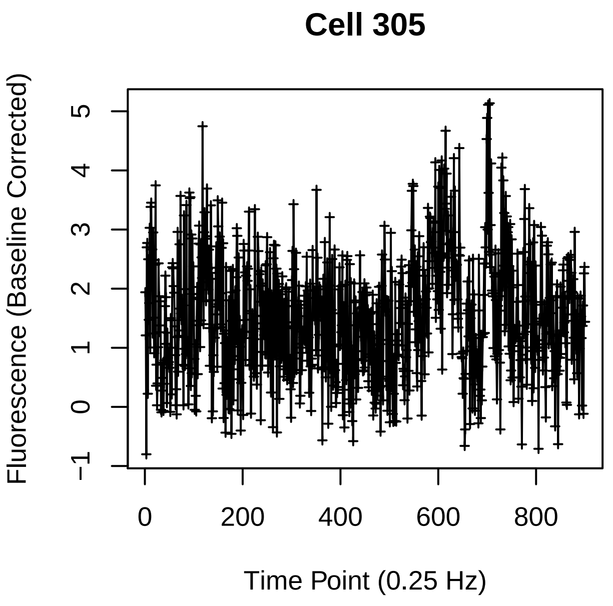
Cell 305
Fluorescence (Baseline Corrected)
−1
0
1
2
3
4
5
0
200
400
600
800
Time Point (0.25 Hz)

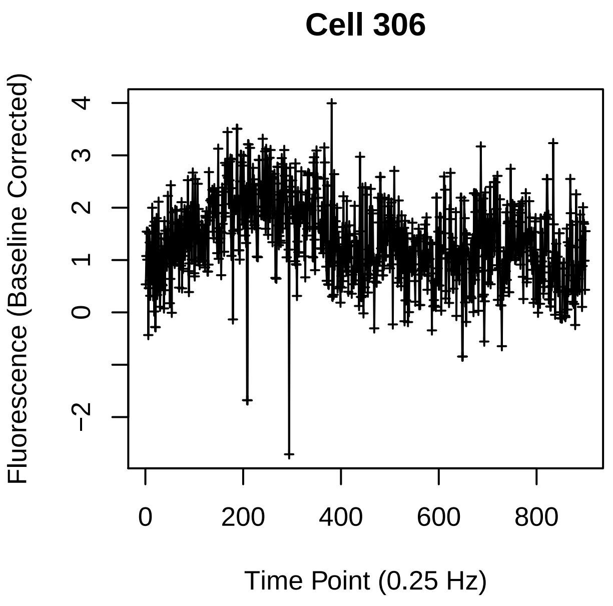
Cell 306
Fluorescence (Baseline Corrected)
−2
0
1
2
3
4
0
200
400
600
800
Time Point (0.25 Hz)

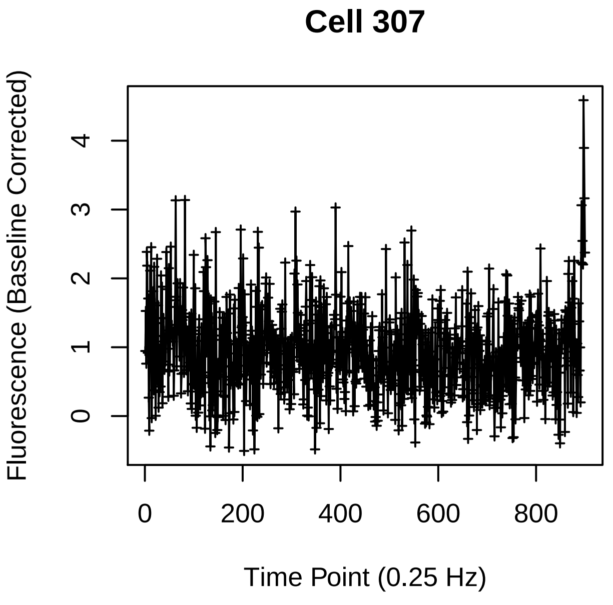
Cell 307
Fluorescence (Baseline Corrected)
0
1
2
3
4
0
200
400
600
800
Time Point (0.25 Hz)

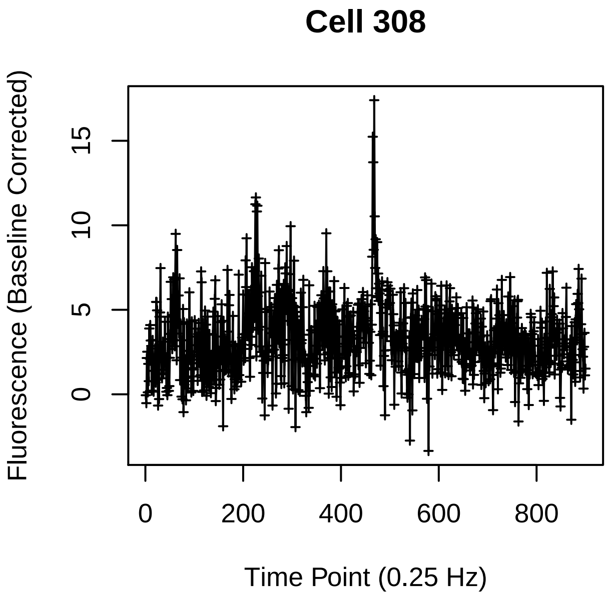
Cell 308
Fluorescence (Baseline Corrected)
0
5
10
15
0
200
400
600
800
Time Point (0.25 Hz)

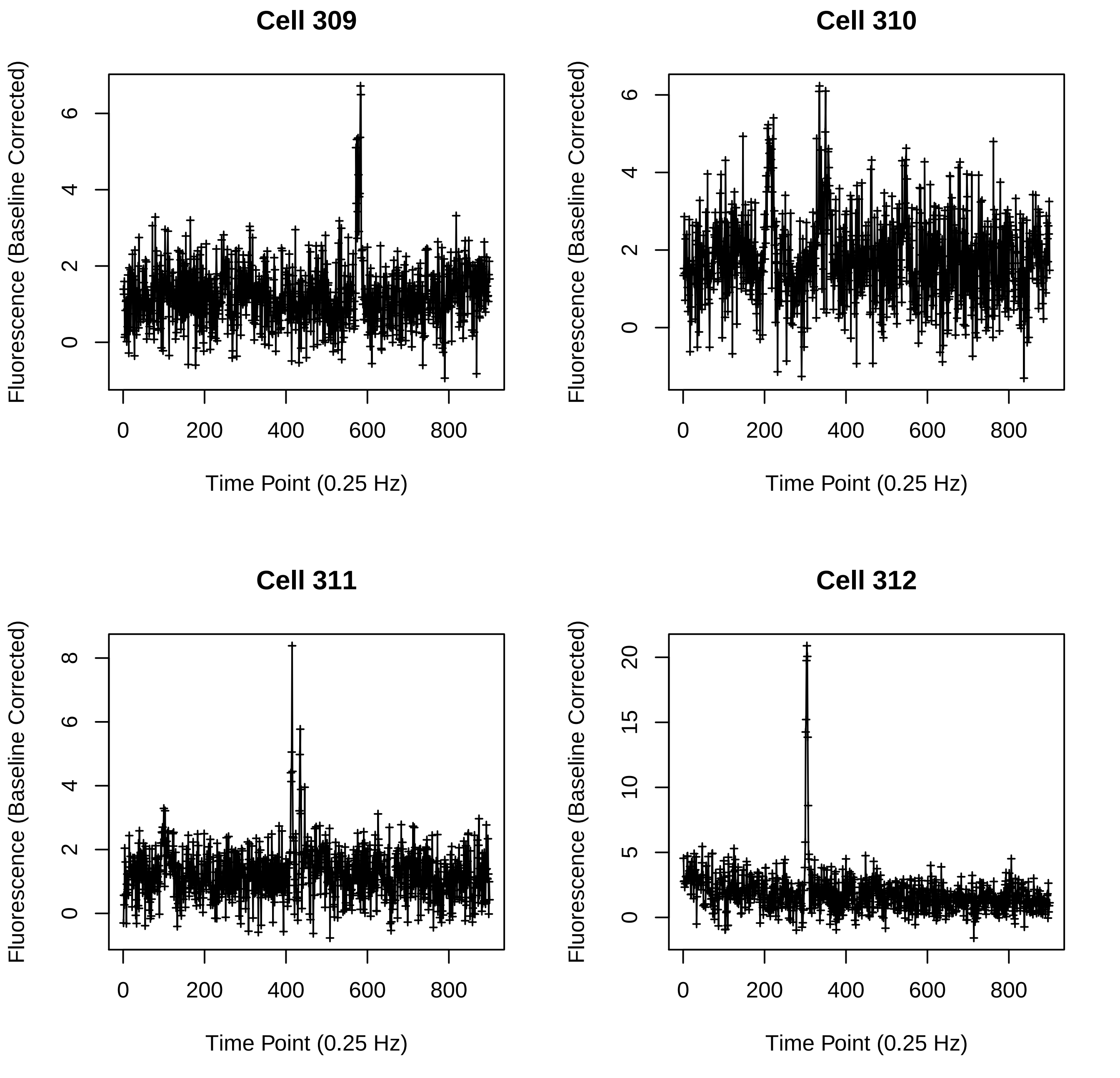
Cell 309
Cell 310
Cell 311
Cell 312
Fluorescence (Baseline Corrected)
Time Point (0.25 Hz)
0
2
4
6
8
5
10
15
20
200
400
600
800

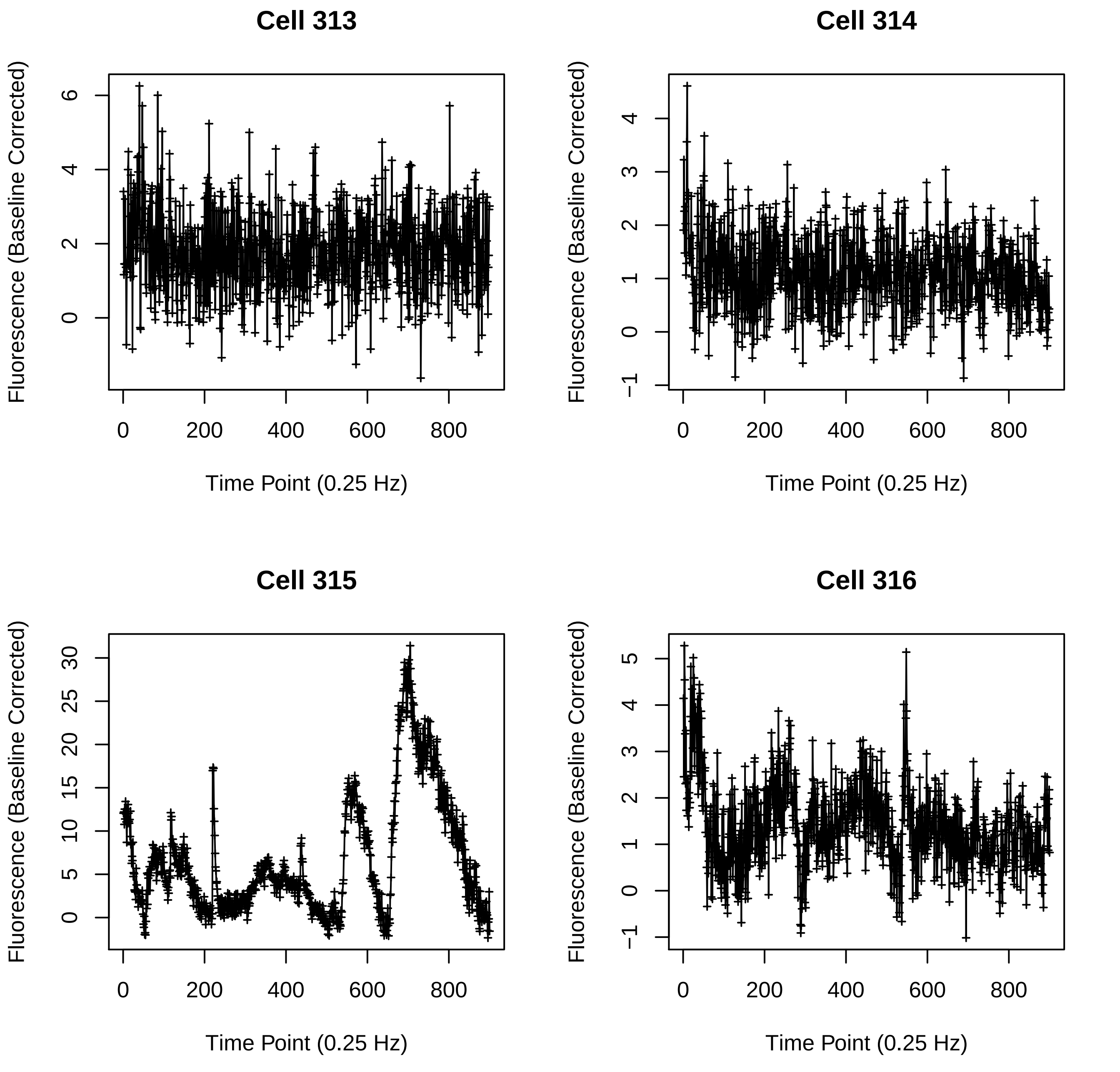

Cell 313
Fluorescence (Baseline Corrected)
Time Point (0.25 Hz)
Cell 314
Fluorescence (Baseline Corrected)
Time Point (0.25 Hz)
Cell 315
Fluorescence (Baseline Corrected)
Time Point (0.25 Hz)
Cell 316
Fluorescence (Baseline Corrected)
Time Point (0.25 Hz)

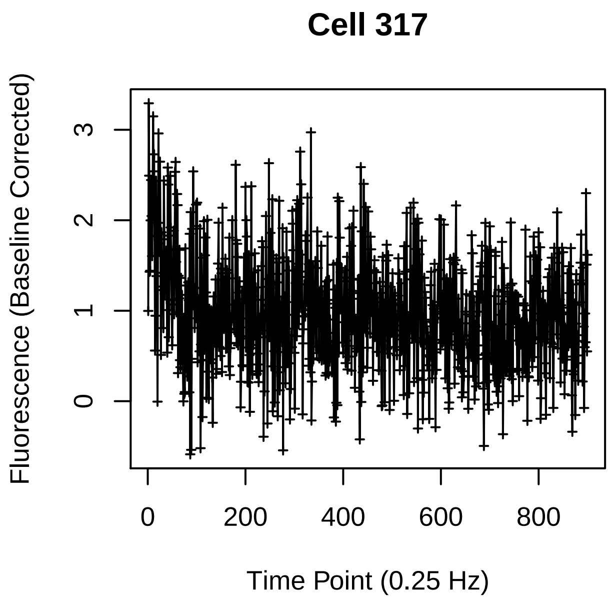
Cell 317
Fluorescence (Baseline Corrected)
0
1
2
3
0
200
400
600
800
Time Point (0.25 Hz)

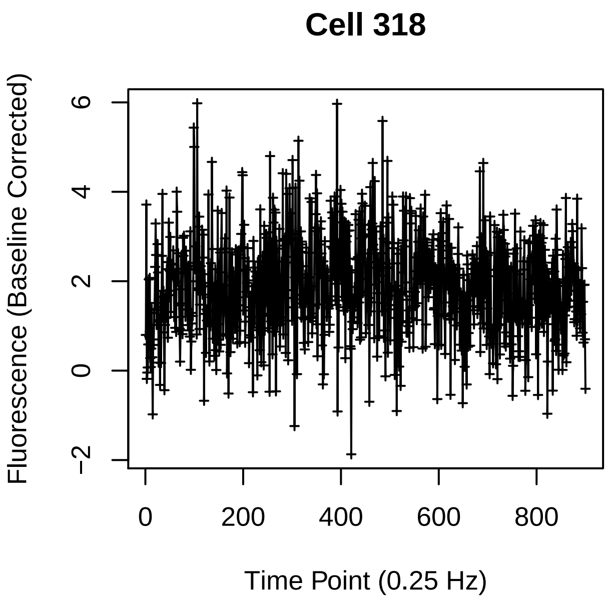
Cell 318
Fluorescence (Baseline Corrected)
−2
0
2
4
6
0
200
400
600
800
Time Point (0.25 Hz)

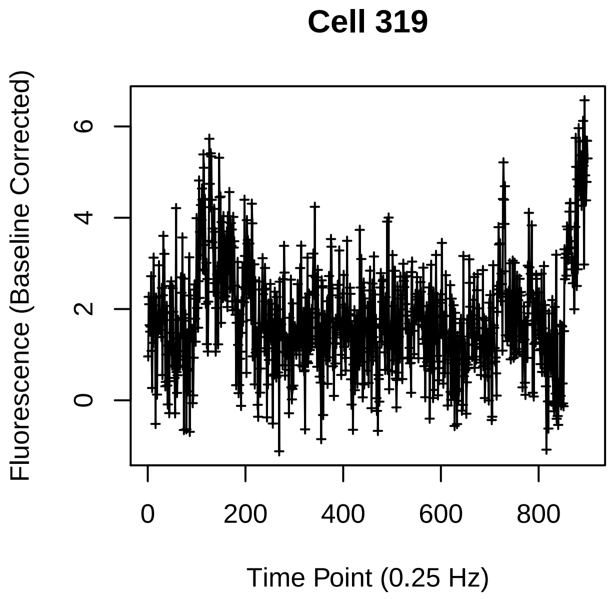
Cell 319
Fluorescence (Baseline Corrected)
0
2
4
6
0
200
400
600
800
Time Point (0.25 Hz)

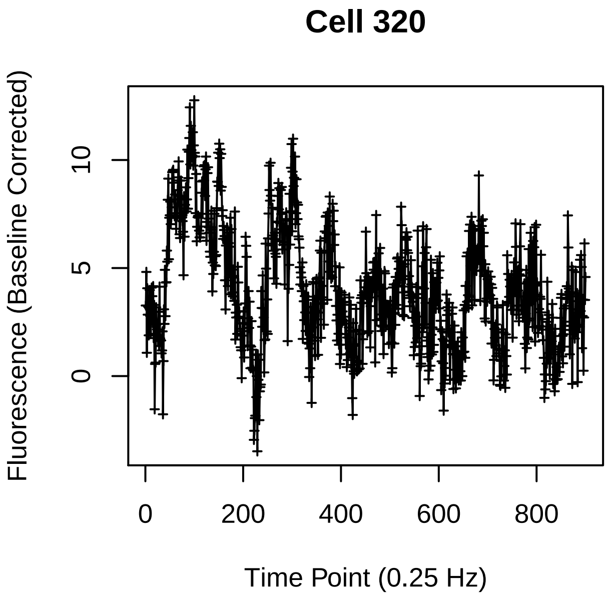
Cell 320
Fluorescence (Baseline Corrected)
0
5
10
0
200
400
600
800
Time Point (0.25 Hz)

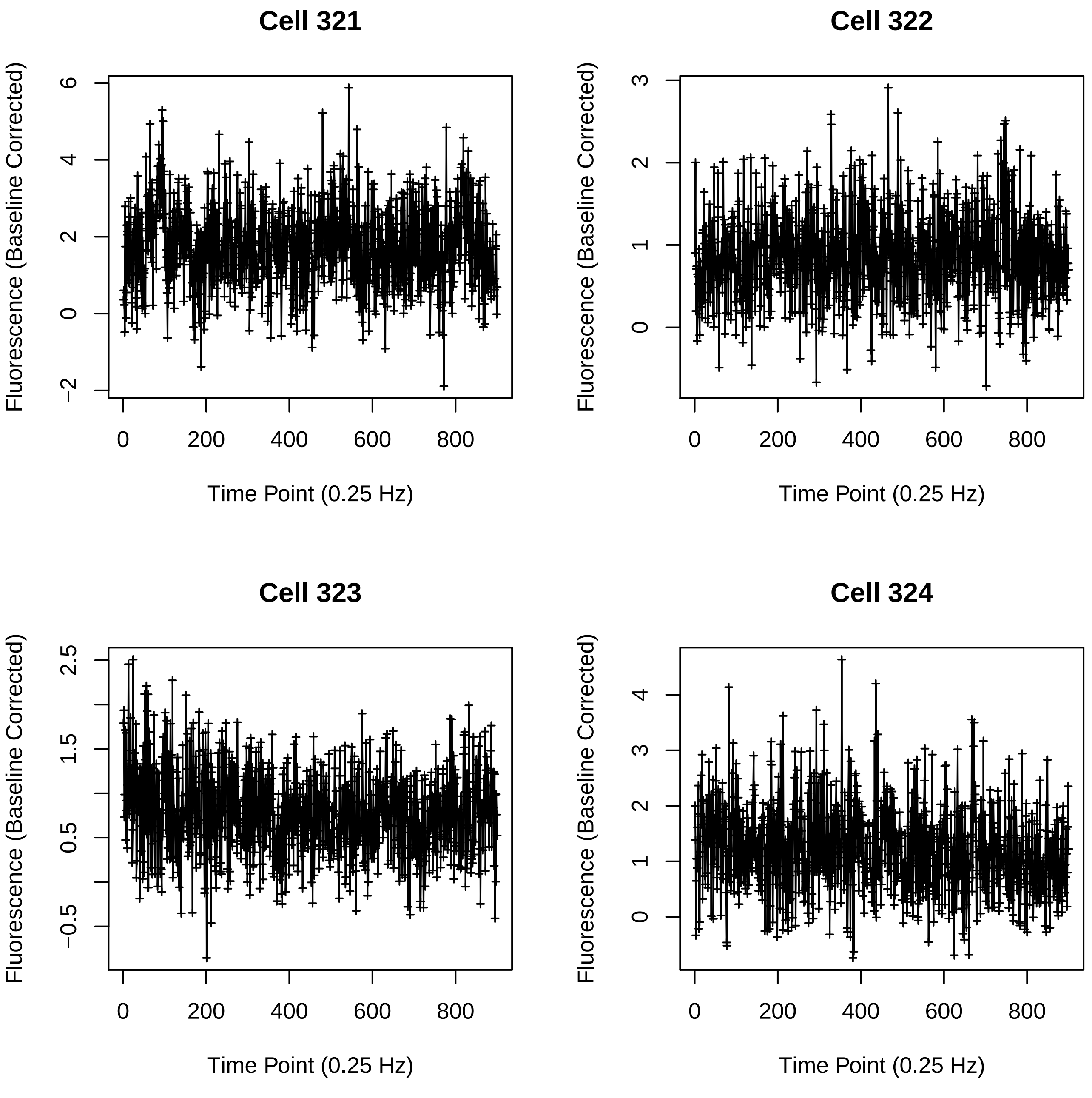
Cell 321
Cell 322
Cell 323
Cell 324
Fluorescence (Baseline Corrected)
Time Point (0.25 Hz)
−2
0
2
4
6
3
1
−0.5
0.5
1.5
2.5
200
400
600
800

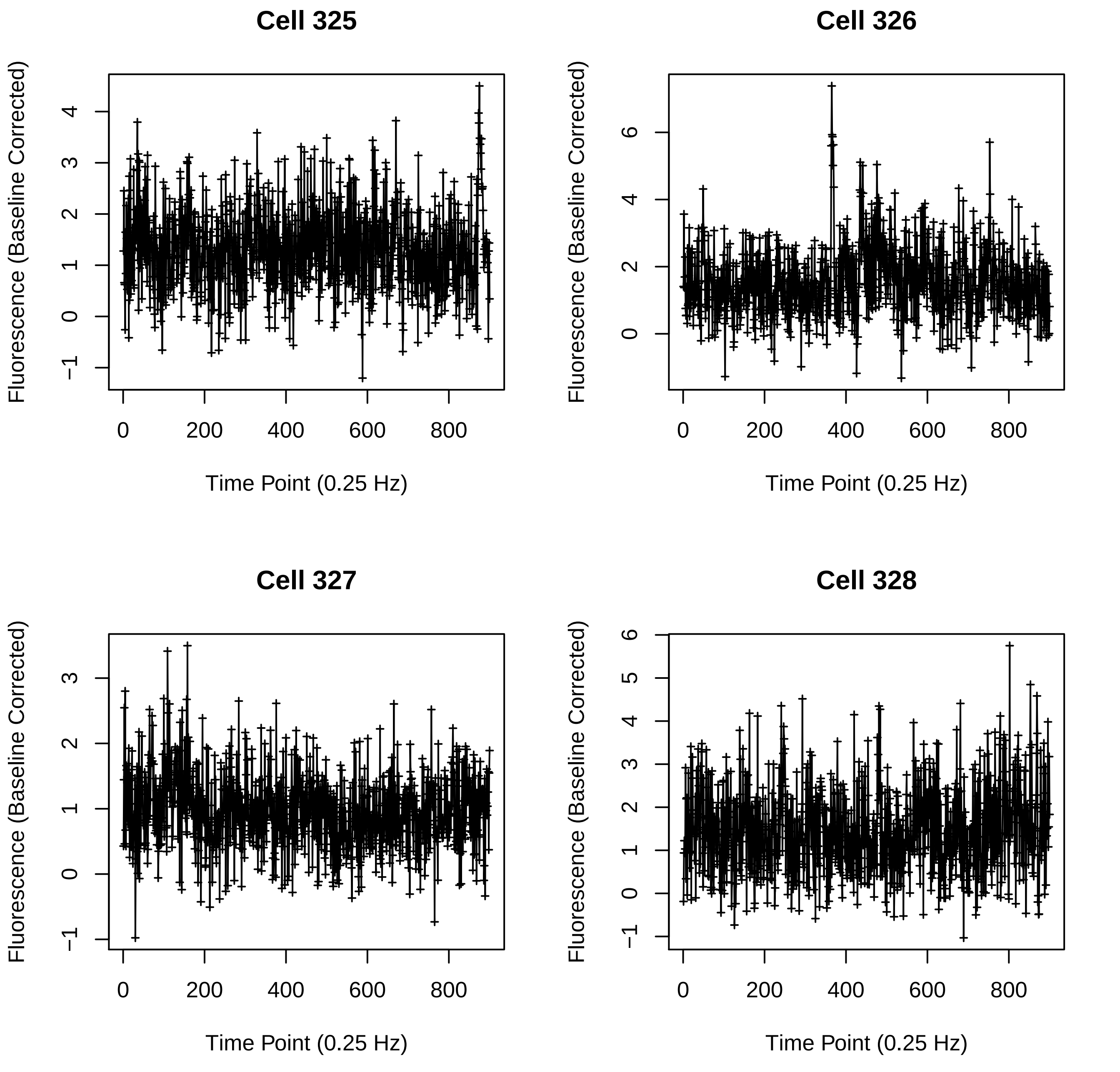
Cell 325
Cell 326
Cell 327
Cell 328
Fluorescence (Baseline Corrected)
Time Point (0.25 Hz)
0
200
400
600
800

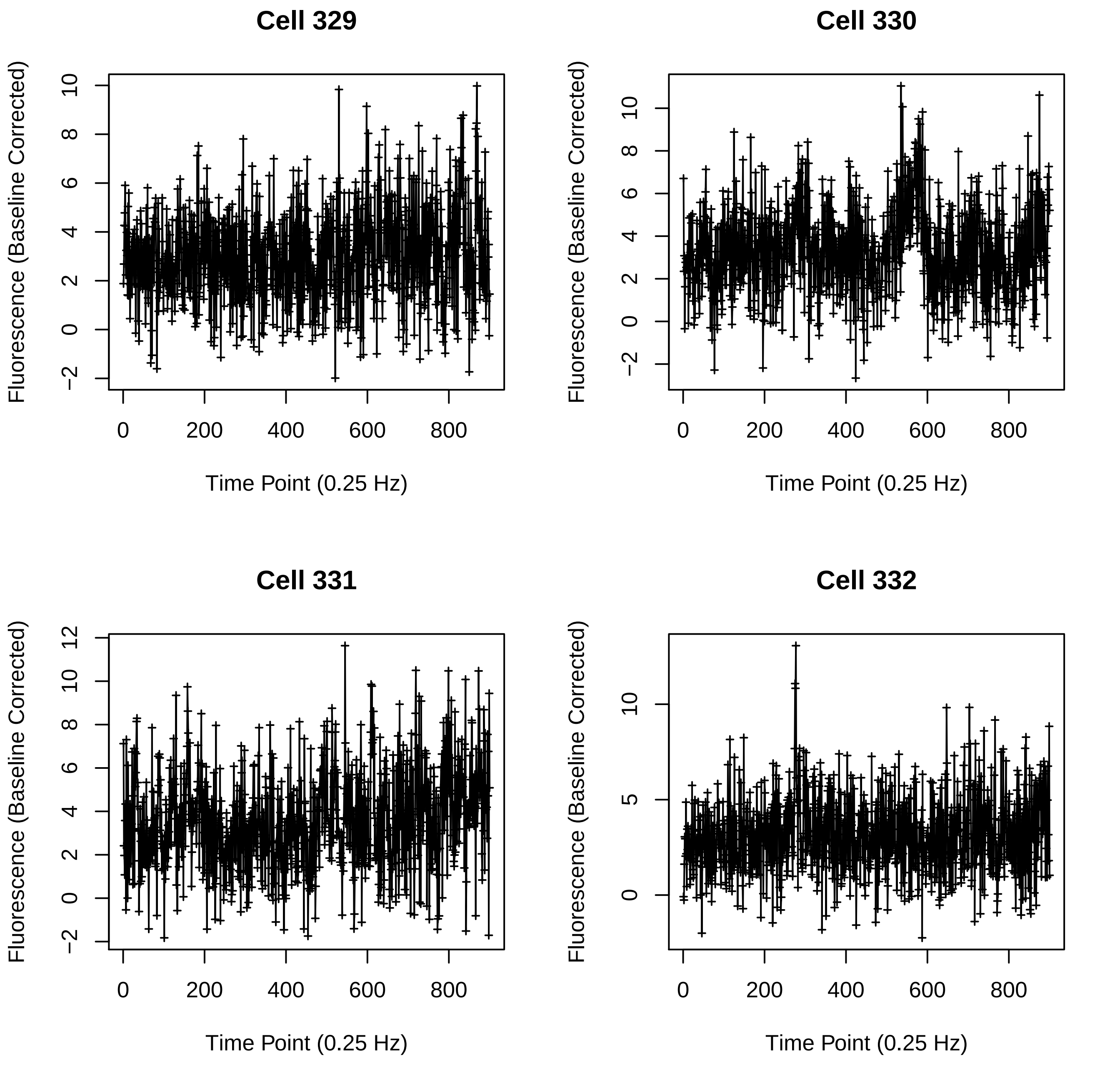
Cell 329
Cell 330
Cell 331
Cell 332
Fluorescence (Baseline Corrected)
Time Point (0.25 Hz)
0
200
400
600
800
−2
0
2
4
6
8
10
12

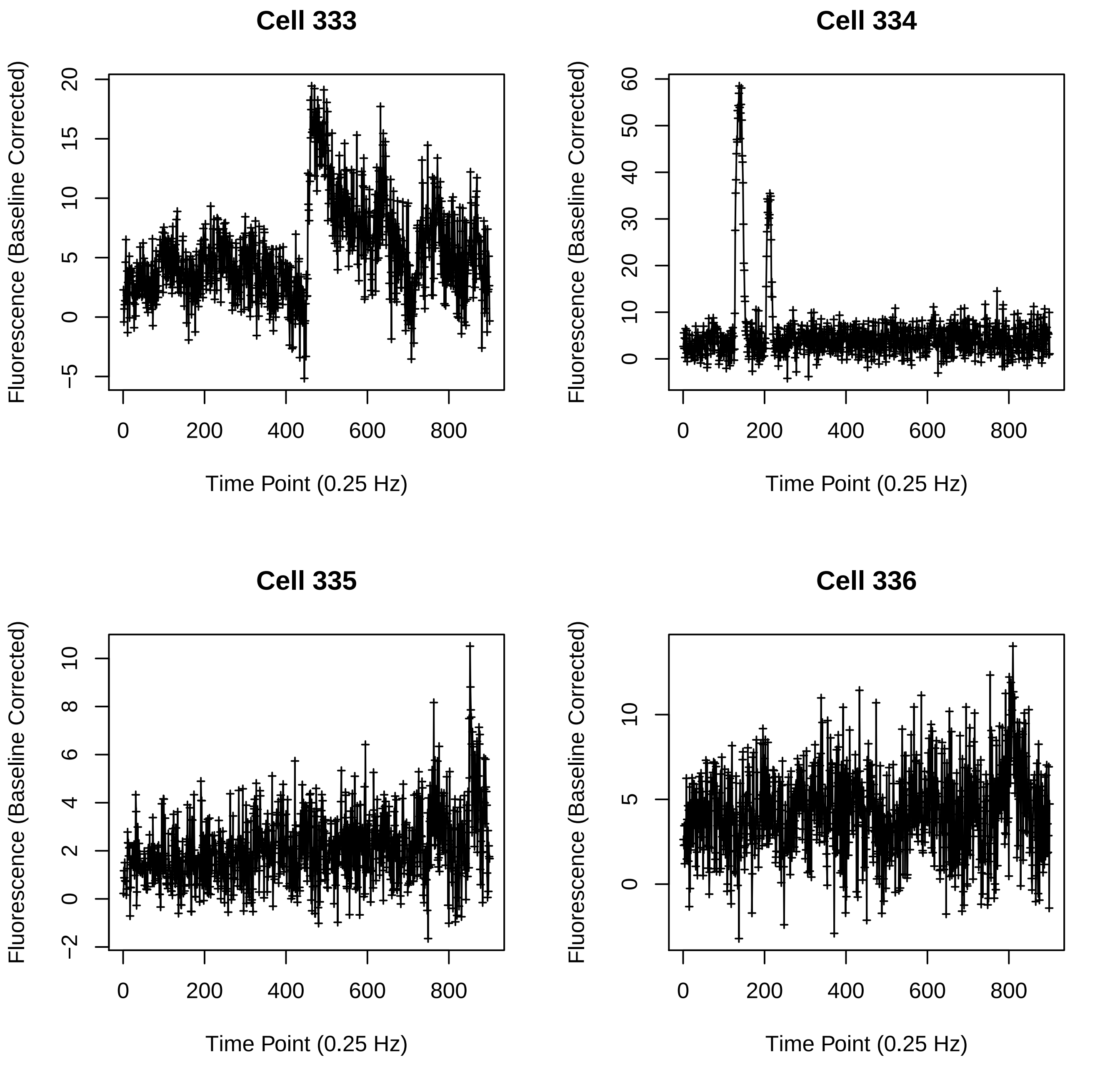

Cell 333
Cell 334
Cell 335
Cell 336
Fluorescence (Baseline Corrected)
Time Point (0.25 Hz)
0
200
400
600
800
−5
5
10
15
20
30
40
50
60
−2
2
4
6
8

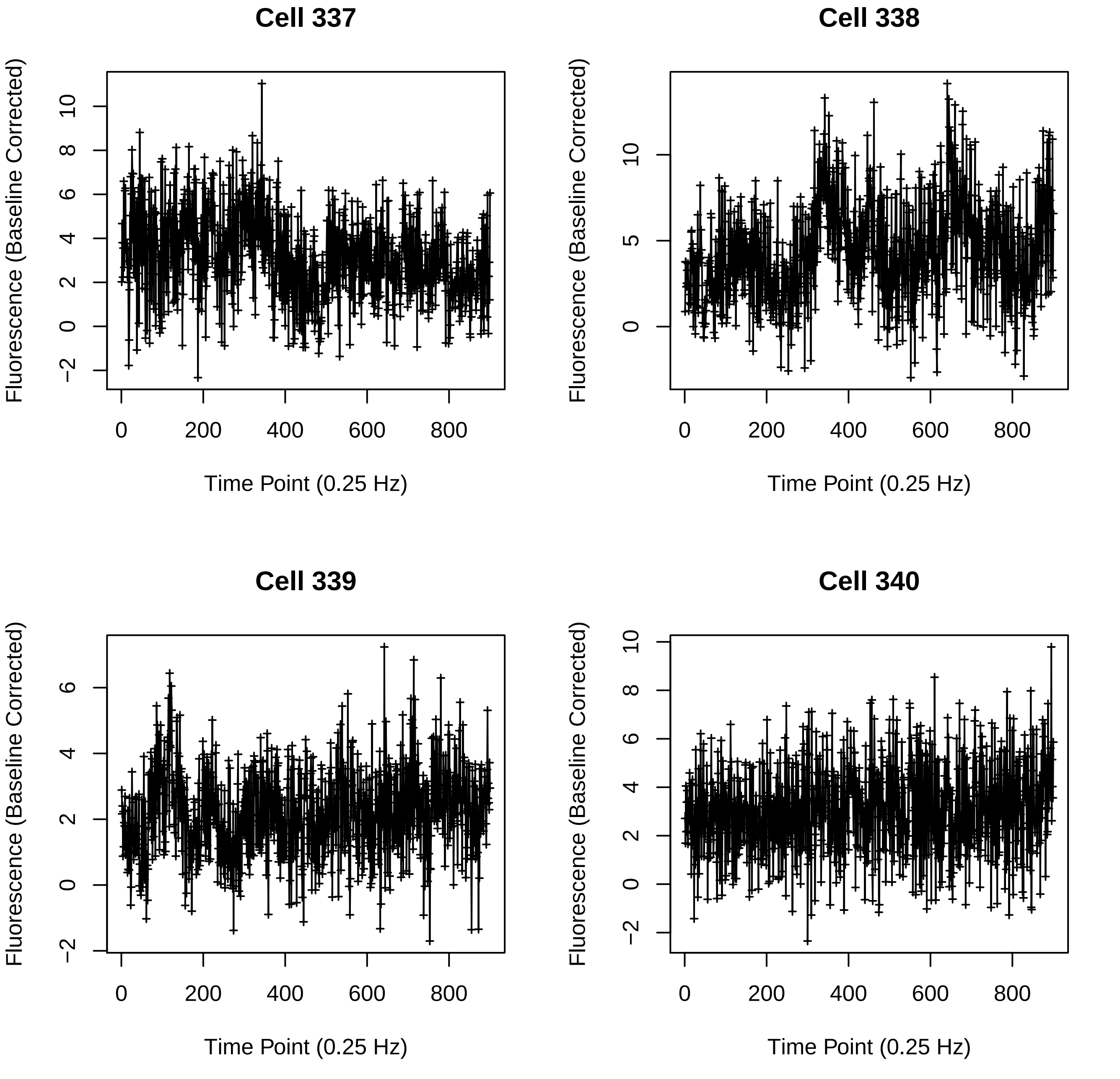
Cell 337
Cell 338
Cell 339
Cell 340
Fluorescence (Baseline Corrected)
Time Point (0.25 Hz)
0
200
400
600
800
−2
2
4
6
8
10
5

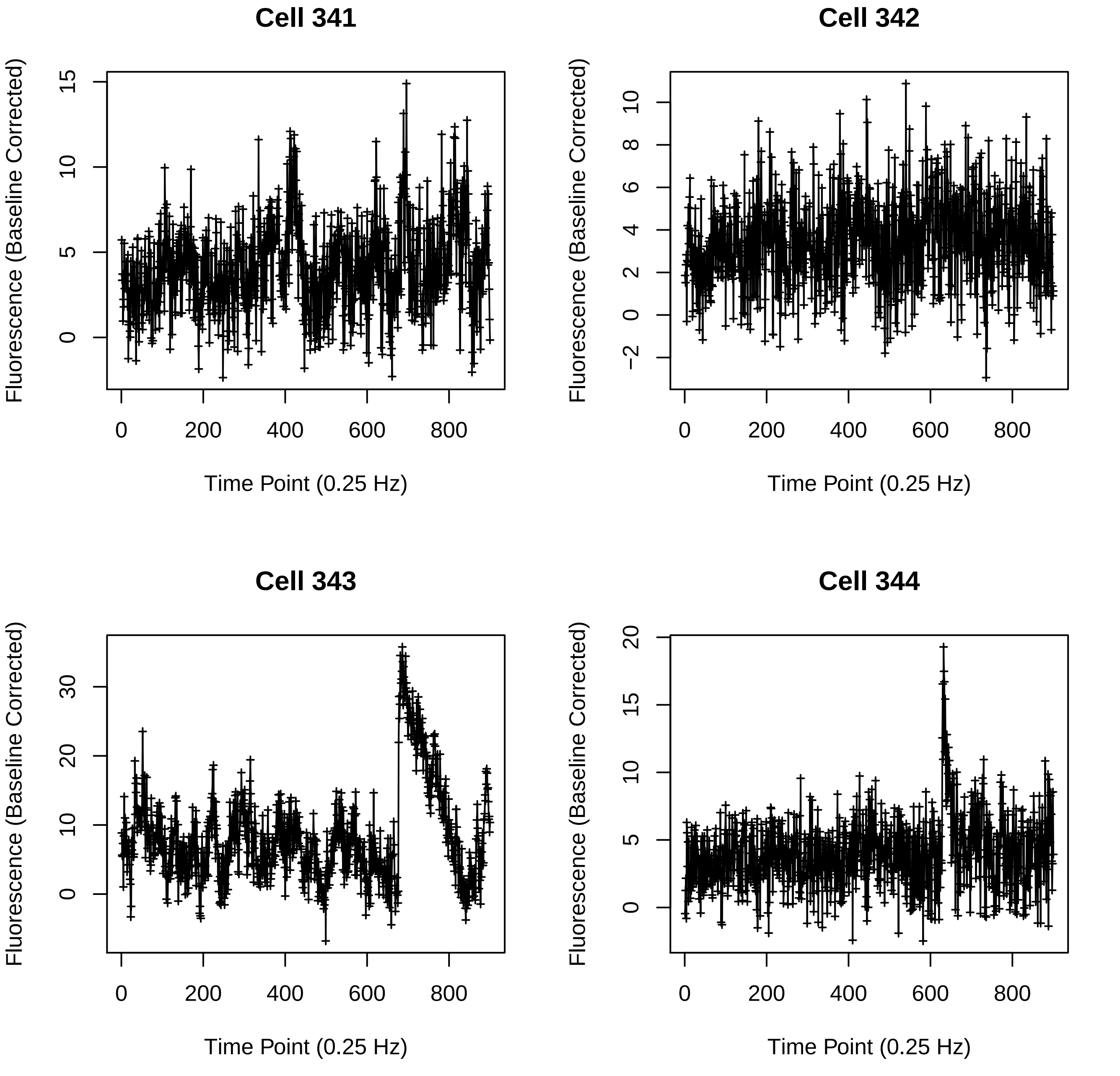
Cell 341
Cell 342
Cell 343
Cell 344
Fluorescence (Baseline Corrected)
Time Point (0.25 Hz)
0
200
400
600
800
0
5
10
15
−2
2
4
6
8
10
20
30

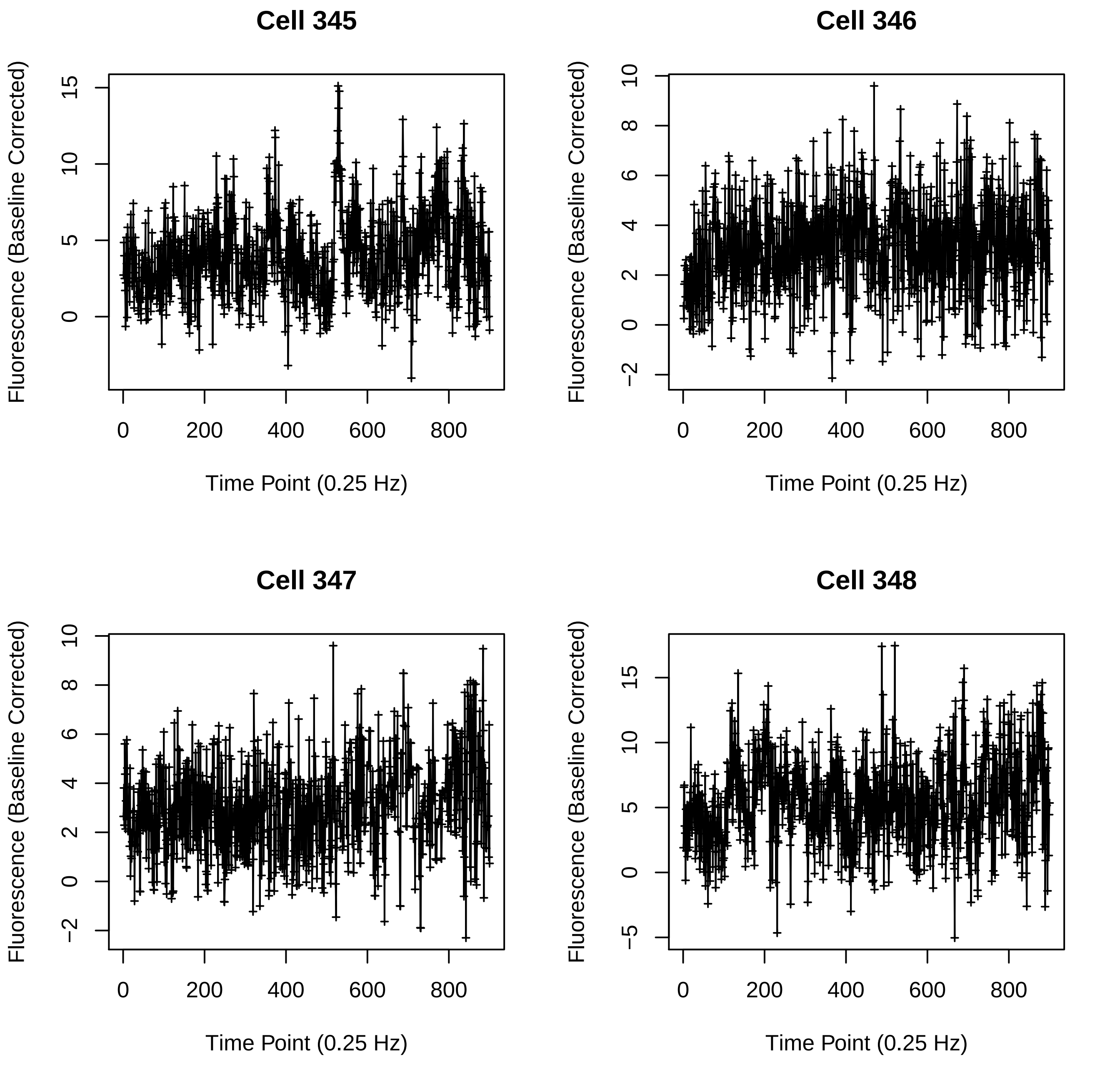
Cell 345
Cell 346
Cell 347
Cell 348
Fluorescence (Baseline Corrected)
Time Point (0.25 Hz)
0
200
400
600
800
−2
0
2
4
6
8
10
−5
5
15

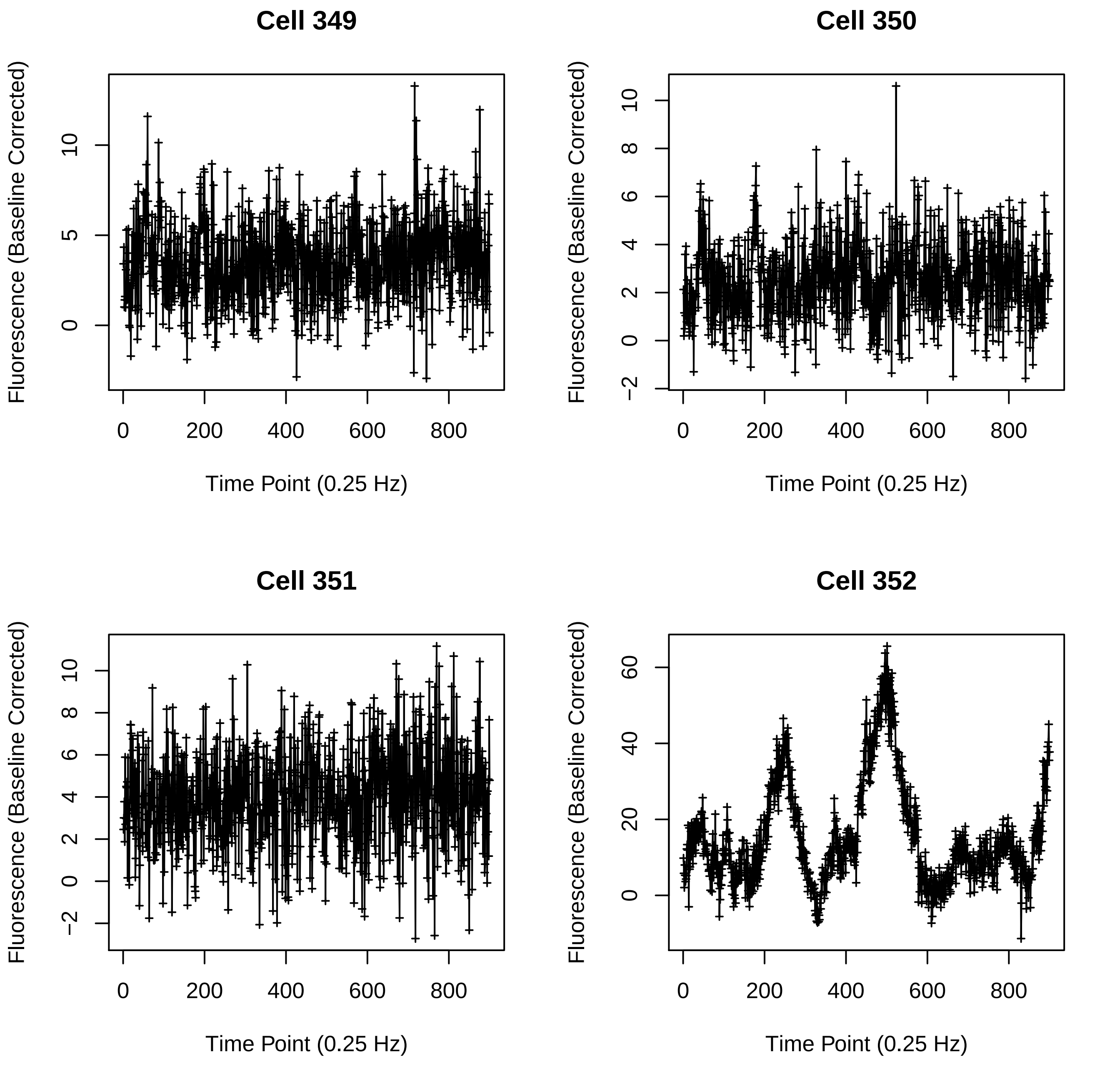
Cell 349
Cell 350
Cell 351
Cell 352
Fluorescence (Baseline Corrected)
Time Point (0.25 Hz)
0
200
400
600
800
−2
0
2
4
5
6
8
10
20
40
60

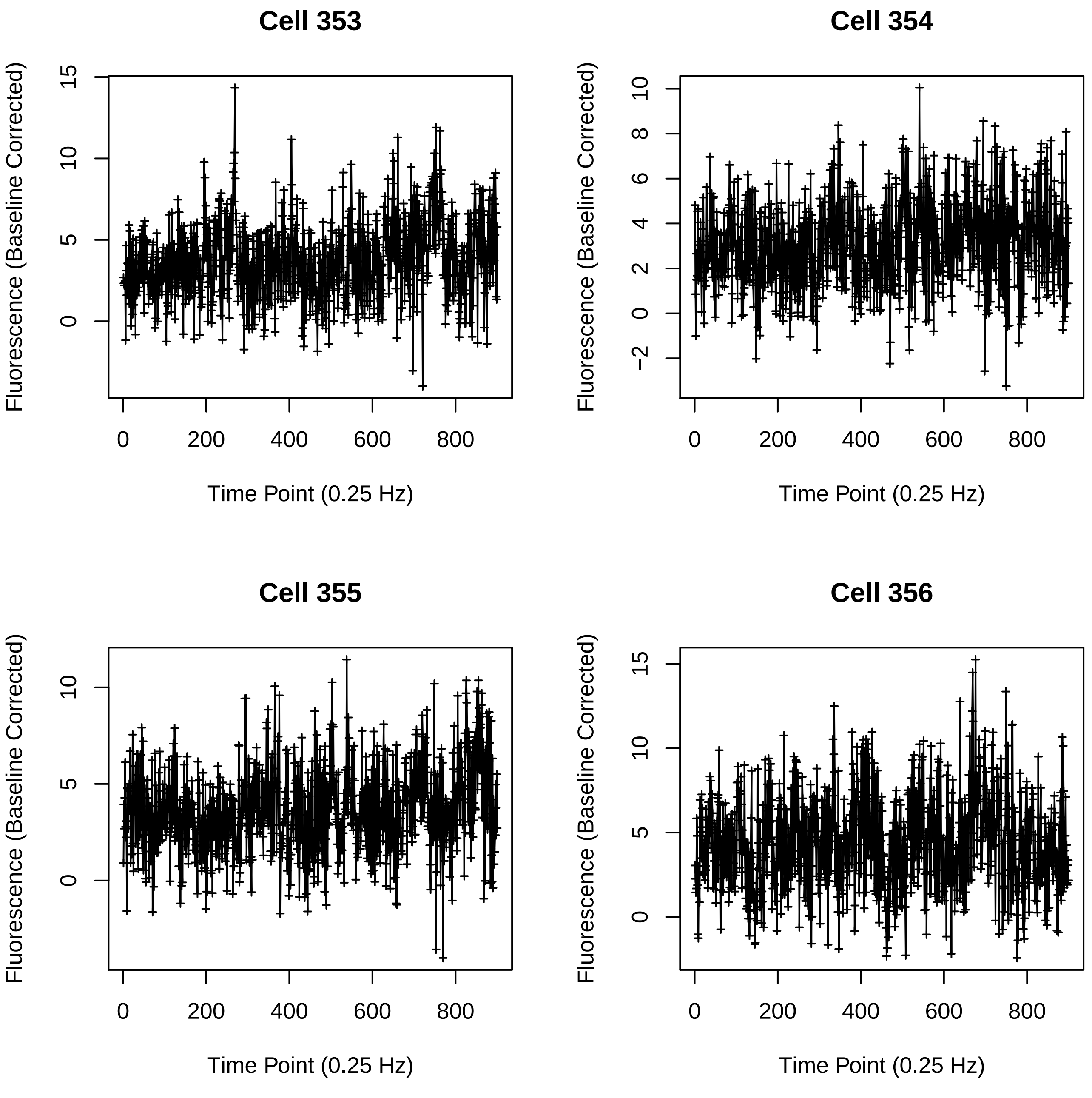

Cell 353
Cell 354
Cell 355
Cell 356
Fluorescence (Baseline Corrected)
Time Point (0.25 Hz)
0
200
400
600
800
−2
0
2
4
6
8
10
5
15

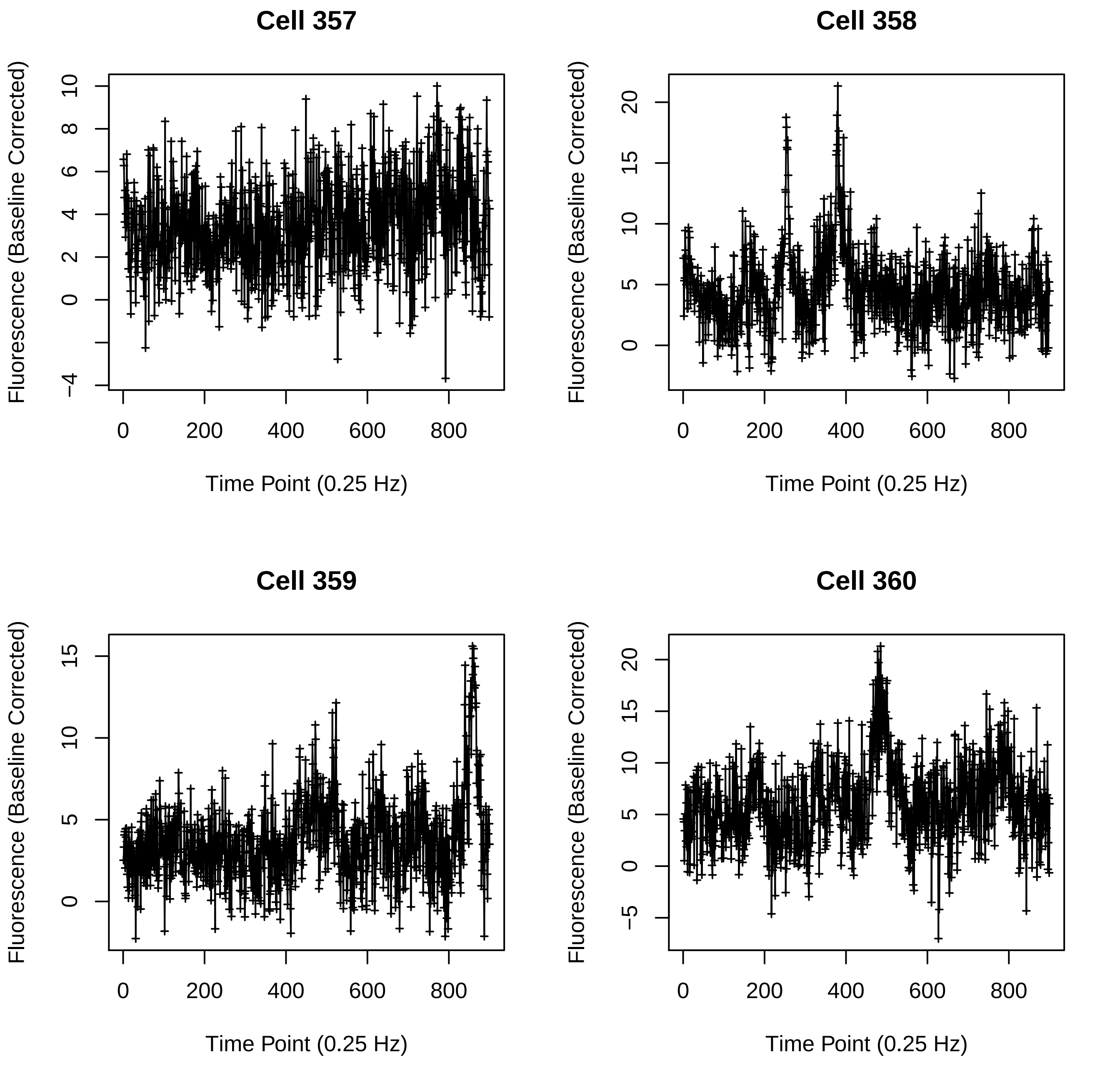
Cell 357
Fluorescence (Baseline Corrected)
Time Point (0.25 Hz)
-4
0
2
4
6
8
10
0
200
400
600
800
Cell 358
Fluorescence (Baseline Corrected)
Time Point (0.25 Hz)
0
5
10
15
20
0
200
400
600
800
Cell 359
Fluorescence (Baseline Corrected)
Time Point (0.25 Hz)
0
5
10
15
0
200
400
600
800
Cell 360
Fluorescence (Baseline Corrected)
Time Point (0.25 Hz)
-5
0
5
10
15
20
0
200
400
600
800

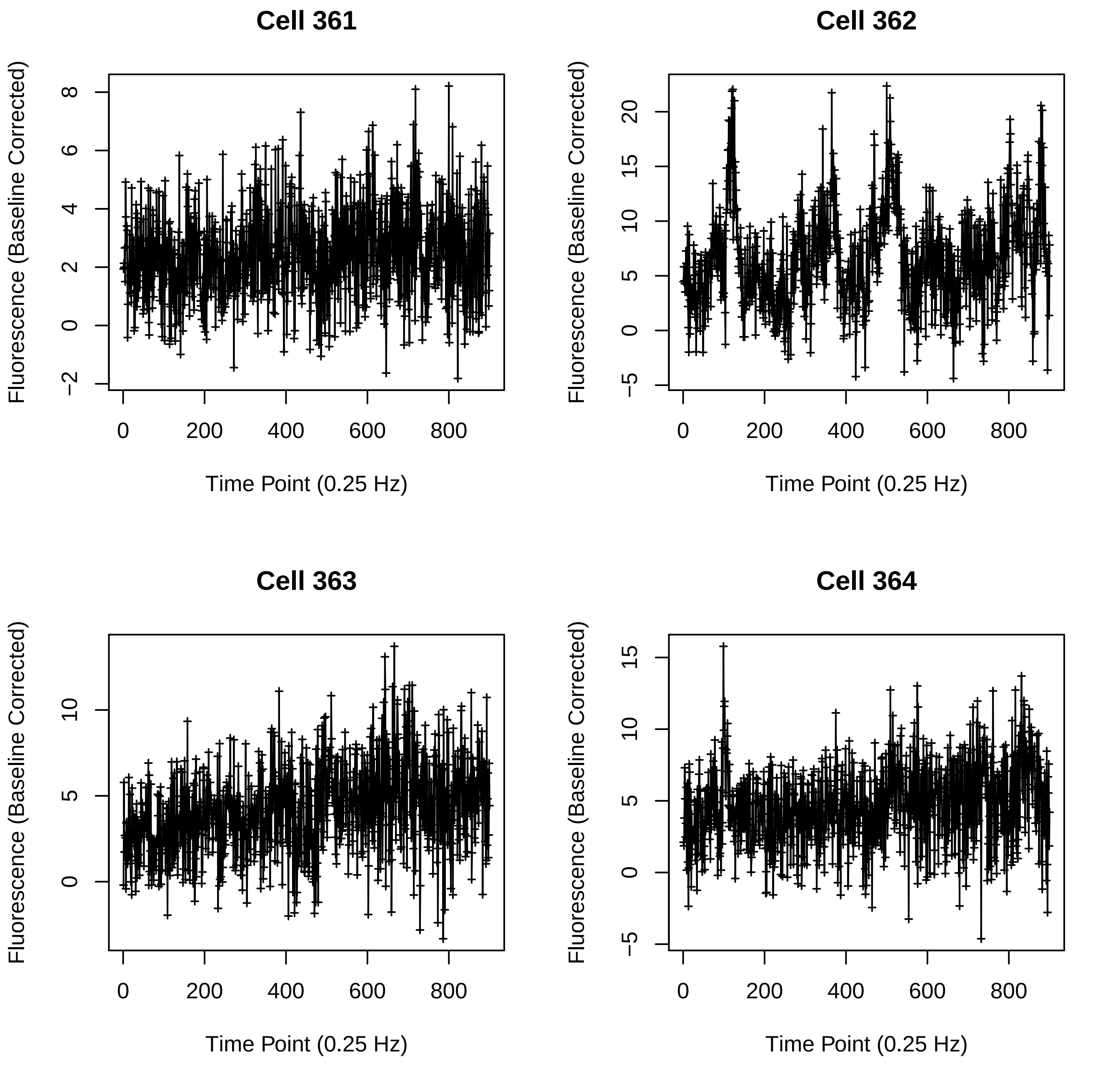

Cell 361
Cell 362
Cell 363
Cell 364
Fluorescence (Baseline Corrected)
Time Point (0.25 Hz)
0
200
400
600
800
−2
2
4
6
8
−5
5
10
15
20

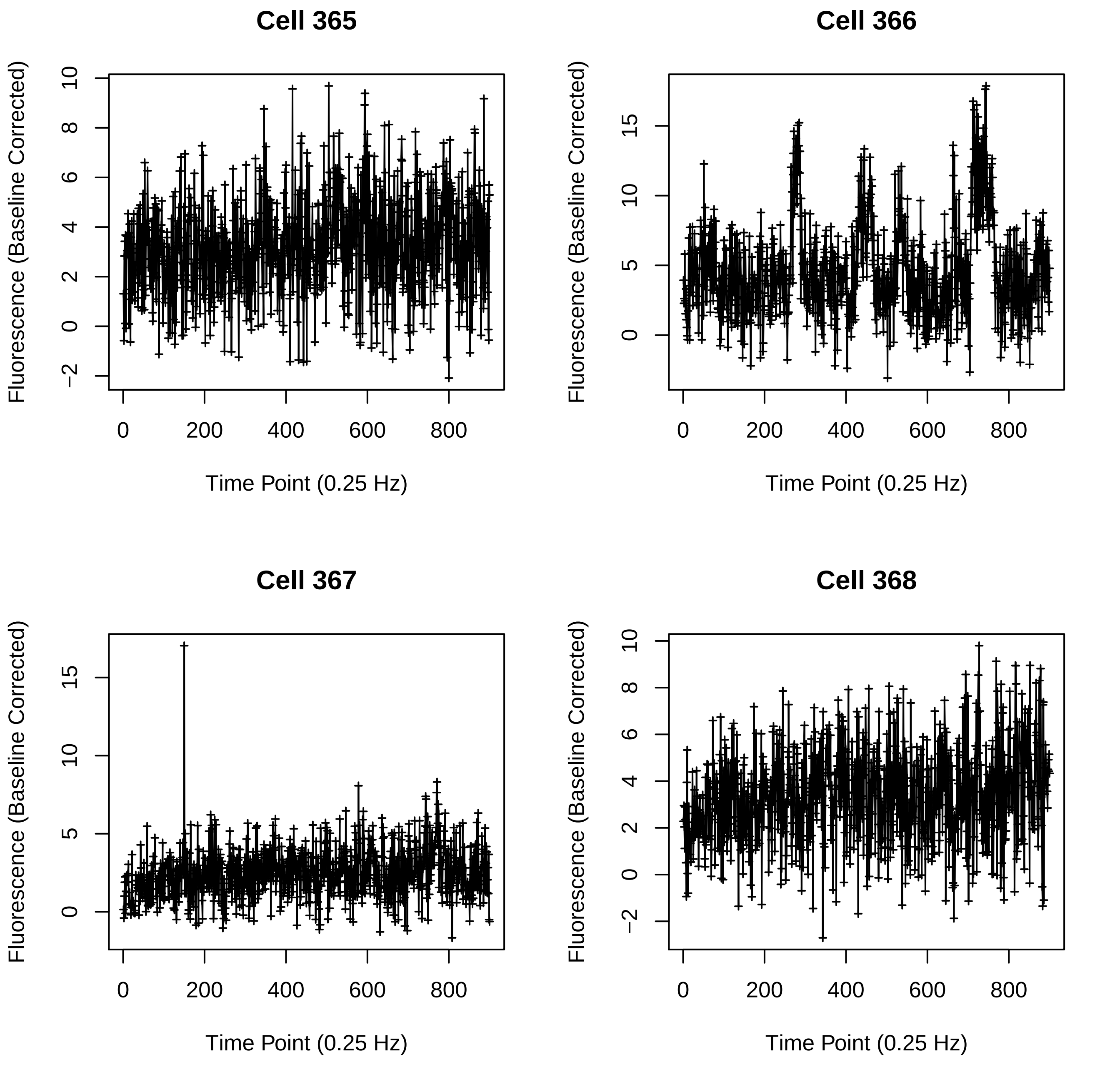

Cell 365
Cell 366
Cell 367
Cell 368
Fluorescence (Baseline Corrected)
Time Point (0.25 Hz)

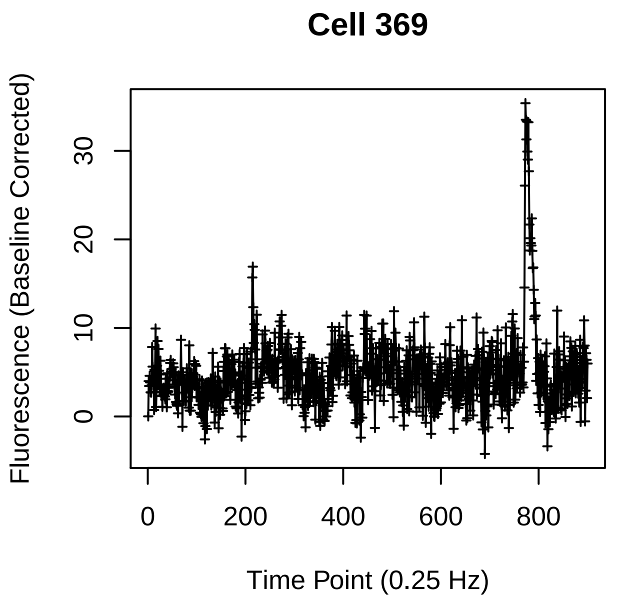
Cell 369
Fluorescence (Baseline Corrected)
0
10
20
30
0
200
400
600
800
Time Point (0.25 Hz)
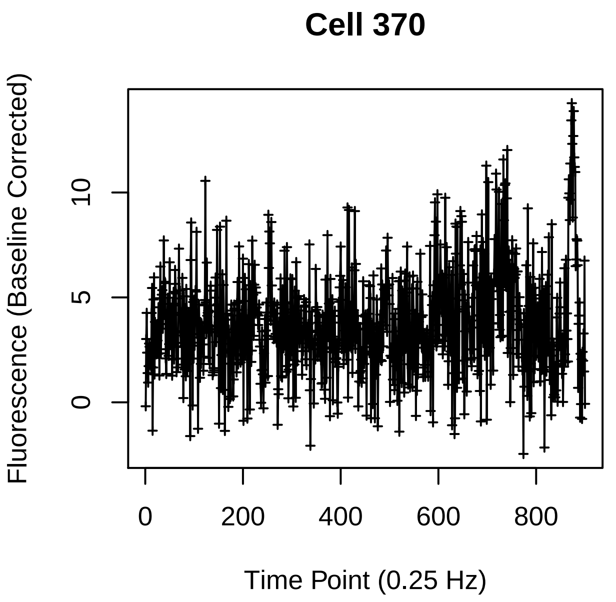
Cell 370
Fluorescence (Baseline Corrected)
0
5
10
0
200
400
600
800
Time Point (0.25 Hz)
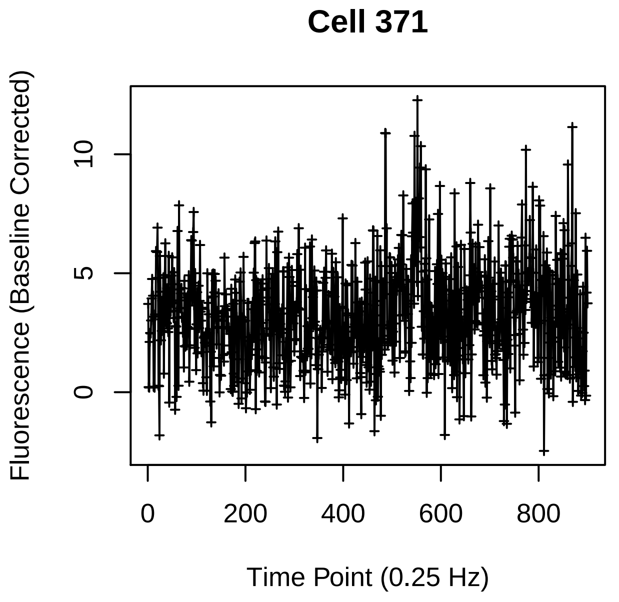
Cell 371
Fluorescence (Baseline Corrected)
0
5
10
0
200
400
600
800
Time Point (0.25 Hz)
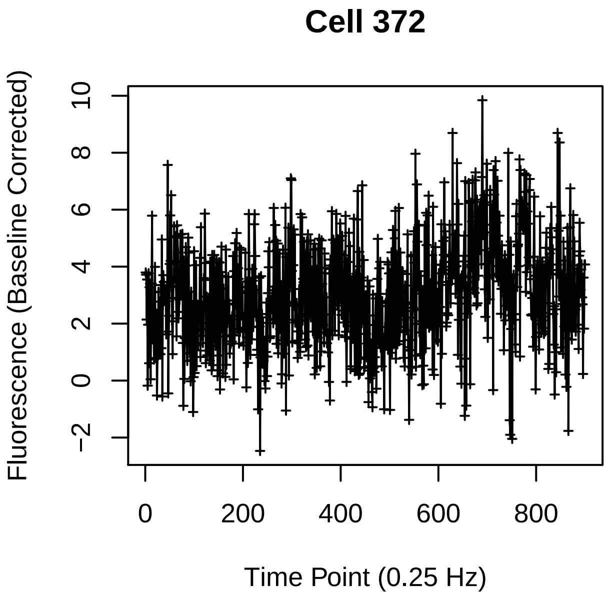
Cell 372
Fluorescence (Baseline Corrected)
−2
0
2
4
6
8
10
0
200
400
600
800
Time Point (0.25 Hz)

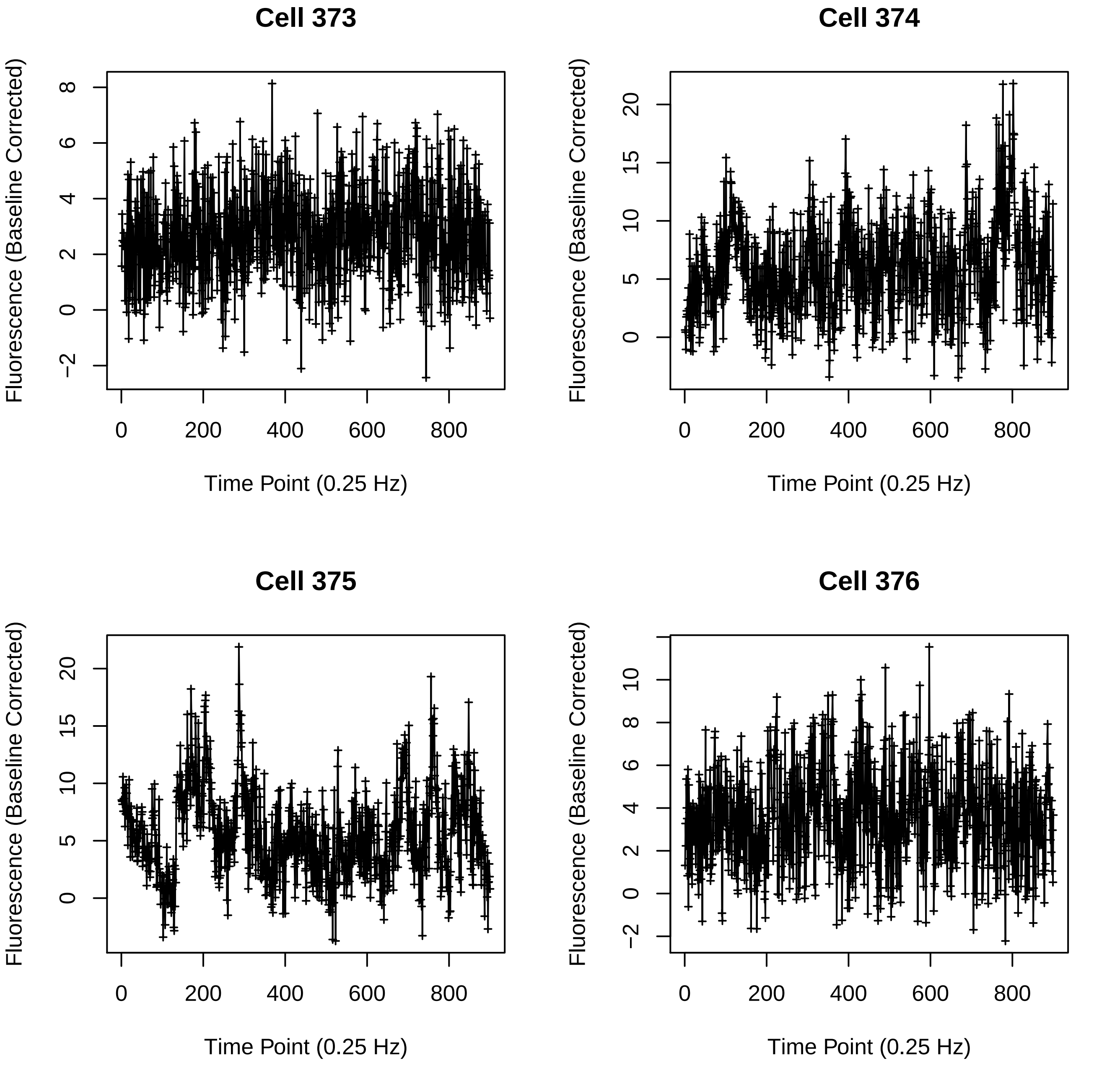

Cell 373
Fluorescence (Baseline Corrected)
Time Point (0.25 Hz)
Cell 374
Fluorescence (Baseline Corrected)
Time Point (0.25 Hz)
Cell 375
Fluorescence (Baseline Corrected)
Time Point (0.25 Hz)
Cell 376
Fluorescence (Baseline Corrected)
Time Point (0.25 Hz)

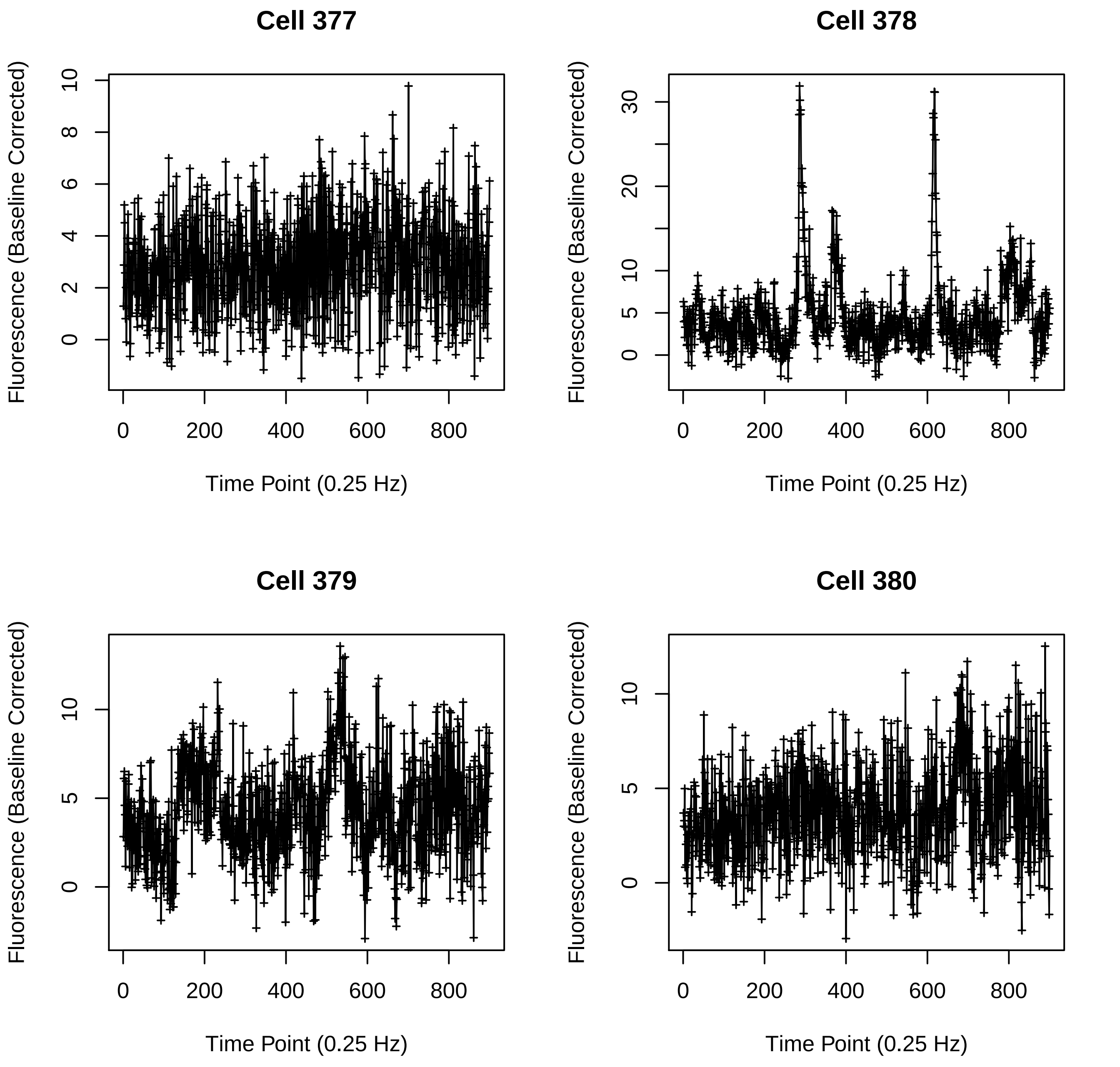

Cell 377
Cell 378
Cell 379
Cell 380
Fluorescence (Baseline Corrected)
Time Point (0.25 Hz)
0
200
400
600
800
2
4
6
8
10
5
10
15
20
25
30

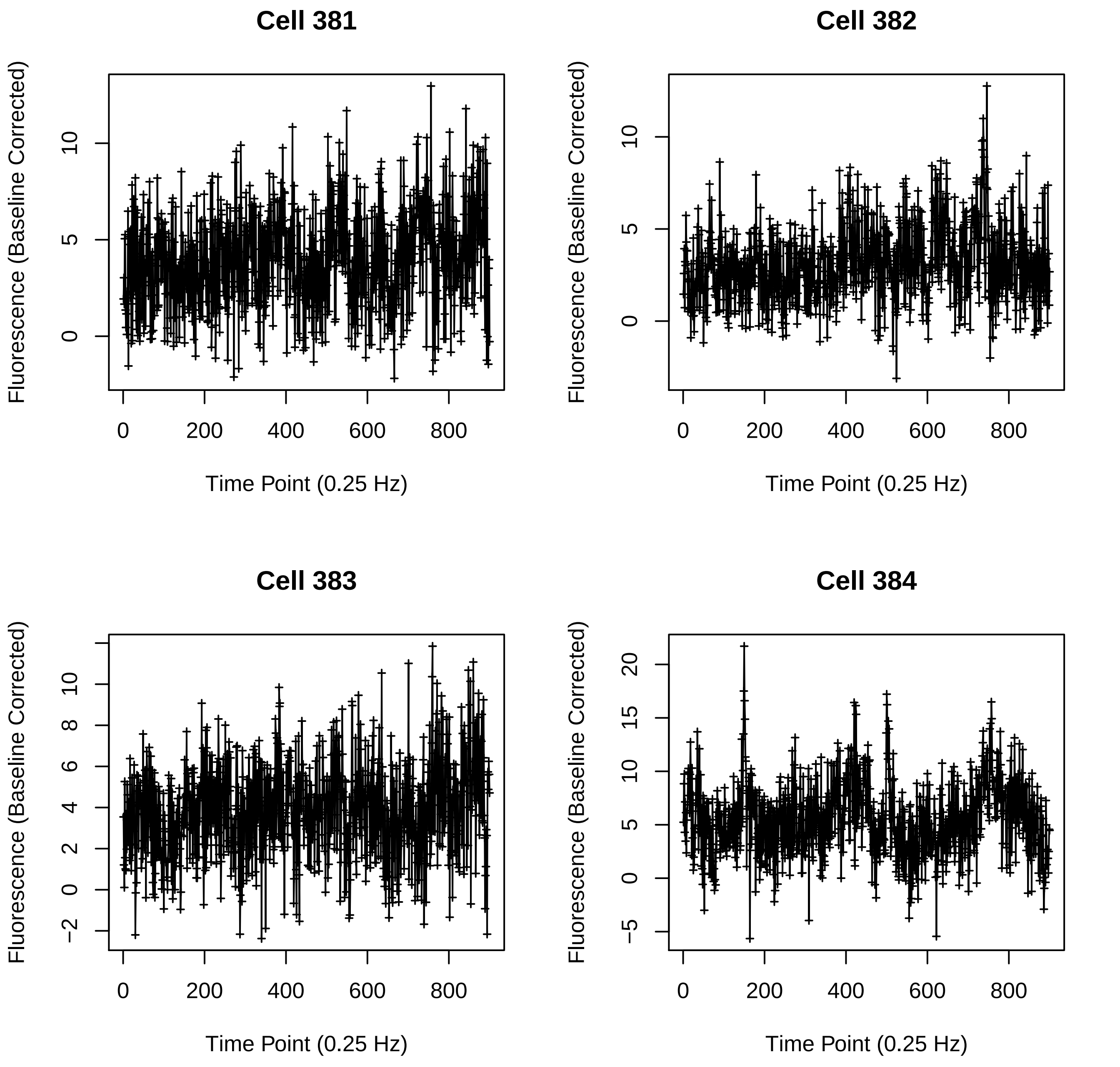

Cell 381
Cell 382
Cell 383
Cell 384
Fluorescence (Baseline Corrected)
Time Point (0.25 Hz)
0
200
400
600
800
-2
0
2
4
5
6
8
10
15
20
-5

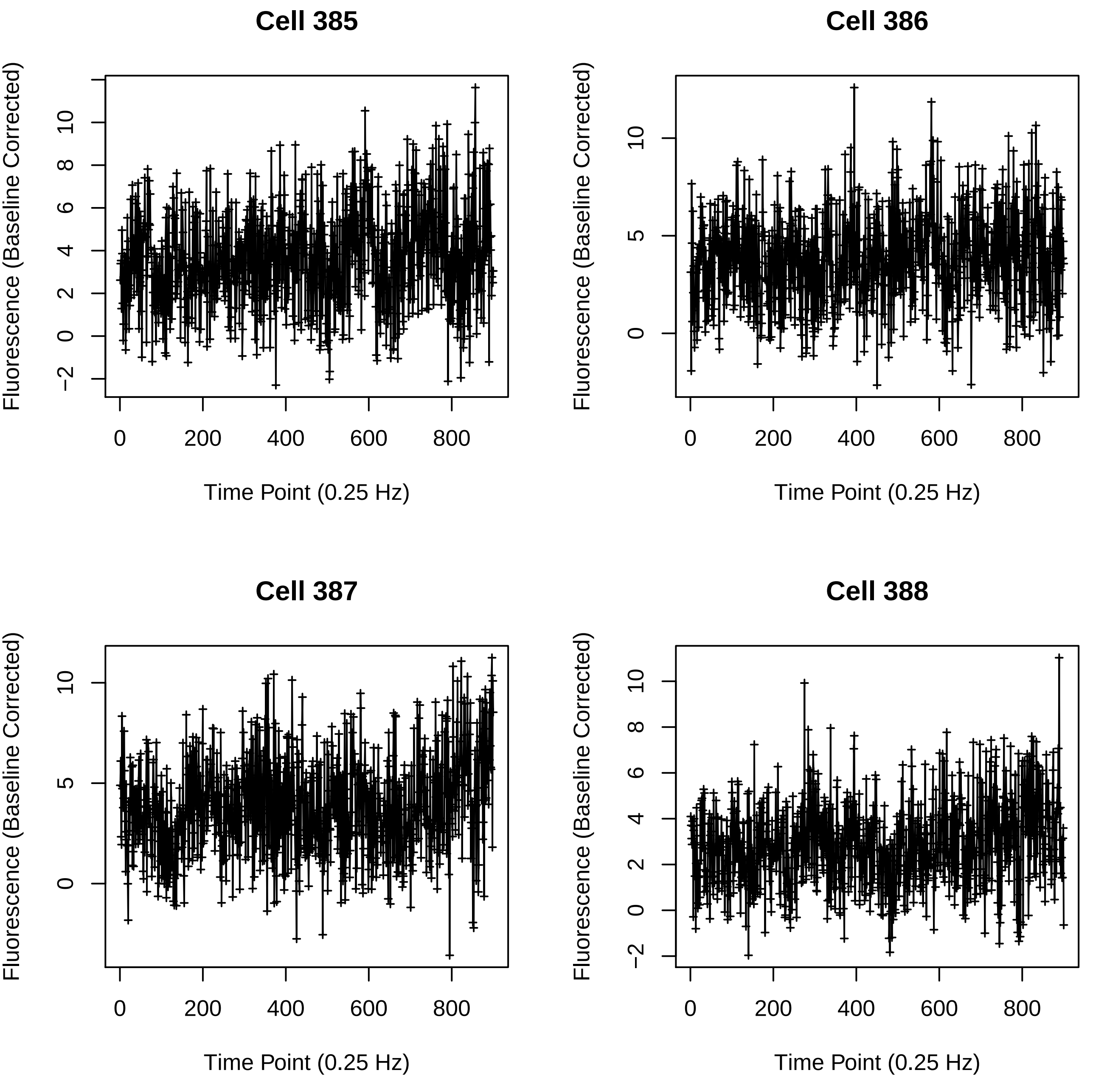
Cell 385
Cell 386
Cell 387
Cell 388
Fluorescence (Baseline Corrected)
Time Point (0.25 Hz)
0
200
400
600
800
−2
0
2
4
6
8
10
5

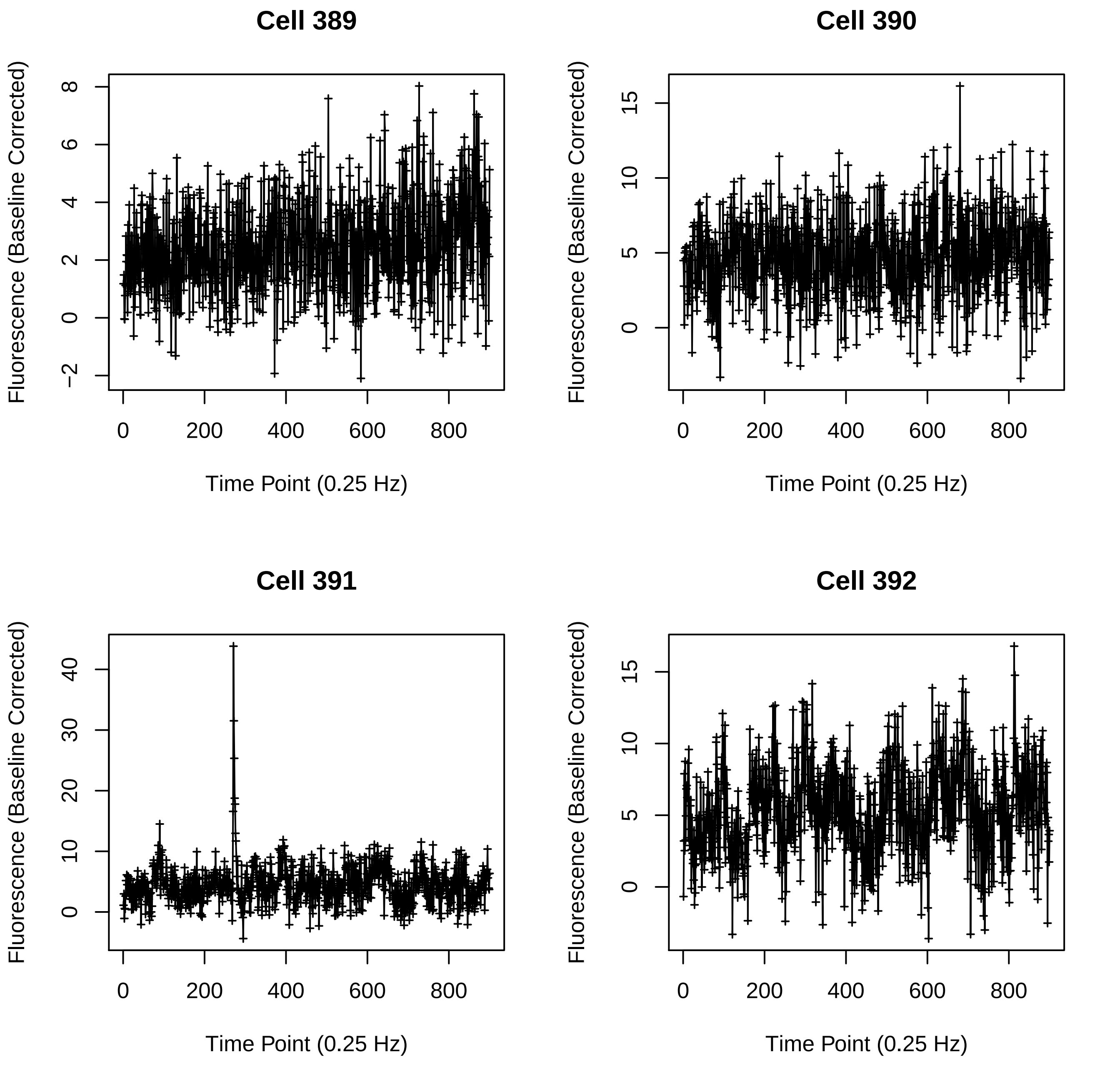
Cell 389
Cell 390
Cell 391
Cell 392
Fluorescence (Baseline Corrected)
Time Point (0.25 Hz)
0
200
400
600
800
−2
0
2
4
6
8
5
10
15
20
30
40

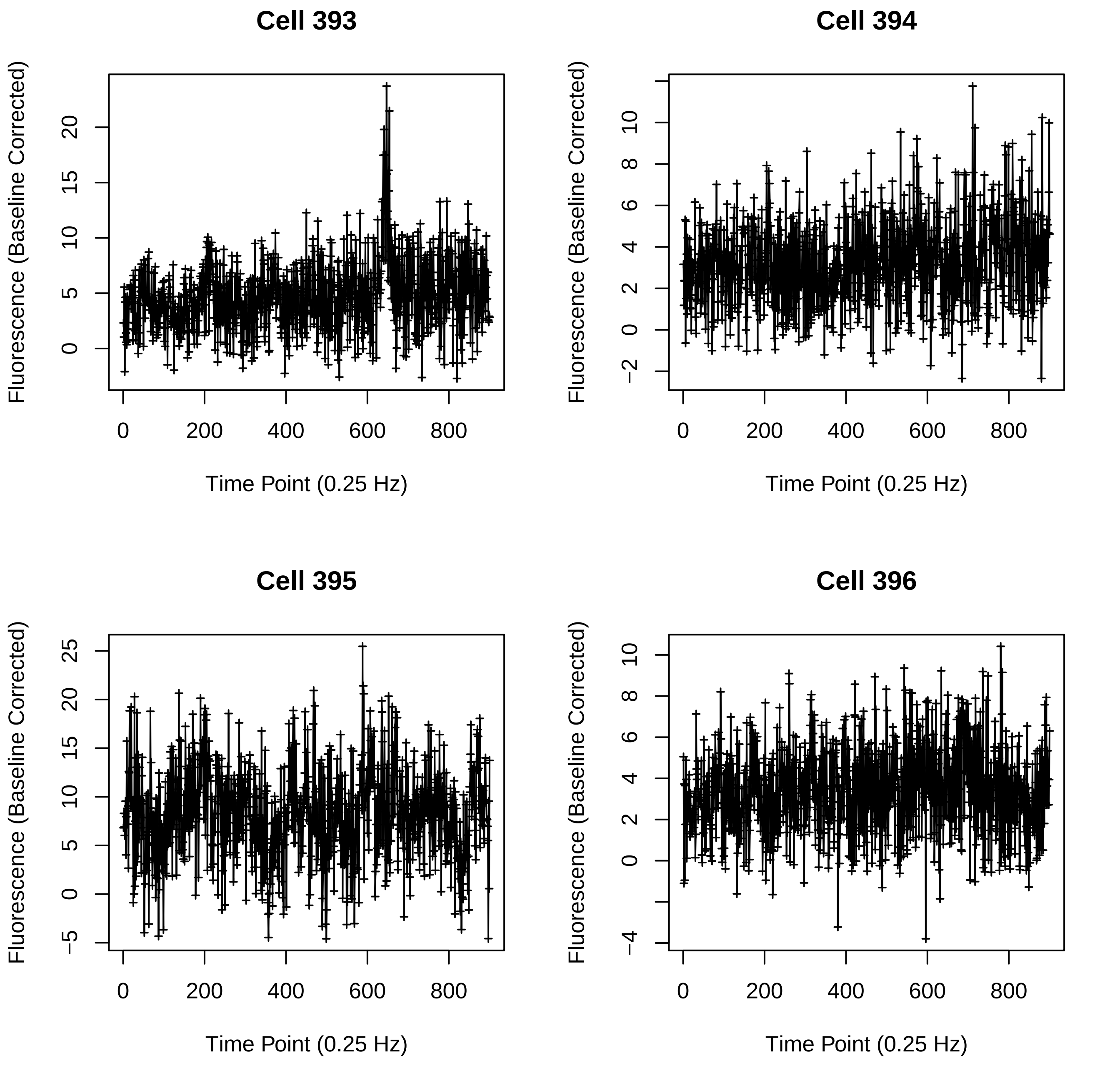
Cell 393
Cell 394
Cell 395
Cell 396
Fluorescence (Baseline Corrected)
Time Point (0.25 Hz)
0
200
400
600
800
−5
0
5
10
15
20
25
−4
−2
2
4
6
8

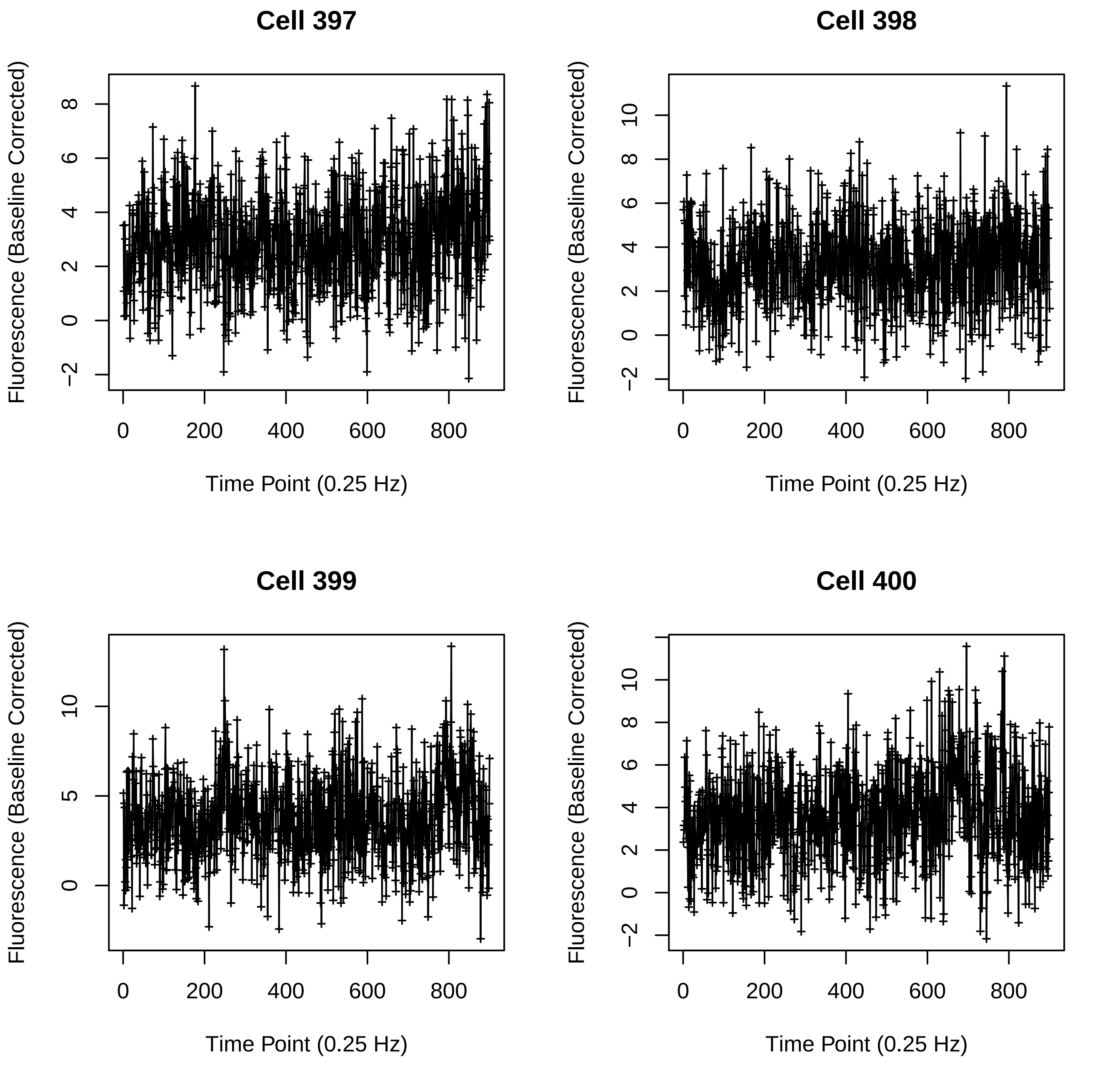
Cell 397
Cell 398
Cell 399
Cell 400
Fluorescence (Baseline Corrected)
Time Point (0.25 Hz)
0
200
400
600
800
−2
0
2
4
6
8
10

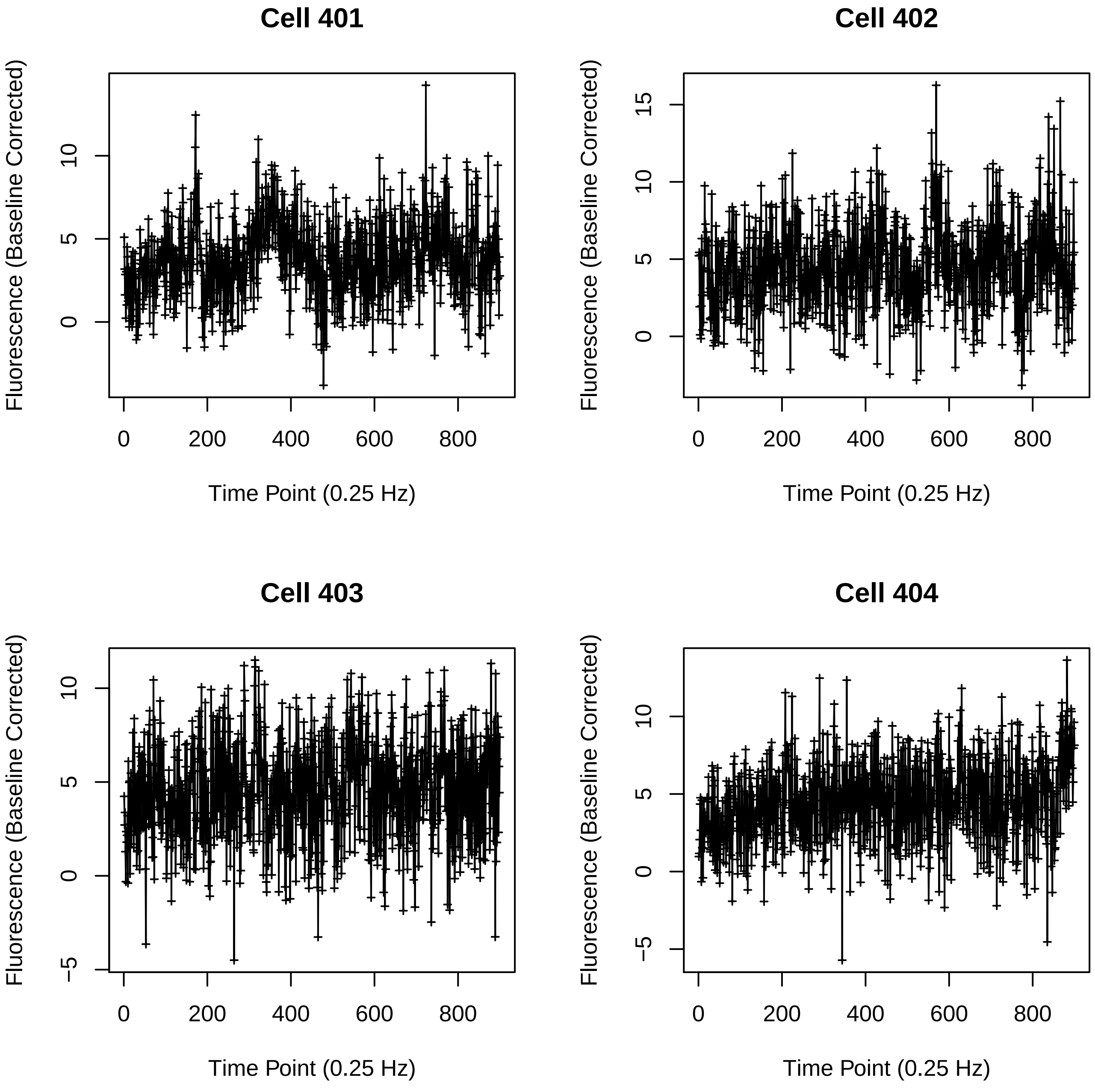
Cell 401
Cell 402
Cell 403
Cell 404
Fluorescence (Baseline Corrected)
Time Point (0.25 Hz)
0
200
400
600
800
-5
0
5
10
15

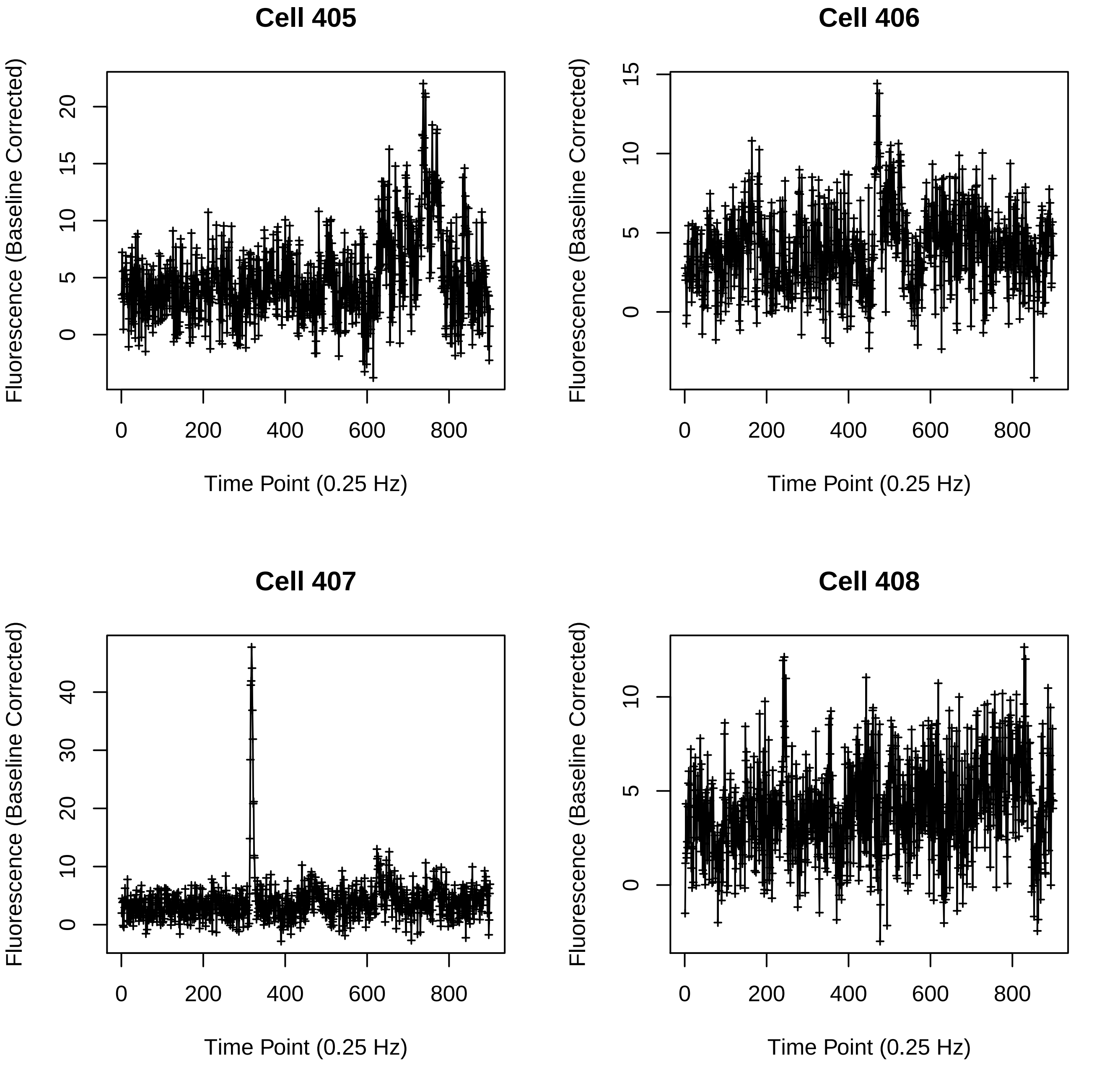

Cell 405
Cell 406
Cell 407
Cell 408
Fluorescence (Baseline Corrected)
Time Point (0.25 Hz)
0
200
400
600
800
0
5
10
15
20
0
5
10
15
0
10
20
30
40
0
5
10

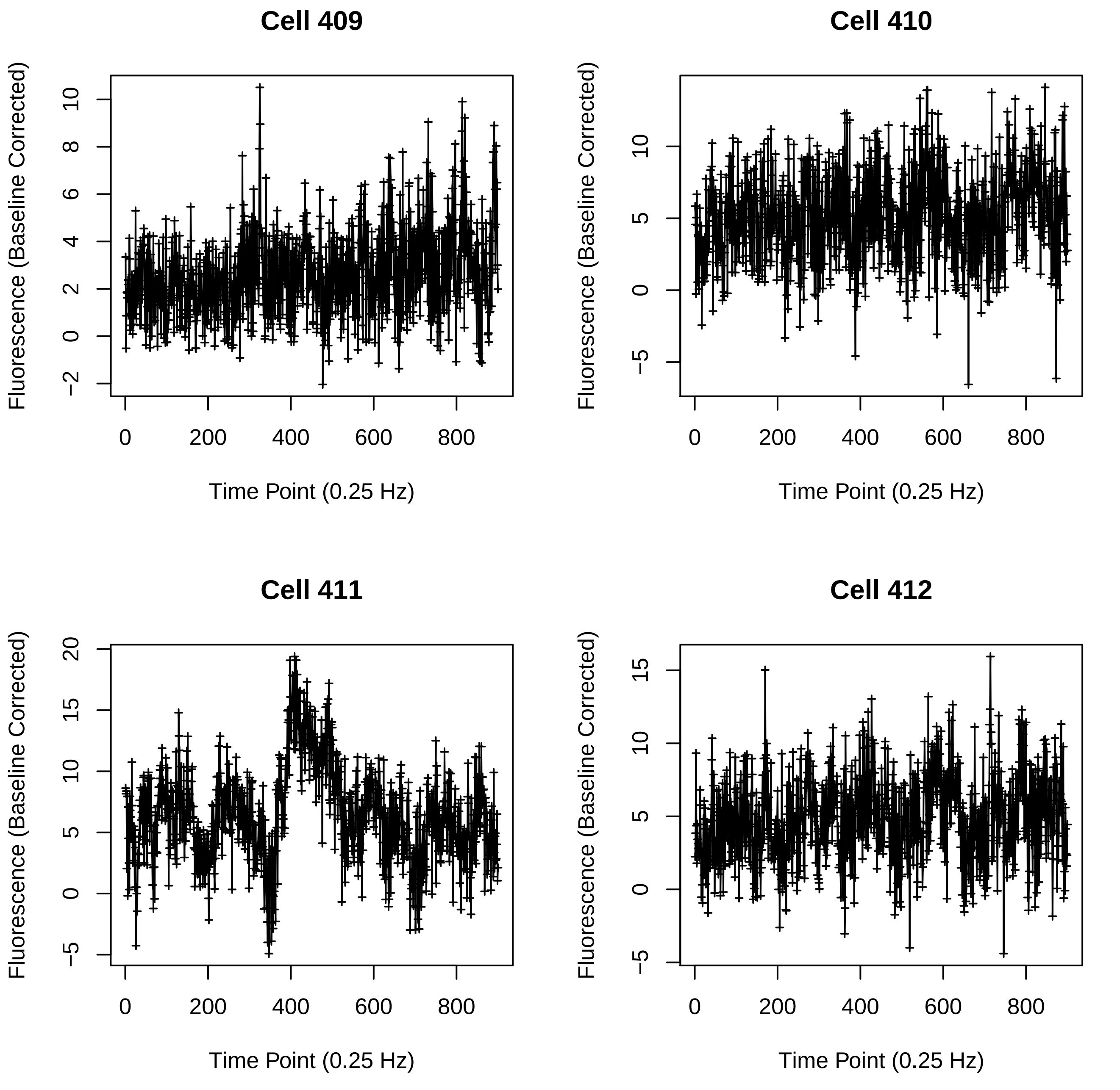

Cell 409
Fluorescence (Baseline Corrected)
−2
0
2
4
6
8
10
Time Point (0.25 Hz)
0
200
400
600
800
Cell 410
Fluorescence (Baseline Corrected)
−5
0
5
10
Time Point (0.25 Hz)
0
200
400
600
800
Cell 411
Fluorescence (Baseline Corrected)
−5
0
5
10
15
20
Time Point (0.25 Hz)
0
200
400
600
800
Cell 412
Fluorescence (Baseline Corrected)
−5
0
5
10
15
Time Point (0.25 Hz)
0
200
400
600
800

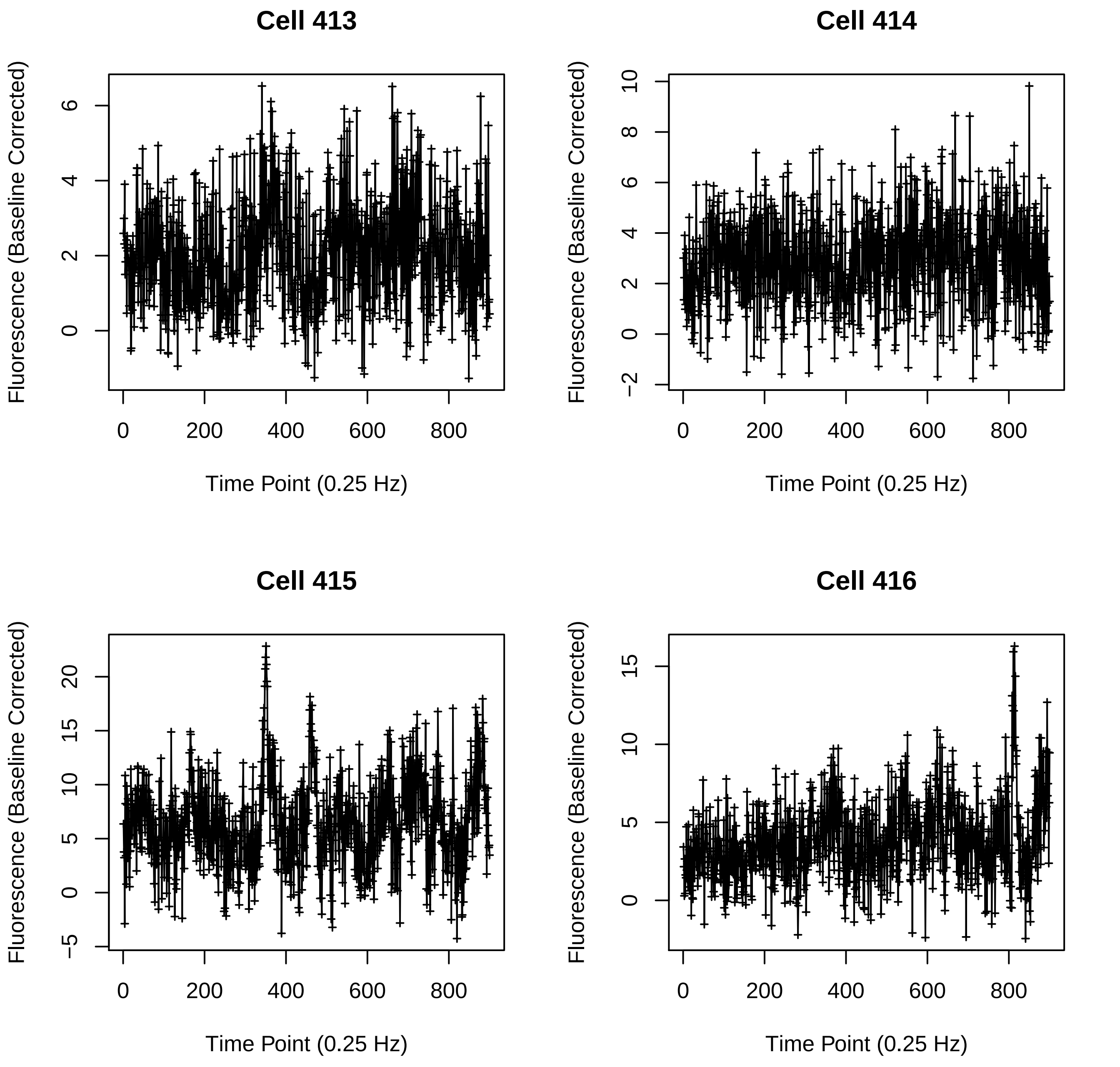
Cell 413
Cell 414
Cell 415
Cell 416
Fluorescence (Baseline Corrected)
Time Point (0.25 Hz)
0
200
400
600
800
−2
2
4
6
8
10
−5
5
15
20

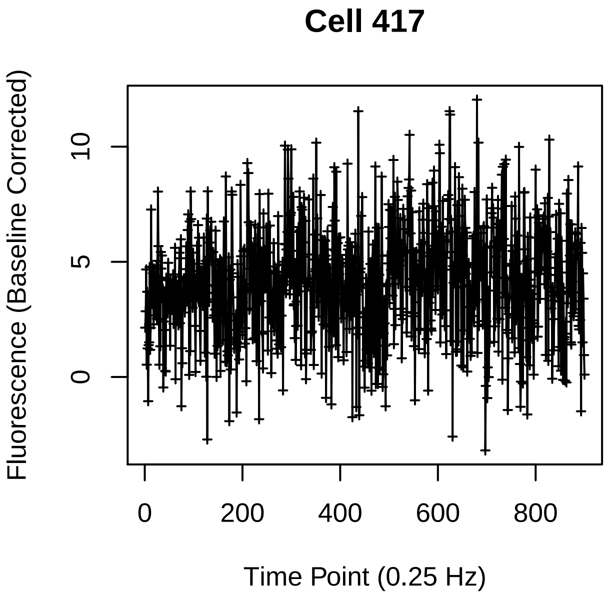
Cell 417
Fluorescence (Baseline Corrected)
0
5
10
0
200
400
600
800
Time Point (0.25 Hz)

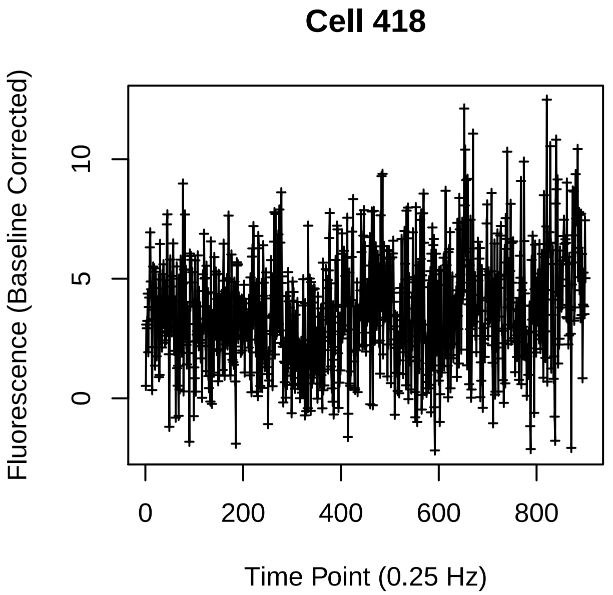
Cell 418
Fluorescence (Baseline Corrected)
0
5
10
0
200
400
600
800
Time Point (0.25 Hz)

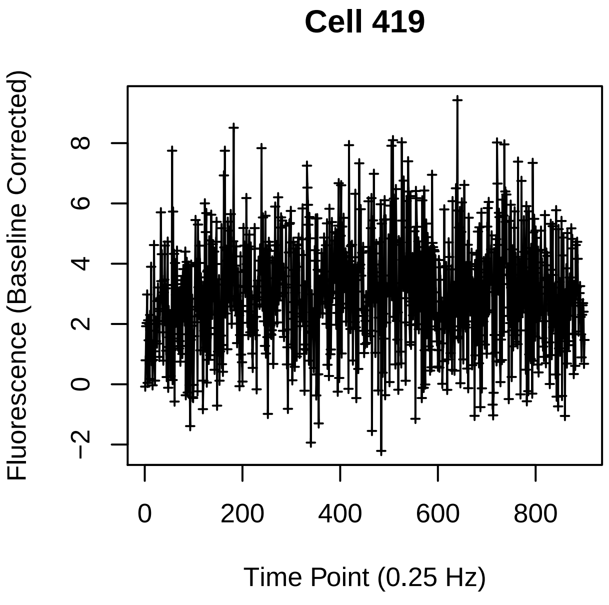
Cell 419
Fluorescence (Baseline Corrected)
−2
0
2
4
6
8
0
200
400
600
800
Time Point (0.25 Hz)

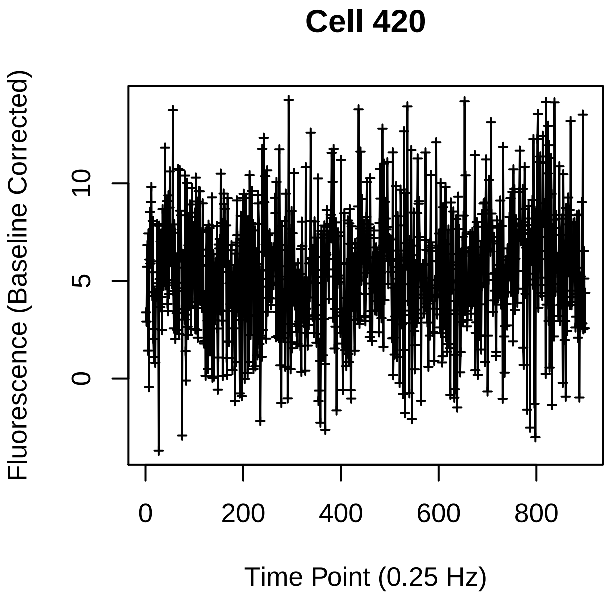
Cell 420
Fluorescence (Baseline Corrected)
0
5
10
0
200
400
600
800
Time Point (0.25 Hz)

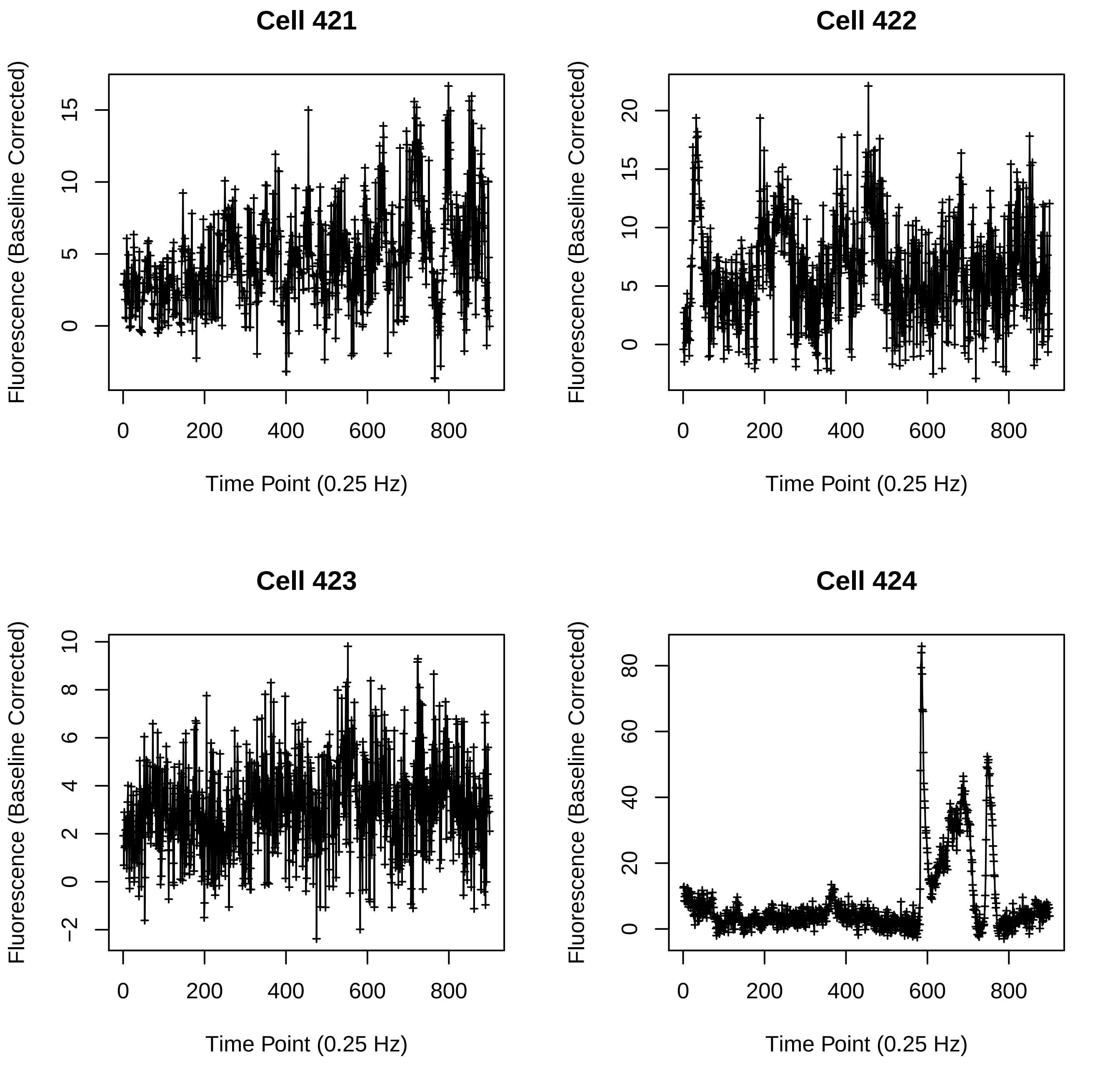

Cell 421
Cell 422
Cell 423
Cell 424
Fluorescence (Baseline Corrected)
Time Point (0.25 Hz)
0
200
400
600
800
-2
0
2
4
5
6
8
10
15
20
40
60
80

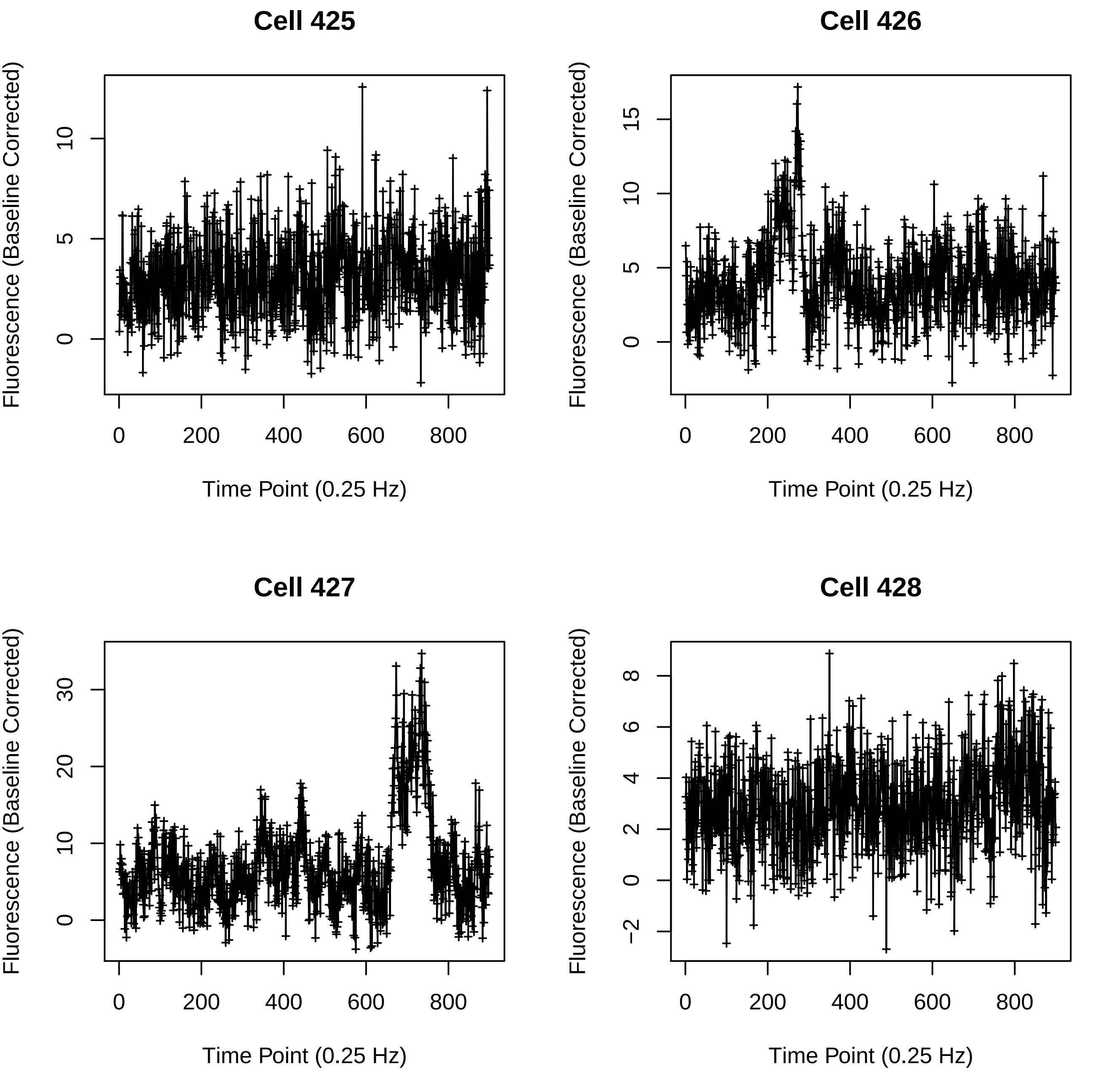

Cell 425
Fluorescence (Baseline Corrected)
0
5
10
0
200
400
600
800
Time Point (0.25 Hz)
Cell 426
Fluorescence (Baseline Corrected)
0
5
10
15
0
200
400
600
800
Time Point (0.25 Hz)
Cell 427
Fluorescence (Baseline Corrected)
0
10
20
30
0
200
400
600
800
Time Point (0.25 Hz)
Cell 428
Fluorescence (Baseline Corrected)
−2
0
2
4
6
8
0
200
400
600
800
Time Point (0.25 Hz)

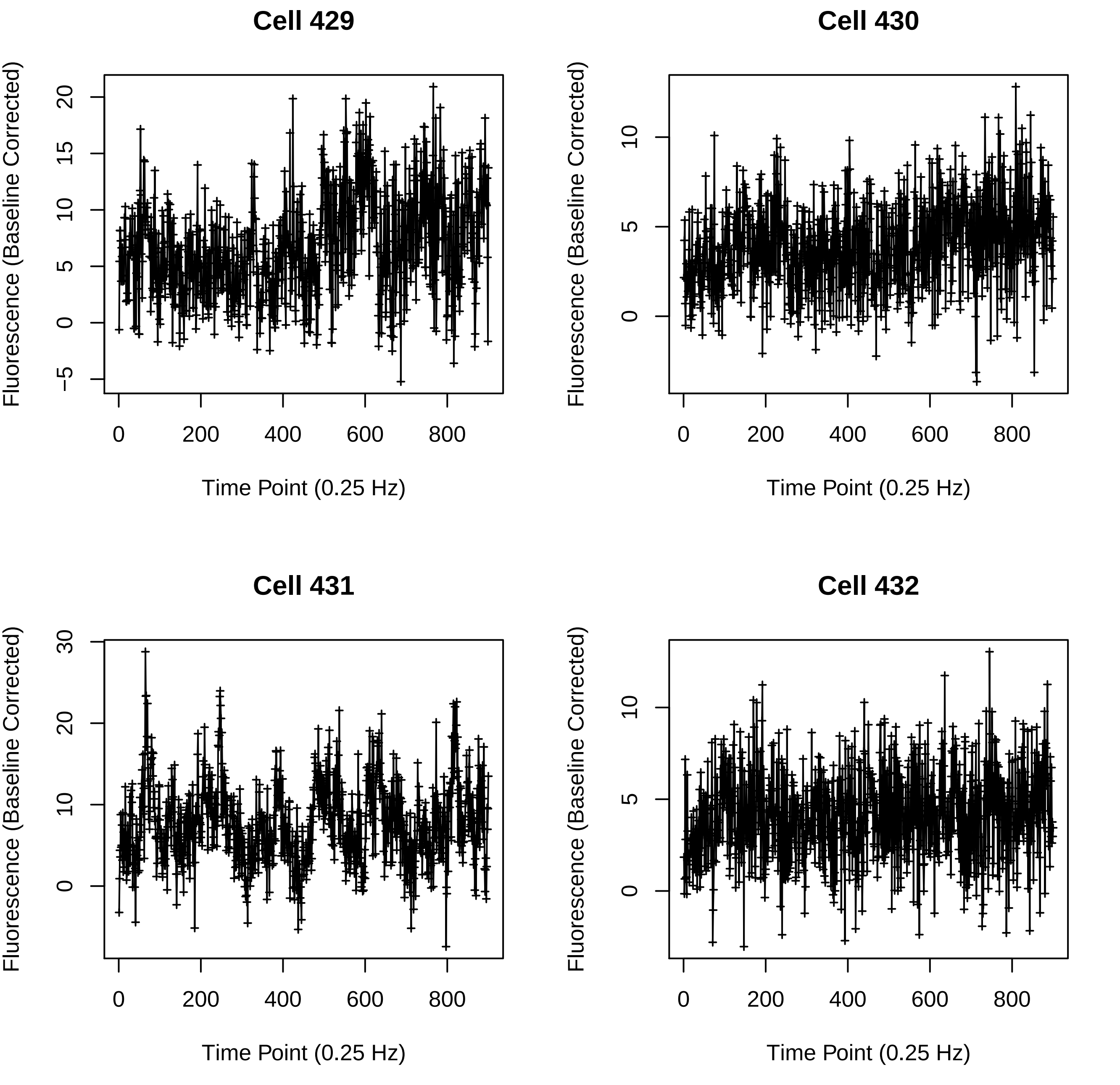
Cell 429
Fluorescence (Baseline Corrected)
Time Point (0.25 Hz)
Cell 430
Fluorescence (Baseline Corrected)
Time Point (0.25 Hz)
Cell 431
Fluorescence (Baseline Corrected)
Time Point (0.25 Hz)
Cell 432
Fluorescence (Baseline Corrected)
Time Point (0.25 Hz)

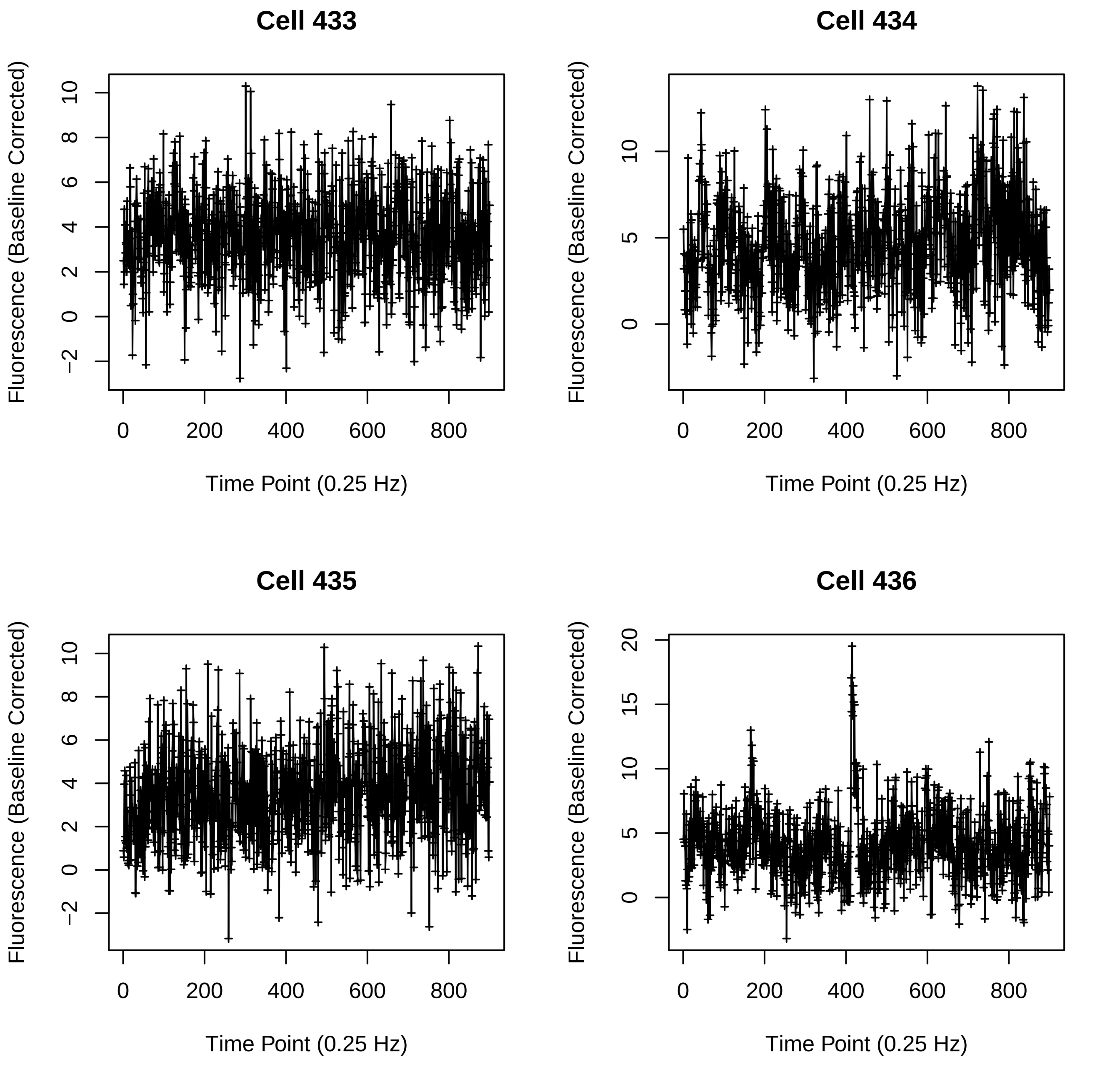

Cell 433
Cell 434
Cell 435
Cell 436
Fluorescence (Baseline Corrected)
Time Point (0.25 Hz)
0
200
400
600
800
−2
0
2
4
6
8
10
5
15
20

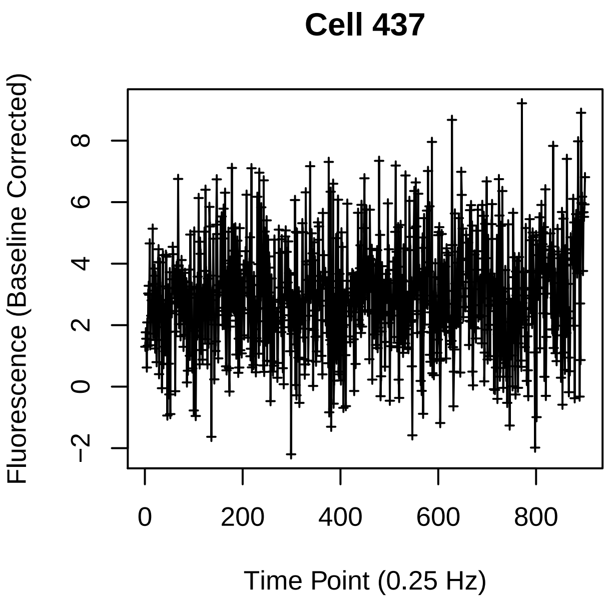
Cell 437
Fluorescence (Baseline Corrected)
Time Point (0.25 Hz)
−2
0
2
4
6
8
0
200
400
600
800
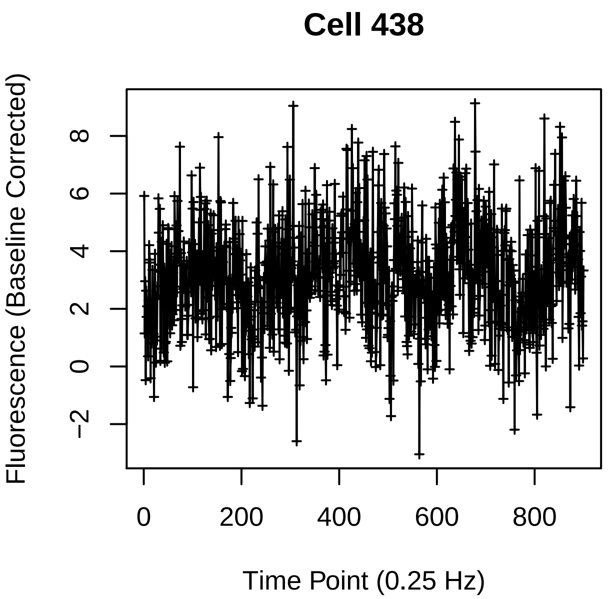
Cell 438
Fluorescence (Baseline Corrected)
Time Point (0.25 Hz)
−2
0
2
4
6
8
0
200
400
600
800
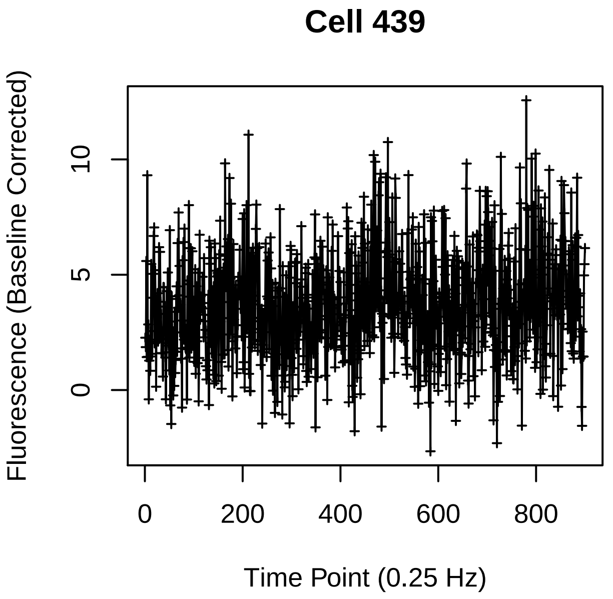
Cell 439
Fluorescence (Baseline Corrected)
Time Point (0.25 Hz)
0
5
10
0
200
400
600
800
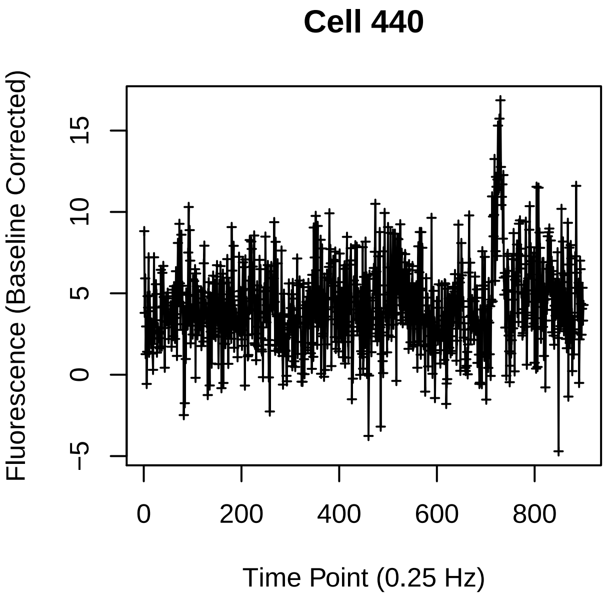
Cell 440
Fluorescence (Baseline Corrected)
Time Point (0.25 Hz)
−5
0
5
10
15
0
200
400
600
800

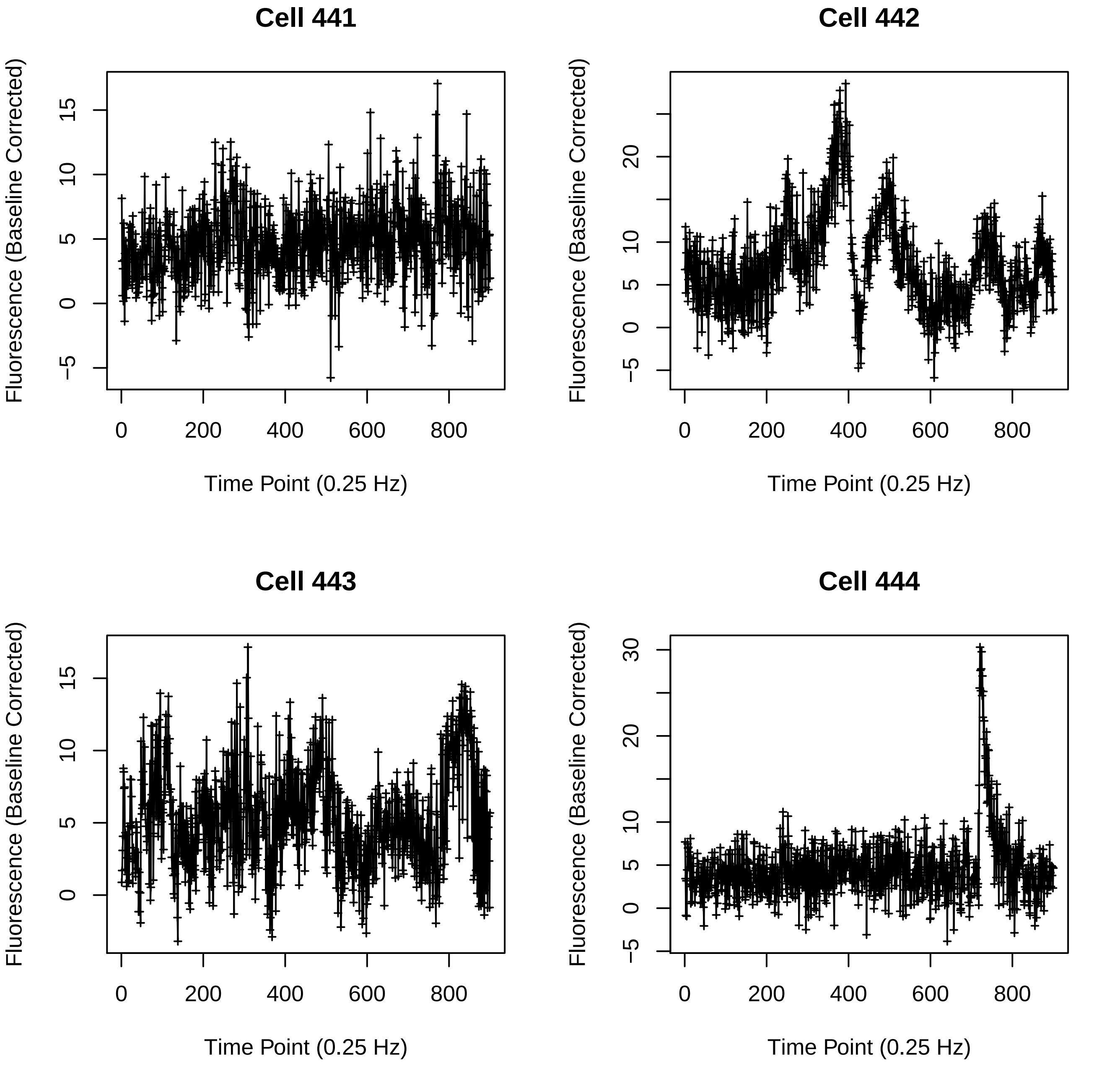

Cell 441
Cell 442
Cell 443
Cell 444
Fluorescence (Baseline Corrected)
Time Point (0.25 Hz)
0
200
400
600
800
−5
0
5
10
15
20
25
30

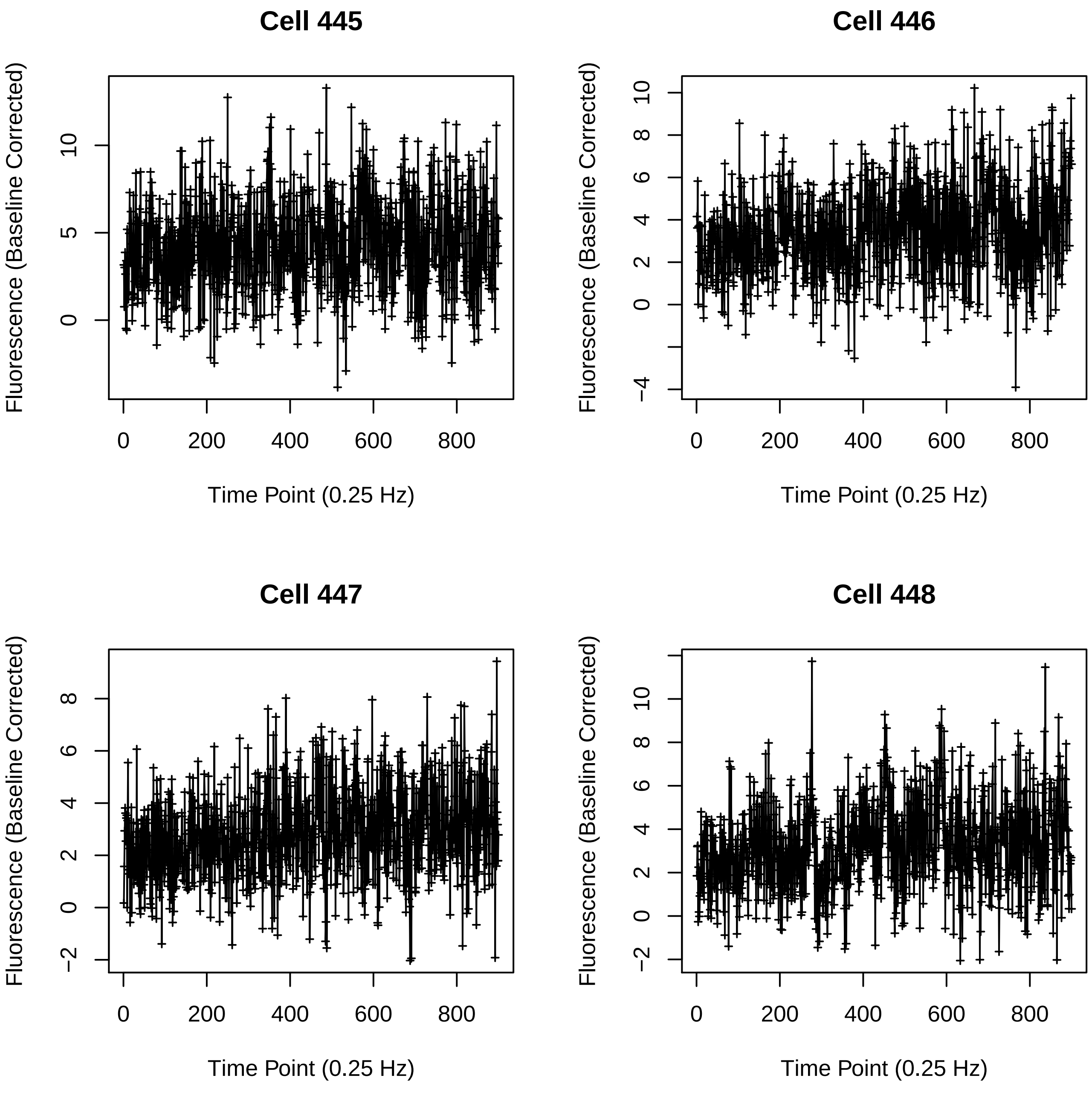
Cell 445
Cell 446
Cell 447
Cell 448
Fluorescence (Baseline Corrected)
Time Point (0.25 Hz)
0
200
400
600
800
-4
-2
0
2
4
5
6
8
10

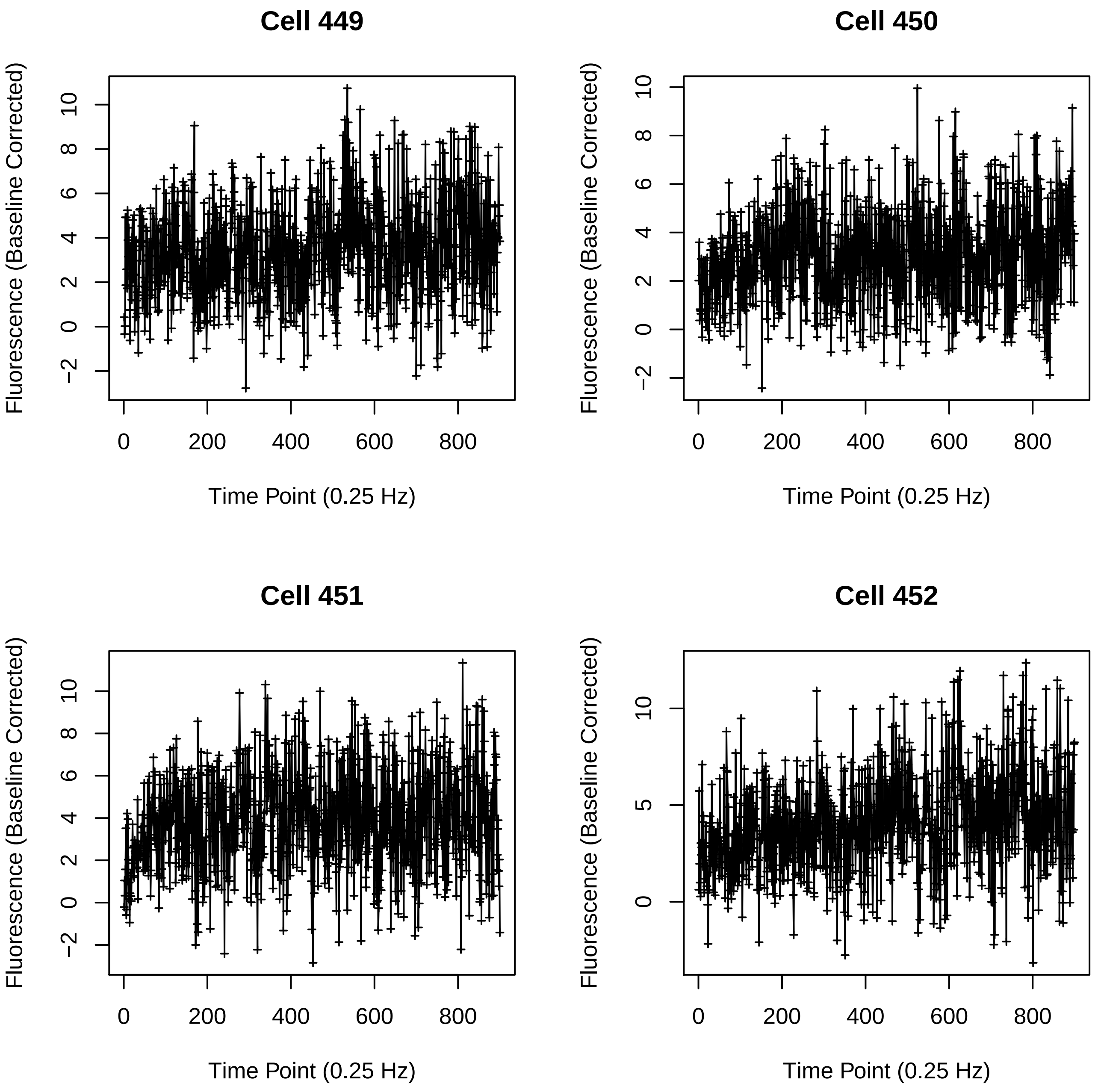
Cell 449
Cell 450
Cell 451
Cell 452
Fluorescence (Baseline Corrected)
Time Point (0.25 Hz)
0
200
400
600
800
−2
0
2
4
6
8
10
5

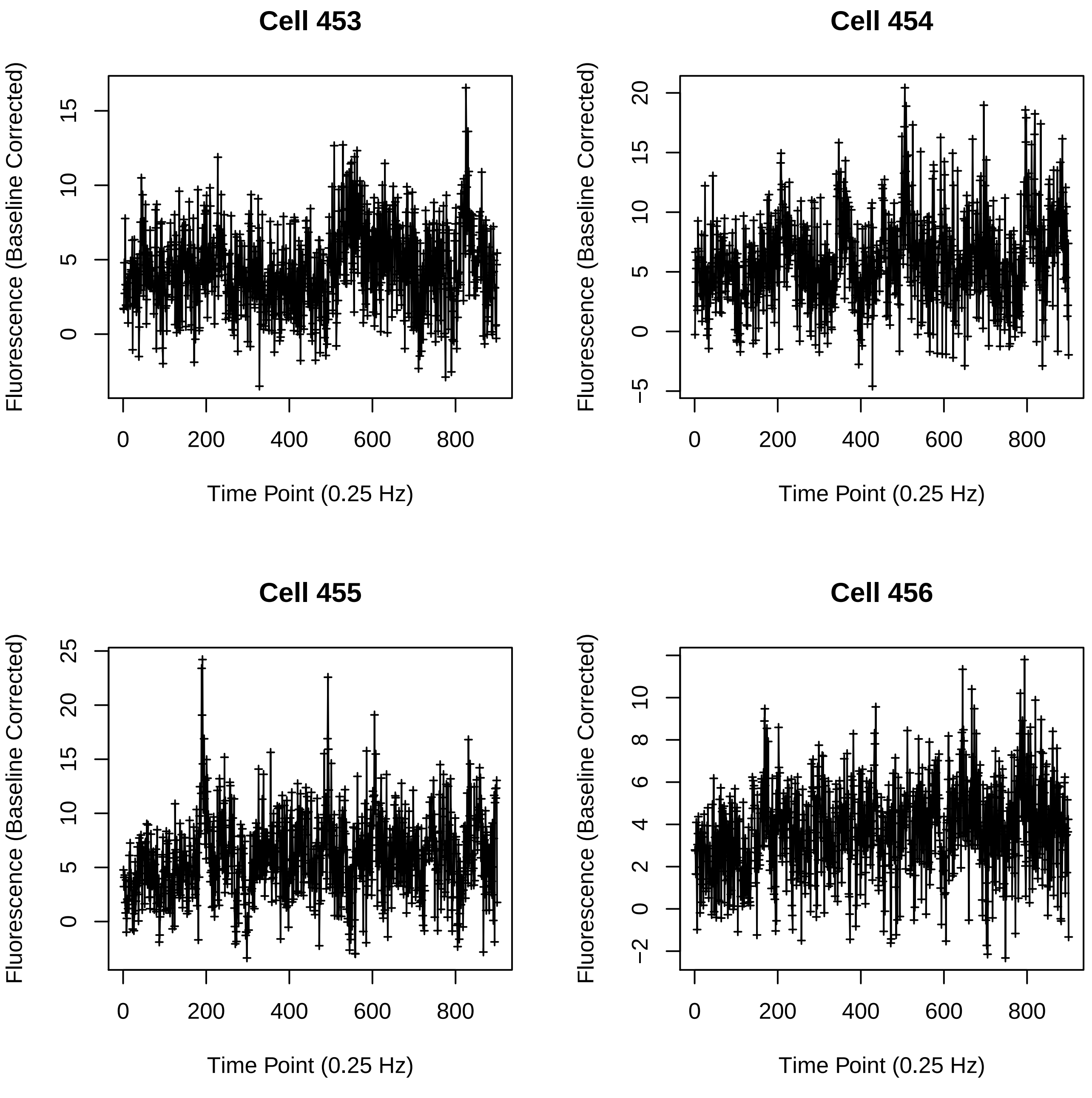
Cell 453
Fluorescence (Baseline Corrected)
0
5
10
15
0
200
400
600
800
Time Point (0.25 Hz)
Cell 454
Fluorescence (Baseline Corrected)
−5
0
5
10
15
20
0
200
400
600
800
Time Point (0.25 Hz)
Cell 455
Fluorescence (Baseline Corrected)
0
5
10
15
20
25
0
200
400
600
800
Time Point (0.25 Hz)
Cell 456
Fluorescence (Baseline Corrected)
−2
0
2
4
6
8
10
0
200
400
600
800
Time Point (0.25 Hz)

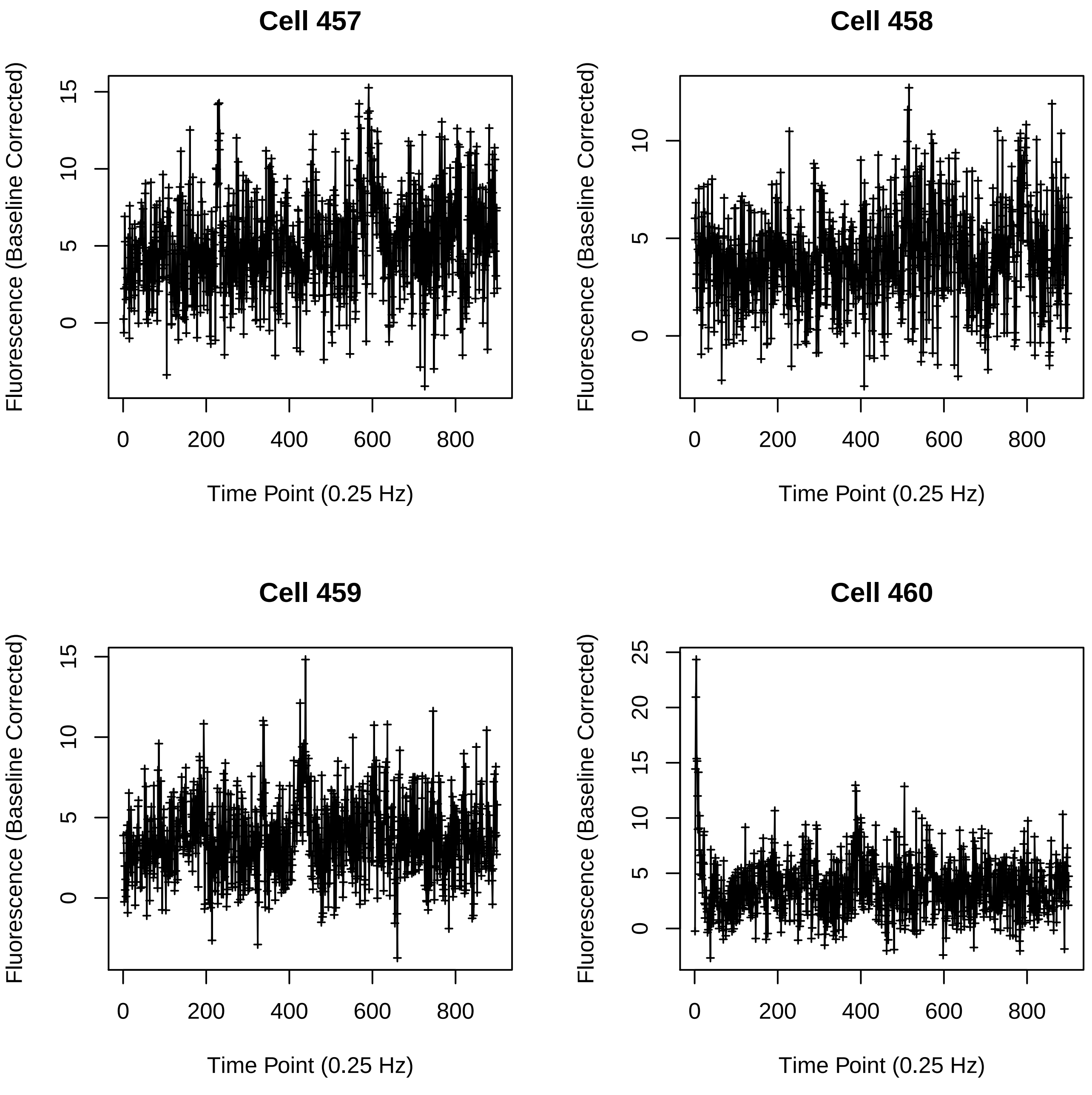
Cell 457
Cell 458
Cell 459
Cell 460
Fluorescence (Baseline Corrected)
Time Point (0.25 Hz)
0
200
400
600
800
5
10
15
20
25

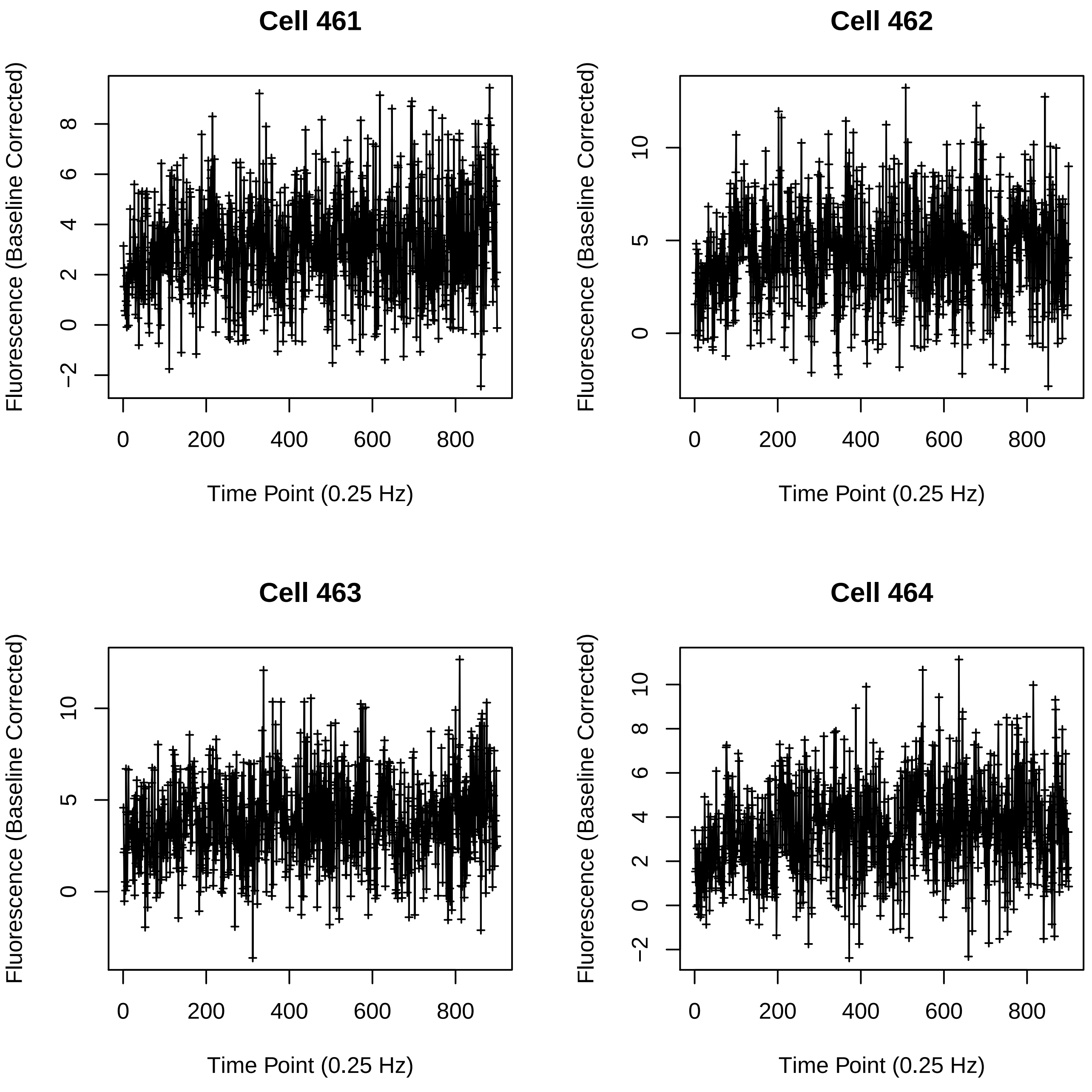
Cell 461
Cell 462
Cell 463
Cell 464
Fluorescence (Baseline Corrected)
Time Point (0.25 Hz)
0
200
400
600
800
−2
2
4
6
8
5
10

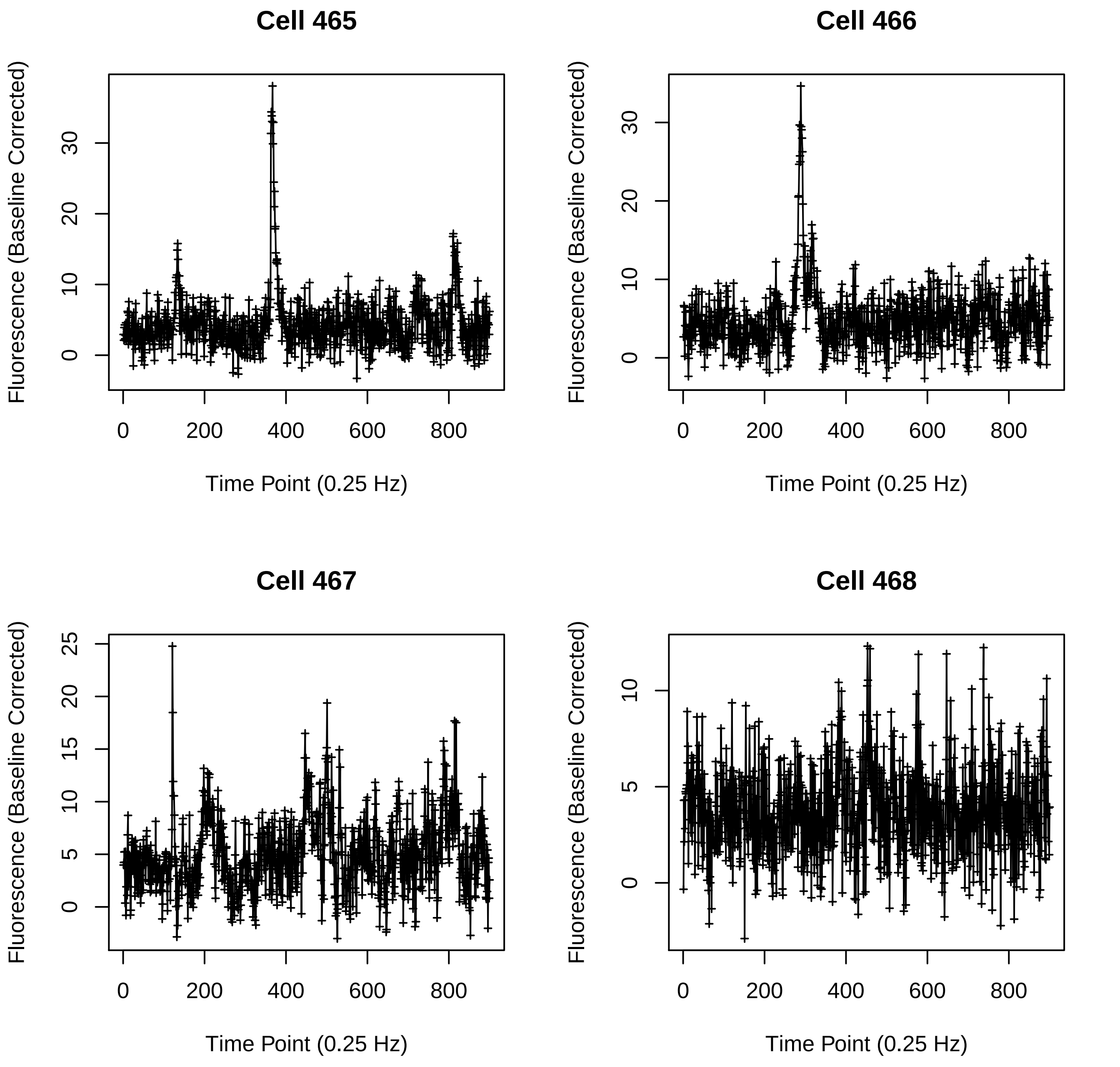
Cell 465
Cell 466
Cell 467
Cell 468
Fluorescence (Baseline Corrected)
Time Point (0.25 Hz)
0
200
400
600
800
10
20
30
5
15
25

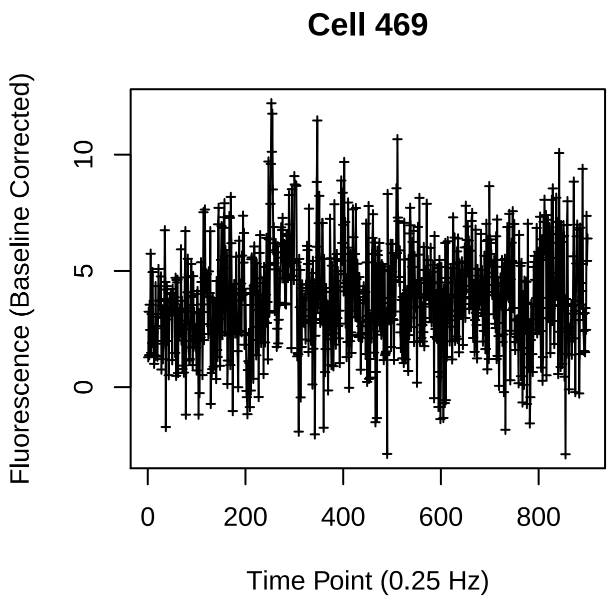
Cell 469
Fluorescence (Baseline Corrected)
0
5
10
0
200
400
600
800
Time Point (0.25 Hz)

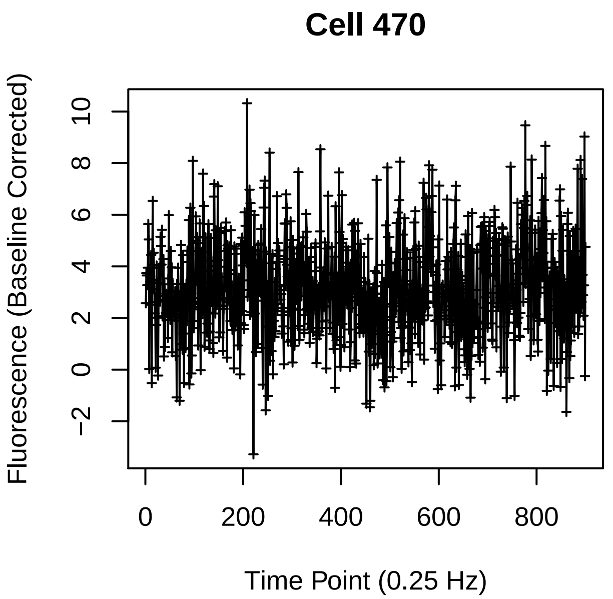
Cell 470
Fluorescence (Baseline Corrected)
−2
0
2
4
6
8
10
0
200
400
600
800
Time Point (0.25 Hz)

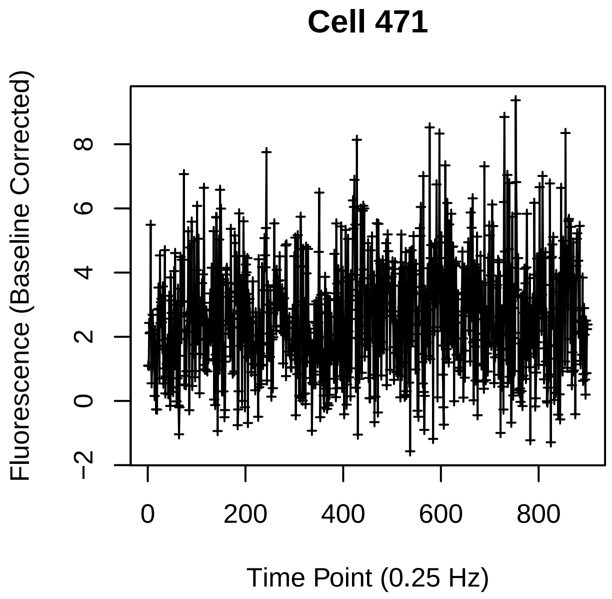
Cell 471
Fluorescence (Baseline Corrected)
−2
0
2
4
6
8
0
200
400
600
800
Time Point (0.25 Hz)

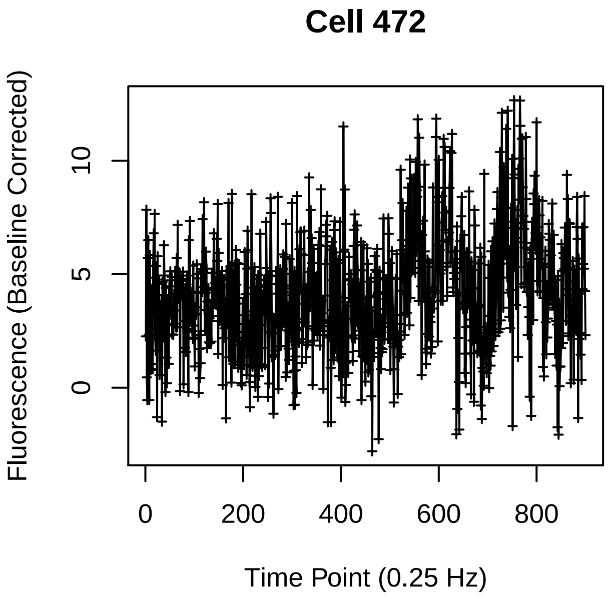
Cell 472
Fluorescence (Baseline Corrected)
0
5
10
0
200
400
600
800
Time Point (0.25 Hz)

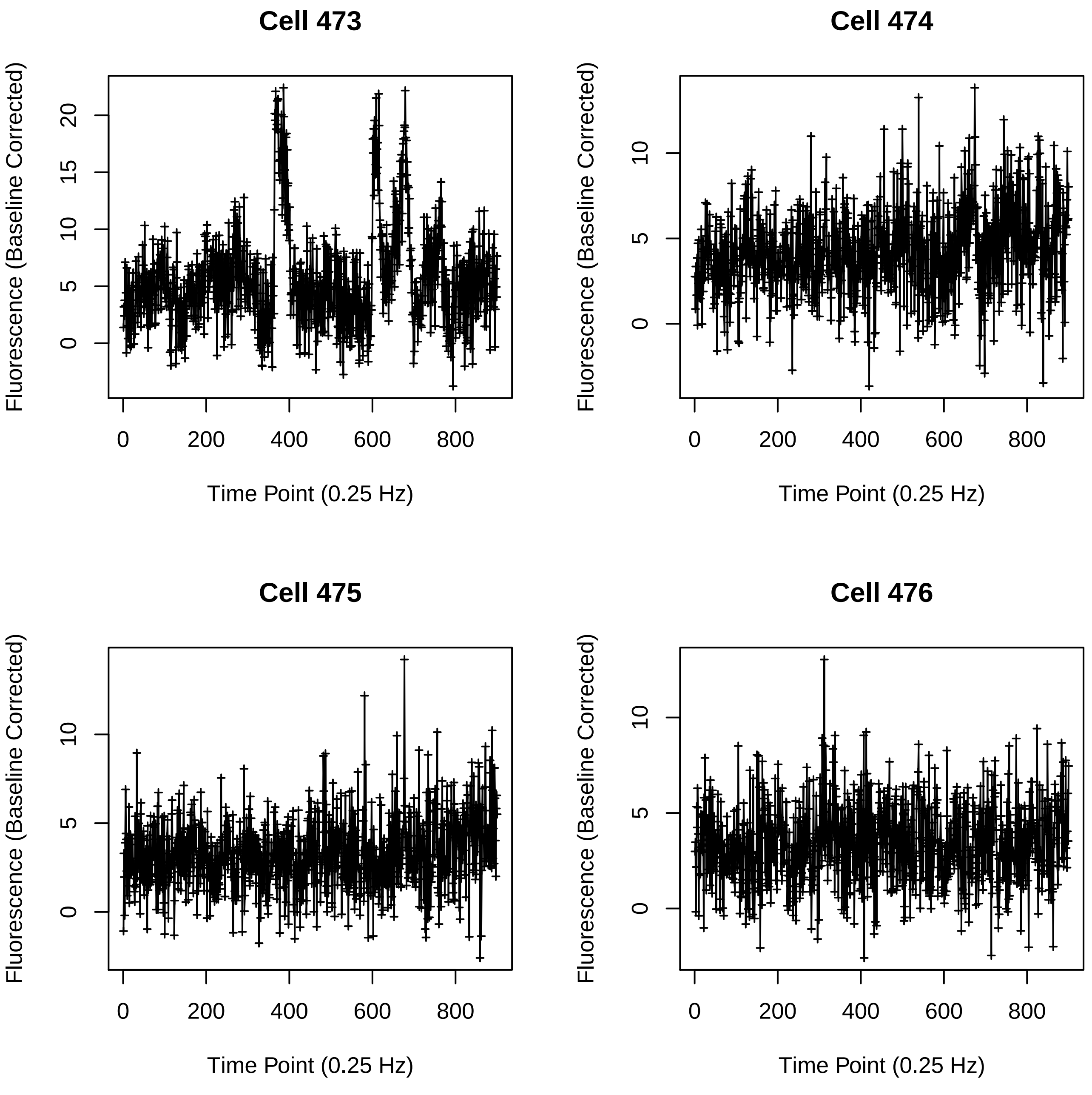
Cell 473
Fluorescence (Baseline Corrected)
0
5
10
15
20
0
200
400
600
800
Time Point (0.25 Hz)
Cell 474
Fluorescence (Baseline Corrected)
0
5
10
0
200
400
600
800
Time Point (0.25 Hz)
Cell 475
Fluorescence (Baseline Corrected)
0
5
10
0
200
400
600
800
Time Point (0.25 Hz)
Cell 476
Fluorescence (Baseline Corrected)
0
5
10
0
200
400
600
800
Time Point (0.25 Hz)

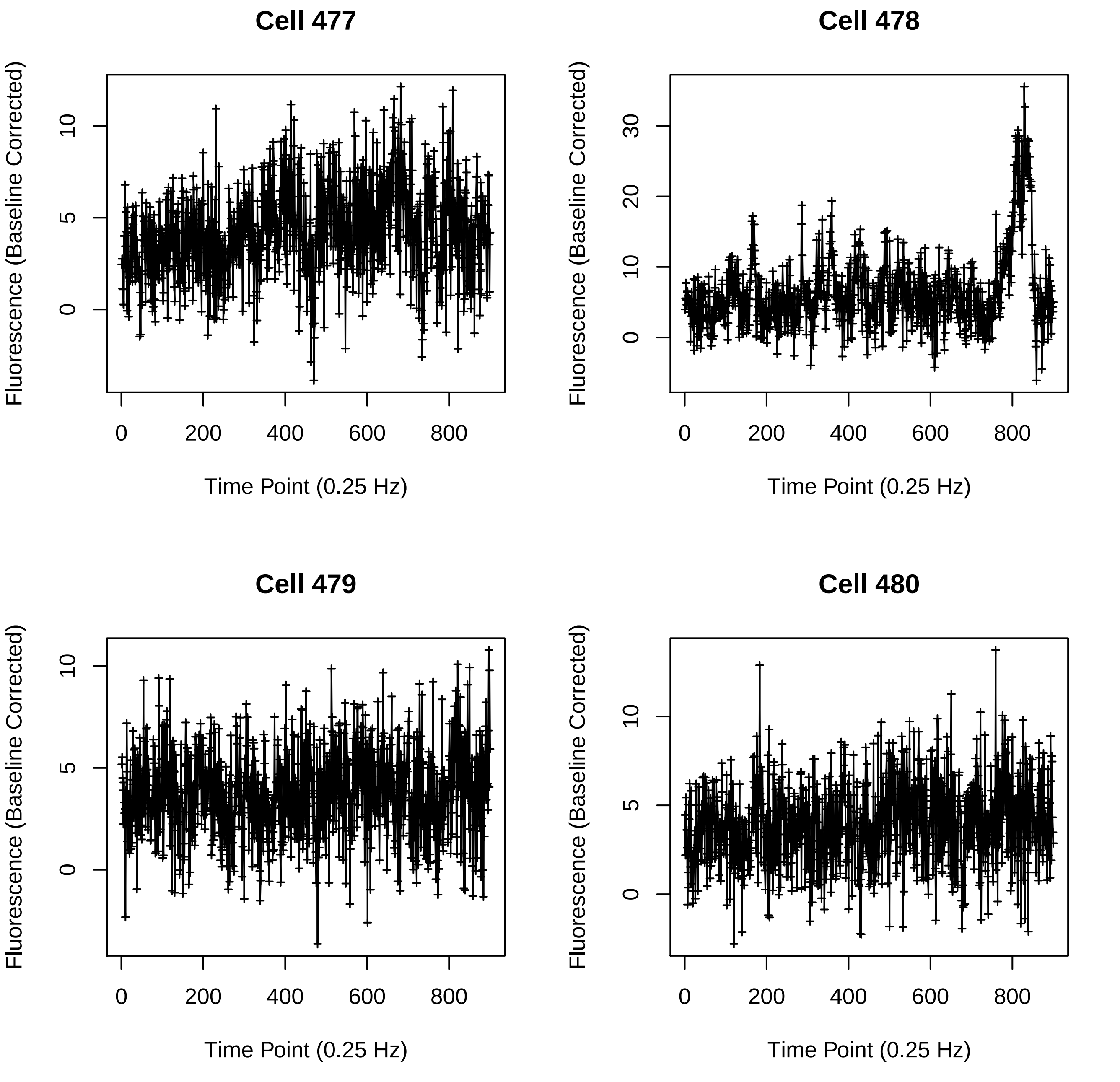
Cell 477
Cell 478
Cell 479
Cell 480
Fluorescence (Baseline Corrected)
Time Point (0.25 Hz)
0
200
400
600
800
5
10
20
30

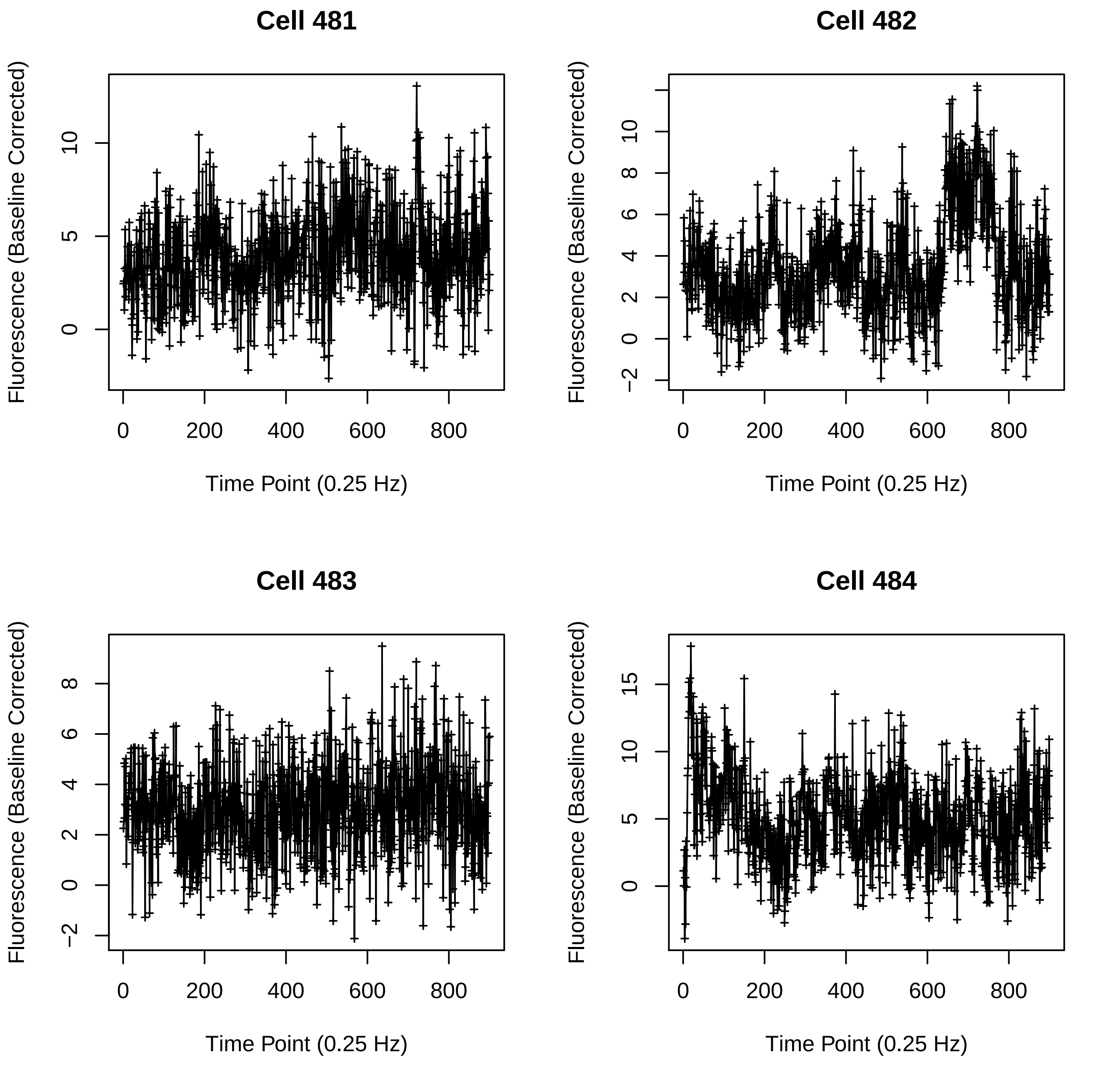
Cell 481
Cell 482
Cell 483
Cell 484
Fluorescence (Baseline Corrected)
Time Point (0.25 Hz)
0
200
400
600
800
0
5
10
15
−2
2
4
6
8

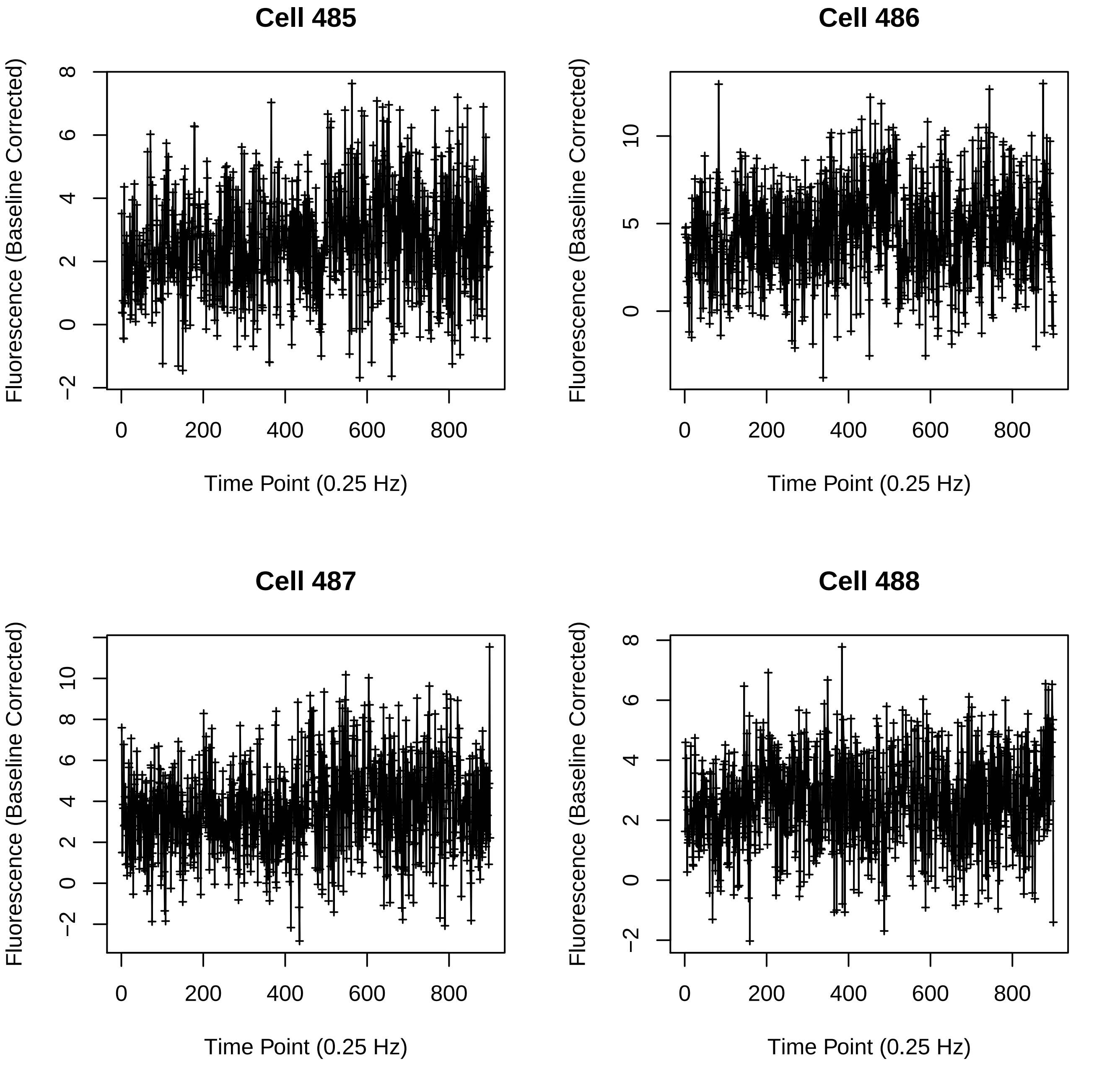
Cell 485
Cell 486
Cell 487
Cell 488
Fluorescence (Baseline Corrected)
Time Point (0.25 Hz)
0
200
400
600
800
−2
2
4
6
8
5
10

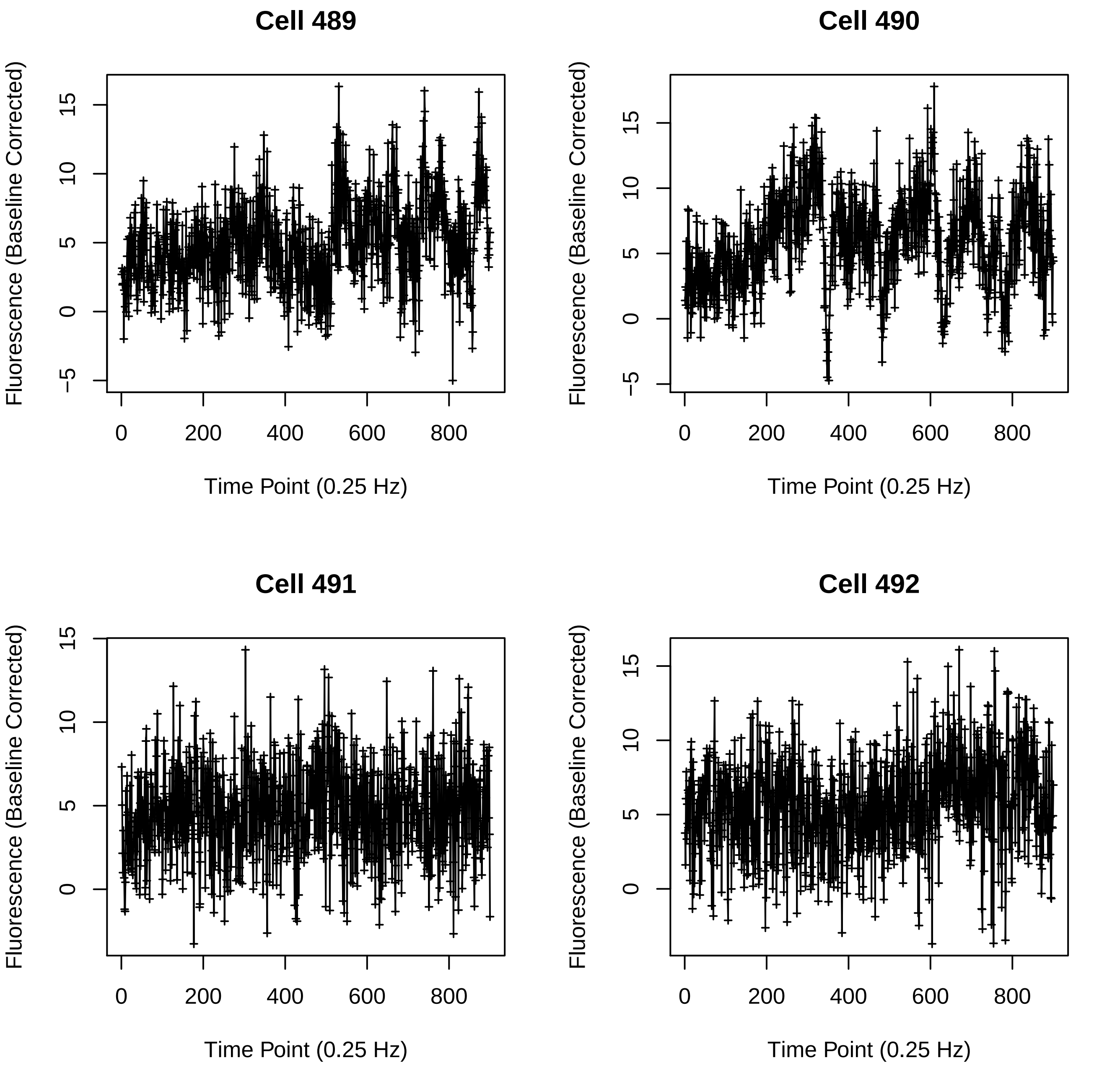
Cell 489
Cell 490
Cell 491
Cell 492
Fluorescence (Baseline Corrected)
Time Point (0.25 Hz)
0
200
400
600
800
−5
0
5
10
15

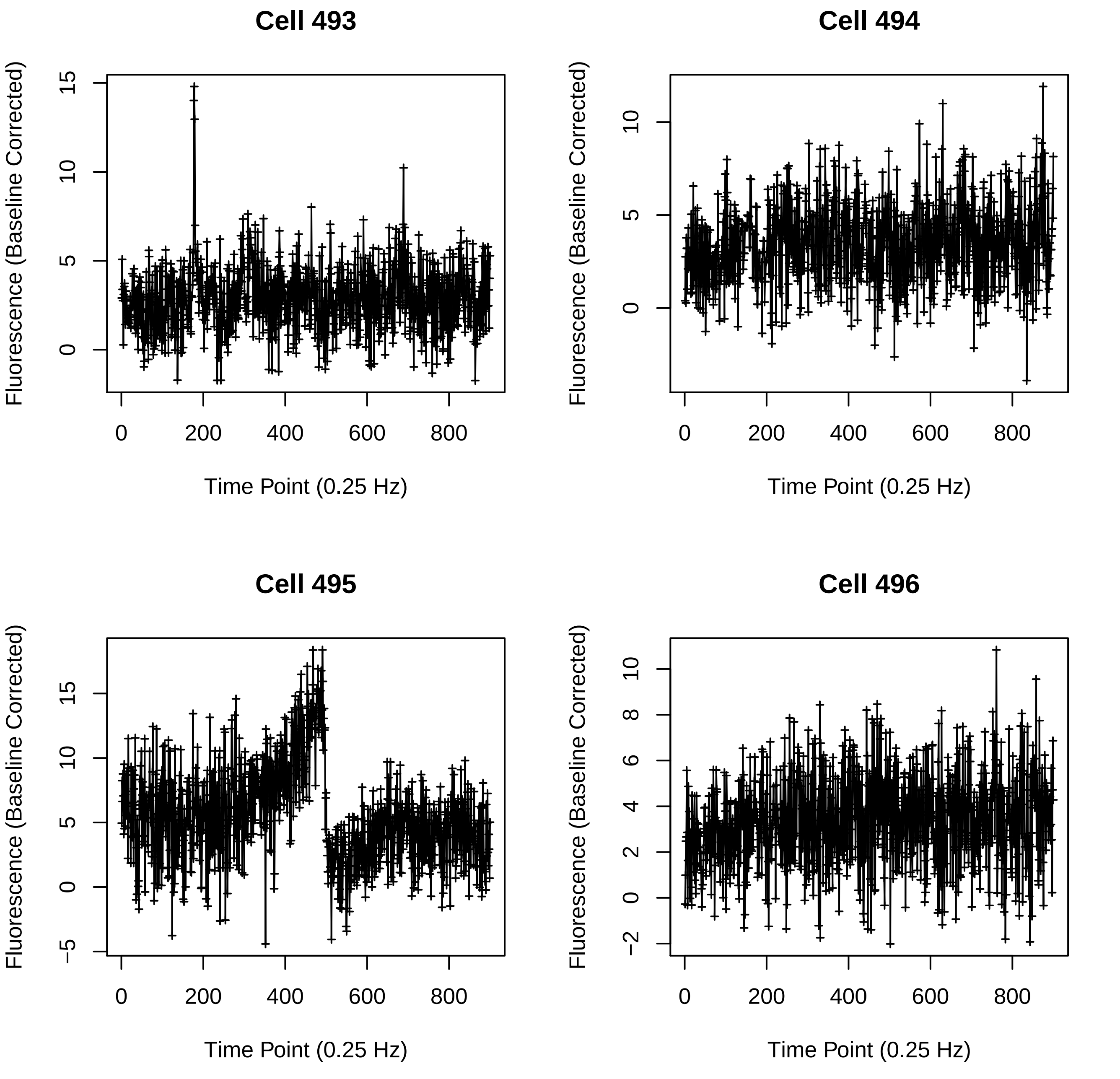
Cell 493
Fluorescence (Baseline Corrected)
Time Point (0.25 Hz)
Cell 494
Fluorescence (Baseline Corrected)
Time Point (0.25 Hz)
Cell 495
Fluorescence (Baseline Corrected)
Time Point (0.25 Hz)
Cell 496
Fluorescence (Baseline Corrected)
Time Point (0.25 Hz)

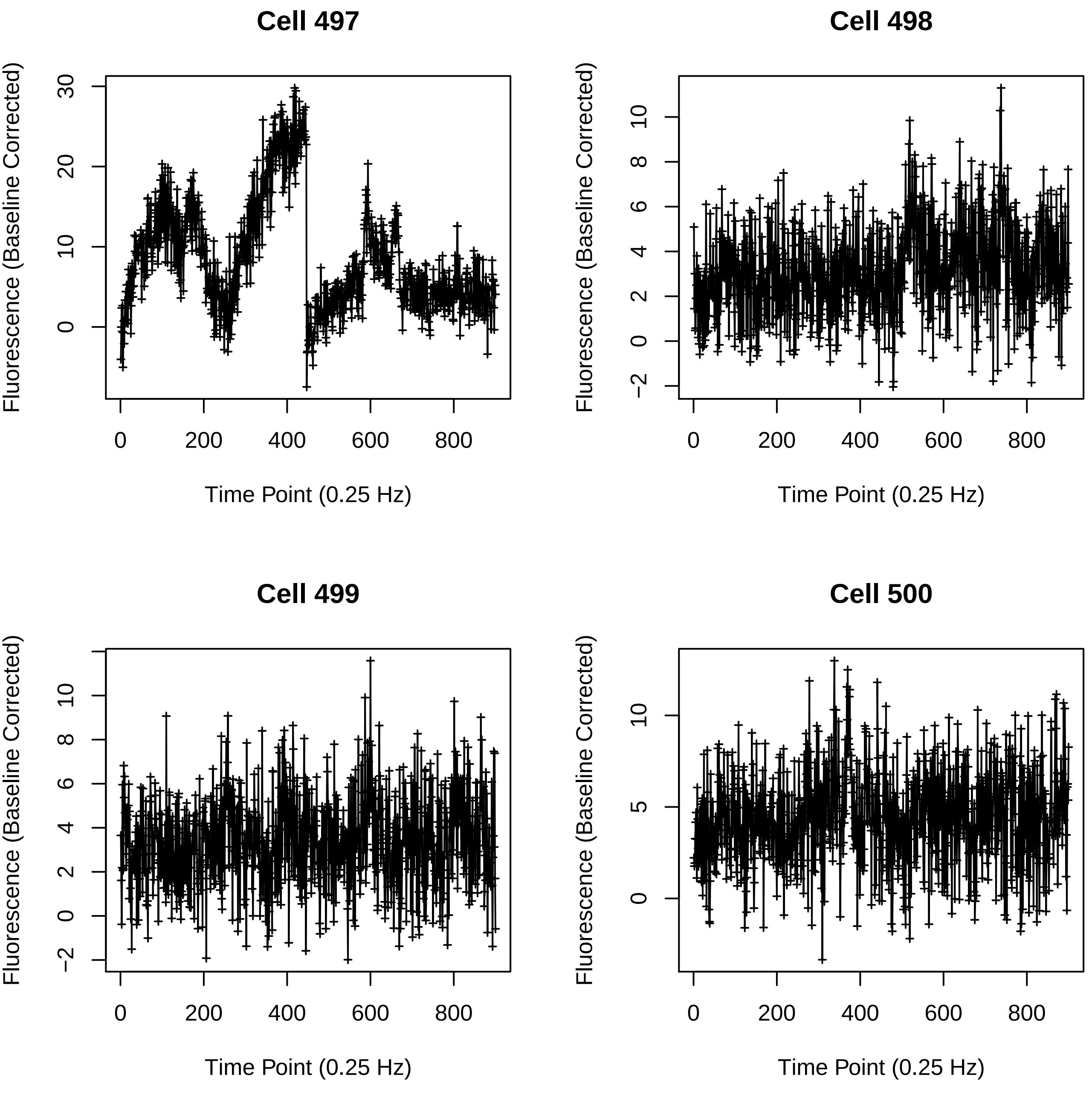
Cell 497
Cell 498
Cell 499
Cell 500
Fluorescence (Baseline Corrected)
Time Point (0.25 Hz)
0
10
20
30
−2
2
4
6
8
5
200
400
600
800

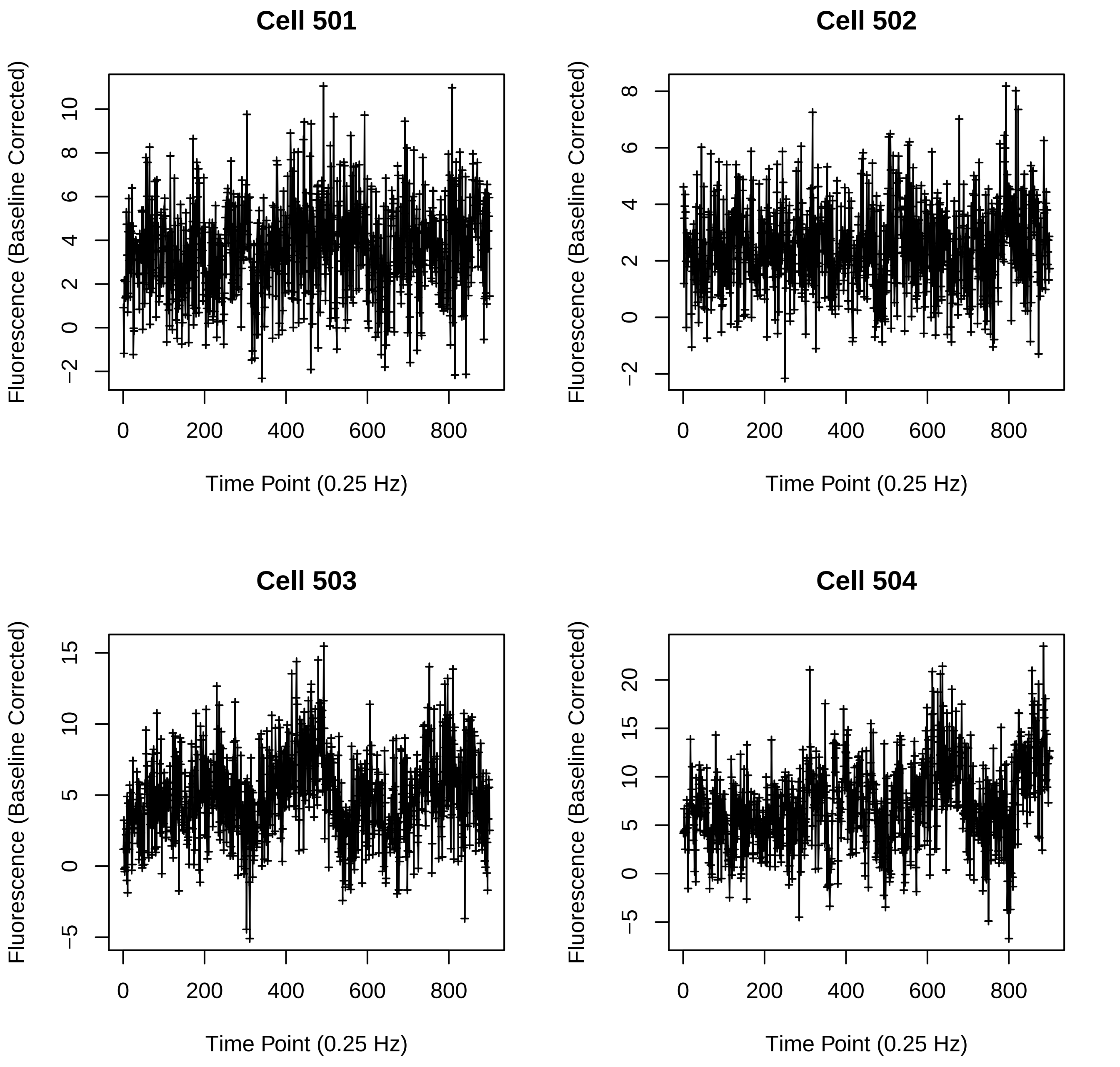

Cell 501
Fluorescence (Baseline Corrected)
−2
0
2
4
6
8
10
0
200
400
600
800
Time Point (0.25 Hz)
Cell 502
Fluorescence (Baseline Corrected)
−2
0
2
4
6
8
0
200
400
600
800
Time Point (0.25 Hz)
Cell 503
Fluorescence (Baseline Corrected)
−5
0
5
10
15
0
200
400
600
800
Time Point (0.25 Hz)
Cell 504
Fluorescence (Baseline Corrected)
−5
0
5
10
15
20
0
200
400
600
800
Time Point (0.25 Hz)

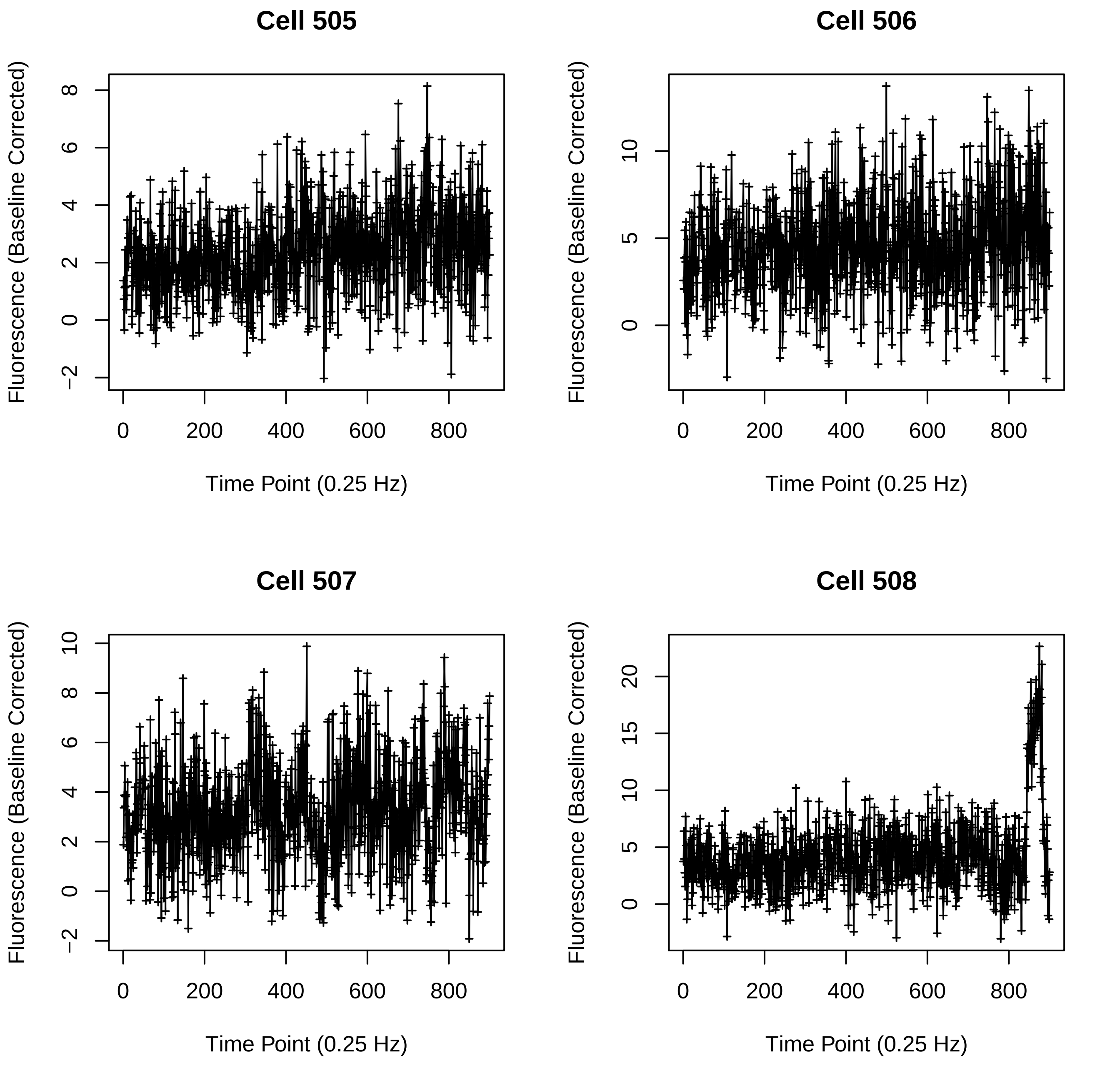

Cell 505
Cell 506
Cell 507
Cell 508
Fluorescence (Baseline Corrected)
Time Point (0.25 Hz)
0
200
400
600
800
-2
2
4
6
8
5
10
15
20

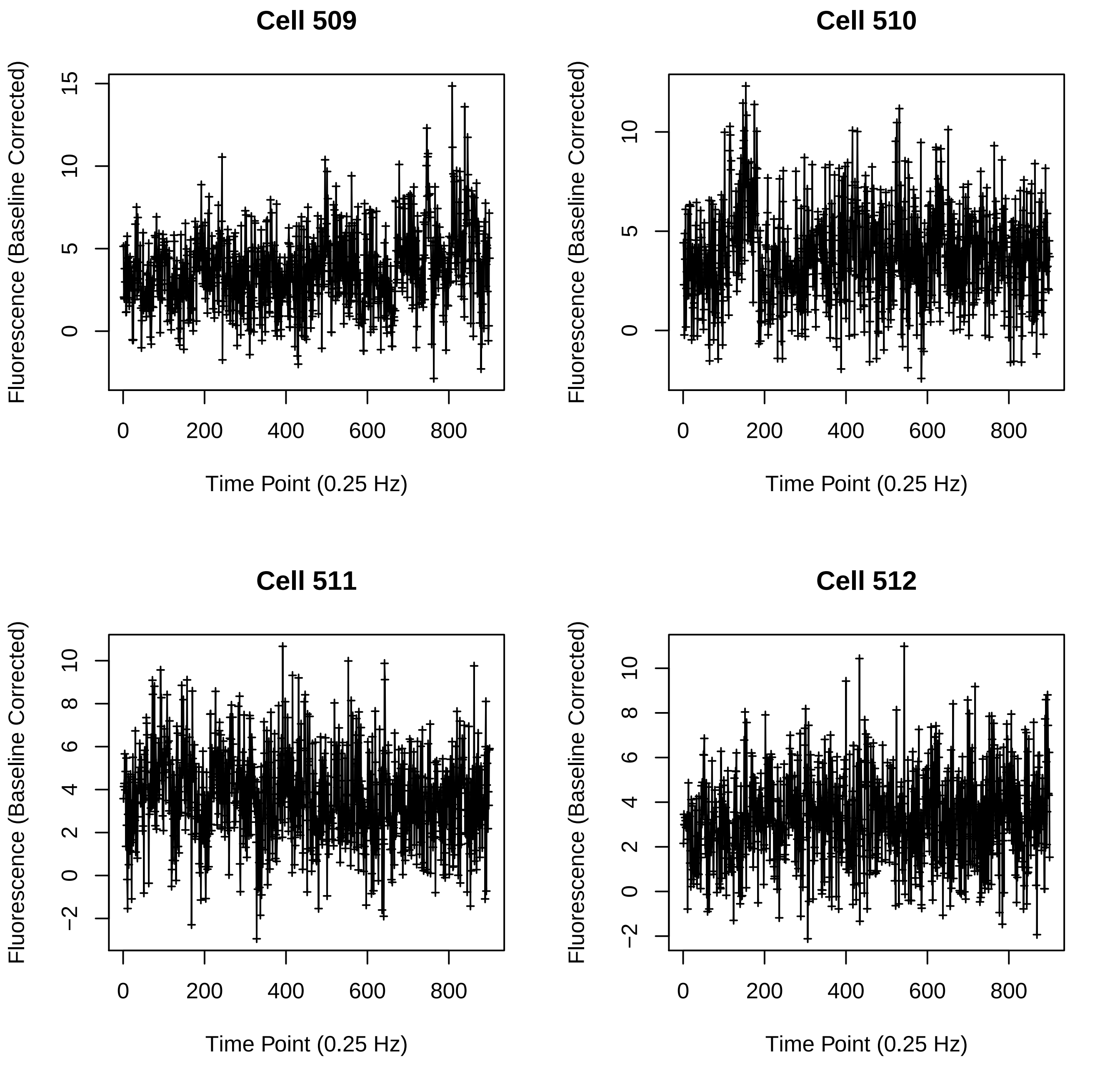
Cell 509
Cell 510
Cell 511
Cell 512
Fluorescence (Baseline Corrected)
Time Point (0.25 Hz)
0
200
400
600
800
−2
0
2
4
5
6
8
10
15

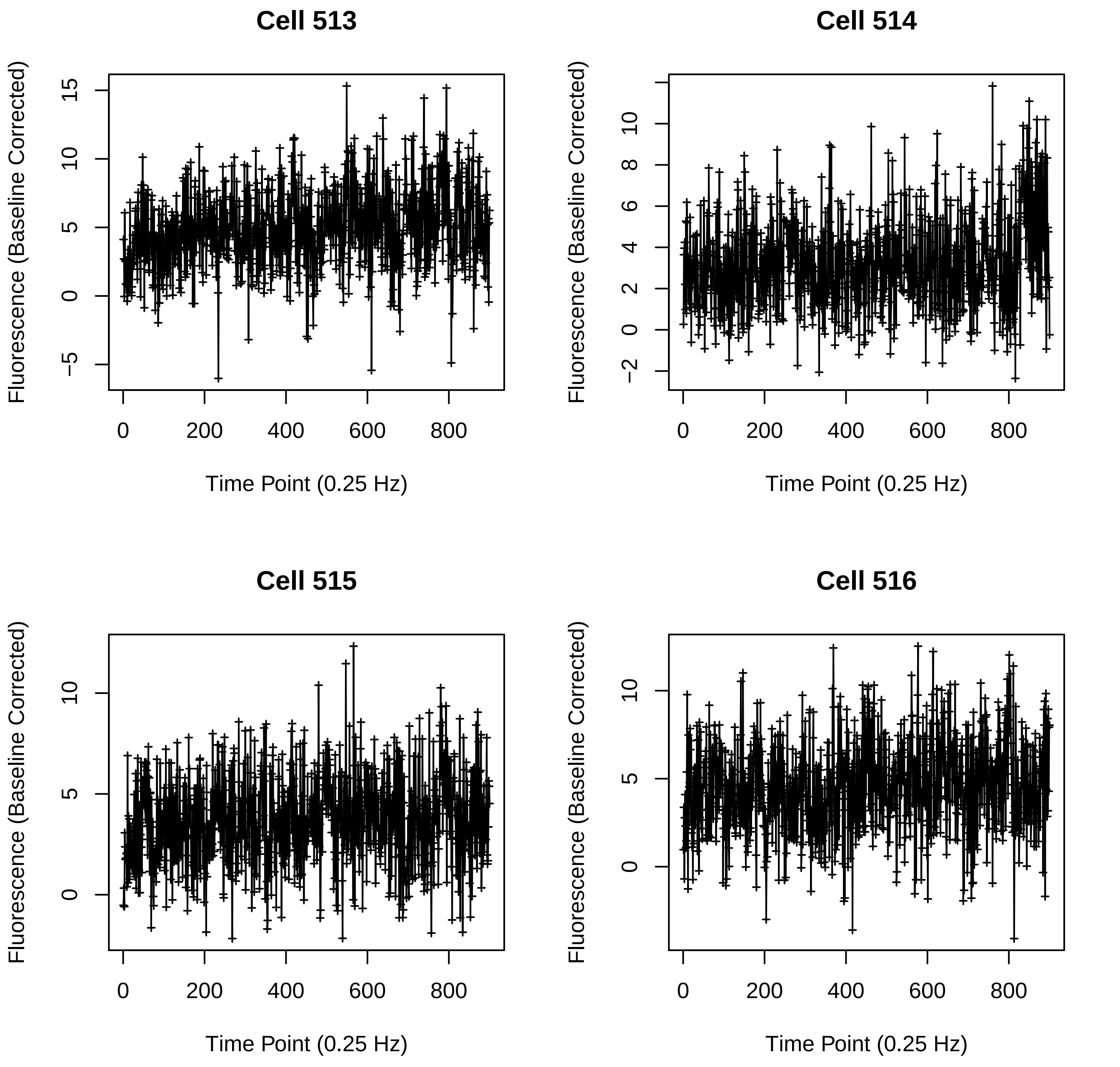
Cell 513
Cell 514
Cell 515
Cell 516
Fluorescence (Baseline Corrected)
Time Point (0.25 Hz)
−5
0
5
10
15
−2
0
2
4
6
8
10
200
400
600
800

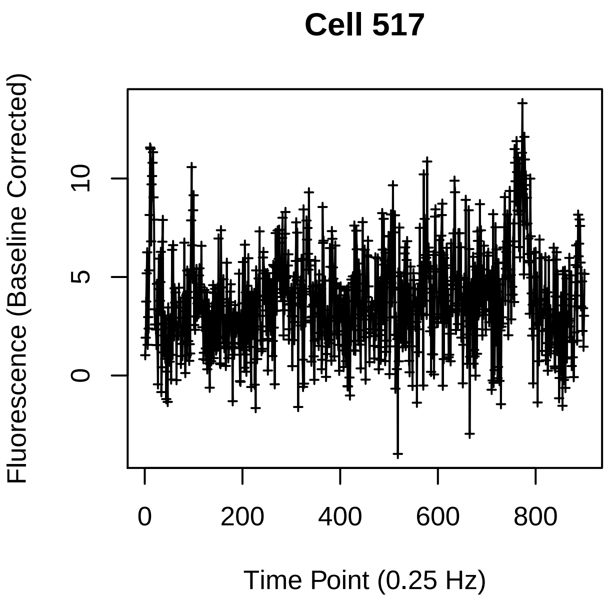
Cell 517
Fluorescence (Baseline Corrected)
0
5
10
0
200
400
600
800
Time Point (0.25 Hz)

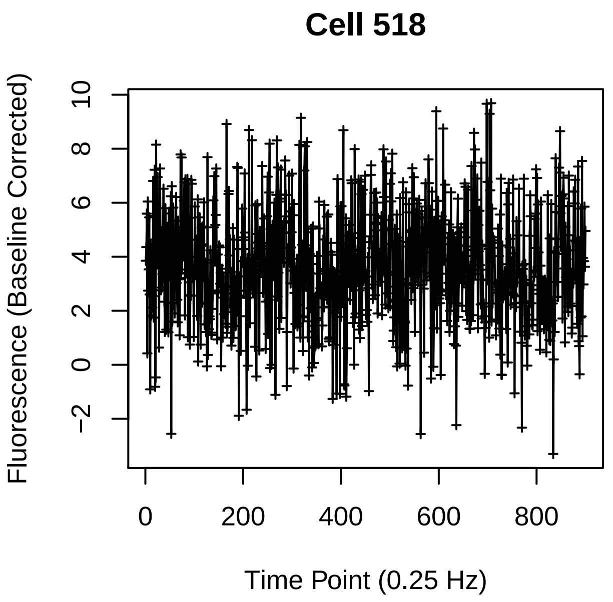
Cell 518
Fluorescence (Baseline Corrected)
−2
0
2
4
6
8
10
0
200
400
600
800
Time Point (0.25 Hz)

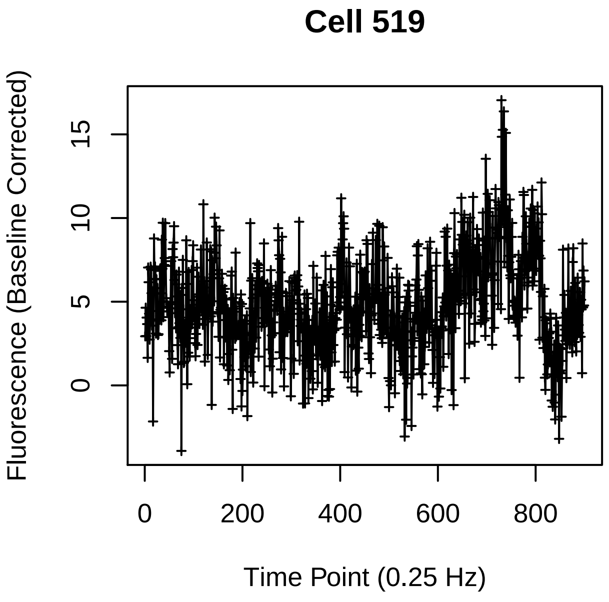
Cell 519
Fluorescence (Baseline Corrected)
0
5
10
15
0
200
400
600
800
Time Point (0.25 Hz)

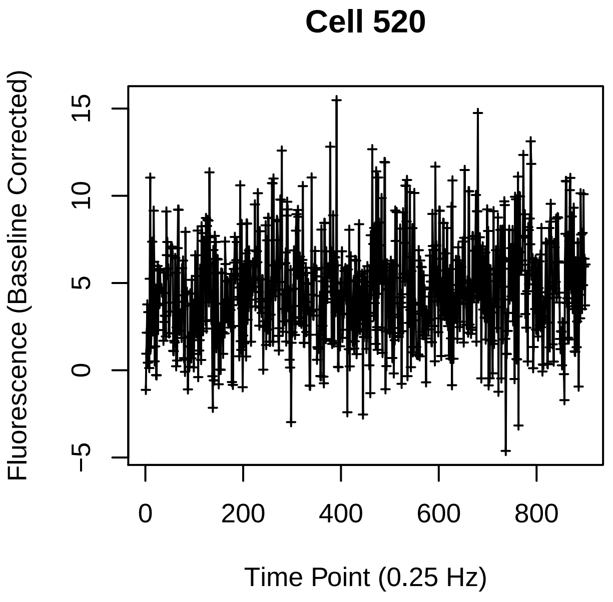
Cell 520
Fluorescence (Baseline Corrected)
−5
0
5
10
15
0
200
400
600
800
Time Point (0.25 Hz)

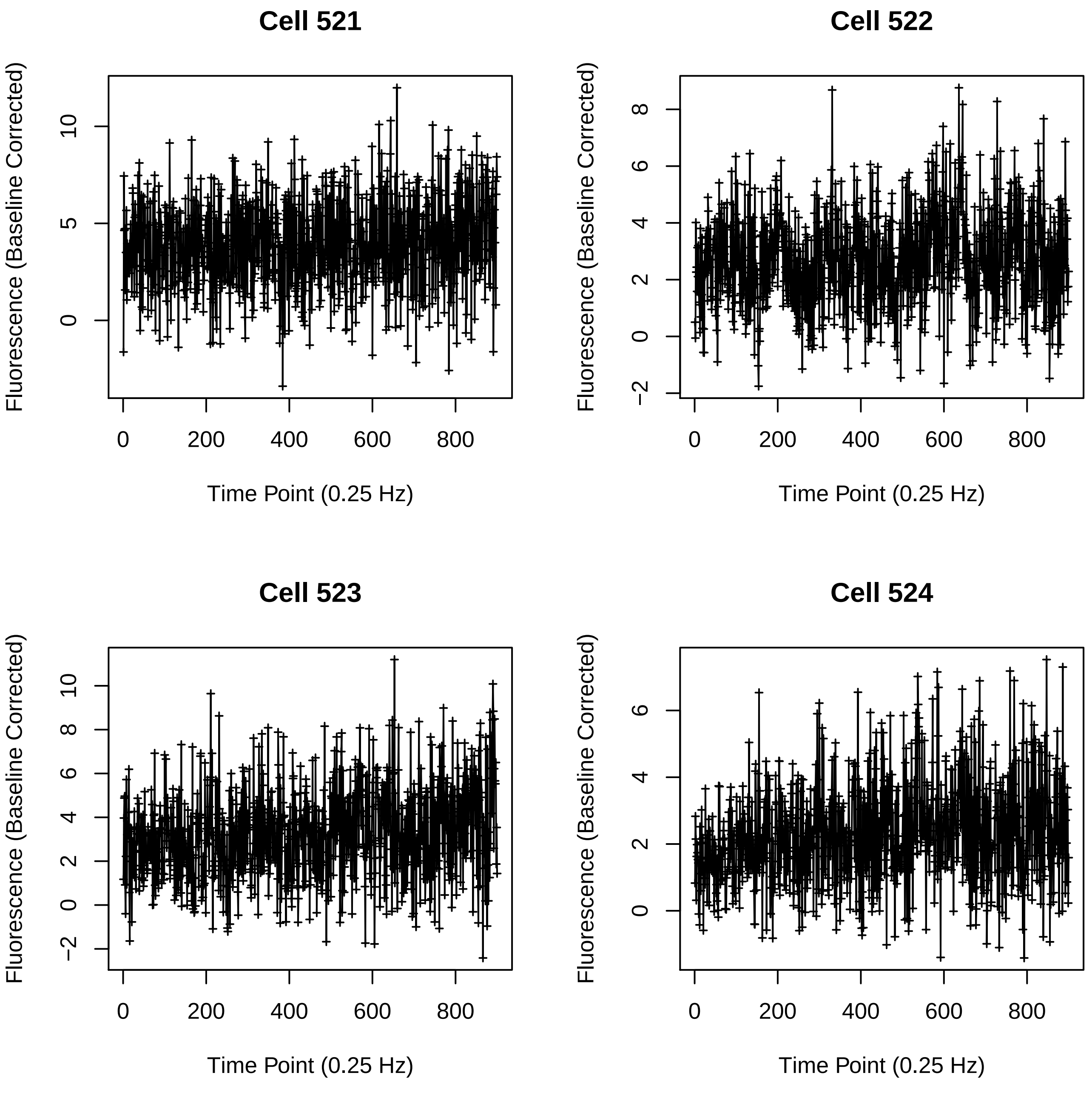
Cell 521
Cell 522
Cell 523
Cell 524
Fluorescence (Baseline Corrected)
Time Point (0.25 Hz)
0
5
10
−2
2
4
6
8
200
400
600
800

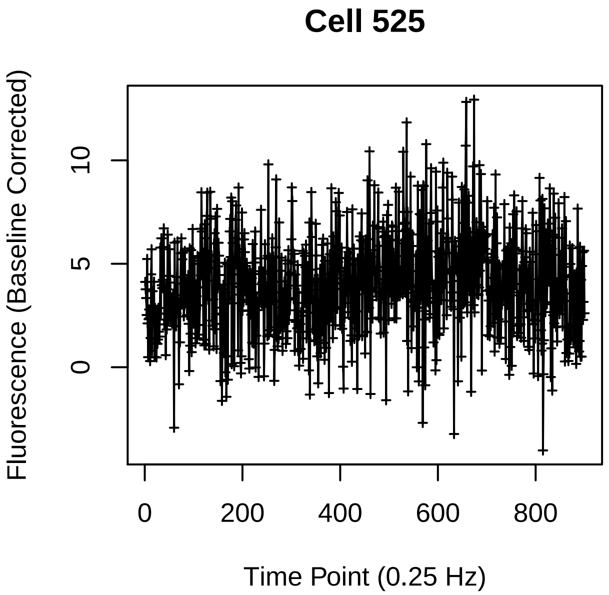
Cell 525
Fluorescence (Baseline Corrected)
Time Point (0.25 Hz)
0
5
10
0
200
400
600
800

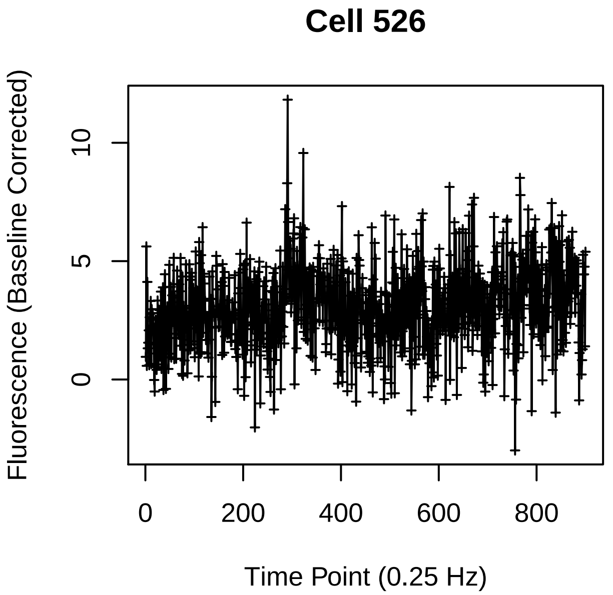
Cell 526
Fluorescence (Baseline Corrected)
Time Point (0.25 Hz)
0
5
10
0
200
400
600
800

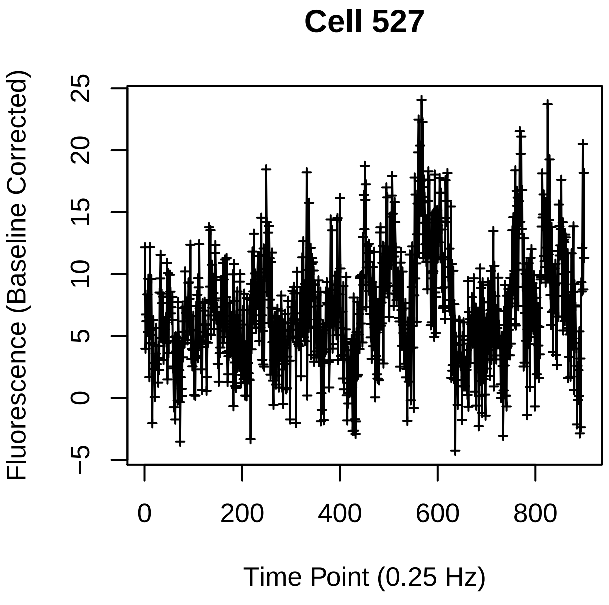
Cell 527
Fluorescence (Baseline Corrected)
Time Point (0.25 Hz)
−5
0
5
10
15
20
25
0
200
400
600
800

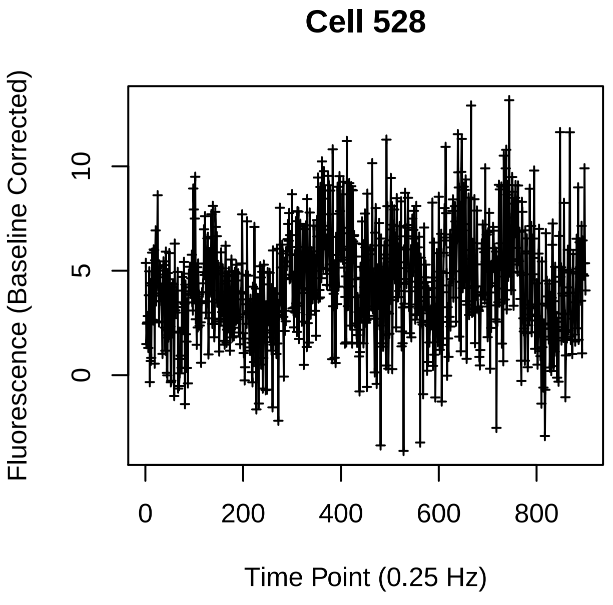
Cell 528
Fluorescence (Baseline Corrected)
Time Point (0.25 Hz)
0
5
10
0
200
400
600
800

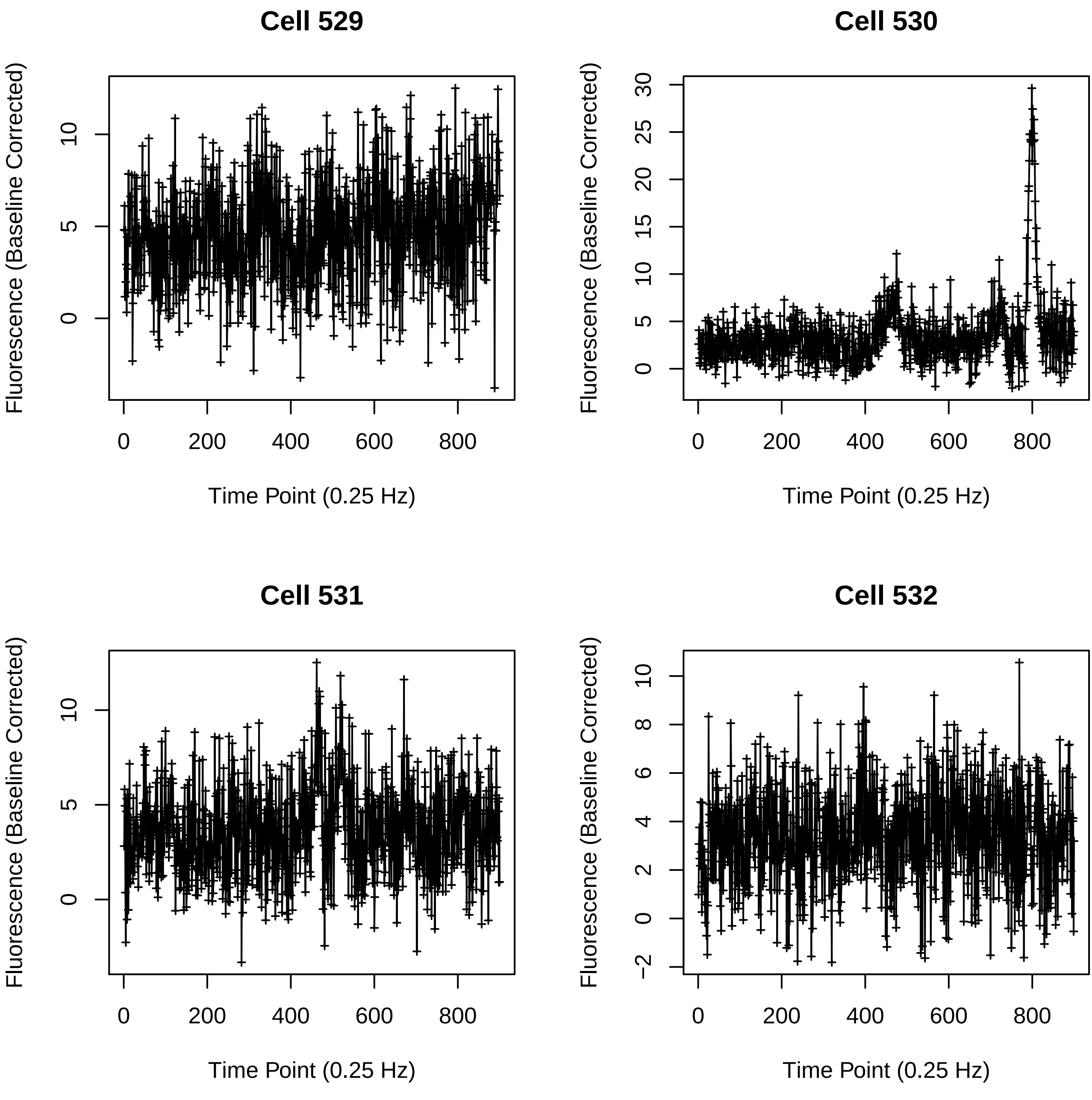

Cell 529
Cell 530
Cell 531
Cell 532
Fluorescence (Baseline Corrected)
Time Point (0.25 Hz)
0
200
400
600
800
-5
0
5
10
15
20
25
30
-2
2
4
6
8

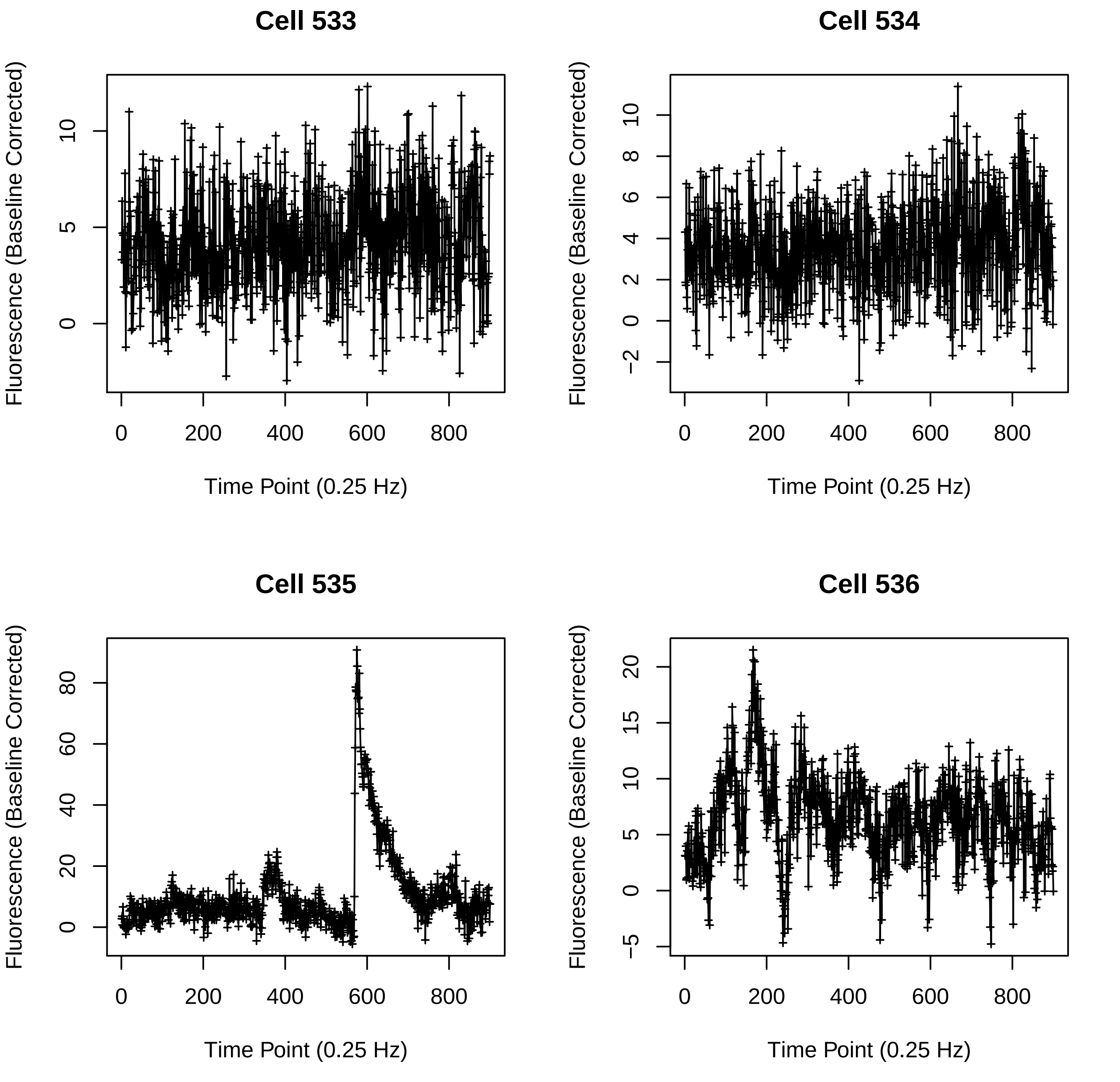
Cell 533
Cell 534
Cell 535
Cell 536
Fluorescence (Baseline Corrected)
Time Point (0.25 Hz)
0
200
400
600
800
-5
0
5
10
15
20
-2
2
4
6
8
40
60
80

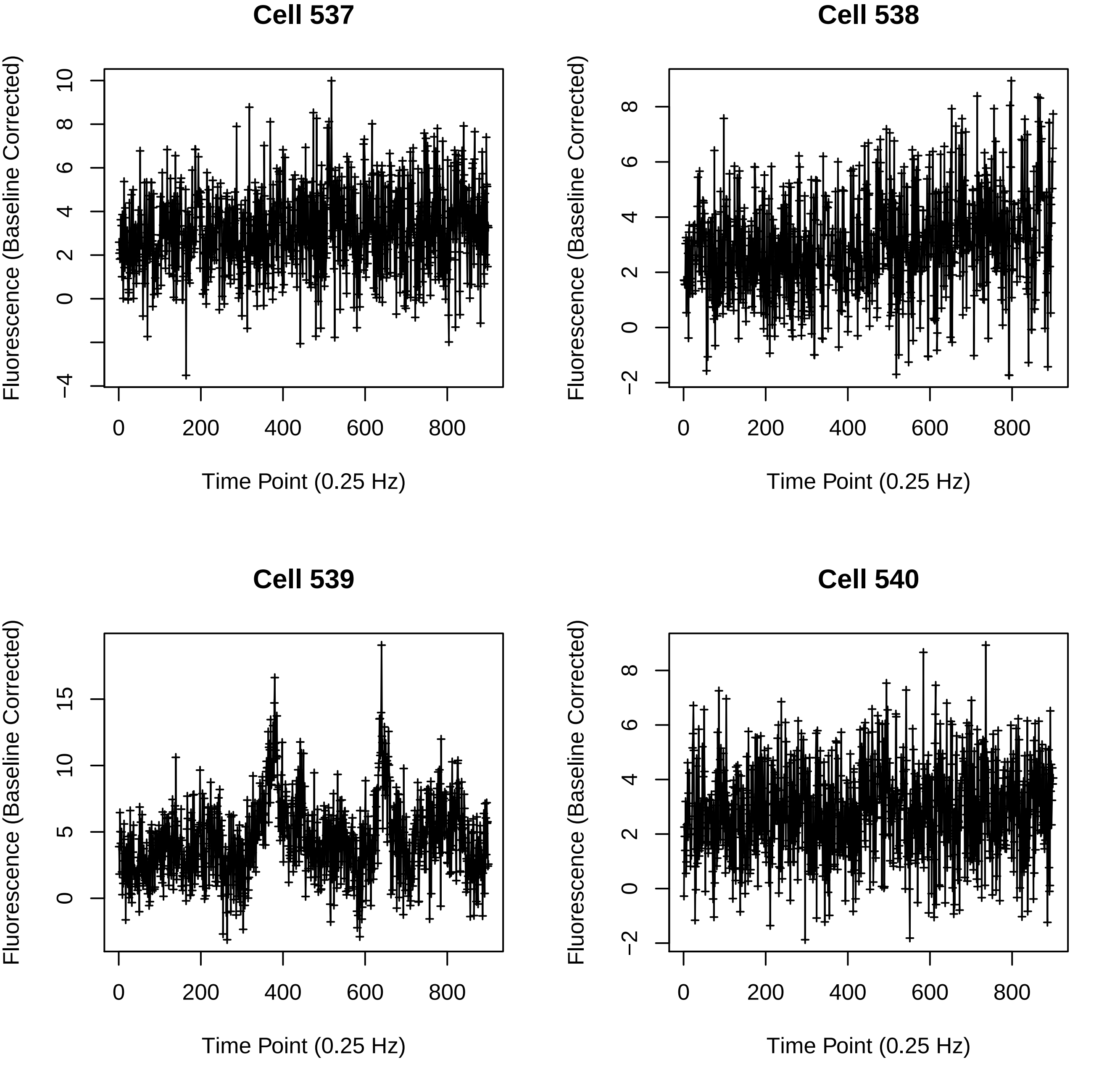
Cell 537
Cell 538
Cell 539
Cell 540
Fluorescence (Baseline Corrected)
Time Point (0.25 Hz)
0
200
400
600
800
−4
0
2
4
6
8
10
−2
5
15

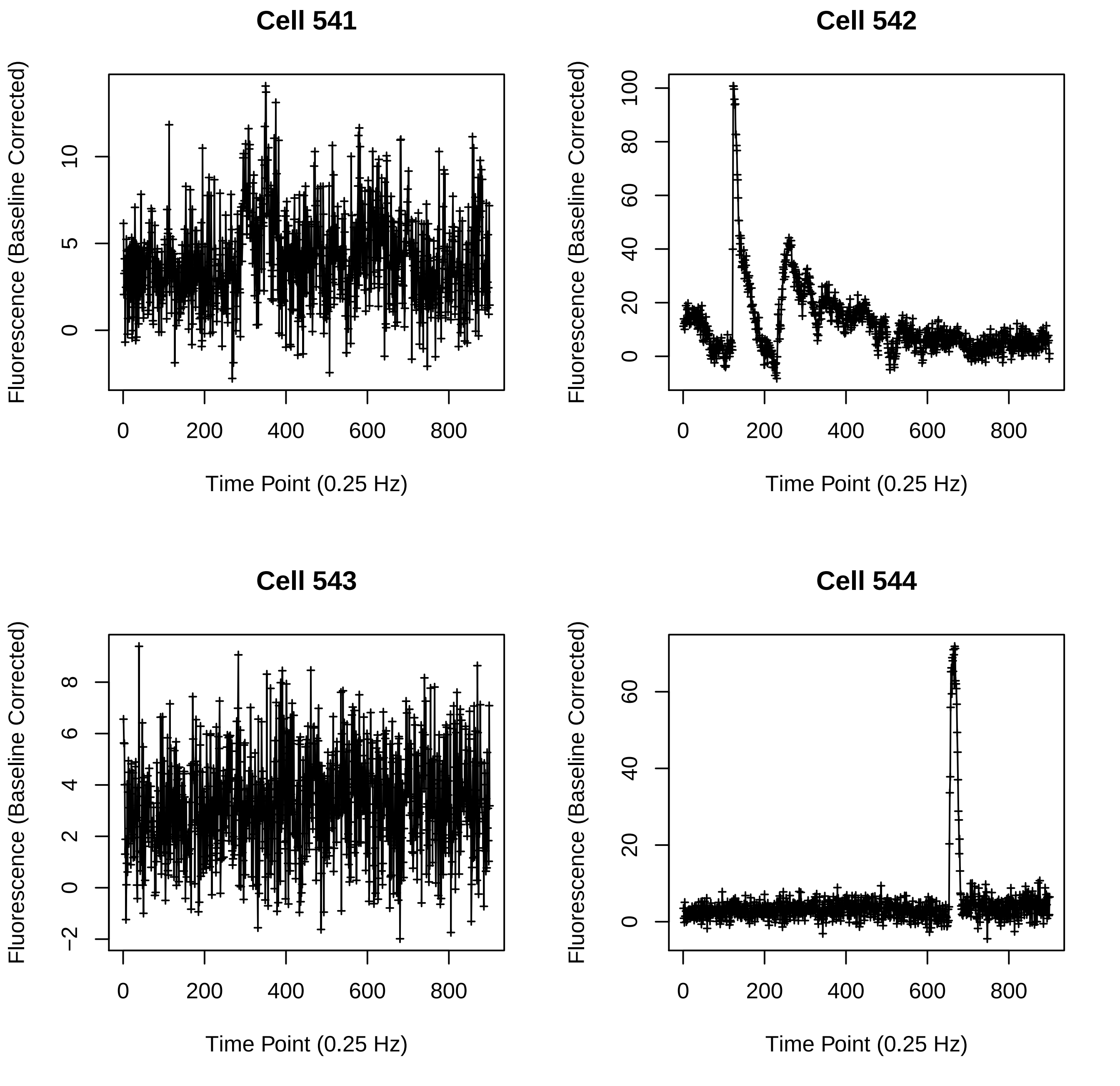
Cell 541
Cell 542
Cell 543
Cell 544
Fluorescence (Baseline Corrected)
Time Point (0.25 Hz)
0
200
400
600
800
0
5
10
0
20
40
60
80
100
-2
0
2
4
6
8
0
20
40
60

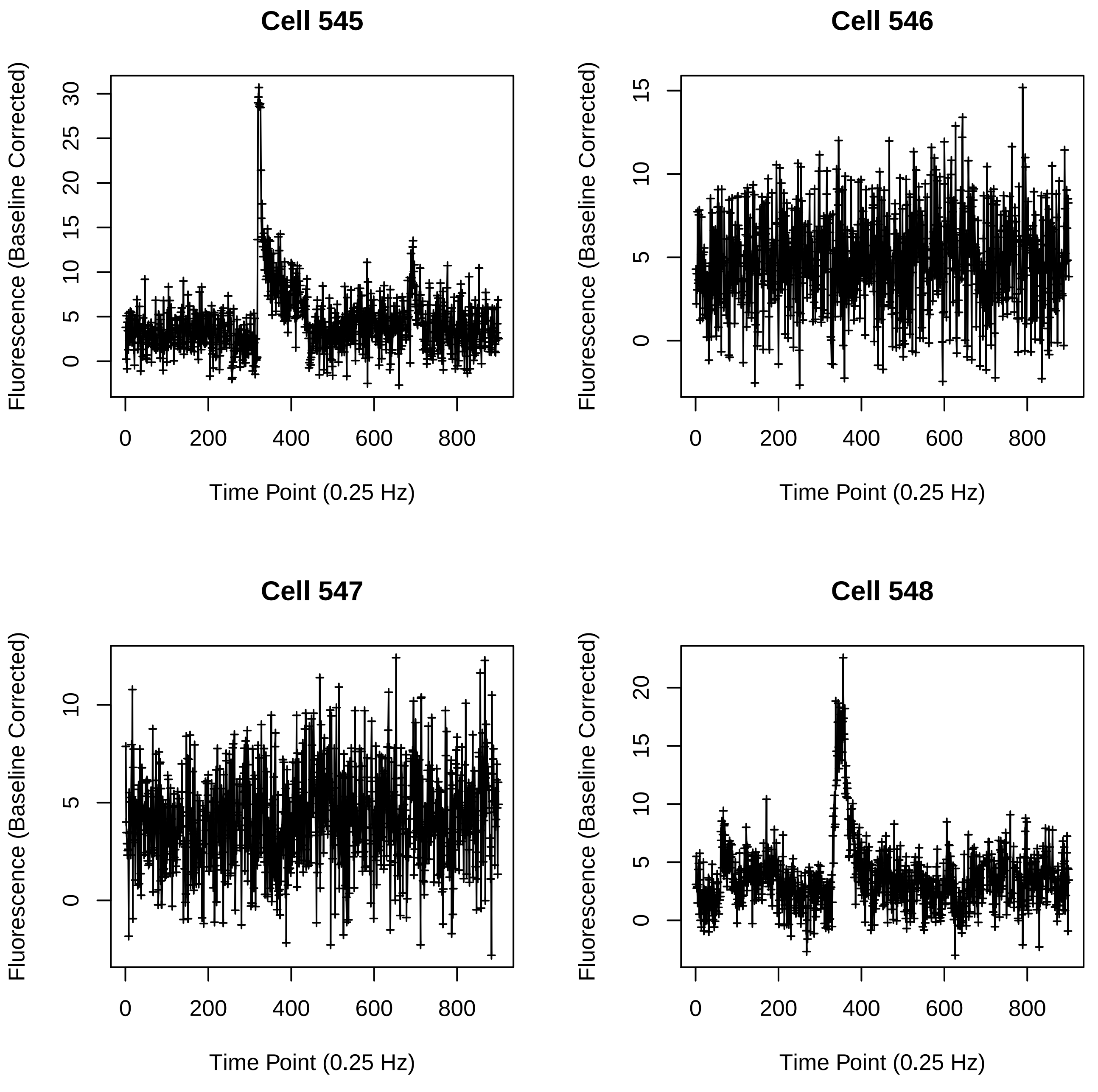
Cell 545
Fluorescence (Baseline Corrected)
Time Point (0.25 Hz)
0
5
10
15
20
25
30
0
200
400
600
800
Cell 546
Fluorescence (Baseline Corrected)
Time Point (0.25 Hz)
0
5
10
15
0
200
400
600
800
Cell 547
Fluorescence (Baseline Corrected)
Time Point (0.25 Hz)
0
5
10
0
200
400
600
800
Cell 548
Fluorescence (Baseline Corrected)
Time Point (0.25 Hz)
0
5
10
15
20
0
200
400
600
800

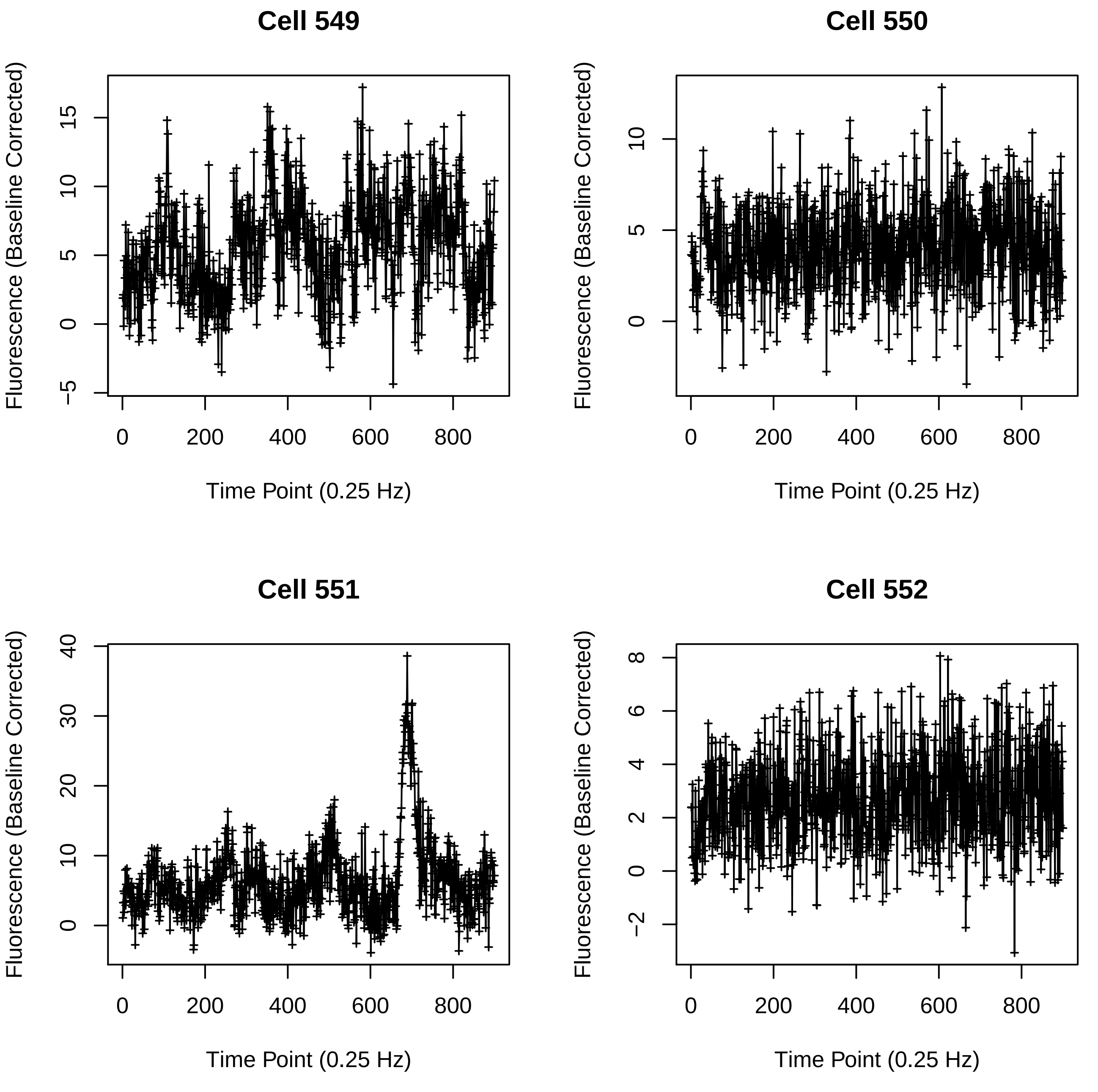

Cell 549
Cell 550
Cell 551
Cell 552
Fluorescence (Baseline Corrected)
Time Point (0.25 Hz)

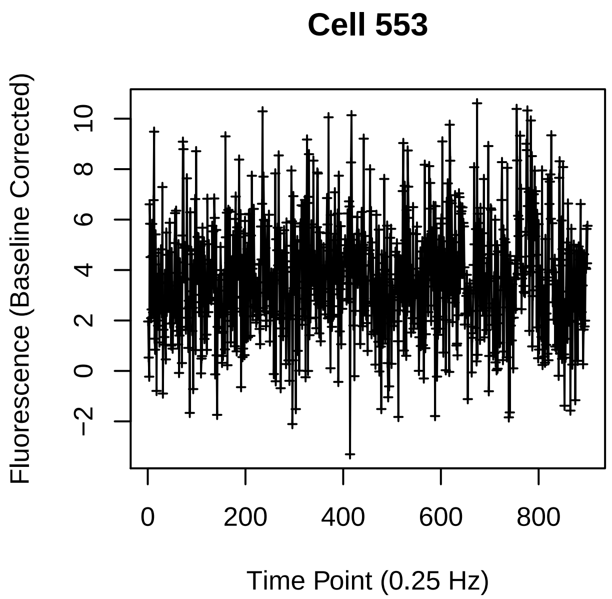
Cell 553
Fluorescence (Baseline Corrected)
−2
0
2
4
6
8
10
0
200
400
600
800
Time Point (0.25 Hz)

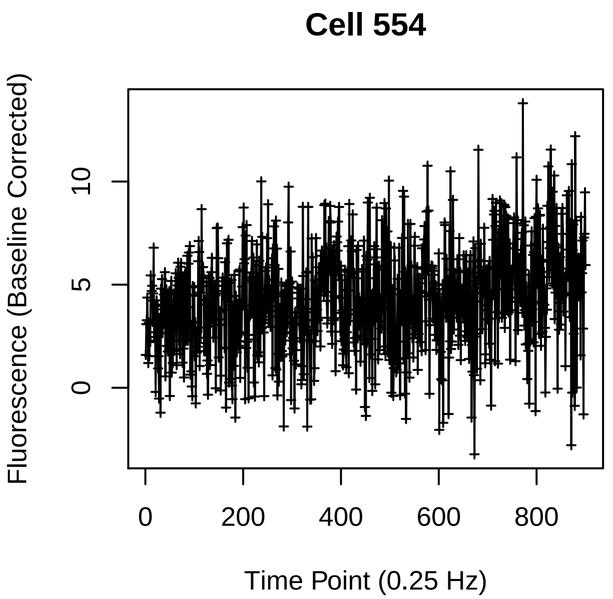
Cell 554
Fluorescence (Baseline Corrected)
0
5
10
0
200
400
600
800
Time Point (0.25 Hz)

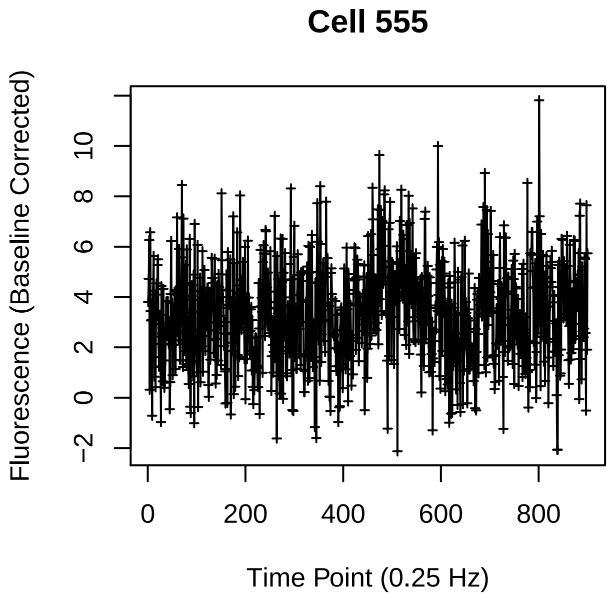
Cell 555
Fluorescence (Baseline Corrected)
−2
0
2
4
6
8
10
0
200
400
600
800
Time Point (0.25 Hz)

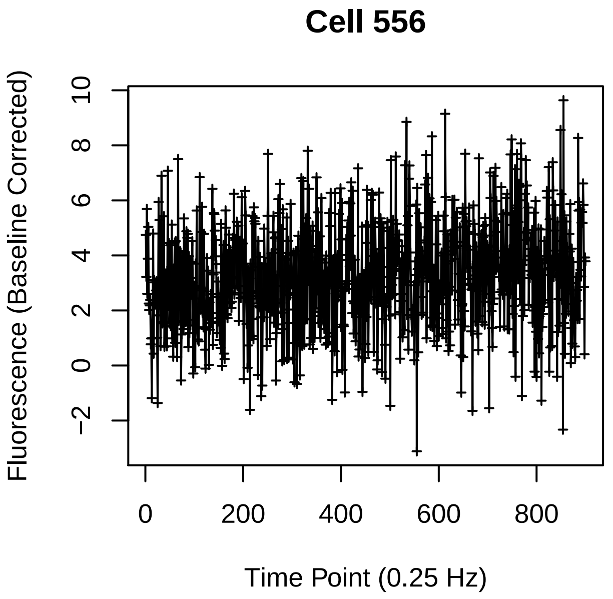
Cell 556
Fluorescence (Baseline Corrected)
−2
0
2
4
6
8
10
0
200
400
600
800
Time Point (0.25 Hz)

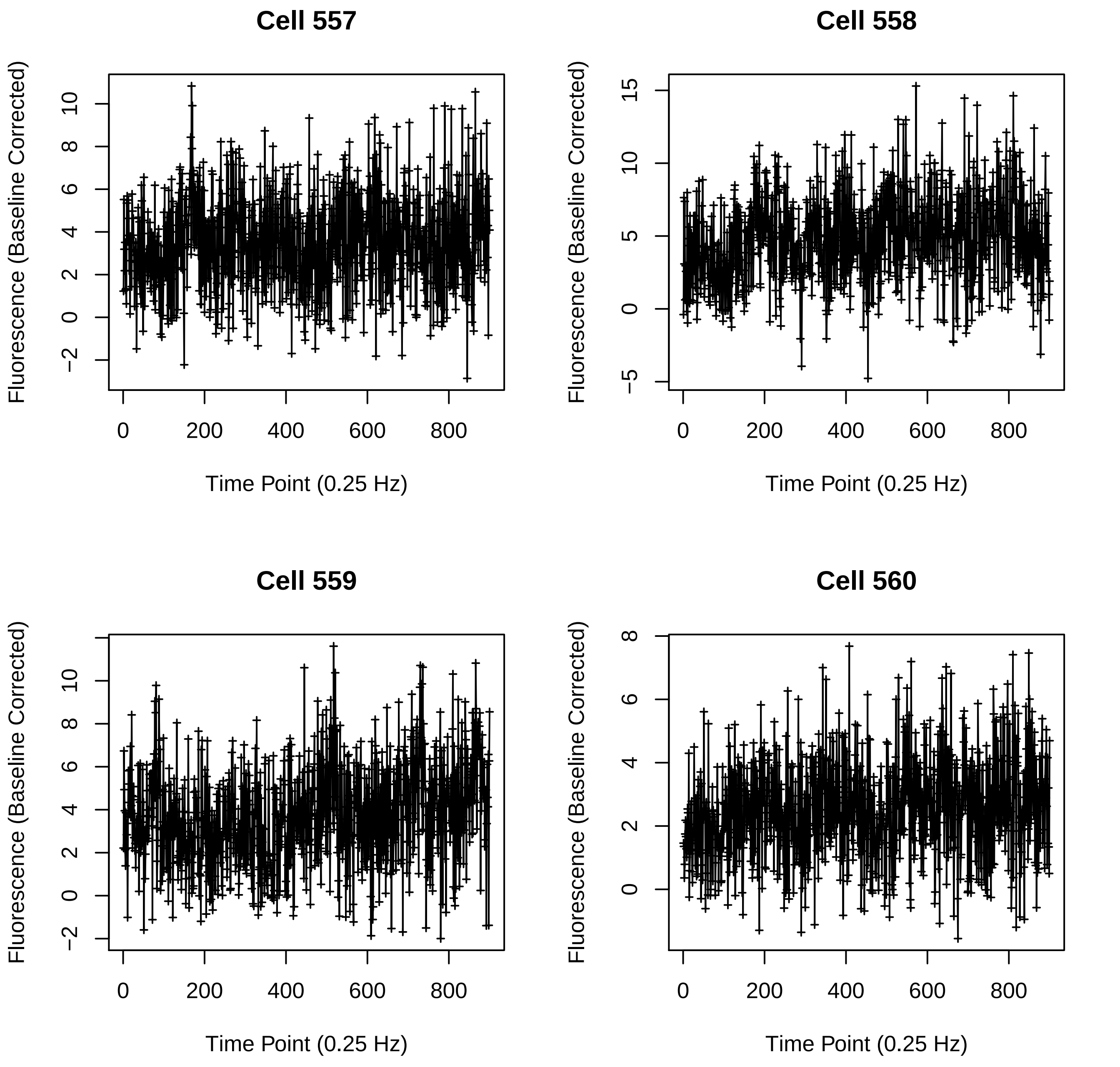
Cell 557
Fluorescence (Baseline Corrected)
Time Point (0.25 Hz)
Cell 558
Fluorescence (Baseline Corrected)
Time Point (0.25 Hz)
Cell 559
Fluorescence (Baseline Corrected)
Time Point (0.25 Hz)
Cell 560
Fluorescence (Baseline Corrected)
Time Point (0.25 Hz)

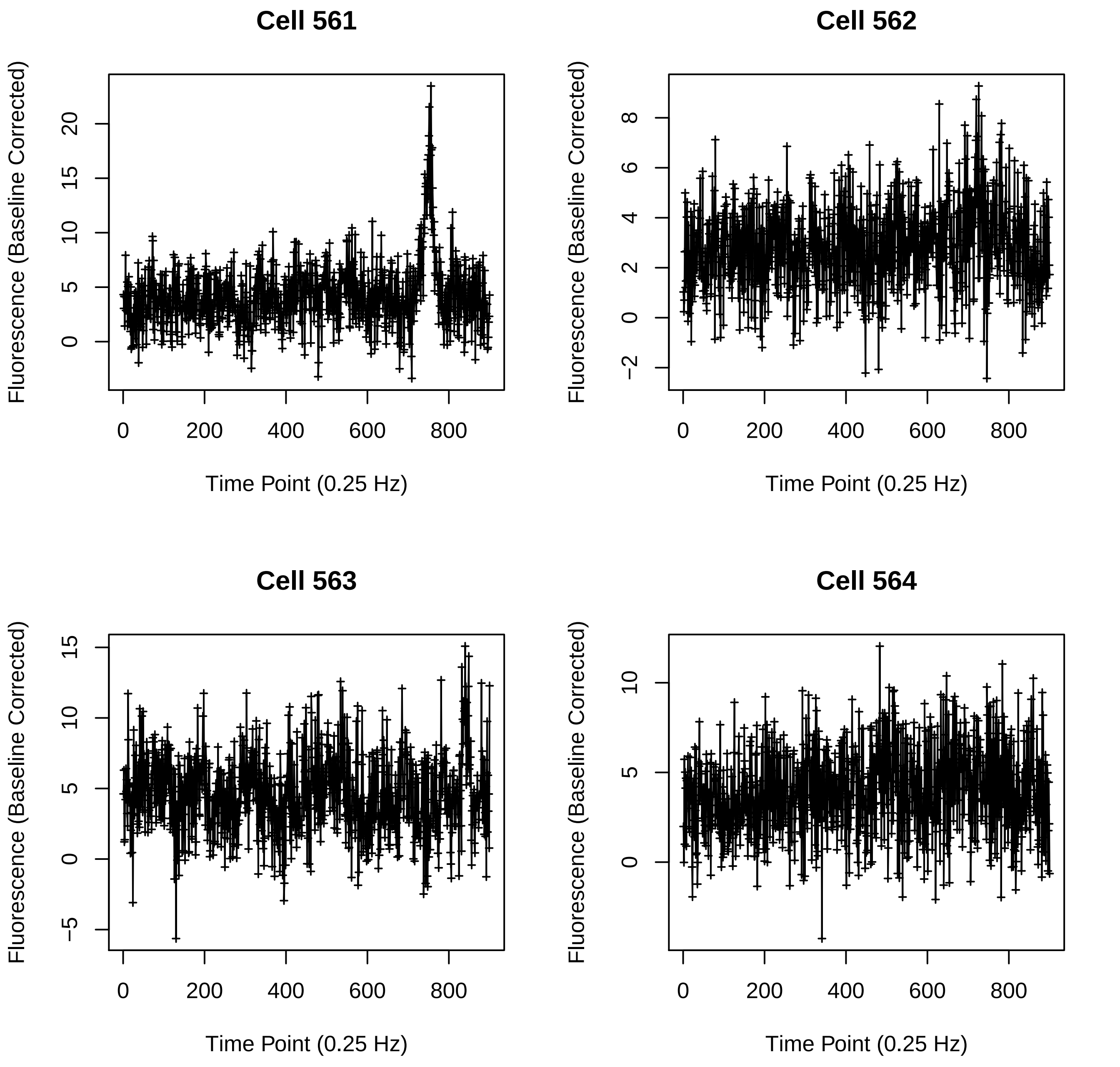
Cell 561
Fluorescence (Baseline Corrected)
0
5
10
15
20
0
200
400
600
800
Time Point (0.25 Hz)
Cell 562
Fluorescence (Baseline Corrected)
−2
0
2
4
6
8
0
200
400
600
800
Time Point (0.25 Hz)
Cell 563
Fluorescence (Baseline Corrected)
−5
0
5
10
15
0
200
400
600
800
Time Point (0.25 Hz)
Cell 564
Fluorescence (Baseline Corrected)
0
5
10
0
200
400
600
800
Time Point (0.25 Hz)

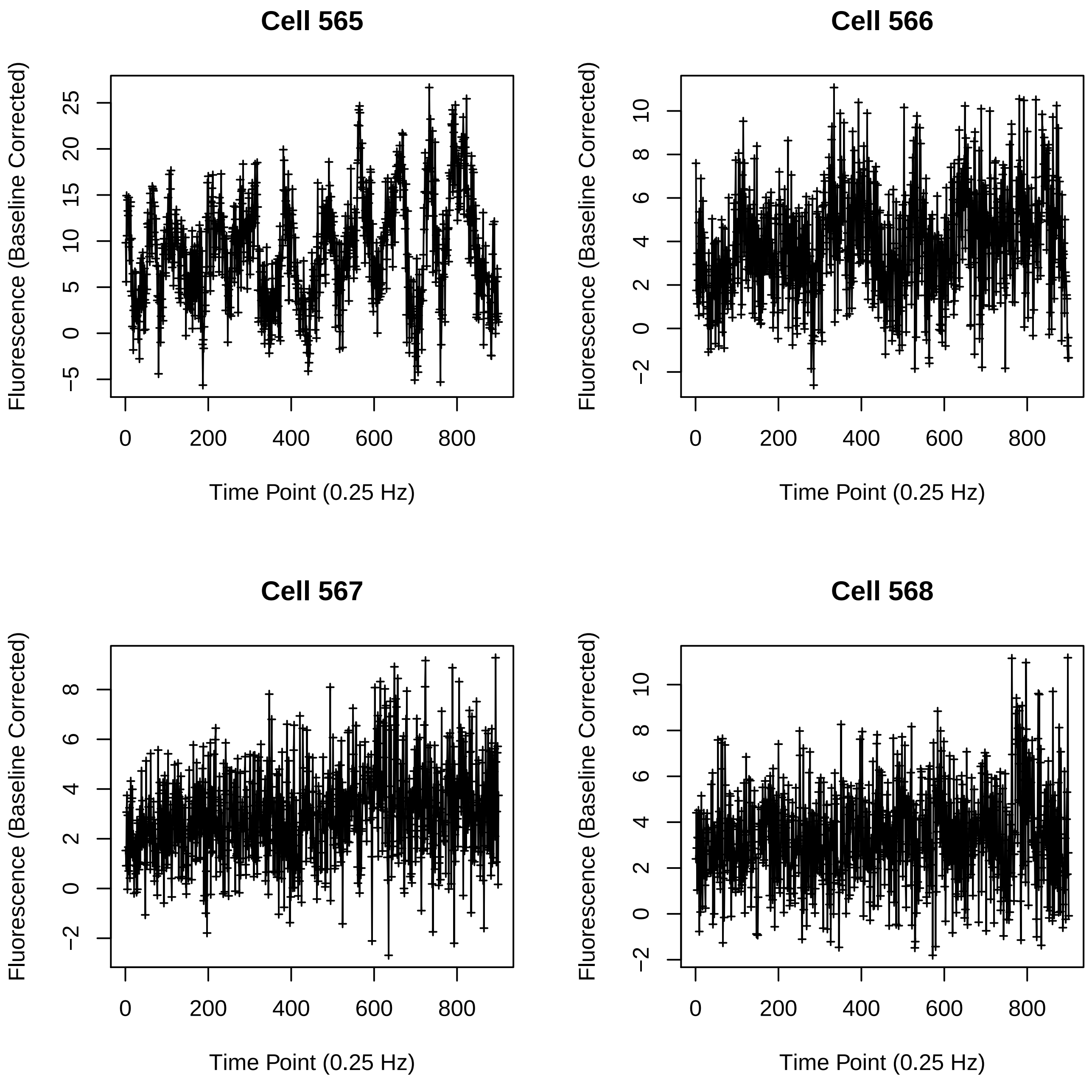

Cell 565
Cell 566
Cell 567
Cell 568
Fluorescence (Baseline Corrected)
Time Point (0.25 Hz)
0
200
400
600
800
-5
0
5
10
15
20
25
-2
2
4
6
8

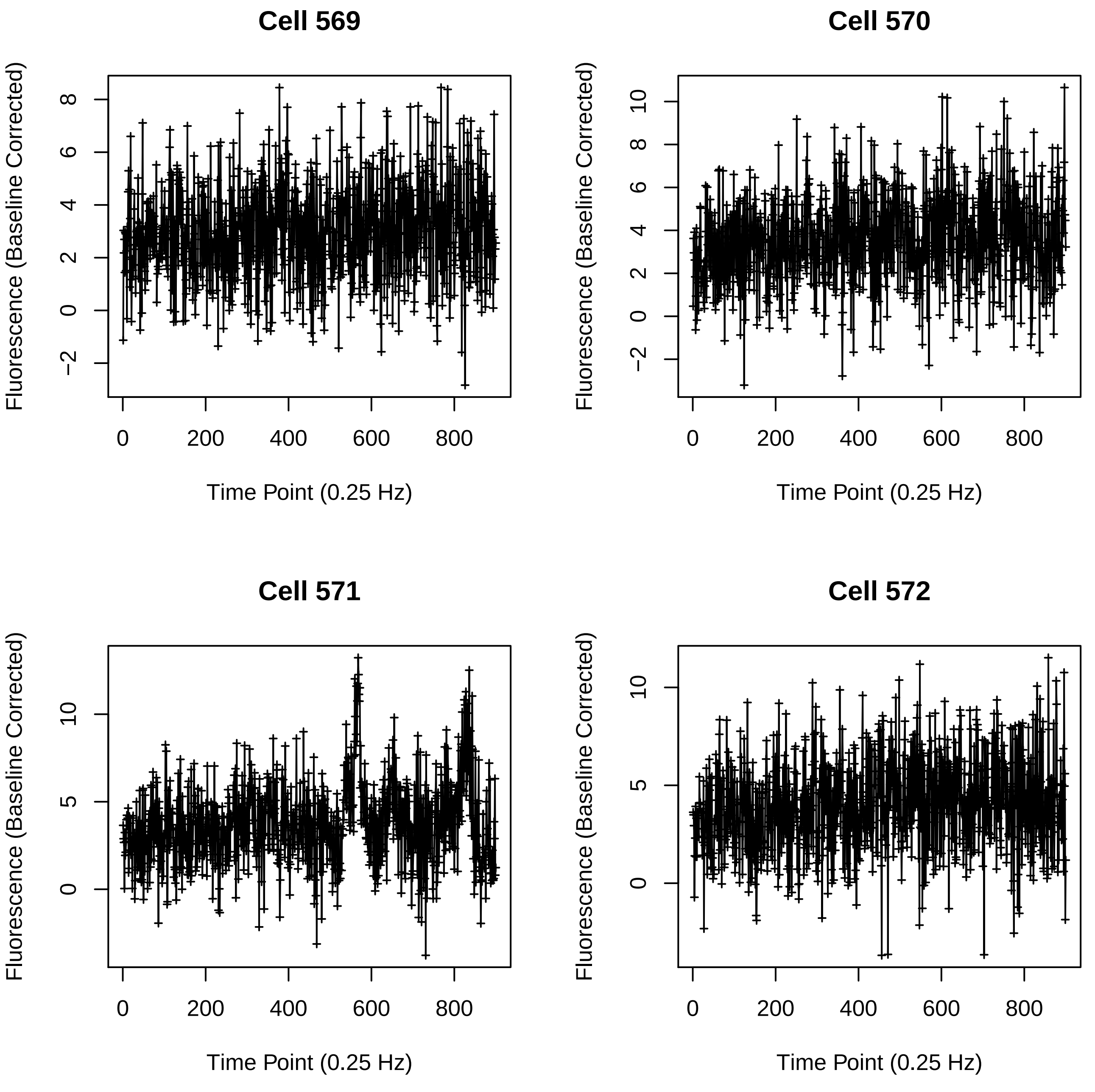
Cell 569
Cell 570
Cell 571
Cell 572
Fluorescence (Baseline Corrected)
Time Point (0.25 Hz)
0
200
400
600
800
−2
2
4
6
8
10
5

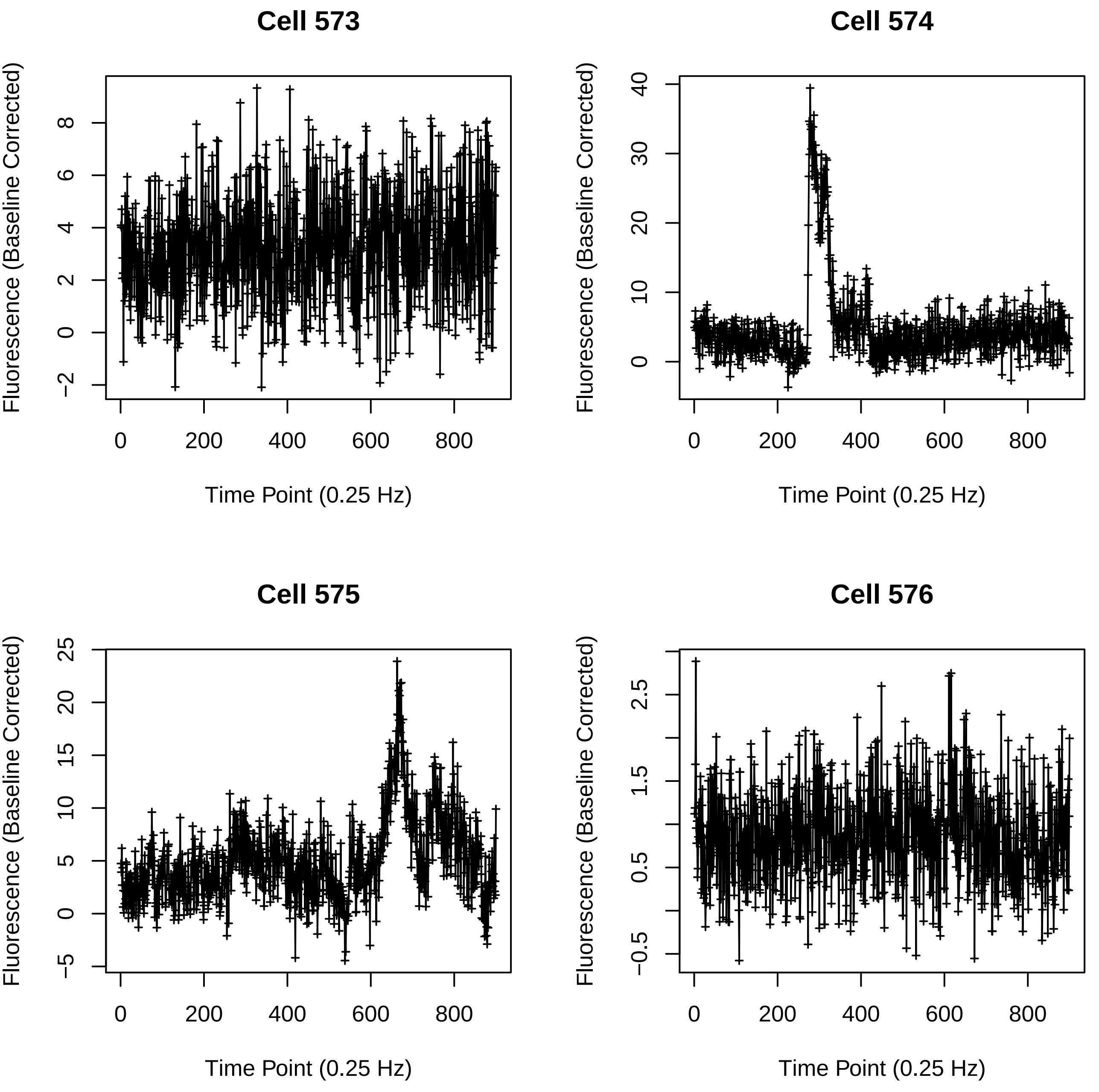
Cell 573
Cell 574
Cell 575
Cell 576
Fluorescence (Baseline Corrected)
Time Point (0.25 Hz)
0
200
400
600
800

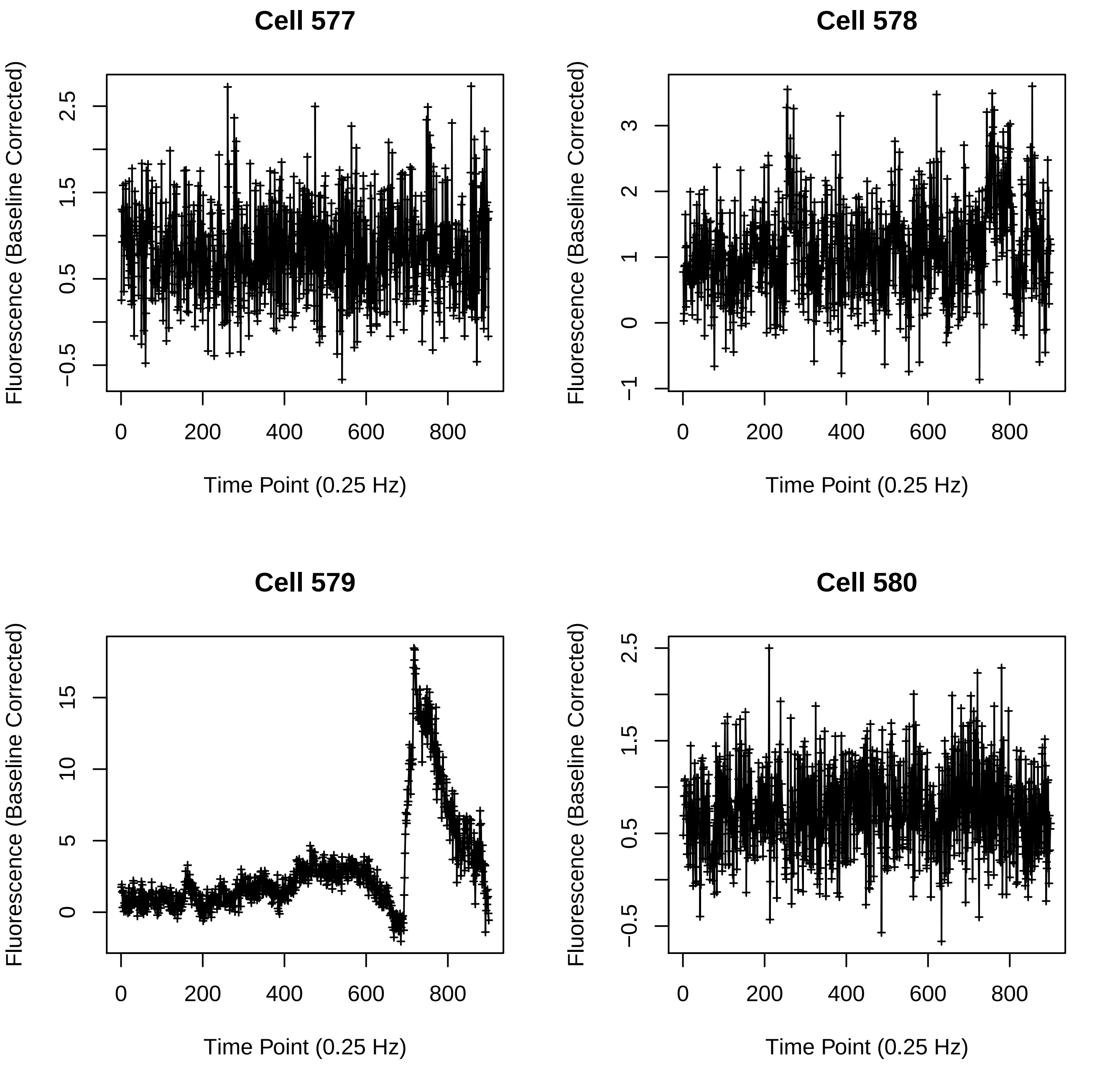
Cell 577
Fluorescence (Baseline Corrected)
−0.5
0.5
1.5
2.5
0
200
400
600
800
Time Point (0.25 Hz)
Cell 578
Fluorescence (Baseline Corrected)
−1
0
1
2
3
0
200
400
600
800
Time Point (0.25 Hz)
Cell 579
Fluorescence (Baseline Corrected)
0
5
10
15
0
200
400
600
800
Time Point (0.25 Hz)
Cell 580
Fluorescence (Baseline Corrected)
−0.5
0.5
1.5
2.5
0
200
400
600
800
Time Point (0.25 Hz)

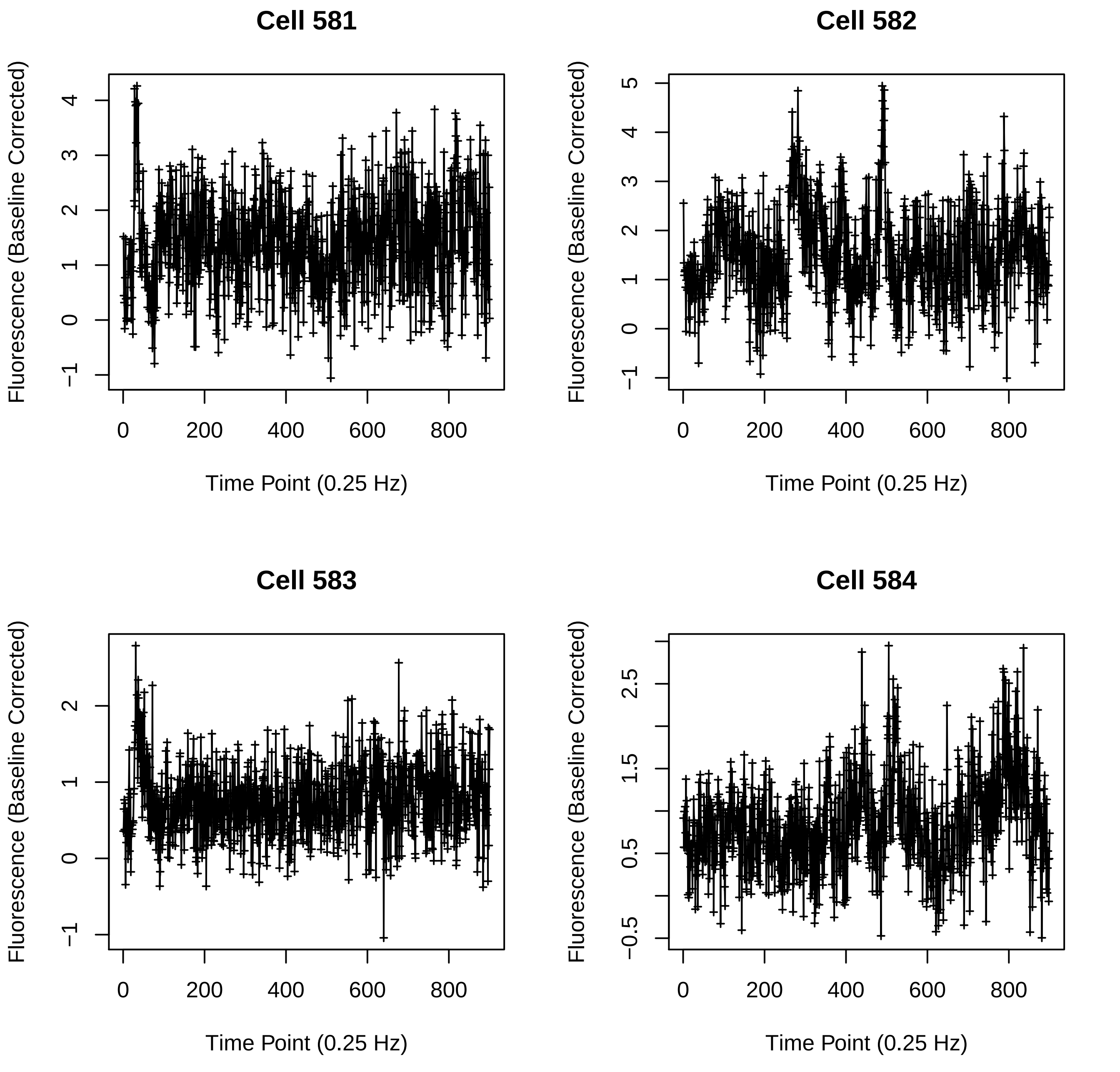
Cell 581
Cell 582
Cell 583
Cell 584
Fluorescence (Baseline Corrected)
Time Point (0.25 Hz)
0
200
400
600
800
−1
0
1
2
3
4
5
−0.5
0.5
1.5
2.5

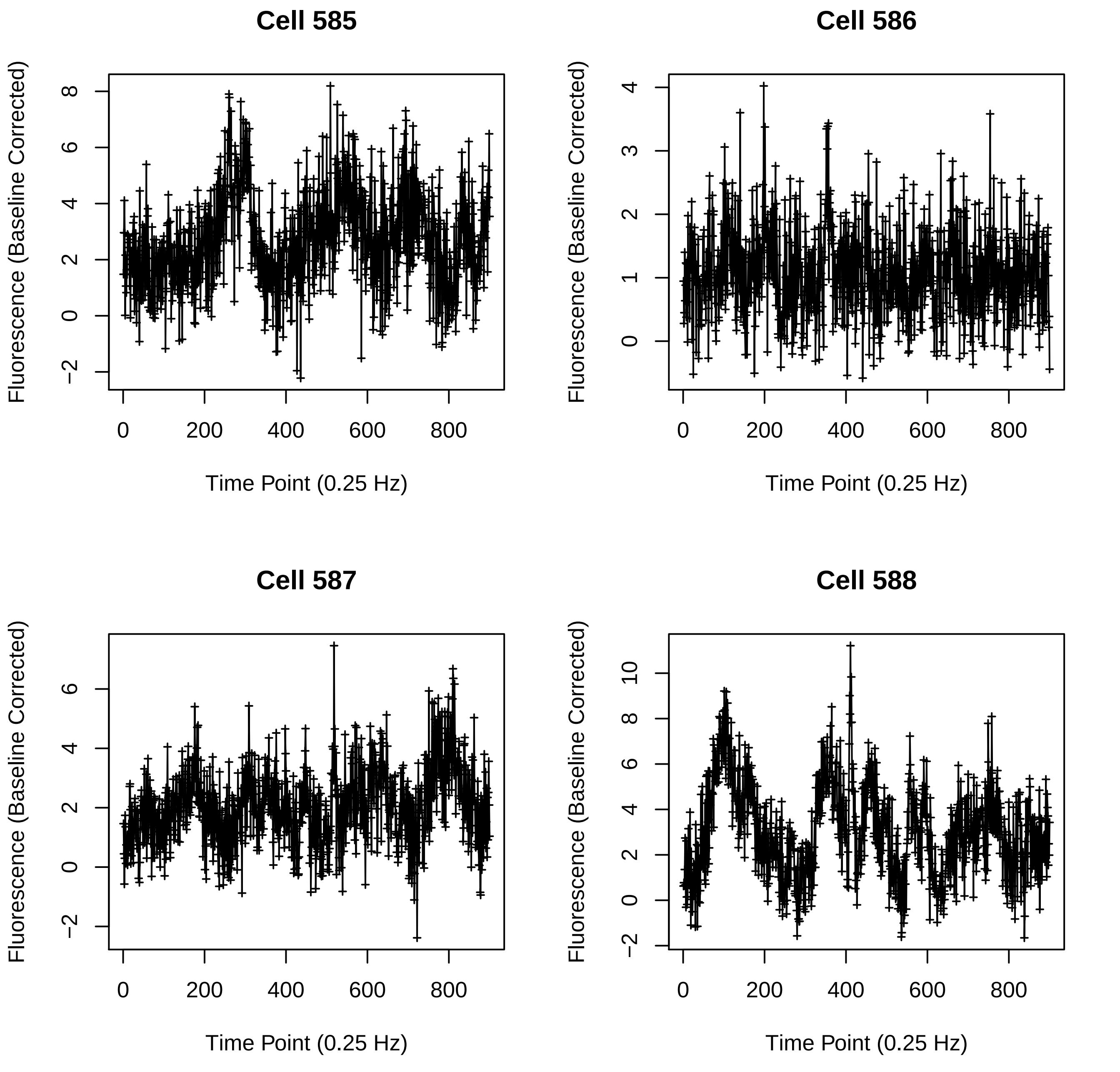
Cell 585
Cell 586
Cell 587
Cell 588
Fluorescence (Baseline Corrected)
Time Point (0.25 Hz)
0
200
400
600
800

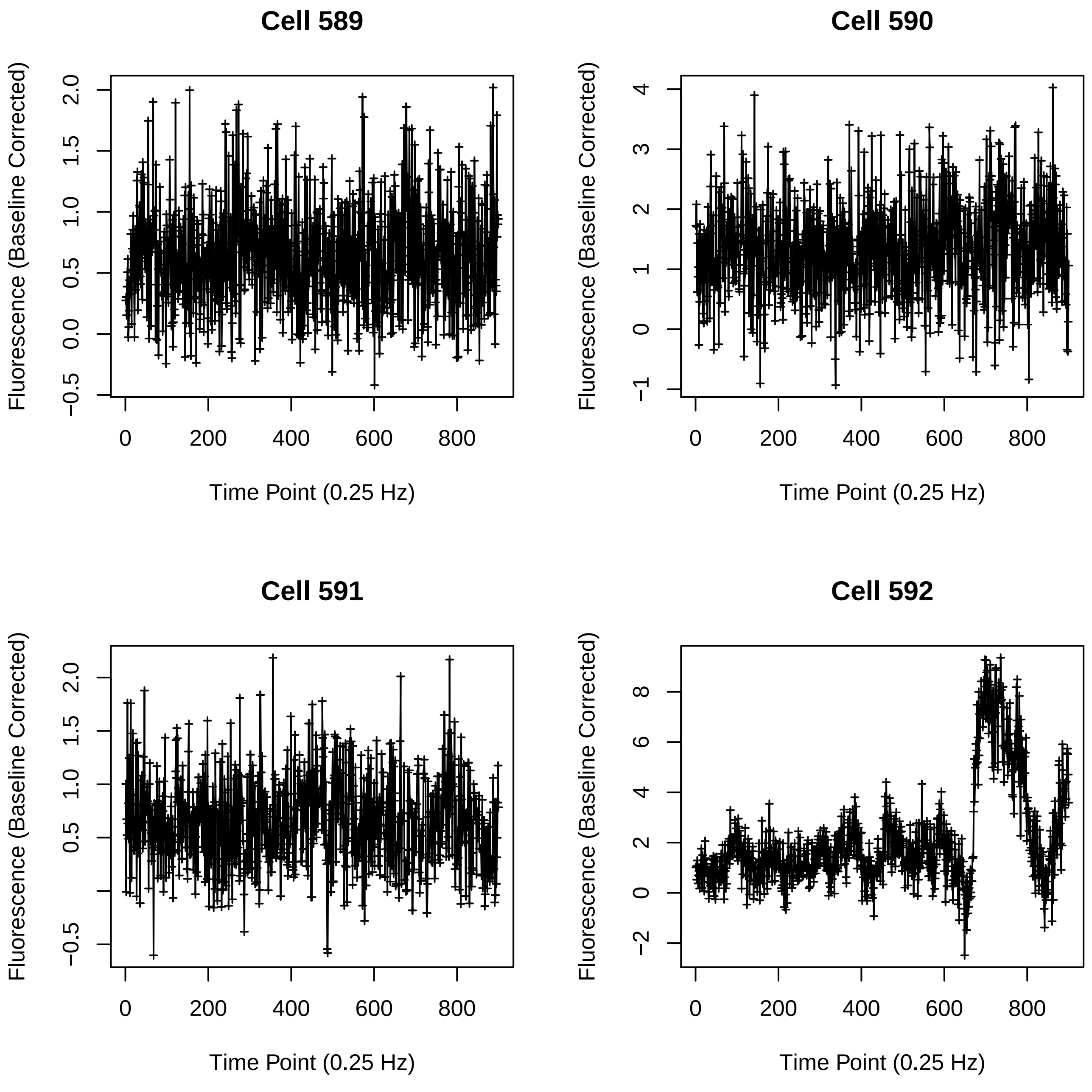
Cell 589
Fluorescence (Baseline Corrected)
−0.5
0.0
0.5
1.0
1.5
2.0
0
200
400
600
800
Time Point (0.25 Hz)
Cell 590
Fluorescence (Baseline Corrected)
−1
0
1
2
3
4
0
200
400
600
800
Time Point (0.25 Hz)
Cell 591
Fluorescence (Baseline Corrected)
−0.5
0.5
1.0
1.5
2.0
0
200
400
600
800
Time Point (0.25 Hz)
Cell 592
Fluorescence (Baseline Corrected)
−2
0
2
4
6
8
0
200
400
600
800
Time Point (0.25 Hz)

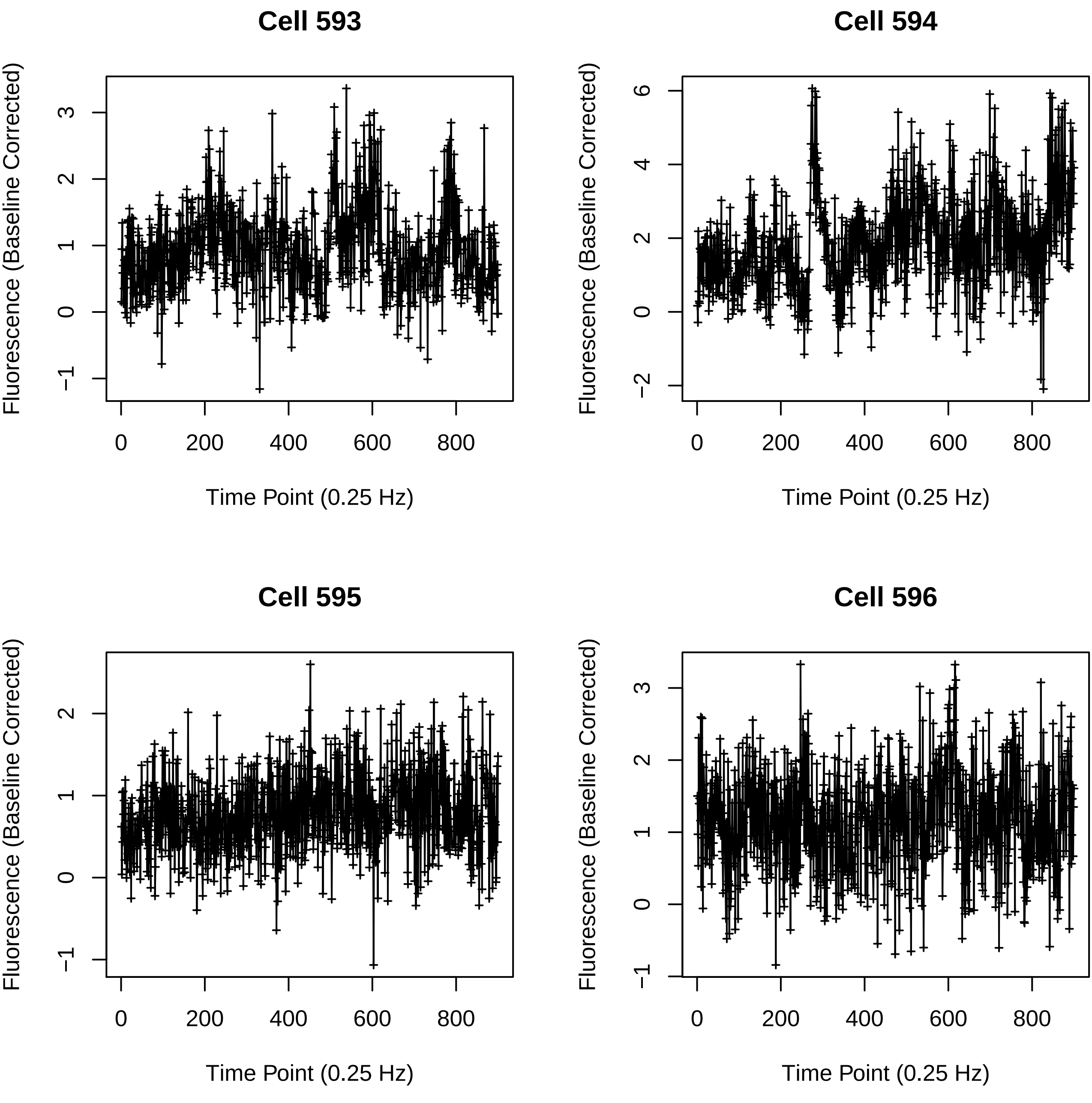

Cell 593
Cell 594
Cell 595
Cell 596
Fluorescence (Baseline Corrected)
Time Point (0.25 Hz)
0
200
400
600
800
−1
0
1
2
3
−2
4
6

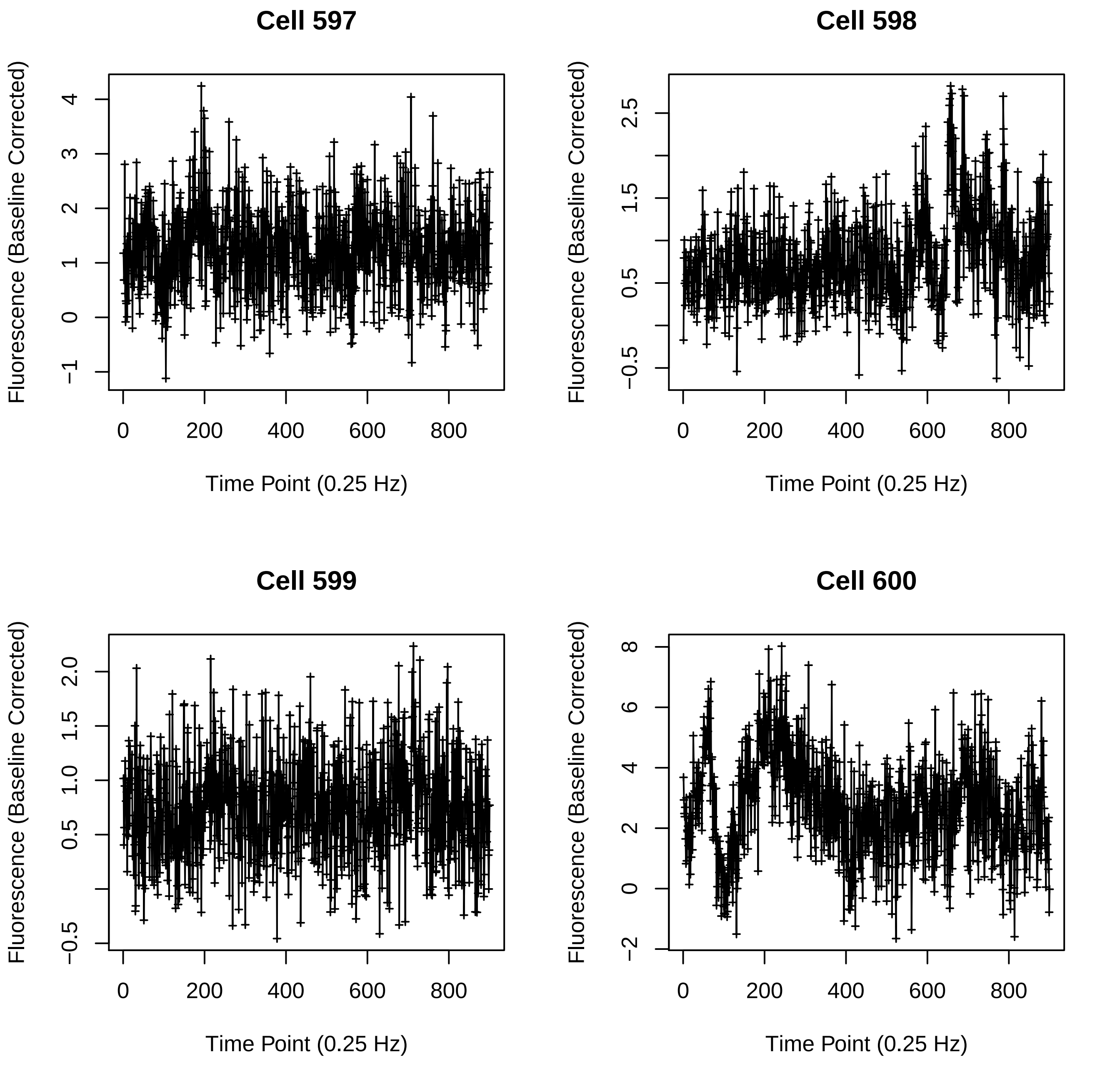
Cell 597
Cell 598
Cell 599
Cell 600
Fluorescence (Baseline Corrected)
Time Point (0.25 Hz)

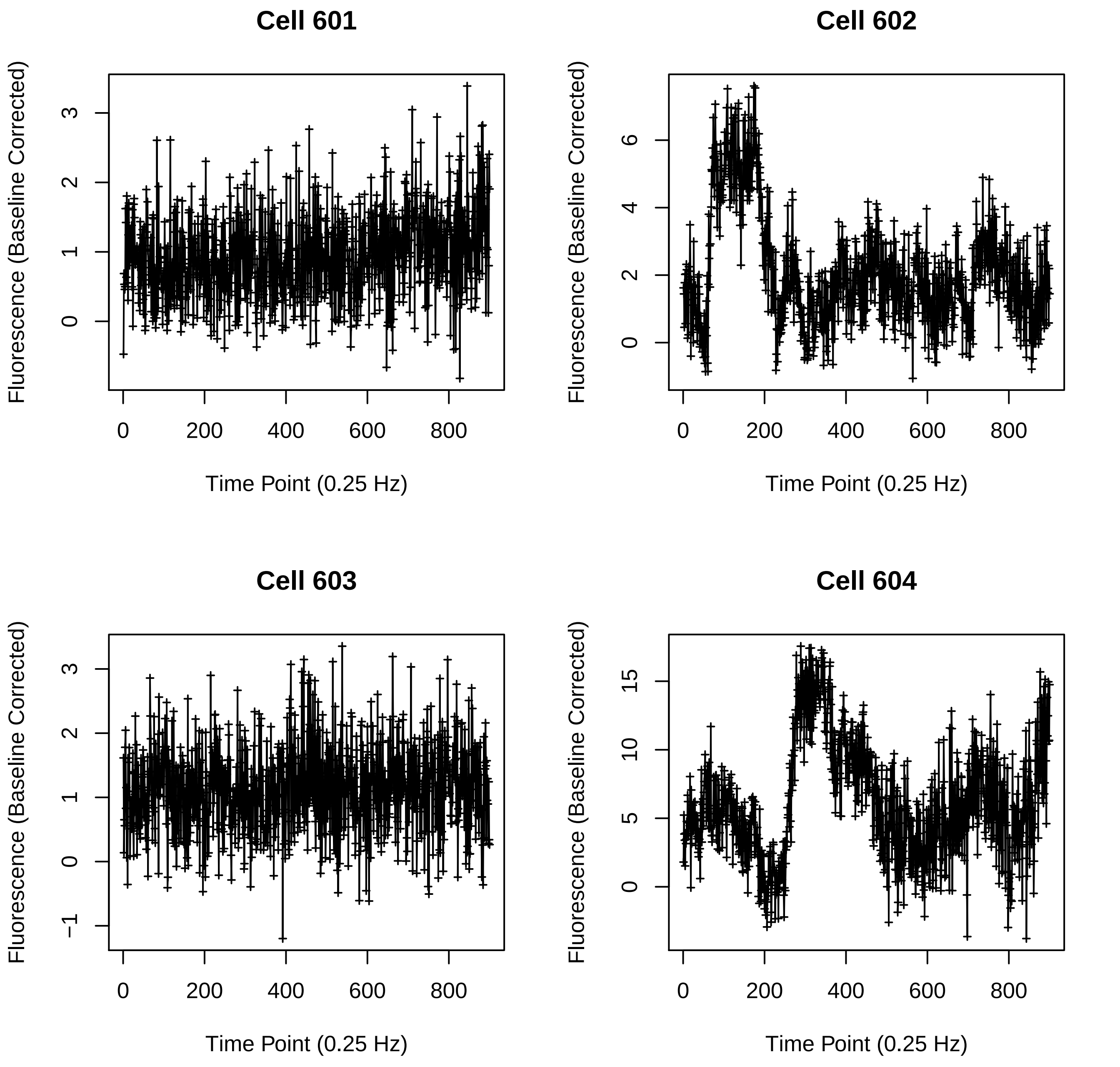

Cell 601
Cell 602
Cell 603
Cell 604
Fluorescence (Baseline Corrected)
Time Point (0.25 Hz)
0
200
400
600
800
−1
1
2
3
4
6
5
10
15

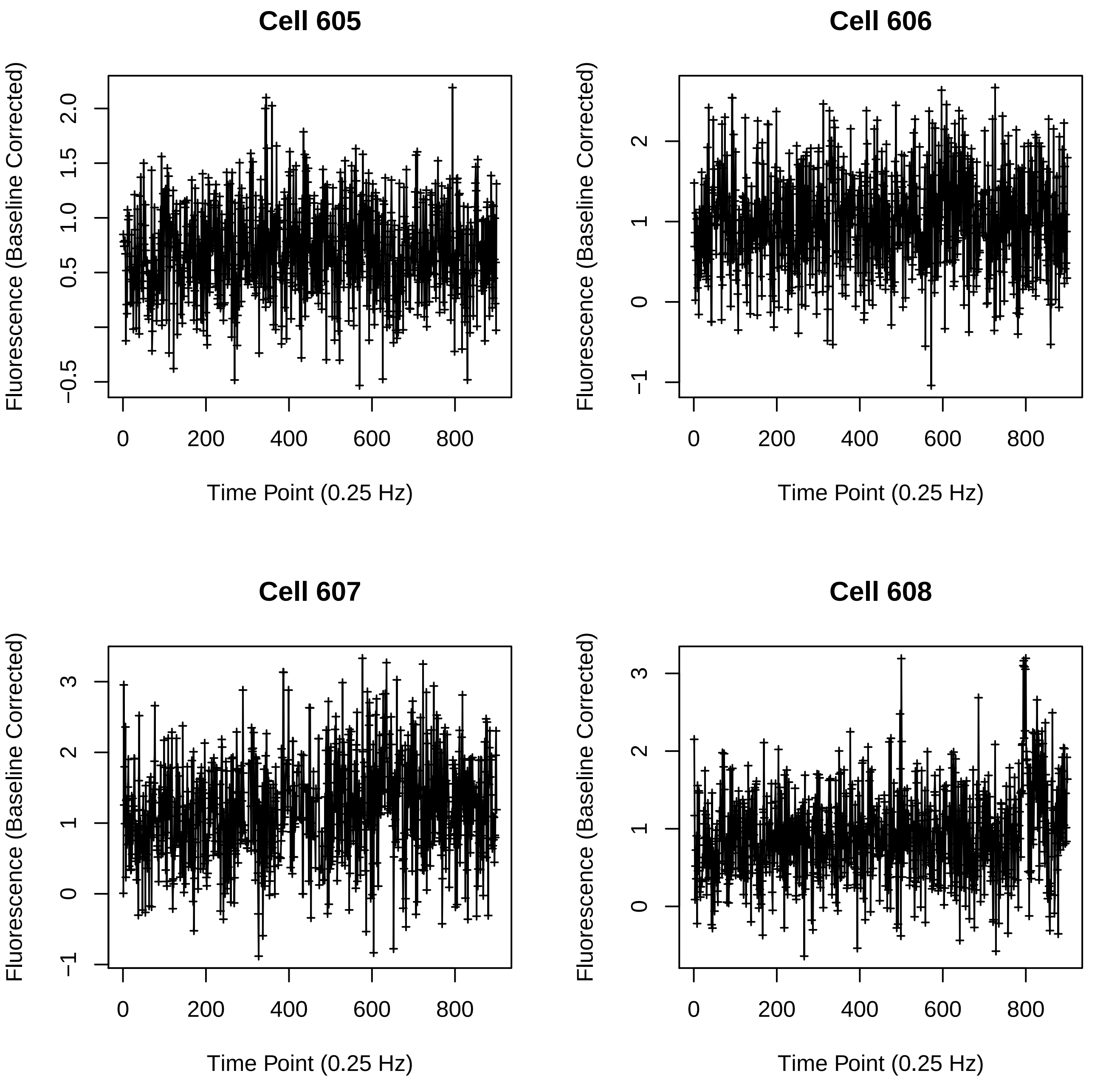
Cell 605
Cell 606
Cell 607
Cell 608
Fluorescence (Baseline Corrected)
Time Point (0.25 Hz)
0
200
400
600
800
−0.5
0.5
1.0
1.5
2.0
−1
0
1
2
3

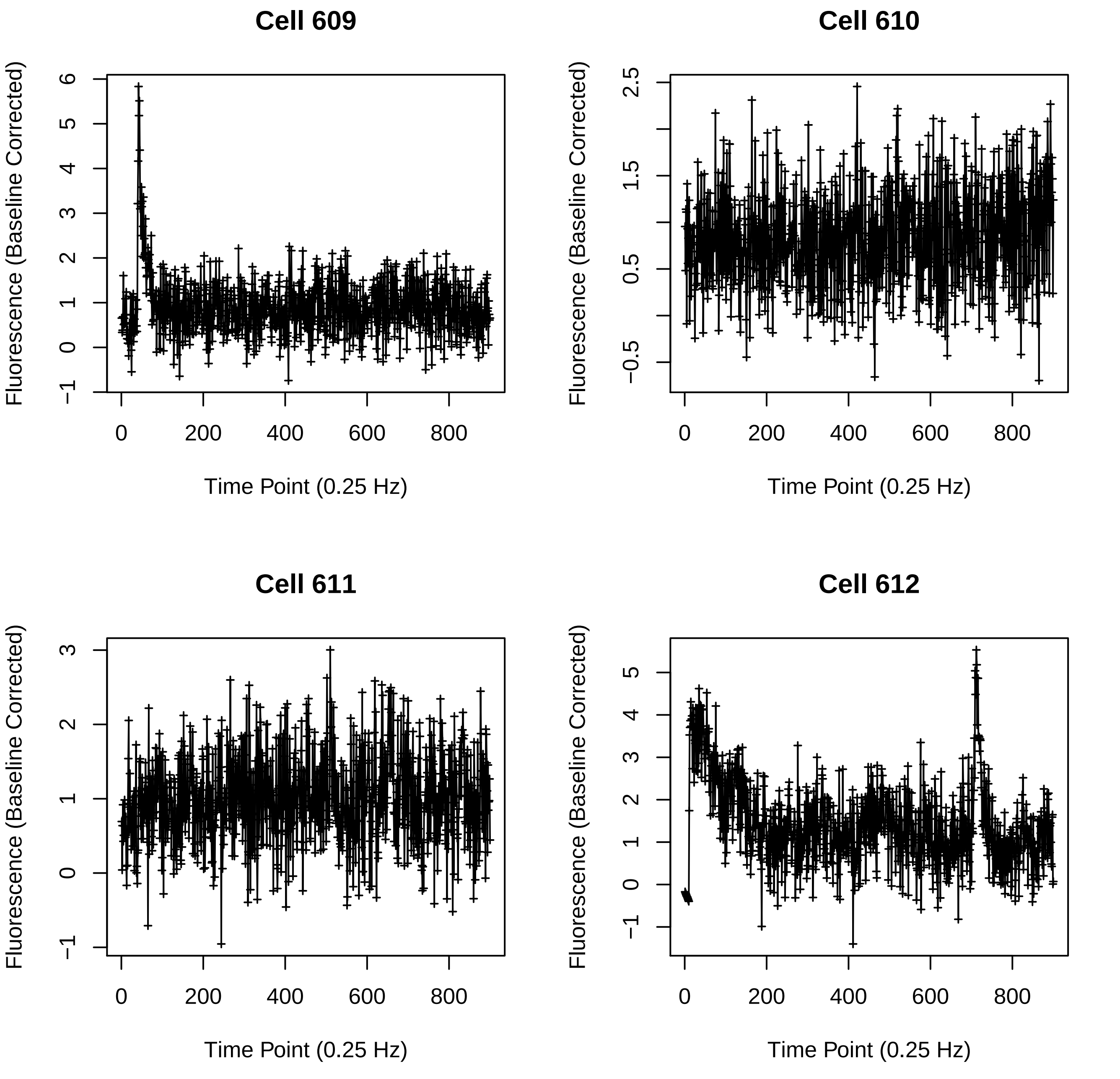
Cell 609
Fluorescence (Baseline Corrected)
−1
0
1
2
3
4
5
6
0
200
400
600
800
Time Point (0.25 Hz)
Cell 610
Fluorescence (Baseline Corrected)
−0.5
0.5
1.5
2.5
0
200
400
600
800
Time Point (0.25 Hz)
Cell 611
Fluorescence (Baseline Corrected)
−1
0
1
2
3
0
200
400
600
800
Time Point (0.25 Hz)
Cell 612
Fluorescence (Baseline Corrected)
−1
0
1
2
3
4
5
0
200
400
600
800
Time Point (0.25 Hz)

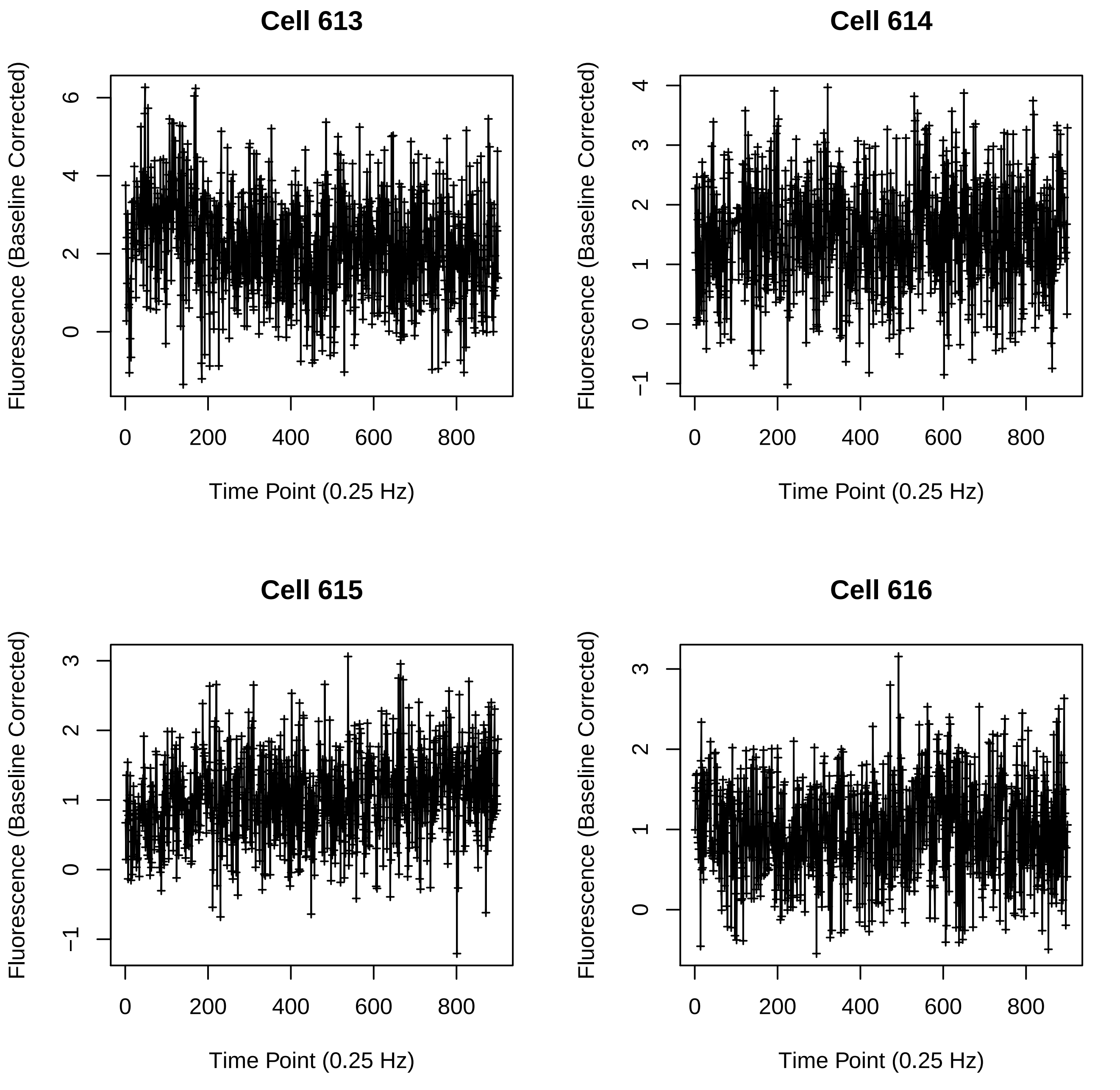
Cell 613
Cell 614
Cell 615
Cell 616
Fluorescence (Baseline Corrected)
Time Point (0.25 Hz)
0
200
400
600
800

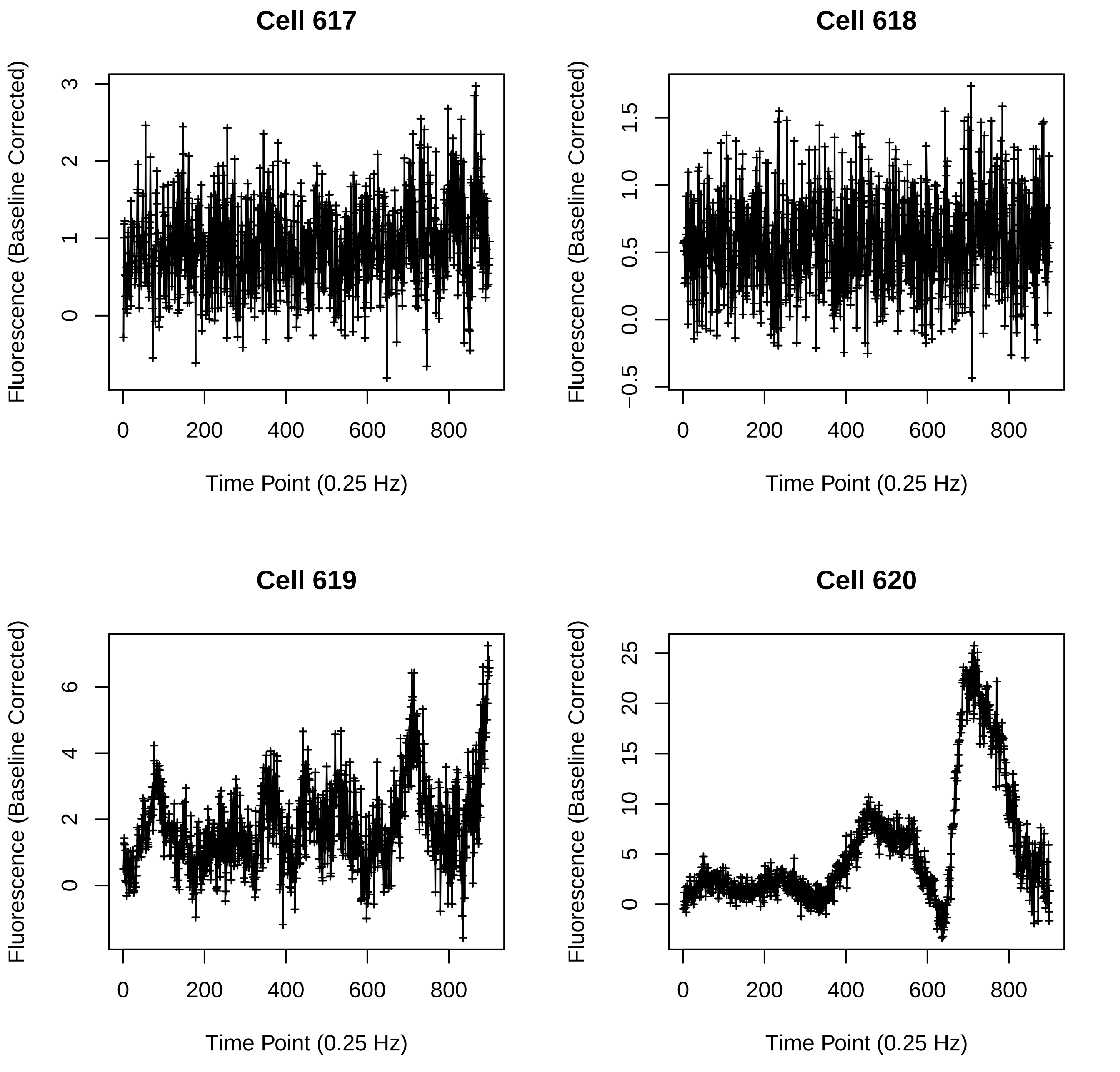
Cell 617
Cell 618
Cell 619
Cell 620
Fluorescence (Baseline Corrected)
Time Point (0.25 Hz)
0
200
400
600
800
-0.5
0.0
0.5
1.0
1.5
1
2
3
4
5
6
10
15
20
25

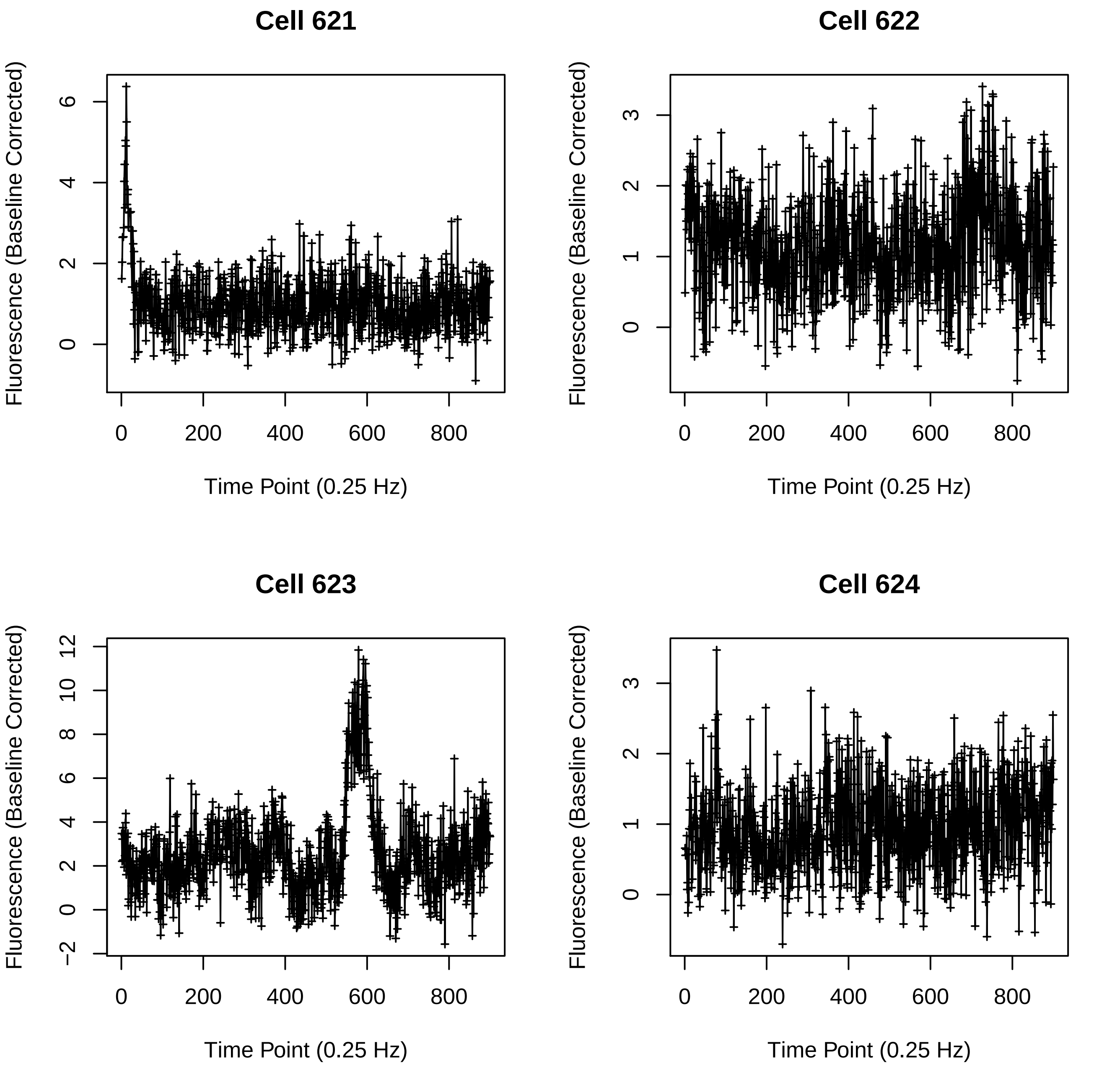

Cell 621
Cell 622
Cell 623
Cell 624
Fluorescence (Baseline Corrected)
Time Point (0.25 Hz)
0
200
400
600
800
−2
2
4
6
8
10
12
3
1

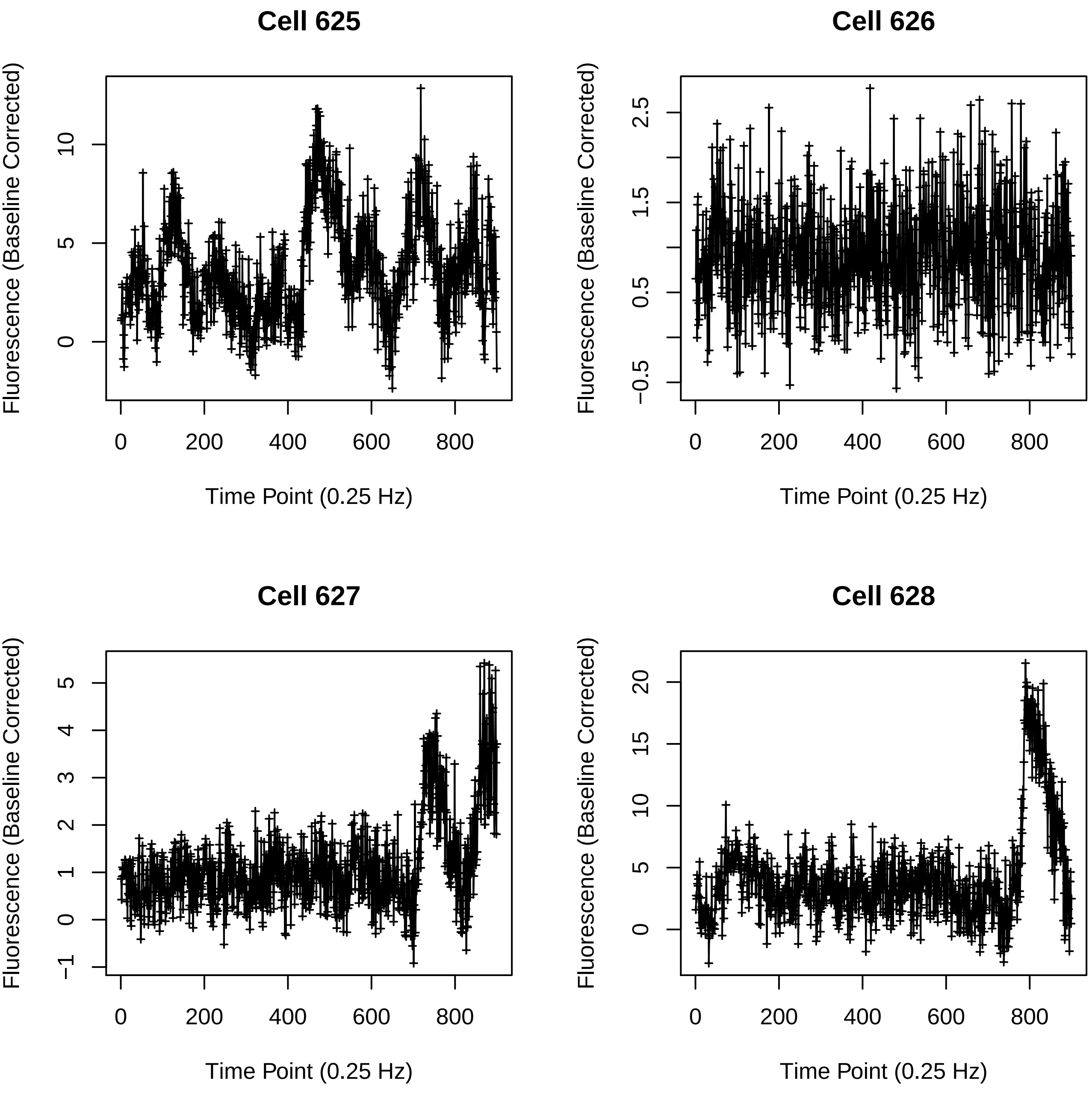
Cell 625
Cell 626
Cell 627
Cell 628
Fluorescence (Baseline Corrected)
Time Point (0.25 Hz)
0
200
400
600
800
0
5
10
−0.5
0.5
1.5
2.5
−1
1
2
3
4
5
15
20

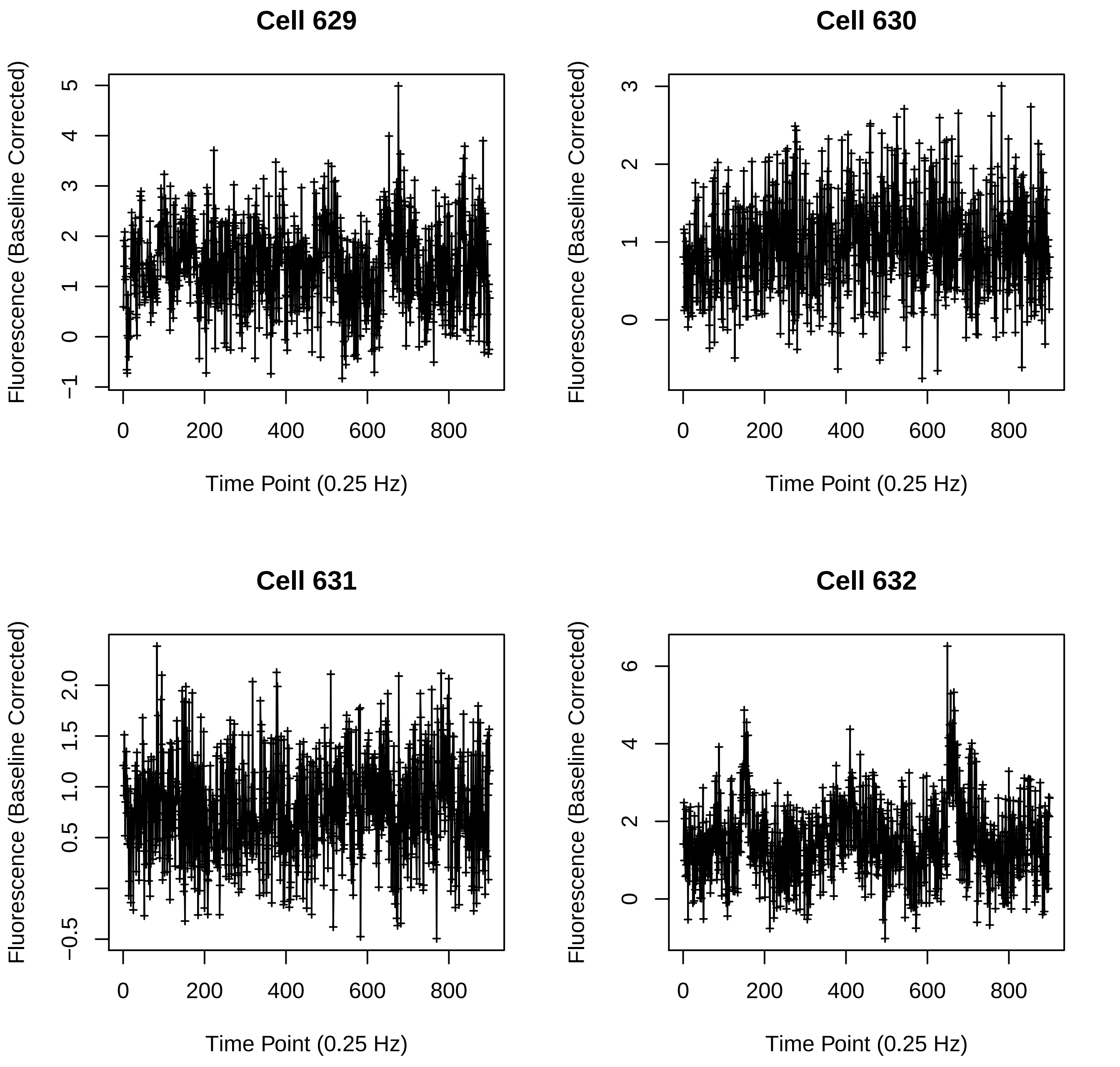

Cell 629
Cell 630
Cell 631
Cell 632
Fluorescence (Baseline Corrected)
Time Point (0.25 Hz)
0
200
400
600
800
−1
1
2
3
4
5
−0.5
0.5
1.0
1.5
2.0
6

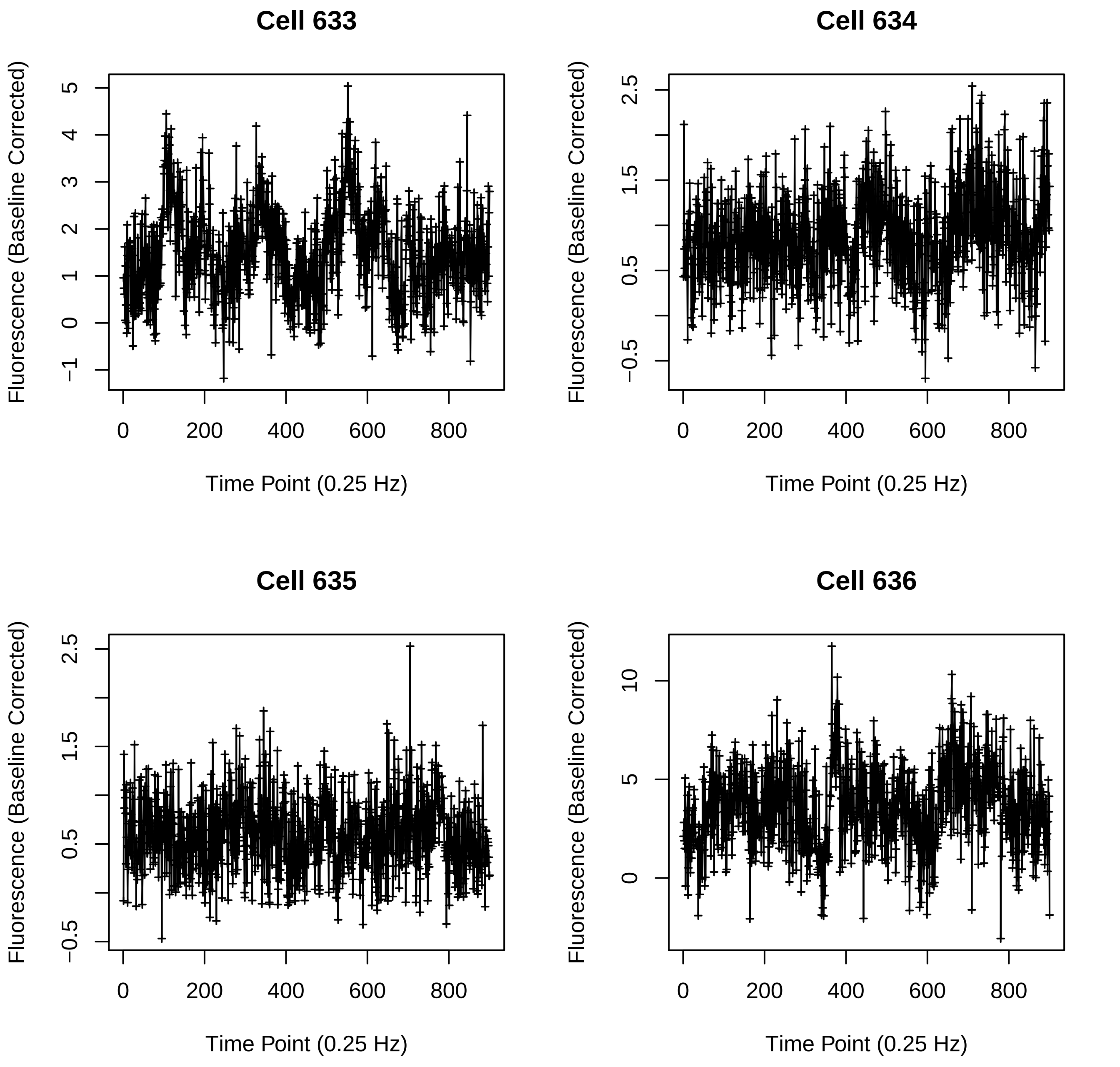
Cell 633
Fluorescence (Baseline Corrected)
−1 0 1 2 3 4 5
0 200 400 600 800
Time Point (0.25 Hz)
Cell 634
Fluorescence (Baseline Corrected)
−0.5 0.5 1.5 2.5
0 200 400 600 800
Time Point (0.25 Hz)
Cell 635
Fluorescence (Baseline Corrected)
−0.5 0.5 1.5 2.5
0 200 400 600 800
Time Point (0.25 Hz)
Cell 636
Fluorescence (Baseline Corrected)
0 5 10
0 200 400 600 800
Time Point (0.25 Hz)

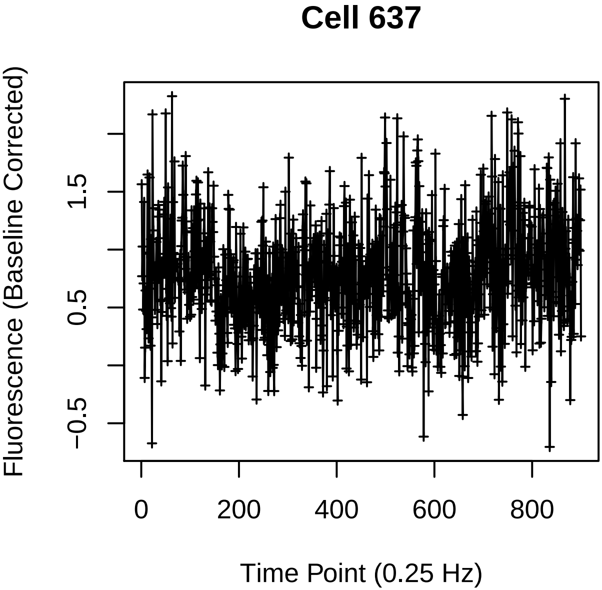
Cell 637
Fluorescence (Baseline Corrected)
−0.5
0.5
1.5
0
200
400
600
800
Time Point (0.25 Hz)

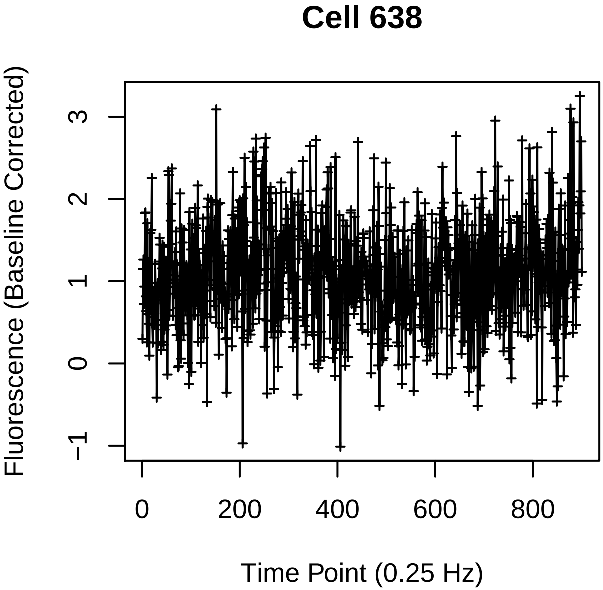
Cell 638
Fluorescence (Baseline Corrected)
−1
0
1
2
3
0
200
400
600
800
Time Point (0.25 Hz)

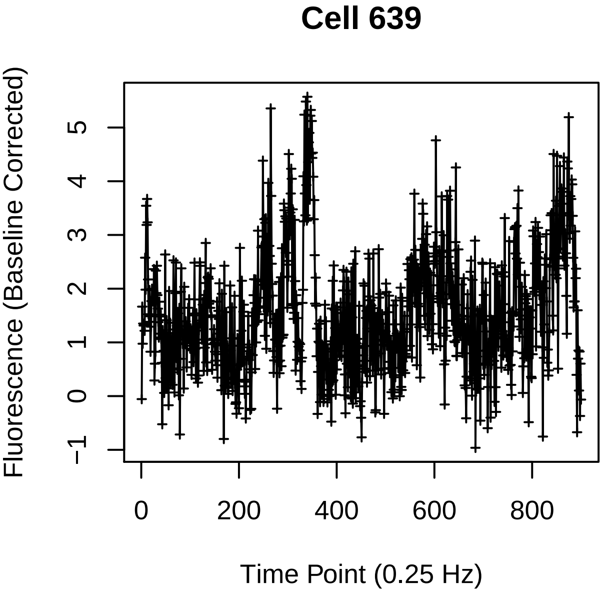
Cell 639
Fluorescence (Baseline Corrected)
−1
0
1
2
3
4
5
0
200
400
600
800
Time Point (0.25 Hz)

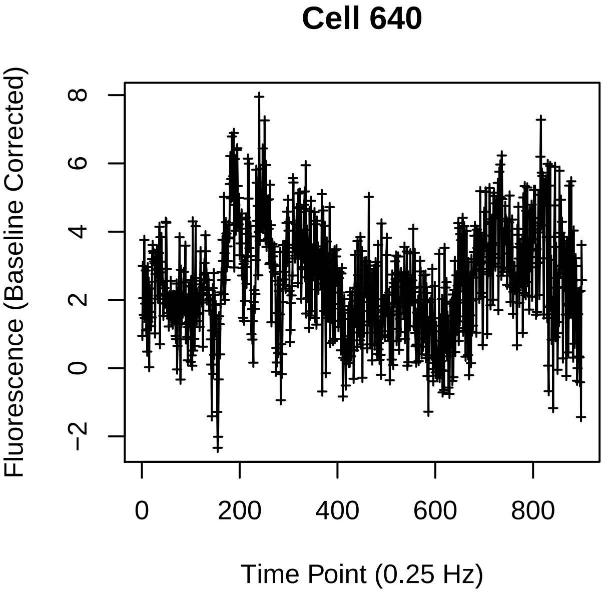
Cell 640
Fluorescence (Baseline Corrected)
−2
0
2
4
6
8
0
200
400
600
800
Time Point (0.25 Hz)

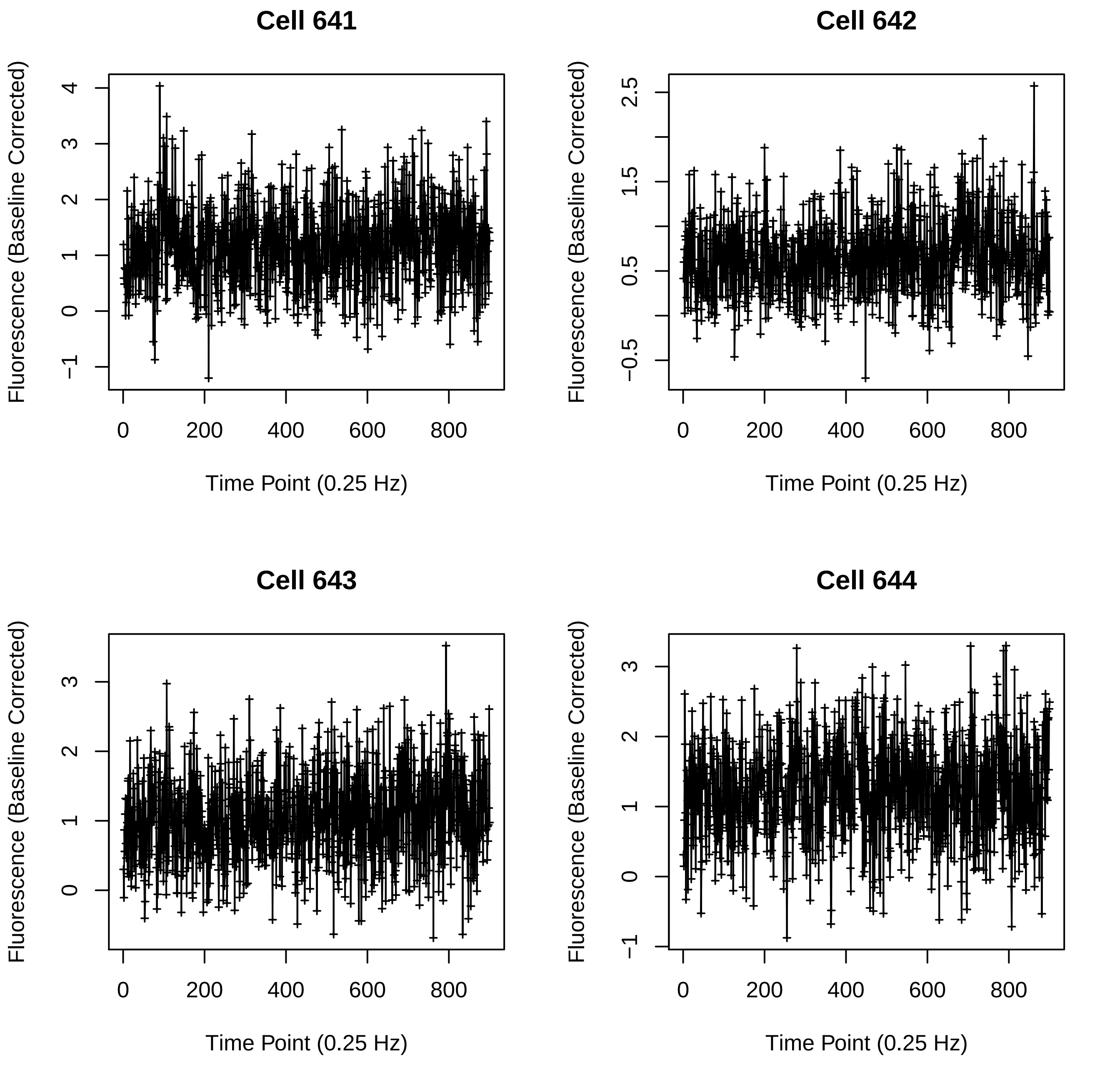

Cell 641
Fluorescence (Baseline Corrected)
Time Point (0.25 Hz)
−1
0
1
2
3
4
0
200
400
600
800
Cell 642
Fluorescence (Baseline Corrected)
Time Point (0.25 Hz)
−0.5
0.5
1.5
2.5
0
200
400
600
800
Cell 643
Fluorescence (Baseline Corrected)
Time Point (0.25 Hz)
0
1
2
3
0
200
400
600
800
Cell 644
Fluorescence (Baseline Corrected)
Time Point (0.25 Hz)
−1
0
1
2
3
0
200
400
600
800

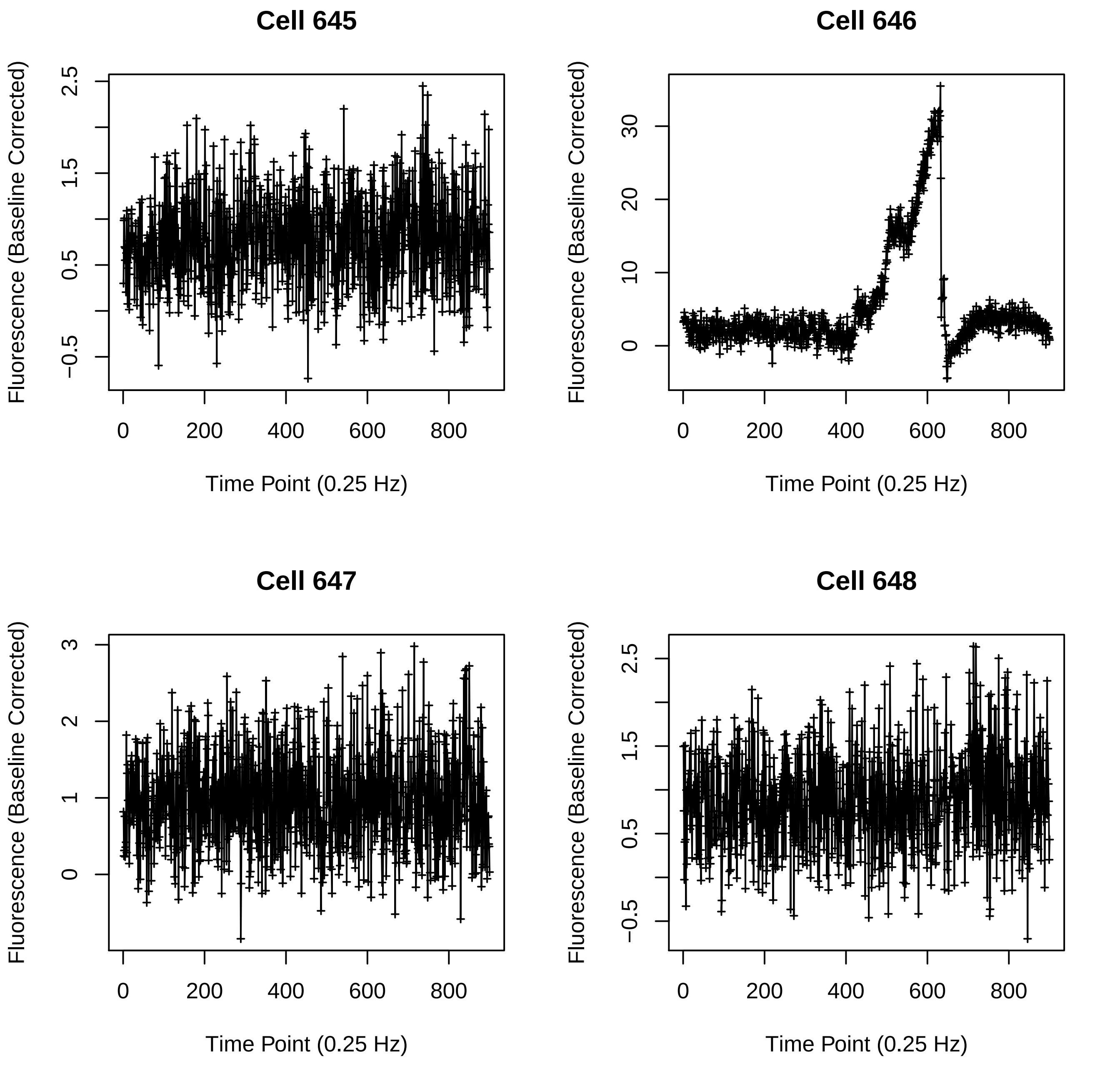
Cell 645
Cell 646
Cell 647
Cell 648
Fluorescence (Baseline Corrected)
Time Point (0.25 Hz)
−0.5
0.5
1.5
2.5
0
10
20
30
0
1
2
3
0
200
400
600
800

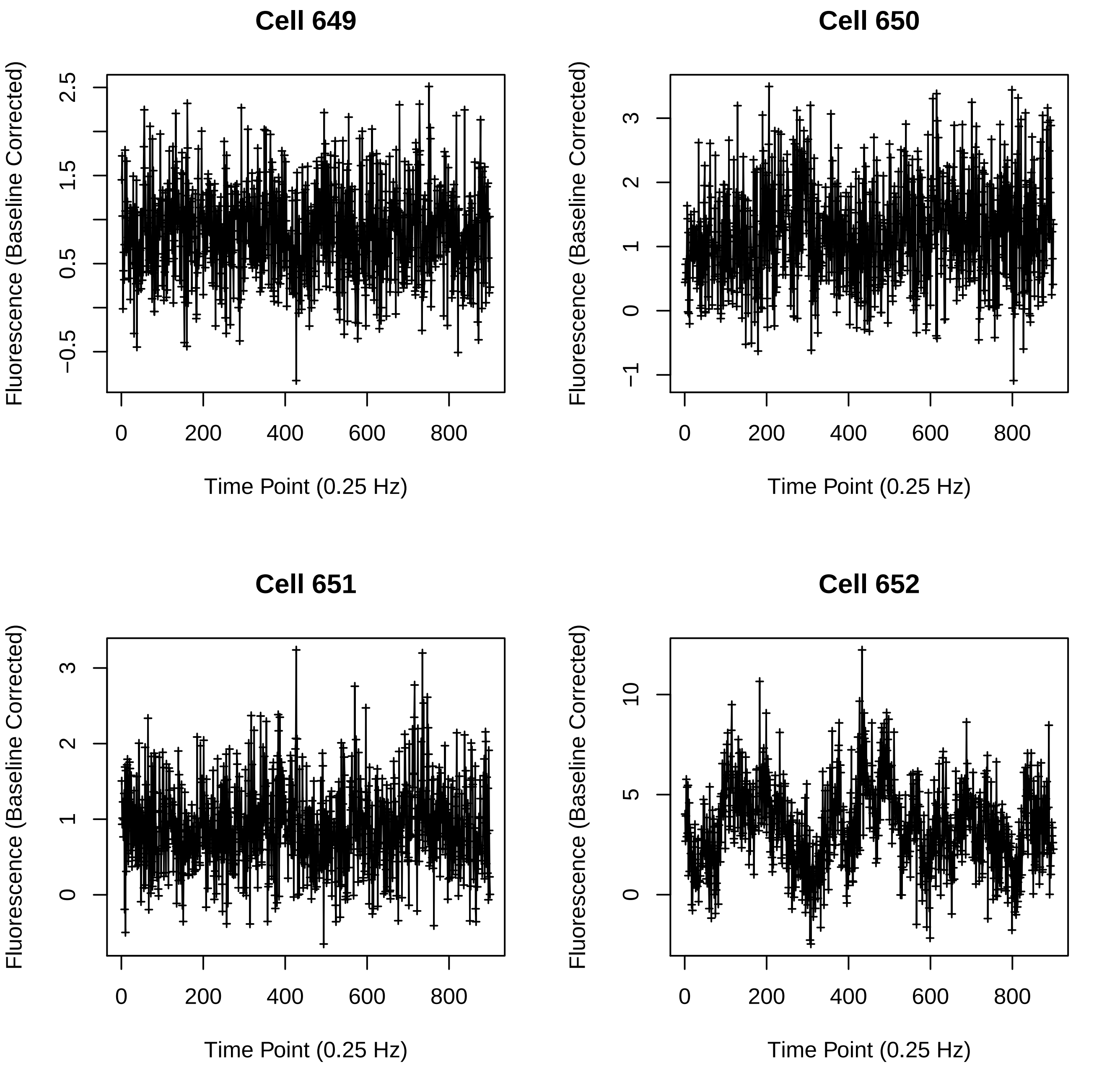
Cell 649
Fluorescence (Baseline Corrected)
-0.5
0.5
1.5
2.5
0
200
400
600
800
Time Point (0.25 Hz)
Cell 650
Fluorescence (Baseline Corrected)
-1
0
1
2
3
0
200
400
600
800
Time Point (0.25 Hz)
Cell 651
Fluorescence (Baseline Corrected)
0
1
2
3
0
200
400
600
800
Time Point (0.25 Hz)
Cell 652
Fluorescence (Baseline Corrected)
0
5
10
0
200
400
600
800
Time Point (0.25 Hz)

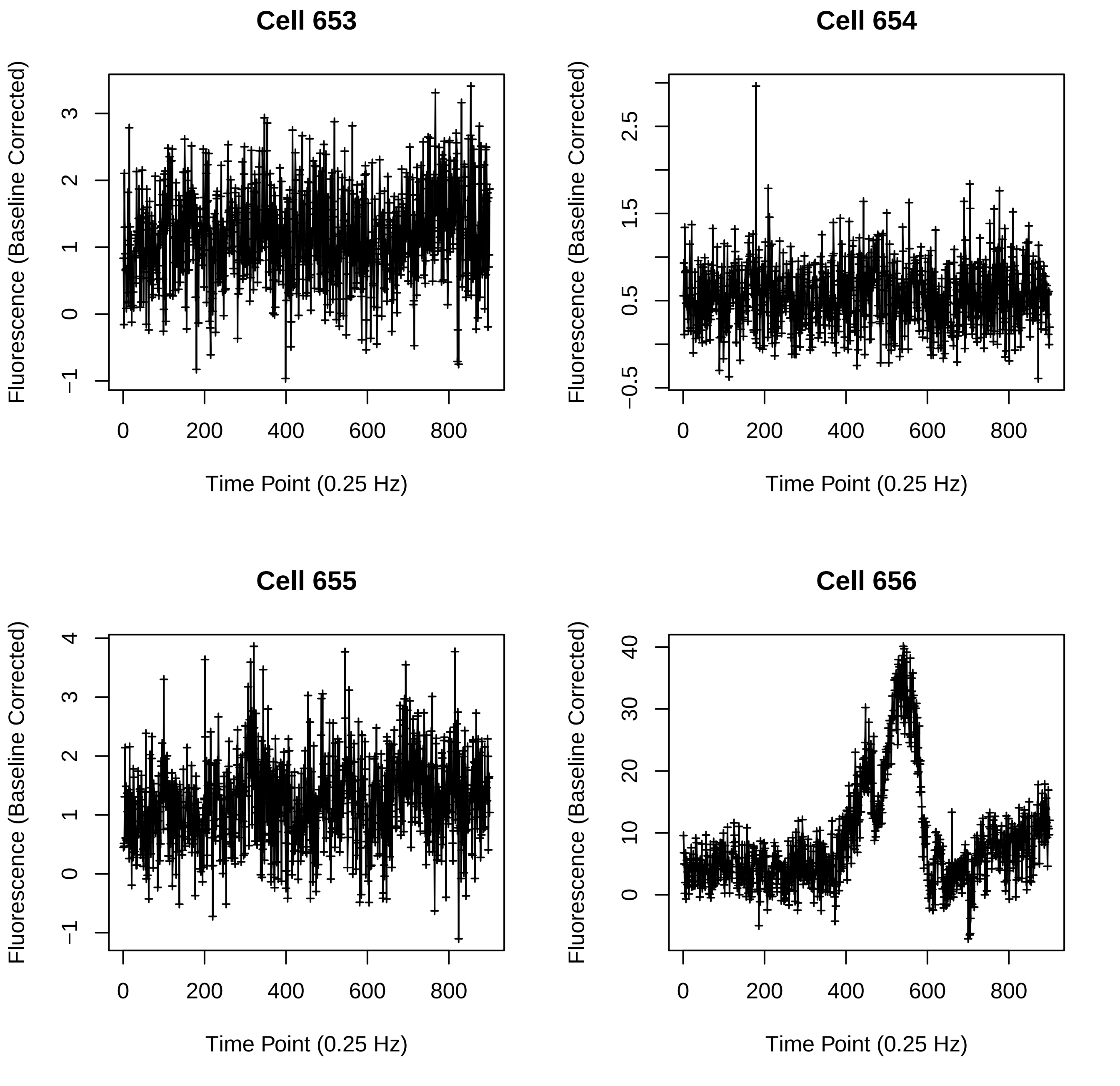
Cell 653
Cell 654
Cell 655
Cell 656
Fluorescence (Baseline Corrected)
Time Point (0.25 Hz)
0
200
400
600
800
−1
0
1
2
3
4
−0.5
0.5
1.5
2.5
10
20
30
40

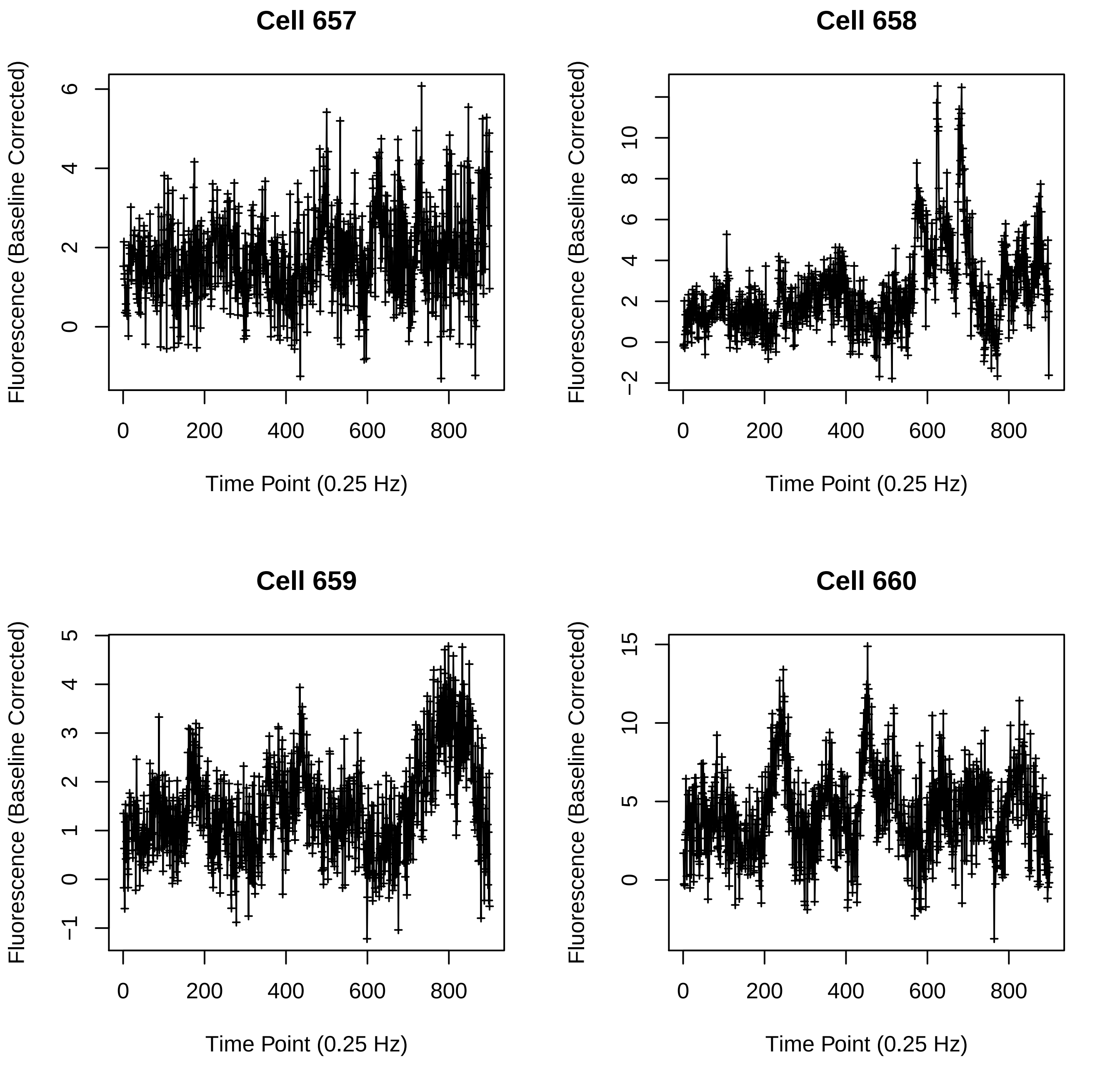
Cell 657
Cell 658
Cell 659
Cell 660
Fluorescence (Baseline Corrected)
Time Point (0.25 Hz)
0
200
400
600
800
−1
0
1
2
3
4
5
6
−2
8
10
15

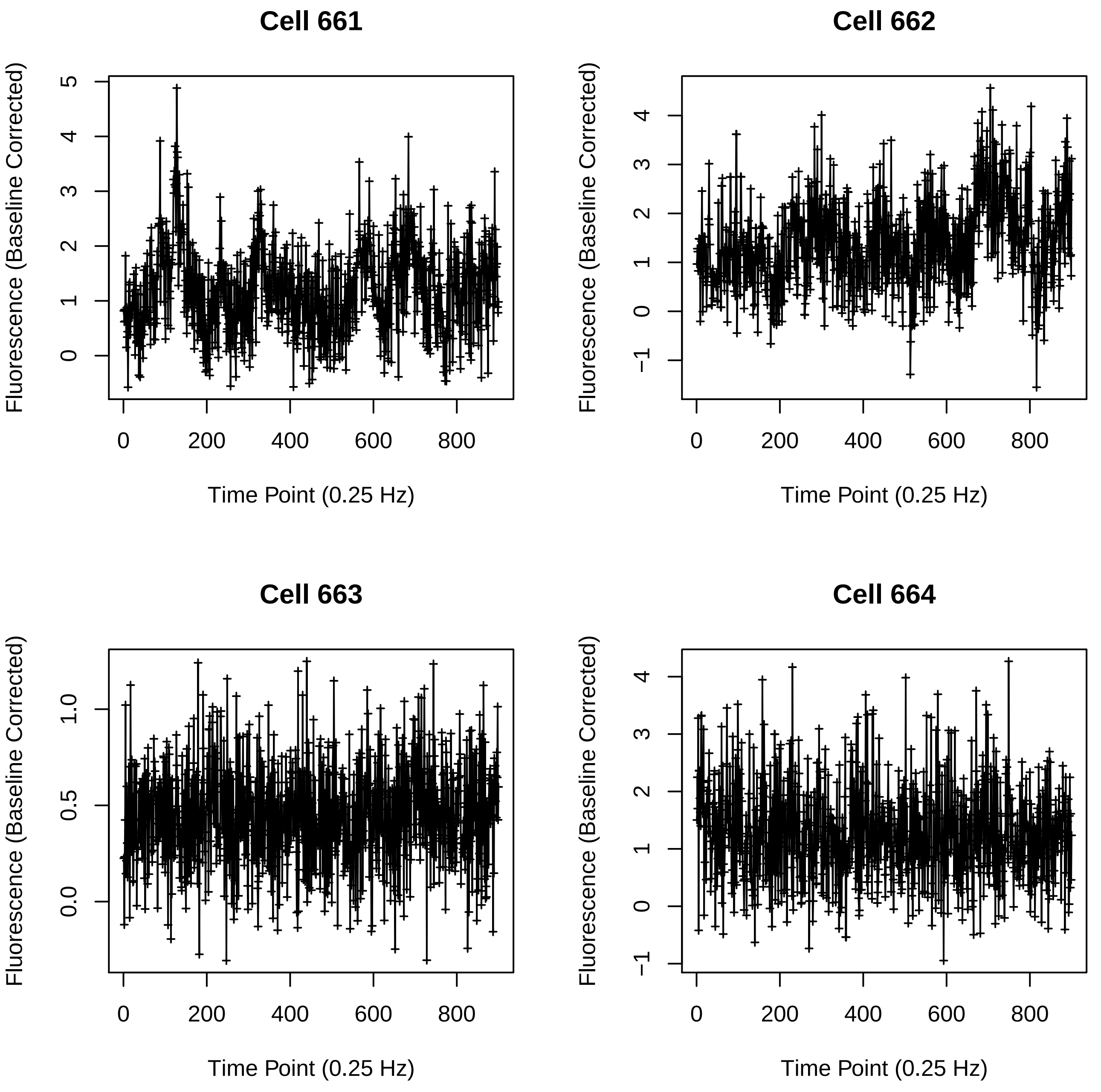

Cell 661
Cell 662
Cell 663
Cell 664
Fluorescence (Baseline Corrected)
Time Point (0.25 Hz)
0
1
2
3
4
5
−1
0.0
0.5
1.0
200
400
600
800

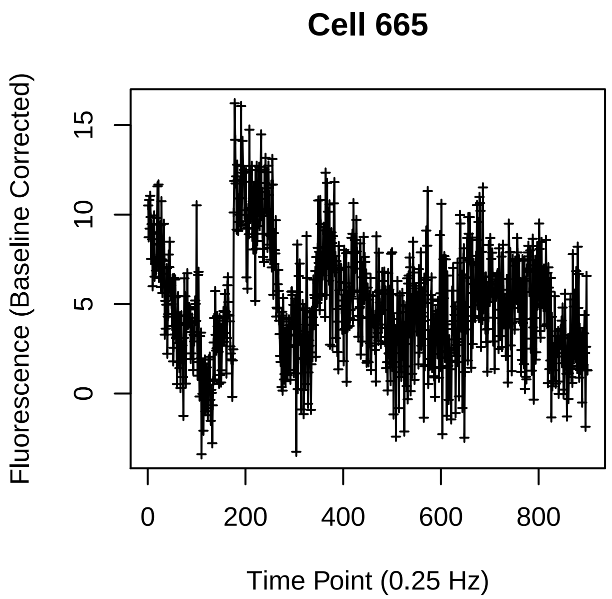
Cell 665
Fluorescence (Baseline Corrected)
Time Point (0.25 Hz)
0
5
10
15
0
200
400
600
800

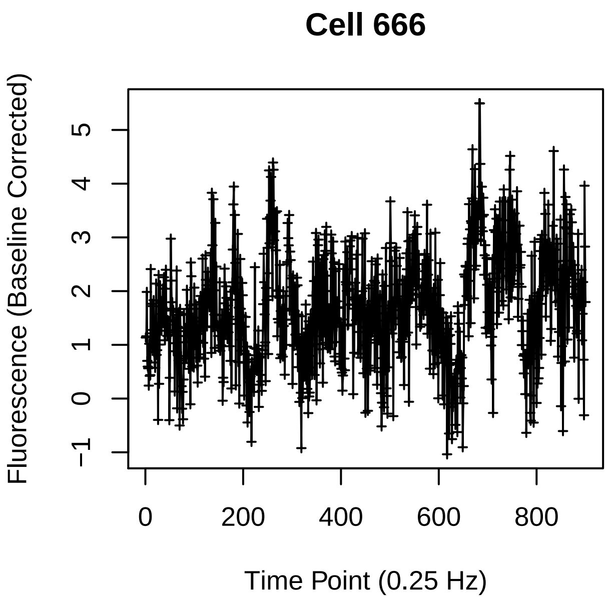
Cell 666
Fluorescence (Baseline Corrected)
Time Point (0.25 Hz)
−1
0
1
2
3
4
5
0
200
400
600
800

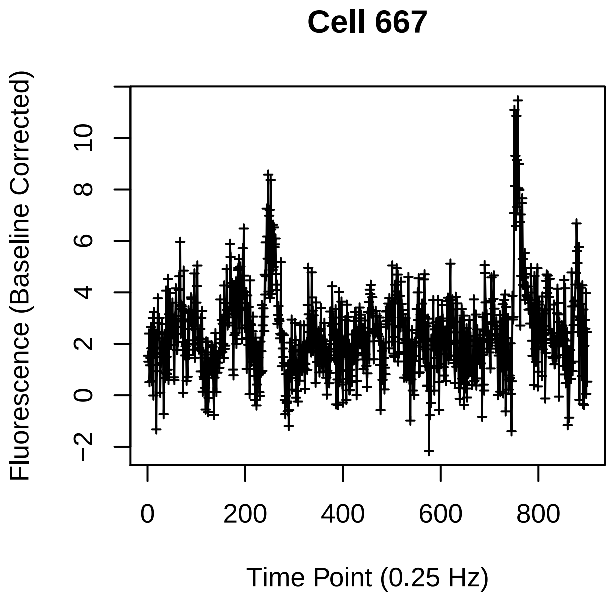
Cell 667
Fluorescence (Baseline Corrected)
Time Point (0.25 Hz)
−2
0
2
4
6
8
10
0
200
400
600
800

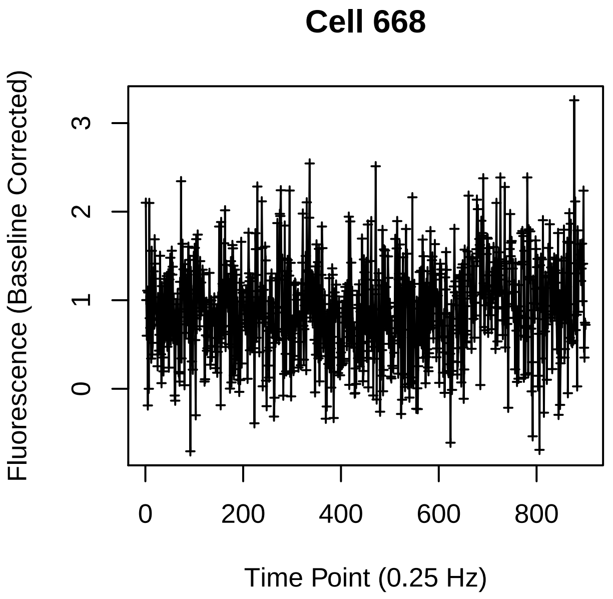
Cell 668
Fluorescence (Baseline Corrected)
Time Point (0.25 Hz)
0
1
2
3
0
200
400
600
800

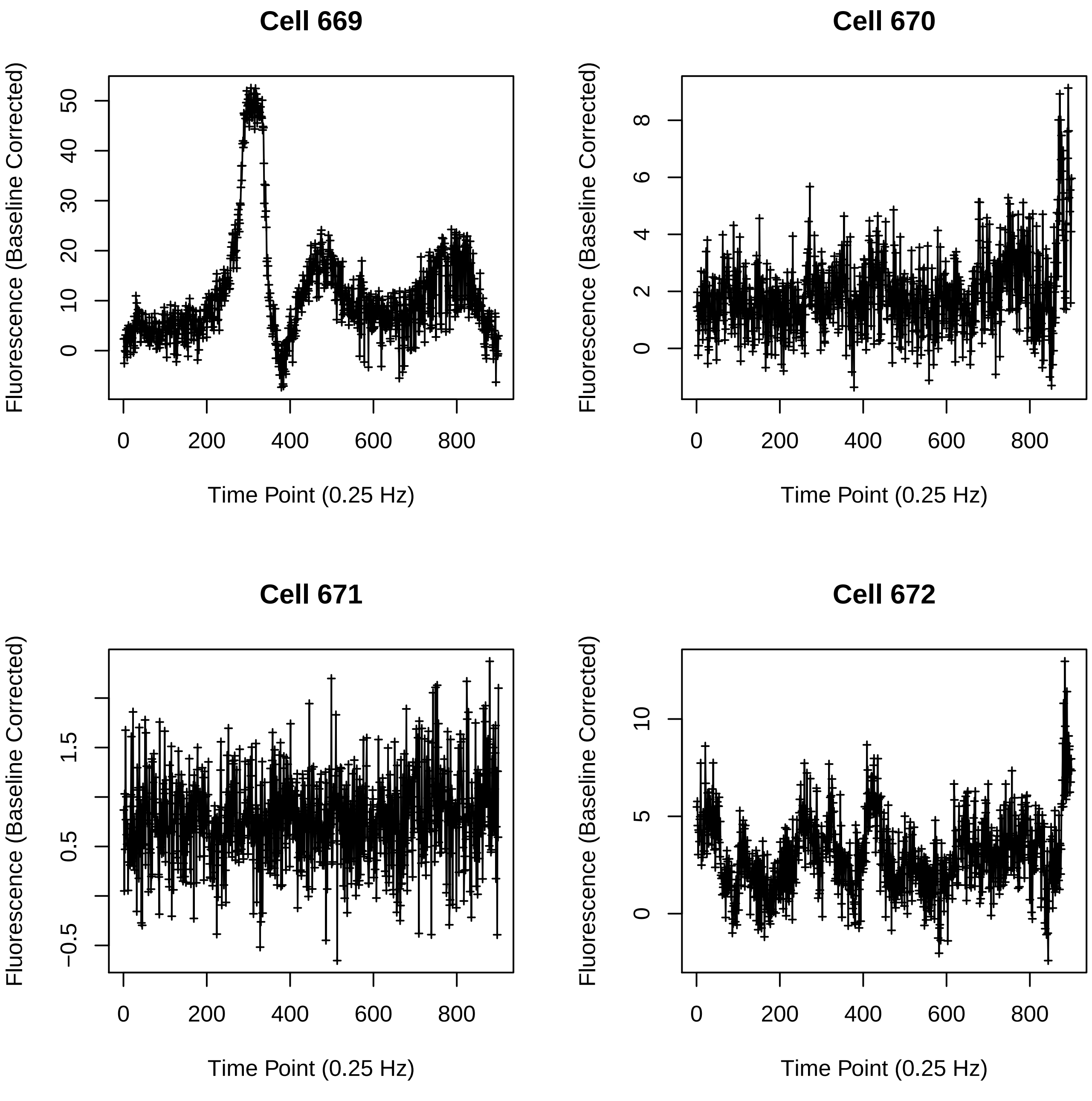
Cell 669
Cell 670
Cell 671
Cell 672
Fluorescence (Baseline Corrected)
Time Point (0.25 Hz)
0
10
20
30
40
50
0
2
4
6
8
−0.5
0.5
1.5
0
5
10
0
200
400
600
800

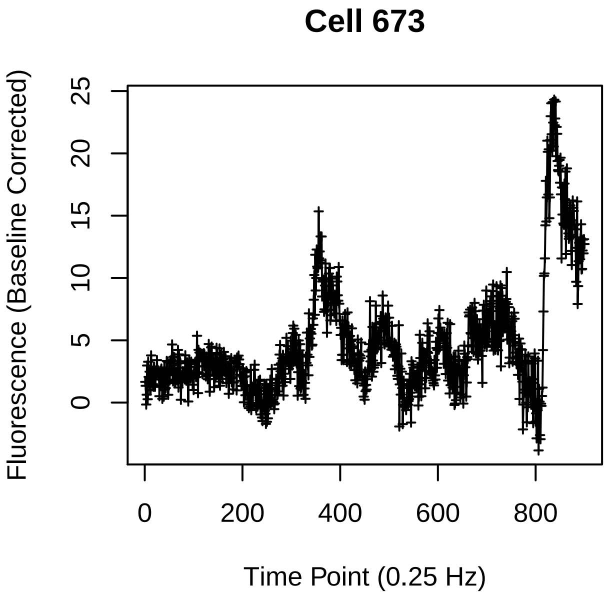
Cell 673
Fluorescence (Baseline Corrected)
0
5
10
15
20
25
0
200
400
600
800
Time Point (0.25 Hz)

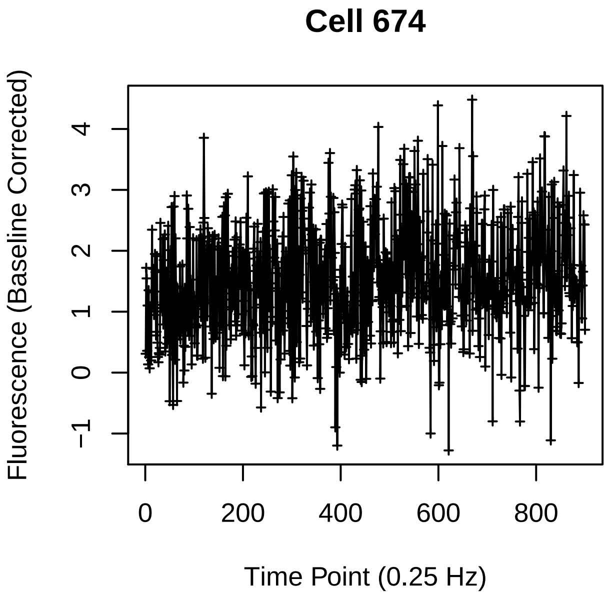
Cell 674
Fluorescence (Baseline Corrected)
−1
0
1
2
3
4
0
200
400
600
800
Time Point (0.25 Hz)

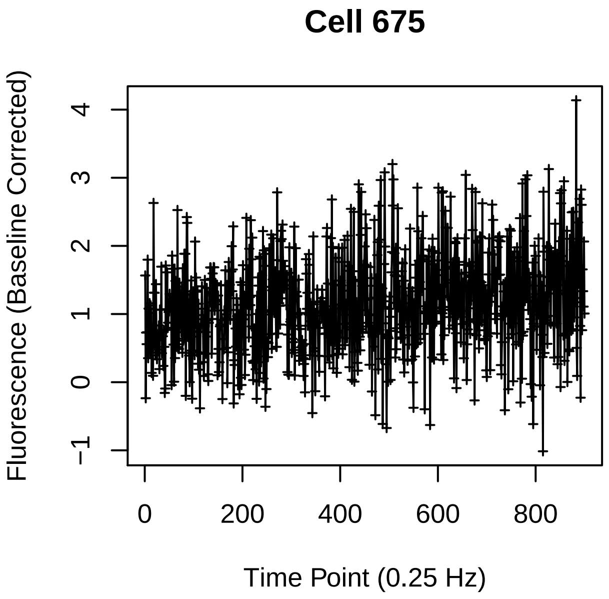
Cell 675
Fluorescence (Baseline Corrected)
−1
0
1
2
3
4
0
200
400
600
800
Time Point (0.25 Hz)

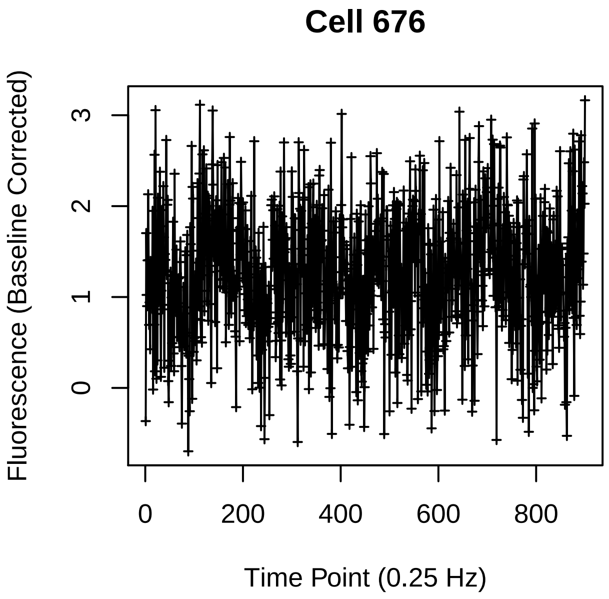
Cell 676
Fluorescence (Baseline Corrected)
0
1
2
3
0
200
400
600
800
Time Point (0.25 Hz)

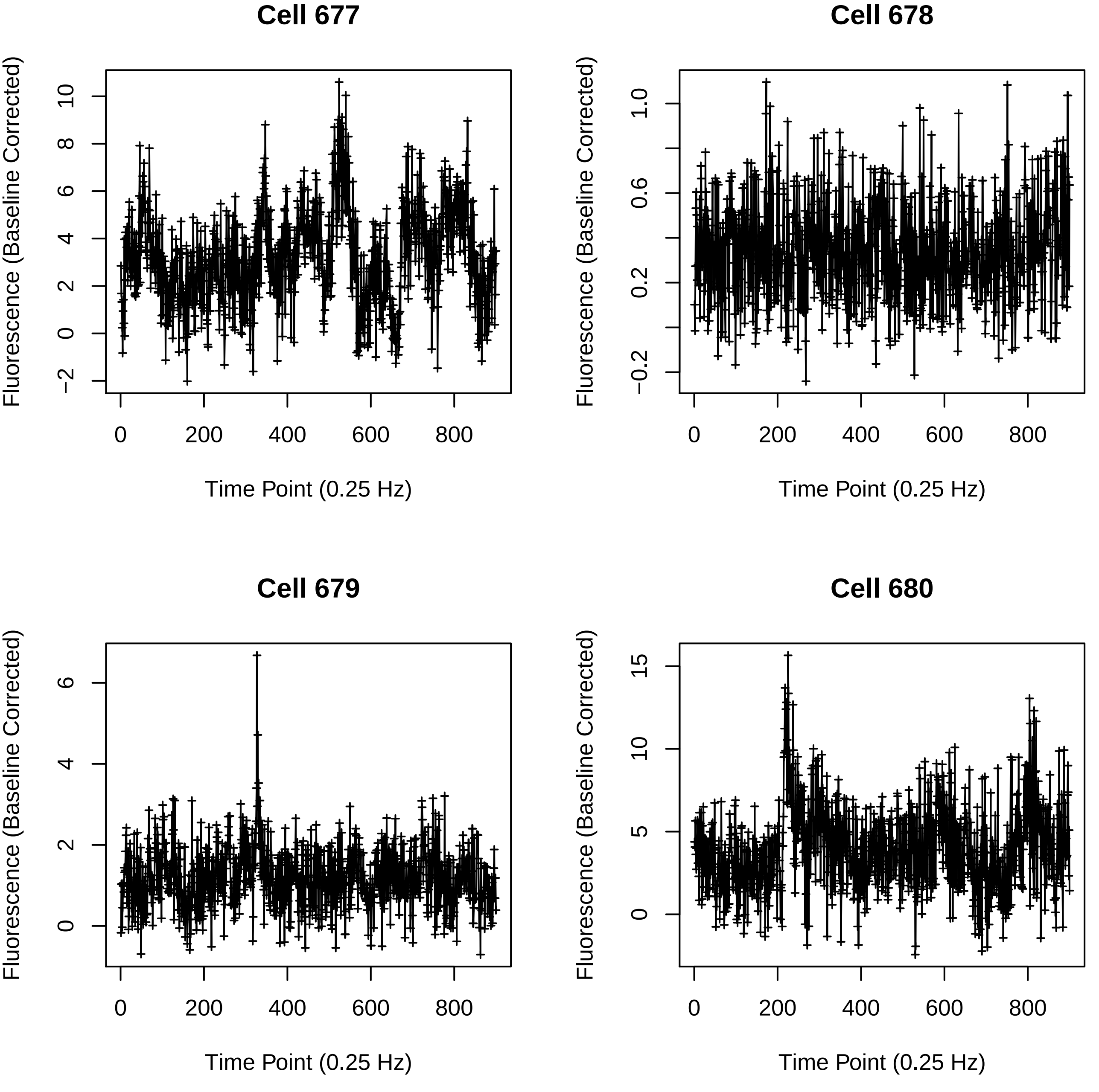

Cell 677
Cell 678
Cell 679
Cell 680
Fluorescence (Baseline Corrected)
Time Point (0.25 Hz)
−2
0
2
4
6
8
10
−0.2
0.2
0.6
1.0
0
2
4
6
0
5
10
15
0
200
400
600
800

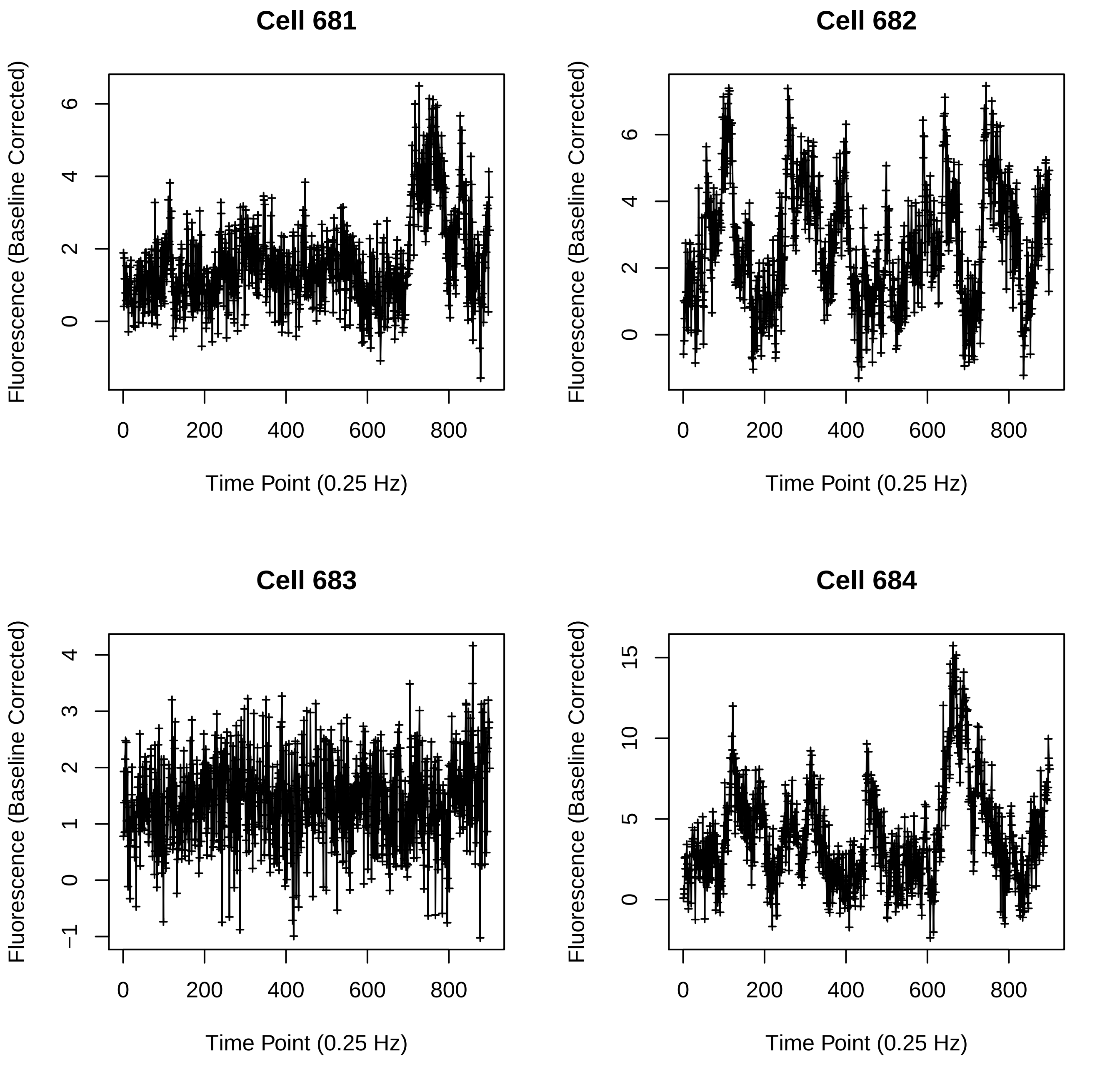
Cell 681
Fluorescence (Baseline Corrected)
0
2
4
6
0
200
400
600
800
Time Point (0.25 Hz)
Cell 682
Fluorescence (Baseline Corrected)
0
2
4
6
0
200
400
600
800
Time Point (0.25 Hz)
Cell 683
Fluorescence (Baseline Corrected)
−1
0
1
2
3
4
0
200
400
600
800
Time Point (0.25 Hz)
Cell 684
Fluorescence (Baseline Corrected)
0
5
10
15
0
200
400
600
800
Time Point (0.25 Hz)

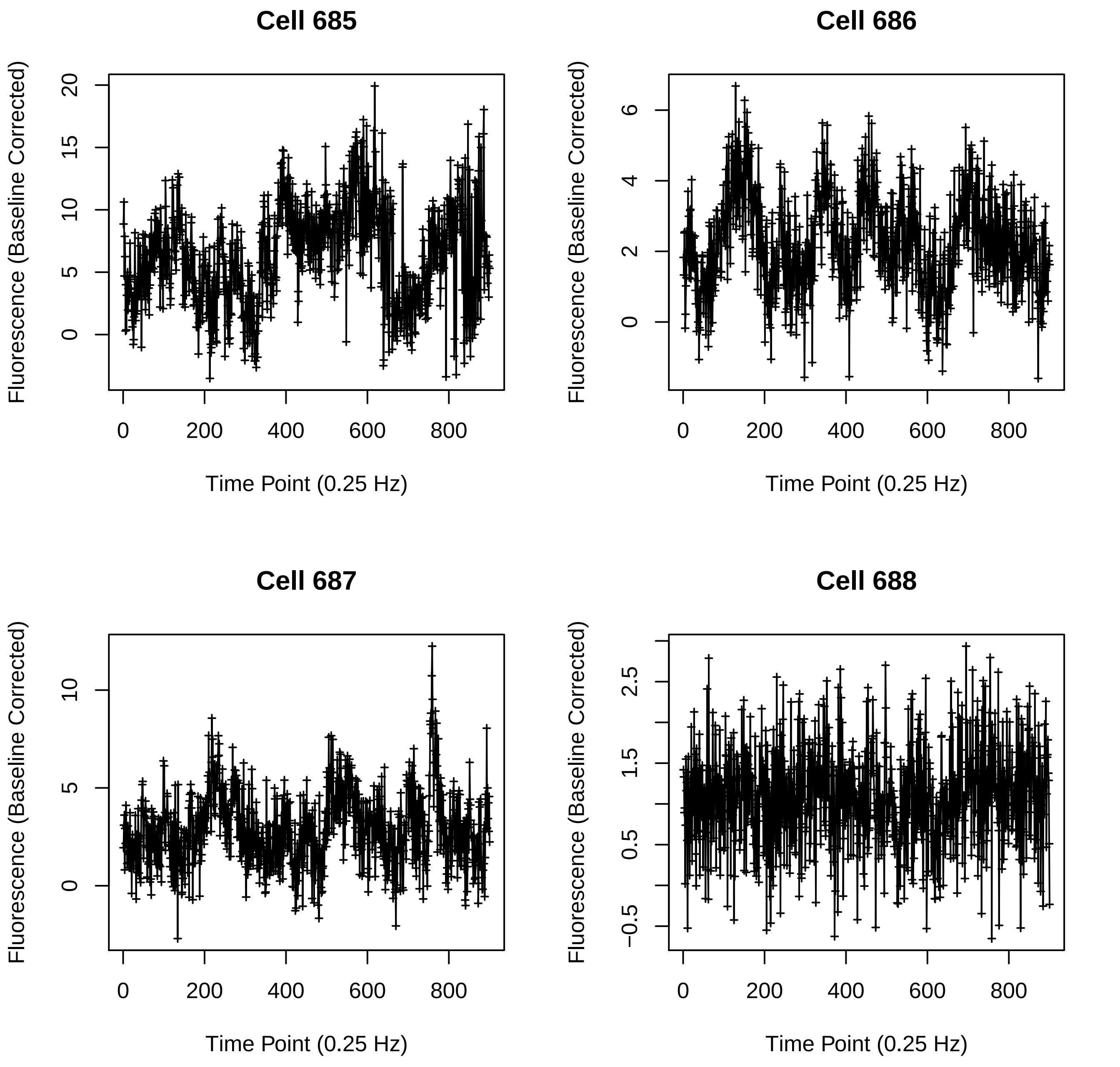
Cell 685
Cell 686
Cell 687
Cell 688
Fluorescence (Baseline Corrected)
Time Point (0.25 Hz)
0
5
10
15
20
0
2
4
6
0
5
10
−0.5
0.5
1.5
2.5
0
200
400
600
800

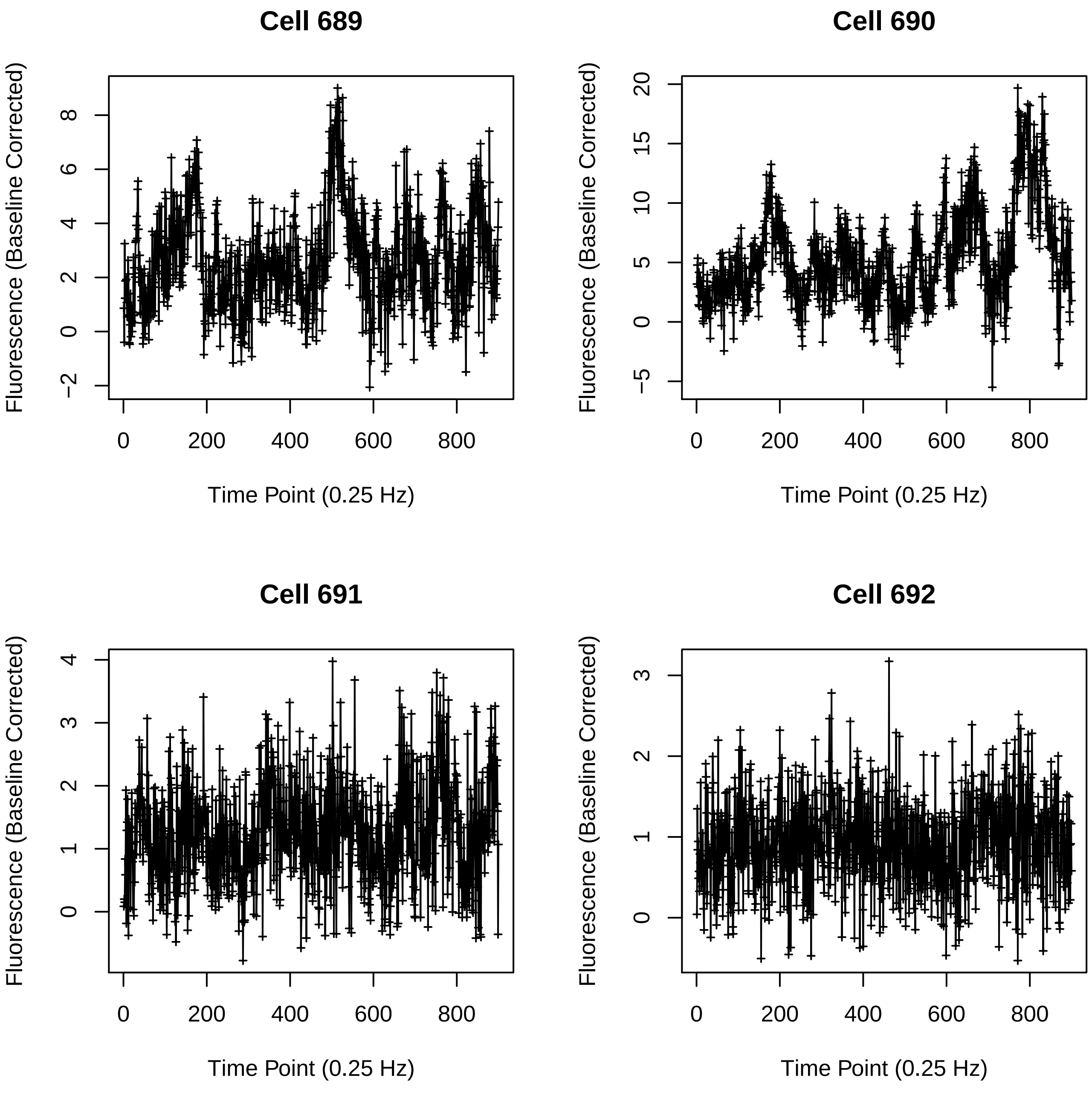
Cell 689
Cell 690
Cell 691
Cell 692
Fluorescence (Baseline Corrected)
Time Point (0.25 Hz)
−2
0
2
4
6
8
−5
0
5
10
15
20
0
1
2
3
4
0
1
2
3
0
200
400
600
800

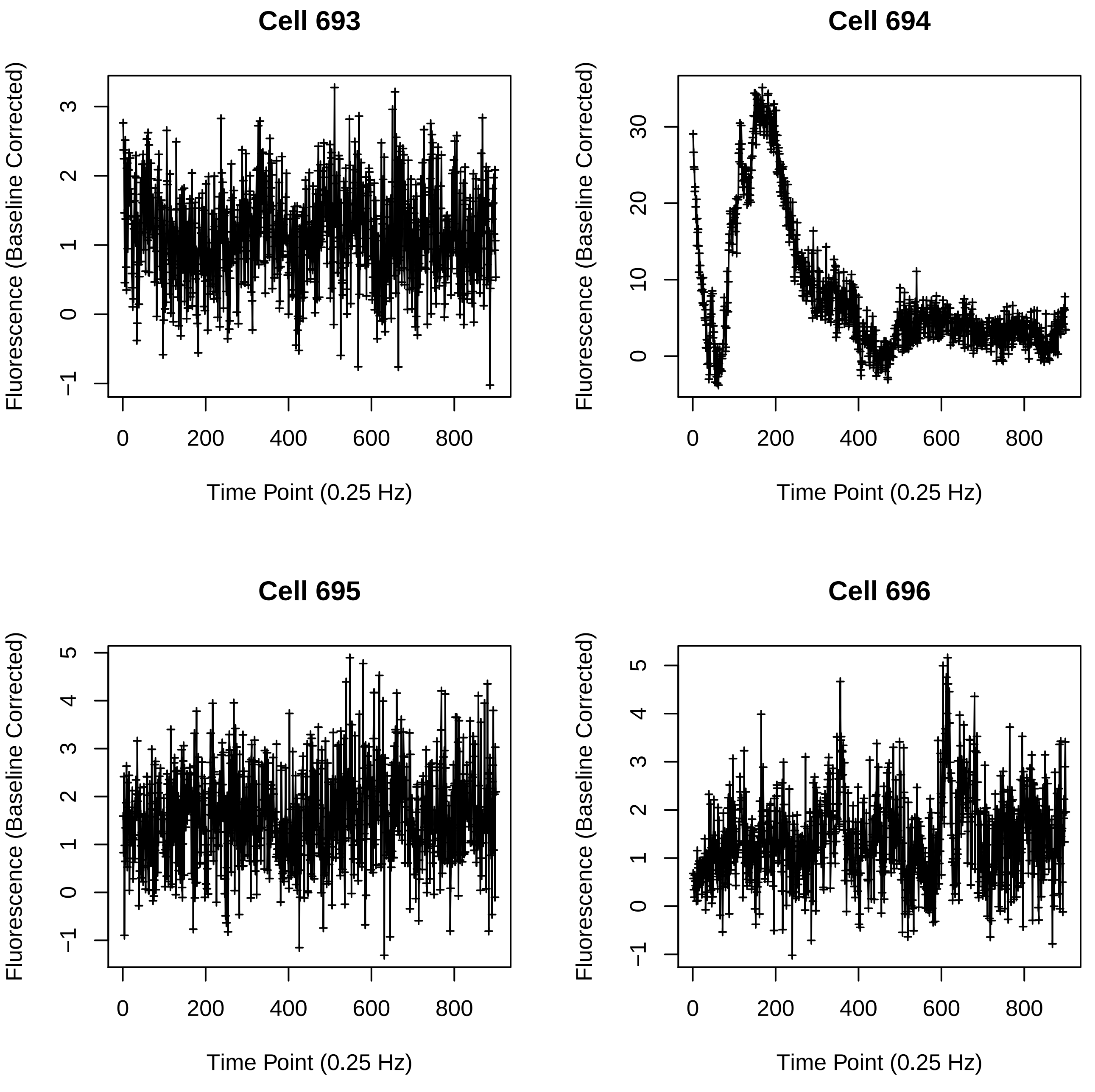
Cell 693
Cell 694
Cell 695
Cell 696
Fluorescence (Baseline Corrected)
Time Point (0.25 Hz)
0
200
400
600
800
−1
1
2
3
4
5
10
20
30

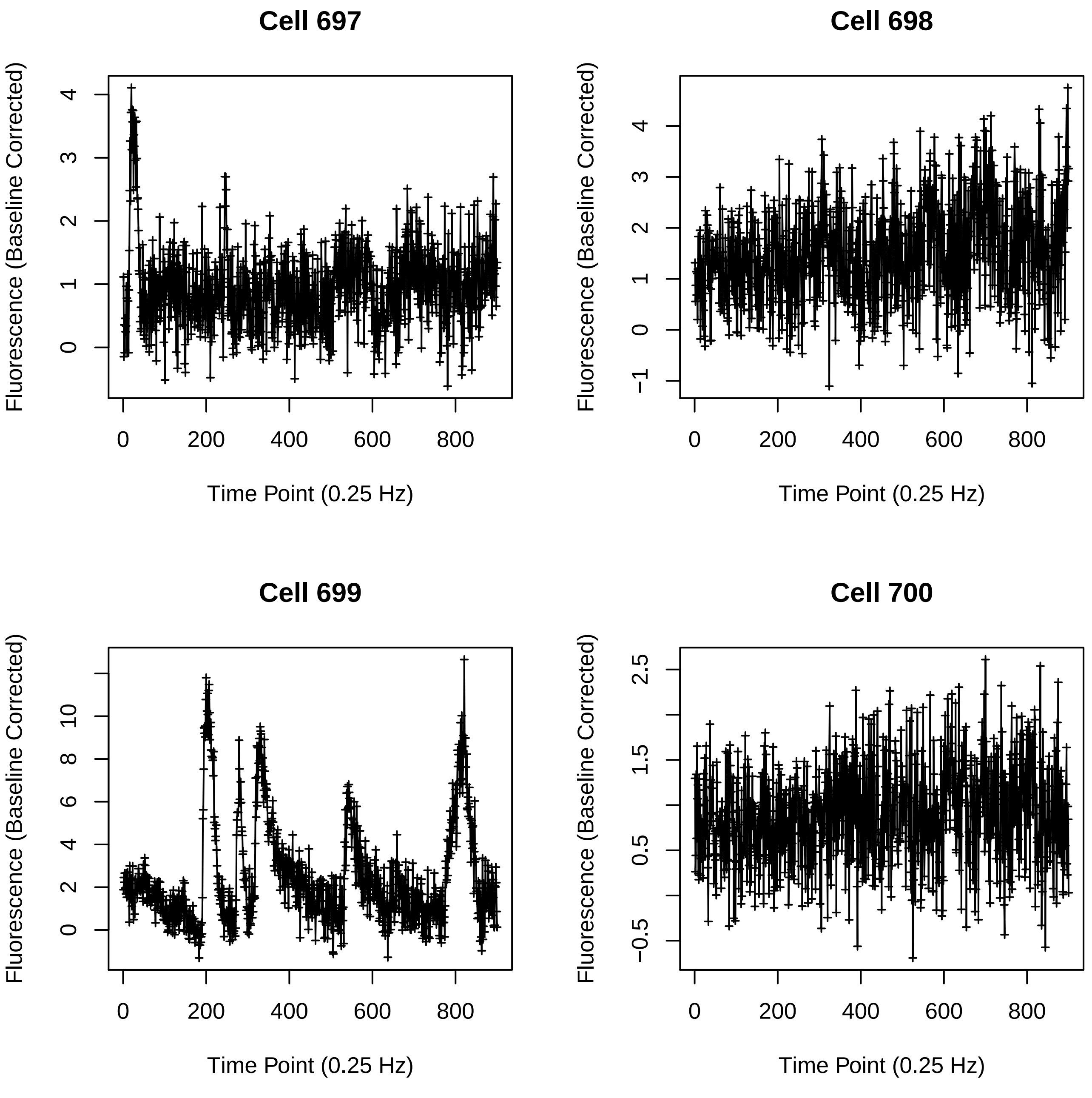

Cell 697
Cell 698
Cell 699
Cell 700
Fluorescence (Baseline Corrected)
Time Point (0.25 Hz)
0
200
400
600
800
1
2
3
4
−1
6
8
10
−0.5
0.5
1.5
2.5

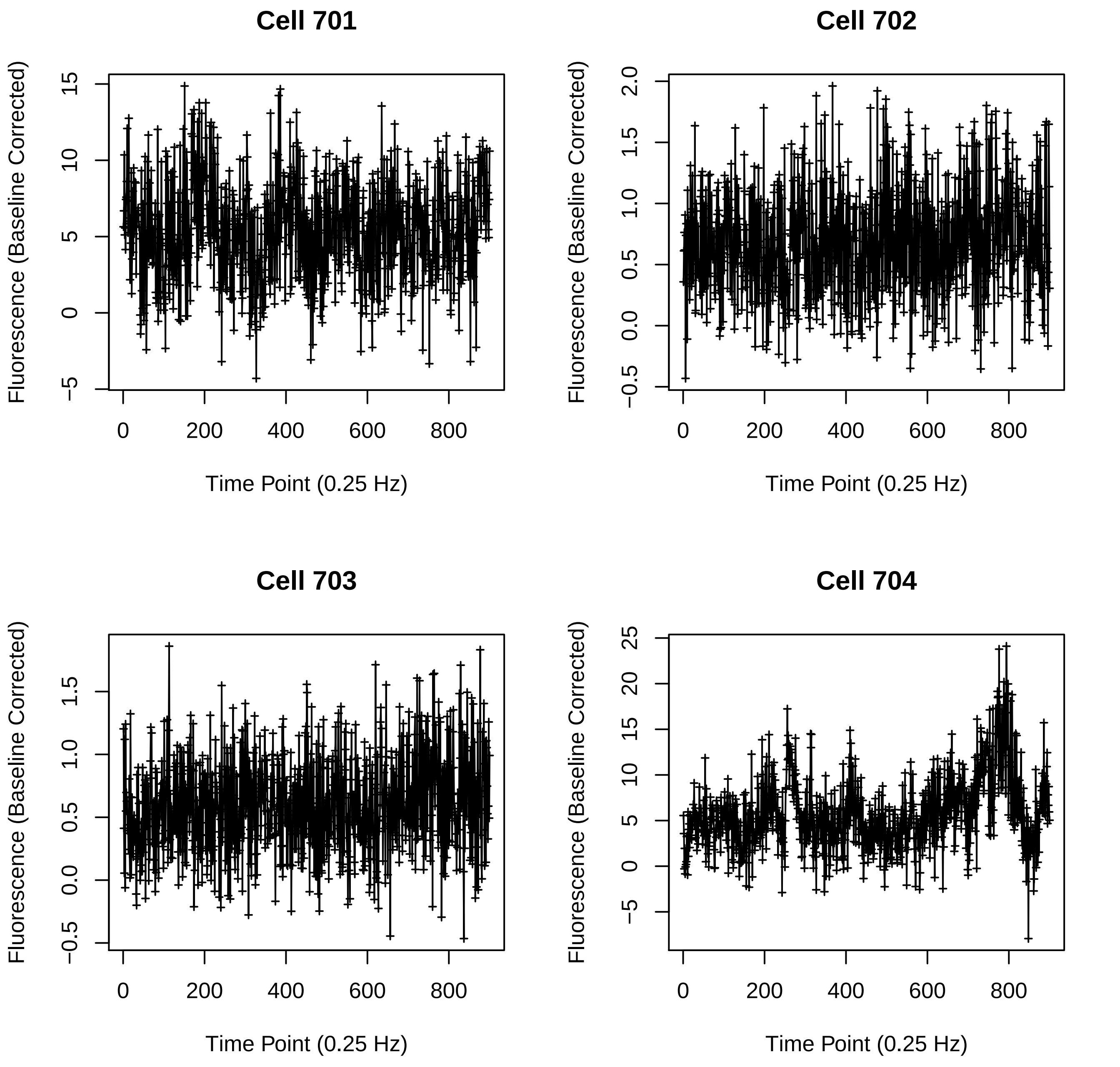
Cell 701
Cell 702
Cell 703
Cell 704
Fluorescence (Baseline Corrected)
Time Point (0.25 Hz)
0
200
400
600
800
−5
0
5
10
15
−0.5
0.0
0.5
1.0
1.5
2.0
20
25

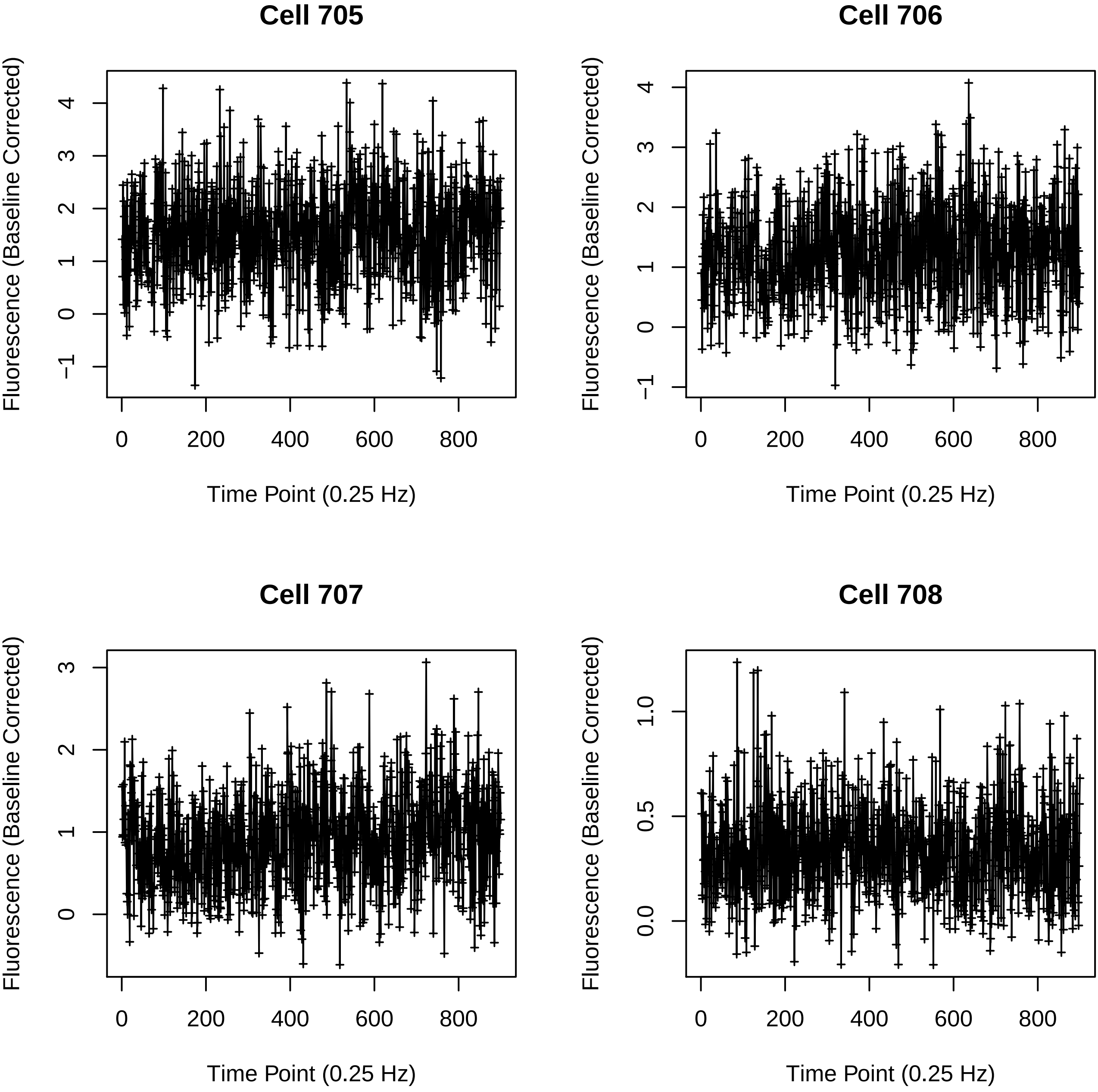
Cell 705
Fluorescence (Baseline Corrected)
−1
0
1
2
3
4
0
200
400
600
800
Time Point (0.25 Hz)
Cell 706
Fluorescence (Baseline Corrected)
−1
0
1
2
3
4
0
200
400
600
800
Time Point (0.25 Hz)
Cell 707
Fluorescence (Baseline Corrected)
0
1
2
3
0
200
400
600
800
Time Point (0.25 Hz)
Cell 708
Fluorescence (Baseline Corrected)
0.0
0.5
1.0
0
200
400
600
800
Time Point (0.25 Hz)

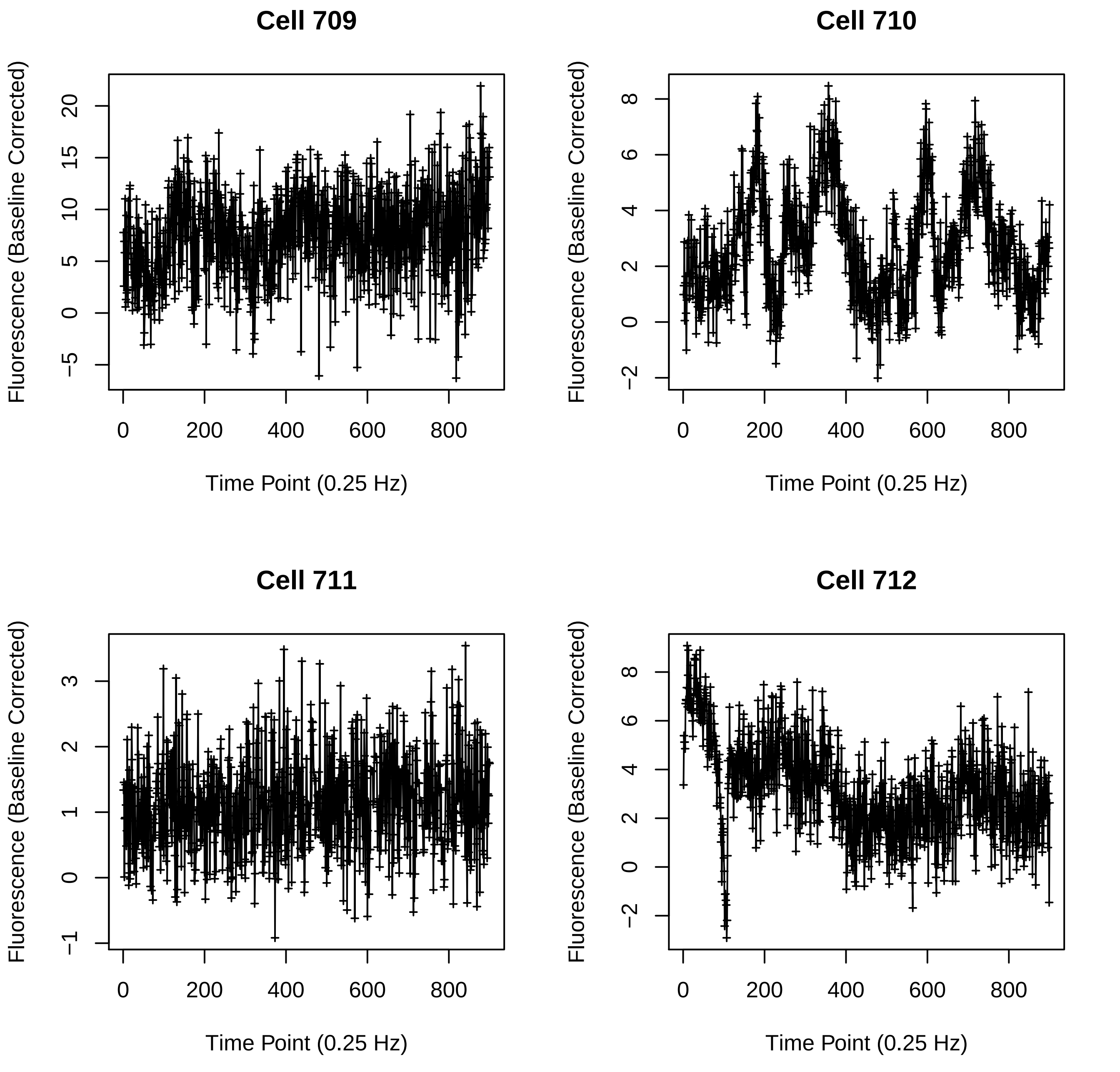
Cell 709
Fluorescence (Baseline Corrected)
Time Point (0.25 Hz)
Cell 710
Fluorescence (Baseline Corrected)
Time Point (0.25 Hz)
Cell 711
Fluorescence (Baseline Corrected)
Time Point (0.25 Hz)
Cell 712
Fluorescence (Baseline Corrected)
Time Point (0.25 Hz)

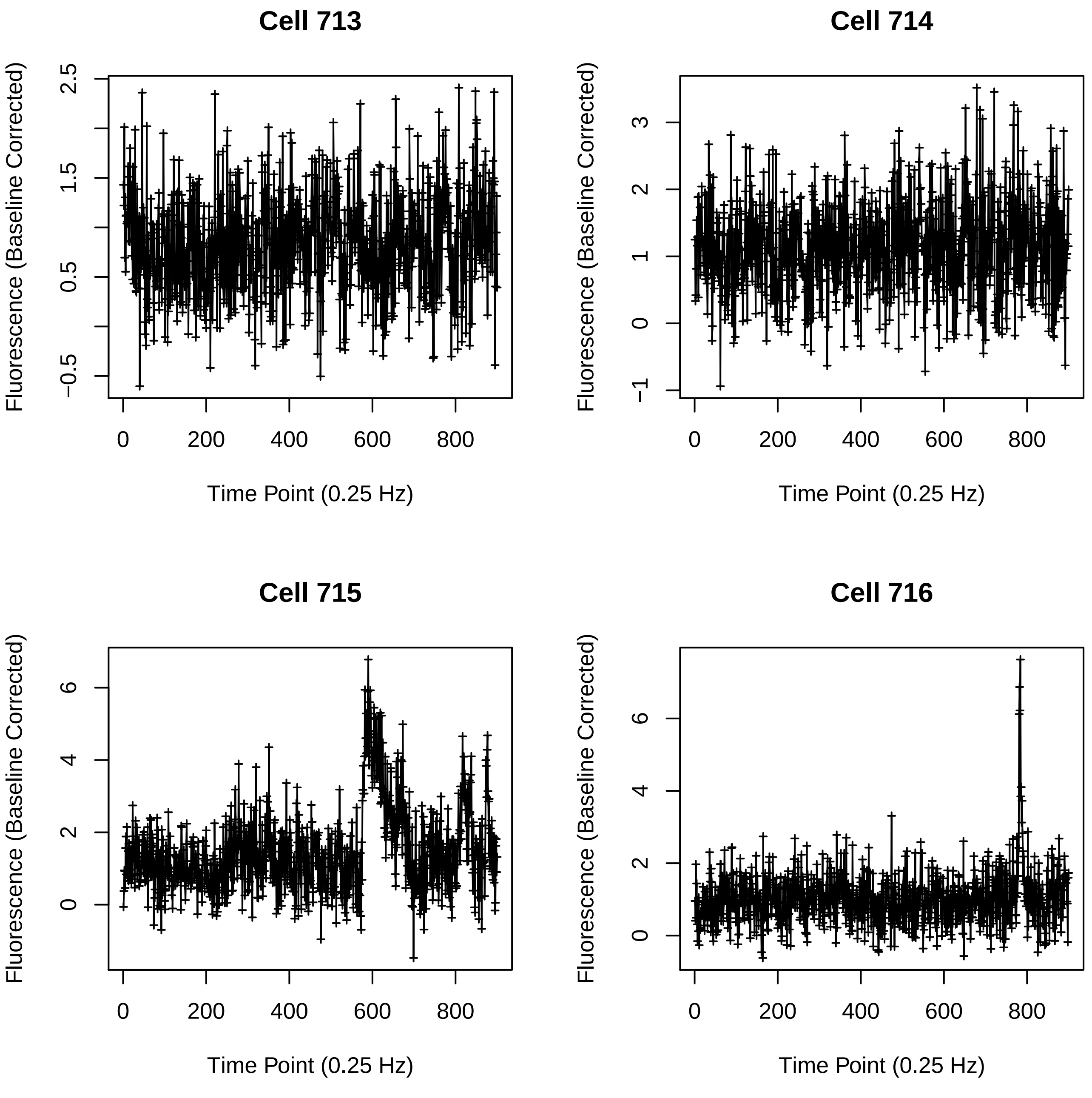
Cell 713
Fluorescence (Baseline Corrected)
2.5
1.5
0.5
−0.5
0
200
400
600
800
Time Point (0.25 Hz)
Cell 714
Fluorescence (Baseline Corrected)
3
2
1
0
−1
0
200
400
600
800
Time Point (0.25 Hz)
Cell 715
Fluorescence (Baseline Corrected)
6
4
2
0
0
200
400
600
800
Time Point (0.25 Hz)
Cell 716
Fluorescence (Baseline Corrected)
6
4
2
0
0
200
400
600
800
Time Point (0.25 Hz)

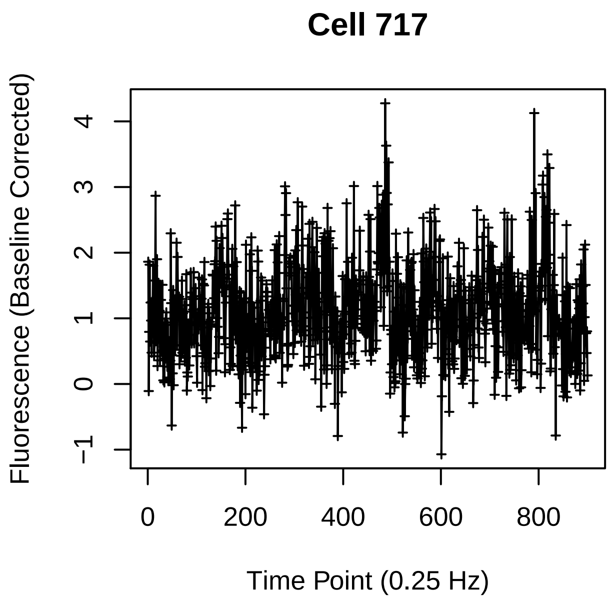
Cell 717
Fluorescence (Baseline Corrected)
Time Point (0.25 Hz)
−1
0
1
2
3
4
0
200
400
600
800

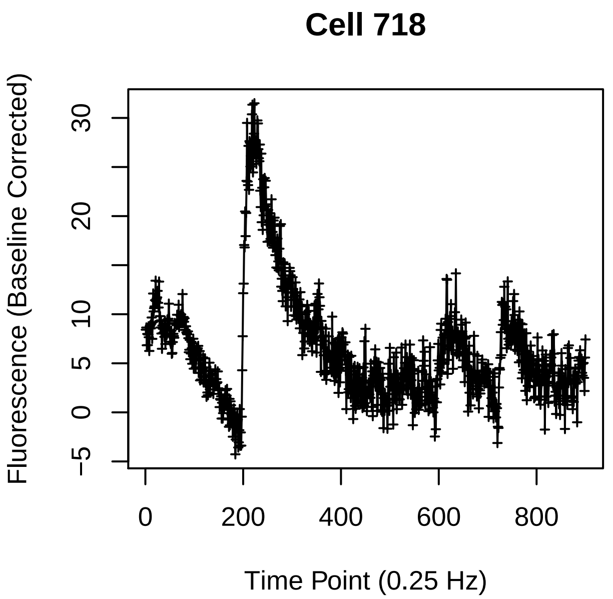
Cell 718
Fluorescence (Baseline Corrected)
Time Point (0.25 Hz)
−5
0
5
10
20
30
0
200
400
600
800

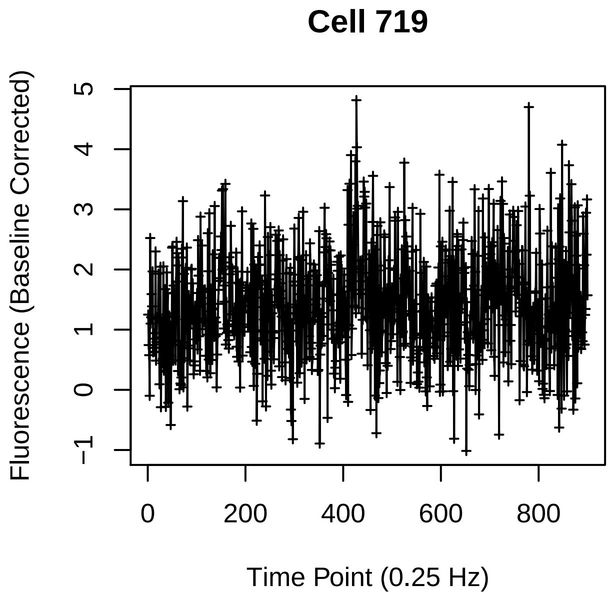
Cell 719
Fluorescence (Baseline Corrected)
Time Point (0.25 Hz)
−1
0
1
2
3
4
5
0
200
400
600
800

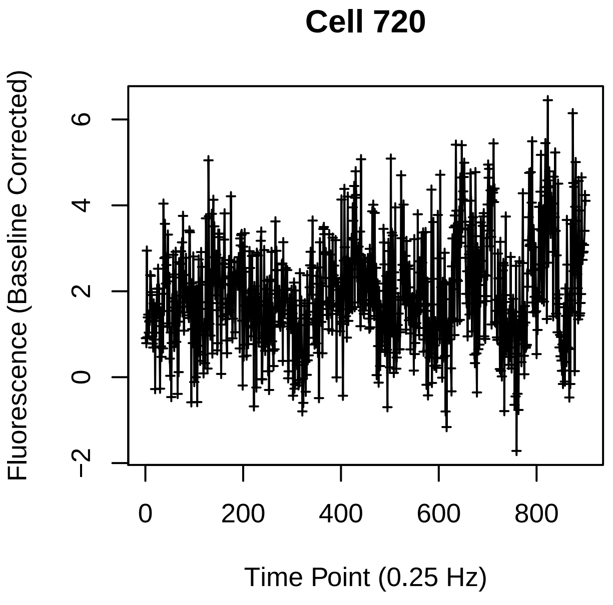
Cell 720
Fluorescence (Baseline Corrected)
Time Point (0.25 Hz)
−2
0
2
4
6
0
200
400
600
800

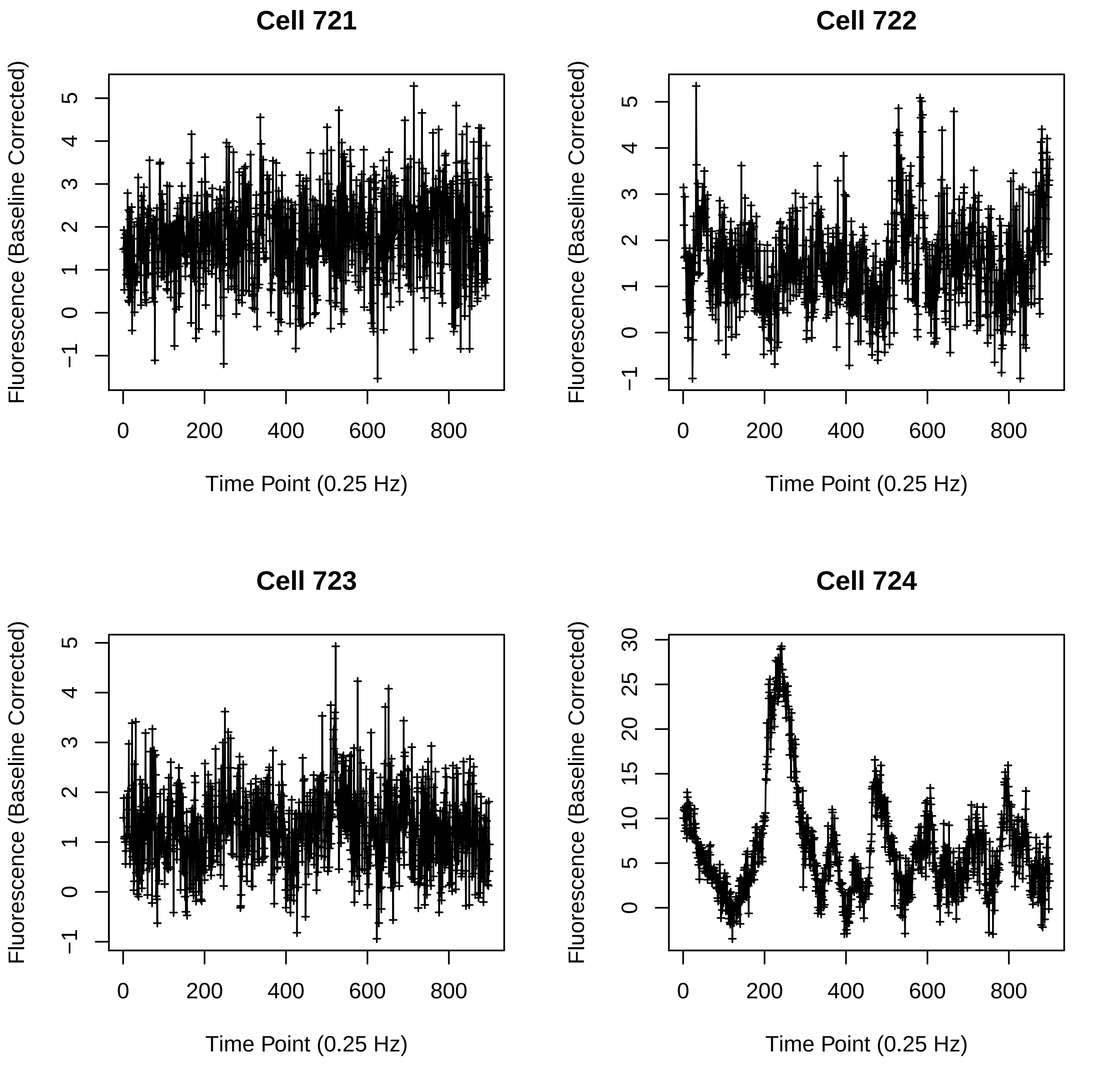
Cell 721
Cell 722
Cell 723
Cell 724
Fluorescence (Baseline Corrected)
Time Point (0.25 Hz)
0
200
400
600
800
−1
0
1
2
3
4
5
10
15
20
25
30

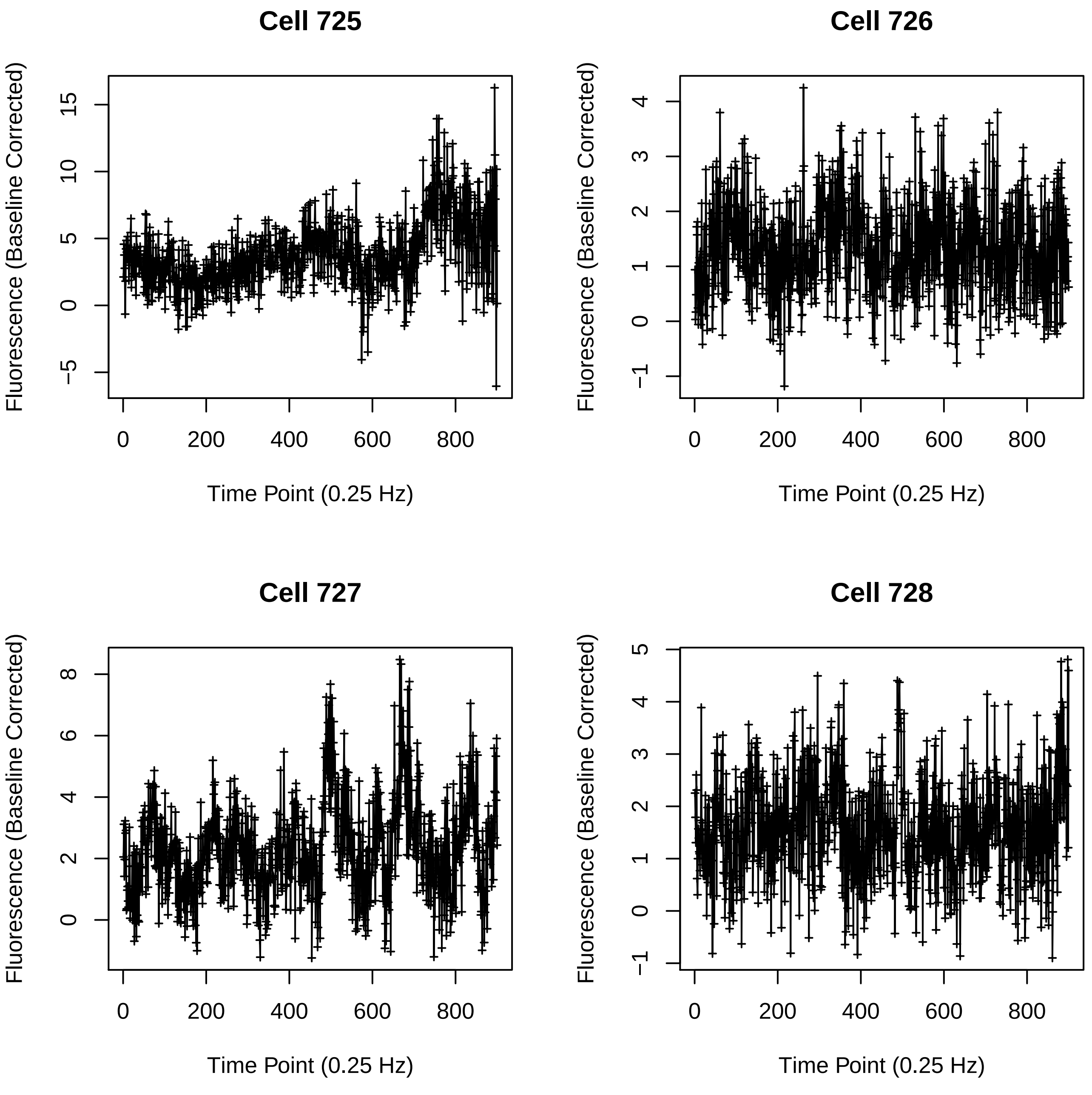
Cell 725
Cell 726
Cell 727
Cell 728
Fluorescence (Baseline Corrected)
Time Point (0.25 Hz)
0
200
400
600
800
−5
0
5
10
15
−1
1
2
3
4
6
8

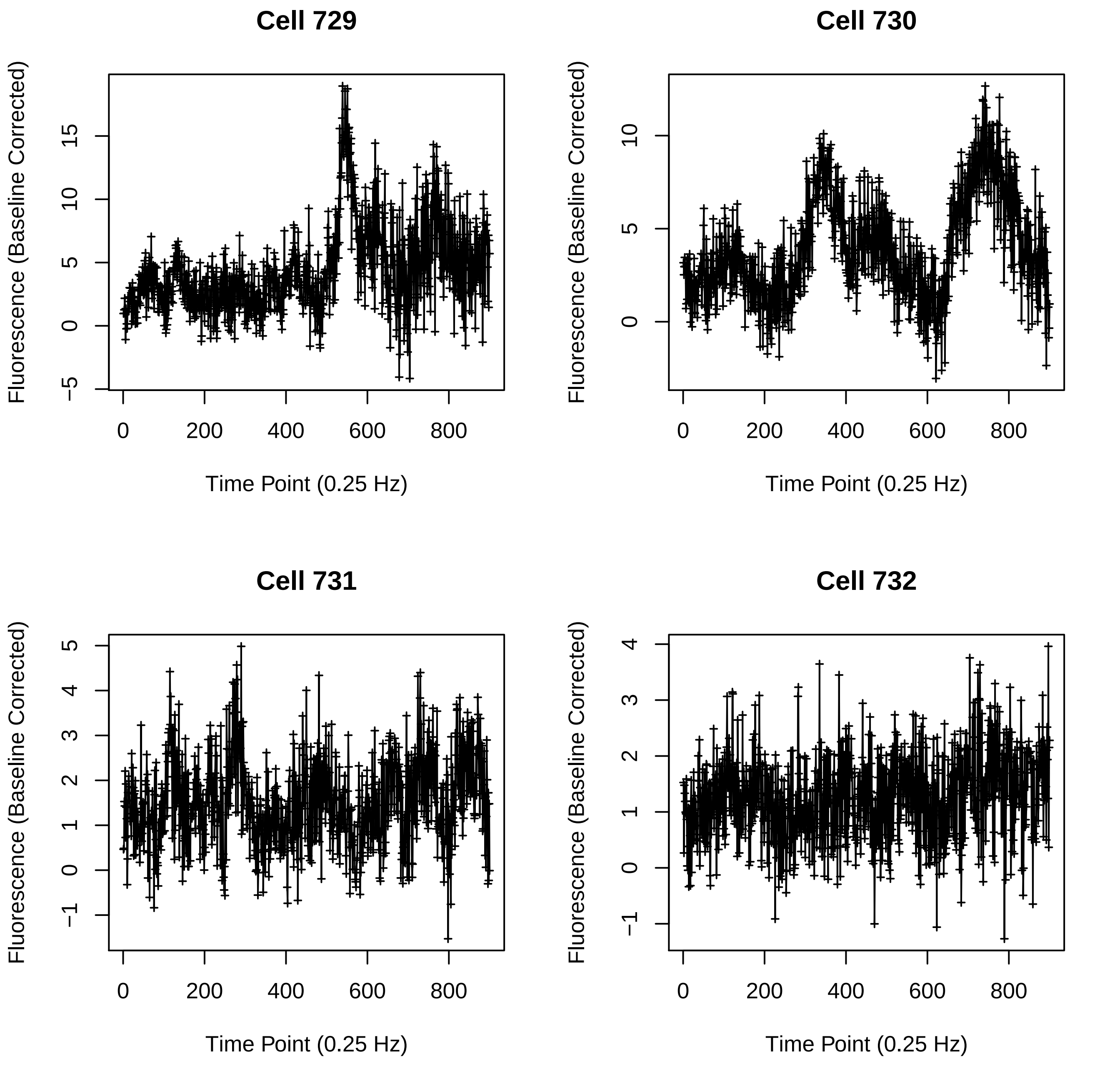
Cell 729
Fluorescence (Baseline Corrected)
−5
0
5
10
15
0
200
400
600
800
Time Point (0.25 Hz)
Cell 730
Fluorescence (Baseline Corrected)
0
5
10
0
200
400
600
800
Time Point (0.25 Hz)
Cell 731
Fluorescence (Baseline Corrected)
−1
0
1
2
3
4
5
0
200
400
600
800
Time Point (0.25 Hz)
Cell 732
Fluorescence (Baseline Corrected)
−1
0
1
2
3
4
0
200
400
600
800
Time Point (0.25 Hz)

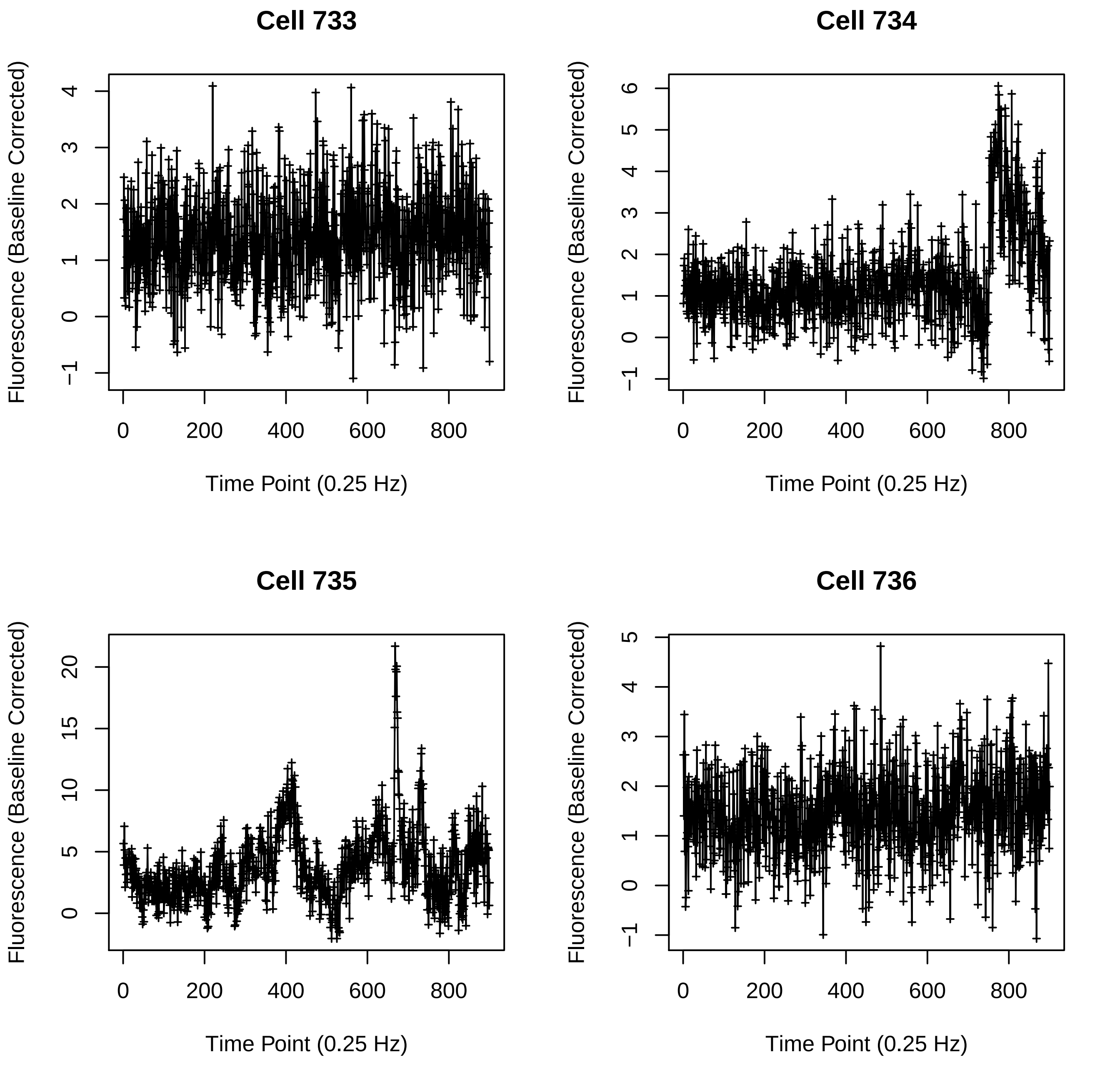

Cell 733
Cell 734
Cell 735
Cell 736
Fluorescence (Baseline Corrected)
Time Point (0.25 Hz)
−1
0
1
2
3
4
5
6
10
15
20
0
200
400
600
800

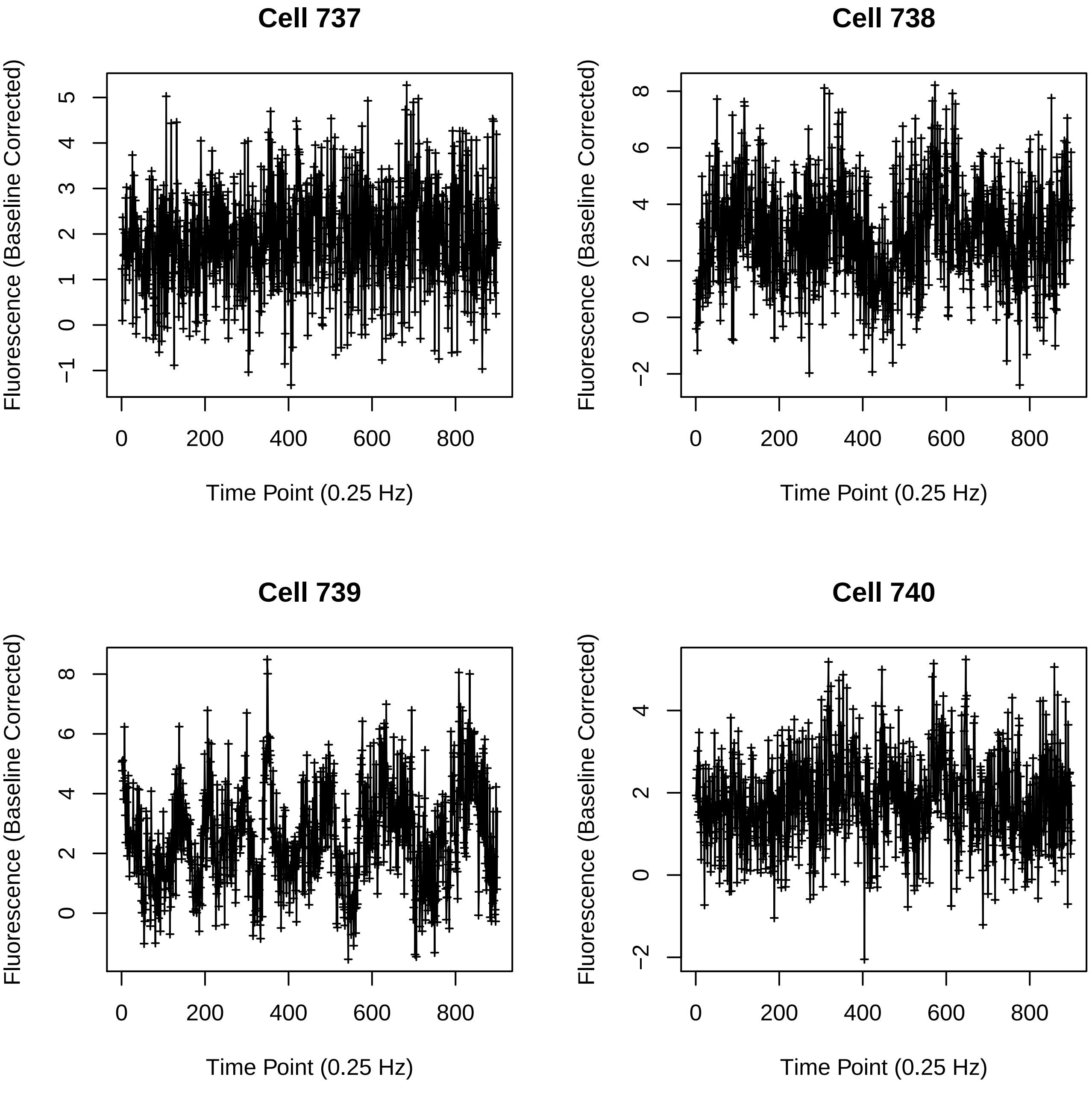
Cell 737
Cell 738
Cell 739
Cell 740
Fluorescence (Baseline Corrected)
Time Point (0.25 Hz)

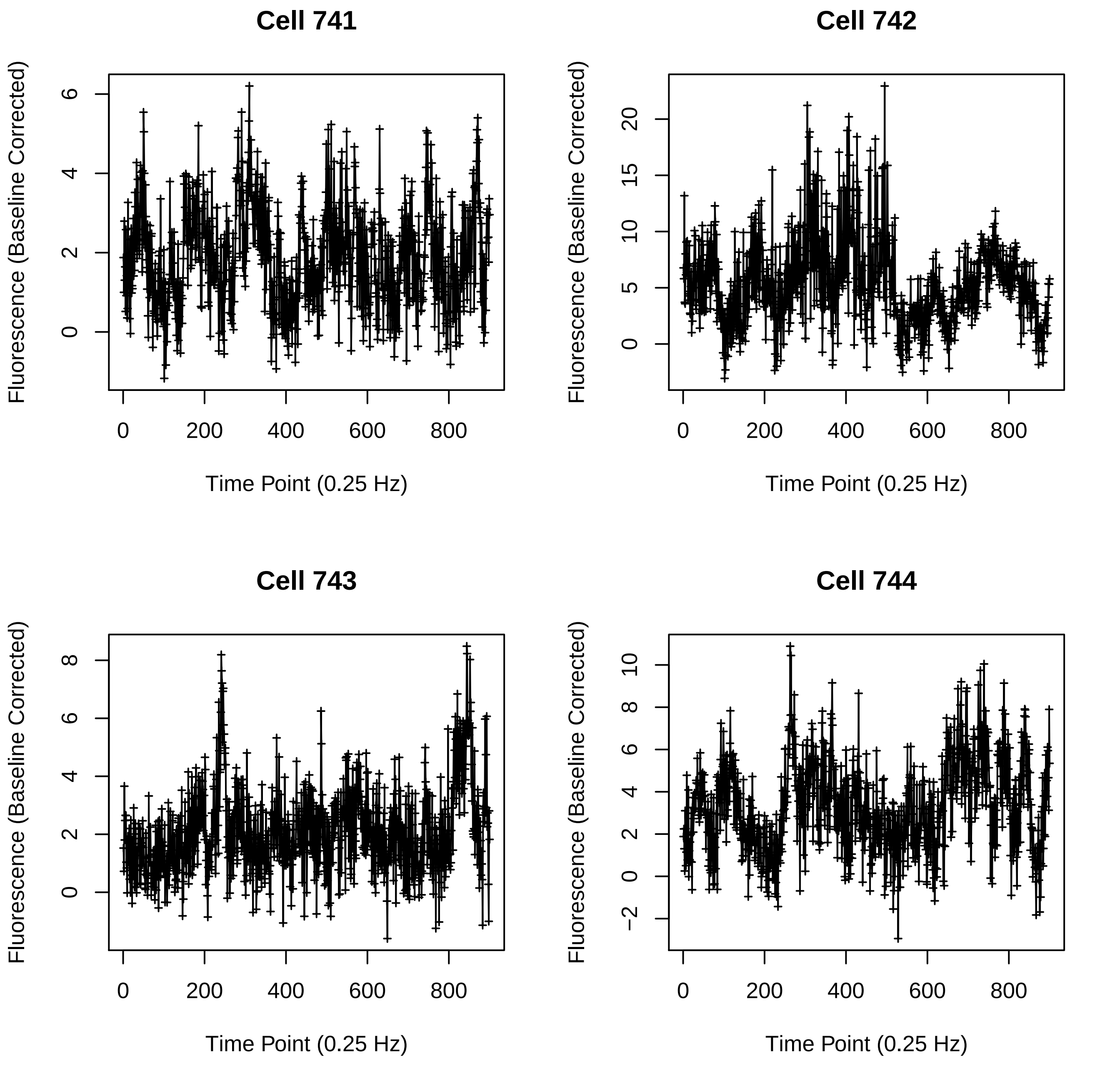

Cell 741
Cell 742
Cell 743
Cell 744
Fluorescence (Baseline Corrected)
Time Point (0.25 Hz)
0
200
400
600
800
-2
2
4
5
6
8
10
15
20

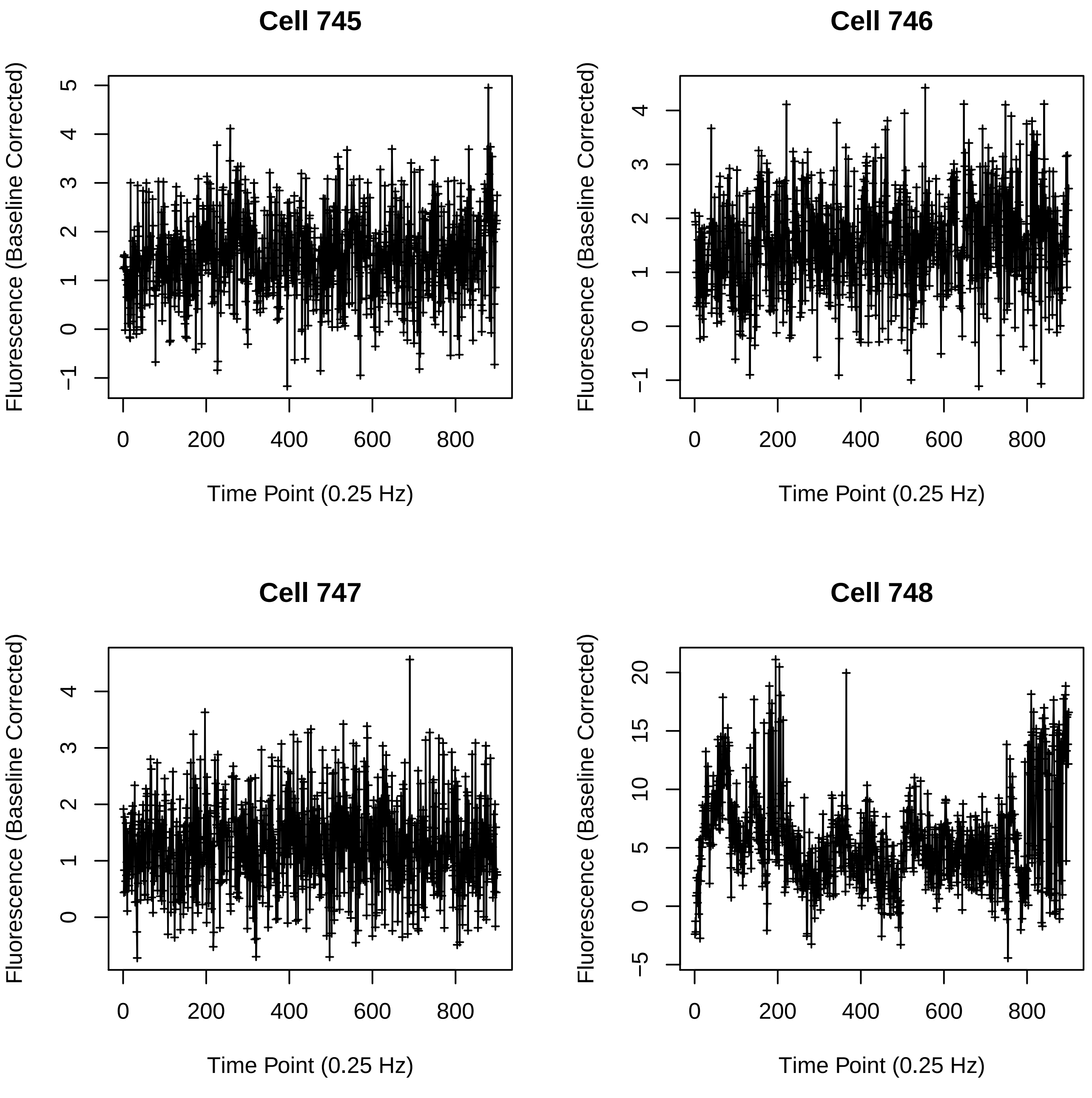

Cell 745
Fluorescence (Baseline Corrected)
−1
0
1
2
3
4
5
0
200
400
600
800
Time Point (0.25 Hz)
Cell 746
Fluorescence (Baseline Corrected)
−1
0
1
2
3
4
0
200
400
600
800
Time Point (0.25 Hz)
Cell 747
Fluorescence (Baseline Corrected)
0
1
2
3
4
0
200
400
600
800
Time Point (0.25 Hz)
Cell 748
Fluorescence (Baseline Corrected)
−5
0
5
10
15
20
0
200
400
600
800
Time Point (0.25 Hz)

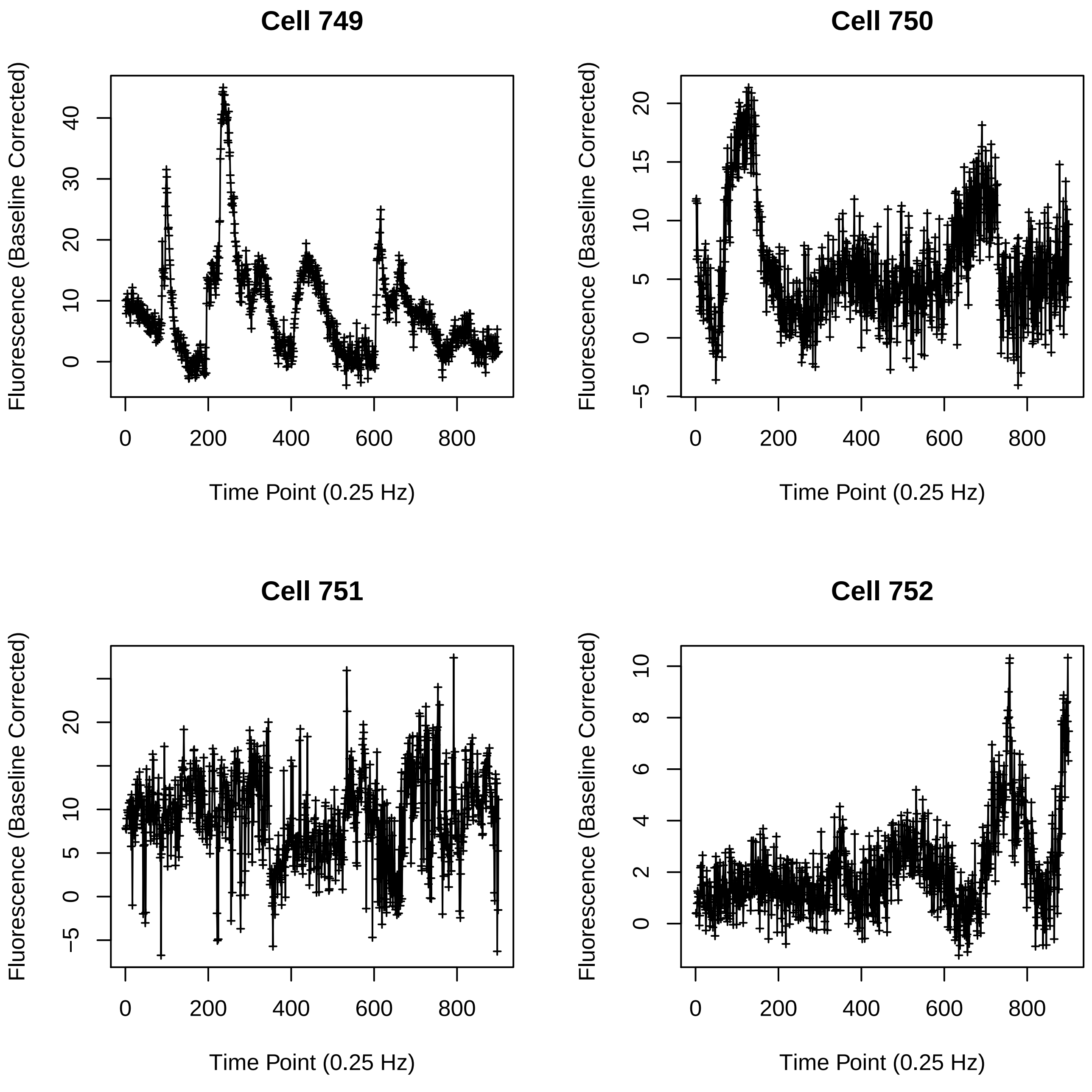

Cell 749
Cell 750
Cell 751
Cell 752
Fluorescence (Baseline Corrected)
Time Point (0.25 Hz)
0
200
400
600
800
40
30
20
10
15
5
−5
10
8
6
4
2

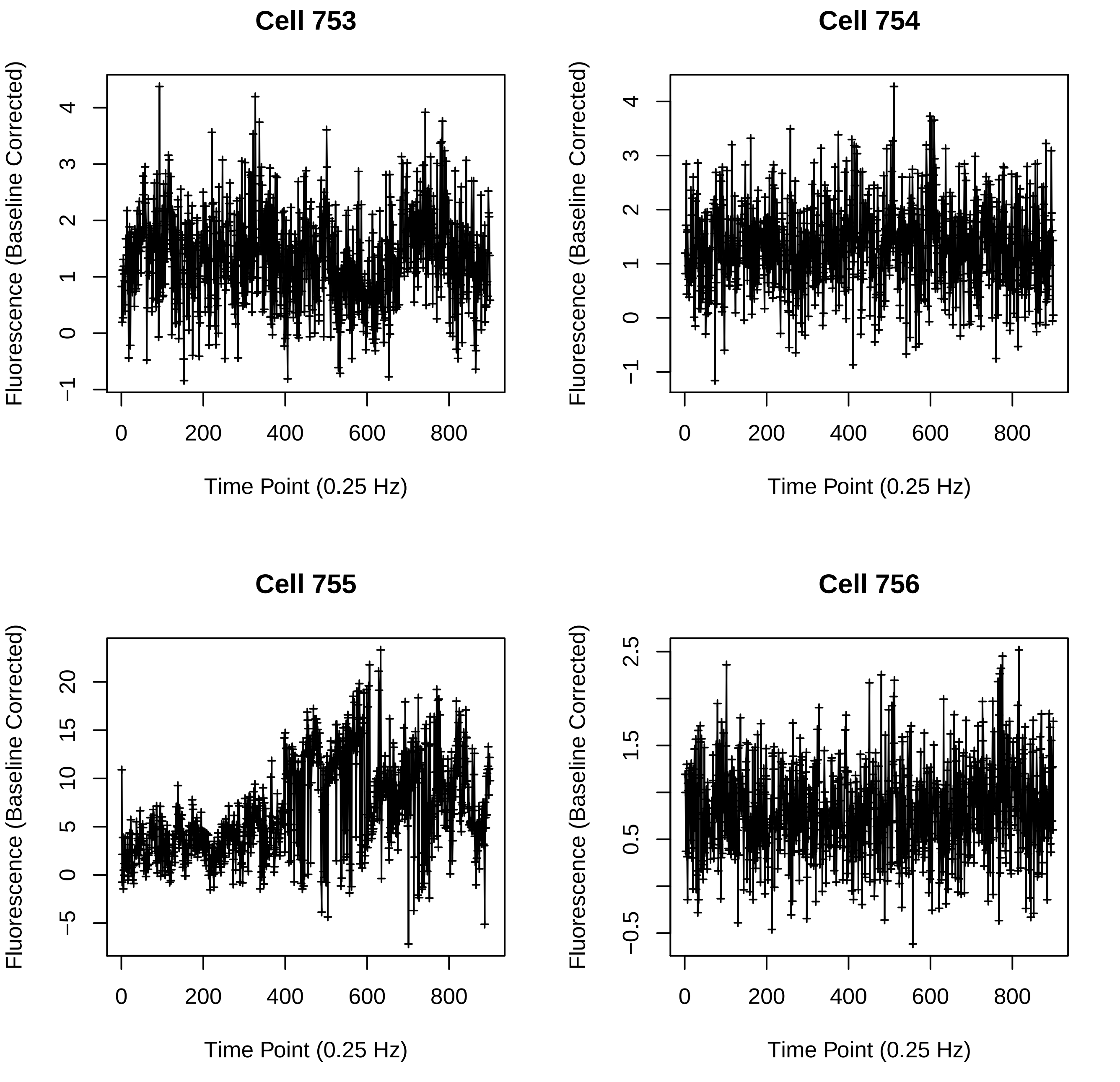
Cell 753
Cell 754
Cell 755
Cell 756
Fluorescence (Baseline Corrected)
Time Point (0.25 Hz)
−1
0
1
2
3
4
−5
5
10
15
20
−0.5
0.5
1.5
2.5
200
400
600
800

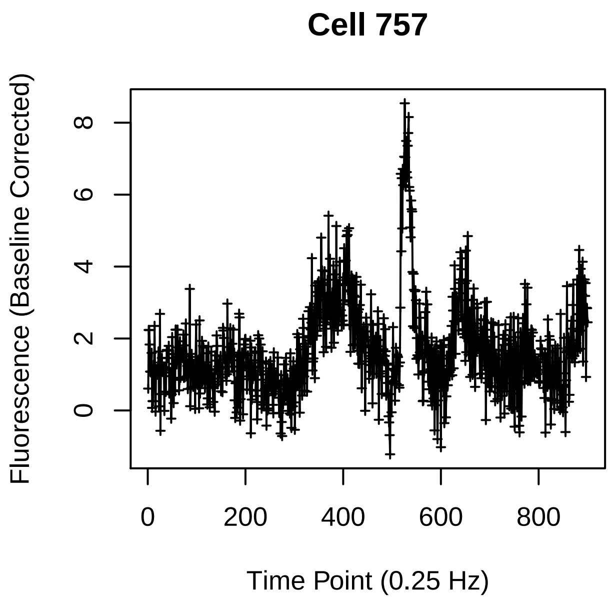
Cell 757
Fluorescence (Baseline Corrected)
0
2
4
6
8
0
200
400
600
800
Time Point (0.25 Hz)

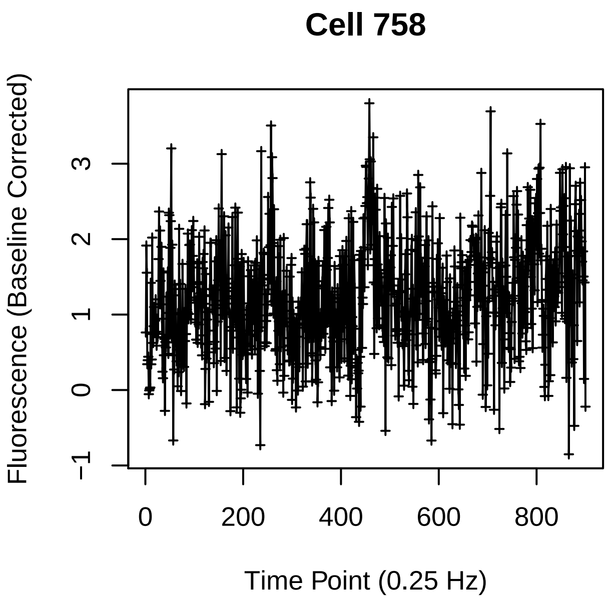
Cell 758
Fluorescence (Baseline Corrected)
−1
0
1
2
3
0
200
400
600
800
Time Point (0.25 Hz)

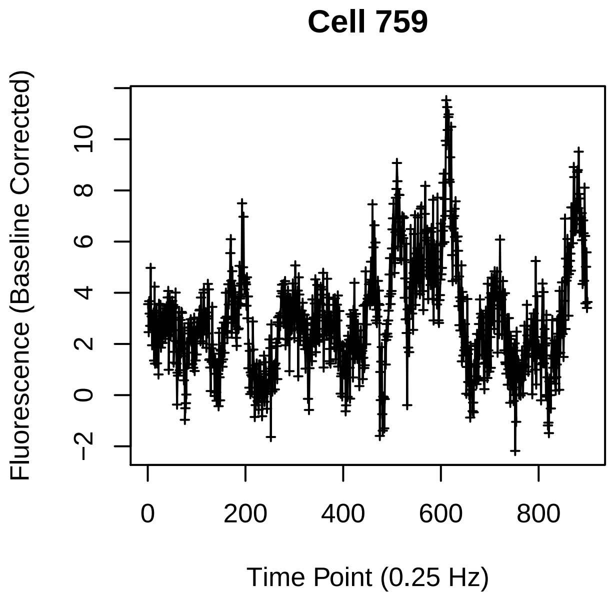
Cell 759
Fluorescence (Baseline Corrected)
−2
0
2
4
6
8
10
0
200
400
600
800
Time Point (0.25 Hz)

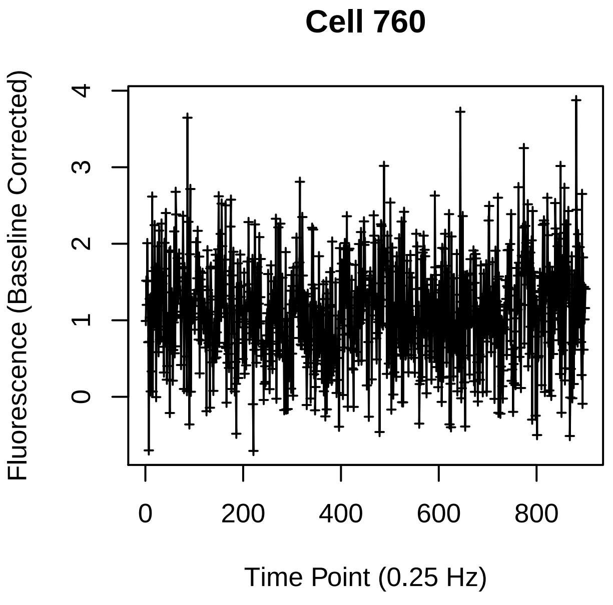
Cell 760
Fluorescence (Baseline Corrected)
0
1
2
3
4
0
200
400
600
800
Time Point (0.25 Hz)

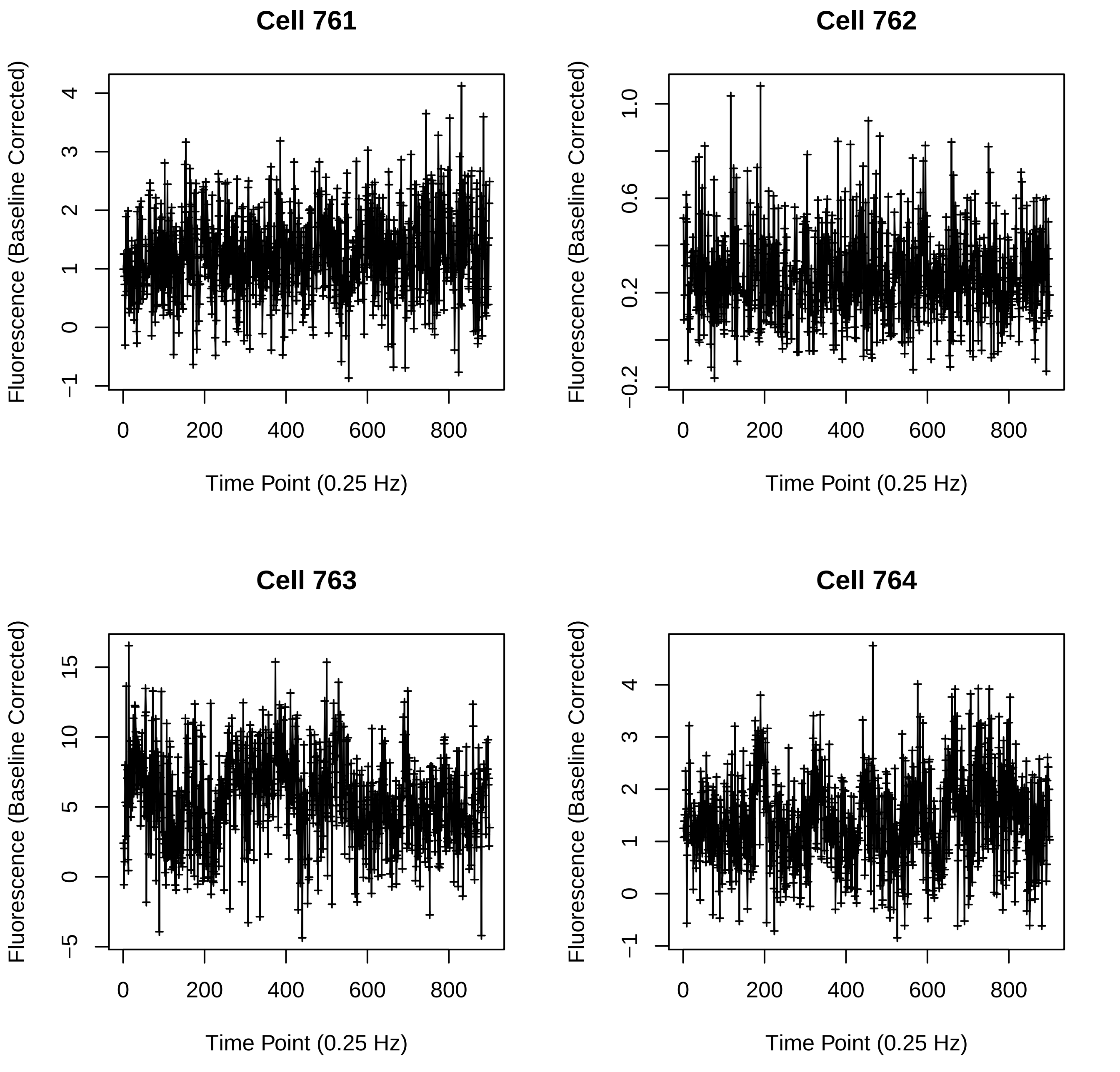

Cell 761
Cell 762
Cell 763
Cell 764
Fluorescence (Baseline Corrected)
Time Point (0.25 Hz)

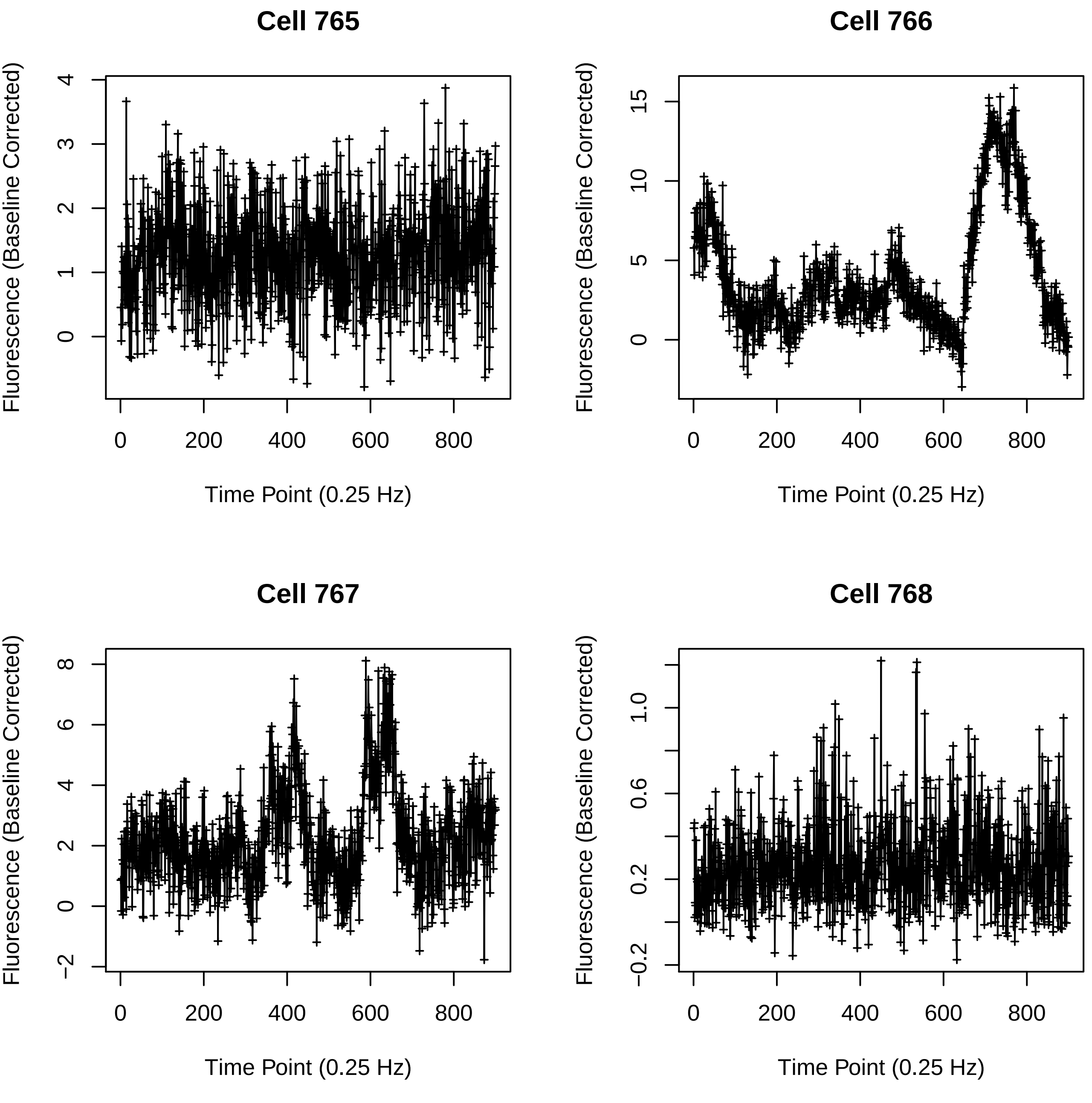

Cell 765
Cell 766
Cell 767
Cell 768
Fluorescence (Baseline Corrected)
Time Point (0.25 Hz)
0
200
400
600
800

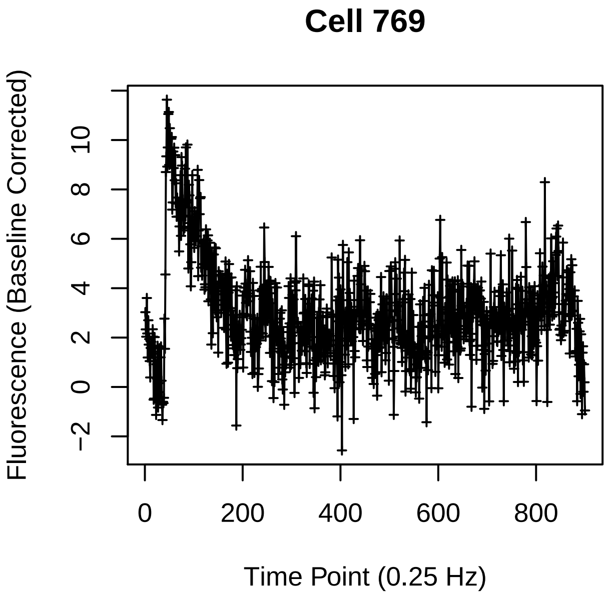
Cell 769
Fluorescence (Baseline Corrected)
Time Point (0.25 Hz)
−2
0
2
4
6
8
10
0
200
400
600
800
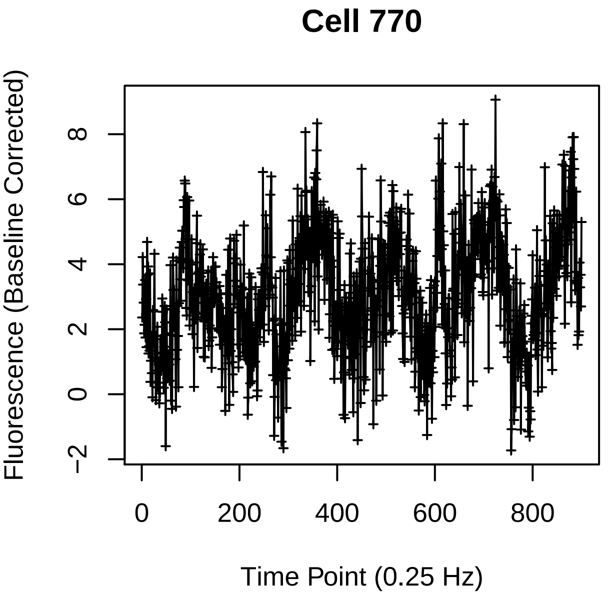
Cell 770
Fluorescence (Baseline Corrected)
Time Point (0.25 Hz)
−2
0
2
4
6
8
0
200
400
600
800
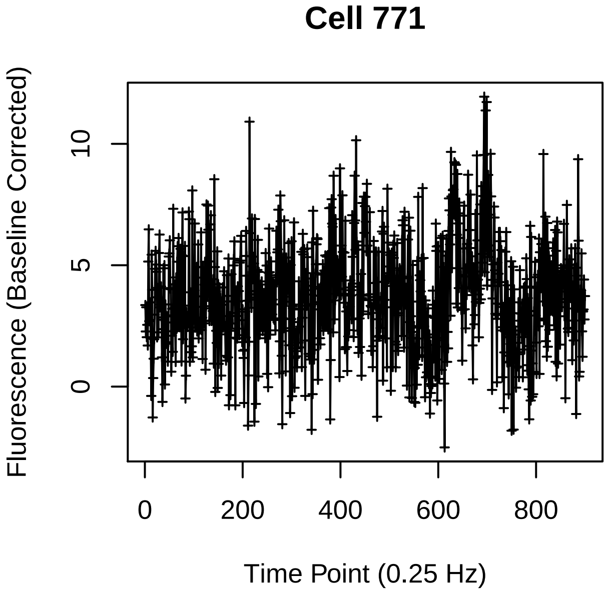
Cell 771
Fluorescence (Baseline Corrected)
Time Point (0.25 Hz)
0
5
10
0
200
400
600
800
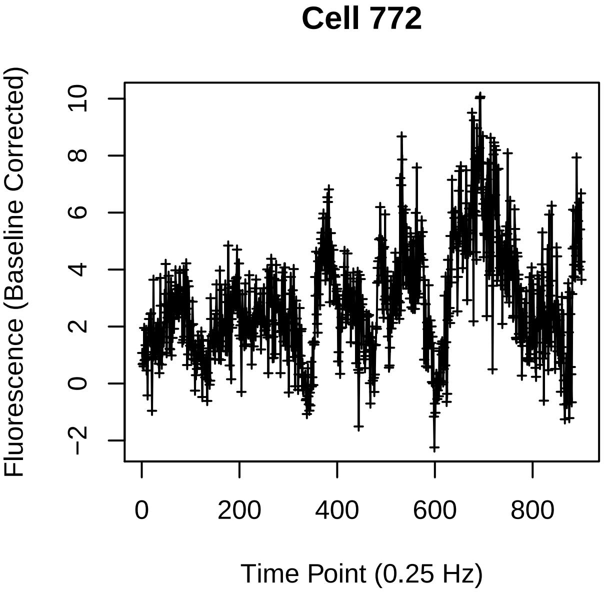
Cell 772
Fluorescence (Baseline Corrected)
Time Point (0.25 Hz)
−2
0
2
4
6
8
10
0
200
400
600
800

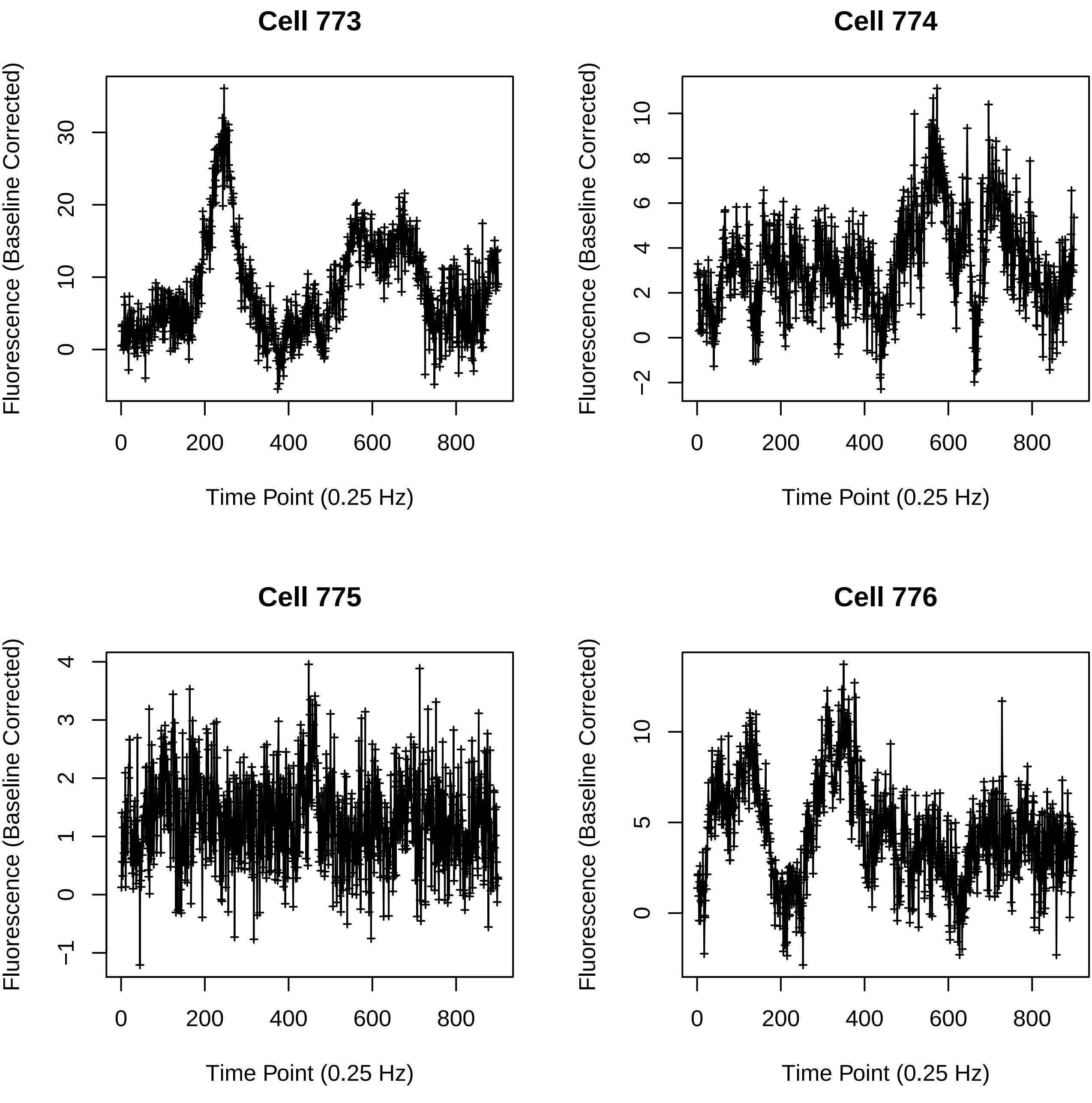

Cell 773
Cell 774
Cell 775
Cell 776
Fluorescence (Baseline Corrected)
Time Point (0.25 Hz)
0
200
400
600
800
0
10
20
30
−2
2
4
6
8
10
−1
1
3
5

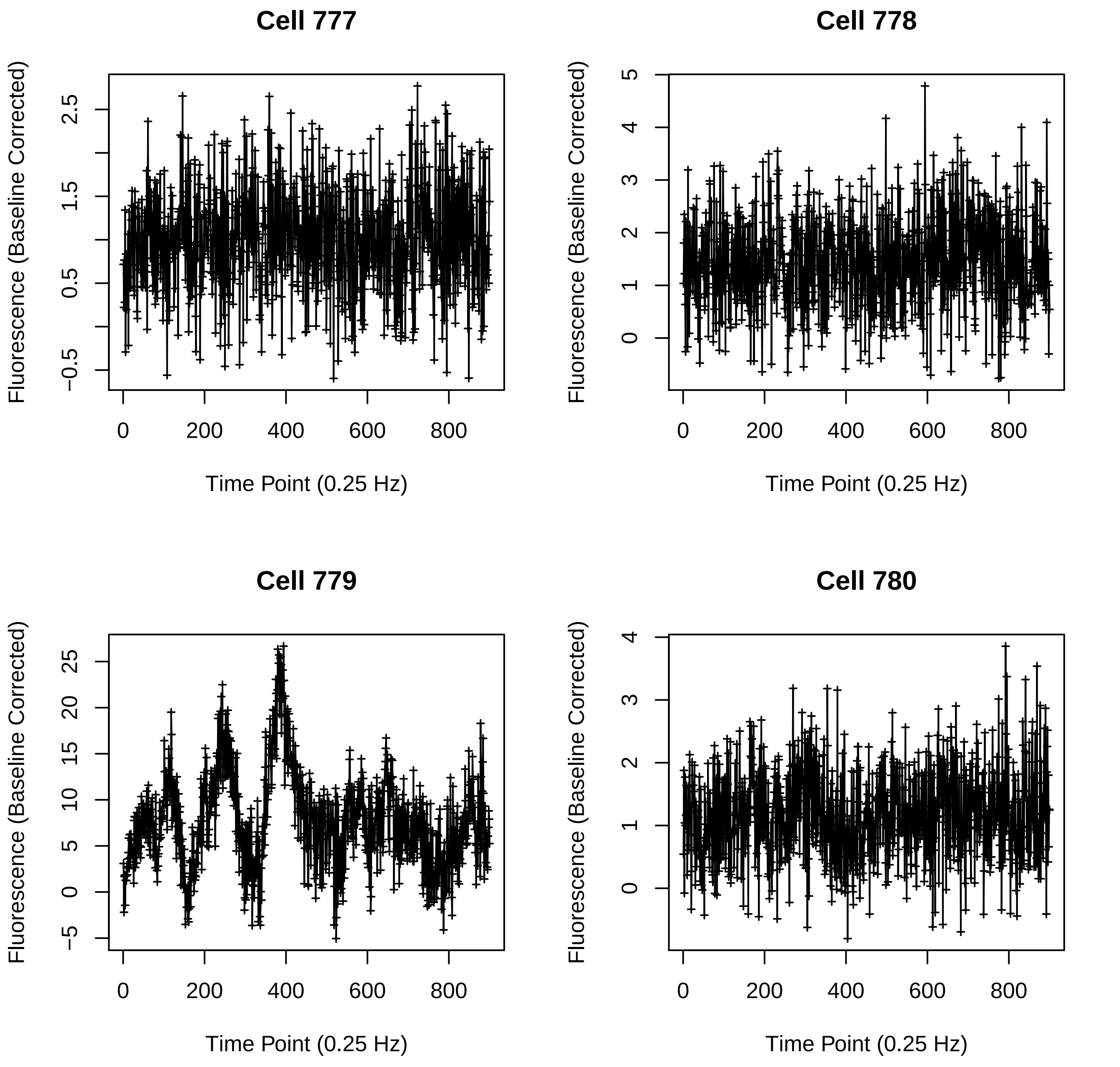

Cell 777
Cell 778
Cell 779
Cell 780
Fluorescence (Baseline Corrected)
Time Point (0.25 Hz)
0
200
400
600
800
−0.5
0.5
1.5
2.5
0
1
2
3
4
5
−5
0
5
10
15
20
25

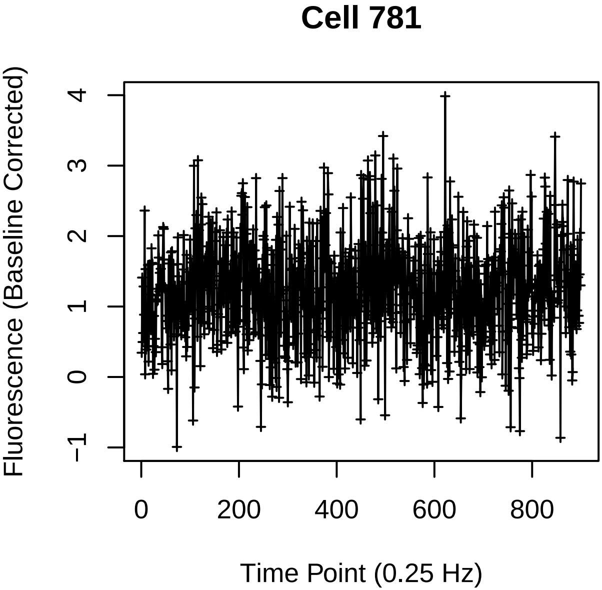
Cell 781
Fluorescence (Baseline Corrected)
Time Point (0.25 Hz)
−1 0 1 2 3 4
0 200 400 600 800

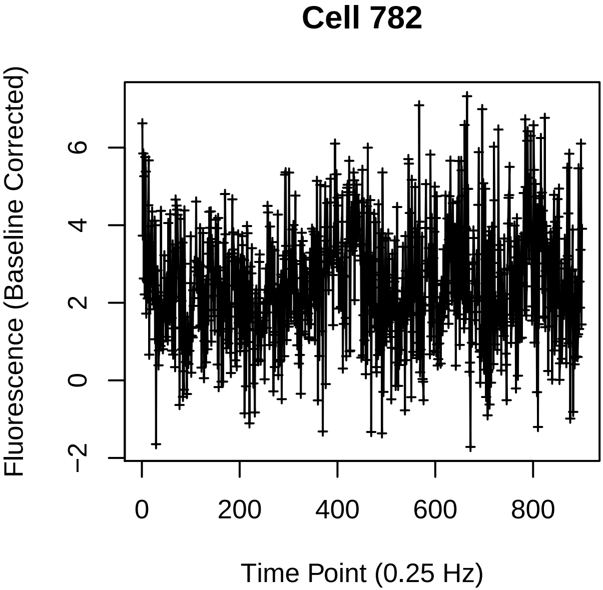
Cell 782
Fluorescence (Baseline Corrected)
Time Point (0.25 Hz)
−2 0 2 4 6
0 200 400 600 800

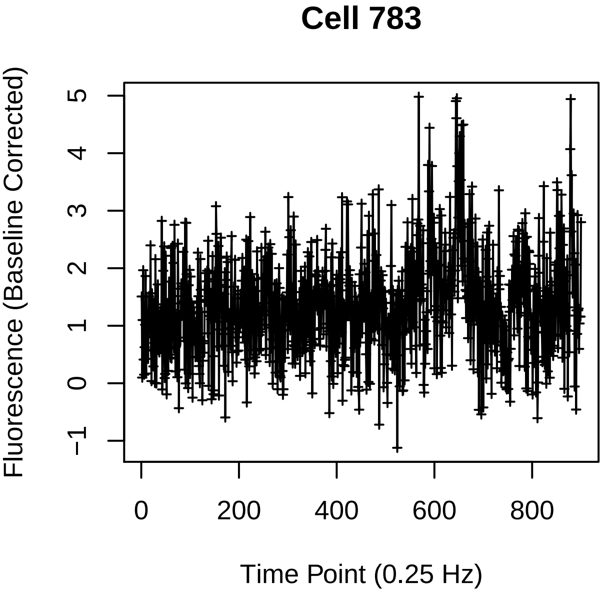
Cell 783
Fluorescence (Baseline Corrected)
Time Point (0.25 Hz)
−1 0 1 2 3 4 5
0 200 400 600 800

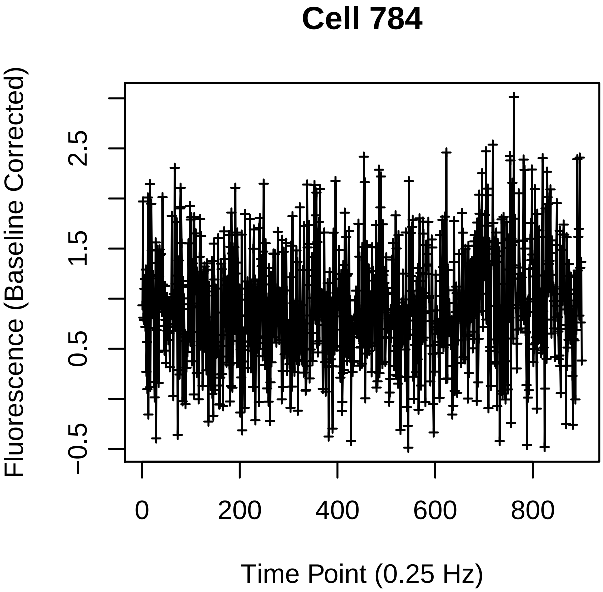
Cell 784
Fluorescence (Baseline Corrected)
Time Point (0.25 Hz)
−0.5 0.5 1.5 2.5
0 200 400 600 800

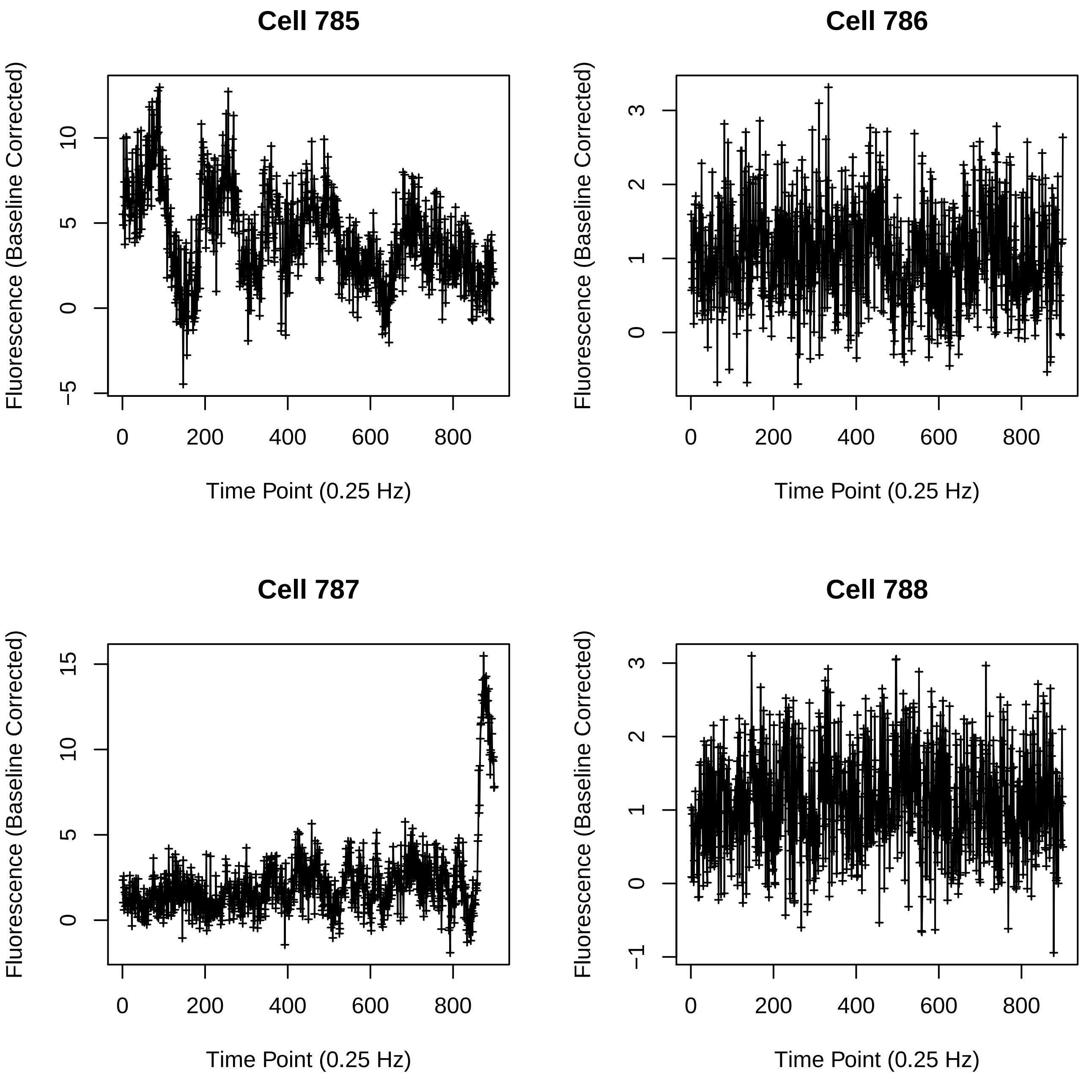

Cell 785
Cell 786
Cell 787
Cell 788
Fluorescence (Baseline Corrected)
Time Point (0.25 Hz)

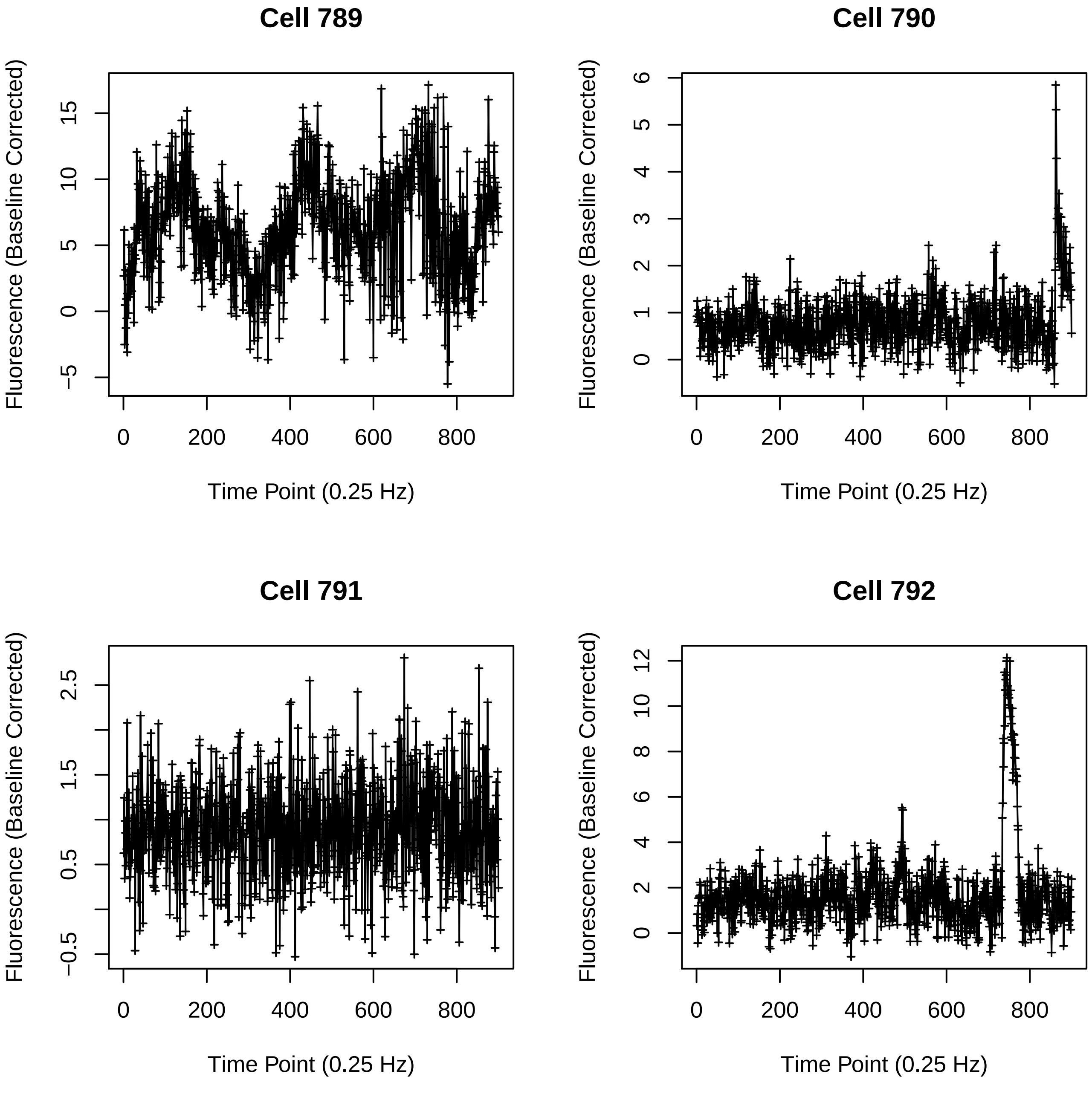

Cell 789
Cell 790
Cell 791
Cell 792
Fluorescence (Baseline Corrected)
Time Point (0.25 Hz)
0
200
400
600
800
−5
0
5
10
15
1
2
3
4
5
6
−0.5
0.5
1.5
2.5
2
4
6
8
10
12

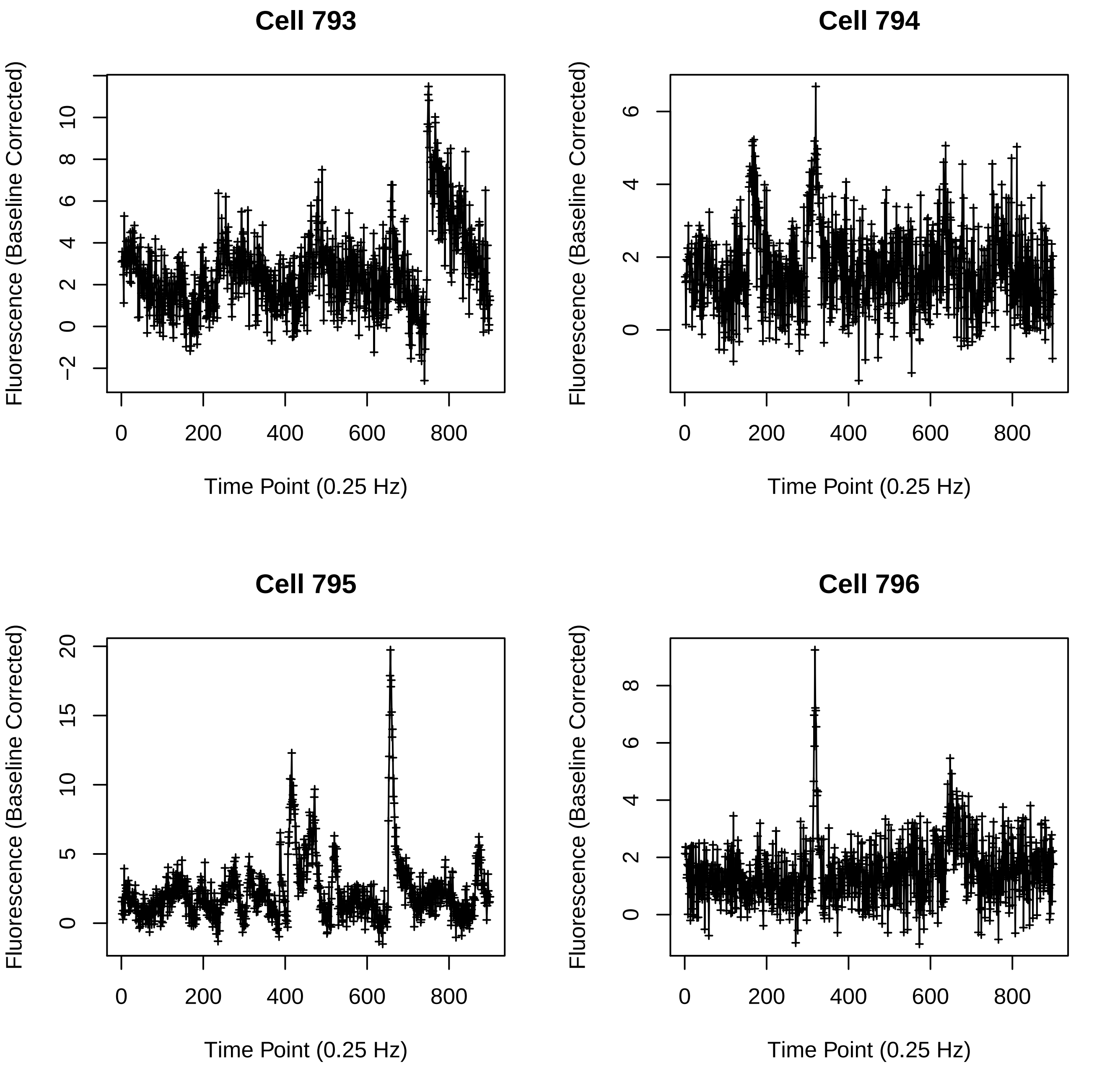

Cell 793
Fluorescence (Baseline Corrected)
−2
0
2
4
6
8
10
0
200
400
600
800
Time Point (0.25 Hz)
Cell 794
Fluorescence (Baseline Corrected)
0
2
4
6
0
200
400
600
800
Time Point (0.25 Hz)
Cell 795
Fluorescence (Baseline Corrected)
0
5
10
15
20
0
200
400
600
800
Time Point (0.25 Hz)
Cell 796
Fluorescence (Baseline Corrected)
0
2
4
6
8
0
200
400
600
800
Time Point (0.25 Hz)

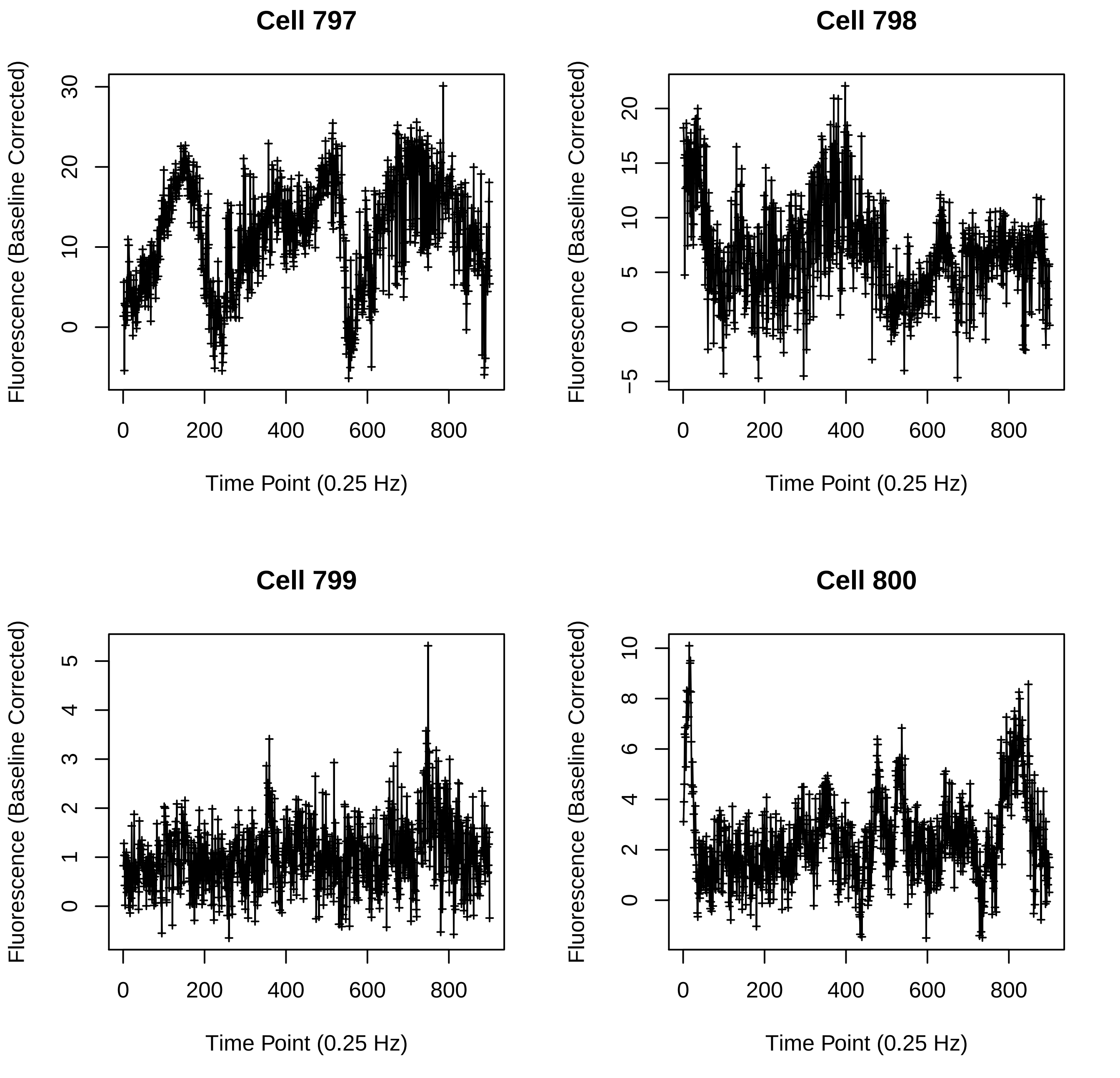
Cell 797
Cell 798
Cell 799
Cell 800
Fluorescence (Baseline Corrected)
Time Point (0.25 Hz)
0
200
400
600
800
-5
5
10
15
20
30
1
2
3
4
6
8

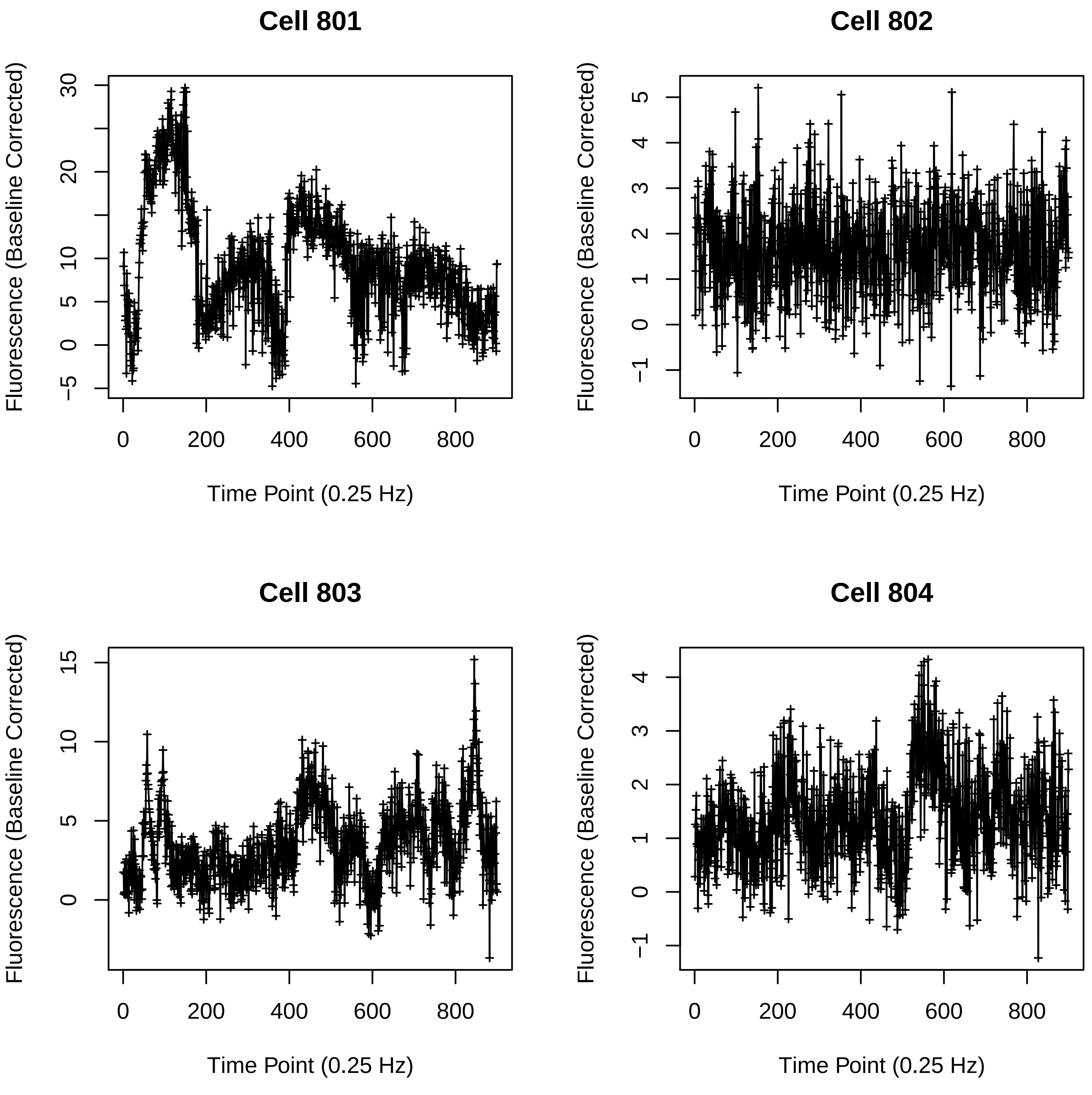
Cell 801
Cell 802
Cell 803
Cell 804
Fluorescence (Baseline Corrected)
Time Point (0.25 Hz)
0
200
400
600
800
−5
5
10
15
20
25
30
−1
1
2
3
4

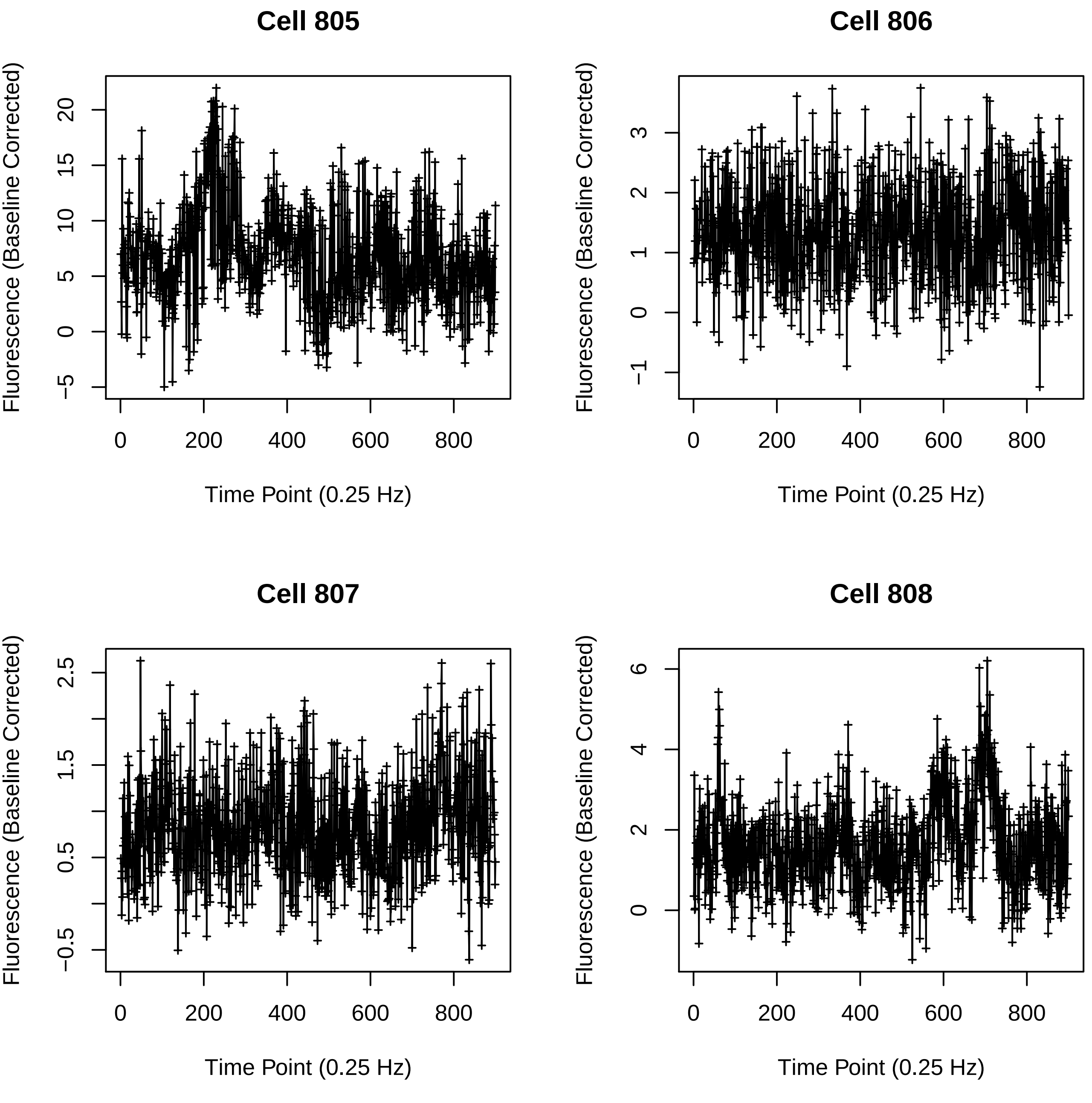
Cell 805
Cell 806
Cell 807
Cell 808
Fluorescence (Baseline Corrected)
Time Point (0.25 Hz)
0
200
400
600
800
−5
0
5
10
15
20
−1
1
2
3
−0.5
0.5
1.5
2.5
4
6

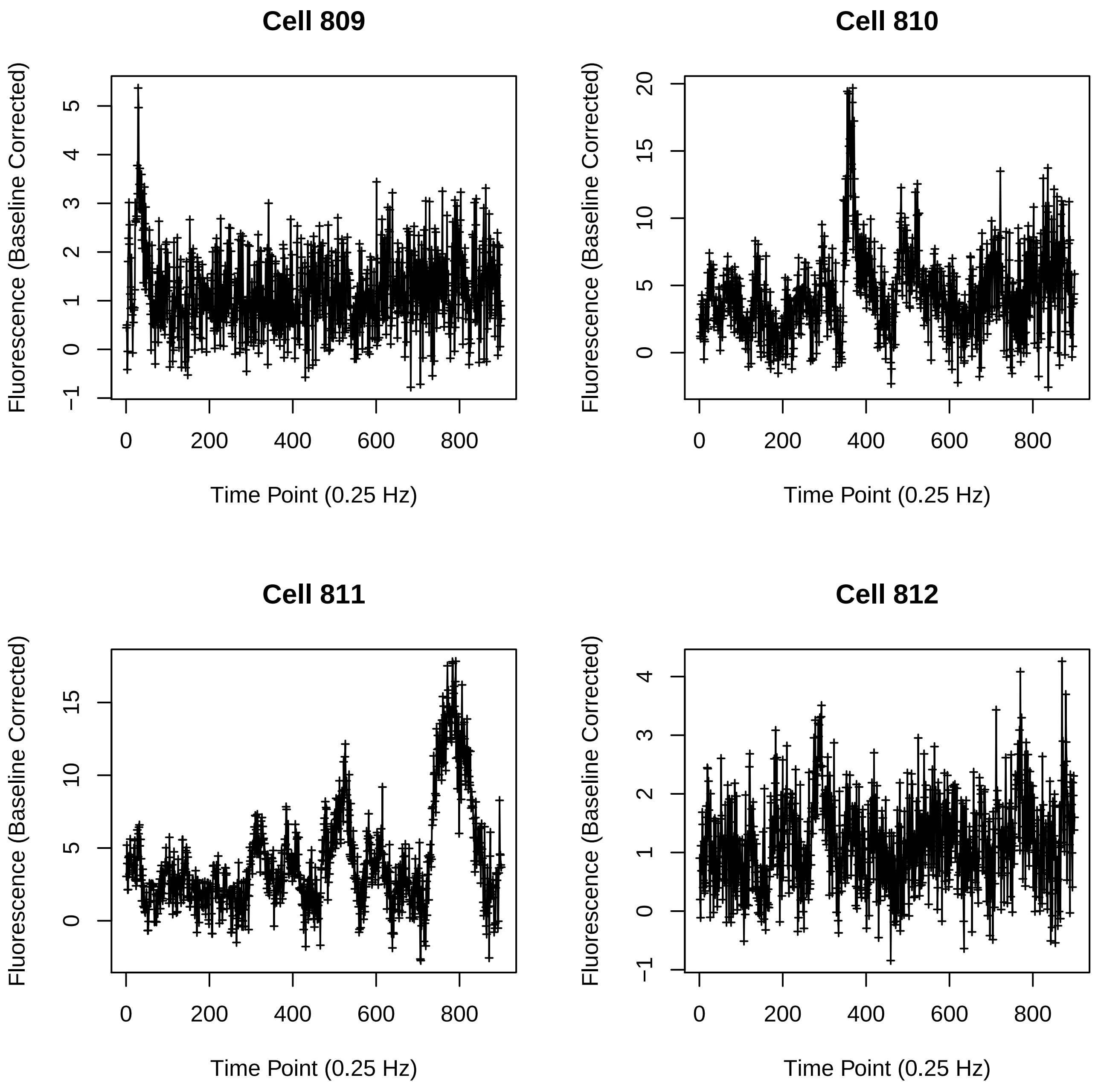
Cell 809
Cell 810
Cell 811
Cell 812
Fluorescence (Baseline Corrected)
Time Point (0.25 Hz)
−1
0
1
2
3
4
5
10
15
20
200
400
600
800

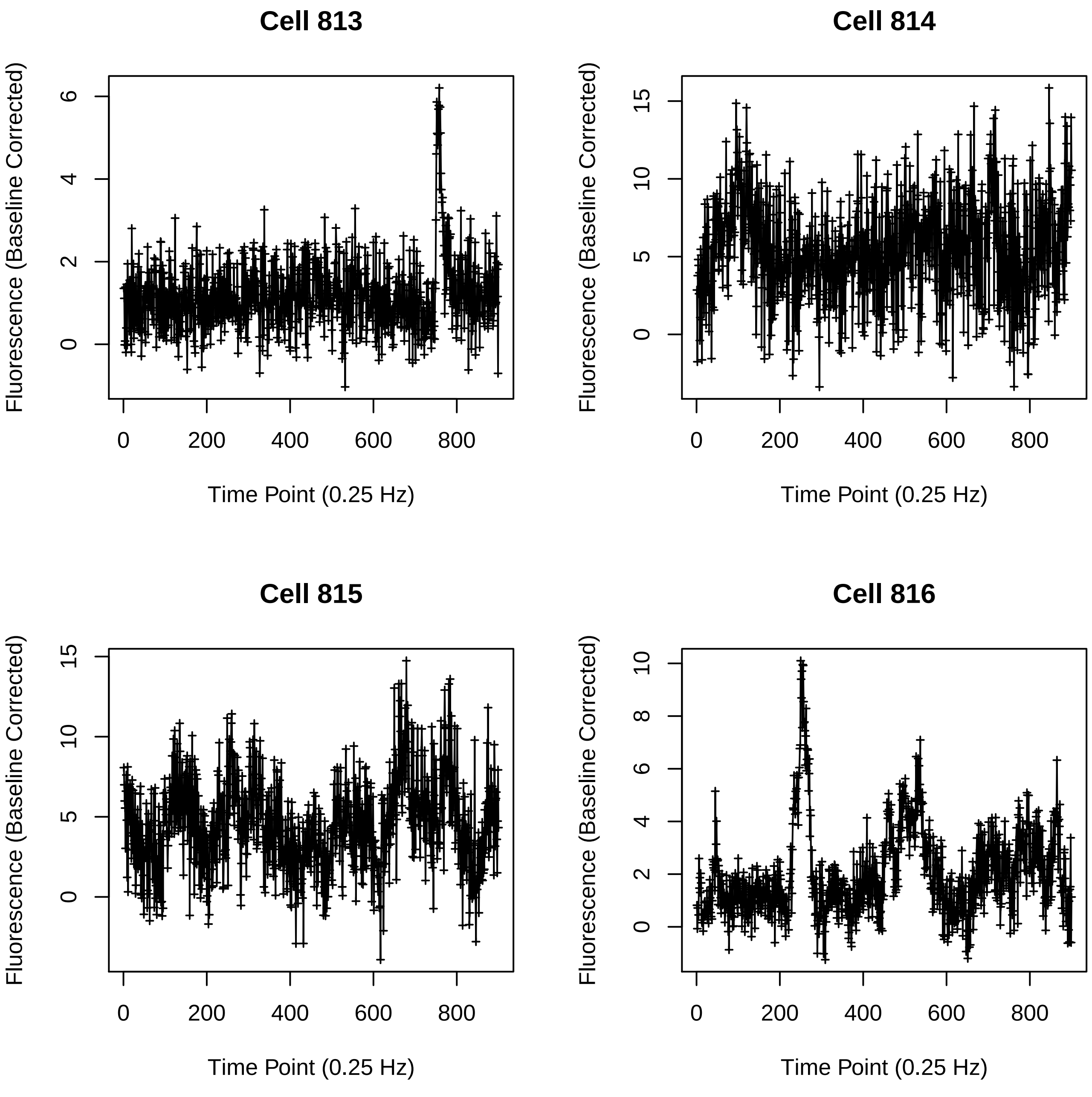
Cell 813
Fluorescence (Baseline Corrected)
Time Point (0.25 Hz)
0
2
4
6
0
200
400
600
800
Cell 814
Fluorescence (Baseline Corrected)
Time Point (0.25 Hz)
0
5
10
15
0
200
400
600
800
Cell 815
Fluorescence (Baseline Corrected)
Time Point (0.25 Hz)
0
5
10
15
0
200
400
600
800
Cell 816
Fluorescence (Baseline Corrected)
Time Point (0.25 Hz)
0
2
4
6
8
10
0
200
400
600
800

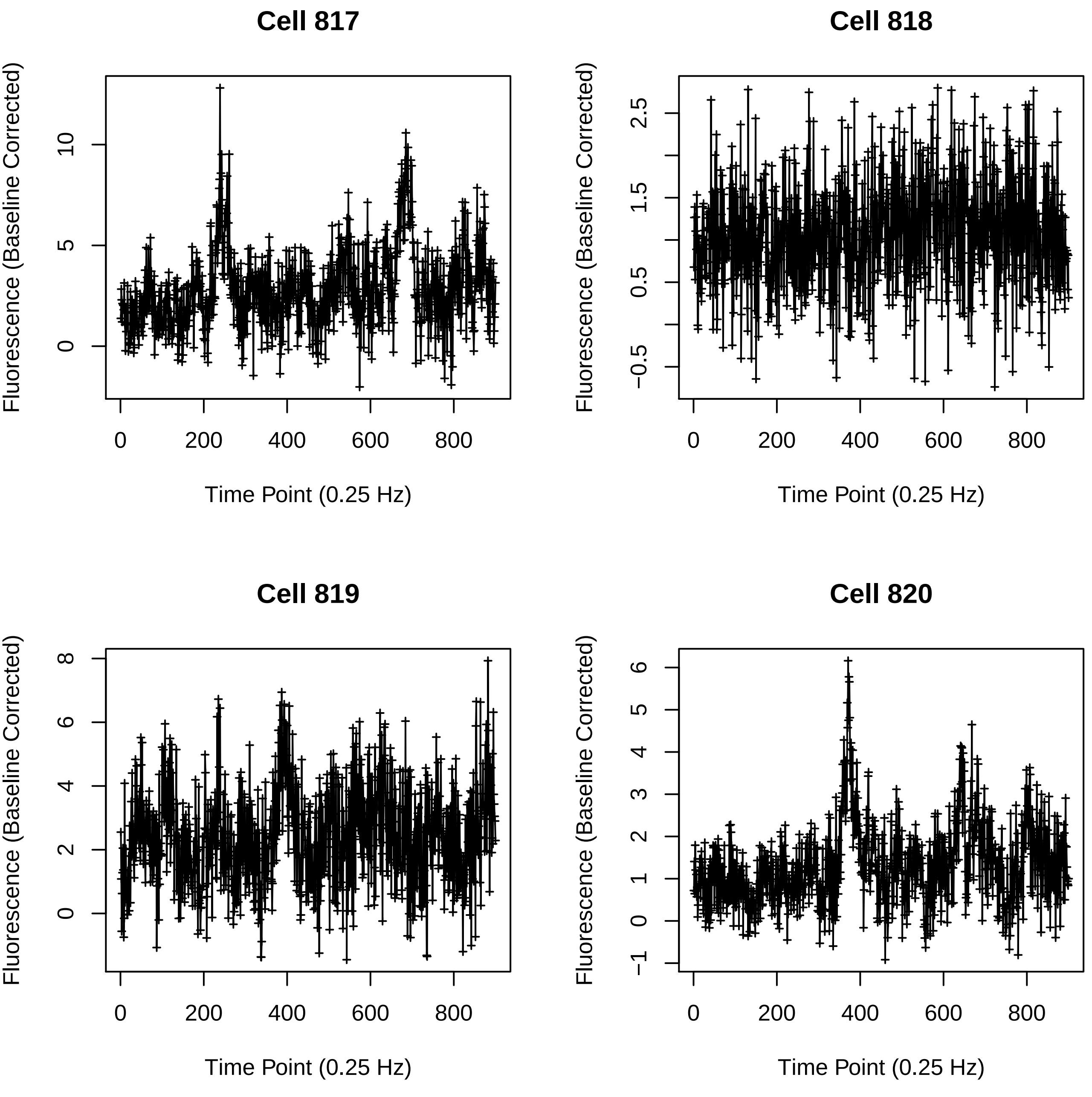
Cell 817
Cell 818
Cell 819
Cell 820
Fluorescence (Baseline Corrected)
Time Point (0.25 Hz)
0
5
10
−0.5
0.5
1.5
2.5
2
4
6
8
−1
1
3
200
400
600
800

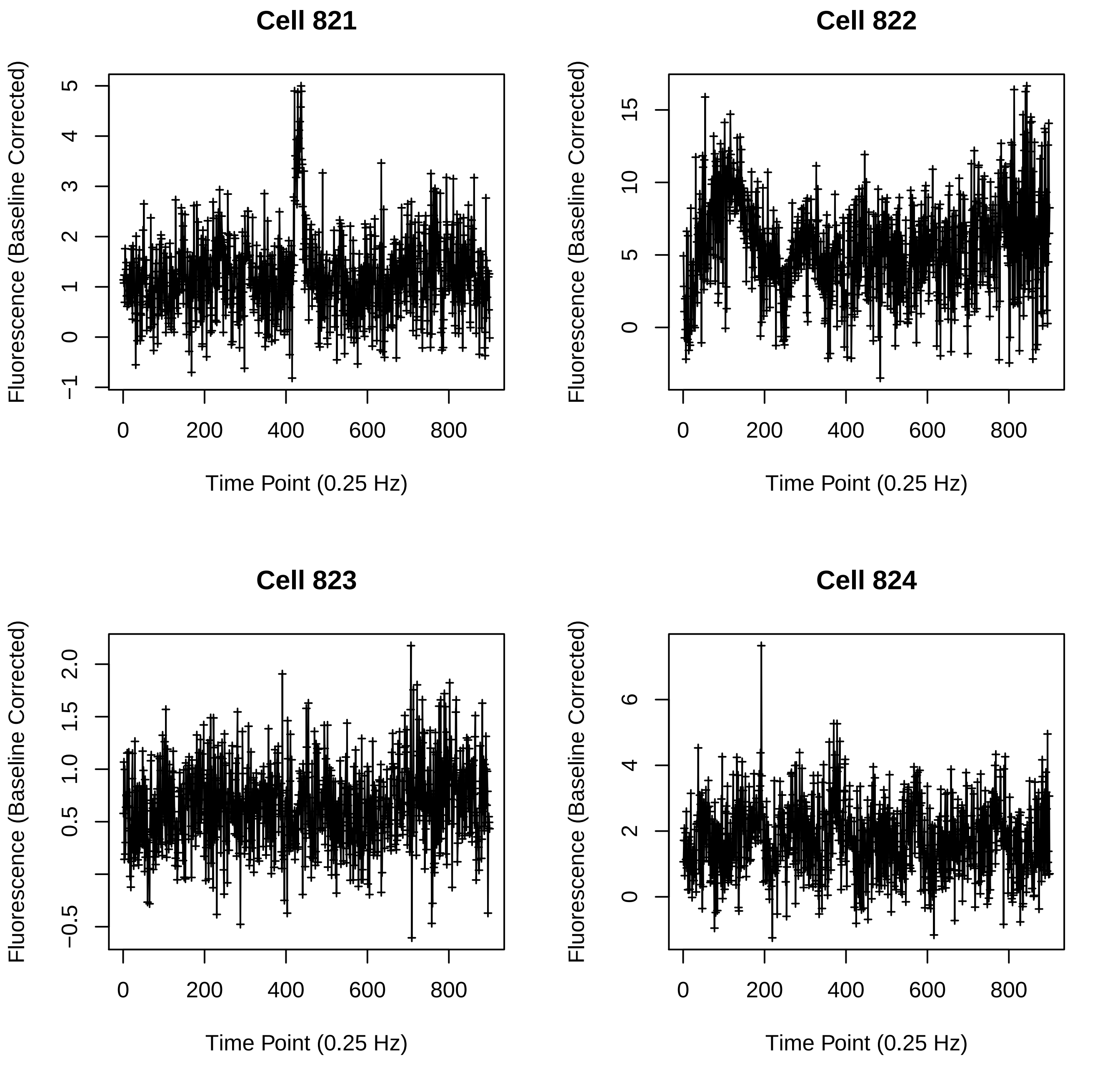
Cell 821
Cell 822
Cell 823
Cell 824
Fluorescence (Baseline Corrected)
Time Point (0.25 Hz)
−1
0
1
2
3
4
5
15
10
−0.5
0.5
1.0
1.5
2.0
6
200
400
600
800

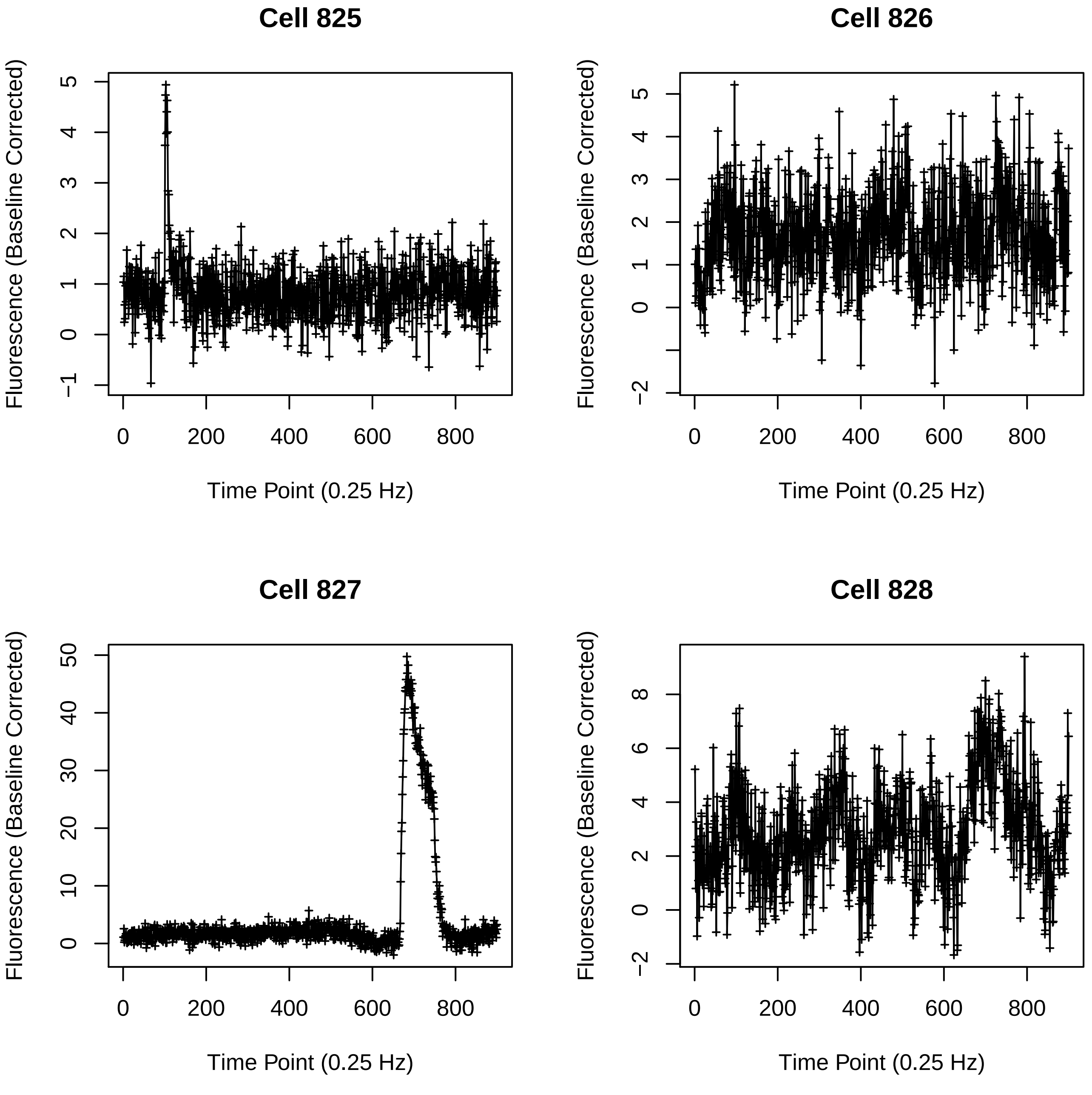

Cell 825
Cell 826
Cell 827
Cell 828
Fluorescence (Baseline Corrected)
Time Point (0.25 Hz)
−1
0
1
2
3
4
5
−2
8
6
10
20
30
40
50
200
400
600
800

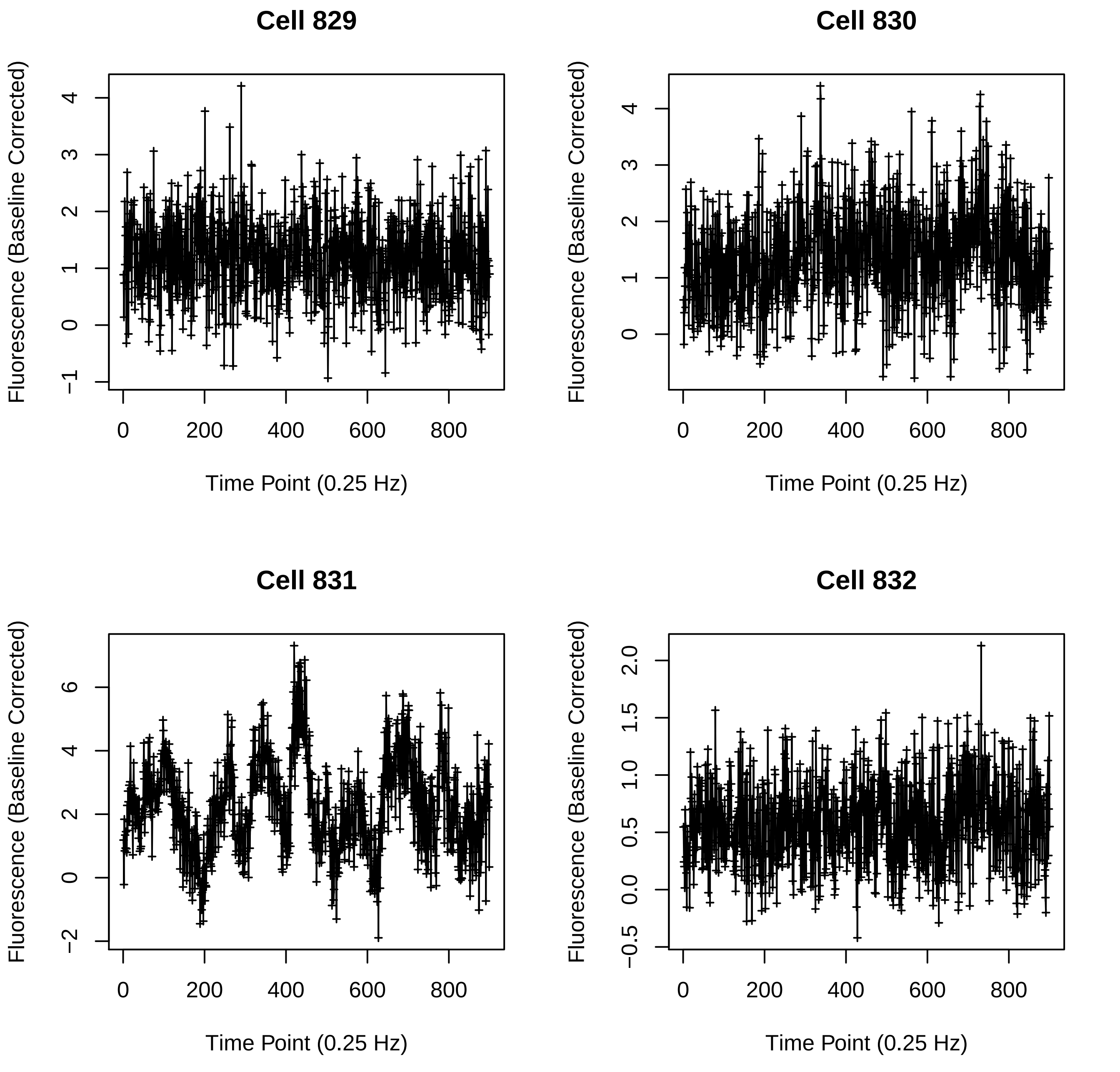
Cell 829
Cell 830
Cell 831
Cell 832
Fluorescence (Baseline Corrected)
Time Point (0.25 Hz)
0
200
400
600
800
−1
0
1
2
3
4
−2
6
−0.5
0.0
0.5
1.0
1.5
2.0

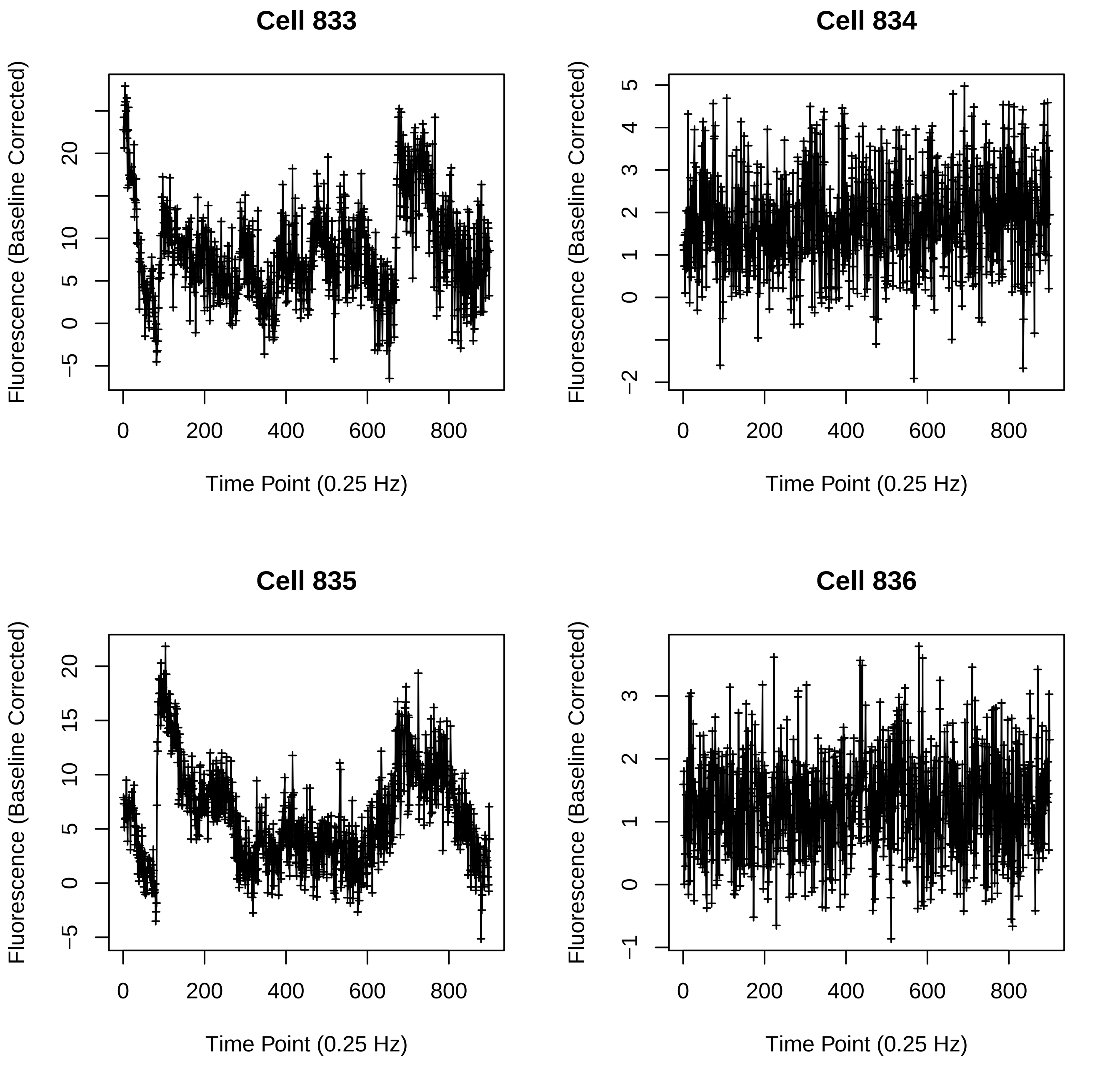

Cell 833
Cell 834
Cell 835
Cell 836
Fluorescence (Baseline Corrected)
Time Point (0.25 Hz)

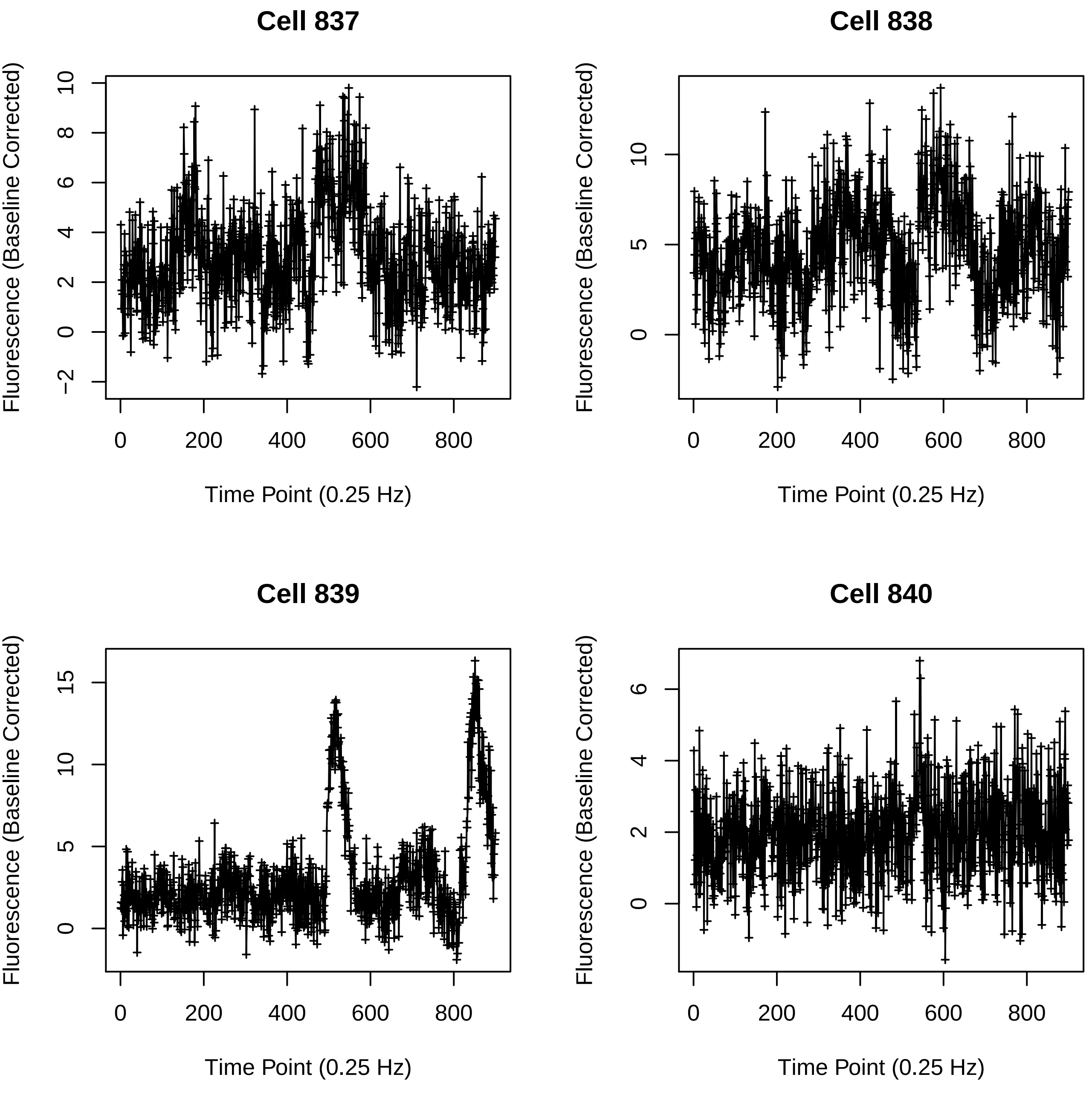
Cell 837
Cell 838
Cell 839
Cell 840
Fluorescence (Baseline Corrected)
Time Point (0.25 Hz)
0
200
400
600
800
−2
2
4
6
8
10
5
15

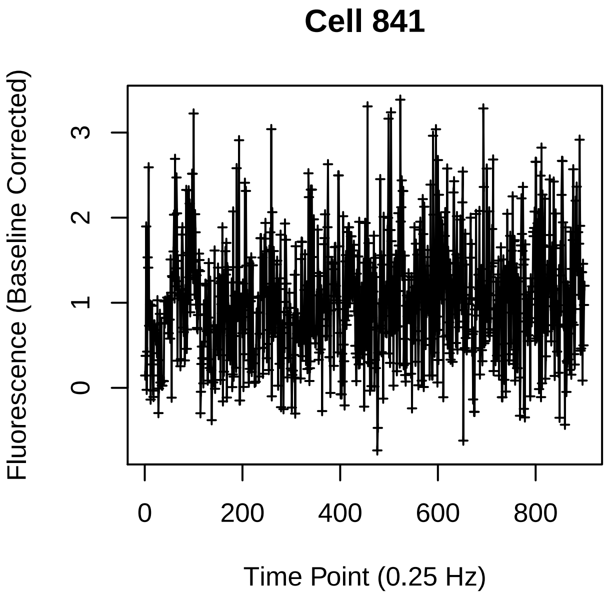
Cell 841
Fluorescence (Baseline Corrected)
0
1
2
3
0
200
400
600
800
Time Point (0.25 Hz)

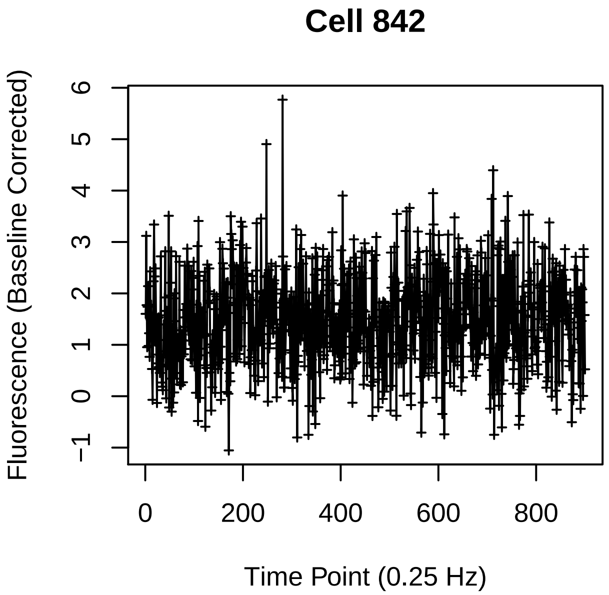
Cell 842
Fluorescence (Baseline Corrected)
−1
0
1
2
3
4
5
6
0
200
400
600
800
Time Point (0.25 Hz)

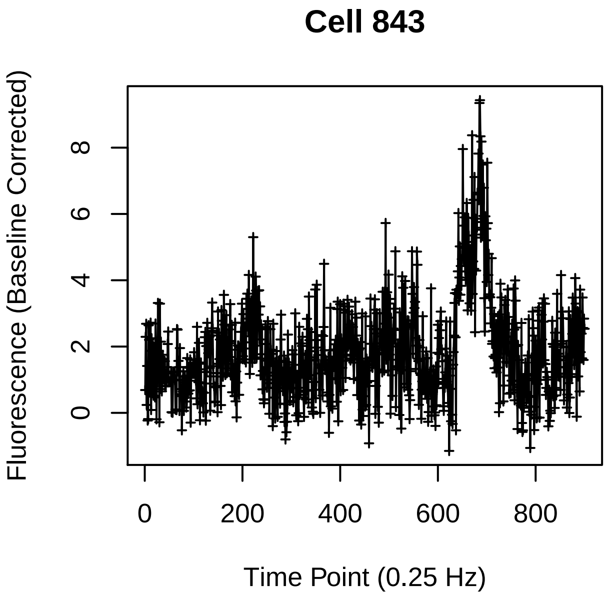
Cell 843
Fluorescence (Baseline Corrected)
0
2
4
6
8
0
200
400
600
800
Time Point (0.25 Hz)

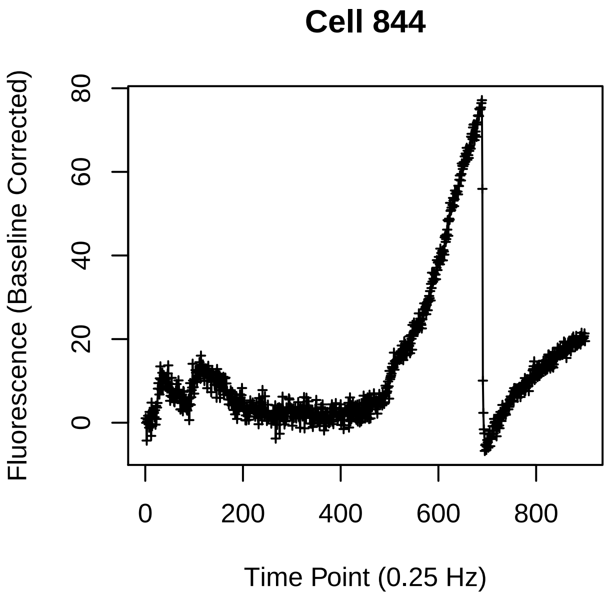
Cell 844
Fluorescence (Baseline Corrected)
0
20
40
60
80
0
200
400
600
800
Time Point (0.25 Hz)

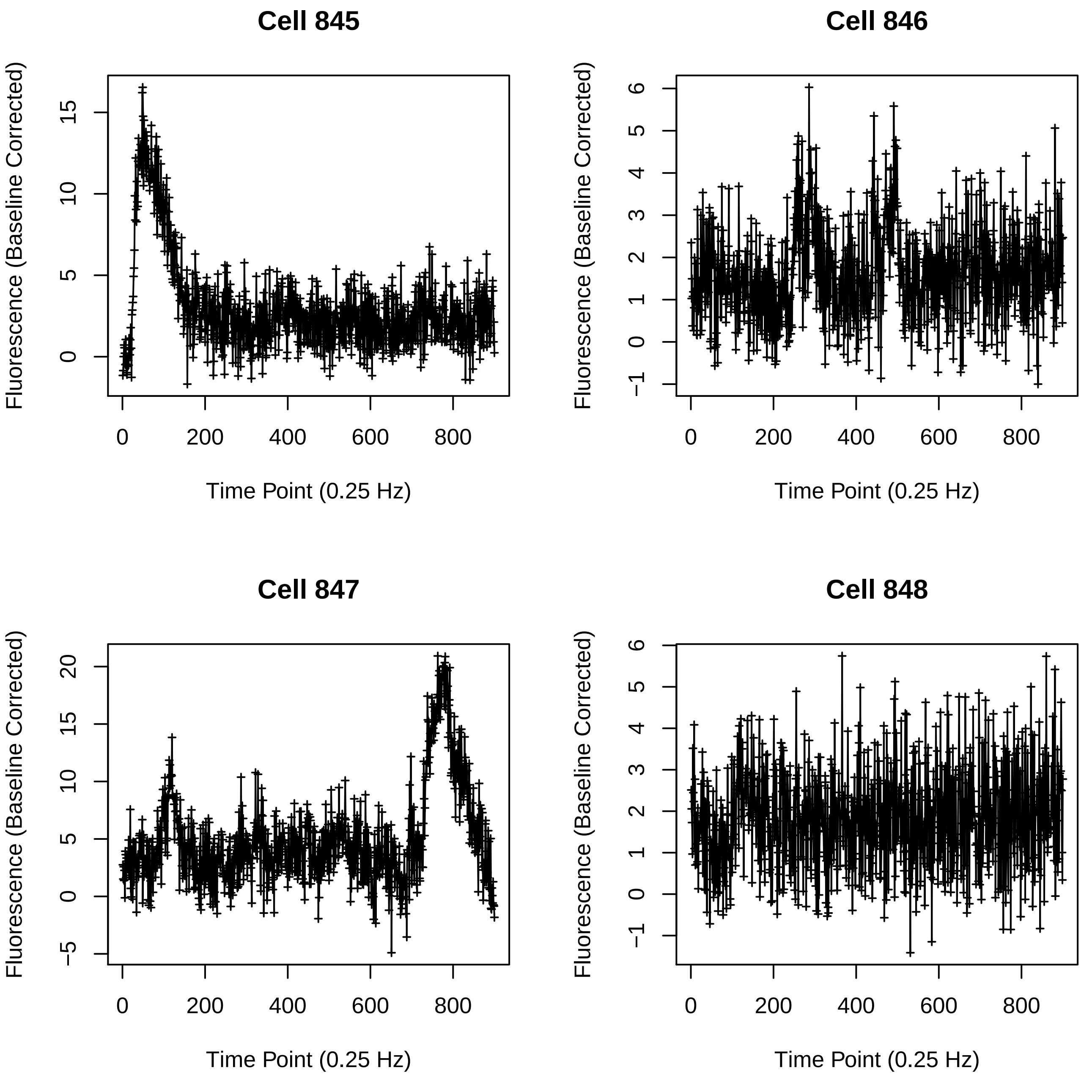
Cell 845
Fluorescence (Baseline Corrected)
Time Point (0.25 Hz)
Cell 846
Fluorescence (Baseline Corrected)
Time Point (0.25 Hz)
Cell 847
Fluorescence (Baseline Corrected)
Time Point (0.25 Hz)
Cell 848
Fluorescence (Baseline Corrected)
Time Point (0.25 Hz)

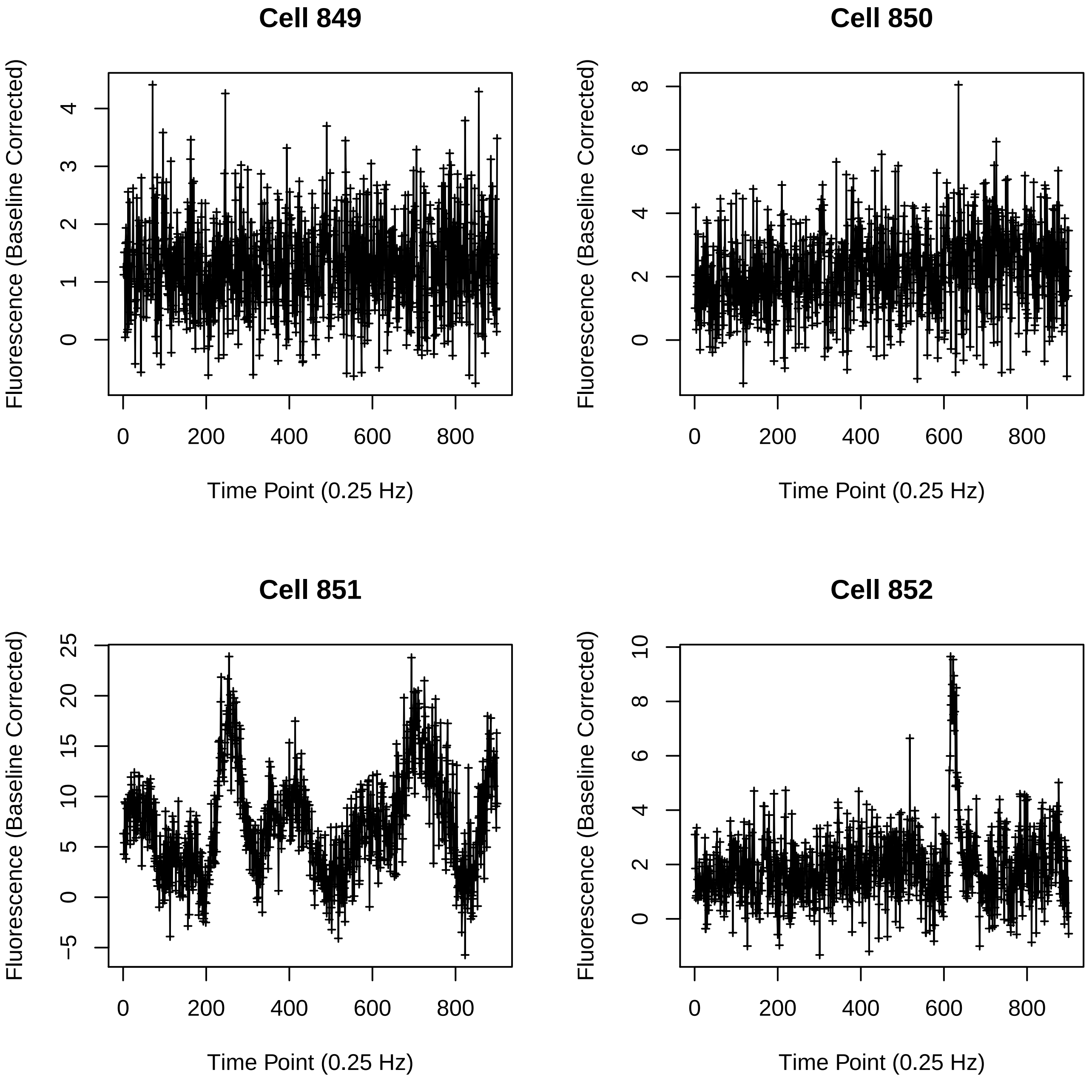
Cell 849
Cell 850
Cell 851
Cell 852
Fluorescence (Baseline Corrected)
Time Point (0.25 Hz)
0
200
400
600
800
1
2
3
4
6
8
10
−5
5
15
20
25

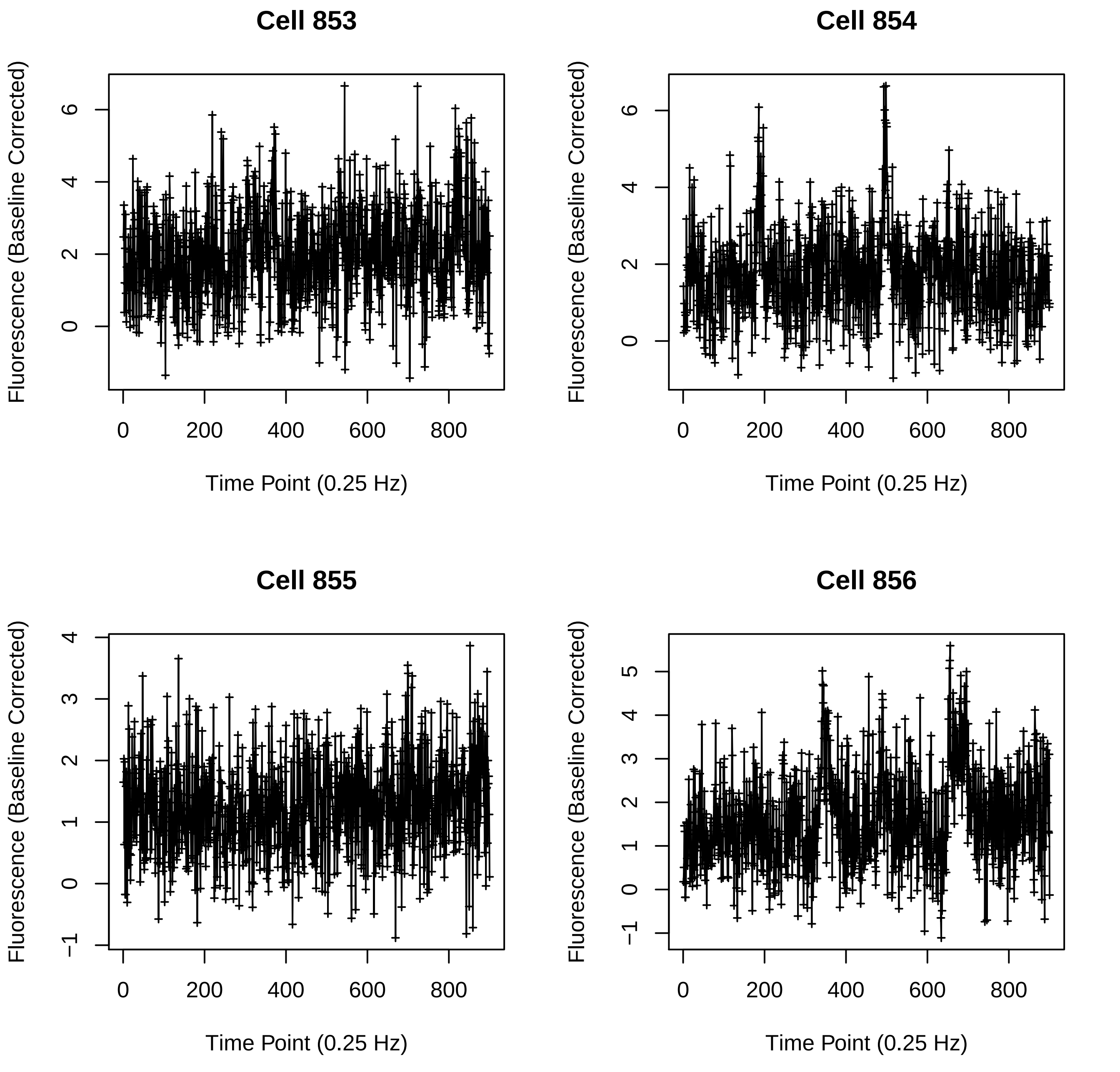
Cell 853
Fluorescence (Baseline Corrected)
Time Point (0.25 Hz)
Cell 854
Fluorescence (Baseline Corrected)
Time Point (0.25 Hz)
Cell 855
Fluorescence (Baseline Corrected)
Time Point (0.25 Hz)
Cell 856
Fluorescence (Baseline Corrected)
Time Point (0.25 Hz)

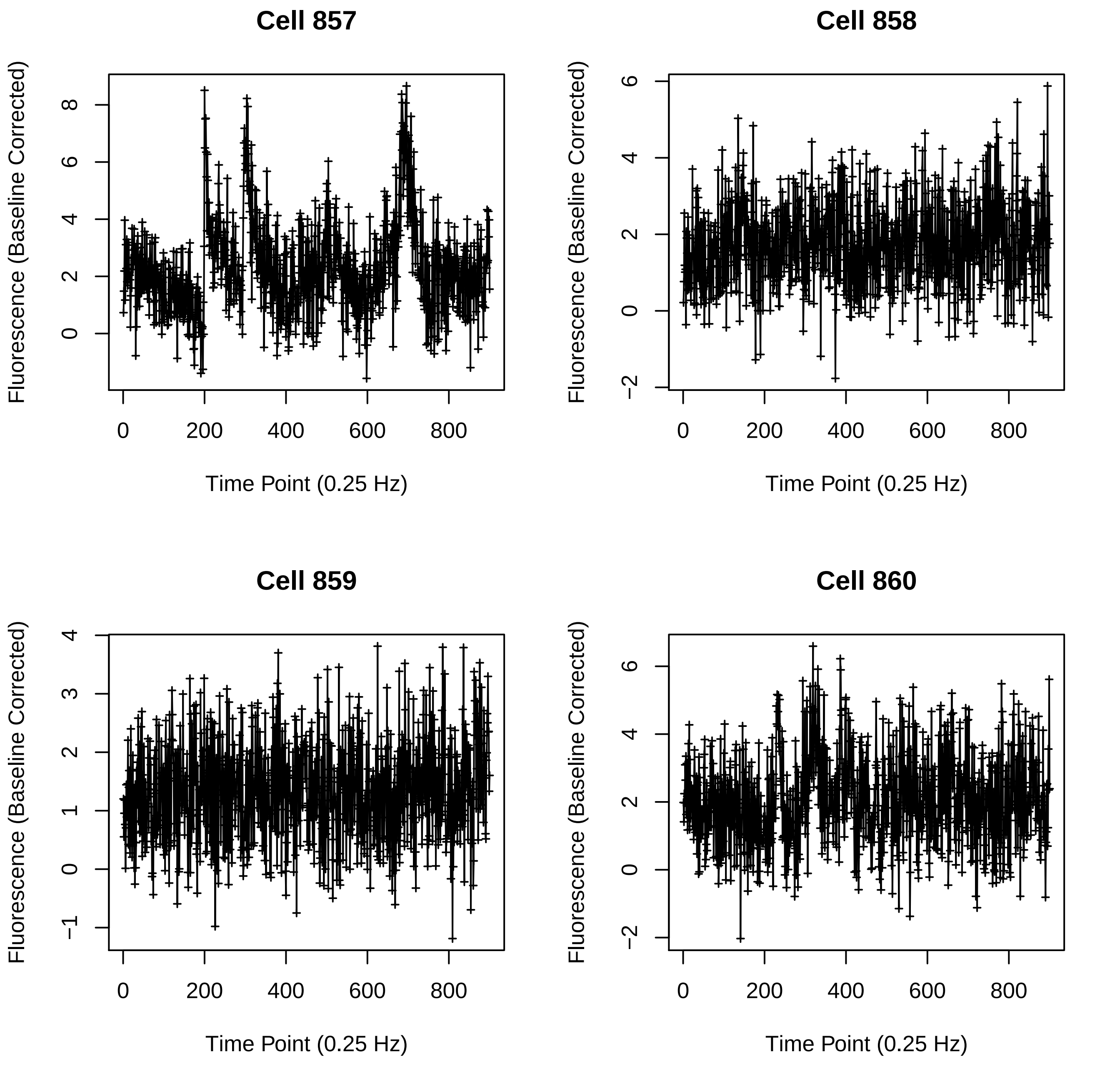
Cell 857
Cell 858
Cell 859
Cell 860
Fluorescence (Baseline Corrected)
Time Point (0.25 Hz)
0
200
400
600
800
0
2
4
6
8
−2
−1
1
3

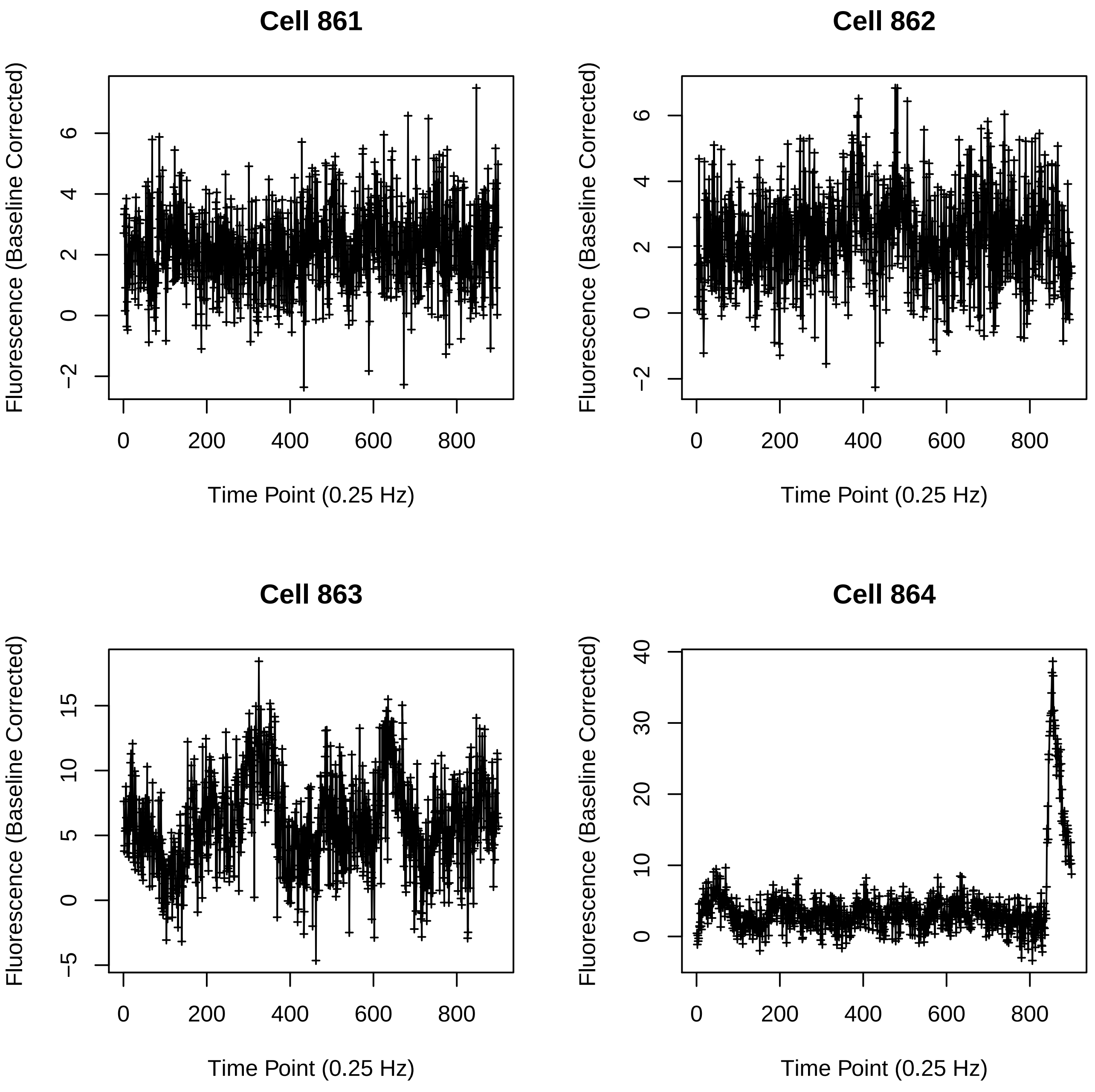
Cell 861
Cell 862
Cell 863
Cell 864
Fluorescence (Baseline Corrected)
Time Point (0.25 Hz)
-2
0
2
4
6
0
200
400
600
800
-5
0
5
10
15
10
20
30
40

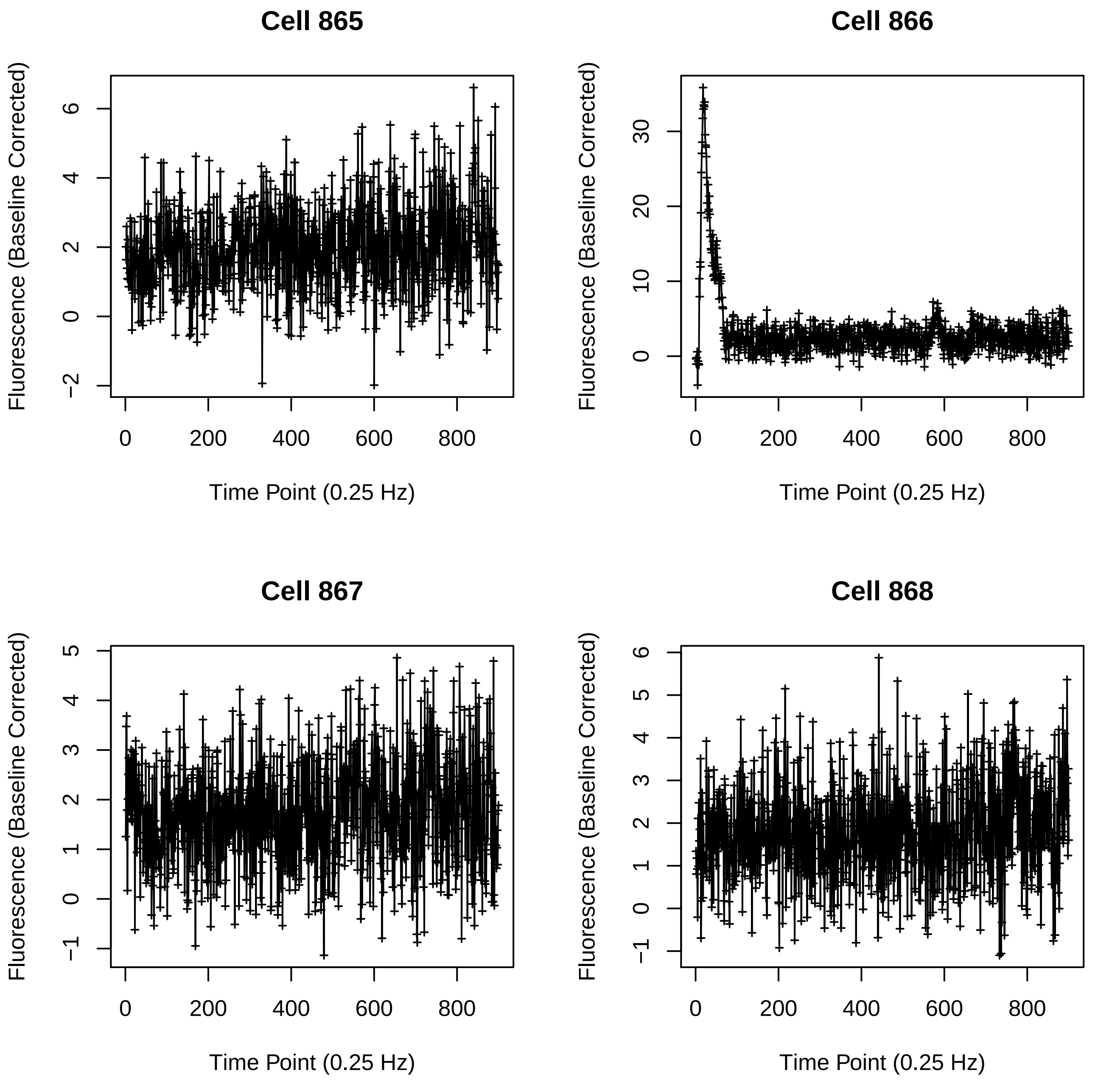

Cell 865
Cell 866
Cell 867
Cell 868
Fluorescence (Baseline Corrected)
Time Point (0.25 Hz)

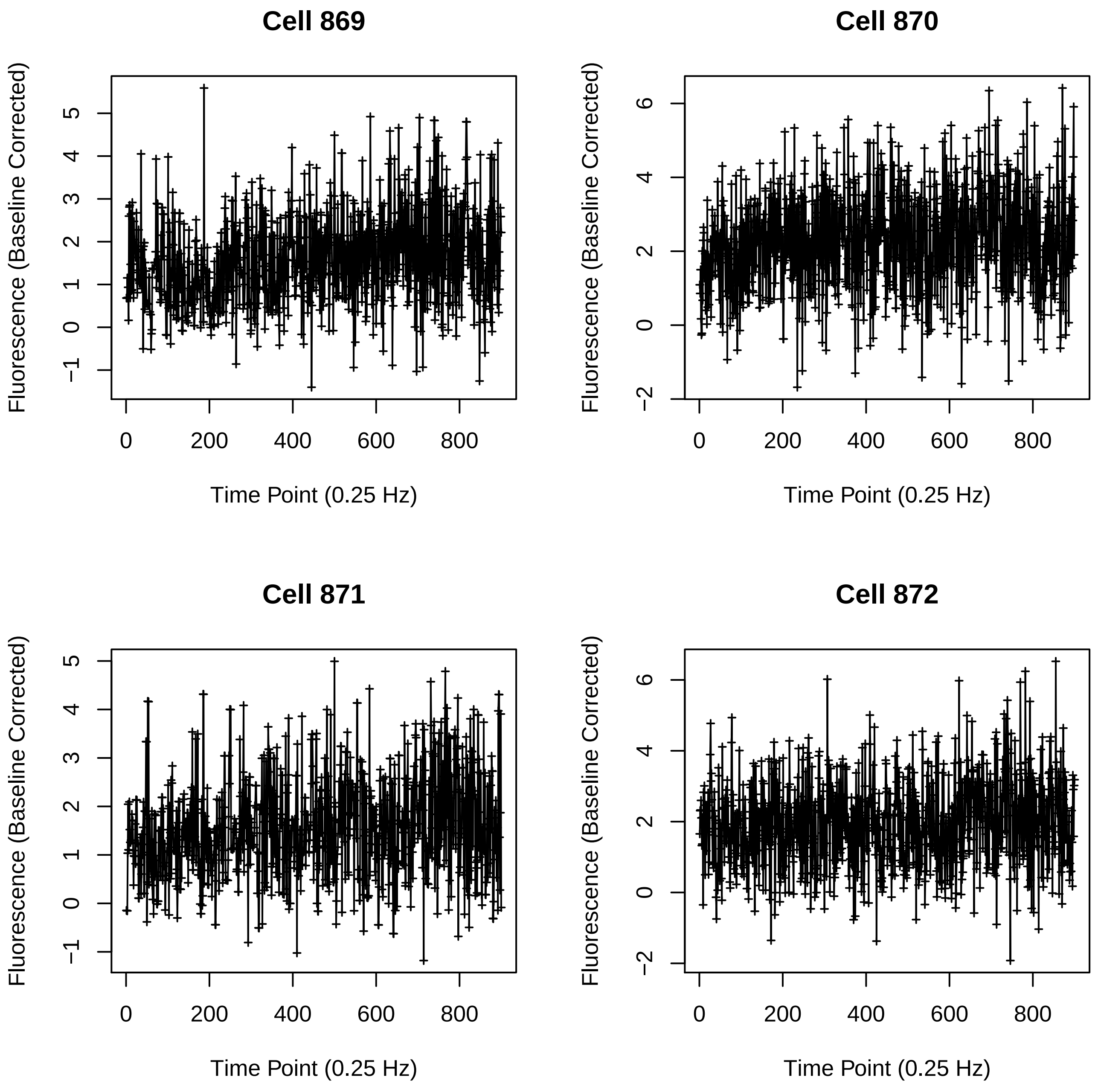
Cell 869
Cell 870
Cell 871
Cell 872
Fluorescence (Baseline Corrected)
Time Point (0.25 Hz)
−1 0 1 2 3 4 5
−2 0 2 4 6
0 200 400 600 800

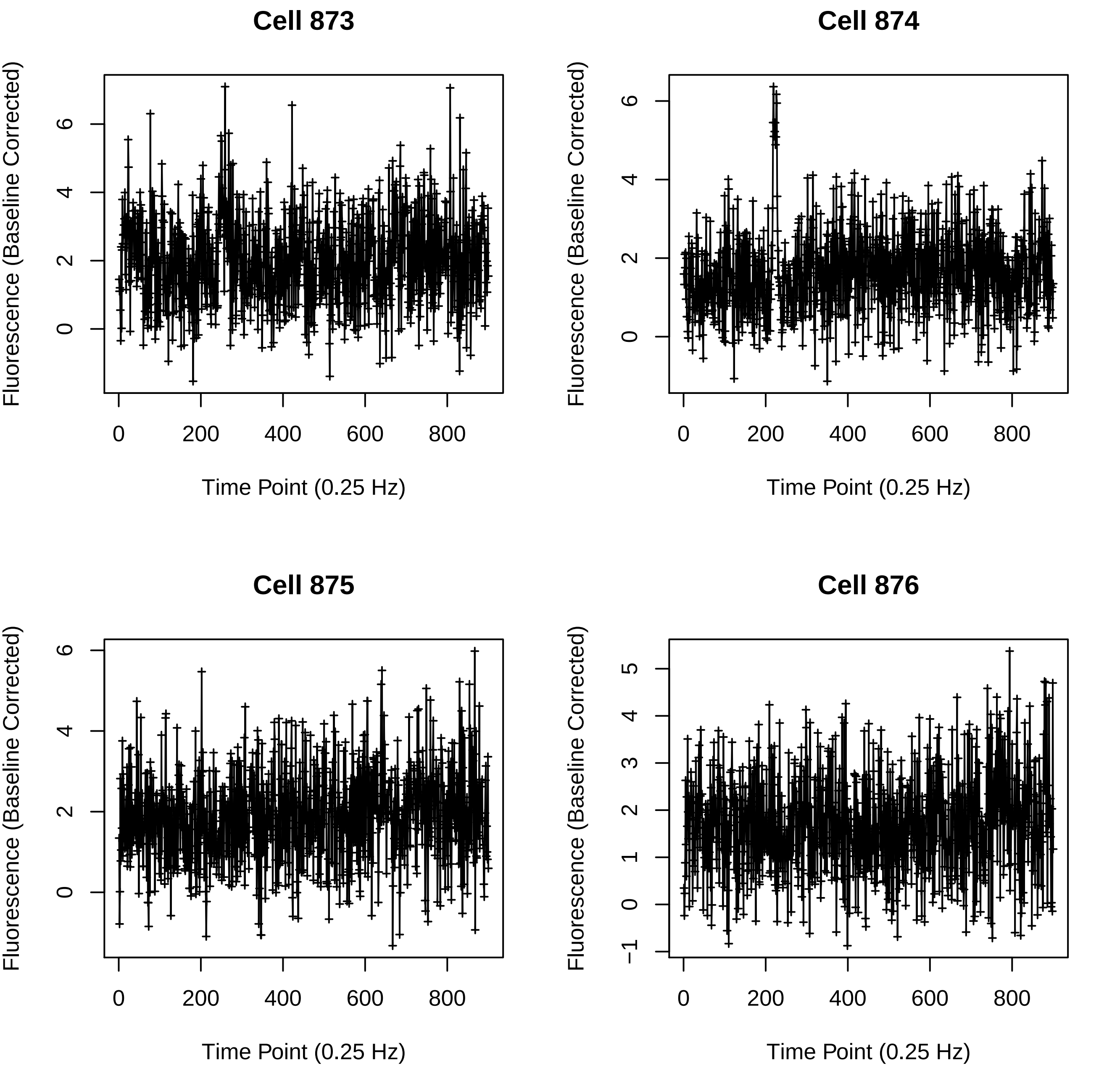
Cell 873
Cell 874
Cell 875
Cell 876
Fluorescence (Baseline Corrected)
Time Point (0.25 Hz)
0
200
400
600
800
−1
0
1
2
3
4
5
6

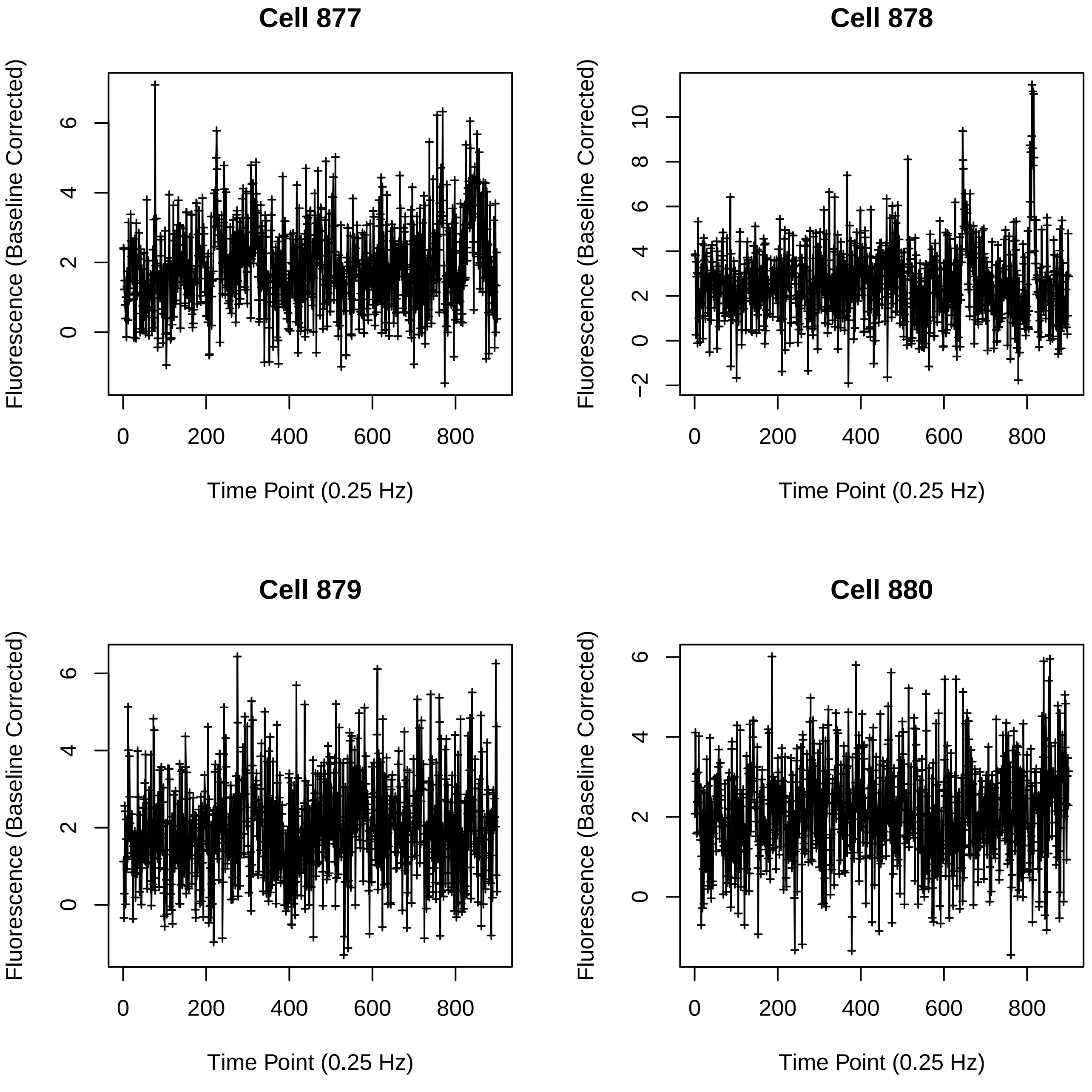
Cell 877
Cell 878
Cell 879
Cell 880
Fluorescence (Baseline Corrected)
Time Point (0.25 Hz)
0
200
400
600
800
−2
2
4
6
8
10

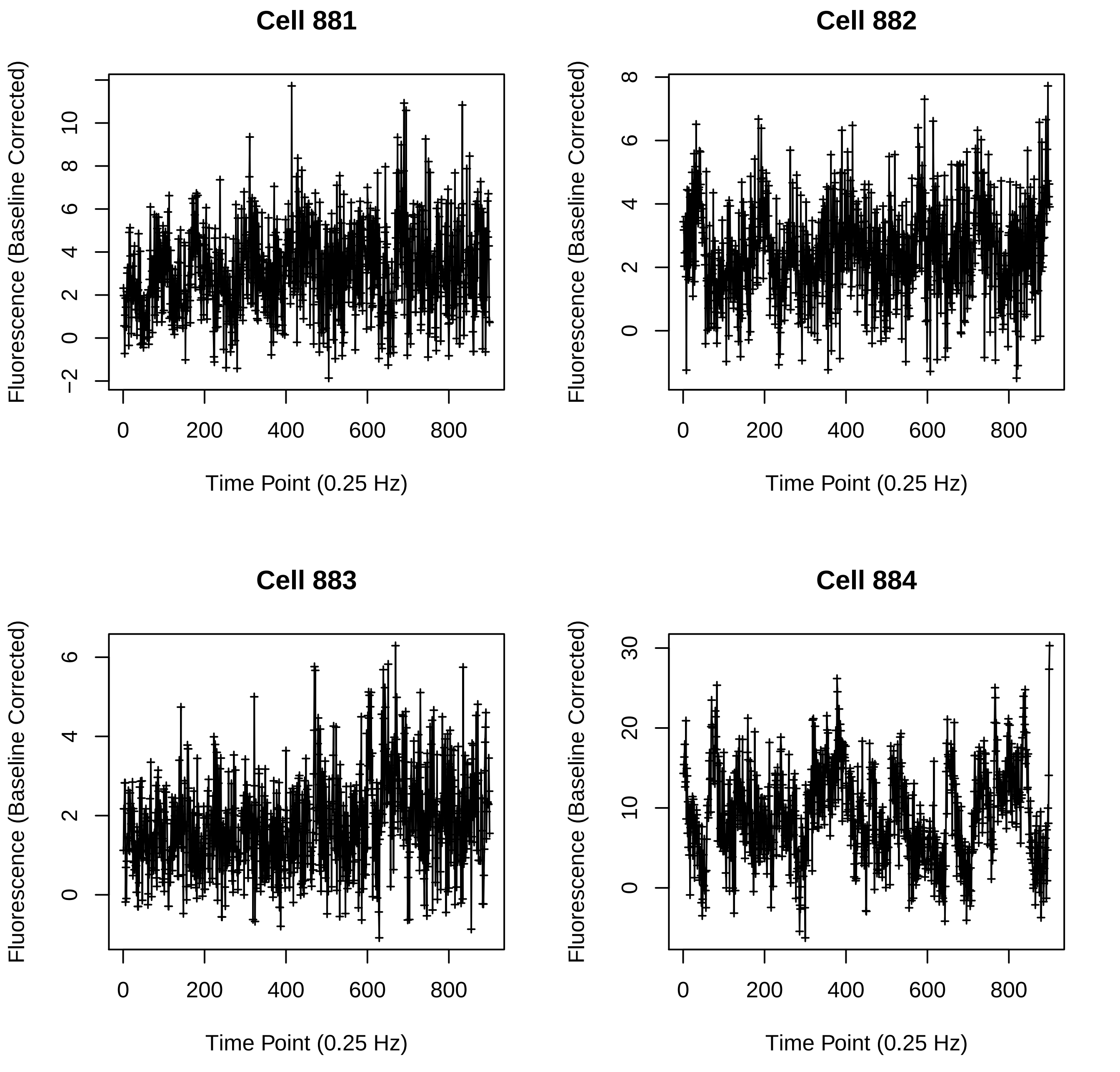

Cell 881
Fluorescence (Baseline Corrected)
−2
0
2
4
6
8
10
0
200
400
600
800
Time Point (0.25 Hz)
Cell 882
Fluorescence (Baseline Corrected)
0
2
4
6
8
0
200
400
600
800
Time Point (0.25 Hz)
Cell 883
Fluorescence (Baseline Corrected)
0
2
4
6
0
200
400
600
800
Time Point (0.25 Hz)
Cell 884
Fluorescence (Baseline Corrected)
0
10
20
30
0
200
400
600
800
Time Point (0.25 Hz)

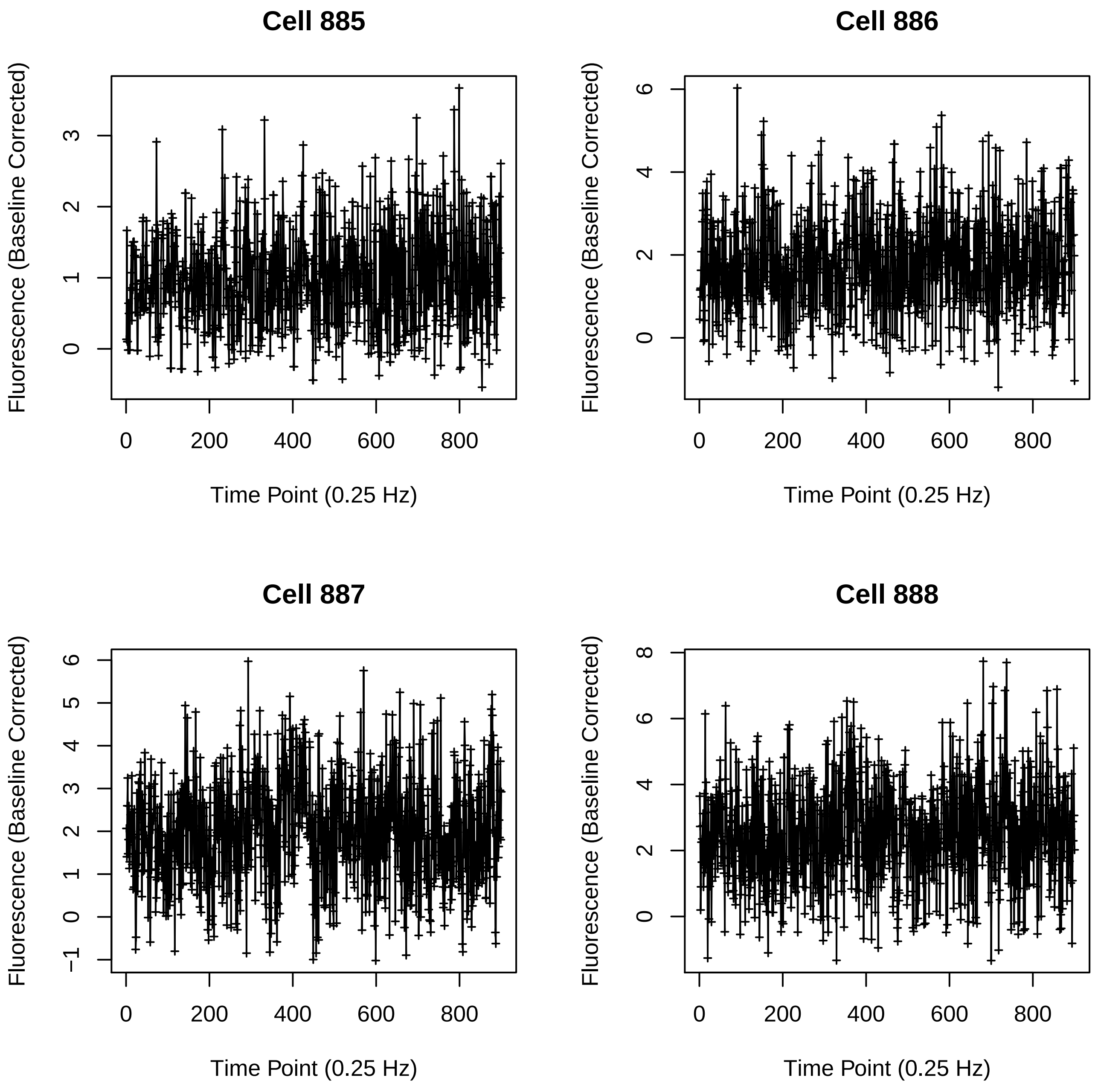
Cell 885
Cell 886
Cell 887
Cell 888
Fluorescence (Baseline Corrected)
Time Point (0.25 Hz)
0
200
400
600
800
−1
0
1
2
3
4
5
6
8

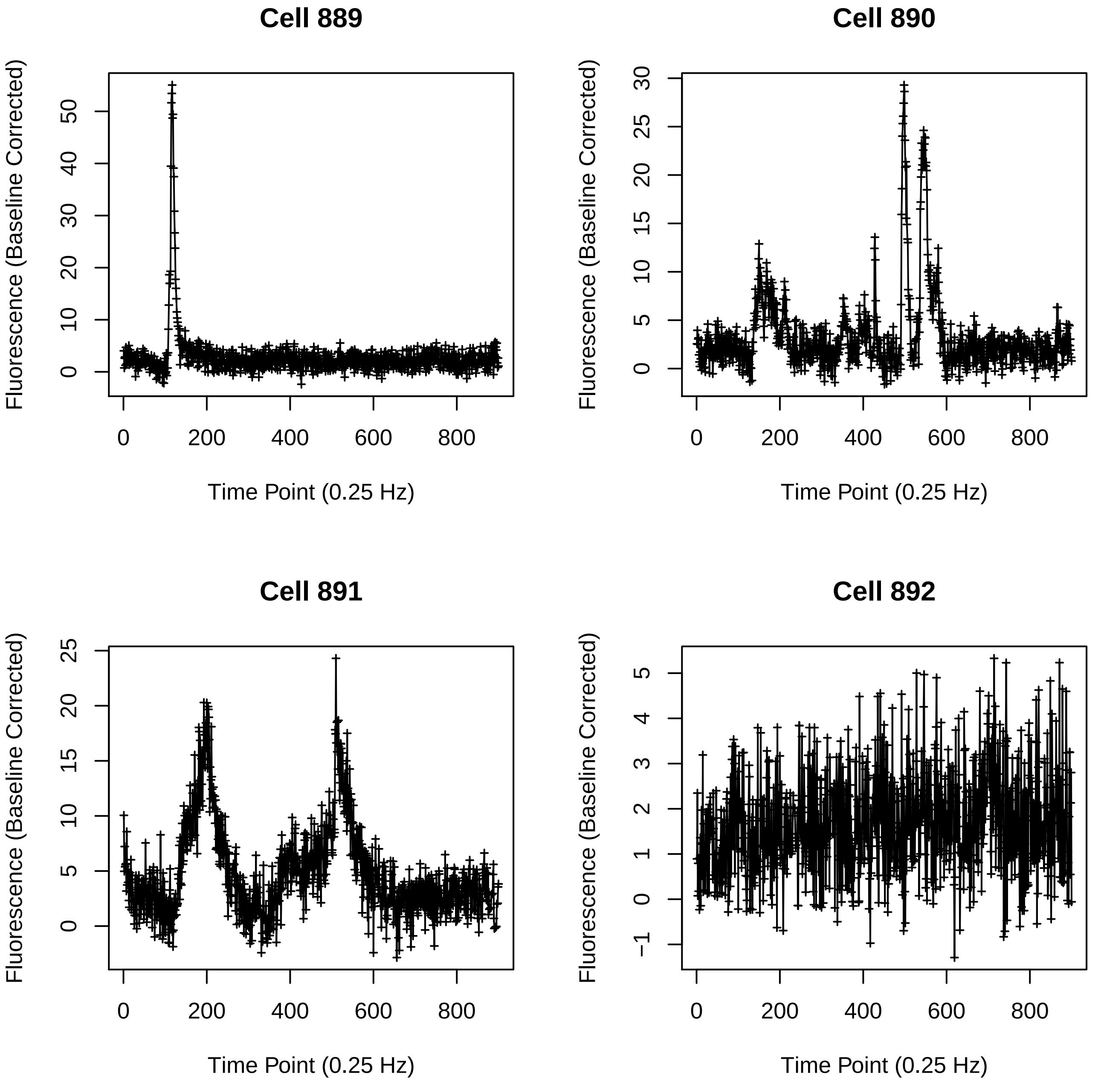
Cell 889
Fluorescence (Baseline Corrected)
0
10
20
30
40
50
Time Point (0.25 Hz)
0
200
400
600
800
Cell 890
Fluorescence (Baseline Corrected)
0
5
10
15
20
25
30
Time Point (0.25 Hz)
0
200
400
600
800
Cell 891
Fluorescence (Baseline Corrected)
0
5
10
15
20
25
Time Point (0.25 Hz)
0
200
400
600
800
Cell 892
Fluorescence (Baseline Corrected)
−1
0
1
2
3
4
5
Time Point (0.25 Hz)
0
200
400
600
800

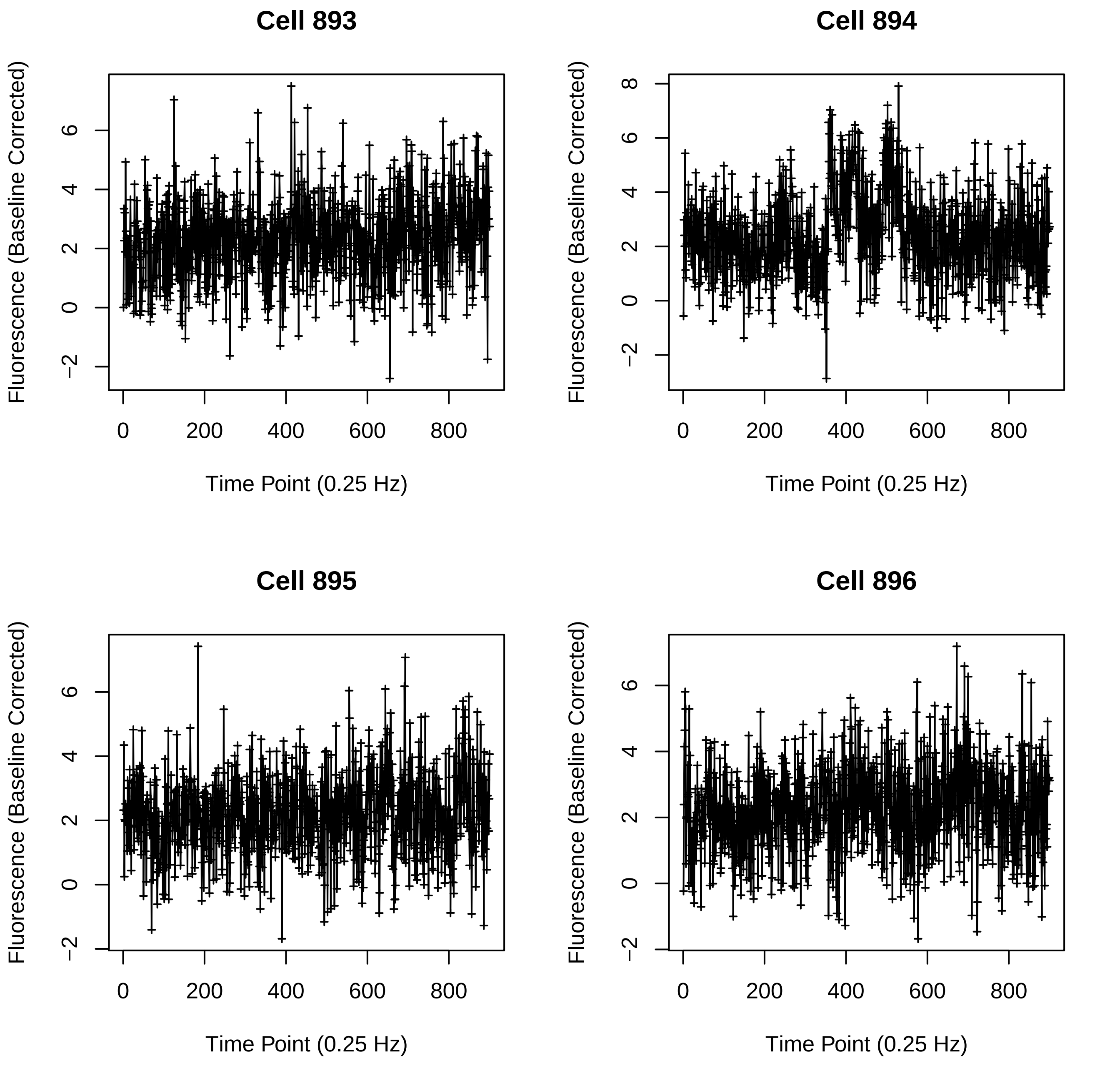

Cell 893
Cell 894
Cell 895
Cell 896
Fluorescence (Baseline Corrected)
Time Point (0.25 Hz)
0
200
400
600
800
−2
2
4
6
8

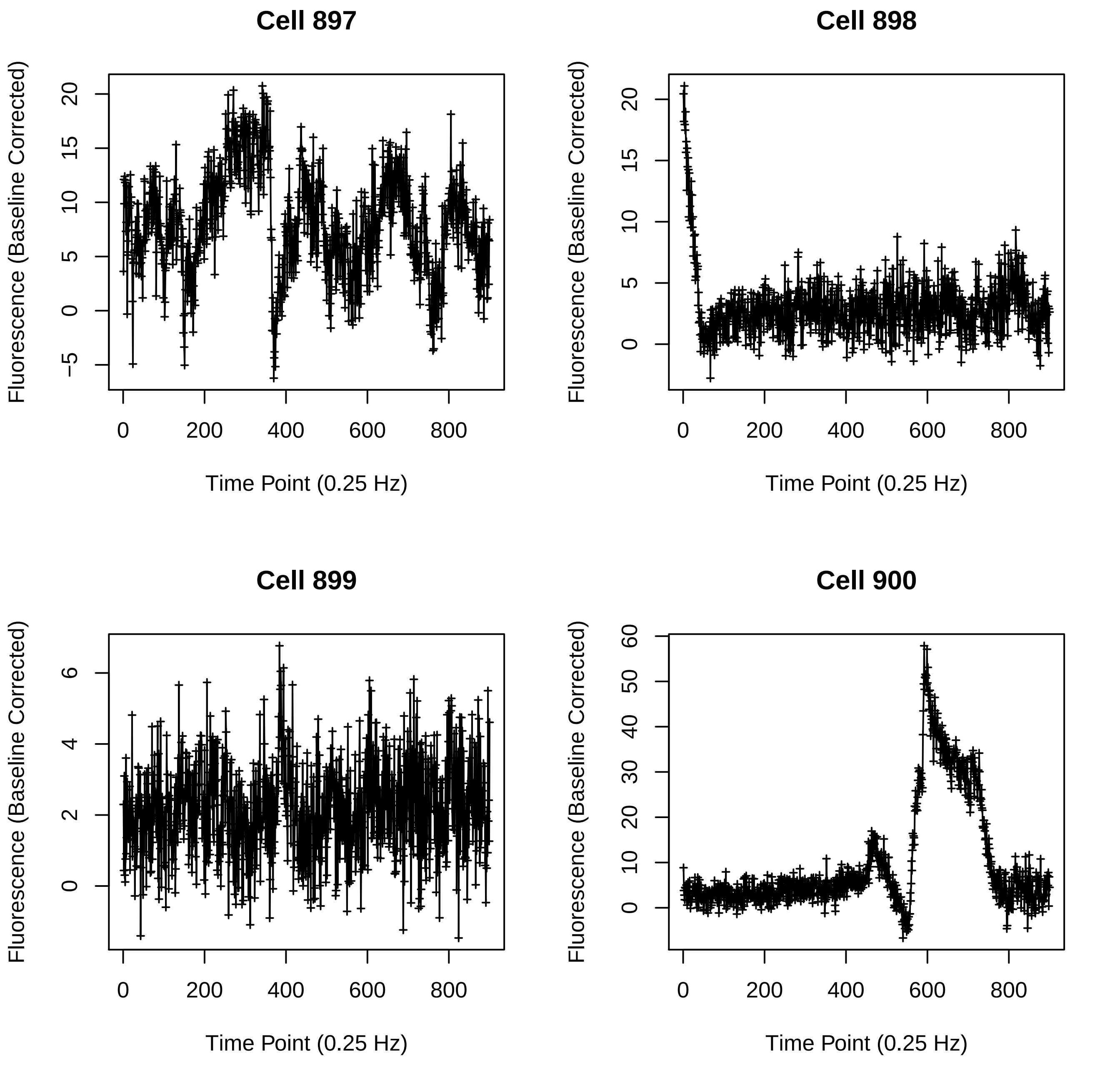

Cell 897
Cell 898
Cell 899
Cell 900
Fluorescence (Baseline Corrected)
Time Point (0.25 Hz)

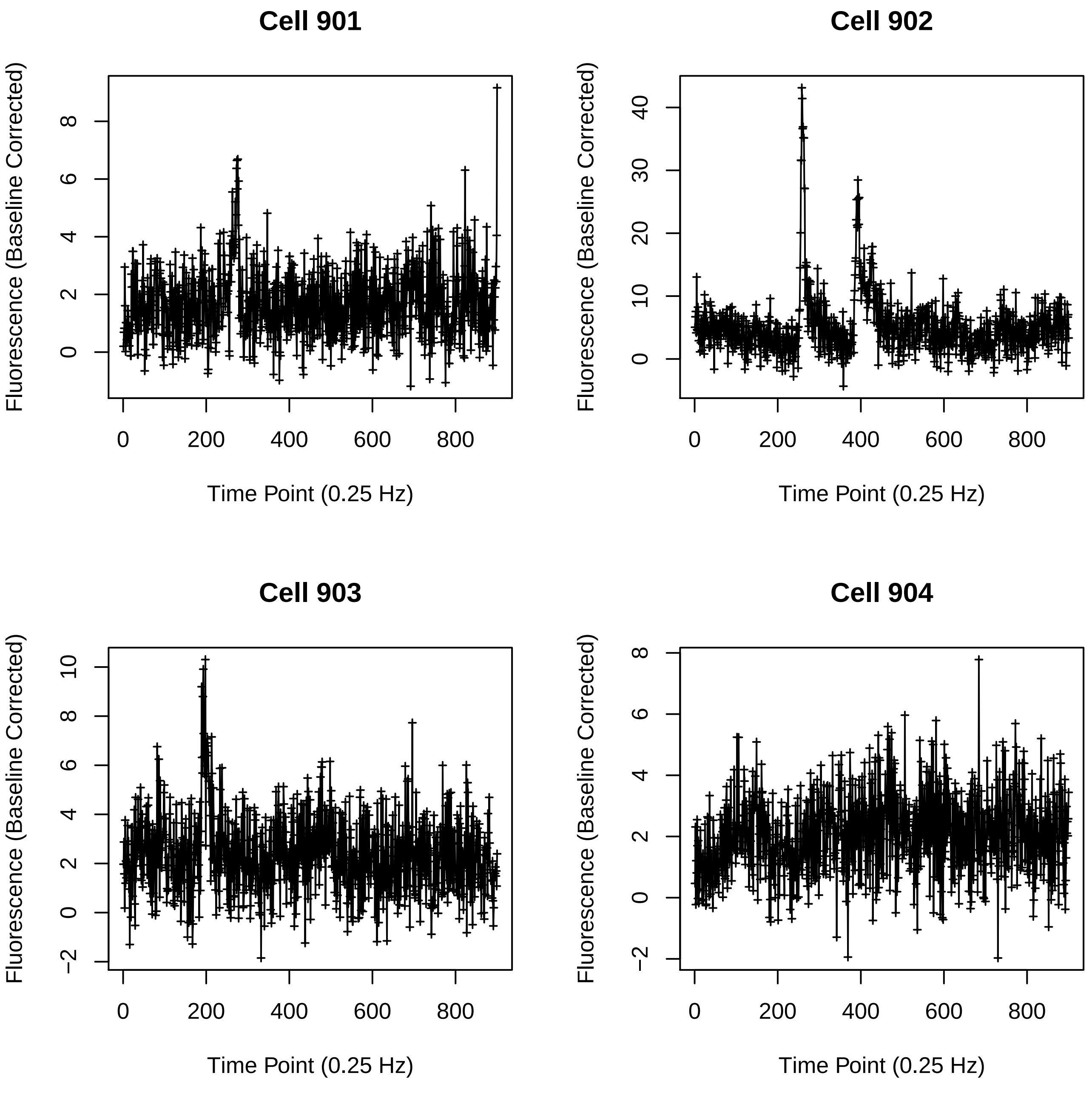
Cell 901
Cell 902
Cell 903
Cell 904
Fluorescence (Baseline Corrected)
Time Point (0.25 Hz)
0
200
400
600
800
-2
0
2
4
6
8
10
20
30
40

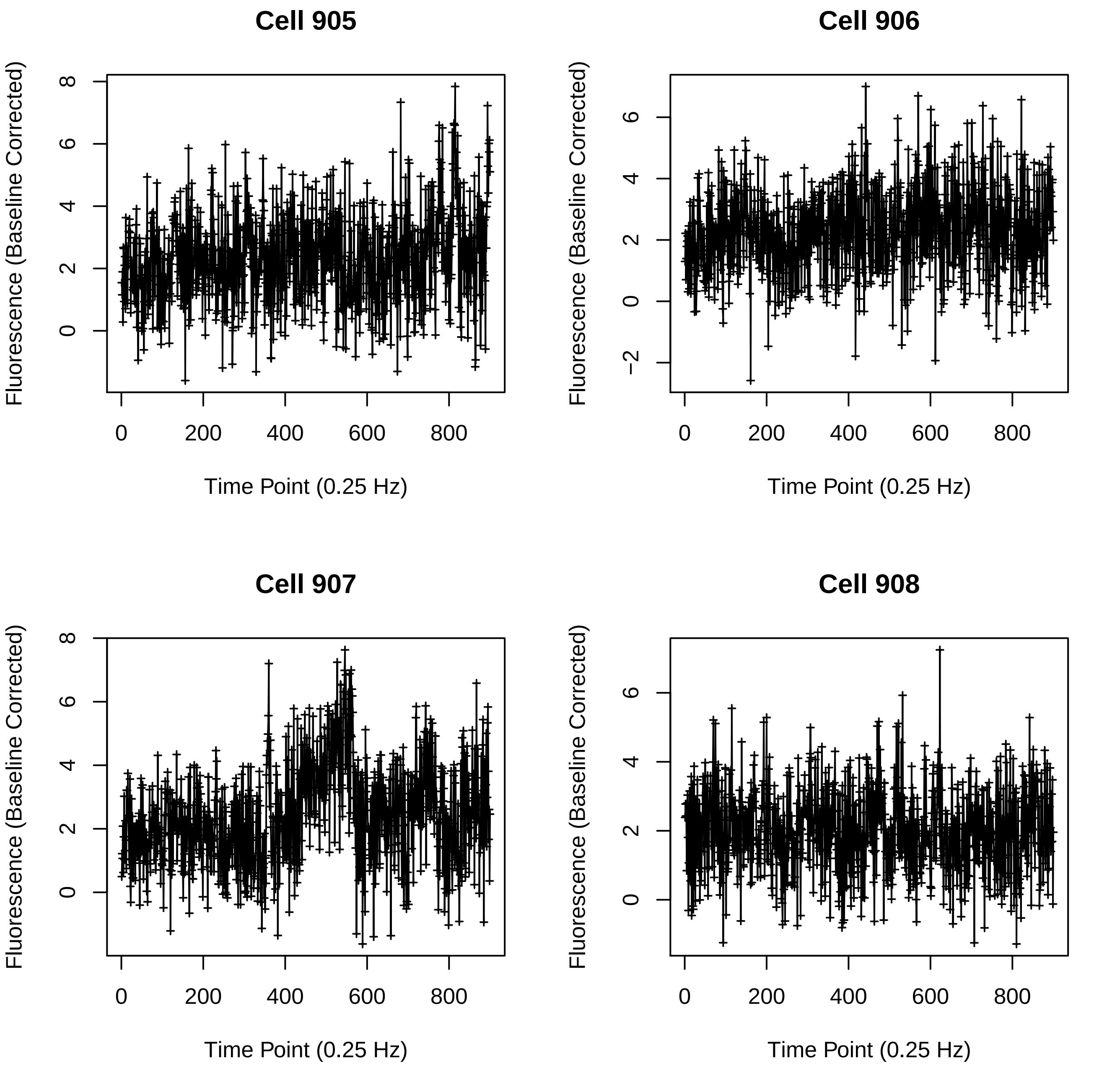

Cell 905
Cell 906
Cell 907
Cell 908
Fluorescence (Baseline Corrected)
Time Point (0.25 Hz)
0
200
400
600
800
−2
2
4
6
8

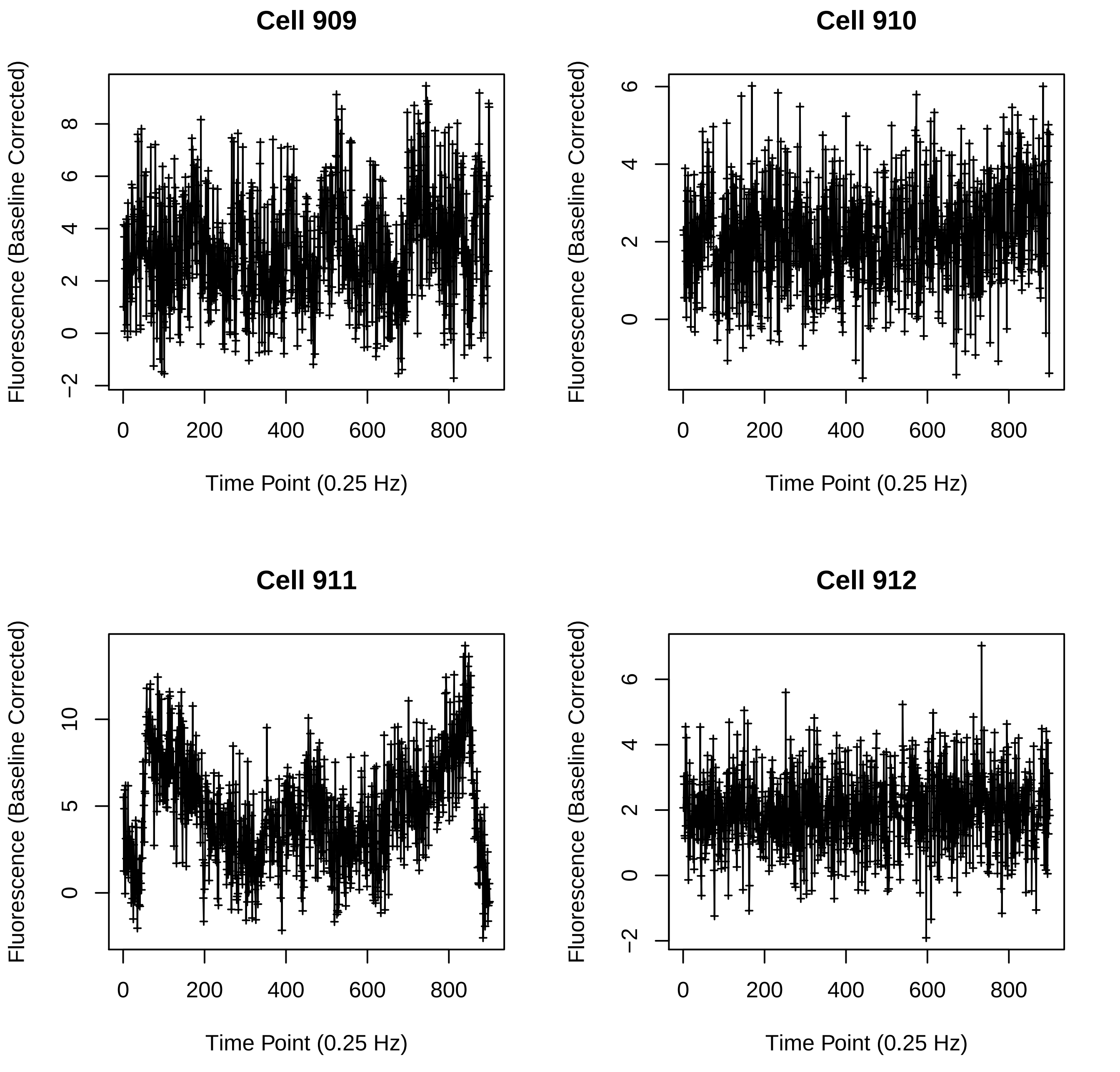

Cell 909
Cell 910
Cell 911
Cell 912
Fluorescence (Baseline Corrected)
Time Point (0.25 Hz)
0
200
400
600
800
-2
2
4
6
8
10
5

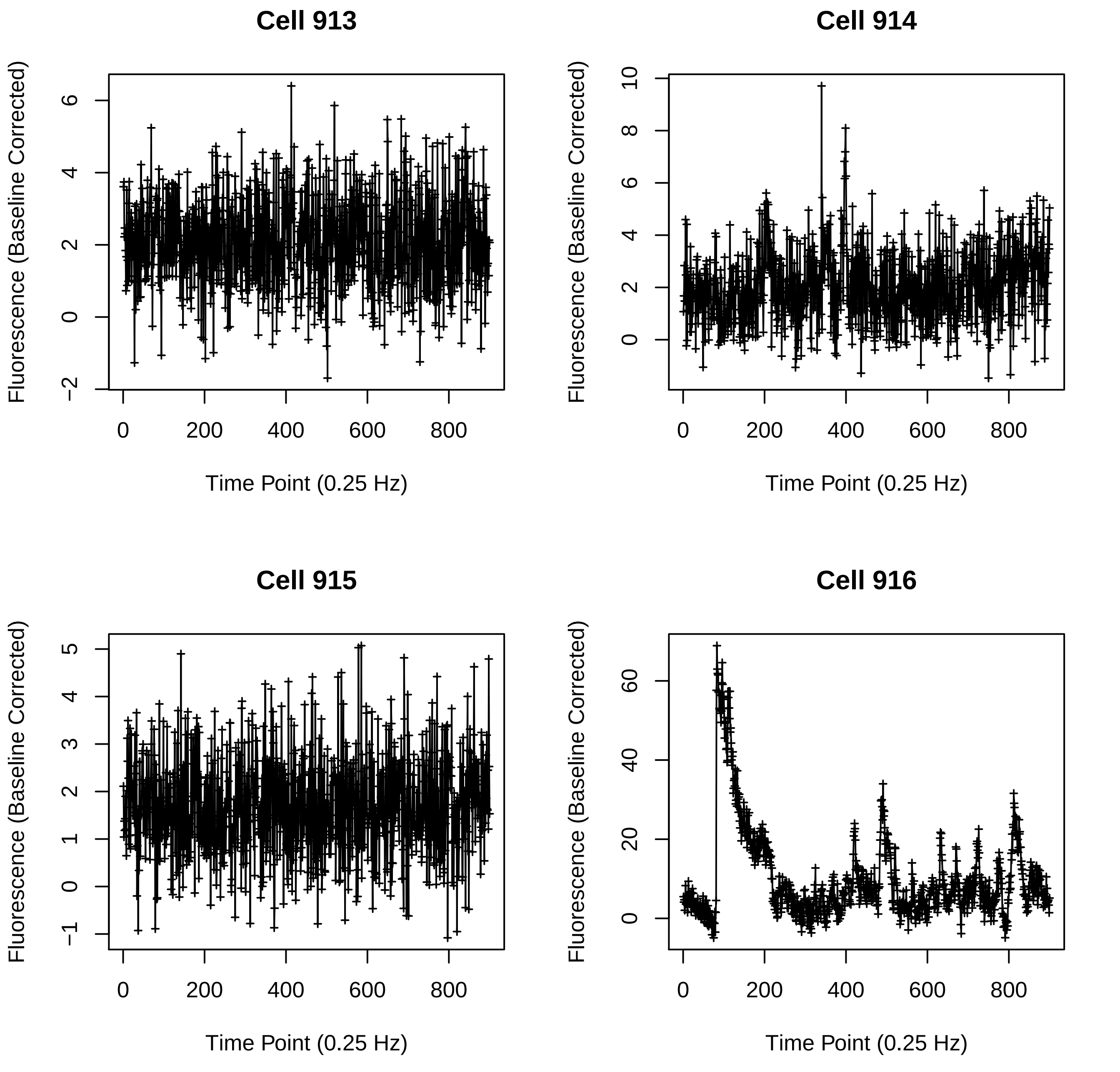
Cell 913
Cell 914
Cell 915
Cell 916
Fluorescence (Baseline Corrected)
Time Point (0.25 Hz)
−2
0
2
4
6
8
10
−1
1
3
5
20
40
60
200
400
600
800

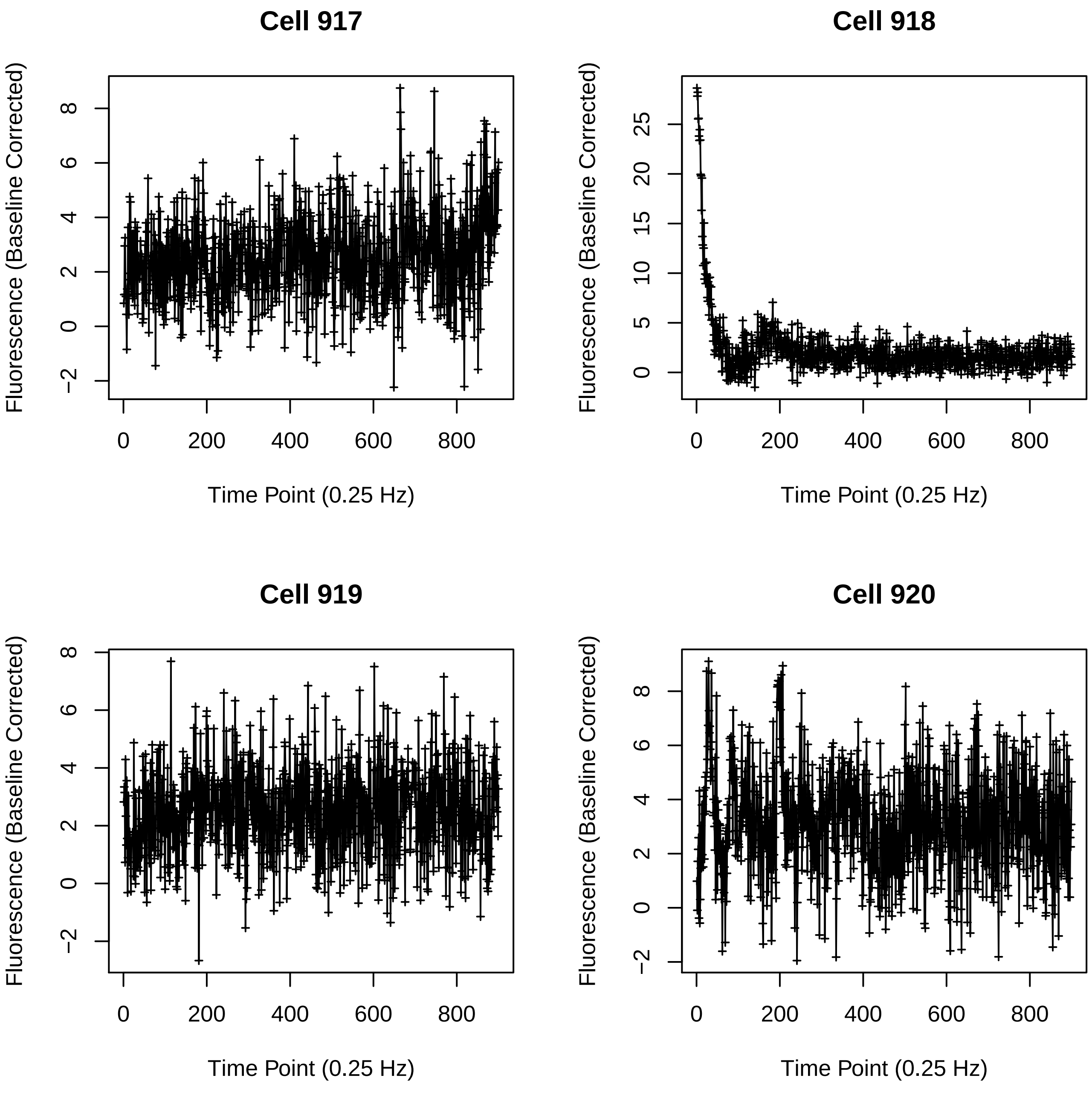
Cell 917
Cell 918
Cell 919
Cell 920
Fluorescence (Baseline Corrected)
Time Point (0.25 Hz)
0
200
400
600
800
−2
2
4
6
8
5
10
15
20
25

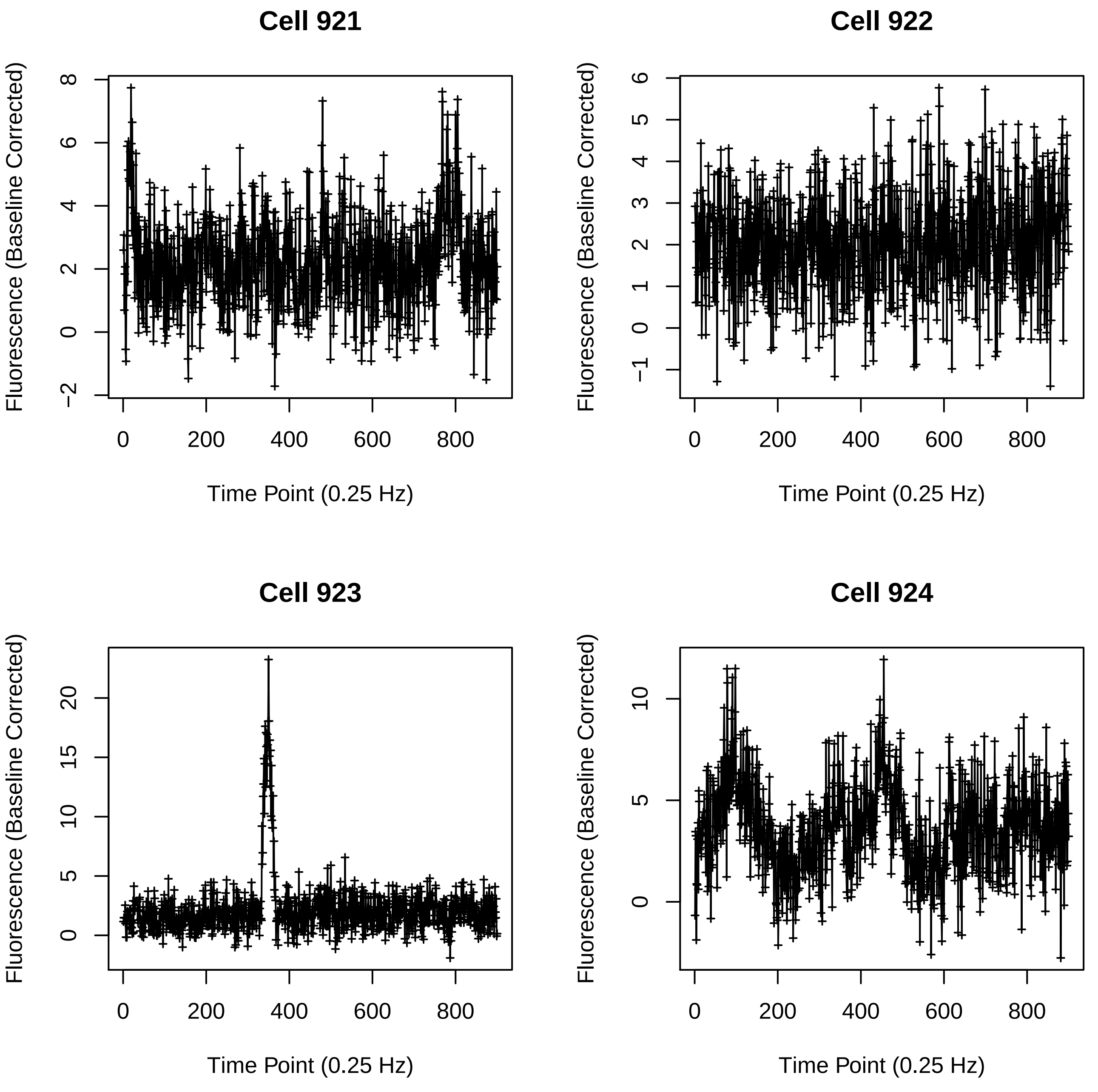
Cell 921
Cell 922
Cell 923
Cell 924
Fluorescence (Baseline Corrected)
Time Point (0.25 Hz)
0
200
400
600
800

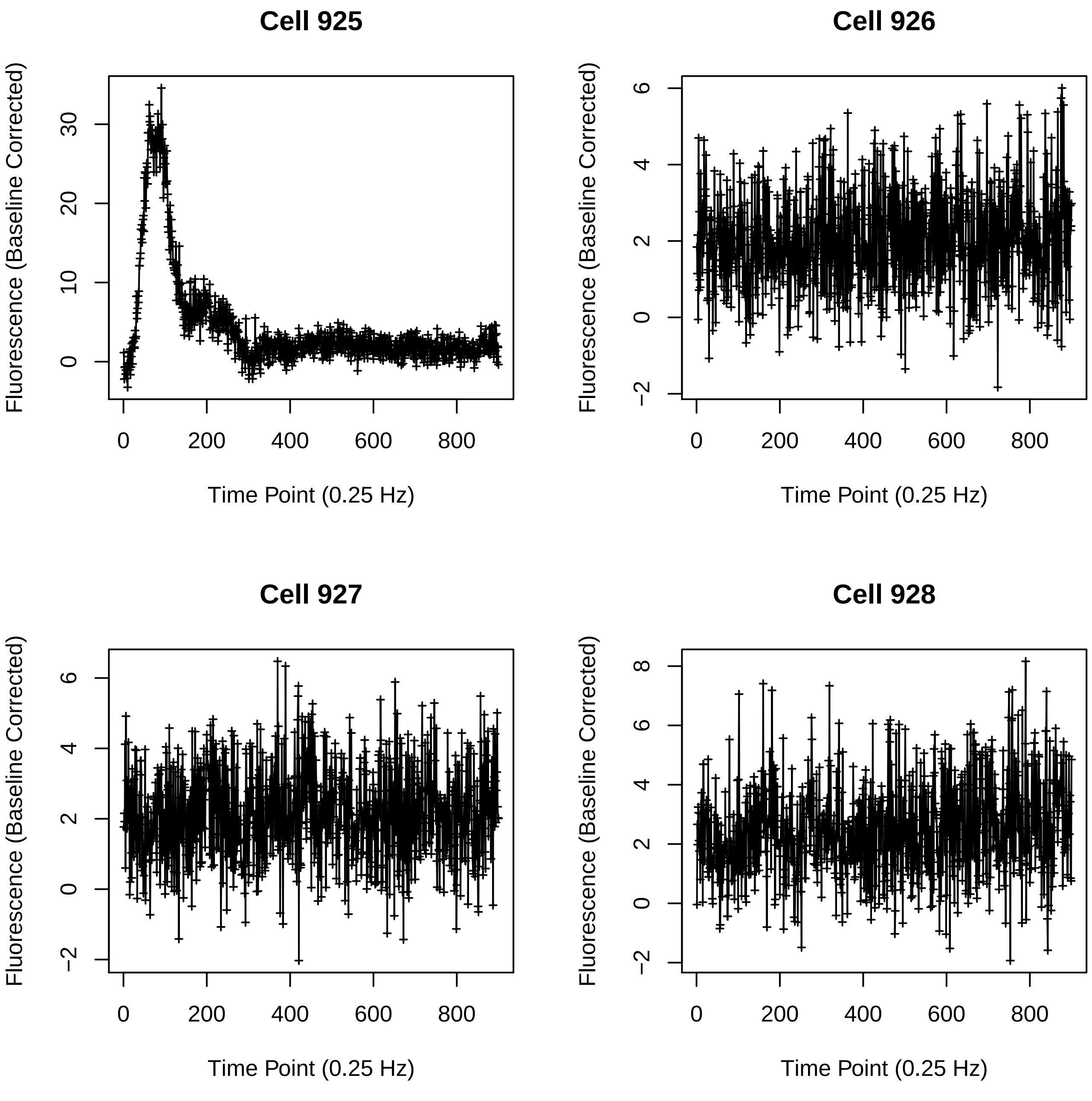
Cell 925
Cell 926
Cell 927
Cell 928
Fluorescence (Baseline Corrected)
Time Point (0.25 Hz)
0
10
20
30
−2
2
4
6
8
200
400
600
800

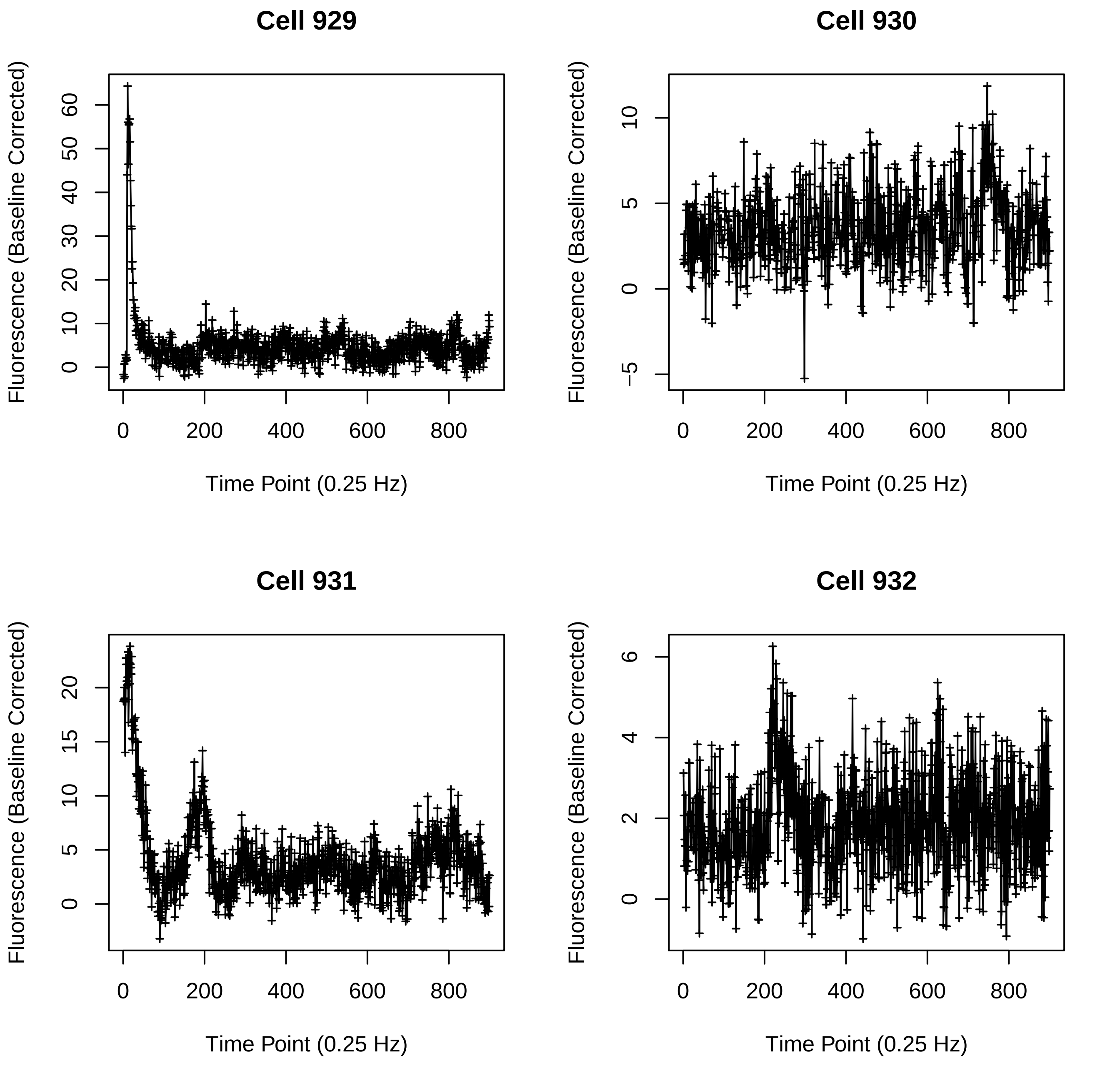
Cell 929
Cell 930
Cell 931
Cell 932
Fluorescence (Baseline Corrected)
Time Point (0.25 Hz)
0
10
20
30
40
50
60
5
10
15
20
−5
2
4
6
200
400
600
800

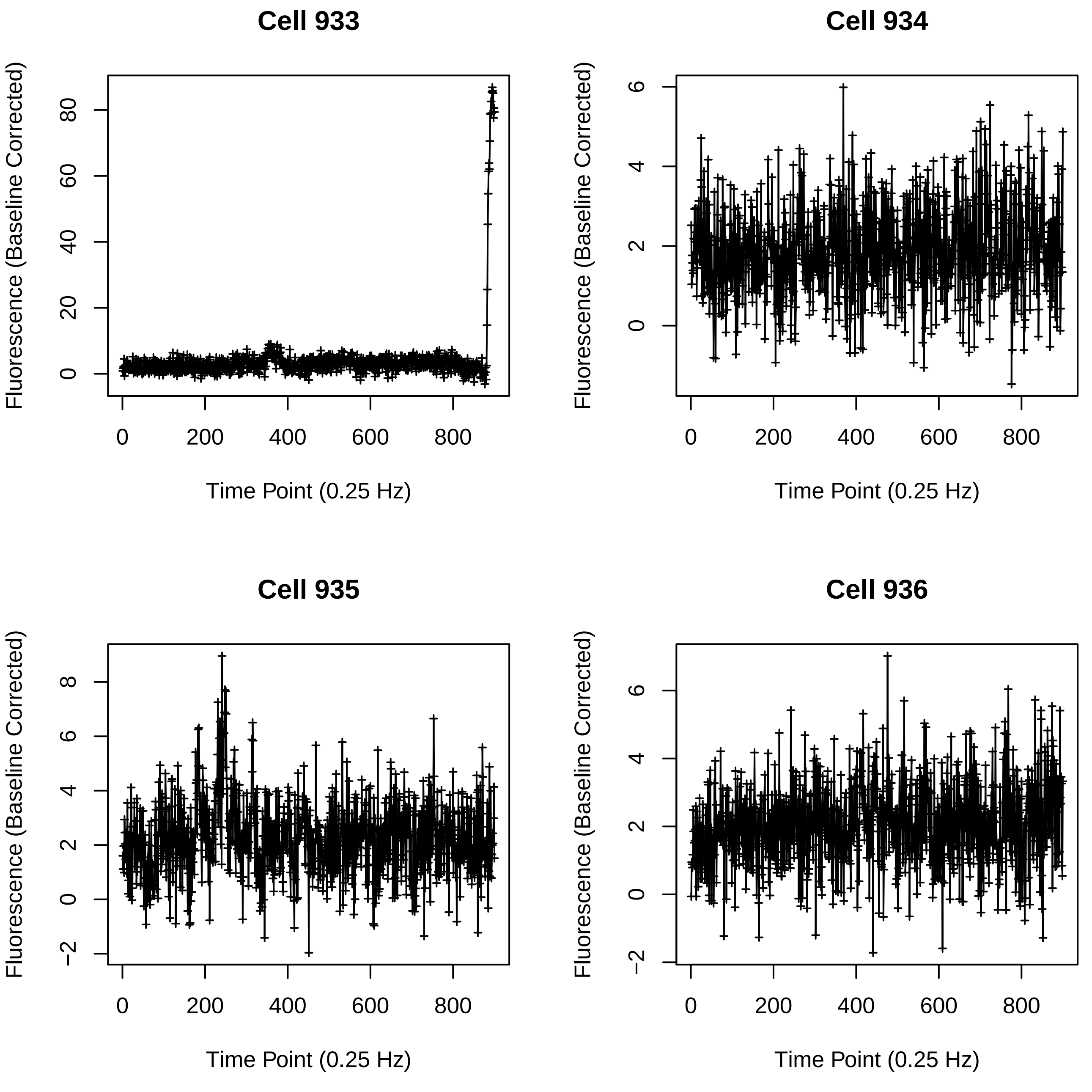
Cell 933
Cell 934
Cell 935
Cell 936
Fluorescence (Baseline Corrected)
Time Point (0.25 Hz)
0
20
40
60
80
0
2
4
6
−2
8
200
400
600
800

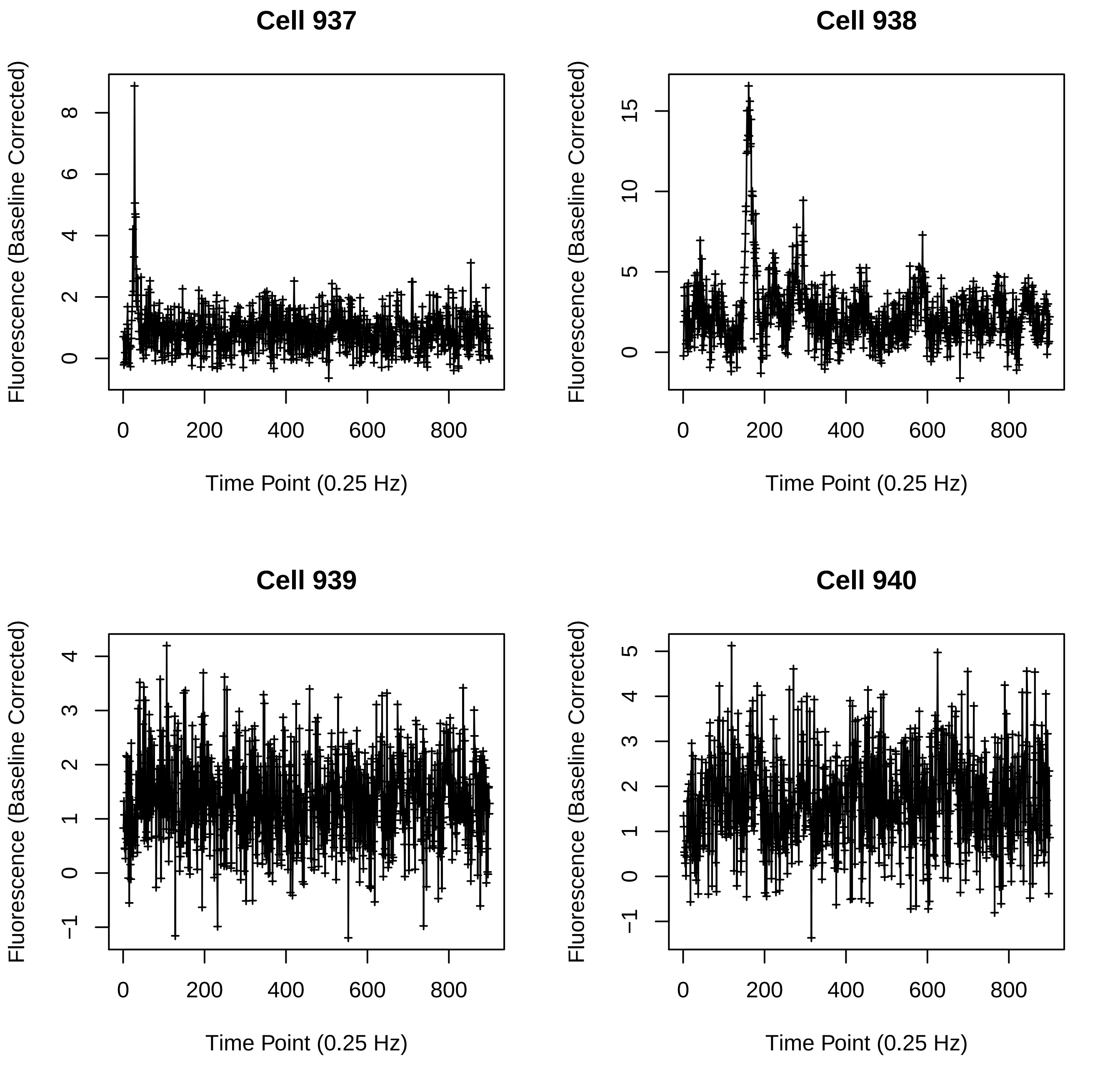
Cell 937
Cell 938
Cell 939
Cell 940
Fluorescence (Baseline Corrected)
Time Point (0.25 Hz)
0
200
400
600
800
2
4
6
8
5
10
15
−1
1
3

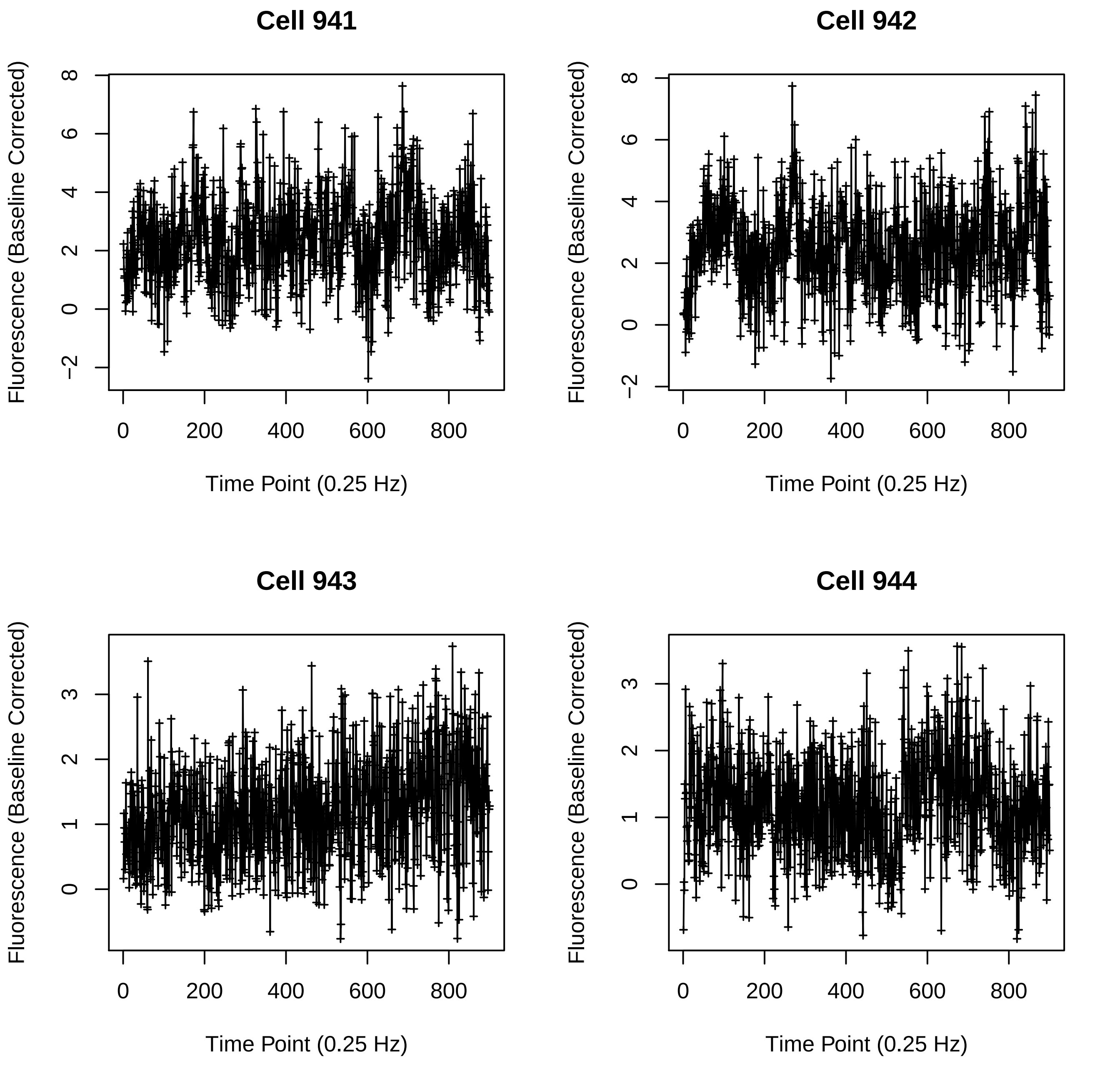

Cell 941
Cell 942
Cell 943
Cell 944
Fluorescence (Baseline Corrected)
Time Point (0.25 Hz)
0
200
400
600
800
−2
0
2
4
6
8
1
3

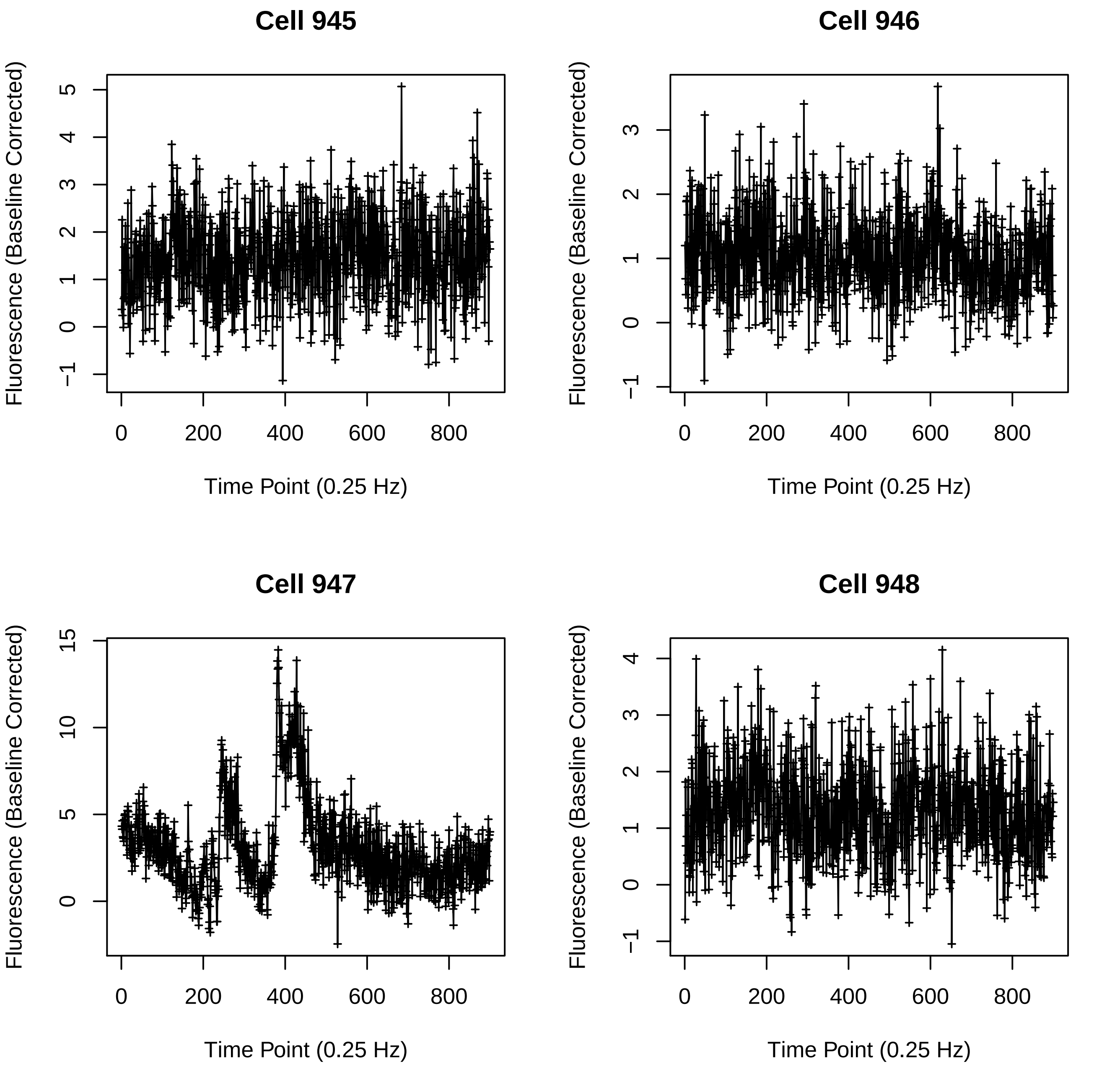
Cell 945
Cell 946
Cell 947
Cell 948
Fluorescence (Baseline Corrected)
Time Point (0.25 Hz)
0
200
400
600
800
−1
1
2
3
4
5
10
15

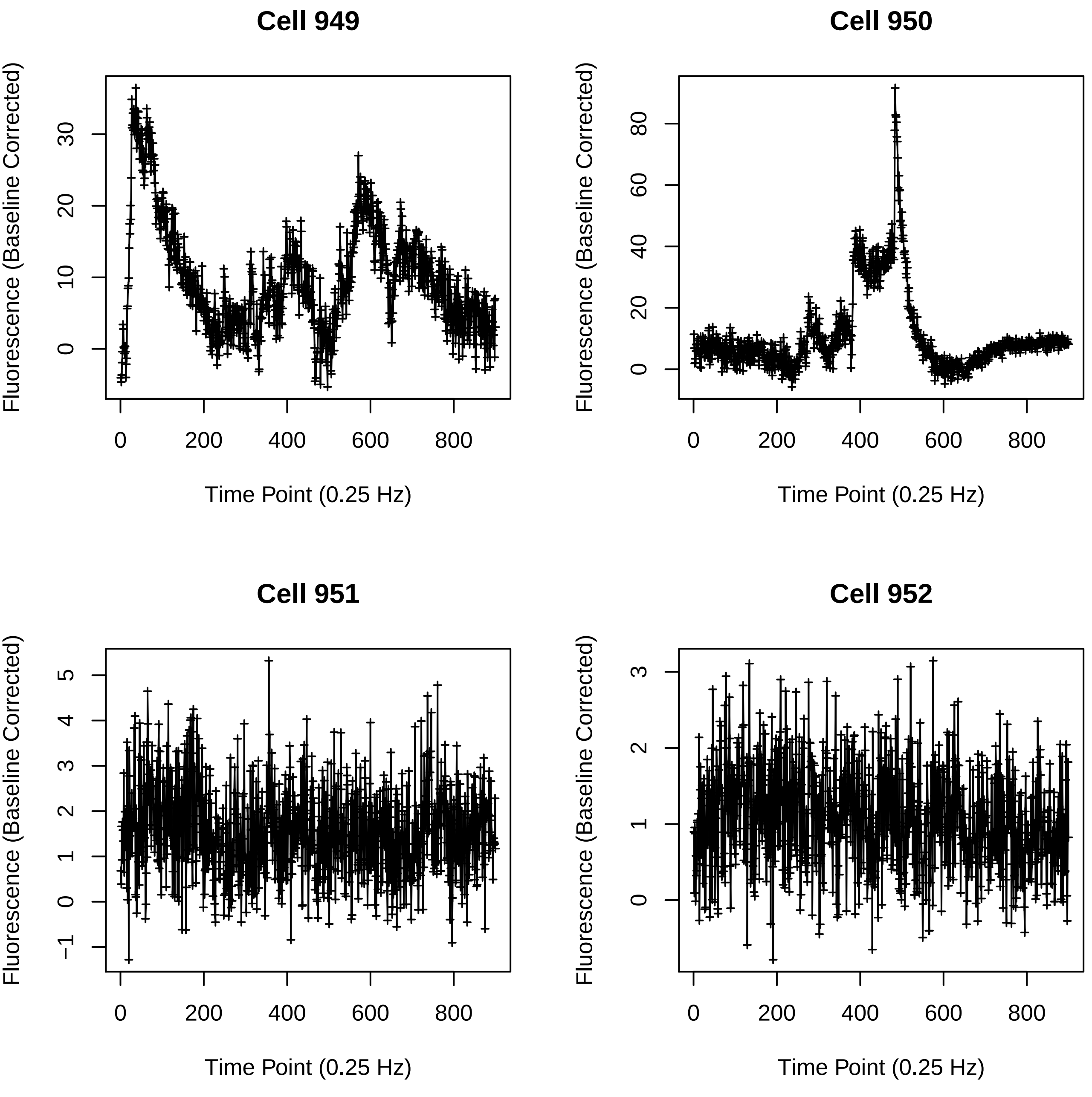

Cell 949
Cell 950
Cell 951
Cell 952
Fluorescence (Baseline Corrected)
Time Point (0.25 Hz)
0
200
400
600
800

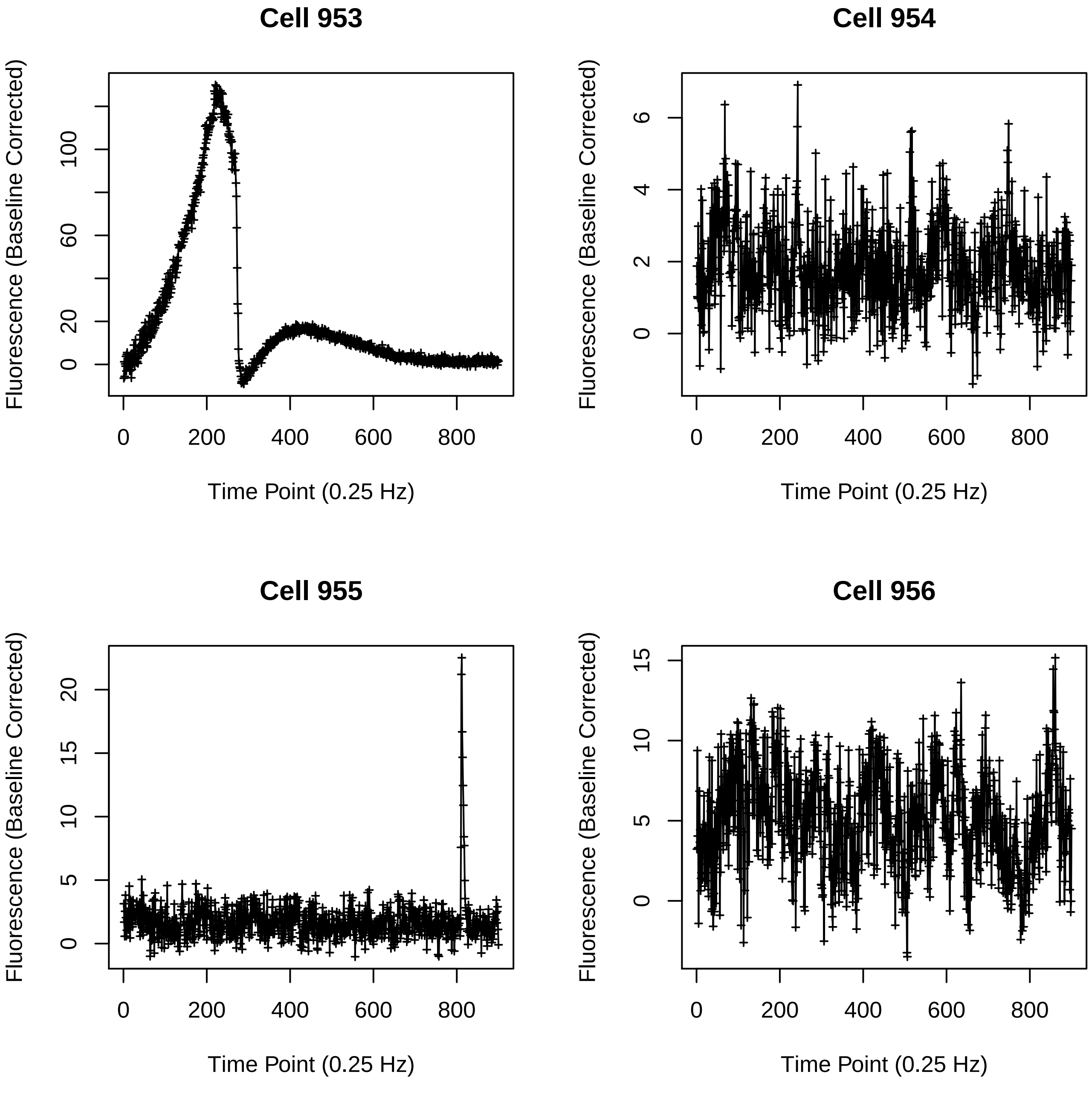
Cell 953
Cell 954
Cell 955
Cell 956
Fluorescence (Baseline Corrected)
Time Point (0.25 Hz)
0
20
60
100
2
4
6
5
10
15
200
400
600
800

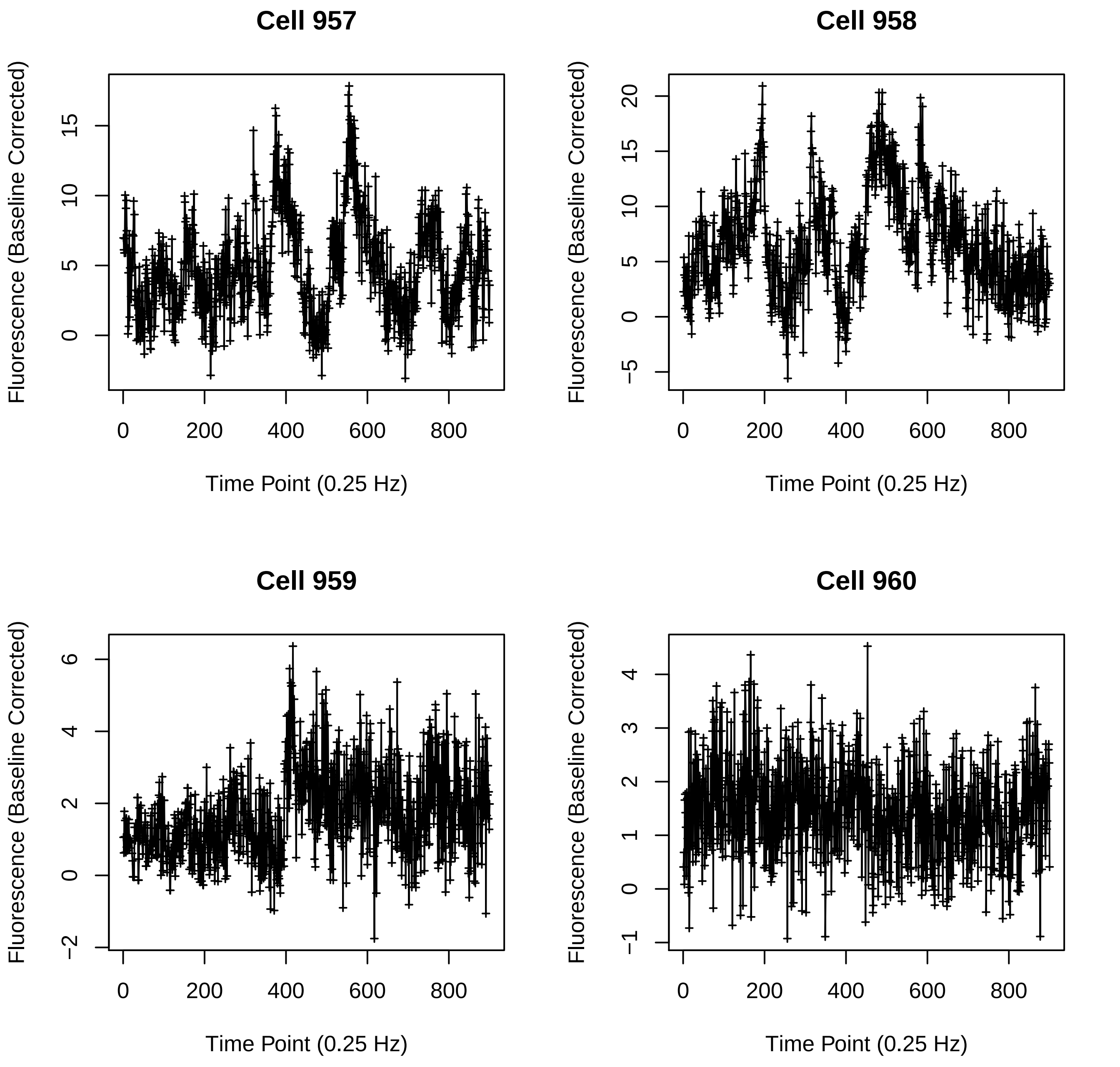

Cell 957
Cell 958
Cell 959
Cell 960
Fluorescence (Baseline Corrected)
Time Point (0.25 Hz)
0
200
400
600
800
15
10
5
0
20
−5
6
4
2
−2
3
1
−1

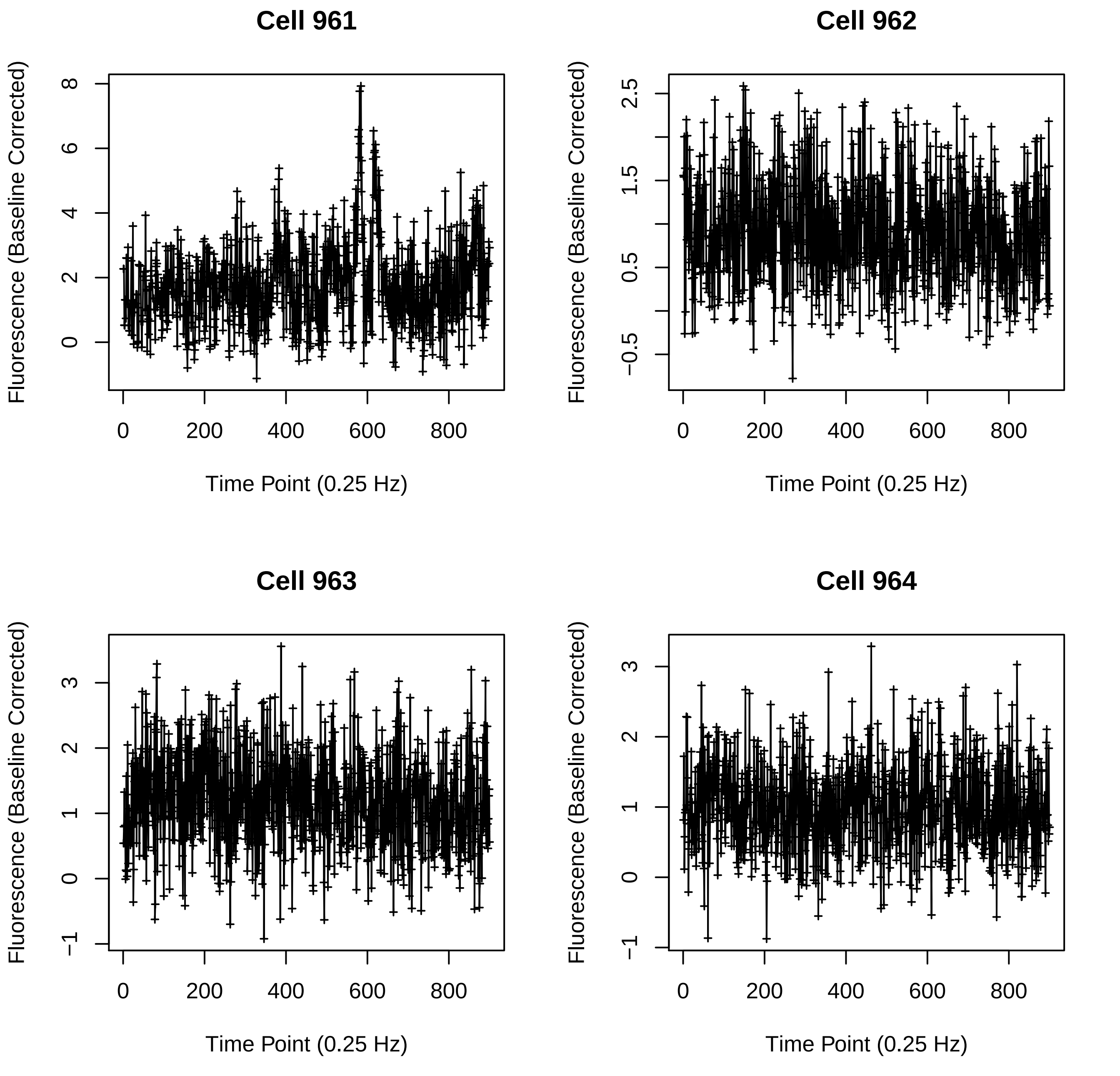
Cell 961
Fluorescence (Baseline Corrected)
Time Point (0.25 Hz)
0
2
4
6
8
0
200
400
600
800
Cell 962
Fluorescence (Baseline Corrected)
Time Point (0.25 Hz)
−0.5
0.5
1.5
2.5
0
200
400
600
800
Cell 963
Fluorescence (Baseline Corrected)
Time Point (0.25 Hz)
−1
0
1
2
3
0
200
400
600
800
Cell 964
Fluorescence (Baseline Corrected)
Time Point (0.25 Hz)
−1
0
1
2
3
0
200
400
600
800

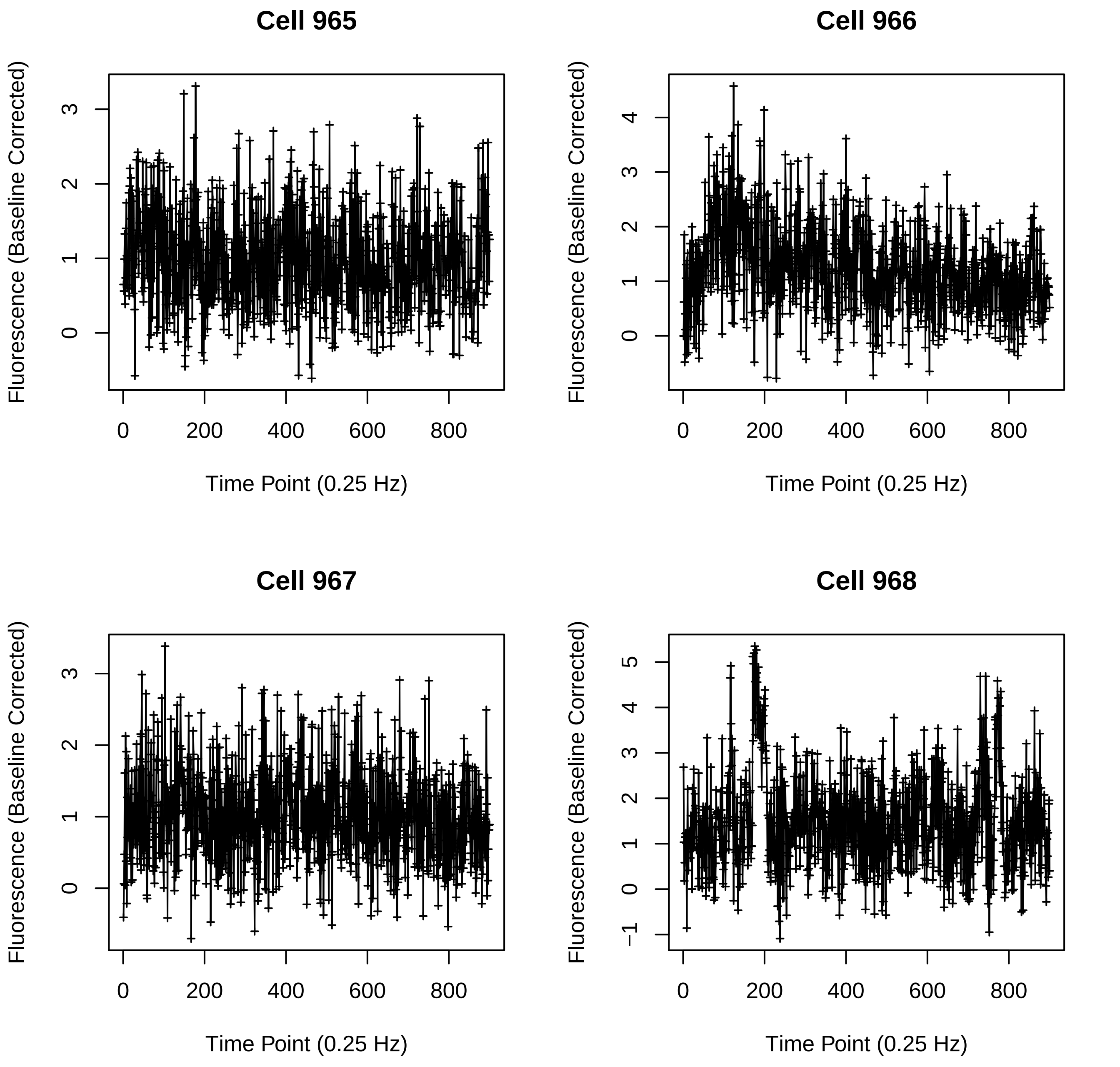
Cell 965
Cell 966
Cell 967
Cell 968
Fluorescence (Baseline Corrected)
Time Point (0.25 Hz)
0
200
400
600
800
-1
1
2
3
4
5

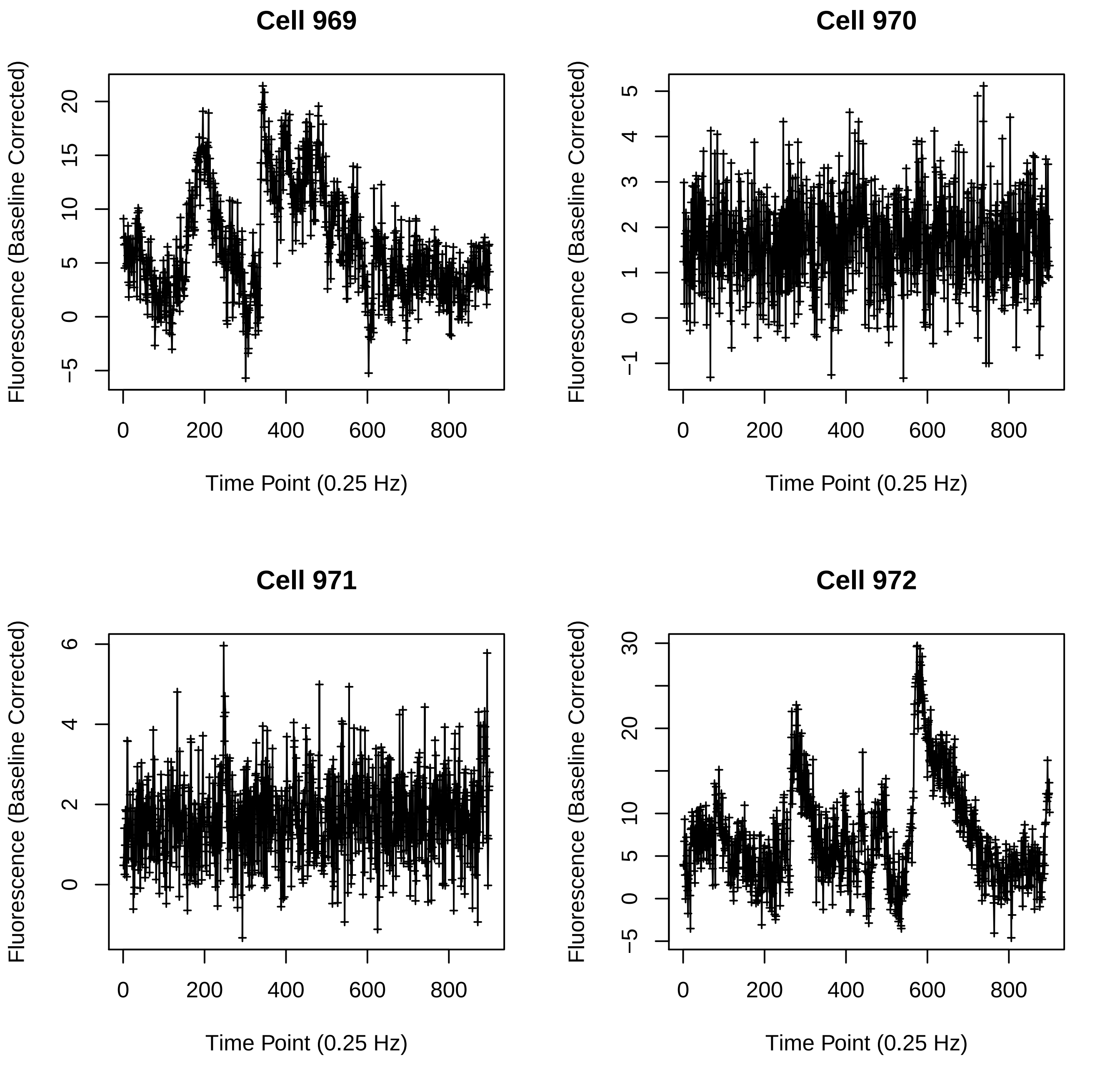
Cell 969
Cell 970
Cell 971
Cell 972
Fluorescence (Baseline Corrected)
Time Point (0.25 Hz)
0
200
400
600
800
−5
0
5
10
15
20
−1
1
2
3
4
6
25
30

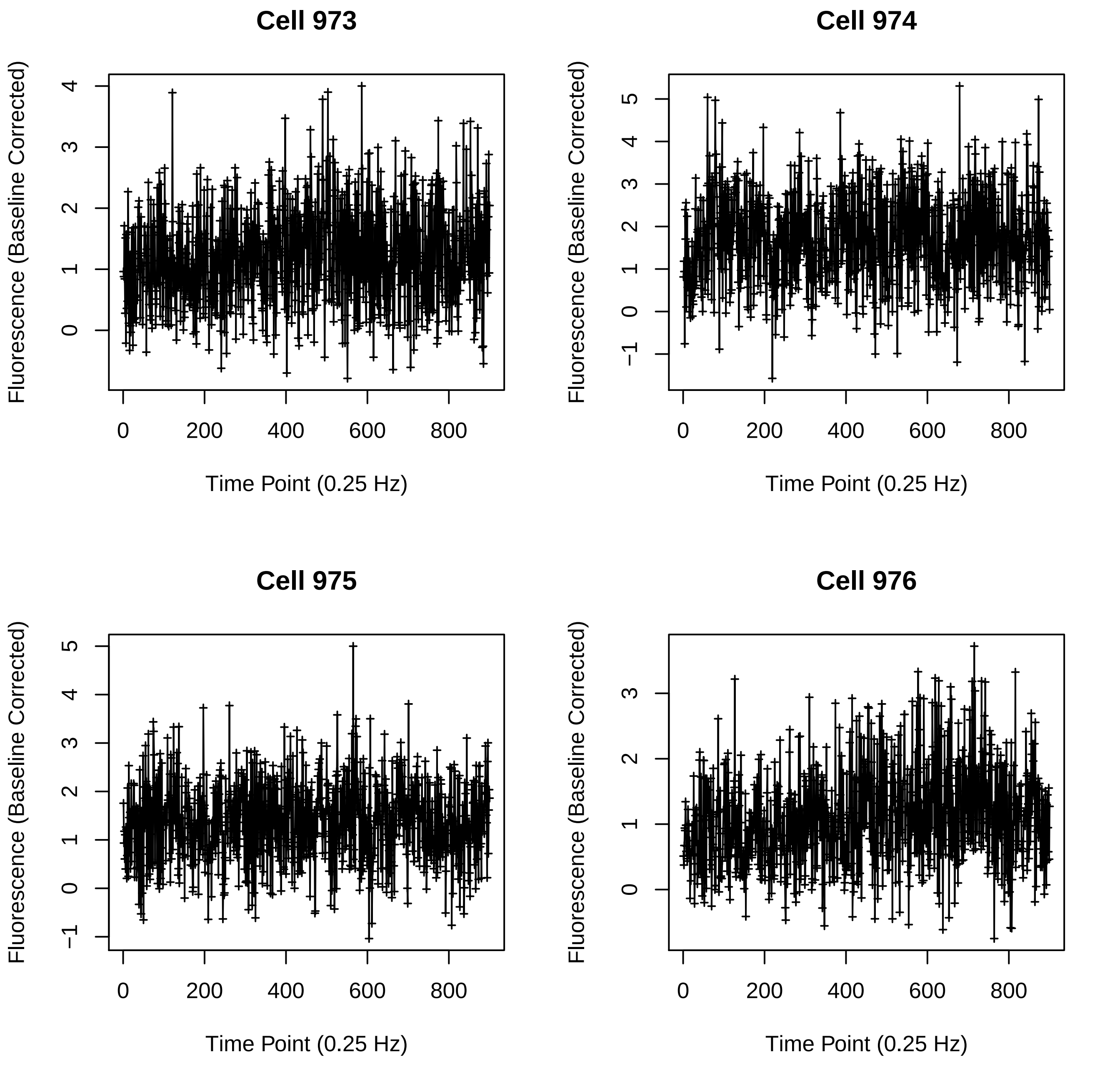
Cell 973
Cell 974
Cell 975
Cell 976
Fluorescence (Baseline Corrected)
Time Point (0.25 Hz)
0
200
400
600
800
-1
0
1
2
3
4
5

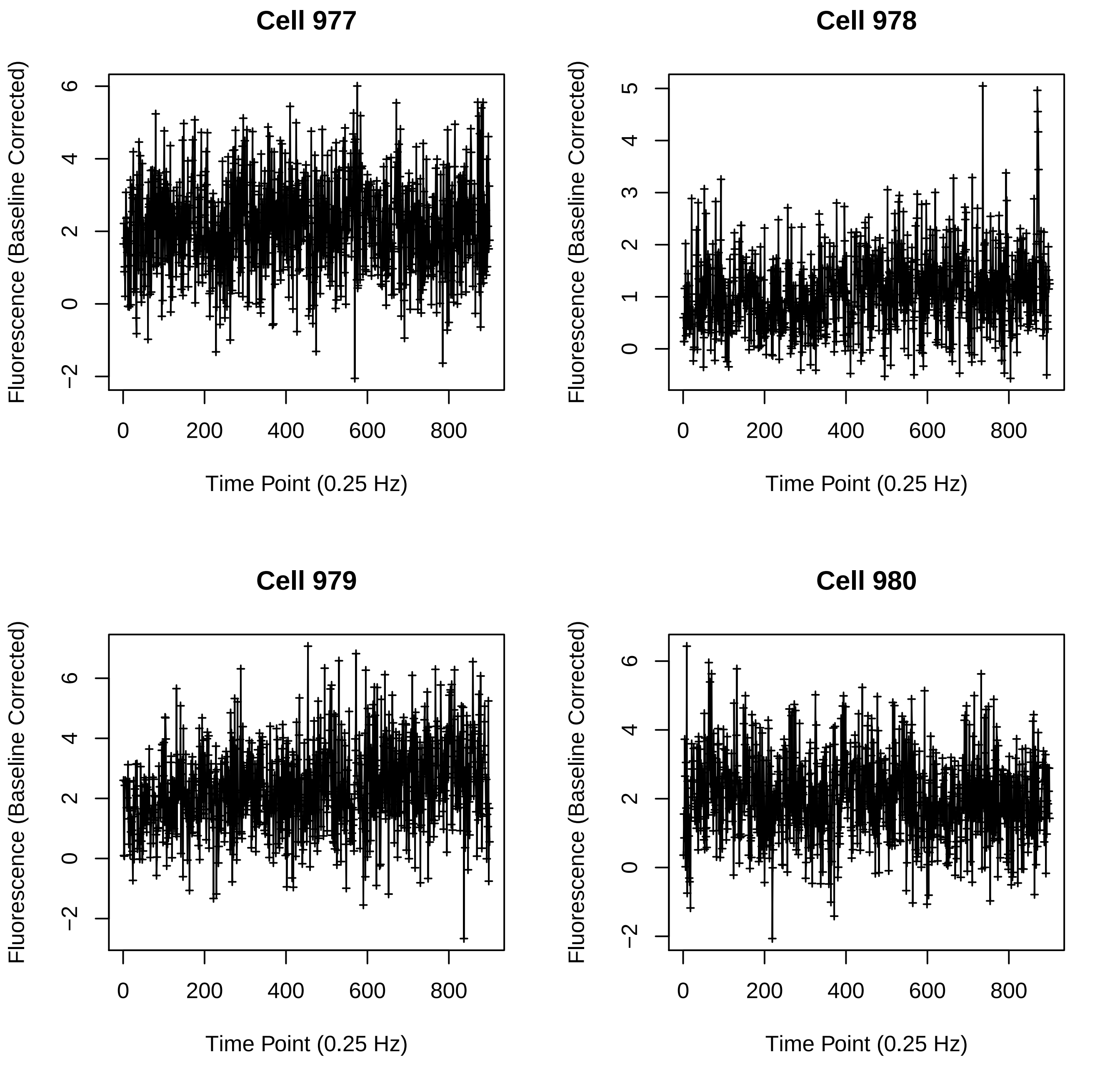

Cell 977
Cell 978
Cell 979
Cell 980
Fluorescence (Baseline Corrected)
Time Point (0.25 Hz)
0
200
400
600
800
−2
0
2
4
6
1
3
5

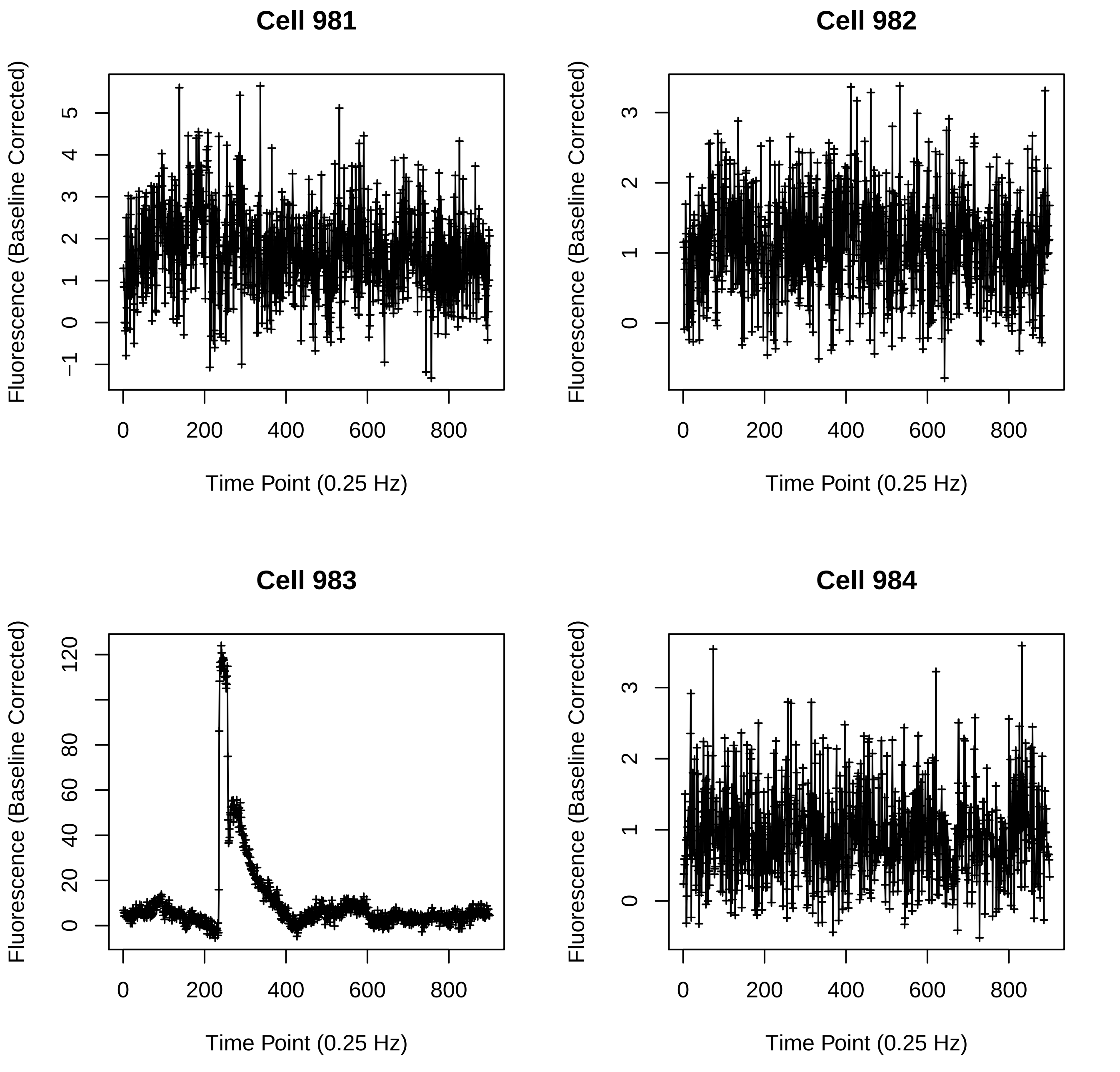
Cell 981
Cell 982
Cell 983
Cell 984
Fluorescence (Baseline Corrected)
Time Point (0.25 Hz)
0
200
400
600
800
−1
0
1
2
3
4
5
20
40
60
80
120

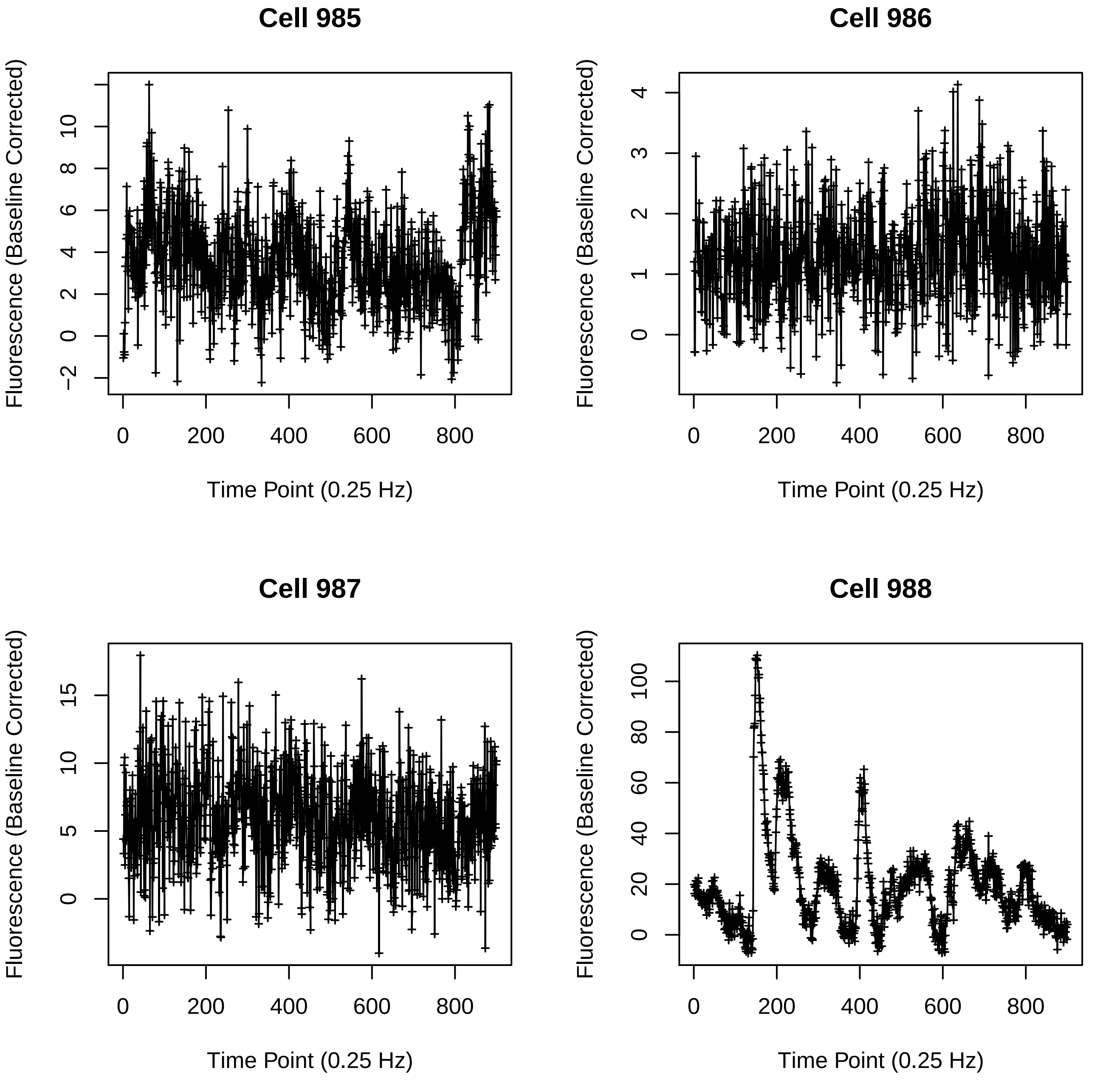
Cell 985
Cell 986
Cell 987
Cell 988
Fluorescence (Baseline Corrected)
Time Point (0.25 Hz)
0
200
400
600
800
−2
2
4
6
8
10
1
3
5
15
20
40
60
80
100

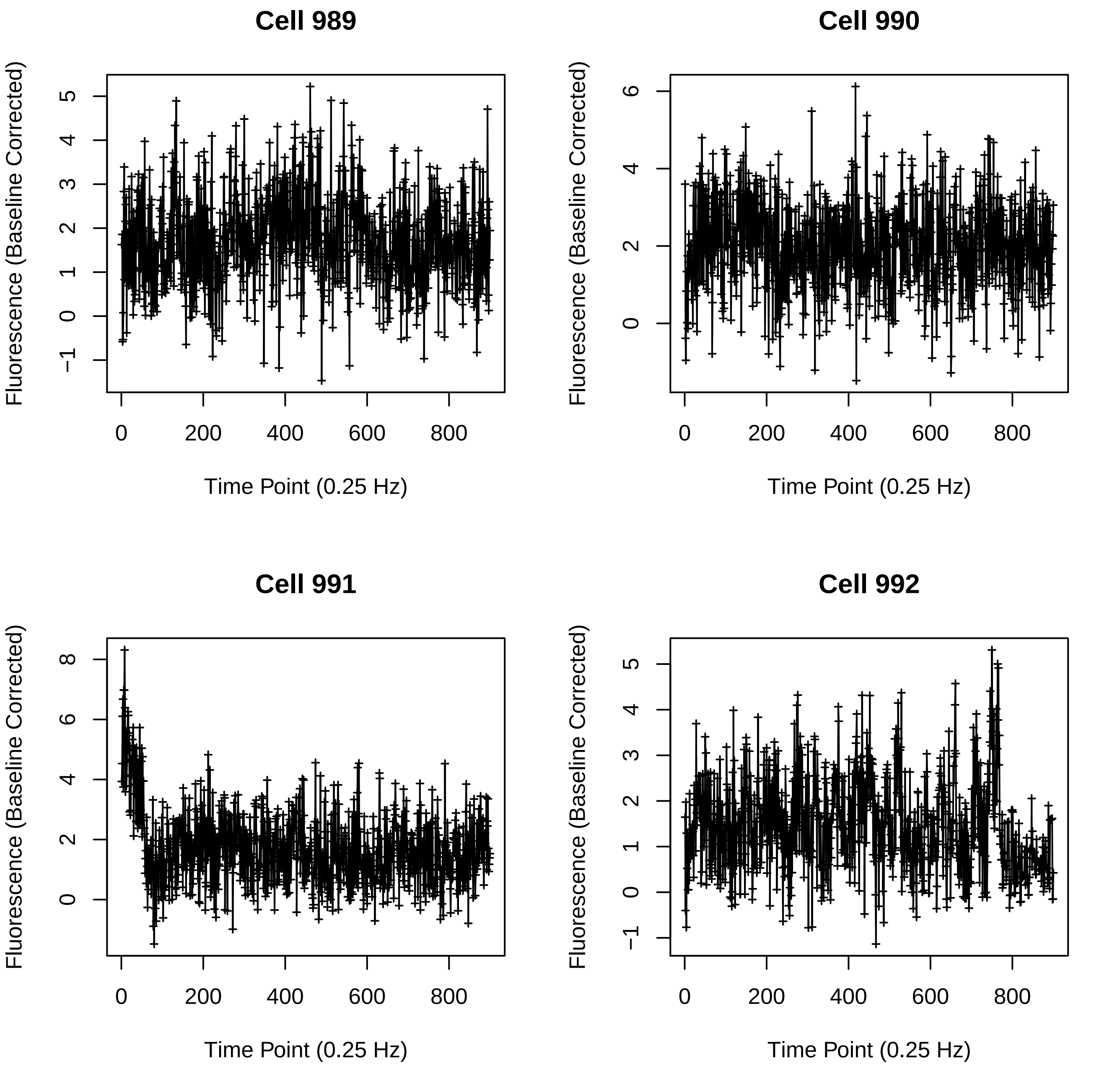
Cell 989
Cell 990
Cell 991
Cell 992
Fluorescence (Baseline Corrected)
Time Point (0.25 Hz)

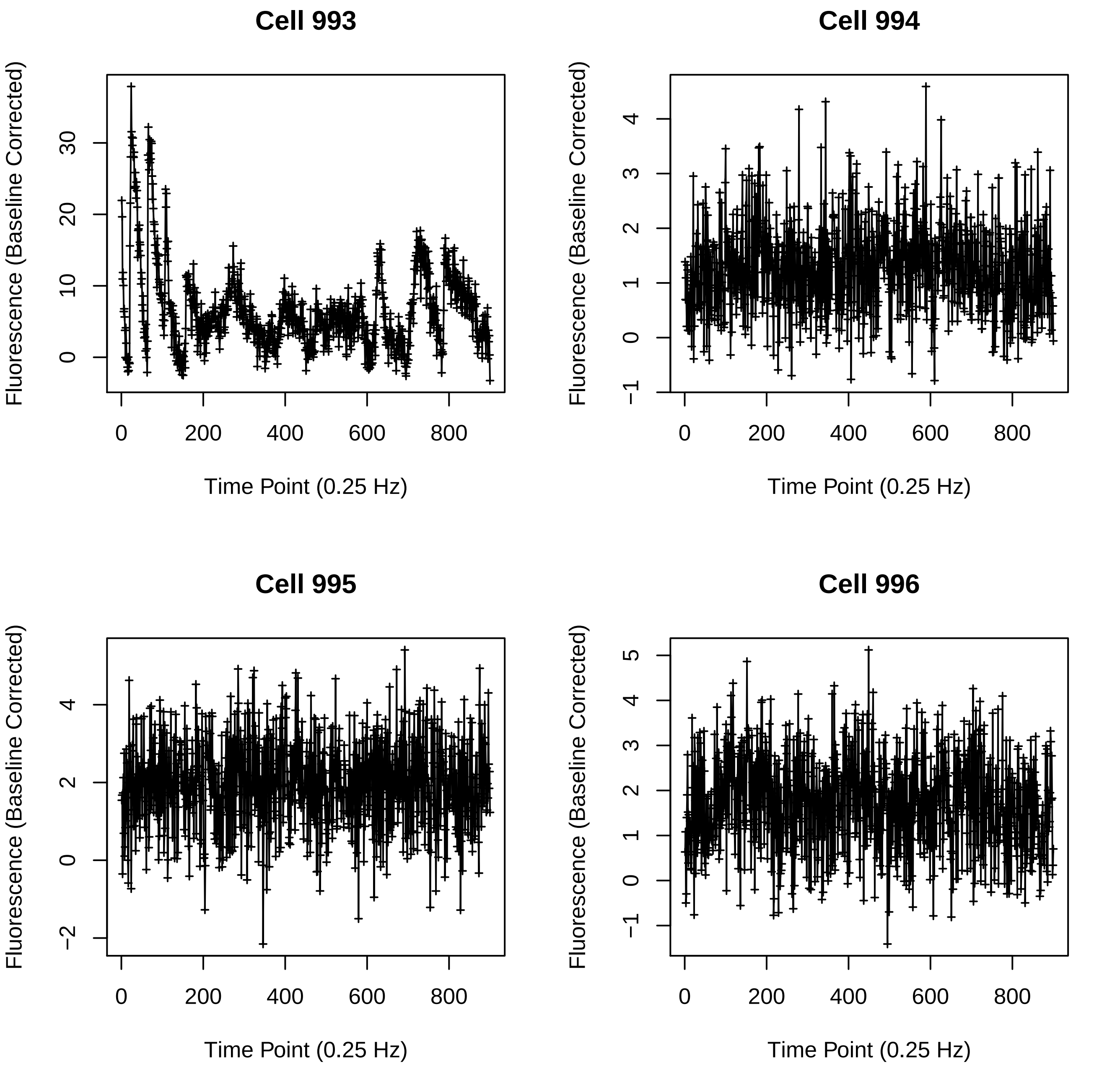
Cell 993
Cell 994
Cell 995
Cell 996
Fluorescence (Baseline Corrected)
Time Point (0.25 Hz)
0
10
20
30
200
400
600
800
−1
1
2
3
4
5
−2

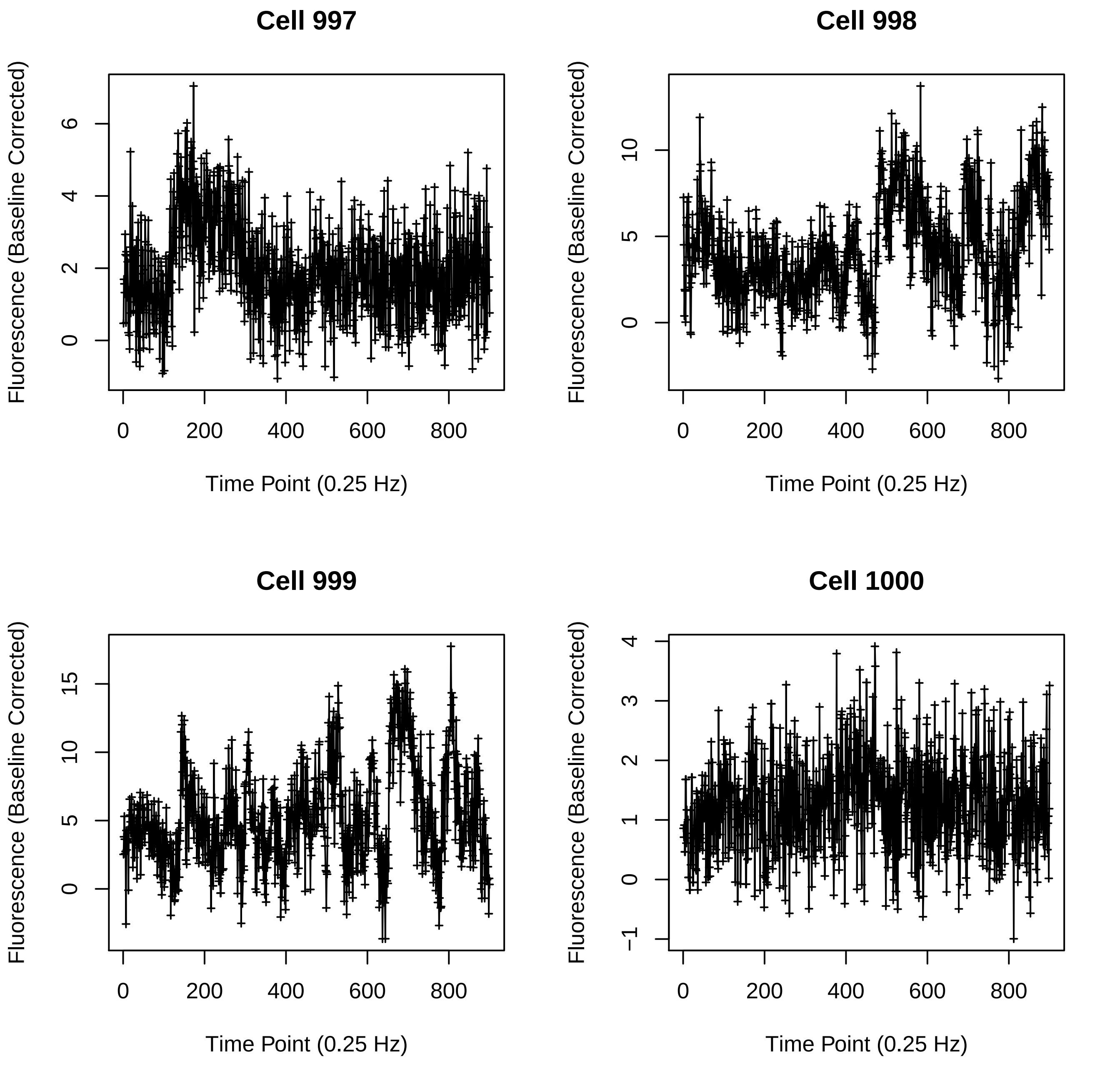
Cell 997
Fluorescence (Baseline Corrected)
Time Point (0.25 Hz)
0
2
4
6
0
200
400
600
800
Cell 998
Fluorescence (Baseline Corrected)
Time Point (0.25 Hz)
0
5
10
0
200
400
600
800
Cell 999
Fluorescence (Baseline Corrected)
Time Point (0.25 Hz)
0
5
10
15
0
200
400
600
800
Cell 1000
Fluorescence (Baseline Corrected)
Time Point (0.25 Hz)
−1
0
1
2
3
4
0
200
400
600
800

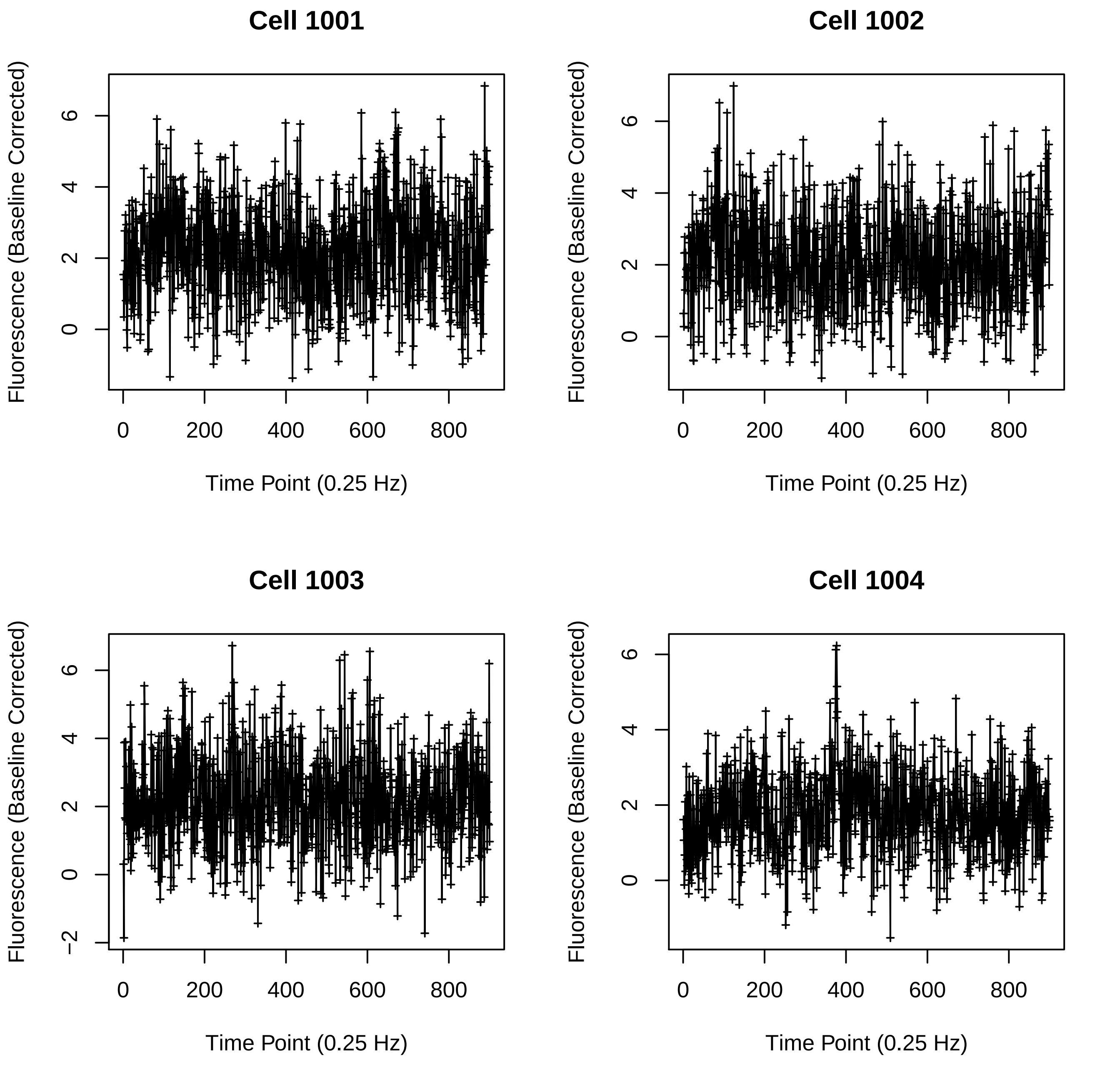

Cell 1001
Cell 1002
Cell 1003
Cell 1004
Fluorescence (Baseline Corrected)
Time Point (0.25 Hz)
0
200
400
600
800
−2
0
2
4
6

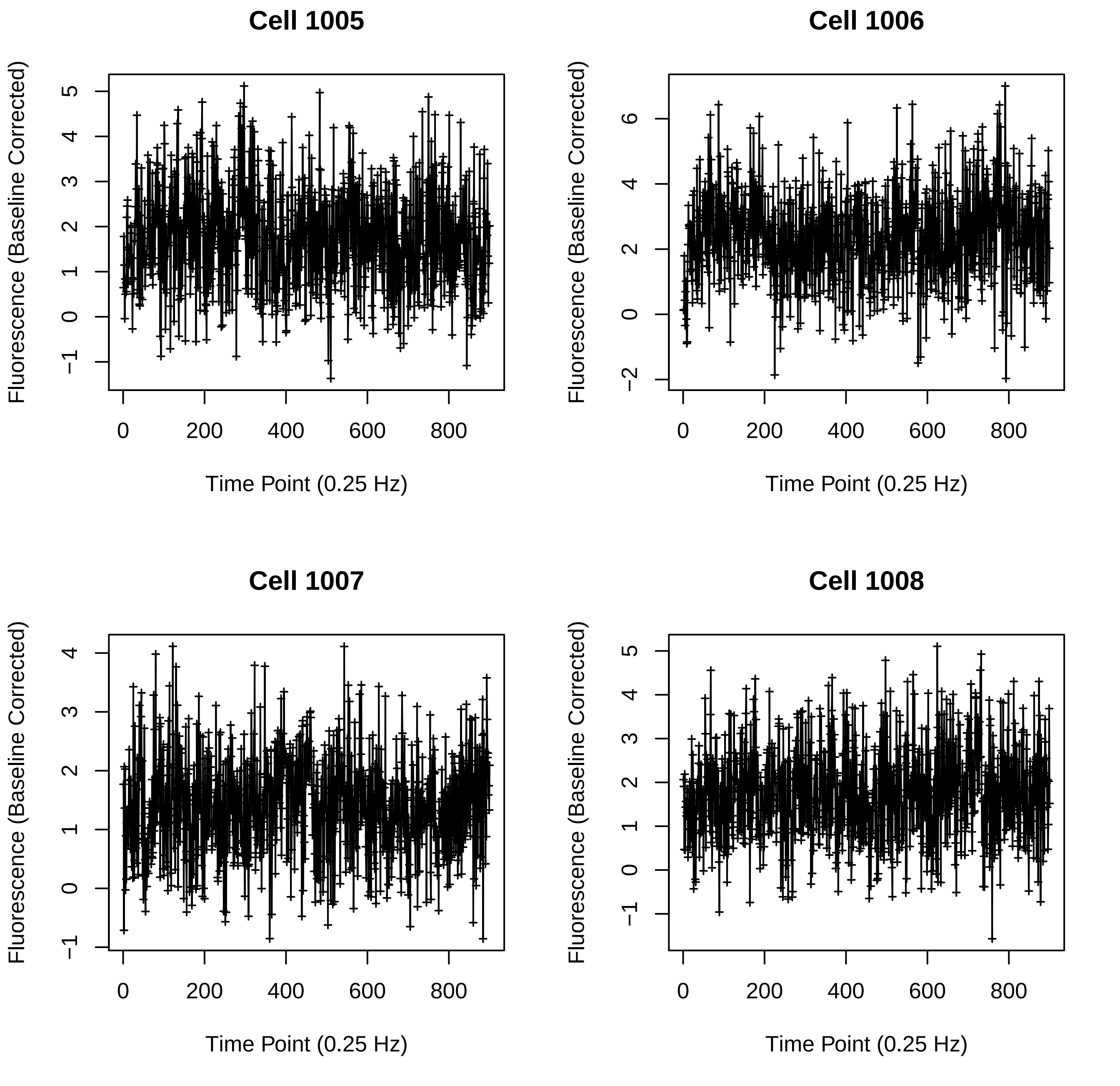
Cell 1005
Cell 1006
Cell 1007
Cell 1008
Fluorescence (Baseline Corrected)
Time Point (0.25 Hz)
0
200
400
600
800

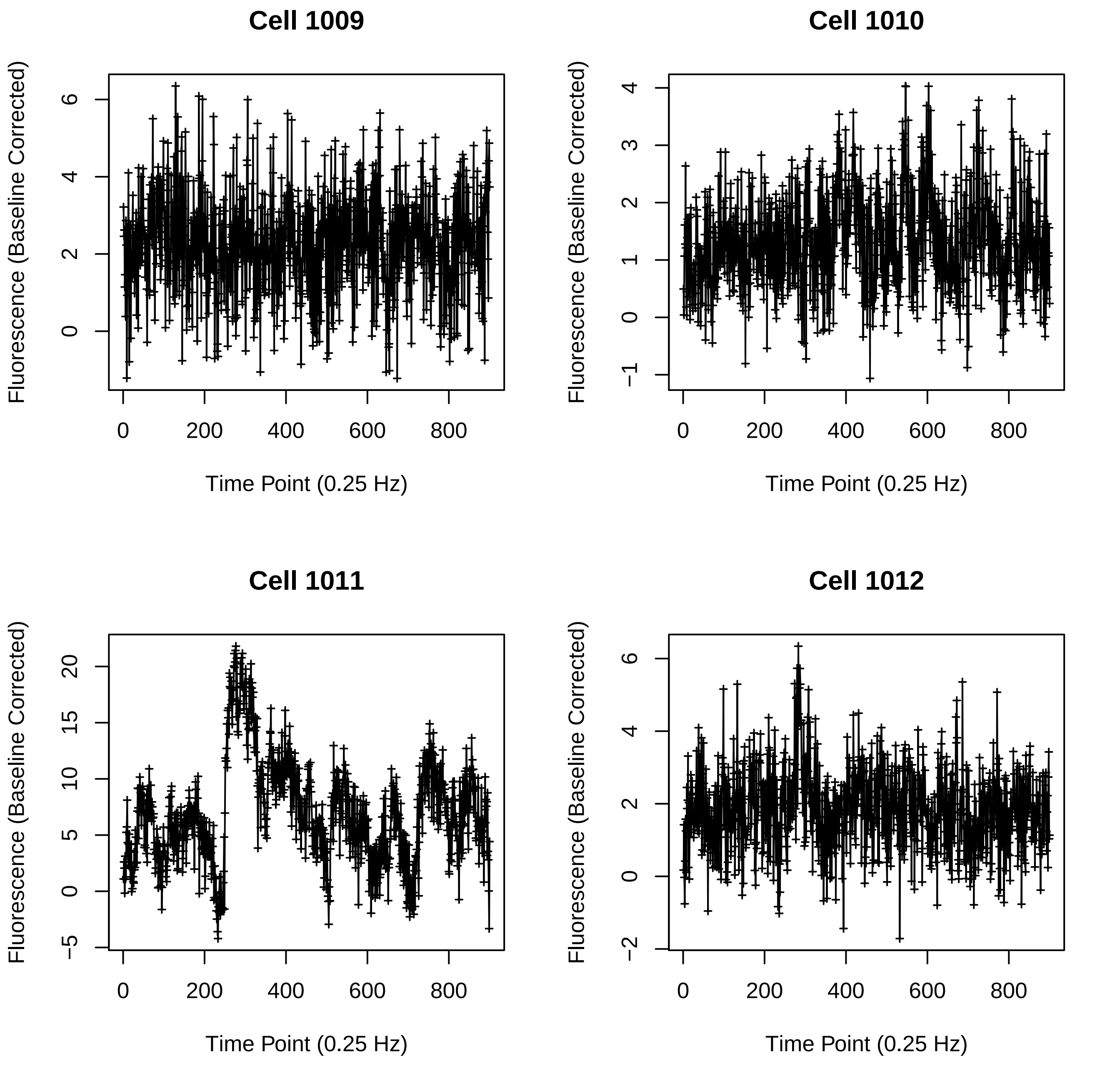
Cell 1009
Cell 1010
Cell 1011
Cell 1012
Fluorescence (Baseline Corrected)
Time Point (0.25 Hz)
0
200
400
600
800
−1
1
2
3
4
6
−5
5
10
15
20
−2

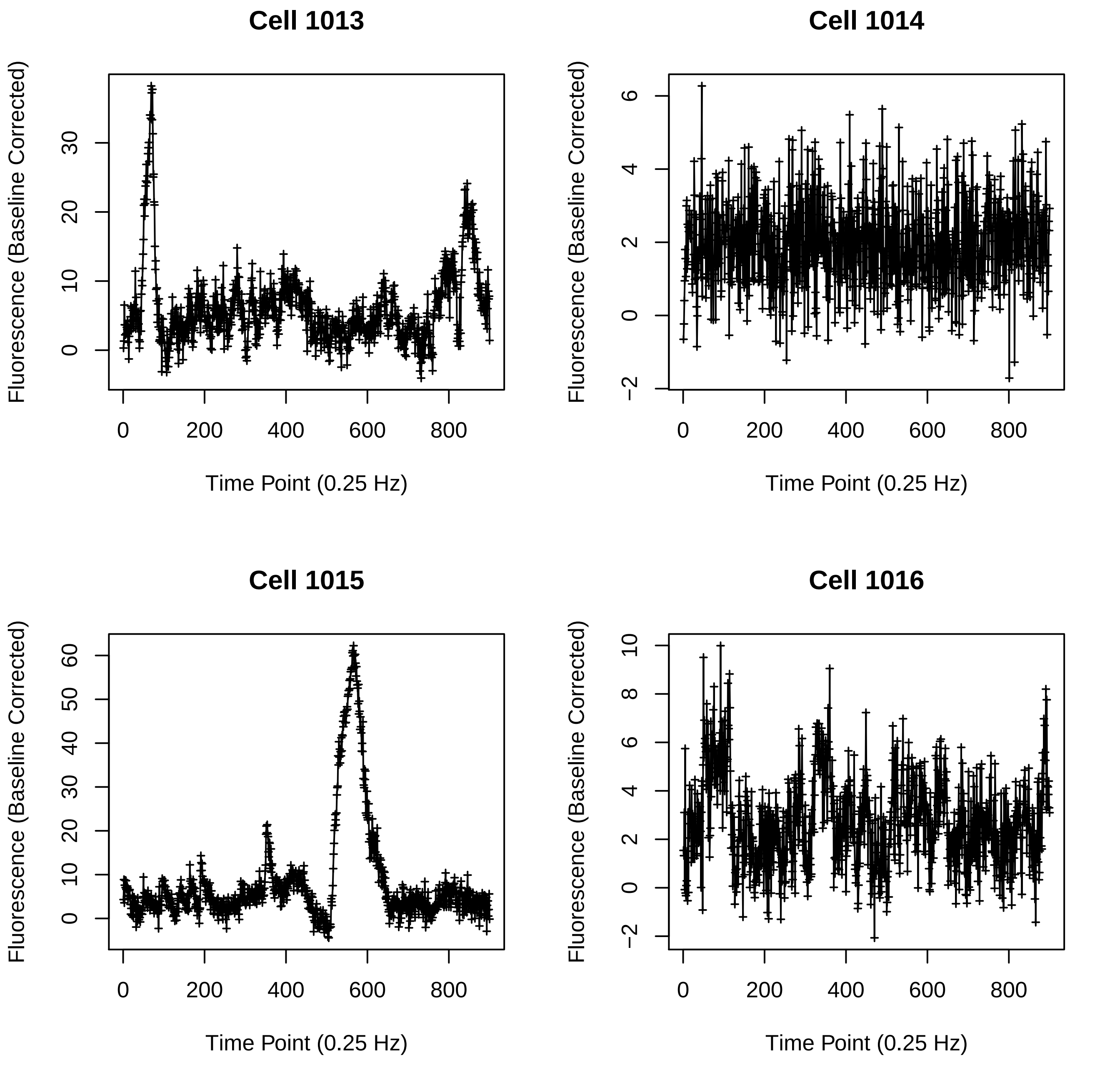
Cell 1013
Fluorescence (Baseline Corrected)
Time Point (0.25 Hz)
0
10
20
30
0
200
400
600
800
Cell 1014
Fluorescence (Baseline Corrected)
Time Point (0.25 Hz)
−2
0
2
4
6
0
200
400
600
800
Cell 1015
Fluorescence (Baseline Corrected)
Time Point (0.25 Hz)
0
10
20
30
40
50
60
0
200
400
600
800
Cell 1016
Fluorescence (Baseline Corrected)
Time Point (0.25 Hz)
−2
0
2
4
6
8
10
0
200
400
600
800

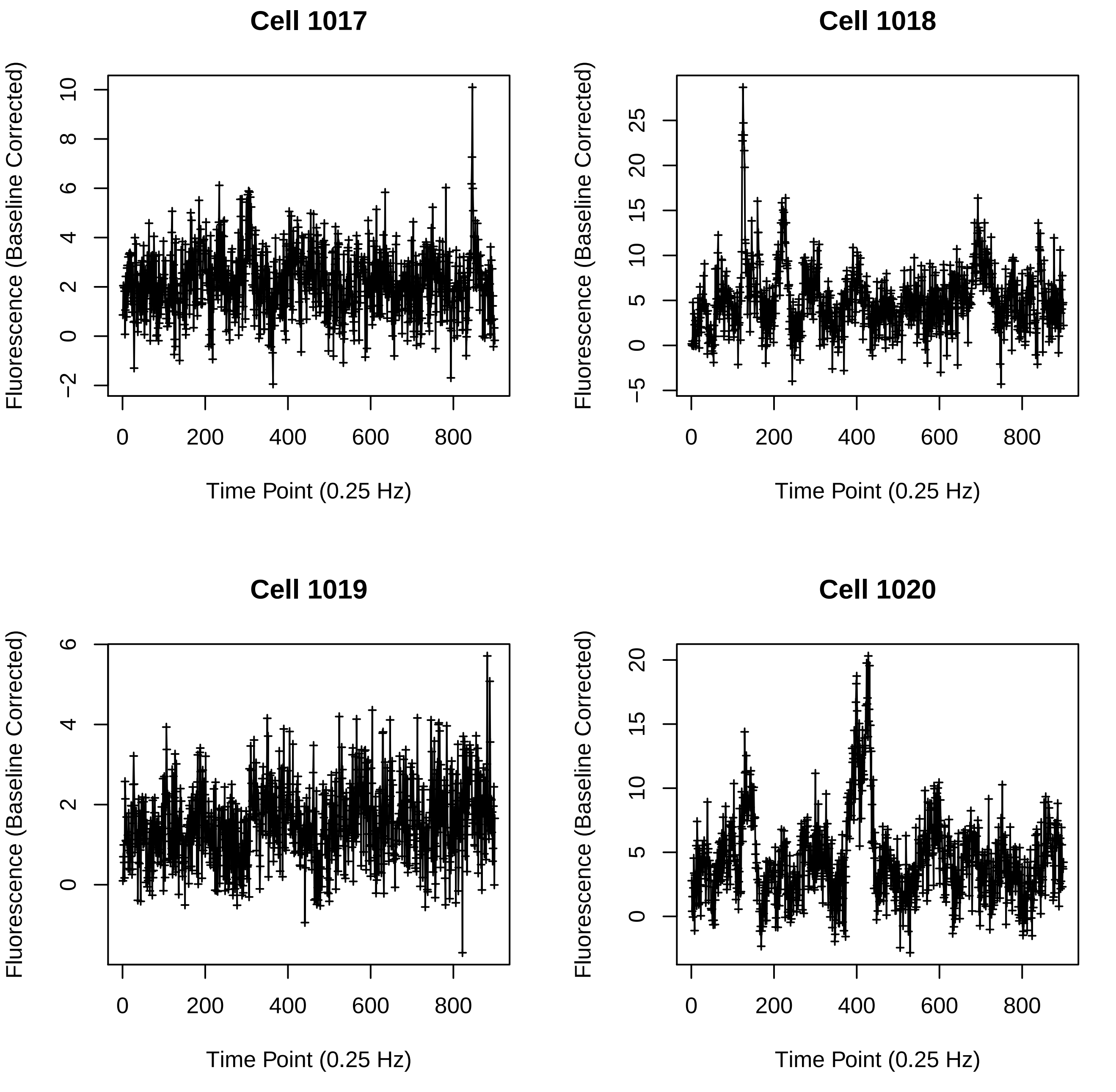

Cell 1017
Fluorescence (Baseline Corrected)
−2
0
2
4
6
8
10
0
200
400
600
800
Time Point (0.25 Hz)
Cell 1018
Fluorescence (Baseline Corrected)
−5
0
5
10
15
20
25
0
200
400
600
800
Time Point (0.25 Hz)
Cell 1019
Fluorescence (Baseline Corrected)
0
2
4
6
0
200
400
600
800
Time Point (0.25 Hz)
Cell 1020
Fluorescence (Baseline Corrected)
0
5
10
15
20
0
200
400
600
800
Time Point (0.25 Hz)

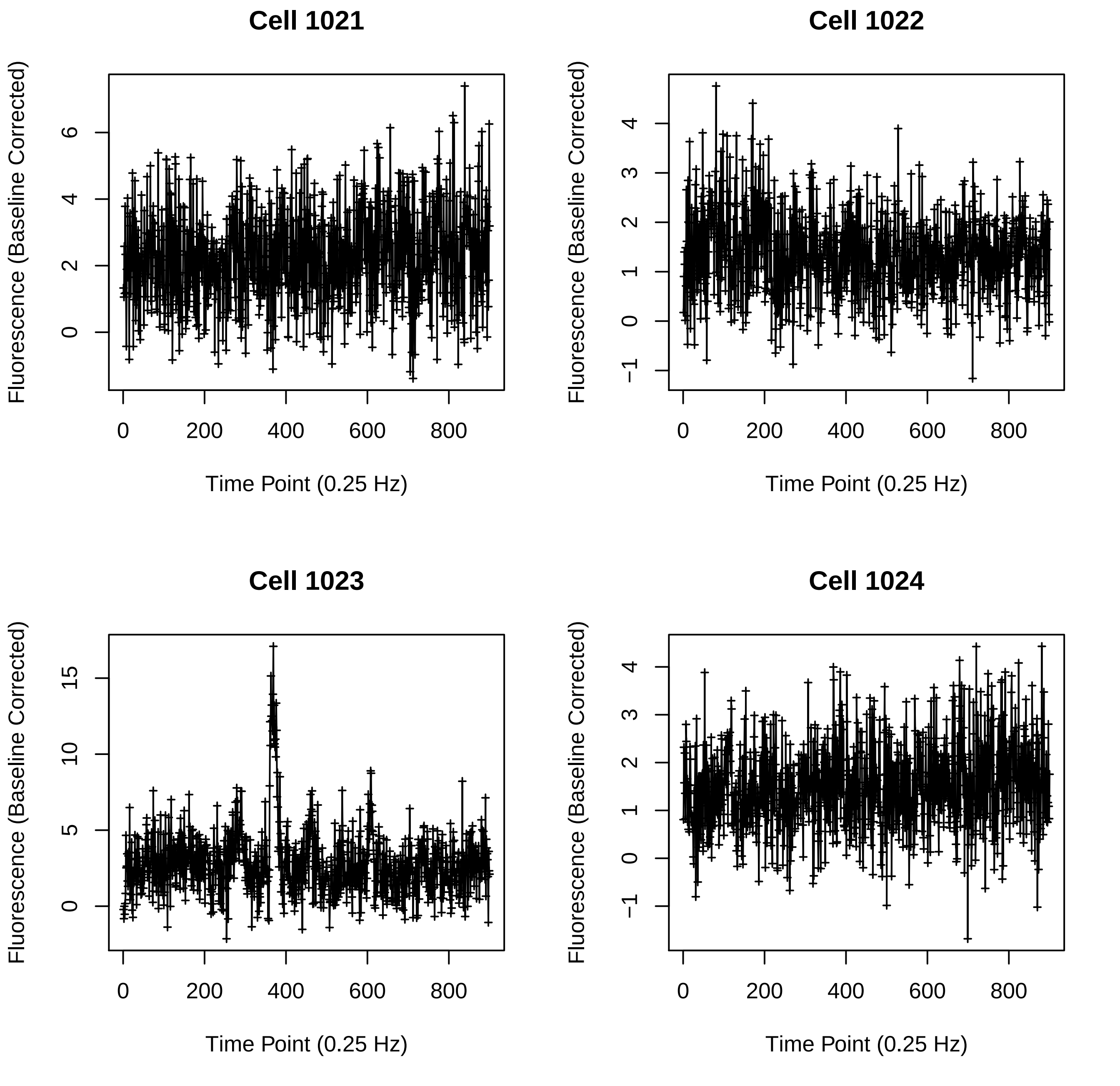
Cell 1021
Cell 1022
Cell 1023
Cell 1024
Fluorescence (Baseline Corrected)
Time Point (0.25 Hz)
0
2
4
6
−1
1
3
5
10
15
200
400
600
800

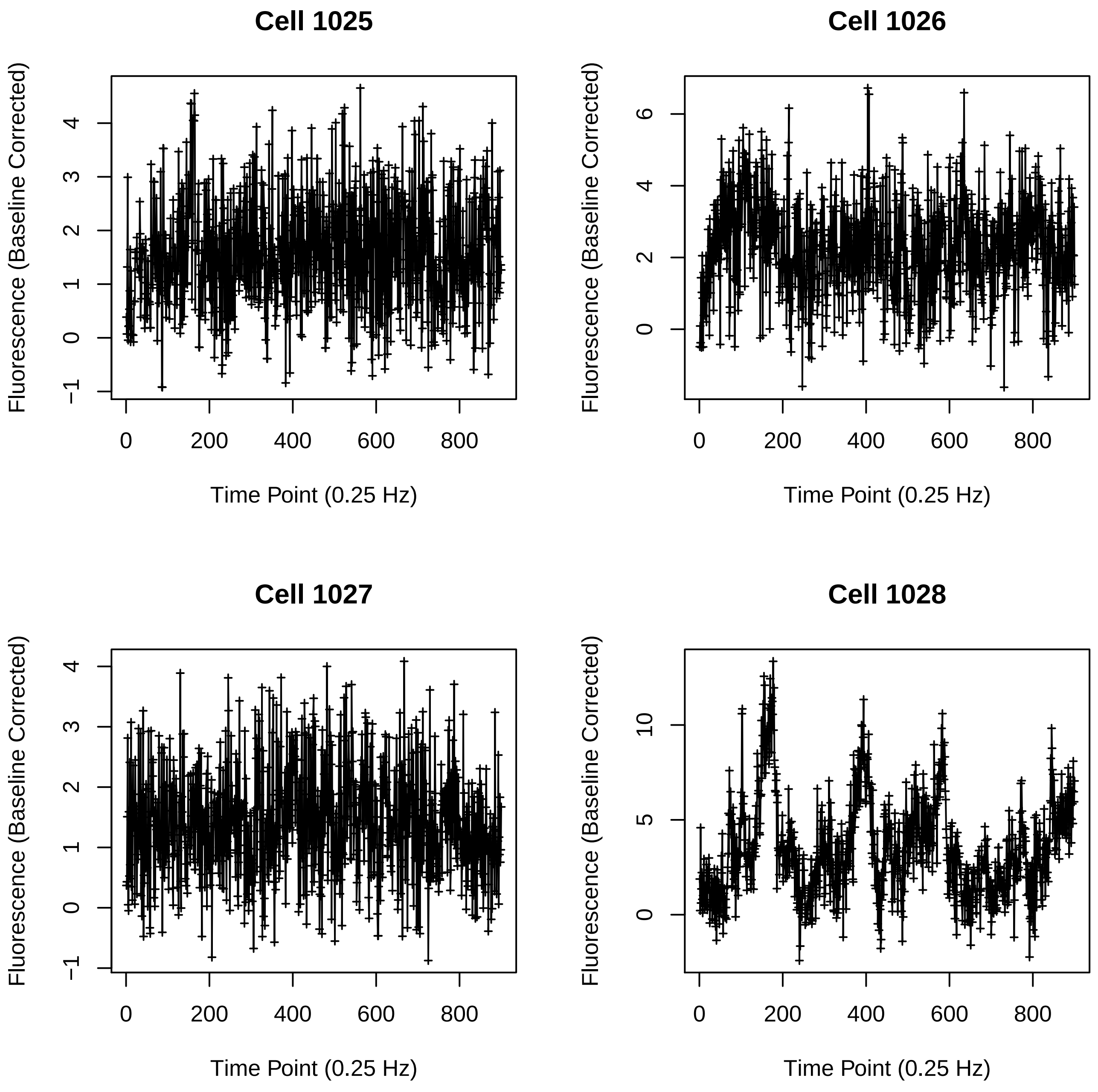

Cell 1025
Cell 1026
Cell 1027
Cell 1028
Fluorescence (Baseline Corrected)
Time Point (0.25 Hz)
0
200
400
600
800
−1
1
2
3
4
6
5
10

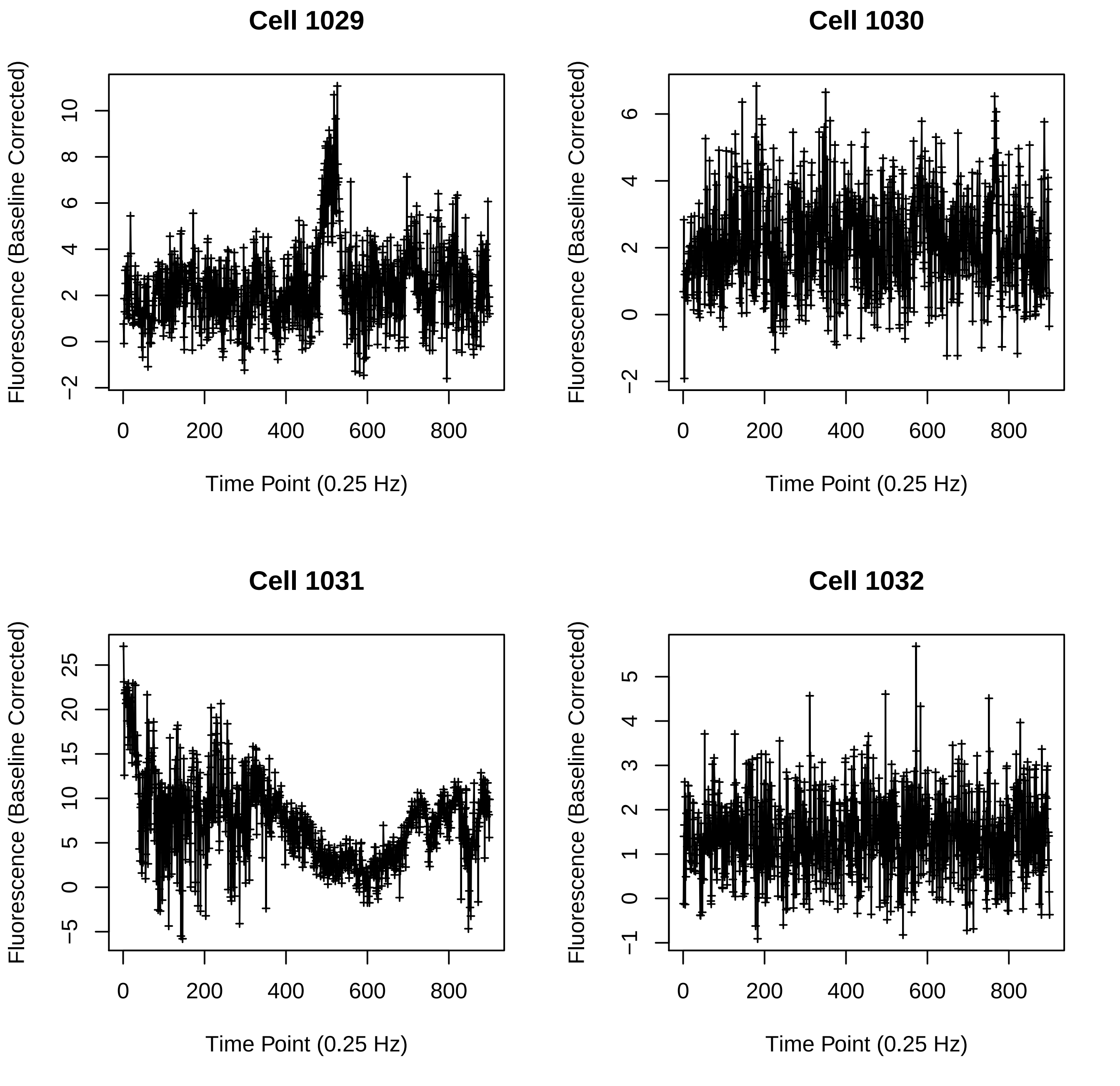

Cell 1029
Fluorescence (Baseline Corrected)
−2
0
2
4
6
8
10
0
200
400
600
800
Time Point (0.25 Hz)
Cell 1030
Fluorescence (Baseline Corrected)
−2
0
2
4
6
0
200
400
600
800
Time Point (0.25 Hz)
Cell 1031
Fluorescence (Baseline Corrected)
−5
0
5
10
15
20
25
0
200
400
600
800
Time Point (0.25 Hz)
Cell 1032
Fluorescence (Baseline Corrected)
−1
0
1
2
3
4
5
0
200
400
600
800
Time Point (0.25 Hz)

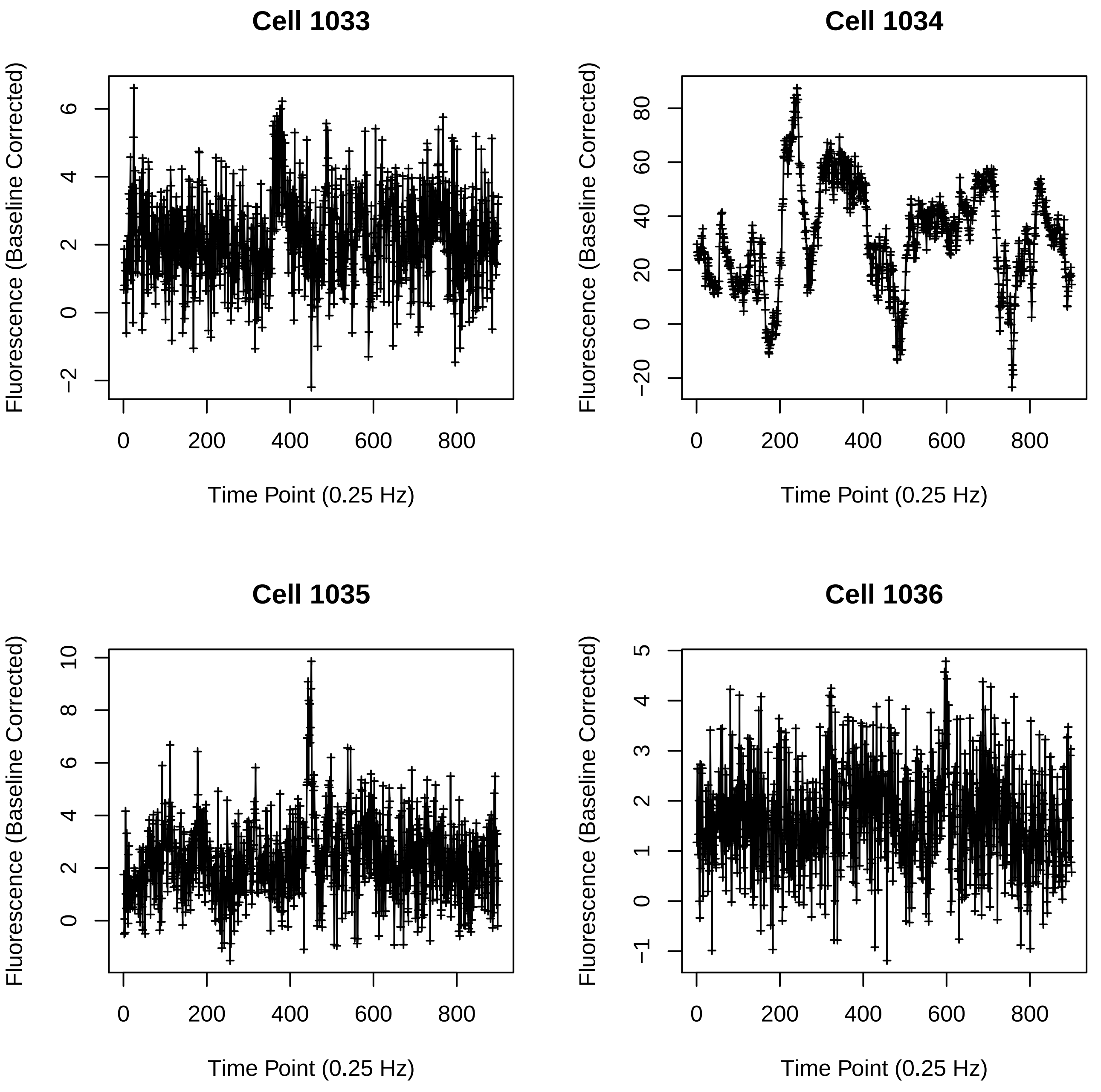
Cell 1033
Fluorescence (Baseline Corrected)
−2
0
2
4
6
0
200
400
600
800
Time Point (0.25 Hz)
Cell 1034
Fluorescence (Baseline Corrected)
−20
0
20
40
60
80
0
200
400
600
800
Time Point (0.25 Hz)
Cell 1035
Fluorescence (Baseline Corrected)
0
2
4
6
8
10
0
200
400
600
800
Time Point (0.25 Hz)
Cell 1036
Fluorescence (Baseline Corrected)
−1
0
1
2
3
4
5
0
200
400
600
800
Time Point (0.25 Hz)

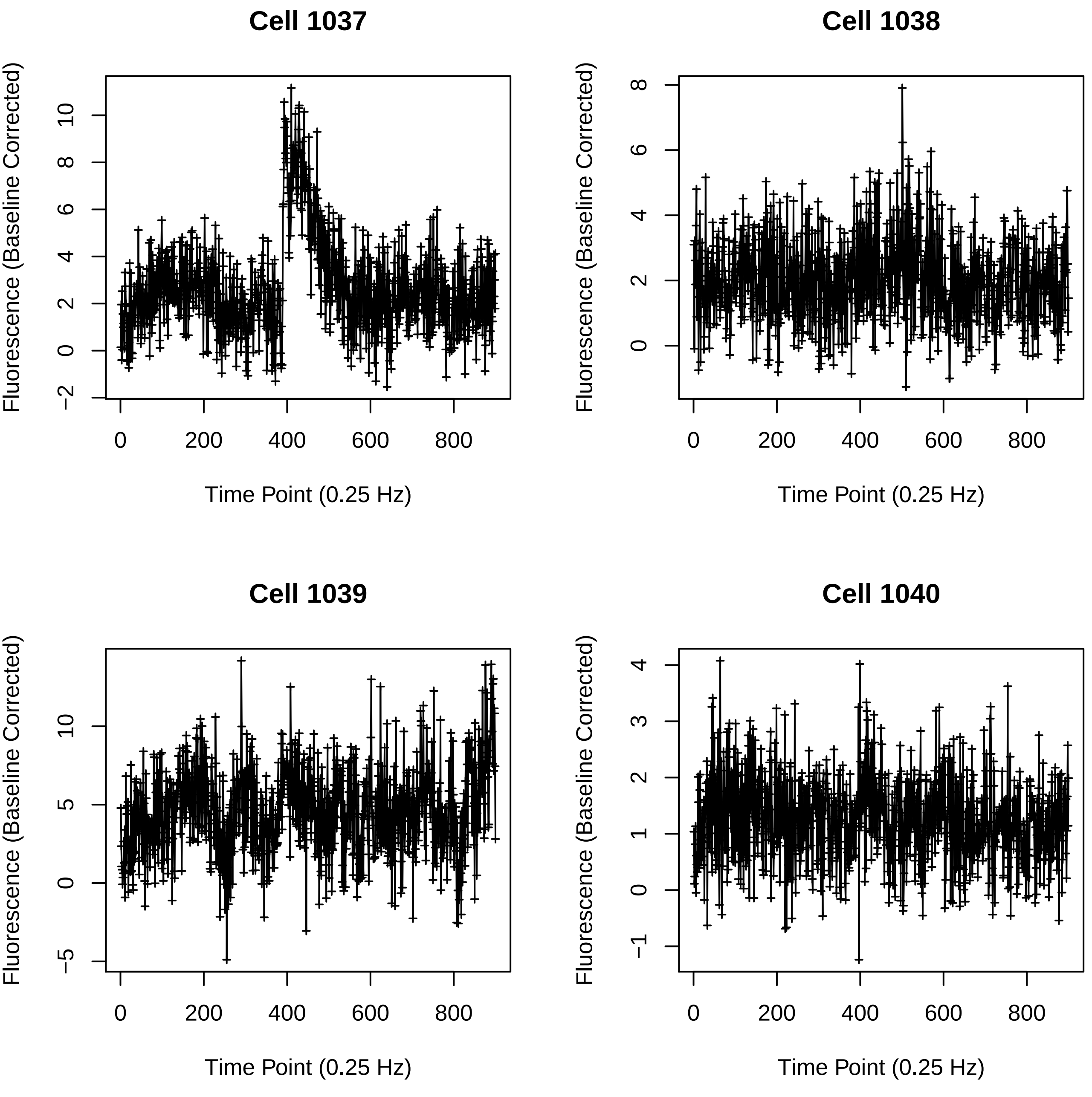
Cell 1037
Cell 1038
Cell 1039
Cell 1040
Fluorescence (Baseline Corrected)
Time Point (0.25 Hz)

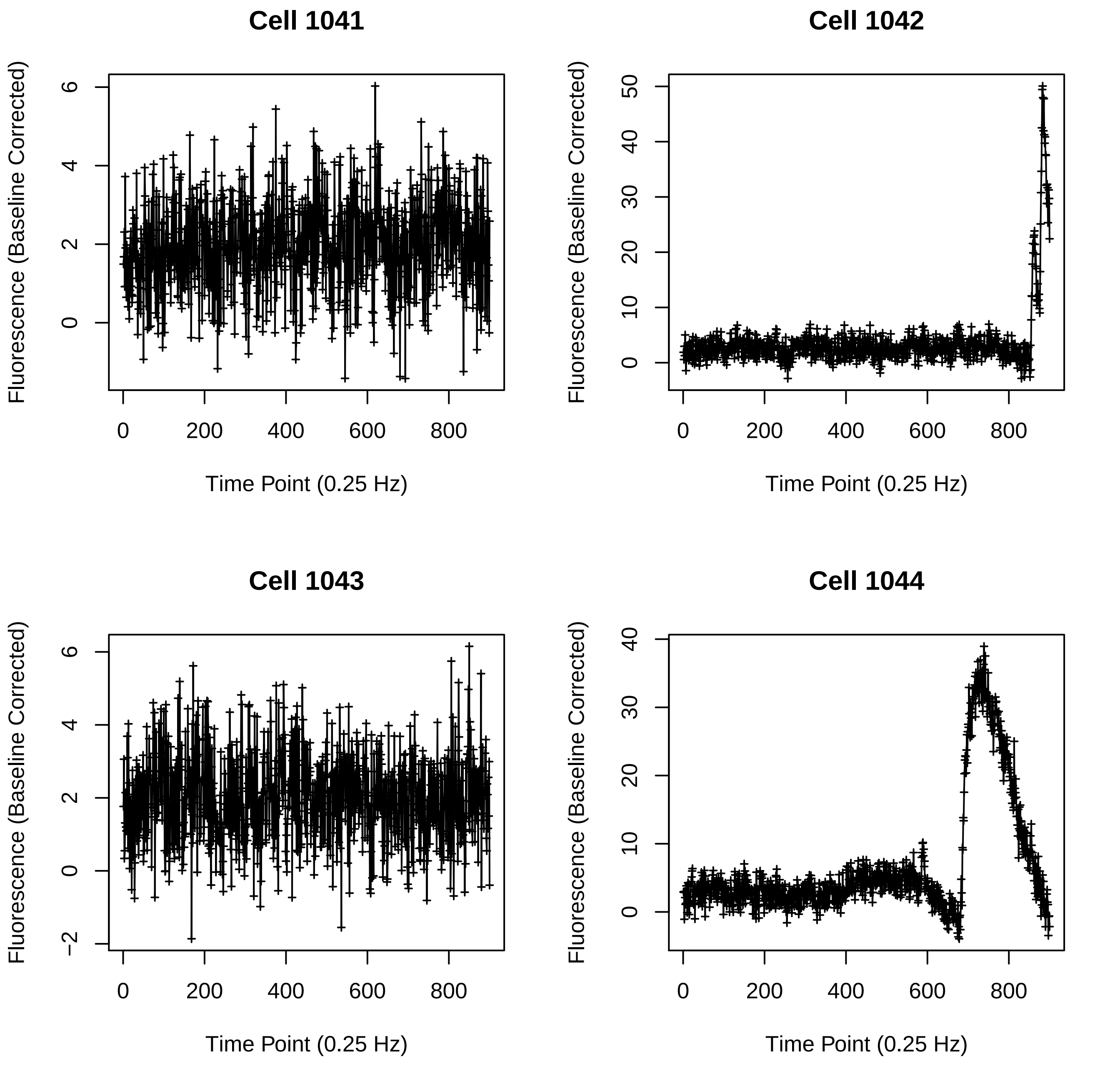
Cell 1041
Cell 1042
Cell 1043
Cell 1044
Fluorescence (Baseline Corrected)
Time Point (0.25 Hz)
0
200
400
600
800
-2
2
4
6
10
20
30
40
50

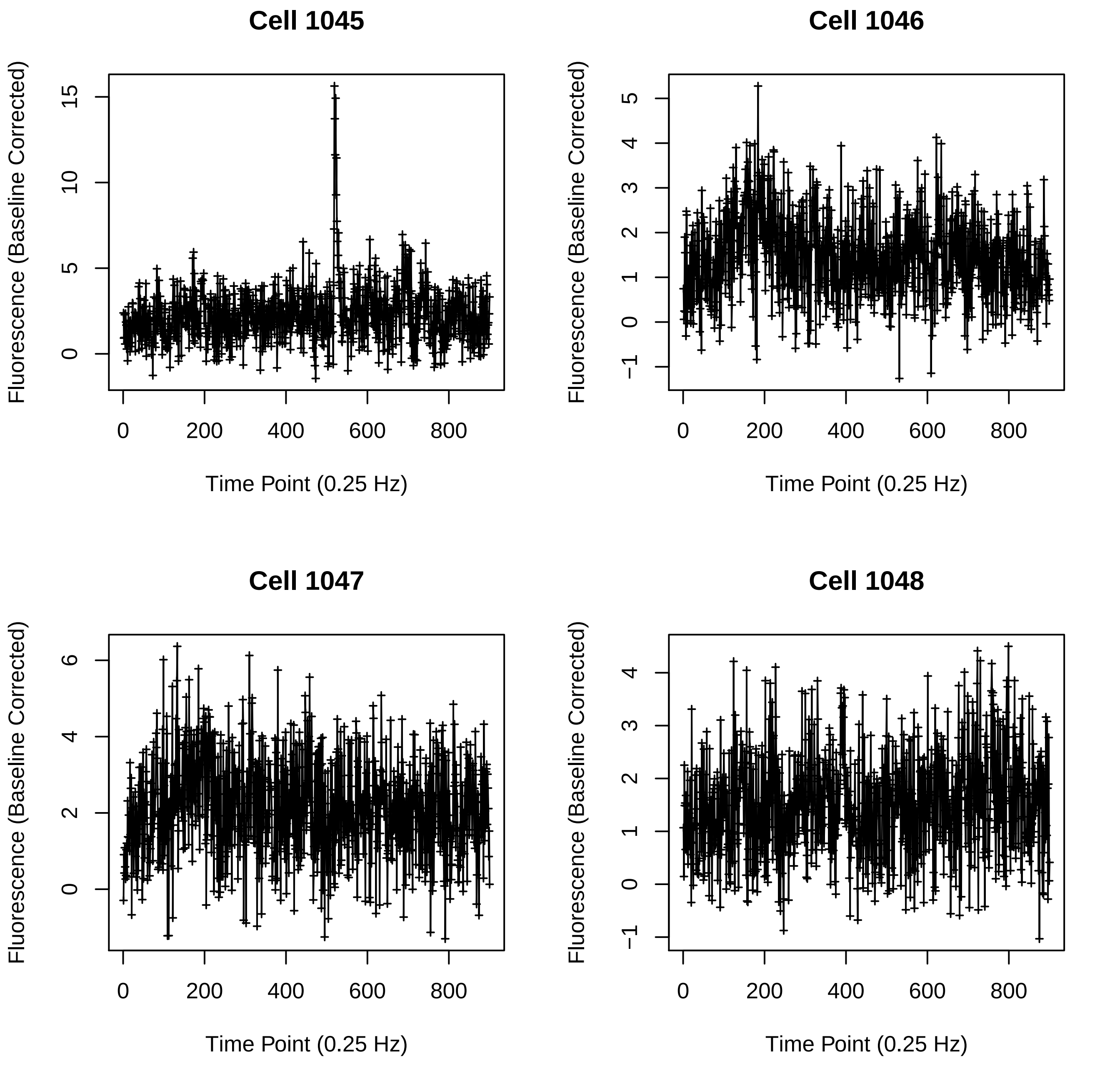
Cell 1045
Cell 1046
Cell 1047
Cell 1048
Fluorescence (Baseline Corrected)
Time Point (0.25 Hz)
0
200
400
600
800
5
10
15
−1
1
2
3
4
6

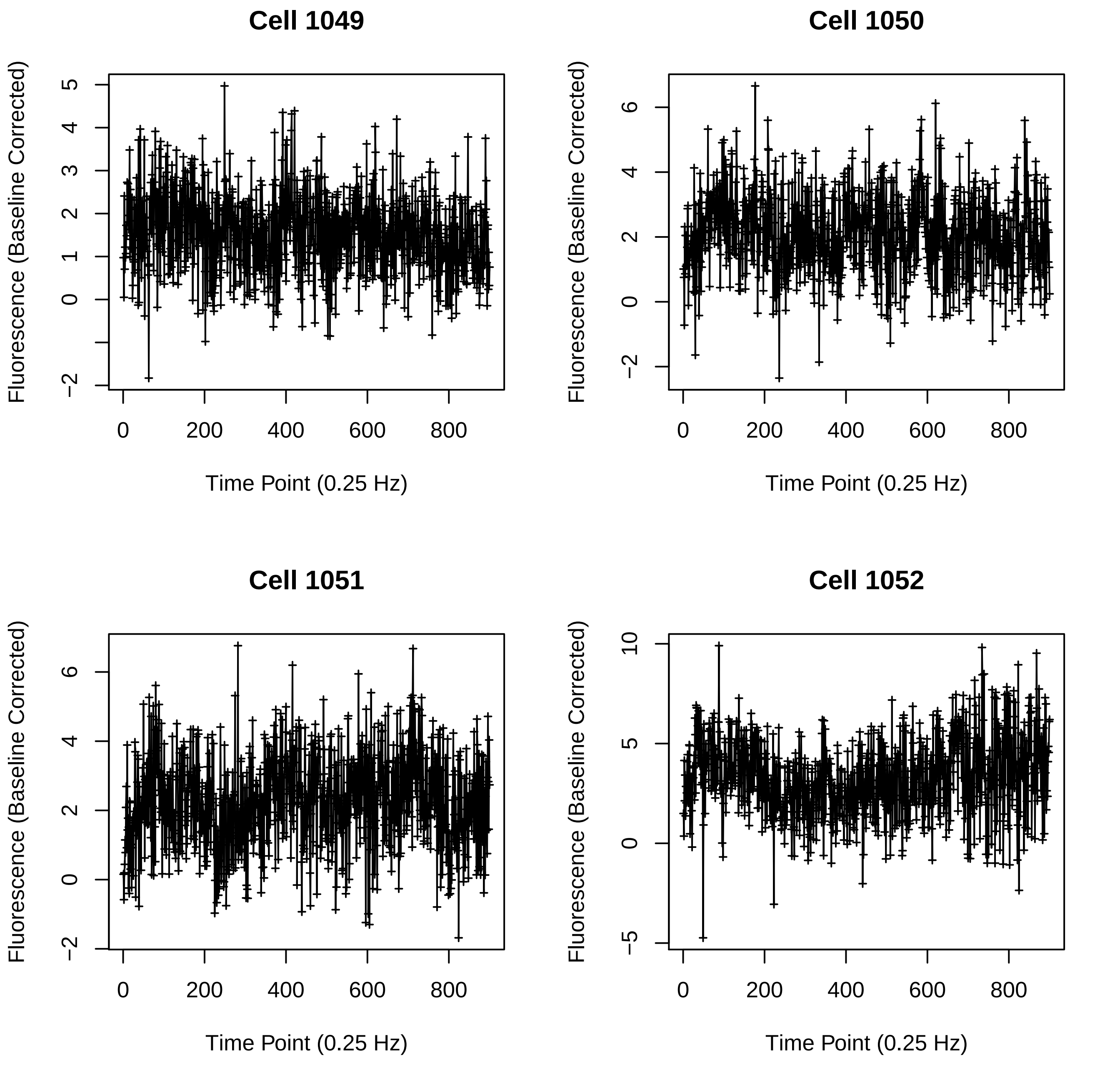
Cell 1049
Cell 1050
Cell 1051
Cell 1052
Fluorescence (Baseline Corrected)
Time Point (0.25 Hz)
0
200
400
600
800
−2
0
1
2
3
4
5
6
−5
10

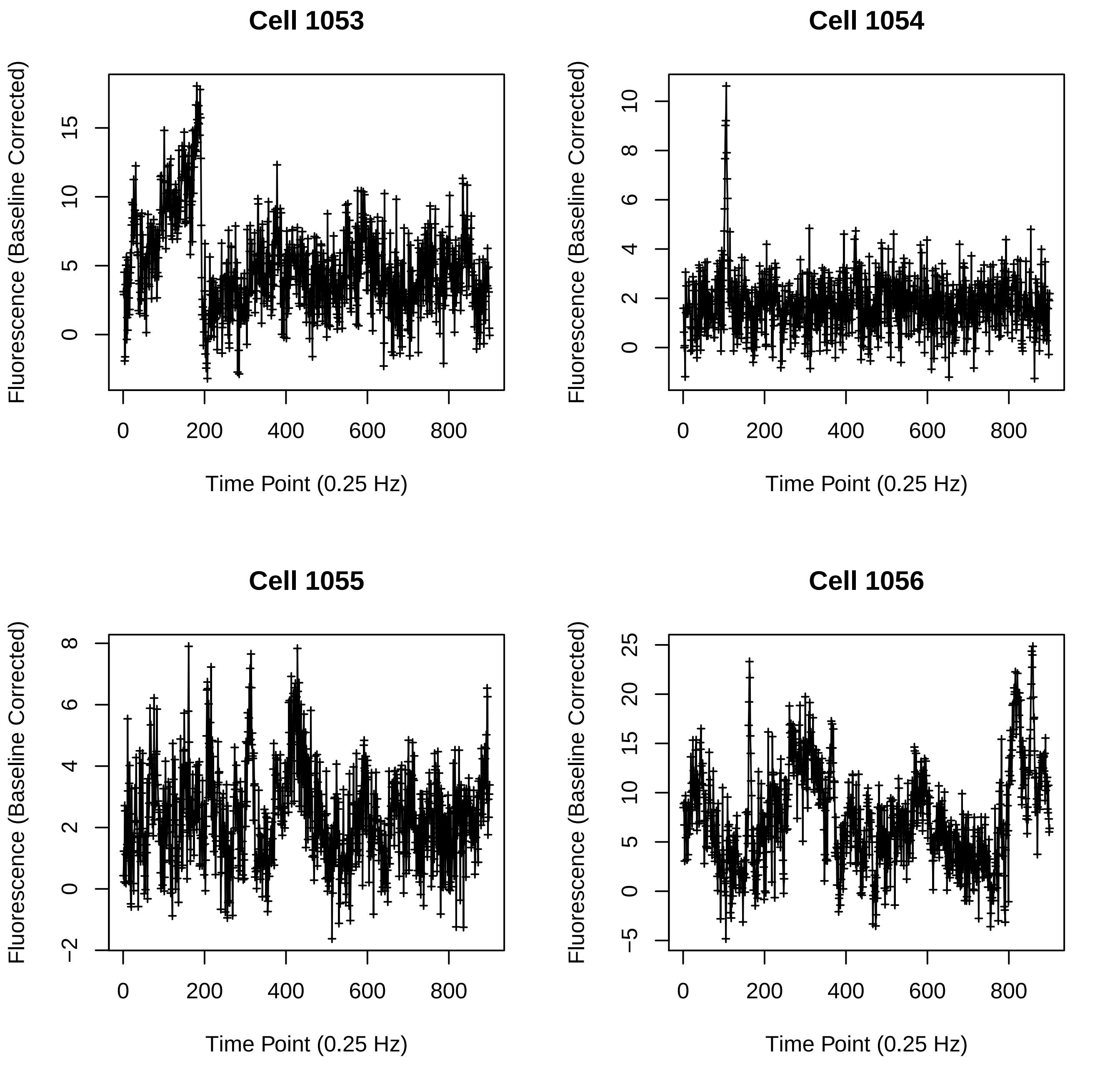
Cell 1053
Cell 1054
Cell 1055
Cell 1056
Fluorescence (Baseline Corrected)
Time Point (0.25 Hz)
0
200
400
600
800
-2
0
2
4
6
8
10
15
20
25
-5
5

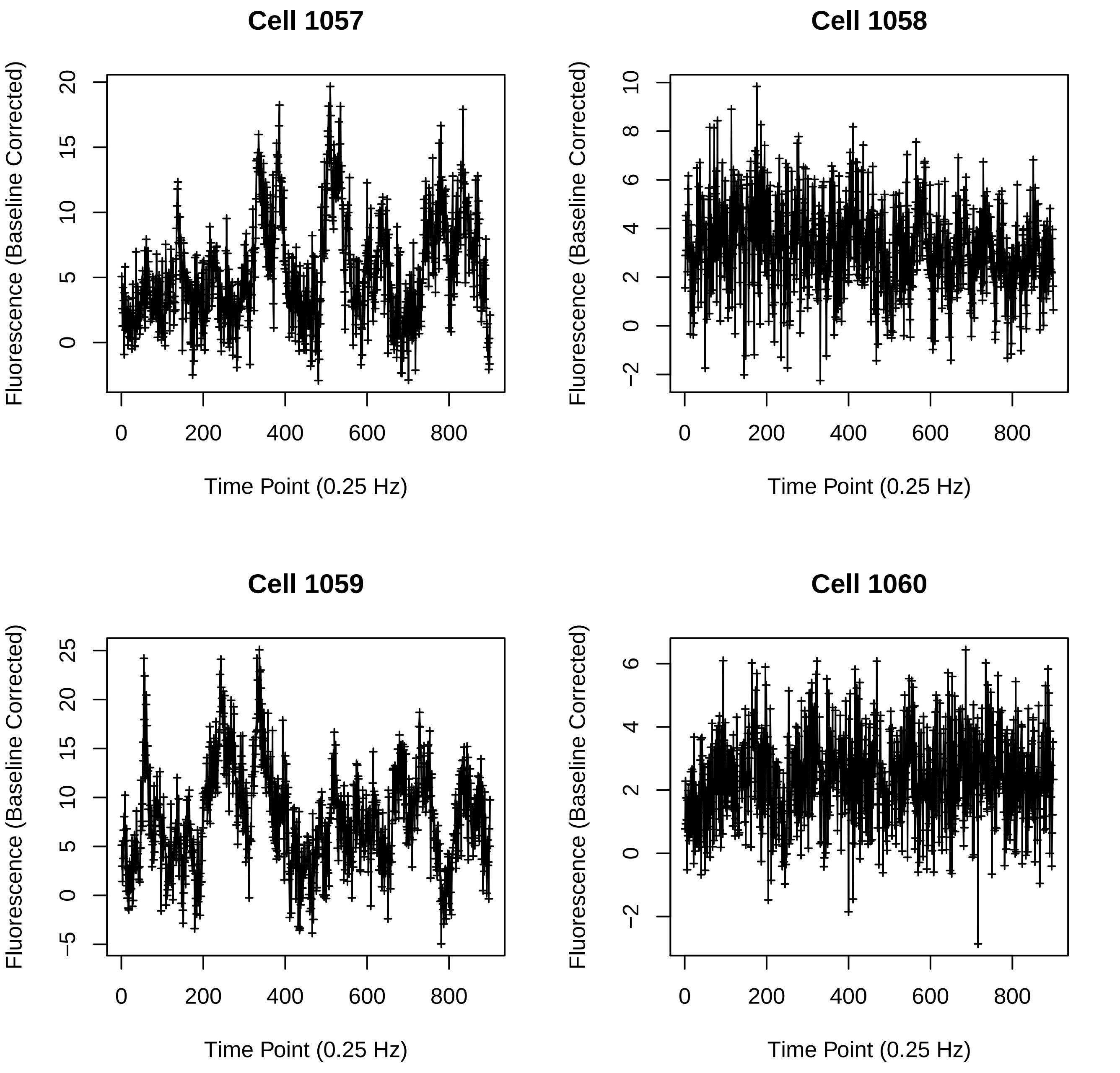

Cell 1057
Cell 1058
Cell 1059
Cell 1060
Fluorescence (Baseline Corrected)
Time Point (0.25 Hz)
0
200
400
600
800

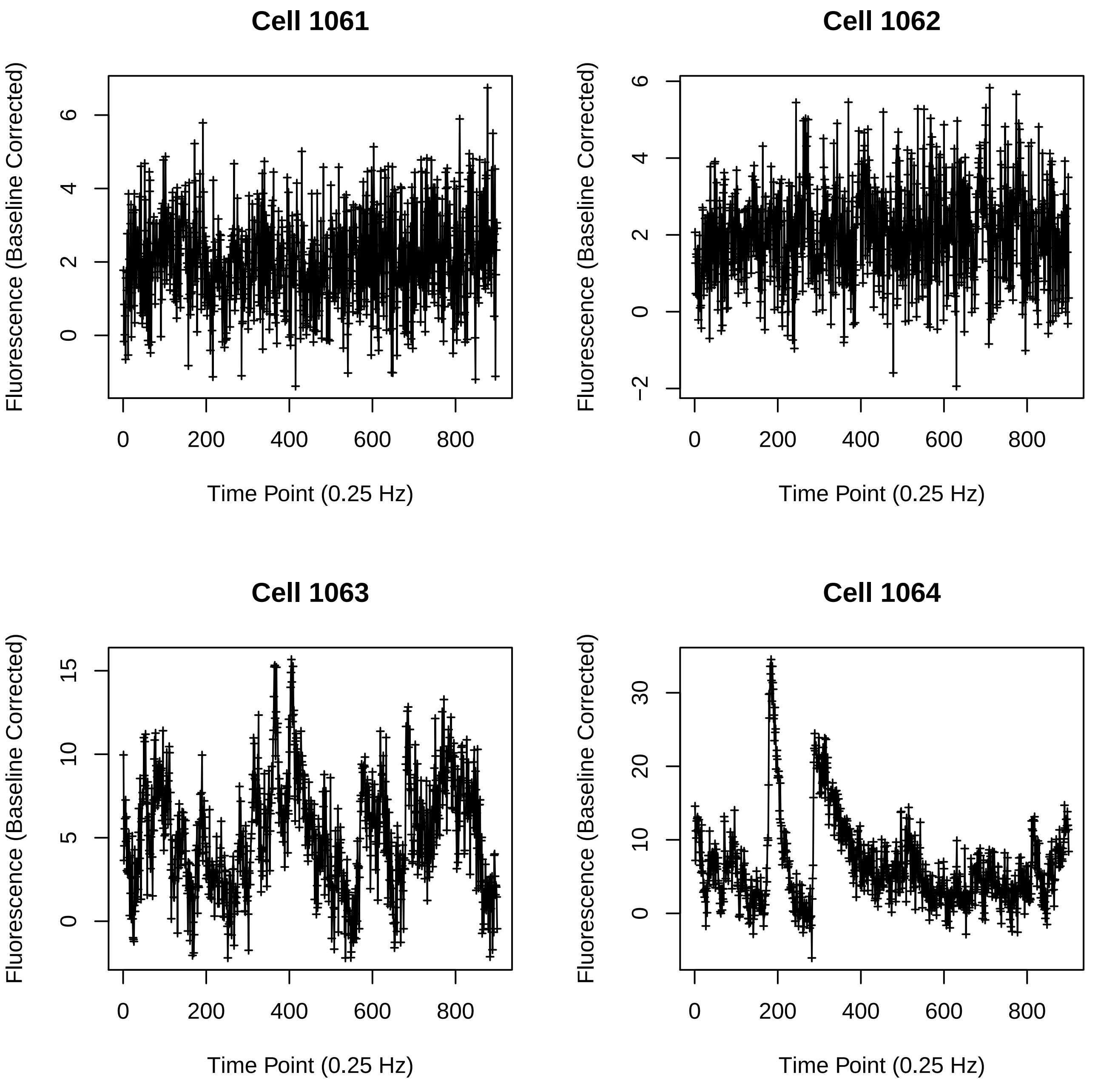
Cell 1061
Cell 1062
Cell 1063
Cell 1064
Fluorescence (Baseline Corrected)
Time Point (0.25 Hz)
0
200
400
600
800
−2
2
4
6
5
10
15
20
30

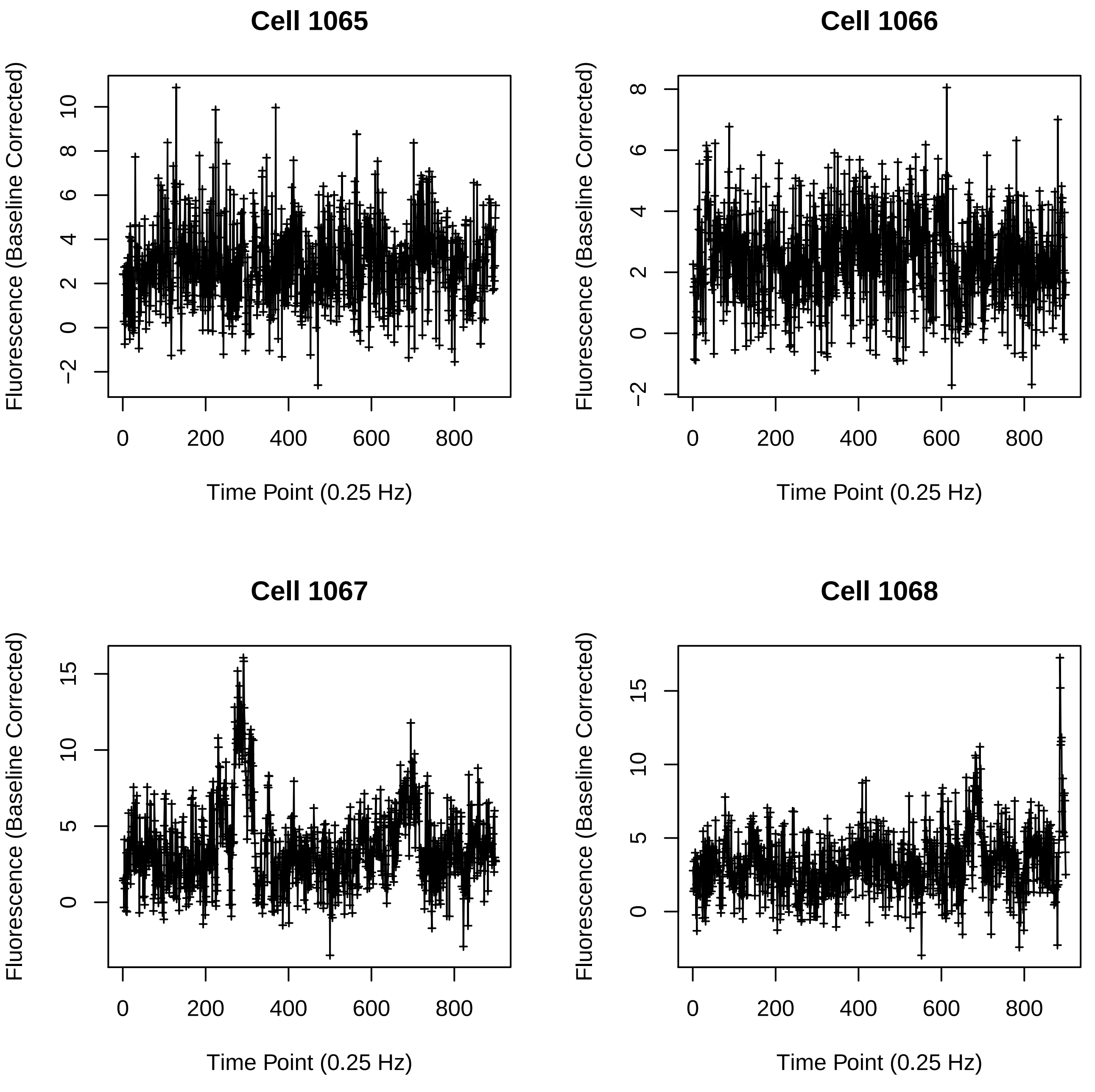
Cell 1065
Cell 1066
Cell 1067
Cell 1068
Fluorescence (Baseline Corrected)
Time Point (0.25 Hz)
0
200
400
600
800
−2
2
4
6
8
10
15
5

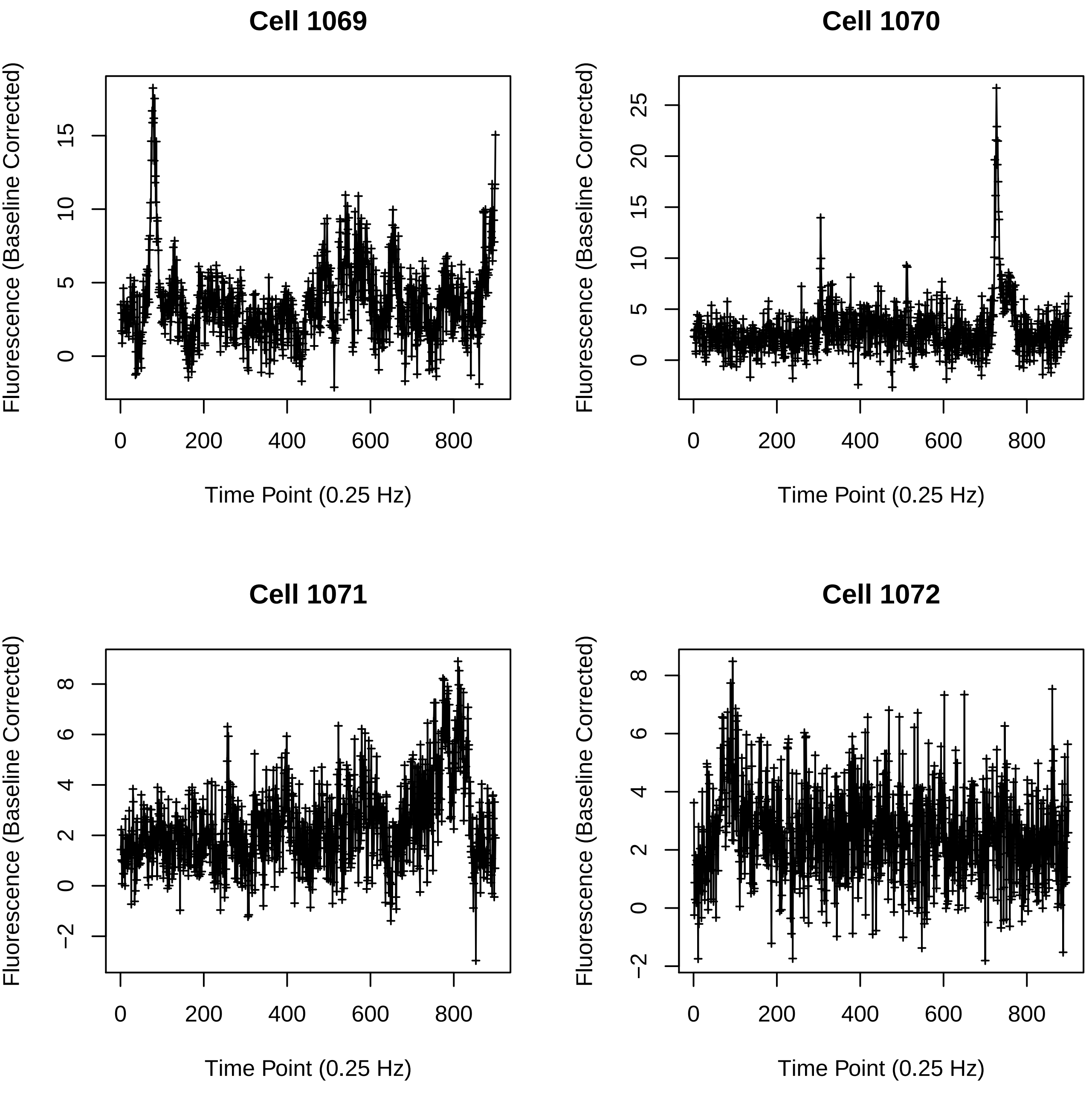
Cell 1069
Cell 1070
Cell 1071
Cell 1072
Fluorescence (Baseline Corrected)
Time Point (0.25 Hz)
0
200
400
600
800
0
5
10
15
20
25
−2
2
4
6
8

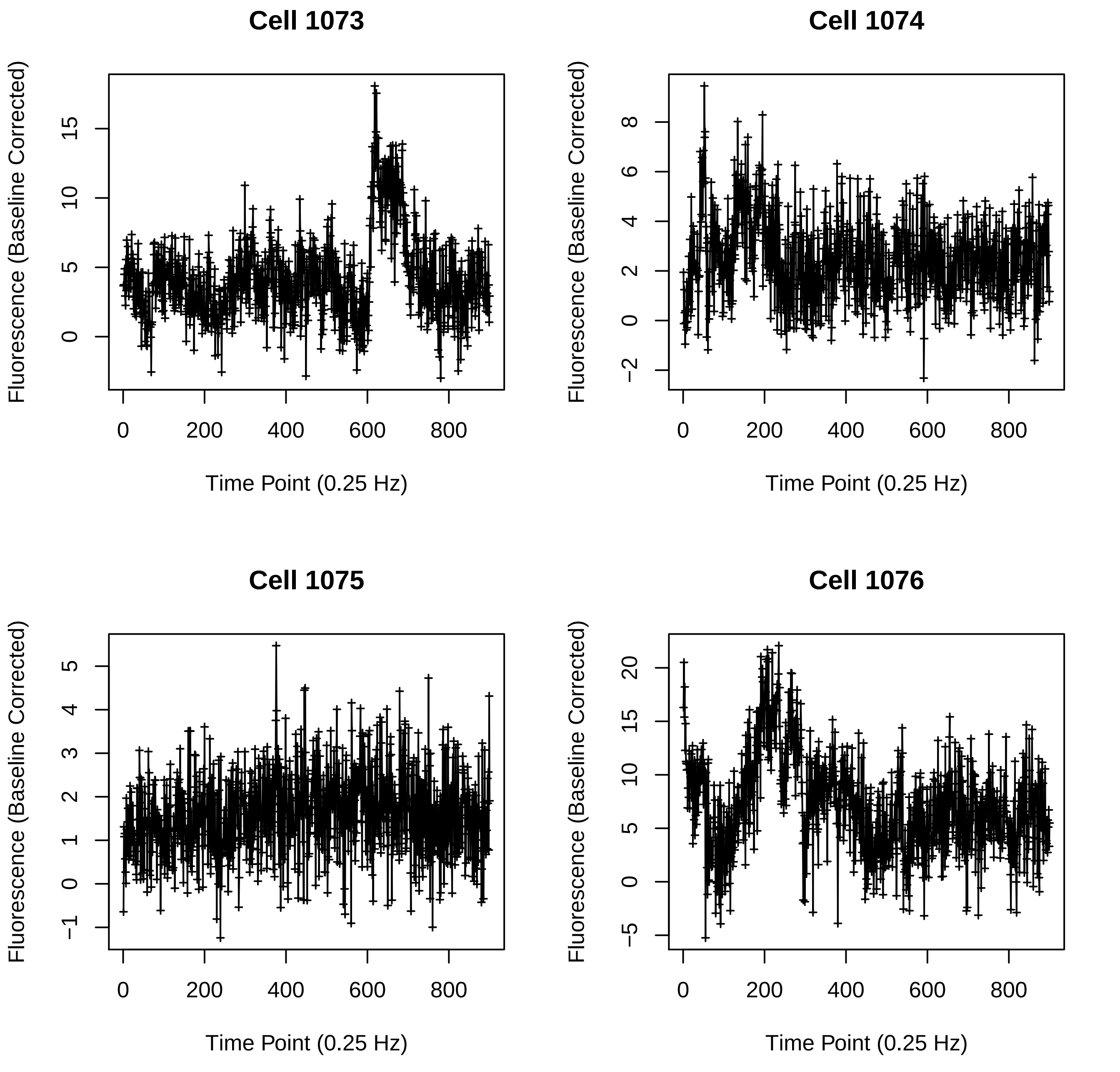
Cell 1073
Cell 1074
Cell 1075
Cell 1076
Fluorescence (Baseline Corrected)
Time Point (0.25 Hz)
0
200
400
600
800
−5
0
5
10
15
20
−2
2
4
6
8
−1
1
3

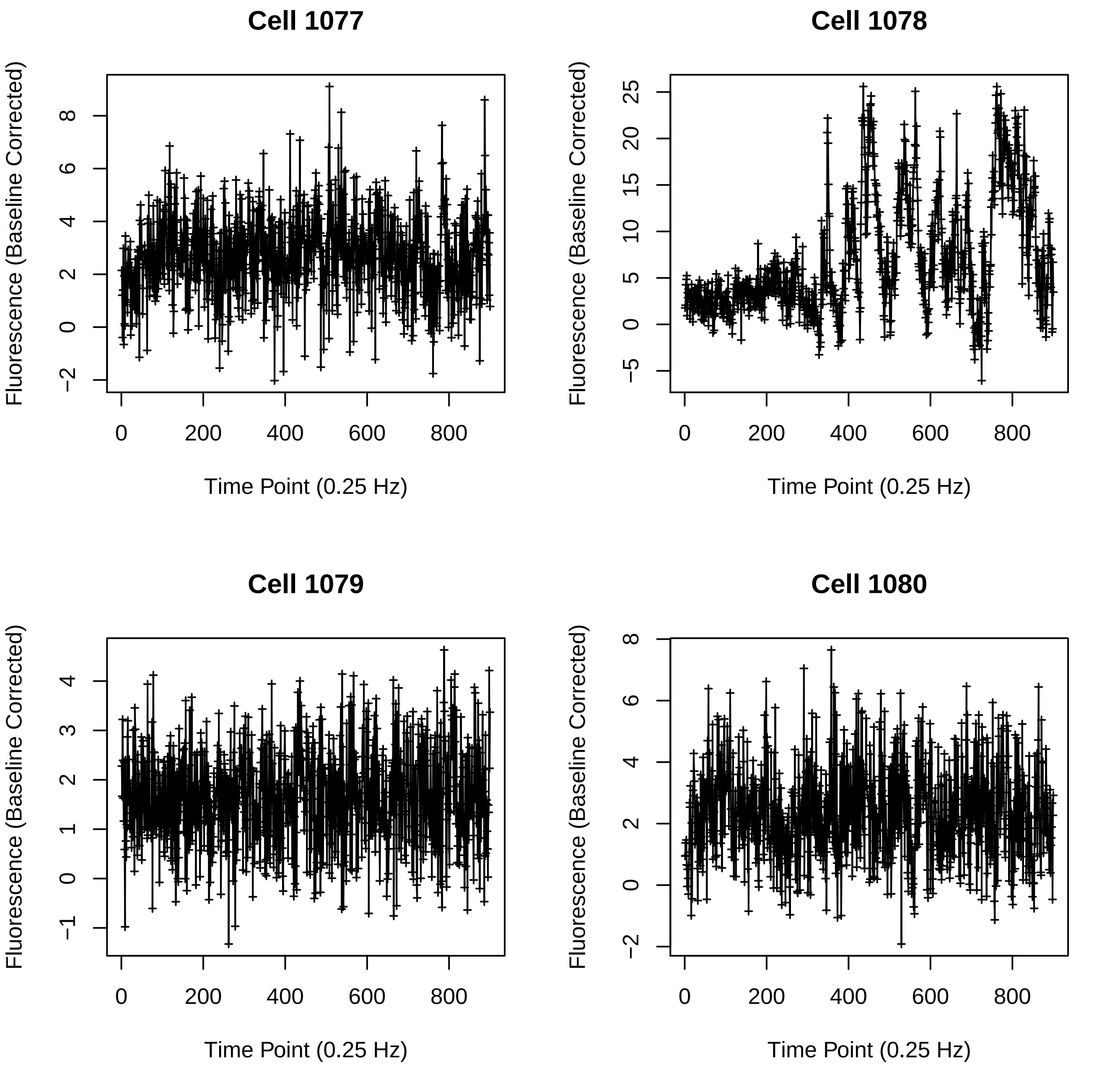
Cell 1077
Cell 1078
Cell 1079
Cell 1080
Fluorescence (Baseline Corrected)
Time Point (0.25 Hz)
0
200
400
600
800
−2
2
4
6
8
−5
5
10
15
20
25
−1
1
3

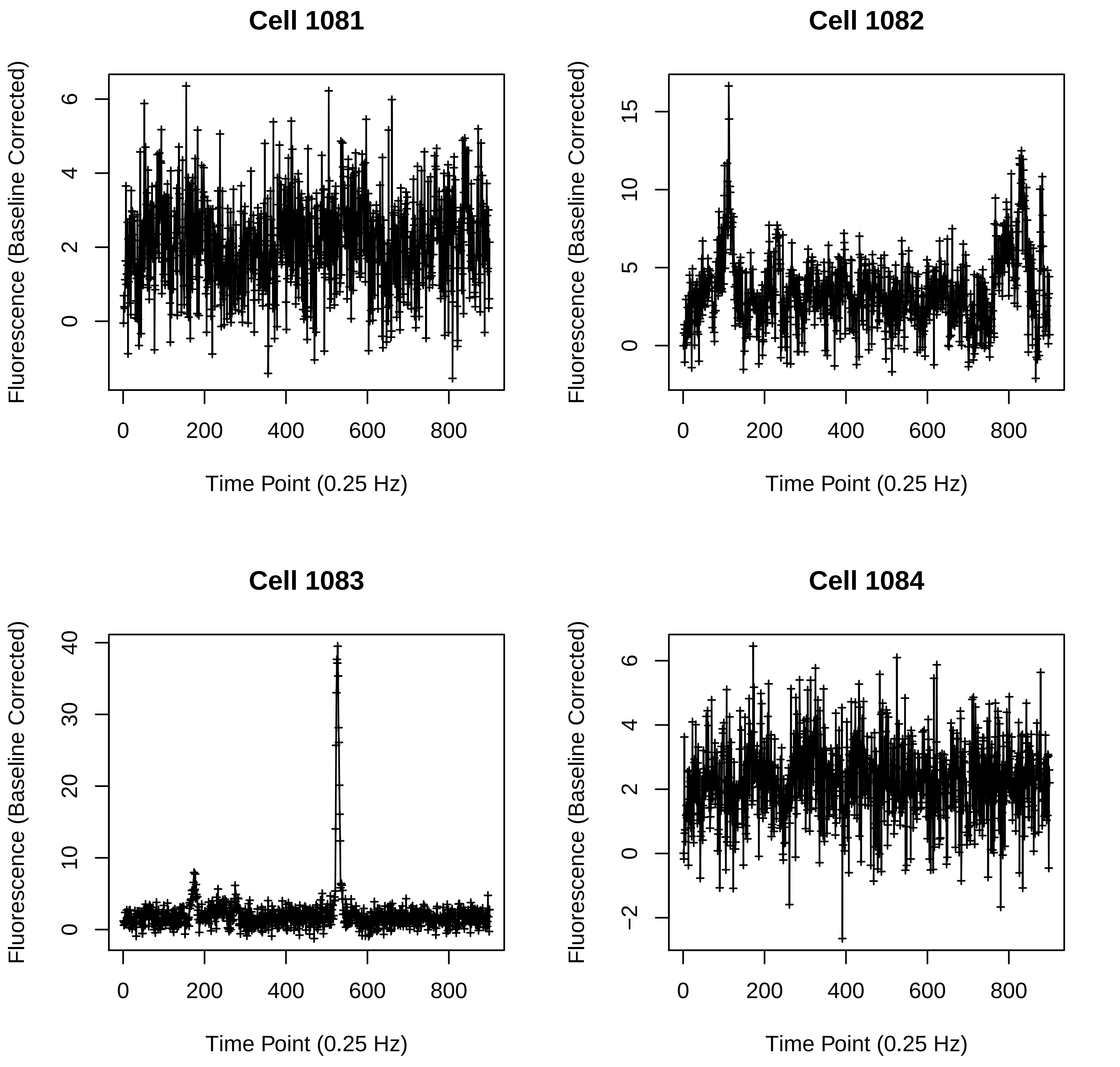
Cell 1081
Fluorescence (Baseline Corrected)
Time Point (0.25 Hz)
Cell 1082
Fluorescence (Baseline Corrected)
Time Point (0.25 Hz)
Cell 1083
Fluorescence (Baseline Corrected)
Time Point (0.25 Hz)
Cell 1084
Fluorescence (Baseline Corrected)
Time Point (0.25 Hz)

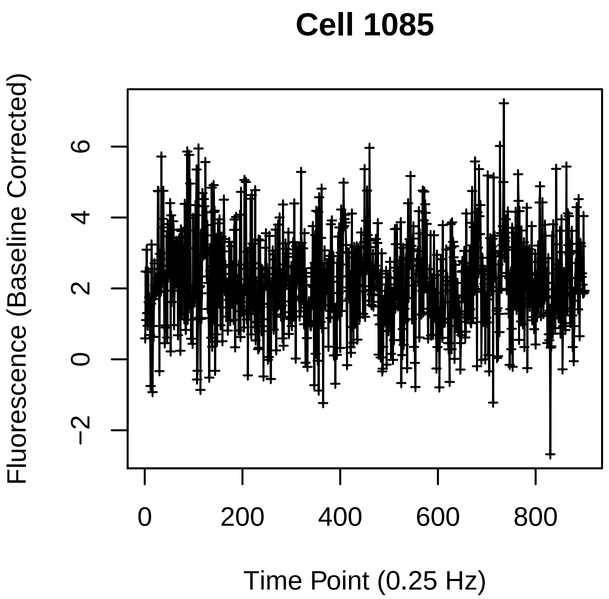

Cell 1085
Fluorescence (Baseline Corrected)
Time Point (0.25 Hz)

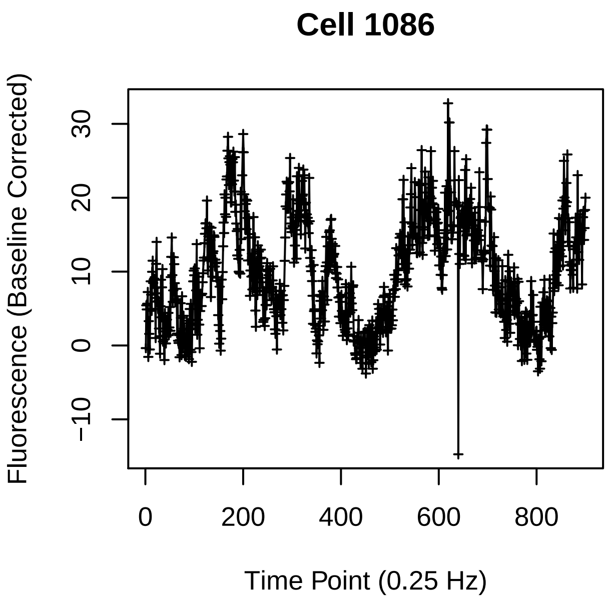

Cell 1086
Fluorescence (Baseline Corrected)
Time Point (0.25 Hz)

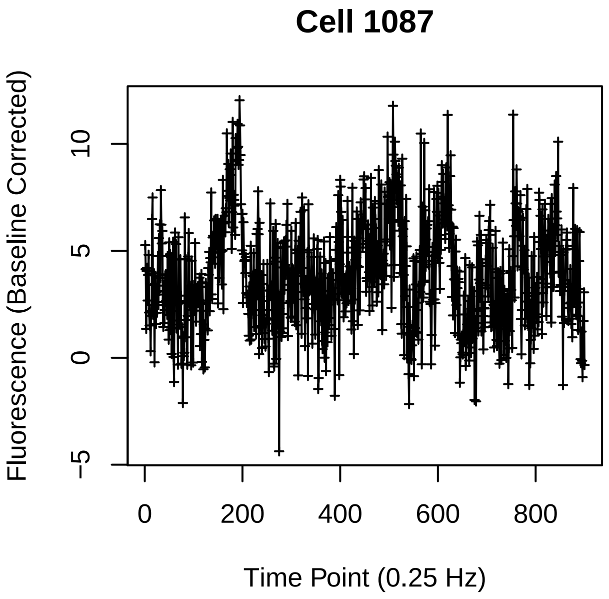

Cell 1087
Fluorescence (Baseline Corrected)
Time Point (0.25 Hz)

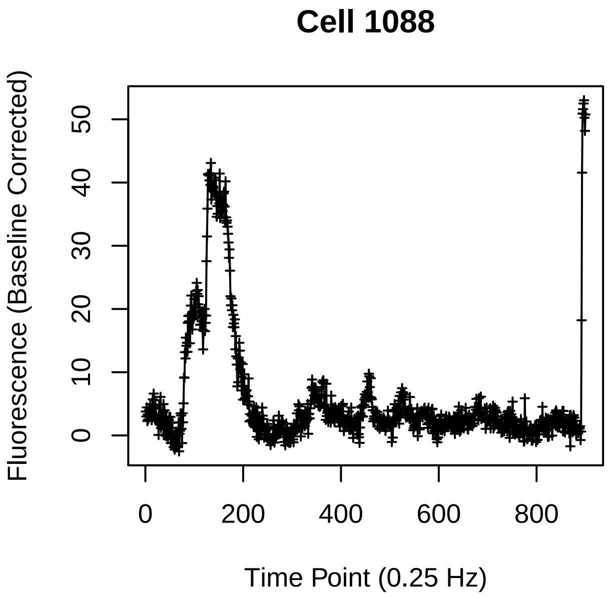

Cell 1088
Fluorescence (Baseline Corrected)
Time Point (0.25 Hz)

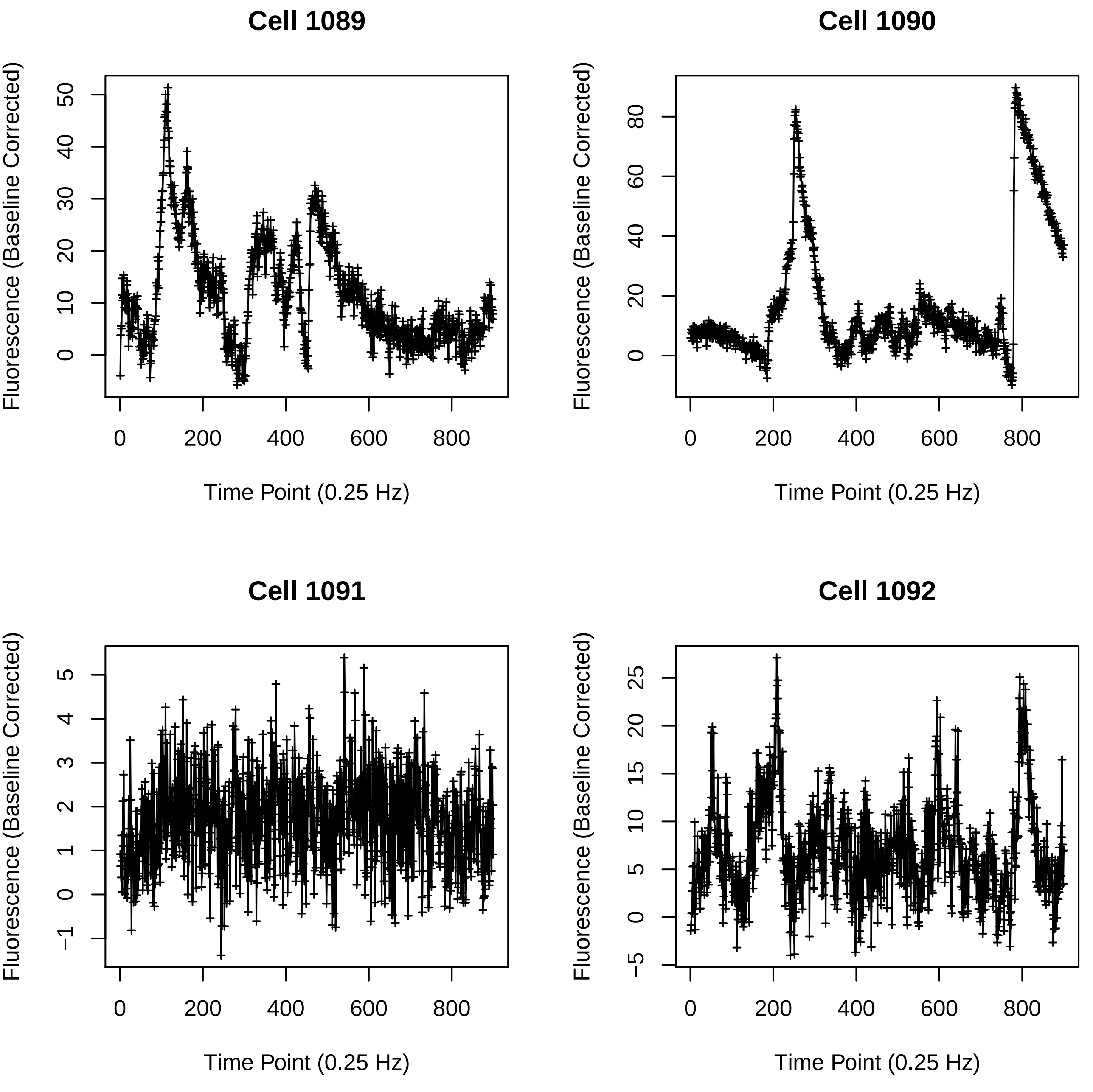

Cell 1089
Cell 1090
Cell 1091
Cell 1092
Fluorescence (Baseline Corrected)
Time Point (0.25 Hz)
0
200
400
600
800
-5
0
5
10
15
20
25
30
40
50
60
80
-1
1
2
3
4

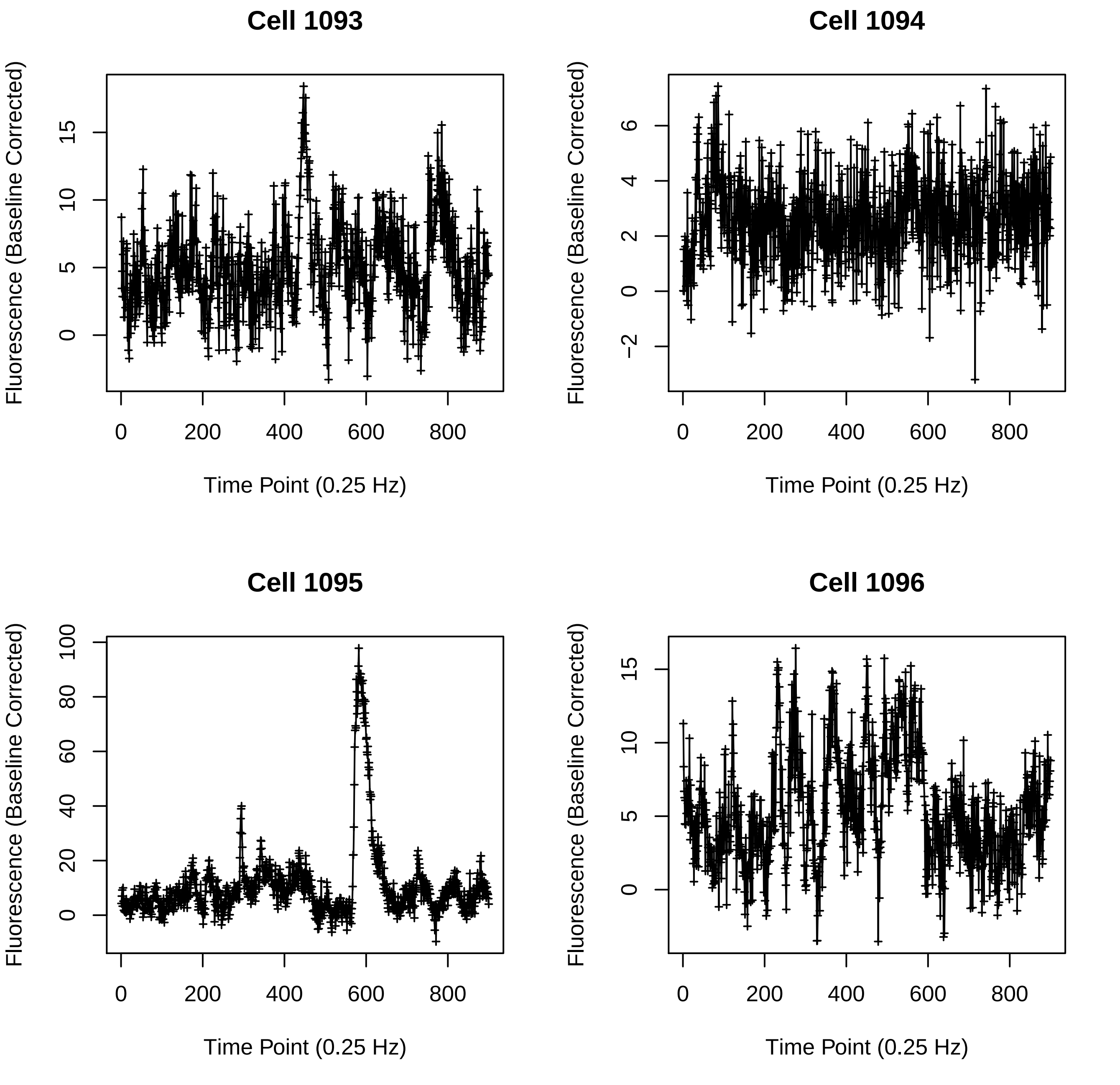
Cell 1093
Cell 1094
Cell 1095
Cell 1096
Fluorescence (Baseline Corrected)
Time Point (0.25 Hz)
0
5
10
15
−2
2
4
6
20
40
60
80
100
200
400
600
800

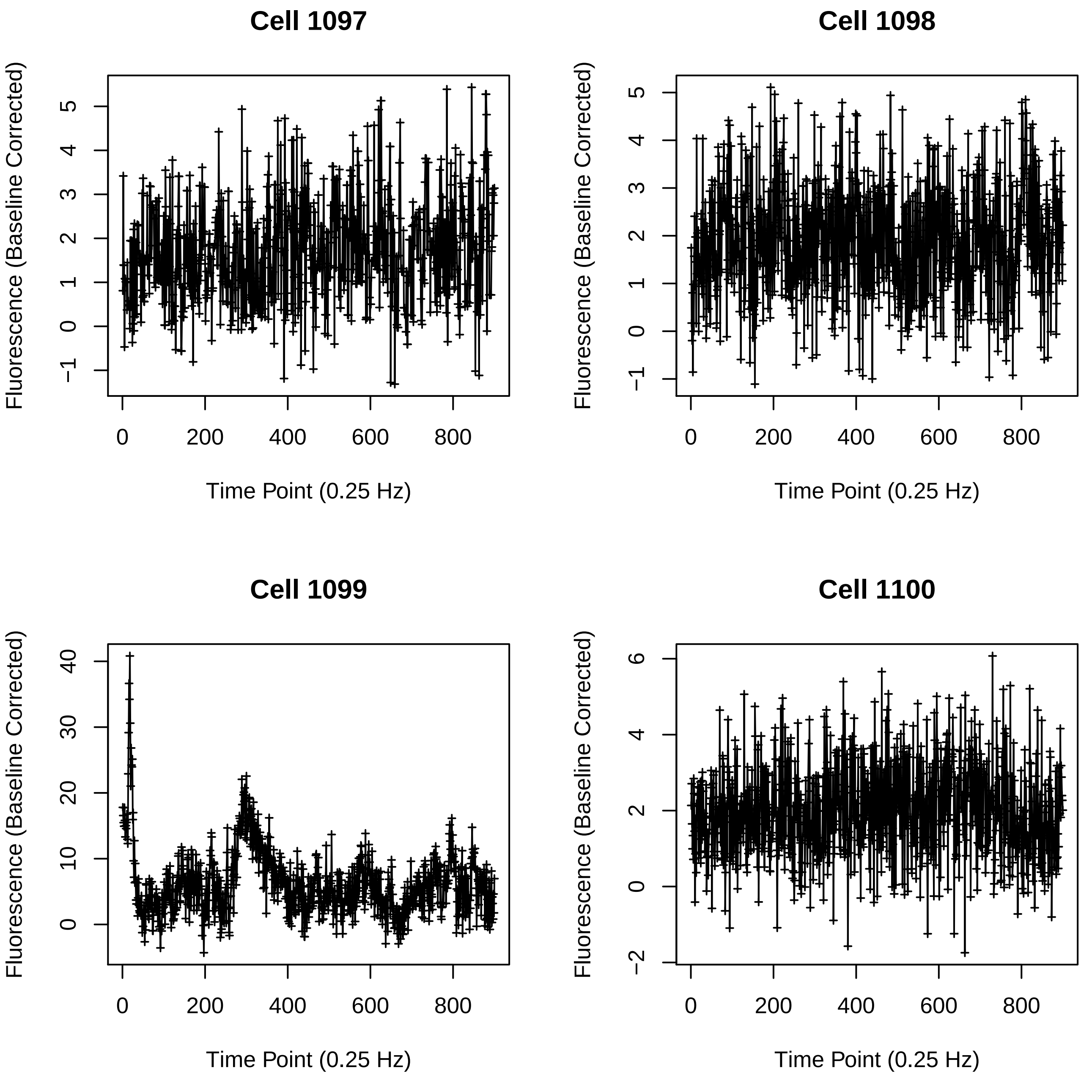

Cell 1097
Fluorescence (Baseline Corrected)
Time Point (0.25 Hz)
Cell 1098
Fluorescence (Baseline Corrected)
Time Point (0.25 Hz)
Cell 1099
Fluorescence (Baseline Corrected)
Time Point (0.25 Hz)
Cell 1100
Fluorescence (Baseline Corrected)
Time Point (0.25 Hz)

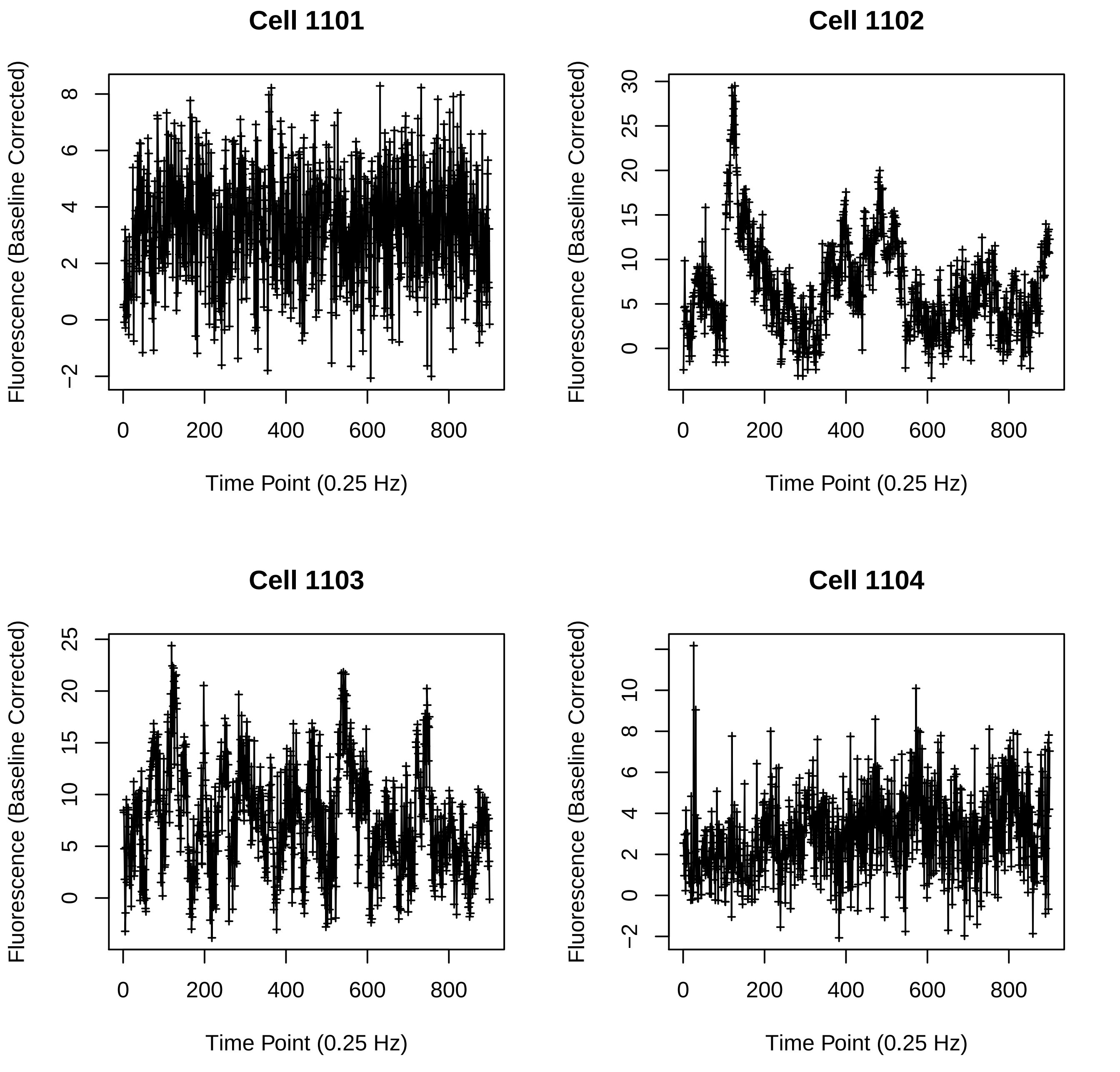

Cell 1101
Cell 1102
Cell 1103
Cell 1104
Fluorescence (Baseline Corrected)
Time Point (0.25 Hz)
0
200
400
600
800
−2
0
2
4
6
8
5
10
15
20
25
30

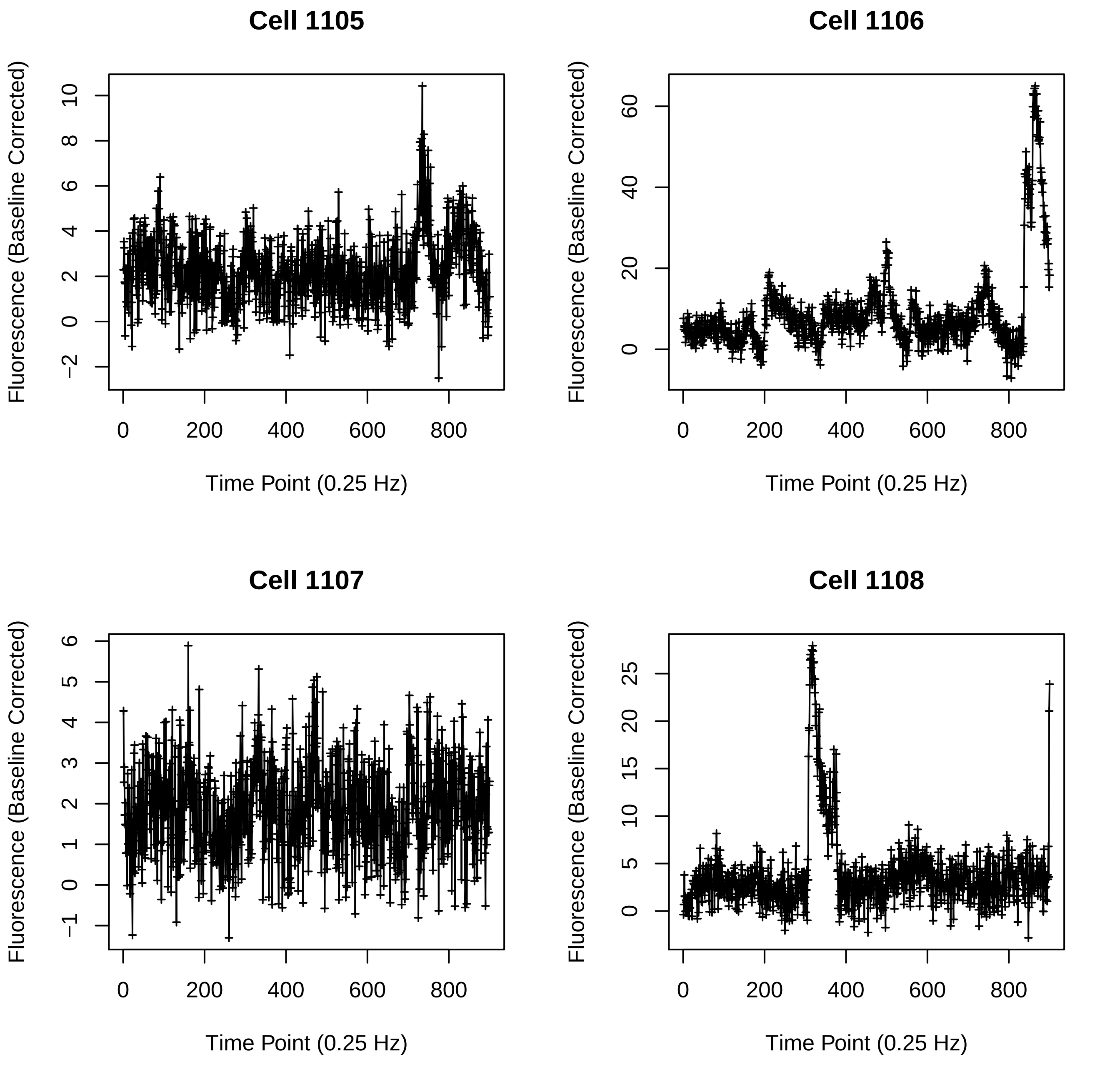
Cell 1105
Fluorescence (Baseline Corrected)
−2
0
2
4
6
8
10
0
200
400
600
800
Time Point (0.25 Hz)
Cell 1106
Fluorescence (Baseline Corrected)
0
20
40
60
0
200
400
600
800
Time Point (0.25 Hz)
Cell 1107
Fluorescence (Baseline Corrected)
−1
0
1
2
3
4
5
6
0
200
400
600
800
Time Point (0.25 Hz)
Cell 1108
Fluorescence (Baseline Corrected)
0
5
10
15
20
25
0
200
400
600
800
Time Point (0.25 Hz)

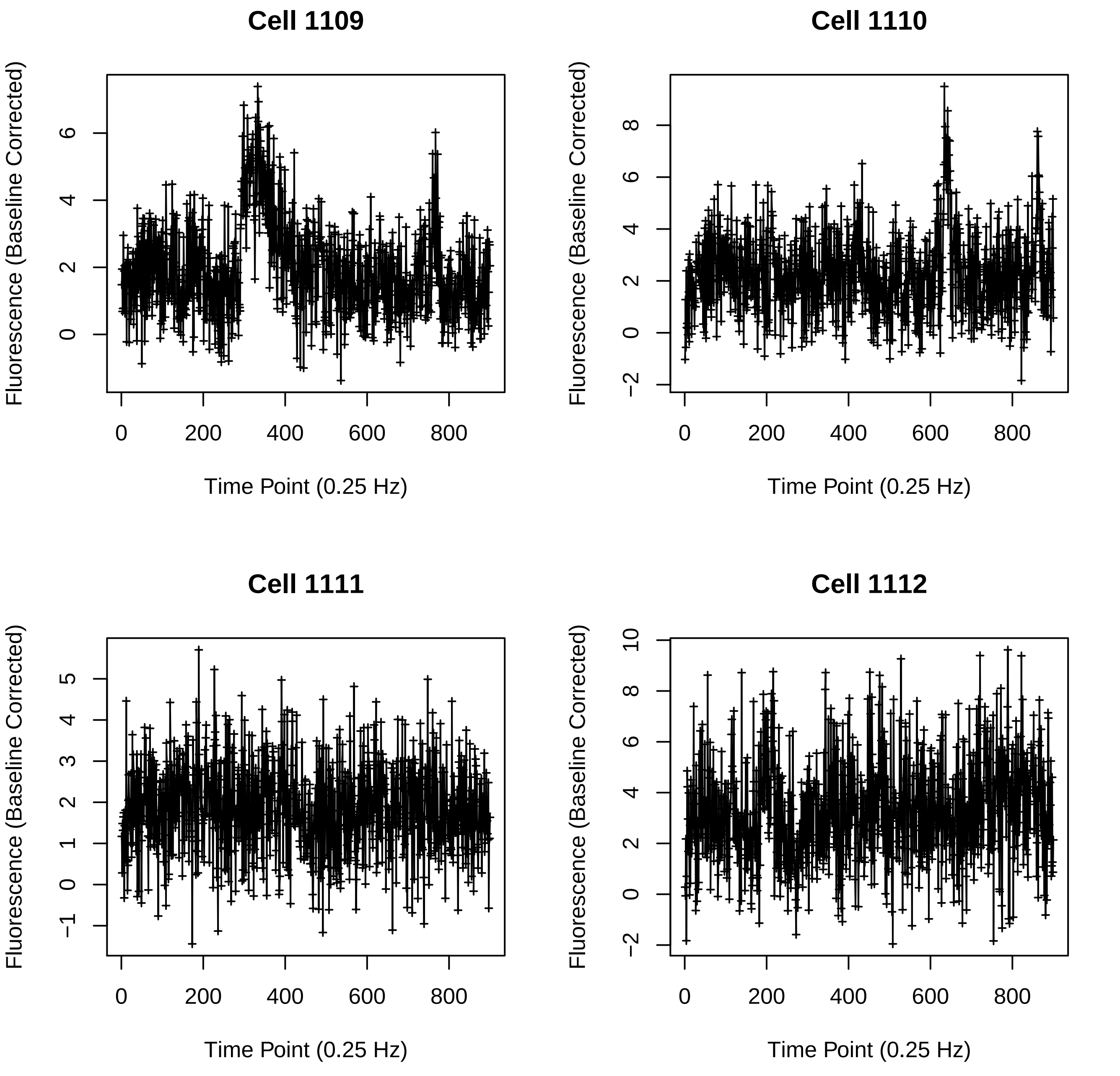
Cell 1109
Cell 1110
Cell 1111
Cell 1112
Fluorescence (Baseline Corrected)
Time Point (0.25 Hz)
0
200
400
600
800
−2
0
2
4
6
8
10
−1
1
3
5

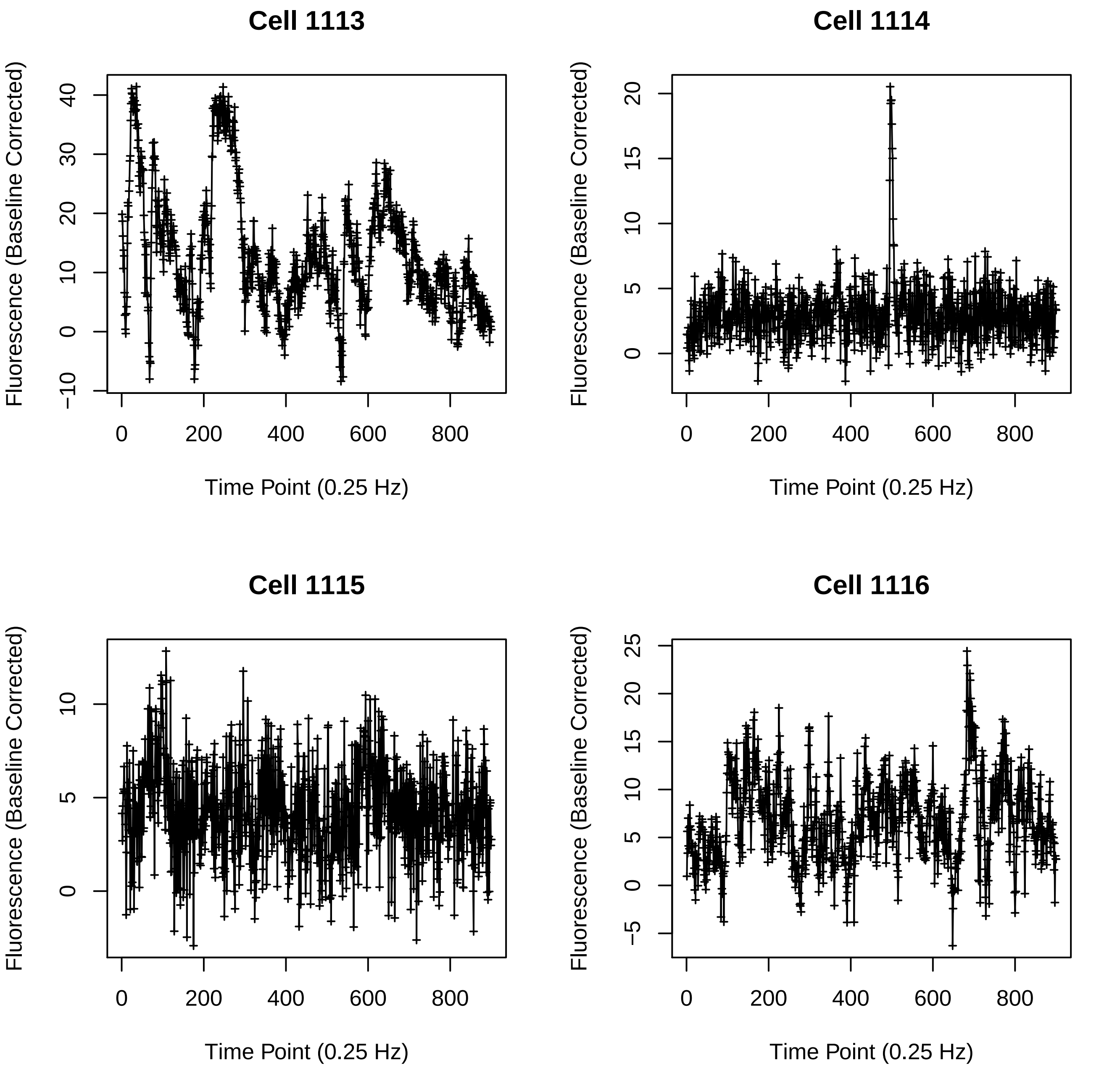

Cell 1113
Cell 1114
Cell 1115
Cell 1116
Fluorescence (Baseline Corrected)
Time Point (0.25 Hz)
−10
0
10
20
30
40
0
5
10
15
20
25
−5
200
400
600
800

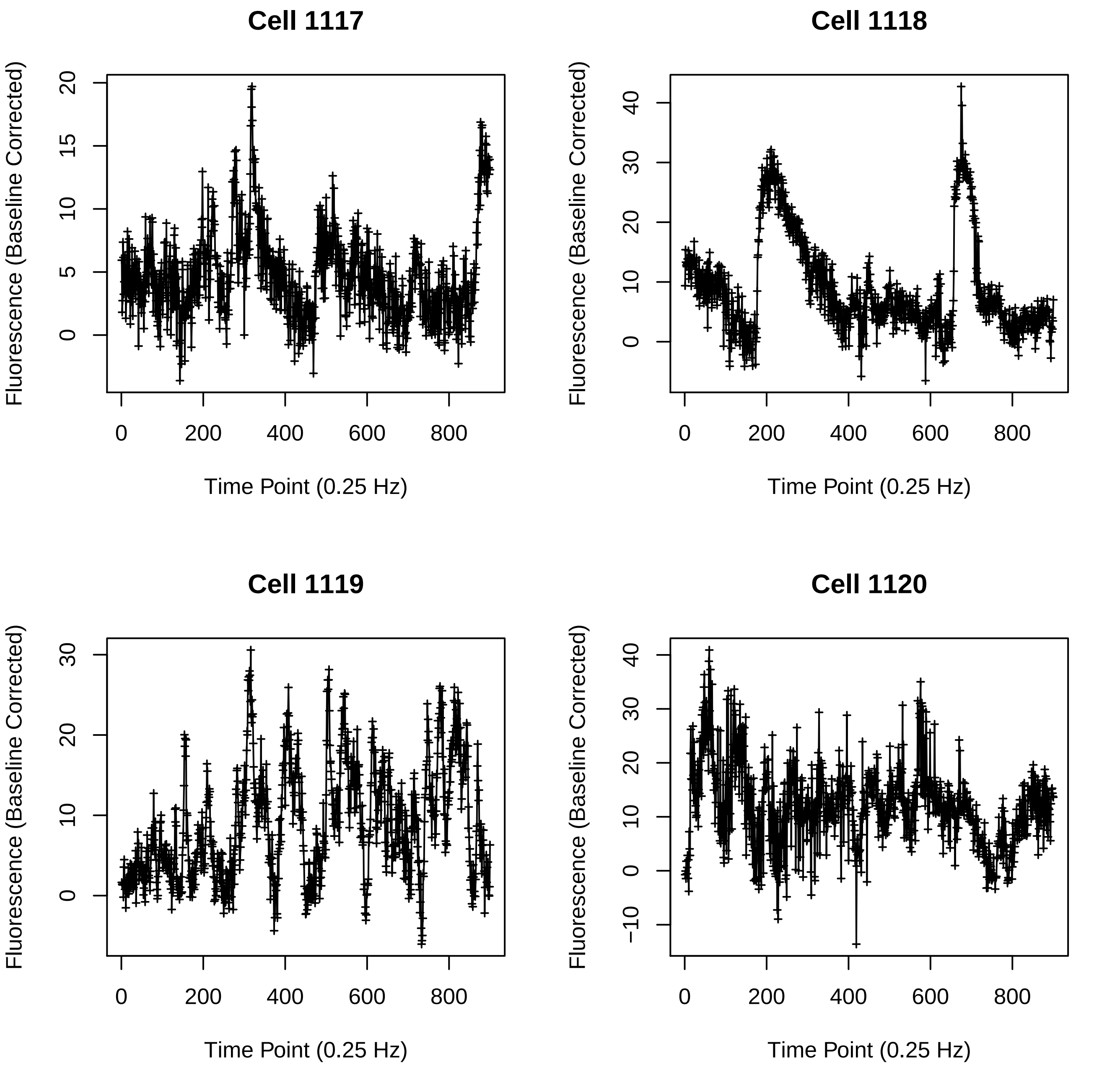

Cell 1117
Cell 1118
Cell 1119
Cell 1120
Fluorescence (Baseline Corrected)
Time Point (0.25 Hz)

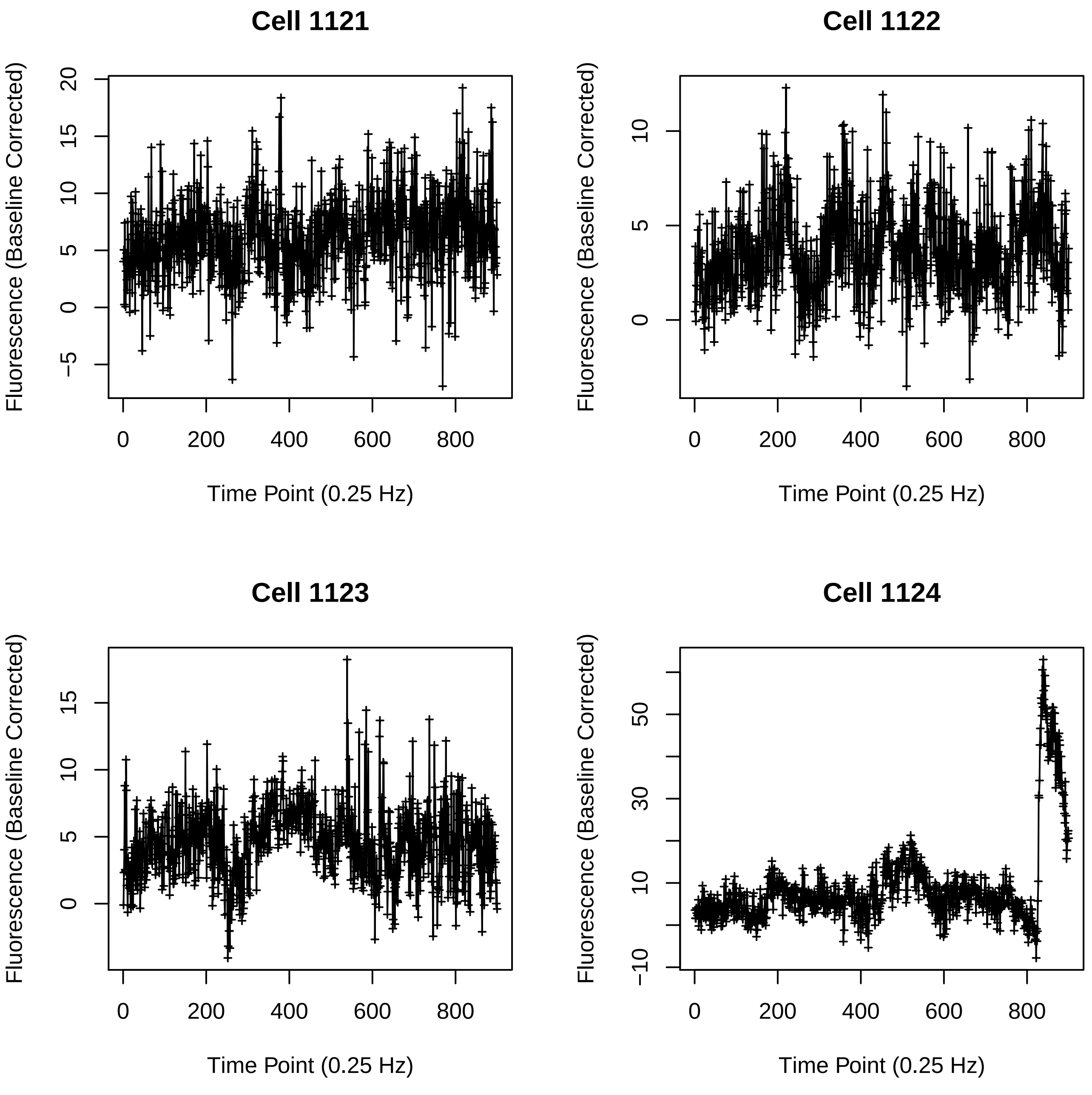

Cell 1121
Cell 1122
Cell 1123
Cell 1124
Fluorescence (Baseline Corrected)
Time Point (0.25 Hz)
0
200
400
600
800
−5
5
10
15
20
−10
30
50

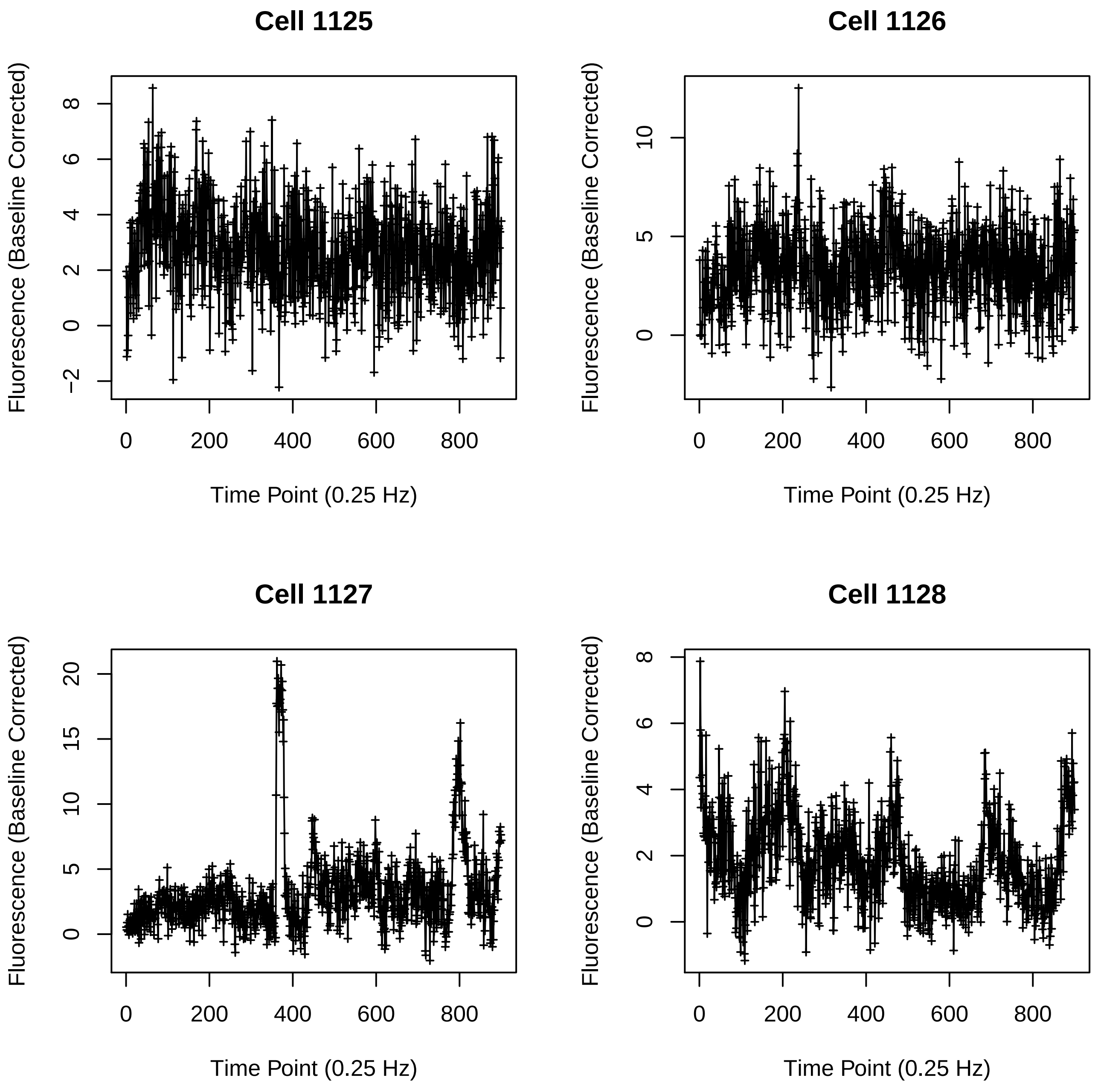
Cell 1125
Fluorescence (Baseline Corrected)
−2
0
2
4
6
8
0
200
400
600
800
Time Point (0.25 Hz)
Cell 1126
Fluorescence (Baseline Corrected)
0
5
10
0
200
400
600
800
Time Point (0.25 Hz)
Cell 1127
Fluorescence (Baseline Corrected)
0
5
10
15
20
0
200
400
600
800
Time Point (0.25 Hz)
Cell 1128
Fluorescence (Baseline Corrected)
0
2
4
6
8
0
200
400
600
800
Time Point (0.25 Hz)

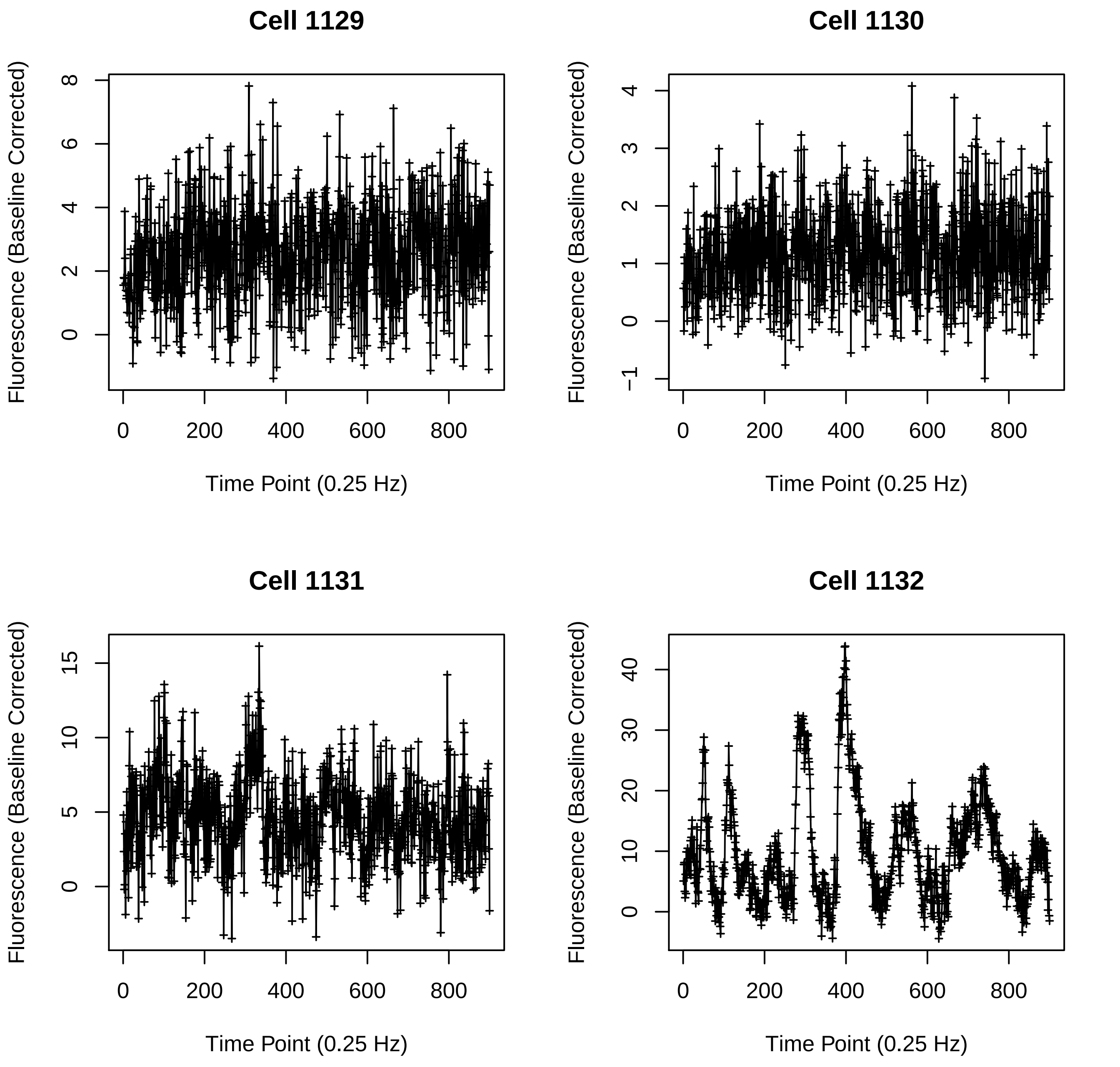
Cell 1129
Cell 1130
Cell 1131
Cell 1132
Fluorescence (Baseline Corrected)
Time Point (0.25 Hz)
0
200
400
600
800

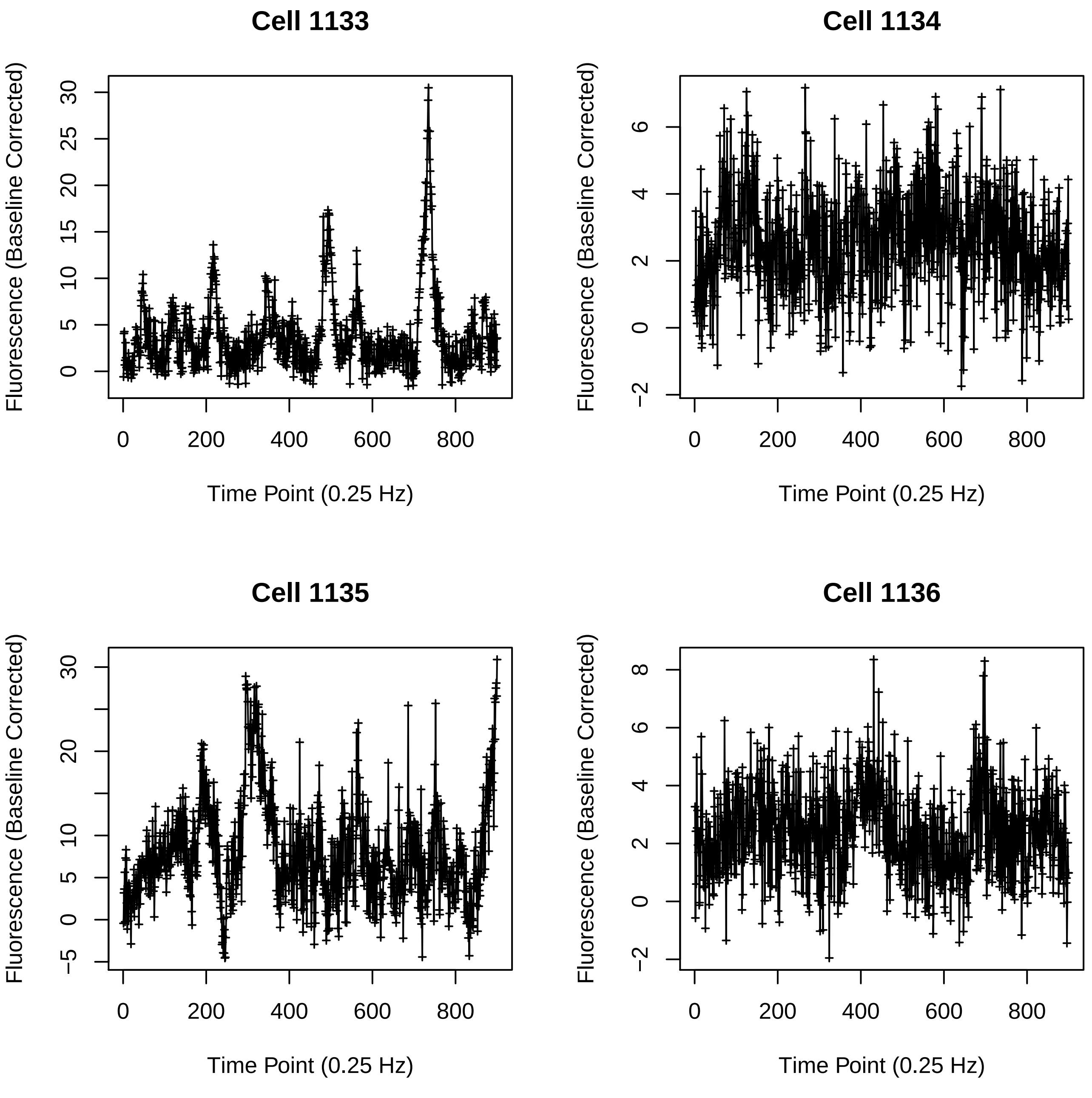

Cell 1133
Cell 1134
Cell 1135
Cell 1136
Fluorescence (Baseline Corrected)
Time Point (0.25 Hz)
0
5
10
15
20
25
30
-5
-2
2
4
6
8
200
400
600
800

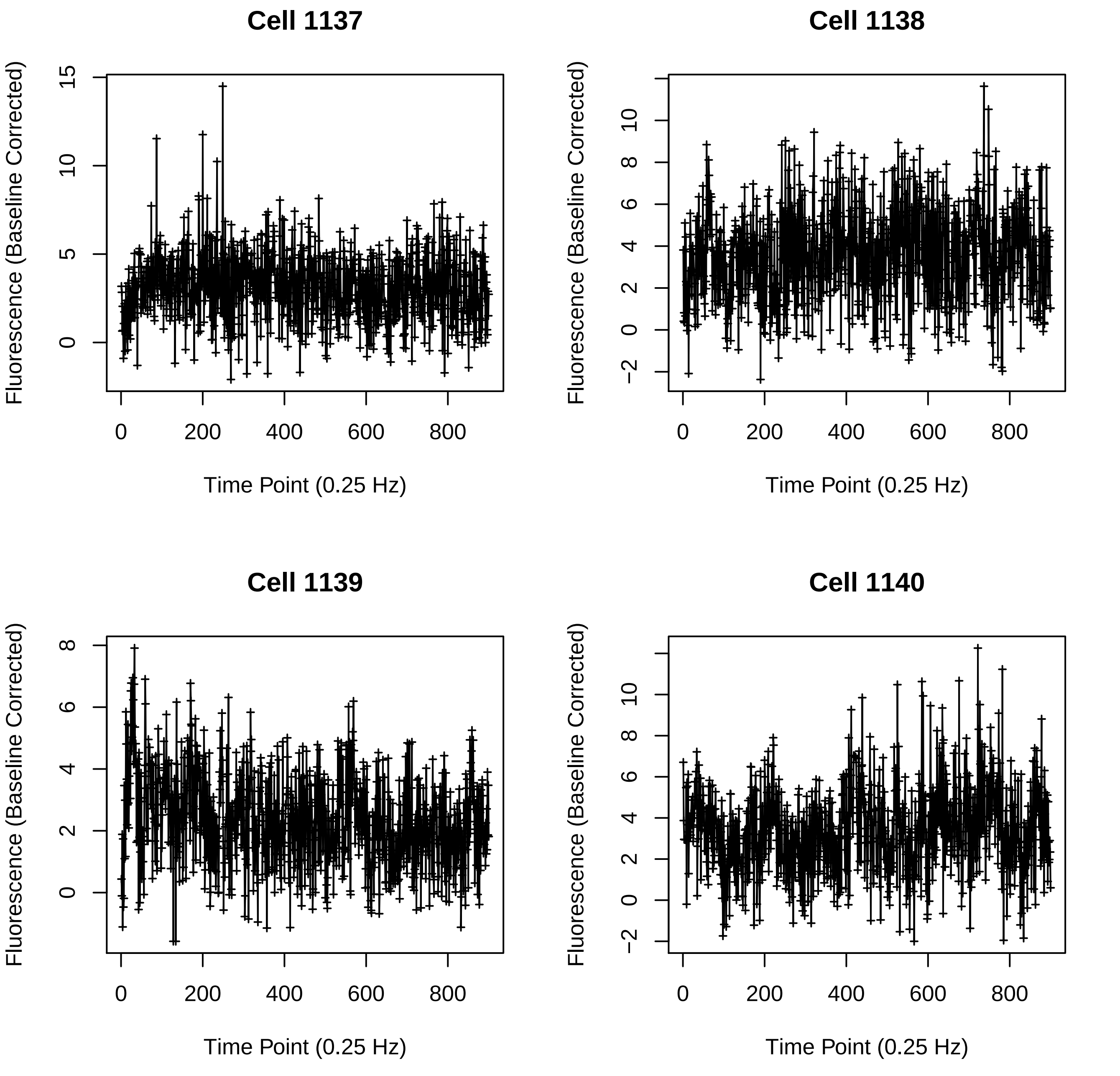
Cell 1137
Cell 1138
Cell 1139
Cell 1140
Fluorescence (Baseline Corrected)
Time Point (0.25 Hz)
0
5
10
15
−2
2
4
6
8
200
400
600
800

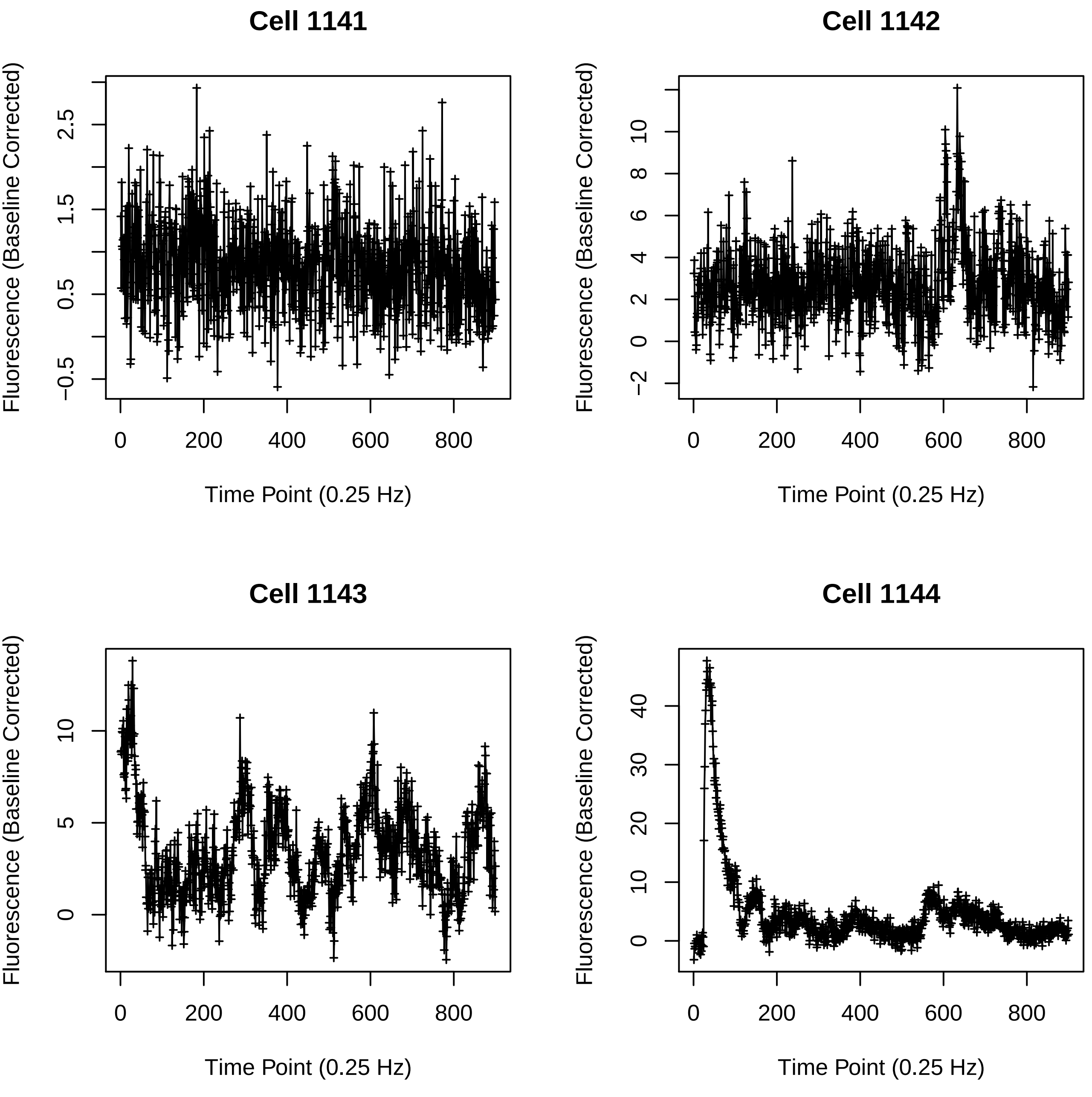

Cell 1141
Cell 1142
Cell 1143
Cell 1144
Fluorescence (Baseline Corrected)
Time Point (0.25 Hz)
−0.5
0.5
1.5
2.5
−2
0
2
4
6
8
10
5
20
30
40
200
400
600
800

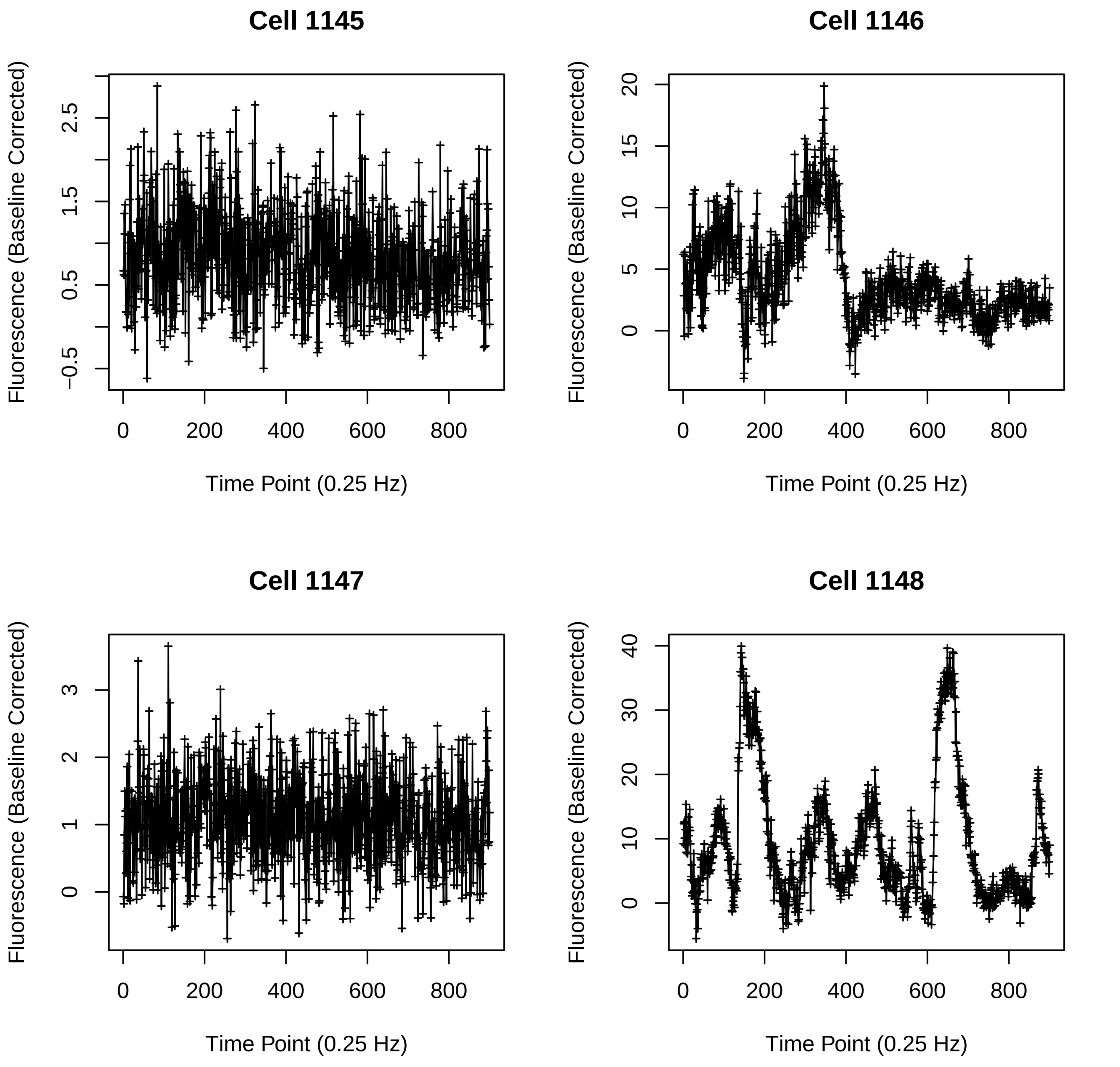
Cell 1145
Fluorescence (Baseline Corrected)
−0.5
0.5
1.5
2.5
0
200
400
600
800
Time Point (0.25 Hz)
Cell 1146
Fluorescence (Baseline Corrected)
0
5
10
15
20
0
200
400
600
800
Time Point (0.25 Hz)
Cell 1147
Fluorescence (Baseline Corrected)
0
1
2
3
0
200
400
600
800
Time Point (0.25 Hz)
Cell 1148
Fluorescence (Baseline Corrected)
0
10
20
30
40
0
200
400
600
800
Time Point (0.25 Hz)

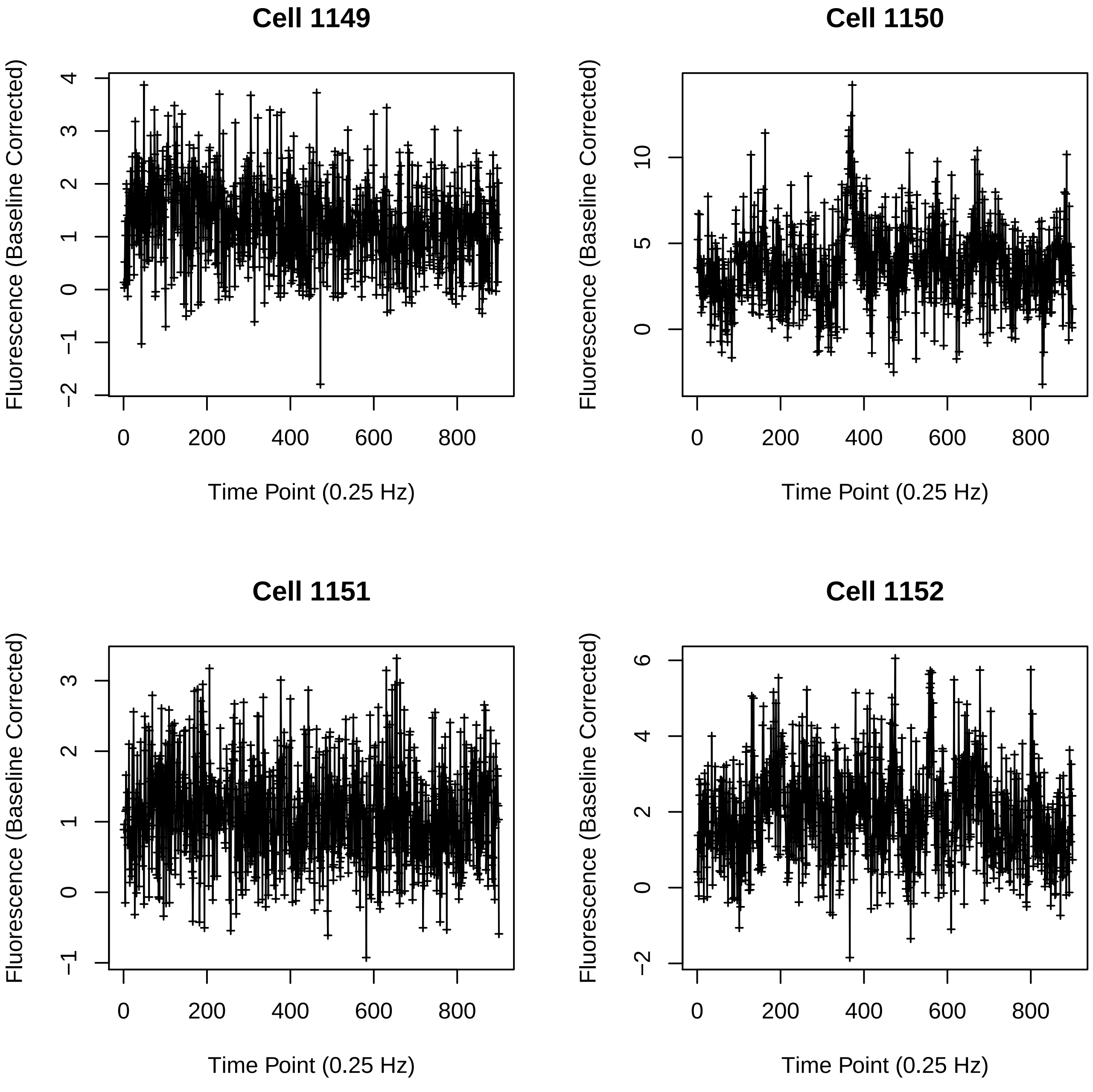
Cell 1149
Cell 1150
Cell 1151
Cell 1152
Fluorescence (Baseline Corrected)
Time Point (0.25 Hz)
−2
−1
0
1
2
3
4
5
6
10
200
400
600
800

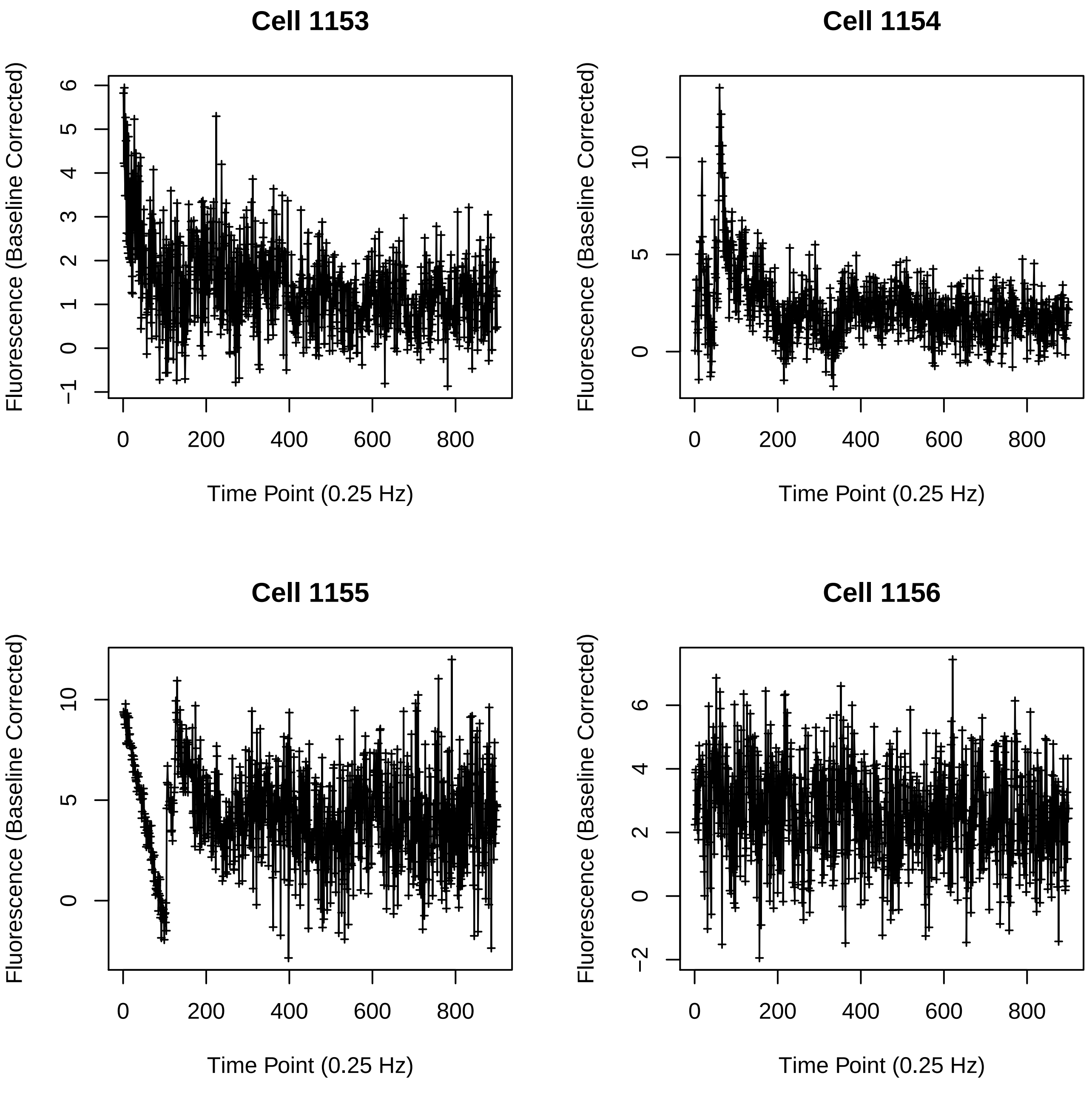
Cell 1153
Cell 1154
Cell 1155
Cell 1156
Fluorescence (Baseline Corrected)
Time Point (0.25 Hz)
0
200
400
600
800

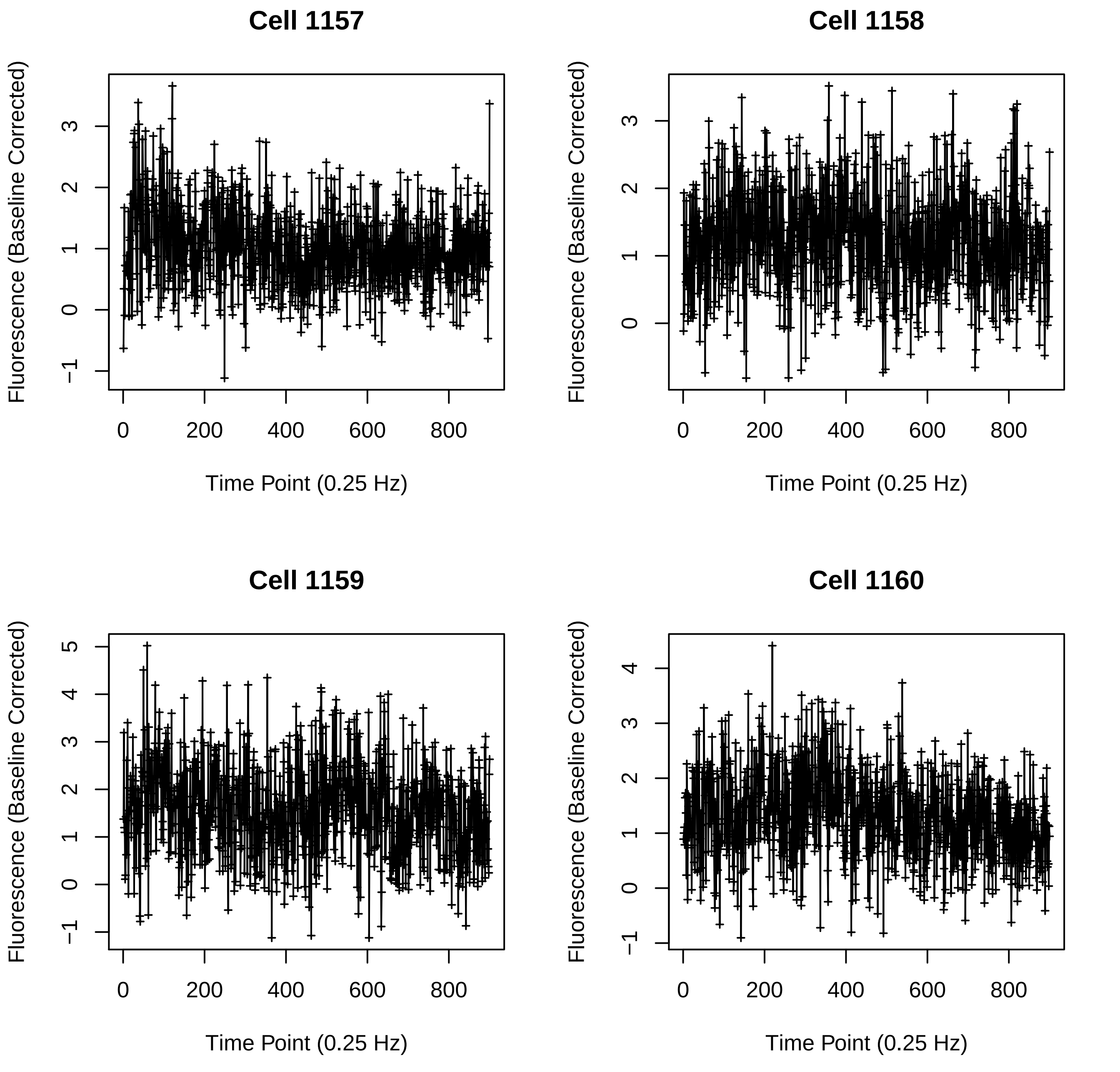

Cell 1157
Cell 1158
Cell 1159
Cell 1160
Fluorescence (Baseline Corrected)
Time Point (0.25 Hz)

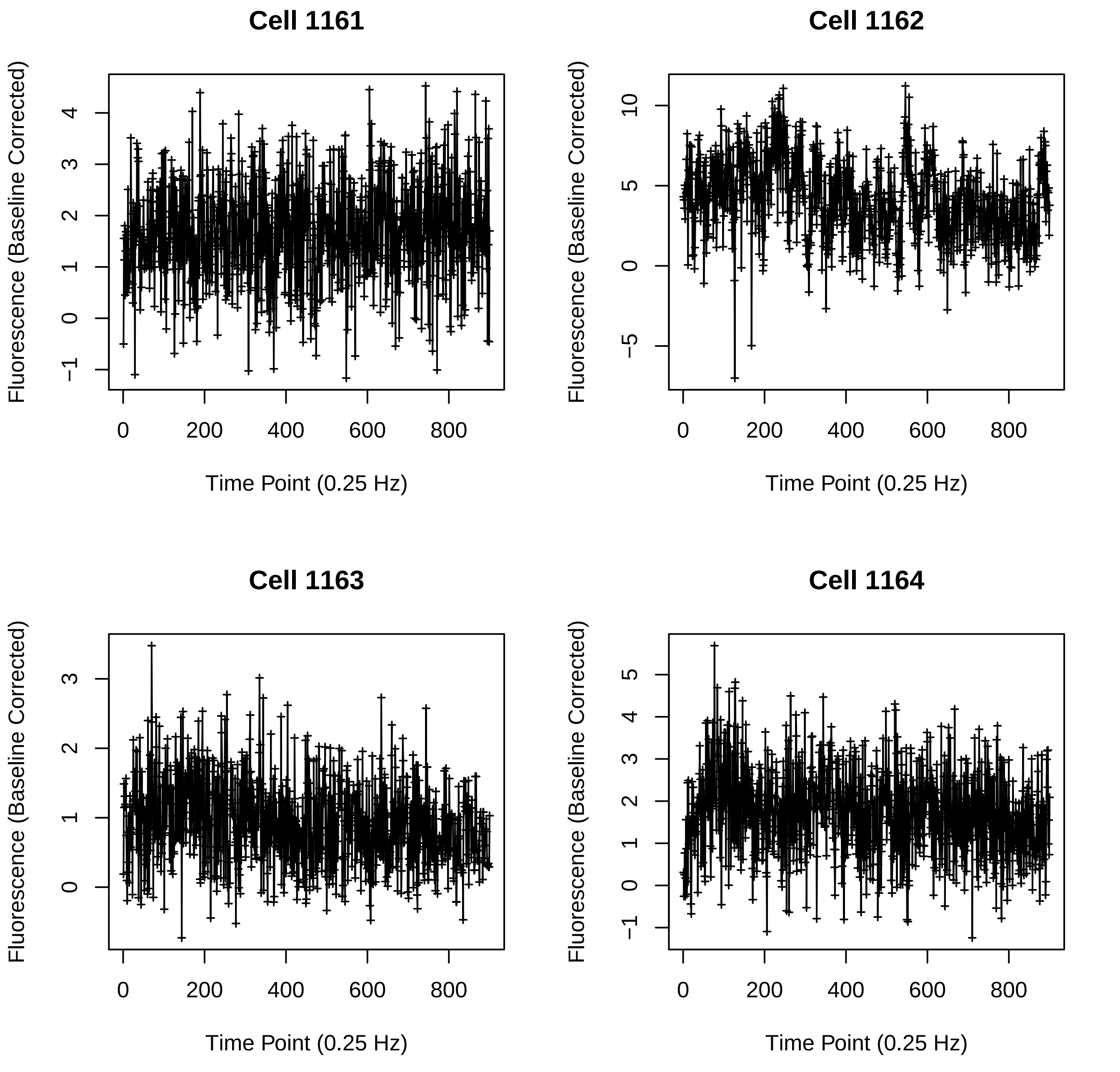

Cell 1161
Cell 1162
Cell 1163
Cell 1164
Fluorescence (Baseline Corrected)
Time Point (0.25 Hz)
0
200
400
600
800
−1
0
1
2
3
4
5
10
−5

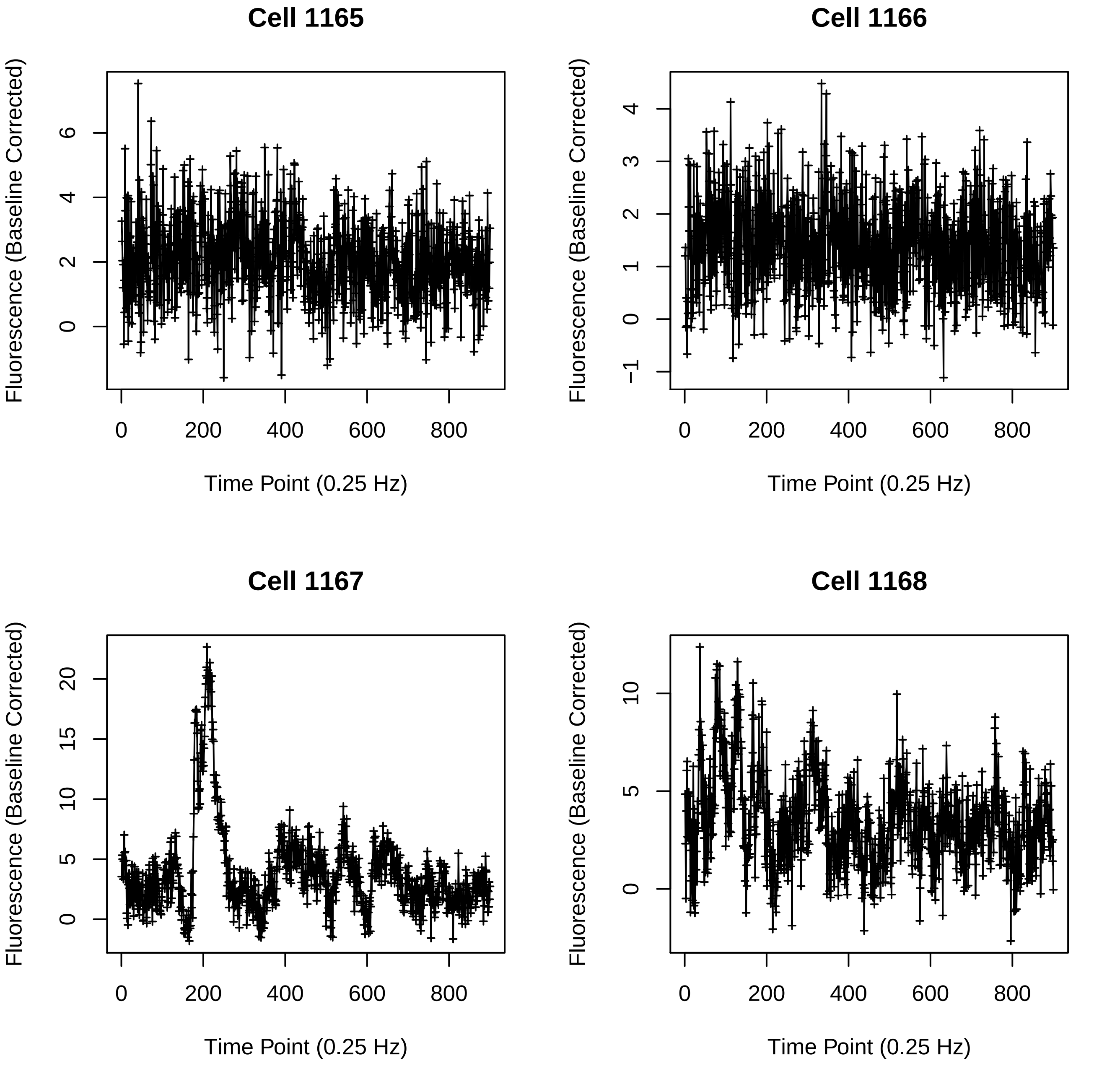
Cell 1165
Fluorescence (Baseline Corrected)
0
2
4
6
0
200
400
600
800
Time Point (0.25 Hz)
Cell 1166
Fluorescence (Baseline Corrected)
−1
0
1
2
3
4
0
200
400
600
800
Time Point (0.25 Hz)
Cell 1167
Fluorescence (Baseline Corrected)
0
5
10
15
20
0
200
400
600
800
Time Point (0.25 Hz)
Cell 1168
Fluorescence (Baseline Corrected)
0
5
10
0
200
400
600
800
Time Point (0.25 Hz)

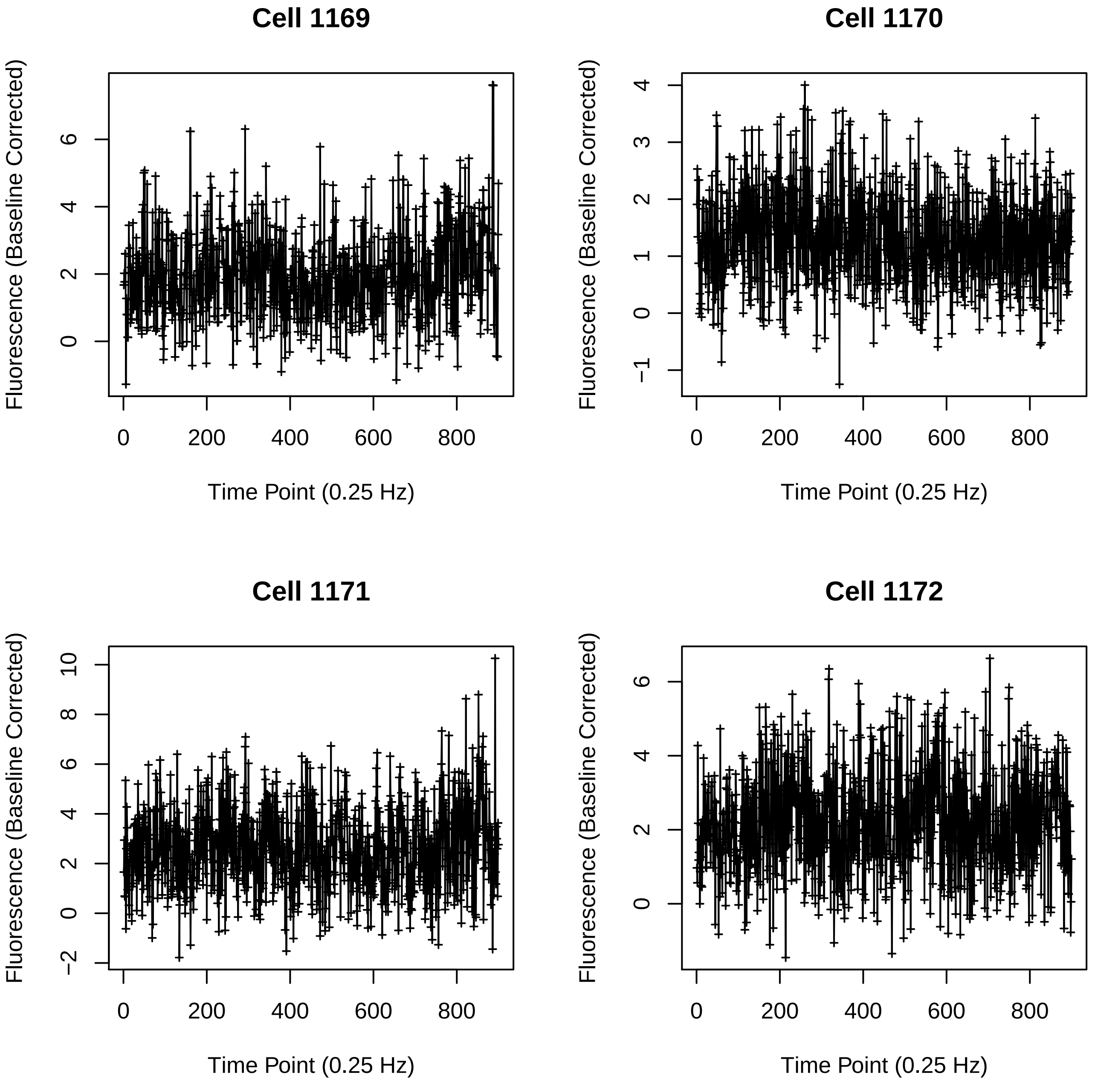
Cell 1169
Cell 1170
Cell 1171
Cell 1172
Fluorescence (Baseline Corrected)
Time Point (0.25 Hz)
0
200
400
600
800
-2
0
2
4
6
8
10
-1
1
3

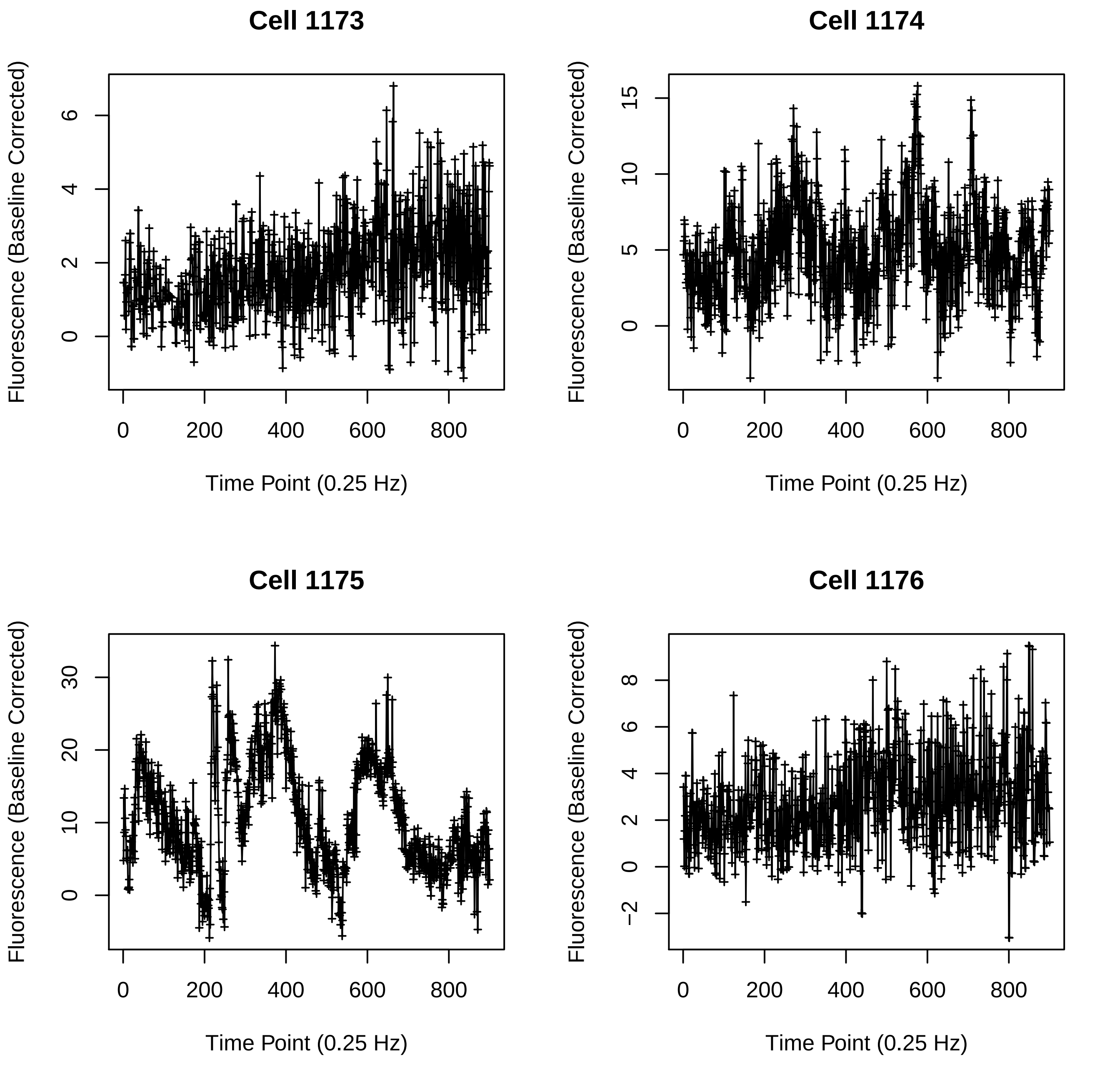
Cell 1173
Cell 1174
Cell 1175
Cell 1176
Fluorescence (Baseline Corrected)
Time Point (0.25 Hz)

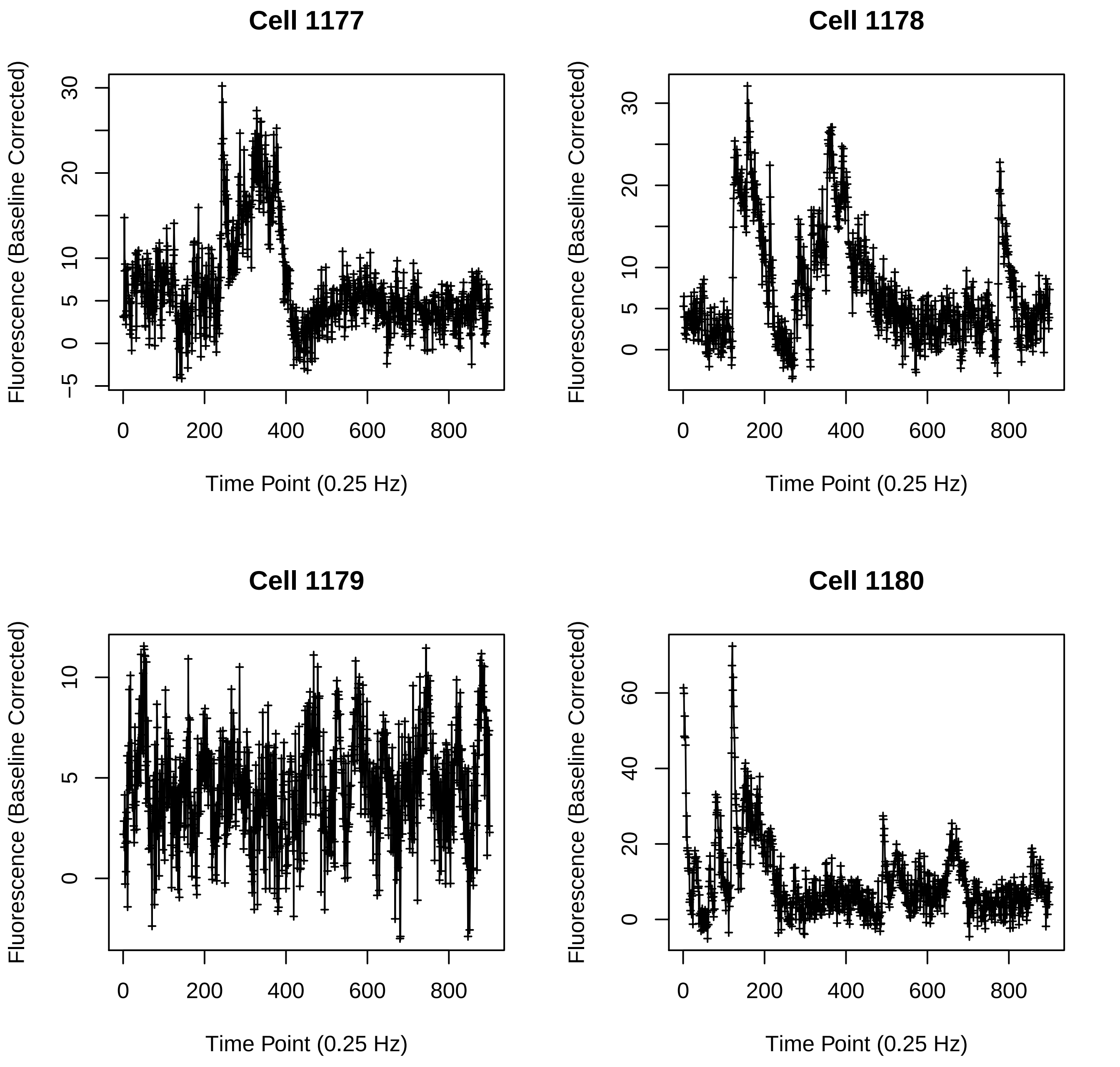
Cell 1177
Cell 1178
Cell 1179
Cell 1180
Fluorescence (Baseline Corrected)
Time Point (0.25 Hz)
0
200
400
600
800
−5
0
5
10
20
30
40
60

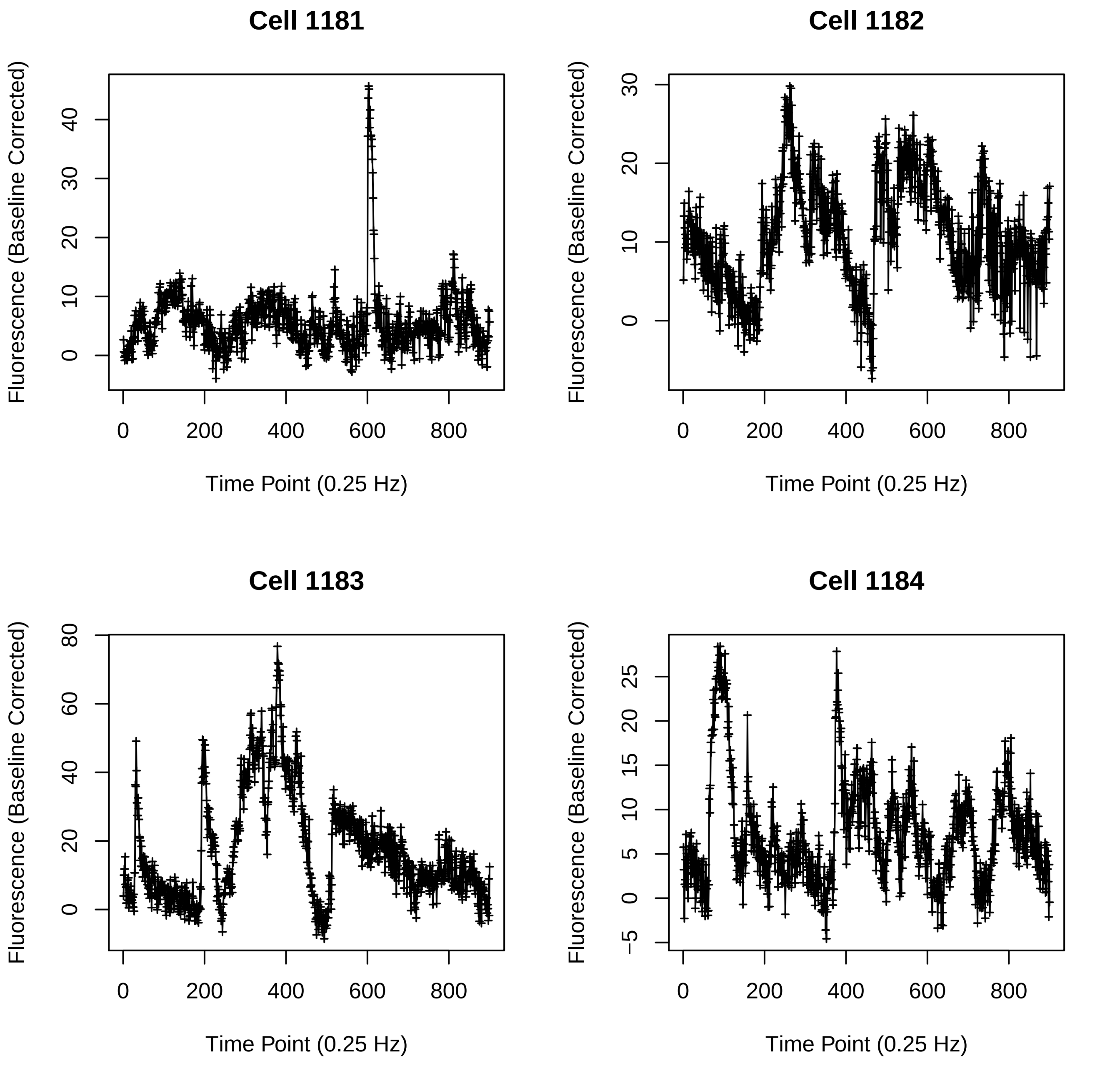

Cell 1181
Cell 1182
Cell 1183
Cell 1184
Fluorescence (Baseline Corrected)
Time Point (0.25 Hz)
0
200
400
600
800
-5
0
5
10
15
20
25
30
40
60
80

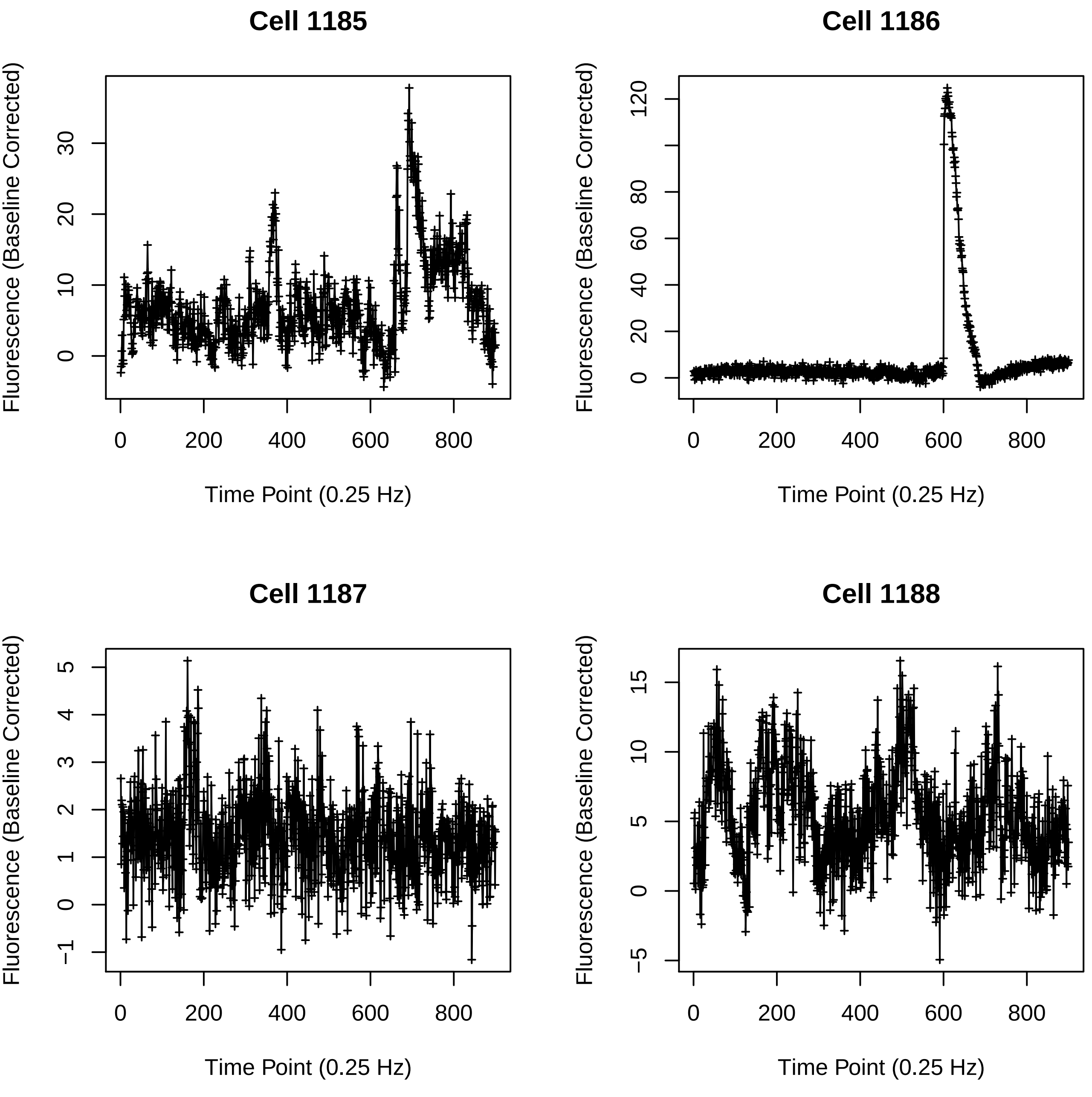

Cell 1185
Cell 1186
Cell 1187
Cell 1188
Fluorescence (Baseline Corrected)
Time Point (0.25 Hz)
0
200
400
600
800
10
20
30
40
60
80
120
−1
1
2
3
4
5
15
−5

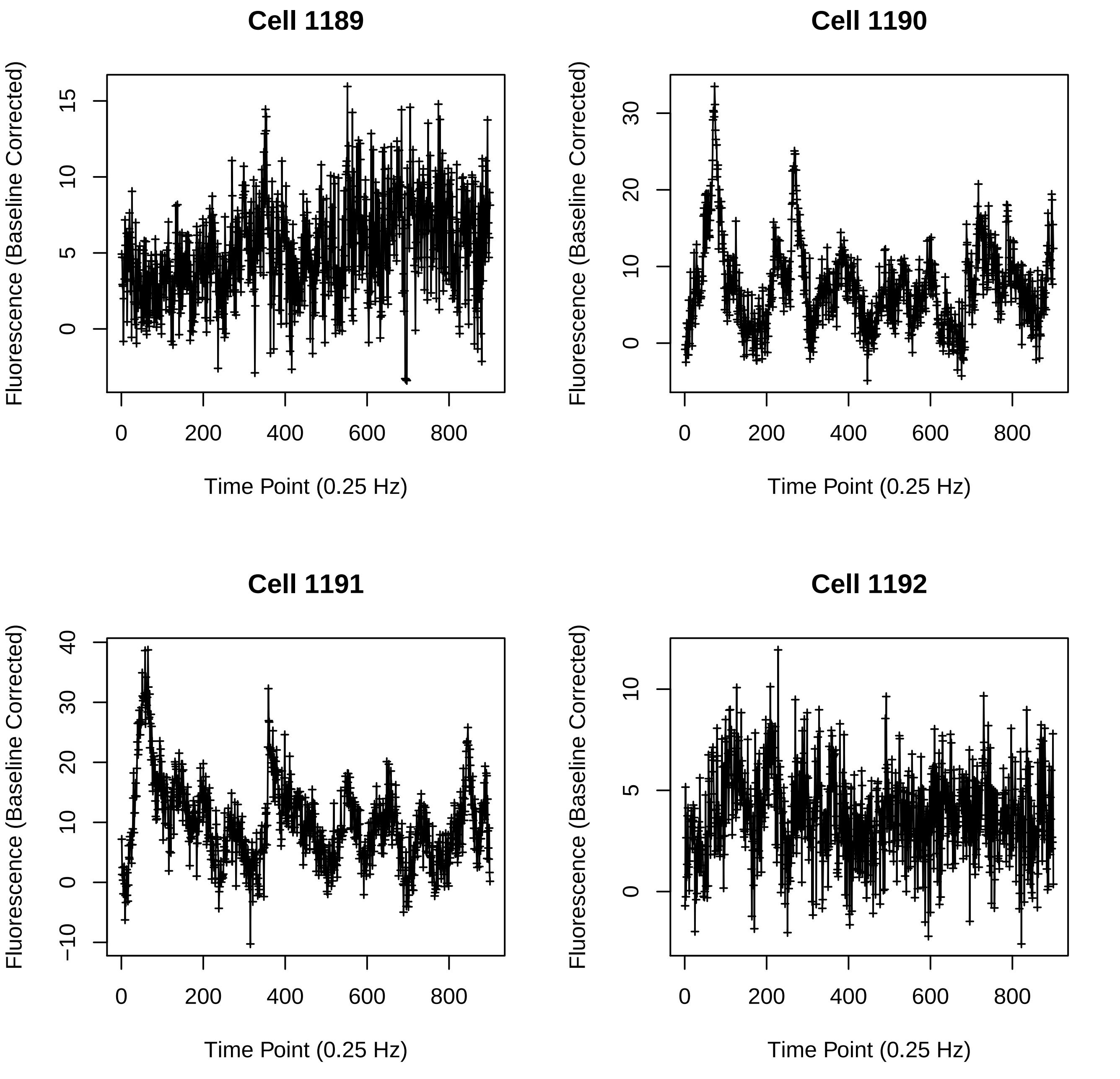
Cell 1189
Cell 1190
Cell 1191
Cell 1192
Fluorescence (Baseline Corrected)
Time Point (0.25 Hz)
0
200
400
600
800
-10
0
5
10
15
20
30
40

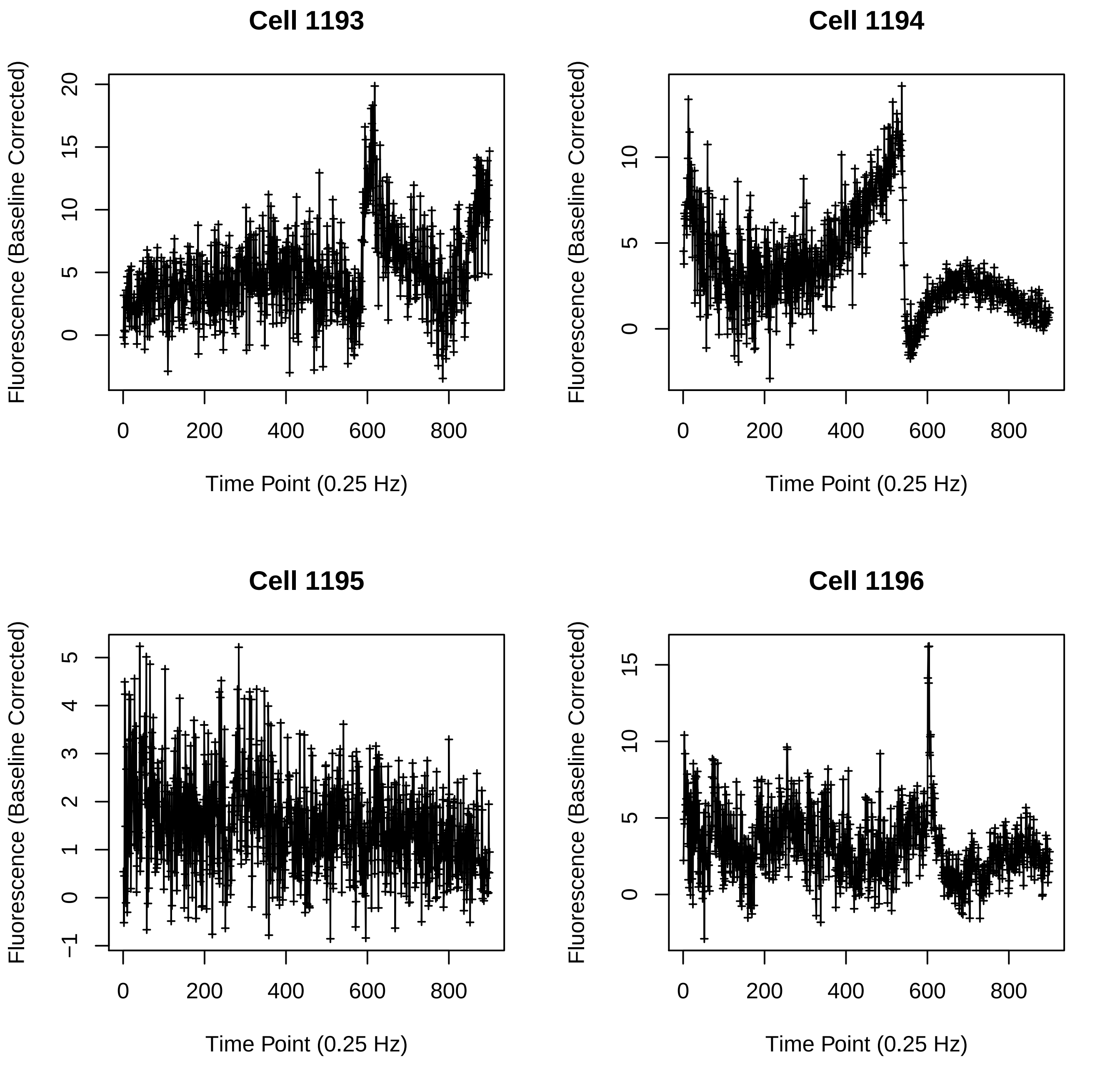

Cell 1193
Cell 1194
Cell 1195
Cell 1196
Fluorescence (Baseline Corrected)
Time Point (0.25 Hz)
0
200
400
600
800

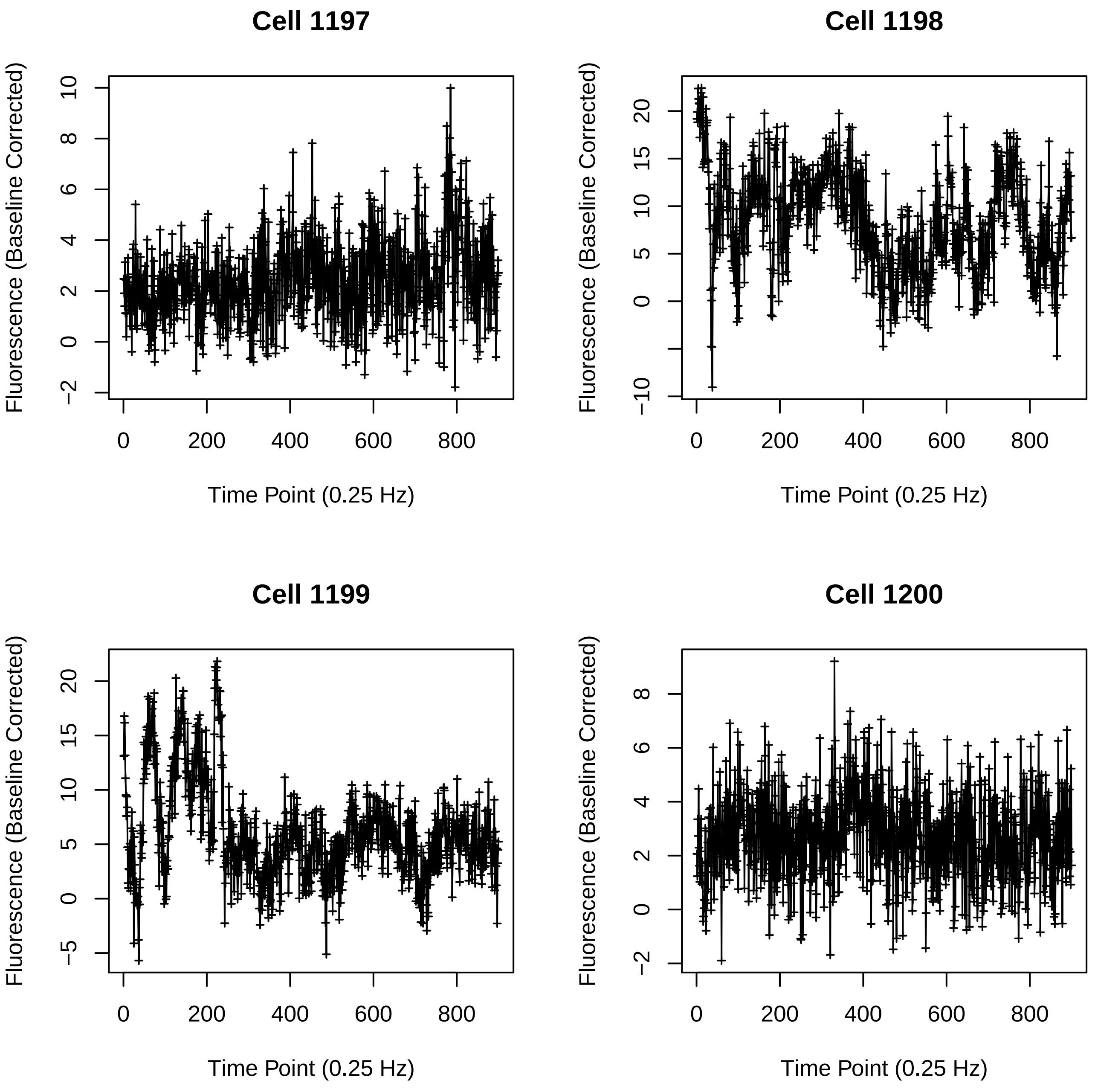
Cell 1197
Cell 1198
Cell 1199
Cell 1200
Fluorescence (Baseline Corrected)
Time Point (0.25 Hz)
0
200
400
600
800
−2
0
2
4
6
8
10
−10
−5
5
15
20

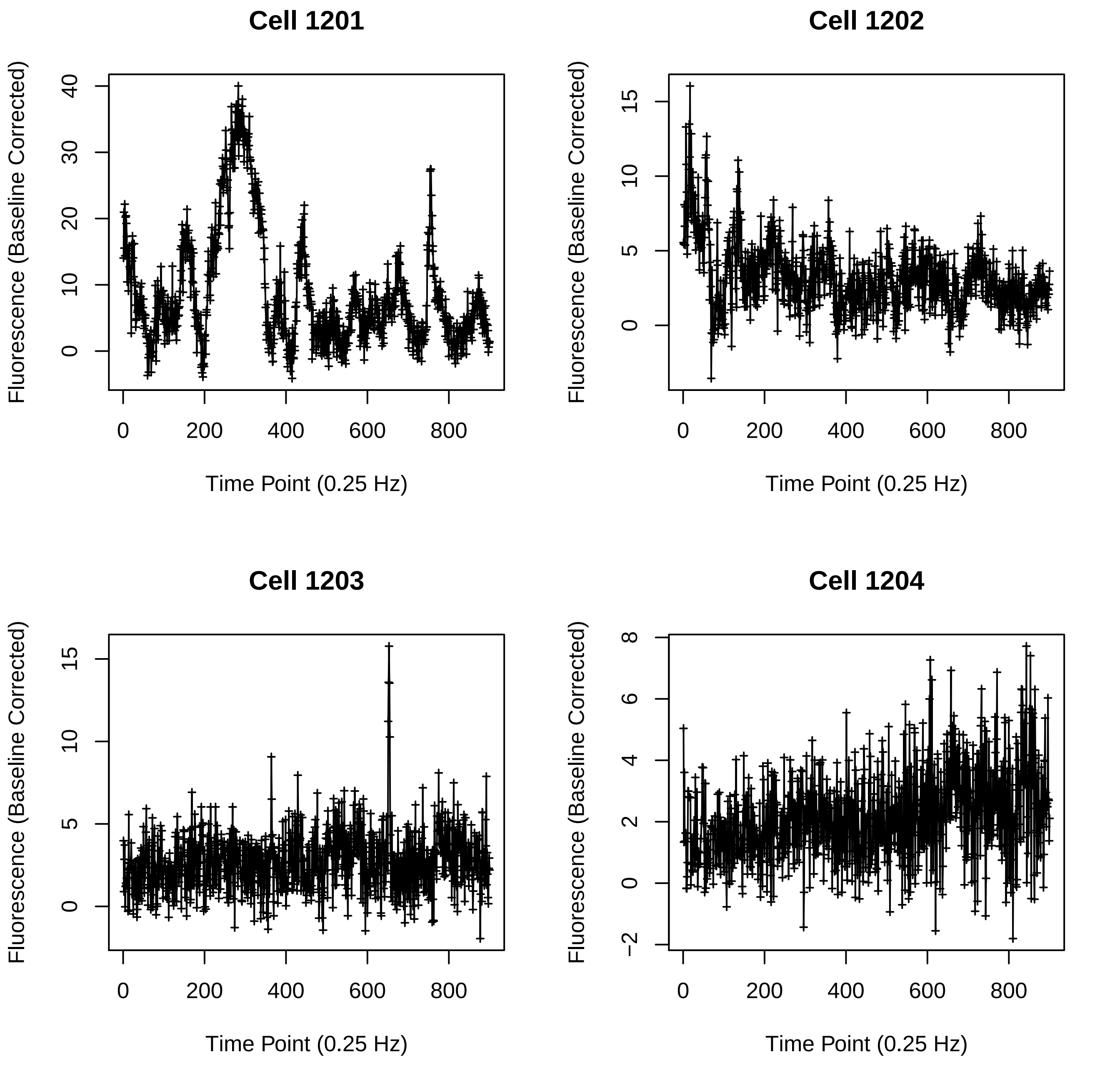

Cell 1201
Cell 1202
Cell 1203
Cell 1204
Fluorescence (Baseline Corrected)
Time Point (0.25 Hz)

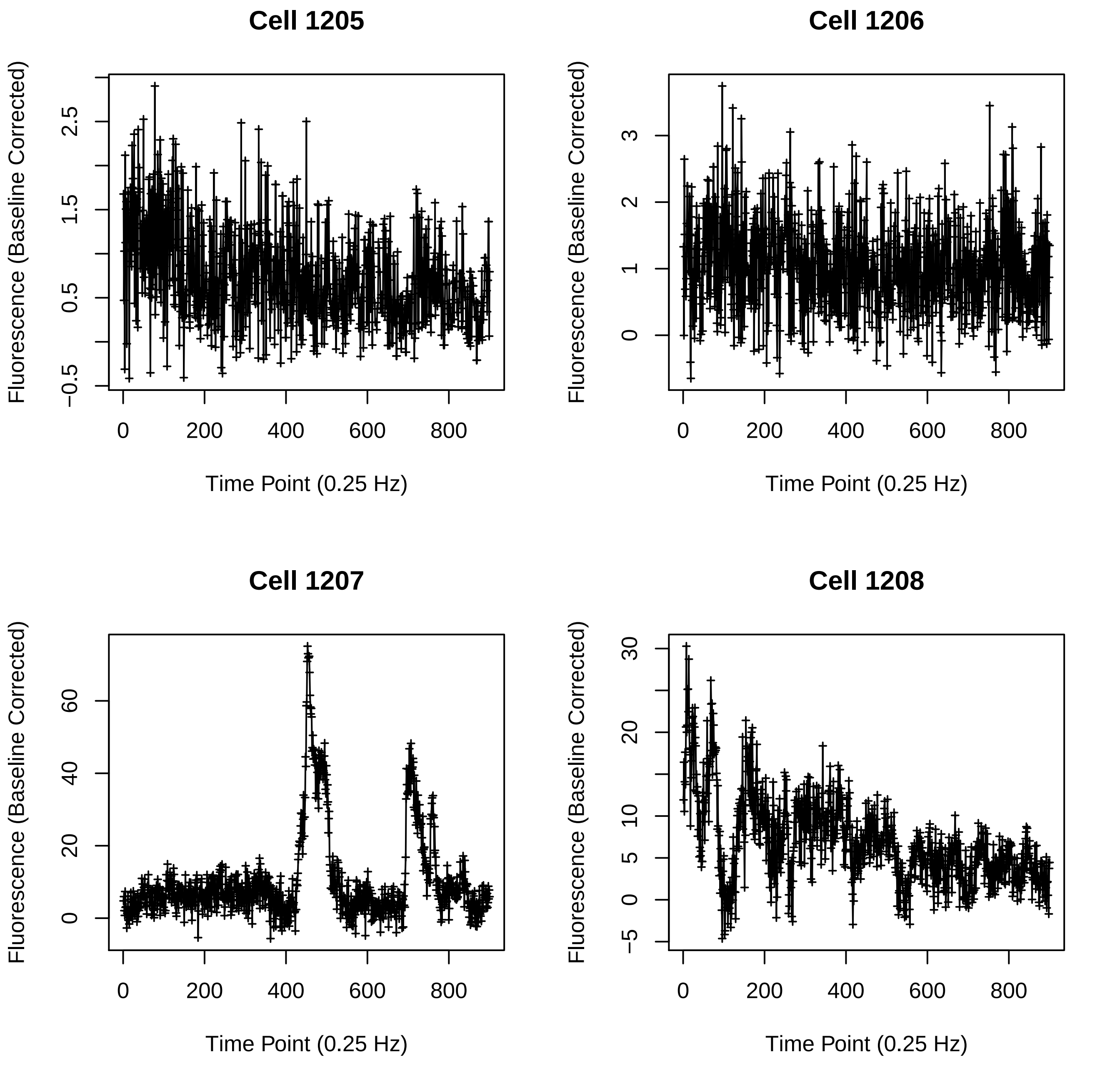
Cell 1205
Cell 1206
Cell 1207
Cell 1208
Fluorescence (Baseline Corrected)
Time Point (0.25 Hz)
−0.5
0.5
1.5
2.5
0
1
2
3
20
40
60
−5
5
10
30
200
400
600
800

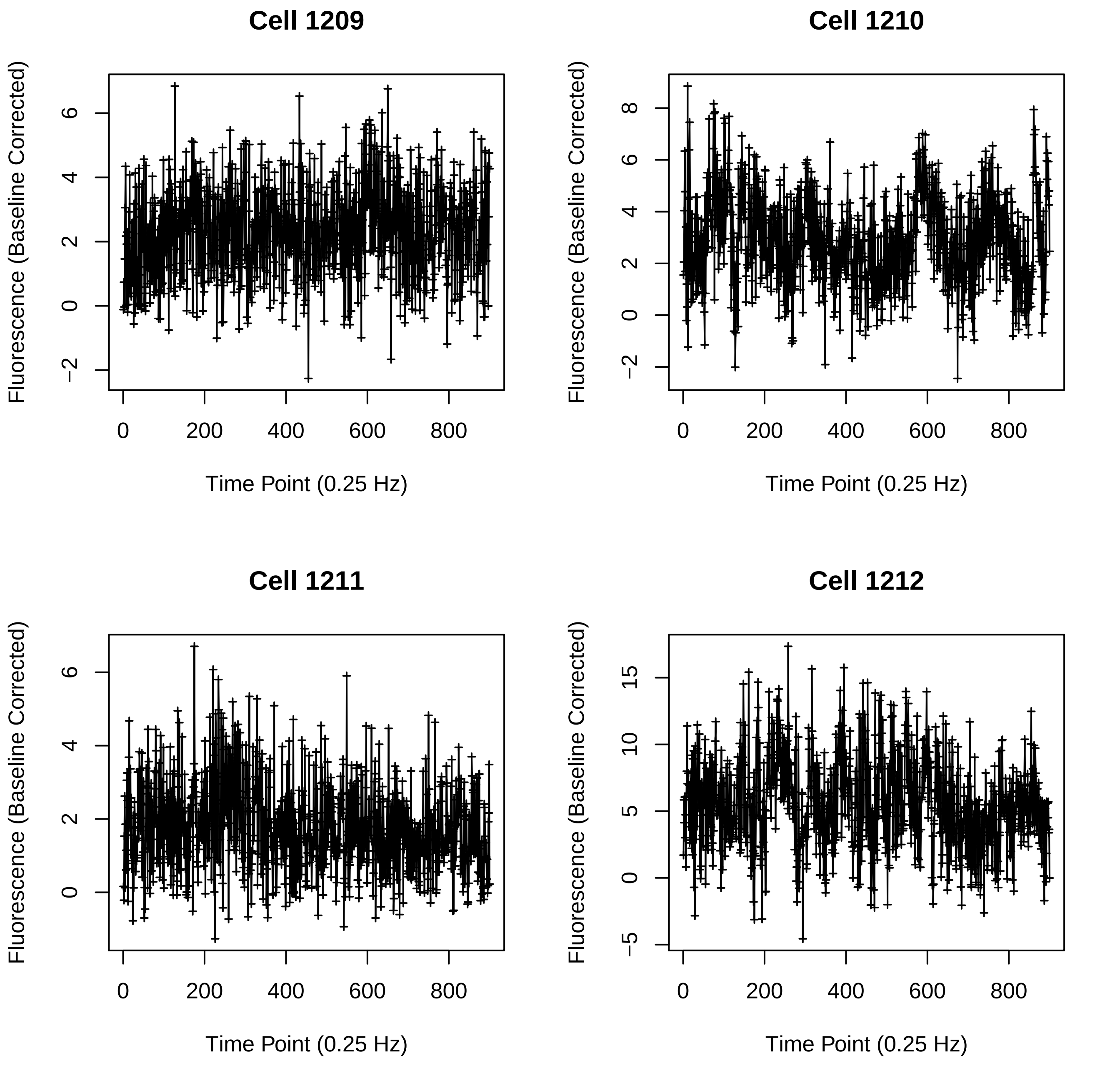
Cell 1209
Cell 1210
Cell 1211
Cell 1212
Fluorescence (Baseline Corrected)
Time Point (0.25 Hz)
0
200
400
600
800
−2
2
4
6
8
−5
5
10
15

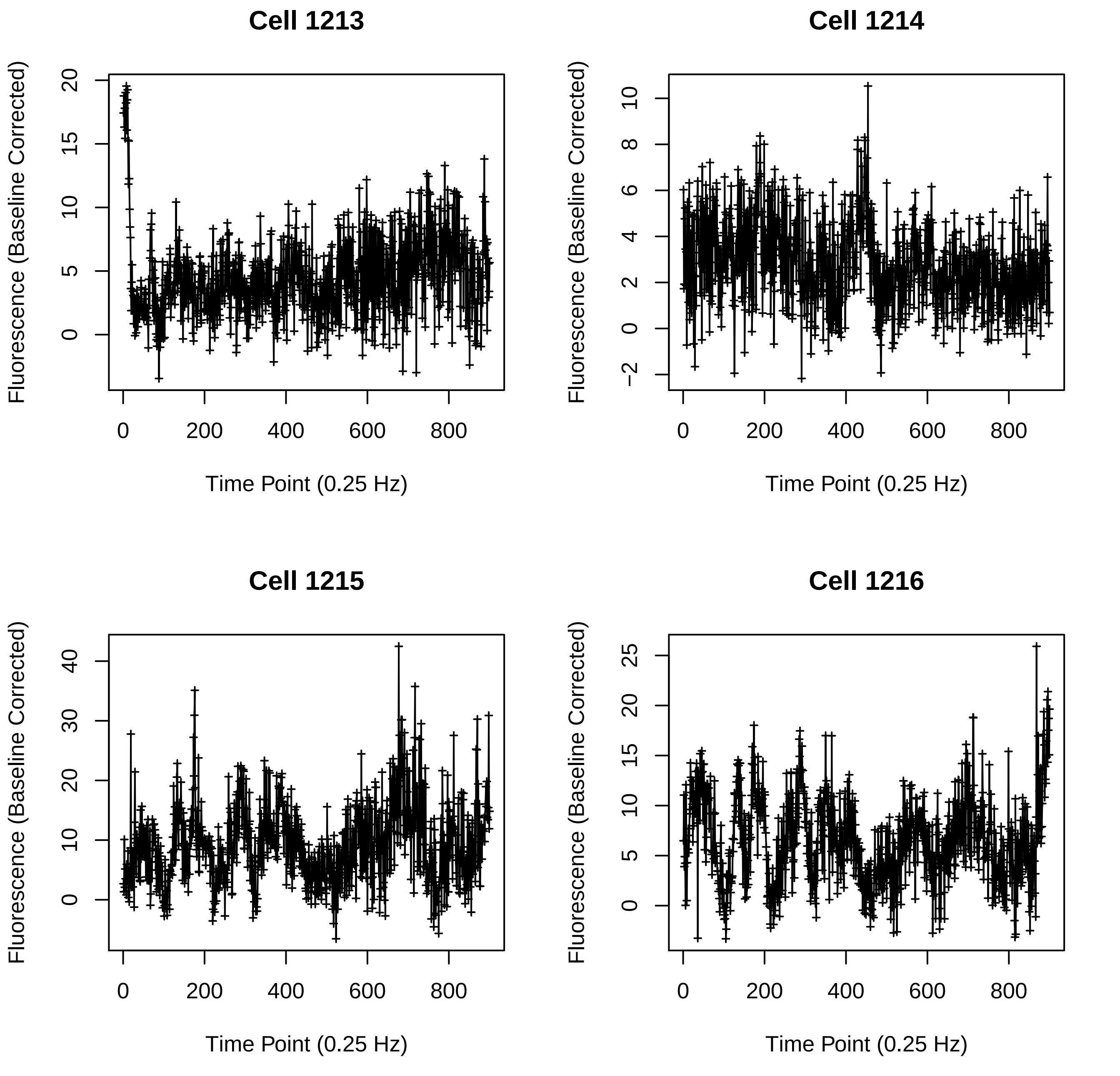
Cell 1213
Cell 1214
Cell 1215
Cell 1216
Fluorescence (Baseline Corrected)
Time Point (0.25 Hz)
0
200
400
600
800
−2
0
2
4
6
8
10
5
15
20
25
30
40

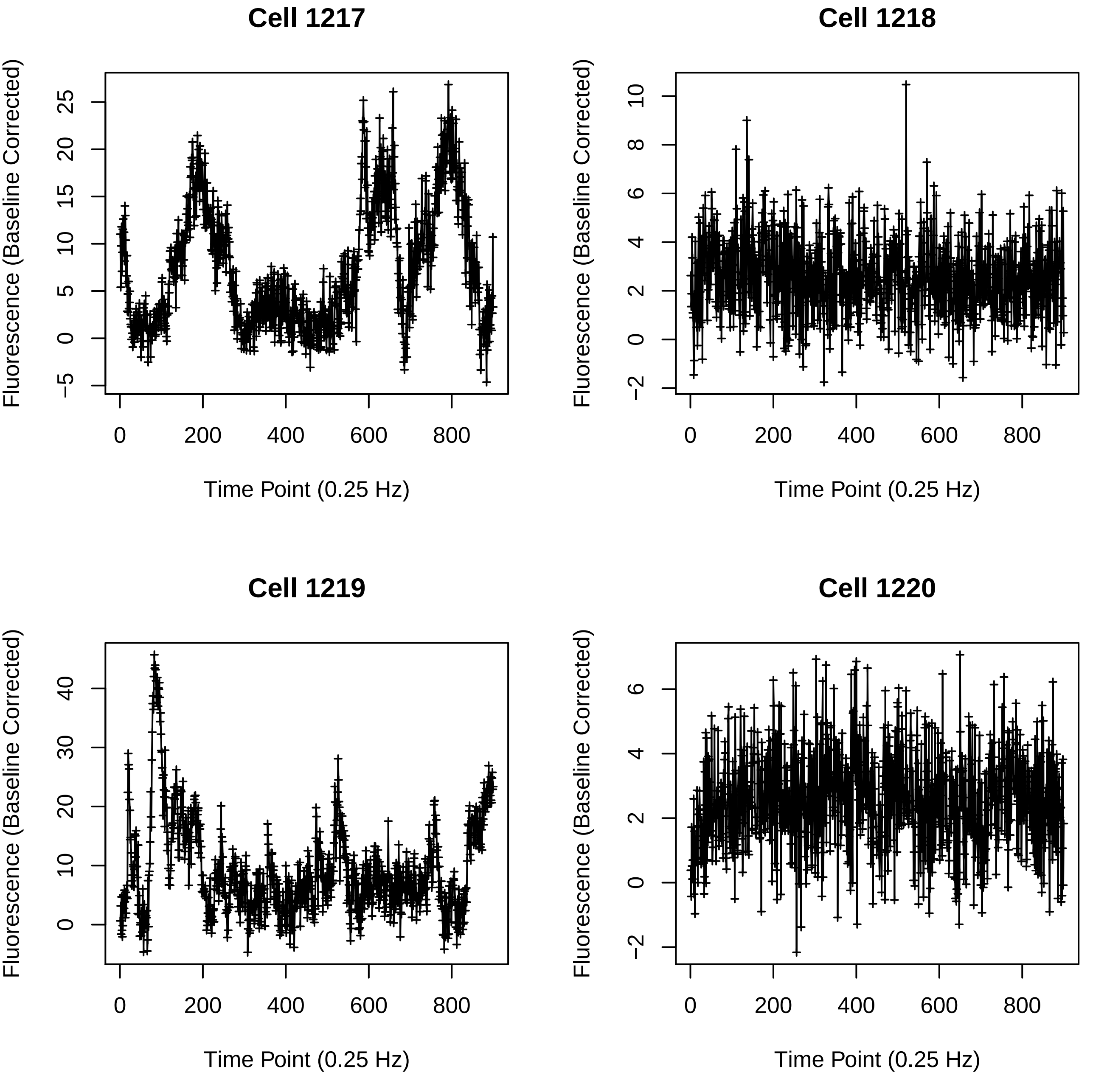

Cell 1217
Cell 1218
Cell 1219
Cell 1220
Fluorescence (Baseline Corrected)
Time Point (0.25 Hz)
0
200
400
600
800
−5
5
10
15
20
25
−2
2
4
6
8
30
40

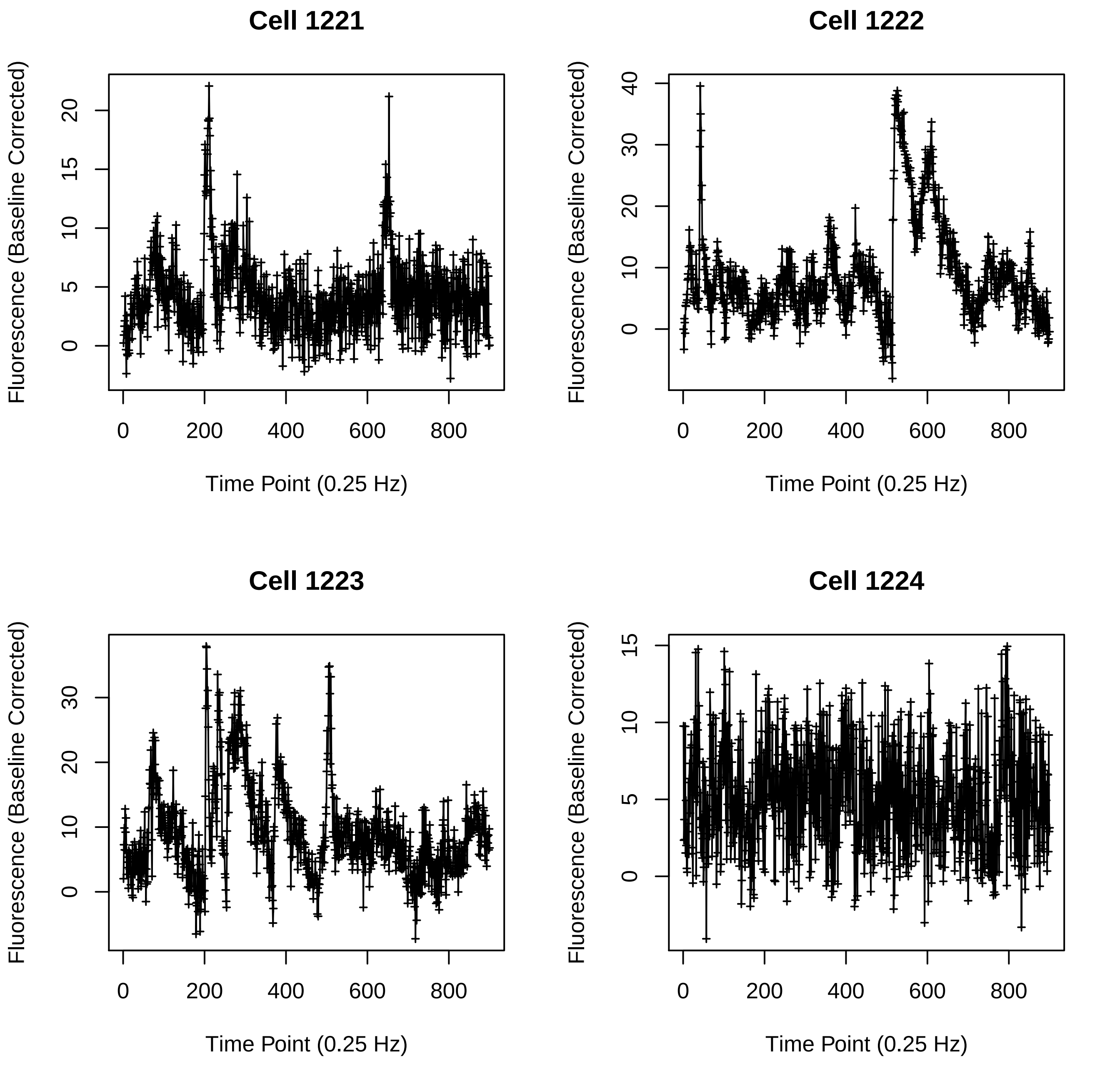

Cell 1221
Cell 1222
Cell 1223
Cell 1224
Fluorescence (Baseline Corrected)
Time Point (0.25 Hz)
0
5
10
15
20
30
40
200
400
600
800

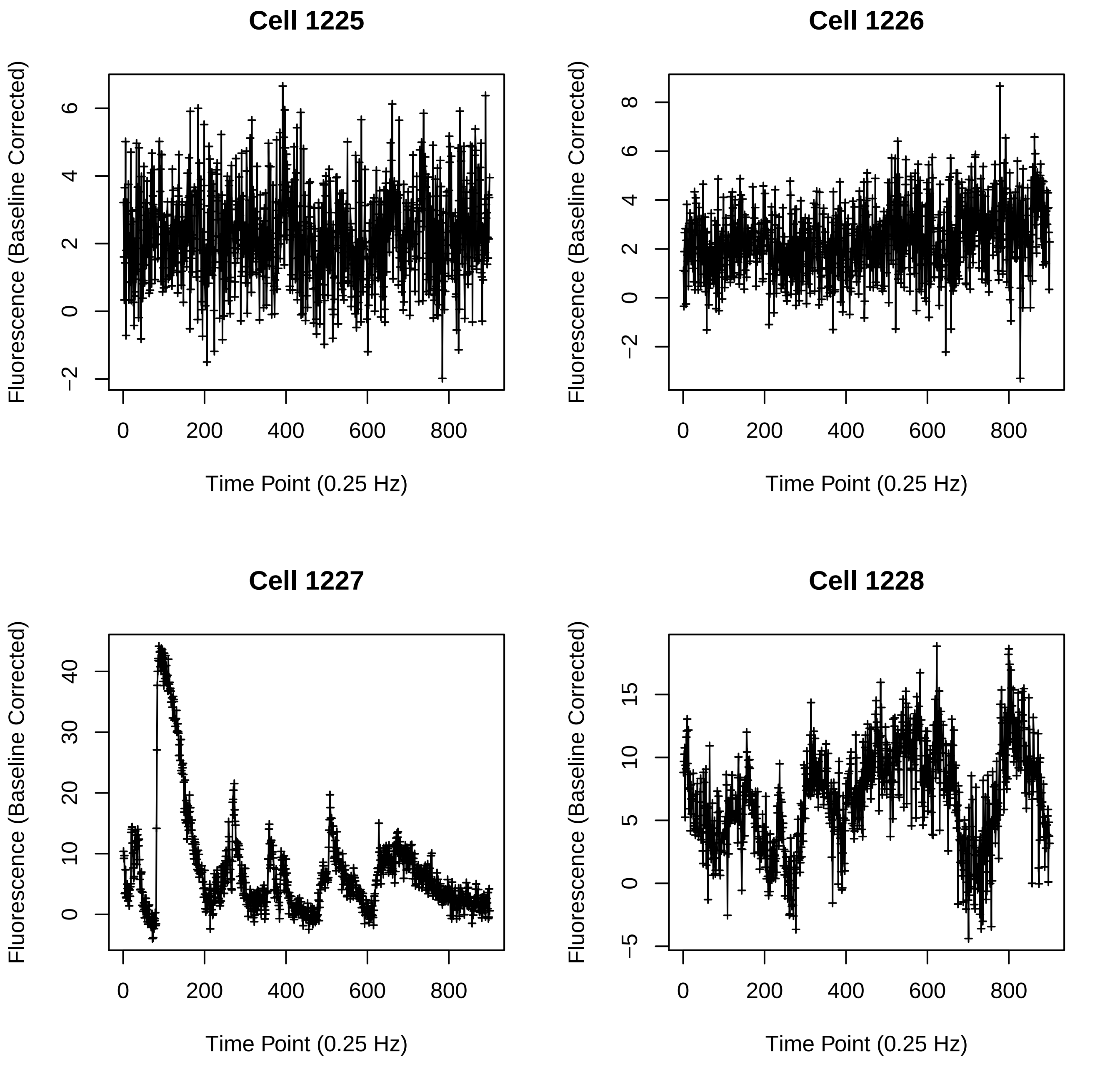
Cell 1225
Cell 1226
Cell 1227
Cell 1228
Fluorescence (Baseline Corrected)
Time Point (0.25 Hz)
0
200
400
600
800
−2
2
4
6
8
10
15
20
30
40
−5
5

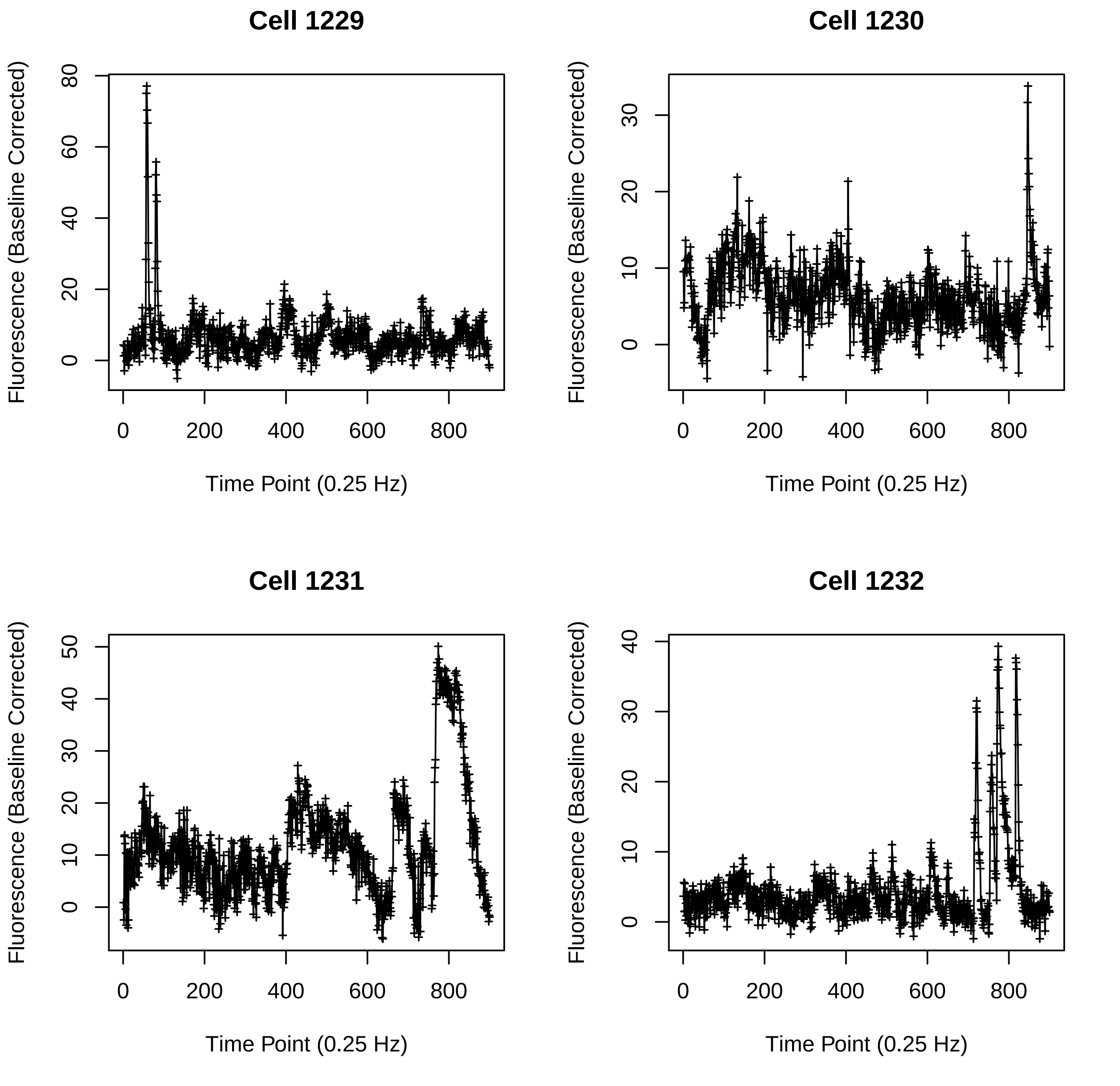

Cell 1229
Cell 1230
Cell 1231
Cell 1232
Fluorescence (Baseline Corrected)
Time Point (0.25 Hz)
0
200
400
600
800
20
40
60
80
10
30
50

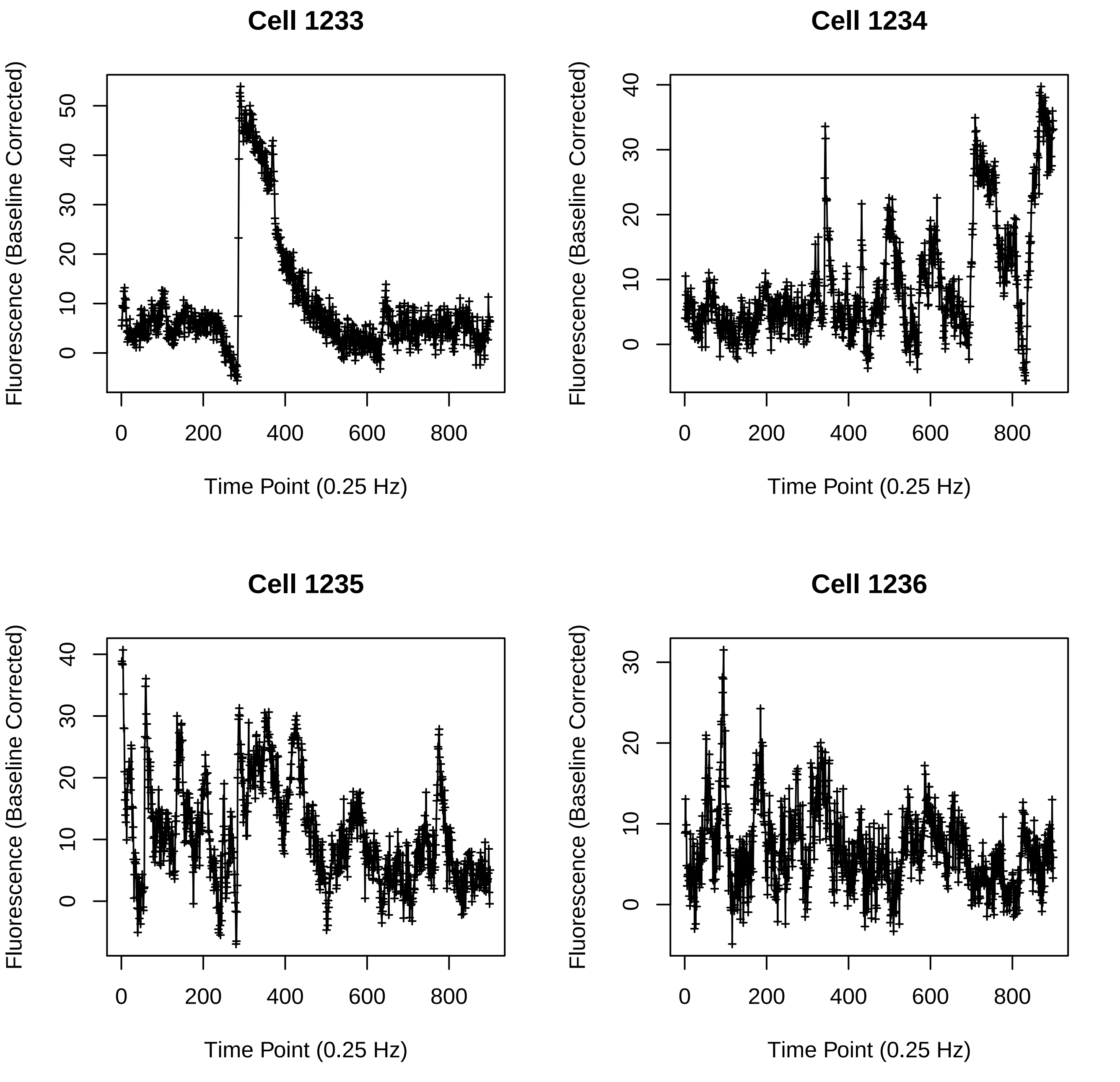
Cell 1233
Cell 1234
Cell 1235
Cell 1236
Fluorescence (Baseline Corrected)
Time Point (0.25 Hz)
0
200
400
600
800
0
10
20
30
40
50

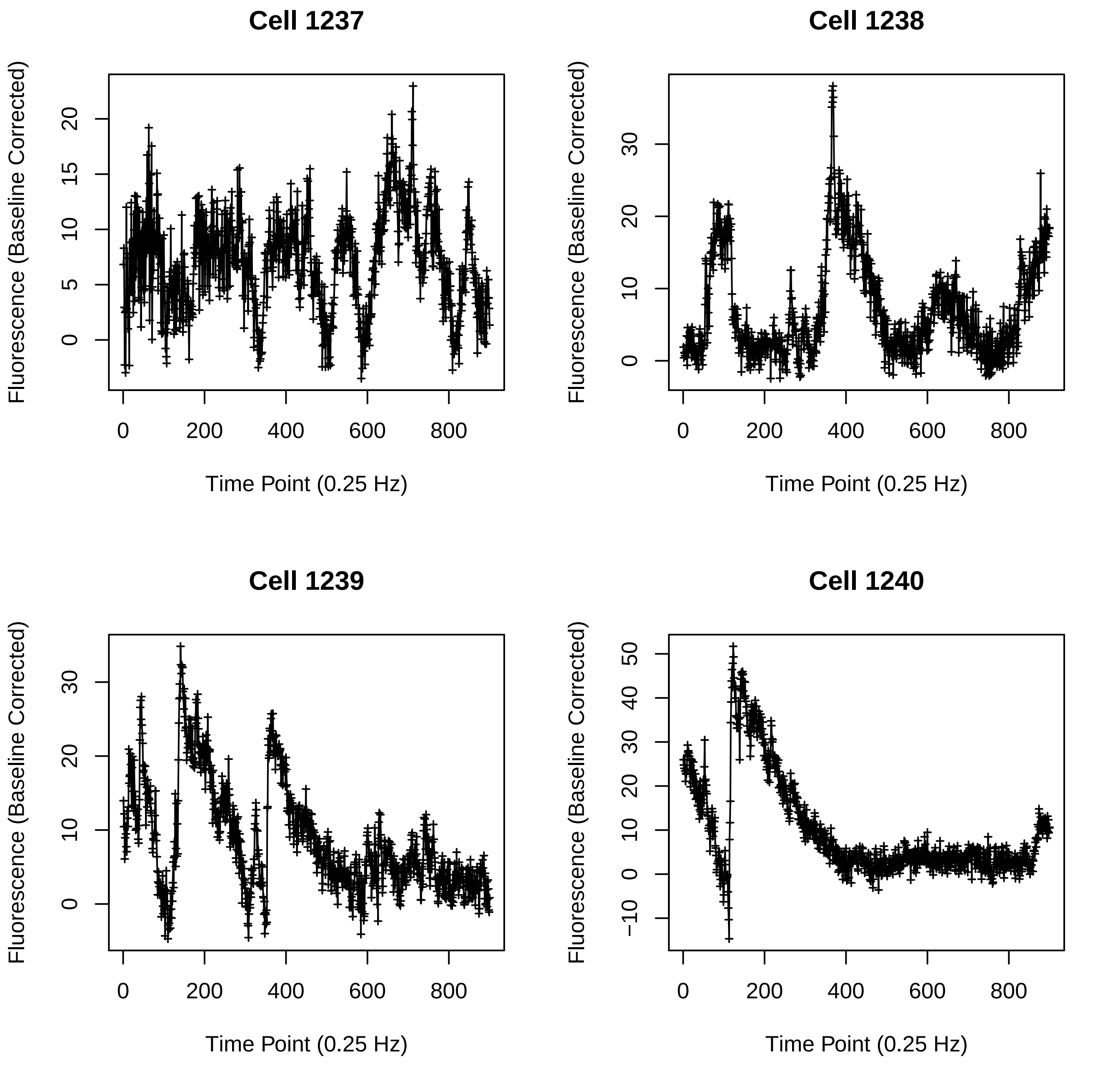

Cell 1237
Cell 1238
Cell 1239
Cell 1240
Fluorescence (Baseline Corrected)
Time Point (0.25 Hz)
0
200
400
600
800

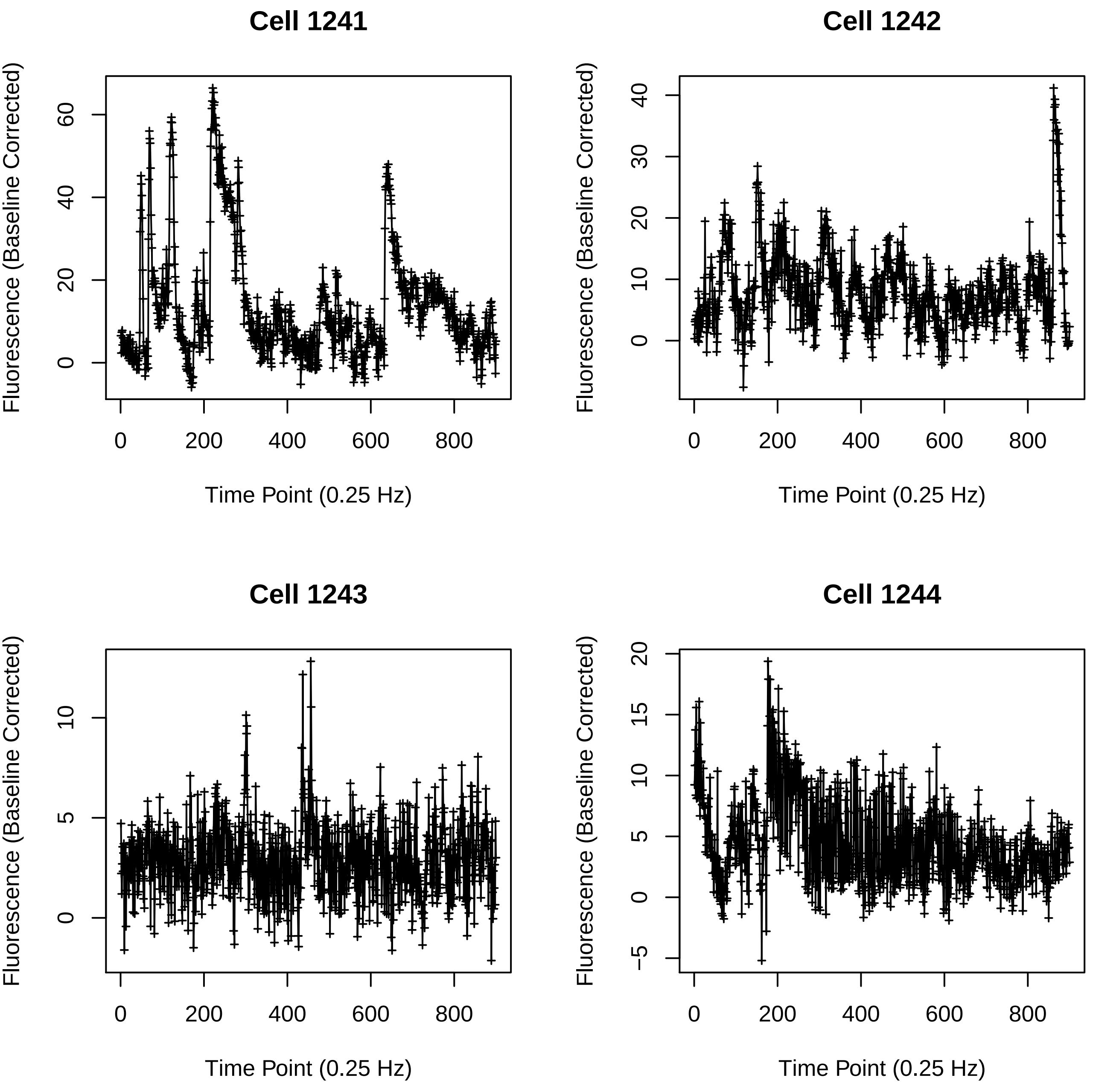
Cell 1241
Cell 1242
Cell 1243
Cell 1244
Fluorescence (Baseline Corrected)
Time Point (0.25 Hz)
0
200
400
600
800
20
40
60
10
30
5
15
−5

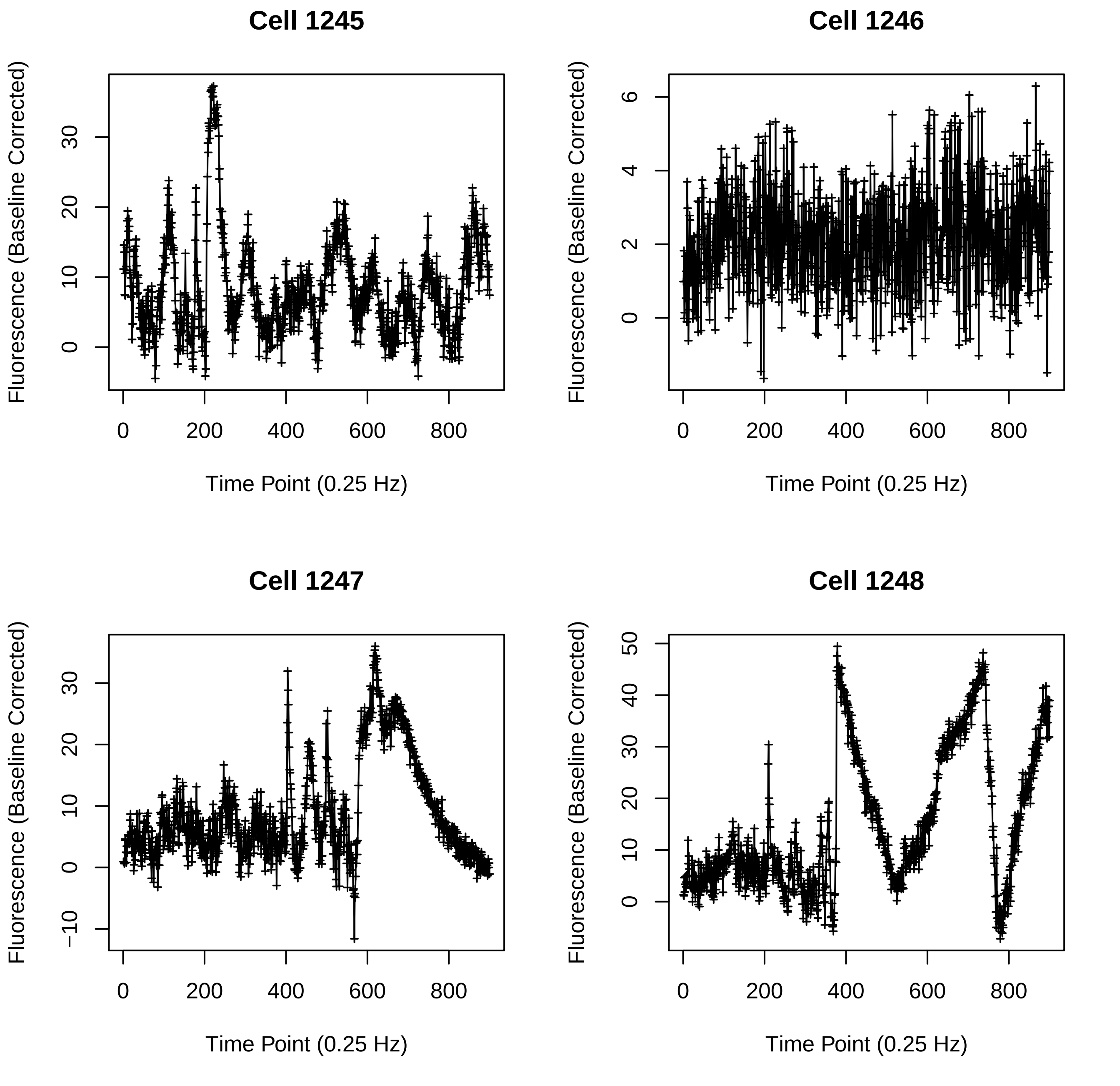

Cell 1245
Cell 1246
Cell 1247
Cell 1248
Fluorescence (Baseline Corrected)
Time Point (0.25 Hz)
0
200
400
600
800

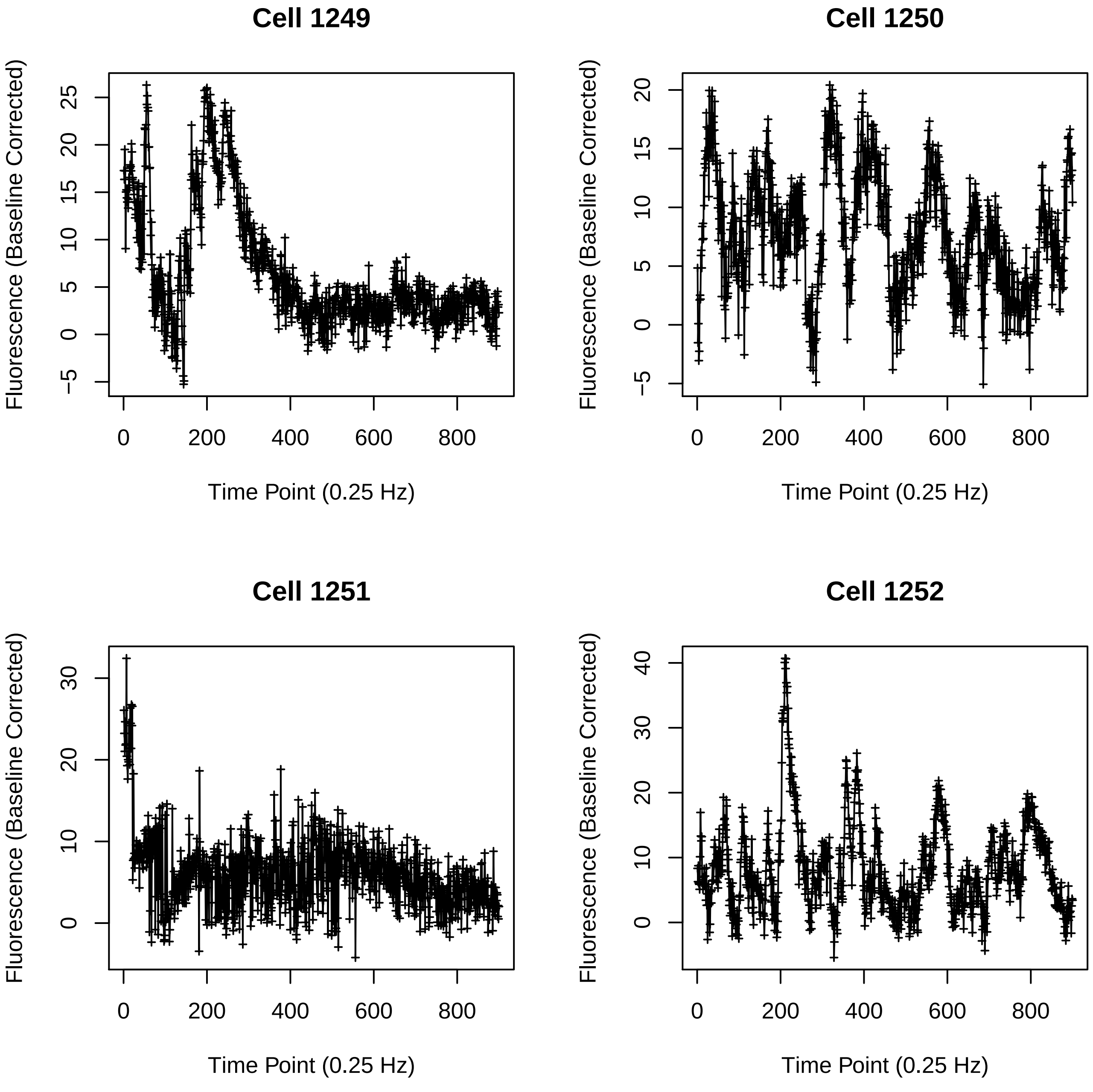
Cell 1249
Cell 1250
Cell 1251
Cell 1252
Fluorescence (Baseline Corrected)
Time Point (0.25 Hz)
0
200
400
600
800
−5
0
5
10
15
20
25
30
40

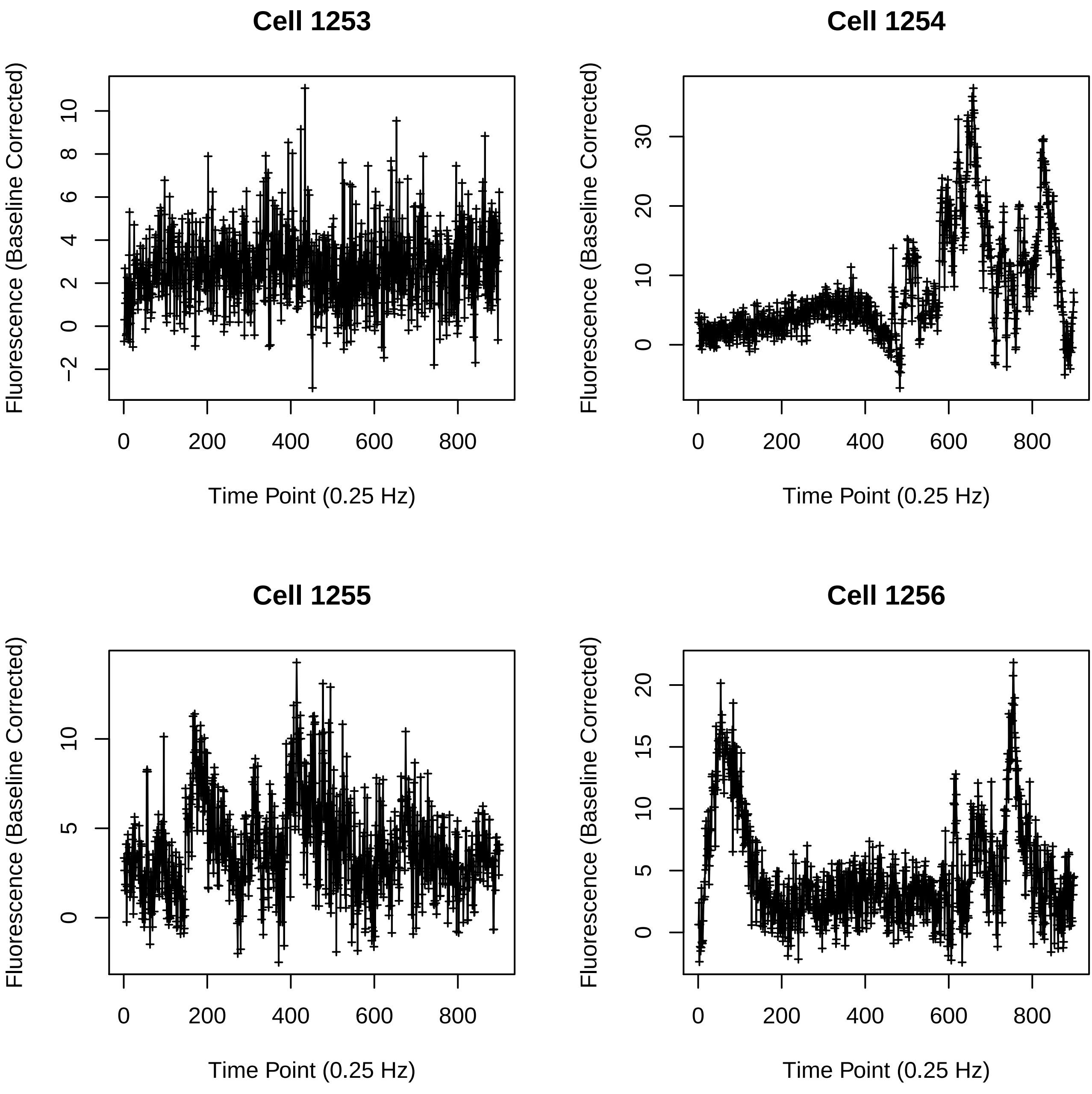

Cell 1253
Cell 1254
Cell 1255
Cell 1256
Fluorescence (Baseline Corrected)
Time Point (0.25 Hz)
−2
0
2
4
6
8
10
20
30
5
15
200
400
600
800

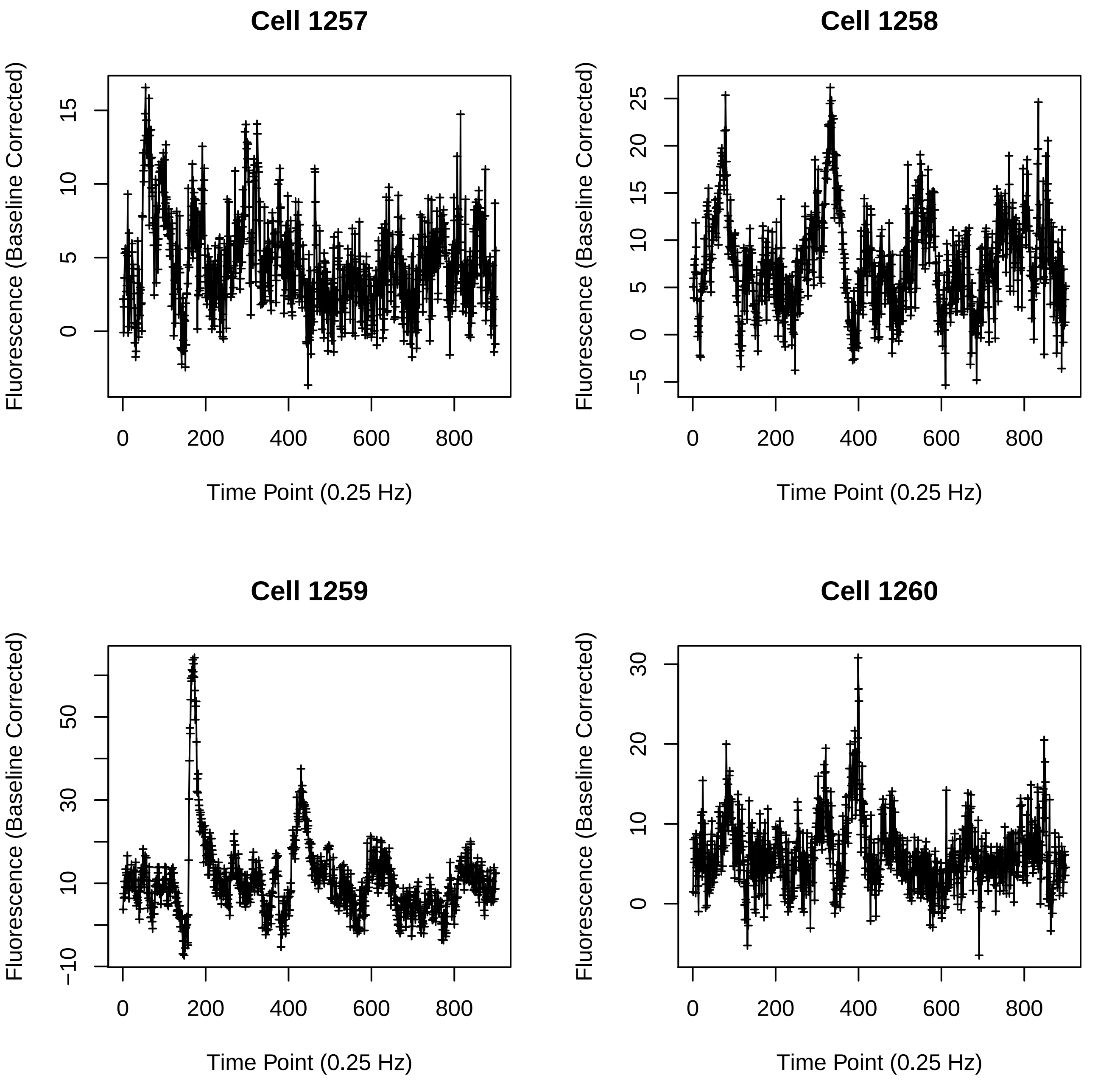
Cell 1257
Cell 1258
Cell 1259
Cell 1260
Fluorescence (Baseline Corrected)
Time Point (0.25 Hz)
0
200
400
600
800
−5
0
5
10
15
20
25
30
50
−10

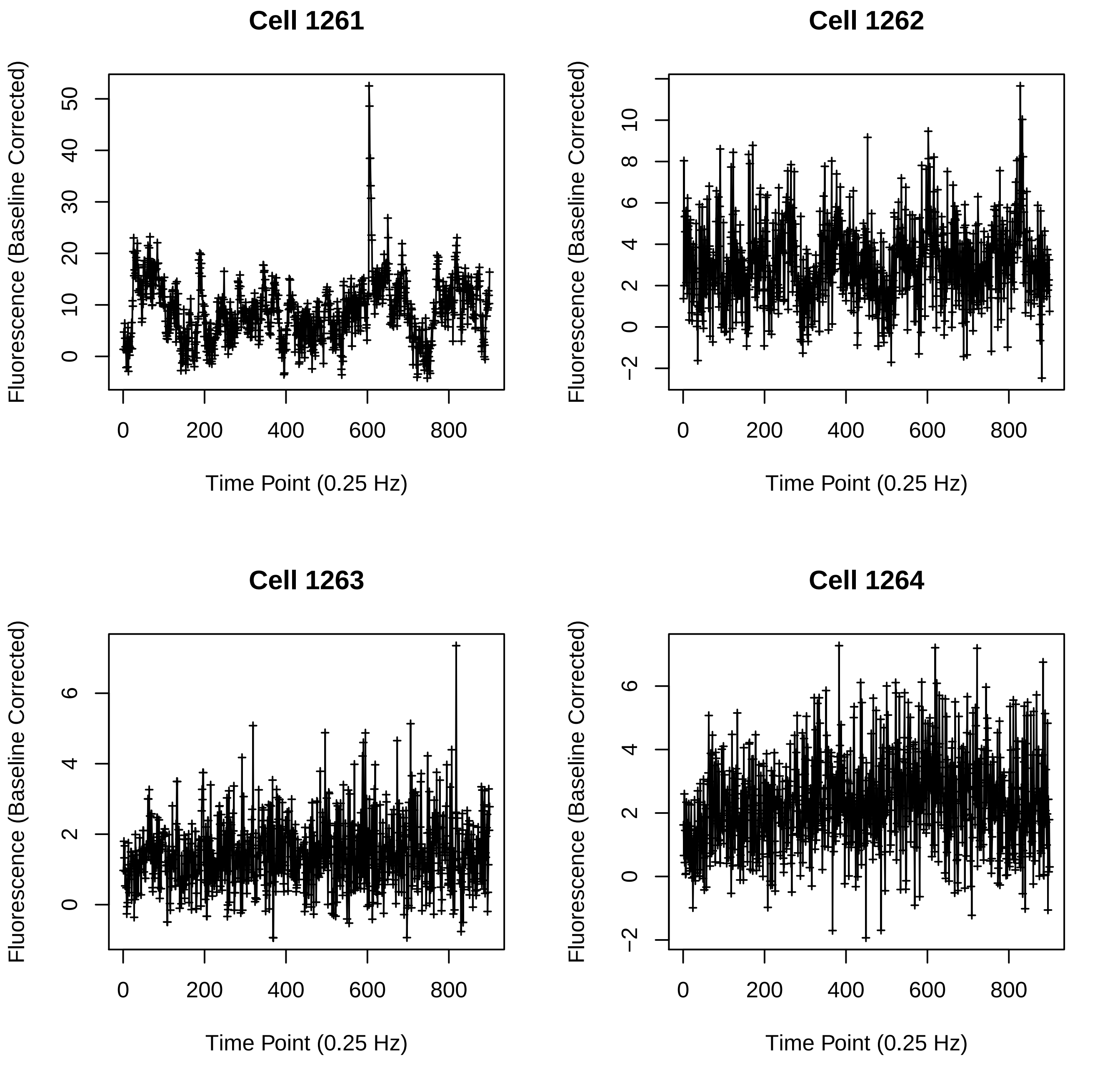
Cell 1261
Cell 1262
Cell 1263
Cell 1264
Fluorescence (Baseline Corrected)
Time Point (0.25 Hz)
0
10
20
30
40
50
−2
2
4
6
8
200
400
600
800

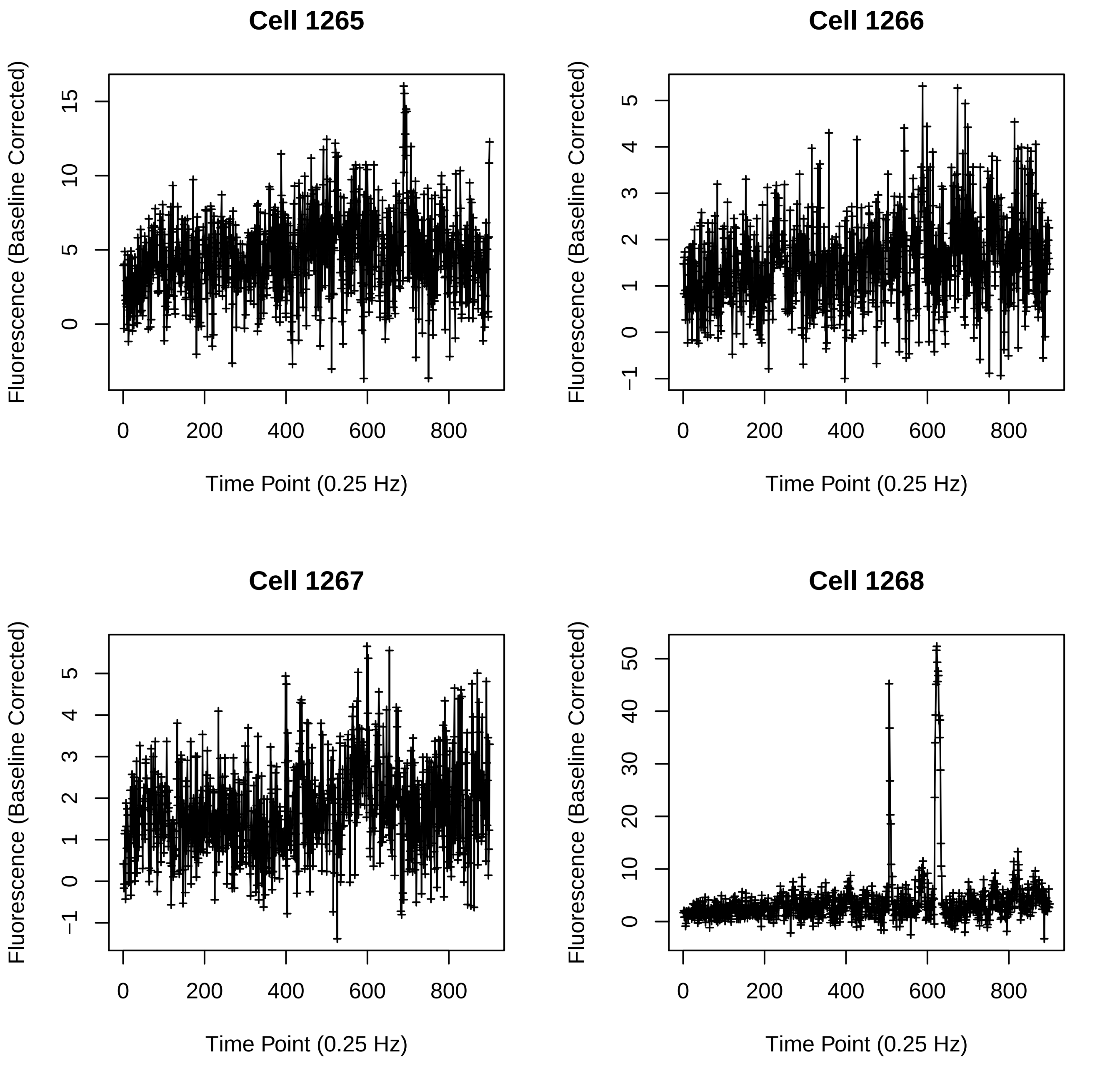
Cell 1265
Cell 1266
Cell 1267
Cell 1268
Fluorescence (Baseline Corrected)
Time Point (0.25 Hz)
0
200
400
600
800
0
5
10
15
−1
1
2
3
4
20
30
40
50

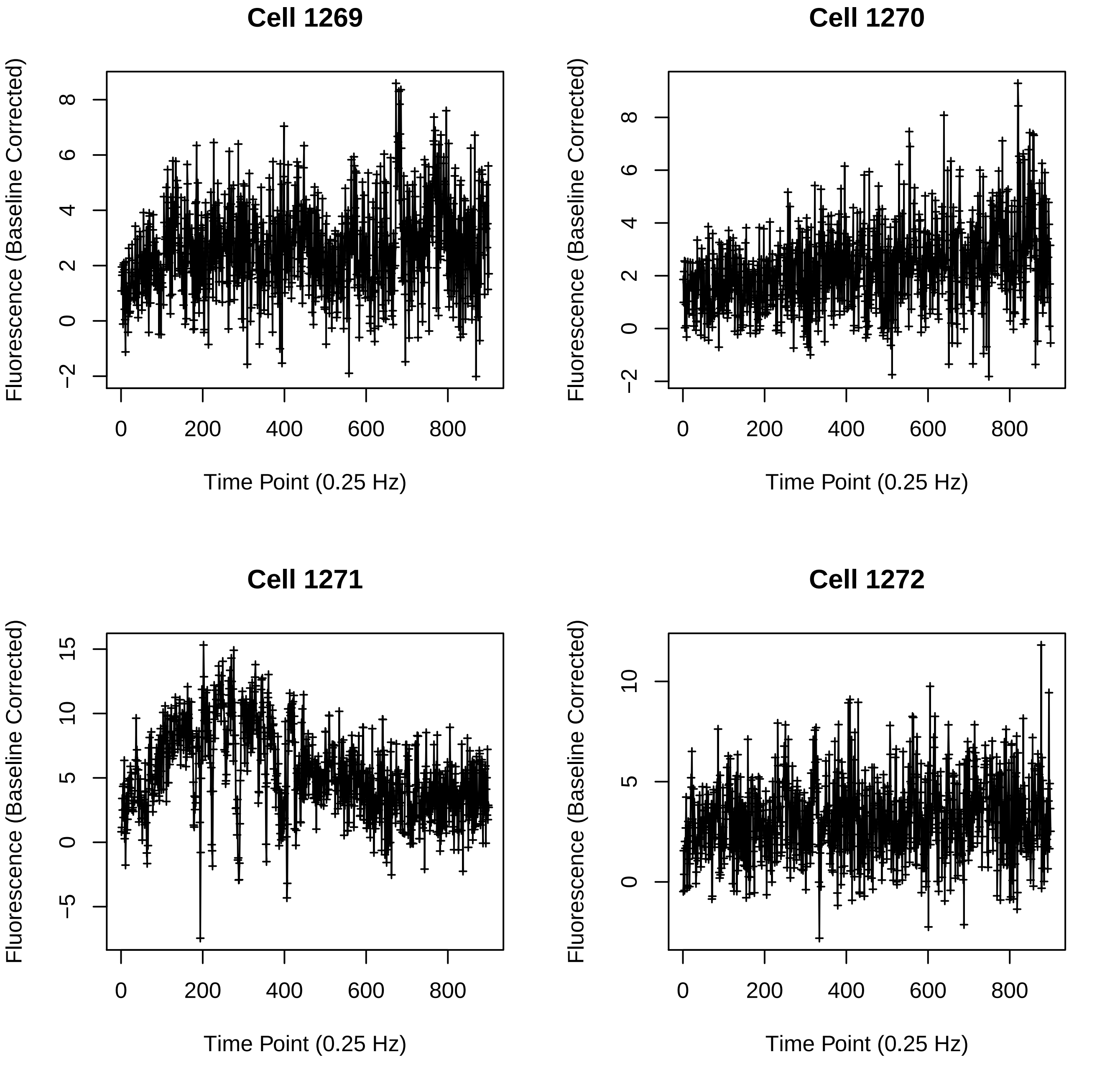

Cell 1269
Cell 1270
Cell 1271
Cell 1272
Fluorescence (Baseline Corrected)
Time Point (0.25 Hz)

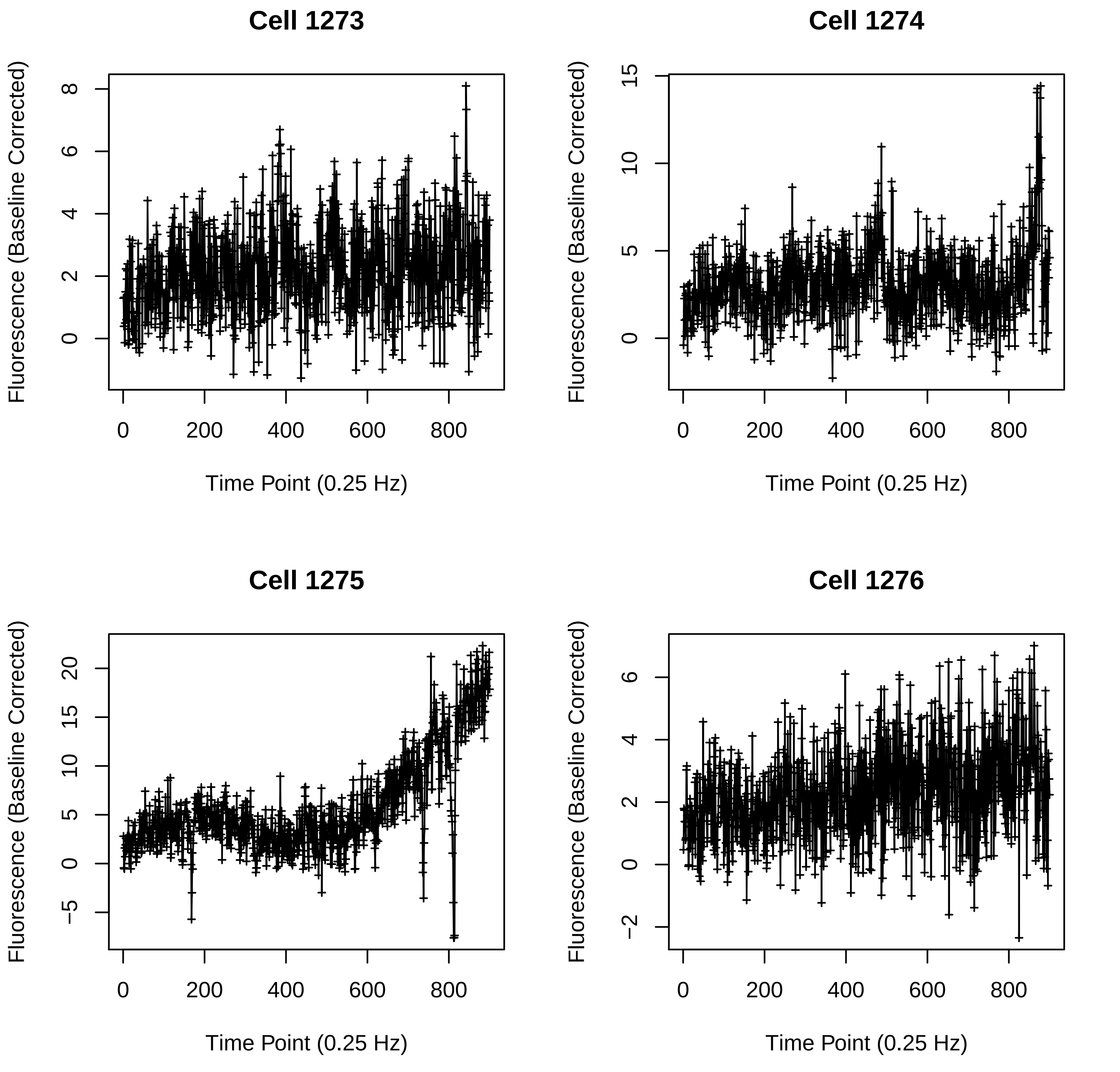
Cell 1273
Cell 1274
Cell 1275
Cell 1276
Fluorescence (Baseline Corrected)
Time Point (0.25 Hz)
0
200
400
600
800
0
2
4
6
8
5
10
15
20
−5
−2

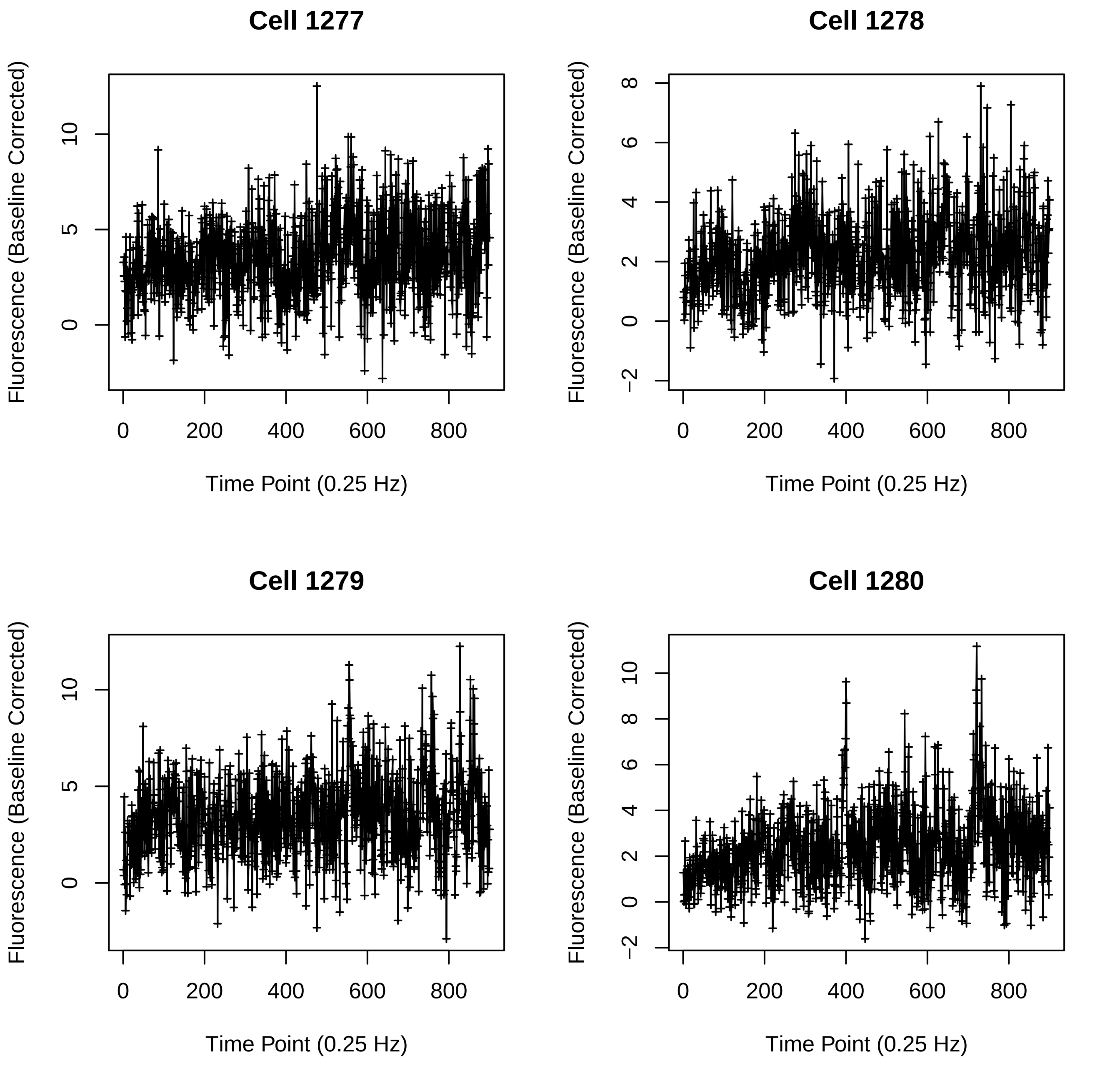

Cell 1277
Cell 1278
Cell 1279
Cell 1280
Fluorescence (Baseline Corrected)
Time Point (0.25 Hz)
0
200
400
600
800
0
5
10
−2
2
4
6
8

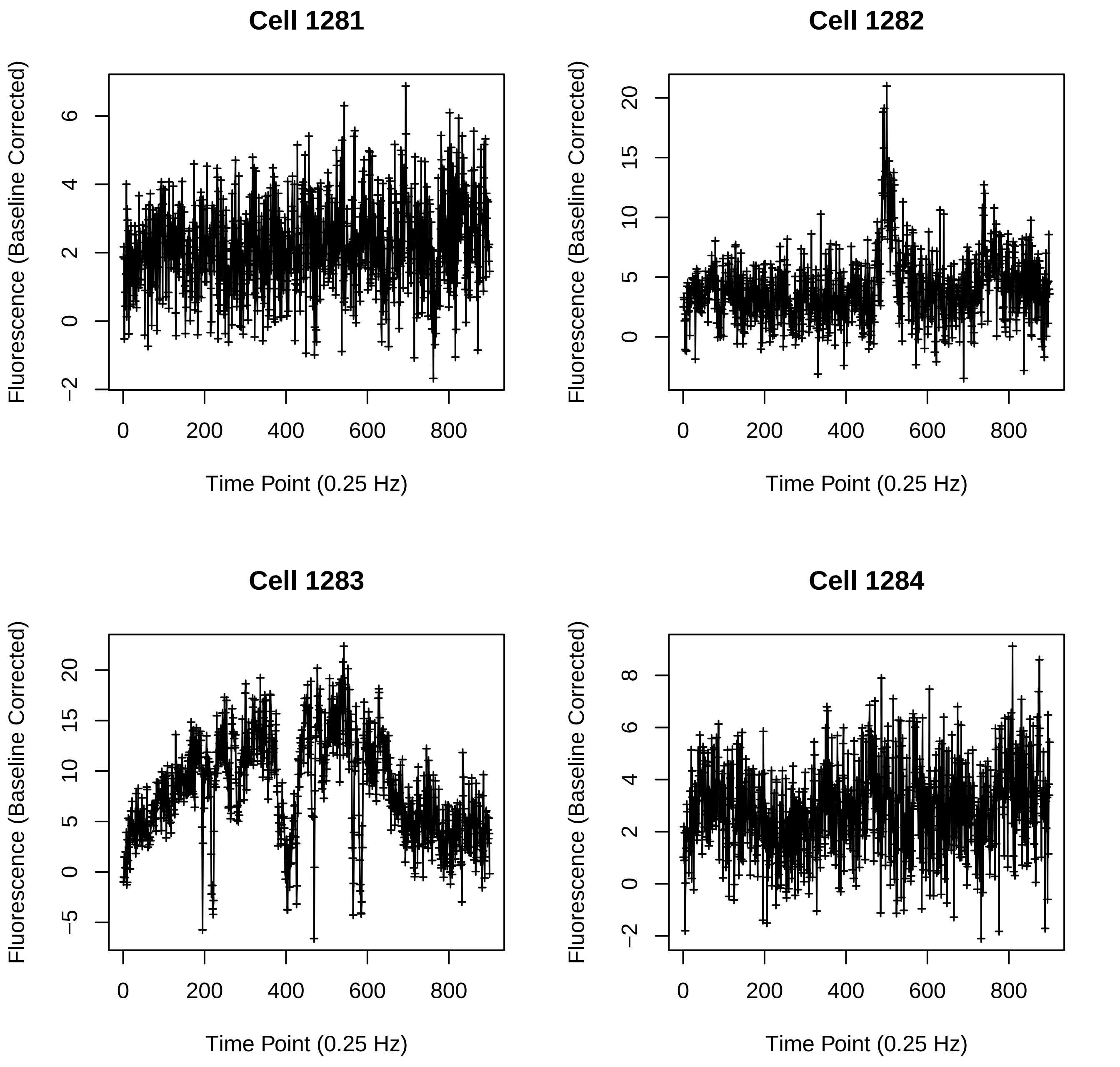

Cell 1281
Cell 1282
Cell 1283
Cell 1284
Fluorescence (Baseline Corrected)
Time Point (0.25 Hz)

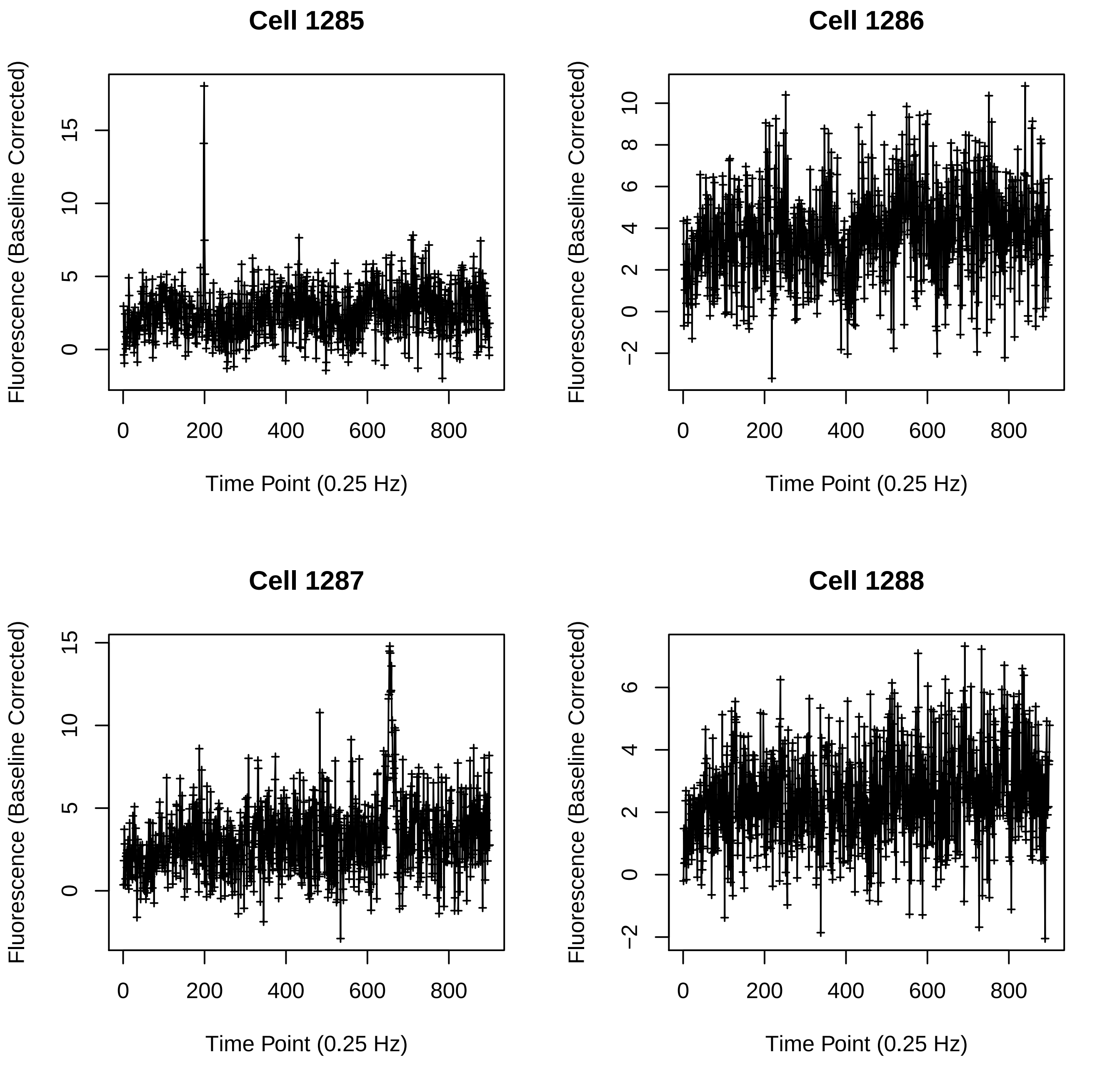

Cell 1285
Cell 1286
Cell 1287
Cell 1288
Fluorescence (Baseline Corrected)
Time Point (0.25 Hz)
0
200
400
600
800
5
10
15
−2
2
4
6
8

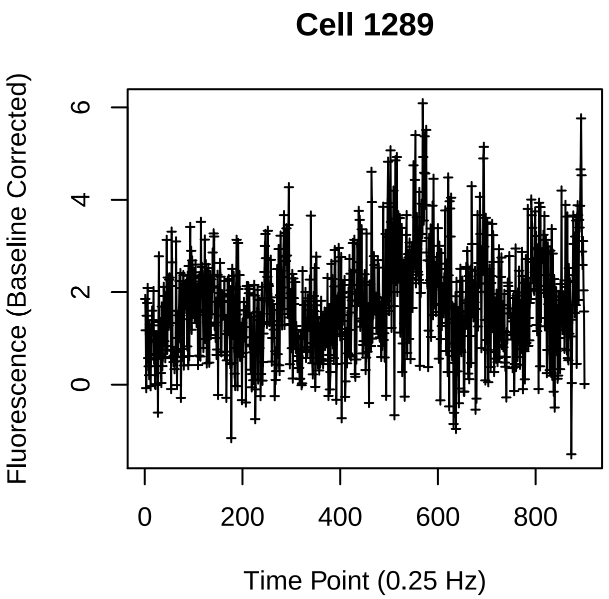
Cell 1289
Fluorescence (Baseline Corrected)
0
2
4
6
0
200
400
600
800
Time Point (0.25 Hz)
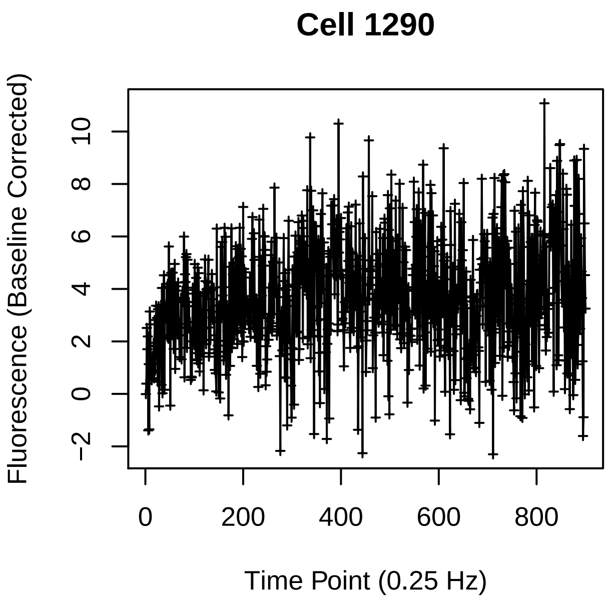
Cell 1290
Fluorescence (Baseline Corrected)
−2
0
2
4
6
8
10
0
200
400
600
800
Time Point (0.25 Hz)
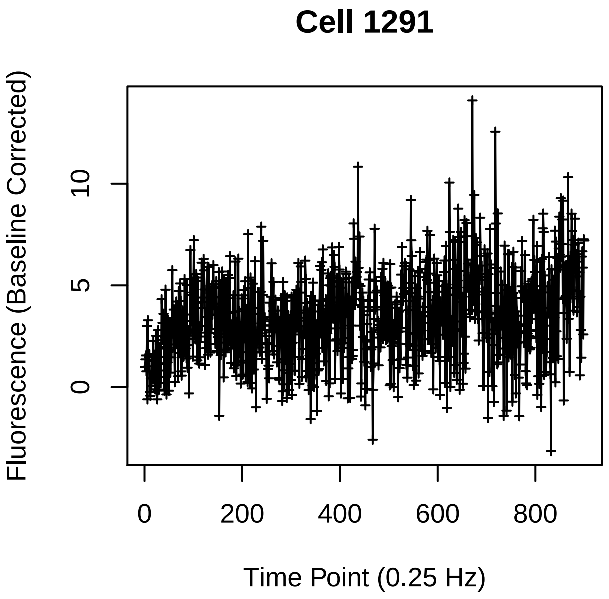
Cell 1291
Fluorescence (Baseline Corrected)
0
5
10
0
200
400
600
800
Time Point (0.25 Hz)
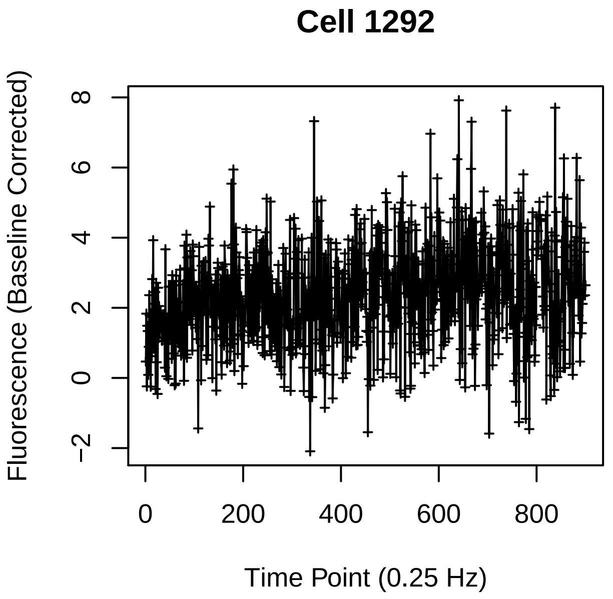
Cell 1292
Fluorescence (Baseline Corrected)
−2
0
2
4
6
8
0
200
400
600
800
Time Point (0.25 Hz)

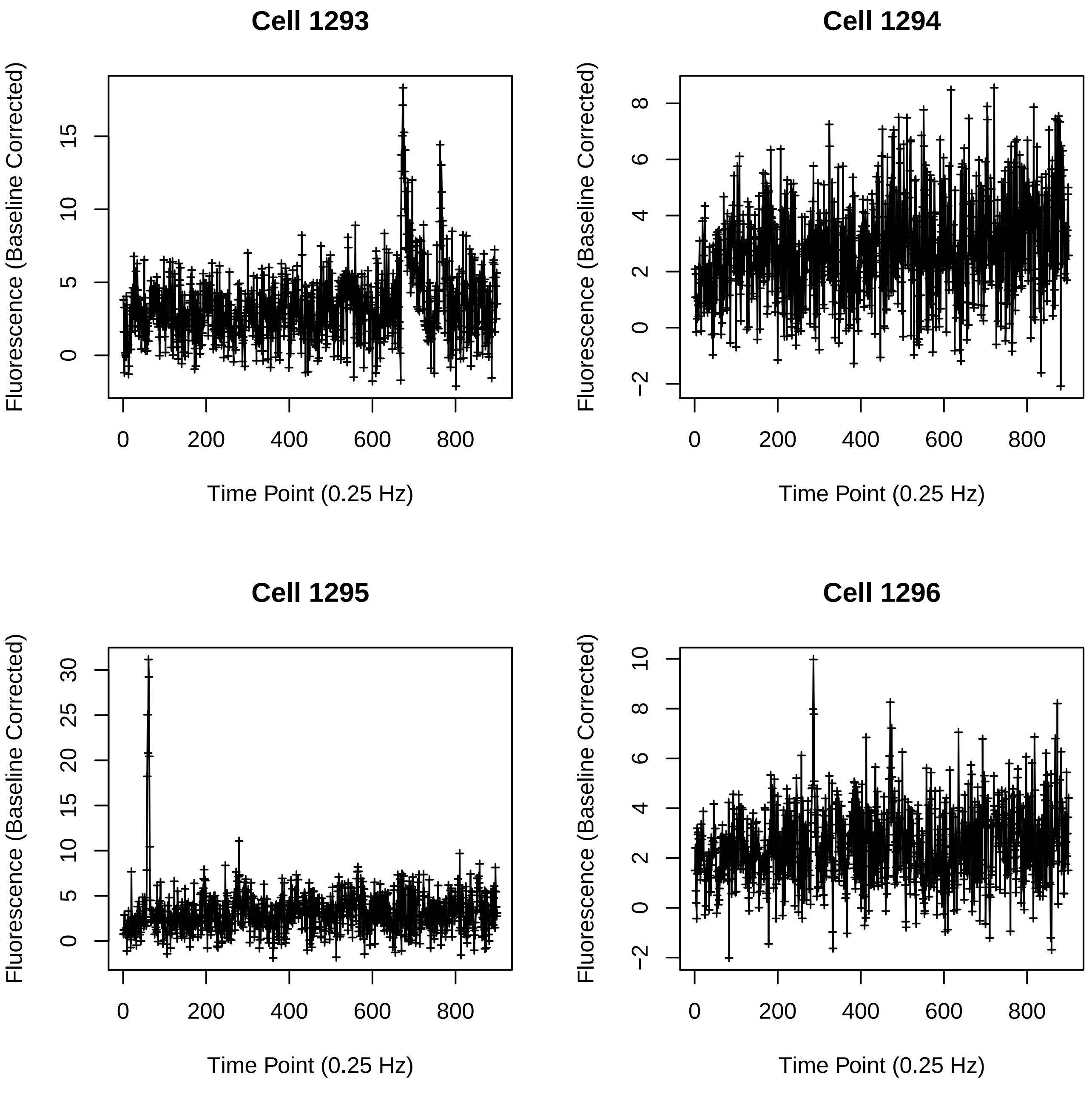
Cell 1293
Fluorescence (Baseline Corrected)
0
5
10
15
0
200
400
600
800
Time Point (0.25 Hz)
Cell 1294
Fluorescence (Baseline Corrected)
−2
0
2
4
6
8
0
200
400
600
800
Time Point (0.25 Hz)
Cell 1295
Fluorescence (Baseline Corrected)
0
5
10
15
20
25
30
0
200
400
600
800
Time Point (0.25 Hz)
Cell 1296
Fluorescence (Baseline Corrected)
−2
0
2
4
6
8
10
0
200
400
600
800
Time Point (0.25 Hz)

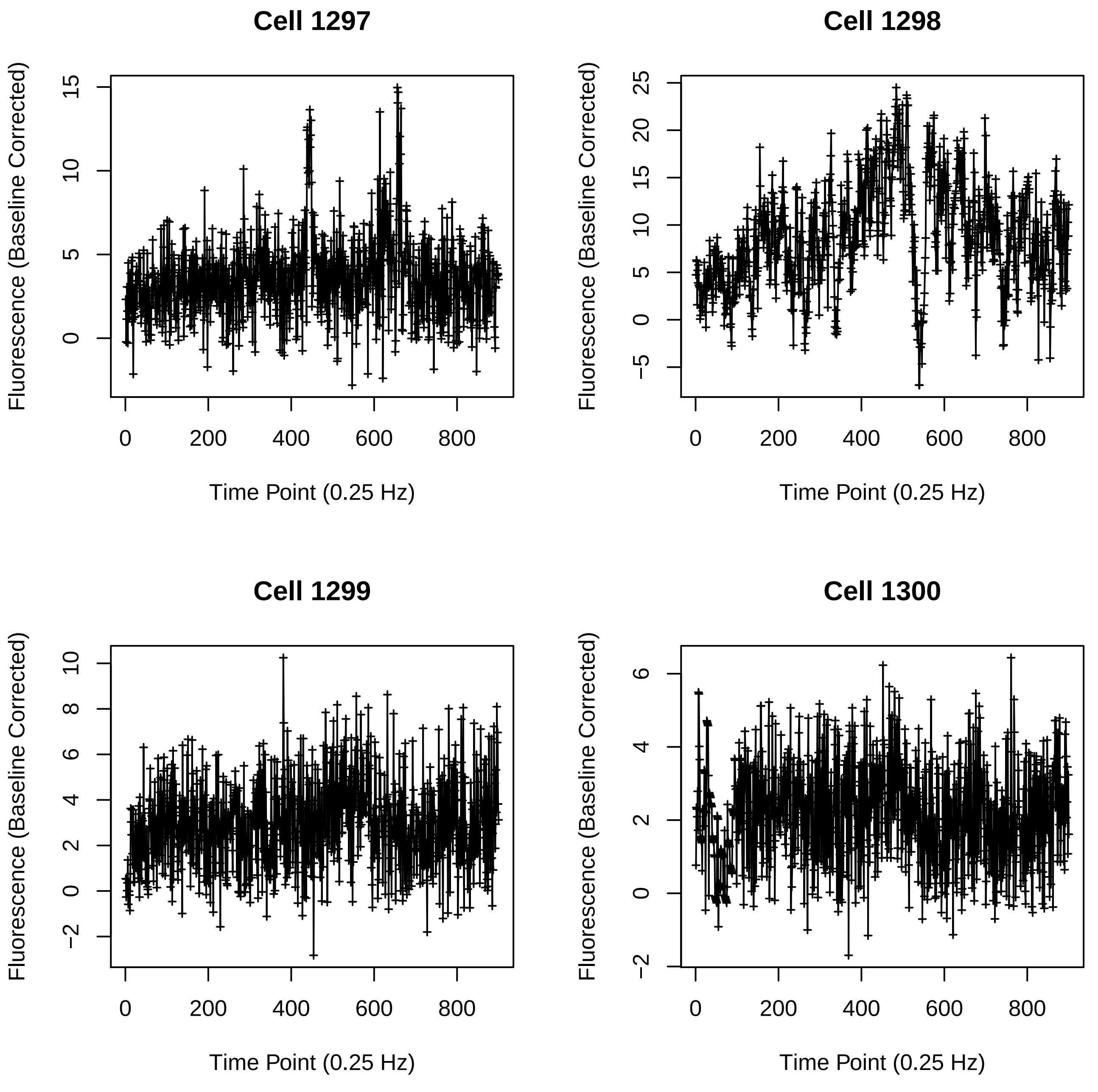
Cell 1297
Fluorescence (Baseline Corrected)
Time Point (0.25 Hz)
0
5
10
15
0
200
400
600
800
Cell 1298
Fluorescence (Baseline Corrected)
Time Point (0.25 Hz)
−5
0
5
10
15
20
25
0
200
400
600
800
Cell 1299
Fluorescence (Baseline Corrected)
Time Point (0.25 Hz)
−2
0
2
4
6
8
10
0
200
400
600
800
Cell 1300
Fluorescence (Baseline Corrected)
Time Point (0.25 Hz)
−2
0
2
4
6
0
200
400
600
800

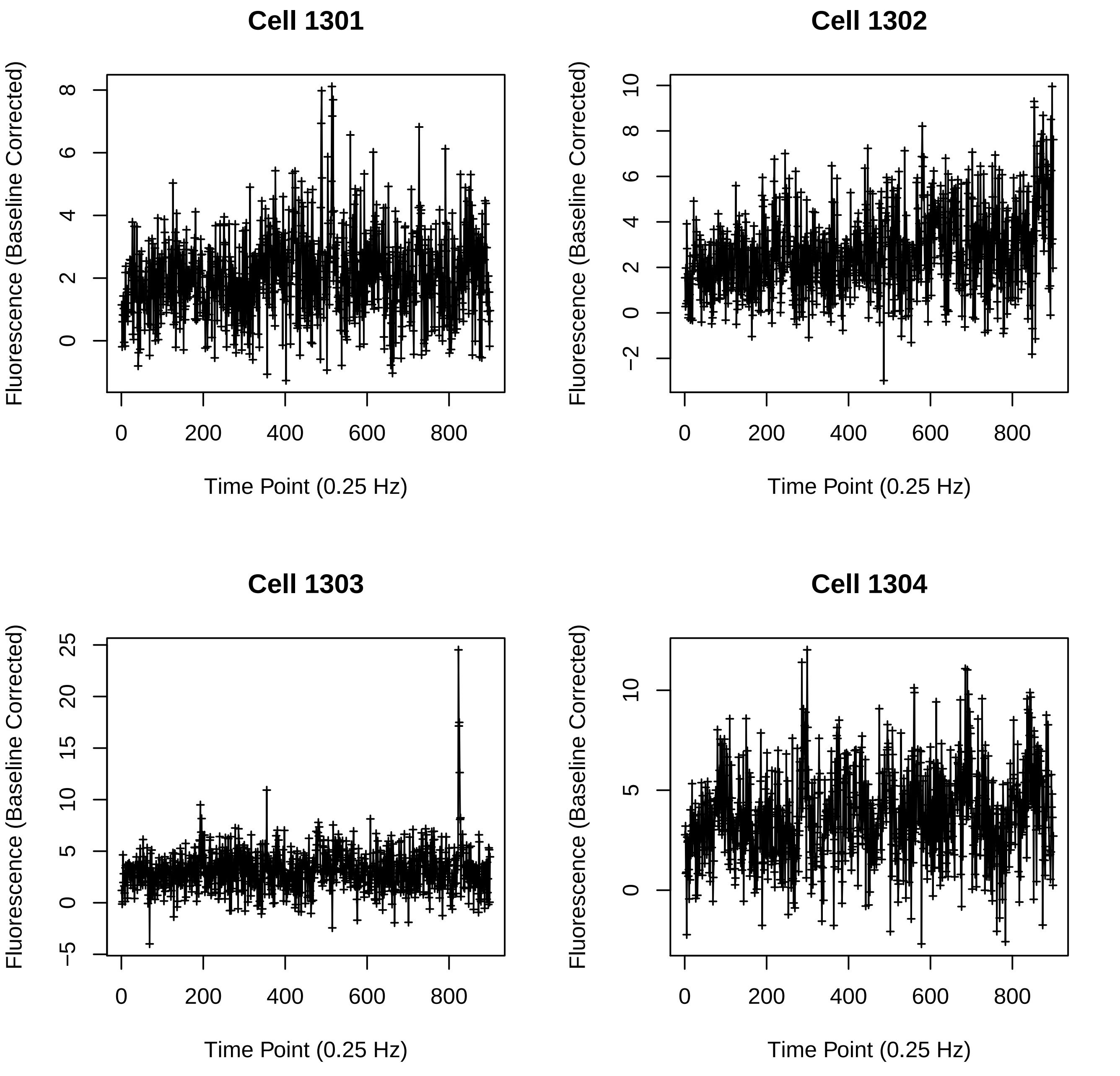
Cell 1301
Cell 1302
Cell 1303
Cell 1304
Fluorescence (Baseline Corrected)
Time Point (0.25 Hz)
0
200
400
600
800
−2
2
4
6
8
10
−5
5
15
20
25

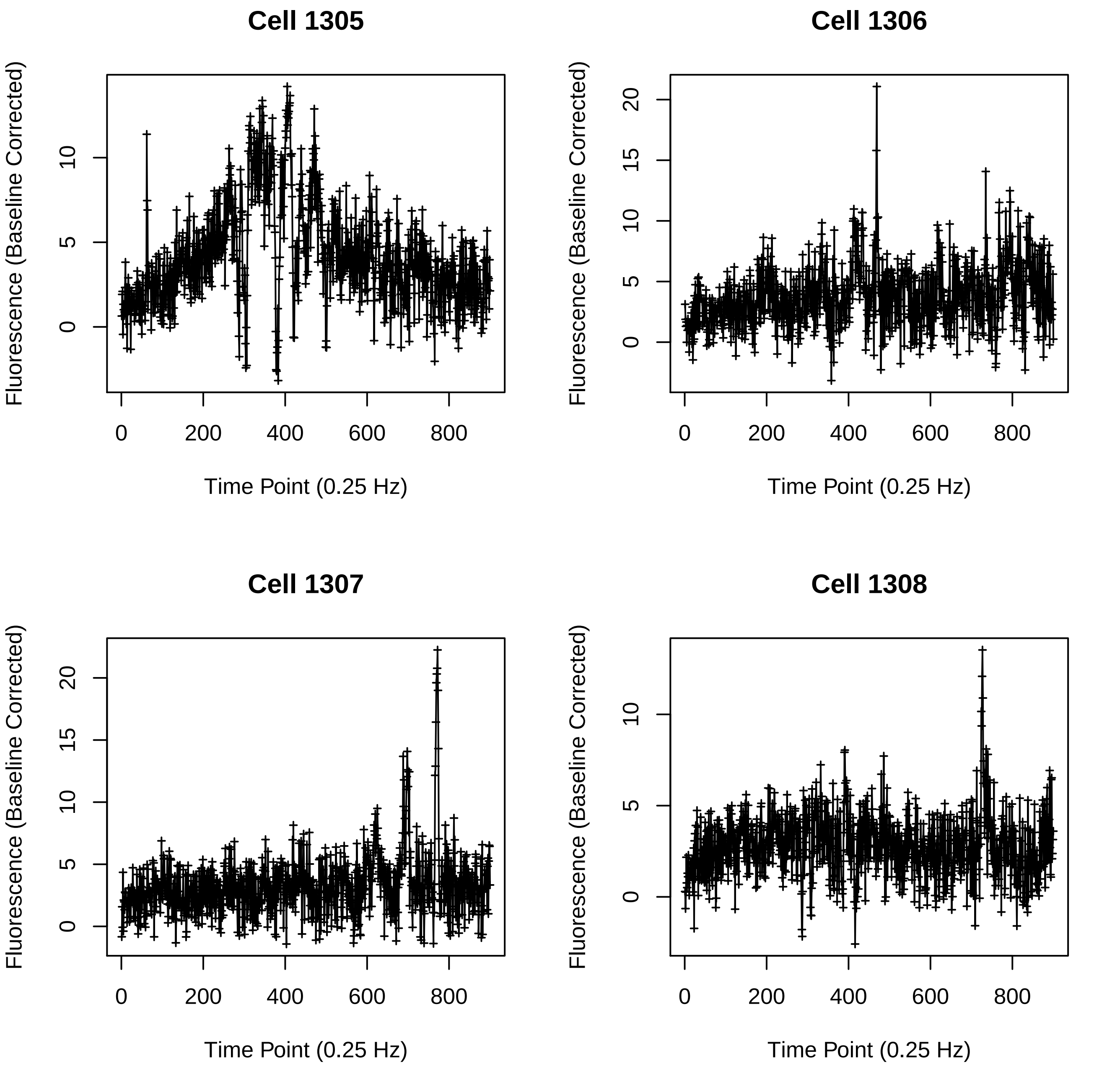
Cell 1305
Cell 1306
Cell 1307
Cell 1308
Fluorescence (Baseline Corrected)
Time Point (0.25 Hz)
0
5
10
15
20
200
400
600
800

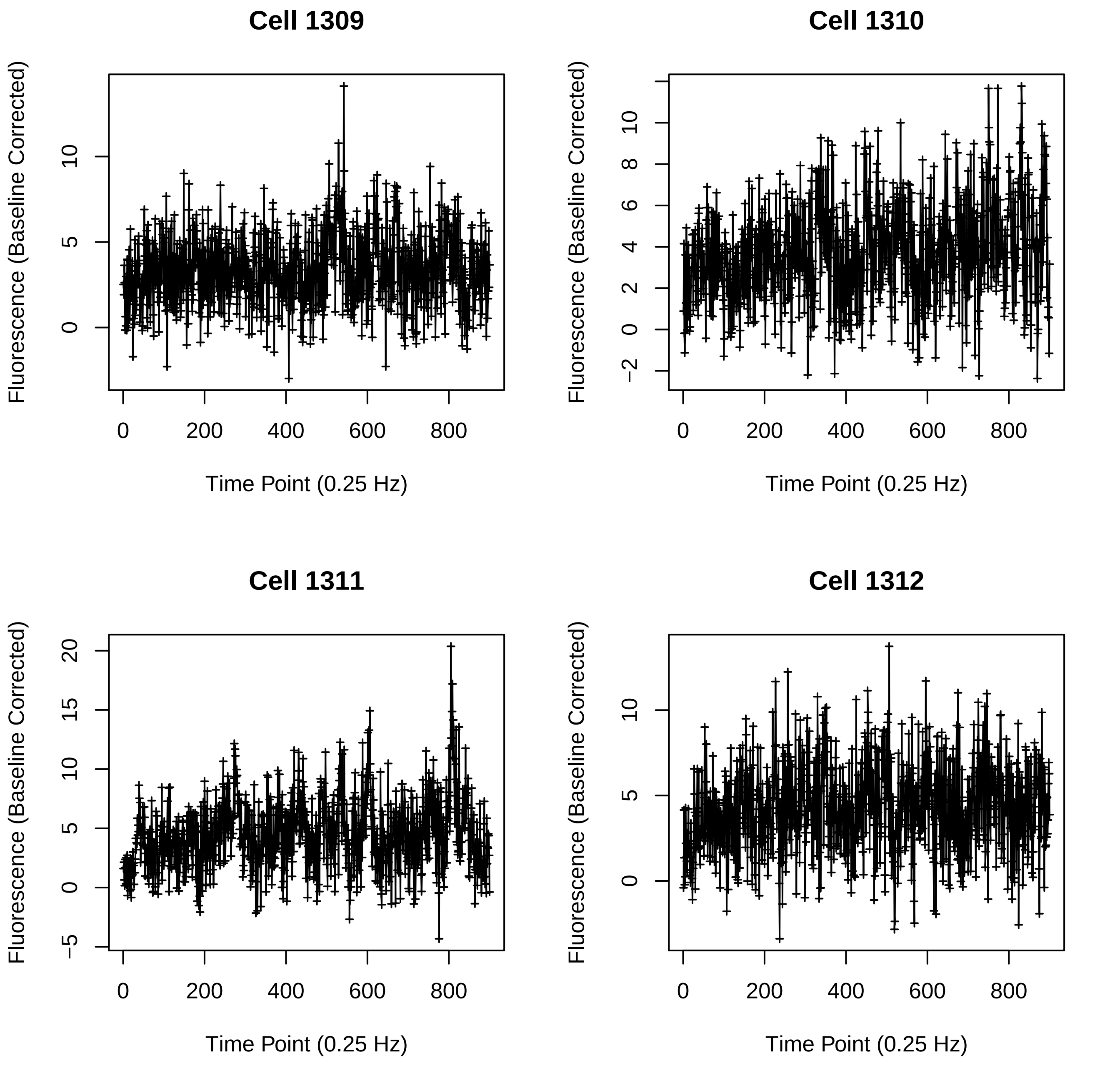
Cell 1309
Cell 1310
Cell 1311
Cell 1312
Fluorescence (Baseline Corrected)
Time Point (0.25 Hz)
0
200
400
600
800
−5
−2
0
2
4
6
8
5
10
15
20

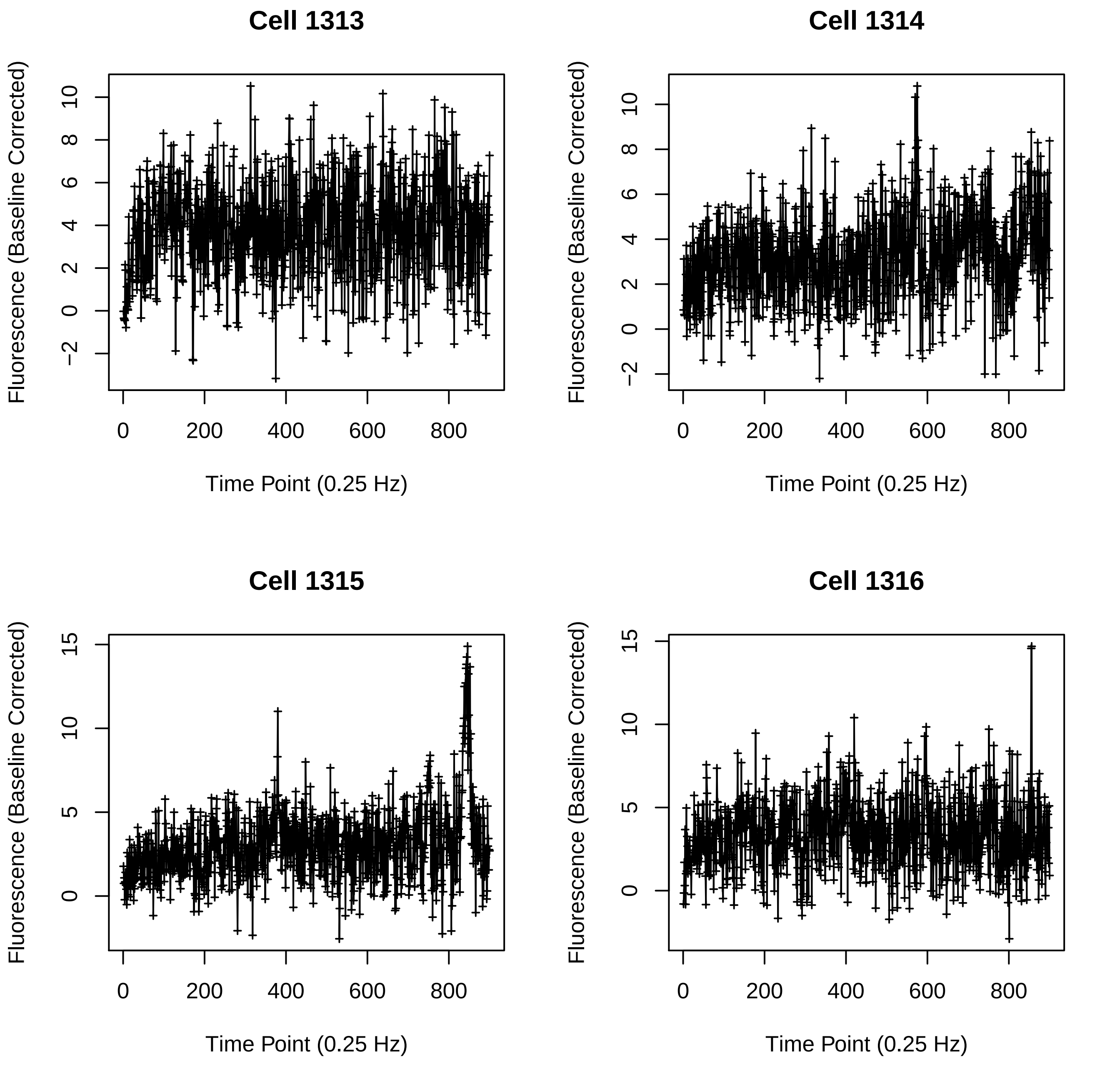

Cell 1313
Cell 1314
Cell 1315
Cell 1316
Fluorescence (Baseline Corrected)
Time Point (0.25 Hz)

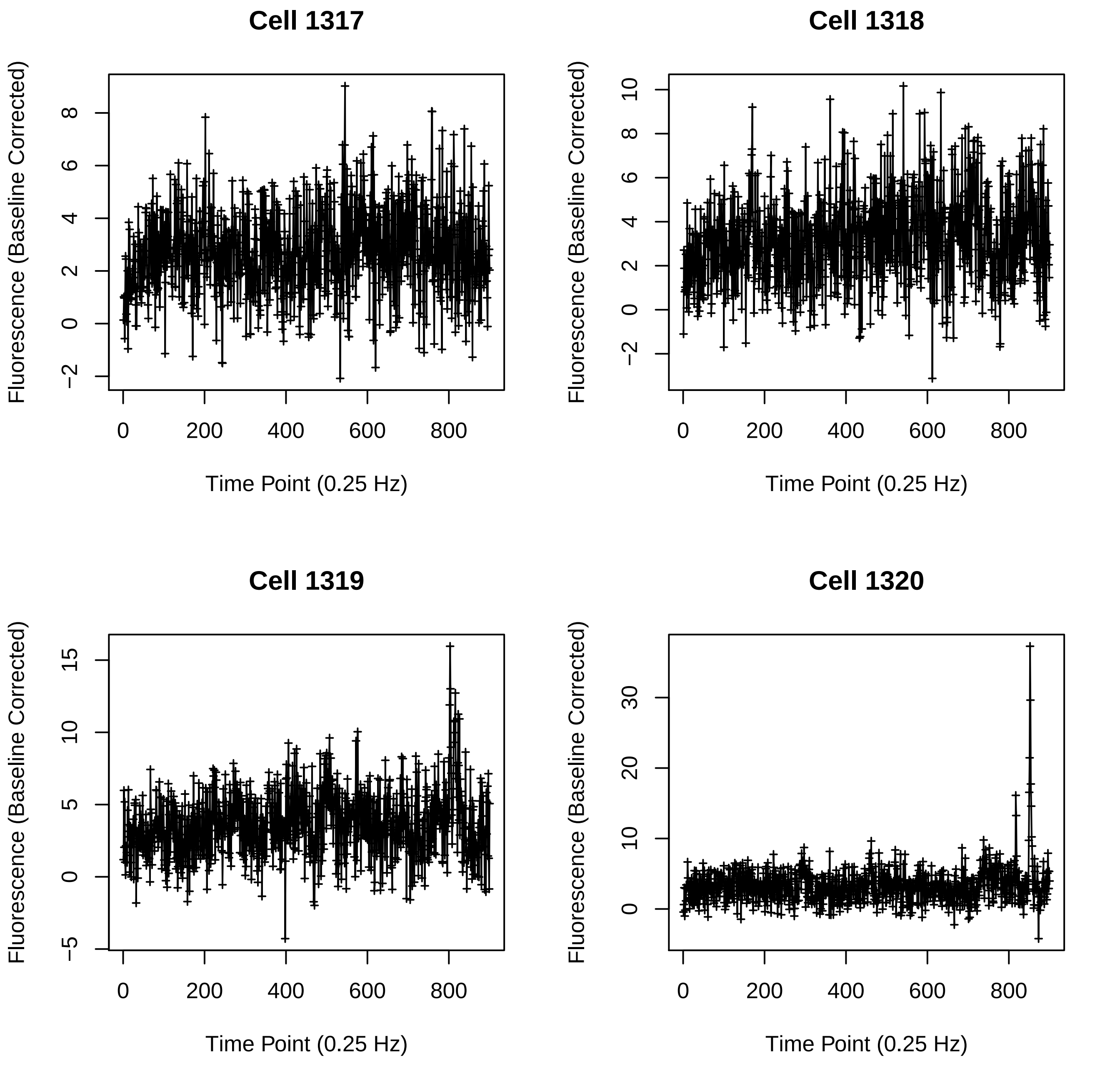
Cell 1317
Cell 1318
Cell 1319
Cell 1320
Fluorescence (Baseline Corrected)
Time Point (0.25 Hz)
−2
0
2
4
6
8
10
−5
5
15
20
30
200
400
600
800

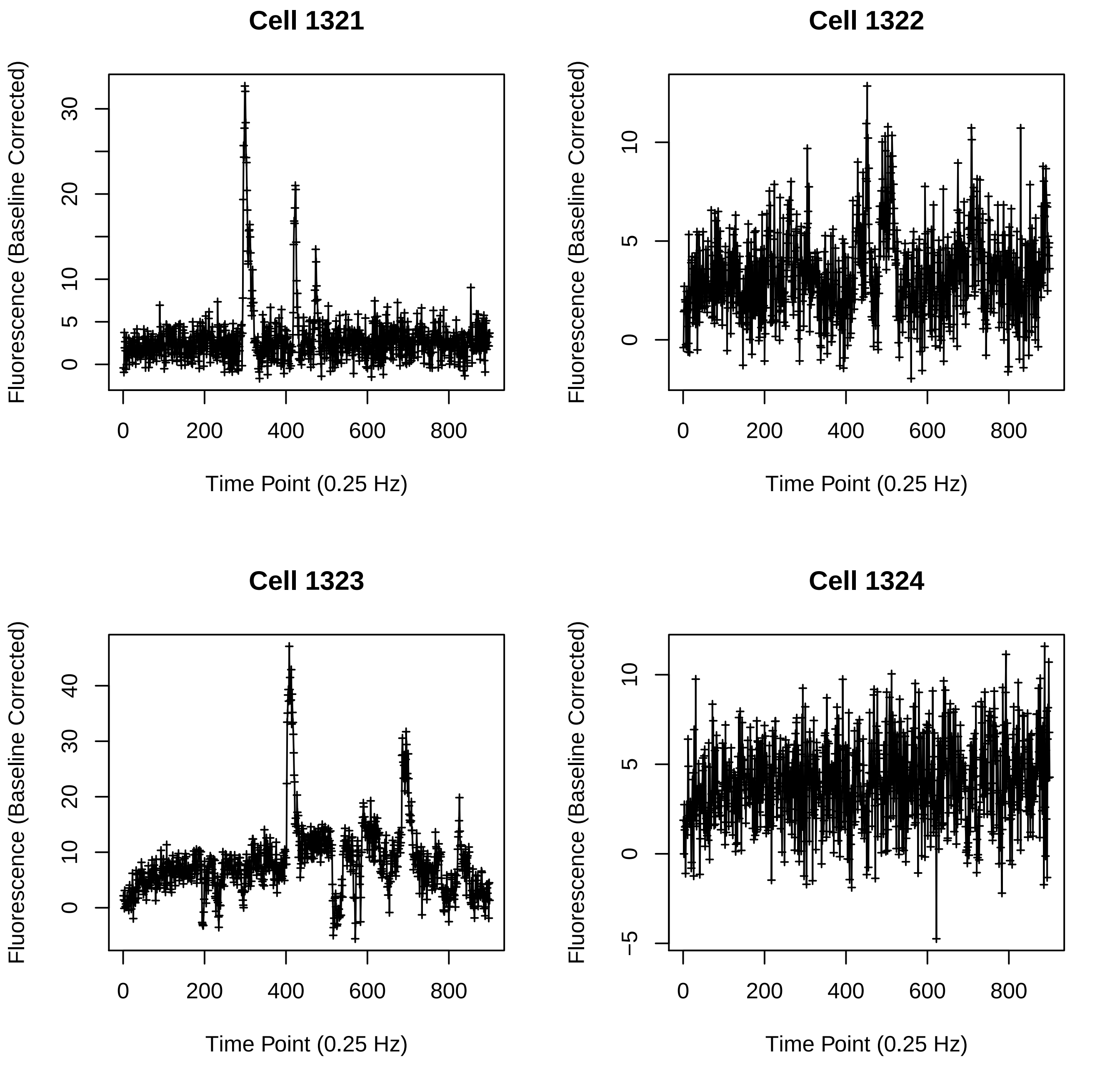

Cell 1321
Cell 1322
Cell 1323
Cell 1324
Fluorescence (Baseline Corrected)
Time Point (0.25 Hz)
0
5
10
20
30
40
−5
200
400
600
800

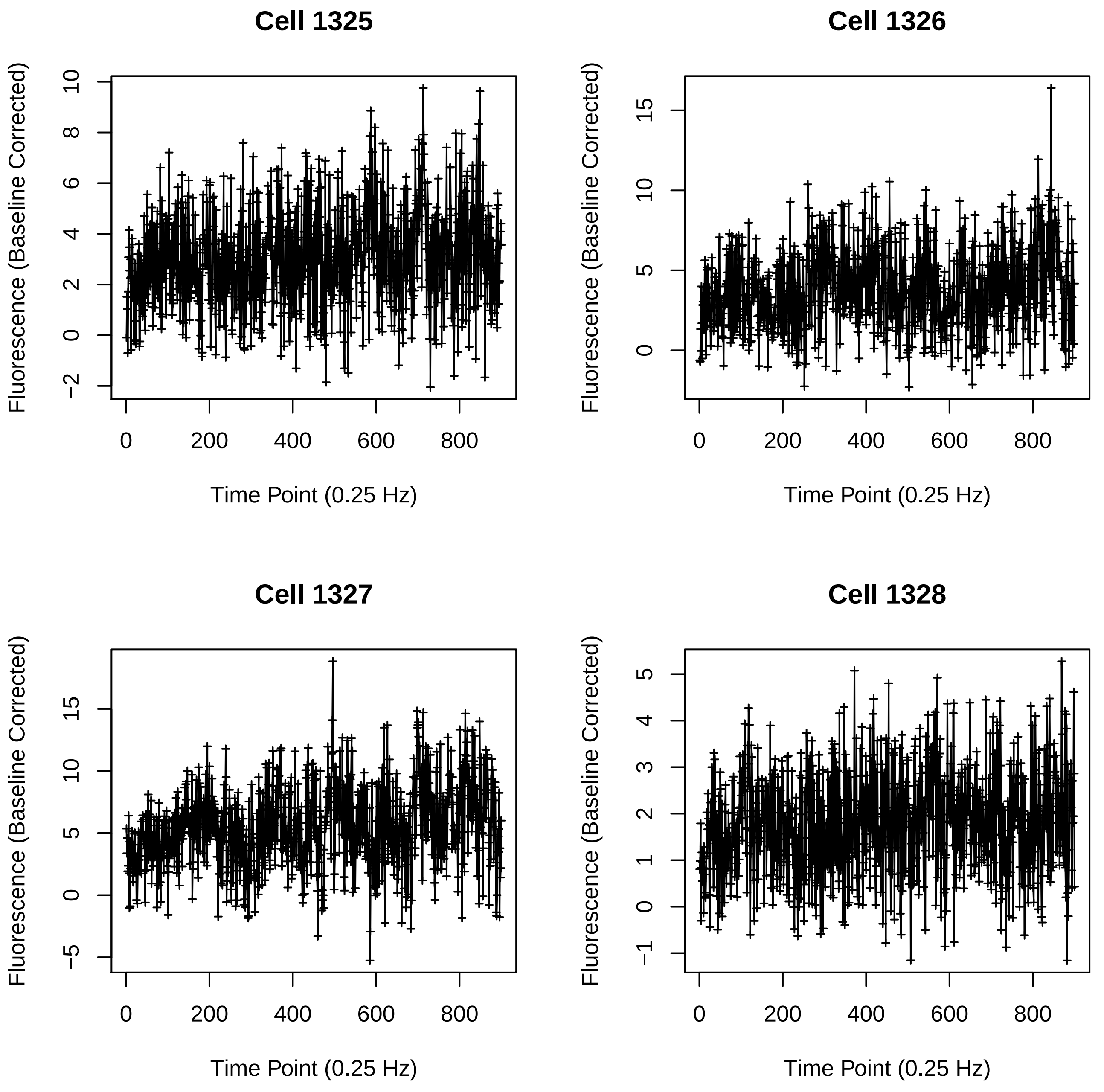

Cell 1325
Cell 1326
Cell 1327
Cell 1328
Fluorescence (Baseline Corrected)
Time Point (0.25 Hz)

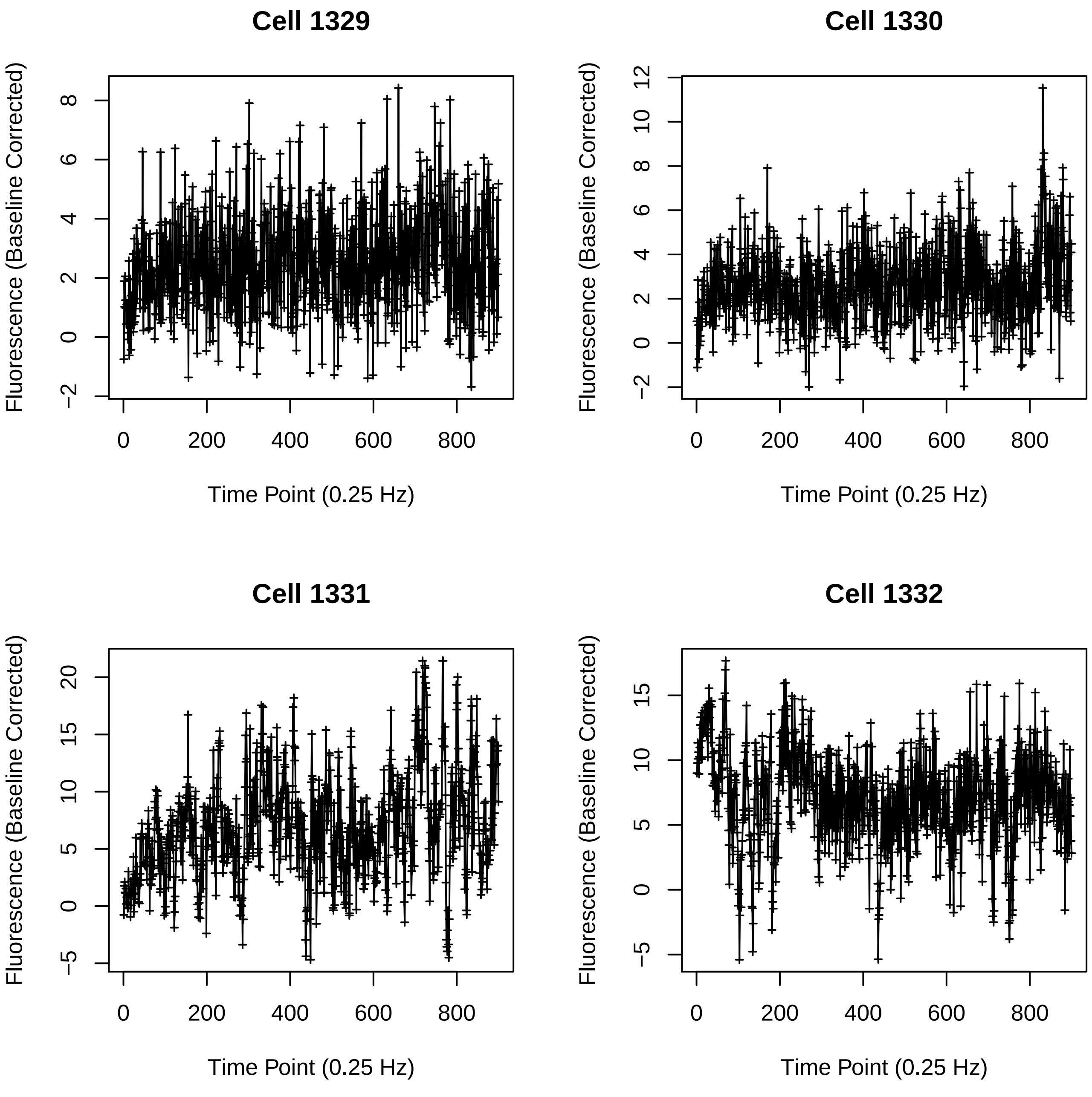
Cell 1329
Cell 1330
Cell 1331
Cell 1332
Fluorescence (Baseline Corrected)
Time Point (0.25 Hz)
0
200
400
600
800

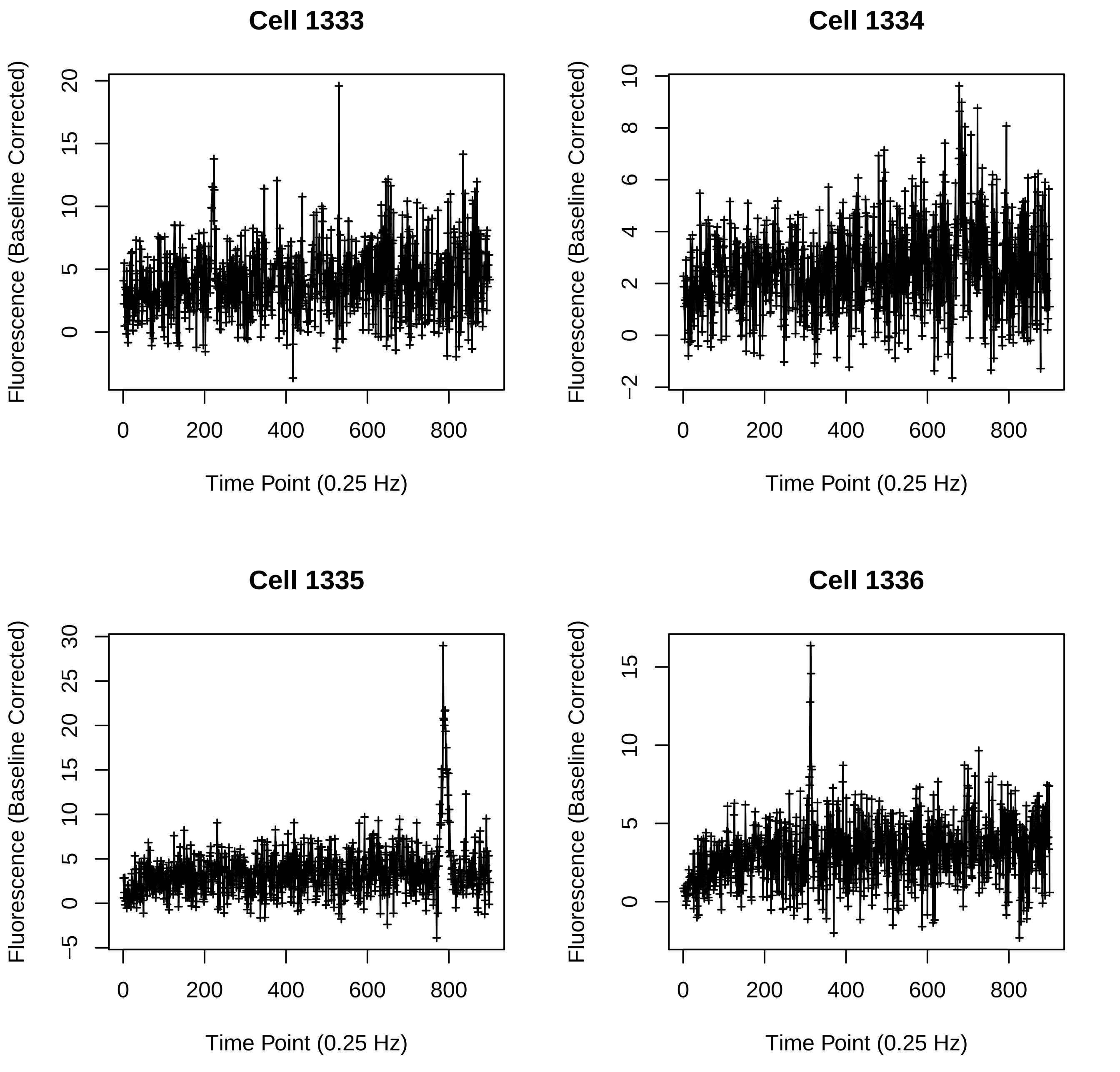
Cell 1333
Cell 1334
Cell 1335
Cell 1336
Fluorescence (Baseline Corrected)
Time Point (0.25 Hz)
0
200
400
600
800
−5
5
10
15
20
25
30
−2
2
4
6
8

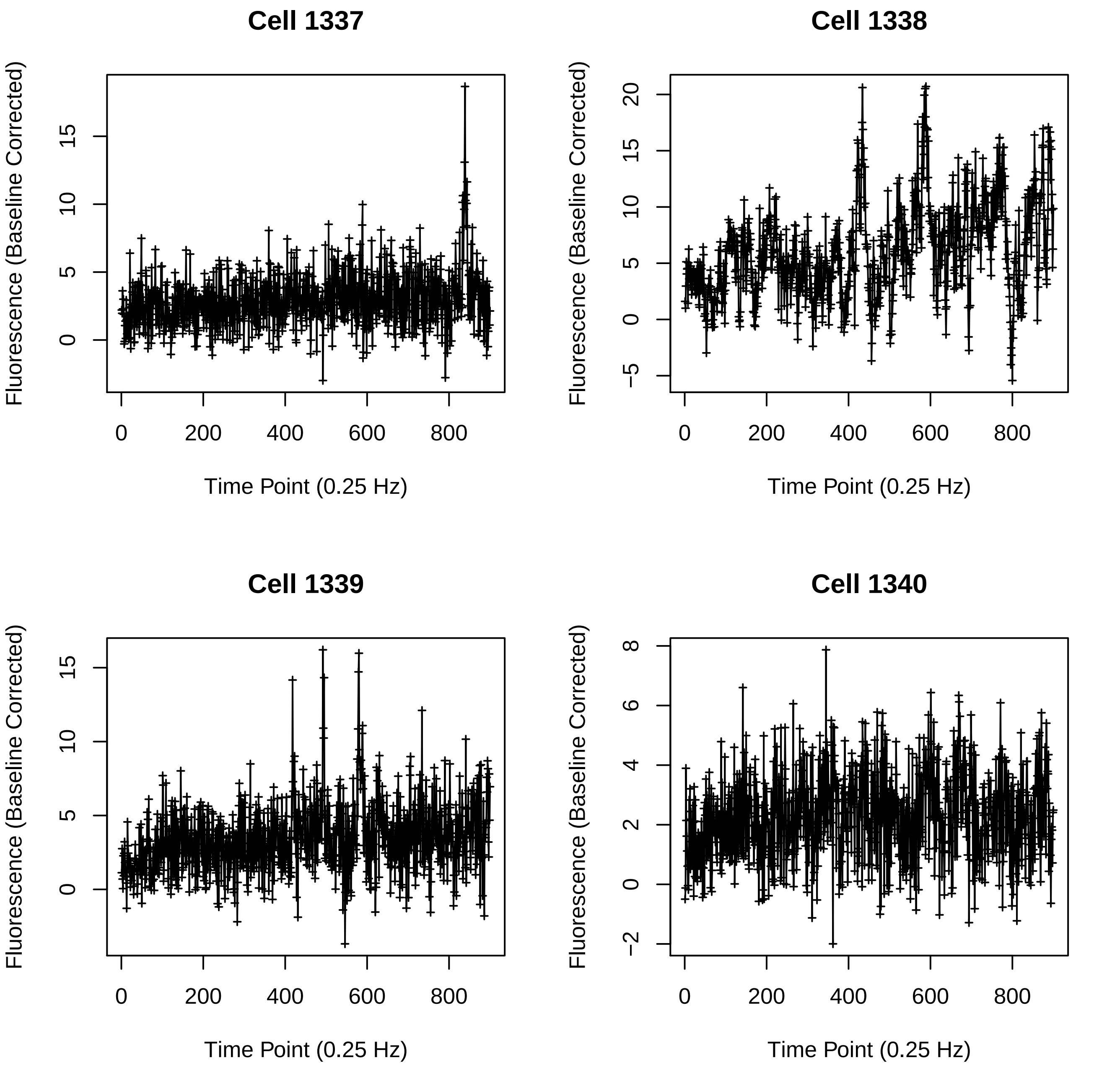
Cell 1337
Cell 1338
Cell 1339
Cell 1340
Fluorescence (Baseline Corrected)
Time Point (0.25 Hz)
0
200
400
600
800
−5
−2
0
2
4
5
6
8
10
15
20

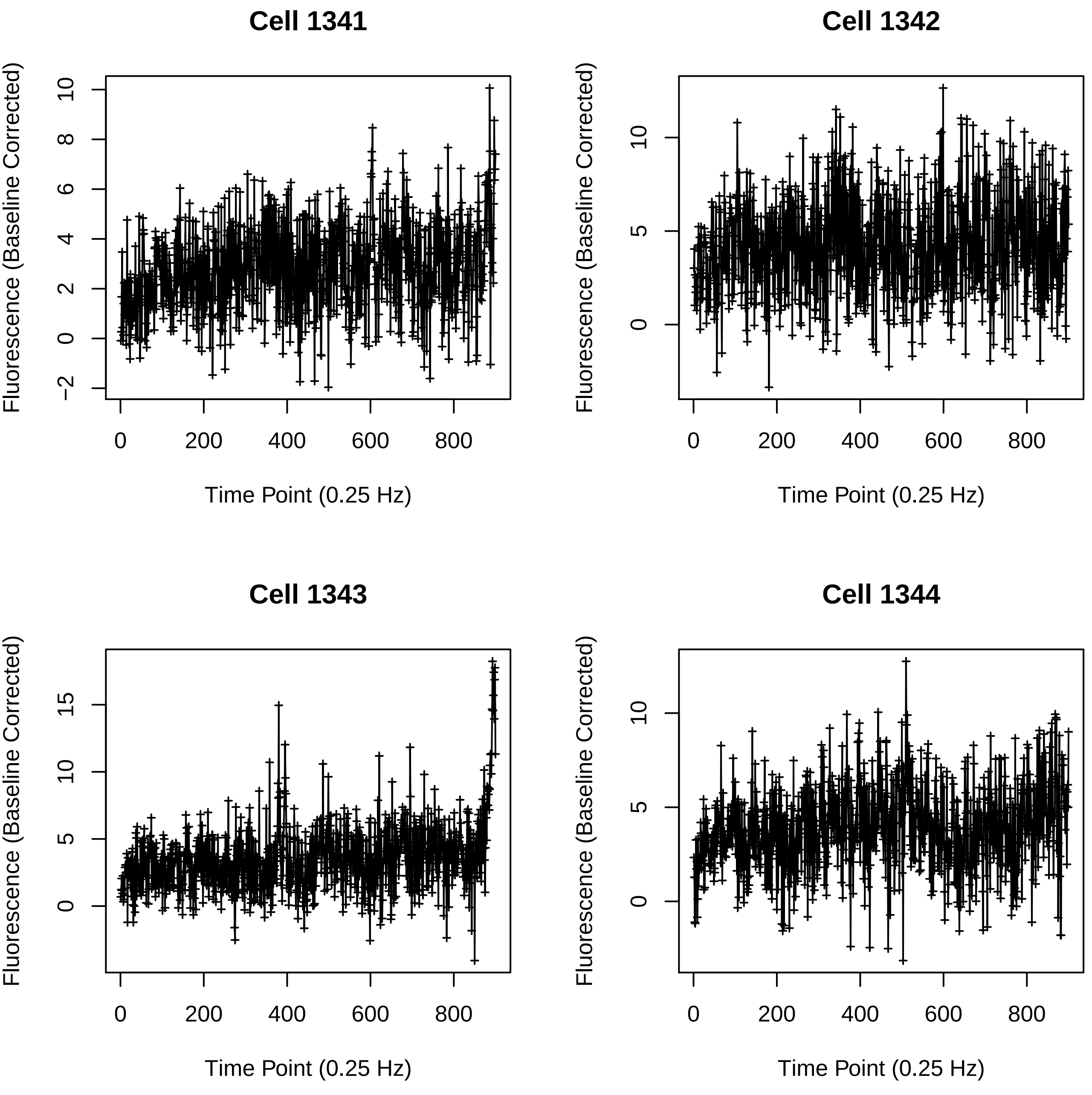

Cell 1341
Cell 1342
Cell 1343
Cell 1344
Fluorescence (Baseline Corrected)
Time Point (0.25 Hz)
0
200
400
600
800
−2
2
4
6
8
10
5
15

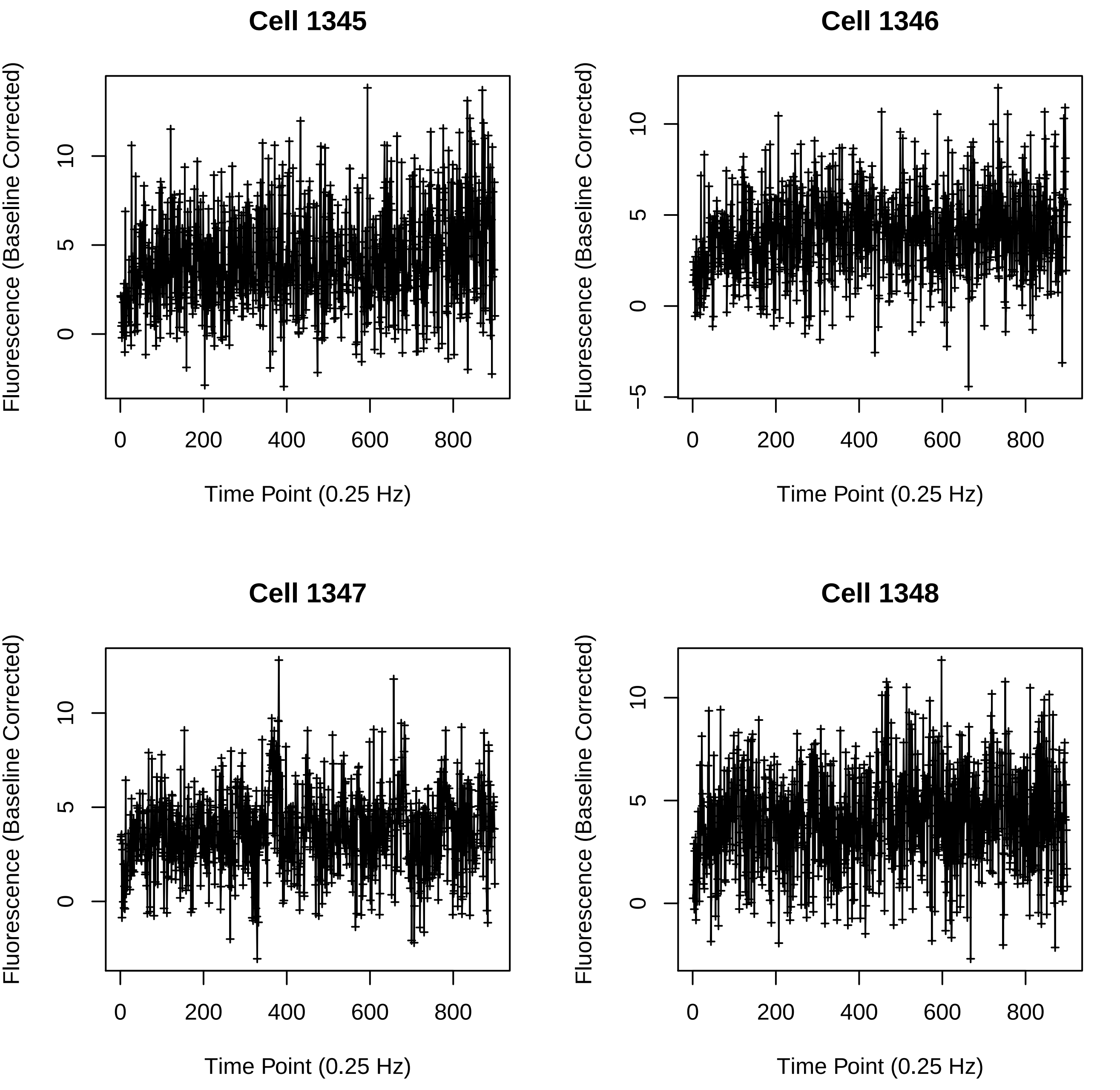

Cell 1345
Cell 1346
Cell 1347
Cell 1348
Fluorescence (Baseline Corrected)
Time Point (0.25 Hz)
0
200
400
600
800
-5
0
5
10

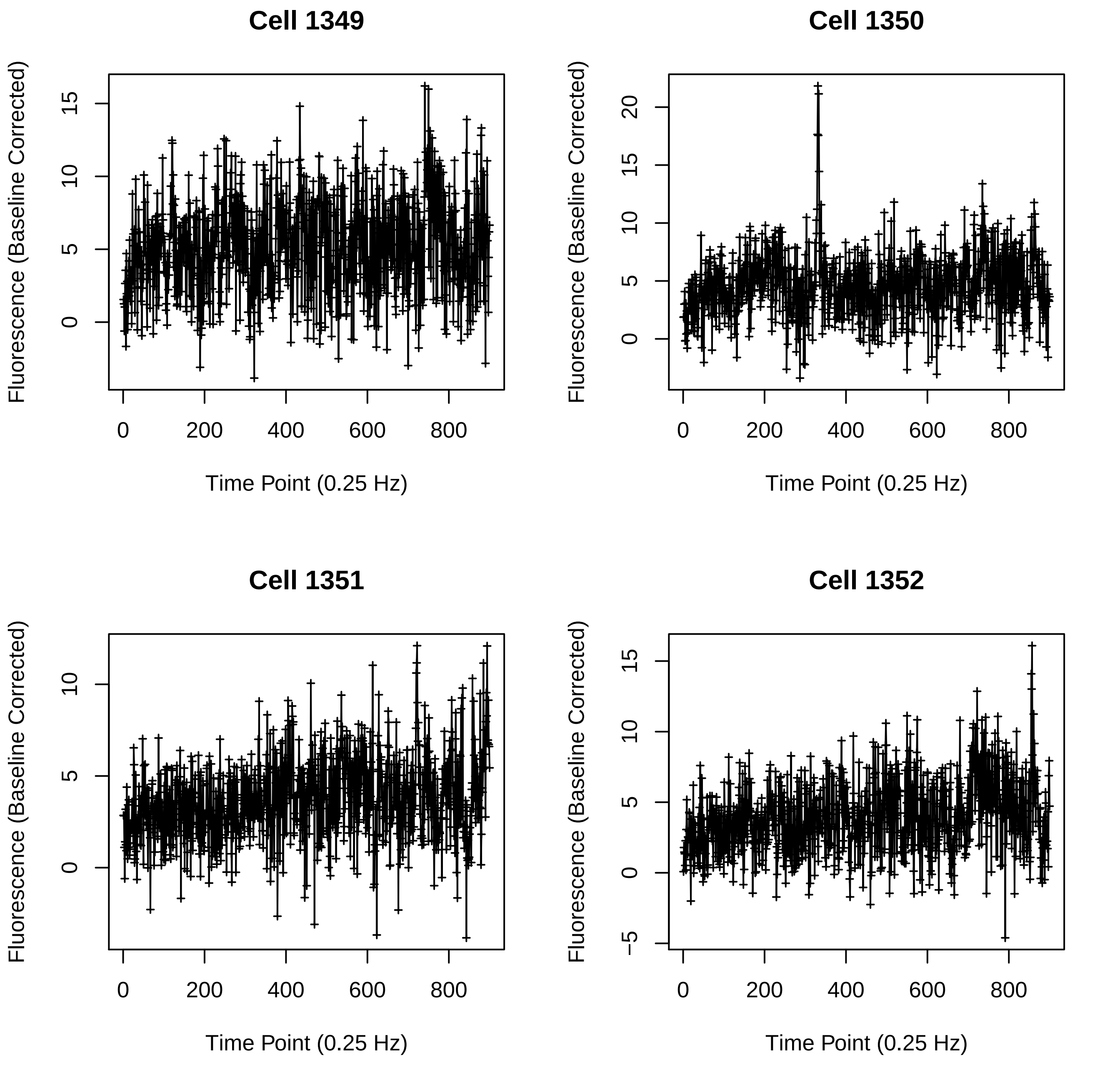
Cell 1349
Cell 1350
Cell 1351
Cell 1352
Fluorescence (Baseline Corrected)
Time Point (0.25 Hz)
0
200
400
600
800
-5
5
10
15
20

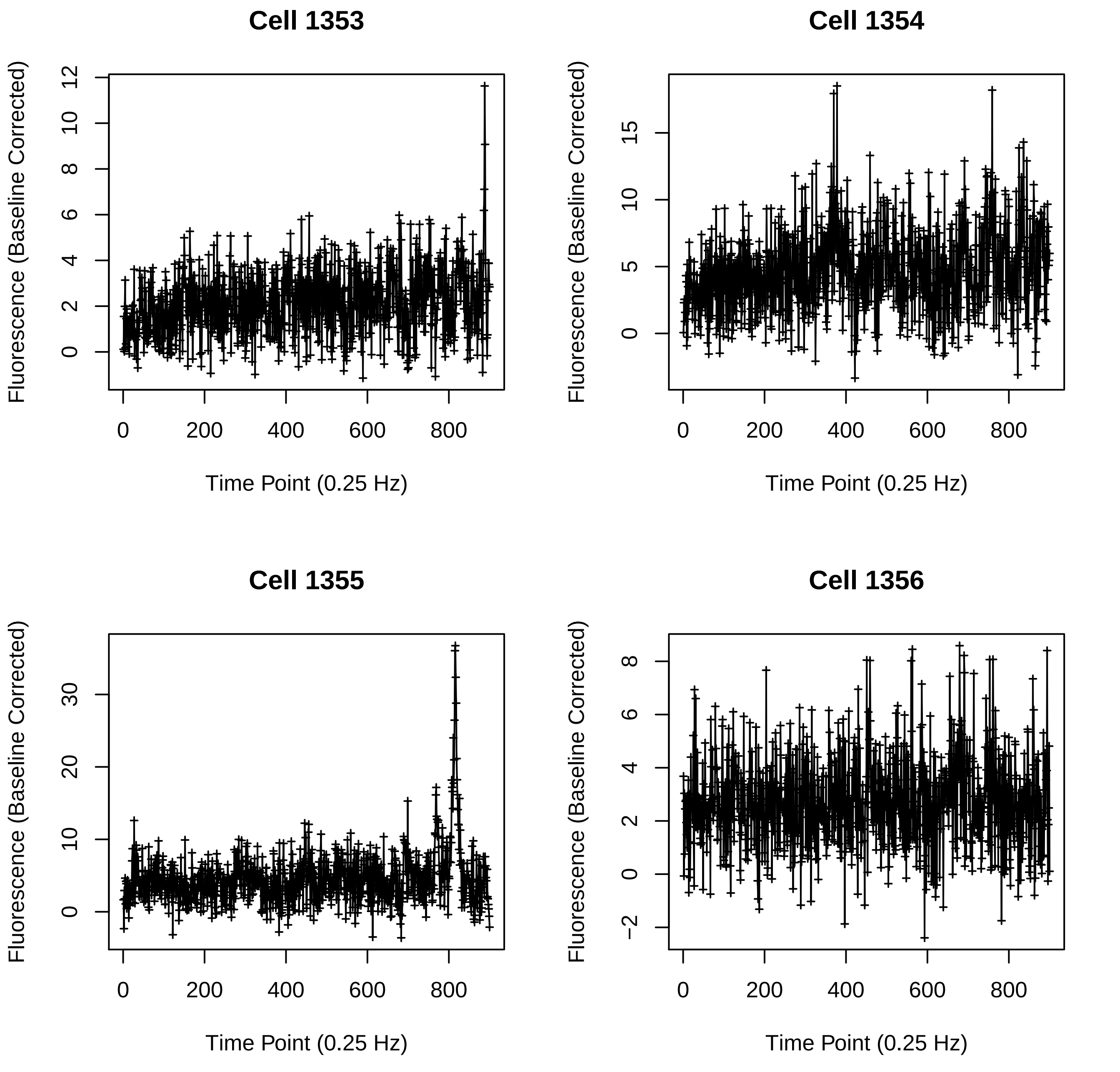
Cell 1353
Cell 1354
Cell 1355
Cell 1356
Fluorescence (Baseline Corrected)
Time Point (0.25 Hz)
0
200
400
600
800
0
2
4
6
8
10
12
0
5
10
15
0
10
20
30
−2
0
2
4
6
8

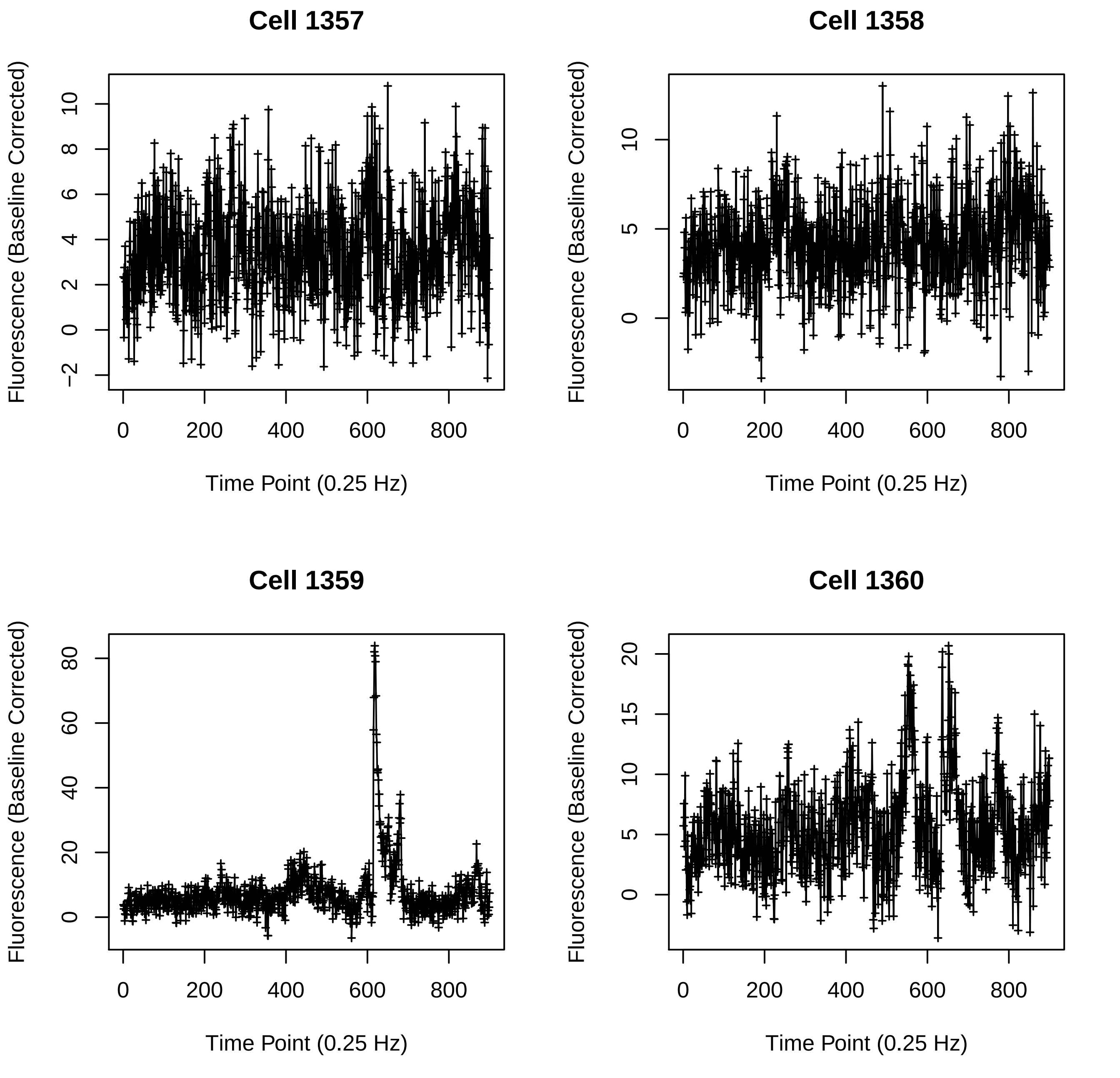
Cell 1357
Cell 1358
Cell 1359
Cell 1360
Fluorescence (Baseline Corrected)
Time Point (0.25 Hz)
0
200
400
600
800

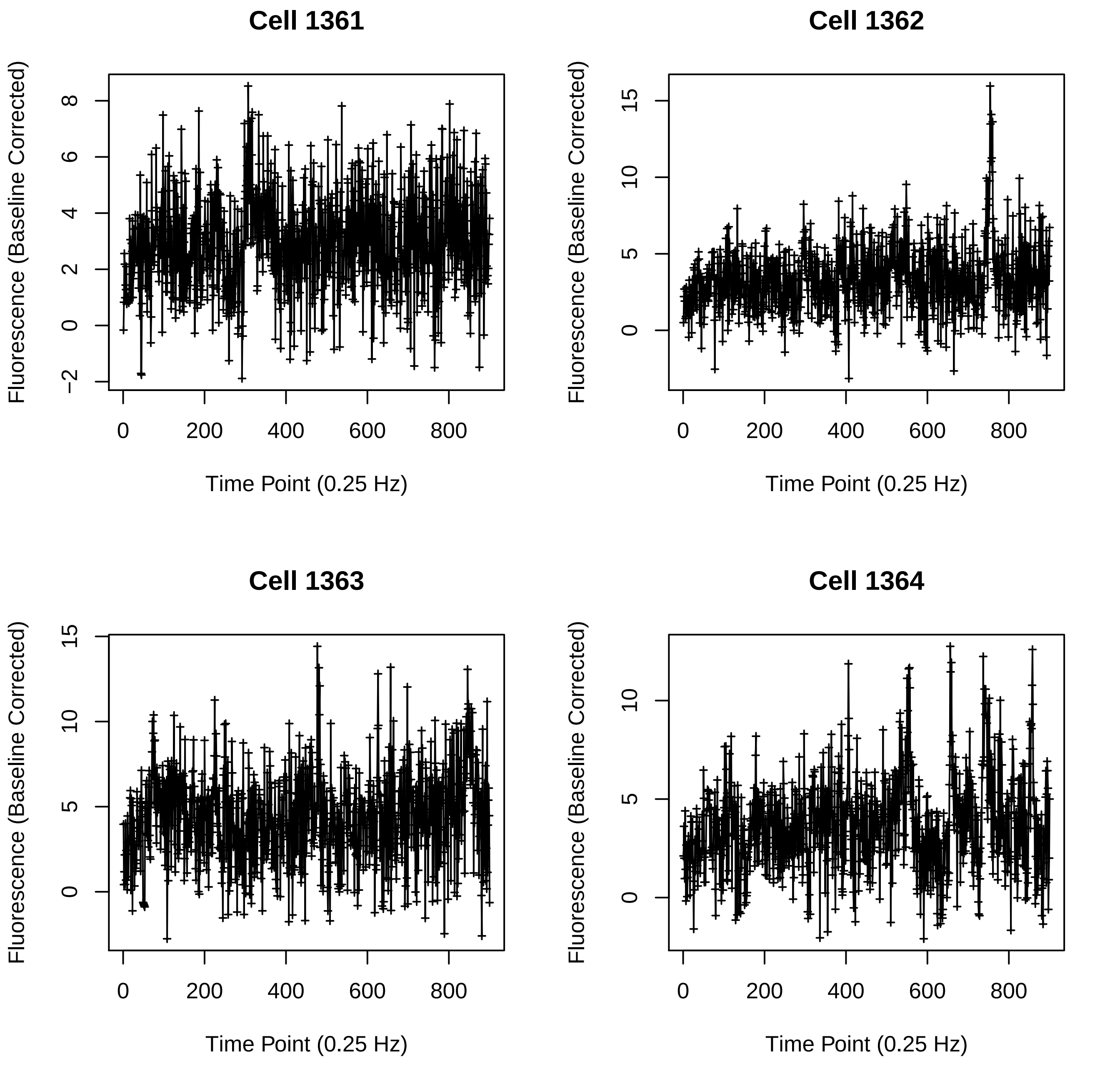
Cell 1361
Cell 1362
Cell 1363
Cell 1364
Fluorescence (Baseline Corrected)
Time Point (0.25 Hz)
0
200
400
600
800
−2
2
4
6
8
5
10
15

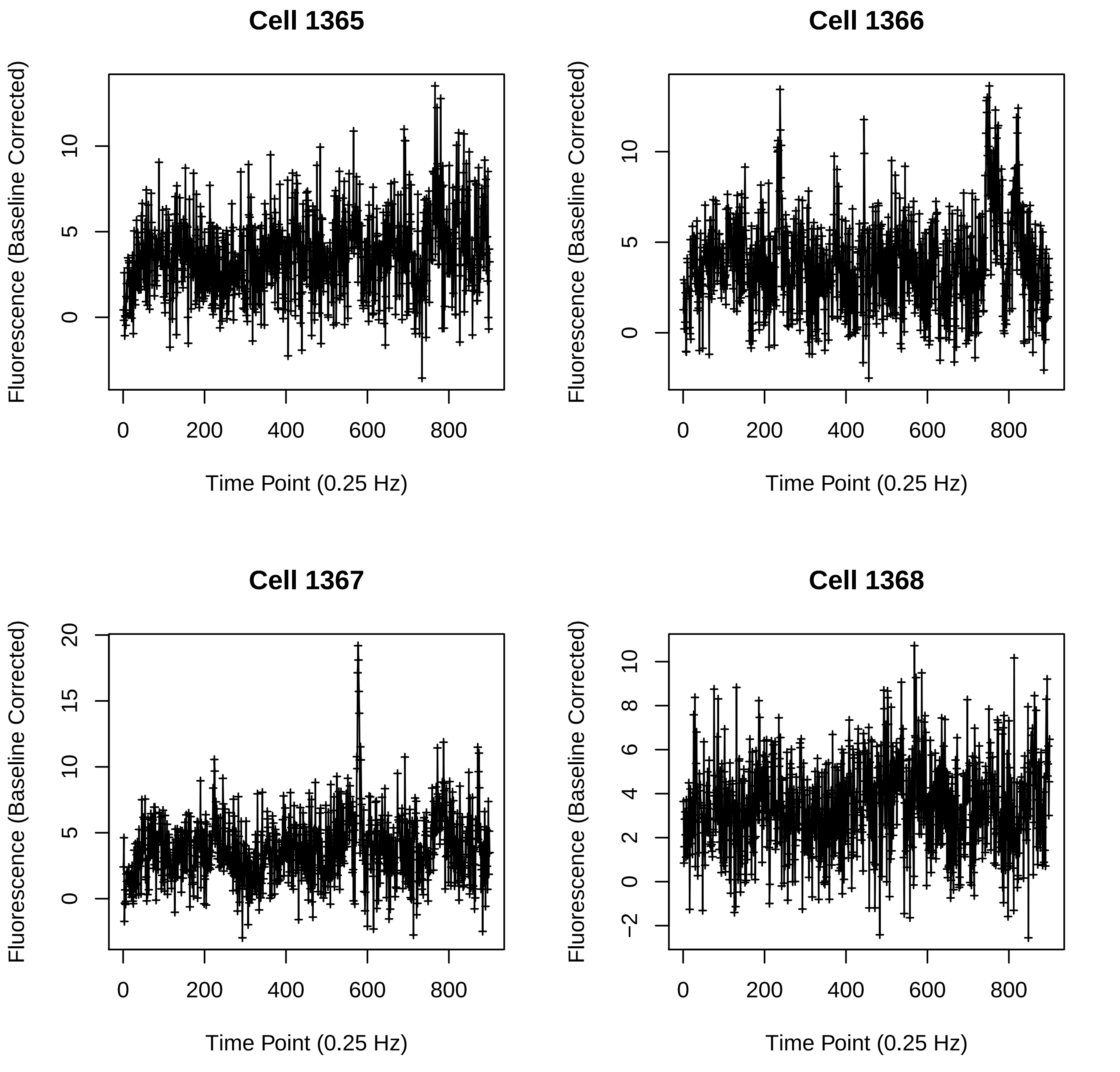

Cell 1365
Cell 1366
Cell 1367
Cell 1368
Fluorescence (Baseline Corrected)
Time Point (0.25 Hz)

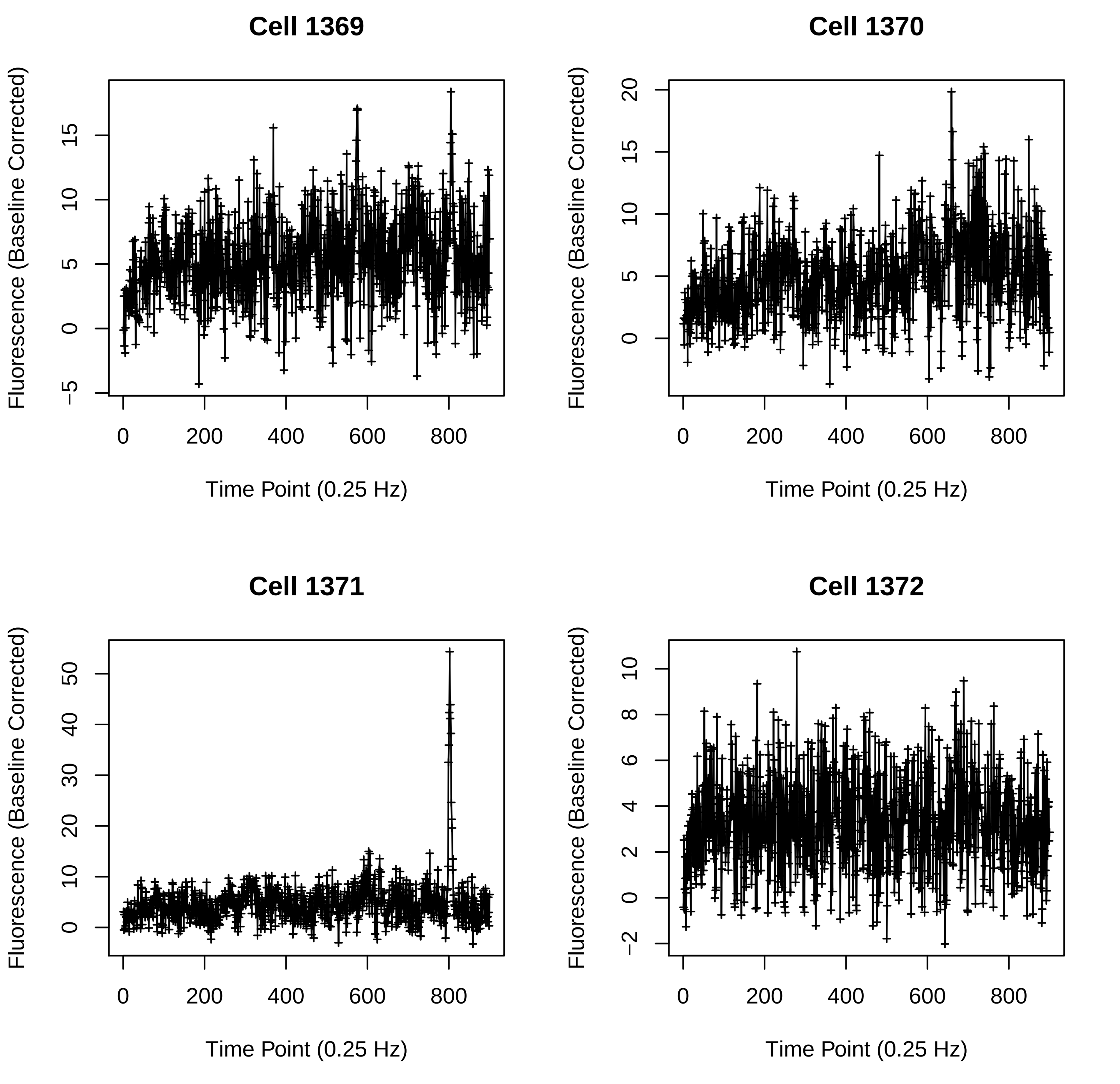
Cell 1369
Cell 1370
Cell 1371
Cell 1372
Fluorescence (Baseline Corrected)
Time Point (0.25 Hz)
0
200
400
600
800
−5
0
5
10
15
20
30
40
50
−2
2
4
6
8

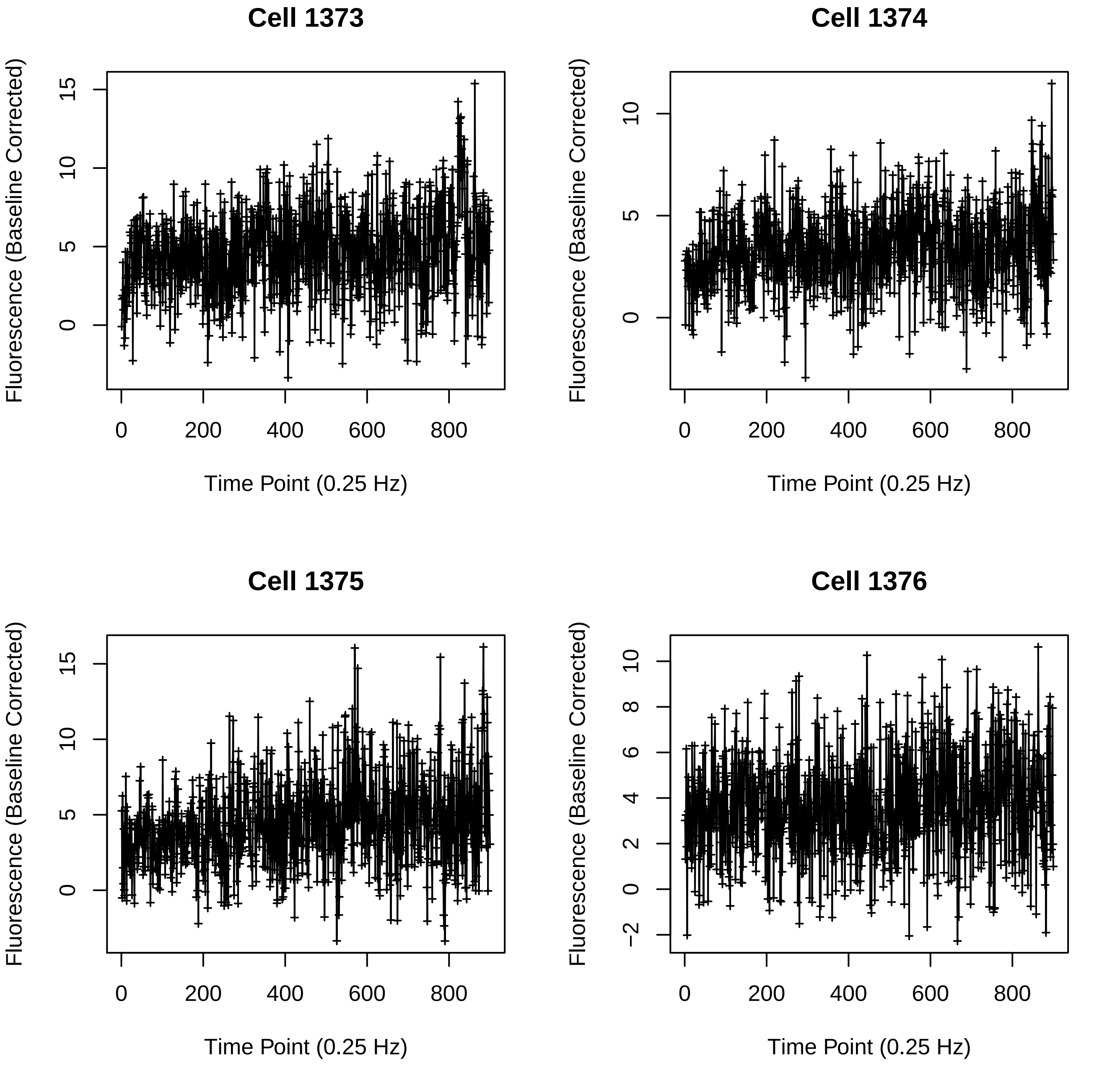

Cell 1373
Cell 1374
Cell 1375
Cell 1376
Fluorescence (Baseline Corrected)
Time Point (0.25 Hz)
0
5
10
15
0
200
400
600
800
−2
2
4
6
8

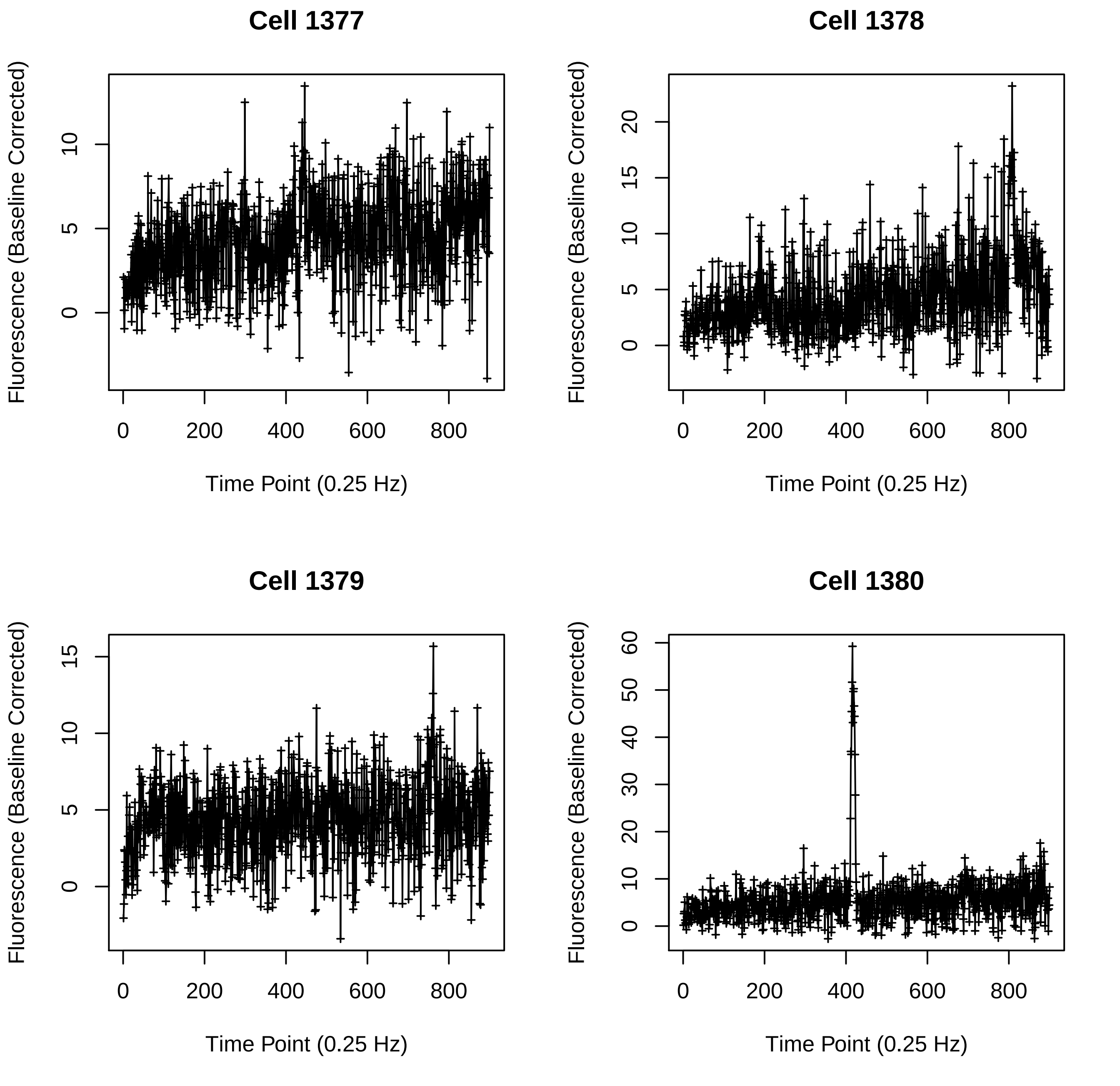

Cell 1377
Cell 1378
Cell 1379
Cell 1380
Fluorescence (Baseline Corrected)
Time Point (0.25 Hz)
0
200
400
600
800
0
5
10
15
20
30
40
50
60

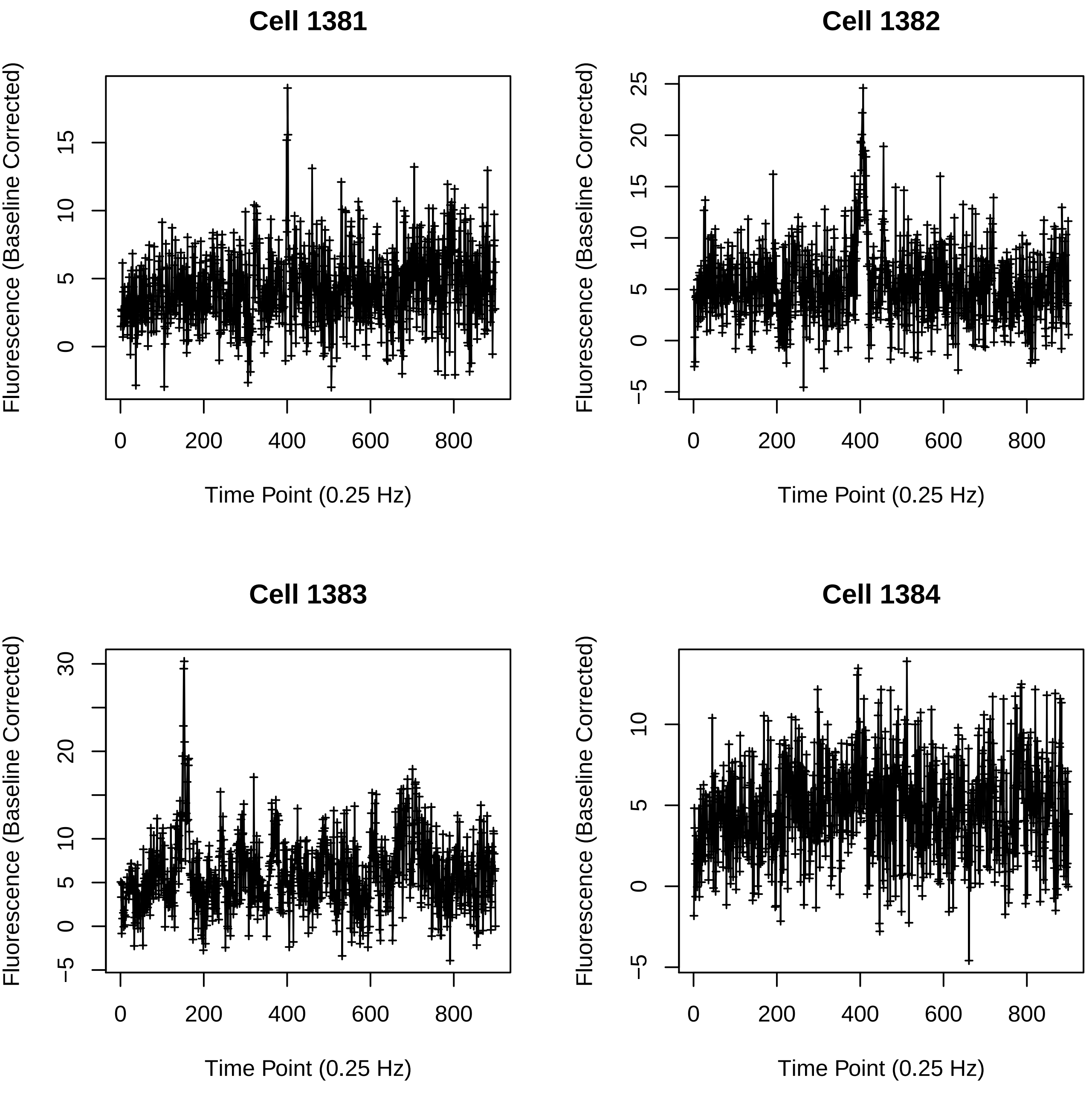
Cell 1381
Cell 1382
Cell 1383
Cell 1384
Fluorescence (Baseline Corrected)
Time Point (0.25 Hz)
0
200
400
600
800
-5
0
5
10
15
20
25
30

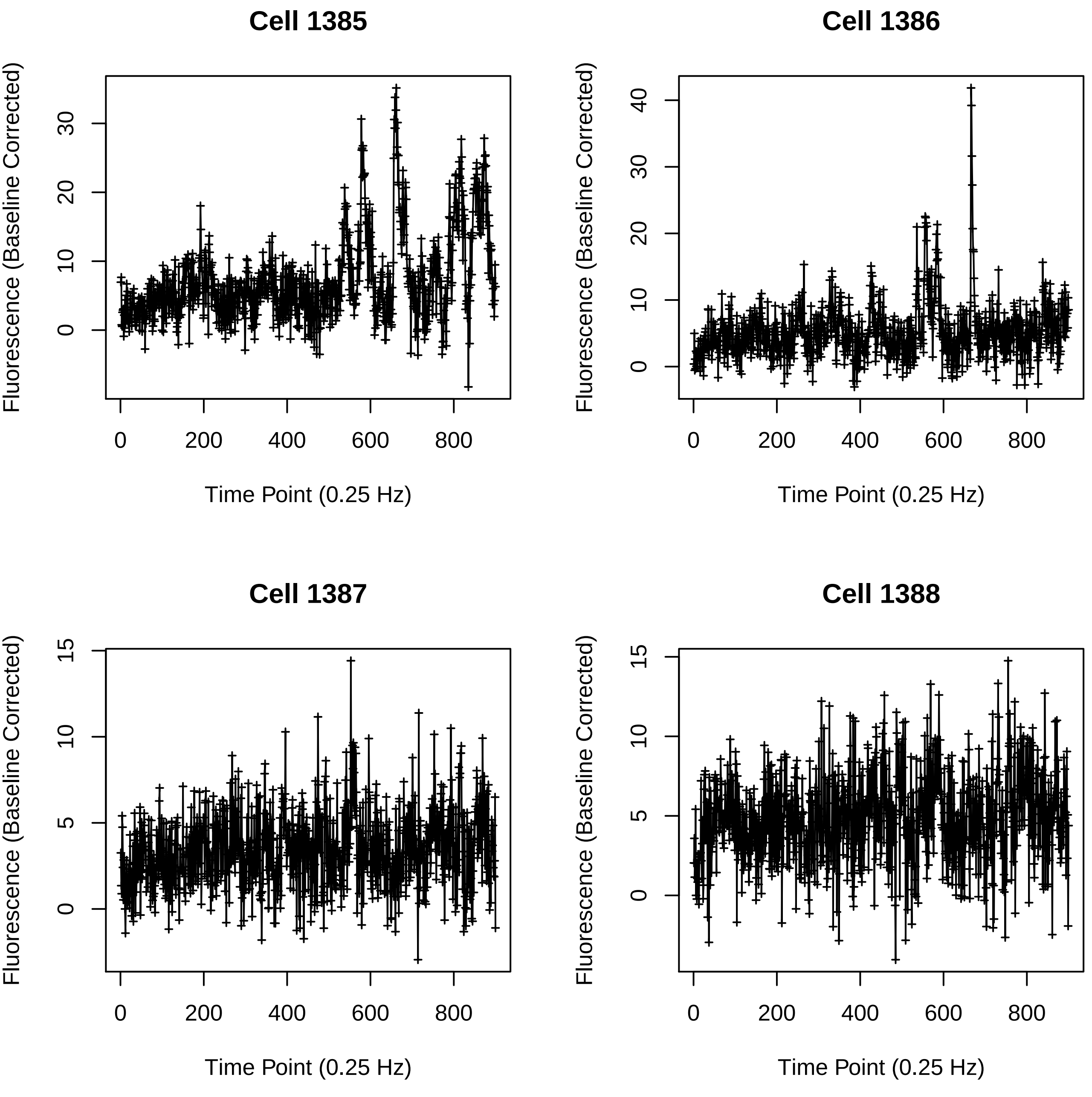

Cell 1385
Cell 1386
Cell 1387
Cell 1388
Fluorescence (Baseline Corrected)
Time Point (0.25 Hz)
0
200
400
600
800
10
20
30
40
5
15

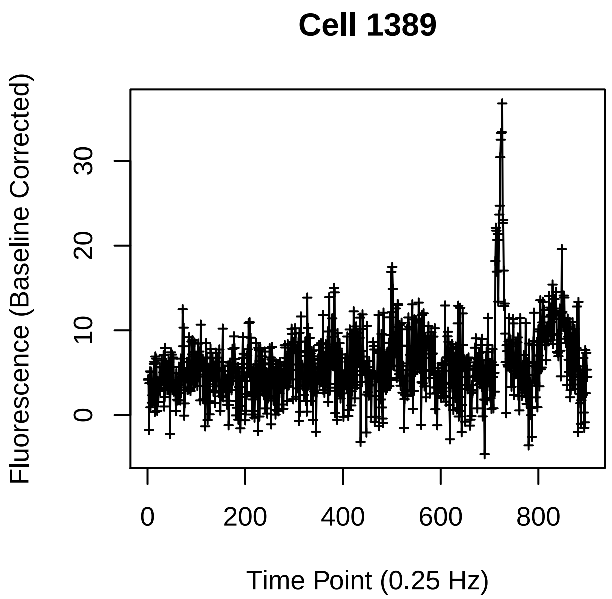
Cell 1389
Fluorescence (Baseline Corrected)
0
10
20
30
0
200
400
600
800
Time Point (0.25 Hz)

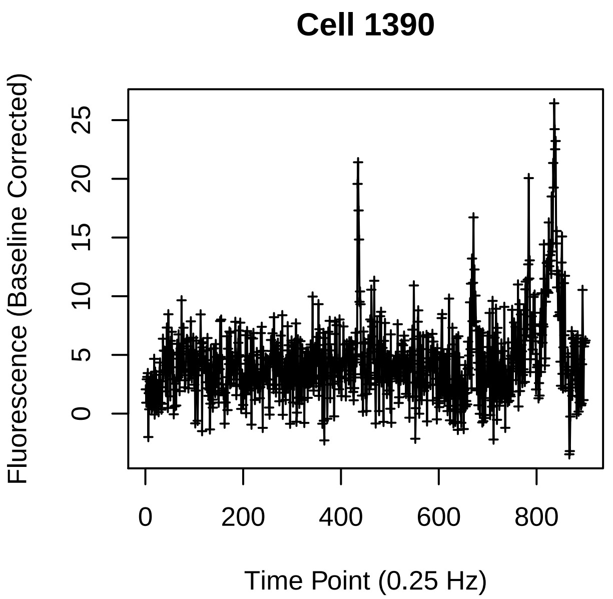
Cell 1390
Fluorescence (Baseline Corrected)
0
5
10
15
20
25
0
200
400
600
800
Time Point (0.25 Hz)

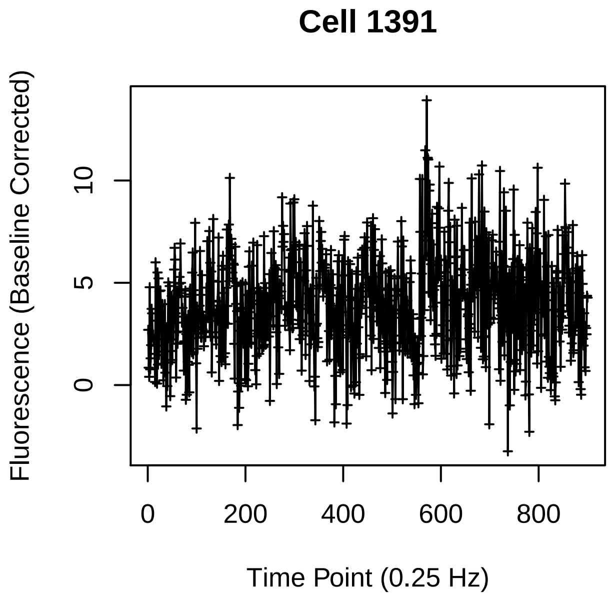
Cell 1391
Fluorescence (Baseline Corrected)
0
5
10
0
200
400
600
800
Time Point (0.25 Hz)

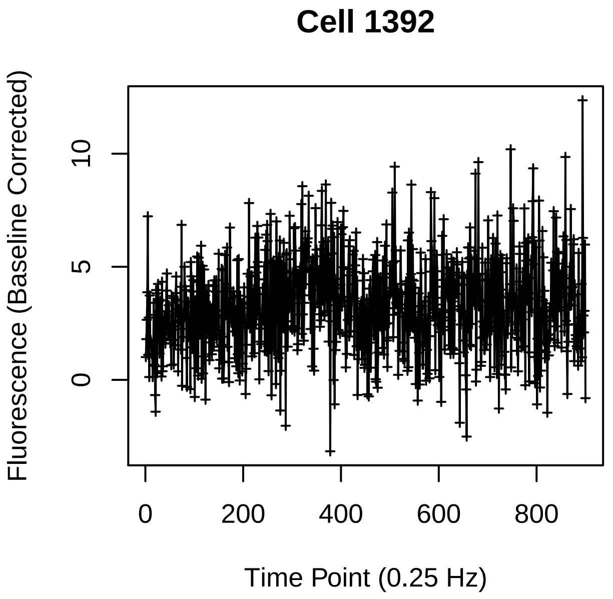
Cell 1392
Fluorescence (Baseline Corrected)
0
5
10
0
200
400
600
800
Time Point (0.25 Hz)

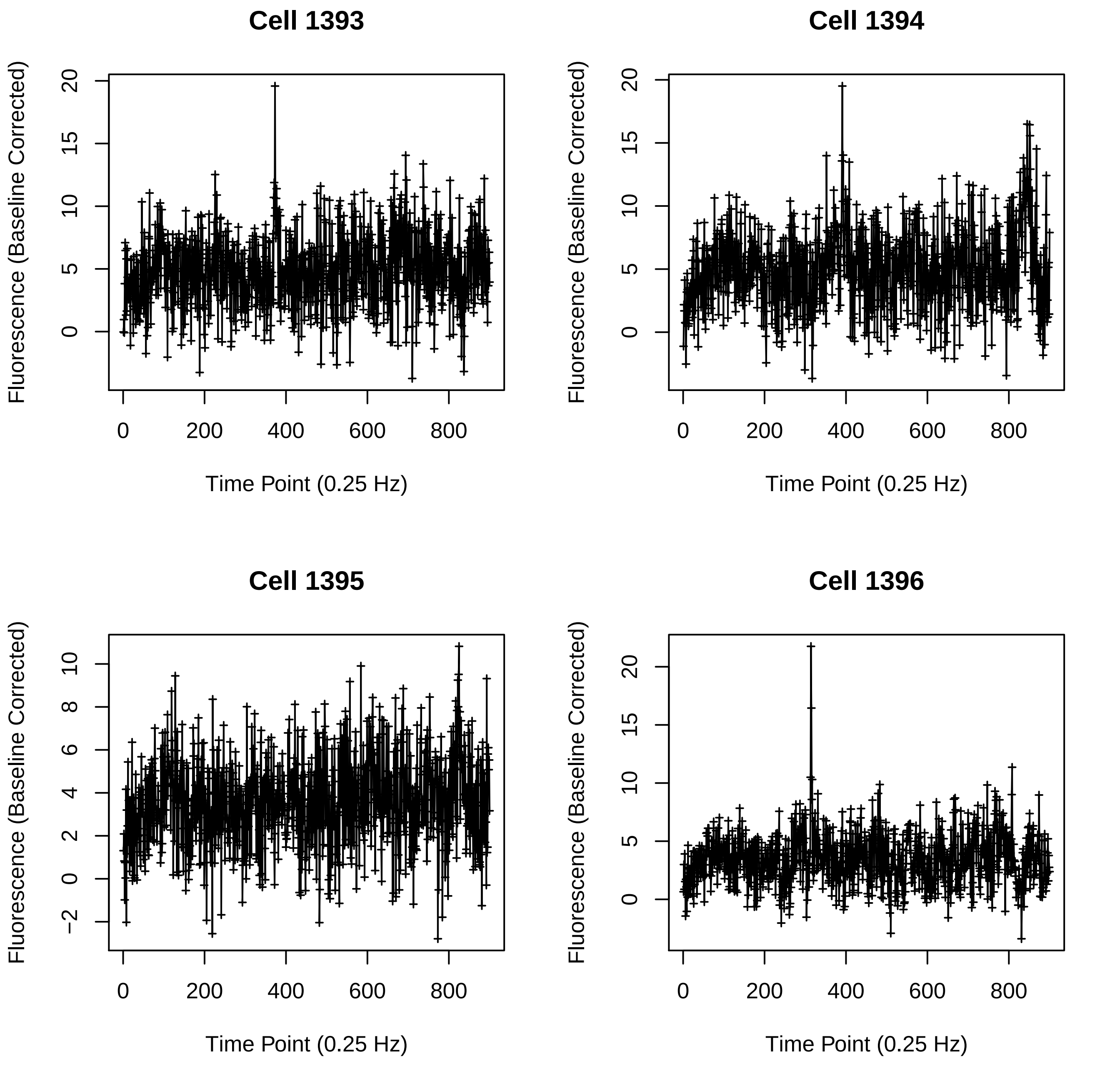

Cell 1393
Cell 1394
Cell 1395
Cell 1396
Fluorescence (Baseline Corrected)
Time Point (0.25 Hz)
0
200
400
600
800
5
10
15
20
−2
2
4
6
8

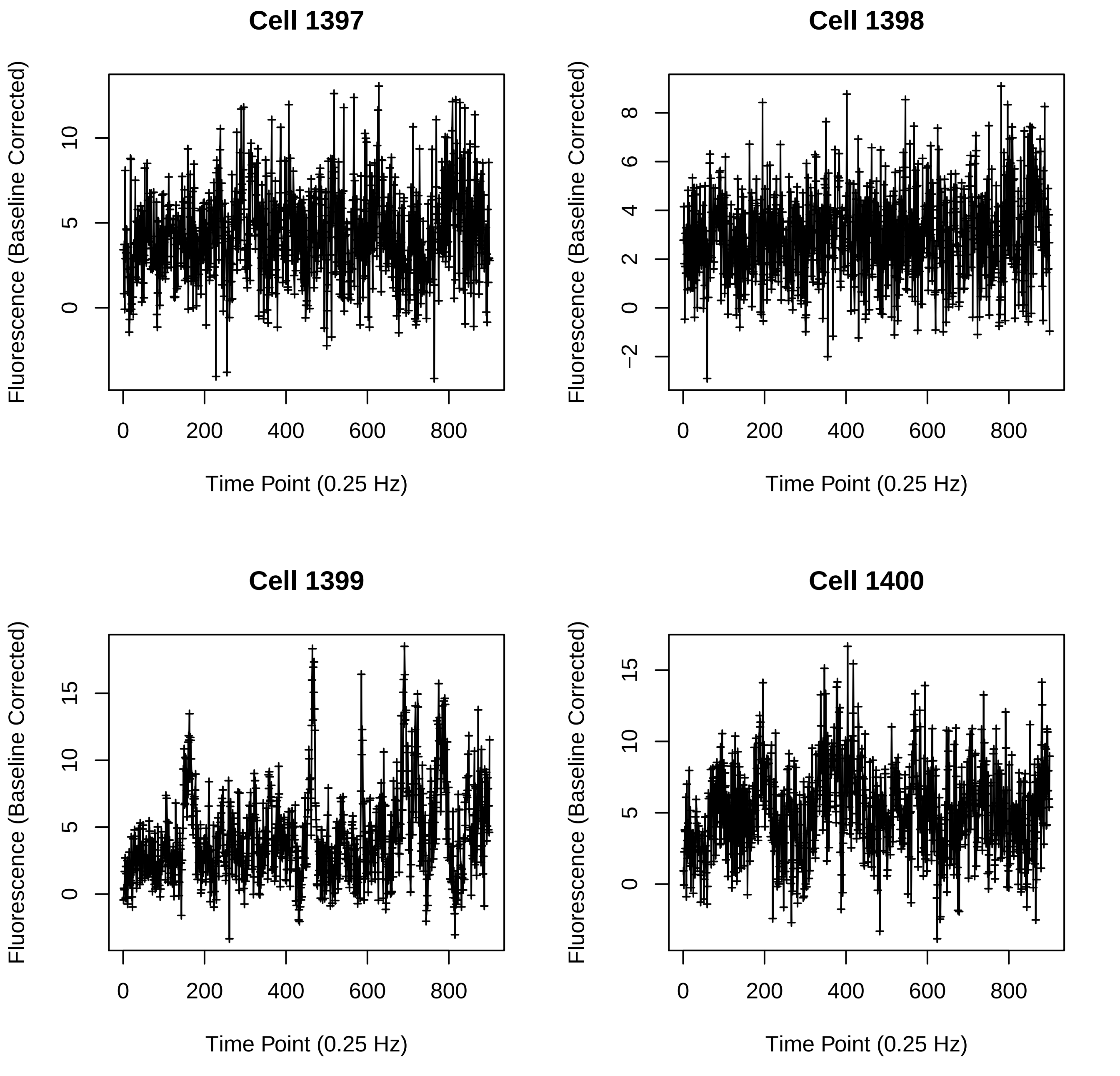

Cell 1397
Cell 1398
Cell 1399
Cell 1400
Fluorescence (Baseline Corrected)
Time Point (0.25 Hz)
0
200
400
600
800
−2
2
4
5
6
8
10
15

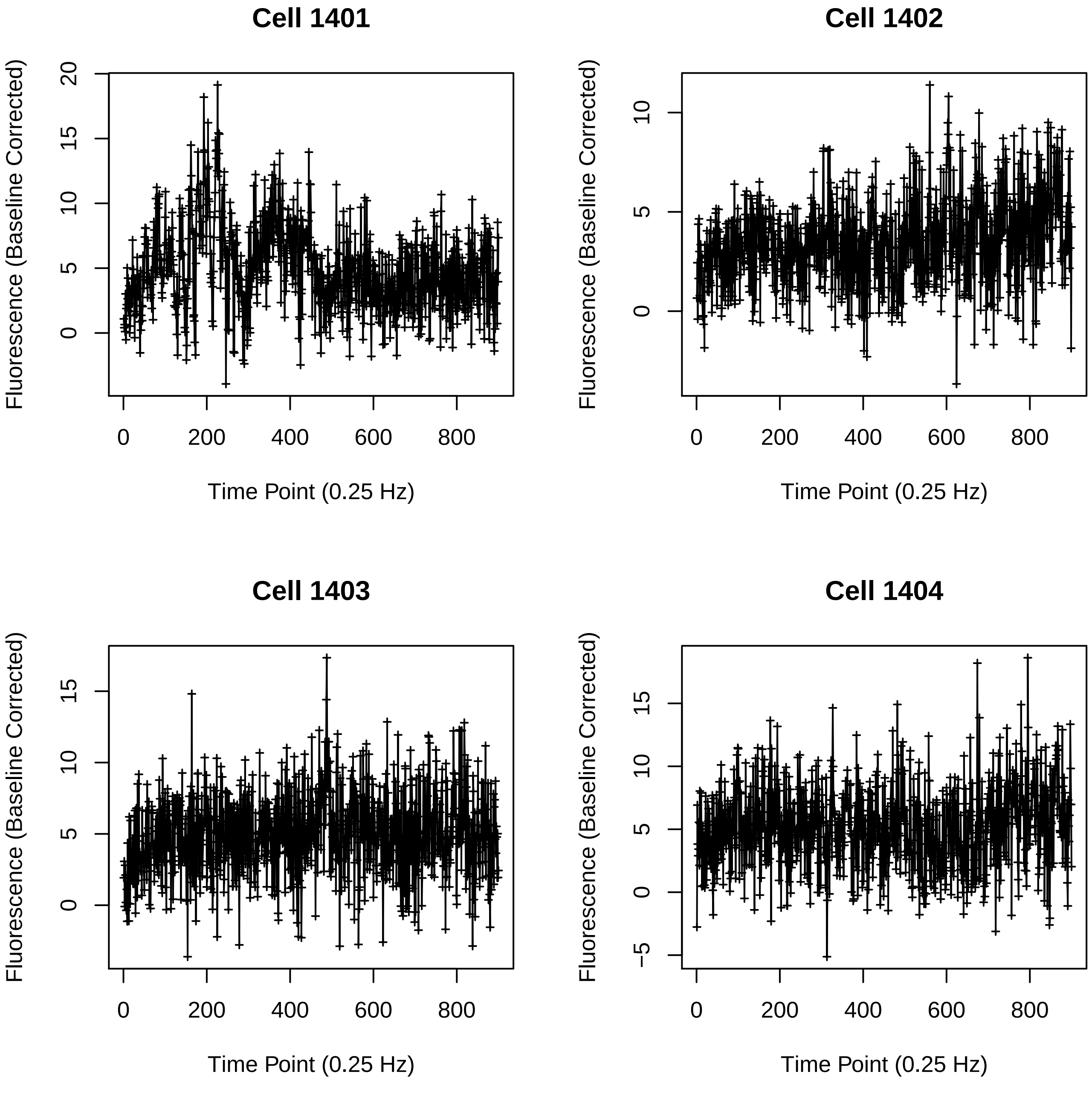
Cell 1401
Cell 1402
Cell 1403
Cell 1404
Fluorescence (Baseline Corrected)
Time Point (0.25 Hz)
0
5
10
15
20
-5
0
200
400
600
800

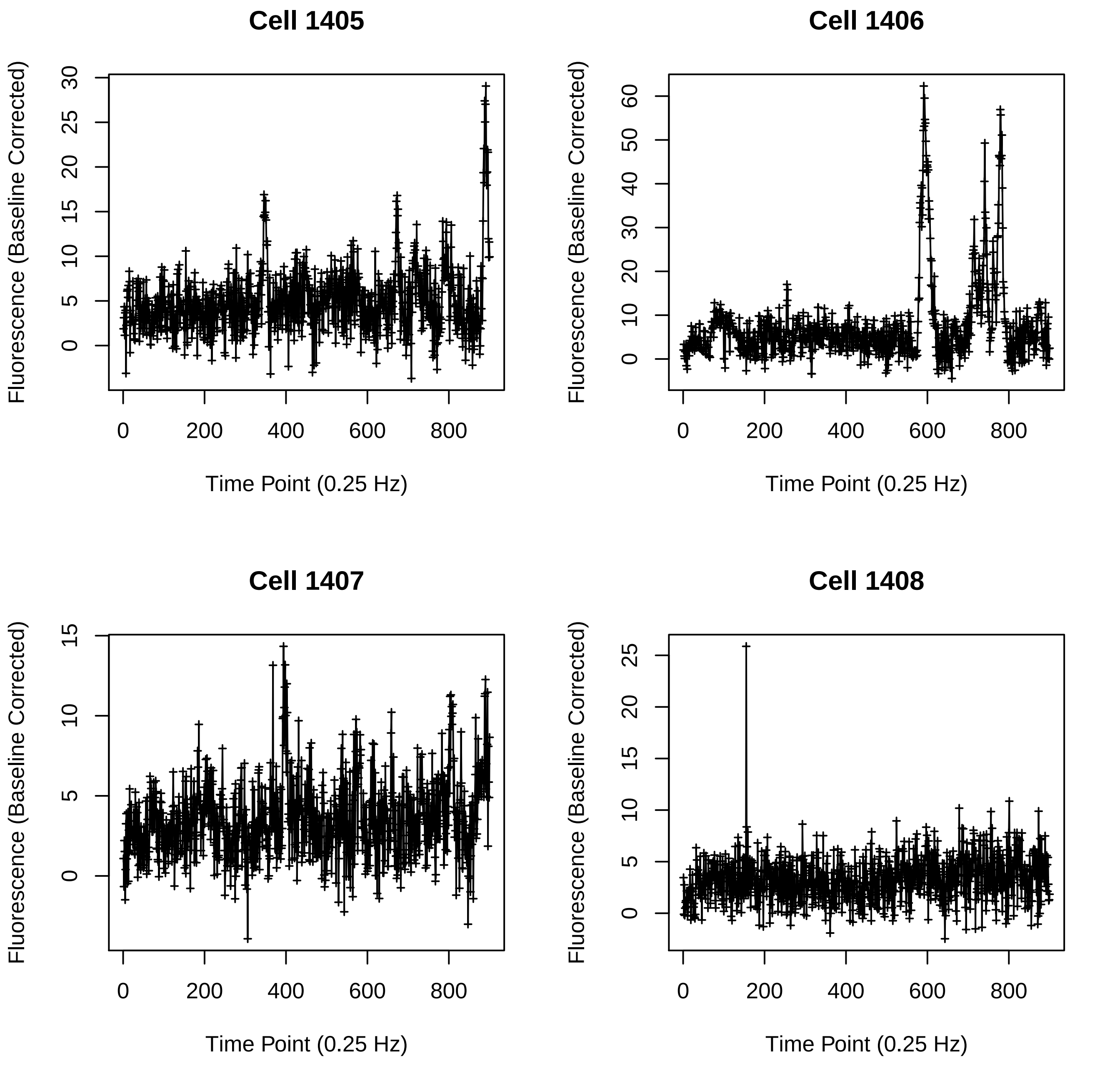

Cell 1405
Cell 1406
Cell 1407
Cell 1408
Fluorescence (Baseline Corrected)
Time Point (0.25 Hz)
0
200
400
600
800

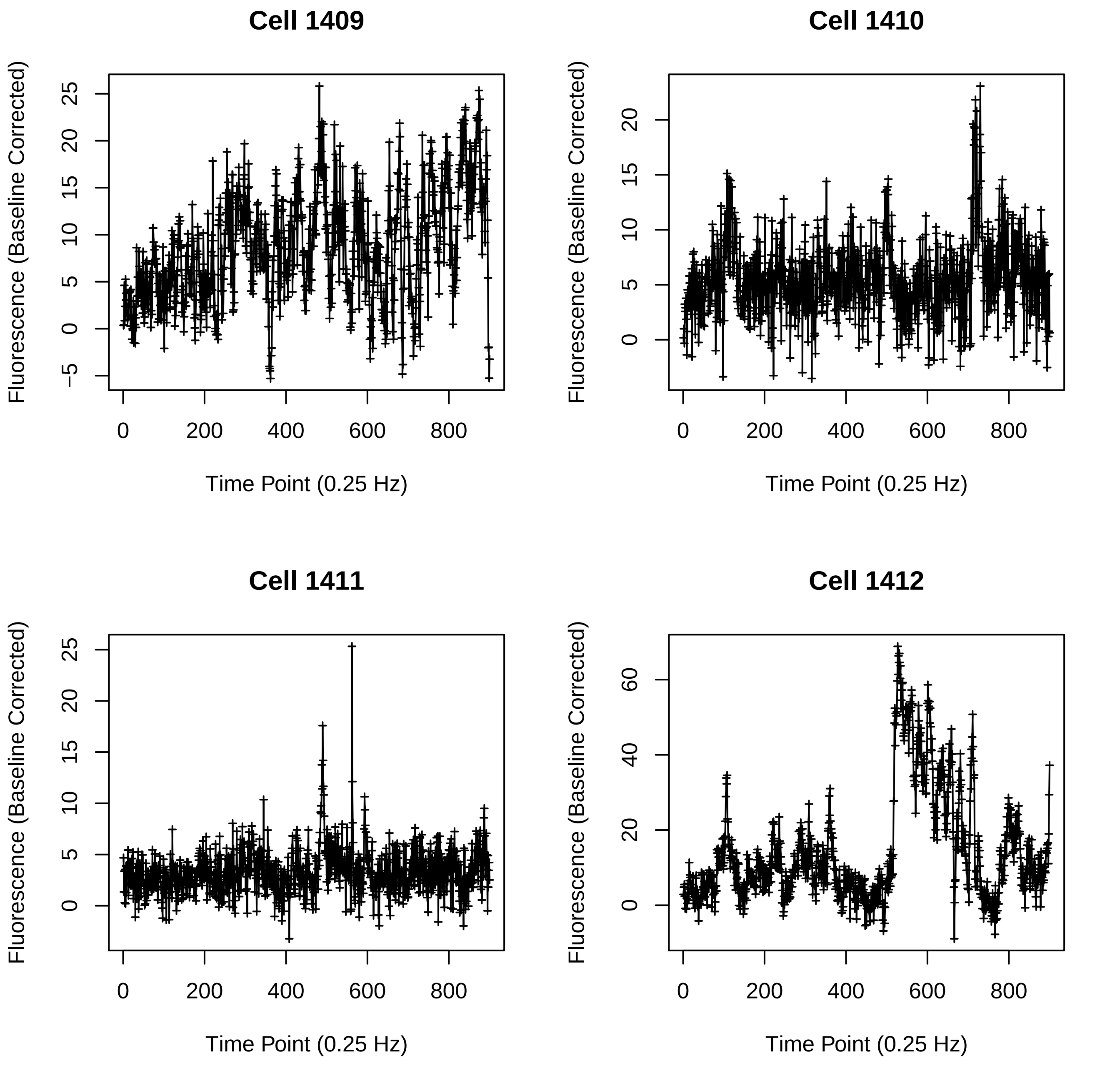

Cell 1409
Cell 1410
Cell 1411
Cell 1412
Fluorescence (Baseline Corrected)
Time Point (0.25 Hz)

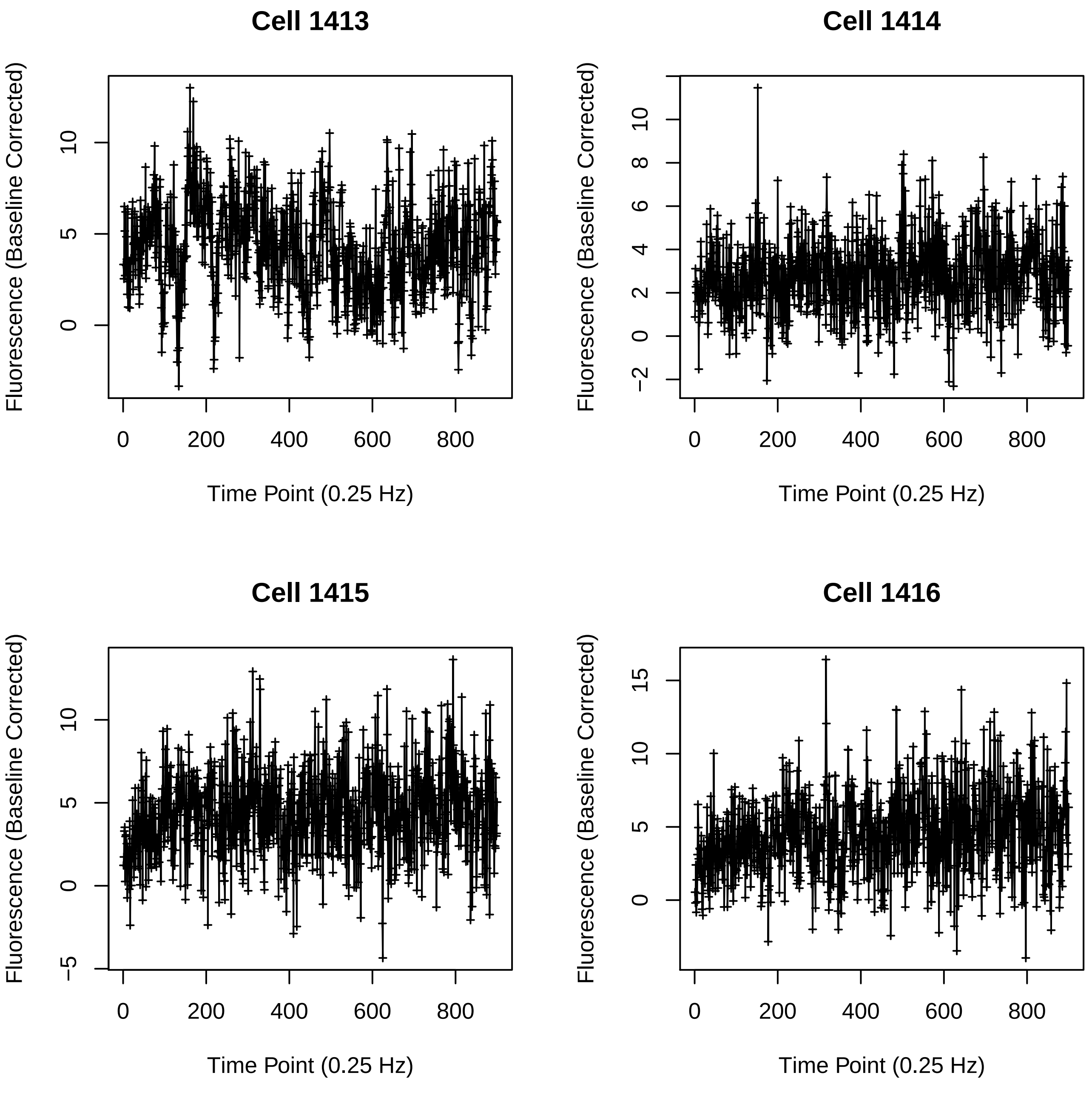
Cell 1413
Fluorescence (Baseline Corrected)
Time Point (0.25 Hz)
0
5
10
0
200
400
600
800
Cell 1414
Fluorescence (Baseline Corrected)
Time Point (0.25 Hz)
−2
0
2
4
6
8
10
0
200
400
600
800
Cell 1415
Fluorescence (Baseline Corrected)
Time Point (0.25 Hz)
−5
0
5
10
0
200
400
600
800
Cell 1416
Fluorescence (Baseline Corrected)
Time Point (0.25 Hz)
0
5
10
15
0
200
400
600
800

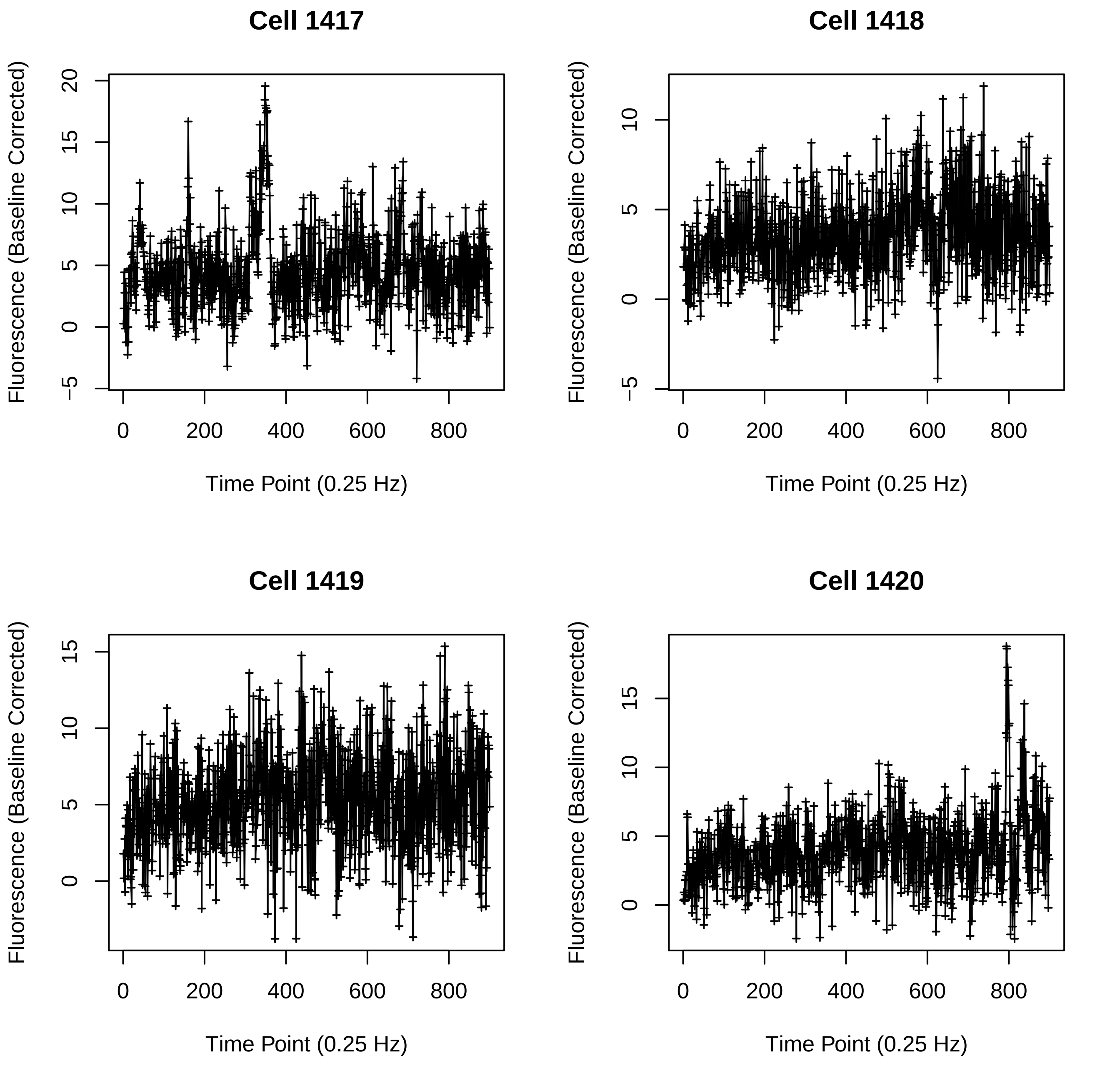

Cell 1417
Cell 1418
Cell 1419
Cell 1420
Fluorescence (Baseline Corrected)
Time Point (0.25 Hz)
−5
0
5
10
15
20
0
200
400
600
800

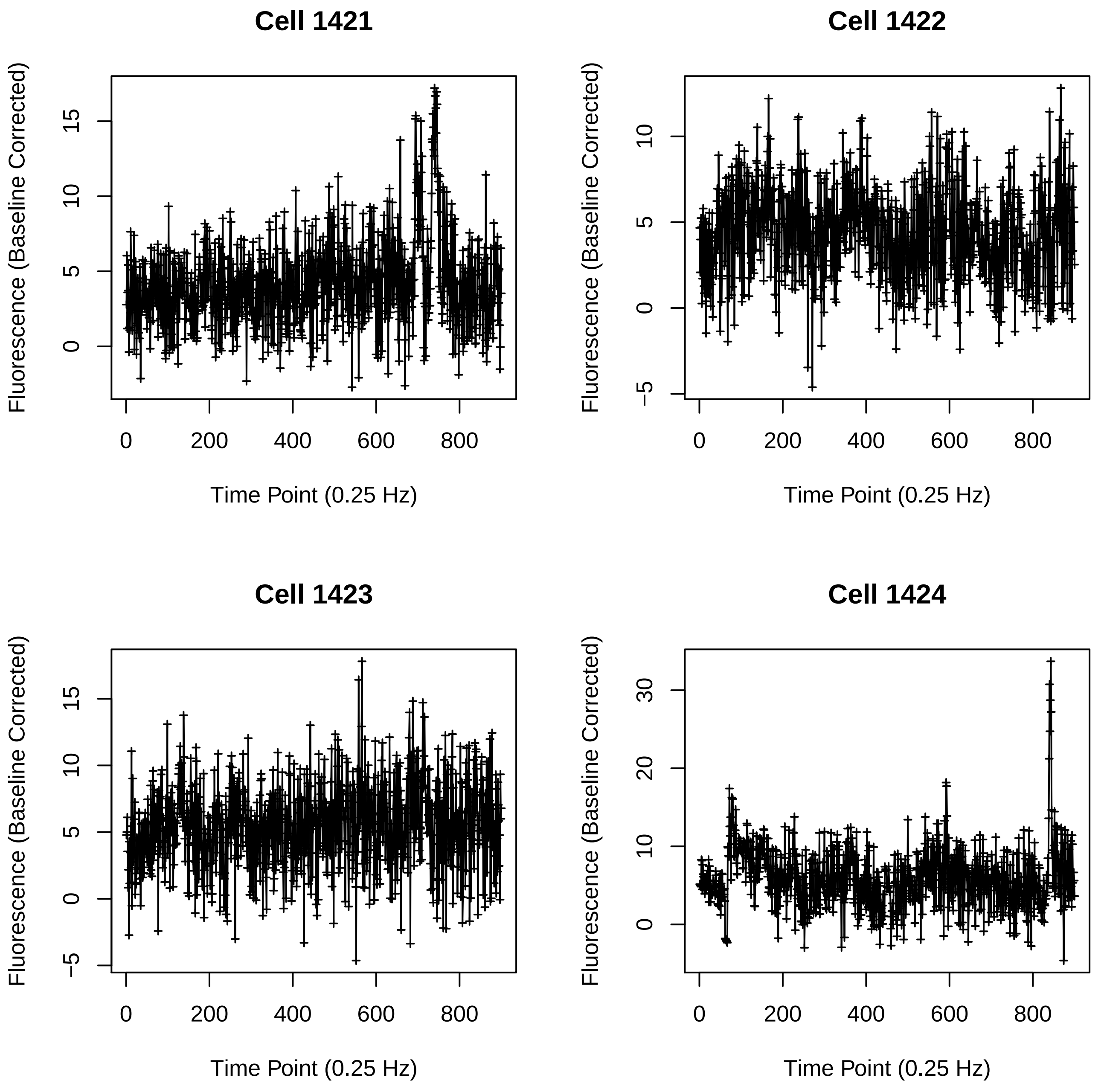

Cell 1421
Cell 1422
Cell 1423
Cell 1424
Fluorescence (Baseline Corrected)
Time Point (0.25 Hz)
0
200
400
600
800
-5
0
5
10
15
20
30

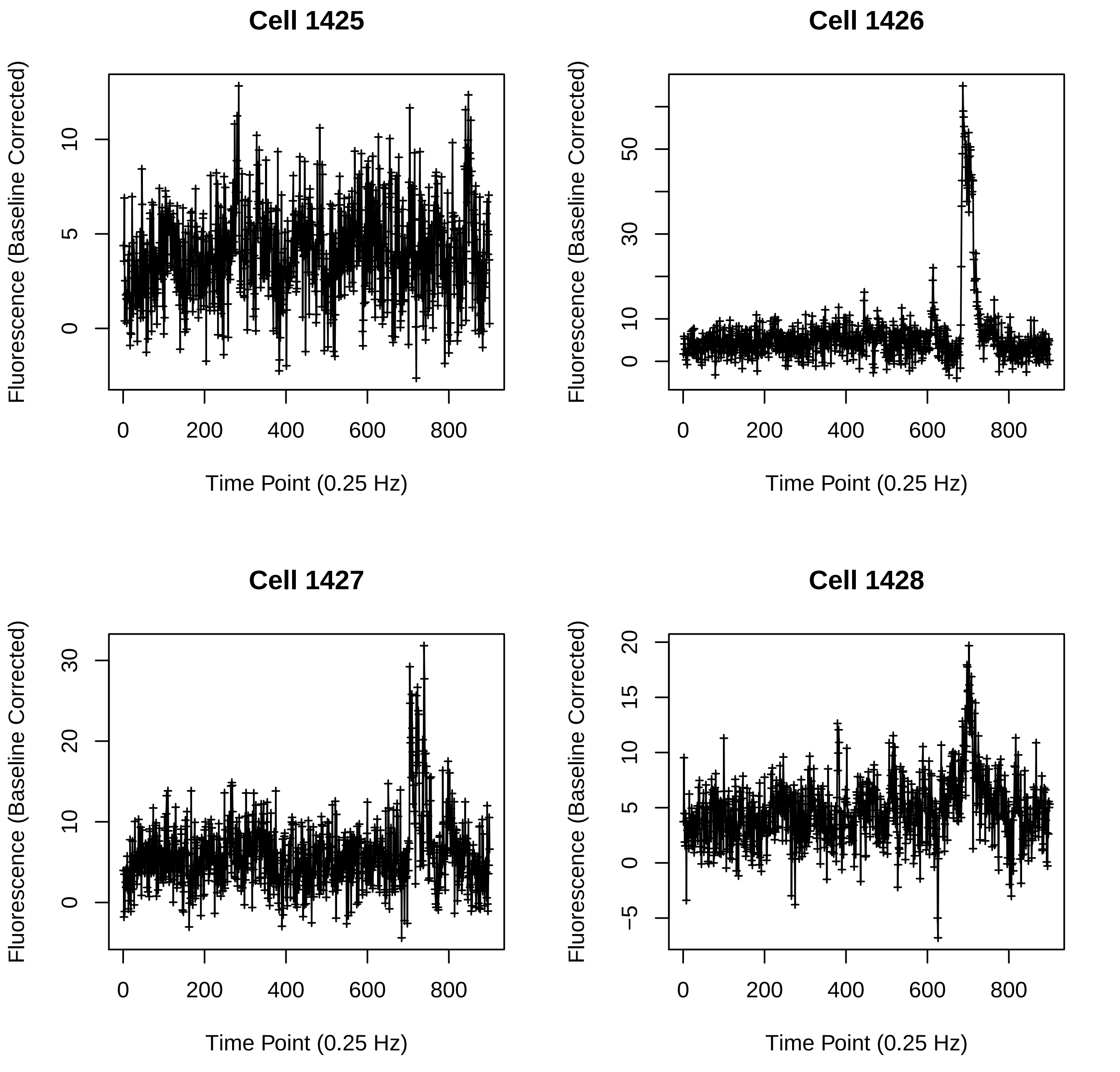
Cell 1425
Cell 1426
Cell 1427
Cell 1428
Fluorescence (Baseline Corrected)
Time Point (0.25 Hz)
0
200
400
600
800
0
5
10
0
10
20
30
50
0
10
20
30
−5
0
5
10
15
20

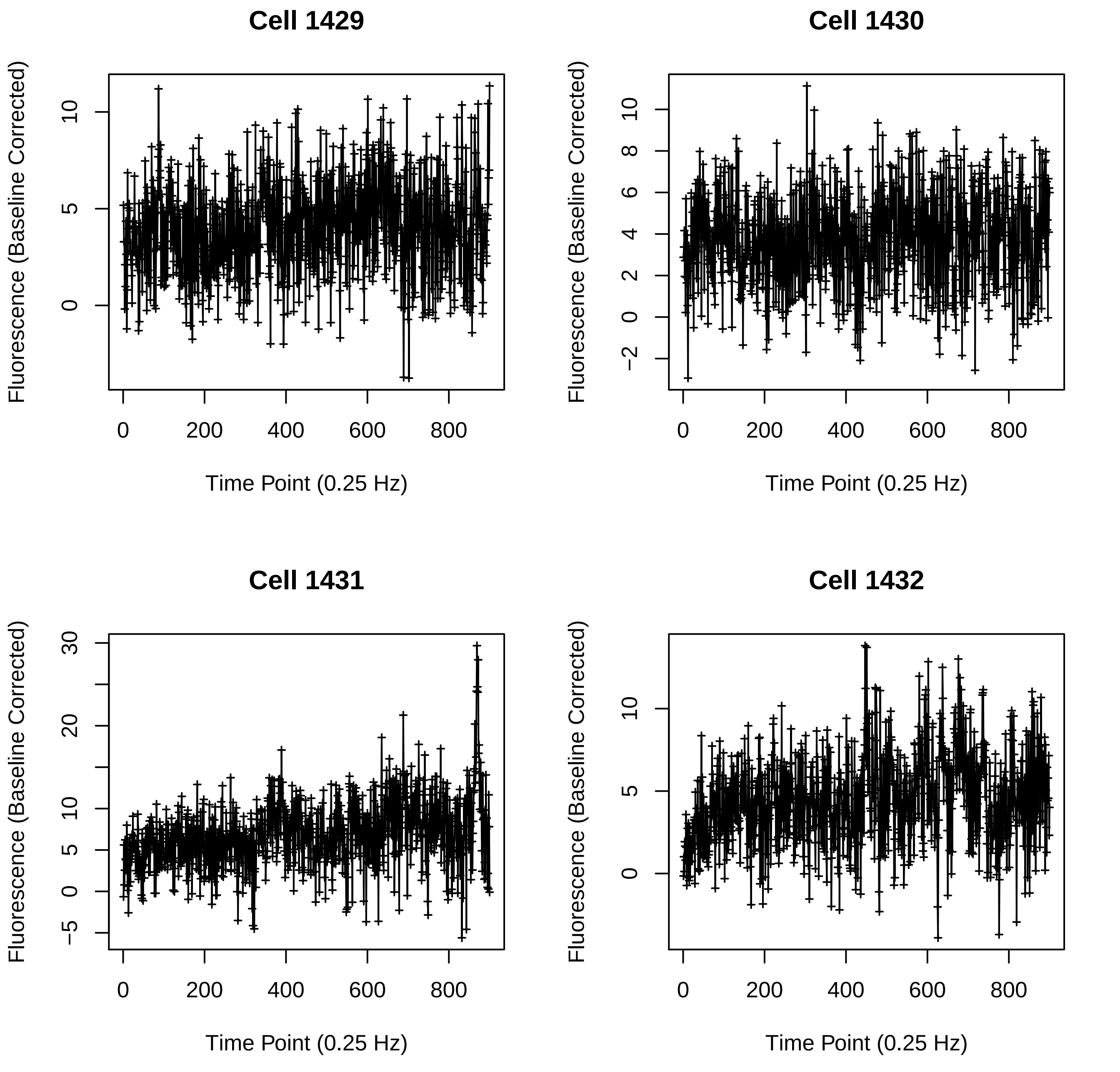
Cell 1429
Cell 1430
Cell 1431
Cell 1432
Fluorescence (Baseline Corrected)
Time Point (0.25 Hz)
0
200
400
600
800
−5
0
5
10
20
30
−2
2
4
6
8

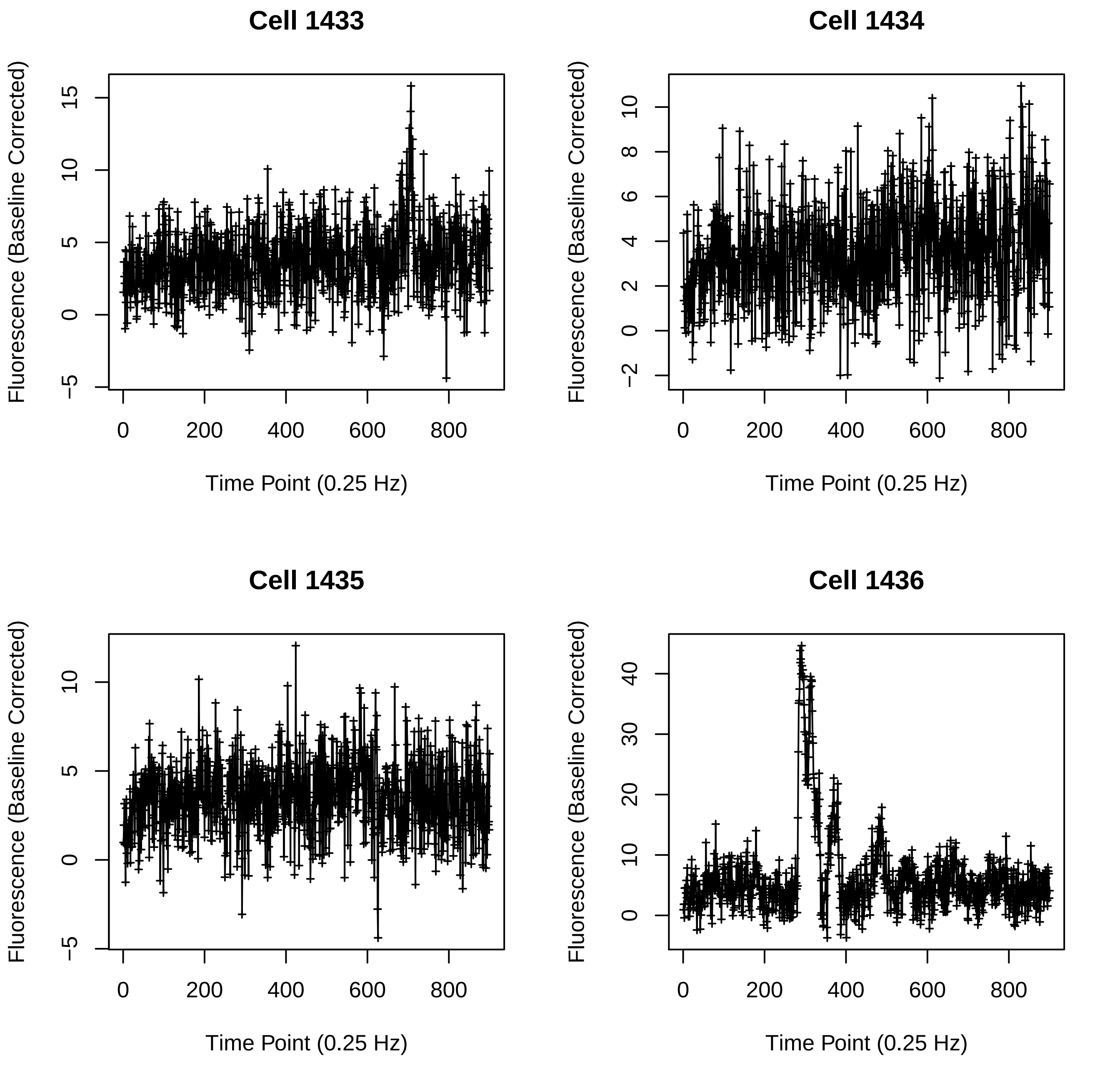
Cell 1433
Cell 1434
Cell 1435
Cell 1436
Fluorescence (Baseline Corrected)
Time Point (0.25 Hz)
−5
0
5
10
15
−2
2
4
6
8
20
30
40
200
400
600
800

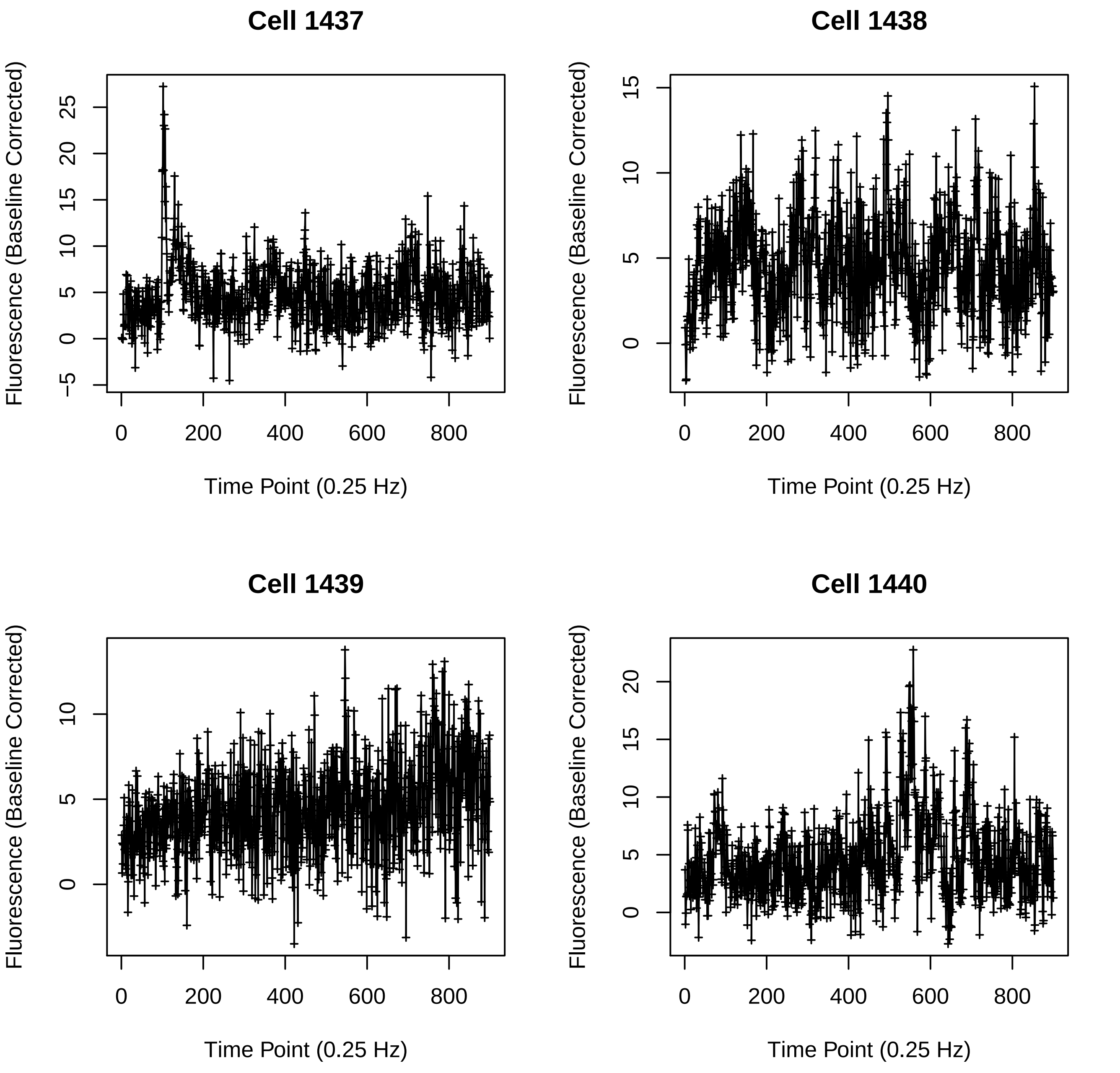
Cell 1437
Fluorescence (Baseline Corrected)
Time Point (0.25 Hz)
Cell 1438
Fluorescence (Baseline Corrected)
Time Point (0.25 Hz)
Cell 1439
Fluorescence (Baseline Corrected)
Time Point (0.25 Hz)
Cell 1440
Fluorescence (Baseline Corrected)
Time Point (0.25 Hz)
0 200 400 600 800

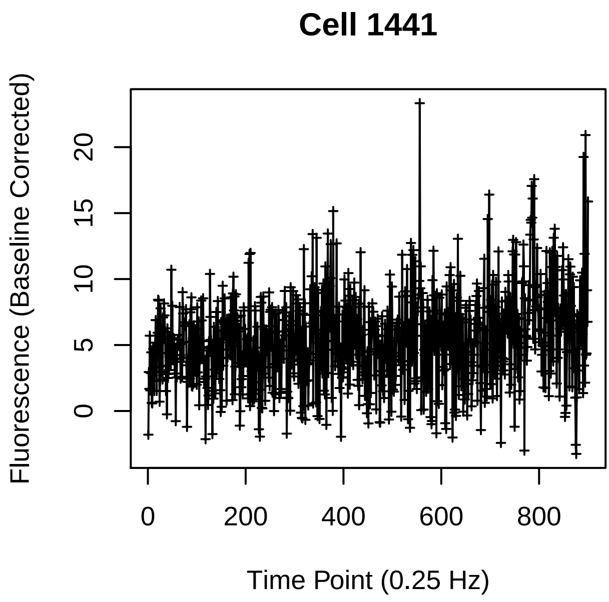
Cell 1441
Fluorescence (Baseline Corrected)
Time Point (0.25 Hz)
0
5
10
15
20
0
200
400
600
800

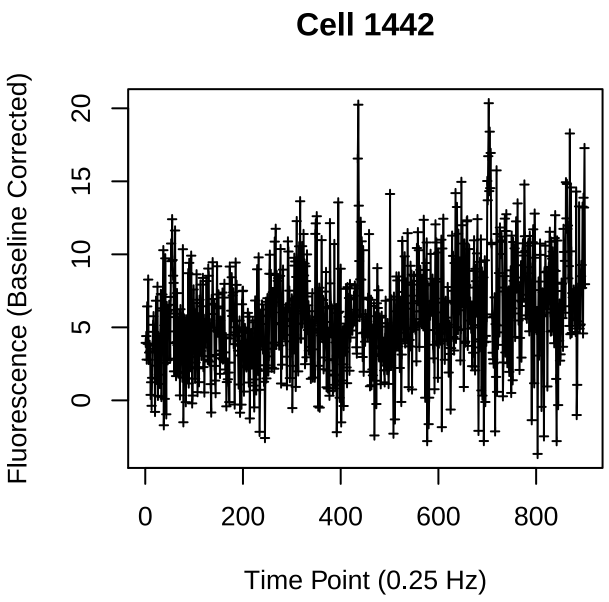
Cell 1442
Fluorescence (Baseline Corrected)
Time Point (0.25 Hz)
0
5
10
15
20
0
200
400
600
800

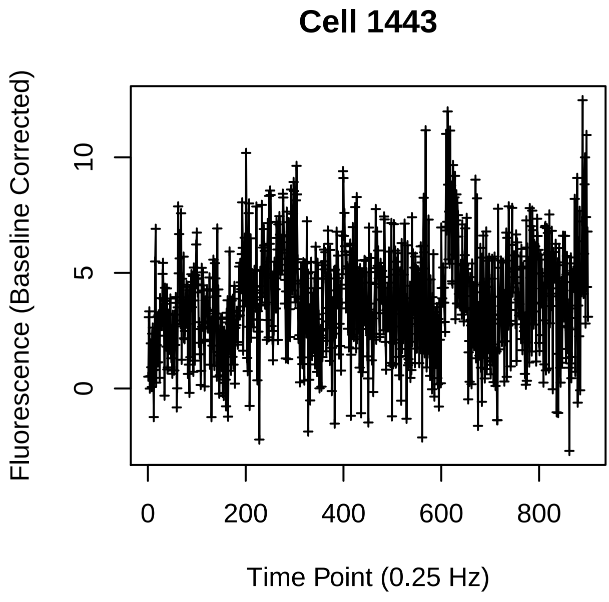
Cell 1443
Fluorescence (Baseline Corrected)
Time Point (0.25 Hz)
0
5
10
0
200
400
600
800

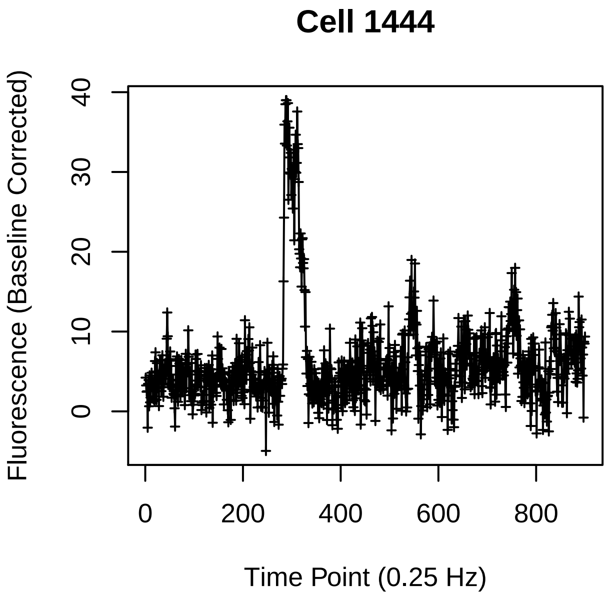
Cell 1444
Fluorescence (Baseline Corrected)
Time Point (0.25 Hz)
0
10
20
30
40
0
200
400
600
800

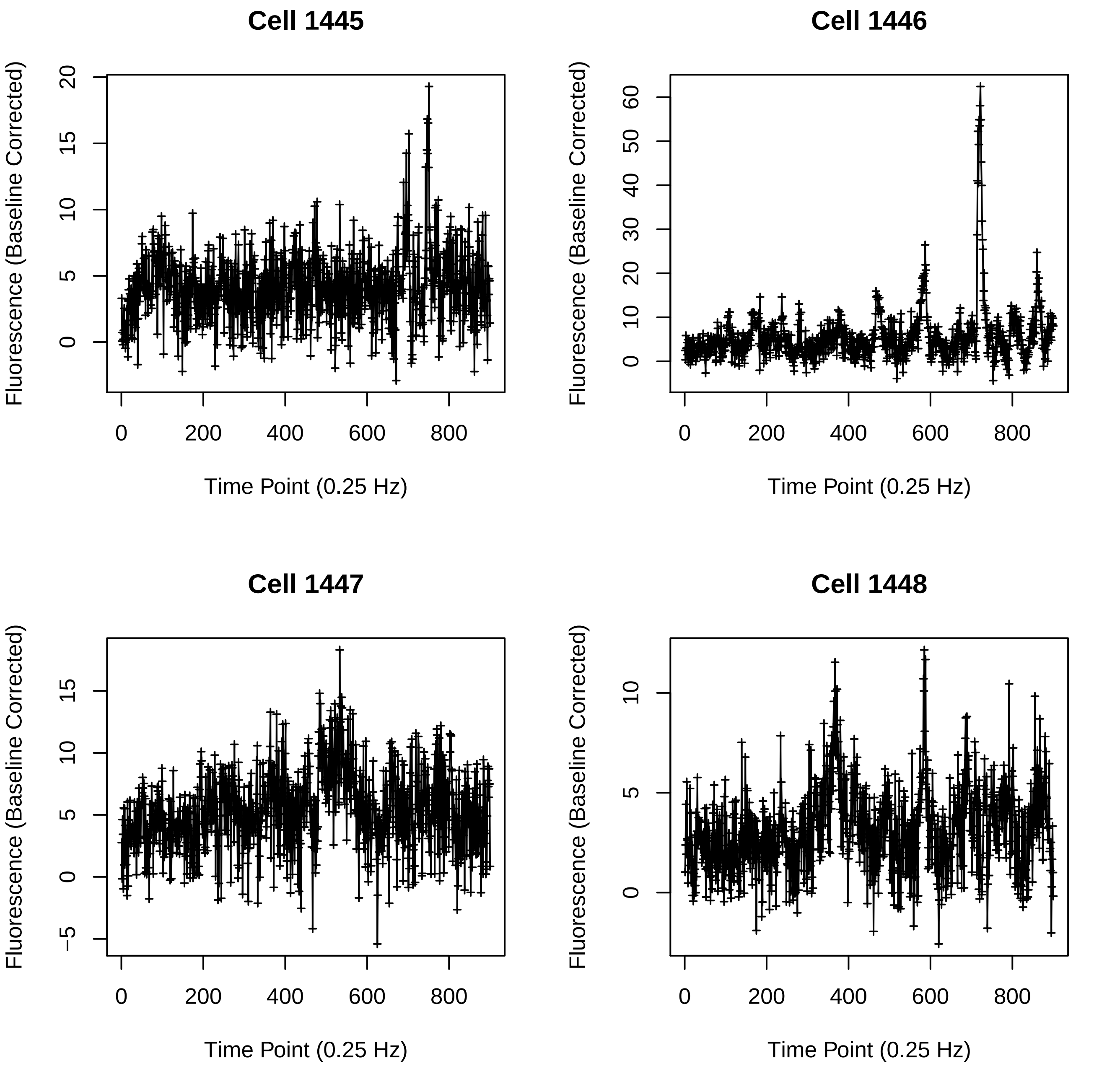
Cell 1445
Fluorescence (Baseline Corrected)
Time Point (0.25 Hz)
Cell 1446
Fluorescence (Baseline Corrected)
Time Point (0.25 Hz)
Cell 1447
Fluorescence (Baseline Corrected)
Time Point (0.25 Hz)
Cell 1448
Fluorescence (Baseline Corrected)
Time Point (0.25 Hz)

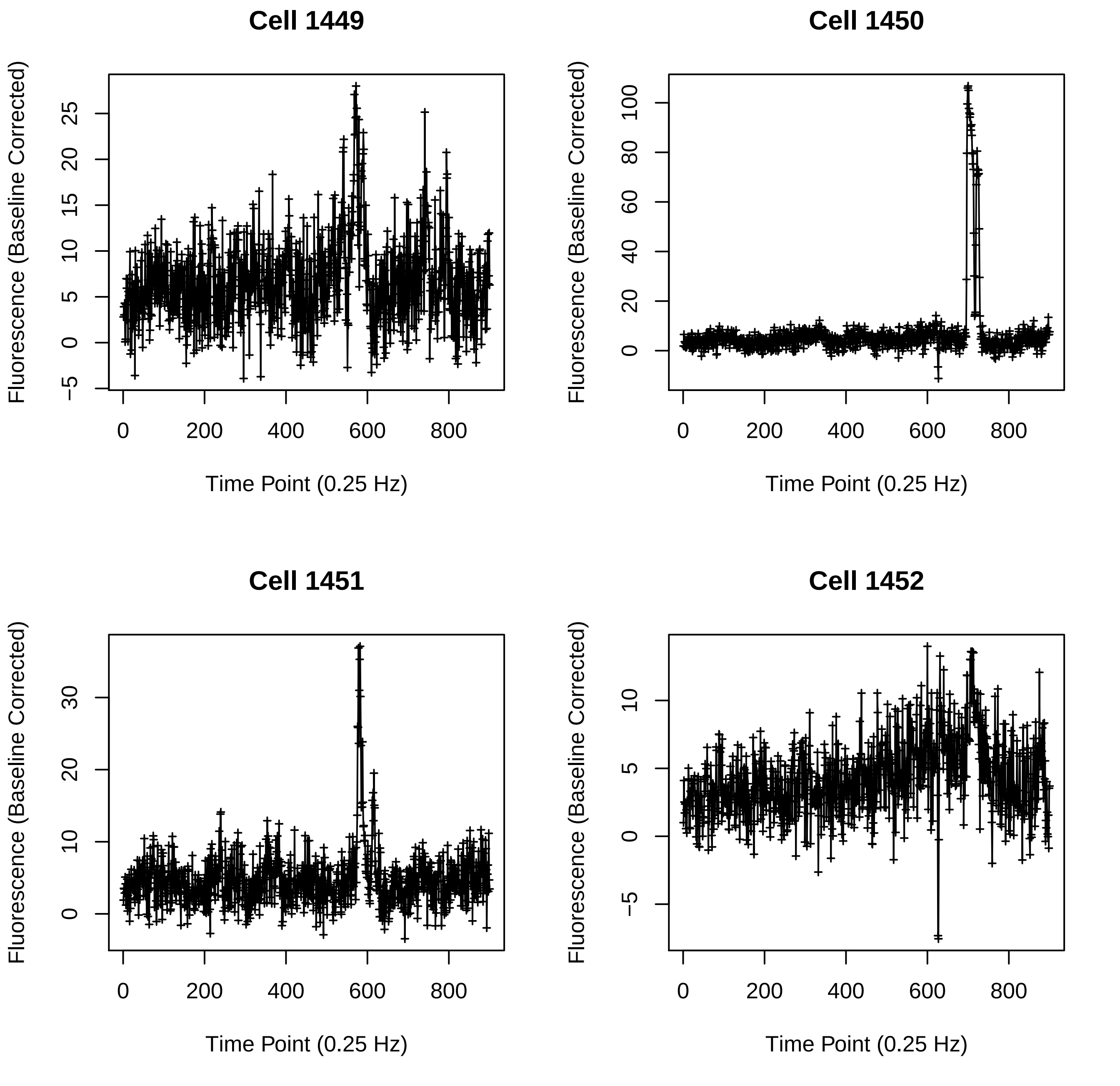

Cell 1449
Cell 1450
Cell 1451
Cell 1452
Fluorescence (Baseline Corrected)
Time Point (0.25 Hz)
0
200
400
600
800
−5
5
10
15
20
25
30
40
60
80
100

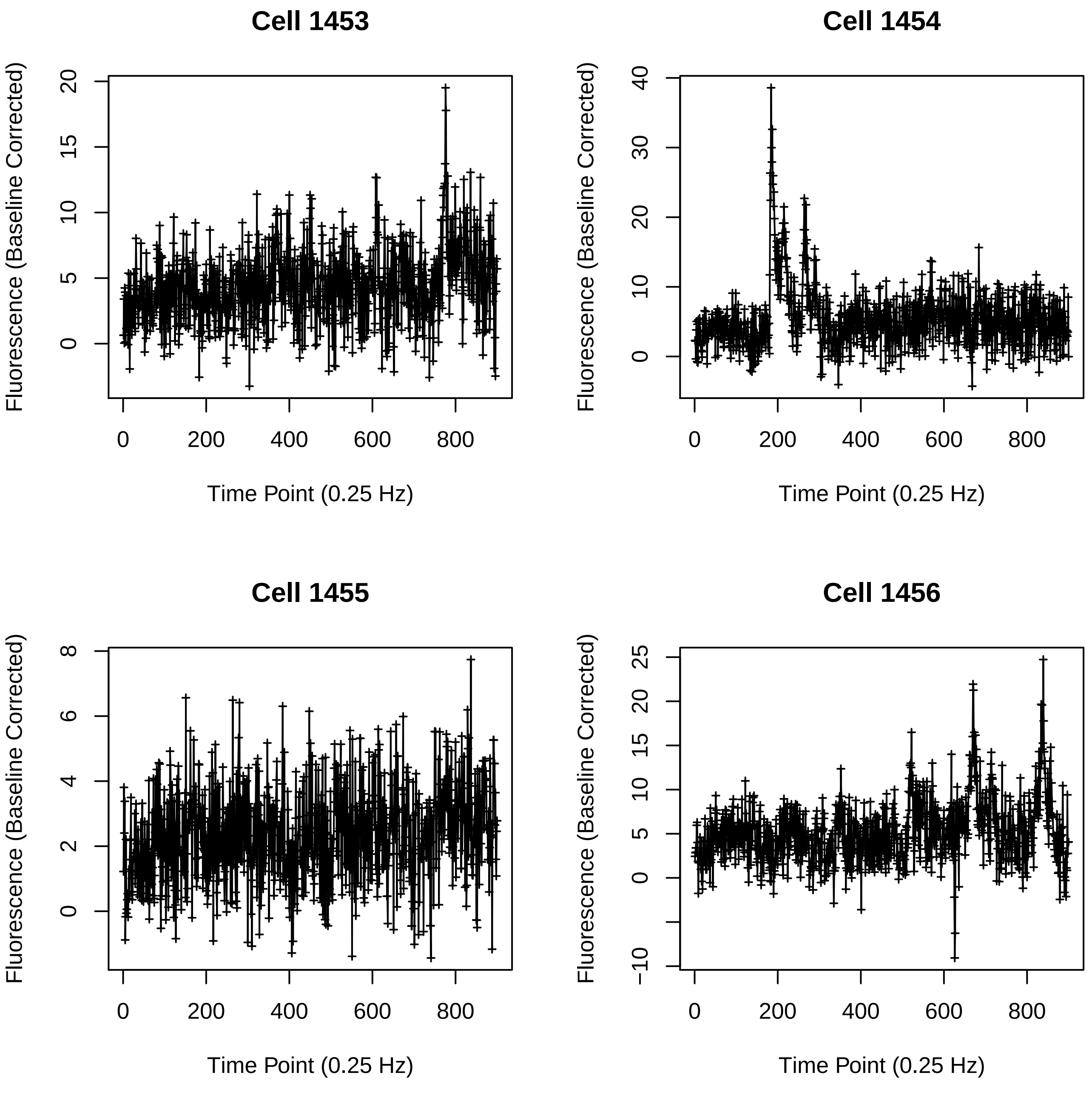
Cell 1453
Cell 1454
Cell 1455
Cell 1456
Fluorescence (Baseline Corrected)
Time Point (0.25 Hz)
0
5
10
15
20
25
30
40
−10
2
4
6
8
200
400
600
800

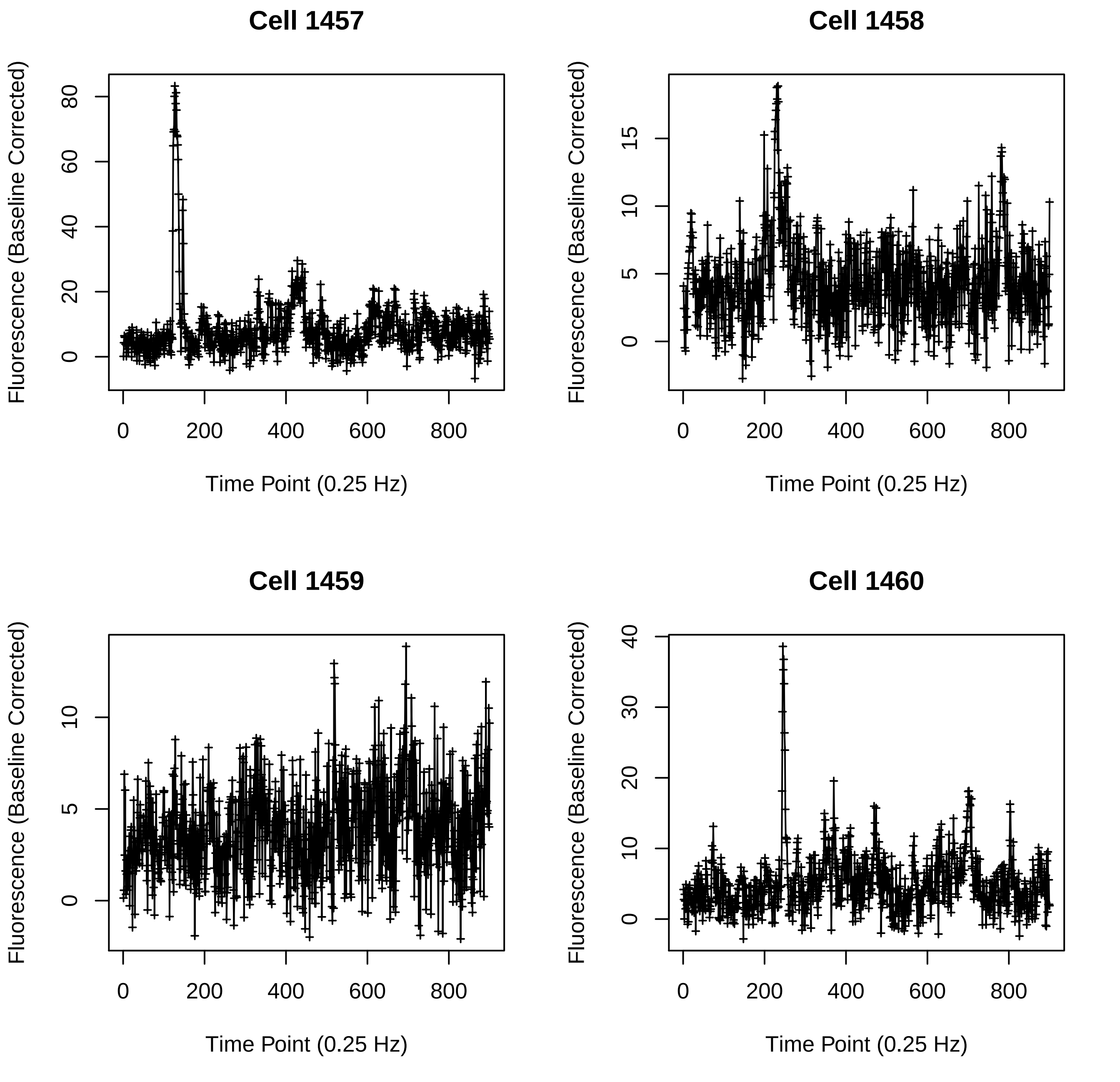
Cell 1457
Cell 1458
Cell 1459
Cell 1460
Fluorescence (Baseline Corrected)
Time Point (0.25 Hz)
0
20
40
60
80
5
10
15
30
200
400
600
800

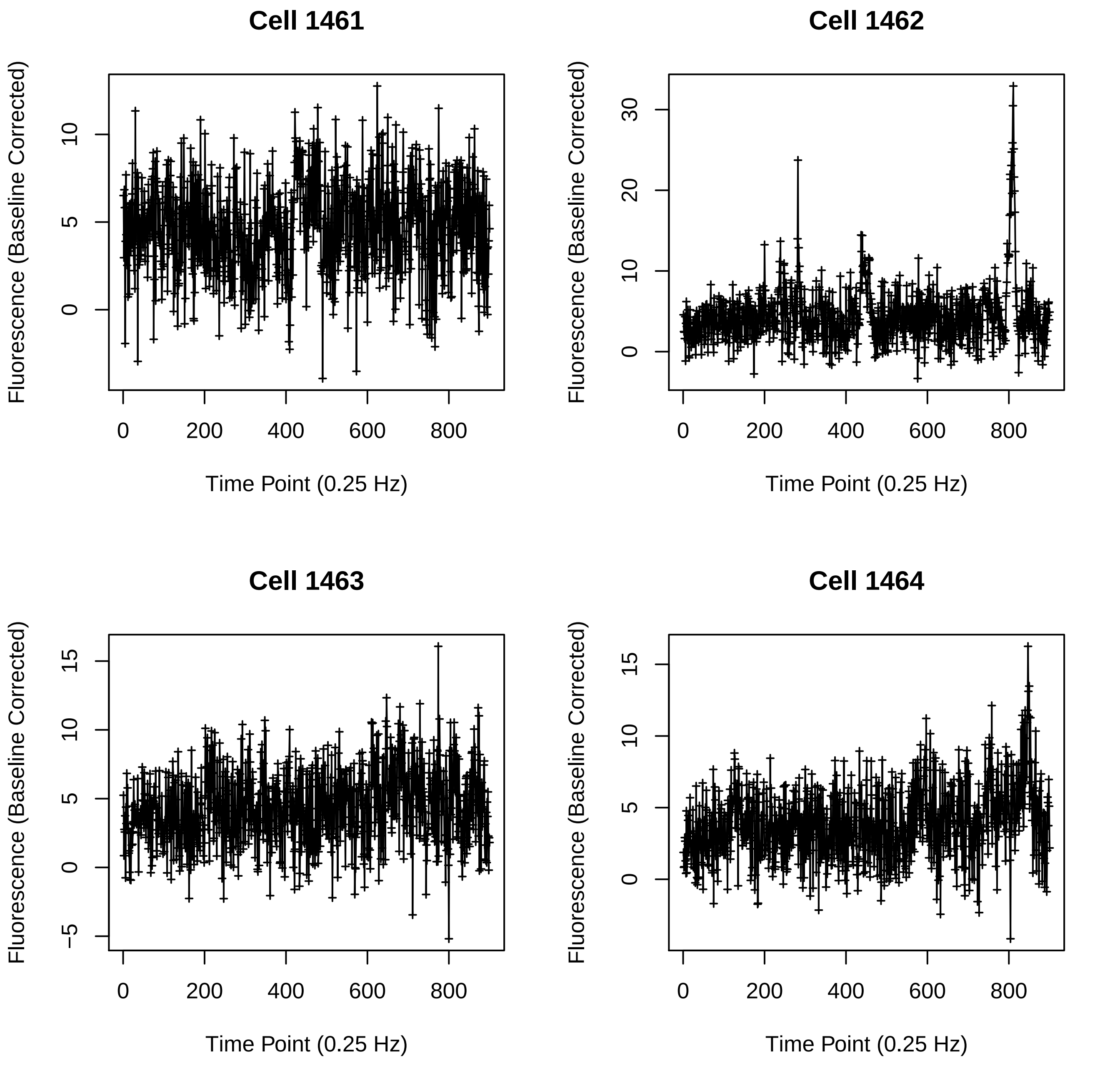
Cell 1461
Cell 1462
Cell 1463
Cell 1464
Fluorescence (Baseline Corrected)
Time Point (0.25 Hz)
0
200
400
600
800
-5
0
5
10
15
20
30

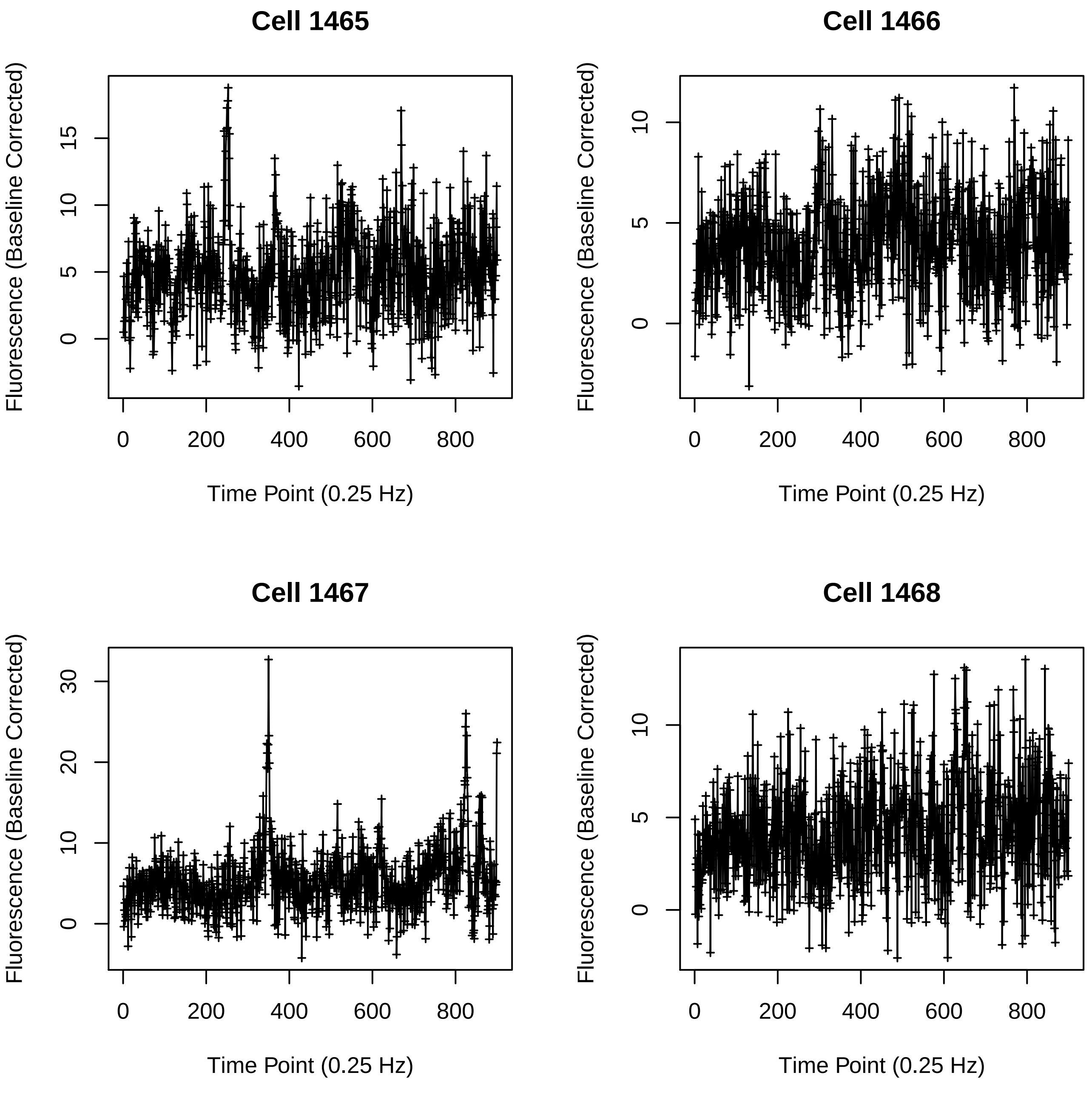
Cell 1465
Cell 1466
Cell 1467
Cell 1468
Fluorescence (Baseline Corrected)
Time Point (0.25 Hz)
0
5
10
15
20
30
200
400
600
800

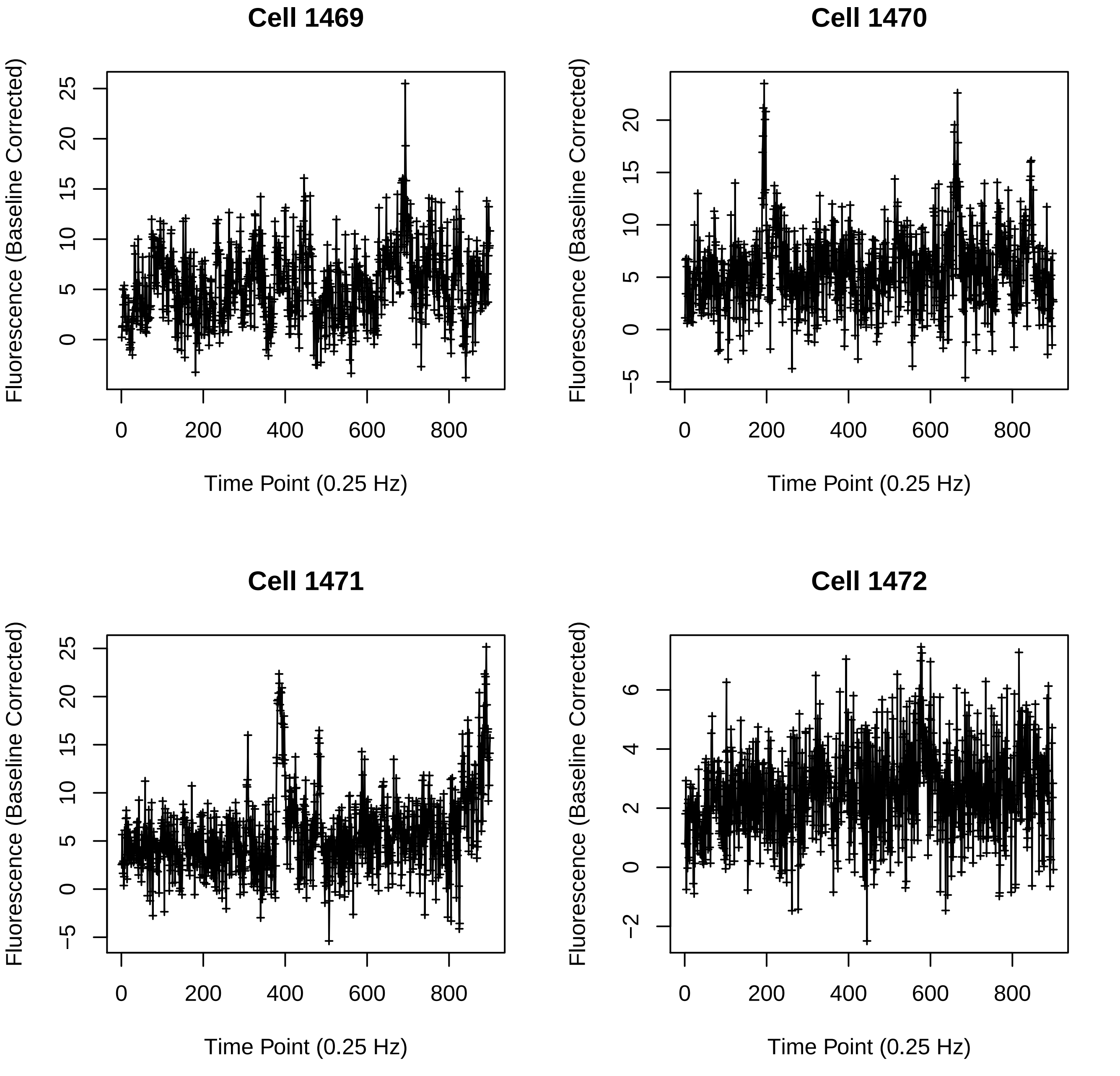
Cell 1469
Fluorescence (Baseline Corrected)
0
5
10
15
20
25
0
200
400
600
800
Time Point (0.25 Hz)
Cell 1470
Fluorescence (Baseline Corrected)
−5
0
5
10
15
20
0
200
400
600
800
Time Point (0.25 Hz)
Cell 1471
Fluorescence (Baseline Corrected)
−5
0
5
10
15
20
25
0
200
400
600
800
Time Point (0.25 Hz)
Cell 1472
Fluorescence (Baseline Corrected)
−2
0
2
4
6
0
200
400
600
800
Time Point (0.25 Hz)

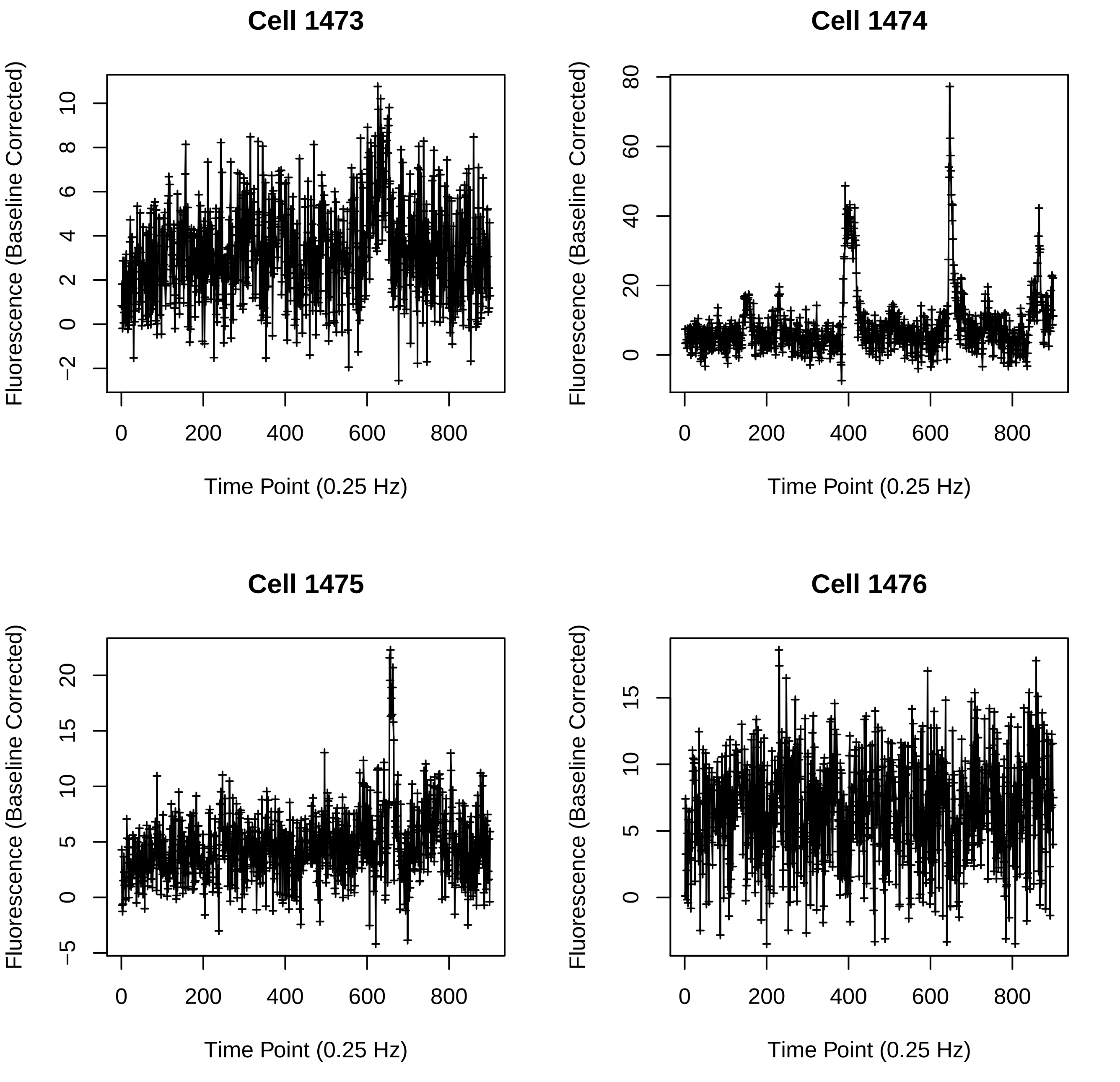
Cell 1473
Fluorescence (Baseline Corrected)
Time Point (0.25 Hz)
Cell 1474
Fluorescence (Baseline Corrected)
Time Point (0.25 Hz)
Cell 1475
Fluorescence (Baseline Corrected)
Time Point (0.25 Hz)
Cell 1476
Fluorescence (Baseline Corrected)
Time Point (0.25 Hz)

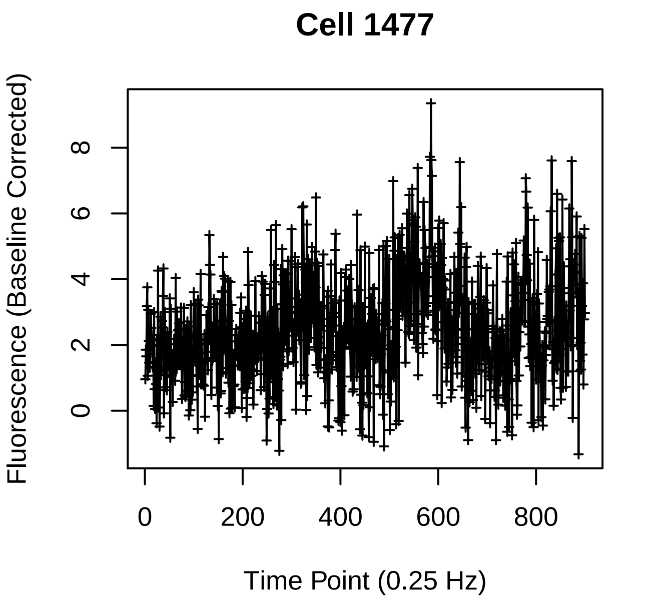
Cell 1477
Fluorescence (Baseline Corrected)
0
2
4
6
8
0
200
400
600
800
Time Point (0.25 Hz)

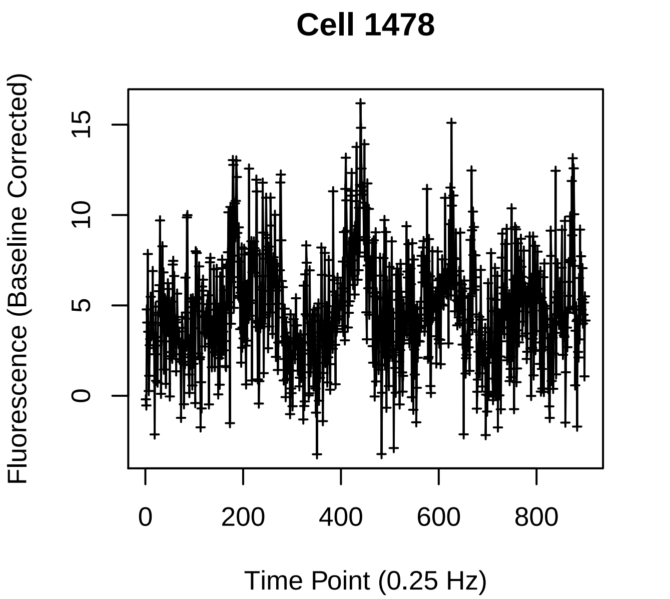
Cell 1478
Fluorescence (Baseline Corrected)
0
5
10
15
0
200
400
600
800
Time Point (0.25 Hz)

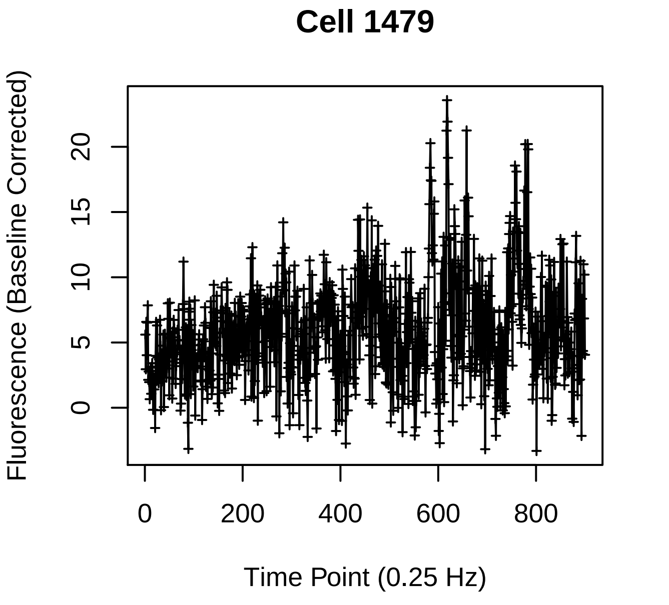
Cell 1479
Fluorescence (Baseline Corrected)
0
5
10
15
20
0
200
400
600
800
Time Point (0.25 Hz)

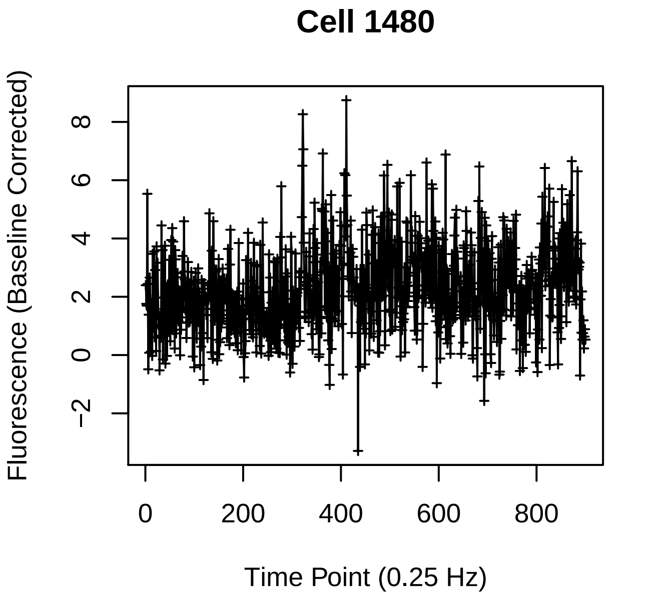
Cell 1480
Fluorescence (Baseline Corrected)
−2
0
2
4
6
8
0
200
400
600
800
Time Point (0.25 Hz)

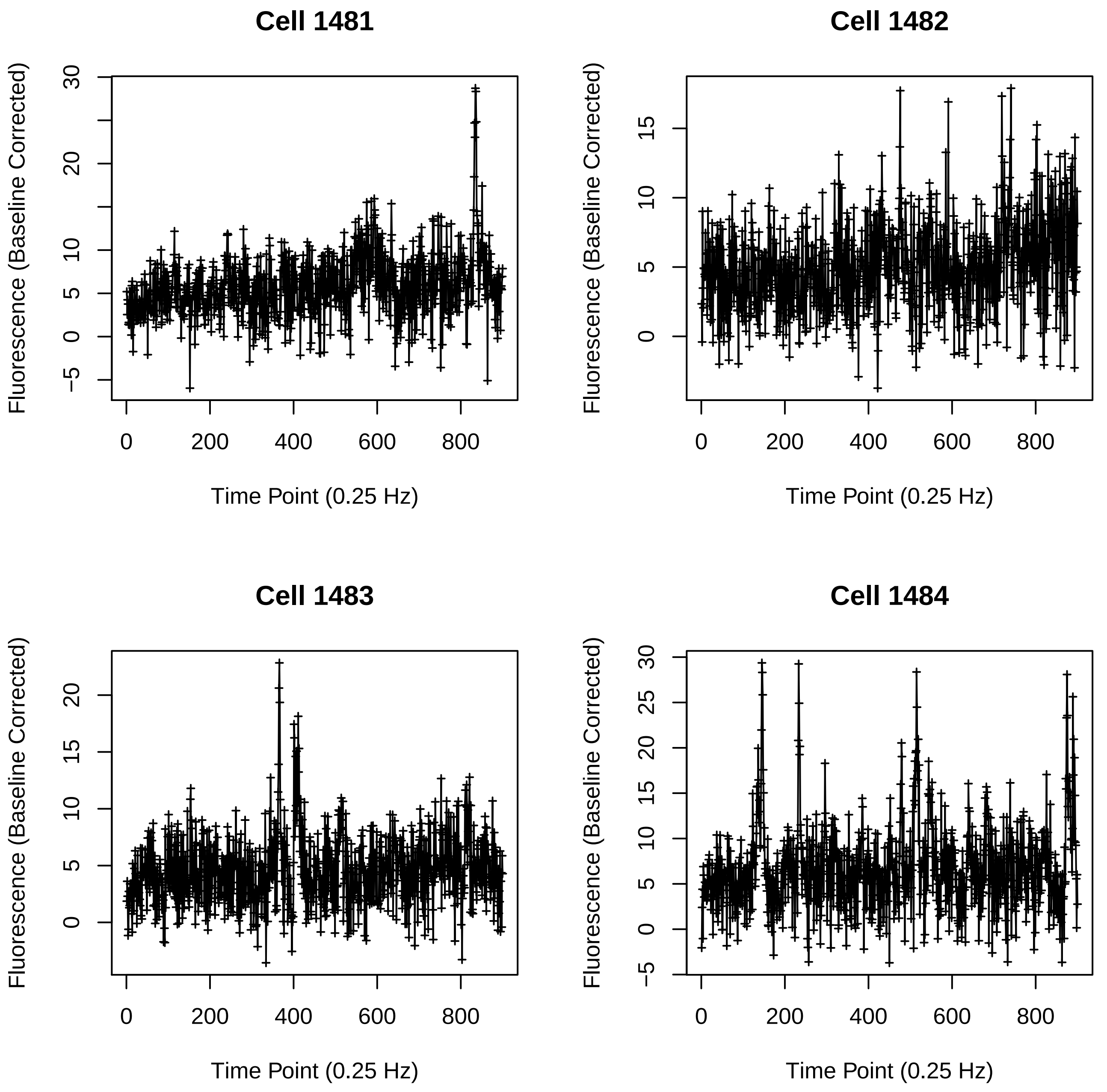
Cell 1481
Cell 1482
Cell 1483
Cell 1484
Fluorescence (Baseline Corrected)
Time Point (0.25 Hz)
−5
0
5
10
15
20
25
30
0
200
400
600
800

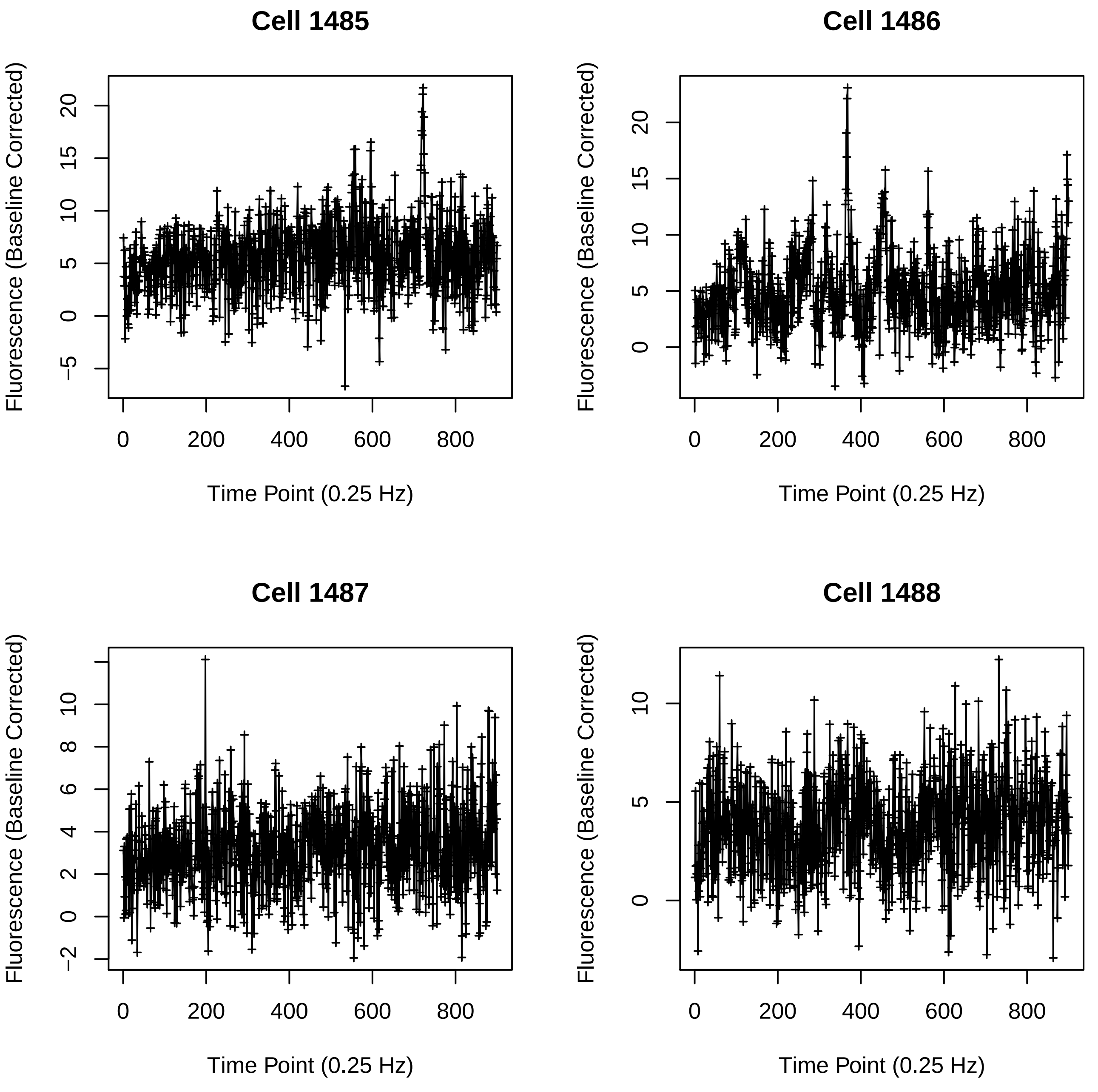

Cell 1485
Cell 1486
Cell 1487
Cell 1488
Fluorescence (Baseline Corrected)
Time Point (0.25 Hz)
0
200
400
600
800
−5
5
10
15
20
−2
2
4
6
8

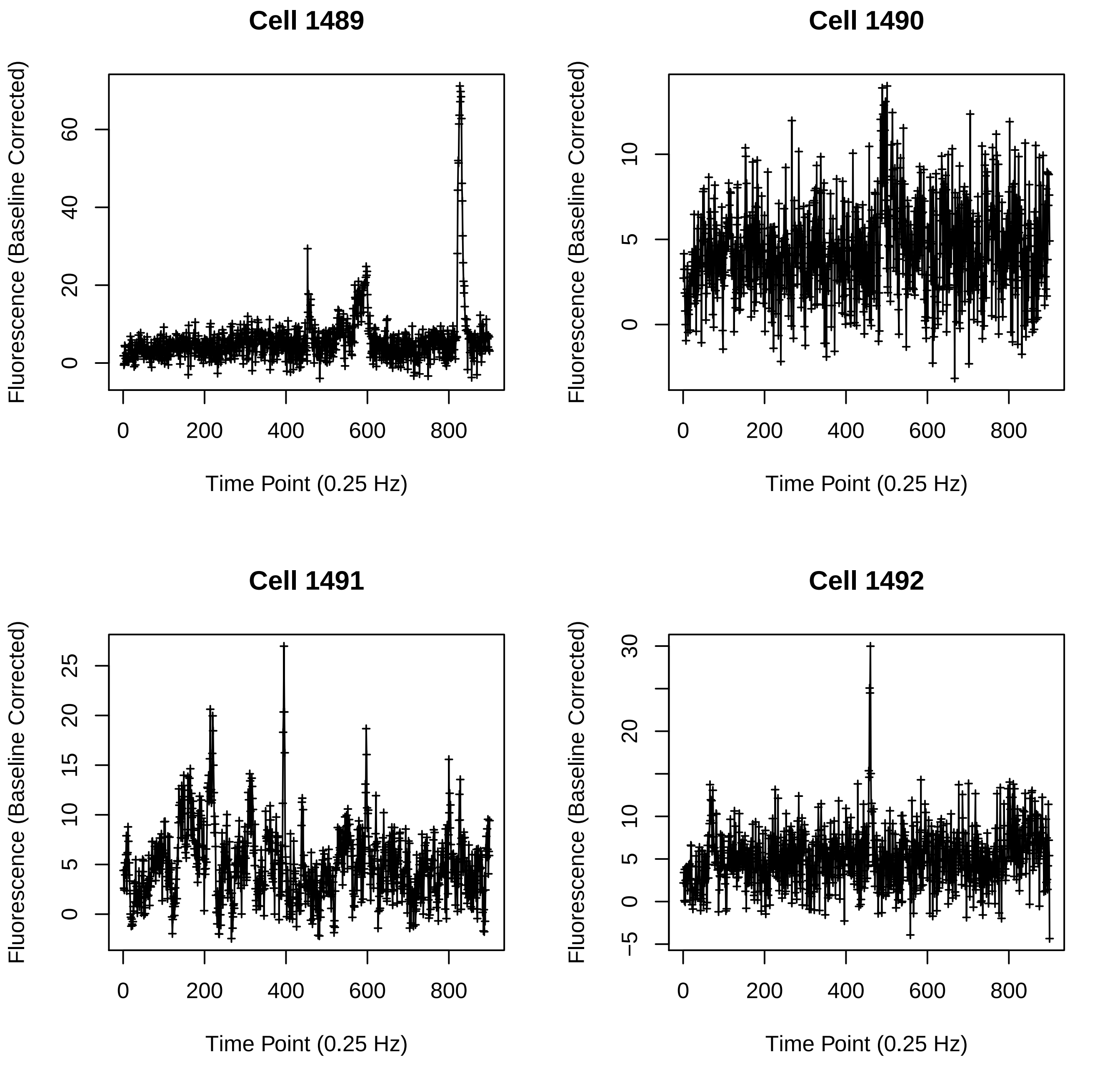

Cell 1489
Cell 1490
Cell 1491
Cell 1492
Fluorescence (Baseline Corrected)
Time Point (0.25 Hz)
0
20
40
60
10
5
-5
15
25
30
200
400
600
800

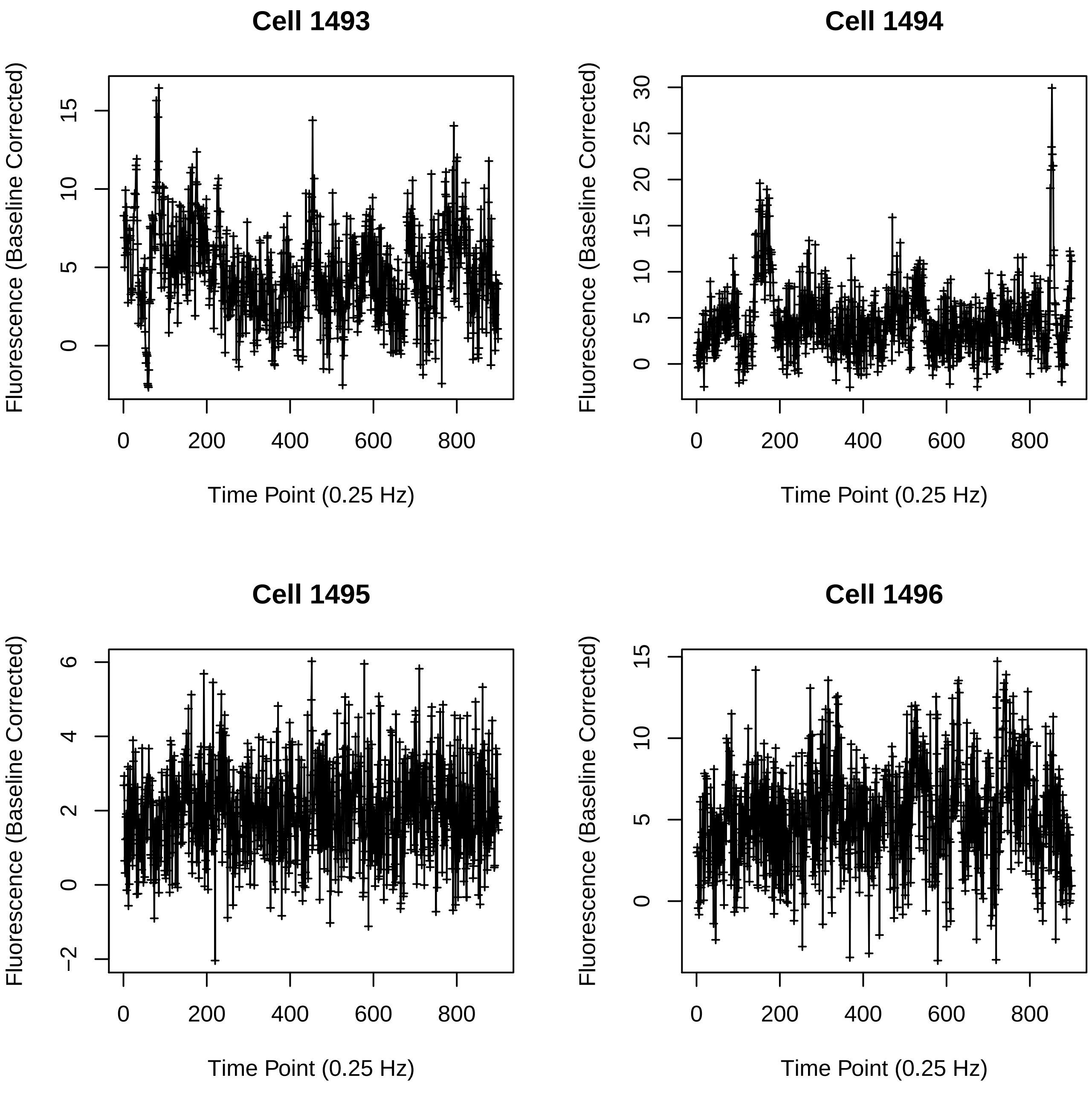
Cell 1493
Cell 1494
Cell 1495
Cell 1496
Fluorescence (Baseline Corrected)
Time Point (0.25 Hz)

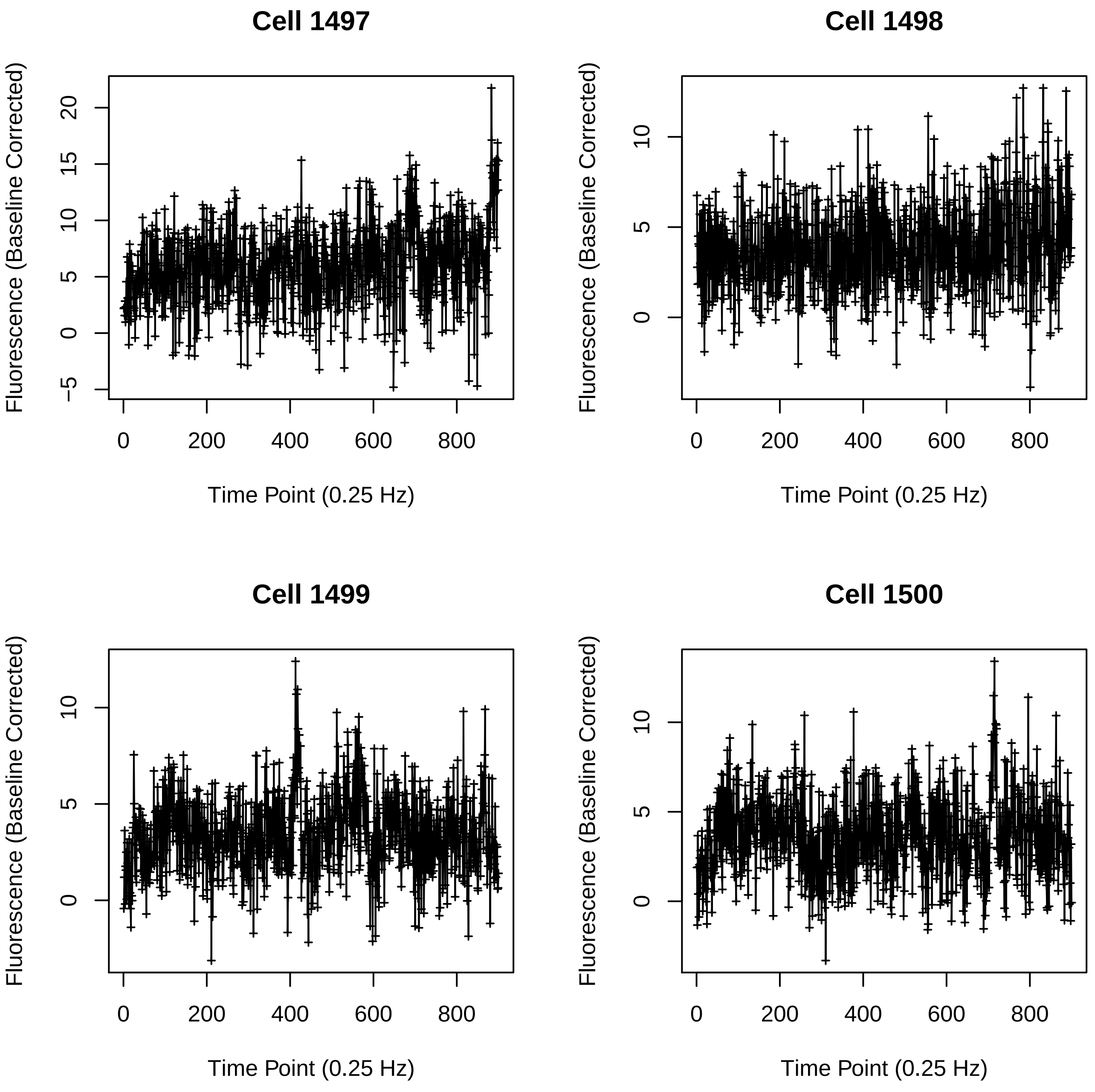
Cell 1497
Cell 1498
Cell 1499
Cell 1500
Fluorescence (Baseline Corrected)
Time Point (0.25 Hz)
−5
0
5
10
15
20
200
400
600
800

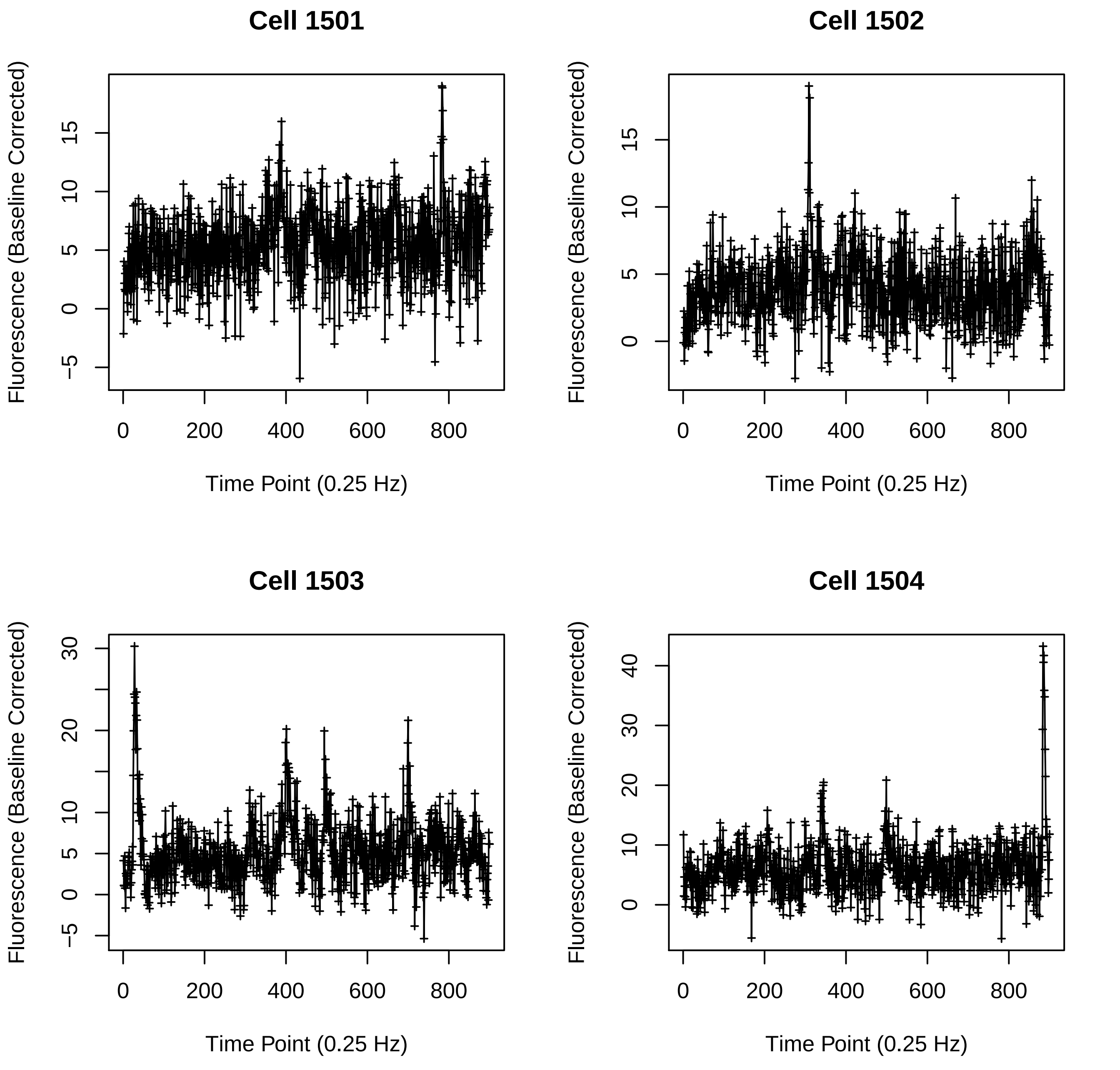
Cell 1501
Fluorescence (Baseline Corrected)
−5
0
5
10
15
0
200
400
600
800
Time Point (0.25 Hz)
Cell 1502
Fluorescence (Baseline Corrected)
0
5
10
15
0
200
400
600
800
Time Point (0.25 Hz)
Cell 1503
Fluorescence (Baseline Corrected)
−5
0
5
10
20
30
0
200
400
600
800
Time Point (0.25 Hz)
Cell 1504
Fluorescence (Baseline Corrected)
0
10
20
30
40
0
200
400
600
800
Time Point (0.25 Hz)

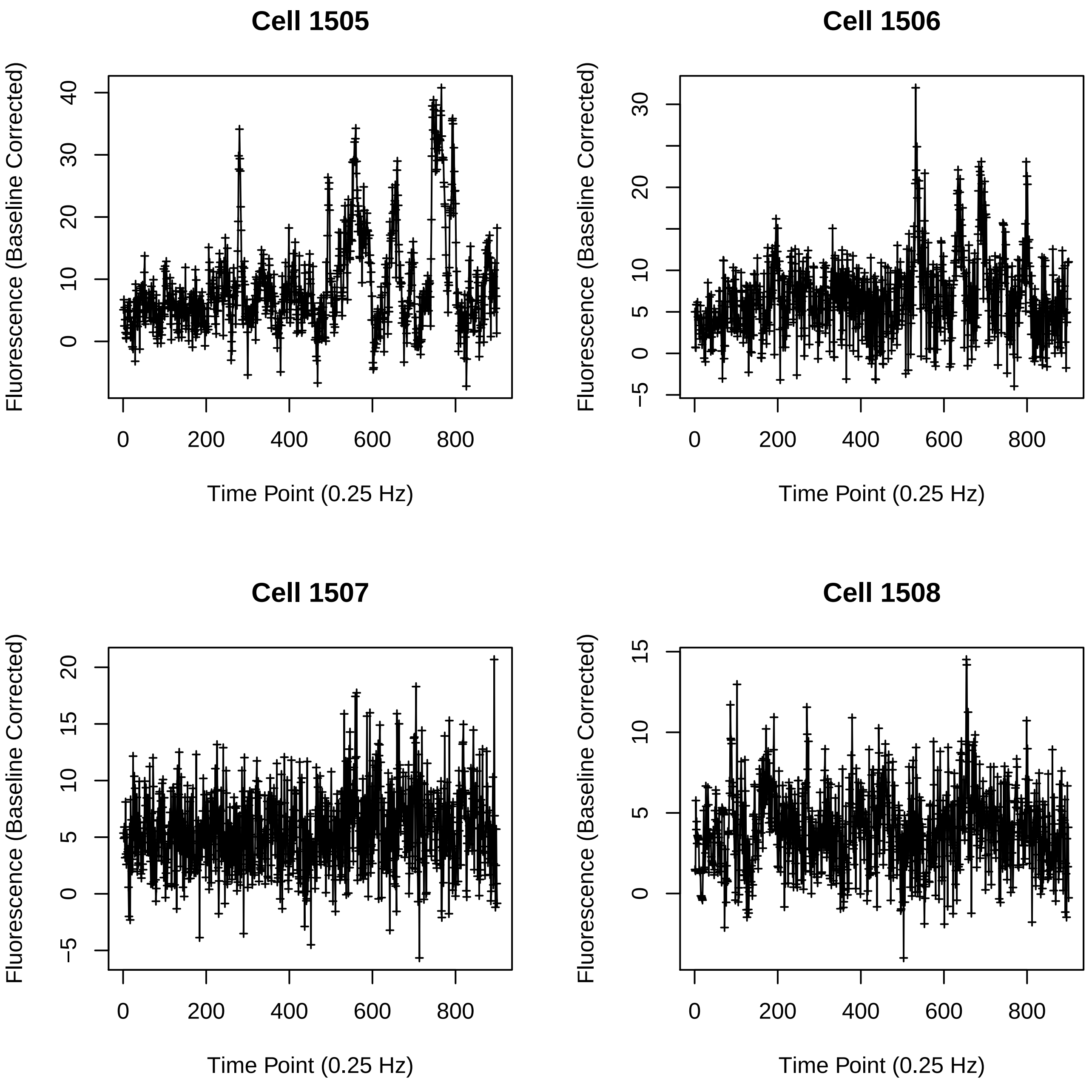
Cell 1505
Cell 1506
Cell 1507
Cell 1508
Fluorescence (Baseline Corrected)
Time Point (0.25 Hz)
0
200
400
600
800
−5
0
5
10
15
20
25
30
40

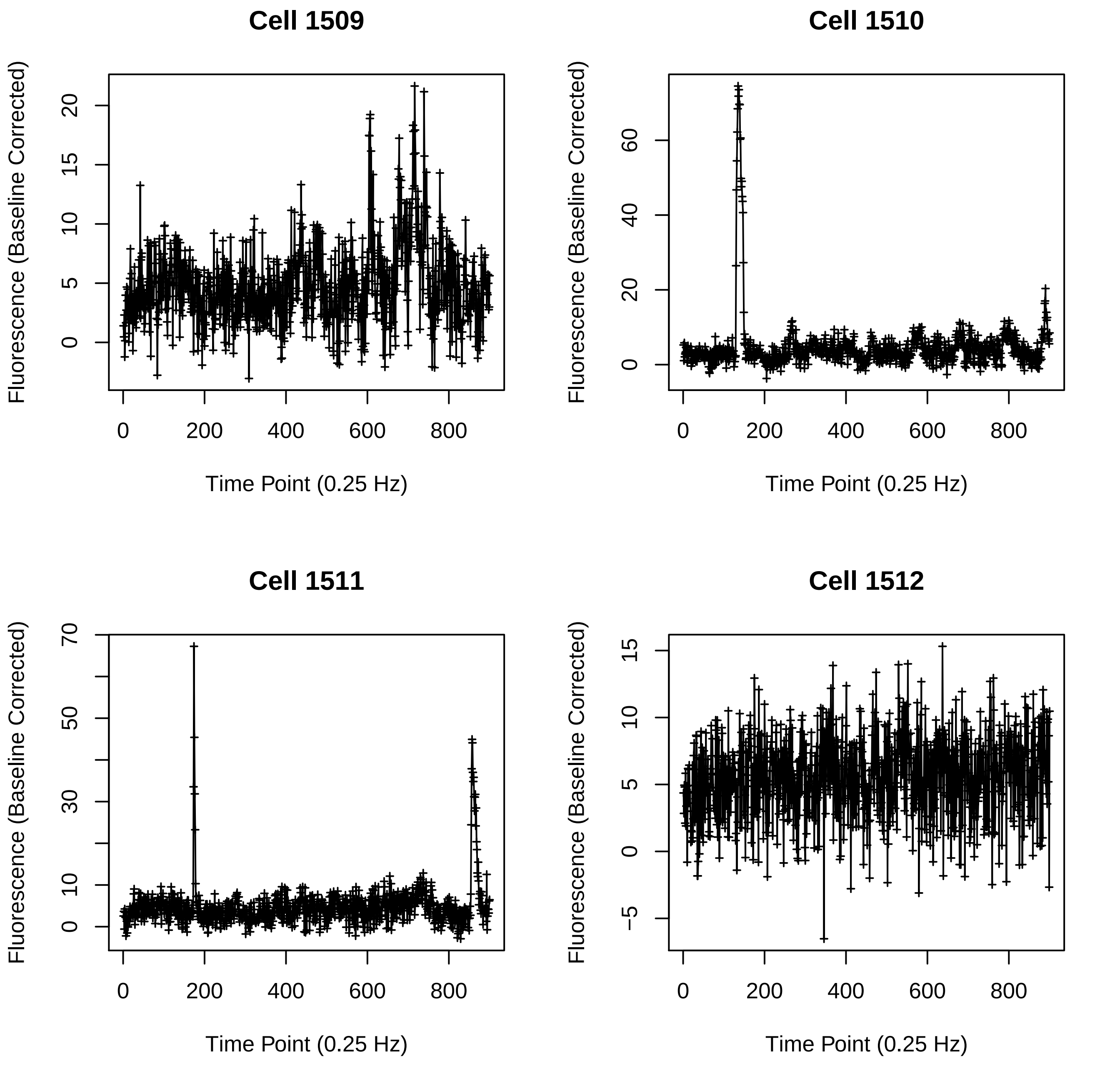

Cell 1509
Cell 1510
Cell 1511
Cell 1512
Fluorescence (Baseline Corrected)
Time Point (0.25 Hz)

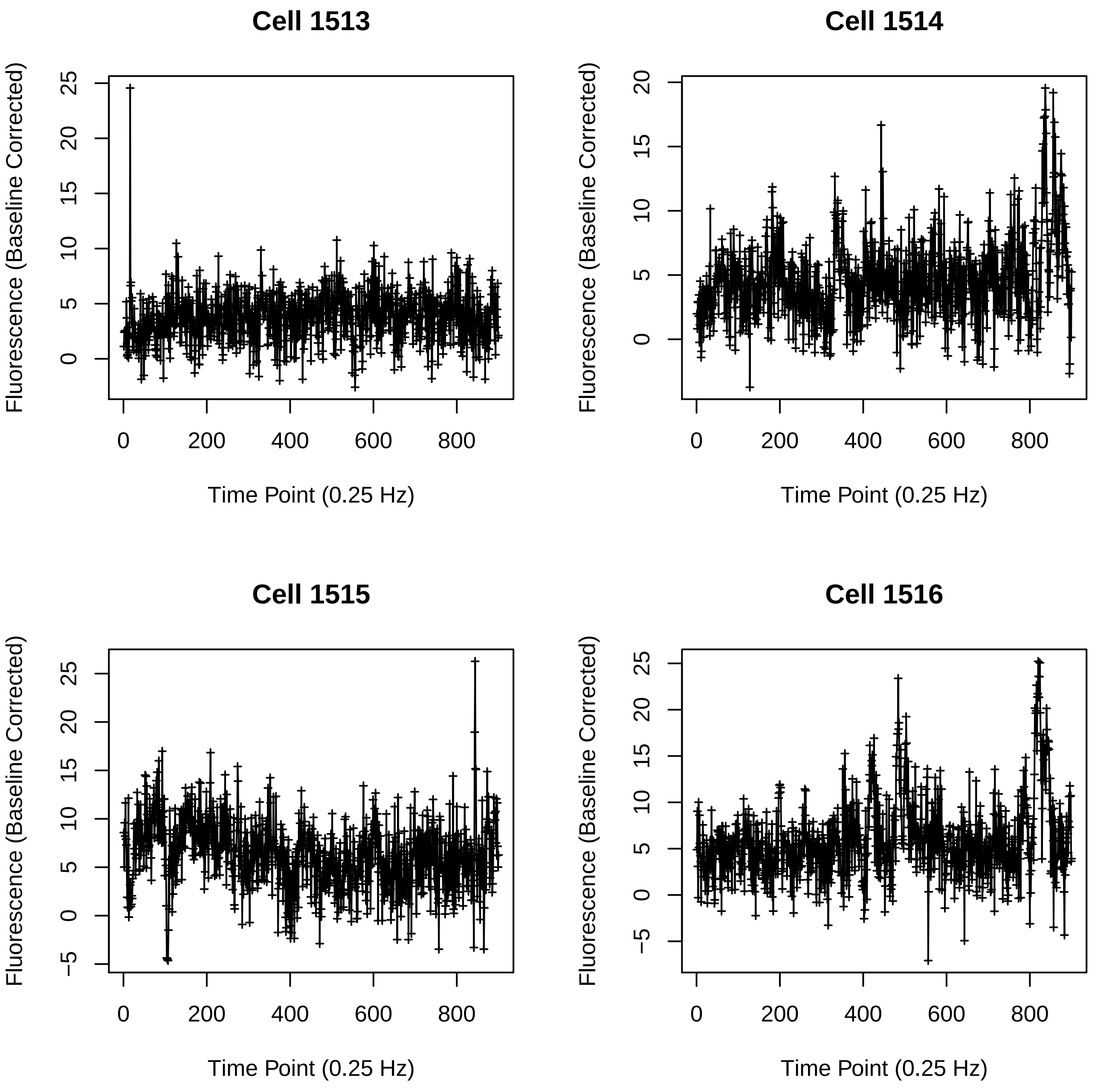
Cell 1513
Cell 1514
Cell 1515
Cell 1516
Fluorescence (Baseline Corrected)
Time Point (0.25 Hz)
0
200
400
600
800
-5
0
5
10
15
20
25

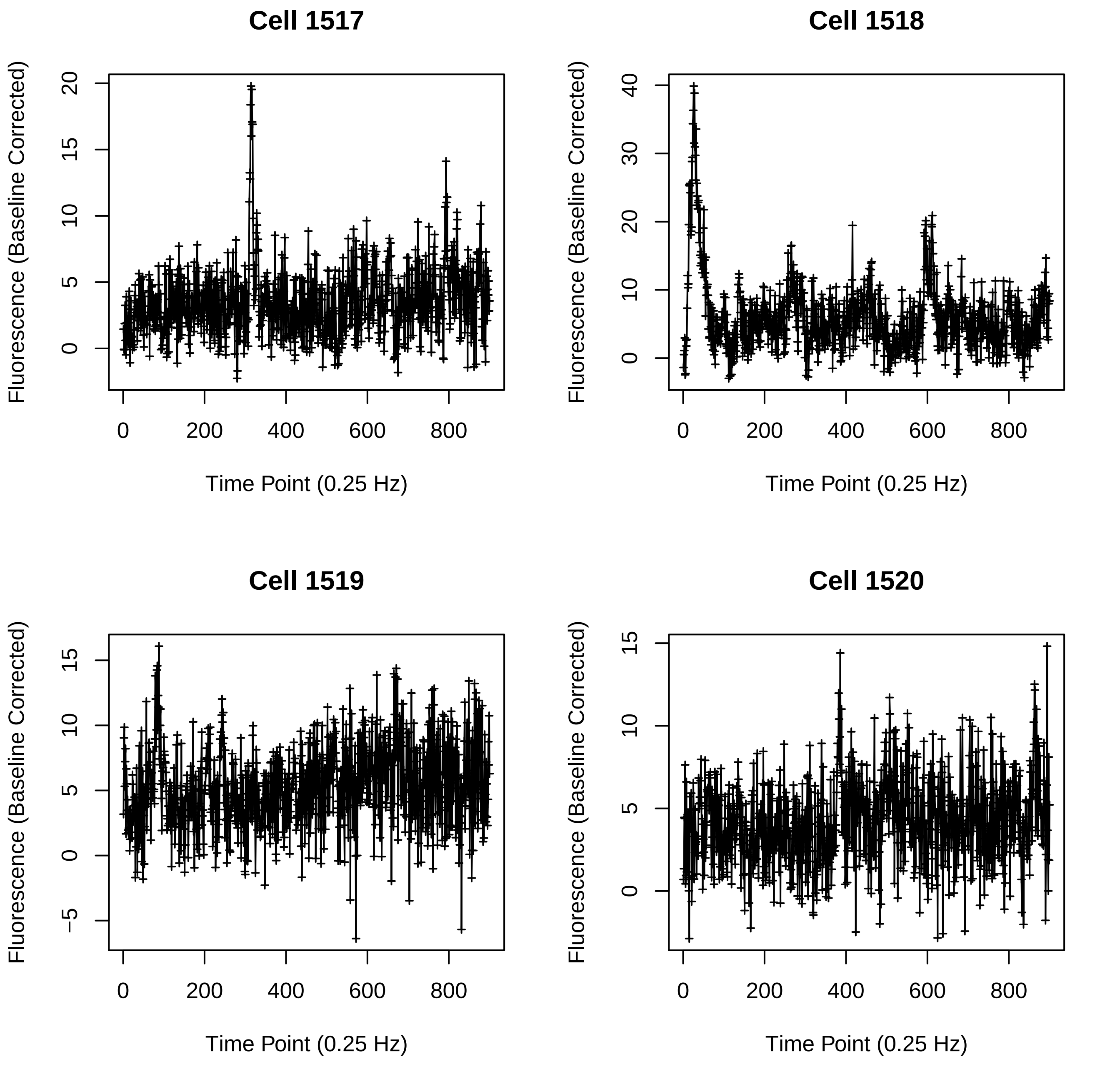
Cell 1517
Cell 1518
Cell 1519
Cell 1520
Fluorescence (Baseline Corrected)
Time Point (0.25 Hz)
0
200
400
600
800
0
5
10
15
20
30
40
−5

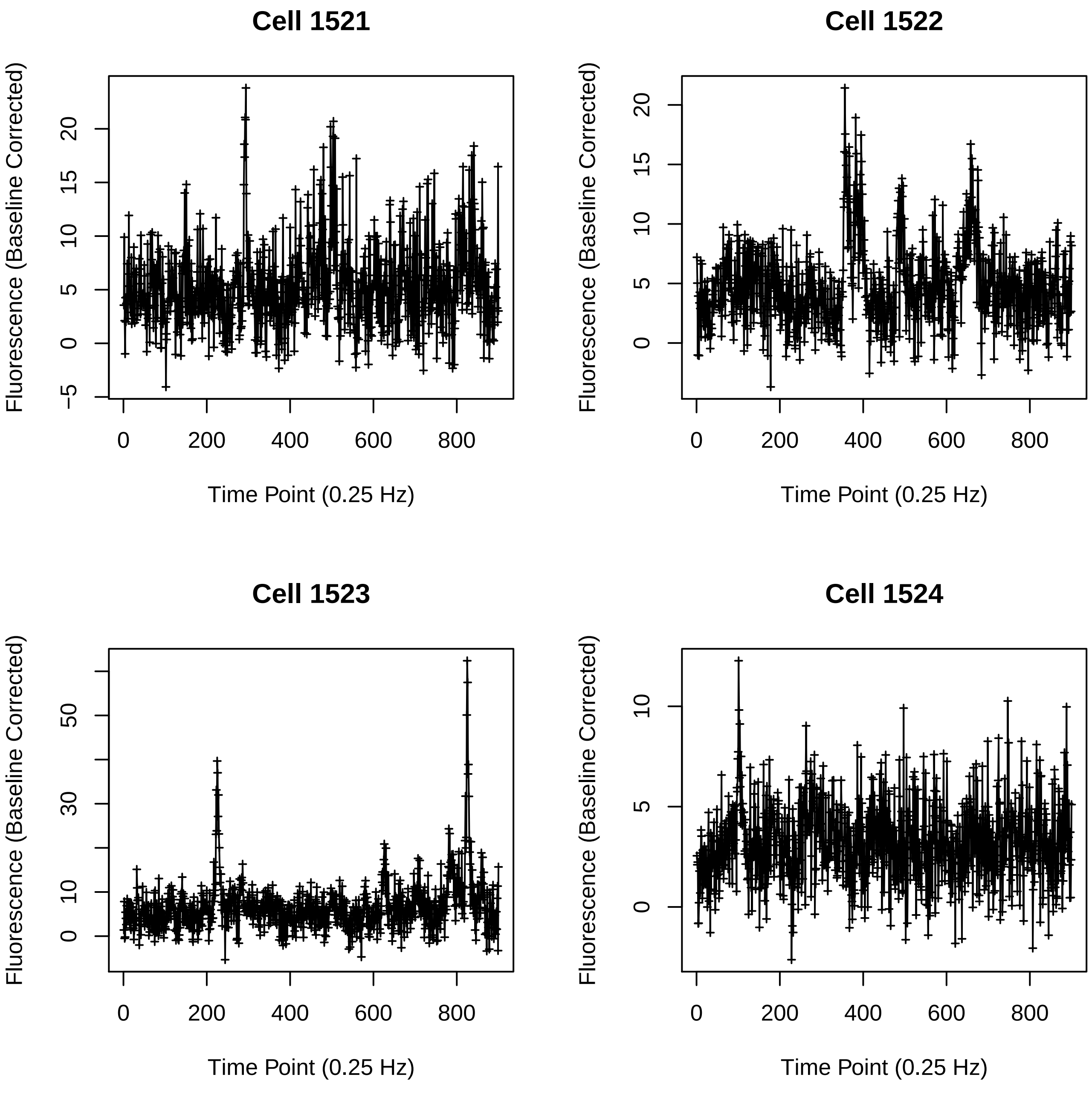

Cell 1521
Cell 1522
Cell 1523
Cell 1524
Fluorescence (Baseline Corrected)
Time Point (0.25 Hz)
−5
0
5
10
15
20
30
50
200
400
600
800

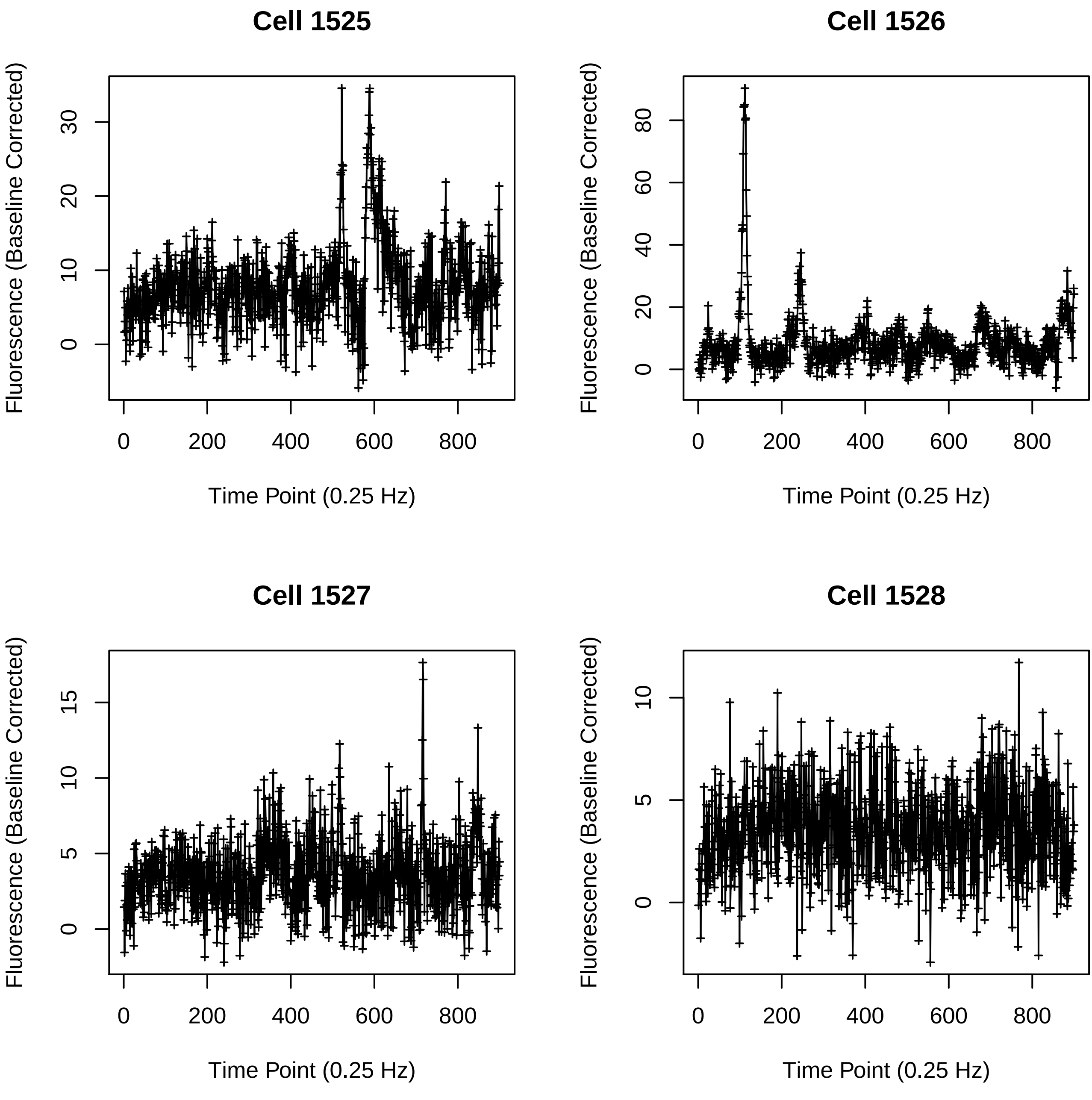
Cell 1525
Cell 1526
Cell 1527
Cell 1528
Fluorescence (Baseline Corrected)
Time Point (0.25 Hz)
0
10
20
30
40
60
80
5
15
200
400
600
800

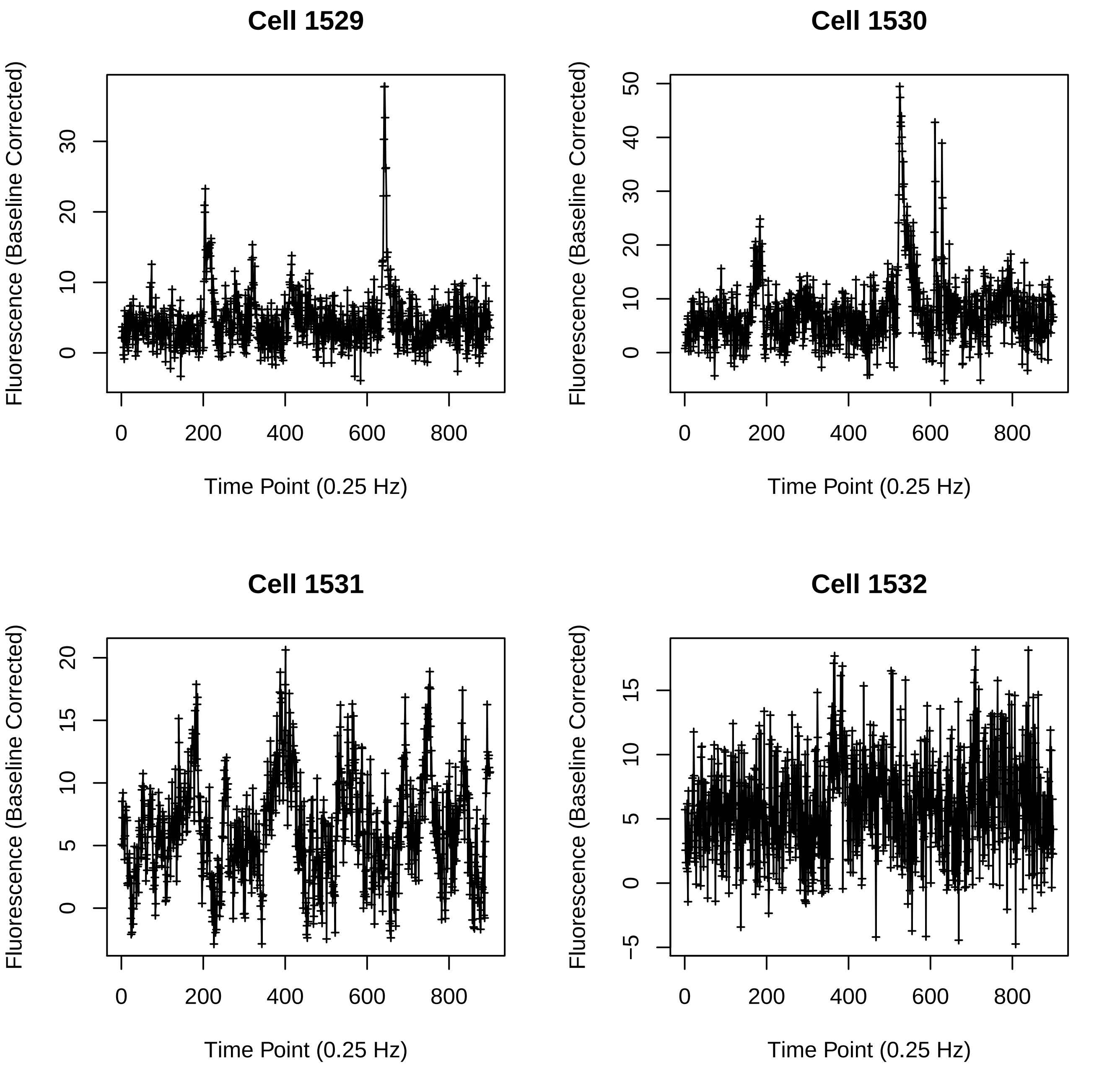
Cell 1529
Cell 1530
Cell 1531
Cell 1532
Fluorescence (Baseline Corrected)
Time Point (0.25 Hz)
0
200
400
600
800
0
10
20
30
40
50
-5
5
15

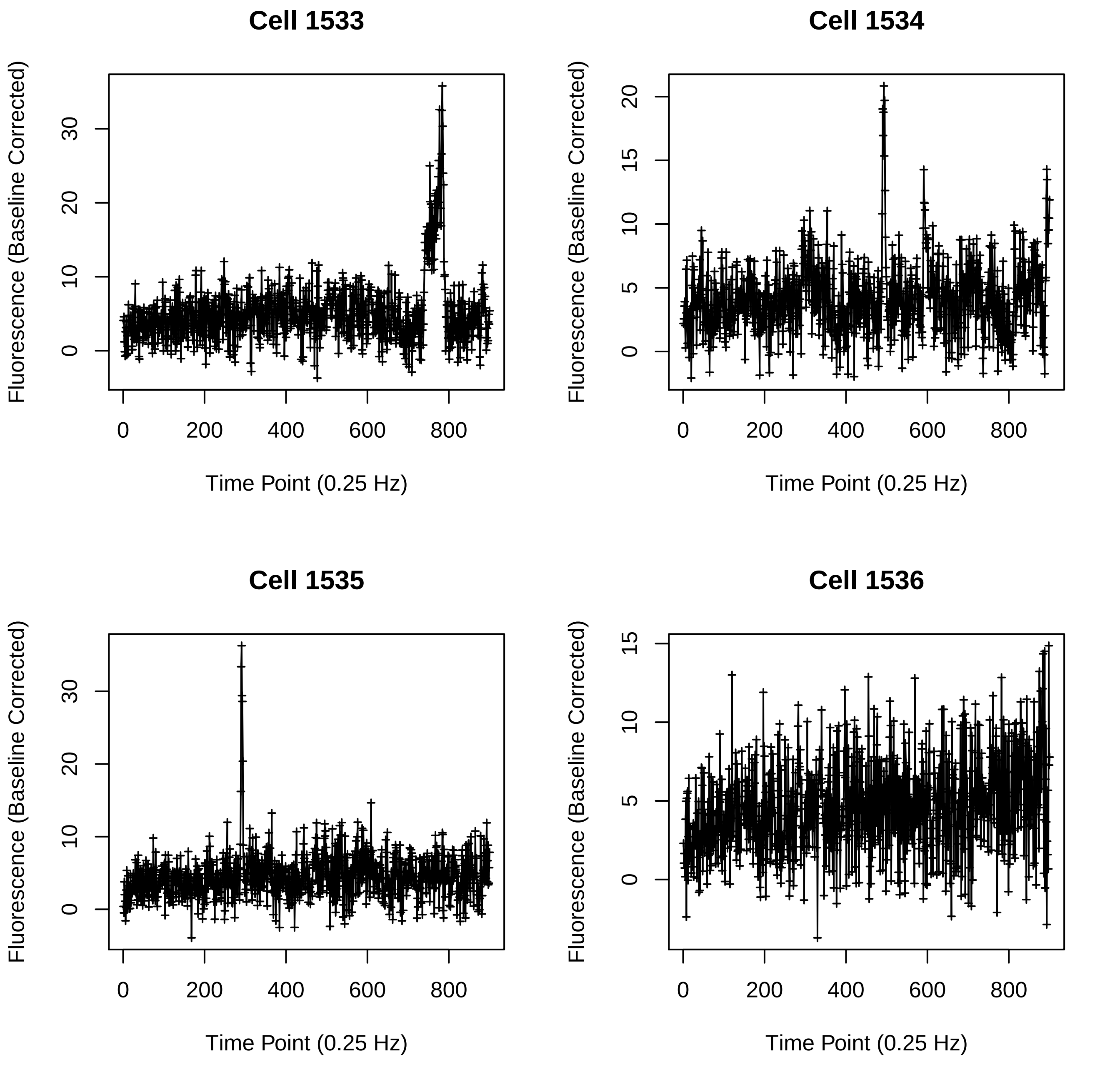
Cell 1533
Fluorescence (Baseline Corrected)
Time Point (0.25 Hz)
0
10
20
30
0
200
400
600
800
Cell 1534
Fluorescence (Baseline Corrected)
Time Point (0.25 Hz)
0
5
10
15
20
0
200
400
600
800
Cell 1535
Fluorescence (Baseline Corrected)
Time Point (0.25 Hz)
0
10
20
30
0
200
400
600
800
Cell 1536
Fluorescence (Baseline Corrected)
Time Point (0.25 Hz)
0
5
10
15
0
200
400
600
800

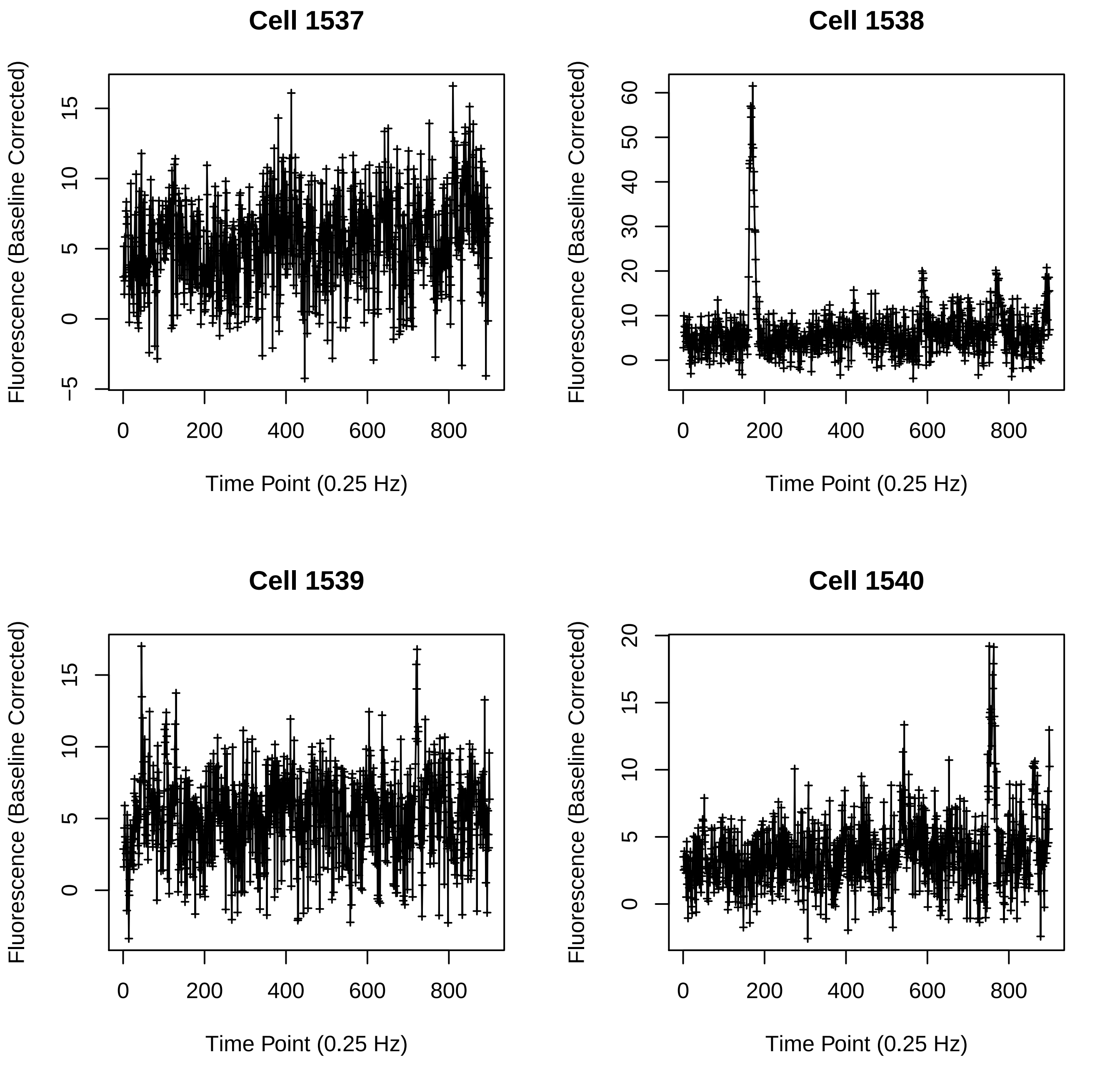
Cell 1537
Cell 1538
Cell 1539
Cell 1540
Fluorescence (Baseline Corrected)
Time Point (0.25 Hz)
−5
0
5
10
15
20
30
40
50
60
200
400
600
800

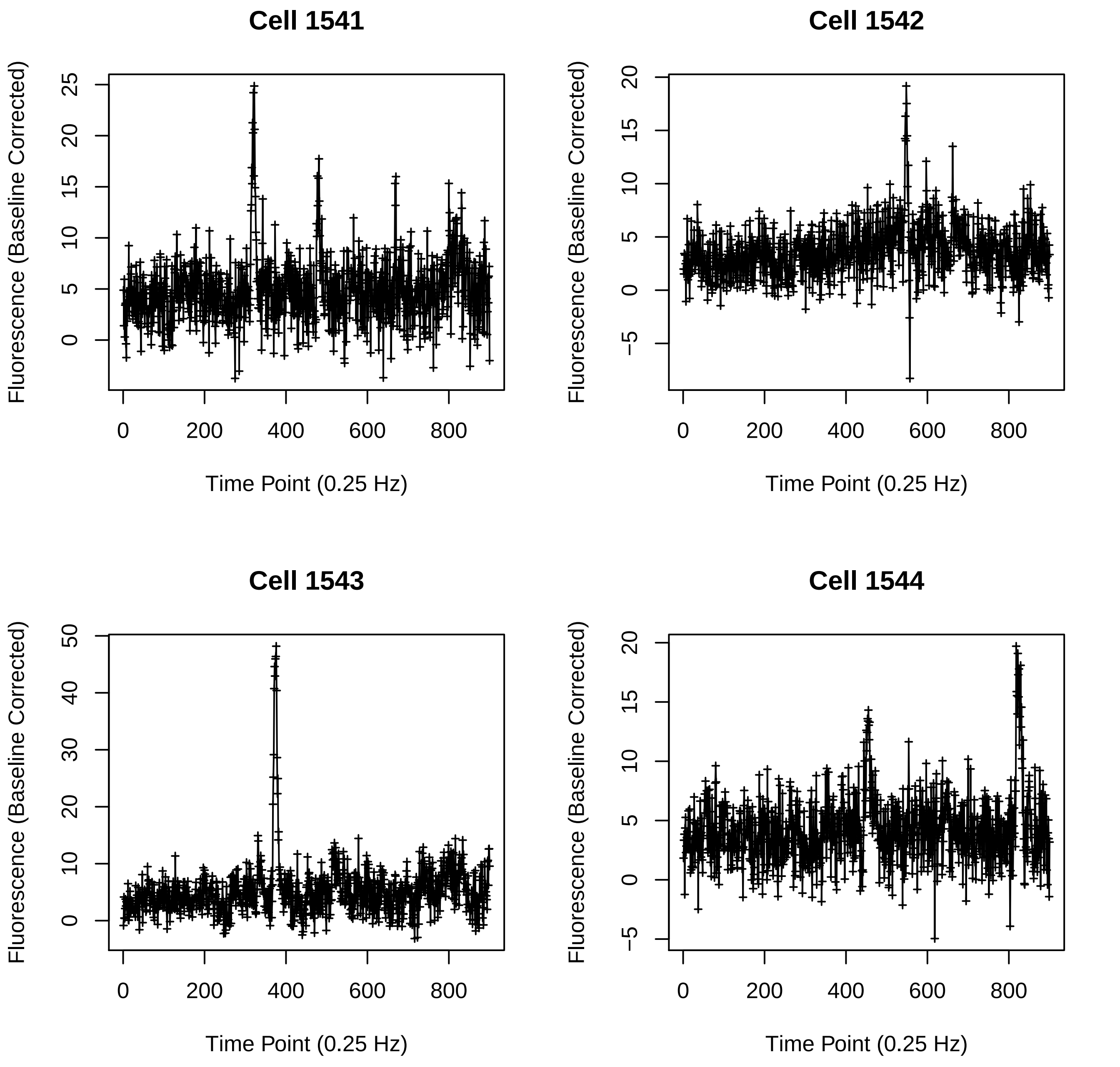

Cell 1541
Cell 1542
Cell 1543
Cell 1544
Fluorescence (Baseline Corrected)
Time Point (0.25 Hz)
0
200
400
600
800
−5
5
10
15
20
25
30
40
50

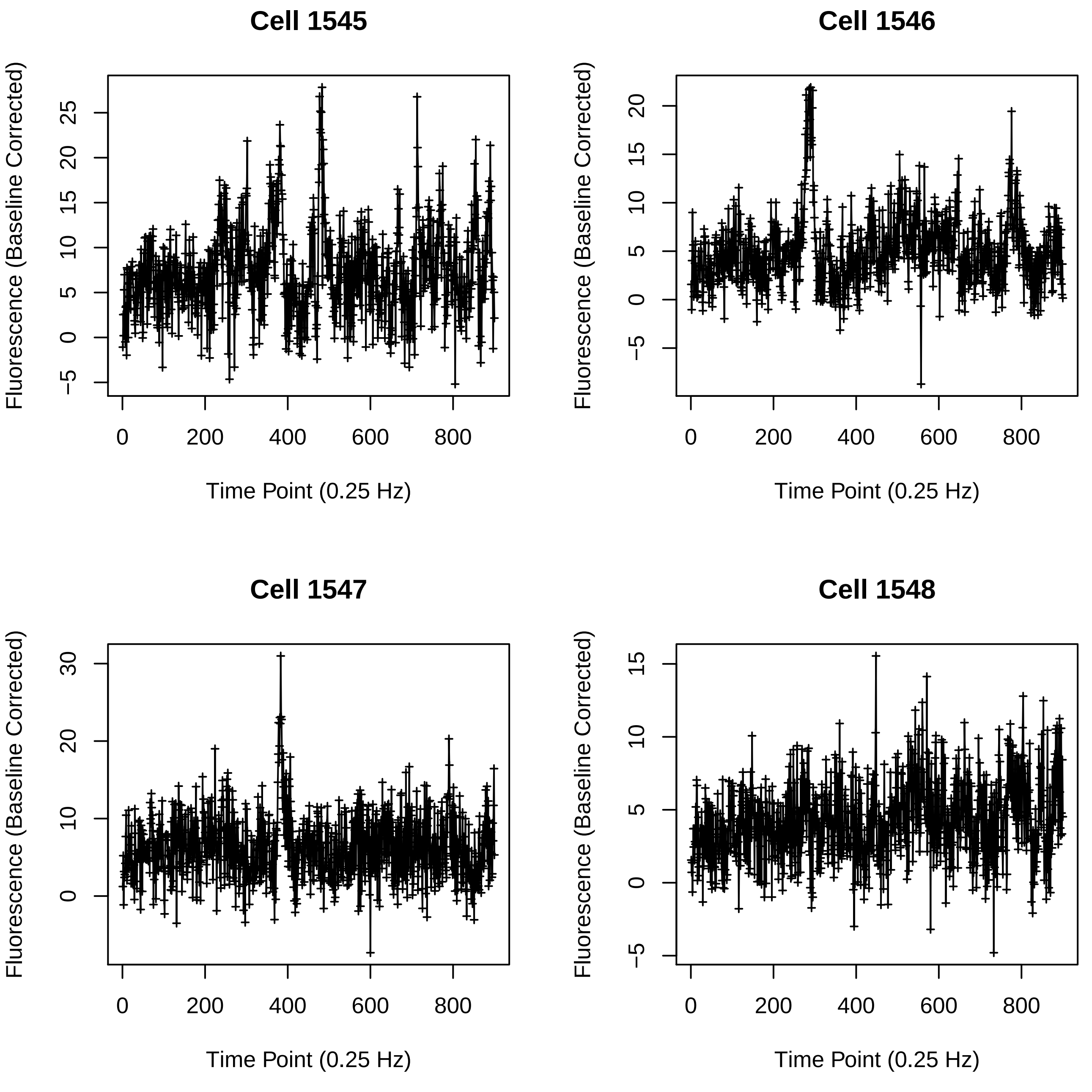
Cell 1545
Cell 1546
Cell 1547
Cell 1548
Fluorescence (Baseline Corrected)
Time Point (0.25 Hz)

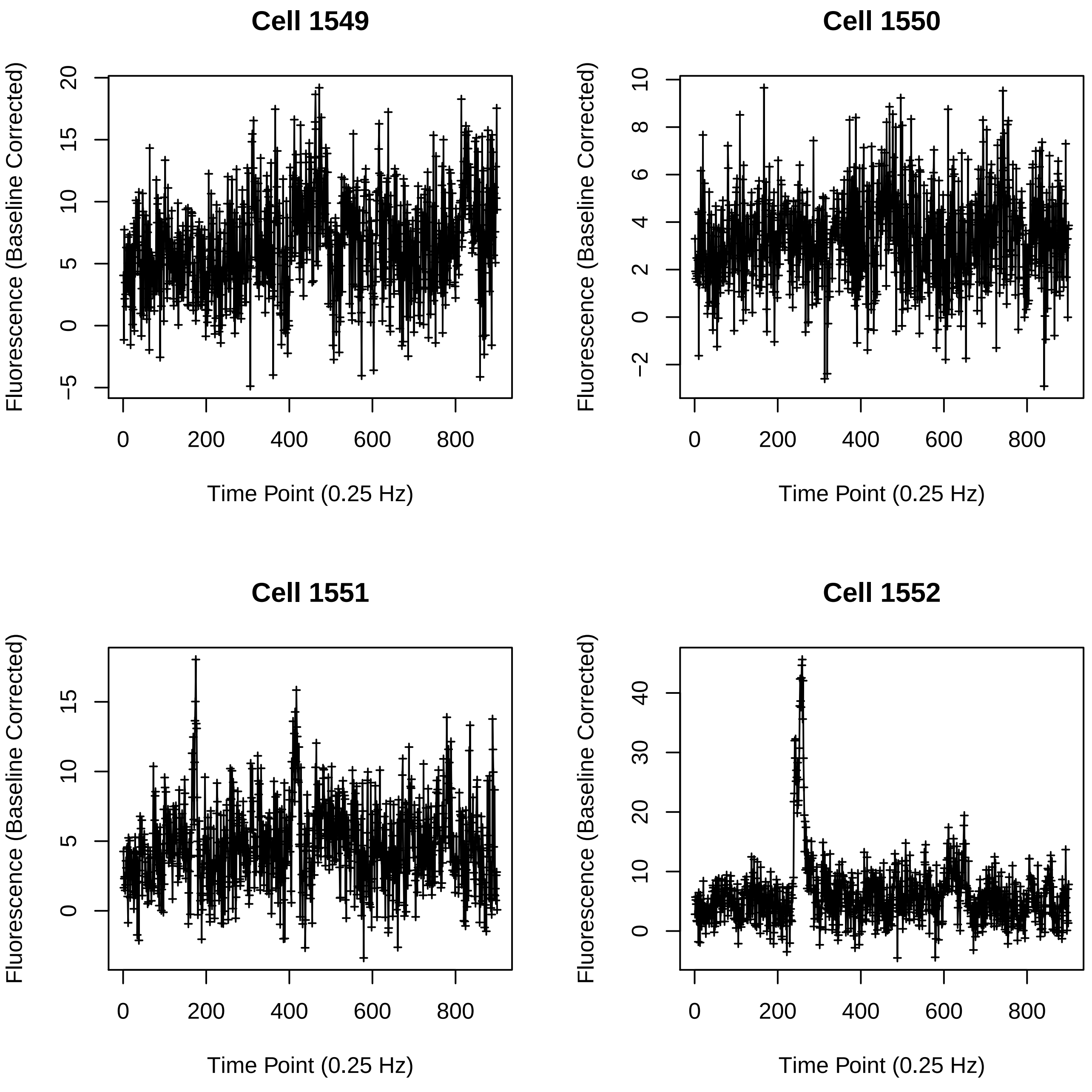
Cell 1549
Cell 1550
Cell 1551
Cell 1552
Fluorescence (Baseline Corrected)
Time Point (0.25 Hz)
0
200
400
600
800
−5
5
10
15
20
−2
2
4
6
8
30
40

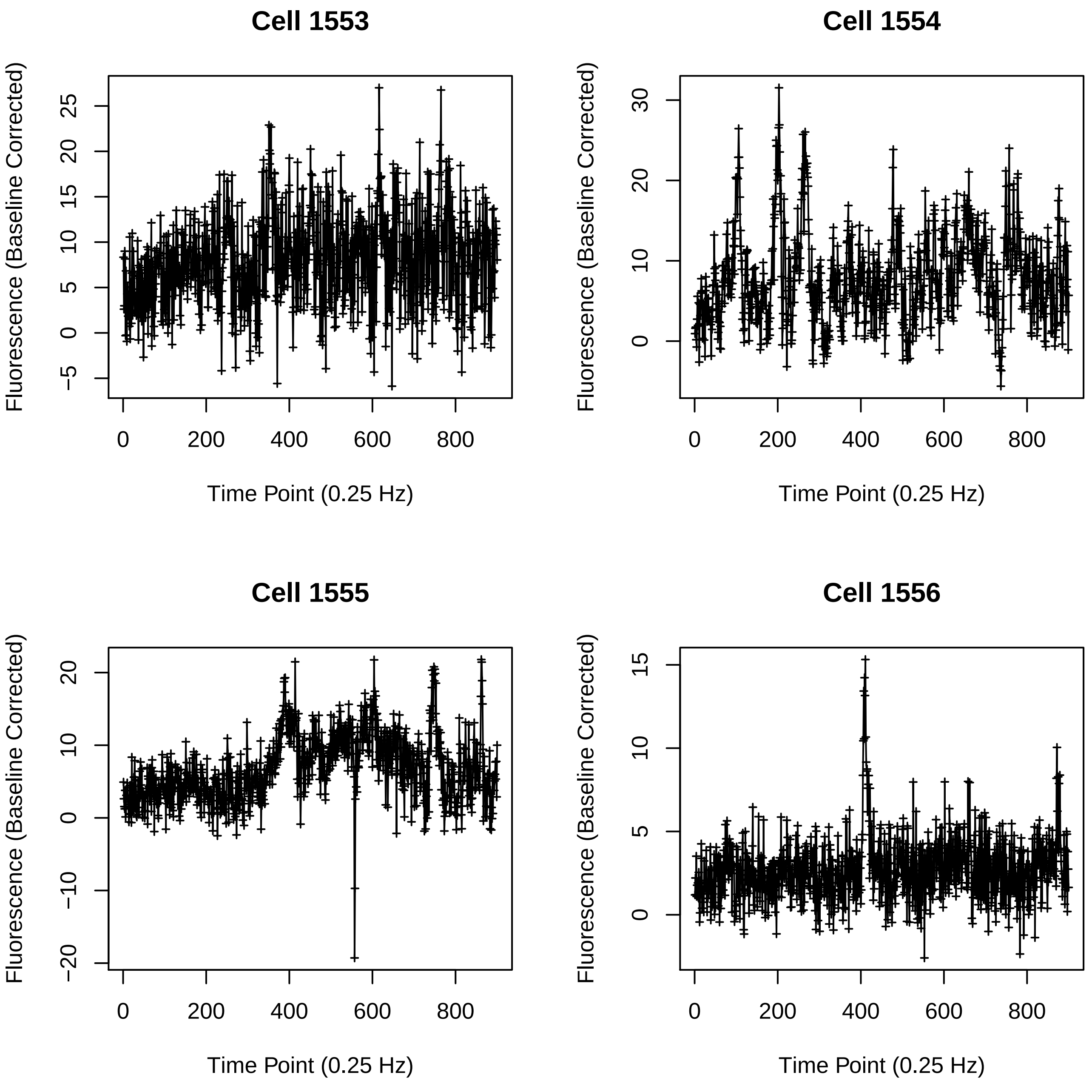

Cell 1553
Cell 1554
Cell 1555
Cell 1556
Fluorescence (Baseline Corrected)
Time Point (0.25 Hz)
−5
0
5
10
15
20
25
30
−20
−10
200
400
600
800

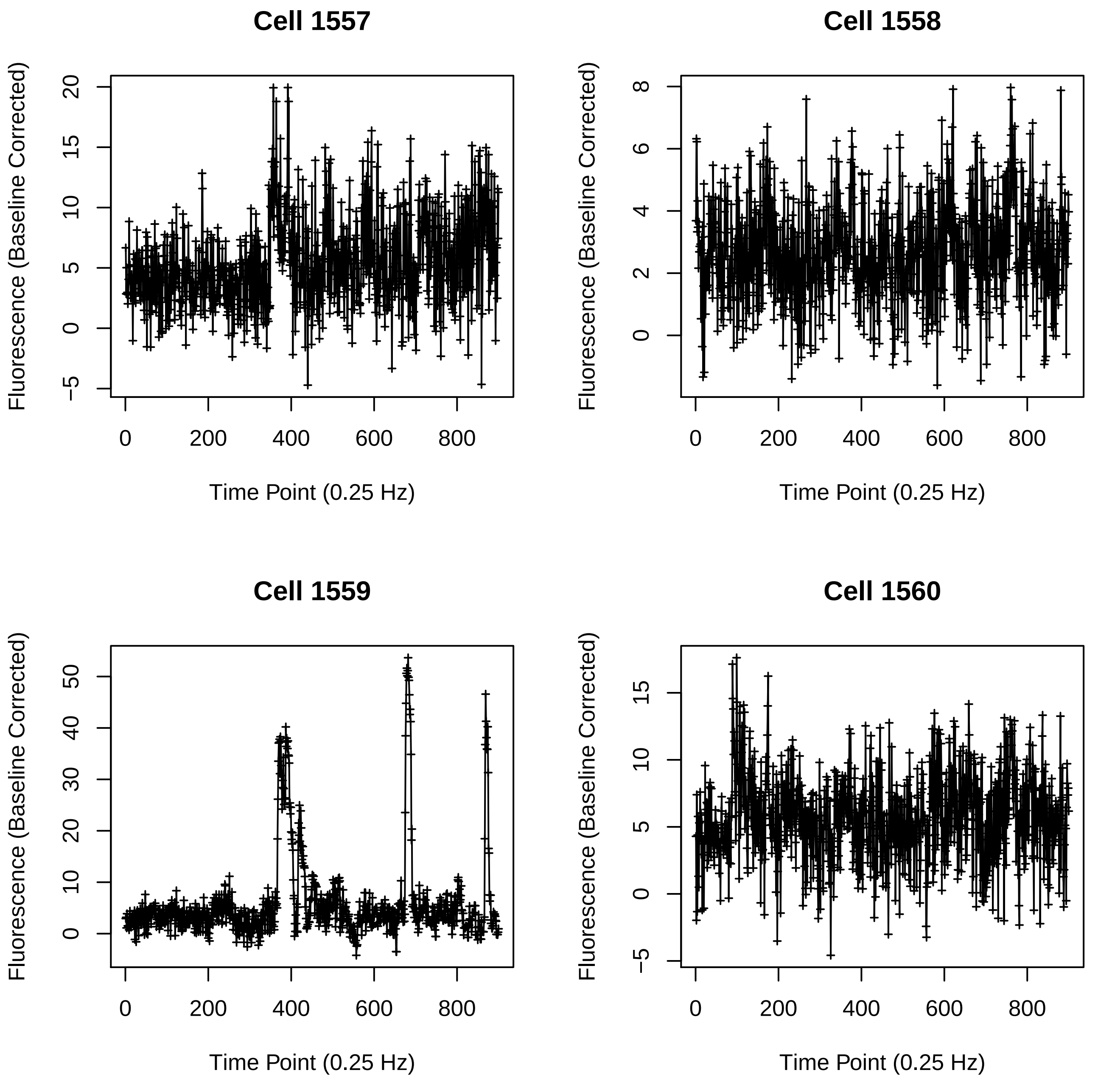
Cell 1557
Cell 1558
Cell 1559
Cell 1560
Fluorescence (Baseline Corrected)
Time Point (0.25 Hz)

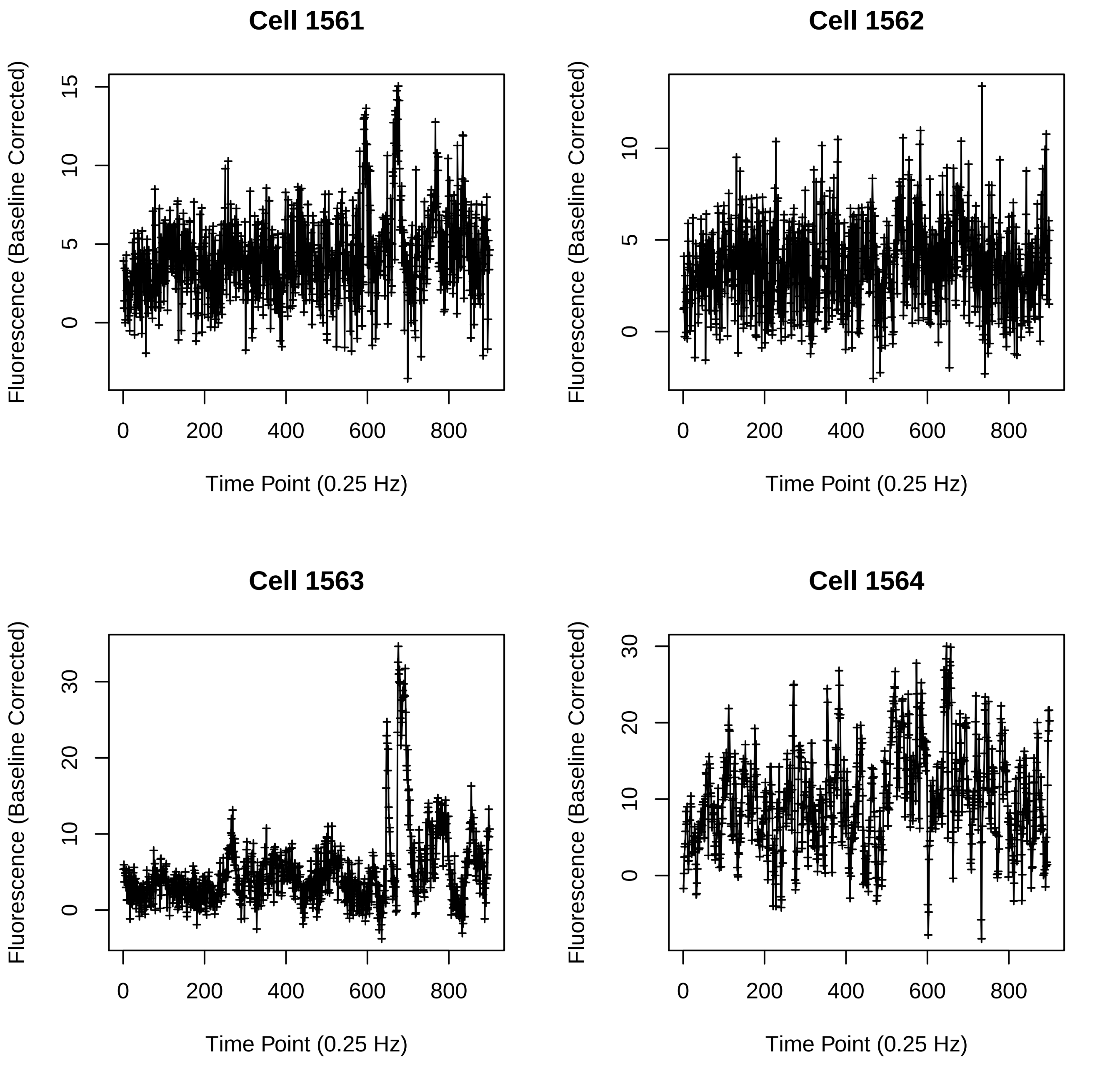
Cell 1561
Cell 1562
Cell 1563
Cell 1564
Fluorescence (Baseline Corrected)
Time Point (0.25 Hz)
0
200
400
600
800
0
5
10
15
10
20
30

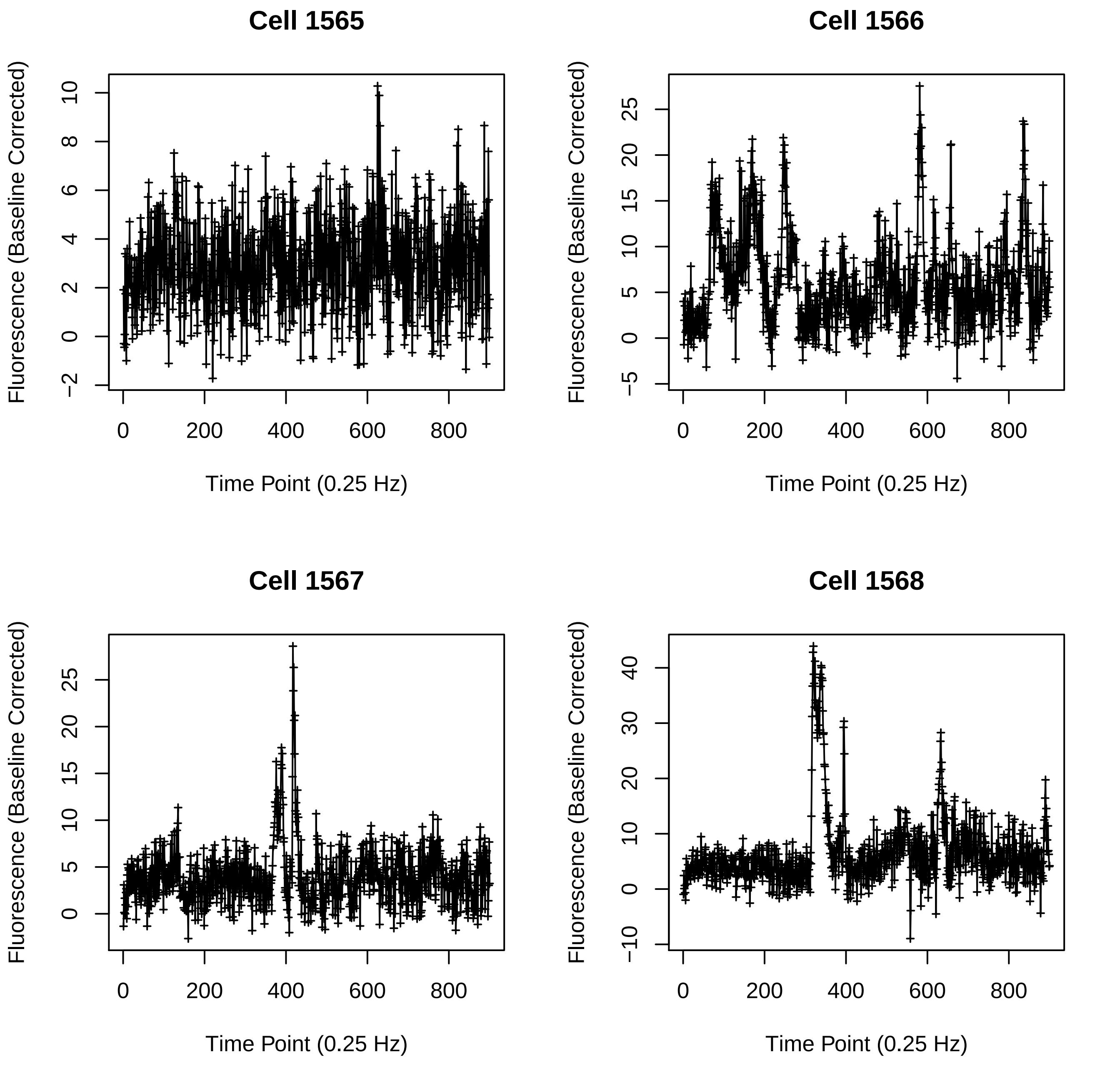
Cell 1565
Cell 1566
Cell 1567
Cell 1568
Fluorescence (Baseline Corrected)
Time Point (0.25 Hz)
0
200
400
600
800
−2
0
2
4
6
8
10
−5
5
10
15
20
25
−10
30
40

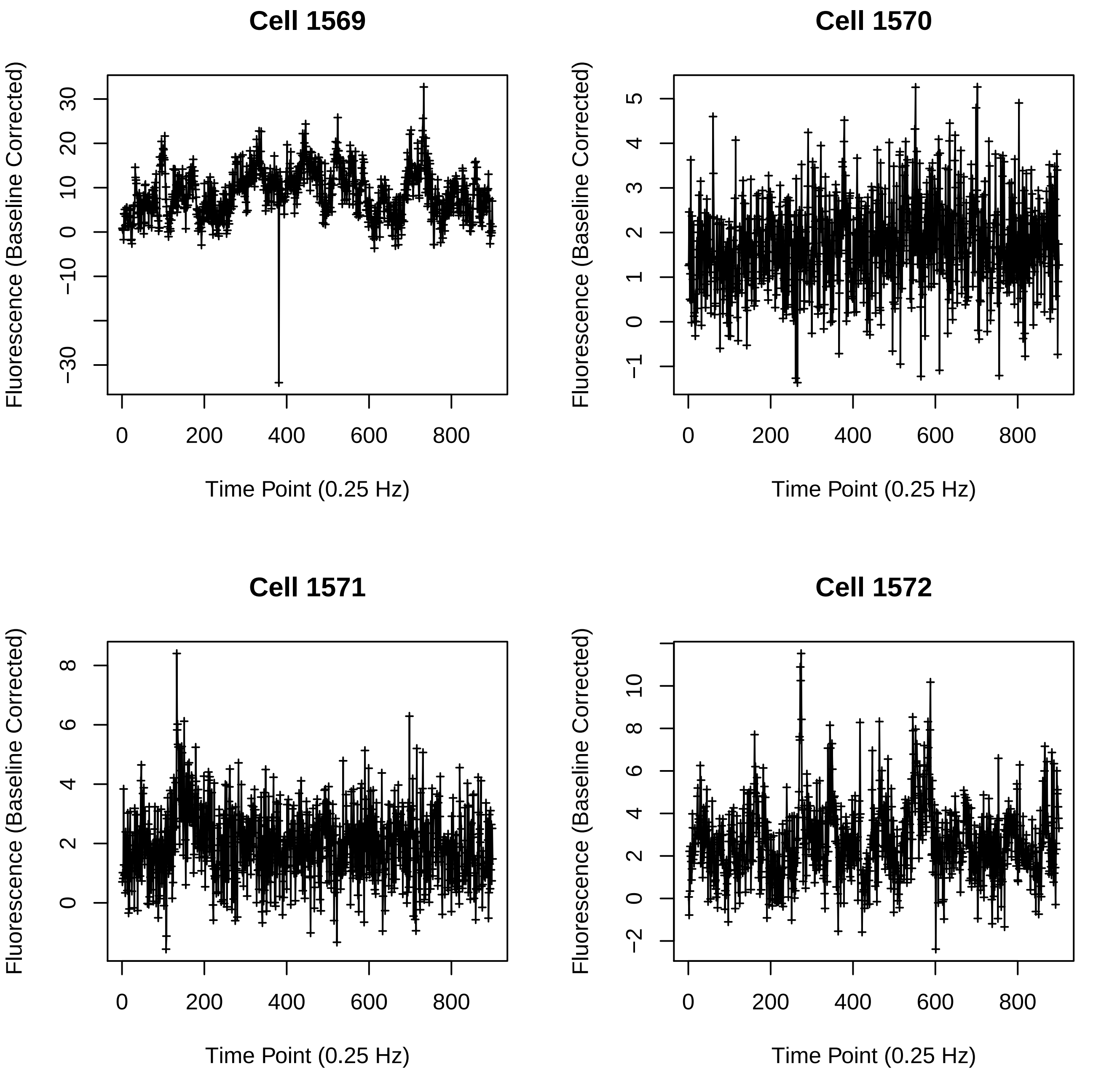
Cell 1569
Cell 1570
Cell 1571
Cell 1572
Fluorescence (Baseline Corrected)
Time Point (0.25 Hz)
0
200
400
600
800
30
20
10
0
−10
−30
5
4
3
2
1
0
−1
8
6
4
2
0
10
8
6
4
2
0
−2

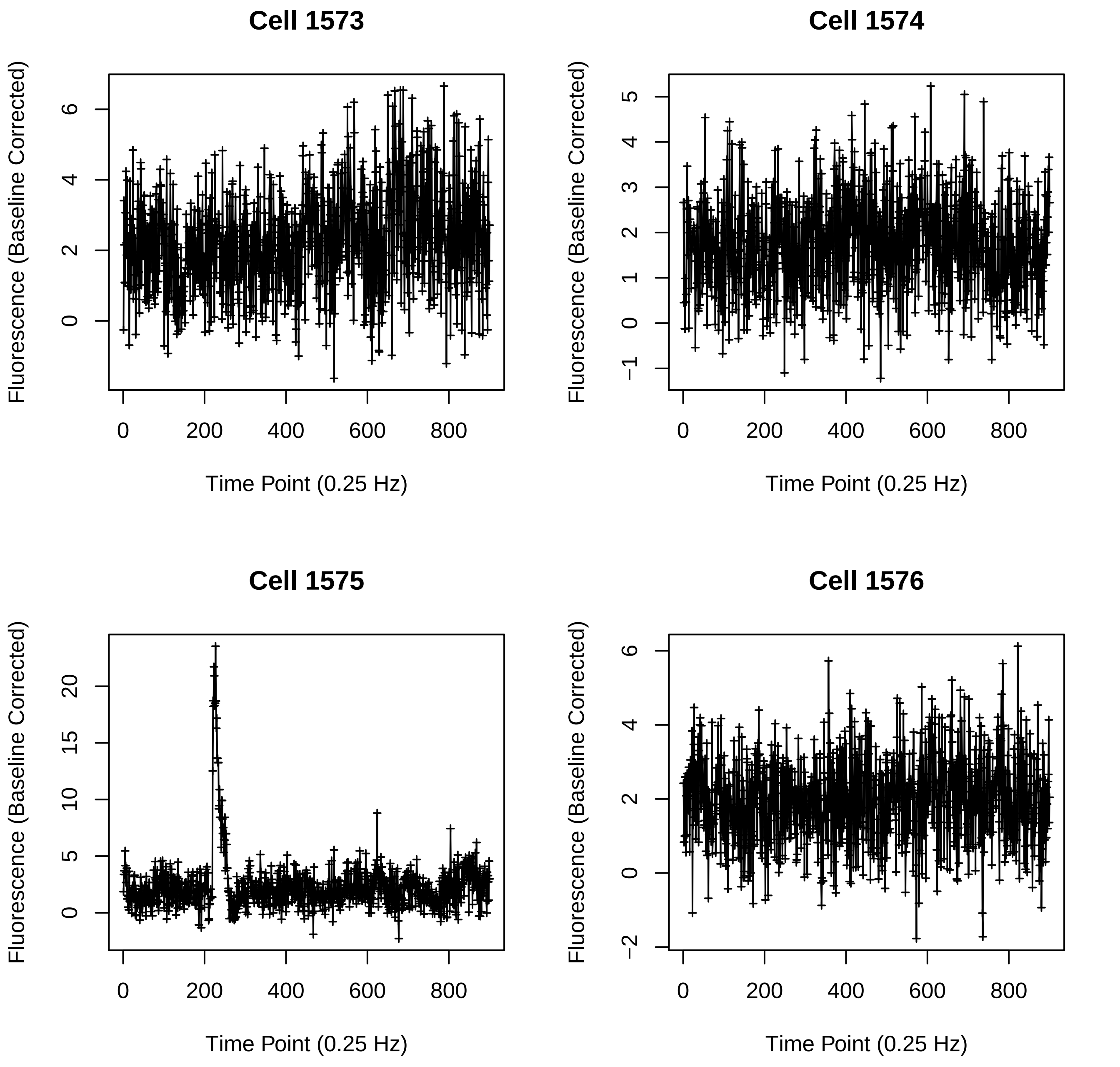
Cell 1573
Cell 1574
Cell 1575
Cell 1576
Fluorescence (Baseline Corrected)
Time Point (0.25 Hz)
0
200
400
600
800
−2
−1
0
1
2
3
4
5
6
10
15
20

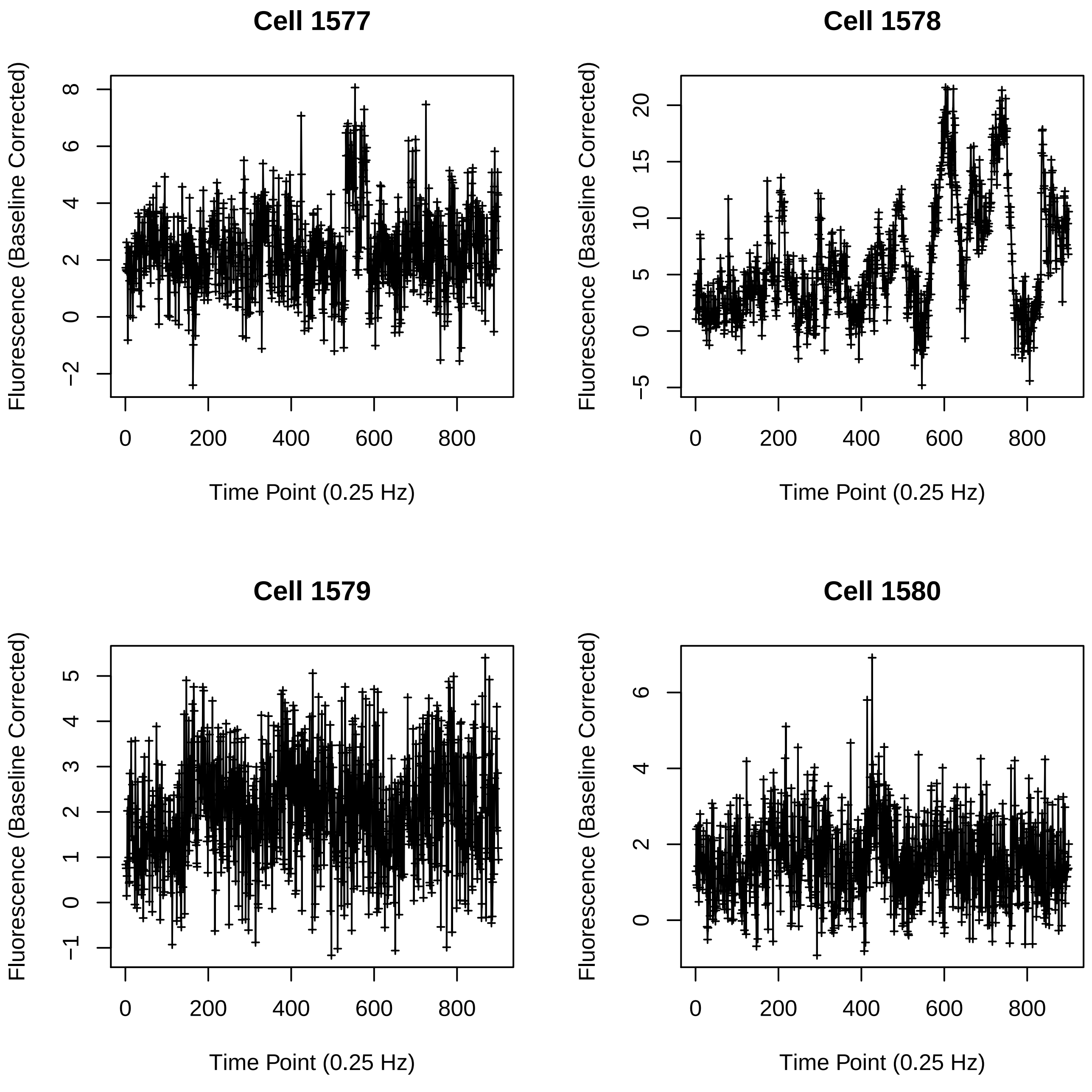
Cell 1577
Cell 1578
Cell 1579
Cell 1580
Fluorescence (Baseline Corrected)
Time Point (0.25 Hz)
0
200
400
600
800
-2
2
4
6
8
5
10
15
20
-5
-1
1
3

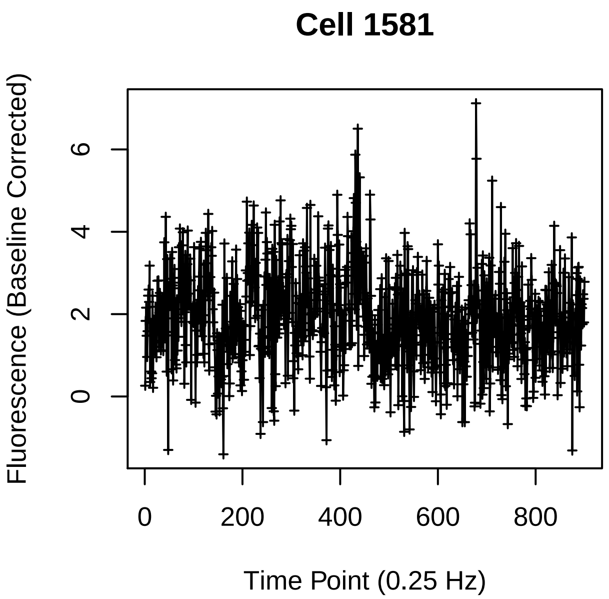
Cell 1581
Fluorescence (Baseline Corrected)
0
2
4
6
0
200
400
600
800
Time Point (0.25 Hz)

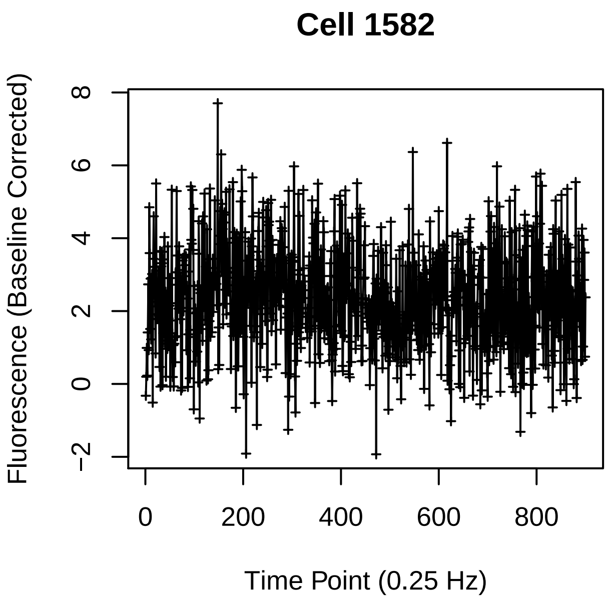
Cell 1582
Fluorescence (Baseline Corrected)
−2
0
2
4
6
8
0
200
400
600
800
Time Point (0.25 Hz)

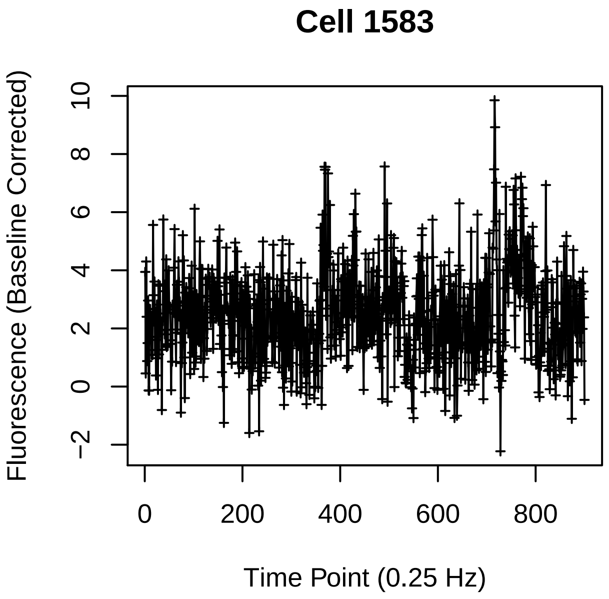
Cell 1583
Fluorescence (Baseline Corrected)
−2
0
2
4
6
8
10
0
200
400
600
800
Time Point (0.25 Hz)

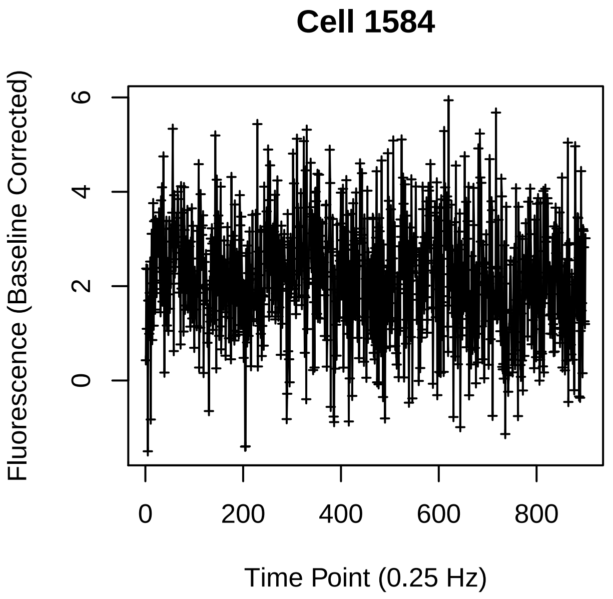
Cell 1584
Fluorescence (Baseline Corrected)
0
2
4
6
0
200
400
600
800
Time Point (0.25 Hz)

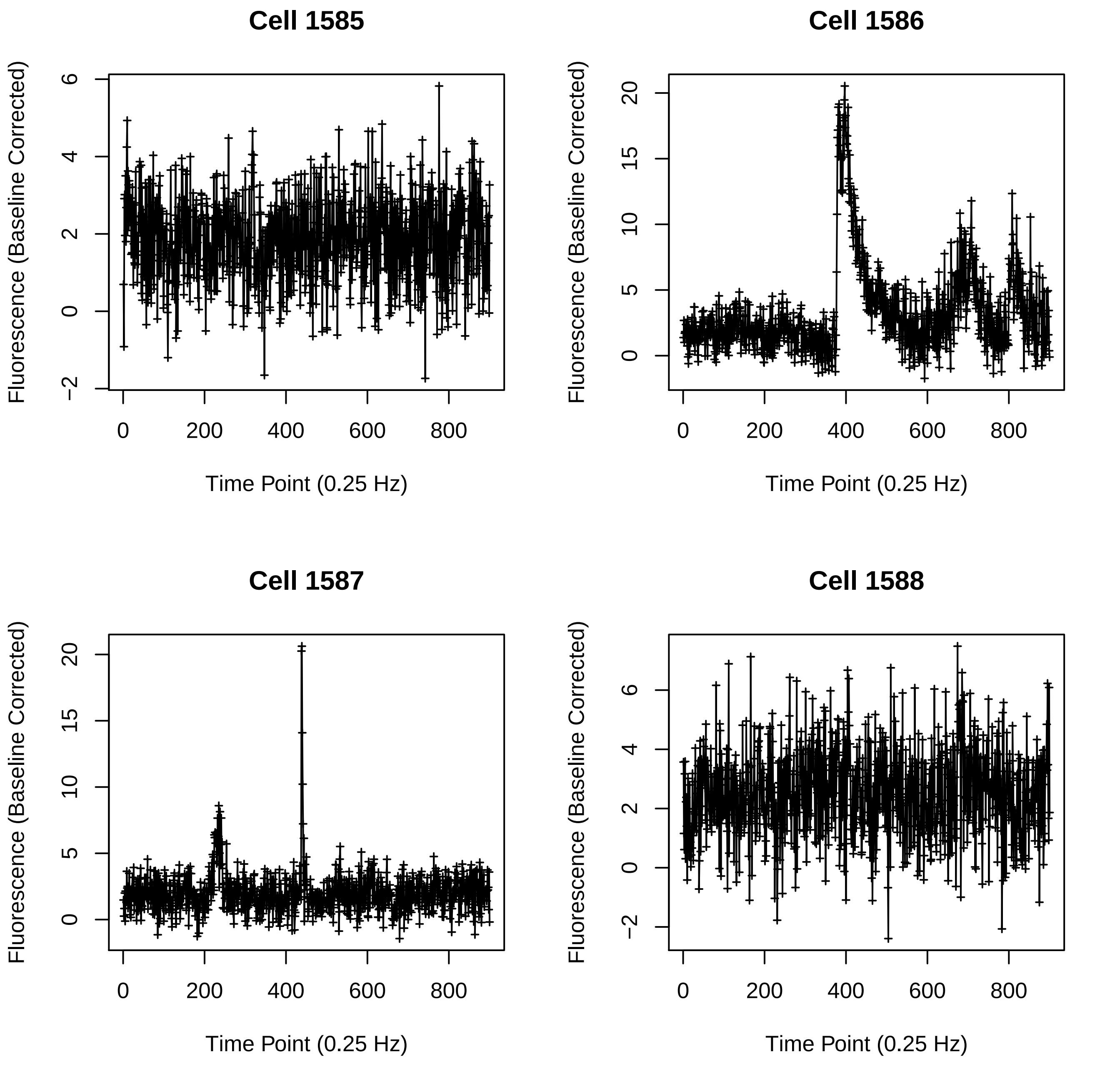

Cell 1585
Cell 1586
Cell 1587
Cell 1588
Fluorescence (Baseline Corrected)
Time Point (0.25 Hz)
0
200
400
600
800
−2
0
2
4
6
5
10
15
20

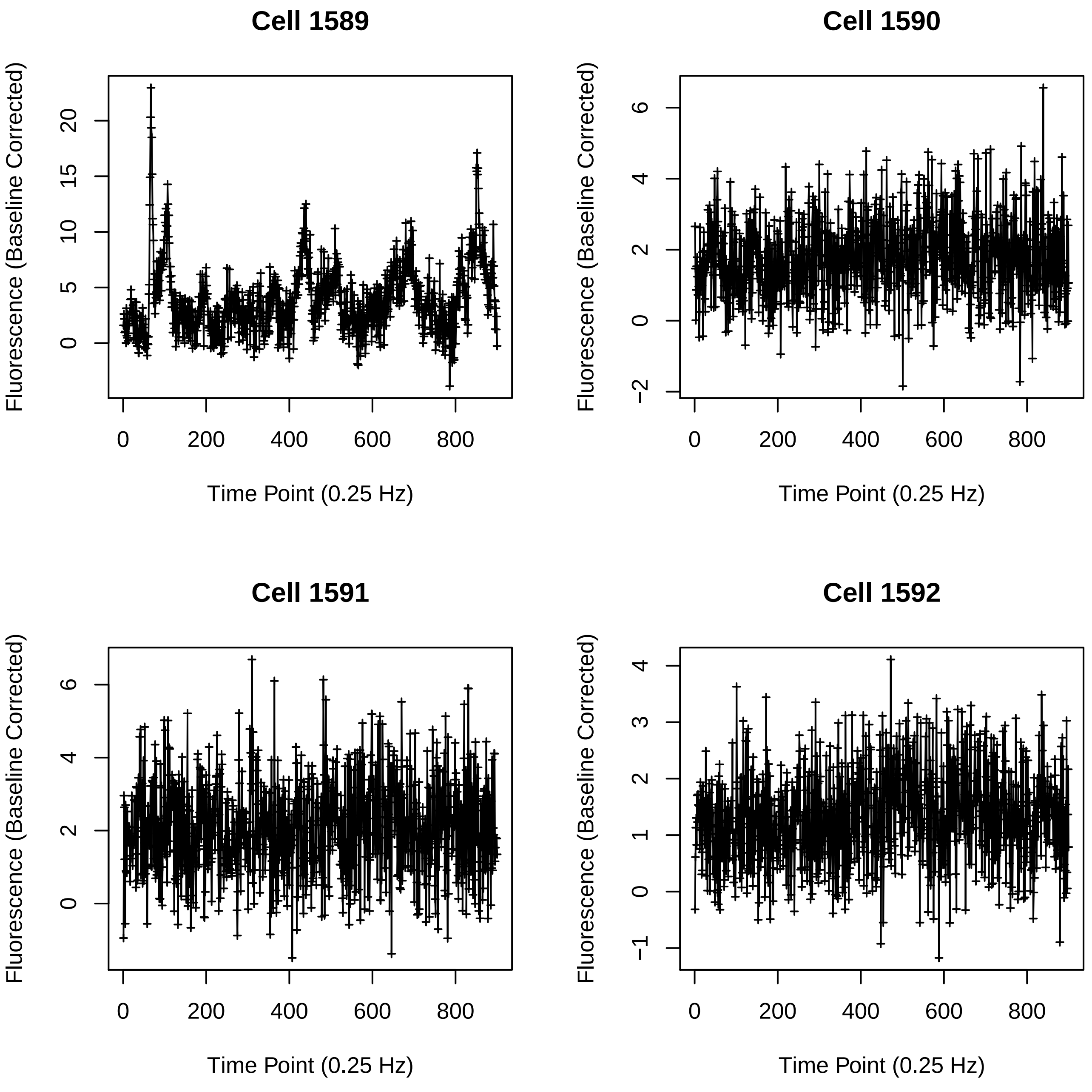

Cell 1589
Cell 1590
Cell 1591
Cell 1592
Fluorescence (Baseline Corrected)
Time Point (0.25 Hz)
0
200
400
600
800
-2
-1
0
1
2
3
4
5
6
10
15
20

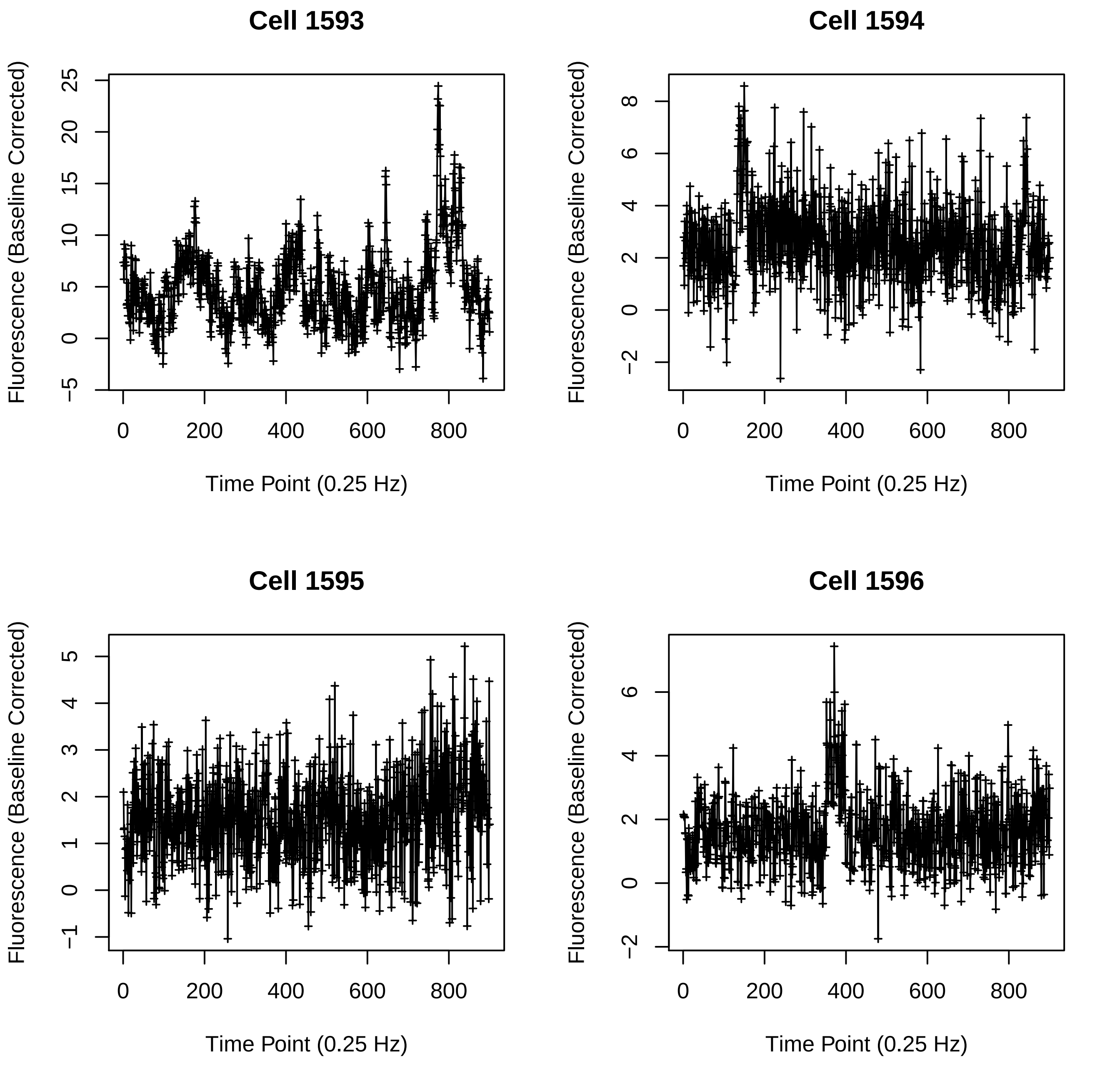
Cell 1593
Cell 1594
Cell 1595
Cell 1596
Fluorescence (Baseline Corrected)
Time Point (0.25 Hz)
0
200
400
600
800
−5
0
5
10
15
20
25
−2
2
4
6
8
−1
1
3

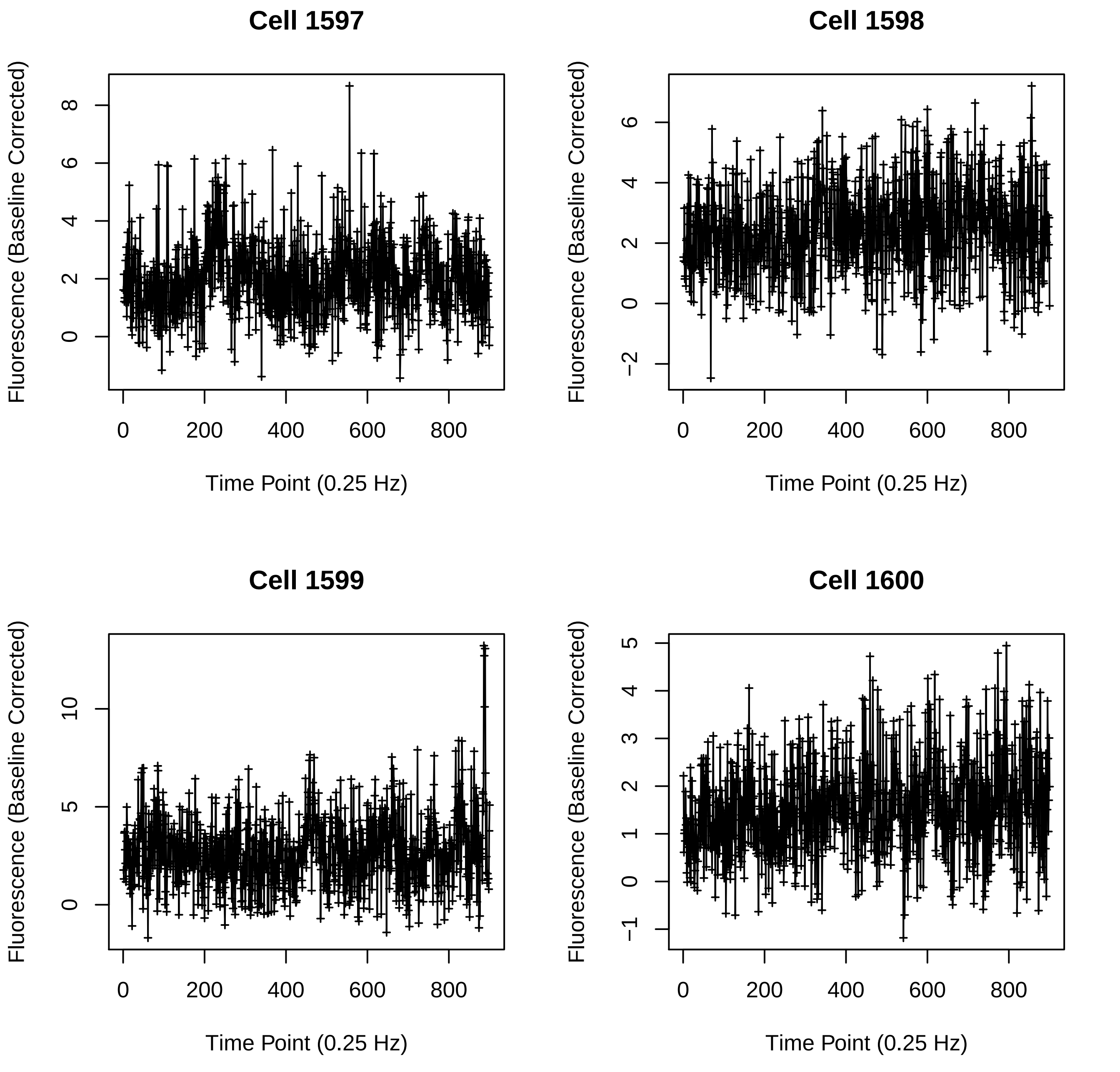

Cell 1597
Cell 1598
Cell 1599
Cell 1600
Fluorescence (Baseline Corrected)
Time Point (0.25 Hz)
0
200
400
600
800

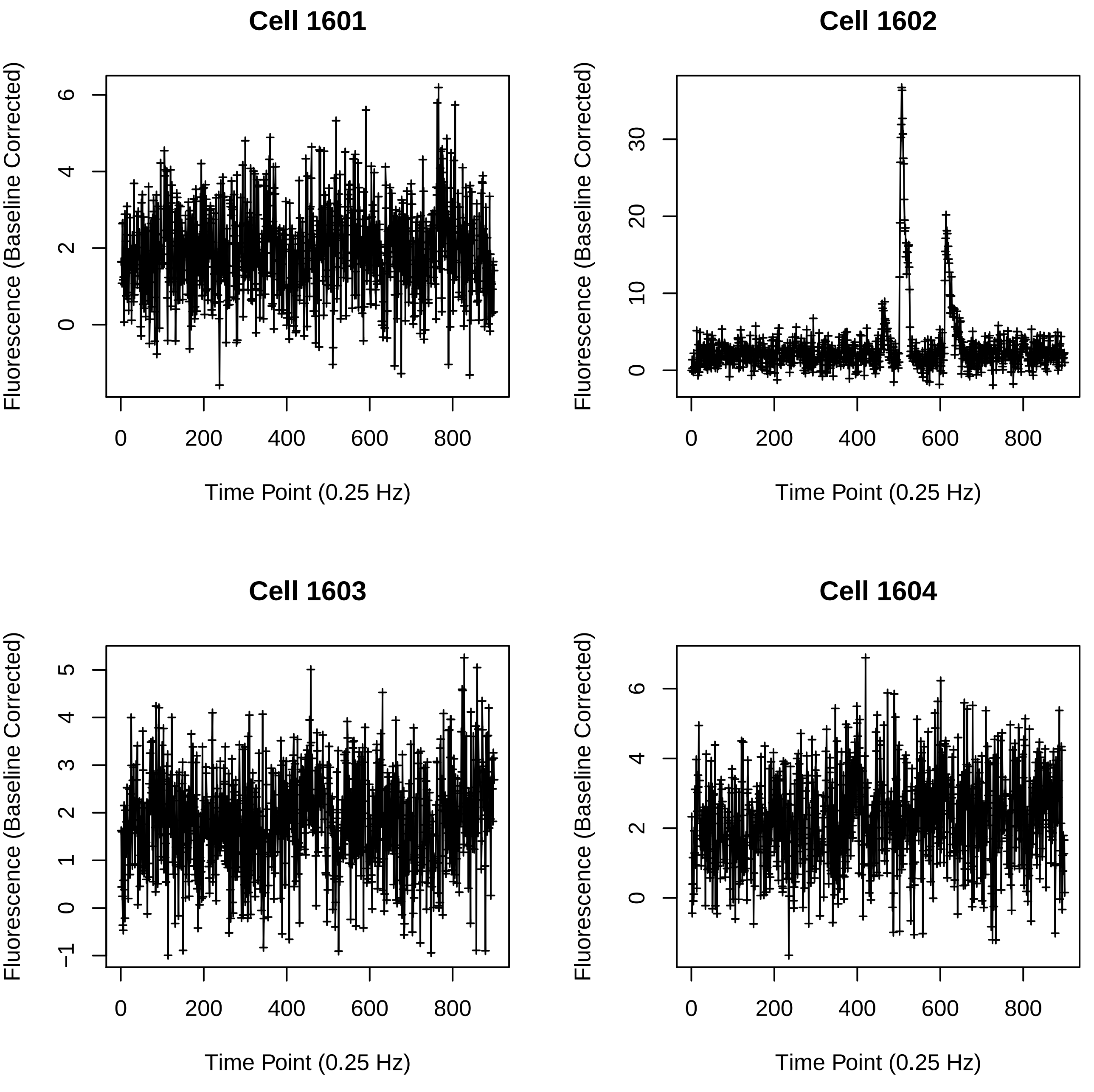
Cell 1601
Cell 1602
Cell 1603
Cell 1604
Fluorescence (Baseline Corrected)
Time Point (0.25 Hz)
0
200
400
600
800
0
2
4
6
10
20
30
−1
1
3
5

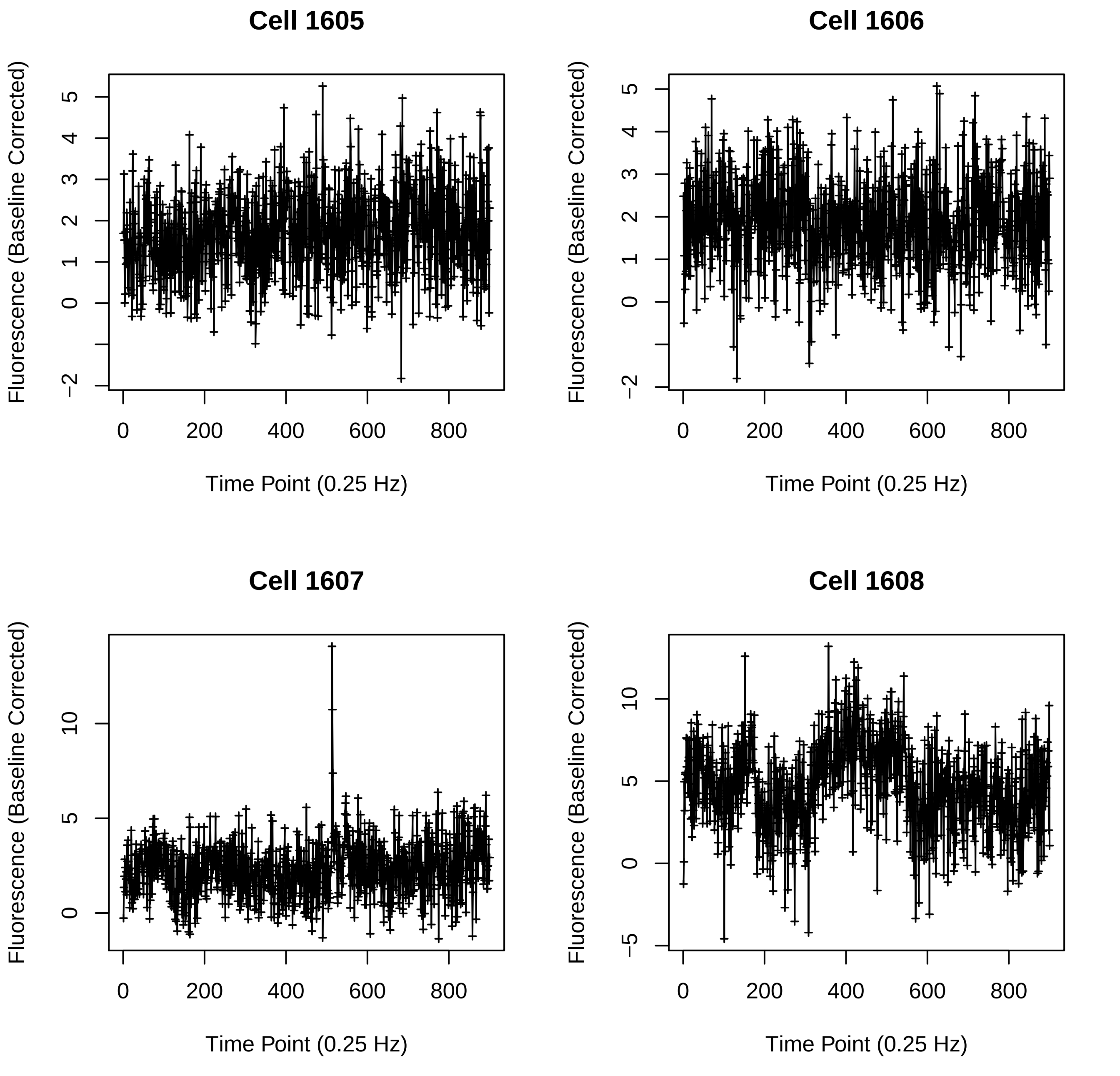
Cell 1605
Cell 1606
Cell 1607
Cell 1608
Fluorescence (Baseline Corrected)
Time Point (0.25 Hz)
0
200
400
600
800
−2
0
1
2
3
4
5
10
−5

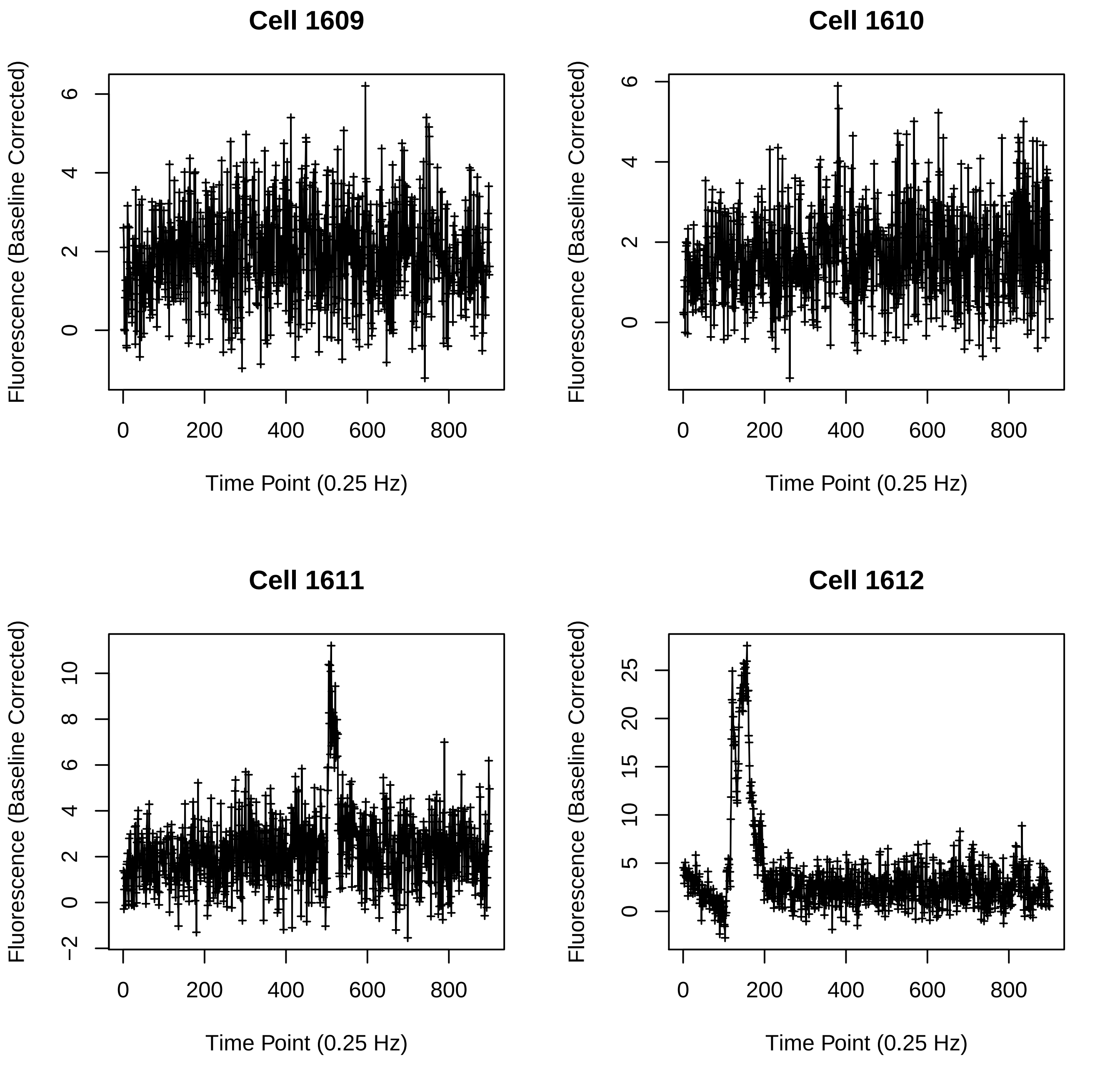
Cell 1609
Cell 1610
Cell 1611
Cell 1612
Fluorescence (Baseline Corrected)
Time Point (0.25 Hz)

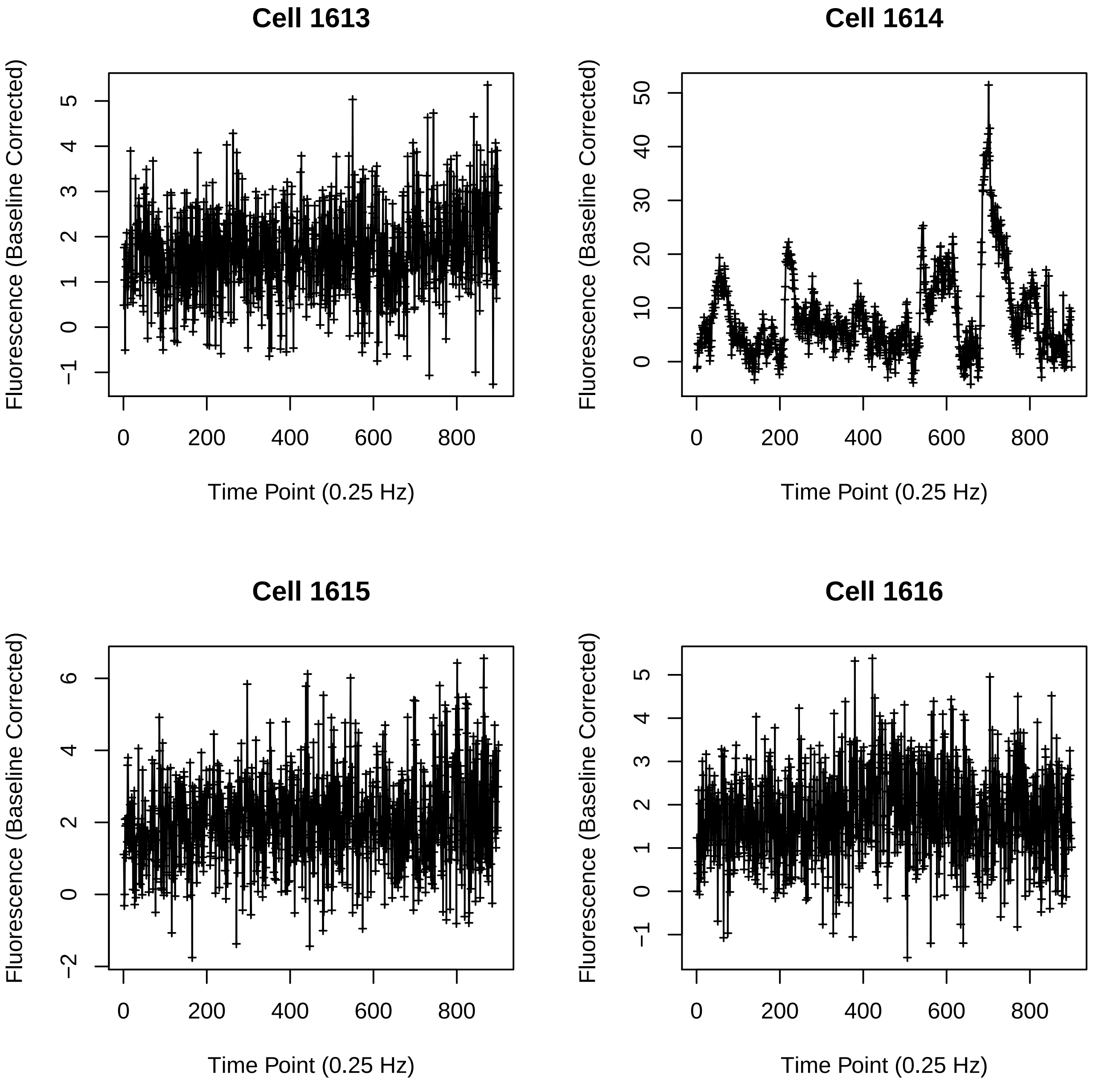
Cell 1613
Fluorescence (Baseline Corrected)
−1
0
1
2
3
4
5
0
200
400
600
800
Time Point (0.25 Hz)
Cell 1614
Fluorescence (Baseline Corrected)
0
10
20
30
40
50
0
200
400
600
800
Time Point (0.25 Hz)
Cell 1615
Fluorescence (Baseline Corrected)
−2
0
2
4
6
0
200
400
600
800
Time Point (0.25 Hz)
Cell 1616
Fluorescence (Baseline Corrected)
−1
0
1
2
3
4
5
0
200
400
600
800
Time Point (0.25 Hz)

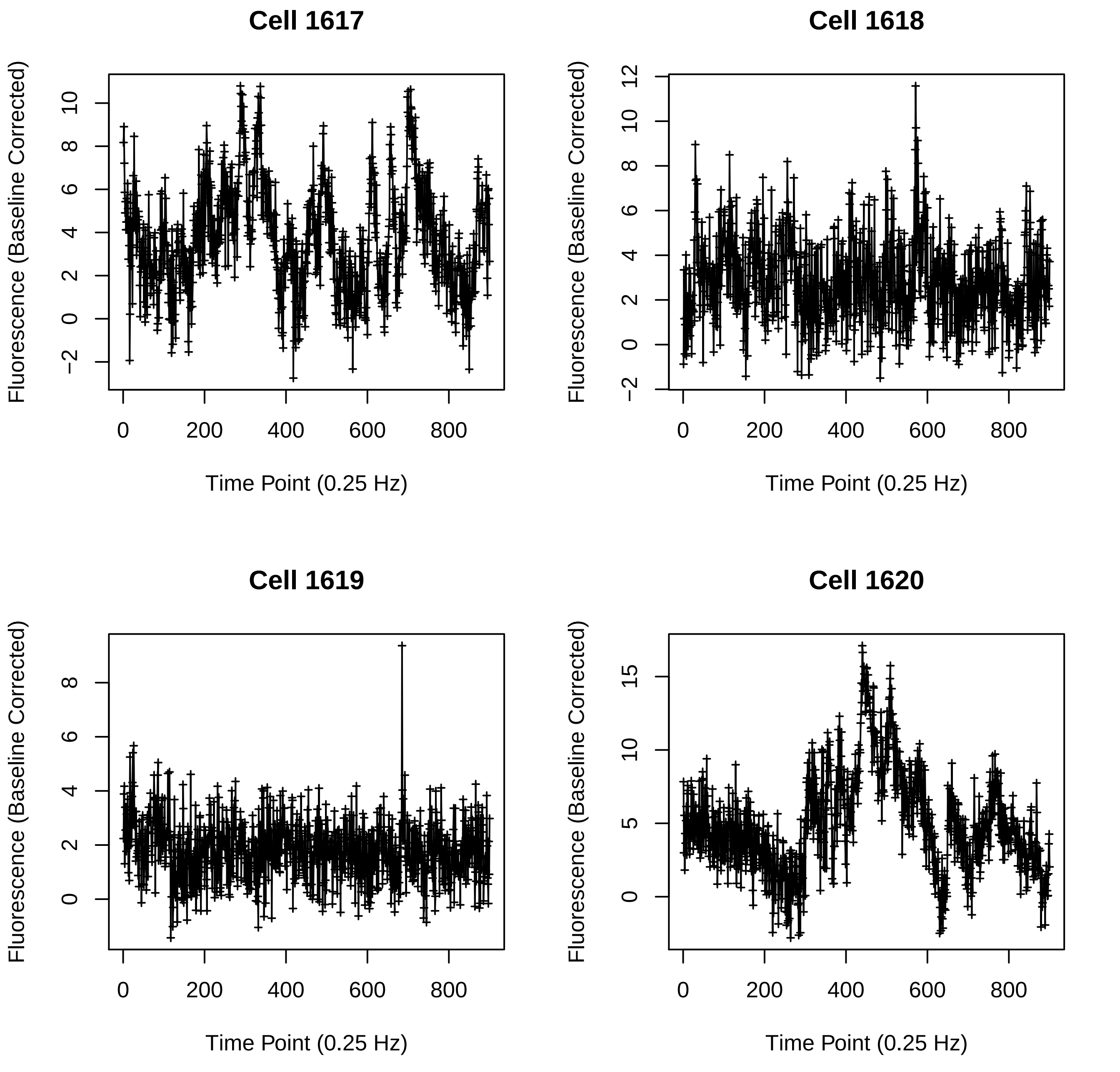
Cell 1617
Fluorescence (Baseline Corrected)
−2
0
2
4
6
8
10
0
200
400
600
800
Time Point (0.25 Hz)
Cell 1618
Fluorescence (Baseline Corrected)
−2
0
2
4
6
8
10
12
0
200
400
600
800
Time Point (0.25 Hz)
Cell 1619
Fluorescence (Baseline Corrected)
0
2
4
6
8
0
200
400
600
800
Time Point (0.25 Hz)
Cell 1620
Fluorescence (Baseline Corrected)
0
5
10
15
0
200
400
600
800
Time Point (0.25 Hz)

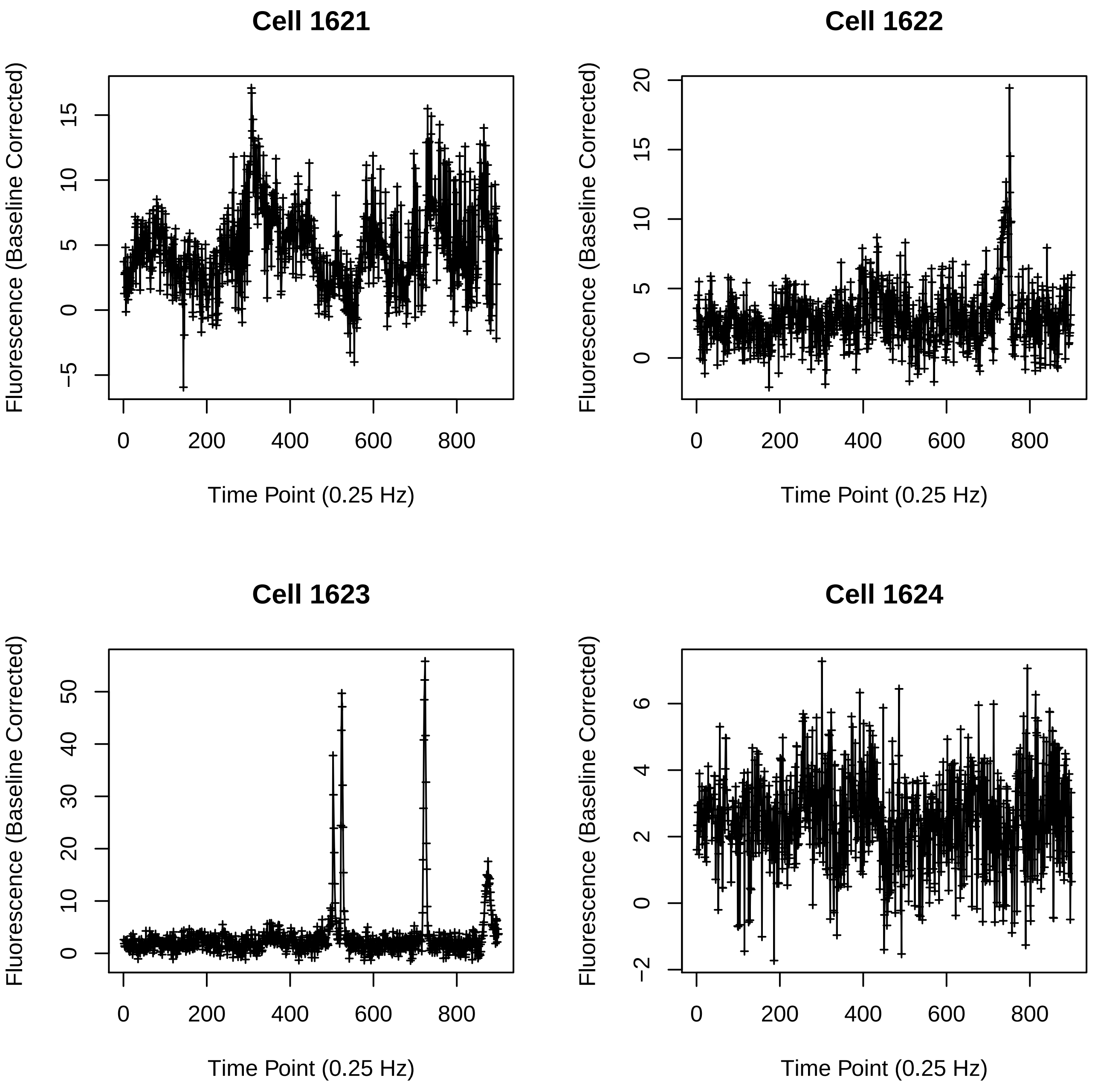

Cell 1621
Cell 1622
Cell 1623
Cell 1624
Fluorescence (Baseline Corrected)
Time Point (0.25 Hz)
0
200
400
600
800
−5
0
5
10
15
20
30
40
50
−2
2
4
6

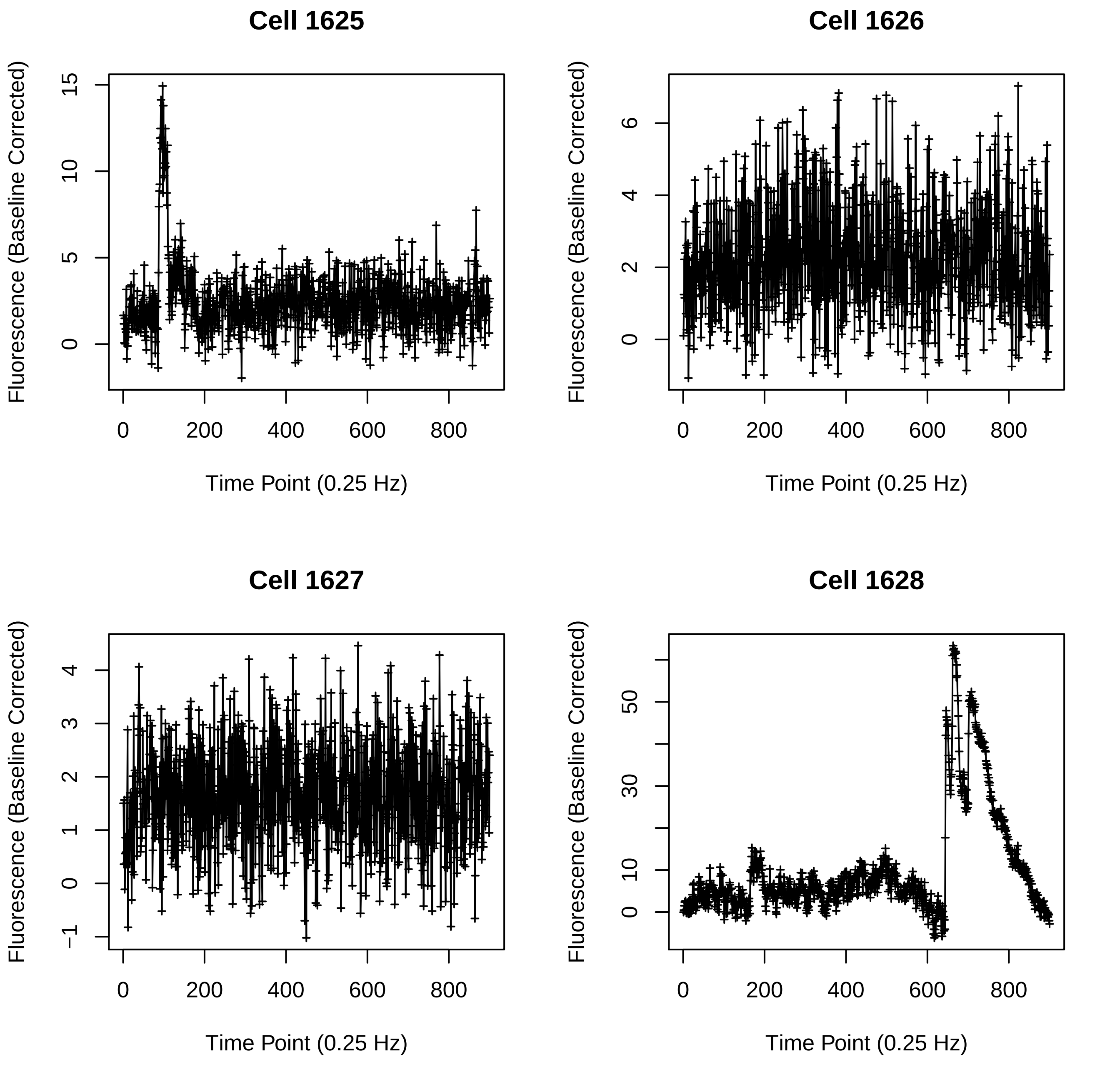
Cell 1625
Cell 1626
Cell 1627
Cell 1628
Fluorescence (Baseline Corrected)
Time Point (0.25 Hz)
0
200
400
600
800
0
5
10
15
0
2
4
6
−1
0
1
2
3
4
0
10
30
50

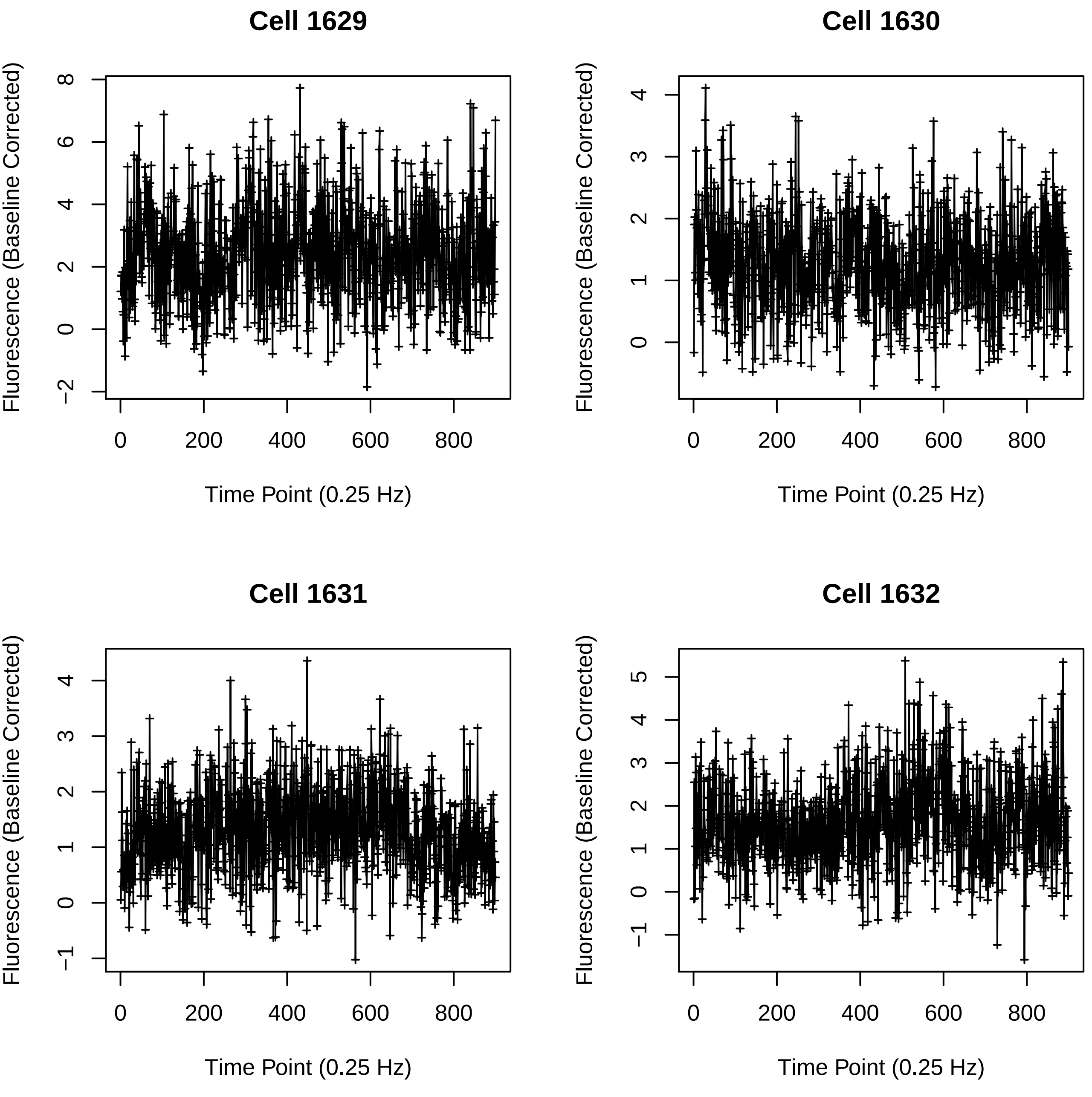

Cell 1629
Cell 1630
Cell 1631
Cell 1632
Fluorescence (Baseline Corrected)
Time Point (0.25 Hz)
−2
0
2
4
6
8
−1
0
1
2
3
4
5
0
200
400
600
800

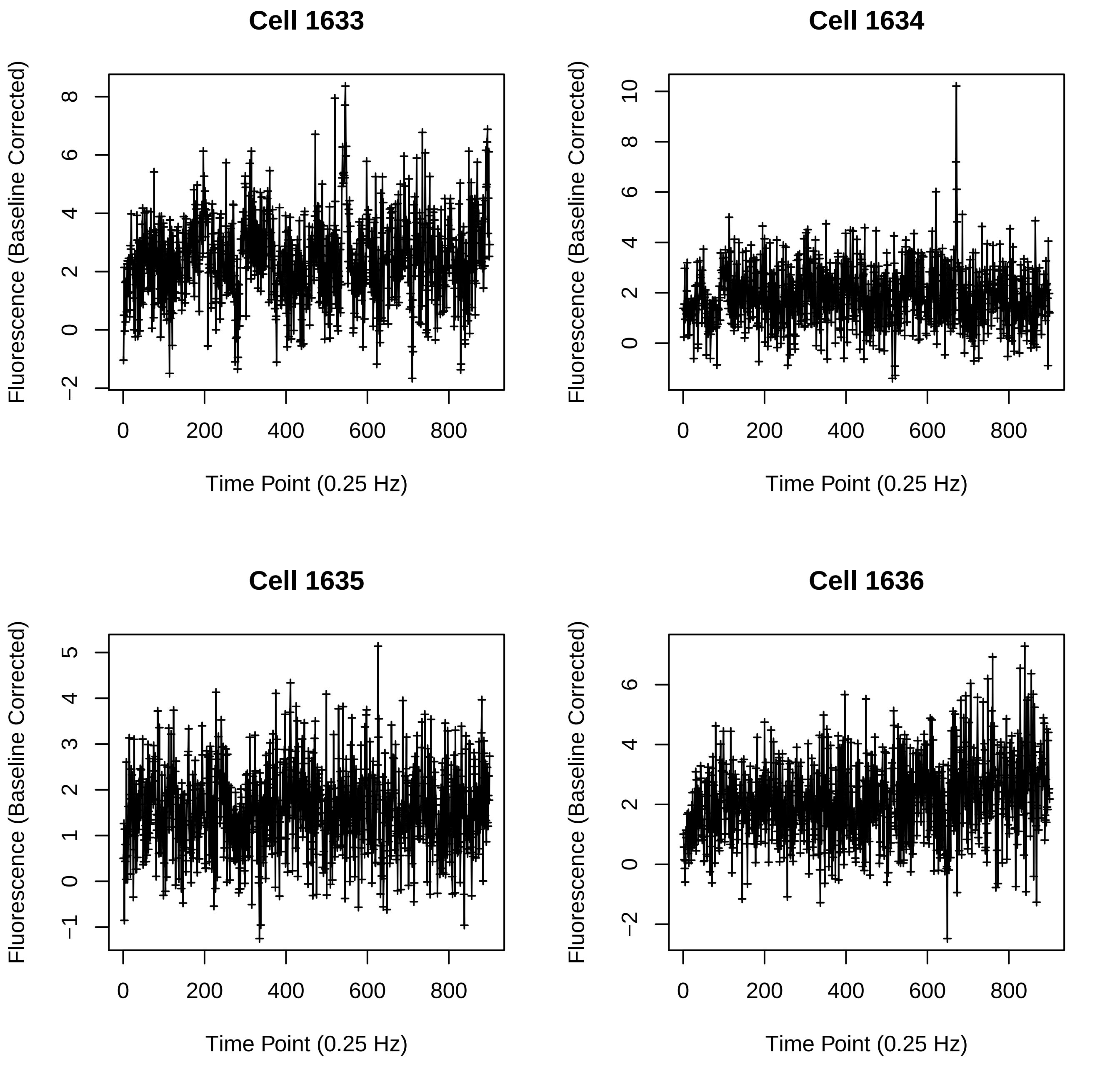

Cell 1633
Cell 1634
Cell 1635
Cell 1636
Fluorescence (Baseline Corrected)
Time Point (0.25 Hz)

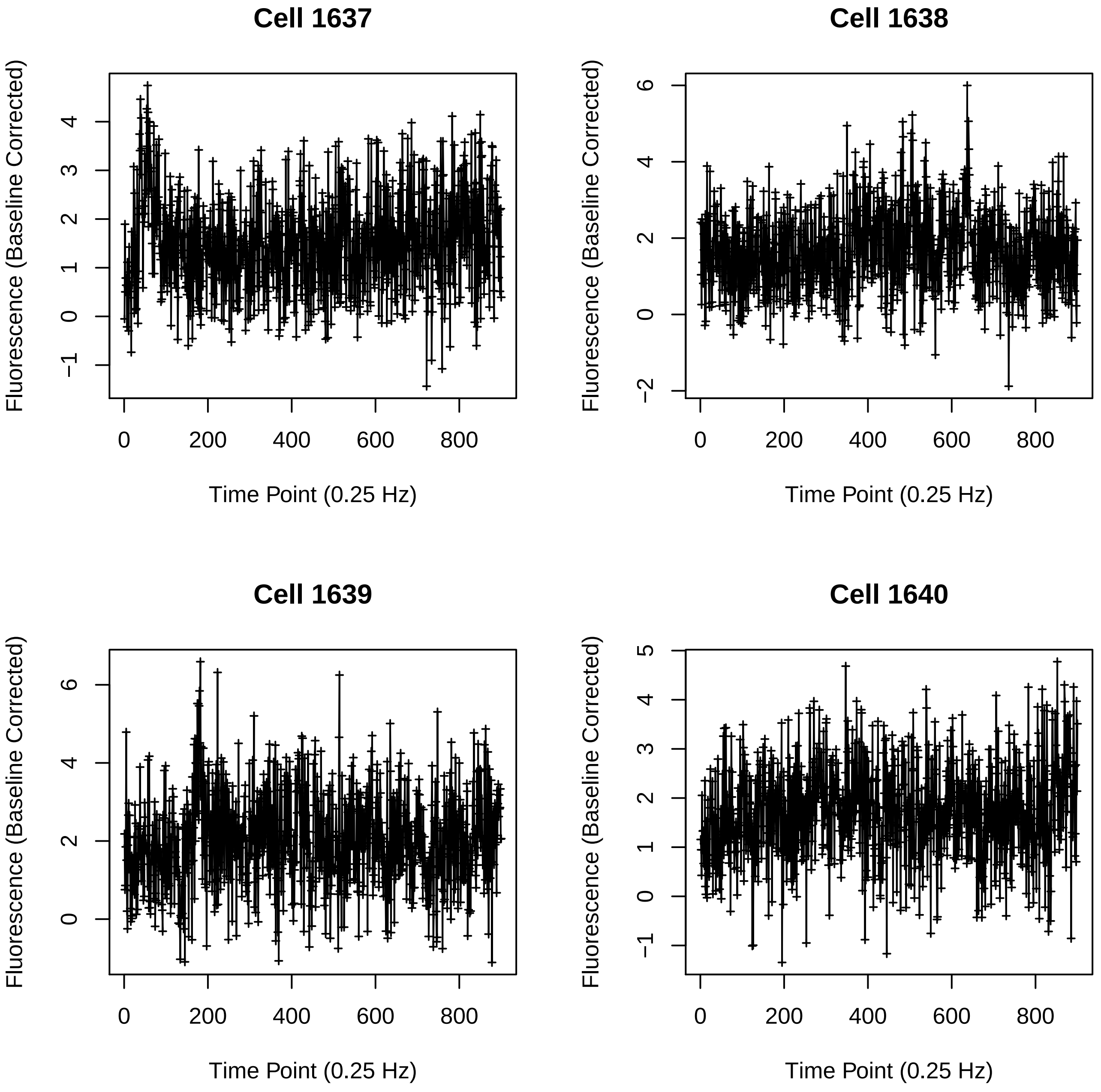

Cell 1637
Cell 1638
Cell 1639
Cell 1640
Fluorescence (Baseline Corrected)
Time Point (0.25 Hz)
0
200
400
600
800
−2
−1
0
1
2
3
4
5
6

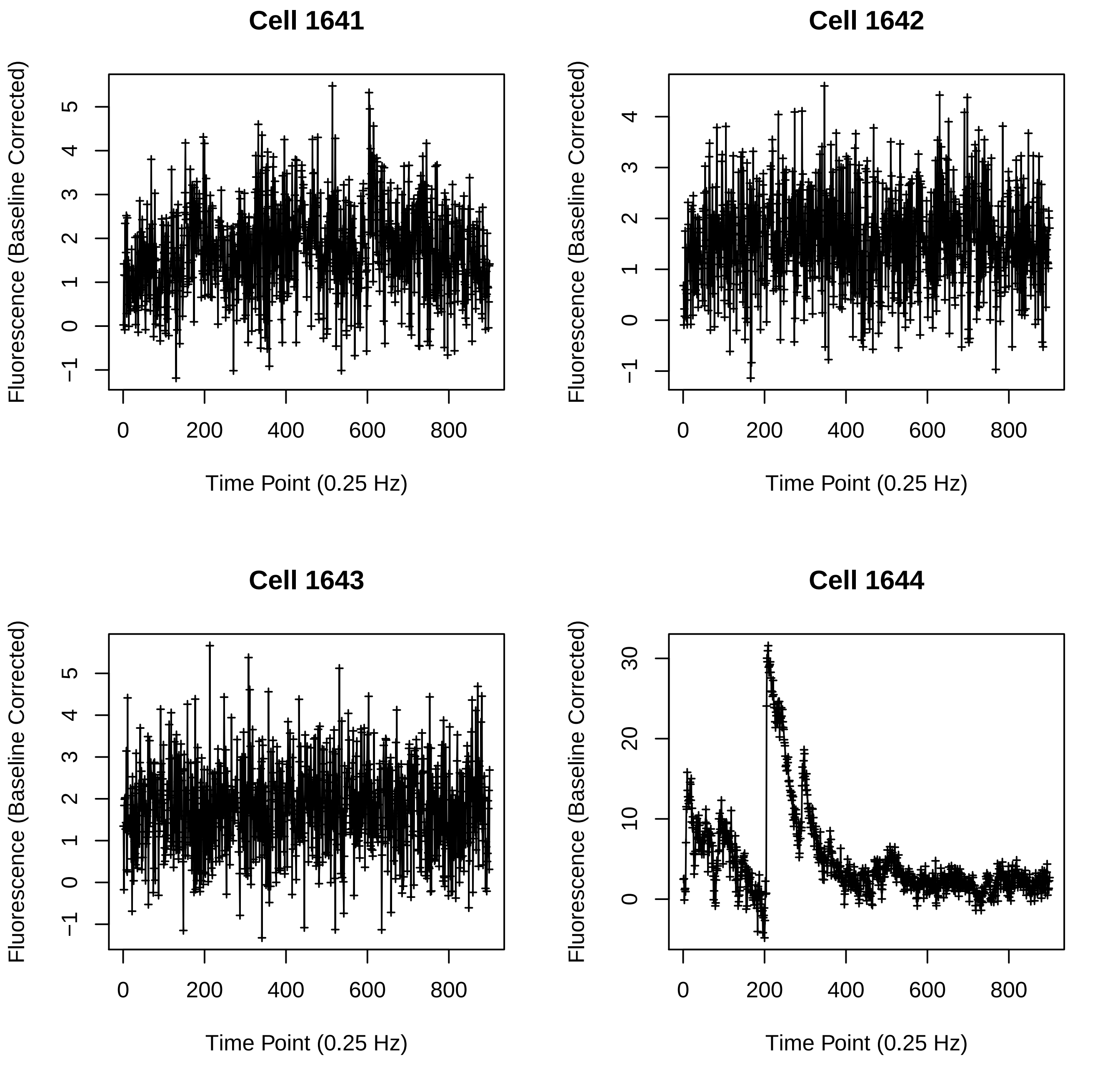
Cell 1641
Cell 1642
Cell 1643
Cell 1644
Fluorescence (Baseline Corrected)
Time Point (0.25 Hz)
0
200
400
600
800
−1
1
2
3
4
5
10
20
30

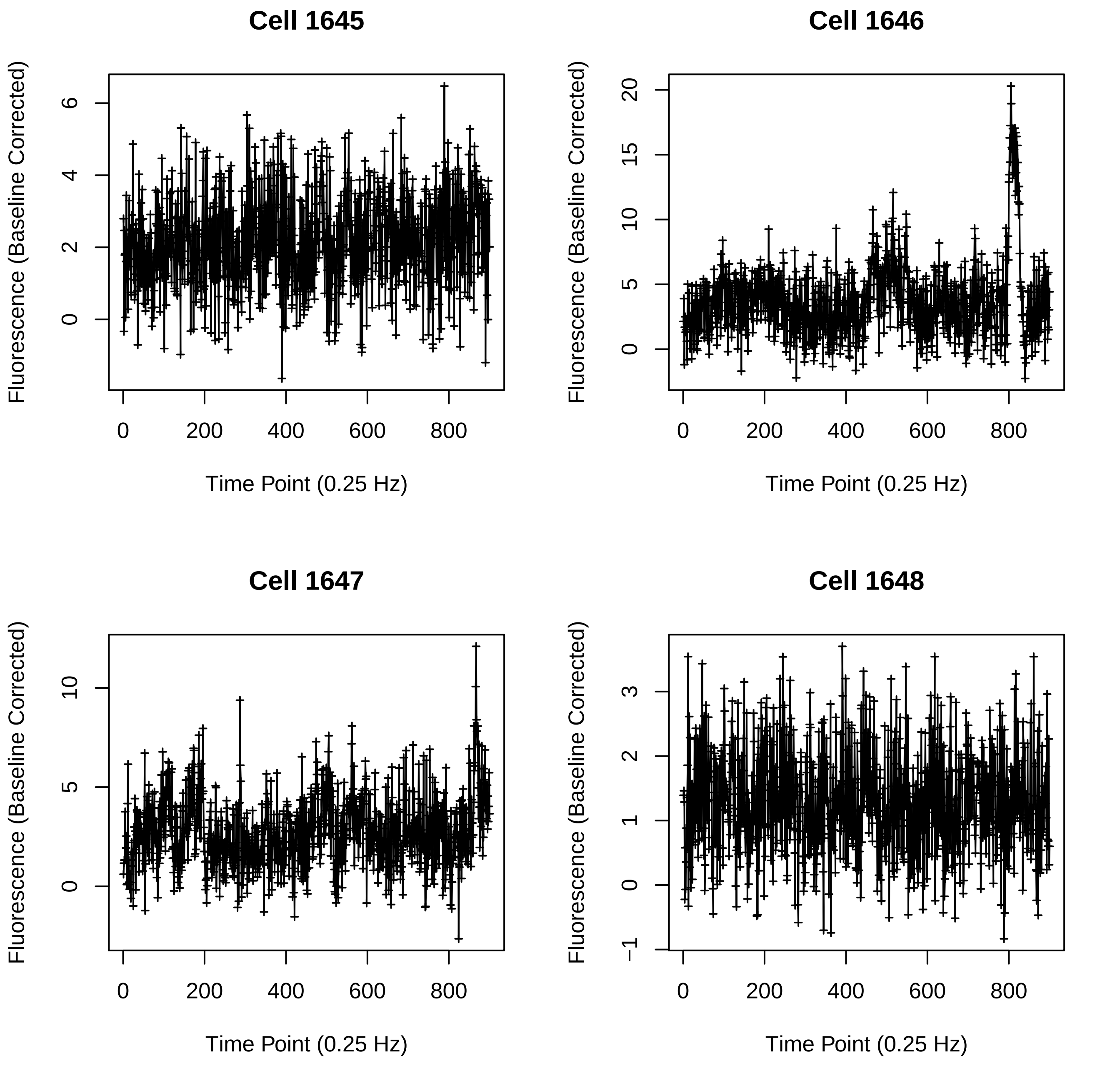
Cell 1645
Cell 1646
Cell 1647
Cell 1648
Fluorescence (Baseline Corrected)
Time Point (0.25 Hz)
0
200
400
600
800
-1
0
1
2
3
4
5
6
10
15
20

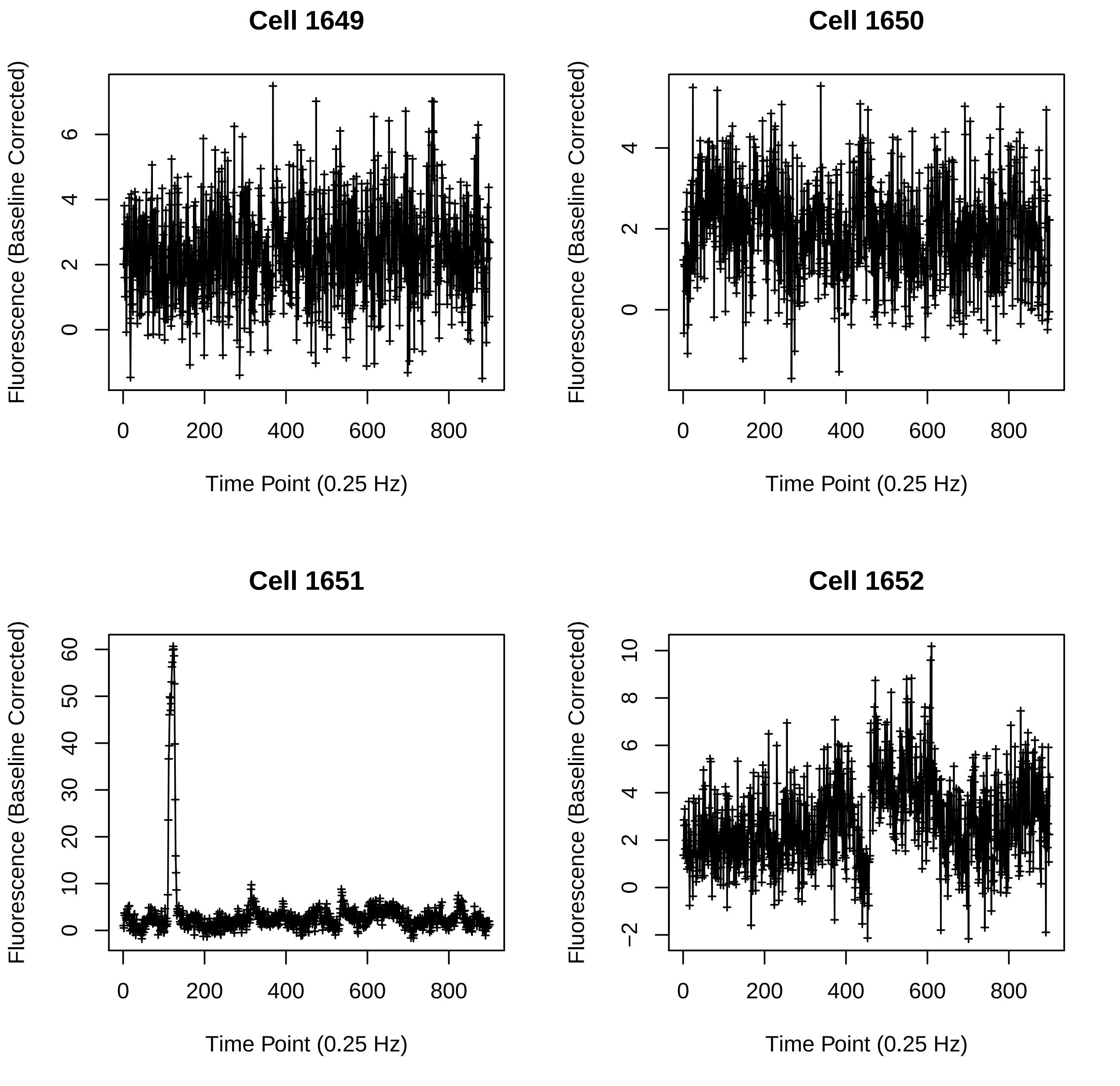

Cell 1649
Cell 1650
Cell 1651
Cell 1652
Fluorescence (Baseline Corrected)
Time Point (0.25 Hz)
0
200
400
600
800
2
4
6
10
20
30
40
50
60
8
−2

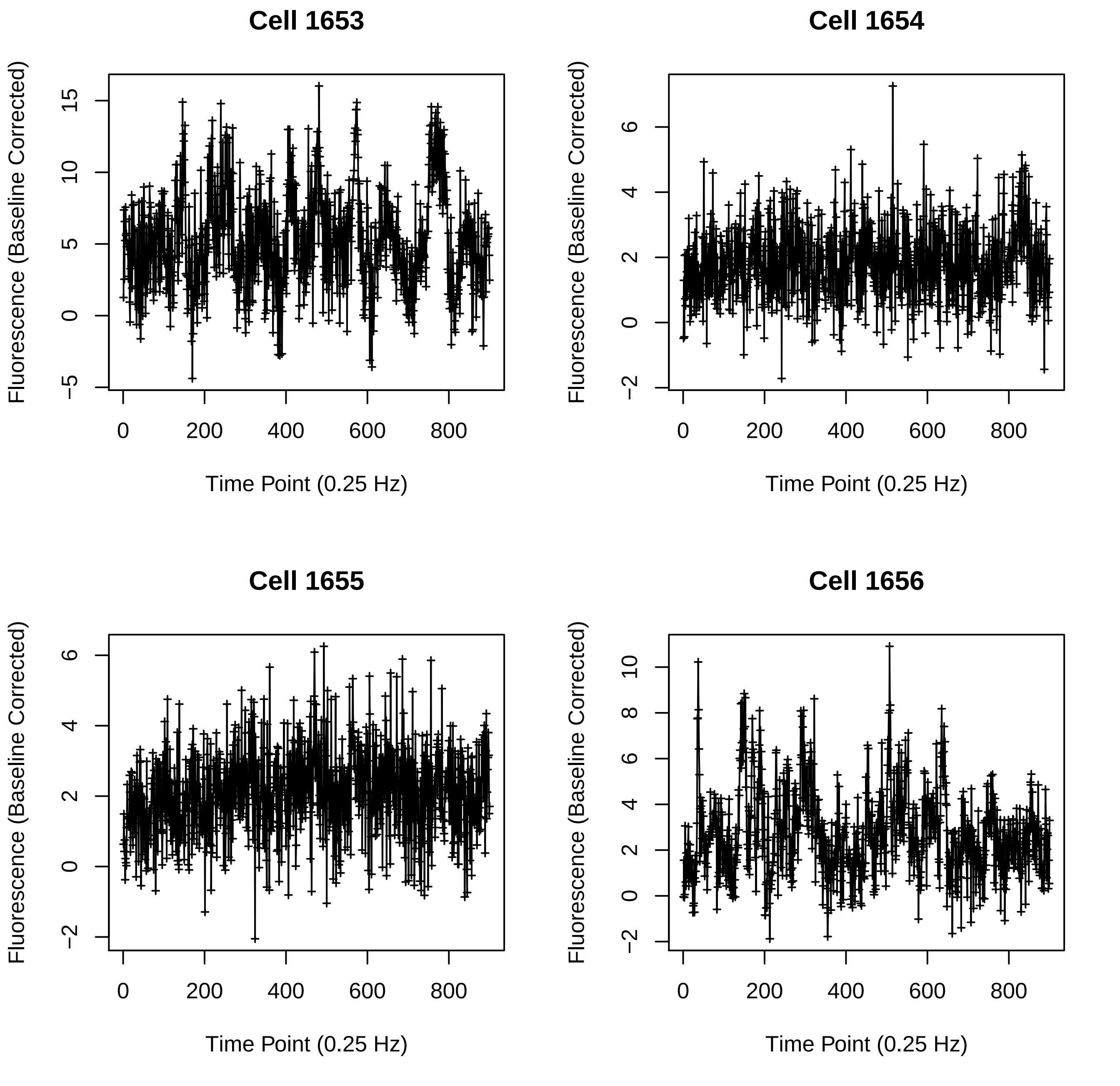

Cell 1653
Cell 1654
Cell 1655
Cell 1656
Fluorescence (Baseline Corrected)
Time Point (0.25 Hz)
0
200
400
600
800
-5
0
5
10
15
-2
2
4
6
8

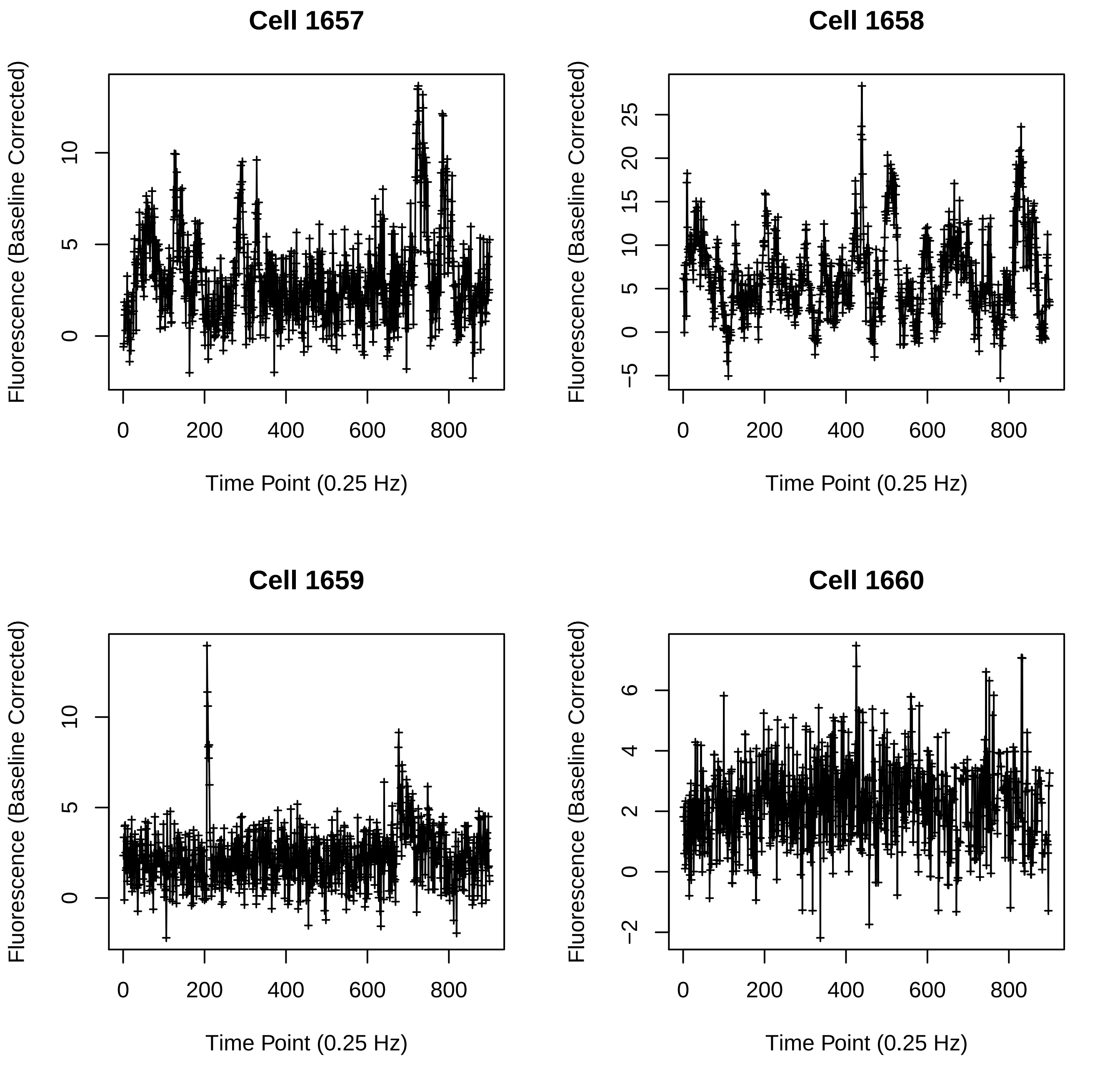
Cell 1657
Cell 1658
Cell 1659
Cell 1660
Fluorescence (Baseline Corrected)
Time Point (0.25 Hz)
0
200
400
600
800
0
5
10
−5
15
20
25
−2
2
4
6

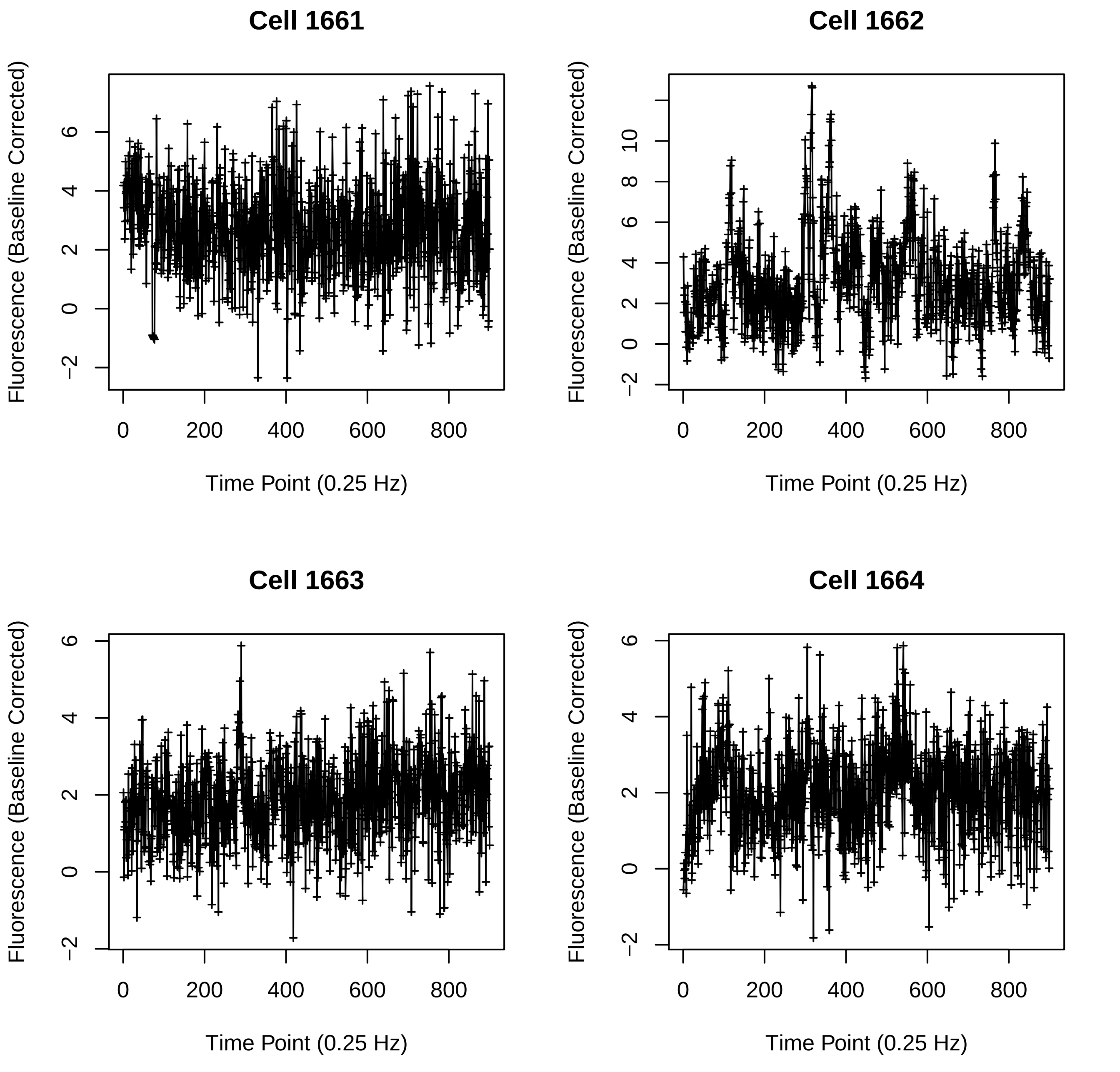
Cell 1661
Fluorescence (Baseline Corrected)
Time Point (0.25 Hz)
0
200
400
600
800
−2
0
2
4
6
Cell 1662
Fluorescence (Baseline Corrected)
Time Point (0.25 Hz)
0
200
400
600
800
−2
0
2
4
6
8
10
Cell 1663
Fluorescence (Baseline Corrected)
Time Point (0.25 Hz)
0
200
400
600
800
−2
0
2
4
6
Cell 1664
Fluorescence (Baseline Corrected)
Time Point (0.25 Hz)
0
200
400
600
800
−2
0
2
4
6

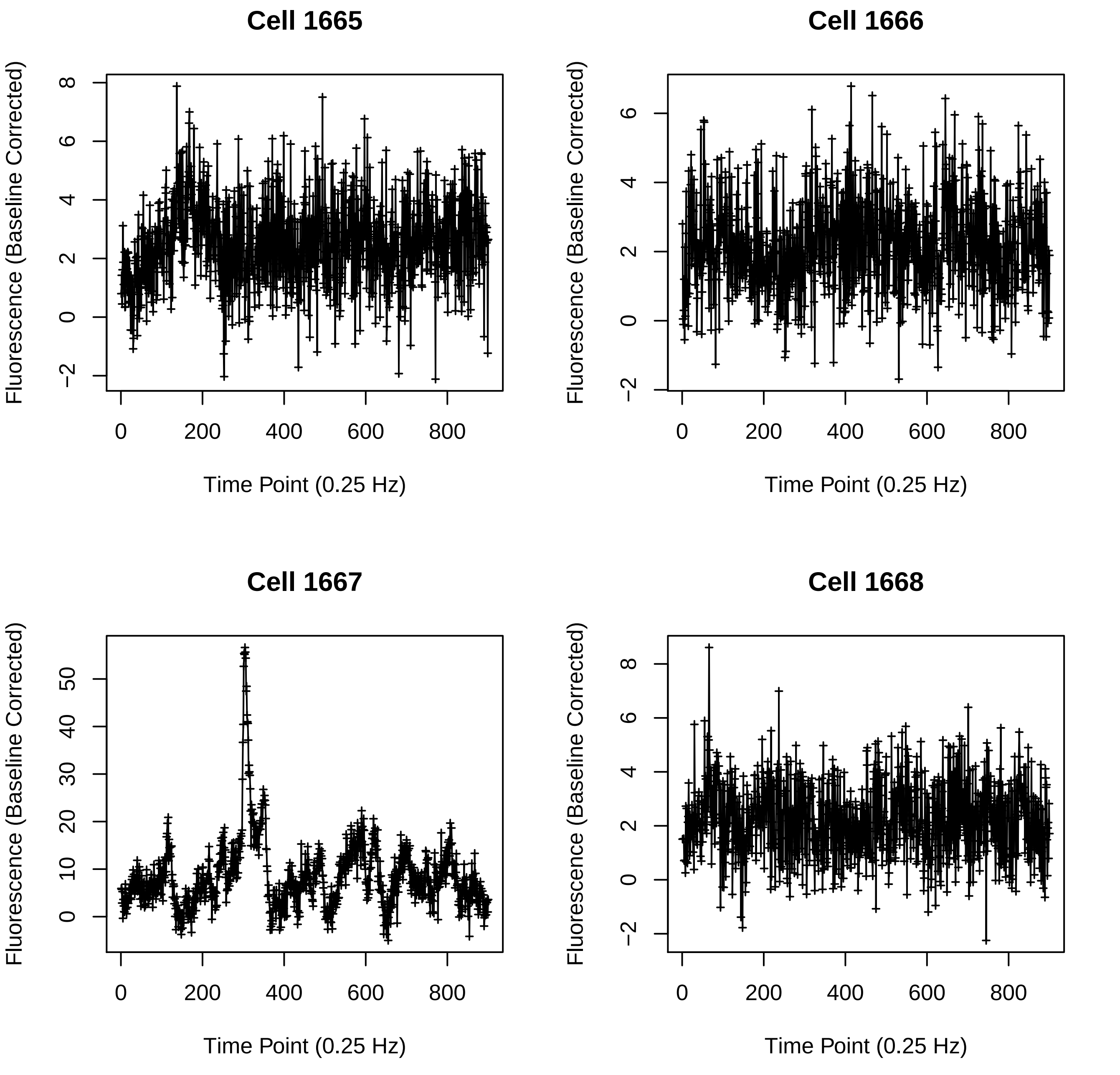
Cell 1665
Cell 1666
Cell 1667
Cell 1668
Fluorescence (Baseline Corrected)
Time Point (0.25 Hz)
0
200
400
600
800
−2
0
2
4
6
8
10
20
30
40
50

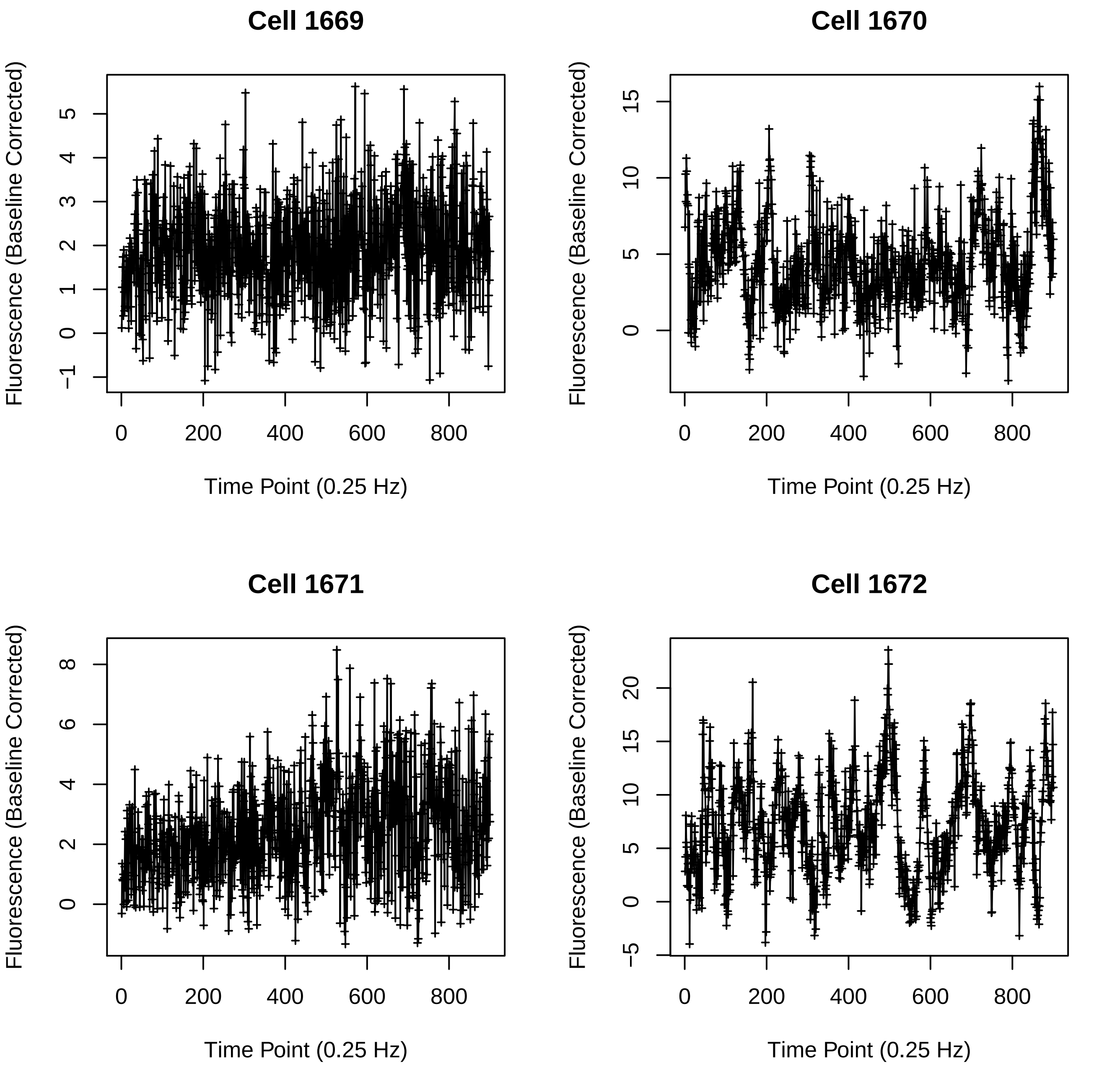
Cell 1669
Cell 1670
Cell 1671
Cell 1672
Fluorescence (Baseline Corrected)
Time Point (0.25 Hz)
0
200
400
600
800
−1
1
2
3
4
5
10
15
20
−5
6
8

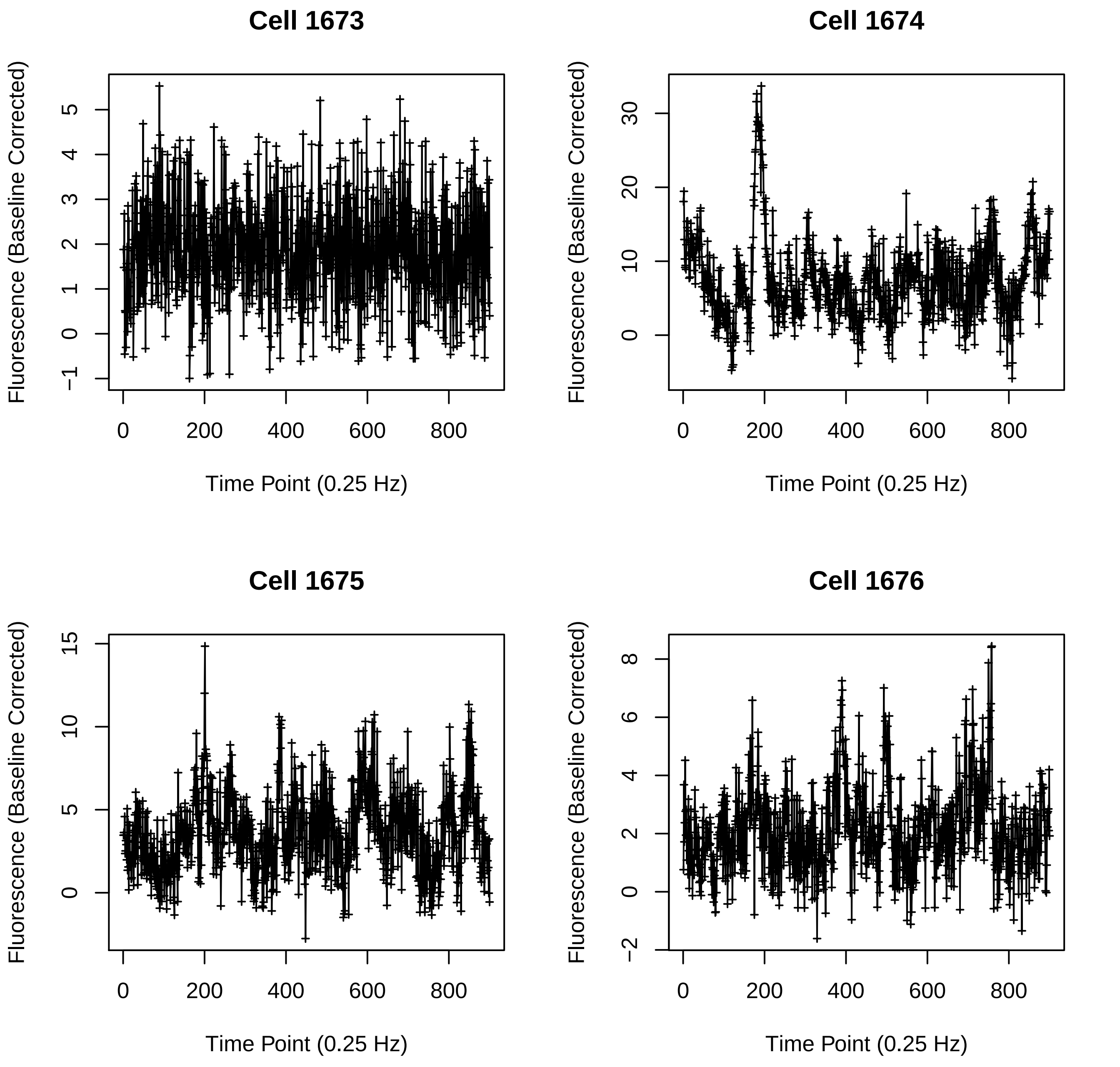
Cell 1673
Cell 1674
Cell 1675
Cell 1676
Fluorescence (Baseline Corrected)
Time Point (0.25 Hz)
0
200
400
600
800
−1
1
2
3
4
5
10
20
30
15
−2
6
8

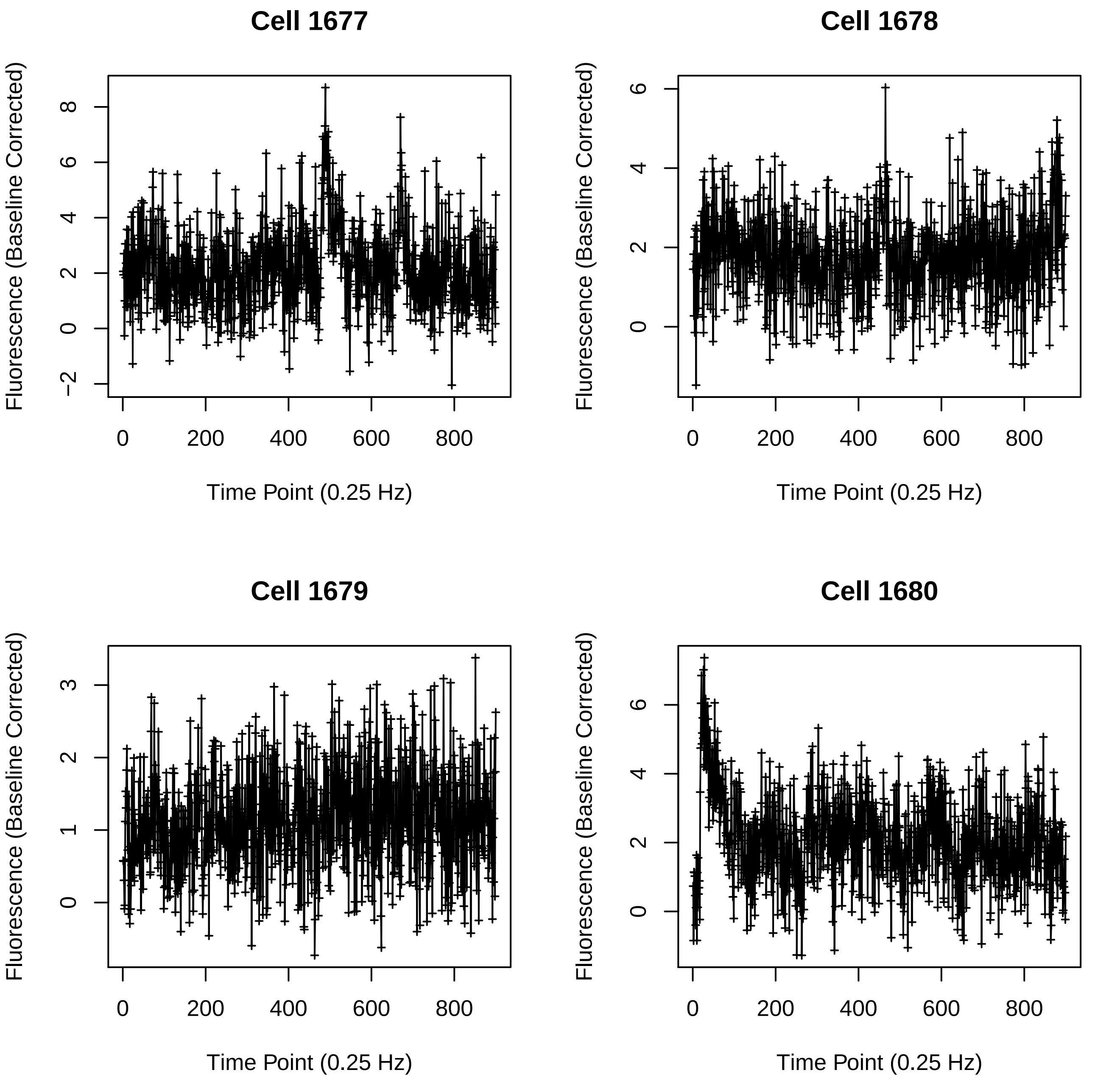
Cell 1677
Fluorescence (Baseline Corrected)
Time Point (0.25 Hz)
Cell 1678
Fluorescence (Baseline Corrected)
Time Point (0.25 Hz)
Cell 1679
Fluorescence (Baseline Corrected)
Time Point (0.25 Hz)
Cell 1680
Fluorescence (Baseline Corrected)
Time Point (0.25 Hz)

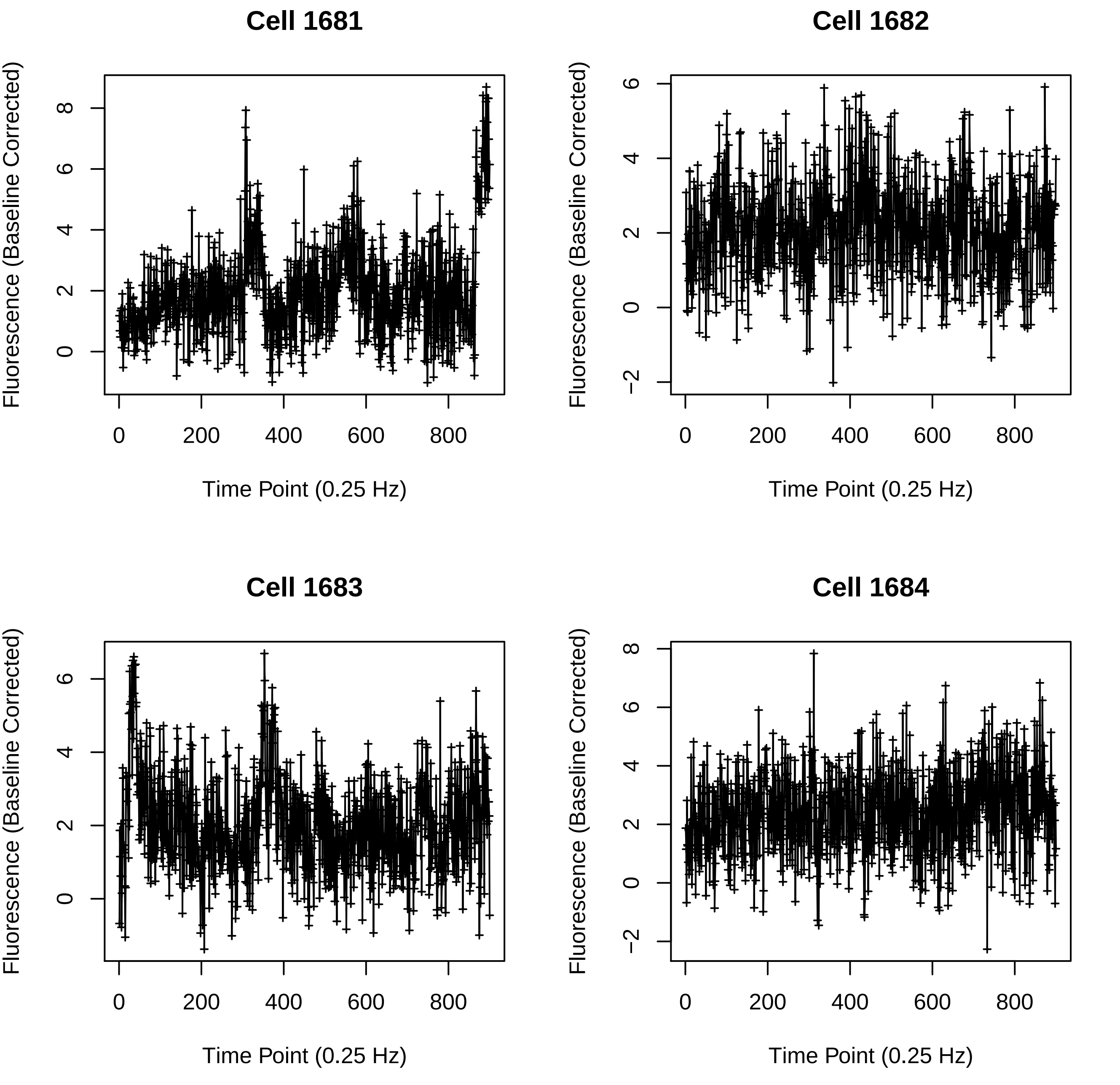
Cell 1681
Cell 1682
Cell 1683
Cell 1684
Fluorescence (Baseline Corrected)
Time Point (0.25 Hz)
0
200
400
600
800
−2
2
4
6
8

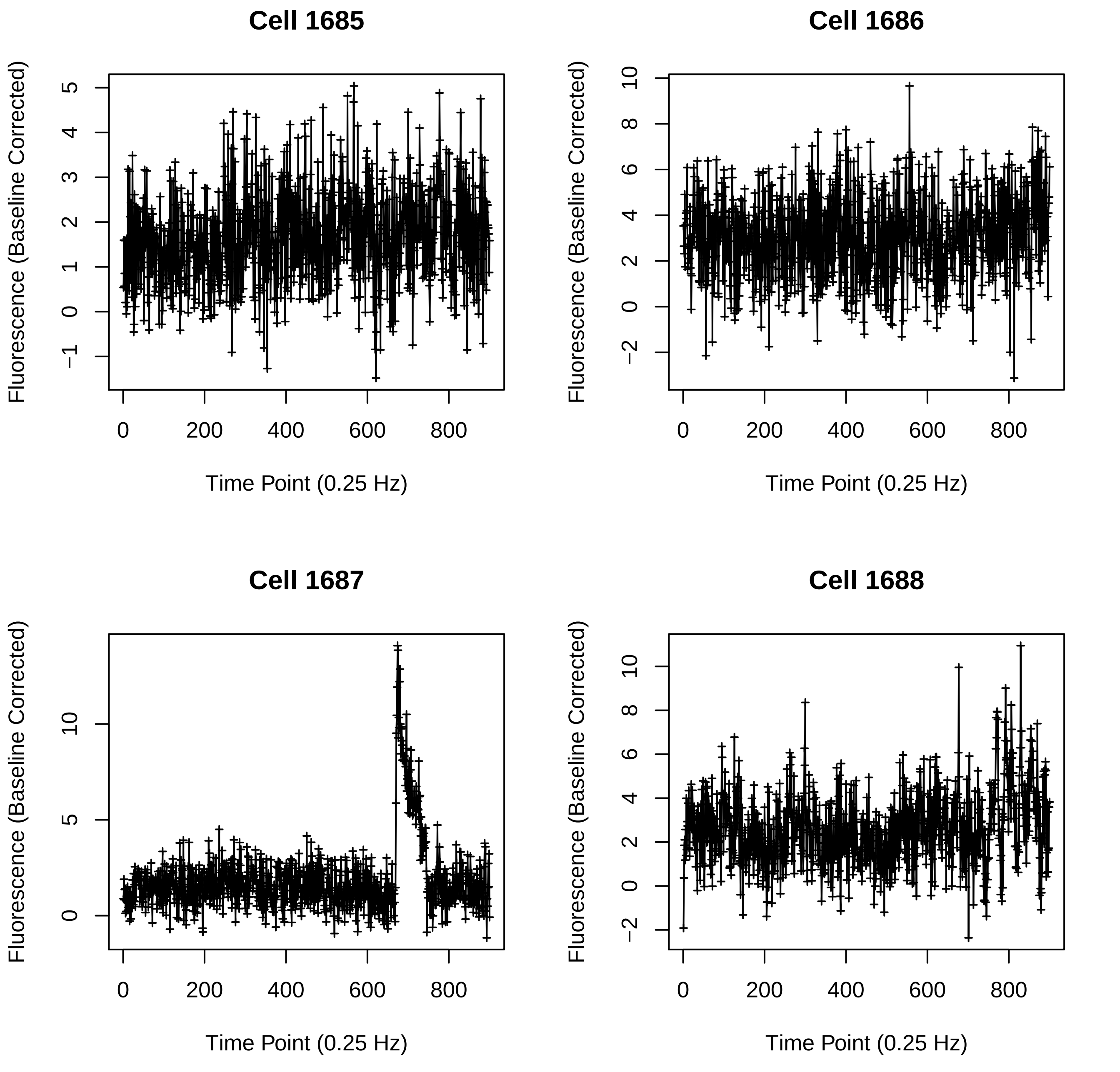
Cell 1685
Cell 1686
Cell 1687
Cell 1688
Fluorescence (Baseline Corrected)
Time Point (0.25 Hz)
0
200
400
600
800
−1
1
2
3
4
5
−2
6
8
10

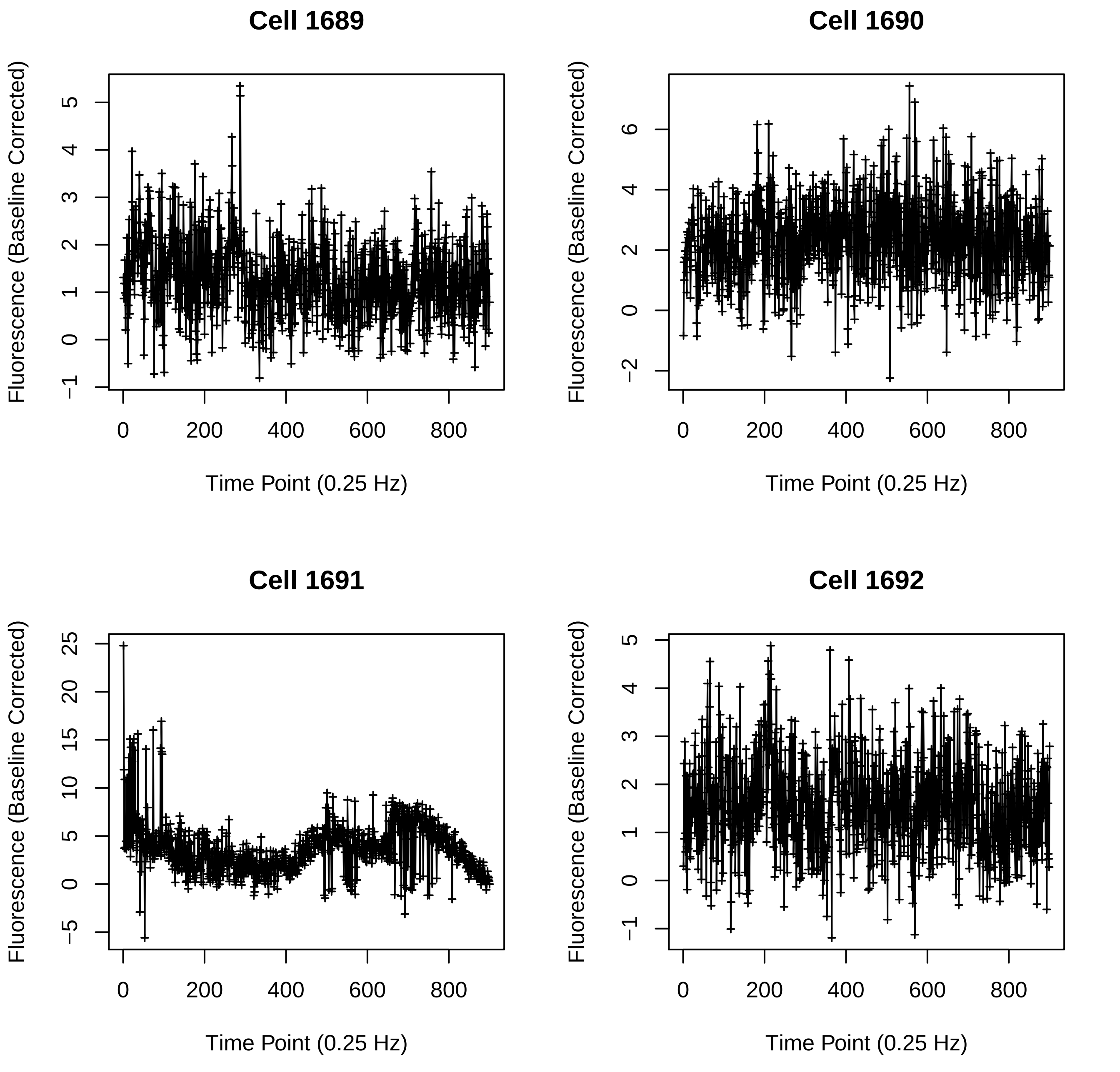
Cell 1689
Cell 1690
Cell 1691
Cell 1692
Fluorescence (Baseline Corrected)
Time Point (0.25 Hz)
0
200
400
600
800

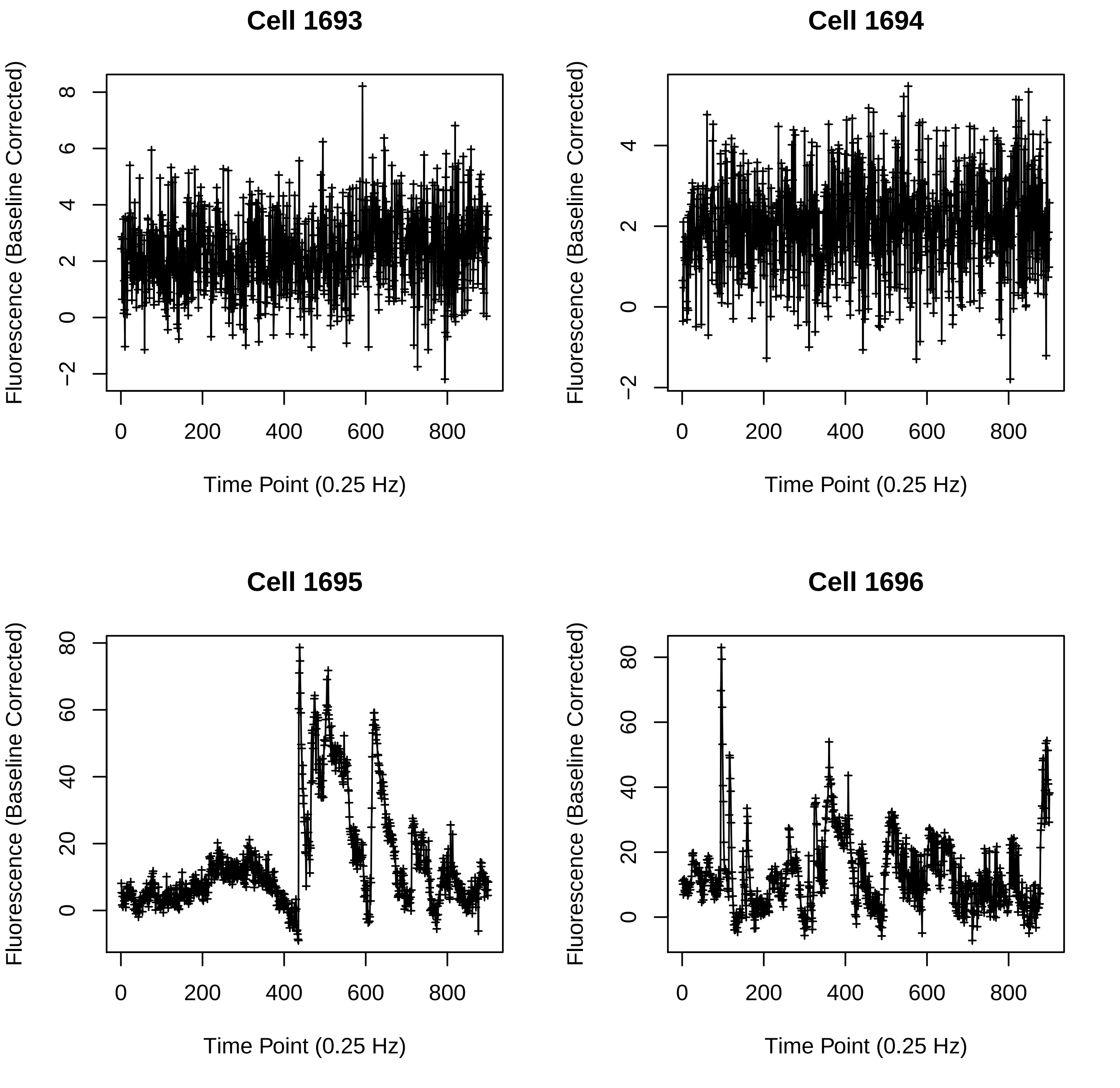
Cell 1693
Cell 1694
Cell 1695
Cell 1696
Fluorescence (Baseline Corrected)
Time Point (0.25 Hz)

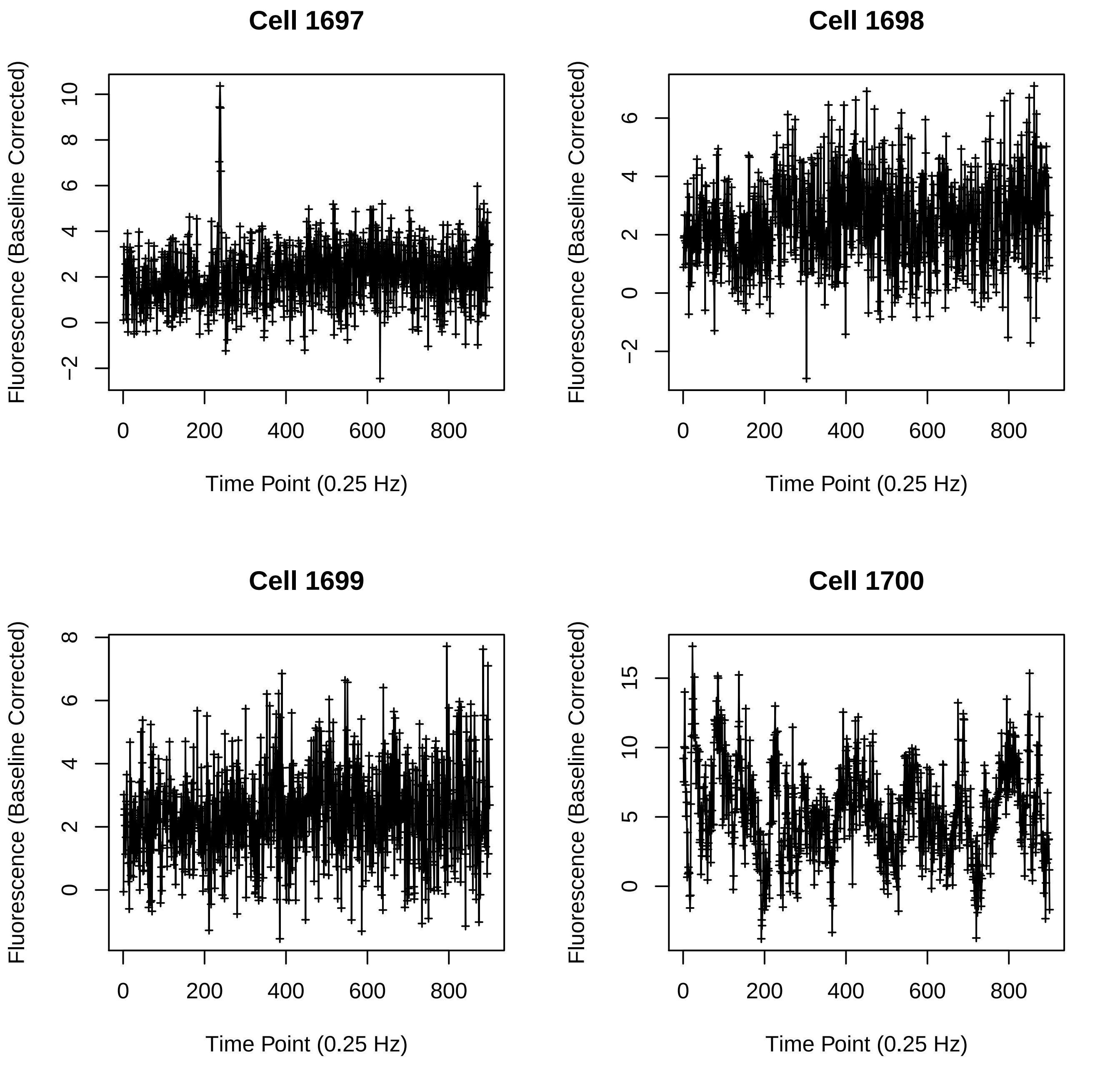

Cell 1697
Cell 1698
Cell 1699
Cell 1700
Fluorescence (Baseline Corrected)
Time Point (0.25 Hz)

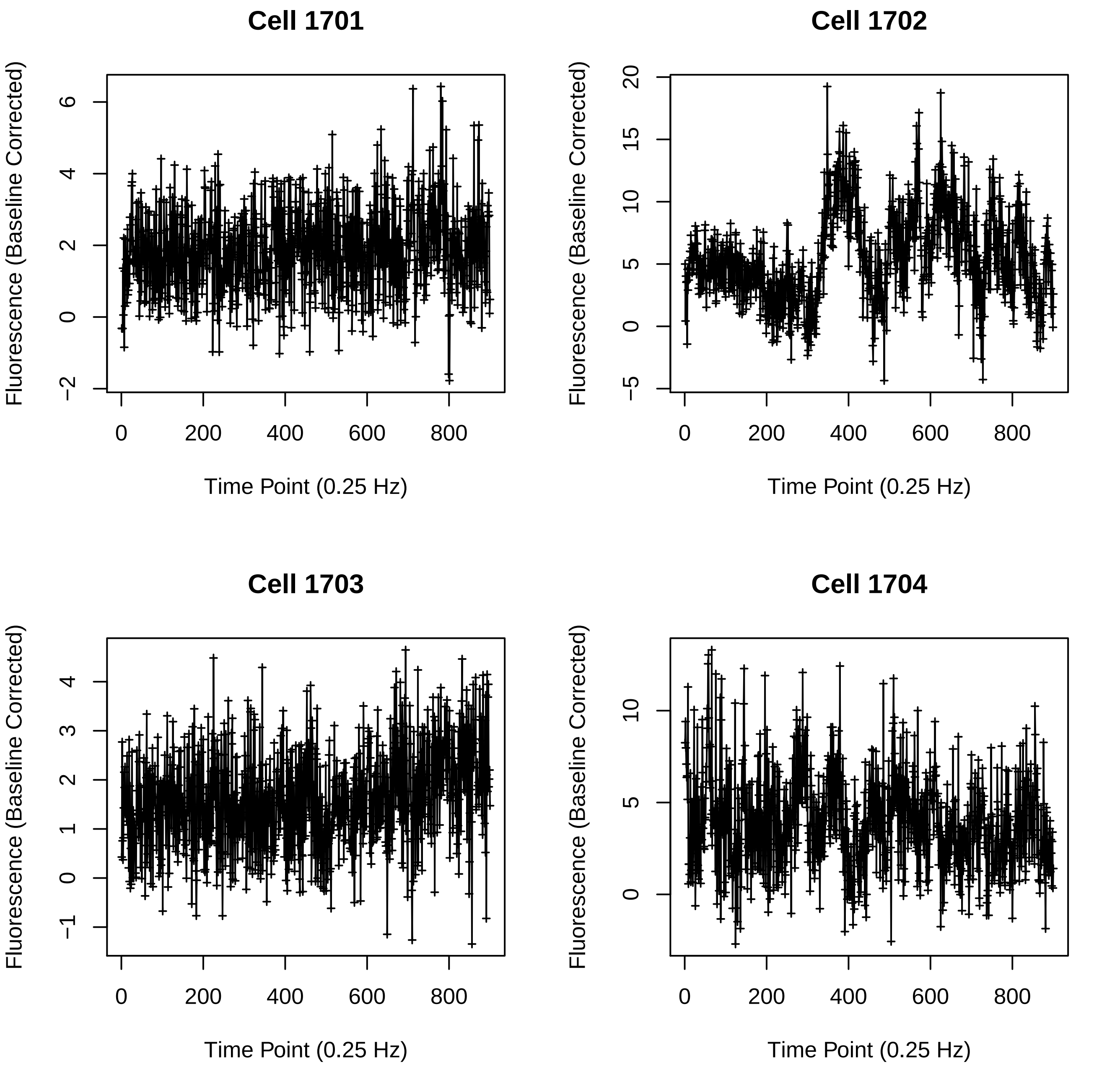
Cell 1701
Cell 1702
Cell 1703
Cell 1704
Fluorescence (Baseline Corrected)
Time Point (0.25 Hz)
0
200
400
600
800
−2
2
4
6
−5
5
10
15
20
−1
1
3

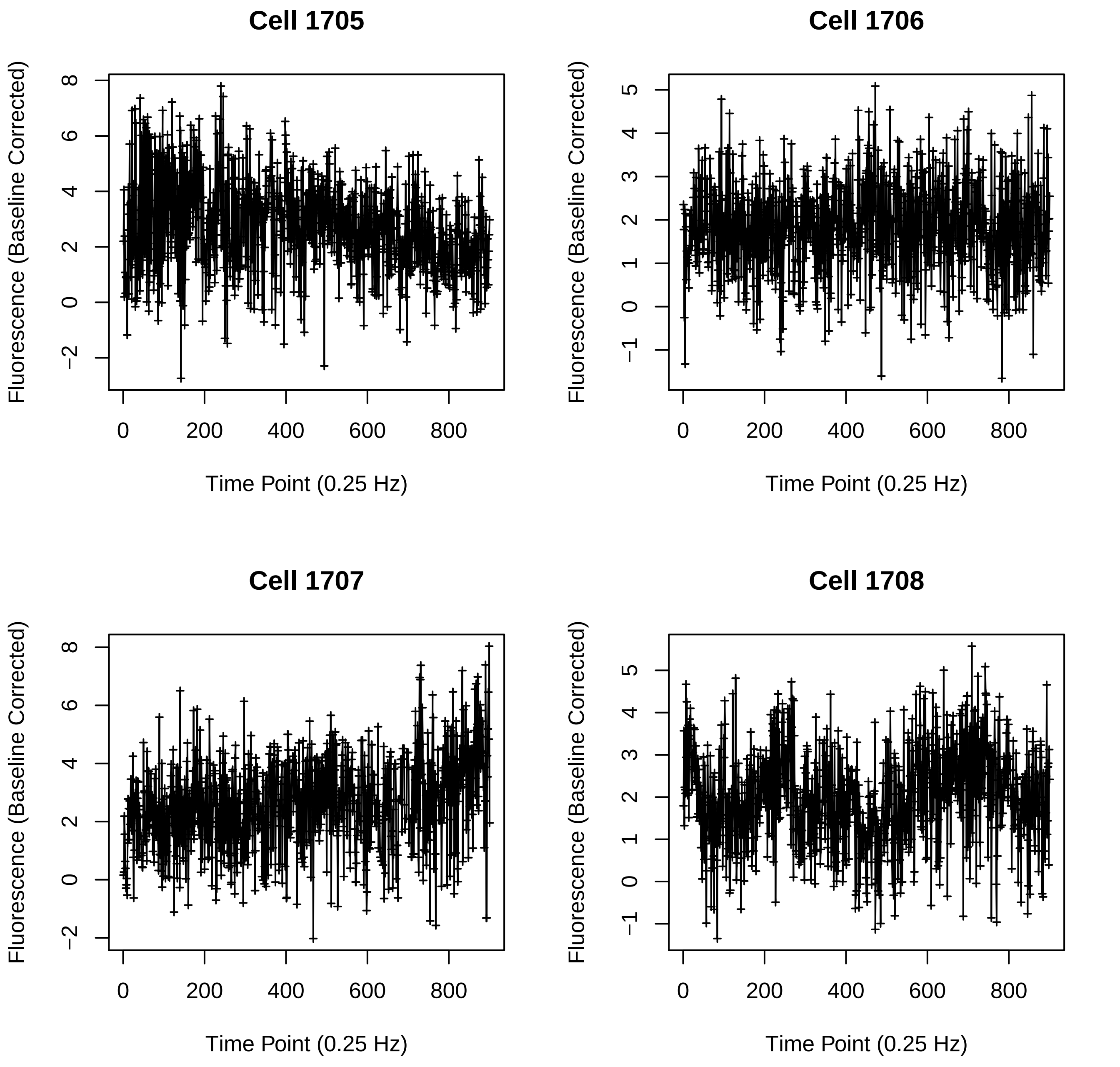
Cell 1705
Cell 1706
Cell 1707
Cell 1708
Fluorescence (Baseline Corrected)
Time Point (0.25 Hz)
−2
0
2
4
6
8
−1
1
3
5
200
400
600
800

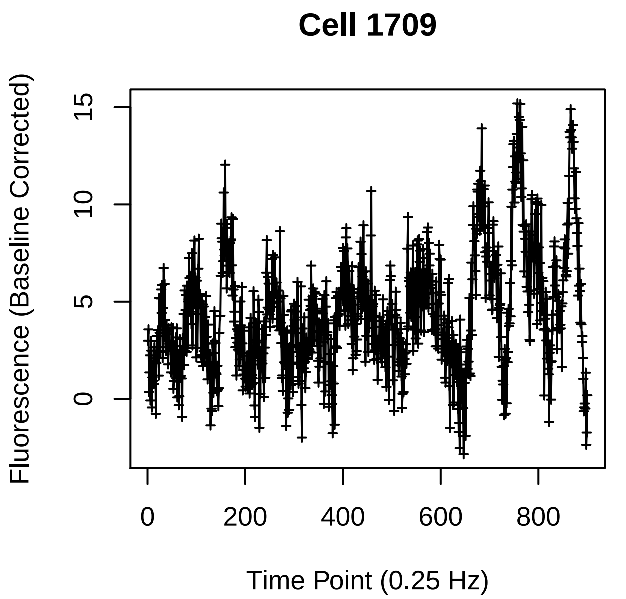
Cell 1709
Fluorescence (Baseline Corrected)
0
5
10
15
0
200
400
600
800
Time Point (0.25 Hz)
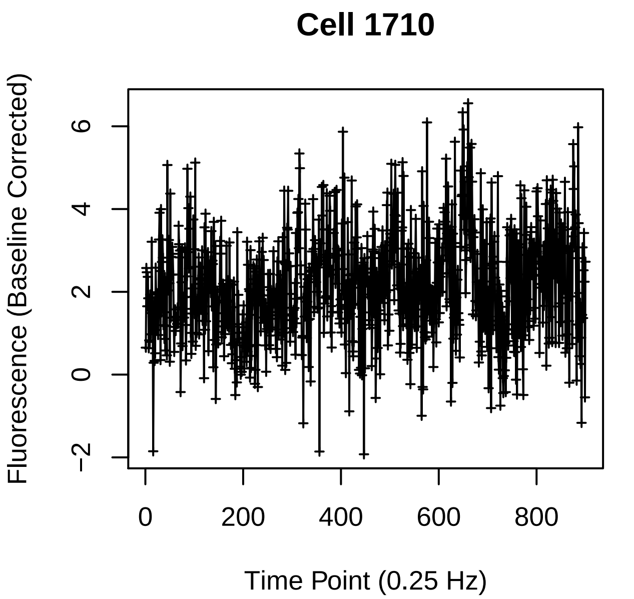
Cell 1710
Fluorescence (Baseline Corrected)
−2
0
2
4
6
0
200
400
600
800
Time Point (0.25 Hz)
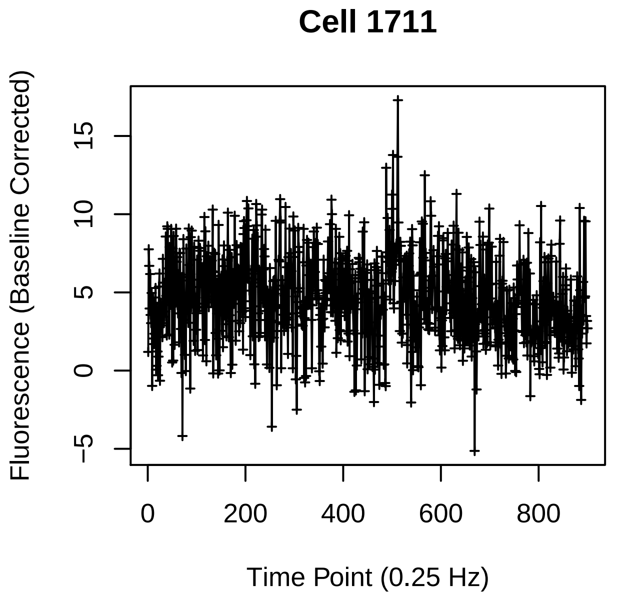
Cell 1711
Fluorescence (Baseline Corrected)
−5
0
5
10
15
0
200
400
600
800
Time Point (0.25 Hz)
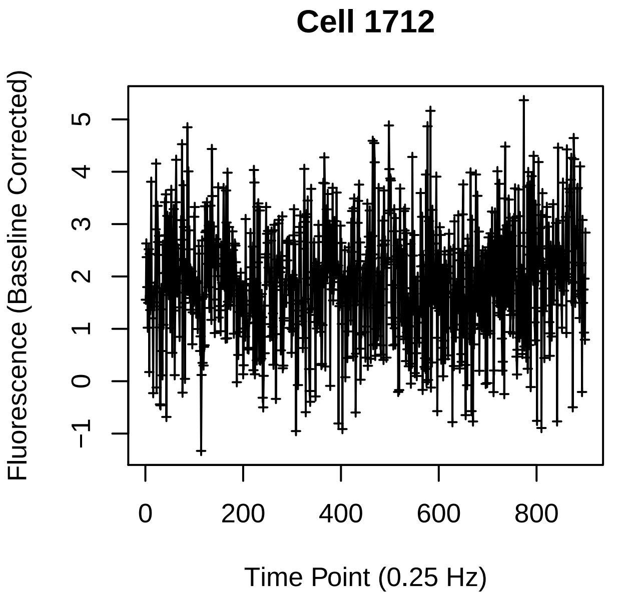
Cell 1712
Fluorescence (Baseline Corrected)
−1
0
1
2
3
4
5
0
200
400
600
800
Time Point (0.25 Hz)

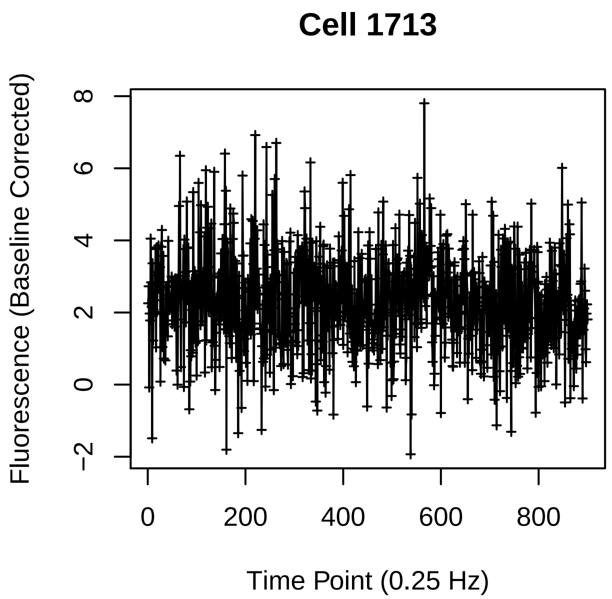
Cell 1713
Fluorescence (Baseline Corrected)
−2
0
2
4
6
8
0
200
400
600
800
Time Point (0.25 Hz)

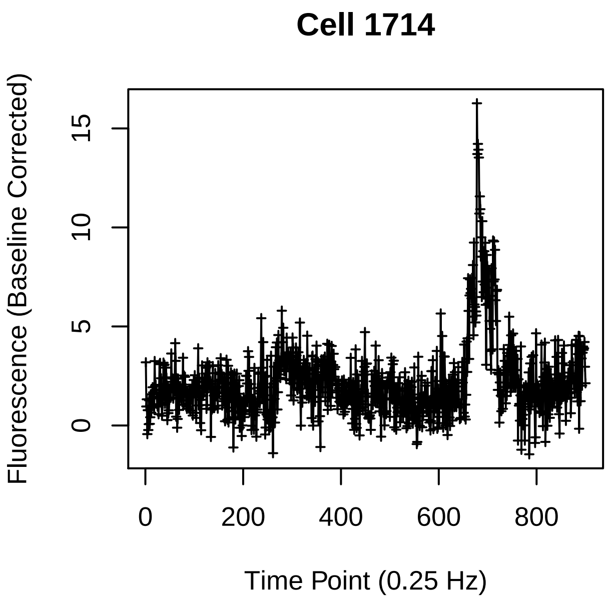
Cell 1714
Fluorescence (Baseline Corrected)
0
5
10
15
0
200
400
600
800
Time Point (0.25 Hz)

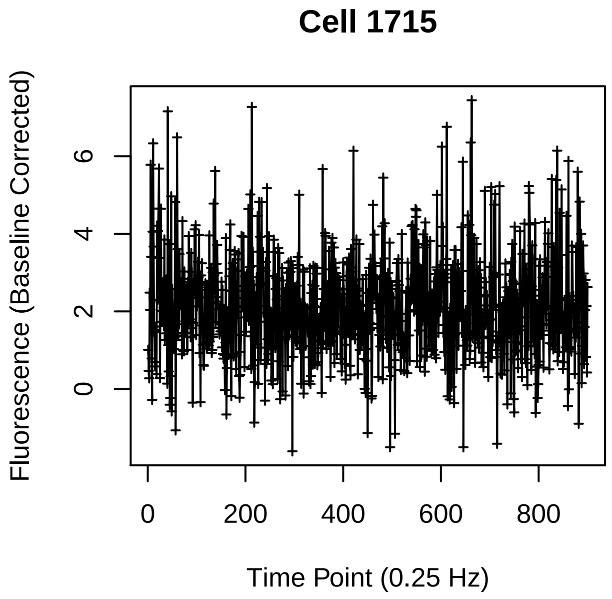
Cell 1715
Fluorescence (Baseline Corrected)
0
2
4
6
0
200
400
600
800
Time Point (0.25 Hz)

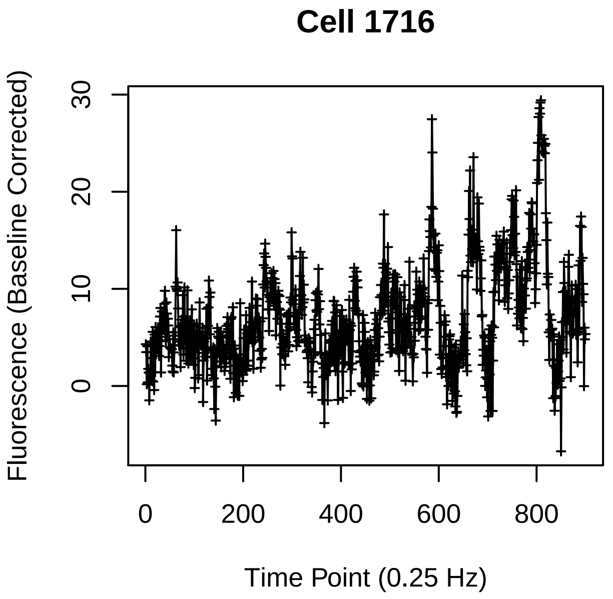
Cell 1716
Fluorescence (Baseline Corrected)
0
10
20
30
0
200
400
600
800
Time Point (0.25 Hz)

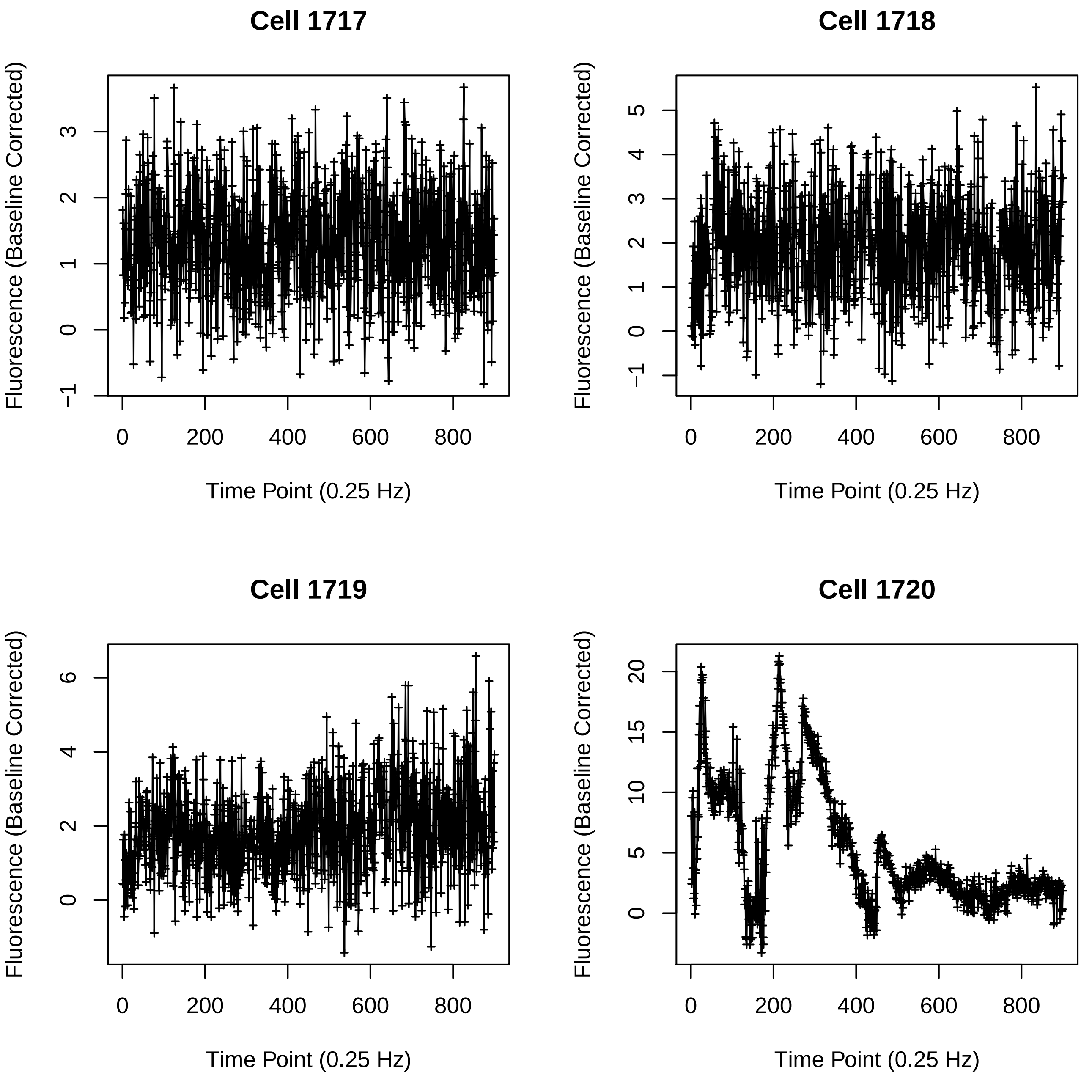
Cell 1717
Cell 1718
Cell 1719
Cell 1720
Fluorescence (Baseline Corrected)
Time Point (0.25 Hz)
0
200
400
600
800
−1
0
1
2
3
4
5
6
10
15
20

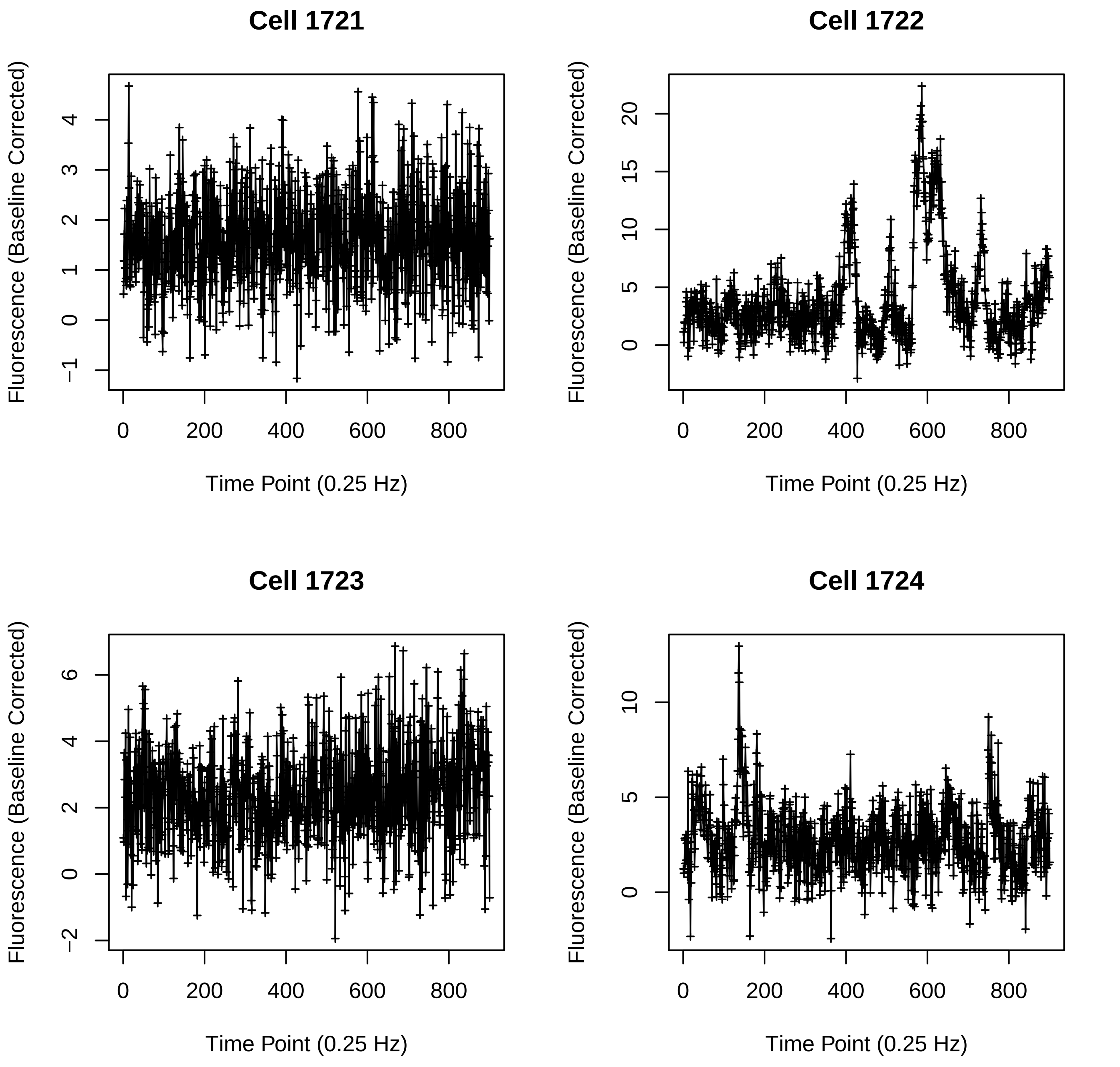
Cell 1721
Fluorescence (Baseline Corrected)
−1 0 1 2 3 4
0 200 400 600 800
Time Point (0.25 Hz)
Cell 1722
Fluorescence (Baseline Corrected)
0 5 10 15 20
0 200 400 600 800
Time Point (0.25 Hz)
Cell 1723
Fluorescence (Baseline Corrected)
−2 0 2 4 6
0 200 400 600 800
Time Point (0.25 Hz)
Cell 1724
Fluorescence (Baseline Corrected)
0 5 10
0 200 400 600 800
Time Point (0.25 Hz)

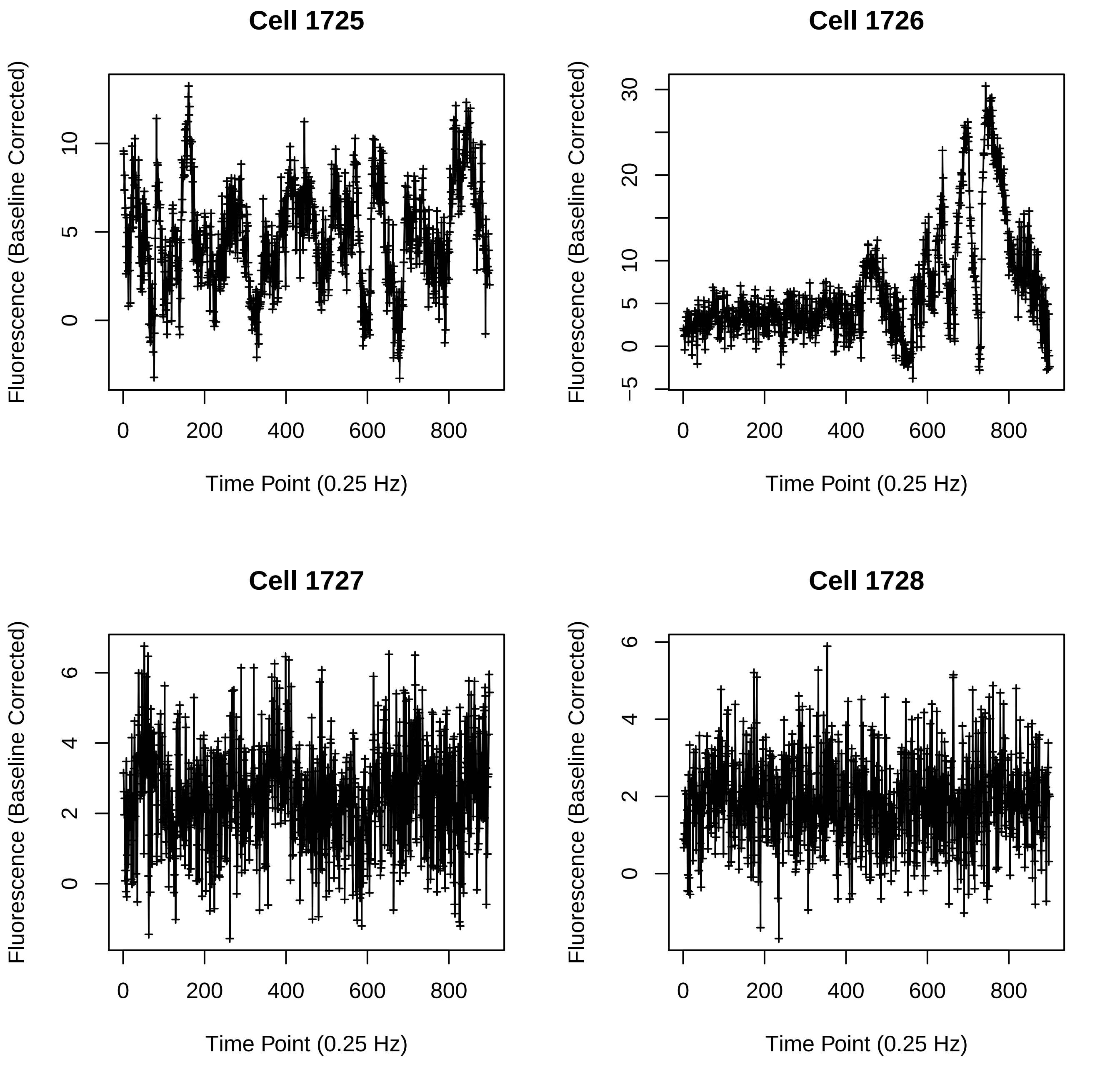
Cell 1725
Cell 1726
Cell 1727
Cell 1728
Fluorescence (Baseline Corrected)
Time Point (0.25 Hz)
0
200
400
600
800
10
5
0
30
20
10
5
0
−5
6
4
2
0

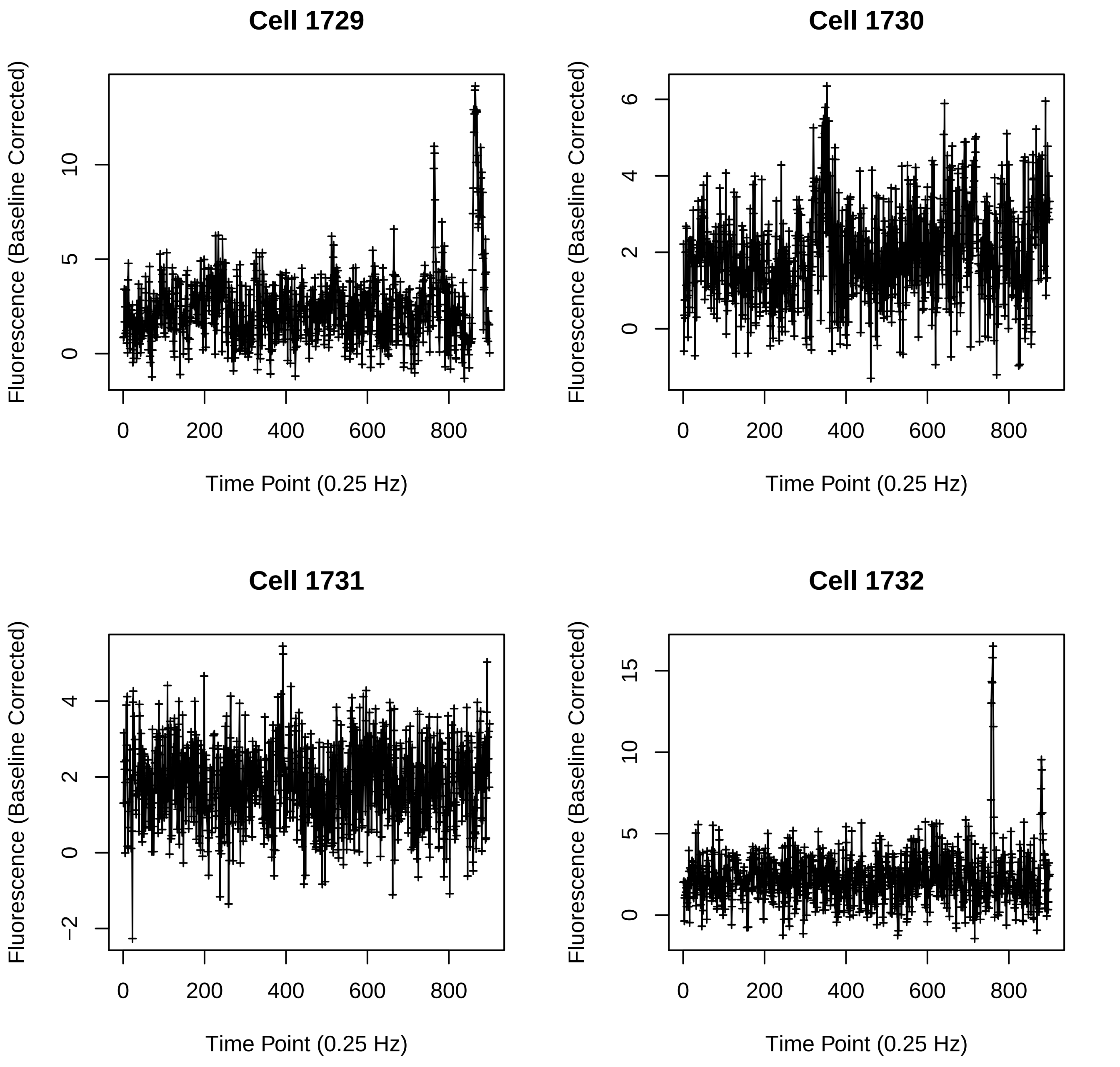

Cell 1729
Cell 1730
Cell 1731
Cell 1732
Fluorescence (Baseline Corrected)
Time Point (0.25 Hz)

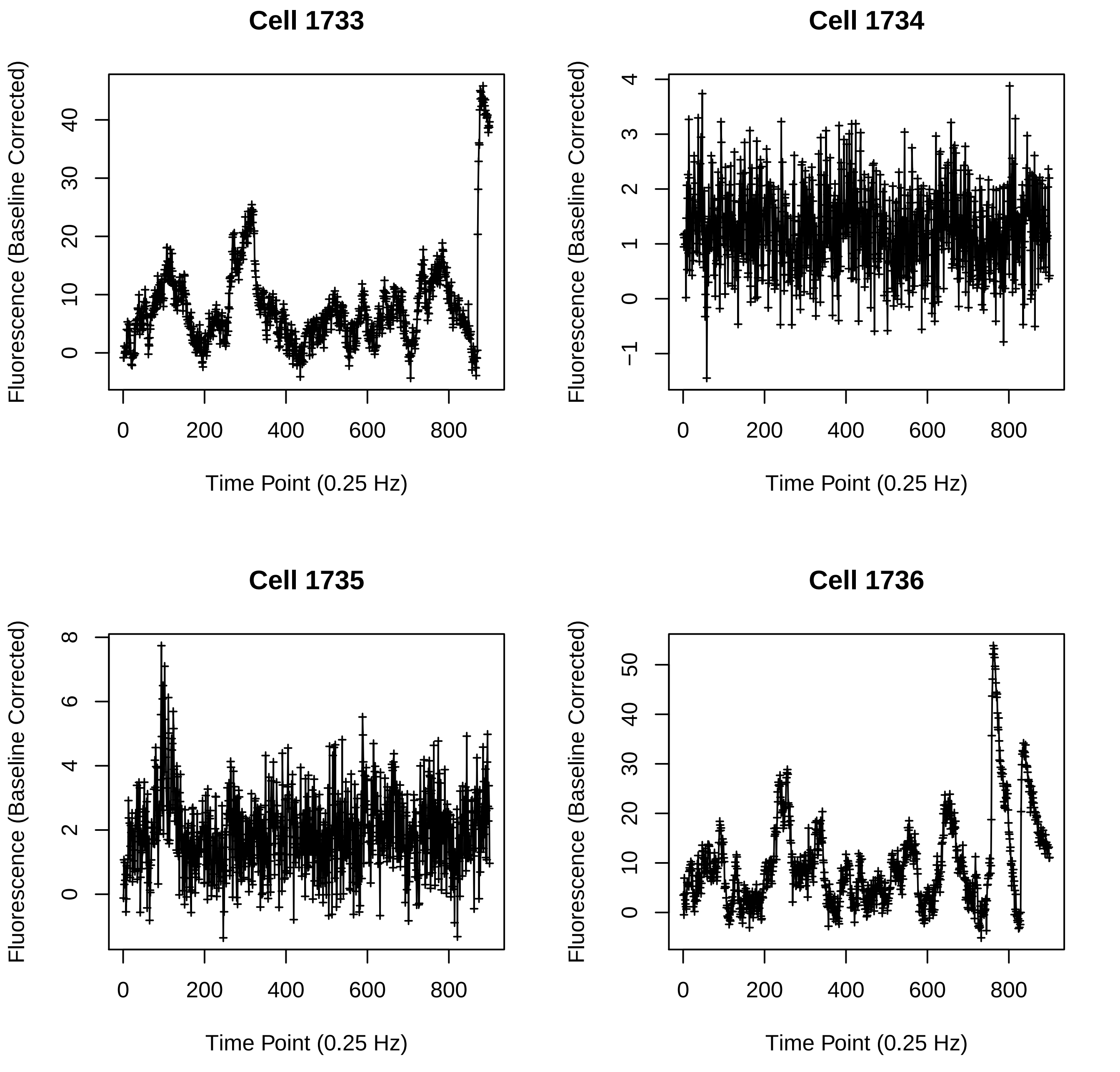

Cell 1733
Cell 1734
Cell 1735
Cell 1736
Fluorescence (Baseline Corrected)
Time Point (0.25 Hz)
0
200
400
600
800
0
10
20
30
40
−1
1
2
3
4
6
8
50

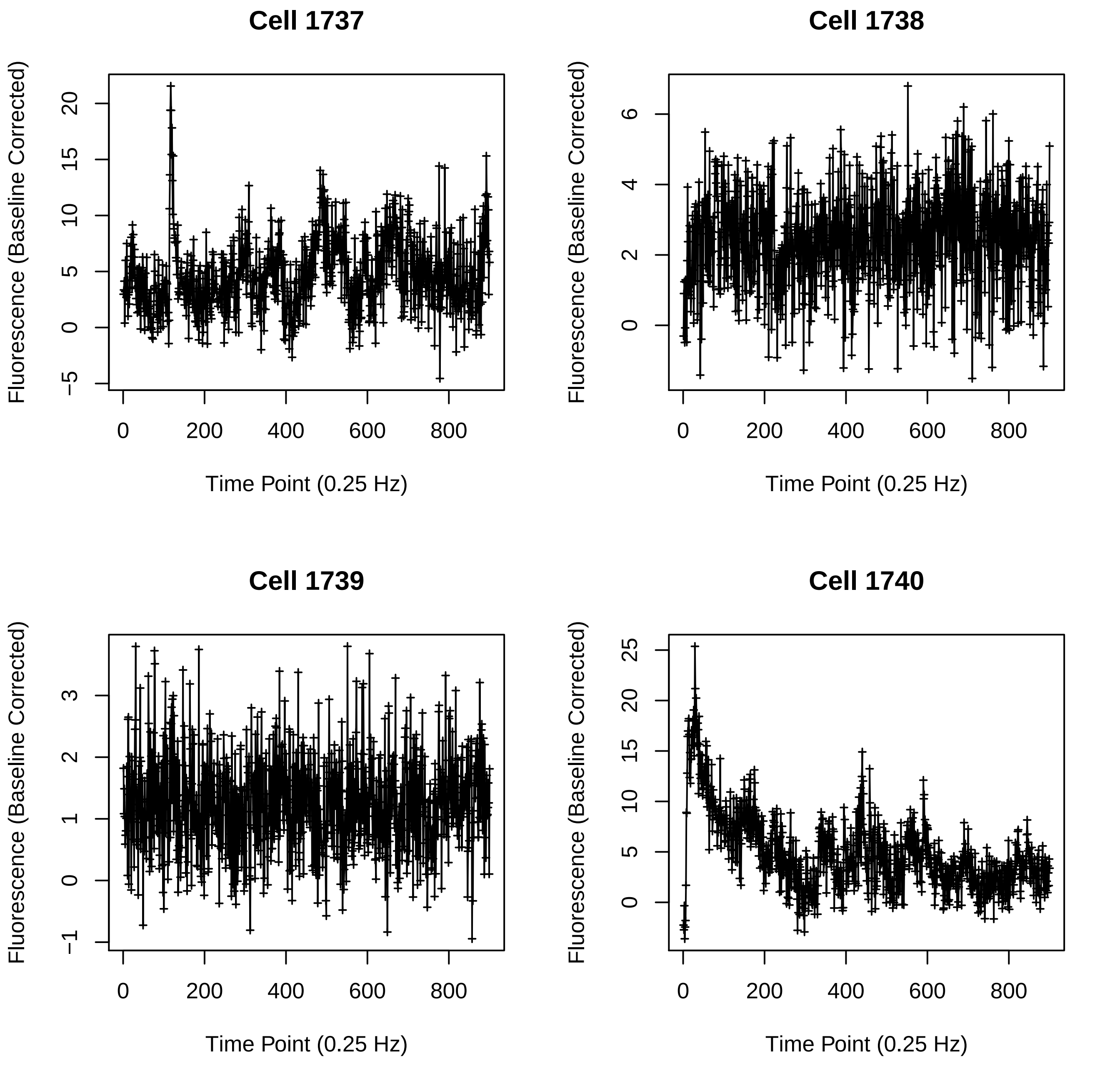

Cell 1737
Cell 1738
Cell 1739
Cell 1740
Fluorescence (Baseline Corrected)
Time Point (0.25 Hz)
-5
0
5
10
15
20
25
0
2
4
6
-1
0
1
2
3
0
200
400
600
800

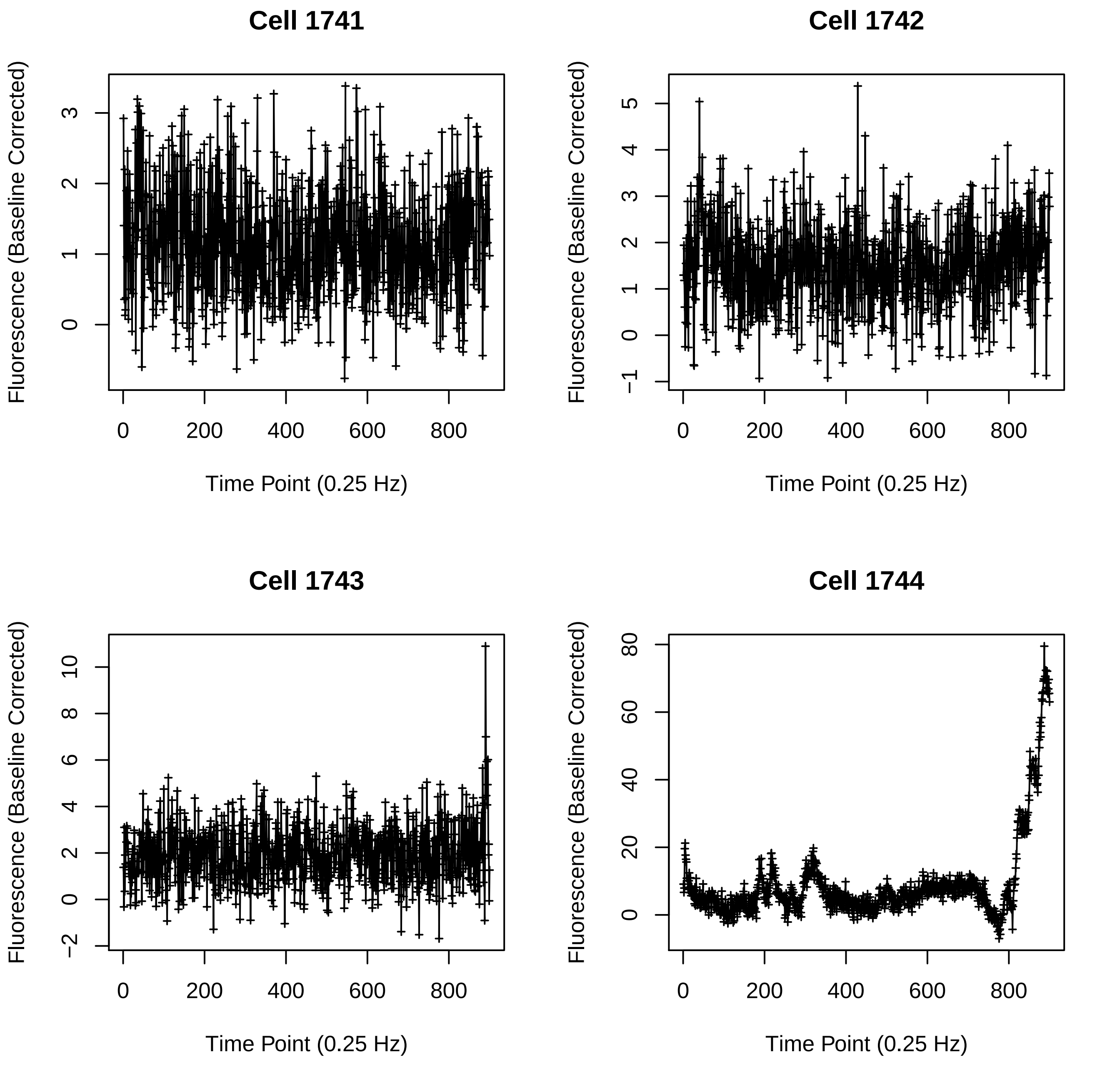
Cell 1741
Fluorescence (Baseline Corrected)
Time Point (0.25 Hz)
0
1
2
3
0
200
400
600
800
Cell 1742
Fluorescence (Baseline Corrected)
Time Point (0.25 Hz)
−1
0
1
2
3
4
5
0
200
400
600
800
Cell 1743
Fluorescence (Baseline Corrected)
Time Point (0.25 Hz)
−2
0
2
4
6
8
10
0
200
400
600
800
Cell 1744
Fluorescence (Baseline Corrected)
Time Point (0.25 Hz)
0
20
40
60
80
0
200
400
600
800

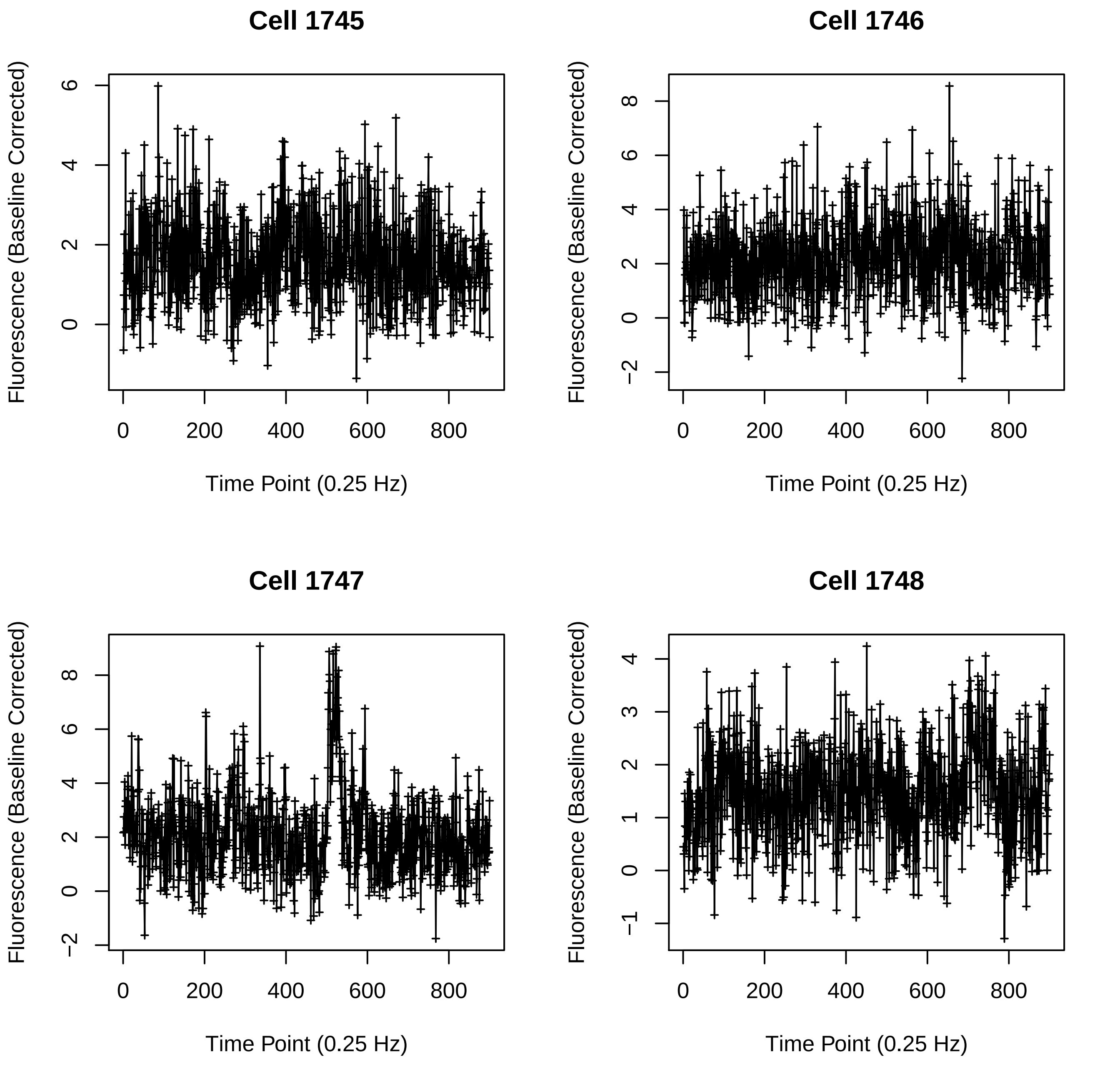

Cell 1745
Cell 1746
Cell 1747
Cell 1748
Fluorescence (Baseline Corrected)
Time Point (0.25 Hz)

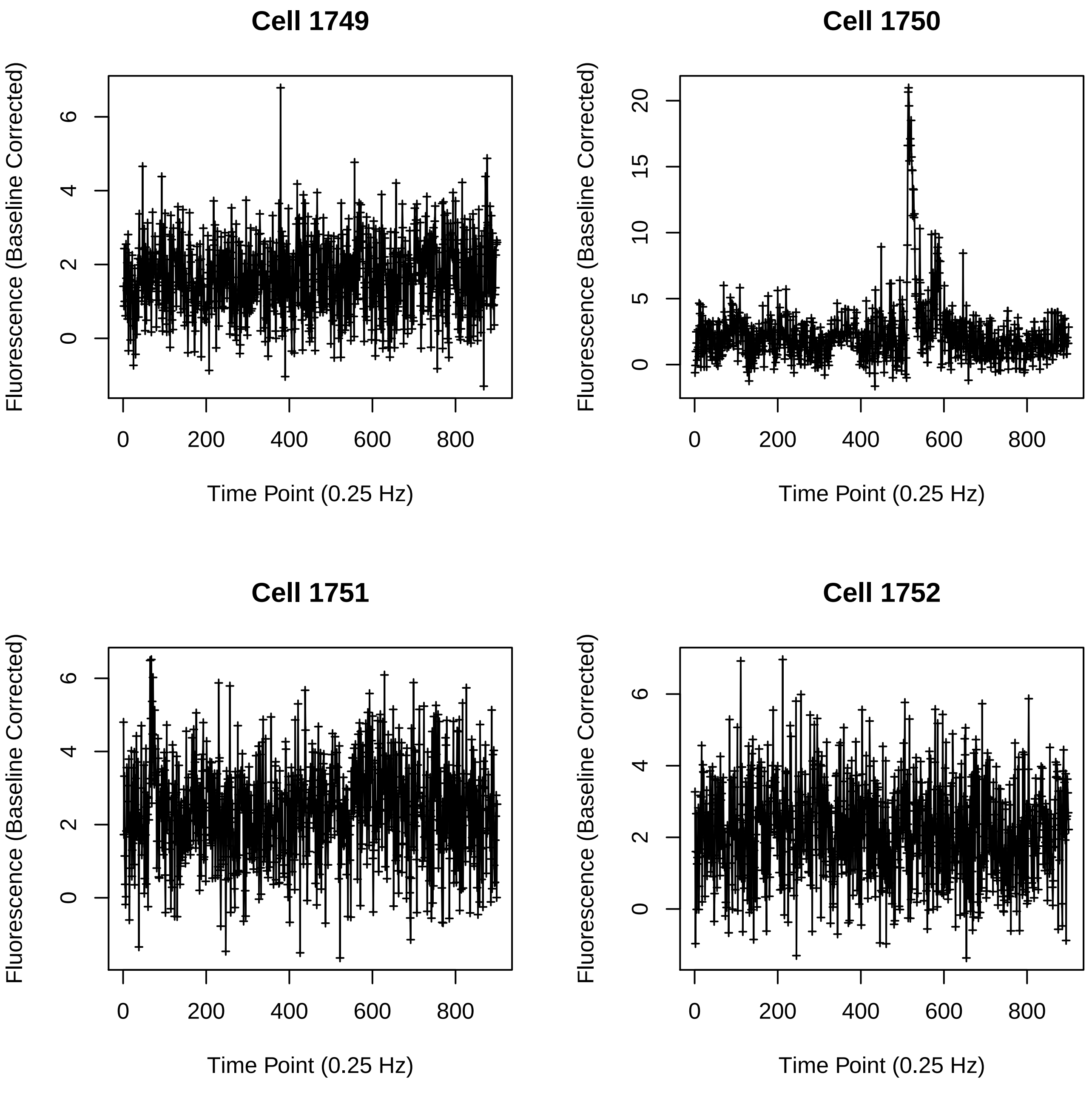
Cell 1749
Cell 1750
Cell 1751
Cell 1752
Fluorescence (Baseline Corrected)
Time Point (0.25 Hz)
0
2
4
6
5
10
15
20
200
400
600
800

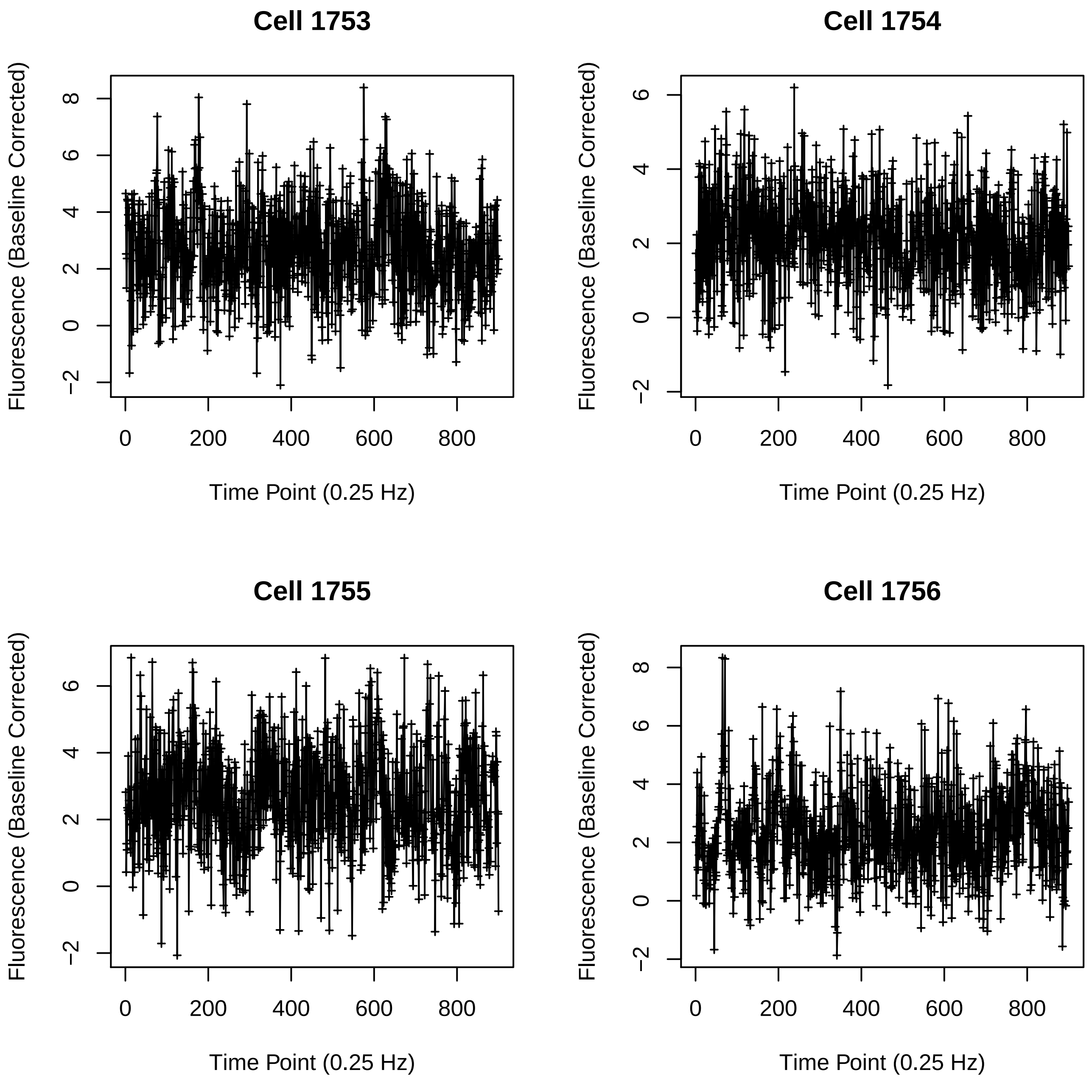
Cell 1753
Cell 1754
Cell 1755
Cell 1756
Fluorescence (Baseline Corrected)
Time Point (0.25 Hz)
−2
0
2
4
6
8
0
200
400
600
800

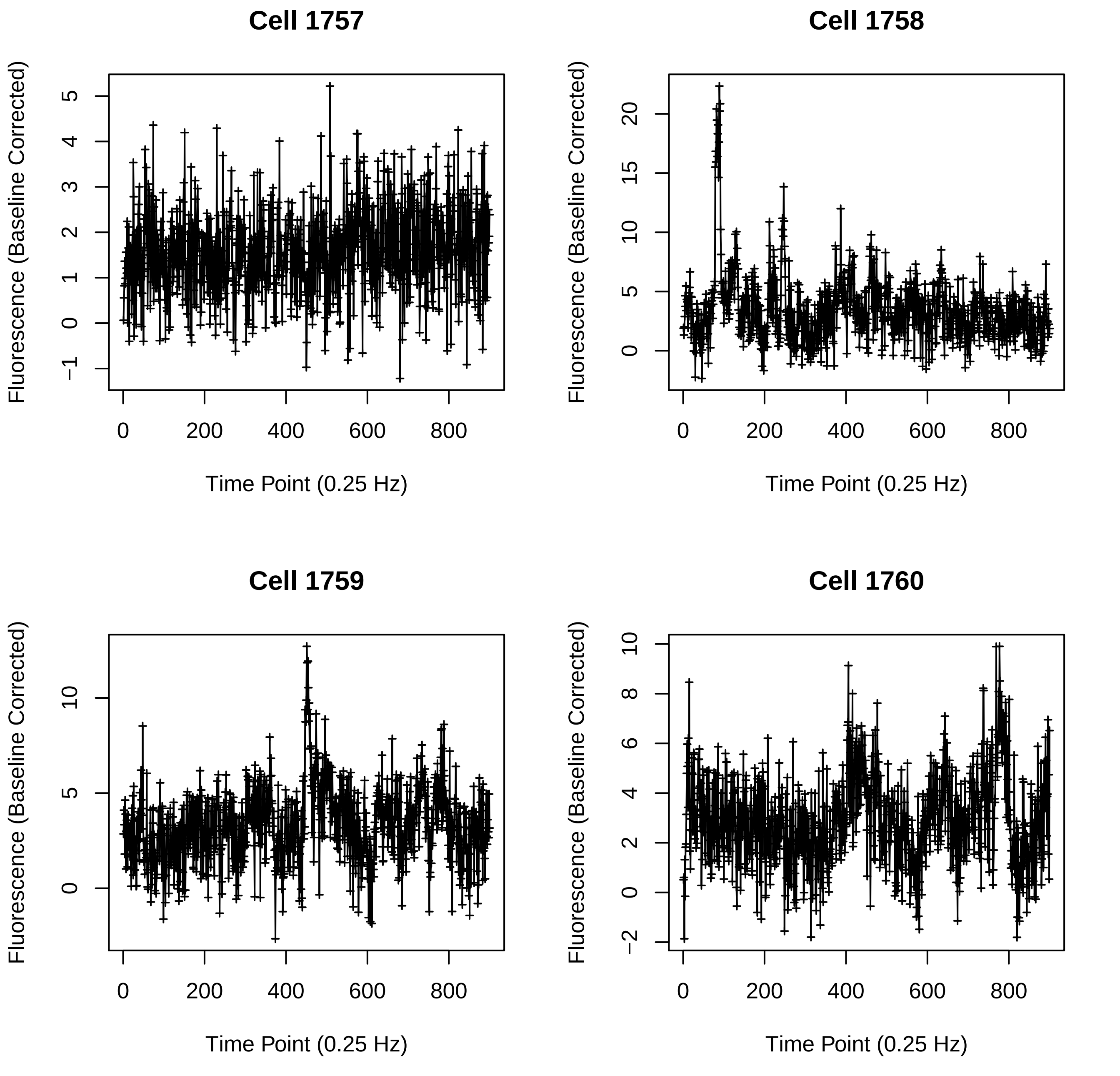
Cell 1757
Cell 1758
Cell 1759
Cell 1760
Fluorescence (Baseline Corrected)
Time Point (0.25 Hz)
0
200
400
600
800
−1
0
1
2
3
4
5
10
15
20
−2
6
8

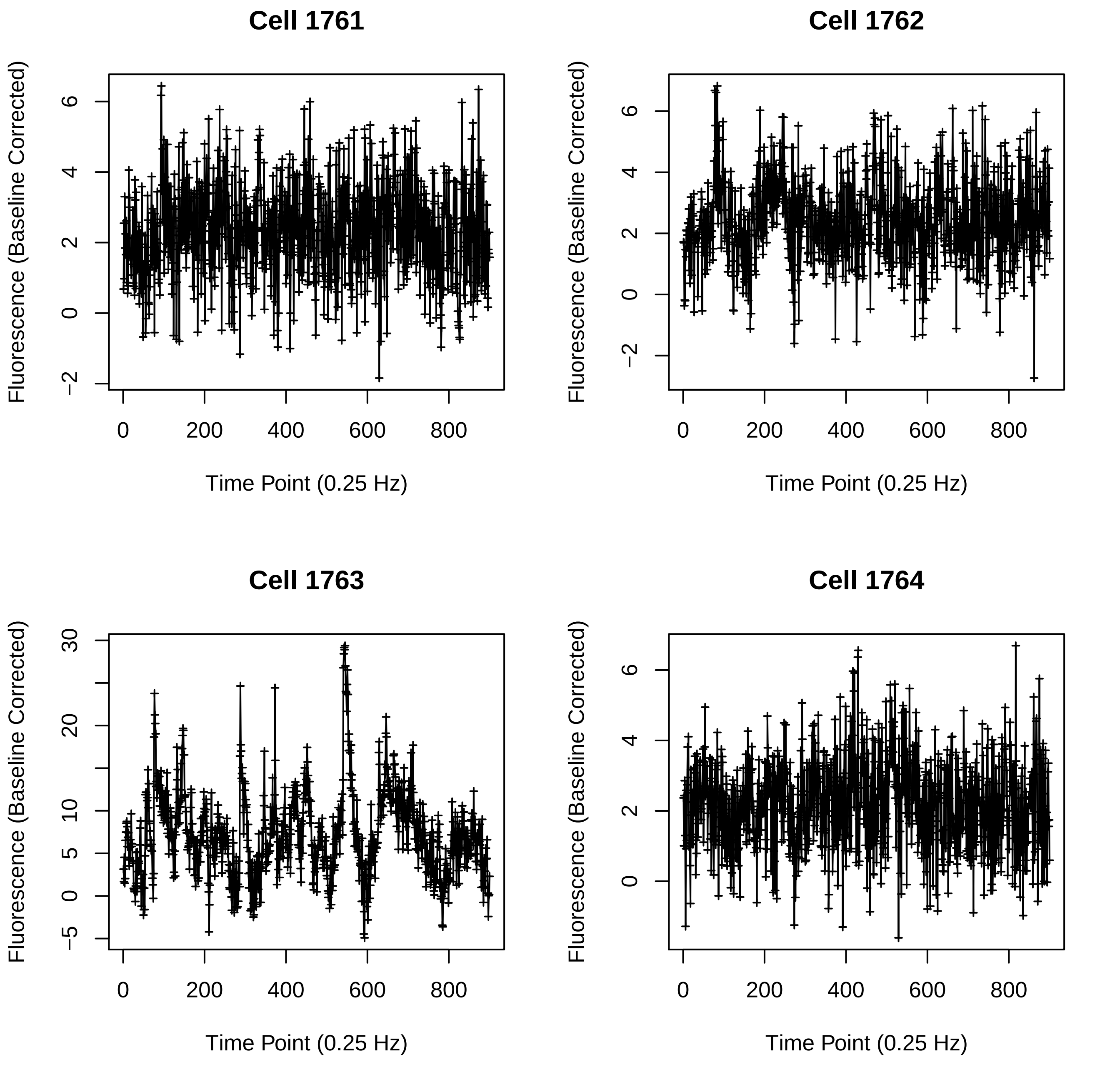

Cell 1761
Cell 1762
Cell 1763
Cell 1764
Fluorescence (Baseline Corrected)
Time Point (0.25 Hz)
0
200
400
600
800
−2
2
4
6
−5
5
10
20
30

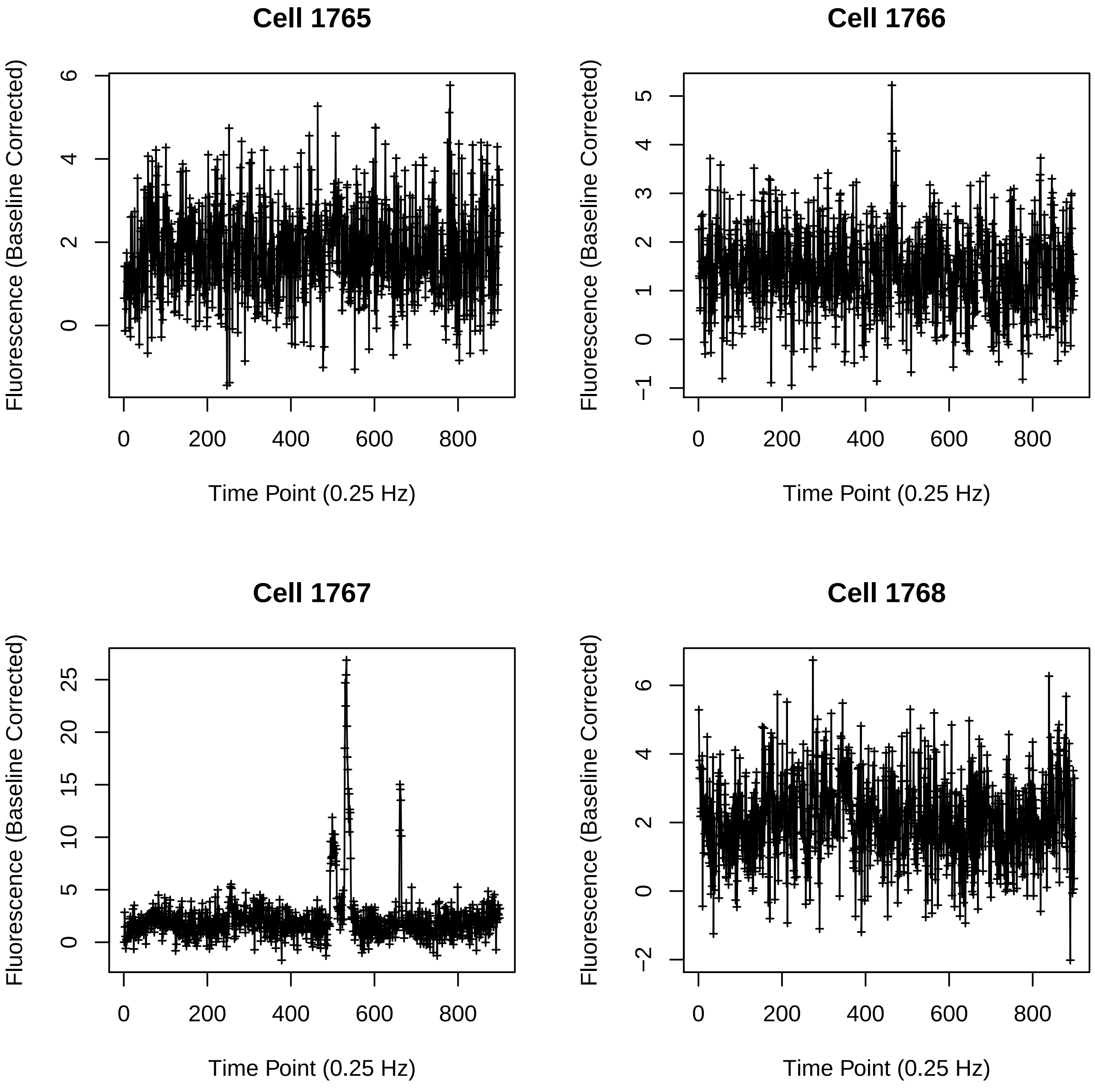
Cell 1765
Cell 1766
Cell 1767
Cell 1768
Fluorescence (Baseline Corrected)
Time Point (0.25 Hz)
0
200
400
600
800
-1
1
2
3
4
5
6
10
15
20
25
-2

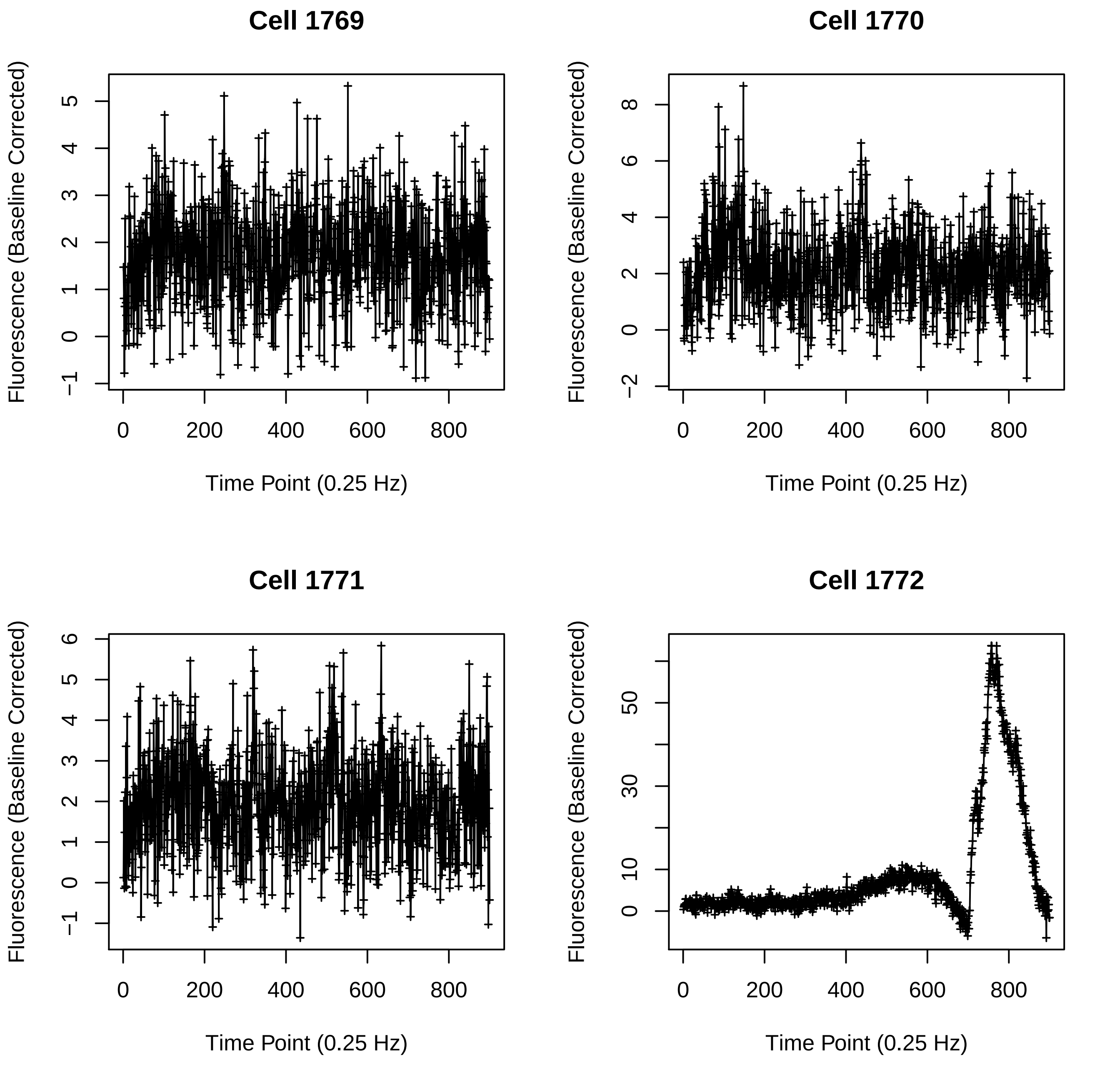
Cell 1769
Cell 1770
Cell 1771
Cell 1772
Fluorescence (Baseline Corrected)
Time Point (0.25 Hz)
0
200
400
600
800
−1
0
1
2
3
4
5
−2
0
2
4
6
8
−1
0
1
2
3
4
5
6
0
10
30
50

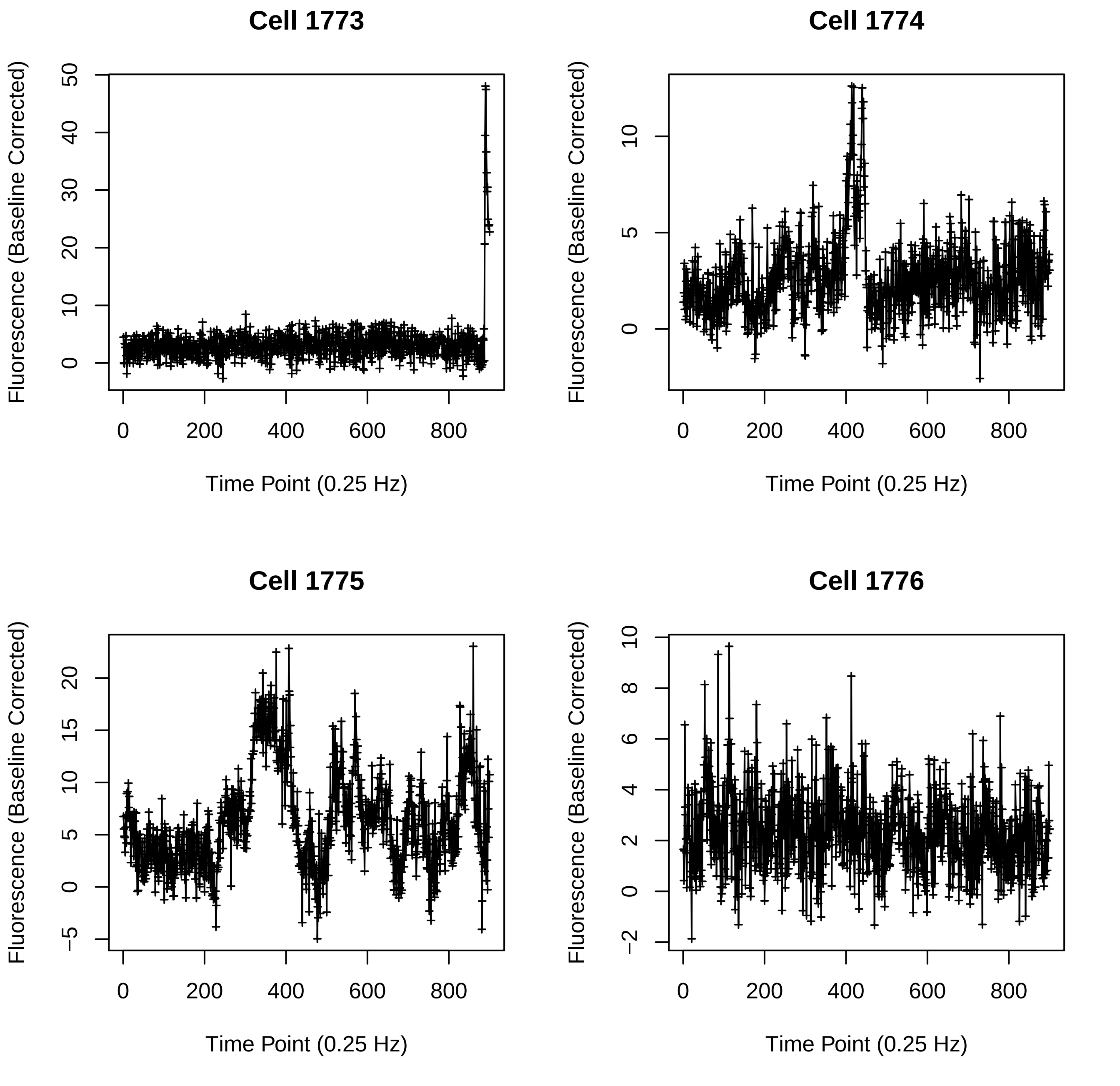

Cell 1773
Cell 1774
Cell 1775
Cell 1776
Fluorescence (Baseline Corrected)
Time Point (0.25 Hz)
0
200
400
600
800
0
10
20
30
40
50
5
15
20
-5
-2
2
4
6
8

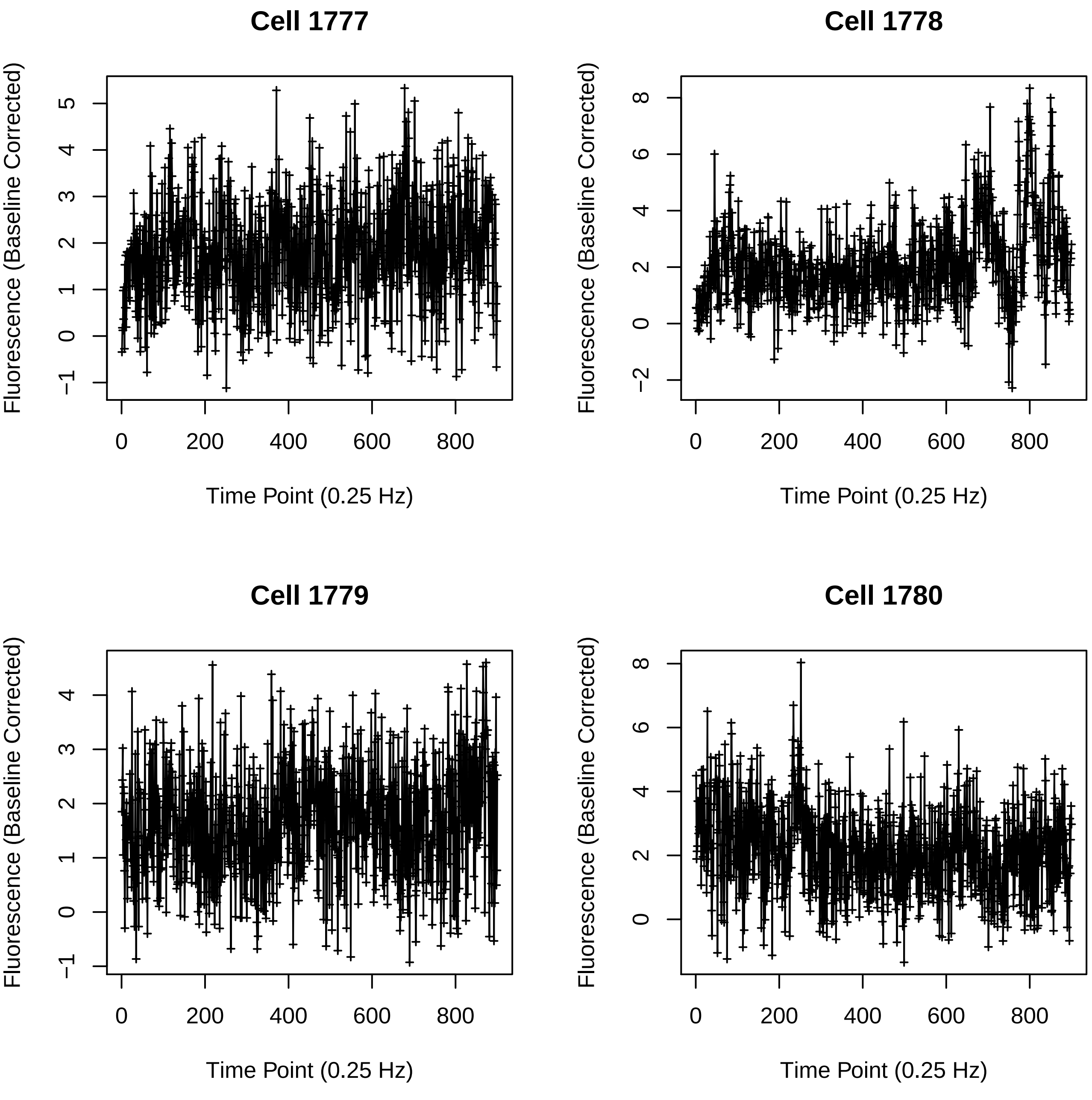

Cell 1777
Cell 1778
Cell 1779
Cell 1780
Fluorescence (Baseline Corrected)
Time Point (0.25 Hz)
0
200
400
600
800
−1
0
1
2
3
4
5
−2
6
8

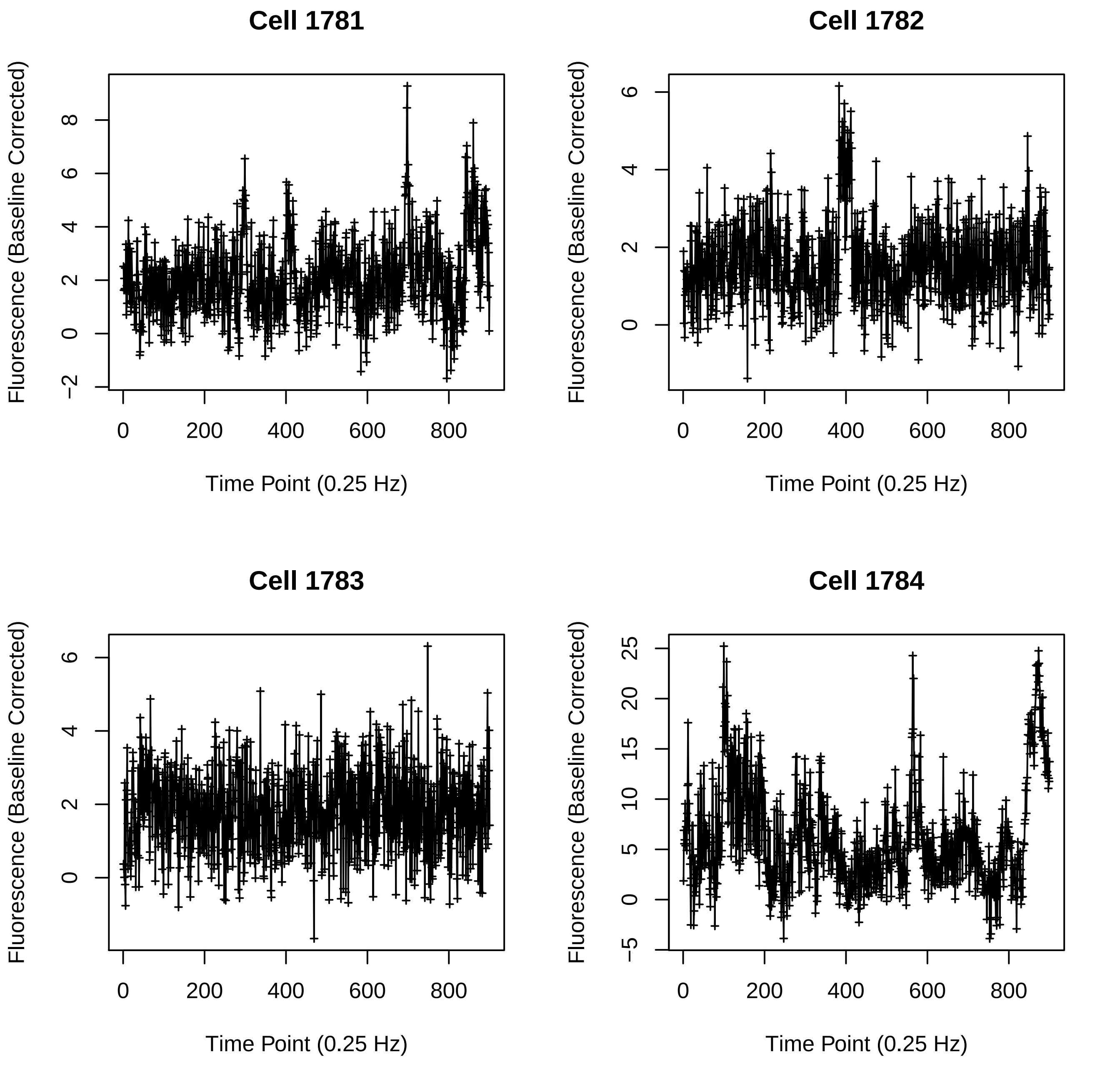
Cell 1781
Cell 1782
Cell 1783
Cell 1784
Fluorescence (Baseline Corrected)
Time Point (0.25 Hz)
−2
0
2
4
6
8
0
200
400
600
800
−5
10
15
20
25

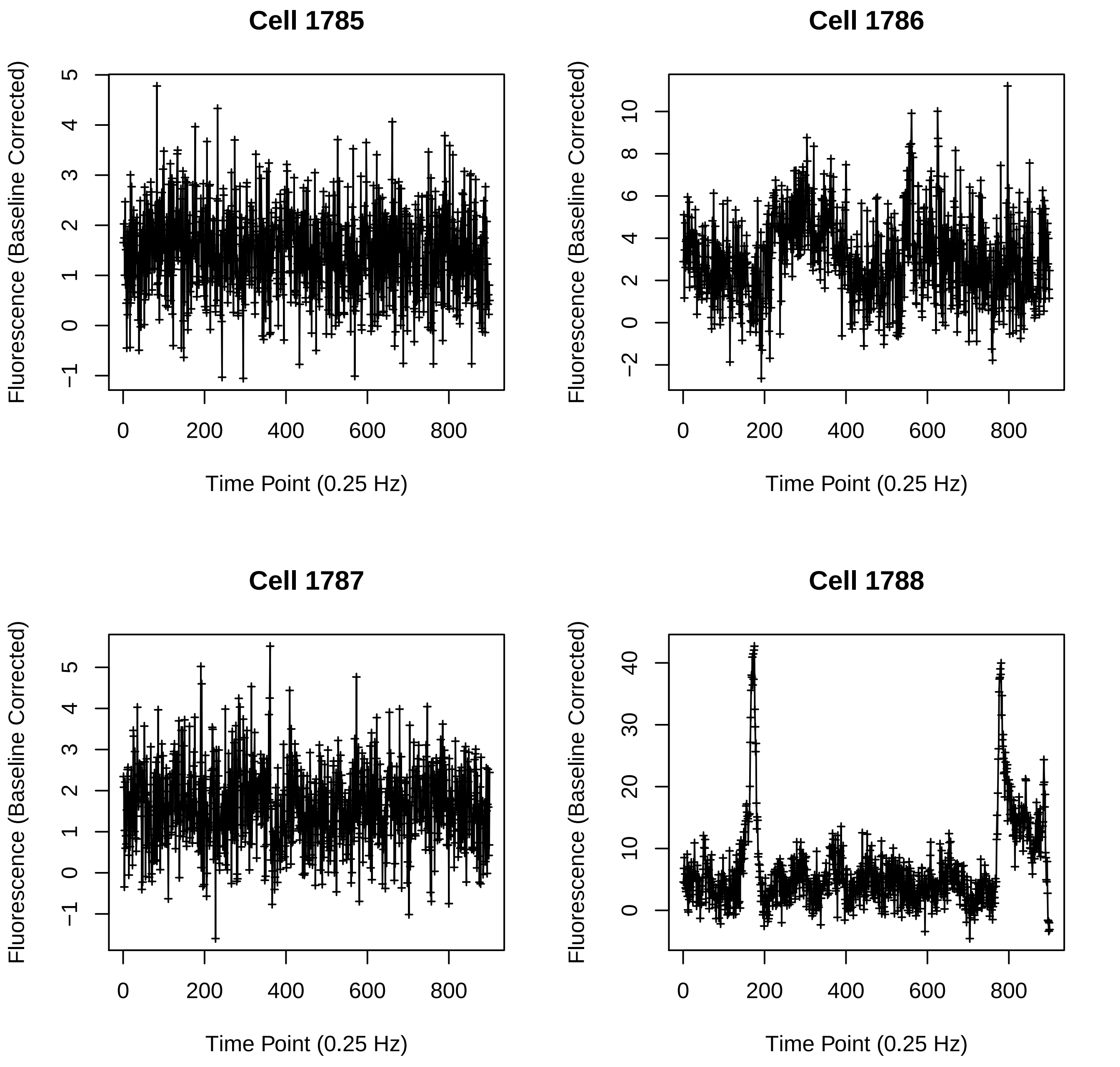
Cell 1785
Cell 1786
Cell 1787
Cell 1788
Fluorescence (Baseline Corrected)
Time Point (0.25 Hz)
0
200
400
600
800

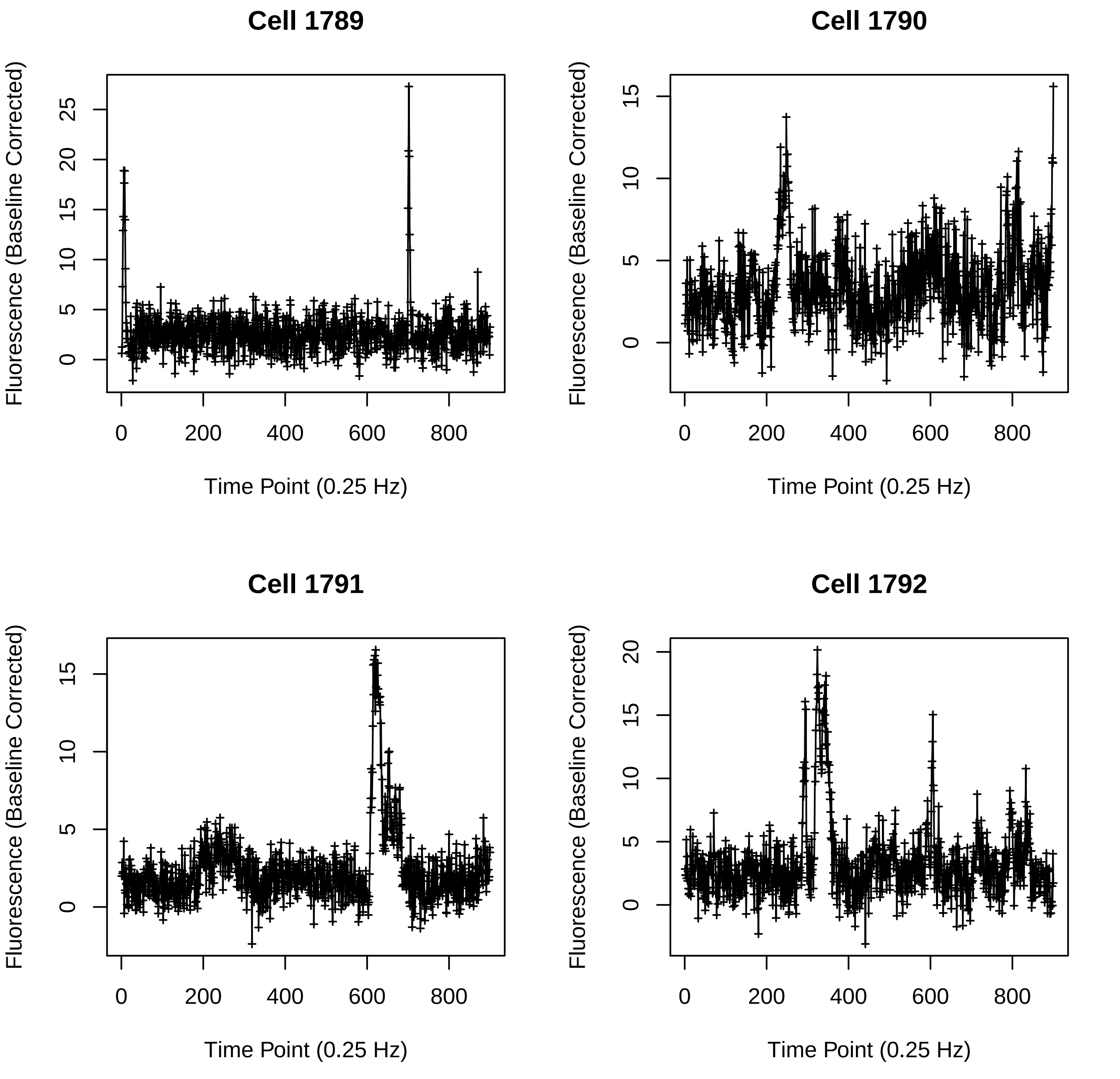
Cell 1789
Fluorescence (Baseline Corrected)
0
5
10
15
20
25
0
200
400
600
800
Time Point (0.25 Hz)
Cell 1790
Fluorescence (Baseline Corrected)
0
5
10
15
0
200
400
600
800
Time Point (0.25 Hz)
Cell 1791
Fluorescence (Baseline Corrected)
0
5
10
15
0
200
400
600
800
Time Point (0.25 Hz)
Cell 1792
Fluorescence (Baseline Corrected)
0
5
10
15
20
0
200
400
600
800
Time Point (0.25 Hz)

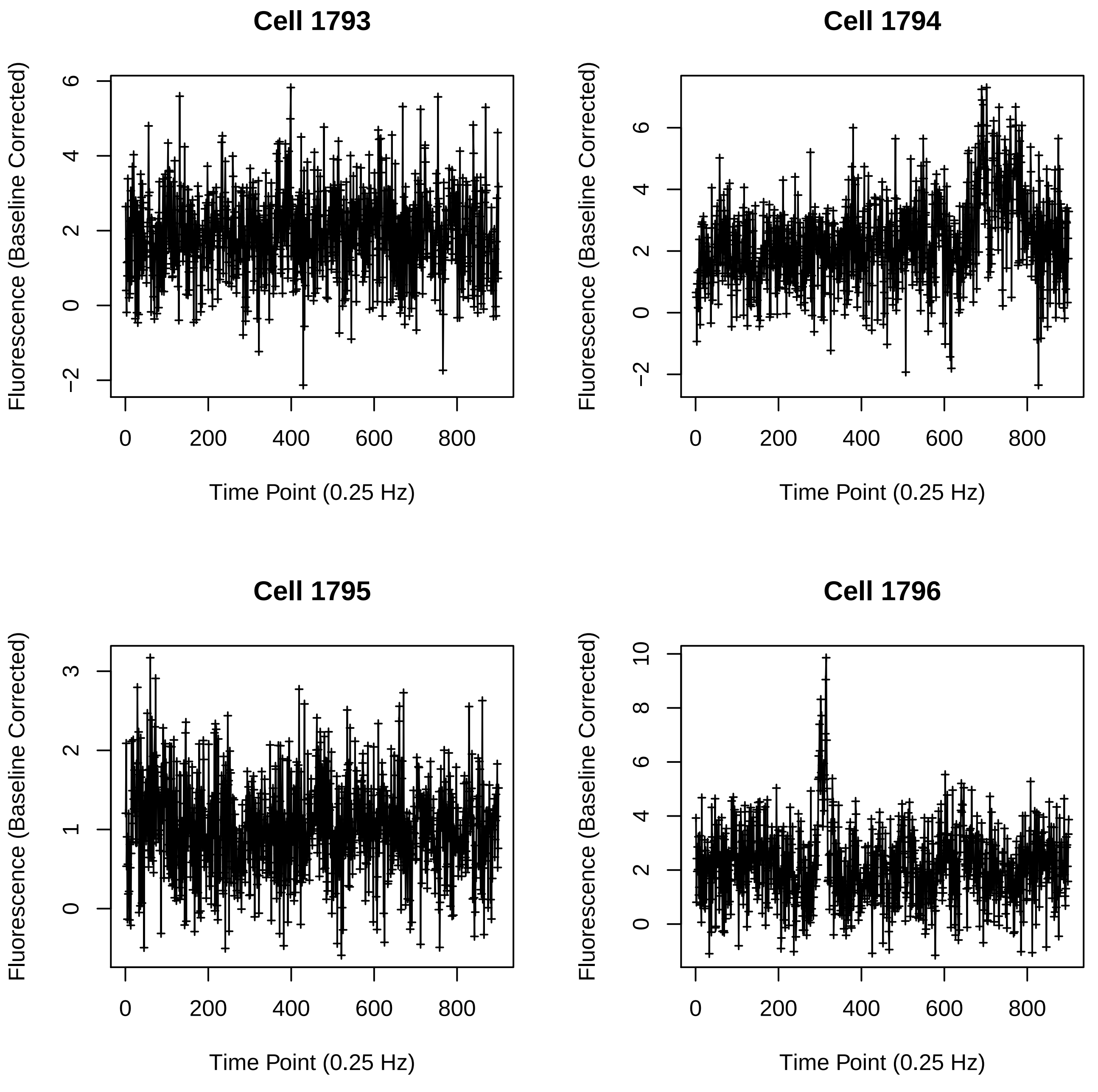

Cell 1793
Cell 1794
Cell 1795
Cell 1796
Fluorescence (Baseline Corrected)
Time Point (0.25 Hz)
0
200
400
600
800
-2
0
2
4
6
0
1
2
3
0
2
4
6
8
10

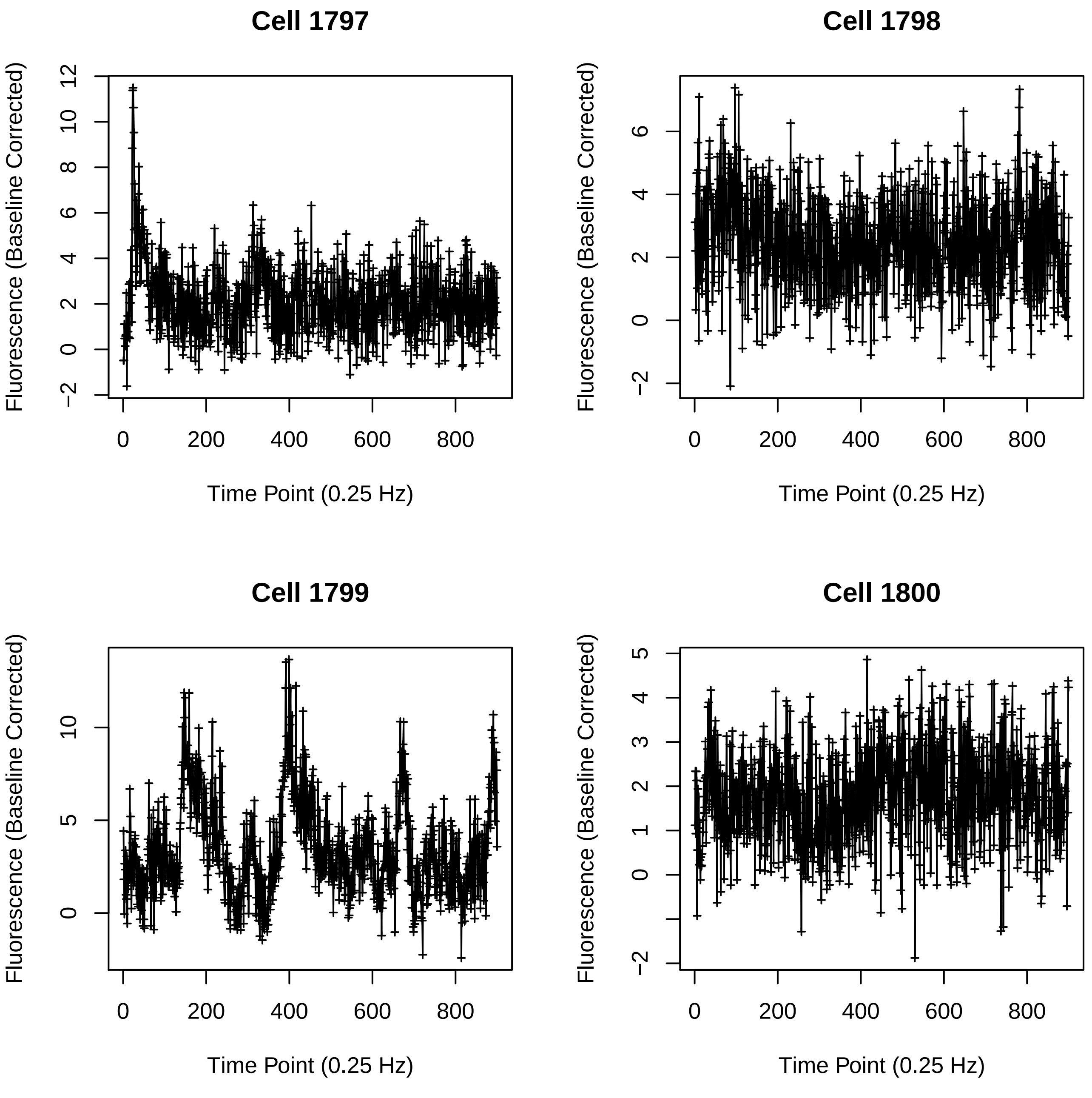
Cell 1797
Cell 1798
Cell 1799
Cell 1800
Fluorescence (Baseline Corrected)
Time Point (0.25 Hz)
−2
0
2
4
6
8
10
12
5
200
400
600
800

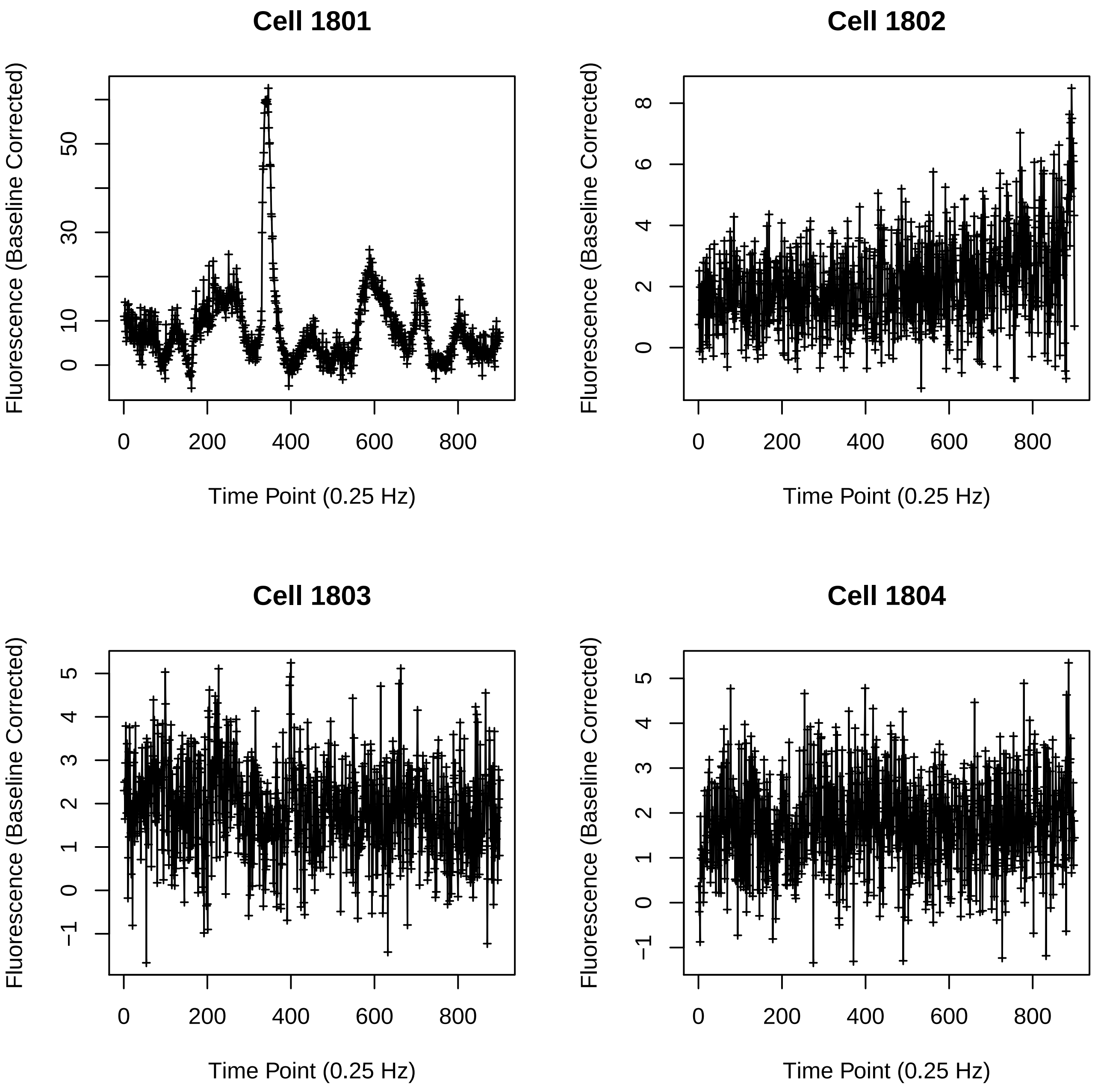
Cell 1801
Cell 1802
Cell 1803
Cell 1804
Fluorescence (Baseline Corrected)
Time Point (0.25 Hz)
0
200
400
600
800
0
10
30
50
2
4
6
8
-1
1
3
5

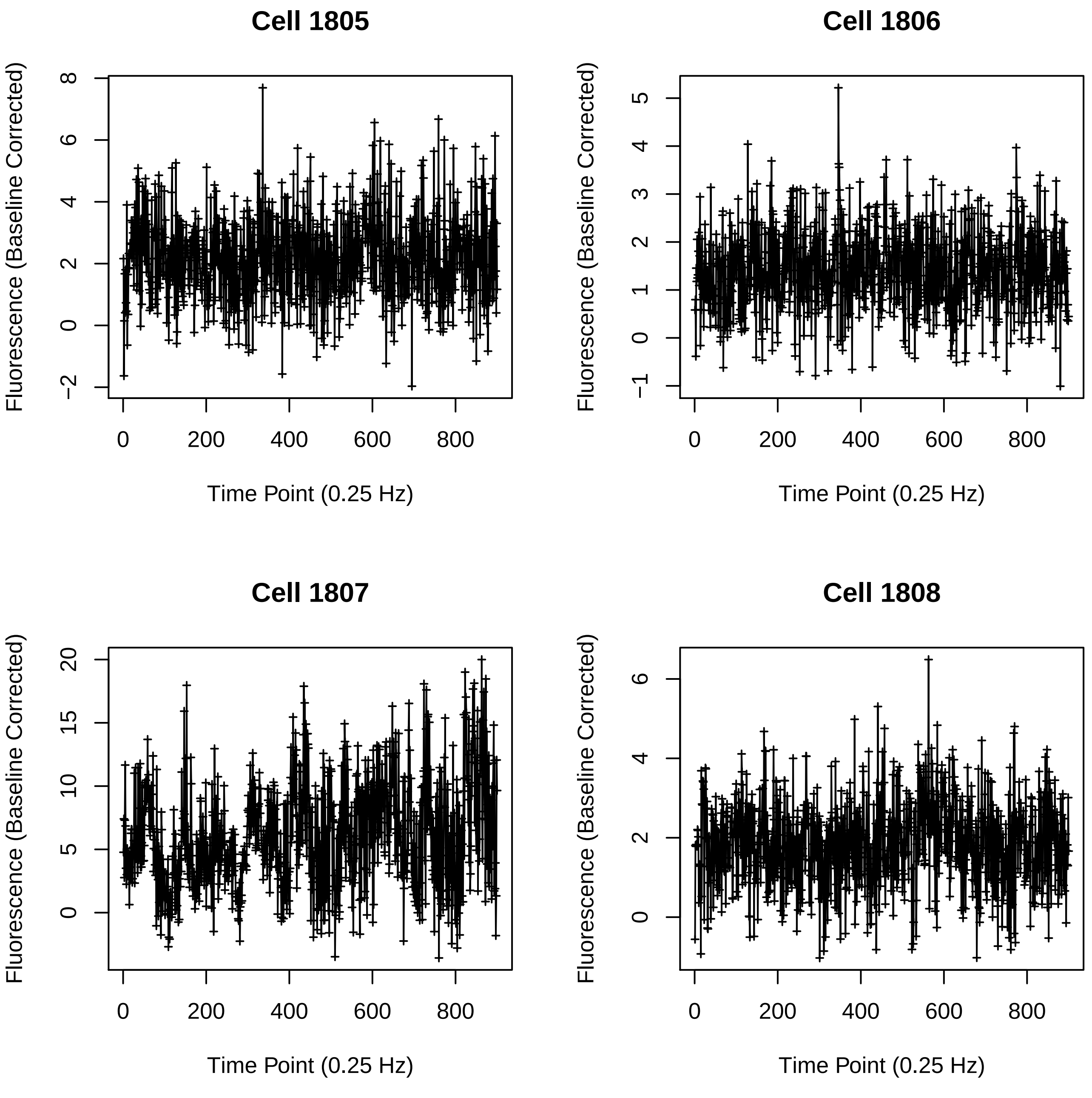
Cell 1805
Cell 1806
Cell 1807
Cell 1808
Fluorescence (Baseline Corrected)
Time Point (0.25 Hz)
−2
0
2
4
6
8
−1
1
3
5
10
15
20
200
400
600
800

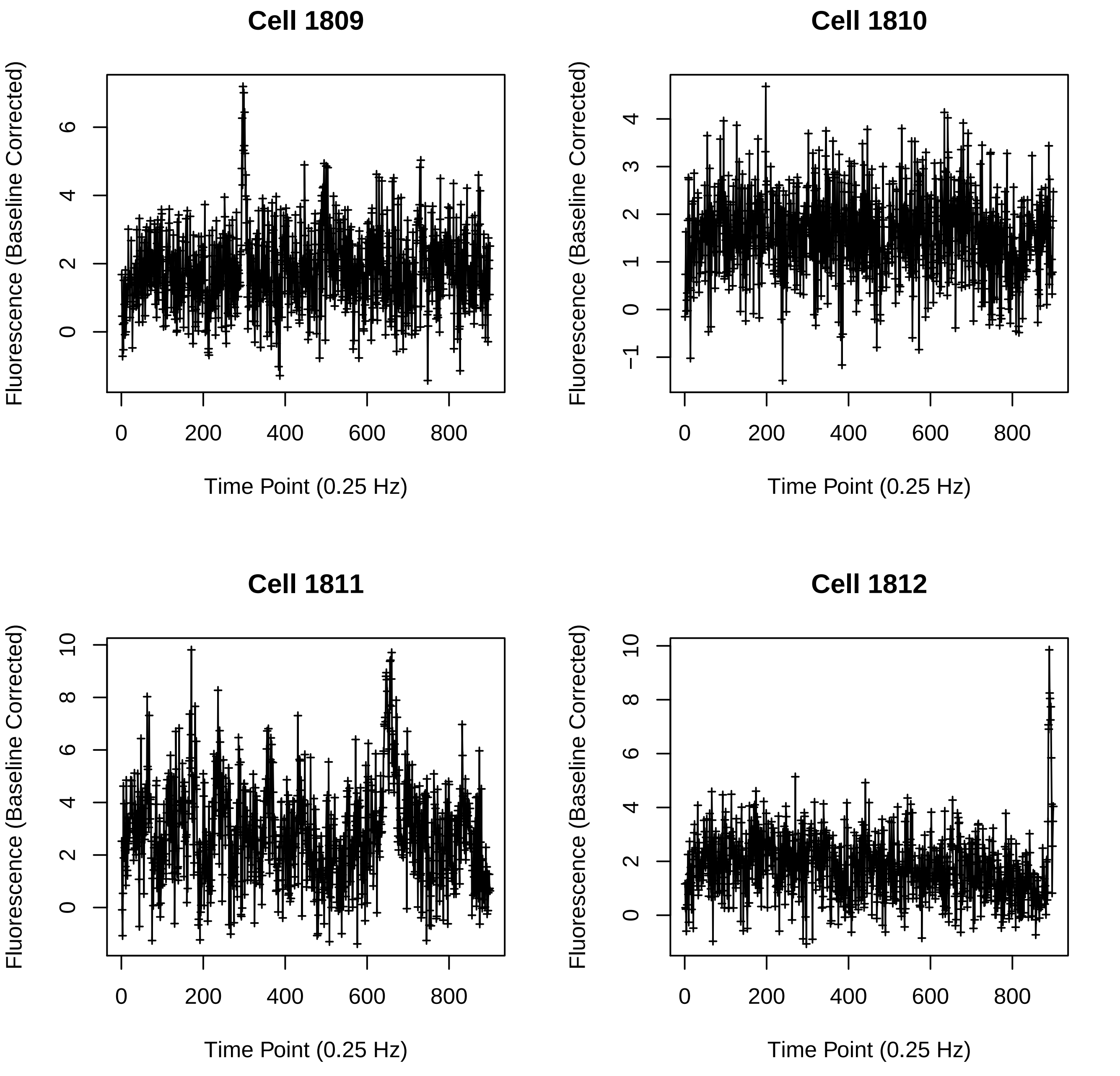
Cell 1809
Cell 1810
Cell 1811
Cell 1812
Fluorescence (Baseline Corrected)
Time Point (0.25 Hz)
0
200
400
600
800
−1
0
1
2
3
4
6
8
10

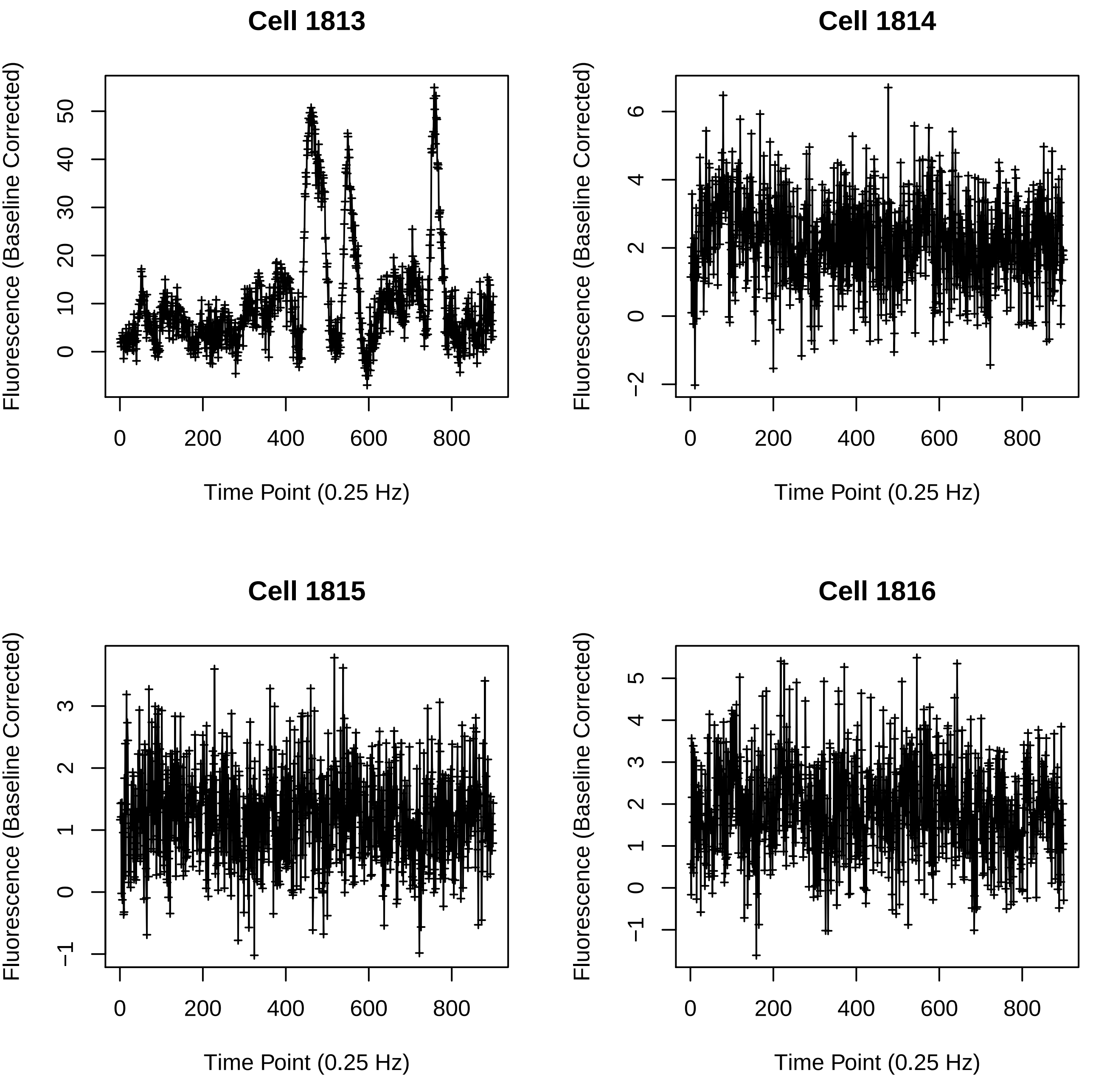
Cell 1813
Cell 1814
Cell 1815
Cell 1816
Fluorescence (Baseline Corrected)
Time Point (0.25 Hz)

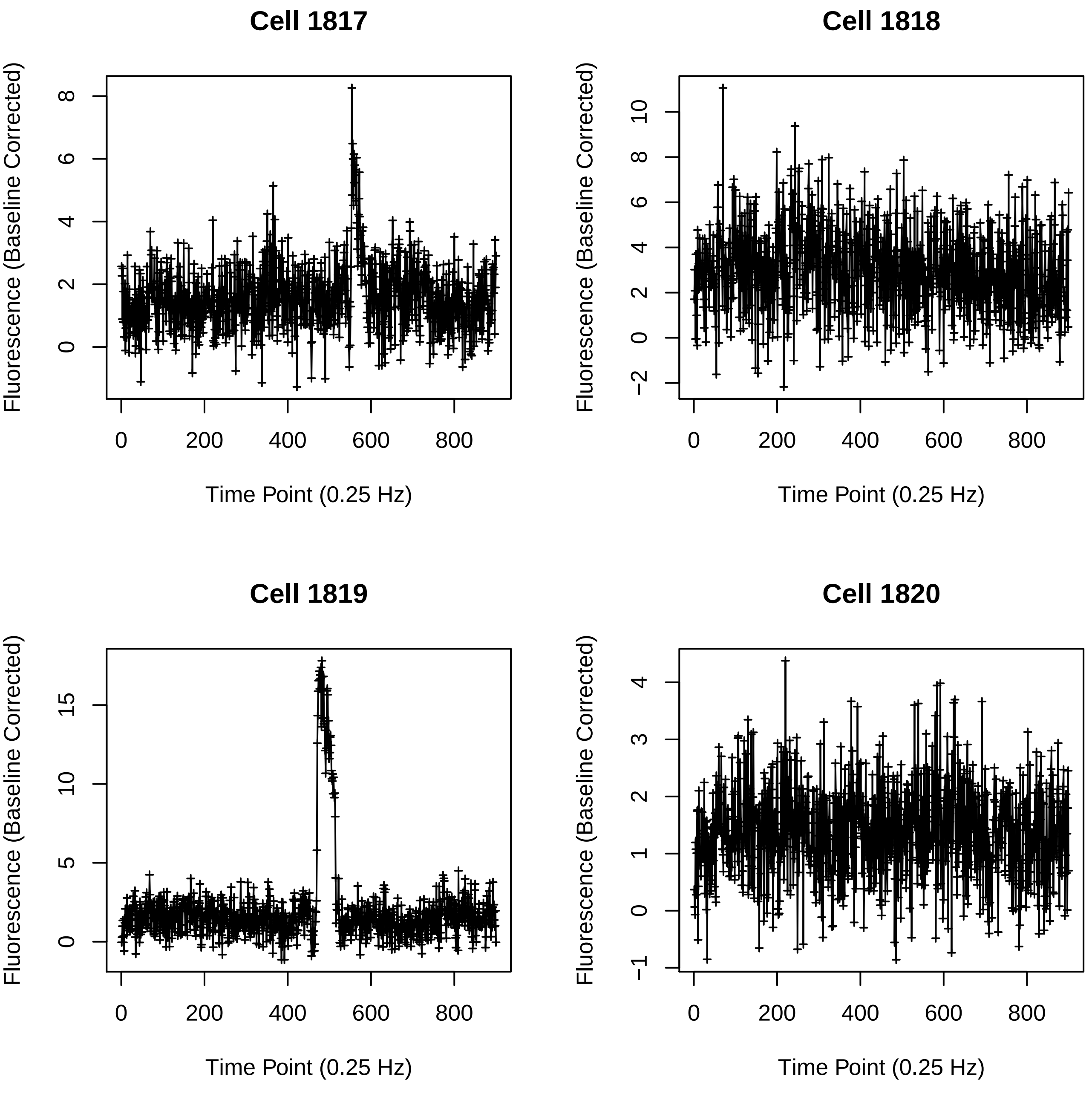
Cell 1817
Cell 1818
Cell 1819
Cell 1820
Fluorescence (Baseline Corrected)
Time Point (0.25 Hz)
0
200
400
600
800
−2
2
4
5
6
8
10
15
−1
1
3

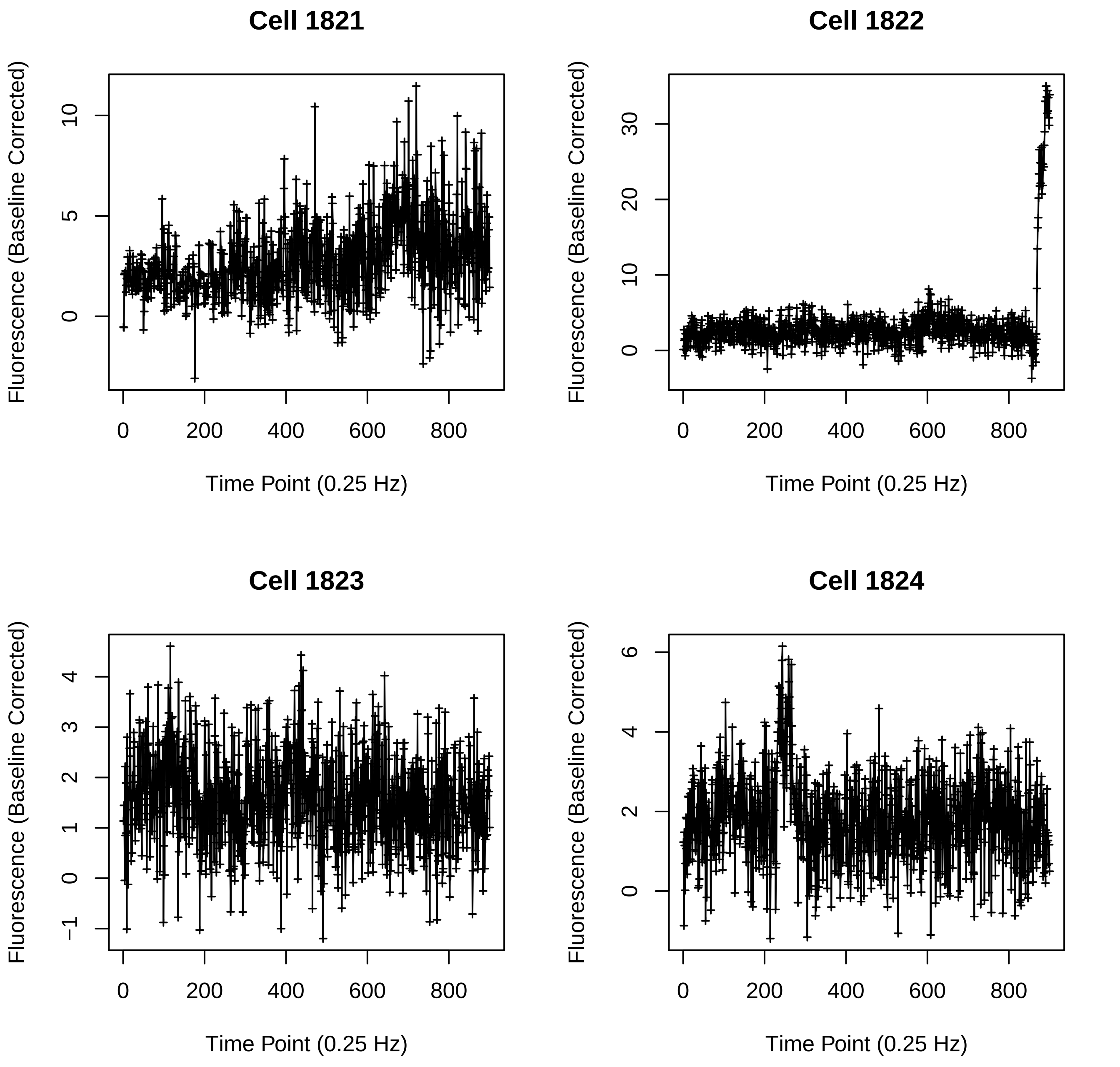
Cell 1821
Cell 1822
Cell 1823
Cell 1824
Fluorescence (Baseline Corrected)
Time Point (0.25 Hz)

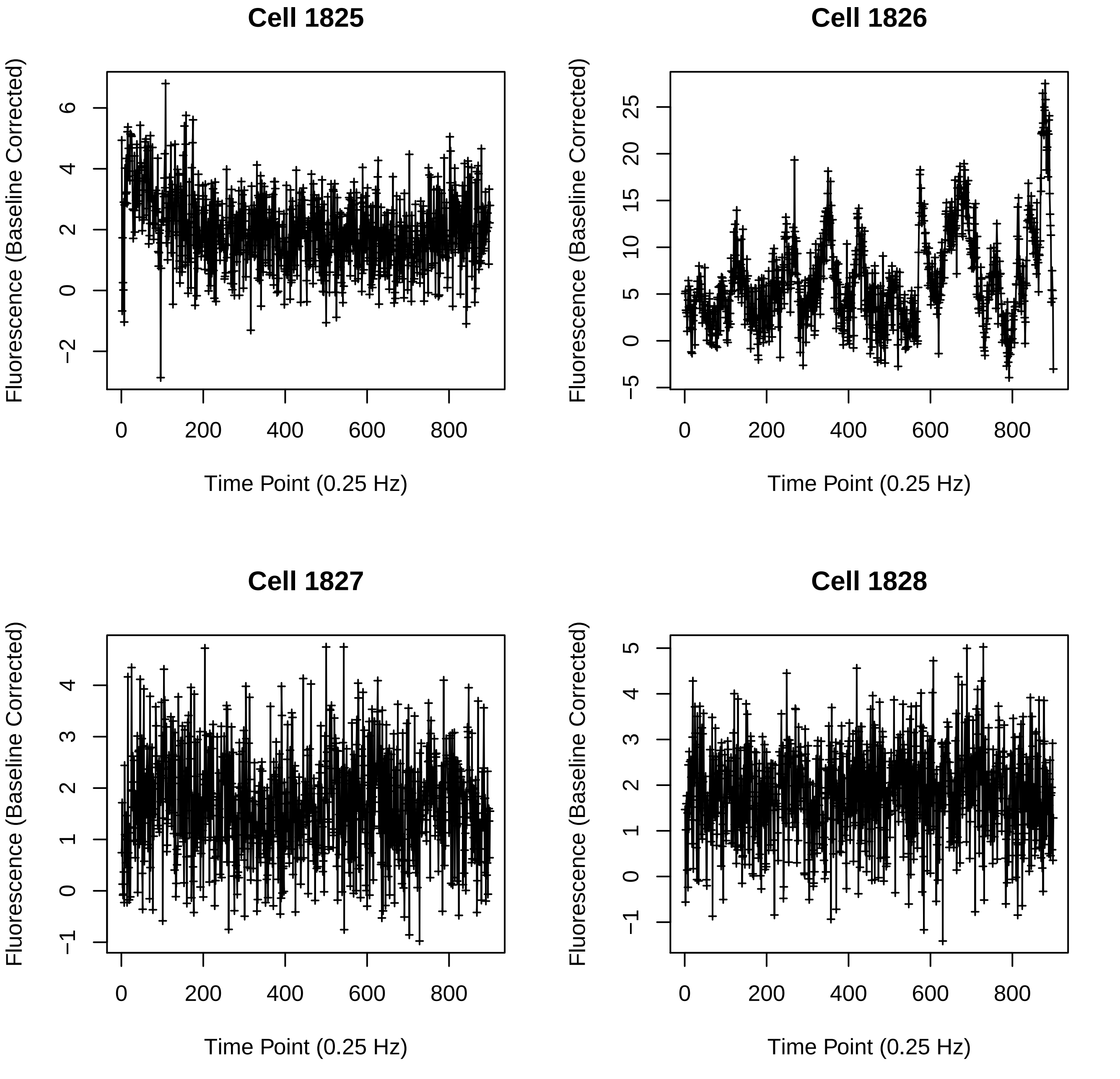
Cell 1825
Cell 1826
Cell 1827
Cell 1828
Fluorescence (Baseline Corrected)
Time Point (0.25 Hz)
−2
0
2
4
6
−5
5
10
15
20
25
−1
1
3
200
400
600
800

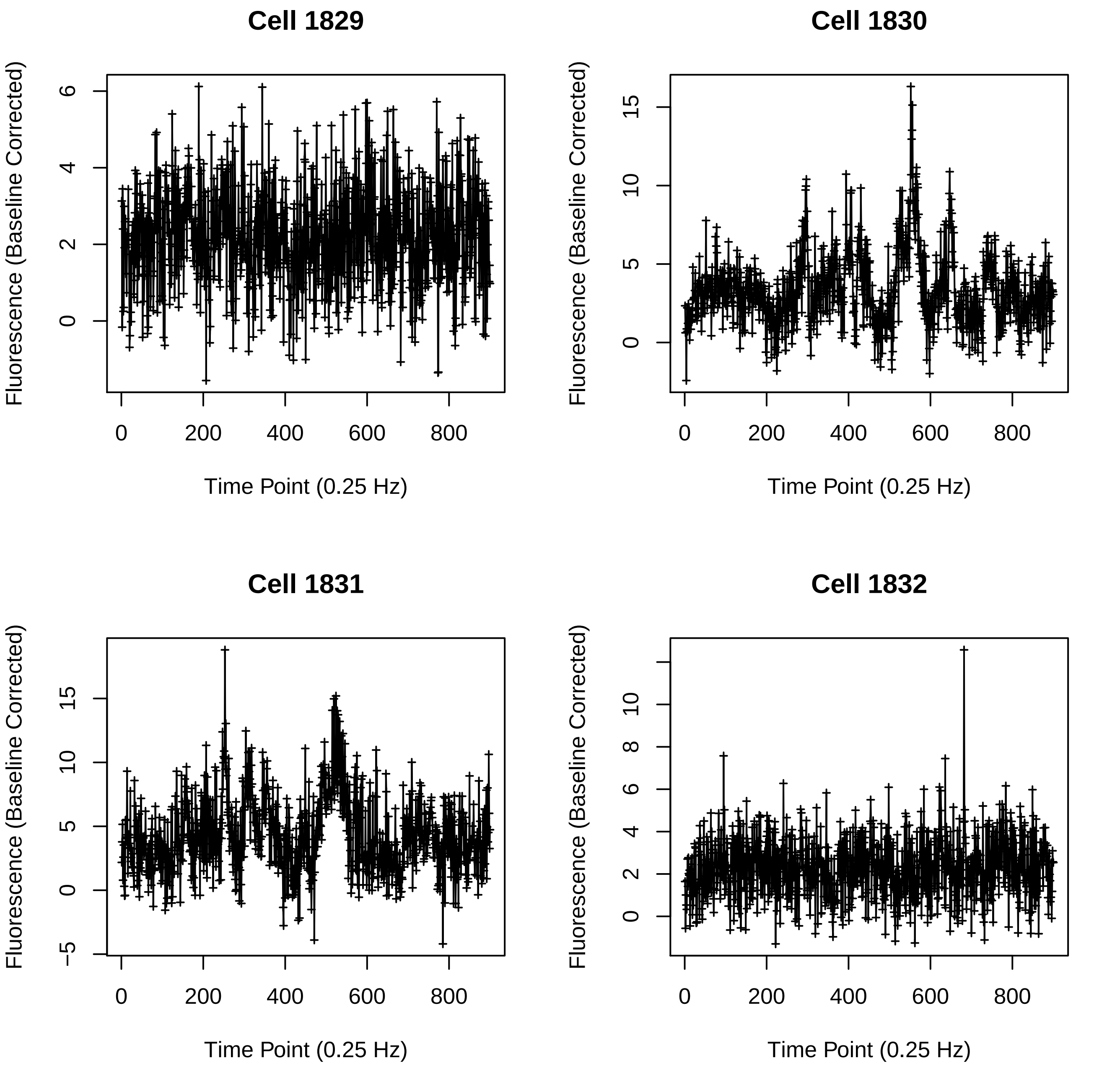
Cell 1829
Fluorescence (Baseline Corrected)
Time Point (0.25 Hz)
0
2
4
6
0
200
400
600
800
Cell 1830
Fluorescence (Baseline Corrected)
Time Point (0.25 Hz)
0
5
10
15
0
200
400
600
800
Cell 1831
Fluorescence (Baseline Corrected)
Time Point (0.25 Hz)
−5
0
5
10
15
0
200
400
600
800
Cell 1832
Fluorescence (Baseline Corrected)
Time Point (0.25 Hz)
0
2
4
6
8
10
0
200
400
600
800

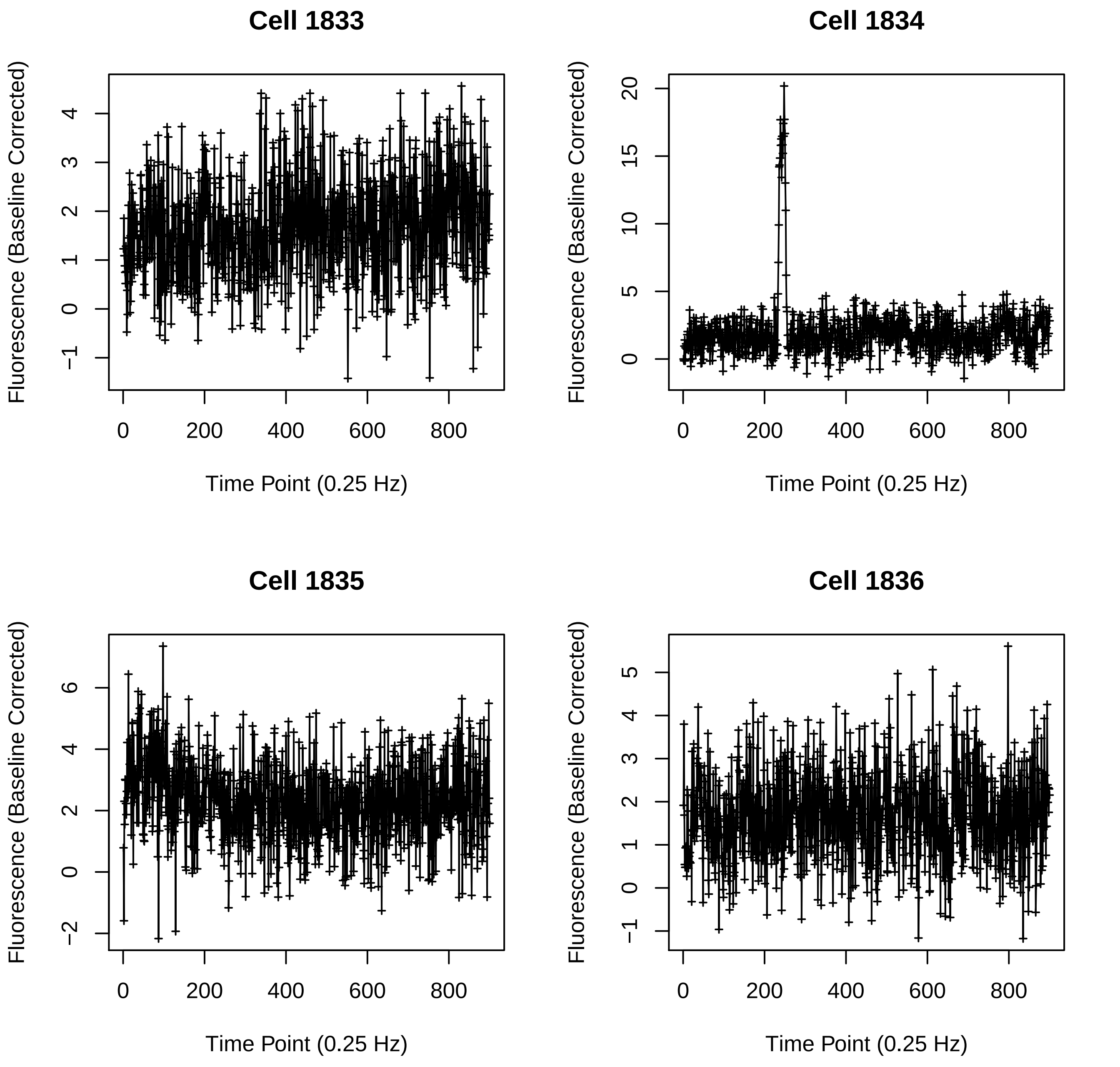

Cell 1833
Cell 1834
Cell 1835
Cell 1836
Fluorescence (Baseline Corrected)
Time Point (0.25 Hz)
−1
0
1
2
3
4
5
6
10
15
20
−2
0
200
400
600
800

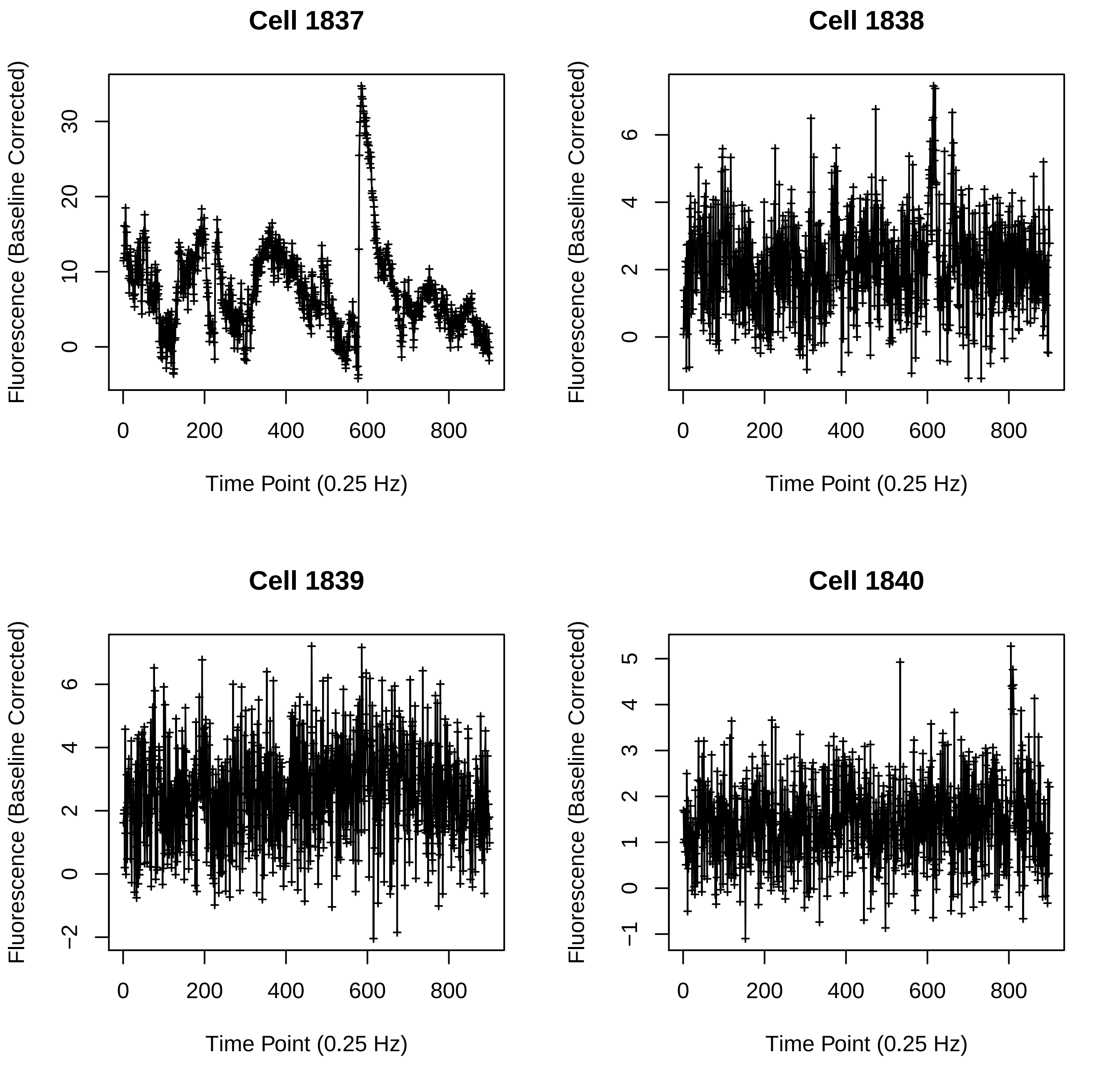

Cell 1837
Fluorescence (Baseline Corrected)
0
10
20
30
0
200
400
600
800
Time Point (0.25 Hz)
Cell 1838
Fluorescence (Baseline Corrected)
0
2
4
6
0
200
400
600
800
Time Point (0.25 Hz)
Cell 1839
Fluorescence (Baseline Corrected)
−2
0
2
4
6
0
200
400
600
800
Time Point (0.25 Hz)
Cell 1840
Fluorescence (Baseline Corrected)
−1
0
1
2
3
4
5
0
200
400
600
800
Time Point (0.25 Hz)

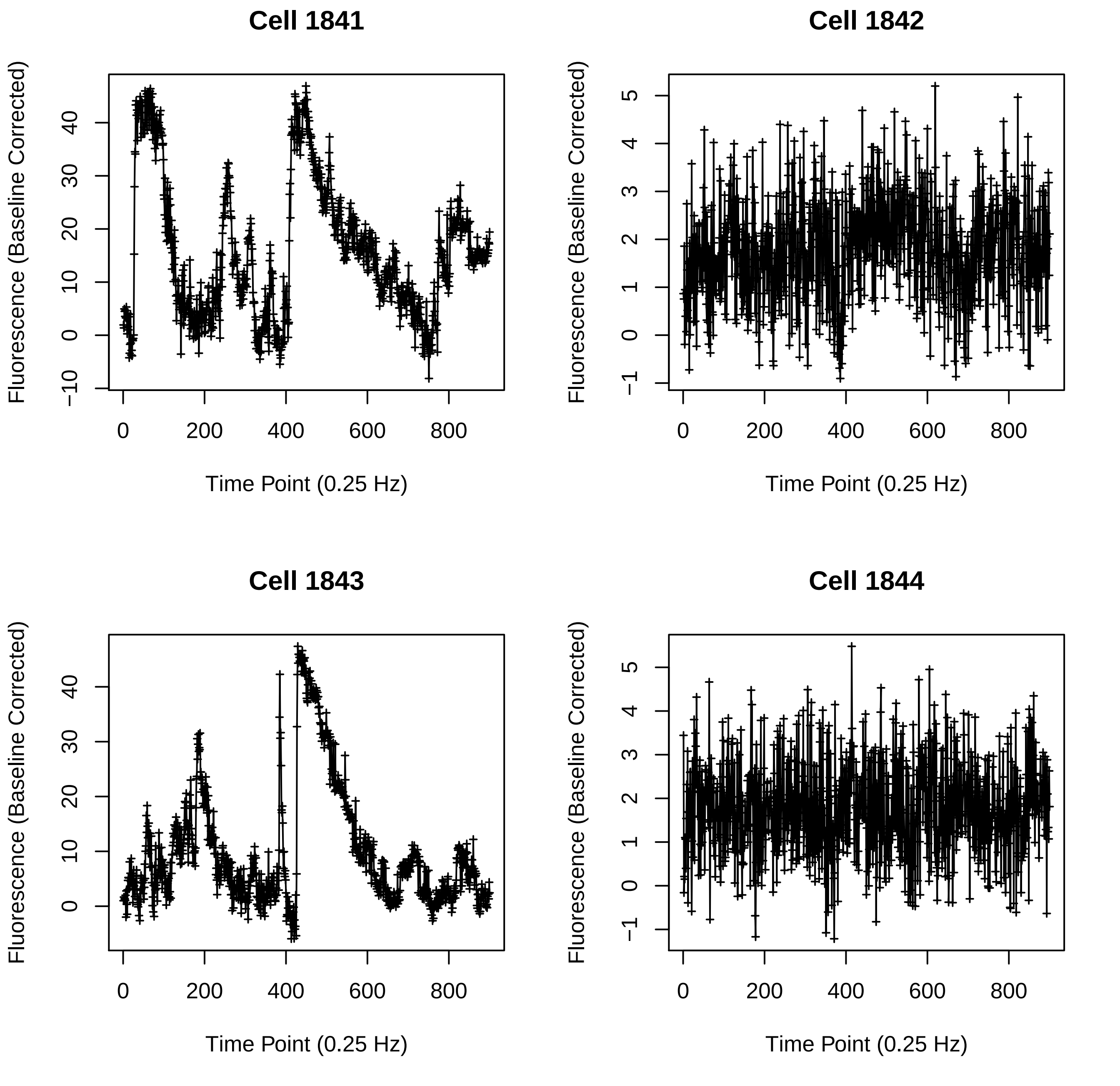
Cell 1841
Cell 1842
Cell 1843
Cell 1844
Fluorescence (Baseline Corrected)
Time Point (0.25 Hz)
−10
0
10
20
30
40
−1
0
1
2
3
4
5
0
200
400
600
800

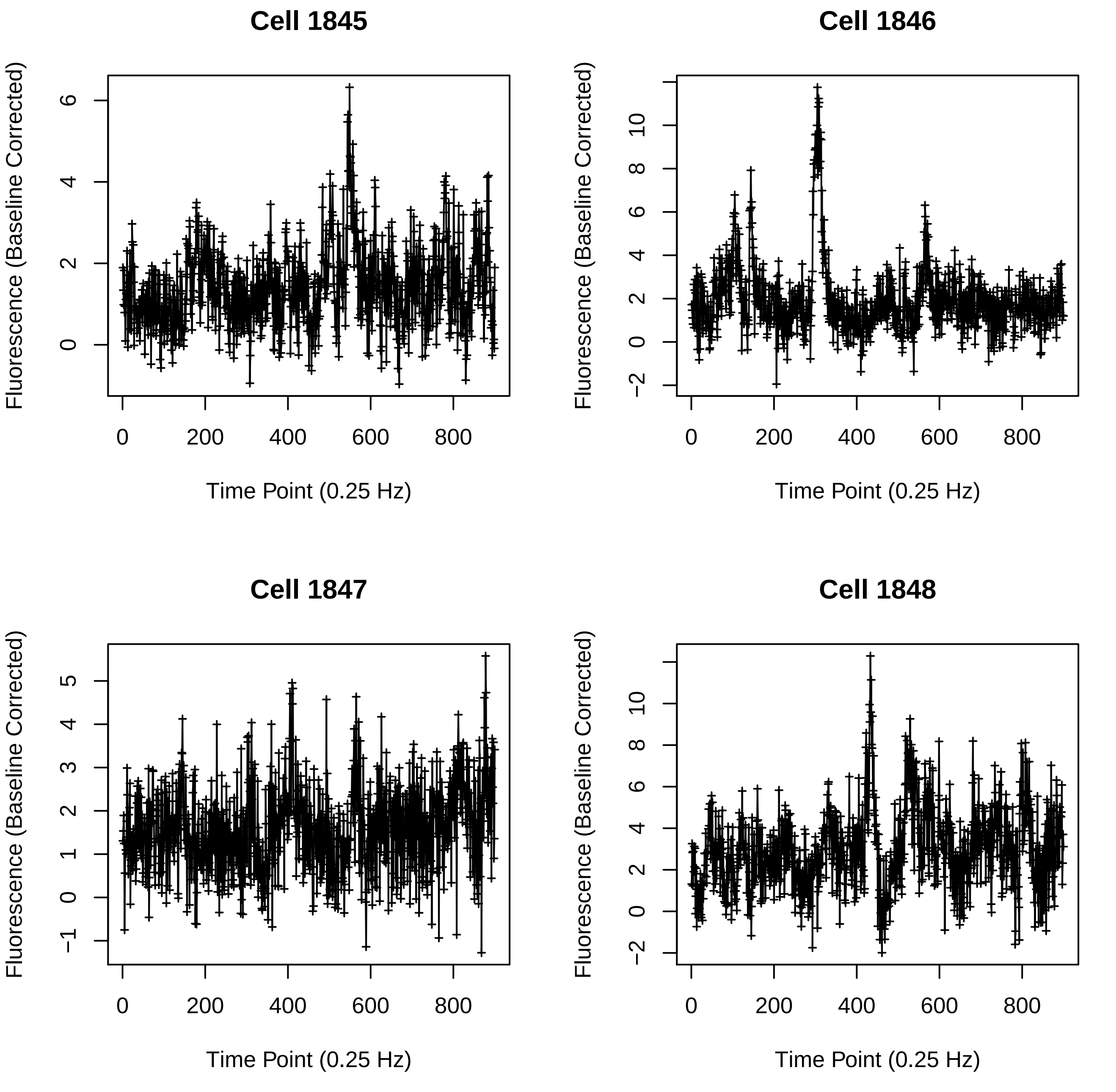

Cell 1845
Cell 1846
Cell 1847
Cell 1848
Fluorescence (Baseline Corrected)
Time Point (0.25 Hz)
0
200
400
600
800
−2
2
4
6
8
10
−1
1
3
5

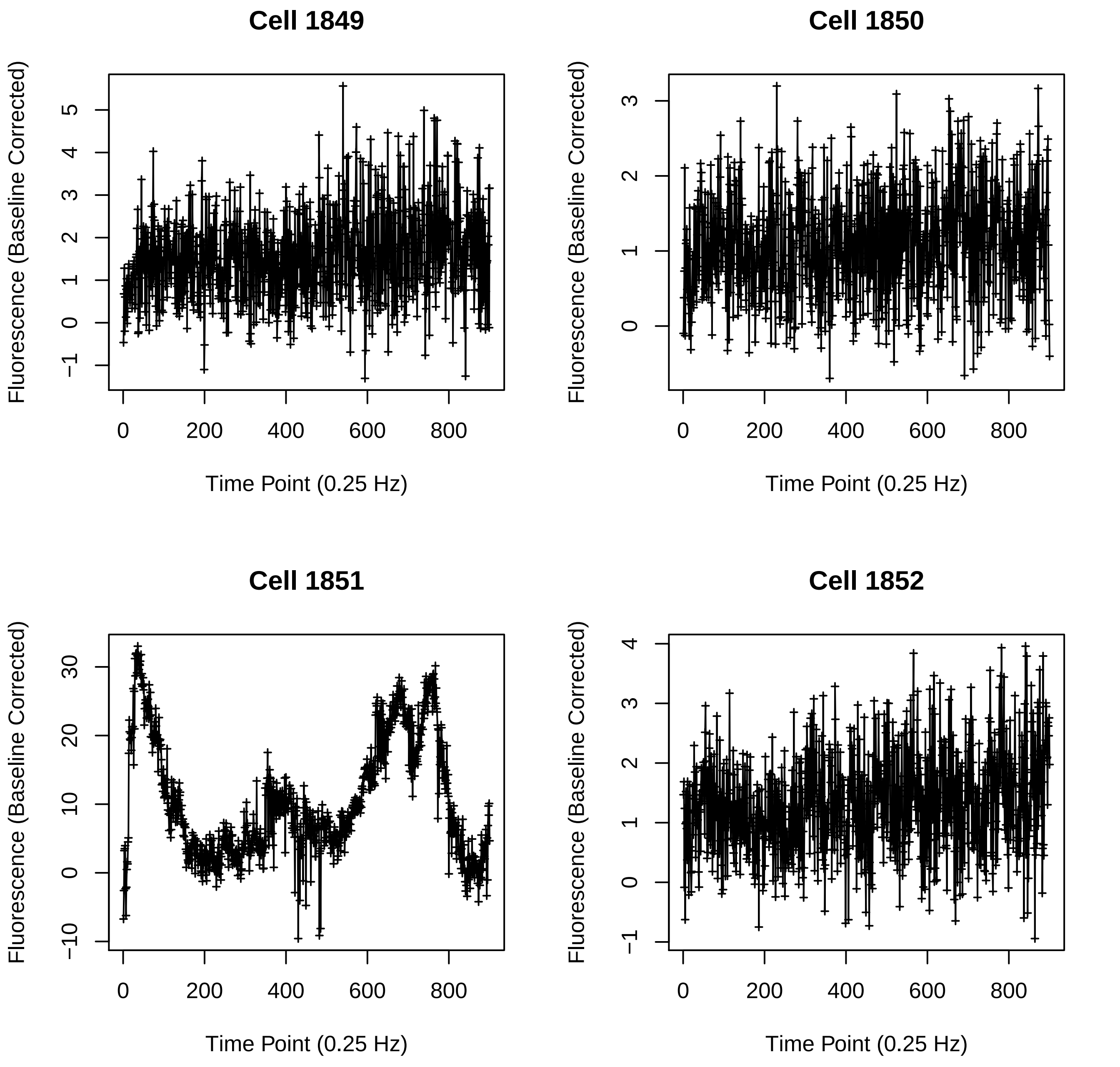
Cell 1849
Cell 1850
Cell 1851
Cell 1852
Fluorescence (Baseline Corrected)
Time Point (0.25 Hz)
0
200
400
600
800

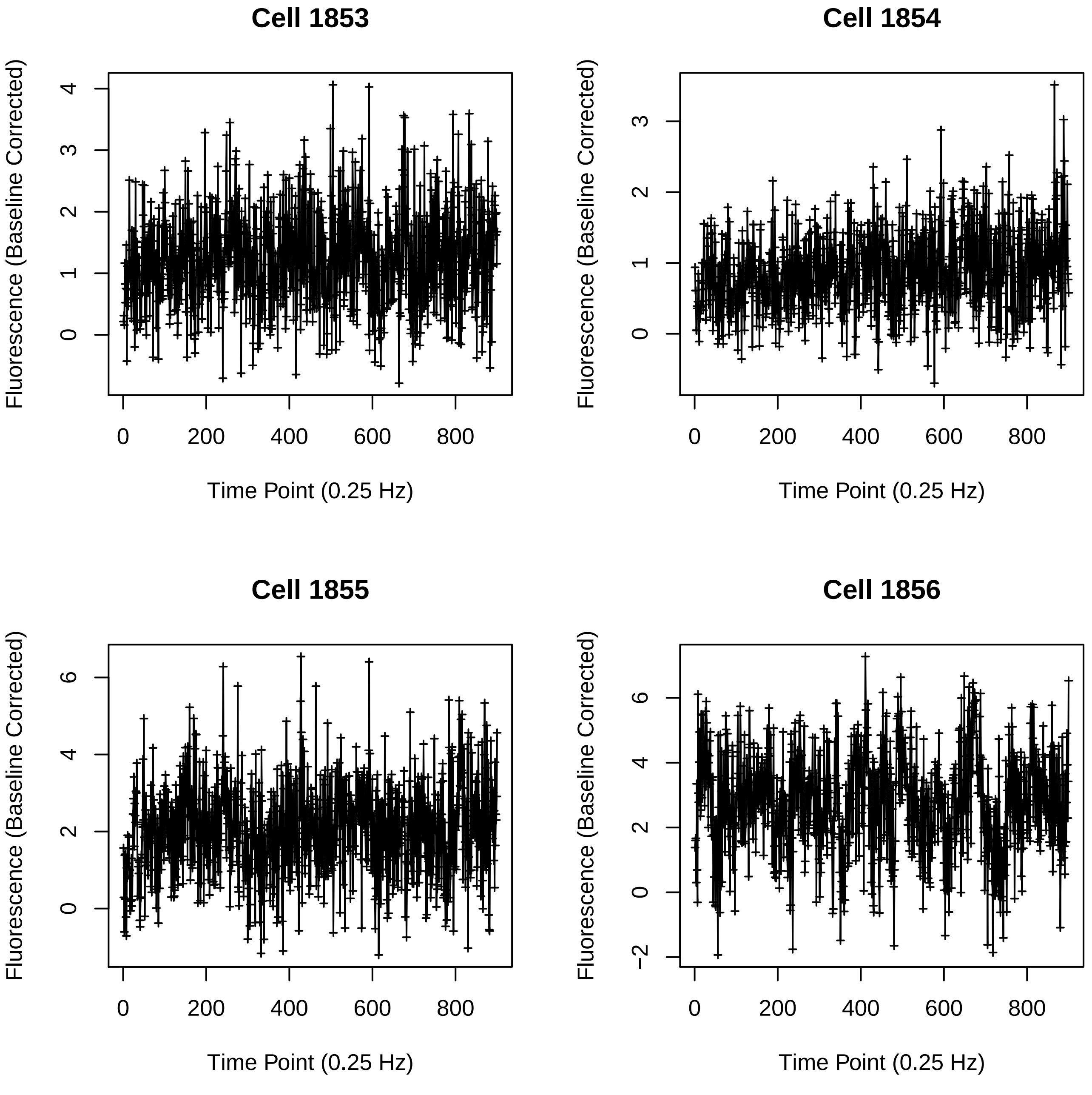

Cell 1853
Cell 1854
Cell 1855
Cell 1856
Fluorescence (Baseline Corrected)
Time Point (0.25 Hz)
0
200
400
600
800
-2
0
1
2
3
4
6

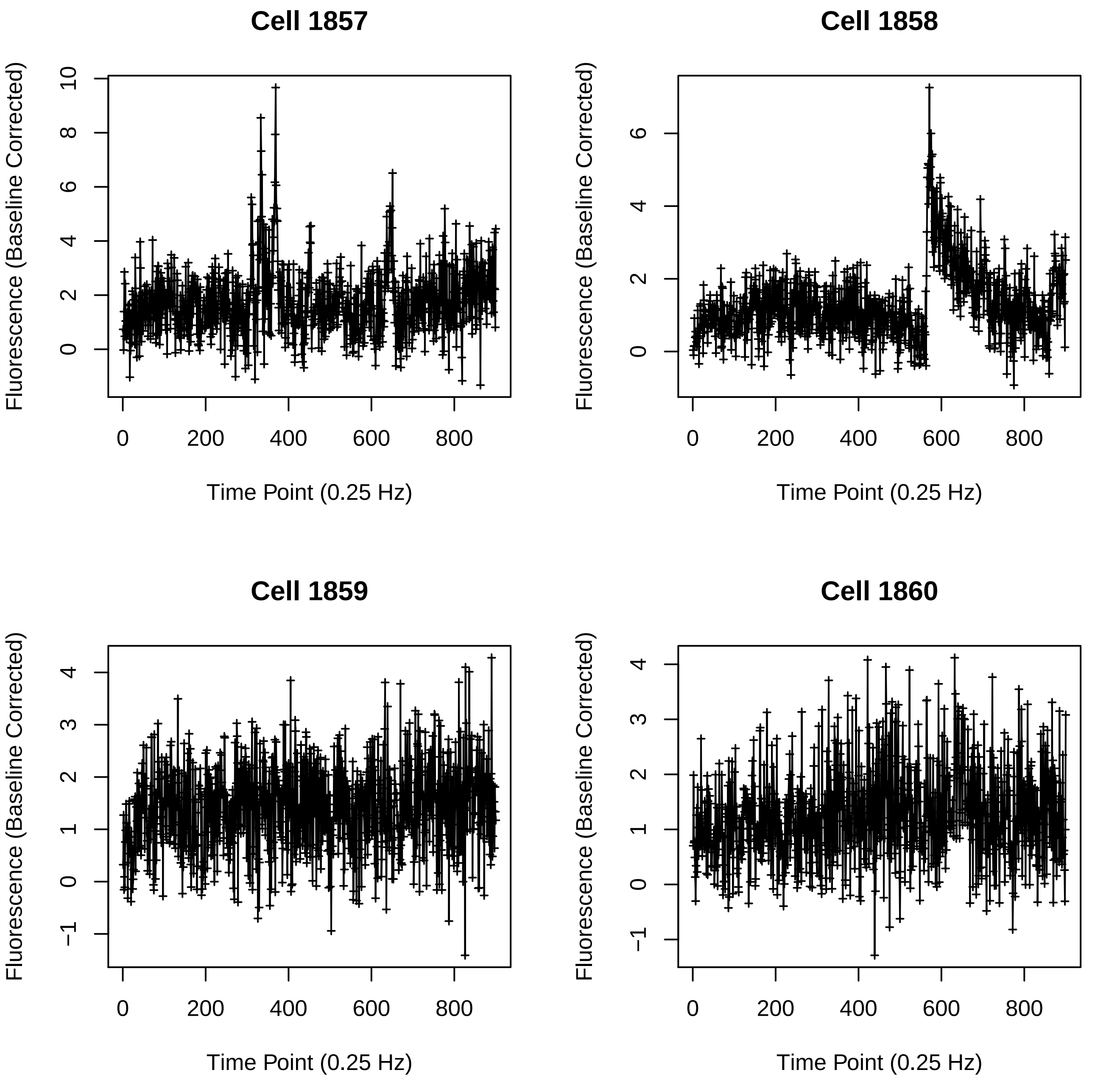
Cell 1857
Cell 1858
Cell 1859
Cell 1860
Fluorescence (Baseline Corrected)
Time Point (0.25 Hz)
0
200
400
600
800
−1
1
2
3
4
6
8
10

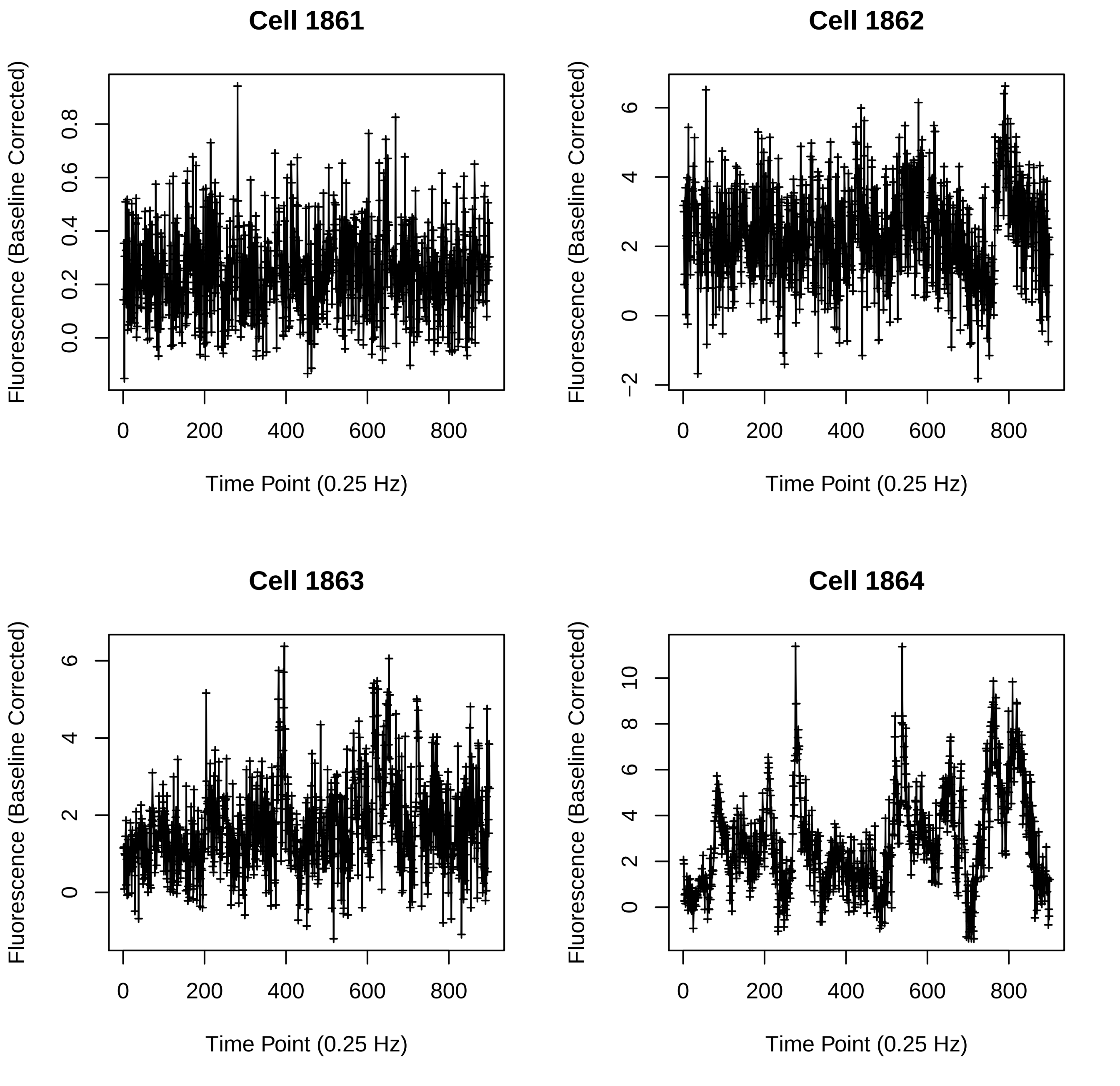

Cell 1861
Cell 1862
Cell 1863
Cell 1864
Fluorescence (Baseline Corrected)
Time Point (0.25 Hz)

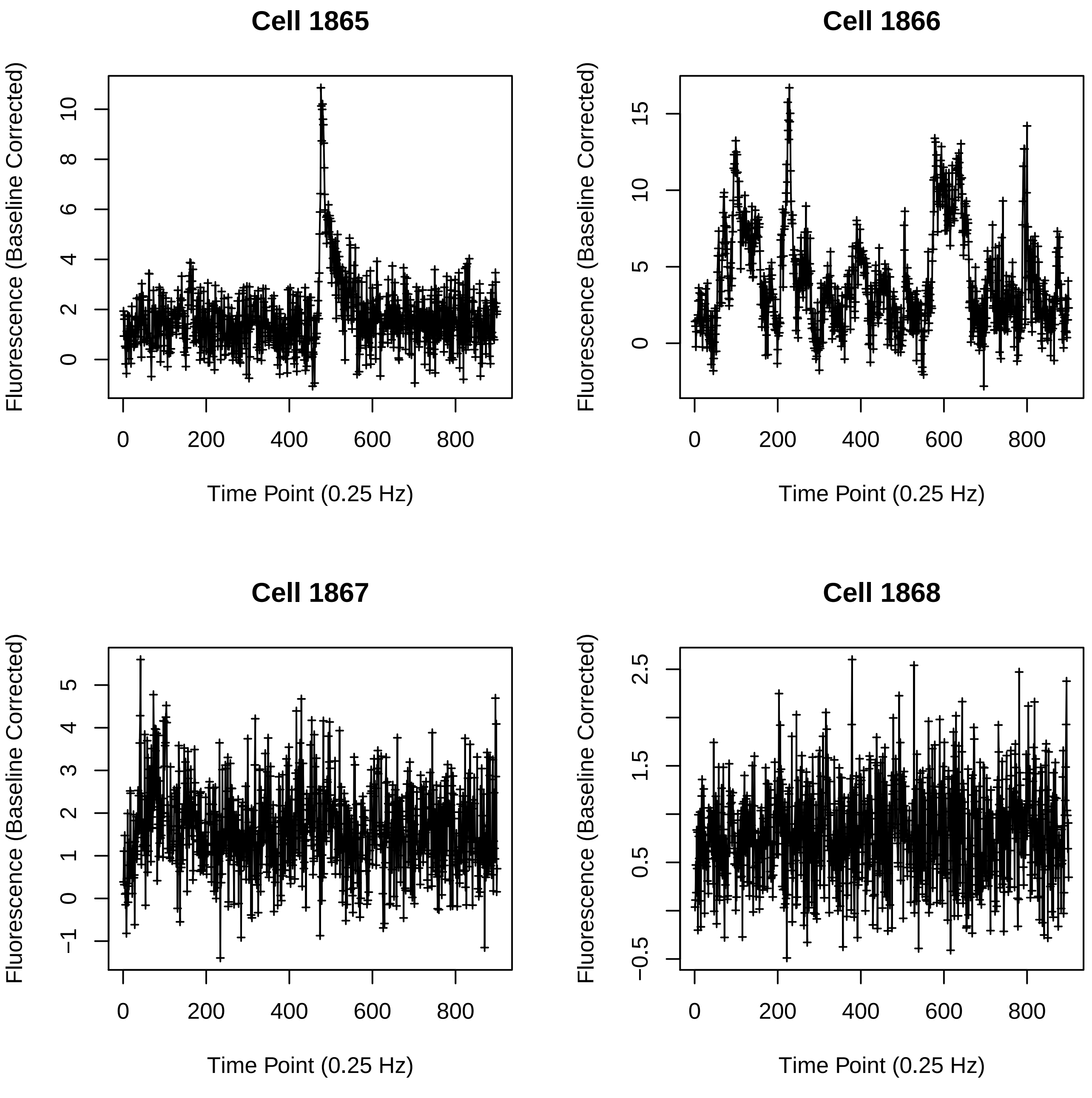

Cell 1865
Cell 1866
Cell 1867
Cell 1868
Fluorescence (Baseline Corrected)
Time Point (0.25 Hz)
0
200
400
600
800
0
2
4
6
8
10
0
5
10
15
−1
0
1
2
3
4
5
−0.5
0.5
1.5
2.5

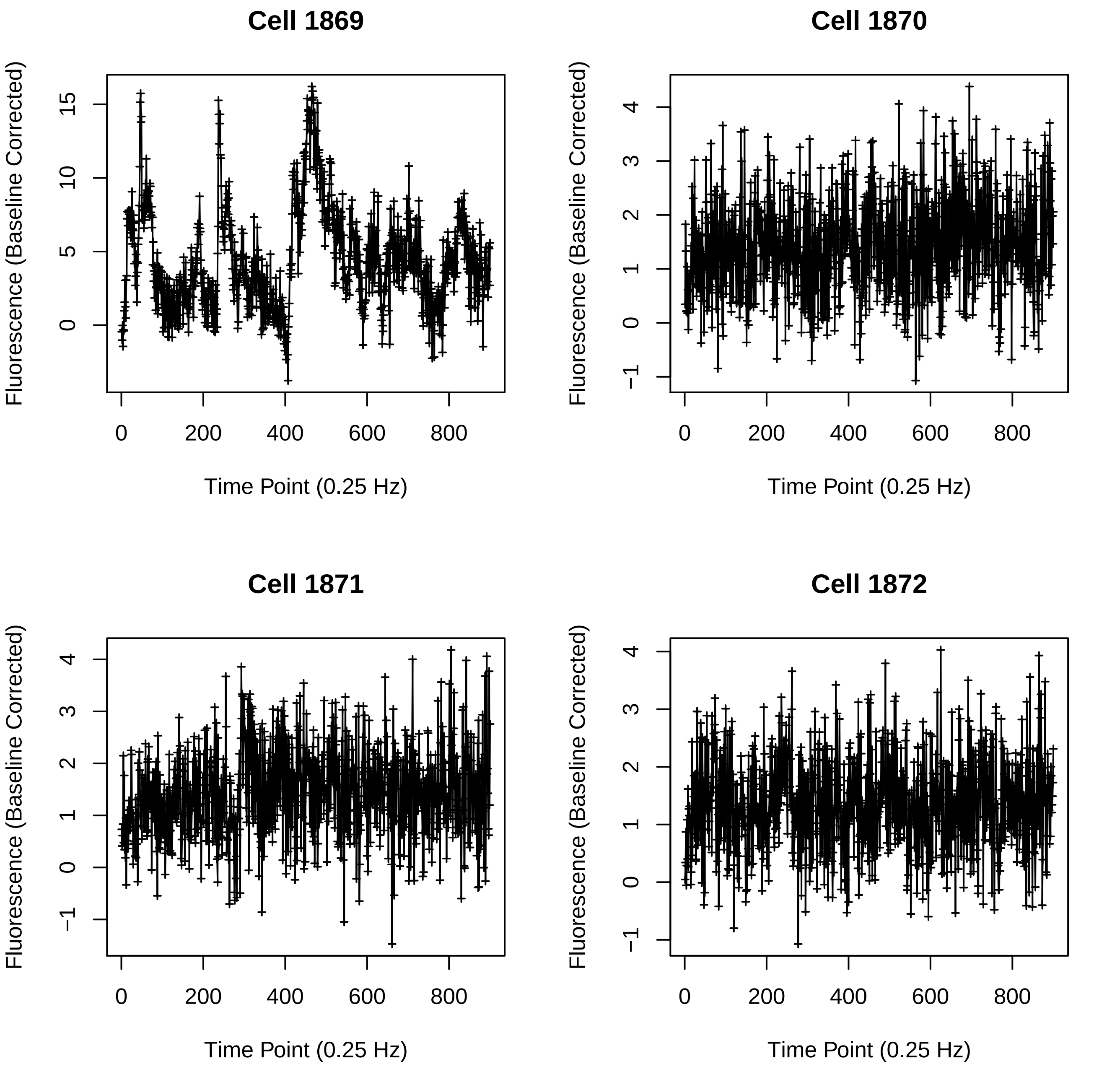
Cell 1869
Cell 1870
Cell 1871
Cell 1872
Fluorescence (Baseline Corrected)
Time Point (0.25 Hz)
0
5
10
15
−1
1
2
3
4
200
400
600
800

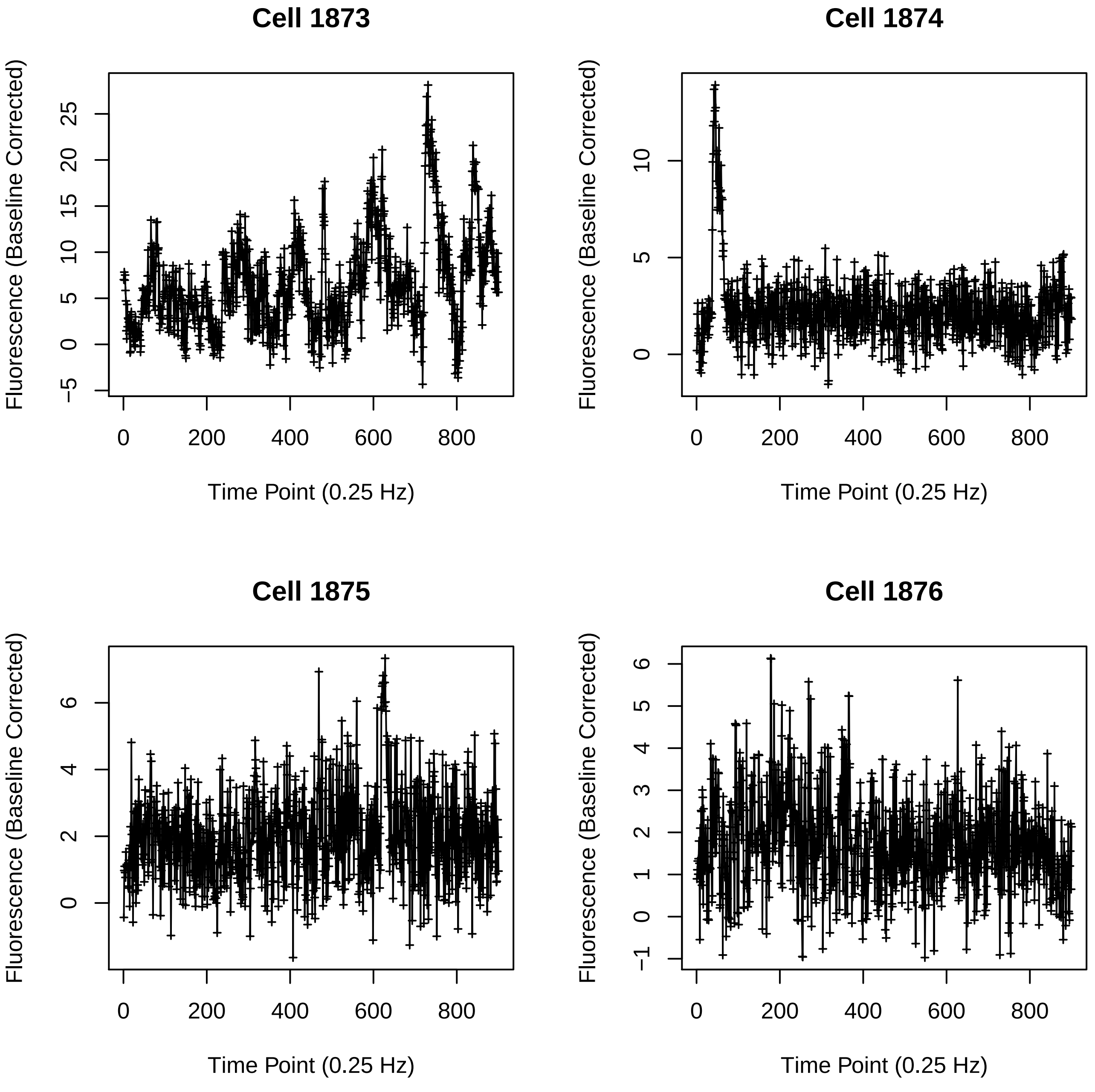
Cell 1873
Cell 1874
Cell 1875
Cell 1876
Fluorescence (Baseline Corrected)
Time Point (0.25 Hz)
0
200
400
600
800
−5
0
5
10
15
20
25
2
4
6
−1
1
3

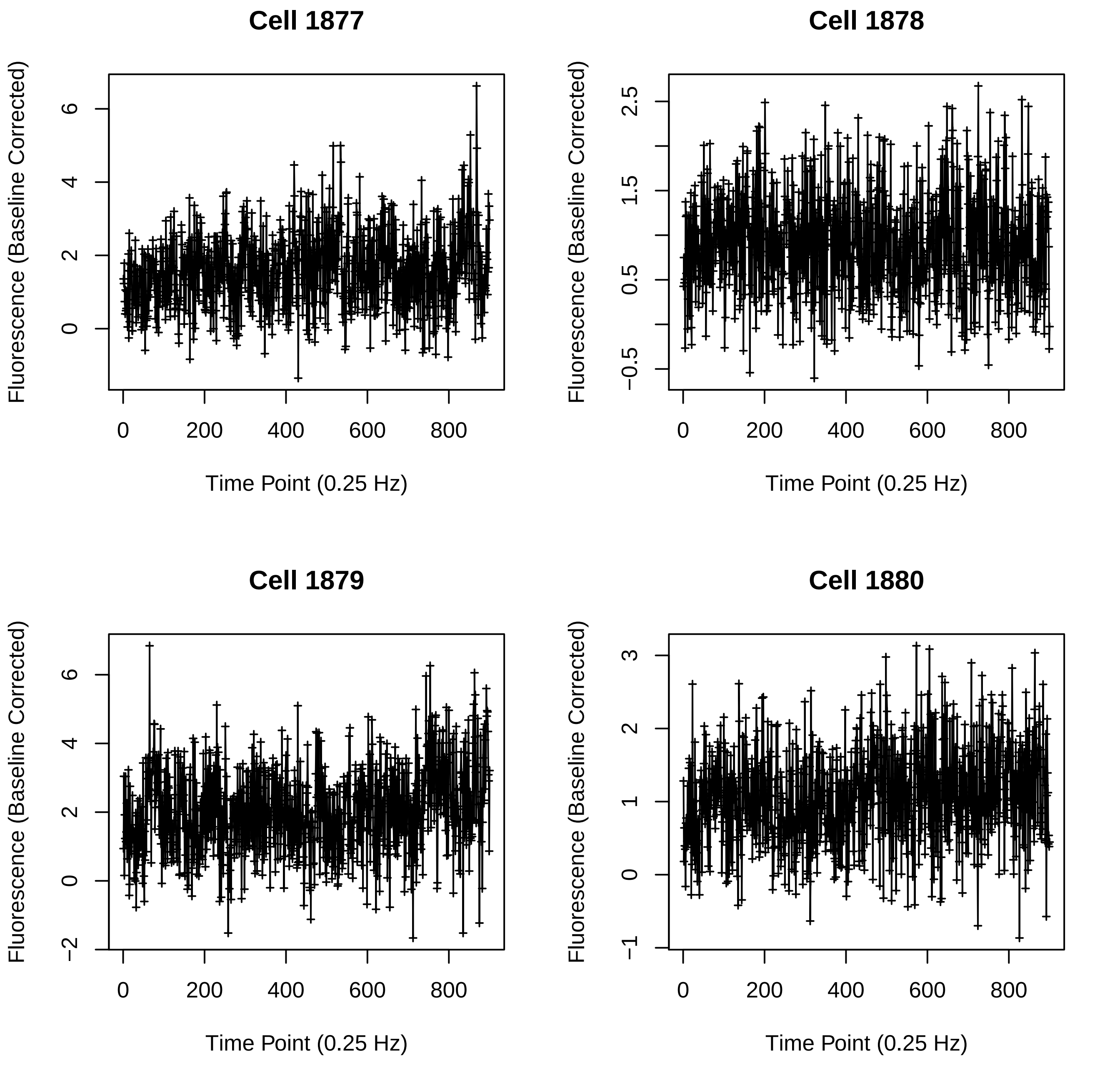
Cell 1877
Cell 1878
Cell 1879
Cell 1880
Fluorescence (Baseline Corrected)
Time Point (0.25 Hz)
0
200
400
600
800
-0.5
0.5
1.5
2.5
-2
-1
0
1
2
3
4
6

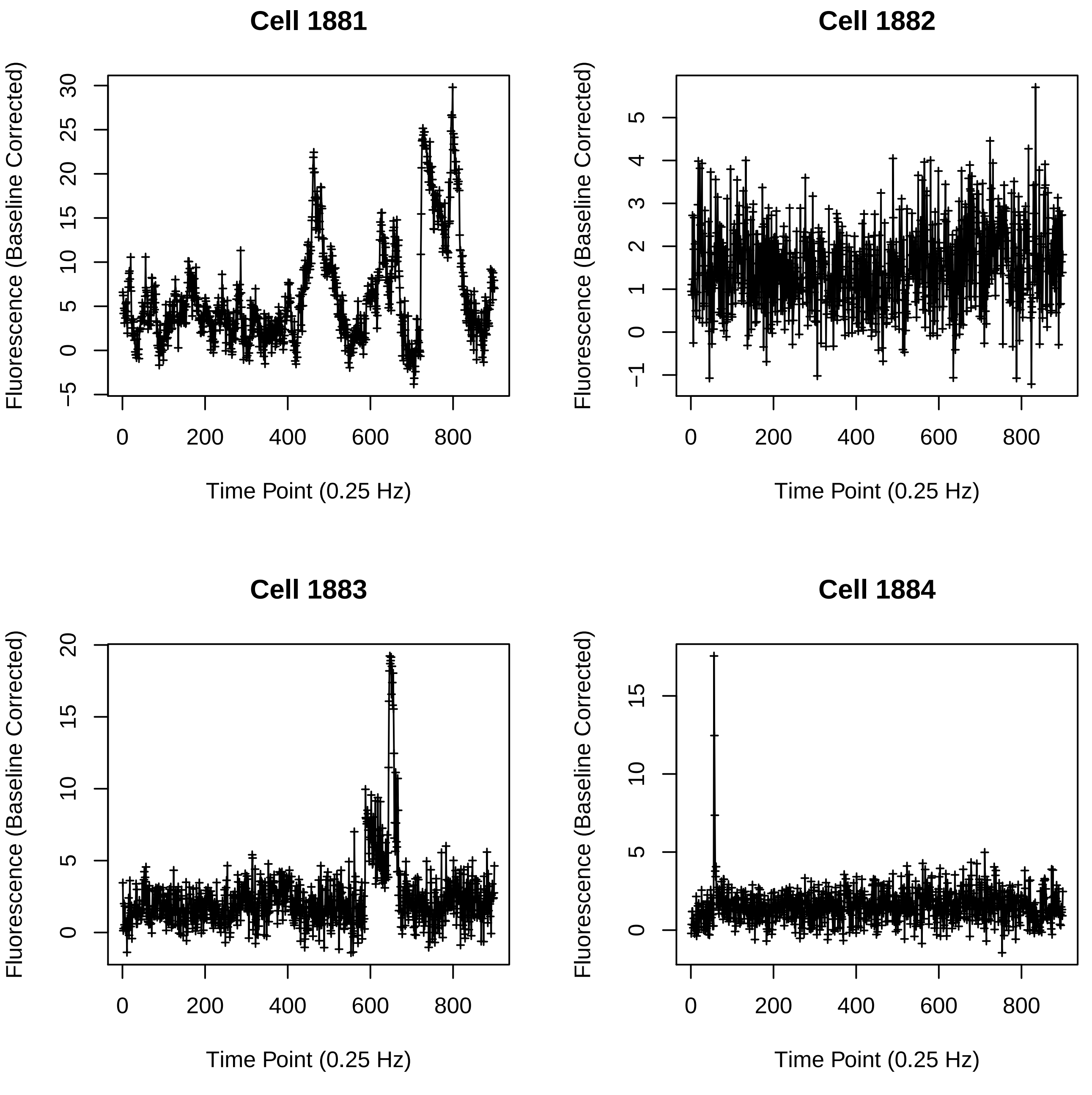
Cell 1881
Cell 1882
Cell 1883
Cell 1884
Fluorescence (Baseline Corrected)
Time Point (0.25 Hz)
0
200
400
600
800
−5
0
5
10
15
20
25
30
−1
1
2
3
4

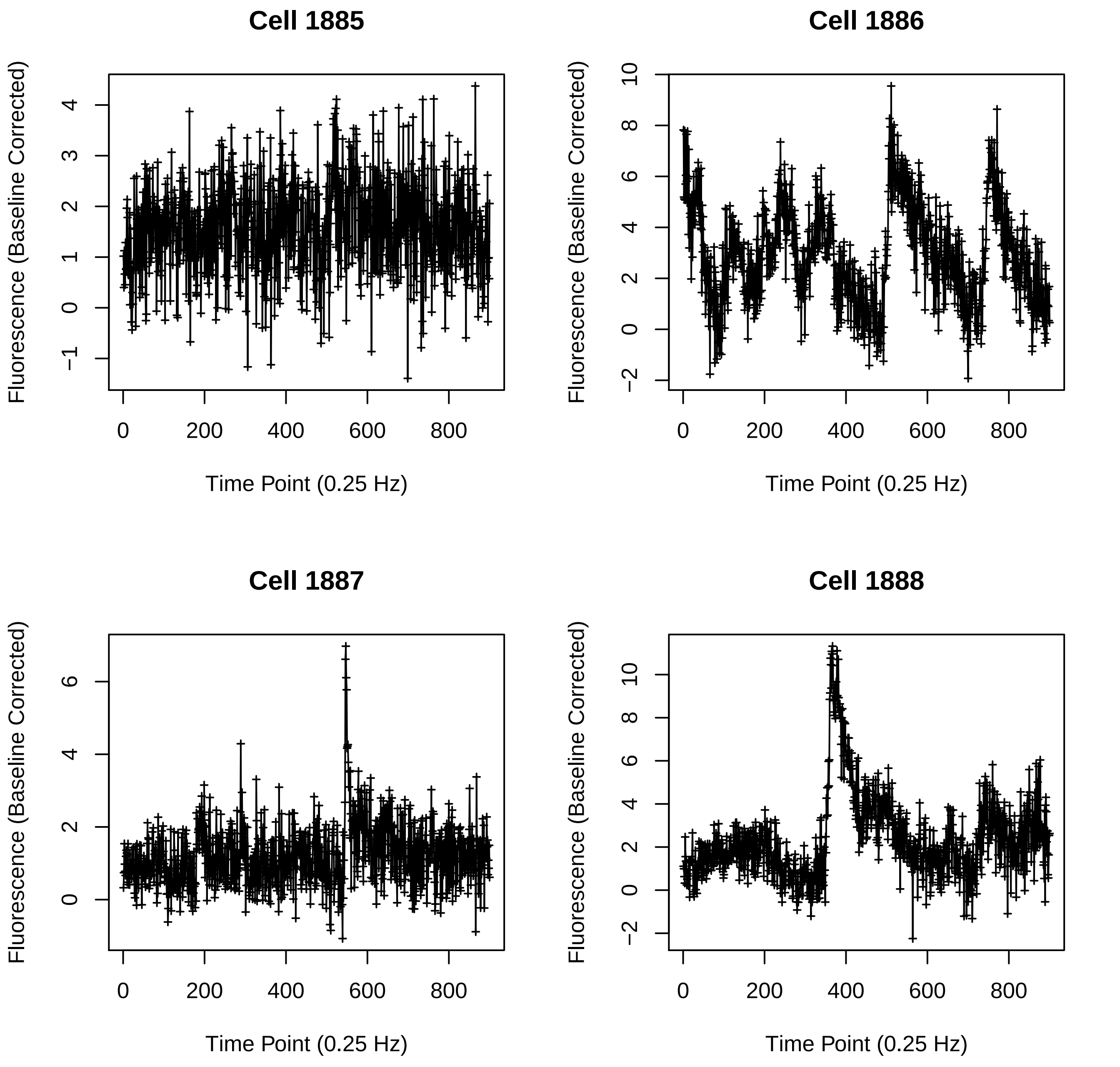
Cell 1885
Cell 1886
Cell 1887
Cell 1888
Fluorescence (Baseline Corrected)
Time Point (0.25 Hz)
−1
0
1
2
3
4
−2
0
2
4
6
8
10
0
200
400
600
800

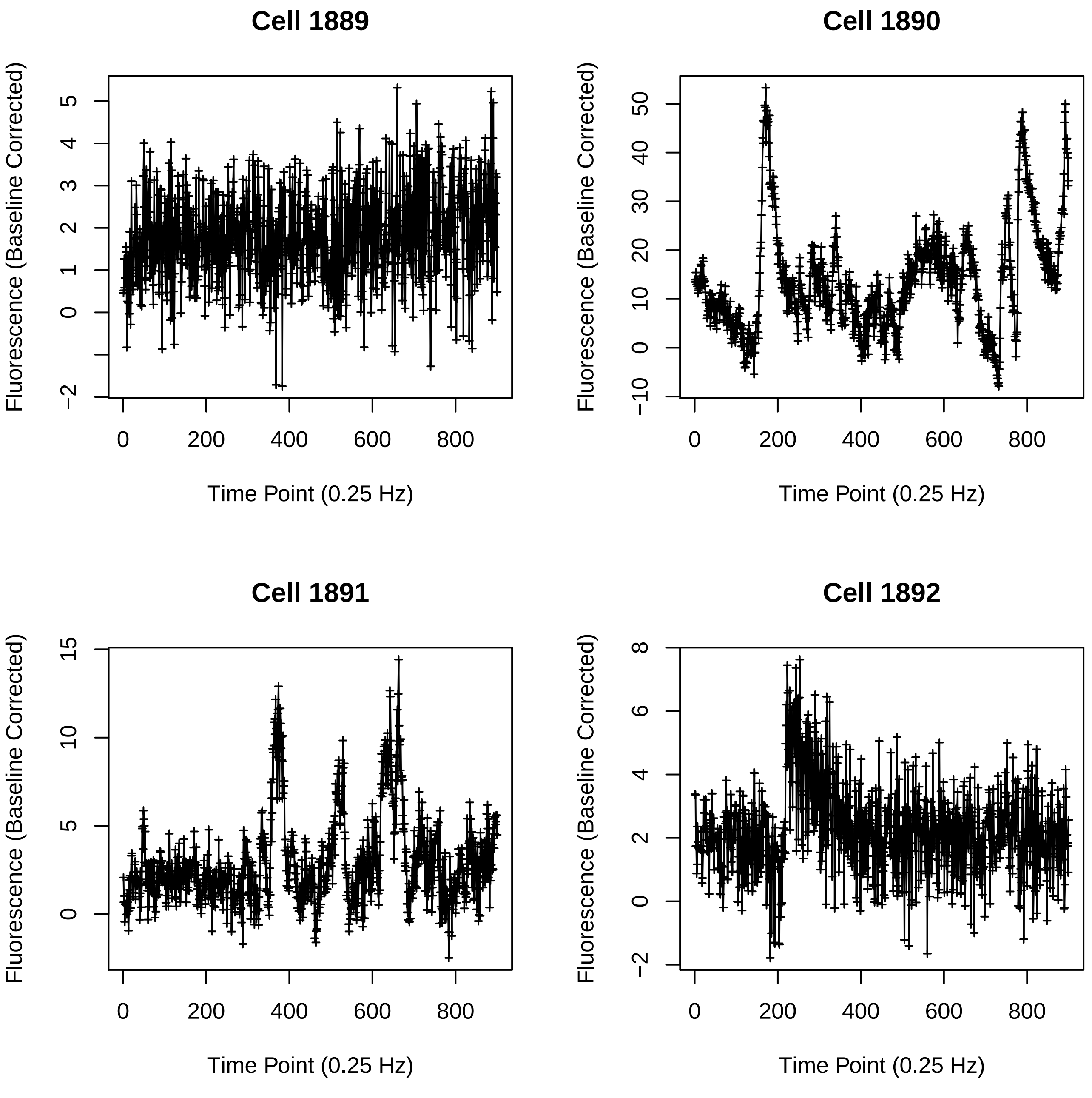

Cell 1889
Fluorescence (Baseline Corrected)
Time Point (0.25 Hz)
−2
0
1
2
3
4
5
0
200
400
600
800
Cell 1890
Fluorescence (Baseline Corrected)
Time Point (0.25 Hz)
−10
0
10
20
30
40
50
0
200
400
600
800
Cell 1891
Fluorescence (Baseline Corrected)
Time Point (0.25 Hz)
0
5
10
15
0
200
400
600
800
Cell 1892
Fluorescence (Baseline Corrected)
Time Point (0.25 Hz)
−2
0
2
4
6
8
0
200
400
600
800

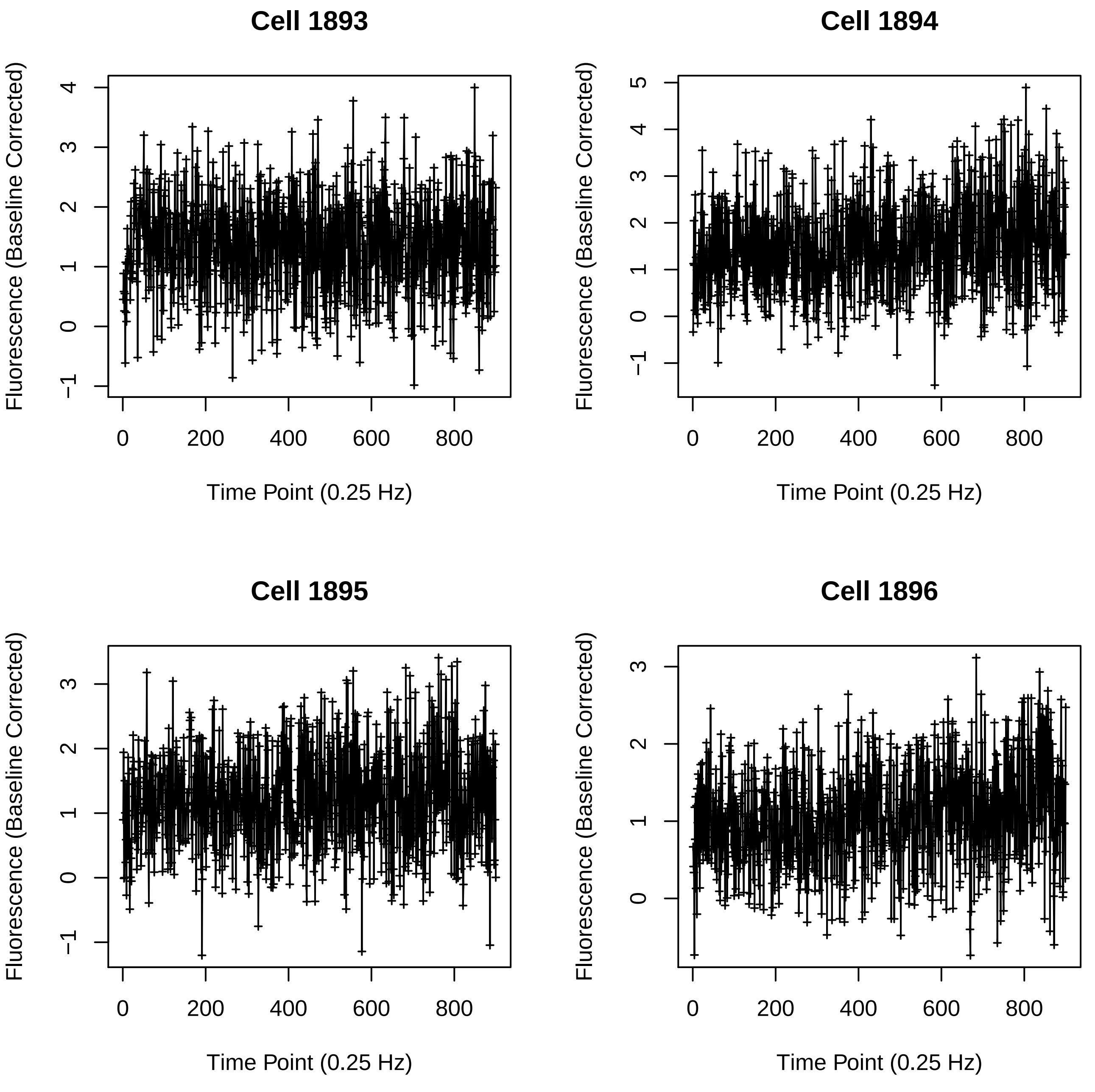
Cell 1893
Cell 1894
Cell 1895
Cell 1896
Fluorescence (Baseline Corrected)
Time Point (0.25 Hz)
0
200
400
600
800
−1
0
1
2
3
4
5

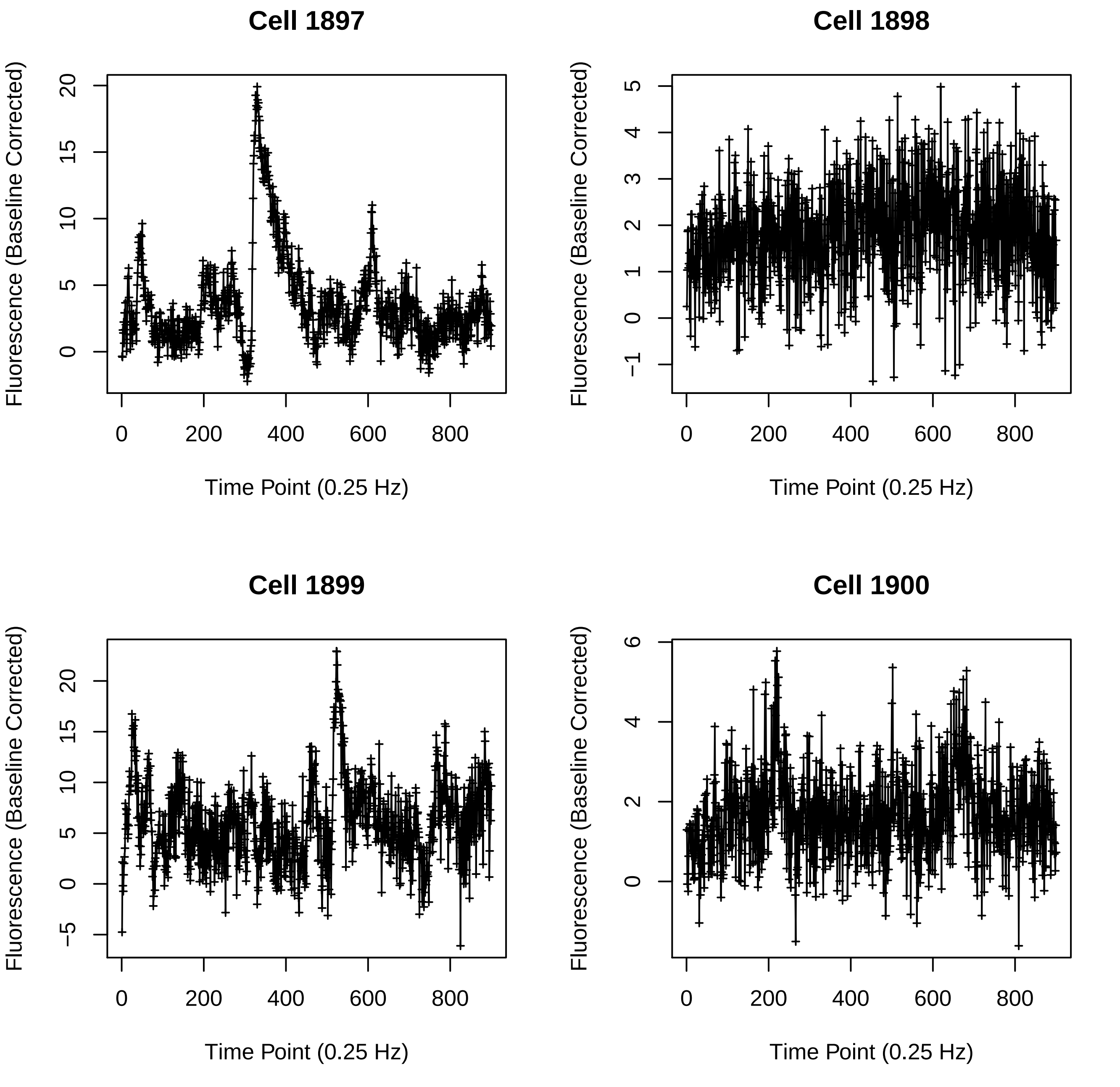
Cell 1897
Cell 1898
Cell 1899
Cell 1900
Fluorescence (Baseline Corrected)
Time Point (0.25 Hz)
0
200
400
600
800
0
5
10
15
20
−1
1
2
3
4
5
−5
0
2
4
6

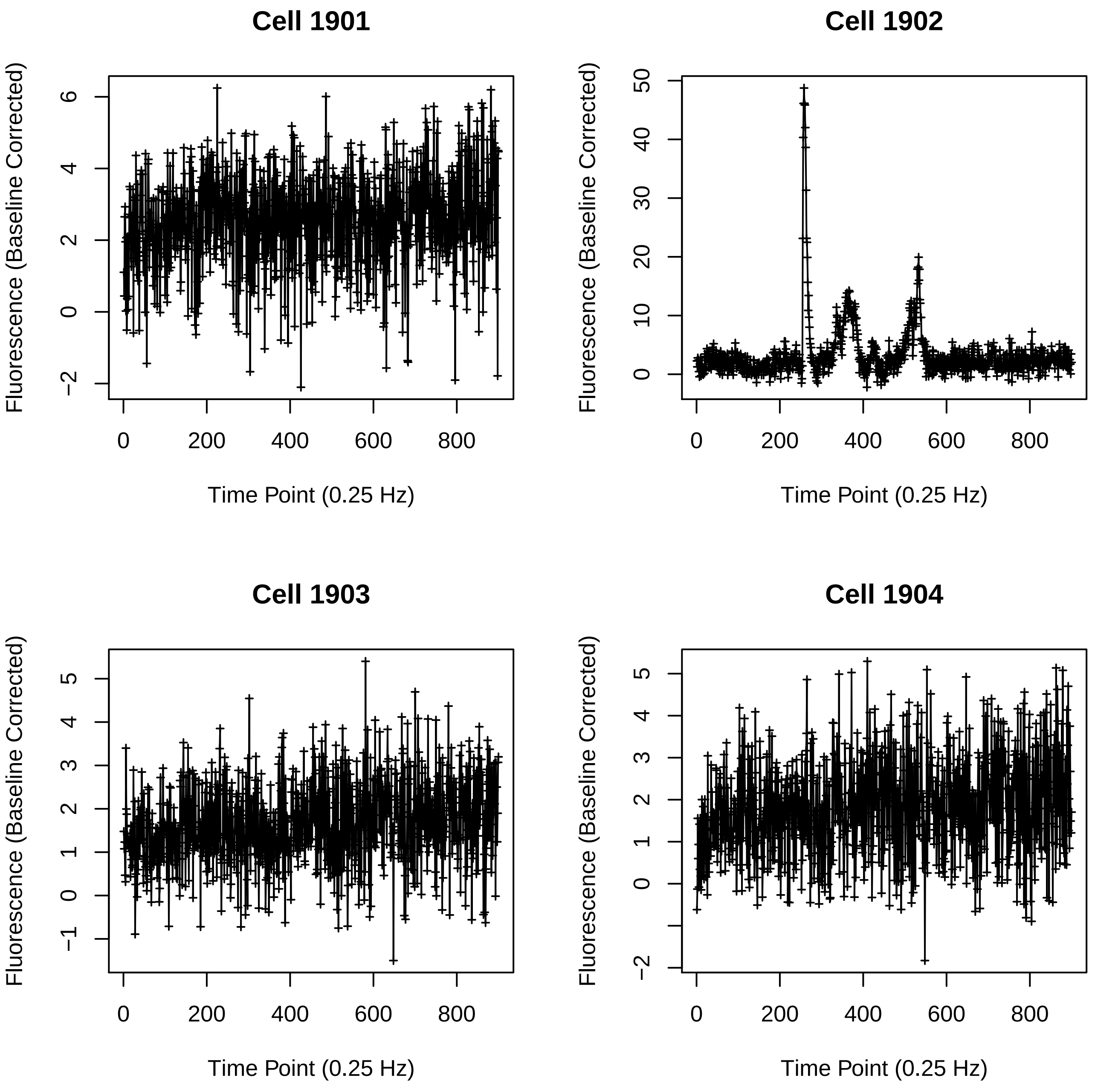
Cell 1901
Cell 1902
Cell 1903
Cell 1904
Fluorescence (Baseline Corrected)
Time Point (0.25 Hz)
−2
0
2
4
6
10
20
30
40
50
−1
1
3
5
200
400
600
800

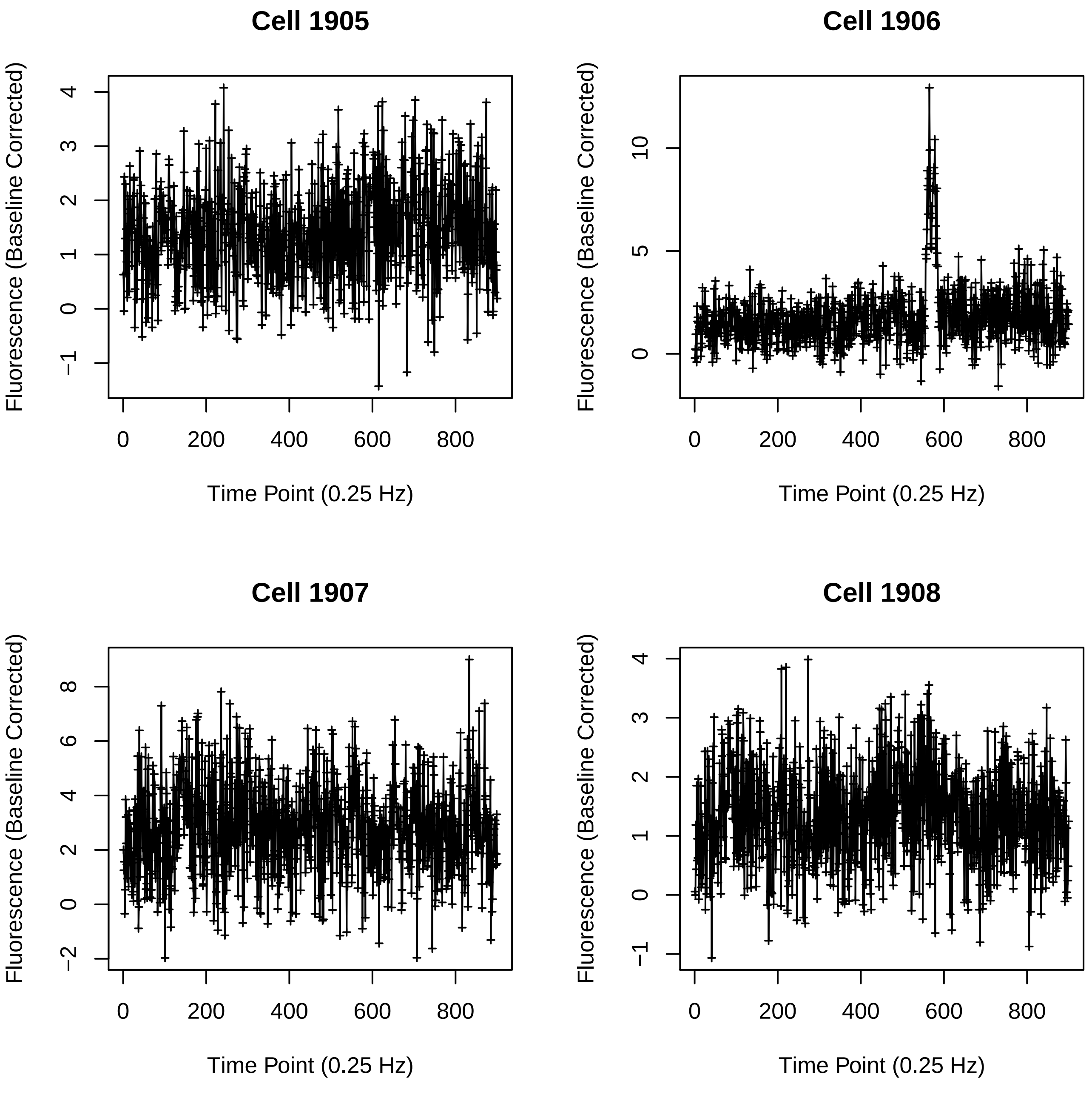
Cell 1905
Cell 1906
Cell 1907
Cell 1908
Fluorescence (Baseline Corrected)
Time Point (0.25 Hz)
0
200
400
600
800
−1
1
2
3
4
5
10
−2
6
8

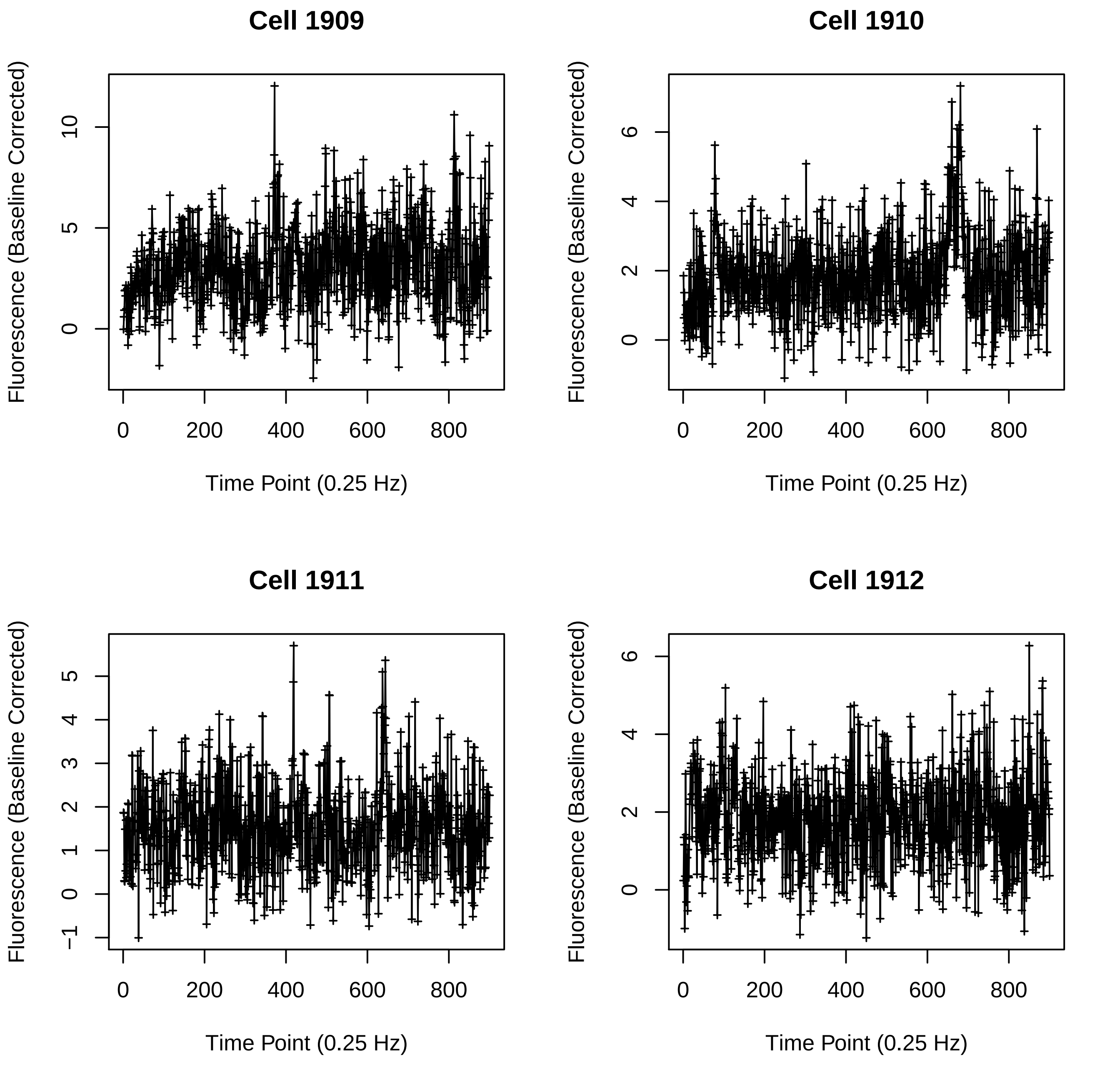

Cell 1909
Cell 1910
Cell 1911
Cell 1912
Fluorescence (Baseline Corrected)
Time Point (0.25 Hz)
0
200
400
600
800
0
5
10
2
4
6
−1
1
3

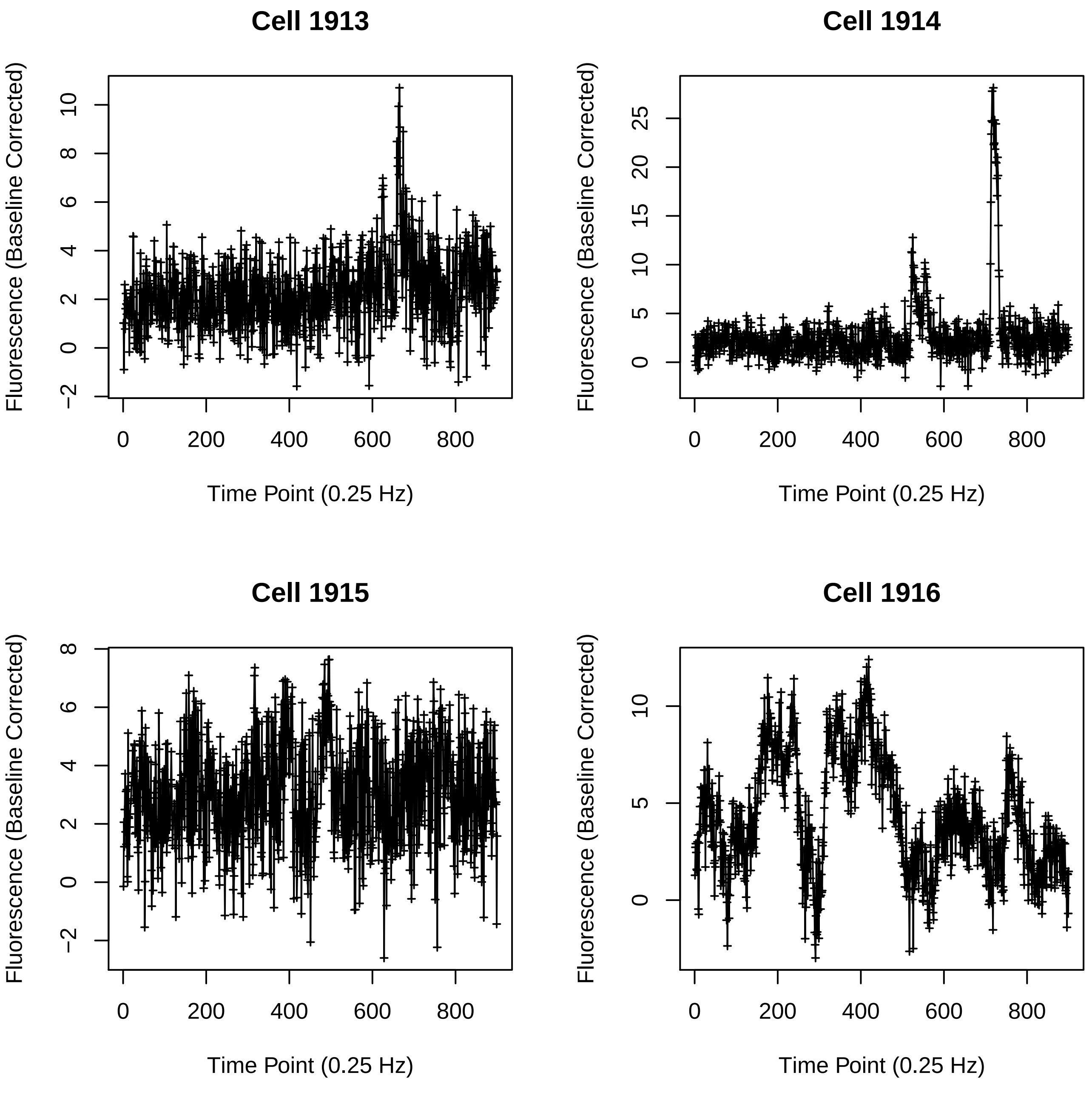
Cell 1913
Cell 1914
Cell 1915
Cell 1916
Fluorescence (Baseline Corrected)
Time Point (0.25 Hz)
−2
0
2
4
6
8
10
5
15
20
25
200
400
600
800

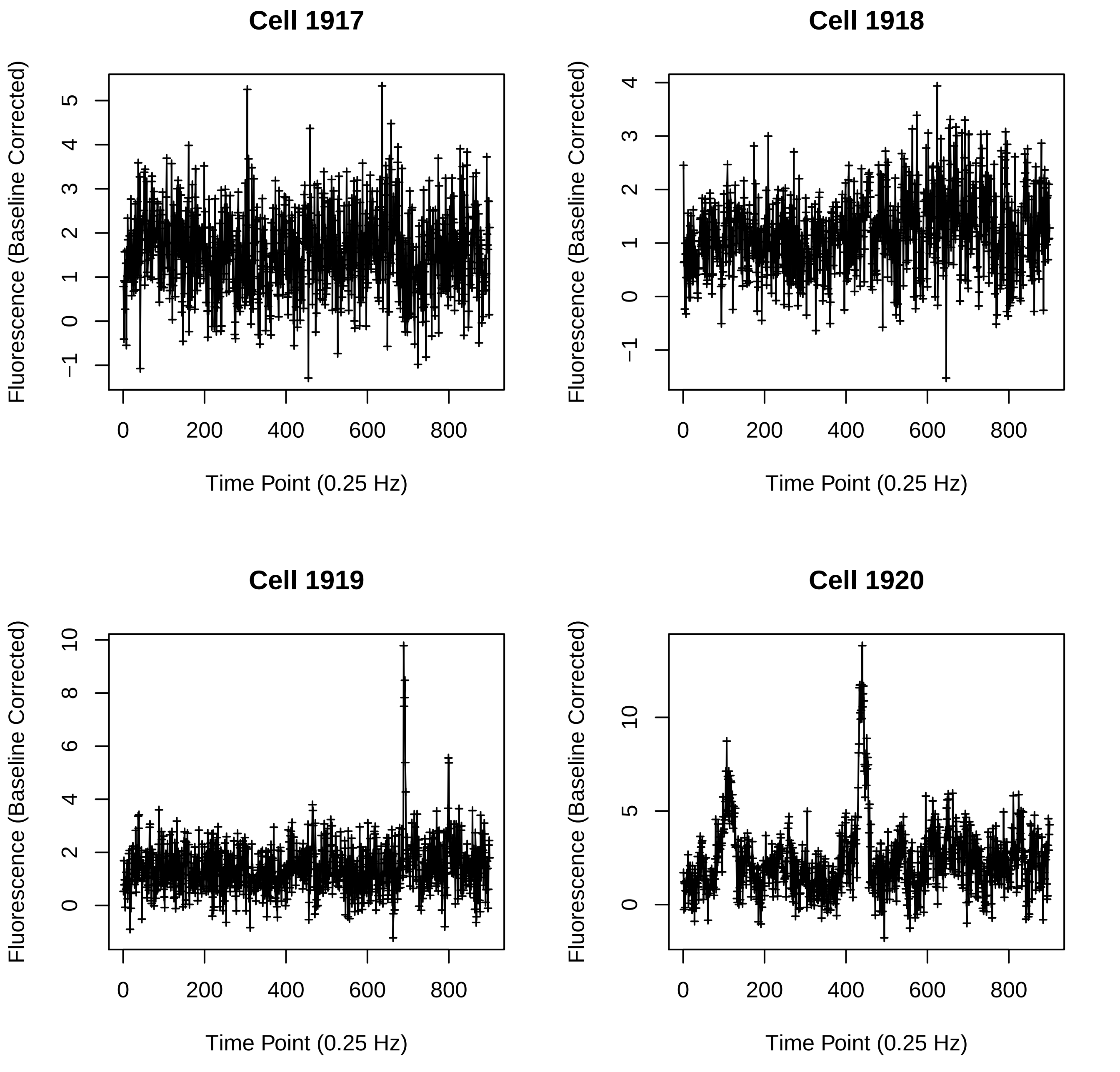
Cell 1917
Cell 1918
Cell 1919
Cell 1920
Fluorescence (Baseline Corrected)
Time Point (0.25 Hz)

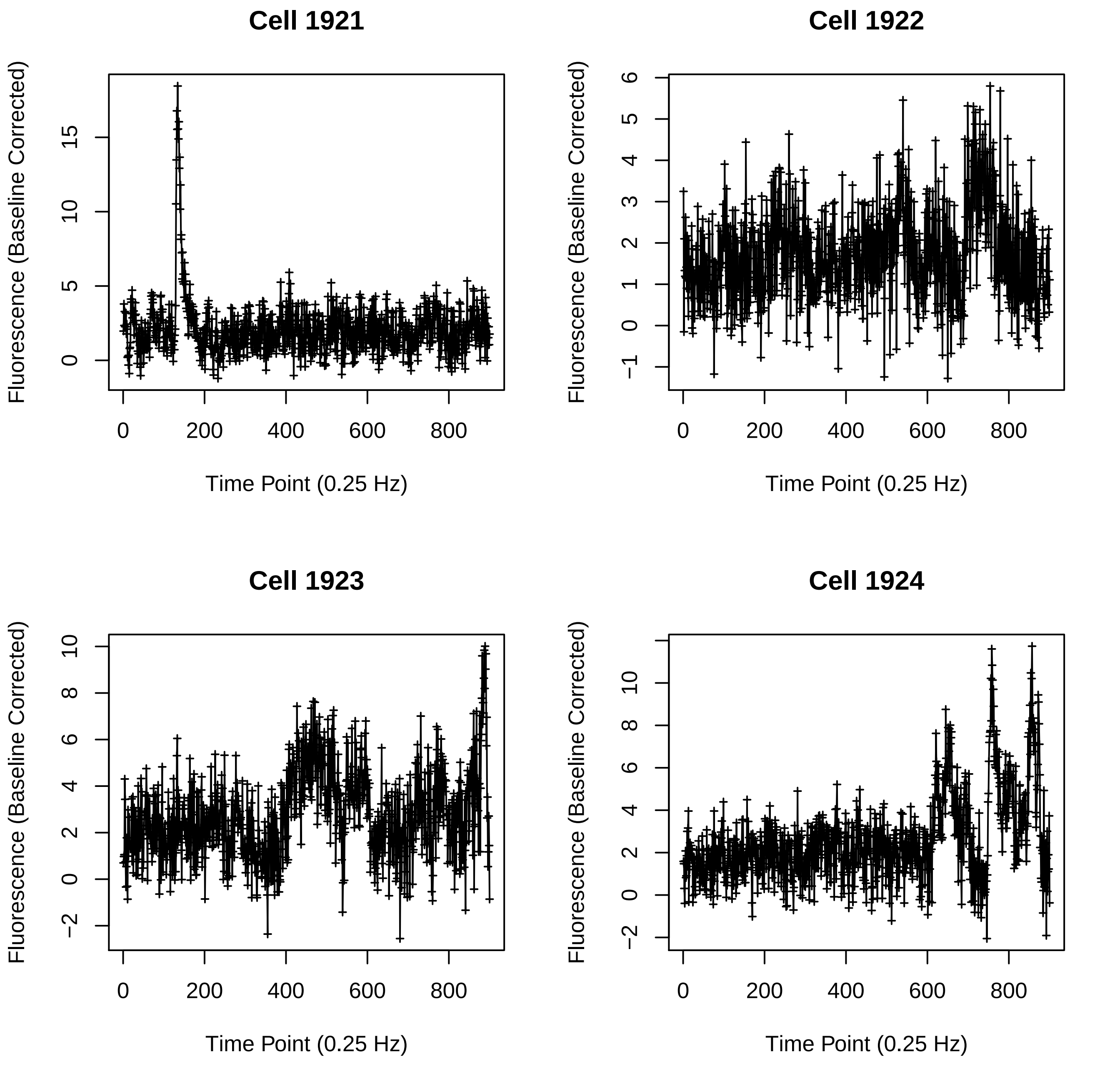

Cell 1921
Cell 1922
Cell 1923
Cell 1924
Fluorescence (Baseline Corrected)
Time Point (0.25 Hz)

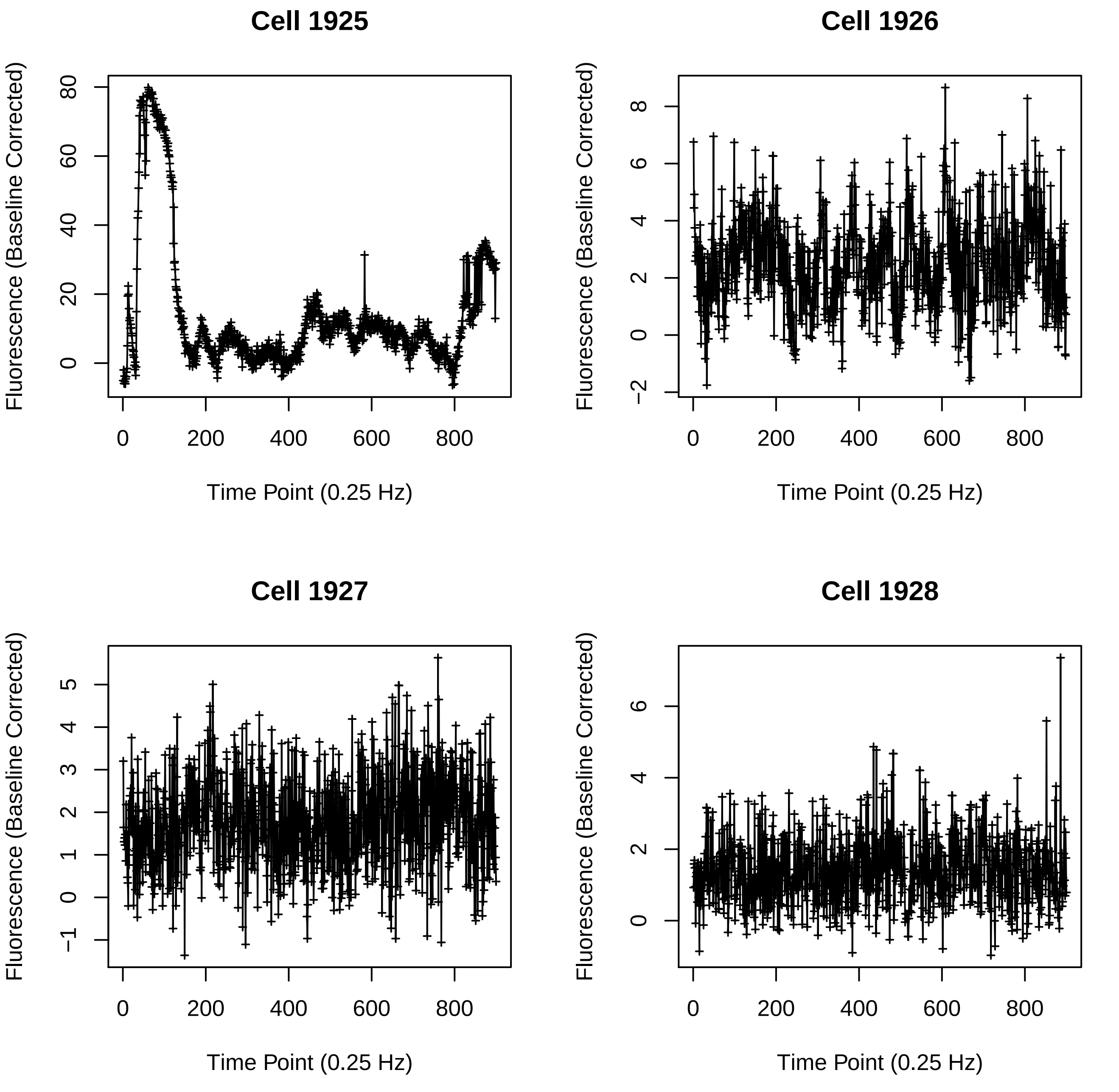
Cell 1925
Cell 1926
Cell 1927
Cell 1928
Fluorescence (Baseline Corrected)
Time Point (0.25 Hz)
0
200
400
600
800
20
40
60
80
−2
2
4
6
8
−1
1
3
5

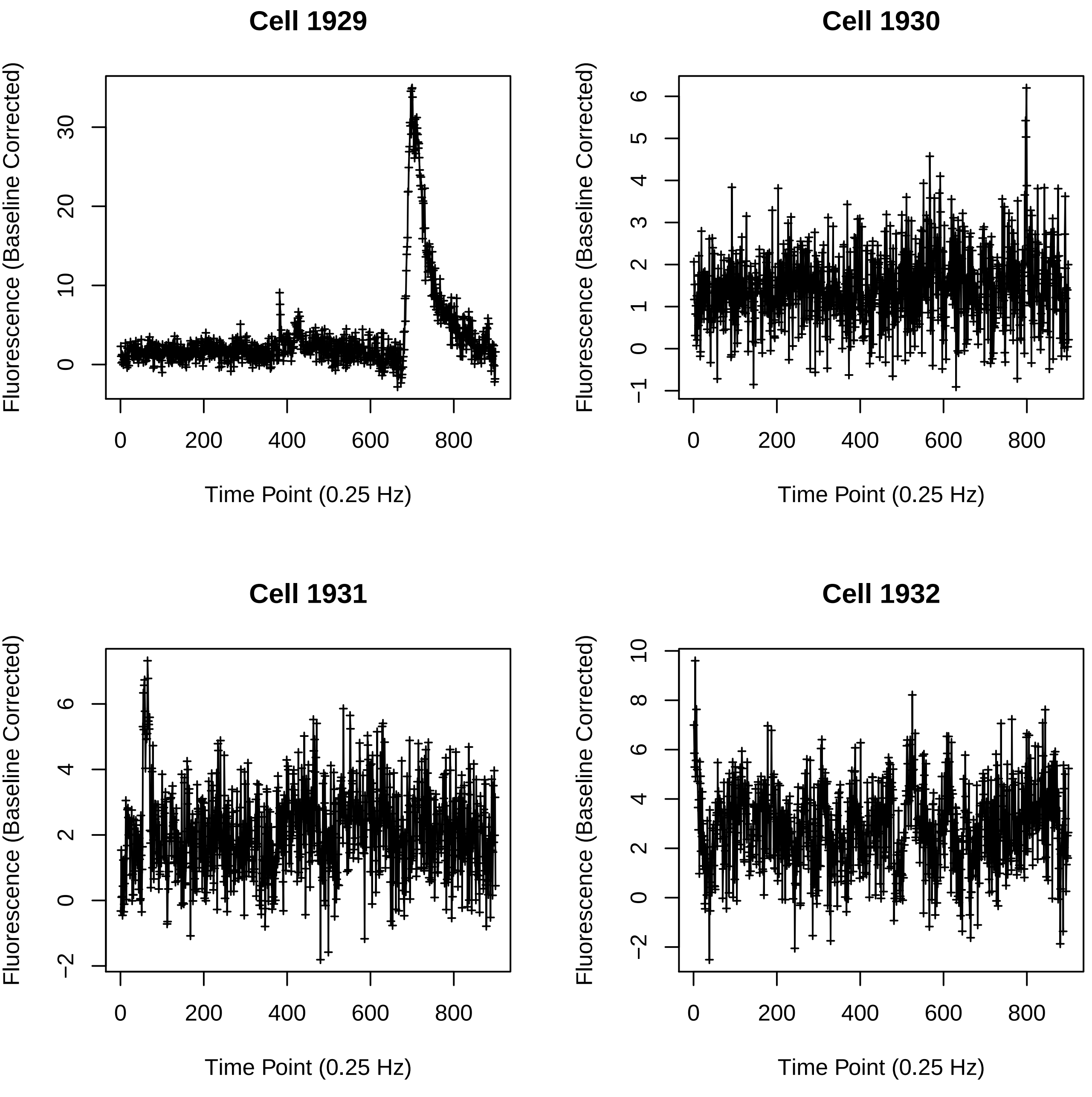
Cell 1929
Fluorescence (Baseline Corrected)
0
10
20
30
0
200
400
600
800
Time Point (0.25 Hz)
Cell 1930
Fluorescence (Baseline Corrected)
−1
0
1
2
3
4
5
6
0
200
400
600
800
Time Point (0.25 Hz)
Cell 1931
Fluorescence (Baseline Corrected)
−2
0
2
4
6
0
200
400
600
800
Time Point (0.25 Hz)
Cell 1932
Fluorescence (Baseline Corrected)
−2
0
2
4
6
8
10
0
200
400
600
800
Time Point (0.25 Hz)

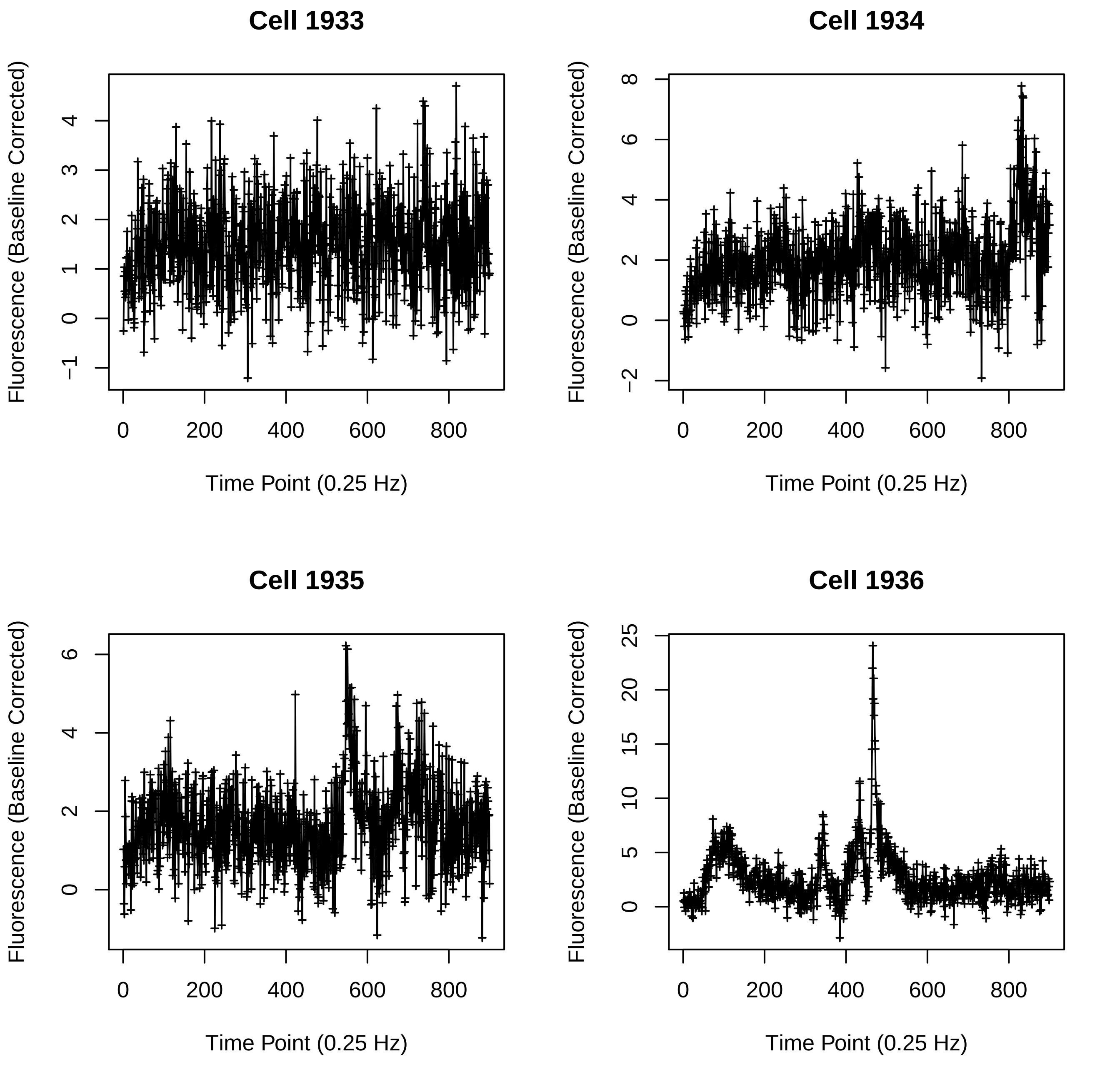
Cell 1933
Cell 1934
Cell 1935
Cell 1936
Fluorescence (Baseline Corrected)
Time Point (0.25 Hz)
−1
0
1
2
3
4
−2
0
2
4
6
8
0
2
4
6
0
5
10
15
20
25
0
200
400
600
800

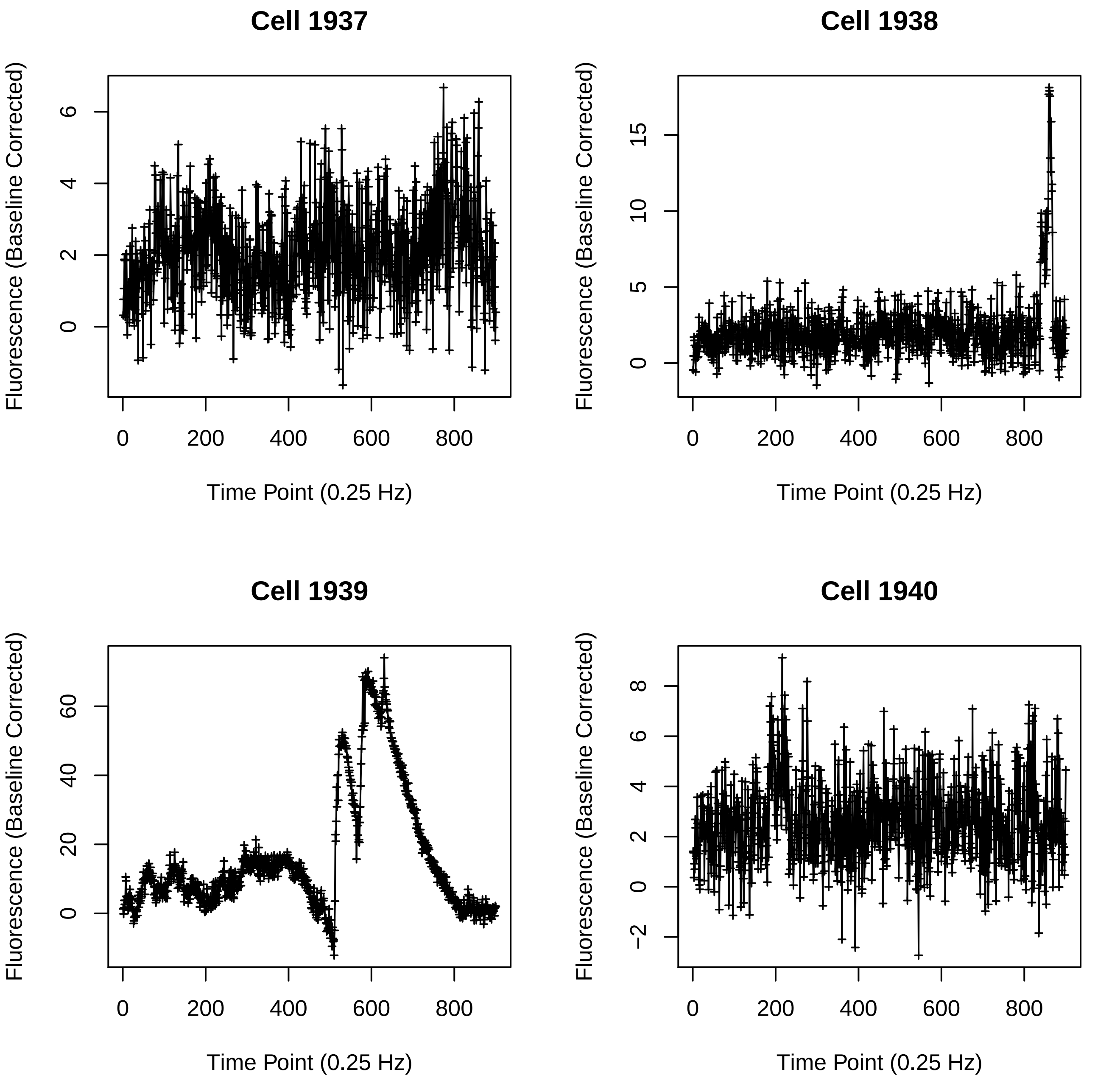
Cell 1937
Fluorescence (Baseline Corrected)
0
2
4
6
0
200
400
600
800
Time Point (0.25 Hz)
Cell 1938
Fluorescence (Baseline Corrected)
0
5
10
15
0
200
400
600
800
Time Point (0.25 Hz)
Cell 1939
Fluorescence (Baseline Corrected)
0
20
40
60
0
200
400
600
800
Time Point (0.25 Hz)
Cell 1940
Fluorescence (Baseline Corrected)
−2
0
2
4
6
8
0
200
400
600
800
Time Point (0.25 Hz)

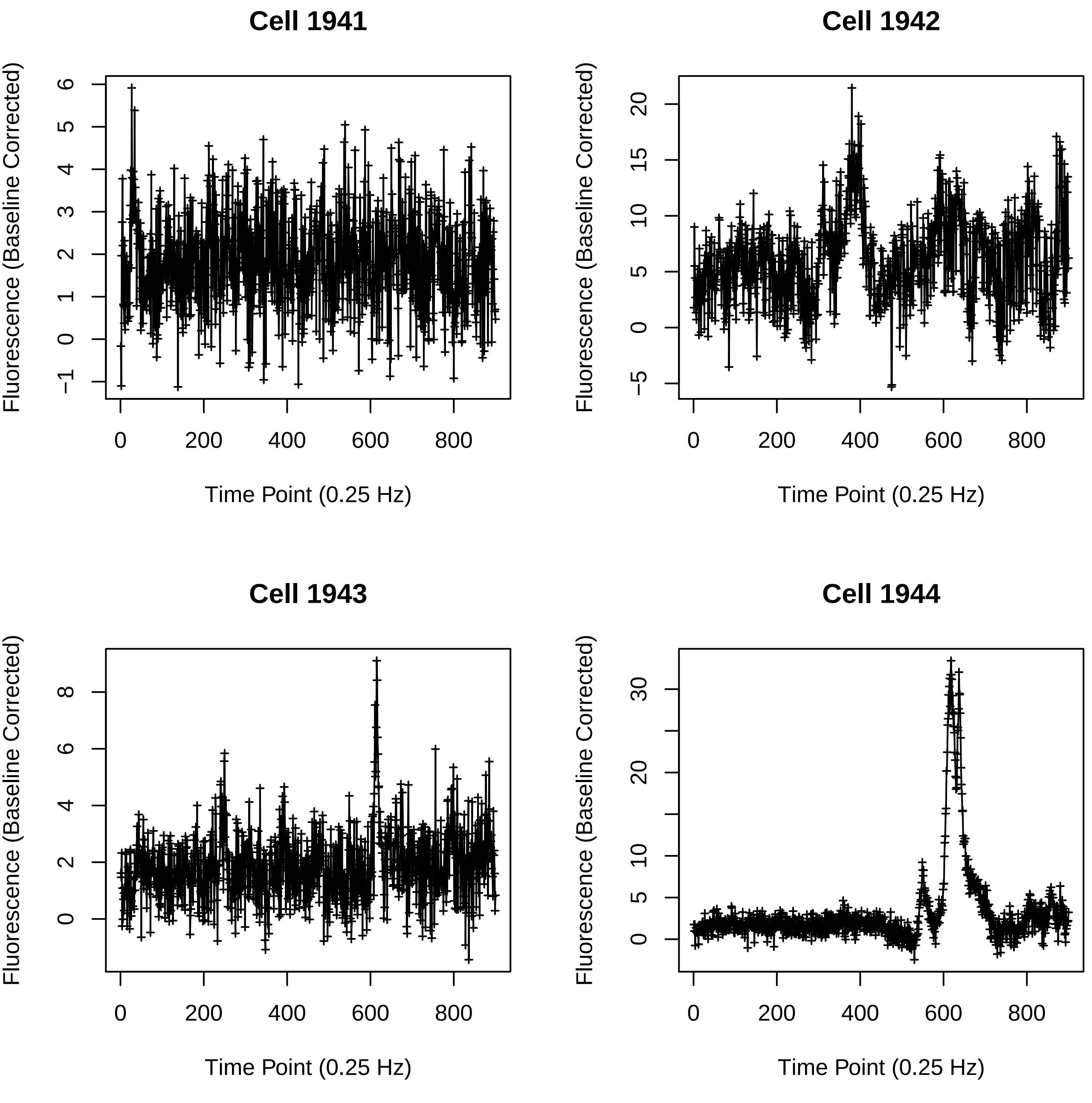

Cell 1941
Cell 1942
Cell 1943
Cell 1944
Fluorescence (Baseline Corrected)
Time Point (0.25 Hz)

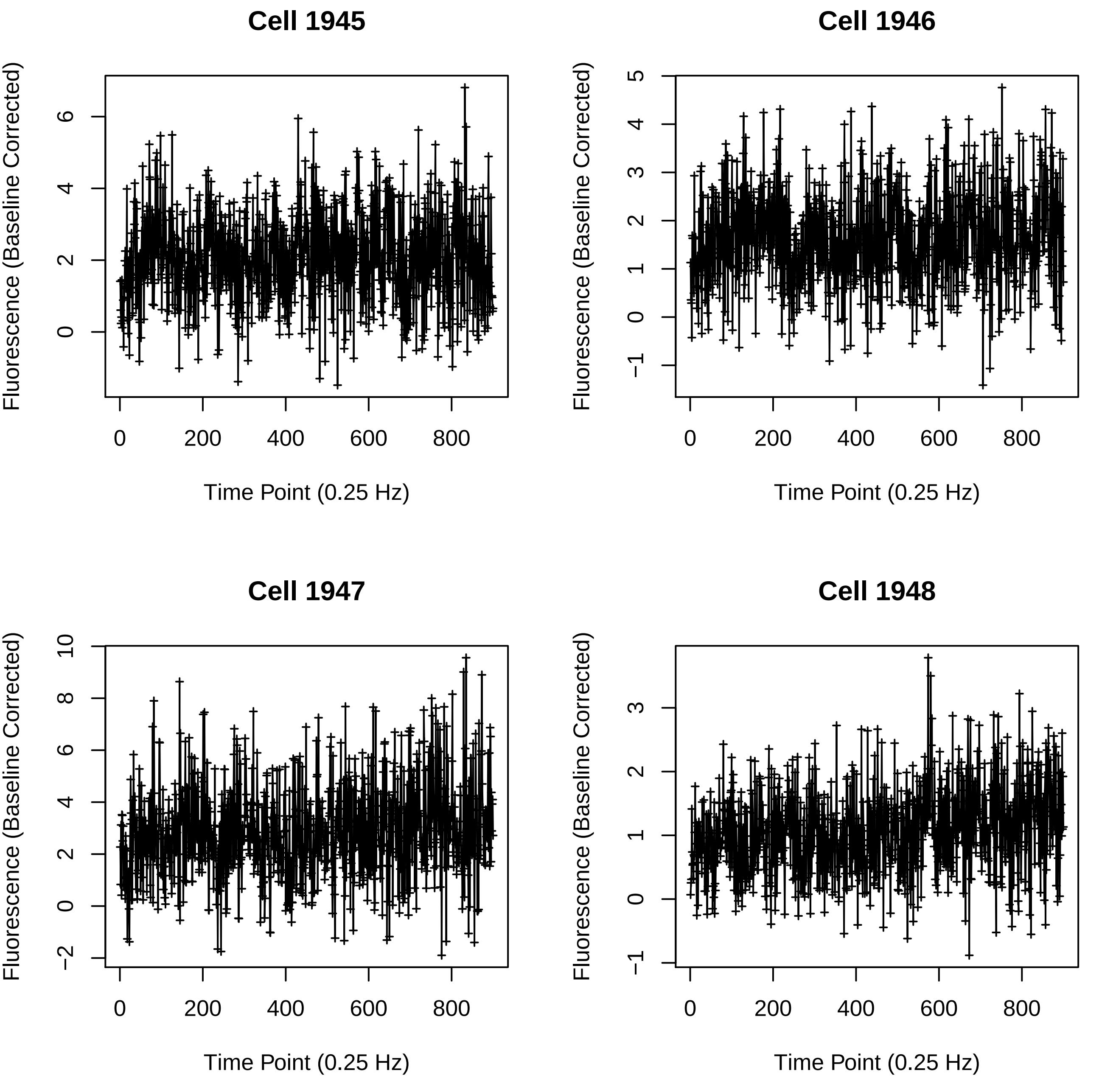
Cell 1945
Cell 1946
Cell 1947
Cell 1948
Fluorescence (Baseline Corrected)
Time Point (0.25 Hz)
0
200
400
600
800
−2
−1
0
1
2
3
4
5
6
8
10

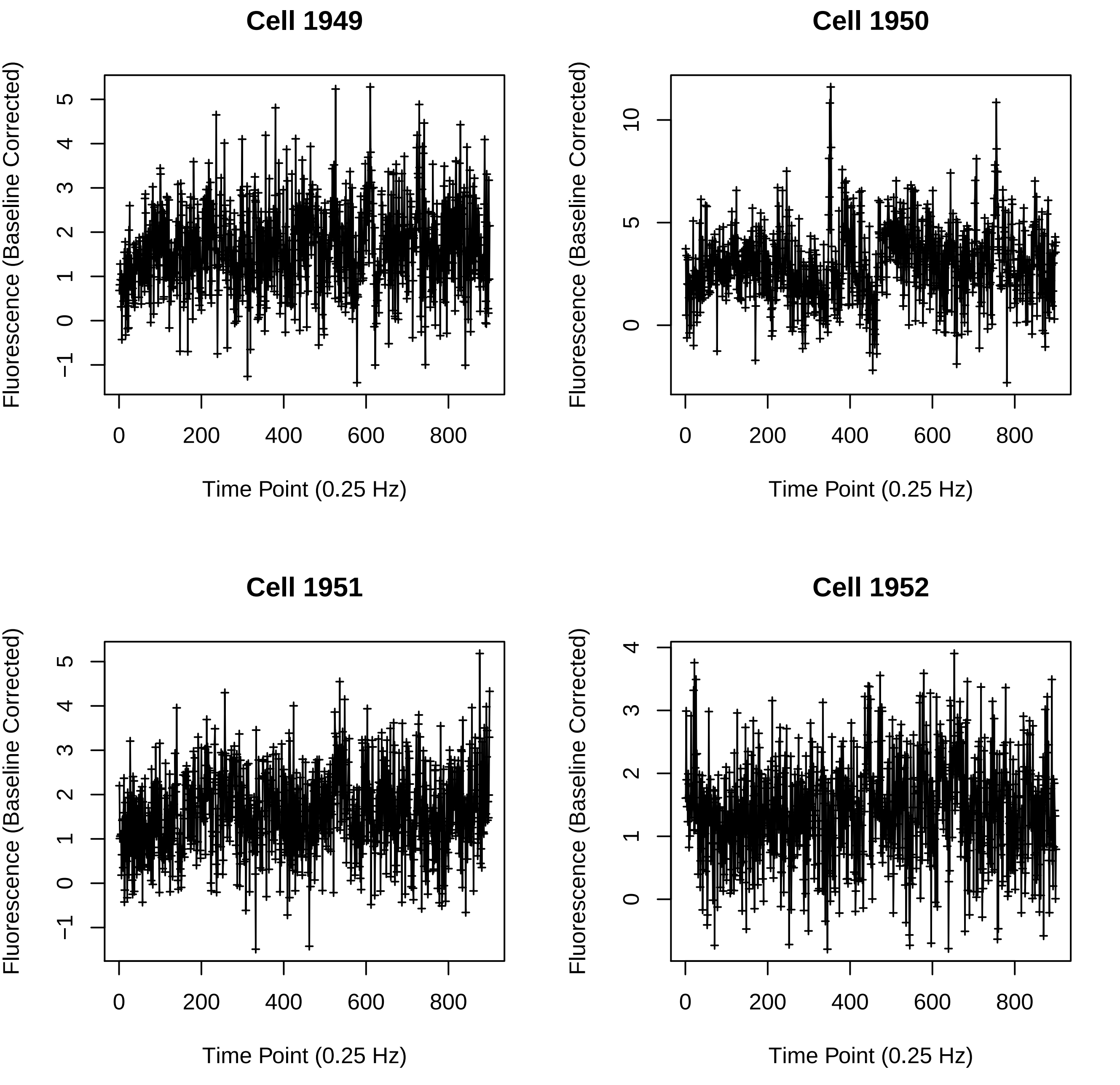
Cell 1949
Cell 1950
Cell 1951
Cell 1952
Fluorescence (Baseline Corrected)
Time Point (0.25 Hz)
−1
0
1
2
3
4
5
10
200
400
600
800

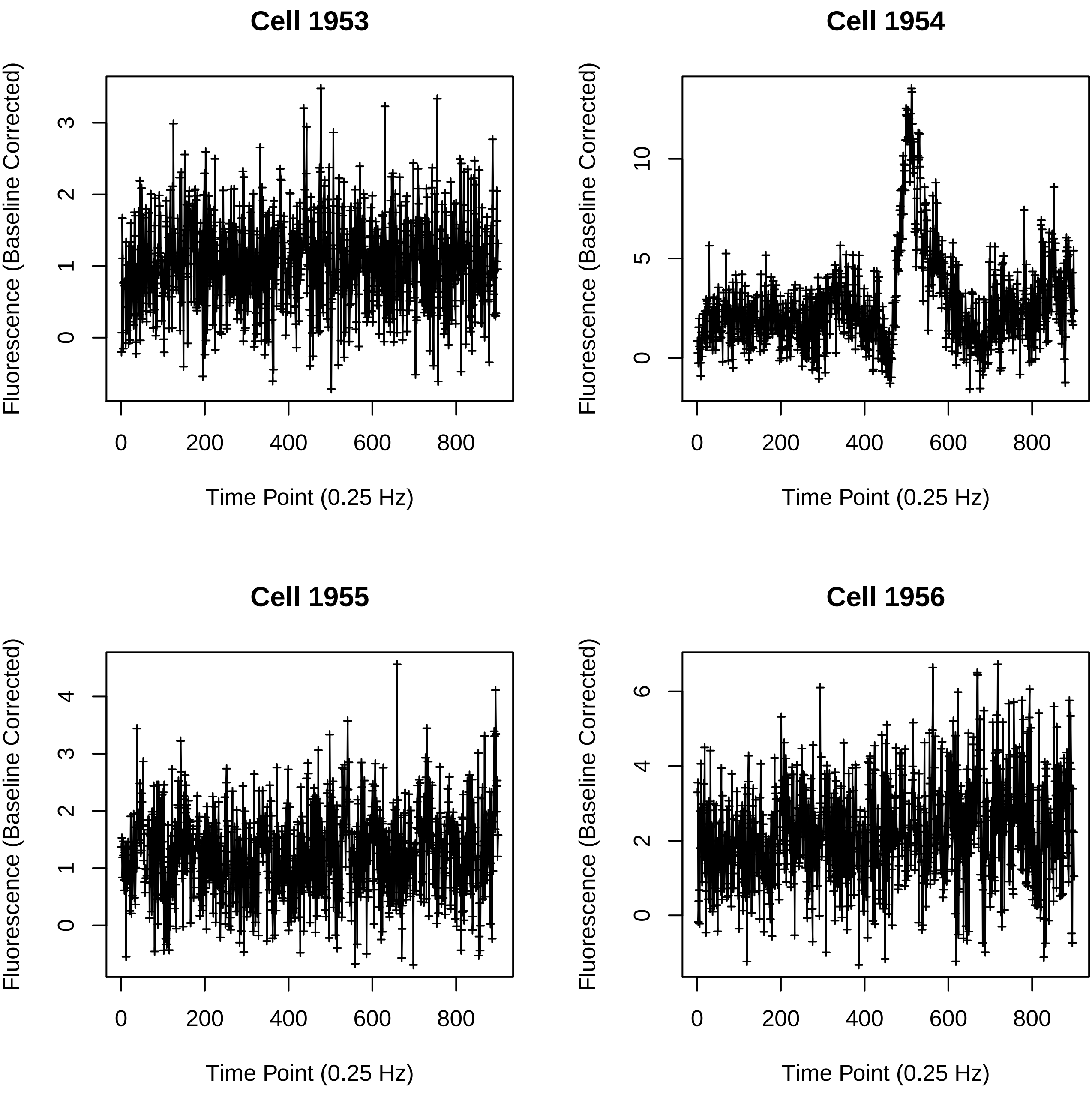

Cell 1953
Cell 1954
Cell 1955
Cell 1956
Fluorescence (Baseline Corrected)
Time Point (0.25 Hz)
0
200
400
600
800
1
2
3
5
10
4
6

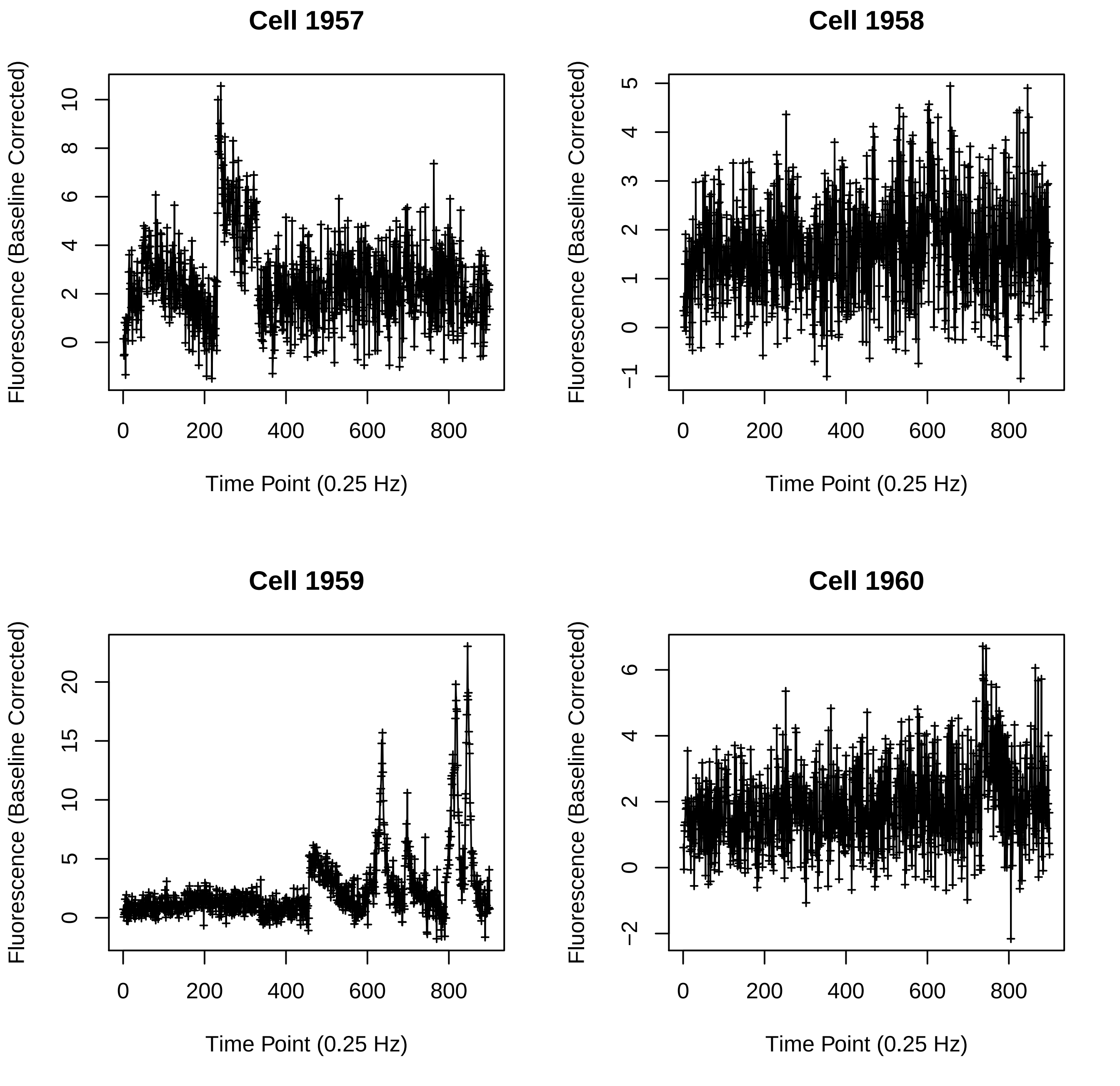

Cell 1957
Cell 1958
Cell 1959
Cell 1960
Fluorescence (Baseline Corrected)
Time Point (0.25 Hz)

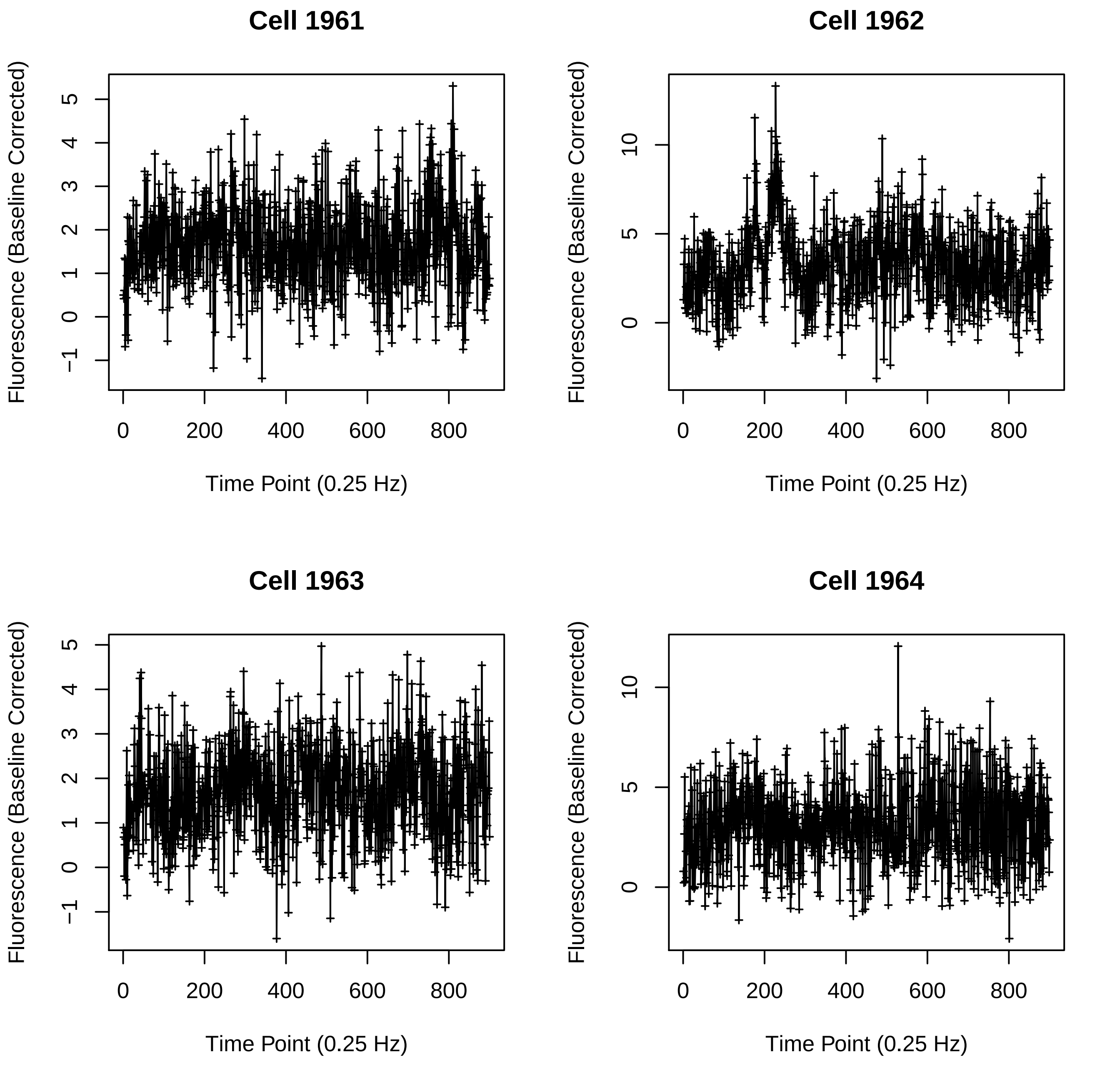

Cell 1961
Cell 1962
Cell 1963
Cell 1964
Fluorescence (Baseline Corrected)
Time Point (0.25 Hz)
0
200
400
600
800
−1
1
2
3
4
5
10

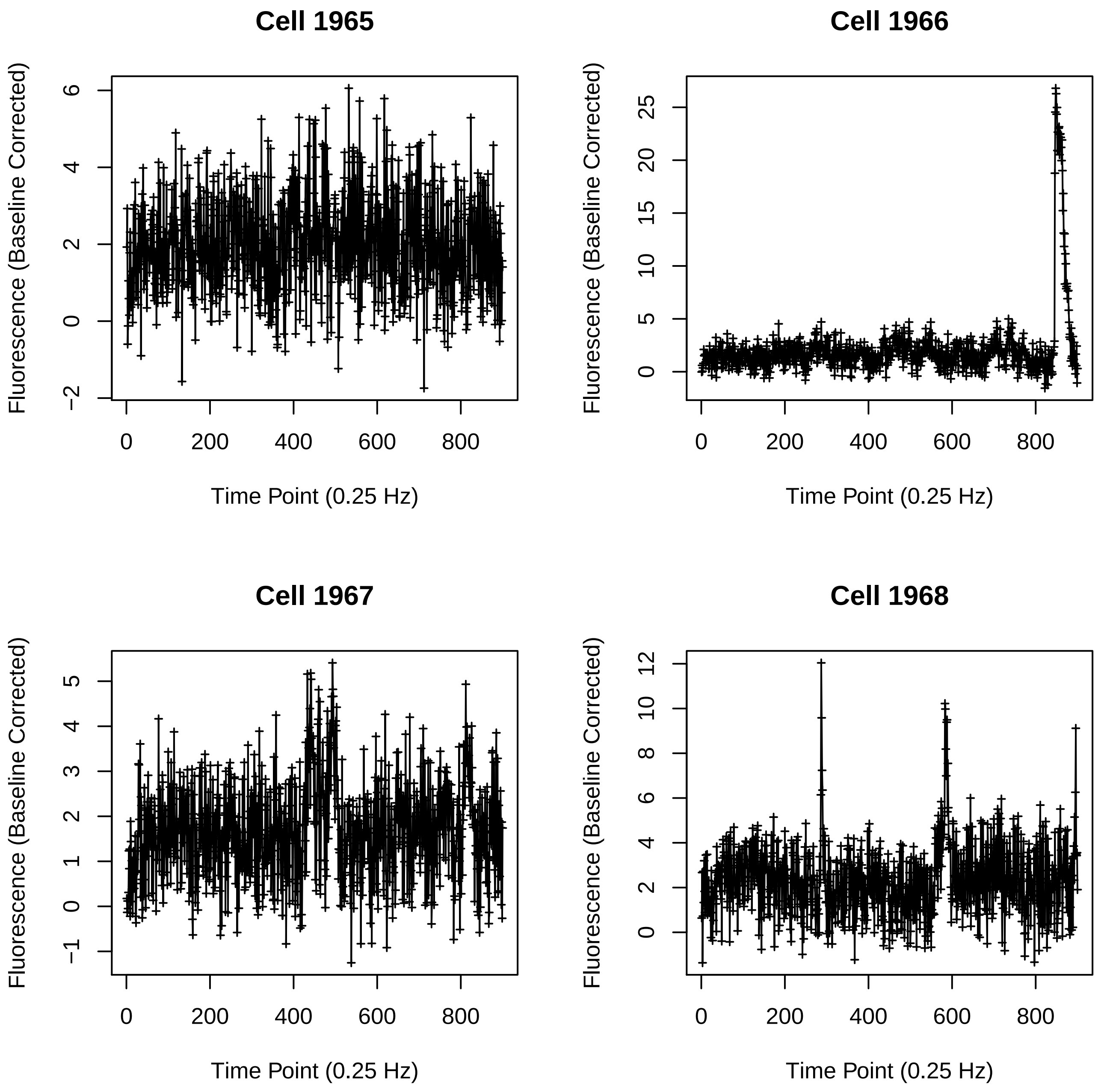
Cell 1965
Fluorescence (Baseline Corrected)
−2
0
2
4
6
0
200
400
600
800
Time Point (0.25 Hz)
Cell 1966
Fluorescence (Baseline Corrected)
0
5
10
15
20
25
0
200
400
600
800
Time Point (0.25 Hz)
Cell 1967
Fluorescence (Baseline Corrected)
−1
0
1
2
3
4
5
0
200
400
600
800
Time Point (0.25 Hz)
Cell 1968
Fluorescence (Baseline Corrected)
0
2
4
6
8
10
12
0
200
400
600
800
Time Point (0.25 Hz)

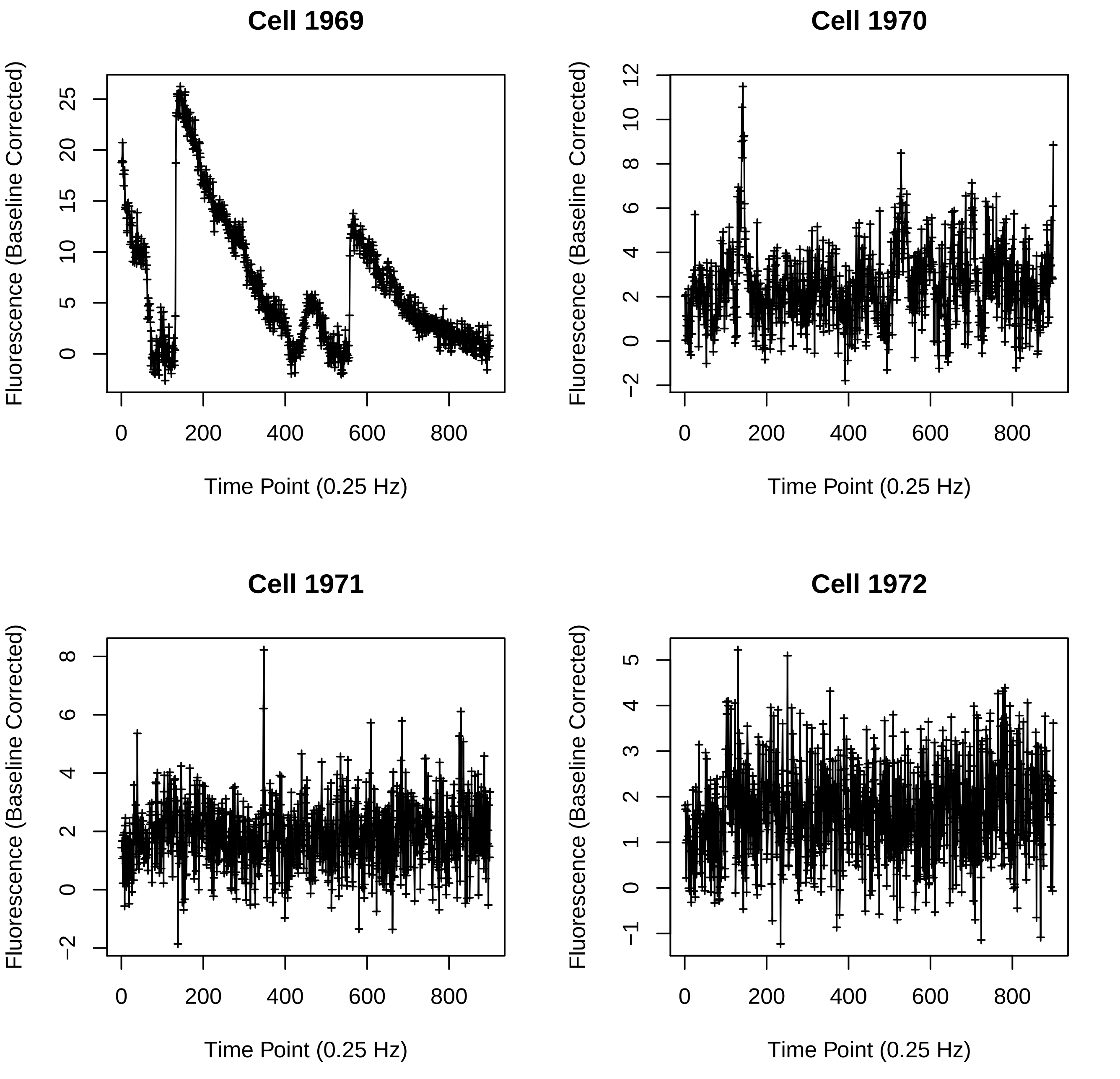
Cell 1969
Cell 1970
Cell 1971
Cell 1972
Fluorescence (Baseline Corrected)
Time Point (0.25 Hz)
0
200
400
600
800
0
5
10
15
20
25
−2
0
2
4
6
8
10
12
−2
0
2
4
6
8
−1
0
1
2
3
4
5

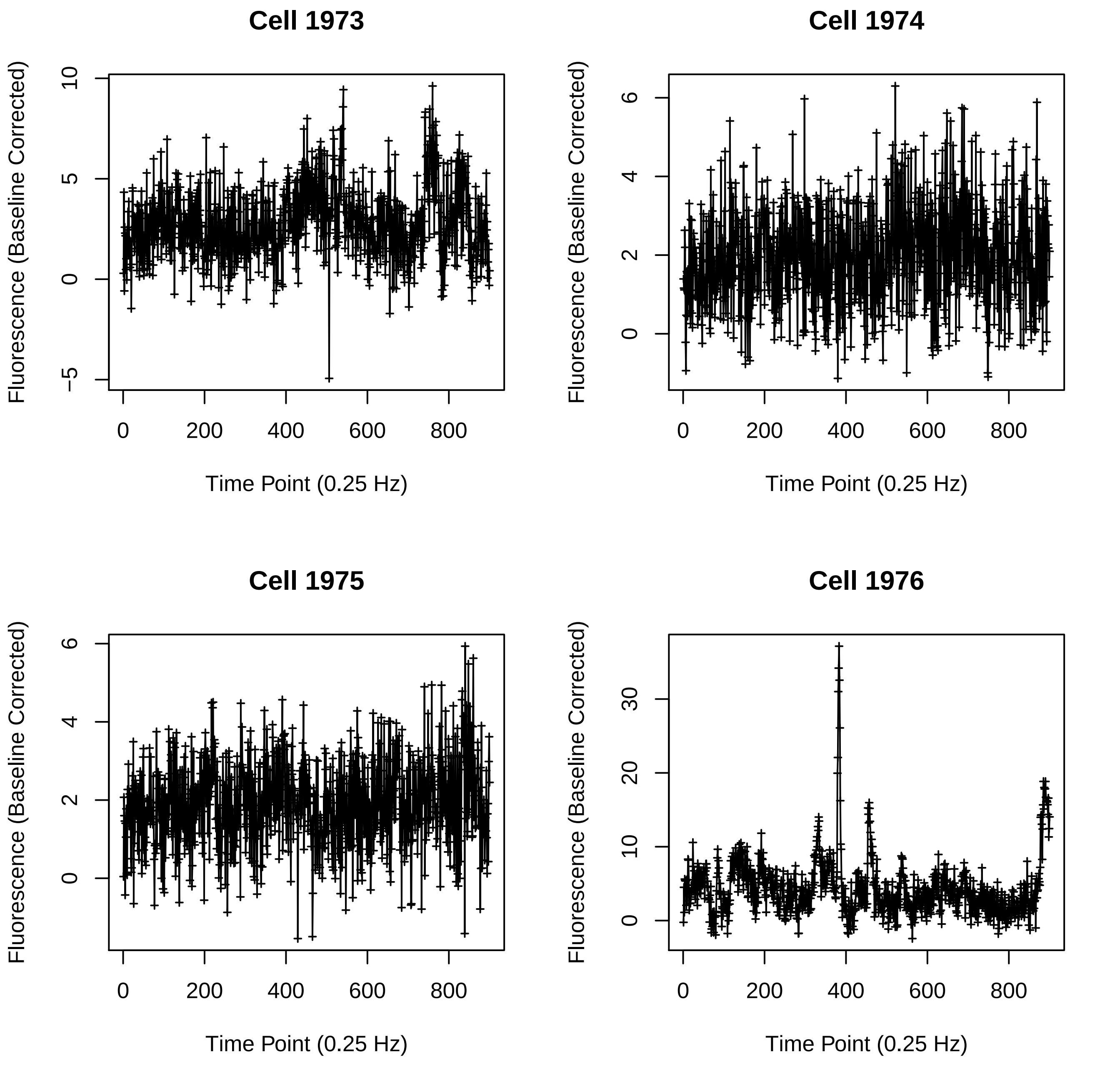
Cell 1973
Cell 1974
Cell 1975
Cell 1976
Fluorescence (Baseline Corrected)
Time Point (0.25 Hz)
0
200
400
600
800
−5
5
10
2
4
6
20
30

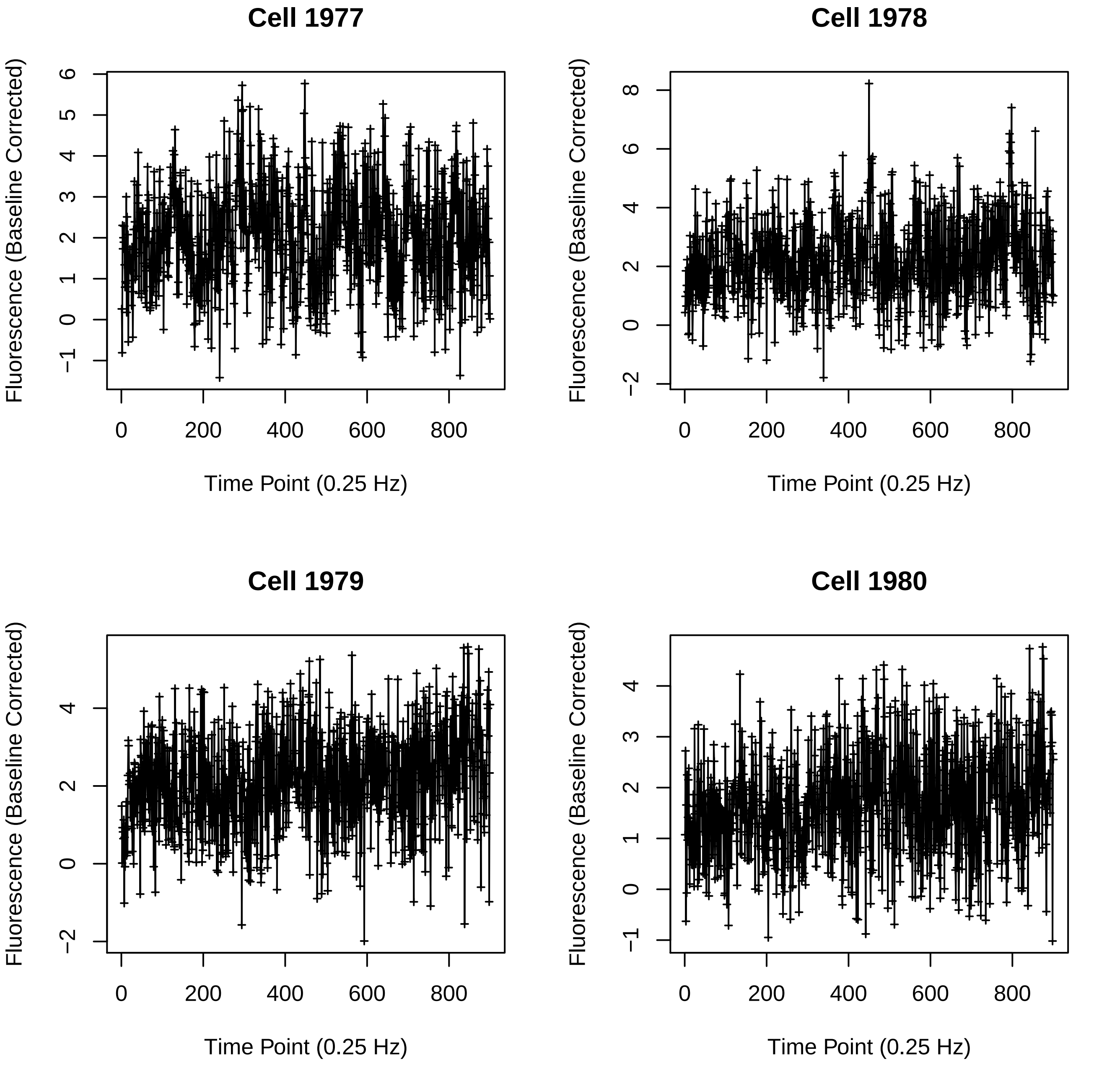
Cell 1977
Cell 1978
Cell 1979
Cell 1980
Fluorescence (Baseline Corrected)
Time Point (0.25 Hz)
0
200
400
600
800
−2
−1
0
1
2
3
4
5
6
8

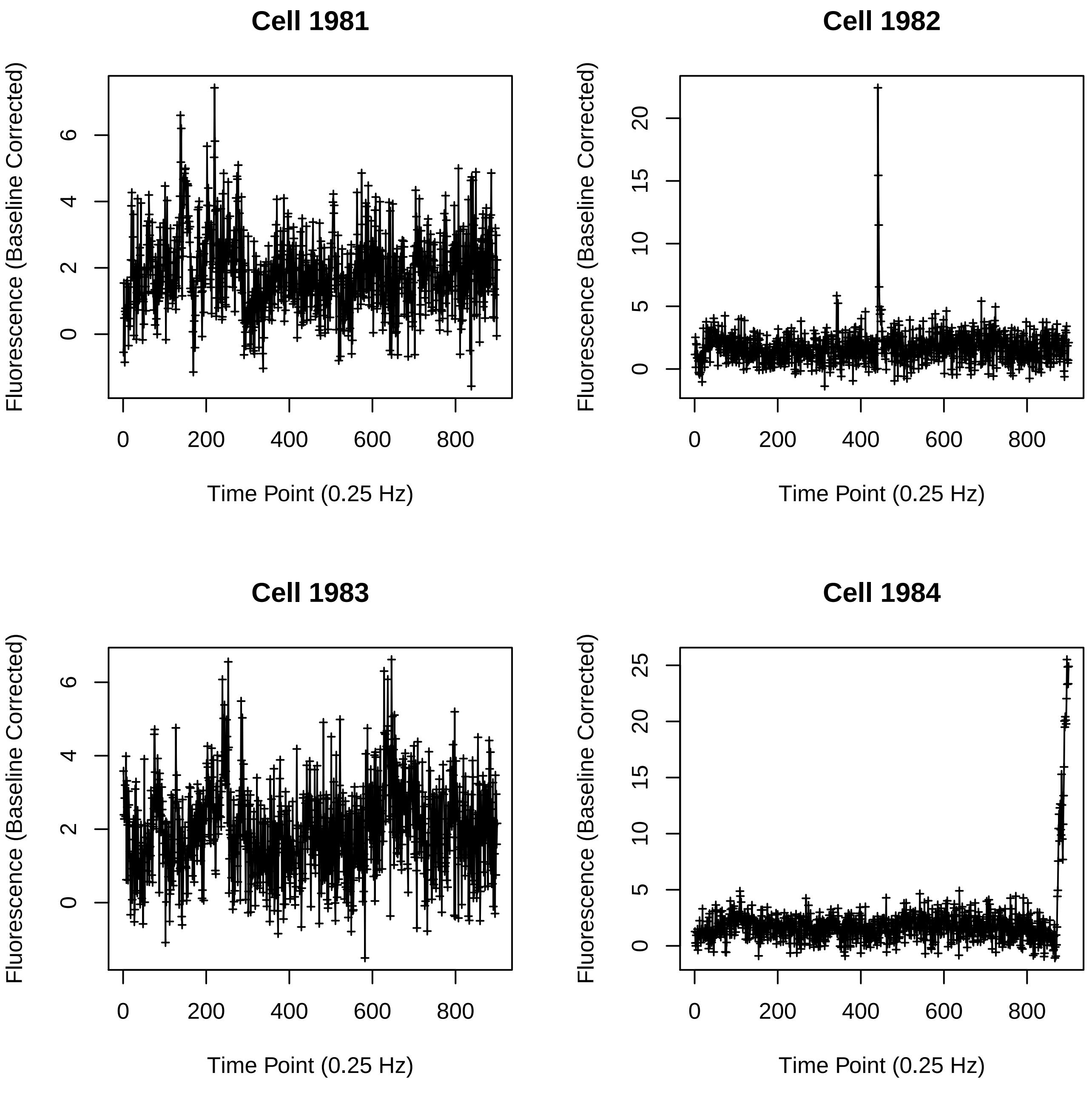
Cell 1981
Fluorescence (Baseline Corrected)
Time Point (0.25 Hz)
0
2
4
6
0
200
400
600
800
Cell 1982
Fluorescence (Baseline Corrected)
Time Point (0.25 Hz)
0
5
10
15
20
0
200
400
600
800
Cell 1983
Fluorescence (Baseline Corrected)
Time Point (0.25 Hz)
0
2
4
6
0
200
400
600
800
Cell 1984
Fluorescence (Baseline Corrected)
Time Point (0.25 Hz)
0
5
10
15
20
25
0
200
400
600
800

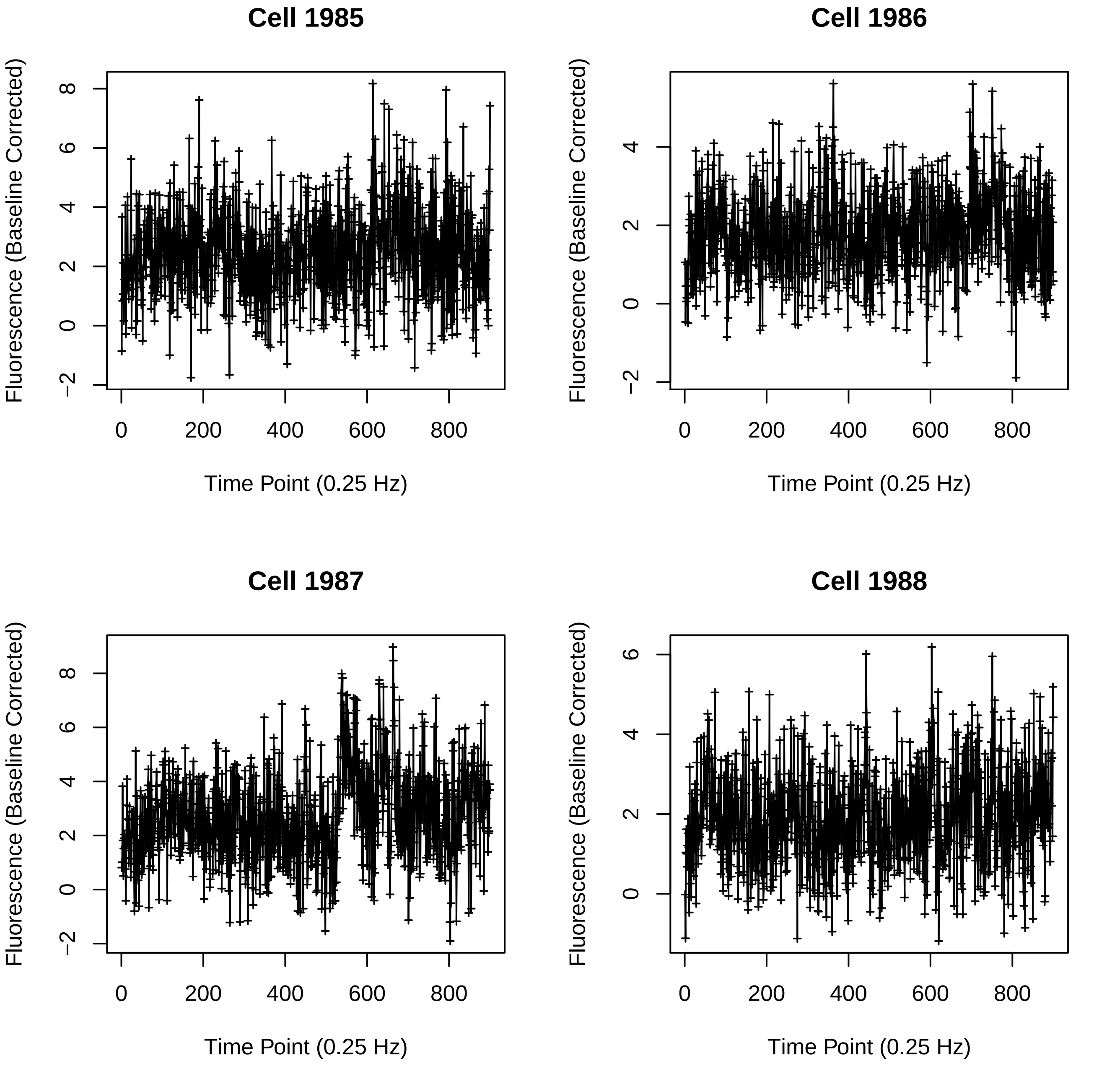
Cell 1985
Cell 1986
Cell 1987
Cell 1988
Fluorescence (Baseline Corrected)
Time Point (0.25 Hz)
−2
0
2
4
6
8
0
200
400
600
800

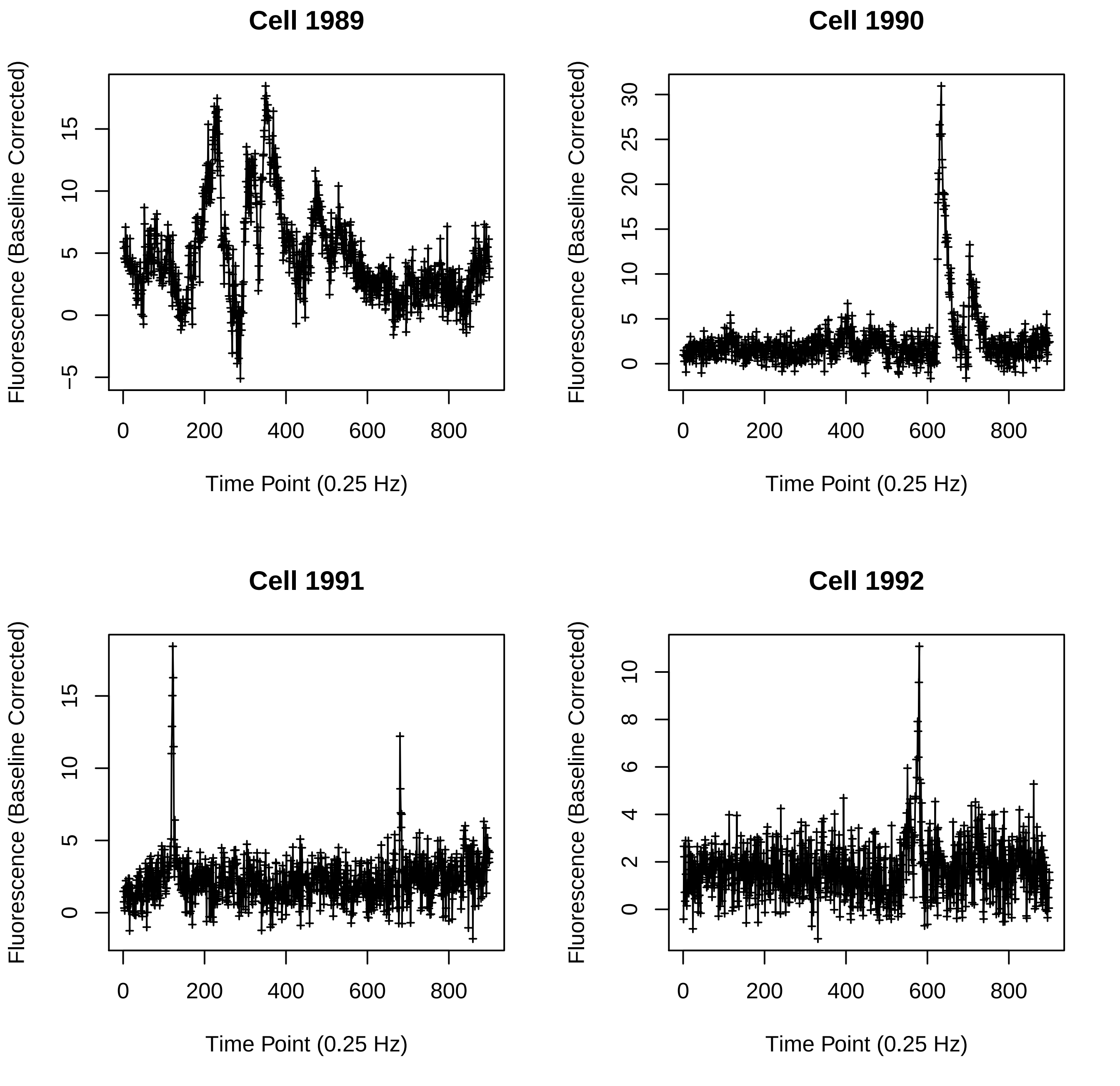

Cell 1989
Cell 1990
Cell 1991
Cell 1992
Fluorescence (Baseline Corrected)
Time Point (0.25 Hz)

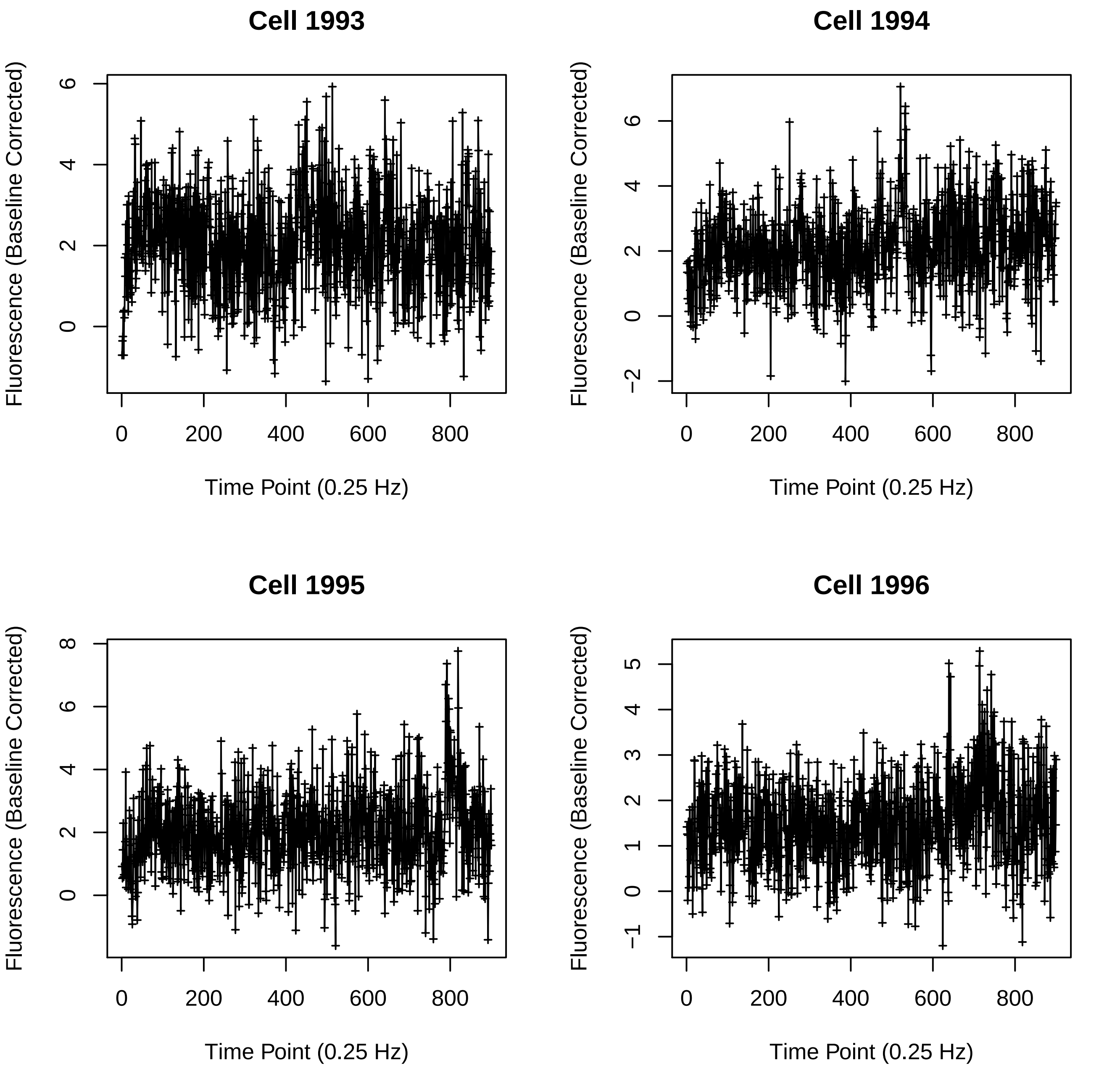
Cell 1993
Cell 1994
Cell 1995
Cell 1996
Fluorescence (Baseline Corrected)
Time Point (0.25 Hz)
0
200
400
600
800
-2
-1
0
1
2
3
4
5
6
8

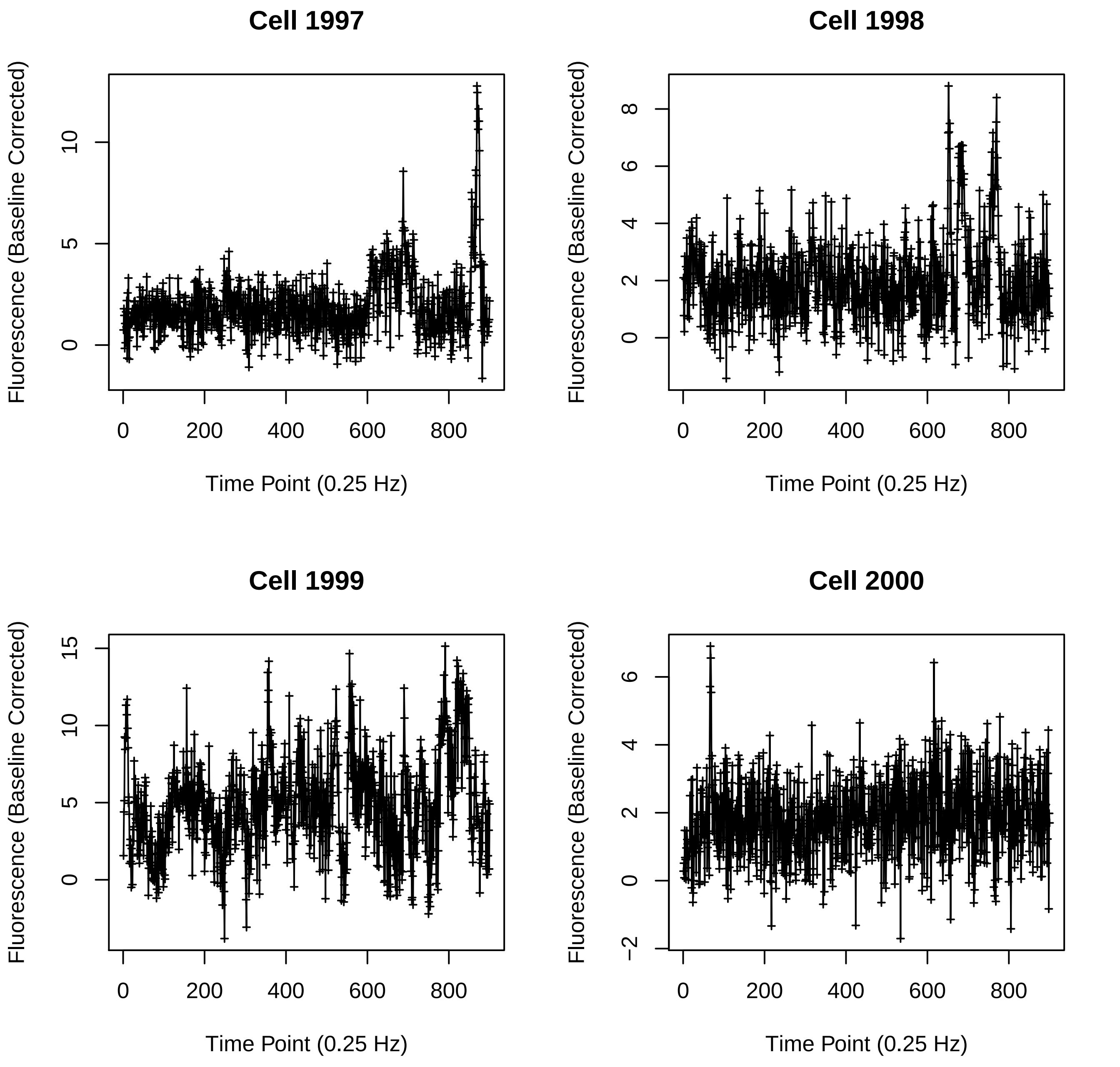
Cell 1997
Fluorescence (Baseline Corrected)
0
5
10
0
200
400
600
800
Time Point (0.25 Hz)
Cell 1998
Fluorescence (Baseline Corrected)
0
2
4
6
8
0
200
400
600
800
Time Point (0.25 Hz)
Cell 1999
Fluorescence (Baseline Corrected)
0
5
10
15
0
200
400
600
800
Time Point (0.25 Hz)
Cell 2000
Fluorescence (Baseline Corrected)
−2
0
2
4
6
0
200
400
600
800
Time Point (0.25 Hz)

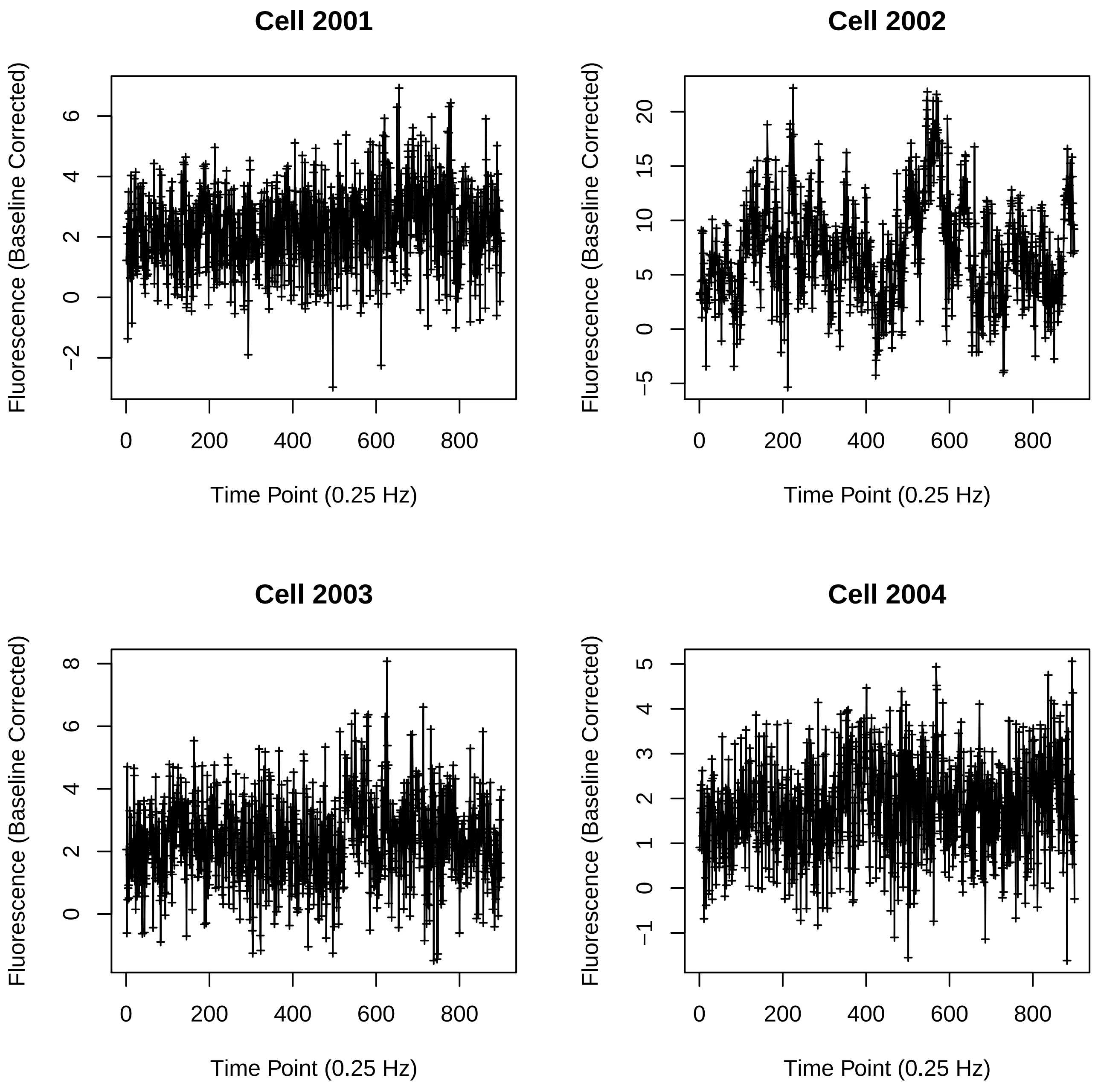
Cell 2001
Cell 2002
Cell 2003
Cell 2004
Fluorescence (Baseline Corrected)
Time Point (0.25 Hz)
0
200
400
600
800
−2
2
4
6
−5
5
10
15
20
8
−1
1
3

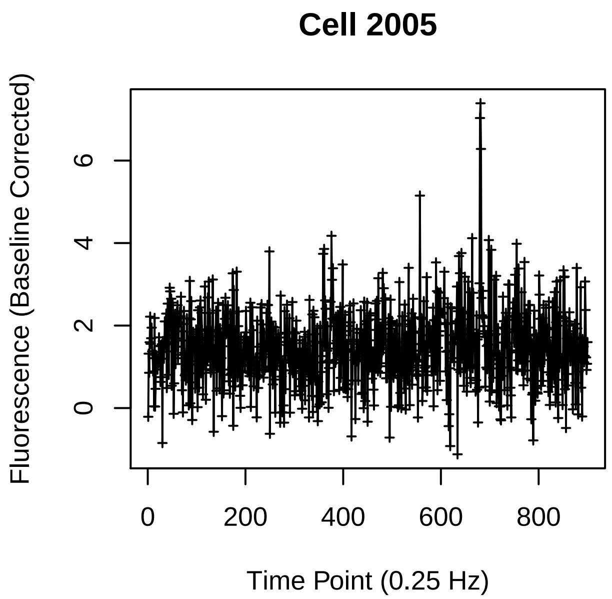
Cell 2005
Fluorescence (Baseline Corrected)
0
2
4
6
0
200
400
600
800
Time Point (0.25 Hz)

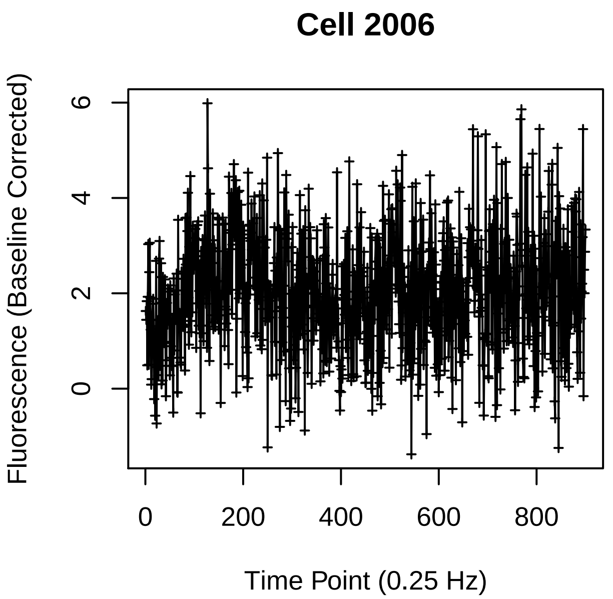
Cell 2006
Fluorescence (Baseline Corrected)
0
2
4
6
0
200
400
600
800
Time Point (0.25 Hz)

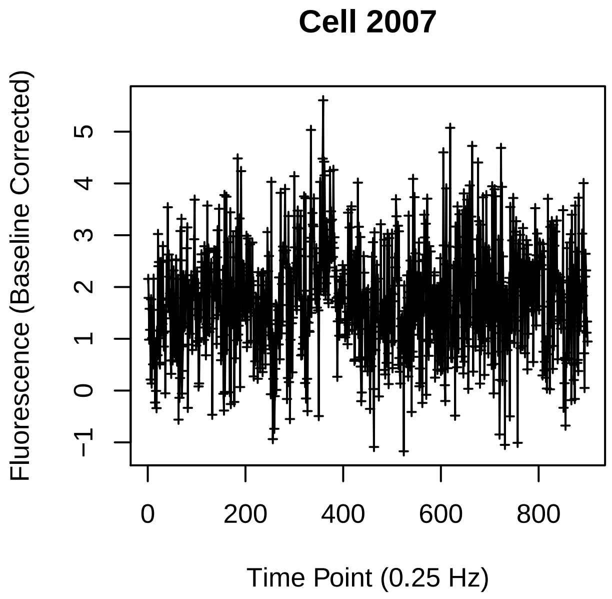
Cell 2007
Fluorescence (Baseline Corrected)
−1
0
1
2
3
4
5
0
200
400
600
800
Time Point (0.25 Hz)

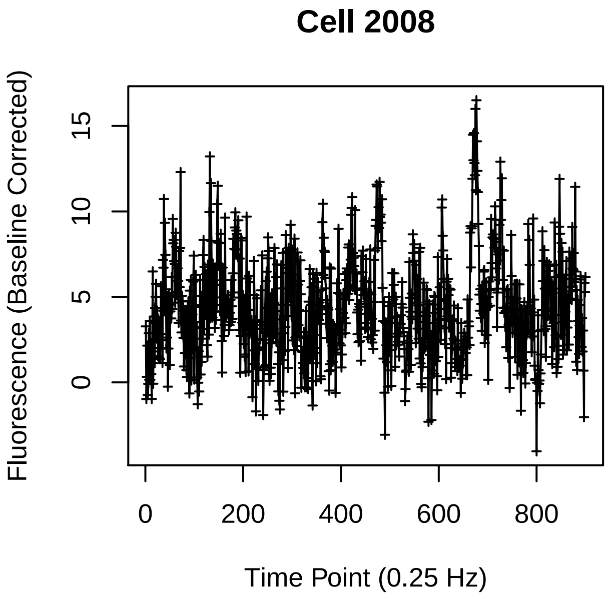
Cell 2008
Fluorescence (Baseline Corrected)
0
5
10
15
0
200
400
600
800
Time Point (0.25 Hz)

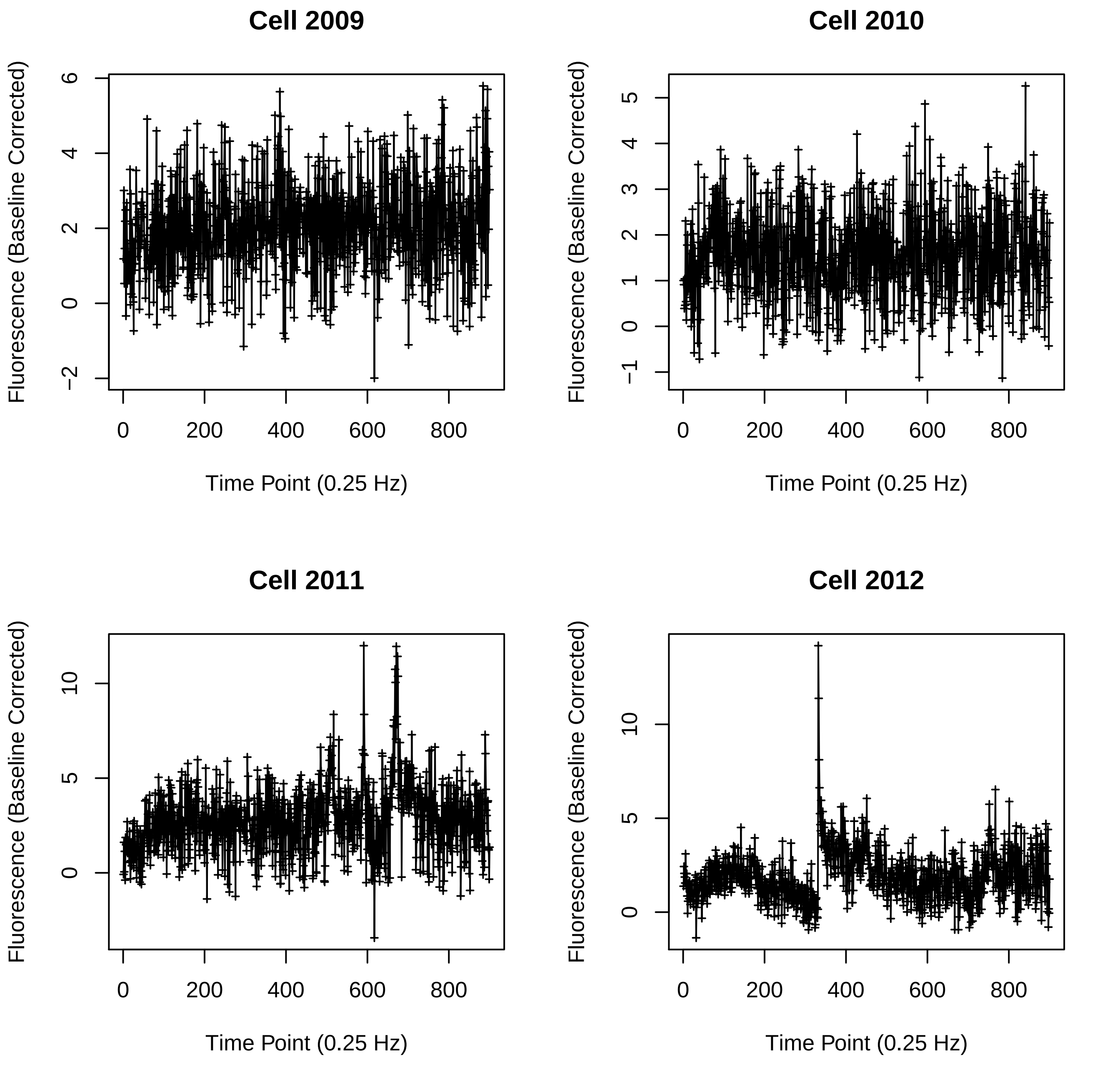
Cell 2009
Cell 2010
Cell 2011
Cell 2012
Fluorescence (Baseline Corrected)
Time Point (0.25 Hz)
0
200
400
600
800
−2
0
2
4
6
−1
1
3
5
10

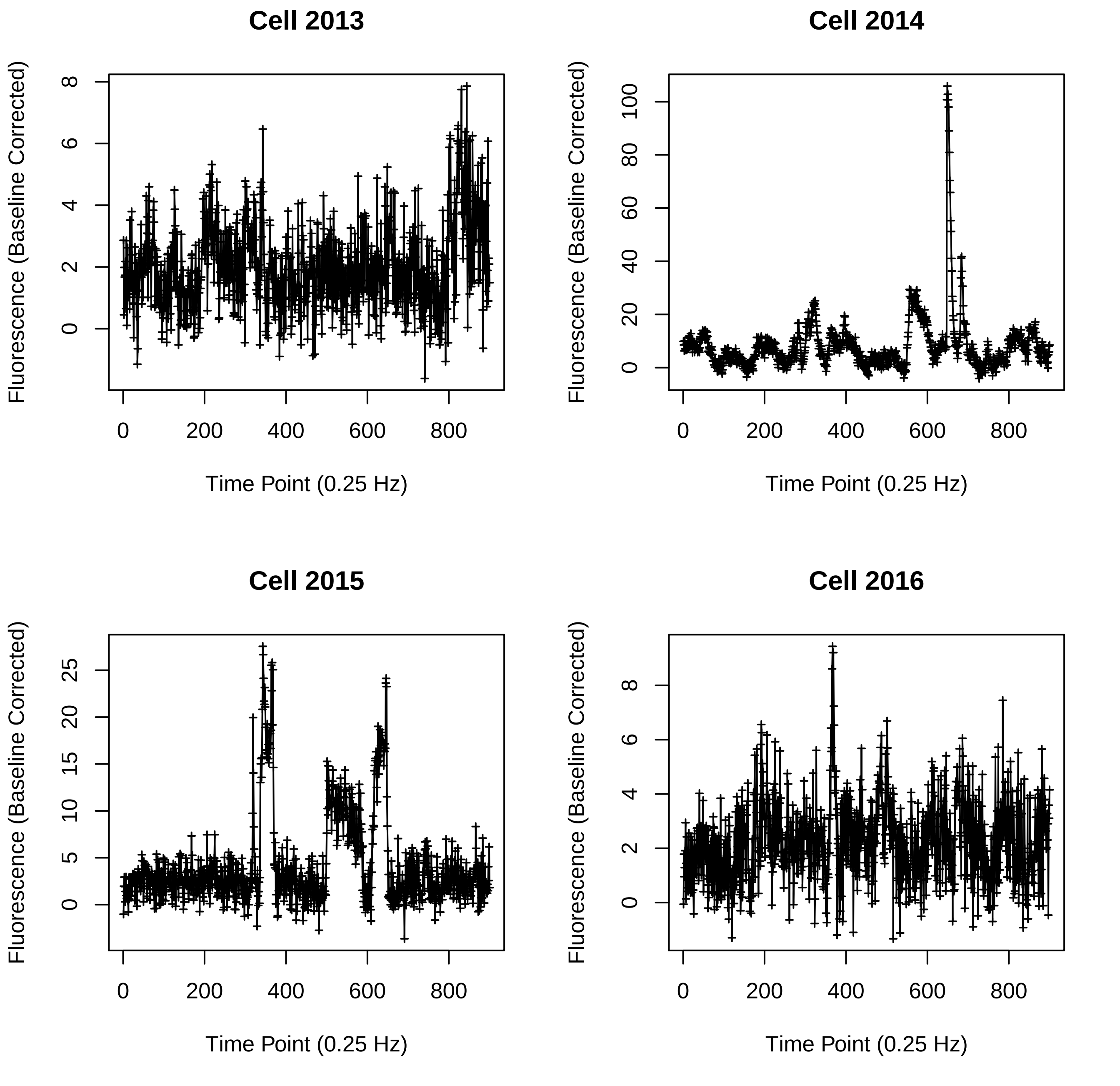

Cell 2013
Cell 2014
Cell 2015
Cell 2016
Fluorescence (Baseline Corrected)
Time Point (0.25 Hz)
0
200
400
600
800
2
4
6
8
20
40
60
80
100
5
10
15
20
25

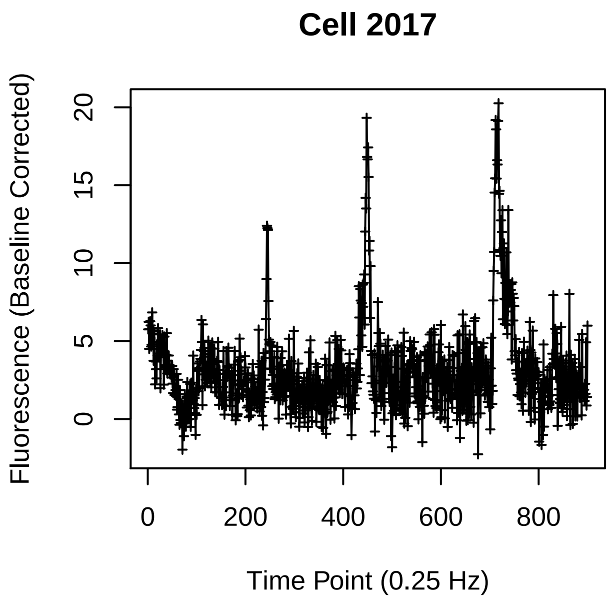
Cell 2017
Fluorescence (Baseline Corrected)
Time Point (0.25 Hz)
0
5
10
15
20
0
200
400
600
800
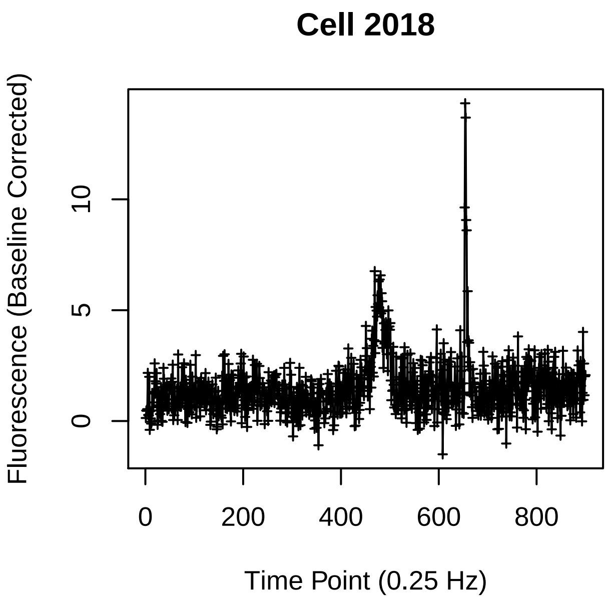
Cell 2018
Fluorescence (Baseline Corrected)
Time Point (0.25 Hz)
0
5
10
0
200
400
600
800
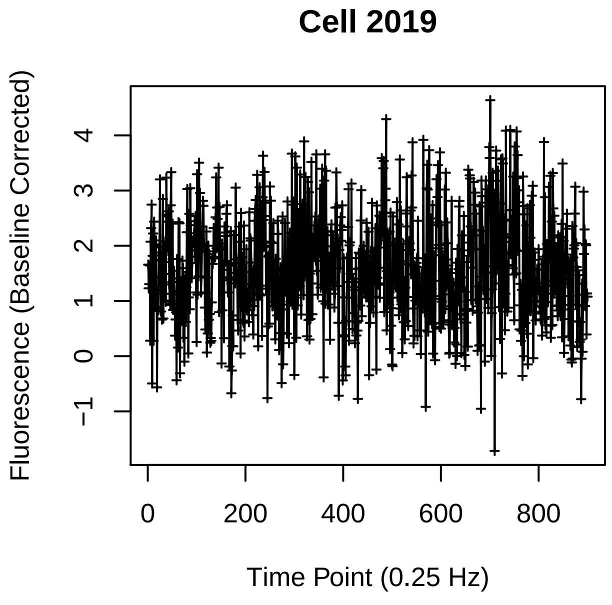
Cell 2019
Fluorescence (Baseline Corrected)
Time Point (0.25 Hz)
−1
0
1
2
3
4
0
200
400
600
800
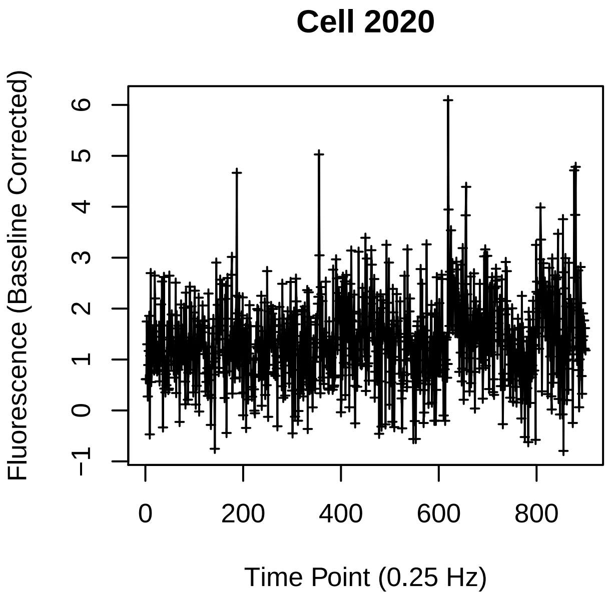
Cell 2020
Fluorescence (Baseline Corrected)
Time Point (0.25 Hz)
−1
0
1
2
3
4
5
6
0
200
400
600
800

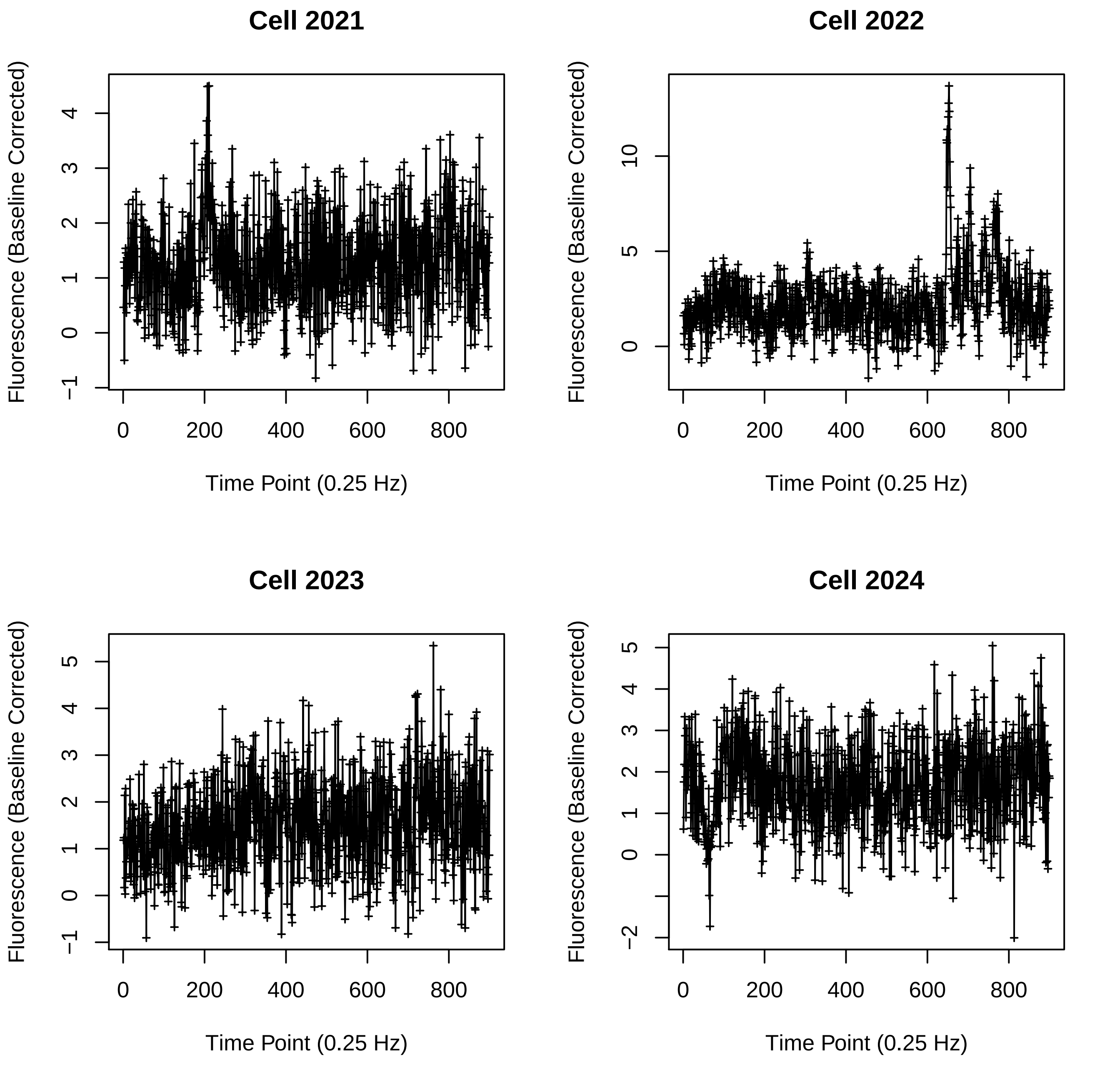
Cell 2021
Cell 2022
Cell 2023
Cell 2024
Fluorescence (Baseline Corrected)
Time Point (0.25 Hz)
0
200
400
600
800
−1
0
1
2
3
4
5
10
−2

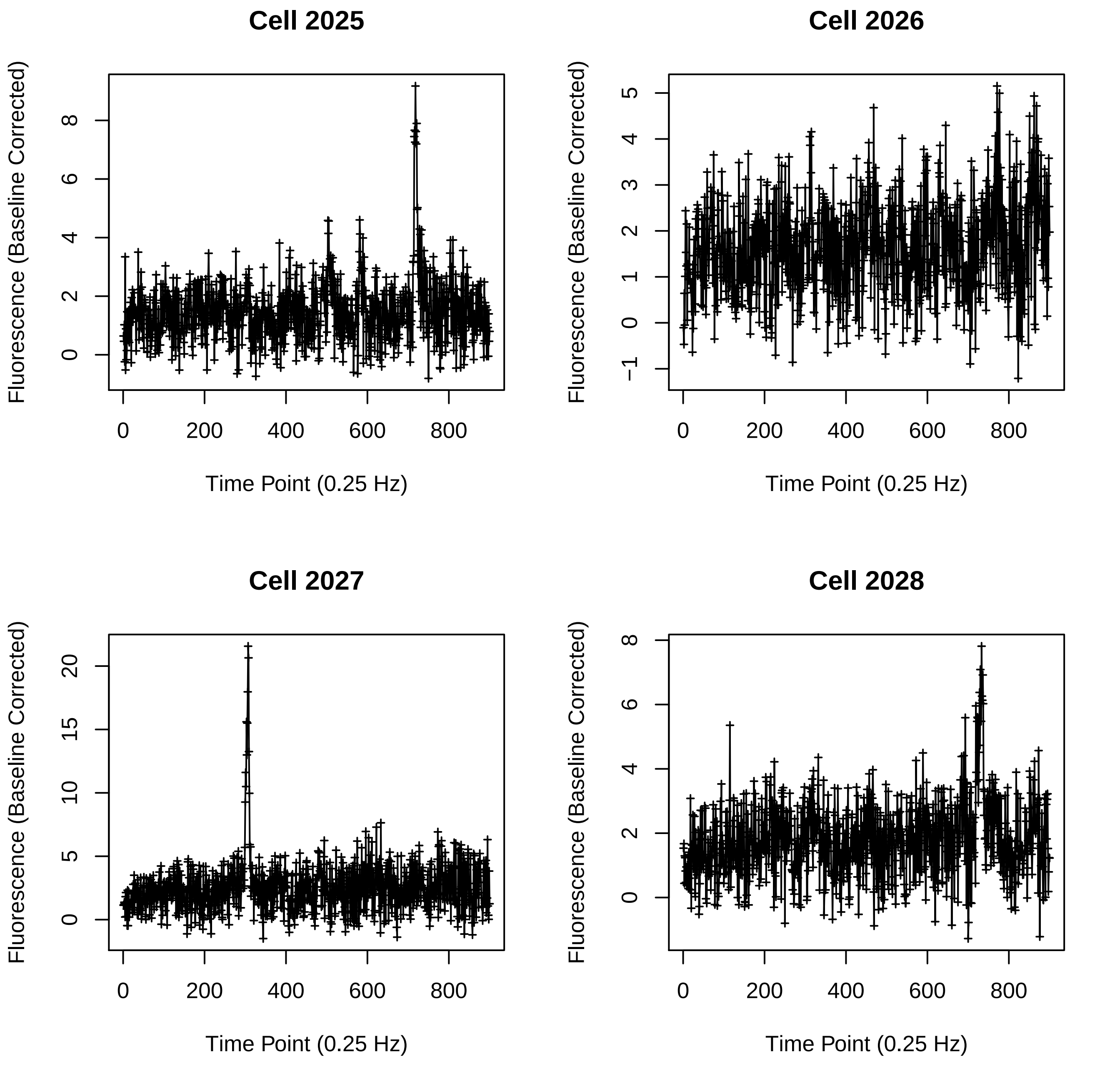
Cell 2025
Cell 2026
Cell 2027
Cell 2028
Fluorescence (Baseline Corrected)
Time Point (0.25 Hz)
0
200
400
600
800
0
2
4
6
8
−1
1
3
5
10
15
20

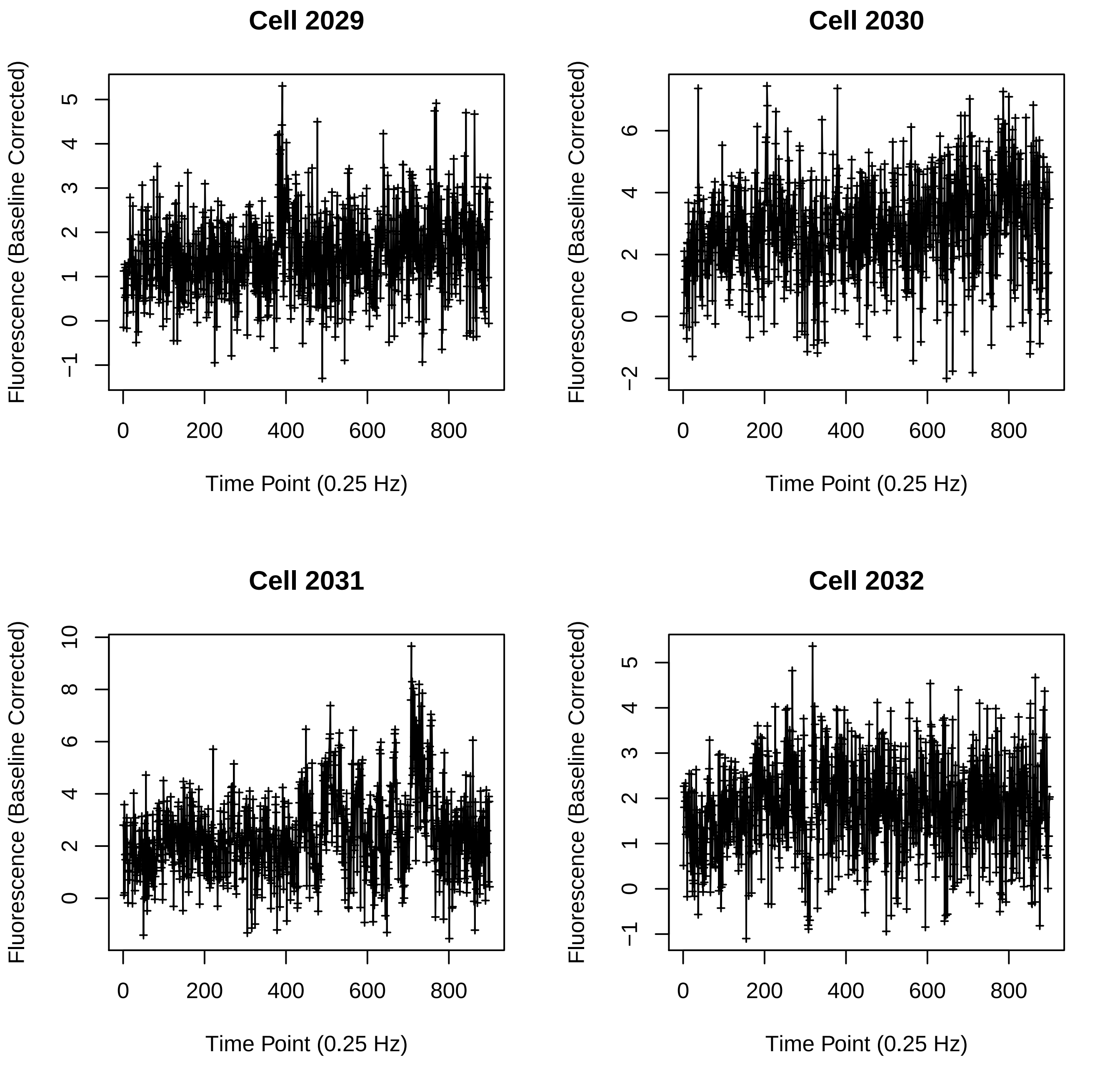
Cell 2029
Cell 2030
Cell 2031
Cell 2032
Fluorescence (Baseline Corrected)
Time Point (0.25 Hz)
0
200
400
600
800
−1
1
2
3
4
5
−2
6
8
10

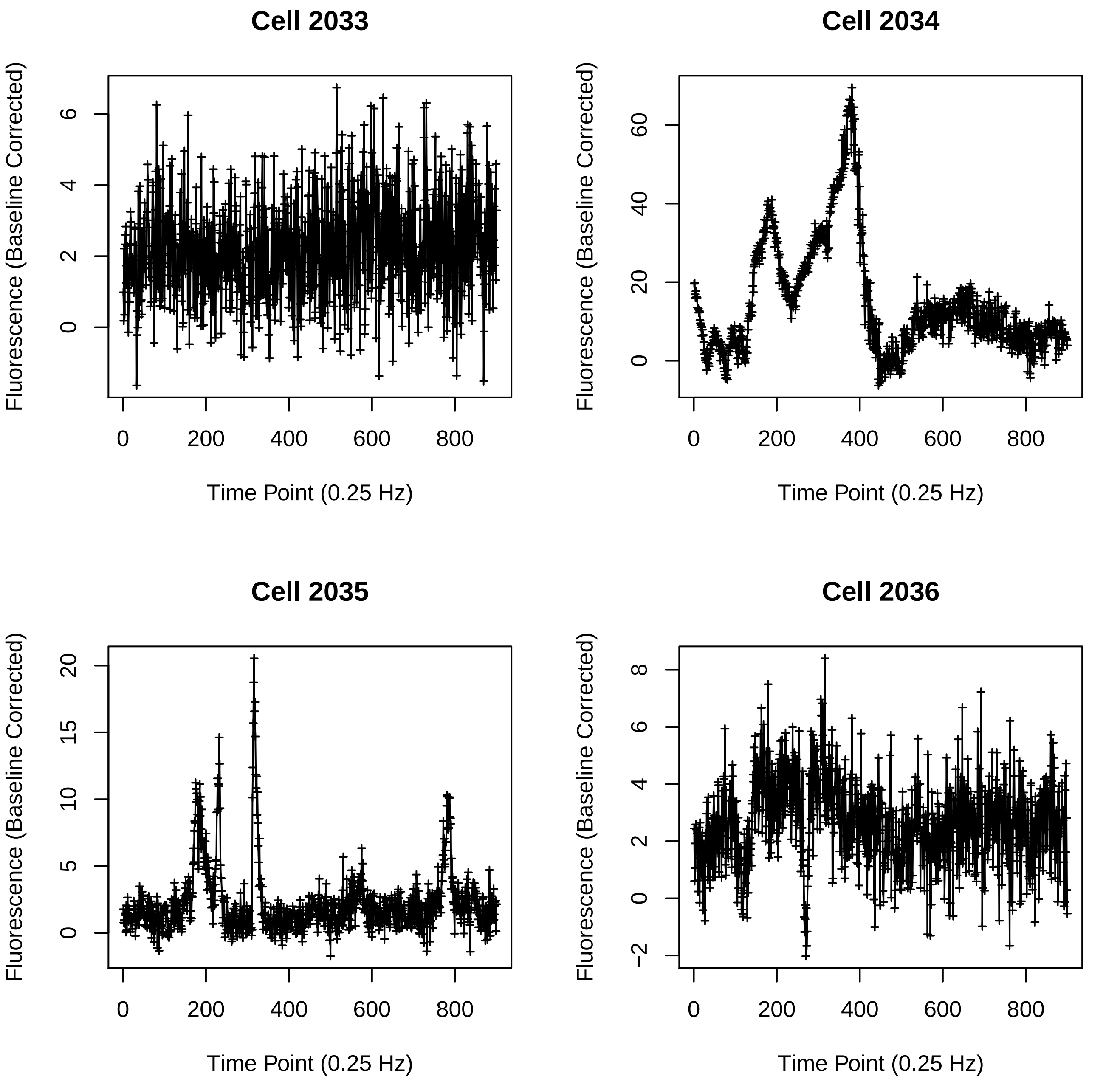
Cell 2033
Cell 2034
Cell 2035
Cell 2036
Fluorescence (Baseline Corrected)
Time Point (0.25 Hz)
0
2
4
6
20
40
60
5
10
15
−2
8
200
400
600
800

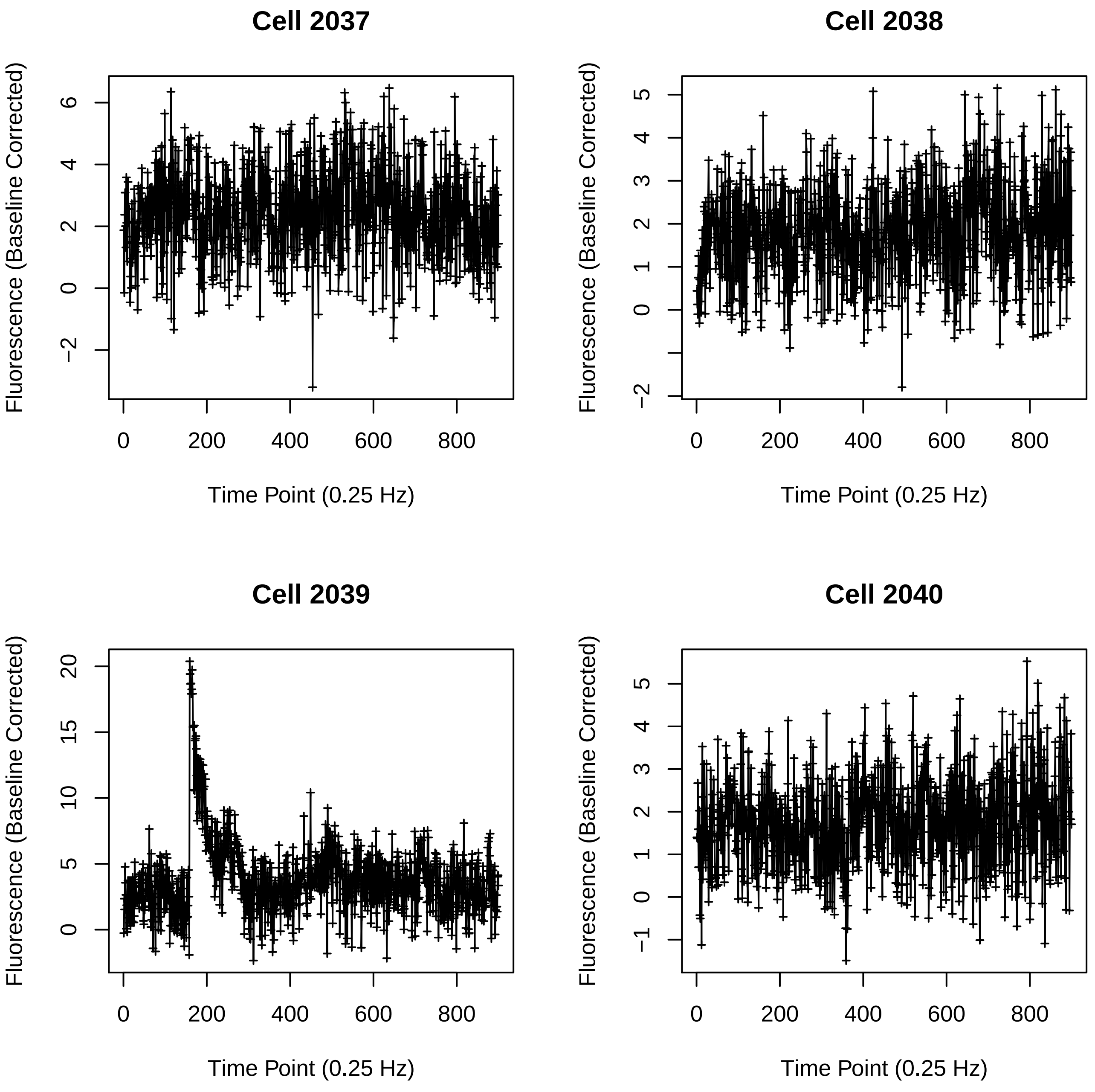
Cell 2037
Cell 2038
Cell 2039
Cell 2040
Fluorescence (Baseline Corrected)
Time Point (0.25 Hz)
0
200
400
600
800
−2
2
4
6
1
3
5
10
15
20
−1

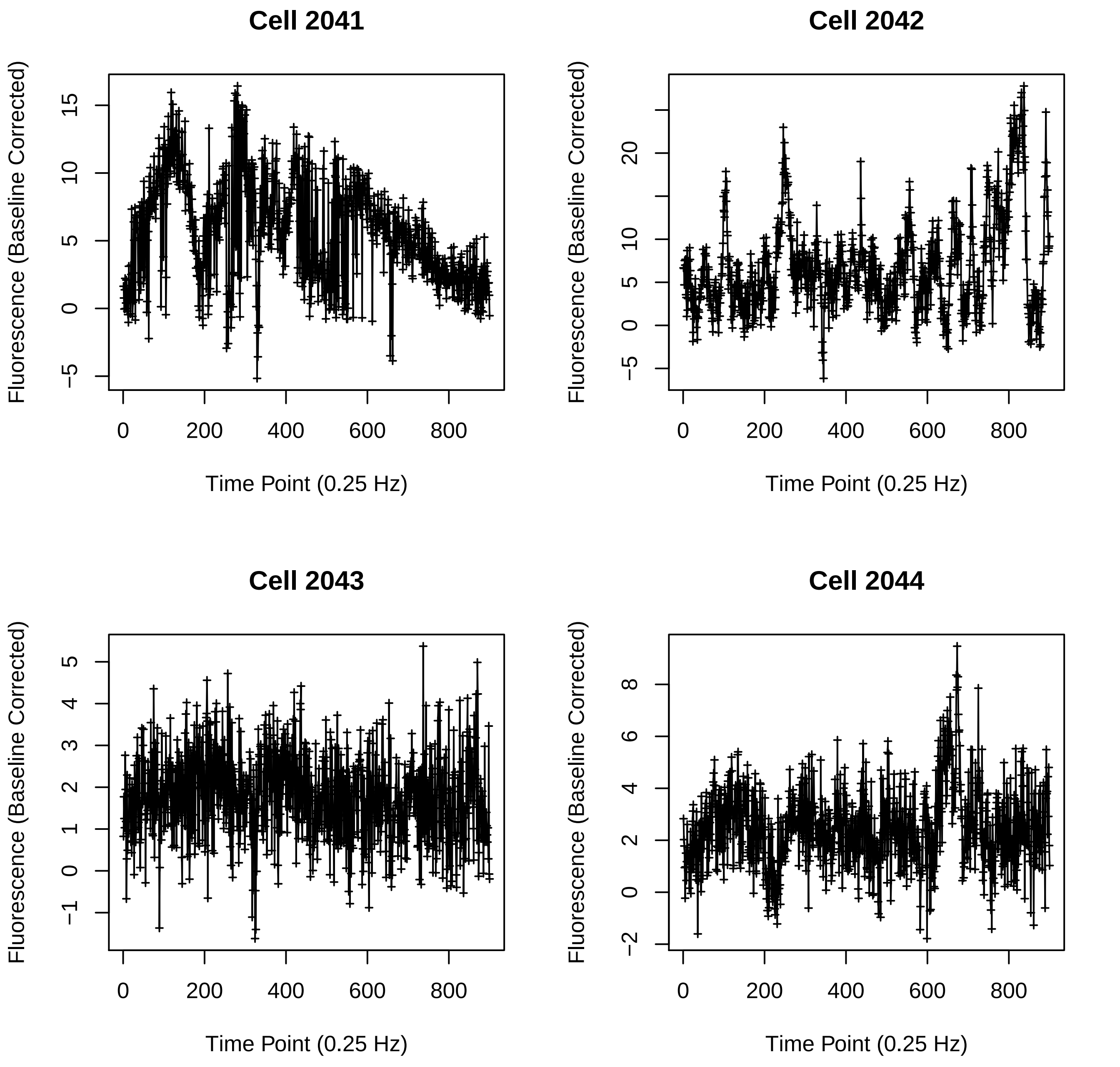
Cell 2041
Cell 2042
Cell 2043
Cell 2044
Fluorescence (Baseline Corrected)
Time Point (0.25 Hz)

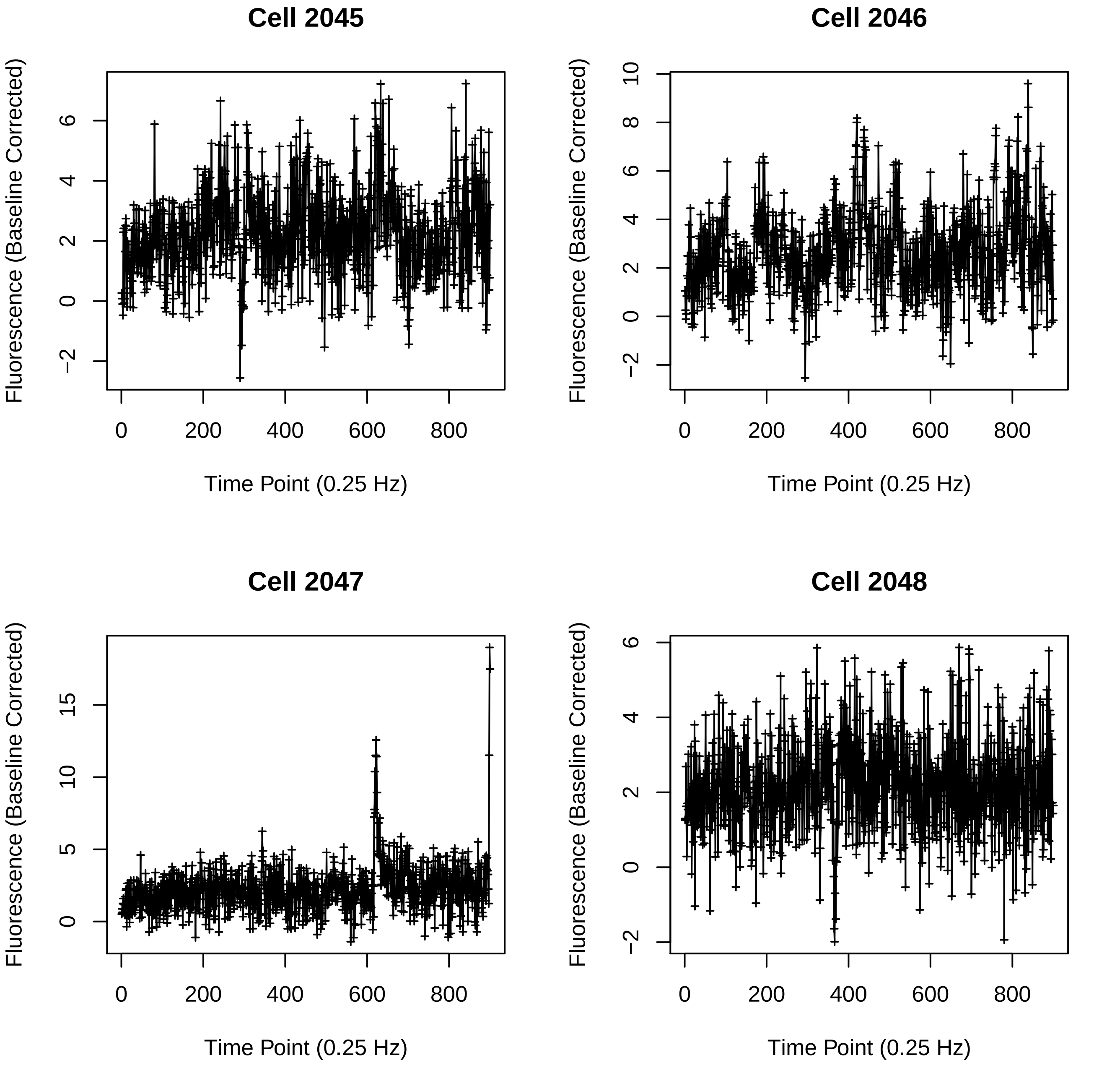

Cell 2045
Cell 2046
Cell 2047
Cell 2048
Fluorescence (Baseline Corrected)
Time Point (0.25 Hz)
0
200
400
600
800
−2
2
4
6
8
10
5
15

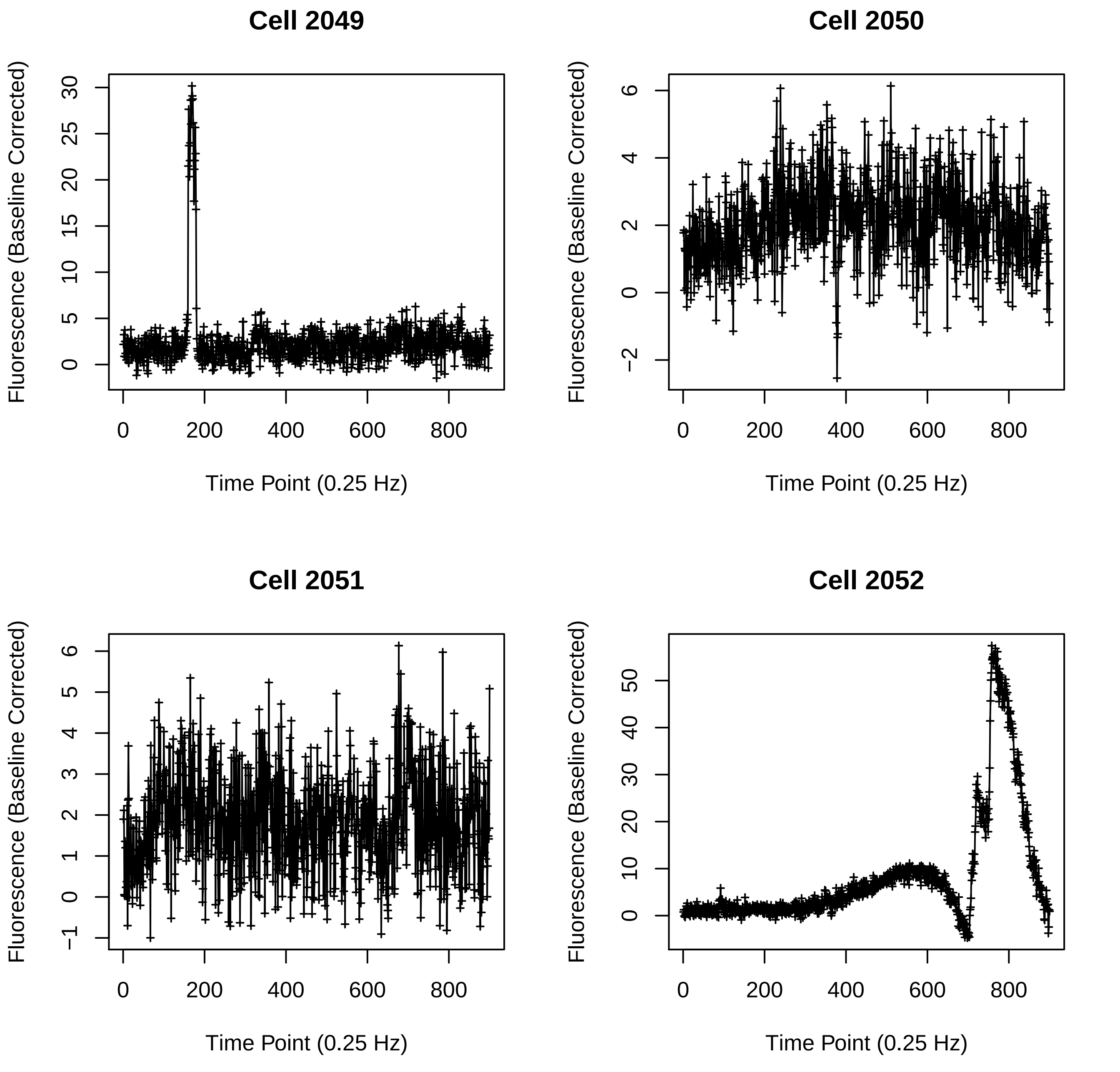
Cell 2049
Cell 2050
Cell 2051
Cell 2052
Fluorescence (Baseline Corrected)
Time Point (0.25 Hz)
0
5
10
15
20
25
30
−2
2
4
6
−1
1
3
40
50
200
400
600
800

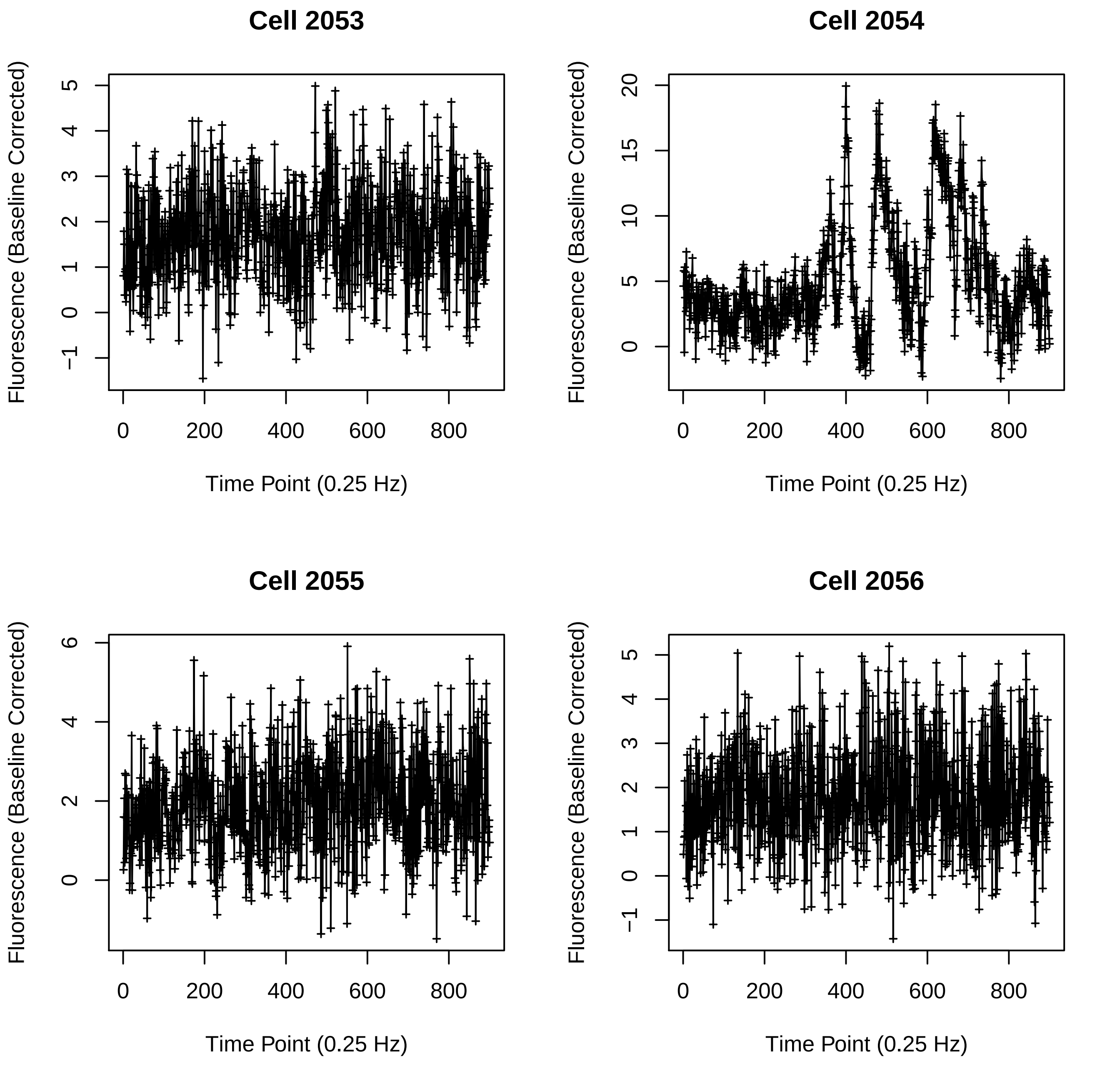
Cell 2053
Cell 2054
Cell 2055
Cell 2056
Fluorescence (Baseline Corrected)
Time Point (0.25 Hz)
0
200
400
600
800

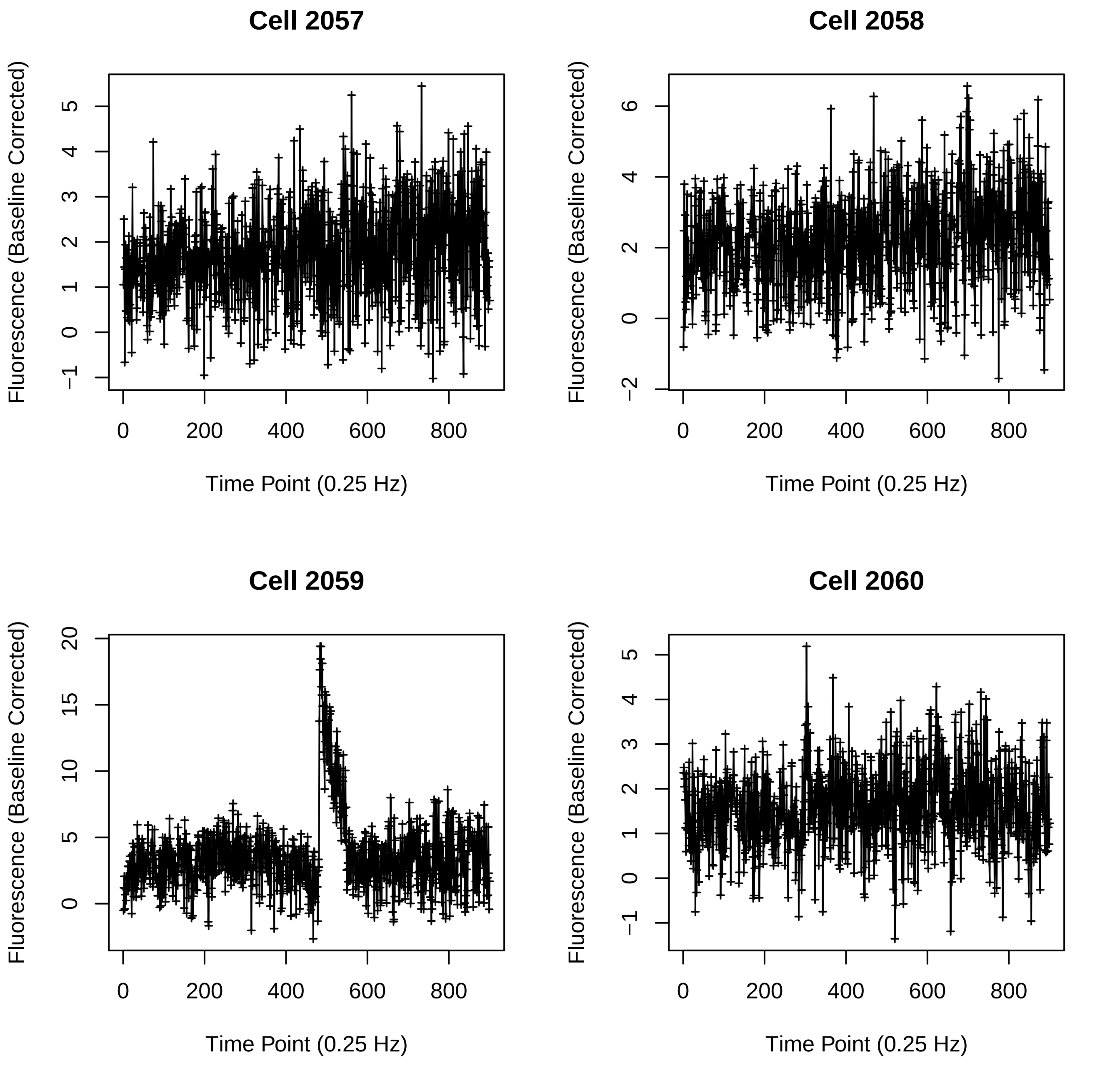
Cell 2057
Cell 2058
Cell 2059
Cell 2060
Fluorescence (Baseline Corrected)
Time Point (0.25 Hz)
0
200
400
600
800

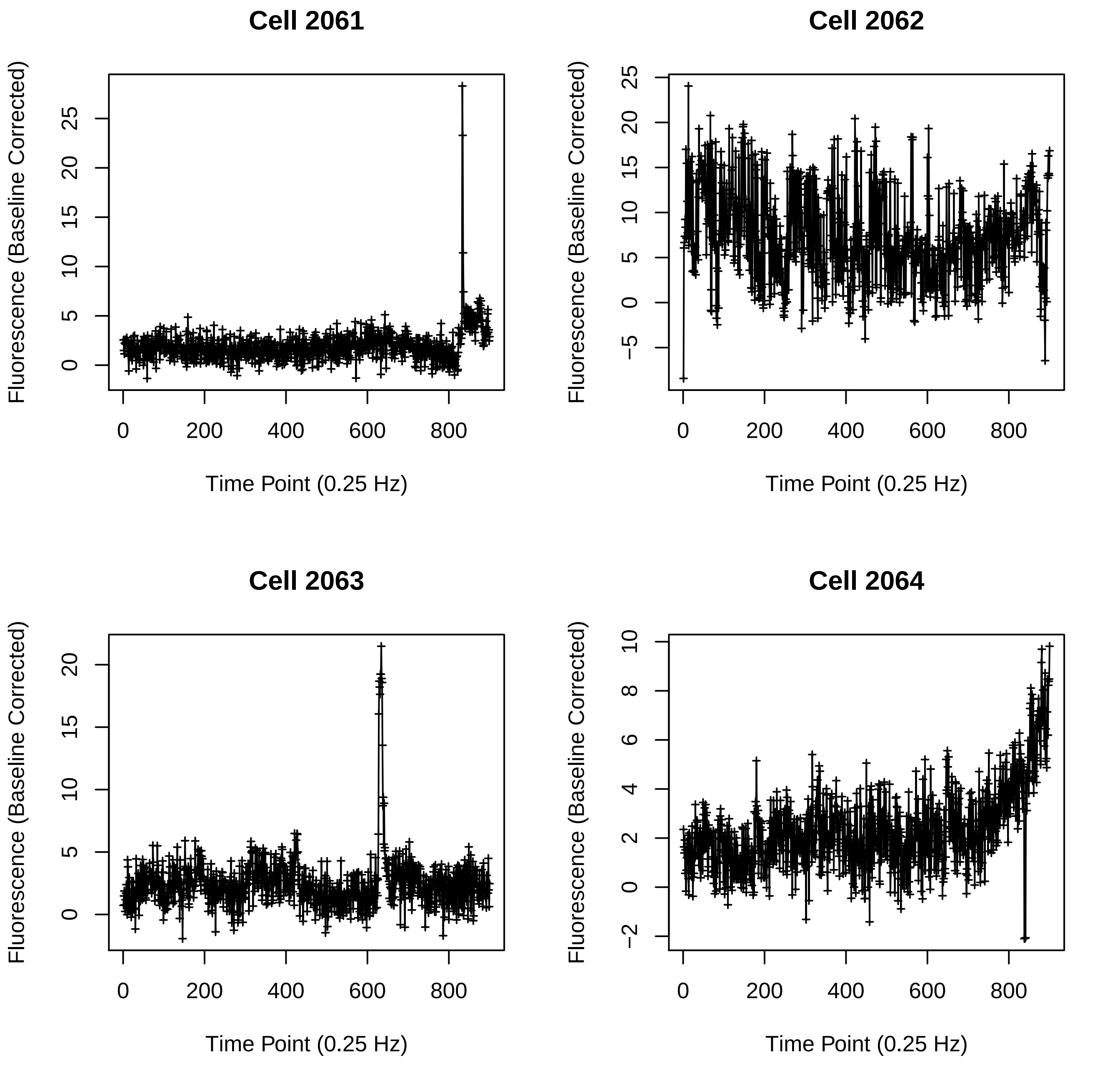

Cell 2061
Cell 2062
Cell 2063
Cell 2064
Fluorescence (Baseline Corrected)
Time Point (0.25 Hz)
0
200
400
600
800
0
5
10
15
20
25
−5
−2
2
4
6
8

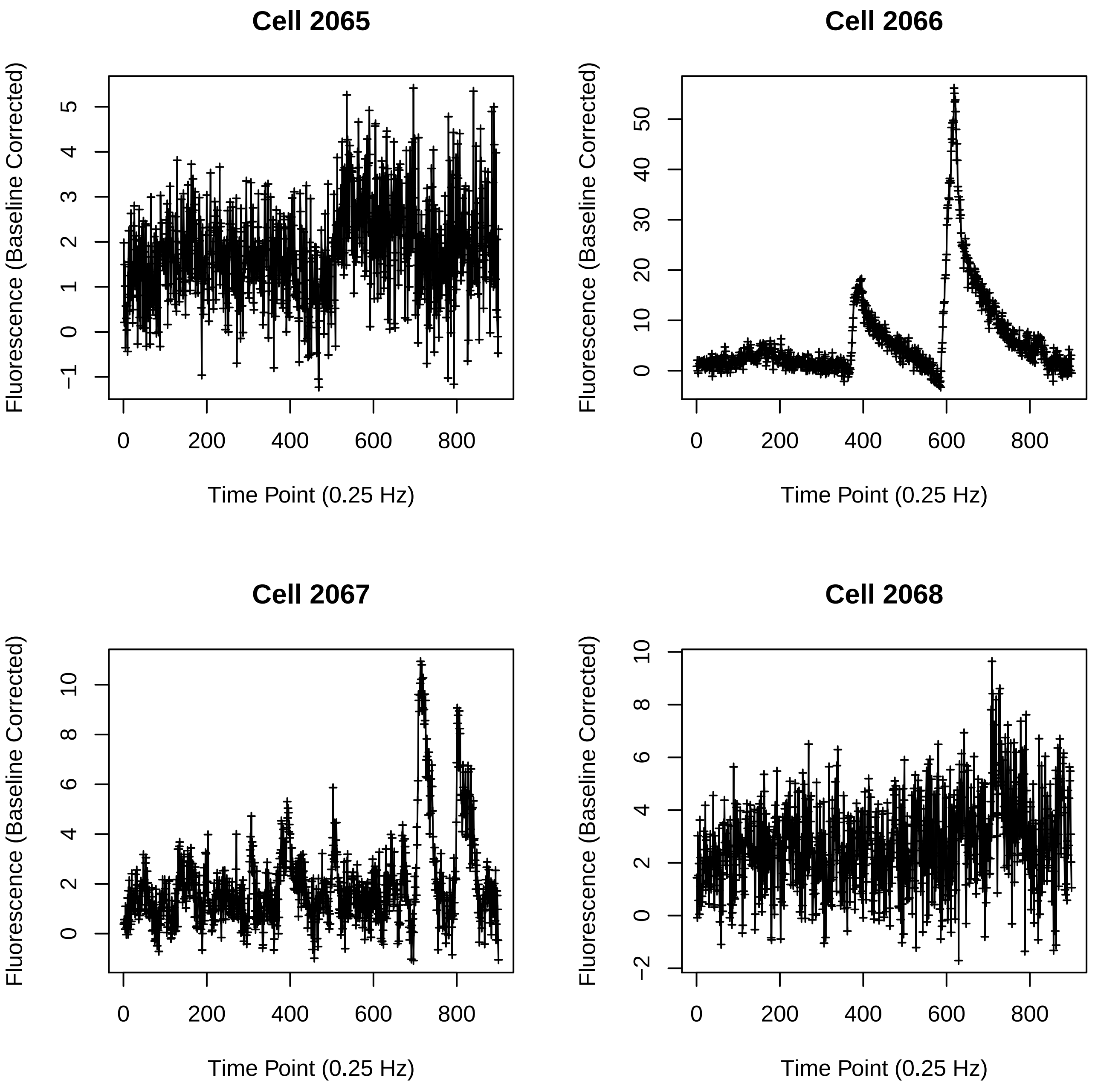
Cell 2065
Cell 2066
Cell 2067
Cell 2068
Fluorescence (Baseline Corrected)
Time Point (0.25 Hz)
−1
0
1
2
3
4
5
10
20
30
40
50
6
8
−2
200
400
600
800

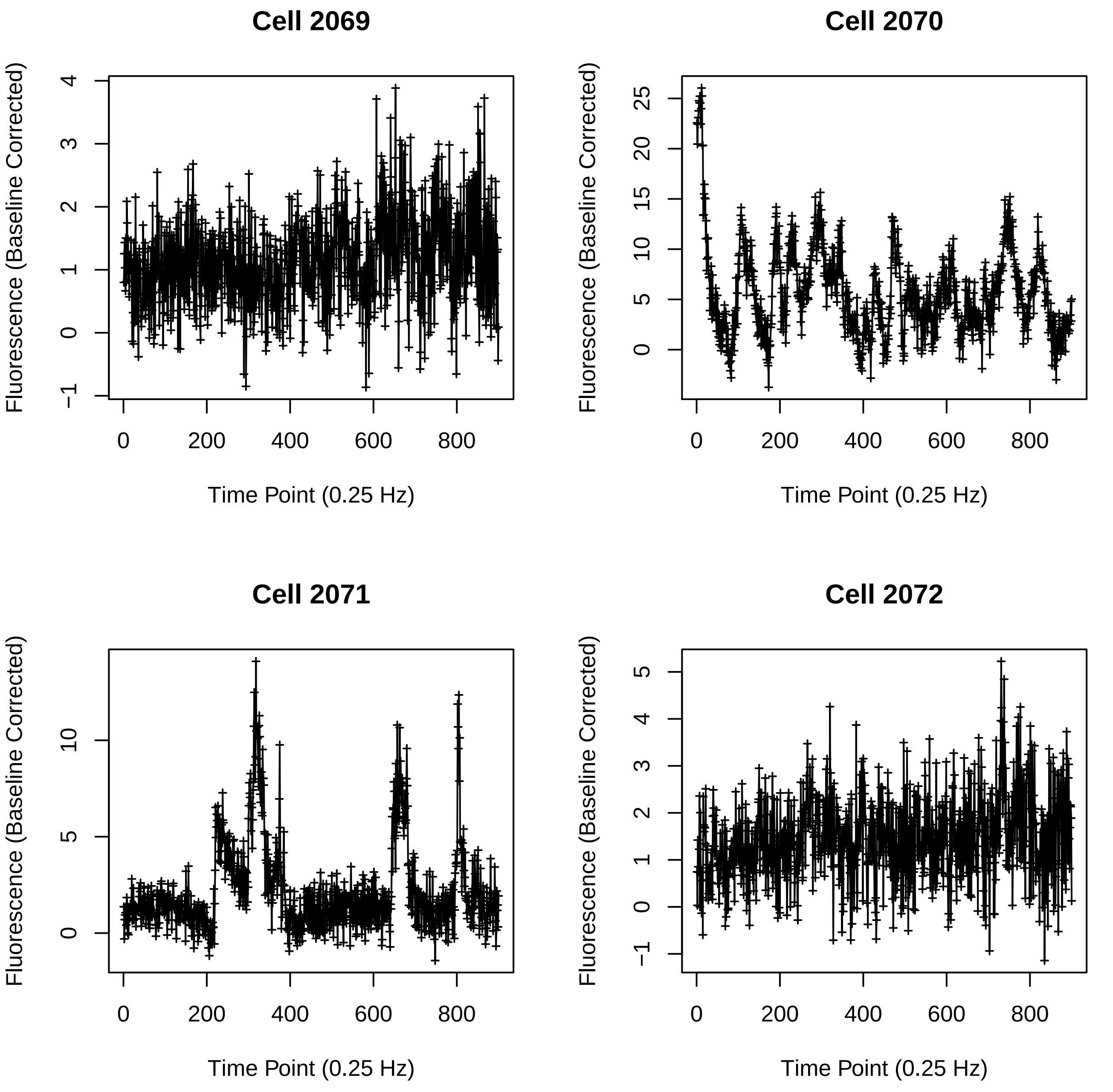
Cell 2069
Fluorescence (Baseline Corrected)
Time Point (0.25 Hz)
Cell 2070
Fluorescence (Baseline Corrected)
Time Point (0.25 Hz)
Cell 2071
Fluorescence (Baseline Corrected)
Time Point (0.25 Hz)
Cell 2072
Fluorescence (Baseline Corrected)
Time Point (0.25 Hz)

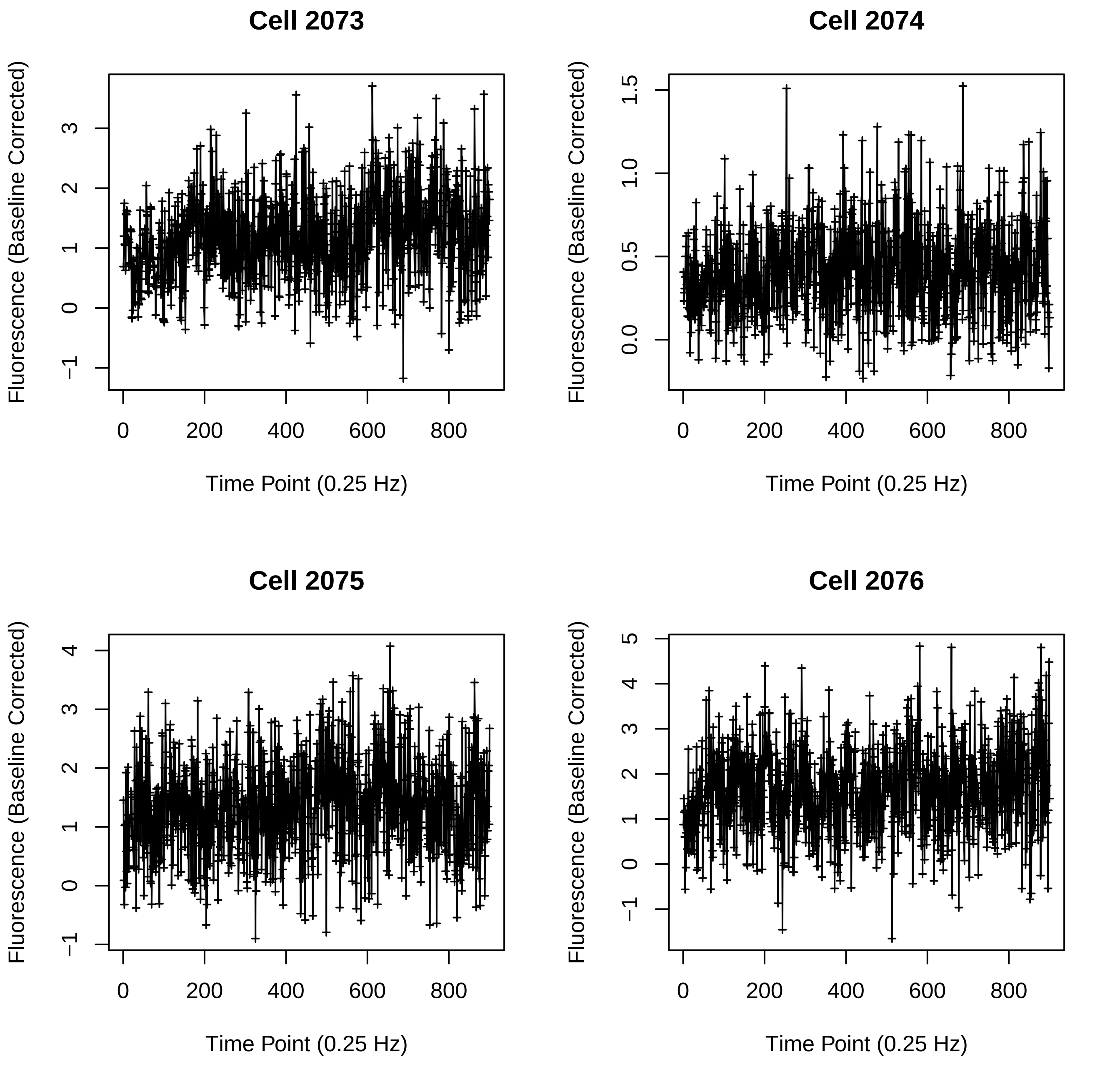

Cell 2073
Cell 2074
Cell 2075
Cell 2076
Fluorescence (Baseline Corrected)
Time Point (0.25 Hz)
0
200
400
600
800
-1
0
1
2
3
4
5
0.0
0.5
1.0
1.5

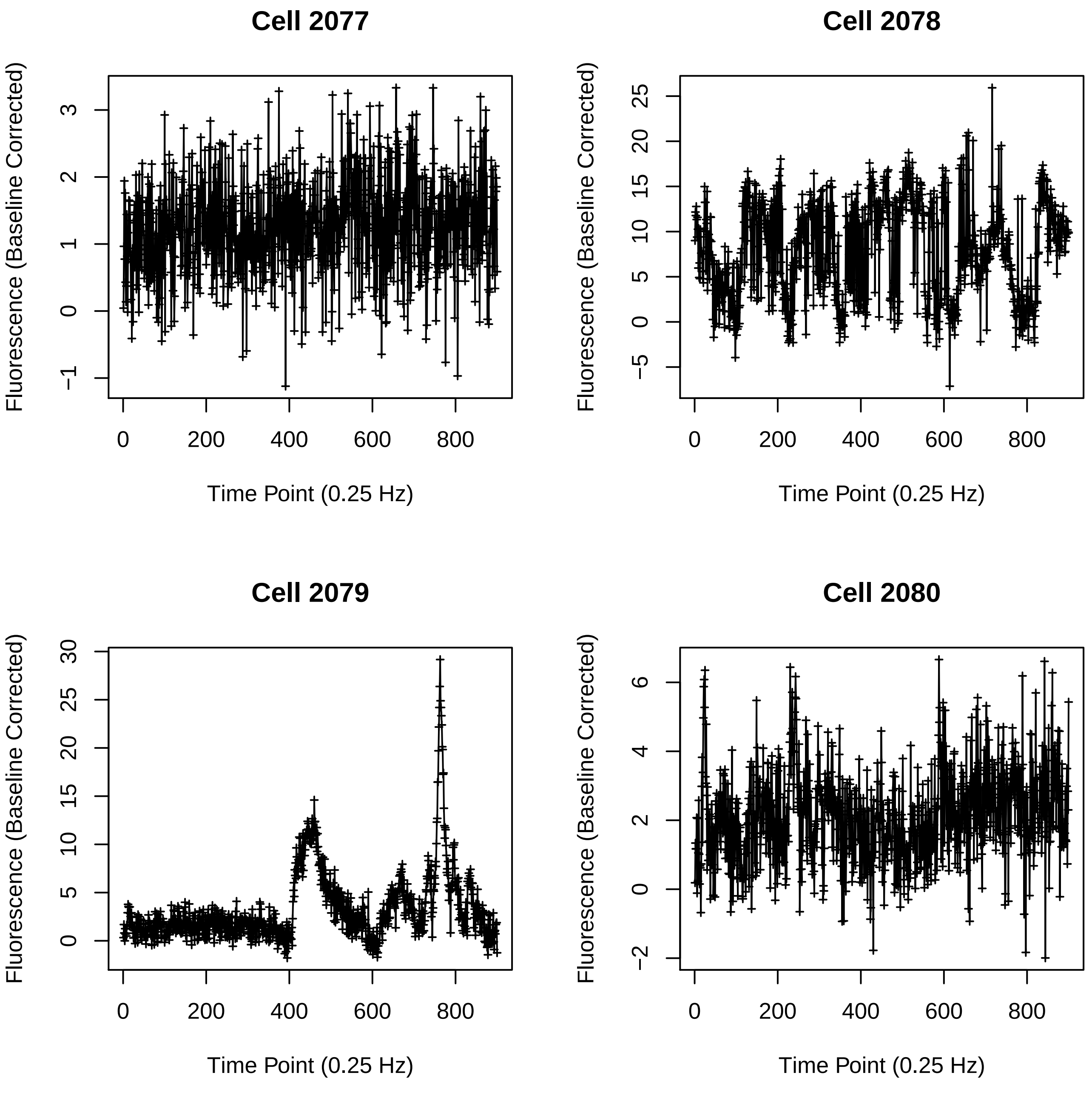
Cell 2077
Fluorescence (Baseline Corrected)
−1
0
1
2
3
0
200
400
600
800
Time Point (0.25 Hz)
Cell 2078
Fluorescence (Baseline Corrected)
−5
0
5
10
15
20
25
0
200
400
600
800
Time Point (0.25 Hz)
Cell 2079
Fluorescence (Baseline Corrected)
0
5
10
15
20
25
30
0
200
400
600
800
Time Point (0.25 Hz)
Cell 2080
Fluorescence (Baseline Corrected)
−2
0
2
4
6
0
200
400
600
800
Time Point (0.25 Hz)

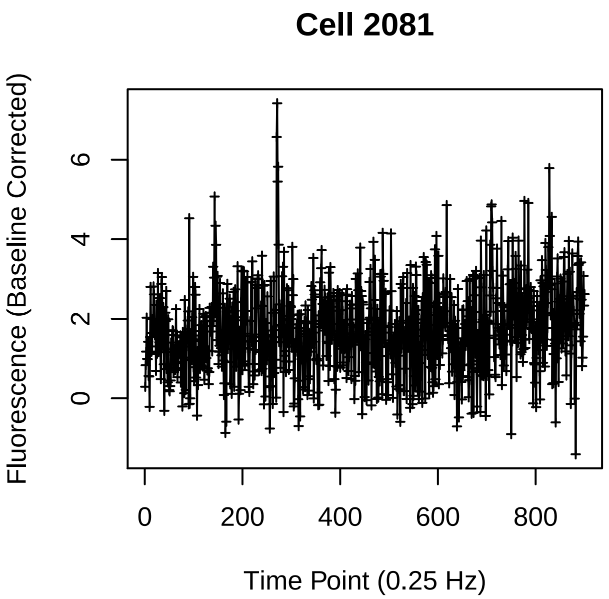
Cell 2081
Fluorescence (Baseline Corrected)
Time Point (0.25 Hz)
0
2
4
6
0
200
400
600
800

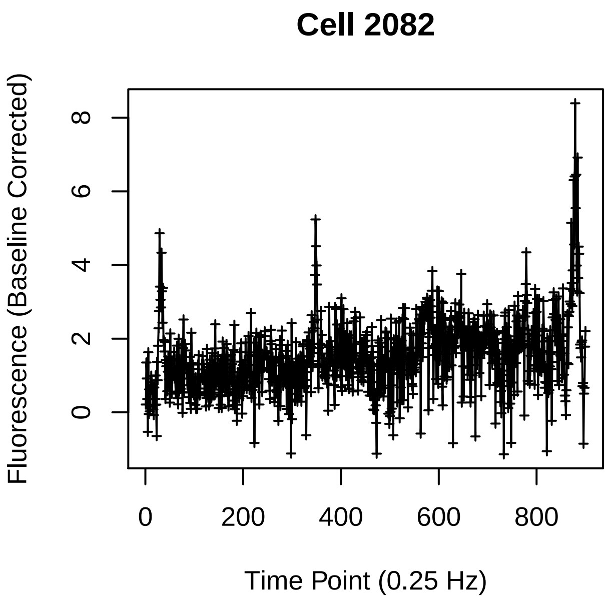
Cell 2082
Fluorescence (Baseline Corrected)
Time Point (0.25 Hz)
0
2
4
6
8
0
200
400
600
800

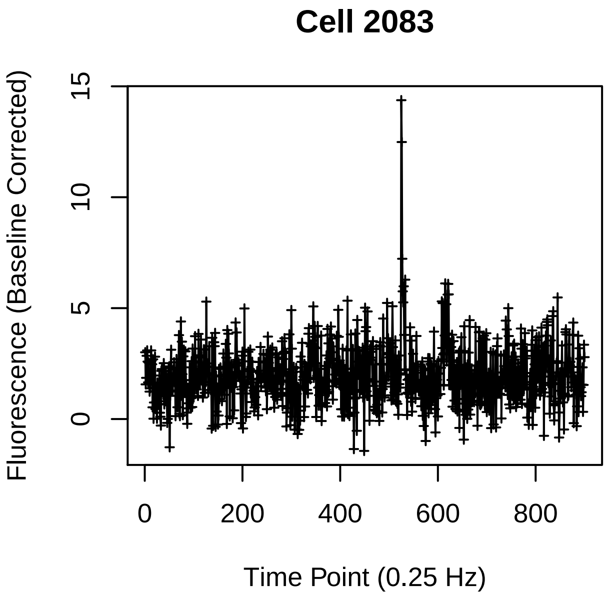
Cell 2083
Fluorescence (Baseline Corrected)
Time Point (0.25 Hz)
0
5
10
15
0
200
400
600
800

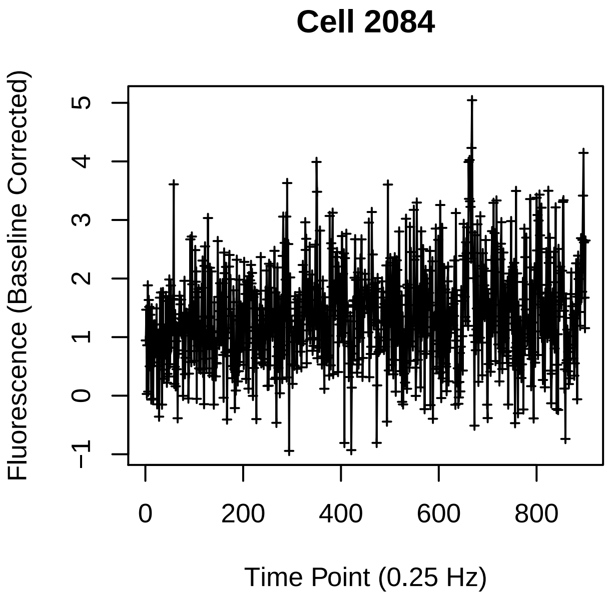
Cell 2084
Fluorescence (Baseline Corrected)
Time Point (0.25 Hz)
−1
0
1
2
3
4
5
0
200
400
600
800

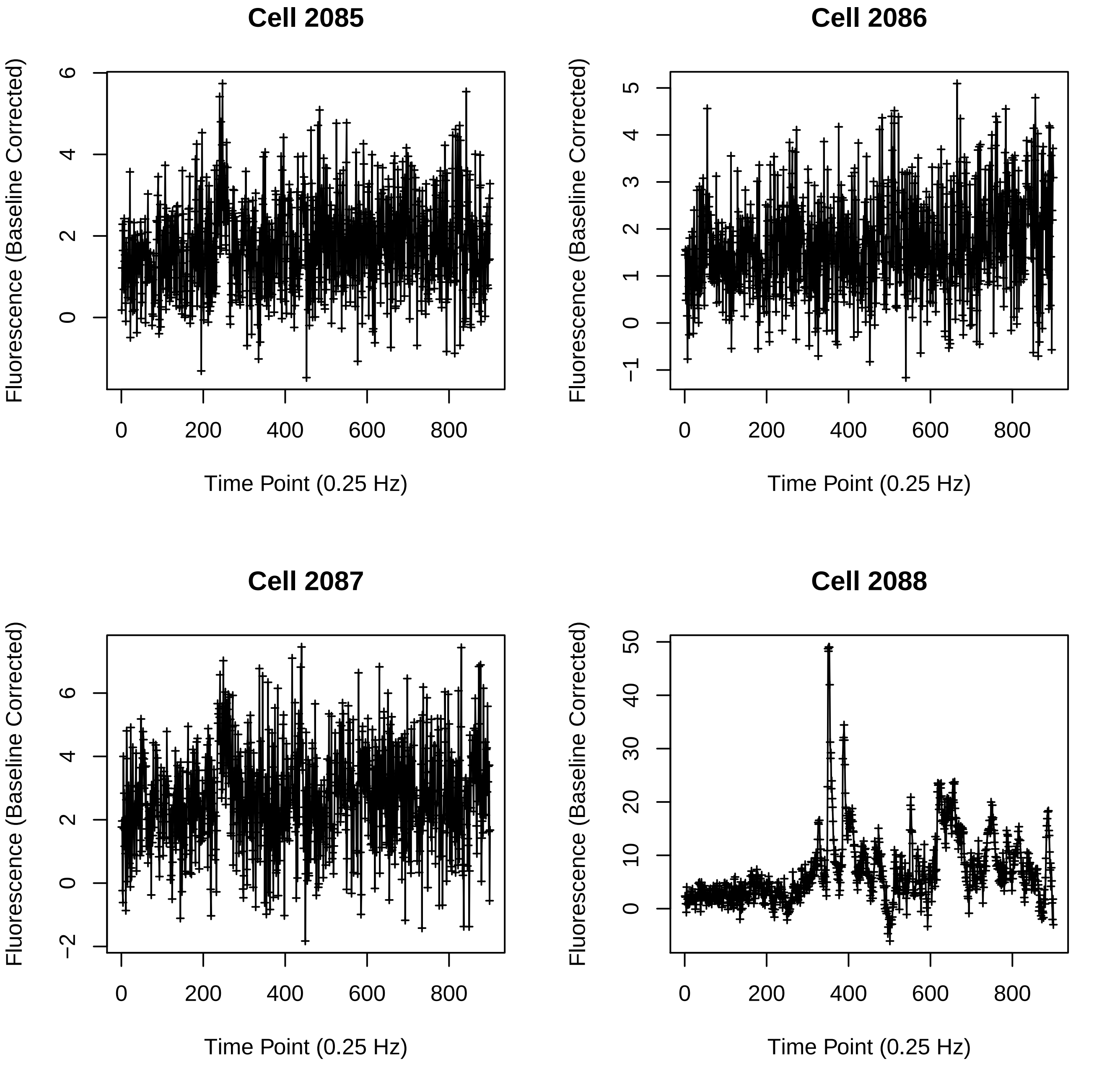

Cell 2085
Cell 2086
Cell 2087
Cell 2088
Fluorescence (Baseline Corrected)
Time Point (0.25 Hz)
0
200
400
600
800
-2
0
2
4
6
-1
1
3
5
10
20
30
40
50

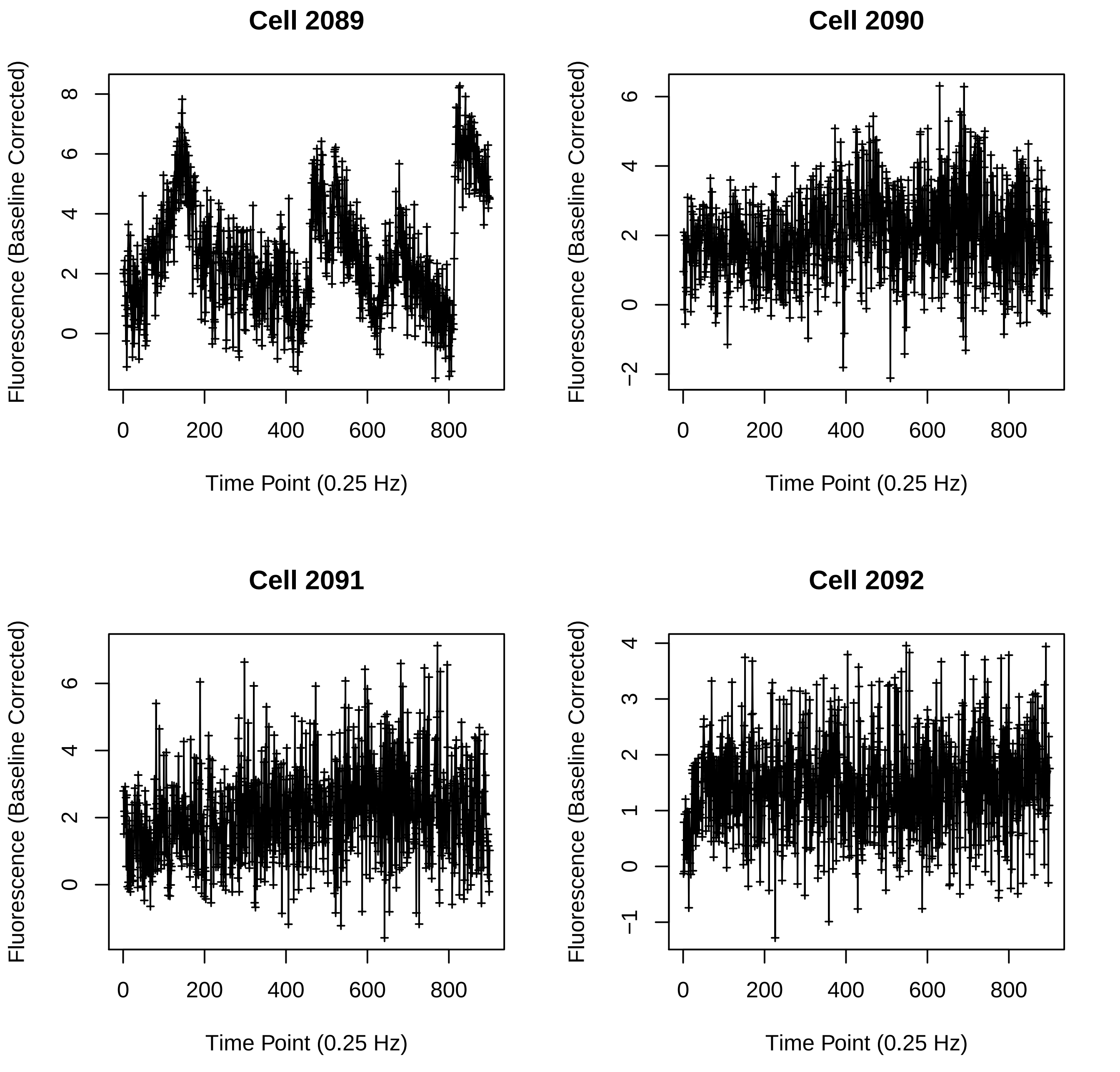
Cell 2089
Cell 2090
Cell 2091
Cell 2092
Fluorescence (Baseline Corrected)
Time Point (0.25 Hz)
0
200
400
600
800
−2
0
2
4
6
8
−1
1
3

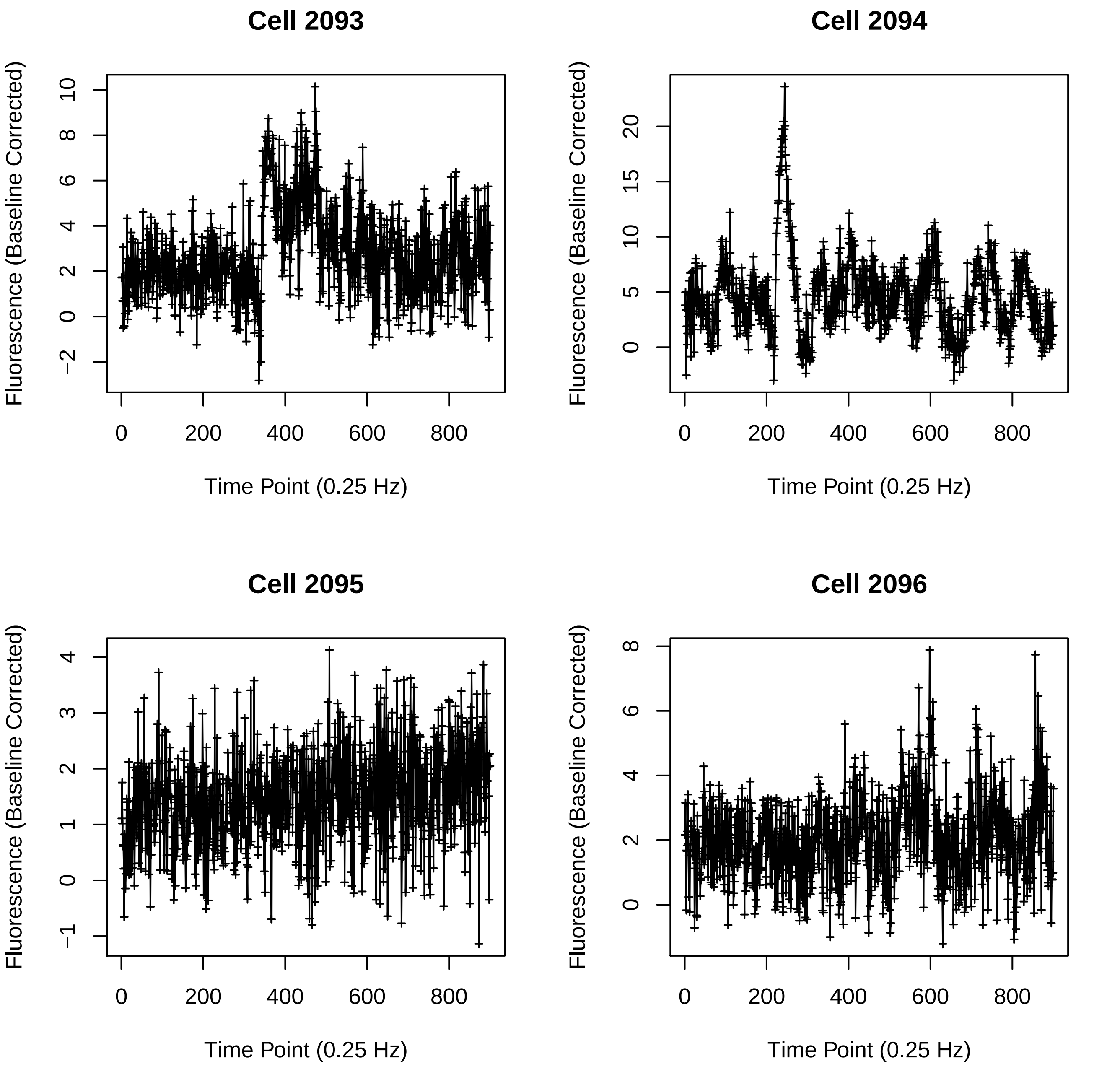

Cell 2093
Cell 2094
Cell 2095
Cell 2096
Fluorescence (Baseline Corrected)
Time Point (0.25 Hz)

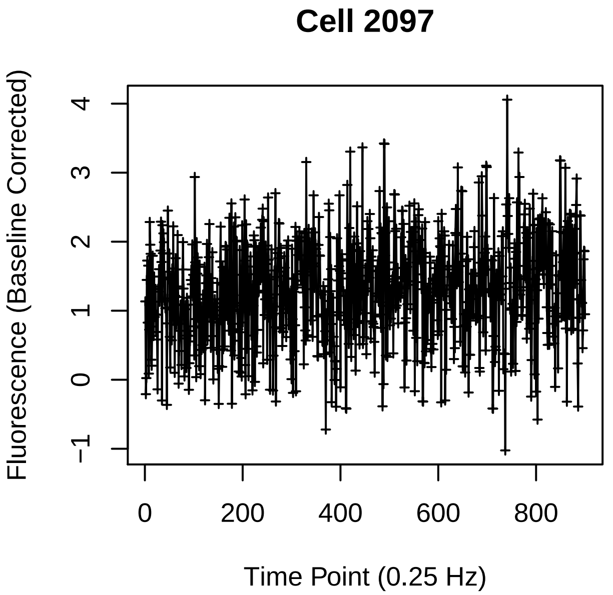
Cell 2097
Fluorescence (Baseline Corrected)
−1
0
1
2
3
4
0
200
400
600
800
Time Point (0.25 Hz)

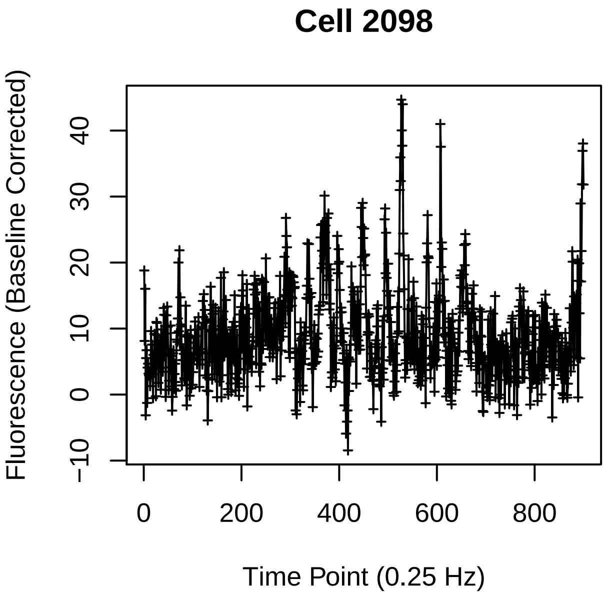
Cell 2098
Fluorescence (Baseline Corrected)
−10
0
10
20
30
40
0
200
400
600
800
Time Point (0.25 Hz)

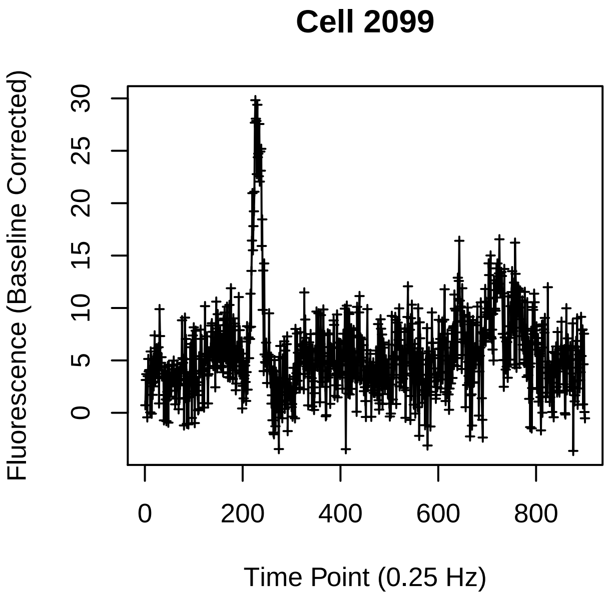
Cell 2099
Fluorescence (Baseline Corrected)
0
5
10
15
20
25
30
0
200
400
600
800
Time Point (0.25 Hz)

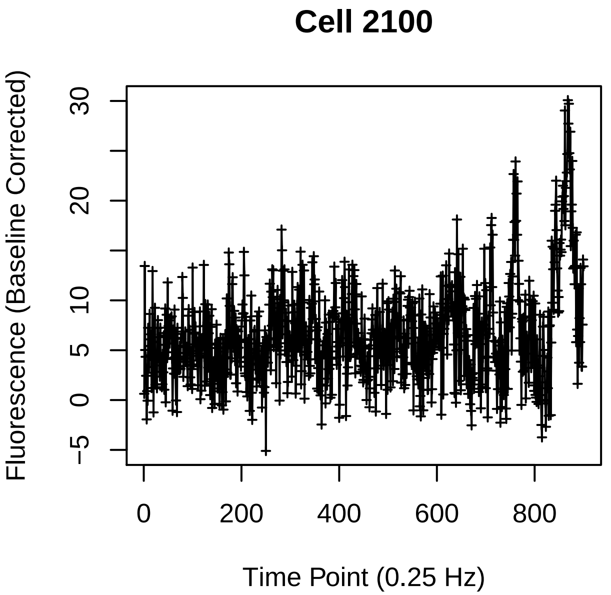
Cell 2100
Fluorescence (Baseline Corrected)
−5
0
5
10
20
30
0
200
400
600
800
Time Point (0.25 Hz)

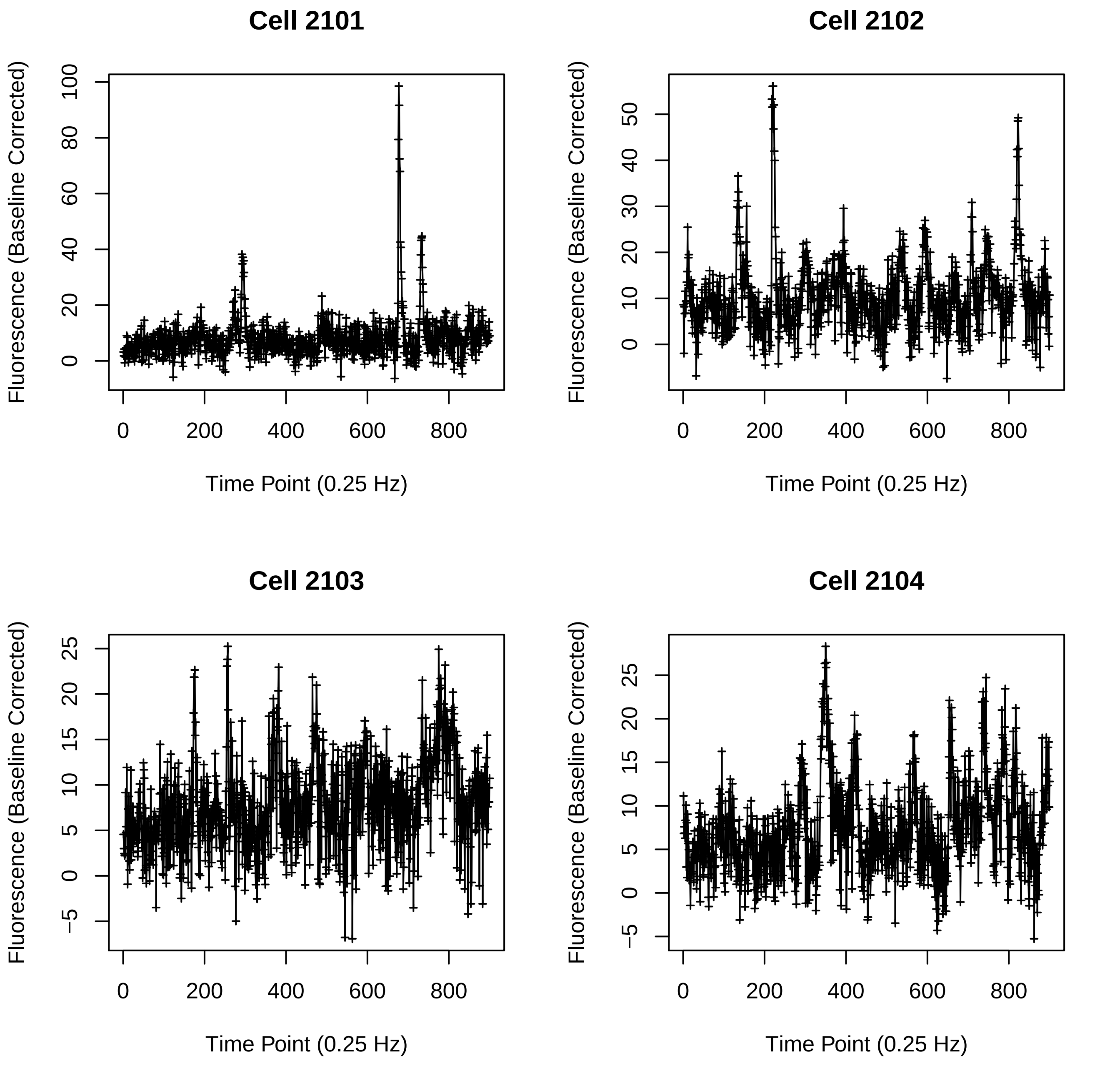

Cell 2101
Cell 2102
Cell 2103
Cell 2104
Fluorescence (Baseline Corrected)
Time Point (0.25 Hz)
0
200
400
600
800
100
80
60
40
20
50
30
10
25
15
5
−5

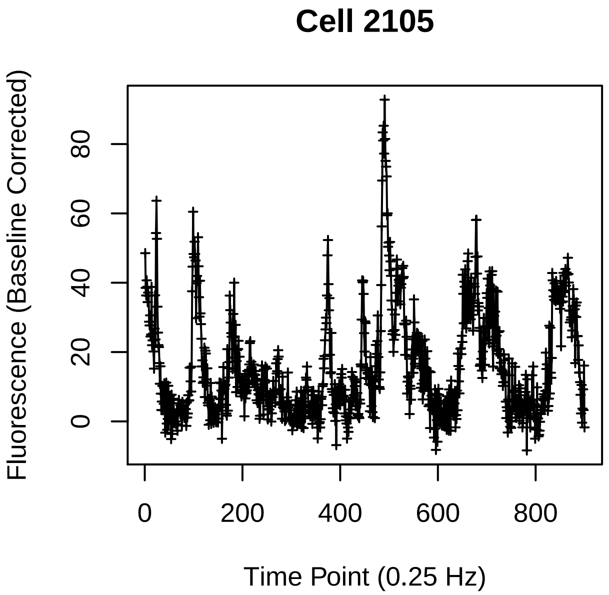
Cell 2105
Fluorescence (Baseline Corrected)
0
20
40
60
80
0
200
400
600
800
Time Point (0.25 Hz)

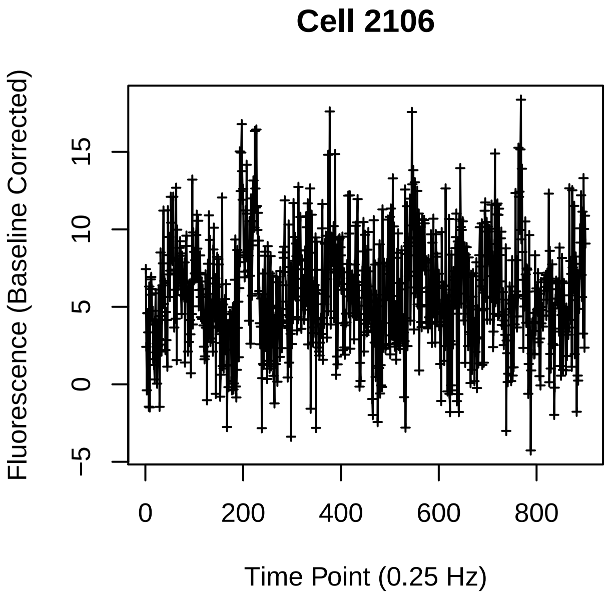
Cell 2106
Fluorescence (Baseline Corrected)
−5
0
5
10
15
0
200
400
600
800
Time Point (0.25 Hz)

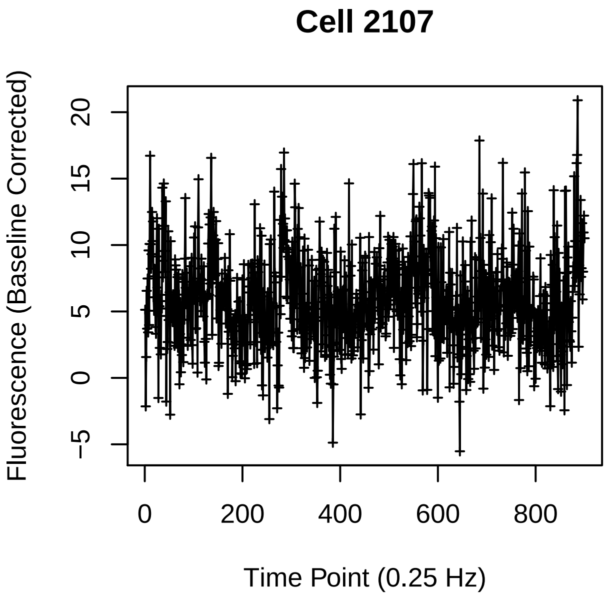
Cell 2107
Fluorescence (Baseline Corrected)
−5
0
5
10
15
20
0
200
400
600
800
Time Point (0.25 Hz)

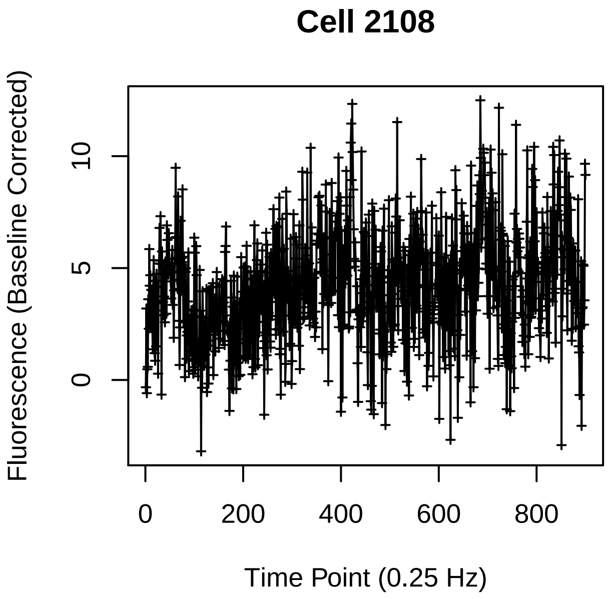
Cell 2108
Fluorescence (Baseline Corrected)
0
5
10
0
200
400
600
800
Time Point (0.25 Hz)

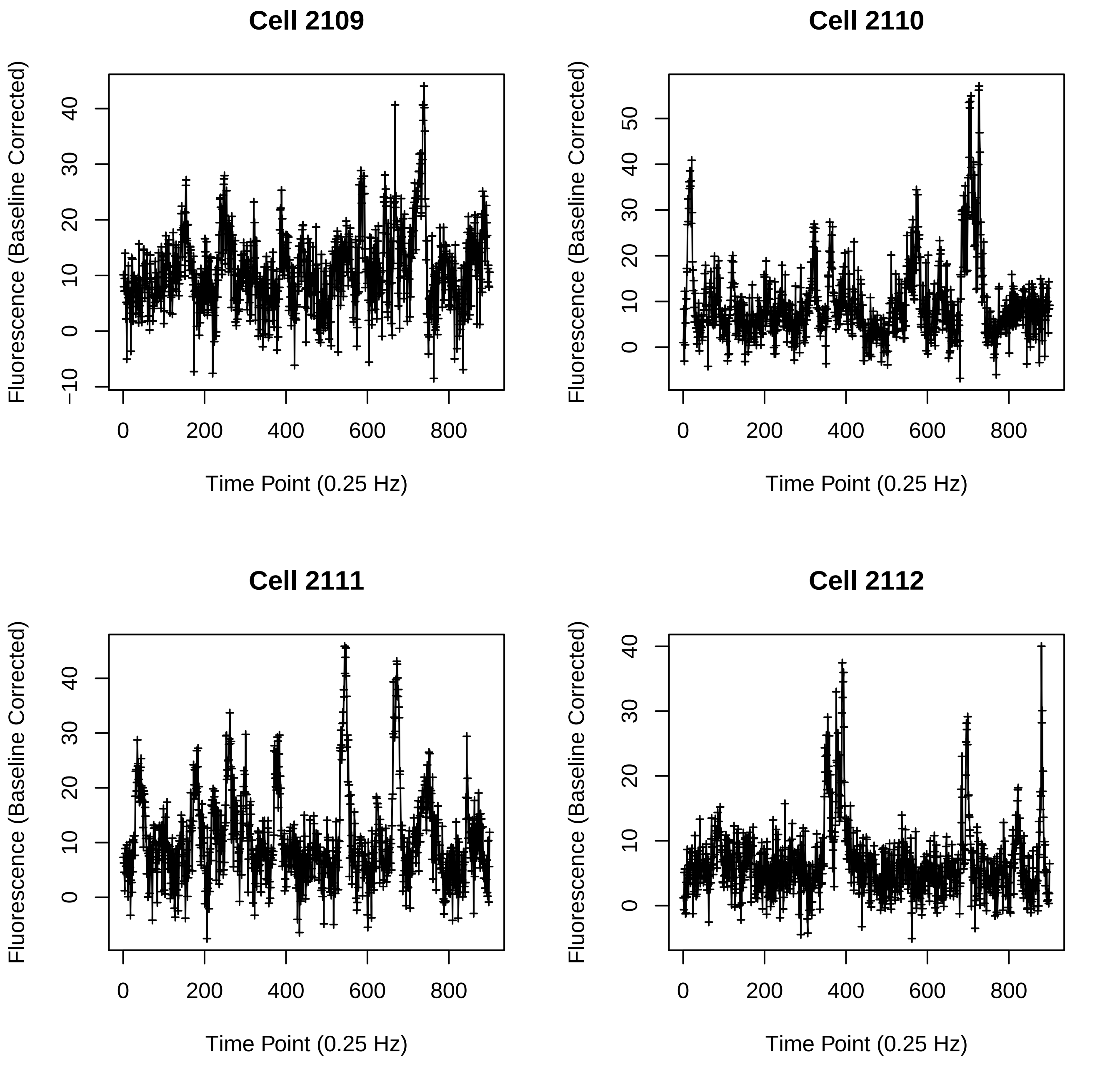

Cell 2109
Cell 2110
Cell 2111
Cell 2112
Fluorescence (Baseline Corrected)
Time Point (0.25 Hz)

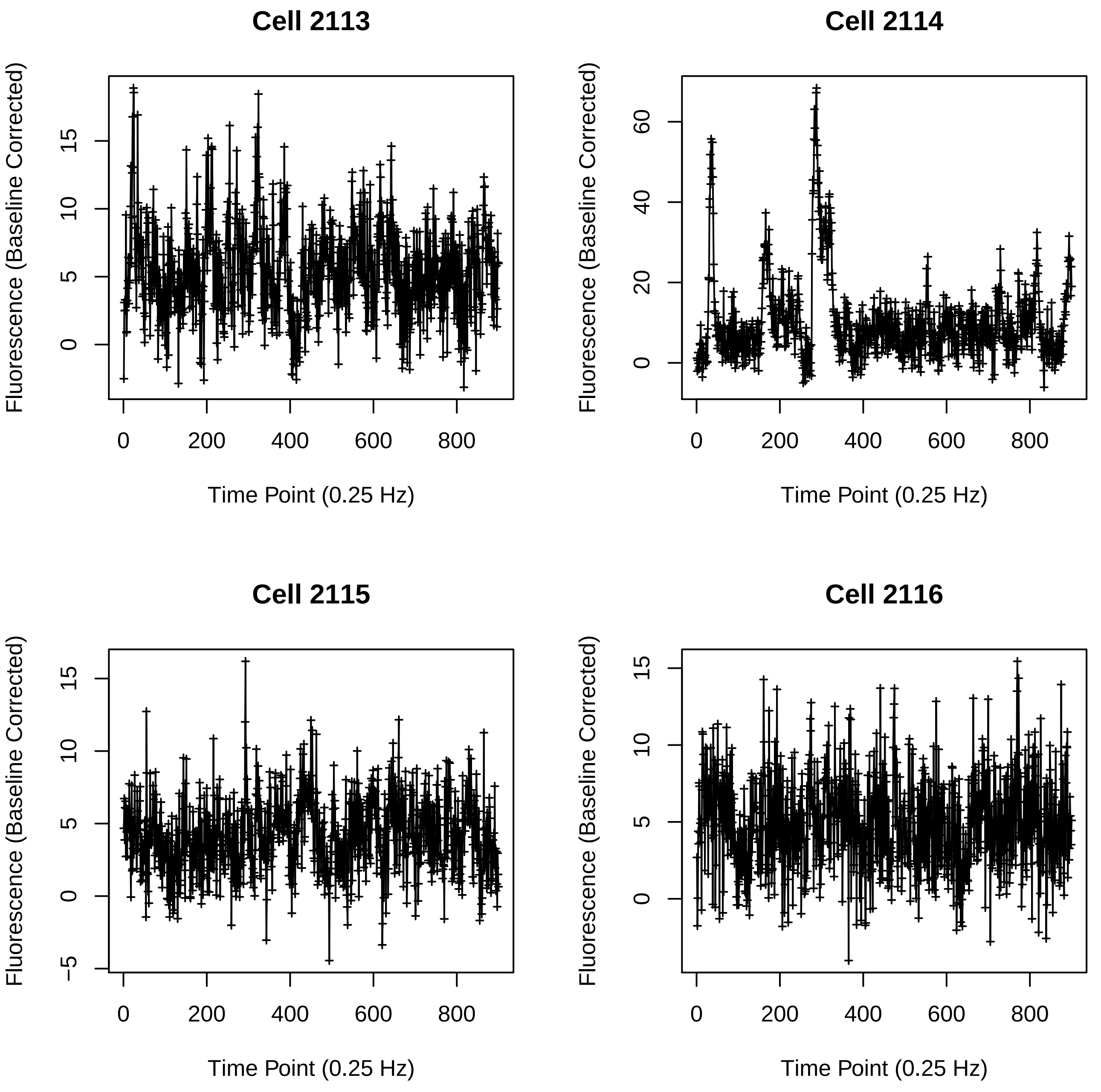
Cell 2113
Fluorescence (Baseline Corrected)
0
5
10
15
0
200
400
600
800
Time Point (0.25 Hz)
Cell 2114
Fluorescence (Baseline Corrected)
0
20
40
60
0
200
400
600
800
Time Point (0.25 Hz)
Cell 2115
Fluorescence (Baseline Corrected)
−5
0
5
10
15
0
200
400
600
800
Time Point (0.25 Hz)
Cell 2116
Fluorescence (Baseline Corrected)
0
5
10
15
0
200
400
600
800
Time Point (0.25 Hz)

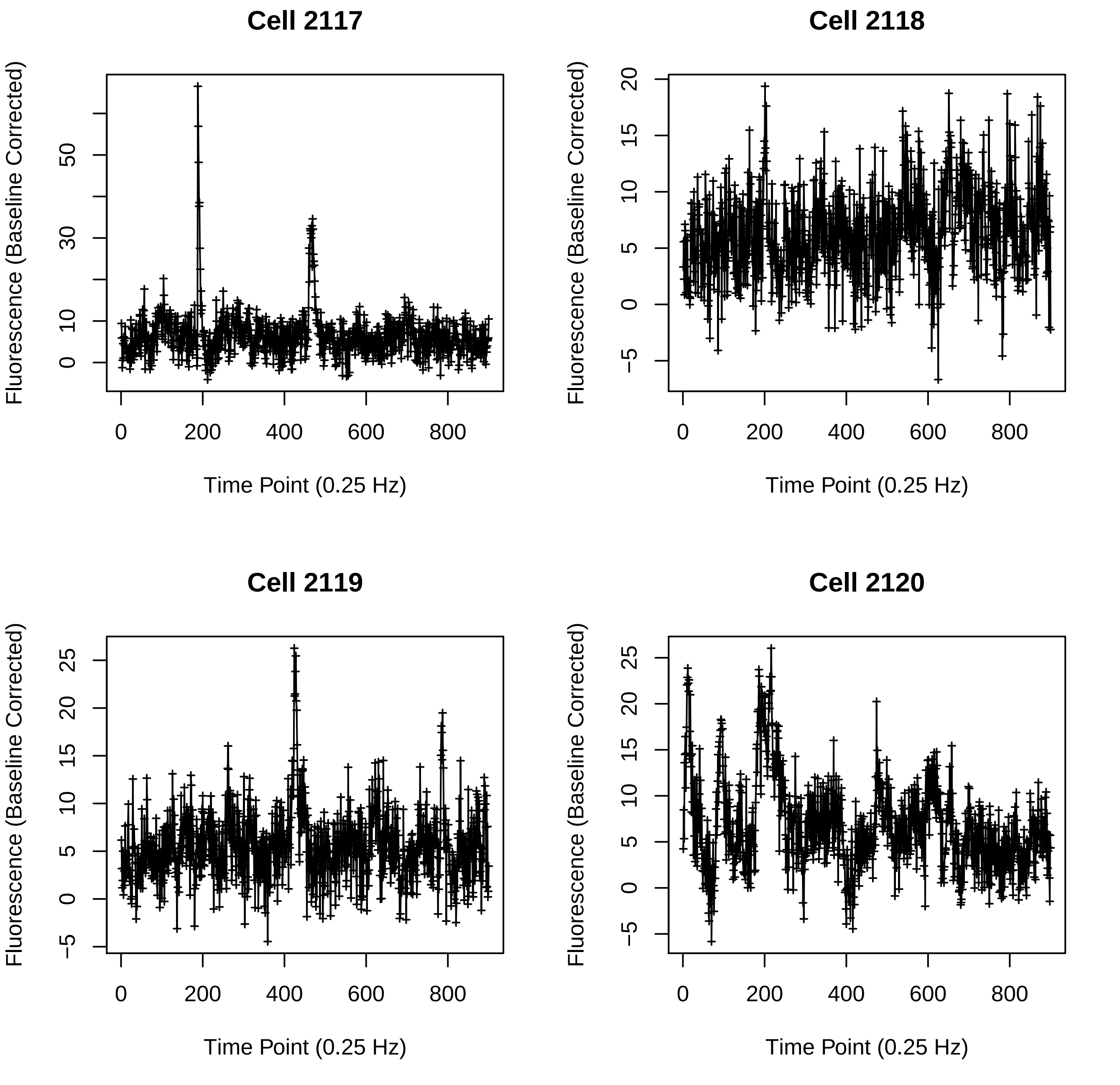
Cell 2117
Cell 2118
Cell 2119
Cell 2120
Fluorescence (Baseline Corrected)
Time Point (0.25 Hz)
0
10
30
50
−5
5
15
20
25
200
400
600
800

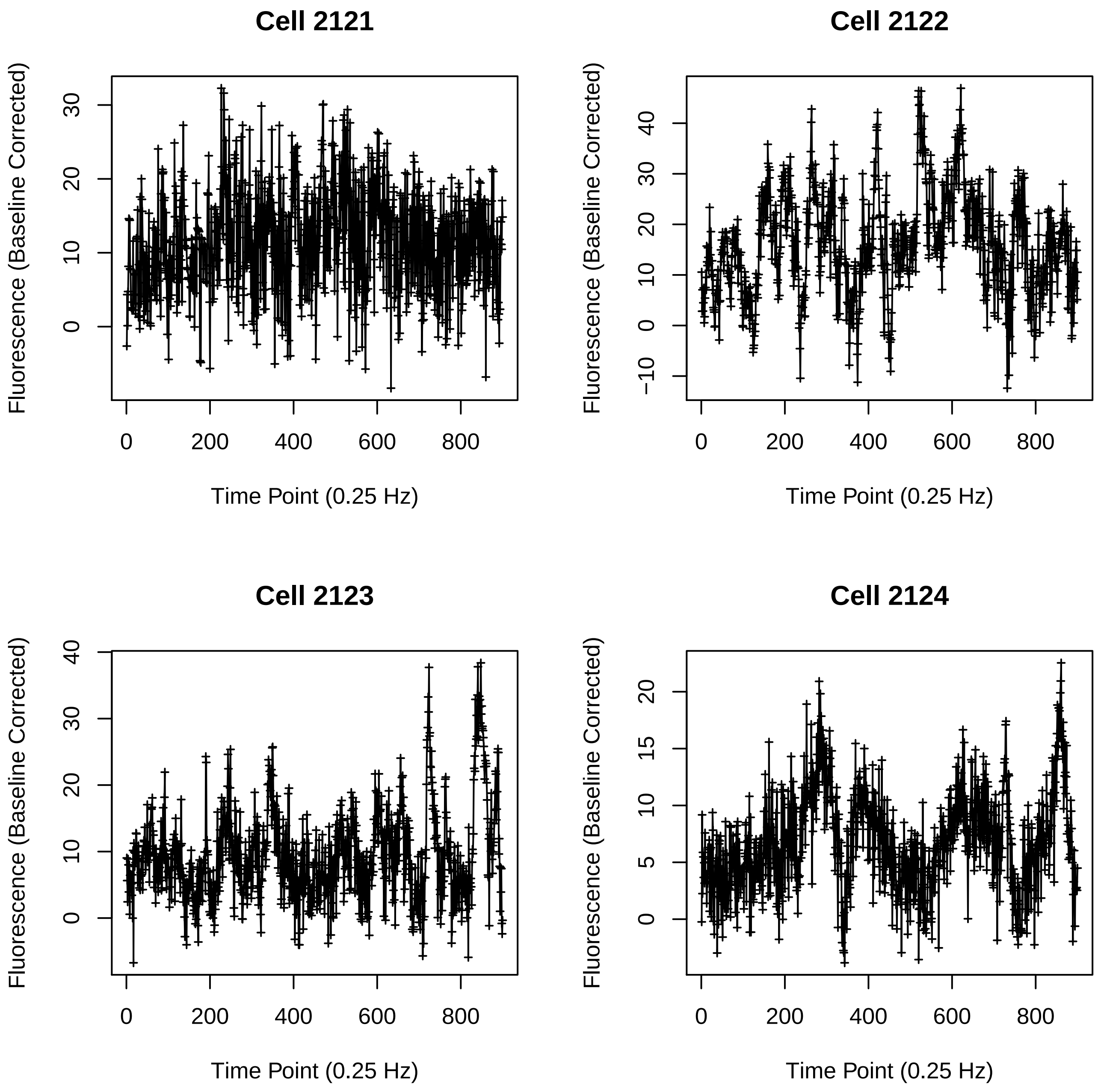

Cell 2121
Cell 2122
Cell 2123
Cell 2124
Fluorescence (Baseline Corrected)
Time Point (0.25 Hz)
0
200
400
600
800
-10
10
20
30
40
5
15

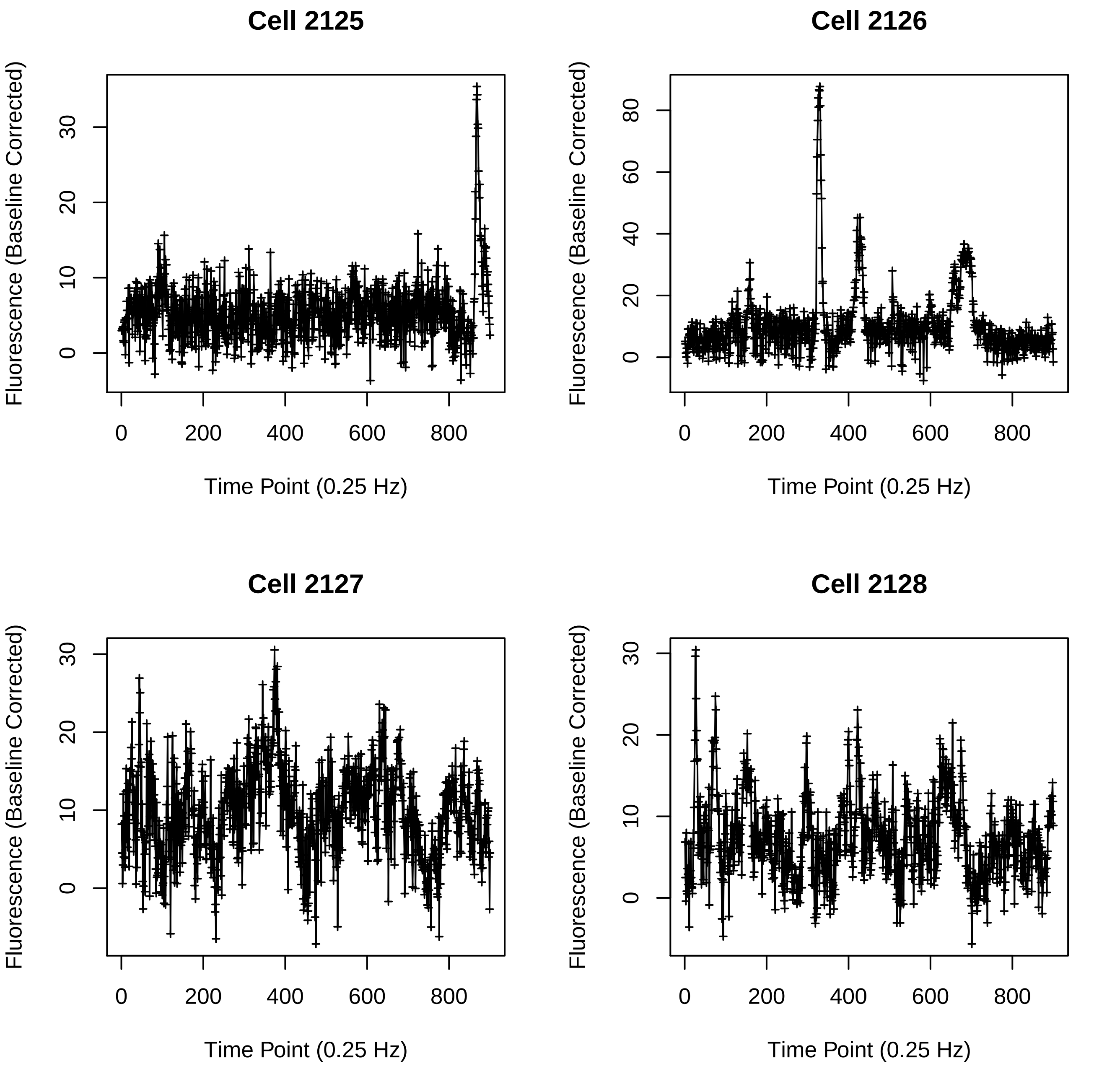
Cell 2125
Cell 2126
Cell 2127
Cell 2128
Fluorescence (Baseline Corrected)
Time Point (0.25 Hz)
0
200
400
600
800
10
20
30
40
60
80

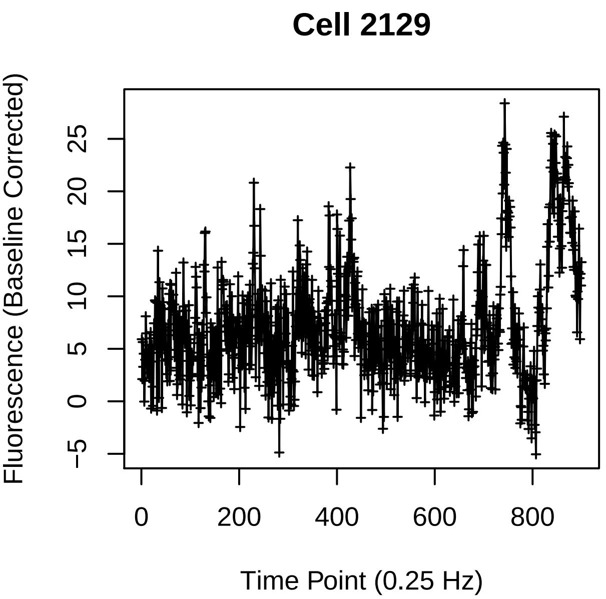
Cell 2129
Fluorescence (Baseline Corrected)
Time Point (0.25 Hz)
−5
0
5
10
15
20
25
0
200
400
600
800

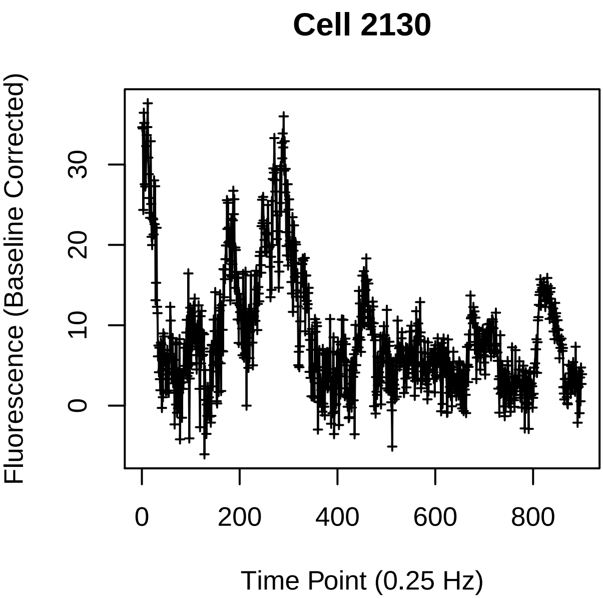
Cell 2130
Fluorescence (Baseline Corrected)
Time Point (0.25 Hz)
0
10
20
30
0
200
400
600
800

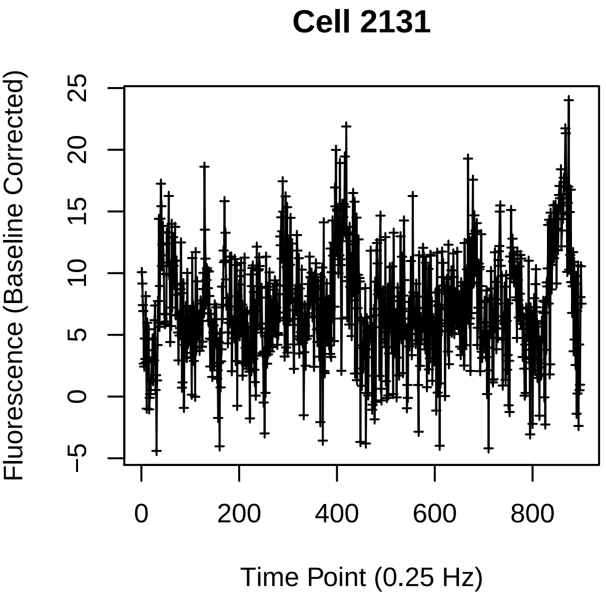
Cell 2131
Fluorescence (Baseline Corrected)
Time Point (0.25 Hz)
−5
0
5
10
15
20
25
0
200
400
600
800

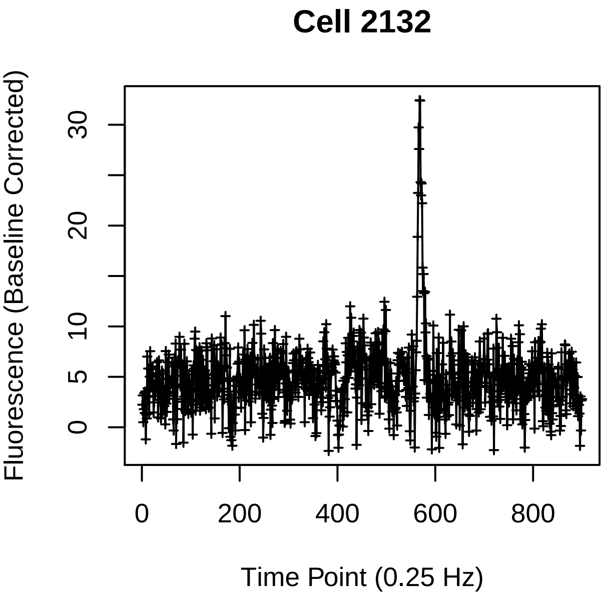
Cell 2132
Fluorescence (Baseline Corrected)
Time Point (0.25 Hz)
0
5
10
20
30
0
200
400
600
800

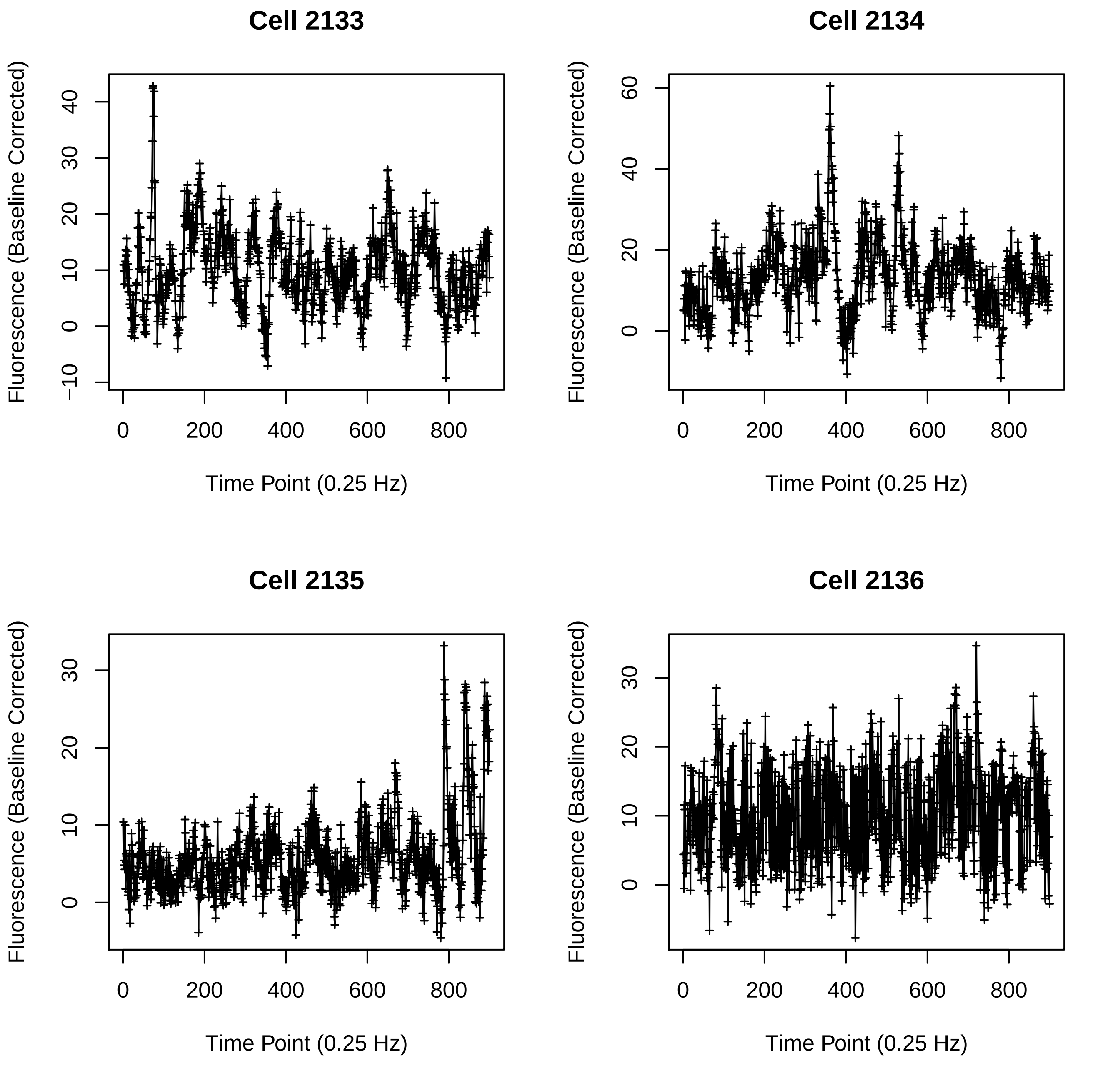
Cell 2133
Cell 2134
Cell 2135
Cell 2136
Fluorescence (Baseline Corrected)
Time Point (0.25 Hz)
−10
0
10
20
30
40
60
0
200
400
600
800

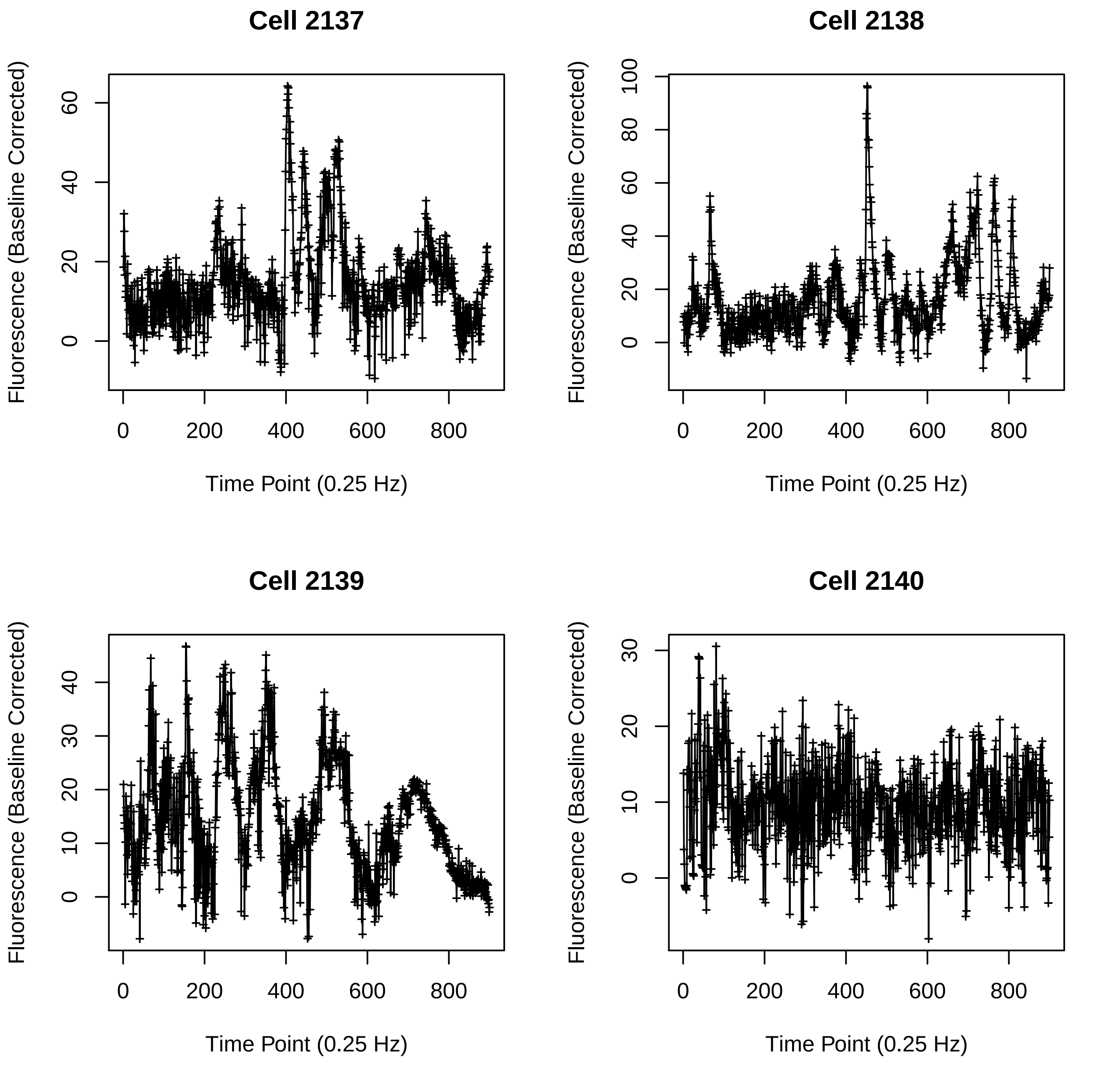
Cell 2137
Cell 2138
Cell 2139
Cell 2140
Fluorescence (Baseline Corrected)
Time Point (0.25 Hz)
0
20
40
60
80
100
200
400
600
800
10
30

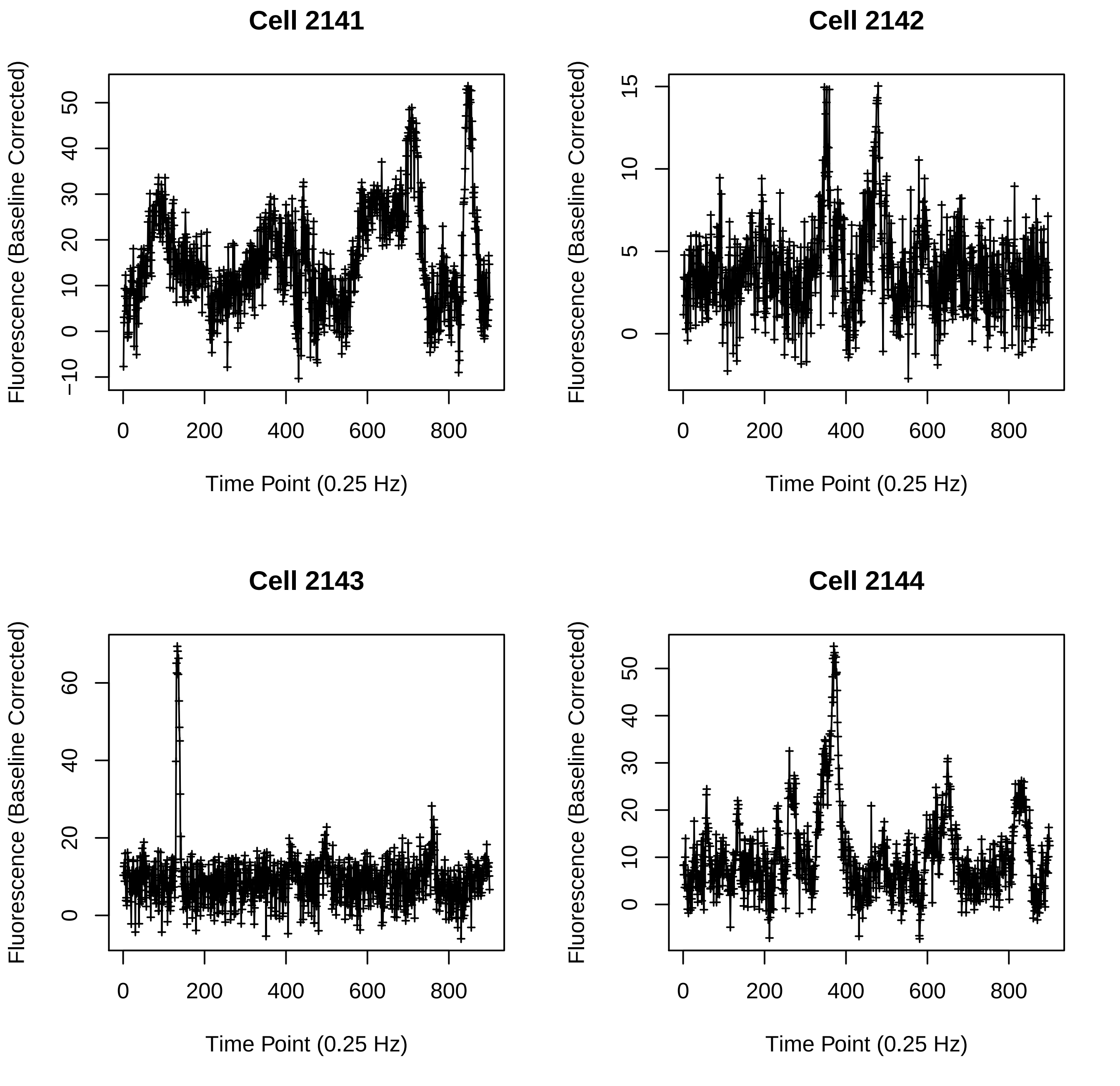
Cell 2141
Fluorescence (Baseline Corrected)
−10
0
10
20
30
40
50
0
200
400
600
800
Time Point (0.25 Hz)
Cell 2142
Fluorescence (Baseline Corrected)
0
5
10
15
0
200
400
600
800
Time Point (0.25 Hz)
Cell 2143
Fluorescence (Baseline Corrected)
0
20
40
60
0
200
400
600
800
Time Point (0.25 Hz)
Cell 2144
Fluorescence (Baseline Corrected)
0
10
20
30
40
50
0
200
400
600
800
Time Point (0.25 Hz)

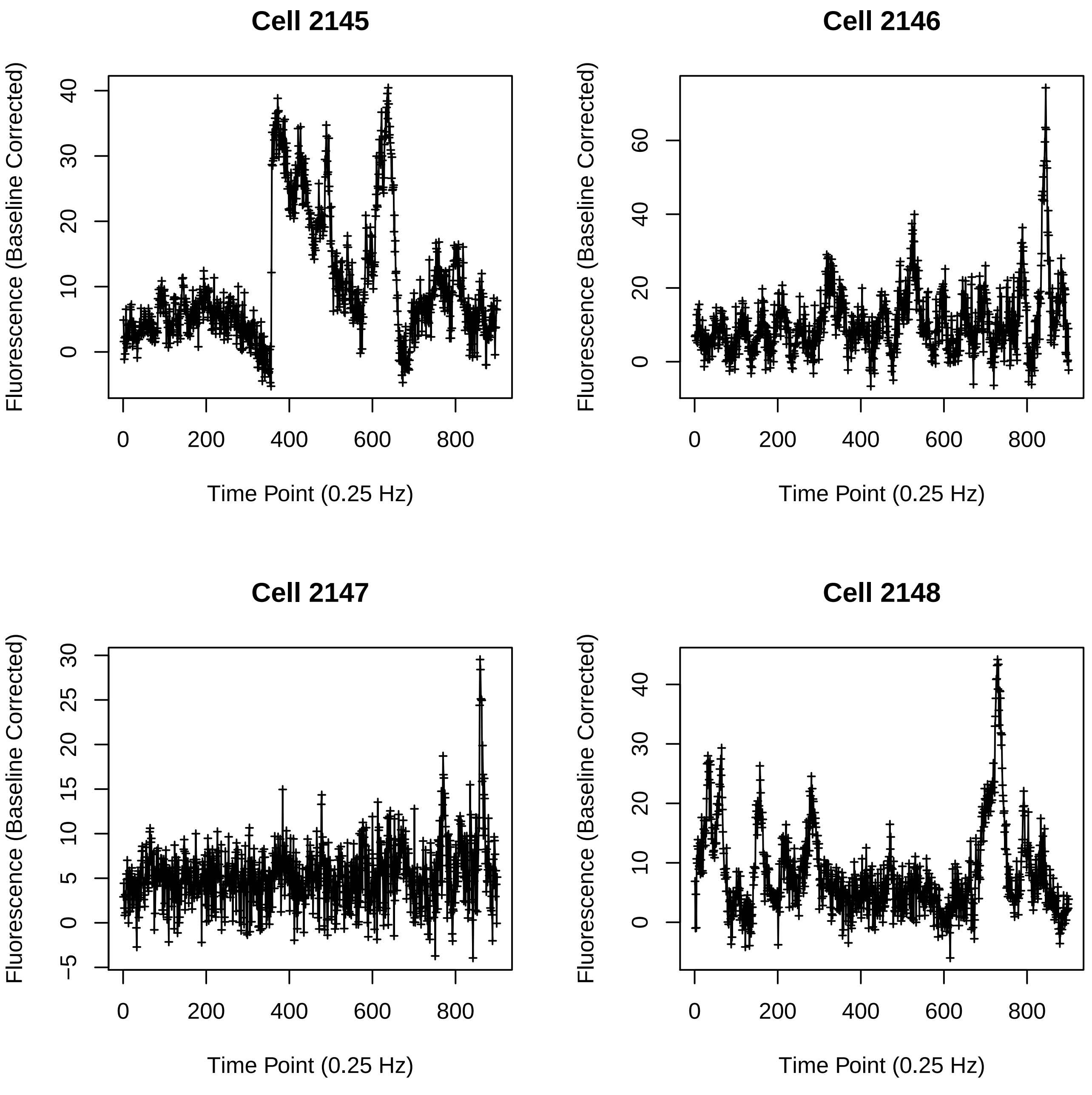
Cell 2145
Fluorescence (Baseline Corrected)
Time Point (0.25 Hz)
Cell 2146
Fluorescence (Baseline Corrected)
Time Point (0.25 Hz)
Cell 2147
Fluorescence (Baseline Corrected)
Time Point (0.25 Hz)
Cell 2148
Fluorescence (Baseline Corrected)
Time Point (0.25 Hz)

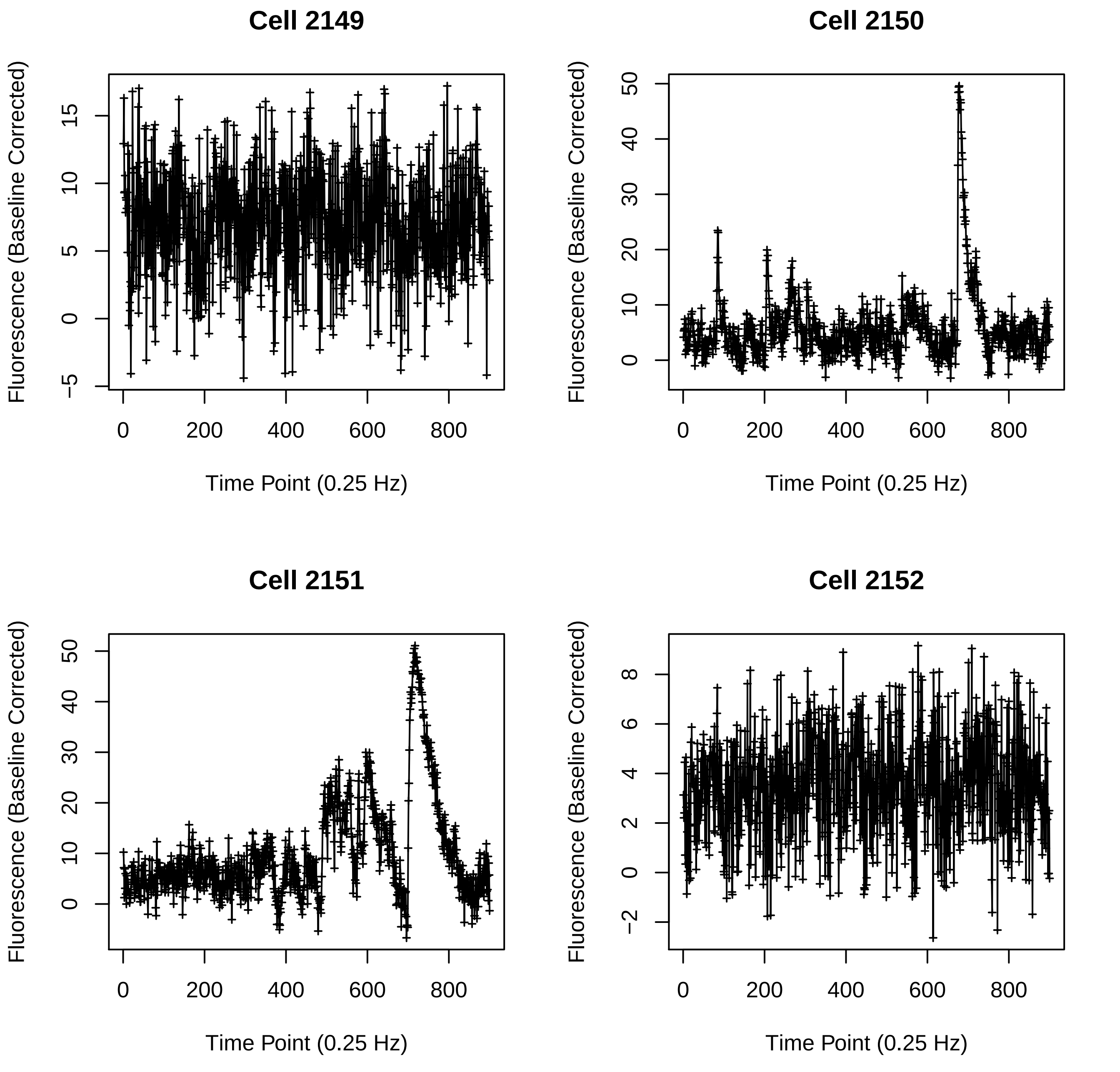
Cell 2149
Cell 2150
Cell 2151
Cell 2152
Fluorescence (Baseline Corrected)
Time Point (0.25 Hz)
−5
0
5
10
15
20
30
40
50
−2
2
4
6
8
200
400
600
800

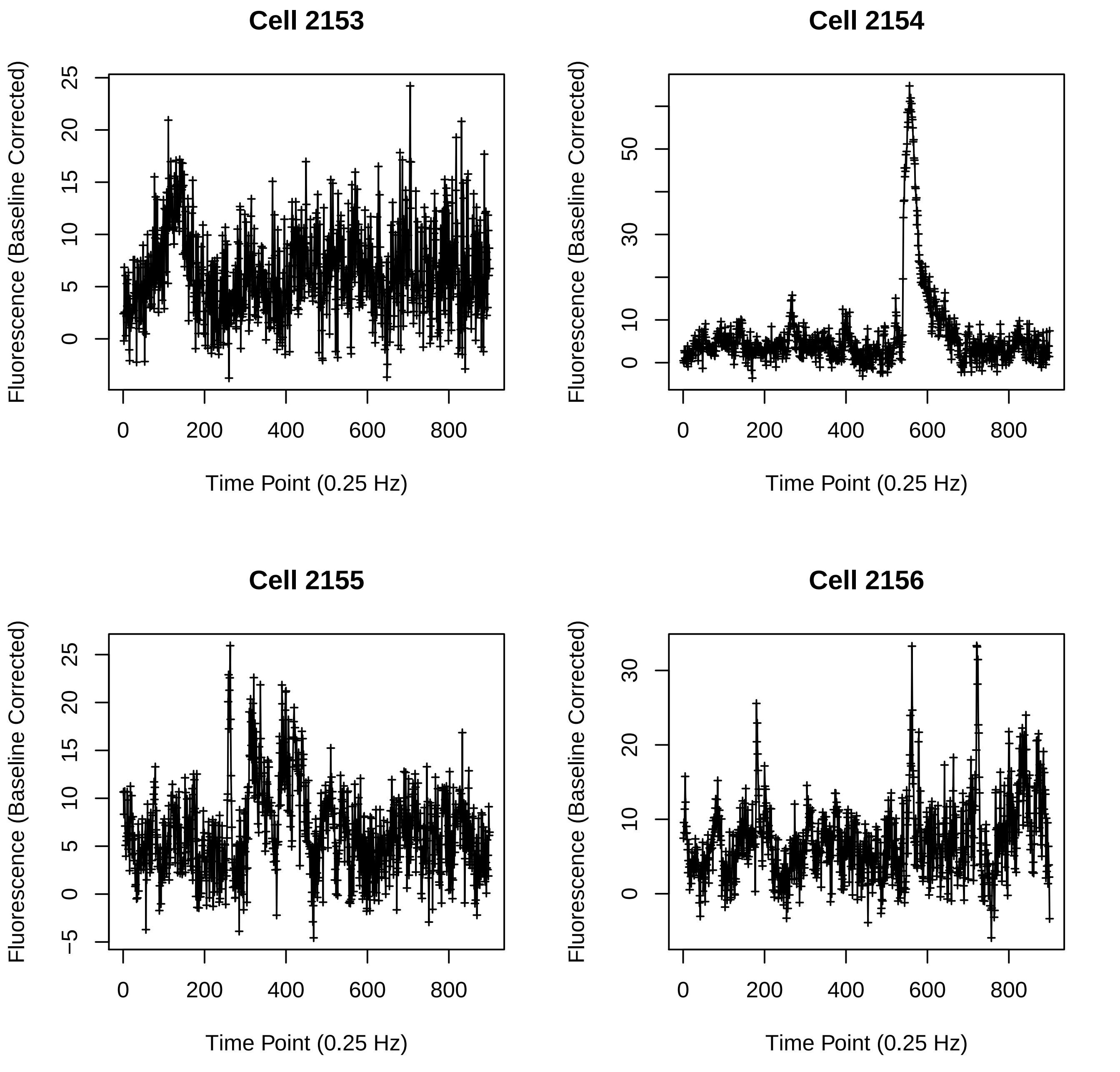

Cell 2153
Cell 2154
Cell 2155
Cell 2156
Fluorescence (Baseline Corrected)
Time Point (0.25 Hz)
0
200
400
600
800
-5
0
5
10
15
20
25
30
50

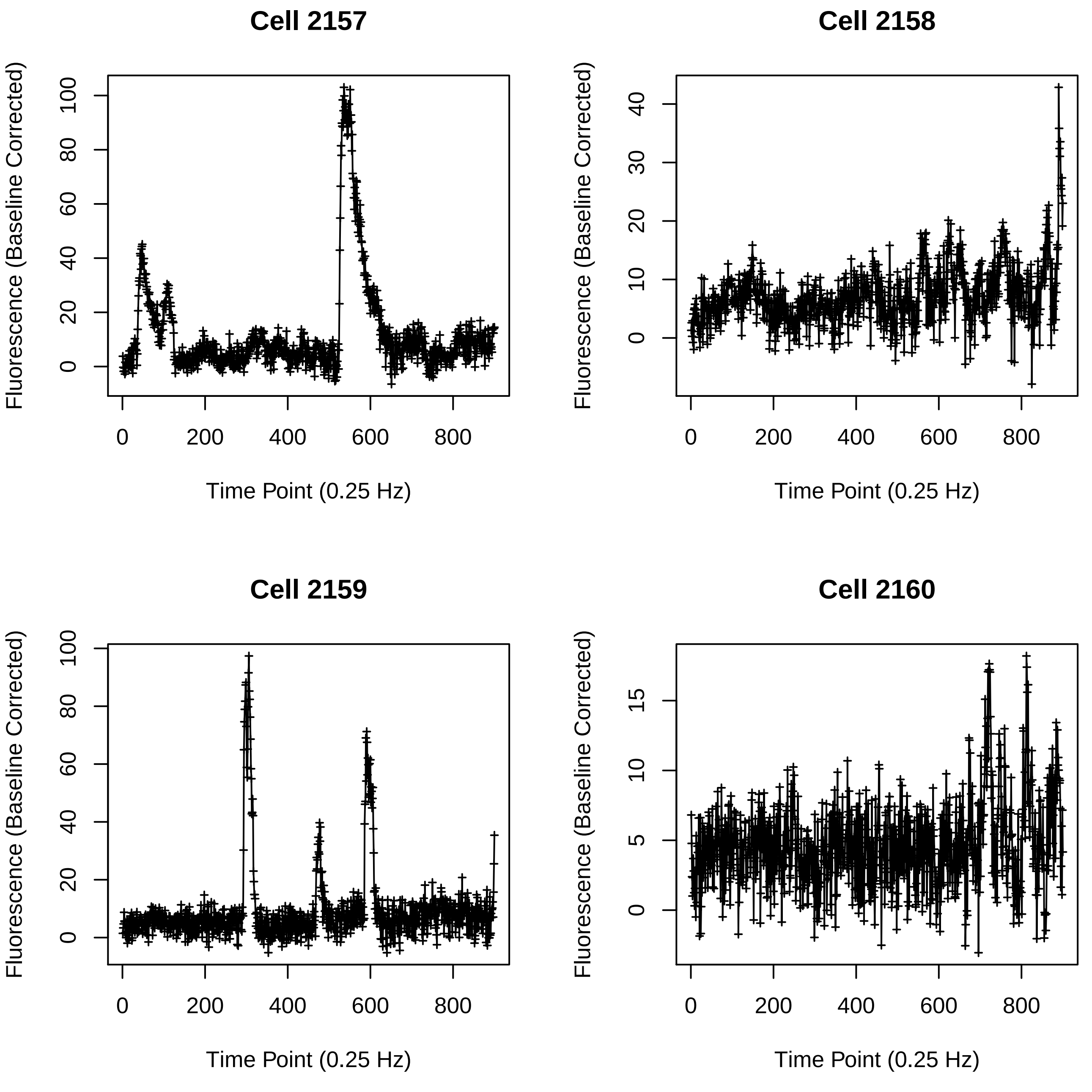
Cell 2157
Fluorescence (Baseline Corrected)
0
20
40
60
80
100
0
200
400
600
800
Time Point (0.25 Hz)
Cell 2158
Fluorescence (Baseline Corrected)
0
10
20
30
40
0
200
400
600
800
Time Point (0.25 Hz)
Cell 2159
Fluorescence (Baseline Corrected)
0
20
40
60
80
100
0
200
400
600
800
Time Point (0.25 Hz)
Cell 2160
Fluorescence (Baseline Corrected)
0
5
10
15
0
200
400
600
800
Time Point (0.25 Hz)

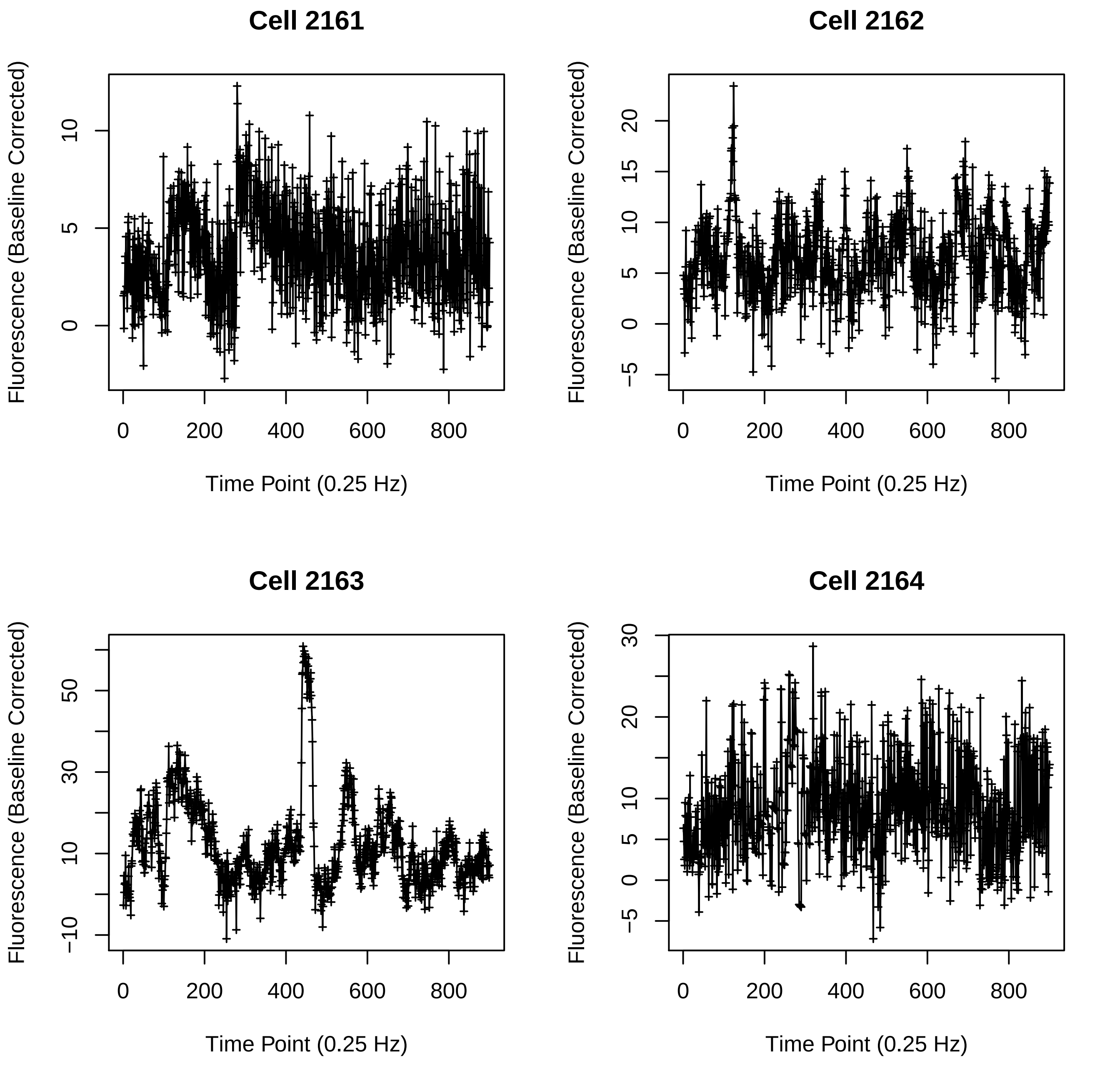

Cell 2161
Cell 2162
Cell 2163
Cell 2164
Fluorescence (Baseline Corrected)
Time Point (0.25 Hz)

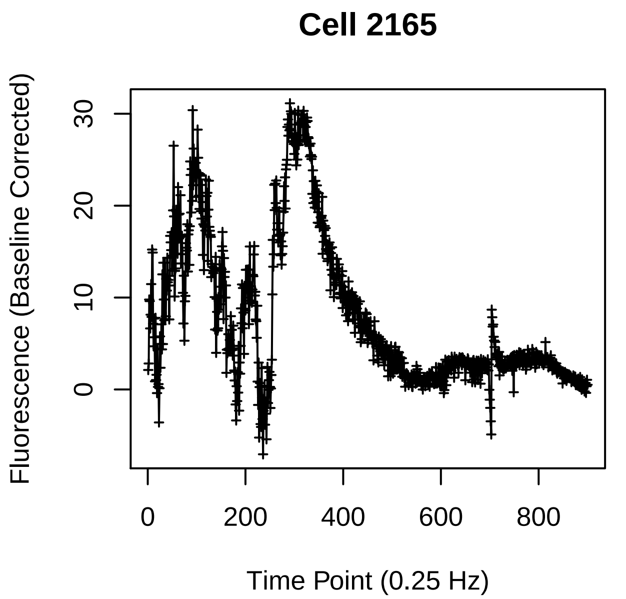
Cell 2165
Fluorescence (Baseline Corrected)
0
10
20
30
0
200
400
600
800
Time Point (0.25 Hz)

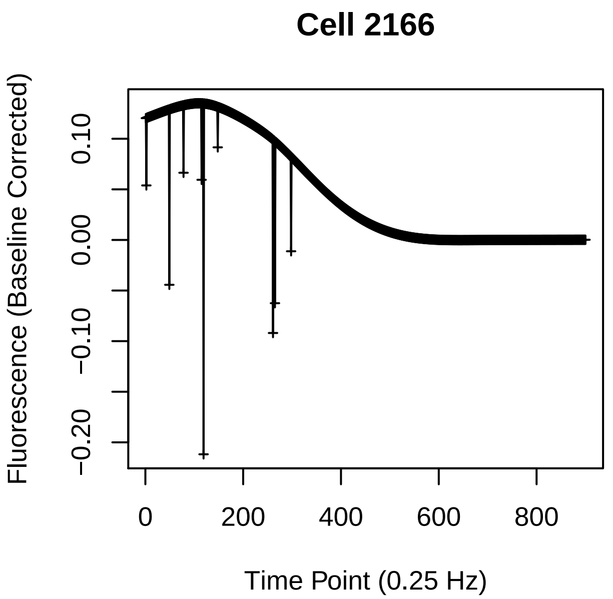
Cell 2166
Fluorescence (Baseline Corrected)
−0.20
−0.10
0.00
0.10
0
200
400
600
800
Time Point (0.25 Hz)

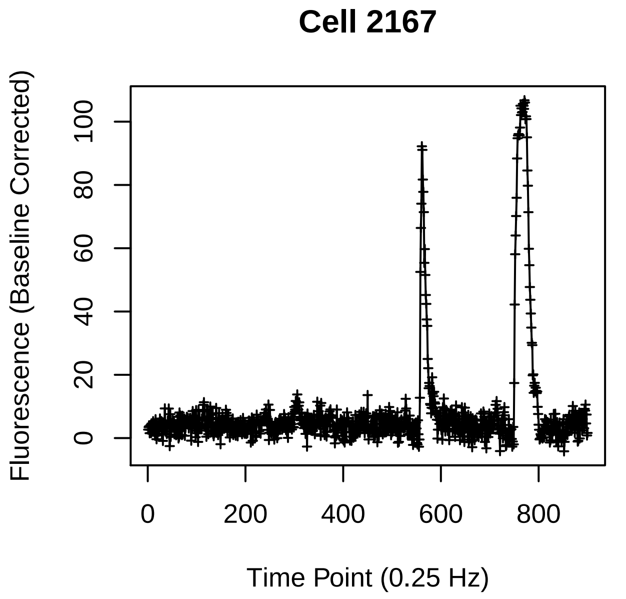
Cell 2167
Fluorescence (Baseline Corrected)
0
20
40
60
80
100
0
200
400
600
800
Time Point (0.25 Hz)

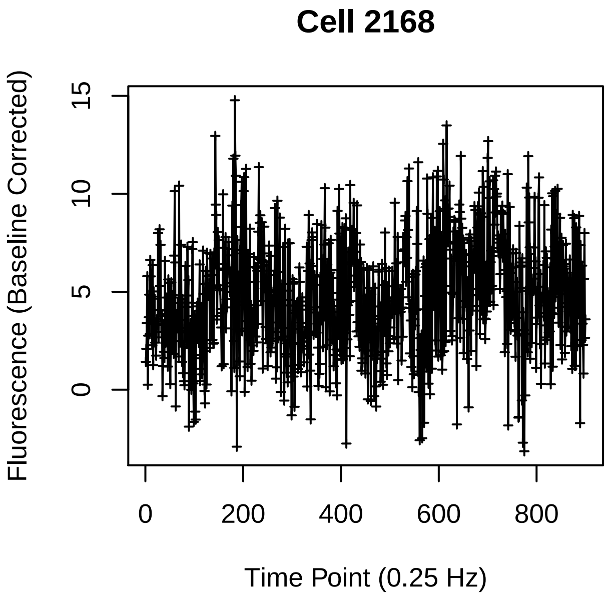
Cell 2168
Fluorescence (Baseline Corrected)
0
5
10
15
0
200
400
600
800
Time Point (0.25 Hz)

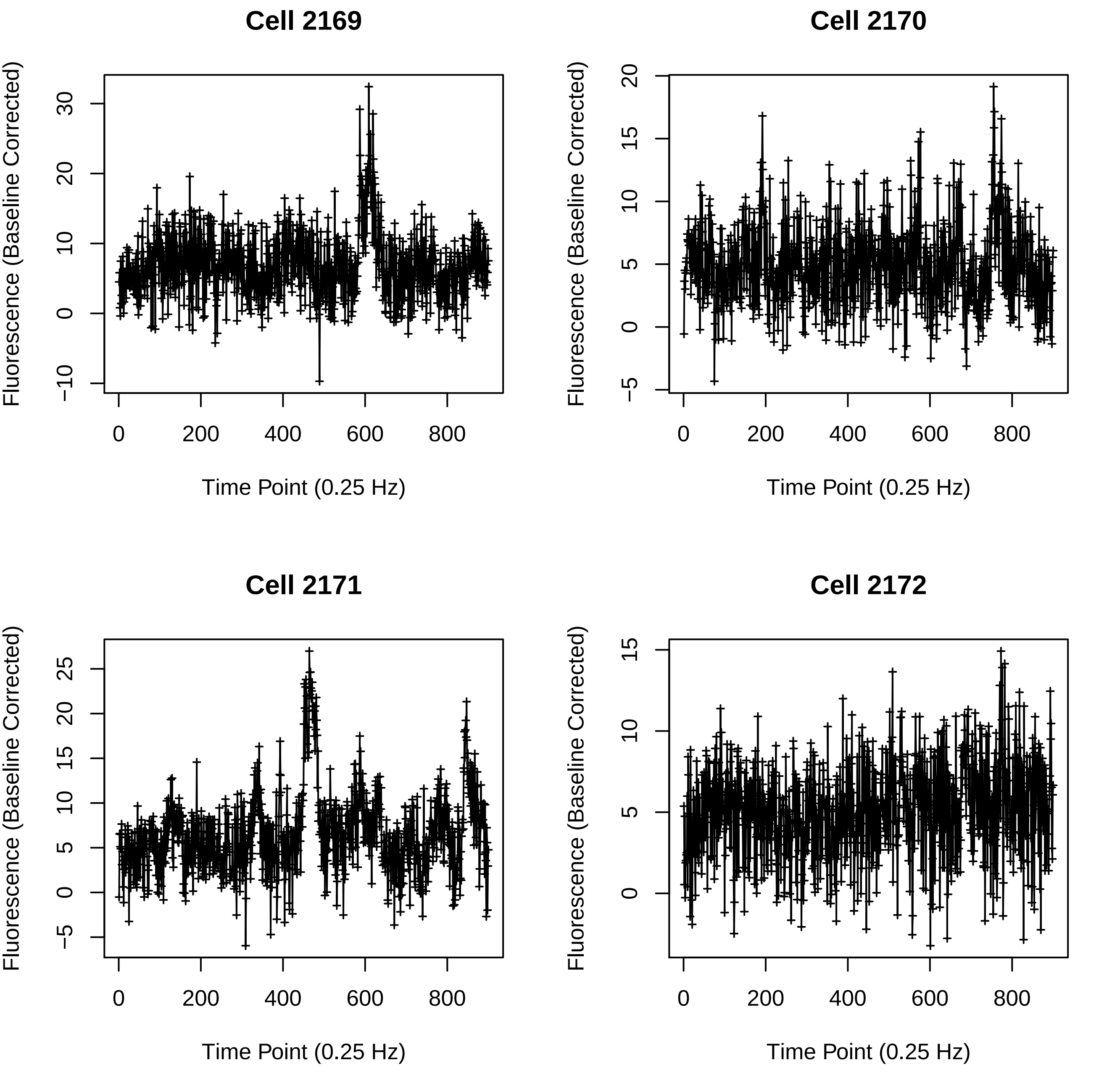
Cell 2169
Cell 2170
Cell 2171
Cell 2172
Fluorescence (Baseline Corrected)
Time Point (0.25 Hz)
0
200
400
600
800
−10
0
10
20
30
−5
5
15
25

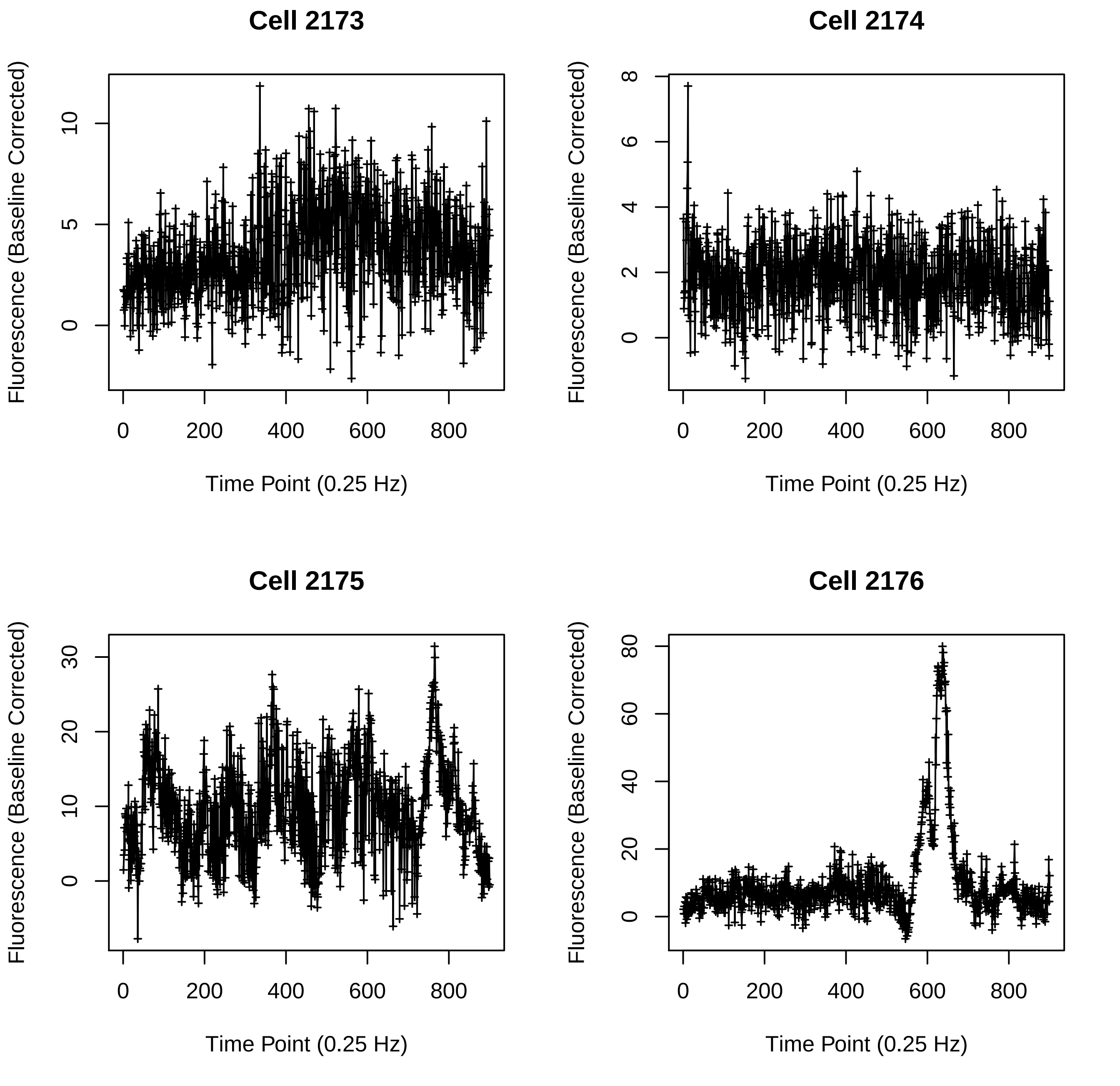
Cell 2173
Fluorescence (Baseline Corrected)
Time Point (0.25 Hz)
0
5
10
0
200
400
600
800
Cell 2174
Fluorescence (Baseline Corrected)
Time Point (0.25 Hz)
0
2
4
6
8
0
200
400
600
800
Cell 2175
Fluorescence (Baseline Corrected)
Time Point (0.25 Hz)
0
10
20
30
0
200
400
600
800
Cell 2176
Fluorescence (Baseline Corrected)
Time Point (0.25 Hz)
0
20
40
60
80
0
200
400
600
800
