# Supplementary material for: A Markovian Entropy Measure for the Analysis of Calcium Activity Time Series
Source: PLoS One. 2016 Dec 15;11(12):e0168342. doi: 10.1371/journal.pone.0168342 (PMC5158058; doi:10.1371/journal.pone.0168342)
Supplement: S2 File — (PDF) [file pone.0168342.s009.pdf]

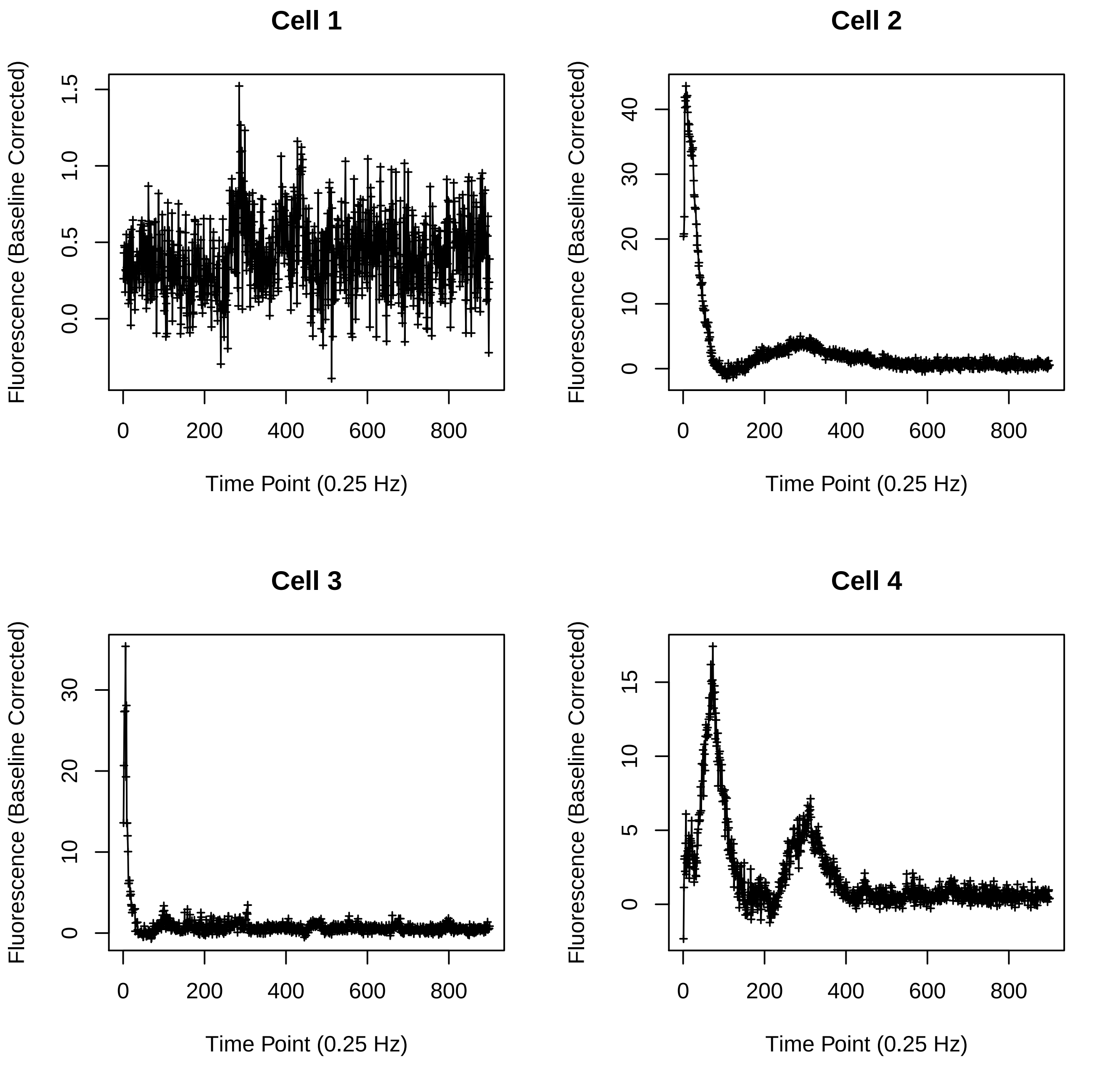

Cell 1
Cell 2
Cell 3
Cell 4
Fluorescence (Baseline Corrected)
Time Point (0.25 Hz)
0
200
400
600
800
0.0
0.5
1.0
1.5
0
10
20
30
40
5
15

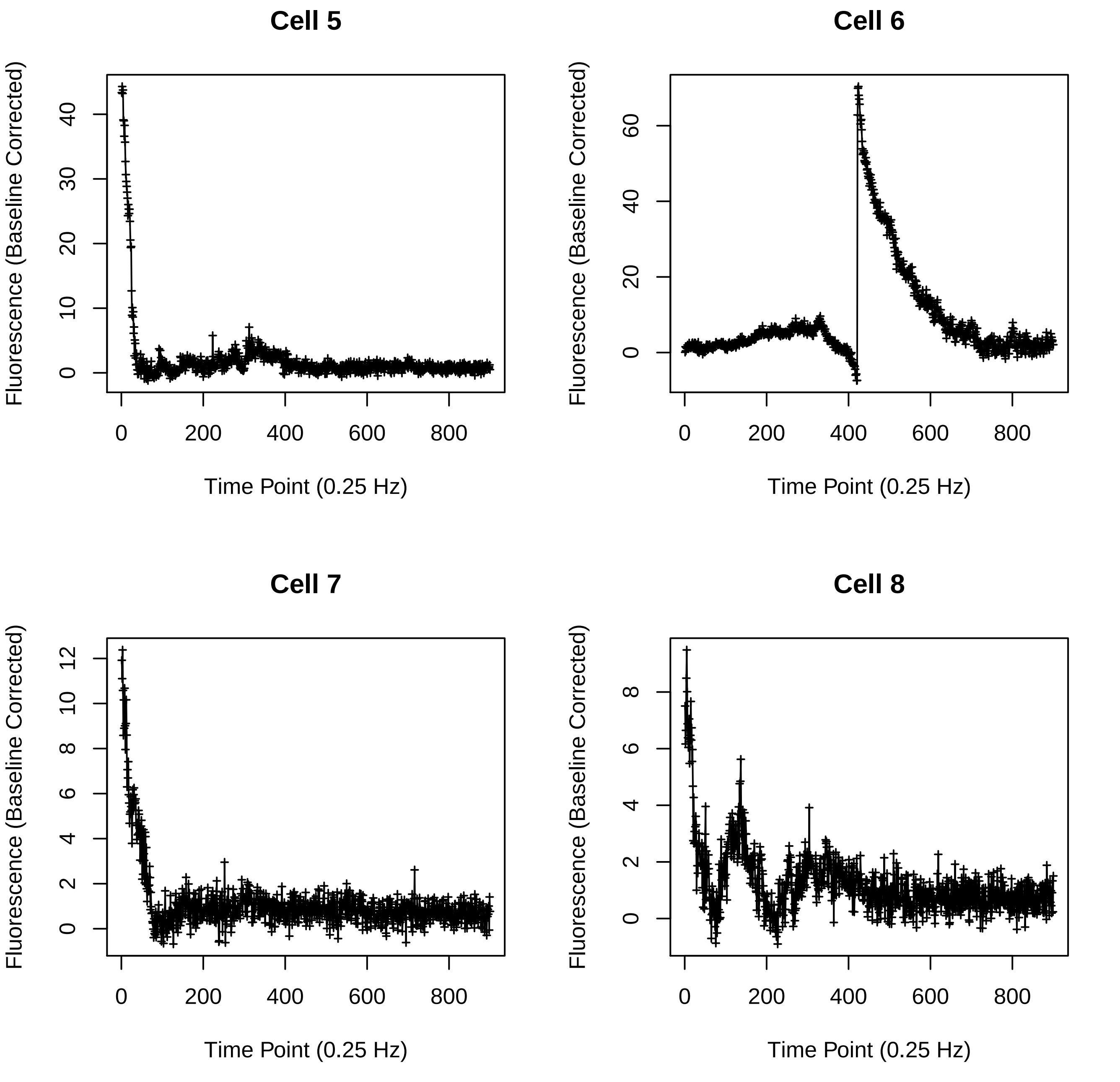
Cell 5
Fluorescence (Baseline Corrected)
0
10
20
30
40
Time Point (0.25 Hz)
0
200
400
600
800
Cell 6
Fluorescence (Baseline Corrected)
0
20
40
60
Time Point (0.25 Hz)
0
200
400
600
800
Cell 7
Fluorescence (Baseline Corrected)
0
2
4
6
8
10
12
Time Point (0.25 Hz)
0
200
400
600
800
Cell 8
Fluorescence (Baseline Corrected)
0
2
4
6
8
Time Point (0.25 Hz)
0
200
400
600
800

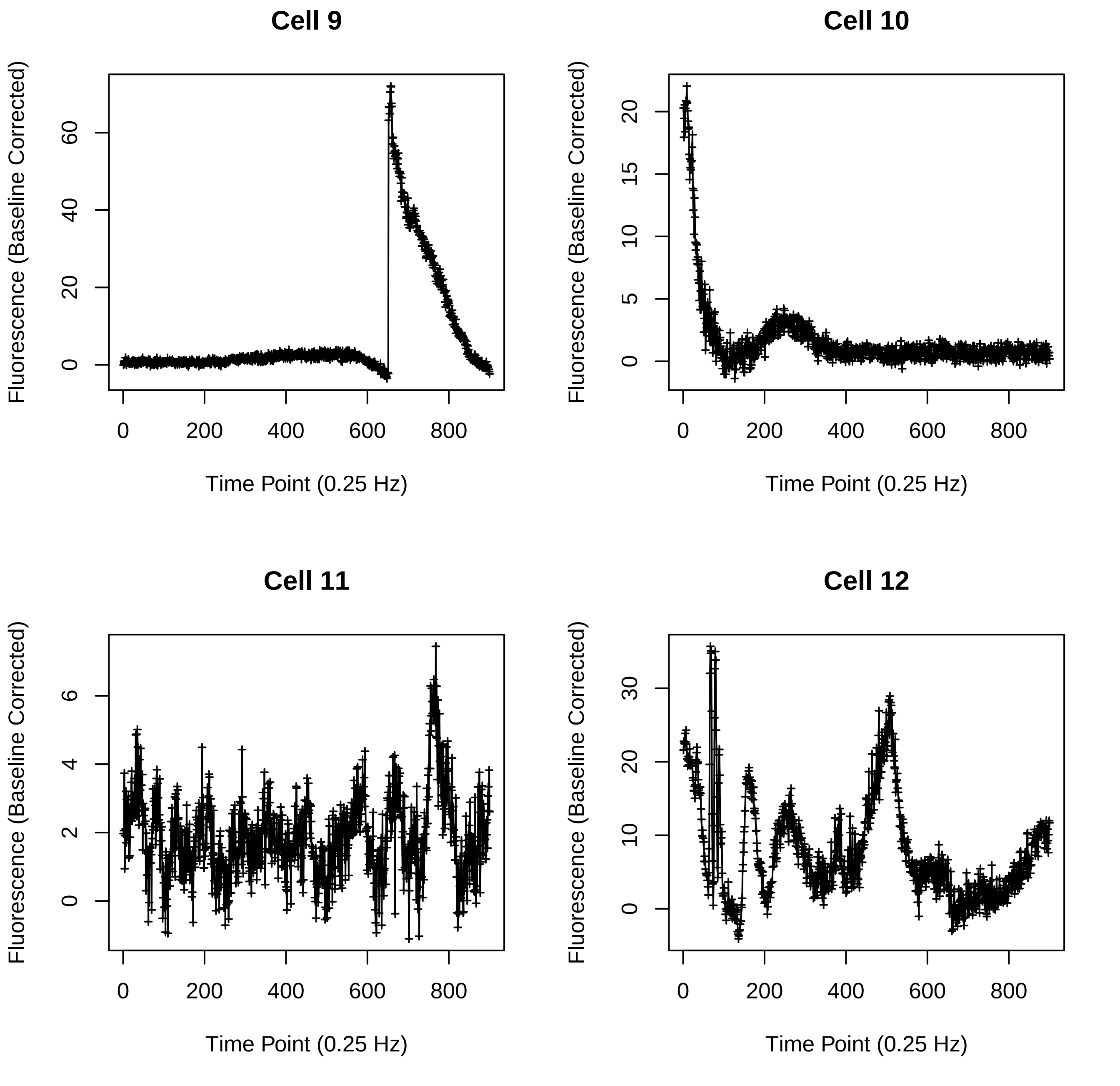
Cell 9
Cell 10
Cell 11
Cell 12
Fluorescence (Baseline Corrected)
Time Point (0.25 Hz)
0
200
400
600
800
20
40
60
5
10
15
2
4
6
30

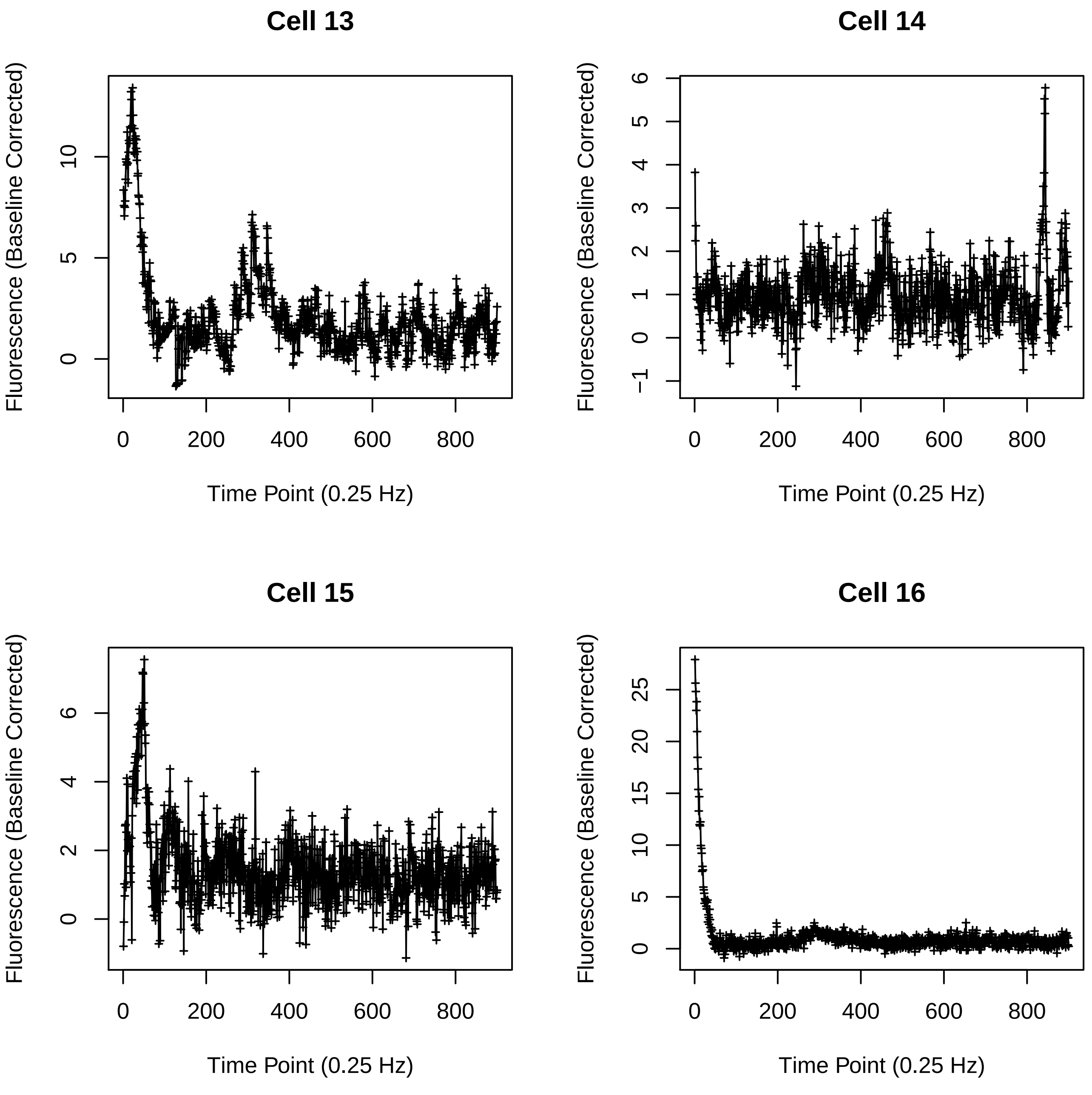
Cell 13
Cell 14
Cell 15
Cell 16
Fluorescence (Baseline Corrected)
Time Point (0.25 Hz)
0
200
400
600
800
0
5
10
−1
1
2
3
4
5
6
15
20
25

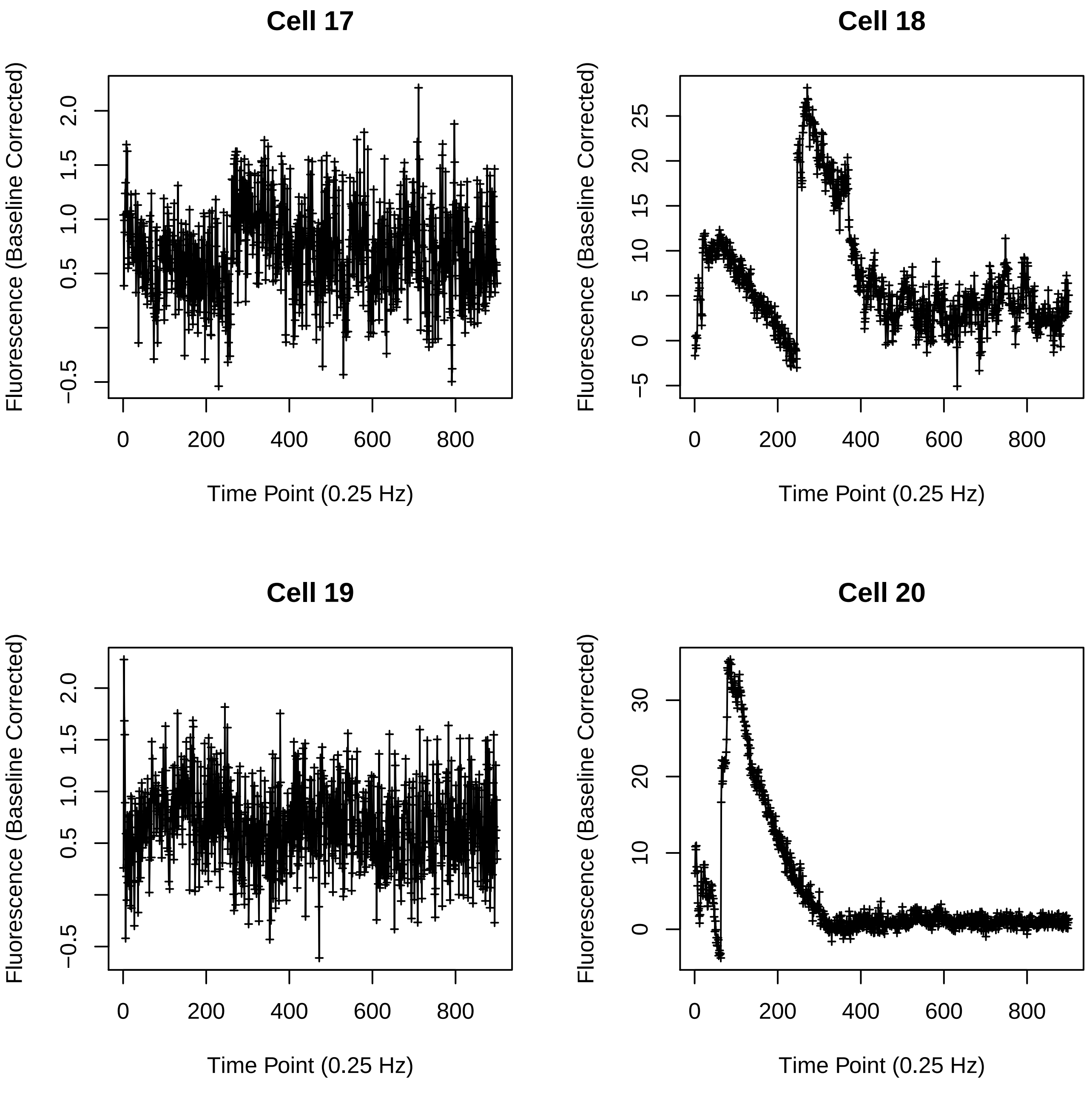
Cell 17
Cell 18
Cell 19
Cell 20
Fluorescence (Baseline Corrected)
Time Point (0.25 Hz)
0
200
400
600
800
−0.5
0.5
1.0
1.5
2.0
−5
0
5
10
15
20
25
30

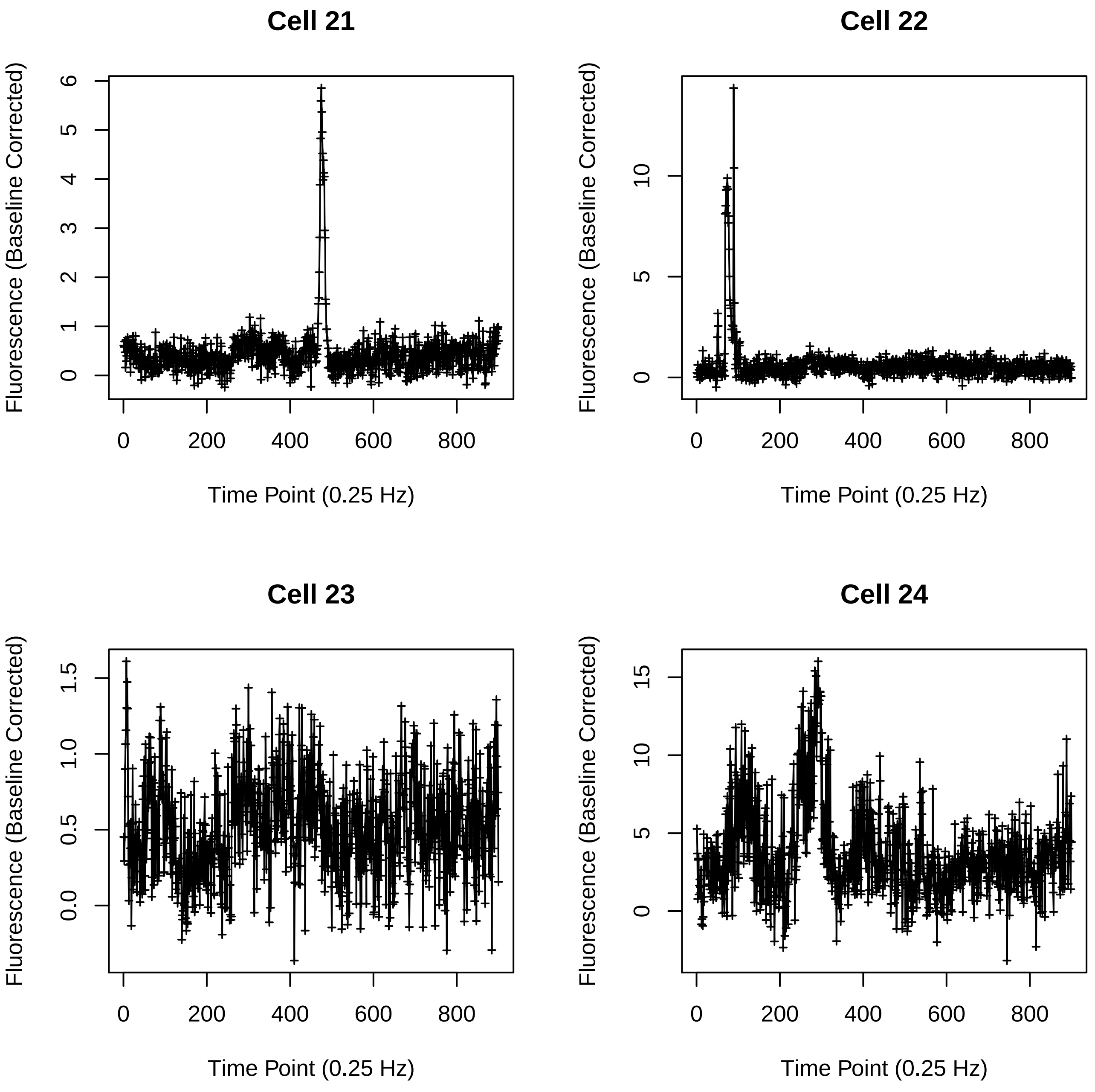
Cell 21
Fluorescence (Baseline Corrected)
Time Point (0.25 Hz)
0
1
2
3
4
5
6
0
200
400
600
800
Cell 22
Fluorescence (Baseline Corrected)
Time Point (0.25 Hz)
0
5
10
0
200
400
600
800
Cell 23
Fluorescence (Baseline Corrected)
Time Point (0.25 Hz)
0.0
0.5
1.0
1.5
0
200
400
600
800
Cell 24
Fluorescence (Baseline Corrected)
Time Point (0.25 Hz)
0
5
10
15
0
200
400
600
800

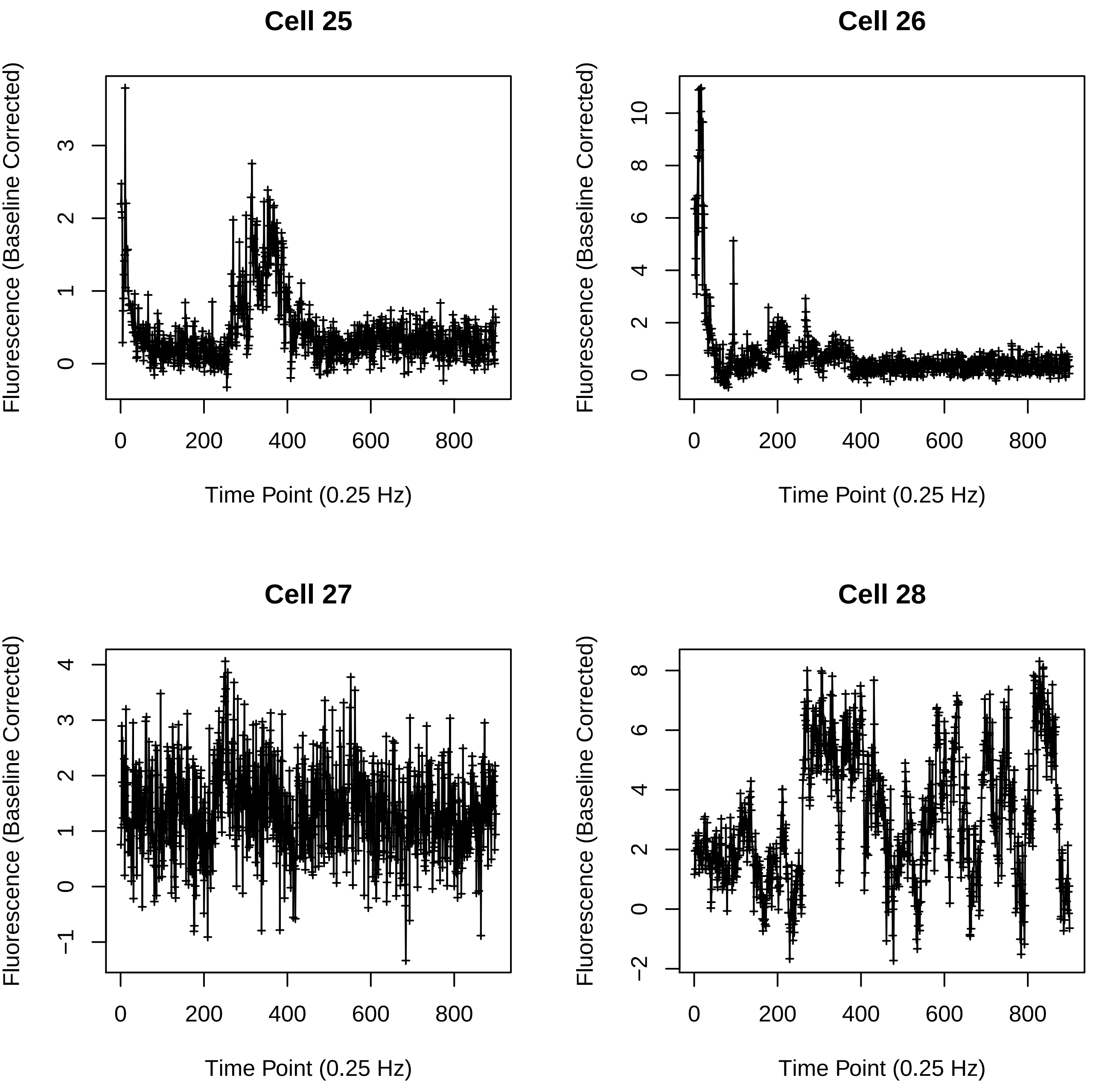
Cell 25
Cell 26
Cell 27
Cell 28
Fluorescence (Baseline Corrected)
Time Point (0.25 Hz)
0
1
2
3
0
2
4
6
8
10
−1
0
1
2
3
4
−2
0
2
4
6
8
0
200
400
600
800

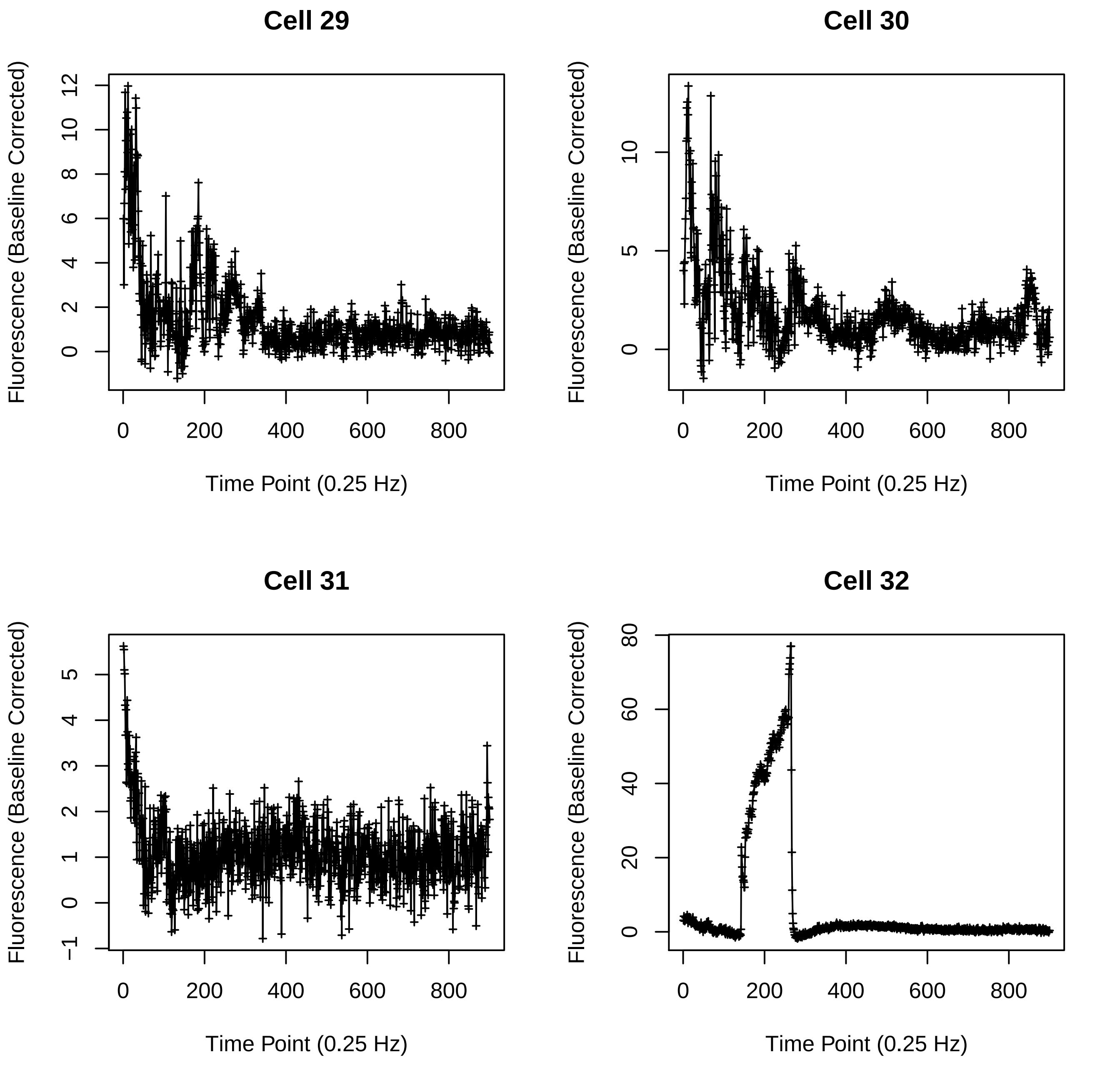
Cell 29
Cell 30
Cell 31
Cell 32
Fluorescence (Baseline Corrected)
Time Point (0.25 Hz)
0
200
400
600
800
0
2
4
6
8
10
12
5
−1
1
3
20
40
60
80

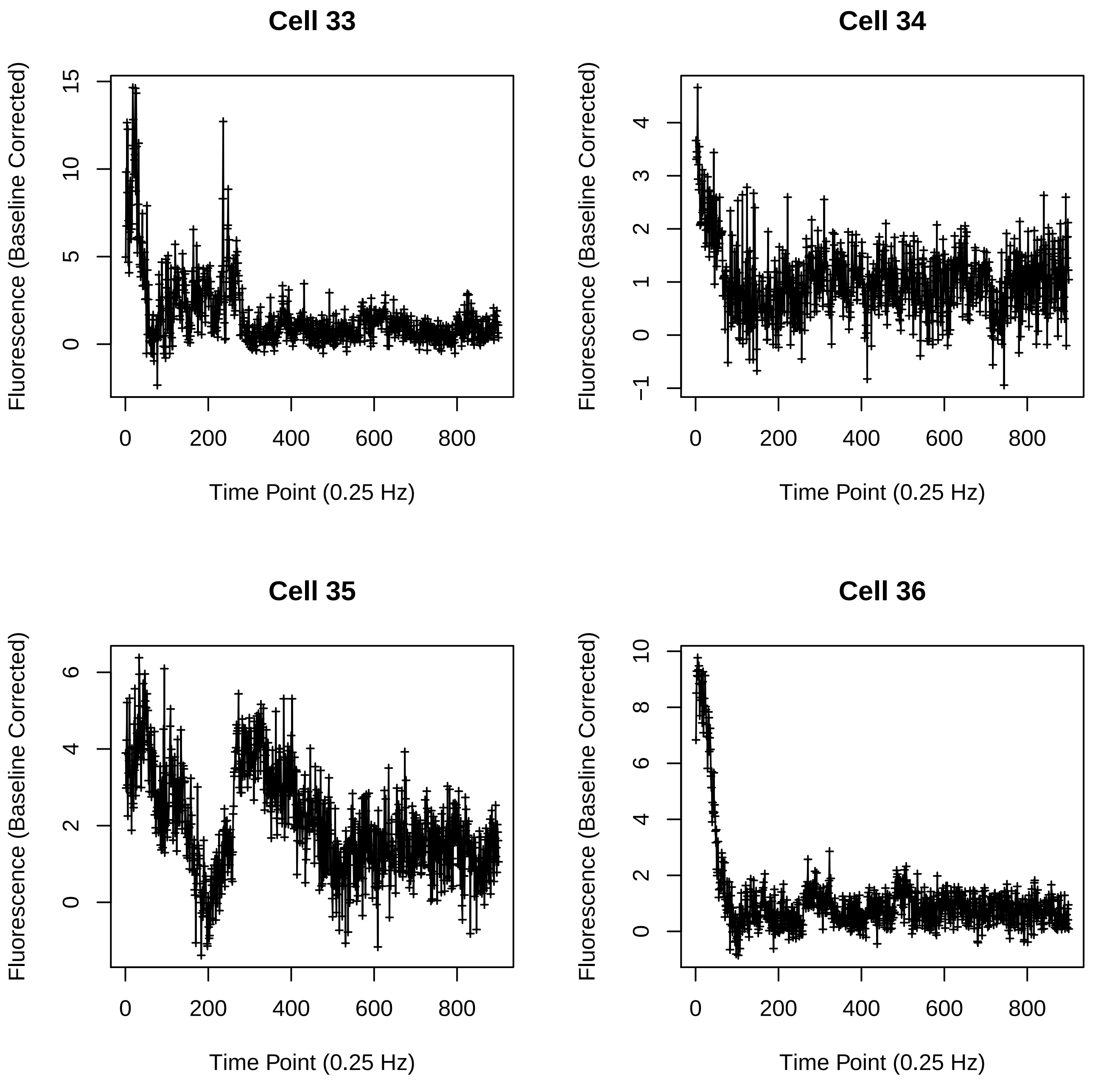
Cell 33
Cell 34
Cell 35
Cell 36
Fluorescence (Baseline Corrected)
Time Point (0.25 Hz)
0
5
10
15
−1
0
1
2
3
4
0
2
4
6
0
2
4
6
8
10
0
200
400
600
800

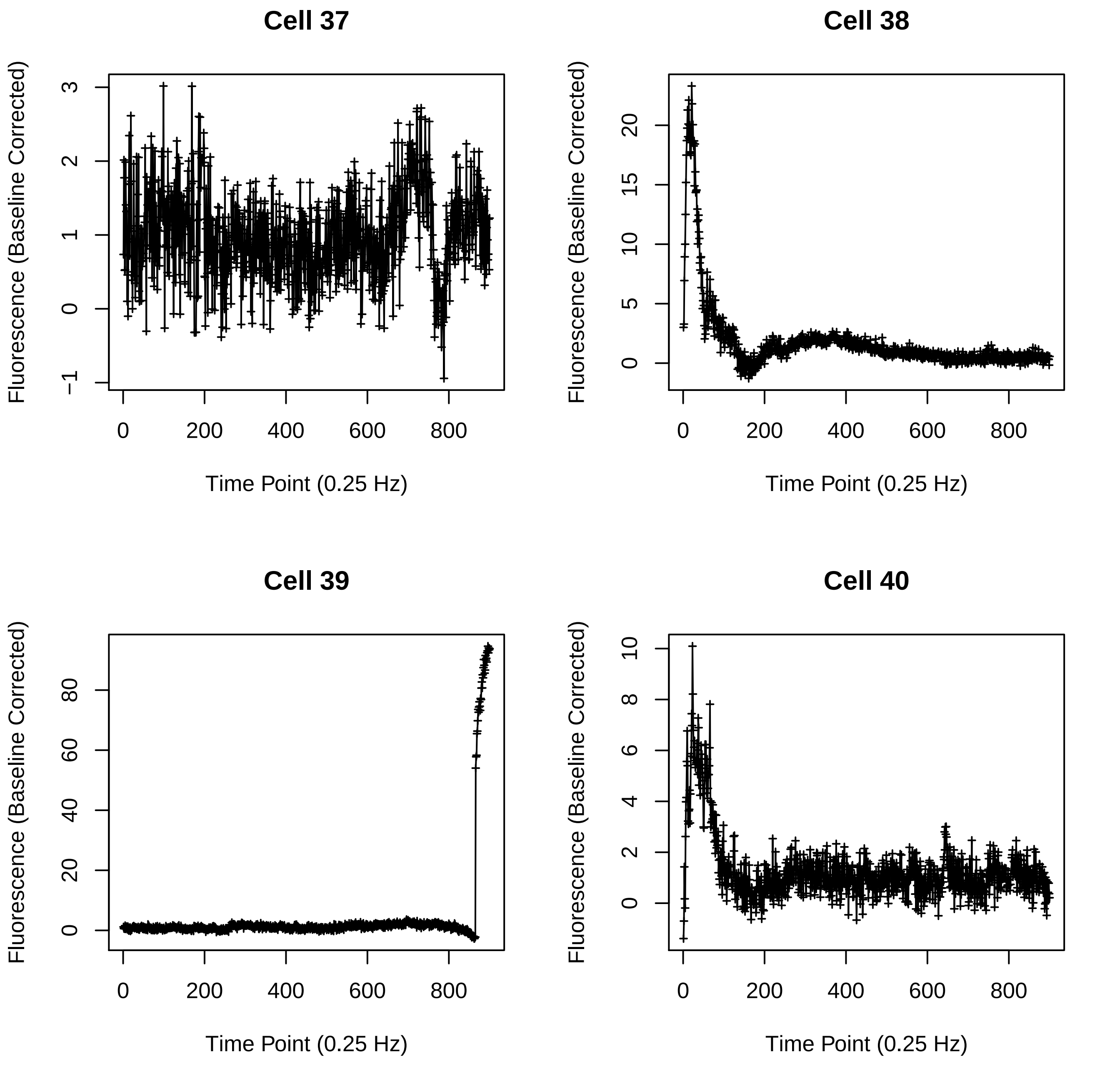
Cell 37
Cell 38
Cell 39
Cell 40
Fluorescence (Baseline Corrected)
Time Point (0.25 Hz)
−1
0
1
2
3
5
10
15
20
40
60
80
4
6
8
200
400
600
800

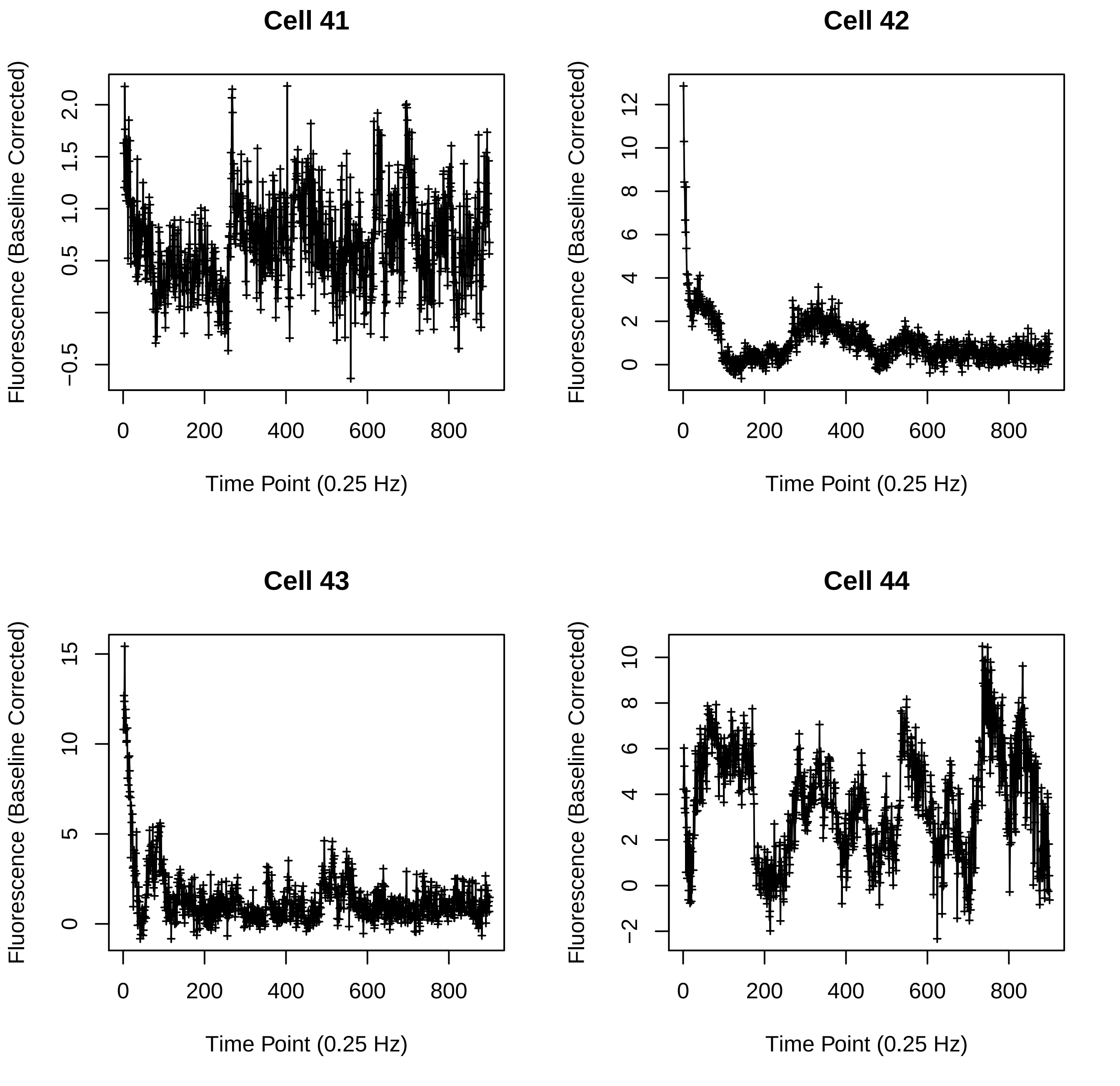
Cell 41
Cell 42
Cell 43
Cell 44
Fluorescence (Baseline Corrected)
Time Point (0.25 Hz)
0
200
400
600
800
−0.5
0.5
1.0
1.5
2.0
2
4
6
8
10
12
5
15
−2

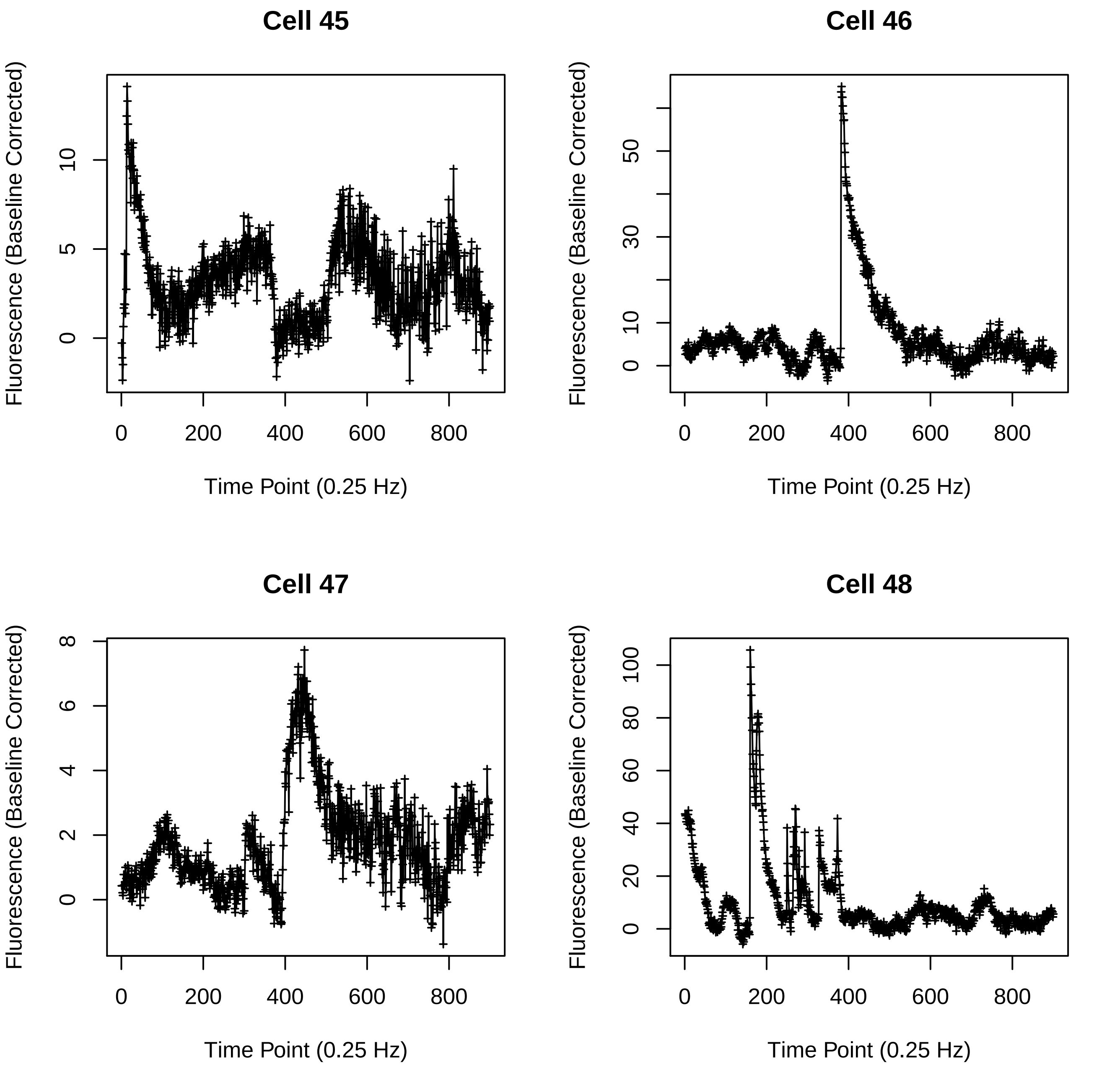
Cell 45
Cell 46
Cell 47
Cell 48
Fluorescence (Baseline Corrected)
Time Point (0.25 Hz)
0
200
400
600
800
10
5
50
30
8
6
4
2
100
80
60
40
20

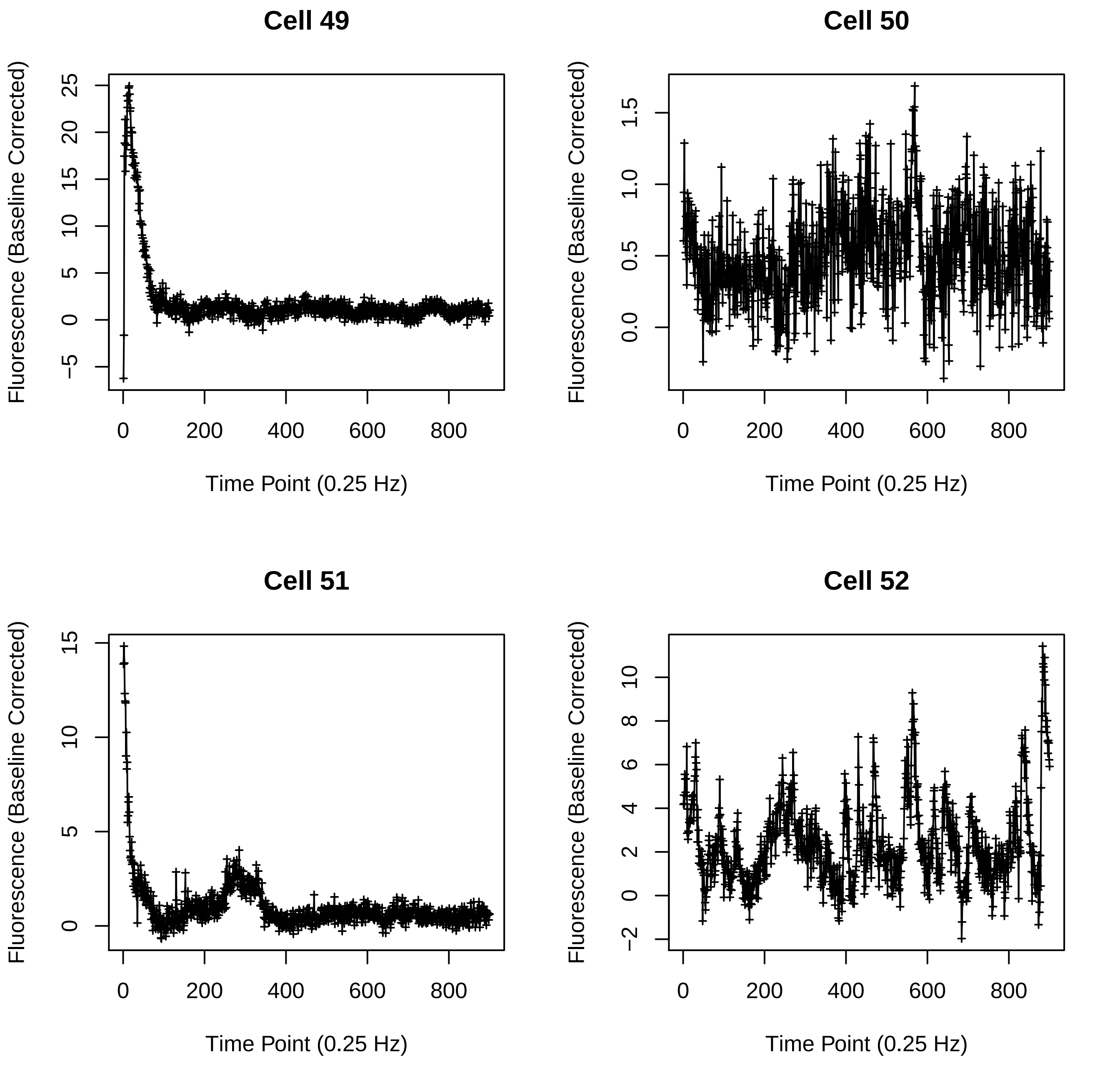
Cell 49
Fluorescence (Baseline Corrected)
−5
0
5
10
15
20
25
0
200
400
600
800
Time Point (0.25 Hz)
Cell 50
Fluorescence (Baseline Corrected)
0.0
0.5
1.0
1.5
0
200
400
600
800
Time Point (0.25 Hz)
Cell 51
Fluorescence (Baseline Corrected)
0
5
10
15
0
200
400
600
800
Time Point (0.25 Hz)
Cell 52
Fluorescence (Baseline Corrected)
−2
0
2
4
6
8
10
0
200
400
600
800
Time Point (0.25 Hz)

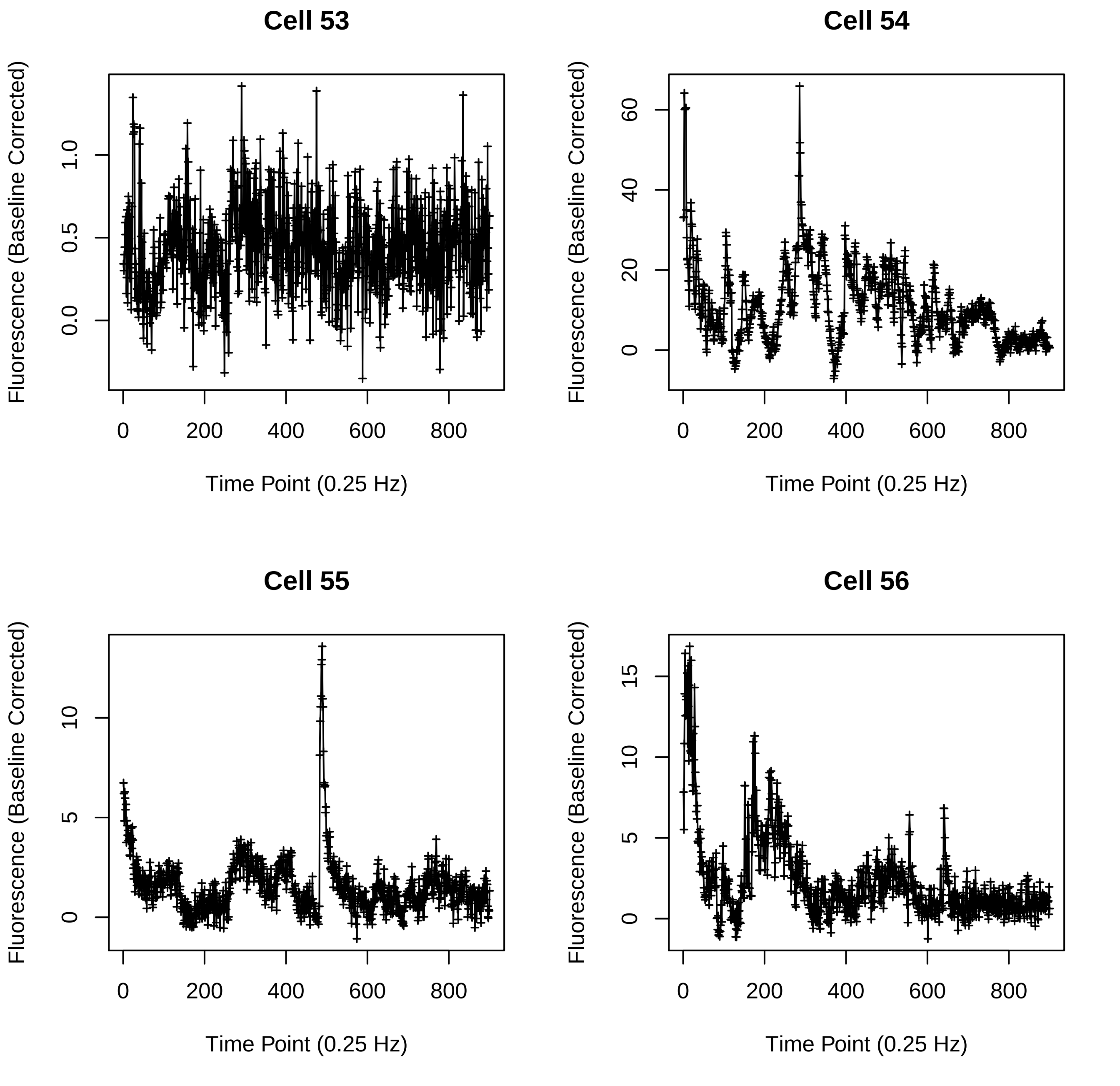
Cell 53
Cell 54
Cell 55
Cell 56
Fluorescence (Baseline Corrected)
Time Point (0.25 Hz)
0.0
0.5
1.0
0
20
40
60
0
5
10
15
0
200
400
600
800

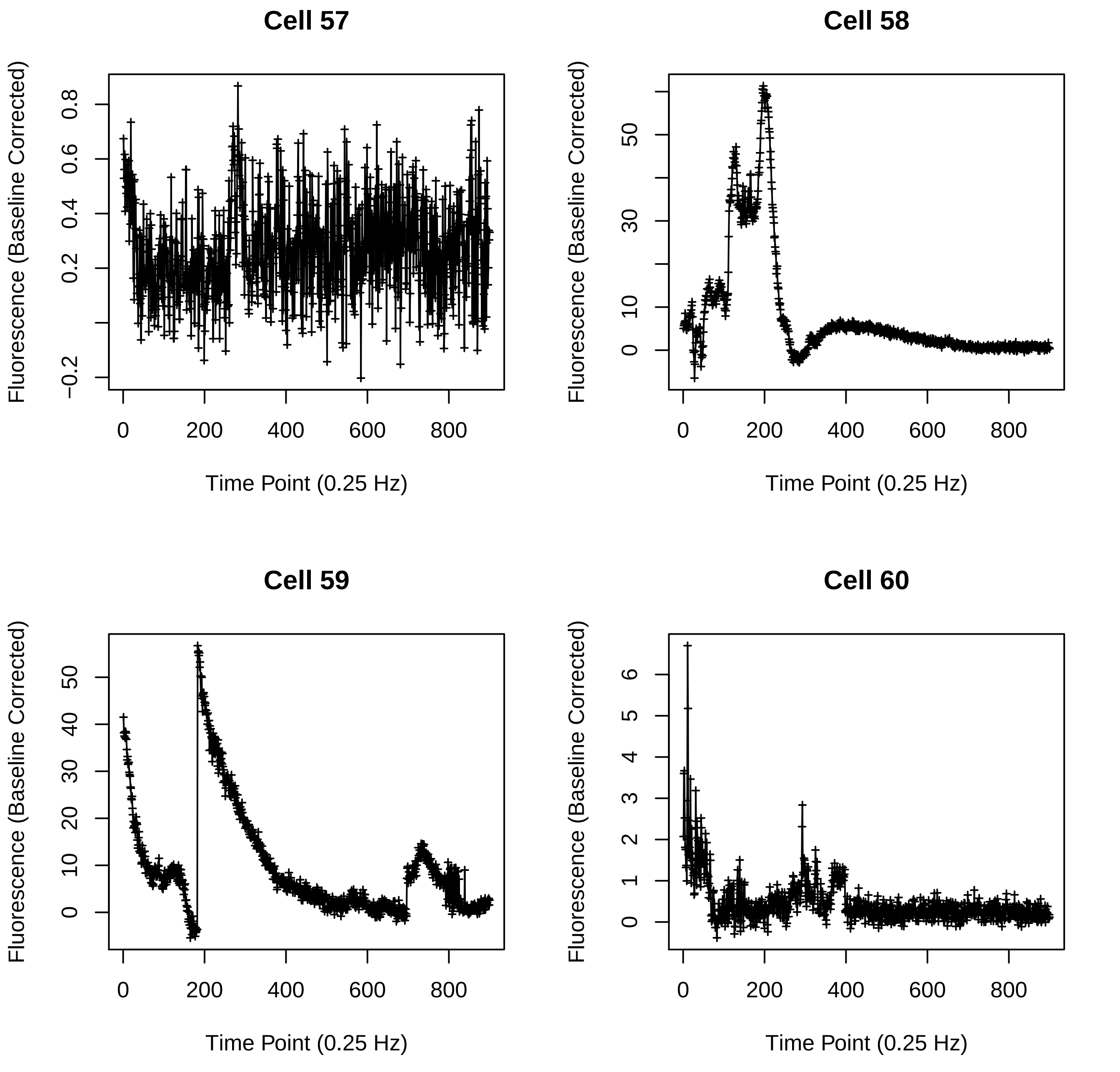
Cell 57
Fluorescence (Baseline Corrected)
−0.2
0.2
0.4
0.6
0.8
0
200
400
600
800
Time Point (0.25 Hz)
Cell 58
Fluorescence (Baseline Corrected)
0
10
30
50
0
200
400
600
800
Time Point (0.25 Hz)
Cell 59
Fluorescence (Baseline Corrected)
0
10
20
30
40
50
0
200
400
600
800
Time Point (0.25 Hz)
Cell 60
Fluorescence (Baseline Corrected)
0
1
2
3
4
5
6
0
200
400
600
800
Time Point (0.25 Hz)

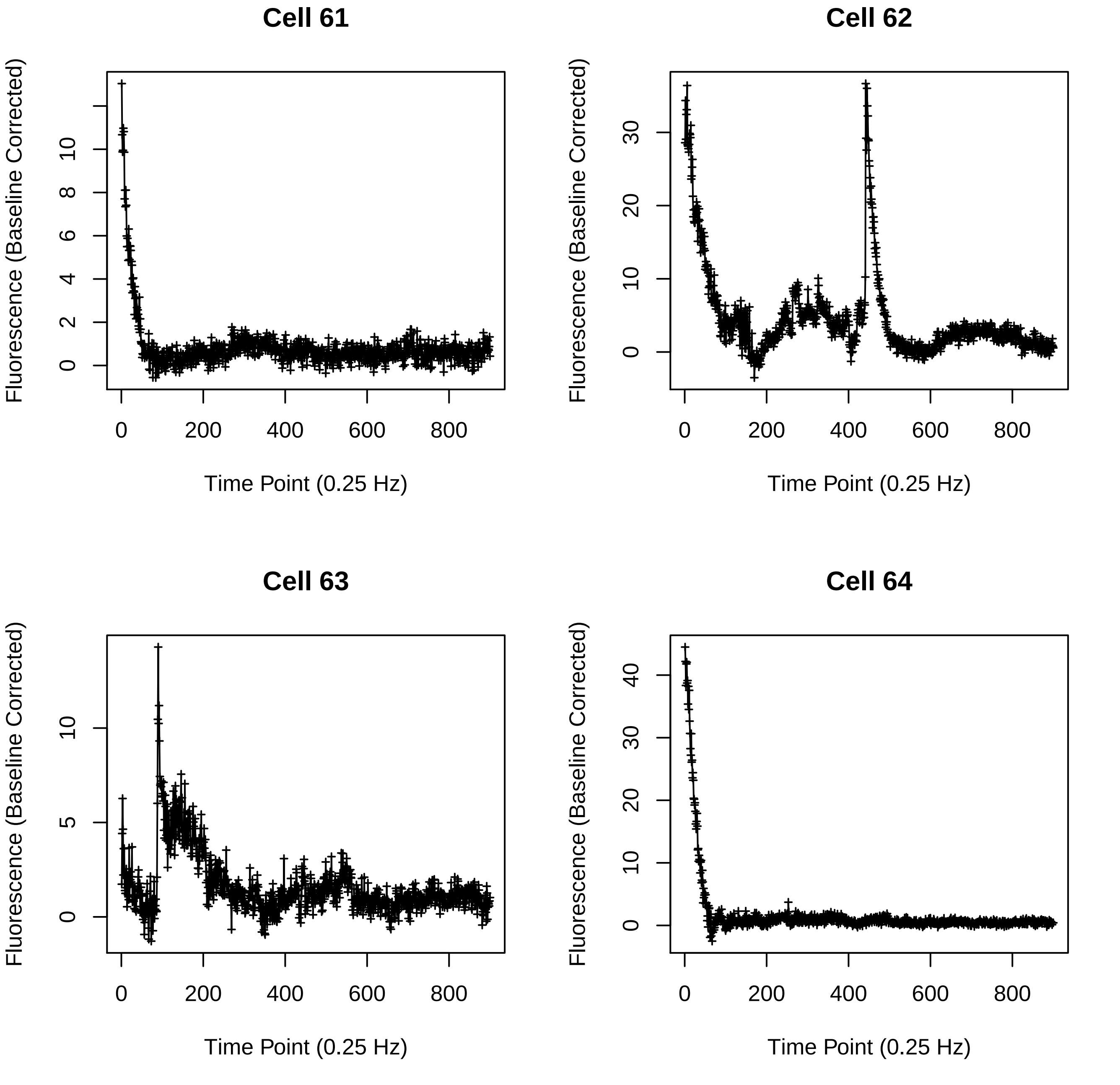
Cell 61
Cell 62
Cell 63
Cell 64
Fluorescence (Baseline Corrected)
Time Point (0.25 Hz)
0
2
4
6
8
10
20
30
40
5
200
400
600
800

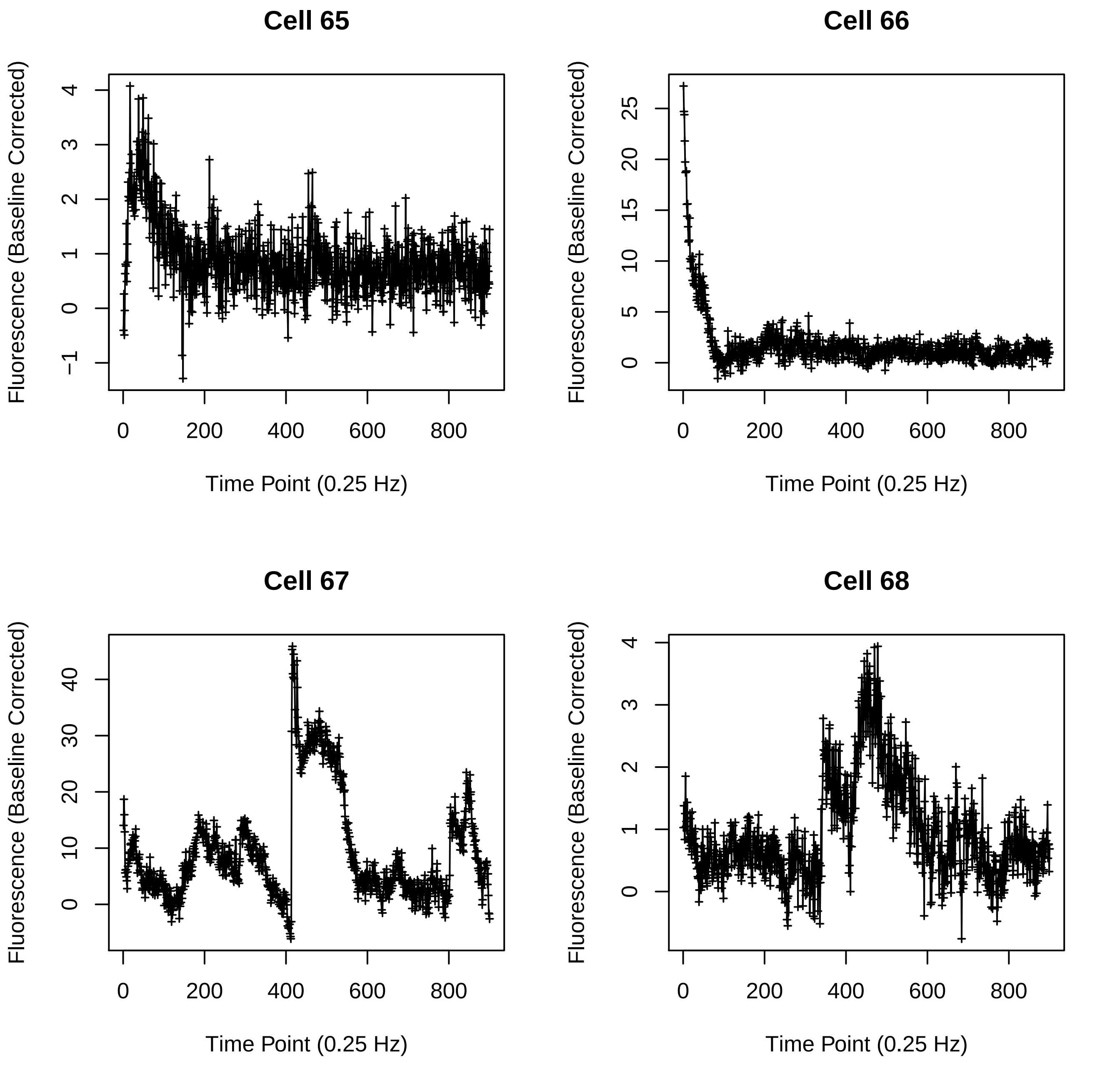
Cell 65
Cell 66
Cell 67
Cell 68
Fluorescence (Baseline Corrected)
Time Point (0.25 Hz)
0
200
400
600
800
−1
1
2
3
4
5
10
15
20
25
30
40

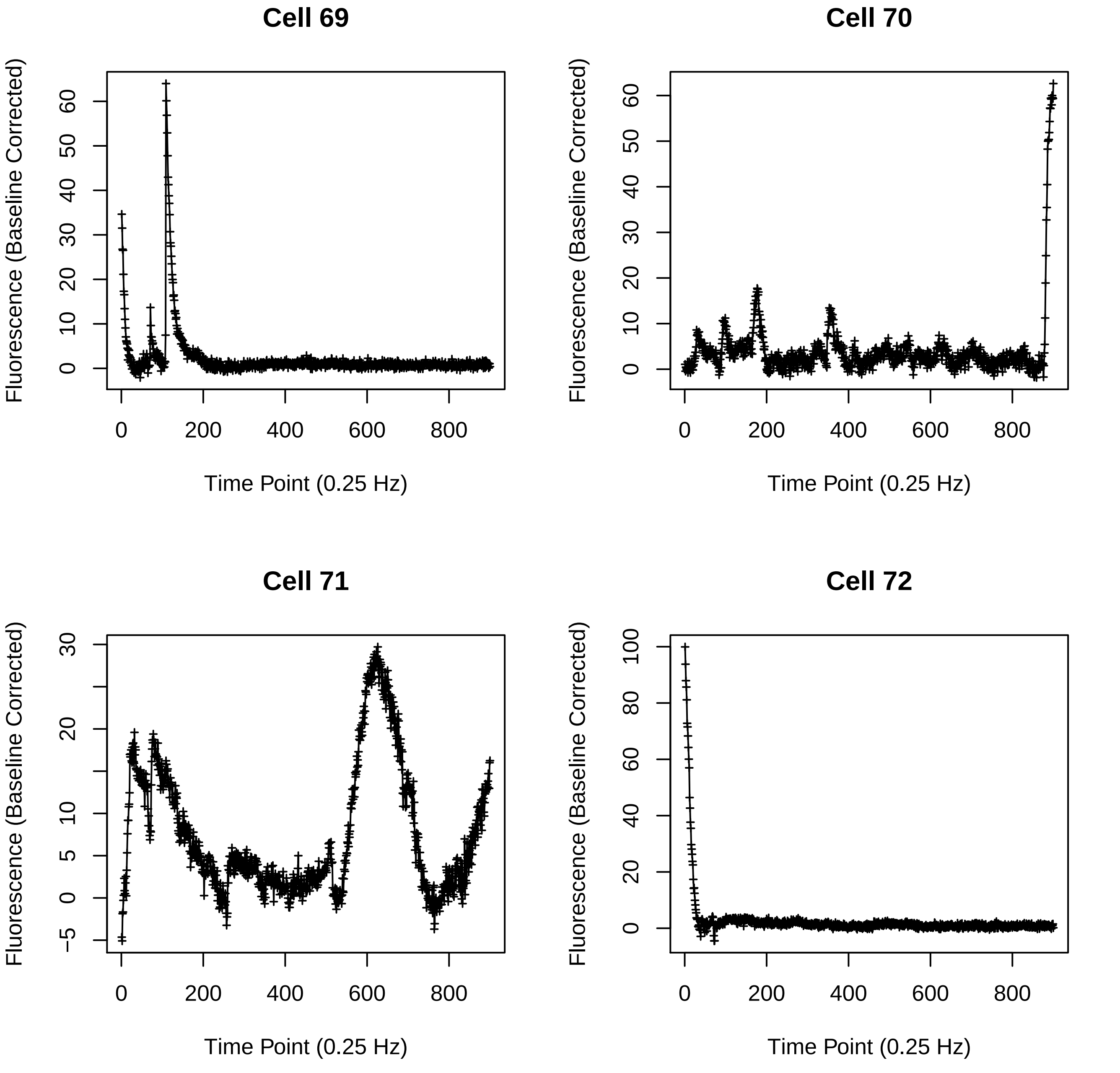
Cell 69
Cell 70
Cell 71
Cell 72
Fluorescence (Baseline Corrected)
Time Point (0.25 Hz)
0
200
400
600
800
0
10
20
30
40
50
60
-5
5
15
25
100
80

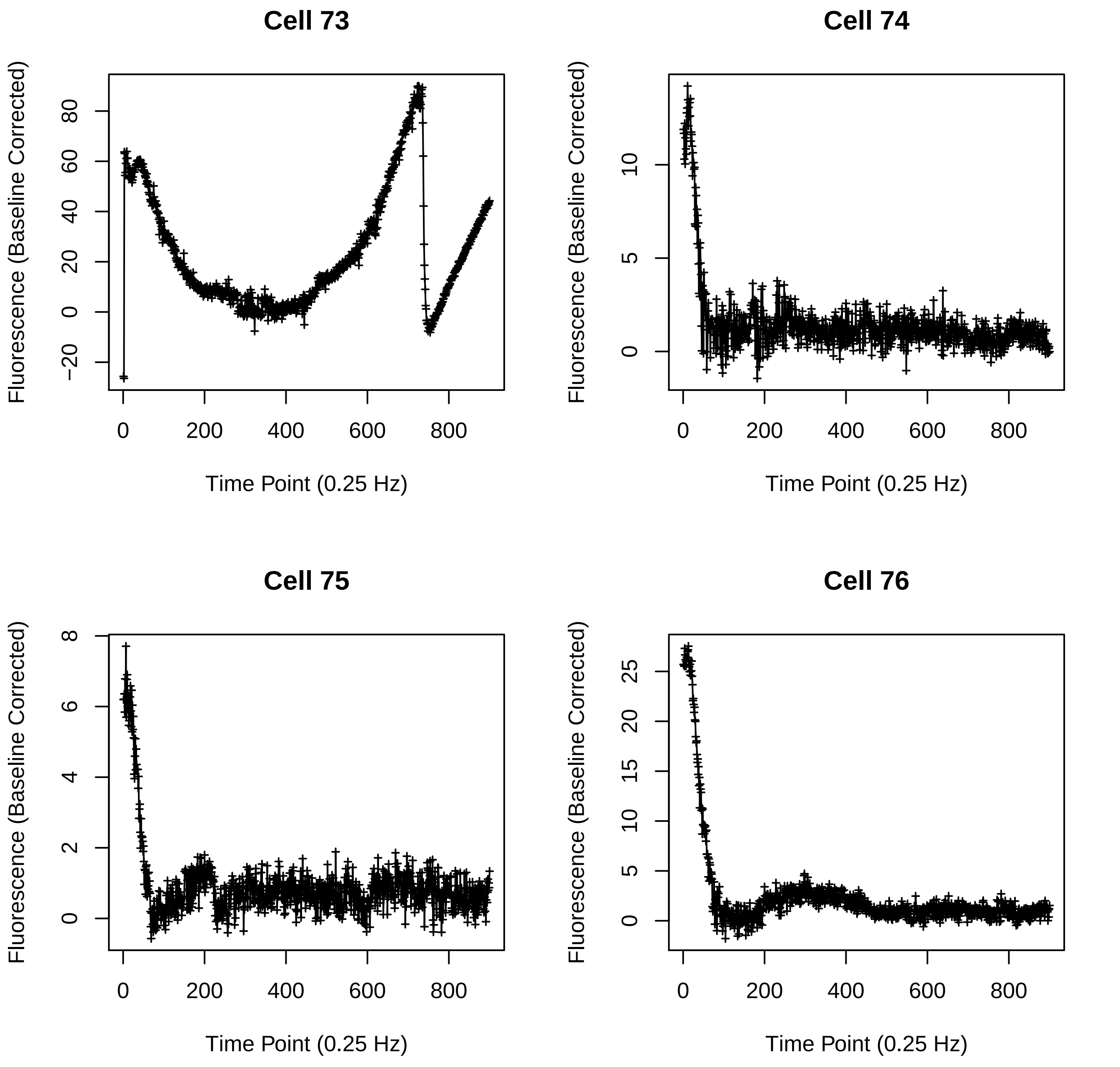
Cell 73
Cell 74
Cell 75
Cell 76
Fluorescence (Baseline Corrected)
Time Point (0.25 Hz)
−20
0
20
40
60
80
0
5
10
0
2
4
6
8
0
5
10
15
20
25
0
200
400
600
800

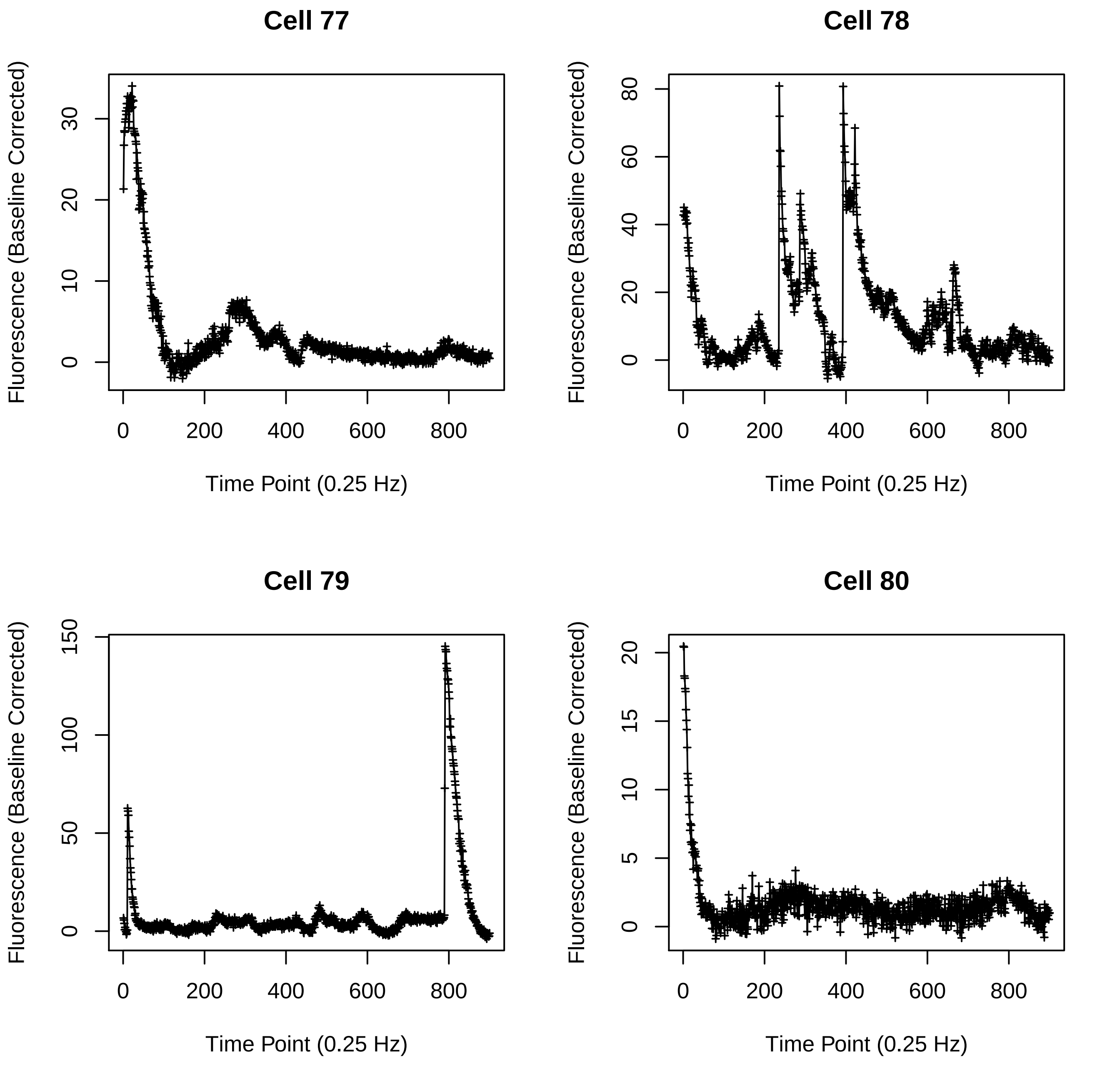
Cell 77
Cell 78
Cell 79
Cell 80
Fluorescence (Baseline Corrected)
Time Point (0.25 Hz)
0
10
20
30
0
20
40
60
80
0
50
100
150
0
5
10
15
20
0
200
400
600
800

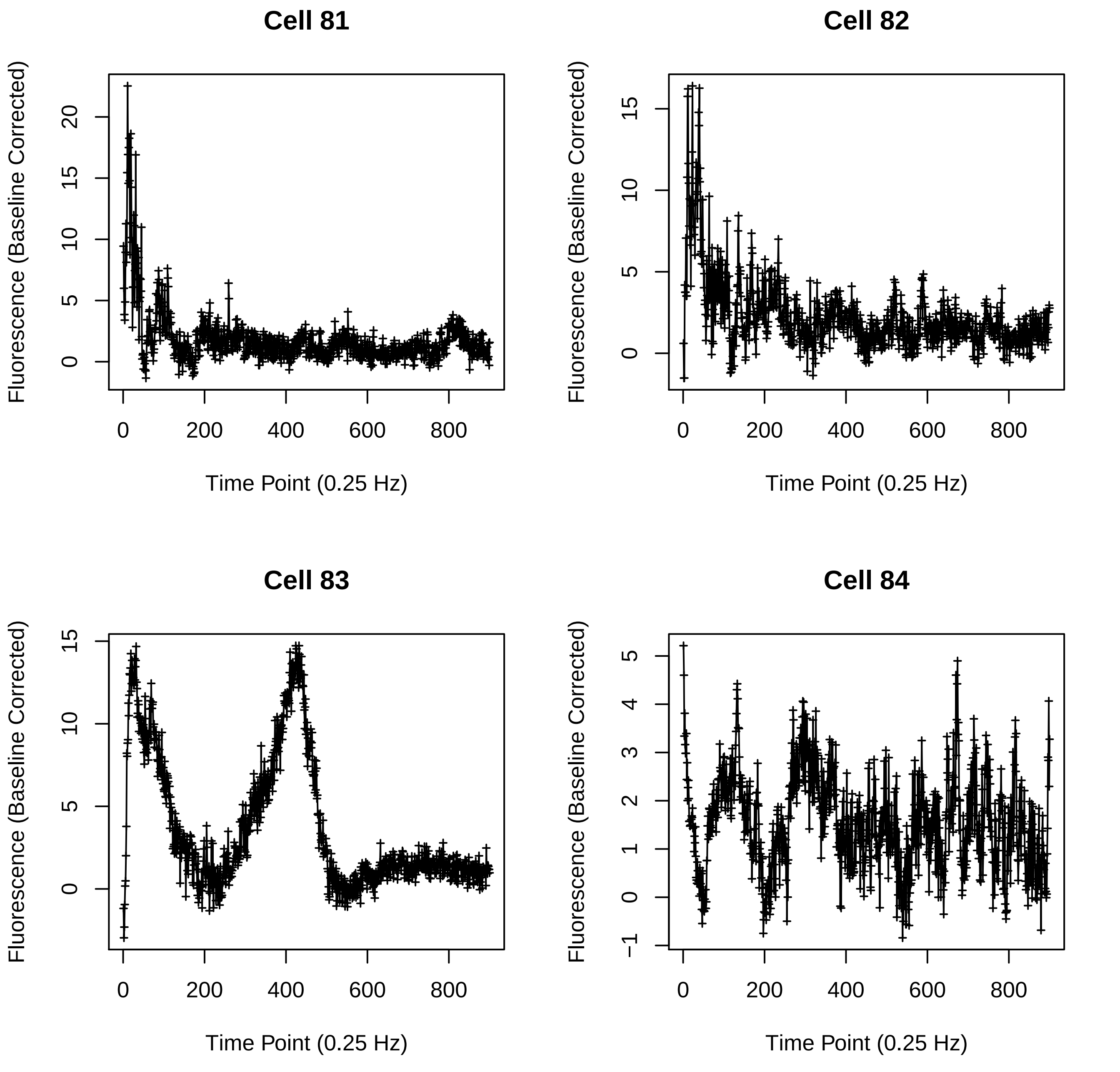
Cell 81
Cell 82
Cell 83
Cell 84
Fluorescence (Baseline Corrected)
Time Point (0.25 Hz)
0
200
400
600
800
5
10
15
20
-1
1
2
3
4

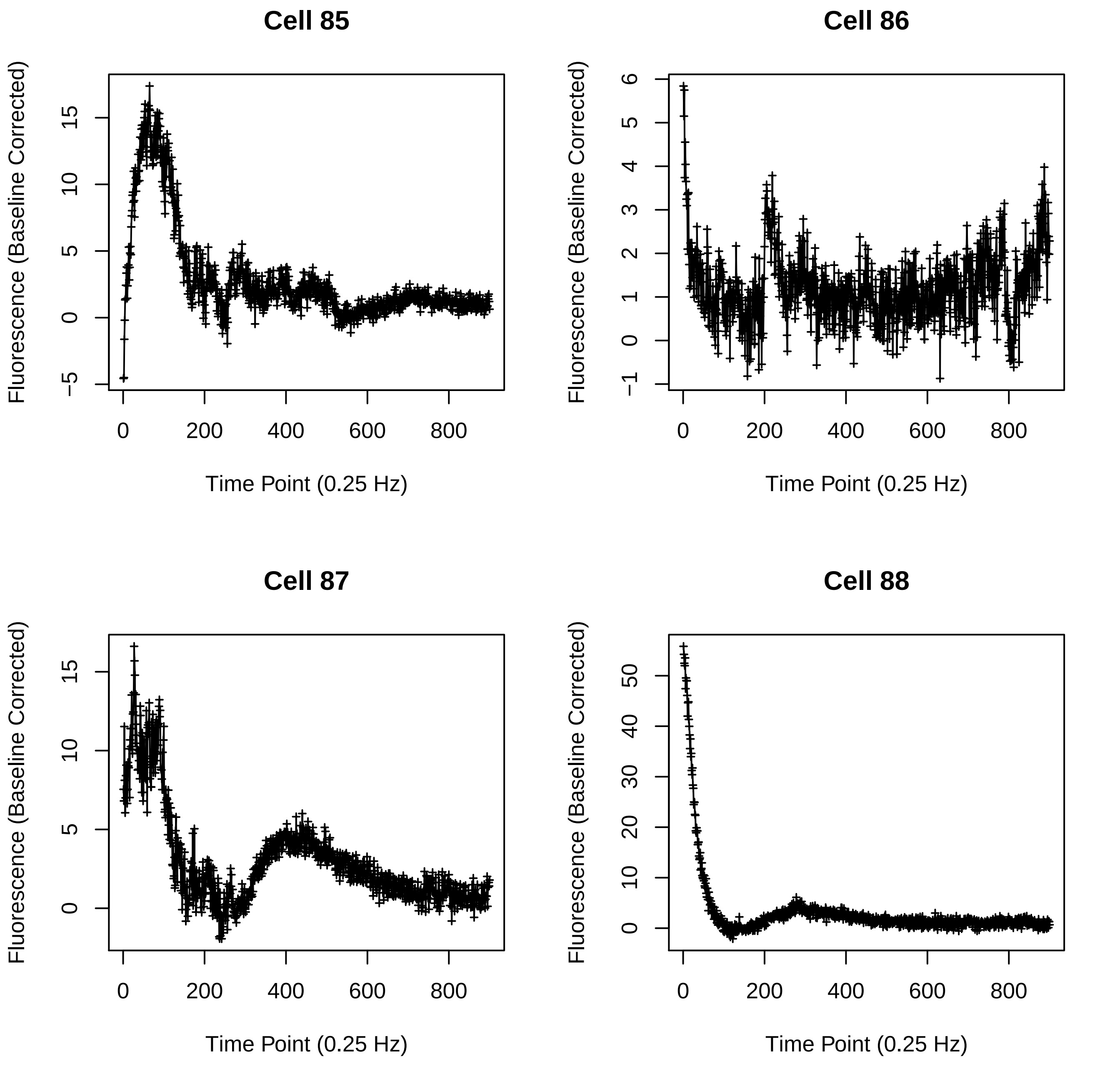
Cell 85
Cell 86
Cell 87
Cell 88
Fluorescence (Baseline Corrected)
Time Point (0.25 Hz)
0
200
400
600
800

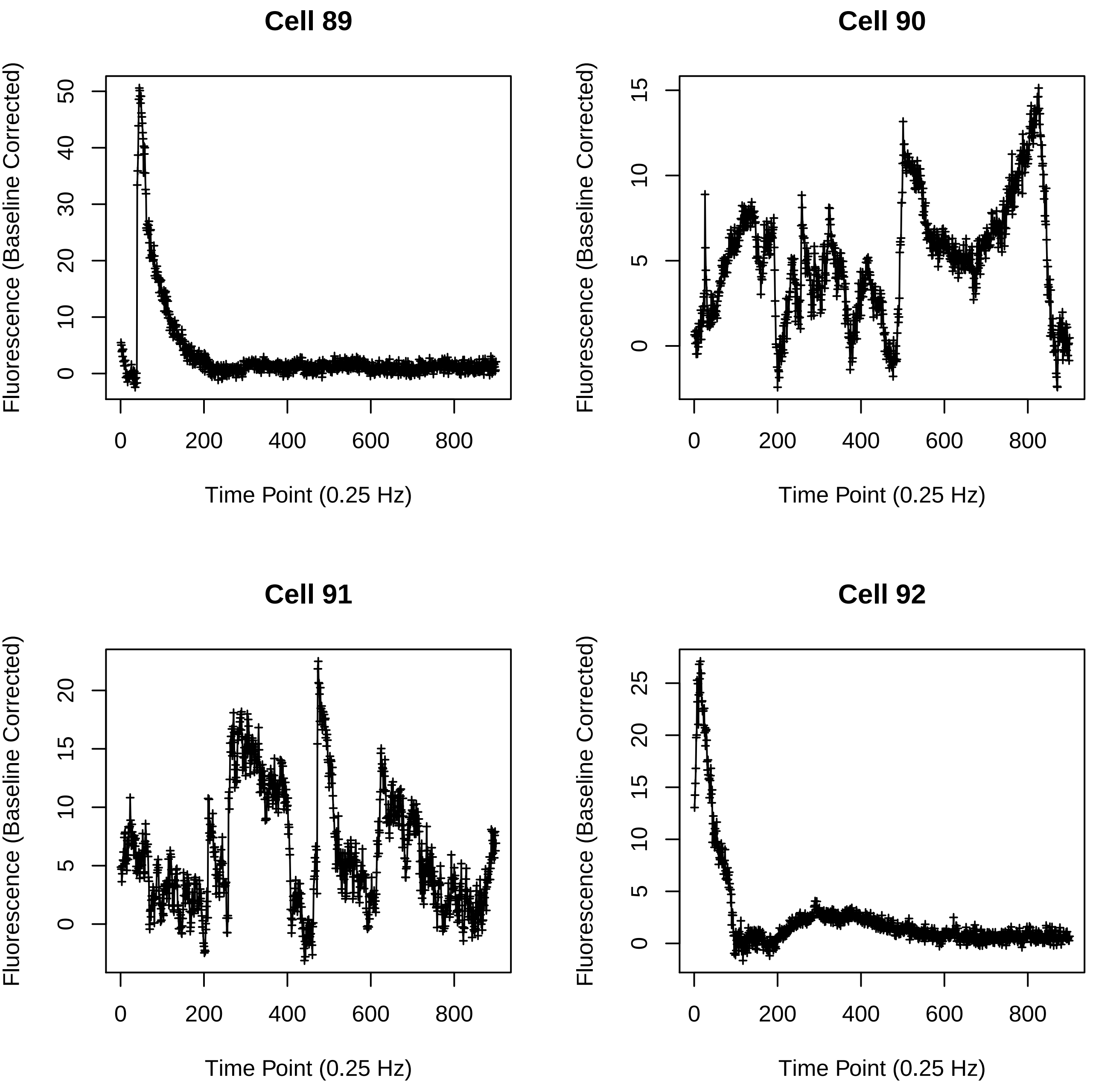
Cell 89
Cell 90
Cell 91
Cell 92
Fluorescence (Baseline Corrected)
Time Point (0.25 Hz)
0
200
400
600
800
0
10
20
30
40
50
5
15
25

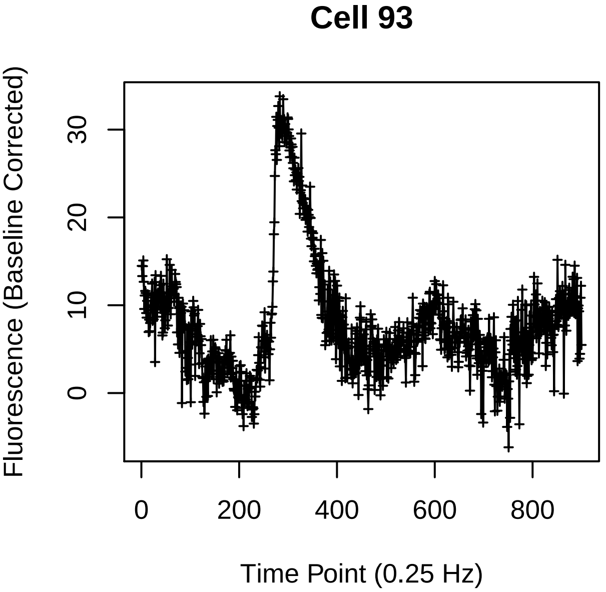
Cell 93
Fluorescence (Baseline Corrected)
0
10
20
30
0
200
400
600
800
Time Point (0.25 Hz)

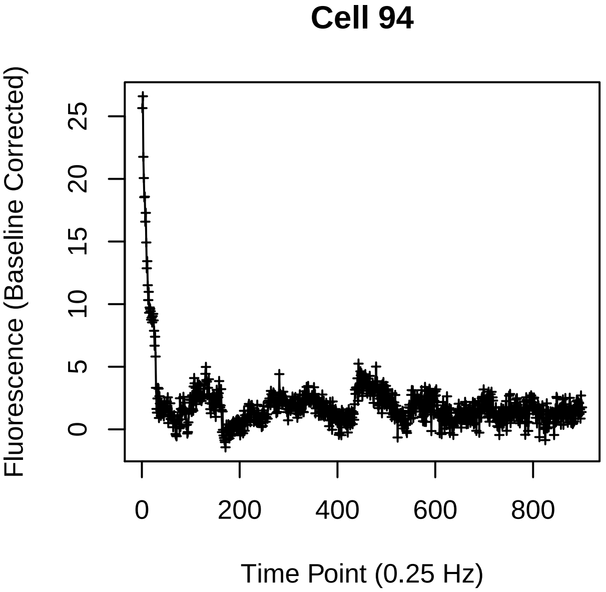
Cell 94
Fluorescence (Baseline Corrected)
0
5
10
15
20
25
0
200
400
600
800
Time Point (0.25 Hz)

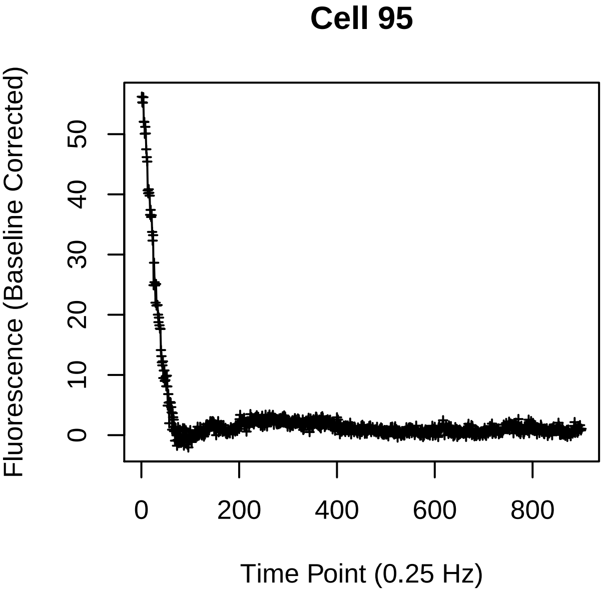
Cell 95
Fluorescence (Baseline Corrected)
0
10
20
30
40
50
0
200
400
600
800
Time Point (0.25 Hz)

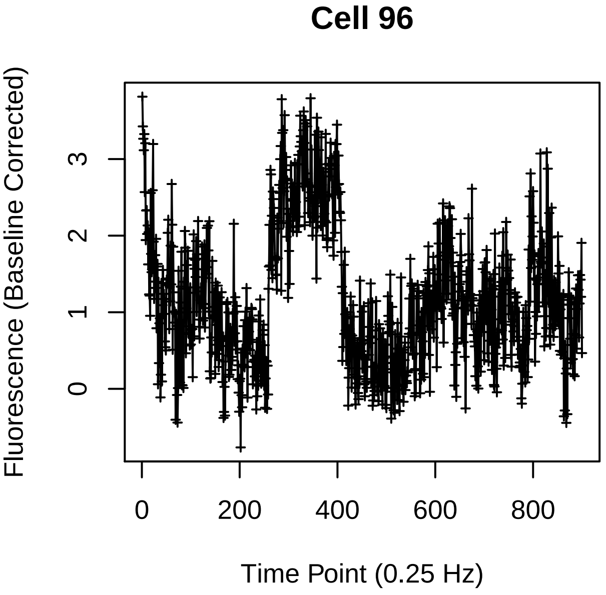
Cell 96
Fluorescence (Baseline Corrected)
0
1
2
3
0
200
400
600
800
Time Point (0.25 Hz)

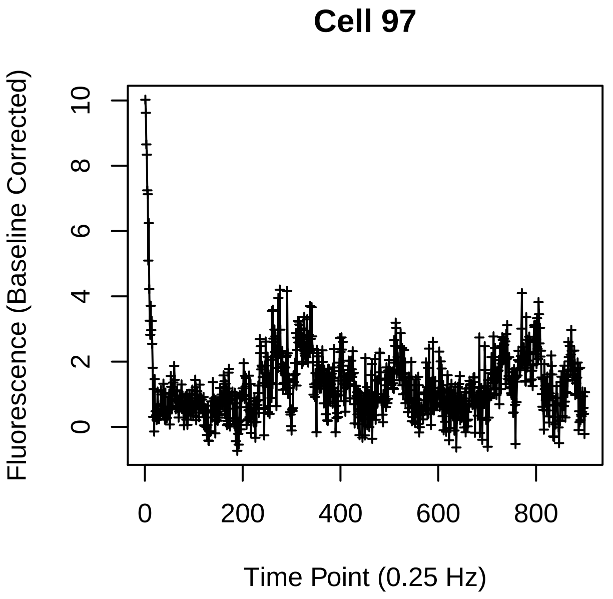
Cell 97
Fluorescence (Baseline Corrected)
0
2
4
6
8
10
0
200
400
600
800
Time Point (0.25 Hz)

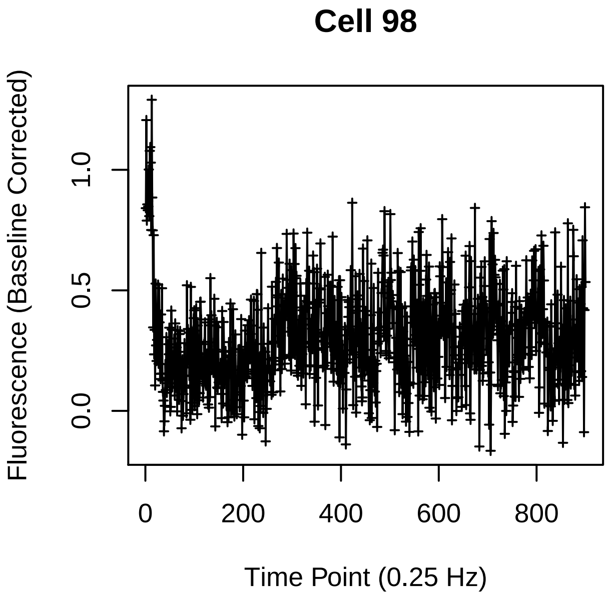
Cell 98
Fluorescence (Baseline Corrected)
0.0
0.5
1.0
0
200
400
600
800
Time Point (0.25 Hz)

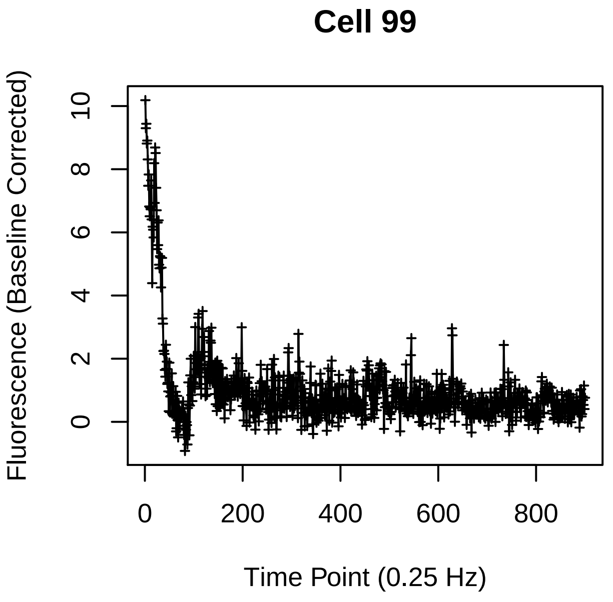
Cell 99
Fluorescence (Baseline Corrected)
0
2
4
6
8
10
0
200
400
600
800
Time Point (0.25 Hz)

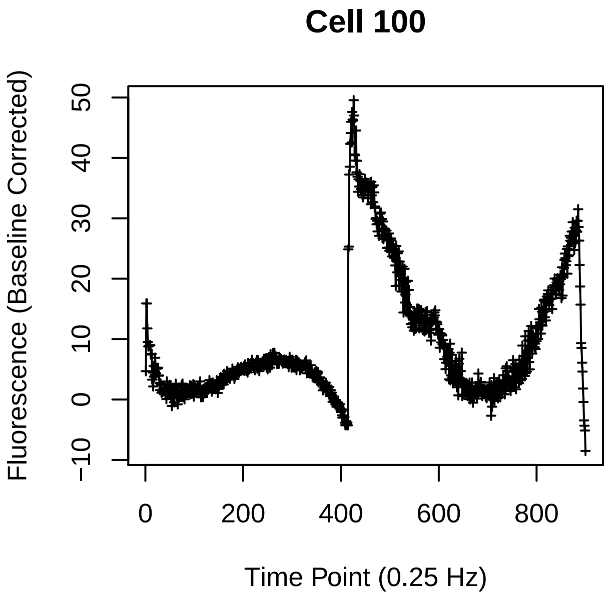
Cell 100
Fluorescence (Baseline Corrected)
−10
0
10
20
30
40
50
0
200
400
600
800
Time Point (0.25 Hz)

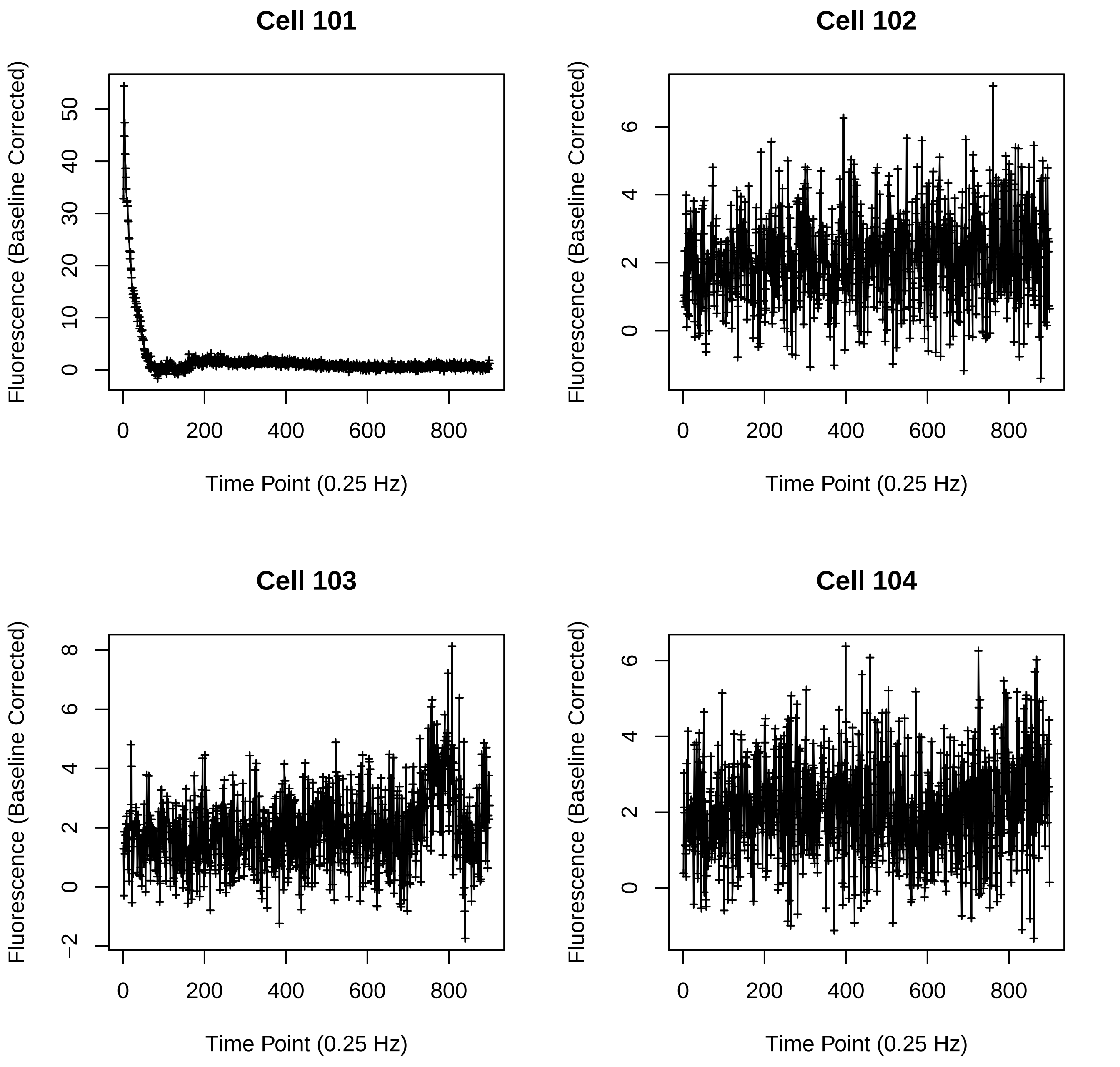
Cell 101
Cell 102
Cell 103
Cell 104
Fluorescence (Baseline Corrected)
Time Point (0.25 Hz)
0
10
20
30
40
50
2
4
6
8
−2
200
400
600
800

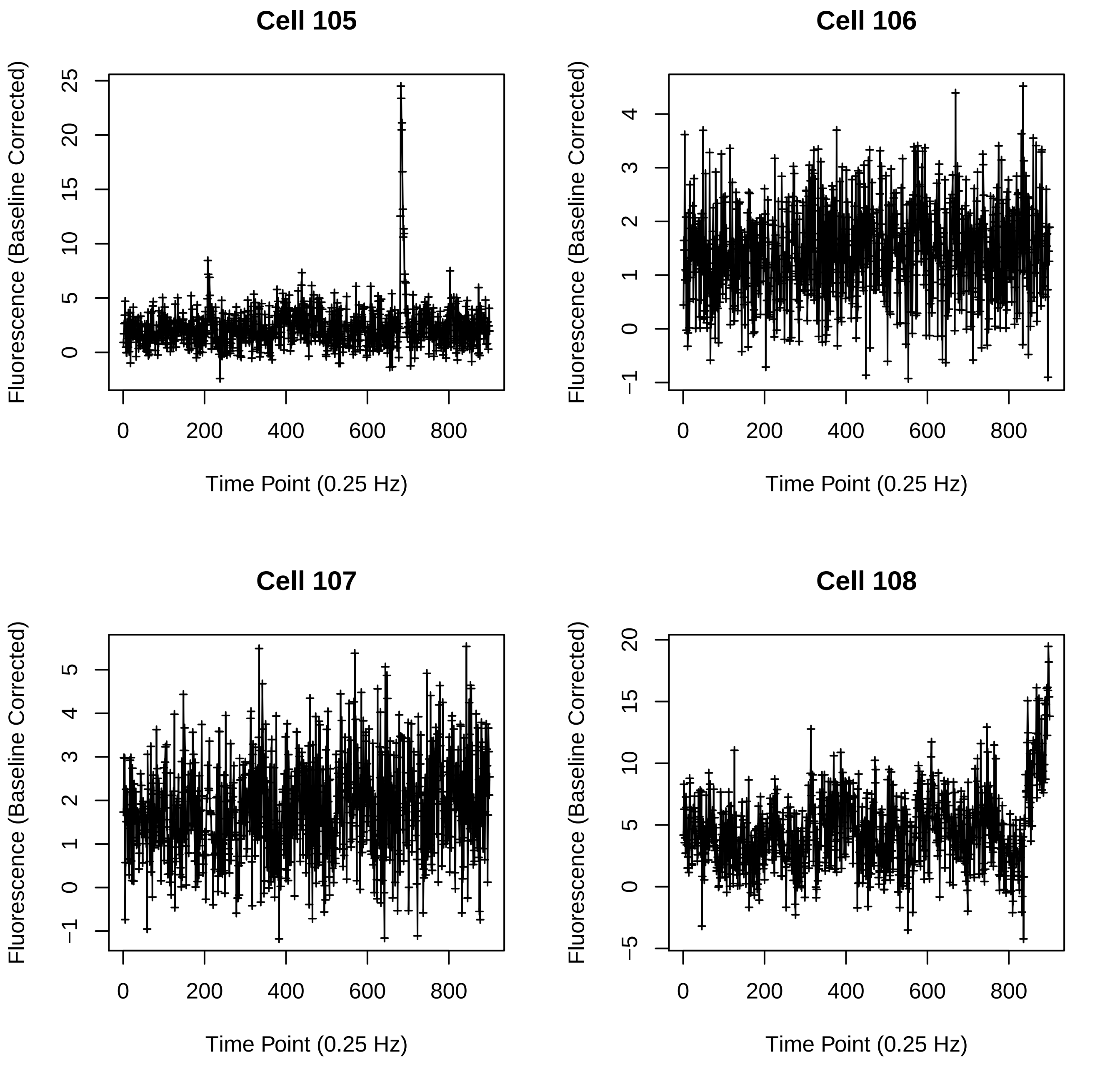
Cell 105
Cell 106
Cell 107
Cell 108
Fluorescence (Baseline Corrected)
Time Point (0.25 Hz)
0
200
400
600
800
-5
0
5
10
15
20
25
-1
1
2
3
4
5

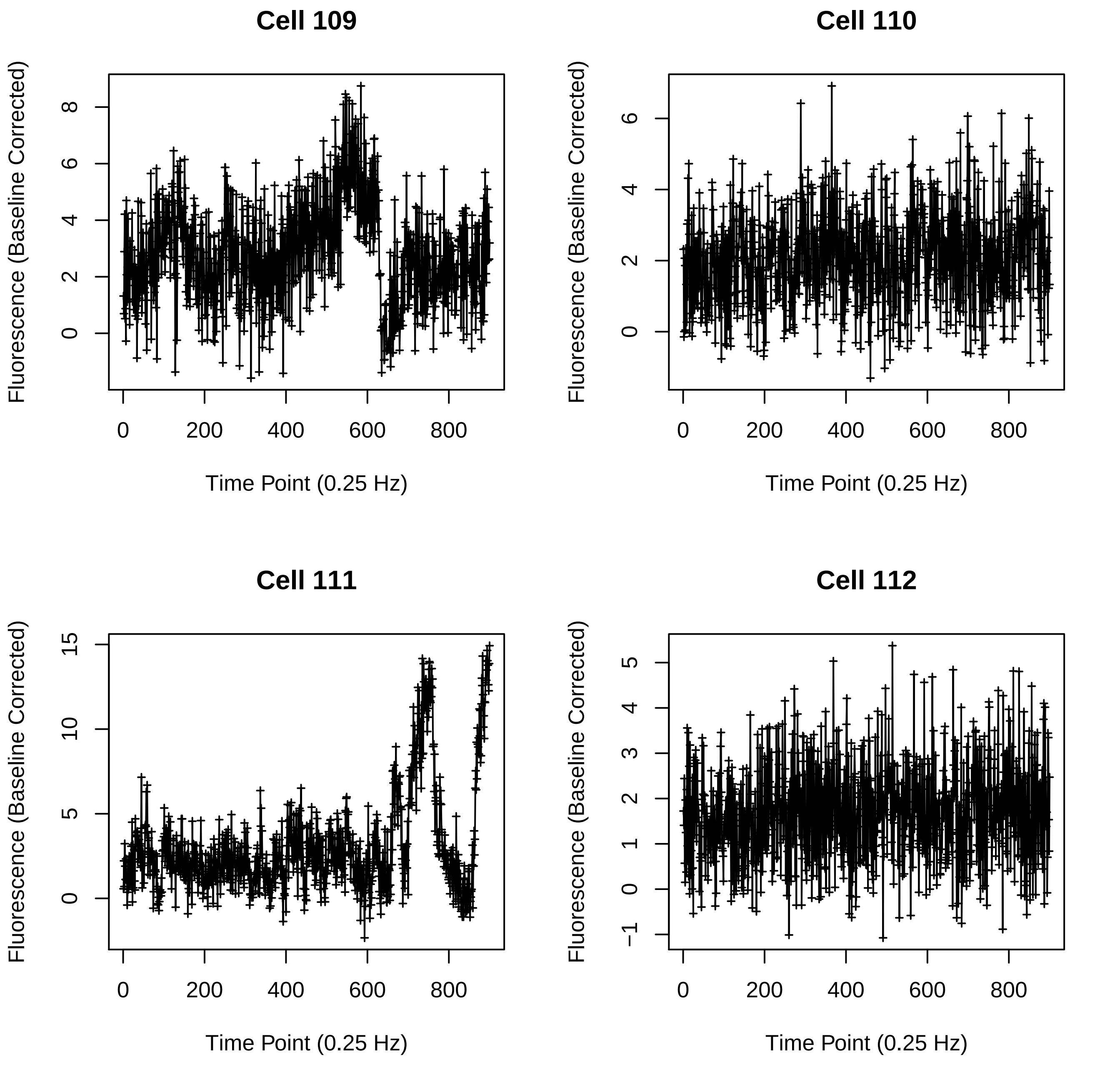

Cell 109
Fluorescence (Baseline Corrected)
0
2
4
6
8
0
200
400
600
800
Time Point (0.25 Hz)
Cell 110
Fluorescence (Baseline Corrected)
0
2
4
6
0
200
400
600
800
Time Point (0.25 Hz)
Cell 111
Fluorescence (Baseline Corrected)
0
5
10
15
0
200
400
600
800
Time Point (0.25 Hz)
Cell 112
Fluorescence (Baseline Corrected)
−1
0
1
2
3
4
5
0
200
400
600
800
Time Point (0.25 Hz)

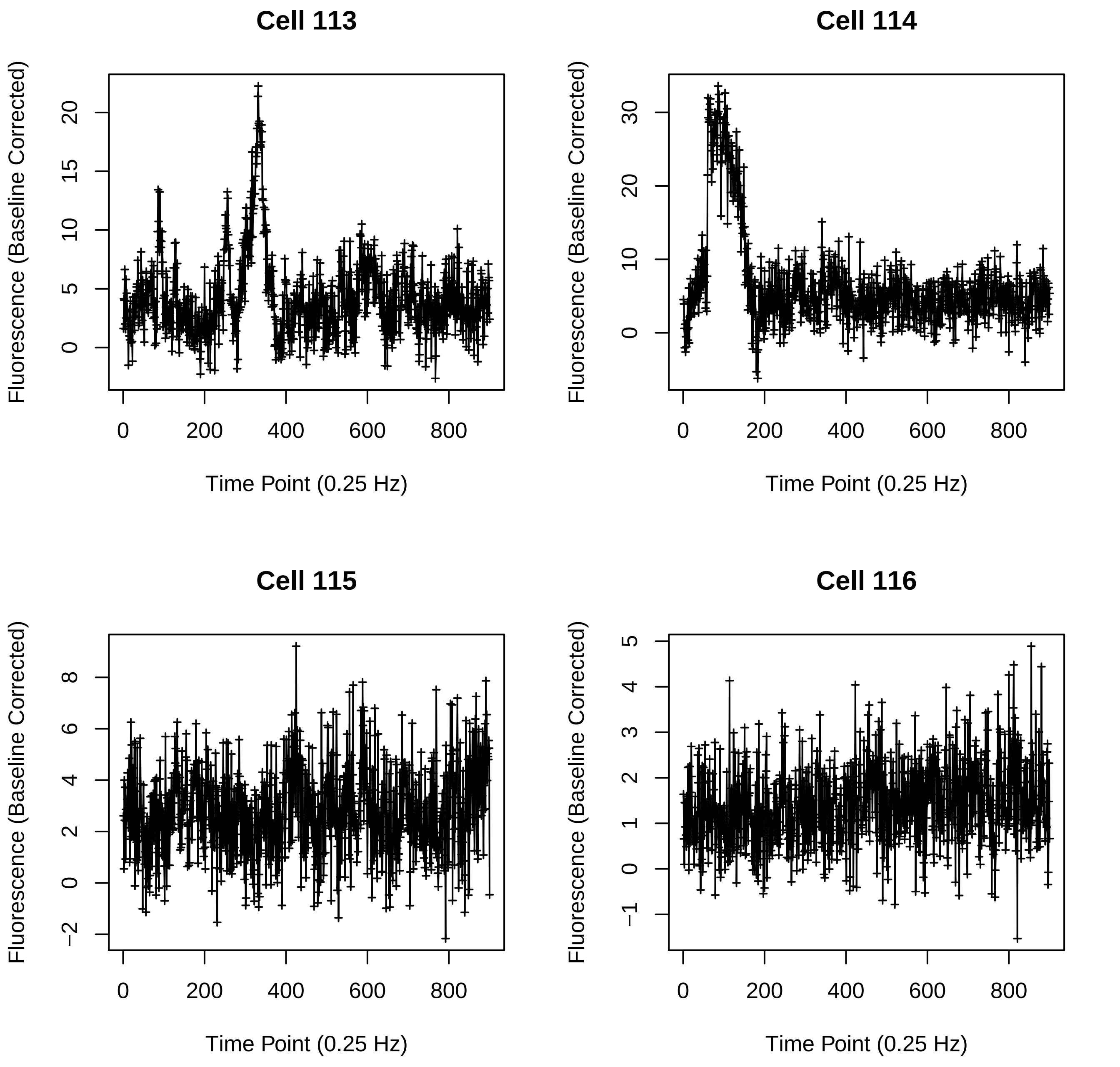
Cell 113
Cell 114
Cell 115
Cell 116
Fluorescence (Baseline Corrected)
Time Point (0.25 Hz)
0
200
400
600
800
0
5
10
15
20
0
10
20
30
−2
0
2
4
6
8
−1
0
1
2
3
4
5

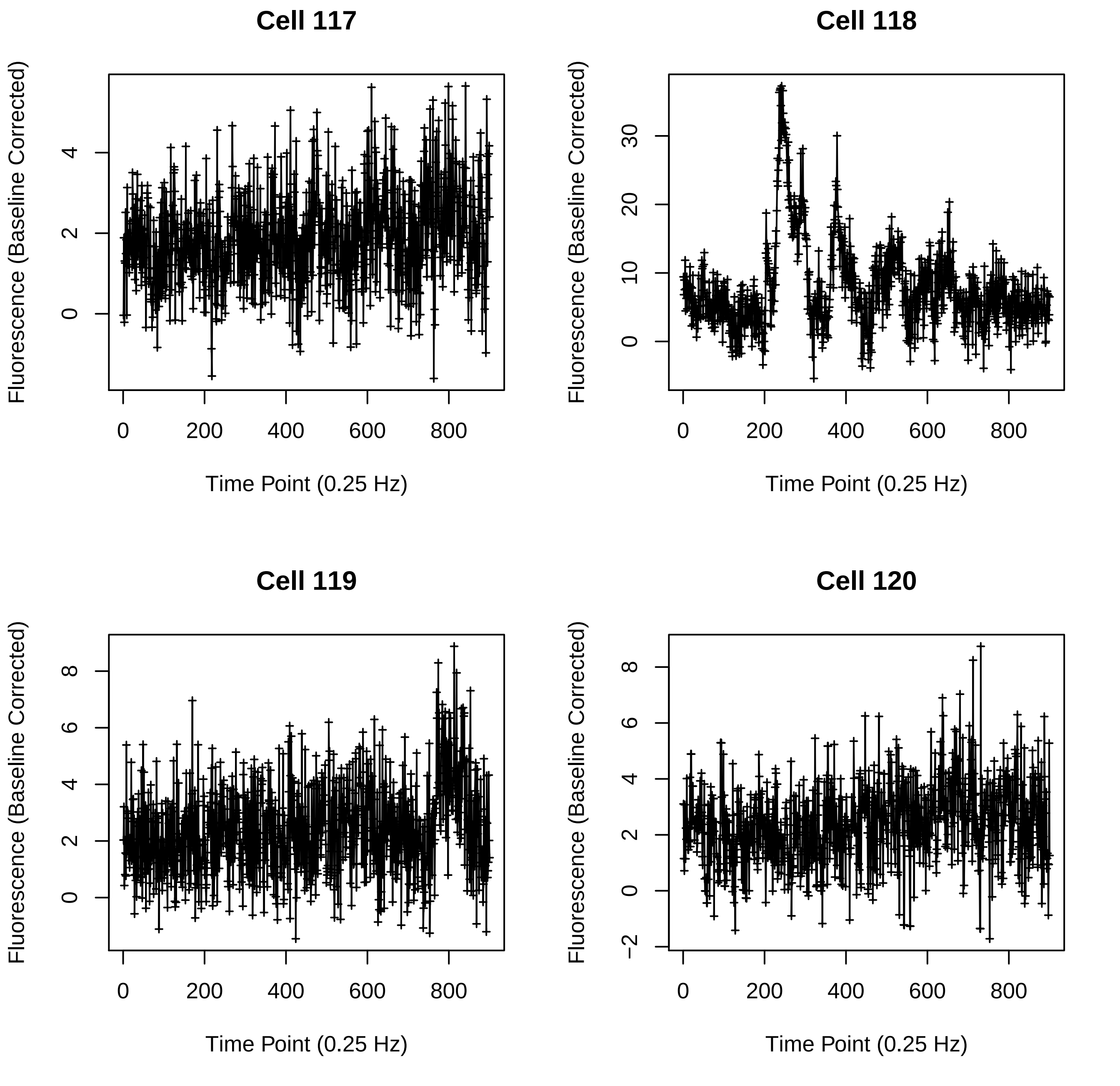
Cell 117
Cell 118
Cell 119
Cell 120
Fluorescence (Baseline Corrected)
Time Point (0.25 Hz)
0
200
400
600
800
−2
2
4
6
8
10
20
30

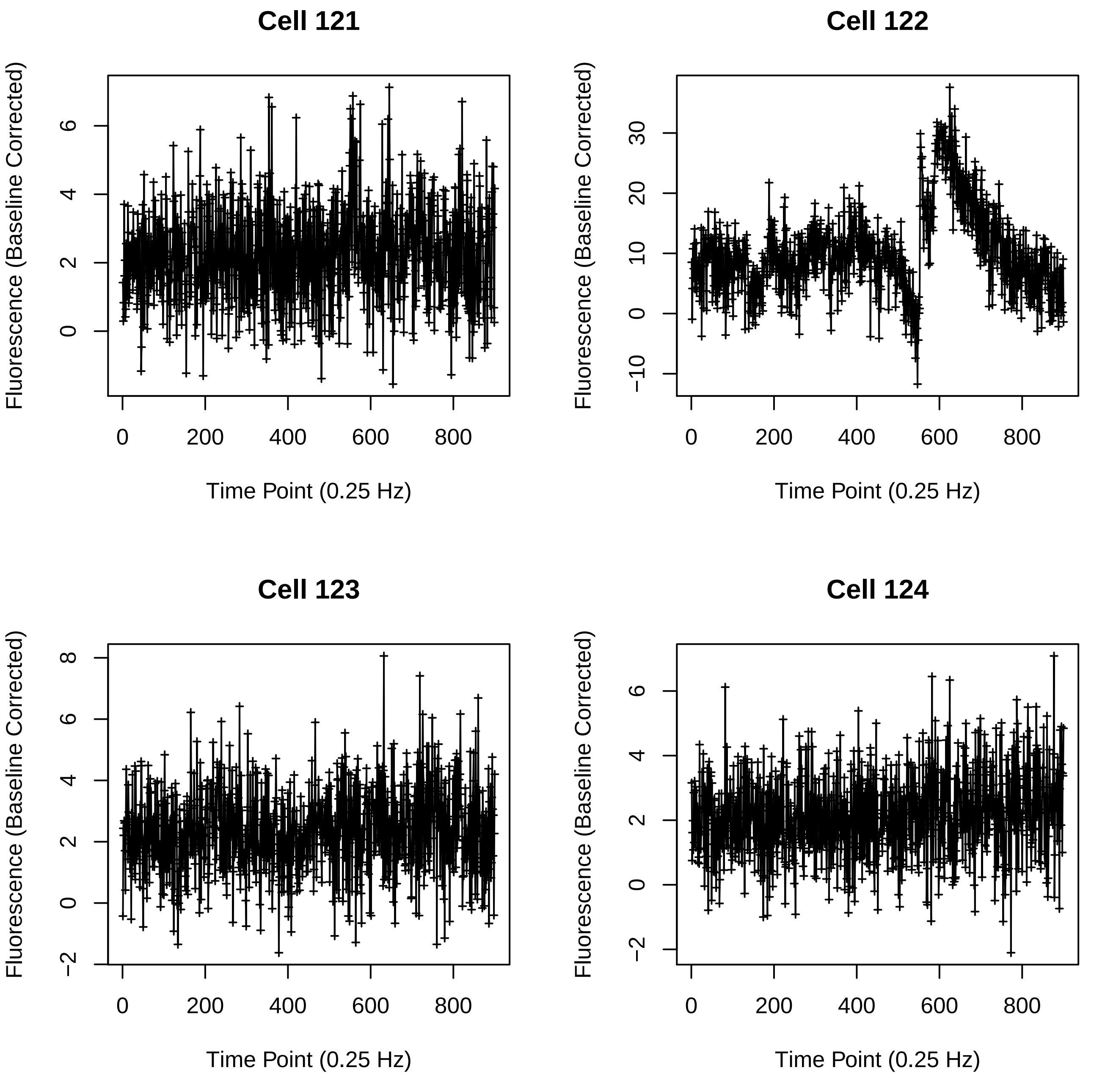
Cell 121
Cell 122
Cell 123
Cell 124
Fluorescence (Baseline Corrected)
Time Point (0.25 Hz)
0
200
400
600
800
−10
10
20
30
−2
2
4
6
8

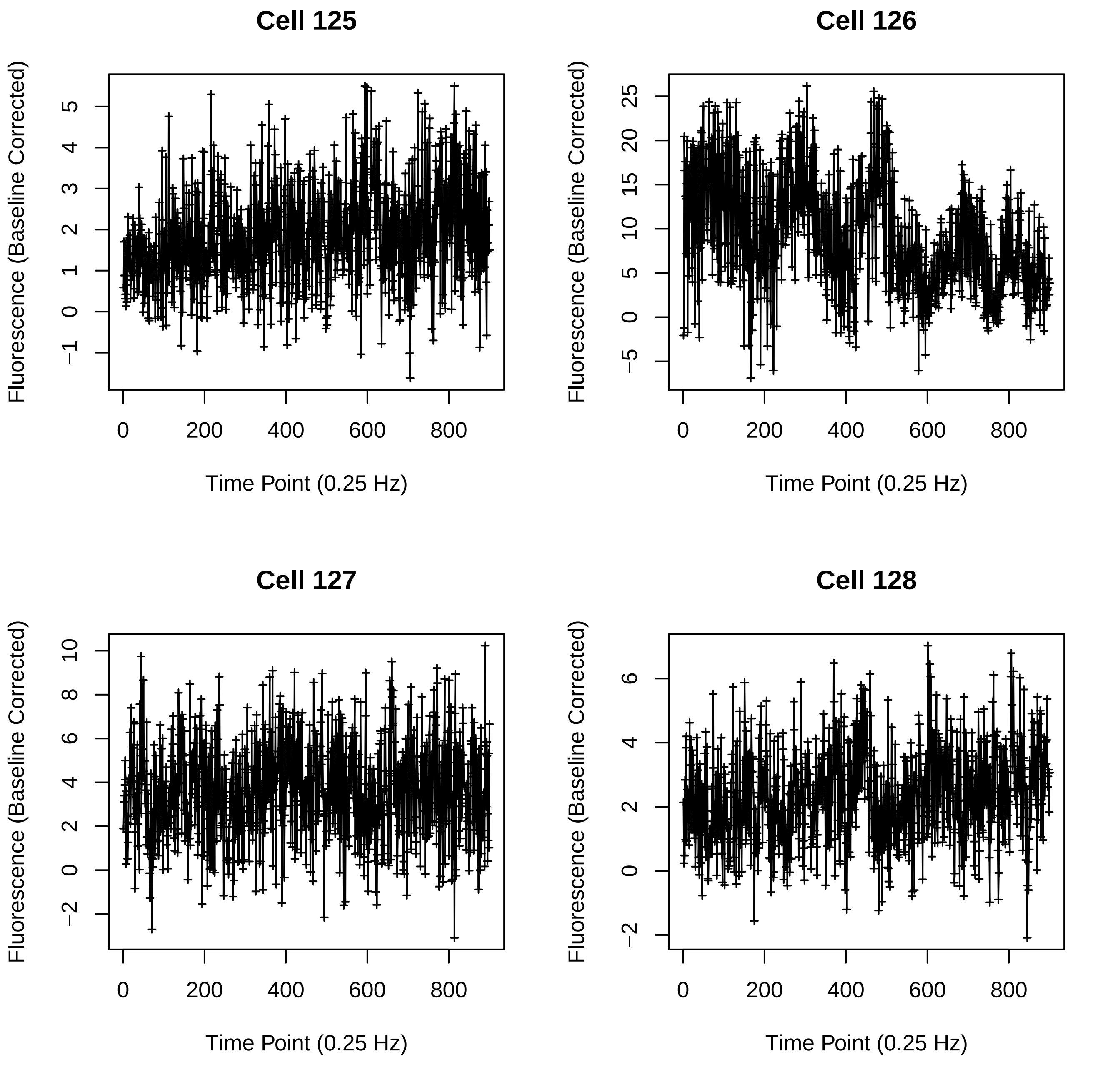
Cell 125
Fluorescence (Baseline Corrected)
Time Point (0.25 Hz)
Cell 126
Fluorescence (Baseline Corrected)
Time Point (0.25 Hz)
Cell 127
Fluorescence (Baseline Corrected)
Time Point (0.25 Hz)
Cell 128
Fluorescence (Baseline Corrected)
Time Point (0.25 Hz)

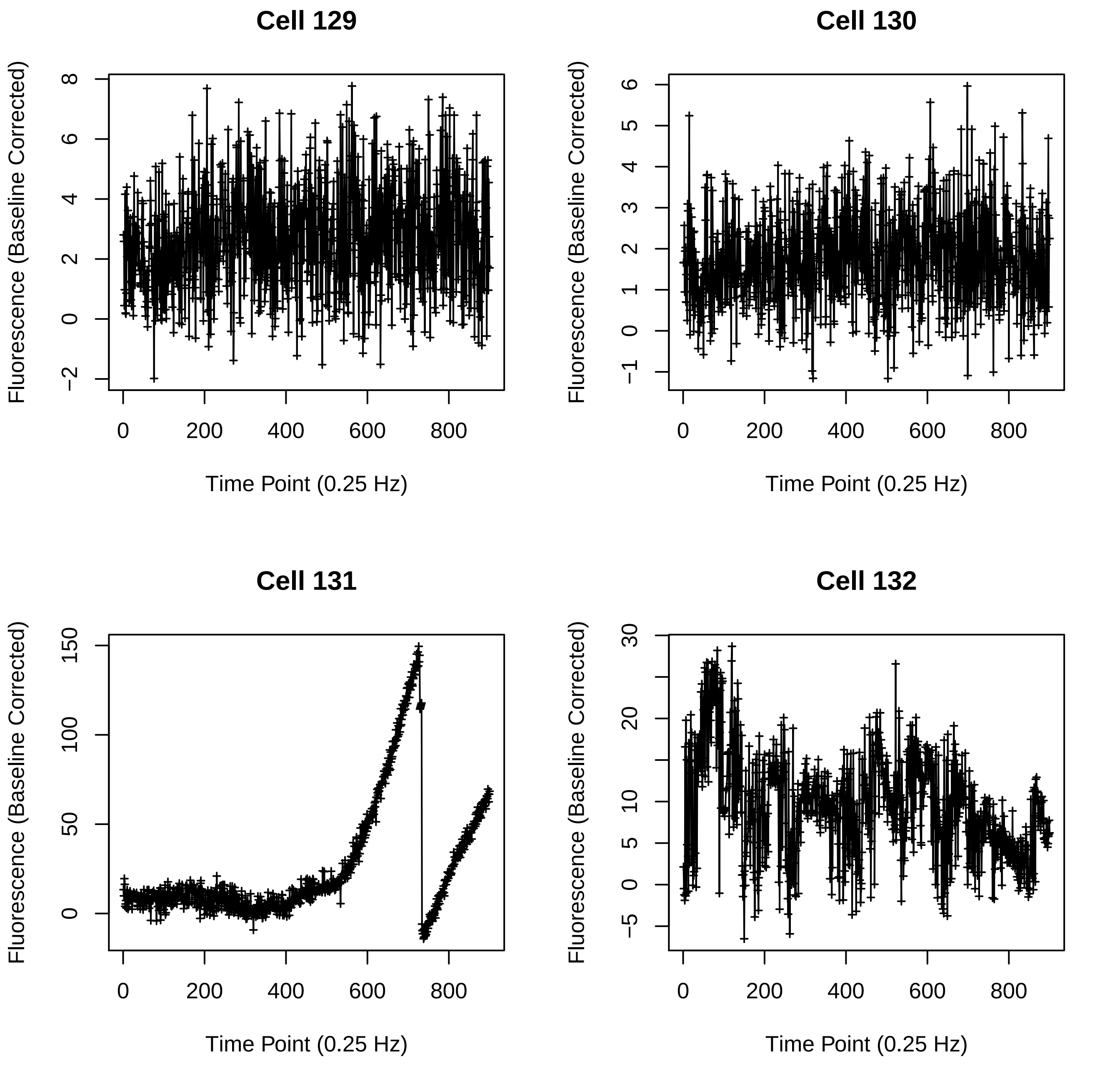
Cell 129
Cell 130
Cell 131
Cell 132
Fluorescence (Baseline Corrected)
Time Point (0.25 Hz)
−2
0
2
4
6
8
−1
0
1
2
3
4
5
6
0
50
100
150
−5
0
5
10
20
30
0
200
400
600
800

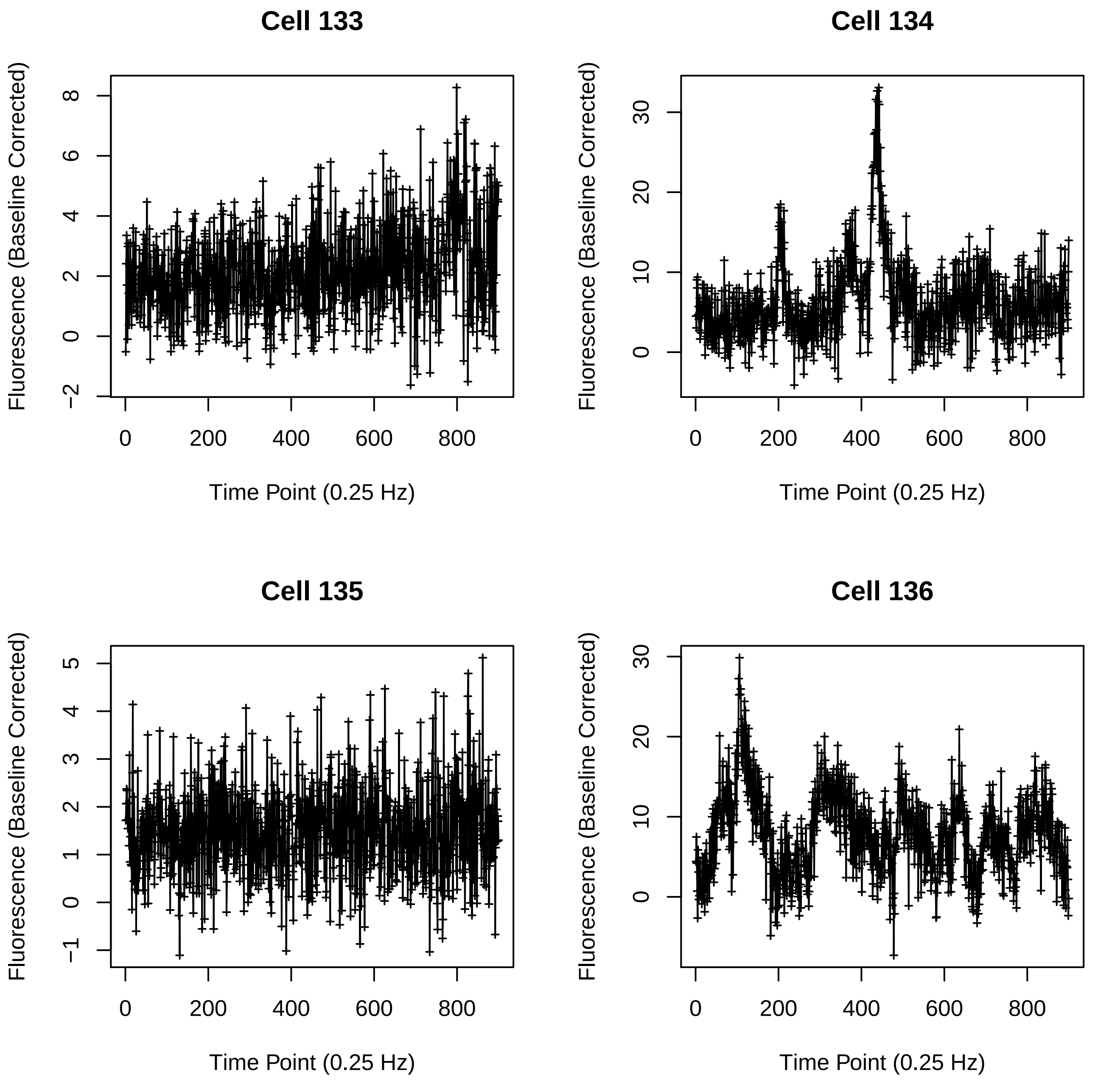
Cell 133
Cell 134
Cell 135
Cell 136
Fluorescence (Baseline Corrected)
Time Point (0.25 Hz)
−2
0
2
4
6
8
10
20
30
−1
1
3
5
200
400
600
800

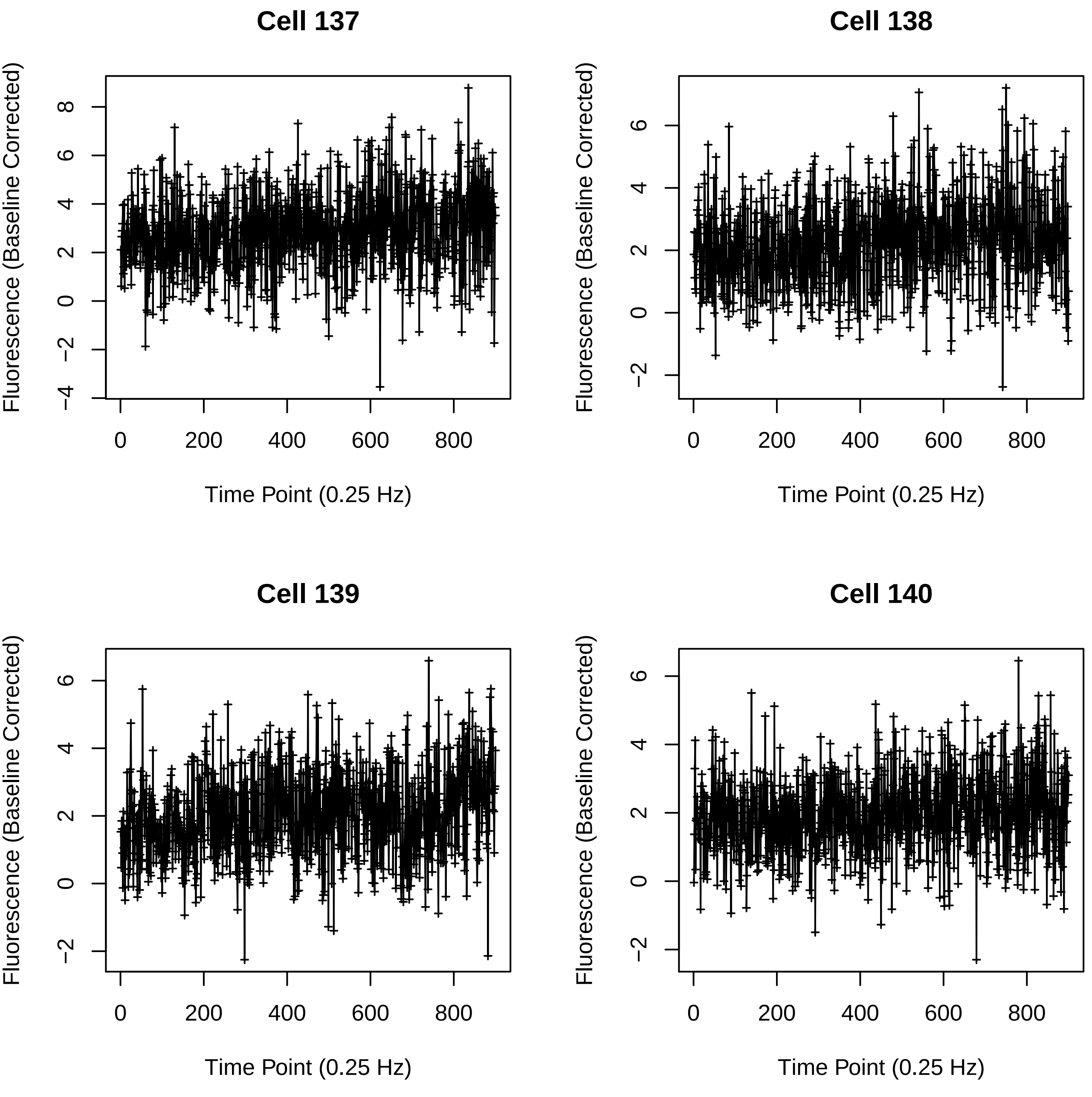

Cell 137
Cell 138
Cell 139
Cell 140
Fluorescence (Baseline Corrected)
Time Point (0.25 Hz)

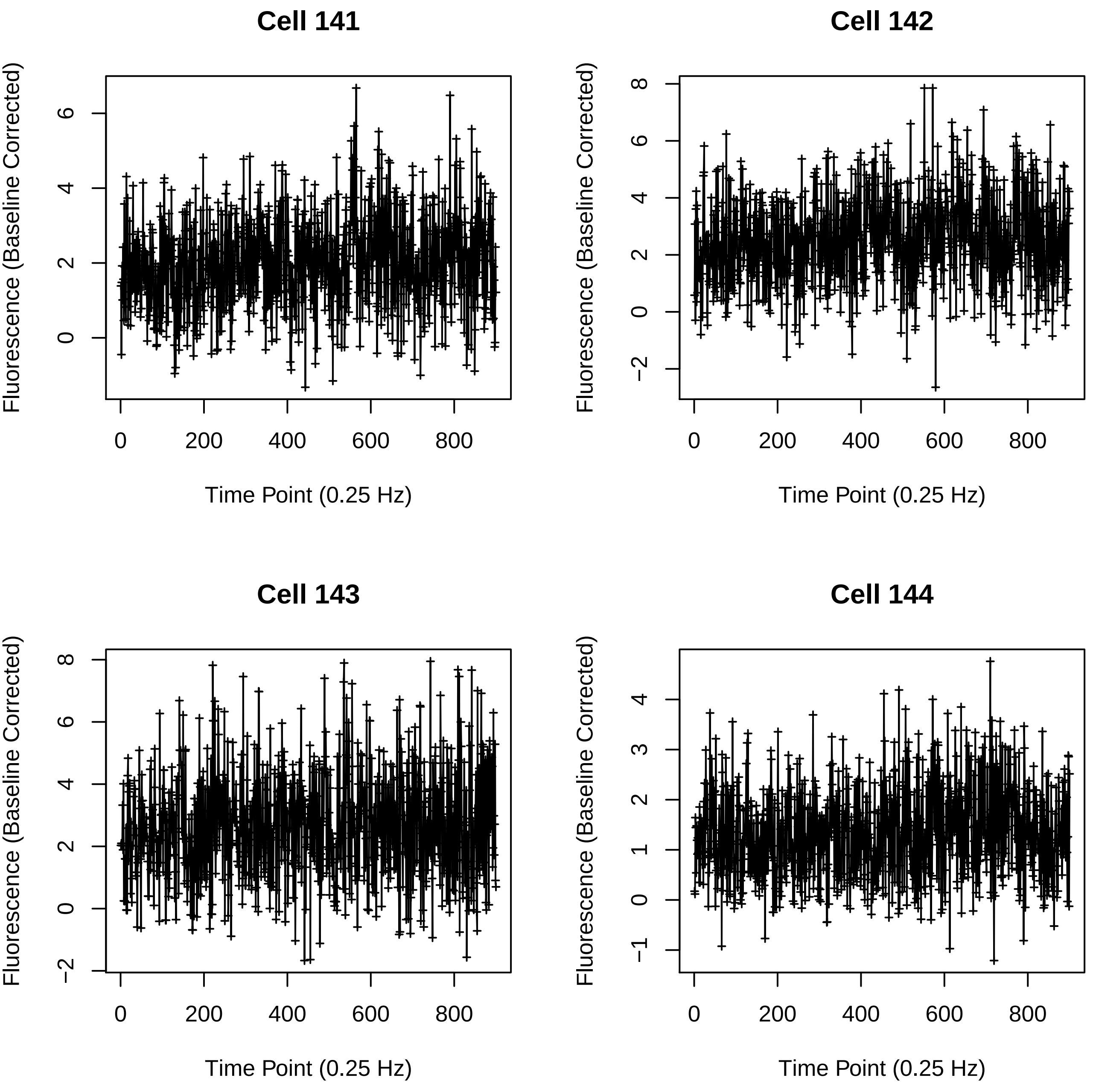
Cell 141
Cell 142
Cell 143
Cell 144
Fluorescence (Baseline Corrected)
Time Point (0.25 Hz)
0
200
400
600
800
−2
0
2
4
6
8
−1
1
3

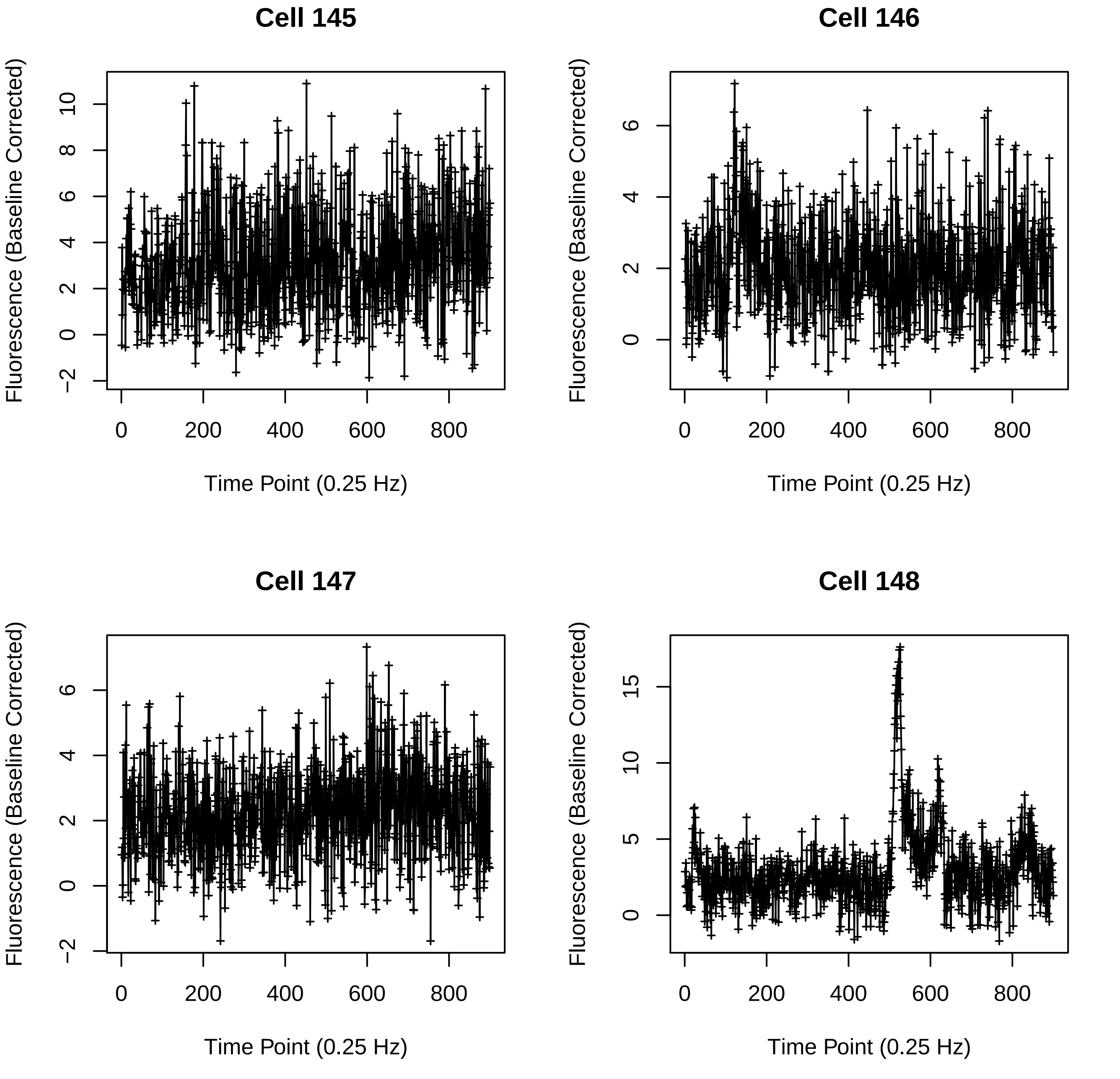
Cell 145
Cell 146
Cell 147
Cell 148
Fluorescence (Baseline Corrected)
Time Point (0.25 Hz)
0
200
400
600
800
−2
0
2
4
6
8
10
15
5

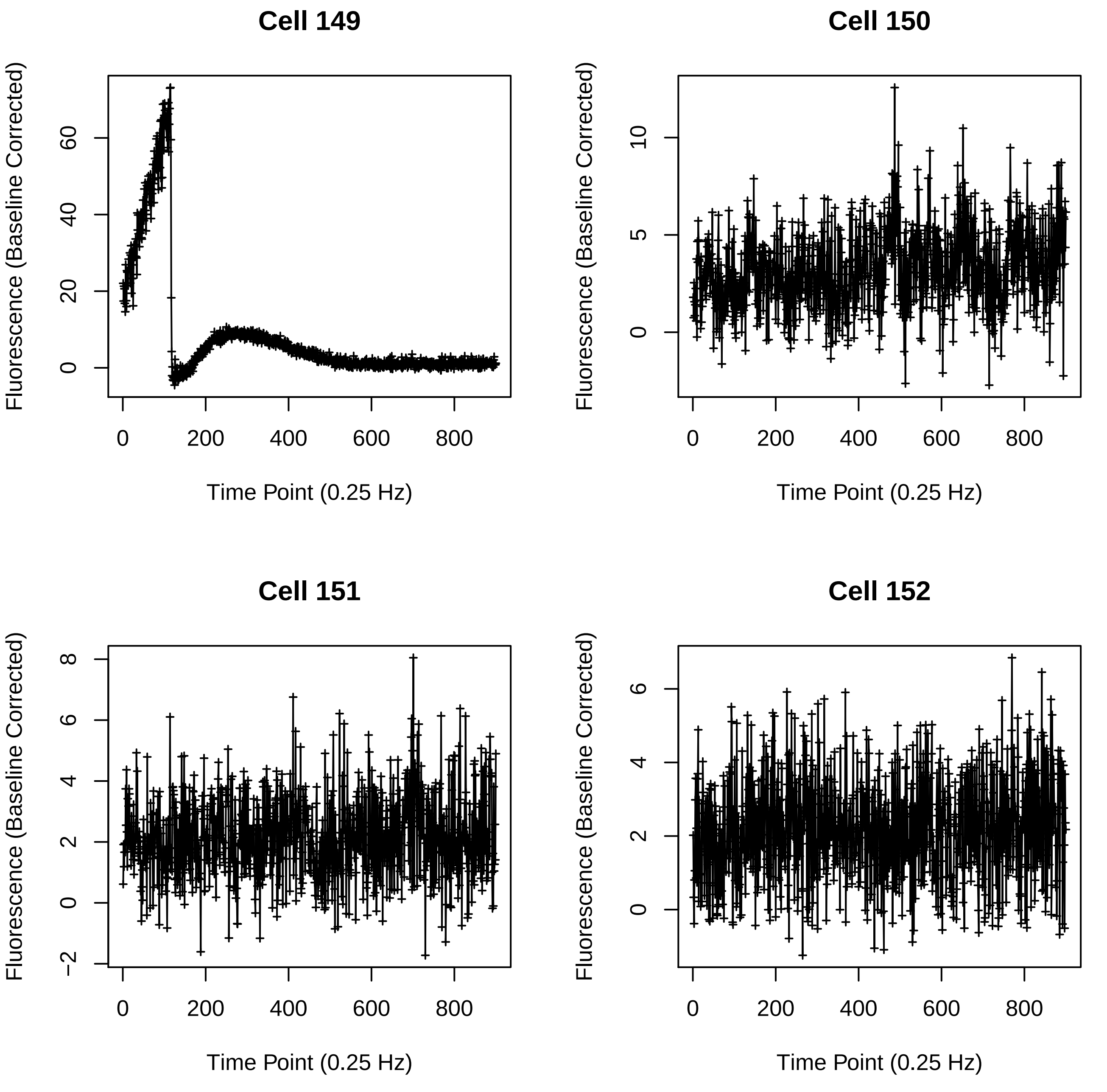
Cell 149
Fluorescence (Baseline Corrected)
0
20
40
60
0
200
400
600
800
Time Point (0.25 Hz)
Cell 150
Fluorescence (Baseline Corrected)
0
5
10
0
200
400
600
800
Time Point (0.25 Hz)
Cell 151
Fluorescence (Baseline Corrected)
−2
0
2
4
6
8
0
200
400
600
800
Time Point (0.25 Hz)
Cell 152
Fluorescence (Baseline Corrected)
0
2
4
6
0
200
400
600
800
Time Point (0.25 Hz)

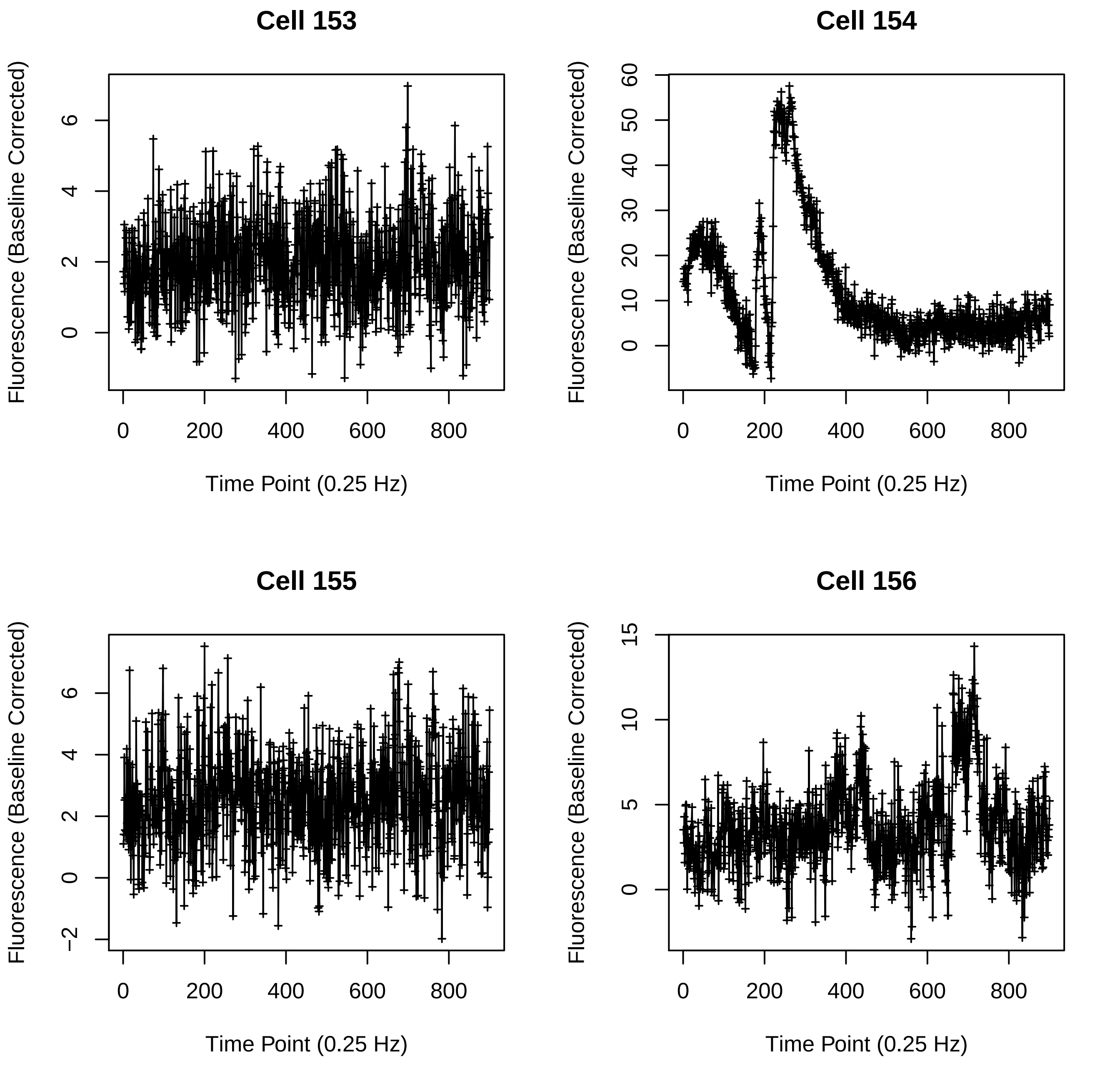
Cell 153
Fluorescence (Baseline Corrected)
0
2
4
6
0
200
400
600
800
Time Point (0.25 Hz)
Cell 154
Fluorescence (Baseline Corrected)
0
10
20
30
40
50
60
0
200
400
600
800
Time Point (0.25 Hz)
Cell 155
Fluorescence (Baseline Corrected)
−2
0
2
4
6
0
200
400
600
800
Time Point (0.25 Hz)
Cell 156
Fluorescence (Baseline Corrected)
0
5
10
15
0
200
400
600
800
Time Point (0.25 Hz)

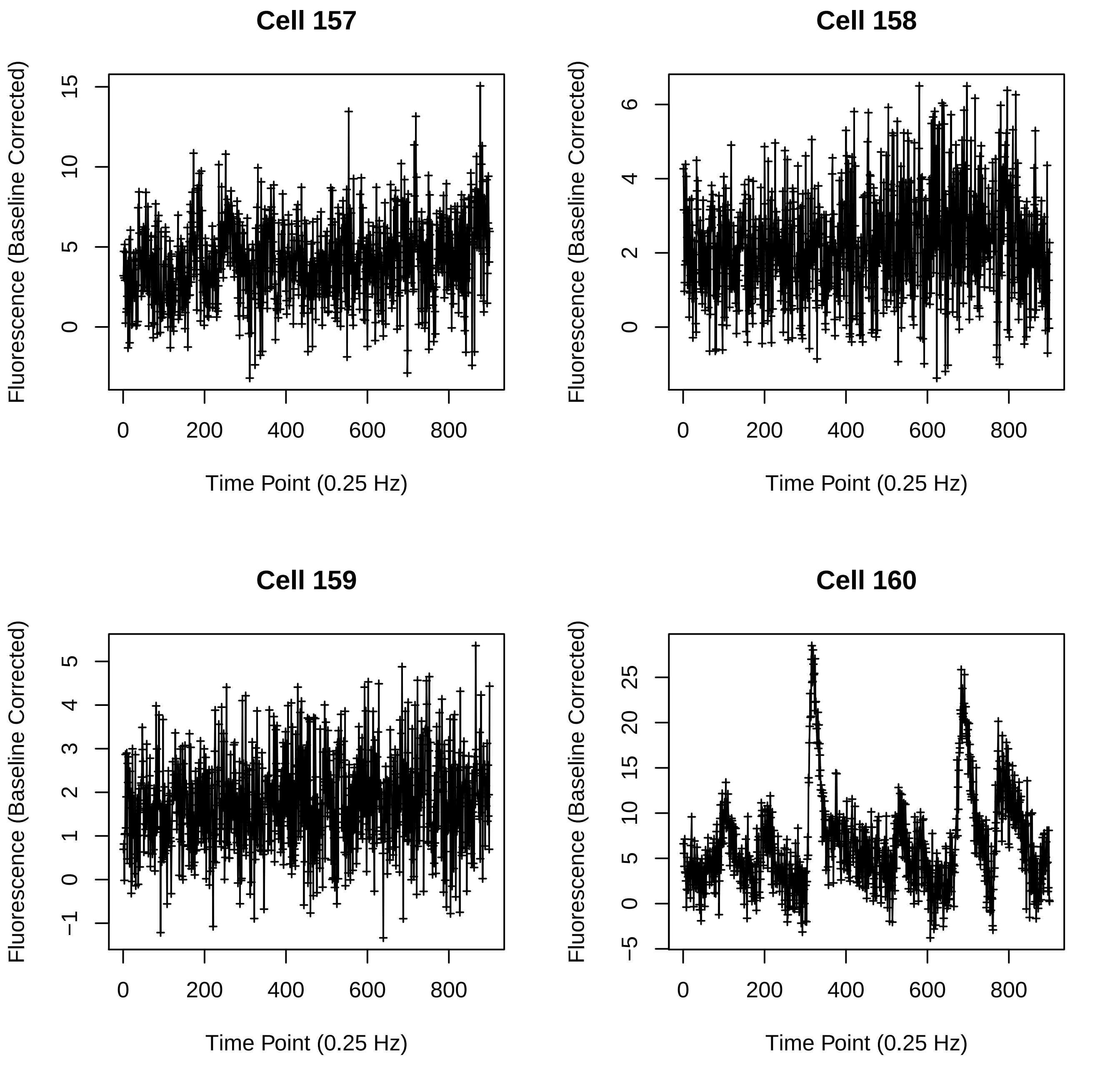
Cell 157
Cell 158
Cell 159
Cell 160
Fluorescence (Baseline Corrected)
Time Point (0.25 Hz)
0
200
400
600
800
-5
0
5
10
15
20
25
-1
1
2
3
4
6

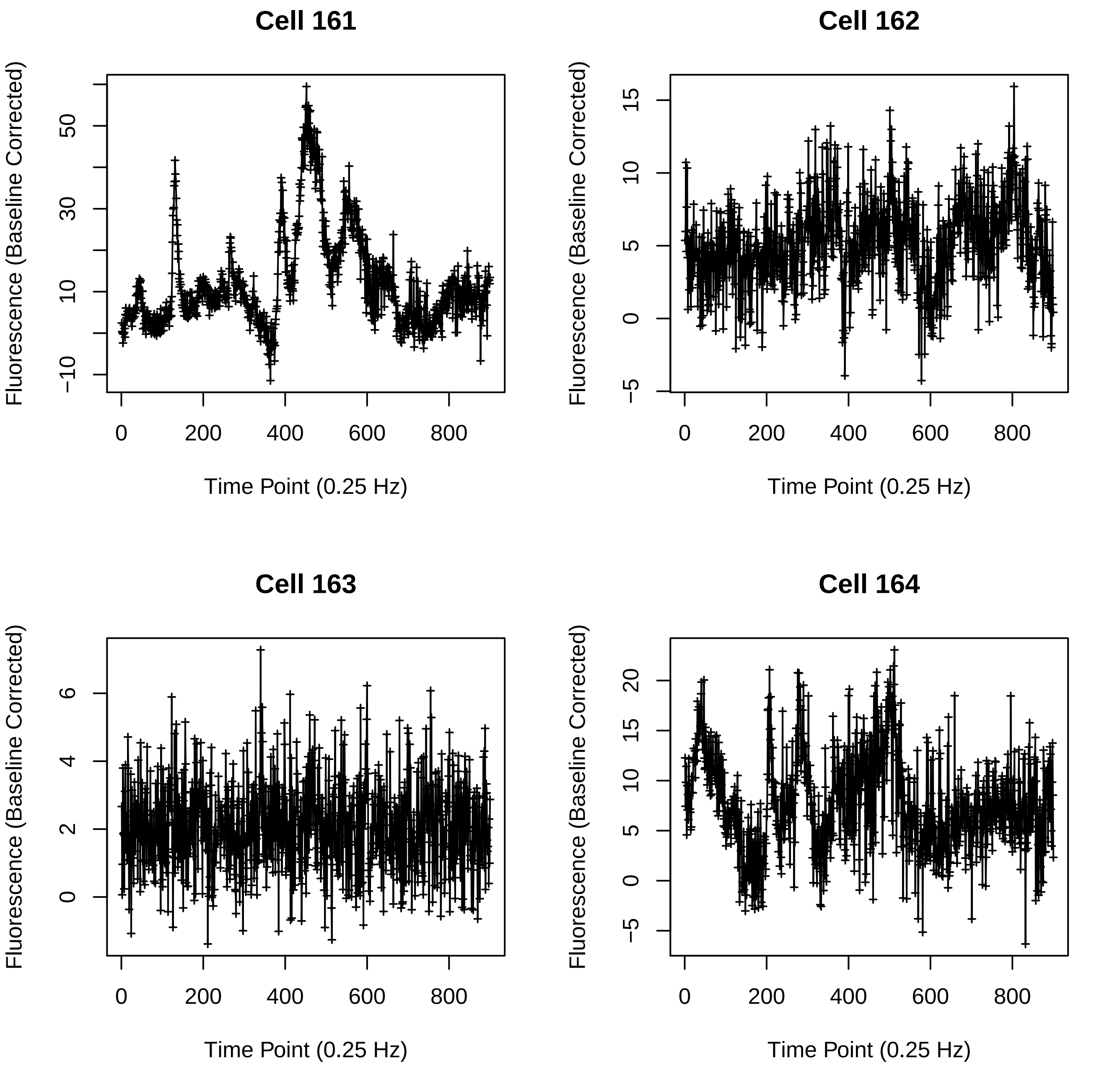
Cell 161
Cell 162
Cell 163
Cell 164
Fluorescence (Baseline Corrected)
Time Point (0.25 Hz)
−10
10
30
50
−5
0
5
10
15
0
2
4
6
−5
0
5
10
15
20
0
200
400
600
800

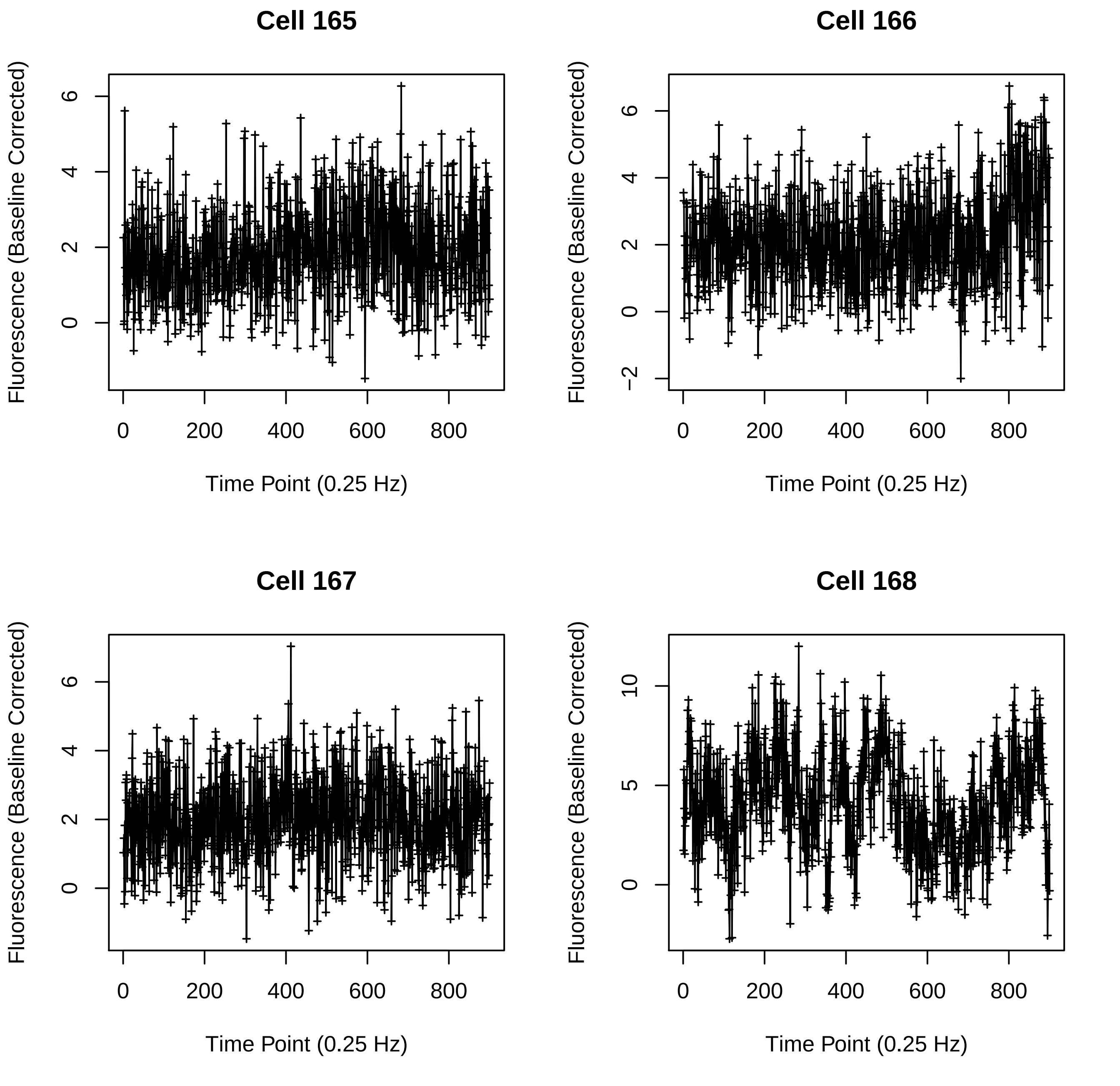
Cell 165
Cell 166
Cell 167
Cell 168
Fluorescence (Baseline Corrected)
Time Point (0.25 Hz)
0
200
400
600
800
−2
2
4
6
5
10

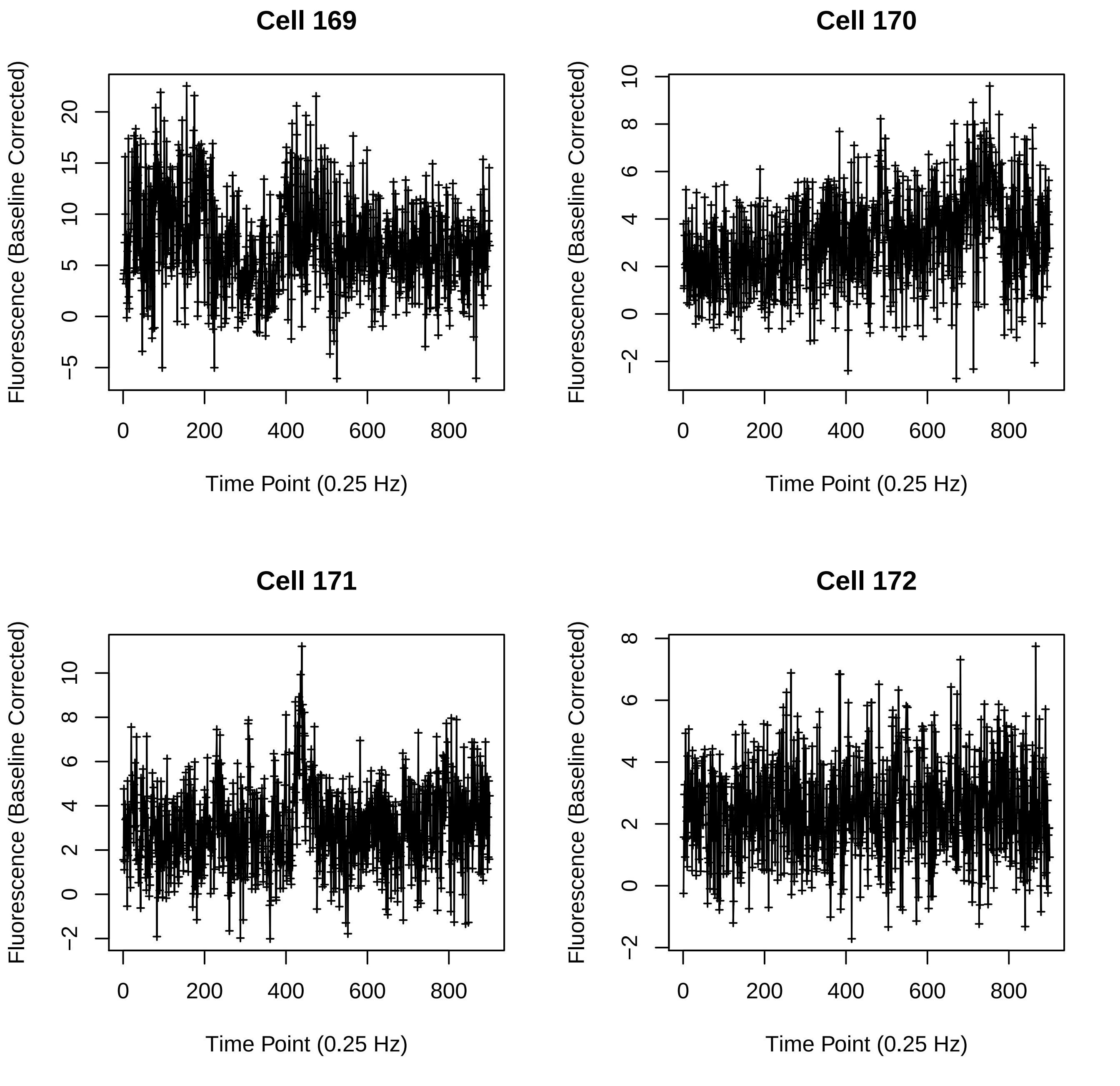
Cell 169
Cell 170
Cell 171
Cell 172
Fluorescence (Baseline Corrected)
Time Point (0.25 Hz)

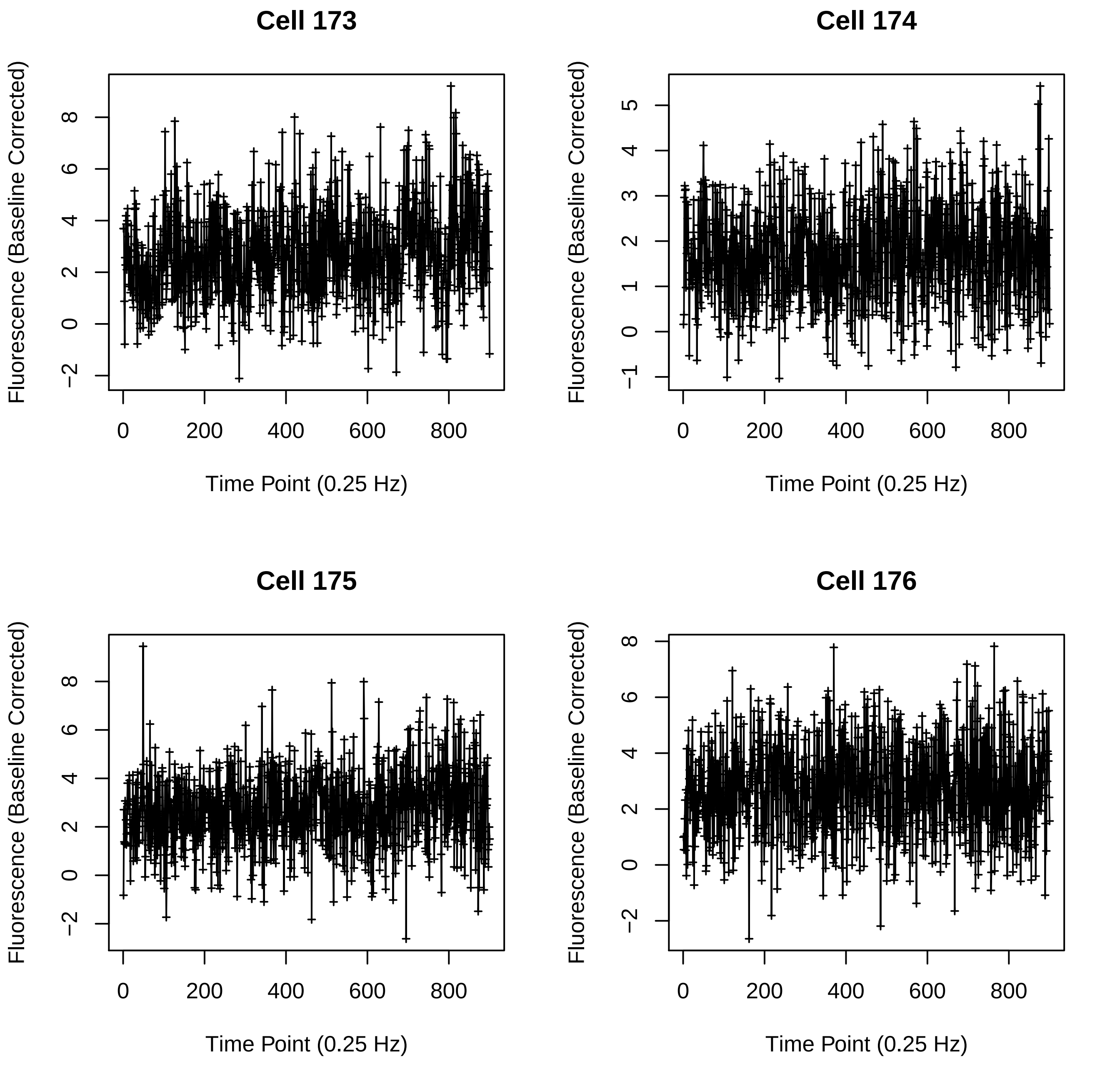

Cell 173
Cell 174
Cell 175
Cell 176
Fluorescence (Baseline Corrected)
Time Point (0.25 Hz)
0
200
400
600
800
−2
0
2
4
6
8
−1
1
3
5

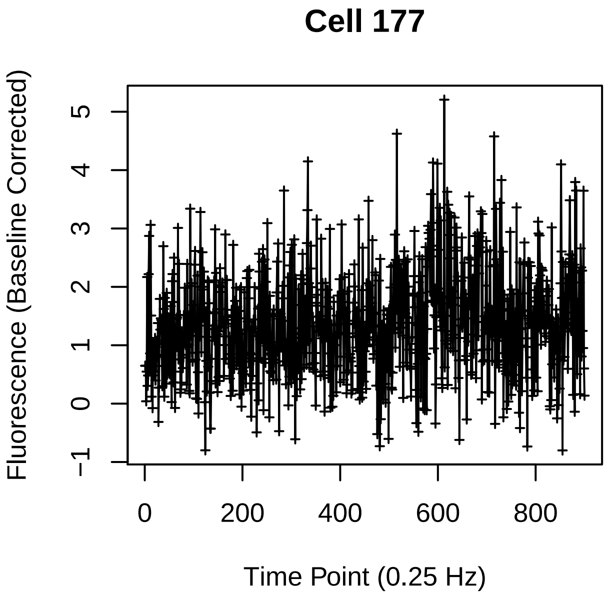
Cell 177
Fluorescence (Baseline Corrected)
Time Point (0.25 Hz)
−1
0
1
2
3
4
5
0
200
400
600
800

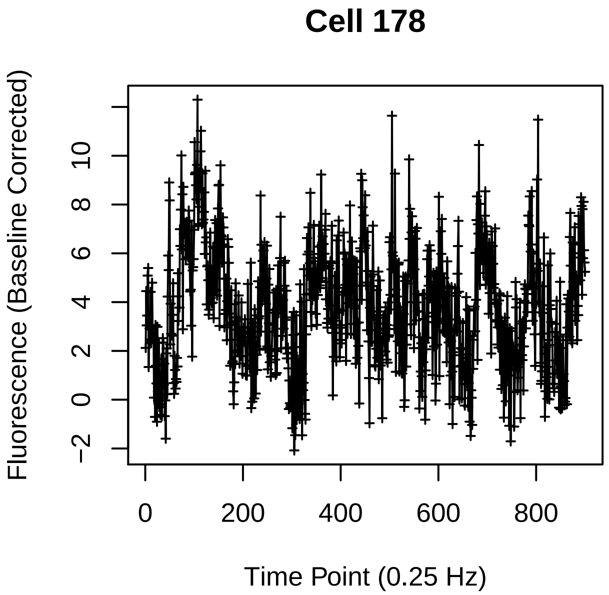
Cell 178
Fluorescence (Baseline Corrected)
Time Point (0.25 Hz)
−2
0
2
4
6
8
10
0
200
400
600
800

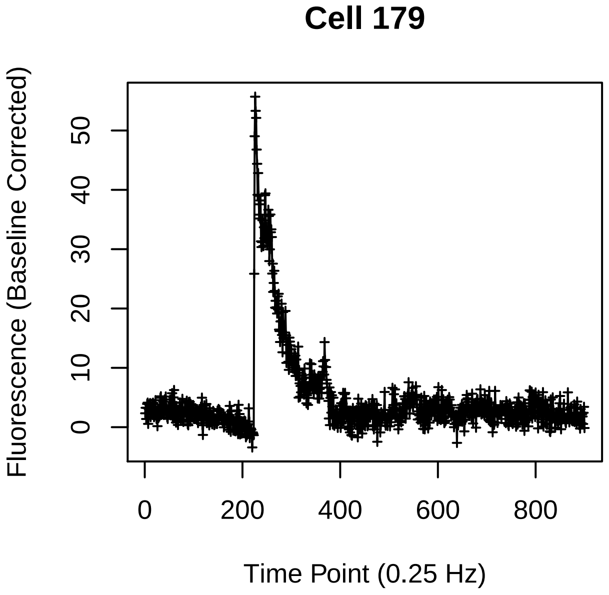
Cell 179
Fluorescence (Baseline Corrected)
Time Point (0.25 Hz)
0
10
20
30
40
50
0
200
400
600
800

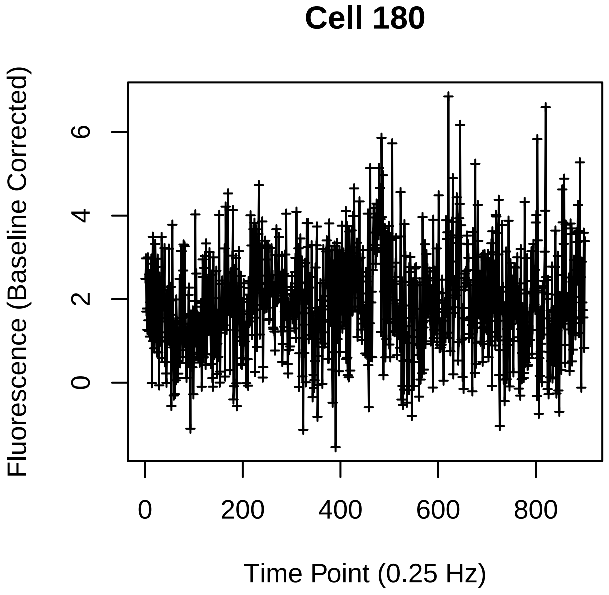
Cell 180
Fluorescence (Baseline Corrected)
Time Point (0.25 Hz)
0
2
4
6
0
200
400
600
800

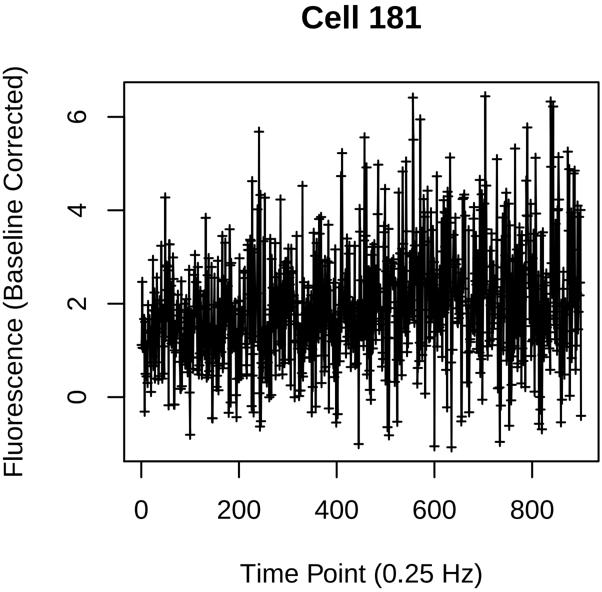
Cell 181
Fluorescence (Baseline Corrected)
0
2
4
6
0
200
400
600
800
Time Point (0.25 Hz)

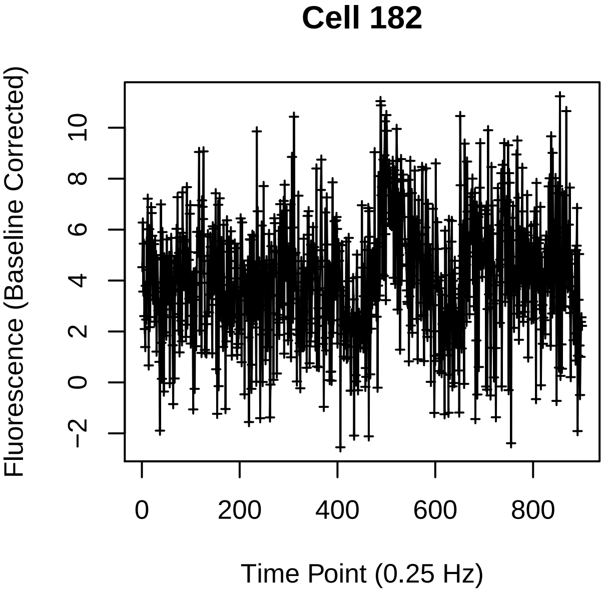
Cell 182
Fluorescence (Baseline Corrected)
−2
0
2
4
6
8
10
0
200
400
600
800
Time Point (0.25 Hz)

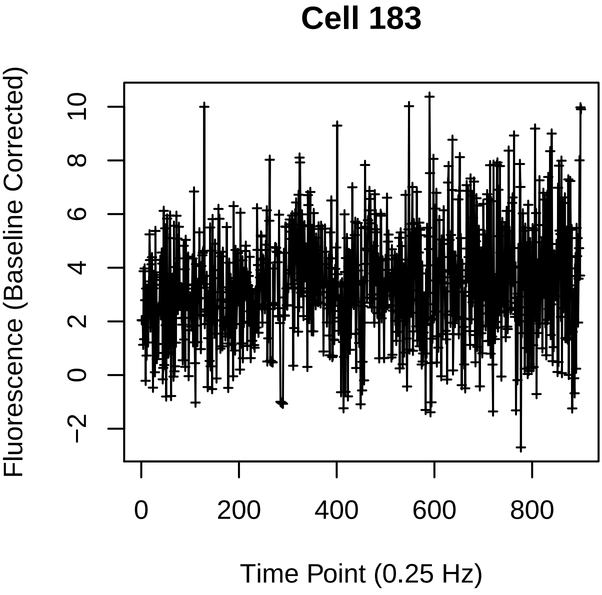
Cell 183
Fluorescence (Baseline Corrected)
−2
0
2
4
6
8
10
0
200
400
600
800
Time Point (0.25 Hz)

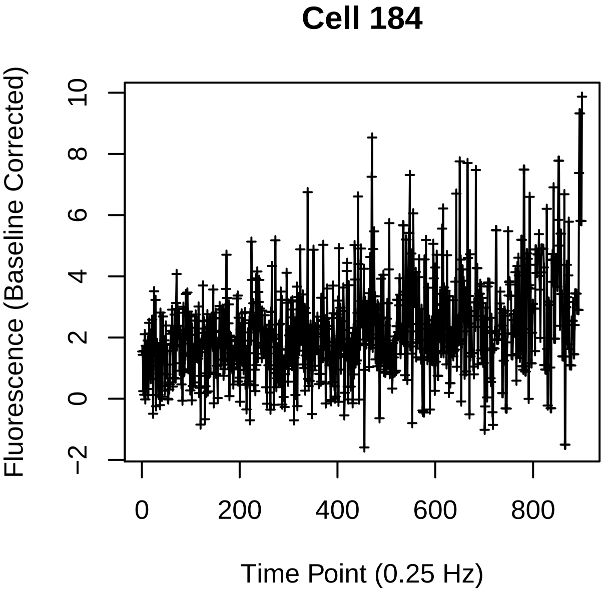
Cell 184
Fluorescence (Baseline Corrected)
−2
0
2
4
6
8
10
0
200
400
600
800
Time Point (0.25 Hz)

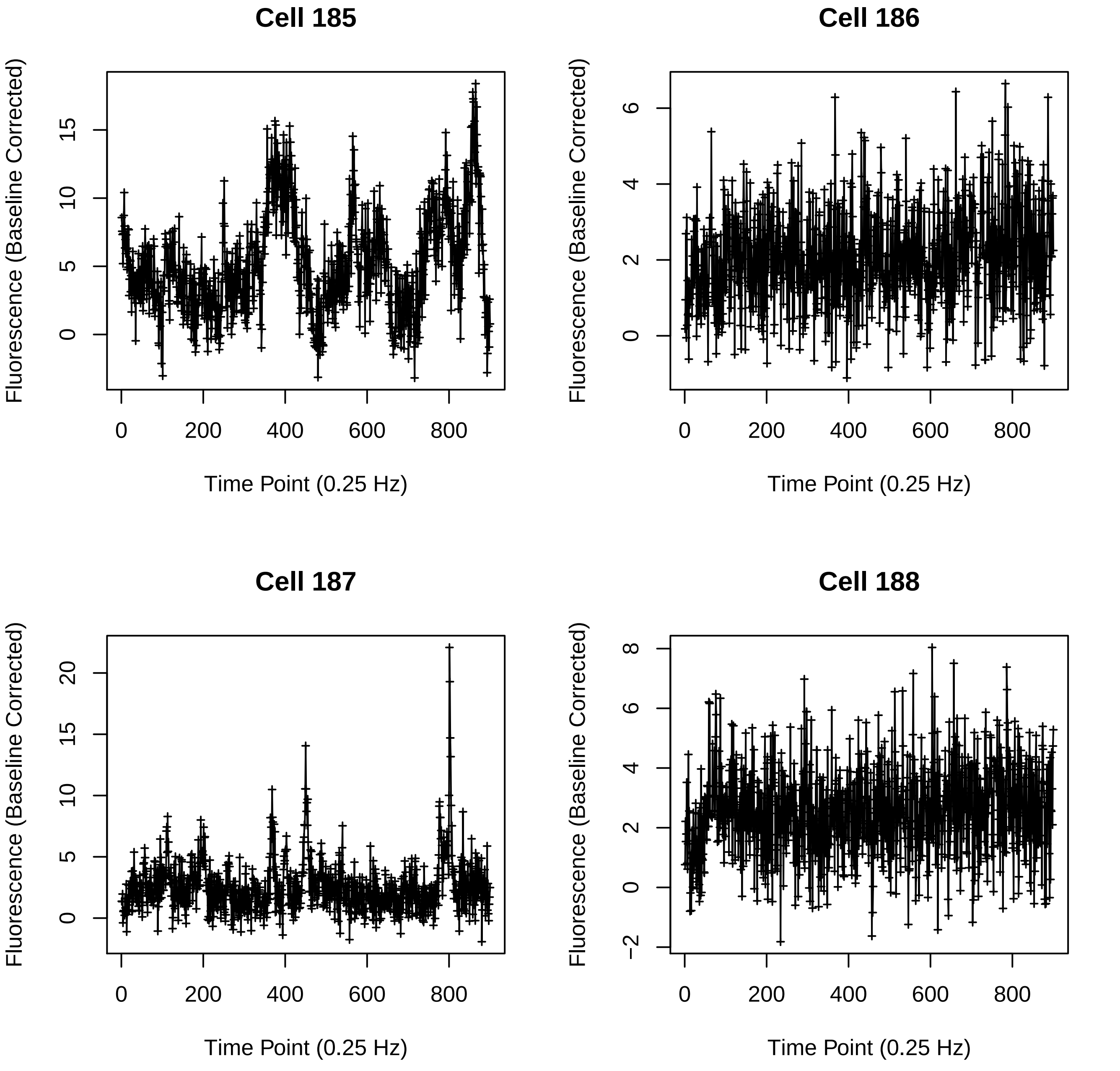
Cell 185
Cell 186
Cell 187
Cell 188
Fluorescence (Baseline Corrected)
Time Point (0.25 Hz)
0
200
400
600
800
-2
0
2
4
5
6
8
10
15
20

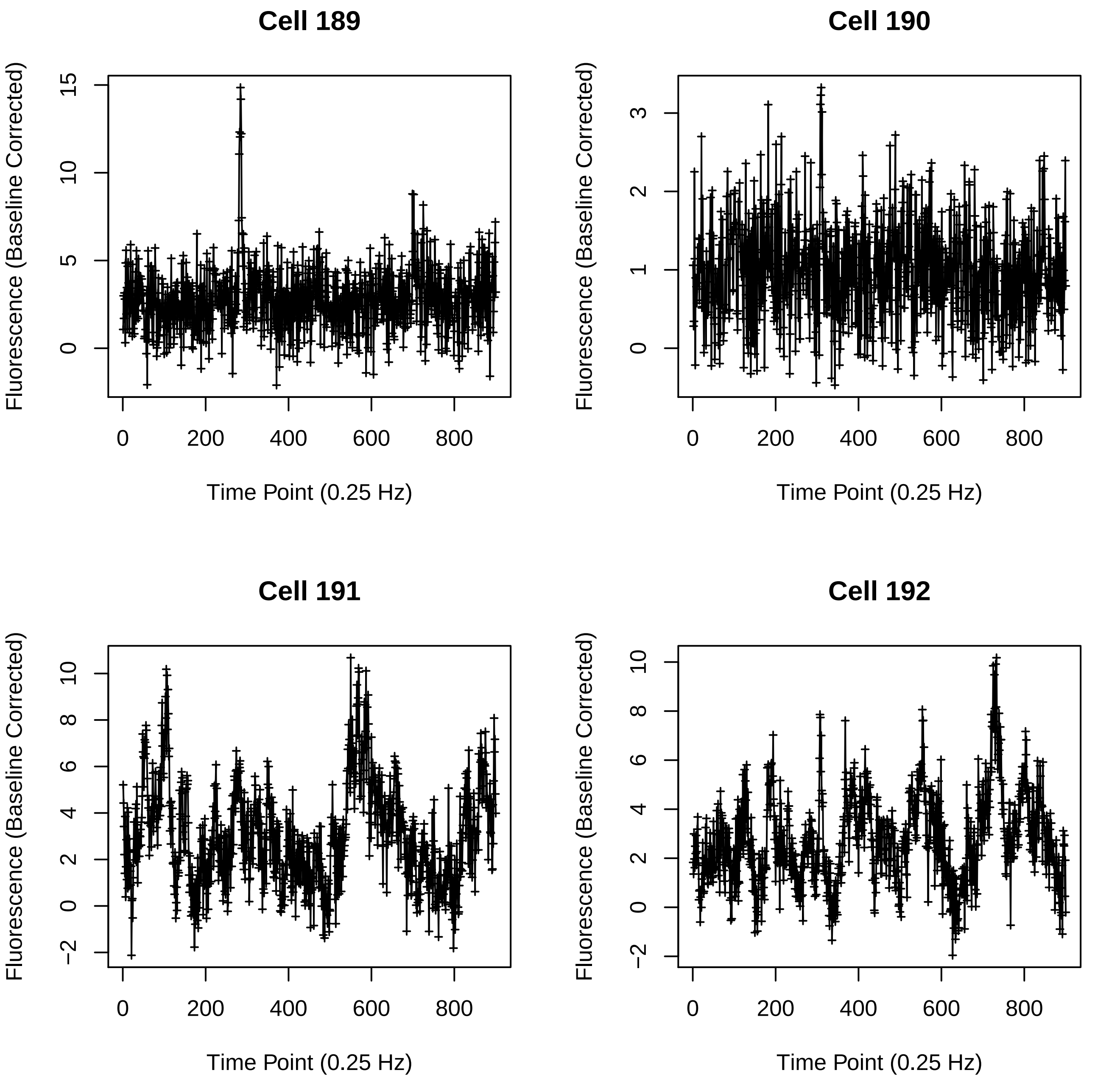

Cell 189
Fluorescence (Baseline Corrected)
Time Point (0.25 Hz)
0
5
10
15
0
200
400
600
800
Cell 190
Fluorescence (Baseline Corrected)
Time Point (0.25 Hz)
0
1
2
3
Cell 191
Fluorescence (Baseline Corrected)
Time Point (0.25 Hz)
−2
0
2
4
6
8
10
Cell 192
Fluorescence (Baseline Corrected)
Time Point (0.25 Hz)

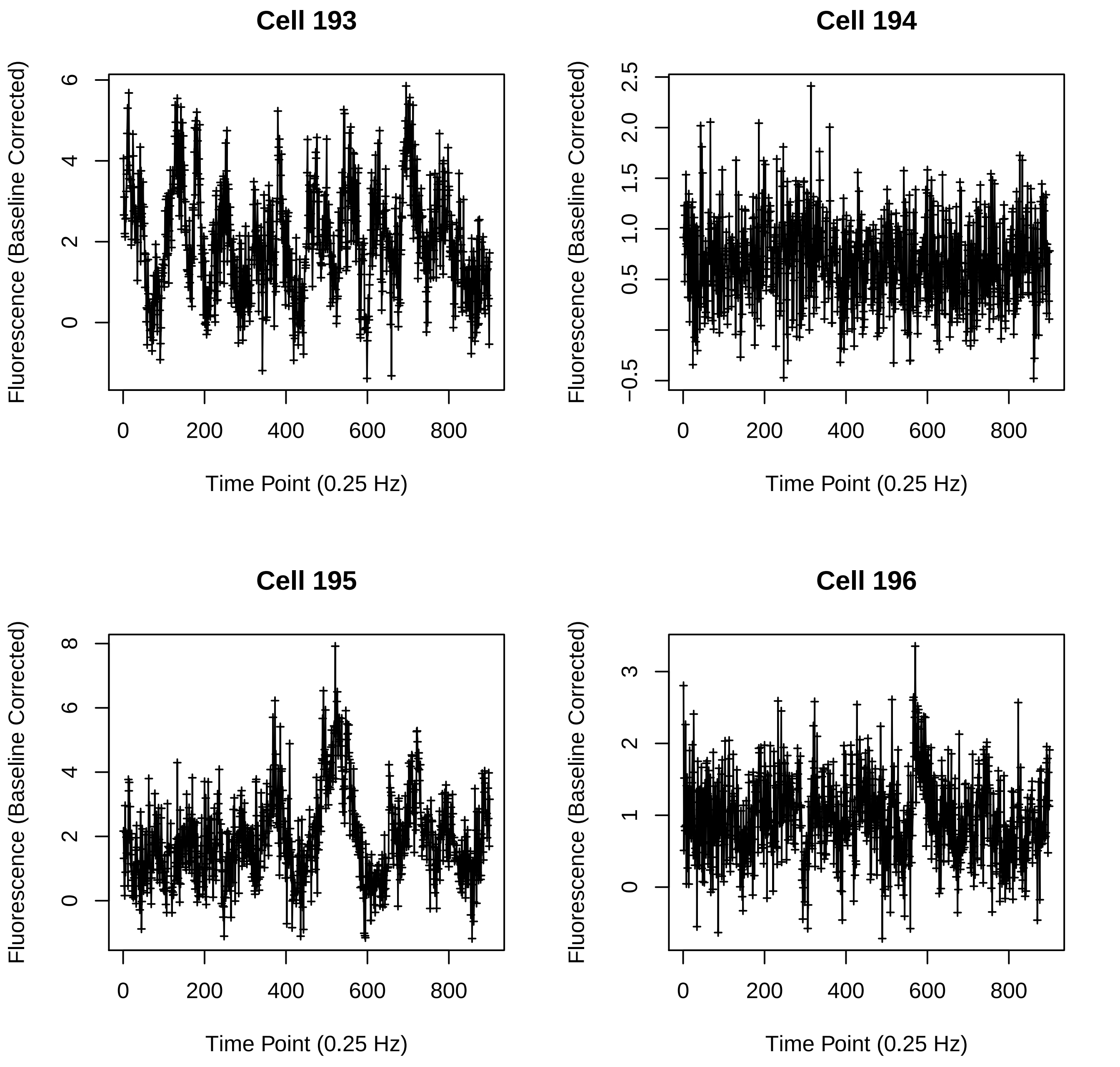
Cell 193
Cell 194
Cell 195
Cell 196
Fluorescence (Baseline Corrected)
Time Point (0.25 Hz)
0
200
400
600
800
-0.5
0.5
1.0
1.5
2.0
2.5
0
1
2
3
4
6
8

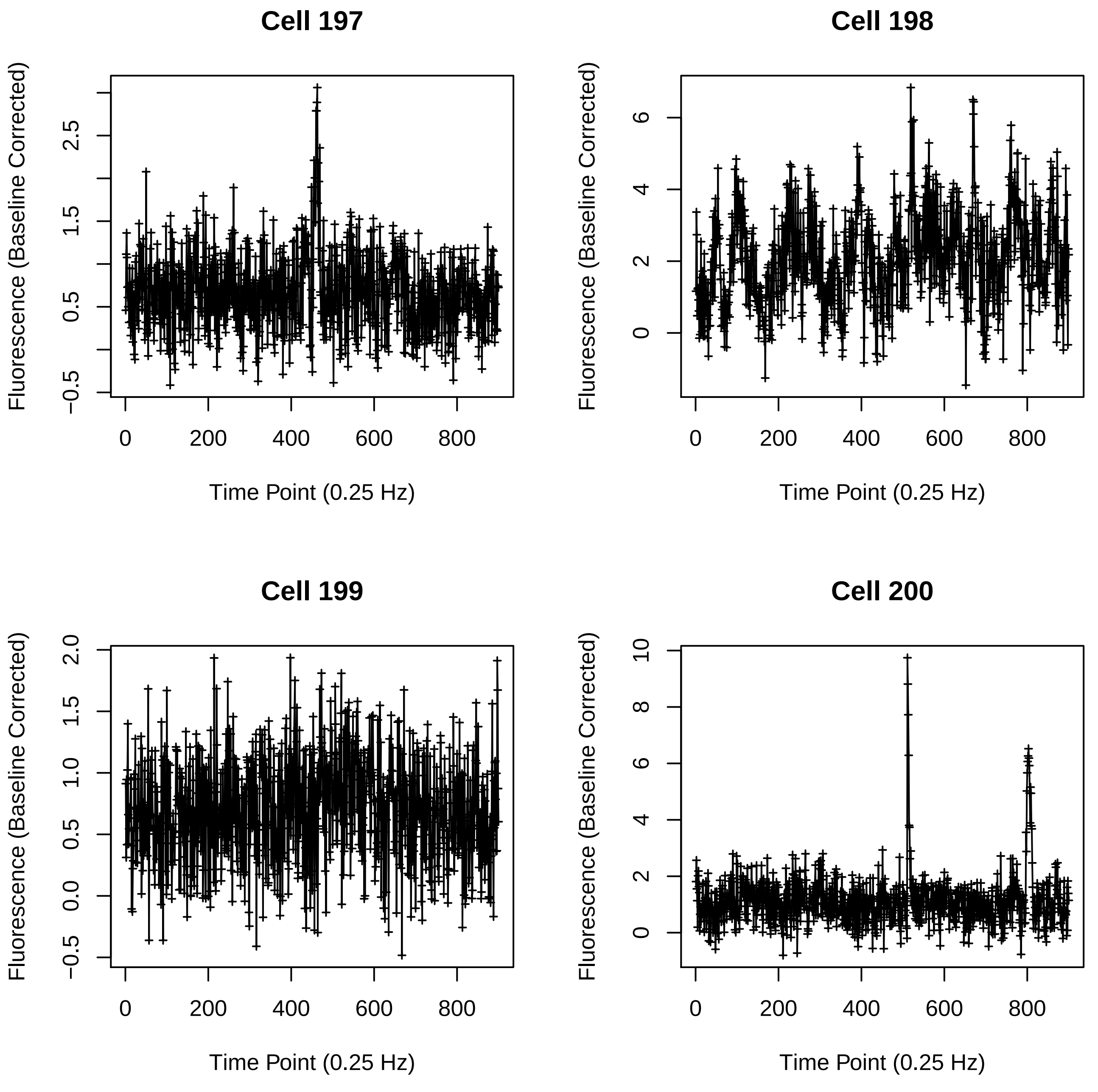
Cell 197
Cell 198
Cell 199
Cell 200
Fluorescence (Baseline Corrected)
Time Point (0.25 Hz)
−0.5
0.5
1.5
2.5
0
2
4
6
8
10
−0.5
0.0
0.5
1.0
1.5
2.0
0
200
400
600
800

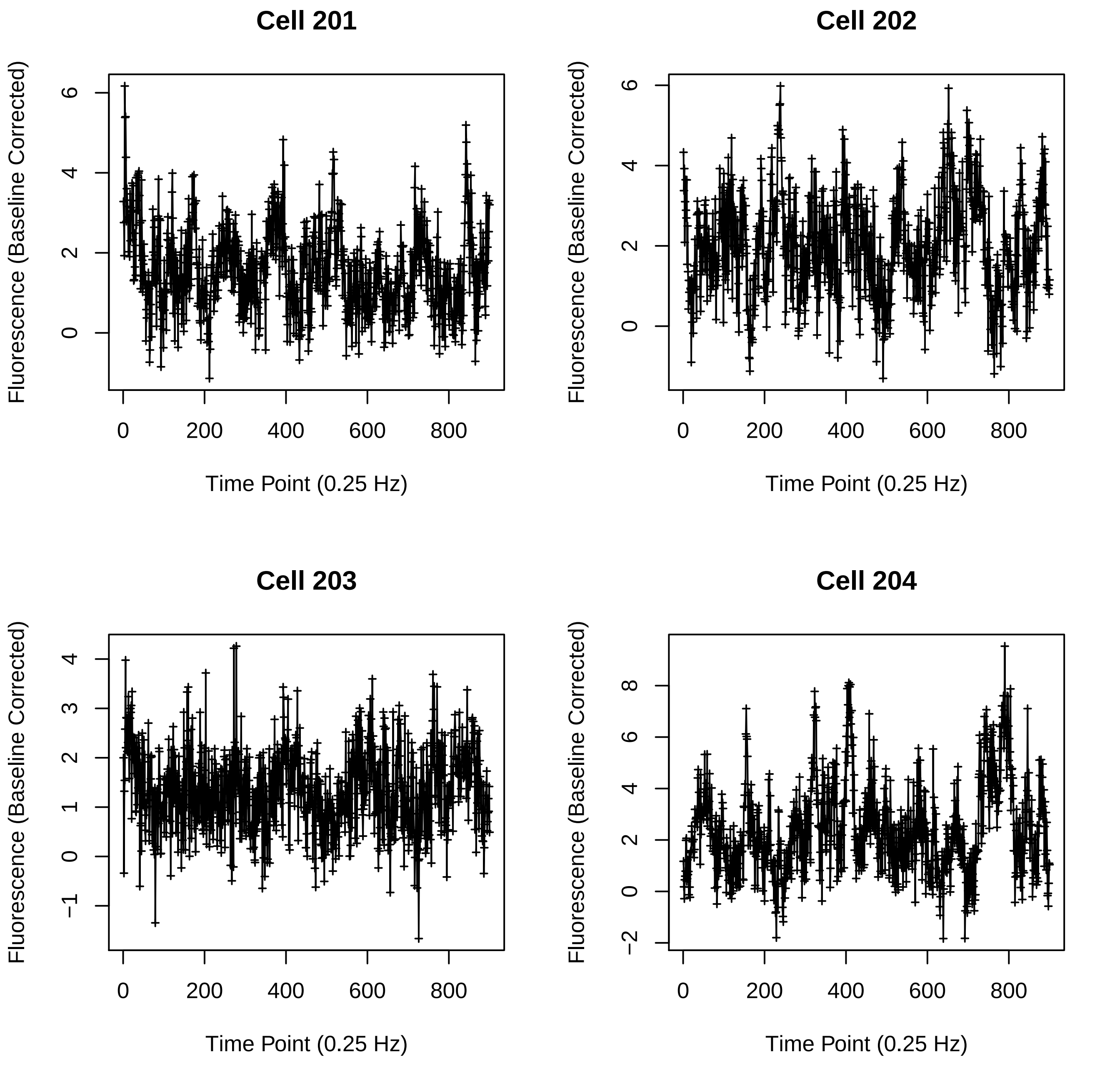
Cell 201
Cell 202
Cell 203
Cell 204
Fluorescence (Baseline Corrected)
Time Point (0.25 Hz)

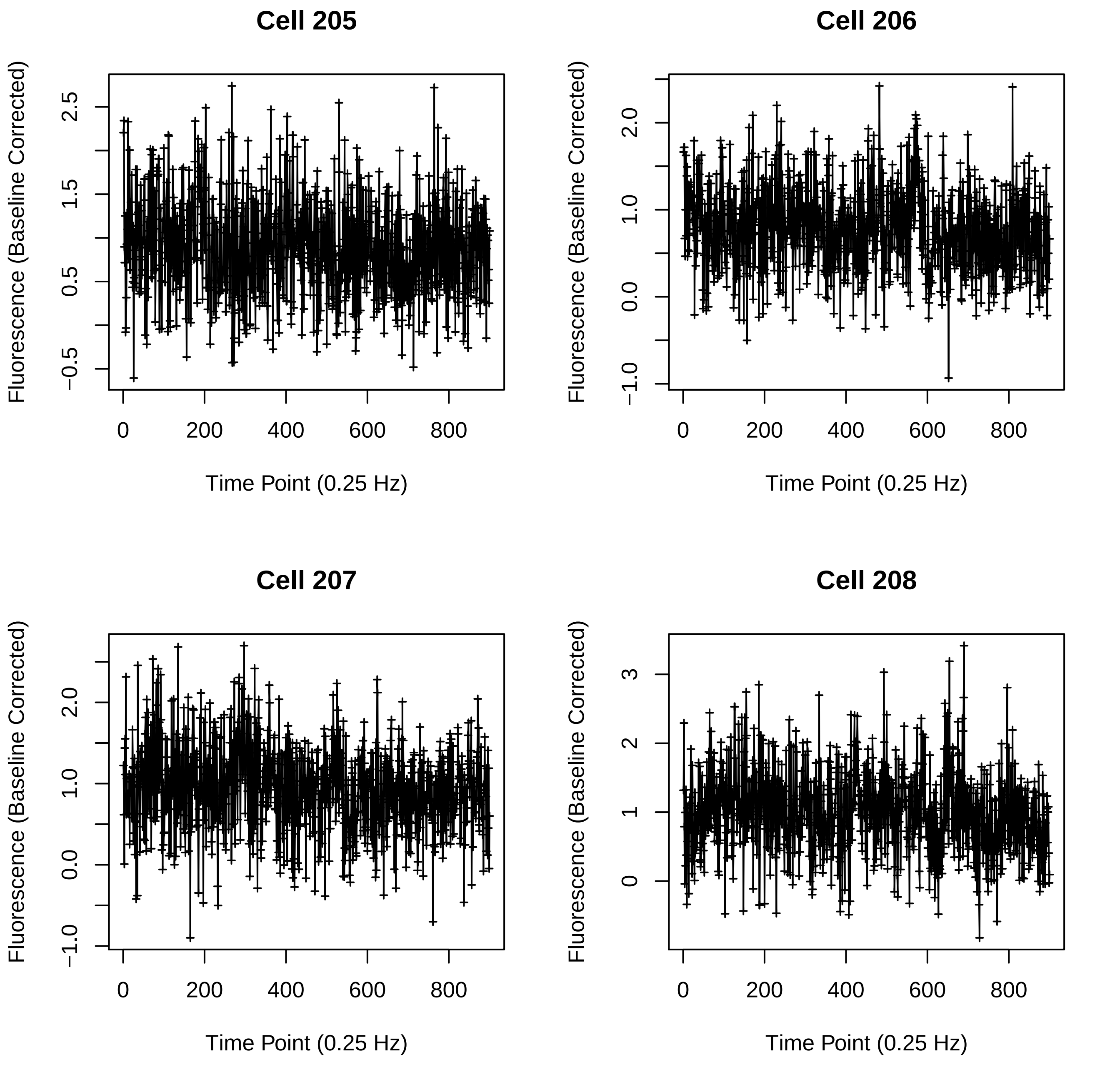
Cell 205
Cell 206
Cell 207
Cell 208
Fluorescence (Baseline Corrected)
Time Point (0.25 Hz)
−0.5
0.5
1.5
2.5
−1.0
0.0
1.0
2.0
0
1
2
3
0
200
400
600
800

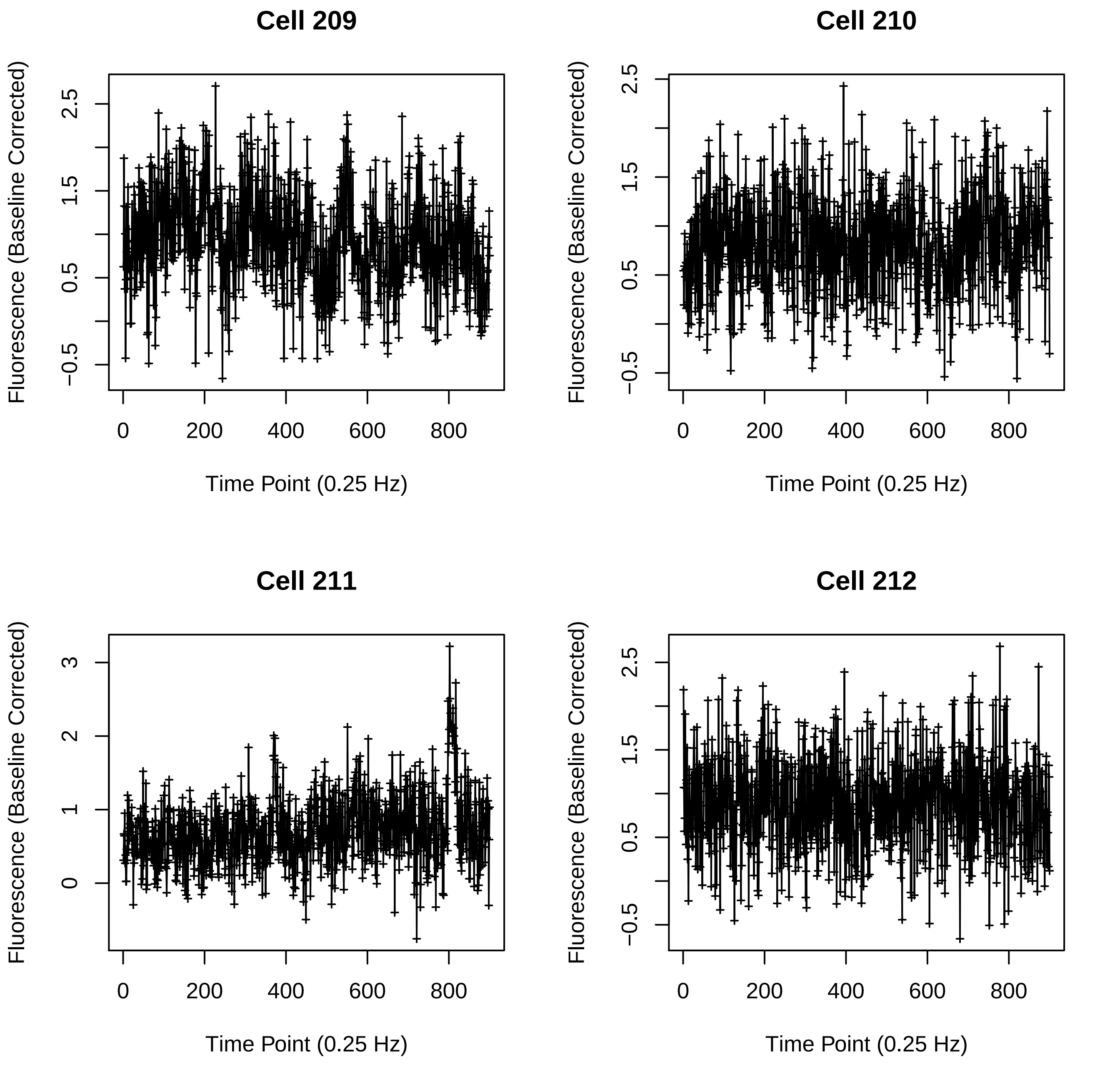

Cell 209
Cell 210
Cell 211
Cell 212
Fluorescence (Baseline Corrected)
Time Point (0.25 Hz)
−0.5
0.5
1.5
2.5
0
1
2
3
0
200
400
600
800

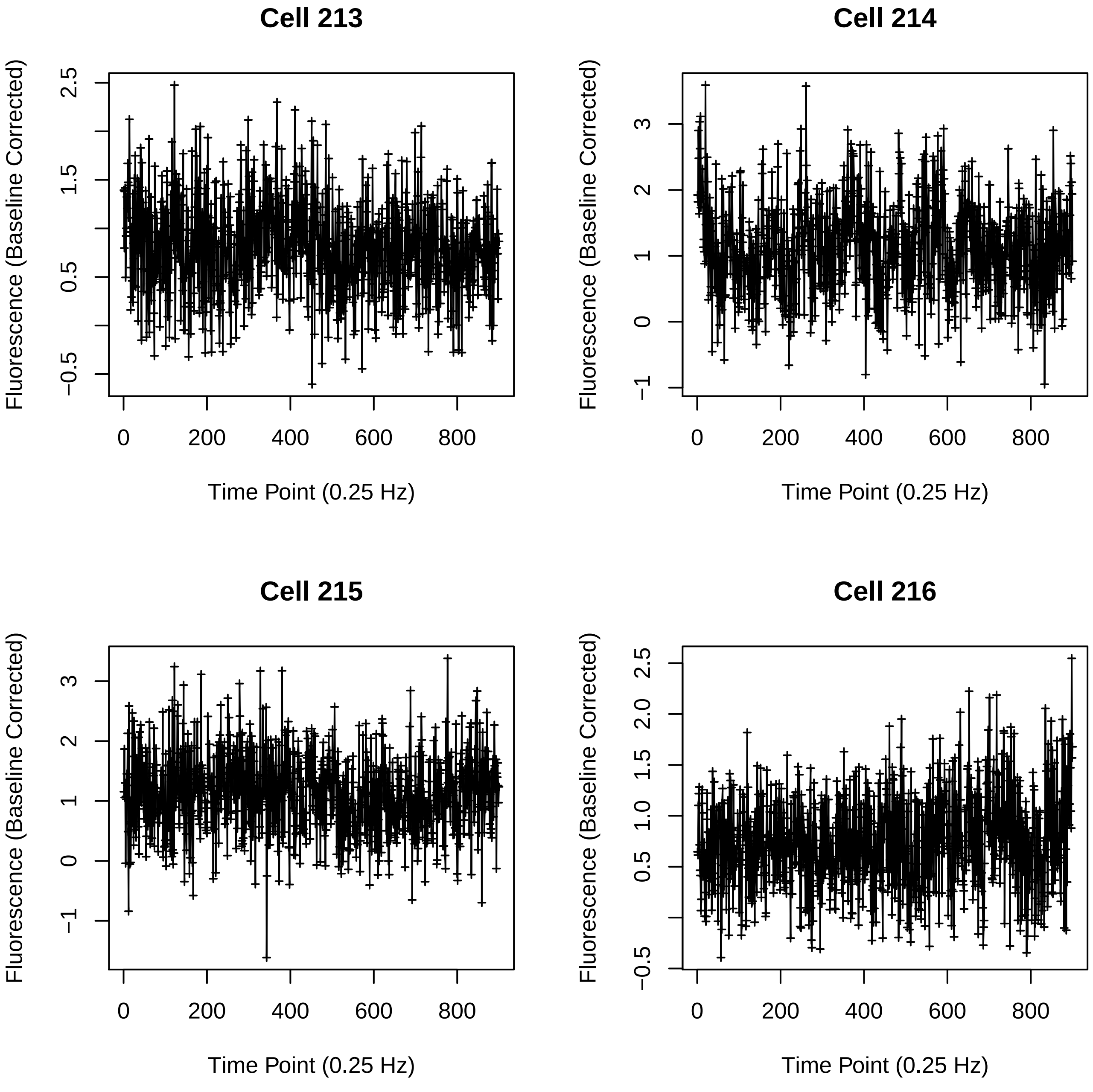
Cell 213
Cell 214
Cell 215
Cell 216
Fluorescence (Baseline Corrected)
Time Point (0.25 Hz)
0
200
400
600
800
−0.5
0.5
1.5
2.5
−1
0
1
2
3
0.0
1.0
2.0

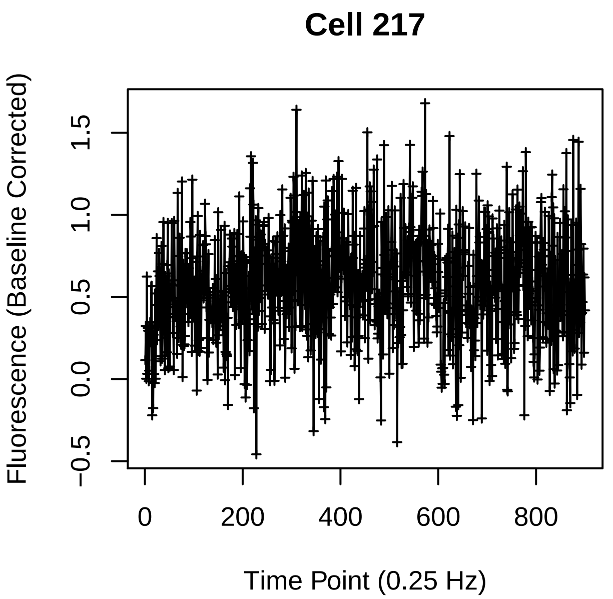
Cell 217
Fluorescence (Baseline Corrected)
−0.5
0.0
0.5
1.0
1.5
0
200
400
600
800
Time Point (0.25 Hz)

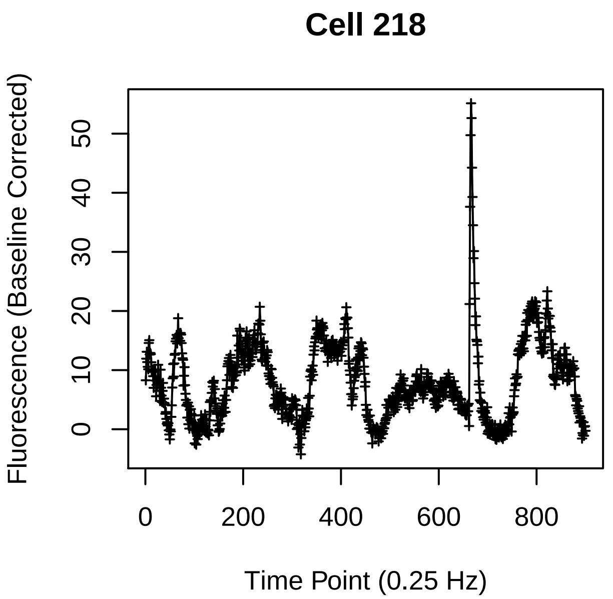
Cell 218
Fluorescence (Baseline Corrected)
0
10
20
30
40
50
0
200
400
600
800
Time Point (0.25 Hz)

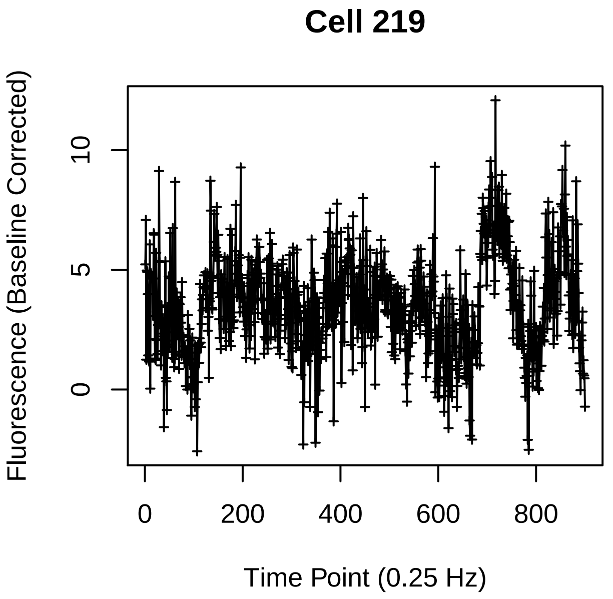
Cell 219
Fluorescence (Baseline Corrected)
0
5
10
0
200
400
600
800
Time Point (0.25 Hz)

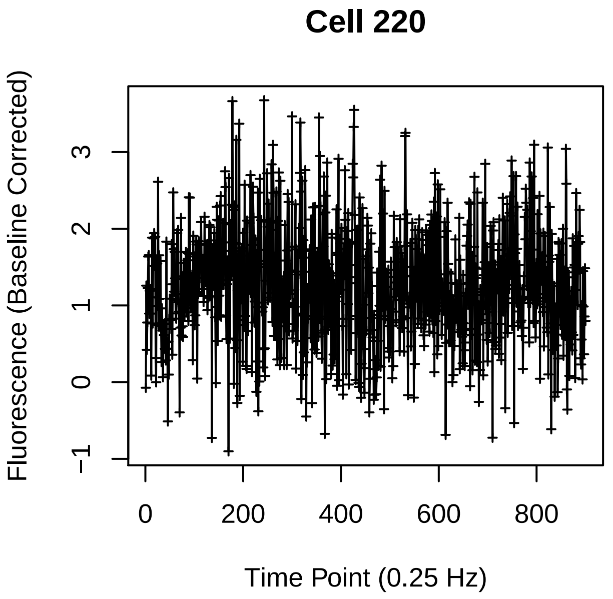
Cell 220
Fluorescence (Baseline Corrected)
−1
0
1
2
3
0
200
400
600
800
Time Point (0.25 Hz)

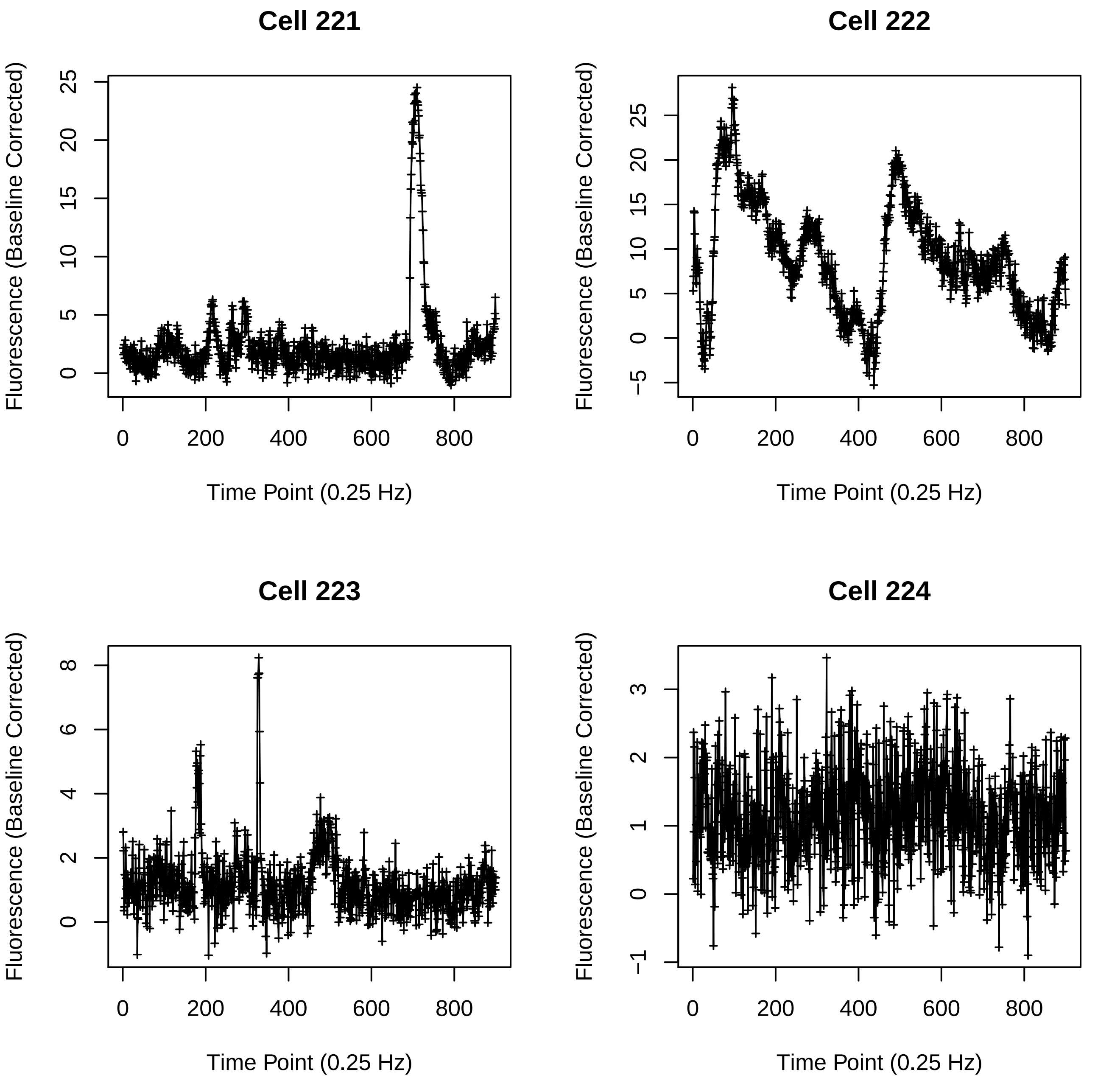
Cell 221
Cell 222
Cell 223
Cell 224
Fluorescence (Baseline Corrected)
Time Point (0.25 Hz)
0
200
400
600
800
0
5
10
15
20
25
−5
2
4
6
8
−1
1
3

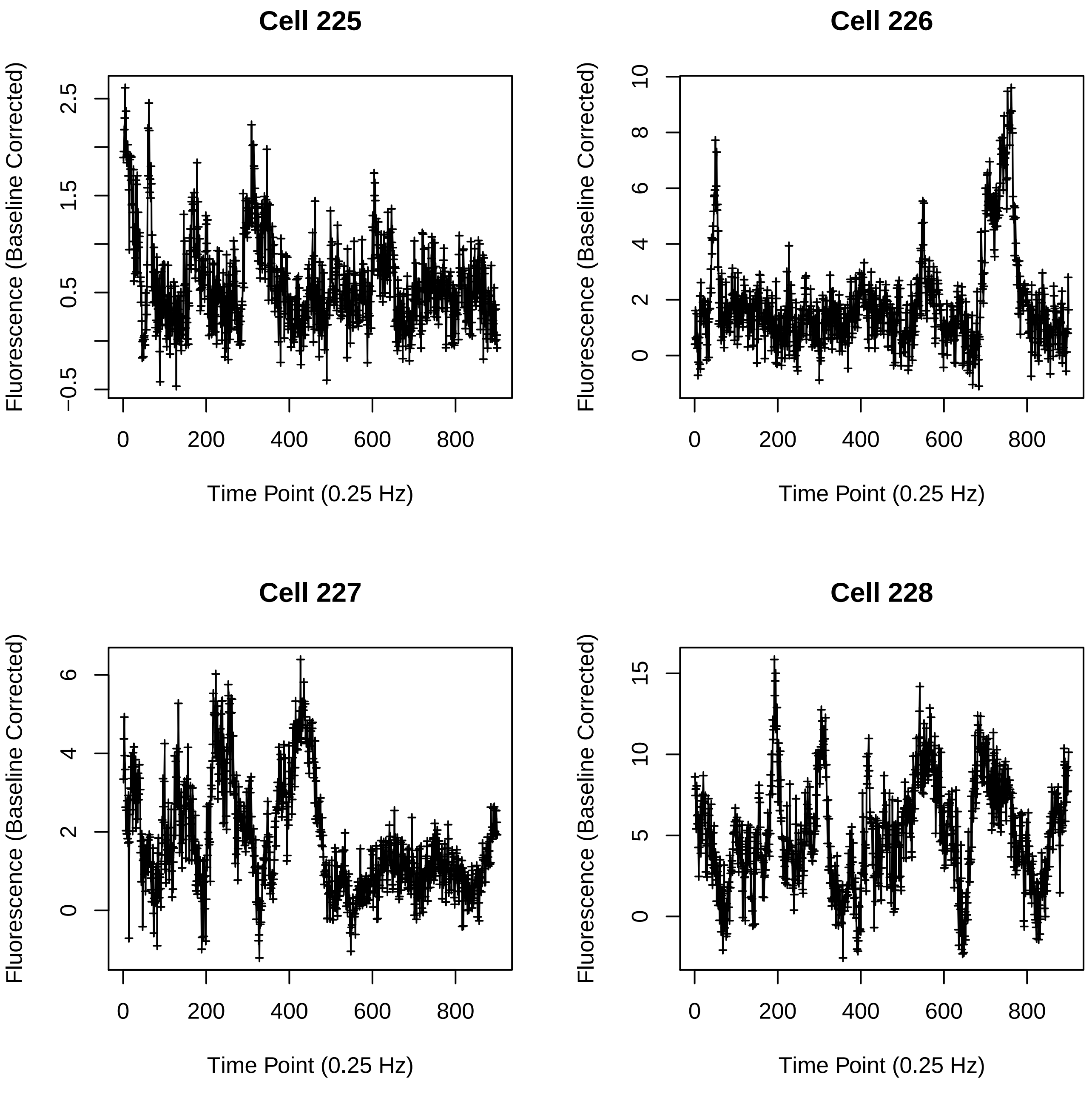
Cell 225
Cell 226
Cell 227
Cell 228
Fluorescence (Baseline Corrected)
Time Point (0.25 Hz)
−0.5
0.5
1.5
2.5
0
2
4
6
8
10
15
200
400
600
800

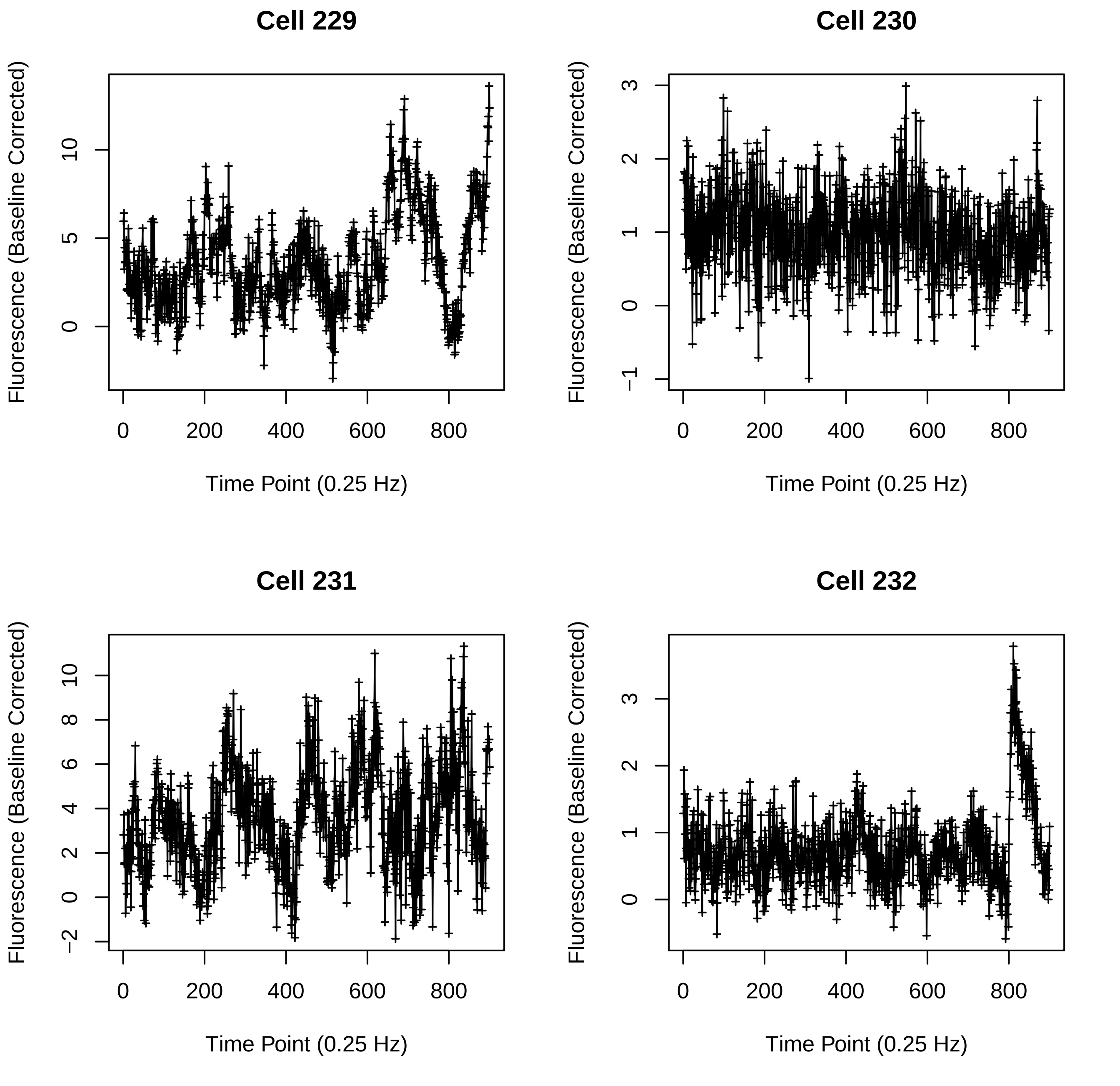
Cell 229
Fluorescence (Baseline Corrected)
0
5
10
0
200
400
600
800
Time Point (0.25 Hz)
Cell 230
Fluorescence (Baseline Corrected)
−1
0
1
2
3
0
200
400
600
800
Time Point (0.25 Hz)
Cell 231
Fluorescence (Baseline Corrected)
−2
0
2
4
6
8
10
0
200
400
600
800
Time Point (0.25 Hz)
Cell 232
Fluorescence (Baseline Corrected)
0
1
2
3
0
200
400
600
800
Time Point (0.25 Hz)

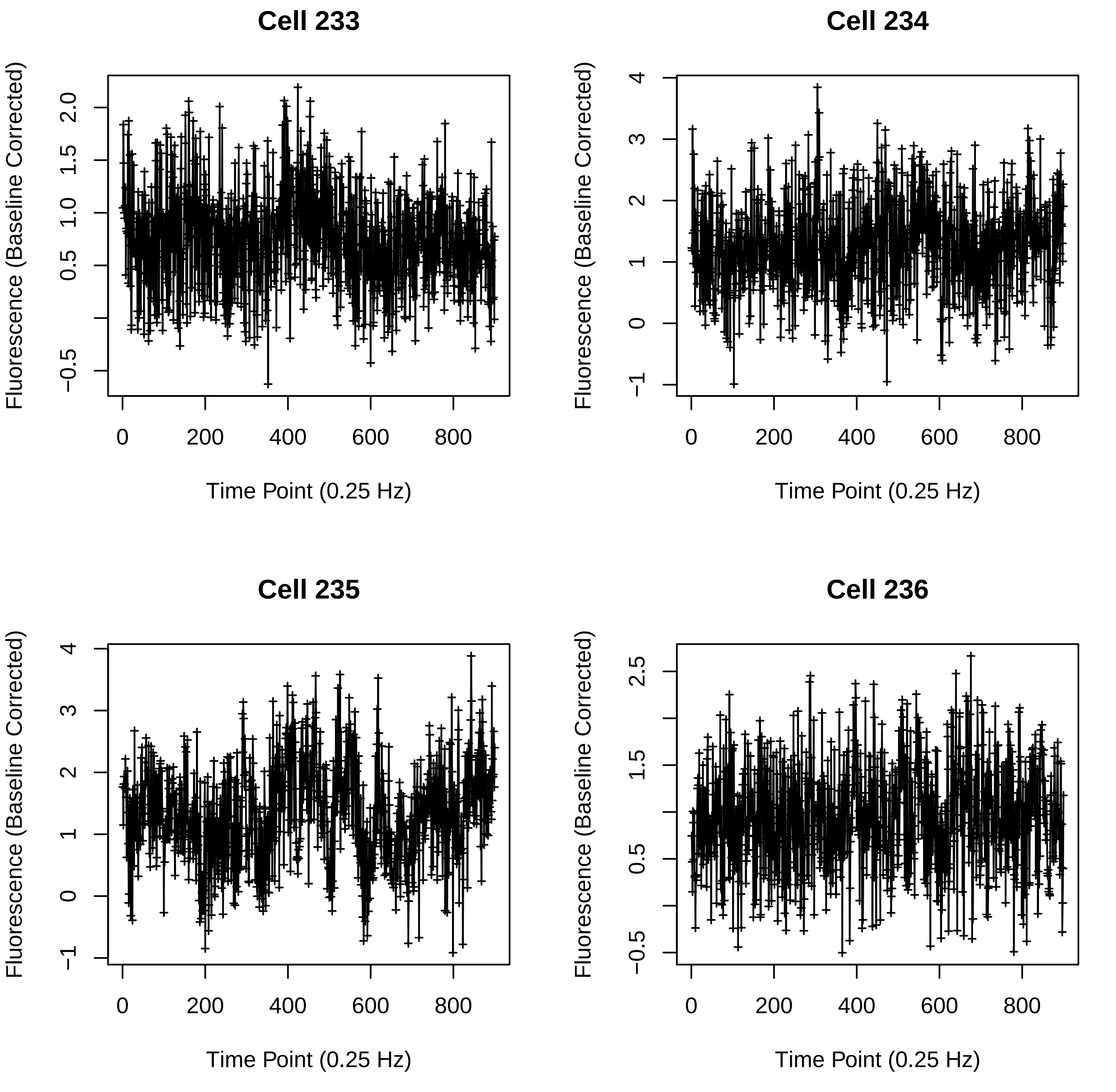
Cell 233
Cell 234
Cell 235
Cell 236
Fluorescence (Baseline Corrected)
Time Point (0.25 Hz)
0
200
400
600
800
−0.5
0.5
1.0
1.5
2.0
−1
1
2
3
4
2.5

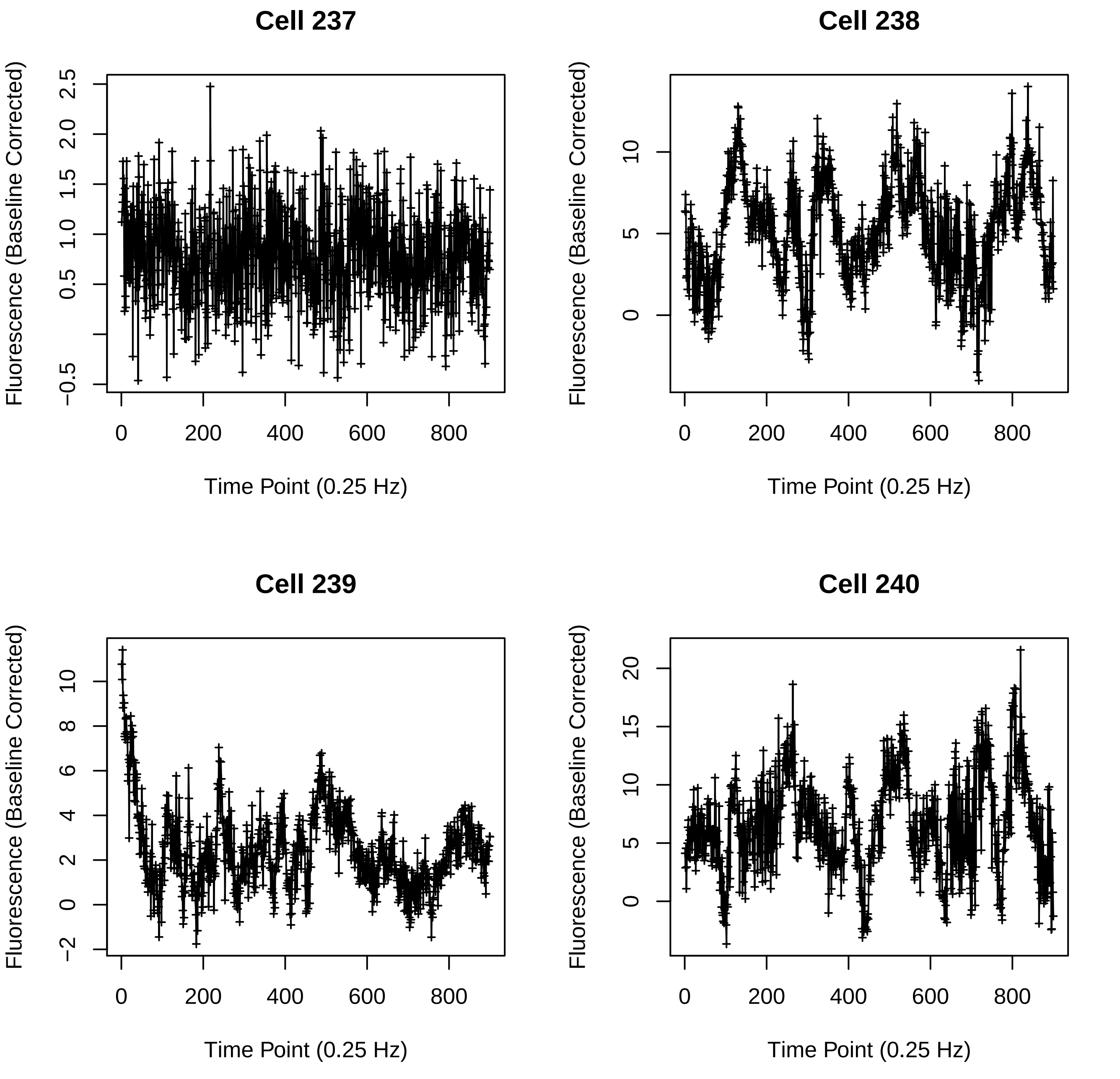
Cell 237
Fluorescence (Baseline Corrected)
−0.5
0.5
1.0
1.5
2.0
2.5
0
200
400
600
800
Time Point (0.25 Hz)
Cell 238
Fluorescence (Baseline Corrected)
0
5
10
0
200
400
600
800
Time Point (0.25 Hz)
Cell 239
Fluorescence (Baseline Corrected)
−2
0
2
4
6
8
10
0
200
400
600
800
Time Point (0.25 Hz)
Cell 240
Fluorescence (Baseline Corrected)
0
5
10
15
20
0
200
400
600
800
Time Point (0.25 Hz)

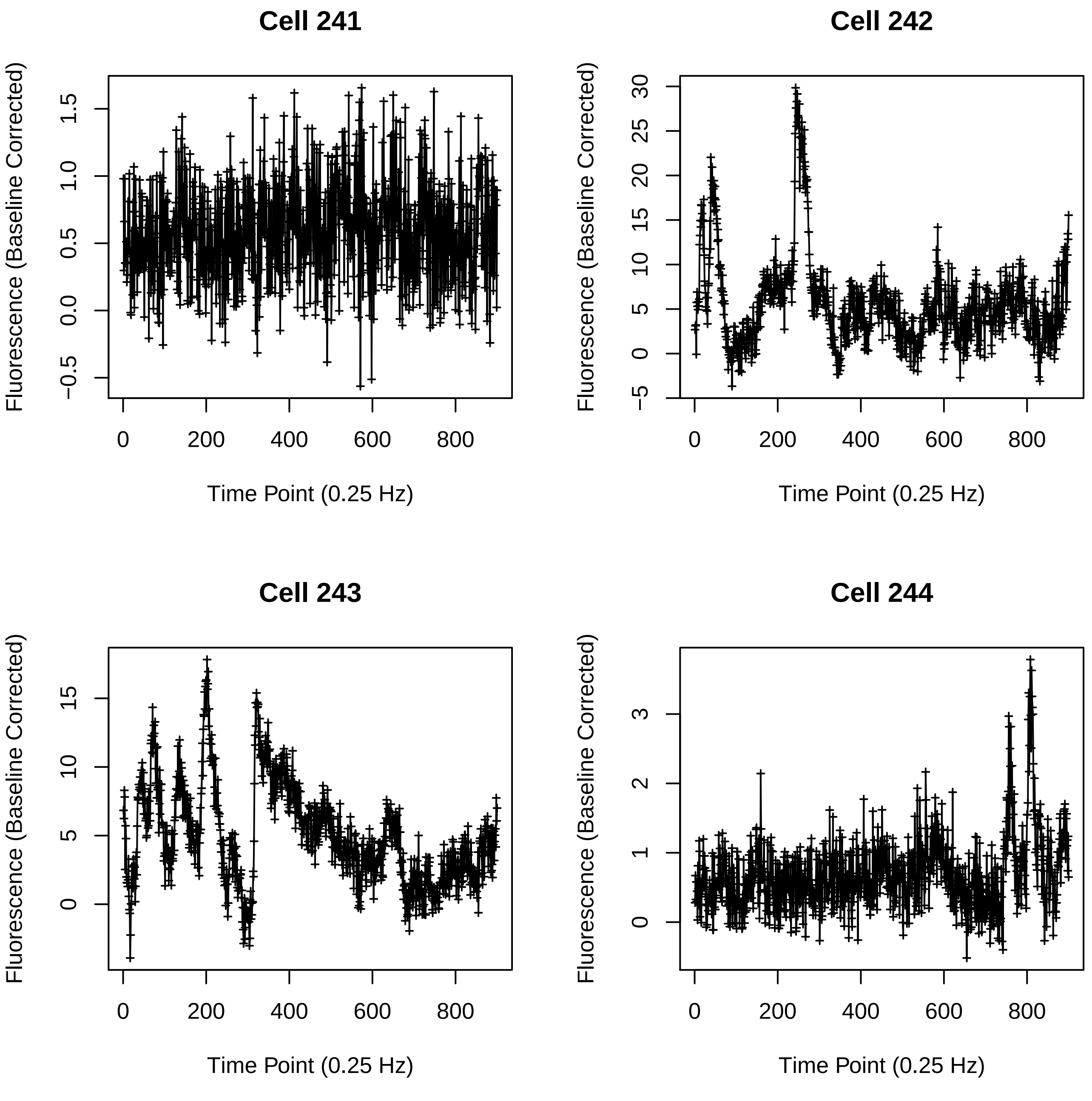
Cell 241
Cell 242
Cell 243
Cell 244
Fluorescence (Baseline Corrected)
Time Point (0.25 Hz)
0
200
400
600
800
−0.5
0.0
0.5
1.0
1.5
−5
5
10
15
20
25
30
1
2
3

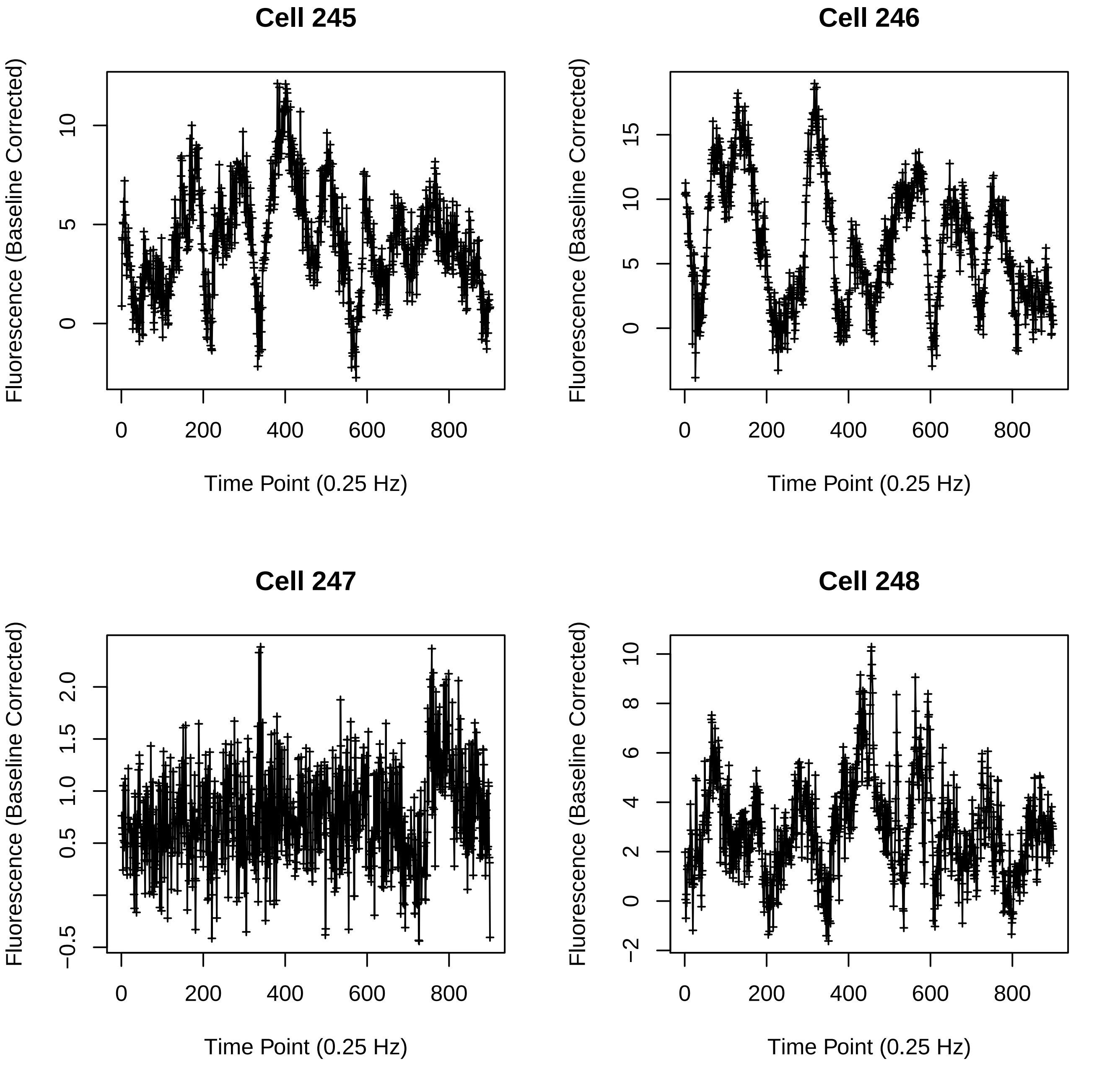
Cell 245
Cell 246
Cell 247
Cell 248
Fluorescence (Baseline Corrected)
Time Point (0.25 Hz)
0
200
400
600
800
−0.5
0.5
1.0
1.5
2.0
−2
0
2
4
6
8
10
5
15

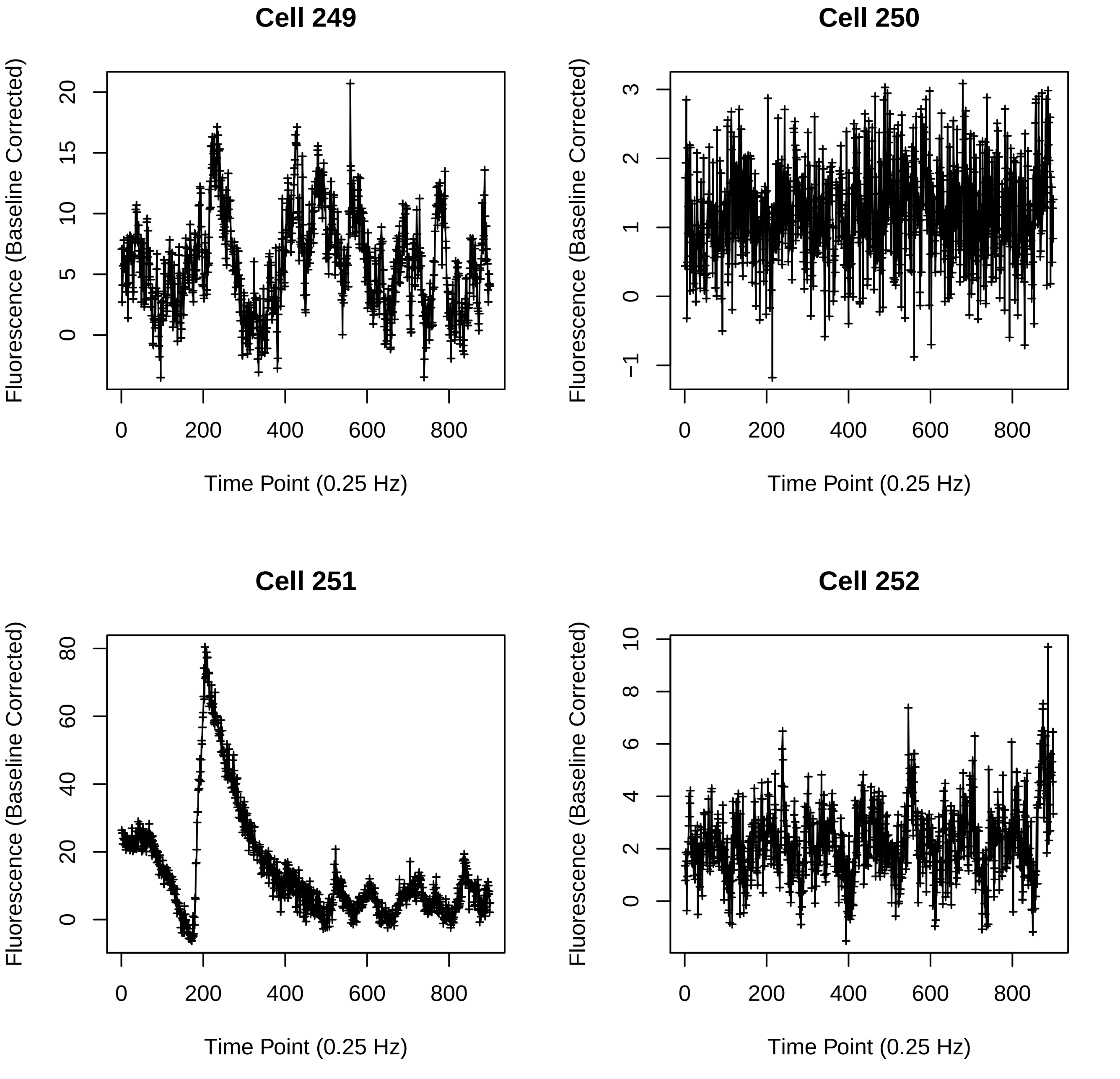
Cell 249
Fluorescence (Baseline Corrected)
0
5
10
15
20
0
200
400
600
800
Time Point (0.25 Hz)
Cell 250
Fluorescence (Baseline Corrected)
−1
0
1
2
3
0
200
400
600
800
Time Point (0.25 Hz)
Cell 251
Fluorescence (Baseline Corrected)
0
20
40
60
80
0
200
400
600
800
Time Point (0.25 Hz)
Cell 252
Fluorescence (Baseline Corrected)
0
2
4
6
8
10
0
200
400
600
800
Time Point (0.25 Hz)

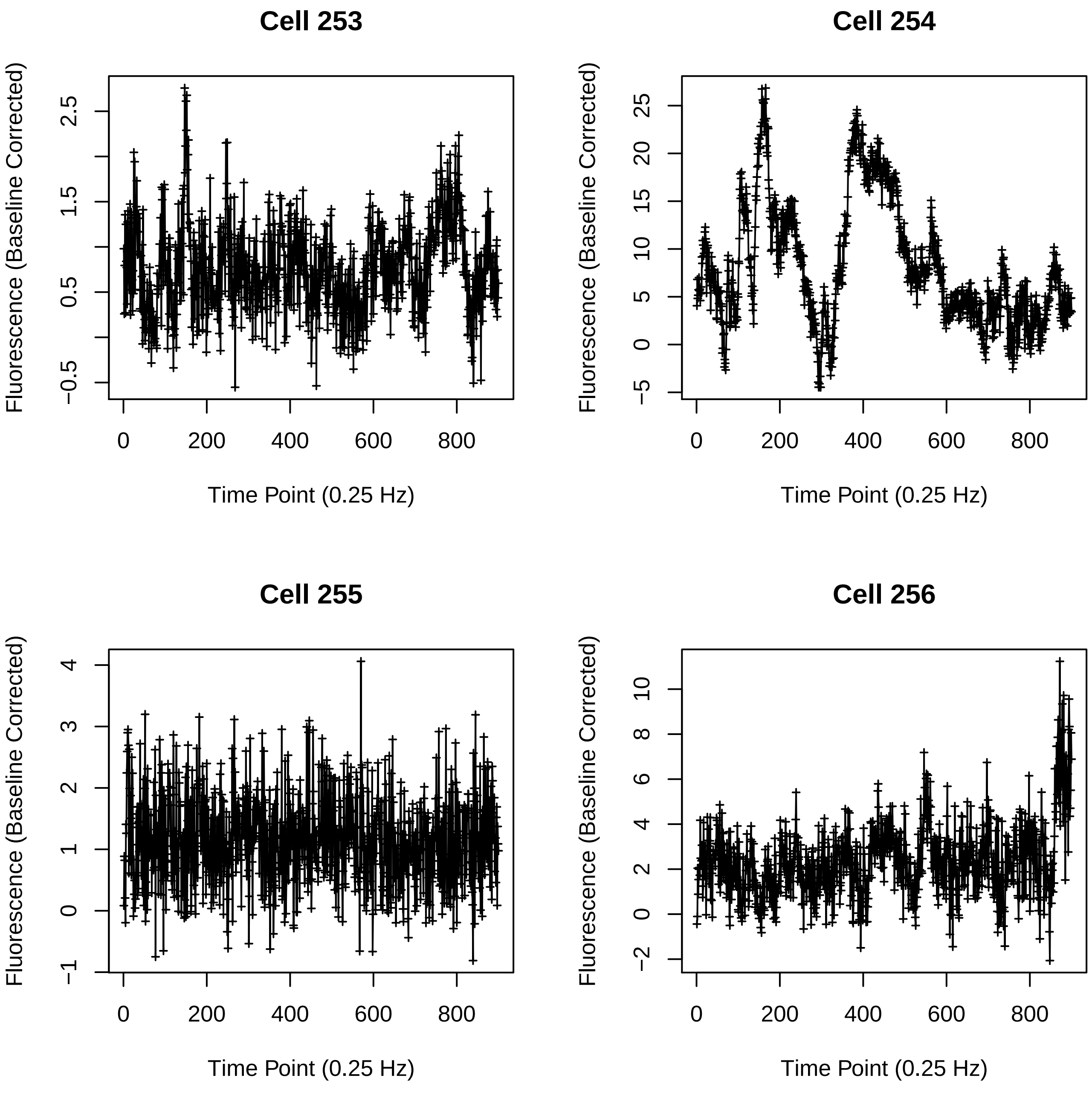
Cell 253
Fluorescence (Baseline Corrected)
−0.5
0.5
1.5
2.5
0
200
400
600
800
Time Point (0.25 Hz)
Cell 254
Fluorescence (Baseline Corrected)
−5
0
5
10
15
20
25
0
200
400
600
800
Time Point (0.25 Hz)
Cell 255
Fluorescence (Baseline Corrected)
−1
0
1
2
3
4
0
200
400
600
800
Time Point (0.25 Hz)
Cell 256
Fluorescence (Baseline Corrected)
−2
0
2
4
6
8
10
0
200
400
600
800
Time Point (0.25 Hz)

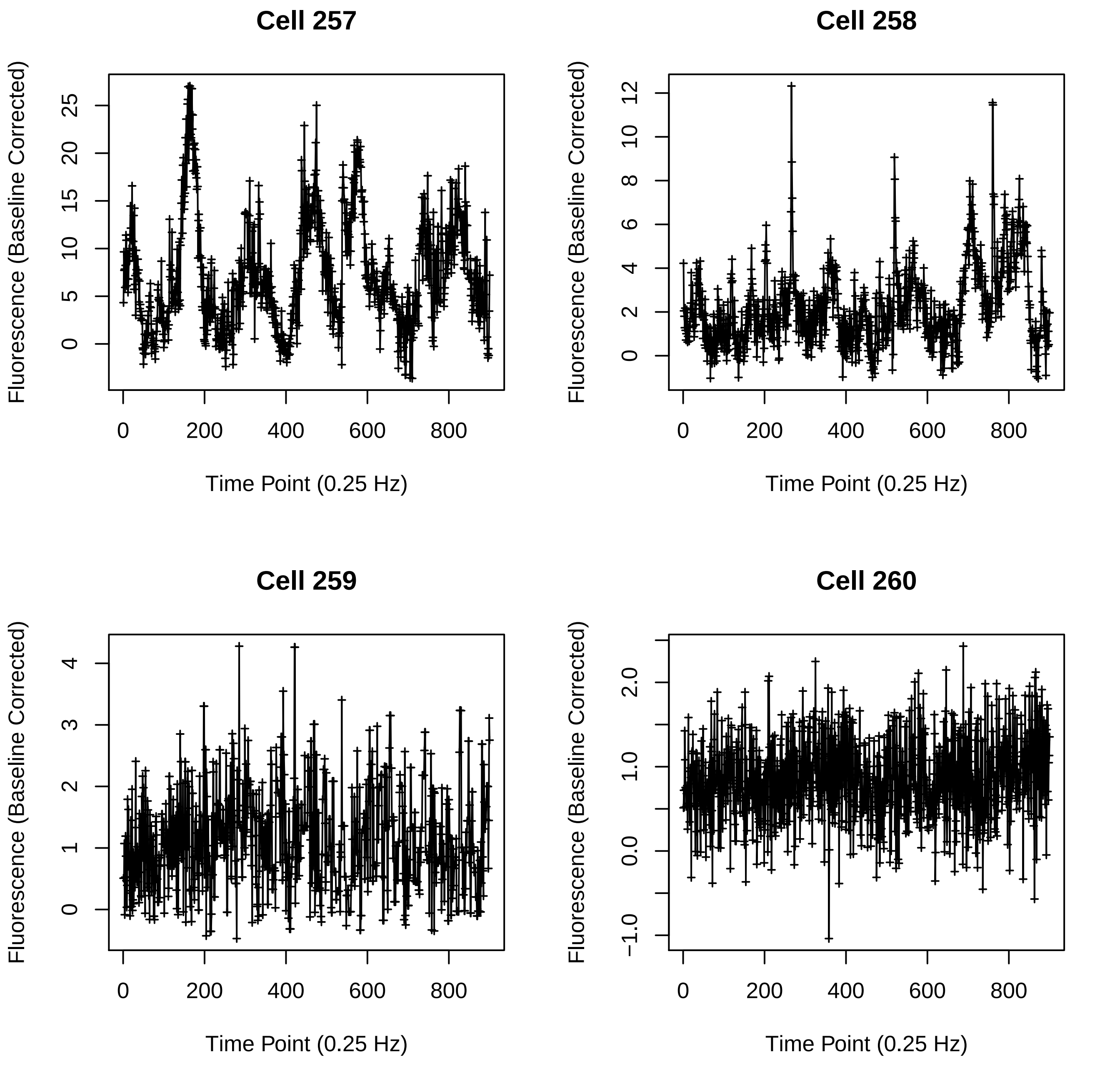
Cell 257
Cell 258
Cell 259
Cell 260
Fluorescence (Baseline Corrected)
Time Point (0.25 Hz)
0
200
400
600
800
25
20
15
10
5
12
8
6
4
2
4
3
1
2.0
1.0
0.0
−1.0

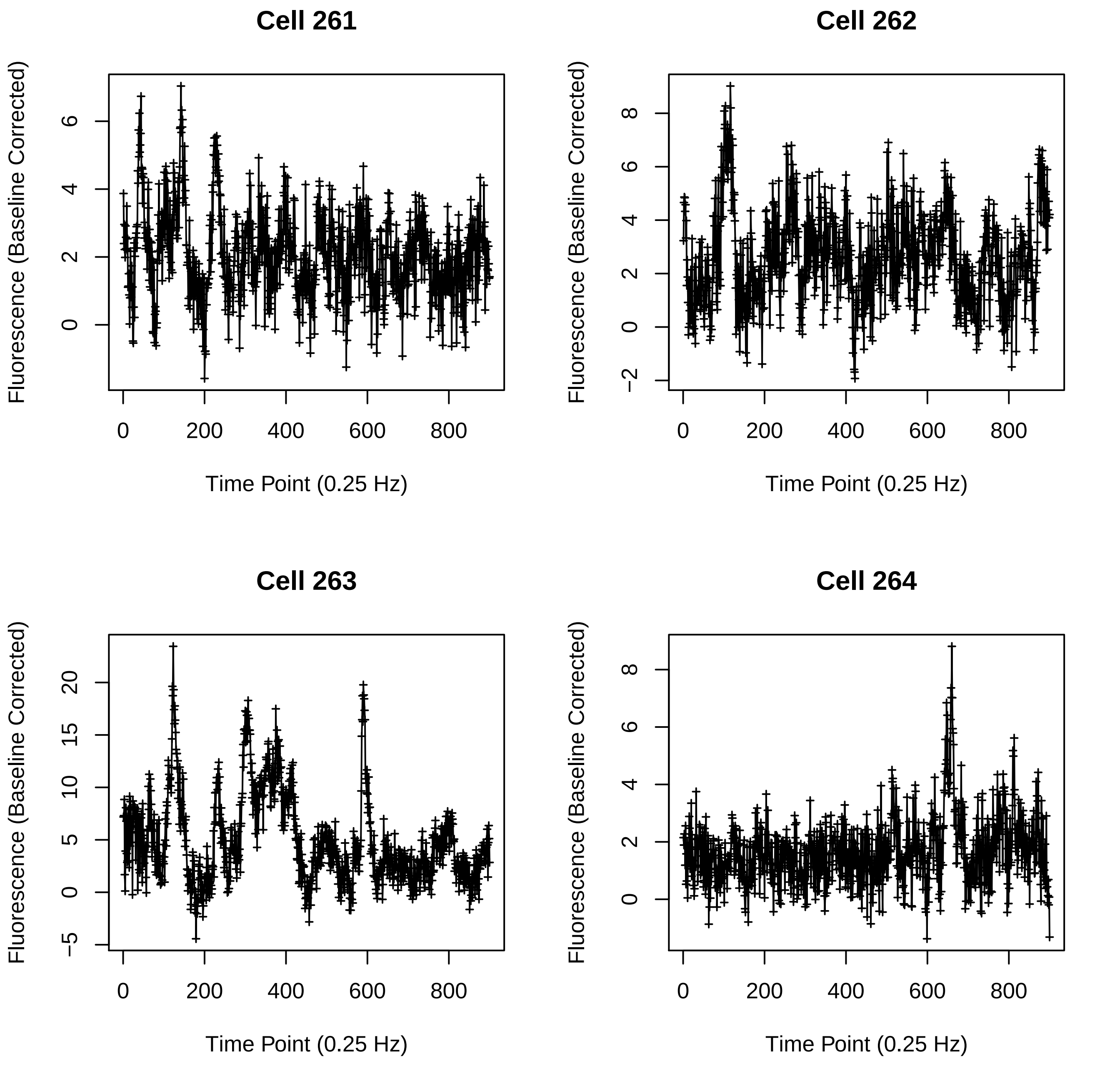

Cell 261
Cell 262
Cell 263
Cell 264
Fluorescence (Baseline Corrected)
Time Point (0.25 Hz)
0
200
400
600
800
0
2
4
6
8
−2
−5
5
10
15
20

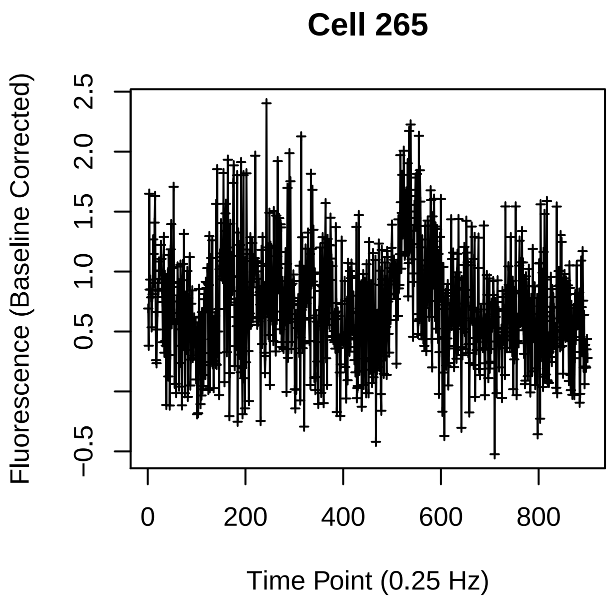
Cell 265
Fluorescence (Baseline Corrected)
−0.5
0.5
1.0
1.5
2.0
2.5
0
200
400
600
800
Time Point (0.25 Hz)

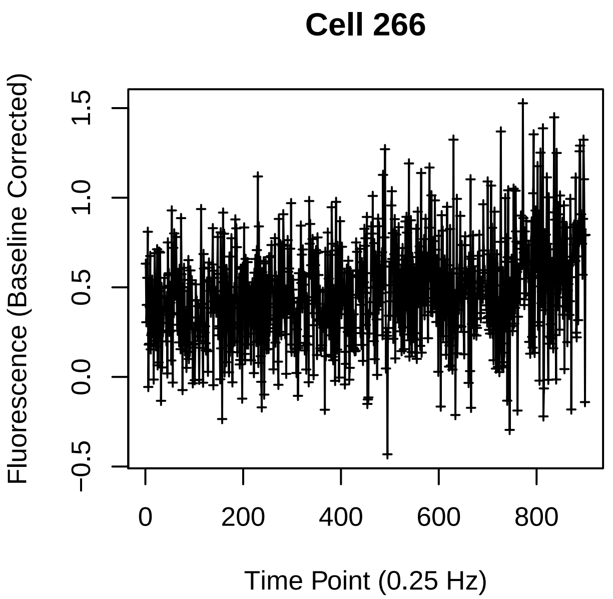
Cell 266
Fluorescence (Baseline Corrected)
−0.5
0.0
0.5
1.0
1.5
0
200
400
600
800
Time Point (0.25 Hz)

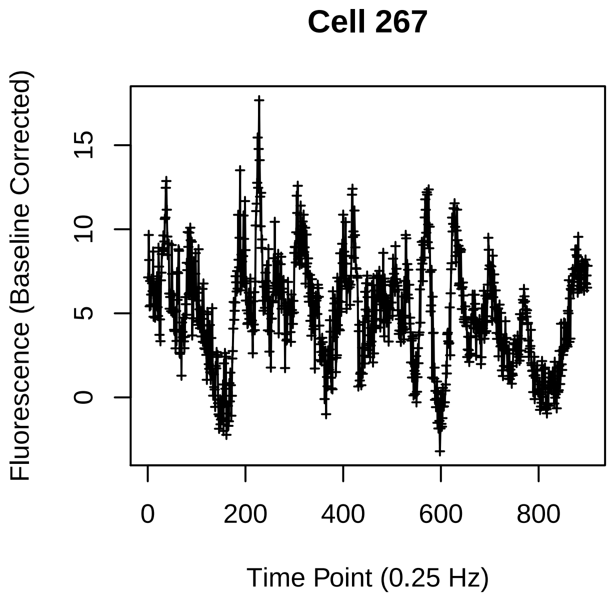
Cell 267
Fluorescence (Baseline Corrected)
0
5
10
15
0
200
400
600
800
Time Point (0.25 Hz)

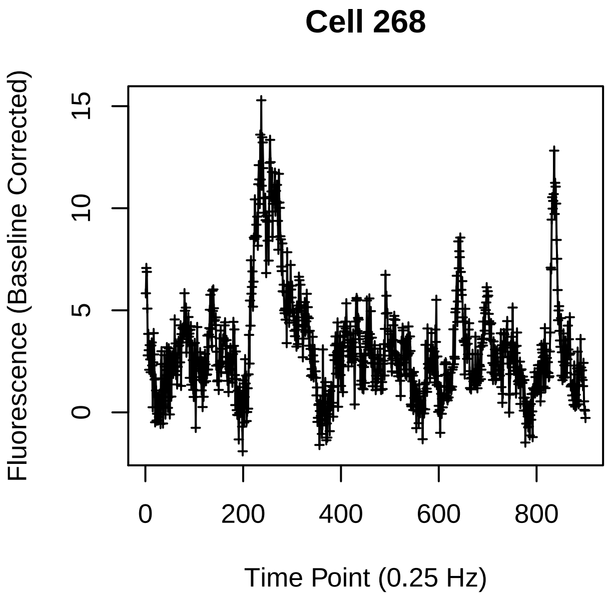
Cell 268
Fluorescence (Baseline Corrected)
0
5
10
15
0
200
400
600
800
Time Point (0.25 Hz)

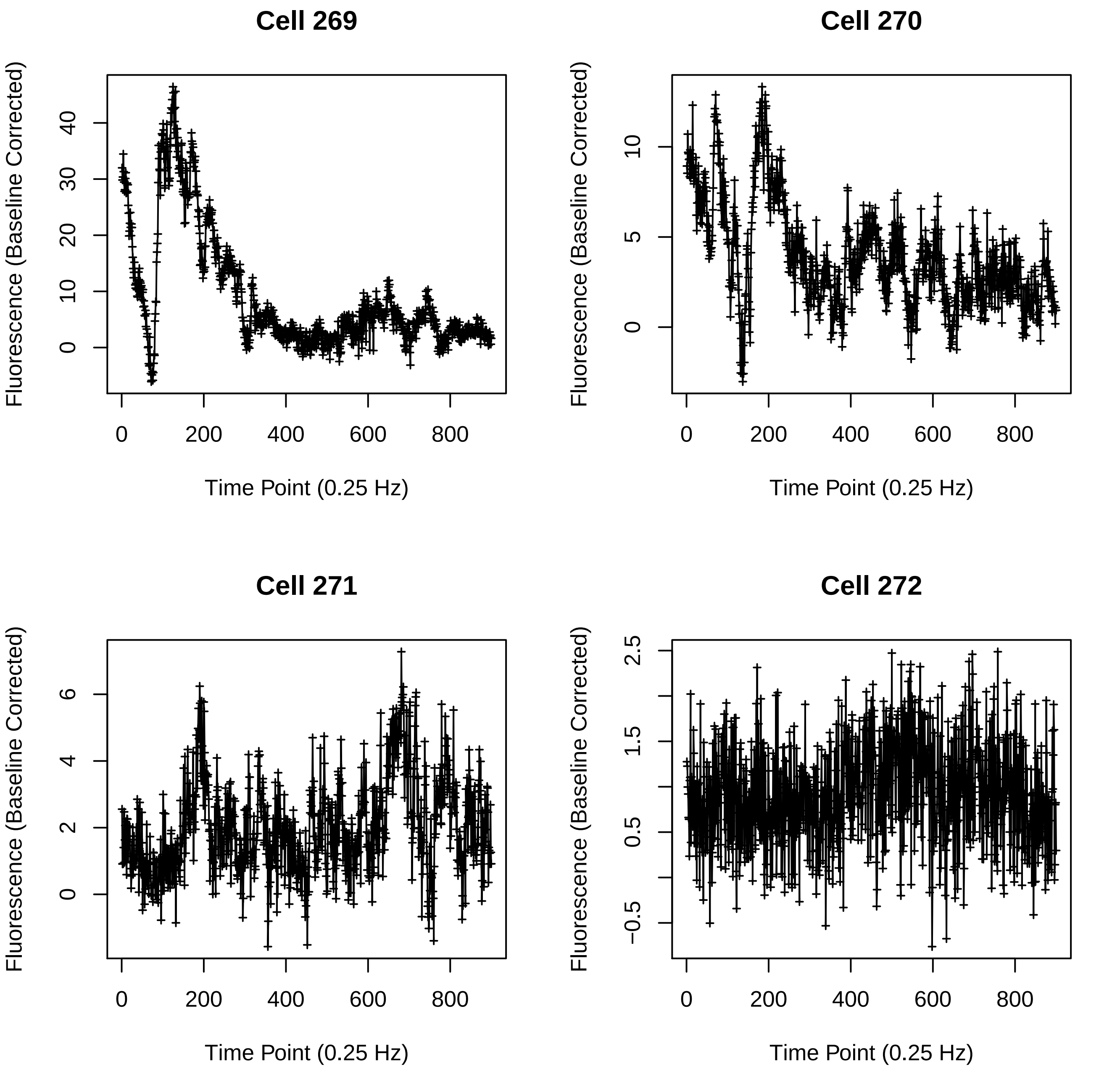
Cell 269
Fluorescence (Baseline Corrected)
Time Point (0.25 Hz)
0
10
20
30
40
0
200
400
600
800
Cell 270
Fluorescence (Baseline Corrected)
Time Point (0.25 Hz)
0
5
10
0
200
400
600
800
Cell 271
Fluorescence (Baseline Corrected)
Time Point (0.25 Hz)
0
2
4
6
0
200
400
600
800
Cell 272
Fluorescence (Baseline Corrected)
Time Point (0.25 Hz)
−0.5
0.5
1.5
2.5
0
200
400
600
800

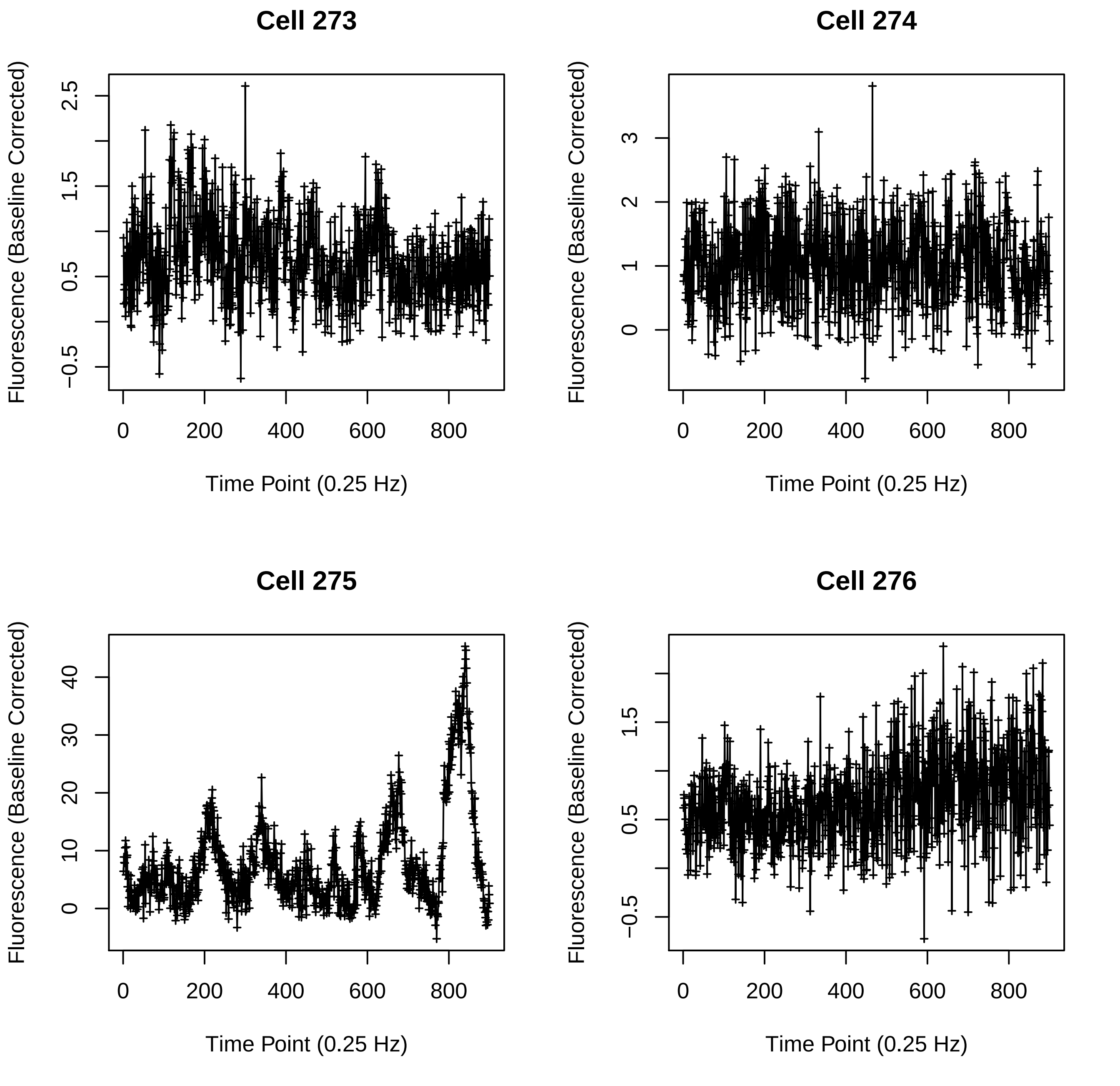
Cell 273
Cell 274
Cell 275
Cell 276
Fluorescence (Baseline Corrected)
Time Point (0.25 Hz)
−0.5
0.5
1.5
2.5
0
1
2
3
10
20
30
40
200
400
600
800

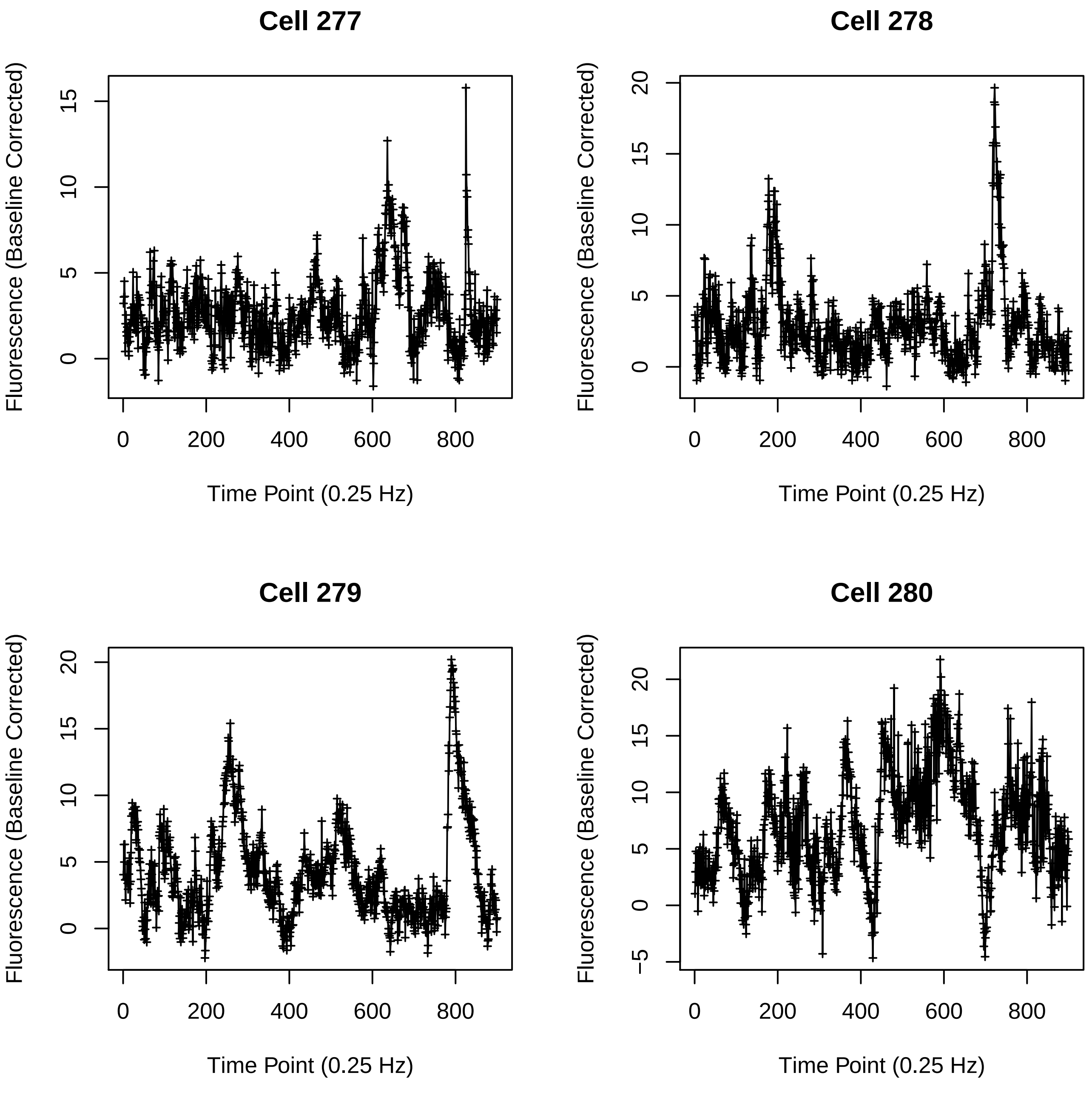
Cell 277
Cell 278
Cell 279
Cell 280
Fluorescence (Baseline Corrected)
Time Point (0.25 Hz)
0
200
400
600
800
-5
5
10
15
20

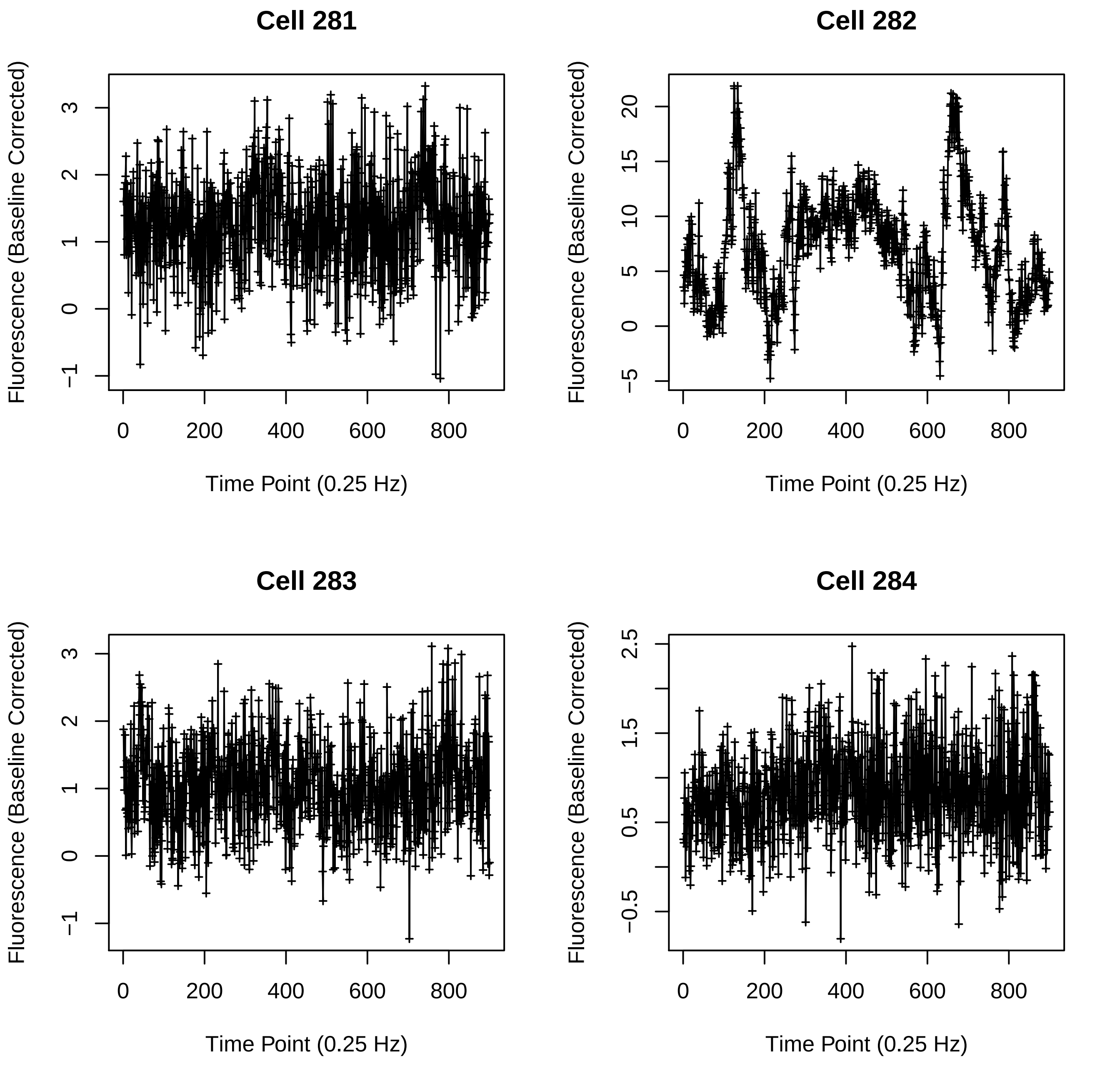

Cell 281
Cell 282
Cell 283
Cell 284
Fluorescence (Baseline Corrected)
Time Point (0.25 Hz)
0
200
400
600
800
−1
1
2
3
−5
5
10
15
20
−0.5
0.5
1.5
2.5

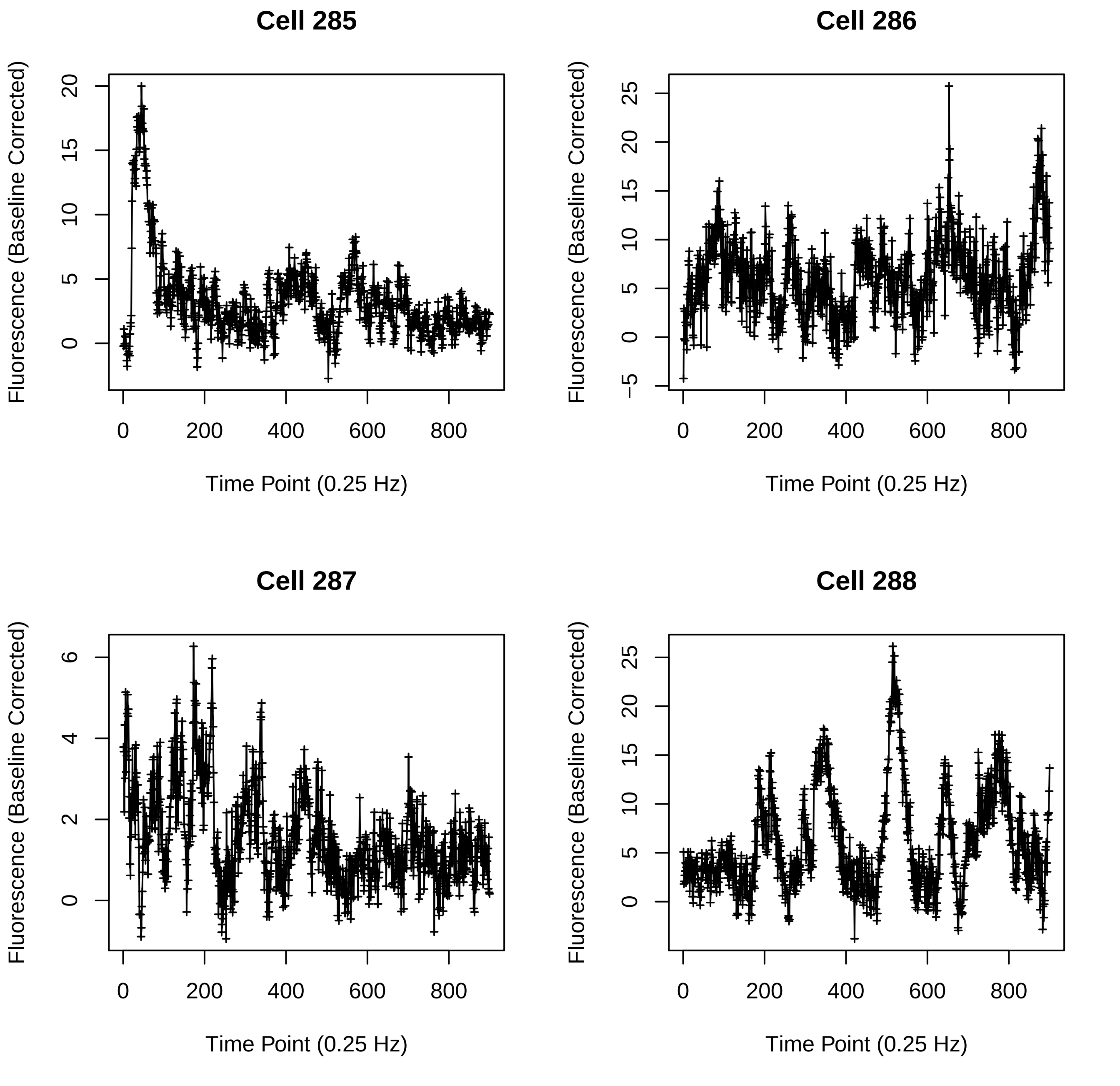
Cell 285
Cell 286
Cell 287
Cell 288
Fluorescence (Baseline Corrected)
Time Point (0.25 Hz)
0
200
400
600
800
-5
5
10
15
20
25
0
2
4
6

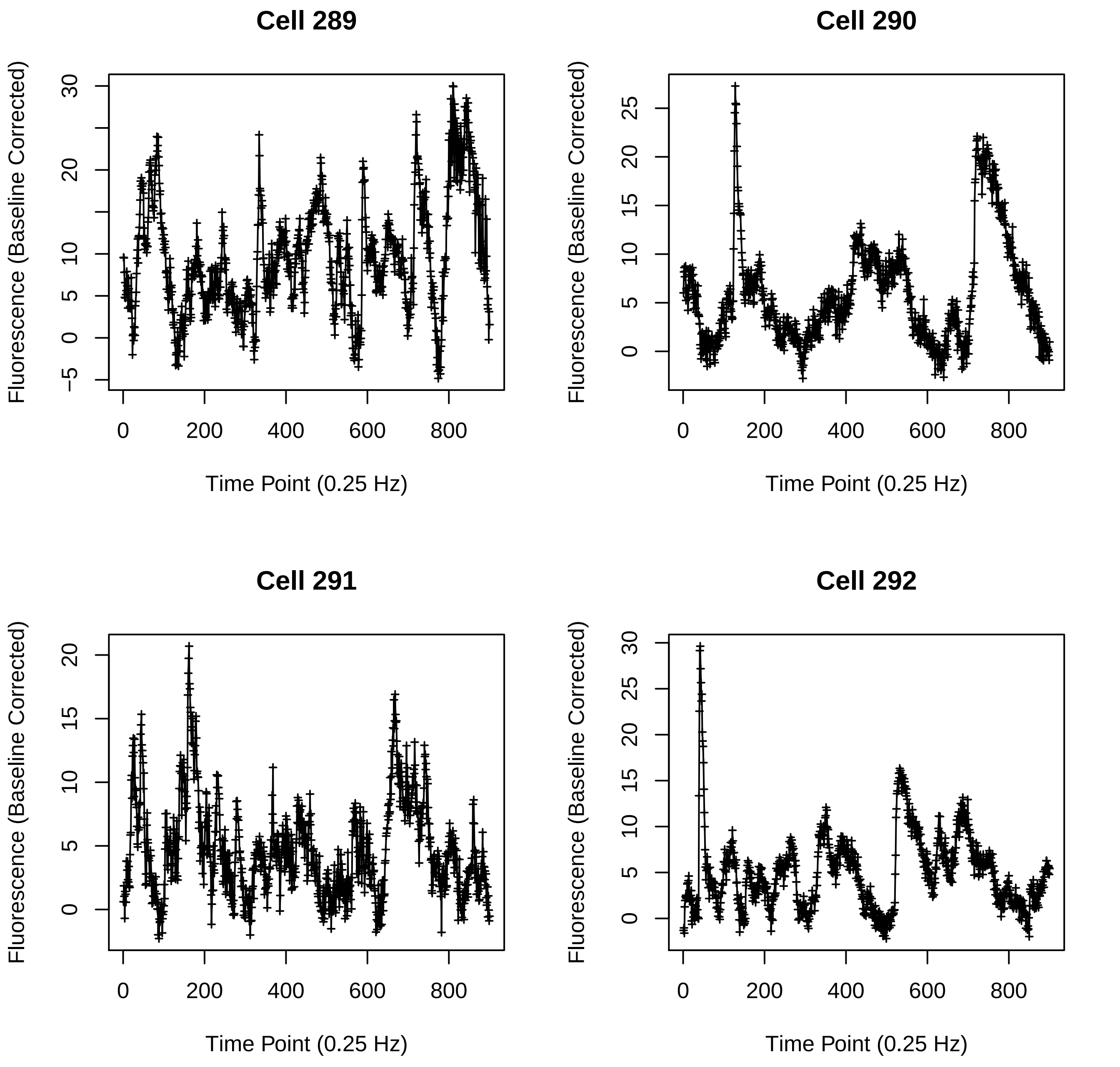
Cell 289
Cell 290
Cell 291
Cell 292
Fluorescence (Baseline Corrected)
Time Point (0.25 Hz)
0
200
400
600
800
−5
0
5
10
15
20
25
30

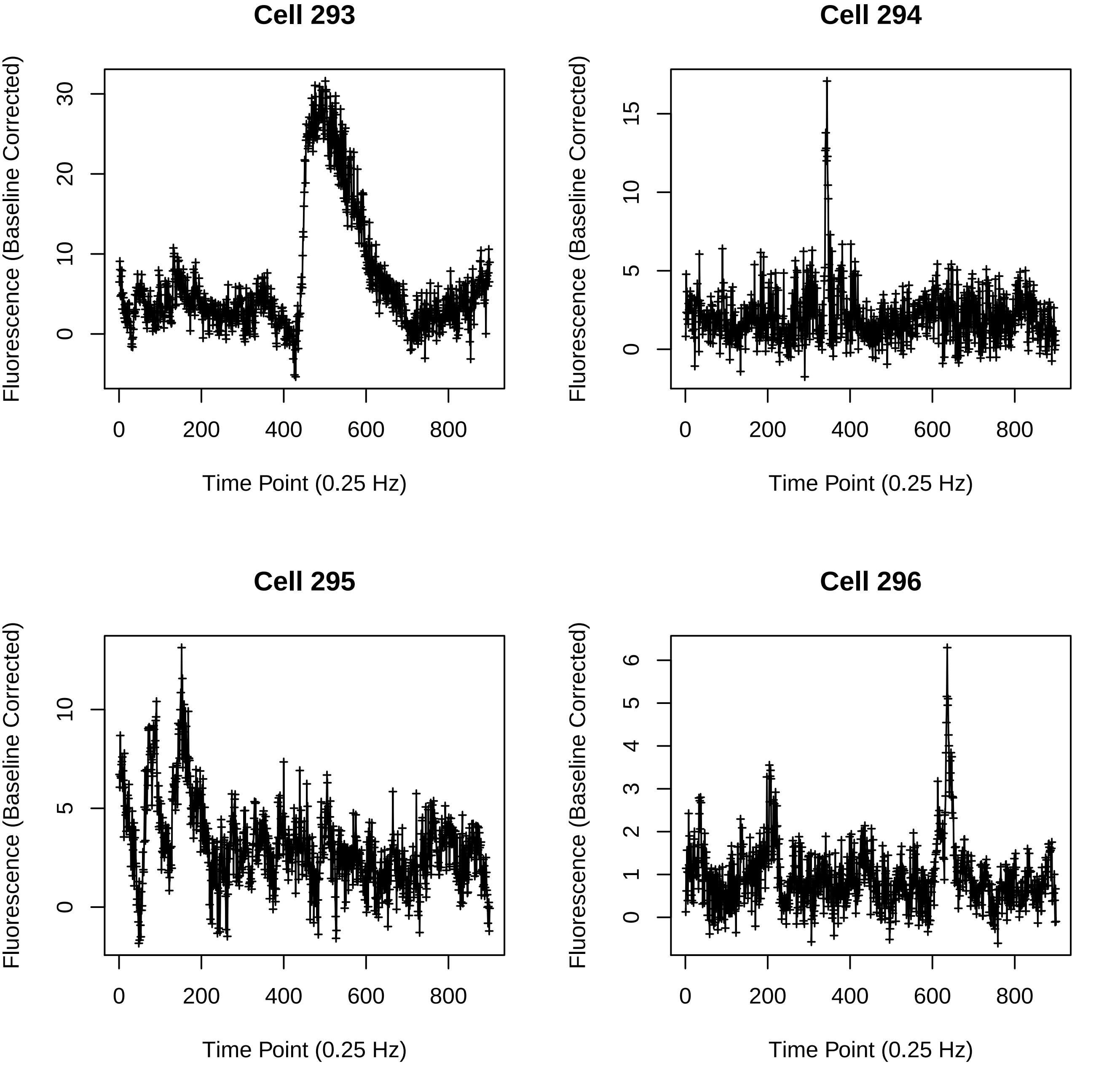
Cell 293
Cell 294
Cell 295
Cell 296
Fluorescence (Baseline Corrected)
Time Point (0.25 Hz)
0
200
400
600
800
-5
0
5
10
15
20
30
1
2
3
4
6

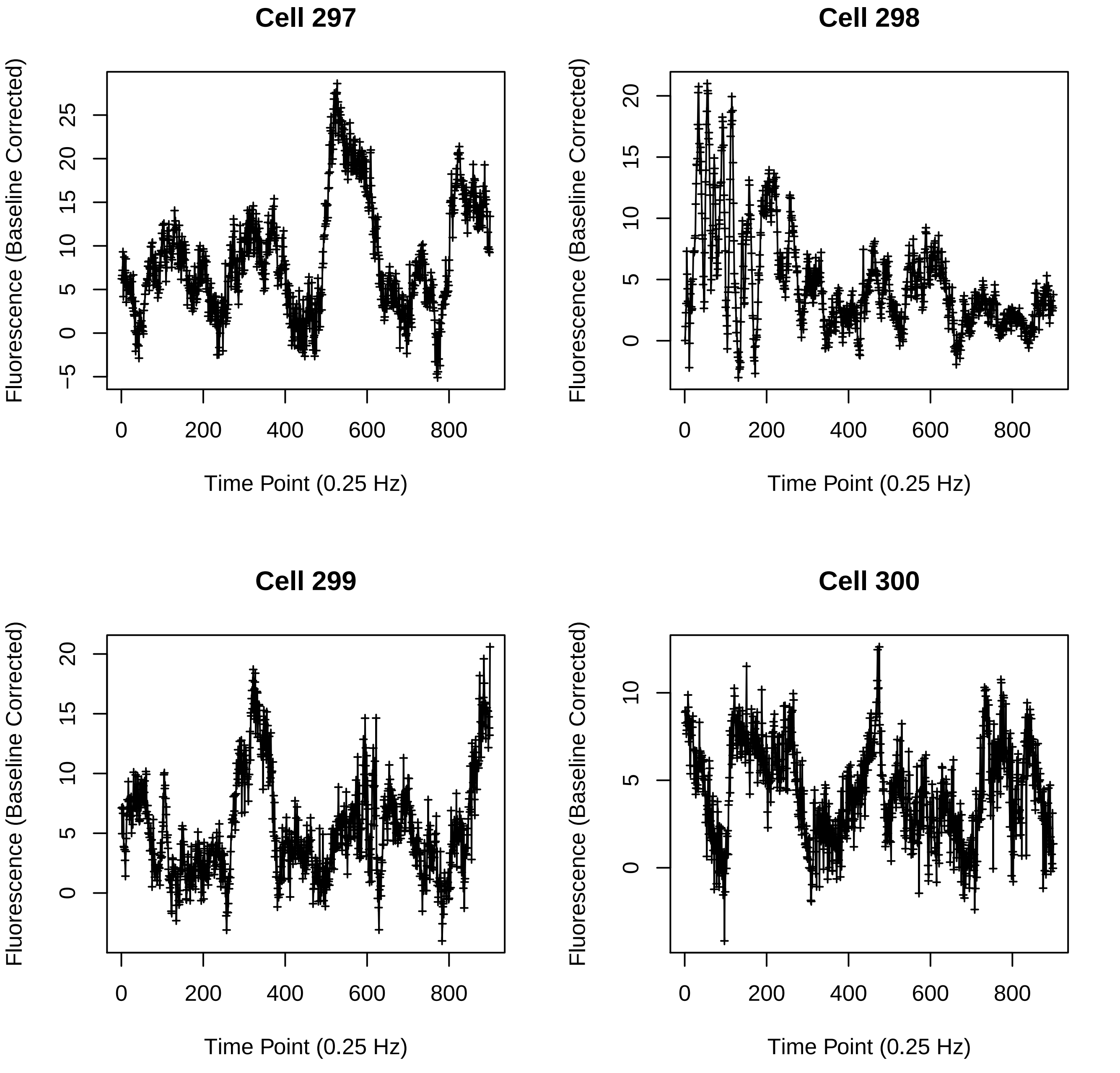

Cell 297
Cell 298
Cell 299
Cell 300
Fluorescence (Baseline Corrected)
Time Point (0.25 Hz)
−5
0
5
10
15
20
25
0
200
400
600
800

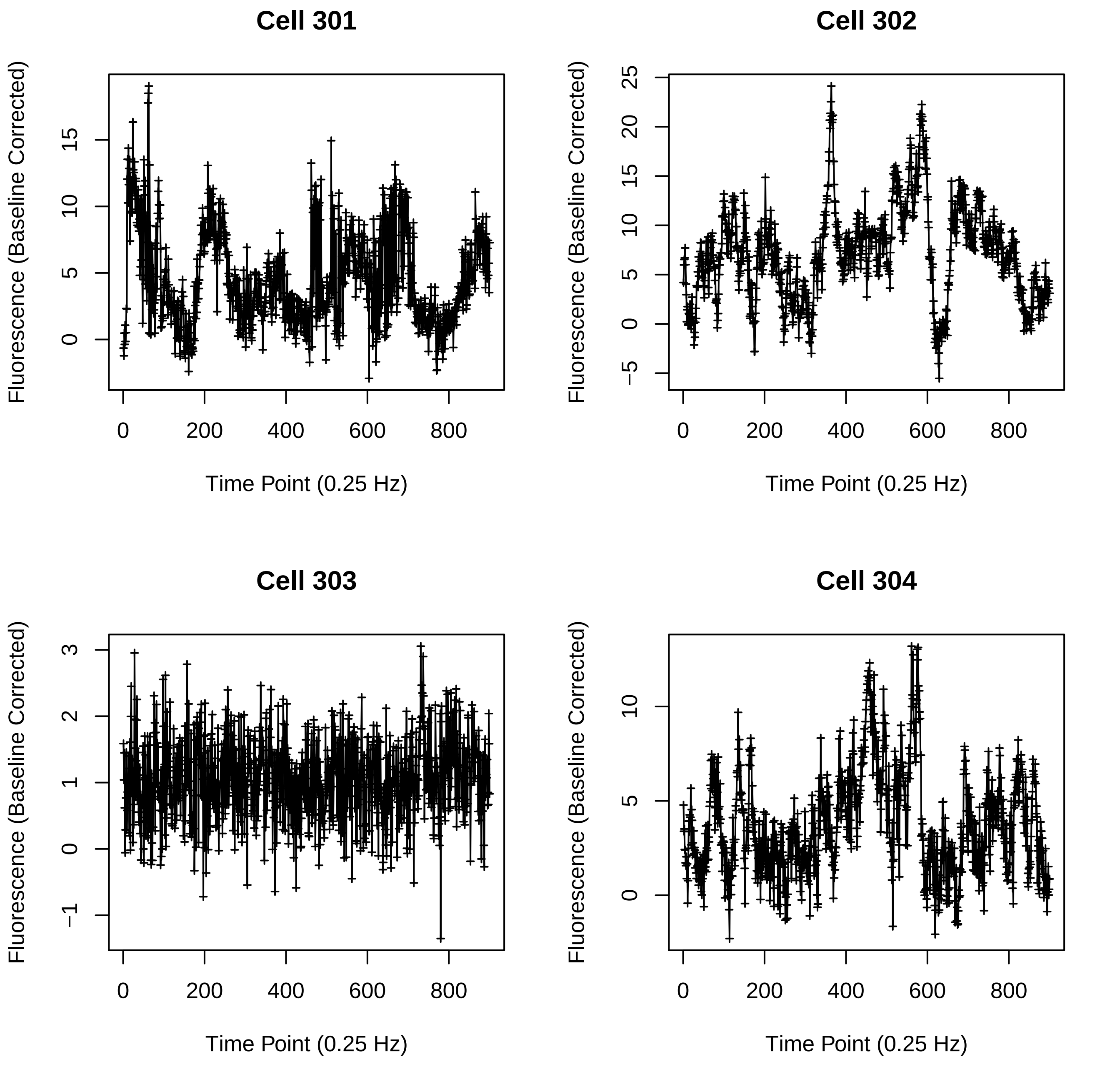
Cell 301
Cell 302
Cell 303
Cell 304
Fluorescence (Baseline Corrected)
Time Point (0.25 Hz)
0
200
400
600
800
−5
5
10
15
20
25
−1
1
2
3

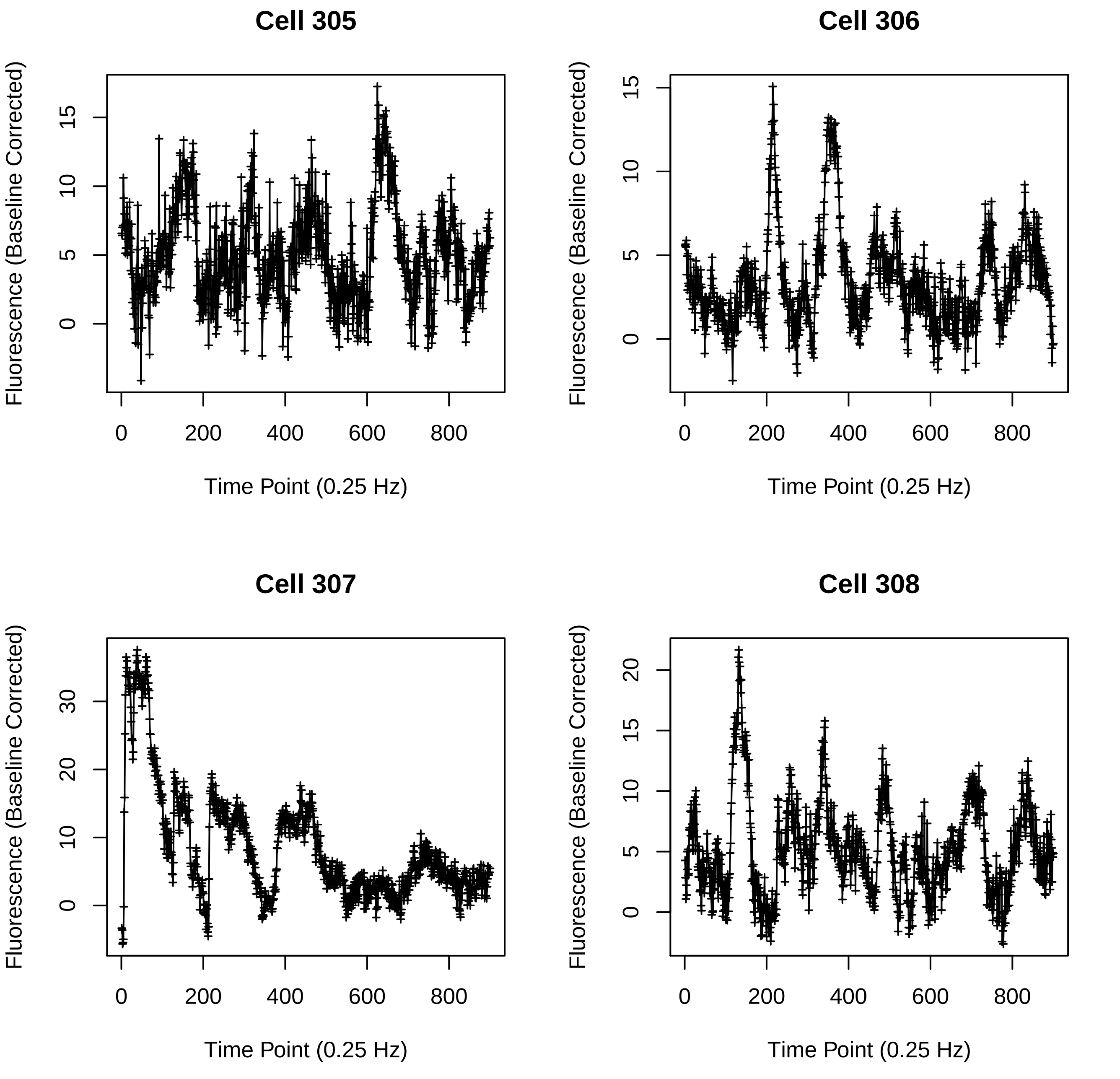
Cell 305
Cell 306
Cell 307
Cell 308
Fluorescence (Baseline Corrected)
Time Point (0.25 Hz)
0
5
10
15
20
30
200
400
600
800

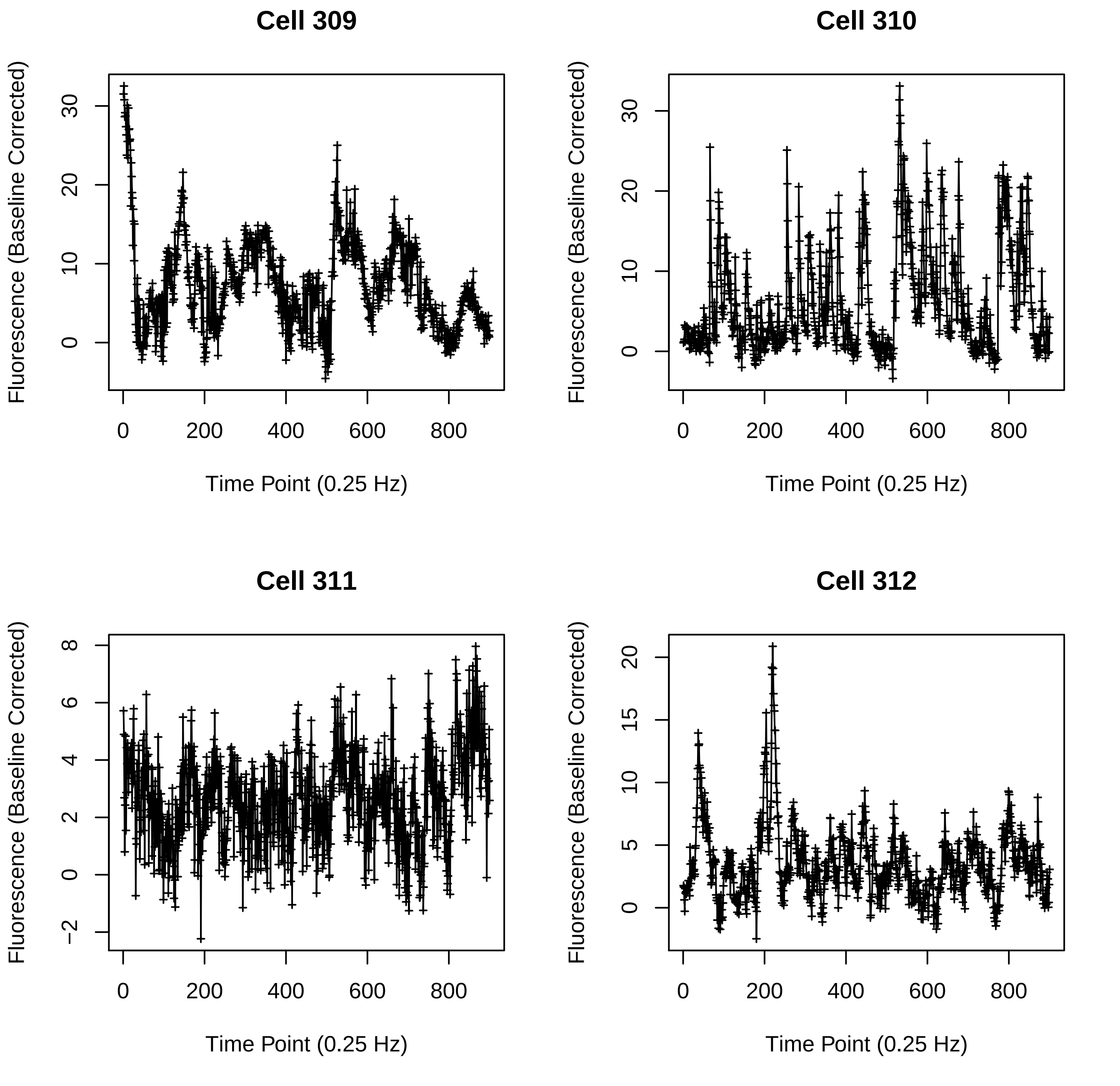
Cell 309
Cell 310
Cell 311
Cell 312
Fluorescence (Baseline Corrected)
Time Point (0.25 Hz)
0
200
400
600
800
10
20
30
-2
2
4
6
8
5
15

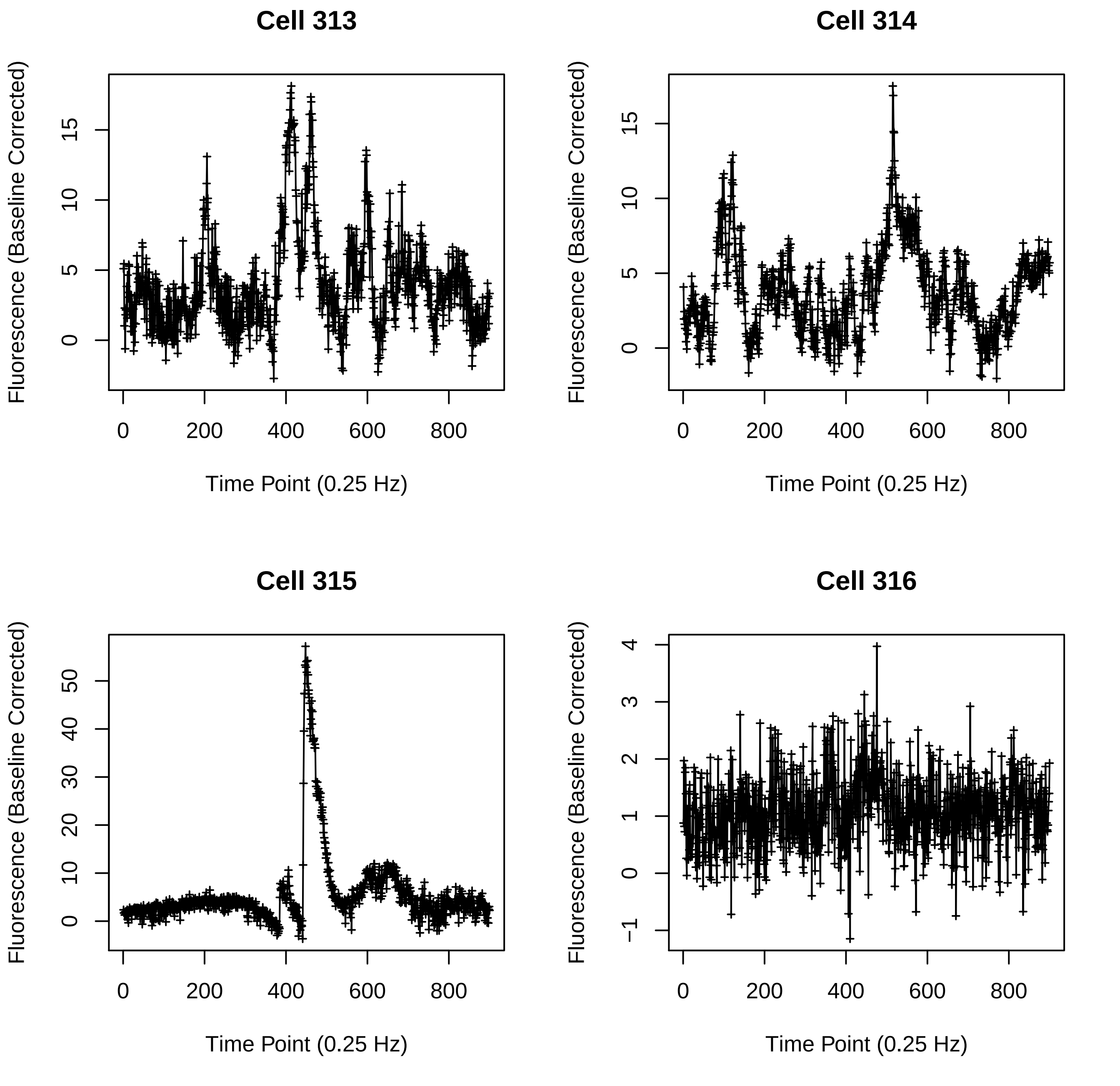
Cell 313
Cell 314
Cell 315
Cell 316
Fluorescence (Baseline Corrected)
Time Point (0.25 Hz)
0
200
400
600
800
15
10
5
0
50
40
30
20
4
3
2
1
−1

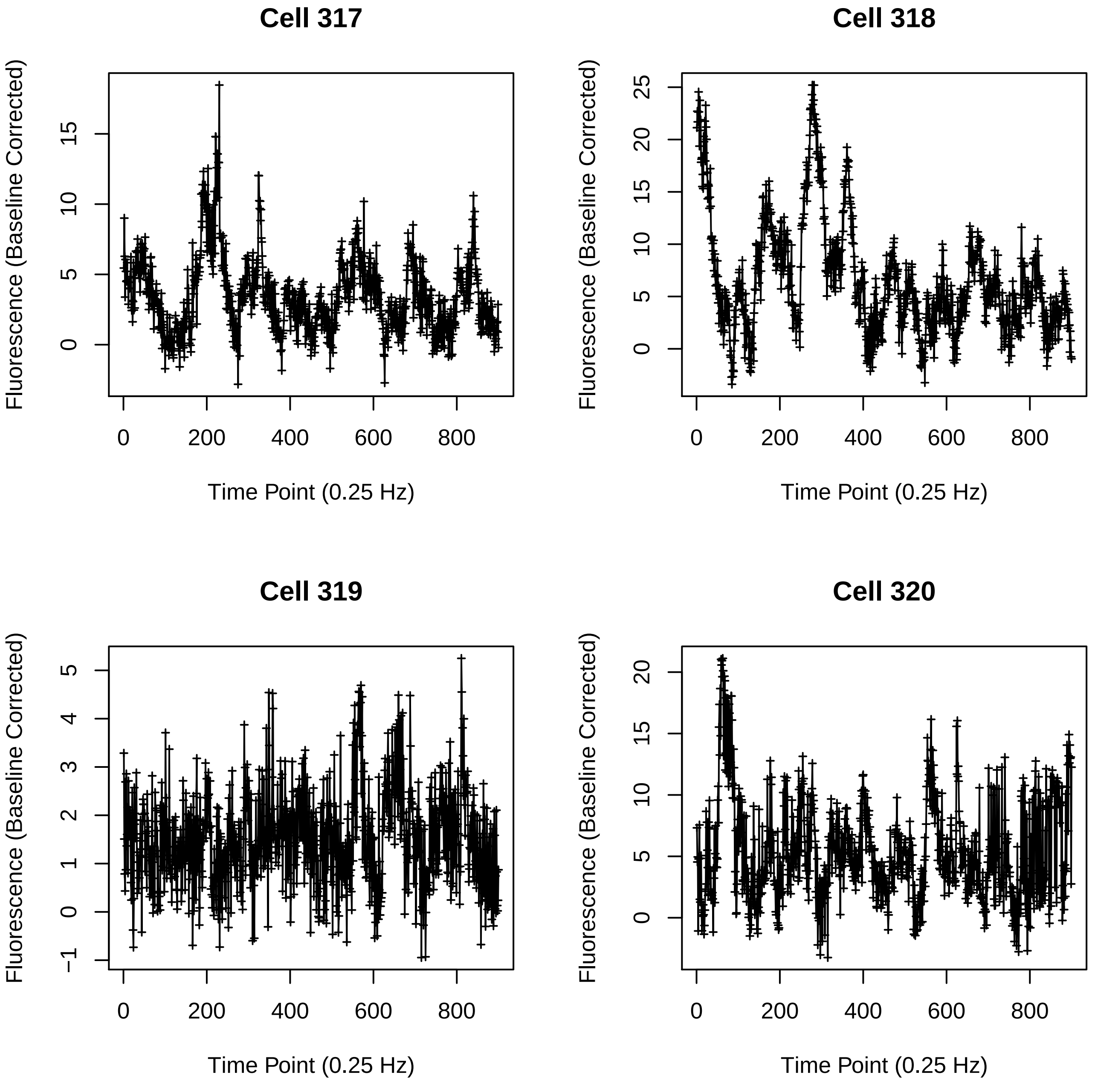
Cell 317
Cell 318
Cell 319
Cell 320
Fluorescence (Baseline Corrected)
Time Point (0.25 Hz)
0
200
400
600
800

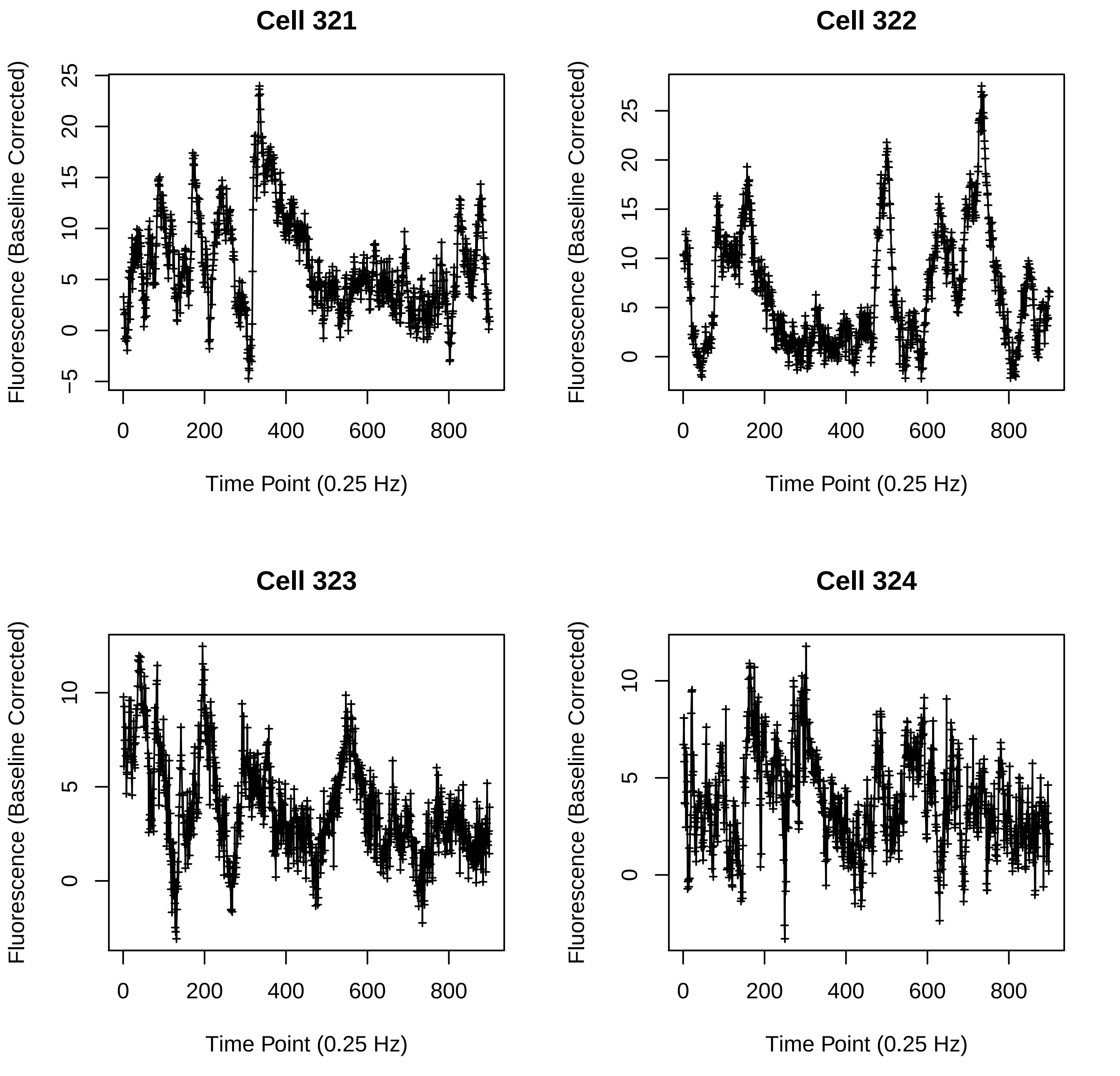
Cell 321
Cell 322
Cell 323
Cell 324
Fluorescence (Baseline Corrected)
Time Point (0.25 Hz)
−5
0
5
10
15
20
25
200
400
600
800

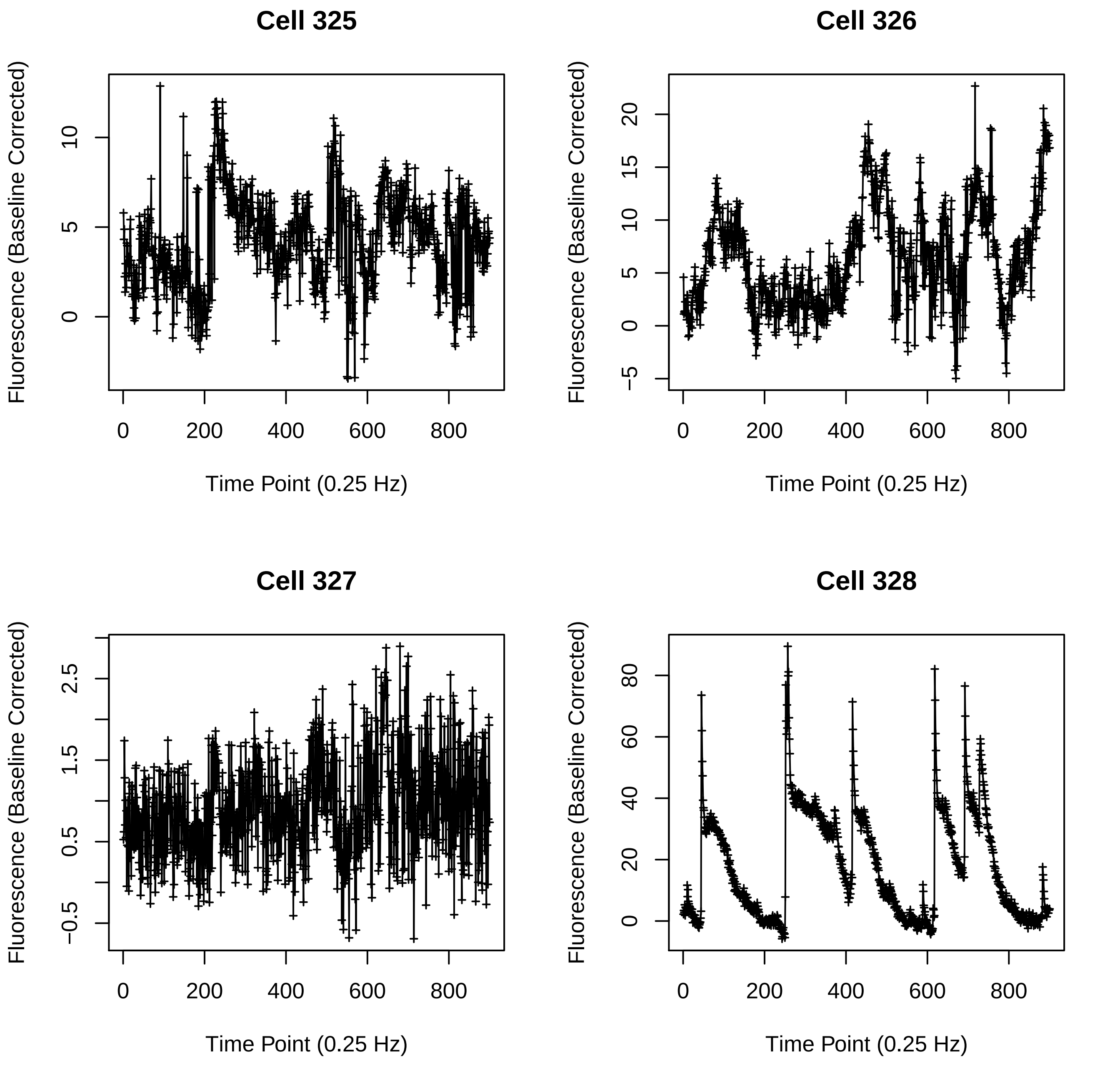
Cell 325
Cell 326
Cell 327
Cell 328
Fluorescence (Baseline Corrected)
Time Point (0.25 Hz)
0
200
400
600
800
10
5
0
20
15
-5
2.5
1.5
0.5
-0.5
80
60
40
20

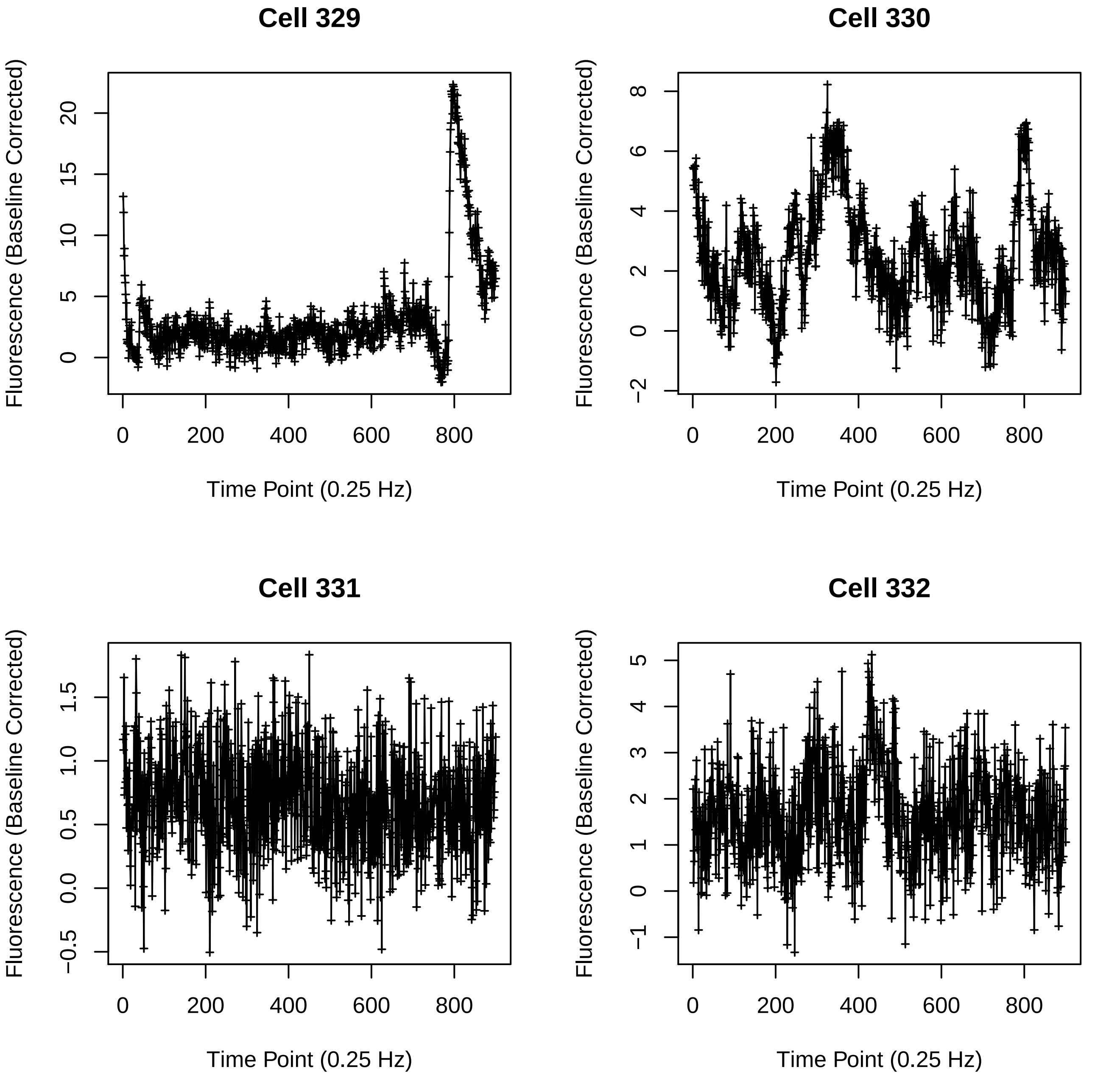
Cell 329
Cell 330
Cell 331
Cell 332
Fluorescence (Baseline Corrected)
Time Point (0.25 Hz)
0
200
400
600
800
0
5
10
15
20
−2
2
4
6
8
−0.5
0.0
0.5
1.0
1.5
−1
1
3

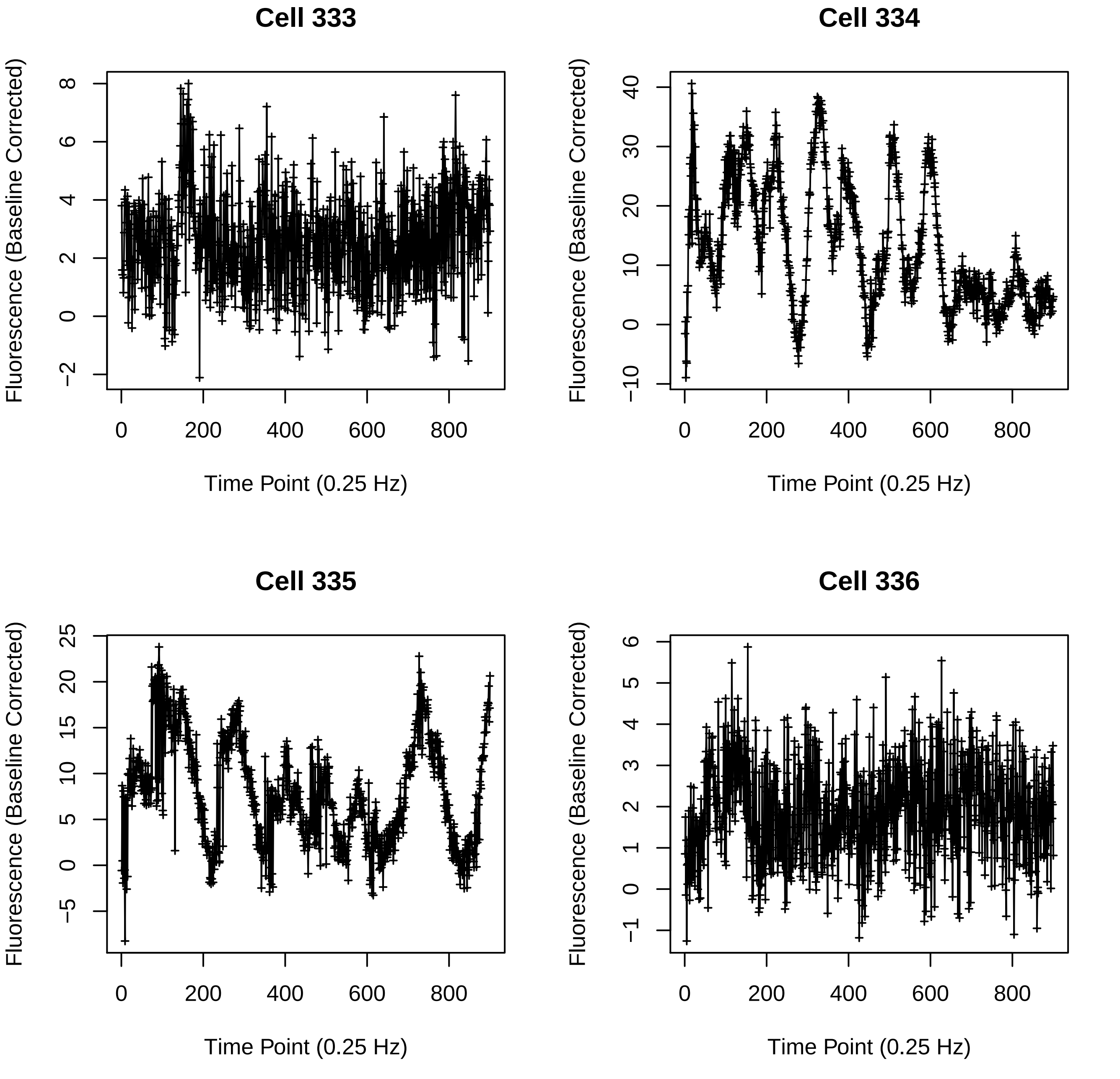
Cell 333
Cell 334
Cell 335
Cell 336
Fluorescence (Baseline Corrected)
Time Point (0.25 Hz)
0
200
400
600
800
−2
2
4
6
8
−10
10
20
30
40
−5
5
15
25
−1
1
3
5

Cell 337

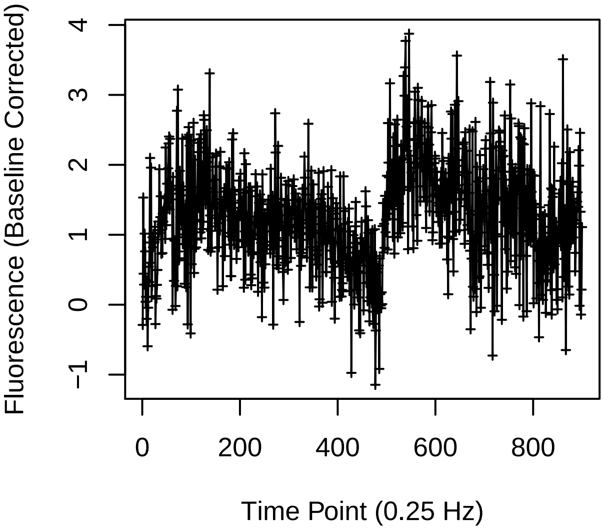

Cell 338

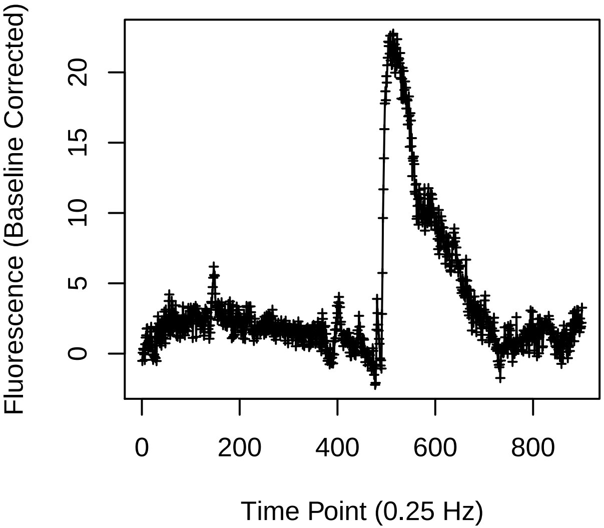

Cell 339

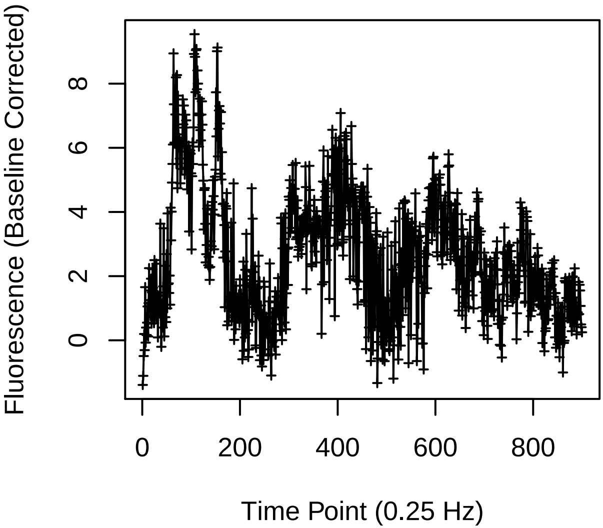

Cell 340

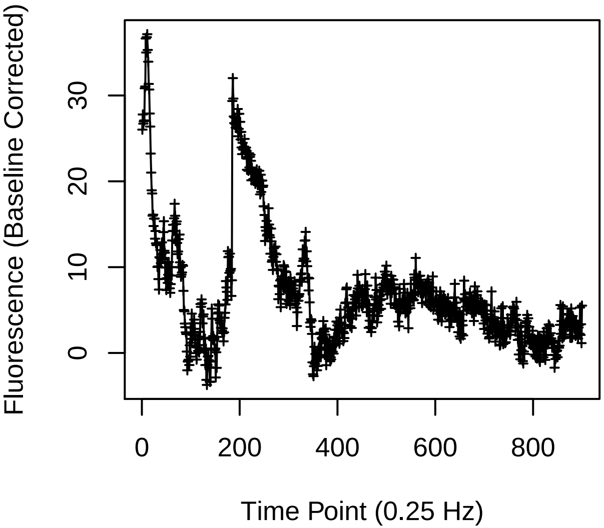

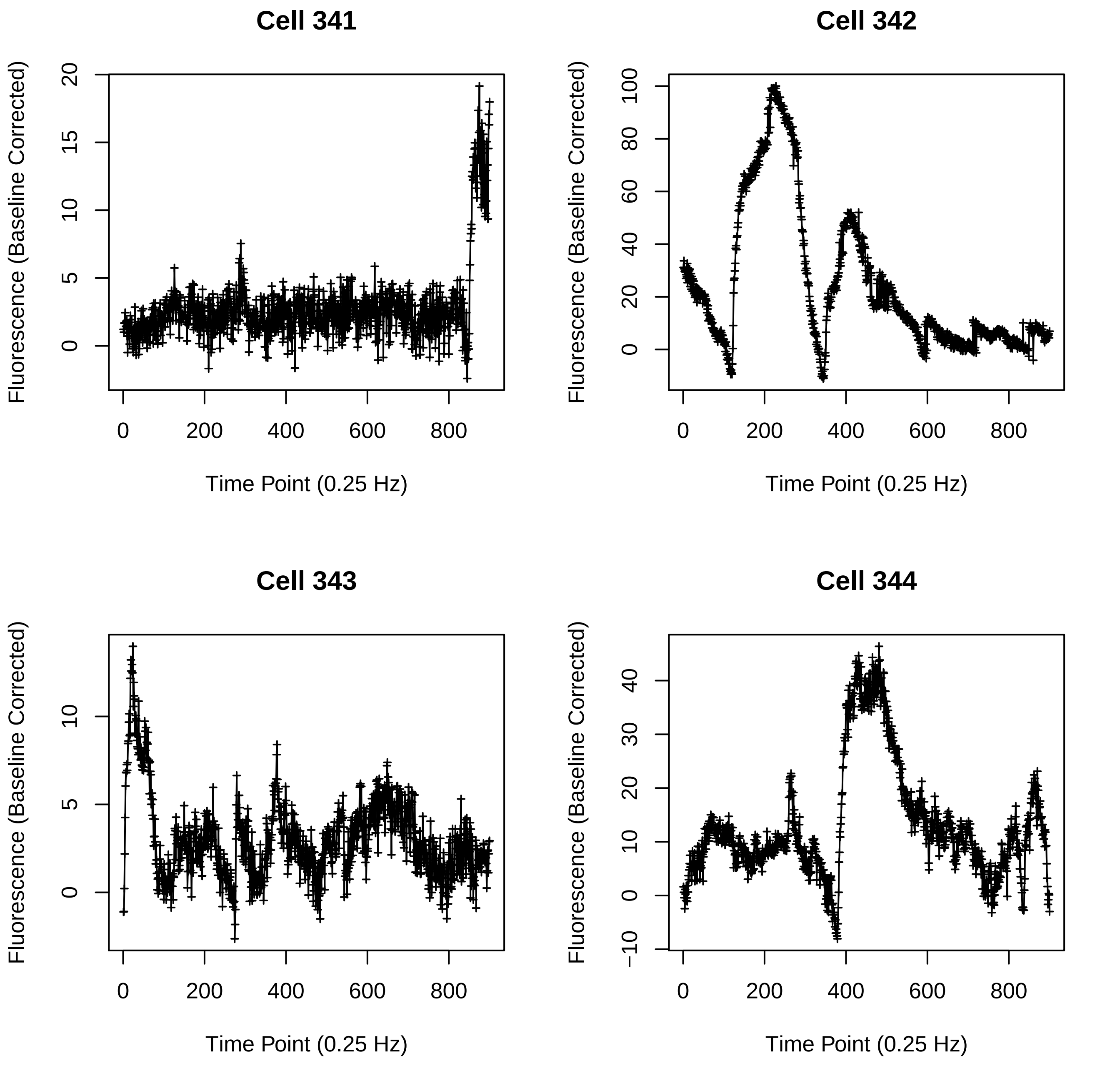
Cell 341
Cell 342
Cell 343
Cell 344
Fluorescence (Baseline Corrected)
Time Point (0.25 Hz)
0
200
400
600
800
-10
0
5
10
15
20
30
40
60
80
100

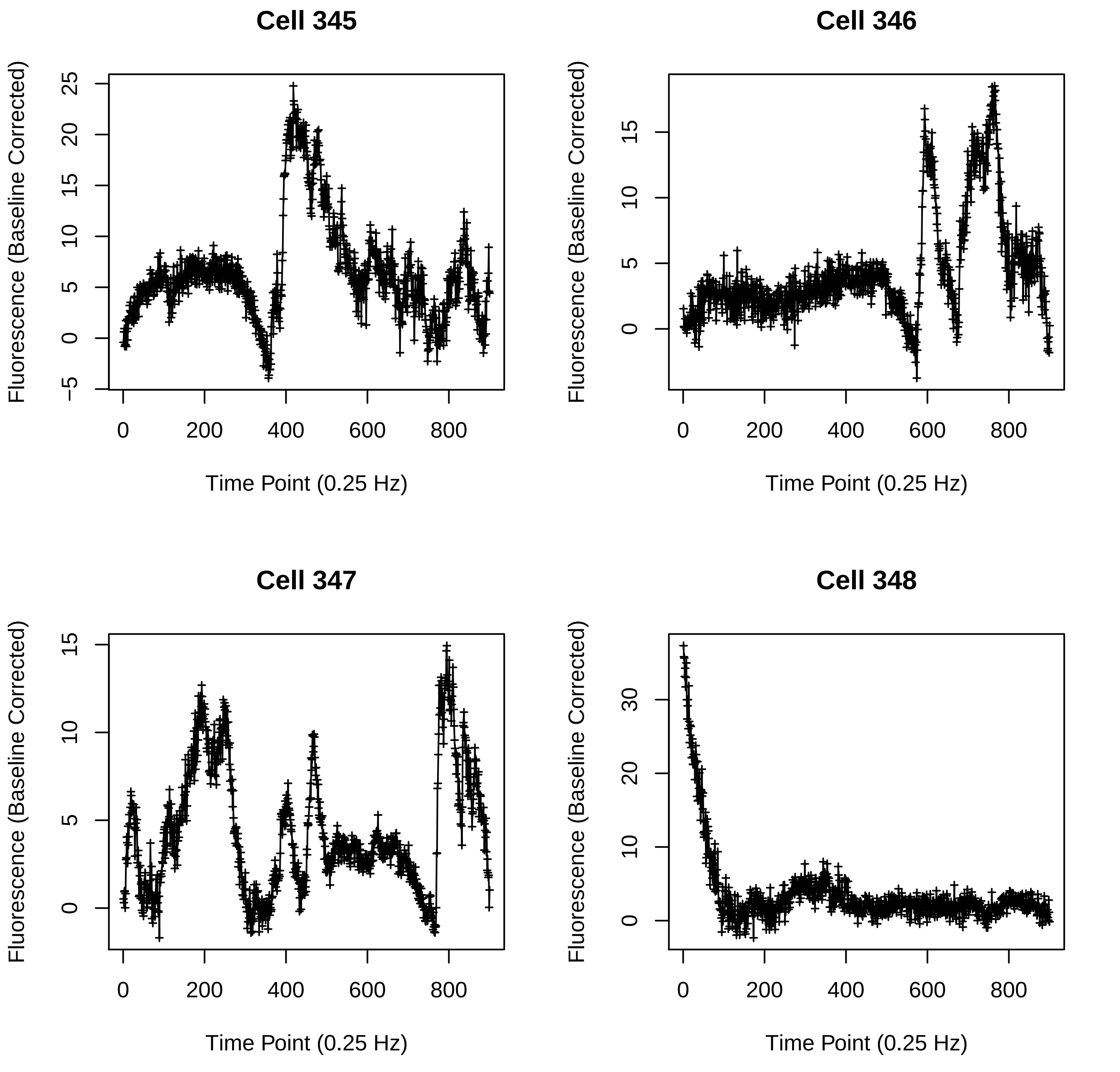

Cell 345
Cell 346
Cell 347
Cell 348
Fluorescence (Baseline Corrected)
Time Point (0.25 Hz)
-5
0
5
10
15
20
25
30
200
400
600
800

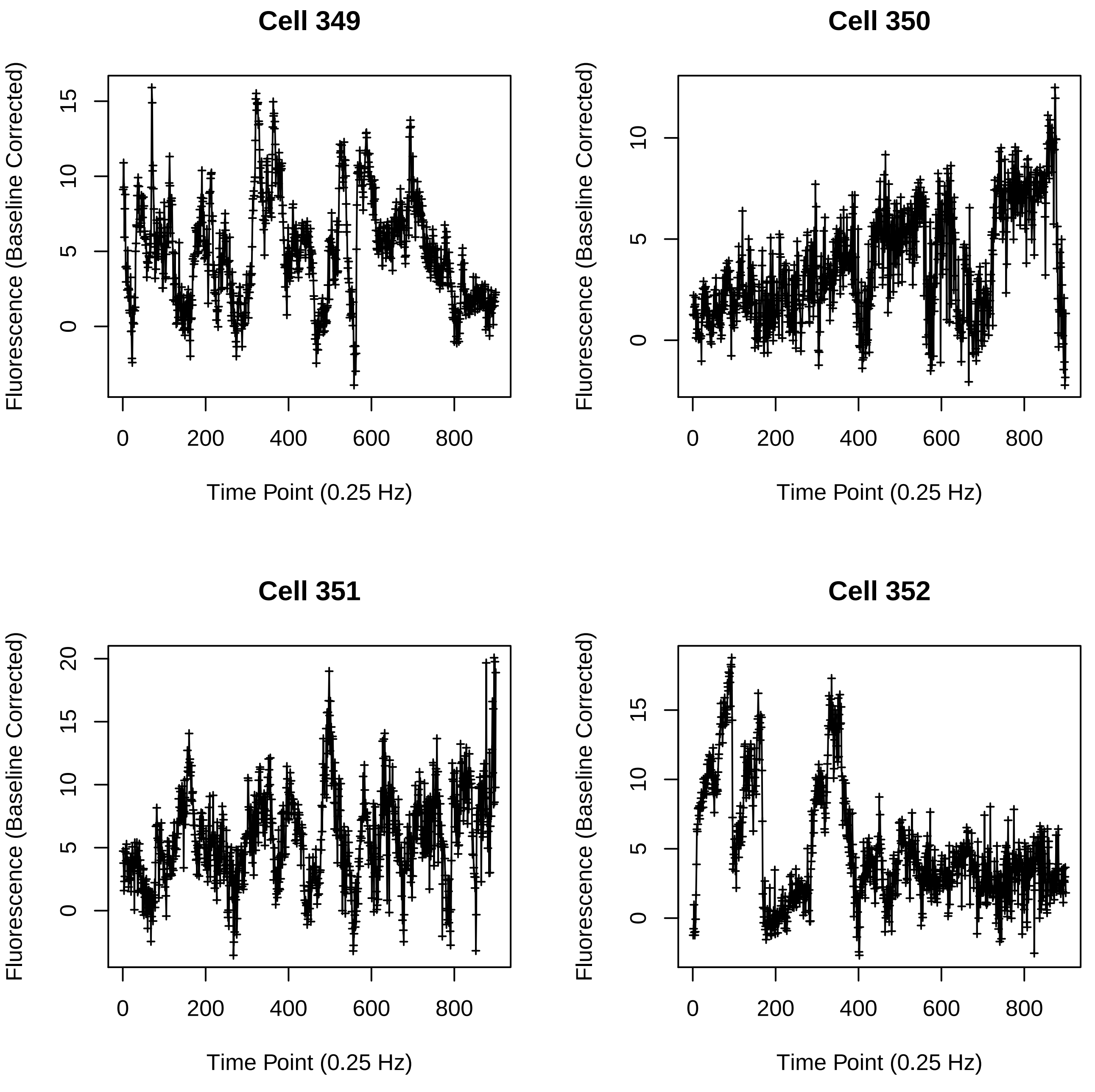
Cell 349
Cell 350
Cell 351
Cell 352
Fluorescence (Baseline Corrected)
Time Point (0.25 Hz)
0
200
400
600
800
5
10
15
20

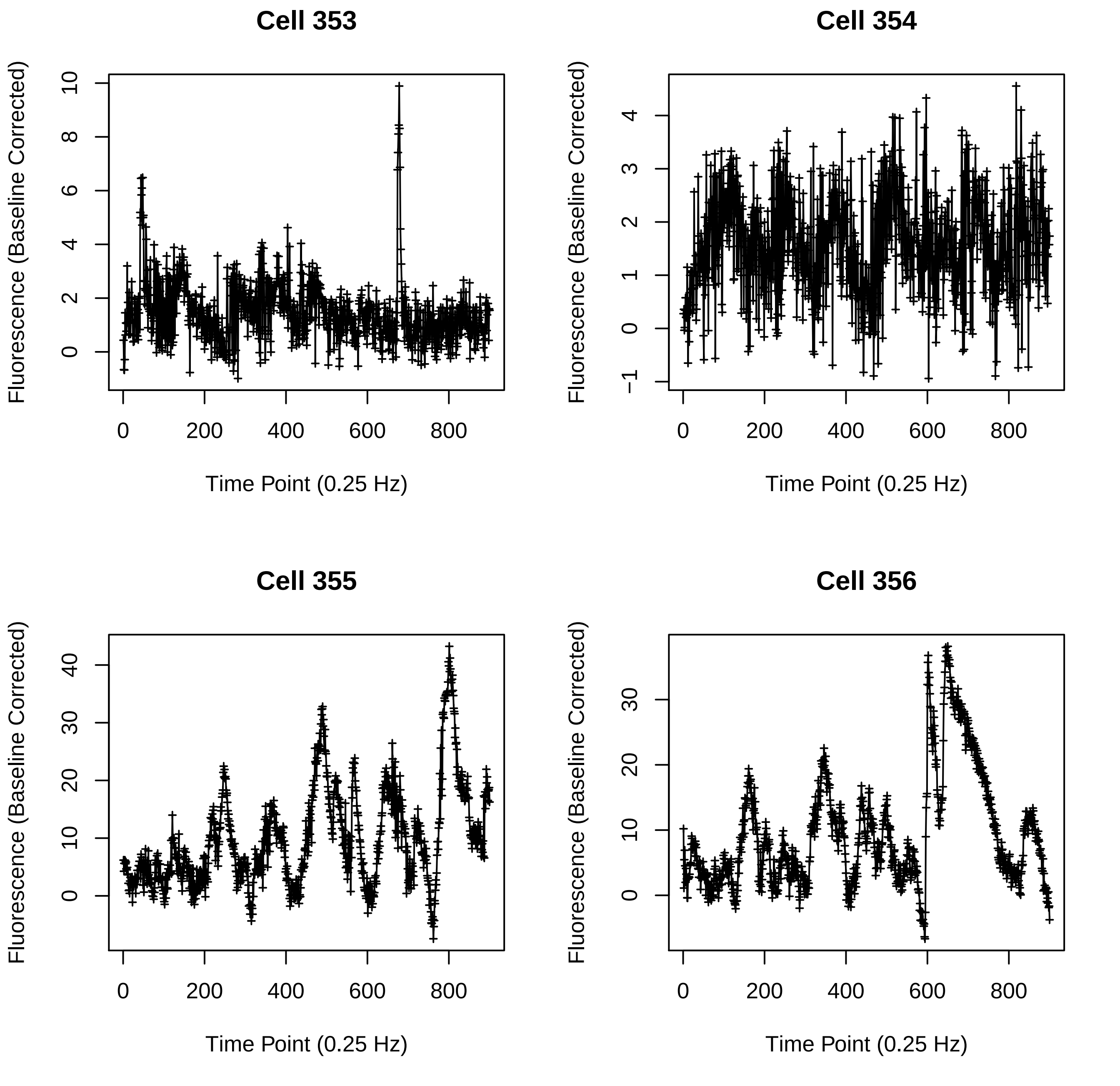
Cell 353
Cell 354
Cell 355
Cell 356
Fluorescence (Baseline Corrected)
Time Point (0.25 Hz)
0
200
400
600
800
10
8
6
4
2
0
4
3
2
1
0
−1
40
30
20
10
0
30
20
10
0

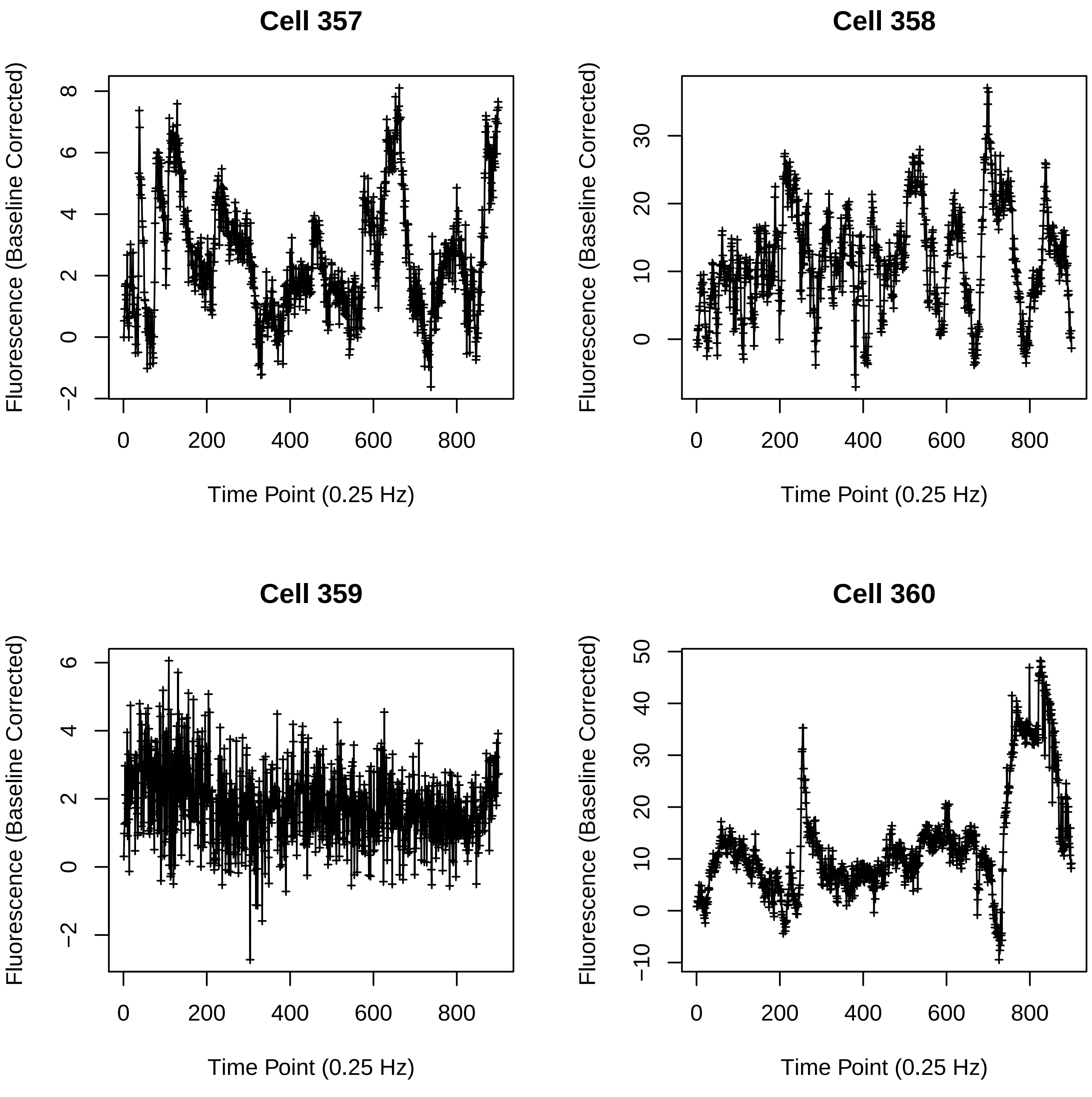
Cell 357
Fluorescence (Baseline Corrected)
8
6
4
2
0
−2
0
200
400
600
800
Time Point (0.25 Hz)
Cell 358
Fluorescence (Baseline Corrected)
30
20
10
0
0
200
400
600
800
Time Point (0.25 Hz)
Cell 359
Fluorescence (Baseline Corrected)
6
4
2
0
−2
0
200
400
600
800
Time Point (0.25 Hz)
Cell 360
Fluorescence (Baseline Corrected)
50
40
30
20
10
0
−10
0
200
400
600
800
Time Point (0.25 Hz)

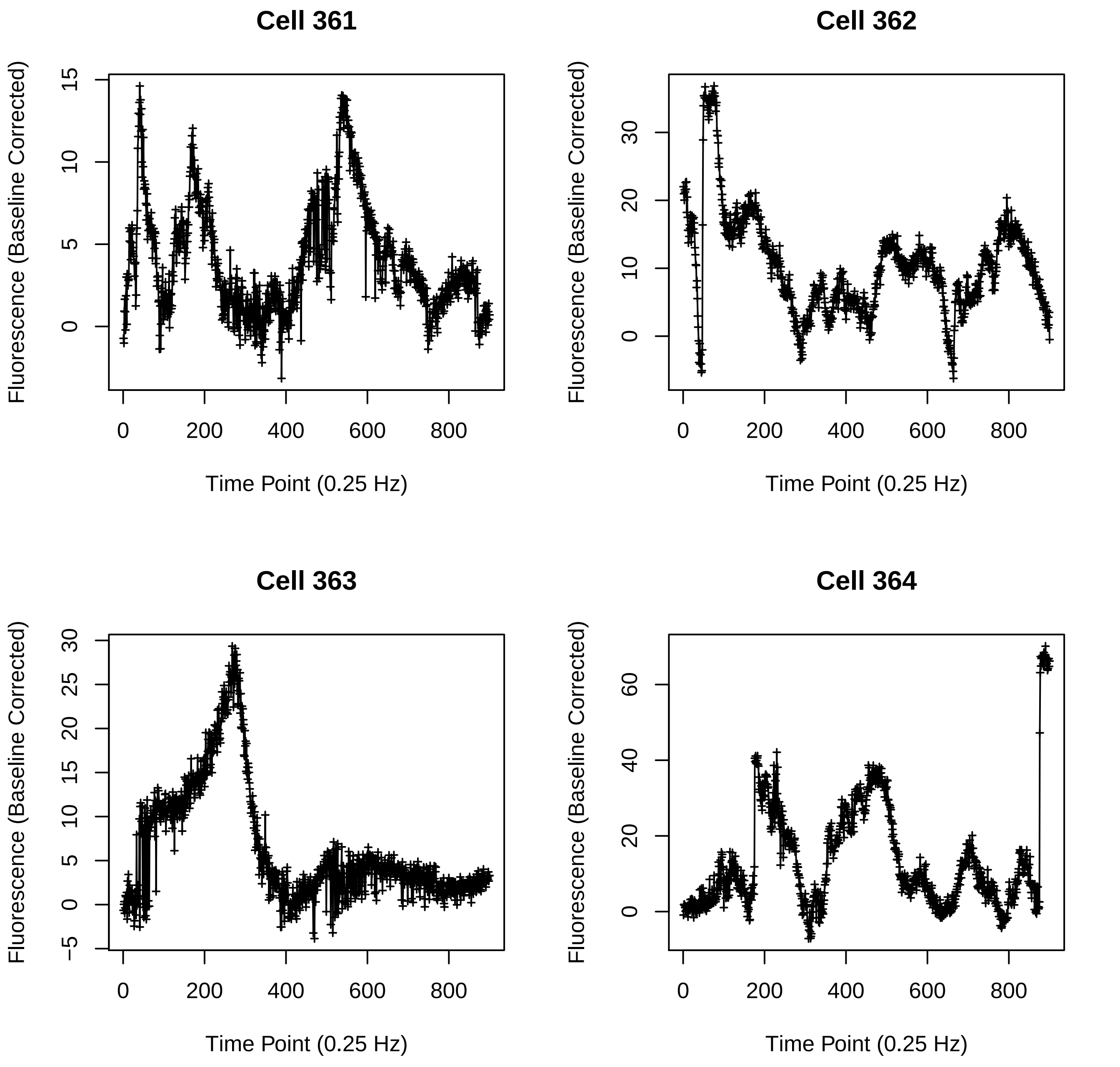
Cell 361
Cell 362
Cell 363
Cell 364
Fluorescence (Baseline Corrected)
Time Point (0.25 Hz)
0
200
400
600
800
-5
5
10
15
20
25
30
40
60

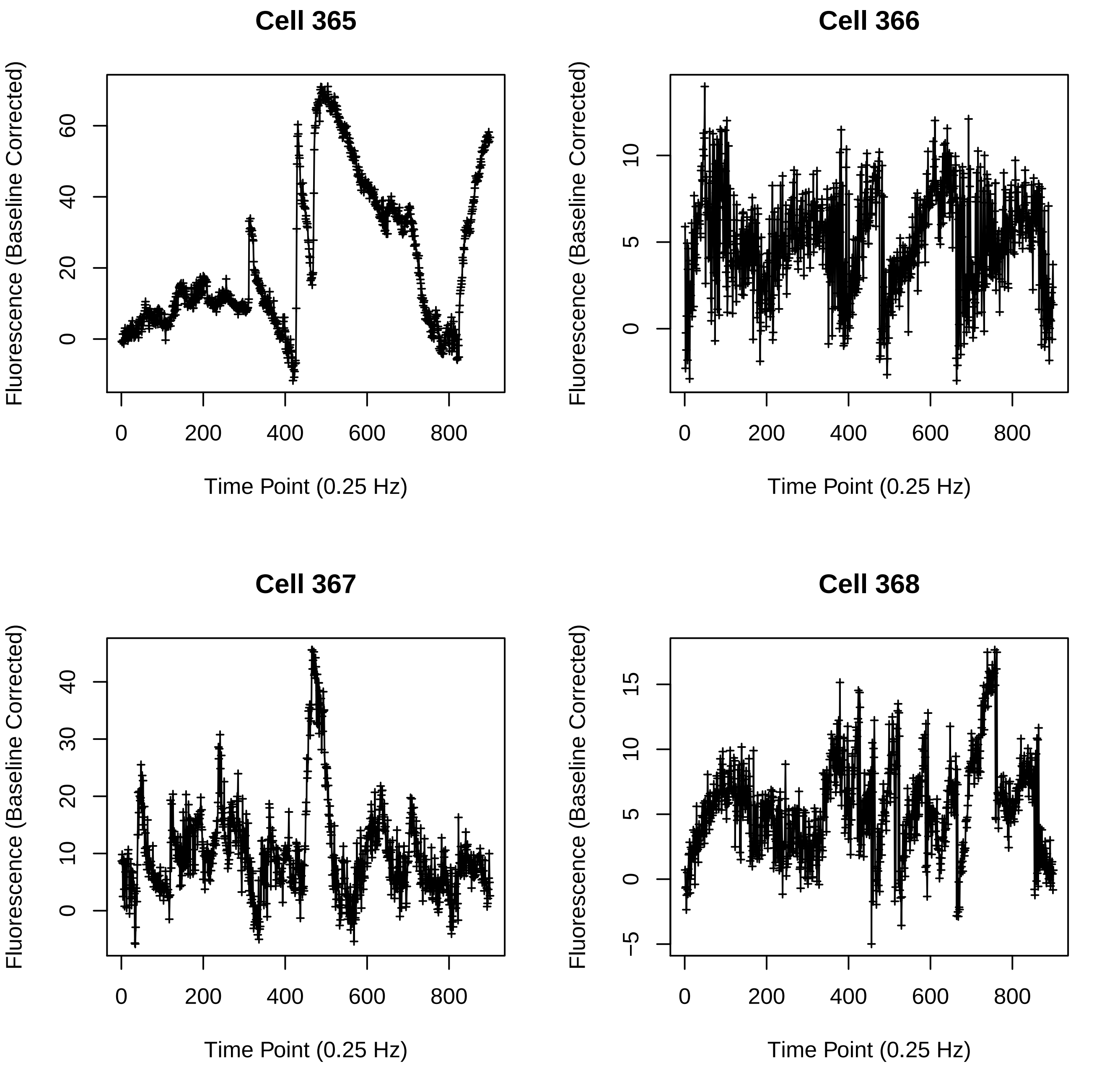
Cell 365
Cell 366
Cell 367
Cell 368
Fluorescence (Baseline Corrected)
Time Point (0.25 Hz)
0
200
400
600
800
-5
5
10
15
20
30
40
60

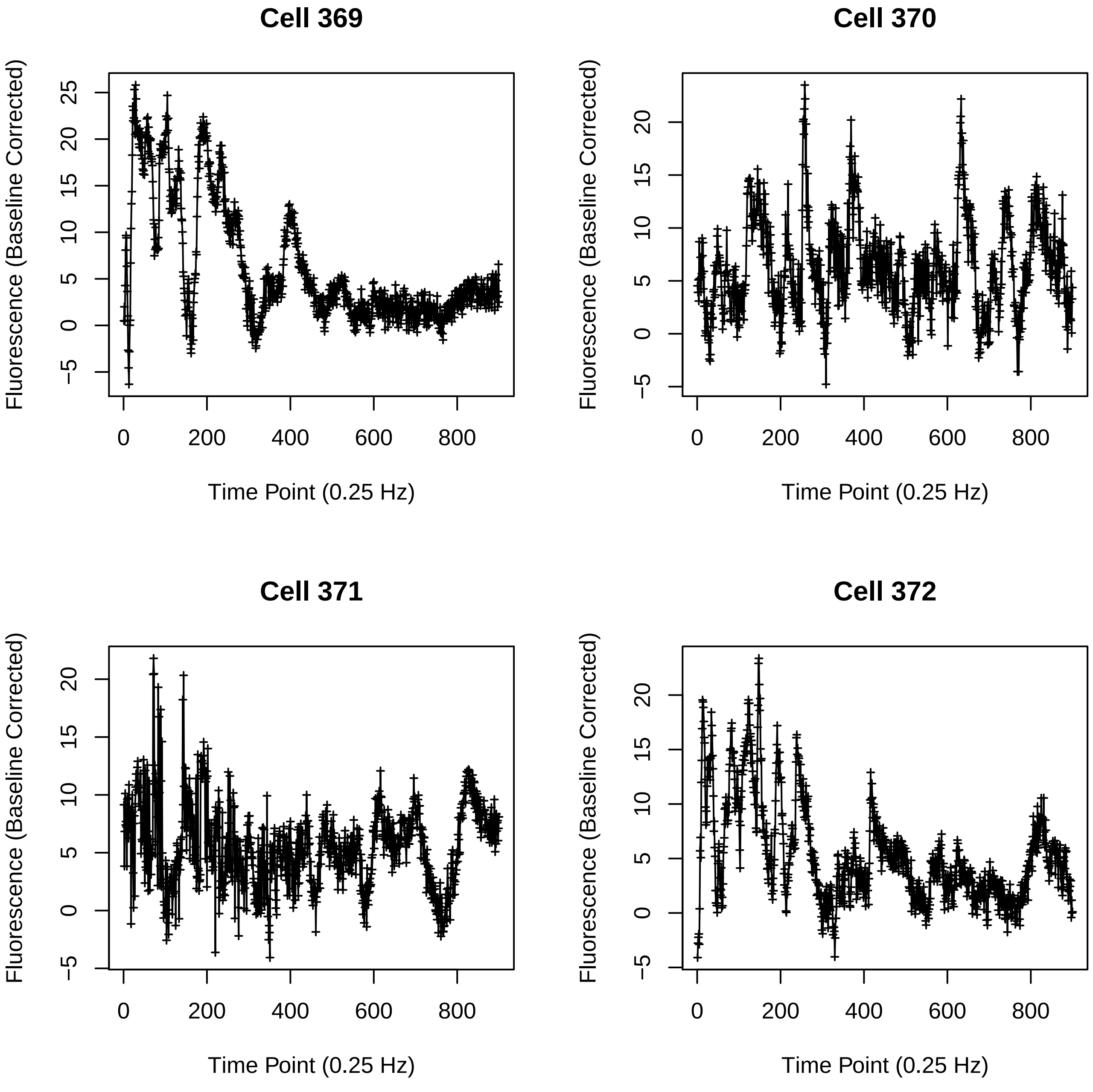
Cell 369
Cell 370
Cell 371
Cell 372
Fluorescence (Baseline Corrected)
Time Point (0.25 Hz)
0
200
400
600
800
−5
0
5
10
15
20
25

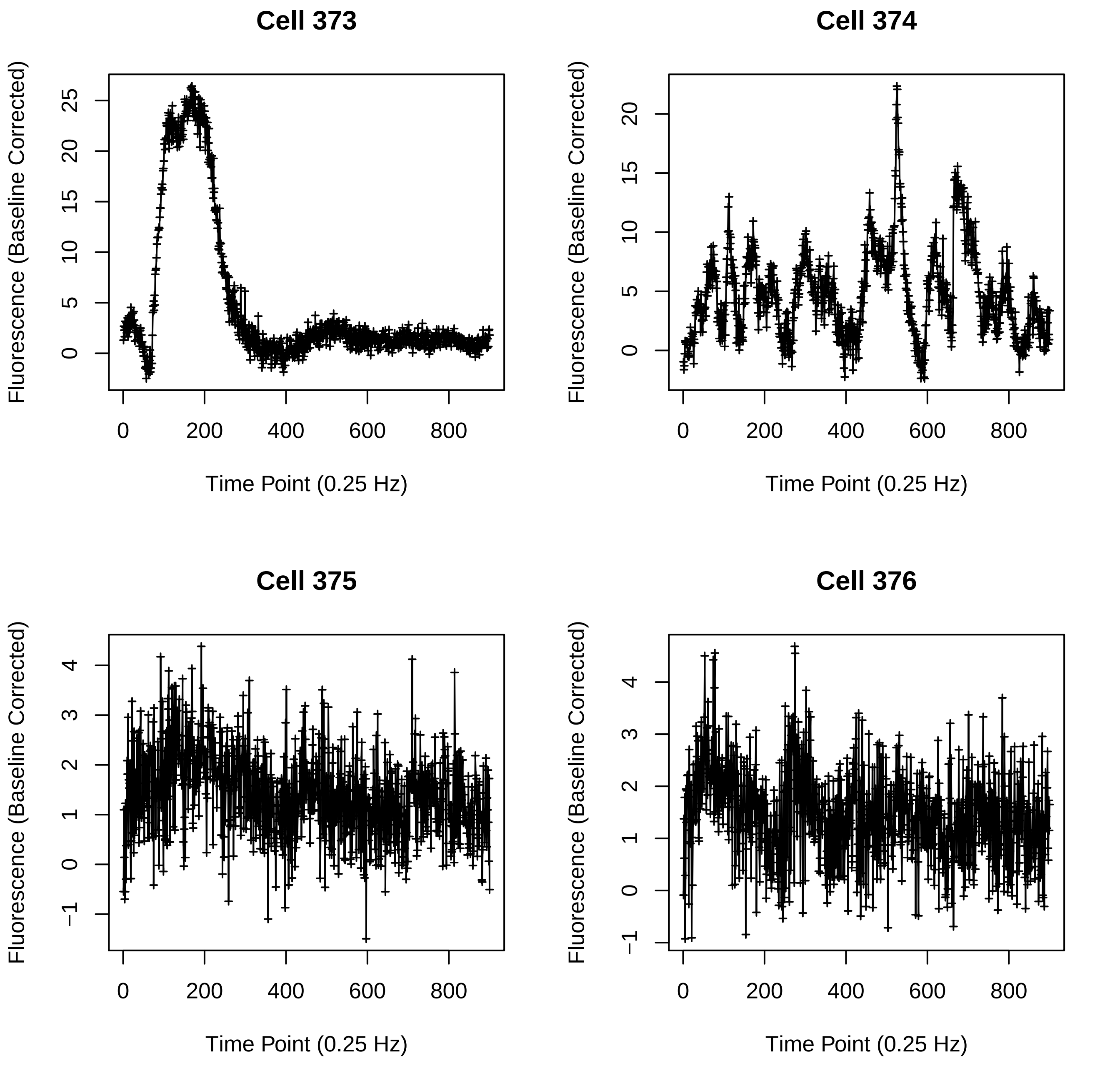
Cell 373
Cell 374
Cell 375
Cell 376
Fluorescence (Baseline Corrected)
Time Point (0.25 Hz)

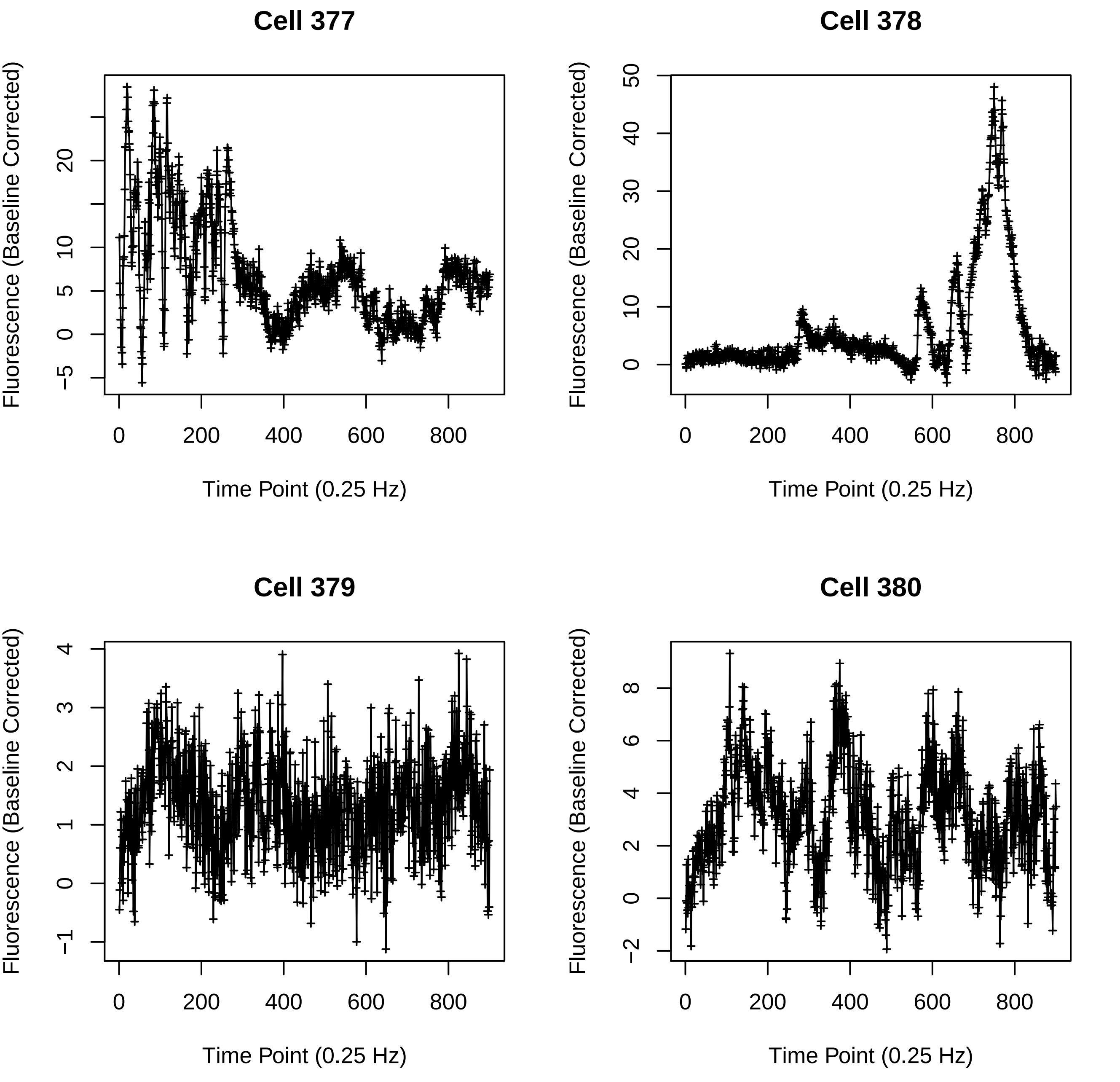
Cell 377
Cell 378
Cell 379
Cell 380
Fluorescence (Baseline Corrected)
Time Point (0.25 Hz)
0
200
400
600
800
−5
0
5
10
20
30
40
50
−1
1
2
3
4
−2
6
8

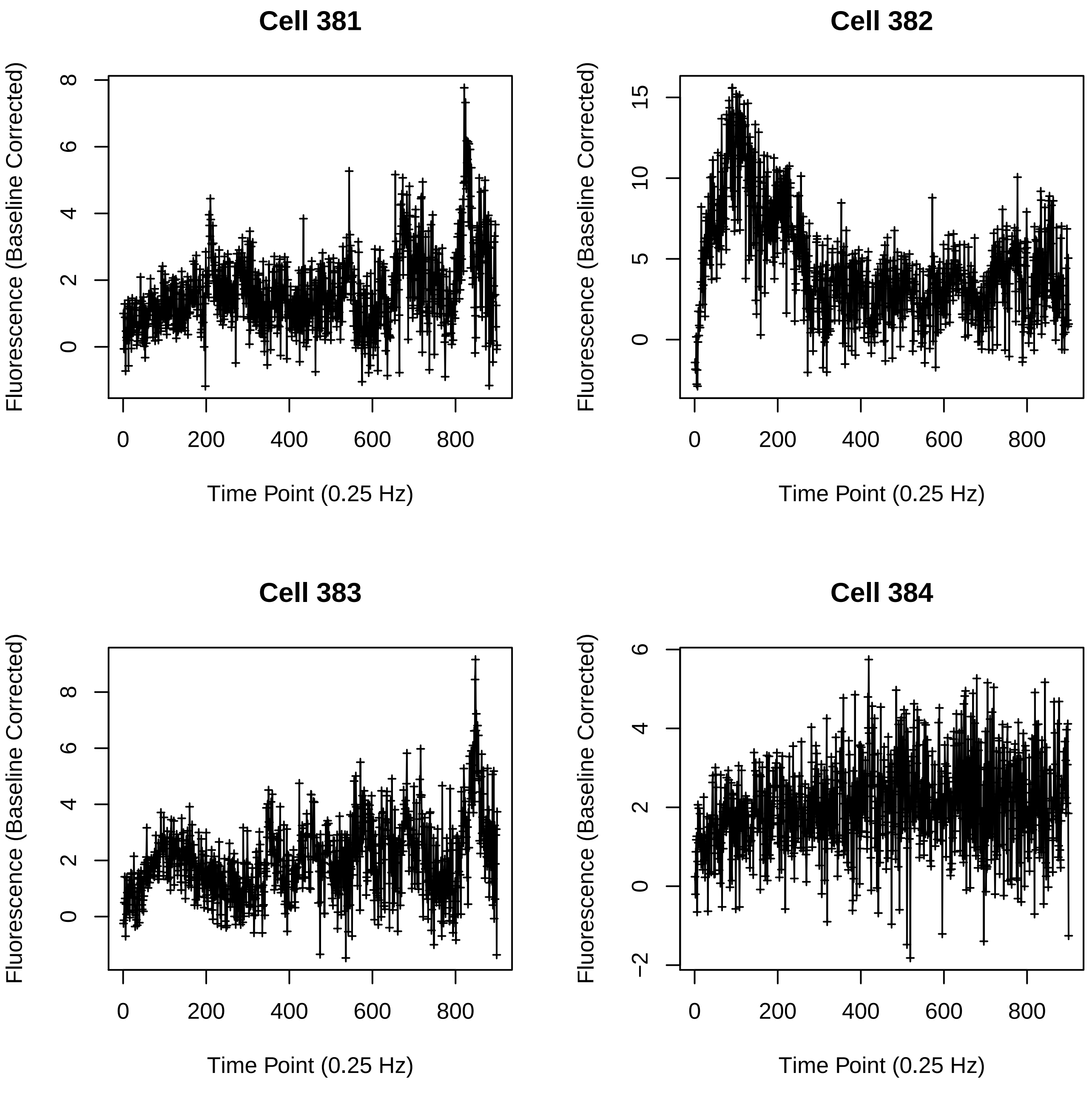

Cell 381
Cell 382
Cell 383
Cell 384
Fluorescence (Baseline Corrected)
Time Point (0.25 Hz)
0
200
400
600
800

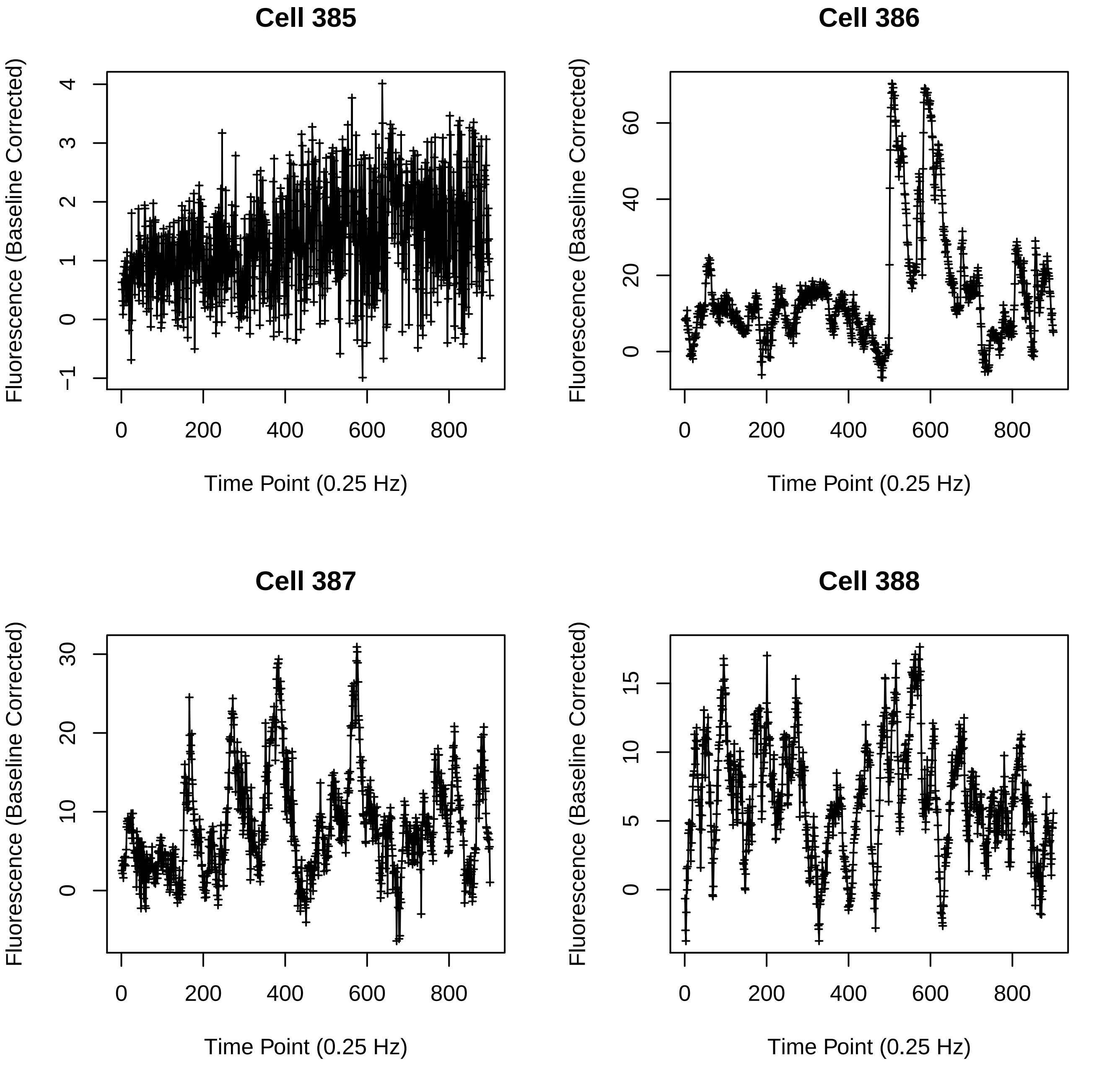
Cell 385
Cell 386
Cell 387
Cell 388
Fluorescence (Baseline Corrected)
Time Point (0.25 Hz)
−1
0
1
2
3
4
0
20
40
60
0
10
20
30
0
5
10
15
0
200
400
600
800

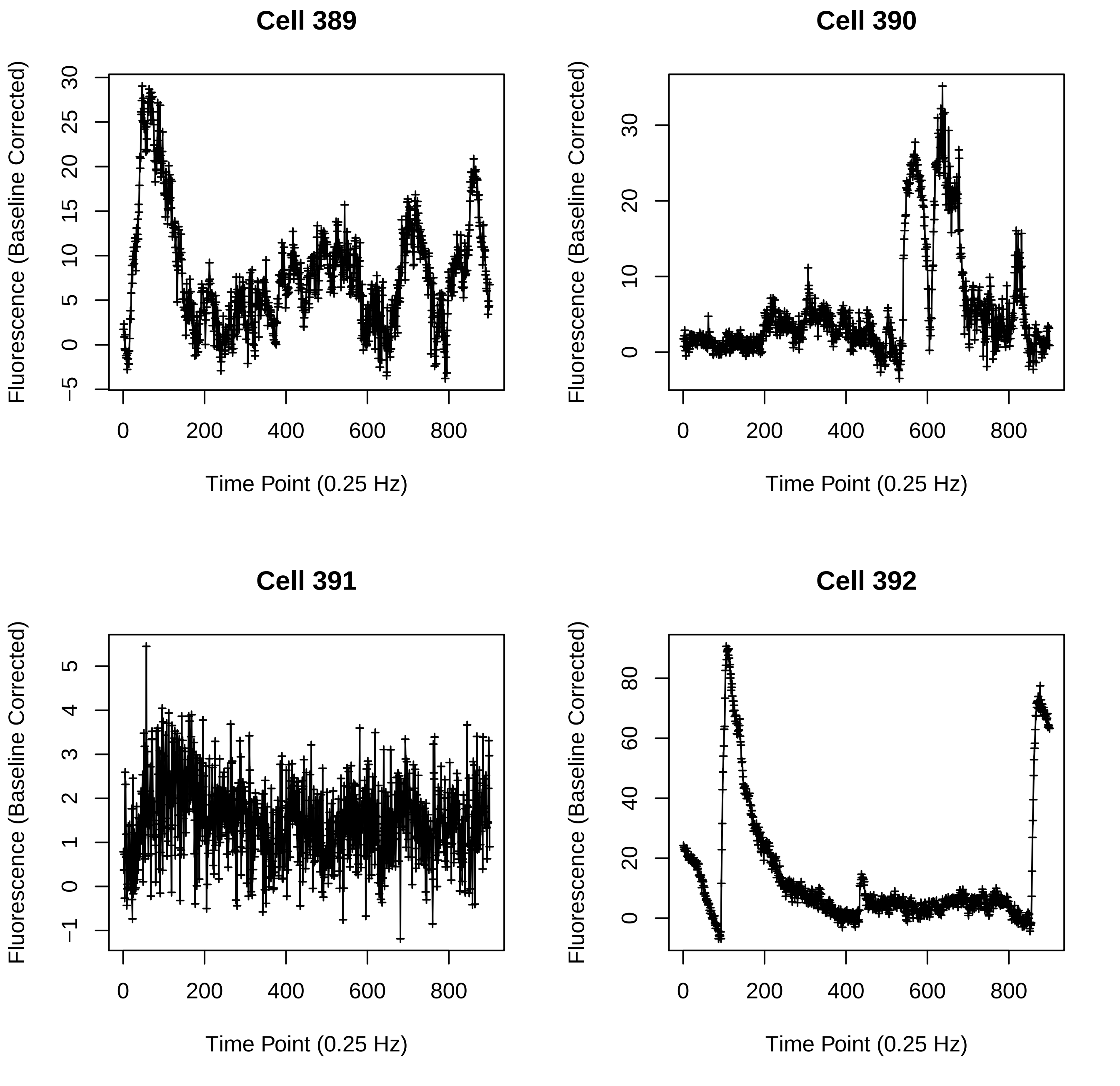
Cell 389
Cell 390
Cell 391
Cell 392
Fluorescence (Baseline Corrected)
Time Point (0.25 Hz)
−5
0
5
10
15
20
25
30
−1
1
2
3
4
40
60
80
200
400
600
800

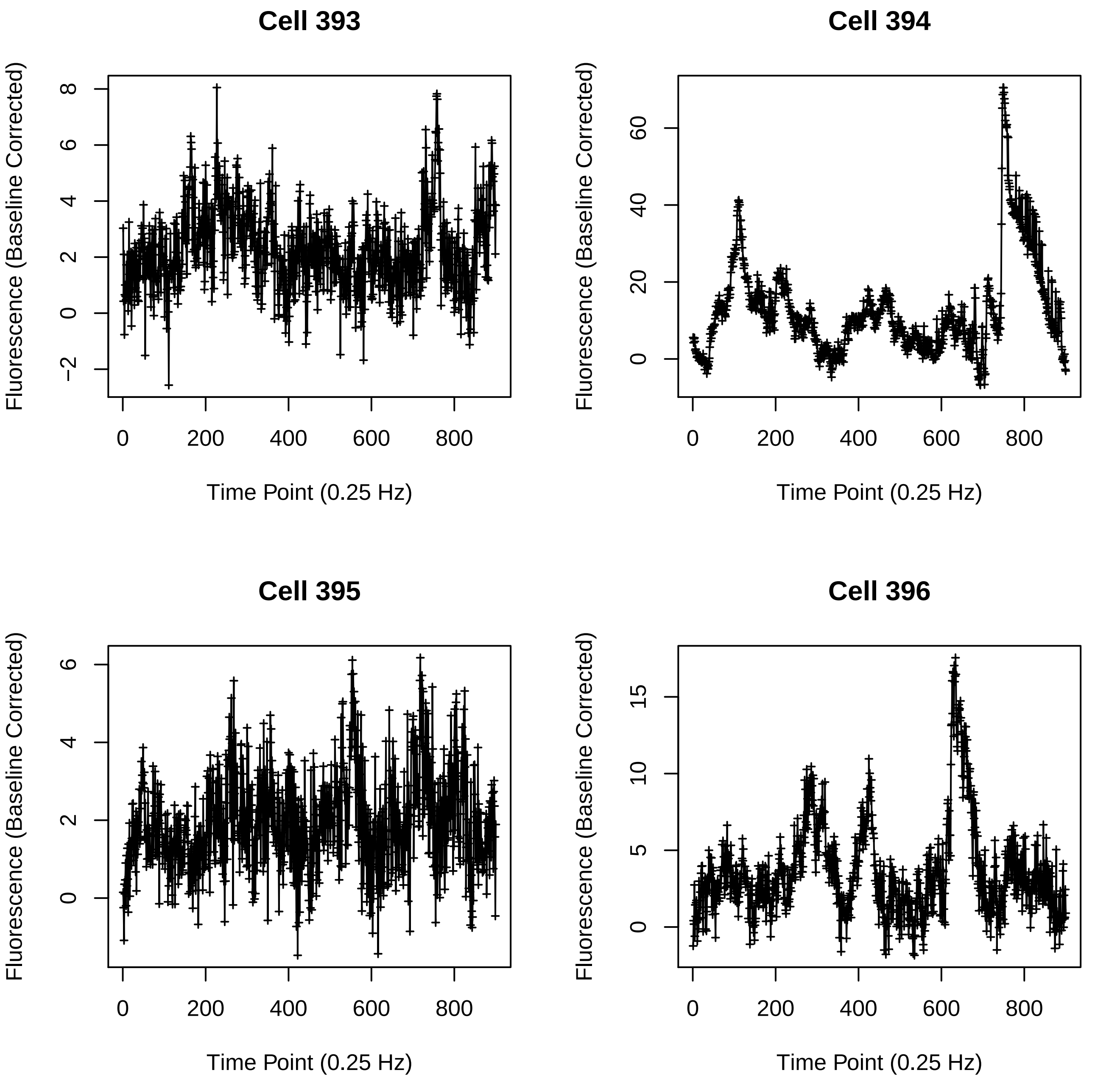
Cell 393
Cell 394
Cell 395
Cell 396
Fluorescence (Baseline Corrected)
Time Point (0.25 Hz)
−2
0
2
4
6
8
20
40
60
5
10
15
0
200
400
600
800

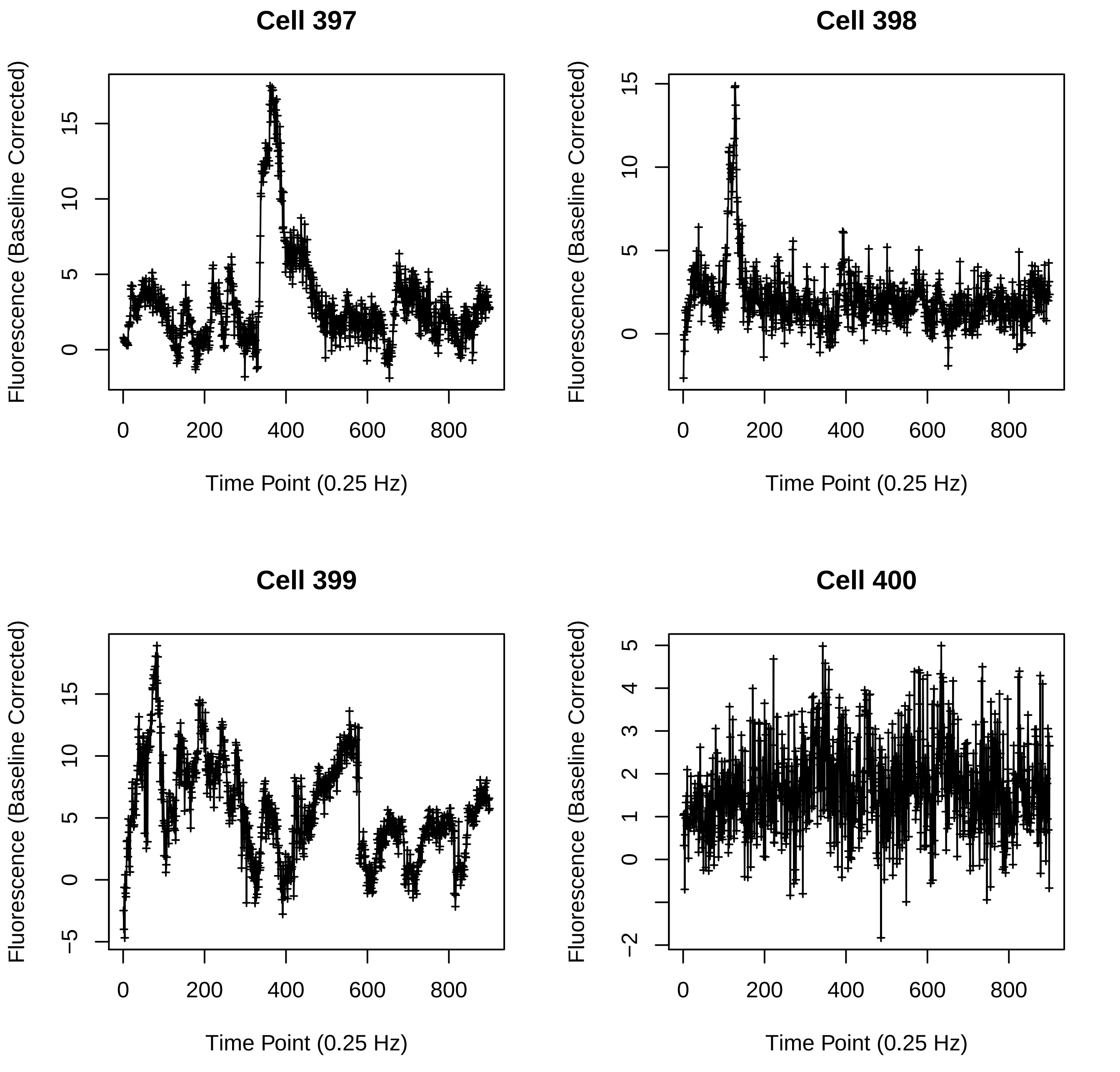
Cell 397
Cell 398
Cell 399
Cell 400
Fluorescence (Baseline Corrected)
Time Point (0.25 Hz)
0
200
400
600
800
−5
5
10
15
−2
0
1
2
3
4

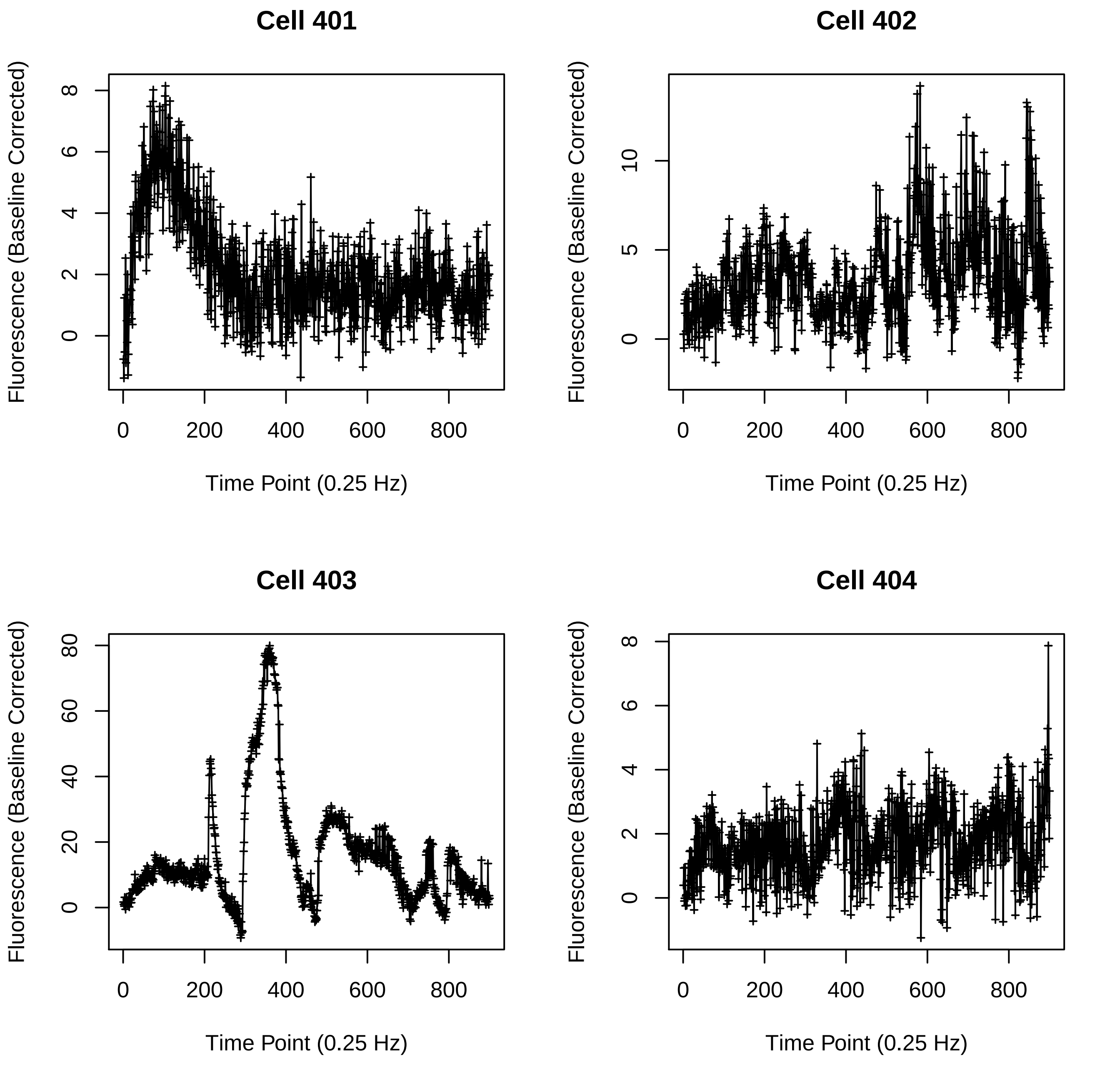
Cell 401
Fluorescence (Baseline Corrected)
0
2
4
6
8
0
200
400
600
800
Time Point (0.25 Hz)
Cell 402
Fluorescence (Baseline Corrected)
0
5
10
0
200
400
600
800
Time Point (0.25 Hz)
Cell 403
Fluorescence (Baseline Corrected)
0
20
40
60
80
0
200
400
600
800
Time Point (0.25 Hz)
Cell 404
Fluorescence (Baseline Corrected)
0
2
4
6
8
0
200
400
600
800
Time Point (0.25 Hz)

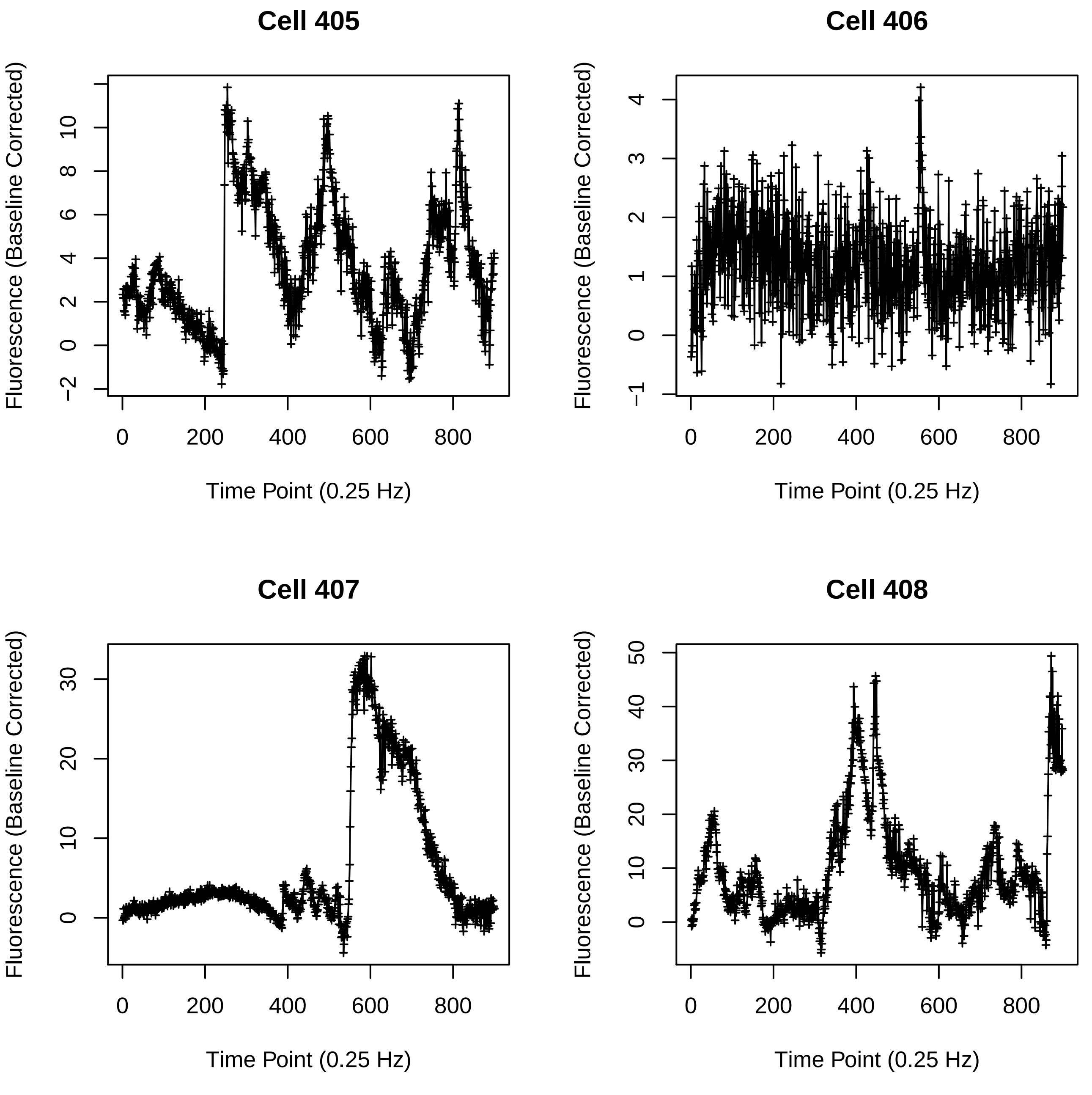
Cell 405
Cell 406
Cell 407
Cell 408
Fluorescence (Baseline Corrected)
Time Point (0.25 Hz)
0
200
400
600
800
−2
2
4
6
8
10
−1
1
3
30
20
10
50
40

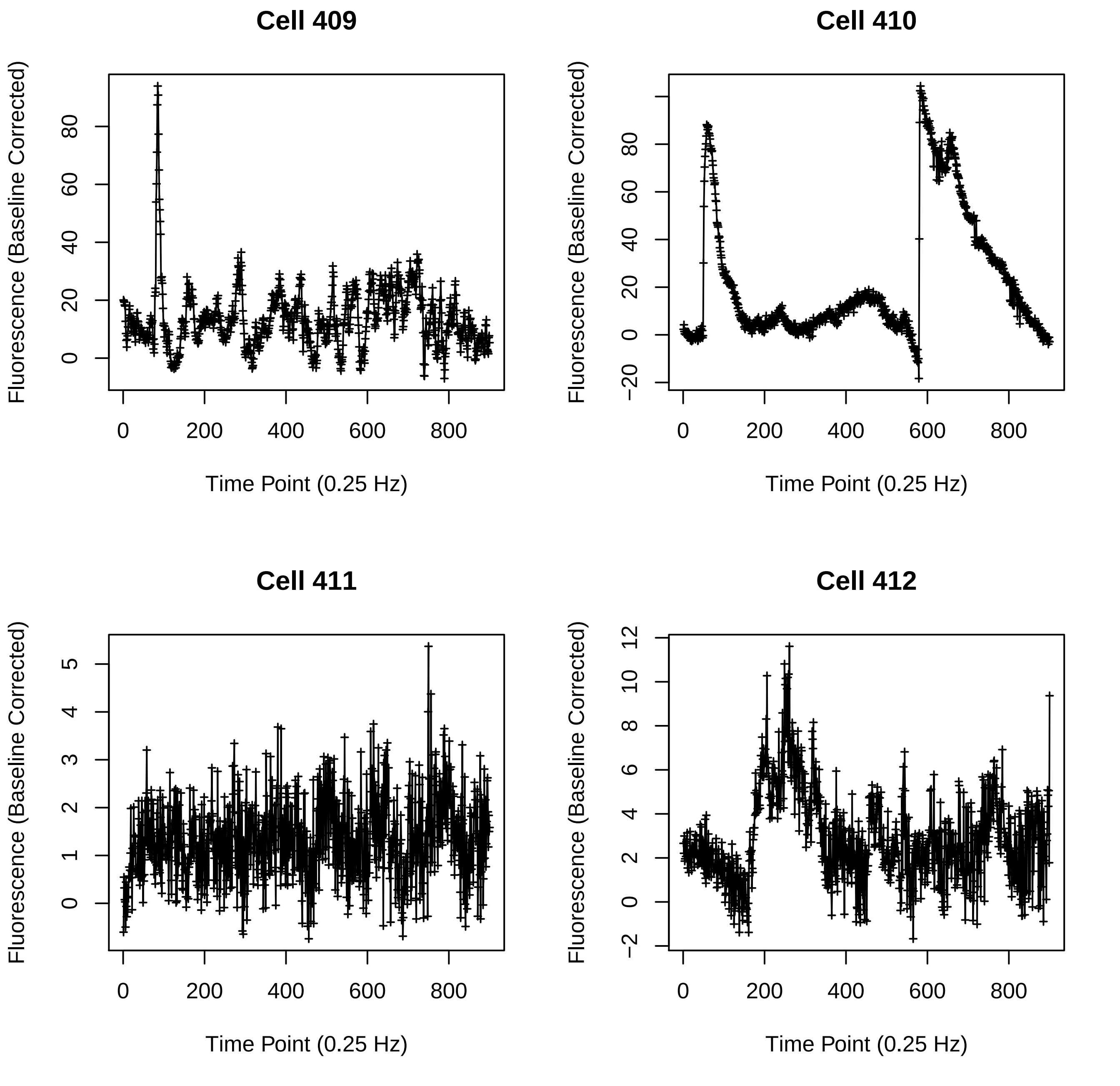
Cell 409
Cell 410
Cell 411
Cell 412
Fluorescence (Baseline Corrected)
Time Point (0.25 Hz)
0
200
400
600
800

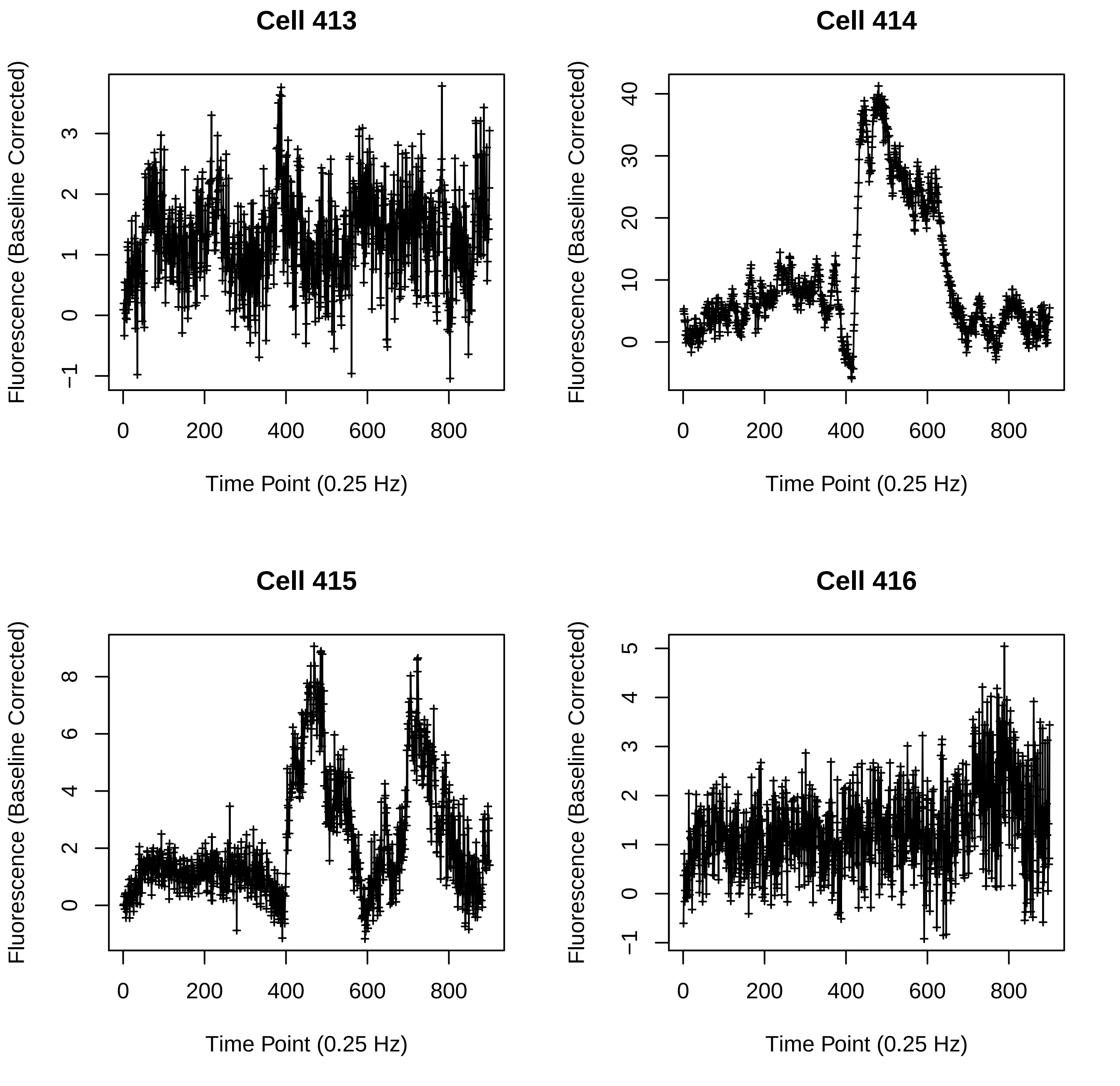
Cell 413
Cell 414
Cell 415
Cell 416
Fluorescence (Baseline Corrected)
Time Point (0.25 Hz)
0
200
400
600
800

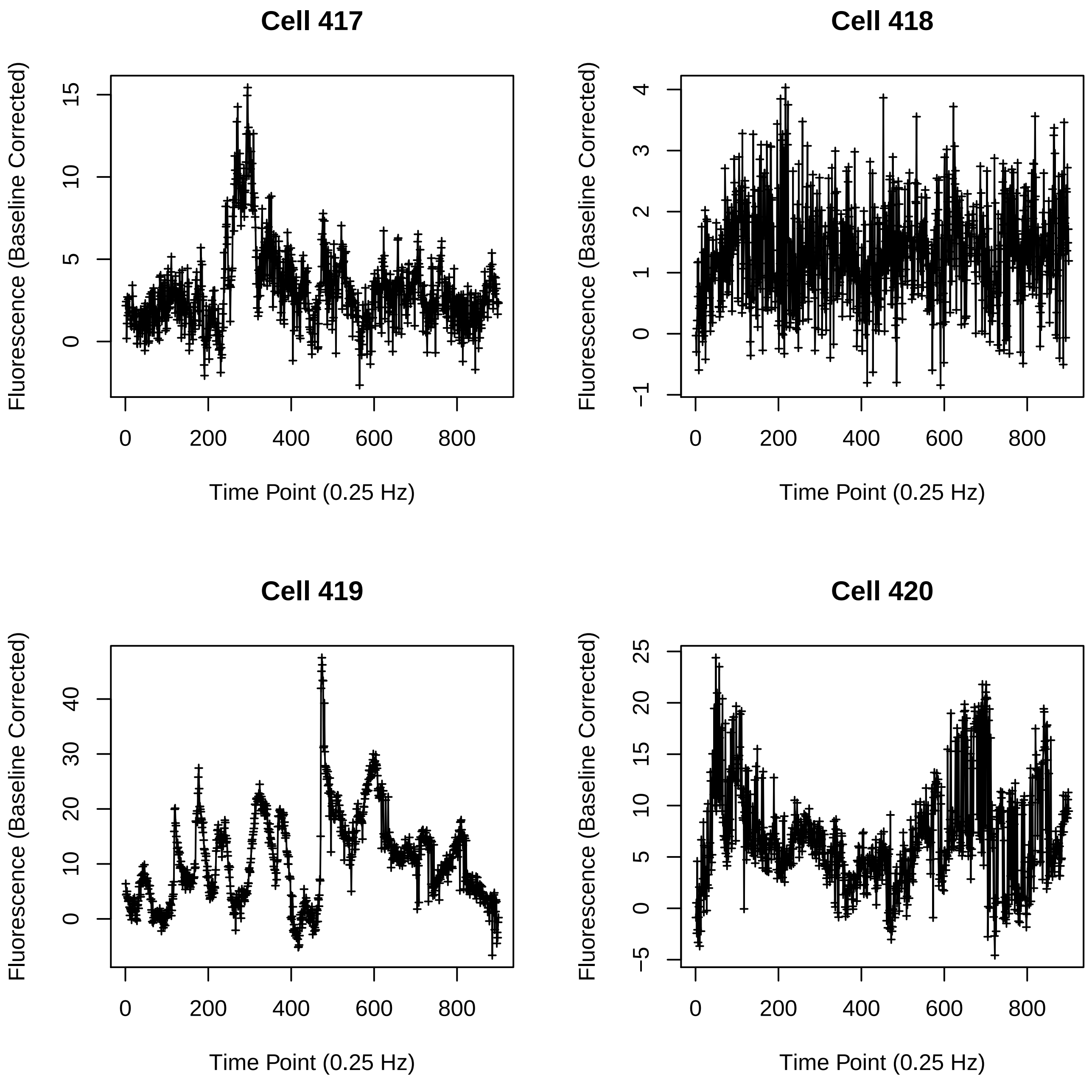
Cell 417
Cell 418
Cell 419
Cell 420
Fluorescence (Baseline Corrected)
Time Point (0.25 Hz)
0
200
400
600
800
0
5
10
15
−1
0
1
2
3
4
0
10
20
30
40
−5
0
5
10
15
20
25

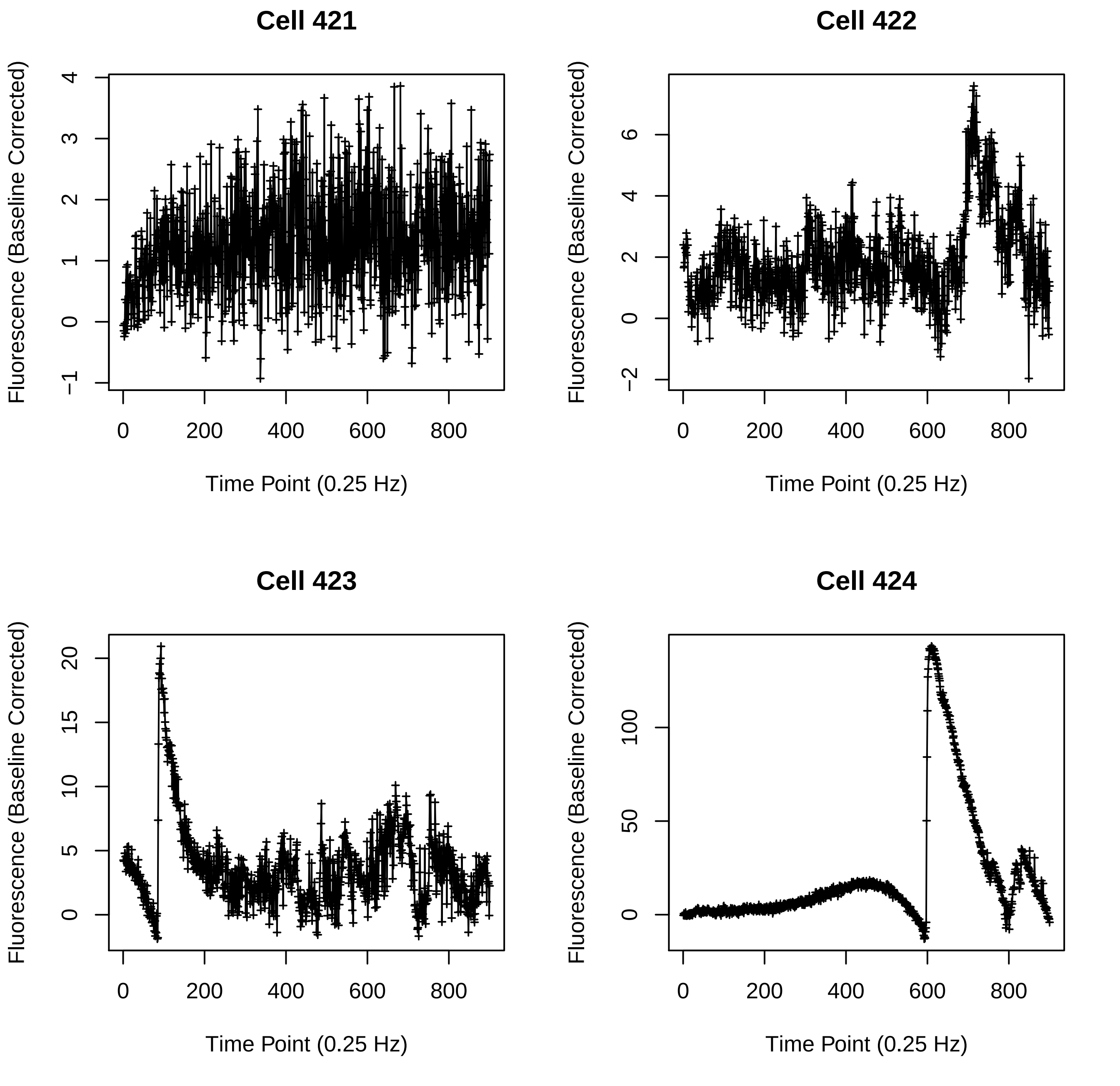

Cell 421
Cell 422
Cell 423
Cell 424
Fluorescence (Baseline Corrected)
Time Point (0.25 Hz)
−1
0
1
2
3
4
−2
6
5
10
15
20
50
100
200
400
600
800

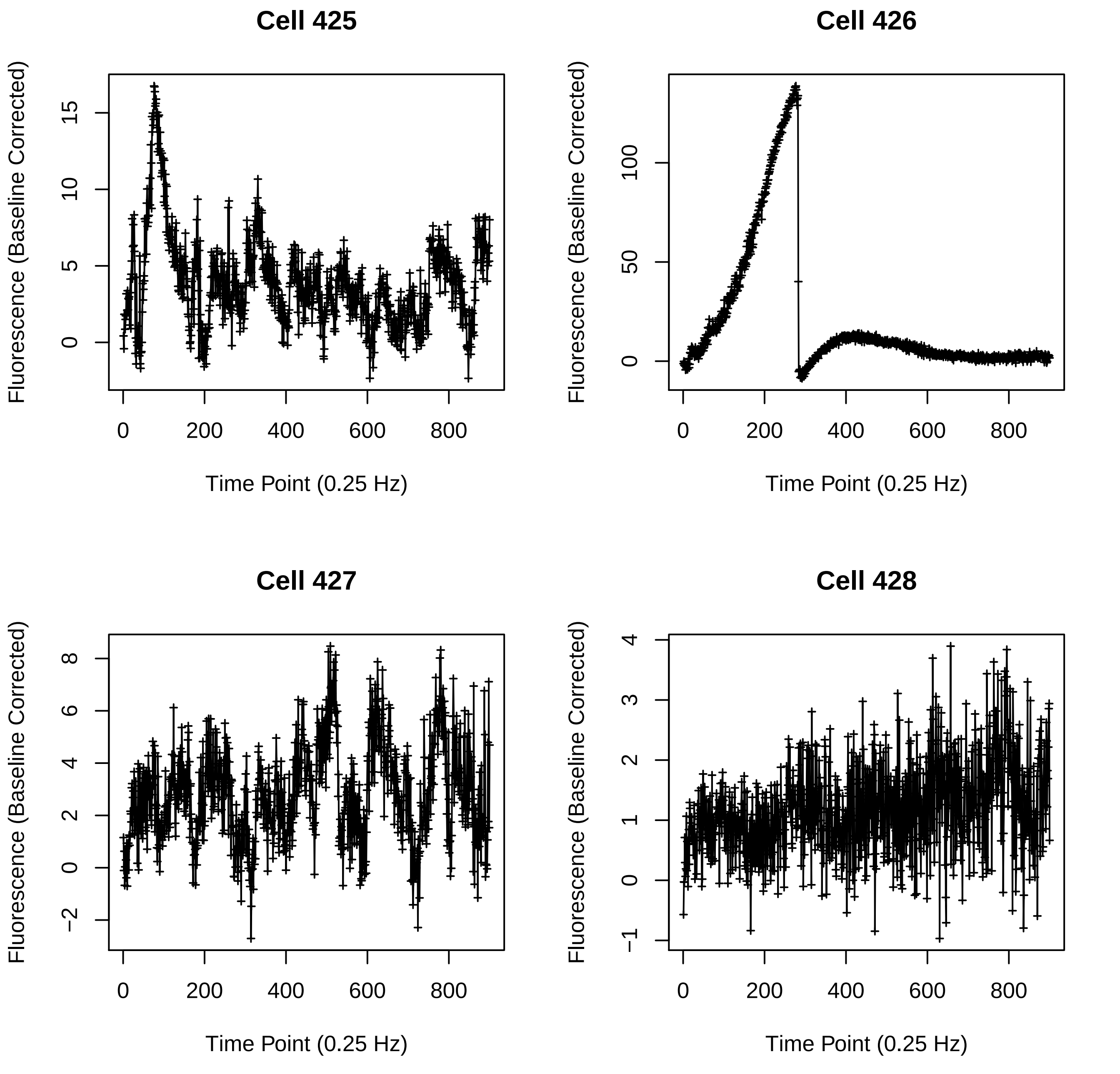
Cell 425
Cell 426
Cell 427
Cell 428
Fluorescence (Baseline Corrected)
Time Point (0.25 Hz)
0
200
400
600
800
-2
2
4
5
6
8
10
15
50
100
-1
1
3

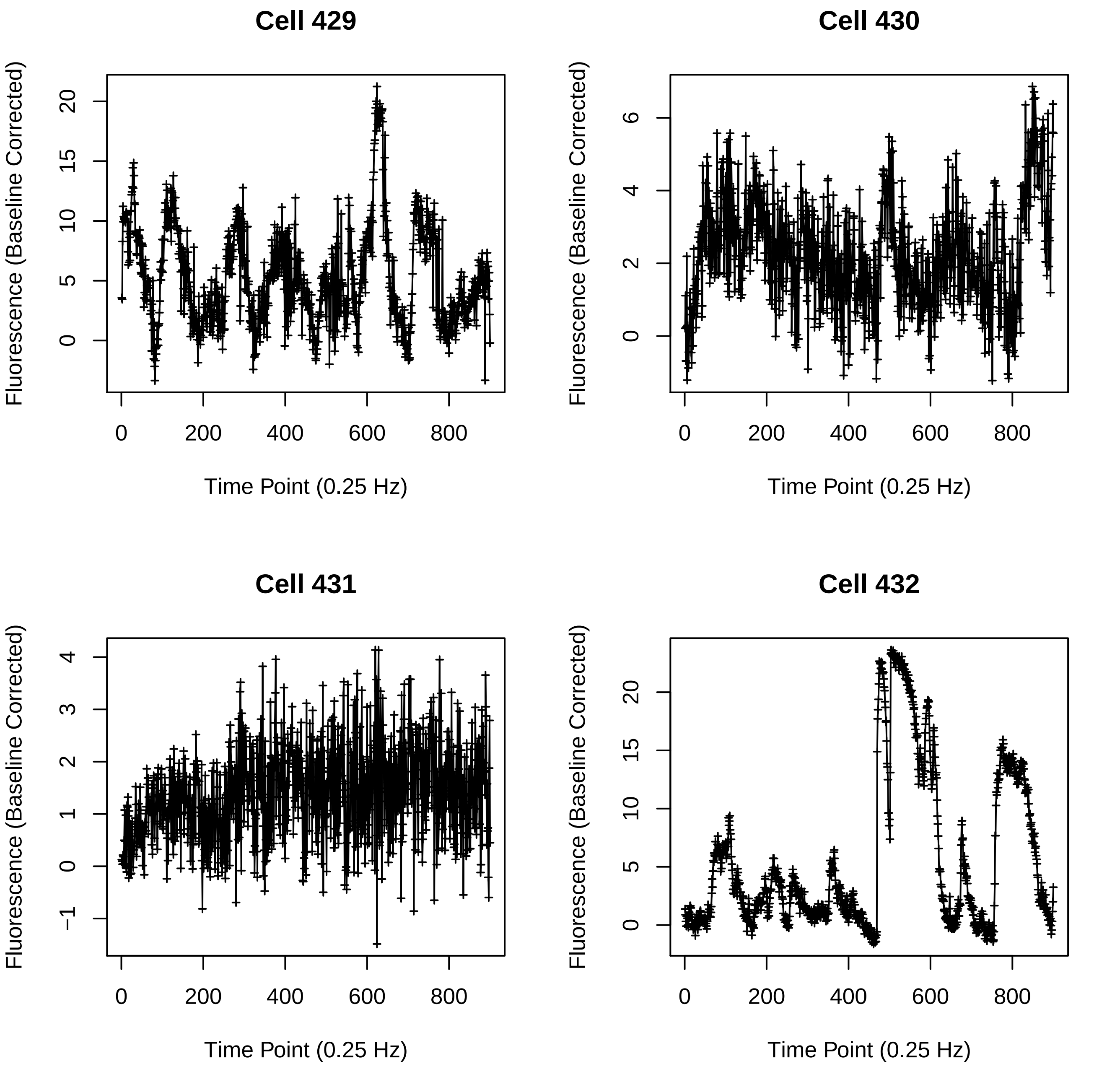
Cell 429
Fluorescence (Baseline Corrected)
Time Point (0.25 Hz)
Cell 430
Fluorescence (Baseline Corrected)
Time Point (0.25 Hz)
Cell 431
Fluorescence (Baseline Corrected)
Time Point (0.25 Hz)
Cell 432
Fluorescence (Baseline Corrected)
Time Point (0.25 Hz)

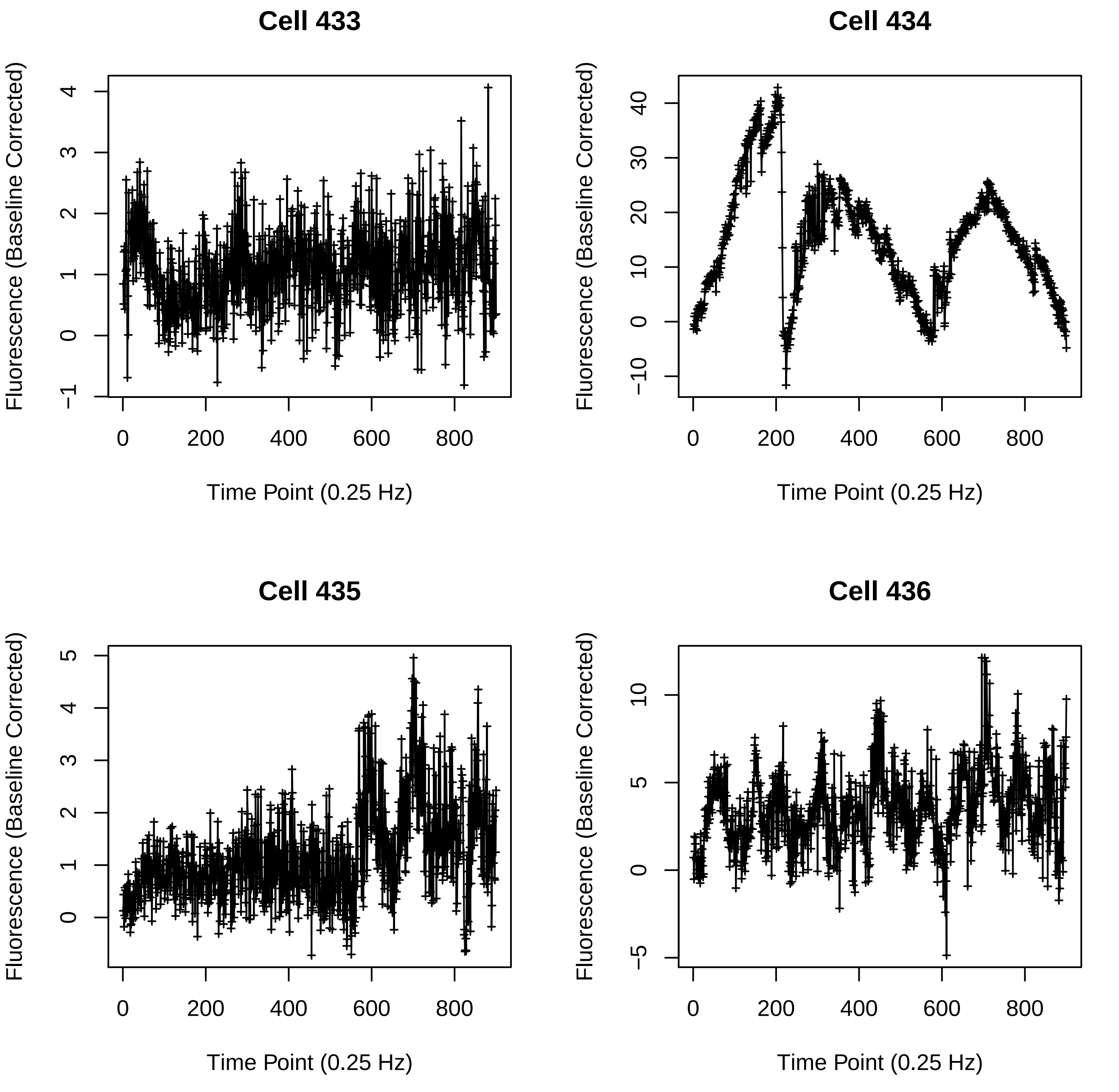
Cell 433
Fluorescence (Baseline Corrected)
Time Point (0.25 Hz)
Cell 434
Fluorescence (Baseline Corrected)
Time Point (0.25 Hz)
Cell 435
Fluorescence (Baseline Corrected)
Time Point (0.25 Hz)
Cell 436
Fluorescence (Baseline Corrected)
Time Point (0.25 Hz)

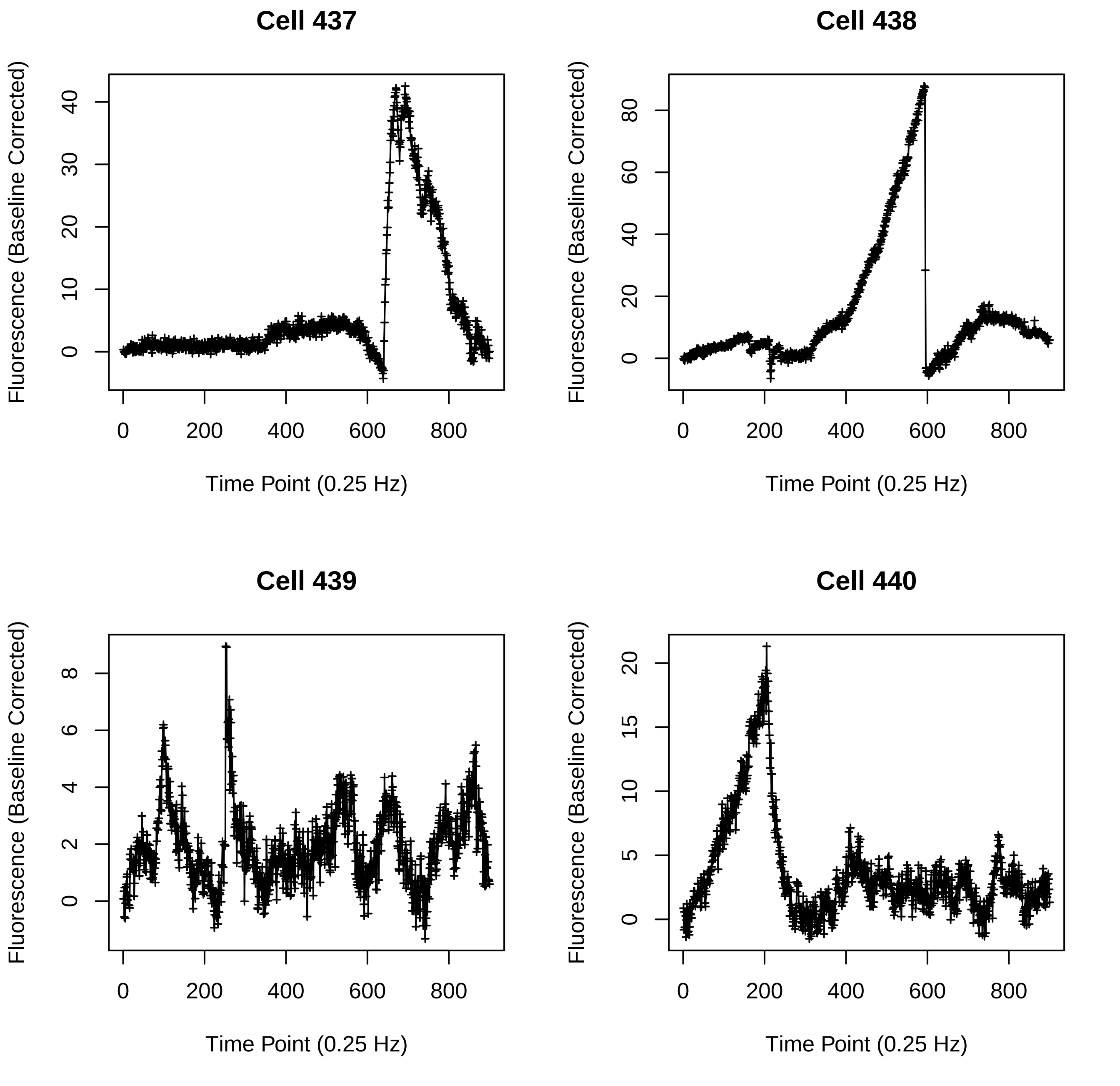

Cell 437
Cell 438
Cell 439
Cell 440
Fluorescence (Baseline Corrected)
Time Point (0.25 Hz)

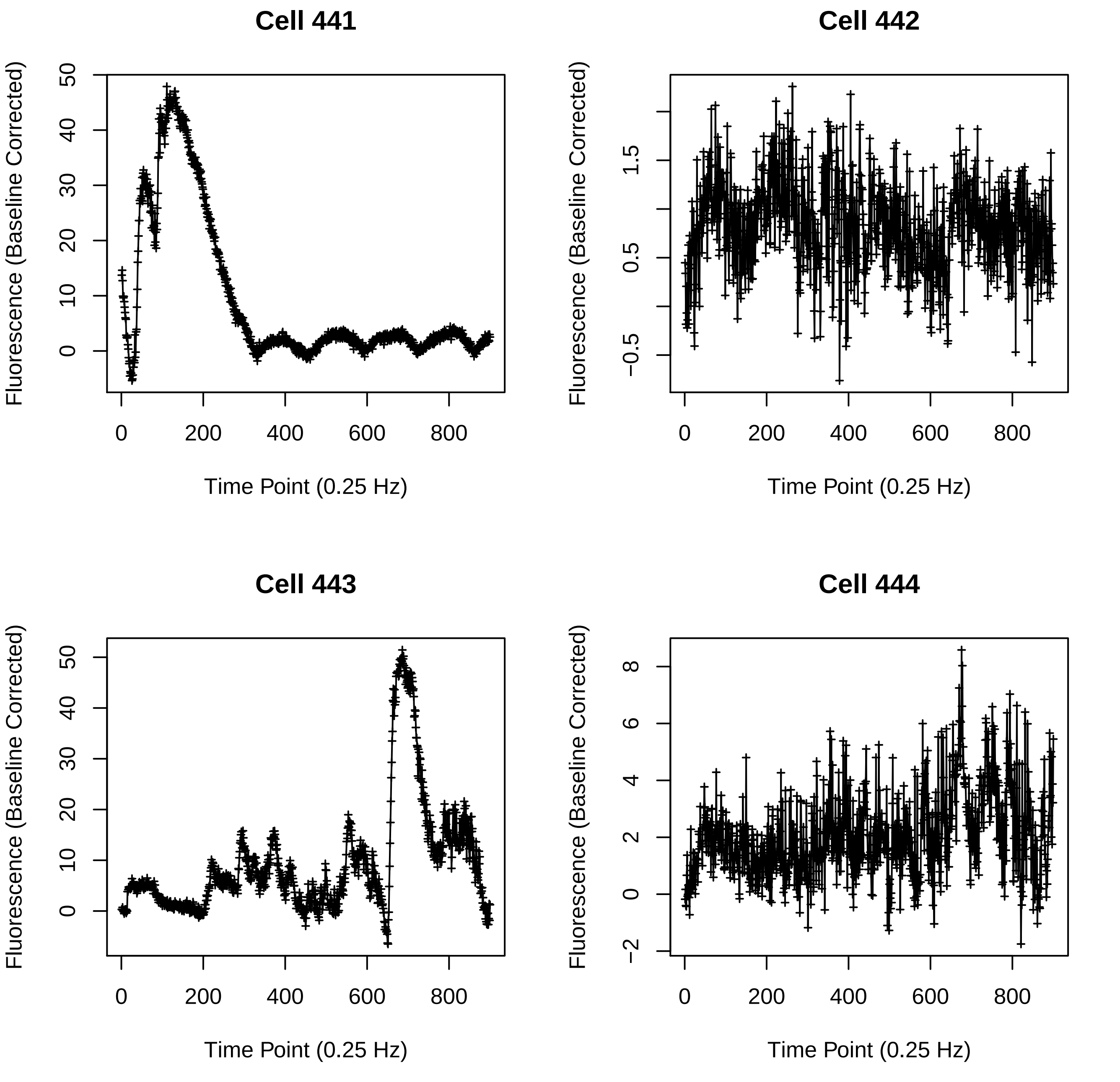
Cell 441
Fluorescence (Baseline Corrected)
0
10
20
30
40
50
0
200
400
600
800
Time Point (0.25 Hz)
Cell 442
Fluorescence (Baseline Corrected)
−0.5
0.5
1.5
0
200
400
600
800
Time Point (0.25 Hz)
Cell 443
Fluorescence (Baseline Corrected)
0
10
20
30
40
50
0
200
400
600
800
Time Point (0.25 Hz)
Cell 444
Fluorescence (Baseline Corrected)
−2
0
2
4
6
8
0
200
400
600
800
Time Point (0.25 Hz)

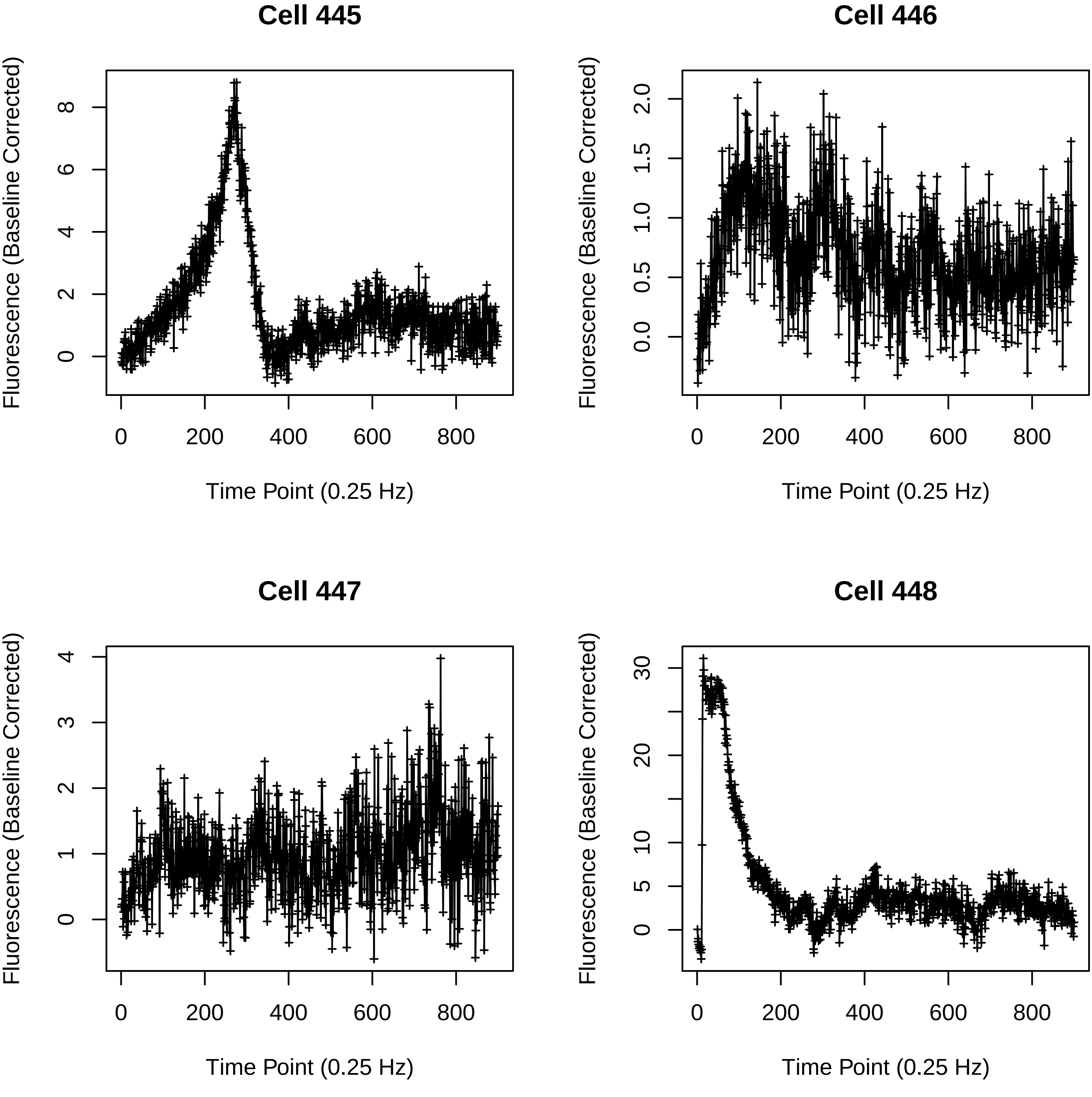
Cell 445
Cell 446
Cell 447
Cell 448
Fluorescence (Baseline Corrected)
Time Point (0.25 Hz)
0
200
400
600
800
0
2
4
6
8
0.0
0.5
1.0
1.5
2.0
0
1
2
3
4
0
5
10
20
30

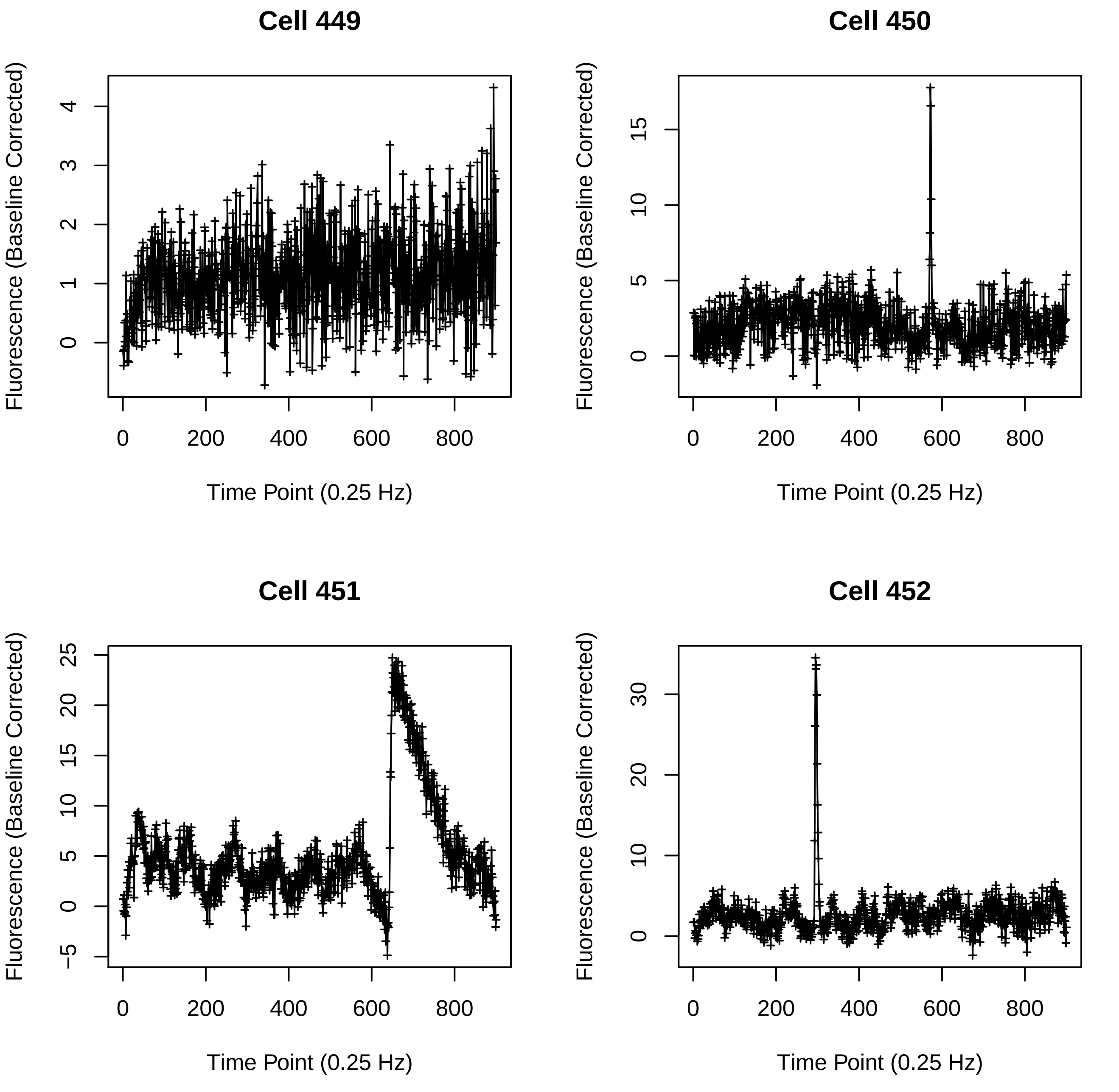
Cell 449
Fluorescence (Baseline Corrected)
0
1
2
3
4
0
200
400
600
800
Time Point (0.25 Hz)
Cell 450
Fluorescence (Baseline Corrected)
0
5
10
15
0
200
400
600
800
Time Point (0.25 Hz)
Cell 451
Fluorescence (Baseline Corrected)
−5
0
5
10
15
20
25
0
200
400
600
800
Time Point (0.25 Hz)
Cell 452
Fluorescence (Baseline Corrected)
0
10
20
30
0
200
400
600
800
Time Point (0.25 Hz)

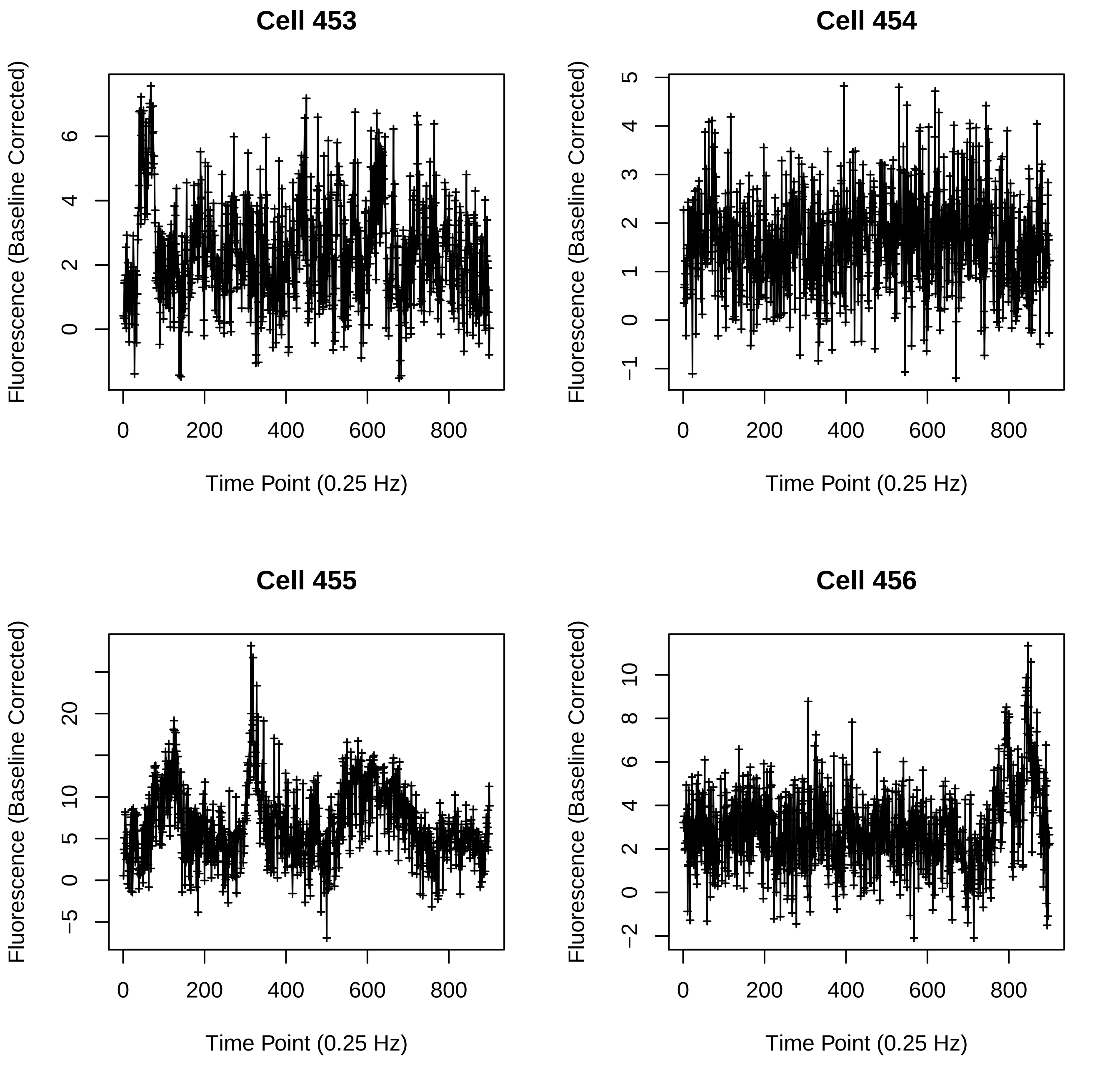
Cell 453
Cell 454
Cell 455
Cell 456
Fluorescence (Baseline Corrected)
Time Point (0.25 Hz)
0
200
400
600
800
−1
2
4
6
8
10
−2
−5
5
20
1
3

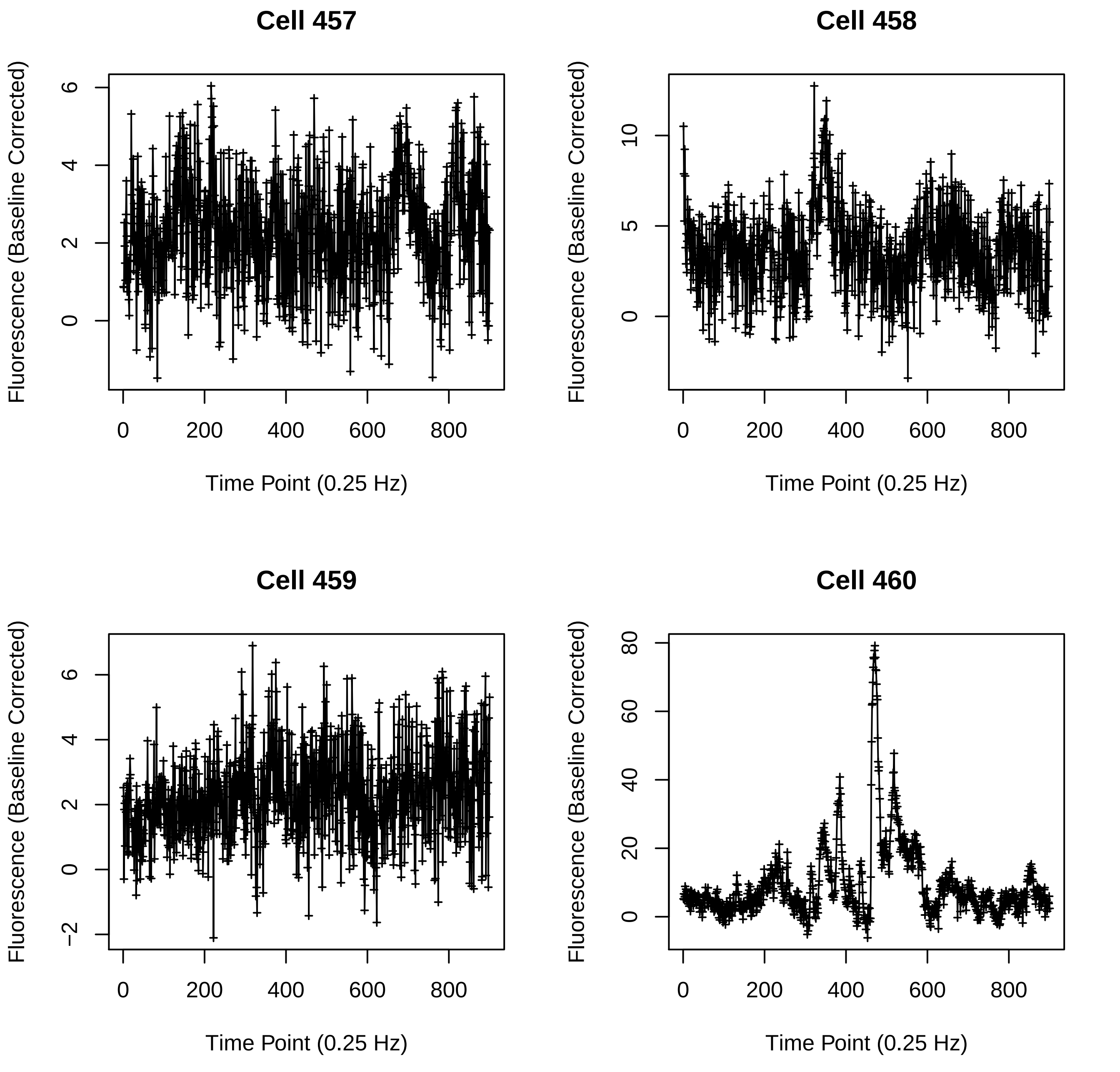
Cell 457
Cell 458
Cell 459
Cell 460
Fluorescence (Baseline Corrected)
Time Point (0.25 Hz)
0
200
400
600
800
−2
2
4
6
5
10
20
40
60
80

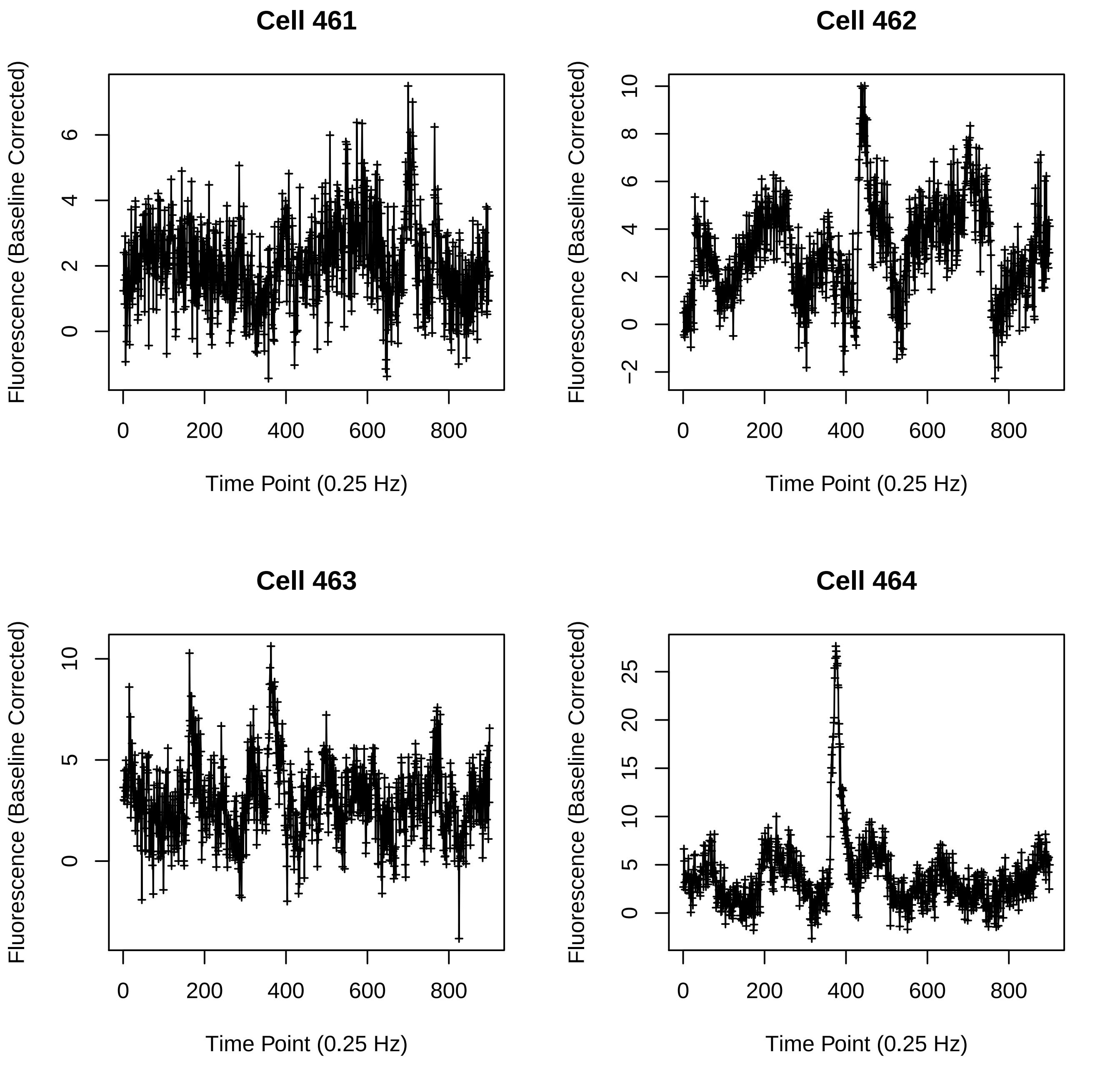
Cell 461
Cell 462
Cell 463
Cell 464
Fluorescence (Baseline Corrected)
Time Point (0.25 Hz)
0
200
400
600
800
0
2
4
6
−2
8
10
5
15
20
25

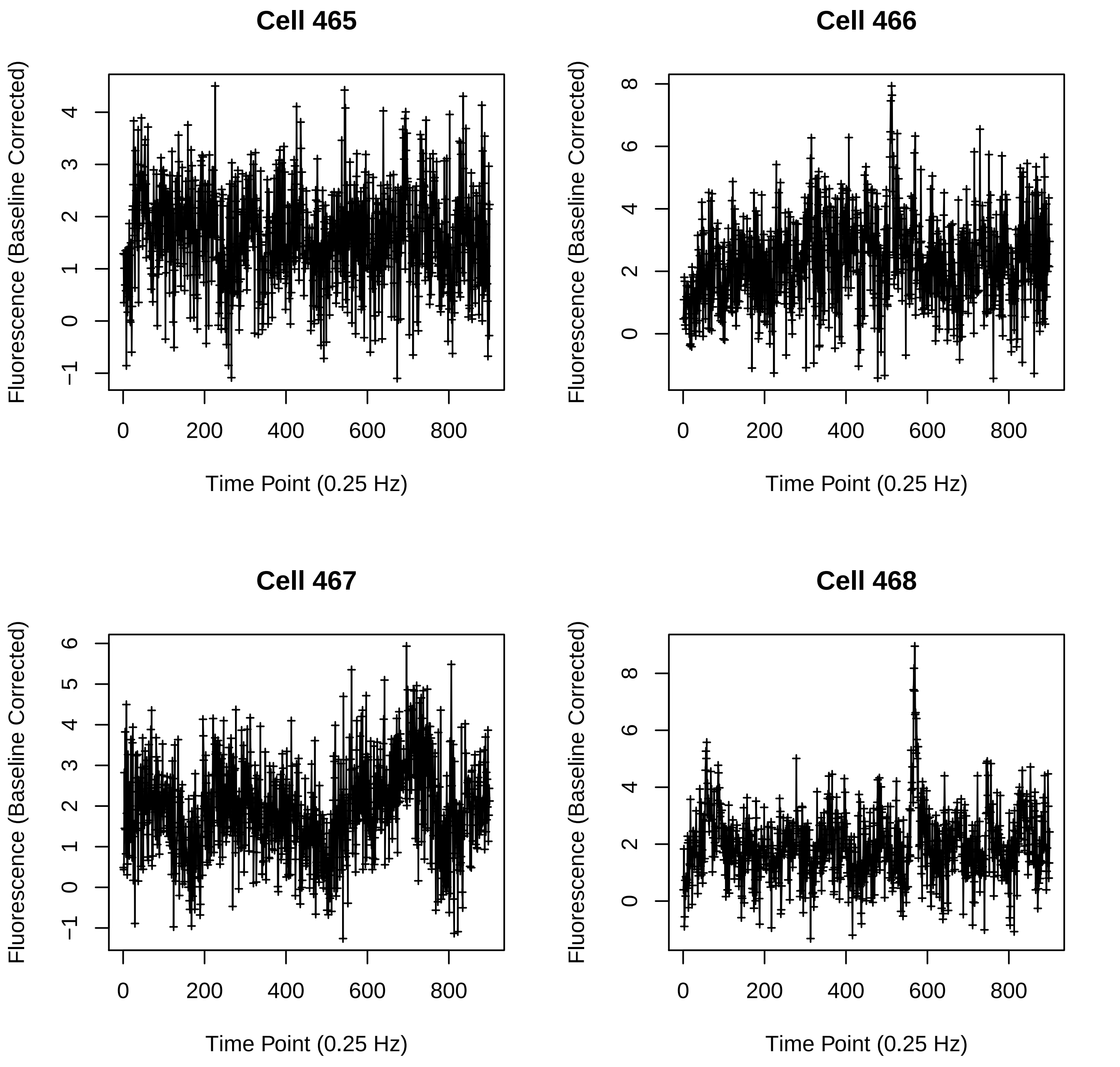

Cell 465
Cell 466
Cell 467
Cell 468
Fluorescence (Baseline Corrected)
Time Point (0.25 Hz)
0
200
400
600
800
−1
0
1
2
3
4
5
6
8

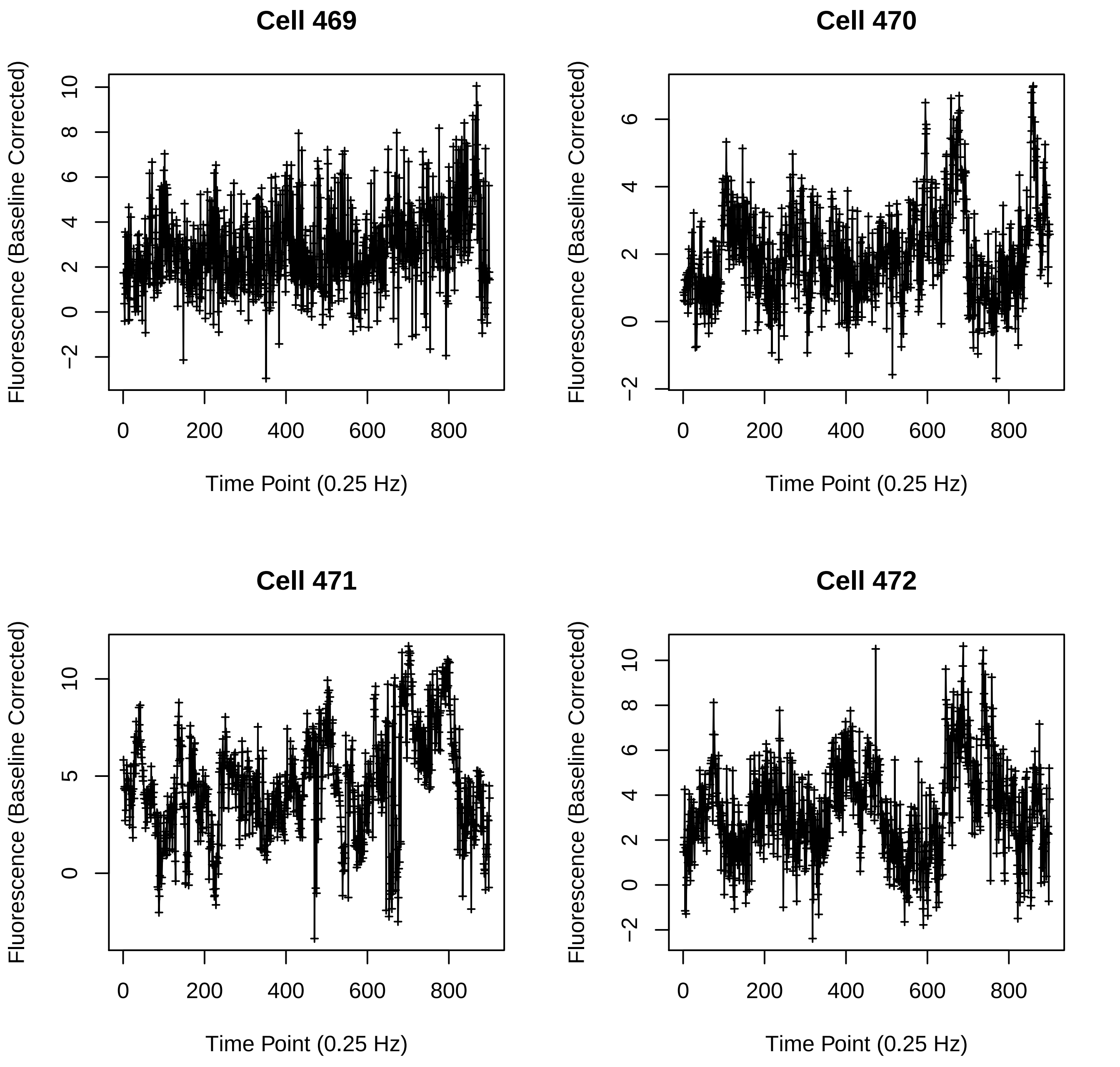

Cell 469
Cell 470
Cell 471
Cell 472
Fluorescence (Baseline Corrected)
Time Point (0.25 Hz)

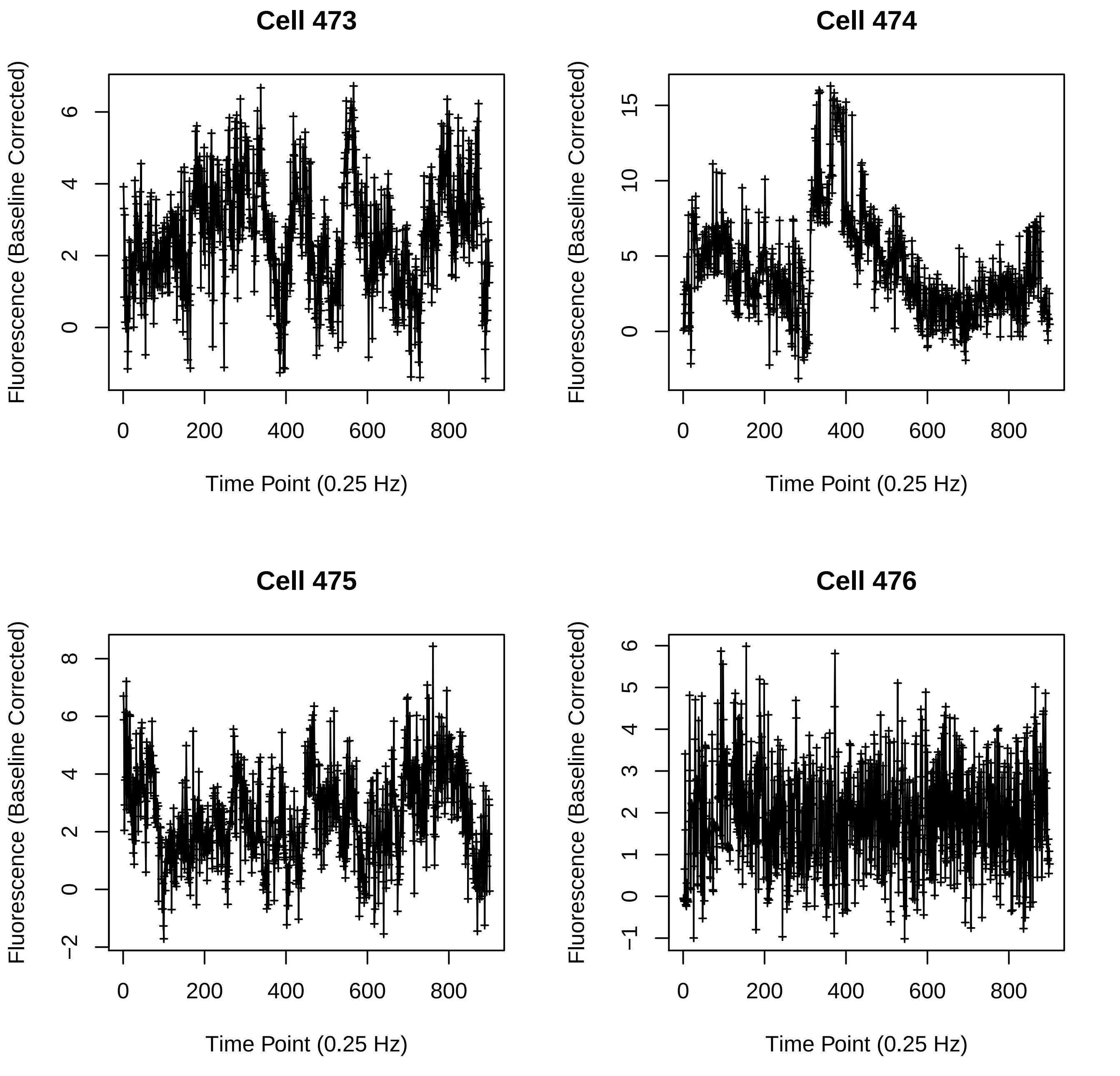

Cell 473
Cell 474
Cell 475
Cell 476
Fluorescence (Baseline Corrected)
Time Point (0.25 Hz)
0
200
400
600
800

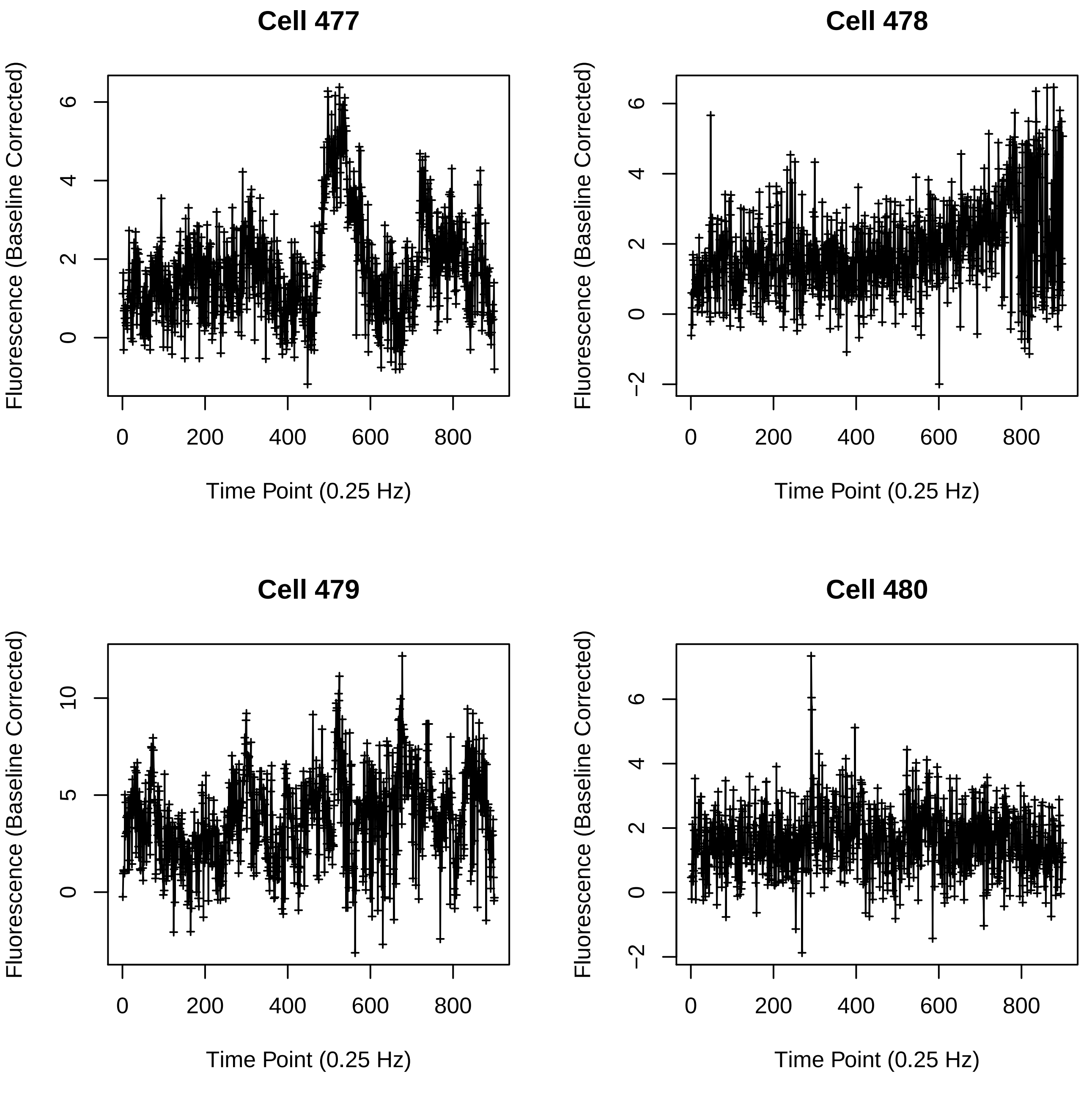
Cell 477
Cell 478
Cell 479
Cell 480
Fluorescence (Baseline Corrected)
Time Point (0.25 Hz)
0
200
400
600
800
−2
0
2
4
6
5
10

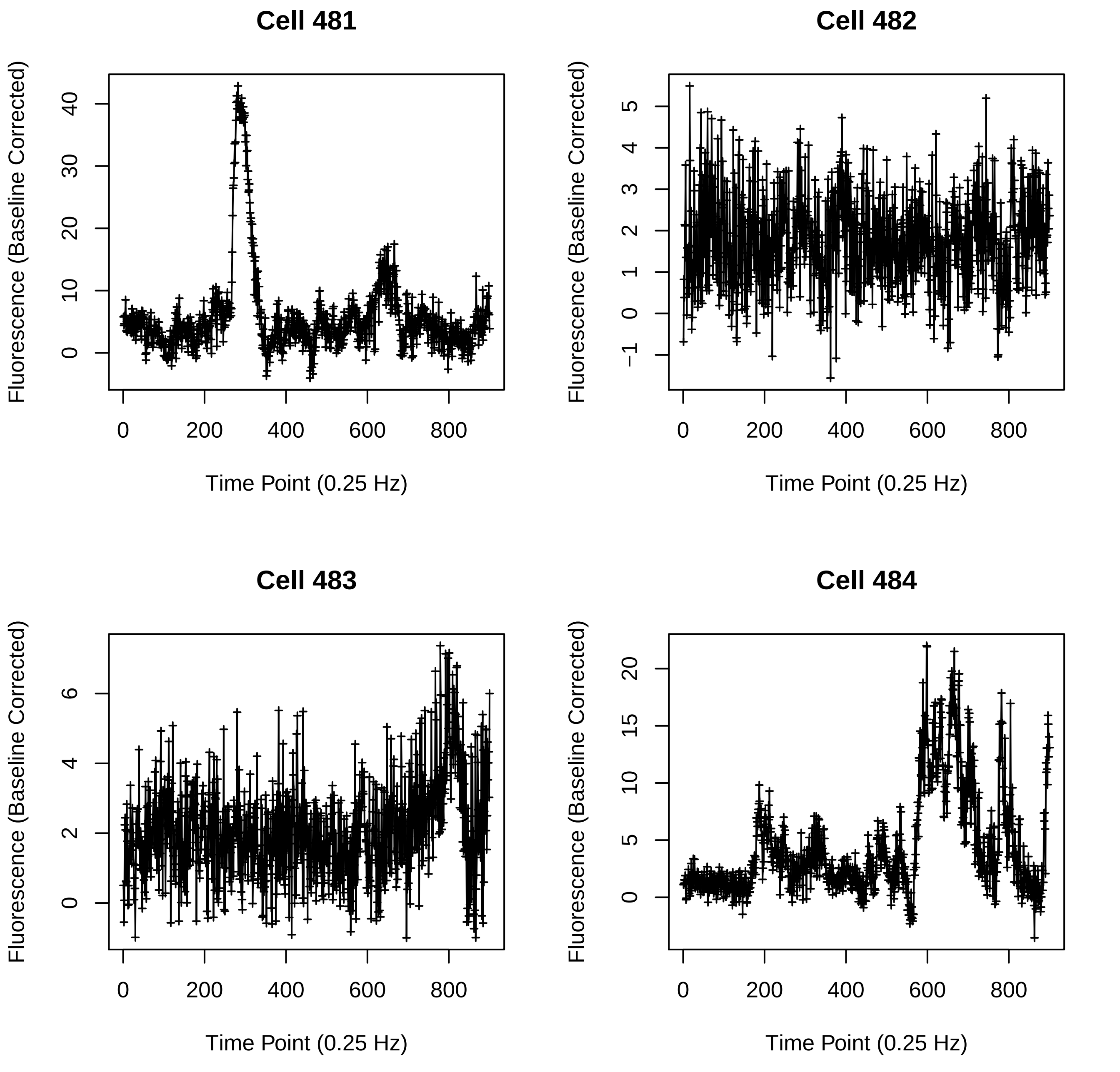
Cell 481
Cell 482
Cell 483
Cell 484
Fluorescence (Baseline Corrected)
Time Point (0.25 Hz)
0
200
400
600
800
0
10
20
30
40
−1
1
2
3
4
5
6
15

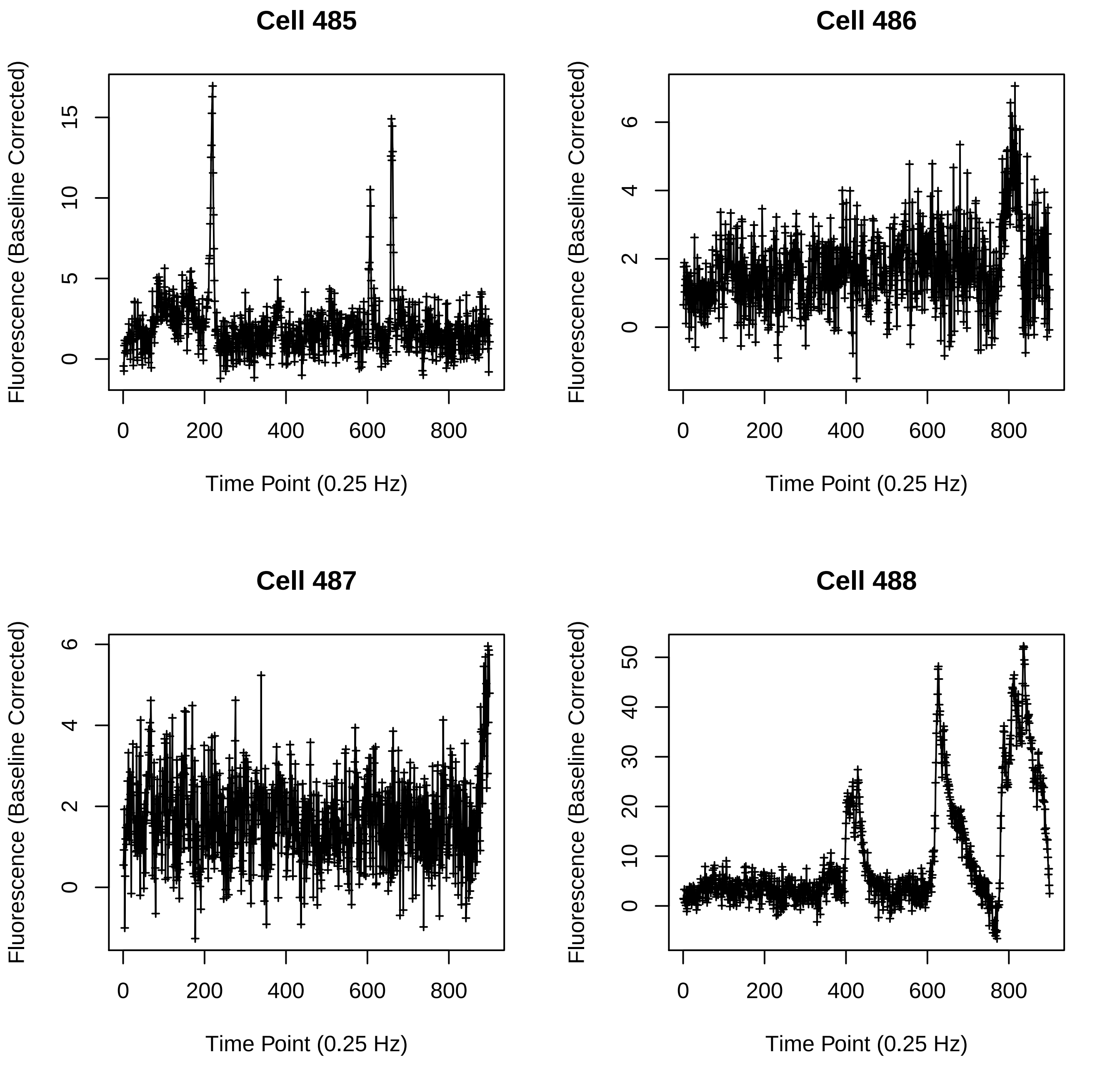

Cell 485
Fluorescence (Baseline Corrected)
0
5
10
15
0
200
400
600
800
Time Point (0.25 Hz)
Cell 486
Fluorescence (Baseline Corrected)
0
2
4
6
0
200
400
600
800
Time Point (0.25 Hz)
Cell 487
Fluorescence (Baseline Corrected)
0
2
4
6
0
200
400
600
800
Time Point (0.25 Hz)
Cell 488
Fluorescence (Baseline Corrected)
0
10
20
30
40
50
0
200
400
600
800
Time Point (0.25 Hz)

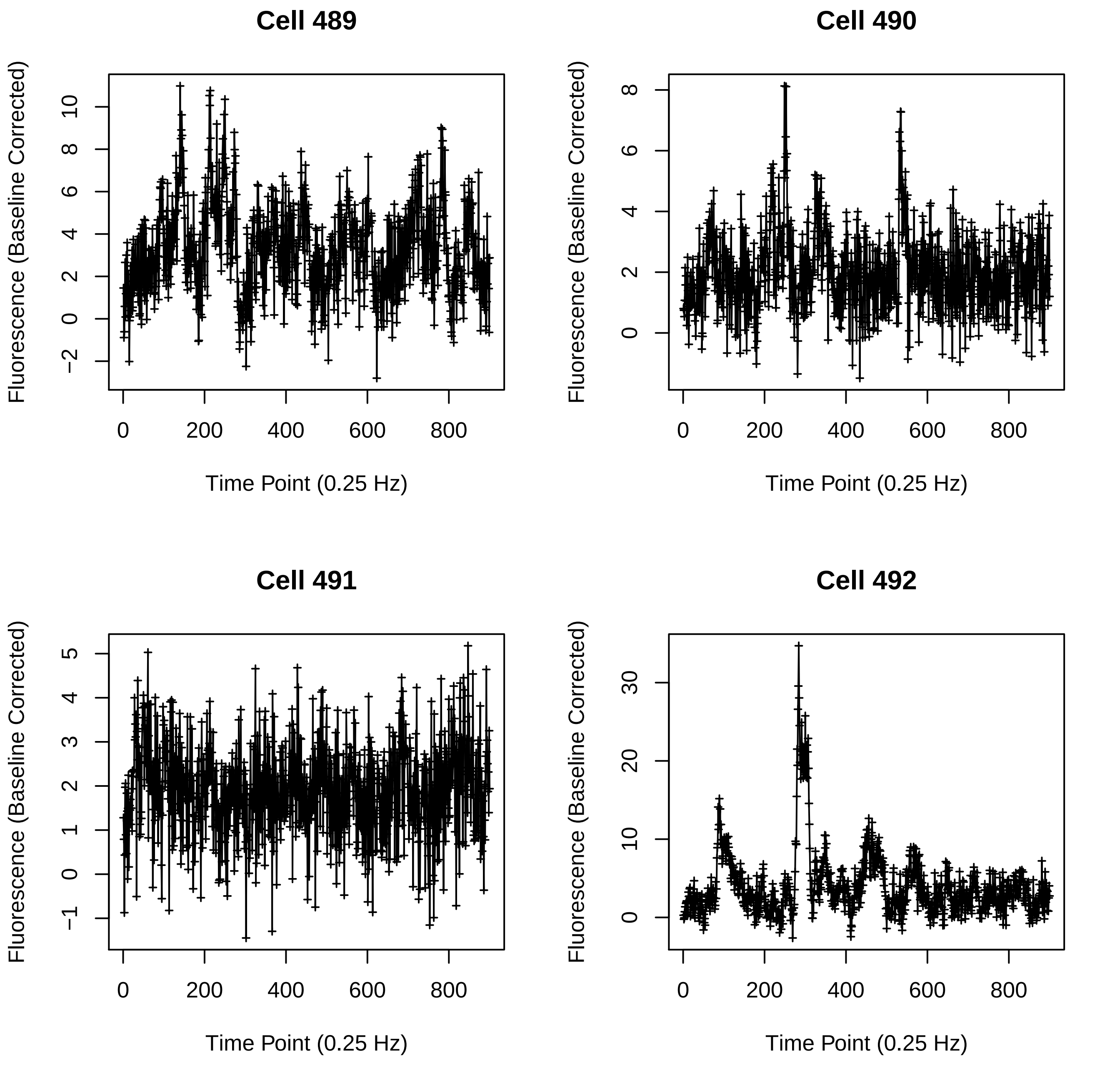
Cell 489
Cell 490
Cell 491
Cell 492
Fluorescence (Baseline Corrected)
Time Point (0.25 Hz)

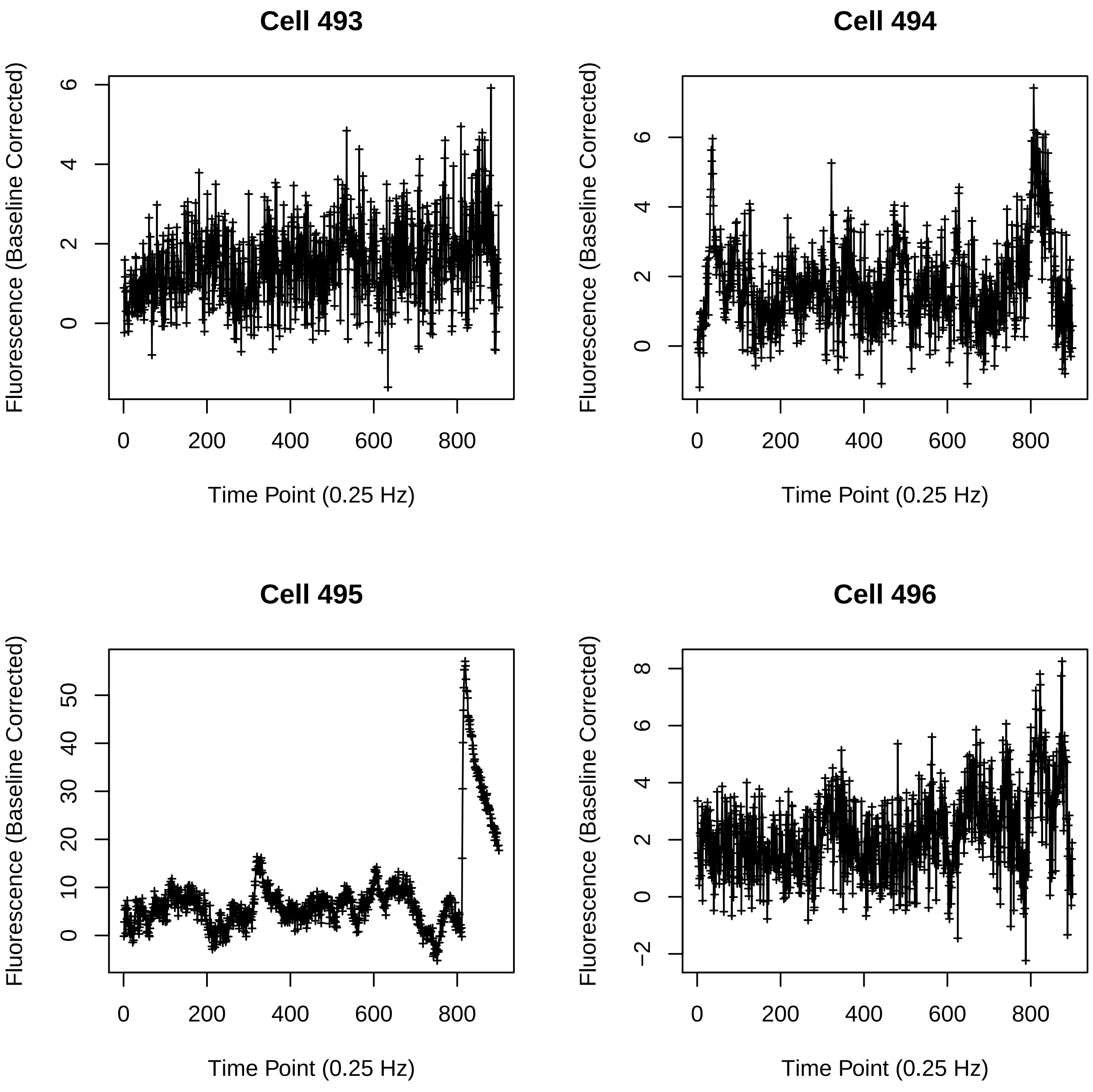
Cell 493
Cell 494
Cell 495
Cell 496
Fluorescence (Baseline Corrected)
Time Point (0.25 Hz)
0
200
400
600
800
−2
0
2
4
6
8
10
20
30
40
50

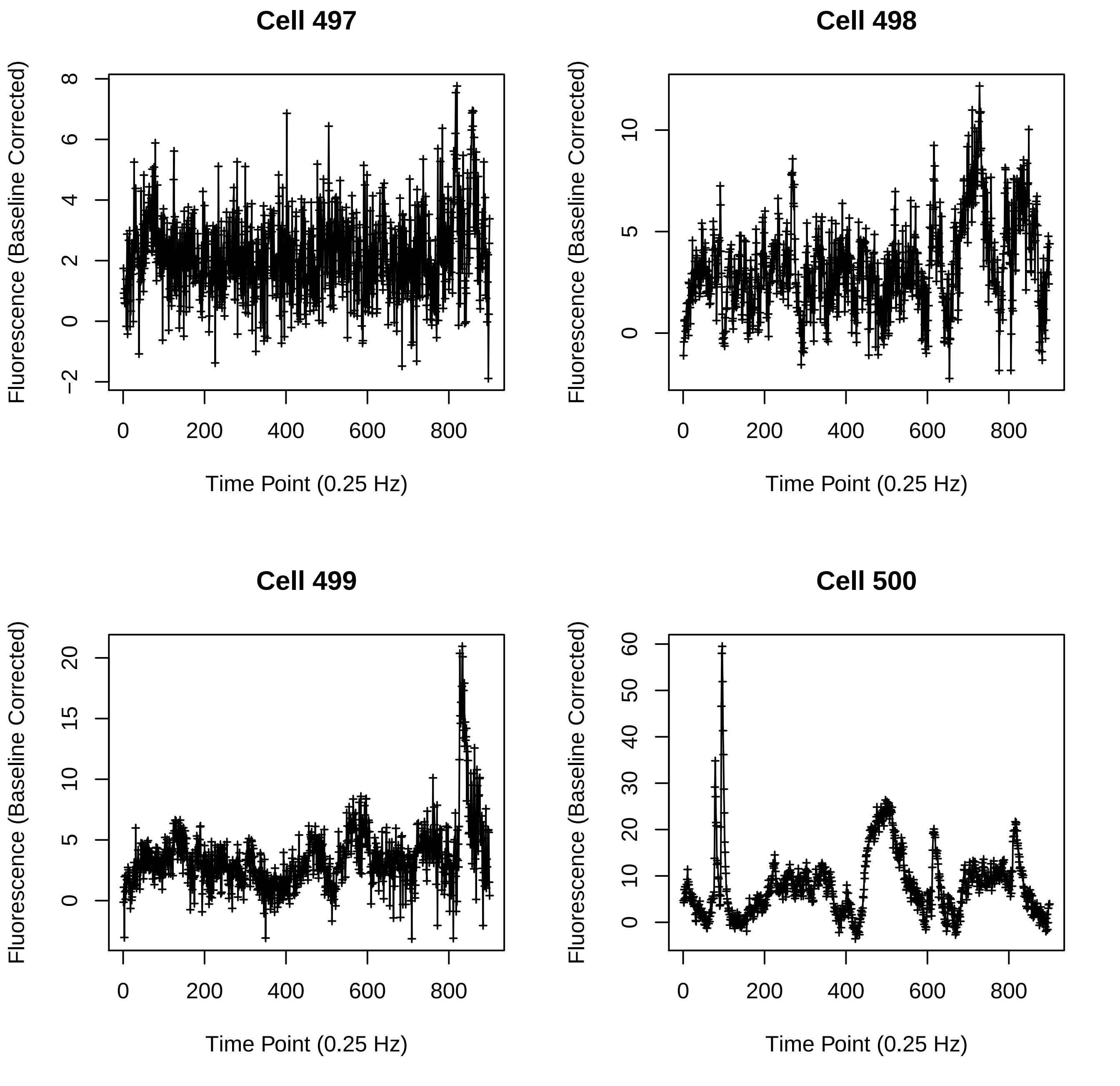
Cell 497
Cell 498
Cell 499
Cell 500
Fluorescence (Baseline Corrected)
Time Point (0.25 Hz)
0
200
400
600
800
−2
0
2
4
6
8
5
10
15
20
30
40
50
60

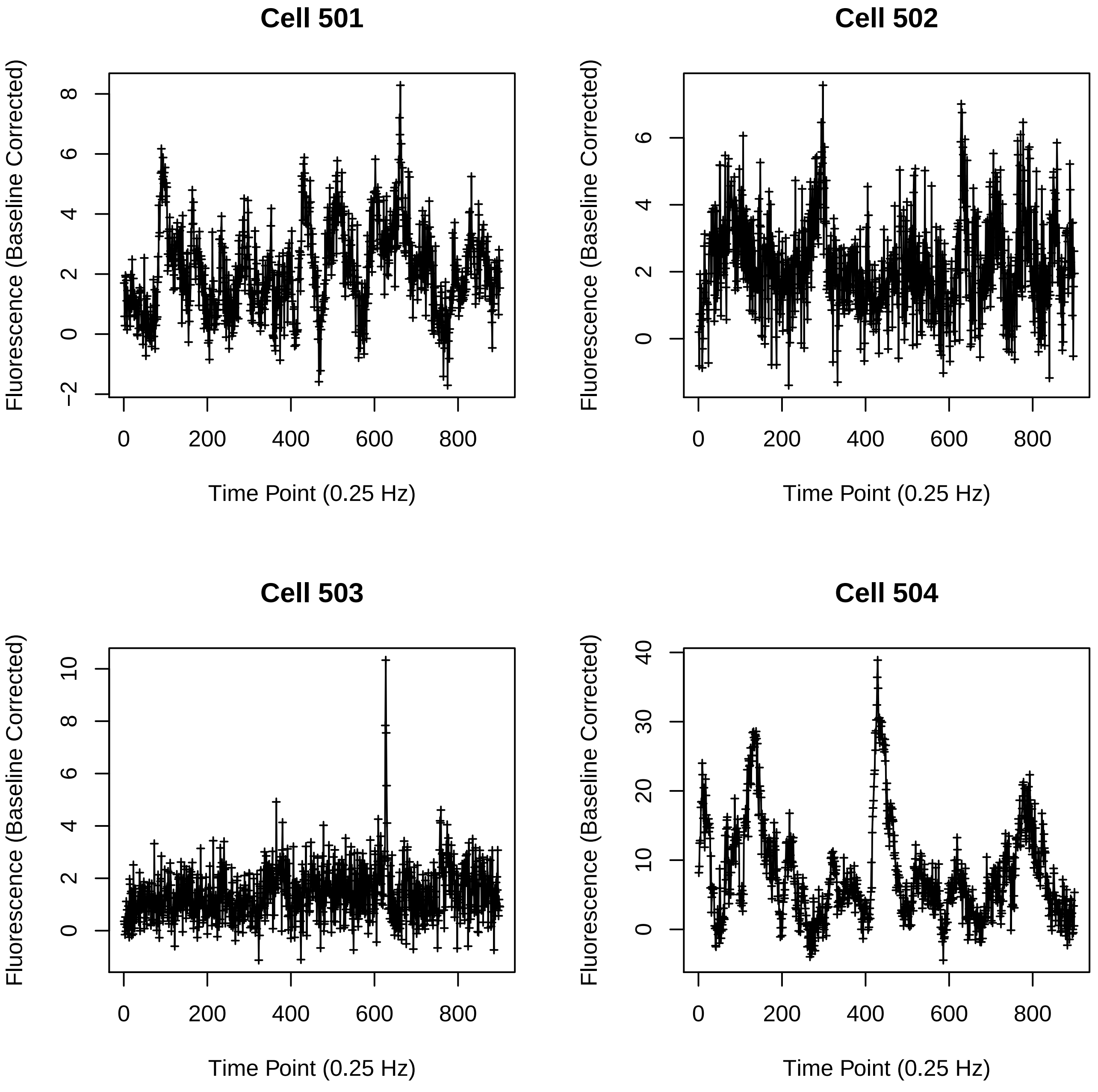
Cell 501
Cell 502
Cell 503
Cell 504
Fluorescence (Baseline Corrected)
Time Point (0.25 Hz)
0
200
400
600
800
−2
2
4
6
8
10
20
30
40

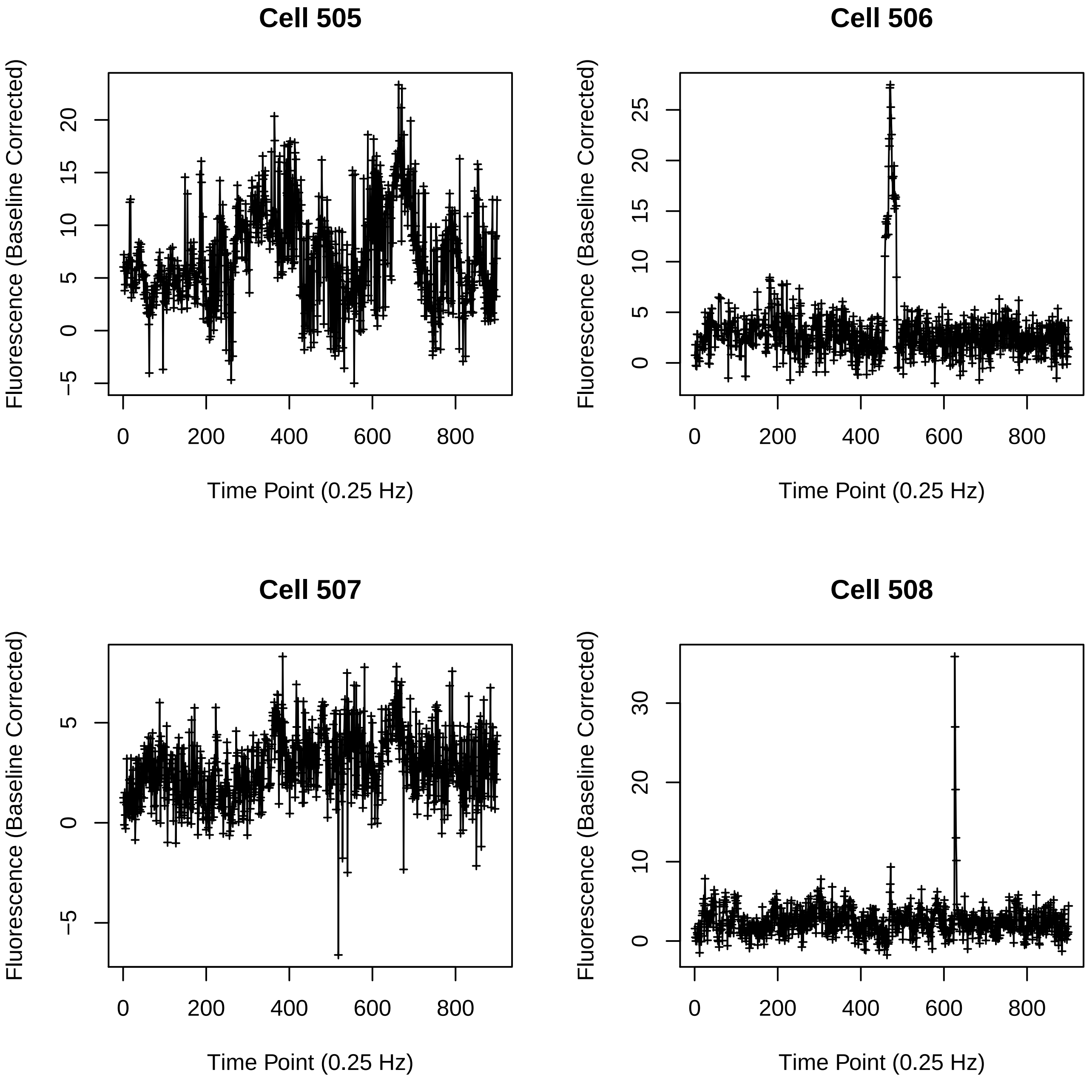
Cell 505
Cell 506
Cell 507
Cell 508
Fluorescence (Baseline Corrected)
Time Point (0.25 Hz)
0
200
400
600
800
−5
5
10
15
20
25
30

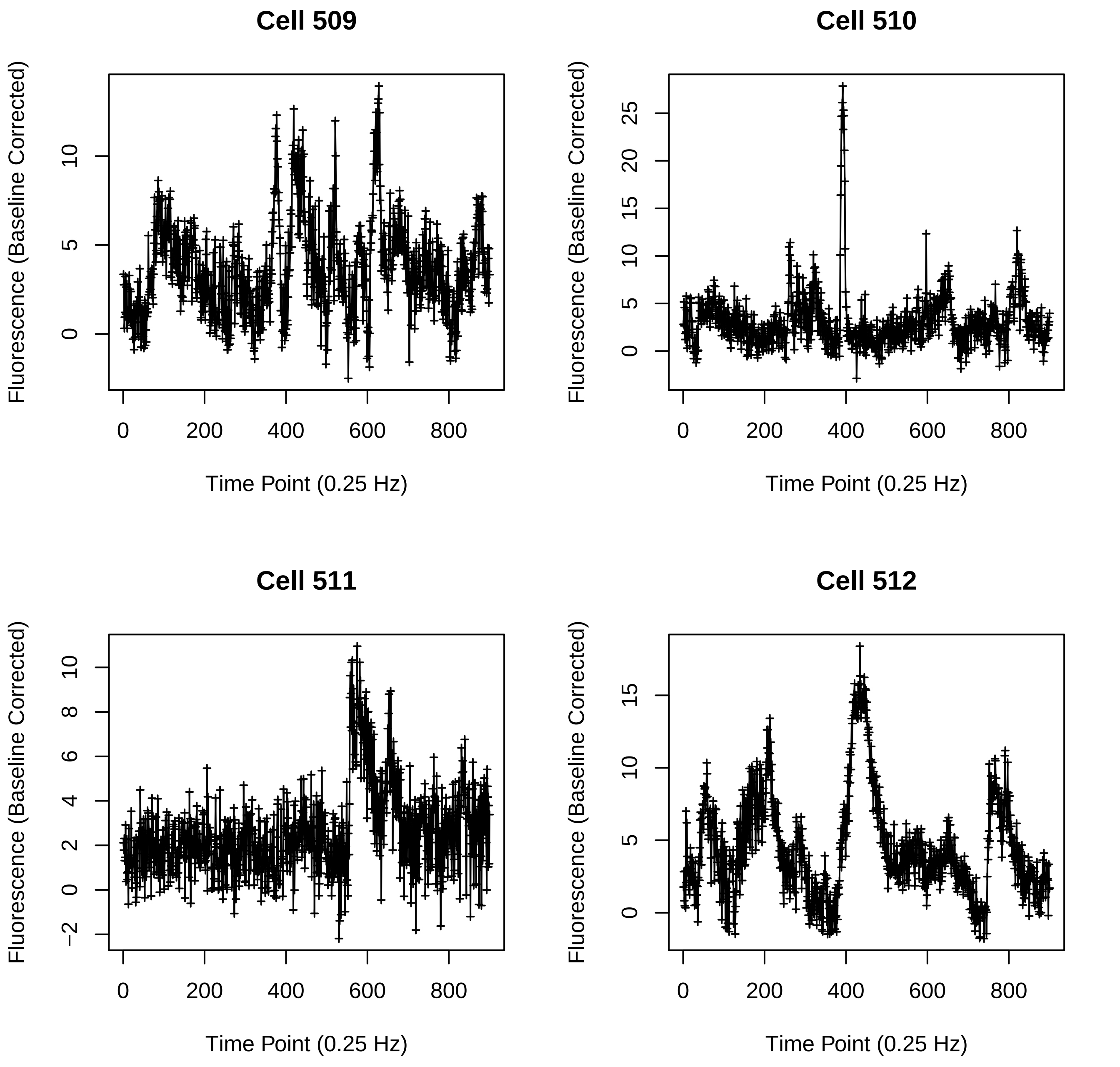

Cell 509
Cell 510
Cell 511
Cell 512
Fluorescence (Baseline Corrected)
Time Point (0.25 Hz)
0
200
400
600
800
0
5
10
15
20
25
−2
2
4
6
8

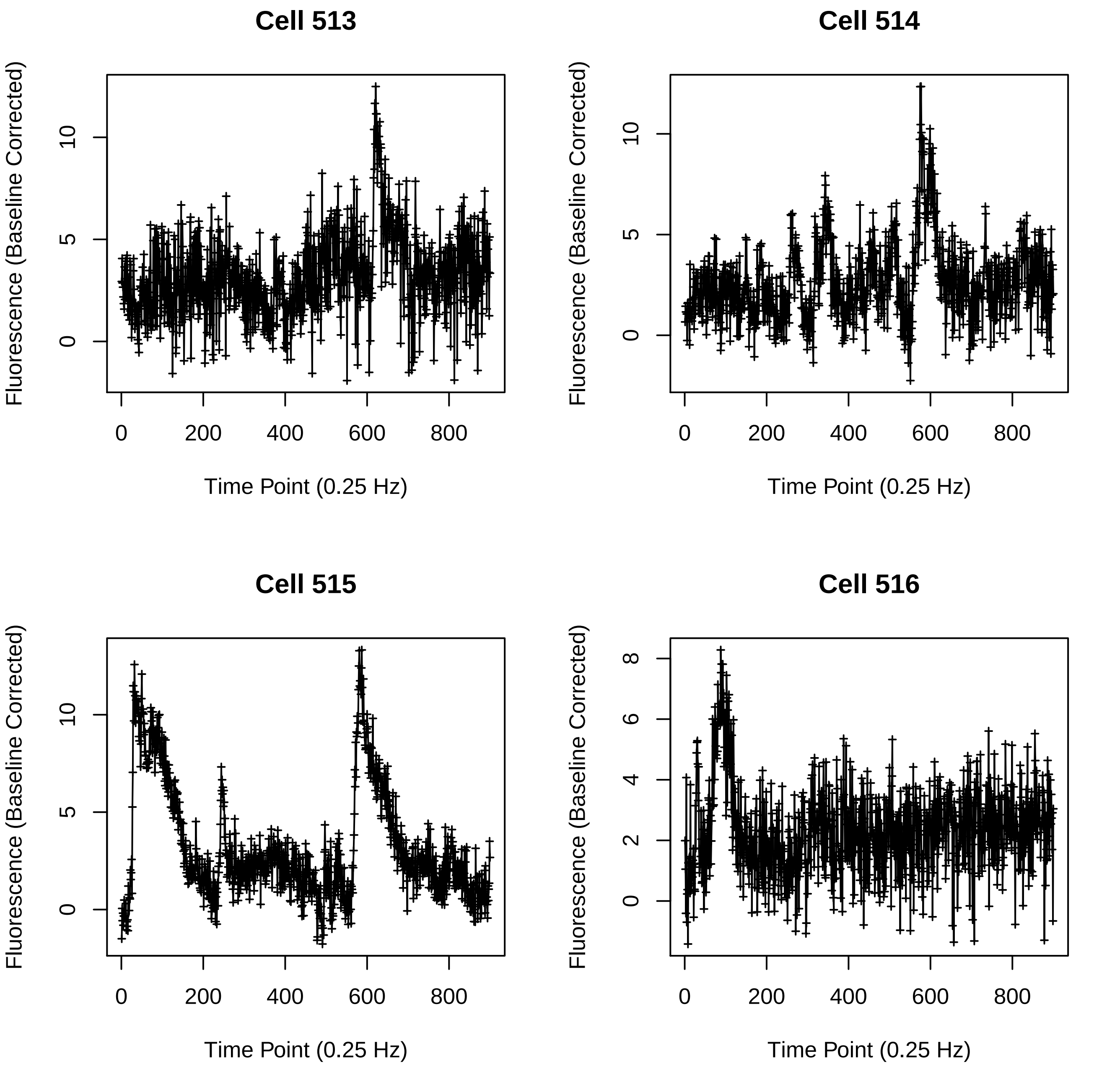
Cell 513
Cell 514
Cell 515
Cell 516
Fluorescence (Baseline Corrected)
Time Point (0.25 Hz)
0
5
10
2
4
6
8
200
400
600
800

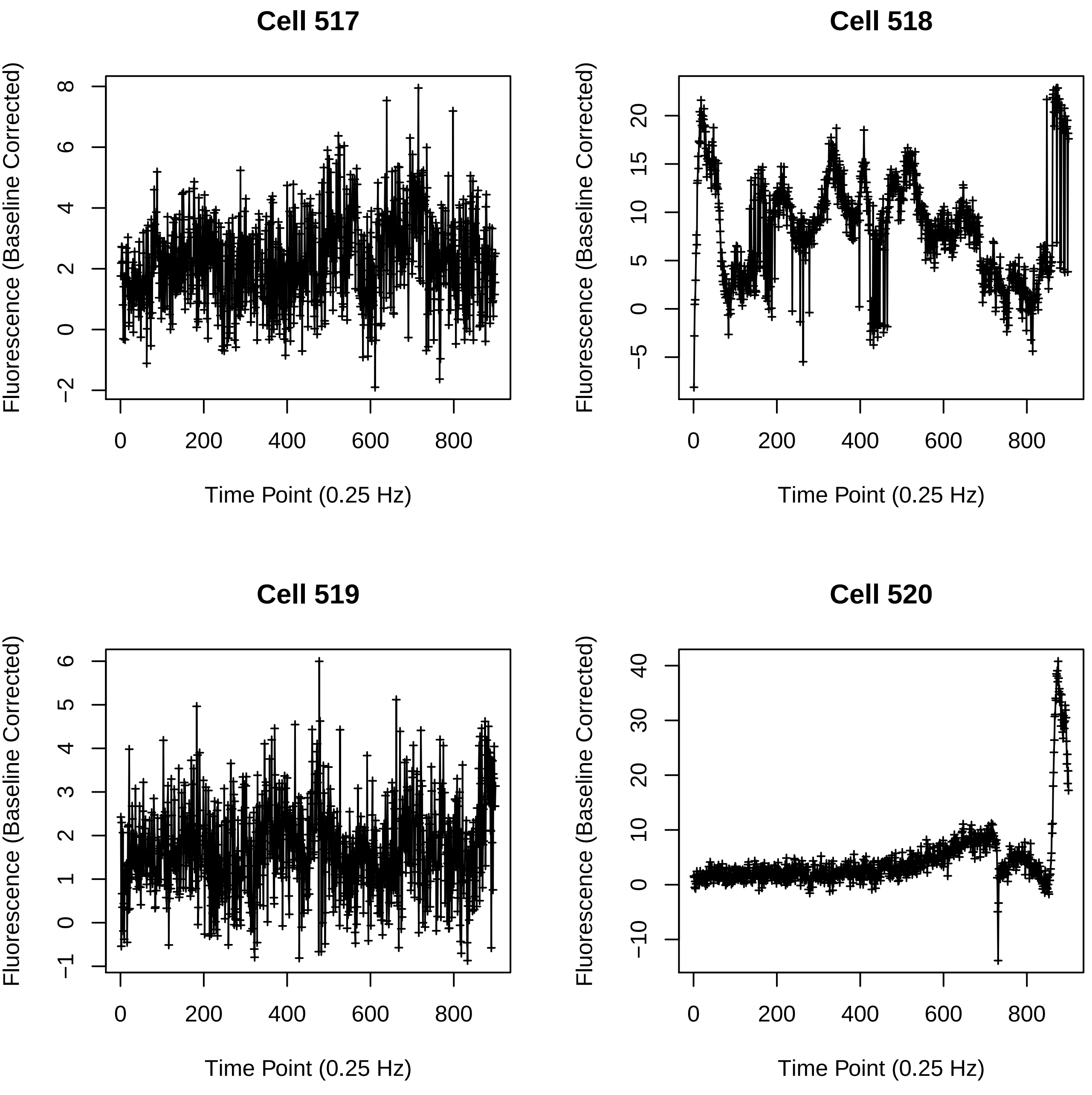

Cell 517
Cell 518
Cell 519
Cell 520
Fluorescence (Baseline Corrected)
Time Point (0.25 Hz)
8
6
4
2
0
−2
20
15
10
5
0
−5
6
5
4
3
2
1
0
−1
40
30
20
10
0
−10
0
200
400
600
800

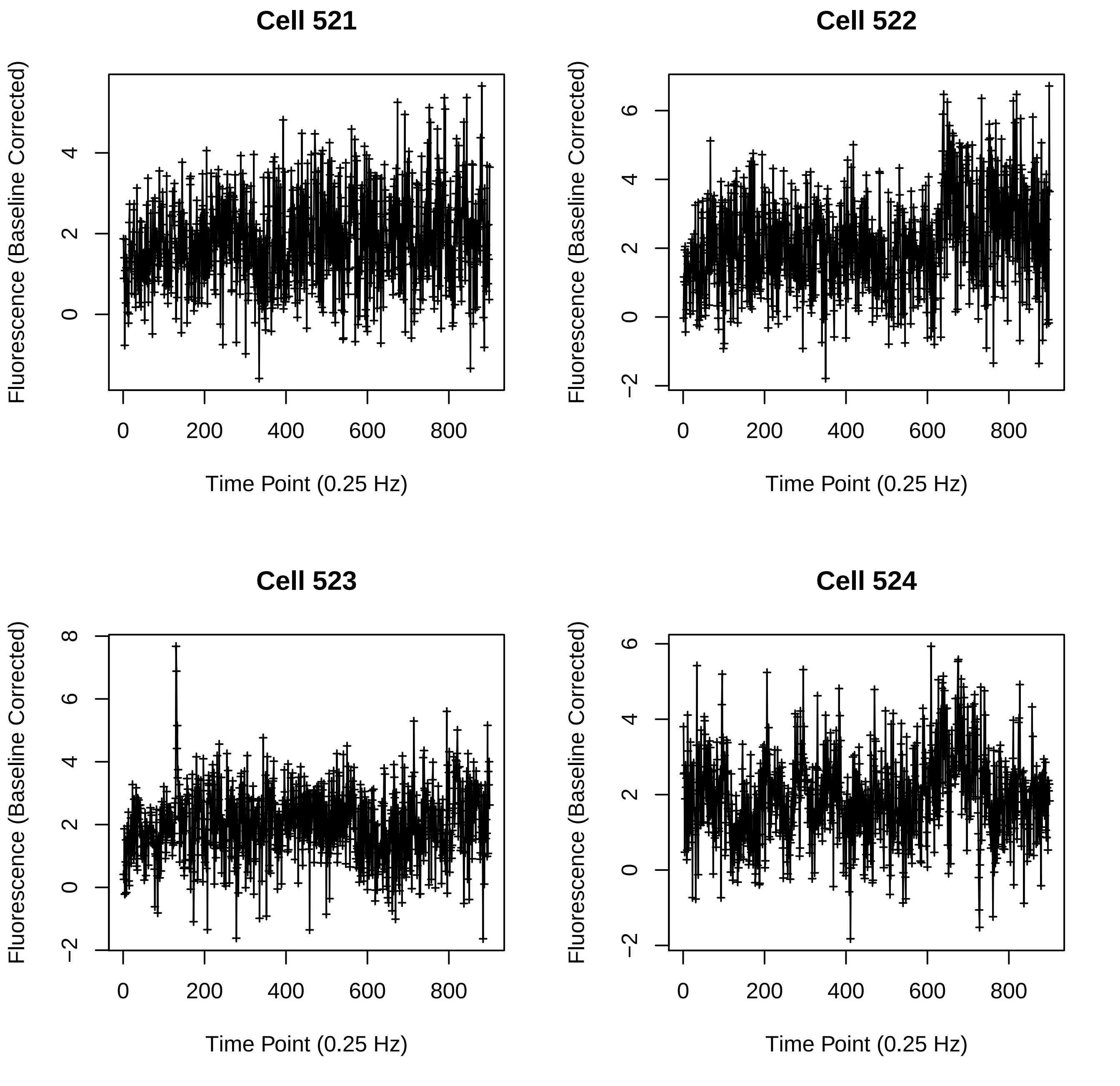

Cell 521
Cell 522
Cell 523
Cell 524
Fluorescence (Baseline Corrected)
Time Point (0.25 Hz)
0
200
400
600
800
-2
2
4
6
8

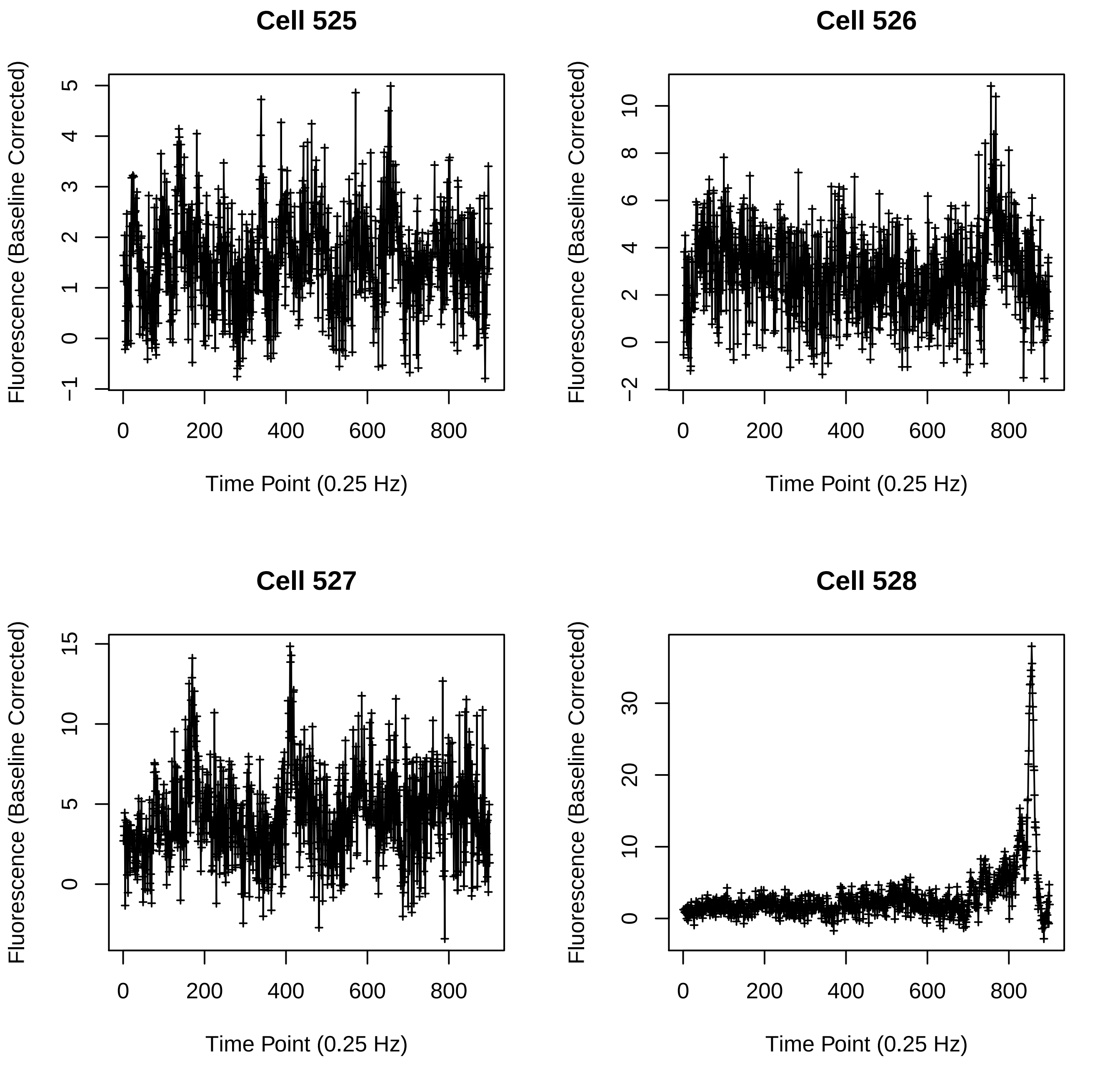

Cell 525
Fluorescence (Baseline Corrected)
Time Point (0.25 Hz)
Cell 526
Fluorescence (Baseline Corrected)
Time Point (0.25 Hz)
Cell 527
Fluorescence (Baseline Corrected)
Time Point (0.25 Hz)
Cell 528
Fluorescence (Baseline Corrected)
Time Point (0.25 Hz)

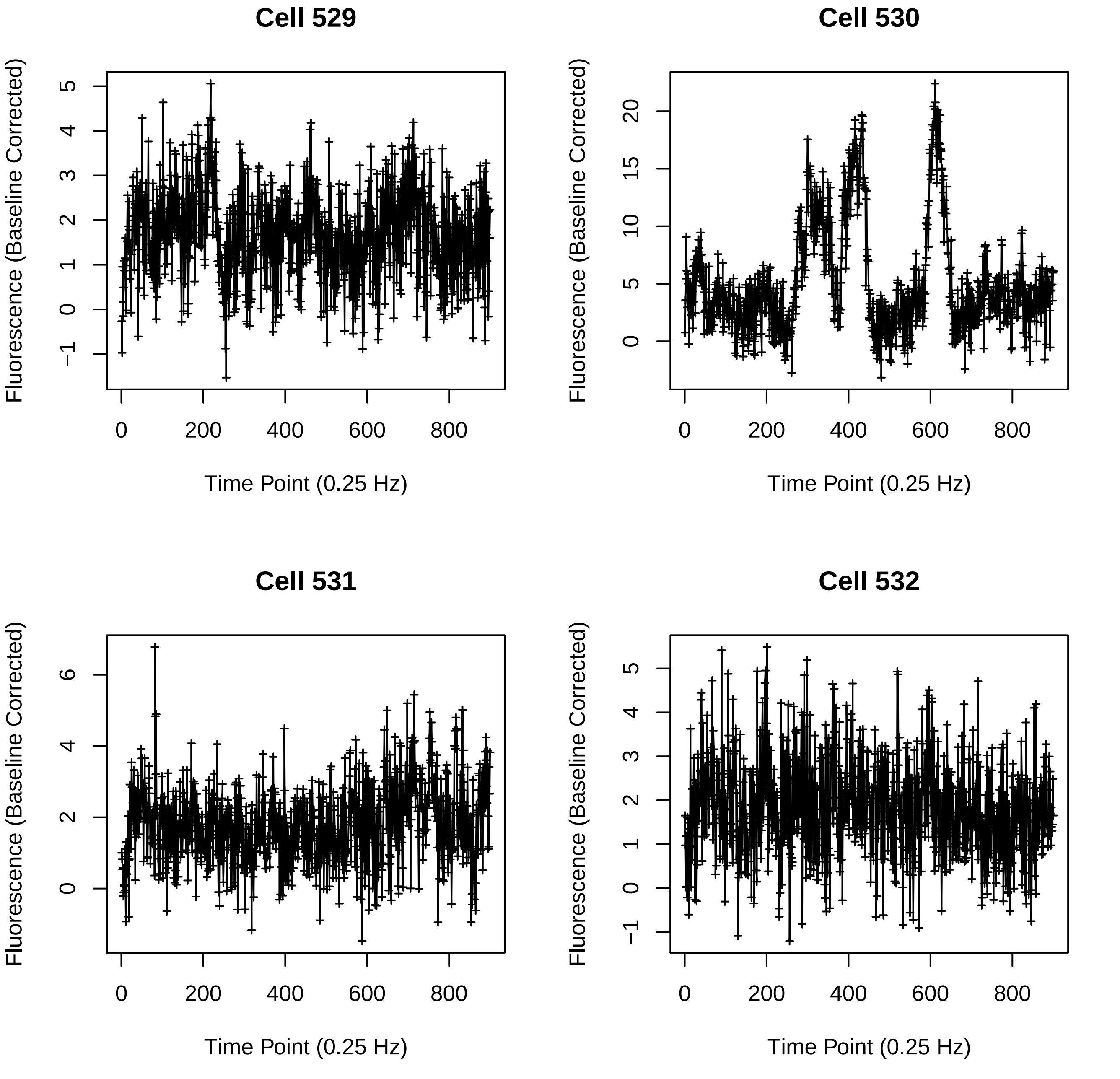
Cell 529
Cell 530
Cell 531
Cell 532
Fluorescence (Baseline Corrected)
Time Point (0.25 Hz)
0
200
400
600
800

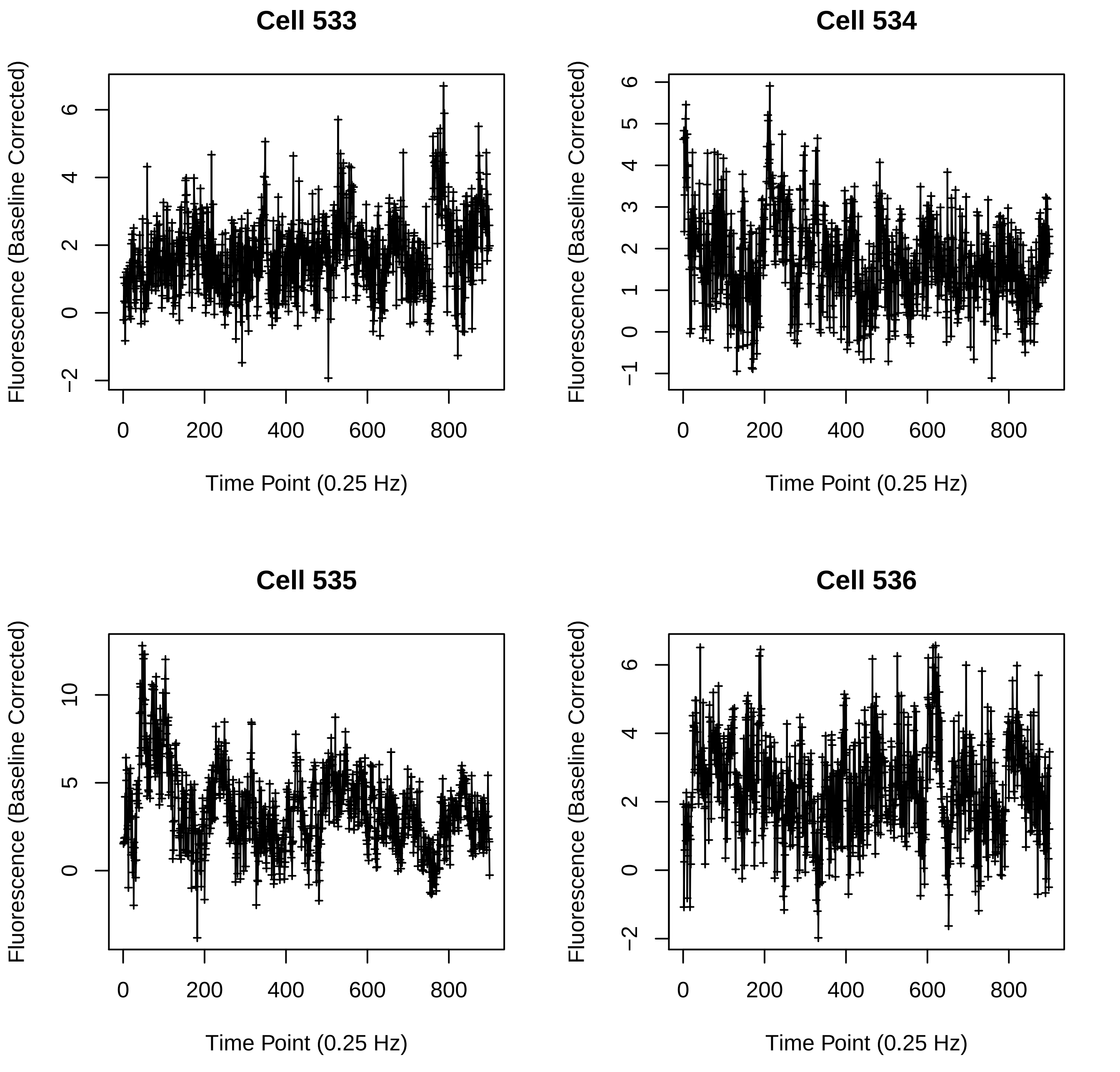
Cell 533
Fluorescence (Baseline Corrected)
Time Point (0.25 Hz)
−2
0
2
4
6
0
200
400
600
800
Cell 534
Fluorescence (Baseline Corrected)
Time Point (0.25 Hz)
−1
0
1
2
3
4
5
6
0
200
400
600
800
Cell 535
Fluorescence (Baseline Corrected)
Time Point (0.25 Hz)
0
5
10
0
200
400
600
800
Cell 536
Fluorescence (Baseline Corrected)
Time Point (0.25 Hz)
−2
0
2
4
6
0
200
400
600
800

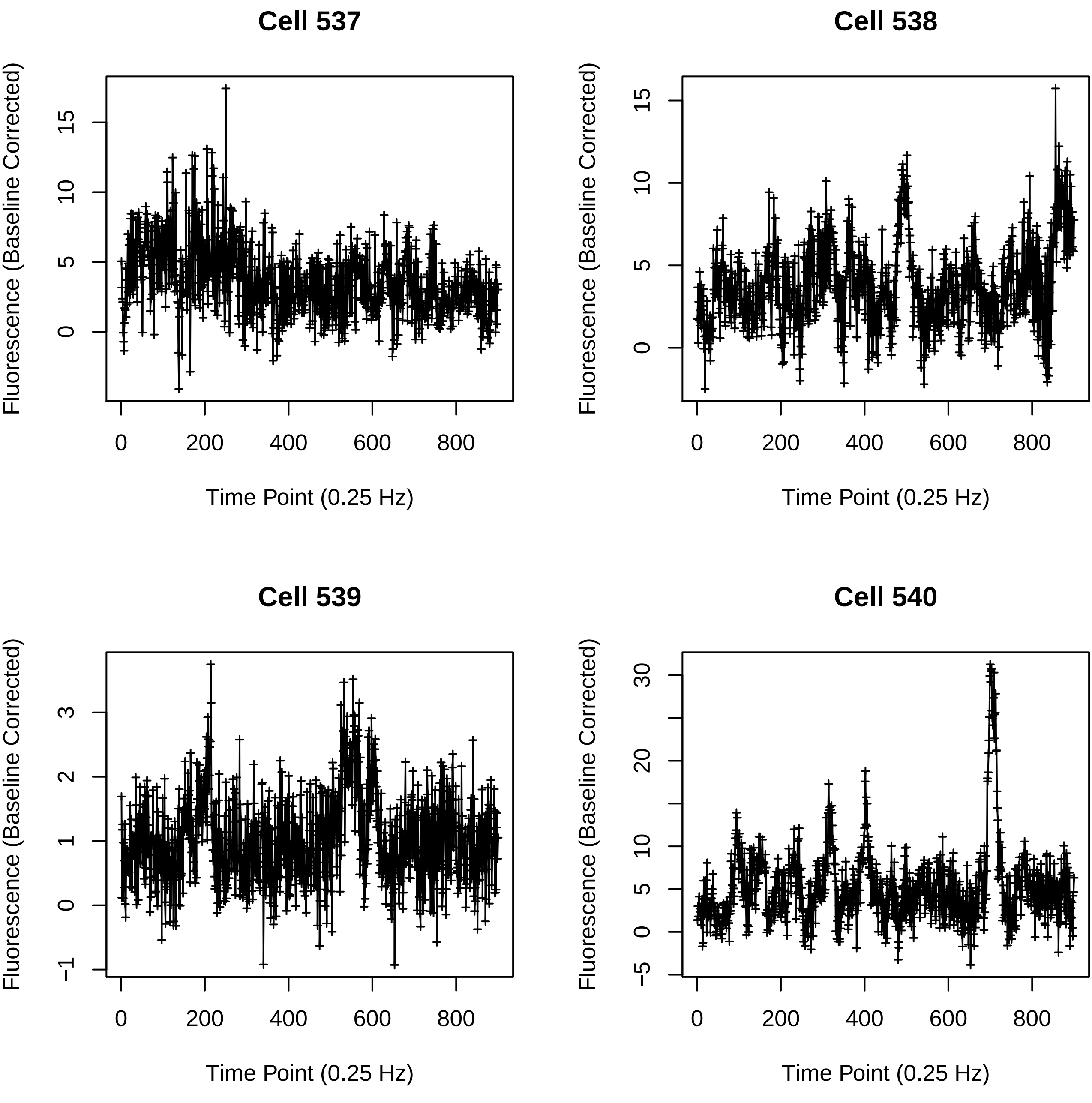
Cell 537
Fluorescence (Baseline Corrected)
0
5
10
15
0
200
400
600
800
Time Point (0.25 Hz)
Cell 538
Fluorescence (Baseline Corrected)
0
5
10
15
0
200
400
600
800
Time Point (0.25 Hz)
Cell 539
Fluorescence (Baseline Corrected)
−1
0
1
2
3
0
200
400
600
800
Time Point (0.25 Hz)
Cell 540
Fluorescence (Baseline Corrected)
−5
0
5
10
20
30
0
200
400
600
800
Time Point (0.25 Hz)

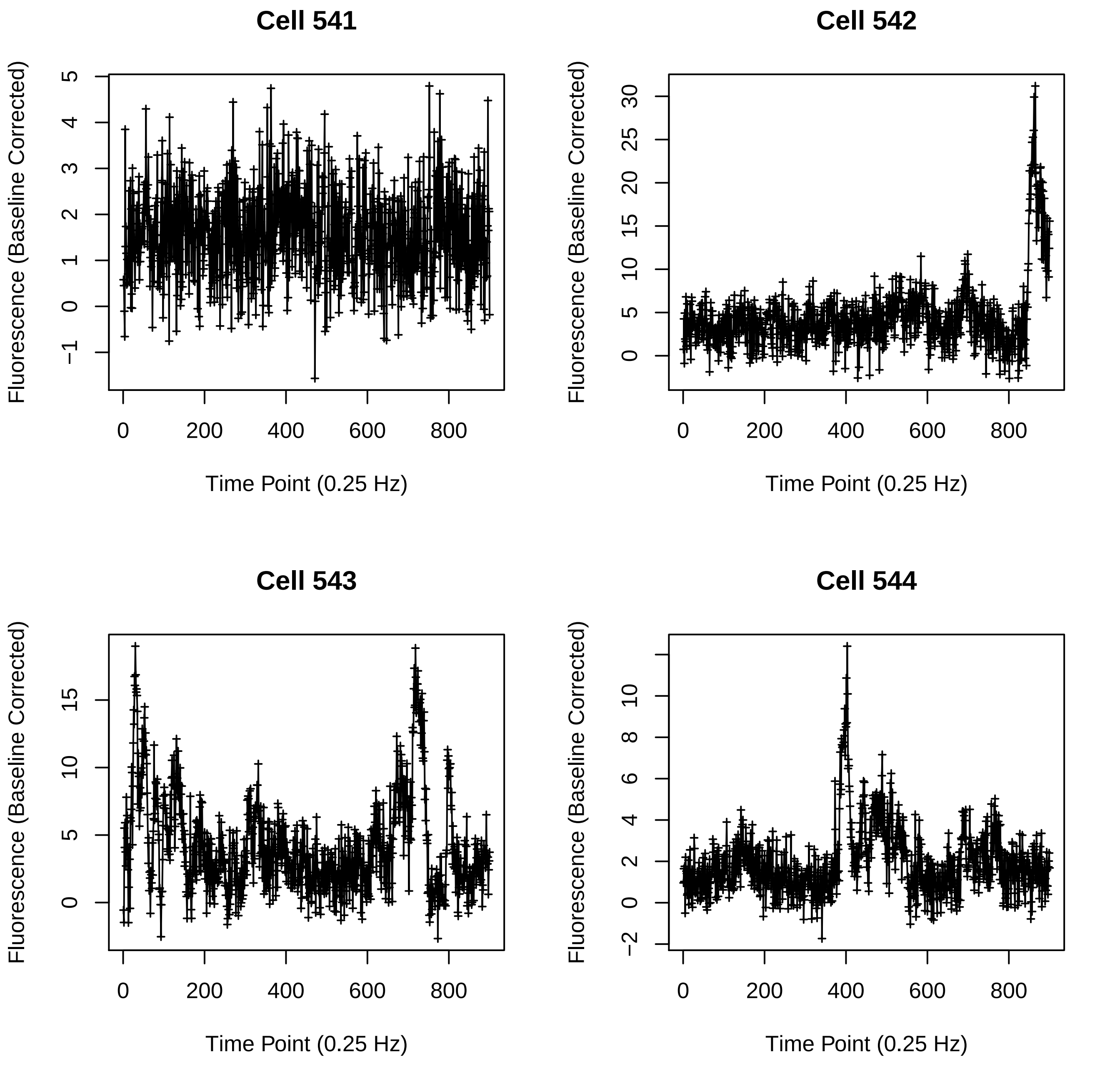

Cell 541
Cell 542
Cell 543
Cell 544
Fluorescence (Baseline Corrected)
Time Point (0.25 Hz)
0
200
400
600
800

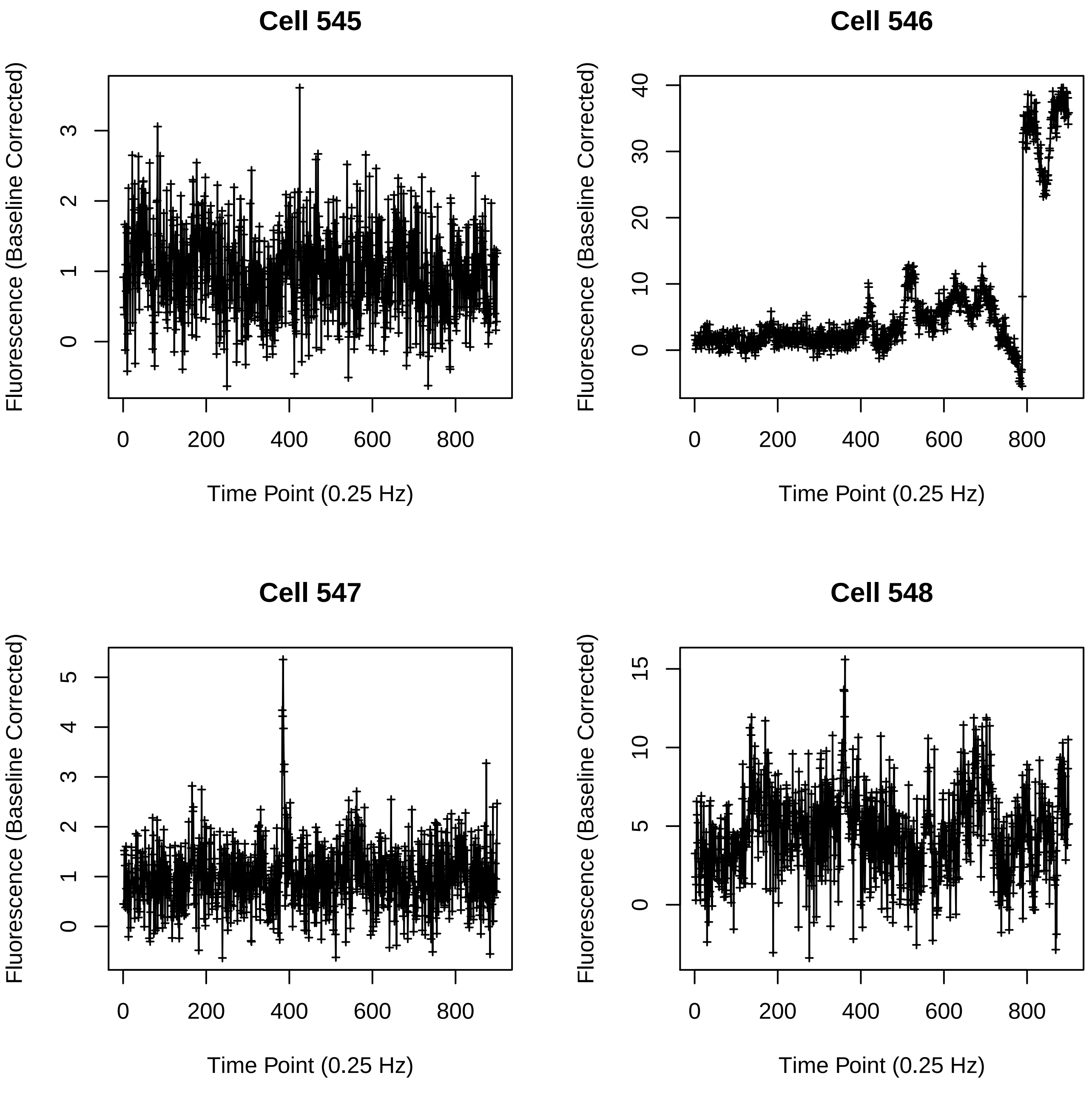
Cell 545
Cell 546
Cell 547
Cell 548
Fluorescence (Baseline Corrected)
Time Point (0.25 Hz)
0
200
400
600
800
-0
1
2
3
10
20
30
40
4
5
15

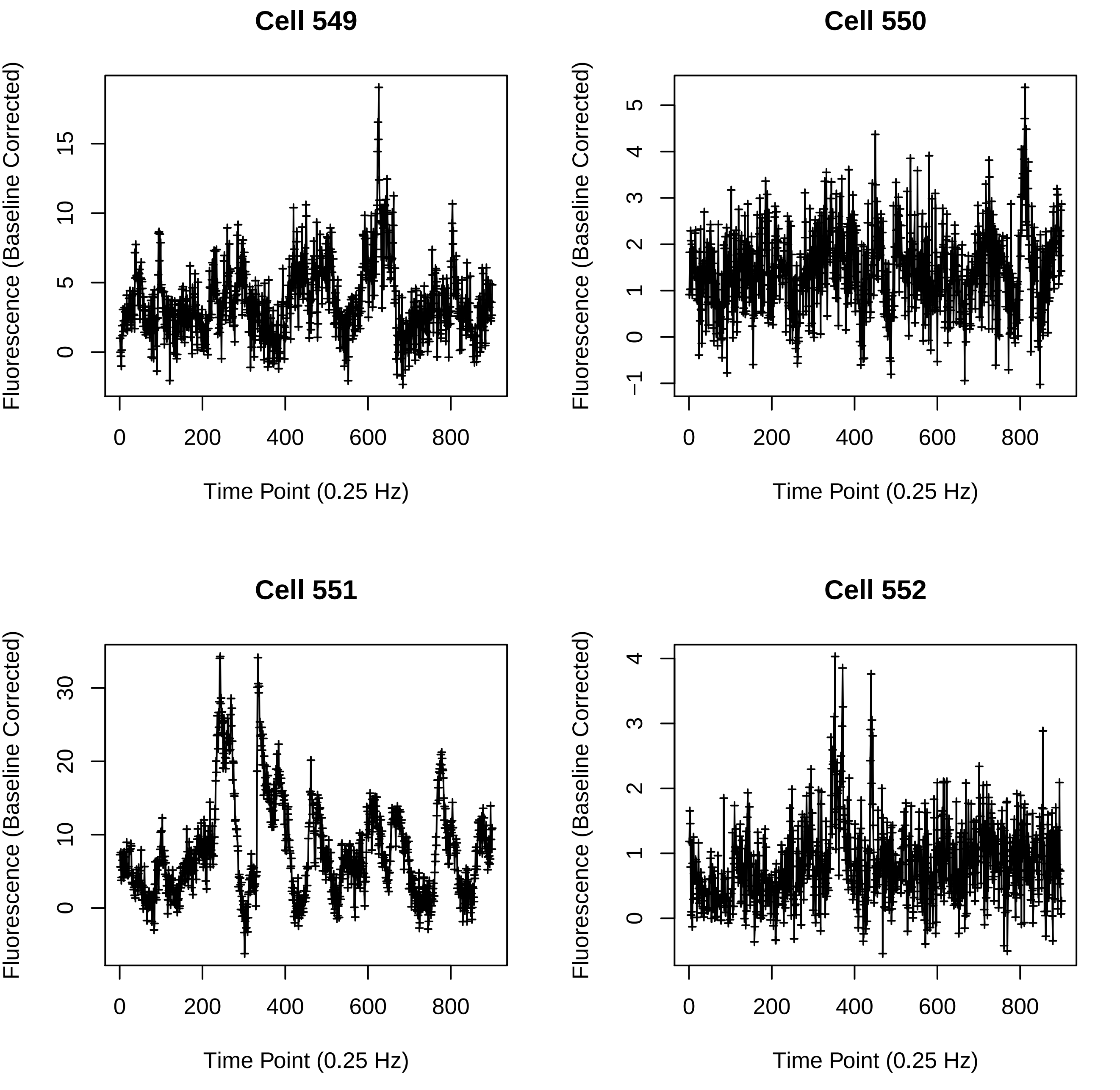
Cell 549
Fluorescence (Baseline Corrected)
0
5
10
15
Time Point (0.25 Hz)
0
200
400
600
800
Cell 550
Fluorescence (Baseline Corrected)
−1
0
1
2
3
4
5
Time Point (0.25 Hz)
0
200
400
600
800
Cell 551
Fluorescence (Baseline Corrected)
0
10
20
30
Time Point (0.25 Hz)
0
200
400
600
800
Cell 552
Fluorescence (Baseline Corrected)
0
1
2
3
4
Time Point (0.25 Hz)
0
200
400
600
800

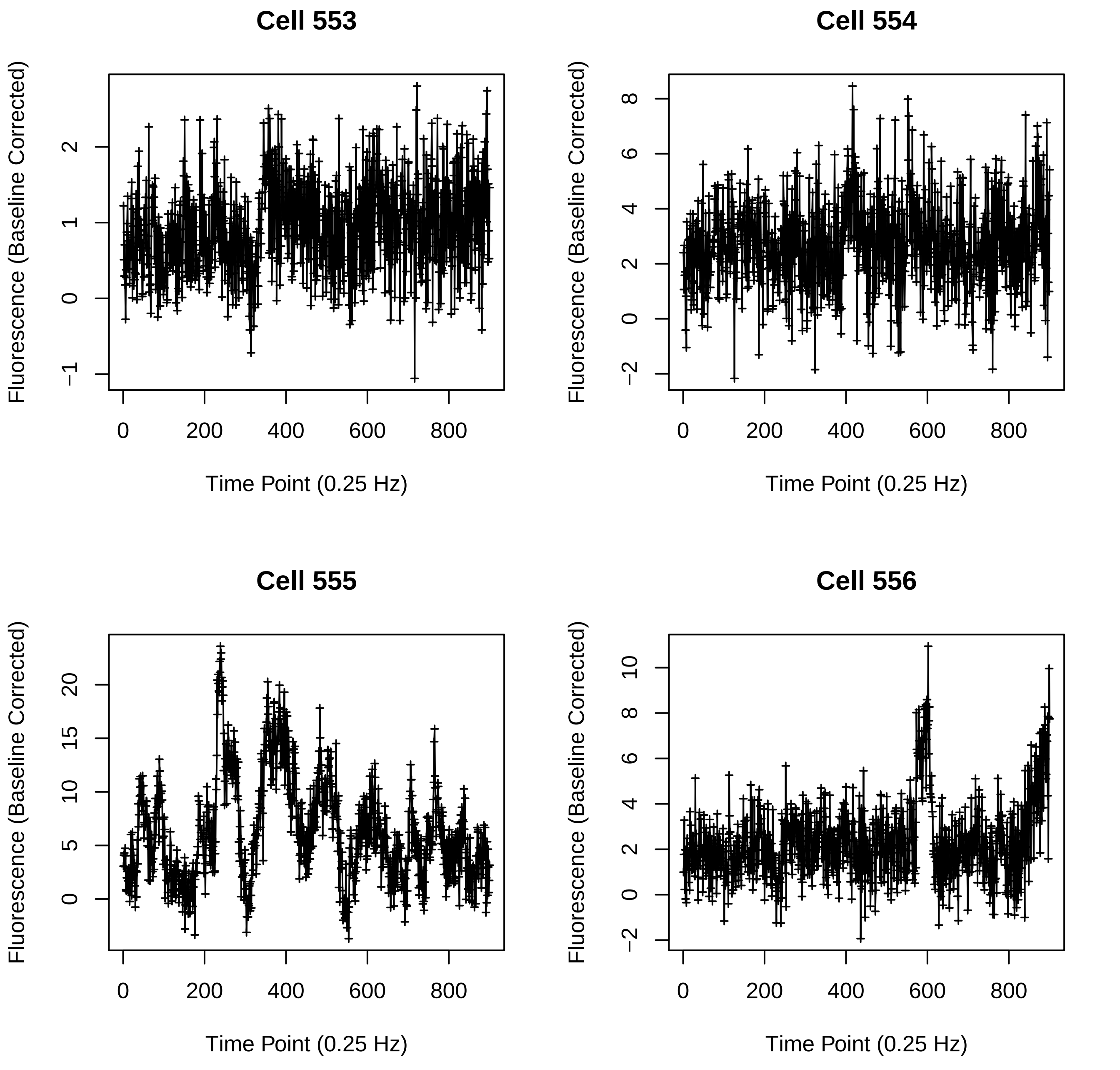
Cell 553
Cell 554
Cell 555
Cell 556
Fluorescence (Baseline Corrected)
Time Point (0.25 Hz)
0
200
400
600
800
−1
1
2
−2
4
6
8
5
10
15
20

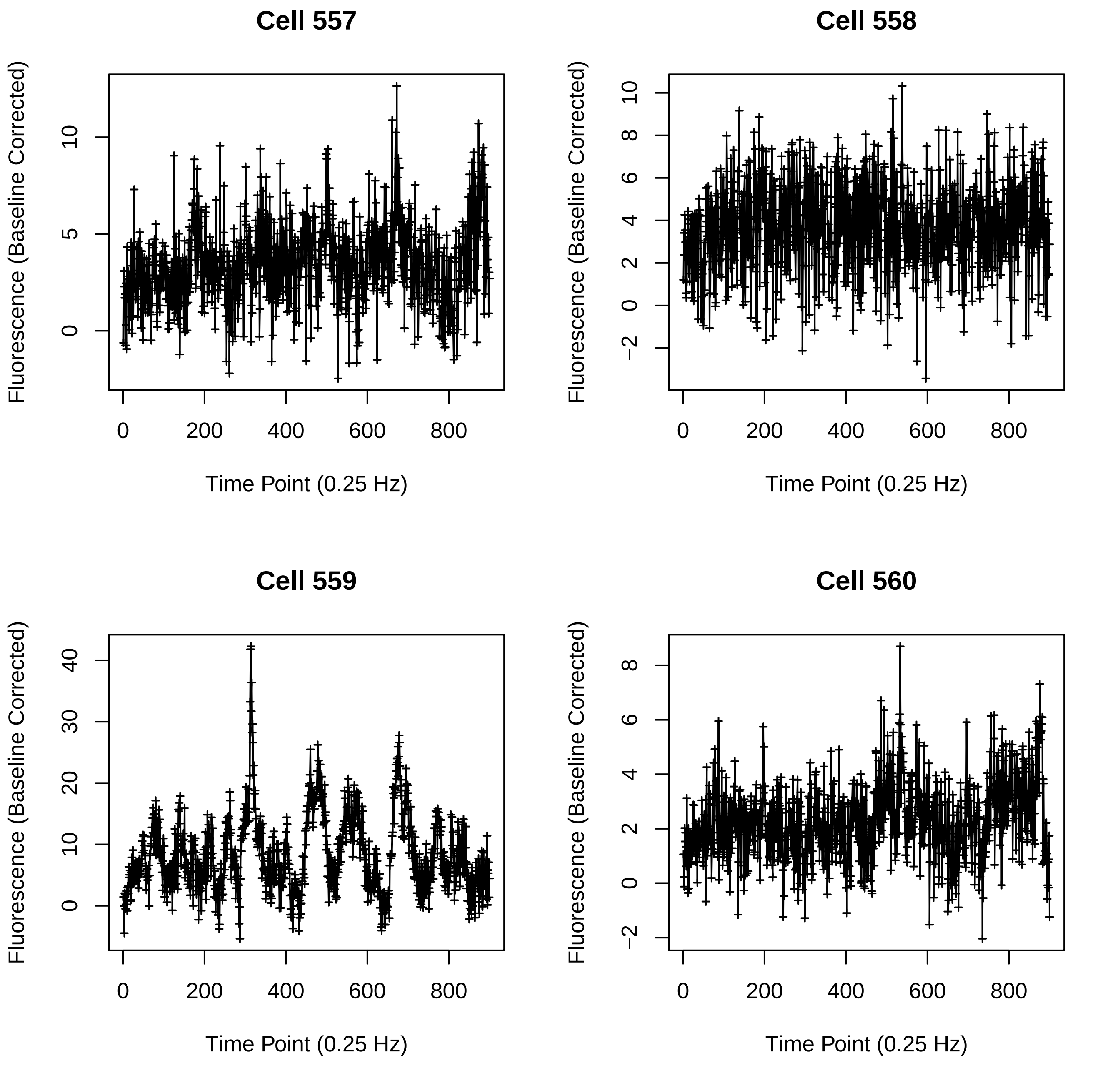
Cell 557
Cell 558
Cell 559
Cell 560
Fluorescence (Baseline Corrected)
Time Point (0.25 Hz)
0
200
400
600
800
0
5
10
−2
2
4
6
8
20
30
40

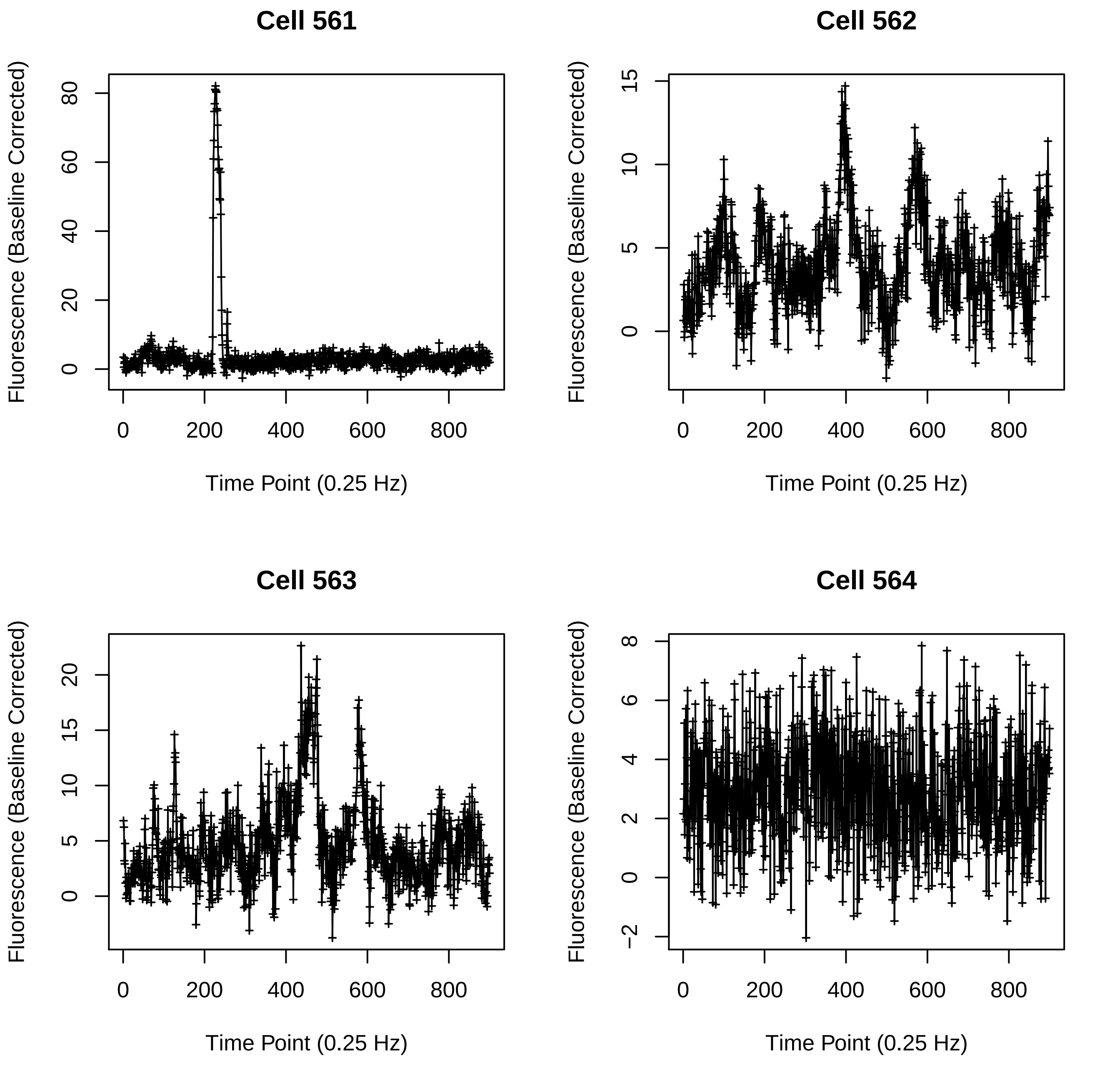
Cell 561
Cell 562
Cell 563
Cell 564
Fluorescence (Baseline Corrected)
Time Point (0.25 Hz)
0
20
40
60
80
5
10
15
−2
2
4
6
8
200
400
600
800

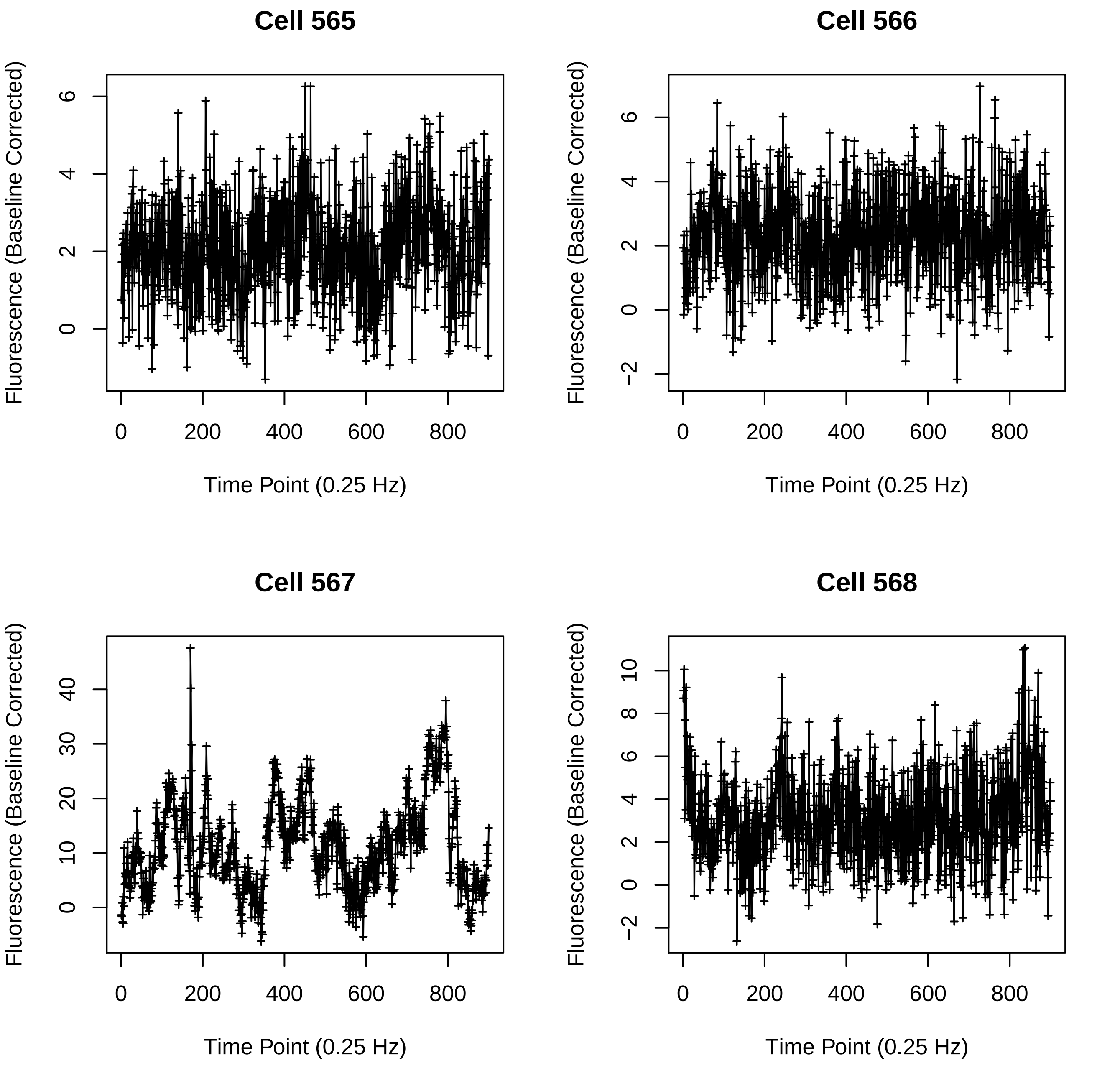
Cell 565
Fluorescence (Baseline Corrected)
0
2
4
6
0
200
400
600
800
Time Point (0.25 Hz)
Cell 566
Fluorescence (Baseline Corrected)
−2
0
2
4
6
0
200
400
600
800
Time Point (0.25 Hz)
Cell 567
Fluorescence (Baseline Corrected)
0
10
20
30
40
0
200
400
600
800
Time Point (0.25 Hz)
Cell 568
Fluorescence (Baseline Corrected)
−2
0
2
4
6
8
10
0
200
400
600
800
Time Point (0.25 Hz)

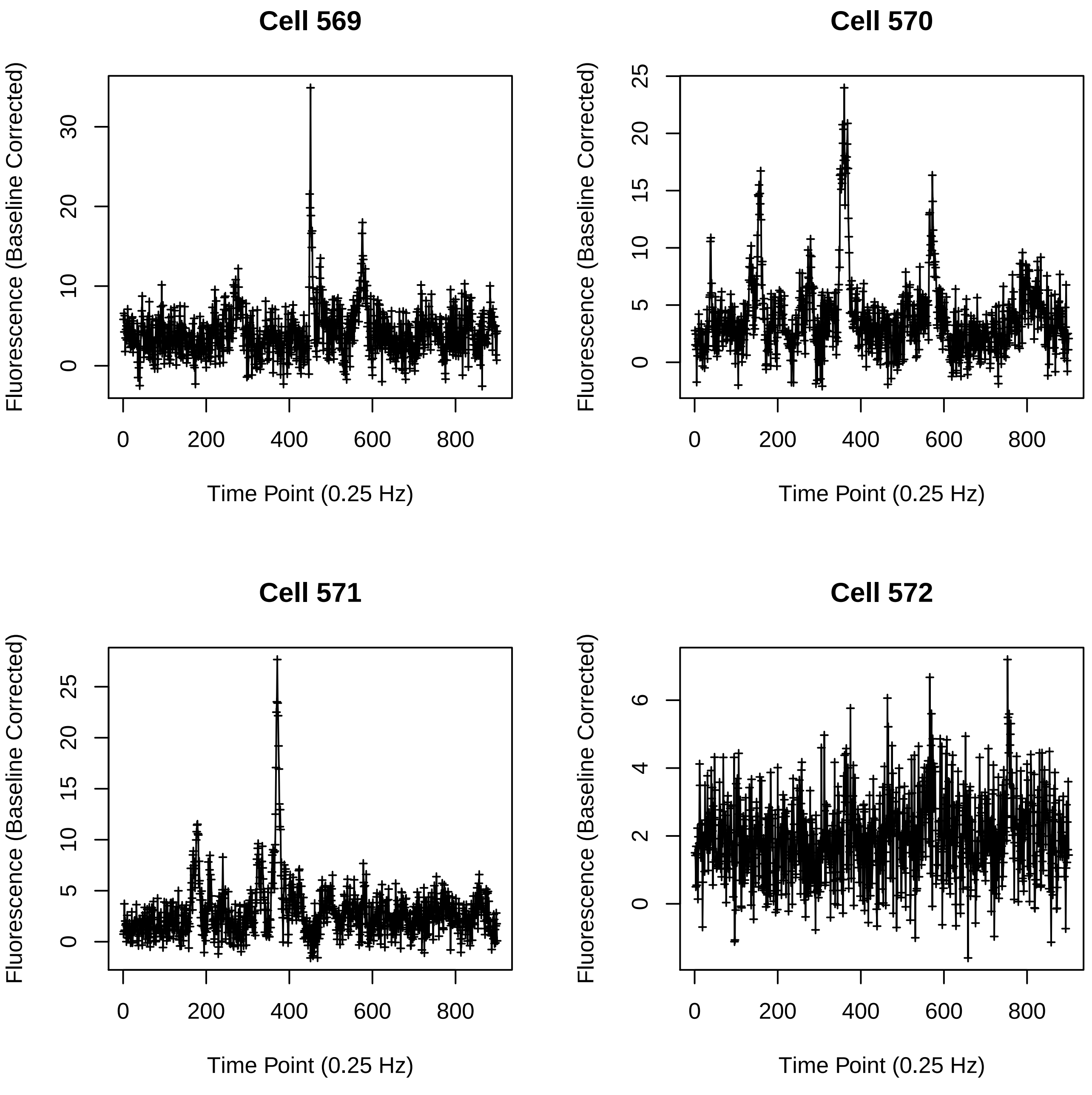
Cell 569
Cell 570
Cell 571
Cell 572
Fluorescence (Baseline Corrected)
Time Point (0.25 Hz)
0
200
400
600
800
0
10
20
30
0
5
10
15
20
25
0
2
4
6

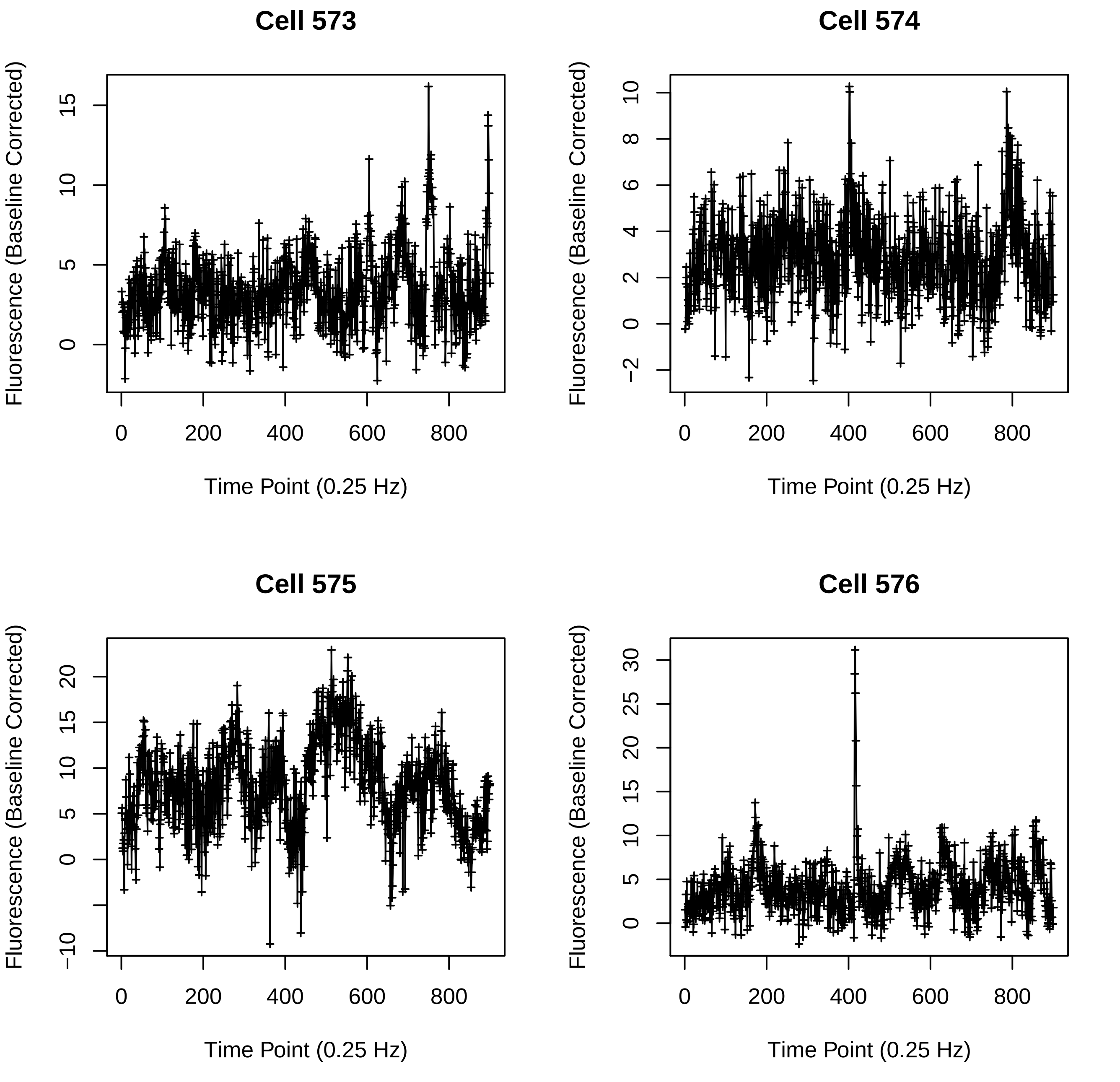
Cell 573
Fluorescence (Baseline Corrected)
0
5
10
15
0
200
400
600
800
Time Point (0.25 Hz)
Cell 574
Fluorescence (Baseline Corrected)
−2
0
2
4
6
8
10
0
200
400
600
800
Time Point (0.25 Hz)
Cell 575
Fluorescence (Baseline Corrected)
−10
0
5
10
15
20
0
200
400
600
800
Time Point (0.25 Hz)
Cell 576
Fluorescence (Baseline Corrected)
0
5
10
15
20
25
30
0
200
400
600
800
Time Point (0.25 Hz)

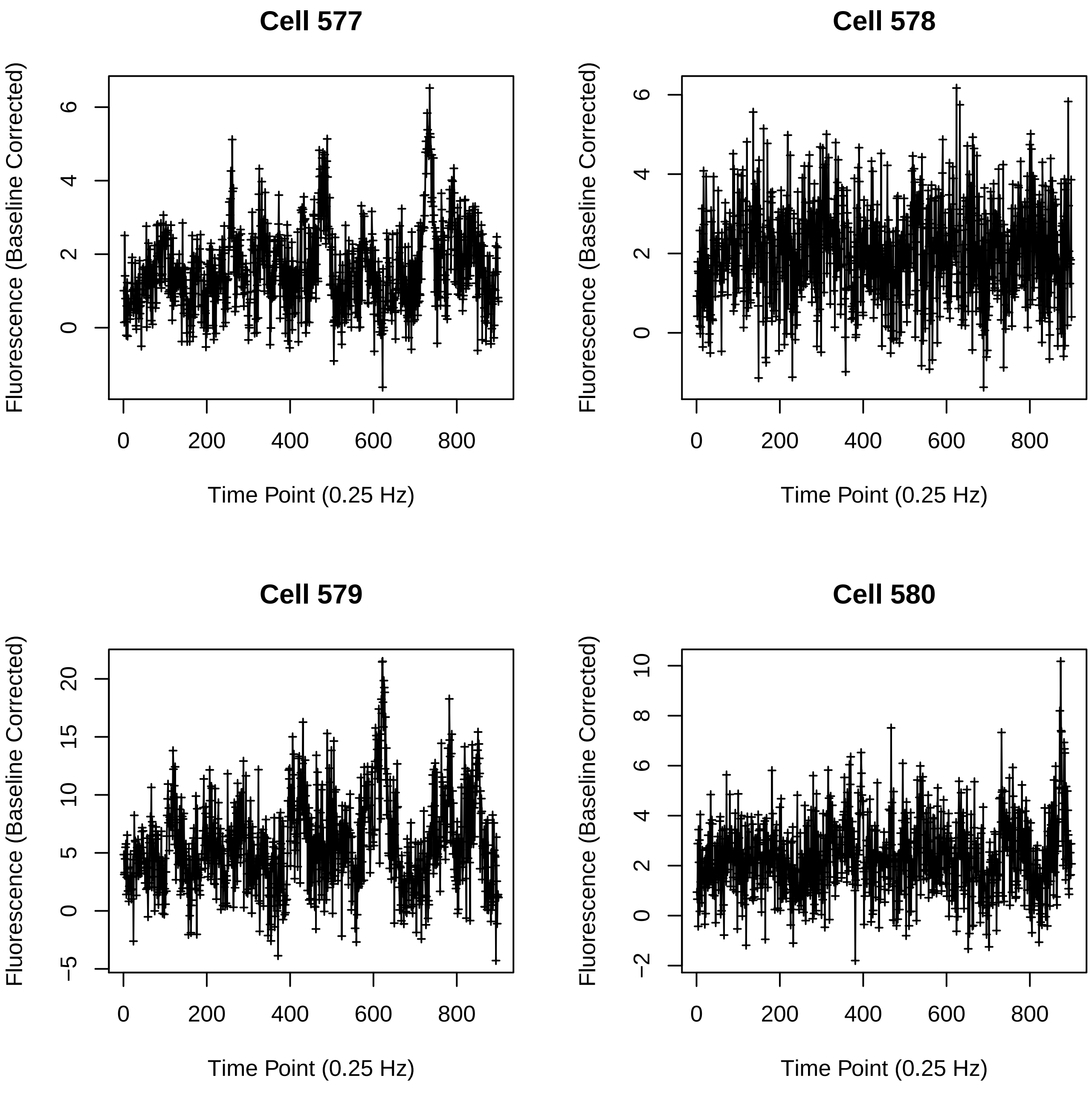
Cell 577
Cell 578
Cell 579
Cell 580
Fluorescence (Baseline Corrected)
Time Point (0.25 Hz)
0
200
400
600
800
0
2
4
6
−2
8
10
−5
5
15
20

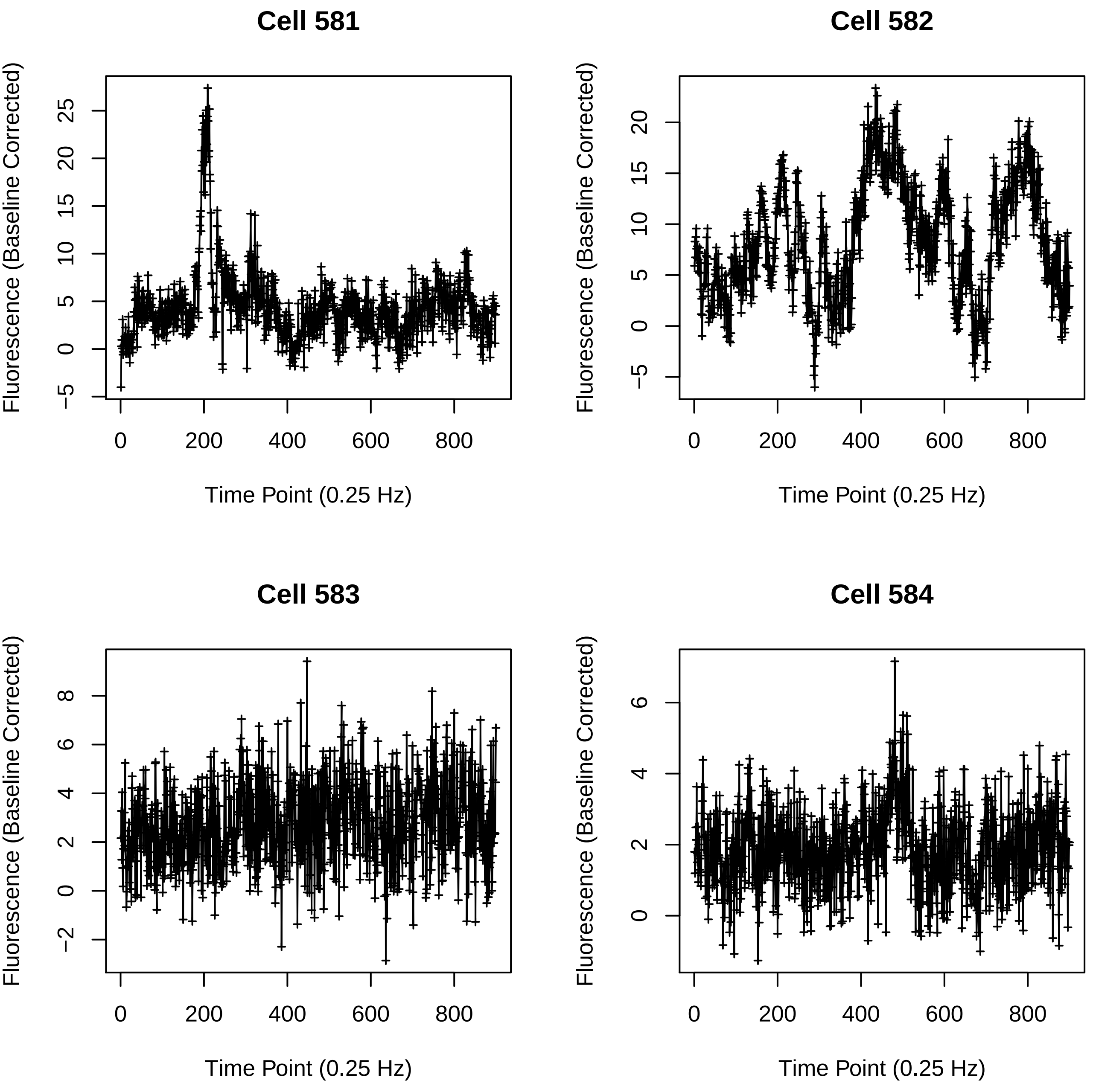

Cell 581
Cell 582
Cell 583
Cell 584
Fluorescence (Baseline Corrected)
Time Point (0.25 Hz)
0
200
400
600
800
−5
5
10
15
20
25
−2
2
4
6
8

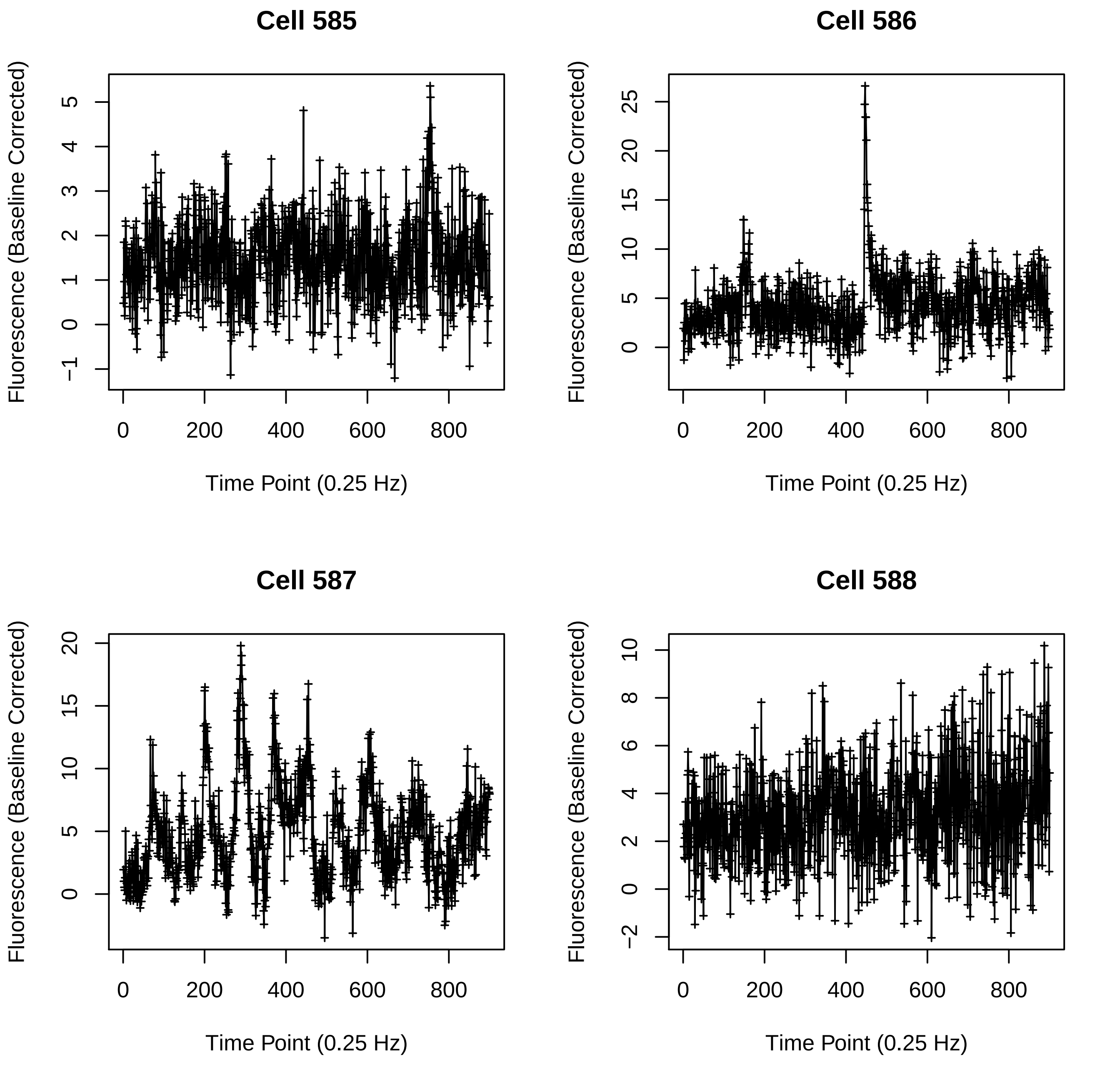

Cell 585
Cell 586
Cell 587
Cell 588
Fluorescence (Baseline Corrected)
Time Point (0.25 Hz)
0
200
400
600
800
−1
0
1
2
3
4
5
10
15
20
25
−2
6
8

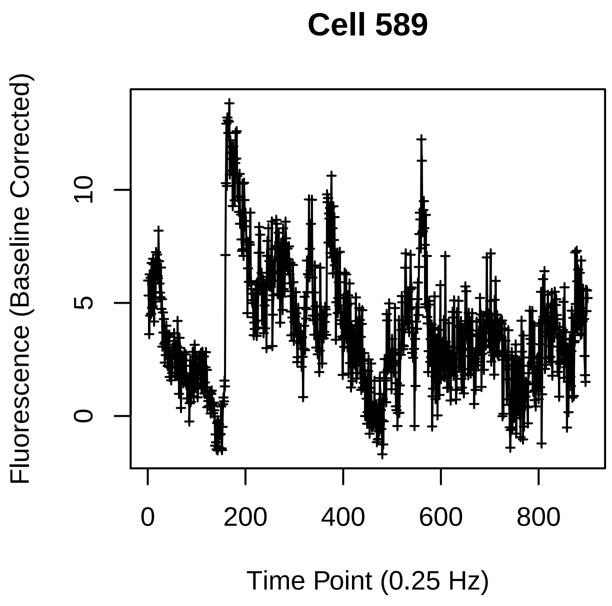
Cell 589
Fluorescence (Baseline Corrected)
0
5
10
0
200
400
600
800
Time Point (0.25 Hz)

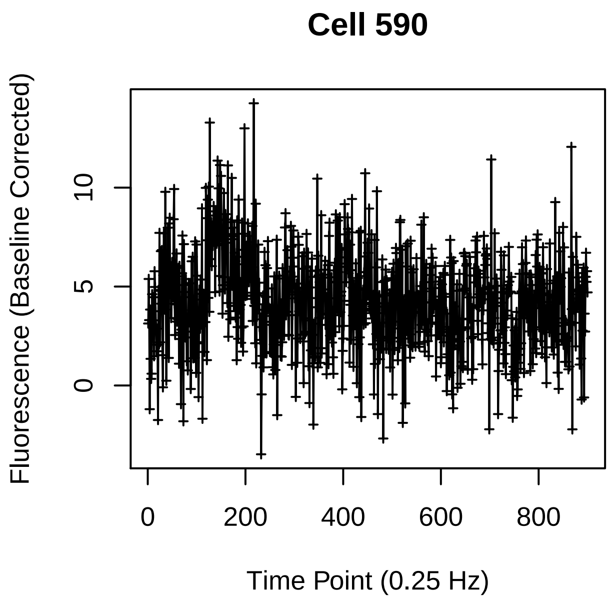
Cell 590
Fluorescence (Baseline Corrected)
0
5
10
0
200
400
600
800
Time Point (0.25 Hz)

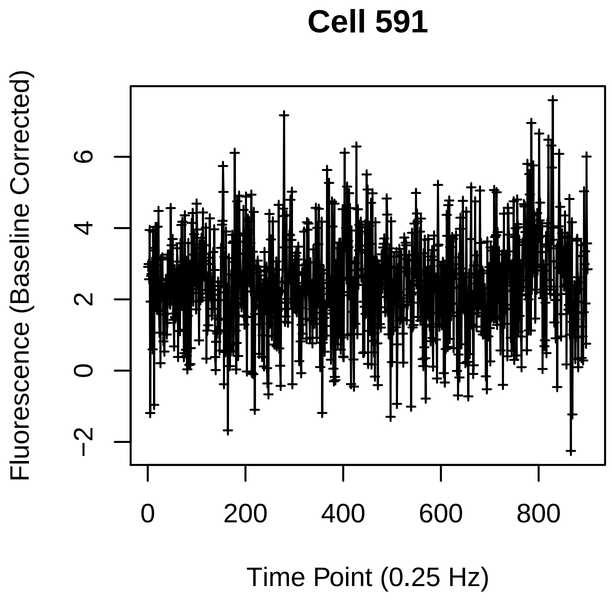
Cell 591
Fluorescence (Baseline Corrected)
−2
0
2
4
6
0
200
400
600
800
Time Point (0.25 Hz)

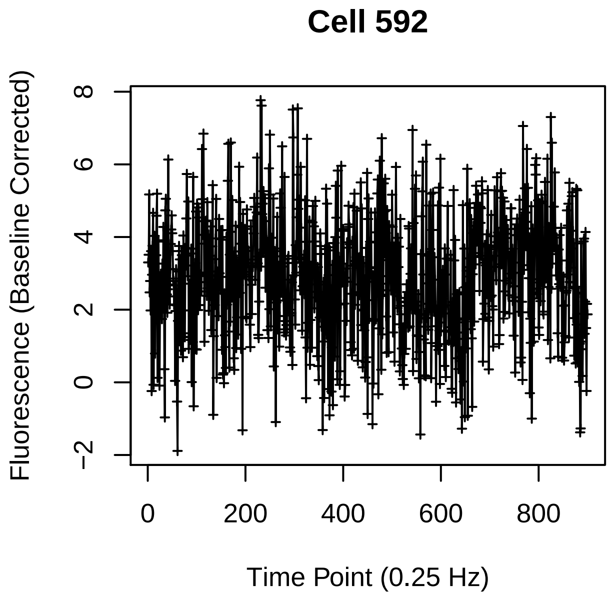
Cell 592
Fluorescence (Baseline Corrected)
−2
0
2
4
6
8
0
200
400
600
800
Time Point (0.25 Hz)

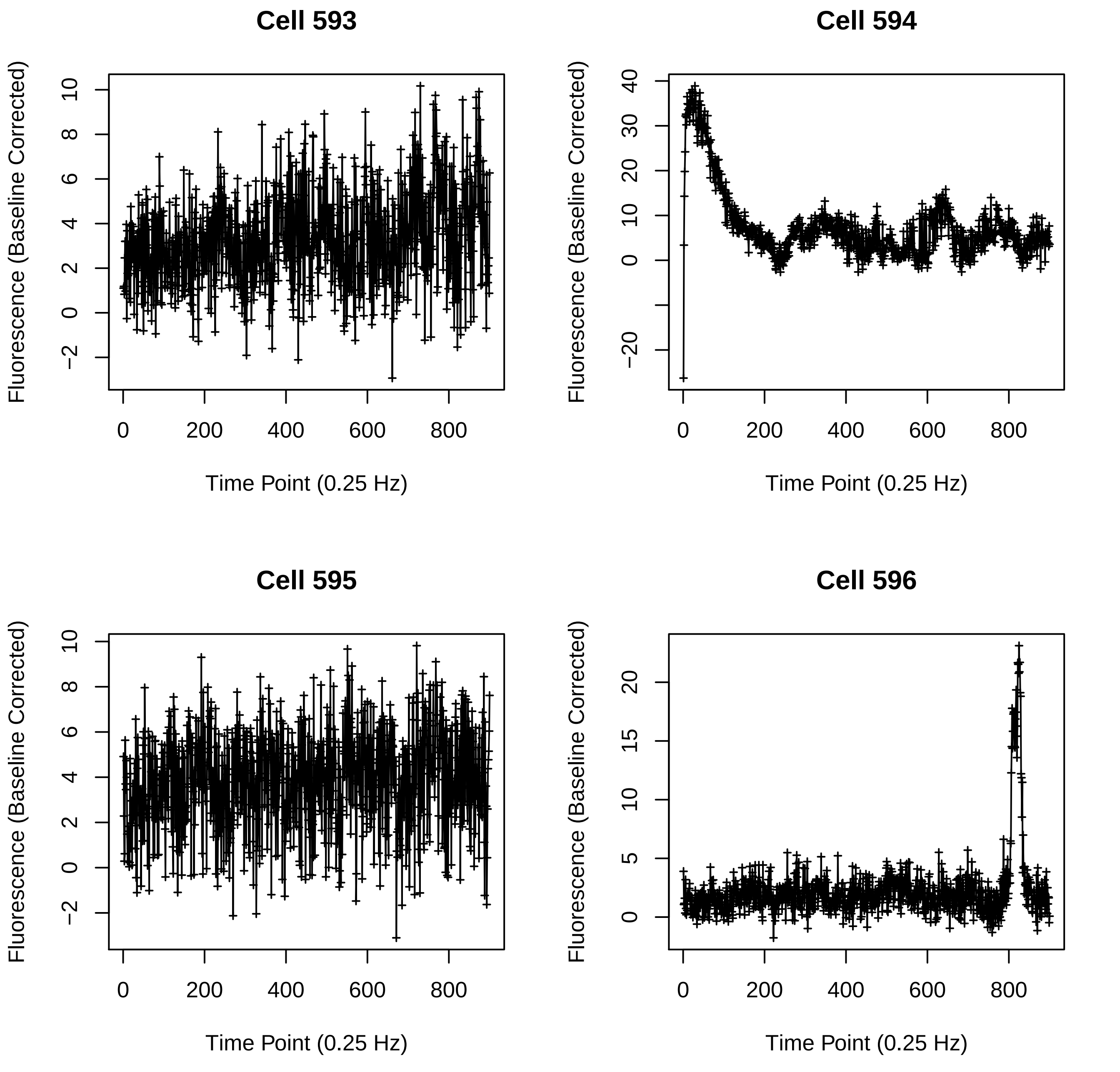

Cell 593
Cell 594
Cell 595
Cell 596
Fluorescence (Baseline Corrected)
Time Point (0.25 Hz)
0
200
400
600
800
−2
2
4
6
8
10
−20
10
20
30
40
5
15

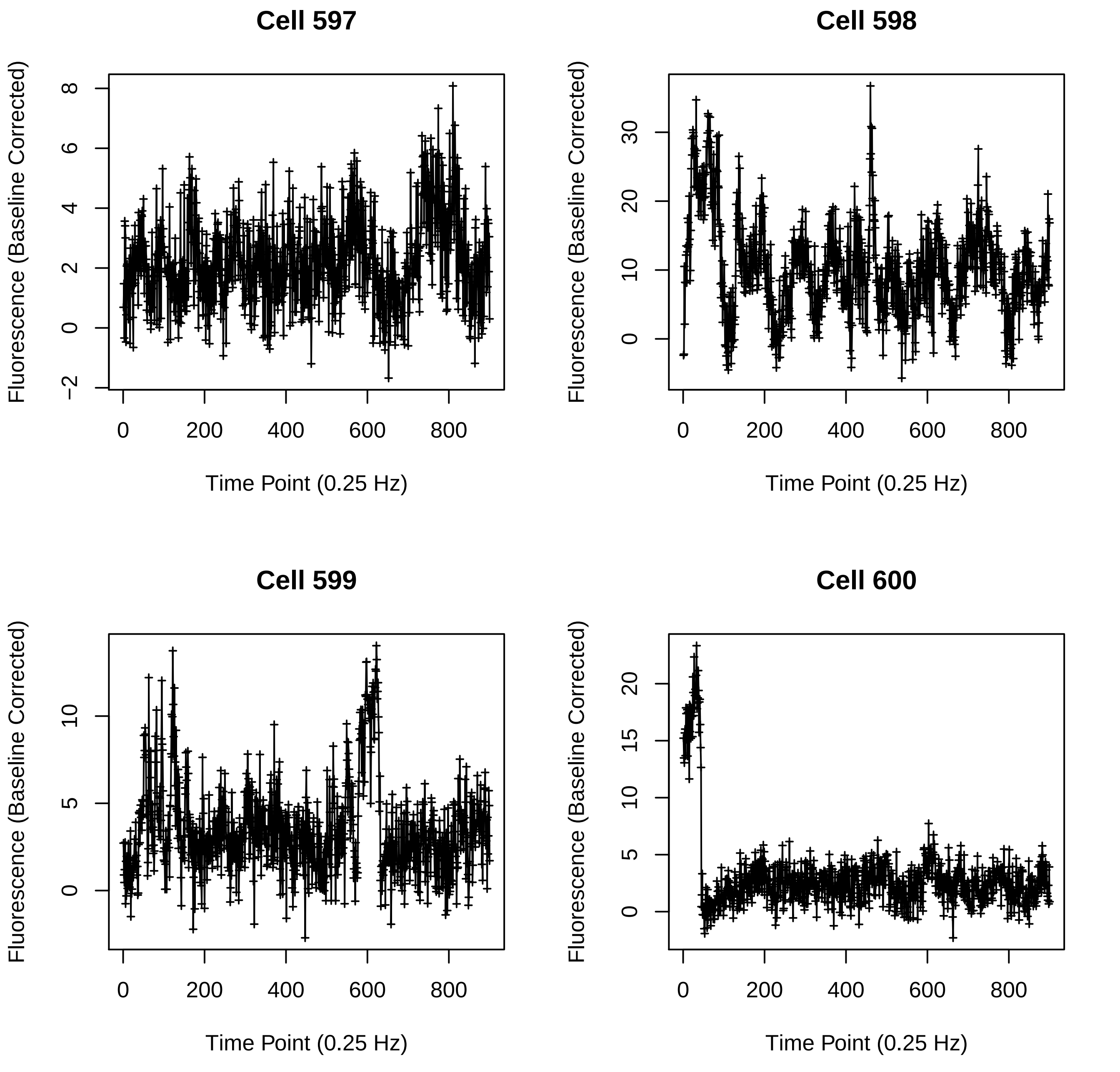
Cell 597
Cell 598
Cell 599
Cell 600
Fluorescence (Baseline Corrected)
Time Point (0.25 Hz)
−2
0
2
4
6
8
10
15
20
30
5
0
200
400
600
800

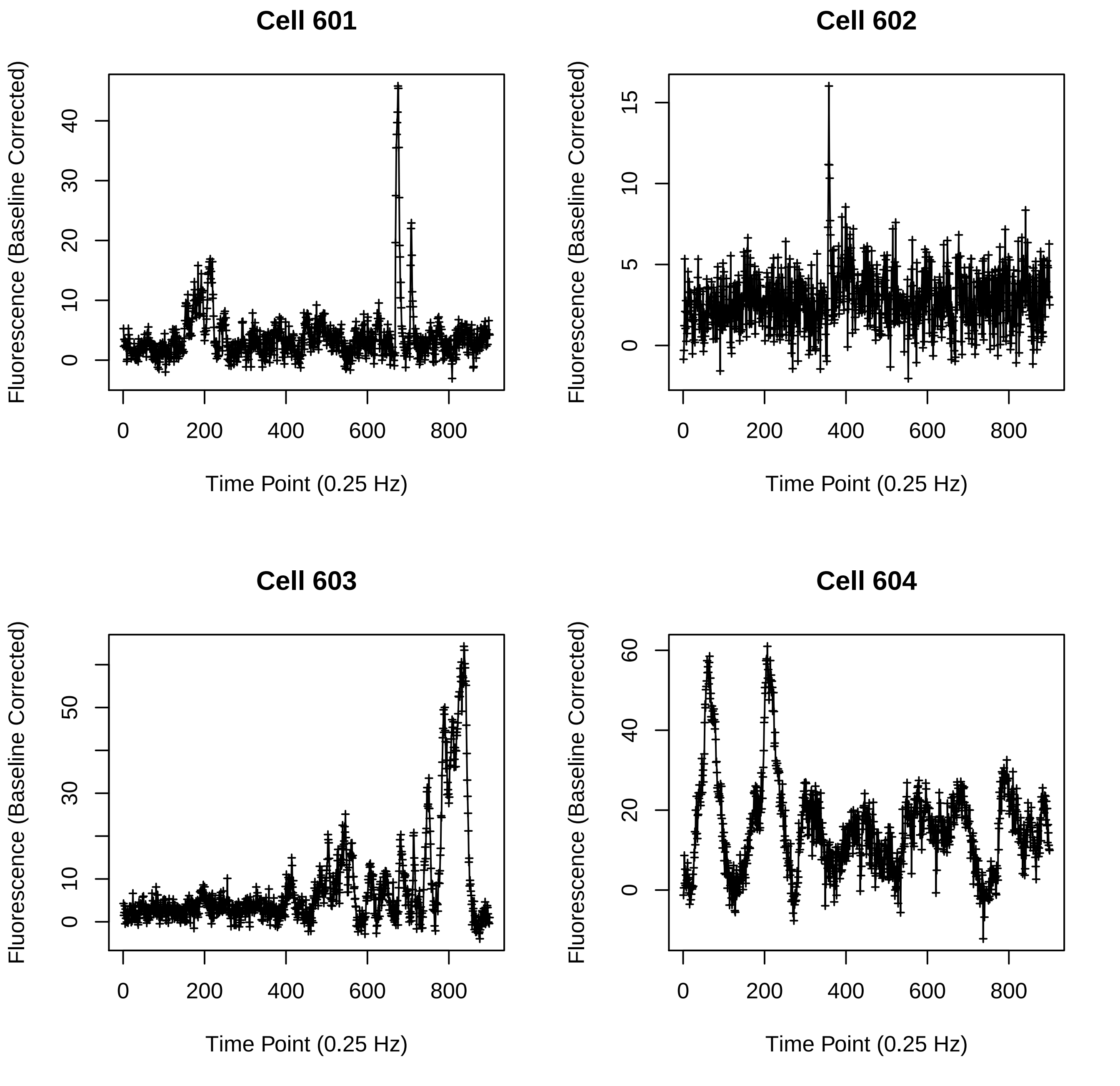
Cell 601
Fluorescence (Baseline Corrected)
Time Point (0.25 Hz)
Cell 602
Fluorescence (Baseline Corrected)
Time Point (0.25 Hz)
Cell 603
Fluorescence (Baseline Corrected)
Time Point (0.25 Hz)
Cell 604
Fluorescence (Baseline Corrected)
Time Point (0.25 Hz)

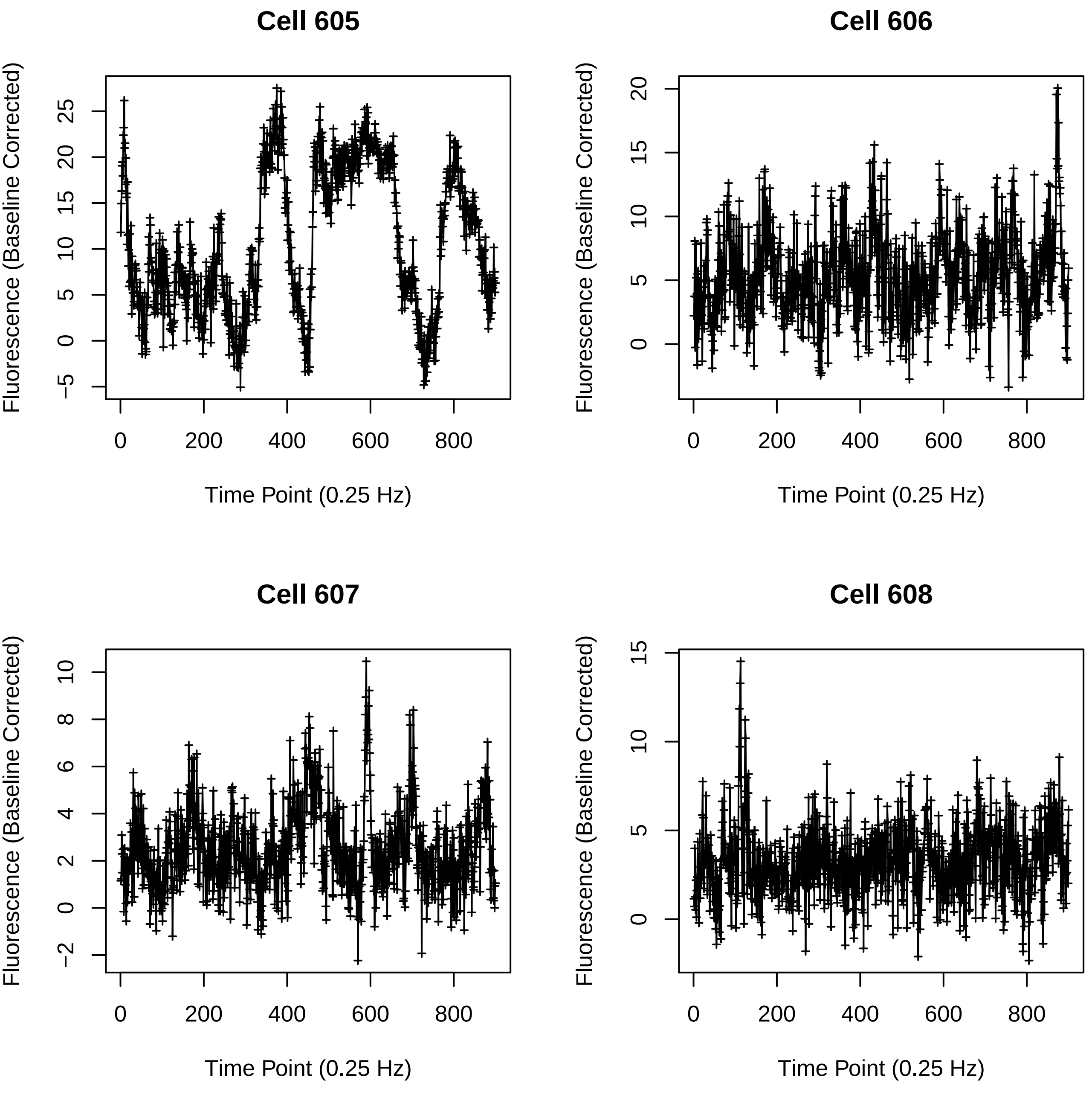

Cell 605
Cell 606
Cell 607
Cell 608
Fluorescence (Baseline Corrected)
Time Point (0.25 Hz)
0
200
400
600
800
-5
0
5
10
15
20
25
-2
2
4
6
8

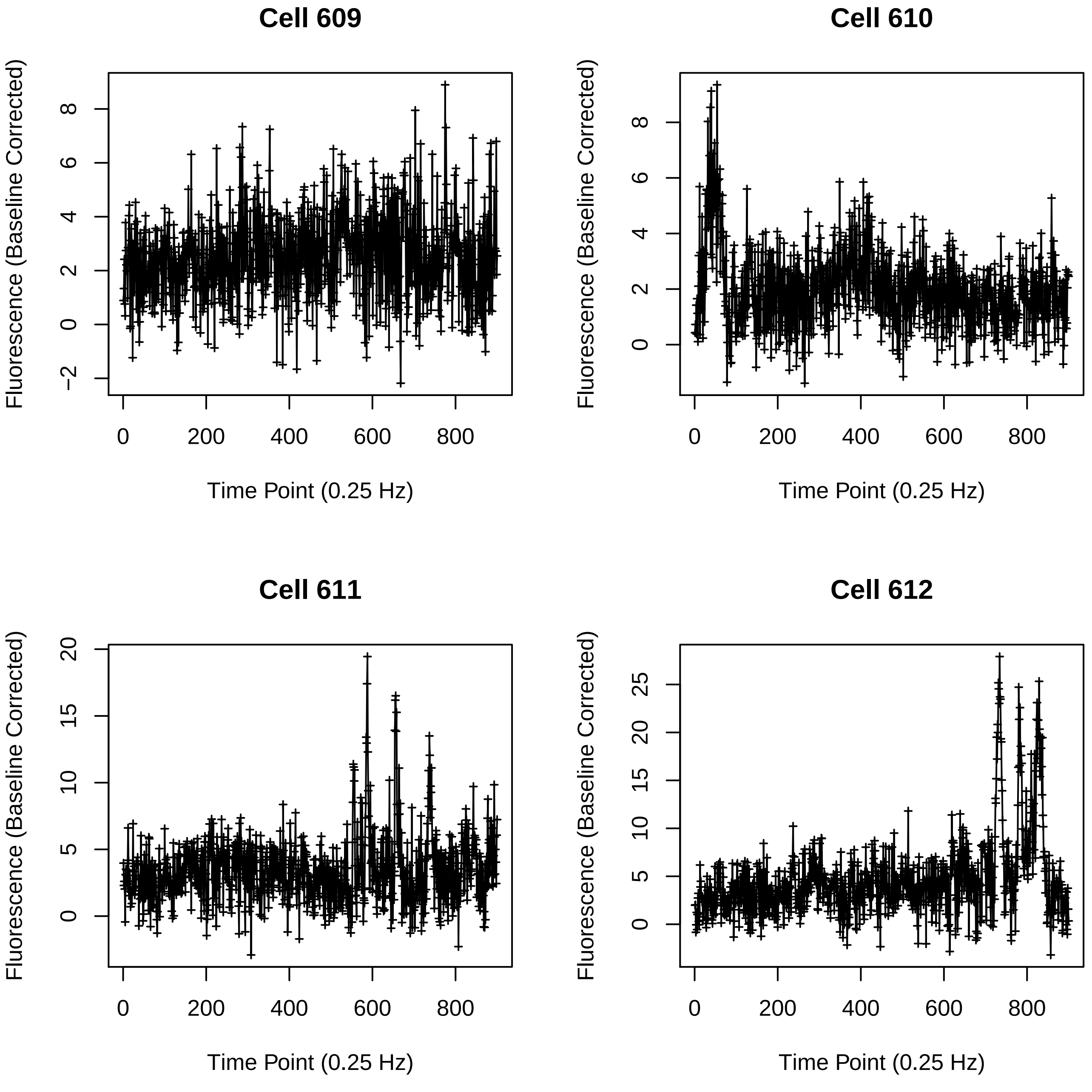

Cell 609
Cell 610
Cell 611
Cell 612
Fluorescence (Baseline Corrected)
Time Point (0.25 Hz)
−2
0
2
4
6
8
10
15
20
25
200
400
600
800

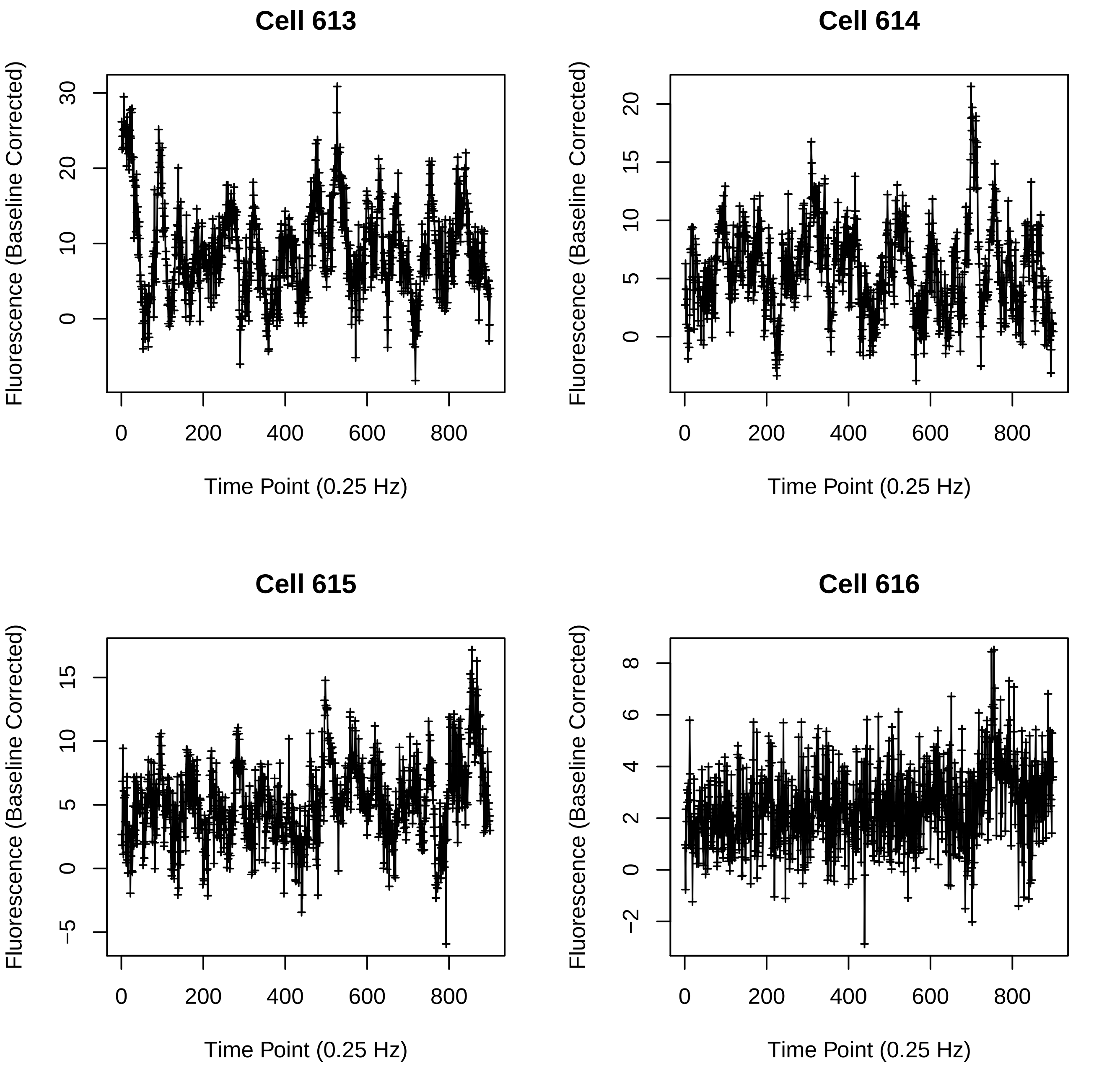
Cell 613
Cell 614
Cell 615
Cell 616
Fluorescence (Baseline Corrected)
Time Point (0.25 Hz)
0
200
400
600
800
30
20
10
0
15
5
−5
8
6
4
2
−2

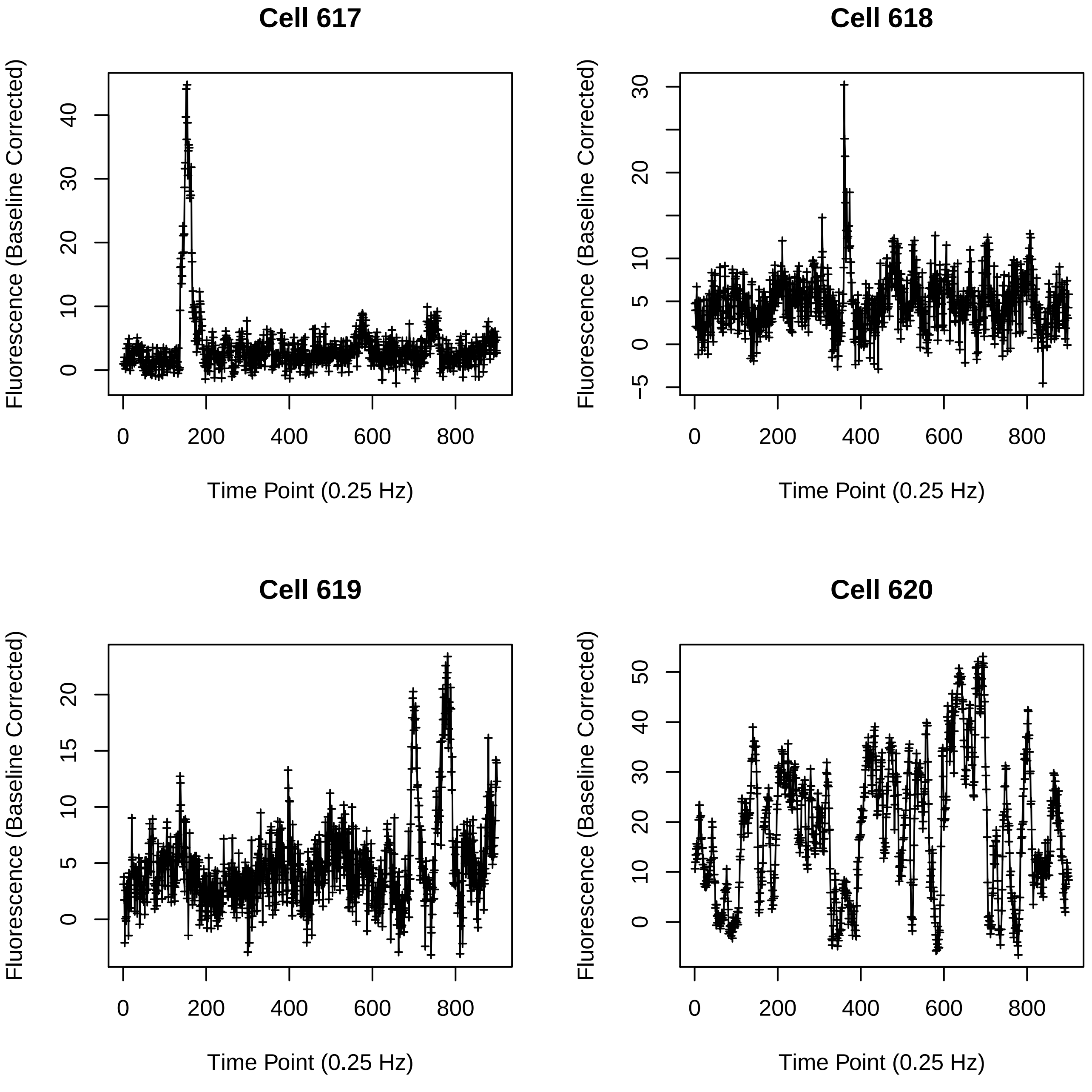
Cell 617
Cell 618
Cell 619
Cell 620
Fluorescence (Baseline Corrected)
Time Point (0.25 Hz)
0
10
20
30
40
-5
5
15
50
200
400
600
800

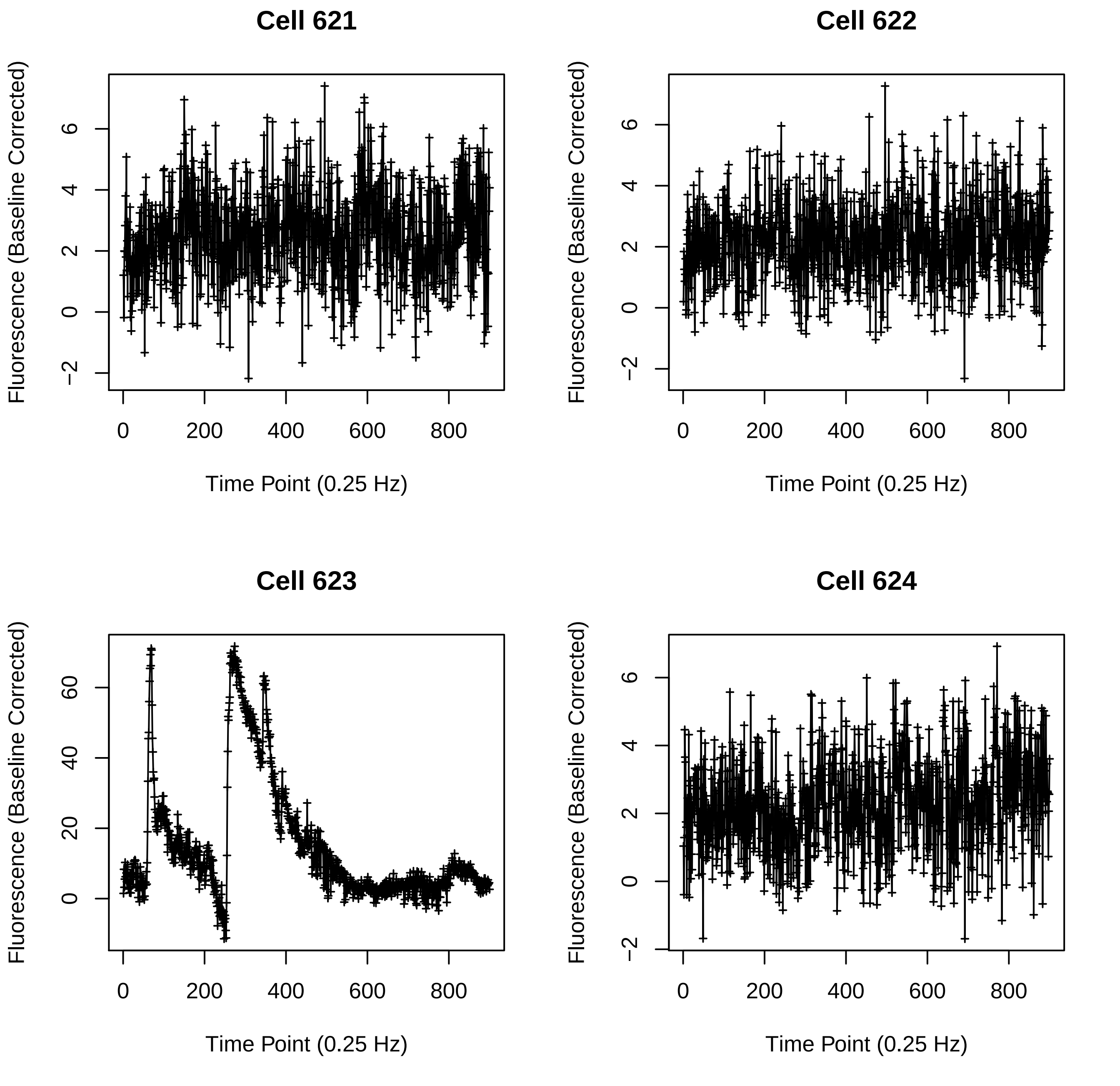

Cell 621
Cell 622
Cell 623
Cell 624
Fluorescence (Baseline Corrected)
Time Point (0.25 Hz)
0
200
400
600
800
−2
0
2
4
6
20
40
60

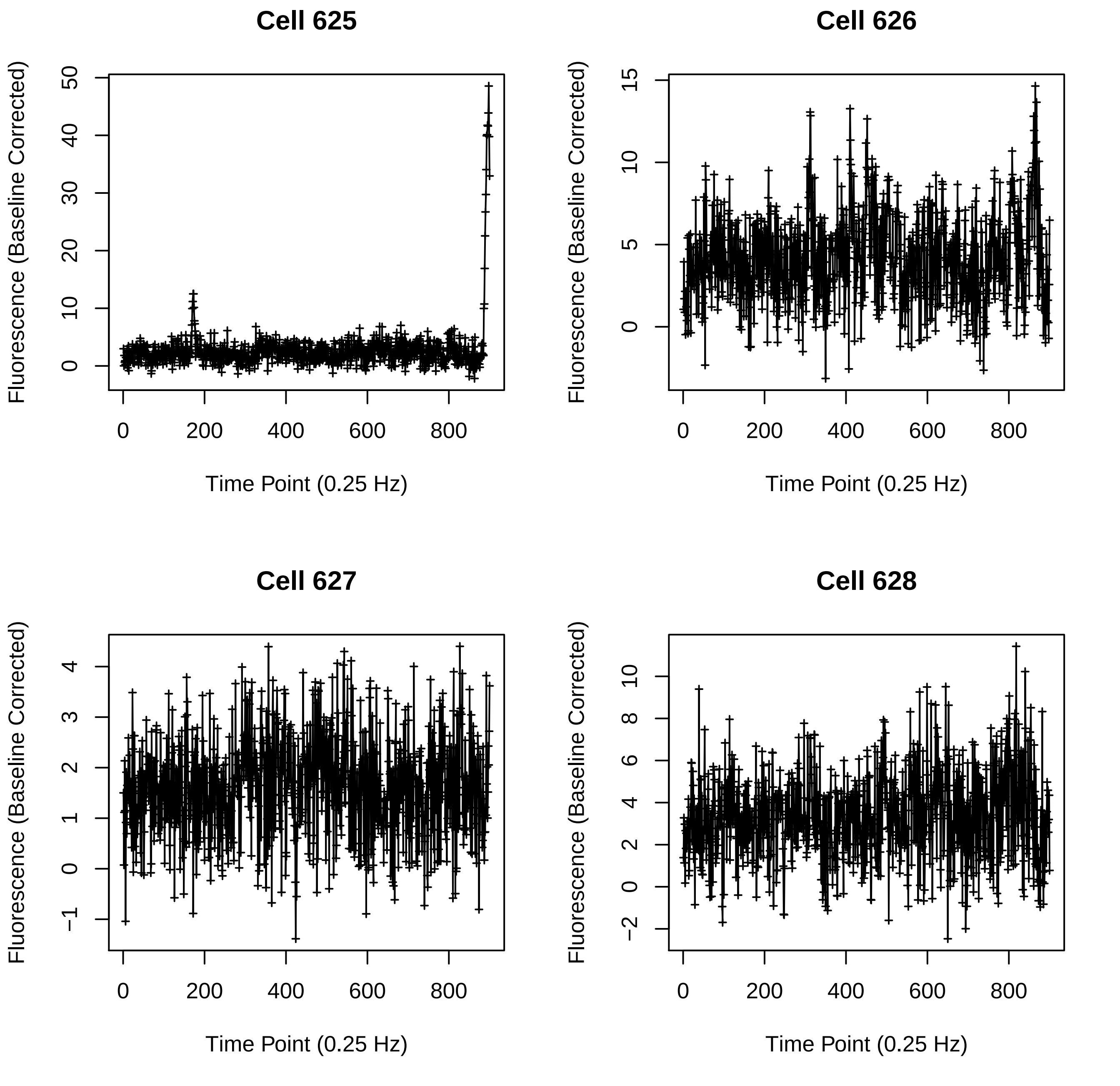
Cell 625
Cell 626
Cell 627
Cell 628
Fluorescence (Baseline Corrected)
Time Point (0.25 Hz)

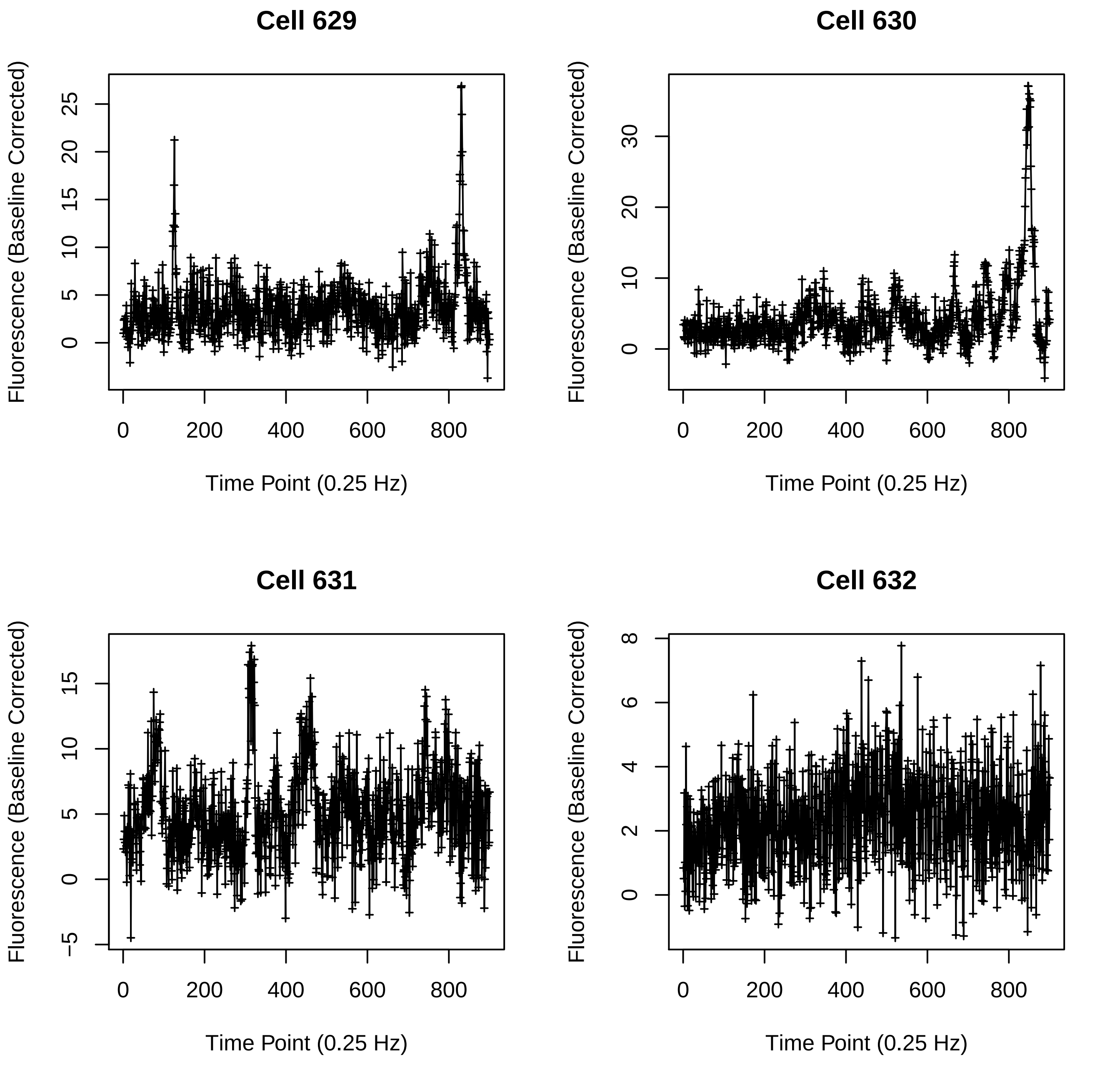
Cell 629
Cell 630
Cell 631
Cell 632
Fluorescence (Baseline Corrected)
Time Point (0.25 Hz)
0
200
400
600
800
5
10
15
20
25
30
−5
2
4
6
8

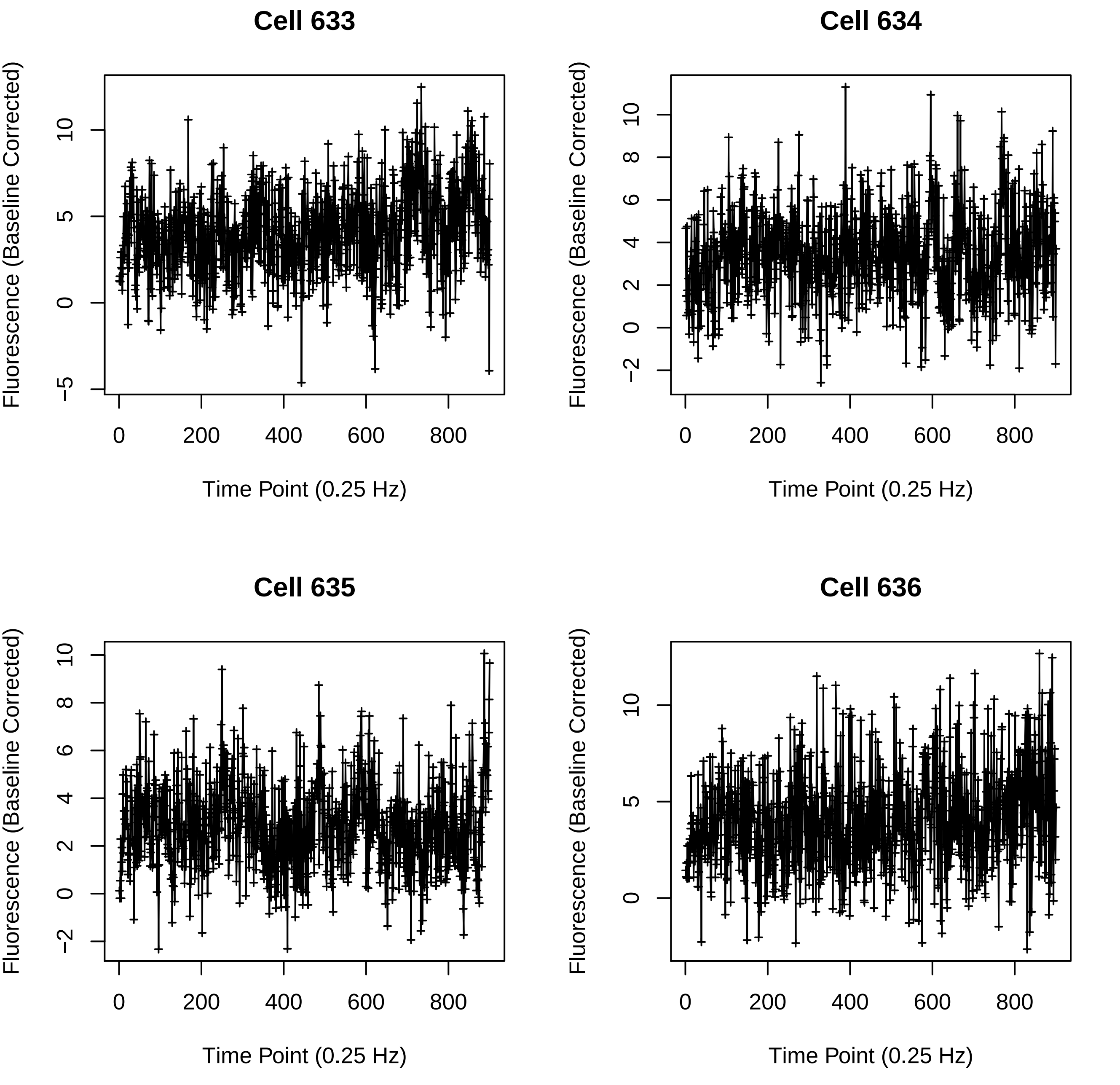
Cell 633
Cell 634
Cell 635
Cell 636
Fluorescence (Baseline Corrected)
Time Point (0.25 Hz)
−5
0
5
10
−2
2
4
6
8
200
400
600
800

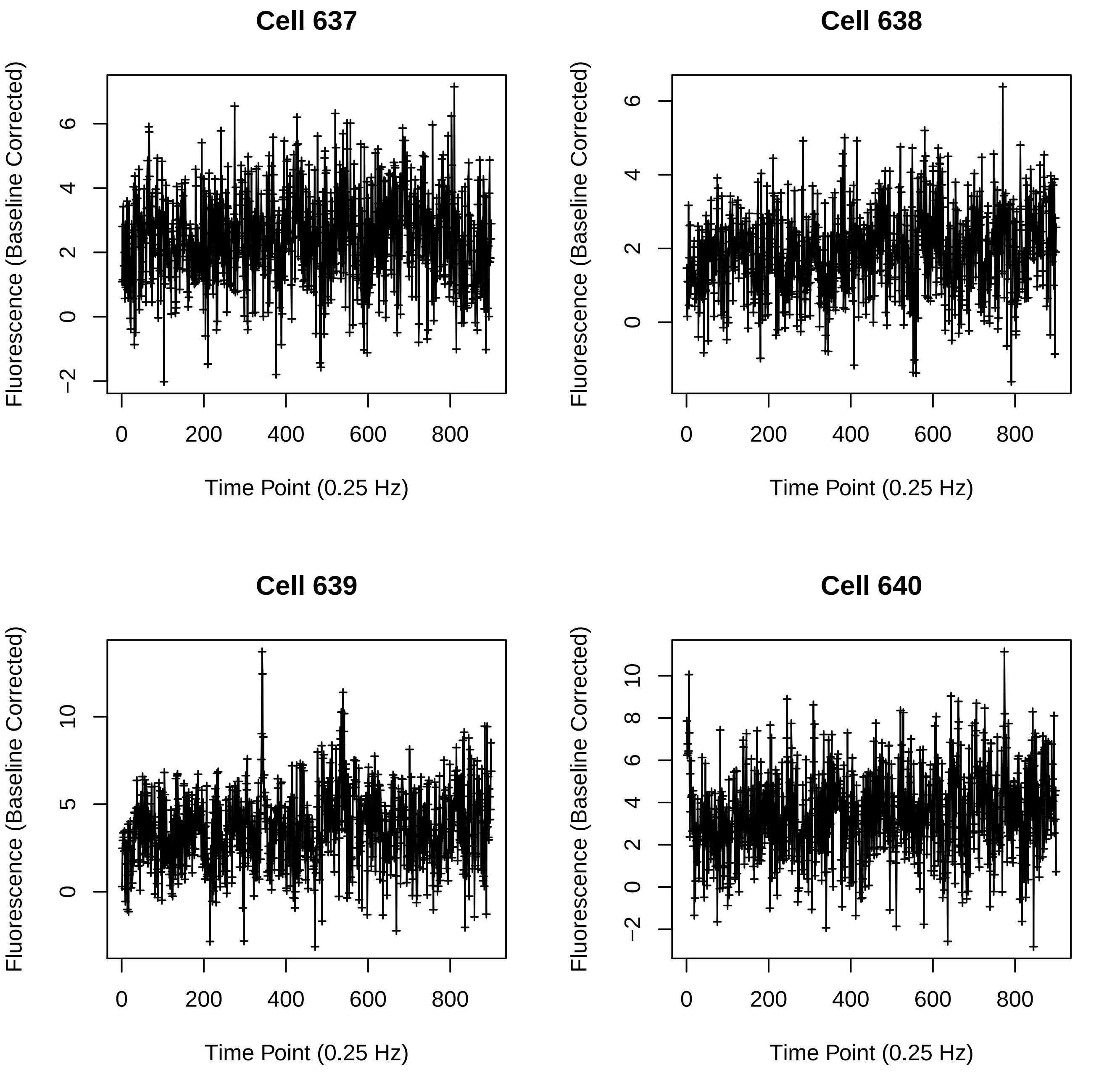
Cell 637
Cell 638
Cell 639
Cell 640
Fluorescence (Baseline Corrected)
Time Point (0.25 Hz)
−2
0
2
4
6
5
10
8
200
400
600
800

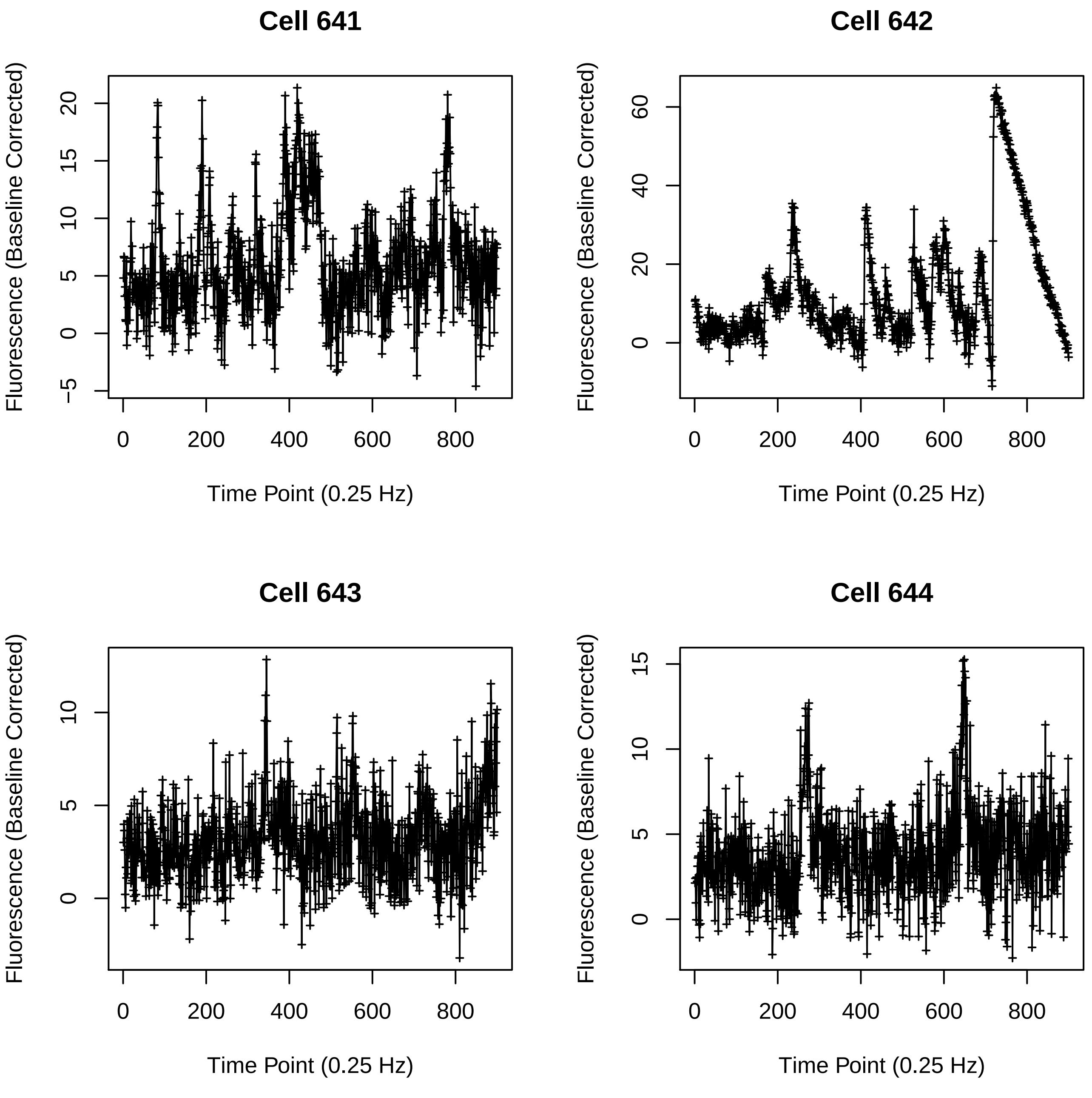

Cell 641
Fluorescence (Baseline Corrected)
−5
0
5
10
15
20
Time Point (0.25 Hz)
0
200
400
600
800
Cell 642
Fluorescence (Baseline Corrected)
0
20
40
60
Time Point (0.25 Hz)
0
200
400
600
800
Cell 643
Fluorescence (Baseline Corrected)
0
5
10
Time Point (0.25 Hz)
0
200
400
600
800
Cell 644
Fluorescence (Baseline Corrected)
0
5
10
15
Time Point (0.25 Hz)
0
200
400
600
800

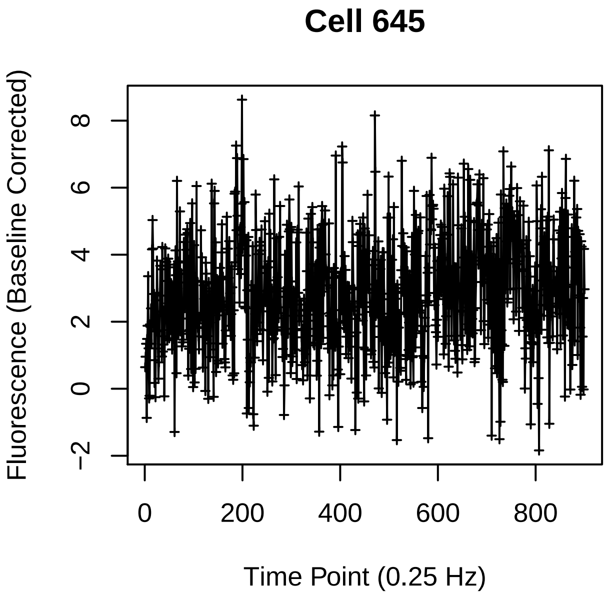
Cell 645
Fluorescence (Baseline Corrected)
−2
0
2
4
6
8
0
200
400
600
800
Time Point (0.25 Hz)
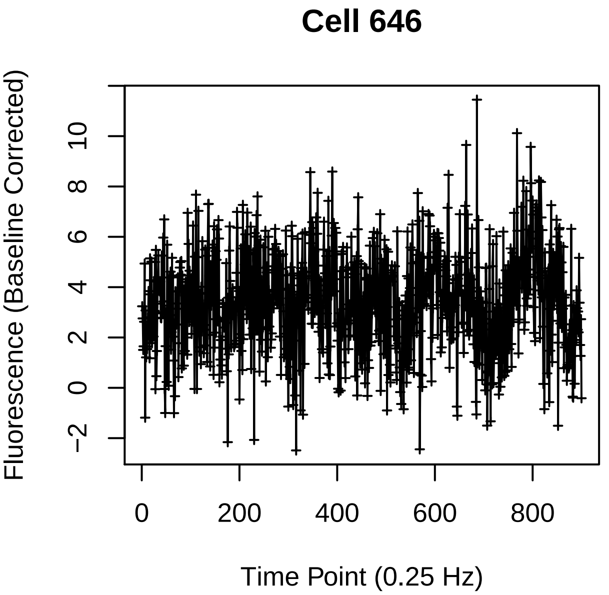
Cell 646
Fluorescence (Baseline Corrected)
−2
0
2
4
6
8
10
0
200
400
600
800
Time Point (0.25 Hz)
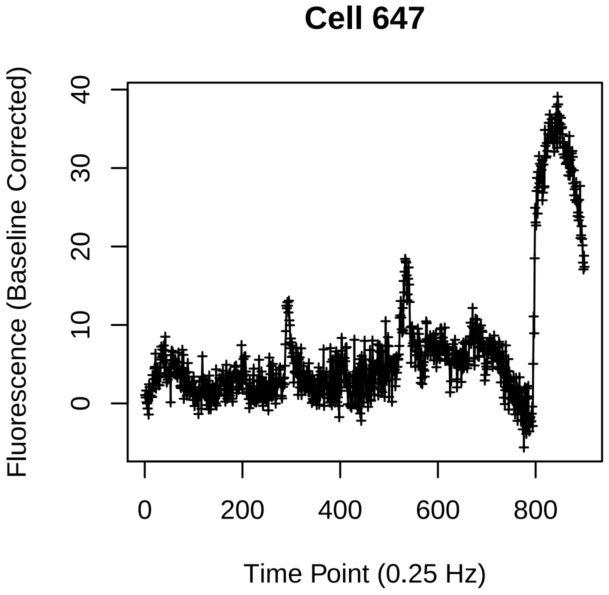
Cell 647
Fluorescence (Baseline Corrected)
0
10
20
30
40
0
200
400
600
800
Time Point (0.25 Hz)
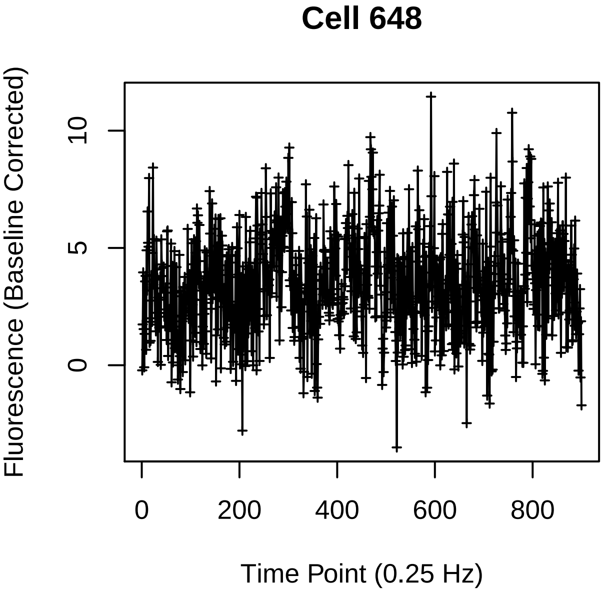
Cell 648
Fluorescence (Baseline Corrected)
0
5
10
0
200
400
600
800
Time Point (0.25 Hz)

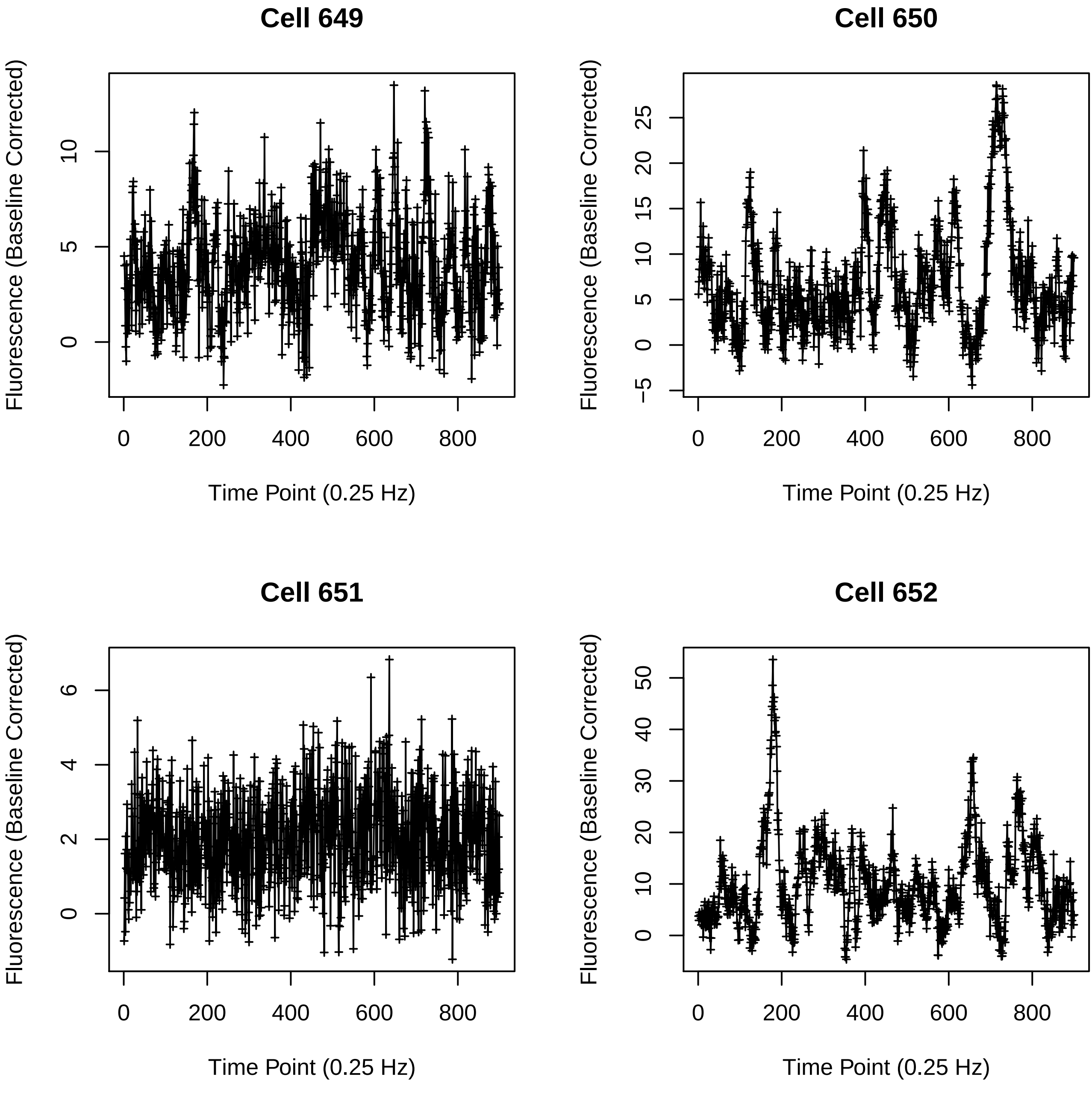
Cell 649
Cell 650
Cell 651
Cell 652
Fluorescence (Baseline Corrected)
Time Point (0.25 Hz)
0
5
10
−5
15
20
25
2
4
6
30
40
50
200
400
600
800

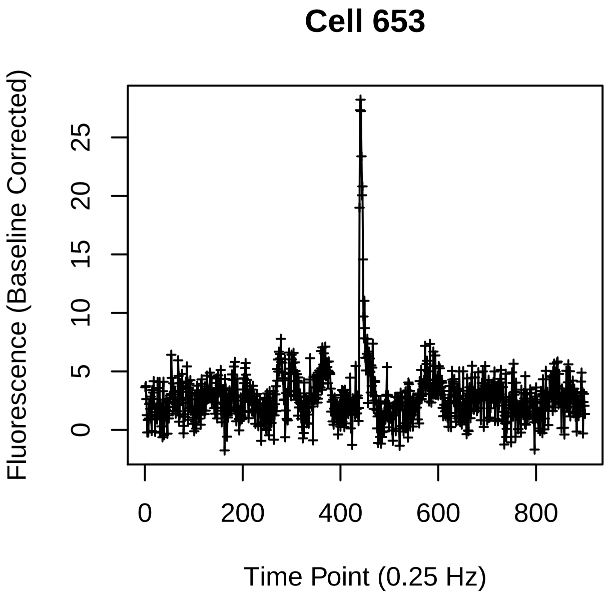
Cell 653
Fluorescence (Baseline Corrected)
0
5
10
15
20
25
0
200
400
600
800
Time Point (0.25 Hz)

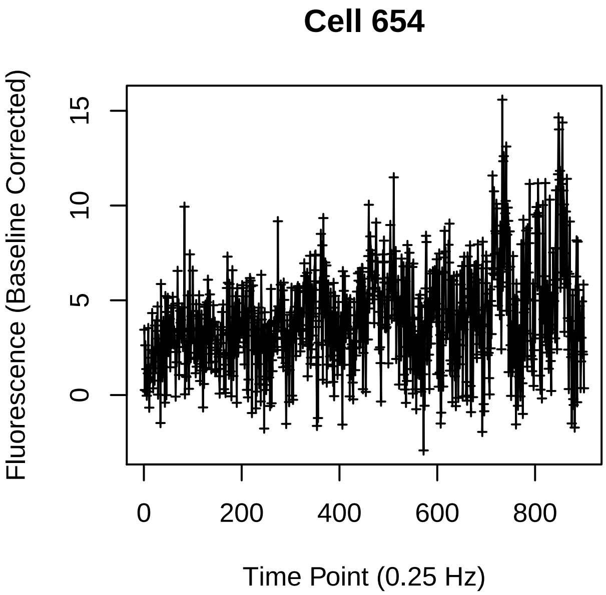
Cell 654
Fluorescence (Baseline Corrected)
0
5
10
15
0
200
400
600
800
Time Point (0.25 Hz)

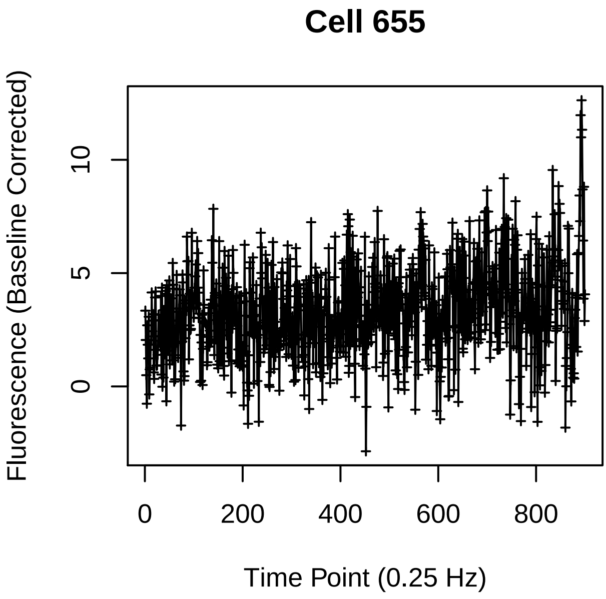
Cell 655
Fluorescence (Baseline Corrected)
0
5
10
0
200
400
600
800
Time Point (0.25 Hz)

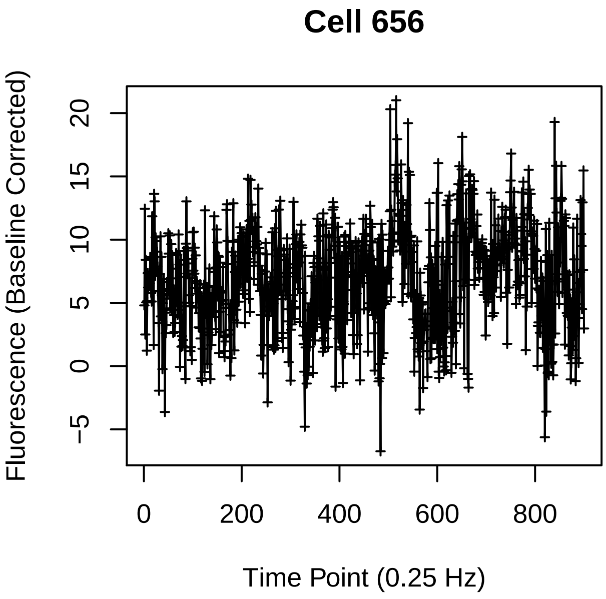
Cell 656
Fluorescence (Baseline Corrected)
−5
0
5
10
15
20
0
200
400
600
800
Time Point (0.25 Hz)

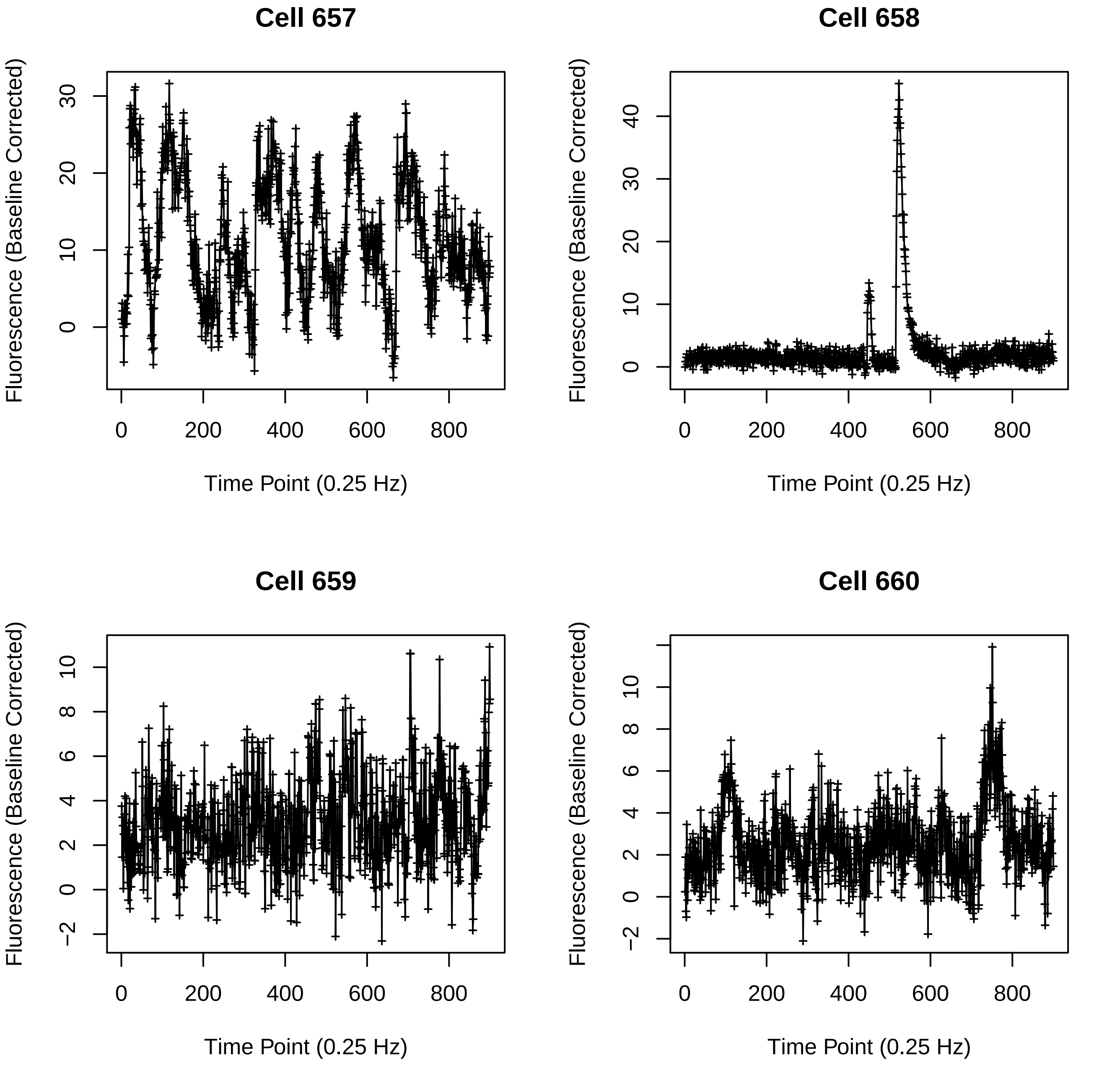
Cell 657
Cell 658
Cell 659
Cell 660
Fluorescence (Baseline Corrected)
Time Point (0.25 Hz)
0
200
400
600
800
30
20
10
0
40
30
20
10
0
10
8
6
4
2
0
−2

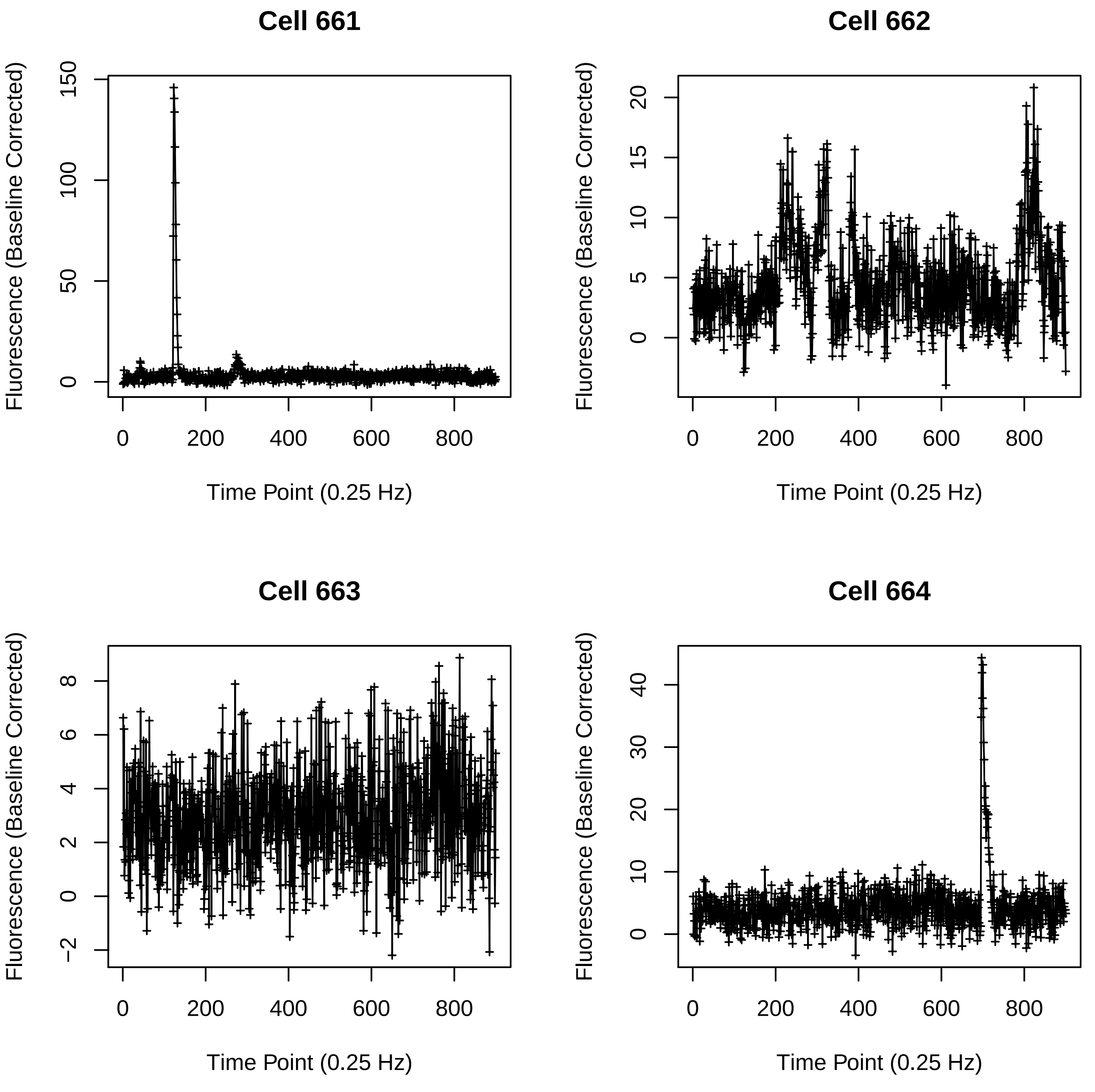
Cell 661
Fluorescence (Baseline Corrected)
0
50
100
150
0
200
400
600
800
Time Point (0.25 Hz)
Cell 662
Fluorescence (Baseline Corrected)
0
5
10
15
20
0
200
400
600
800
Time Point (0.25 Hz)
Cell 663
Fluorescence (Baseline Corrected)
−2
0
2
4
6
8
0
200
400
600
800
Time Point (0.25 Hz)
Cell 664
Fluorescence (Baseline Corrected)
0
10
20
30
40
0
200
400
600
800
Time Point (0.25 Hz)

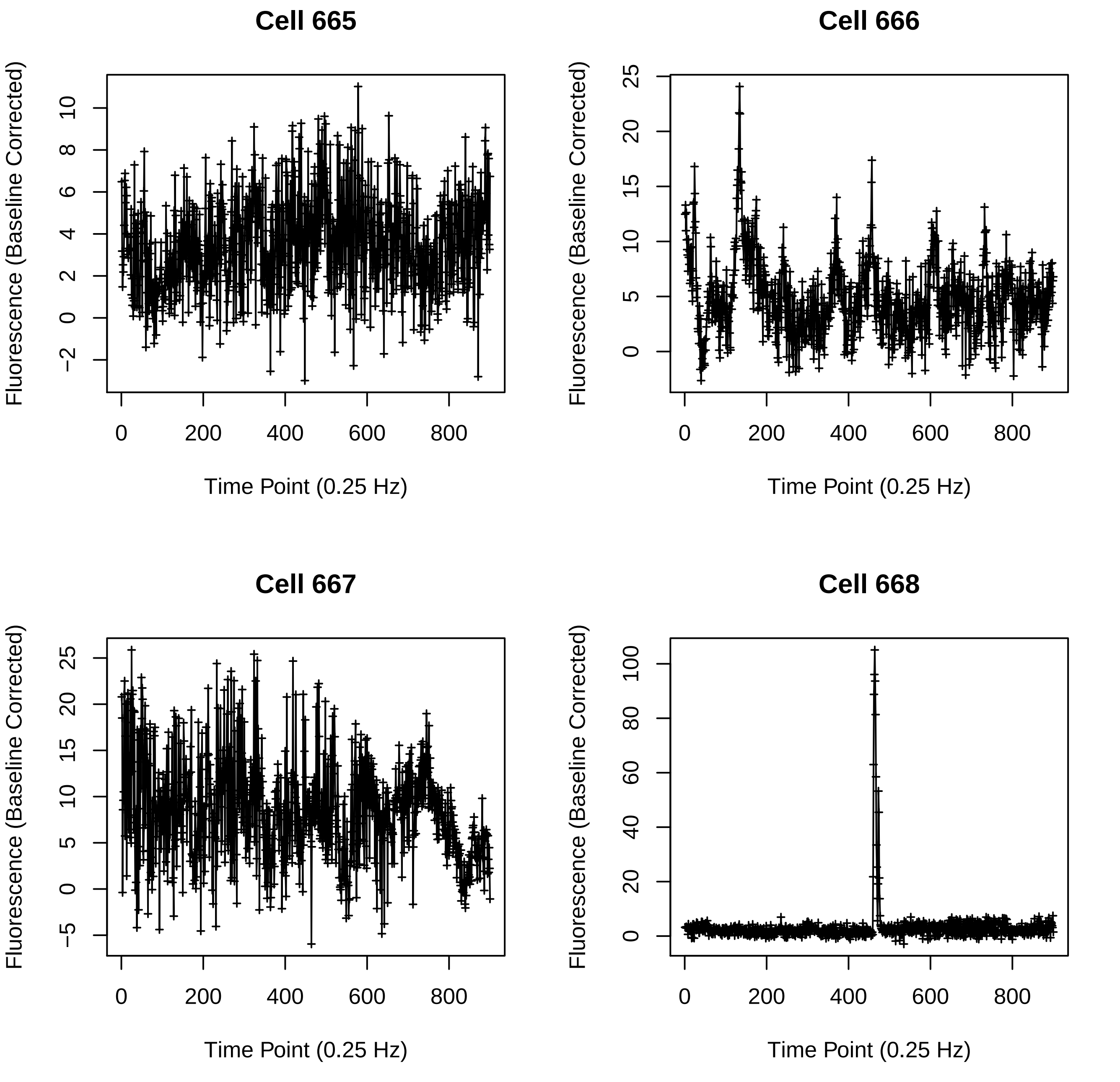
Cell 665
Cell 666
Cell 667
Cell 668
Fluorescence (Baseline Corrected)
Time Point (0.25 Hz)
0
200
400
600
800
−2
2
4
6
8
10
5
15
20
25
−5
20
40
60
80
100

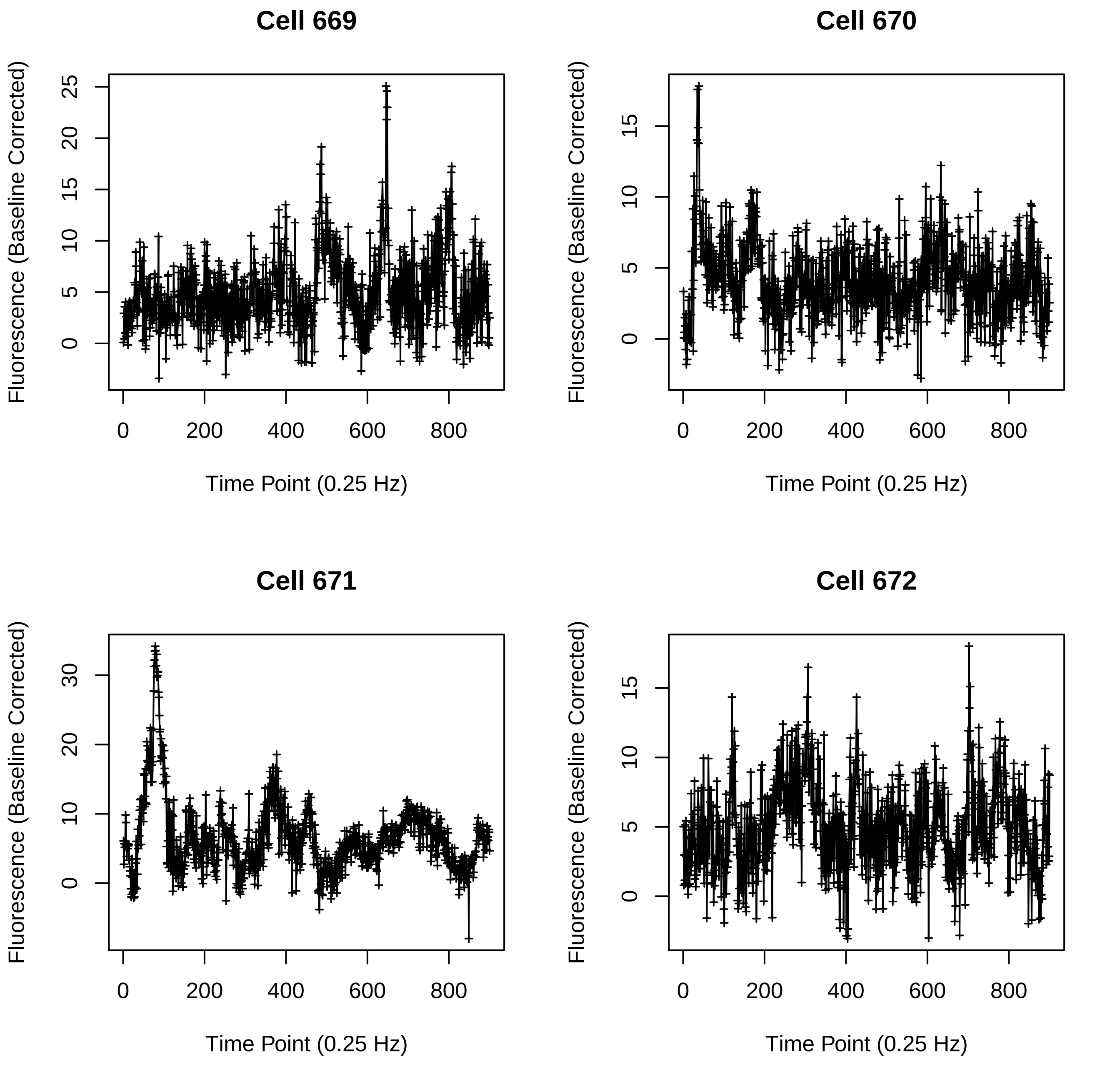
Cell 669
Cell 670
Cell 671
Cell 672
Fluorescence (Baseline Corrected)
Time Point (0.25 Hz)
0
200
400
600
800
5
10
15
20
25
30

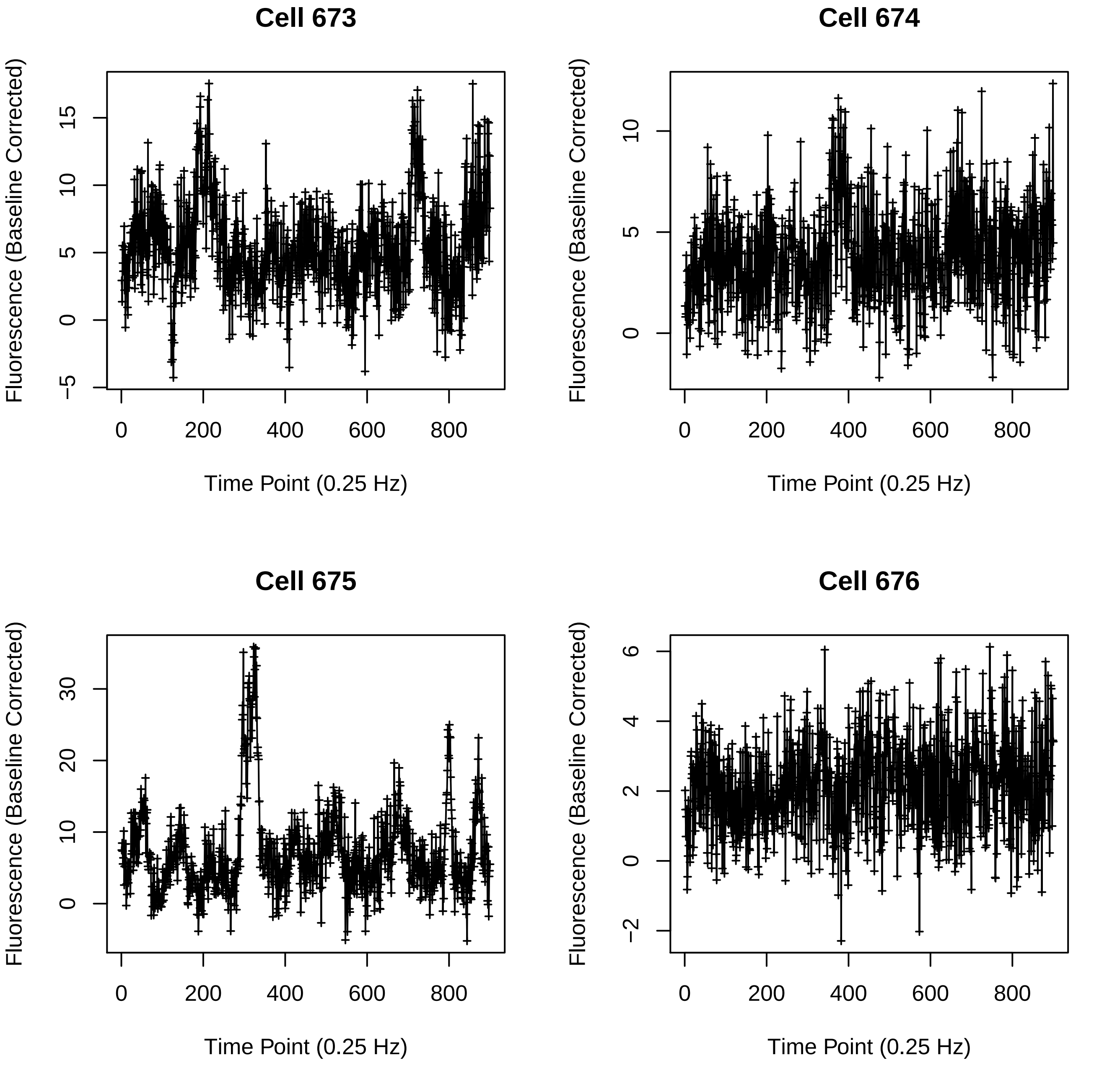
Cell 673
Fluorescence (Baseline Corrected)
−5
0
5
10
15
0
200
400
600
800
Time Point (0.25 Hz)
Cell 674
Fluorescence (Baseline Corrected)
0
5
10
0
200
400
600
800
Time Point (0.25 Hz)
Cell 675
Fluorescence (Baseline Corrected)
0
10
20
30
0
200
400
600
800
Time Point (0.25 Hz)
Cell 676
Fluorescence (Baseline Corrected)
−2
0
2
4
6
0
200
400
600
800
Time Point (0.25 Hz)

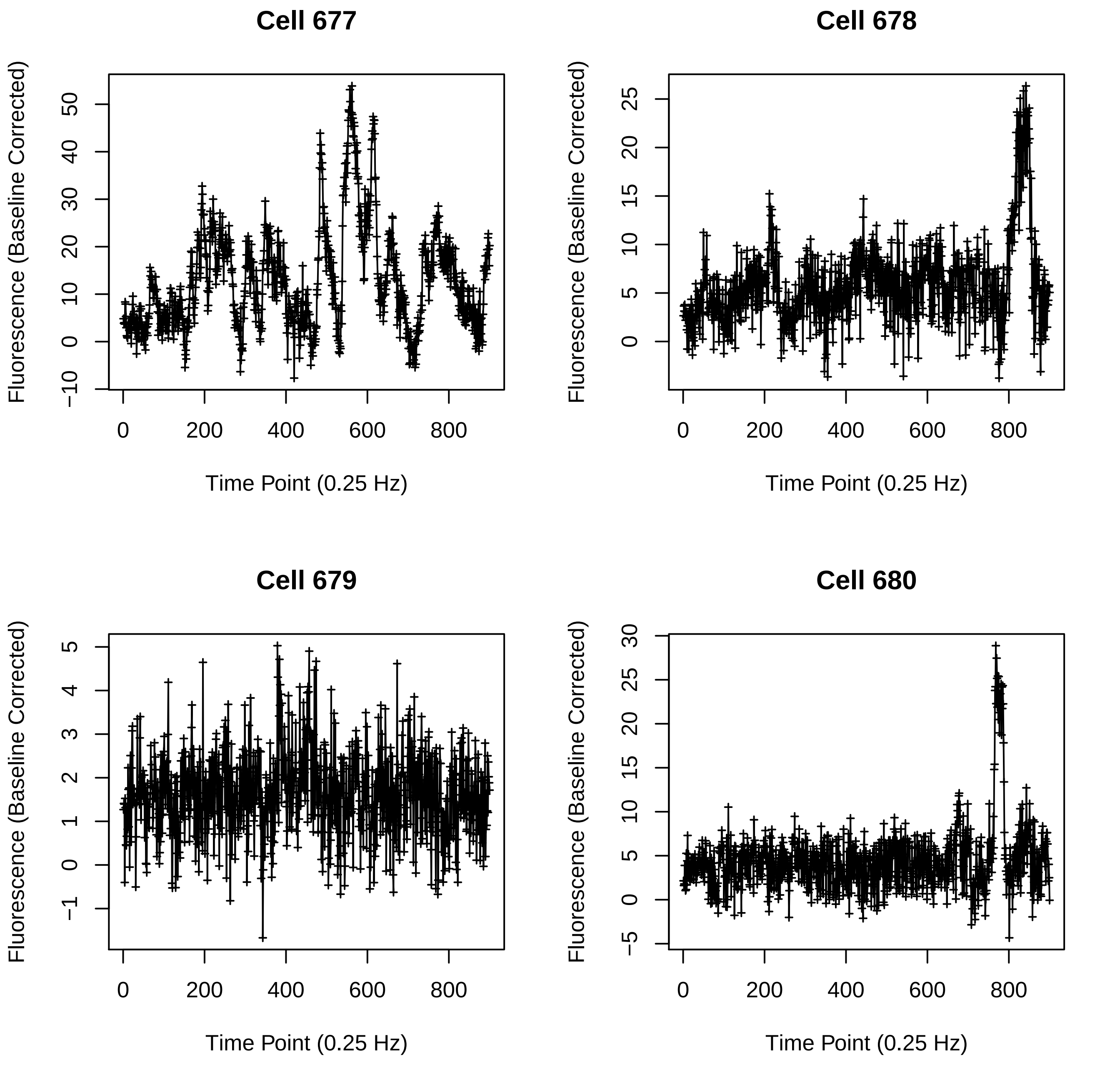
Cell 677
Cell 678
Cell 679
Cell 680
Fluorescence (Baseline Corrected)
Time Point (0.25 Hz)

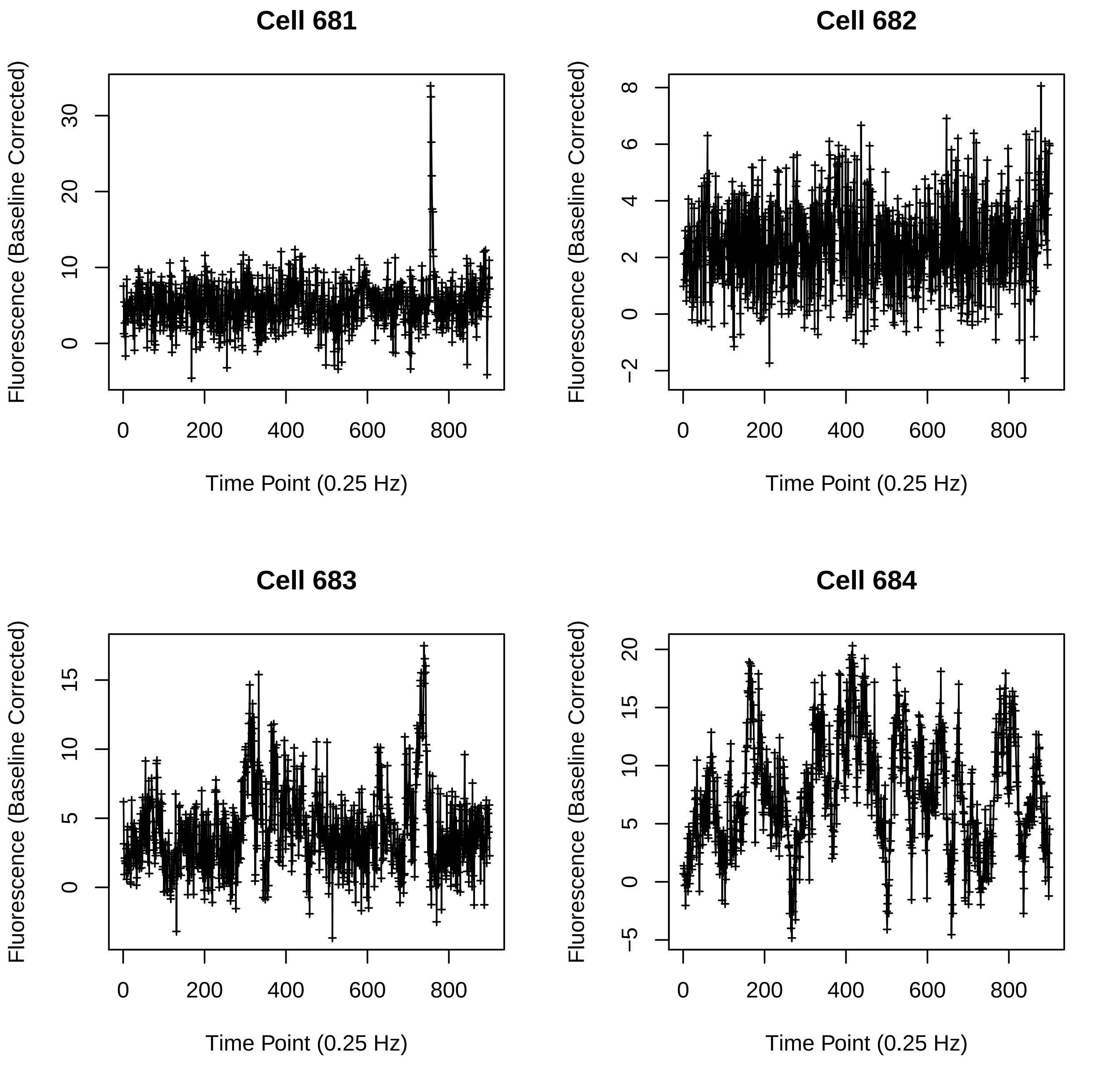
Cell 681
Cell 682
Cell 683
Cell 684
Fluorescence (Baseline Corrected)
Time Point (0.25 Hz)
0
200
400
600
800
30
20
10
8
6
4
2
−2
15
5
−5

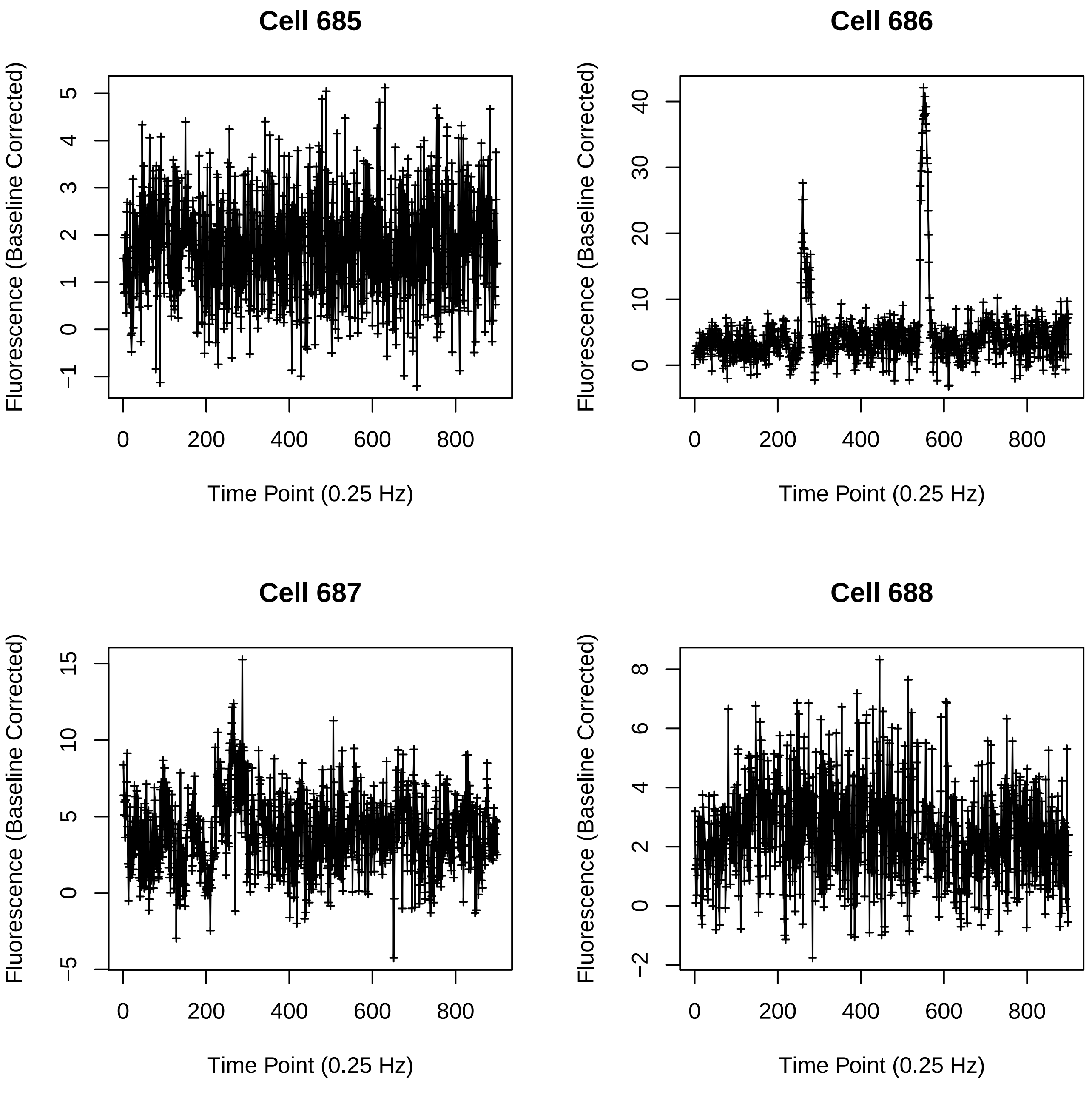
Cell 685
Fluorescence (Baseline Corrected)
Time Point (0.25 Hz)
Cell 686
Fluorescence (Baseline Corrected)
Time Point (0.25 Hz)
Cell 687
Fluorescence (Baseline Corrected)
Time Point (0.25 Hz)
Cell 688
Fluorescence (Baseline Corrected)
Time Point (0.25 Hz)

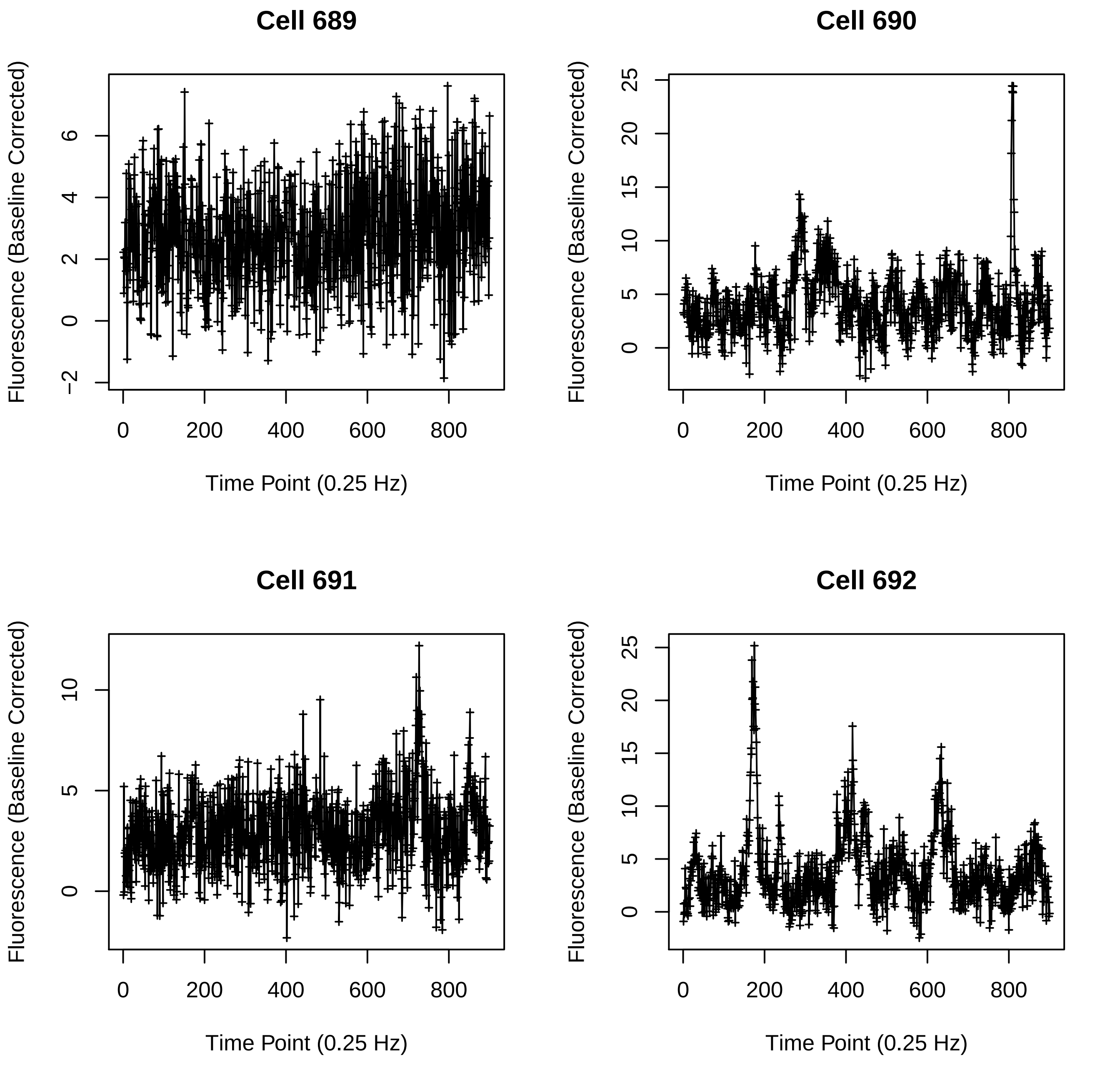

Cell 689
Cell 690
Cell 691
Cell 692
Fluorescence (Baseline Corrected)
Time Point (0.25 Hz)
0
200
400
600
800
−2
2
4
6
5
10
15
20
25

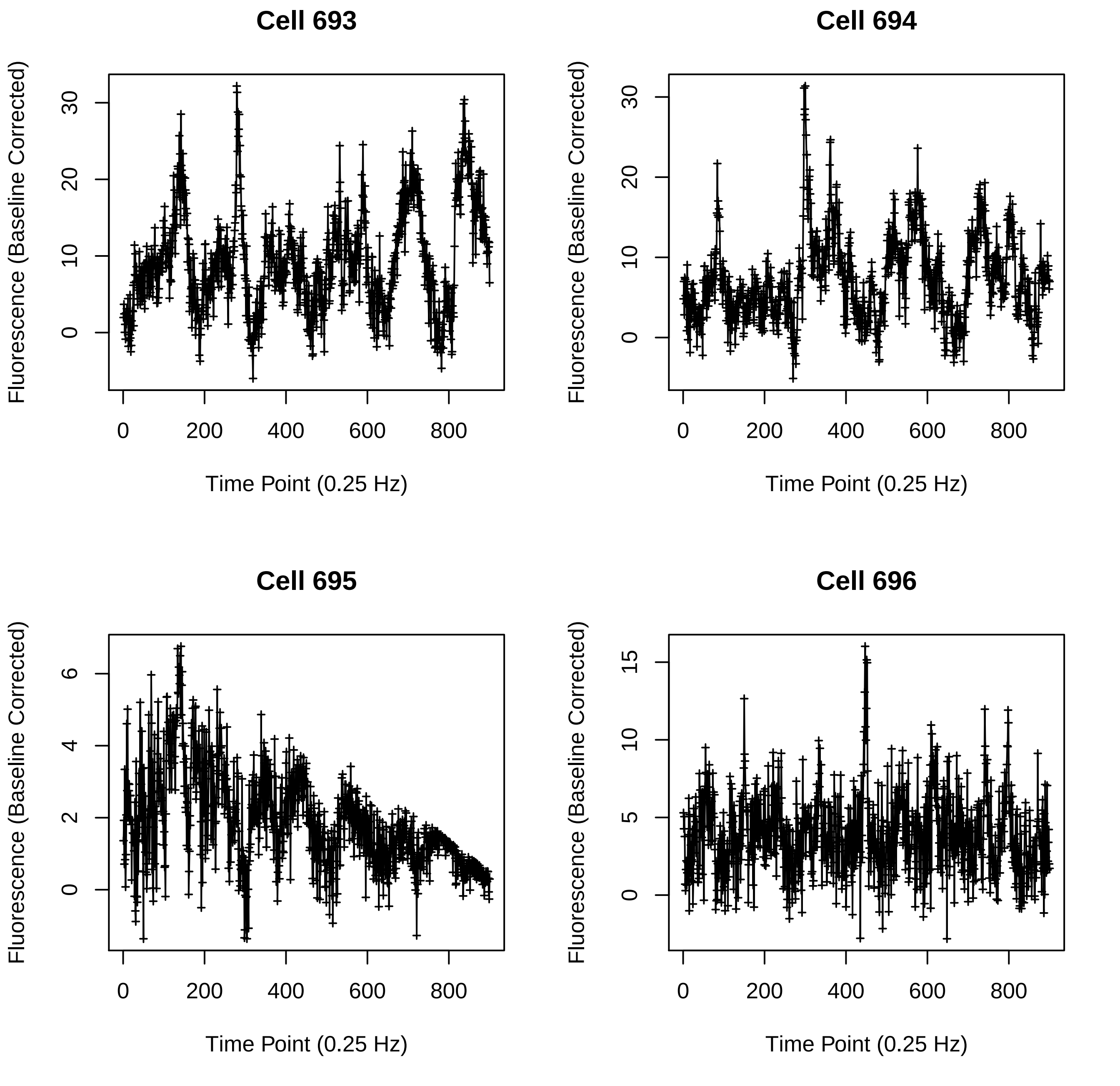
Cell 693
Cell 694
Cell 695
Cell 696
Fluorescence (Baseline Corrected)
Time Point (0.25 Hz)
0
200
400
600
800
-5
0
10
20
30
0
2
4
6
0
5
10
15

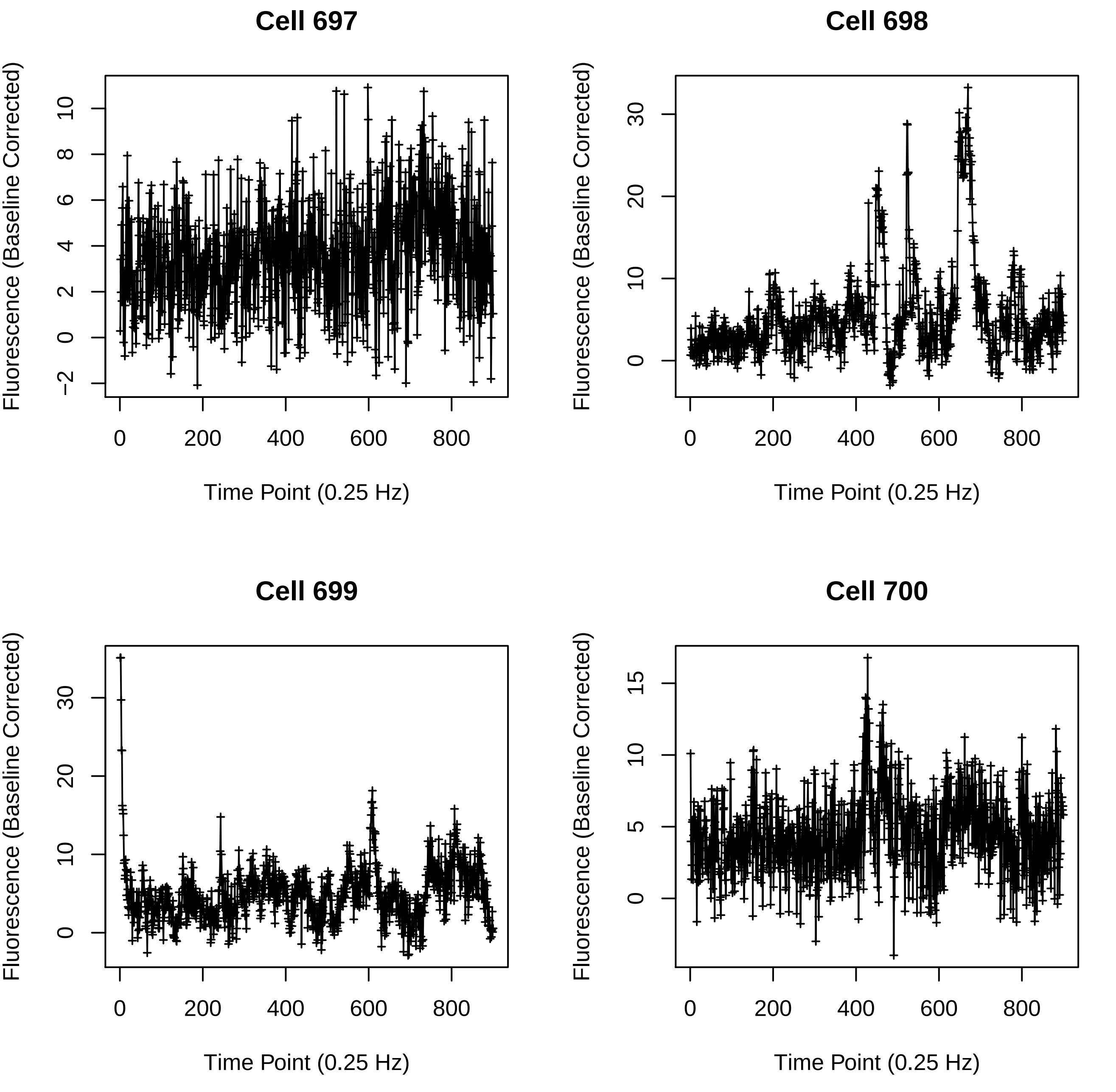
Cell 697
Cell 698
Cell 699
Cell 700
Fluorescence (Baseline Corrected)
Time Point (0.25 Hz)
0
200
400
600
800
−2
2
4
6
8
10
20
30
5
15

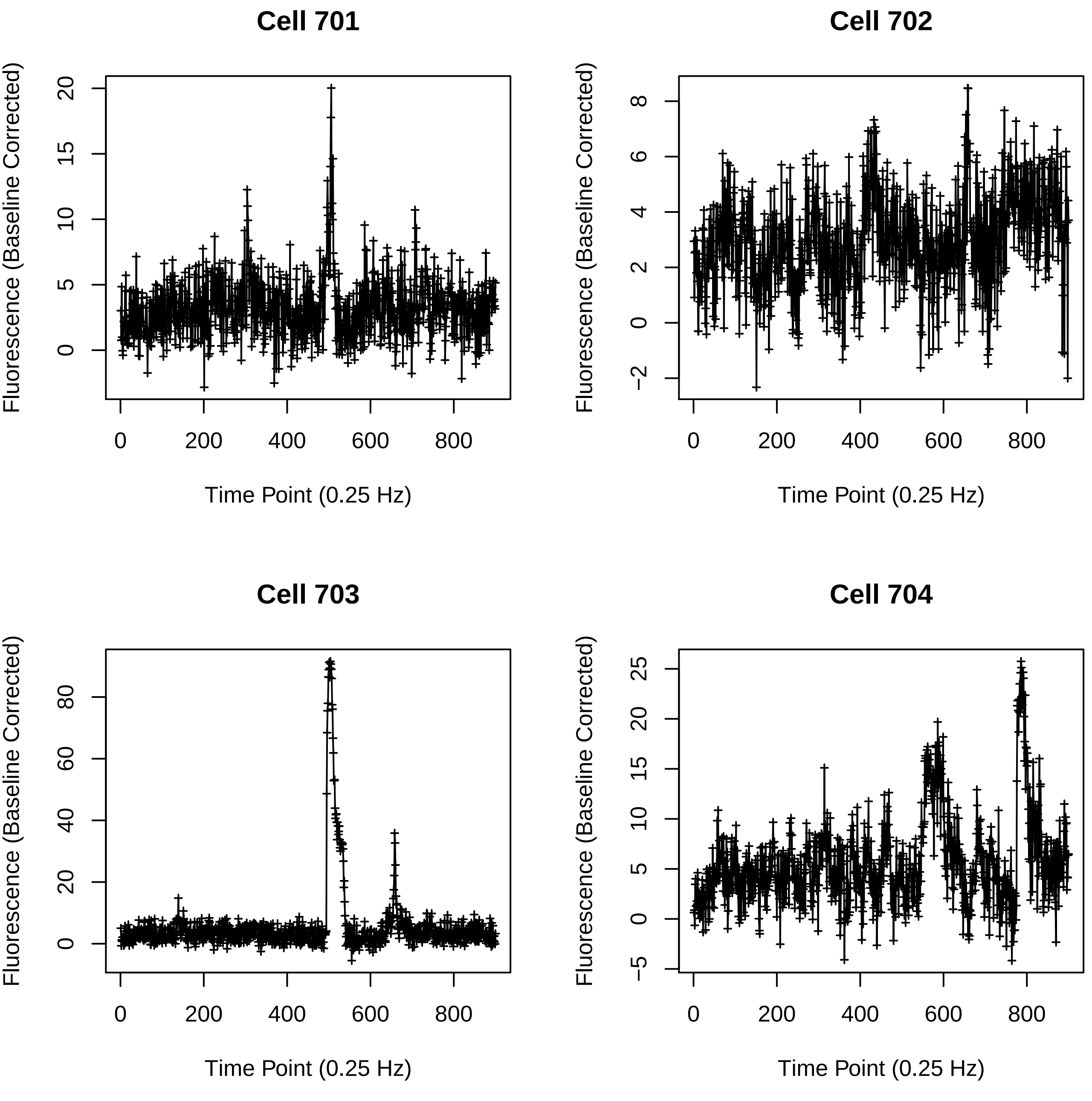
Cell 701
Cell 702
Cell 703
Cell 704
Fluorescence (Baseline Corrected)
Time Point (0.25 Hz)
0
200
400
600
800
-2
2
4
6
8
5
10
15
20
25
-5
20
40
60
80

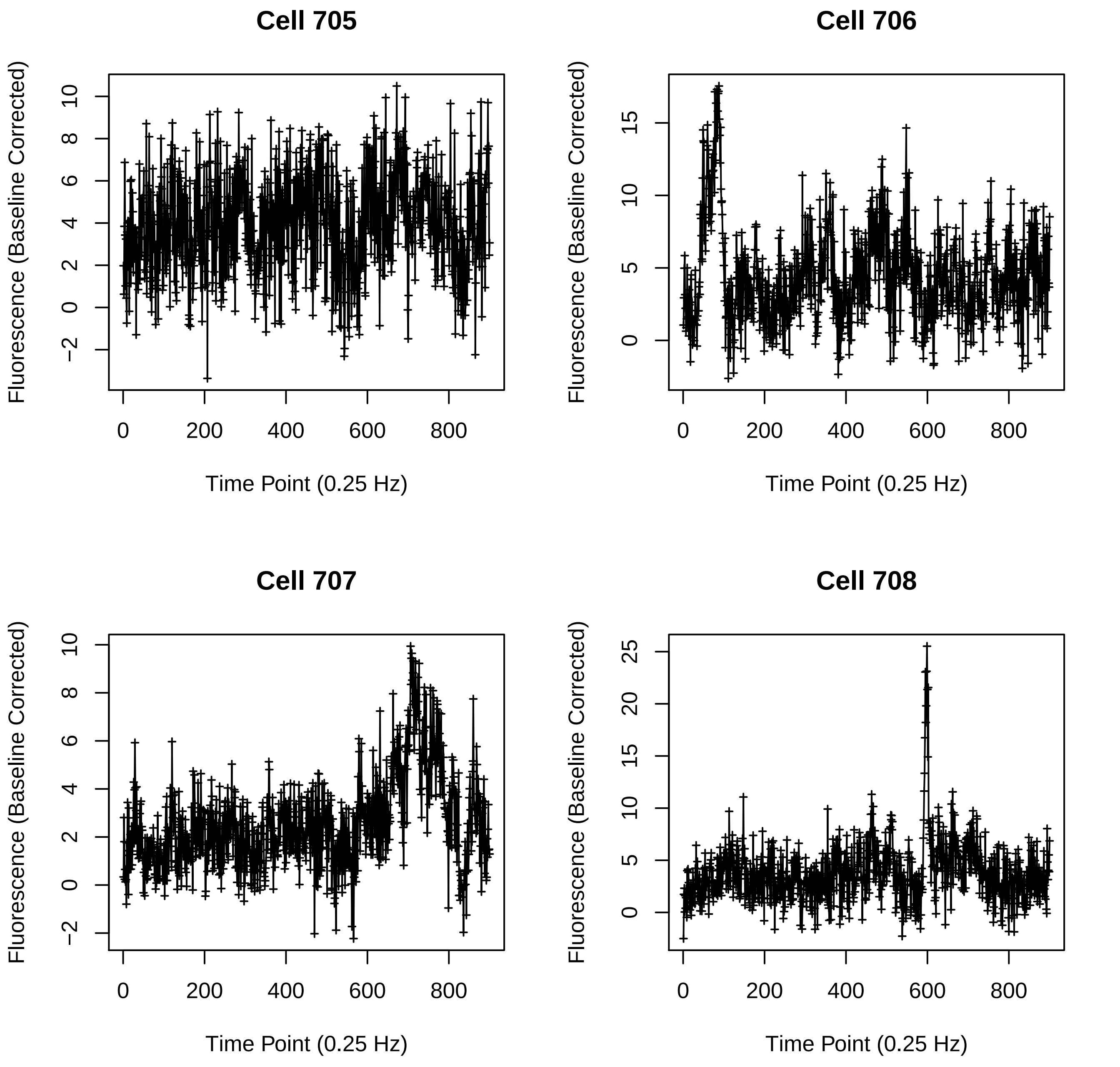
Cell 705
Cell 706
Cell 707
Cell 708
Fluorescence (Baseline Corrected)
Time Point (0.25 Hz)
0
200
400
600
800
−2
2
4
6
8
10
5
15
20
25

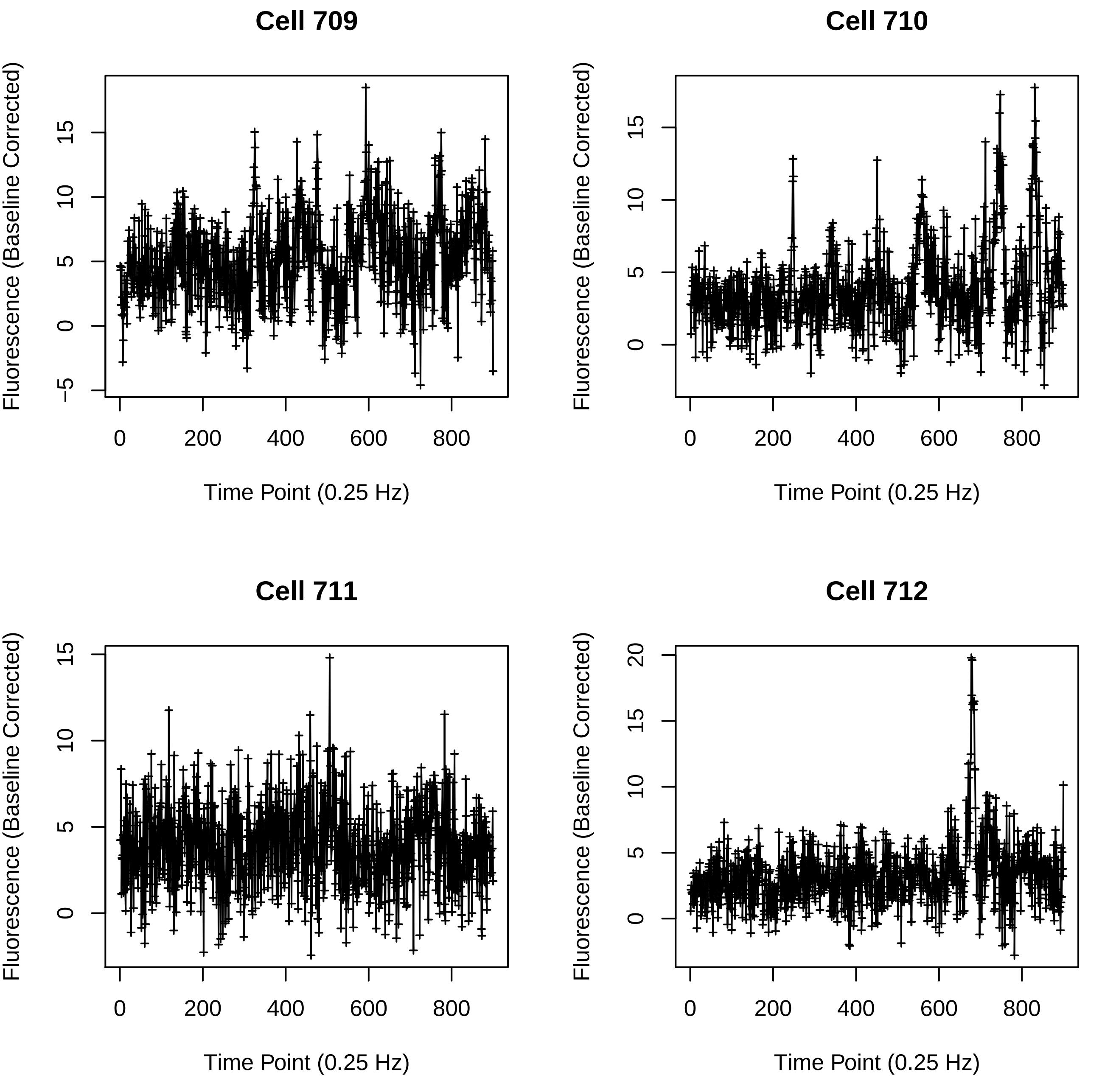
Cell 709
Cell 710
Cell 711
Cell 712
Fluorescence (Baseline Corrected)
Time Point (0.25 Hz)
0
200
400
600
800
−5
5
10
15
20

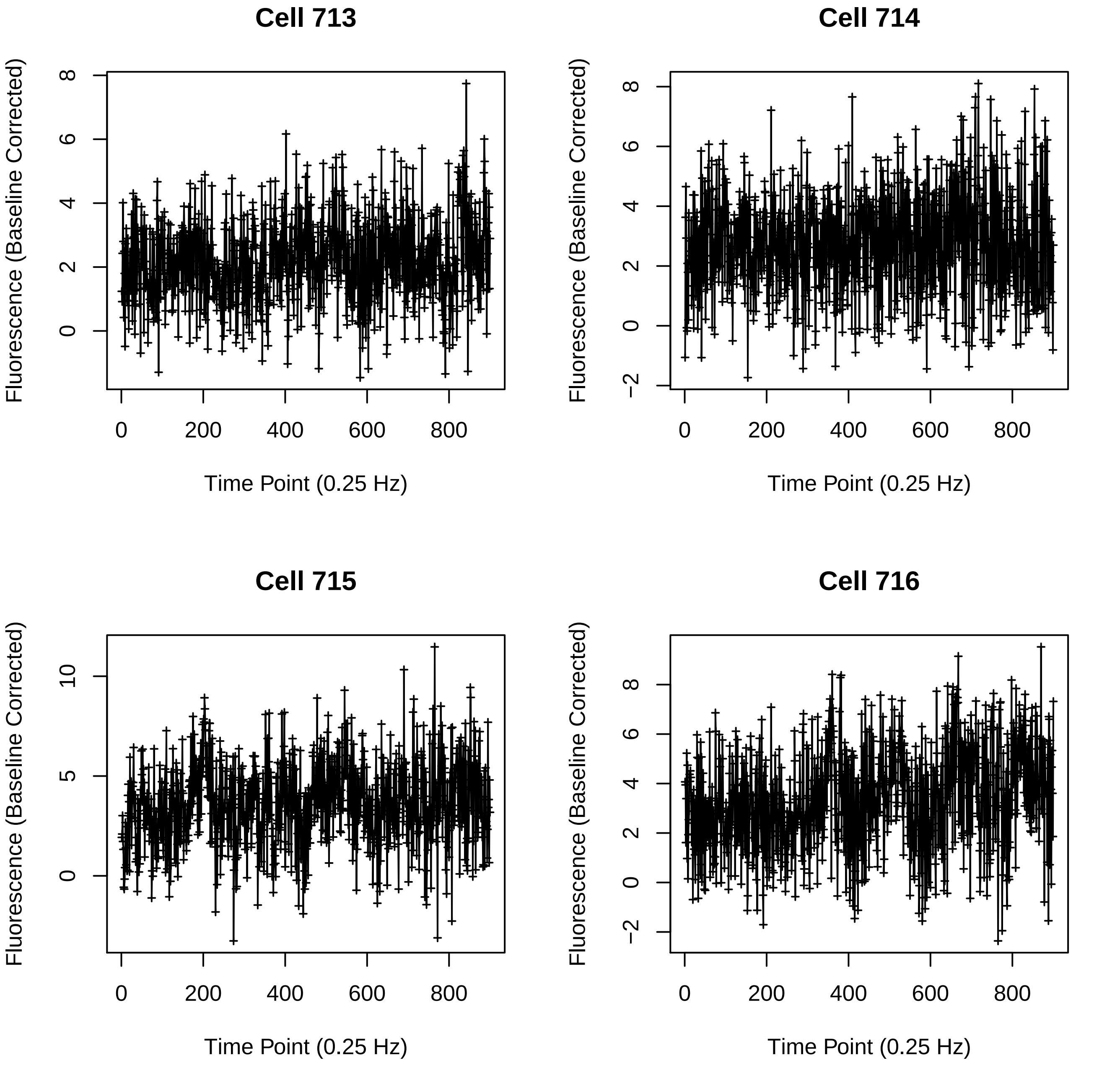

Cell 713
Fluorescence (Baseline Corrected)
Time Point (0.25 Hz)
Cell 714
Fluorescence (Baseline Corrected)
Time Point (0.25 Hz)
Cell 715
Fluorescence (Baseline Corrected)
Time Point (0.25 Hz)
Cell 716
Fluorescence (Baseline Corrected)
Time Point (0.25 Hz)

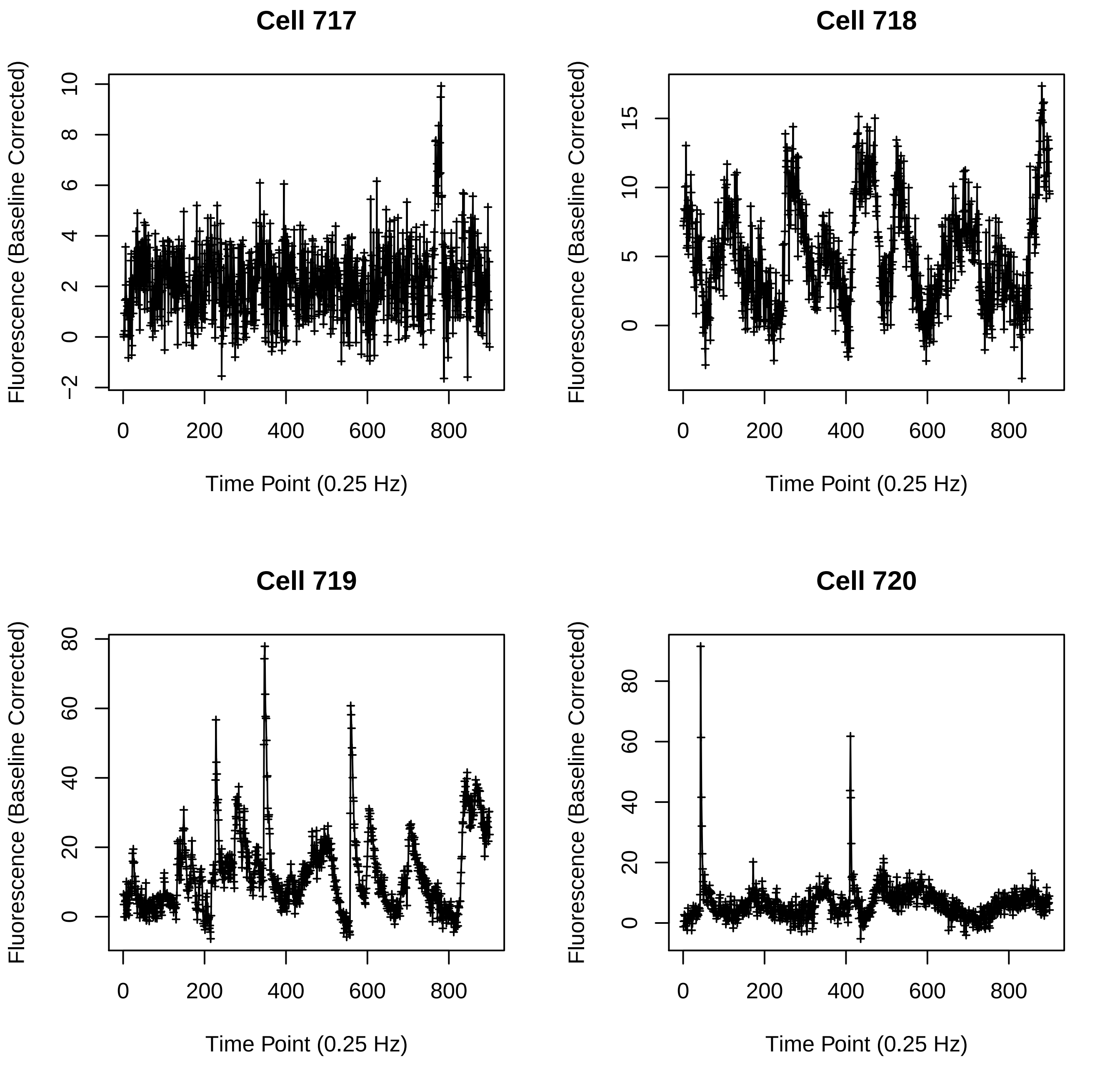

Cell 717
Cell 718
Cell 719
Cell 720
Fluorescence (Baseline Corrected)
Time Point (0.25 Hz)

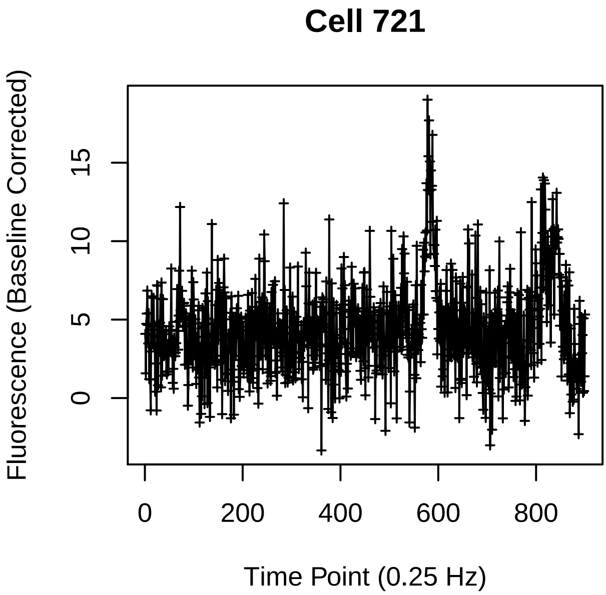
Cell 721
Fluorescence (Baseline Corrected)
0
5
10
15
0
200
400
600
800
Time Point (0.25 Hz)

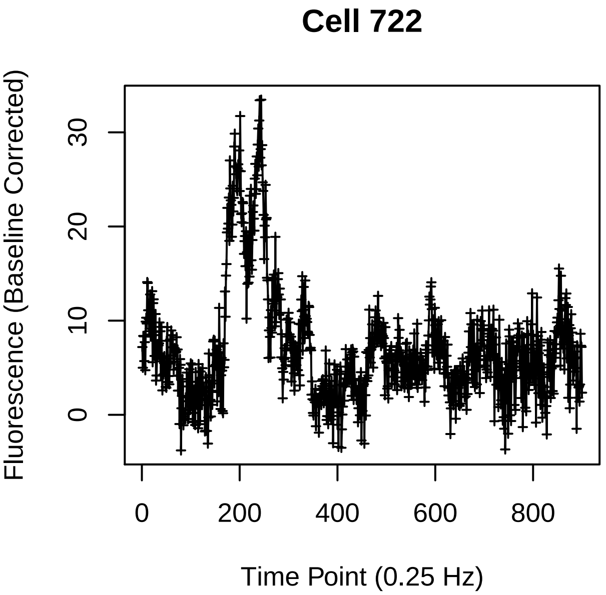
Cell 722
Fluorescence (Baseline Corrected)
0
10
20
30
0
200
400
600
800
Time Point (0.25 Hz)

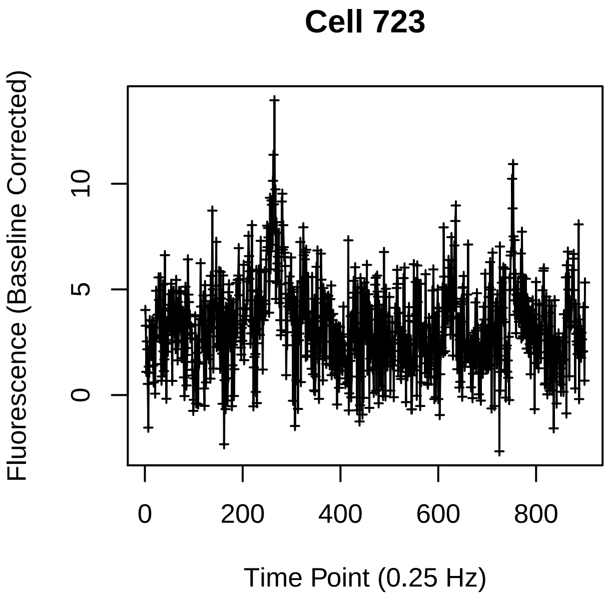
Cell 723
Fluorescence (Baseline Corrected)
0
5
10
0
200
400
600
800
Time Point (0.25 Hz)

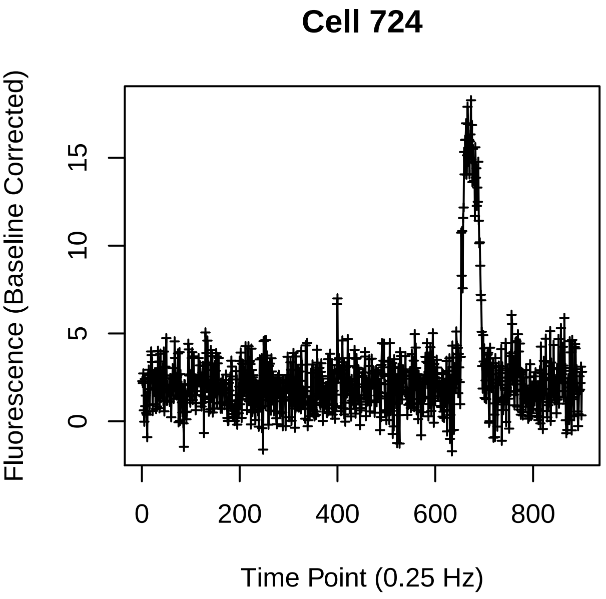
Cell 724
Fluorescence (Baseline Corrected)
0
5
10
15
0
200
400
600
800
Time Point (0.25 Hz)

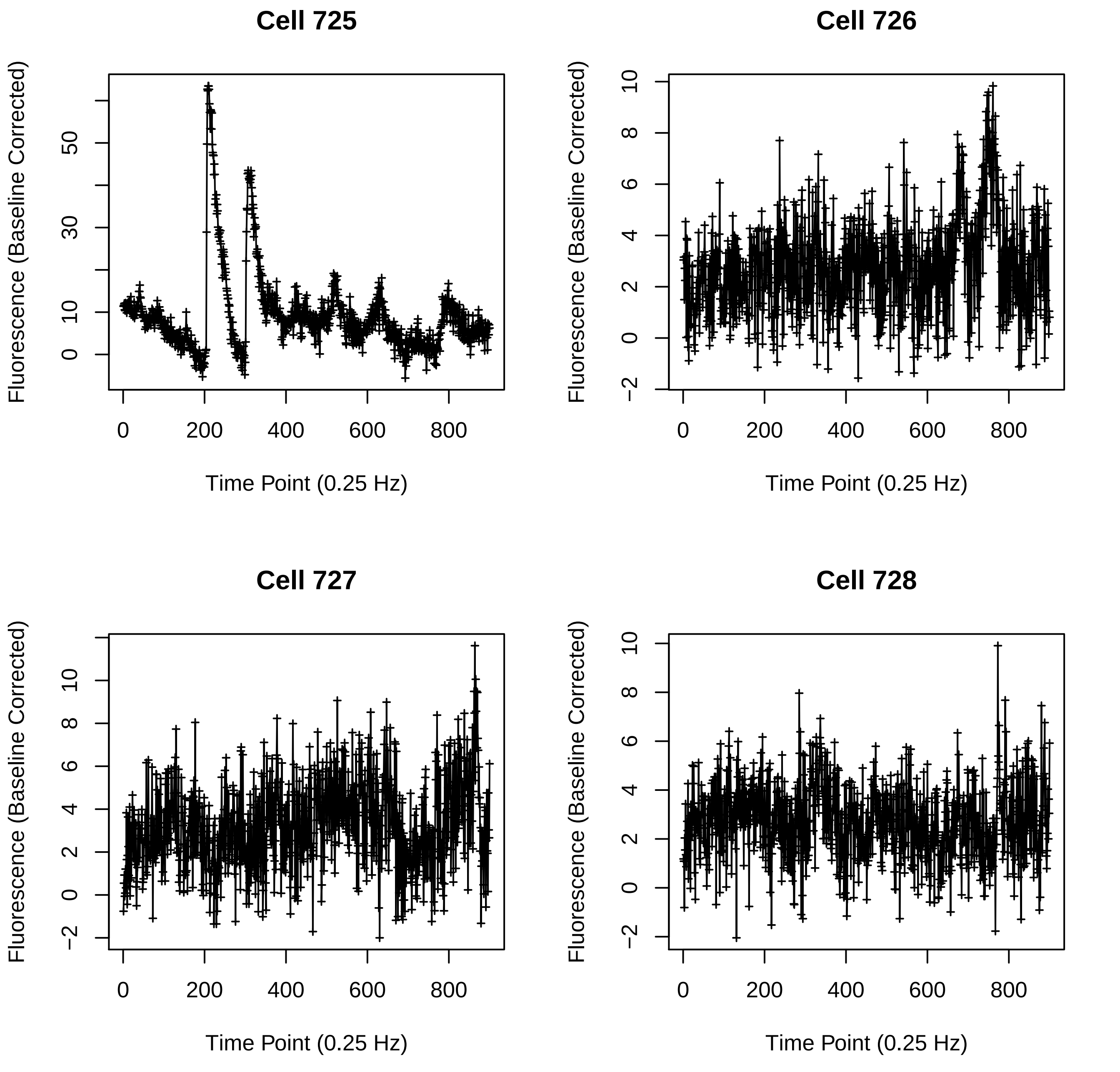
Cell 725
Cell 726
Cell 727
Cell 728
Fluorescence (Baseline Corrected)
Time Point (0.25 Hz)
0
200
400
600
800
0
10
30
50
−2
2
4
6
8
10

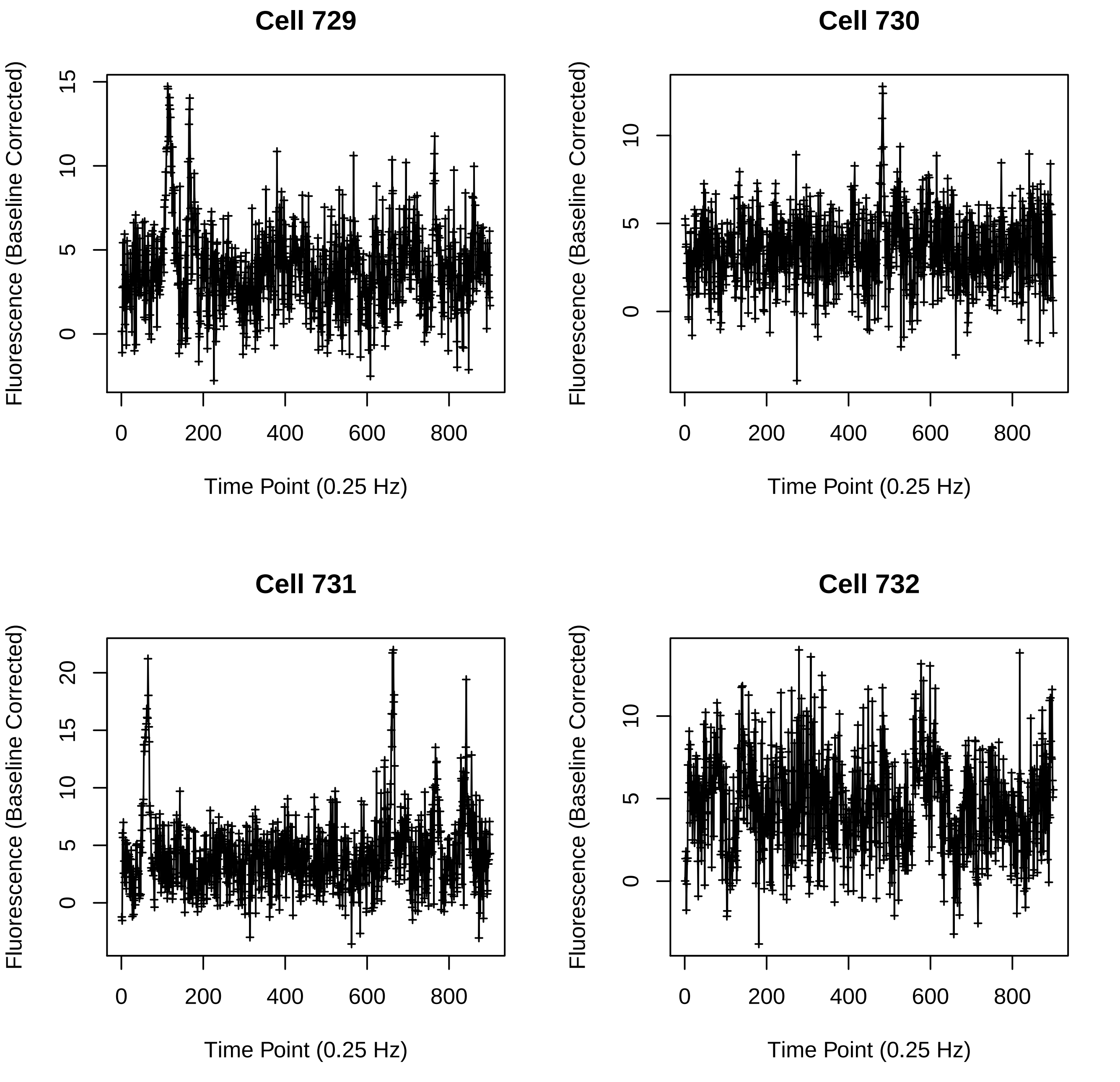
Cell 729
Cell 730
Cell 731
Cell 732
Fluorescence (Baseline Corrected)
Time Point (0.25 Hz)
0
5
10
15
20
200
400
600
800

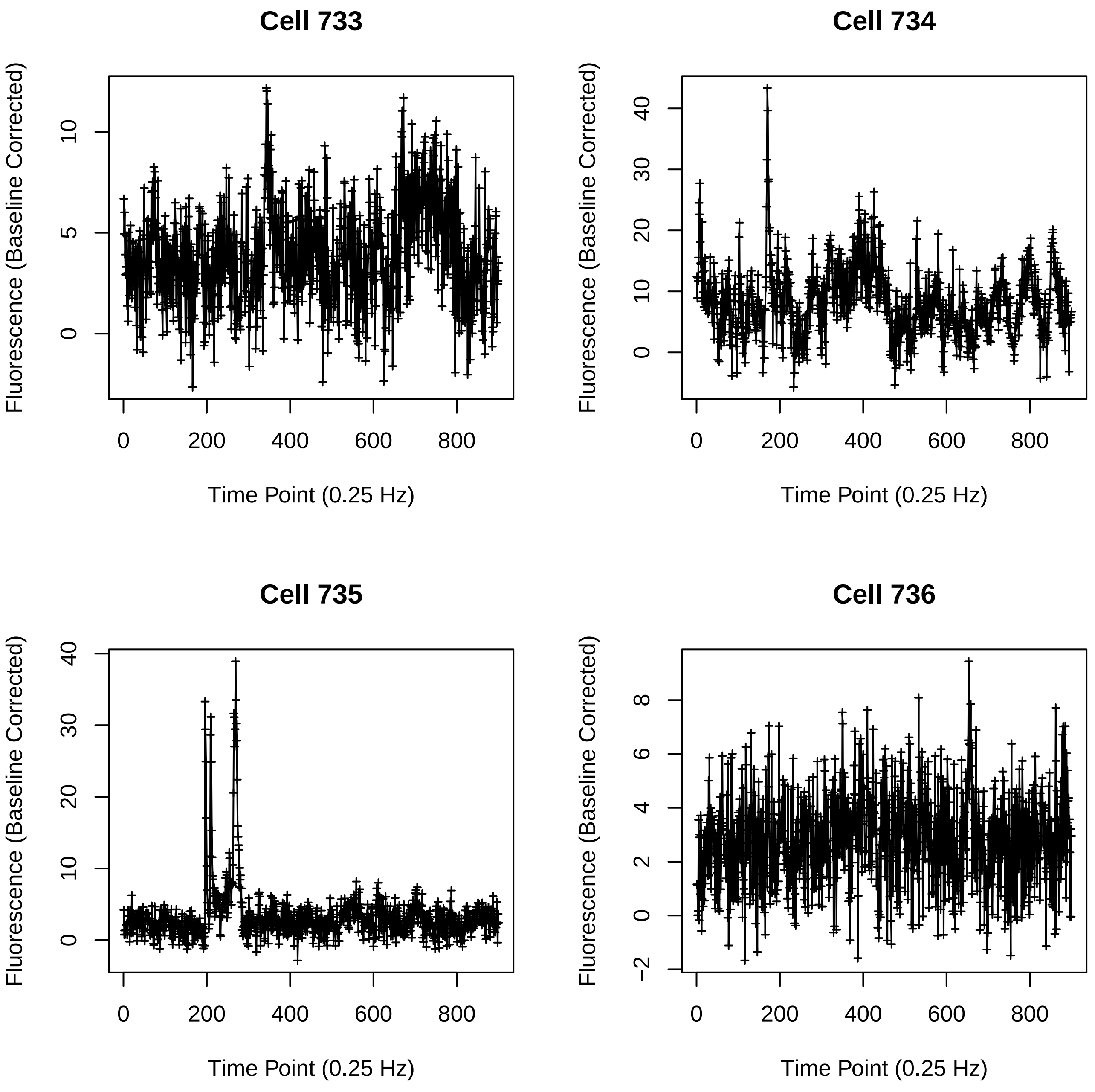
Cell 733
Fluorescence (Baseline Corrected)
Time Point (0.25 Hz)
0
5
10
0
200
400
600
800
Cell 734
Fluorescence (Baseline Corrected)
Time Point (0.25 Hz)
0
10
20
30
40
0
200
400
600
800
Cell 735
Fluorescence (Baseline Corrected)
Time Point (0.25 Hz)
0
10
20
30
40
0
200
400
600
800
Cell 736
Fluorescence (Baseline Corrected)
Time Point (0.25 Hz)
−2
0
2
4
6
8
0
200
400
600
800

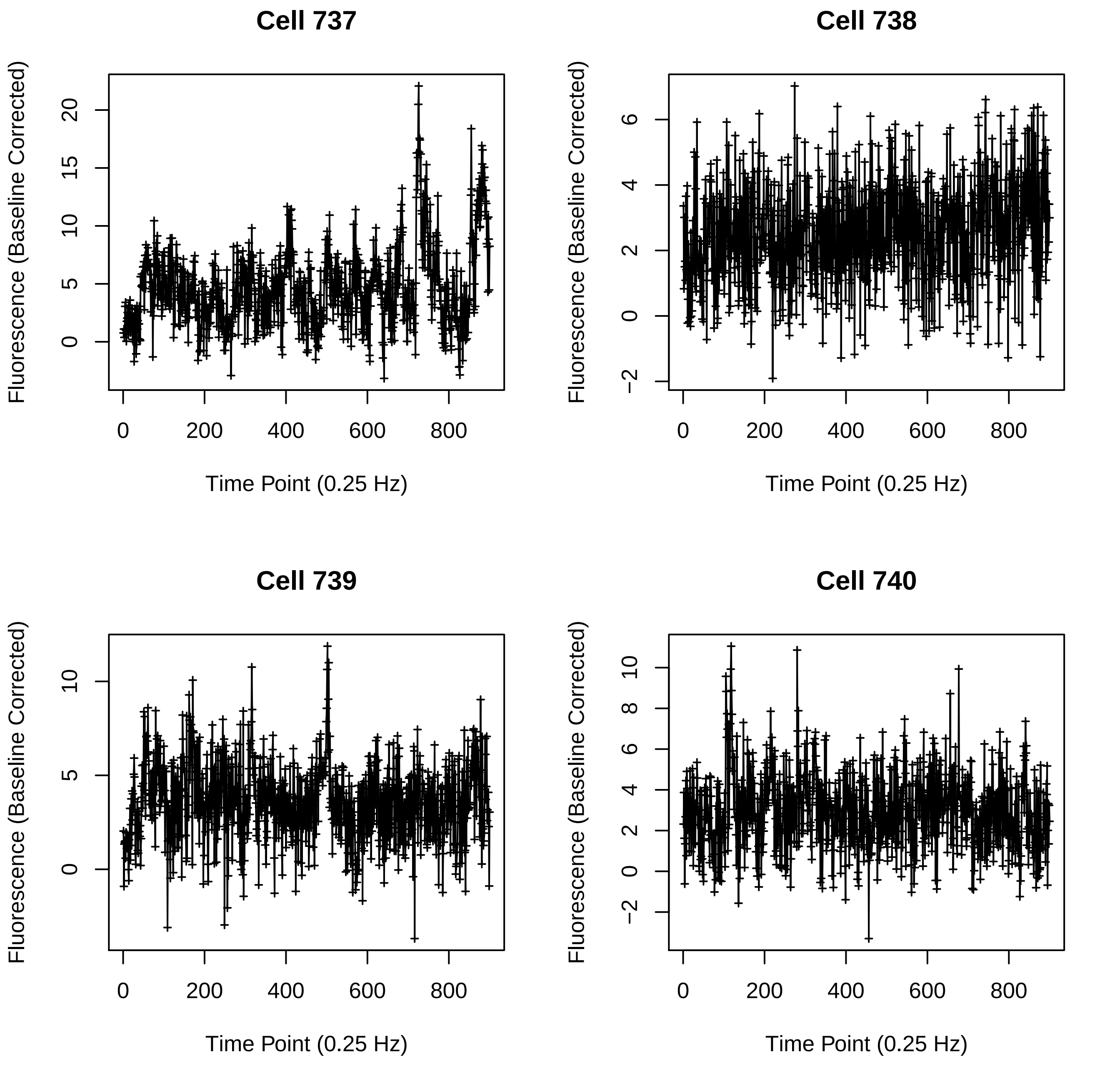
Cell 737
Cell 738
Cell 739
Cell 740
Fluorescence (Baseline Corrected)
Time Point (0.25 Hz)
0
200
400
600
800
−2
2
4
6
8
10
15
20

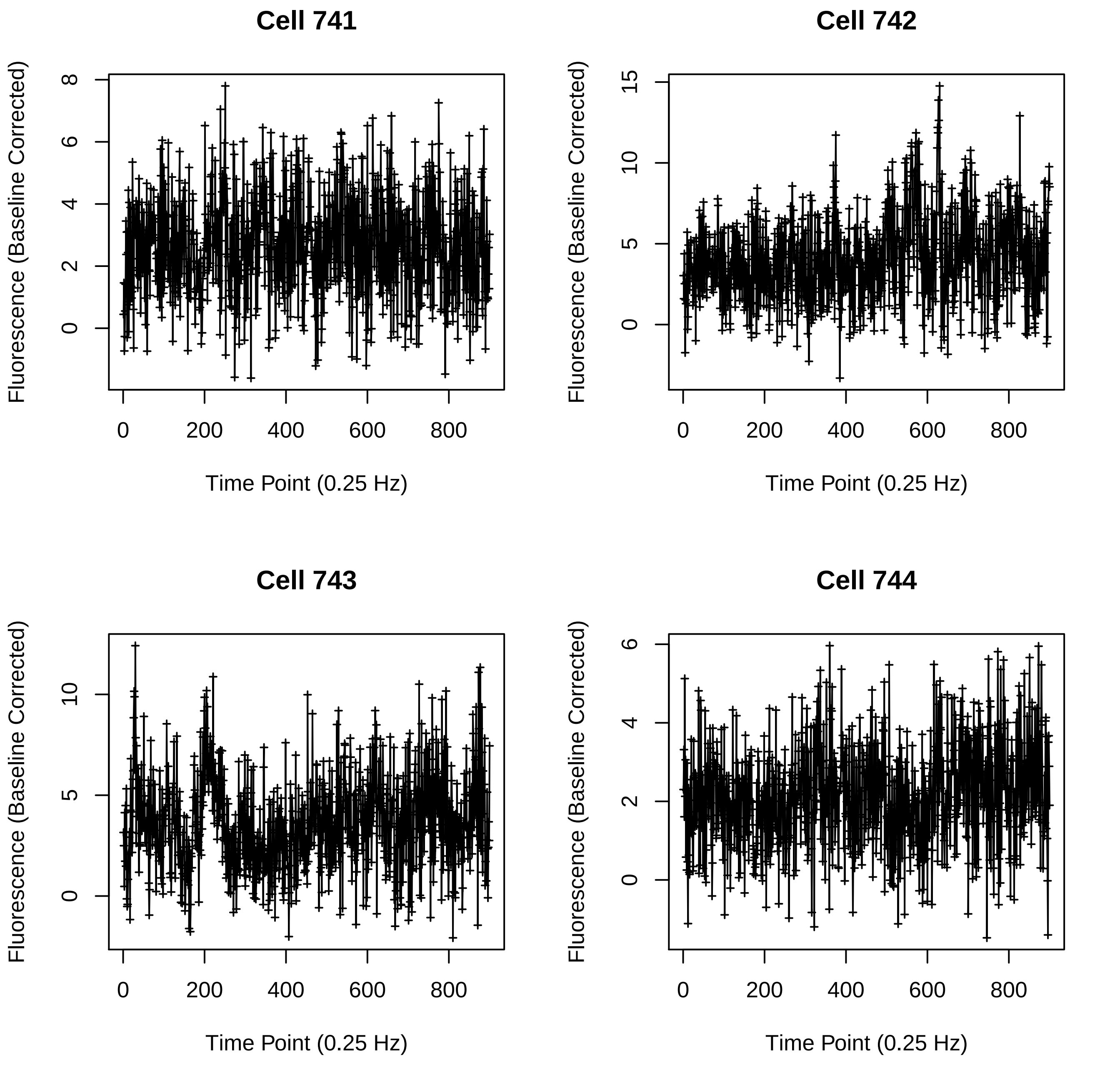

Cell 741
Cell 742
Cell 743
Cell 744
Fluorescence (Baseline Corrected)
Time Point (0.25 Hz)
0
200
400
600
800
2
4
5
6
8
10
15

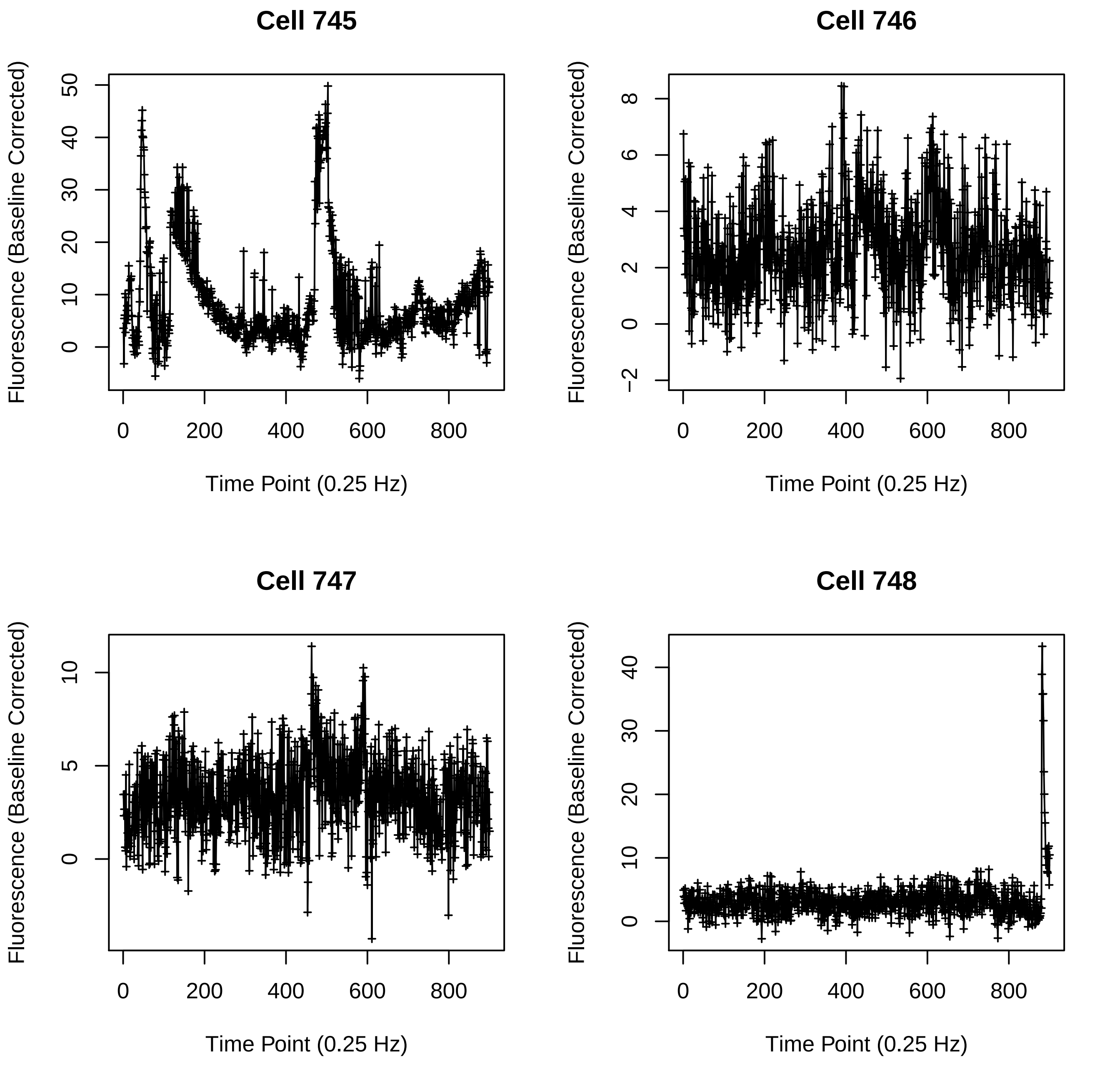
Cell 745
Cell 746
Cell 747
Cell 748
Fluorescence (Baseline Corrected)
Time Point (0.25 Hz)
0
200
400
600
800
−2
0
2
4
6
8
10
20
30
40
50
5

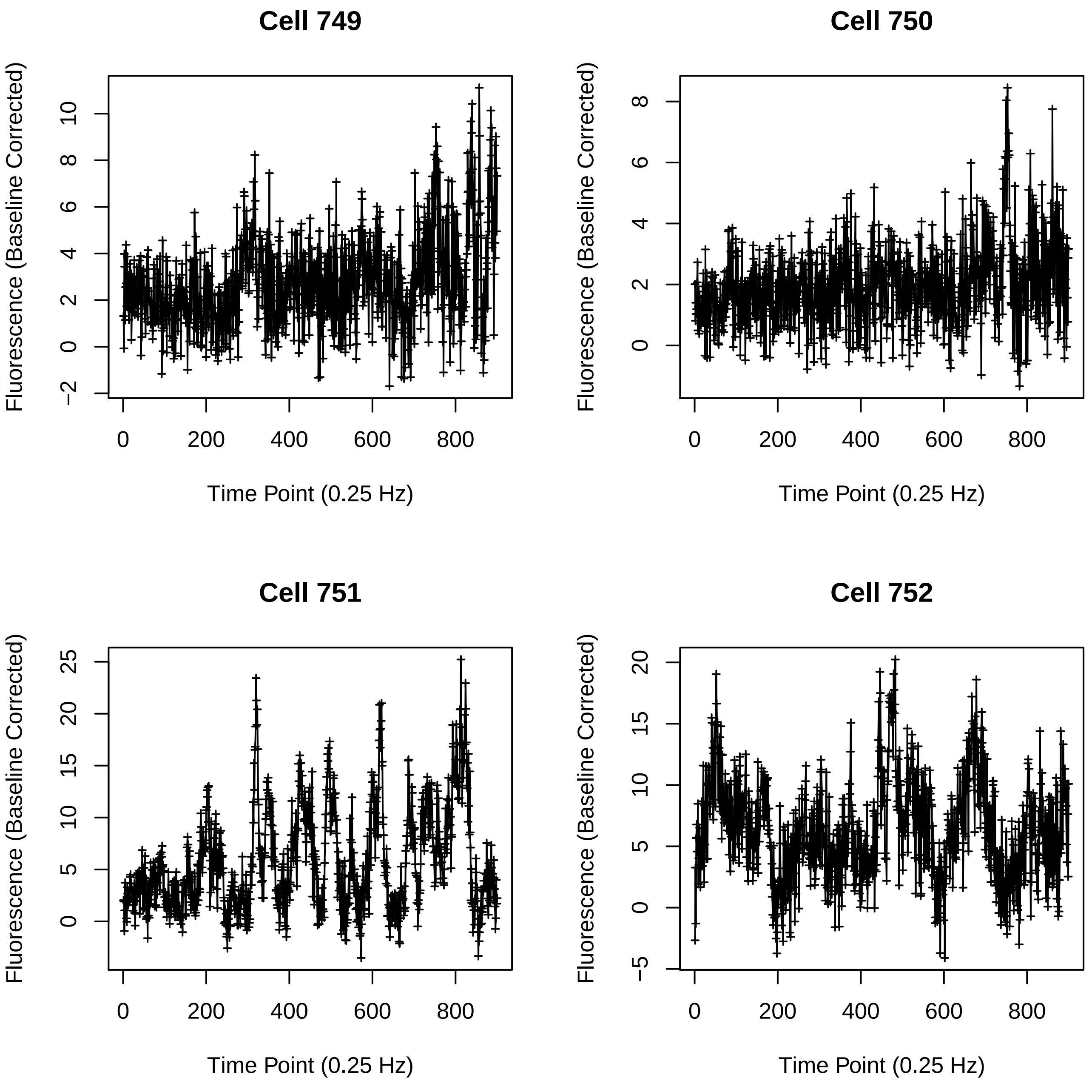
Cell 749
Cell 750
Cell 751
Cell 752
Fluorescence (Baseline Corrected)
Time Point (0.25 Hz)
0
200
400
600
800
−2
0
2
4
6
8
10
−5
5
15
20
25

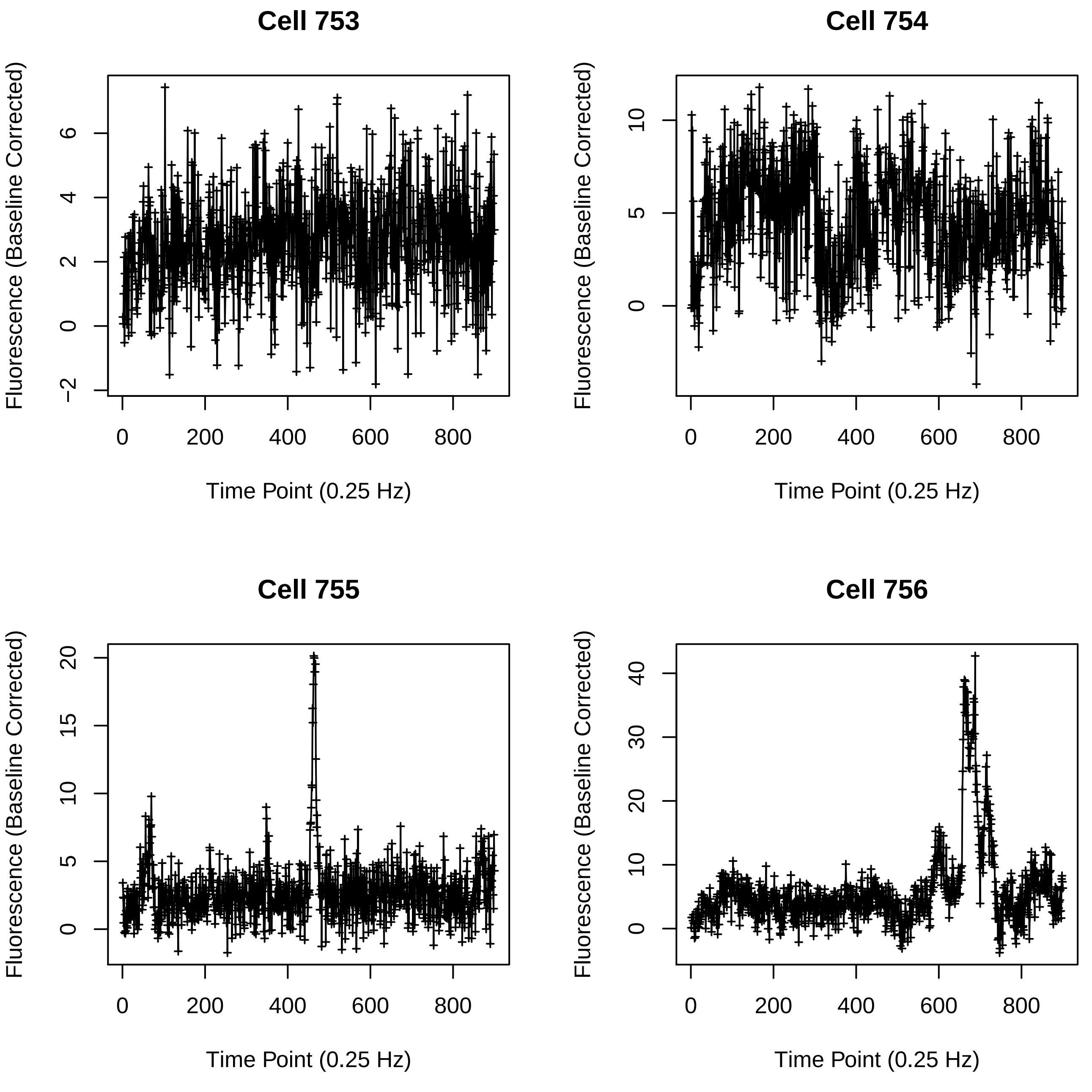
Cell 753
Cell 754
Cell 755
Cell 756
Fluorescence (Baseline Corrected)
Time Point (0.25 Hz)
0
200
400
600
800
−2
2
4
6
5
10
15
20
30
40

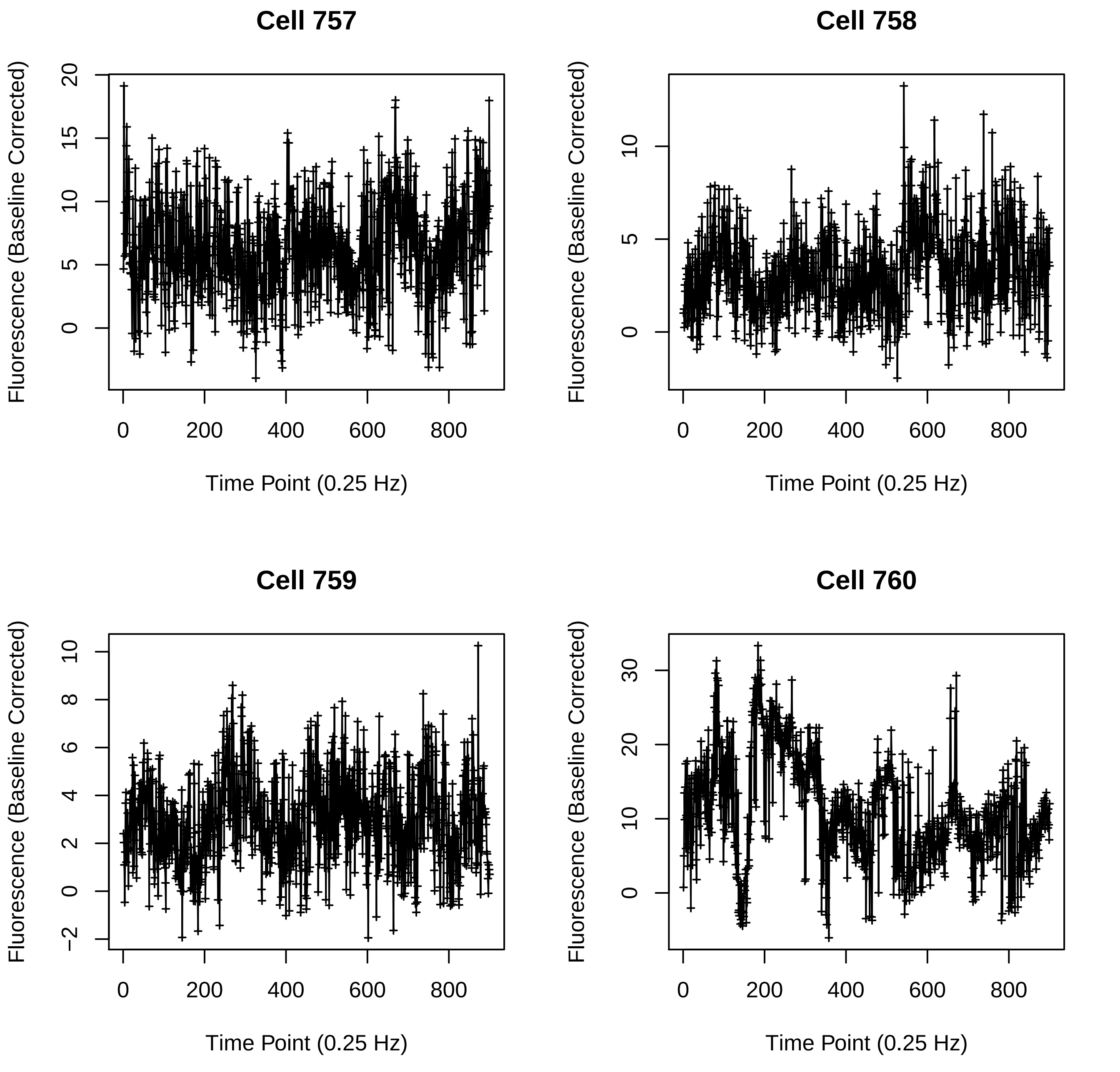
Cell 757
Cell 758
Cell 759
Cell 760
Fluorescence (Baseline Corrected)
Time Point (0.25 Hz)
0
200
400
600
800
−2
0
2
4
5
6
8
10
15
20
30

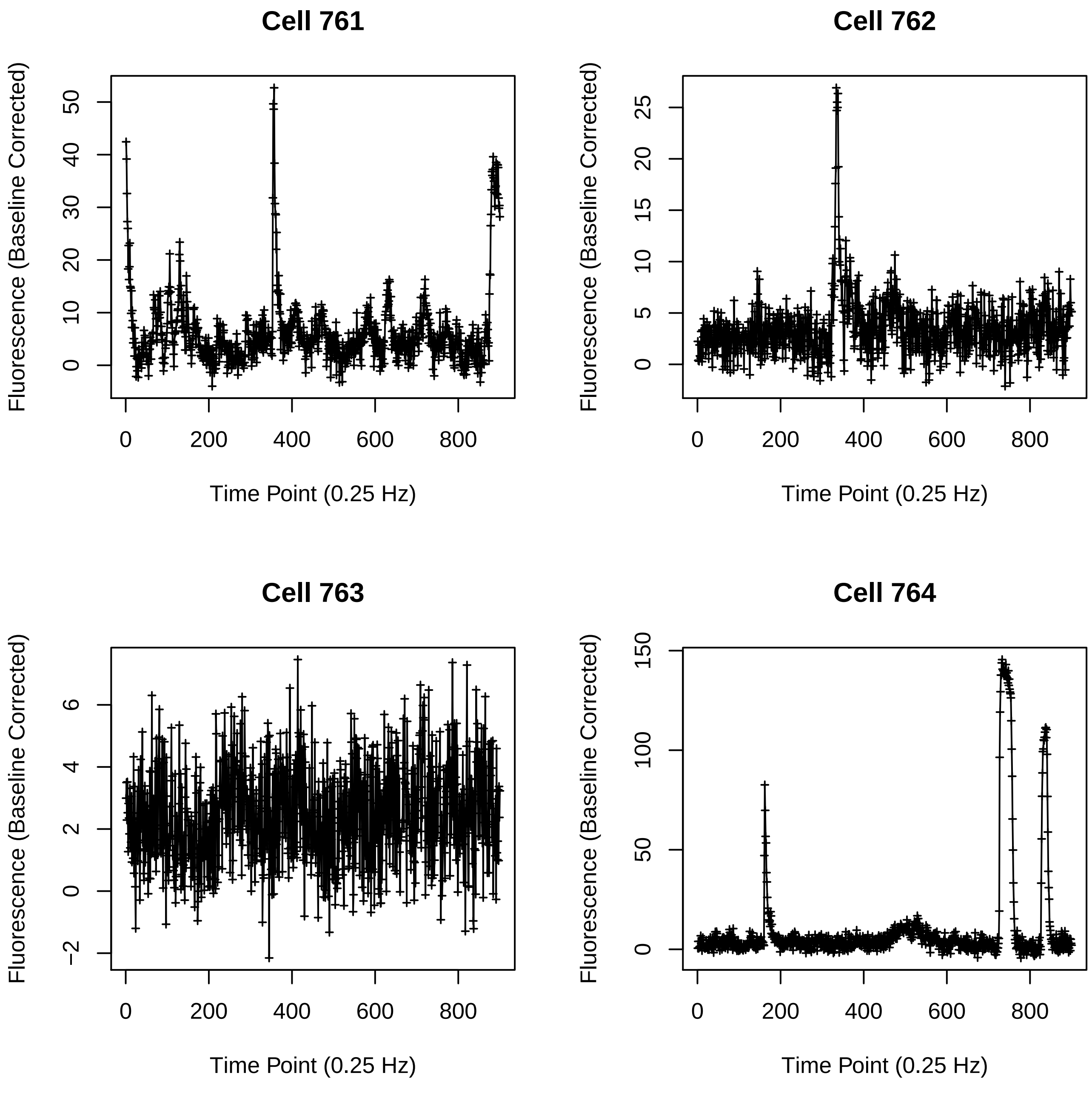
Cell 761
Cell 762
Cell 763
Cell 764
Fluorescence (Baseline Corrected)
Time Point (0.25 Hz)
0
10
20
30
40
50
0
5
10
15
20
25
−2
0
2
4
6
0
50
100
150
0
200
400
600
800

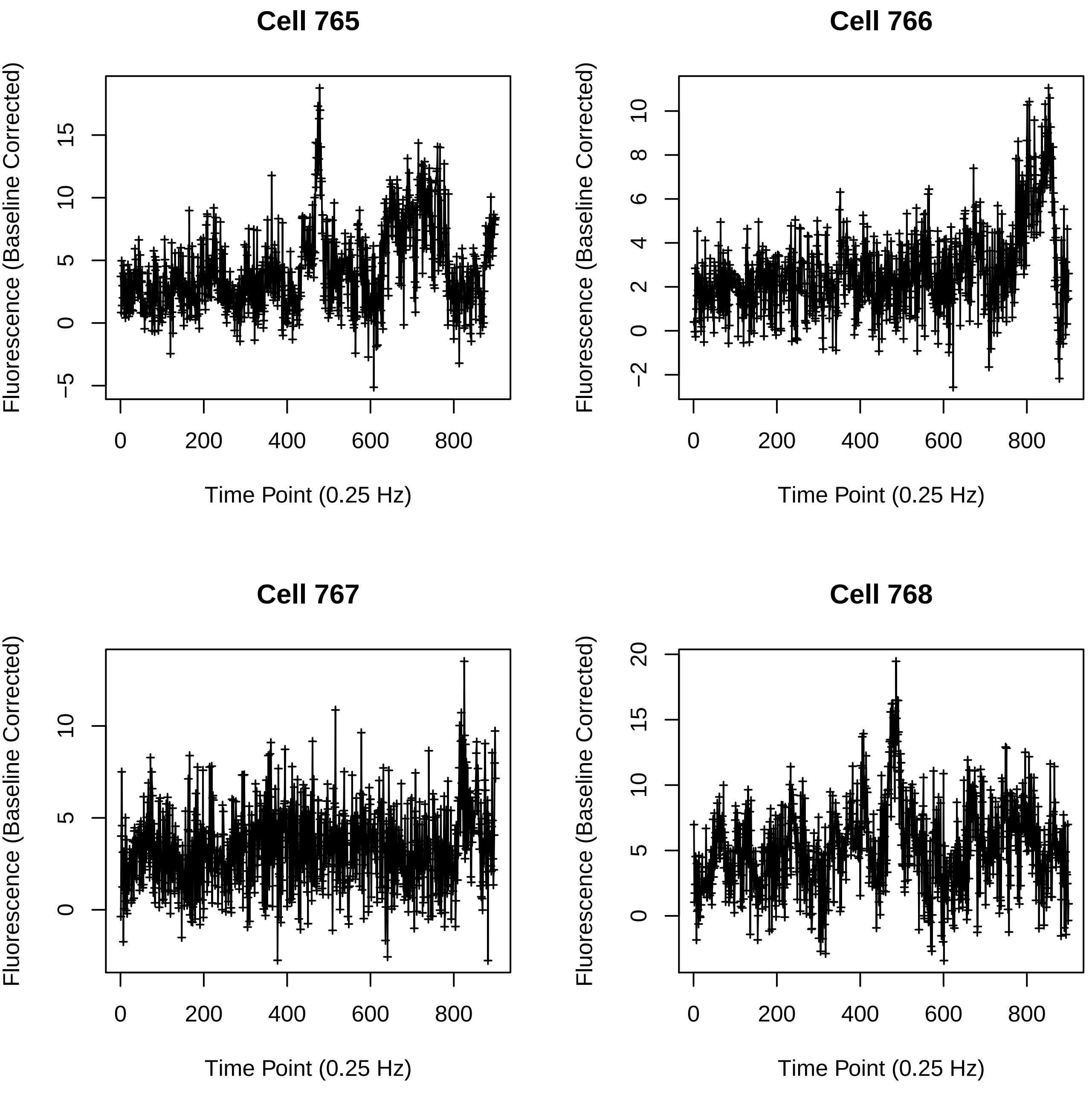
Cell 765
Cell 766
Cell 767
Cell 768
Fluorescence (Baseline Corrected)
Time Point (0.25 Hz)
0
200
400
600
800
−5
0
5
10
15
−2
2
4
6
8
20

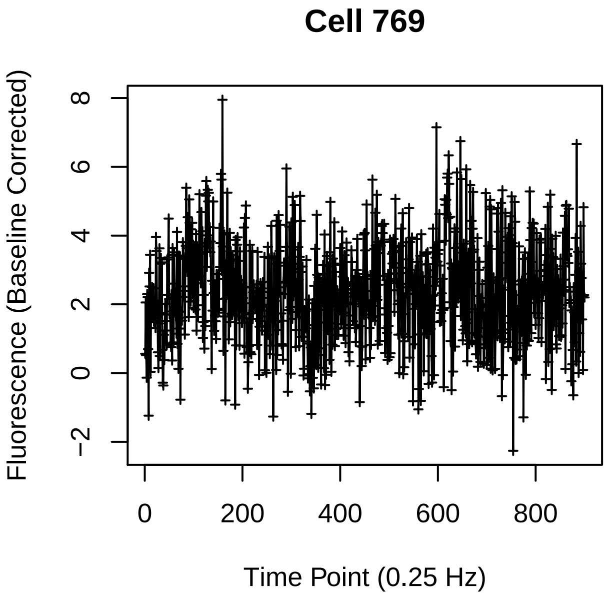
Cell 769
Fluorescence (Baseline Corrected)
Time Point (0.25 Hz)
−2
0
2
4
6
8
0
200
400
600
800
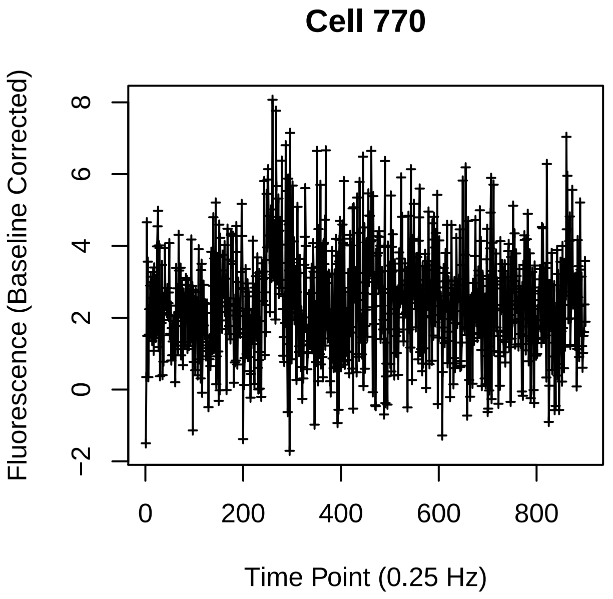
Cell 770
Fluorescence (Baseline Corrected)
Time Point (0.25 Hz)
−2
0
2
4
6
8
0
200
400
600
800
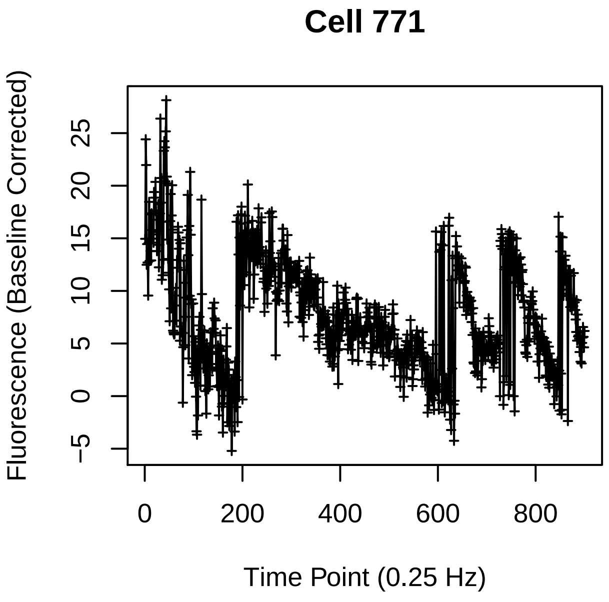
Cell 771
Fluorescence (Baseline Corrected)
Time Point (0.25 Hz)
−5
0
5
10
15
20
25
0
200
400
600
800
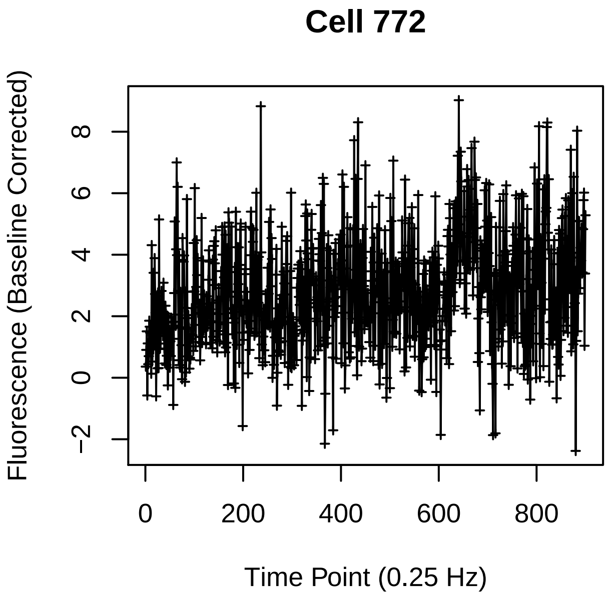
Cell 772
Fluorescence (Baseline Corrected)
Time Point (0.25 Hz)
−2
0
2
4
6
8
0
200
400
600
800

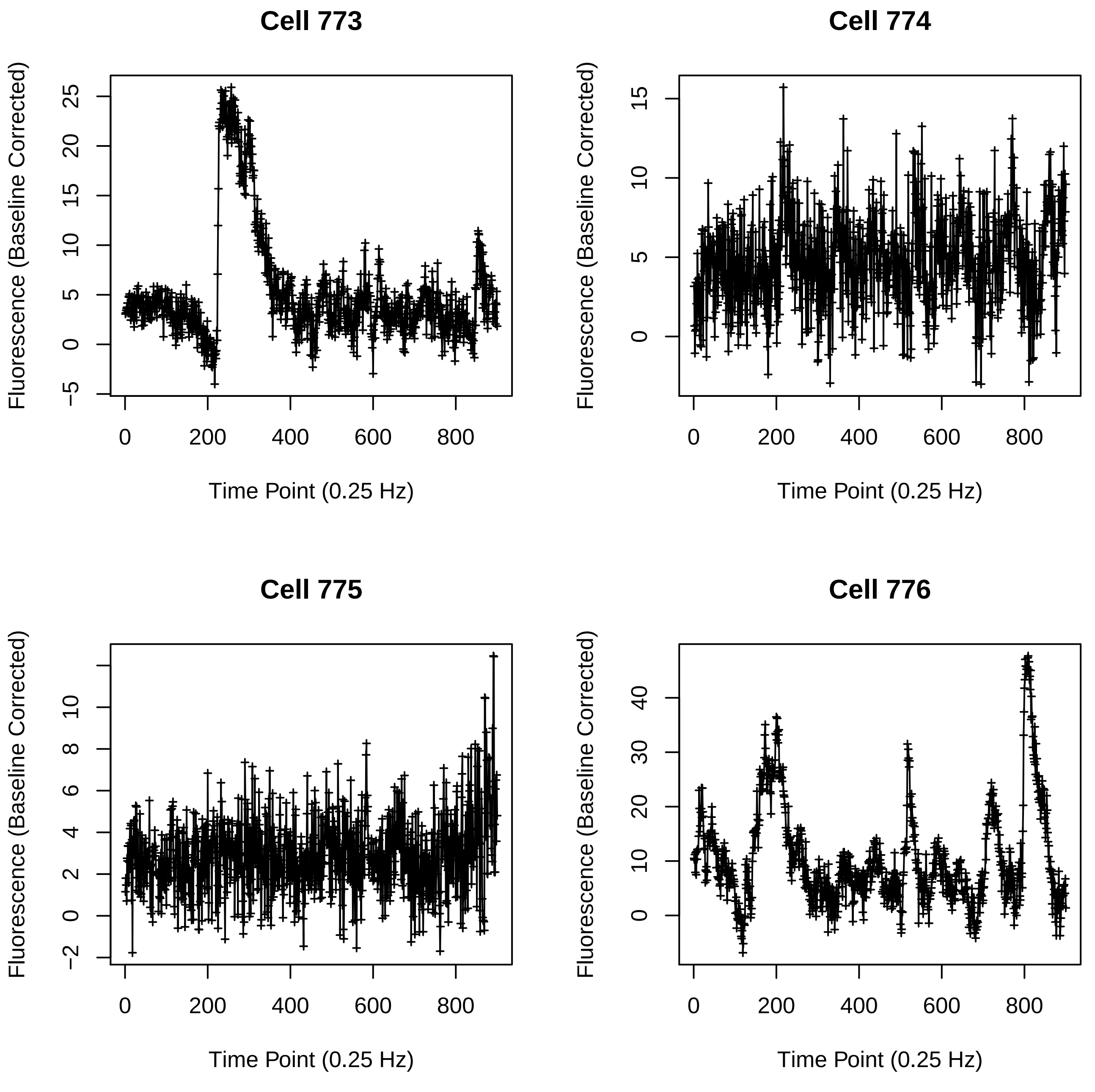
Cell 773
Cell 774
Cell 775
Cell 776
Fluorescence (Baseline Corrected)
Time Point (0.25 Hz)
-5
0
5
10
15
20
25
0
200
400
600
800
-2
0
2
4
6
8
10
20
30
40

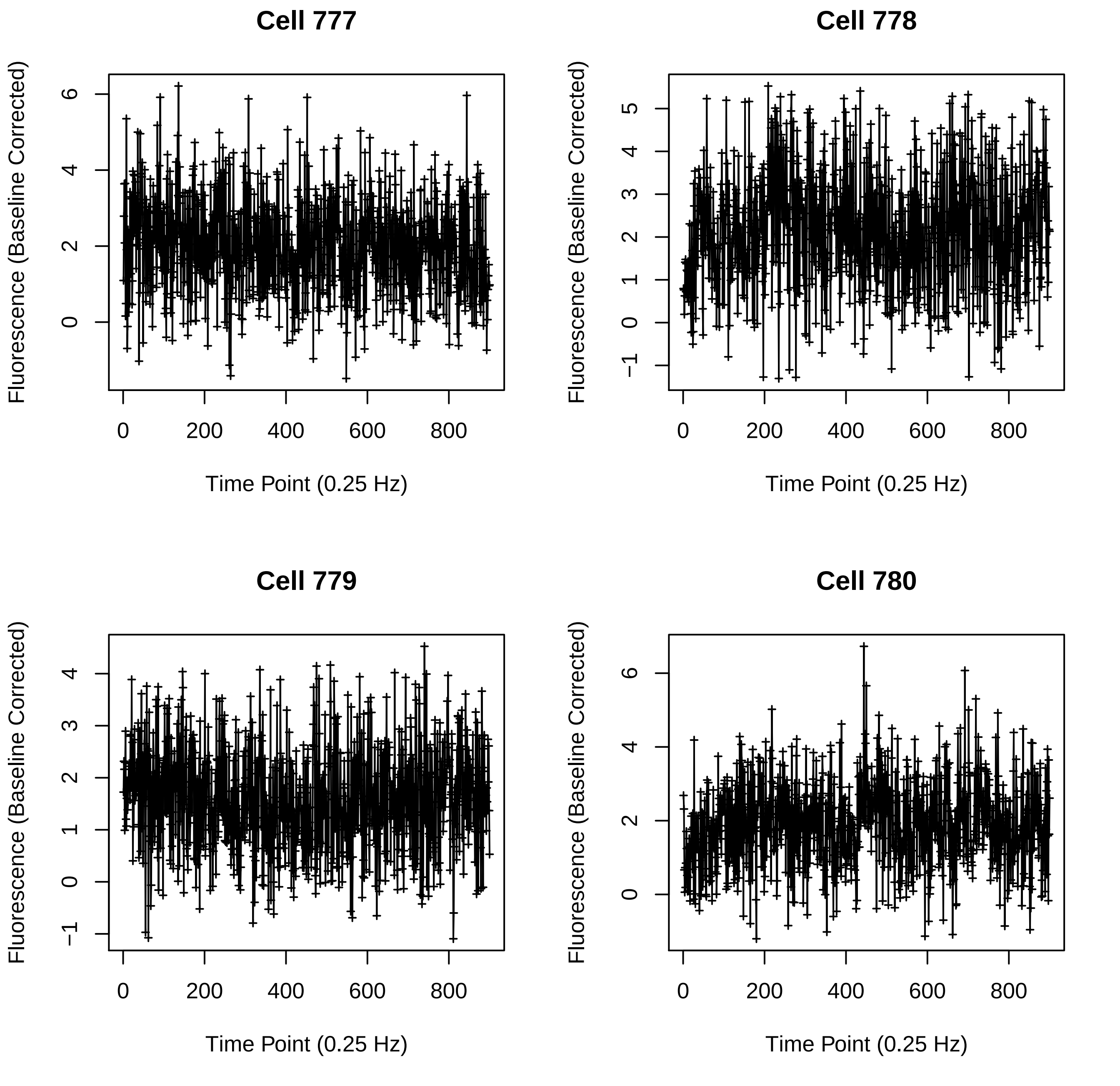
Cell 777
Cell 778
Cell 779
Cell 780
Fluorescence (Baseline Corrected)
Time Point (0.25 Hz)
0
200
400
600
800

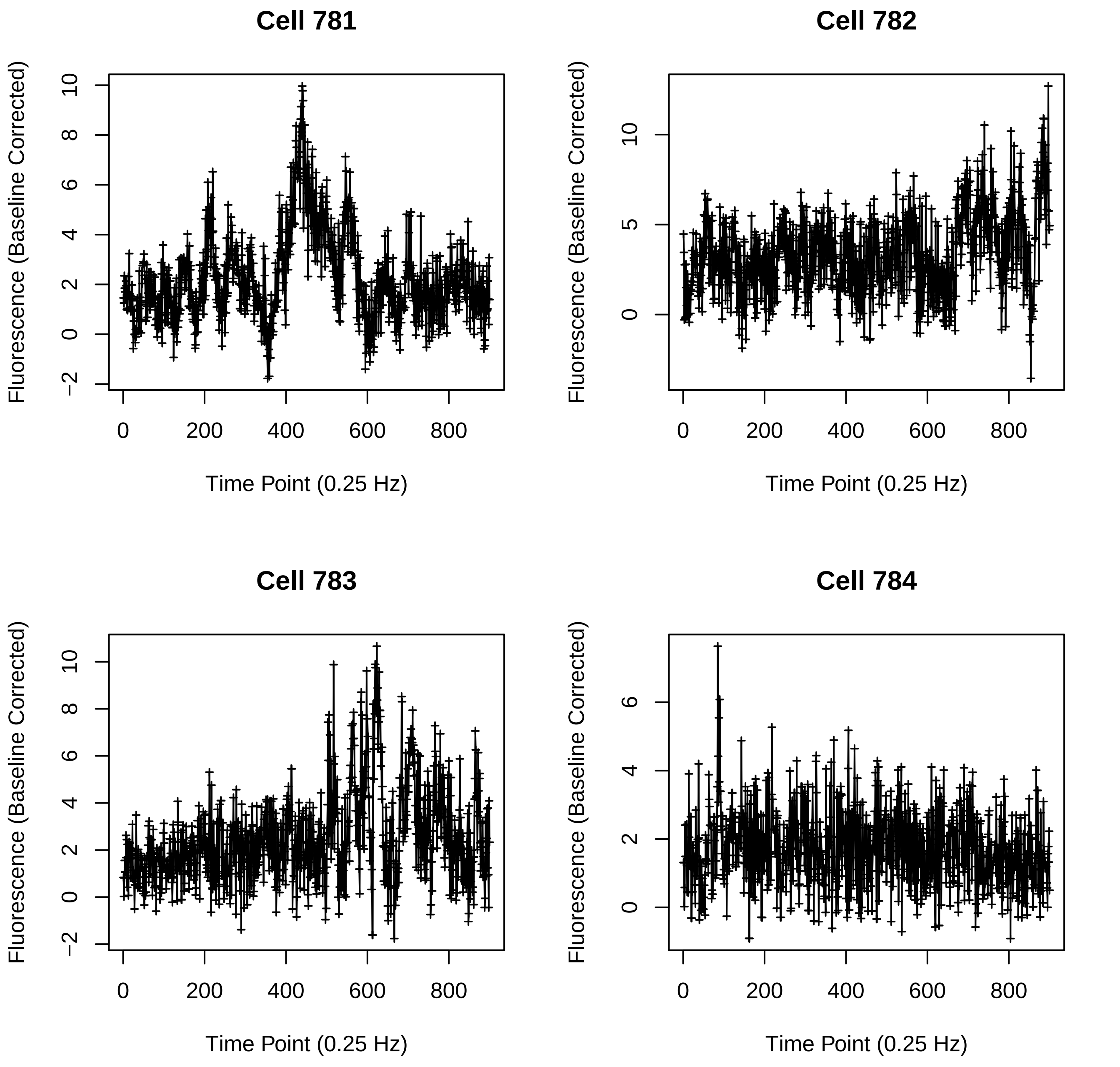

Cell 781
Cell 782
Cell 783
Cell 784
Fluorescence (Baseline Corrected)
Time Point (0.25 Hz)
0
200
400
600
800
−2
0
2
4
6
8
10
5

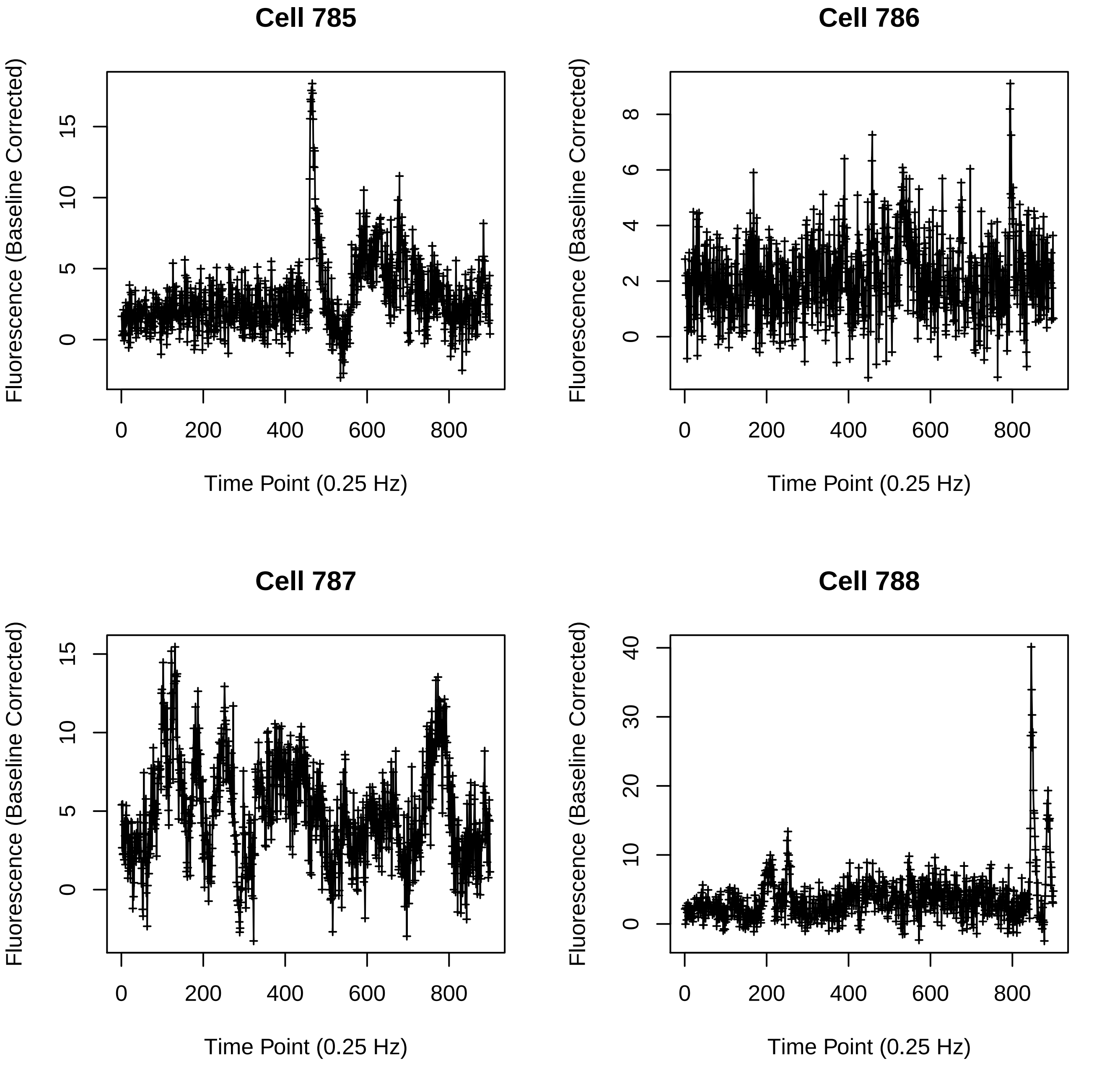
Cell 785
Cell 786
Cell 787
Cell 788
Fluorescence (Baseline Corrected)
Time Point (0.25 Hz)
0
5
10
15
200
400
600
800
2
4
6
8
20
30
40

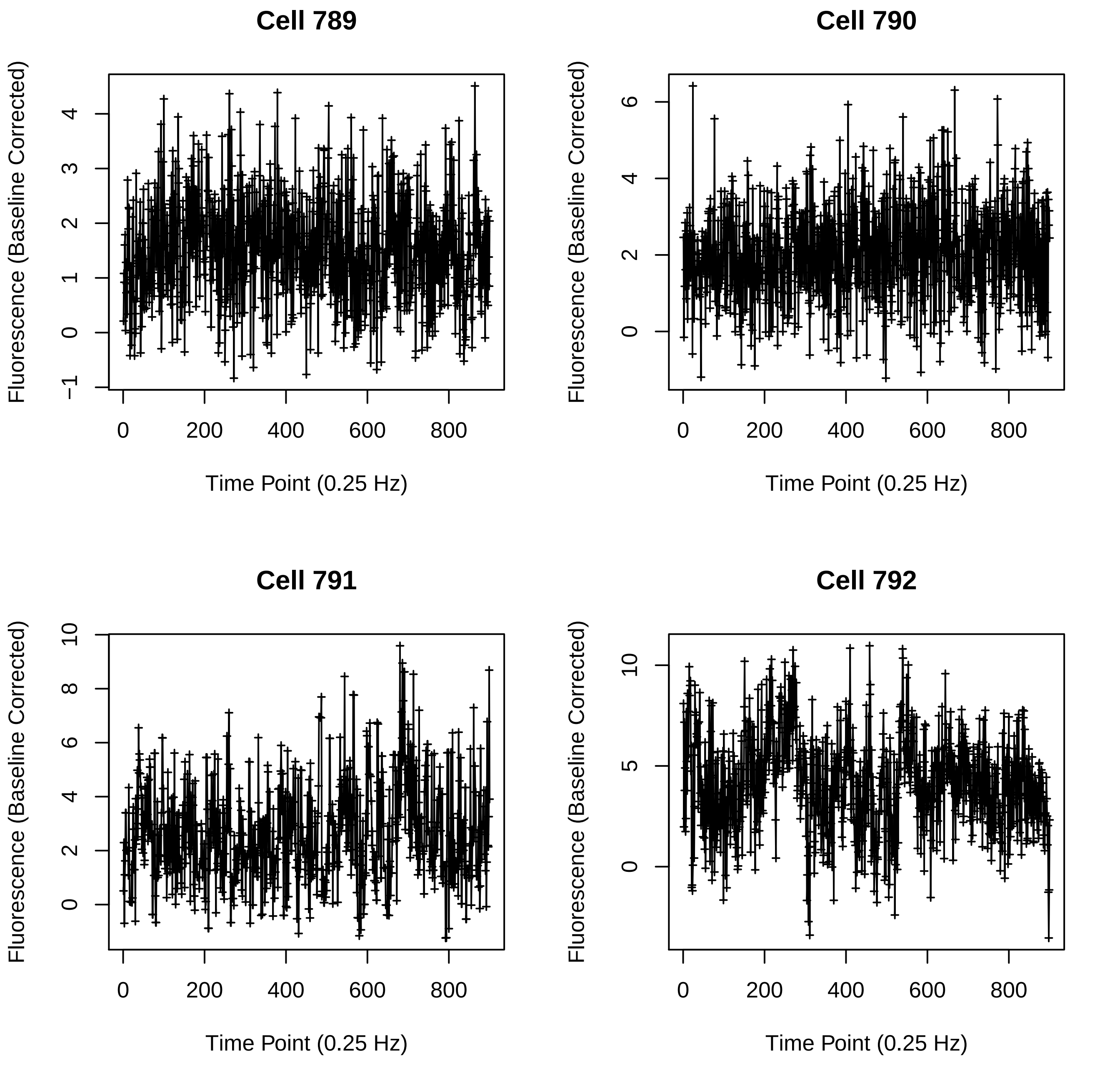

Cell 789
Cell 790
Cell 791
Cell 792
Fluorescence (Baseline Corrected)
Time Point (0.25 Hz)
0
200
400
600
800
−1
0
1
2
3
4
6
8
10
5

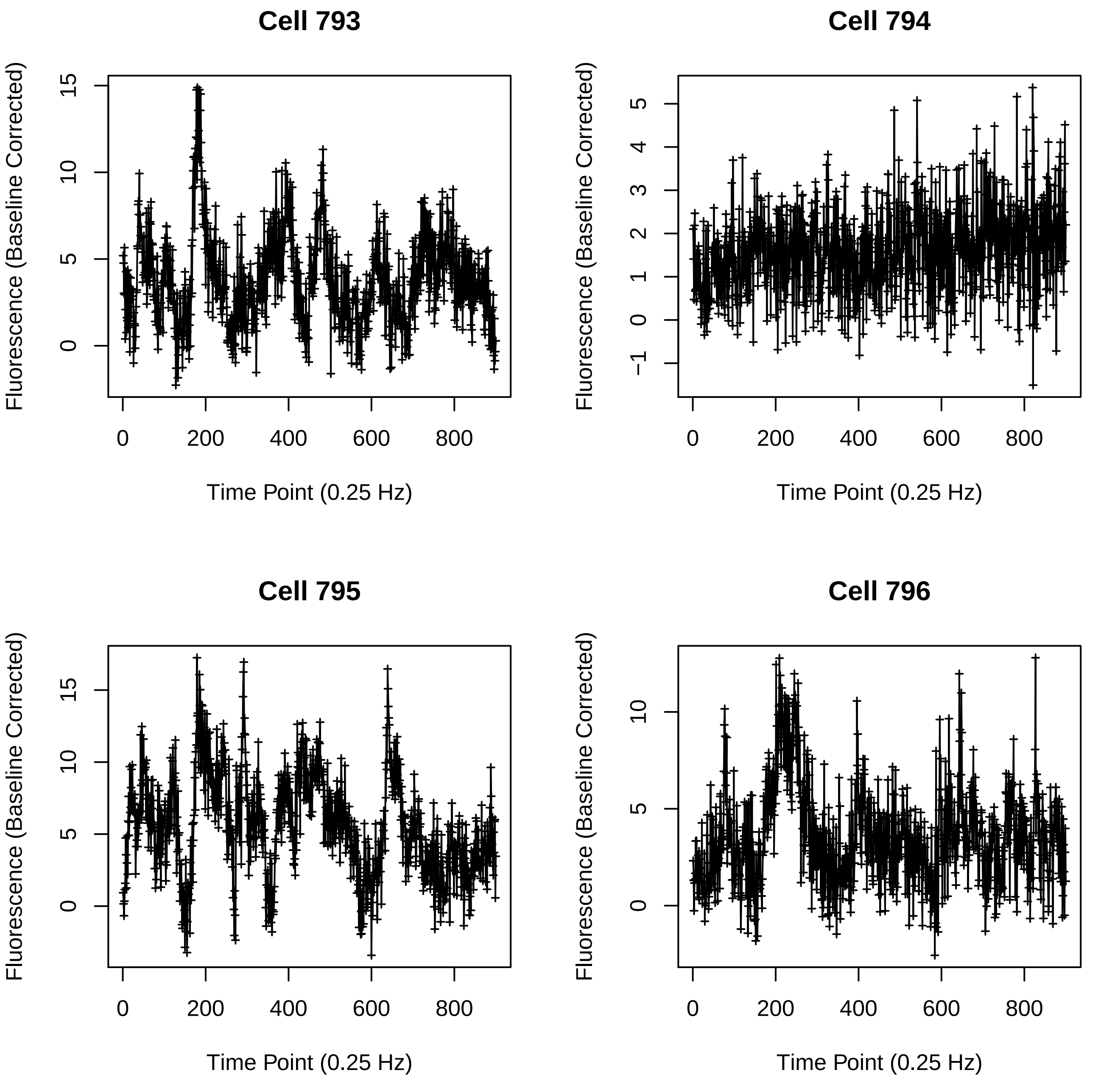

Cell 793
Cell 794
Cell 795
Cell 796
Fluorescence (Baseline Corrected)
Time Point (0.25 Hz)
0
200
400
600
800
-1
0
1
2
3
4
5
10
15

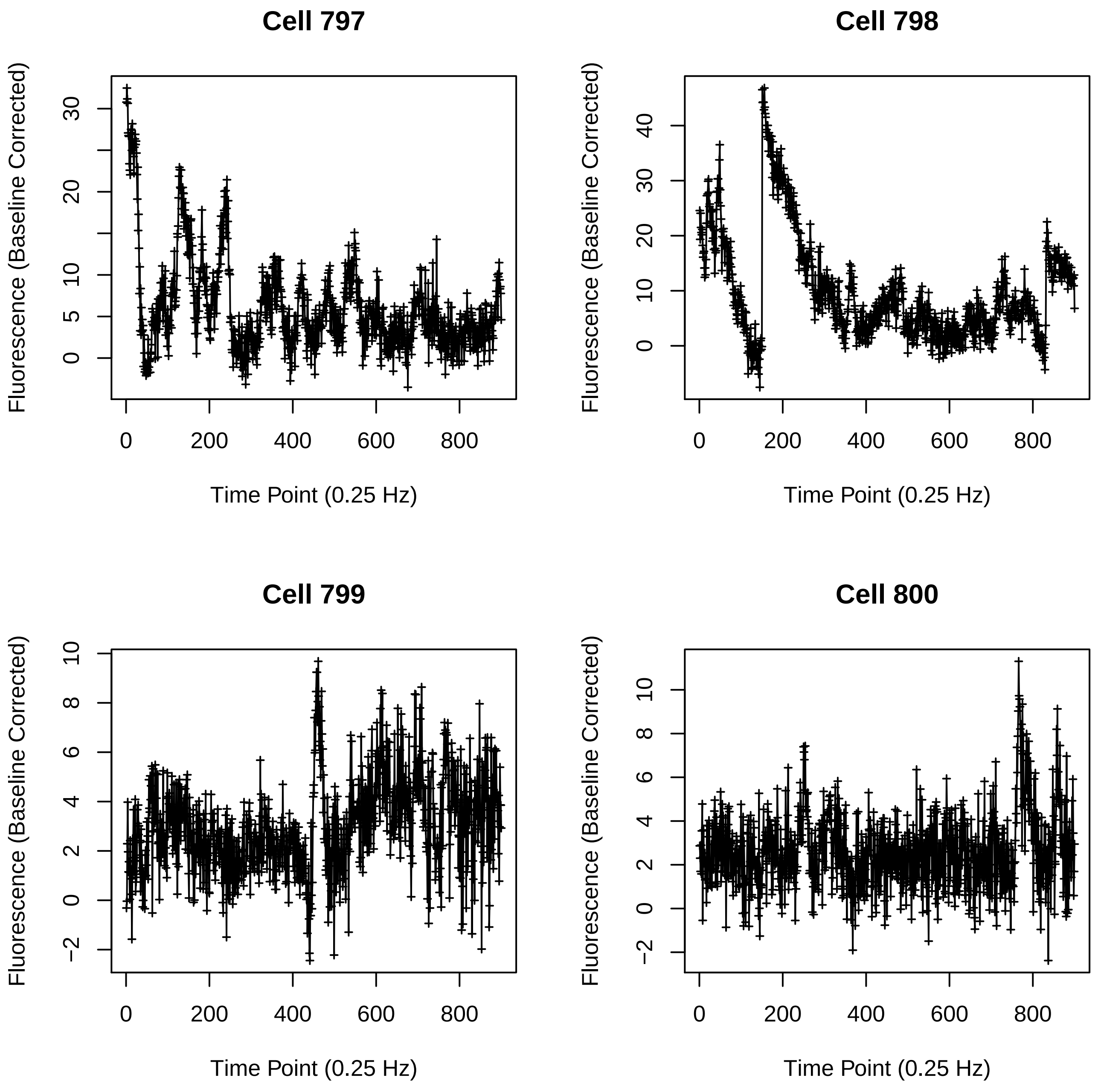
Cell 797
Cell 798
Cell 799
Cell 800
Fluorescence (Baseline Corrected)
Time Point (0.25 Hz)
0
200
400
600
800
0
5
10
20
30
40
−2
2
4
6
8

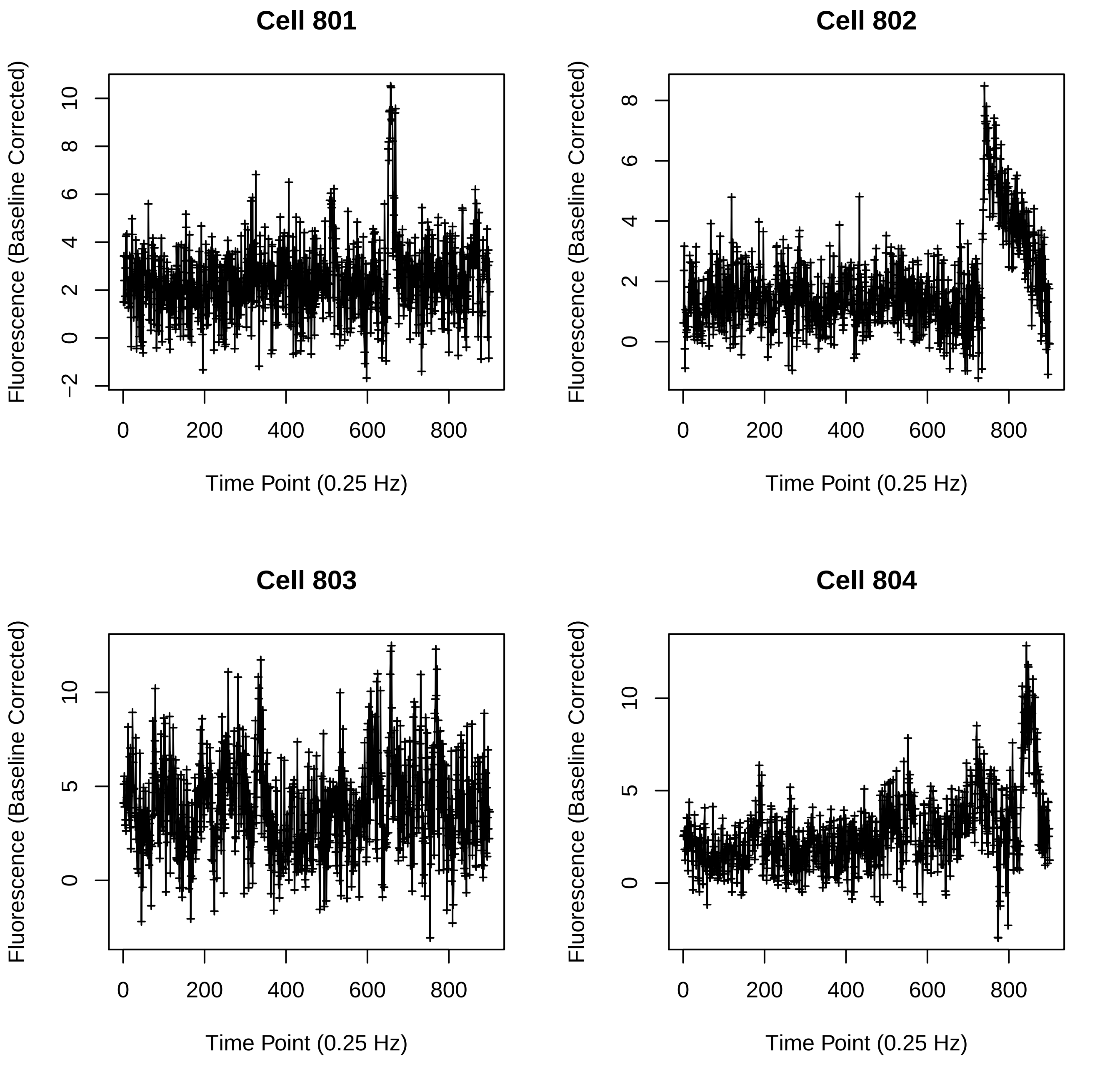
Cell 801
Cell 802
Cell 803
Cell 804
Fluorescence (Baseline Corrected)
Time Point (0.25 Hz)
0
200
400
600
800
−2
2
4
6
8
10
5

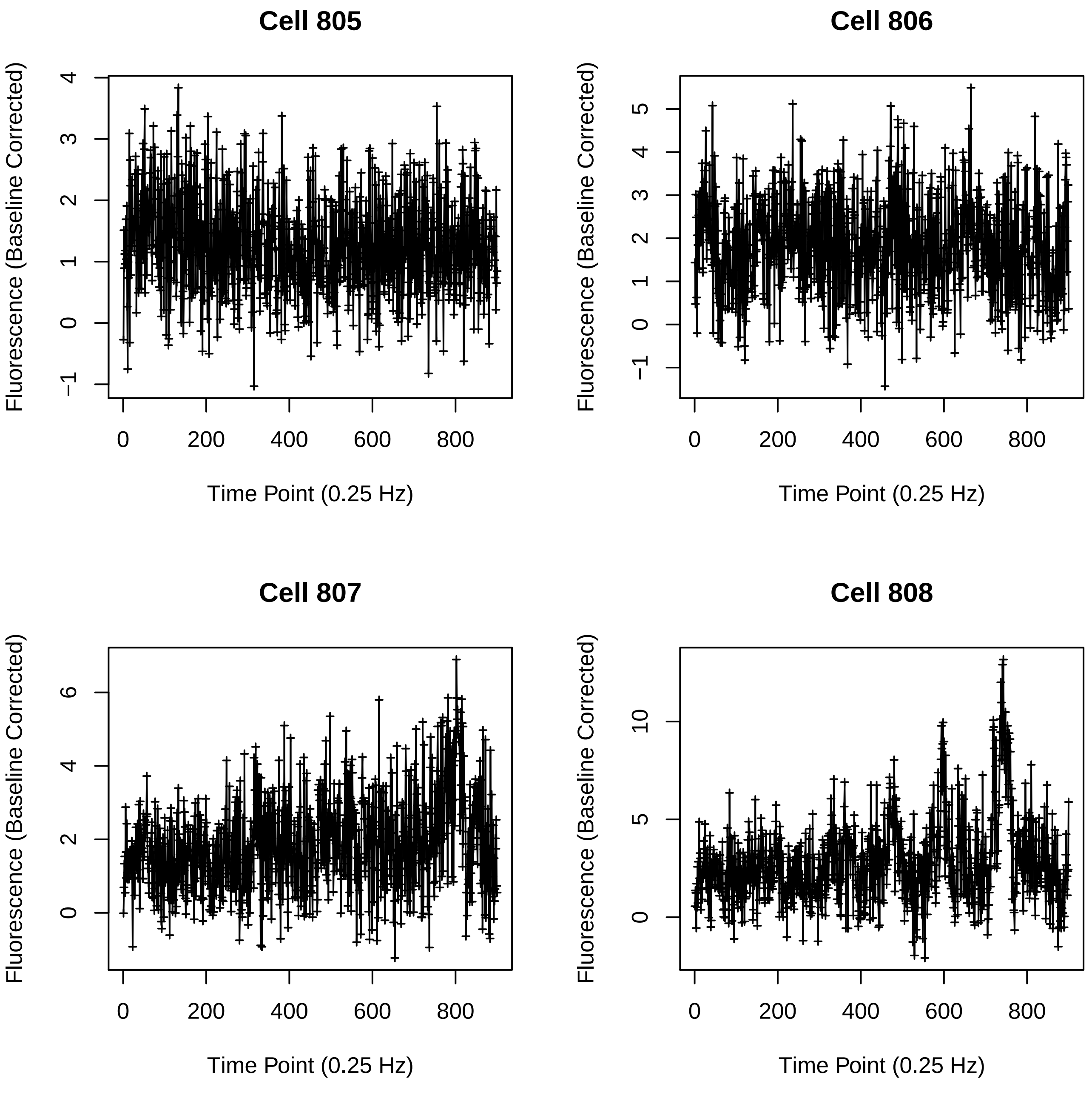
Cell 805
Cell 806
Cell 807
Cell 808
Fluorescence (Baseline Corrected)
Time Point (0.25 Hz)
0
200
400
600
800
−1
1
2
3
4
5
6
10

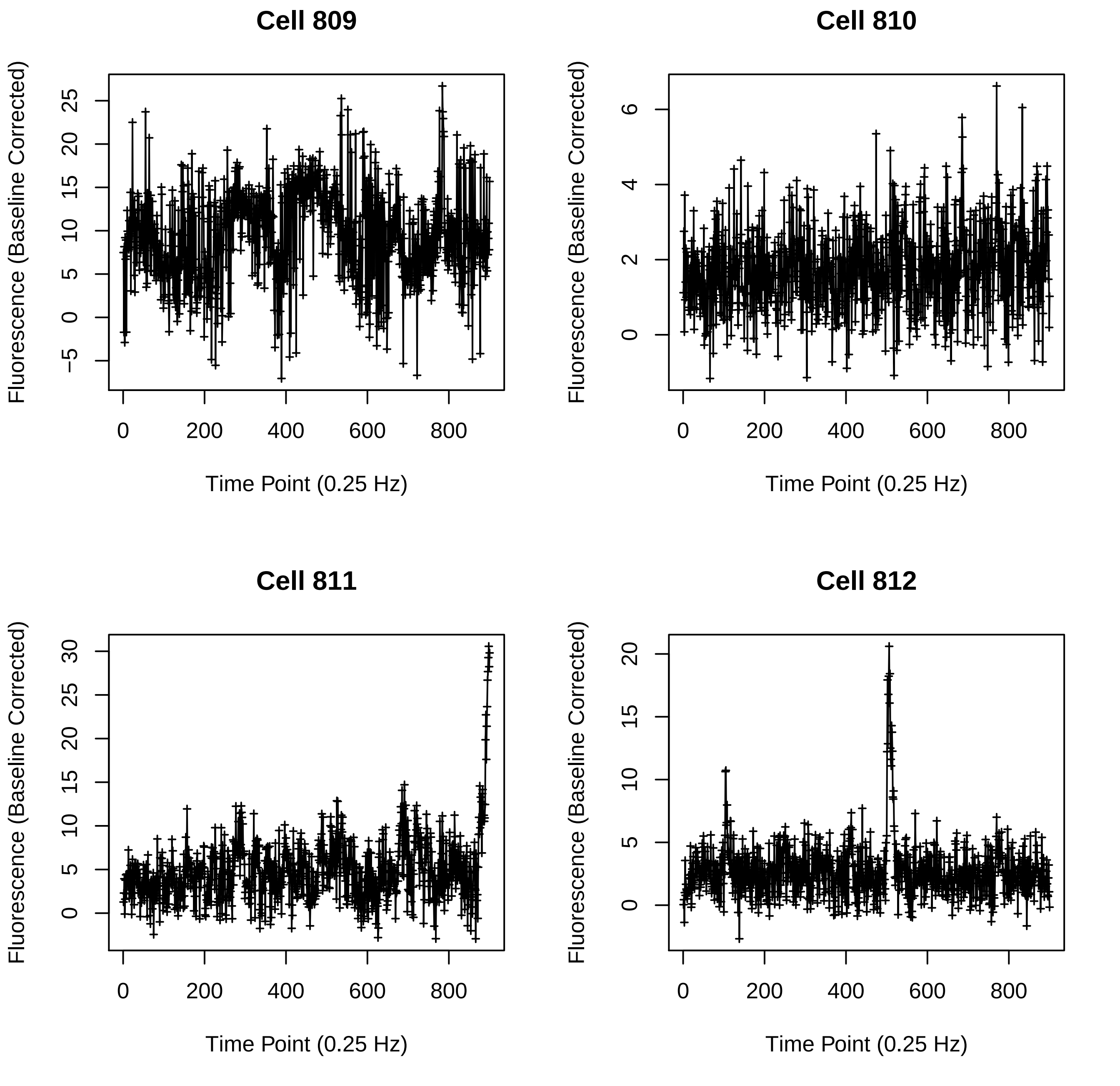
Cell 809
Cell 810
Cell 811
Cell 812
Fluorescence (Baseline Corrected)
Time Point (0.25 Hz)
0
200
400
600
800
−5
5
10
15
20
25
30
6
4
2

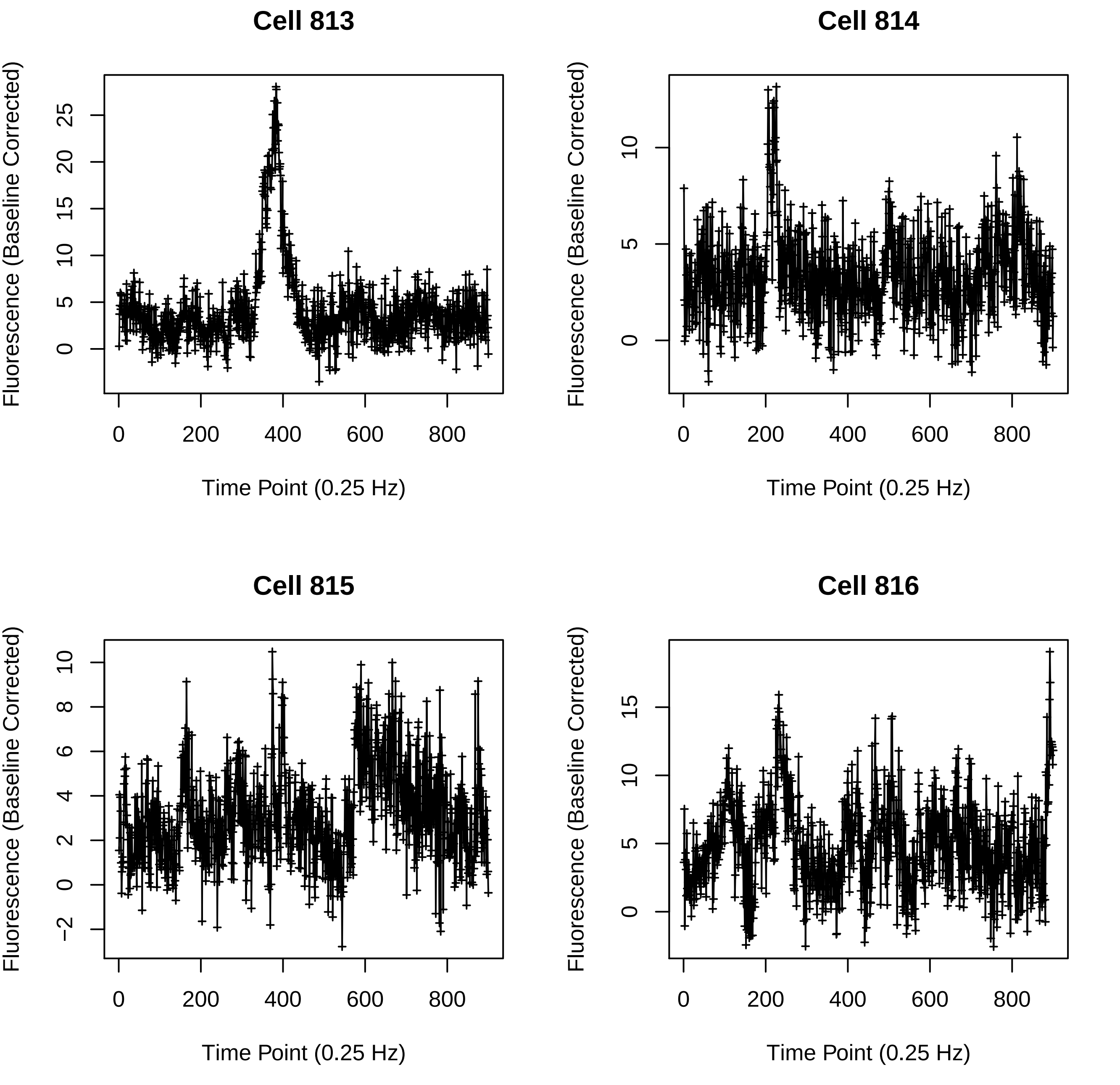

Cell 813
Cell 814
Cell 815
Cell 816
Fluorescence (Baseline Corrected)
Time Point (0.25 Hz)
0
200
400
600
800

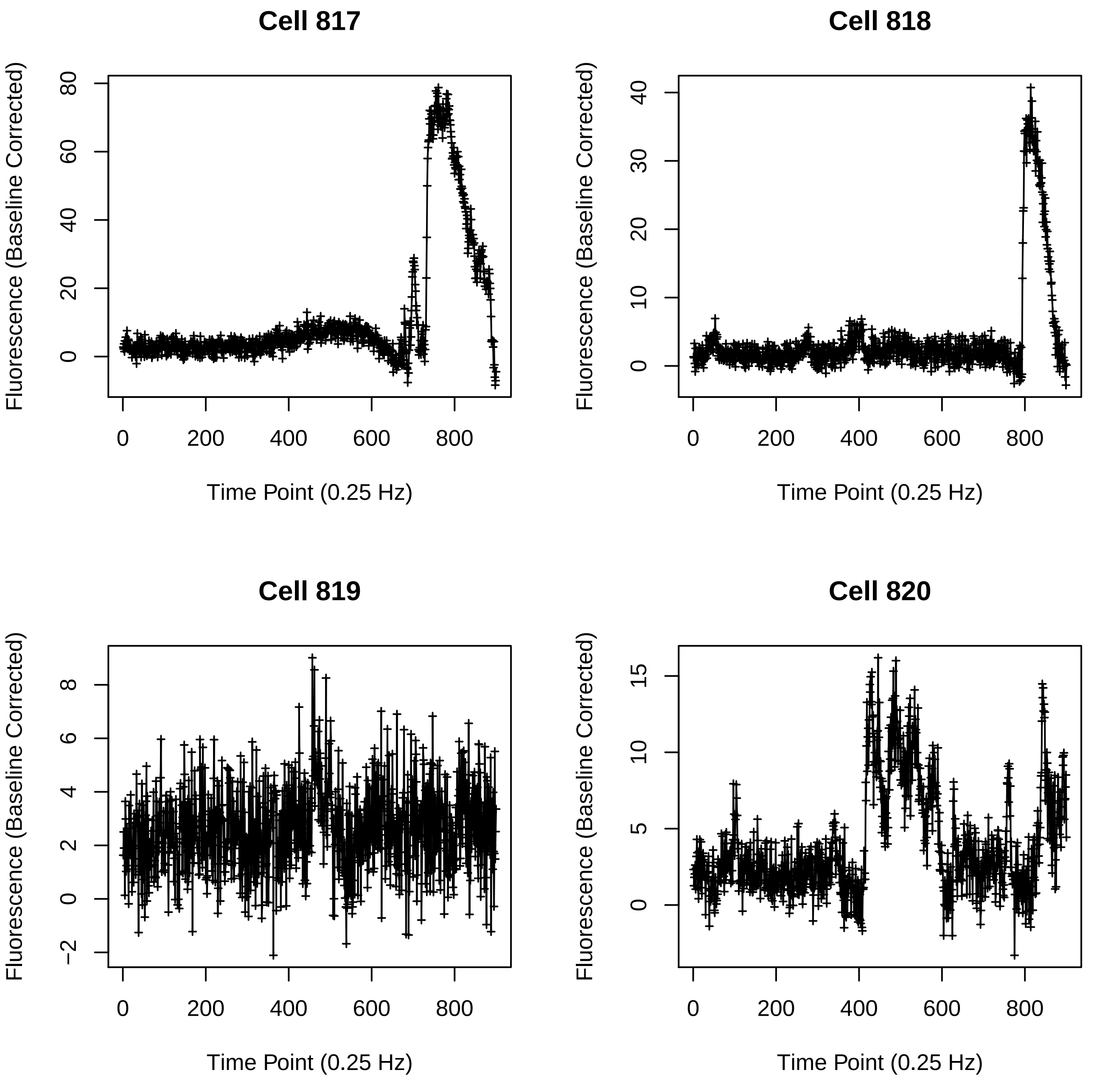
Cell 817
Cell 818
Cell 819
Cell 820
Fluorescence (Baseline Corrected)
Time Point (0.25 Hz)

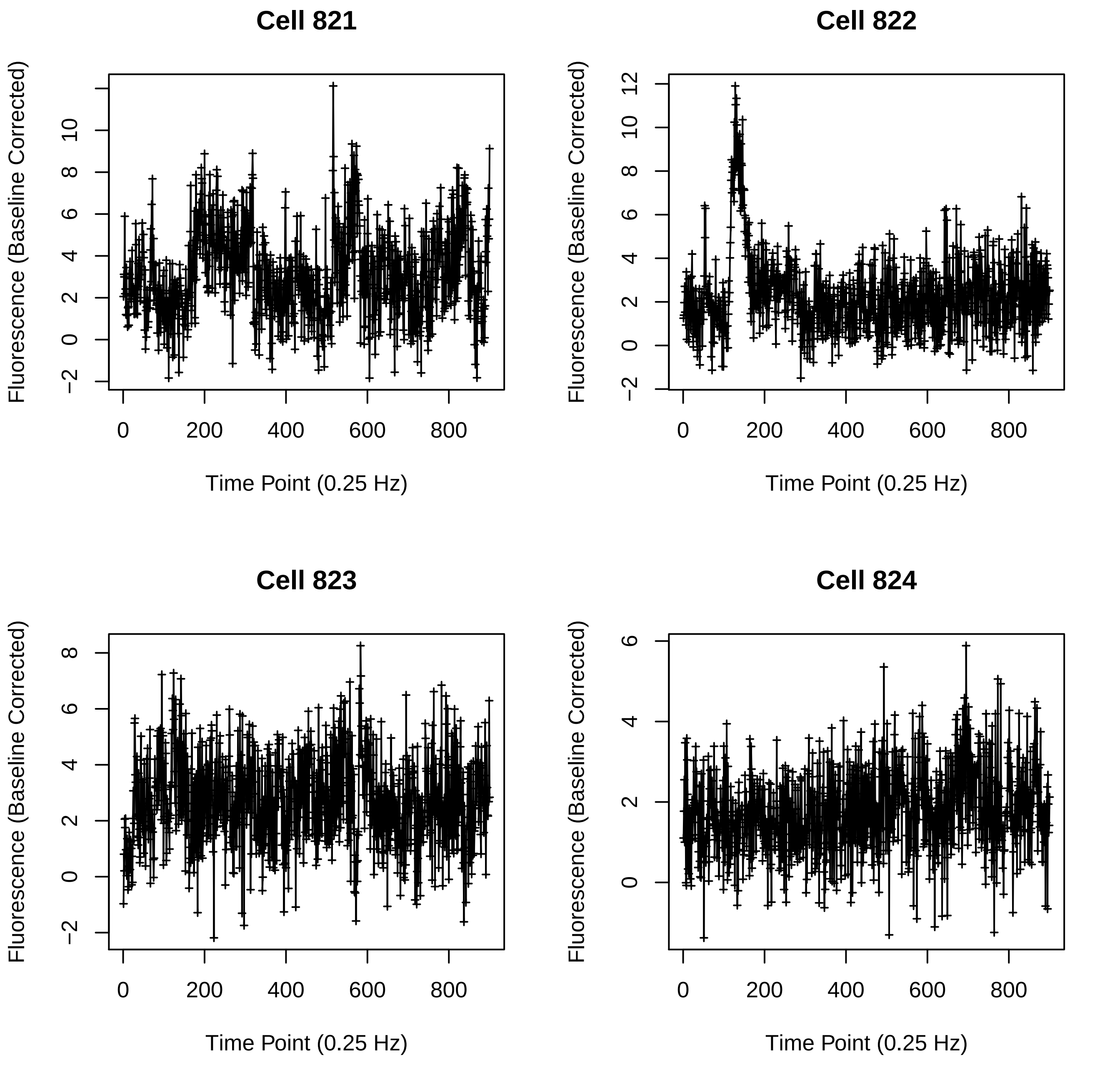
Cell 821
Cell 822
Cell 823
Cell 824
Fluorescence (Baseline Corrected)
Time Point (0.25 Hz)
0
200
400
600
800
−2
0
2
4
6
8
10
12

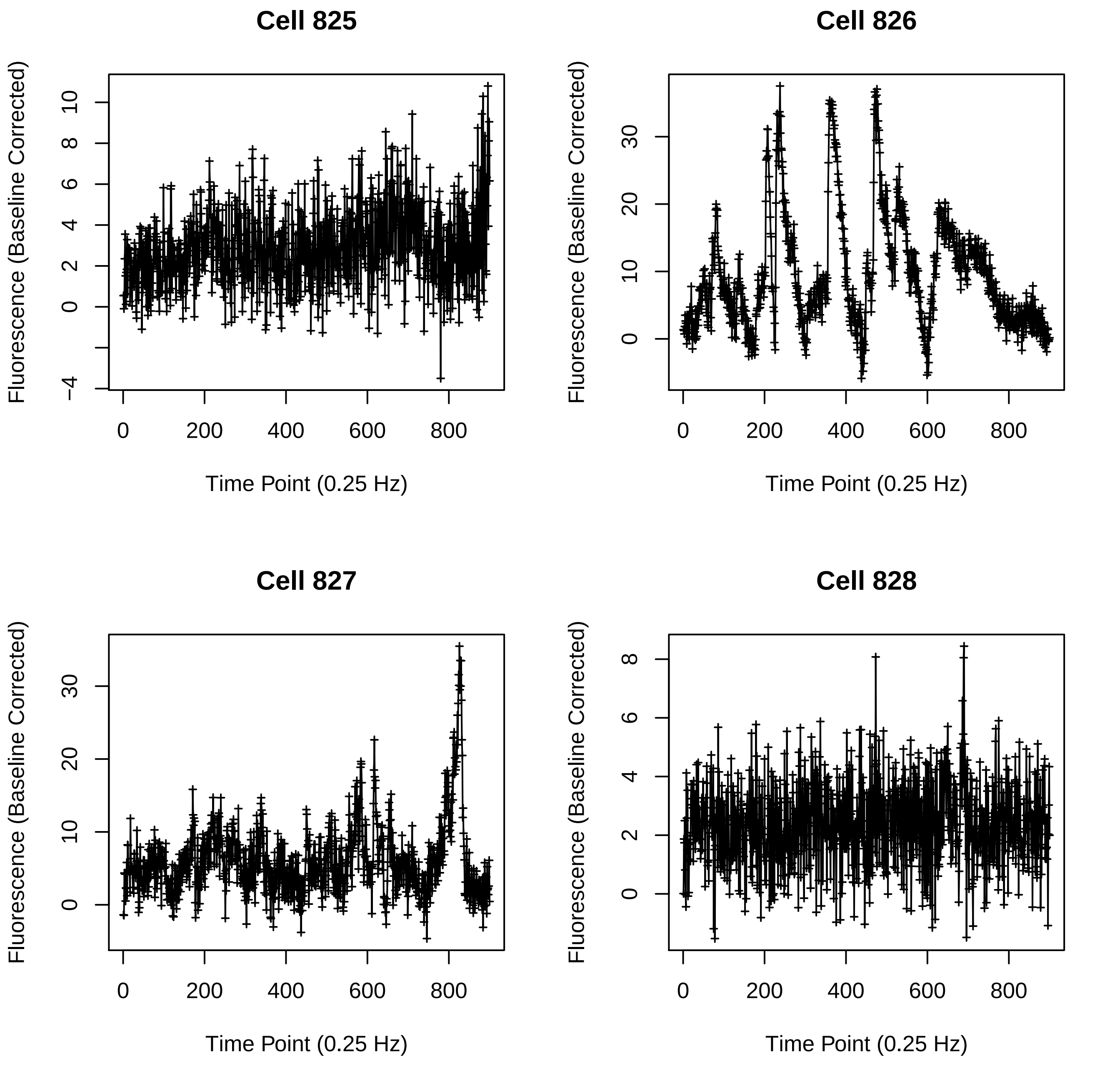
Cell 825
Cell 826
Cell 827
Cell 828
Fluorescence (Baseline Corrected)
Time Point (0.25 Hz)
0
200
400
600
800
−4
2
4
6
8
10
20
30

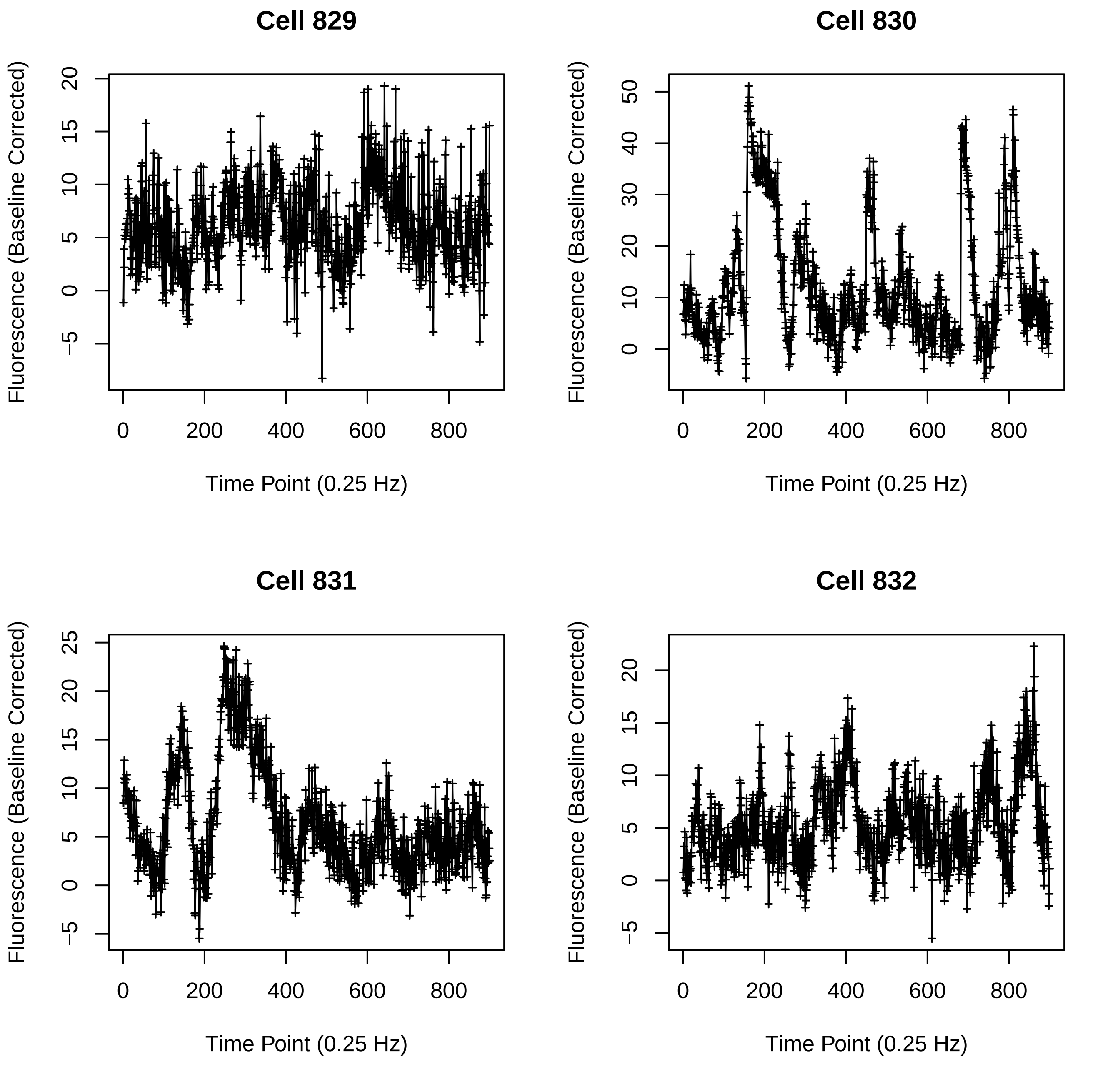

Cell 829
Cell 830
Cell 831
Cell 832
Fluorescence (Baseline Corrected)
Time Point (0.25 Hz)
0
200
400
600
800
−5
0
5
10
15
20
25
30
40
50

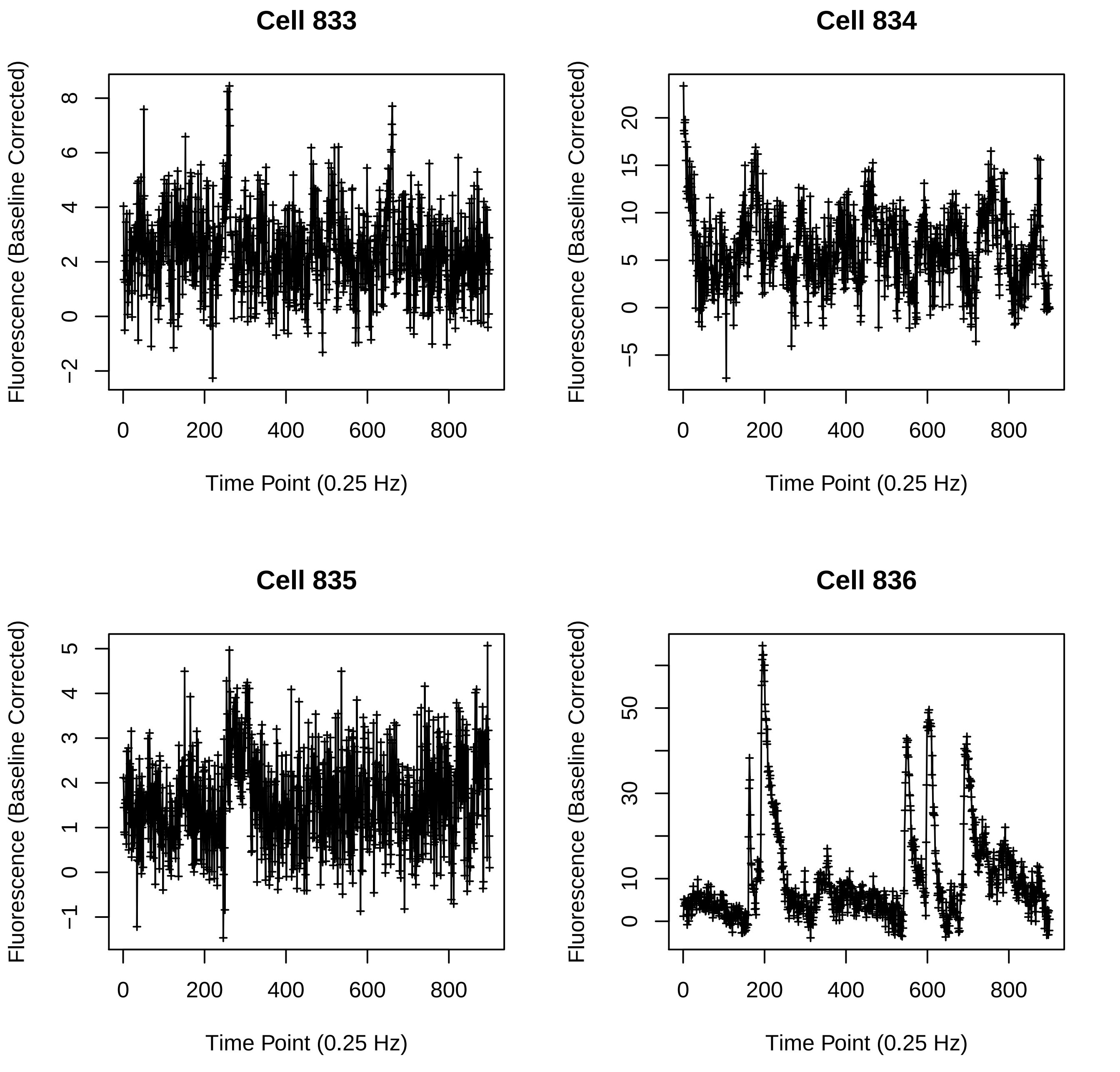
Cell 833
Fluorescence (Baseline Corrected)
−2
0
2
4
6
8
0
200
400
600
800
Time Point (0.25 Hz)
Cell 834
Fluorescence (Baseline Corrected)
−5
0
5
10
15
20
0
200
400
600
800
Time Point (0.25 Hz)
Cell 835
Fluorescence (Baseline Corrected)
−1
0
1
2
3
4
5
0
200
400
600
800
Time Point (0.25 Hz)
Cell 836
Fluorescence (Baseline Corrected)
0
10
30
50
0
200
400
600
800
Time Point (0.25 Hz)

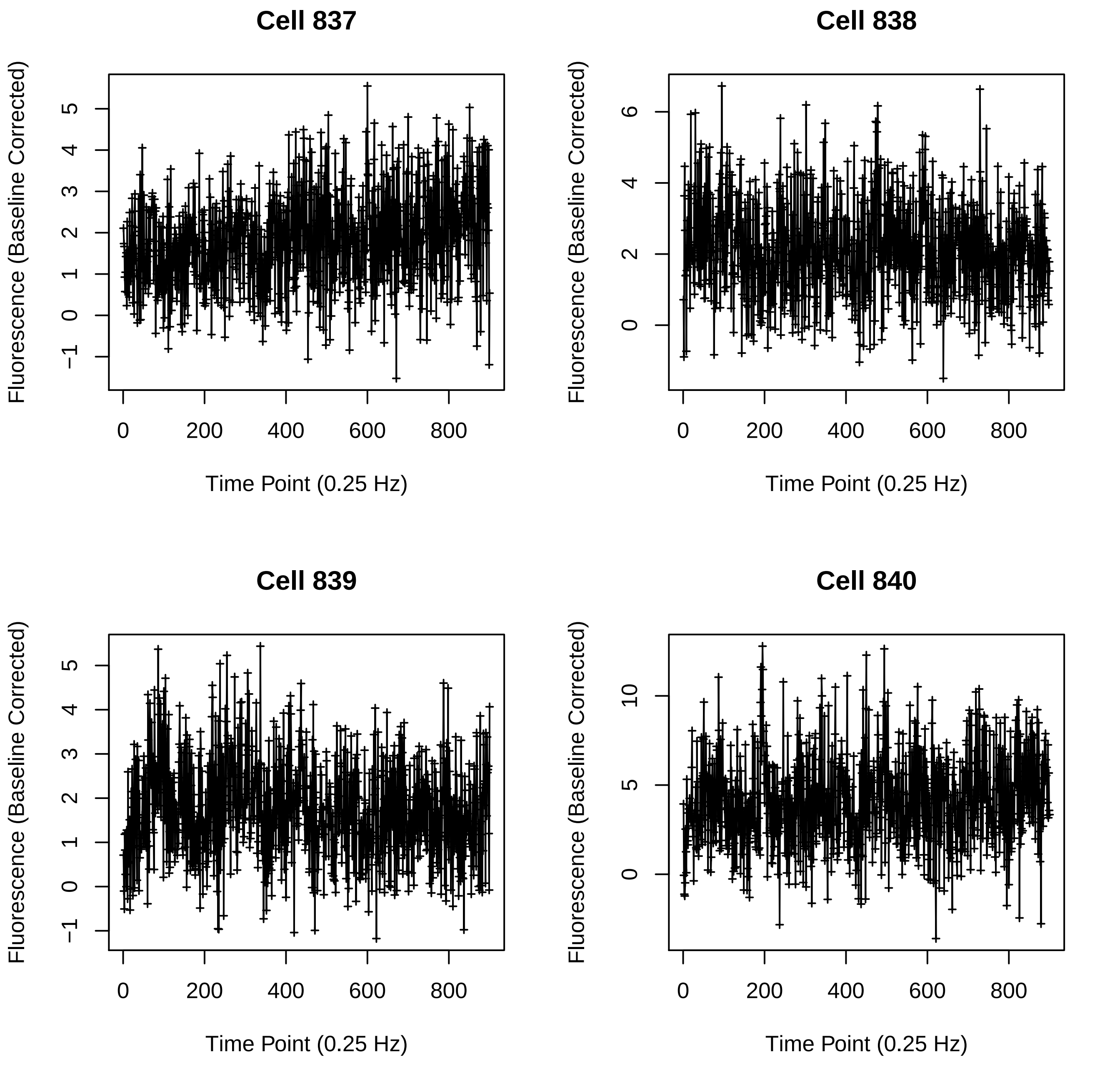
Cell 837
Fluorescence (Baseline Corrected)
−1
0
1
2
3
4
5
0
200
400
600
800
Time Point (0.25 Hz)
Cell 838
Fluorescence (Baseline Corrected)
0
2
4
6
0
200
400
600
800
Time Point (0.25 Hz)
Cell 839
Fluorescence (Baseline Corrected)
−1
0
1
2
3
4
5
0
200
400
600
800
Time Point (0.25 Hz)
Cell 840
Fluorescence (Baseline Corrected)
0
5
10
0
200
400
600
800
Time Point (0.25 Hz)

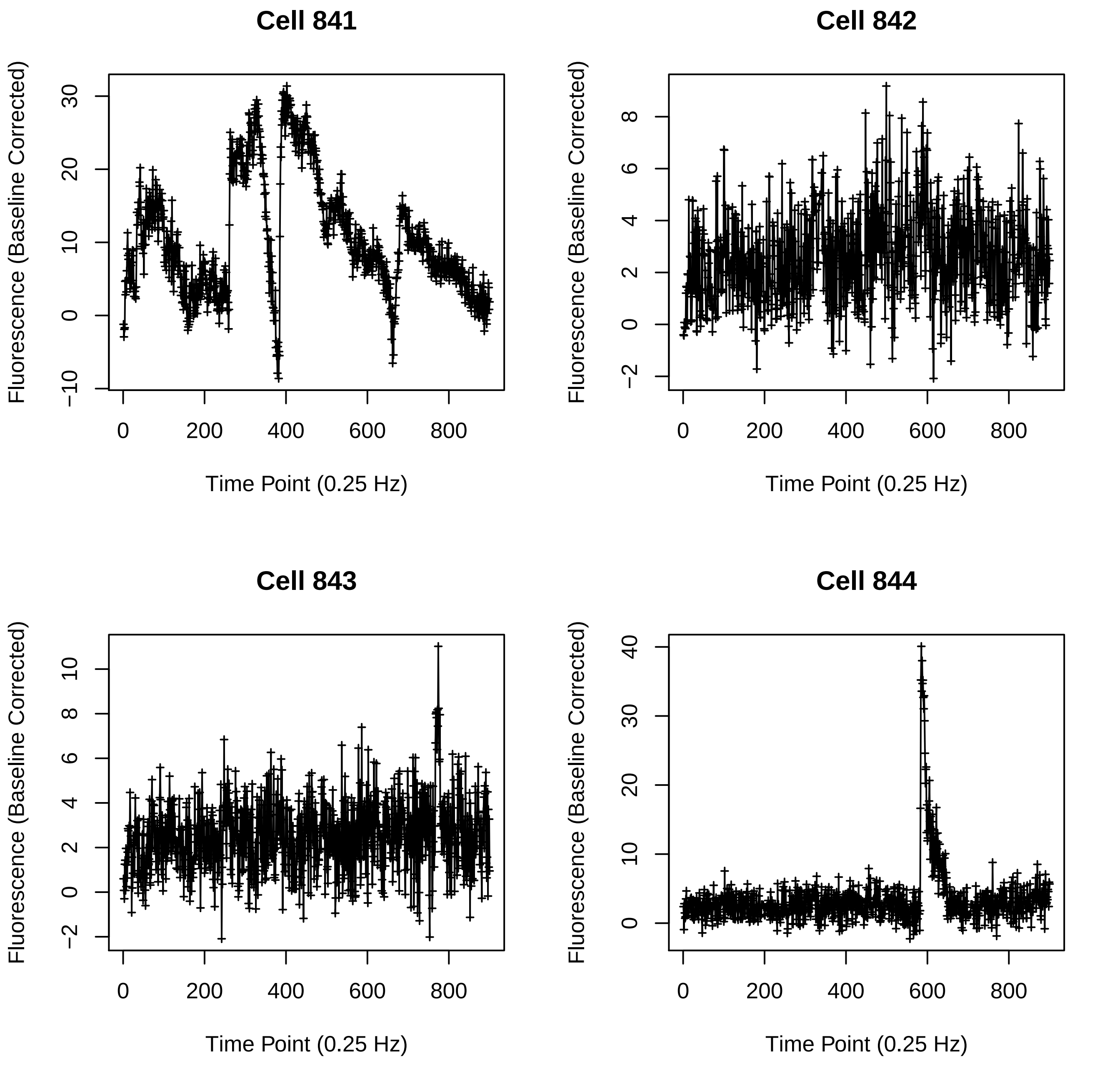

Cell 841
Cell 842
Cell 843
Cell 844
Fluorescence (Baseline Corrected)
Time Point (0.25 Hz)

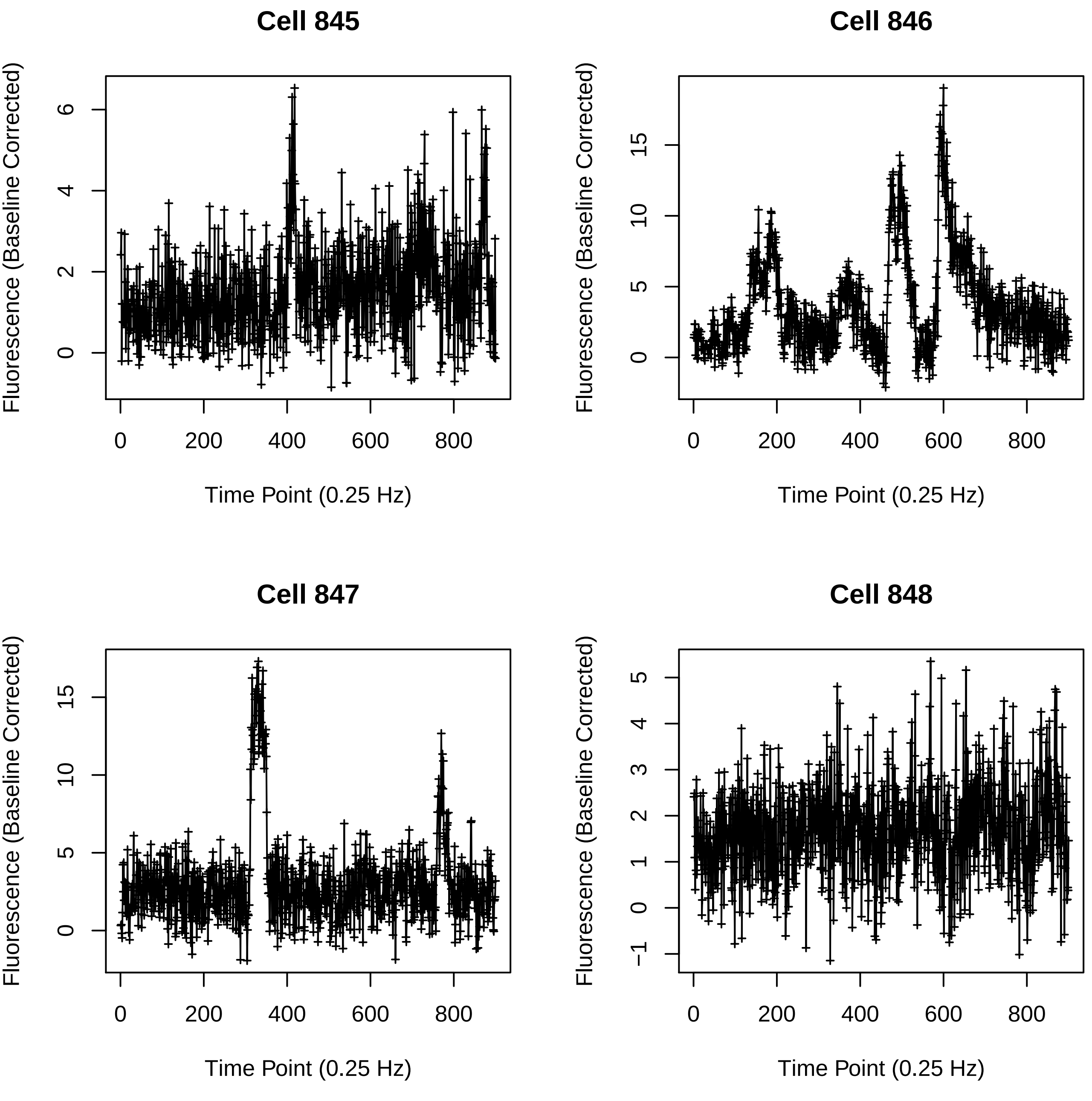
Cell 845
Cell 846
Cell 847
Cell 848
Fluorescence (Baseline Corrected)
Time Point (0.25 Hz)
0
200
400
600
800
-1
0
1
2
3
4
5
6
10
15

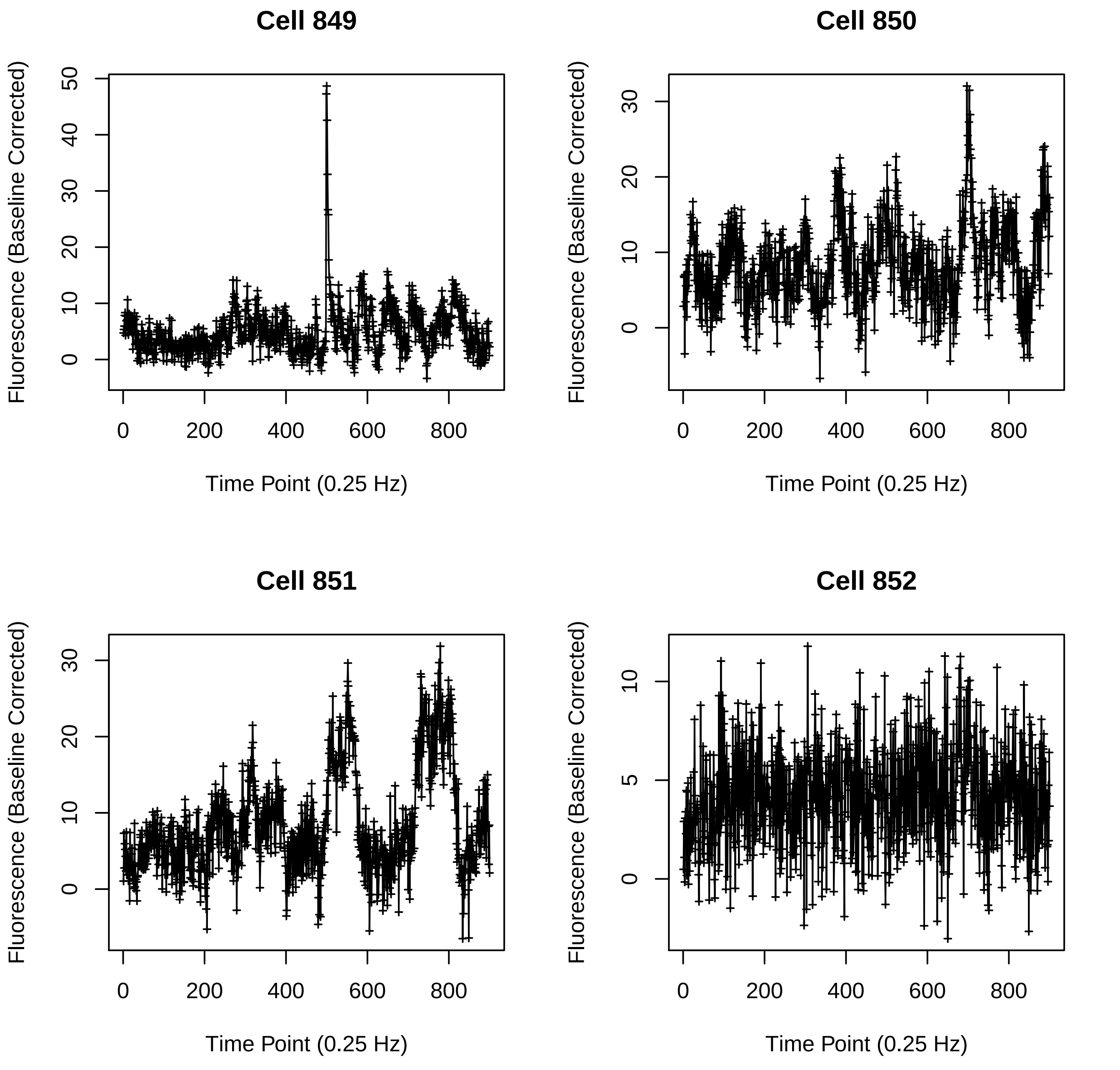
Cell 849
Cell 850
Cell 851
Cell 852
Fluorescence (Baseline Corrected)
Time Point (0.25 Hz)
0
200
400
600
800
-
10
20
30
40
50

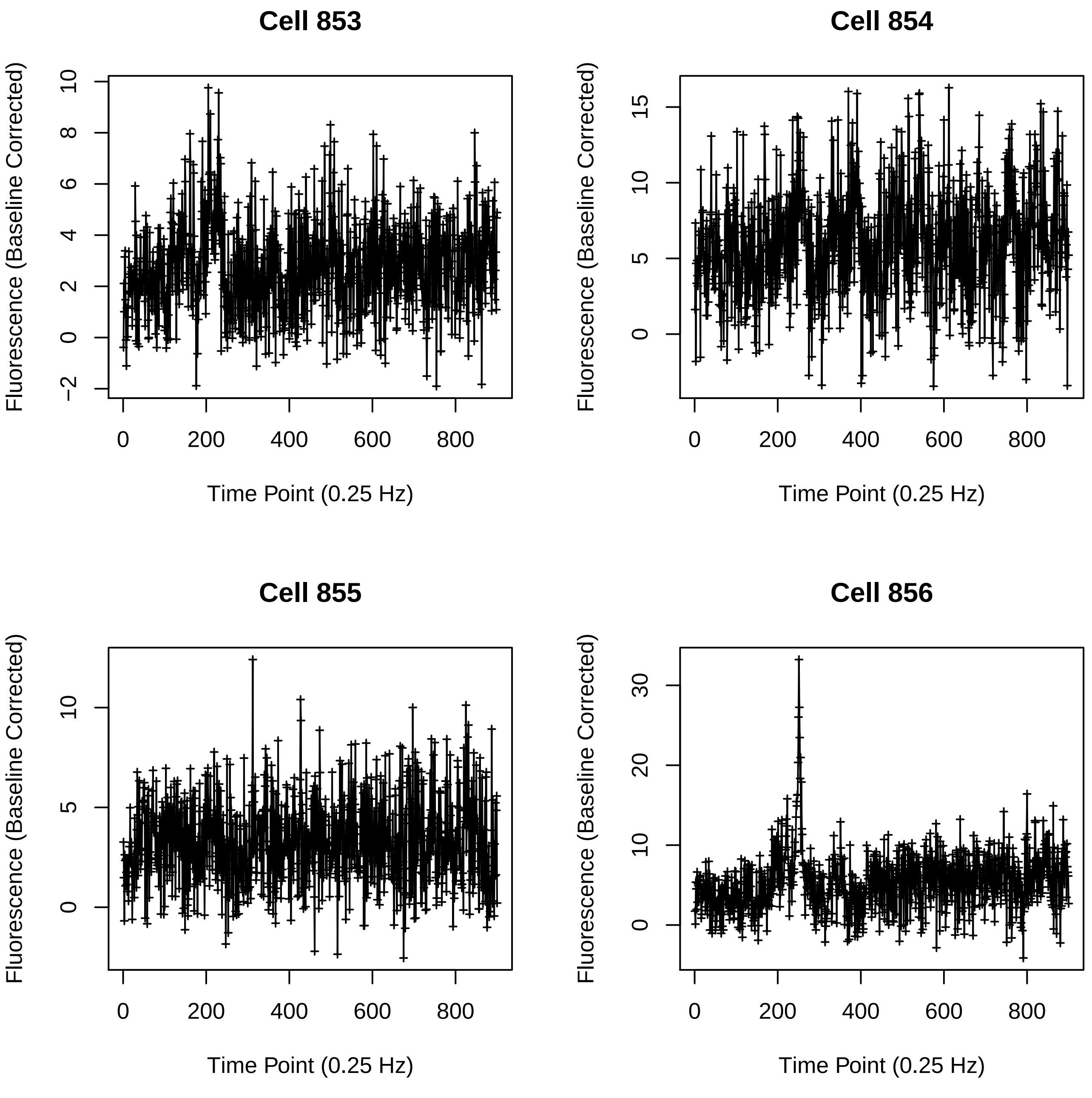
Cell 853
Cell 854
Cell 855
Cell 856
Fluorescence (Baseline Corrected)
Time Point (0.25 Hz)
0
200
400
600
800
−2
0
2
4
6
8
10
5
15
20
30

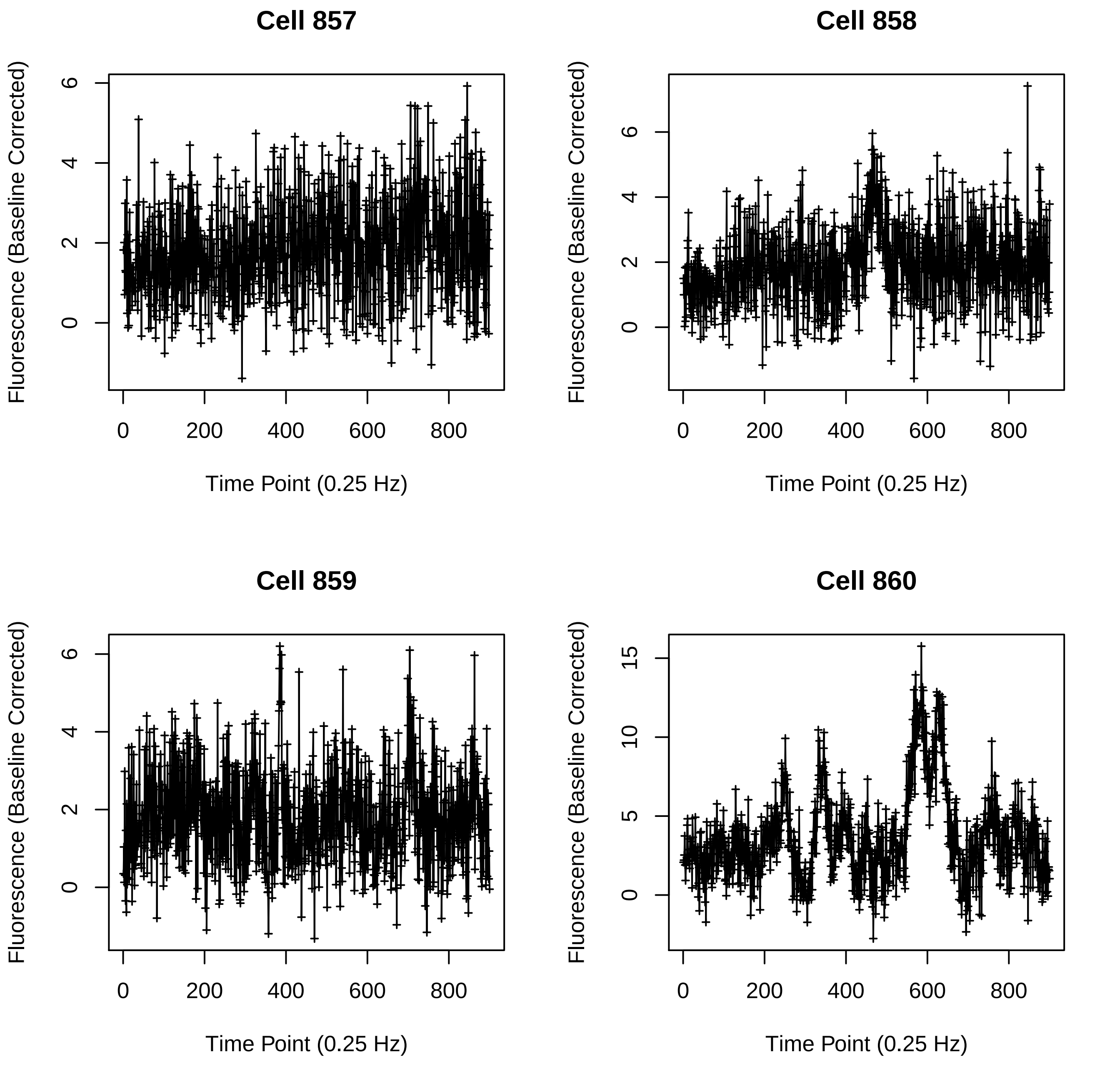
Cell 857
Cell 858
Cell 859
Cell 860
Fluorescence (Baseline Corrected)
Time Point (0.25 Hz)
0
200
400
600
800
2
4
6
5
10
15

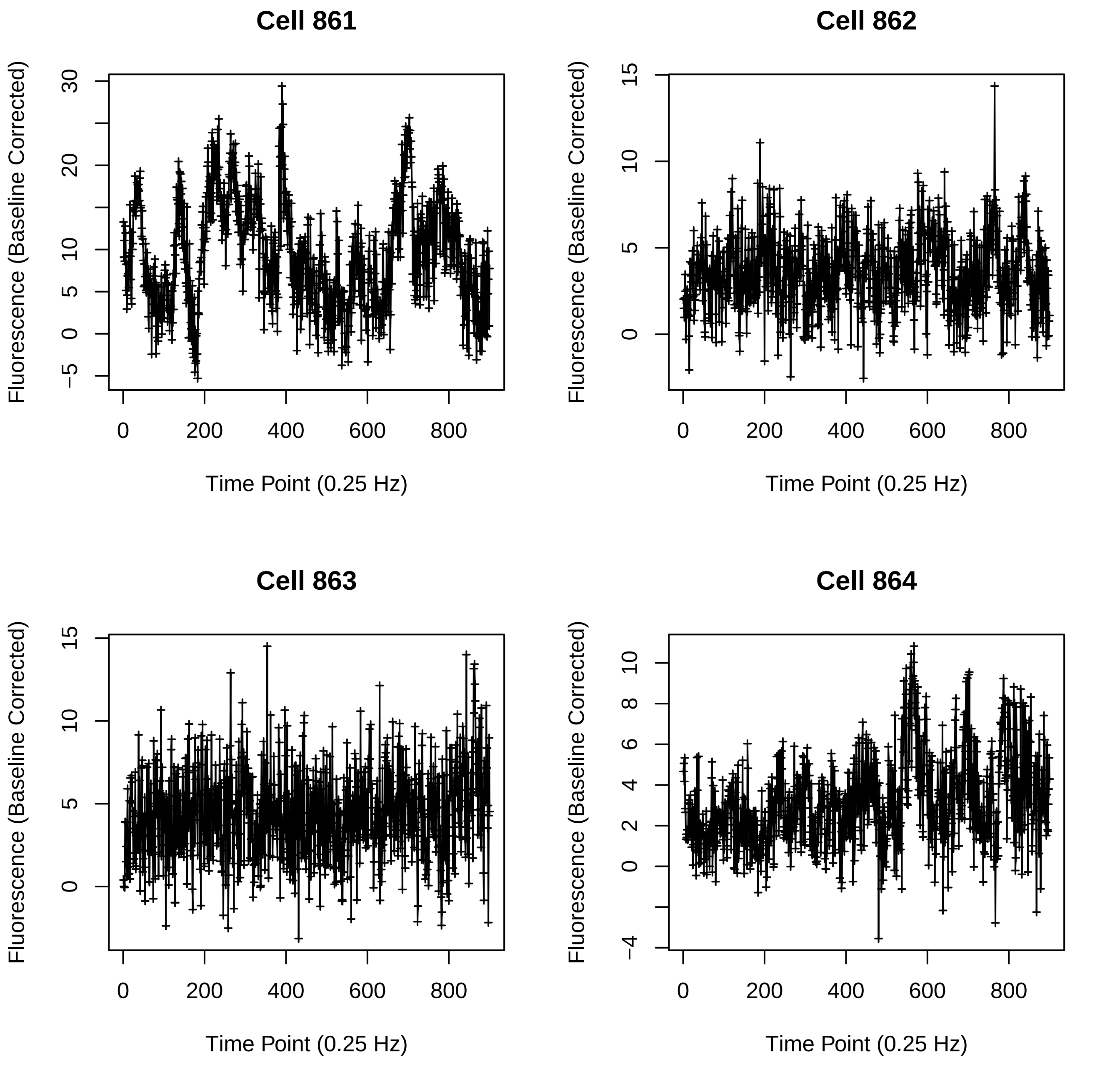
Cell 861
Fluorescence (Baseline Corrected)
Time Point (0.25 Hz)
Cell 862
Fluorescence (Baseline Corrected)
Time Point (0.25 Hz)
Cell 863
Fluorescence (Baseline Corrected)
Time Point (0.25 Hz)
Cell 864
Fluorescence (Baseline Corrected)
Time Point (0.25 Hz)

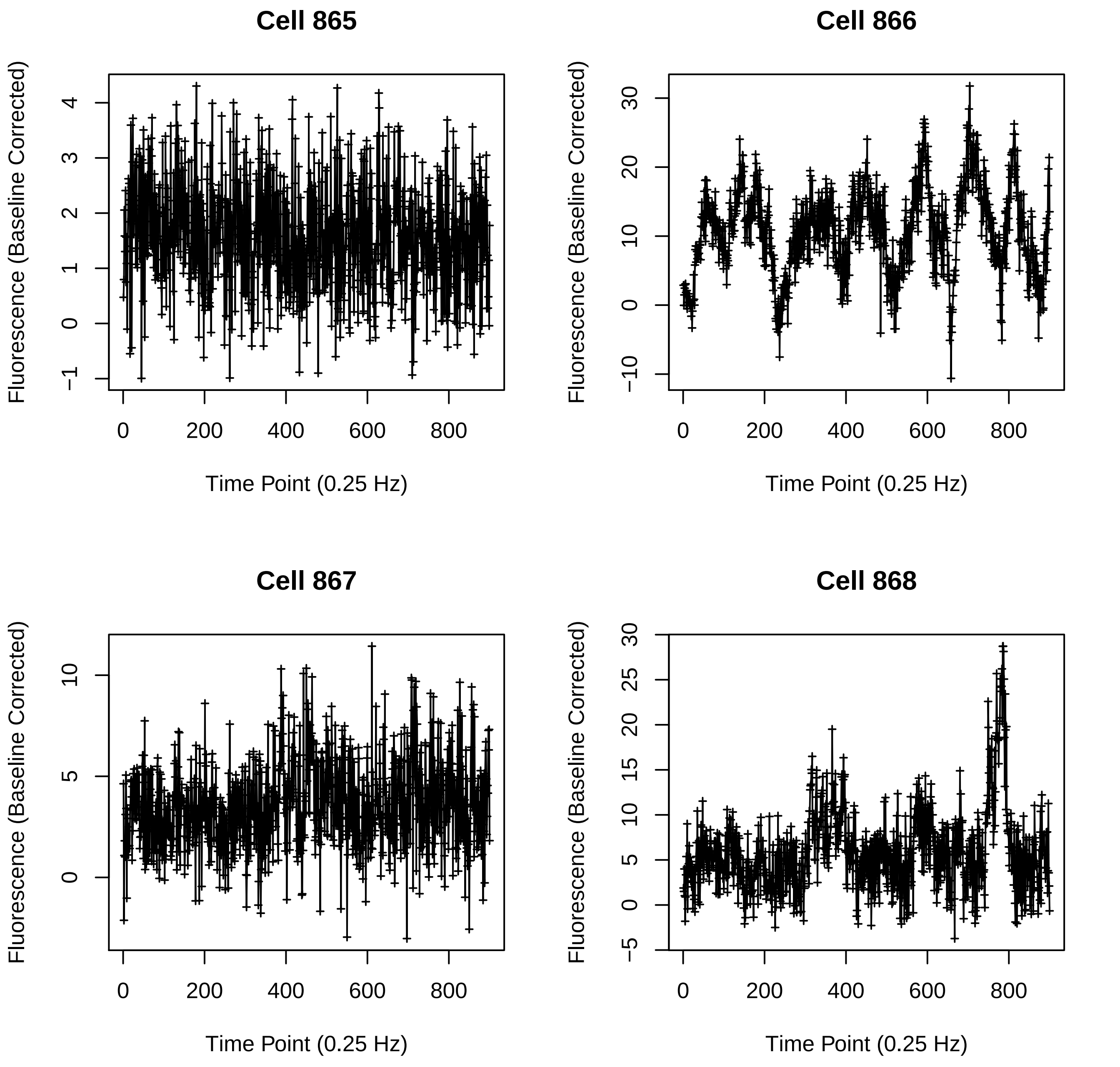
Cell 865
Cell 866
Cell 867
Cell 868
Fluorescence (Baseline Corrected)
Time Point (0.25 Hz)
0
200
400
600
800

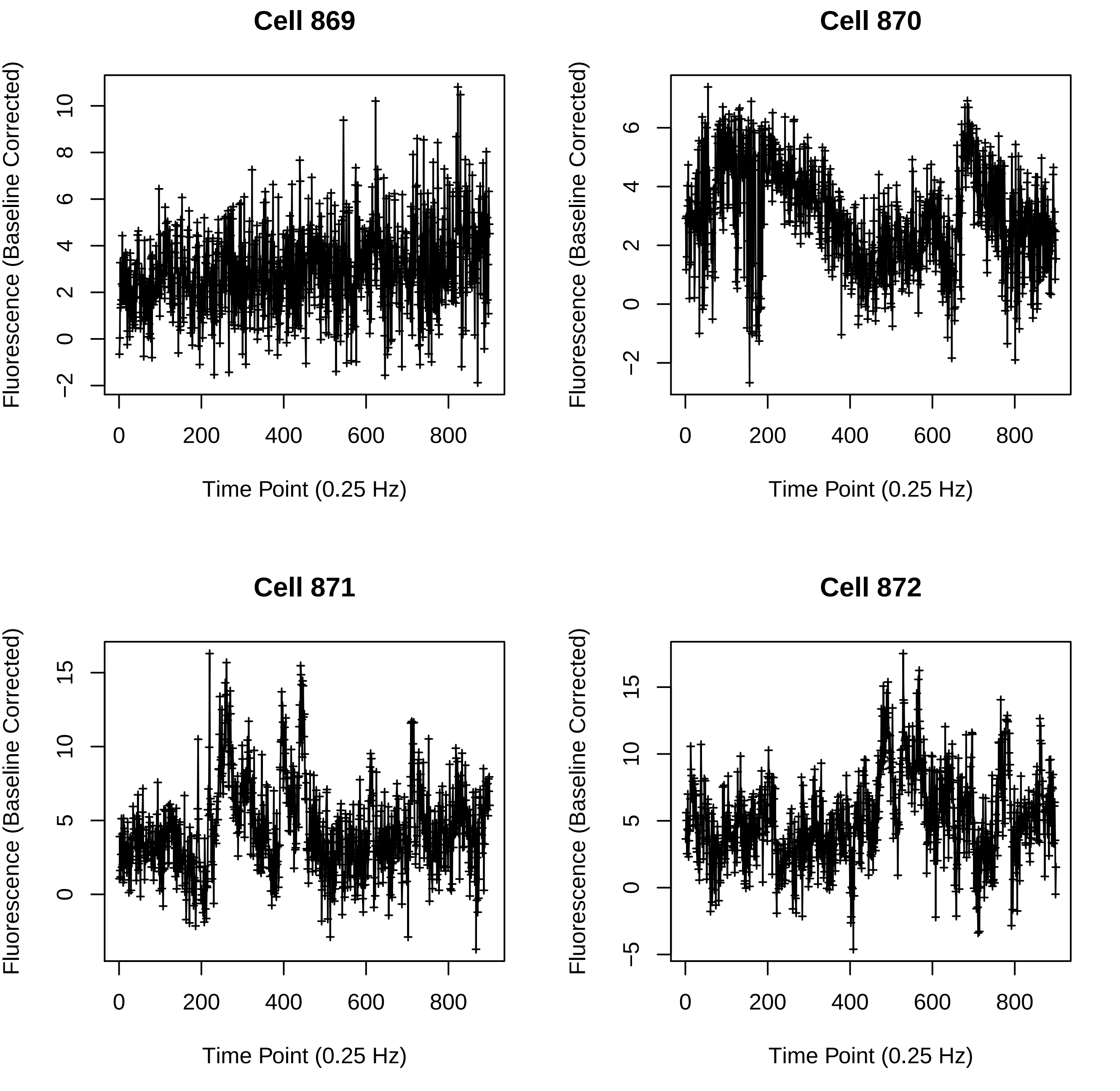

Cell 869
Cell 870
Cell 871
Cell 872
Fluorescence (Baseline Corrected)
Time Point (0.25 Hz)
0
200
400
600
800
−2
0
2
4
6
8
10
−5
5
15

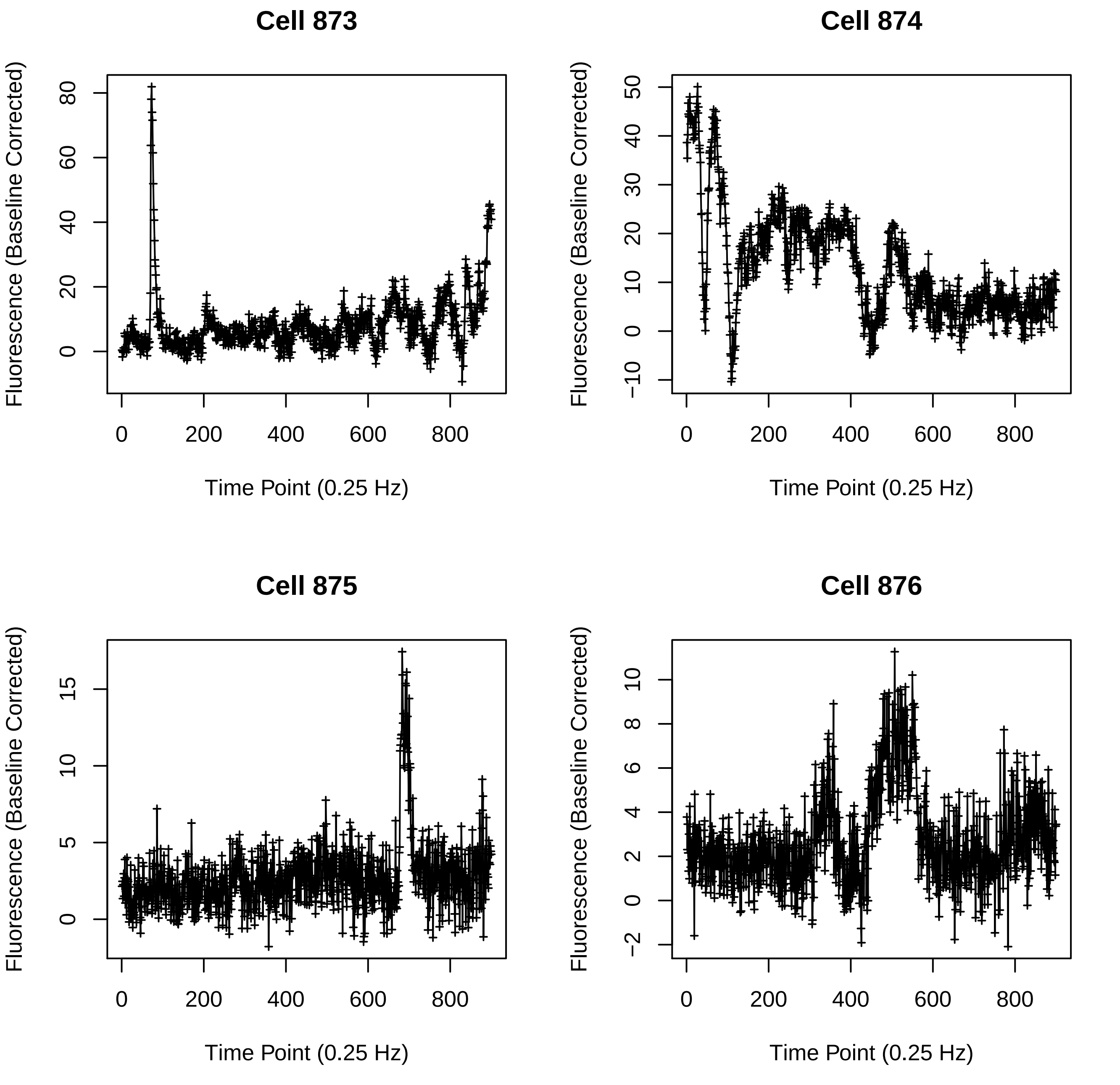

Cell 873
Cell 874
Cell 875
Cell 876
Fluorescence (Baseline Corrected)
Time Point (0.25 Hz)
0
200
400
600
800
20
40
60
80
−10
10
30
50
5
10
15
−2
2
4
6
8

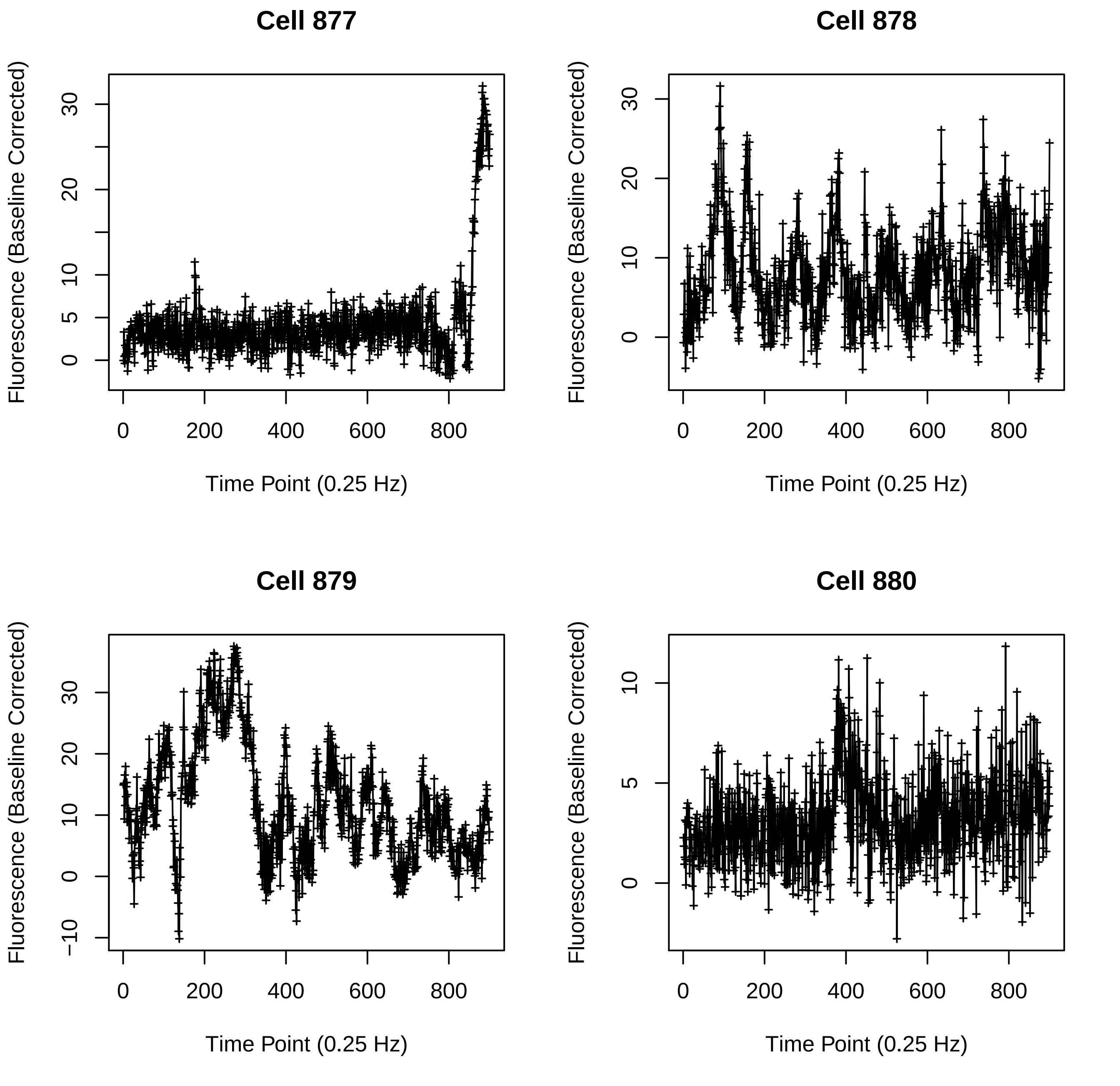
Cell 877
Cell 878
Cell 879
Cell 880
Fluorescence (Baseline Corrected)
Time Point (0.25 Hz)
0
200
400
600
800
-10
0
5
10
20
30

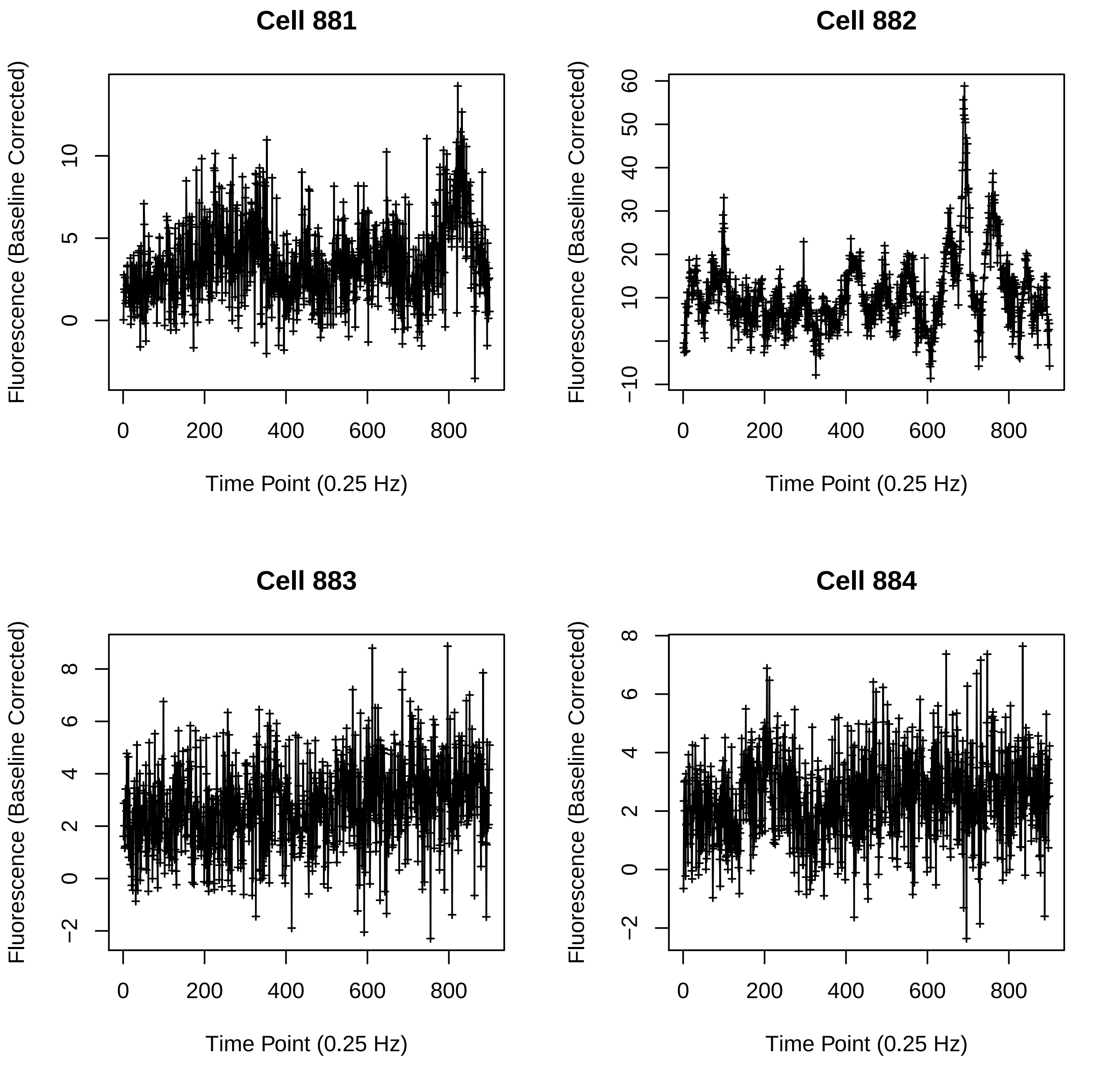
Cell 881
Cell 882
Cell 883
Cell 884
Fluorescence (Baseline Corrected)
Time Point (0.25 Hz)
0
200
400
600
800
−10
0
10
20
30
40
50
60
−2
2
4
5
6
8

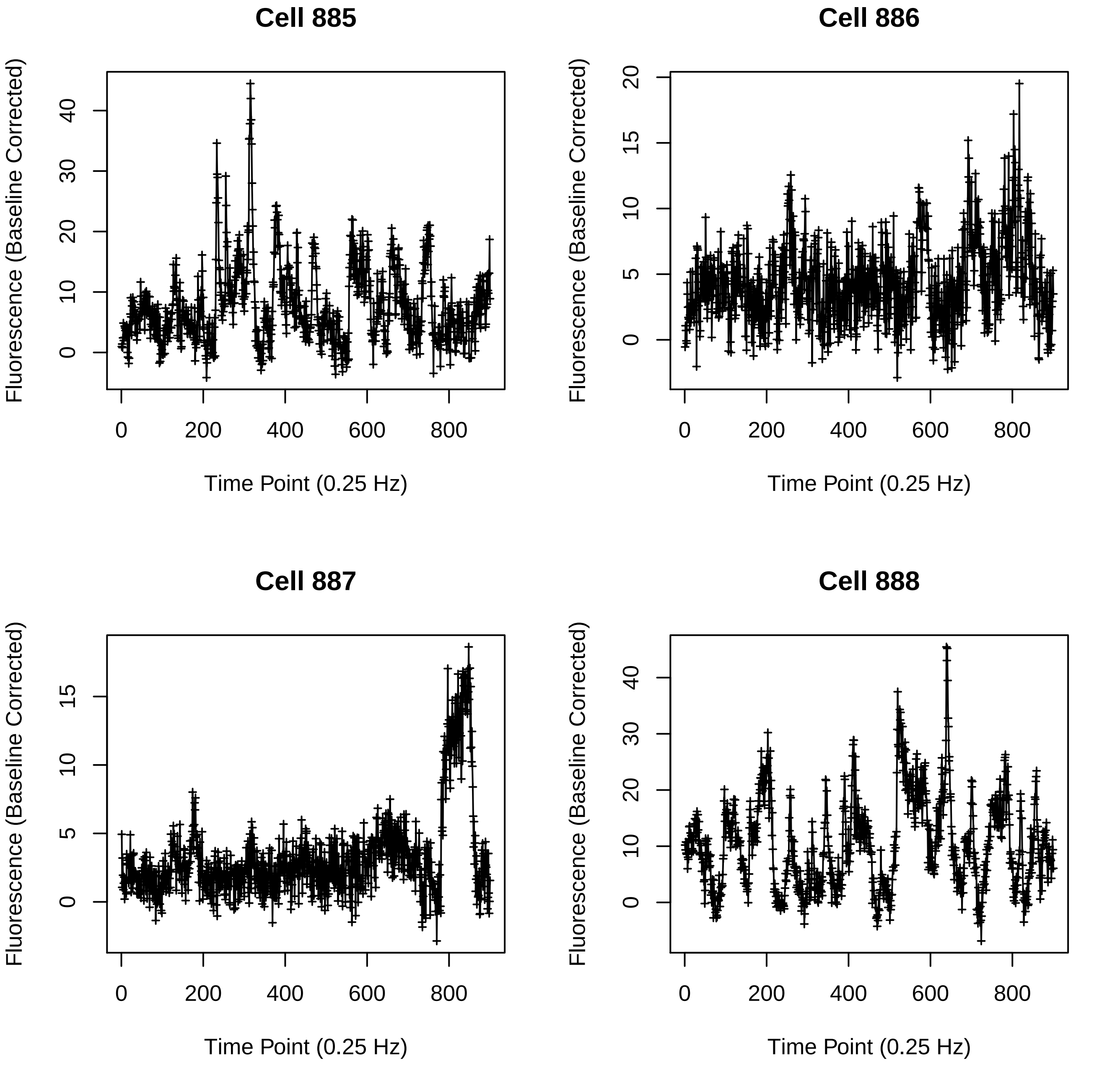

Cell 885
Fluorescence (Baseline Corrected)
Time Point (0.25 Hz)
Cell 886
Fluorescence (Baseline Corrected)
Time Point (0.25 Hz)
Cell 887
Fluorescence (Baseline Corrected)
Time Point (0.25 Hz)
Cell 888
Fluorescence (Baseline Corrected)
Time Point (0.25 Hz)

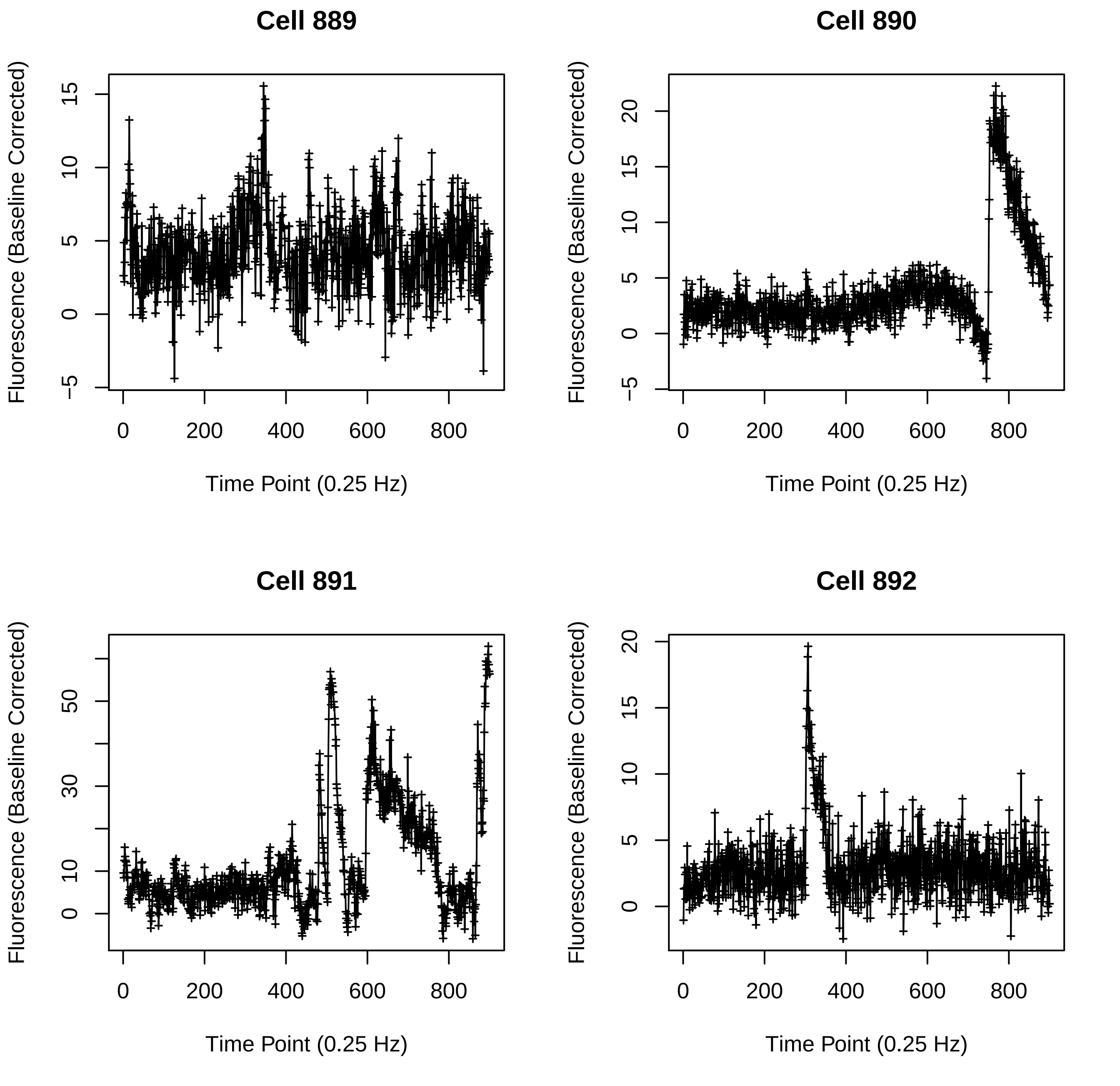

Cell 889
Cell 890
Cell 891
Cell 892
Fluorescence (Baseline Corrected)
Time Point (0.25 Hz)
-5
0
5
10
15
20
30
50
200
400
600
800

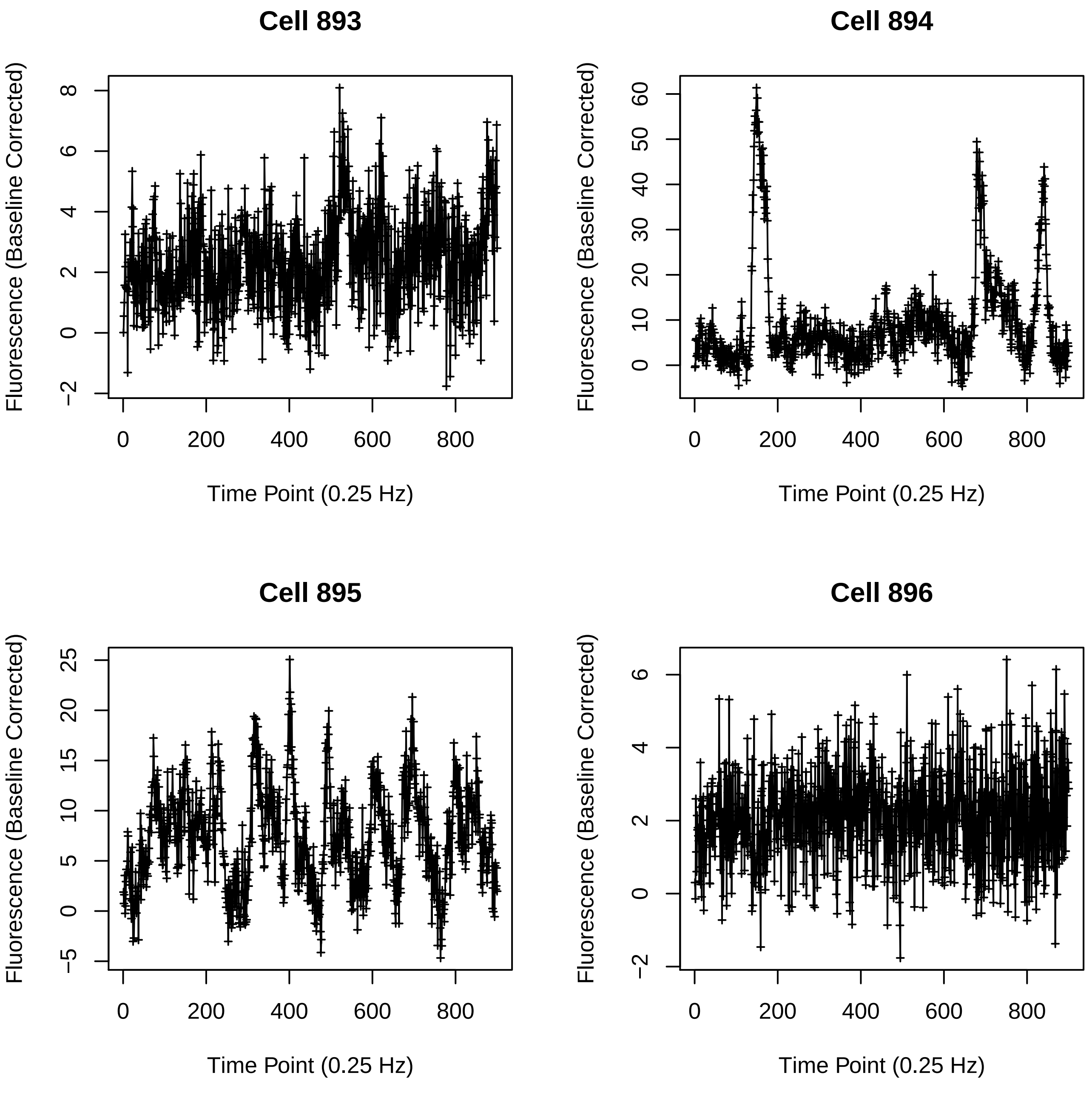
Cell 893
Cell 894
Cell 895
Cell 896
Fluorescence (Baseline Corrected)
Time Point (0.25 Hz)
-2
0
2
4
6
8
0
10
20
30
40
50
60
-5
0
5
10
15
20
25
-2
0
2
4
6
0
200
400
600
800

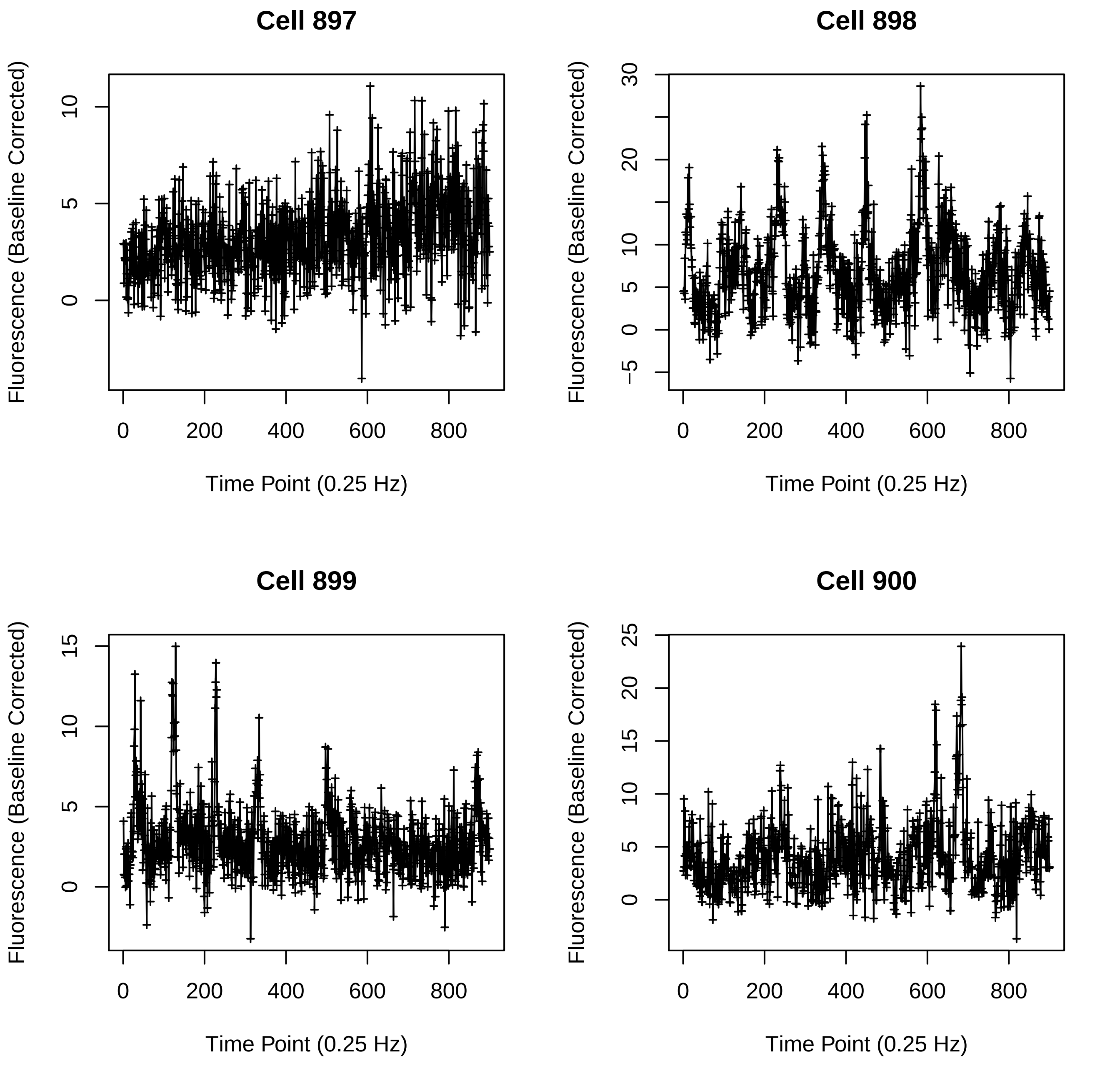

Cell 897
Cell 898
Cell 899
Cell 900
Fluorescence (Baseline Corrected)
Time Point (0.25 Hz)
0
200
400
600
800
-5
5
10
15
20
25
30

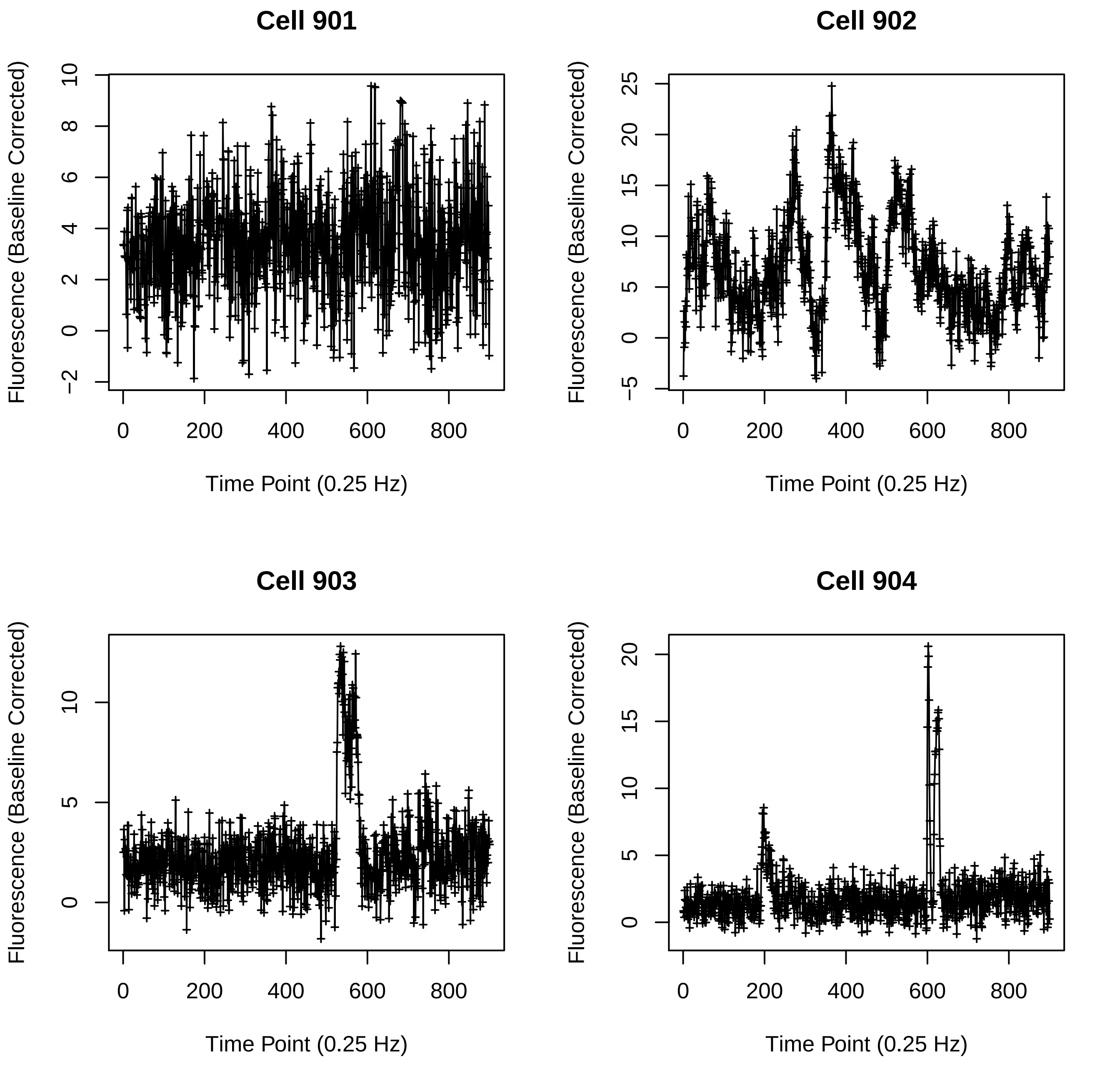

Cell 901
Cell 902
Cell 903
Cell 904
Fluorescence (Baseline Corrected)
Time Point (0.25 Hz)
0
200
400
600
800
−2
0
2
4
6
8
10
−5
5
15
20
25

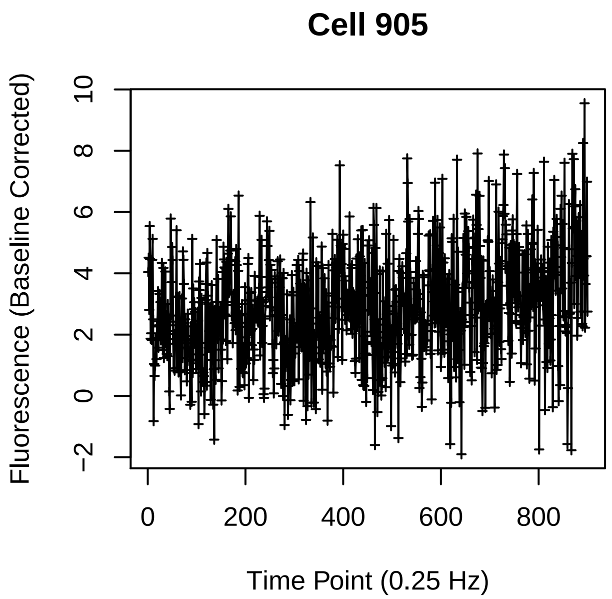
Cell 905
Fluorescence (Baseline Corrected)
Time Point (0.25 Hz)
−2
0
2
4
6
8
10
0
200
400
600
800

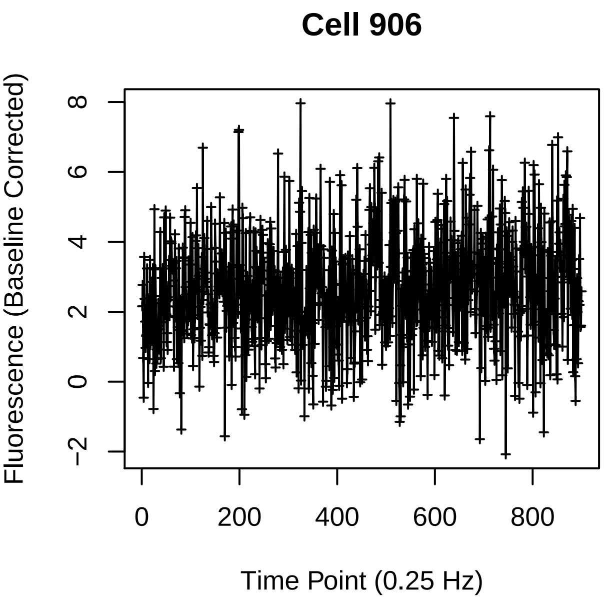
Cell 906
Fluorescence (Baseline Corrected)
Time Point (0.25 Hz)
−2
0
2
4
6
8
0
200
400
600
800

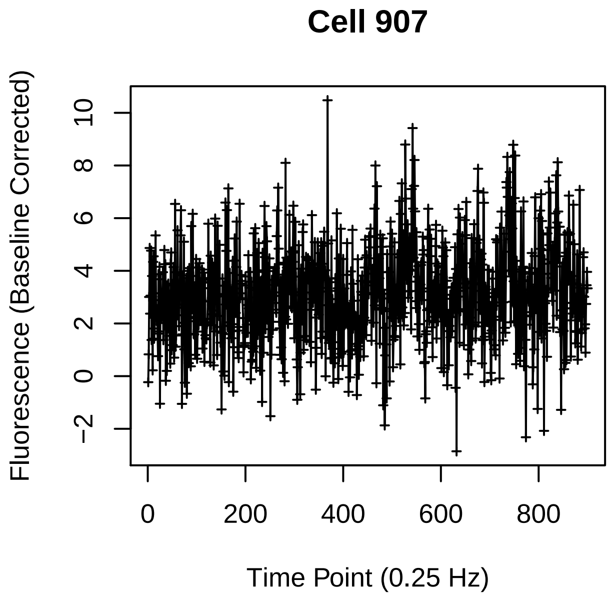
Cell 907
Fluorescence (Baseline Corrected)
Time Point (0.25 Hz)
−2
0
2
4
6
8
10
0
200
400
600
800

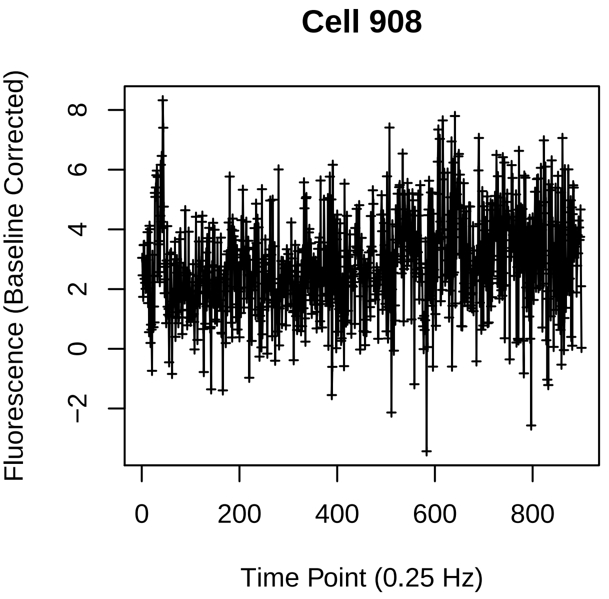
Cell 908
Fluorescence (Baseline Corrected)
Time Point (0.25 Hz)
−2
0
2
4
6
8
0
200
400
600
800

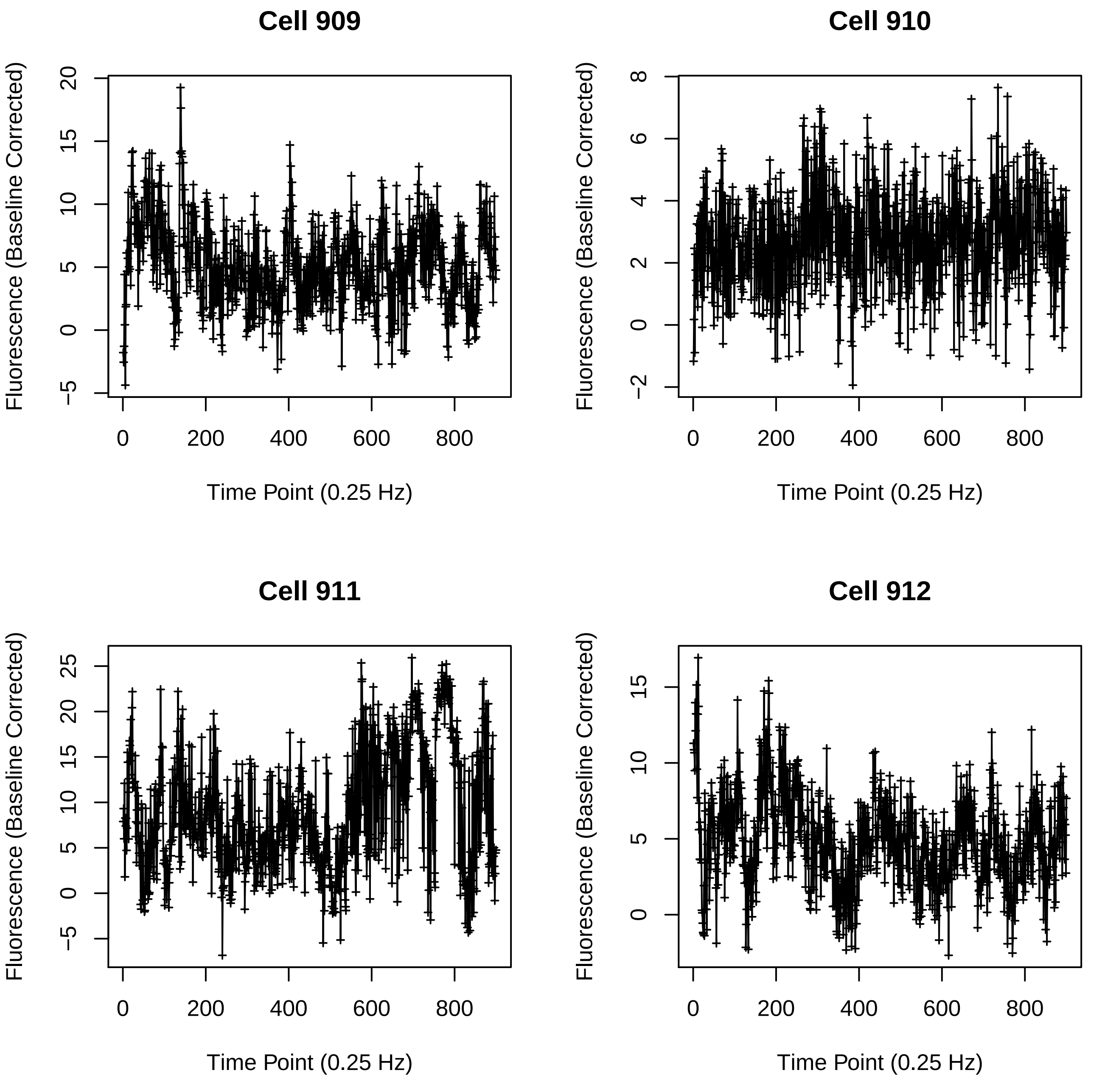
Cell 909
Cell 910
Cell 911
Cell 912
Fluorescence (Baseline Corrected)
Time Point (0.25 Hz)
0
200
400
600
800

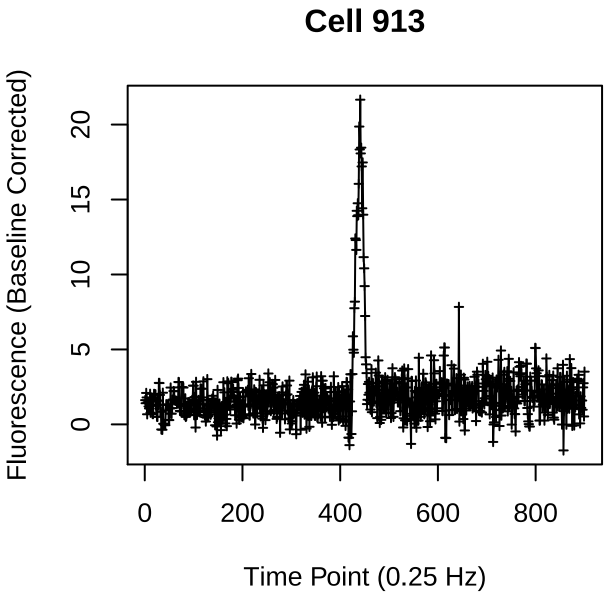
Cell 913
Fluorescence (Baseline Corrected)
0
5
10
15
20
0
200
400
600
800
Time Point (0.25 Hz)

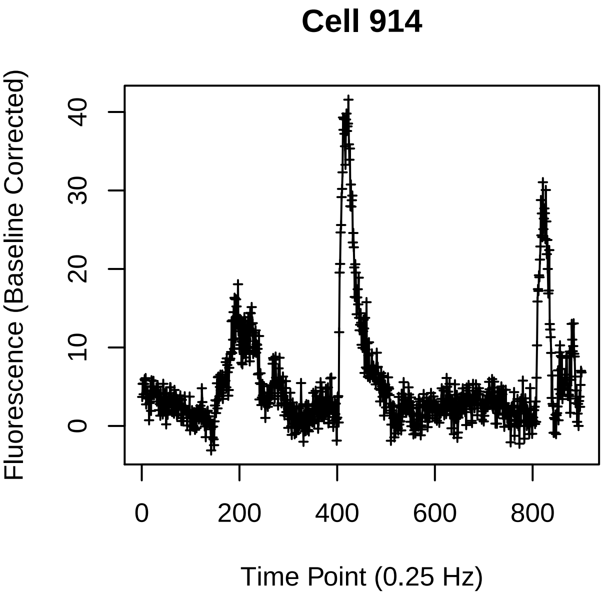
Cell 914
Fluorescence (Baseline Corrected)
0
10
20
30
40
0
200
400
600
800
Time Point (0.25 Hz)

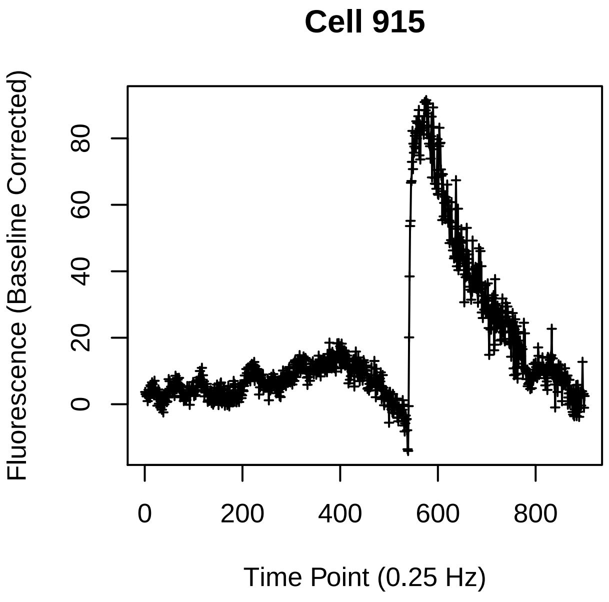
Cell 915
Fluorescence (Baseline Corrected)
0
20
40
60
80
0
200
400
600
800
Time Point (0.25 Hz)

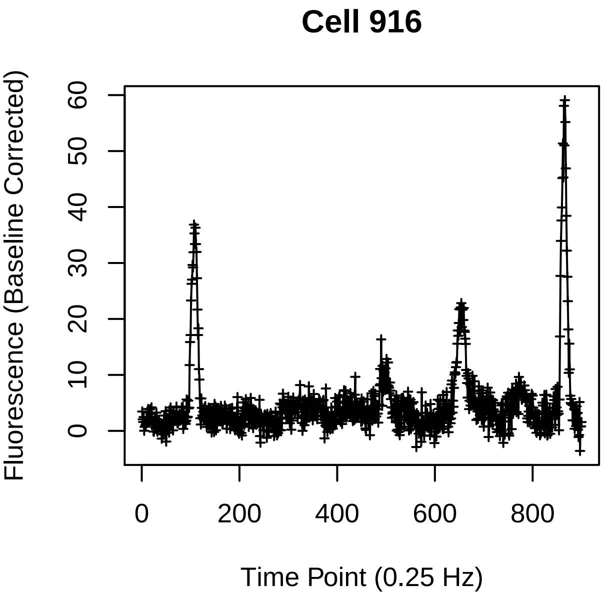
Cell 916
Fluorescence (Baseline Corrected)
0
10
20
30
40
50
60
0
200
400
600
800
Time Point (0.25 Hz)

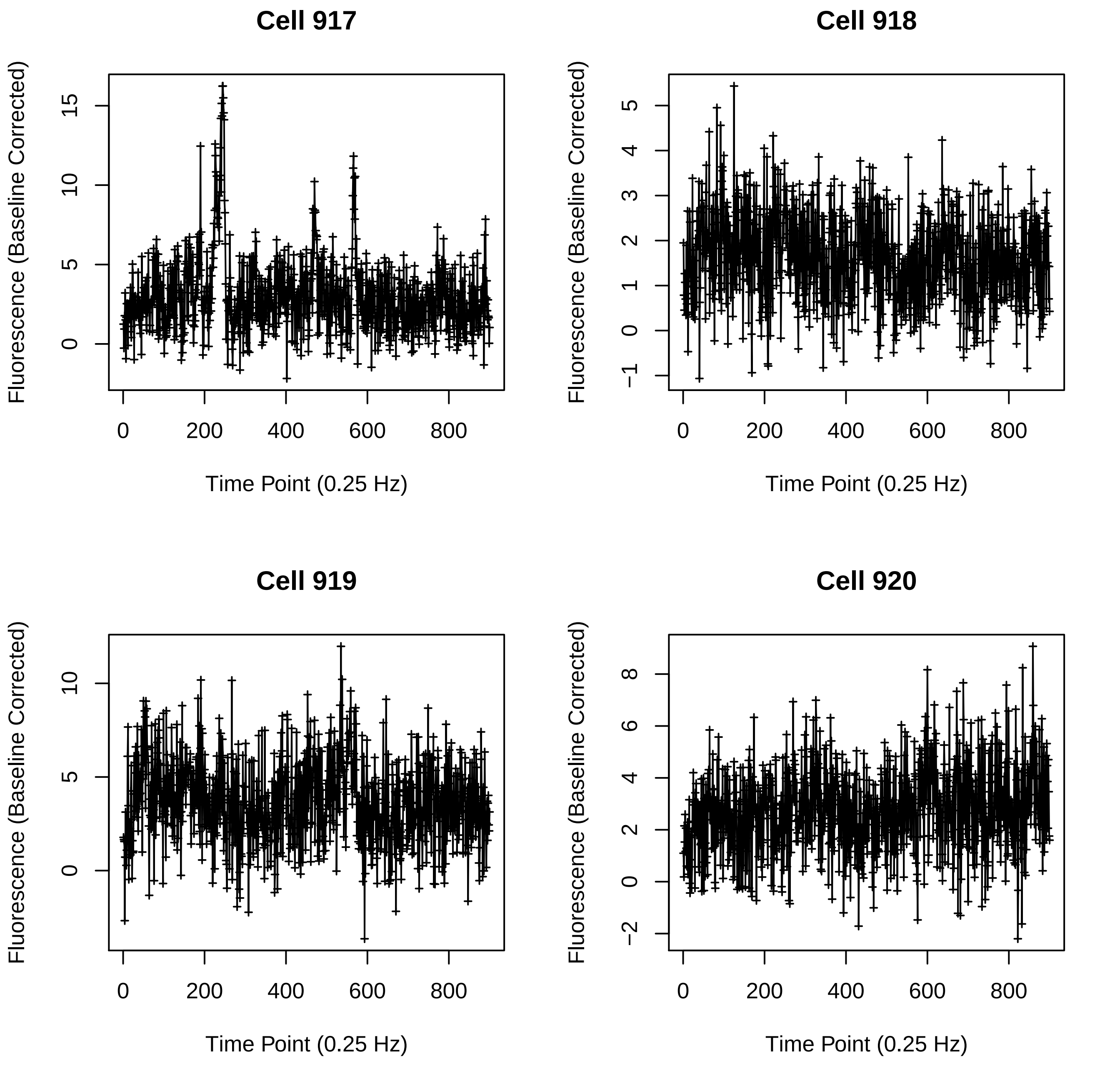

Cell 917
Cell 918
Cell 919
Cell 920
Fluorescence (Baseline Corrected)
Time Point (0.25 Hz)
0
200
400
600
800
0
5
10
15
−1
1
2
3
4
−2
6
8

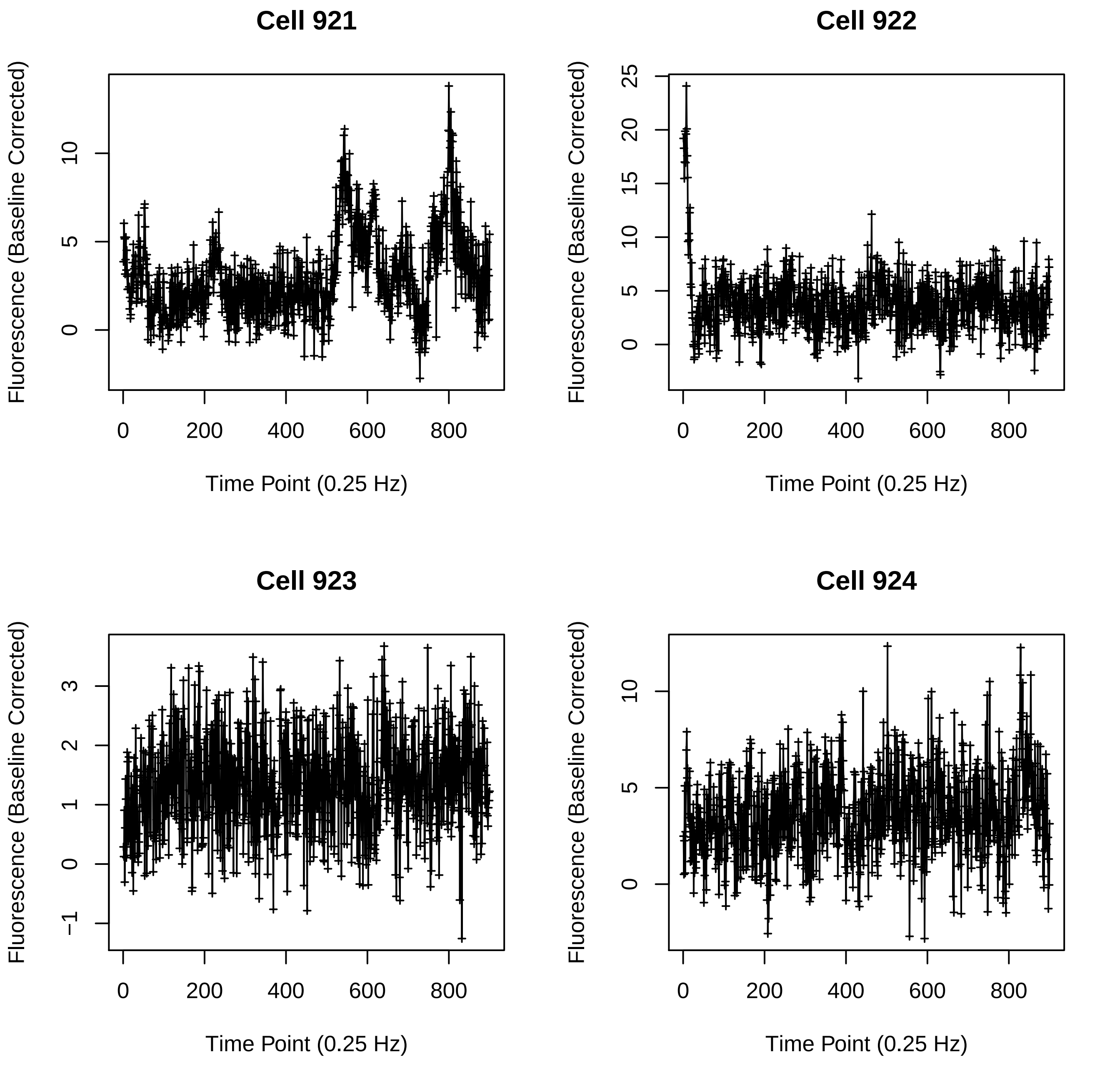
Cell 921
Cell 922
Cell 923
Cell 924
Fluorescence (Baseline Corrected)
Time Point (0.25 Hz)
0
200
400
600
800
10
5
0
25
20
15
3
2
1
−1

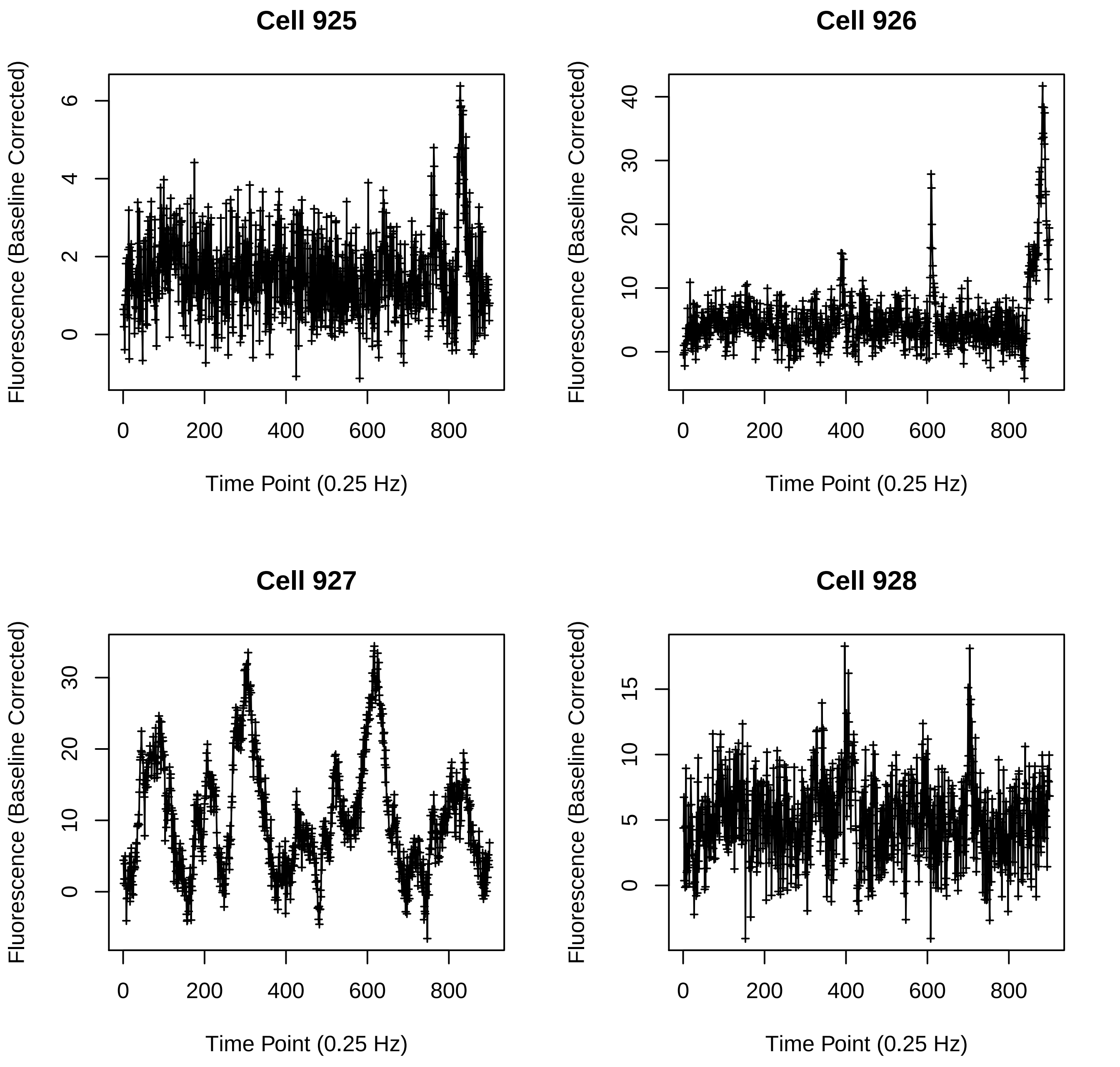
Cell 925
Cell 926
Cell 927
Cell 928
Fluorescence (Baseline Corrected)
Time Point (0.25 Hz)
0
200
400
600
800
-2
0
2
4
6
0
10
20
30
40
15

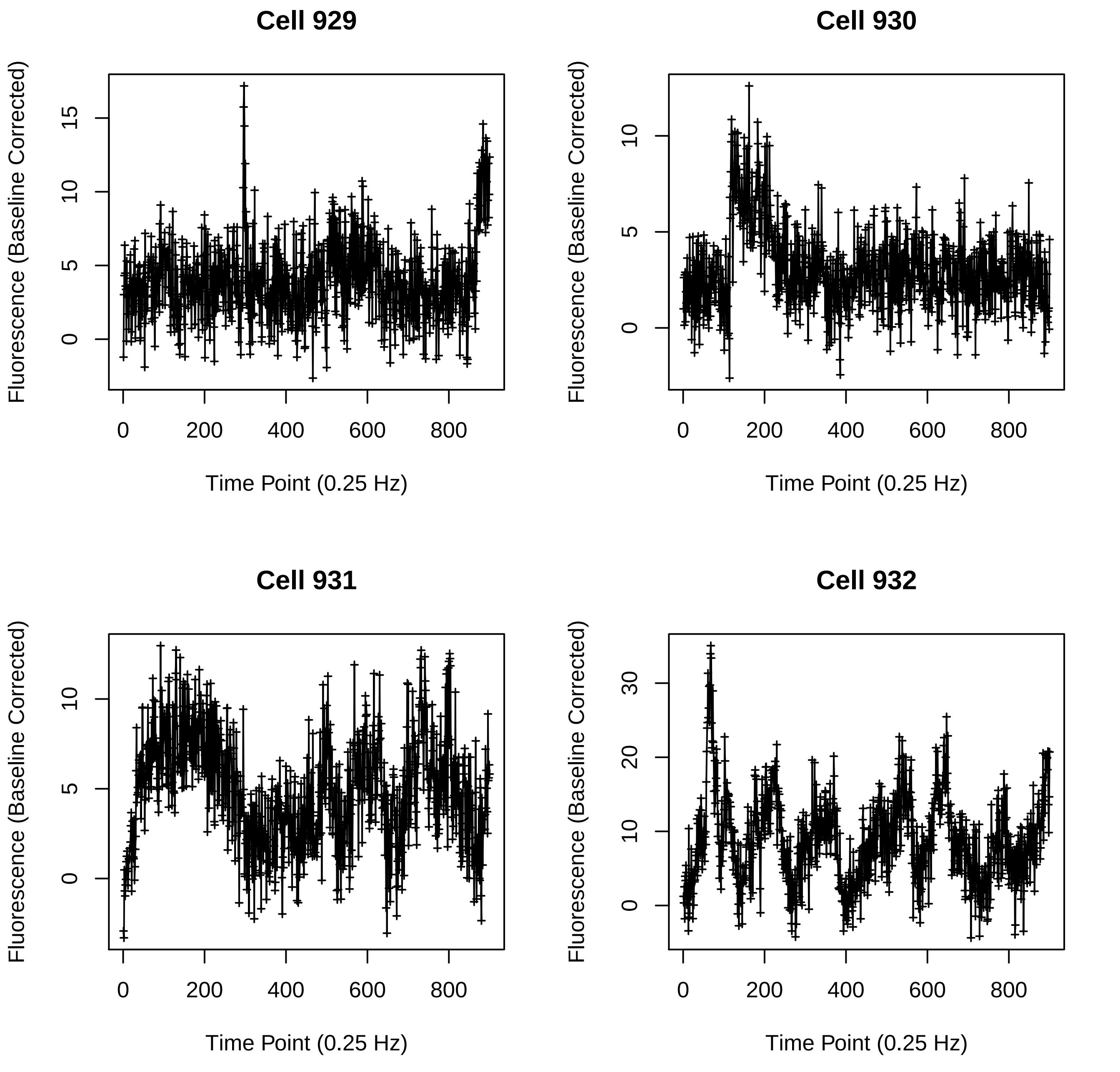
Cell 929
Cell 930
Cell 931
Cell 932
Fluorescence (Baseline Corrected)
Time Point (0.25 Hz)
0
200
400
600
800
0
5
10
15
20
30

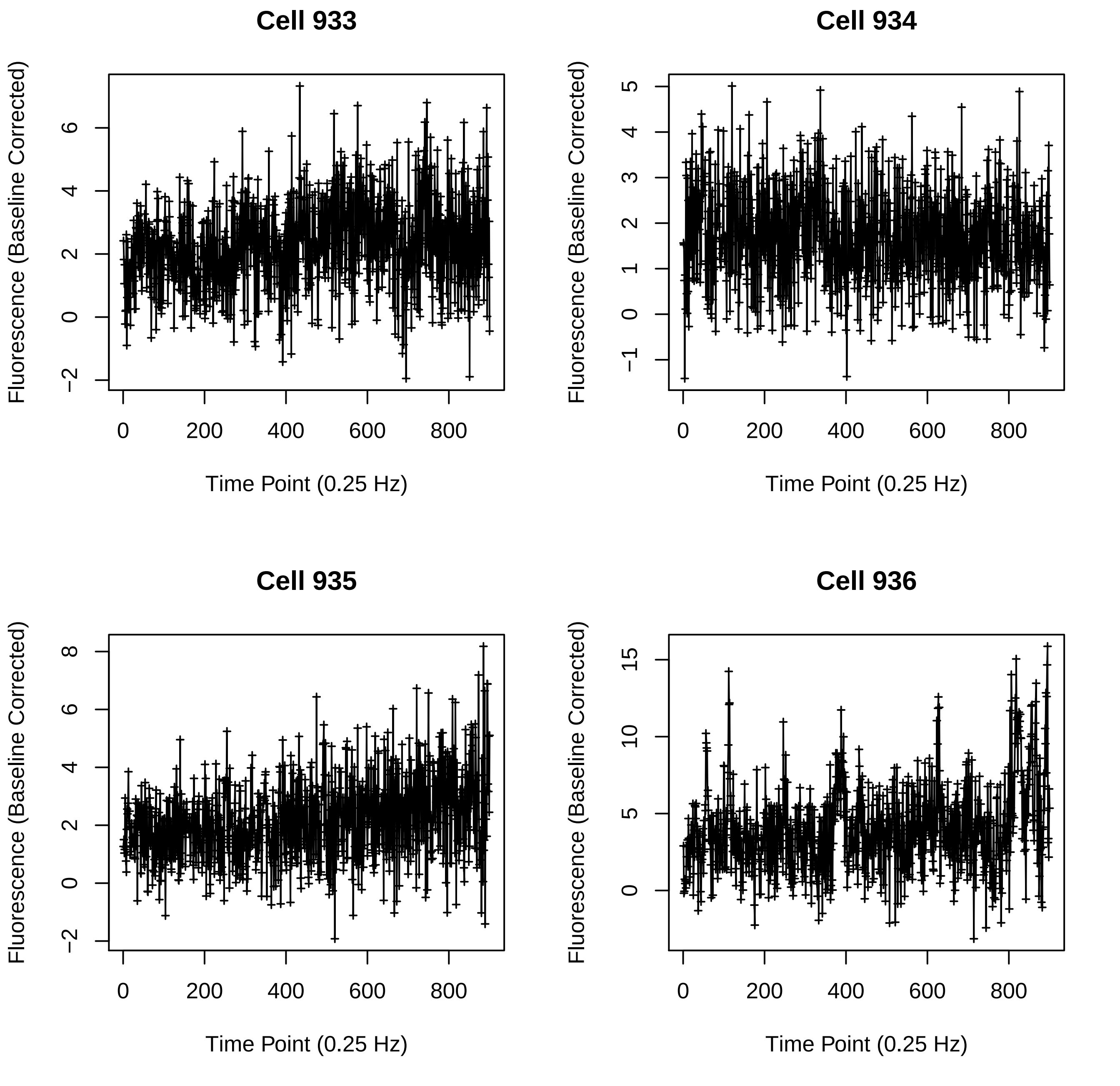

Cell 933
Fluorescence (Baseline Corrected)
Time Point (0.25 Hz)
Cell 934
Fluorescence (Baseline Corrected)
Time Point (0.25 Hz)
Cell 935
Fluorescence (Baseline Corrected)
Time Point (0.25 Hz)
Cell 936
Fluorescence (Baseline Corrected)
Time Point (0.25 Hz)

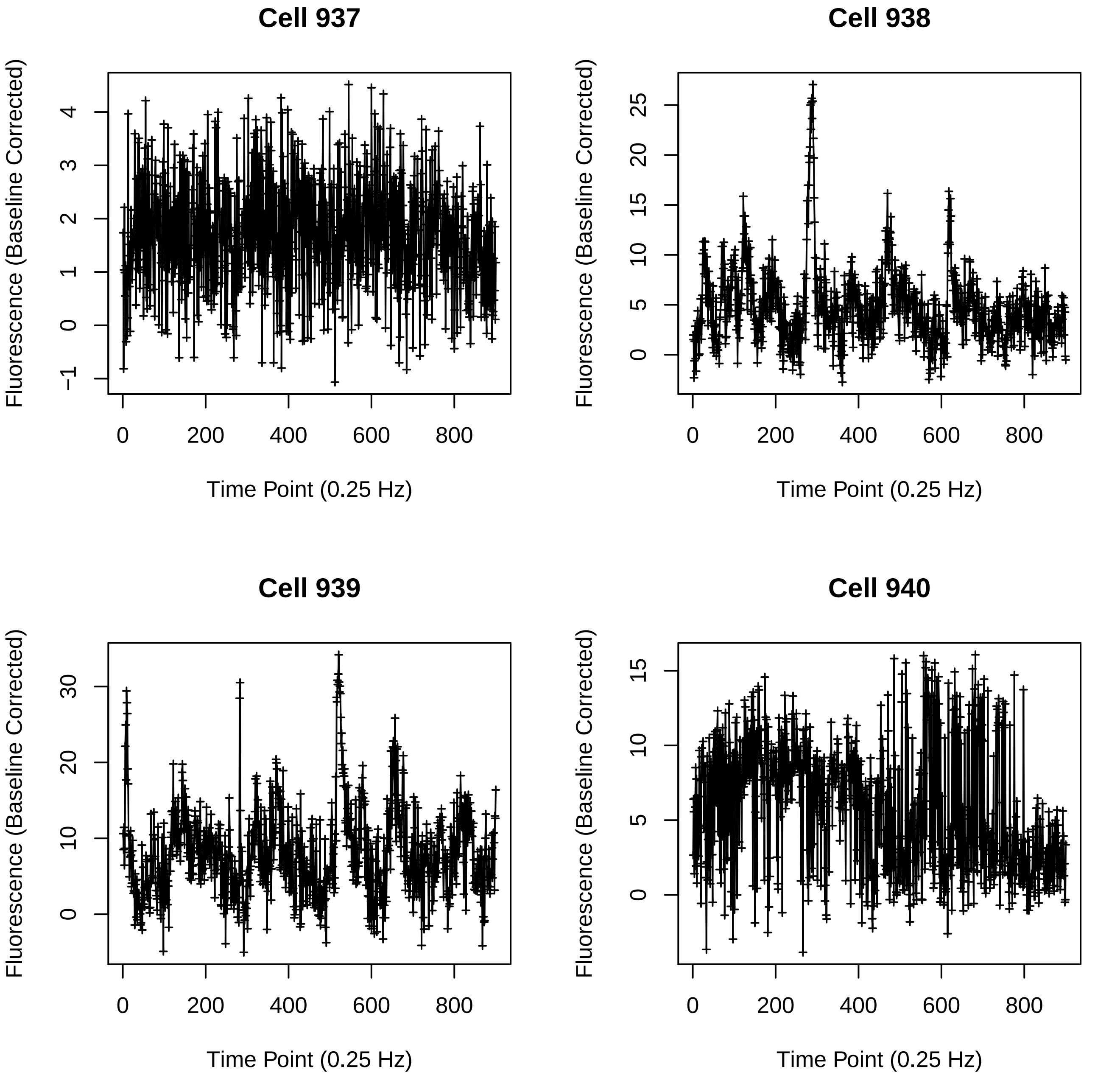
Cell 937
Cell 938
Cell 939
Cell 940
Fluorescence (Baseline Corrected)
Time Point (0.25 Hz)
−1
0
1
2
3
4
5
10
15
20
25
30
200
400
600
800

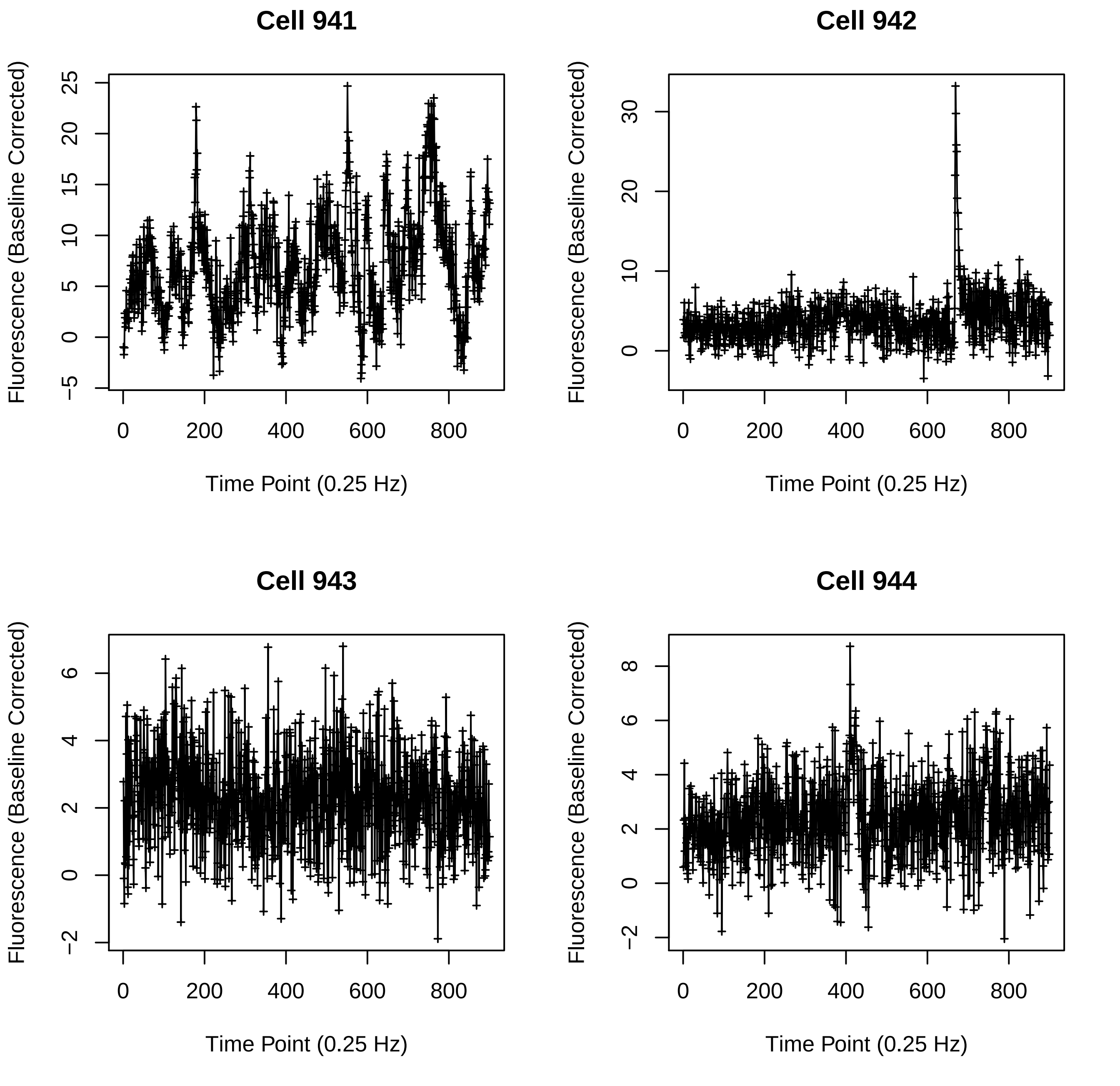
Cell 941
Cell 942
Cell 943
Cell 944
Fluorescence (Baseline Corrected)
Time Point (0.25 Hz)
−5
0
5
10
15
20
25
30
−2
2
4
6
8
200
400
600
800

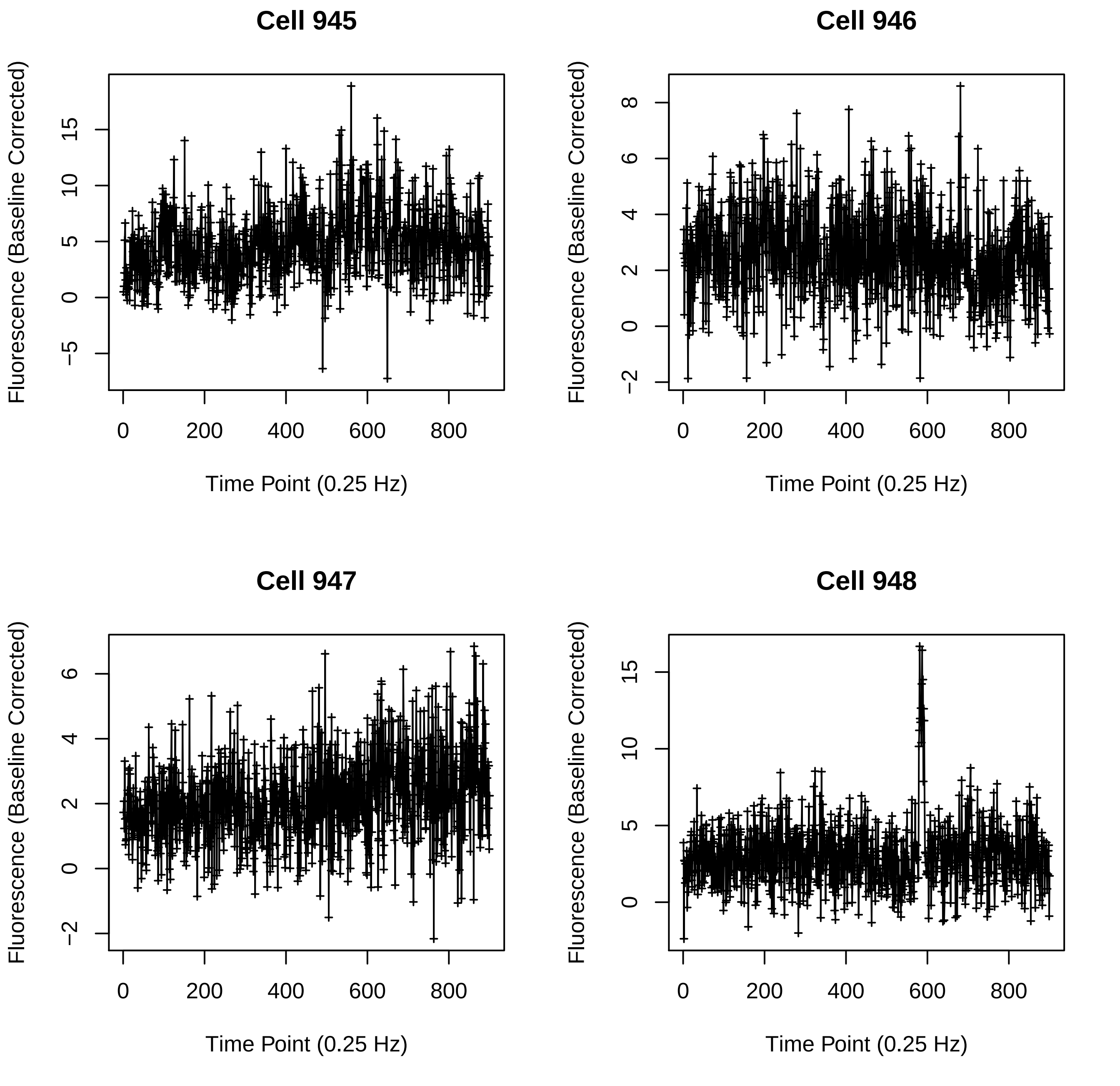
Cell 945
Fluorescence (Baseline Corrected)
−5
0
5
10
15
0
200
400
600
800
Time Point (0.25 Hz)
Cell 946
Fluorescence (Baseline Corrected)
−2
0
2
4
6
8
0
200
400
600
800
Time Point (0.25 Hz)
Cell 947
Fluorescence (Baseline Corrected)
−2
0
2
4
6
0
200
400
600
800
Time Point (0.25 Hz)
Cell 948
Fluorescence (Baseline Corrected)
0
5
10
15
0
200
400
600
800
Time Point (0.25 Hz)

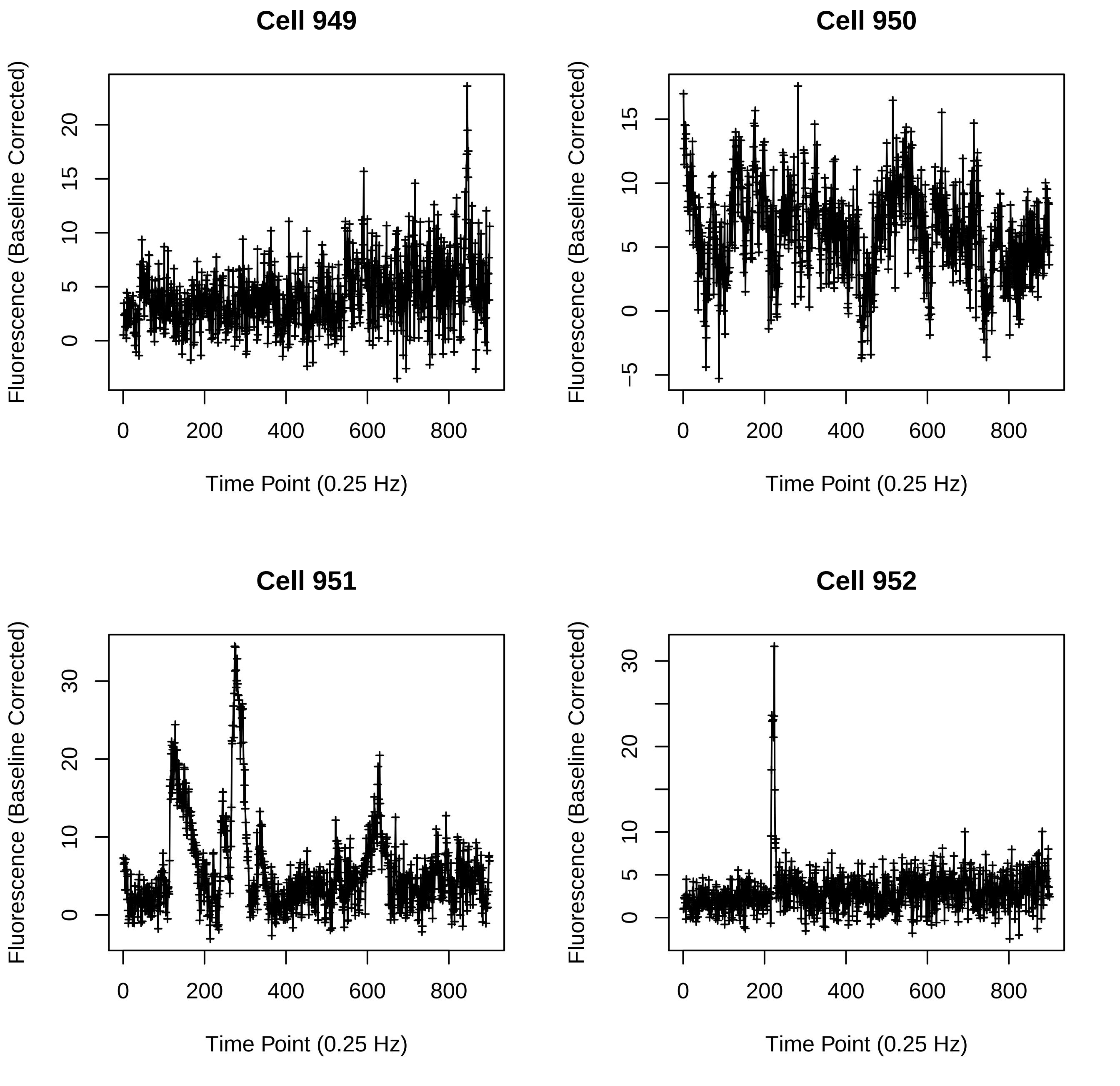
Cell 949
Cell 950
Cell 951
Cell 952
Fluorescence (Baseline Corrected)
Time Point (0.25 Hz)
0
200
400
600
800
5
10
15
20
30
−5

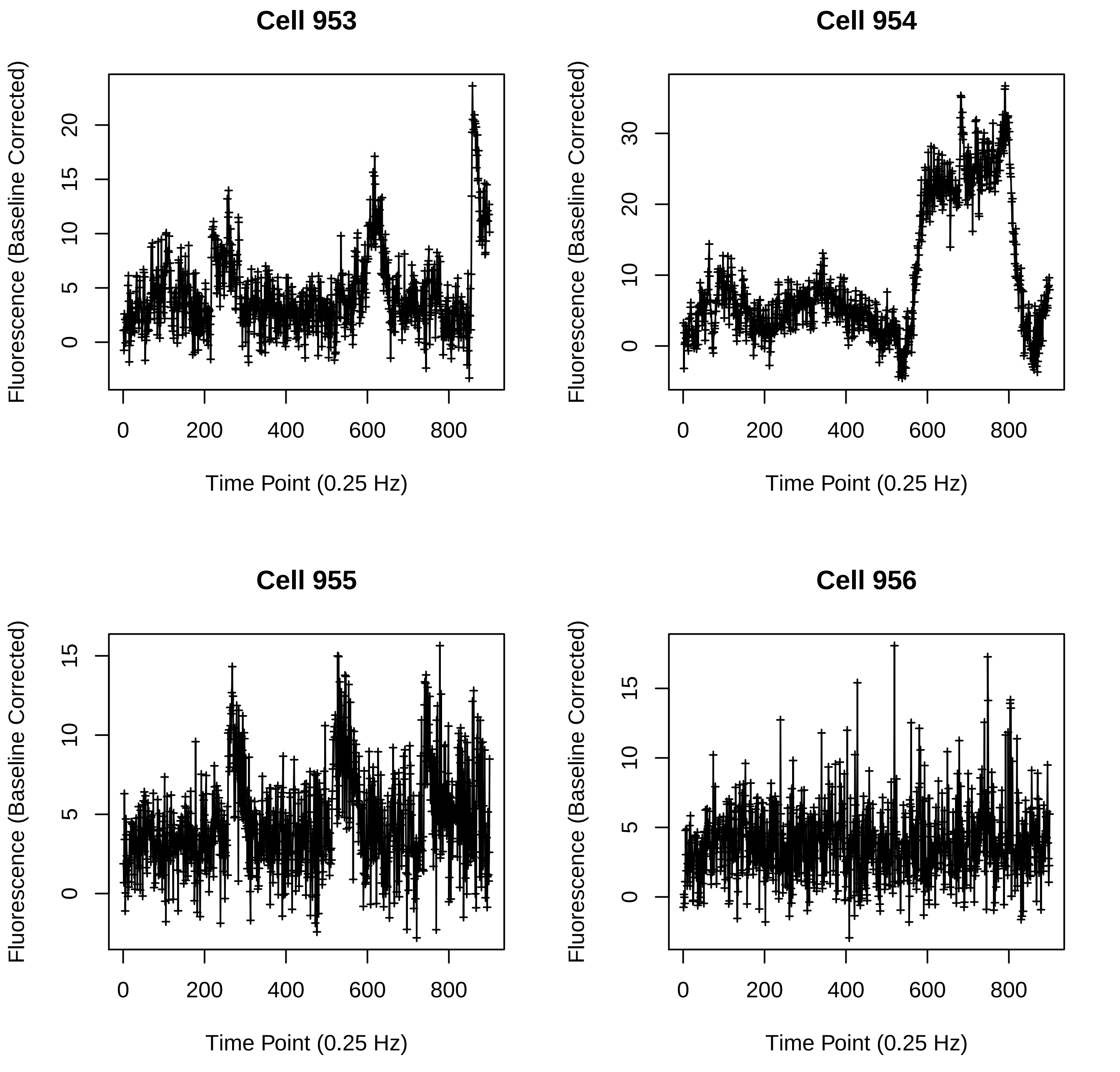

Cell 953
Fluorescence (Baseline Corrected)
Time Point (0.25 Hz)
0
5
10
15
20
0
200
400
600
800
Cell 954
Fluorescence (Baseline Corrected)
Time Point (0.25 Hz)
0
10
20
30
0
200
400
600
800
Cell 955
Fluorescence (Baseline Corrected)
Time Point (0.25 Hz)
0
5
10
15
0
200
400
600
800
Cell 956
Fluorescence (Baseline Corrected)
Time Point (0.25 Hz)
0
5
10
15
0
200
400
600
800

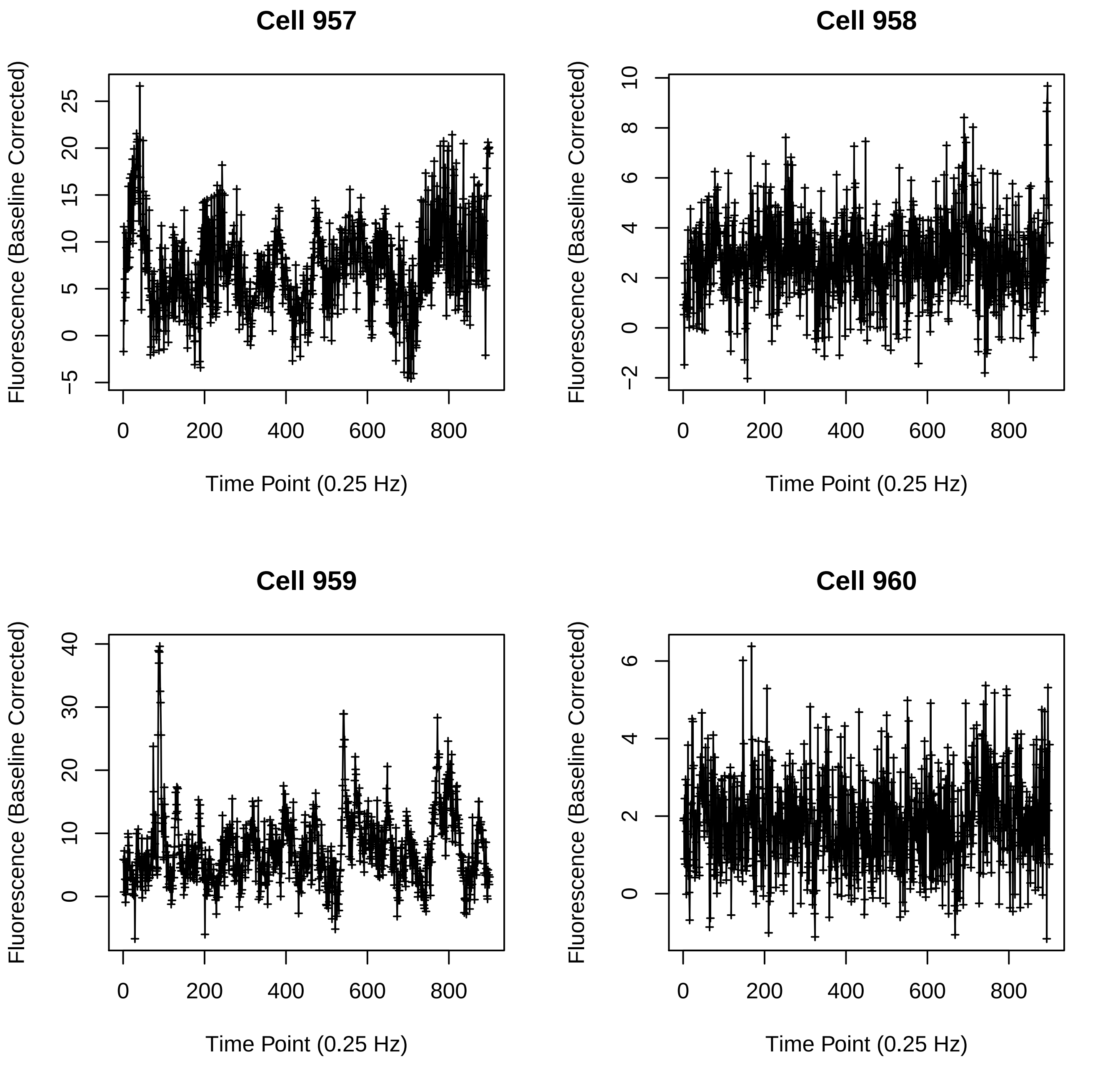
Cell 957
Cell 958
Cell 959
Cell 960
Fluorescence (Baseline Corrected)
Time Point (0.25 Hz)
0
200
400
600
800

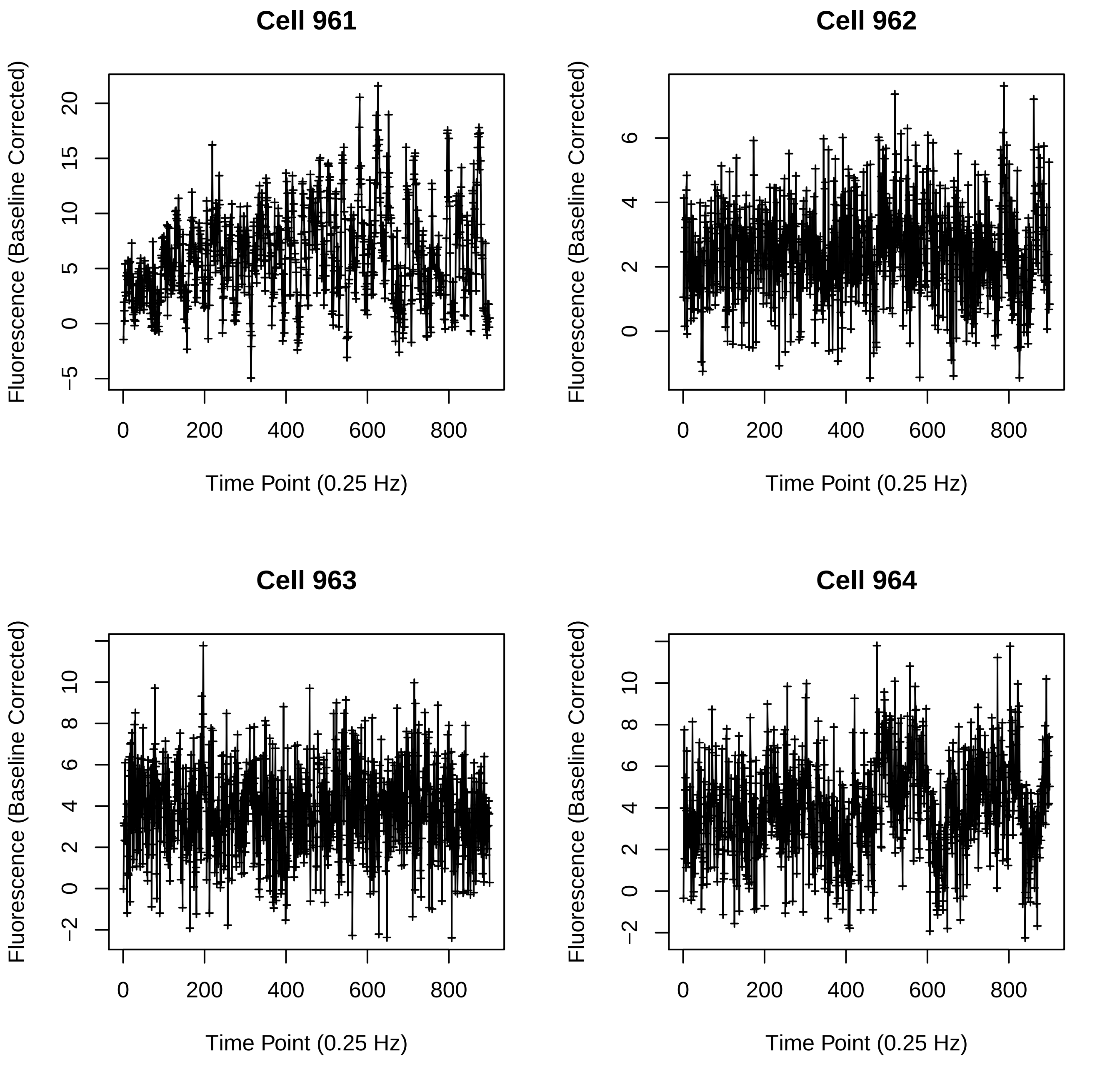
Cell 961
Cell 962
Cell 963
Cell 964
Fluorescence (Baseline Corrected)
Time Point (0.25 Hz)
0
200
400
600
800
-5
0
5
10
15
20
-2
2
4
6
8
10

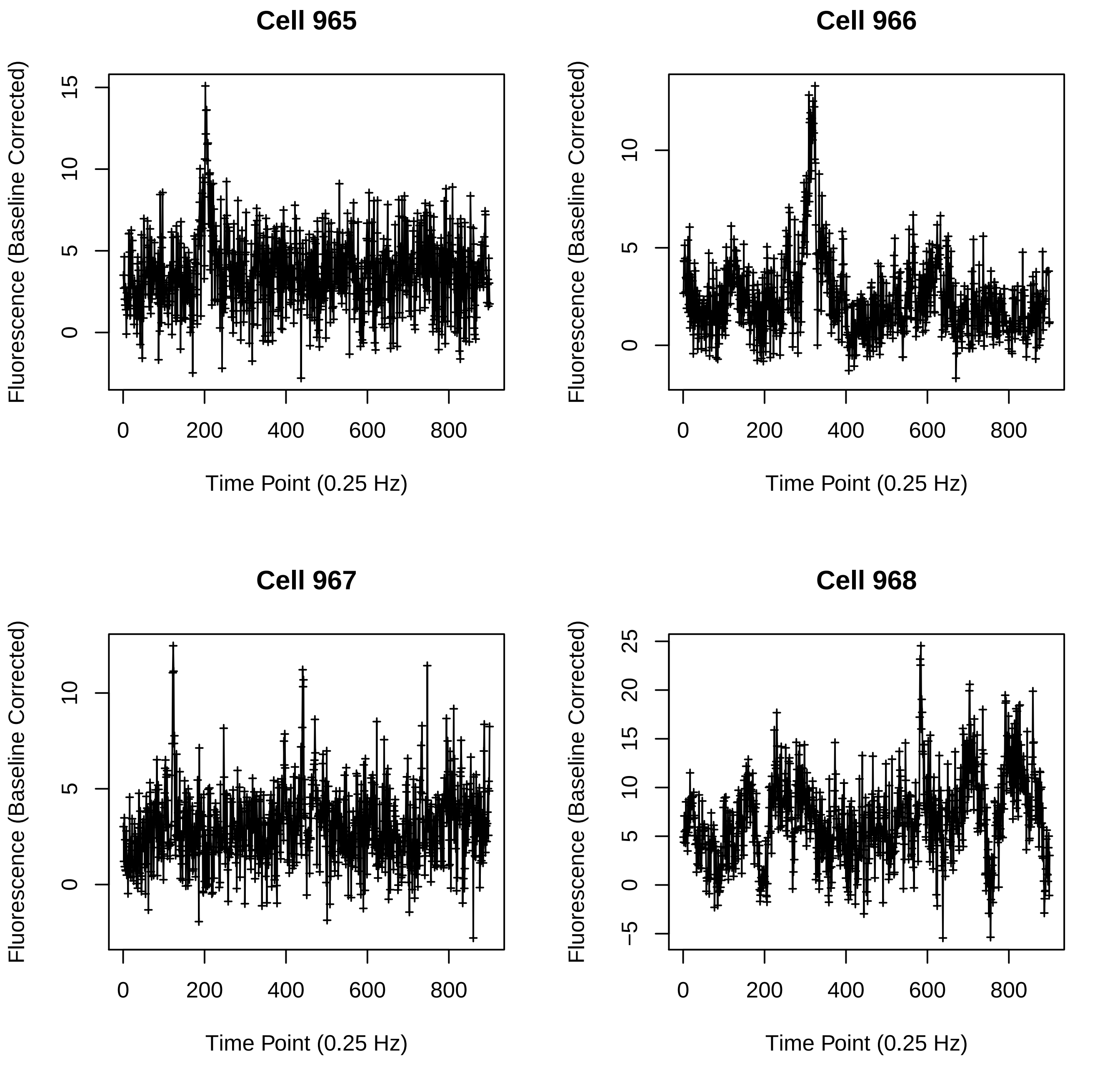

Cell 965
Cell 966
Cell 967
Cell 968
Fluorescence (Baseline Corrected)
Time Point (0.25 Hz)
0
200
400
600
800
15
10
5
25
20
−5

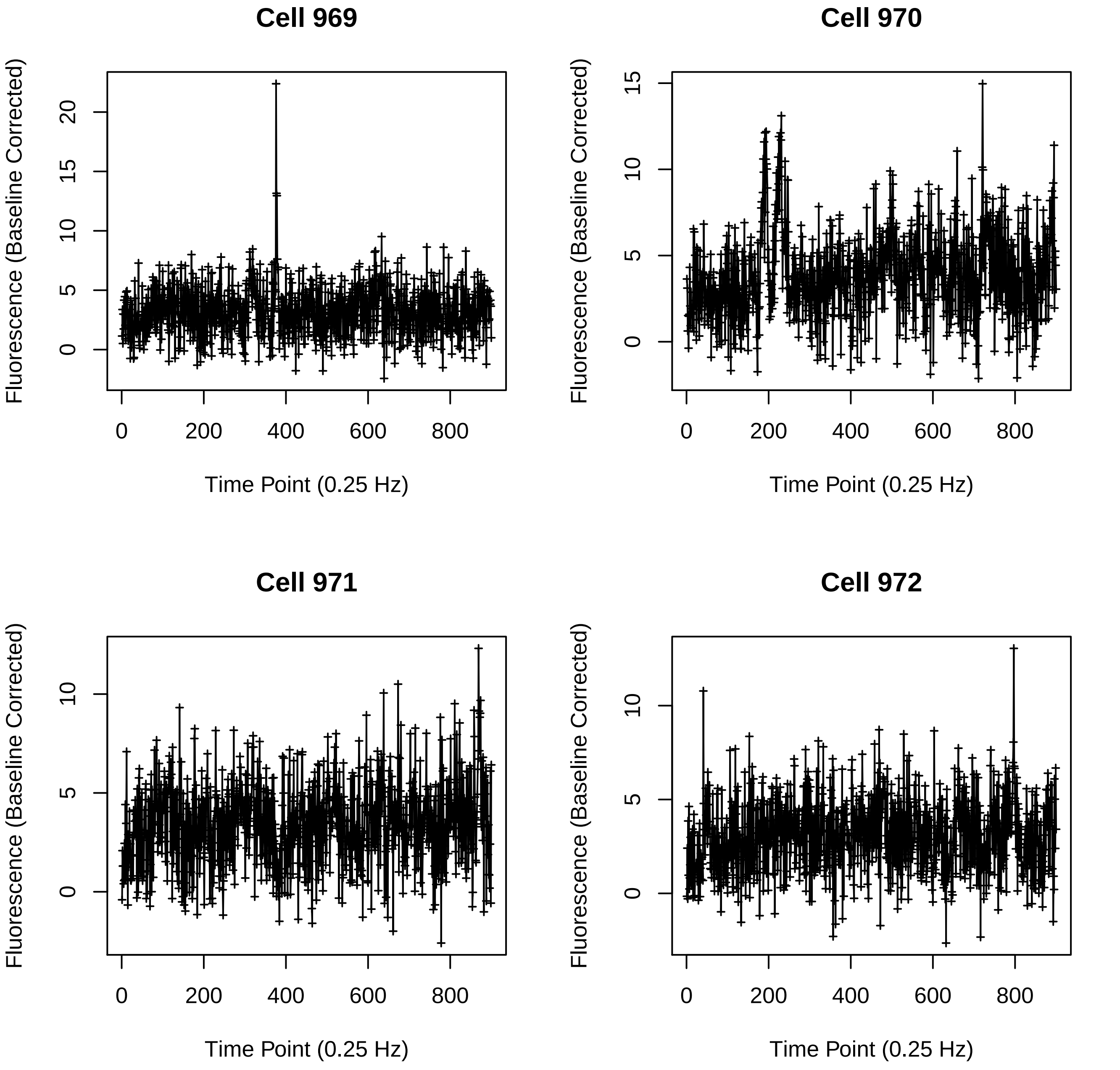
Cell 969
Cell 970
Cell 971
Cell 972
Fluorescence (Baseline Corrected)
Time Point (0.25 Hz)
0
5
10
15
20
200
400
600
800

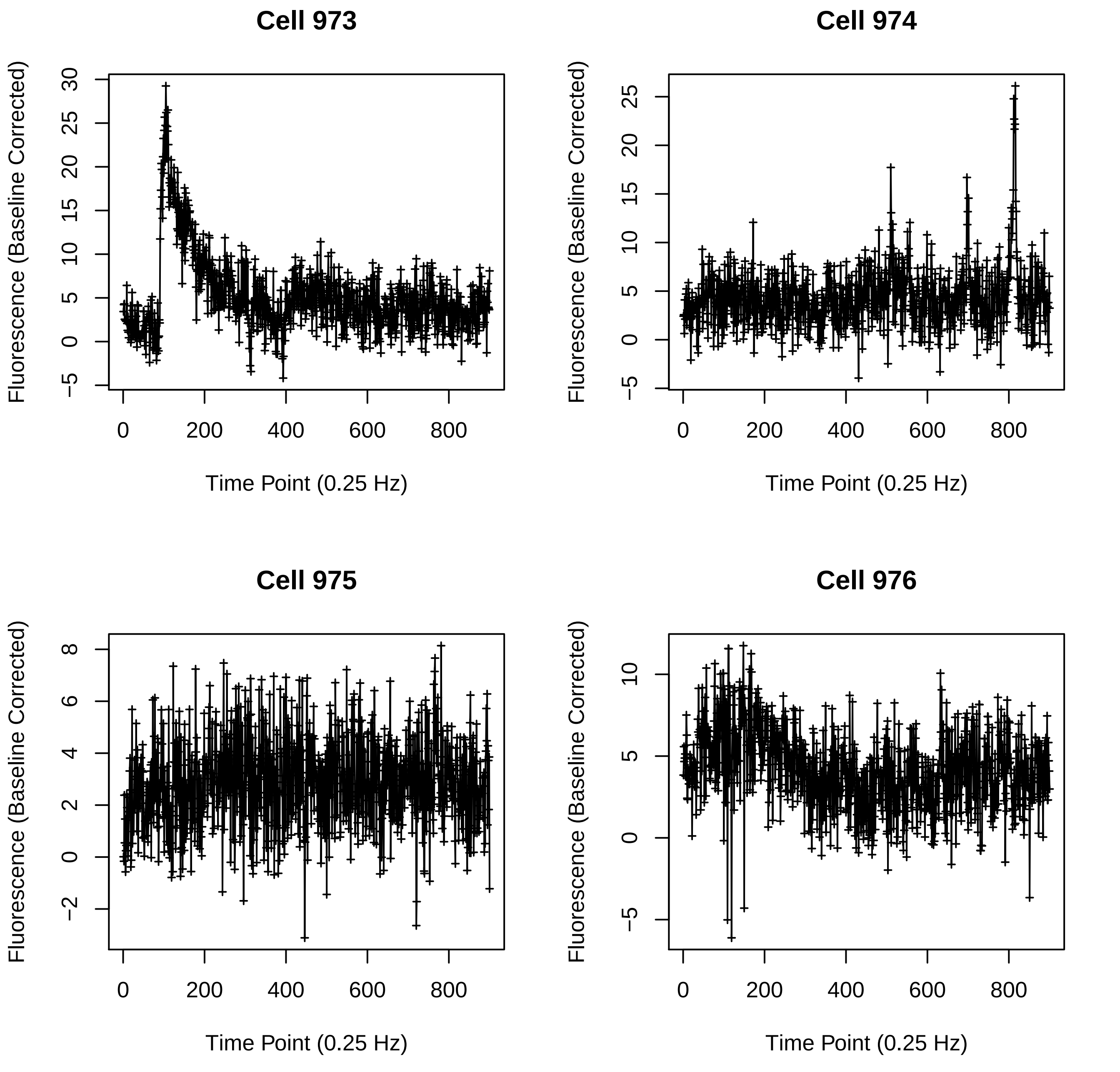

Cell 973
Cell 974
Cell 975
Cell 976
Fluorescence (Baseline Corrected)
Time Point (0.25 Hz)
0
200
400
600
800
-5
0
5
10
15
20
25
30
-2
2
4
6
8

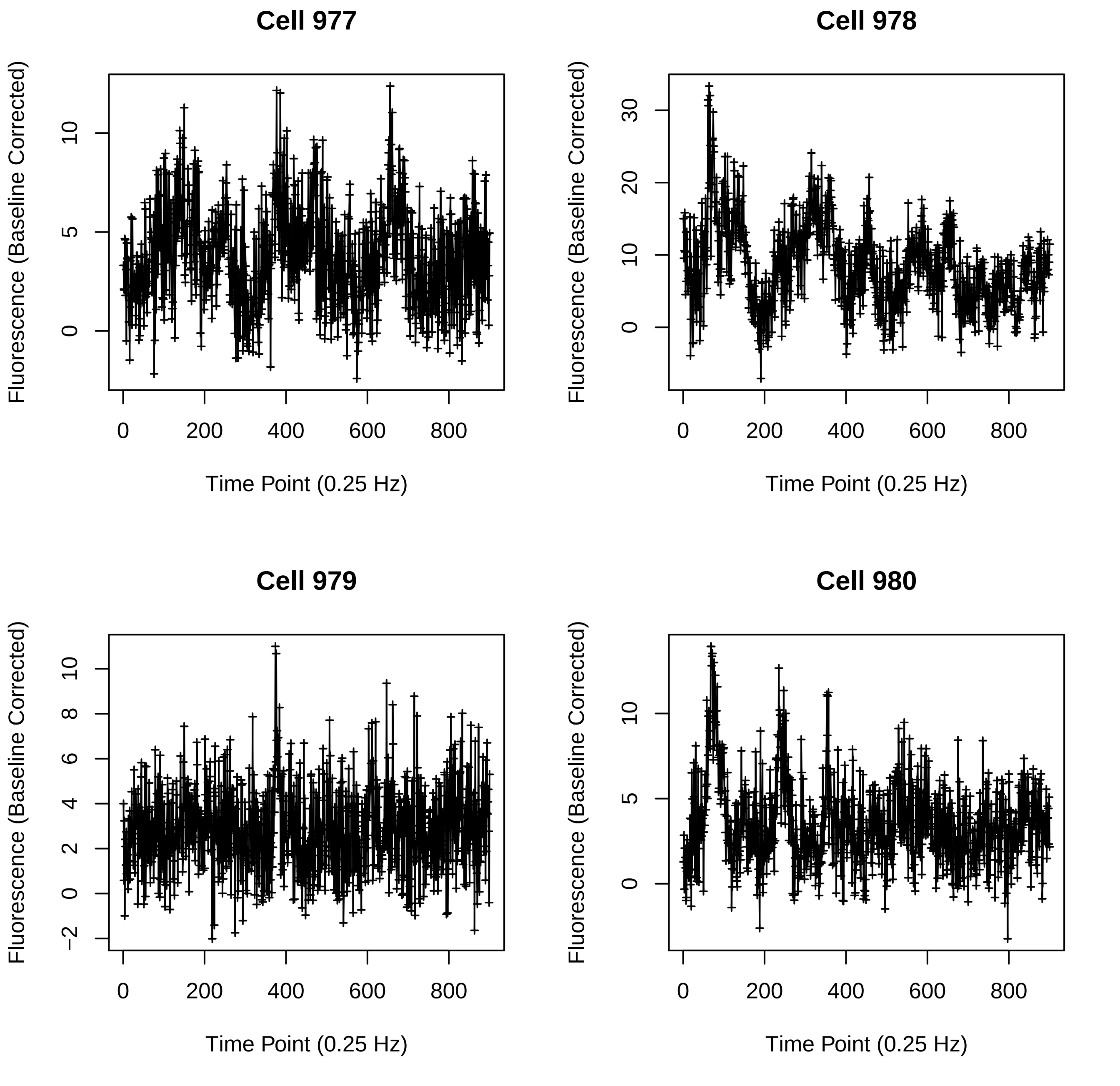
Cell 977
Cell 978
Cell 979
Cell 980
Fluorescence (Baseline Corrected)
Time Point (0.25 Hz)
0
200
400
600
800
−2
0
2
4
5
6
8
10
20
30

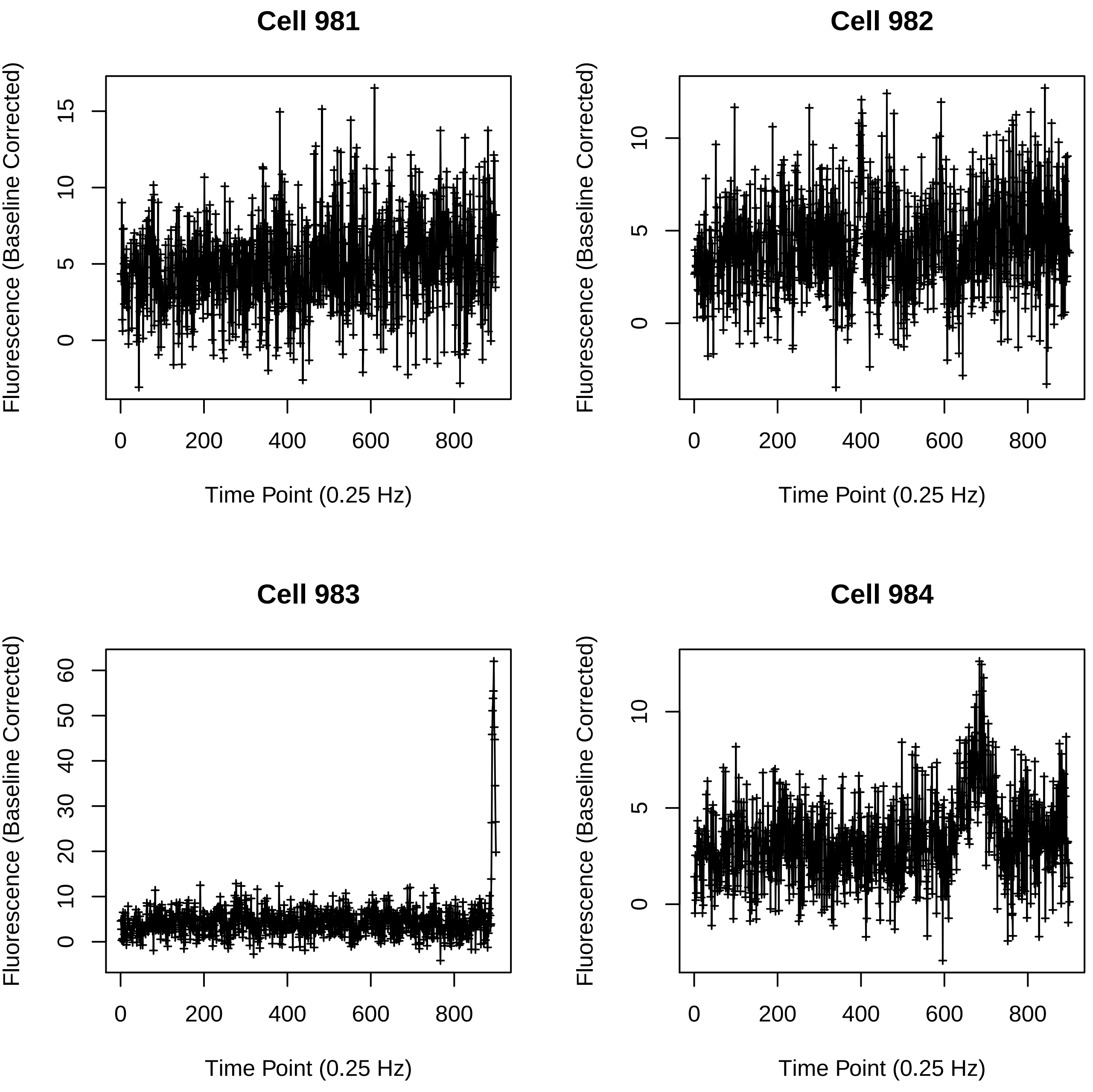

Cell 981
Cell 982
Cell 983
Cell 984
Fluorescence (Baseline Corrected)
Time Point (0.25 Hz)
0
200
400
600
800
0
5
10
15
10
20
30
40
50
60

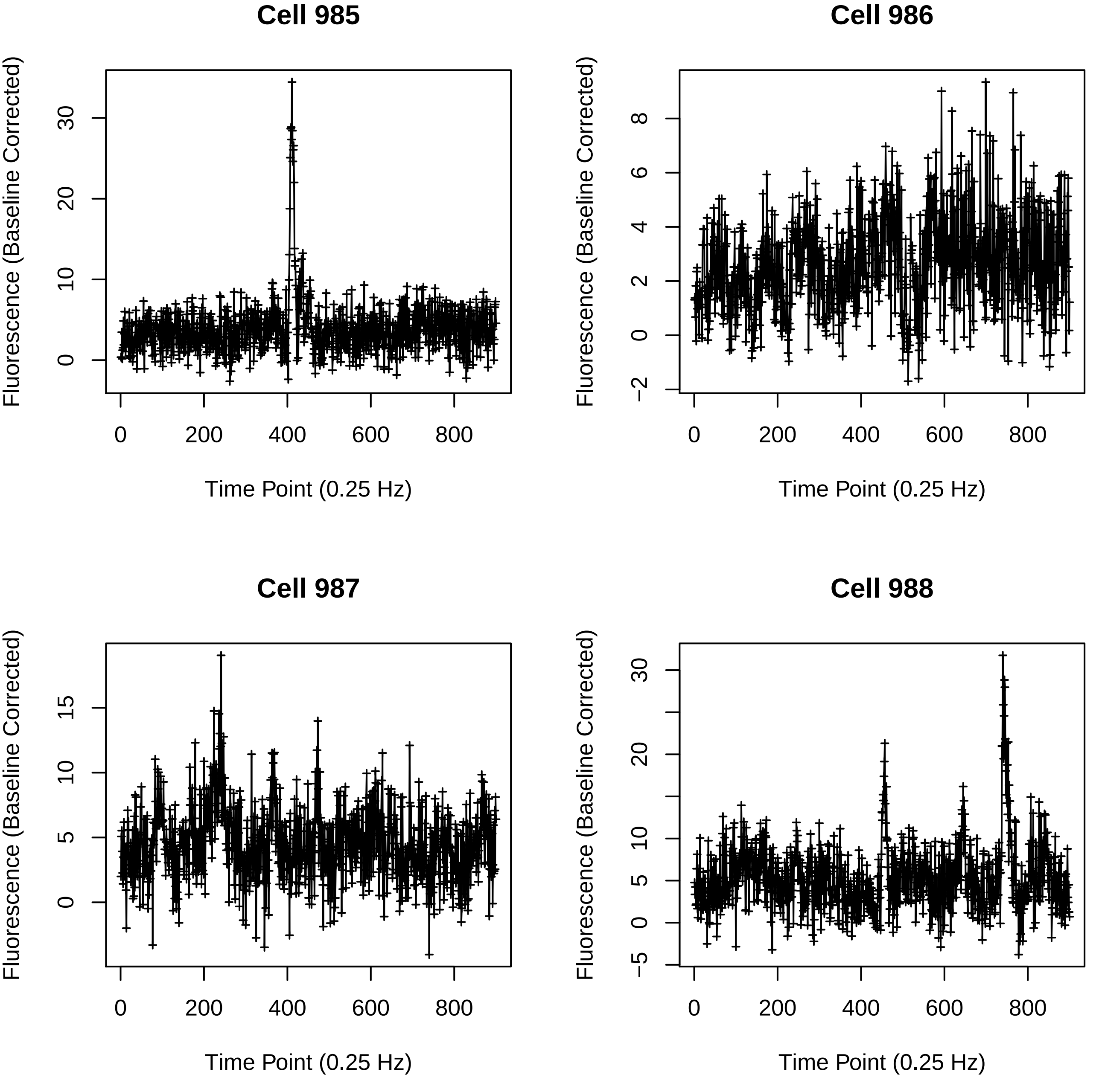

Cell 985
Cell 986
Cell 987
Cell 988
Fluorescence (Baseline Corrected)
Time Point (0.25 Hz)
0
200
400
600
800
-2
0
2
4
6
8
10
15
20
30
-5
5

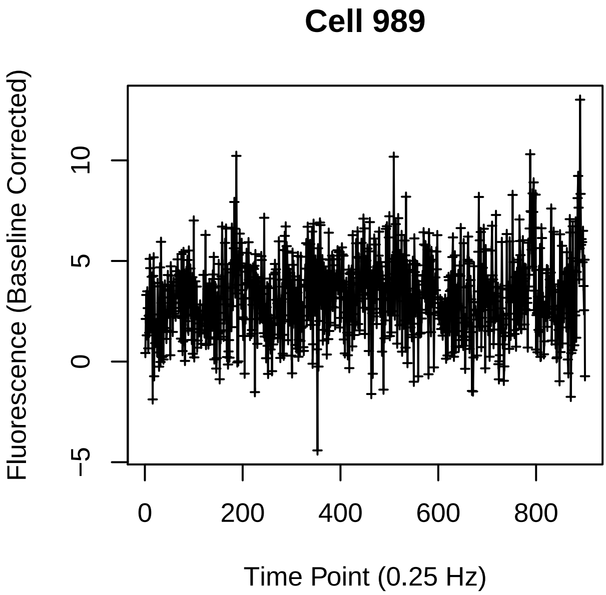
Cell 989
Fluorescence (Baseline Corrected)
Time Point (0.25 Hz)
−5
0
5
10
0
200
400
600
800

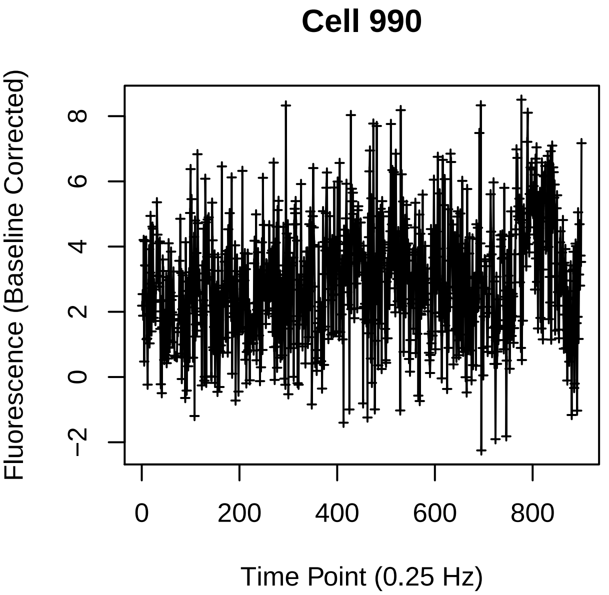
Cell 990
Fluorescence (Baseline Corrected)
Time Point (0.25 Hz)
−2
0
2
4
6
8
0
200
400
600
800

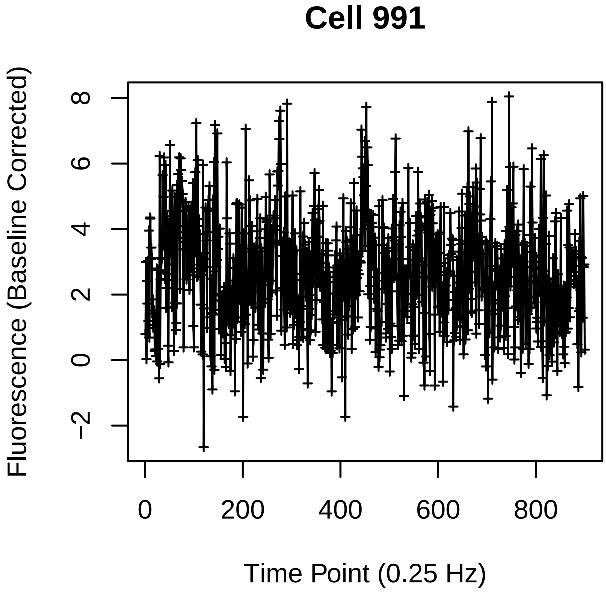
Cell 991
Fluorescence (Baseline Corrected)
Time Point (0.25 Hz)
−2
0
2
4
6
8
0
200
400
600
800

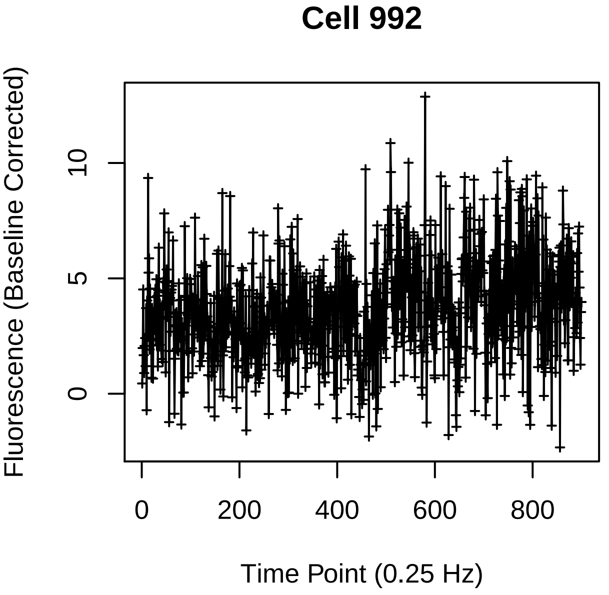
Cell 992
Fluorescence (Baseline Corrected)
Time Point (0.25 Hz)
0
5
10
0
200
400
600
800

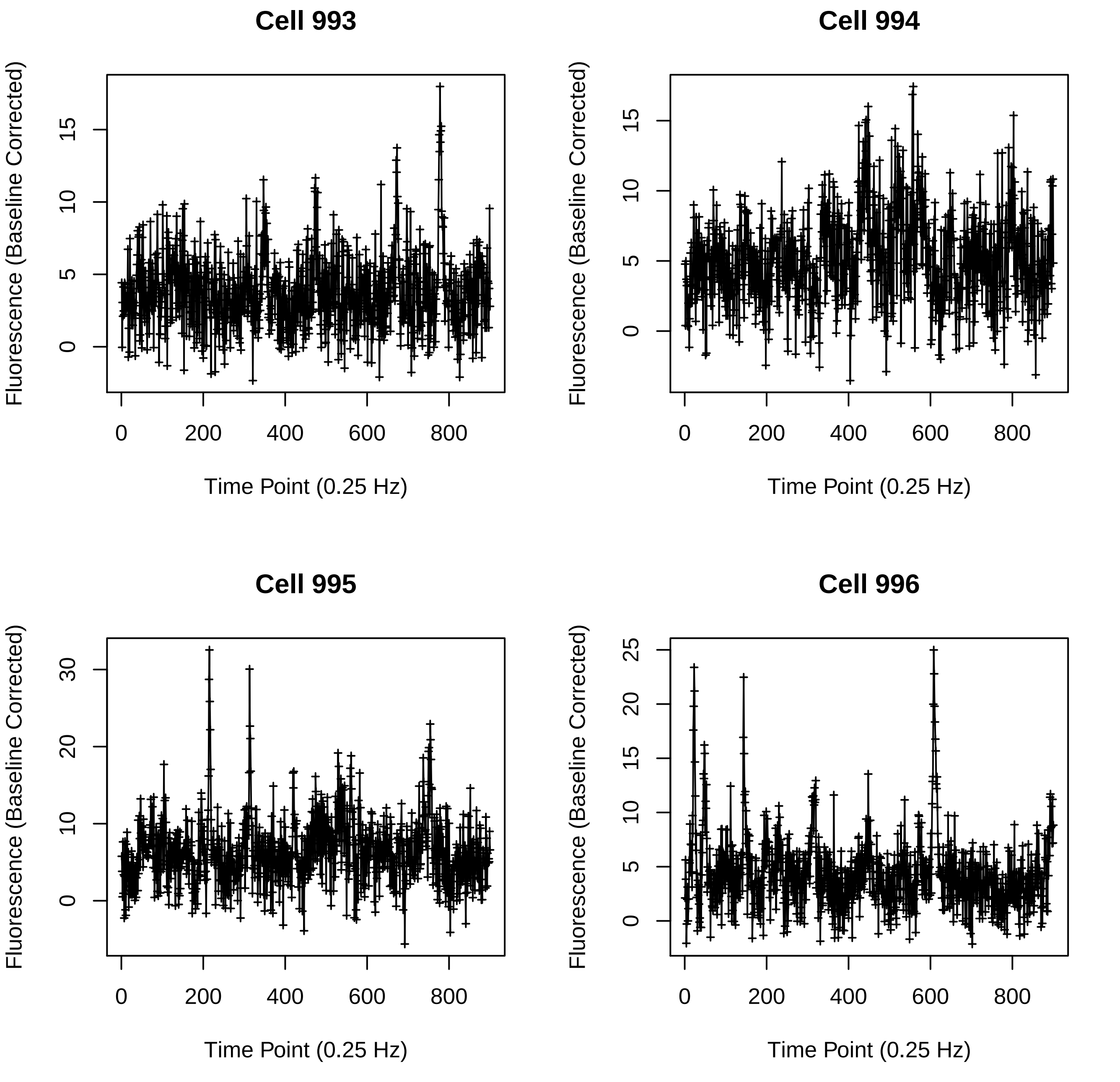
Cell 993
Cell 994
Cell 995
Cell 996
Fluorescence (Baseline Corrected)
Time Point (0.25 Hz)
0
200
400
600
800
0
5
10
15
20
25
30

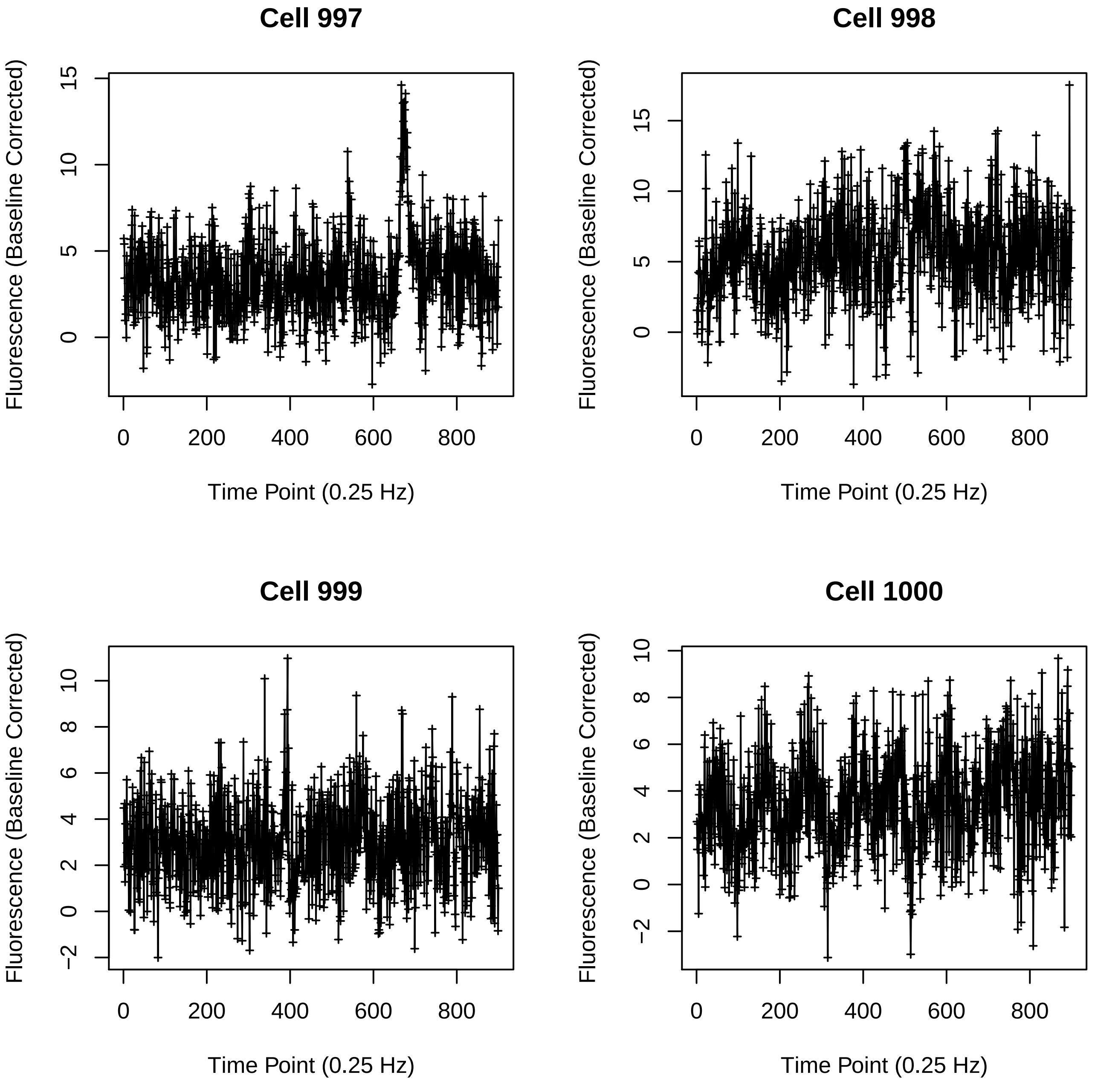
Cell 997
Cell 998
Cell 999
Cell 1000
Fluorescence (Baseline Corrected)
Time Point (0.25 Hz)
0
200
400
600
800
−2
0
2
4
5
6
8
10
15

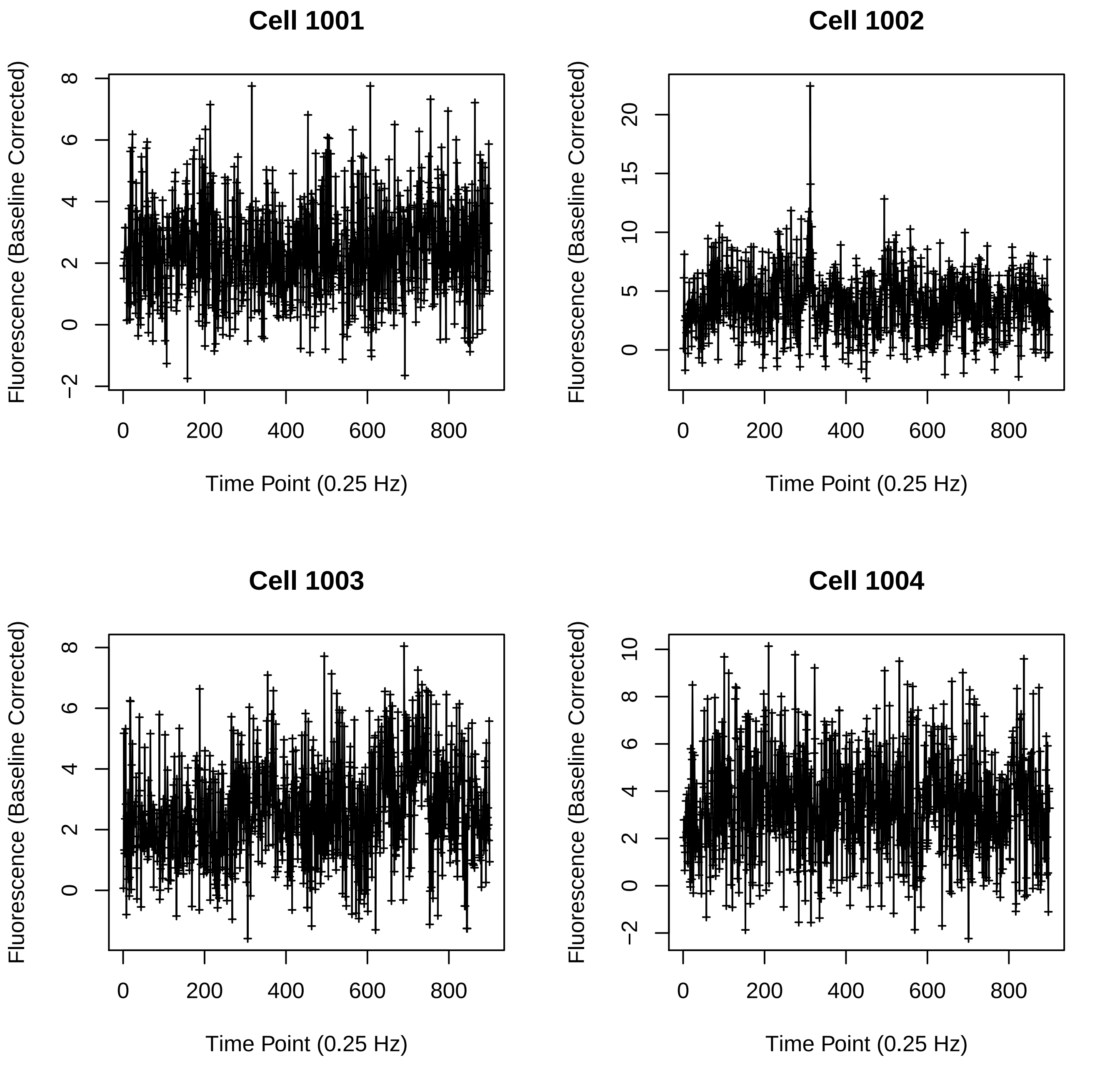

Cell 1001
Cell 1002
Cell 1003
Cell 1004
Fluorescence (Baseline Corrected)
Time Point (0.25 Hz)
0
200
400
600
800
-2
2
4
6
8
5
10
15
20

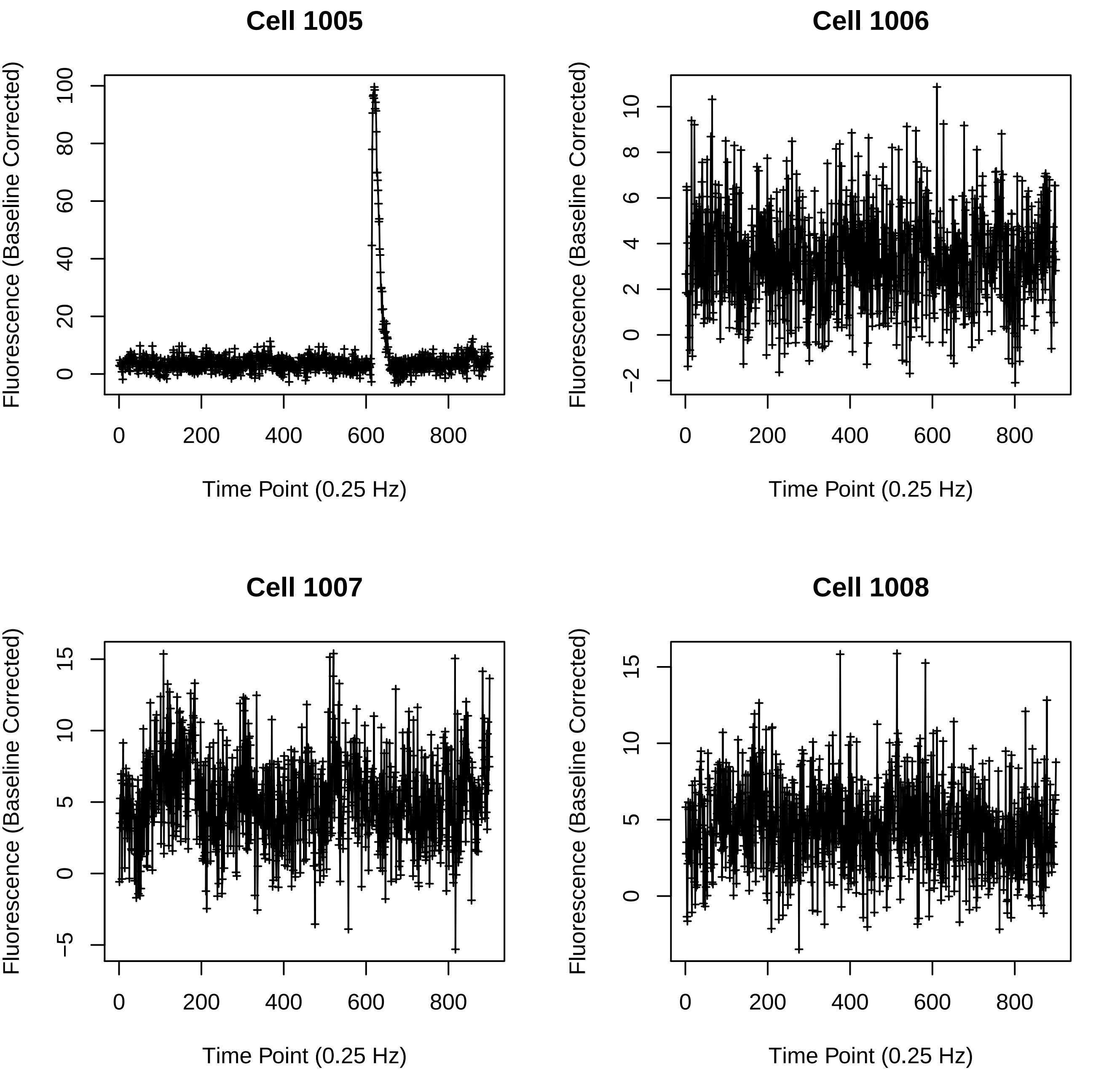
Cell 1005
Fluorescence (Baseline Corrected)
0
20
40
60
80
100
0
200
400
600
800
Time Point (0.25 Hz)
Cell 1006
Fluorescence (Baseline Corrected)
−2
0
2
4
6
8
10
0
200
400
600
800
Time Point (0.25 Hz)
Cell 1007
Fluorescence (Baseline Corrected)
−5
0
5
10
15
0
200
400
600
800
Time Point (0.25 Hz)
Cell 1008
Fluorescence (Baseline Corrected)
0
5
10
15
0
200
400
600
800
Time Point (0.25 Hz)

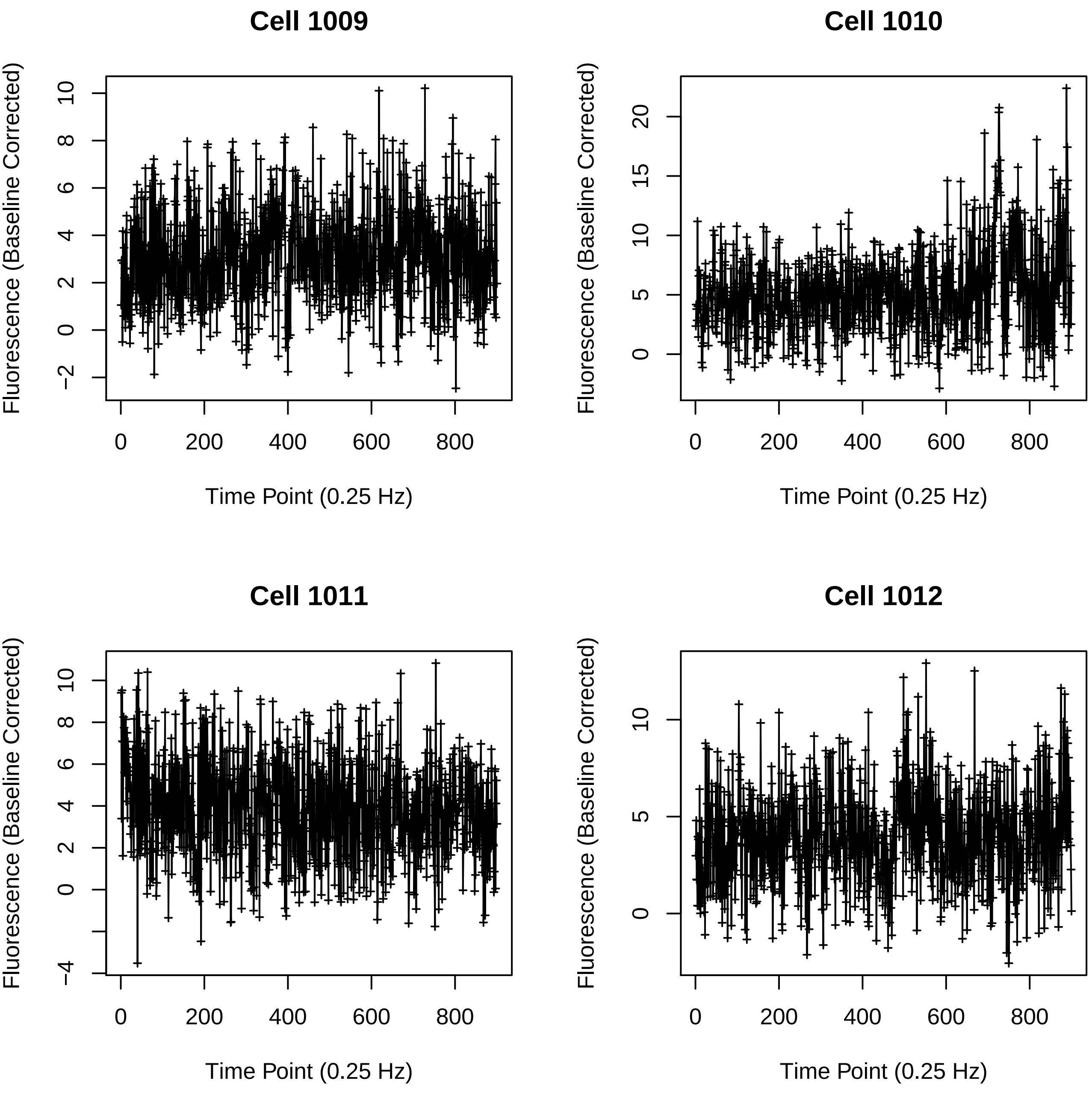

Cell 1009
Cell 1010
Cell 1011
Cell 1012
Fluorescence (Baseline Corrected)
Time Point (0.25 Hz)
0
200
400
600
800
−2
−4
0
2
4
6
8
10
5
15
20

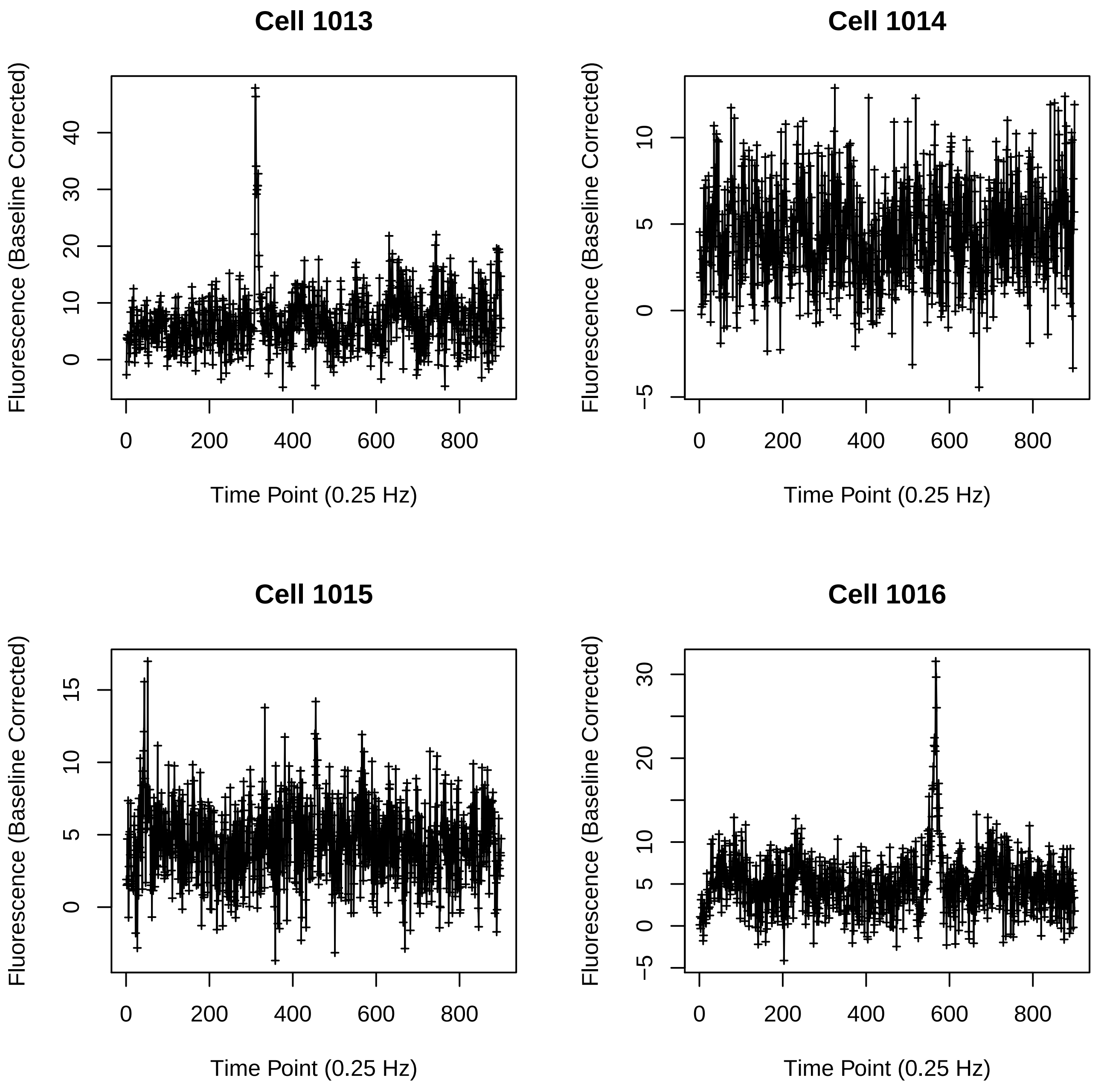
Cell 1013
Cell 1014
Cell 1015
Cell 1016
Fluorescence (Baseline Corrected)
Time Point (0.25 Hz)
0
200
400
600
800
0
10
20
30
40
−5
5
15
25

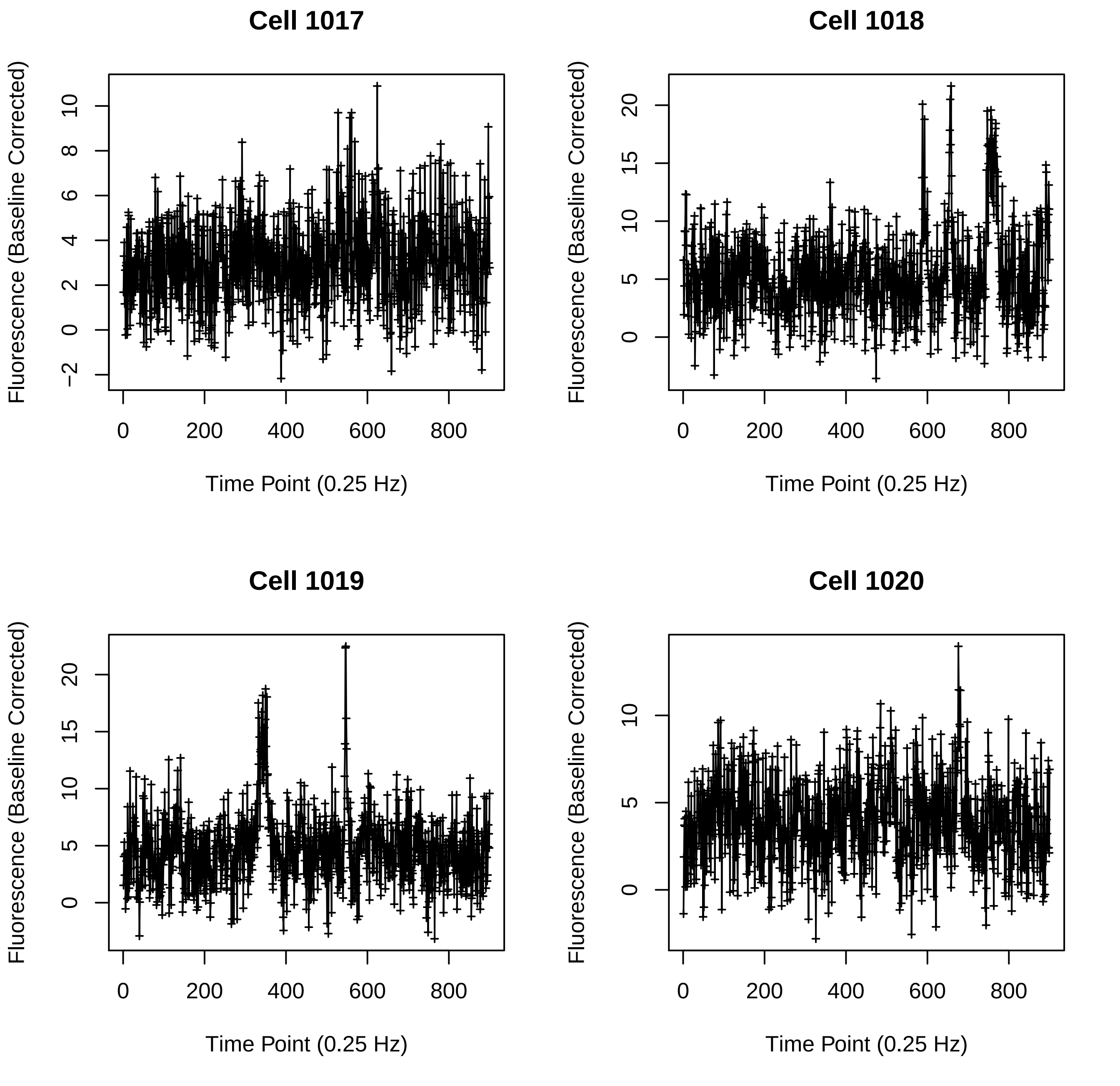
Cell 1017
Cell 1018
Cell 1019
Cell 1020
Fluorescence (Baseline Corrected)
Time Point (0.25 Hz)

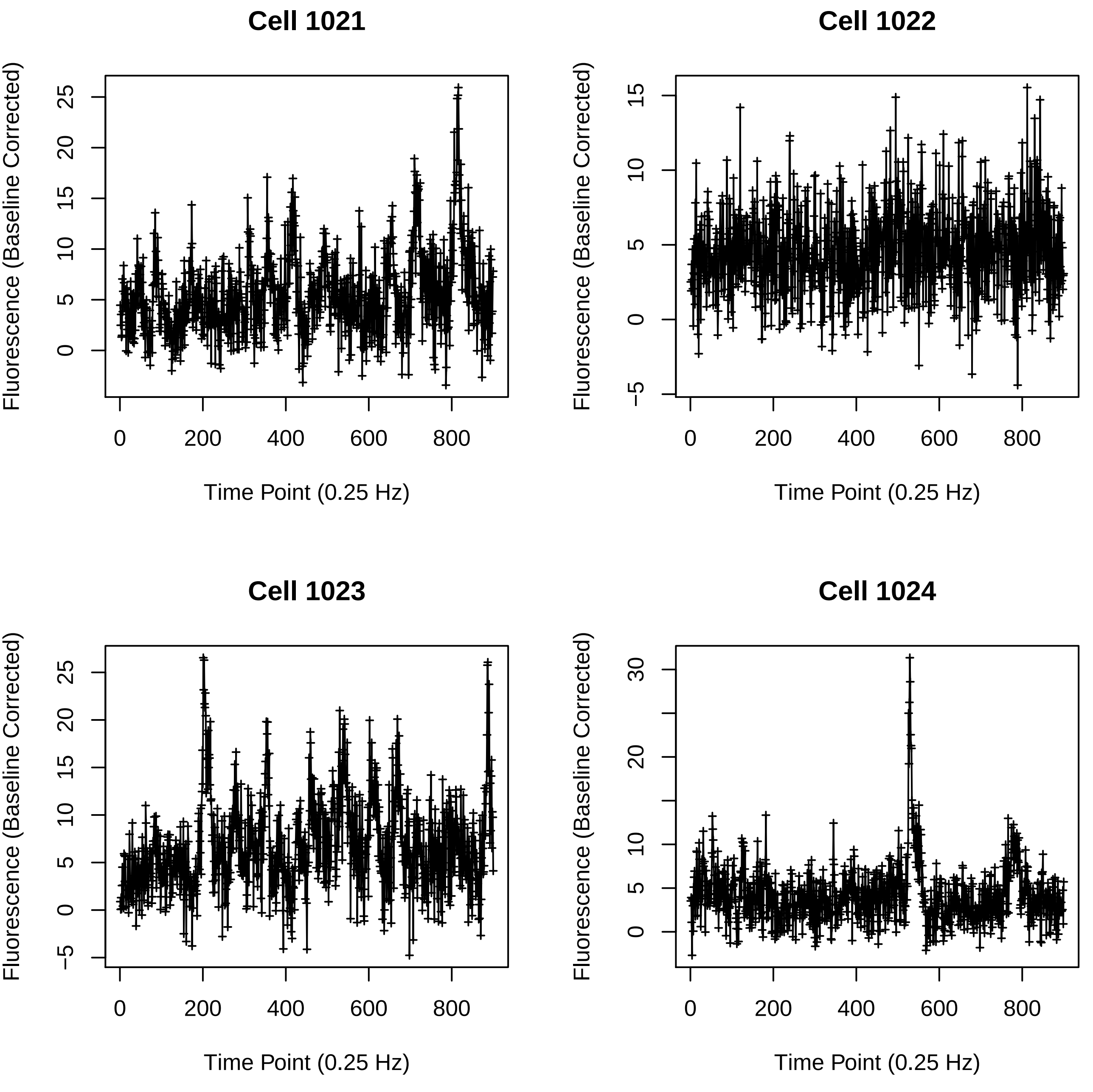
Cell 1021
Cell 1022
Cell 1023
Cell 1024
Fluorescence (Baseline Corrected)
Time Point (0.25 Hz)
0
200
400
600
800
-5
5
10
15
20
25
30

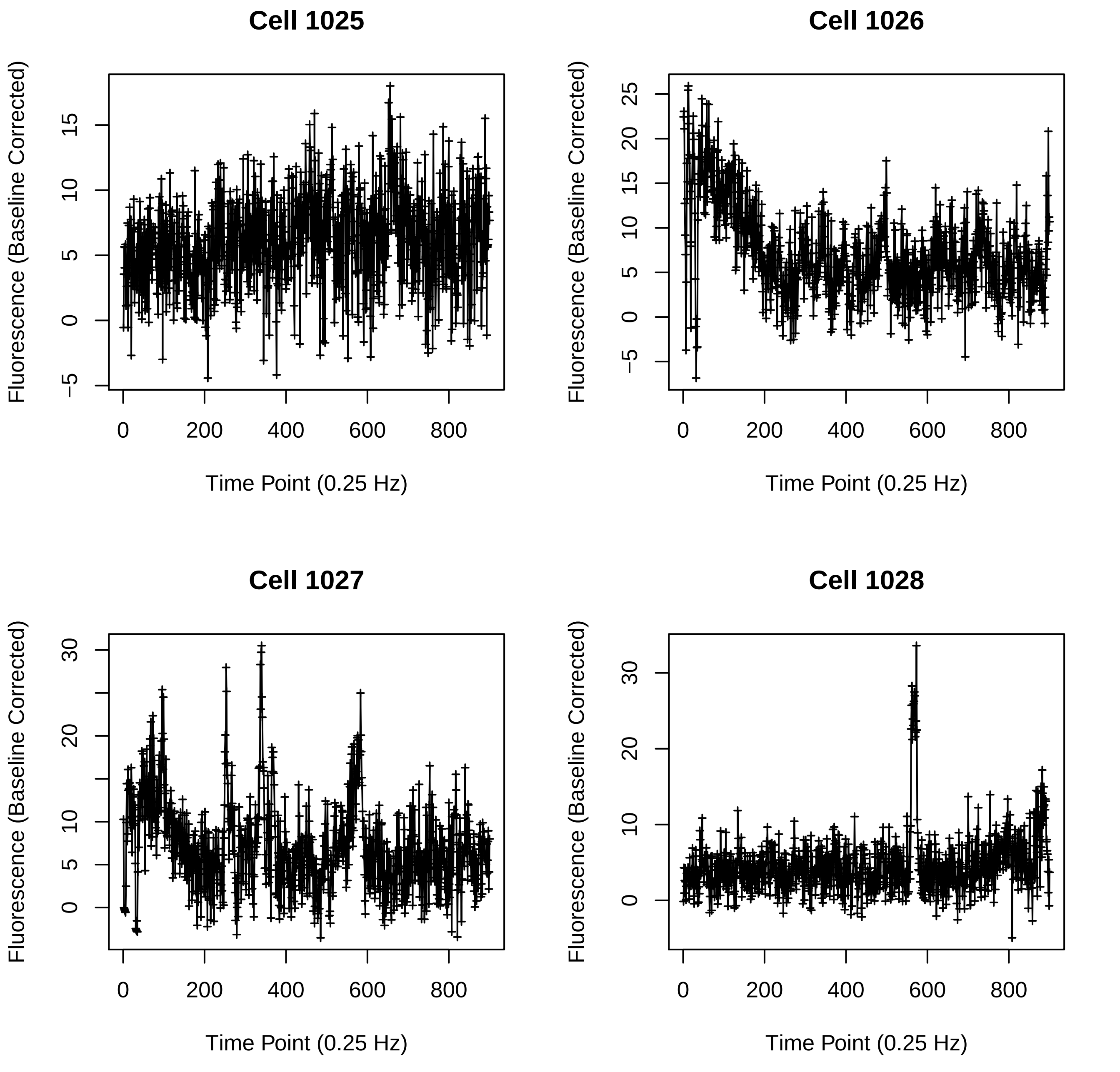

Cell 1025
Cell 1026
Cell 1027
Cell 1028
Fluorescence (Baseline Corrected)
Time Point (0.25 Hz)
0
200
400
600
800
−5
5
10
15
20
25
30

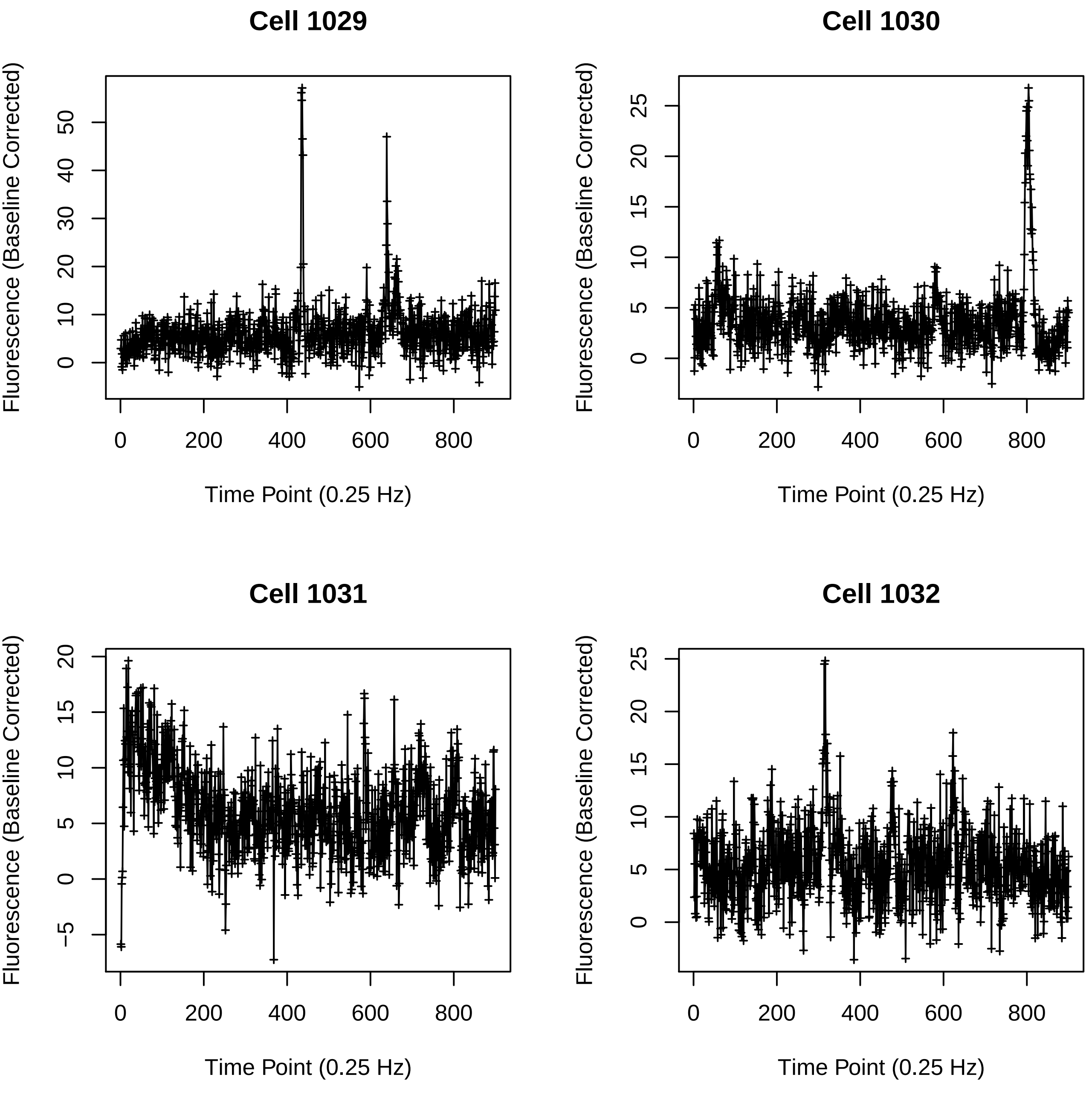
Cell 1029
Cell 1030
Cell 1031
Cell 1032
Fluorescence (Baseline Corrected)
Time Point (0.25 Hz)
0
10
20
30
40
50
5
15
25
−5
200
400
600
800

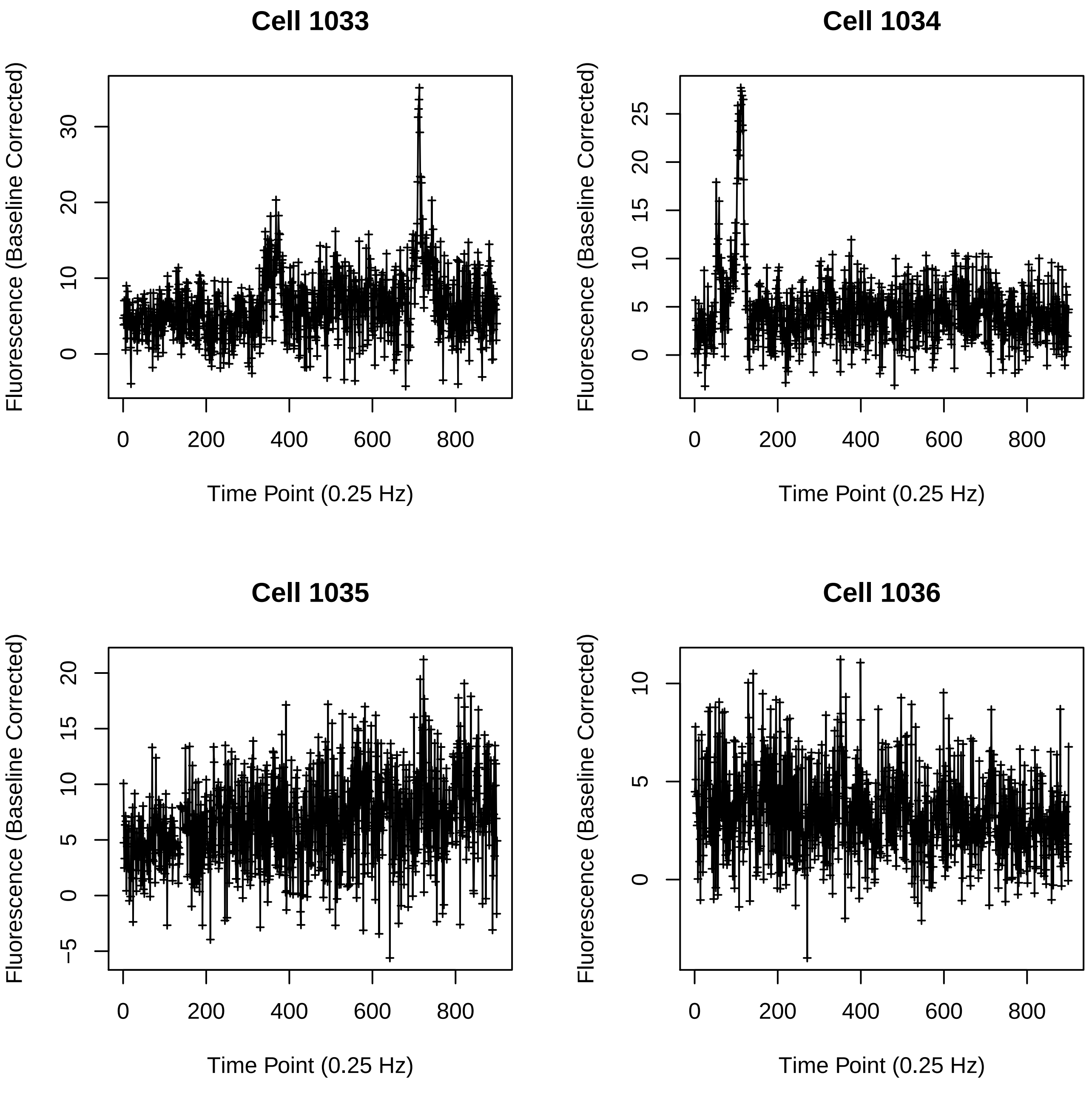
Cell 1033
Cell 1034
Cell 1035
Cell 1036
Fluorescence (Baseline Corrected)
Time Point (0.25 Hz)
0
200
400
600
800
-5
0
5
10
15
20
25
30

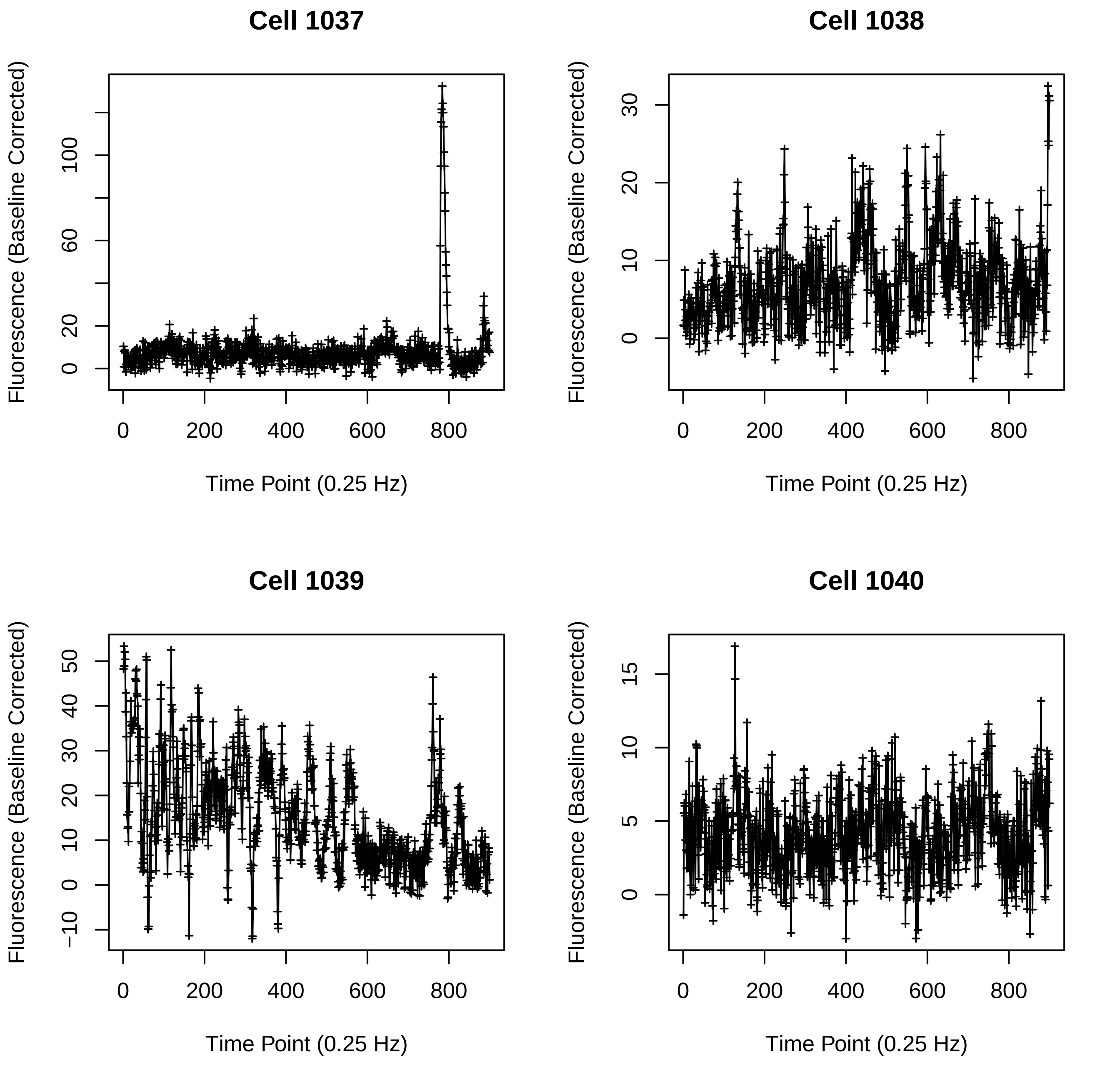

Cell 1037
Cell 1038
Cell 1039
Cell 1040
Fluorescence (Baseline Corrected)
Time Point (0.25 Hz)
0
200
400
600
800
20
60
100
10
30
50
−10
5
15

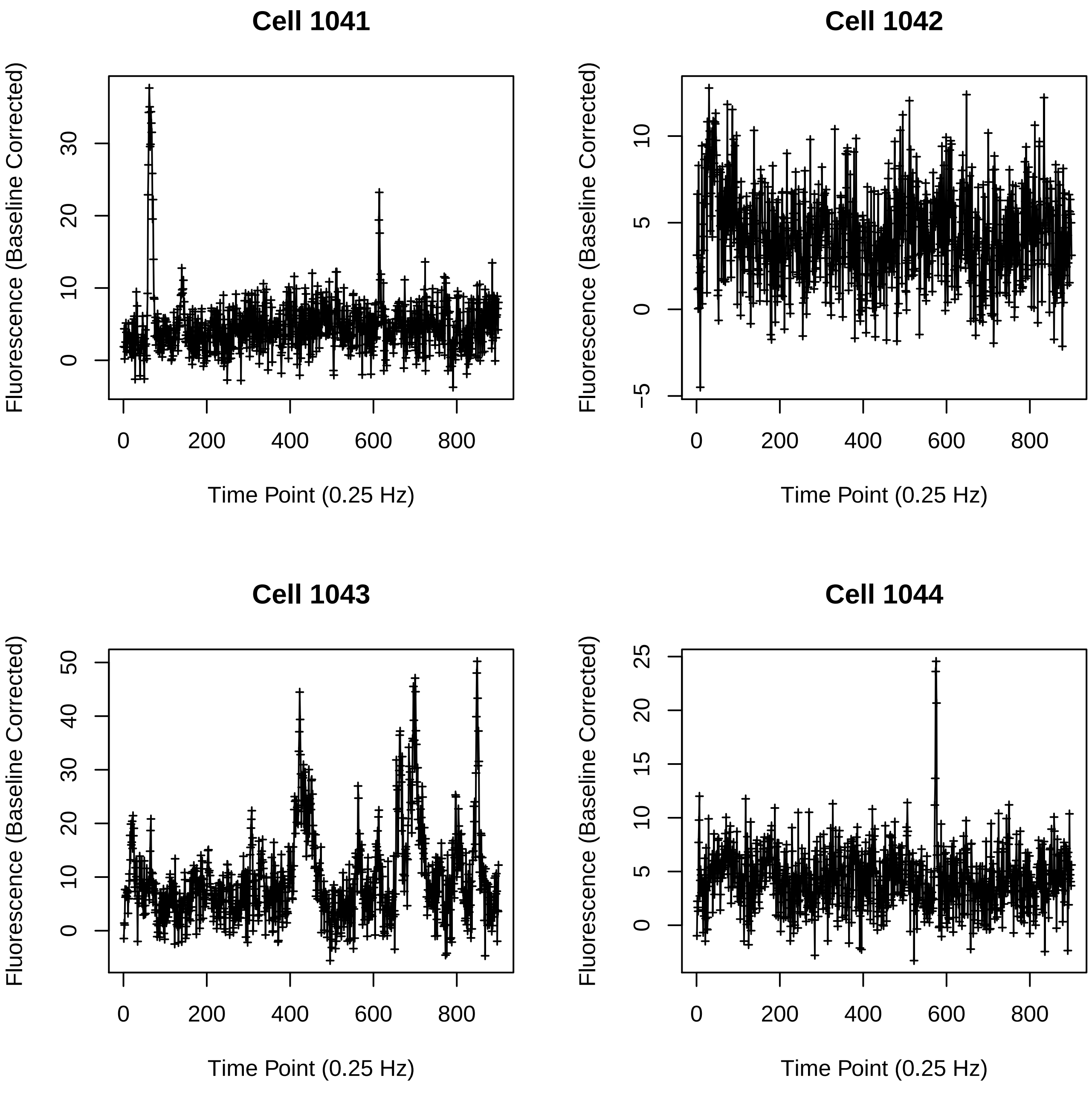
Cell 1041
Cell 1042
Cell 1043
Cell 1044
Fluorescence (Baseline Corrected)
Time Point (0.25 Hz)
0
200
400
600
800
-5
0
5
10
15
20
25
30
40
50

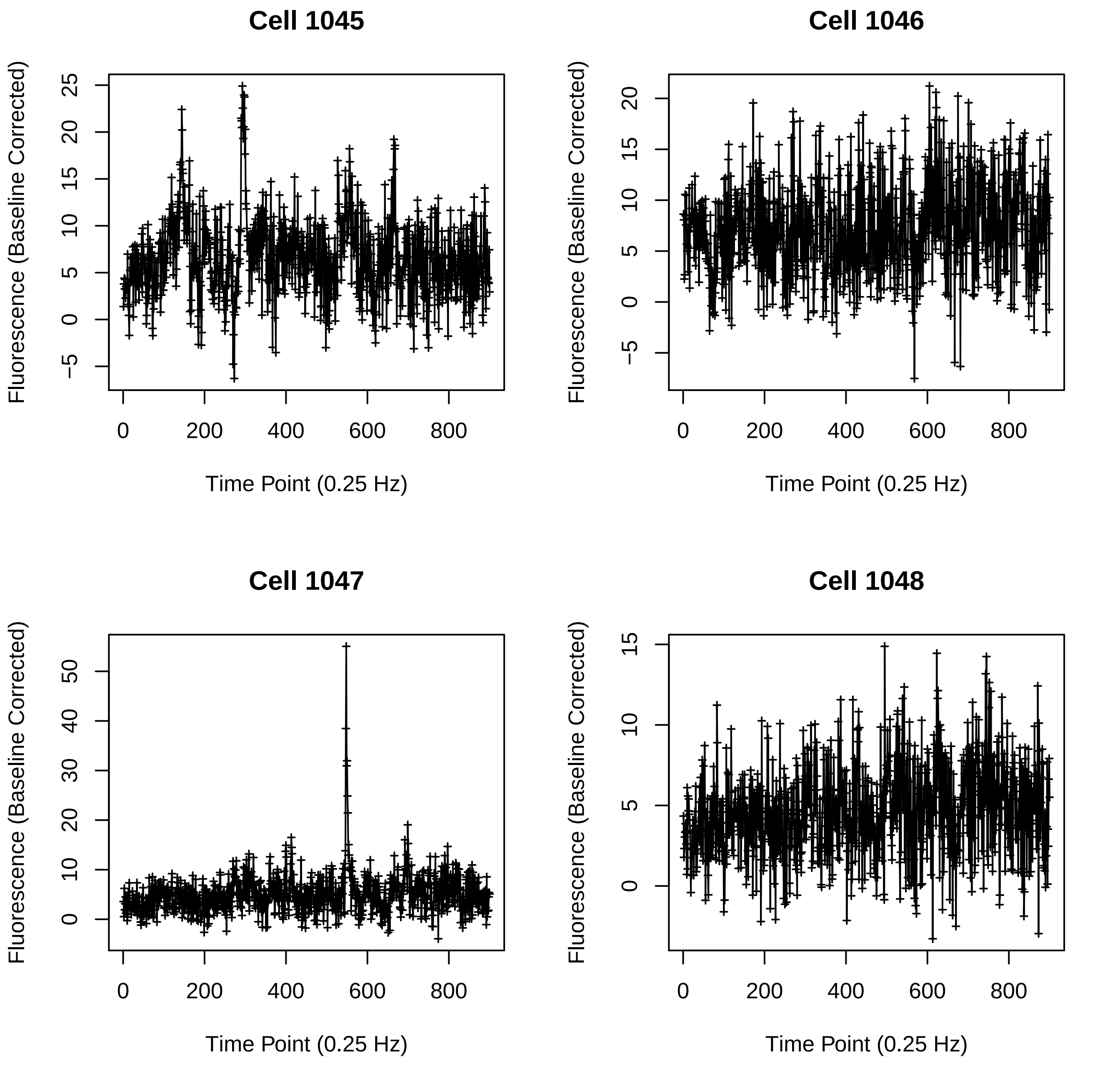
Cell 1045
Cell 1046
Cell 1047
Cell 1048
Fluorescence (Baseline Corrected)
Time Point (0.25 Hz)
0
200
400
600
800
−5
0
5
10
15
20
25
10
20
30
40
50

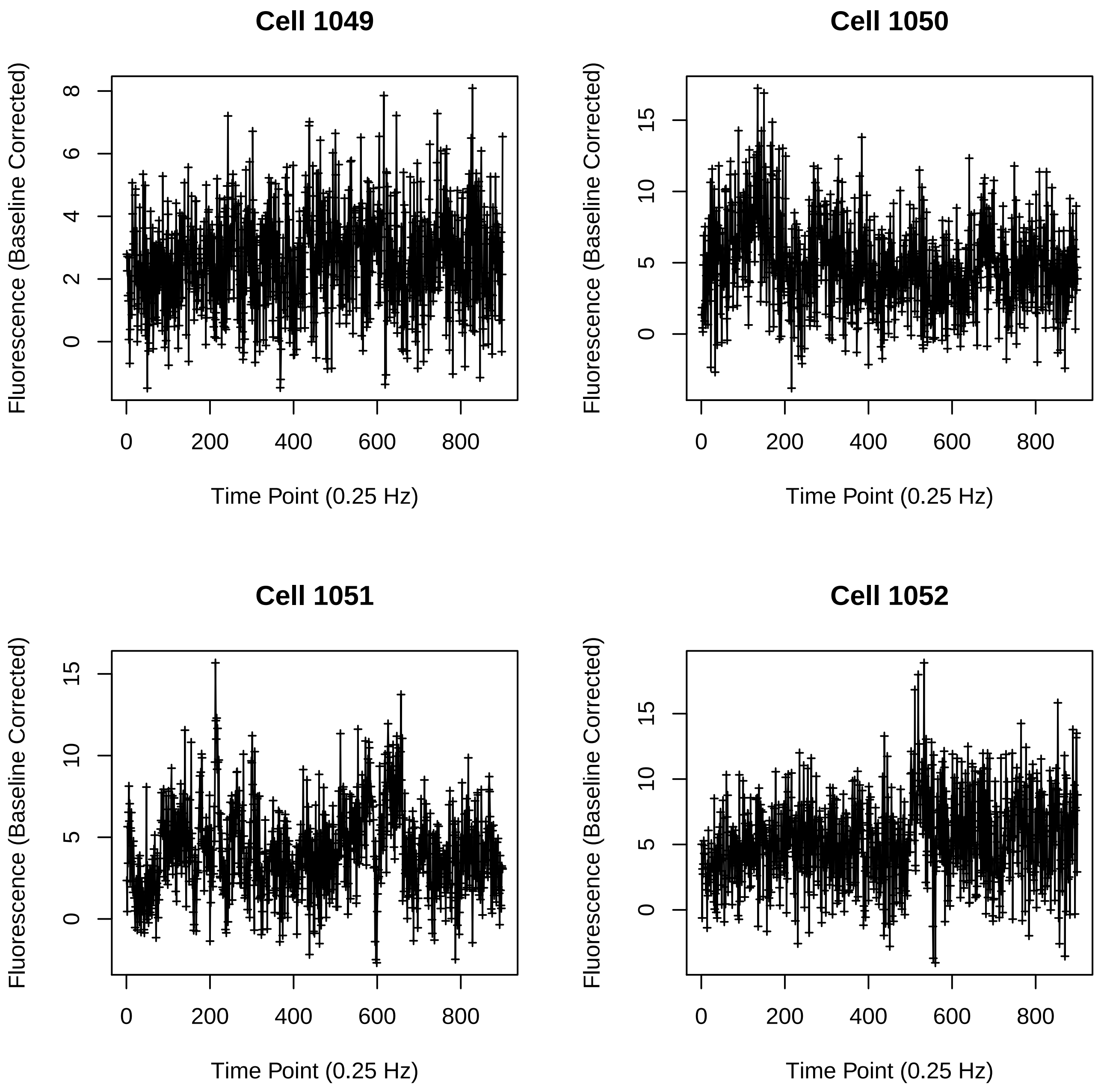
Cell 1049
Cell 1050
Cell 1051
Cell 1052
Fluorescence (Baseline Corrected)
Time Point (0.25 Hz)
0
2
4
6
8
5
10
15
0
200
400
600
800

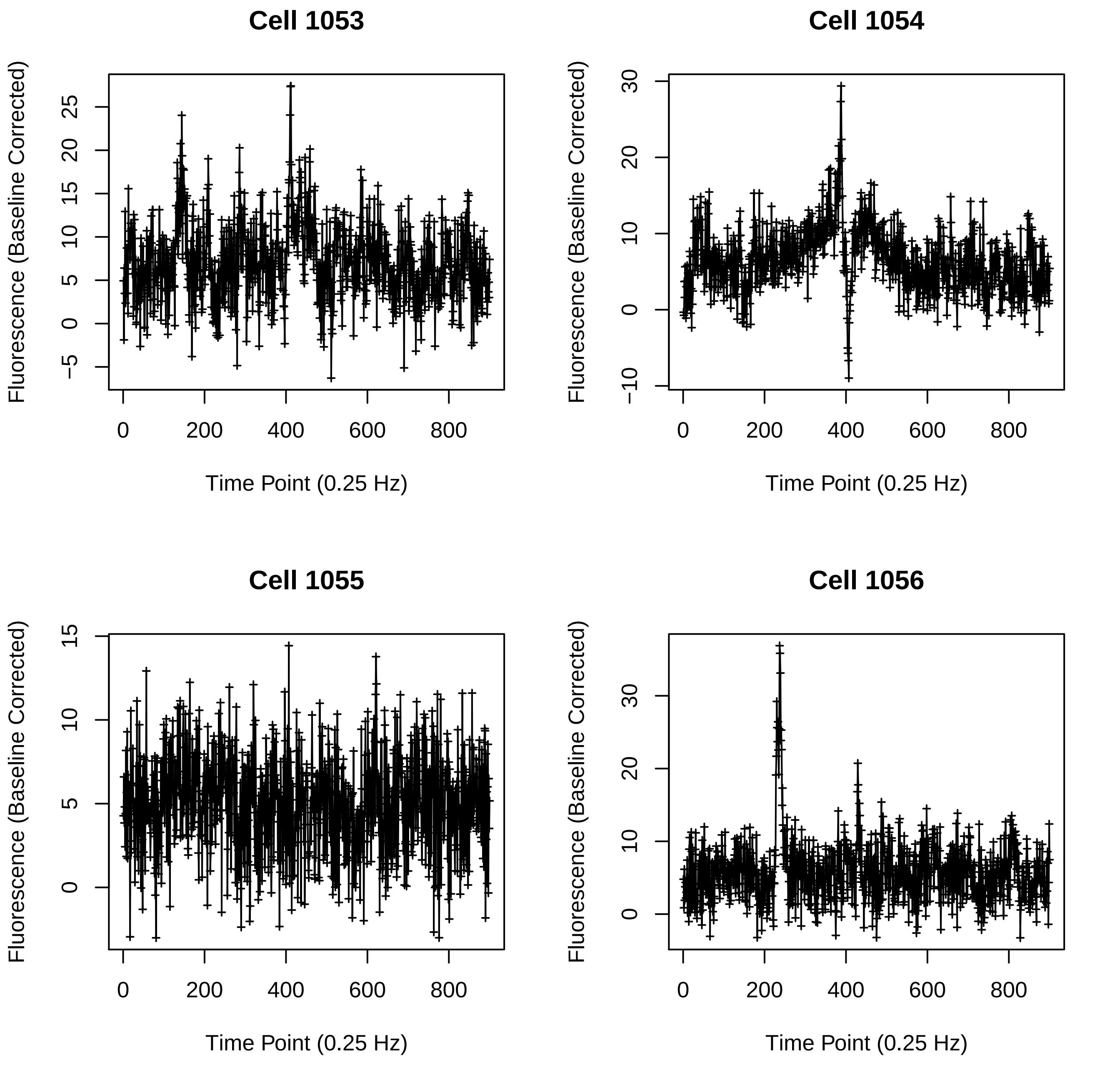
Cell 1053
Fluorescence (Baseline Corrected)
−5
0
5
10
15
20
25
0
200
400
600
800
Time Point (0.25 Hz)
Cell 1054
Fluorescence (Baseline Corrected)
−10
0
10
20
30
0
200
400
600
800
Time Point (0.25 Hz)
Cell 1055
Fluorescence (Baseline Corrected)
0
5
10
15
0
200
400
600
800
Time Point (0.25 Hz)
Cell 1056
Fluorescence (Baseline Corrected)
0
10
20
30
0
200
400
600
800
Time Point (0.25 Hz)

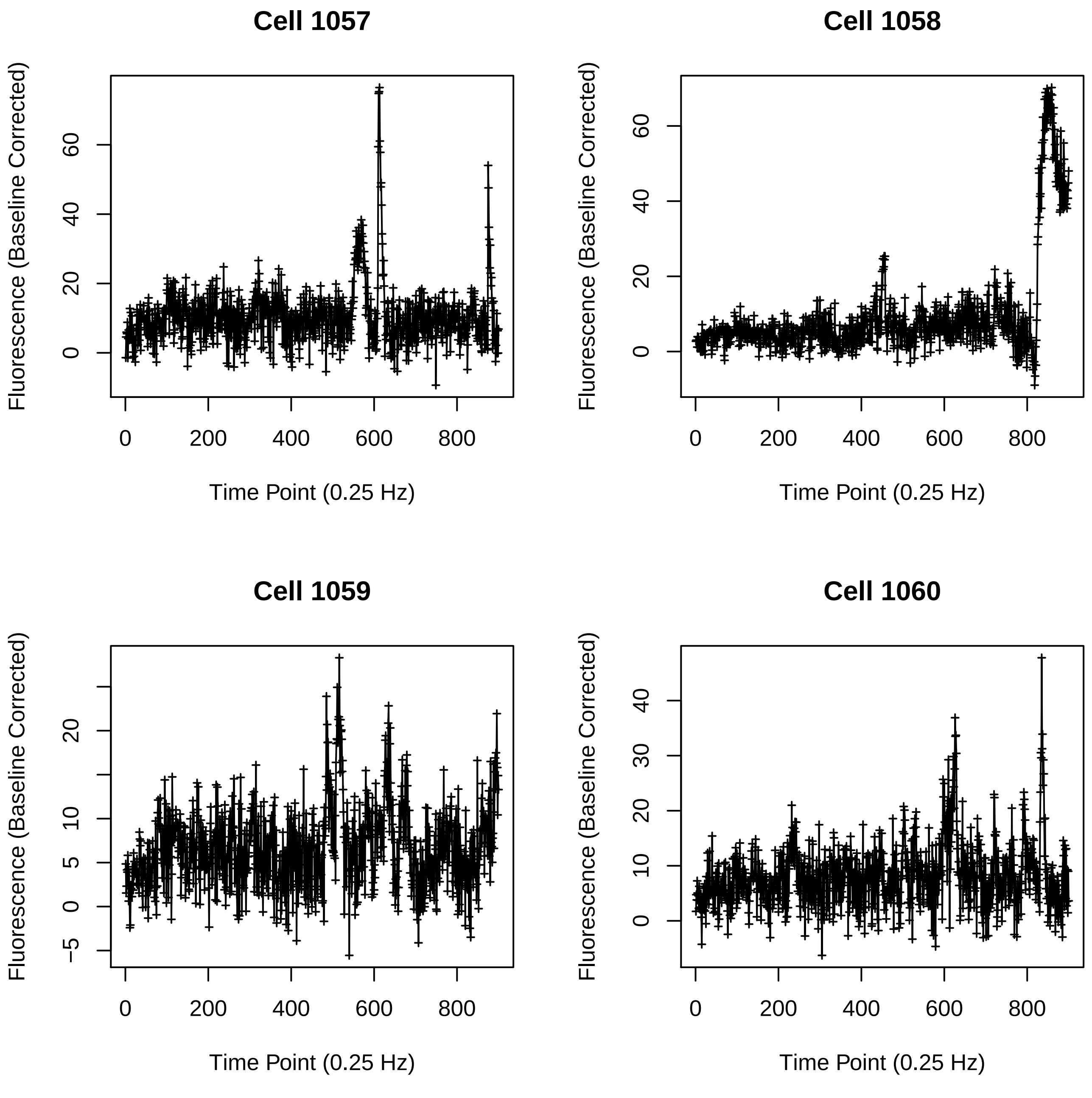
Cell 1057
Cell 1058
Cell 1059
Cell 1060
Fluorescence (Baseline Corrected)
Time Point (0.25 Hz)
0
200
400
600
800
−5
5
10
20
30
40
60

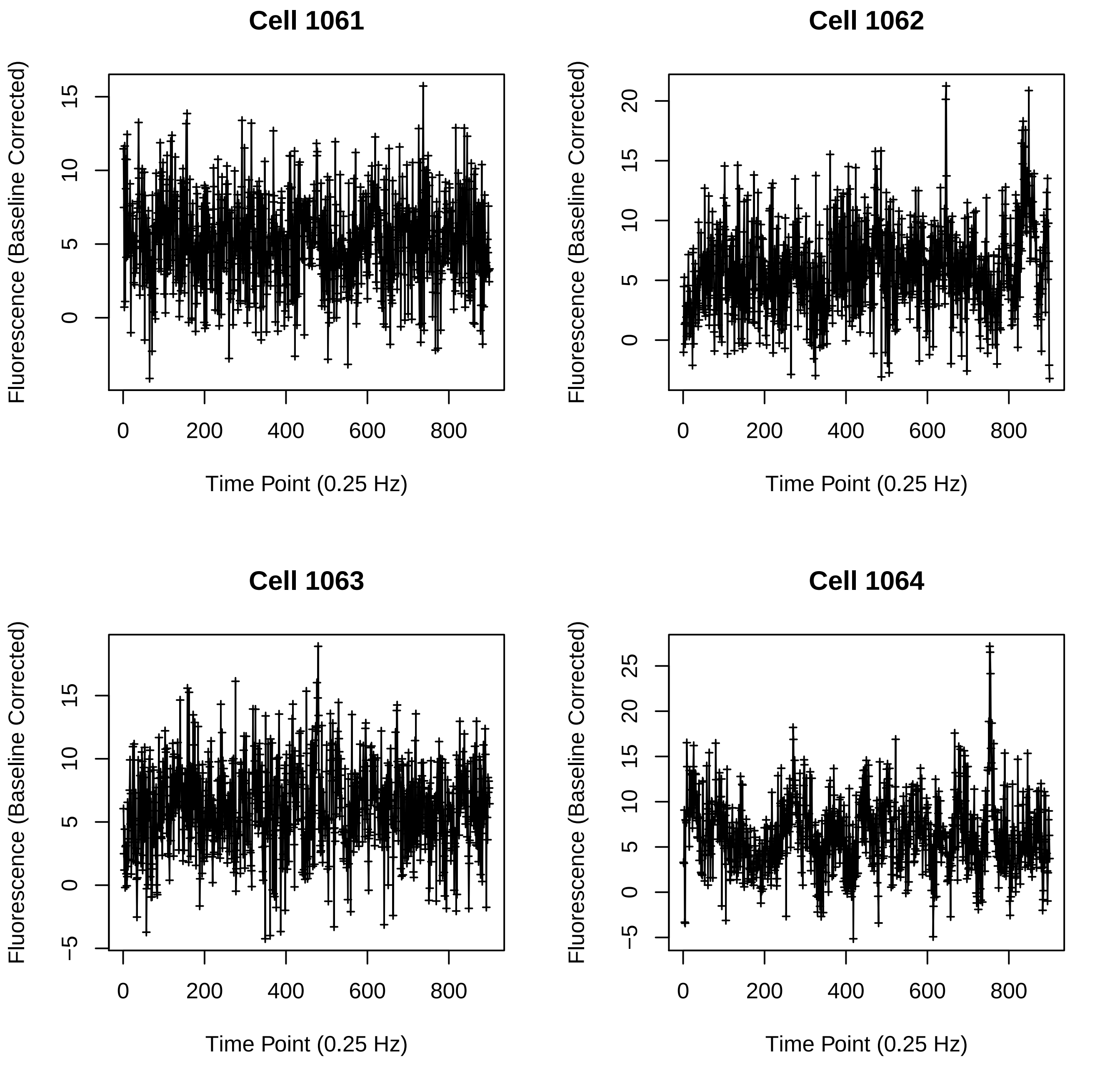

Cell 1061
Cell 1062
Cell 1063
Cell 1064
Fluorescence (Baseline Corrected)
Time Point (0.25 Hz)
0
200
400
600
800
-5
0
5
10
15
20
25

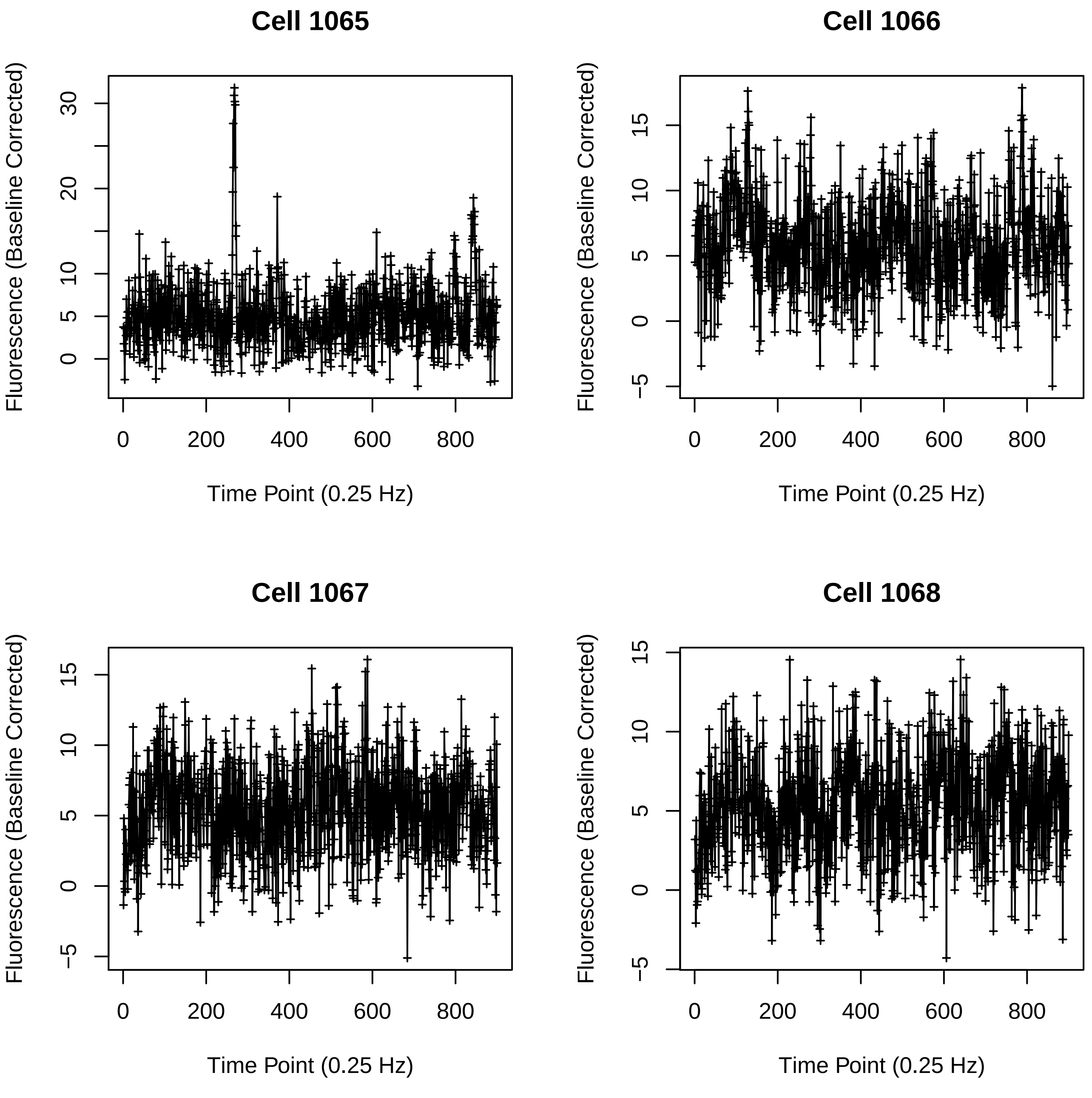
Cell 1065
Cell 1066
Cell 1067
Cell 1068
Fluorescence (Baseline Corrected)
Time Point (0.25 Hz)
0
200
400
600
800
-5
0
5
10
15
20
30

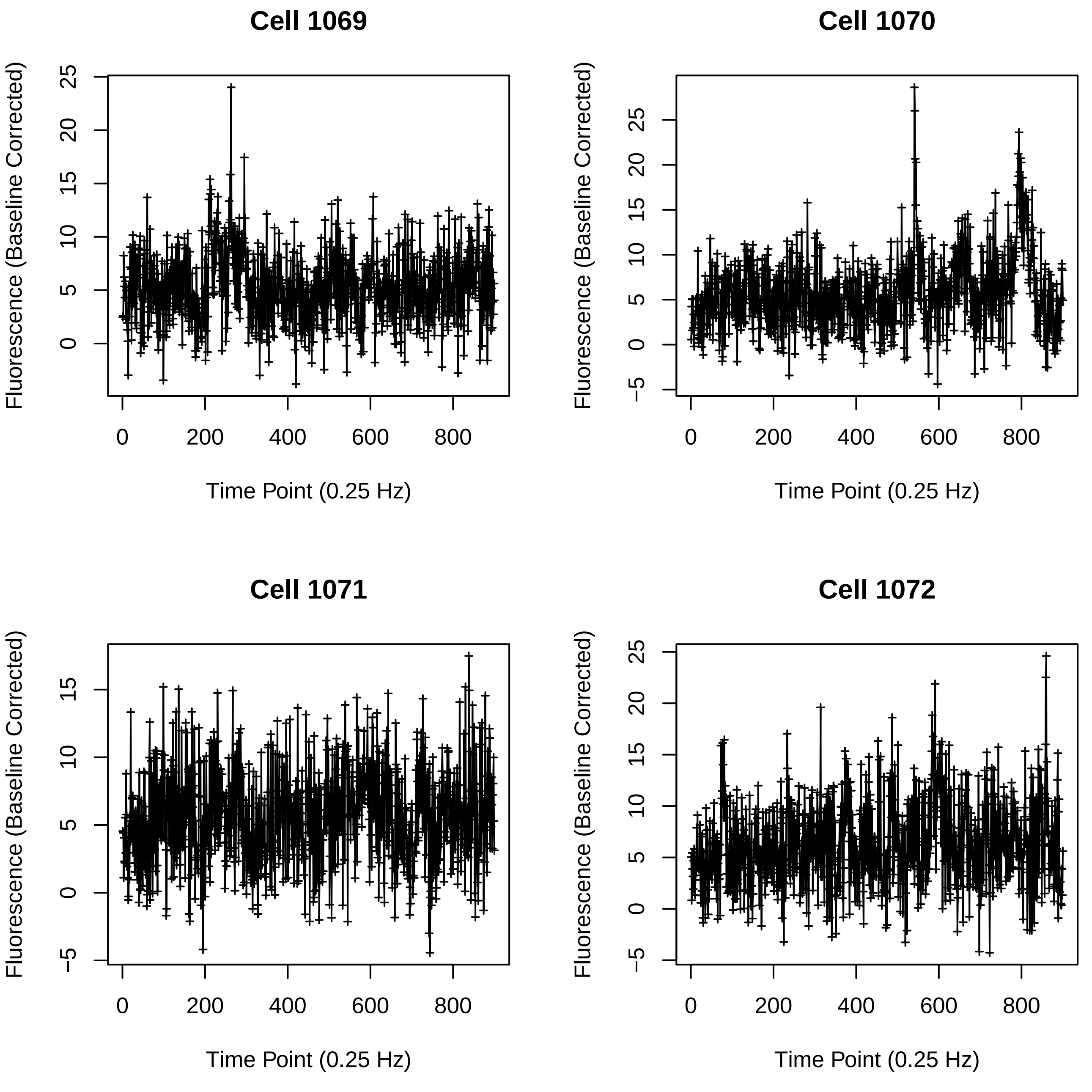

Cell 1069
Cell 1070
Cell 1071
Cell 1072
Fluorescence (Baseline Corrected)
Time Point (0.25 Hz)
0
200
400
600
800
-5
0
5
10
15
20
25

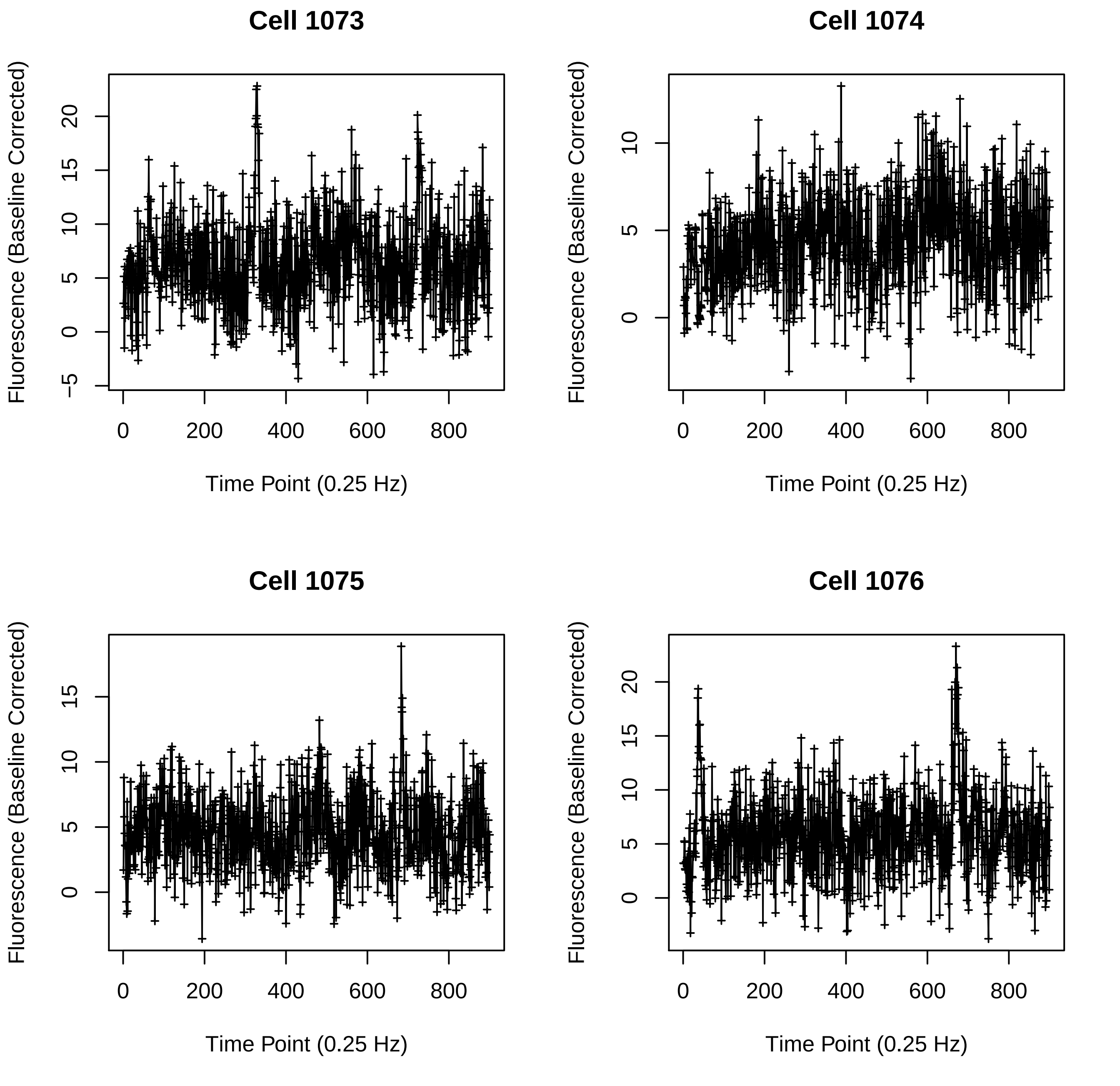
Cell 1073
Cell 1074
Cell 1075
Cell 1076
Fluorescence (Baseline Corrected)
Time Point (0.25 Hz)
0
200
400
600
800
-5
0
5
10
15
20

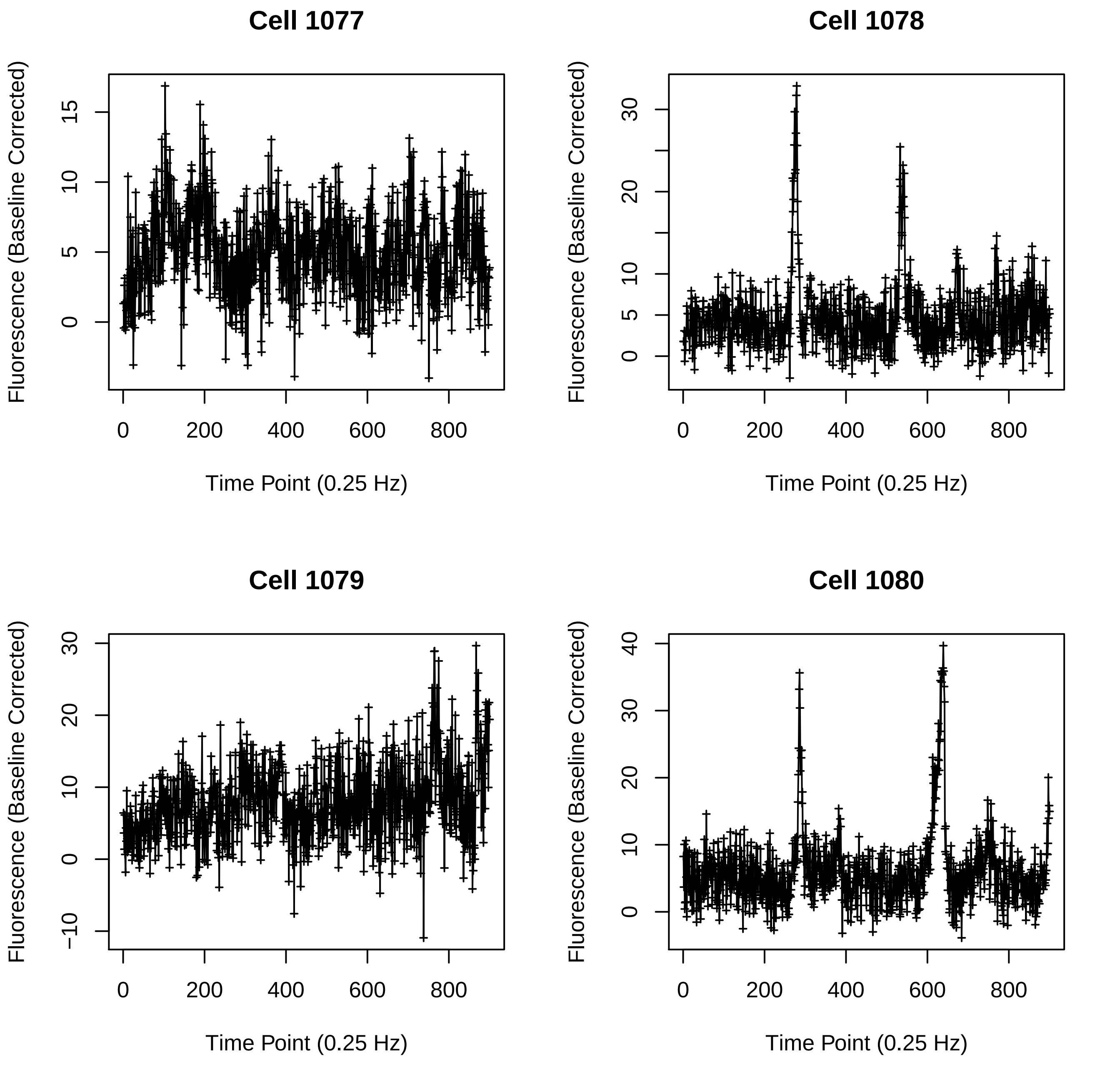
Cell 1077
Cell 1078
Cell 1079
Cell 1080
Fluorescence (Baseline Corrected)
Time Point (0.25 Hz)
0
200
400
600
800
-10
0
5
10
15
20
25
30
35
40

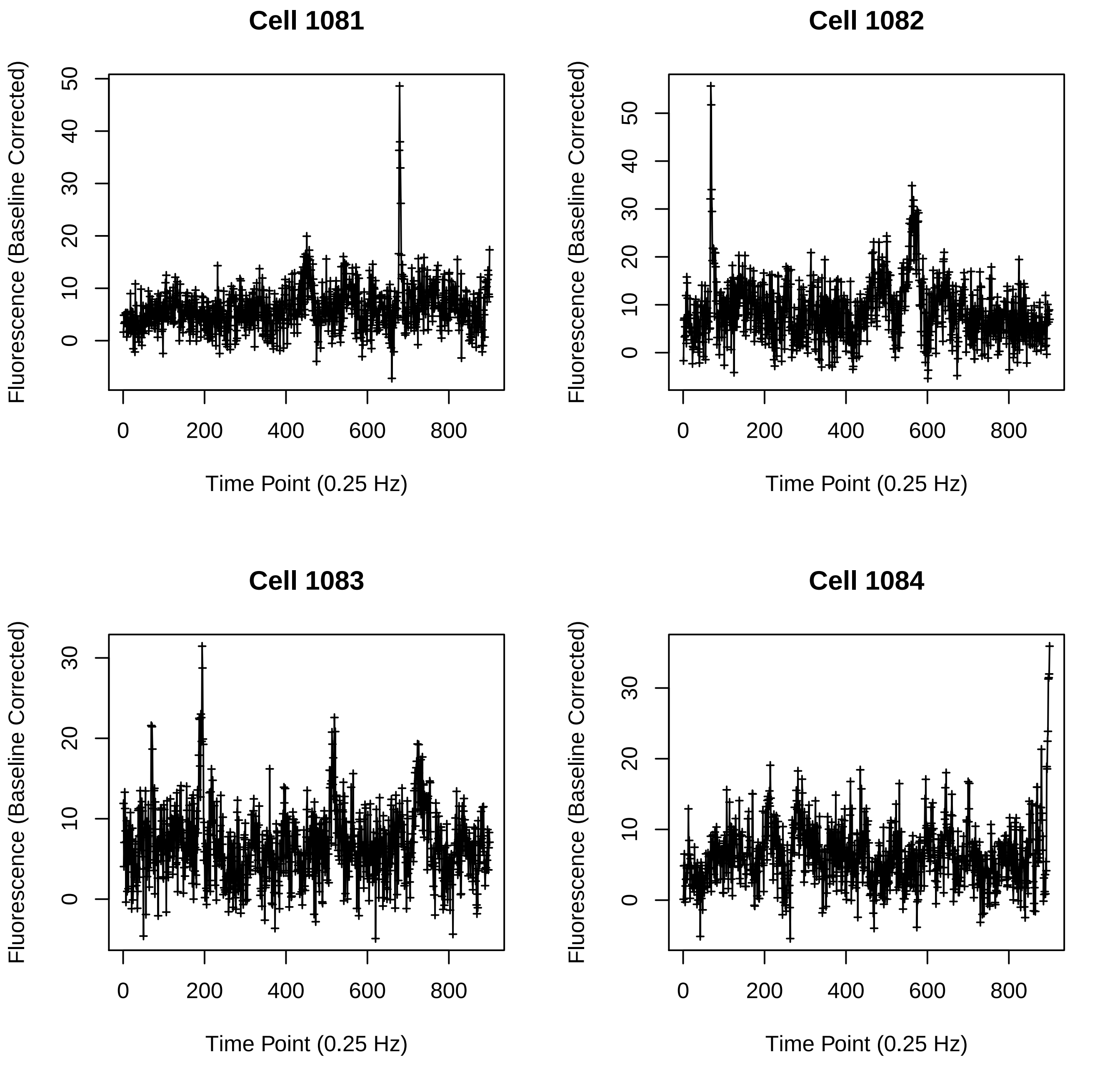
Cell 1081
Cell 1082
Cell 1083
Cell 1084
Fluorescence (Baseline Corrected)
Time Point (0.25 Hz)
0
200
400
600
800
10
20
30
40
50

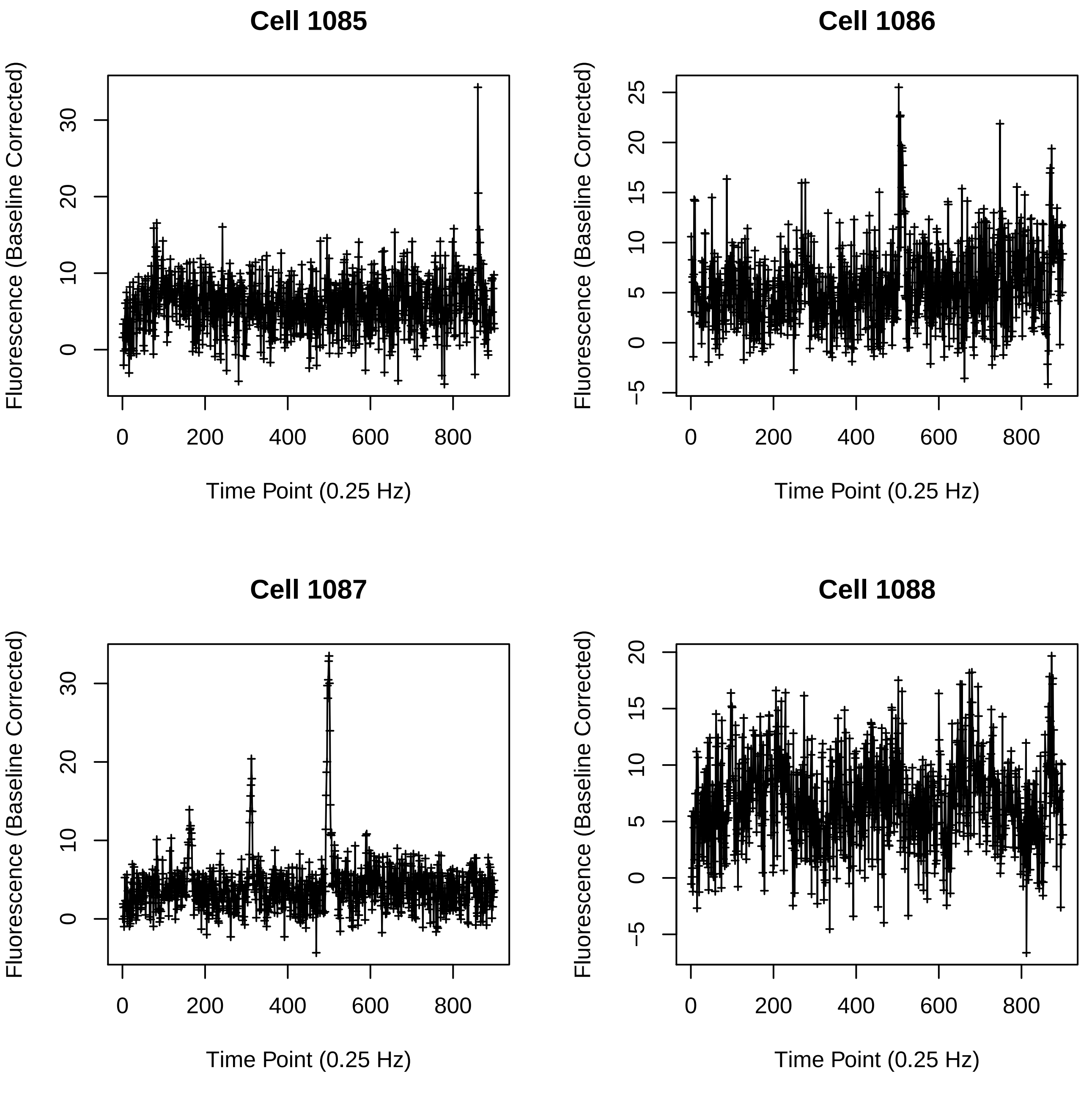
Cell 1085
Fluorescence (Baseline Corrected)
Time Point (0.25 Hz)
Cell 1086
Fluorescence (Baseline Corrected)
Time Point (0.25 Hz)
Cell 1087
Fluorescence (Baseline Corrected)
Time Point (0.25 Hz)
Cell 1088
Fluorescence (Baseline Corrected)
Time Point (0.25 Hz)

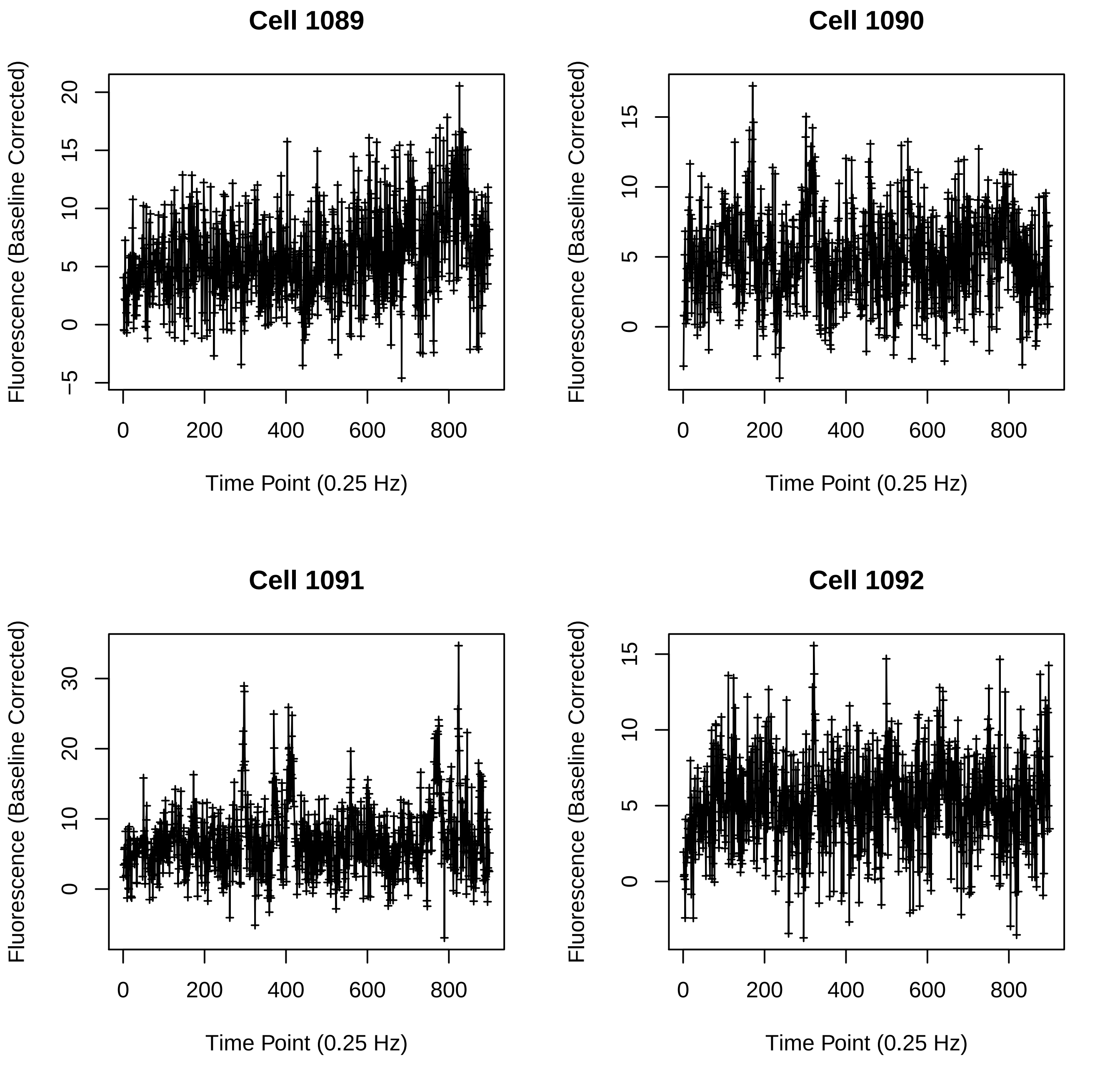
Cell 1089
Cell 1090
Cell 1091
Cell 1092
Fluorescence (Baseline Corrected)
Time Point (0.25 Hz)
0
200
400
600
800
−5
0
5
10
15
20
25
30

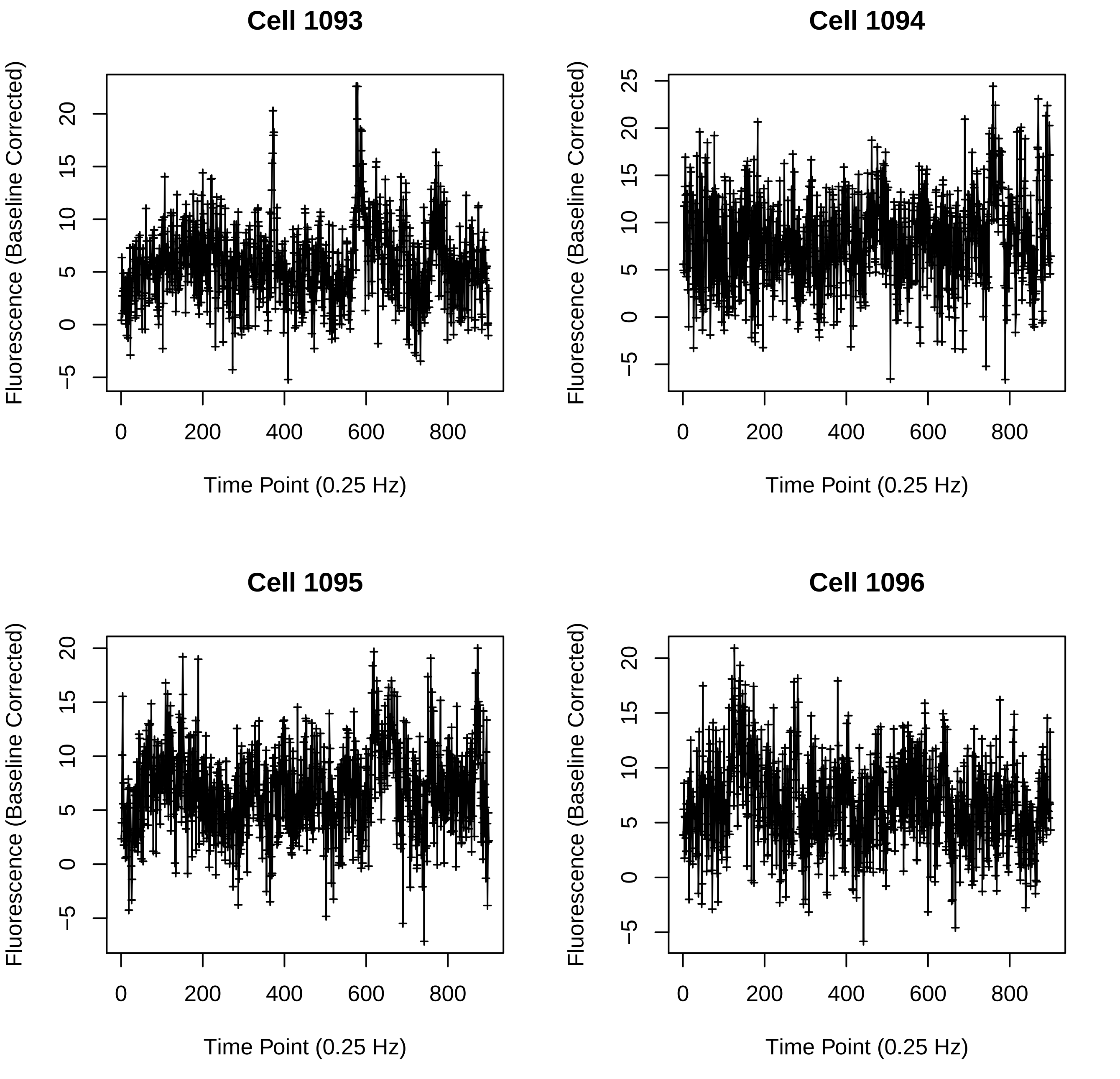

Cell 1093
Cell 1094
Cell 1095
Cell 1096
Fluorescence (Baseline Corrected)
Time Point (0.25 Hz)
0
200
400
600
800
−5
0
5
10
15
20
25

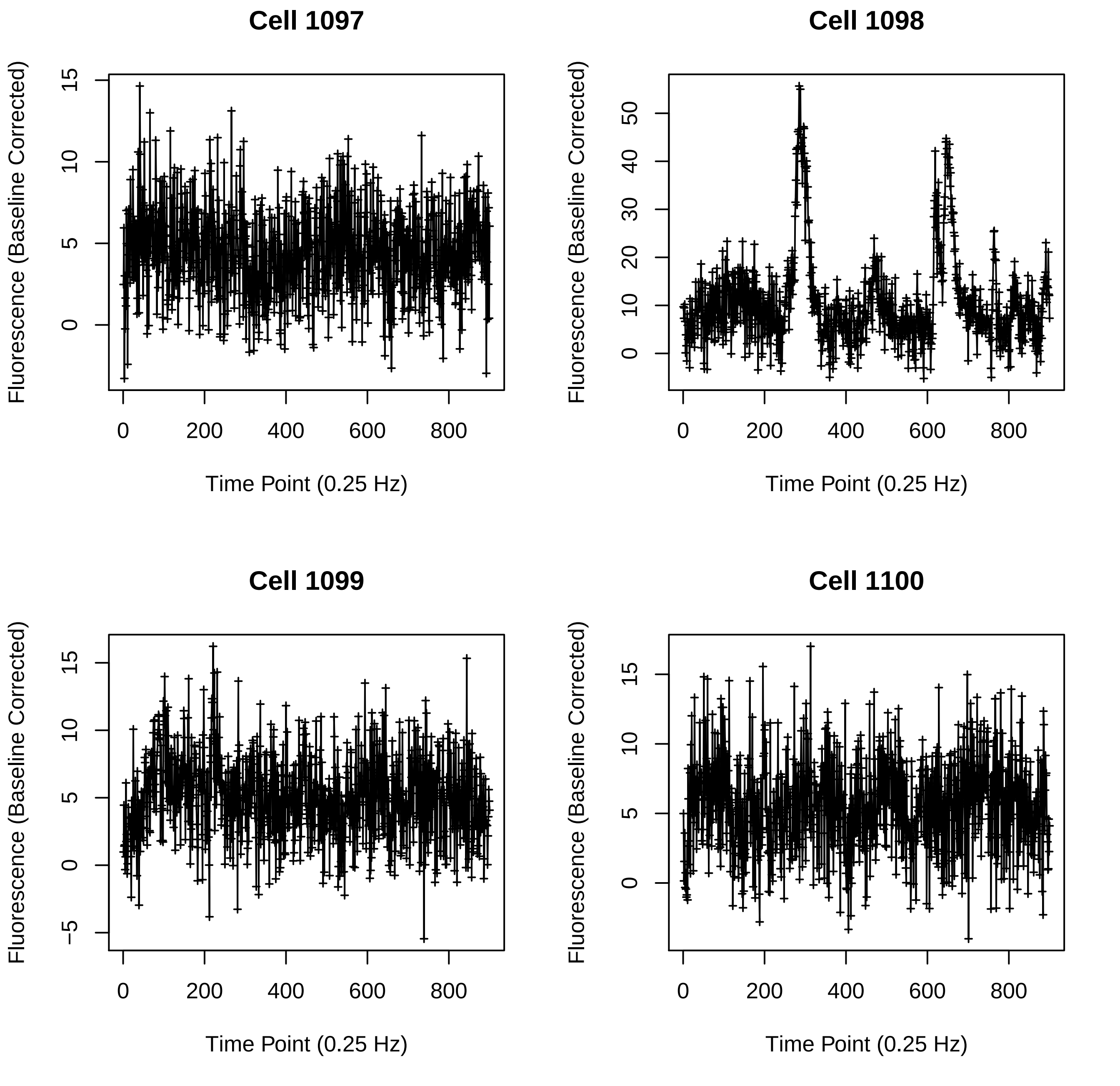

Cell 1097
Cell 1098
Cell 1099
Cell 1100
Fluorescence (Baseline Corrected)
Time Point (0.25 Hz)
0
200
400
600
800
-5
5
10
15
20
30
40
50

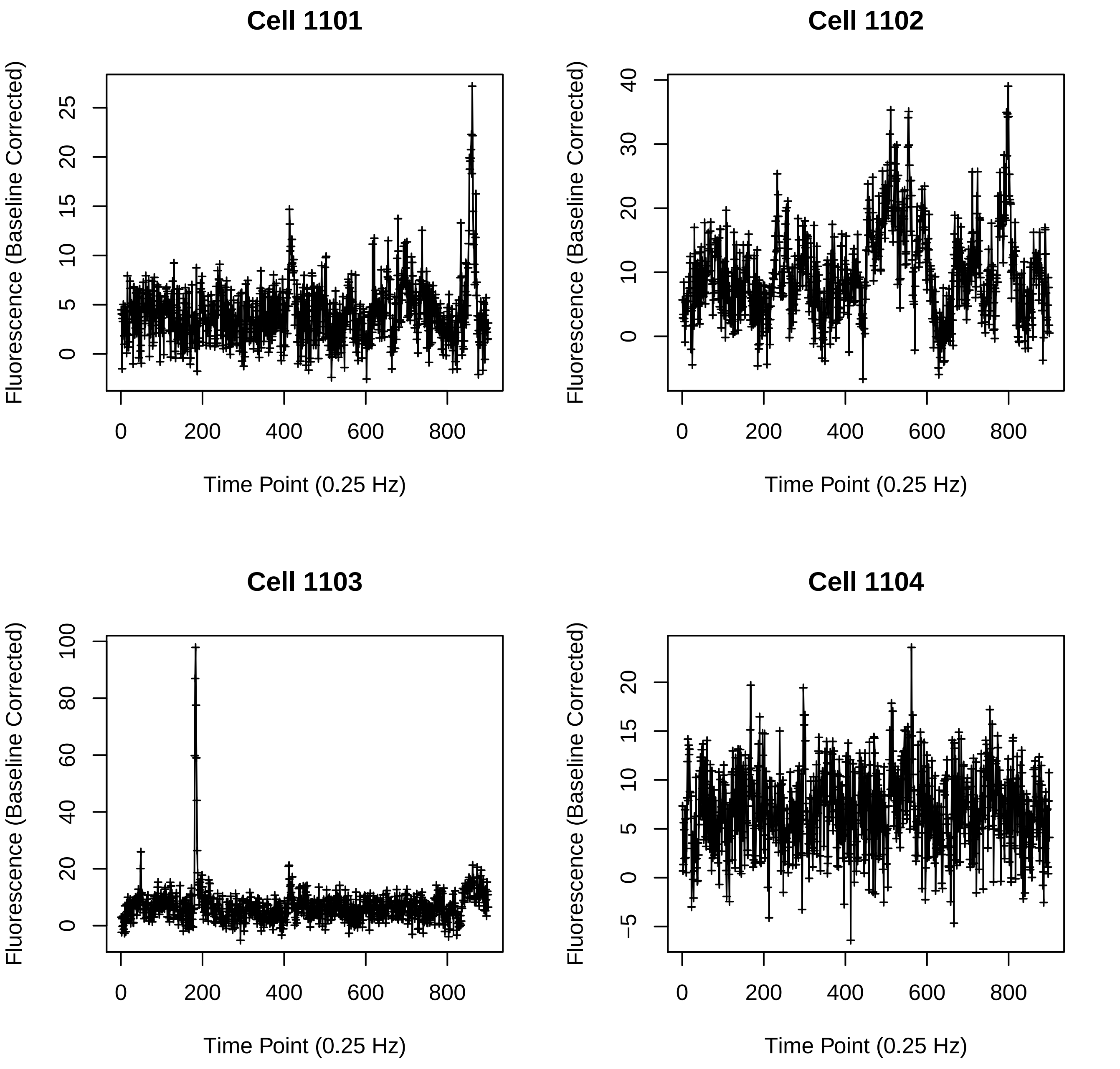
Cell 1101
Cell 1102
Cell 1103
Cell 1104
Fluorescence (Baseline Corrected)
Time Point (0.25 Hz)
0
5
10
15
20
25
30
40
60
80
100
−5
200
400
600
800

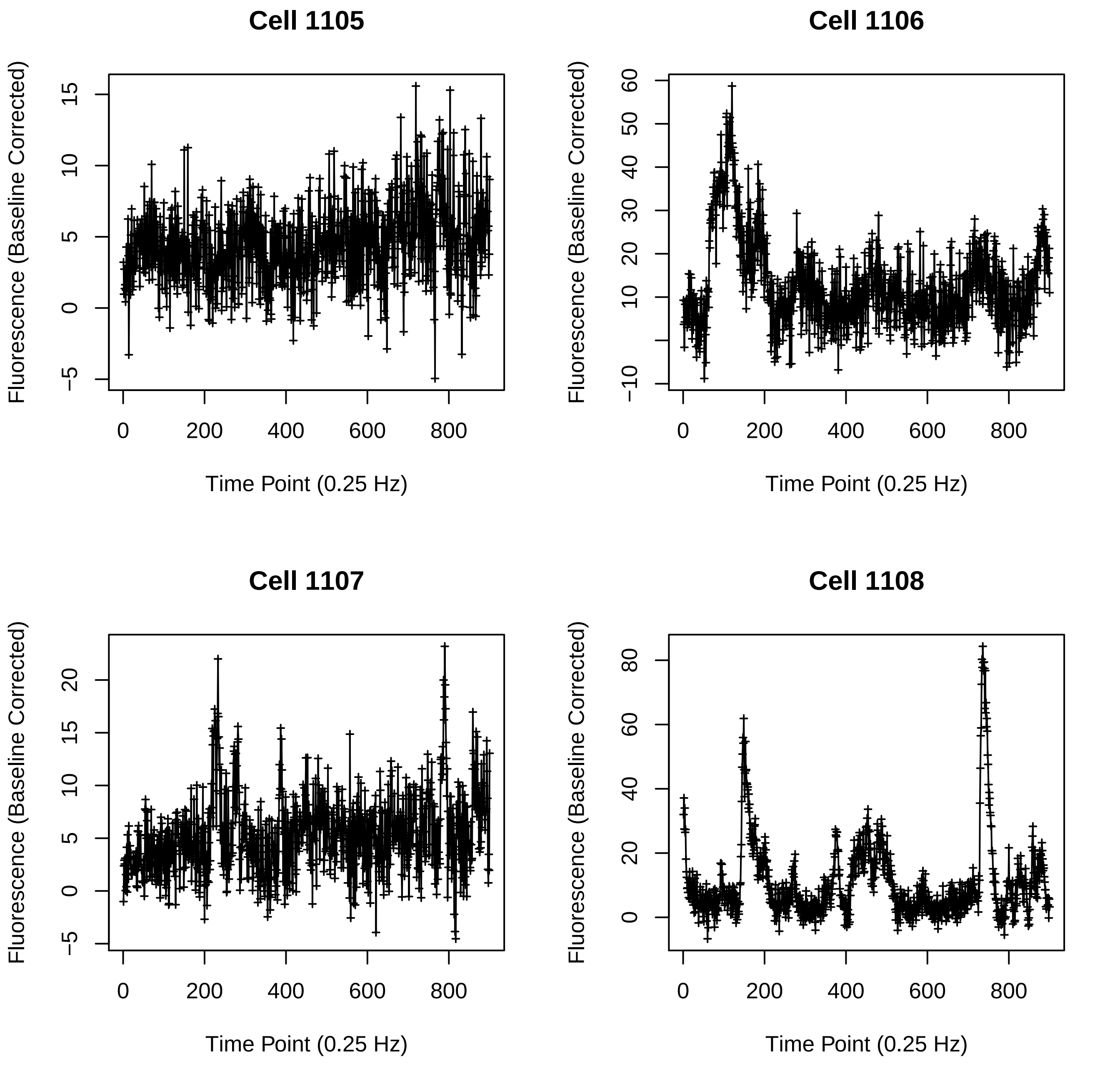
Cell 1105
Cell 1106
Cell 1107
Cell 1108
Fluorescence (Baseline Corrected)
Time Point (0.25 Hz)
0
200
400
600
800
−5
5
10
15
−10
20
30
40
50
60
80

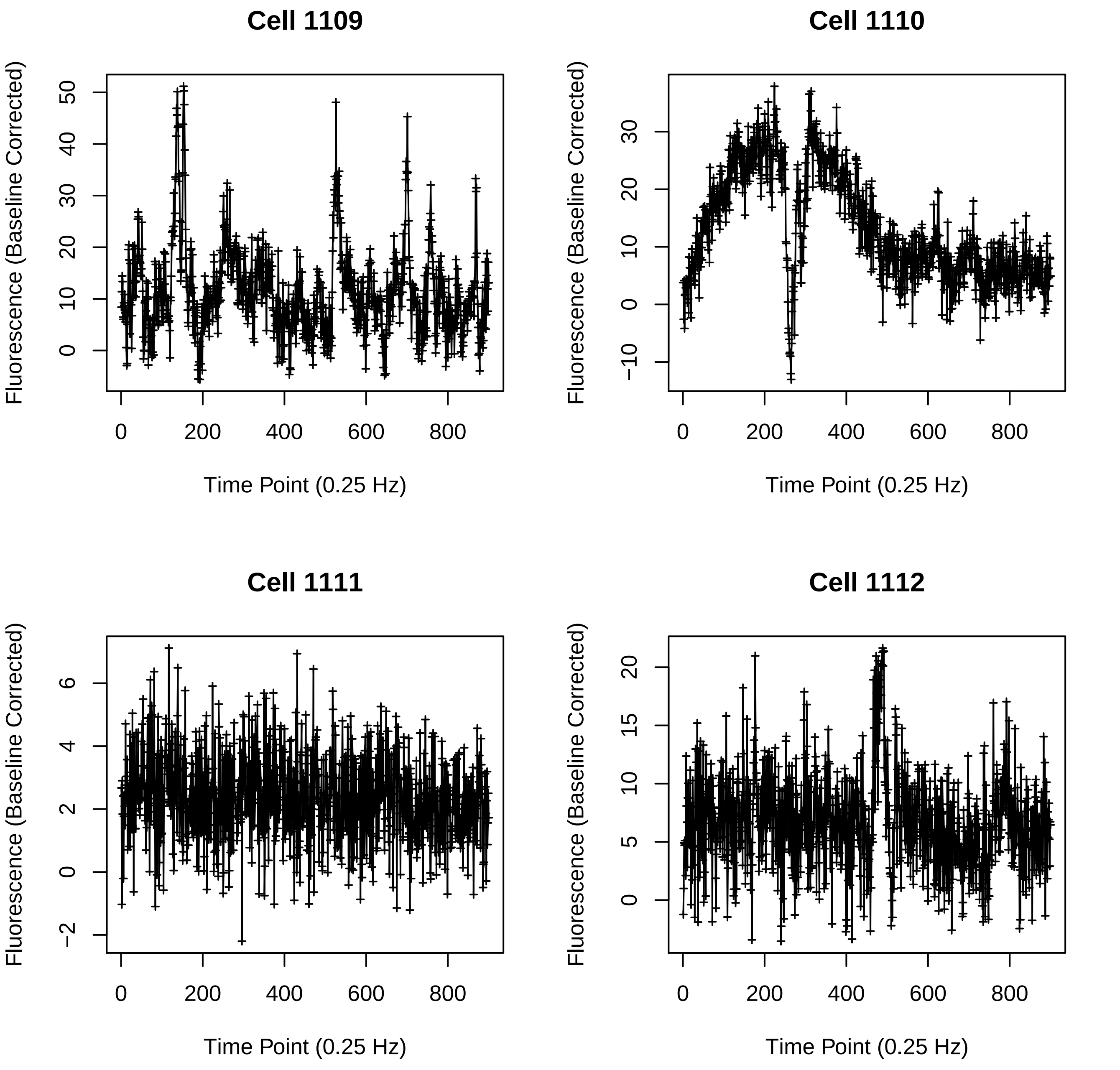

Cell 1109
Cell 1110
Cell 1111
Cell 1112
Fluorescence (Baseline Corrected)
Time Point (0.25 Hz)
0
200
400
600
800

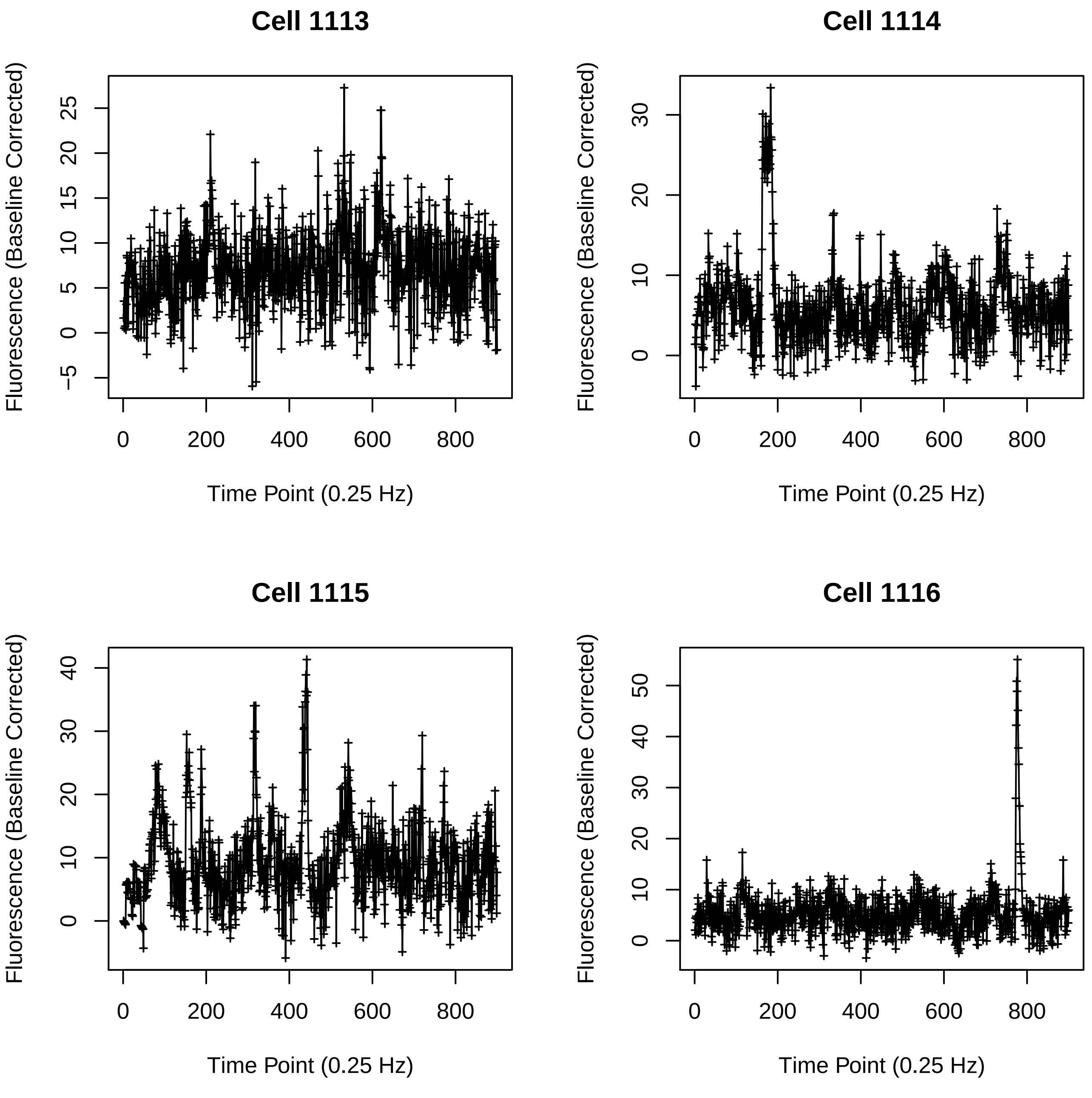

Cell 1113
Fluorescence (Baseline Corrected)
−5
0
5
10
15
20
25
Time Point (0.25 Hz)
0
200
400
600
800
Cell 1114
Fluorescence (Baseline Corrected)
0
10
20
30
Time Point (0.25 Hz)
0
200
400
600
800
Cell 1115
Fluorescence (Baseline Corrected)
0
10
20
30
40
Time Point (0.25 Hz)
0
200
400
600
800
Cell 1116
Fluorescence (Baseline Corrected)
0
10
20
30
40
50
Time Point (0.25 Hz)
0
200
400
600
800

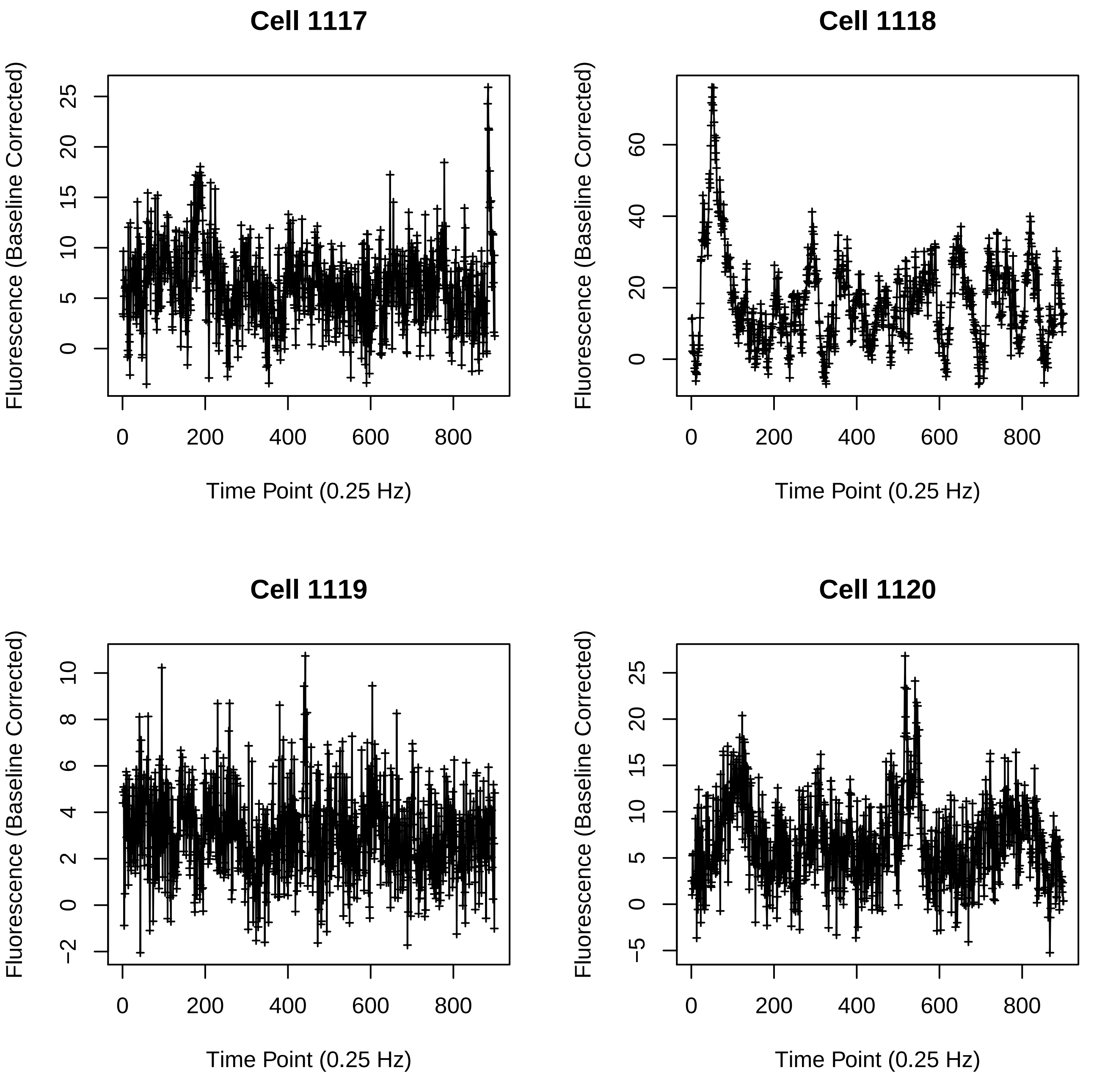
Cell 1117
Cell 1118
Cell 1119
Cell 1120
Fluorescence (Baseline Corrected)
Time Point (0.25 Hz)
0
200
400
600
800
0
5
10
15
20
25
0
20
40
60
−2
0
2
4
6
8
10
−5
0
5
10
15
20
25

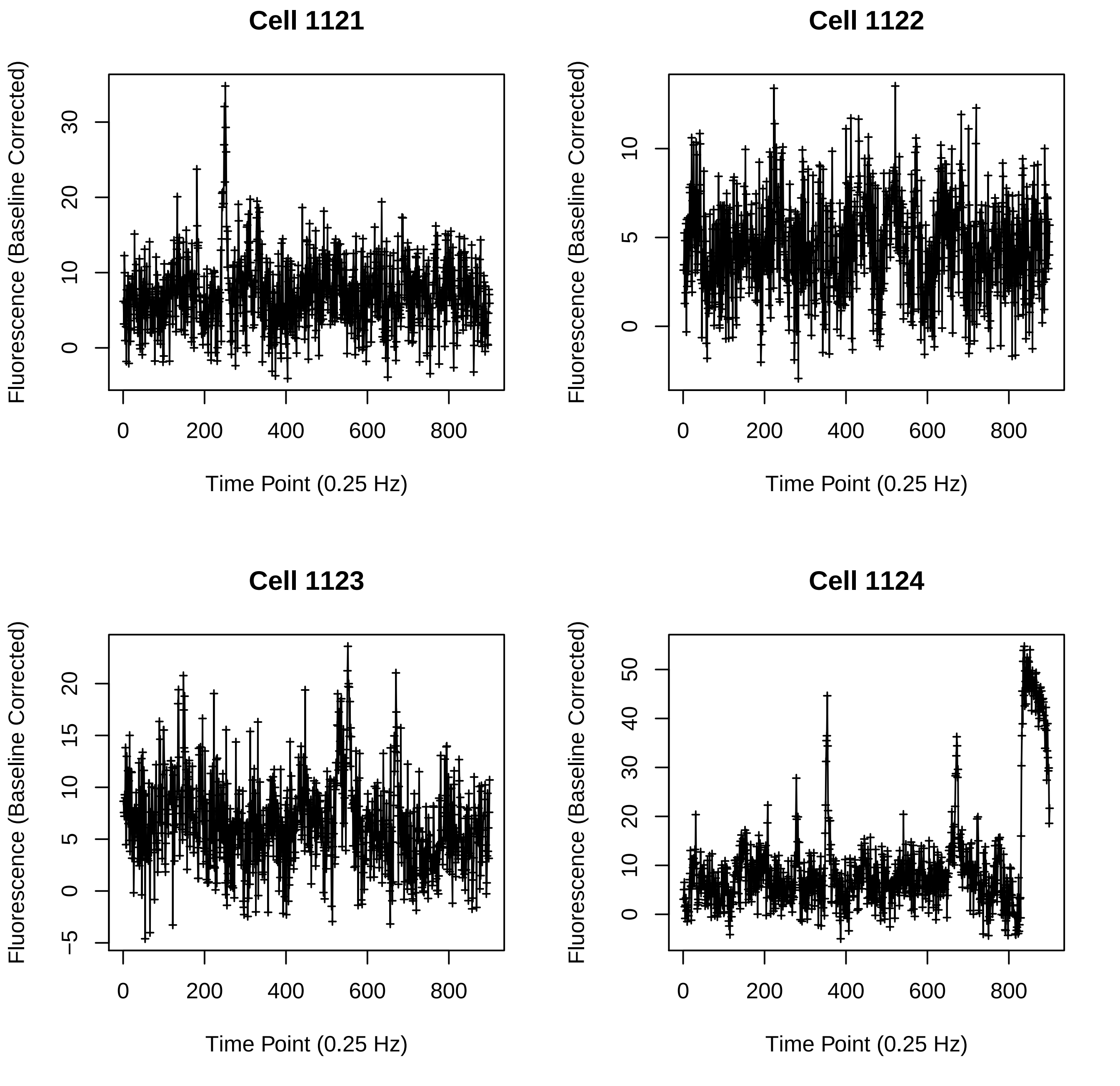

Cell 1121
Cell 1122
Cell 1123
Cell 1124
Fluorescence (Baseline Corrected)
Time Point (0.25 Hz)
0
200
400
600
800

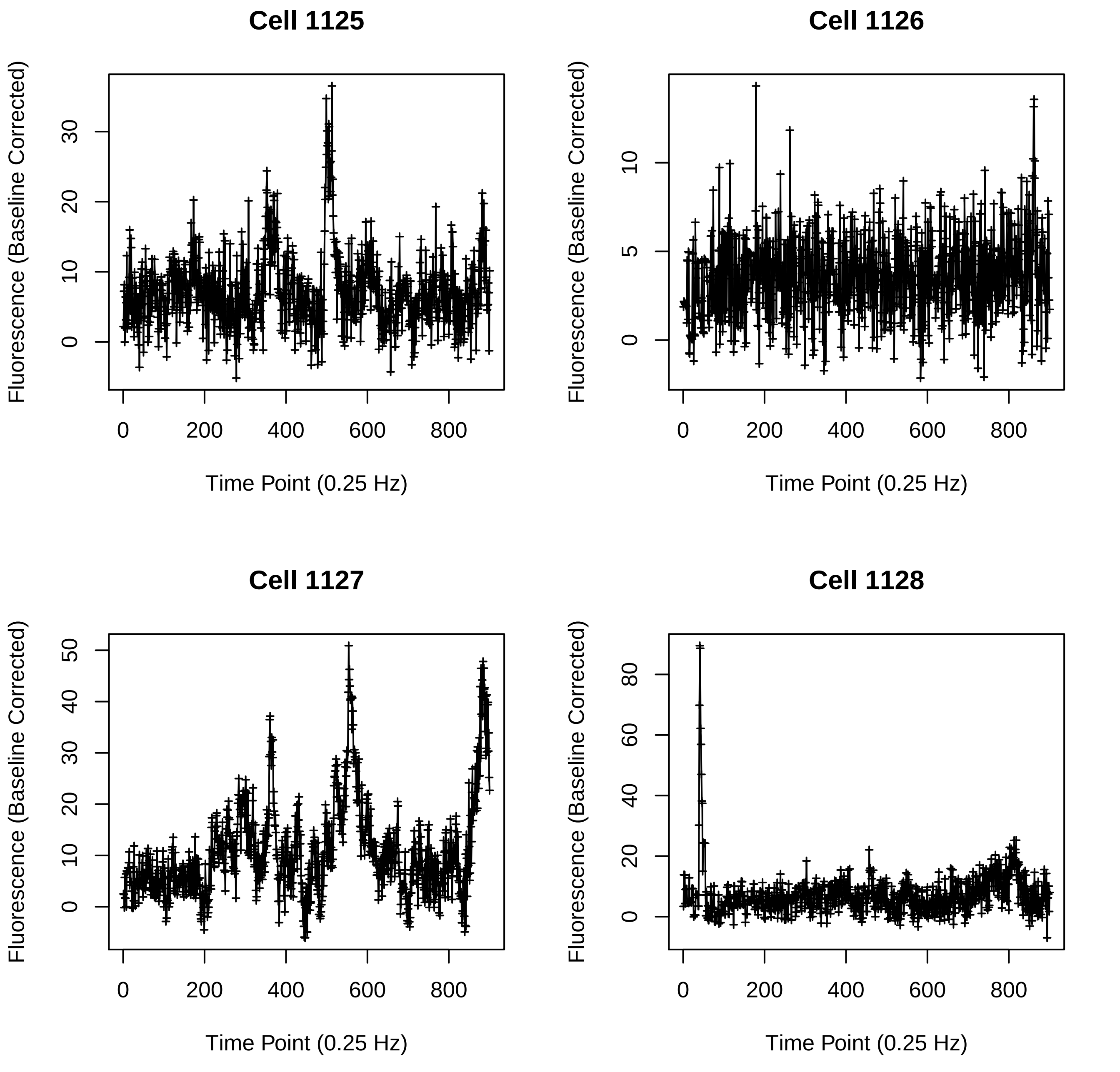
Cell 1125
Cell 1126
Cell 1127
Cell 1128
Fluorescence (Baseline Corrected)
Time Point (0.25 Hz)
0
200
400
600
800
0
10
20
30
5
40
50
60
80

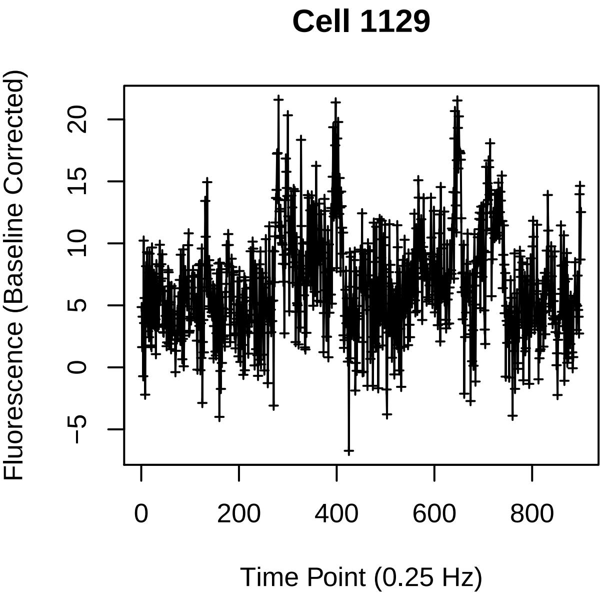
Cell 1129
Fluorescence (Baseline Corrected)
Time Point (0.25 Hz)
−5
0
5
10
15
20
0
200
400
600
800

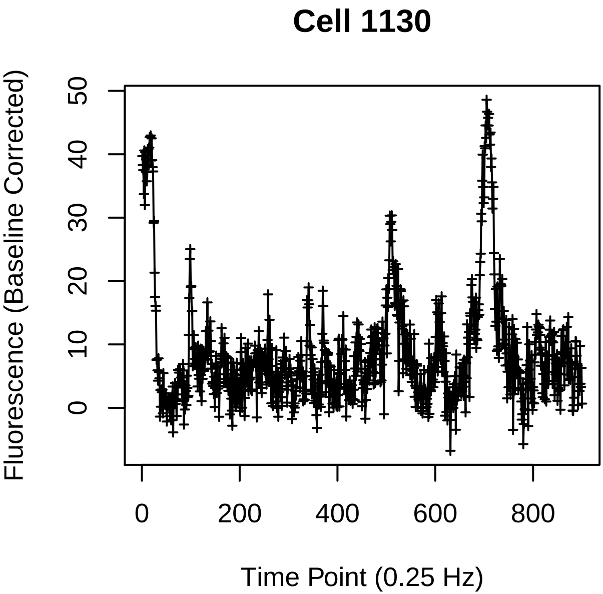
Cell 1130
Fluorescence (Baseline Corrected)
Time Point (0.25 Hz)
0
10
20
30
40
50
0
200
400
600
800

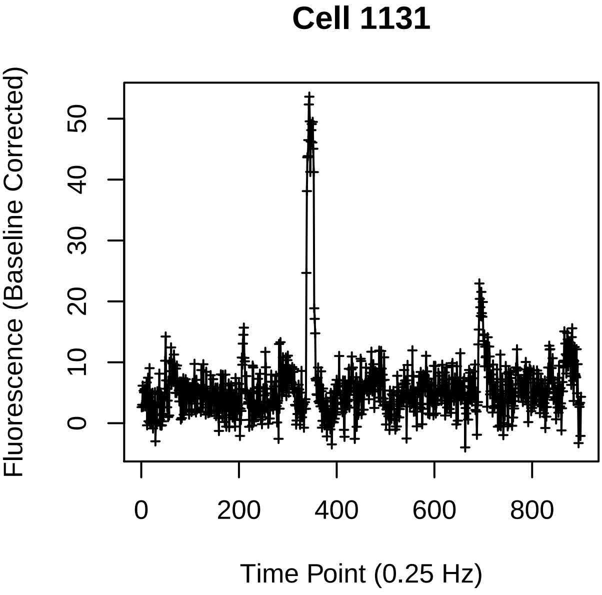
Cell 1131
Fluorescence (Baseline Corrected)
Time Point (0.25 Hz)
0
10
20
30
40
50
0
200
400
600
800

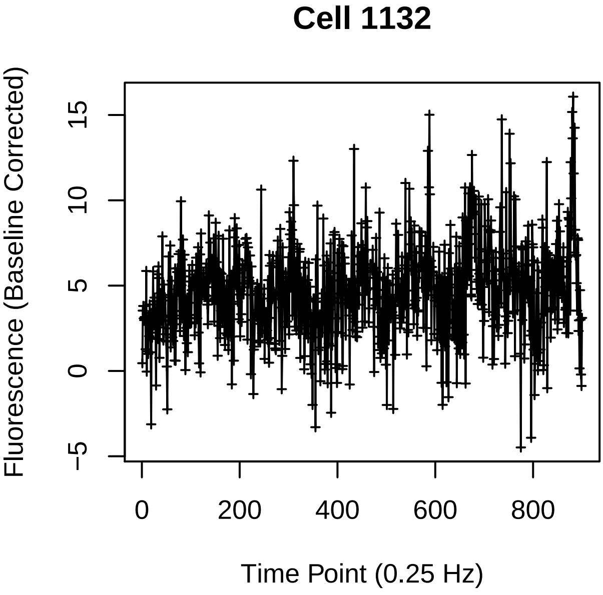
Cell 1132
Fluorescence (Baseline Corrected)
Time Point (0.25 Hz)
−5
0
5
10
15
0
200
400
600
800

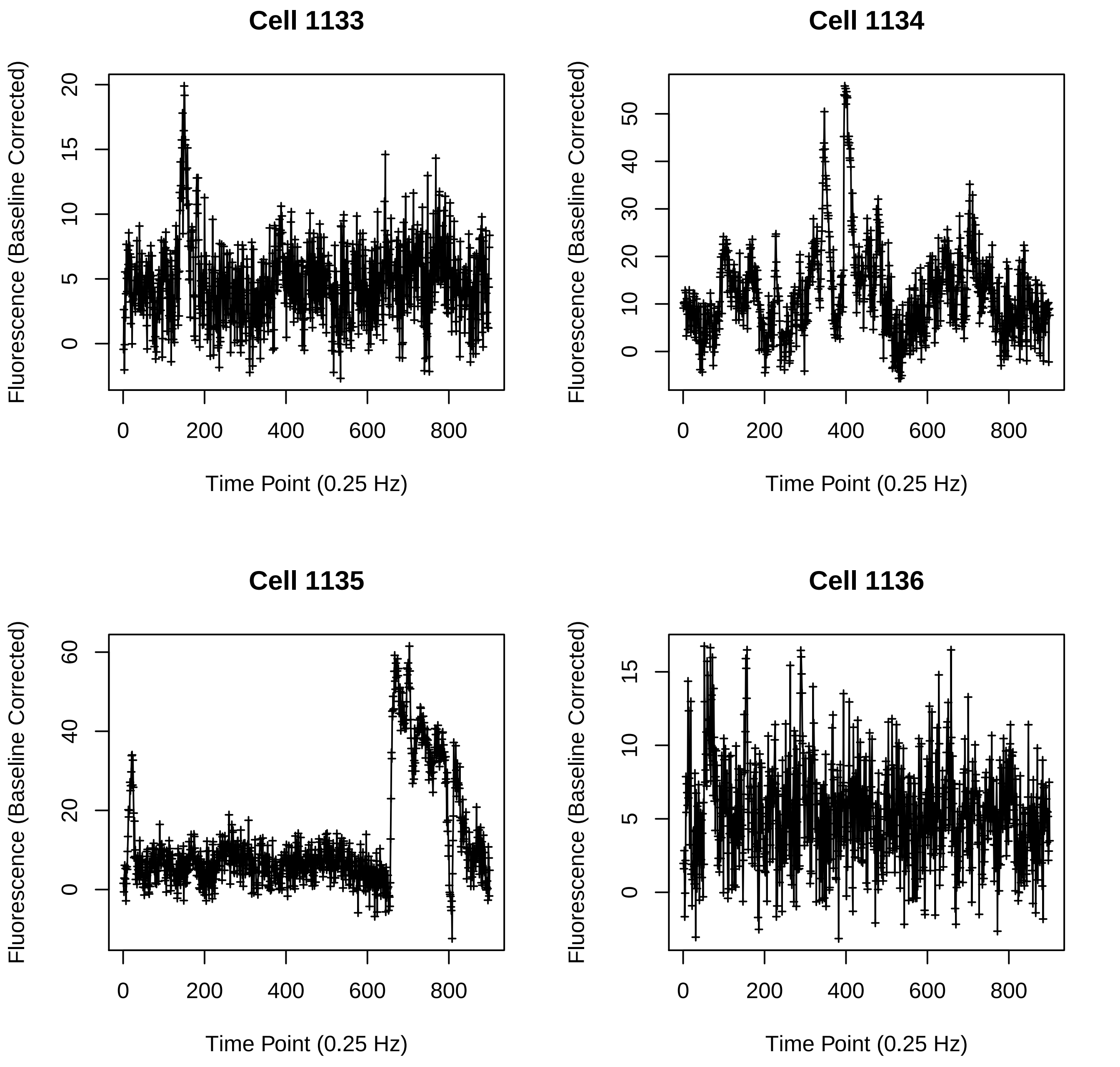
Cell 1133
Cell 1134
Cell 1135
Cell 1136
Fluorescence (Baseline Corrected)
Time Point (0.25 Hz)
0
200
400
600
800
5
10
15
20
30
40
50
60

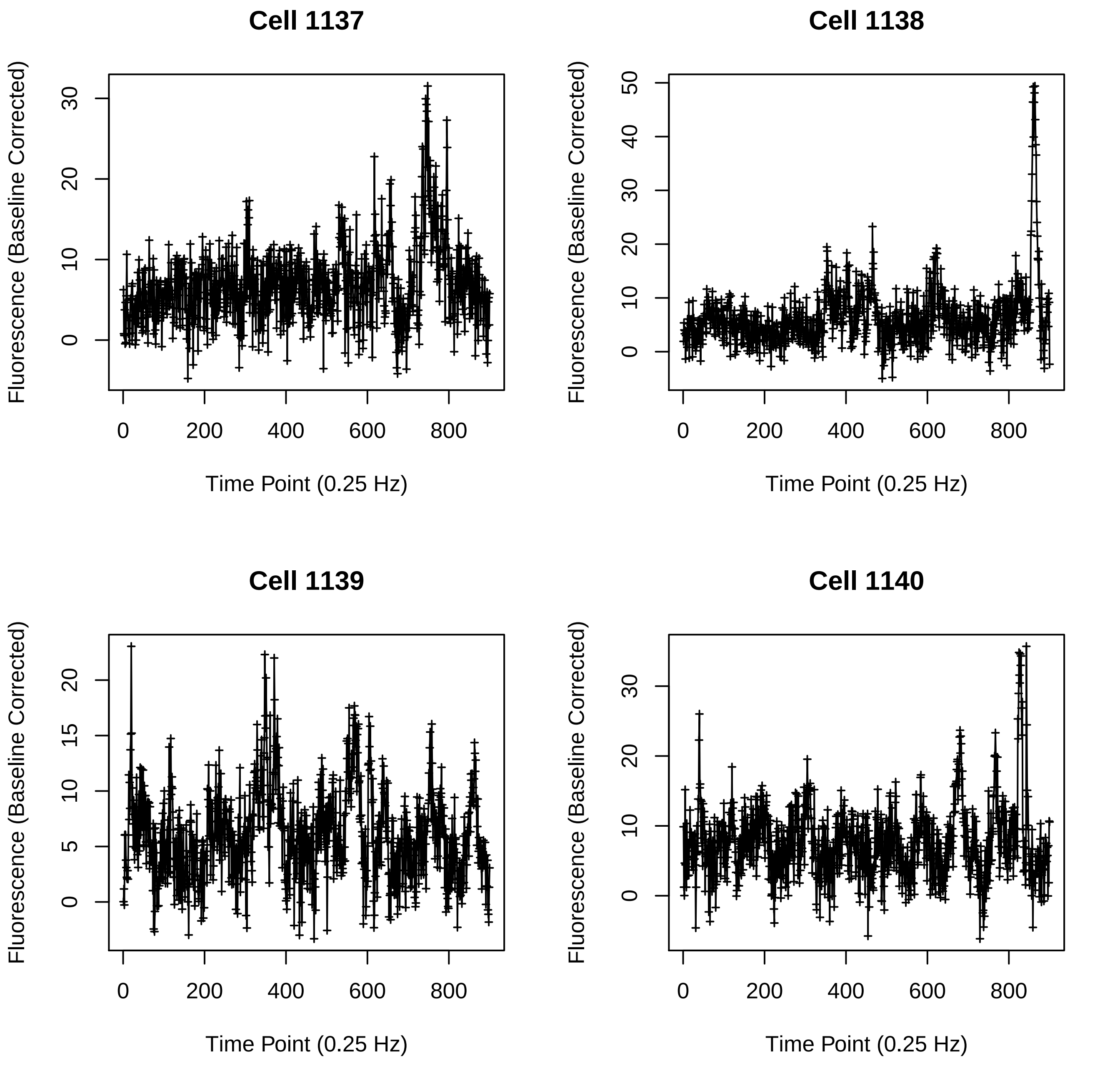
Cell 1137
Cell 1138
Cell 1139
Cell 1140
Fluorescence (Baseline Corrected)
Time Point (0.25 Hz)
0
200
400
600
800
0
10
20
30
40
50
5
15

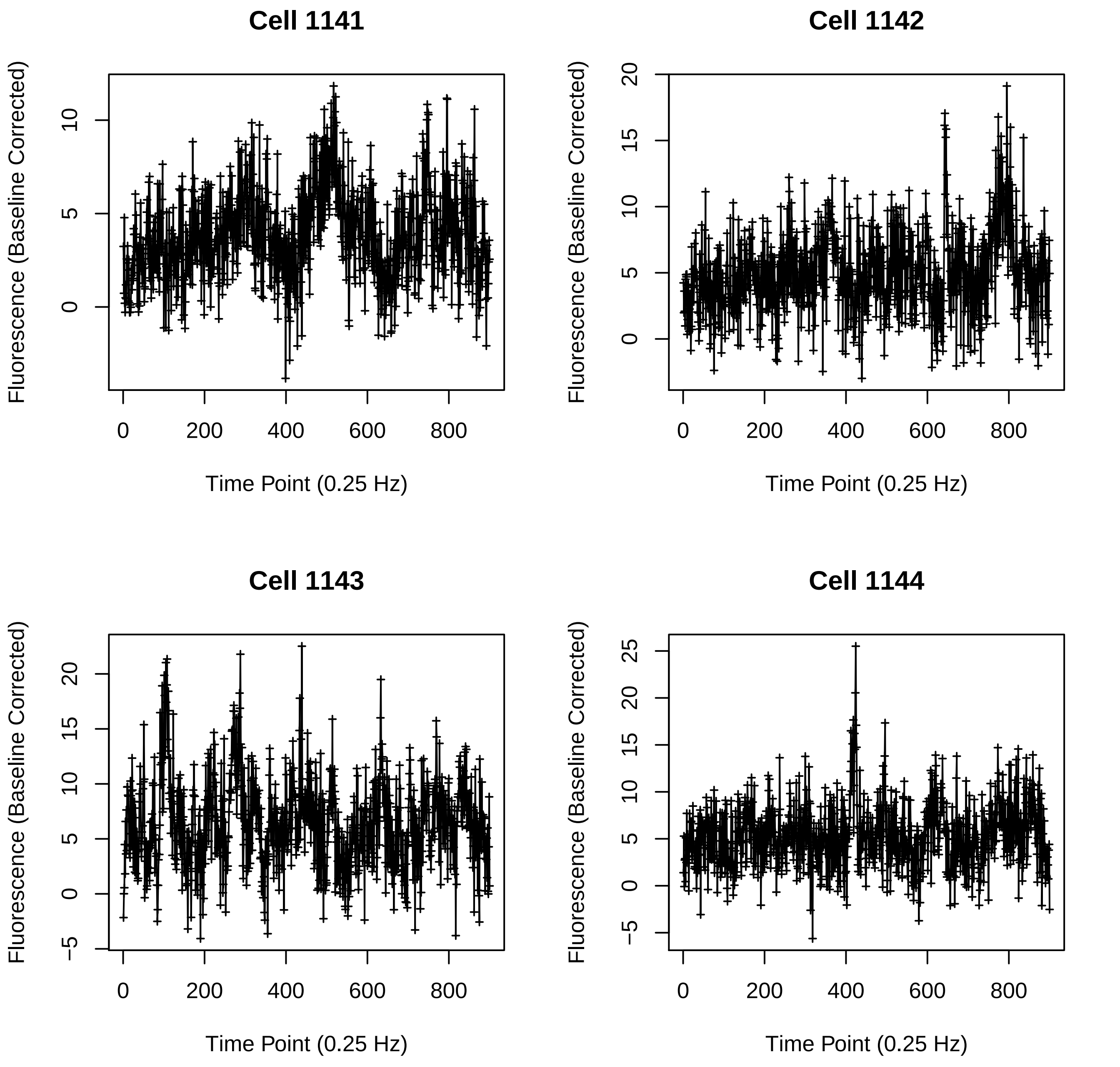
Cell 1141
Cell 1142
Cell 1143
Cell 1144
Fluorescence (Baseline Corrected)
Time Point (0.25 Hz)
0
200
400
600
800
-5
0
5
10
15
20
25

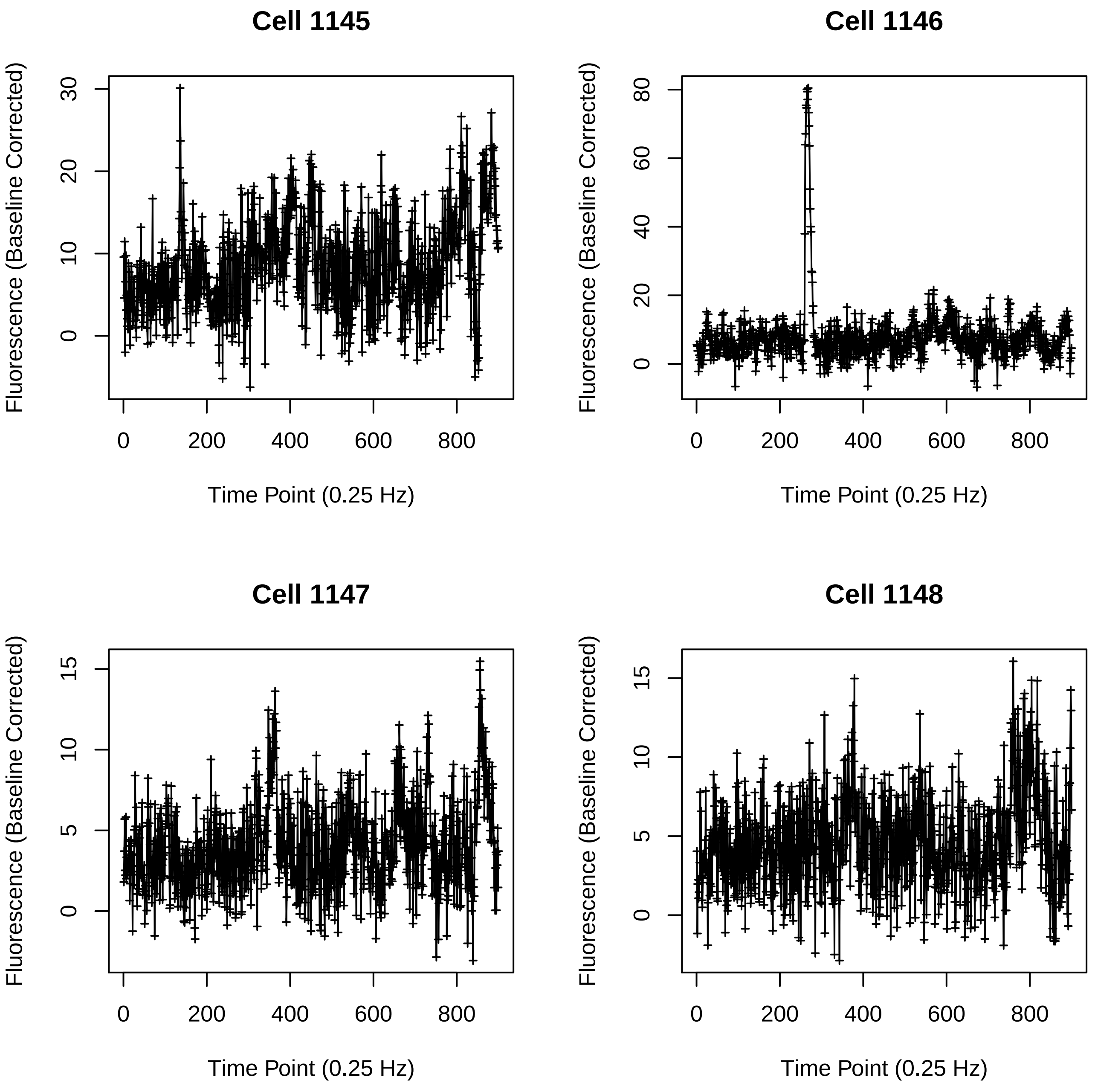

Cell 1145
Cell 1146
Cell 1147
Cell 1148
Fluorescence (Baseline Corrected)
Time Point (0.25 Hz)
0
200
400
600
800
10
20
30
40
60
80
5
15

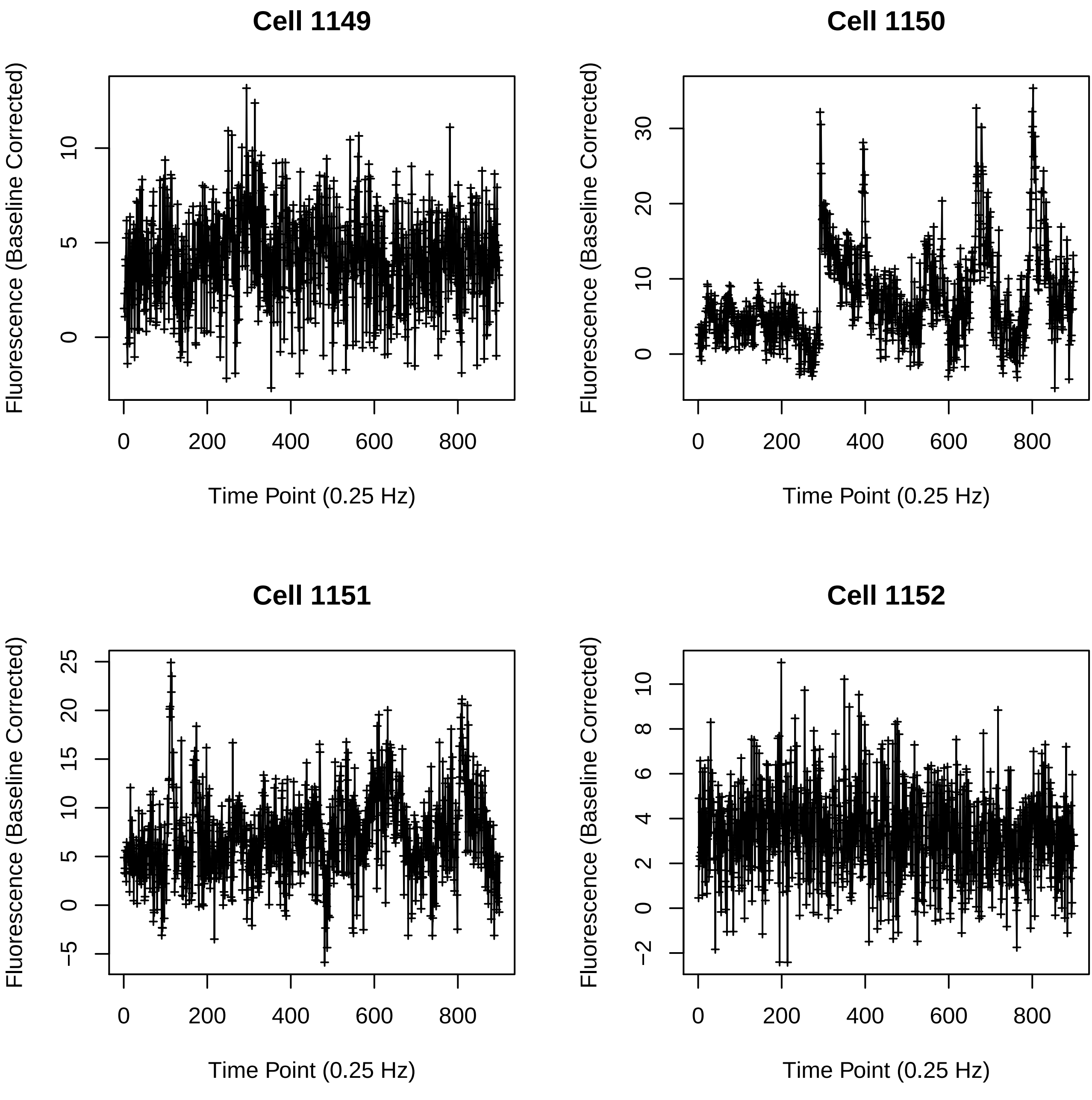
Cell 1149
Cell 1150
Cell 1151
Cell 1152
Fluorescence (Baseline Corrected)
Time Point (0.25 Hz)
0
200
400
600
800
−5
0
5
10
15
20
25
30
−2
2
4
6
8

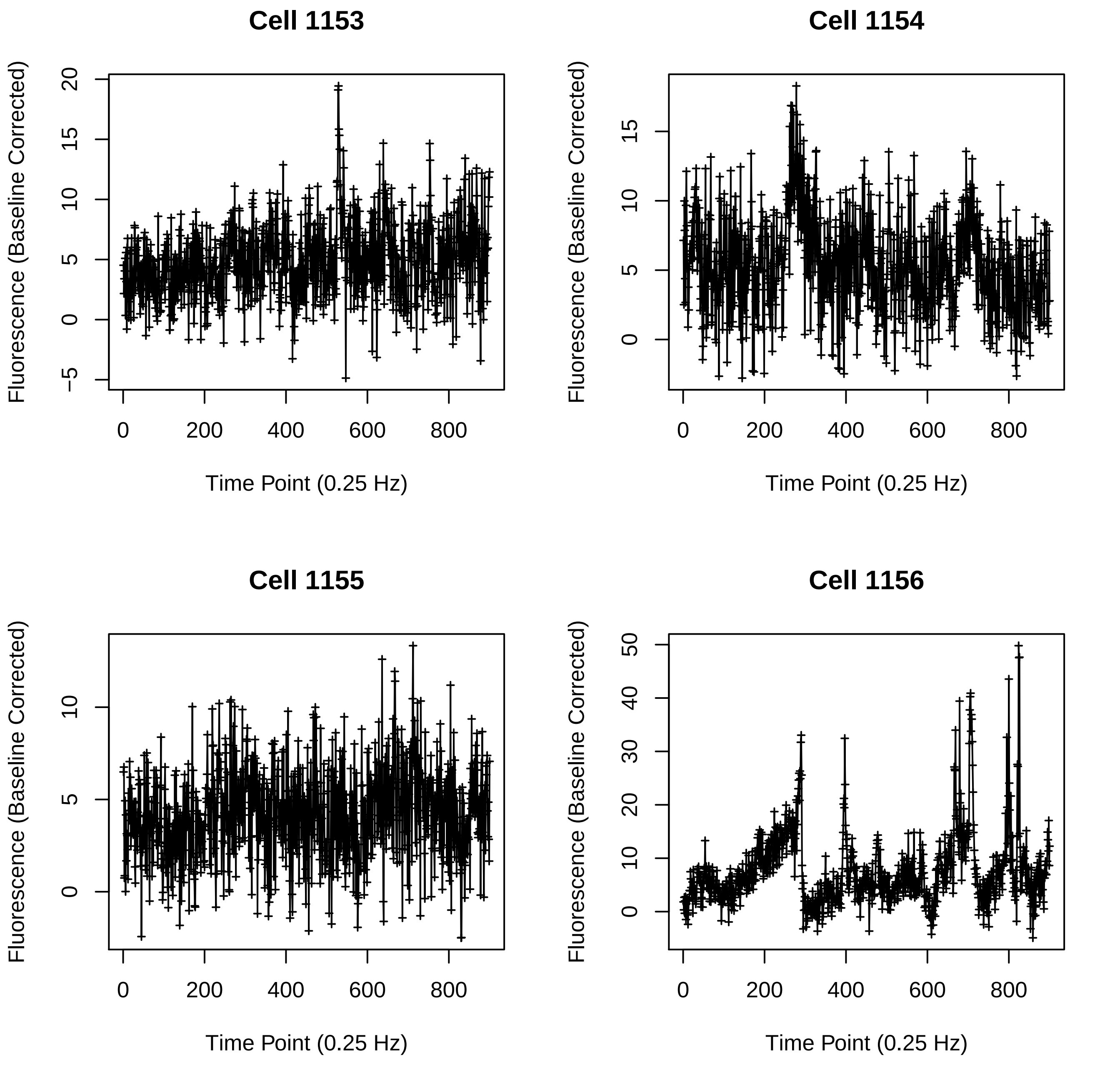
Cell 1153
Fluorescence (Baseline Corrected)
−5
0
5
10
15
20
0
200
400
600
800
Time Point (0.25 Hz)
Cell 1154
Fluorescence (Baseline Corrected)
0
5
10
15
0
200
400
600
800
Time Point (0.25 Hz)
Cell 1155
Fluorescence (Baseline Corrected)
0
5
10
0
200
400
600
800
Time Point (0.25 Hz)
Cell 1156
Fluorescence (Baseline Corrected)
0
10
20
30
40
50
0
200
400
600
800
Time Point (0.25 Hz)

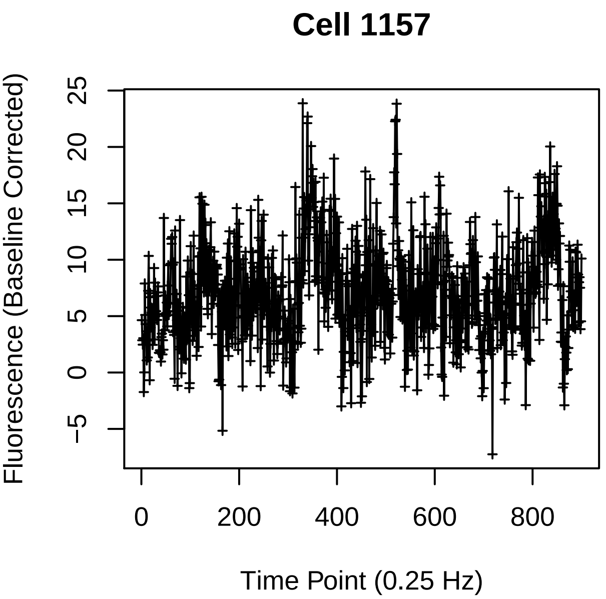
Cell 1157
Fluorescence (Baseline Corrected)
−5 0 5 10 15 20 25
0 200 400 600 800
Time Point (0.25 Hz)

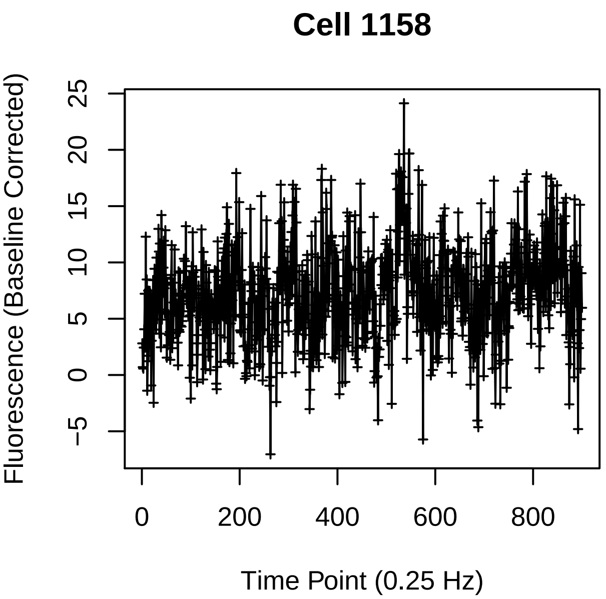
Cell 1158
Fluorescence (Baseline Corrected)
−5 0 5 10 15 20 25
0 200 400 600 800
Time Point (0.25 Hz)

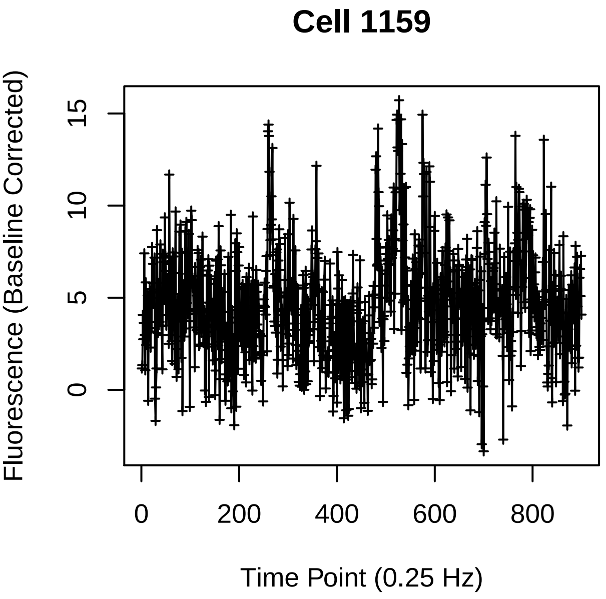
Cell 1159
Fluorescence (Baseline Corrected)
0 5 10 15
0 200 400 600 800
Time Point (0.25 Hz)

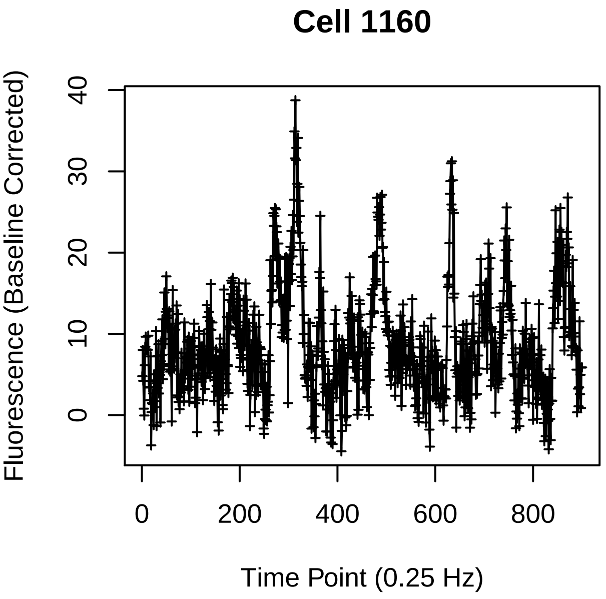
Cell 1160
Fluorescence (Baseline Corrected)
0 10 20 30 40
0 200 400 600 800
Time Point (0.25 Hz)

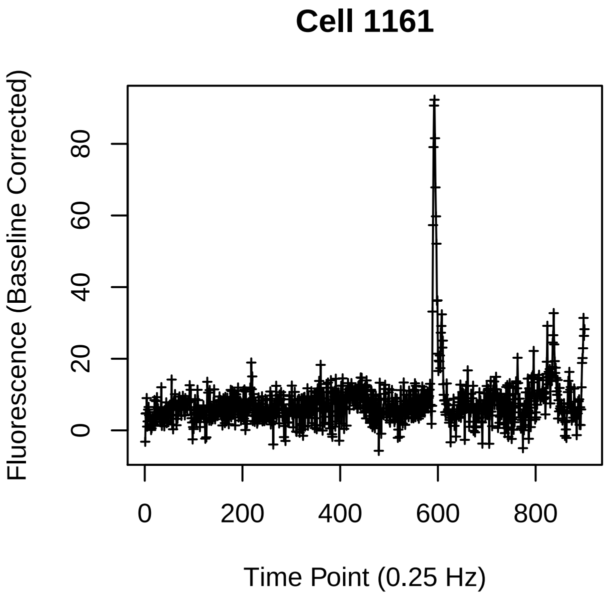
Cell 1161
Fluorescence (Baseline Corrected)
0
20
40
60
80
0
200
400
600
800
Time Point (0.25 Hz)

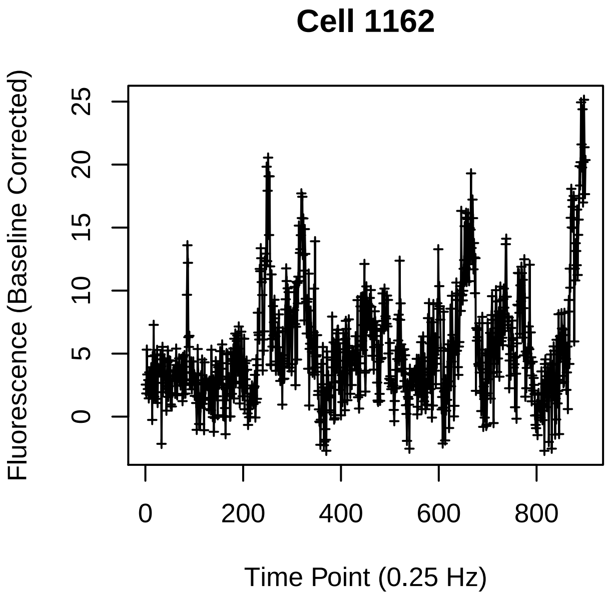
Cell 1162
Fluorescence (Baseline Corrected)
0
5
10
15
20
25
0
200
400
600
800
Time Point (0.25 Hz)

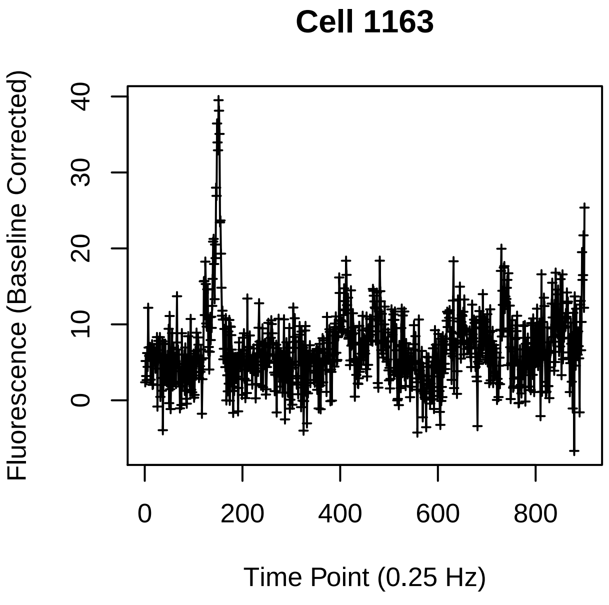
Cell 1163
Fluorescence (Baseline Corrected)
0
10
20
30
40
0
200
400
600
800
Time Point (0.25 Hz)

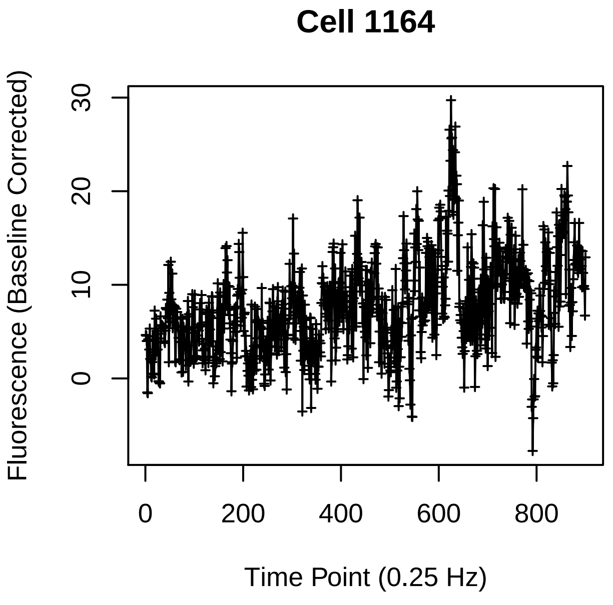
Cell 1164
Fluorescence (Baseline Corrected)
0
10
20
30
0
200
400
600
800
Time Point (0.25 Hz)

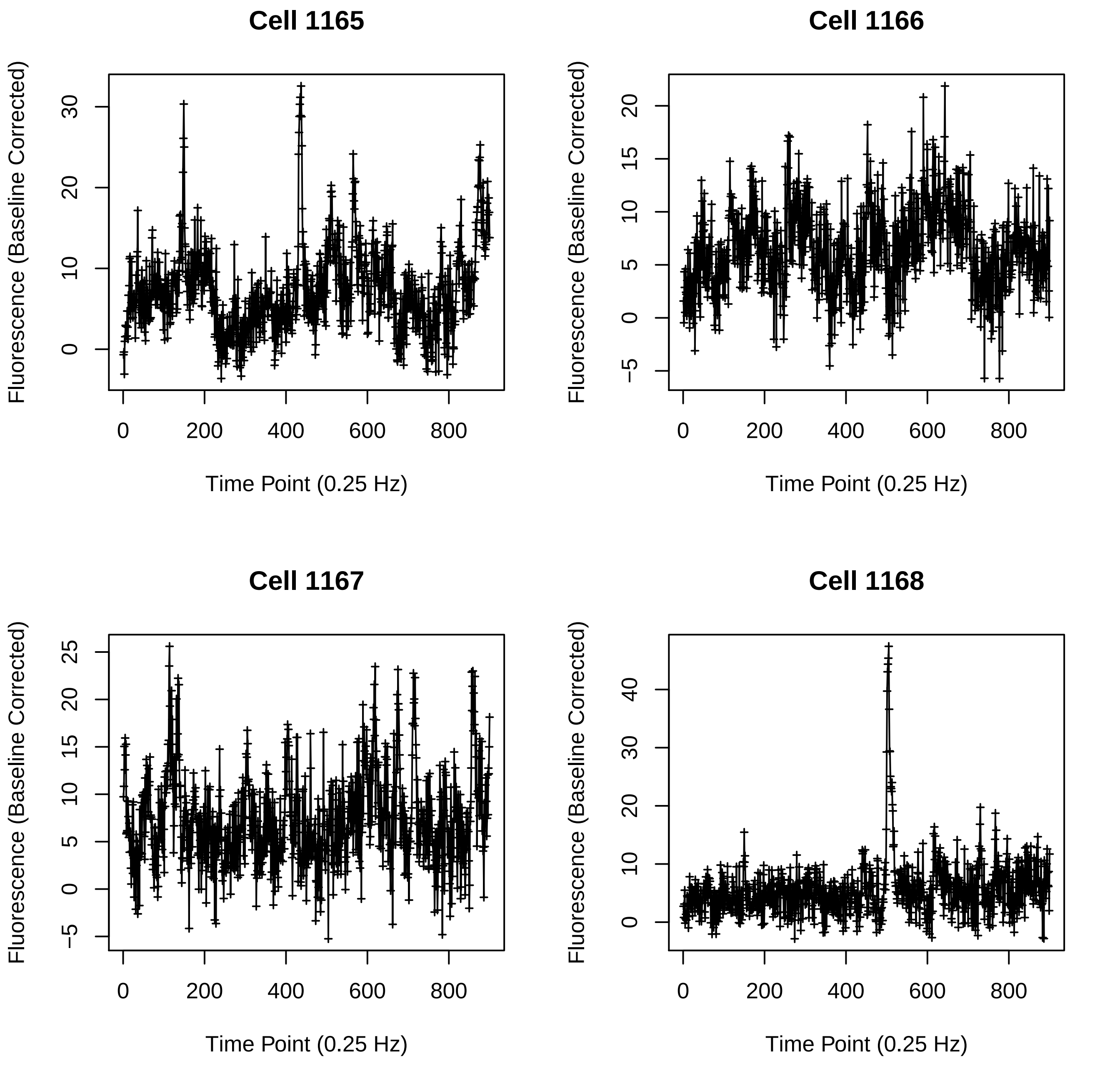
Cell 1165
Cell 1166
Cell 1167
Cell 1168
Fluorescence (Baseline Corrected)
Time Point (0.25 Hz)
0
10
20
30
−5
5
15
25
40
200
400
600
800

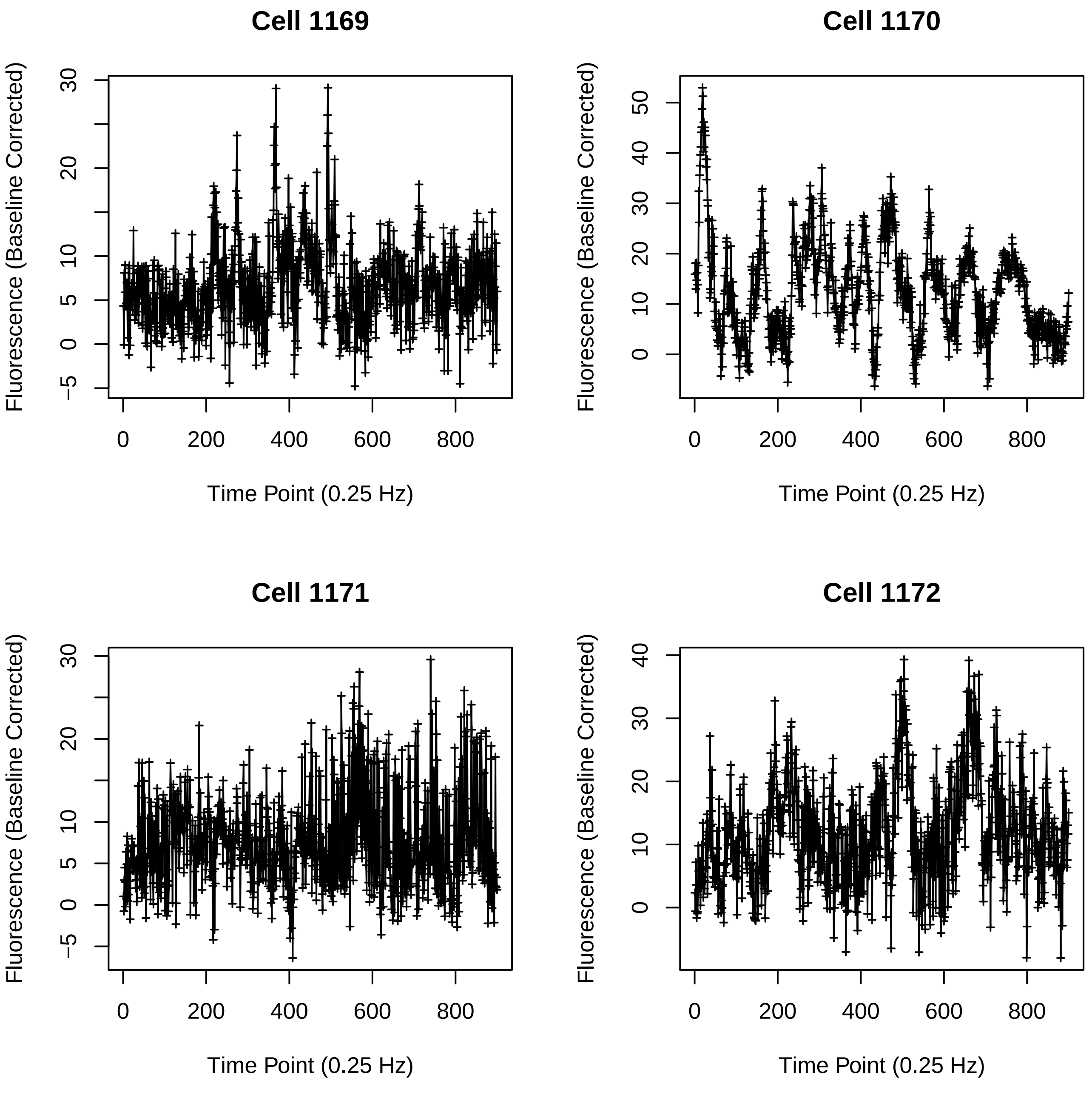
Cell 1169
Cell 1170
Cell 1171
Cell 1172
Fluorescence (Baseline Corrected)
Time Point (0.25 Hz)
−5
0
5
10
20
30
40
50
0
200
400
600
800

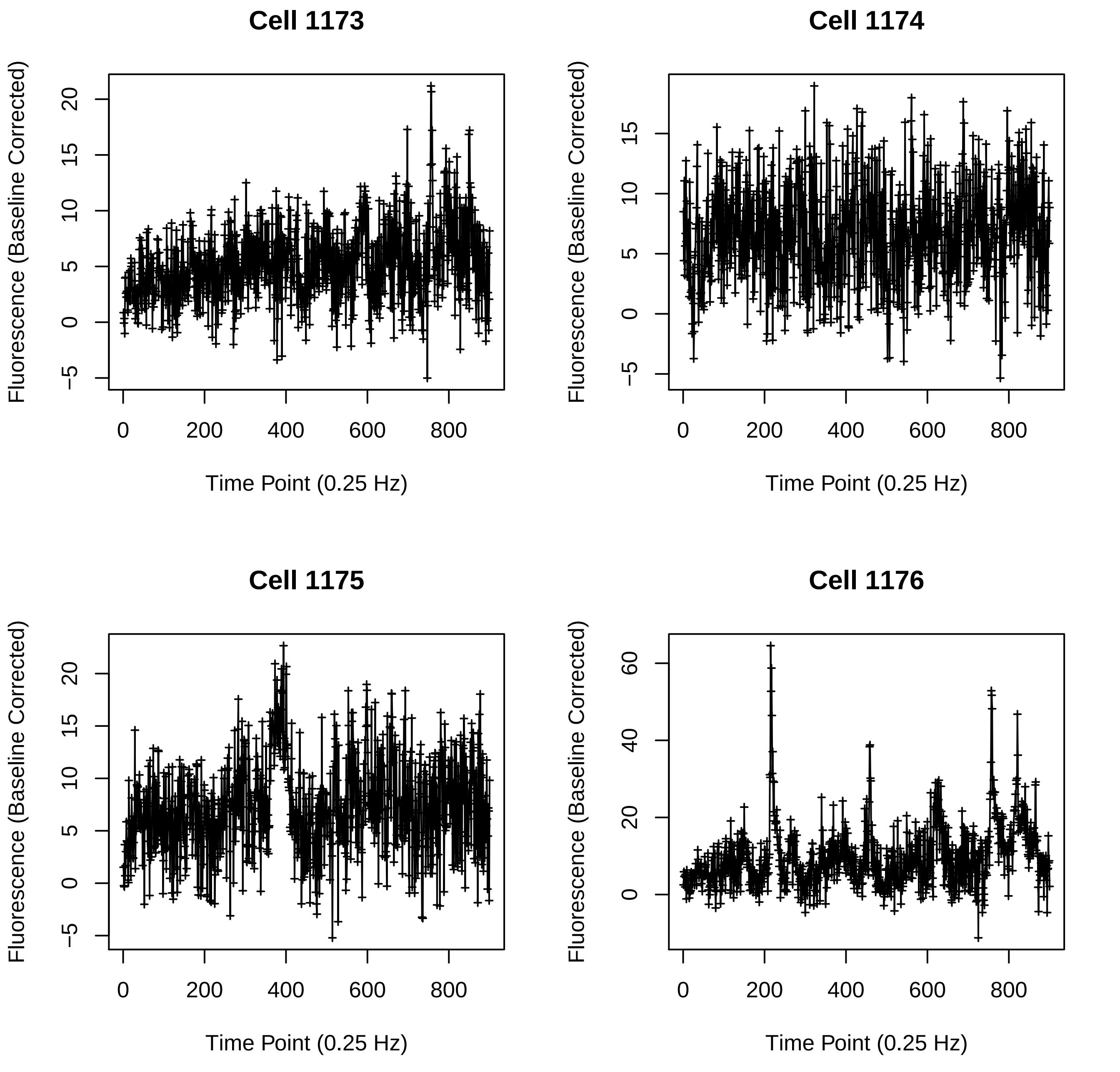

Cell 1173
Cell 1174
Cell 1175
Cell 1176
Fluorescence (Baseline Corrected)
Time Point (0.25 Hz)

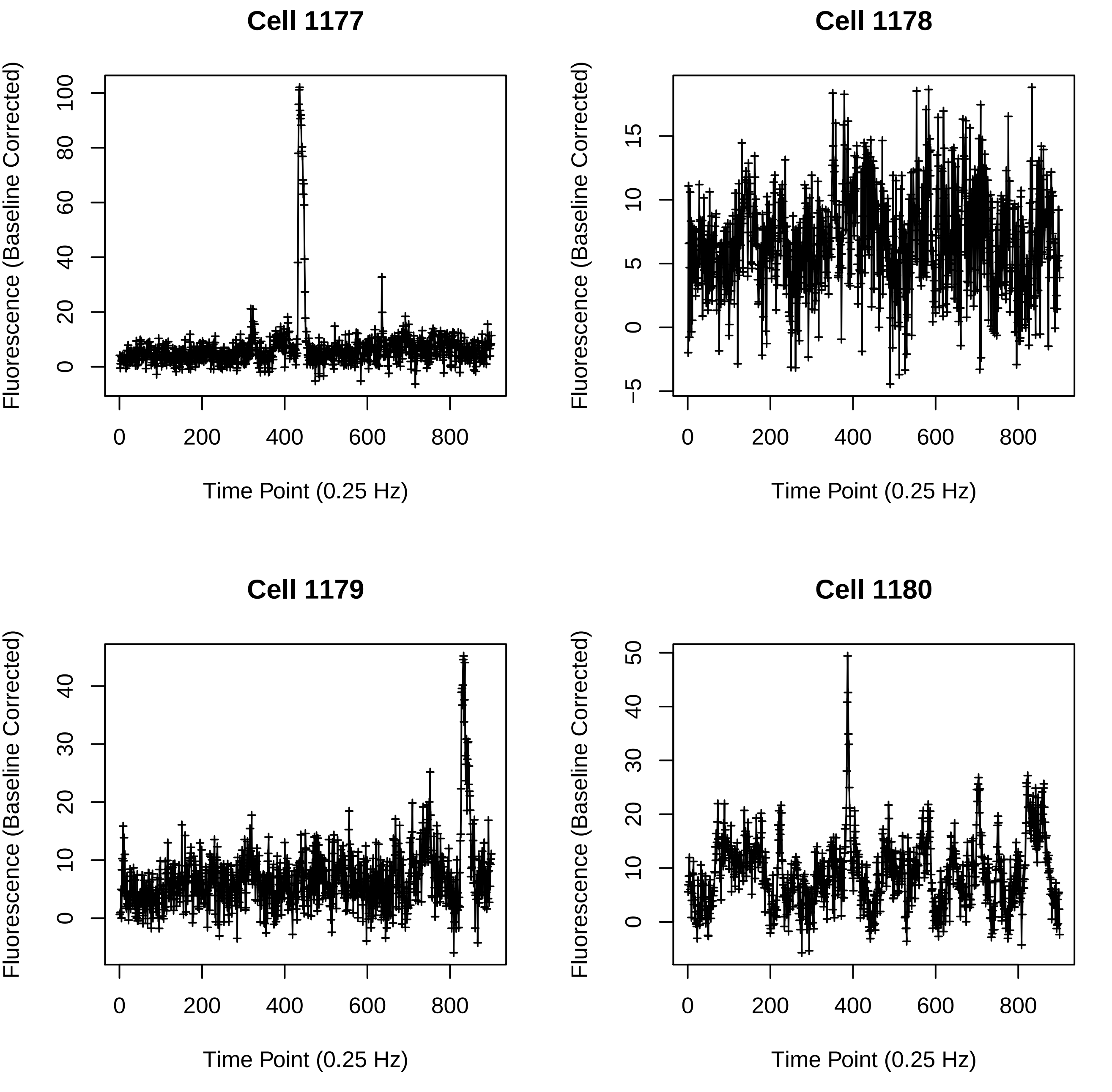

Cell 1177
Cell 1178
Cell 1179
Cell 1180
Fluorescence (Baseline Corrected)
Time Point (0.25 Hz)
0
200
400
600
800
0
20
40
60
80
100
−5
5
10
15
30
50

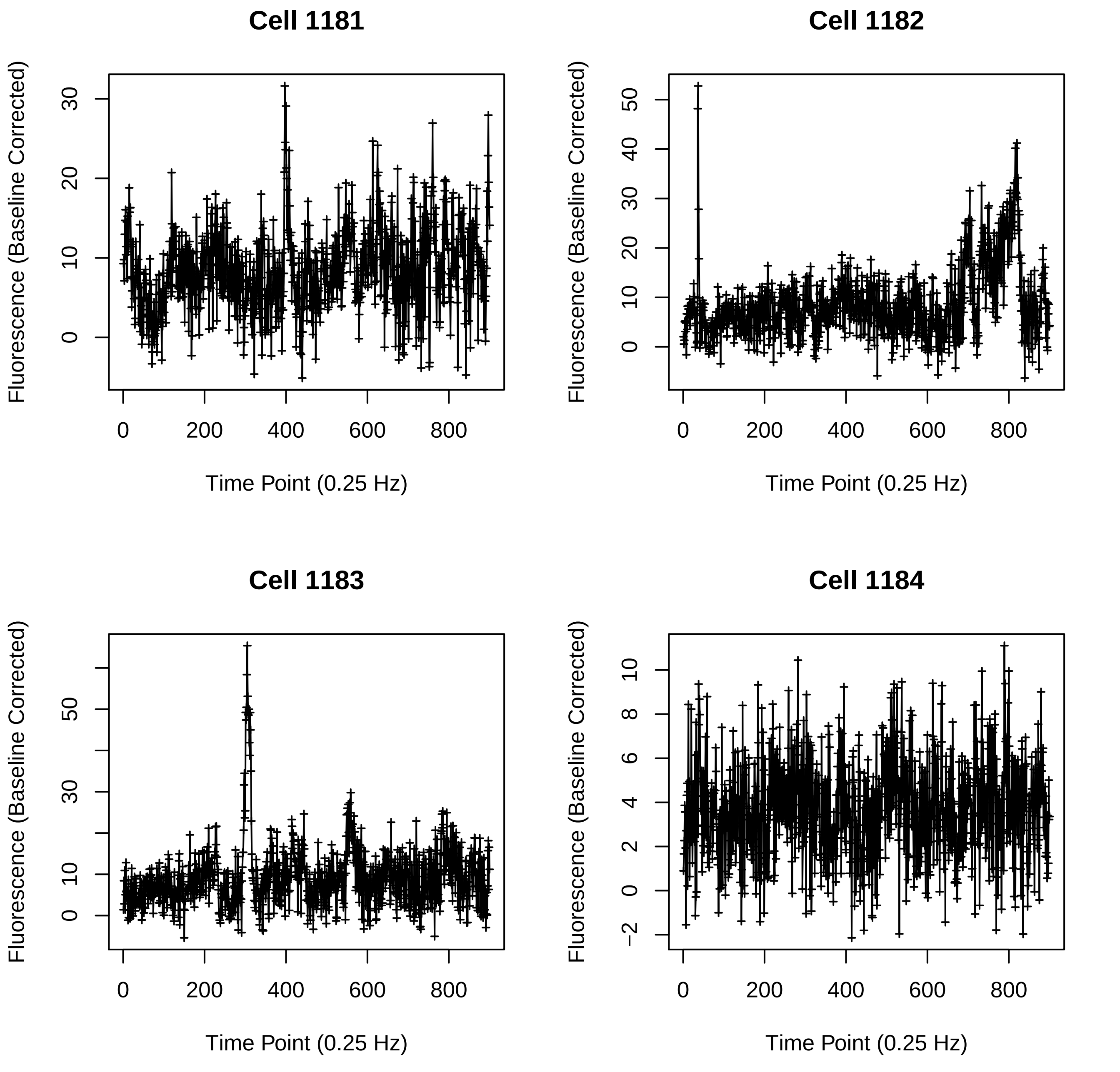
Cell 1181
Cell 1182
Cell 1183
Cell 1184
Fluorescence (Baseline Corrected)
Time Point (0.25 Hz)
0
200
400
600
800
0
10
20
30
40
50
−2
2
4
6
8

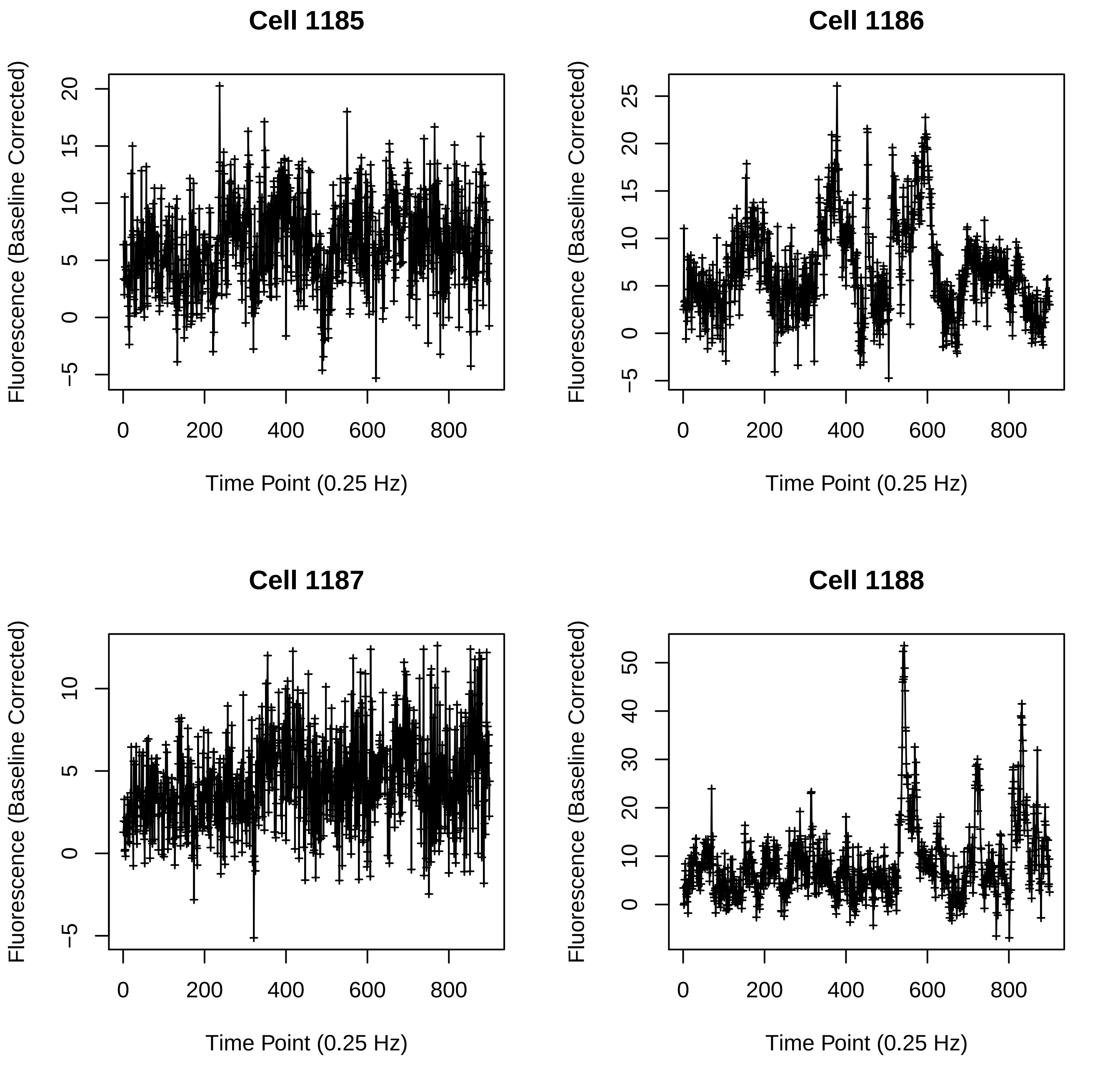
Cell 1185
Cell 1186
Cell 1187
Cell 1188
Fluorescence (Baseline Corrected)
Time Point (0.25 Hz)
−5
0
5
10
15
20
25
30
40
50
0
200
400
600
800

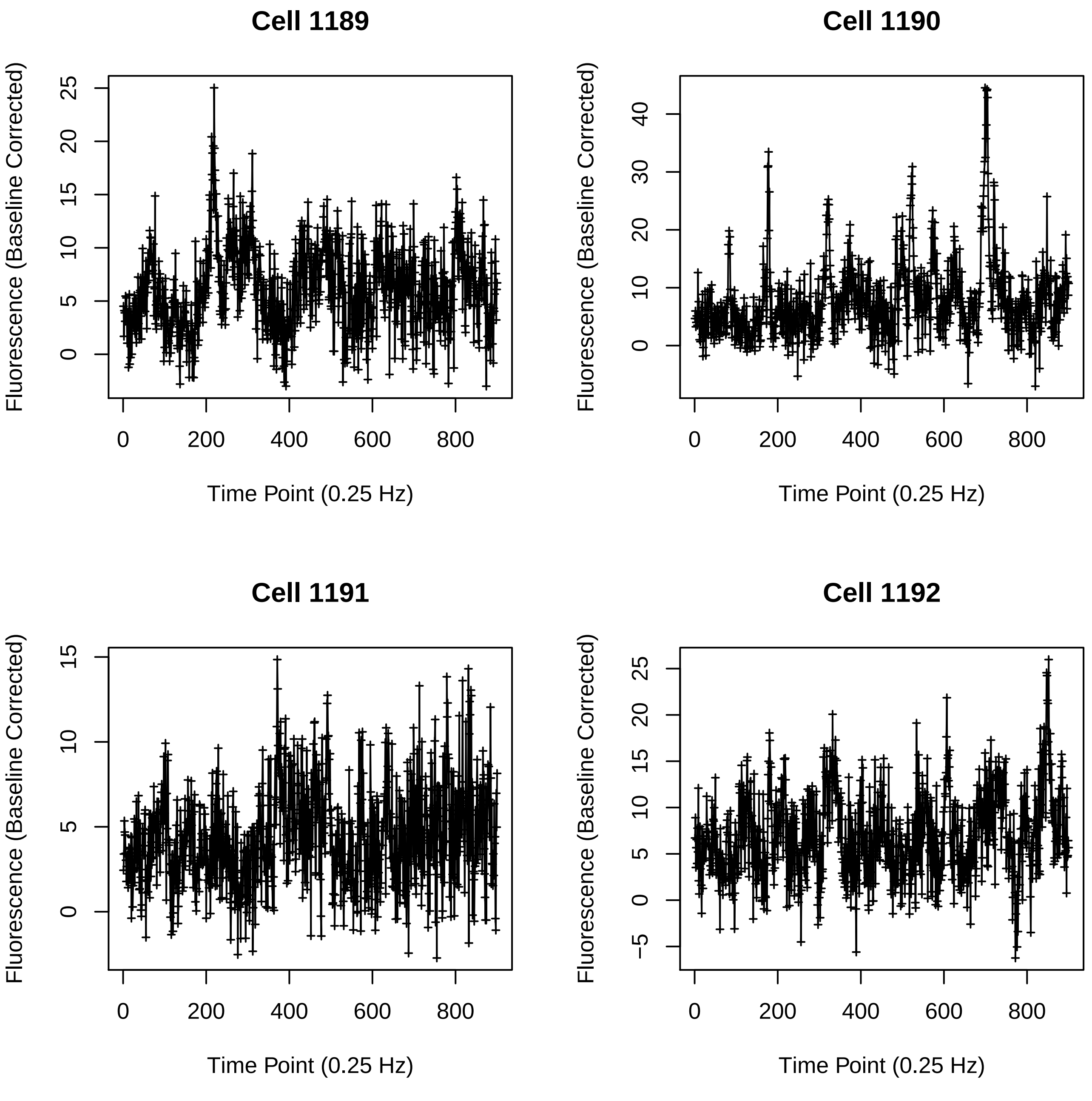
Cell 1189
Fluorescence (Baseline Corrected)
0
5
10
15
20
25
0
200
400
600
800
Time Point (0.25 Hz)
Cell 1190
Fluorescence (Baseline Corrected)
0
10
20
30
40
0
200
400
600
800
Time Point (0.25 Hz)
Cell 1191
Fluorescence (Baseline Corrected)
0
5
10
15
0
200
400
600
800
Time Point (0.25 Hz)
Cell 1192
Fluorescence (Baseline Corrected)
−5
0
5
10
15
20
25
0
200
400
600
800
Time Point (0.25 Hz)

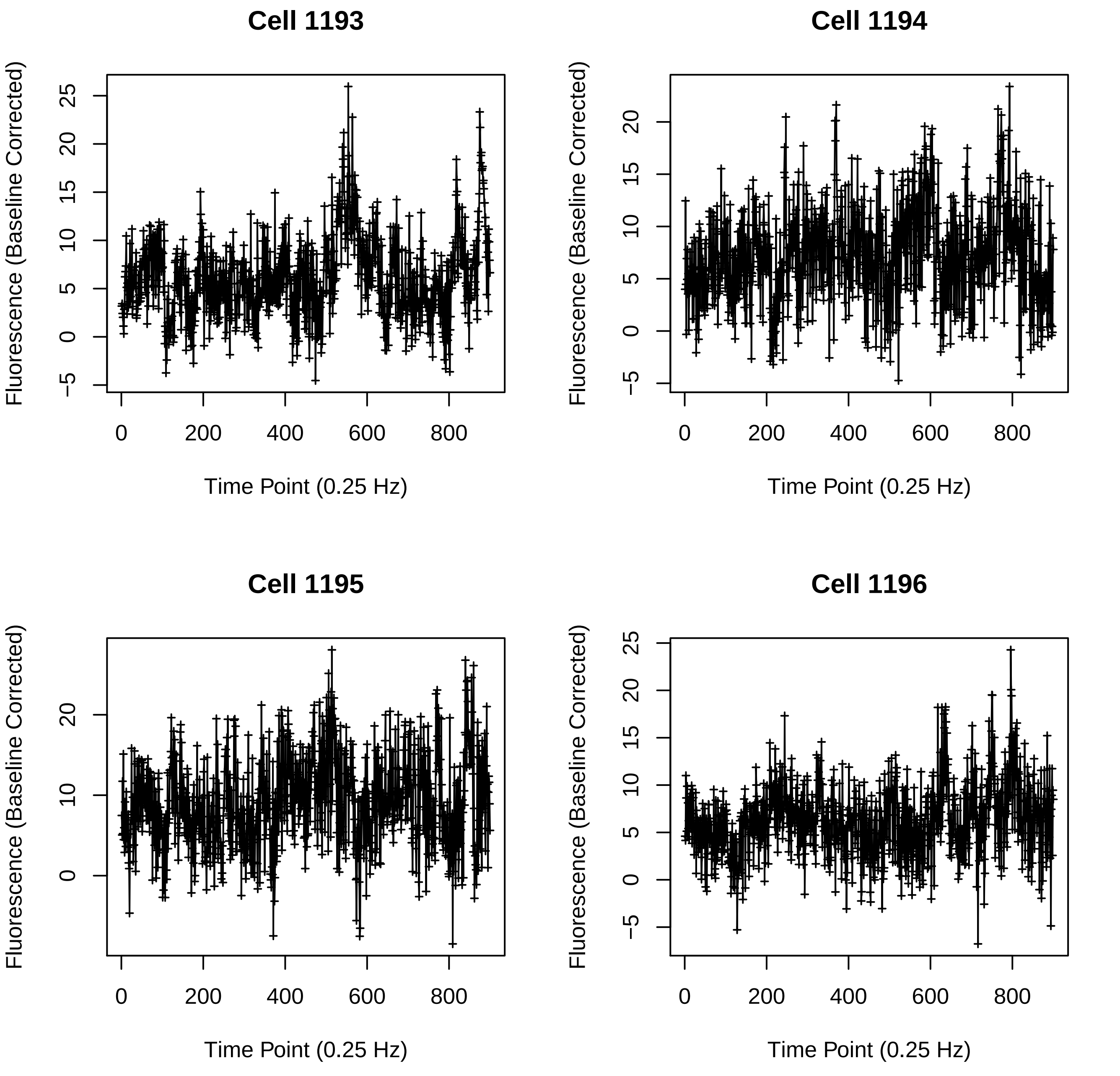
Cell 1193
Cell 1194
Cell 1195
Cell 1196
Fluorescence (Baseline Corrected)
Time Point (0.25 Hz)
-5
0
5
10
15
20
25
0
200
400
600
800

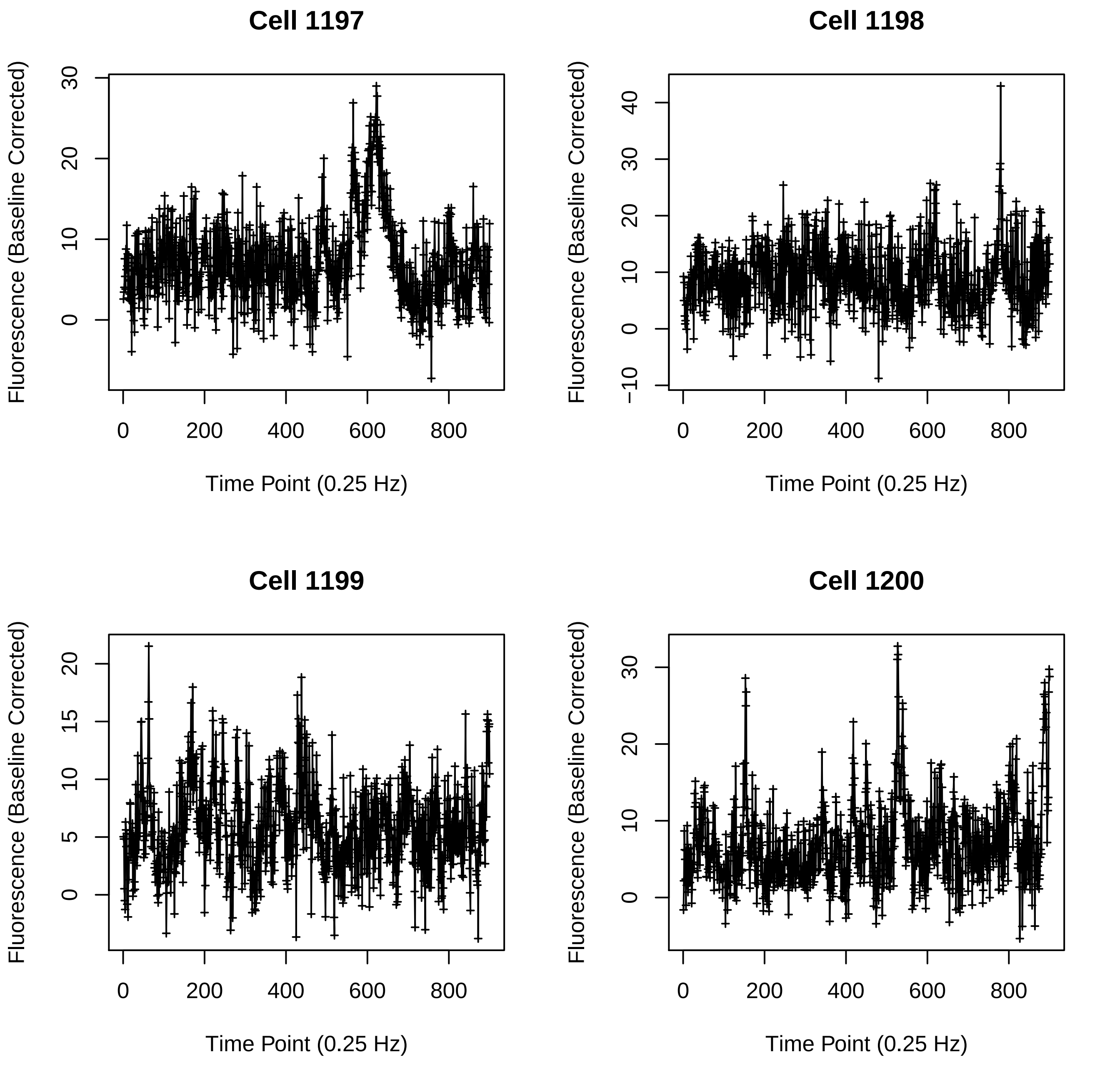

Cell 1197
Cell 1198
Cell 1199
Cell 1200
Fluorescence (Baseline Corrected)
Time Point (0.25 Hz)
0
200
400
600
800
-10
0
5
10
15
20
30
40

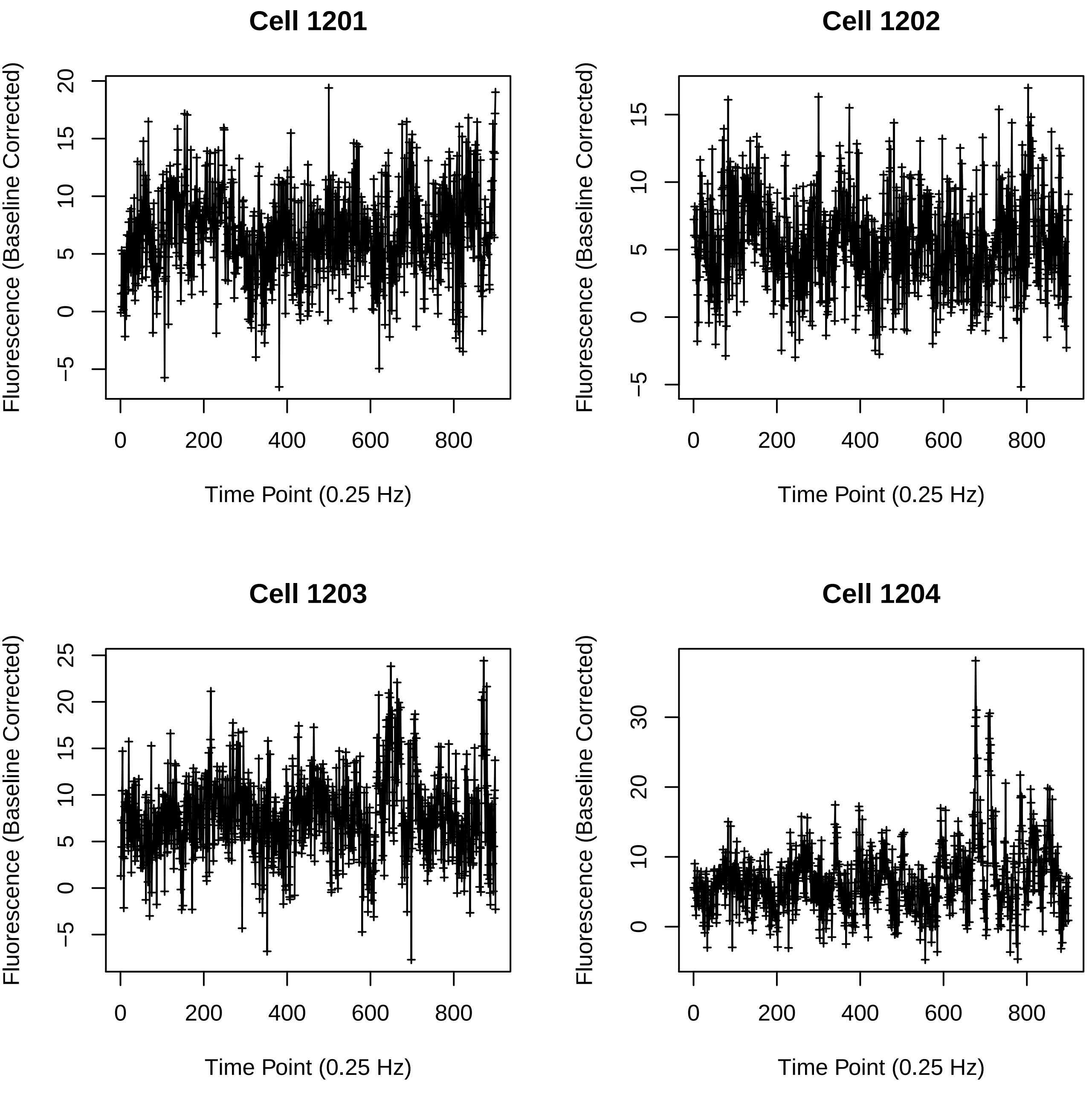
Cell 1201
Cell 1202
Cell 1203
Cell 1204
Fluorescence (Baseline Corrected)
Time Point (0.25 Hz)
0
200
400
600
800
−5
5
10
15
20
25
30

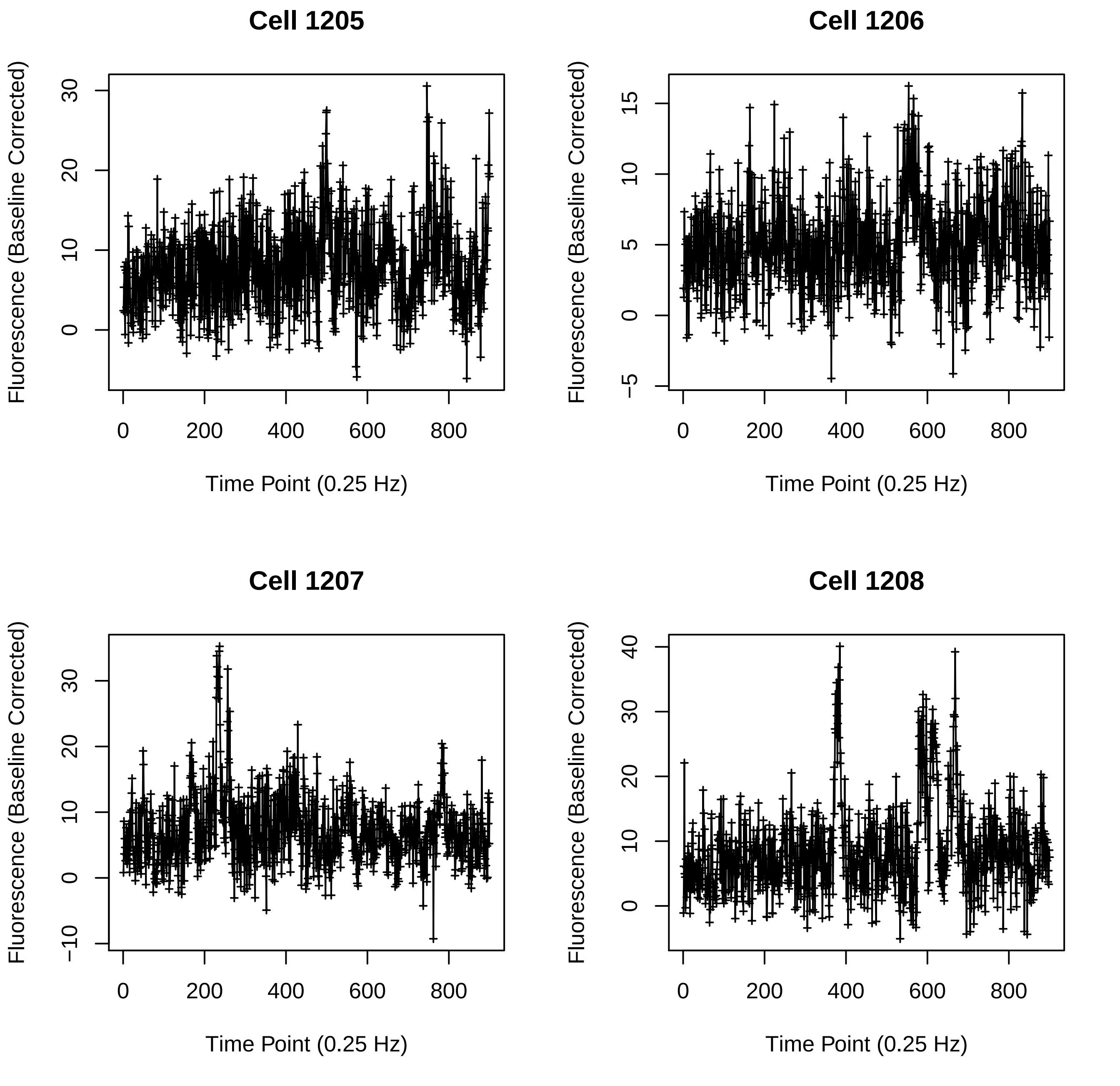
Cell 1205
Cell 1206
Cell 1207
Cell 1208
Fluorescence (Baseline Corrected)
Time Point (0.25 Hz)
0
200
400
600
800
-10
-5
0
5
10
15
20
30
40

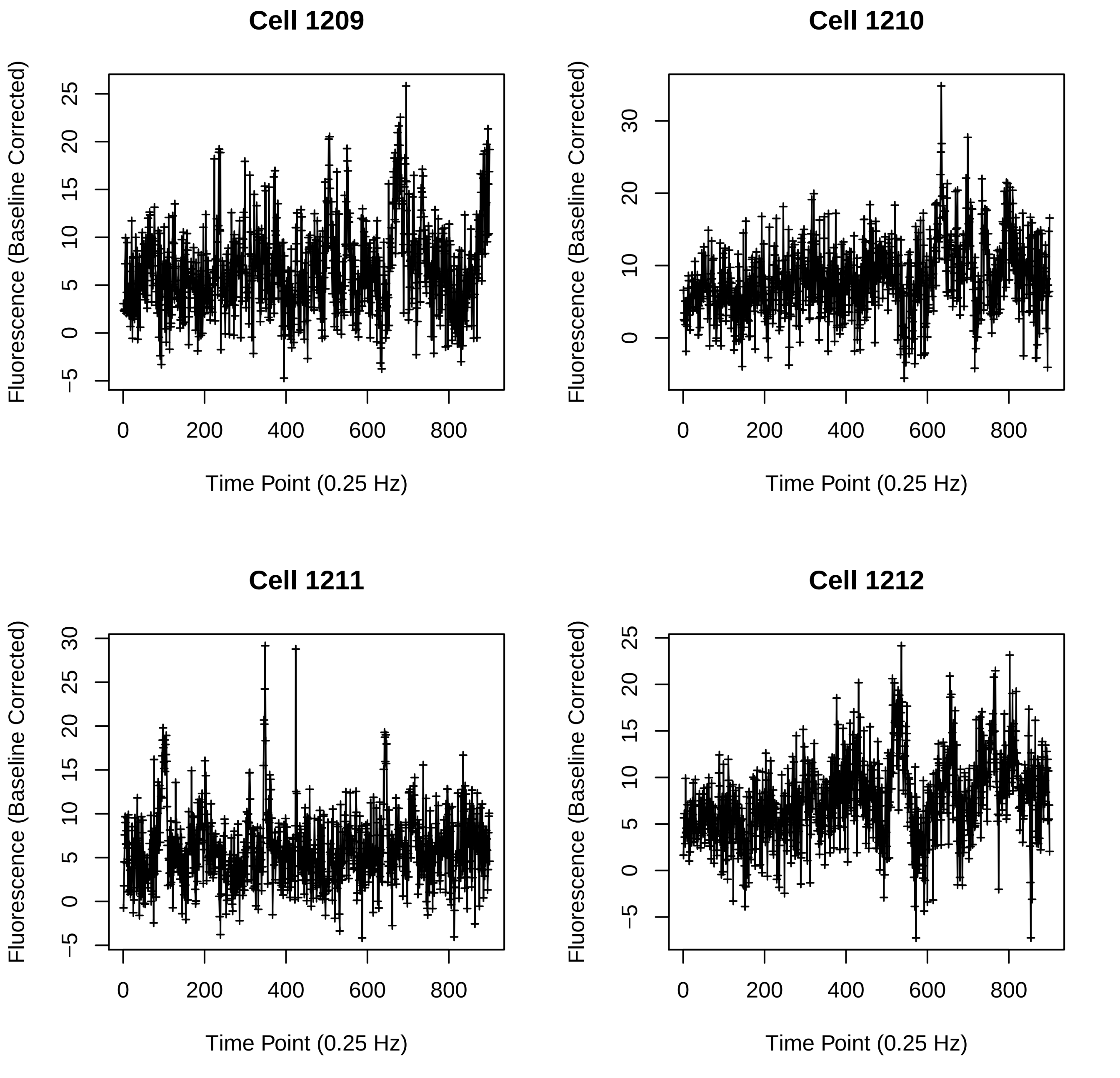
Cell 1209
Cell 1210
Cell 1211
Cell 1212
Fluorescence (Baseline Corrected)
Time Point (0.25 Hz)
0
200
400
600
800
−5
0
5
10
15
20
25
30

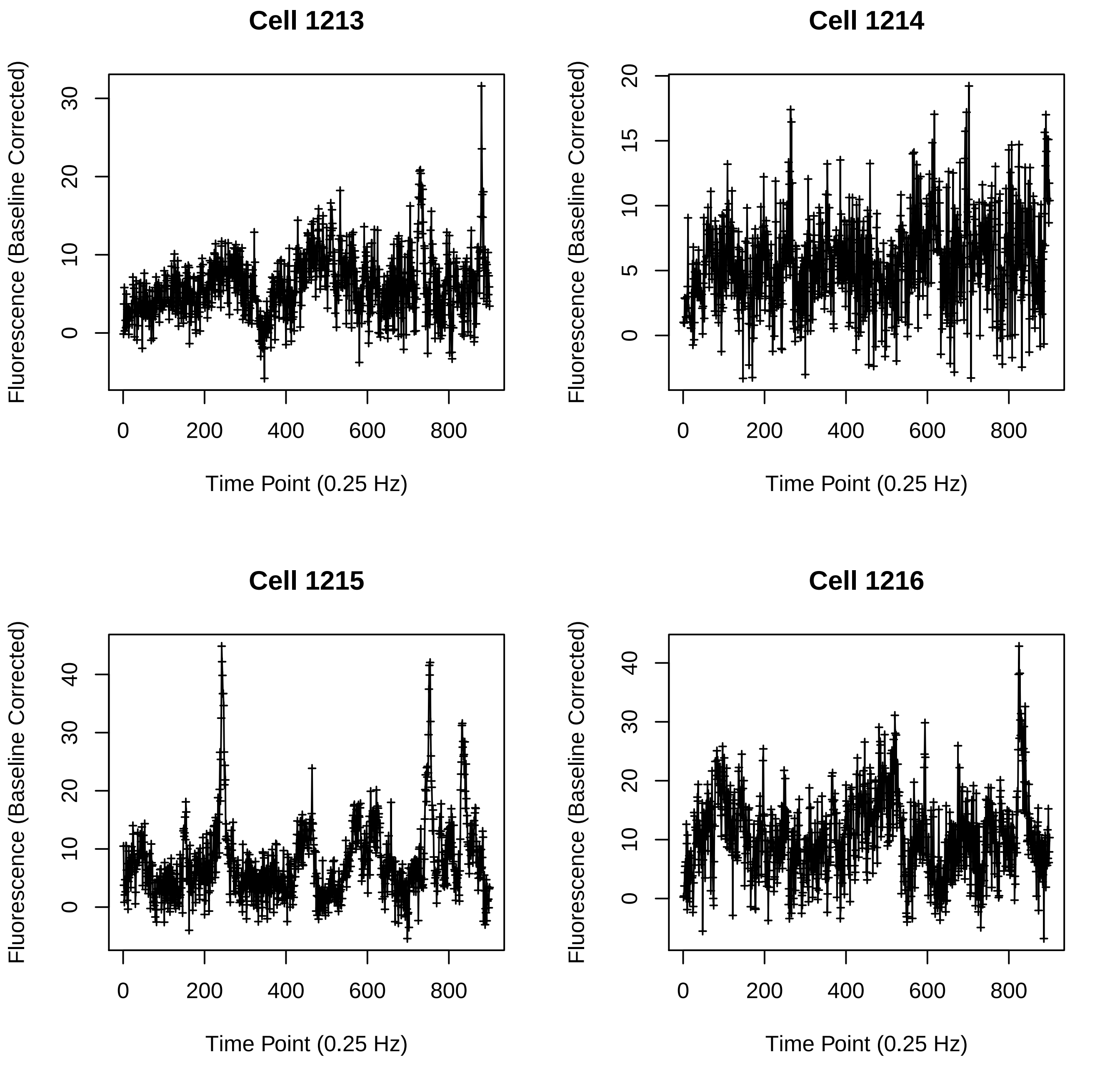
Cell 1213
Fluorescence (Baseline Corrected)
0
10
20
30
0
200
400
600
800
Time Point (0.25 Hz)
Cell 1214
Fluorescence (Baseline Corrected)
0
5
10
15
20
0
200
400
600
800
Time Point (0.25 Hz)
Cell 1215
Fluorescence (Baseline Corrected)
0
10
20
30
40
0
200
400
600
800
Time Point (0.25 Hz)
Cell 1216
Fluorescence (Baseline Corrected)
0
10
20
30
40
0
200
400
600
800
Time Point (0.25 Hz)

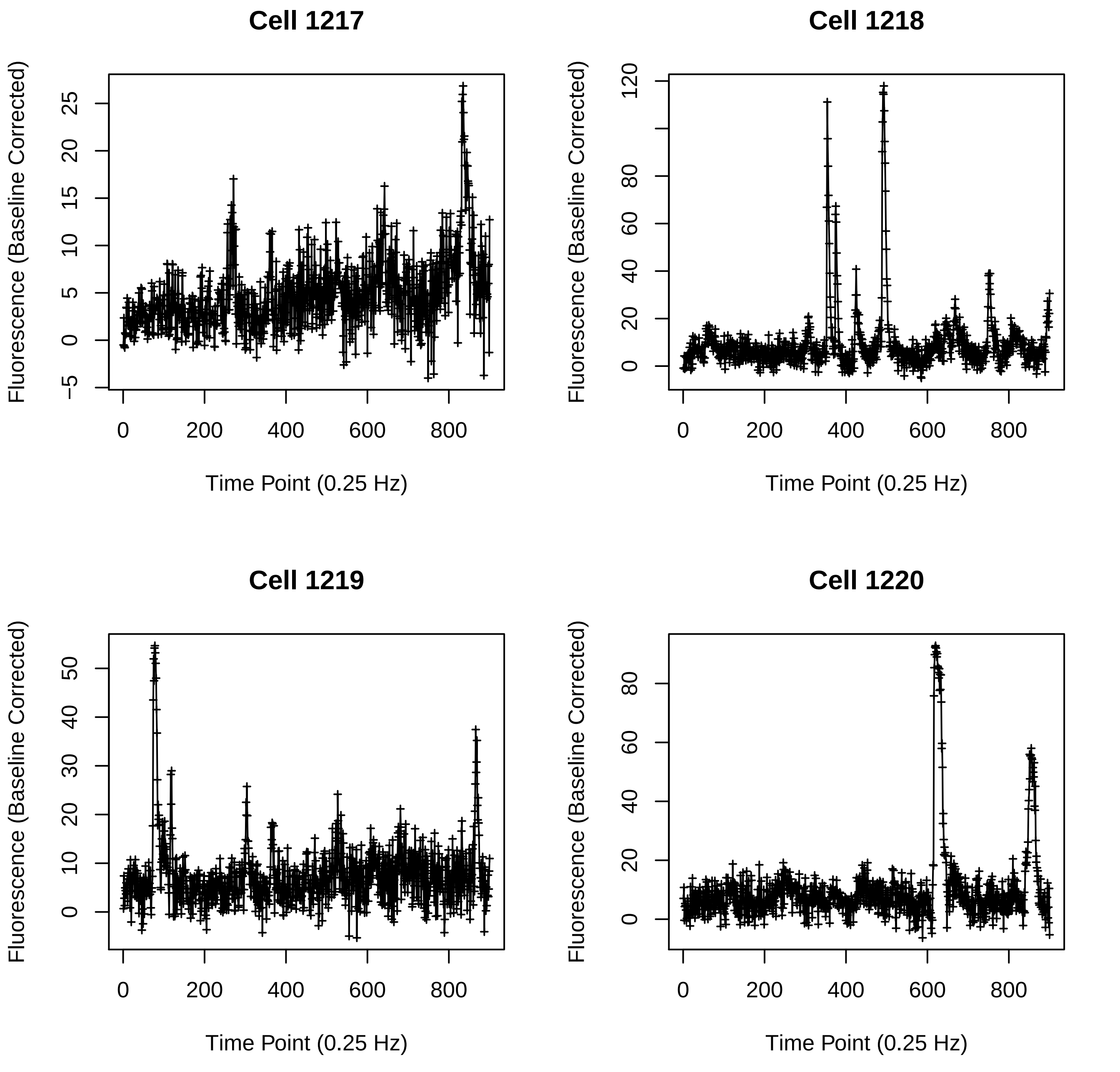

Cell 1217
Cell 1218
Cell 1219
Cell 1220
Fluorescence (Baseline Corrected)
Time Point (0.25 Hz)

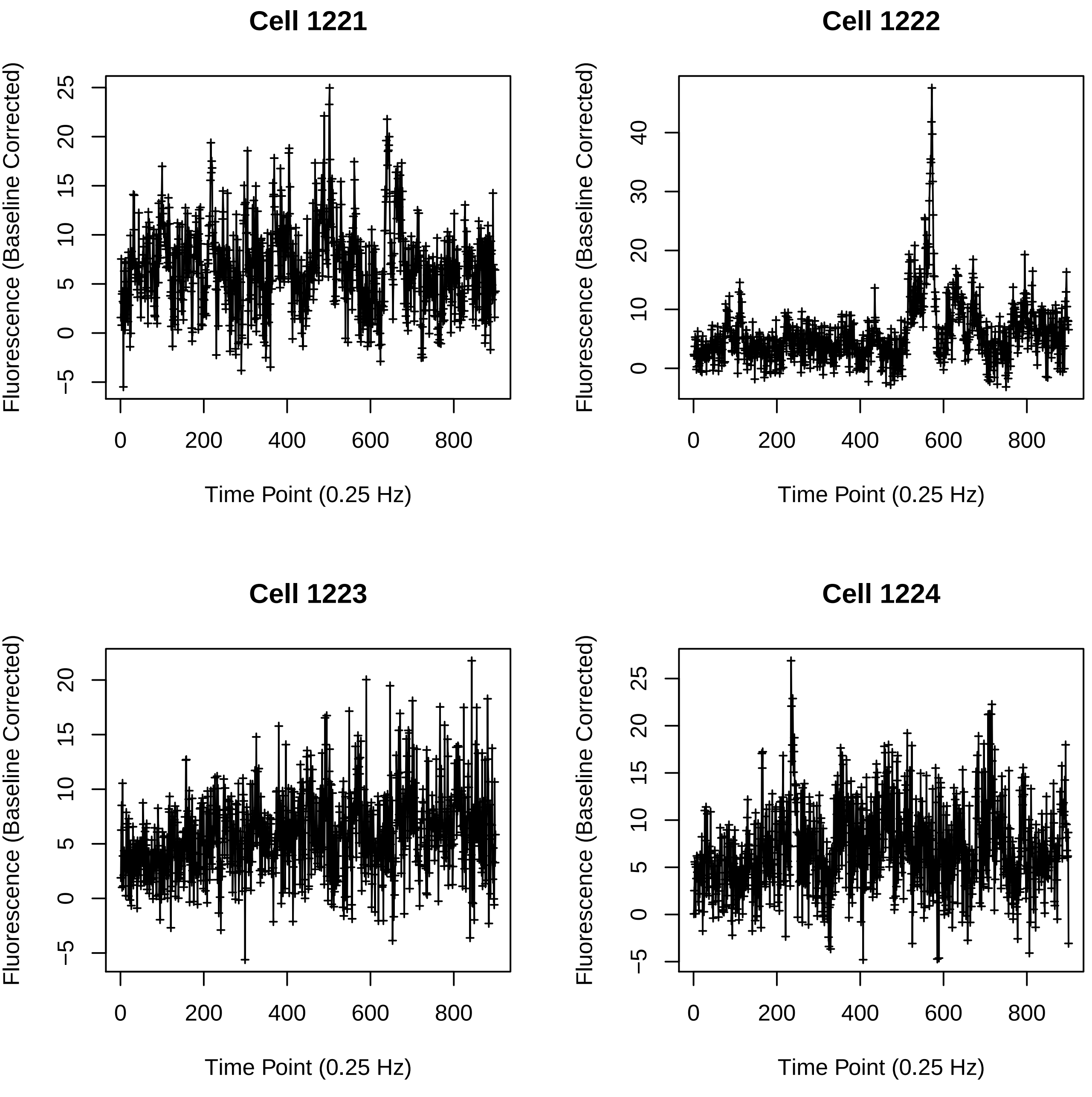

Cell 1221
Cell 1222
Cell 1223
Cell 1224
Fluorescence (Baseline Corrected)
Time Point (0.25 Hz)
-5
0
5
10
15
20
25
30
40
0
200
400
600
800

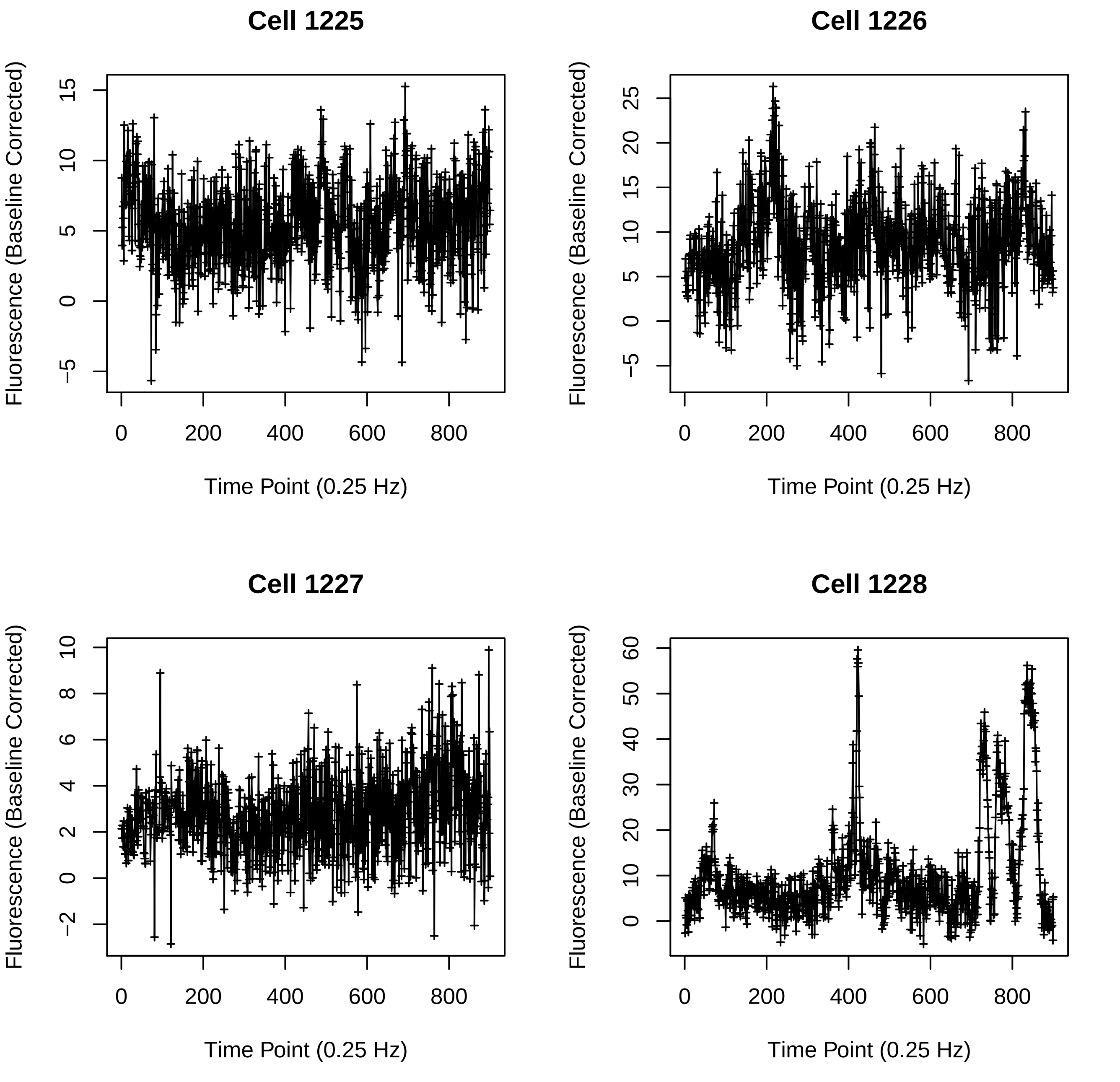
Cell 1225
Cell 1226
Cell 1227
Cell 1228
Fluorescence (Baseline Corrected)
Time Point (0.25 Hz)
0
200
400
600
800
−5
0
5
10
15
20
25
−2
2
4
6
8
30
40
50
60

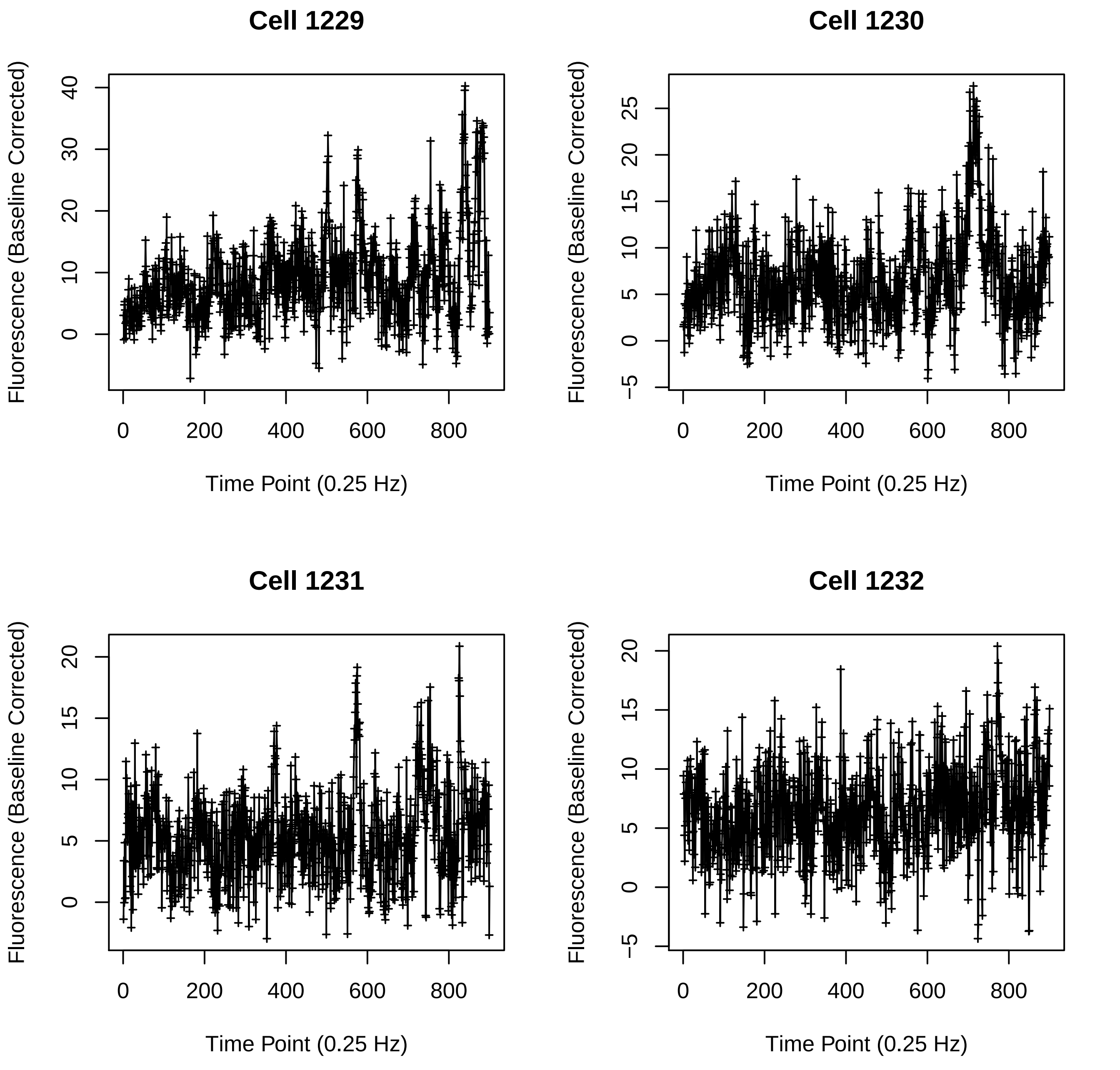

Cell 1229
Cell 1230
Cell 1231
Cell 1232
Fluorescence (Baseline Corrected)
Time Point (0.25 Hz)
0
200
400
600
800
-5
5
10
15
20
25
30
40

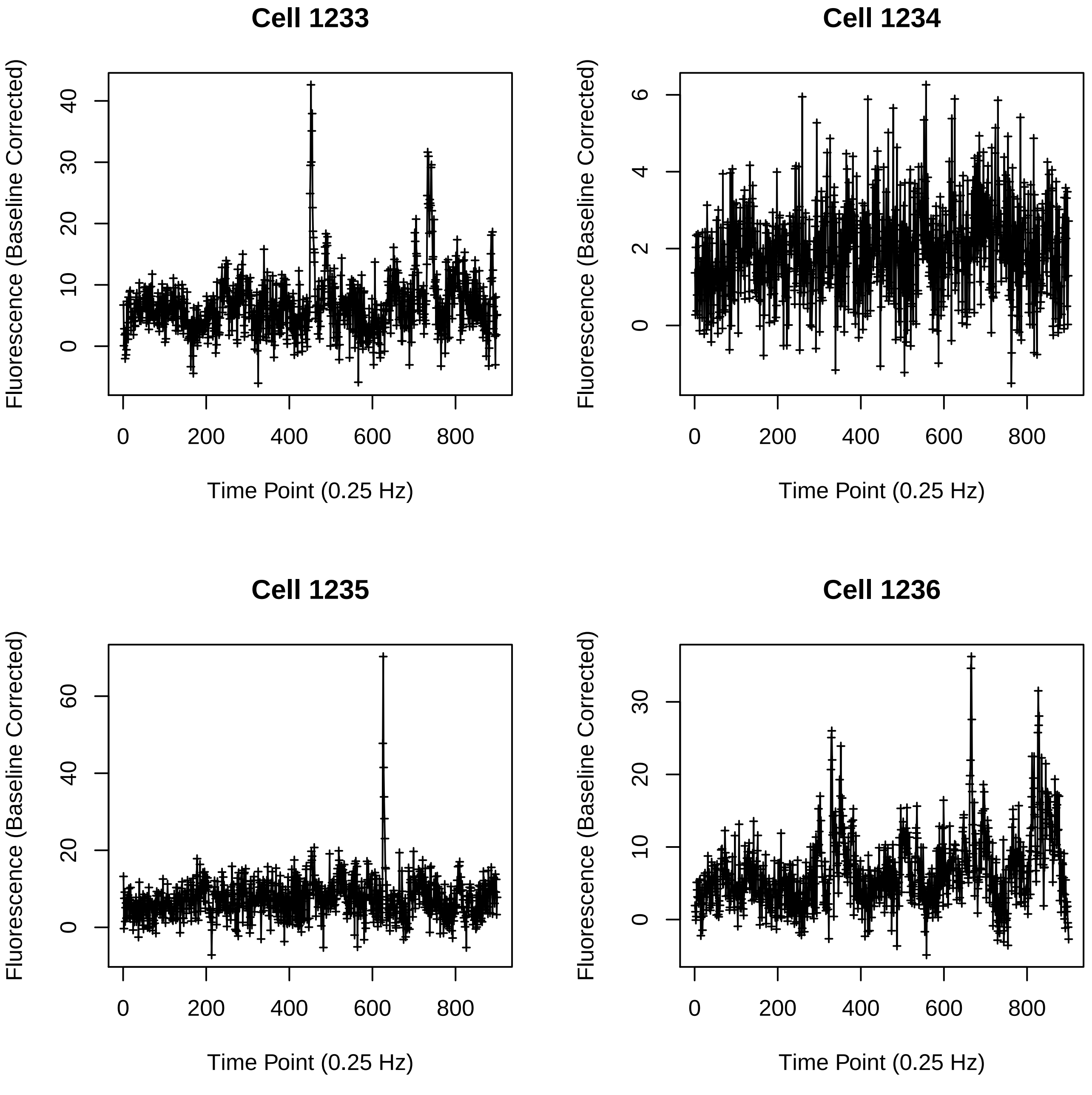

Cell 1233
Cell 1234
Cell 1235
Cell 1236
Fluorescence (Baseline Corrected)
Time Point (0.25 Hz)
0
10
20
30
40
200
400
600
800
2
4
6
60

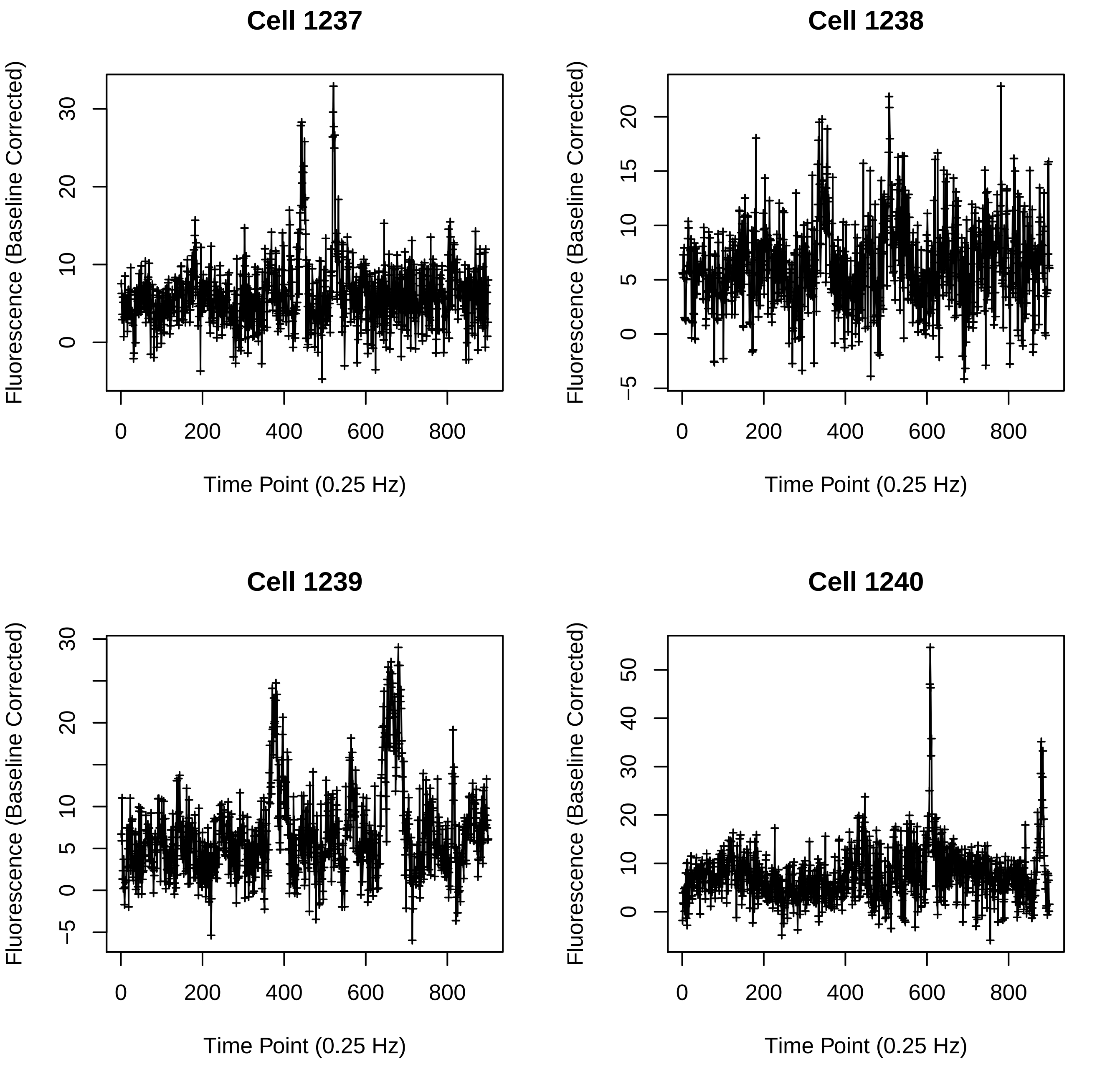
Cell 1237
Cell 1238
Cell 1239
Cell 1240
Fluorescence (Baseline Corrected)
Time Point (0.25 Hz)
0
200
400
600
800
-5
5
10
15
20
30
40
50

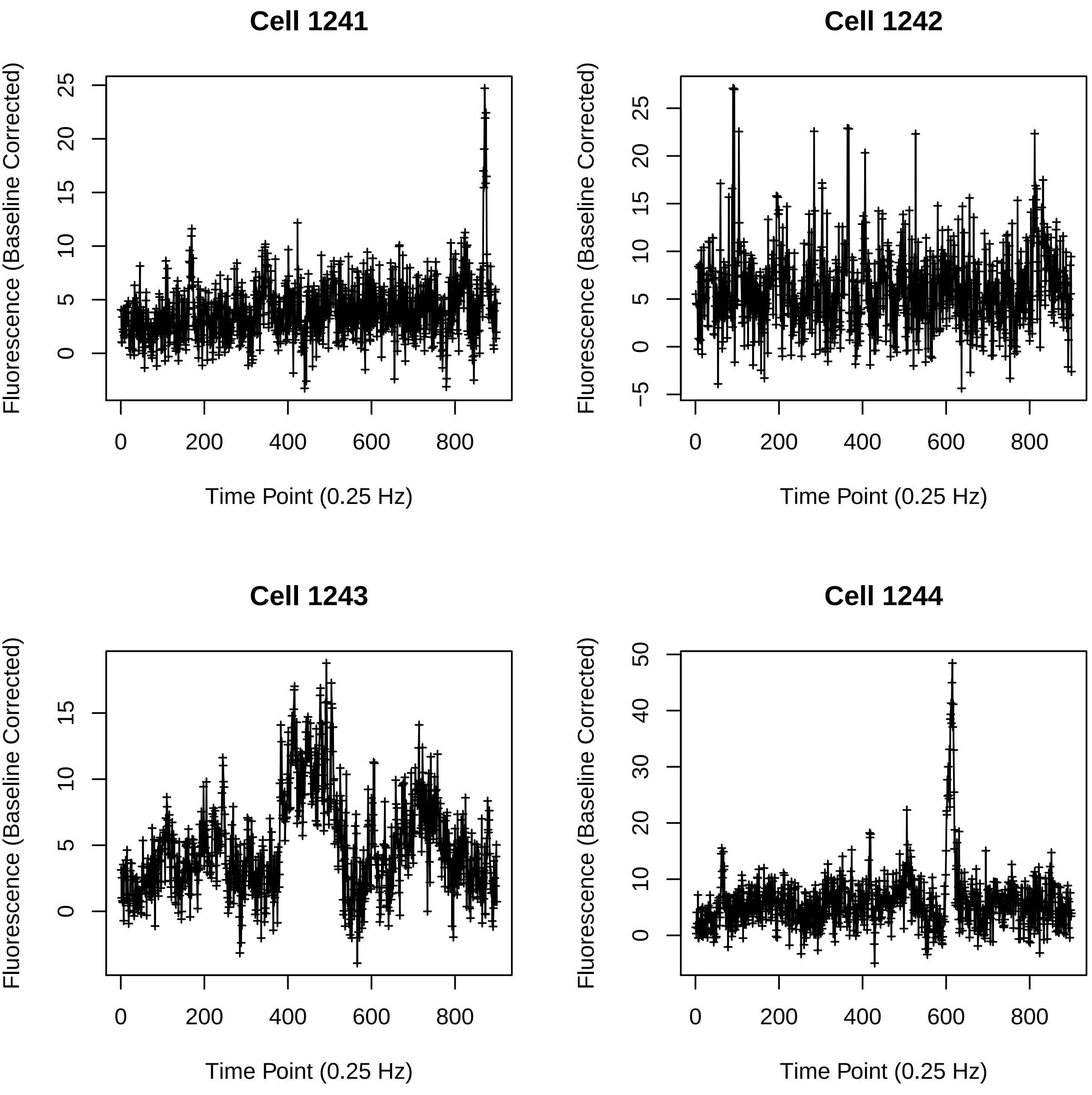

Cell 1241
Fluorescence (Baseline Corrected)
0
5
10
15
20
25
0
200
400
600
800
Time Point (0.25 Hz)
Cell 1242
Fluorescence (Baseline Corrected)
−5
0
5
10
15
20
25
0
200
400
600
800
Time Point (0.25 Hz)
Cell 1243
Fluorescence (Baseline Corrected)
0
5
10
15
0
200
400
600
800
Time Point (0.25 Hz)
Cell 1244
Fluorescence (Baseline Corrected)
0
10
20
30
40
50
0
200
400
600
800
Time Point (0.25 Hz)

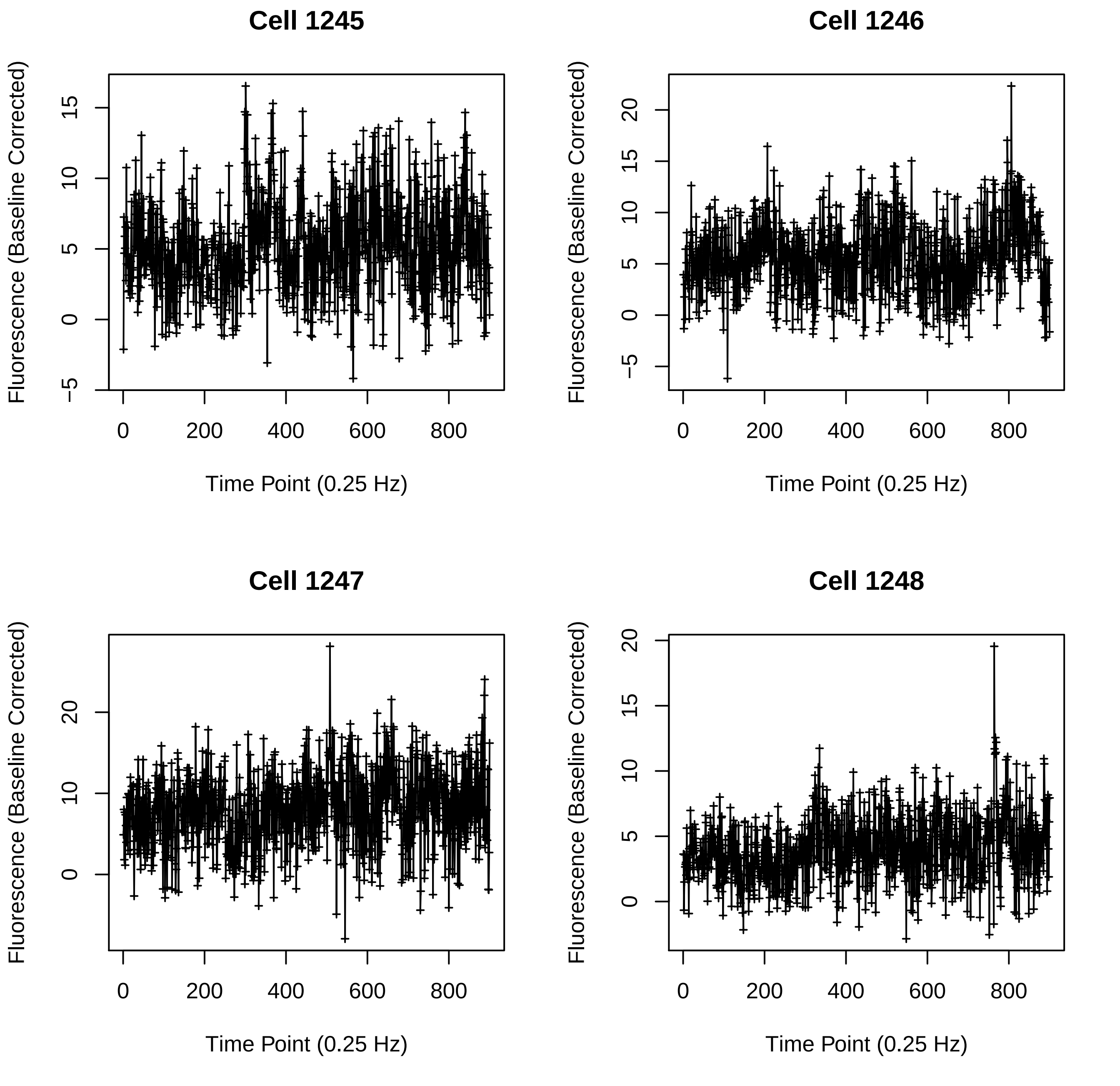
Cell 1245
Fluorescence (Baseline Corrected)
−5
0
5
10
15
0
200
400
600
800
Time Point (0.25 Hz)
Cell 1246
Fluorescence (Baseline Corrected)
−5
0
5
10
15
20
0
200
400
600
800
Time Point (0.25 Hz)
Cell 1247
Fluorescence (Baseline Corrected)
0
10
20
0
200
400
600
800
Time Point (0.25 Hz)
Cell 1248
Fluorescence (Baseline Corrected)
0
5
10
15
20
0
200
400
600
800
Time Point (0.25 Hz)

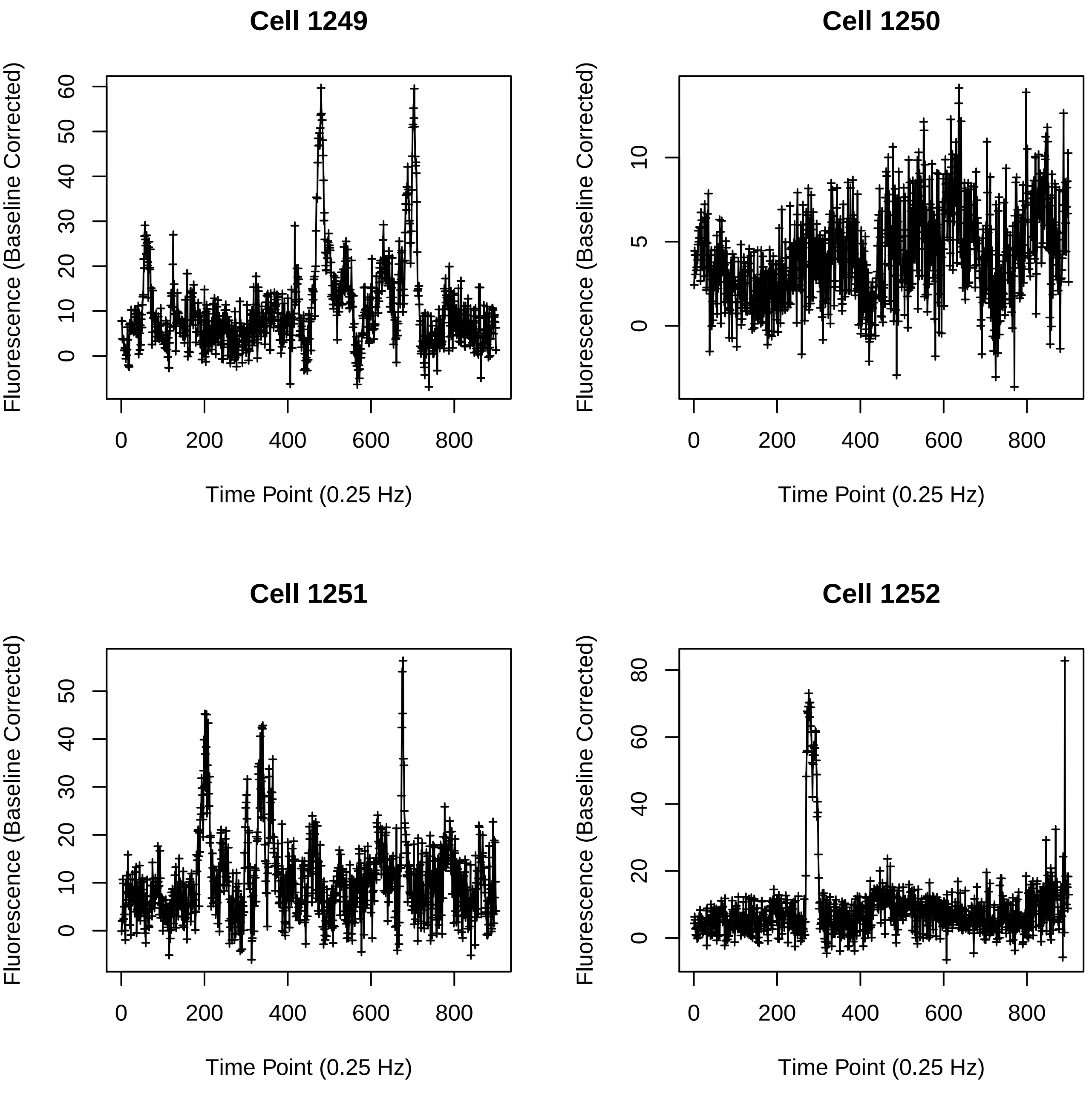

Cell 1249
Cell 1250
Cell 1251
Cell 1252
Fluorescence (Baseline Corrected)
Time Point (0.25 Hz)
0
10
20
30
40
50
60
5
80
200
400
600
800

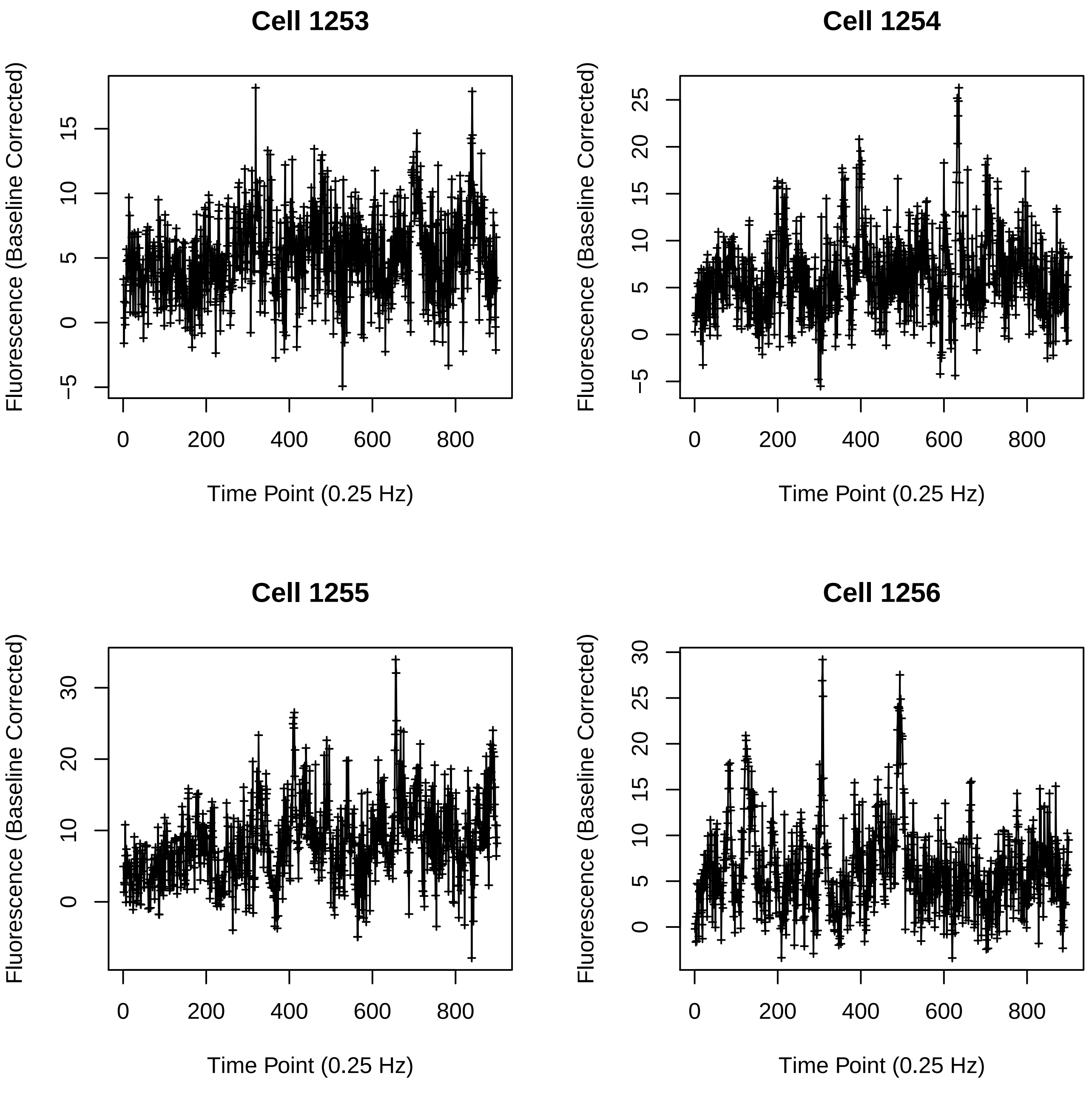
Cell 1253
Cell 1254
Cell 1255
Cell 1256
Fluorescence (Baseline Corrected)
Time Point (0.25 Hz)
−5
0
5
10
15
20
25
30
0
200
400
600
800

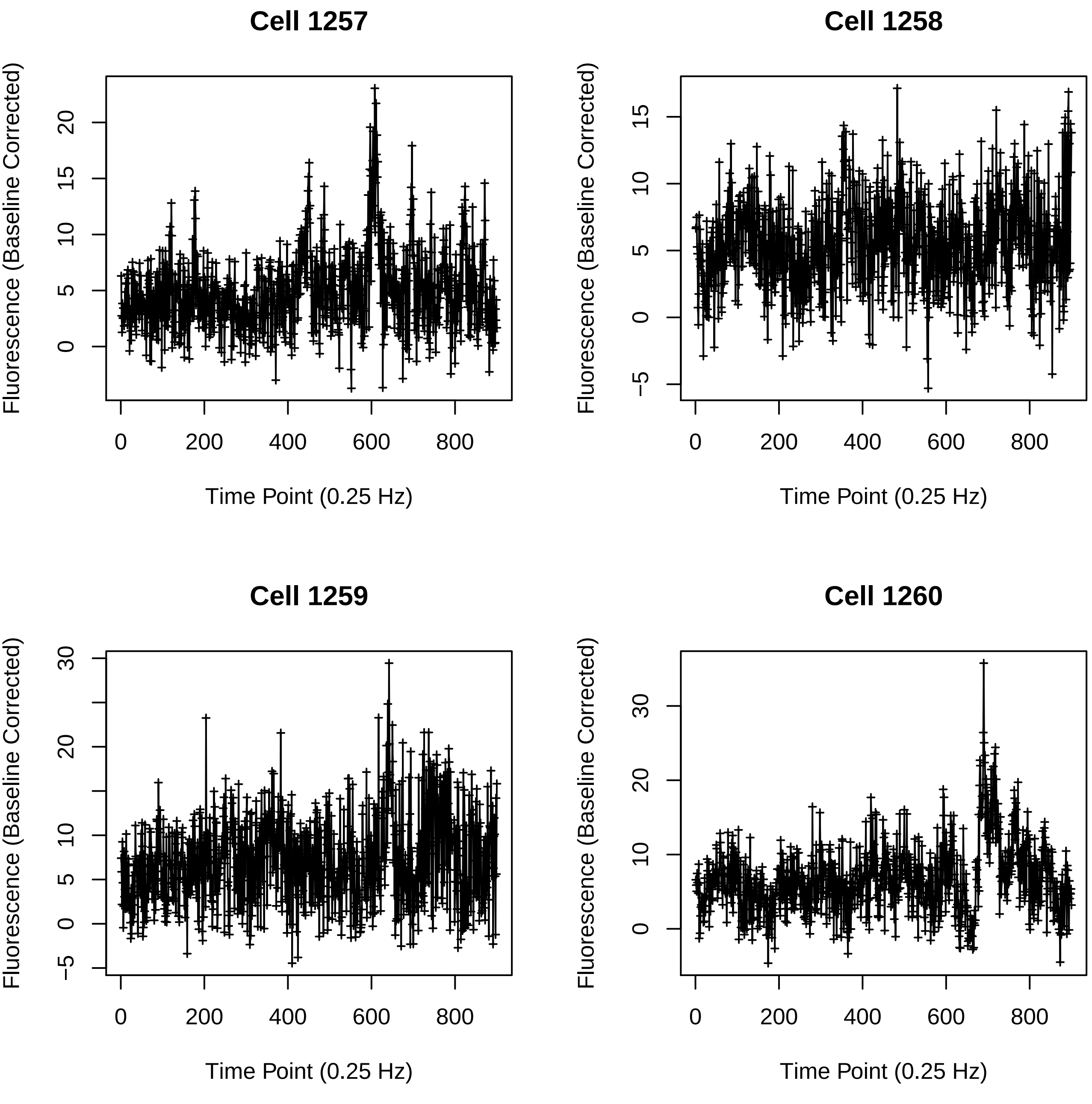
Cell 1257
Cell 1258
Cell 1259
Cell 1260
Fluorescence (Baseline Corrected)
Time Point (0.25 Hz)
0
200
400
600
800
-5
5
10
15
20
25
30

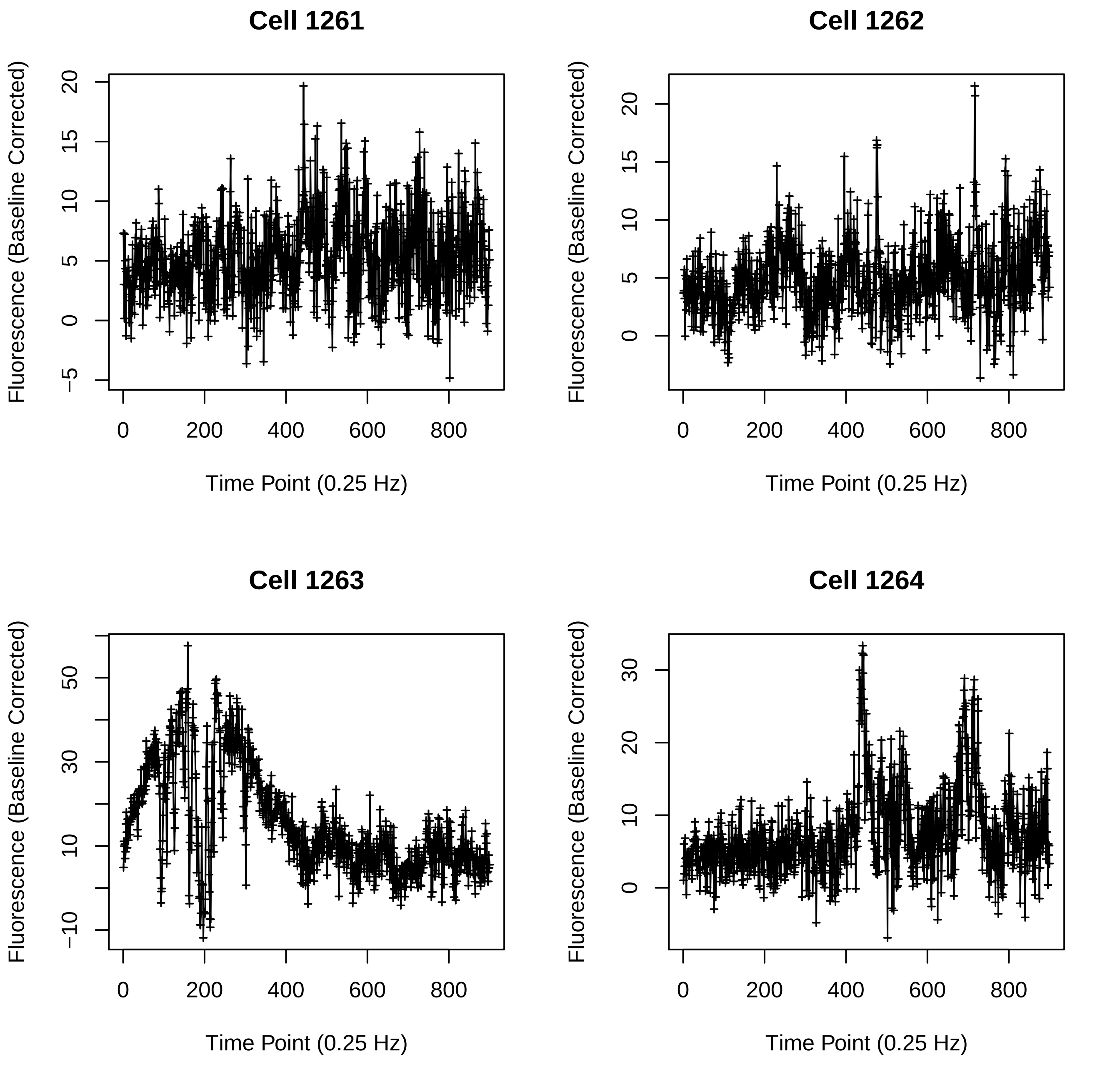
Cell 1261
Fluorescence (Baseline Corrected)
−5
0
5
10
15
20
0
200
400
600
800
Time Point (0.25 Hz)
Cell 1262
Fluorescence (Baseline Corrected)
0
5
10
15
20
0
200
400
600
800
Time Point (0.25 Hz)
Cell 1263
Fluorescence (Baseline Corrected)
−10
10
30
50
0
200
400
600
800
Time Point (0.25 Hz)
Cell 1264
Fluorescence (Baseline Corrected)
0
10
20
30
0
200
400
600
800
Time Point (0.25 Hz)

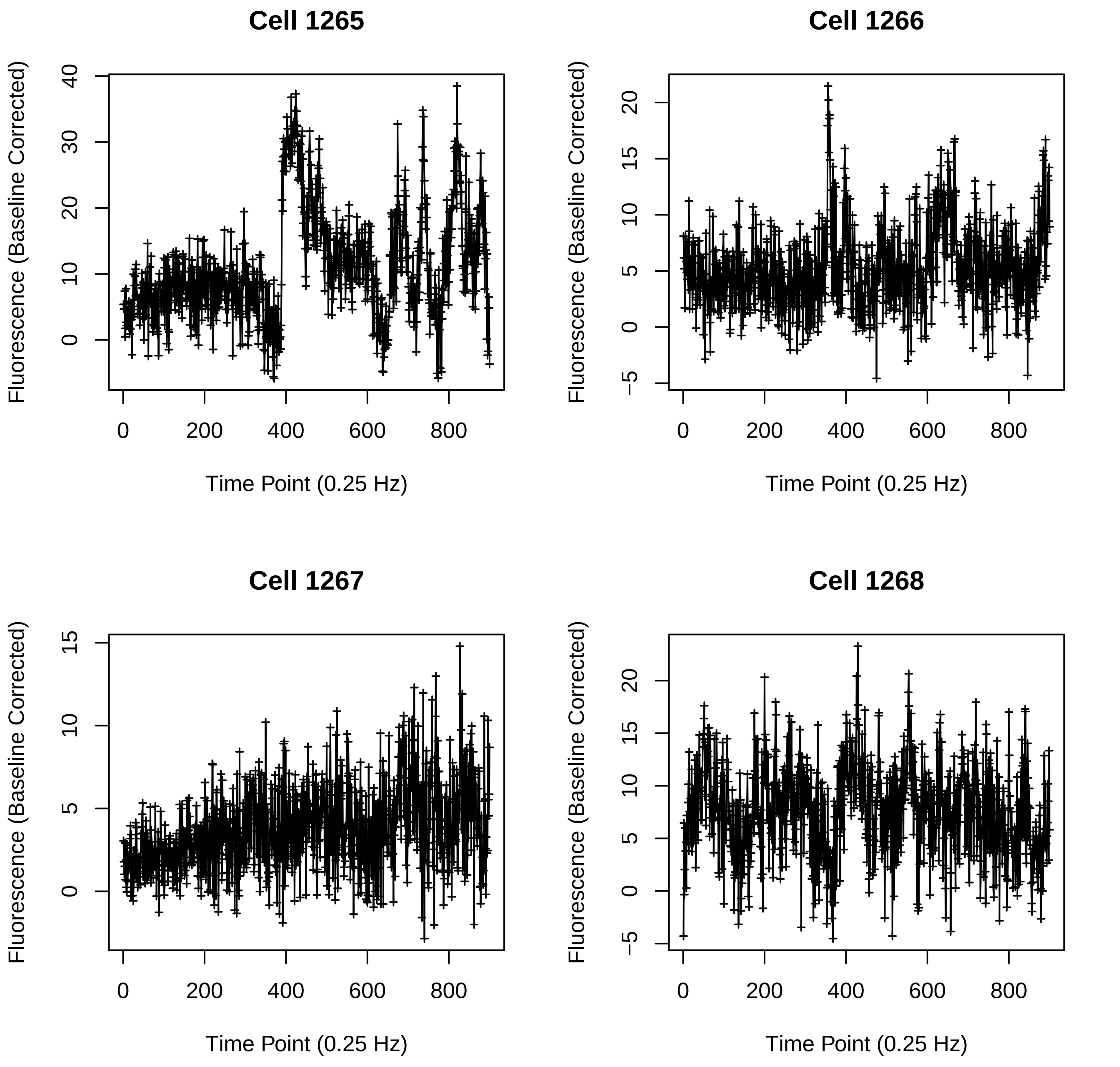
Cell 1265
Cell 1266
Cell 1267
Cell 1268
Fluorescence (Baseline Corrected)
Time Point (0.25 Hz)
0
200
400
600
800
−5
5
10
15
20
30
40

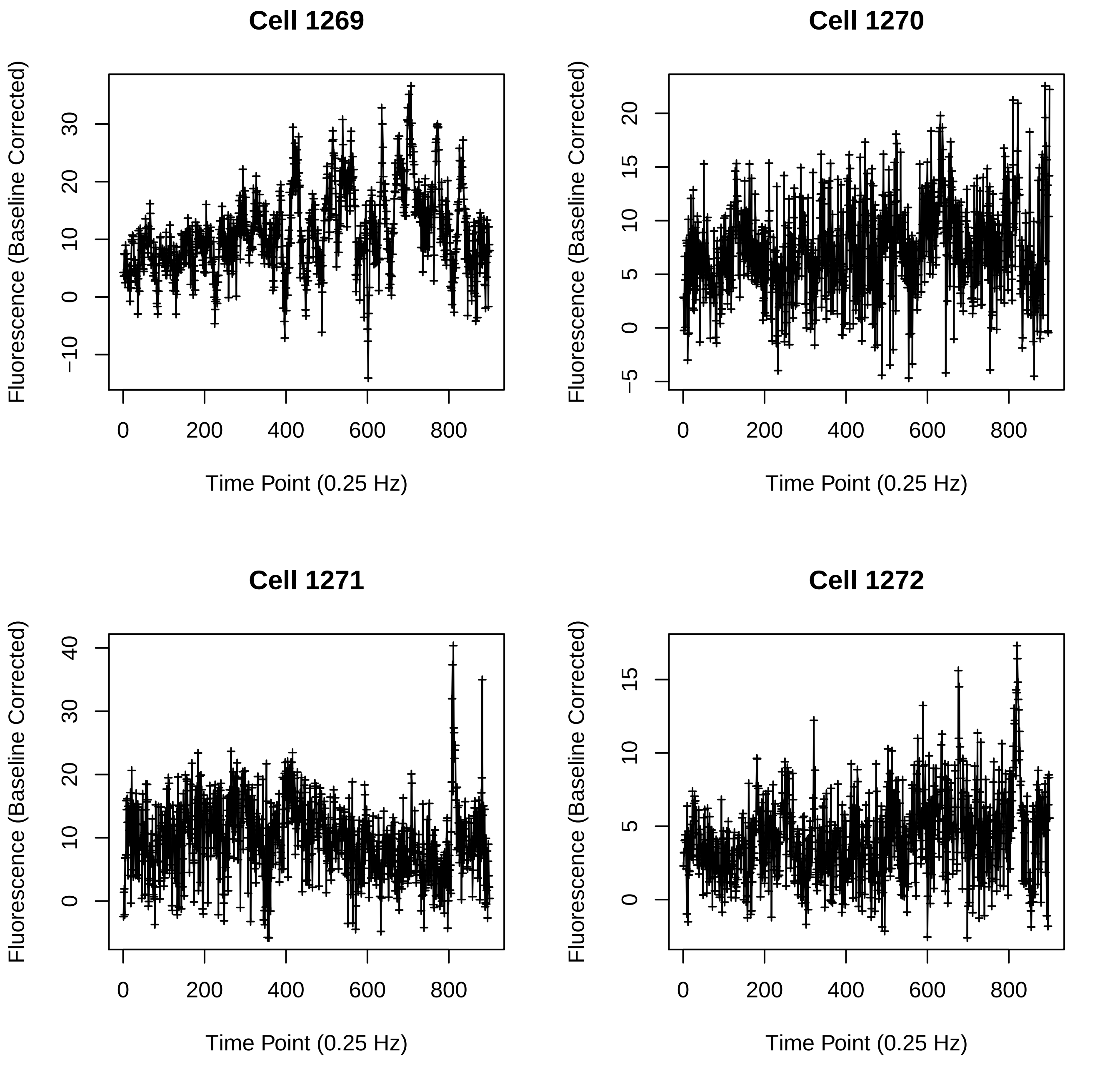
Cell 1269
Cell 1270
Cell 1271
Cell 1272
Fluorescence (Baseline Corrected)
Time Point (0.25 Hz)
0
200
400
600
800
−10
−5
0
5
10
15
20
30
40

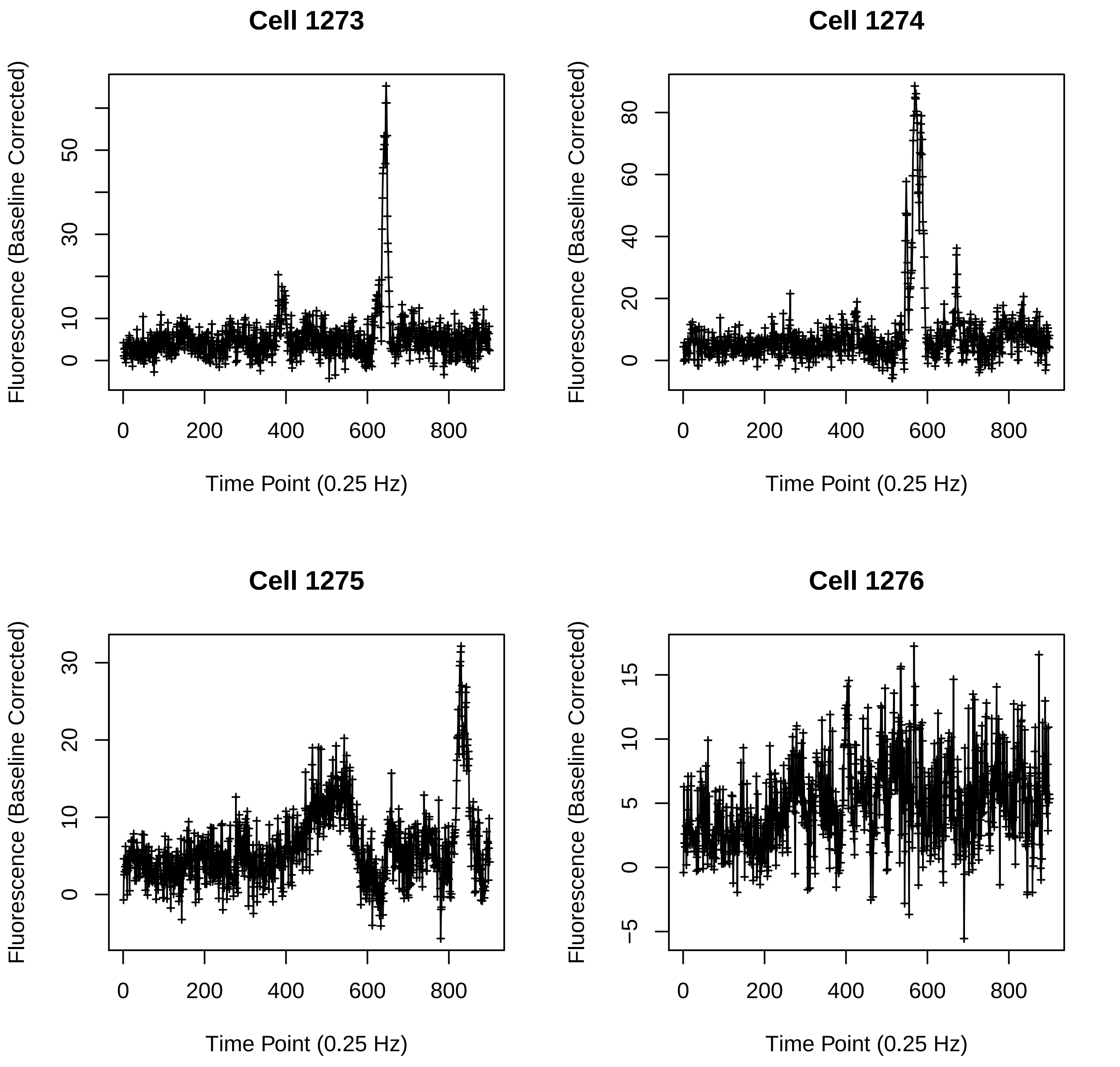
Cell 1273
Cell 1274
Cell 1275
Cell 1276
Fluorescence (Baseline Corrected)
Time Point (0.25 Hz)
0
200
400
600
800
0
10
30
50
20
40
60
80
5
15
-5

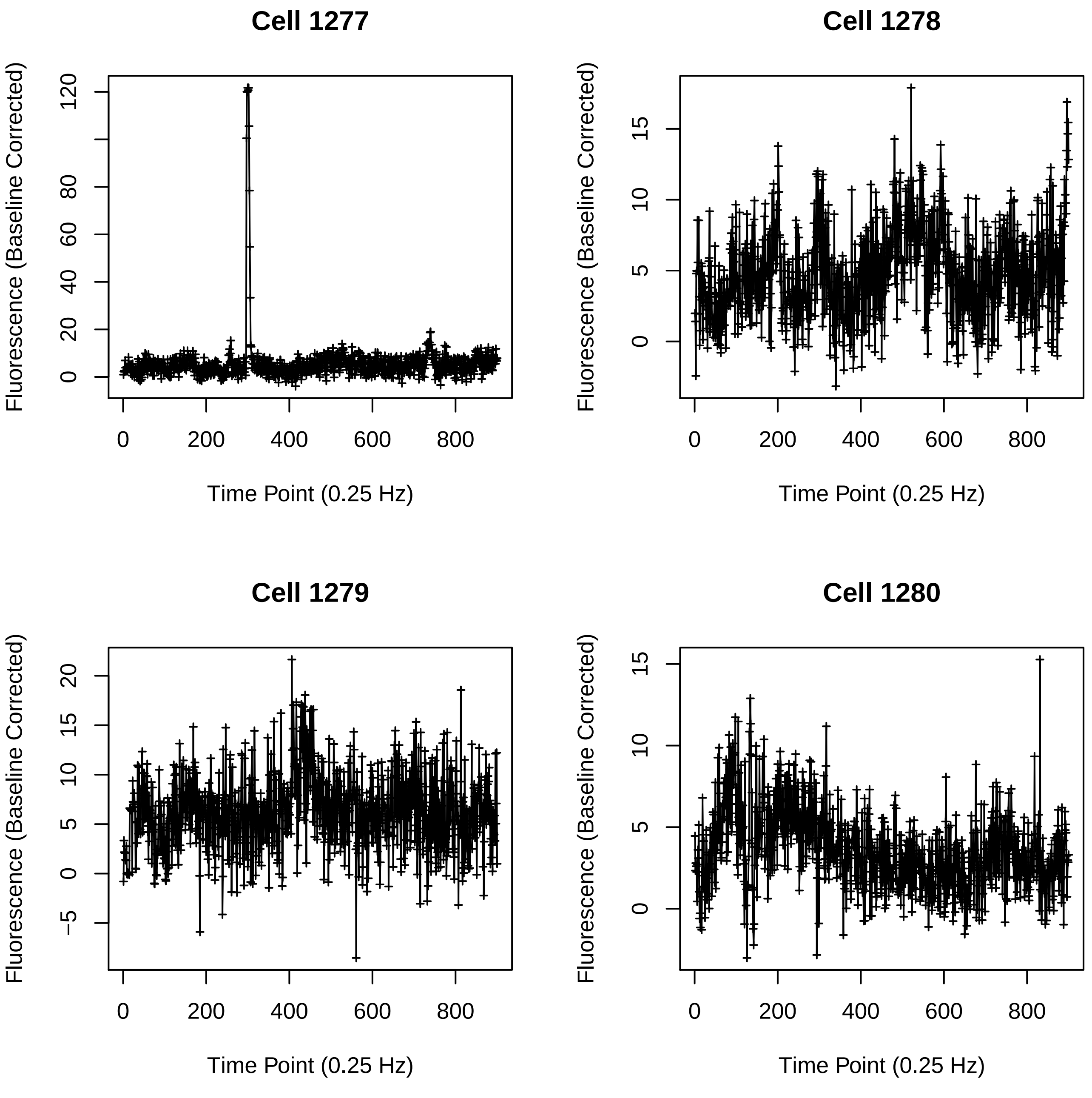
Cell 1277
Fluorescence (Baseline Corrected)
0
20
40
60
80
120
0
200
400
600
800
Time Point (0.25 Hz)
Cell 1278
Fluorescence (Baseline Corrected)
0
5
10
15
0
200
400
600
800
Time Point (0.25 Hz)
Cell 1279
Fluorescence (Baseline Corrected)
−5
0
5
10
15
20
0
200
400
600
800
Time Point (0.25 Hz)
Cell 1280
Fluorescence (Baseline Corrected)
0
5
10
15
0
200
400
600
800
Time Point (0.25 Hz)

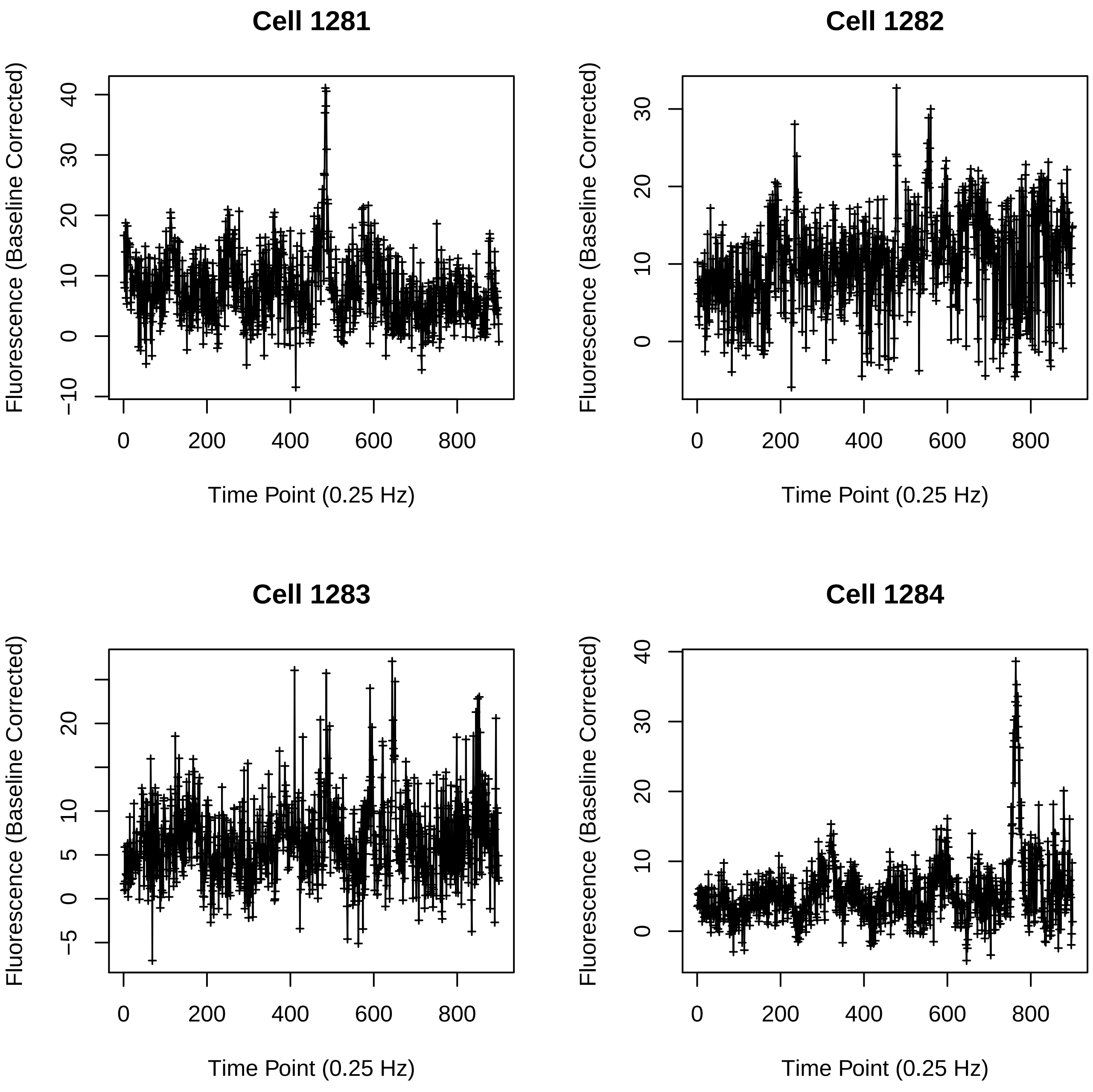
Cell 1281
Fluorescence (Baseline Corrected)
−10
0
10
20
30
40
0
200
400
600
800
Time Point (0.25 Hz)
Cell 1282
Fluorescence (Baseline Corrected)
0
10
20
30
0
200
400
600
800
Time Point (0.25 Hz)
Cell 1283
Fluorescence (Baseline Corrected)
−5
0
5
10
20
0
200
400
600
800
Time Point (0.25 Hz)
Cell 1284
Fluorescence (Baseline Corrected)
0
10
20
30
40
0
200
400
600
800
Time Point (0.25 Hz)

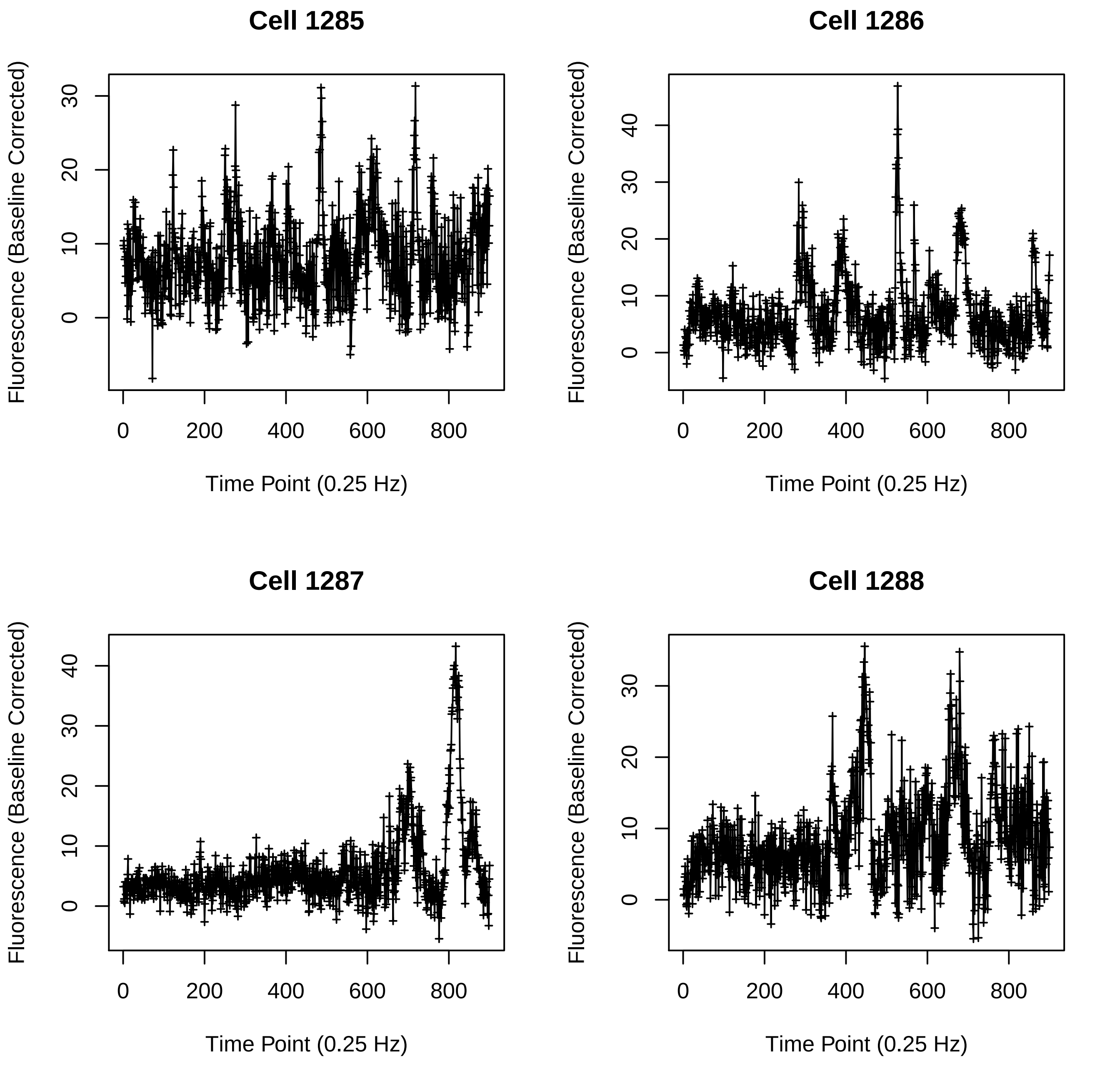
Cell 1285
Cell 1286
Cell 1287
Cell 1288
Fluorescence (Baseline Corrected)
Time Point (0.25 Hz)
0
200
400
600
800
0
10
20
30
40

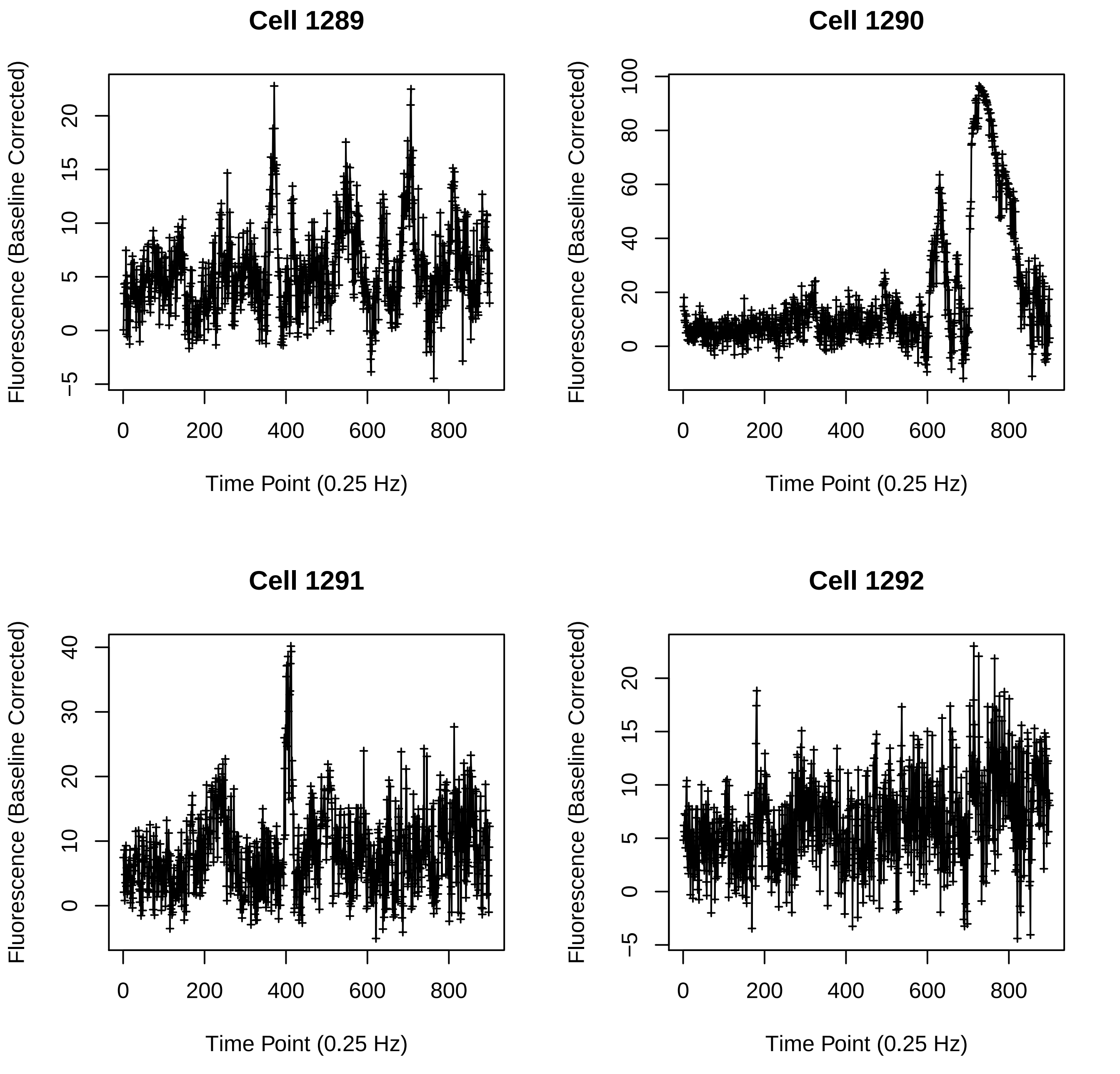
Cell 1289
Cell 1290
Cell 1291
Cell 1292
Fluorescence (Baseline Corrected)
Time Point (0.25 Hz)
0
200
400
600
800
−5
5
10
15
20
30
40
60
80
100

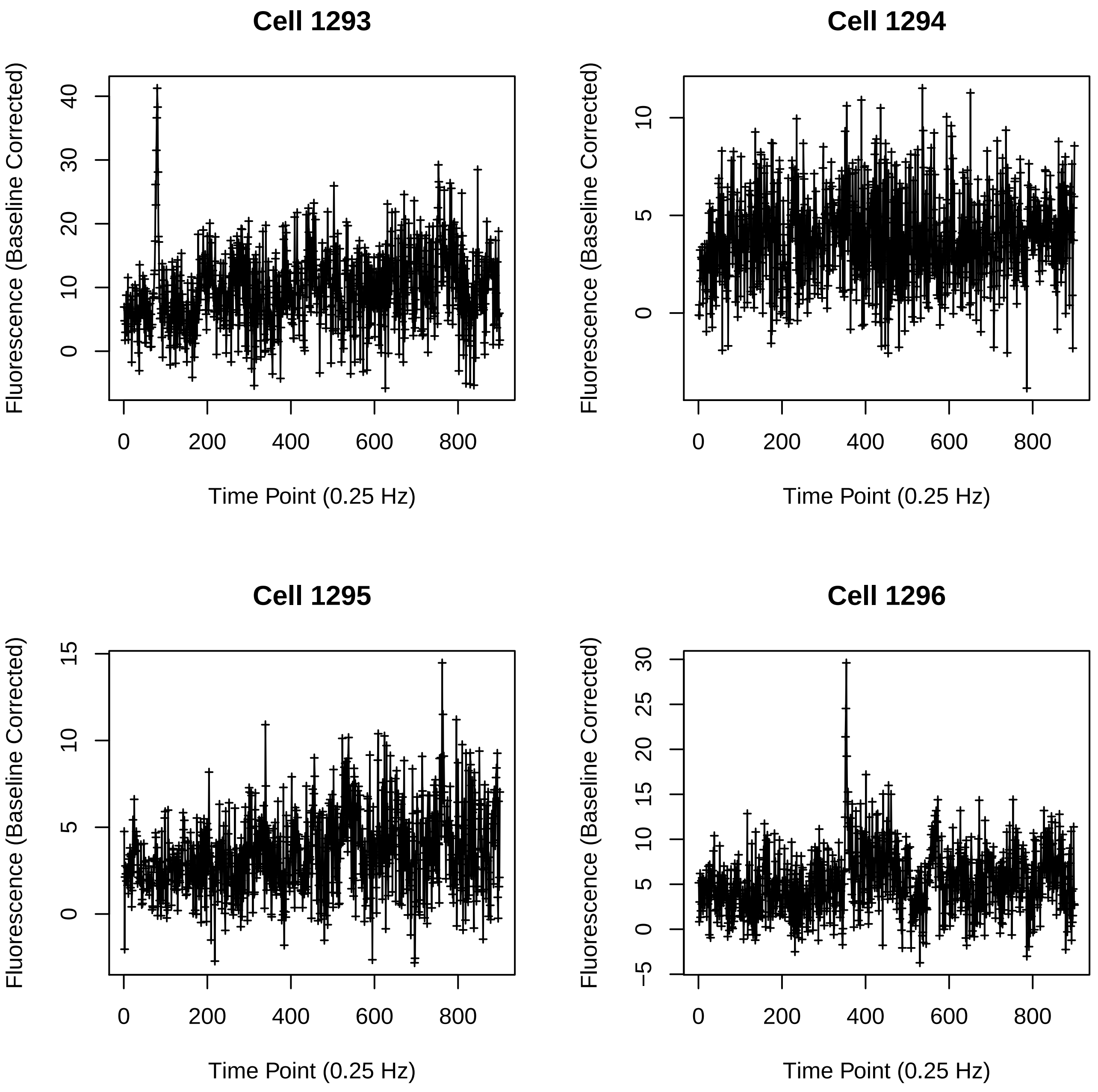
Cell 1293
Fluorescence (Baseline Corrected)
0
10
20
30
40
0
200
400
600
800
Time Point (0.25 Hz)
Cell 1294
Fluorescence (Baseline Corrected)
0
5
10
0
200
400
600
800
Time Point (0.25 Hz)
Cell 1295
Fluorescence (Baseline Corrected)
0
5
10
15
0
200
400
600
800
Time Point (0.25 Hz)
Cell 1296
Fluorescence (Baseline Corrected)
−5
0
5
10
15
20
25
30
0
200
400
600
800
Time Point (0.25 Hz)

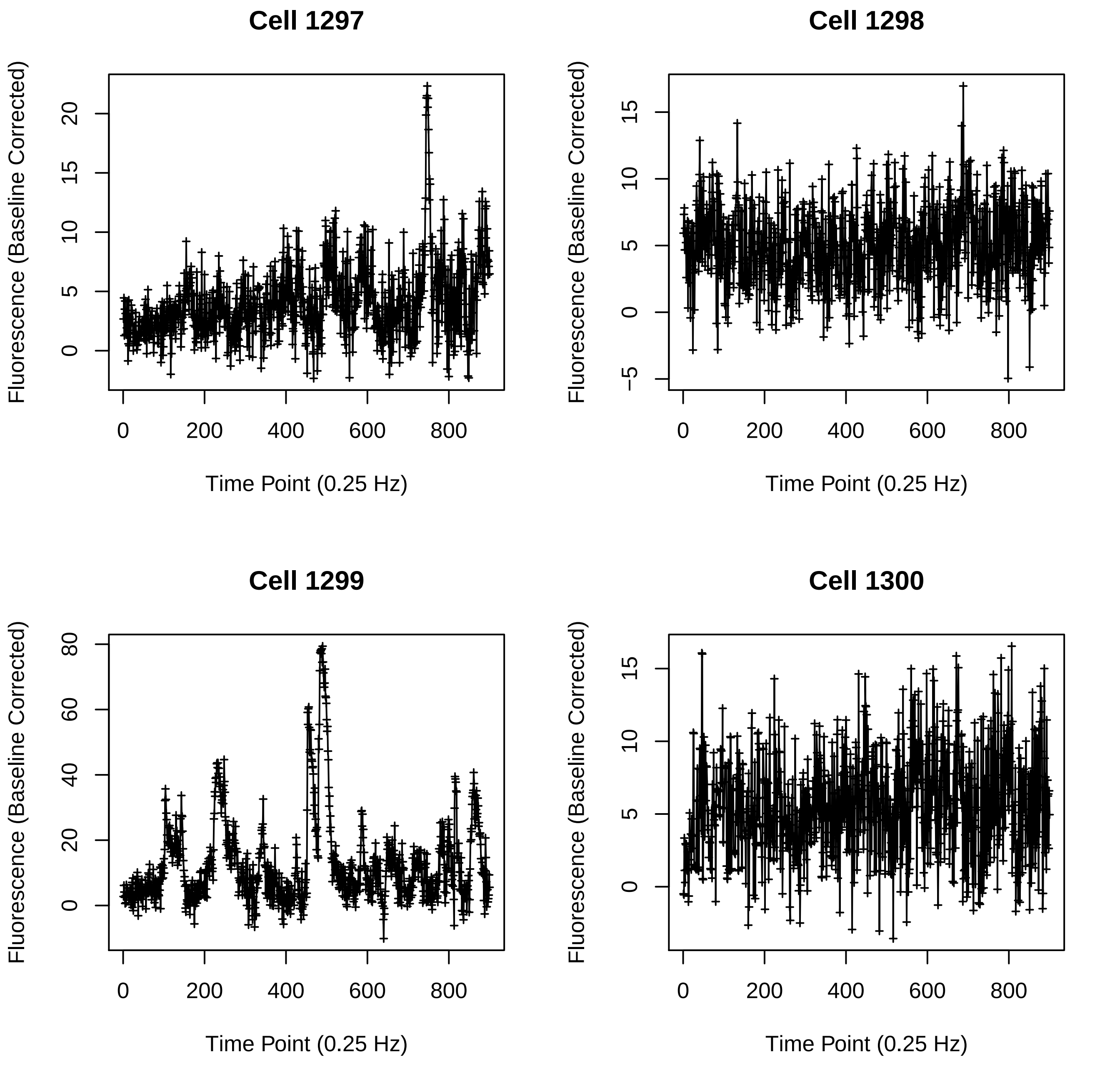
Cell 1297
Cell 1298
Cell 1299
Cell 1300
Fluorescence (Baseline Corrected)
Time Point (0.25 Hz)
0
200
400
600
800
−5
5
10
15
20
40
60
80

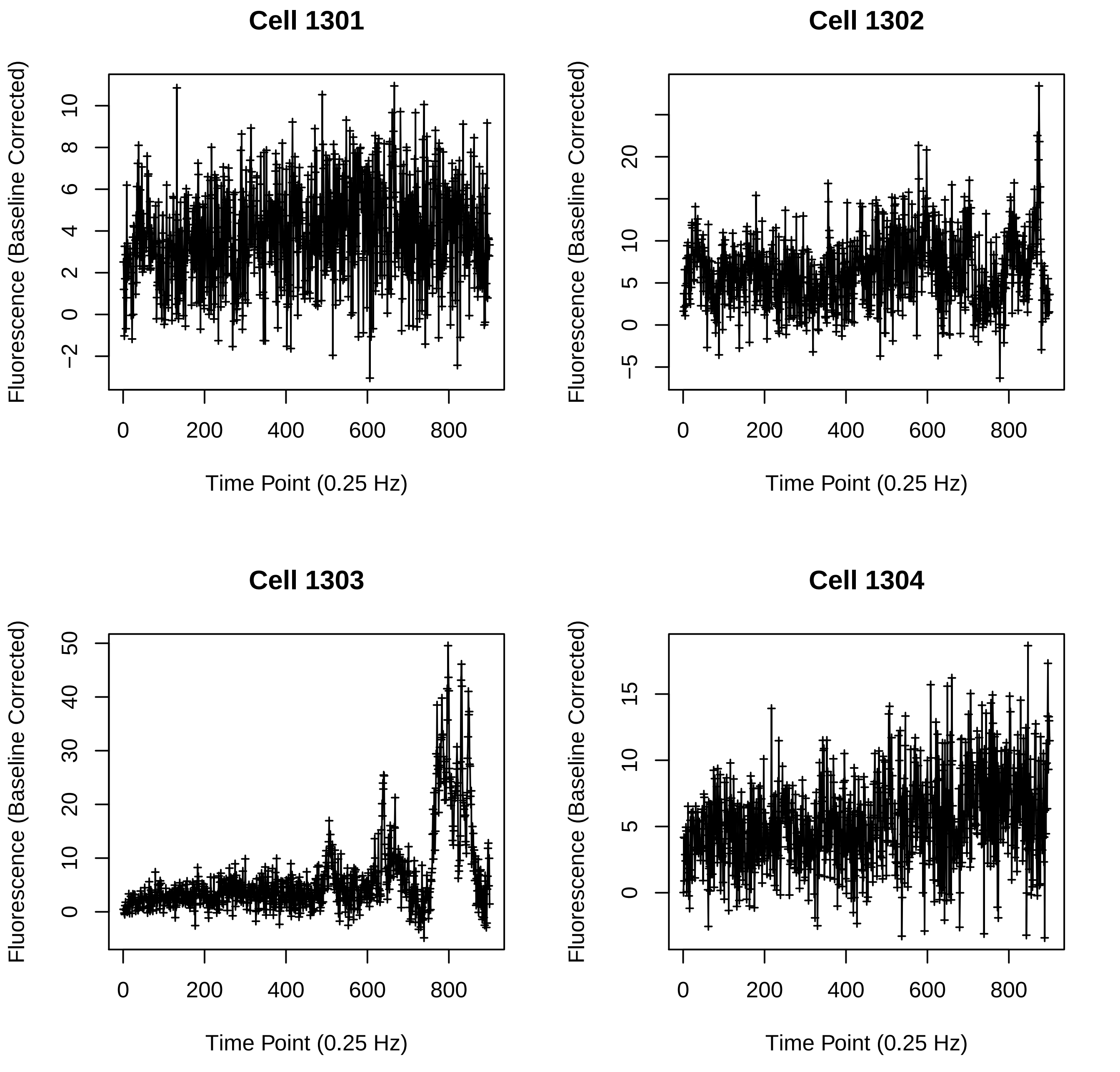

Cell 1301
Cell 1302
Cell 1303
Cell 1304
Fluorescence (Baseline Corrected)
Time Point (0.25 Hz)
0
200
400
600
800
−2
0
2
4
6
8
10
−5
5
15
20
30
40
50

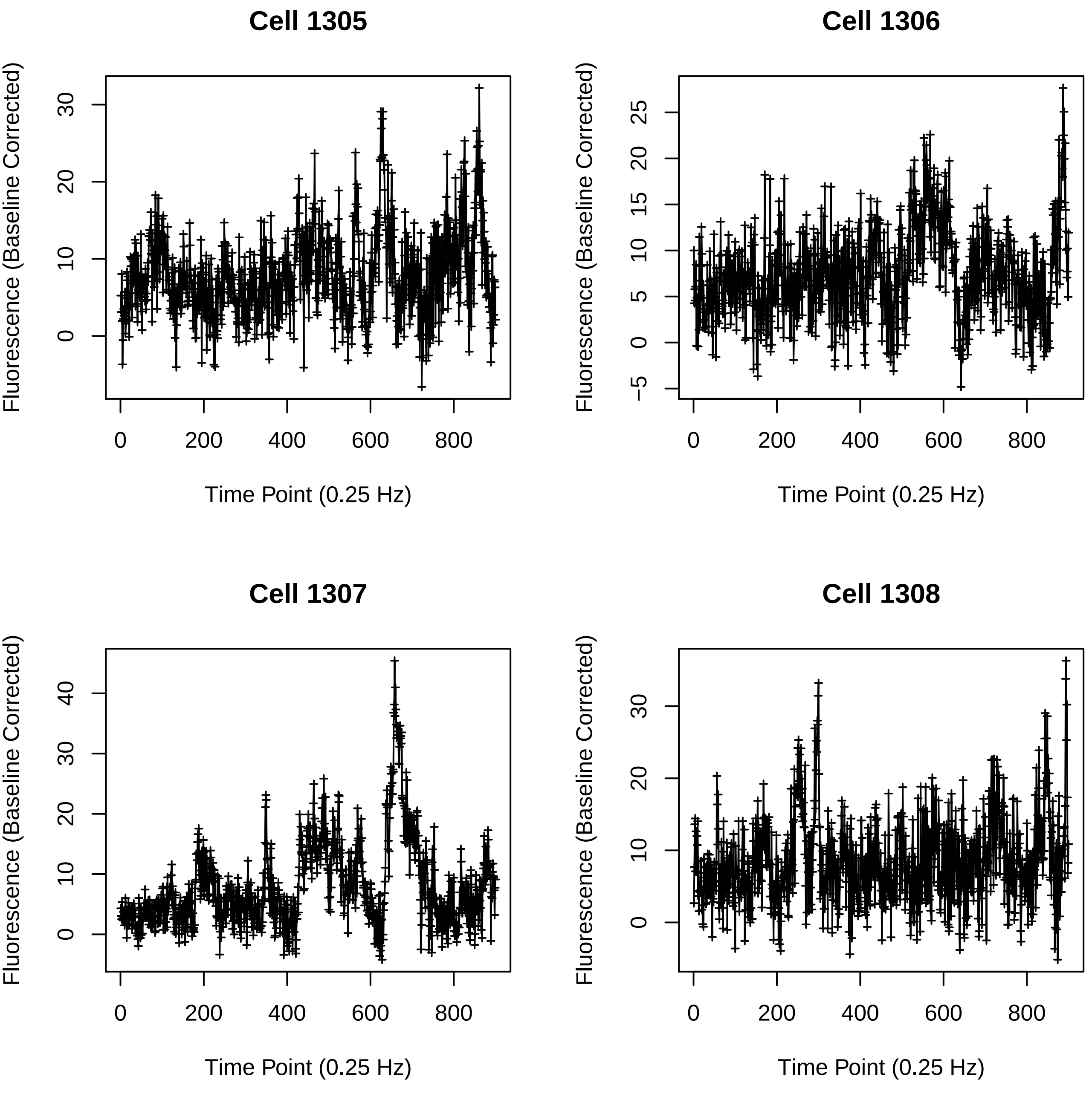
Cell 1305
Fluorescence (Baseline Corrected)
Time Point (0.25 Hz)
0
10
20
30
0
200
400
600
800
Cell 1306
Fluorescence (Baseline Corrected)
Time Point (0.25 Hz)
−5
0
5
10
15
20
25
0
200
400
600
800
Cell 1307
Fluorescence (Baseline Corrected)
Time Point (0.25 Hz)
0
10
20
30
40
0
200
400
600
800
Cell 1308
Fluorescence (Baseline Corrected)
Time Point (0.25 Hz)
0
10
20
30
0
200
400
600
800

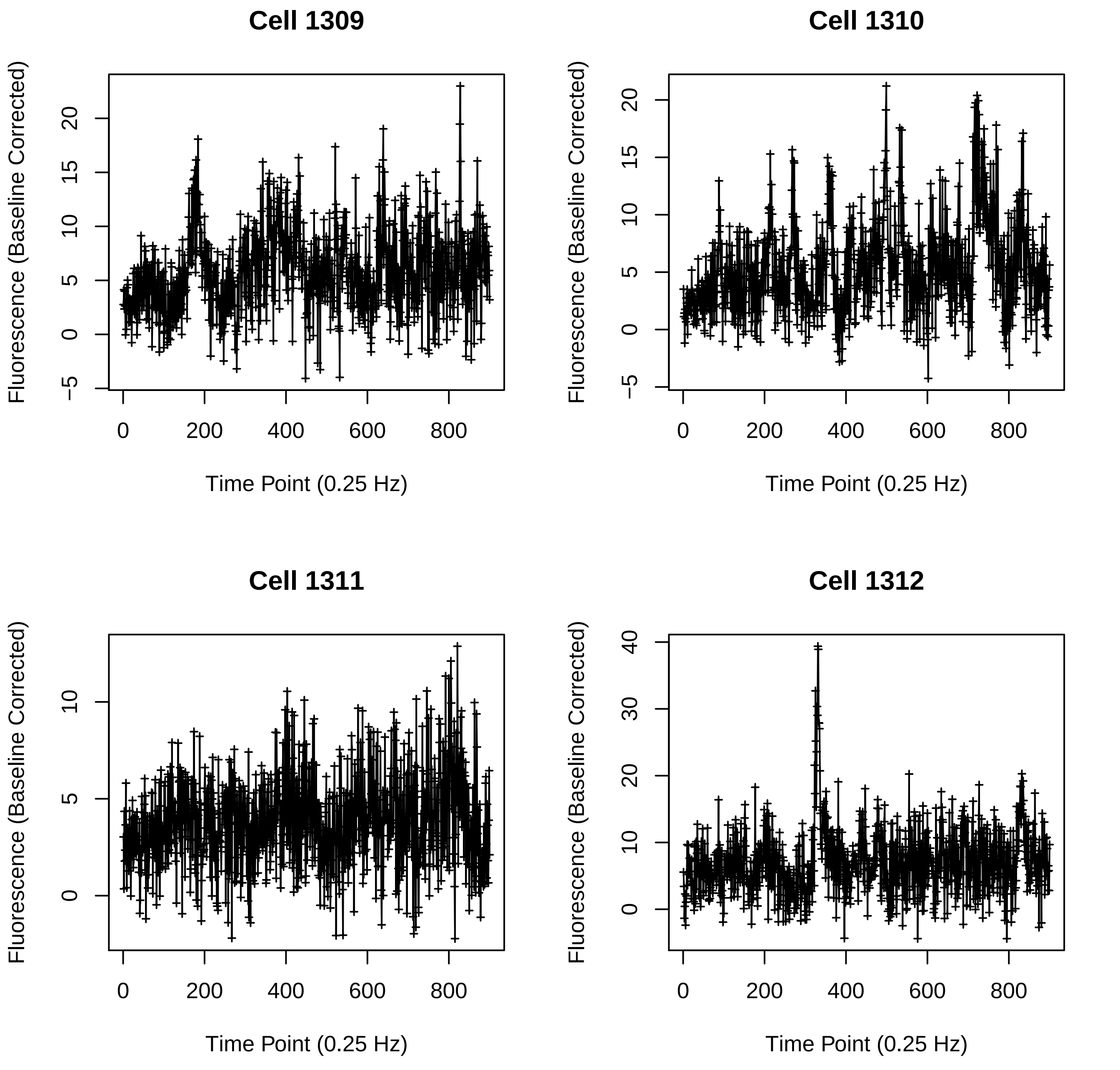
Cell 1309
Cell 1310
Cell 1311
Cell 1312
Fluorescence (Baseline Corrected)
Time Point (0.25 Hz)
0
200
400
600
800
−5
0
5
10
15
20
30
40

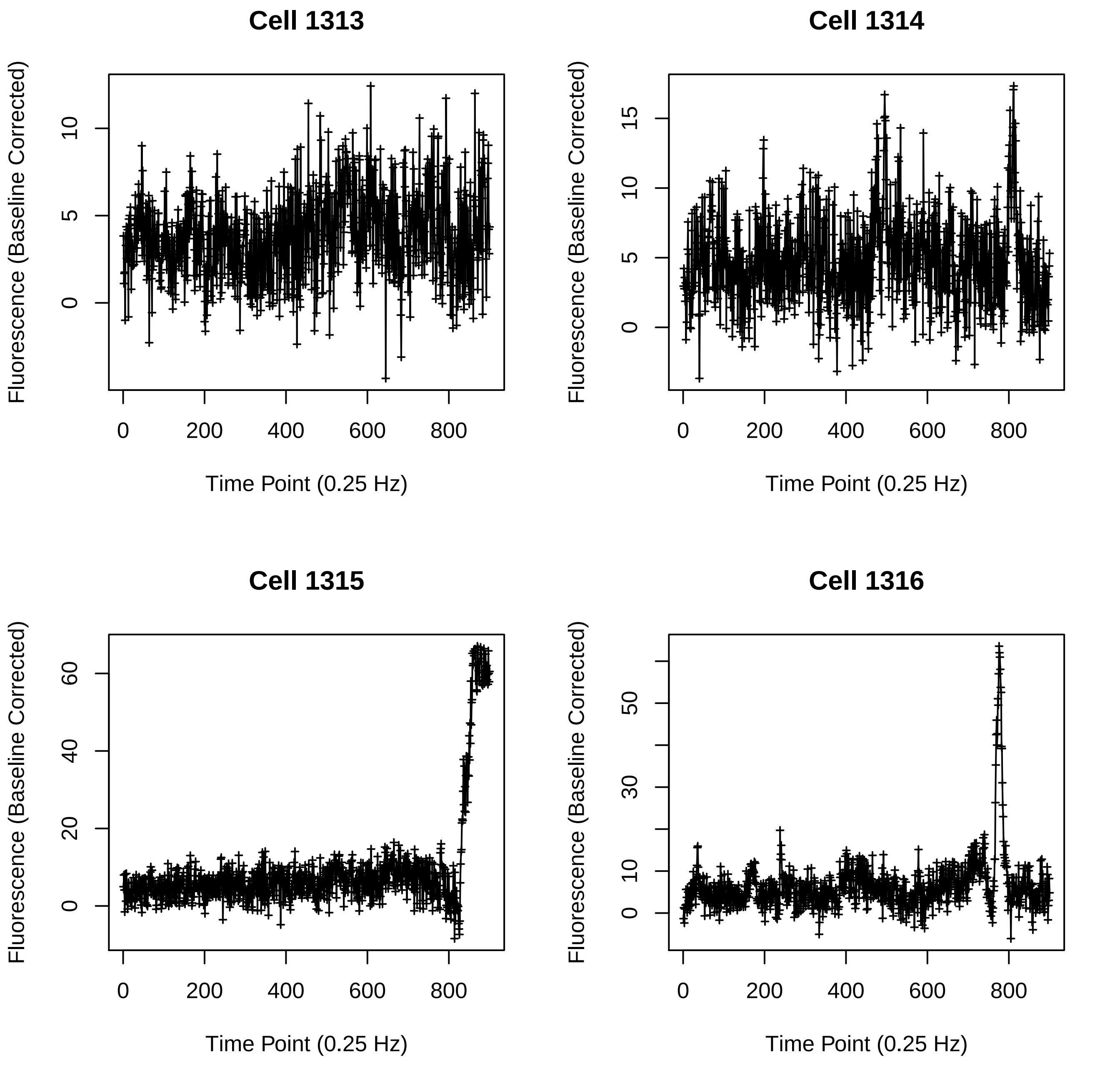
Cell 1313
Cell 1314
Cell 1315
Cell 1316
Fluorescence (Baseline Corrected)
Time Point (0.25 Hz)

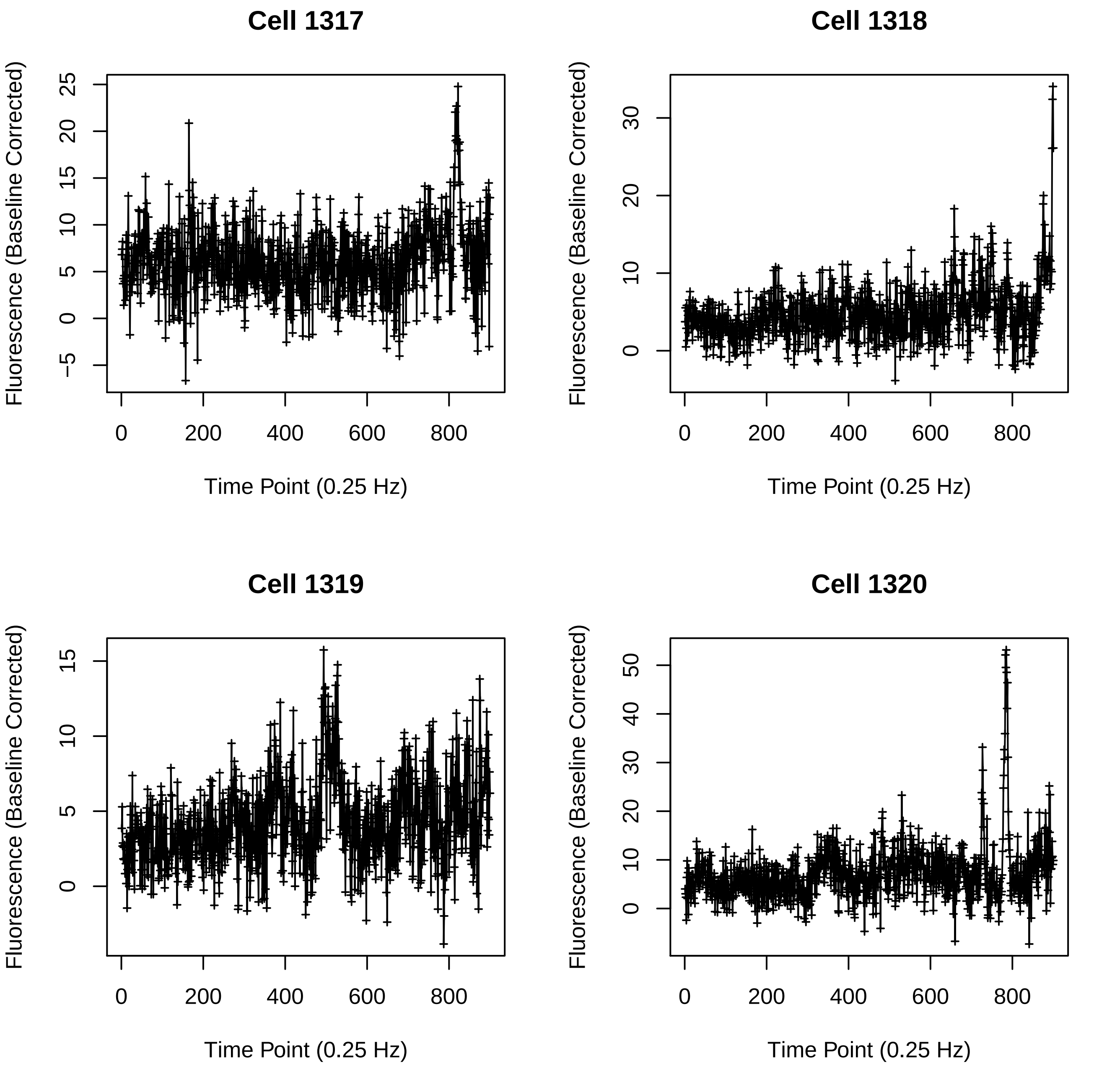
Cell 1317
Cell 1318
Cell 1319
Cell 1320
Fluorescence (Baseline Corrected)
Time Point (0.25 Hz)
0
200
400
600
800
−5
0
5
10
15
20
25
30
40
50

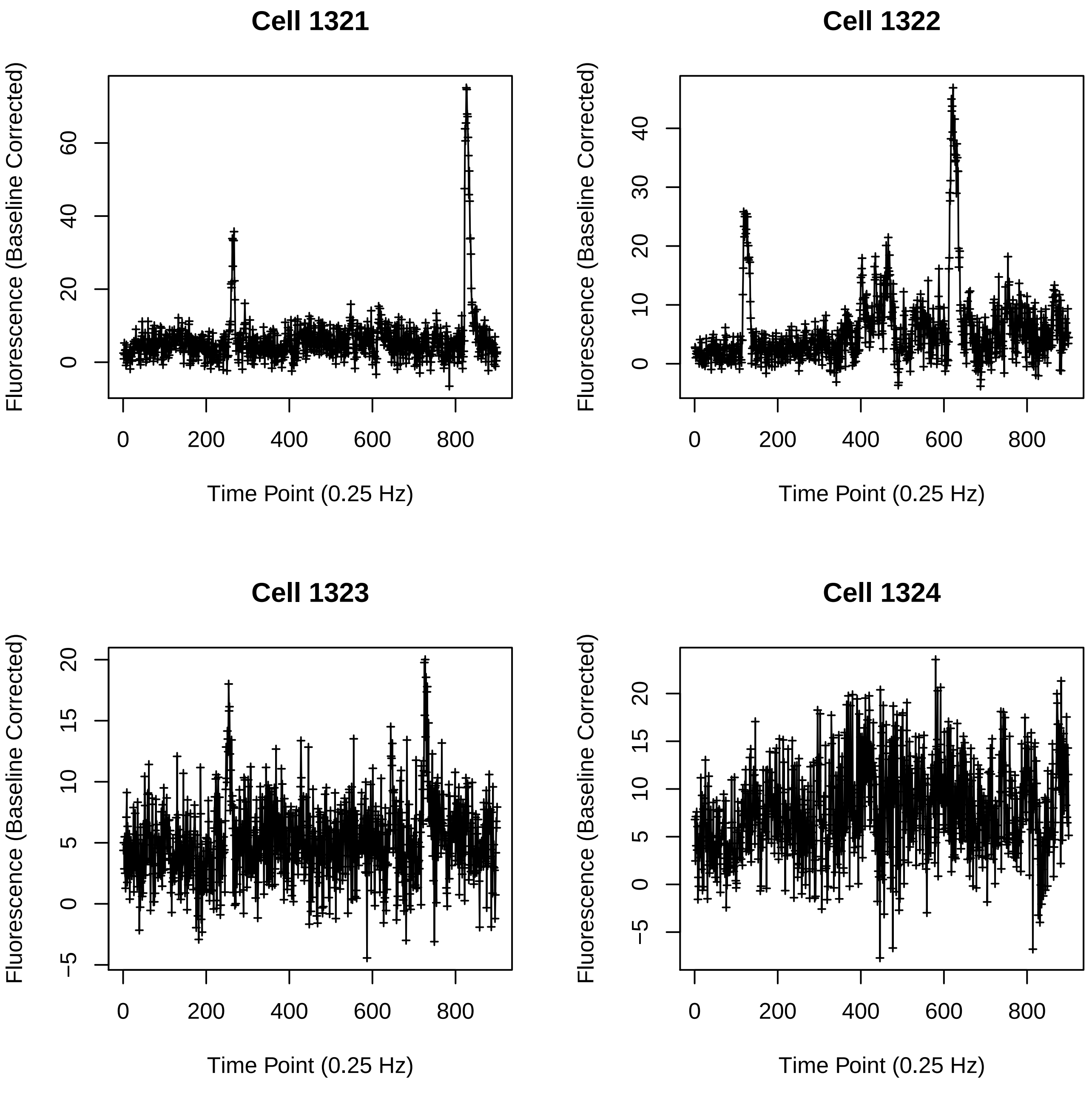
Cell 1321
Cell 1322
Cell 1323
Cell 1324
Fluorescence (Baseline Corrected)
Time Point (0.25 Hz)
0
20
40
60
10
30
5
15
−5
200
400
600
800

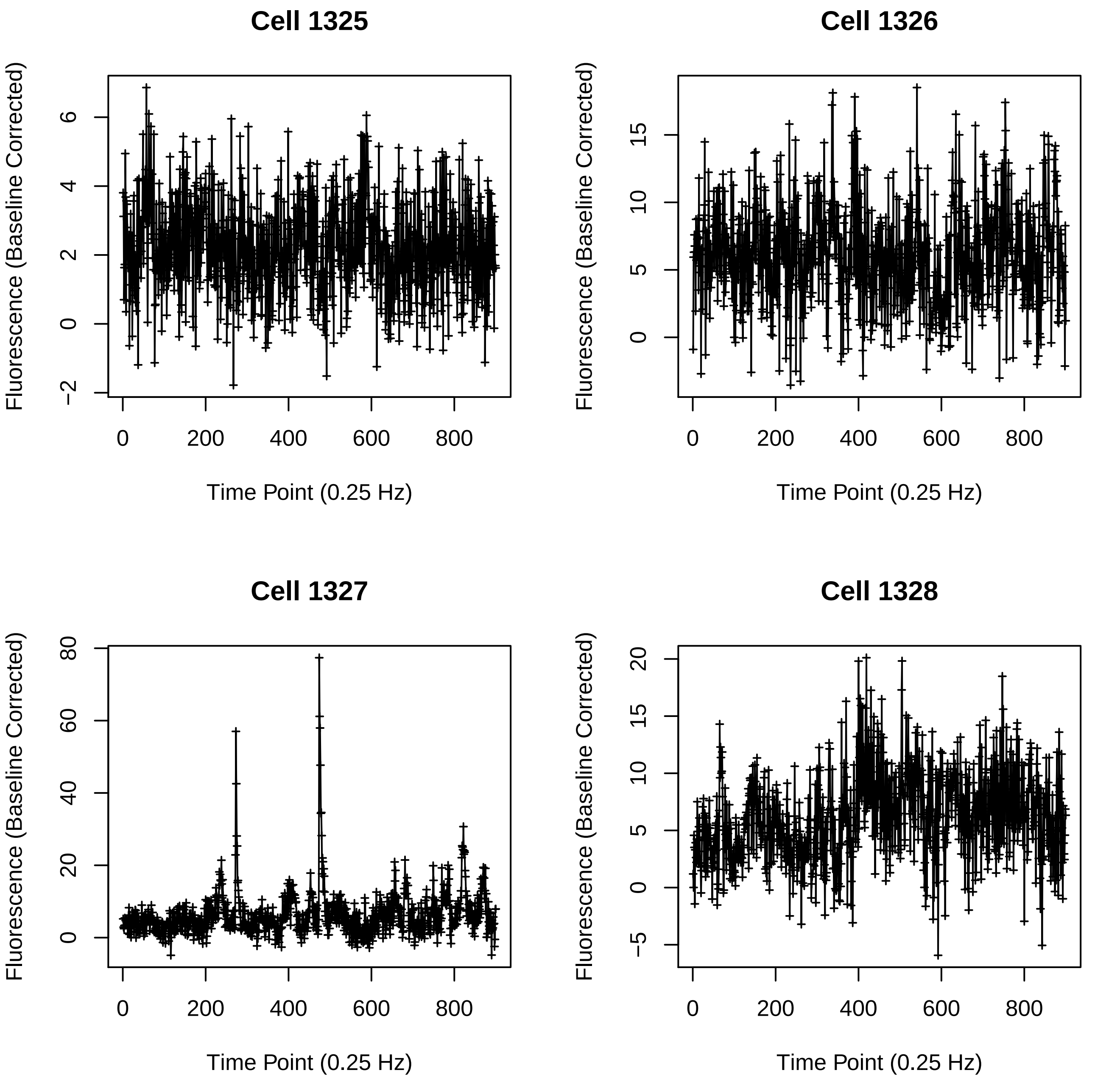
Cell 1325
Cell 1326
Cell 1327
Cell 1328
Fluorescence (Baseline Corrected)
Time Point (0.25 Hz)
0
200
400
600
800
-2
0
2
4
6
5
10
15
20
40
60
80
-5

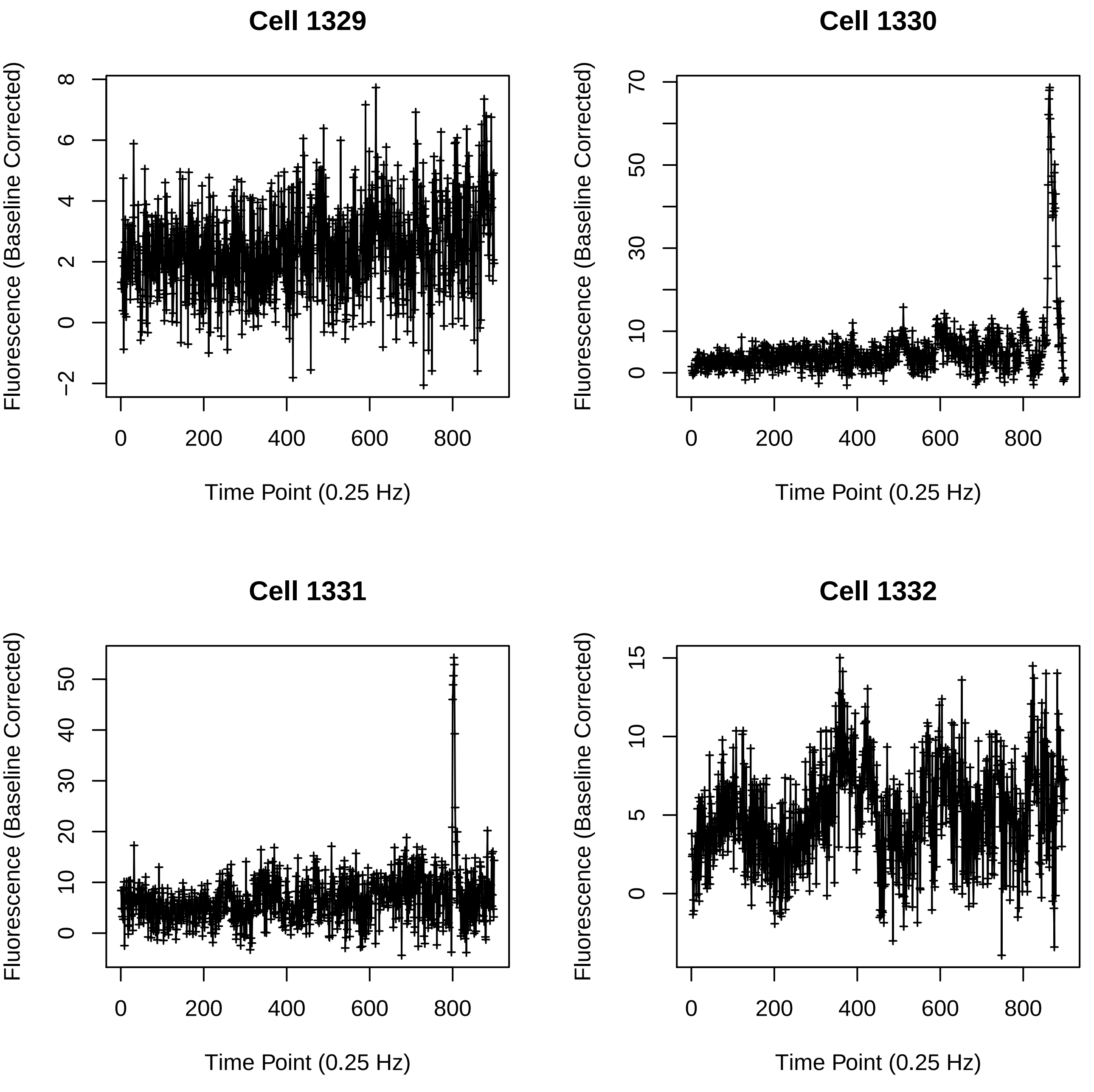
Cell 1329
Cell 1330
Cell 1331
Cell 1332
Fluorescence (Baseline Corrected)
Time Point (0.25 Hz)
−2
0
2
4
6
8
10
20
30
40
50
60
70
5
15
200
400
600
800

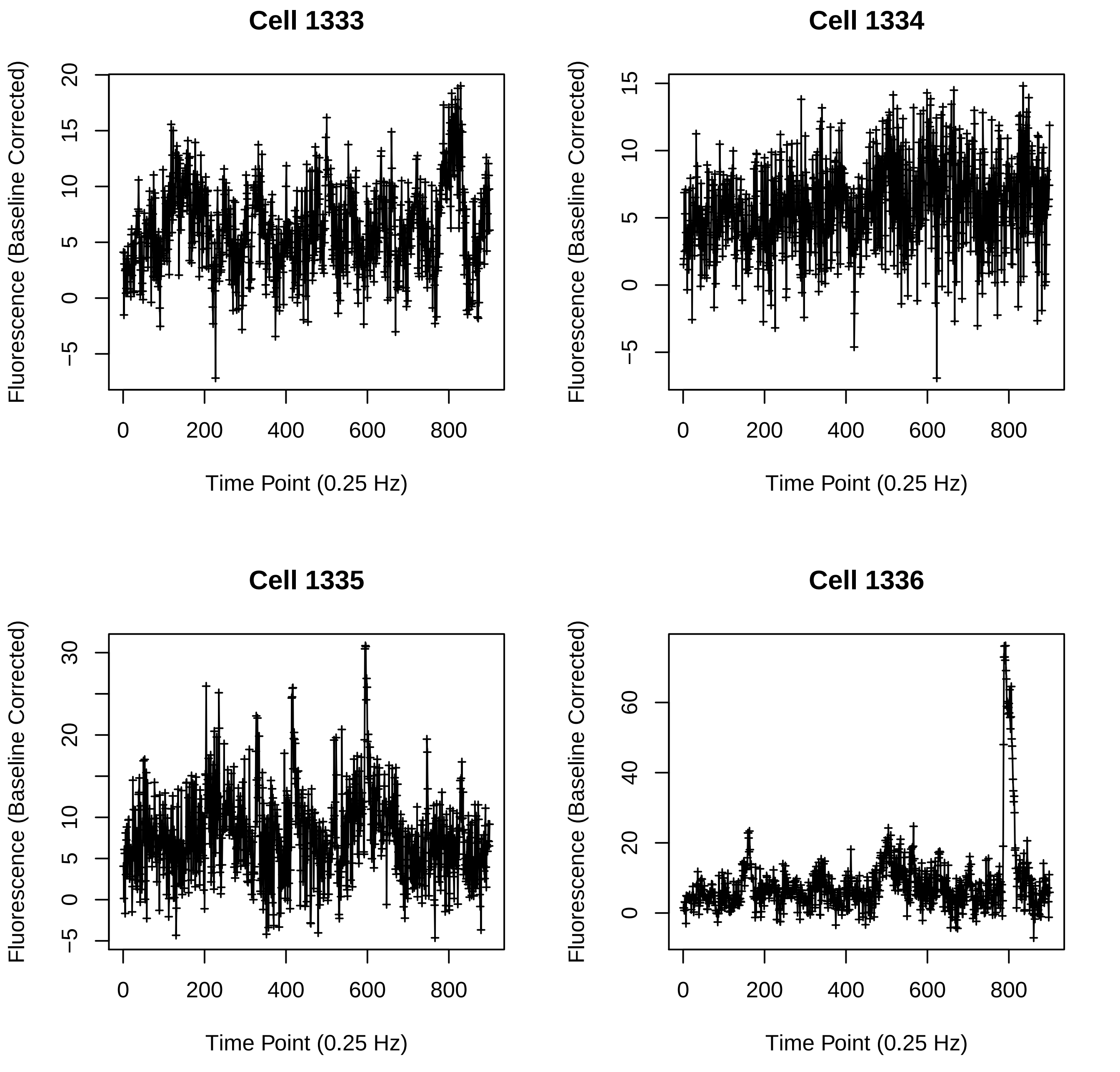

Cell 1333
Fluorescence (Baseline Corrected)
−5
0
5
10
15
20
0
200
400
600
800
Time Point (0.25 Hz)
Cell 1334
Fluorescence (Baseline Corrected)
−5
0
5
10
15
0
200
400
600
800
Time Point (0.25 Hz)
Cell 1335
Fluorescence (Baseline Corrected)
−5
0
5
10
20
30
0
200
400
600
800
Time Point (0.25 Hz)
Cell 1336
Fluorescence (Baseline Corrected)
0
20
40
60
0
200
400
600
800
Time Point (0.25 Hz)

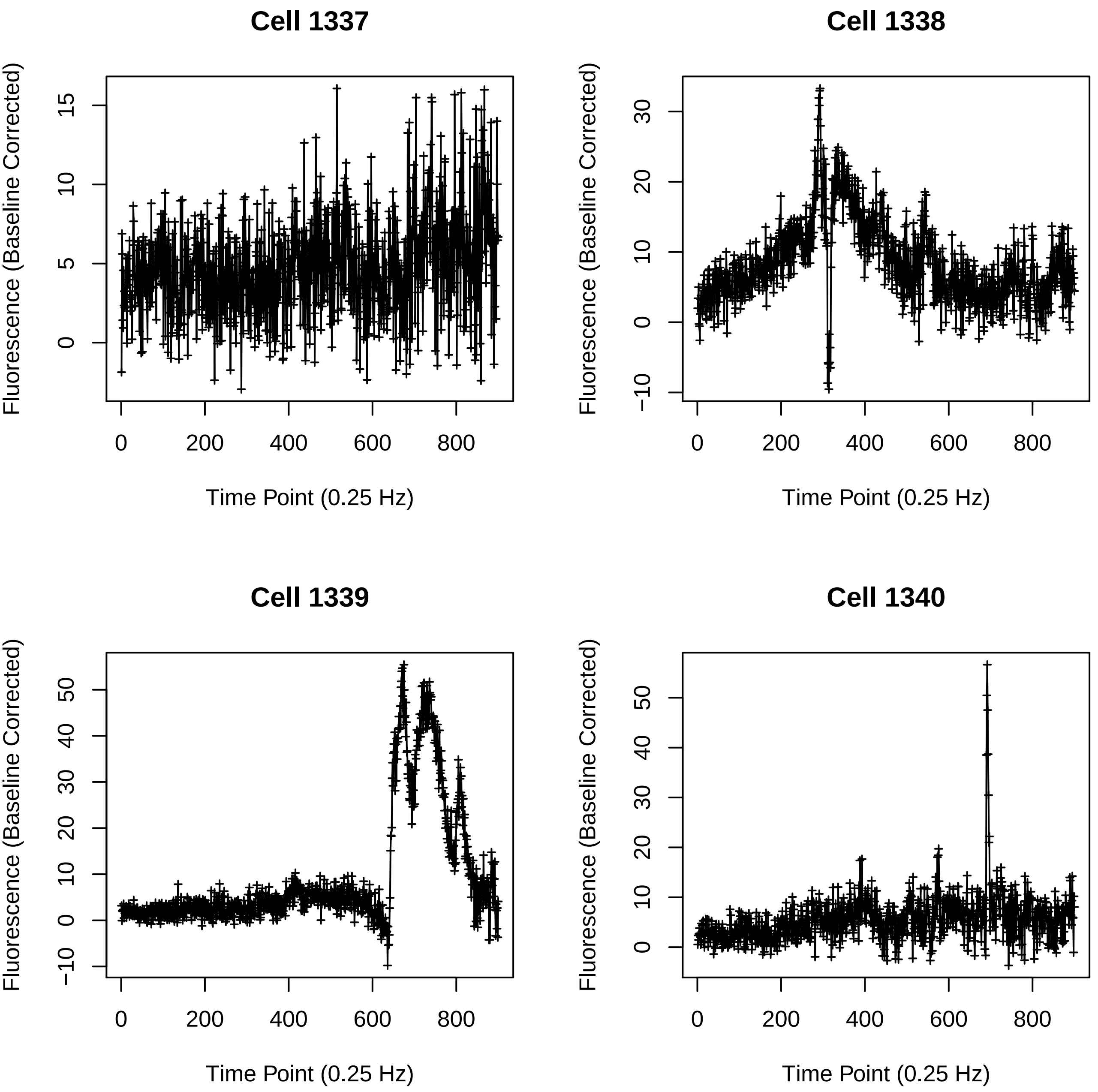
Cell 1337
Cell 1338
Cell 1339
Cell 1340
Fluorescence (Baseline Corrected)
Time Point (0.25 Hz)

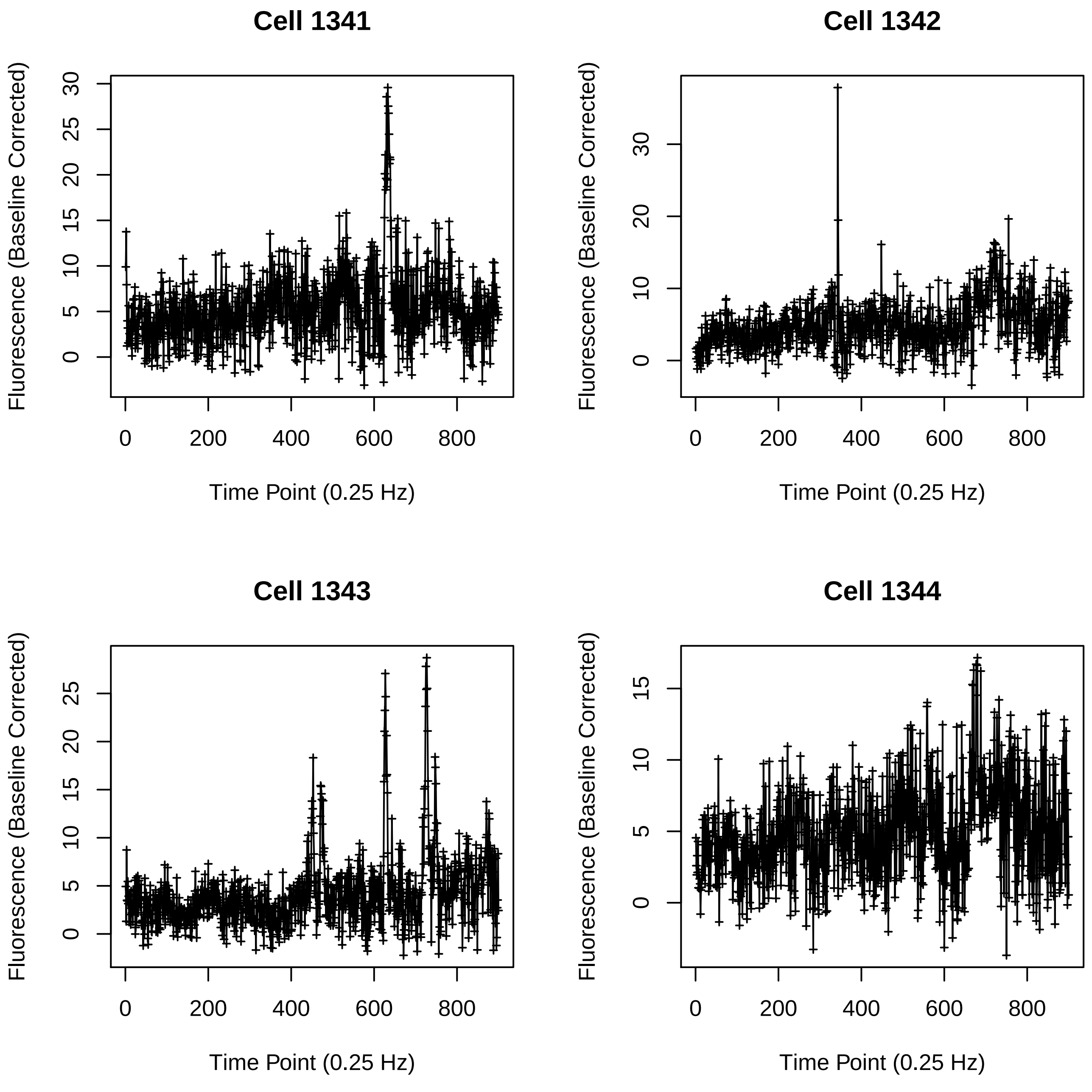

Cell 1341
Cell 1342
Cell 1343
Cell 1344
Fluorescence (Baseline Corrected)
Time Point (0.25 Hz)
0
200
400
600
800
0
5
10
15
20
25
30

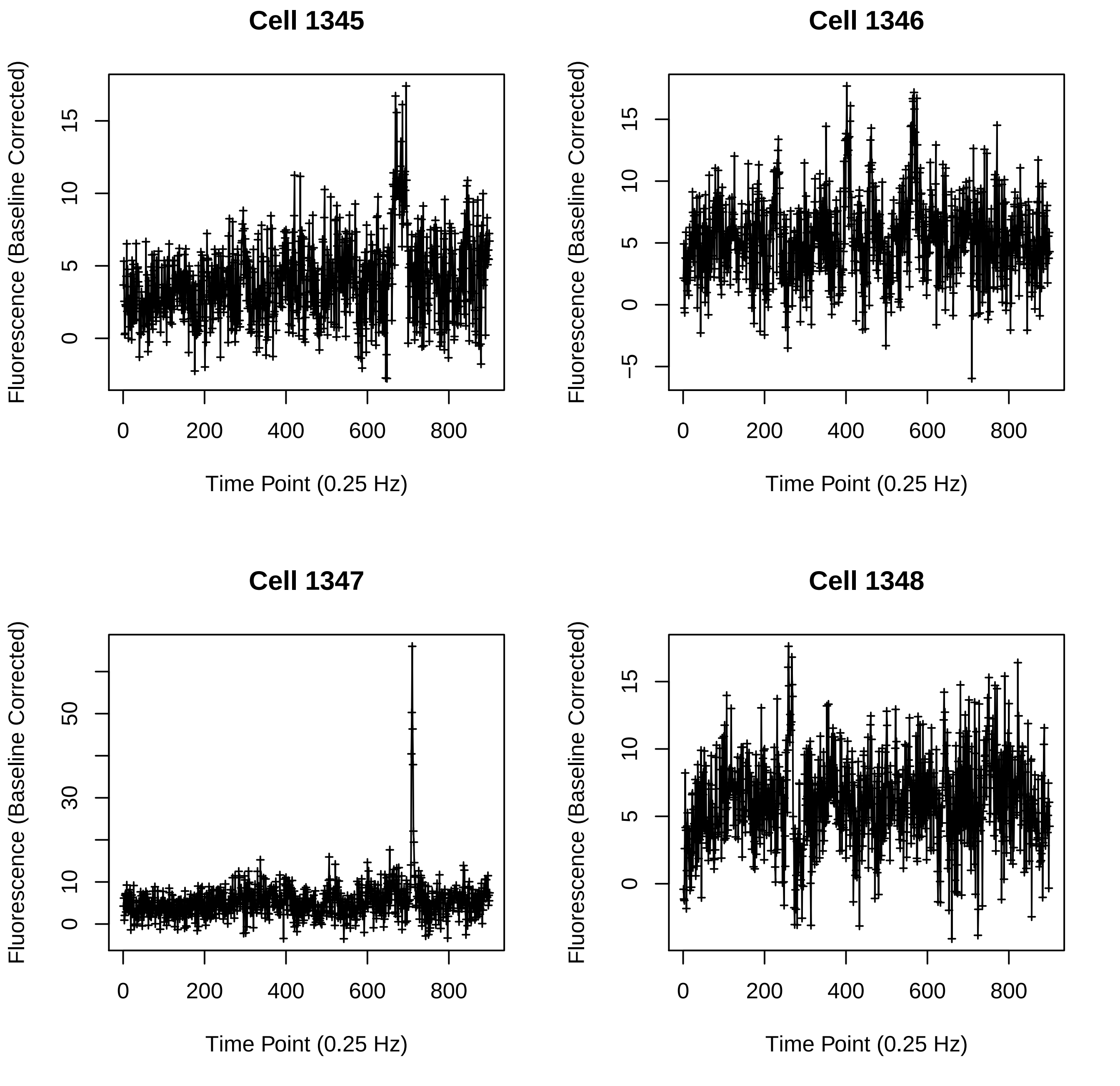
Cell 1345
Cell 1346
Cell 1347
Cell 1348
Fluorescence (Baseline Corrected)
Time Point (0.25 Hz)
0
200
400
600
800
-5
5
10
15
30
50

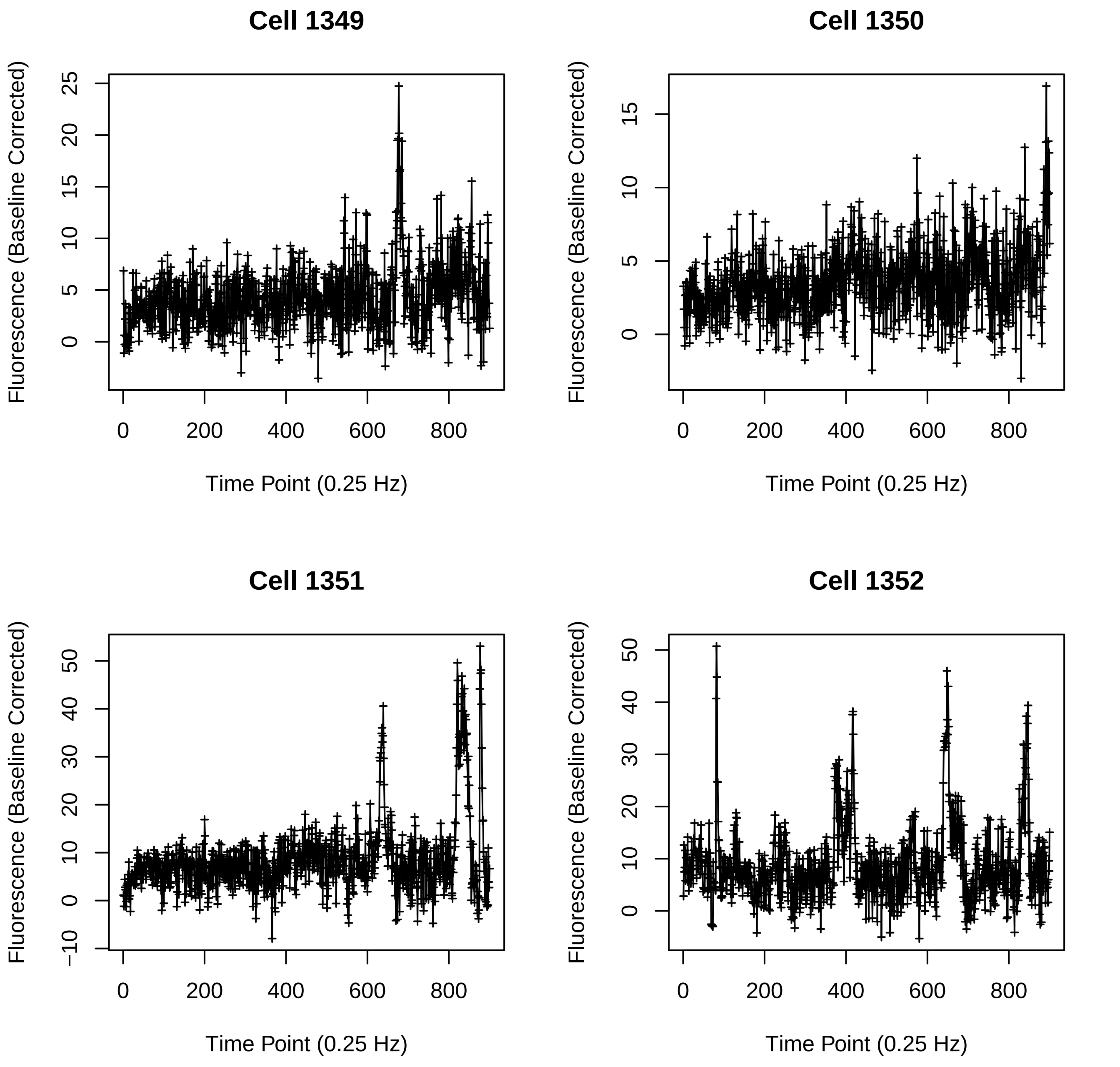

Cell 1349
Cell 1350
Cell 1351
Cell 1352
Fluorescence (Baseline Corrected)
Time Point (0.25 Hz)
0
200
400
600
800

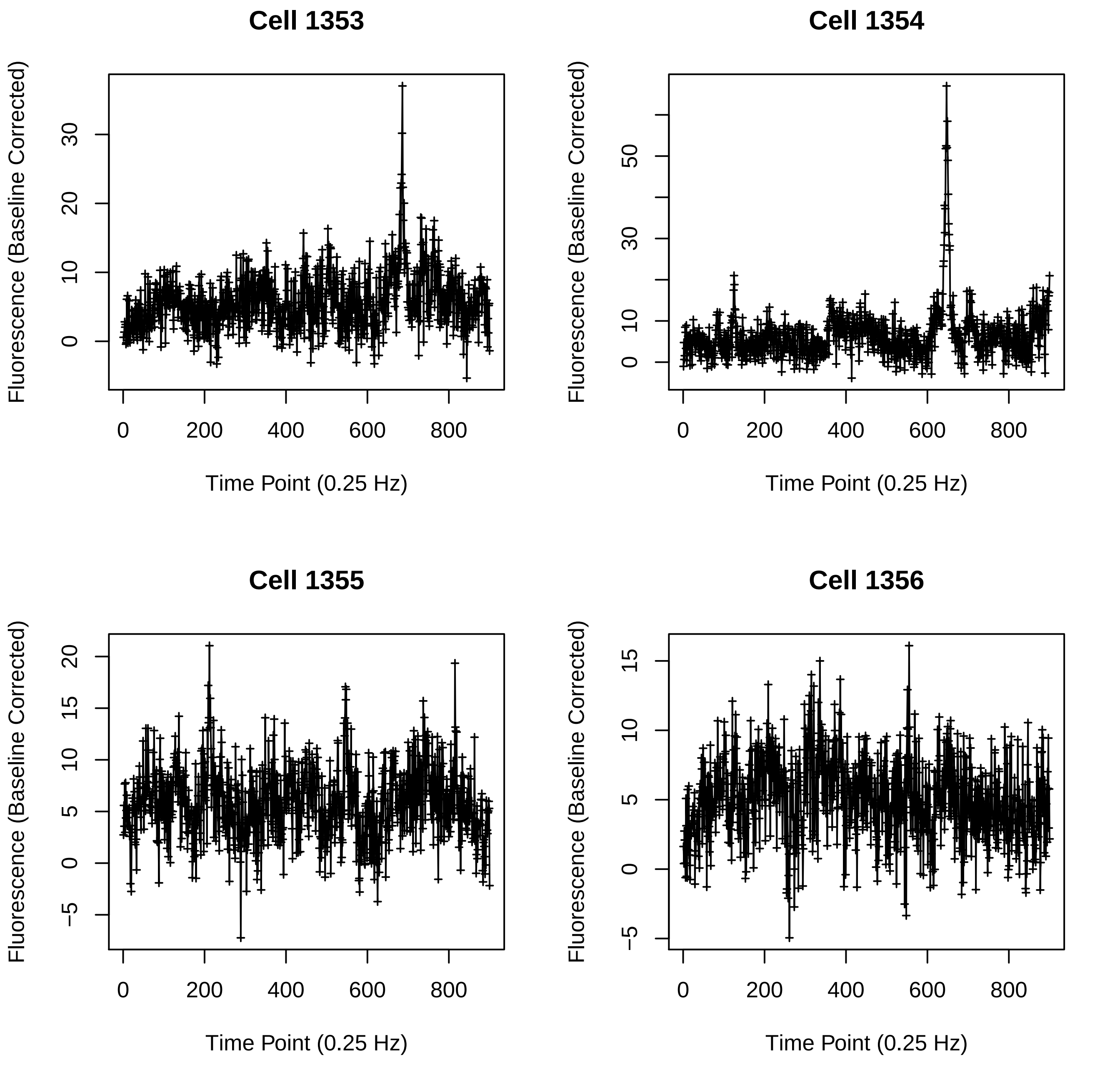

Cell 1353
Cell 1354
Cell 1355
Cell 1356
Fluorescence (Baseline Corrected)
Time Point (0.25 Hz)
0
200
400
600
800
0
10
20
30
50
−5
5
15

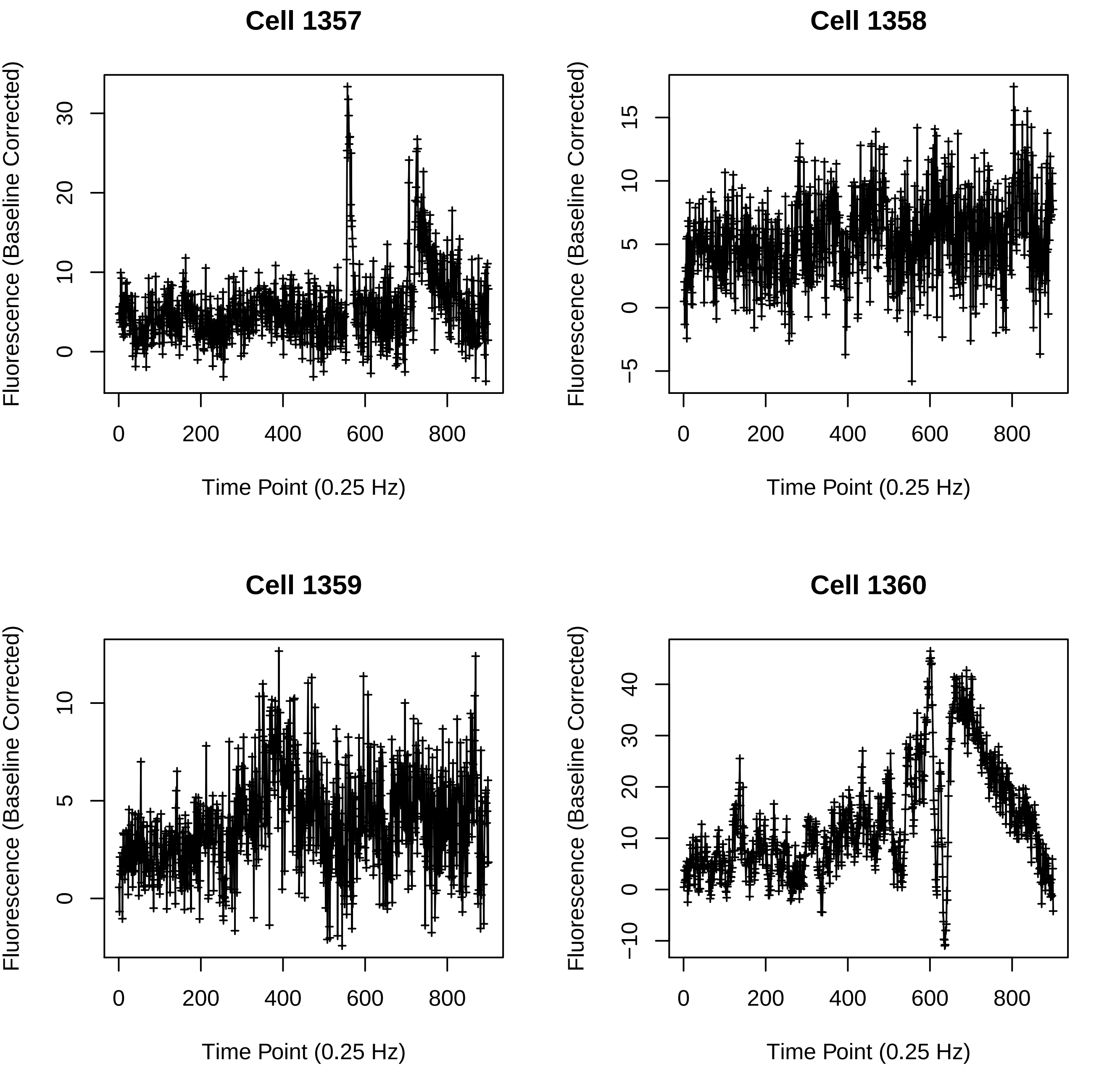
Cell 1357
Cell 1358
Cell 1359
Cell 1360
Fluorescence (Baseline Corrected)
Time Point (0.25 Hz)
0
200
400
600
800
10
20
30
−5
5
15
−10
40

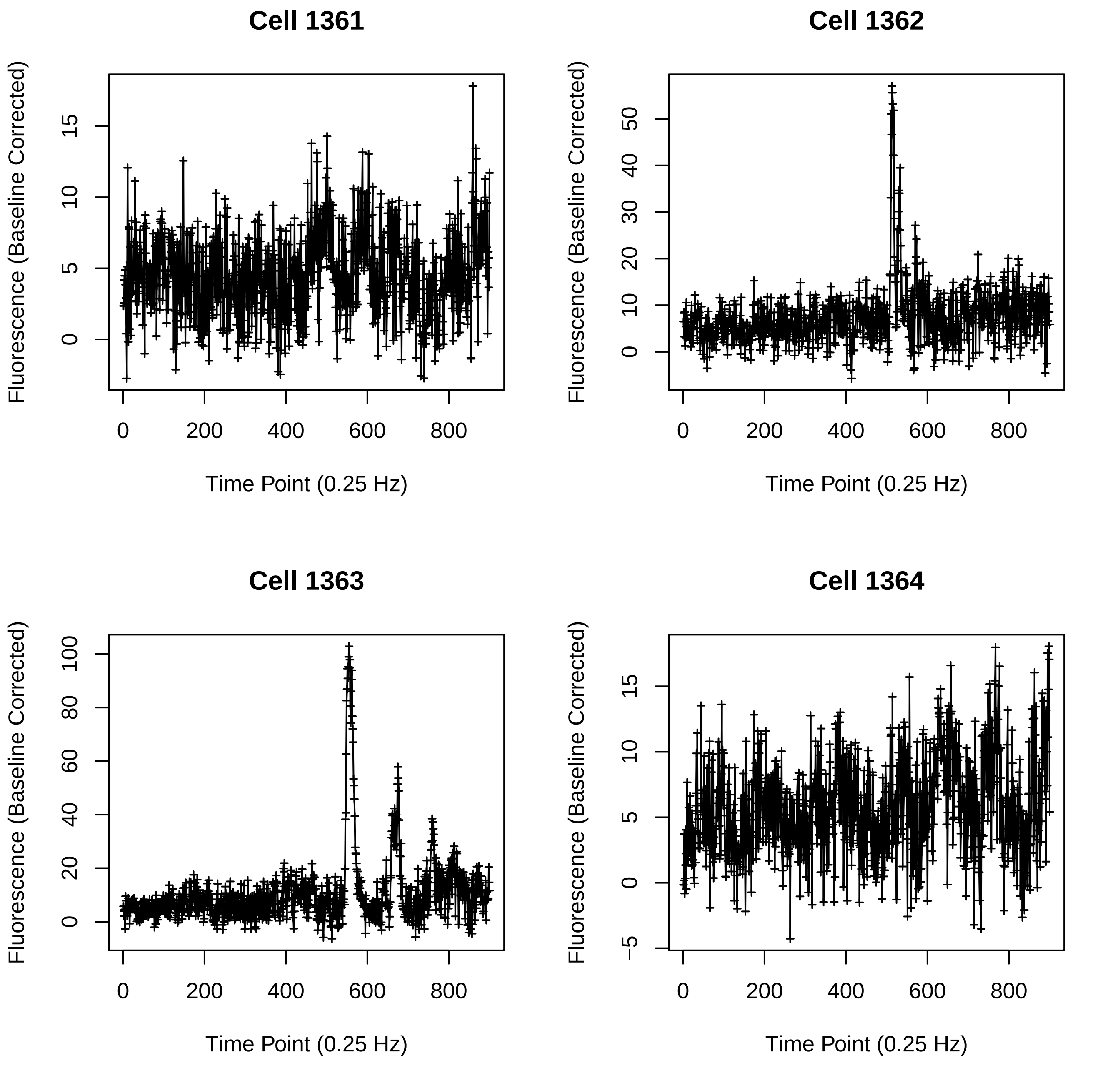
Cell 1361
Cell 1362
Cell 1363
Cell 1364
Fluorescence (Baseline Corrected)
Time Point (0.25 Hz)
0
200
400
600
800
−5
5
10
15
20
30
40
50
60
80
100

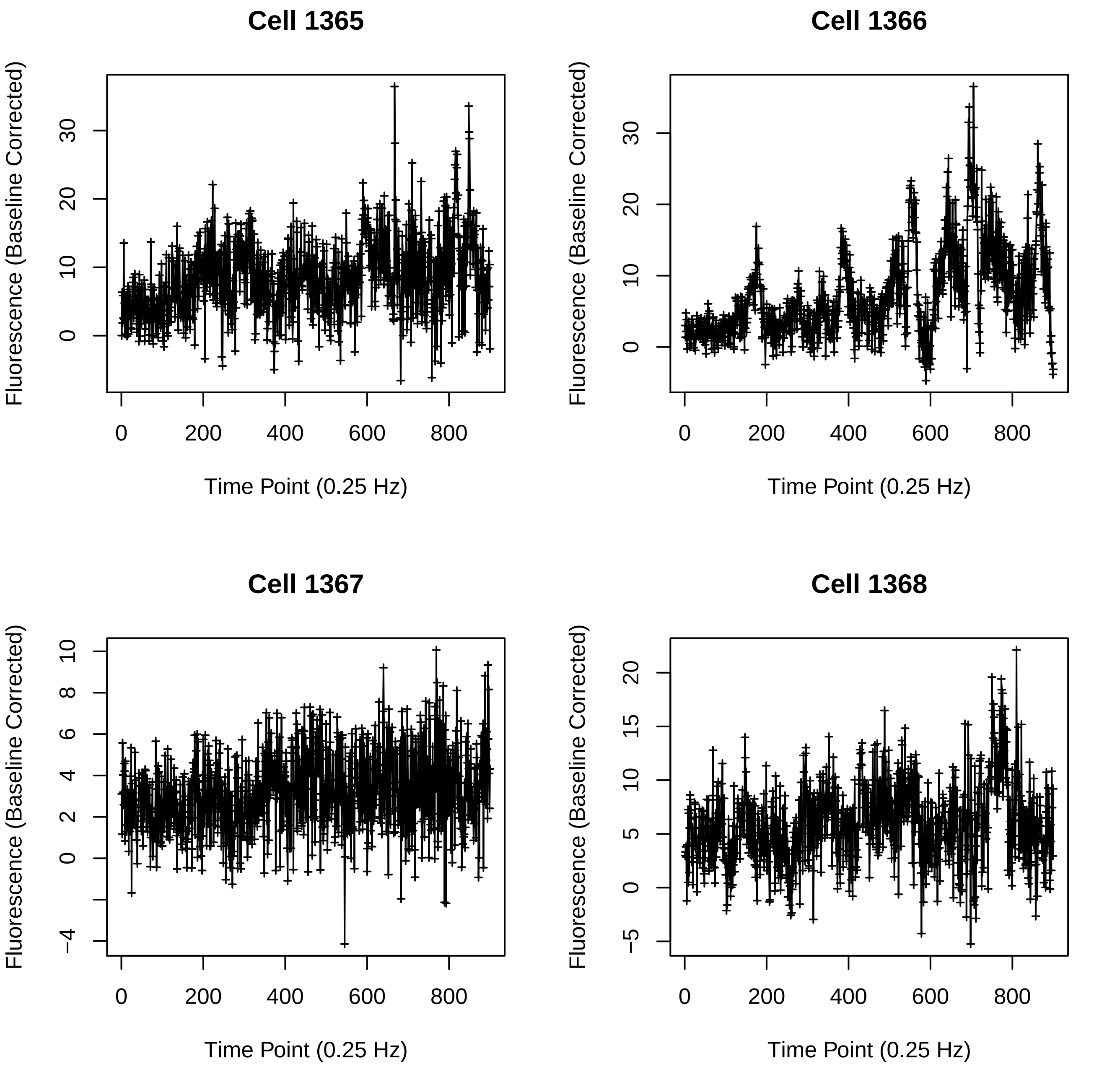

Cell 1365
Cell 1366
Cell 1367
Cell 1368
Fluorescence (Baseline Corrected)
Time Point (0.25 Hz)
0
10
20
30
-4
0
2
4
6
8
10
-5
5
15
20
200
400
600
800

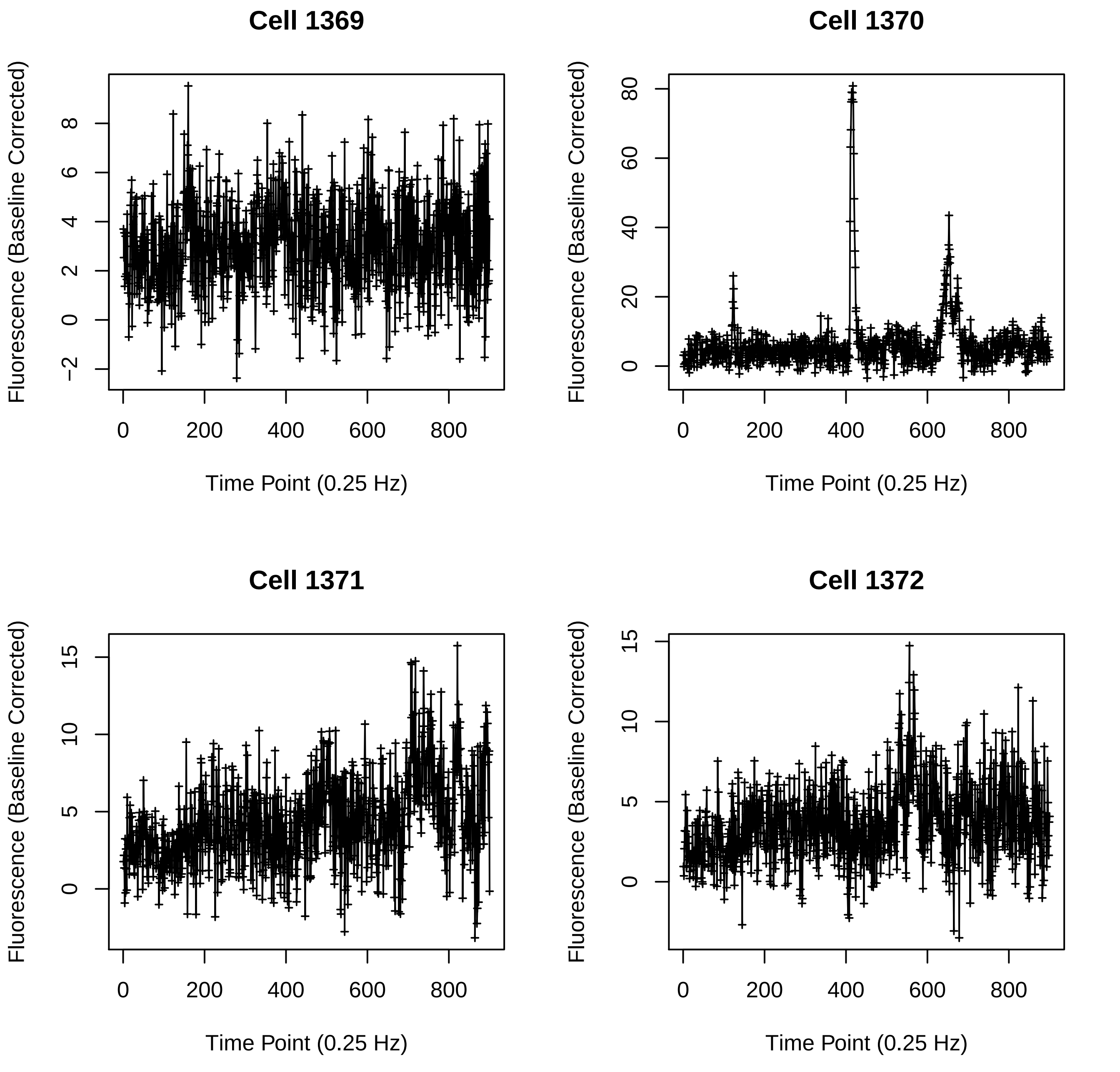
Cell 1369
Cell 1370
Cell 1371
Cell 1372
Fluorescence (Baseline Corrected)
Time Point (0.25 Hz)
−2
0
2
4
6
8
20
40
60
80
5
10
15
200
400
600
800

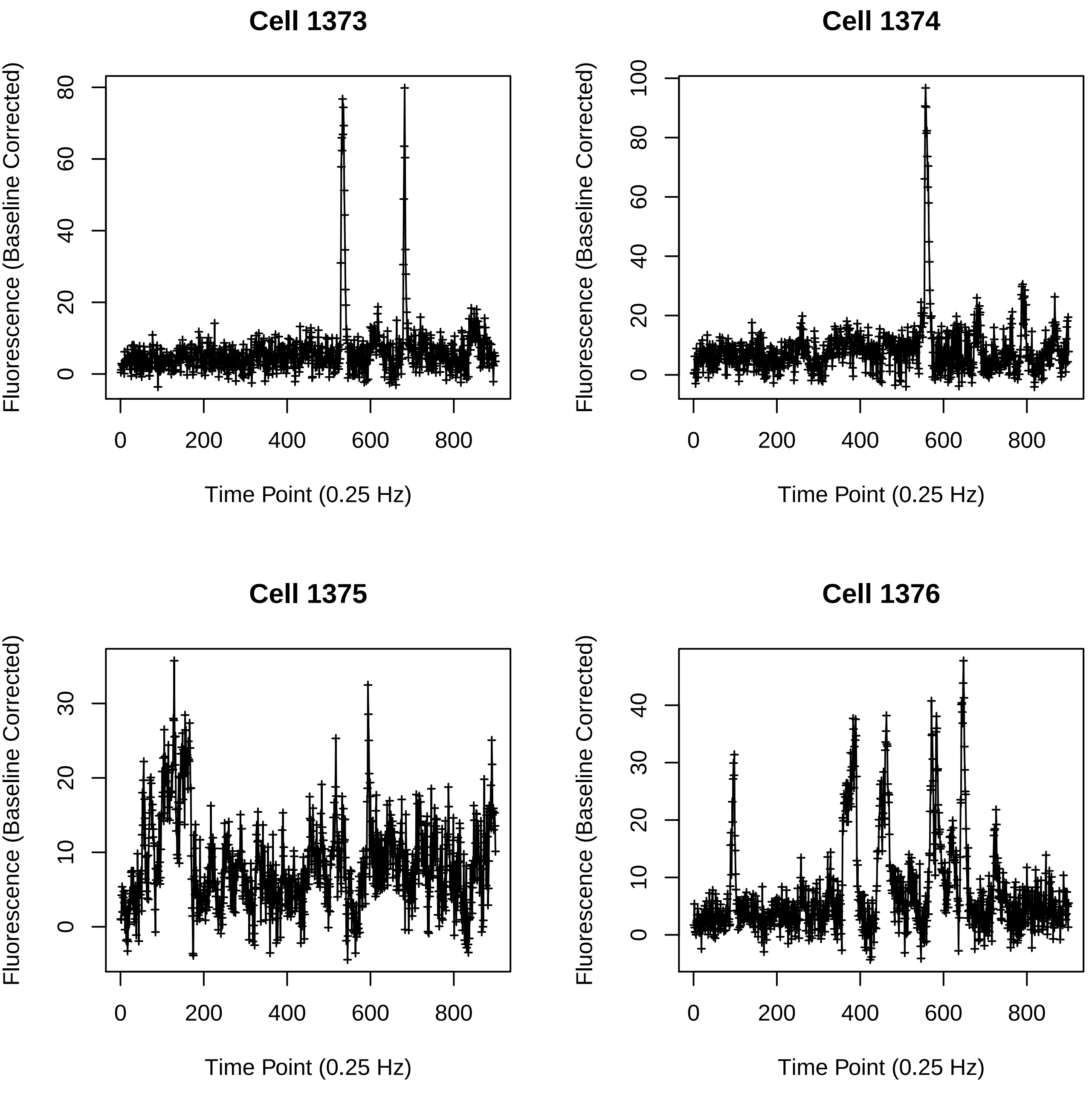
Cell 1373
Cell 1374
Cell 1375
Cell 1376
Fluorescence (Baseline Corrected)
Time Point (0.25 Hz)
0
20
40
60
80
100
10
30
200
400
600
800

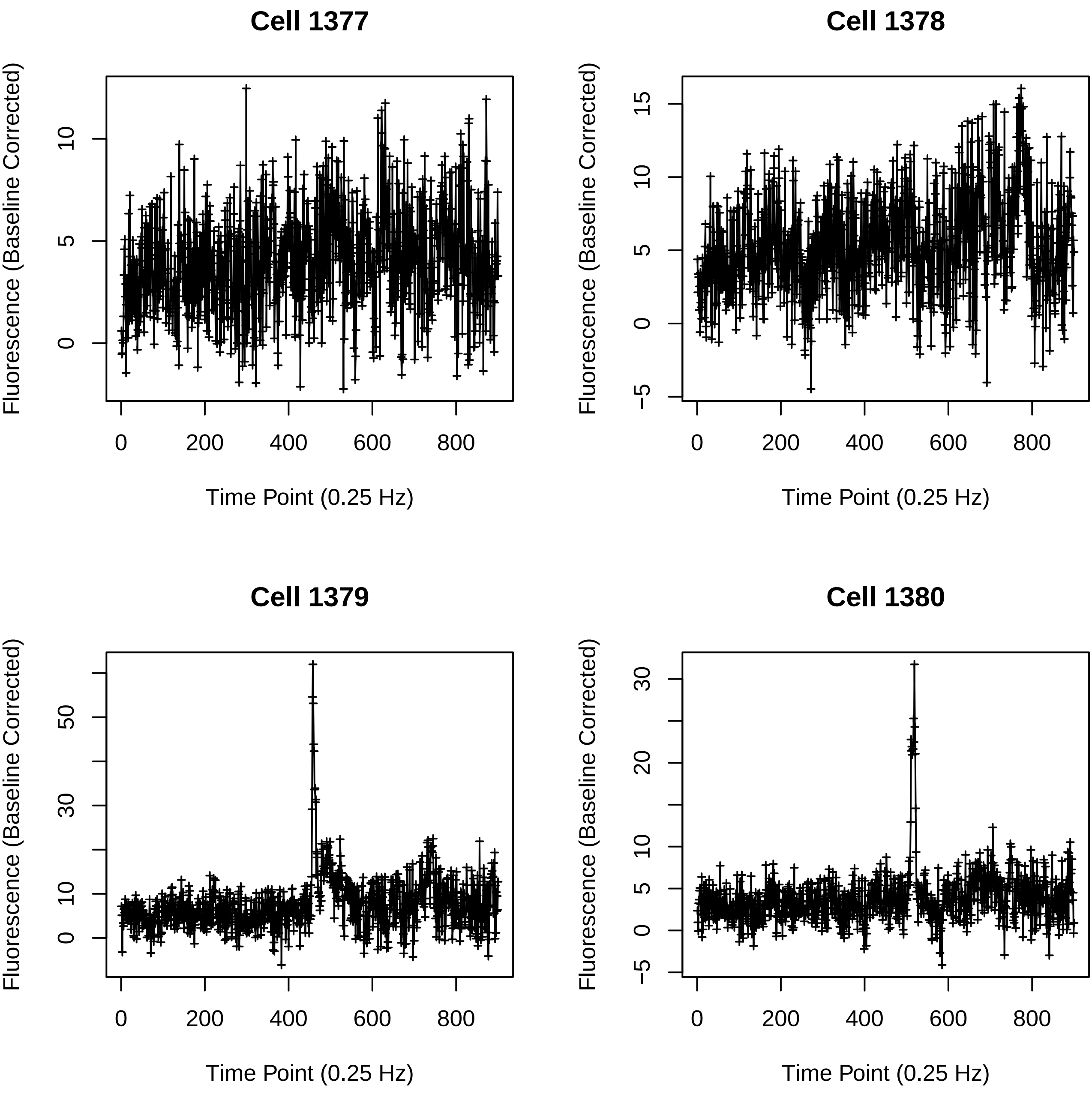
Cell 1377
Fluorescence (Baseline Corrected)
0
5
10
0
200
400
600
800
Time Point (0.25 Hz)
Cell 1378
Fluorescence (Baseline Corrected)
−5
0
5
10
15
0
200
400
600
800
Time Point (0.25 Hz)
Cell 1379
Fluorescence (Baseline Corrected)
0
10
30
50
0
200
400
600
800
Time Point (0.25 Hz)
Cell 1380
Fluorescence (Baseline Corrected)
−5
0
5
10
20
30
0
200
400
600
800
Time Point (0.25 Hz)

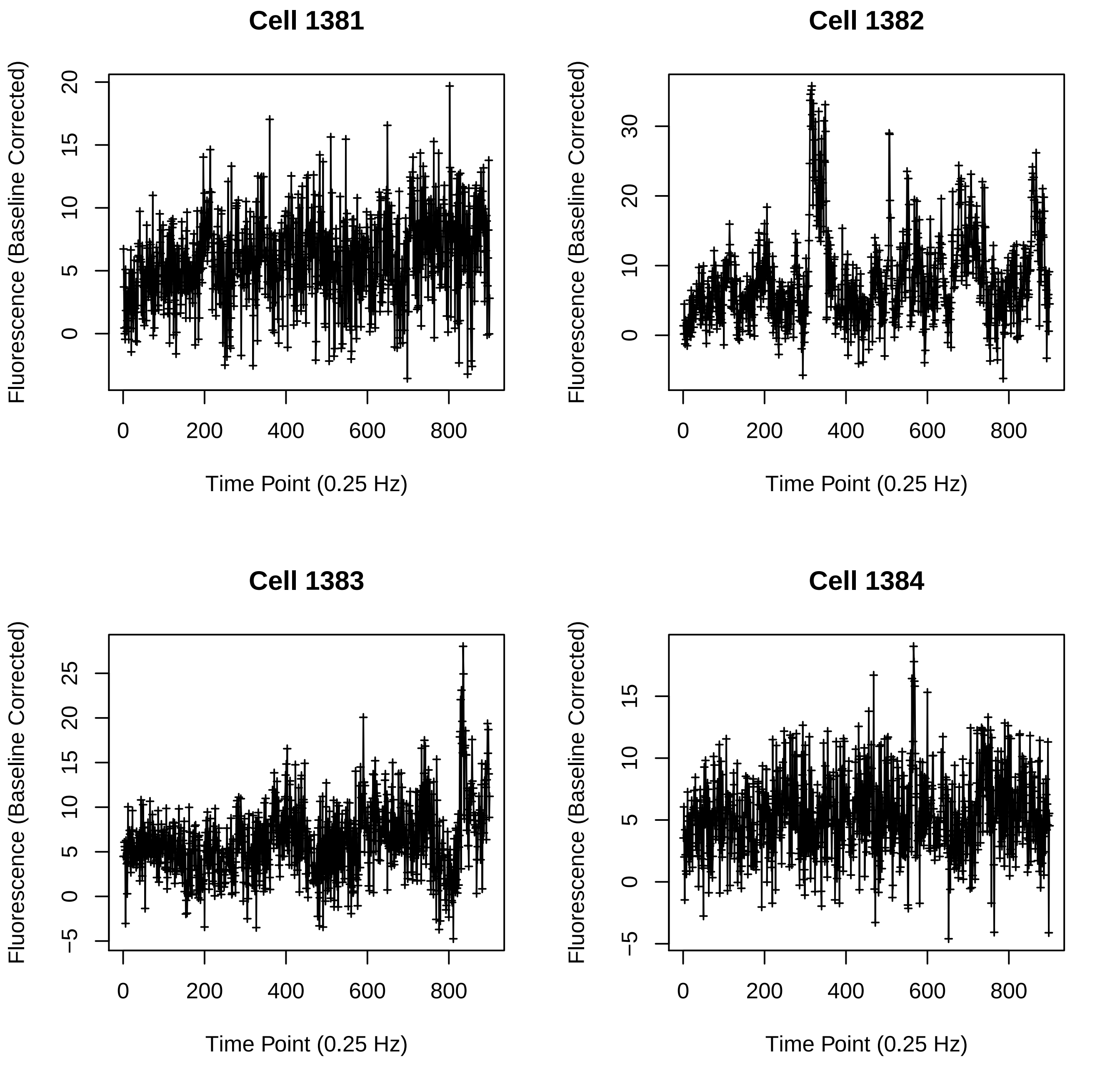
Cell 1381
Cell 1382
Cell 1383
Cell 1384
Fluorescence (Baseline Corrected)
Time Point (0.25 Hz)
0
200
400
600
800
−5
0
5
10
15
20
25
30

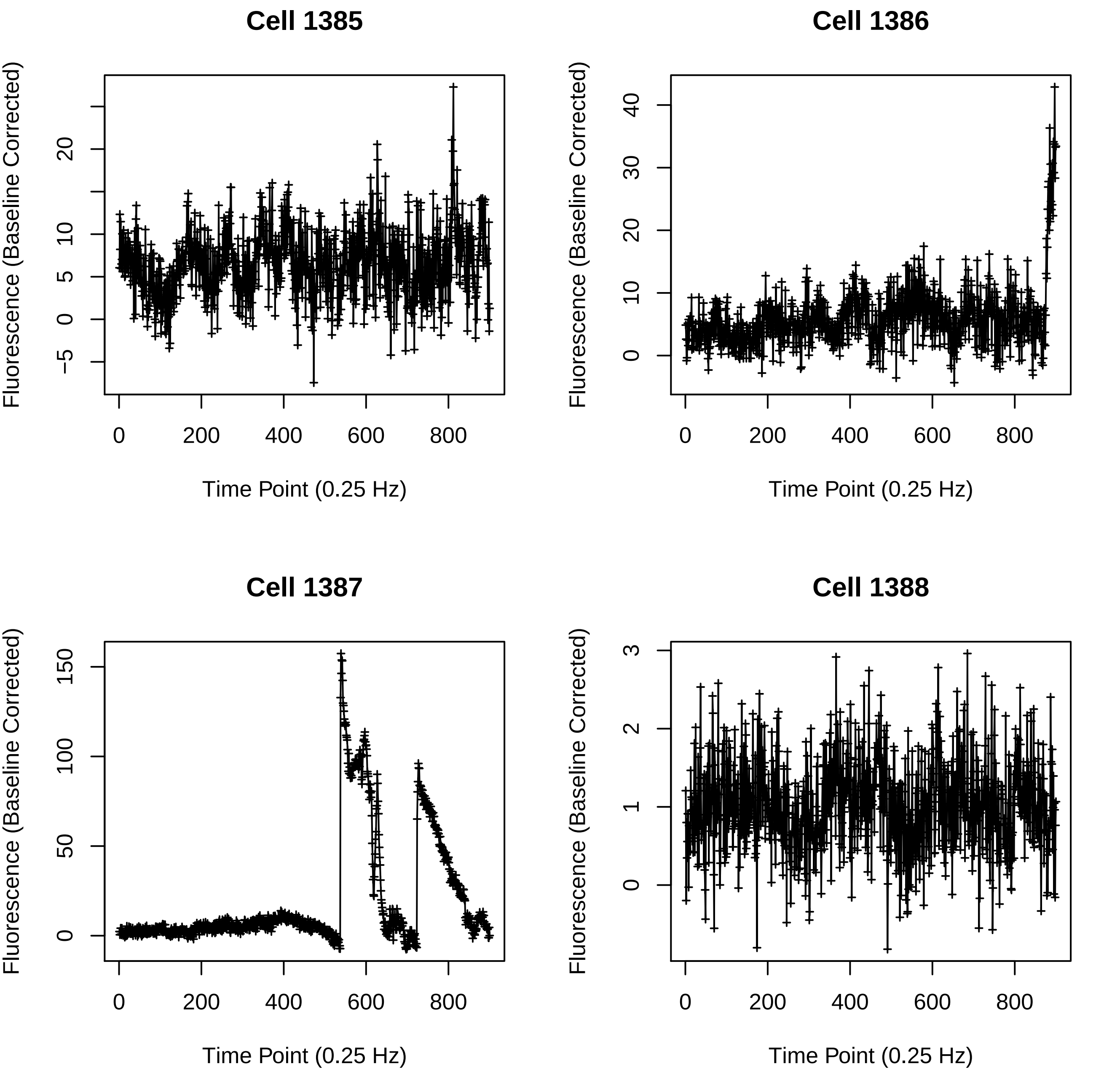
Cell 1385
Fluorescence (Baseline Corrected)
−5
0
5
10
20
0
200
400
600
800
Time Point (0.25 Hz)
Cell 1386
Fluorescence (Baseline Corrected)
0
10
20
30
40
0
200
400
600
800
Time Point (0.25 Hz)
Cell 1387
Fluorescence (Baseline Corrected)
0
50
100
150
0
200
400
600
800
Time Point (0.25 Hz)
Cell 1388
Fluorescence (Baseline Corrected)
0
1
2
3
0
200
400
600
800
Time Point (0.25 Hz)

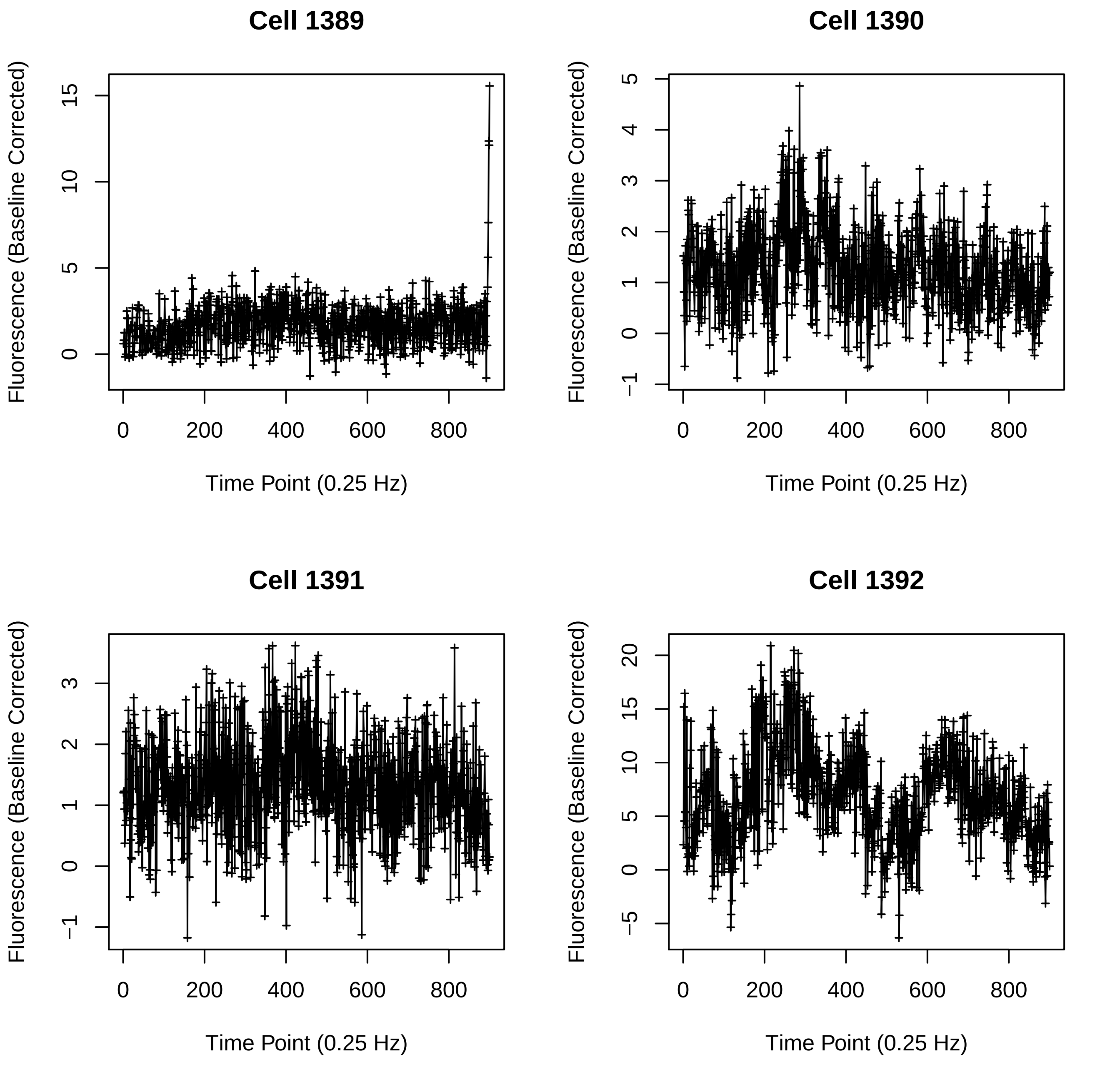
Cell 1389
Cell 1390
Cell 1391
Cell 1392
Fluorescence (Baseline Corrected)
Time Point (0.25 Hz)
0
200
400
600
800
0
5
10
15
−1
1
2
3
4
20
−5

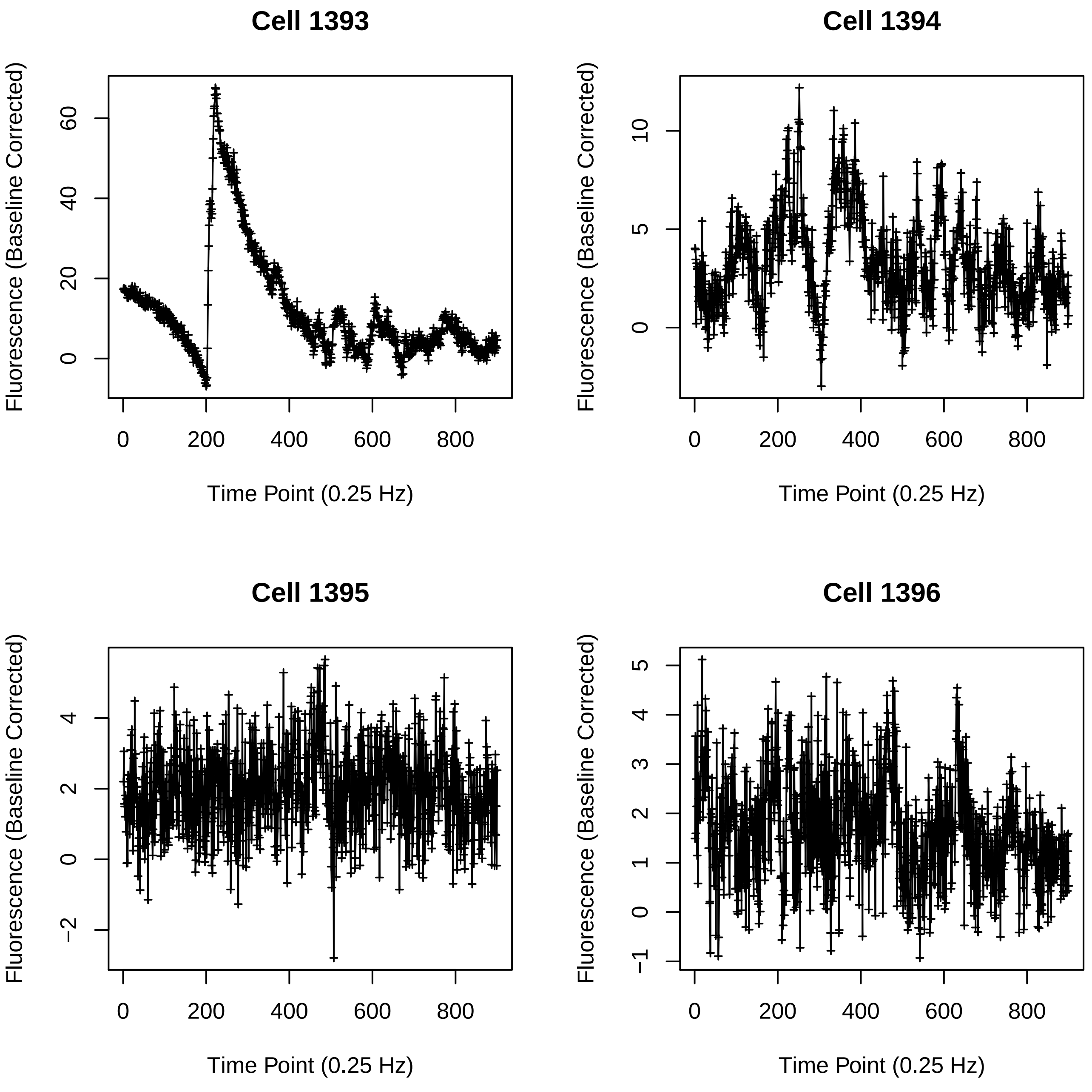
Cell 1393
Fluorescence (Baseline Corrected)
Time Point (0.25 Hz)
0
20
40
60
0
200
400
600
800
Cell 1394
Fluorescence (Baseline Corrected)
Time Point (0.25 Hz)
0
5
10
0
200
400
600
800
Cell 1395
Fluorescence (Baseline Corrected)
Time Point (0.25 Hz)
−2
0
2
4
0
200
400
600
800
Cell 1396
Fluorescence (Baseline Corrected)
Time Point (0.25 Hz)
−1
0
1
2
3
4
5
0
200
400
600
800

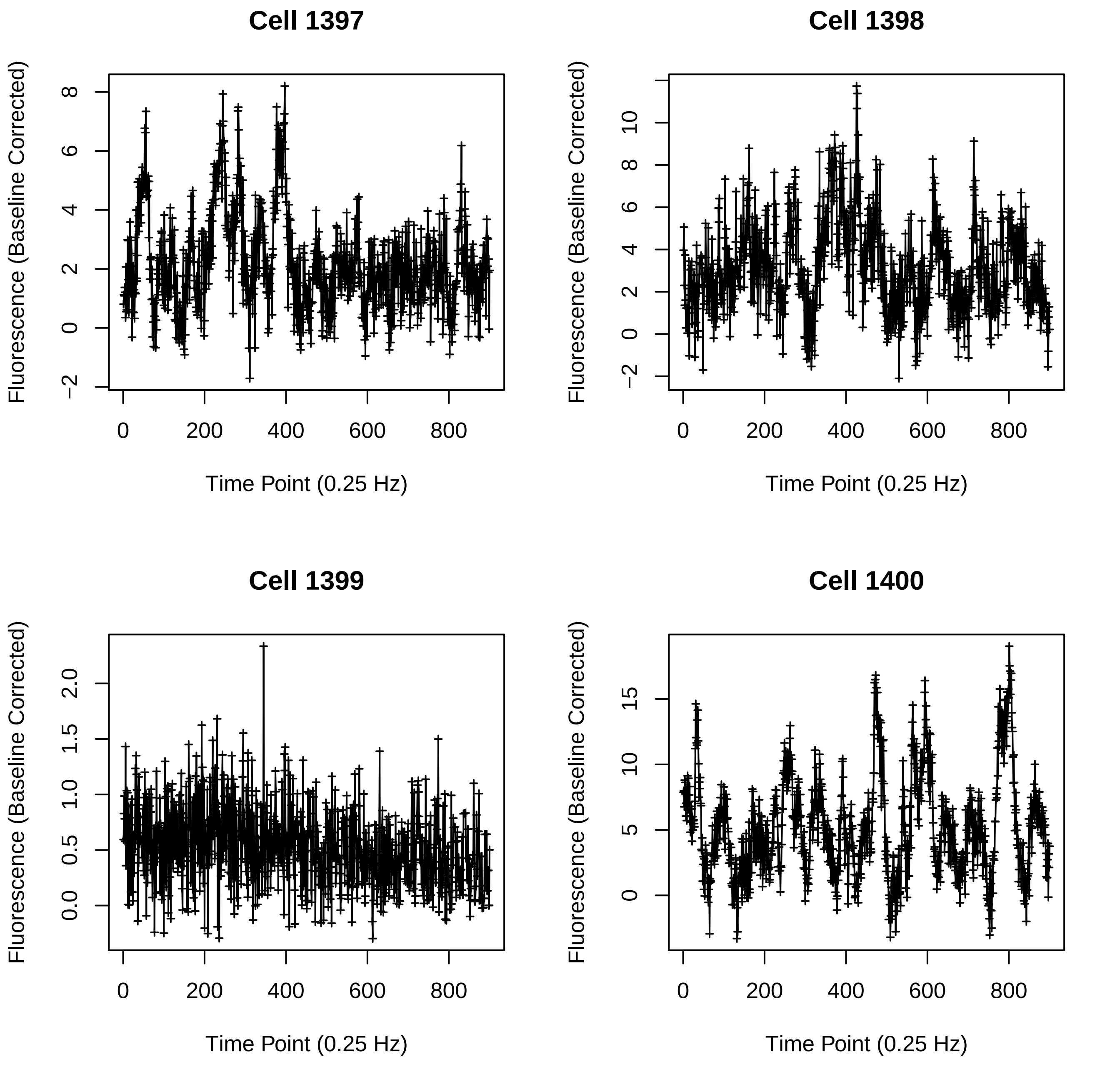

Cell 1397
Cell 1398
Cell 1399
Cell 1400
Fluorescence (Baseline Corrected)
Time Point (0.25 Hz)

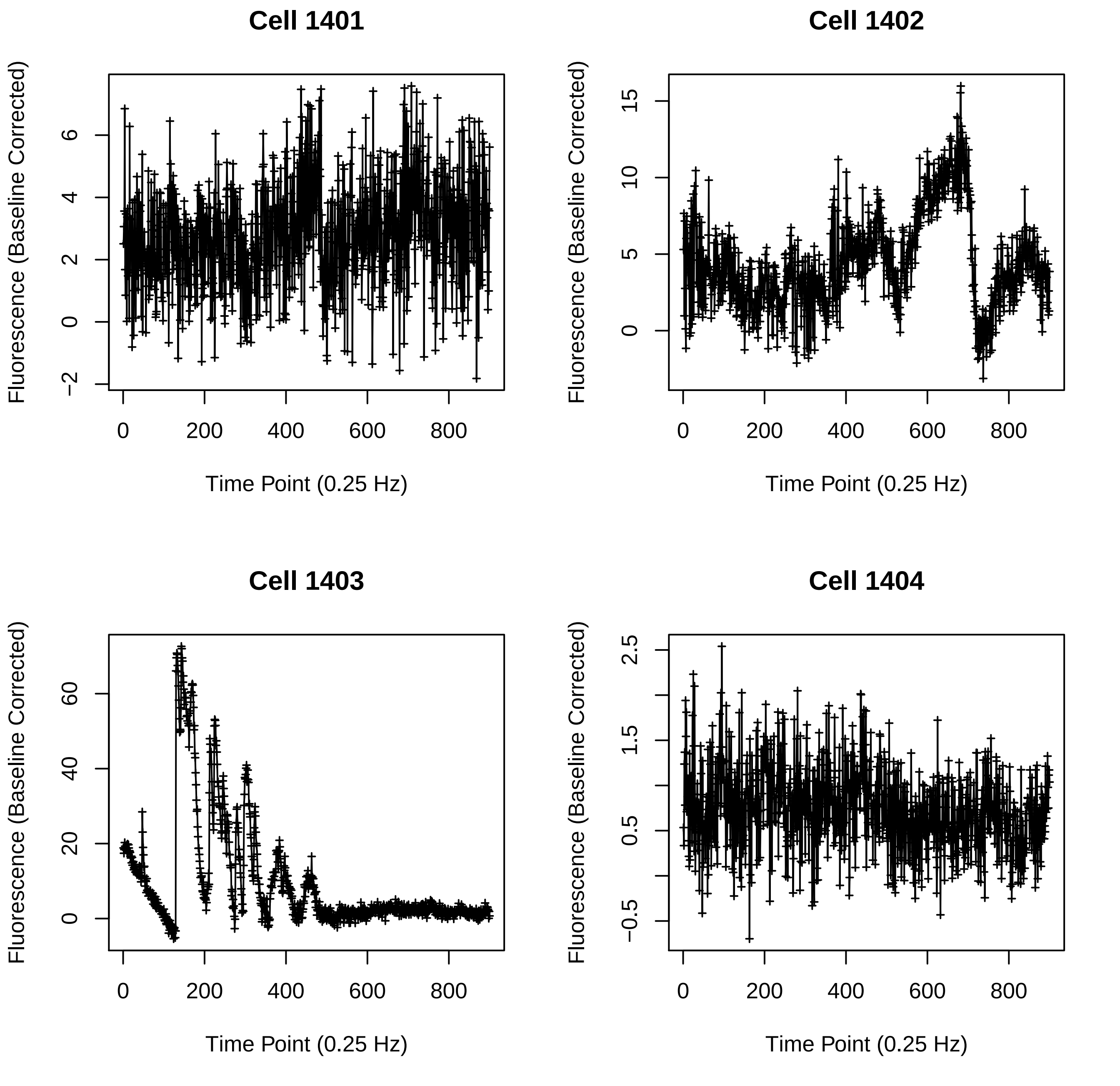

Cell 1401
Cell 1402
Cell 1403
Cell 1404
Fluorescence (Baseline Corrected)
Time Point (0.25 Hz)
0
200
400
600
800
−2
2
4
6
5
10
15
20
40
60
−0.5
0.5
1.5
2.5

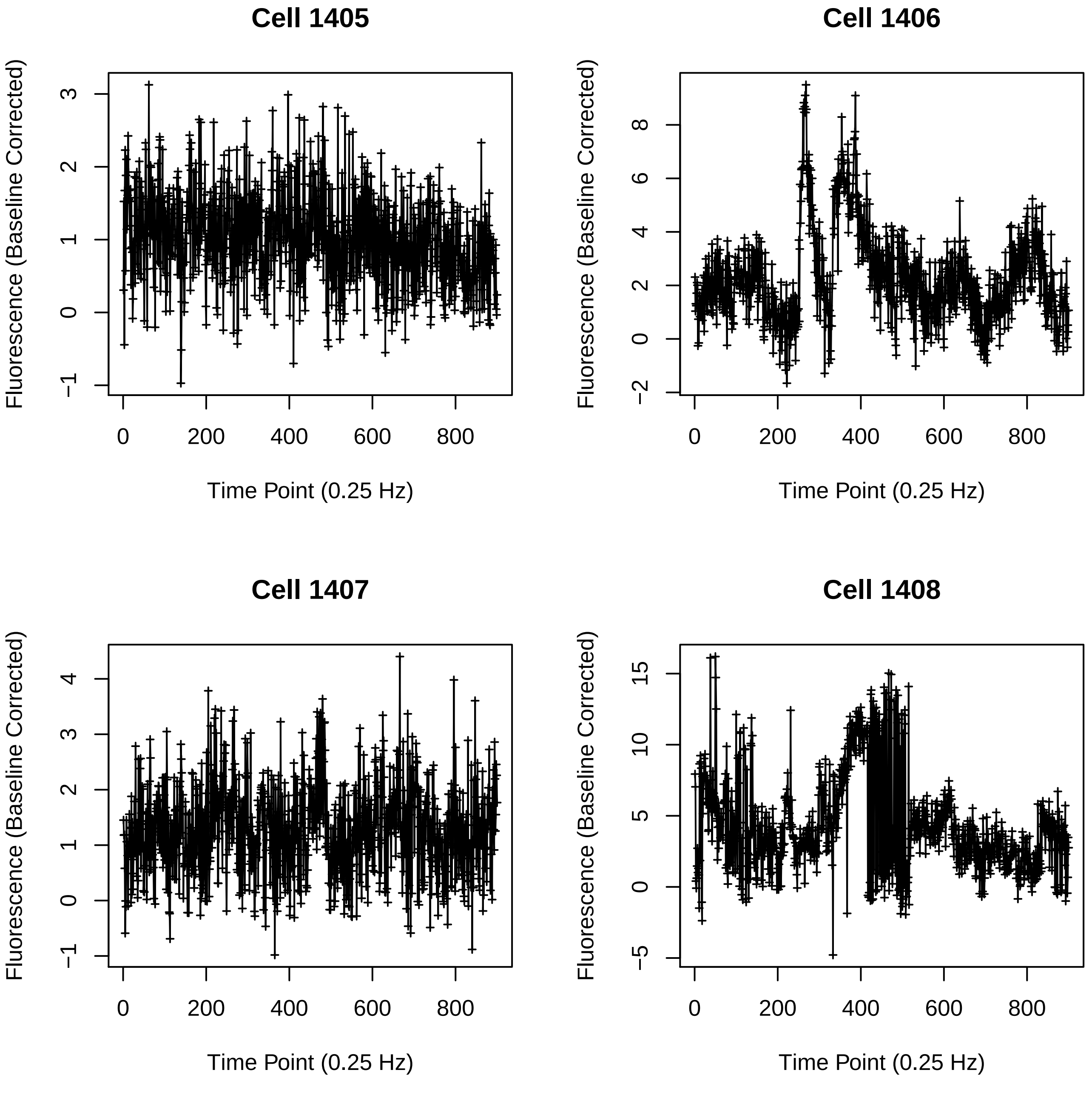
Cell 1405
Cell 1406
Cell 1407
Cell 1408
Fluorescence (Baseline Corrected)
Time Point (0.25 Hz)
0
200
400
600
800
−1
0
1
2
3
4
−2
6
8
−5
5
10
15

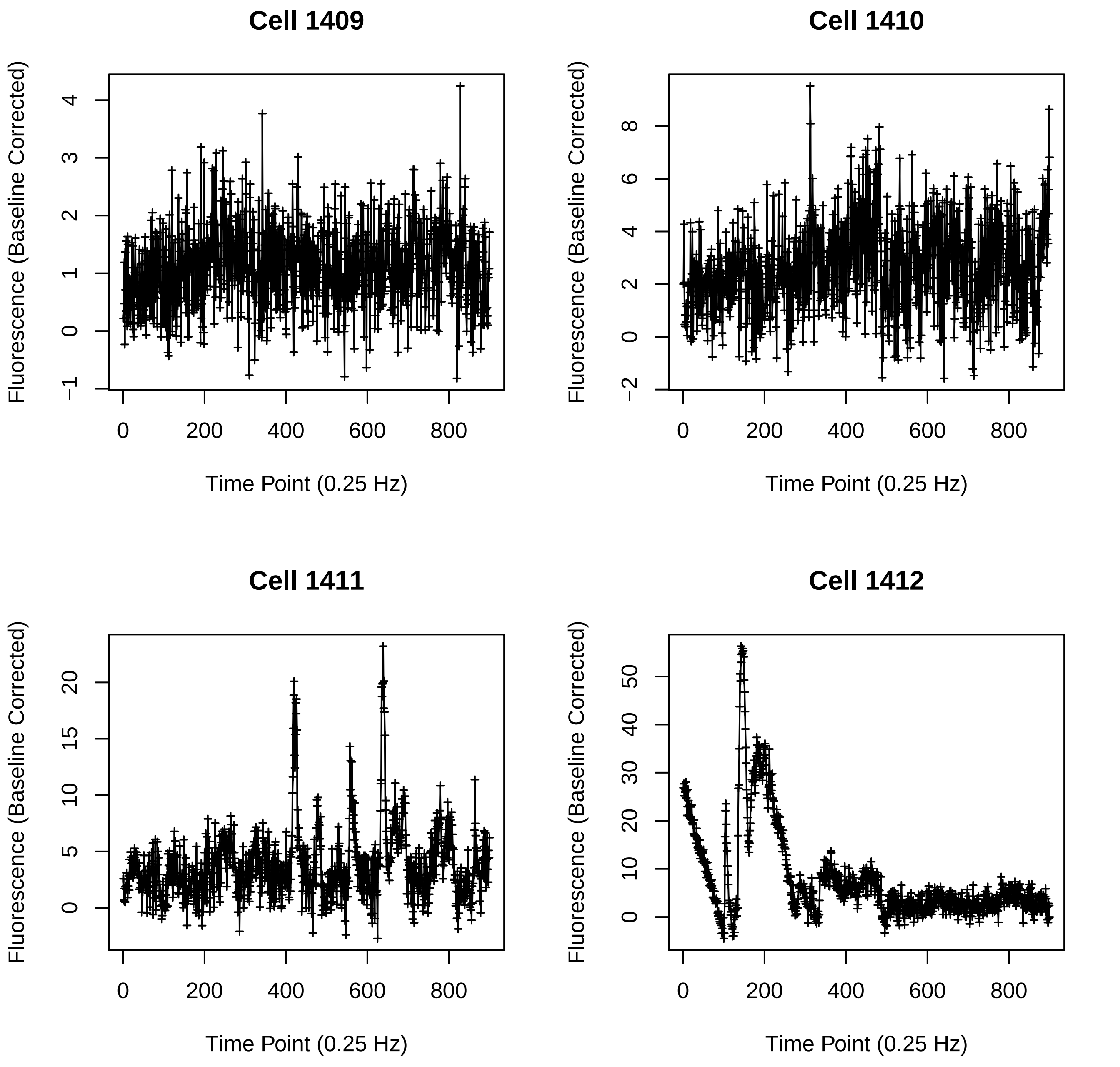
Cell 1409
Cell 1410
Cell 1411
Cell 1412
Fluorescence (Baseline Corrected)
Time Point (0.25 Hz)
0
200
400
600
800

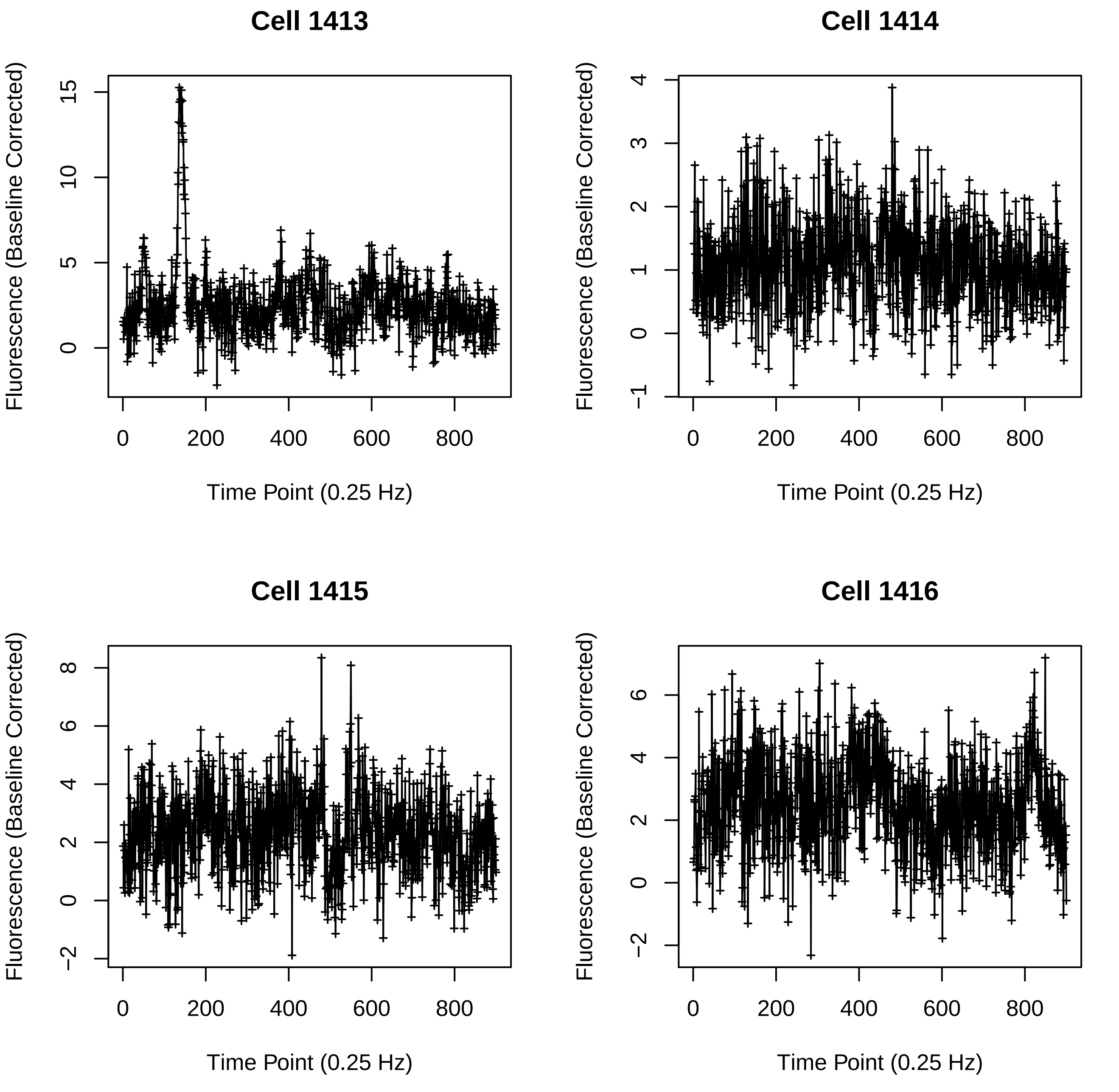
Cell 1413
Fluorescence (Baseline Corrected)
0
5
10
15
0
200
400
600
800
Time Point (0.25 Hz)
Cell 1414
Fluorescence (Baseline Corrected)
−1
0
1
2
3
4
0
200
400
600
800
Time Point (0.25 Hz)
Cell 1415
Fluorescence (Baseline Corrected)
−2
0
2
4
6
8
0
200
400
600
800
Time Point (0.25 Hz)
Cell 1416
Fluorescence (Baseline Corrected)
−2
0
2
4
6
0
200
400
600
800
Time Point (0.25 Hz)

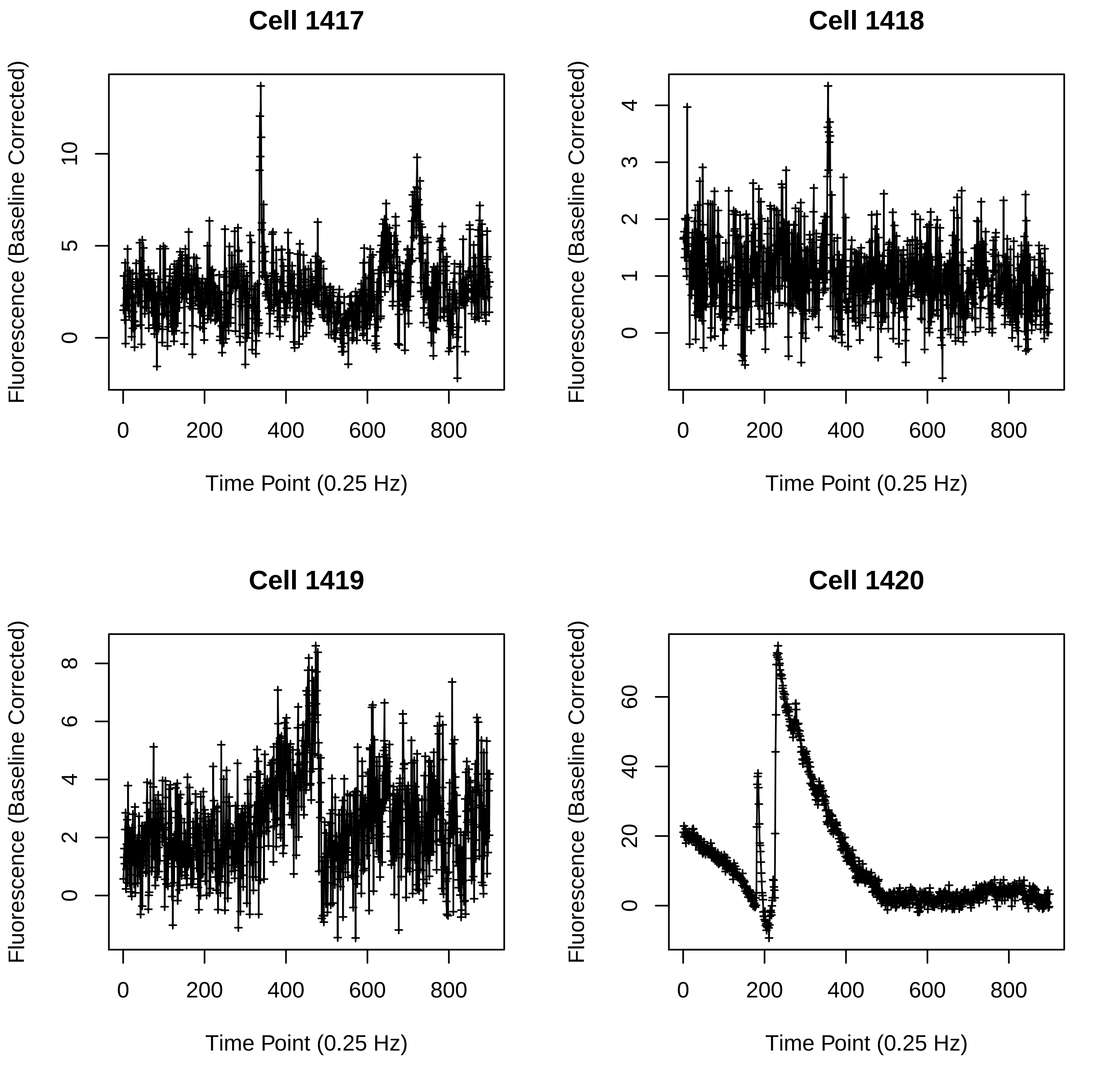
Cell 1417
Cell 1418
Cell 1419
Cell 1420
Fluorescence (Baseline Corrected)
Time Point (0.25 Hz)
0
200
400
600
800
0
5
10
1
2
3
4
6
8
20
40
60

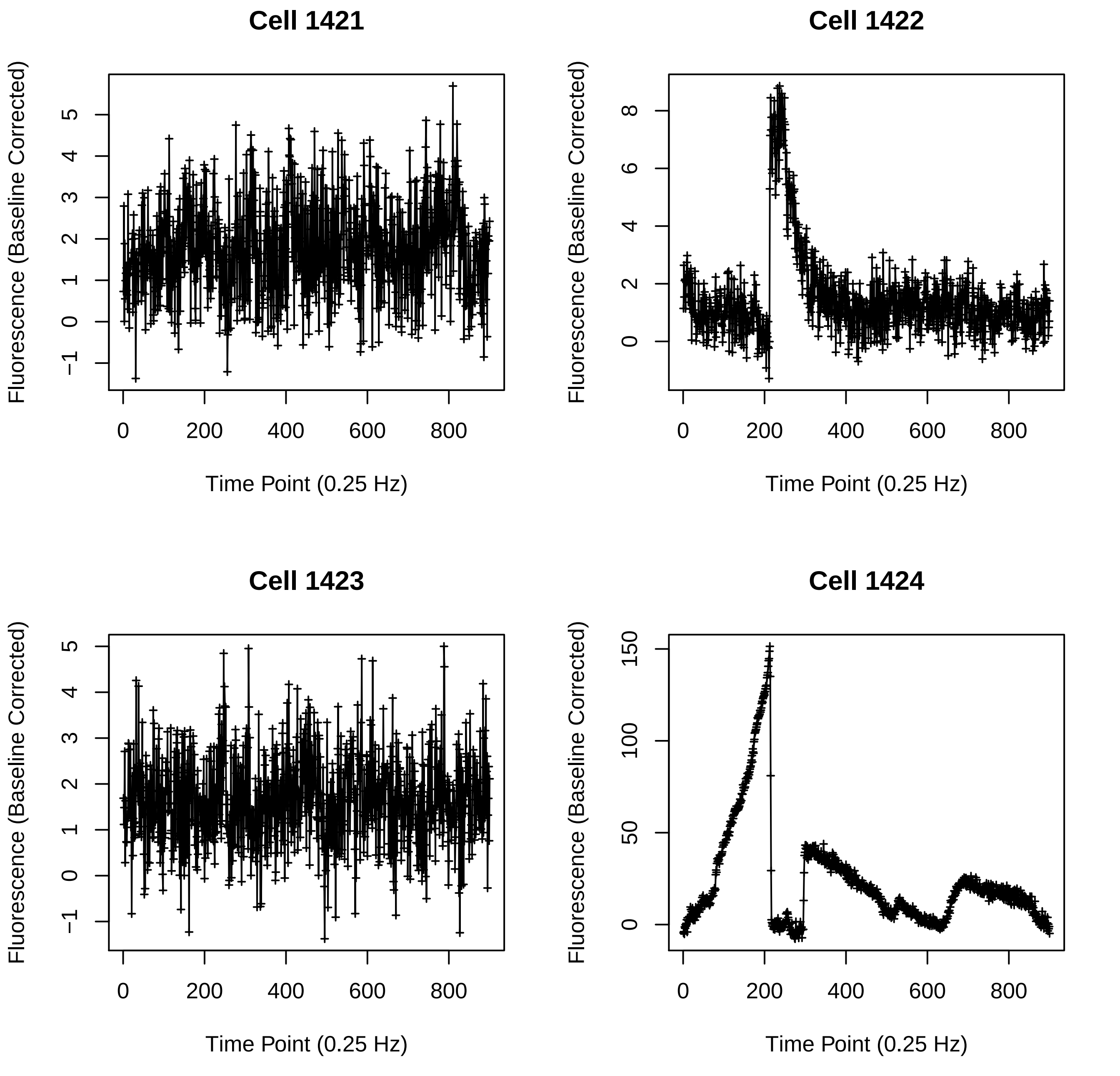
Cell 1421
Cell 1422
Cell 1423
Cell 1424
Fluorescence (Baseline Corrected)
Time Point (0.25 Hz)
0
200
400
600
800
−1
0
1
2
3
4
5
6
8
50
100
150

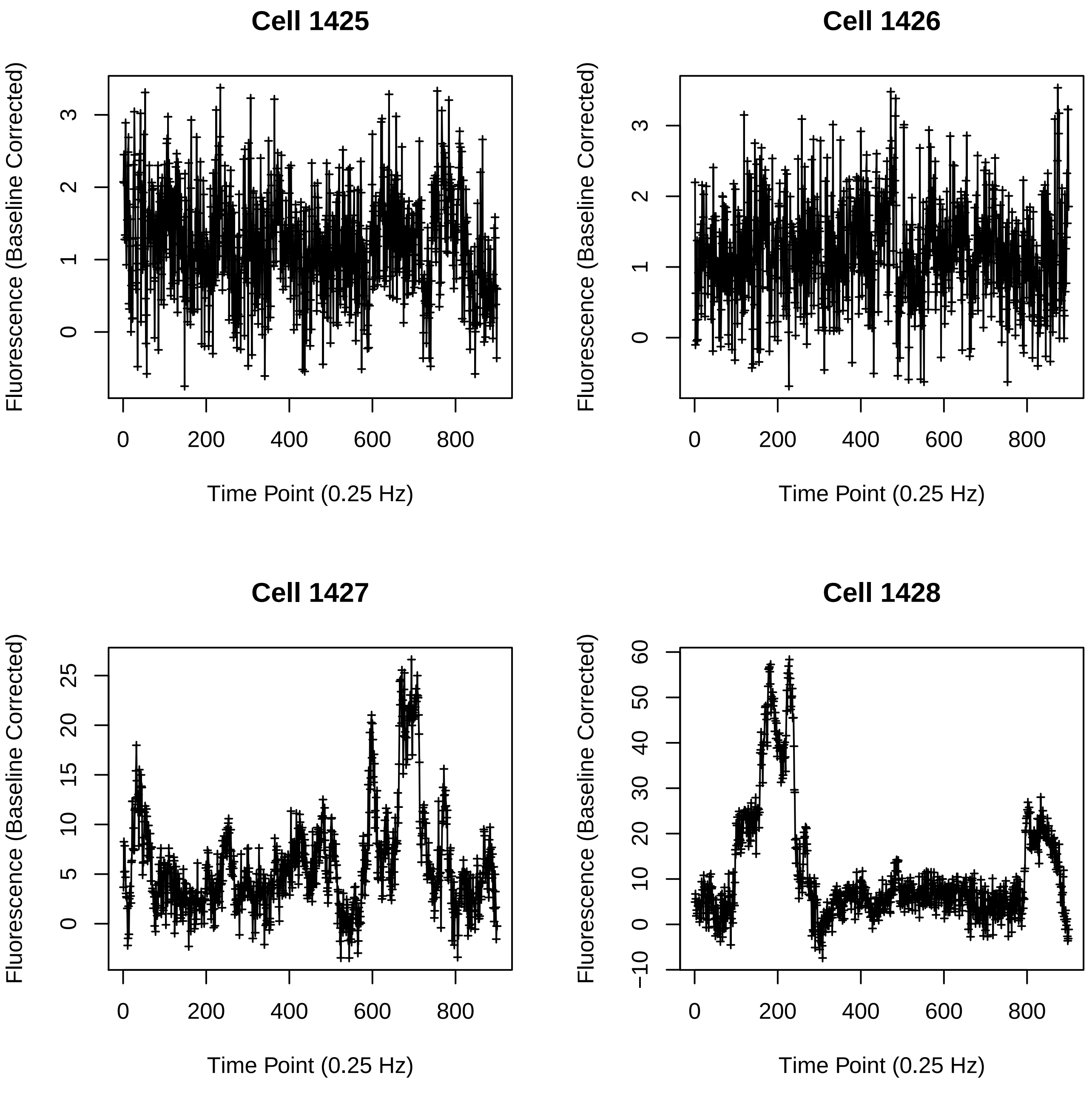
Cell 1425
Cell 1426
Cell 1427
Cell 1428
Fluorescence (Baseline Corrected)
Time Point (0.25 Hz)

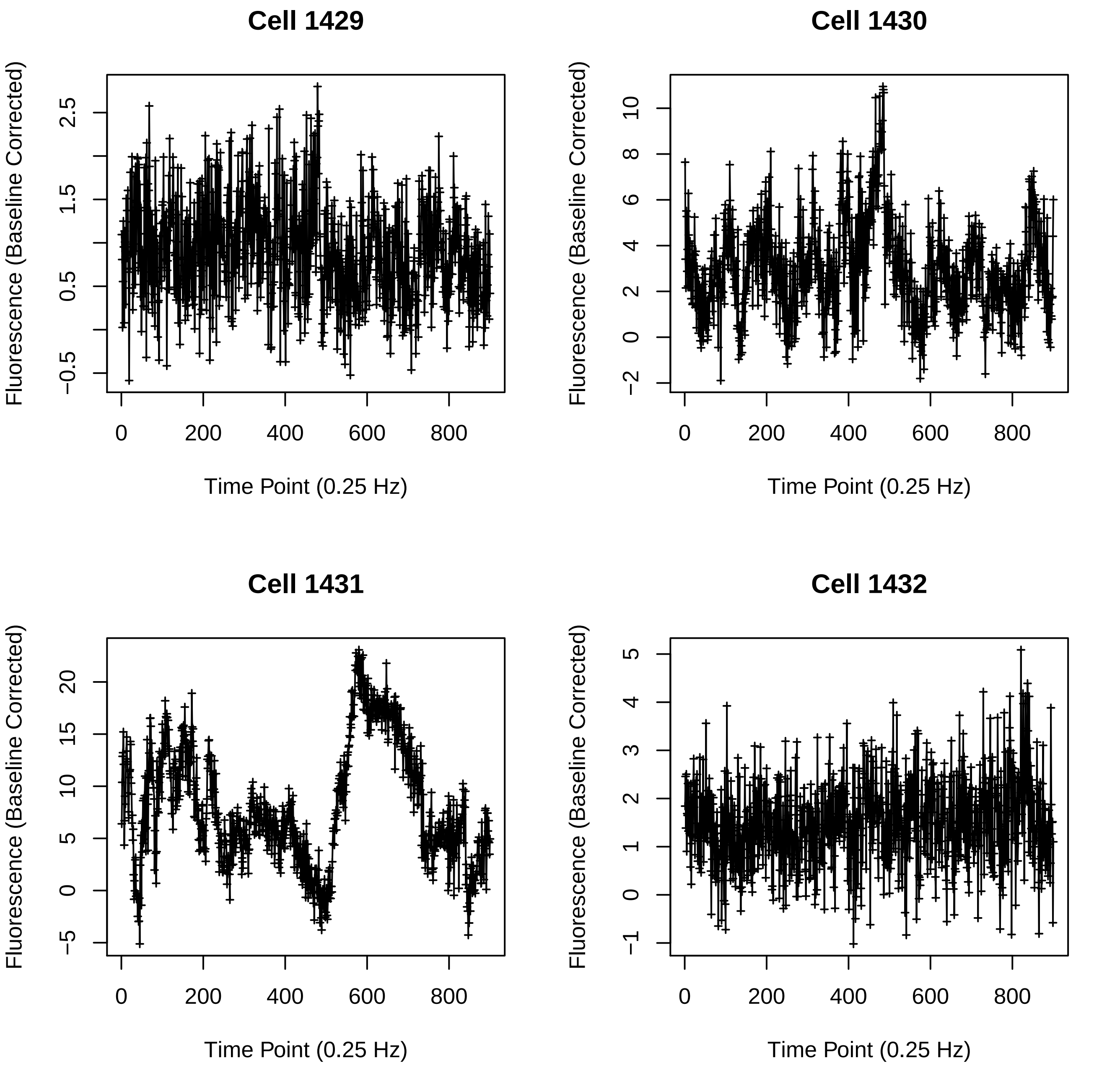
Cell 1429
Fluorescence (Baseline Corrected)
Time Point (0.25 Hz)
Cell 1430
Fluorescence (Baseline Corrected)
Time Point (0.25 Hz)
Cell 1431
Fluorescence (Baseline Corrected)
Time Point (0.25 Hz)
Cell 1432
Fluorescence (Baseline Corrected)
Time Point (0.25 Hz)

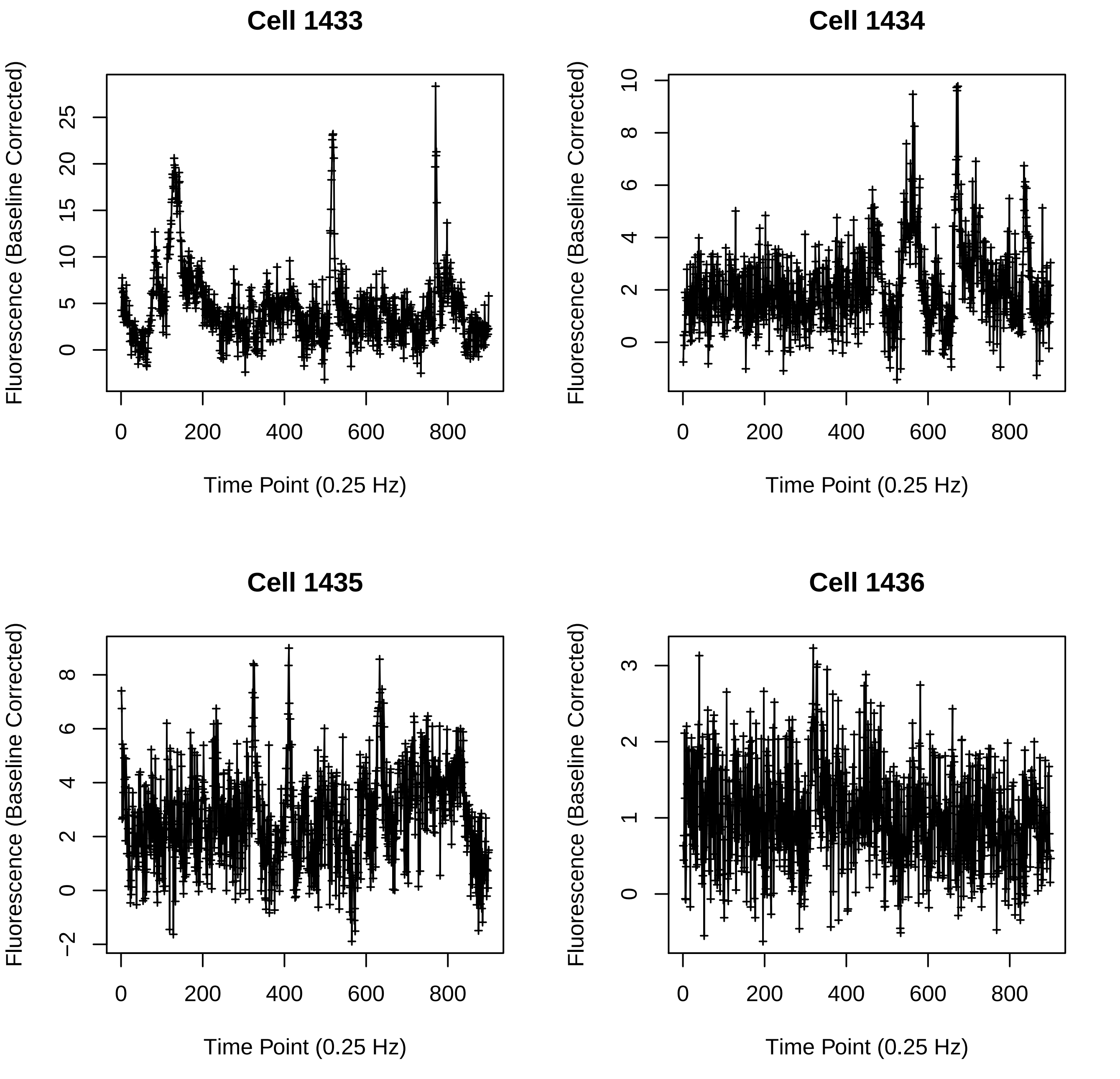
Cell 1433
Cell 1434
Cell 1435
Cell 1436
Fluorescence (Baseline Corrected)
Time Point (0.25 Hz)
0
5
10
15
20
25
2
4
6
8
−2
3
1
200
400
600
800

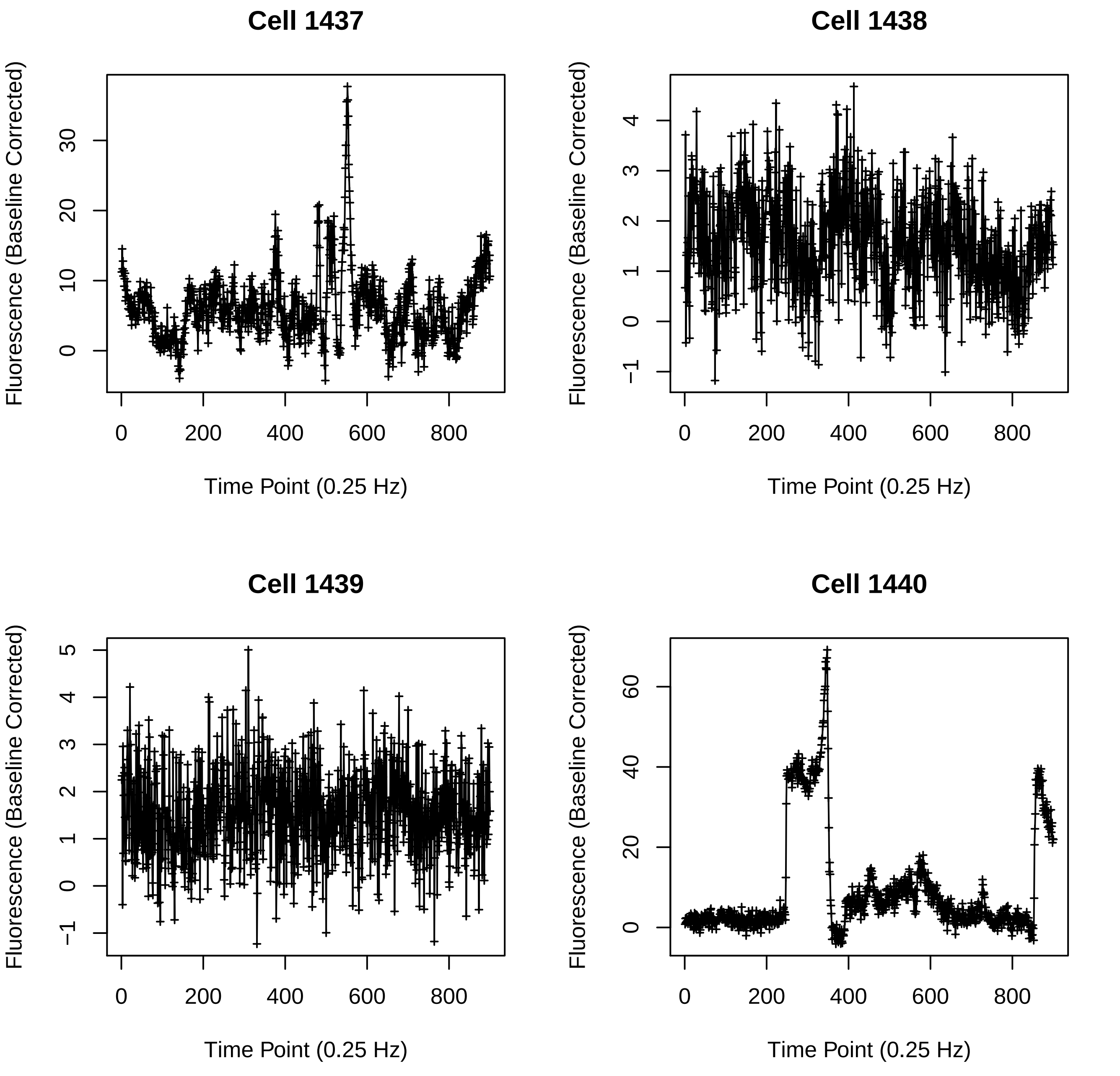
Cell 1437
Fluorescence (Baseline Corrected)
0
10
20
30
0
200
400
600
800
Time Point (0.25 Hz)
Cell 1438
Fluorescence (Baseline Corrected)
−1
0
1
2
3
4
0
200
400
600
800
Time Point (0.25 Hz)
Cell 1439
Fluorescence (Baseline Corrected)
−1
0
1
2
3
4
5
0
200
400
600
800
Time Point (0.25 Hz)
Cell 1440
Fluorescence (Baseline Corrected)
0
20
40
60
0
200
400
600
800
Time Point (0.25 Hz)

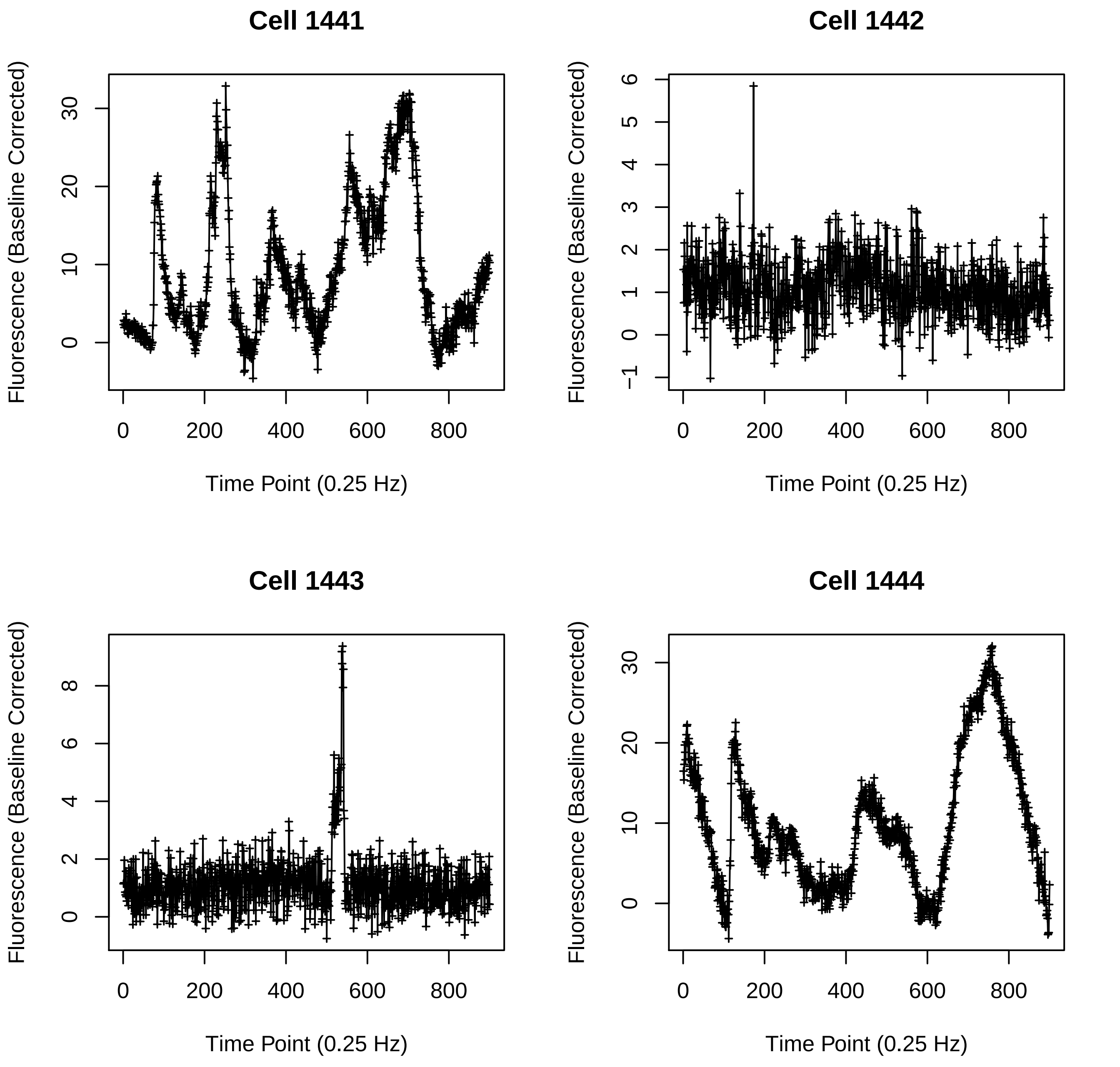
Cell 1441
Cell 1442
Cell 1443
Cell 1444
Fluorescence (Baseline Corrected)
Time Point (0.25 Hz)
0
200
400
600
800
0
10
20
30
−1
1
2
3
4
5
6
8

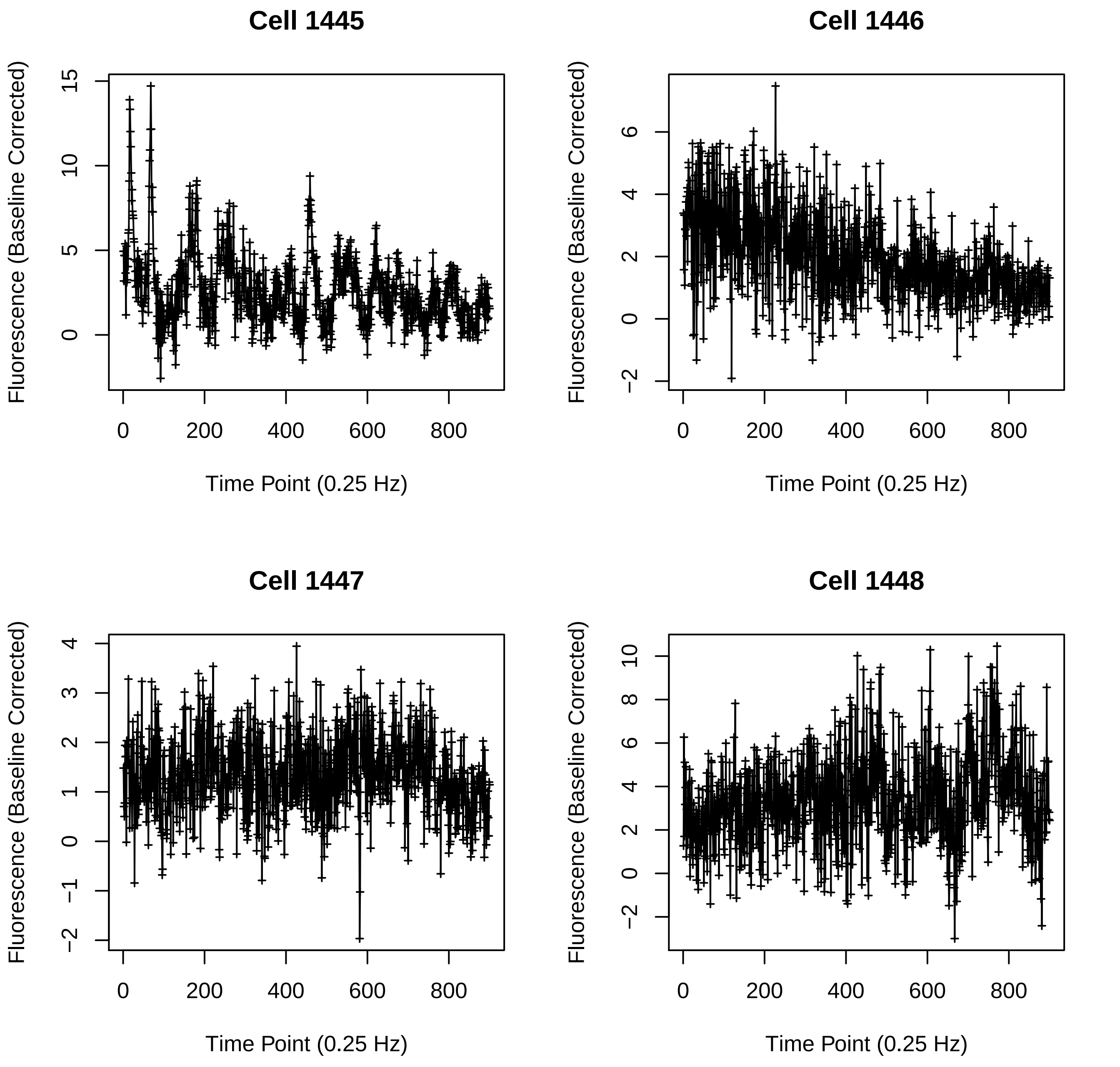
Cell 1445
Cell 1446
Cell 1447
Cell 1448
Fluorescence (Baseline Corrected)
Time Point (0.25 Hz)
0
200
400
600
800
15
10
5
6
4
2
−2
3
1
−1
8

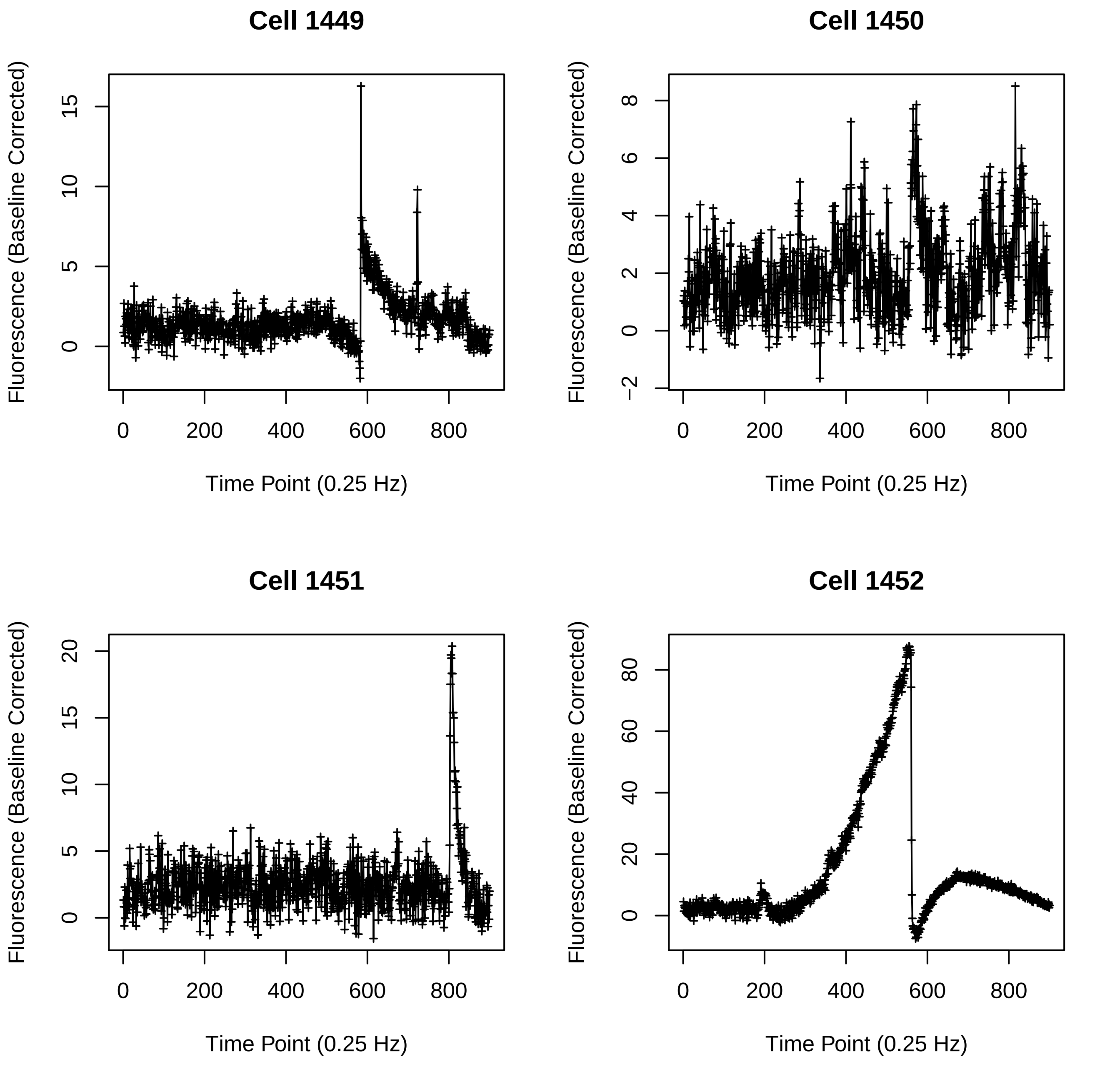

Cell 1449
Cell 1450
Cell 1451
Cell 1452
Fluorescence (Baseline Corrected)
Time Point (0.25 Hz)
0
200
400
600
800

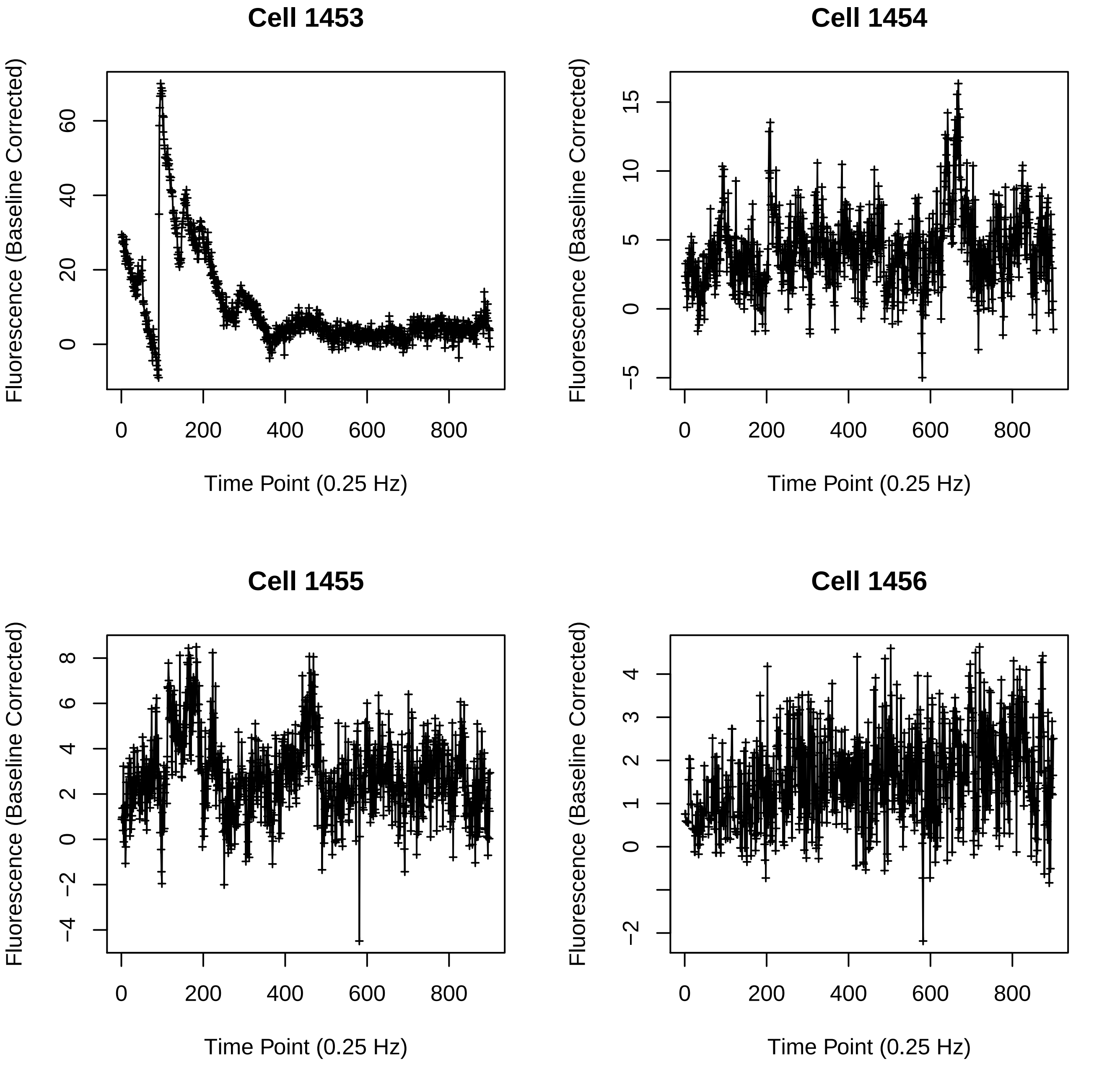
Cell 1453
Cell 1454
Cell 1455
Cell 1456
Fluorescence (Baseline Corrected)
Time Point (0.25 Hz)

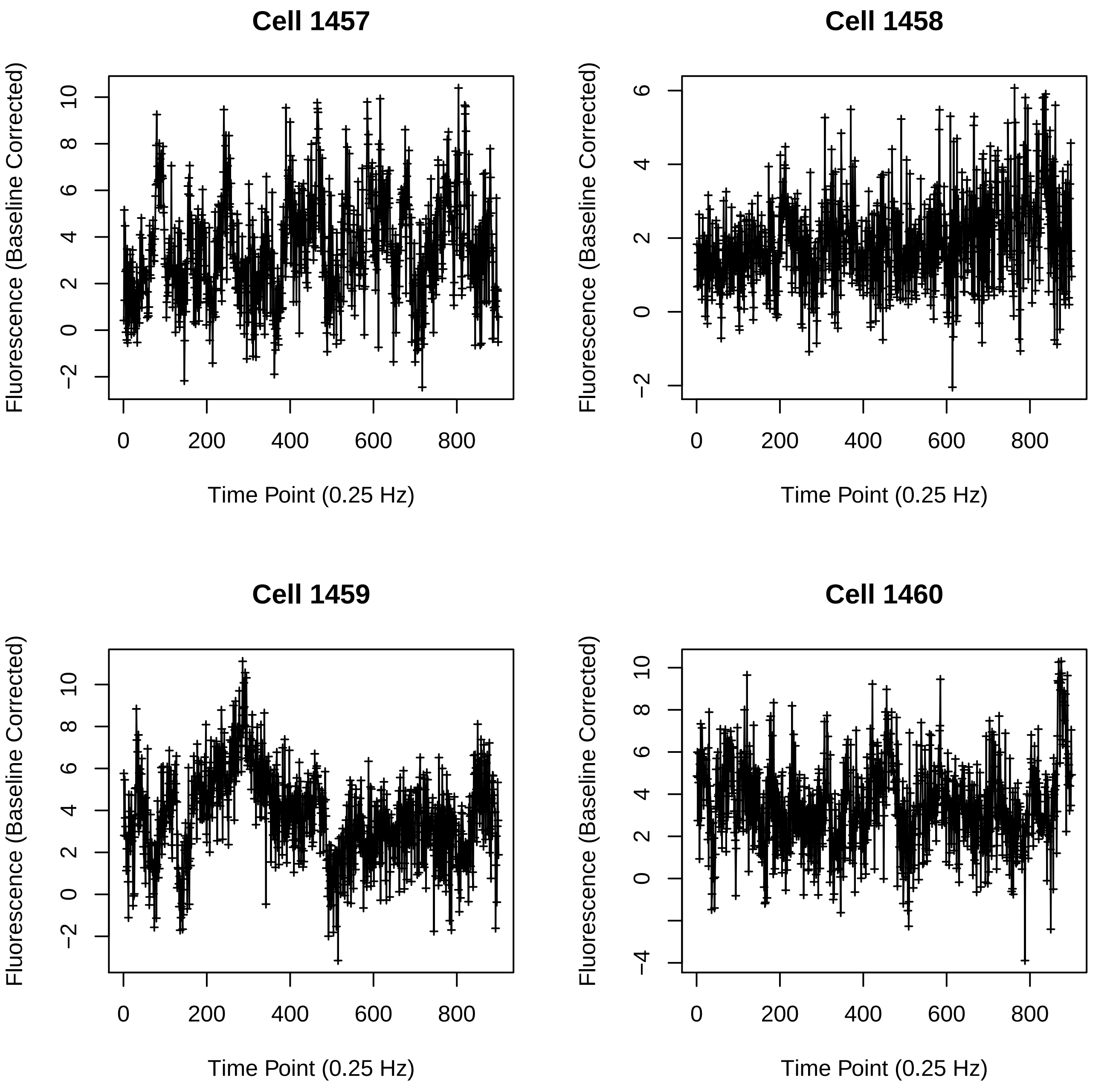

Cell 1457
Cell 1458
Cell 1459
Cell 1460
Fluorescence (Baseline Corrected)
Time Point (0.25 Hz)
0
200
400
600
800
−4
−2
0
2
4
6
8
10

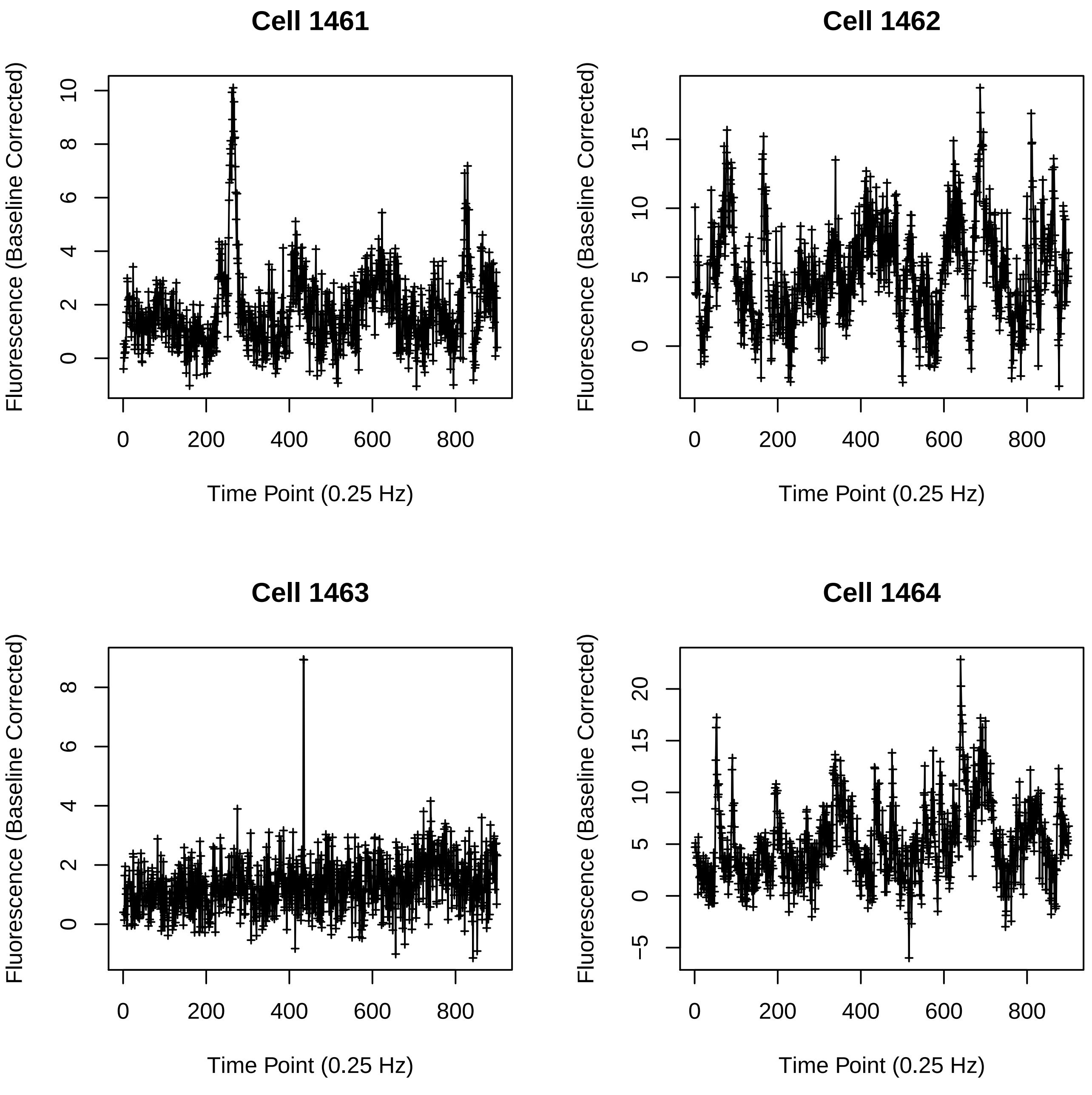
Cell 1461
Cell 1462
Cell 1463
Cell 1464
Fluorescence (Baseline Corrected)
Time Point (0.25 Hz)
0
200
400
600
800
0
2
4
6
8
10
5
15
20
−5

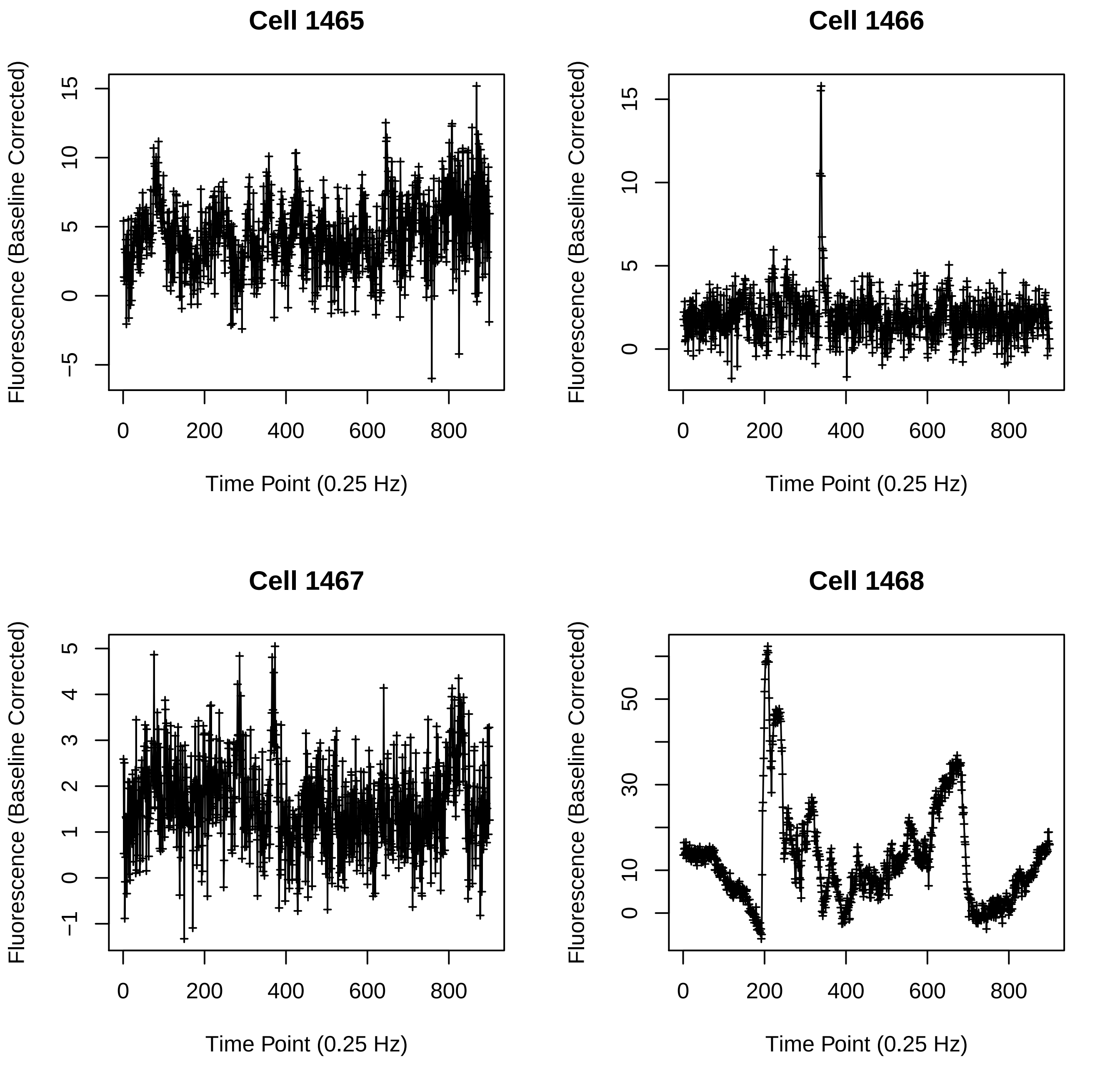

Cell 1465
Cell 1466
Cell 1467
Cell 1468
Fluorescence (Baseline Corrected)
Time Point (0.25 Hz)

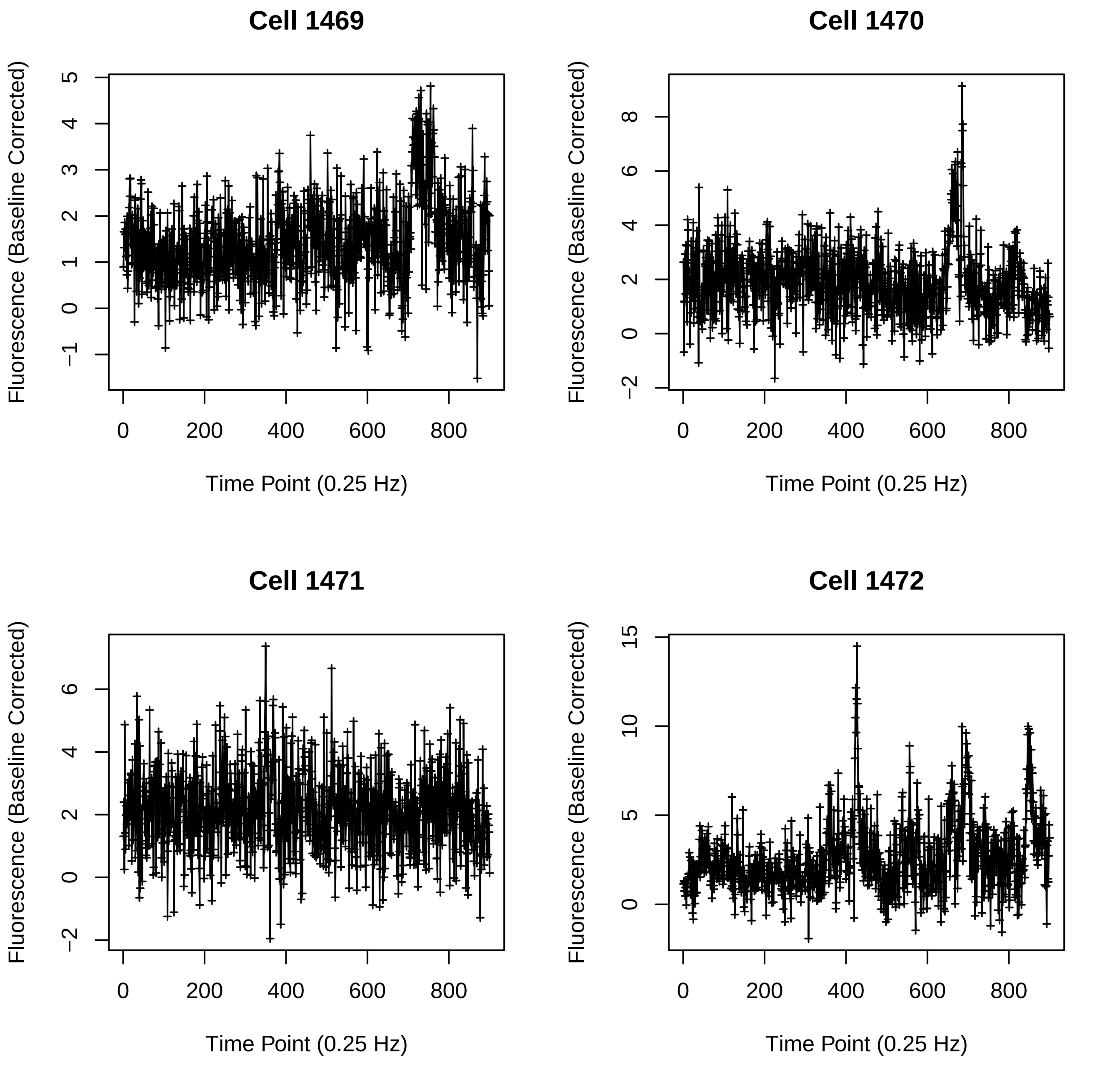
Cell 1469
Cell 1470
Cell 1471
Cell 1472
Fluorescence (Baseline Corrected)
Time Point (0.25 Hz)
0
200
400
600
800

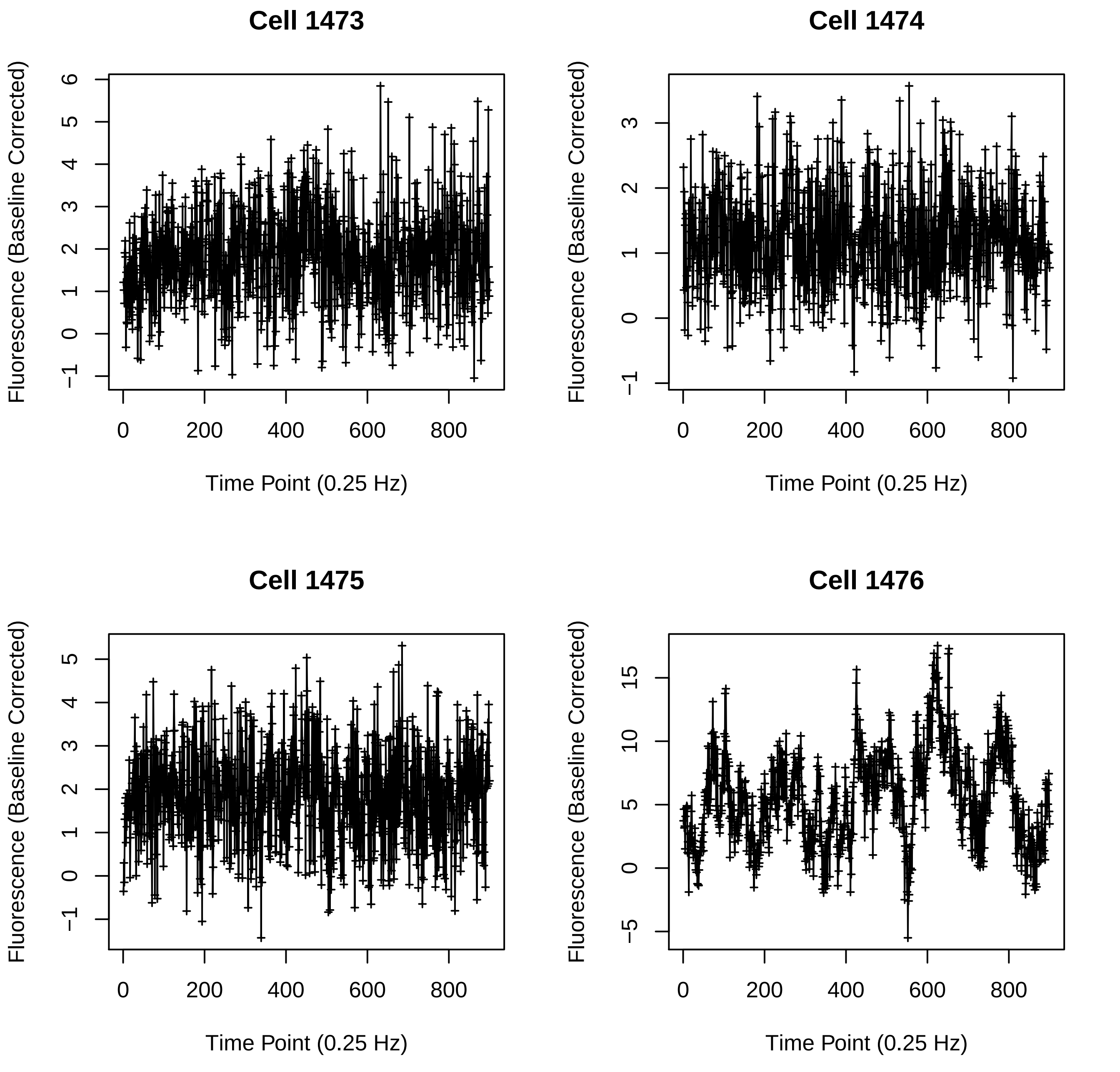
Cell 1473
Fluorescence (Baseline Corrected)
Time Point (0.25 Hz)
Cell 1474
Fluorescence (Baseline Corrected)
Time Point (0.25 Hz)
Cell 1475
Fluorescence (Baseline Corrected)
Time Point (0.25 Hz)
Cell 1476
Fluorescence (Baseline Corrected)
Time Point (0.25 Hz)

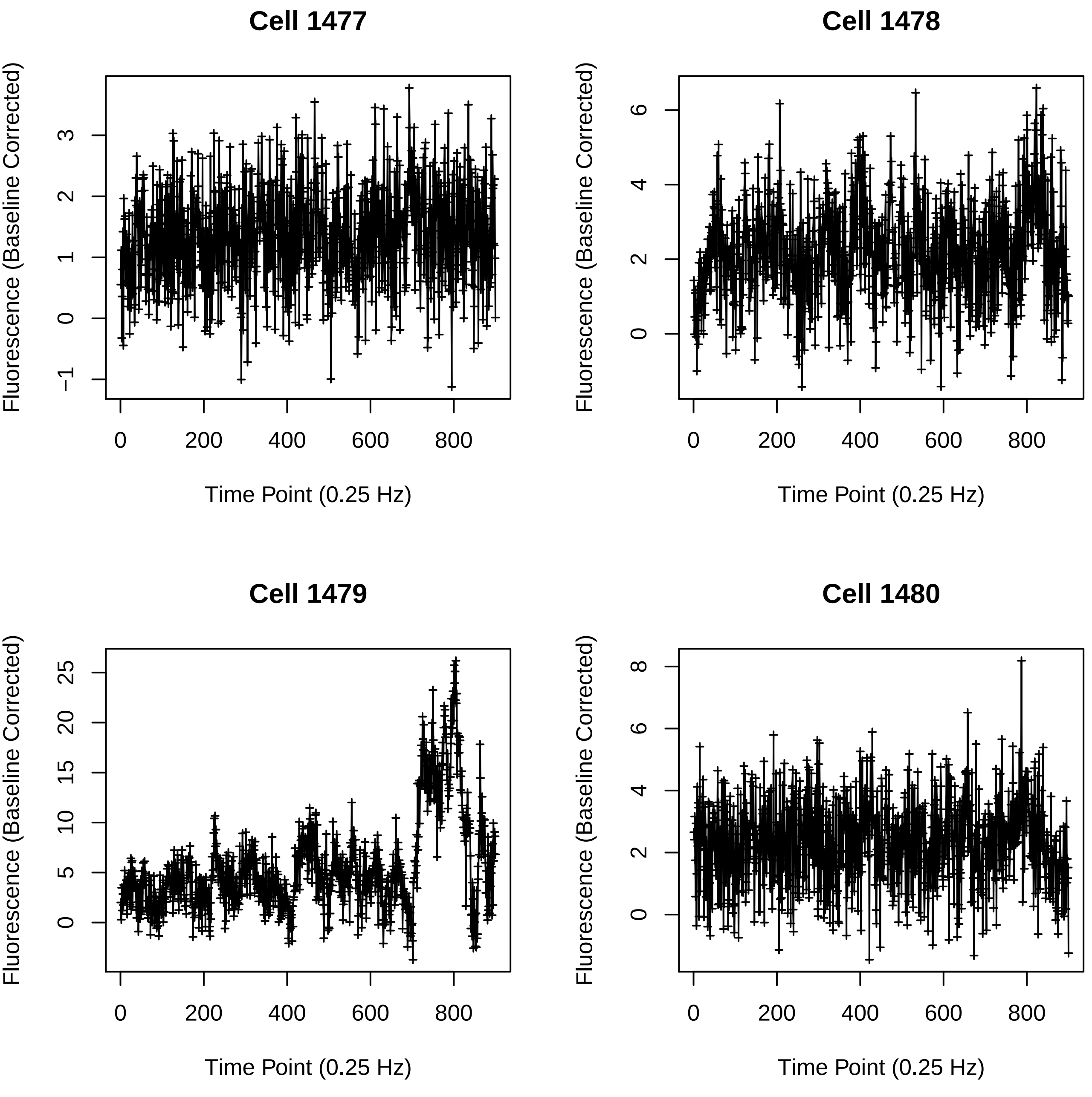
Cell 1477
Cell 1478
Cell 1479
Cell 1480
Fluorescence (Baseline Corrected)
Time Point (0.25 Hz)
−1
0
1
2
3
4
5
6
8
10
15
20
25
0
200
400
600
800

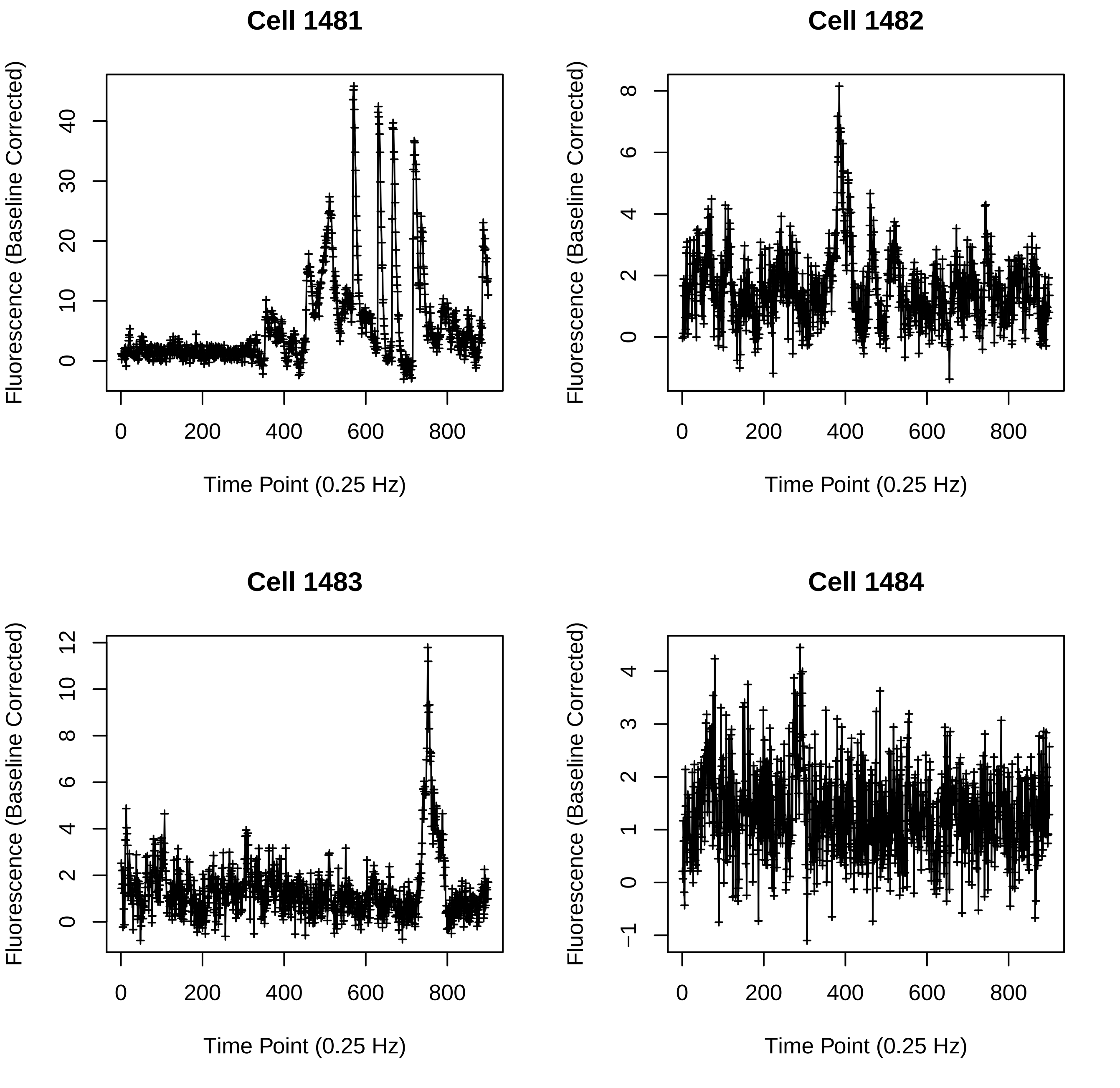
Cell 1481
Fluorescence (Baseline Corrected)
0
10
20
30
40
0
200
400
600
800
Time Point (0.25 Hz)
Cell 1482
Fluorescence (Baseline Corrected)
0
2
4
6
8
0
200
400
600
800
Time Point (0.25 Hz)
Cell 1483
Fluorescence (Baseline Corrected)
0
2
4
6
8
10
12
0
200
400
600
800
Time Point (0.25 Hz)
Cell 1484
Fluorescence (Baseline Corrected)
−1
0
1
2
3
4
0
200
400
600
800
Time Point (0.25 Hz)

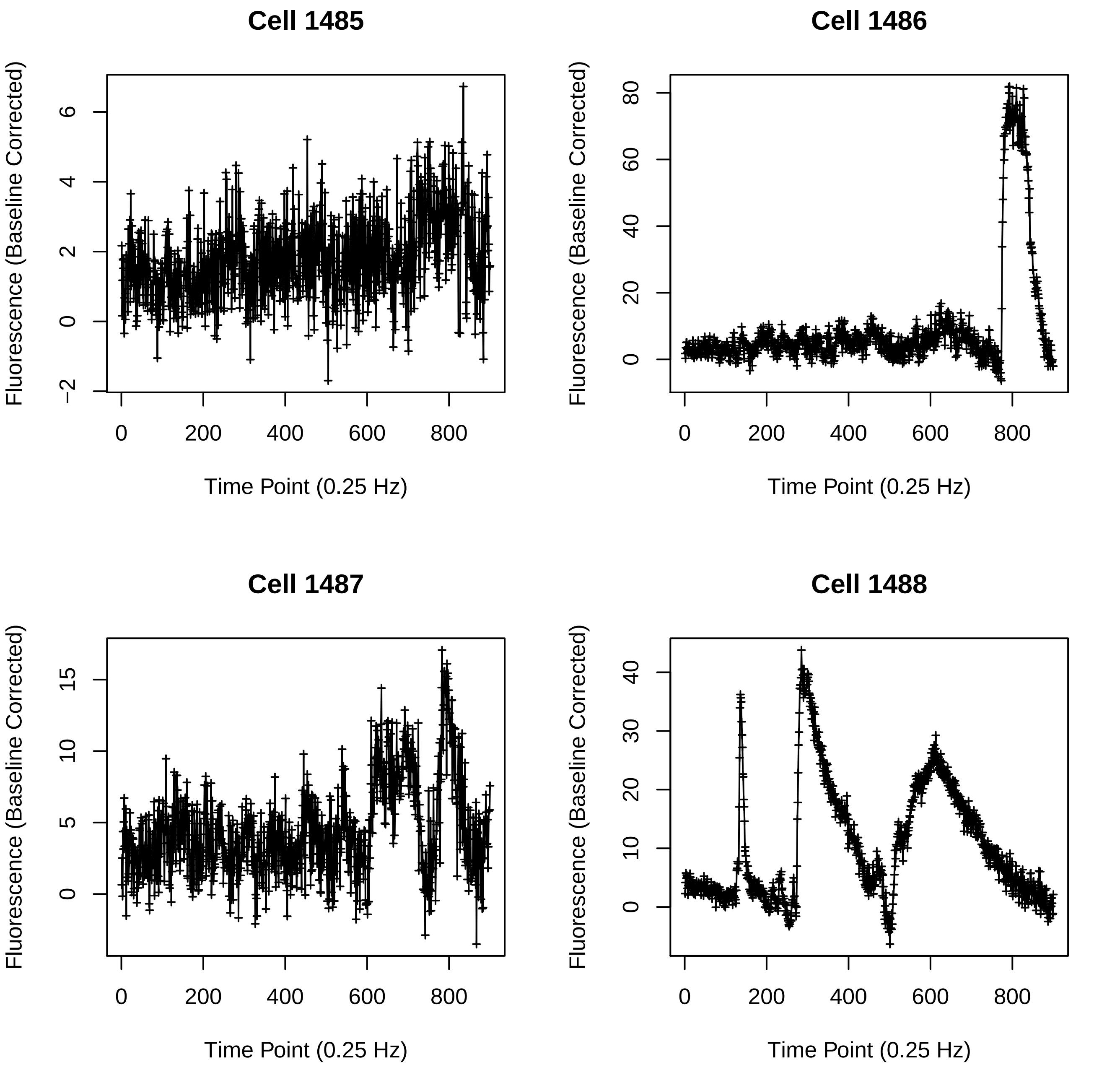

Cell 1485
Cell 1486
Cell 1487
Cell 1488
Fluorescence (Baseline Corrected)
Time Point (0.25 Hz)
−2
0
2
4
6
20
40
60
80
5
10
15
30
200
400
600
800

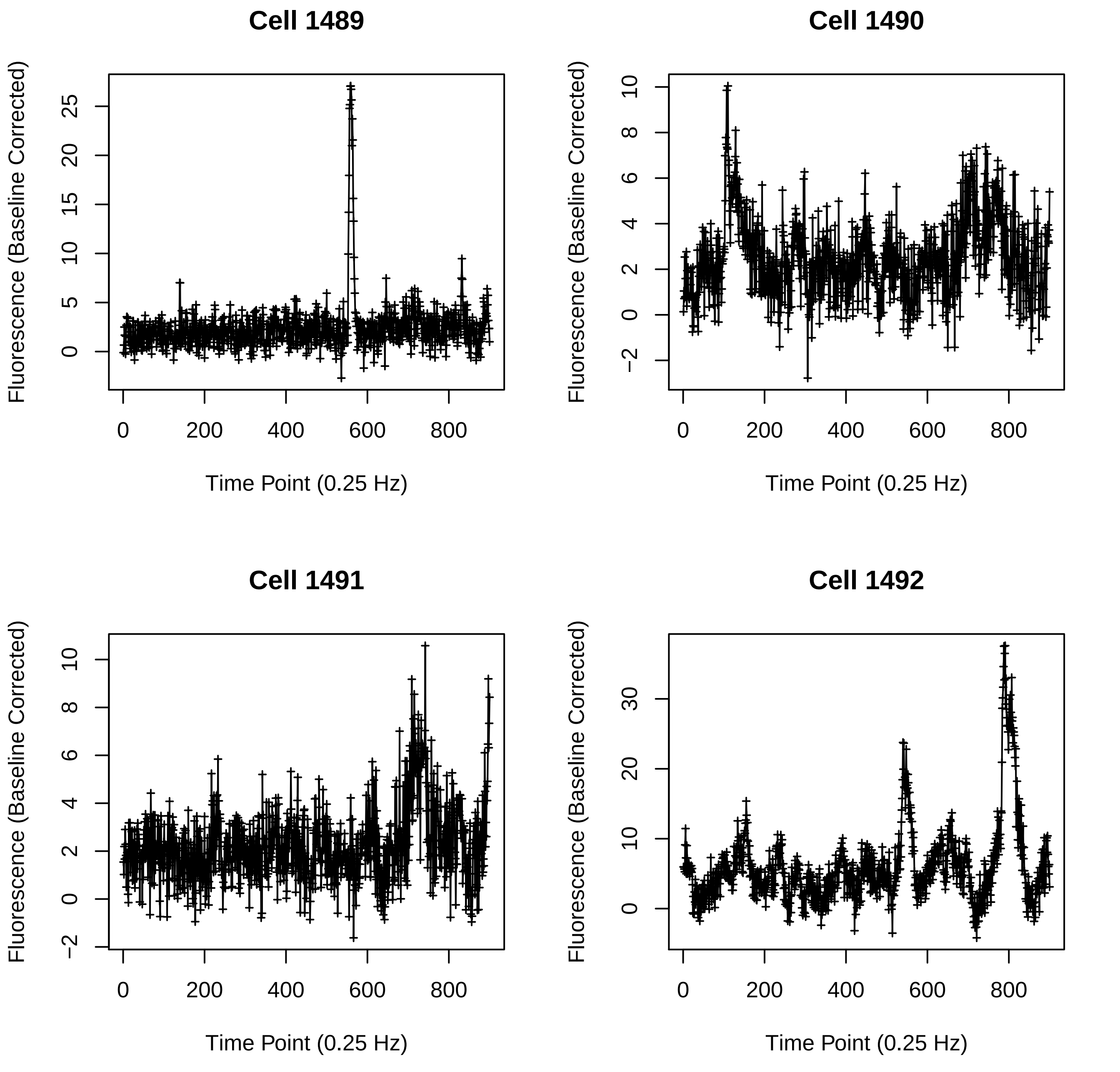

Cell 1489
Cell 1490
Cell 1491
Cell 1492
Fluorescence (Baseline Corrected)
Time Point (0.25 Hz)
0
200
400
600
800
0
5
10
15
20
25
−2
2
4
6
8
10
30

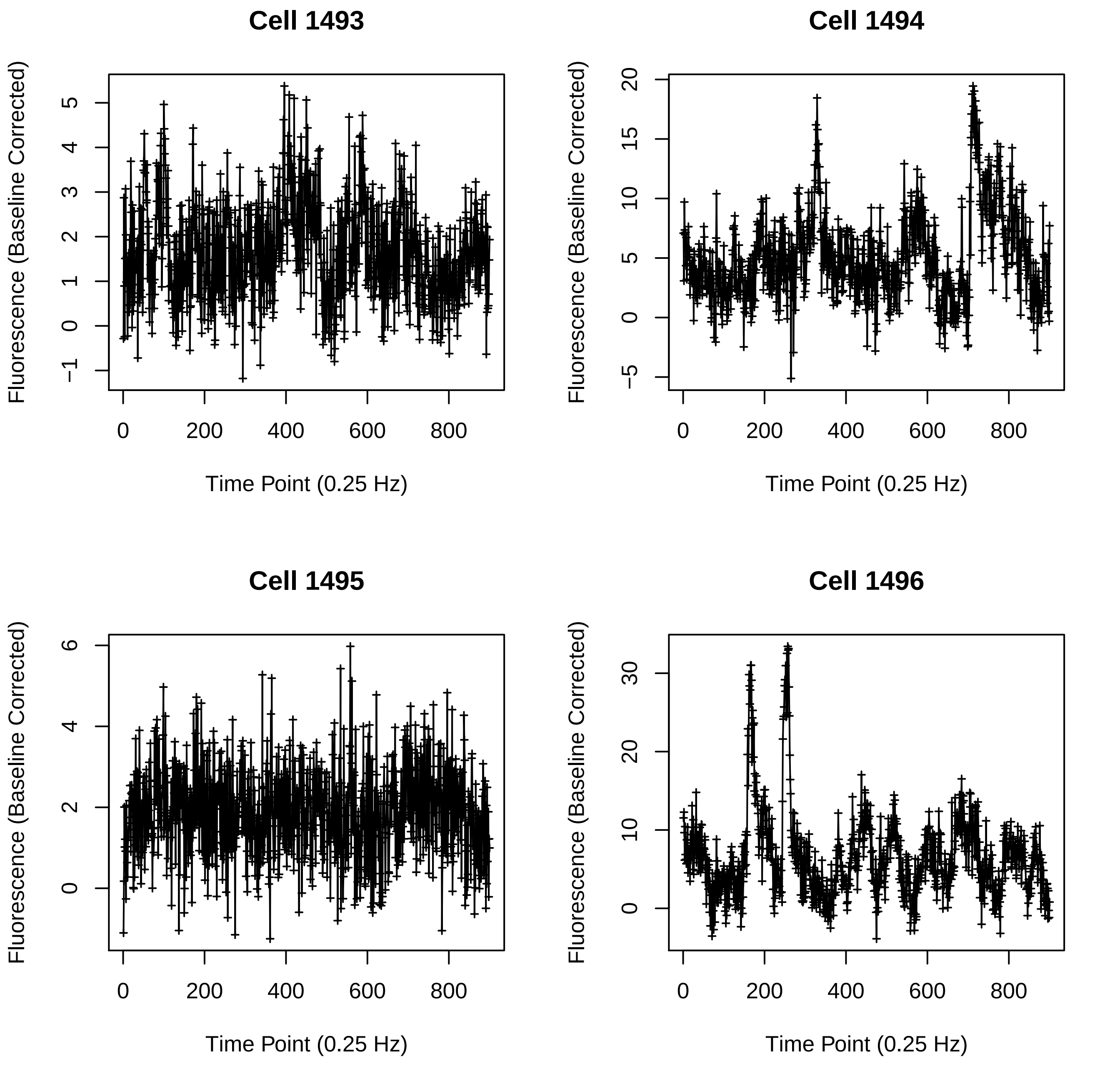
Cell 1493
Cell 1494
Cell 1495
Cell 1496
Fluorescence (Baseline Corrected)
Time Point (0.25 Hz)
0
200
400
600
800
−1
0
1
2
3
4
5
−5
5
10
15
20
6
30

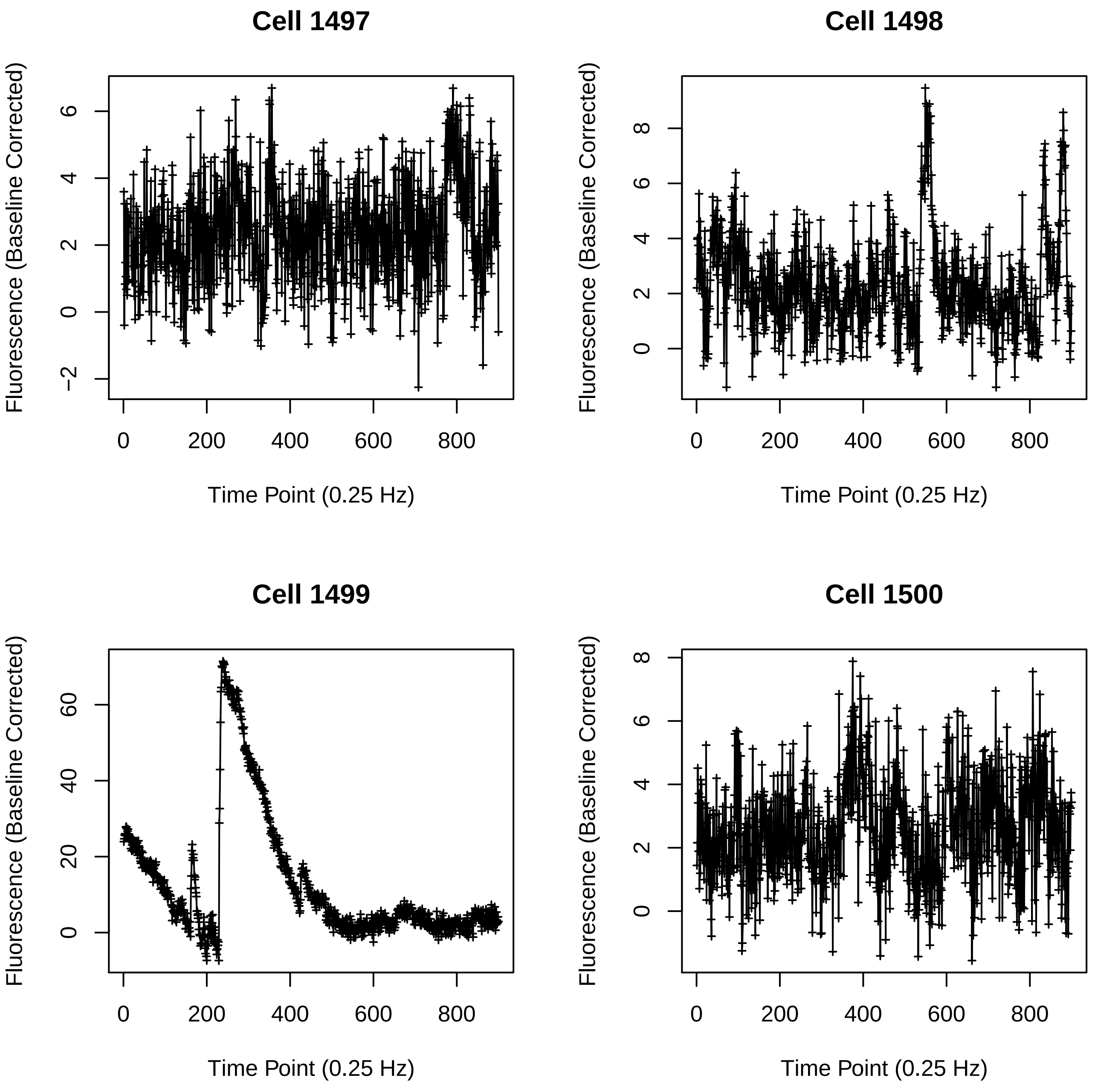
Cell 1497
Fluorescence (Baseline Corrected)
−2
0
2
4
6
0
200
400
600
800
Time Point (0.25 Hz)
Cell 1498
Fluorescence (Baseline Corrected)
0
2
4
6
8
0
200
400
600
800
Time Point (0.25 Hz)
Cell 1499
Fluorescence (Baseline Corrected)
0
20
40
60
0
200
400
600
800
Time Point (0.25 Hz)
Cell 1500
Fluorescence (Baseline Corrected)
0
2
4
6
8
0
200
400
600
800
Time Point (0.25 Hz)

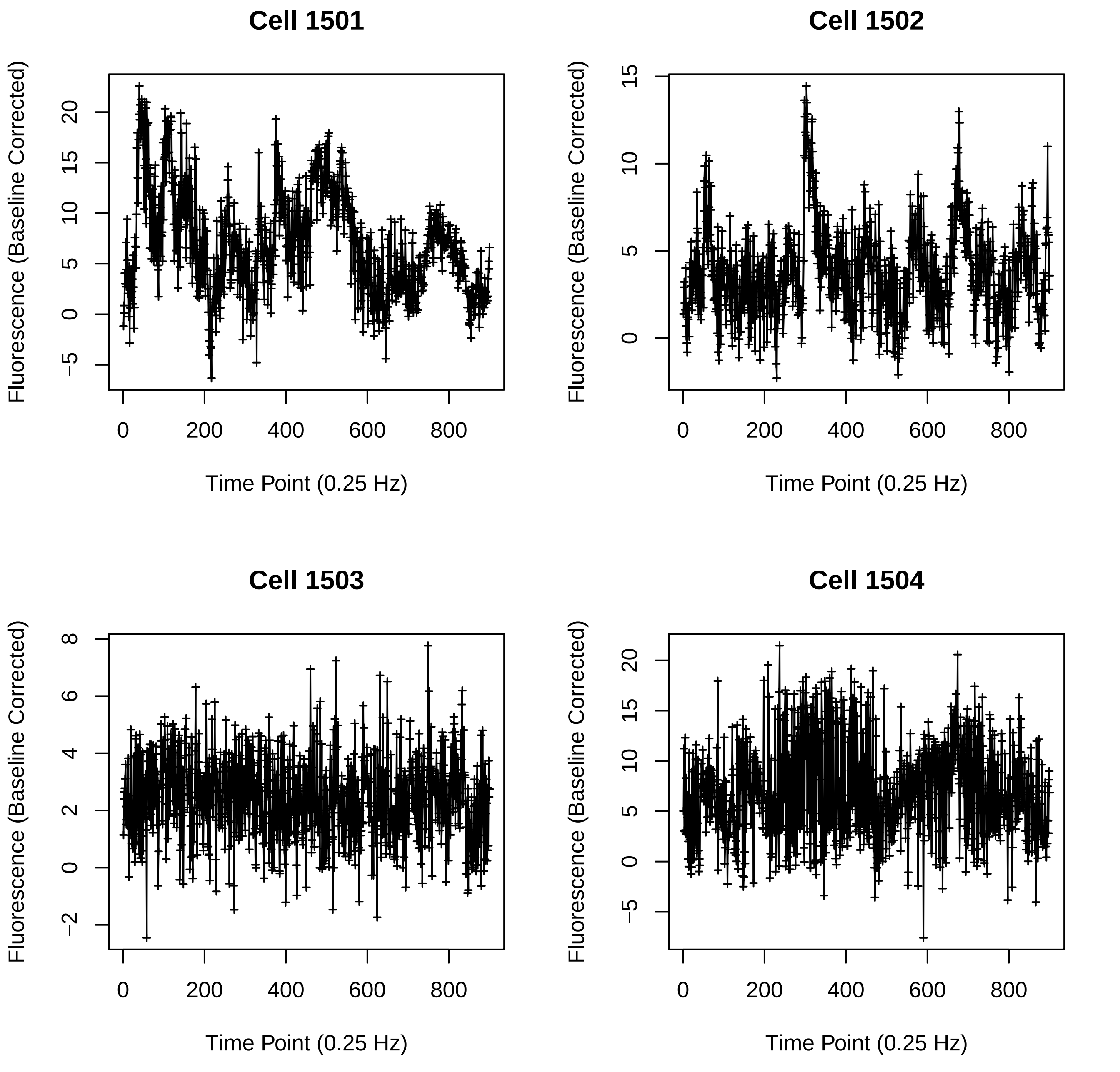

Cell 1501
Cell 1502
Cell 1503
Cell 1504
Fluorescence (Baseline Corrected)
Time Point (0.25 Hz)
0
200
400
600
800

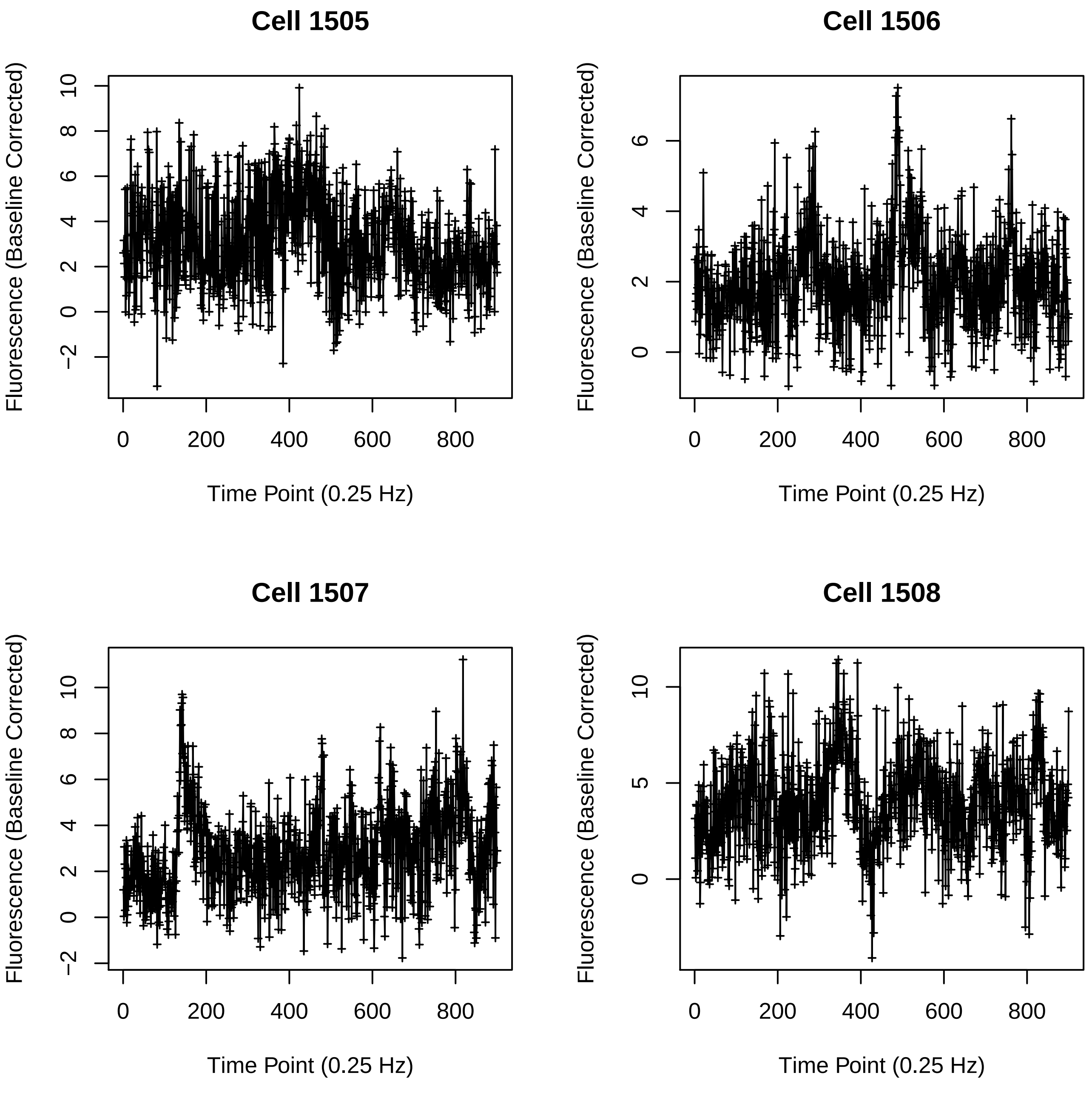
Cell 1505
Cell 1506
Cell 1507
Cell 1508
Fluorescence (Baseline Corrected)
Time Point (0.25 Hz)
0
200
400
600
800
−2
2
4
6
8
10

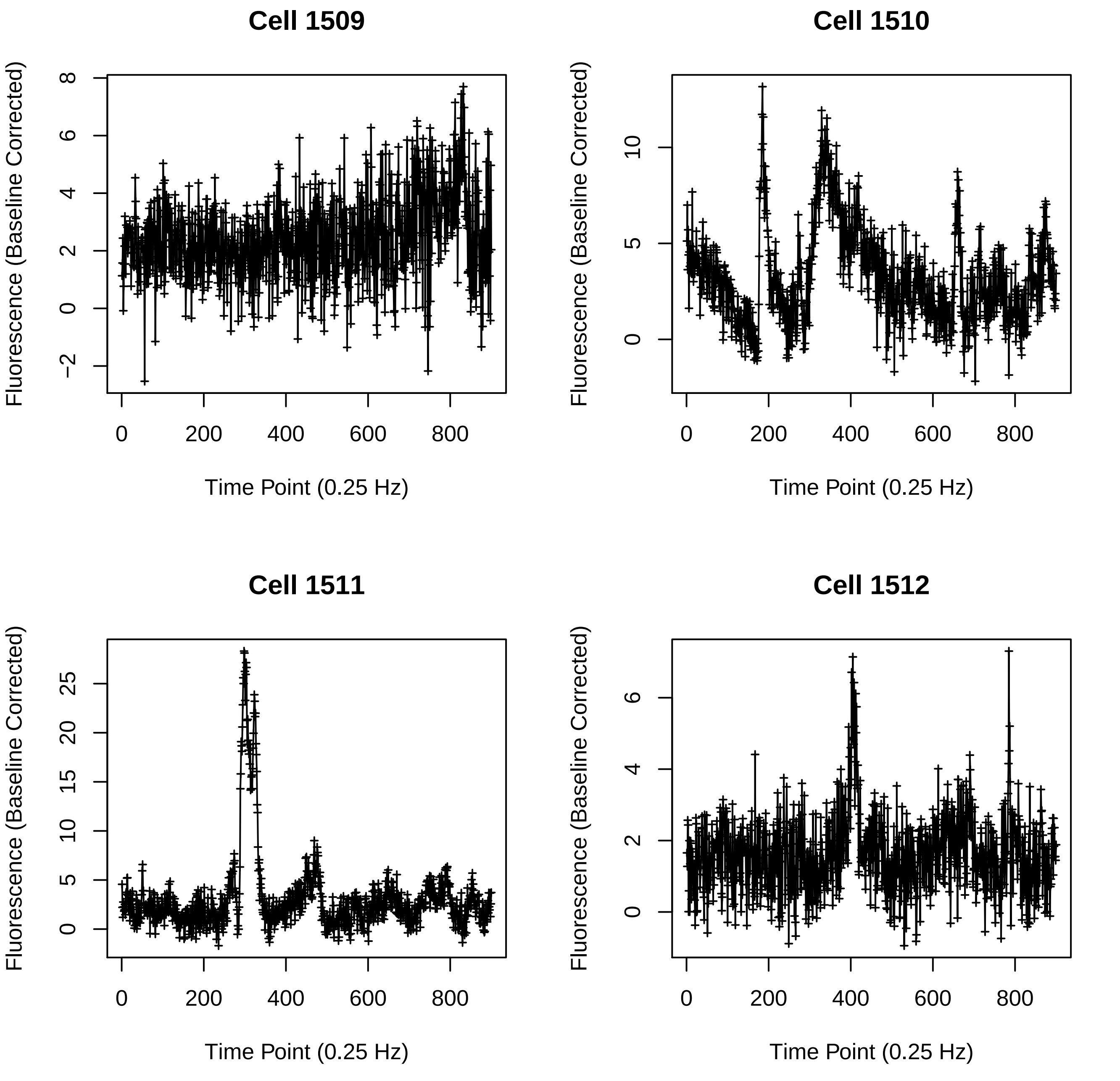
Cell 1509
Cell 1510
Cell 1511
Cell 1512
Fluorescence (Baseline Corrected)
Time Point (0.25 Hz)
−2
0
2
4
6
8
10
5
15
20
25
200
400
600
800

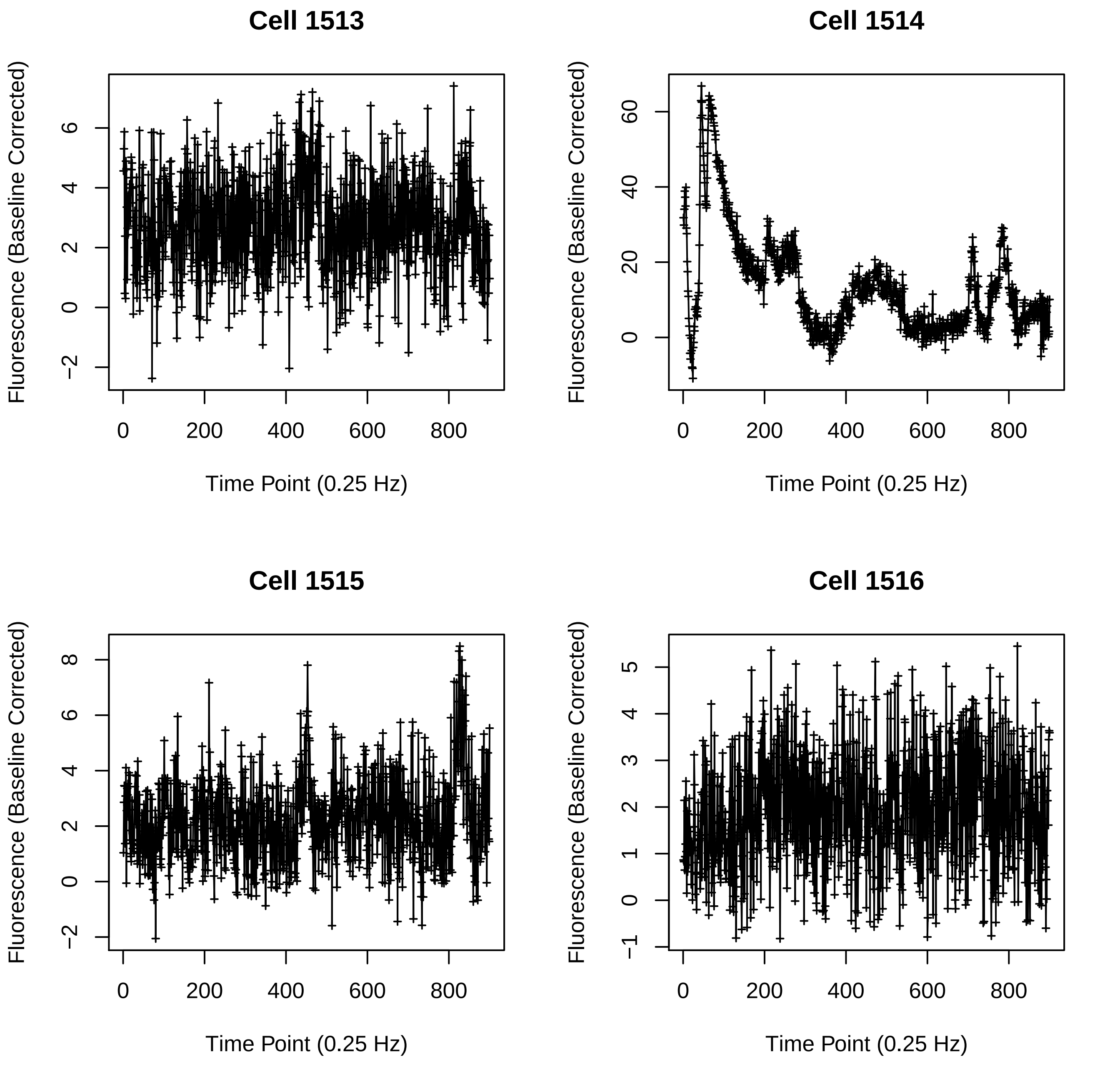

Cell 1513
Cell 1514
Cell 1515
Cell 1516
Fluorescence (Baseline Corrected)
Time Point (0.25 Hz)
0
200
400
600
800

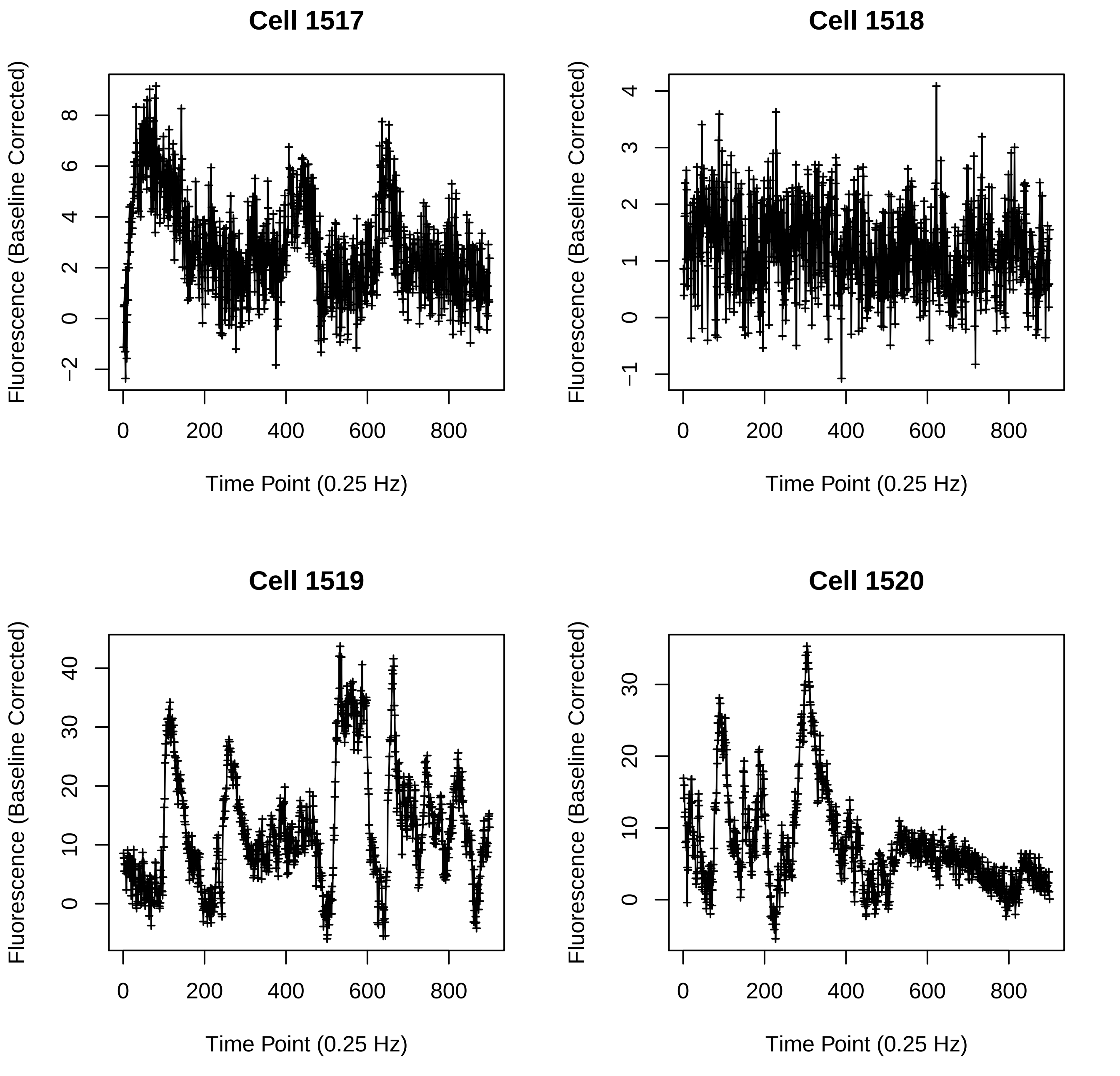
Cell 1517
Fluorescence (Baseline Corrected)
−2
0
2
4
6
8
0
200
400
600
800
Time Point (0.25 Hz)
Cell 1518
Fluorescence (Baseline Corrected)
−1
0
1
2
3
4
0
200
400
600
800
Time Point (0.25 Hz)
Cell 1519
Fluorescence (Baseline Corrected)
0
10
20
30
40
0
200
400
600
800
Time Point (0.25 Hz)
Cell 1520
Fluorescence (Baseline Corrected)
0
10
20
30
0
200
400
600
800
Time Point (0.25 Hz)

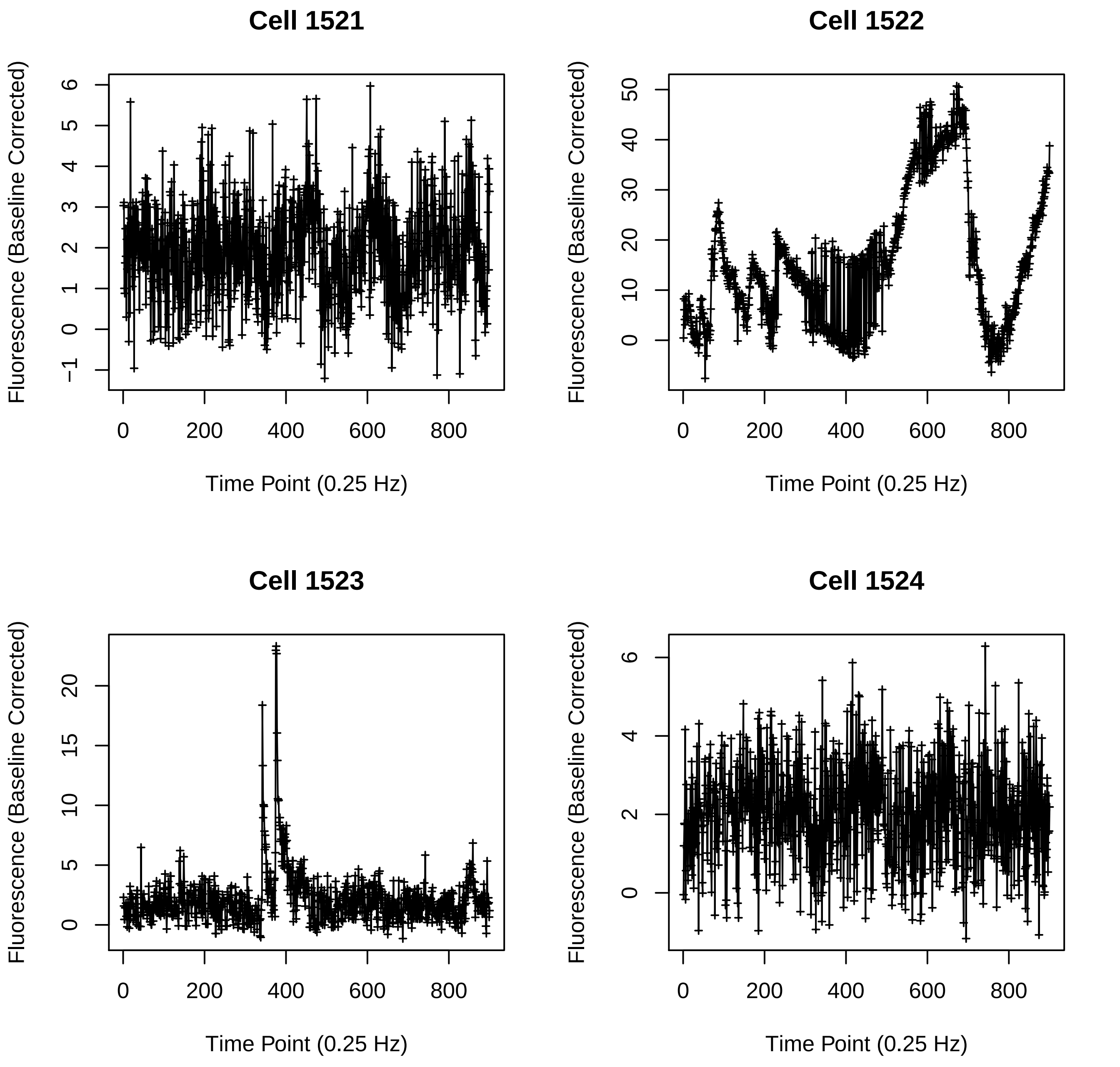

Cell 1521
Cell 1522
Cell 1523
Cell 1524
Fluorescence (Baseline Corrected)
Time Point (0.25 Hz)

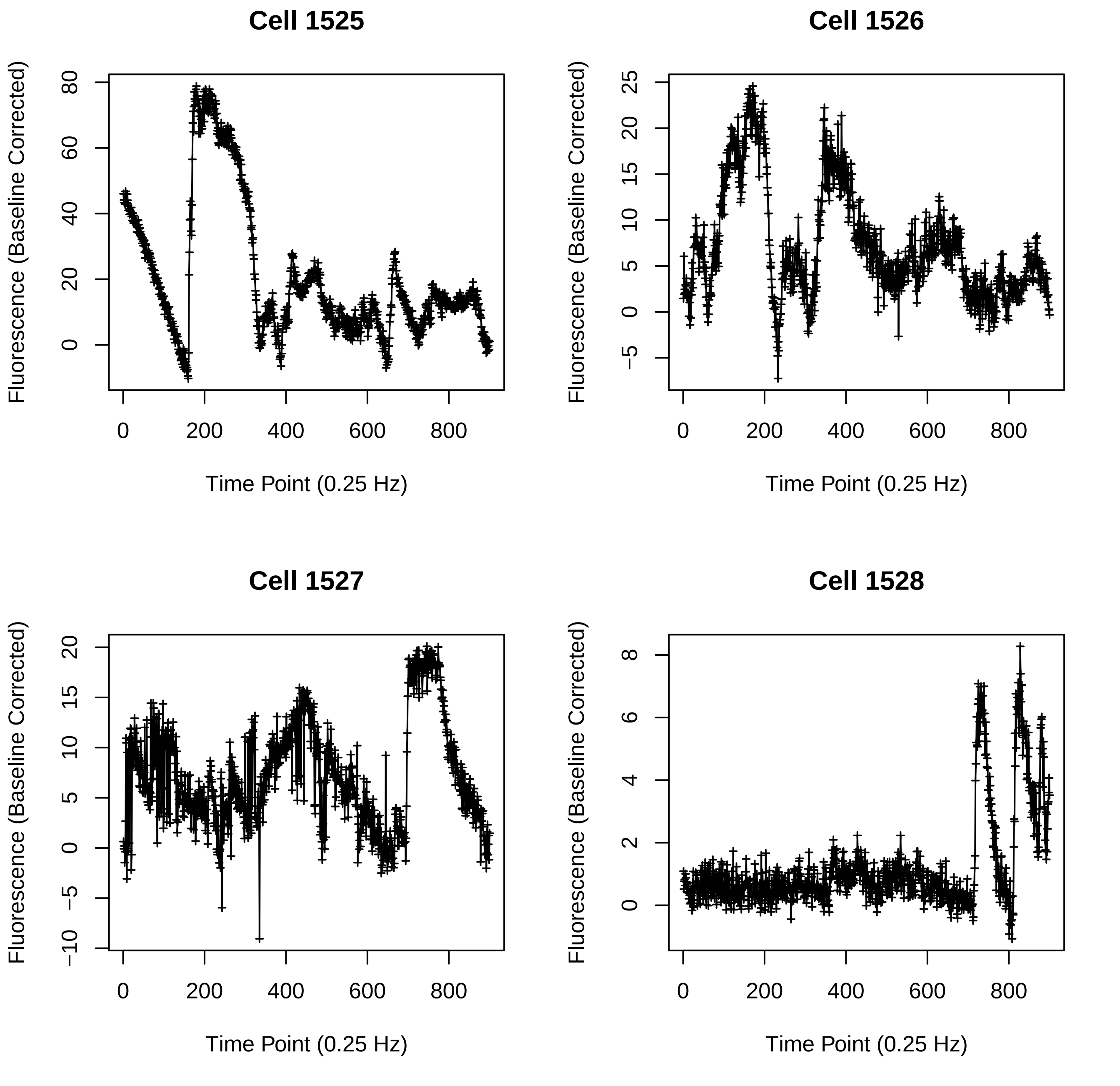
Cell 1525
Cell 1526
Cell 1527
Cell 1528
Fluorescence (Baseline Corrected)
Time Point (0.25 Hz)
0
200
400
600
800
−10
−5
5
10
15
20
25
40
60
80
2
4
6
8

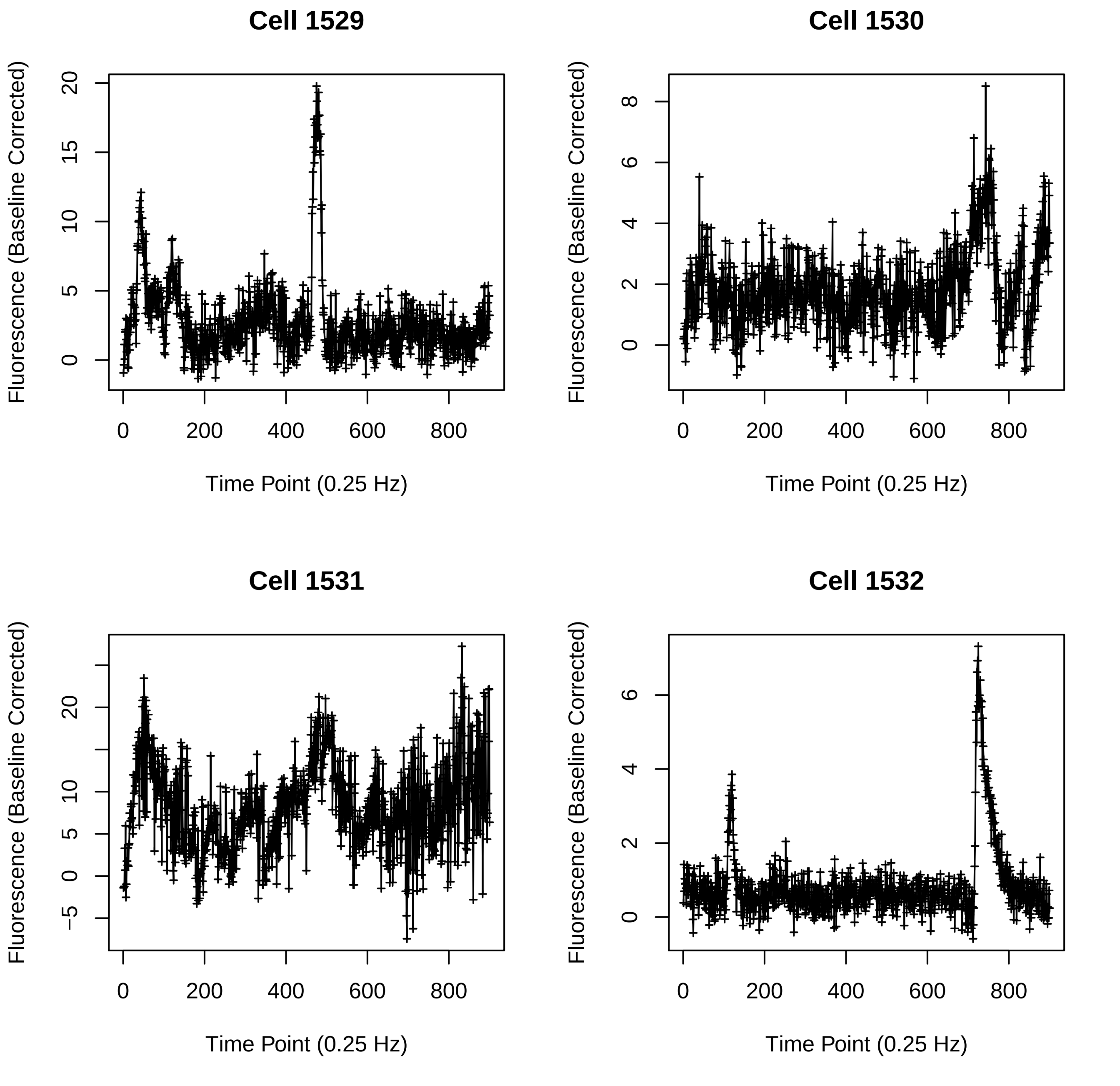
Cell 1529
Cell 1530
Cell 1531
Cell 1532
Fluorescence (Baseline Corrected)
Time Point (0.25 Hz)
0
200
400
600
800
5
10
15
20
2
4
6
8
−5

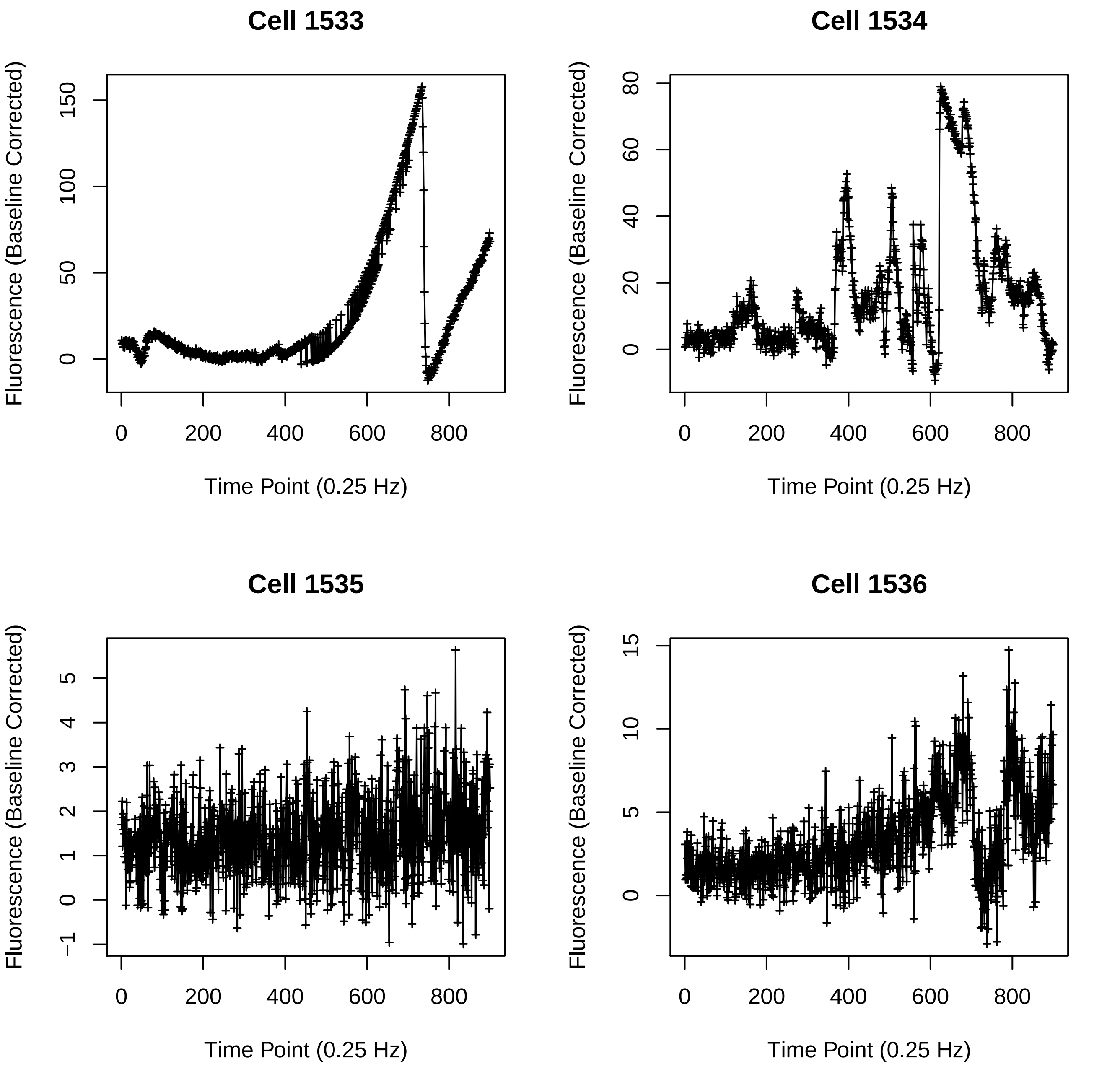
Cell 1533
Cell 1534
Cell 1535
Cell 1536
Fluorescence (Baseline Corrected)
Time Point (0.25 Hz)
0
50
100
150
0
20
40
60
80
−1
0
1
2
3
4
5
0
5
10
15
0
200
400
600
800

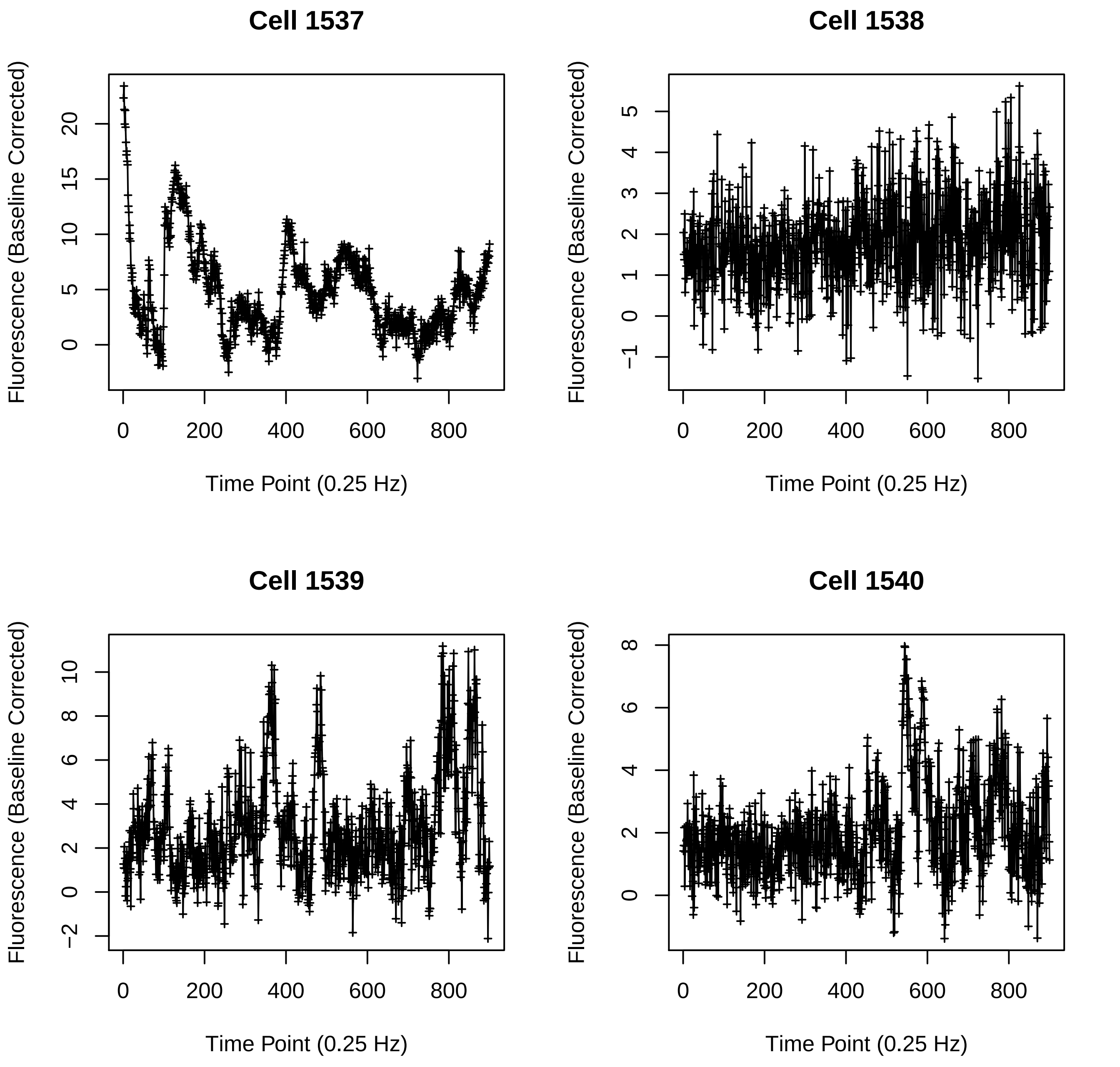
Cell 1537
Cell 1538
Cell 1539
Cell 1540
Fluorescence (Baseline Corrected)
Time Point (0.25 Hz)
0
200
400
600
800

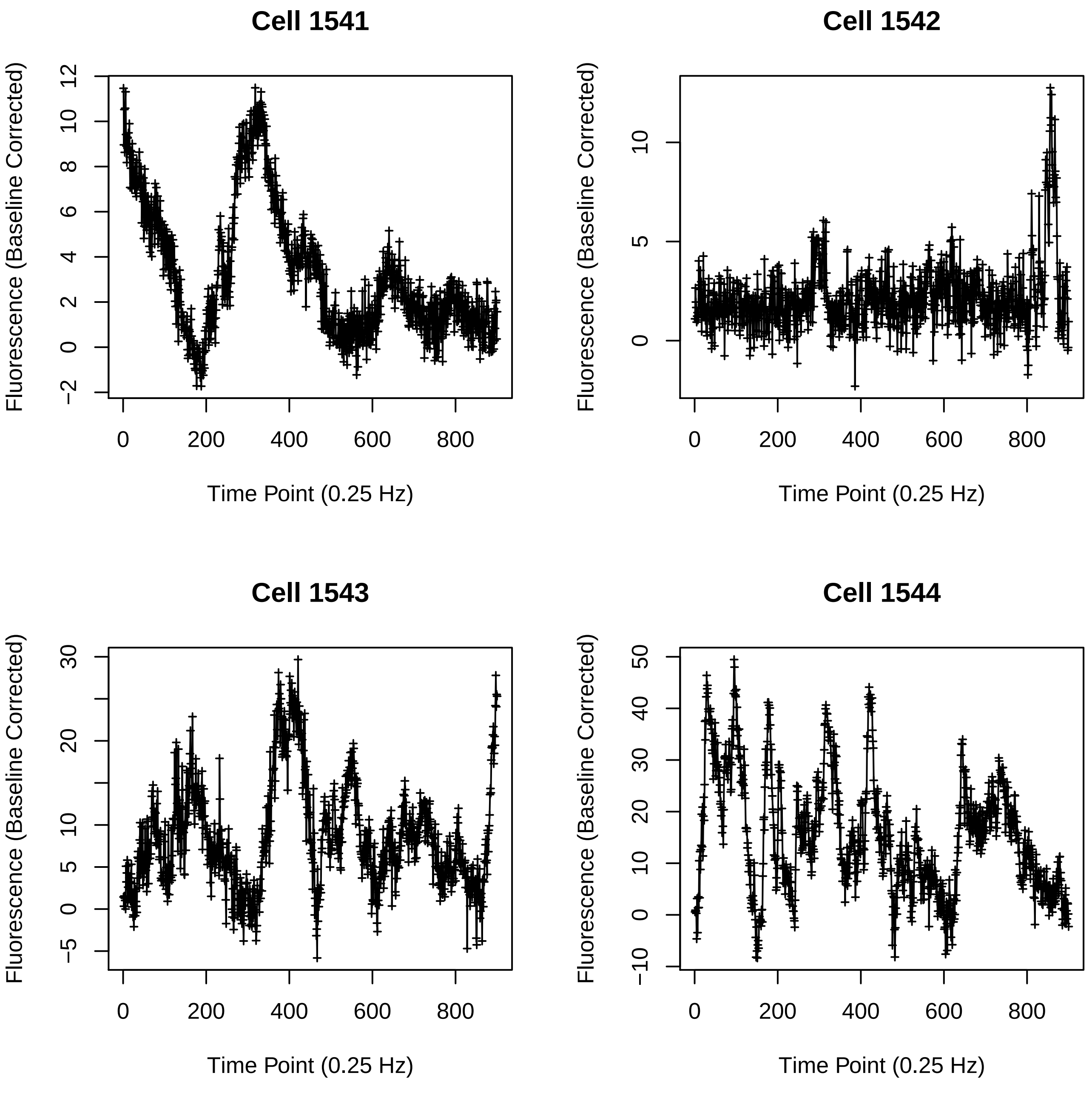

Cell 1541
Fluorescence (Baseline Corrected)
−2
0
2
4
6
8
10
12
0
200
400
600
800
Time Point (0.25 Hz)
Cell 1542
Fluorescence (Baseline Corrected)
0
5
10
0
200
400
600
800
Time Point (0.25 Hz)
Cell 1543
Fluorescence (Baseline Corrected)
−5
0
5
10
20
30
0
200
400
600
800
Time Point (0.25 Hz)
Cell 1544
Fluorescence (Baseline Corrected)
−10
0
10
20
30
40
50
0
200
400
600
800
Time Point (0.25 Hz)

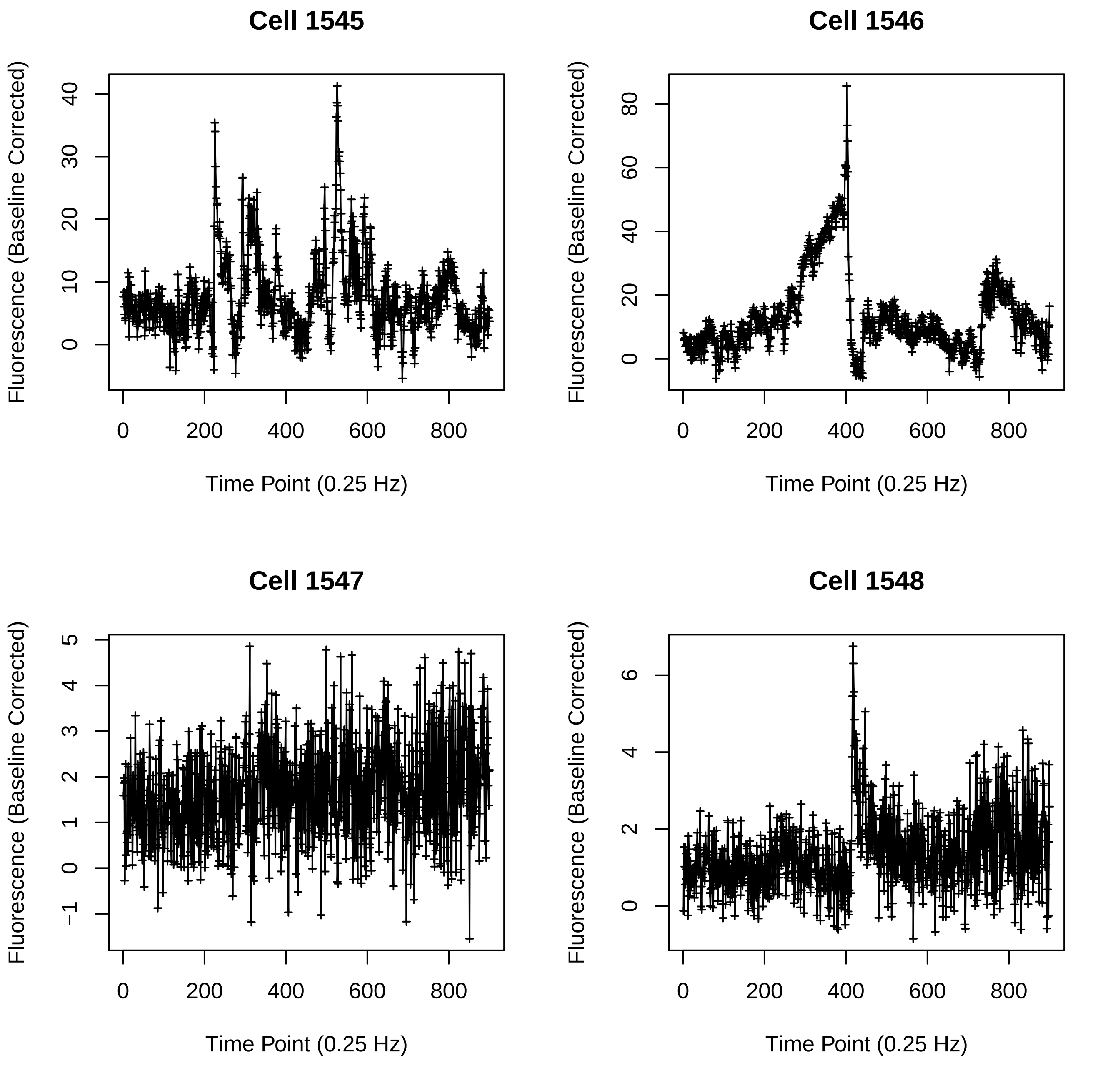
Cell 1545
Cell 1546
Cell 1547
Cell 1548
Fluorescence (Baseline Corrected)
Time Point (0.25 Hz)
0
200
400
600
800
-1
0
1
2
3
4
5
6
10
20
30
40
60
80

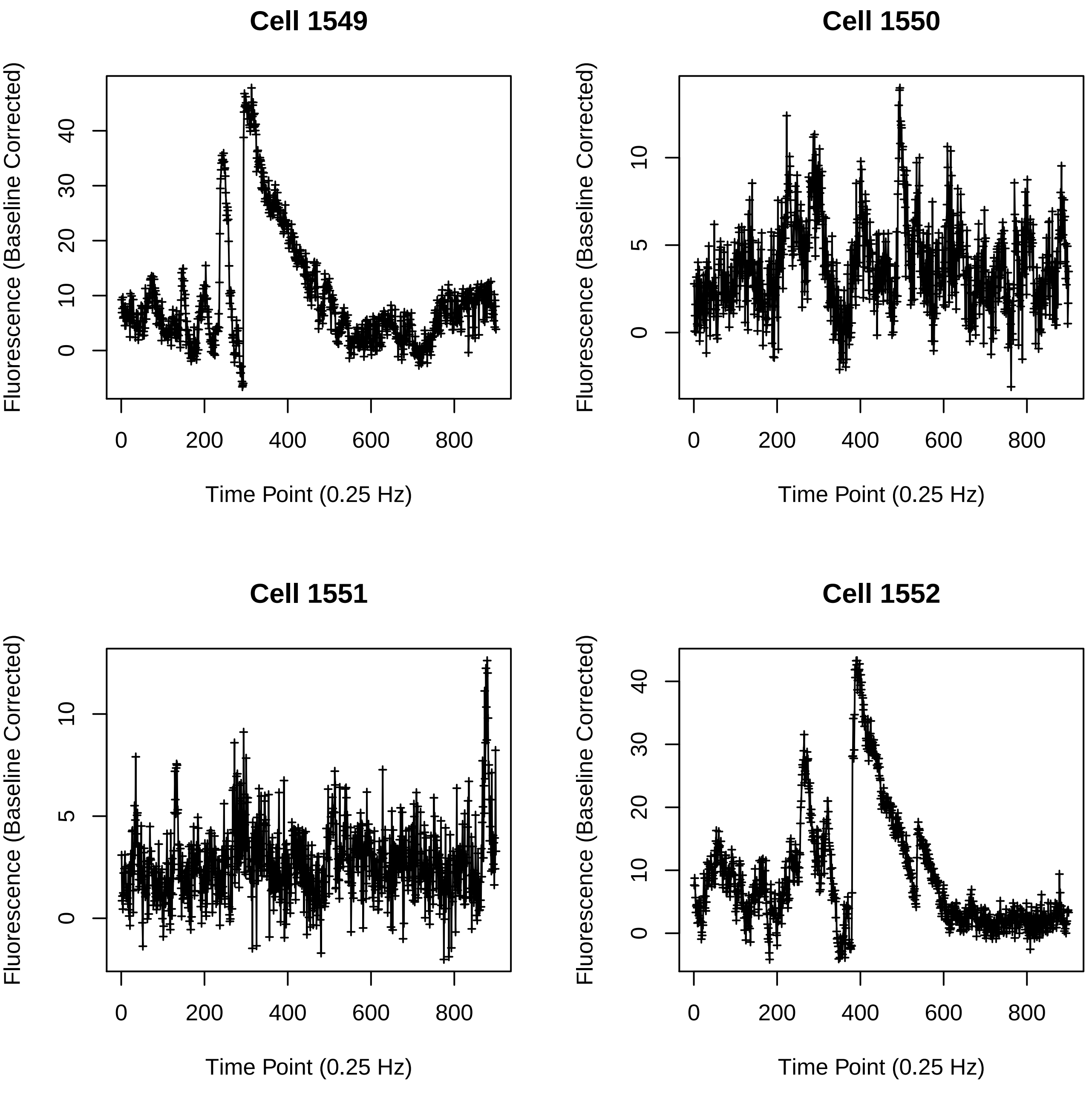
Cell 1549
Cell 1550
Cell 1551
Cell 1552
Fluorescence (Baseline Corrected)
Time Point (0.25 Hz)
0
200
400
600
800
0
10
20
30
40
0
5
10

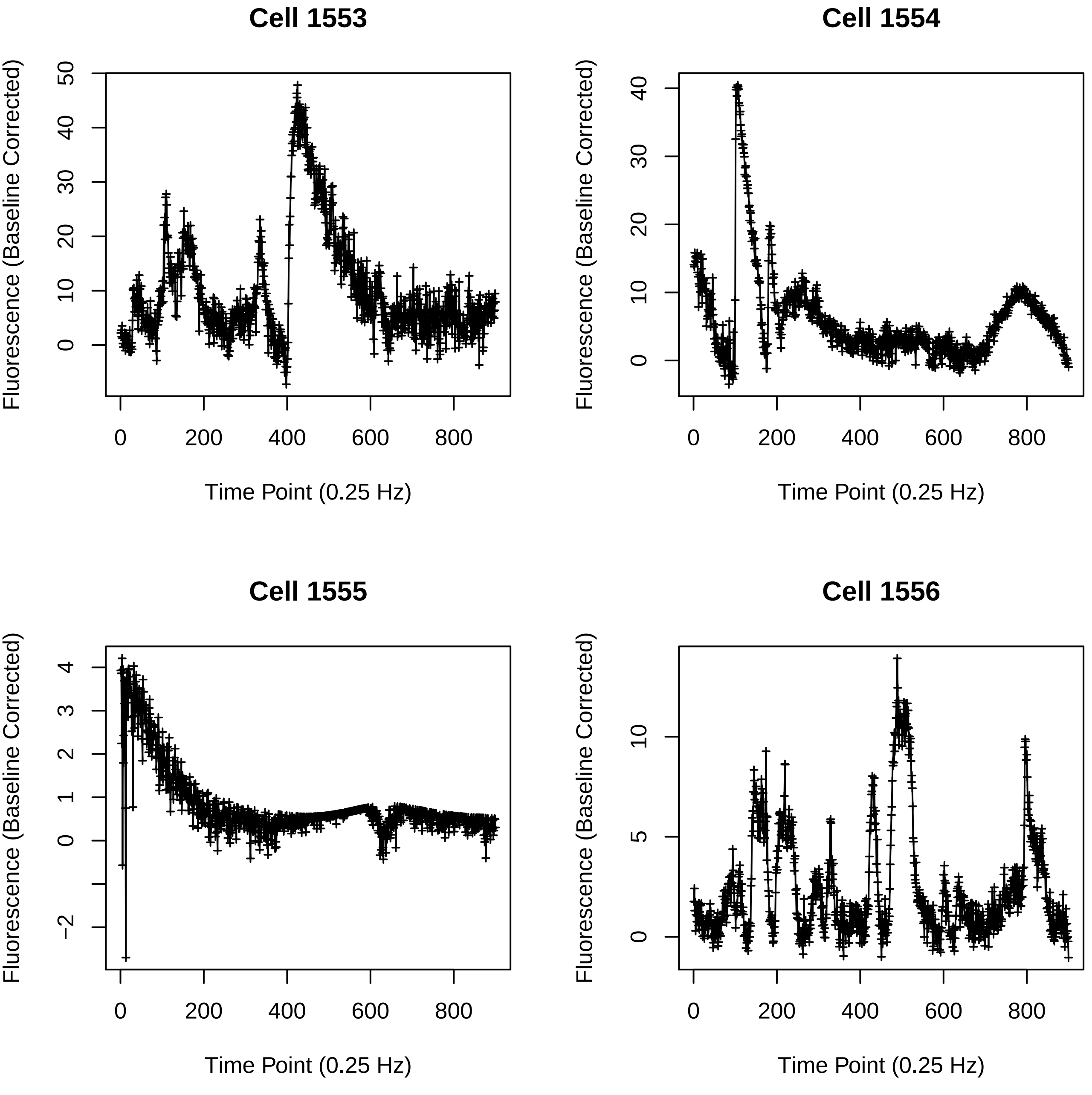
Cell 1553
Cell 1554
Cell 1555
Cell 1556
Fluorescence (Baseline Corrected)
Time Point (0.25 Hz)
0
200
400
600
800
0
10
20
30
40
50
−2
1
2
3
4
5

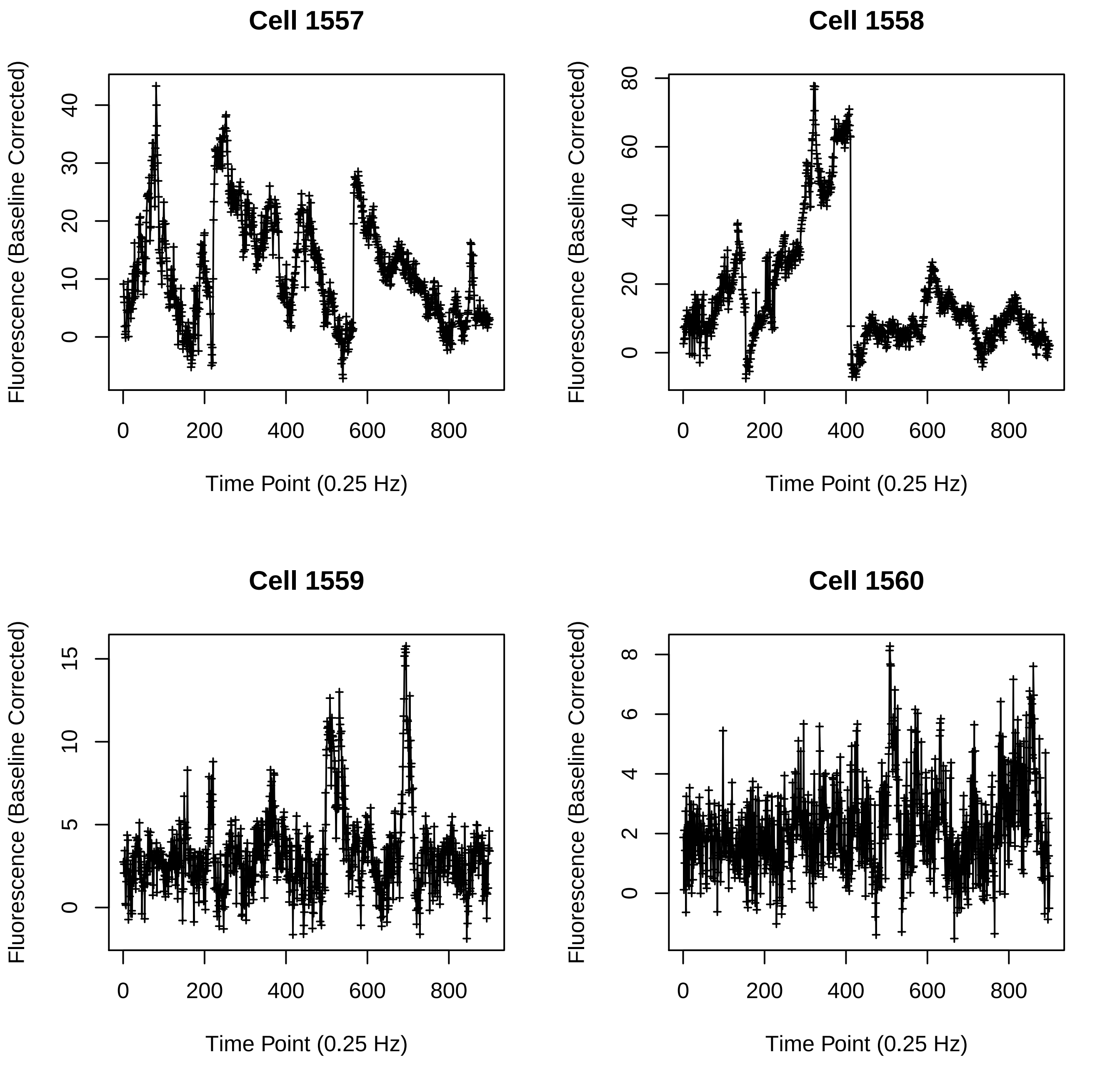
Cell 1557
Cell 1558
Cell 1559
Cell 1560
Fluorescence (Baseline Corrected)
Time Point (0.25 Hz)
0
200
400
600
800
0
10
20
30
40
20
60
80
5
15
2
4
6
8

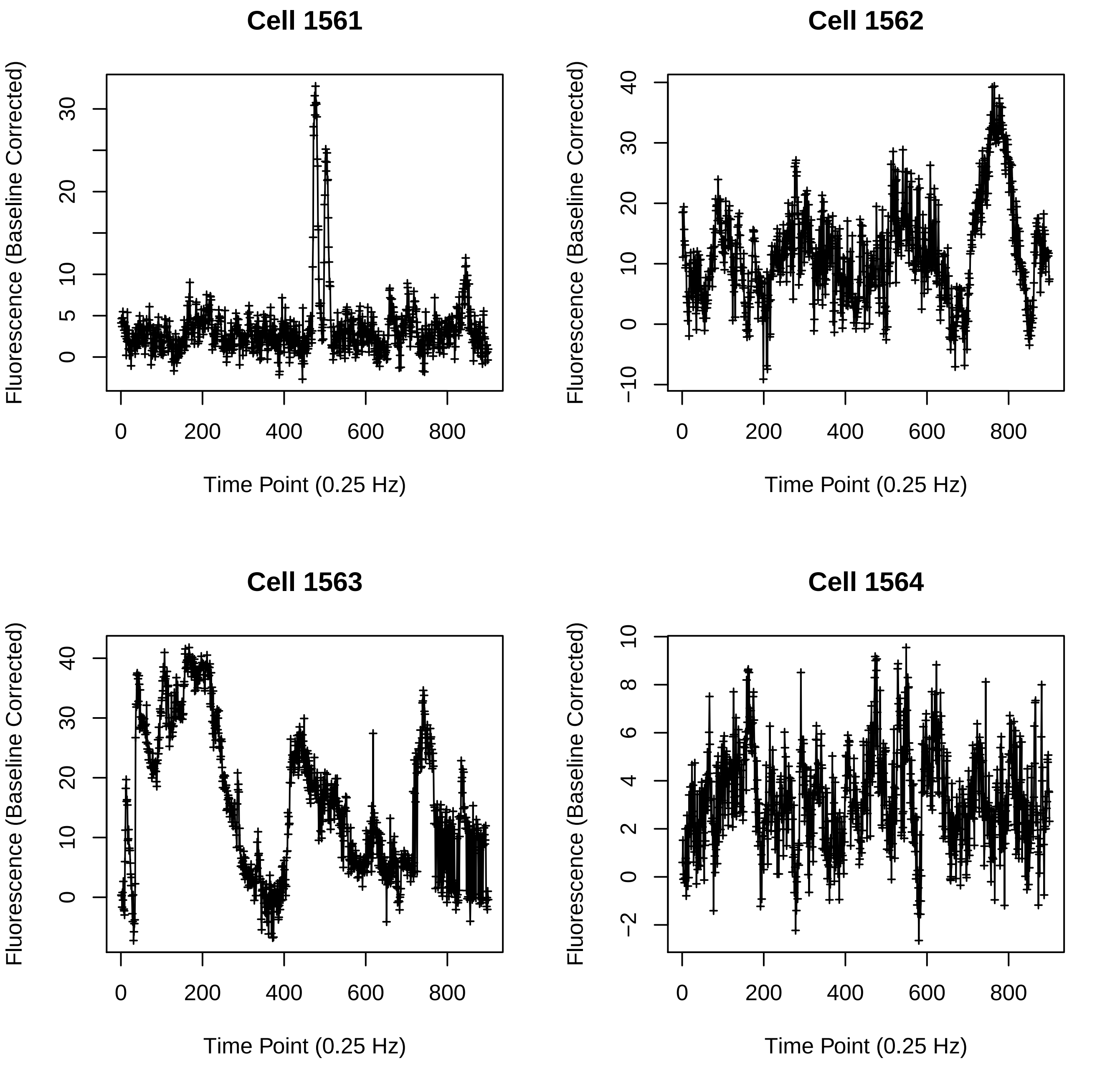
Cell 1561
Fluorescence (Baseline Corrected)
0
5
10
20
30
0
200
400
600
800
Time Point (0.25 Hz)
Cell 1562
Fluorescence (Baseline Corrected)
−10
0
10
20
30
40
0
200
400
600
800
Time Point (0.25 Hz)
Cell 1563
Fluorescence (Baseline Corrected)
0
10
20
30
40
0
200
400
600
800
Time Point (0.25 Hz)
Cell 1564
Fluorescence (Baseline Corrected)
−2
0
2
4
6
8
10
0
200
400
600
800
Time Point (0.25 Hz)

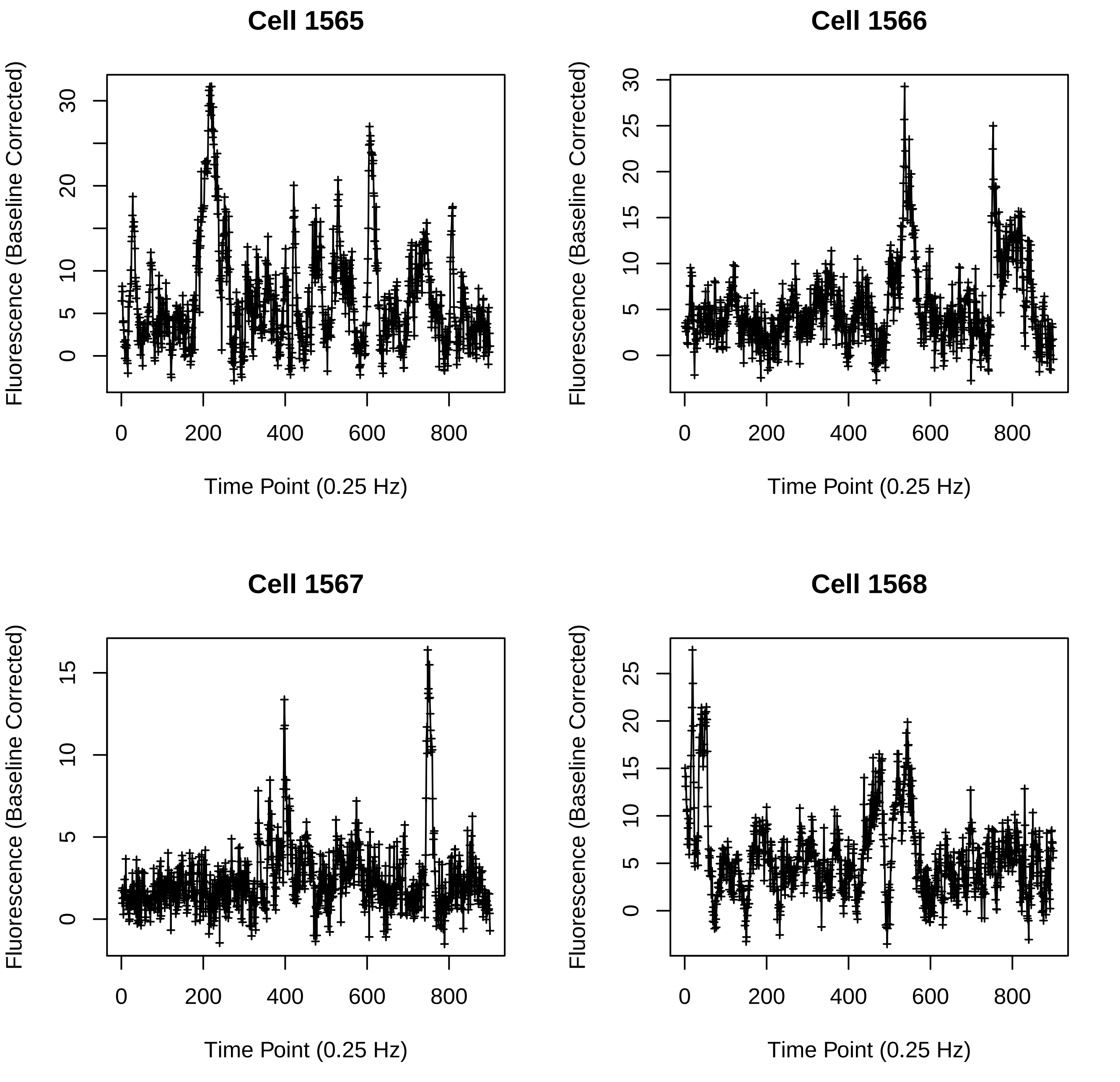
Cell 1565
Cell 1566
Cell 1567
Cell 1568
Fluorescence (Baseline Corrected)
Time Point (0.25 Hz)
0
200
400
600
800
0
5
10
15
20
25
30

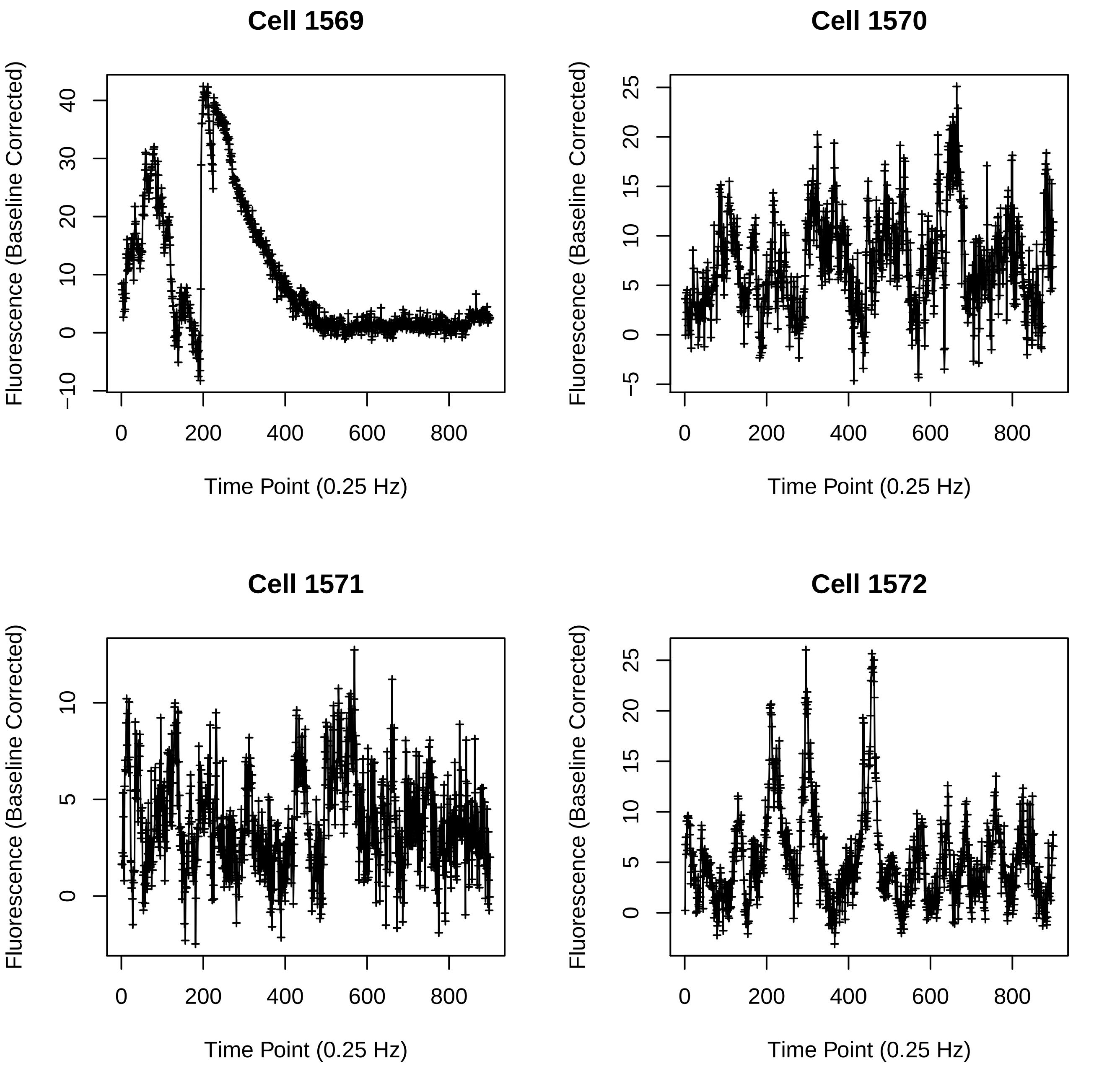
Cell 1569
Cell 1570
Cell 1571
Cell 1572
Fluorescence (Baseline Corrected)
Time Point (0.25 Hz)
−10
0
10
20
30
40
−5
5
15
25
0
200
400
600
800

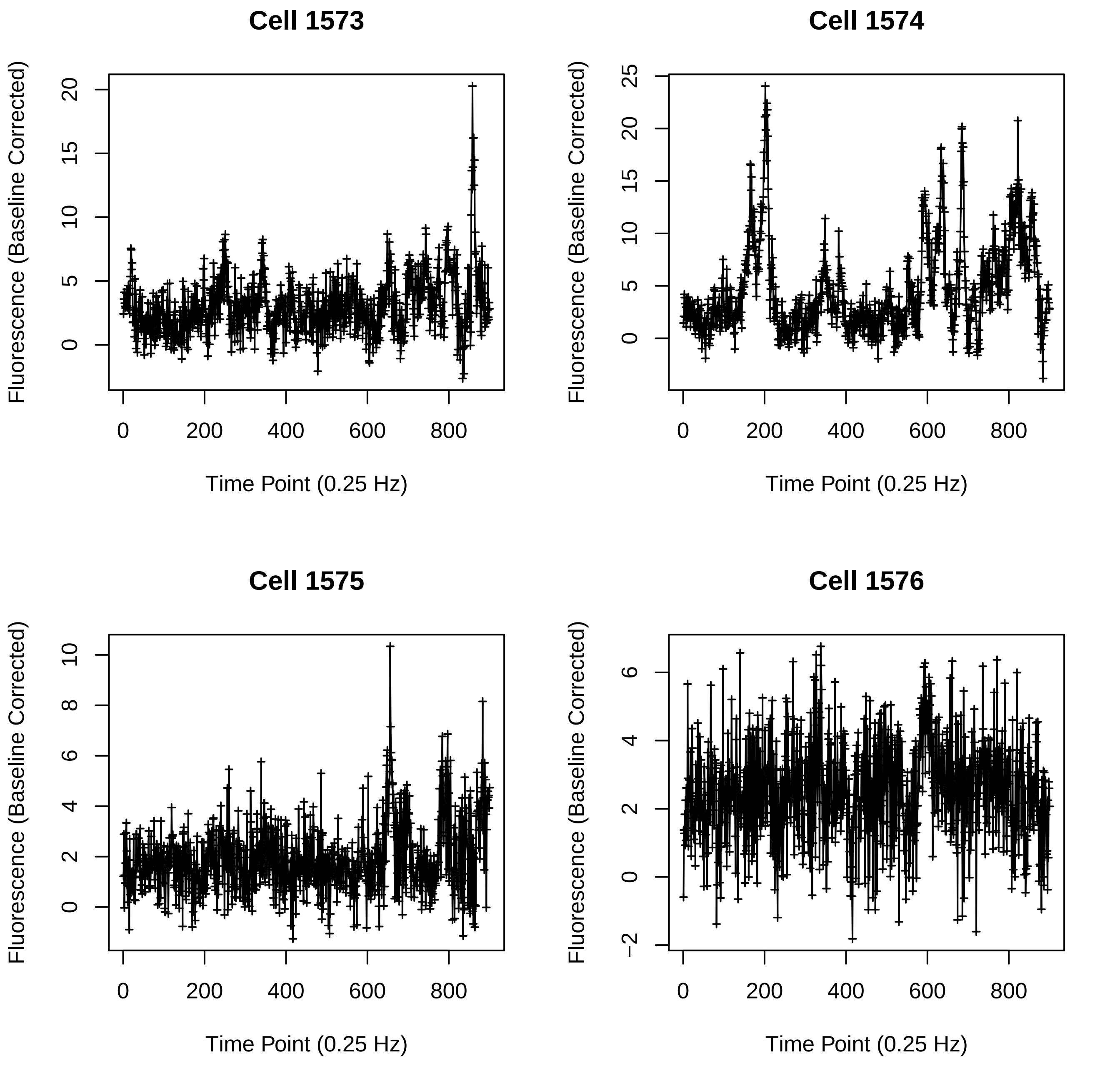
Cell 1573
Cell 1574
Cell 1575
Cell 1576
Fluorescence (Baseline Corrected)
Time Point (0.25 Hz)
0
200
400
600
800
0
5
10
15
20
25
2
4
6
8
−2

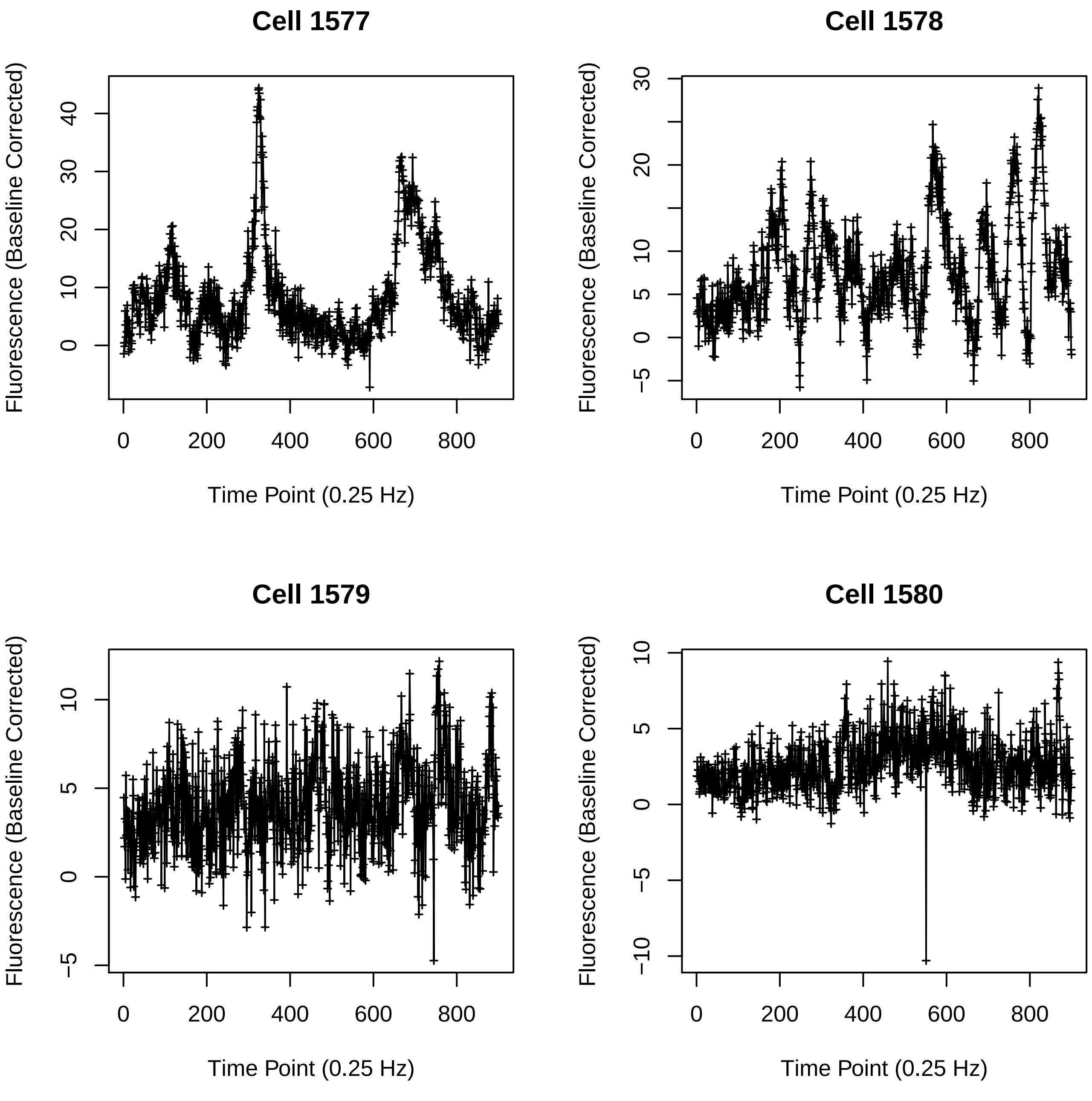
Cell 1577
Cell 1578
Cell 1579
Cell 1580
Fluorescence (Baseline Corrected)
Time Point (0.25 Hz)
0
200
400
600
800
-5
0
5
10
20
25
30
40
-10

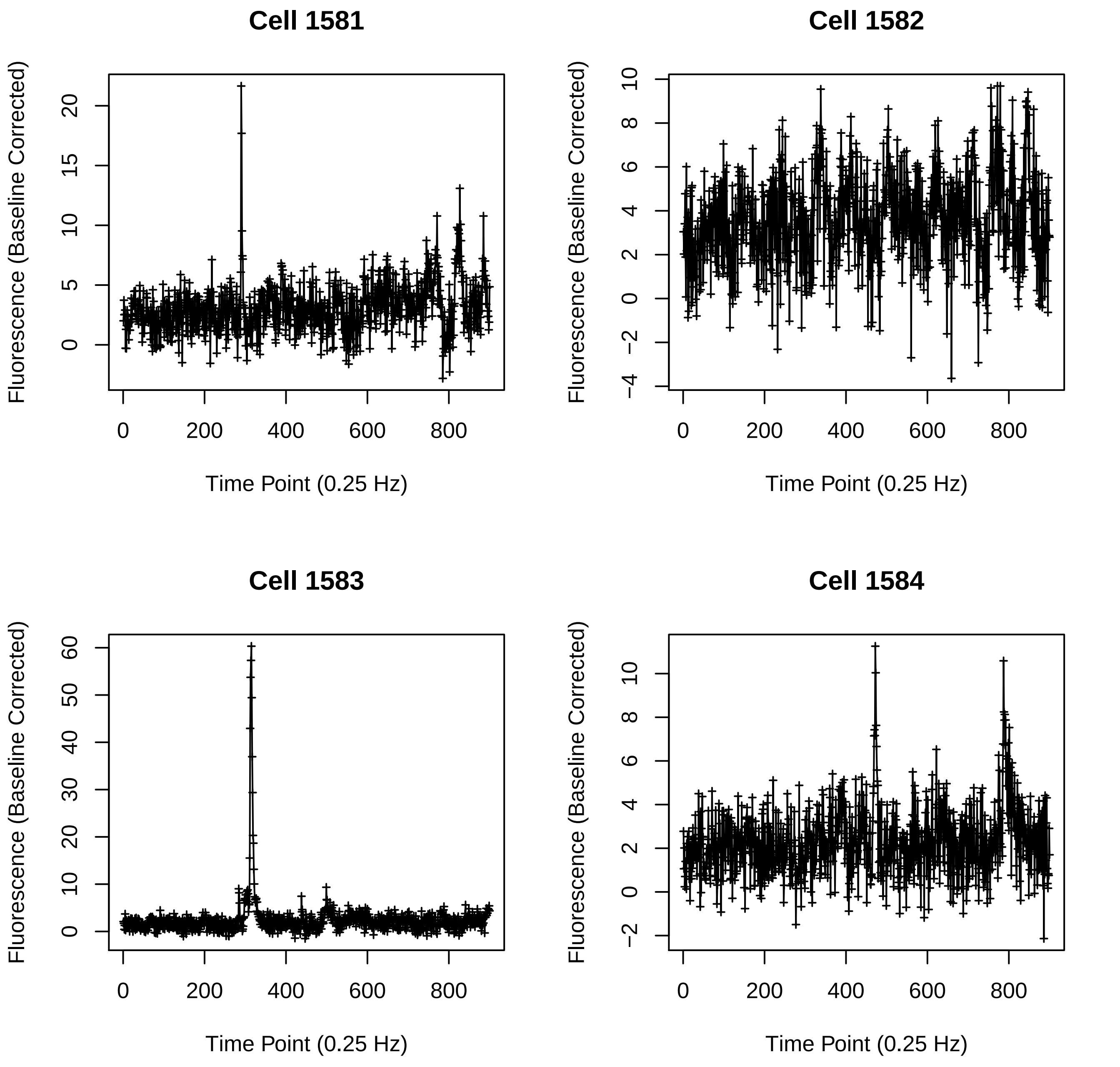
Cell 1581
Cell 1582
Cell 1583
Cell 1584
Fluorescence (Baseline Corrected)
Time Point (0.25 Hz)
0
200
400
600
800
0
5
10
15
20
−4
−2
0
2
4
6
8
10
0
10
20
30
40
50
60

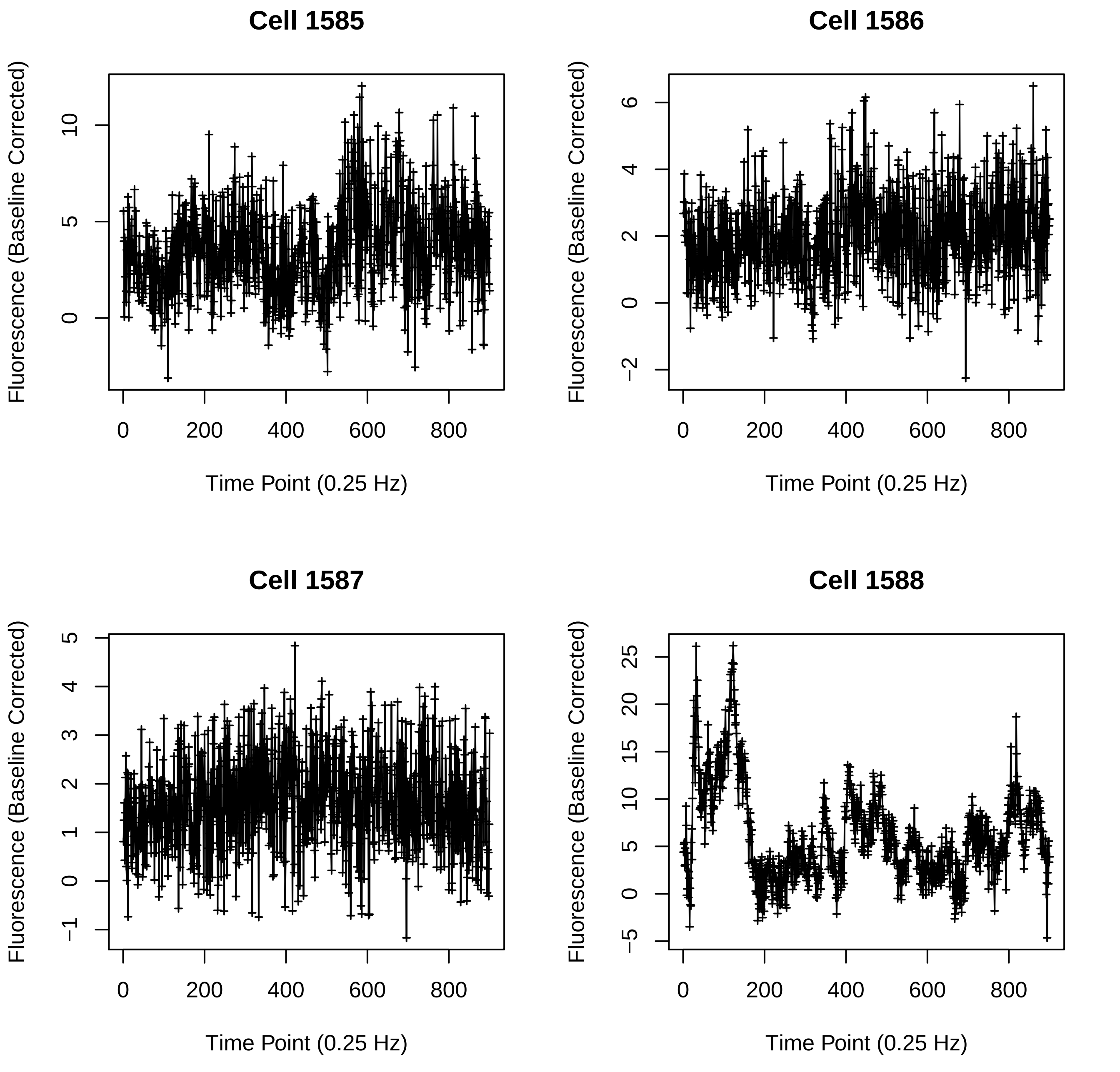
Cell 1585
Cell 1586
Cell 1587
Cell 1588
Fluorescence (Baseline Corrected)
Time Point (0.25 Hz)
0
200
400
600
800
-5
0
5
10
15
20
25
-2
-1
1
2
3
4
6

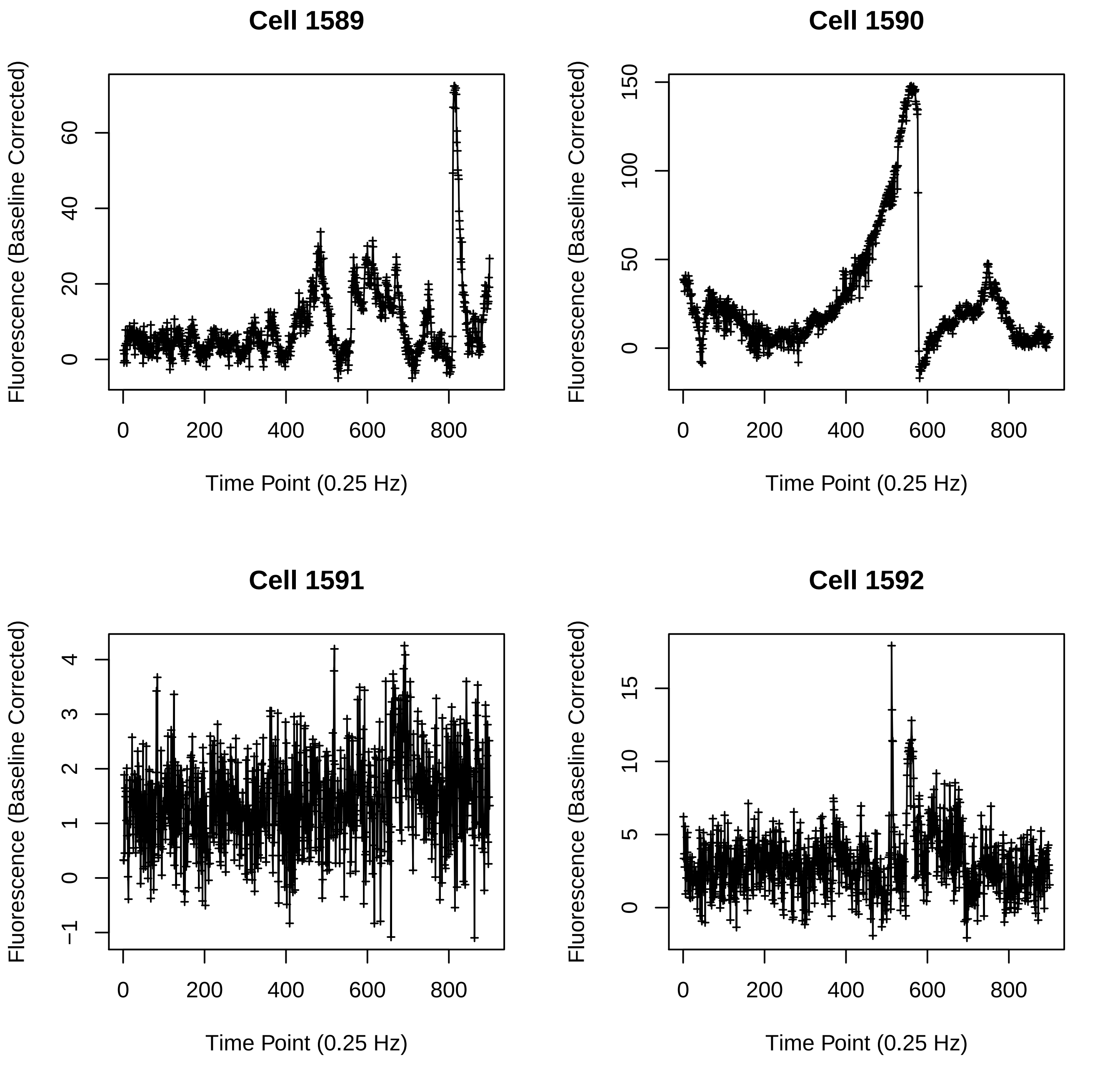

Cell 1589
Cell 1590
Cell 1591
Cell 1592
Fluorescence (Baseline Corrected)
Time Point (0.25 Hz)
0
200
400
600
800
20
40
60
50
100
150
−1
1
2
3
4
5
10
15

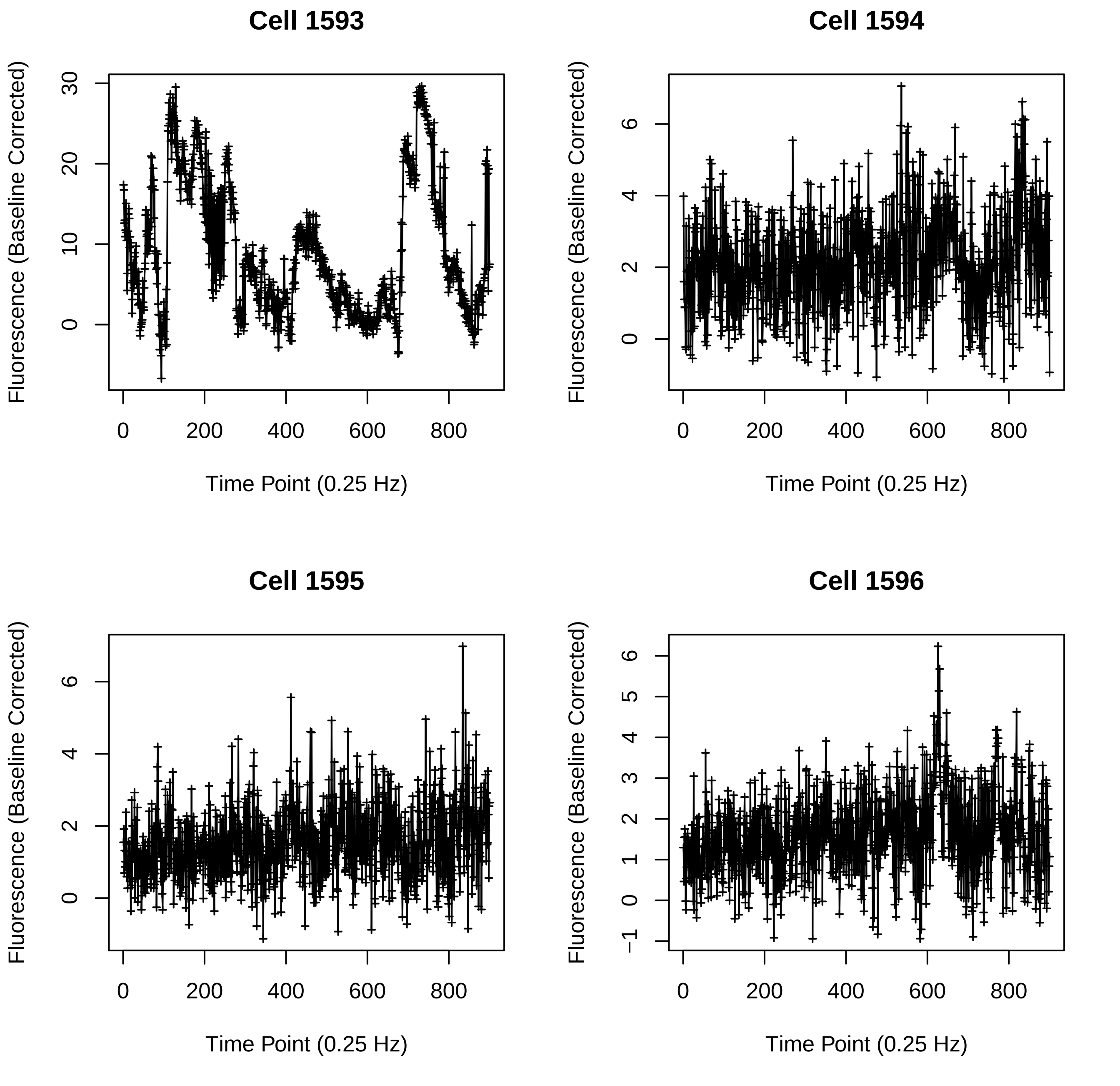
Cell 1593
Cell 1594
Cell 1595
Cell 1596
Fluorescence (Baseline Corrected)
Time Point (0.25 Hz)
0
200
400
600
800
-1
0
1
2
3
4
5
6
10
20
30

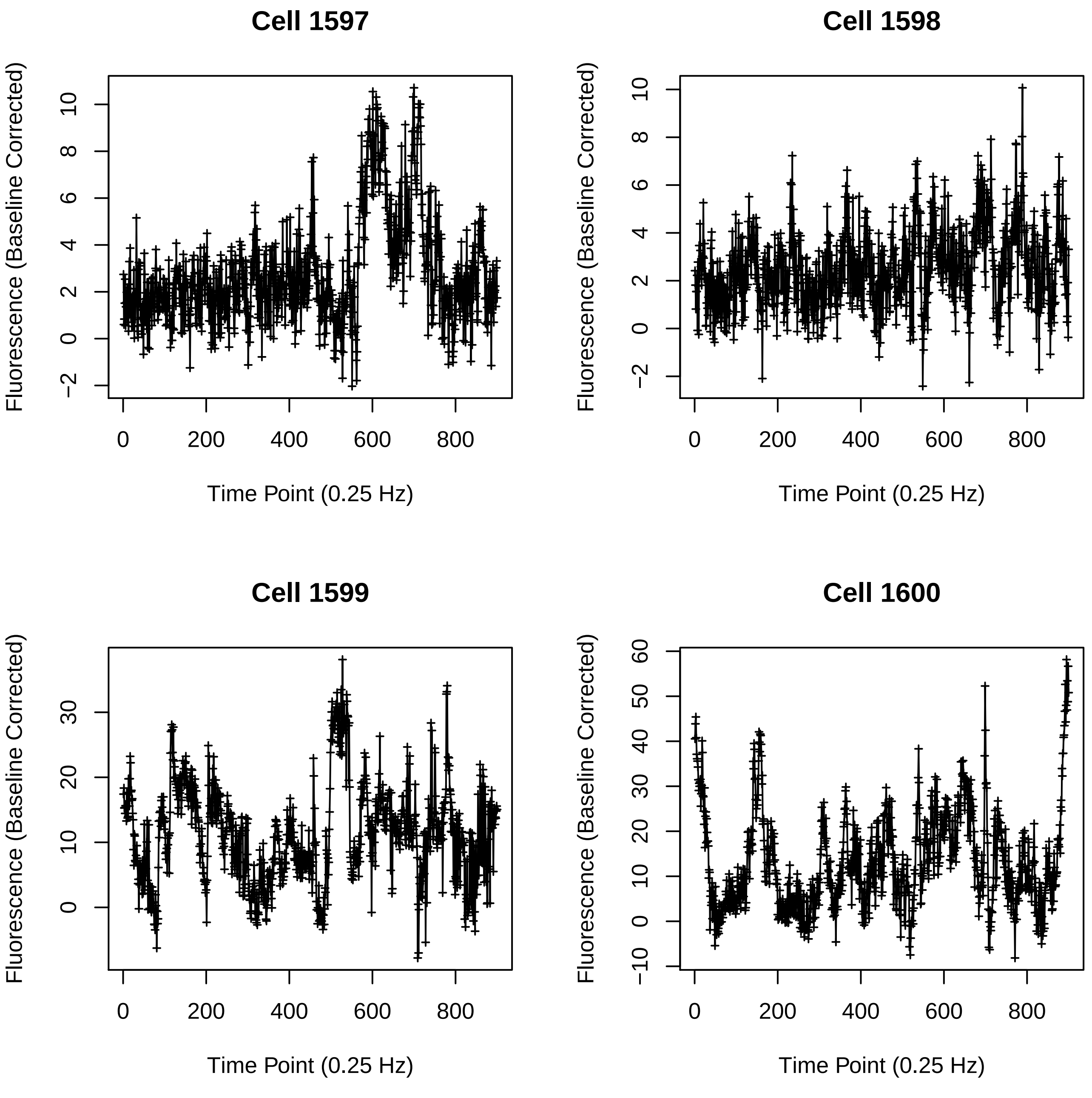
Cell 1597
Cell 1598
Cell 1599
Cell 1600
Fluorescence (Baseline Corrected)
Time Point (0.25 Hz)

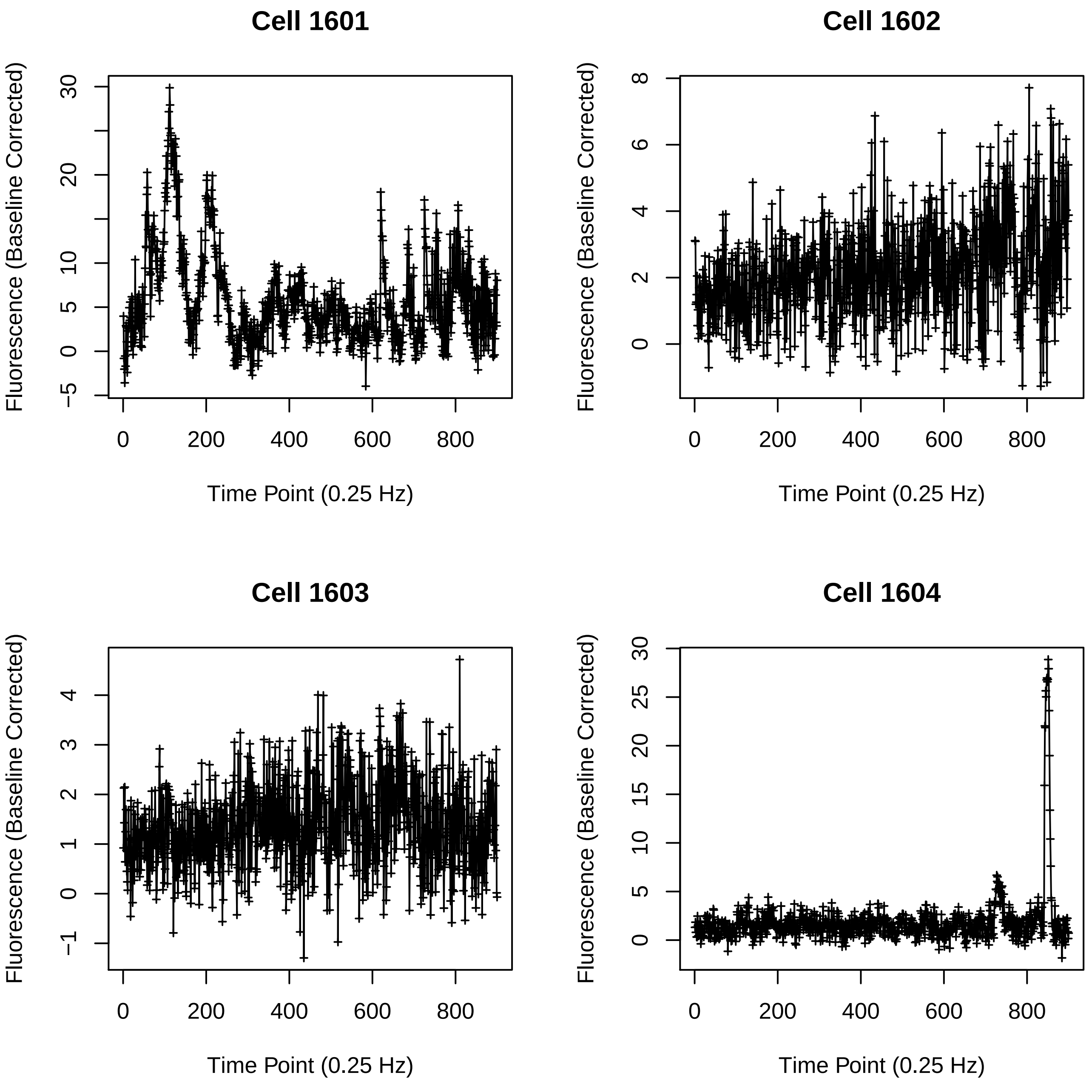
Cell 1601
Cell 1602
Cell 1603
Cell 1604
Fluorescence (Baseline Corrected)
Time Point (0.25 Hz)
0
200
400
600
800
−5
0
5
10
20
30
2
4
6
8
−1
1
3
15
25

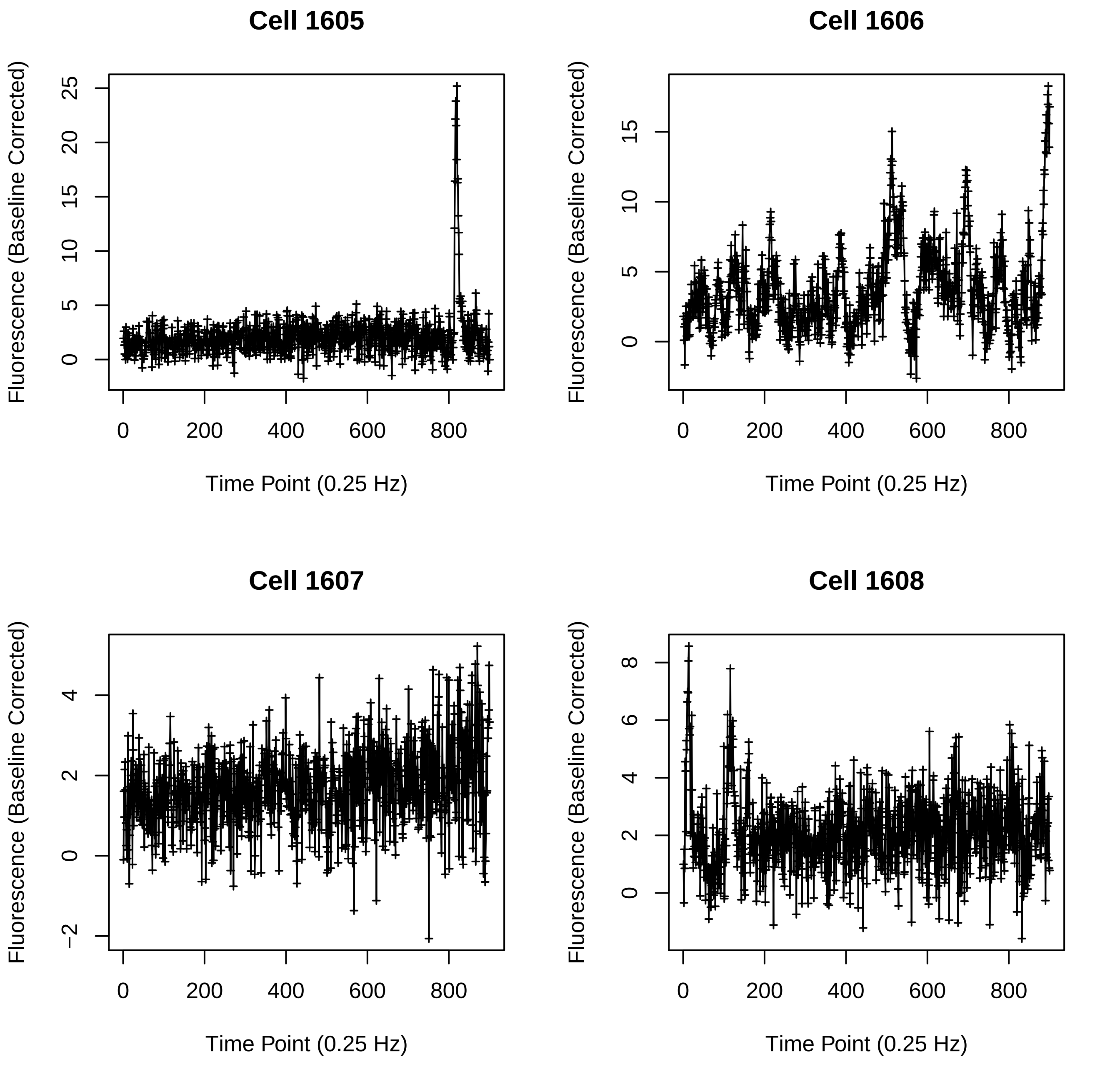
Cell 1605
Cell 1606
Cell 1607
Cell 1608
Fluorescence (Baseline Corrected)
Time Point (0.25 Hz)
0
200
400
600
800
5
10
15
20
25
2
4
6
8
−2

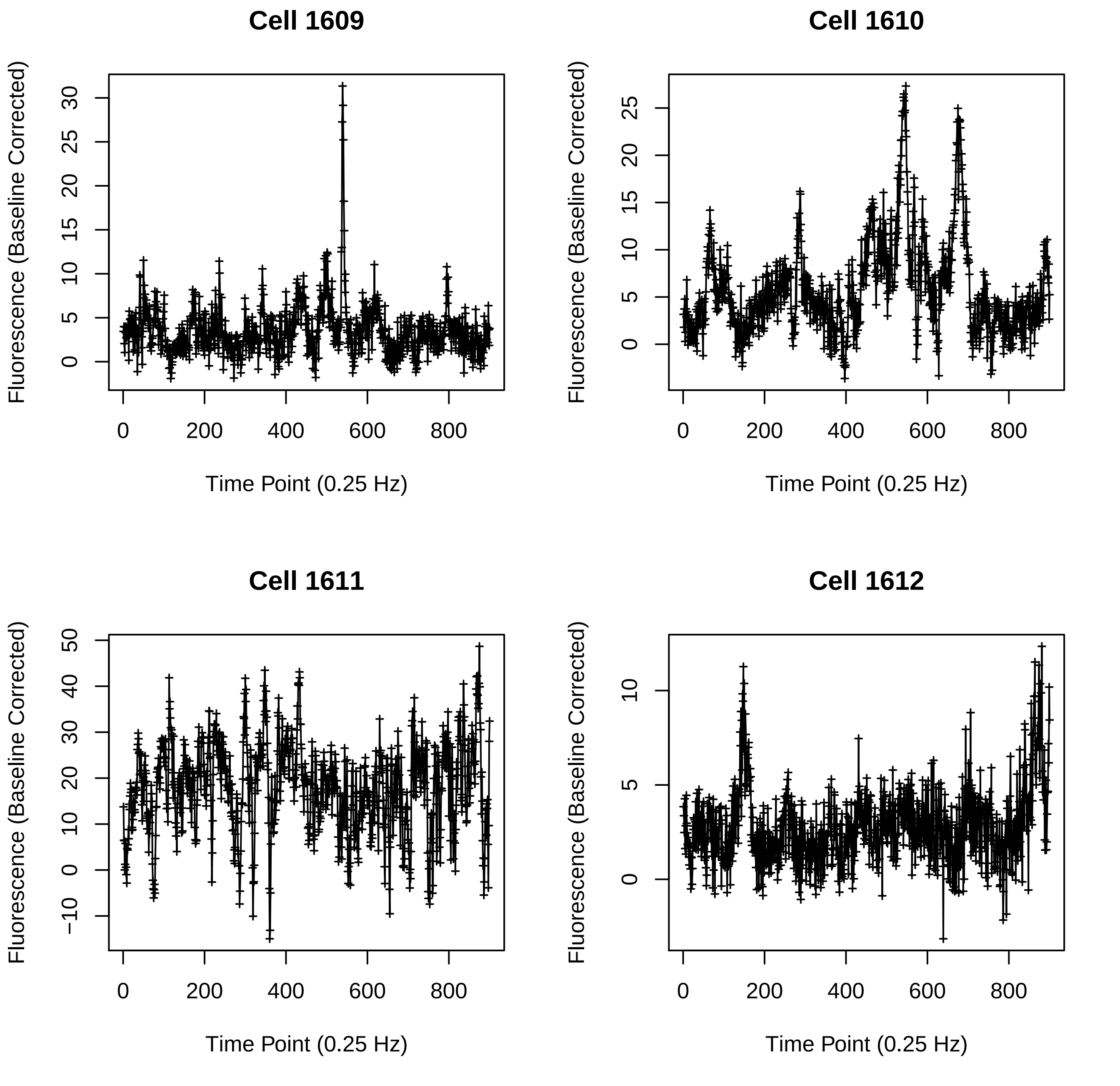
Cell 1609
Cell 1610
Cell 1611
Cell 1612
Fluorescence (Baseline Corrected)
Time Point (0.25 Hz)
0
200
400
600
800

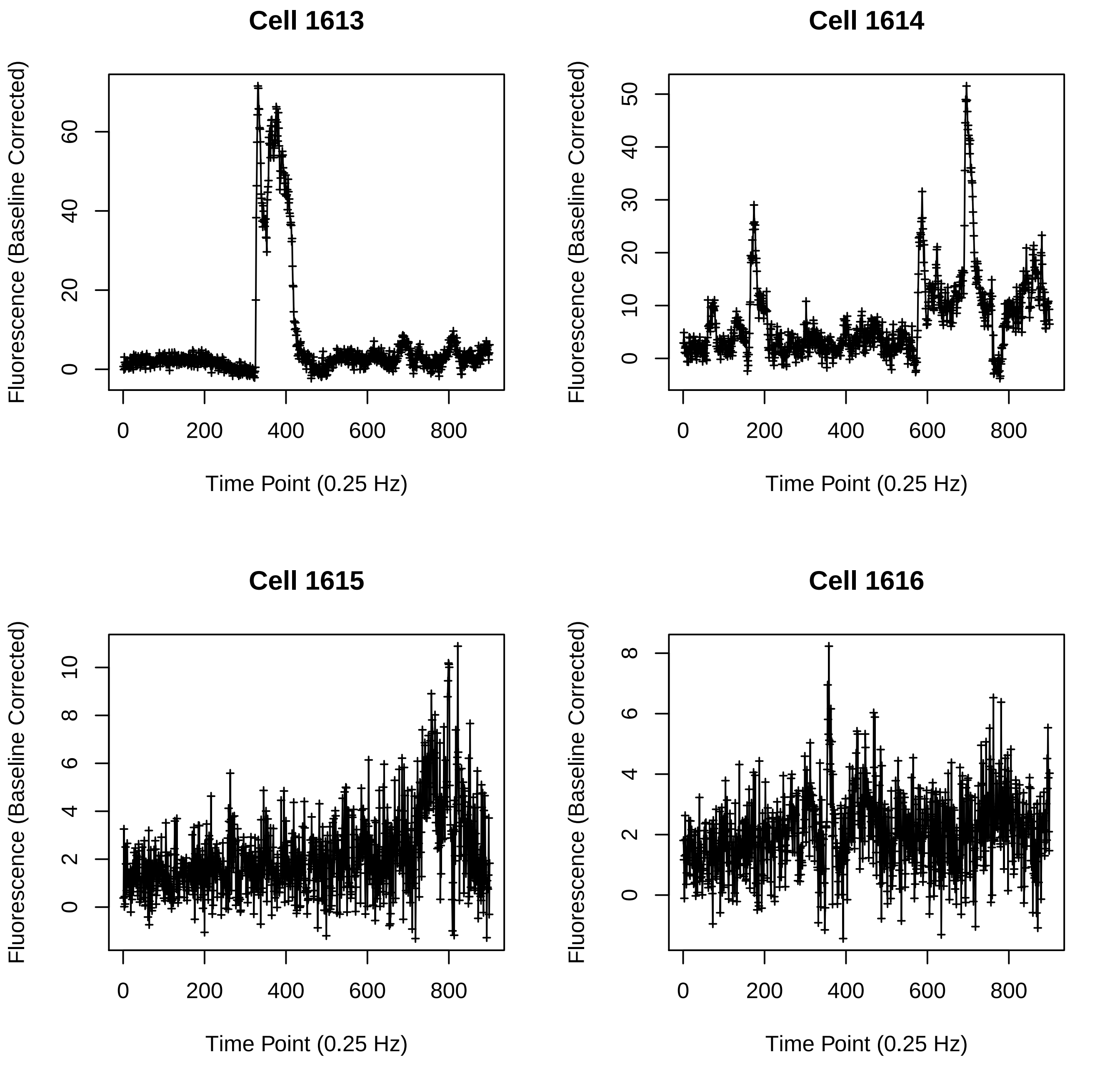
Cell 1613
Cell 1614
Cell 1615
Cell 1616
Fluorescence (Baseline Corrected)
Time Point (0.25 Hz)
0
20
40
60
0
10
20
30
40
50
0
2
4
6
8
10
0
200
400
600
800

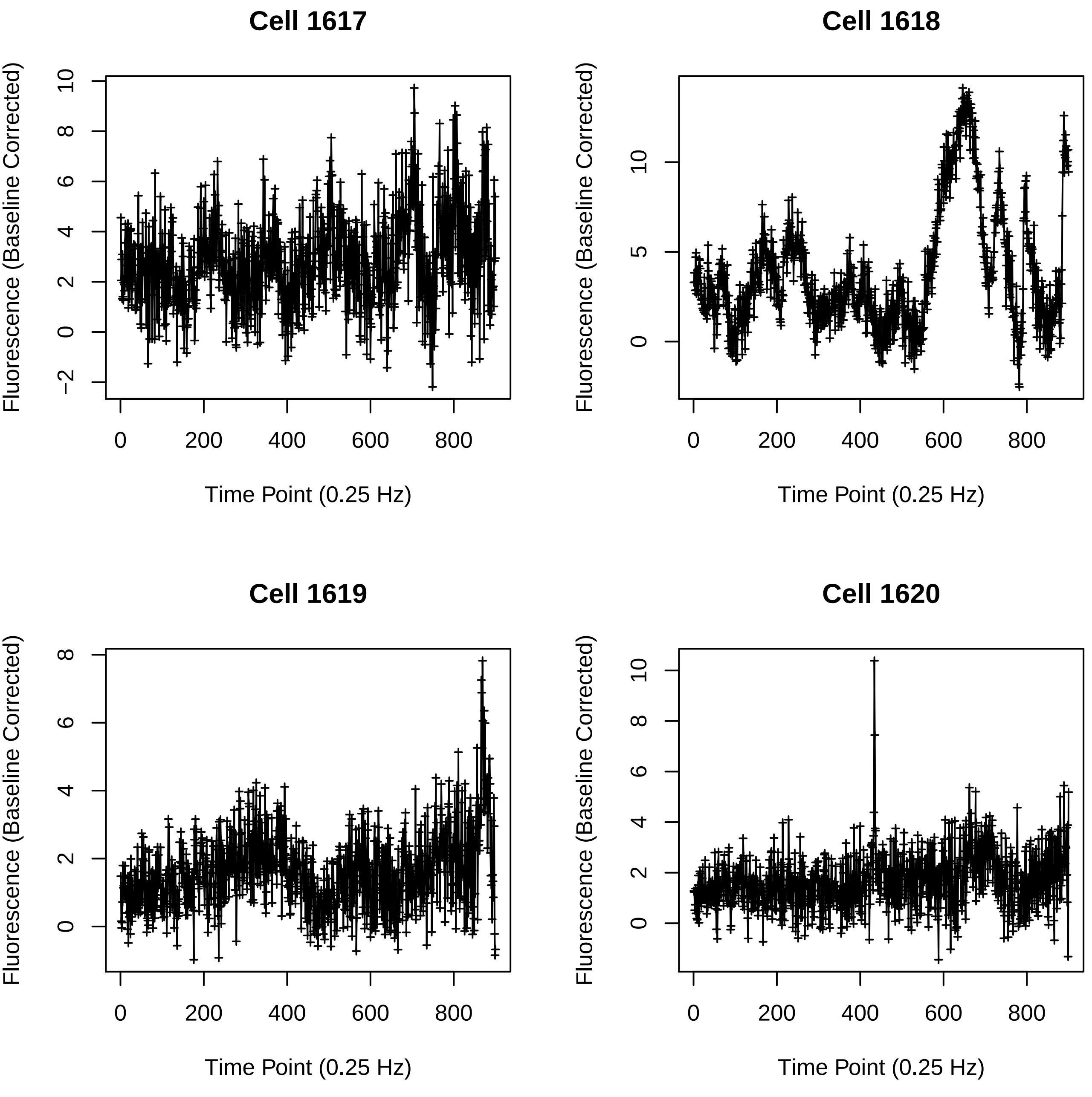

Cell 1617
Cell 1618
Cell 1619
Cell 1620
Fluorescence (Baseline Corrected)
Time Point (0.25 Hz)
0
200
400
600
800

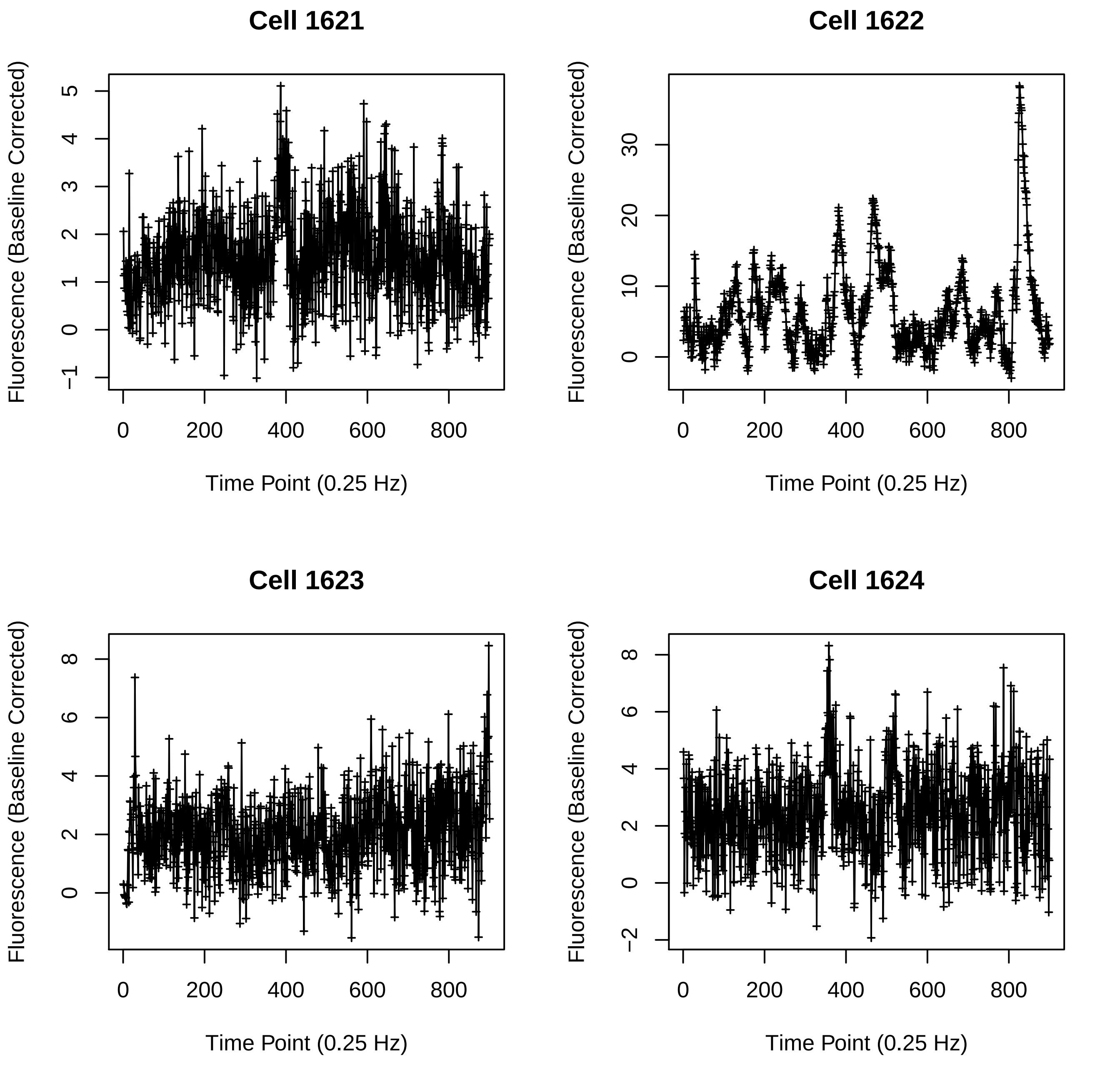

Cell 1621
Cell 1622
Cell 1623
Cell 1624
Fluorescence (Baseline Corrected)
Time Point (0.25 Hz)
0
200
400
600
800

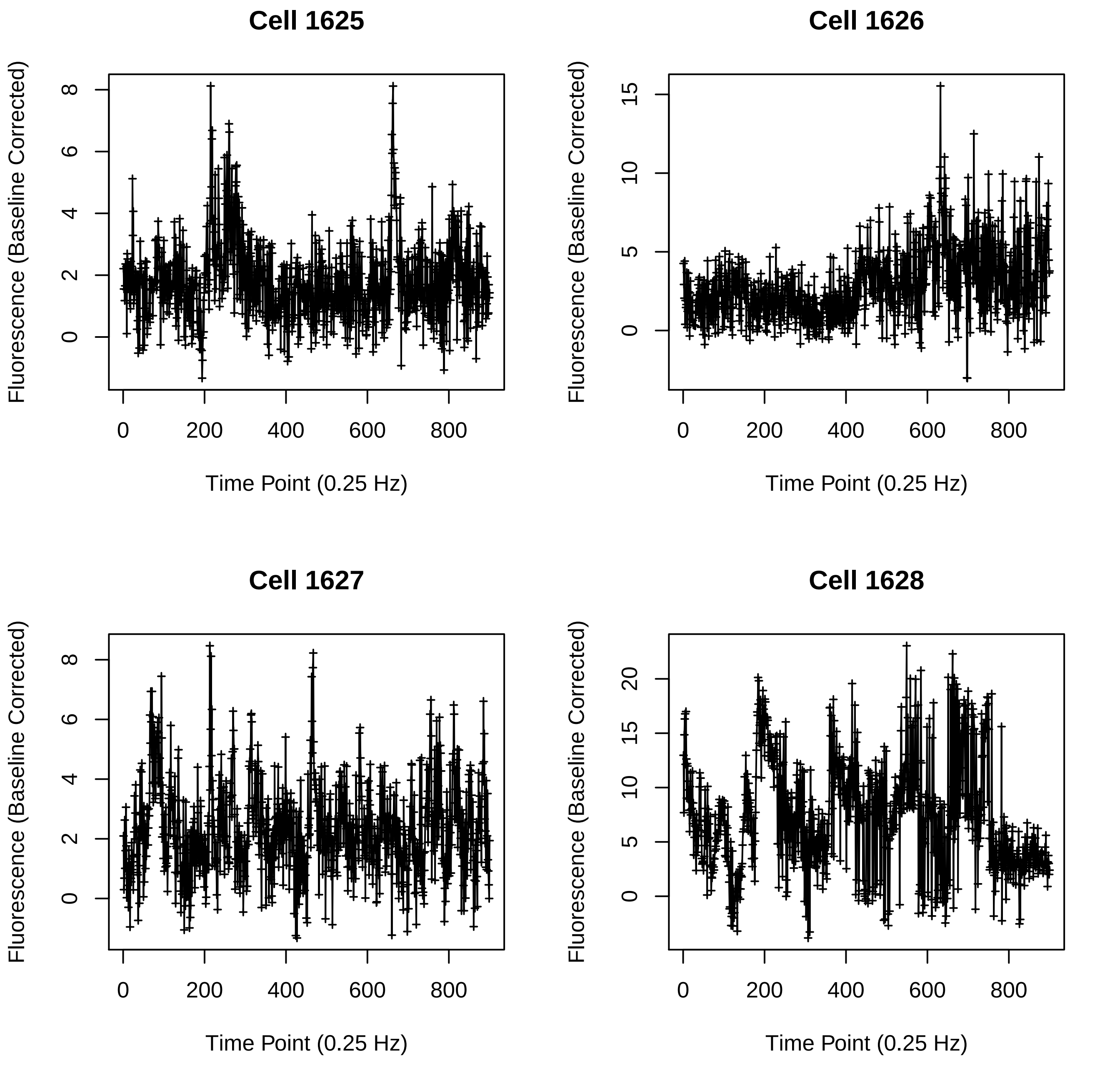
Cell 1625
Cell 1626
Cell 1627
Cell 1628
Fluorescence (Baseline Corrected)
Time Point (0.25 Hz)
0
200
400
600
800
2
4
6
8
5
10
15
20

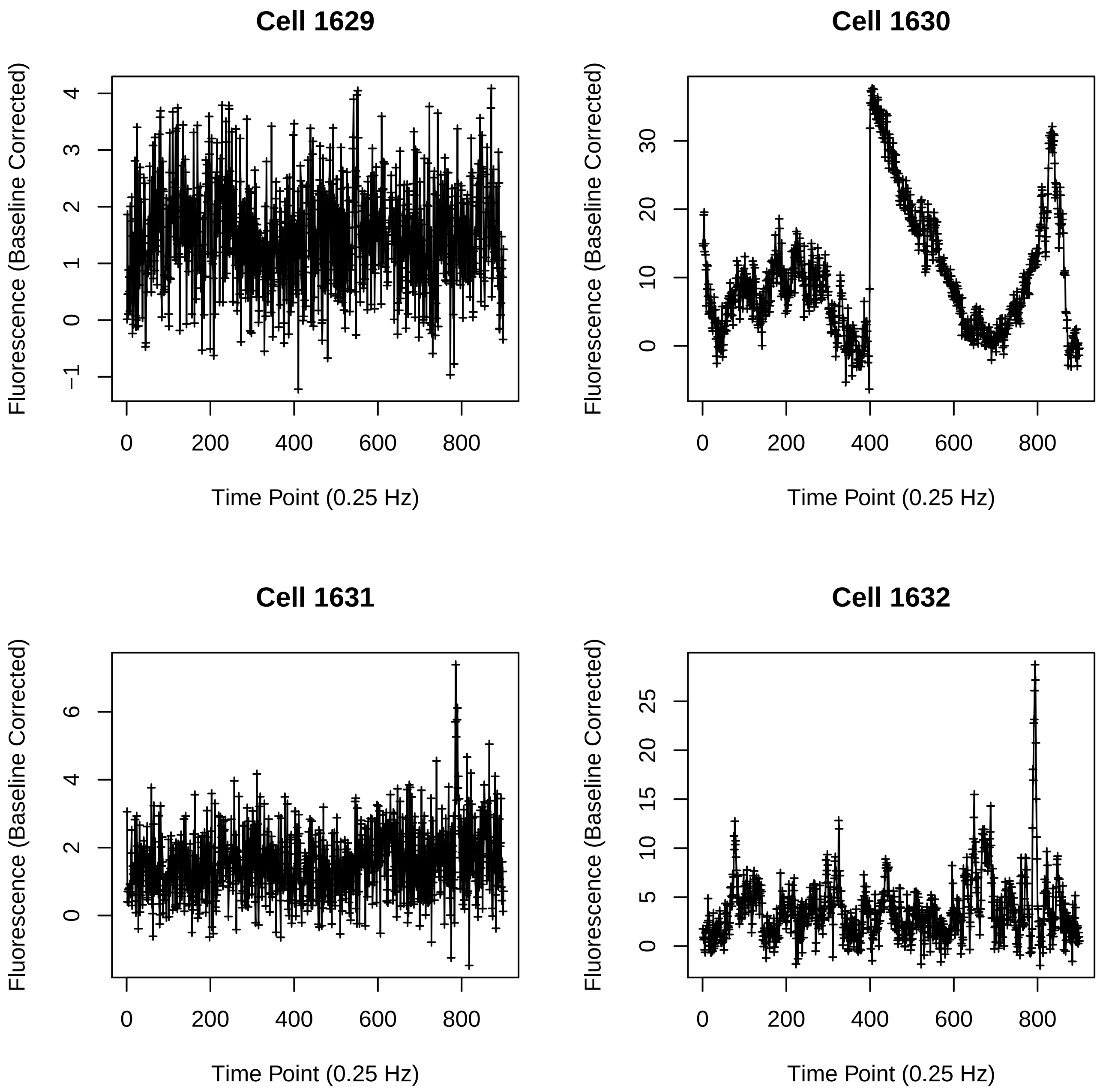

Cell 1629
Cell 1630
Cell 1631
Cell 1632
Fluorescence (Baseline Corrected)
Time Point (0.25 Hz)

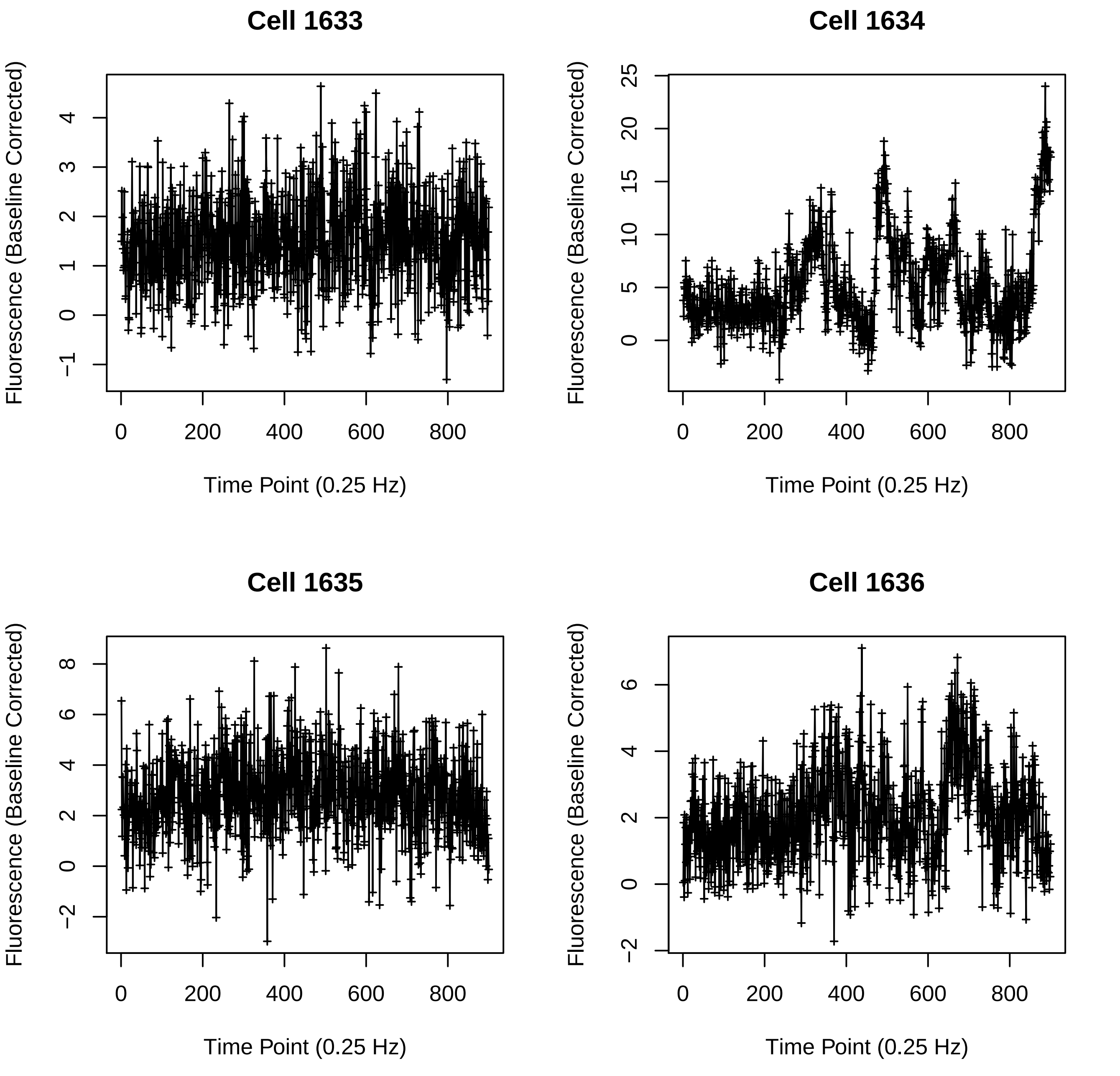

Cell 1633
Cell 1634
Cell 1635
Cell 1636
Fluorescence (Baseline Corrected)
Time Point (0.25 Hz)
0
200
400
600
800
−1
1
2
3
4
5
10
15
20
25
−2
6
8

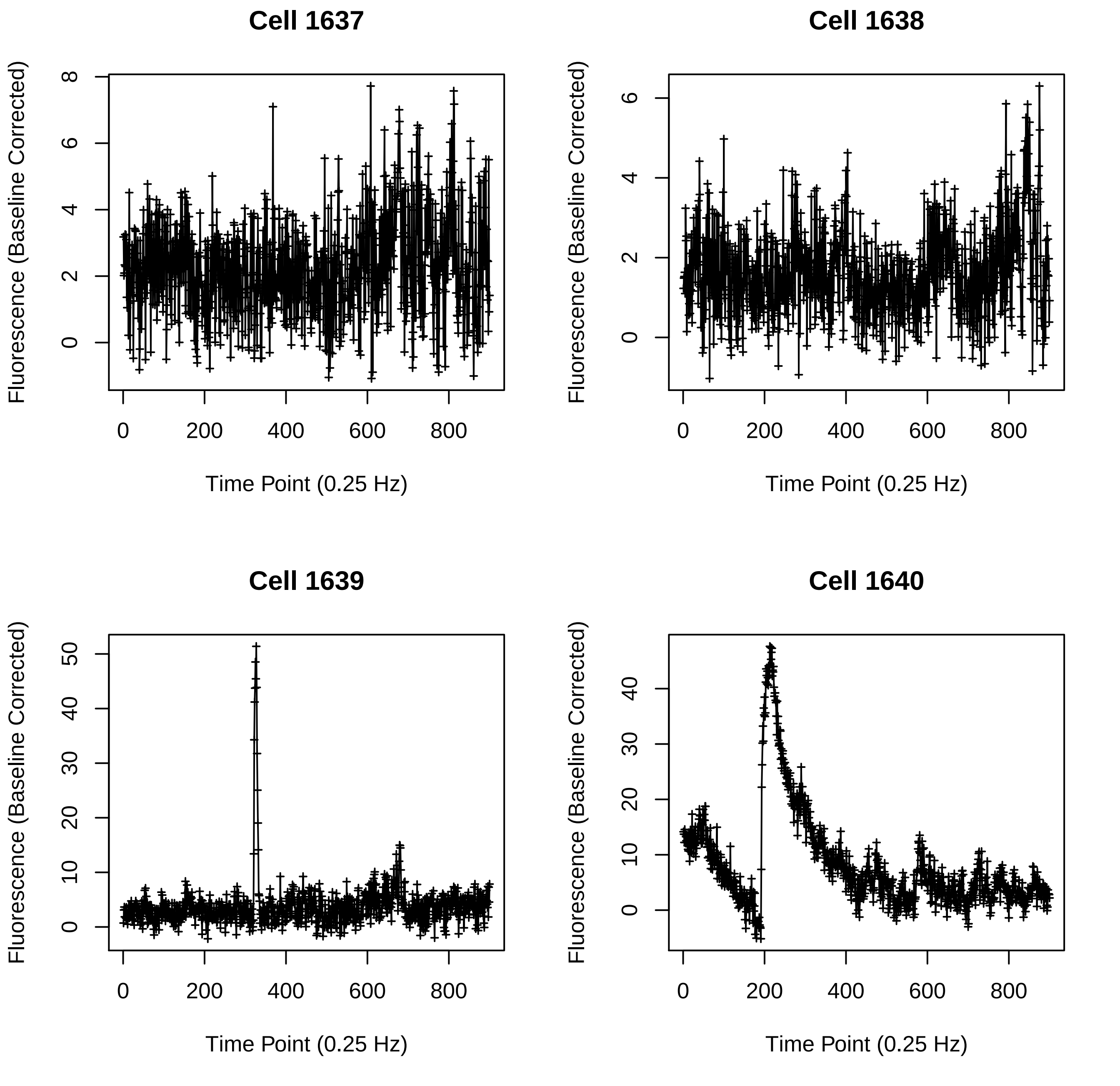

Cell 1637
Cell 1638
Cell 1639
Cell 1640
Fluorescence (Baseline Corrected)
Time Point (0.25 Hz)

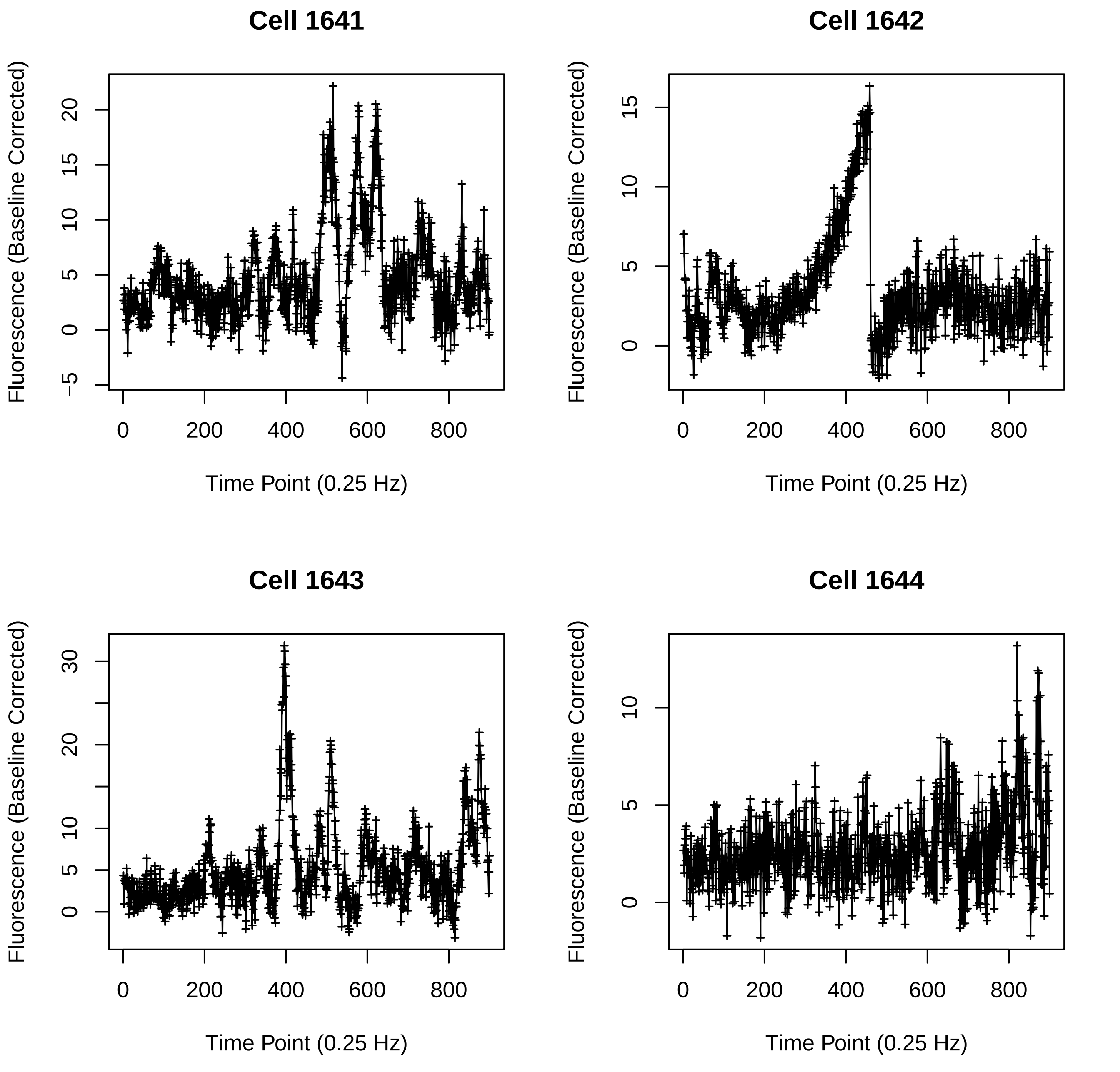

Cell 1641
Cell 1642
Cell 1643
Cell 1644
Fluorescence (Baseline Corrected)
Time Point (0.25 Hz)

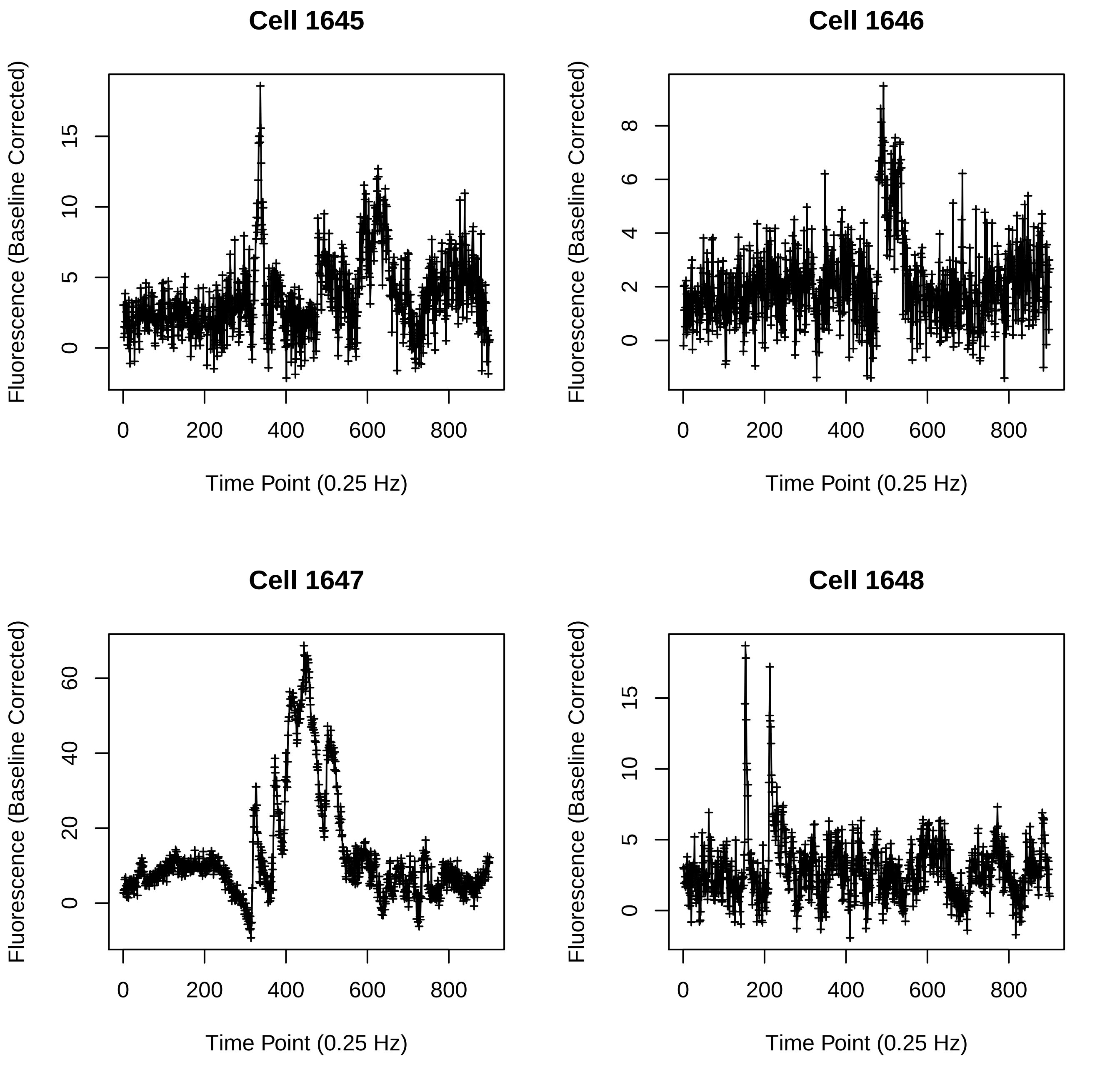
Cell 1645
Fluorescence (Baseline Corrected)
0
5
10
15
0
200
400
600
800
Time Point (0.25 Hz)
Cell 1646
Fluorescence (Baseline Corrected)
0
2
4
6
8
0
200
400
600
800
Time Point (0.25 Hz)
Cell 1647
Fluorescence (Baseline Corrected)
0
20
40
60
0
200
400
600
800
Time Point (0.25 Hz)
Cell 1648
Fluorescence (Baseline Corrected)
0
5
10
15
0
200
400
600
800
Time Point (0.25 Hz)

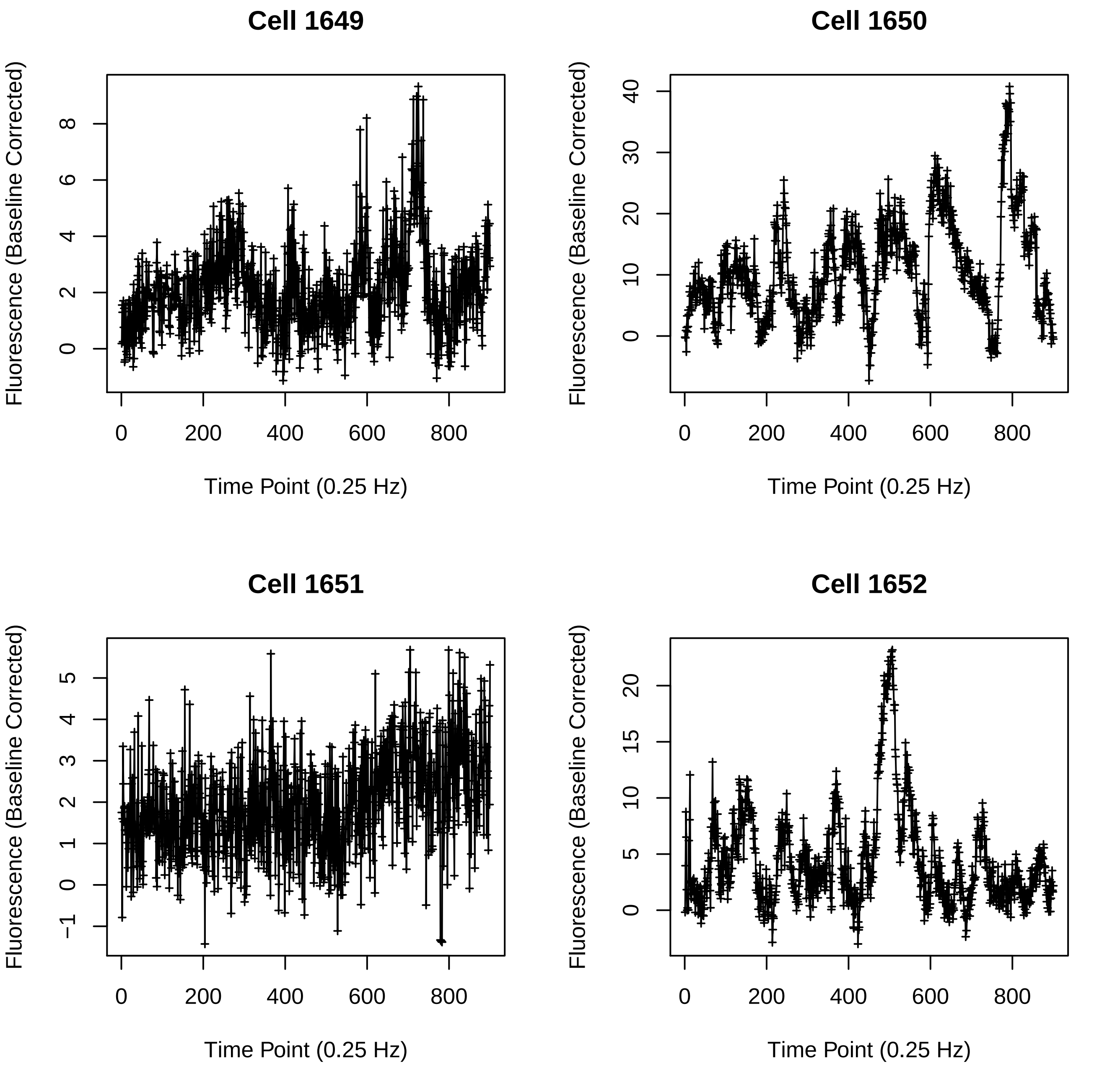
Cell 1649
Fluorescence (Baseline Corrected)
0
2
4
6
8
0
200
400
600
800
Time Point (0.25 Hz)
Cell 1650
Fluorescence (Baseline Corrected)
0
10
20
30
40
0
200
400
600
800
Time Point (0.25 Hz)
Cell 1651
Fluorescence (Baseline Corrected)
−1
0
1
2
3
4
5
0
200
400
600
800
Time Point (0.25 Hz)
Cell 1652
Fluorescence (Baseline Corrected)
0
5
10
15
20
0
200
400
600
800
Time Point (0.25 Hz)

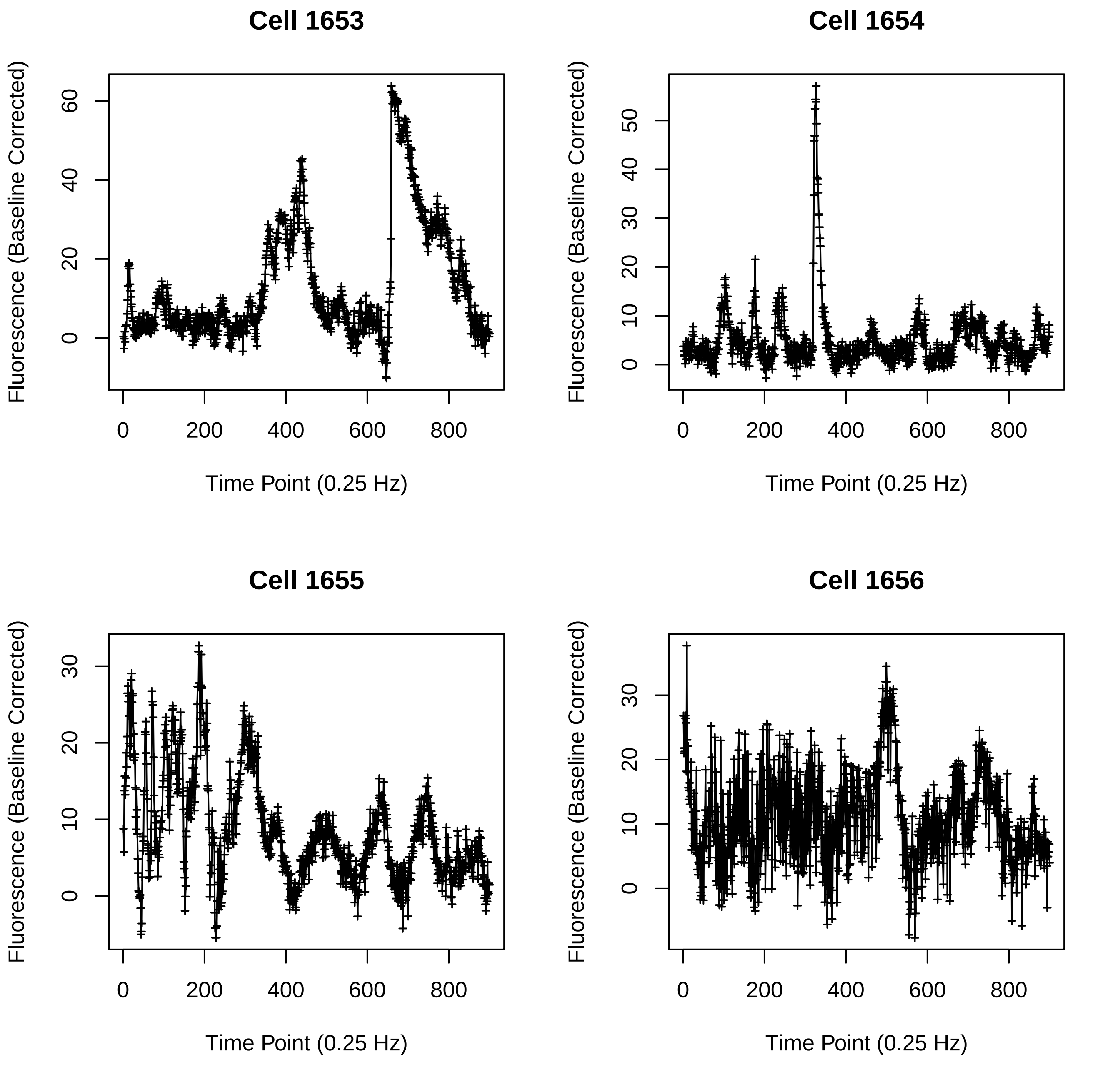

Cell 1653
Cell 1654
Cell 1655
Cell 1656
Fluorescence (Baseline Corrected)
Time Point (0.25 Hz)
0
200
400
600
800

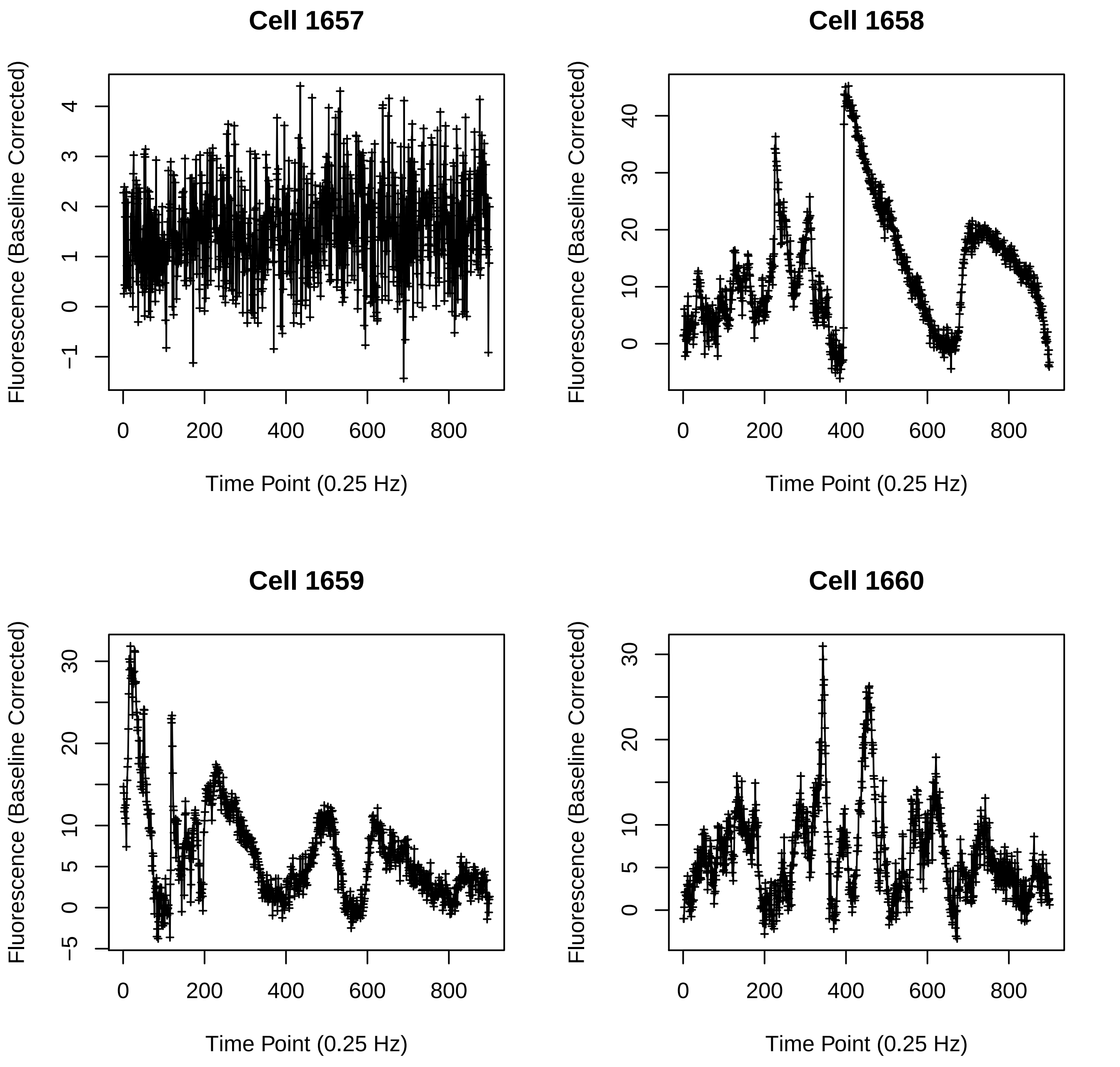
Cell 1657
Cell 1658
Cell 1659
Cell 1660
Fluorescence (Baseline Corrected)
Time Point (0.25 Hz)
0
200
400
600
800

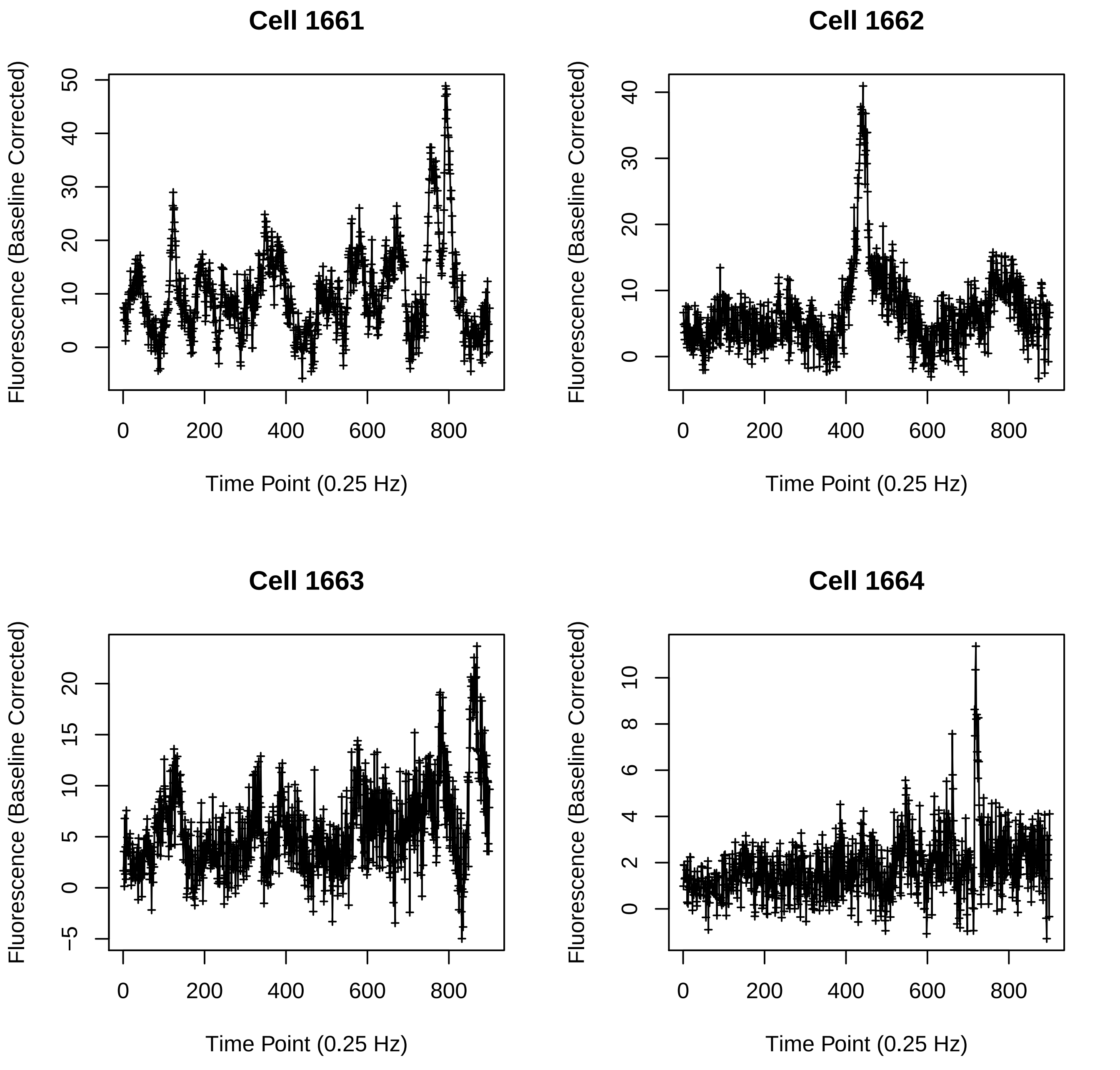

Cell 1661
Cell 1662
Cell 1663
Cell 1664
Fluorescence (Baseline Corrected)
Time Point (0.25 Hz)
0
200
400
600
800
0
10
20
30
40
50
−5
5
15
2
4
6
8

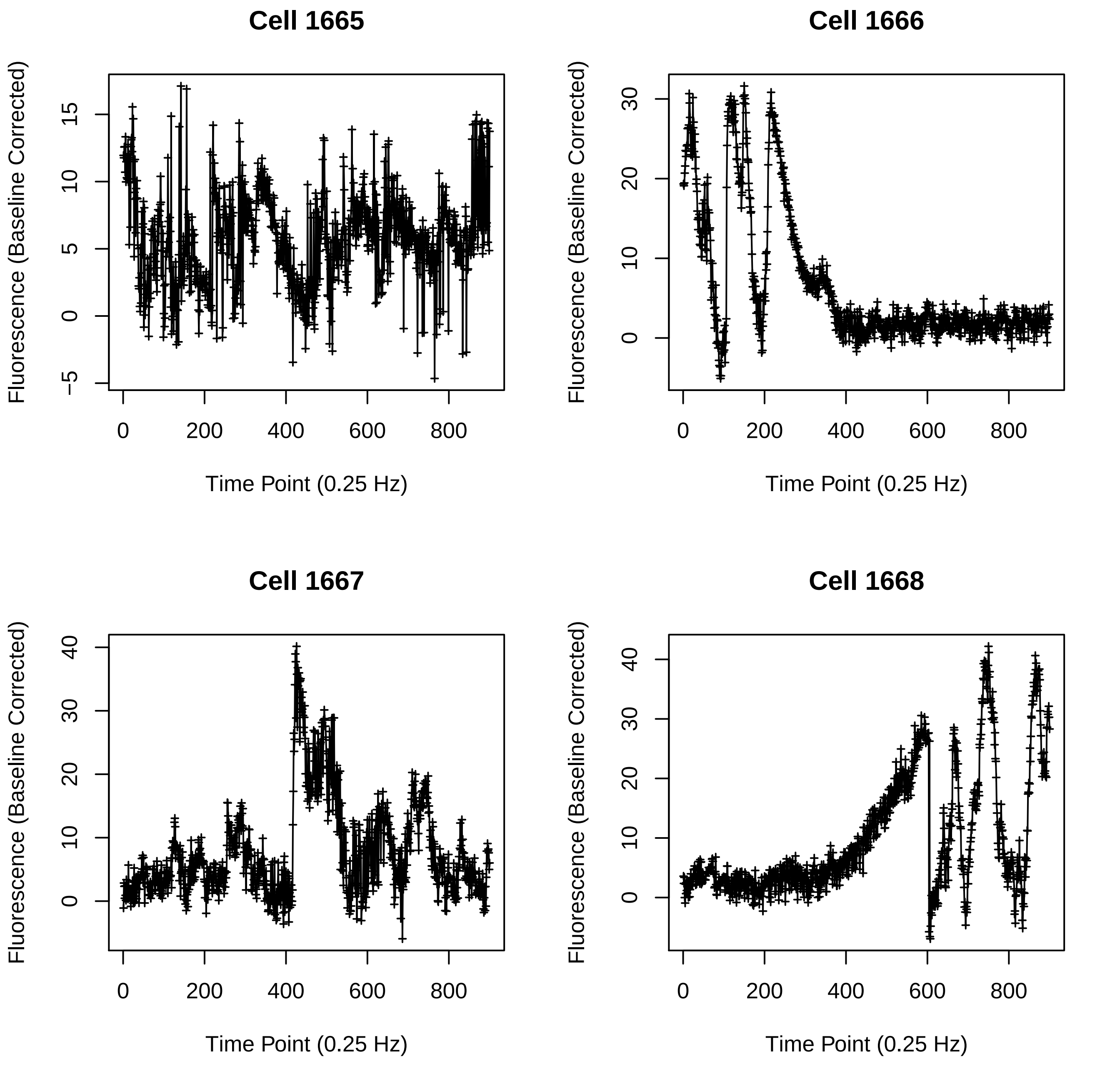
Cell 1665
Cell 1666
Cell 1667
Cell 1668
Fluorescence (Baseline Corrected)
Time Point (0.25 Hz)
−5
0
5
10
15
20
30
40
0
200
400
600
800

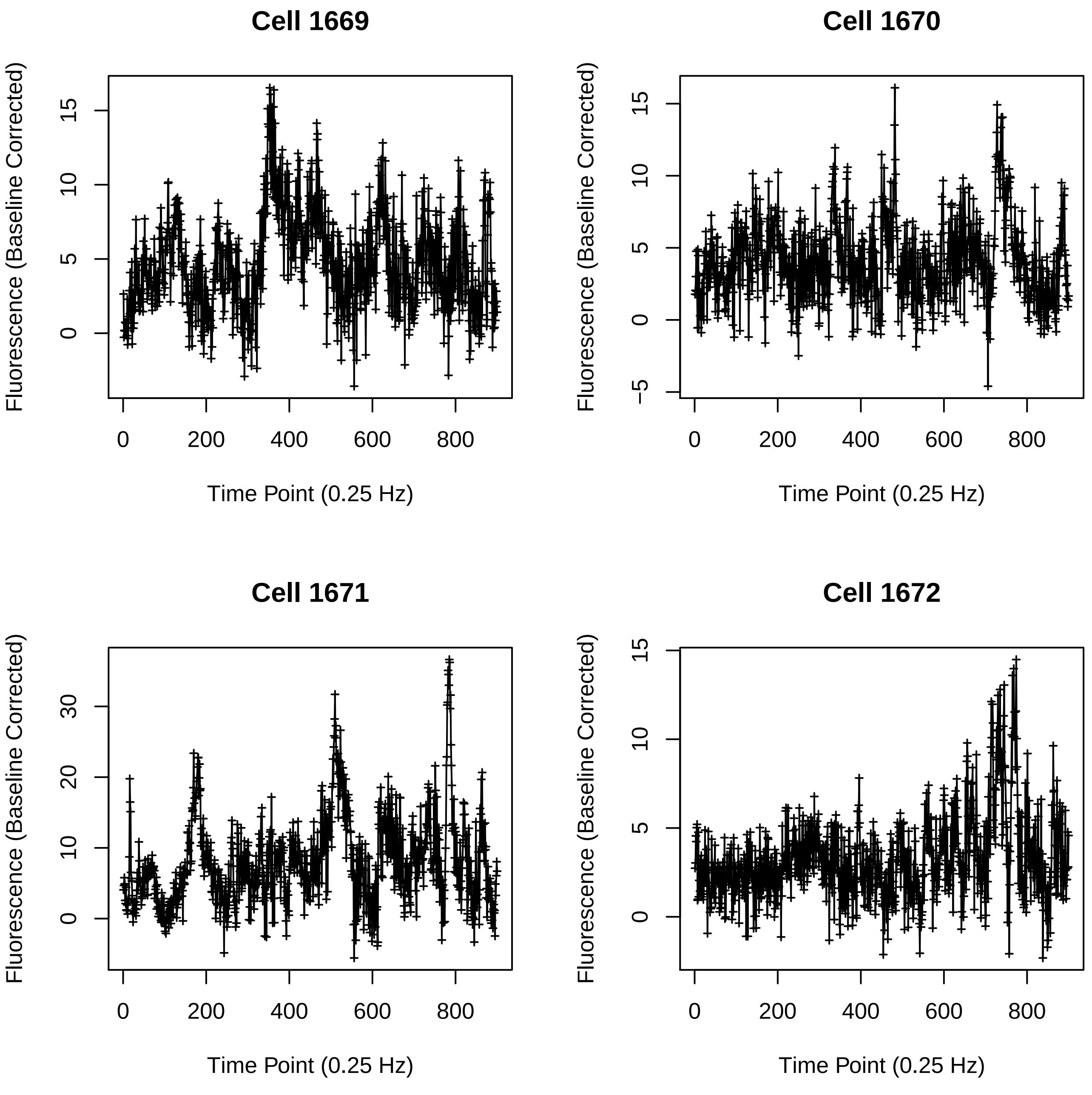
Cell 1669
Cell 1670
Cell 1671
Cell 1672
Fluorescence (Baseline Corrected)
Time Point (0.25 Hz)
0
200
400
600
800
−5
5
10
15
20
30

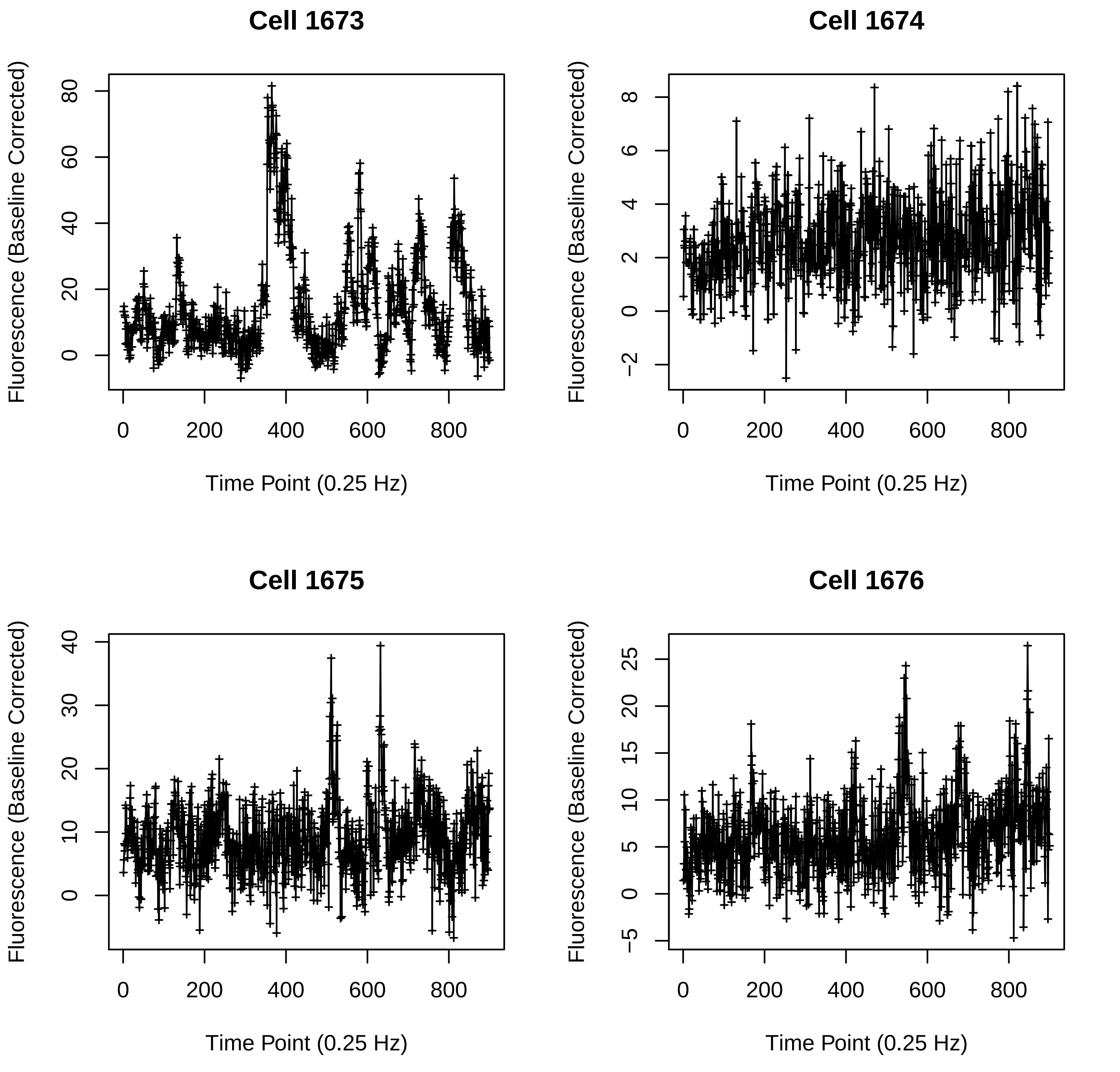

Cell 1673
Cell 1674
Cell 1675
Cell 1676
Fluorescence (Baseline Corrected)
Time Point (0.25 Hz)

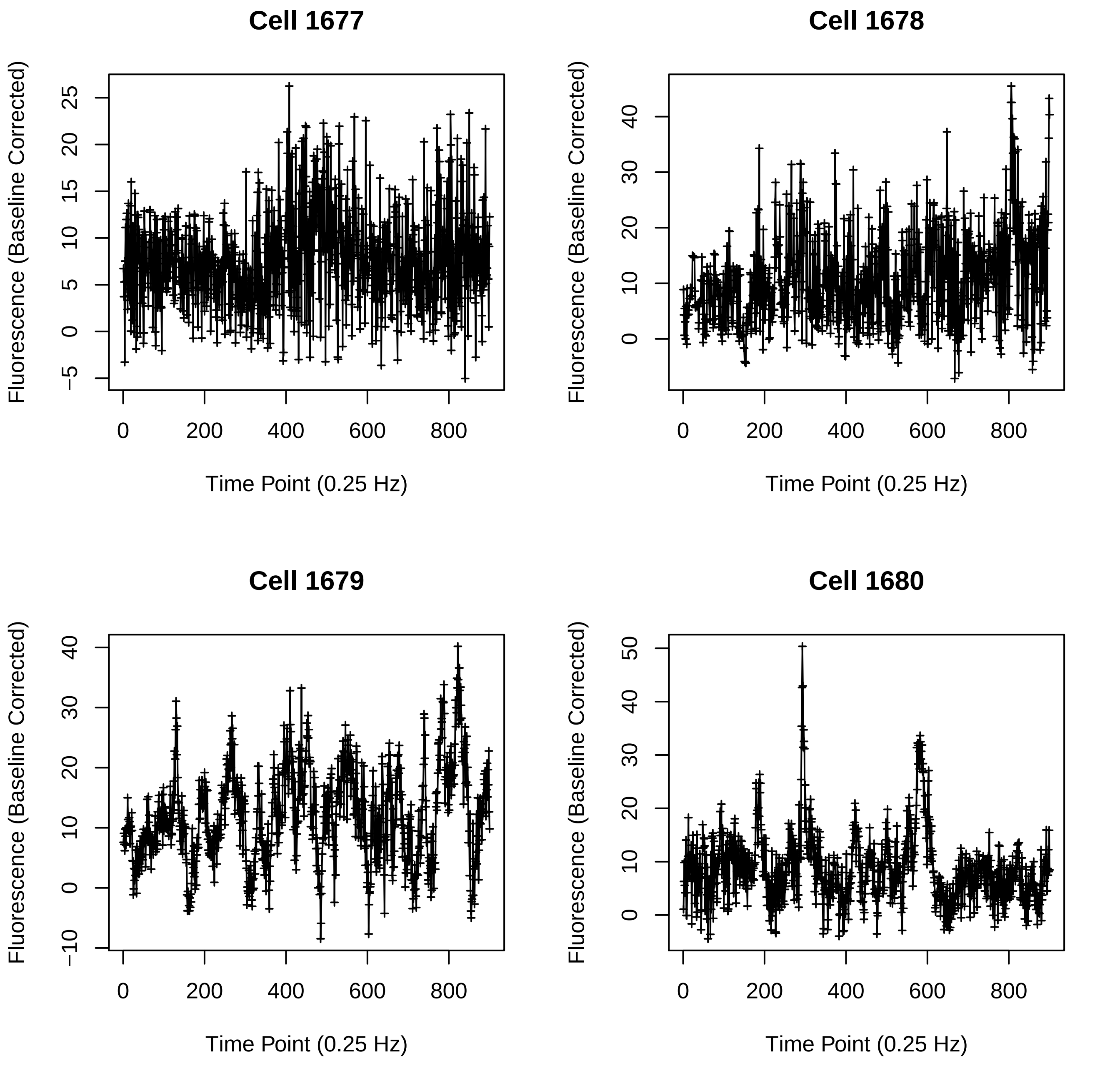
Cell 1677
Cell 1678
Cell 1679
Cell 1680
Fluorescence (Baseline Corrected)
Time Point (0.25 Hz)
−5
0
5
10
15
20
25
30
40
50
−10
0
200
400
600
800

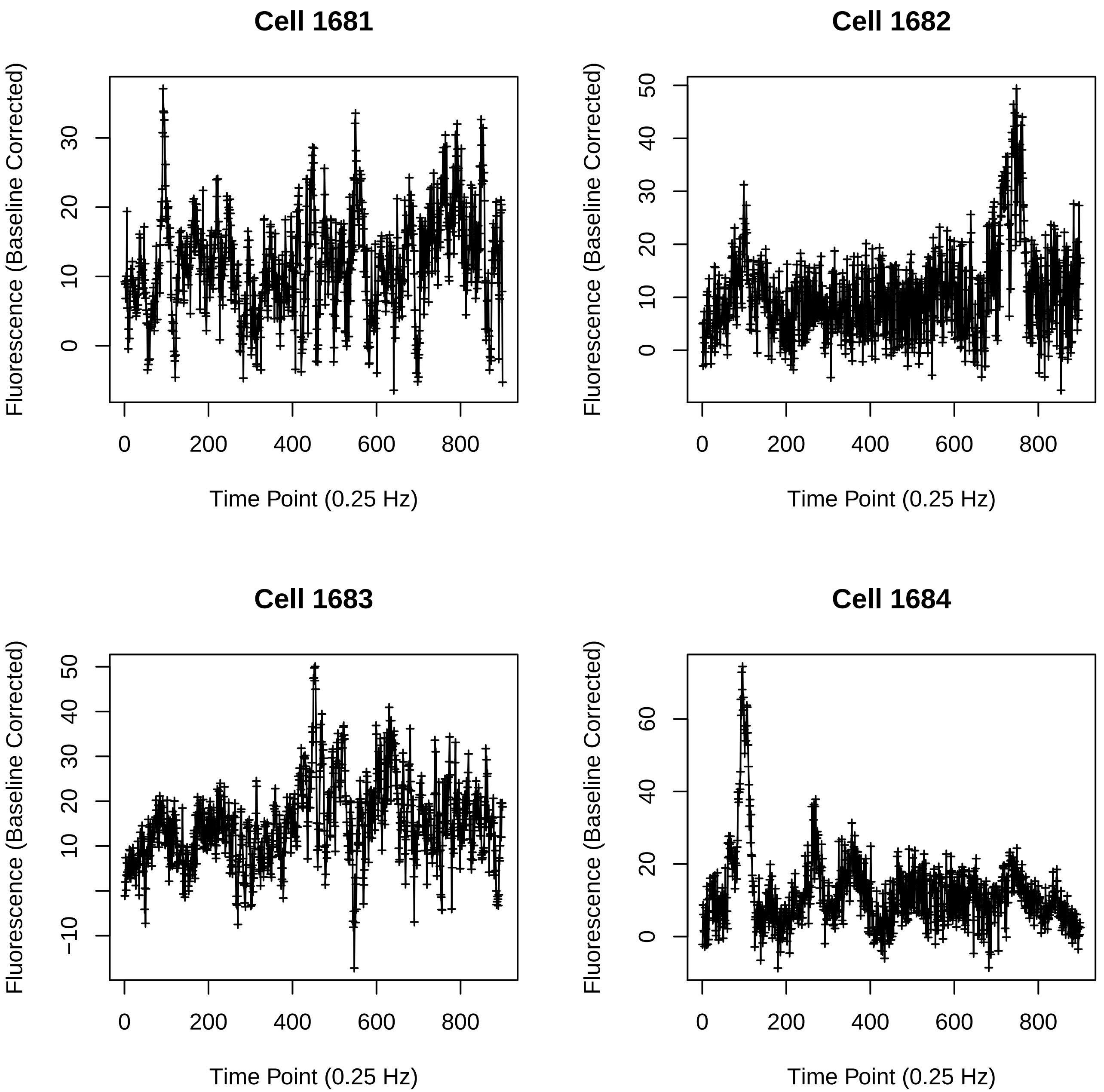
Cell 1681
Cell 1682
Cell 1683
Cell 1684
Fluorescence (Baseline Corrected)
Time Point (0.25 Hz)
0
200
400
600
800
-10
10
20
30
40
50
60

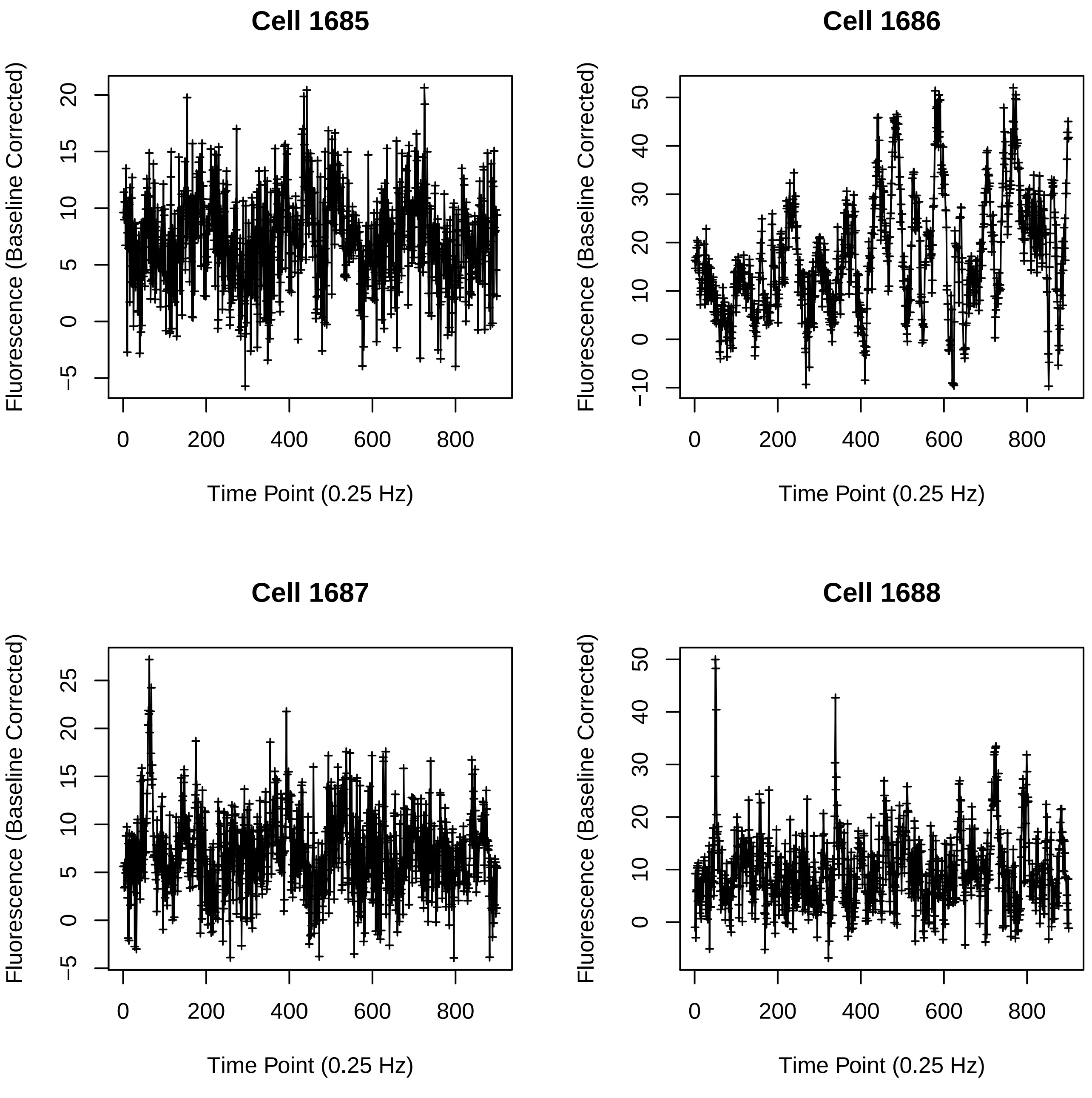
Cell 1685
Fluorescence (Baseline Corrected)
Time Point (0.25 Hz)
Cell 1686
Fluorescence (Baseline Corrected)
Time Point (0.25 Hz)
Cell 1687
Fluorescence (Baseline Corrected)
Time Point (0.25 Hz)
Cell 1688
Fluorescence (Baseline Corrected)
Time Point (0.25 Hz)

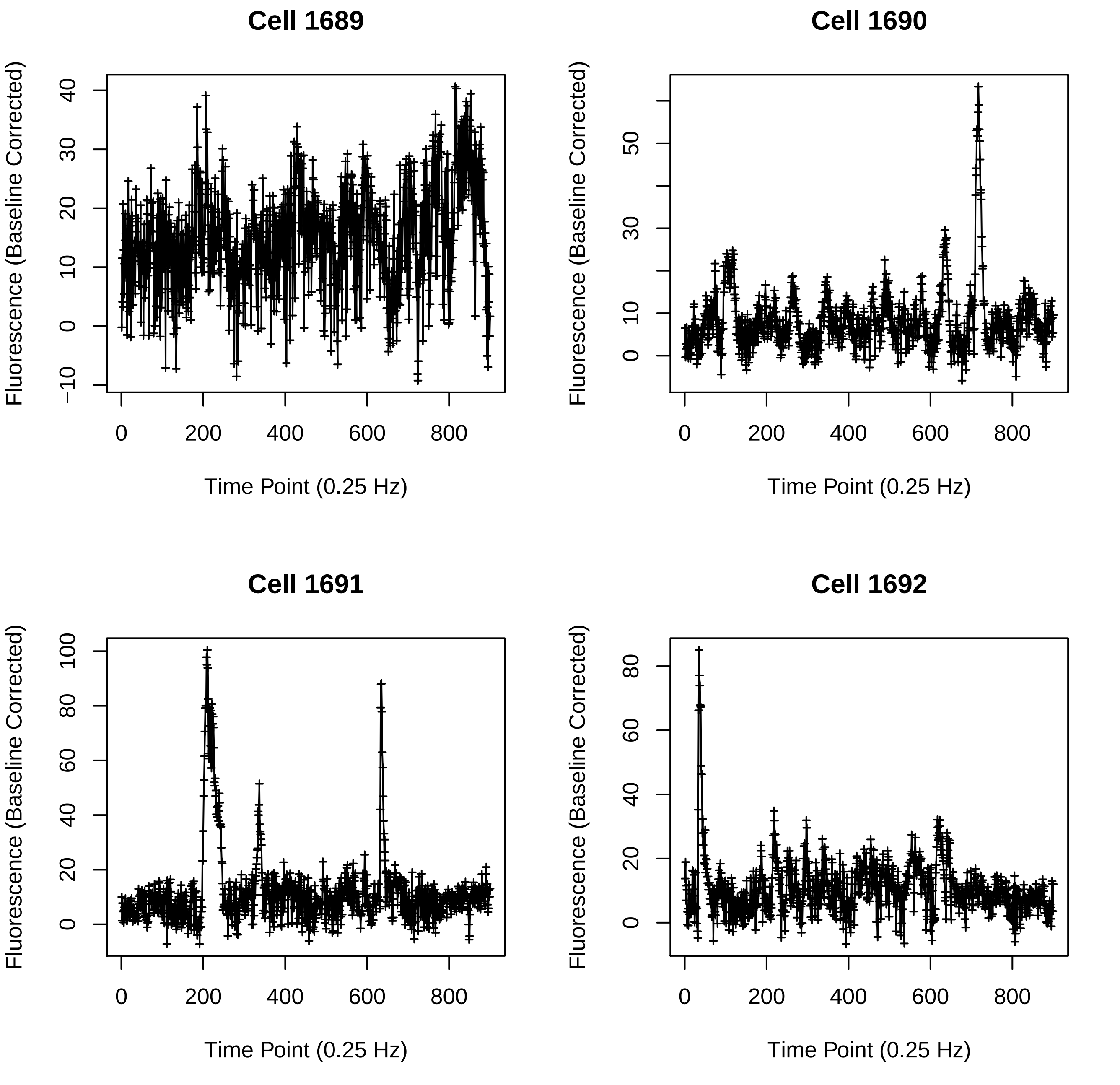

Cell 1689
Fluorescence (Baseline Corrected)
−10
0
10
20
30
40
0
200
400
600
800
Time Point (0.25 Hz)
Cell 1690
Fluorescence (Baseline Corrected)
0
10
30
50
0
200
400
600
800
Time Point (0.25 Hz)
Cell 1691
Fluorescence (Baseline Corrected)
0
20
40
60
80
100
0
200
400
600
800
Time Point (0.25 Hz)
Cell 1692
Fluorescence (Baseline Corrected)
0
20
40
60
80
0
200
400
600
800
Time Point (0.25 Hz)

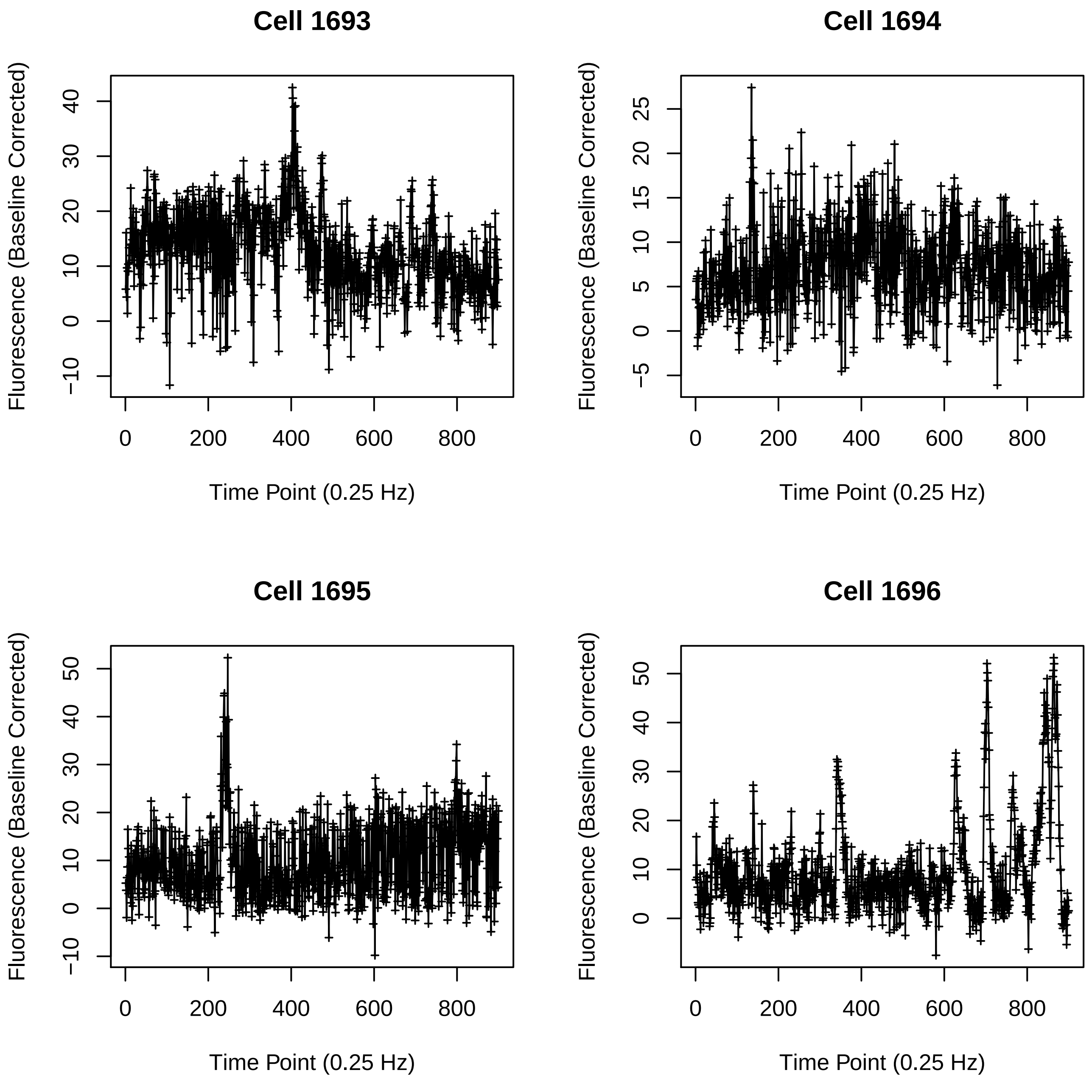
Cell 1693
Cell 1694
Cell 1695
Cell 1696
Fluorescence (Baseline Corrected)
Time Point (0.25 Hz)
0
200
400
600
800
−10
0
10
20
30
40
50
−5
5
15
25

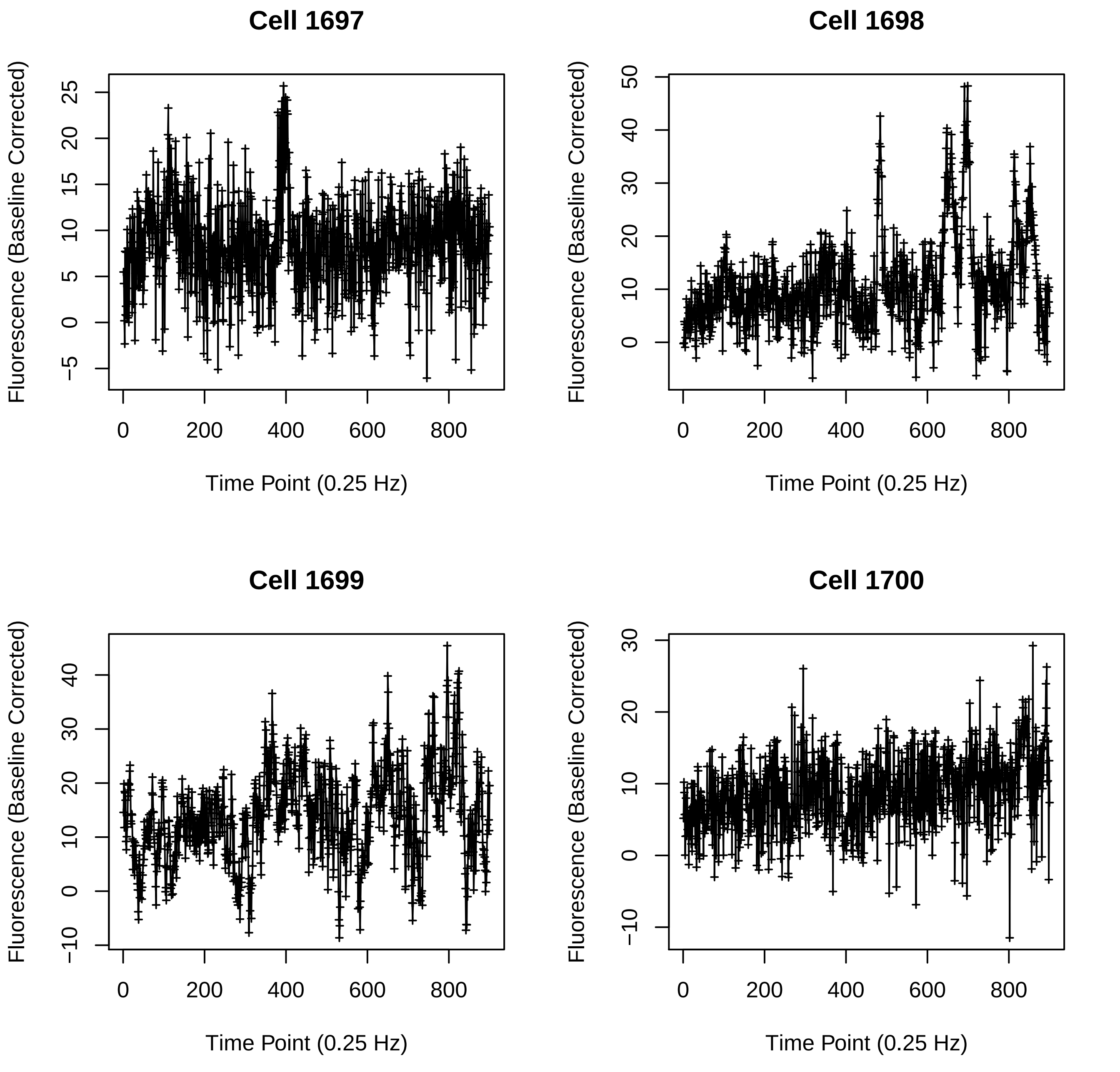
Cell 1697
Cell 1698
Cell 1699
Cell 1700
Fluorescence (Baseline Corrected)
Time Point (0.25 Hz)
−5
0
5
10
15
20
25
30
40
50
−10
0
200
400
600
800

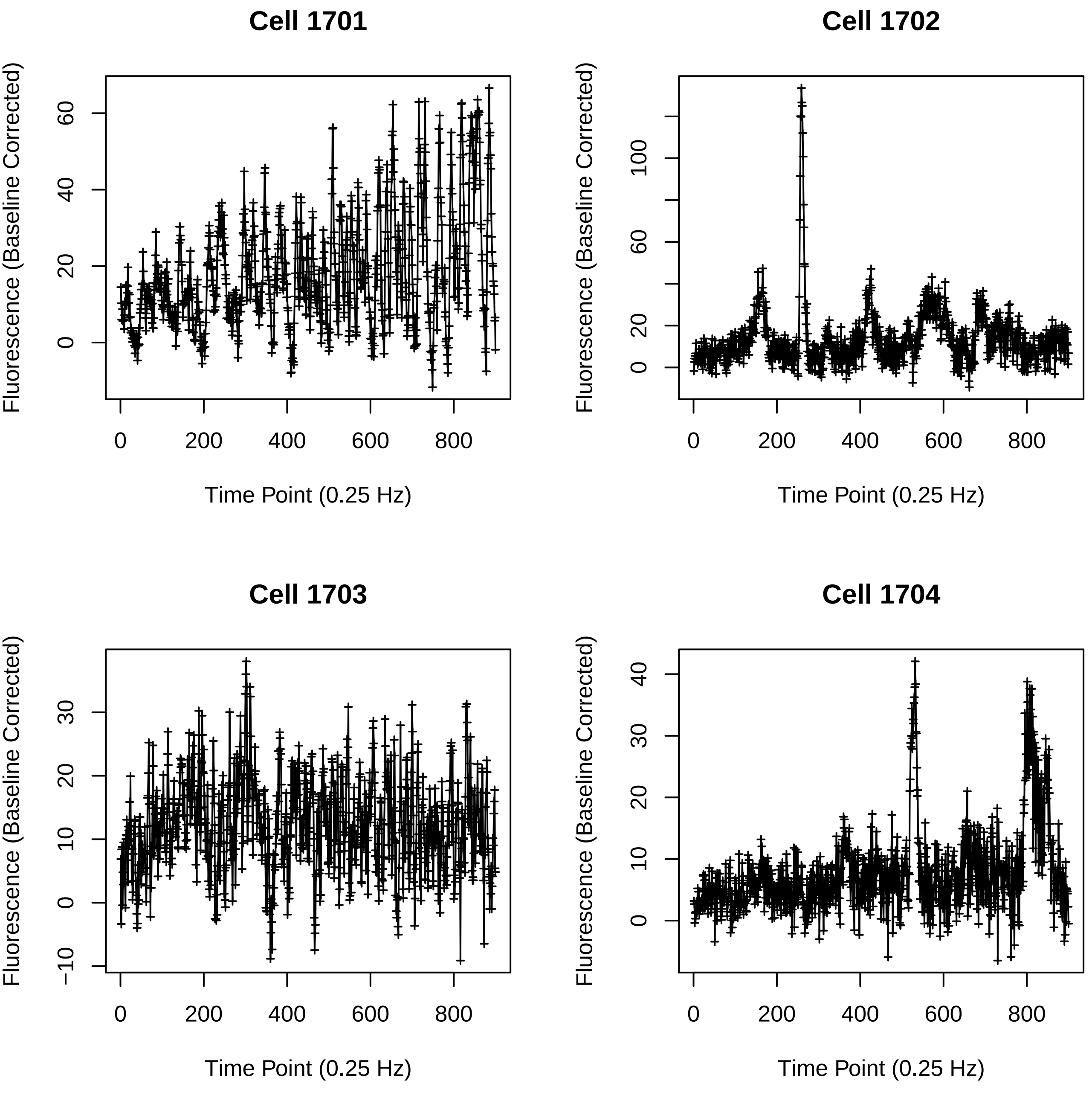

Cell 1701
Cell 1702
Cell 1703
Cell 1704
Fluorescence (Baseline Corrected)
Time Point (0.25 Hz)
0
200
400
600
800
-10
10
20
30
40
60
100

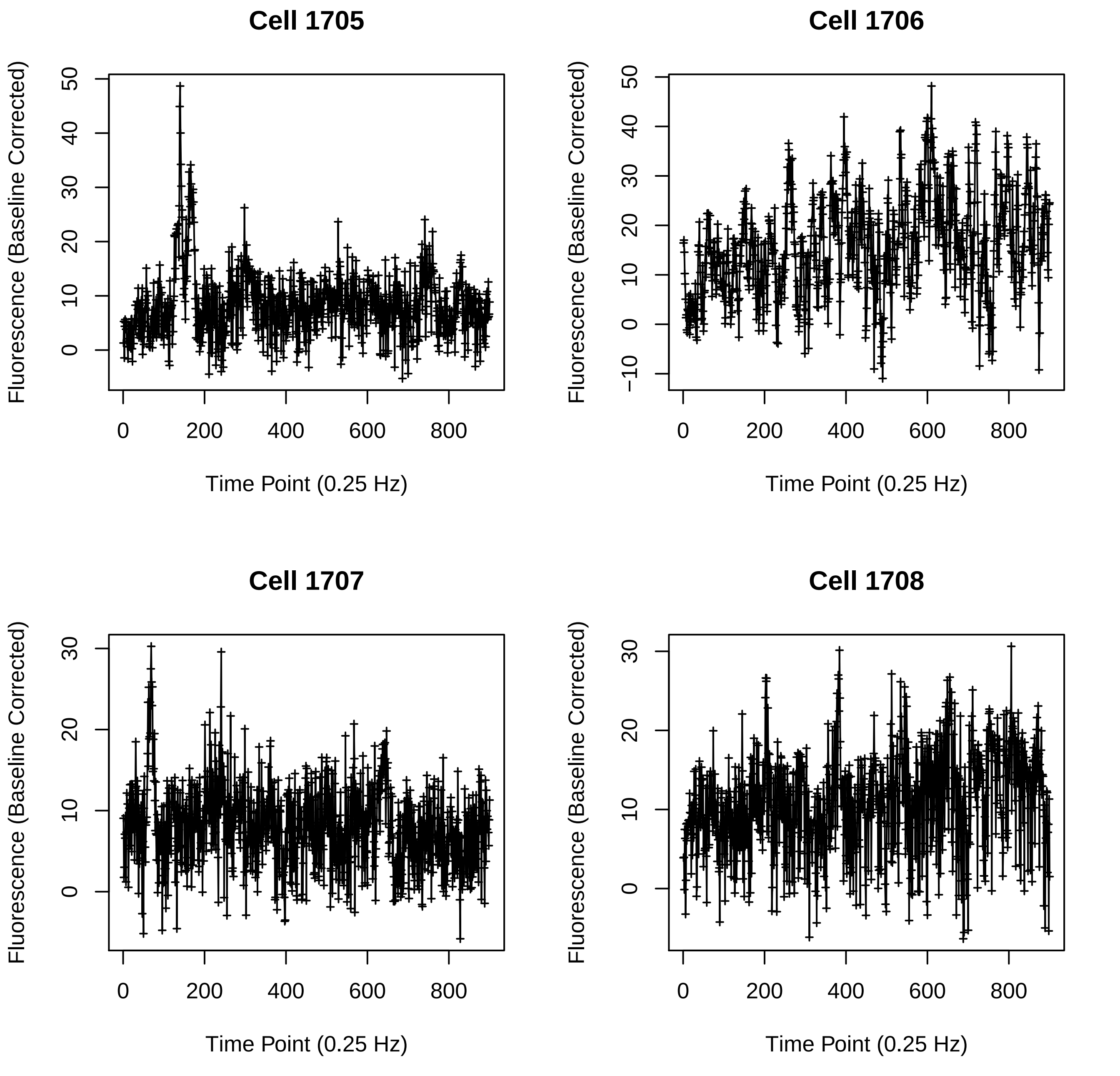
Cell 1705
Cell 1706
Cell 1707
Cell 1708
Fluorescence (Baseline Corrected)
Time Point (0.25 Hz)
0
200
400
600
800
−10
0
10
20
30
40
50

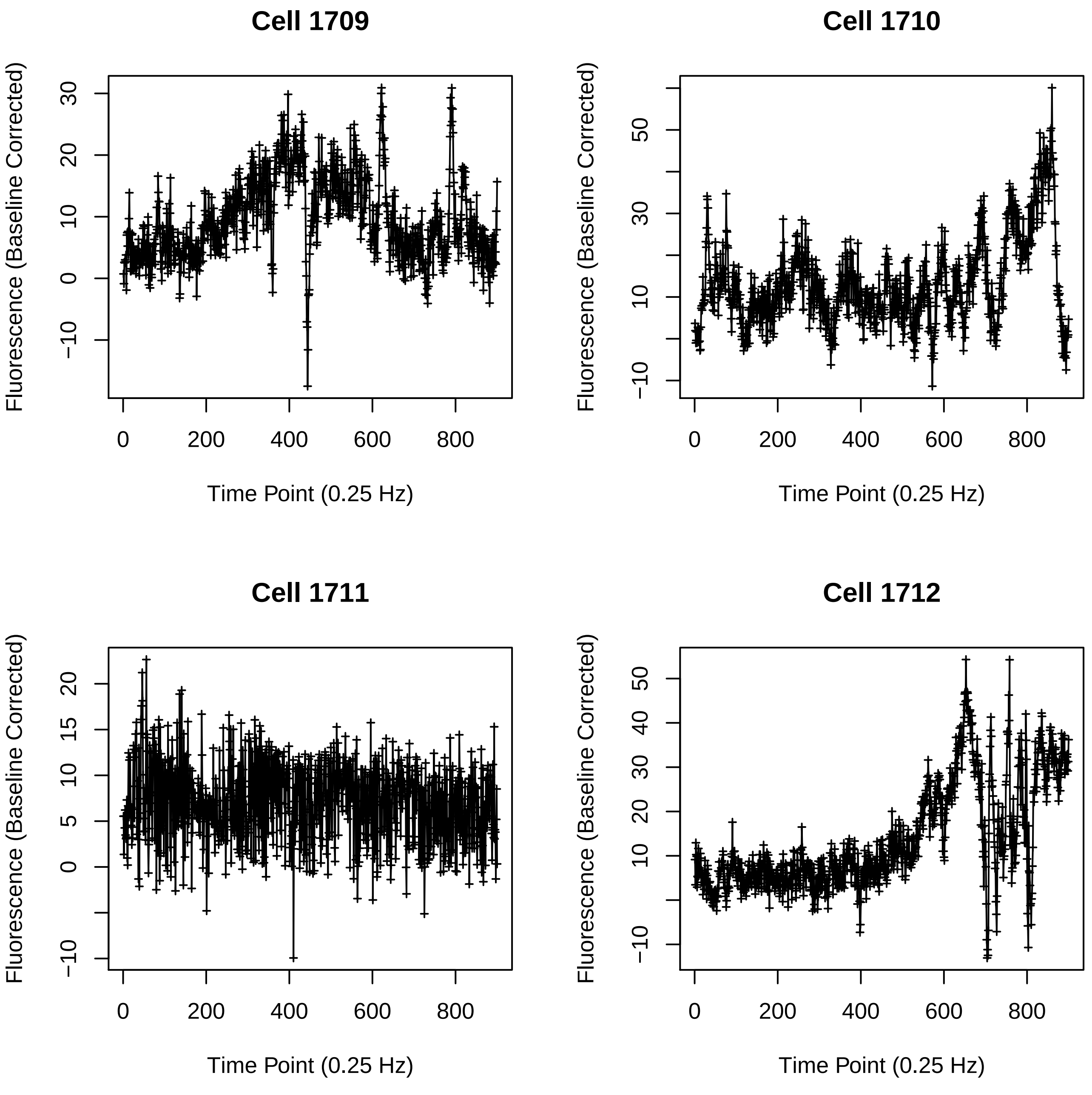
Cell 1709
Cell 1710
Cell 1711
Cell 1712
Fluorescence (Baseline Corrected)
Time Point (0.25 Hz)
0
200
400
600
800
−10
0
10
20
30
50
5
15

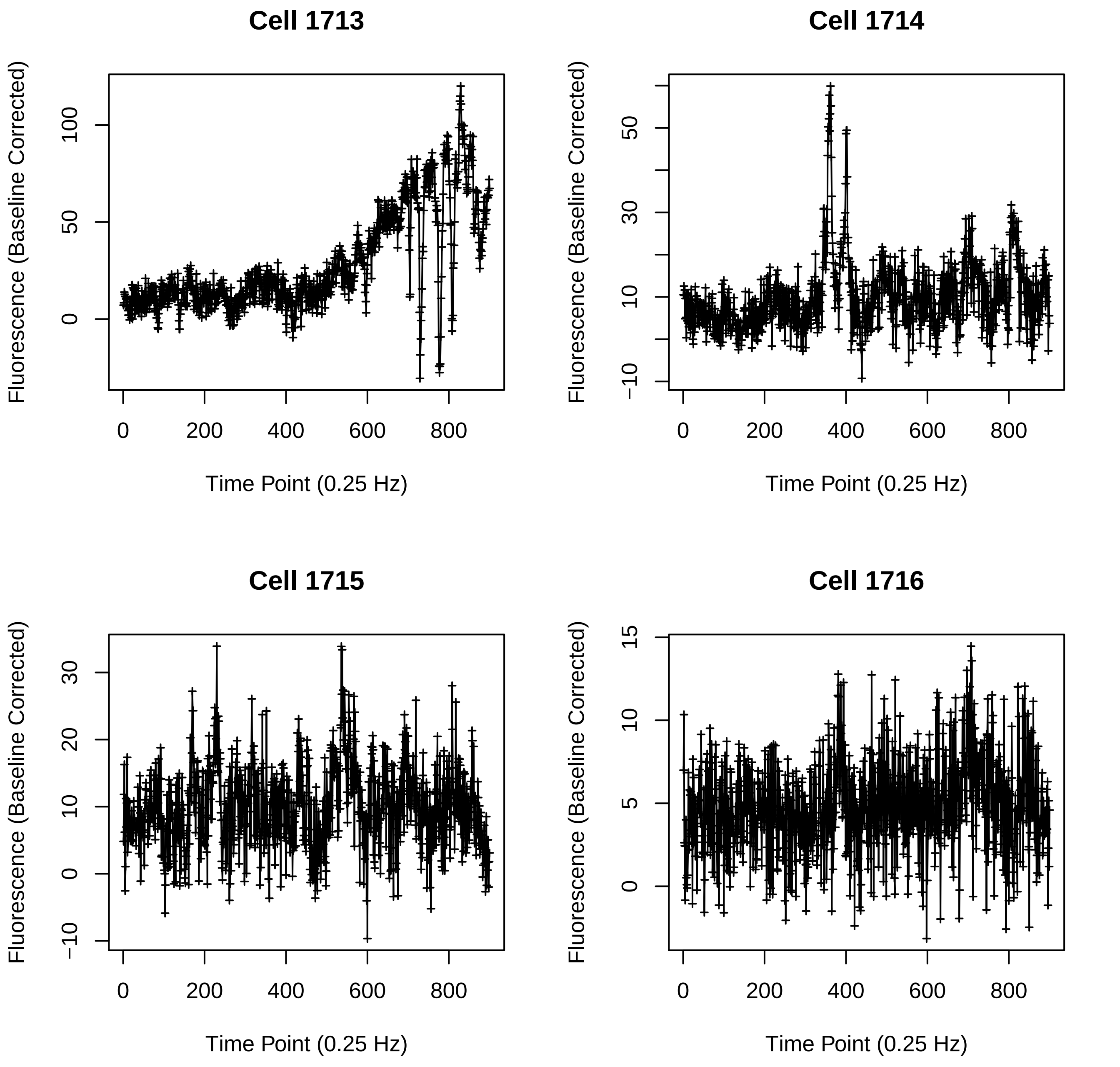
Cell 1713
Cell 1714
Cell 1715
Cell 1716
Fluorescence (Baseline Corrected)
Time Point (0.25 Hz)
0
200
400
600
800
100
50
0
50
30
10
−10
30
20
10
0
−10
15
10
5
0

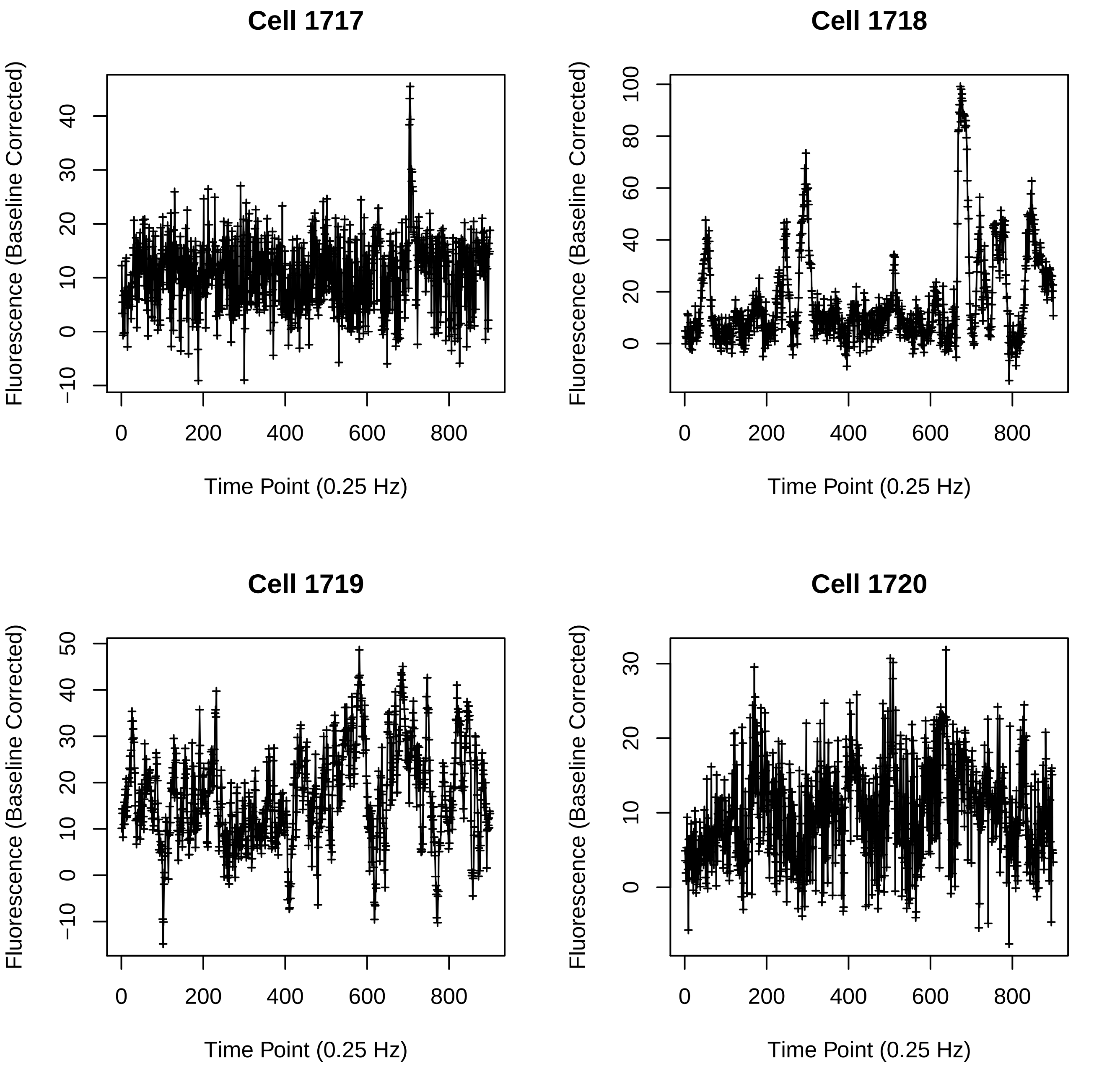
Cell 1717
Fluorescence (Baseline Corrected)
−10
0
10
20
30
40
0
200
400
600
800
Time Point (0.25 Hz)
Cell 1718
Fluorescence (Baseline Corrected)
0
20
40
60
80
100
0
200
400
600
800
Time Point (0.25 Hz)
Cell 1719
Fluorescence (Baseline Corrected)
−10
0
10
20
30
40
50
0
200
400
600
800
Time Point (0.25 Hz)
Cell 1720
Fluorescence (Baseline Corrected)
0
10
20
30
0
200
400
600
800
Time Point (0.25 Hz)

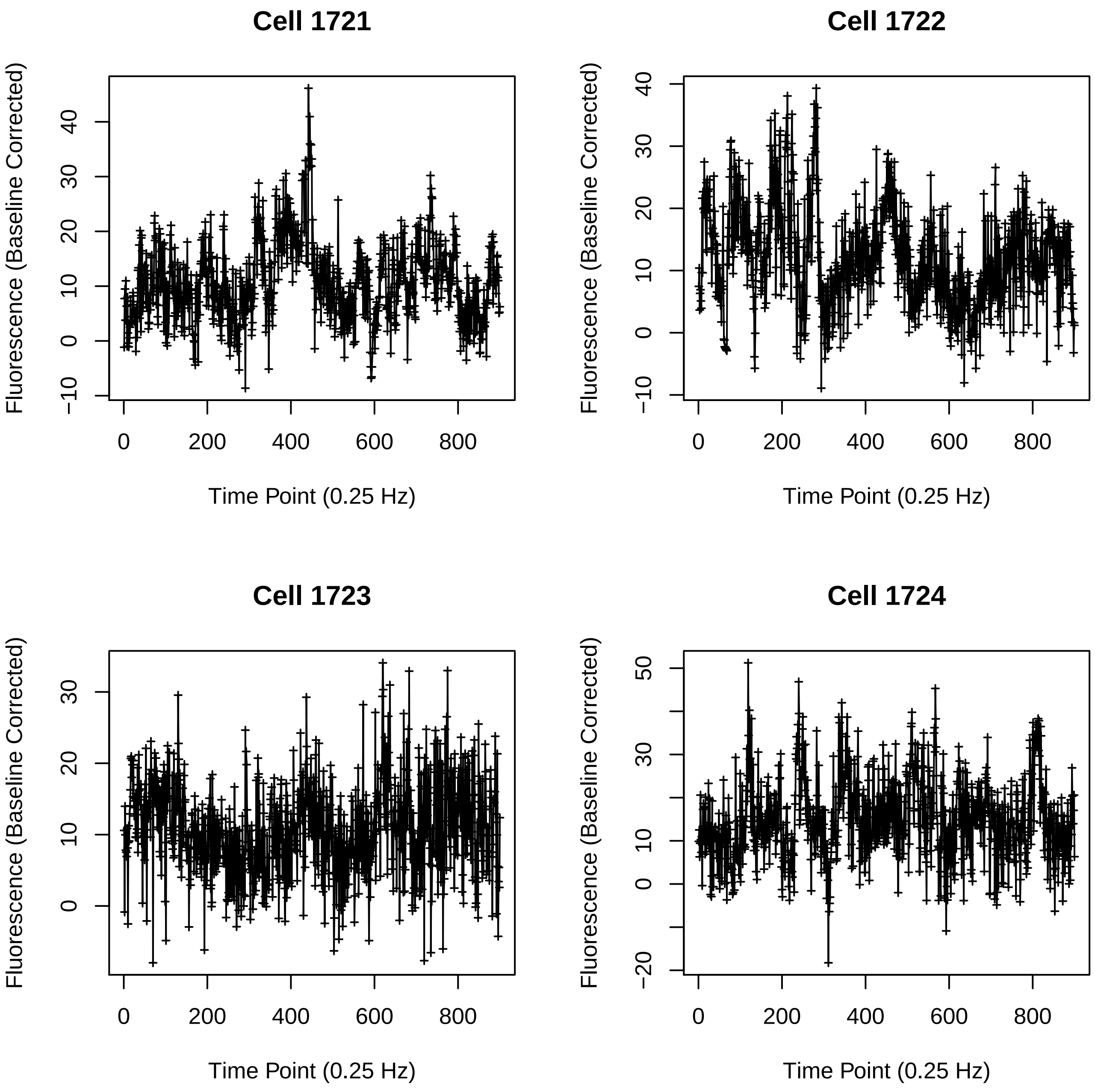
Cell 1721
Cell 1722
Cell 1723
Cell 1724
Fluorescence (Baseline Corrected)
Time Point (0.25 Hz)
0
200
400
600
800
−10
0
10
20
30
40
−20
50

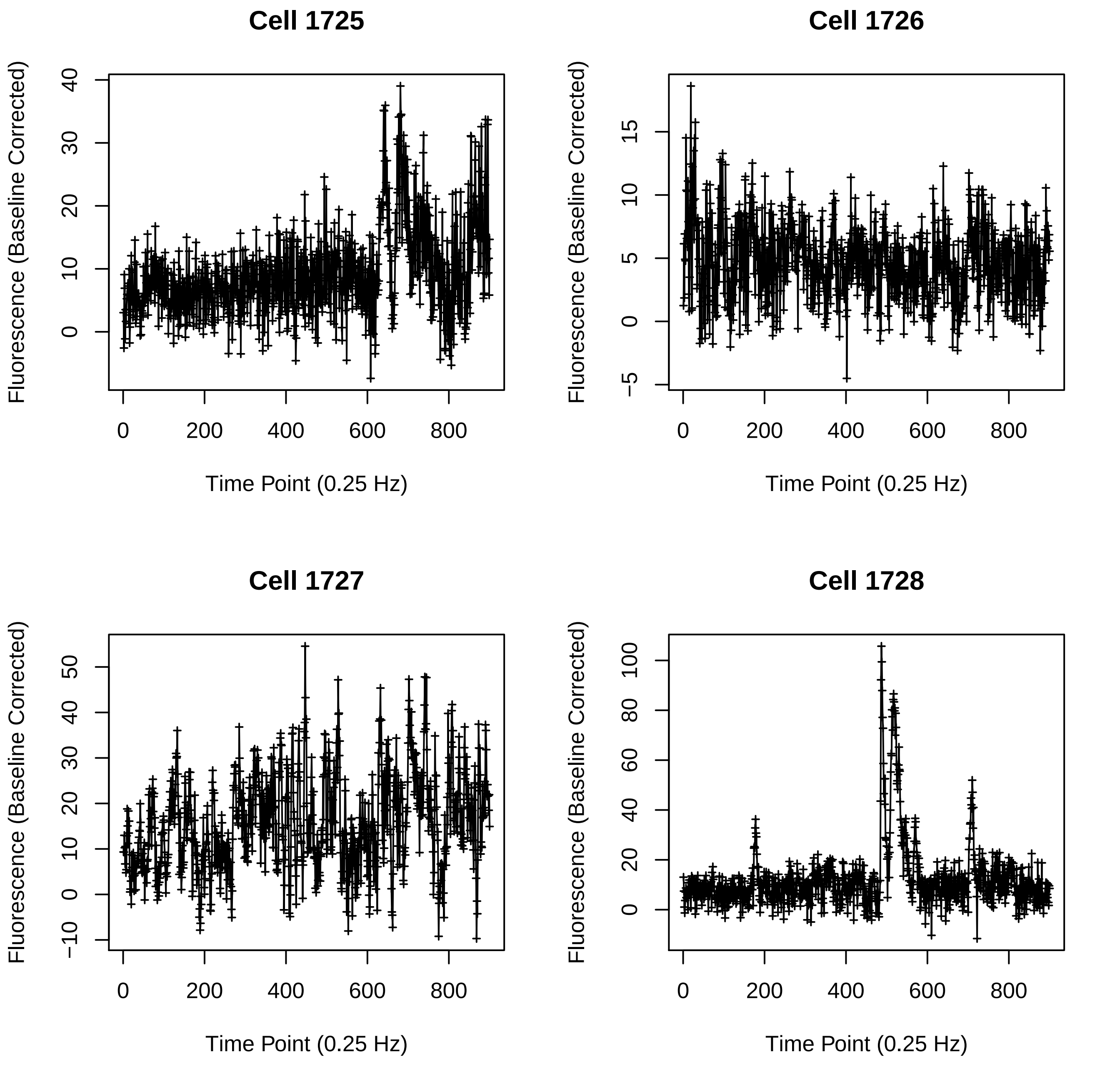

Cell 1725
Fluorescence (Baseline Corrected)
Time Point (0.25 Hz)
0
10
20
30
40
0
200
400
600
800
Cell 1726
Fluorescence (Baseline Corrected)
Time Point (0.25 Hz)
−5
0
5
10
15
0
200
400
600
800
Cell 1727
Fluorescence (Baseline Corrected)
Time Point (0.25 Hz)
−10
0
10
20
30
40
50
0
200
400
600
800
Cell 1728
Fluorescence (Baseline Corrected)
Time Point (0.25 Hz)
0
20
40
60
80
100
0
200
400
600
800

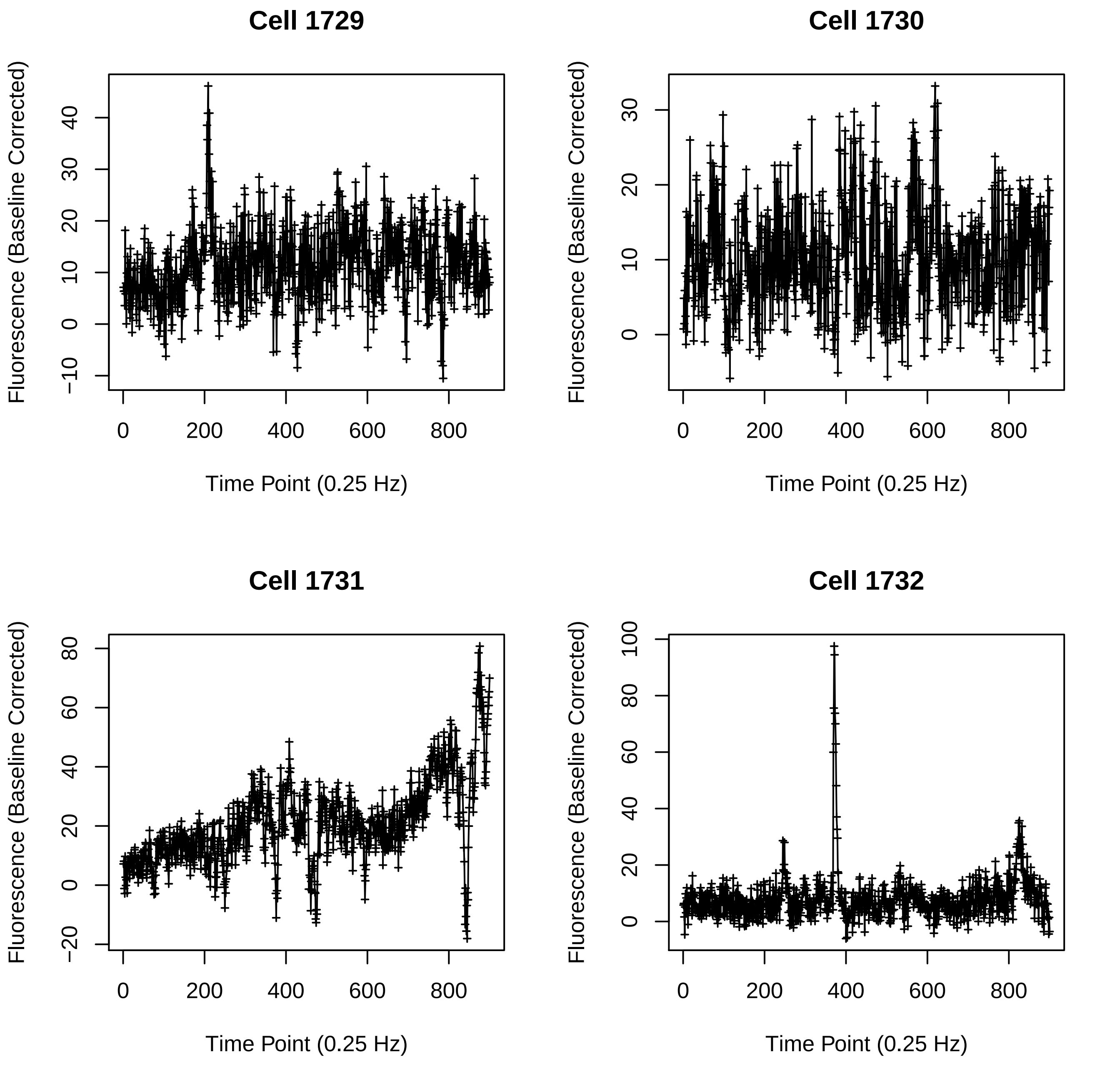

Cell 1729
Cell 1730
Cell 1731
Cell 1732
Fluorescence (Baseline Corrected)
Time Point (0.25 Hz)
−10
0
10
20
30
40
80
60
100
−20
0
200
400
600
800

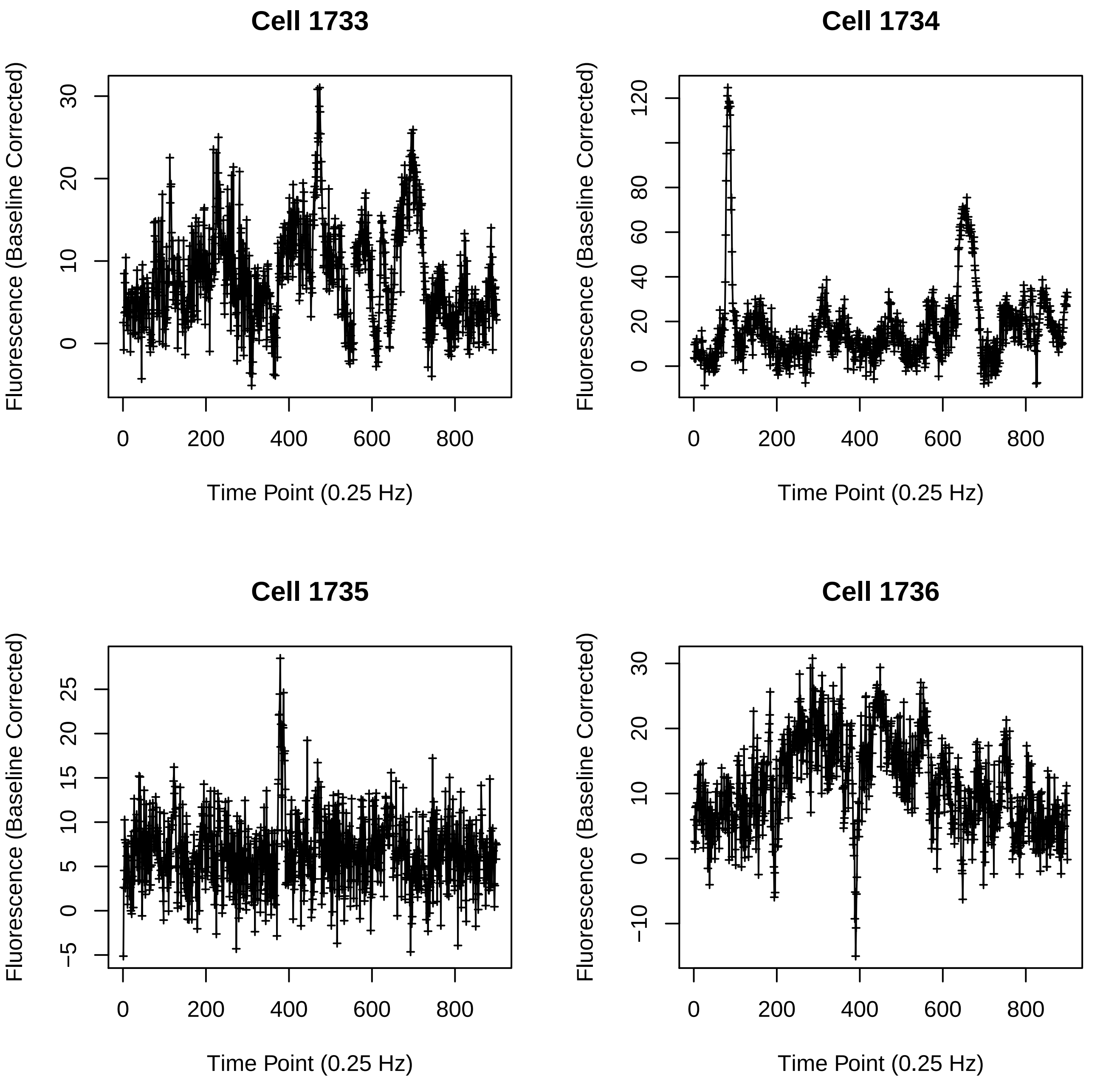
Cell 1733
Fluorescence (Baseline Corrected)
Time Point (0.25 Hz)
0
10
20
30
0
200
400
600
800
Cell 1734
Fluorescence (Baseline Corrected)
Time Point (0.25 Hz)
0
20
40
60
80
120
0
200
400
600
800
Cell 1735
Fluorescence (Baseline Corrected)
Time Point (0.25 Hz)
−5
0
5
10
15
20
25
0
200
400
600
800
Cell 1736
Fluorescence (Baseline Corrected)
Time Point (0.25 Hz)
−10
0
10
20
30
0
200
400
600
800

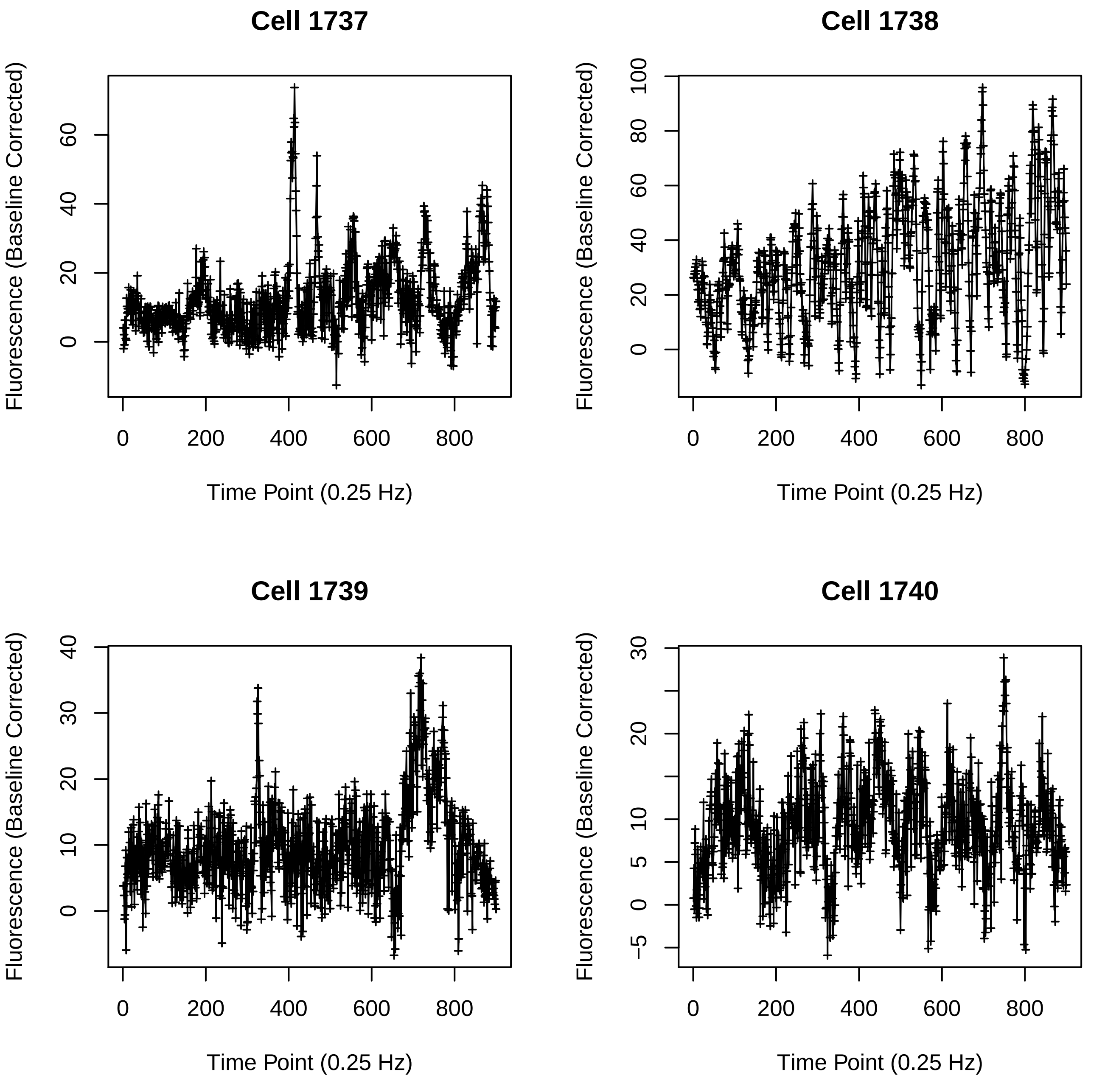
Cell 1737
Cell 1738
Cell 1739
Cell 1740
Fluorescence (Baseline Corrected)
Time Point (0.25 Hz)
0
200
400
600
800
-10 0 20 40 60
0 20 40 60 80 100
0 10 20 30 40
−5 0 5 10 20 30

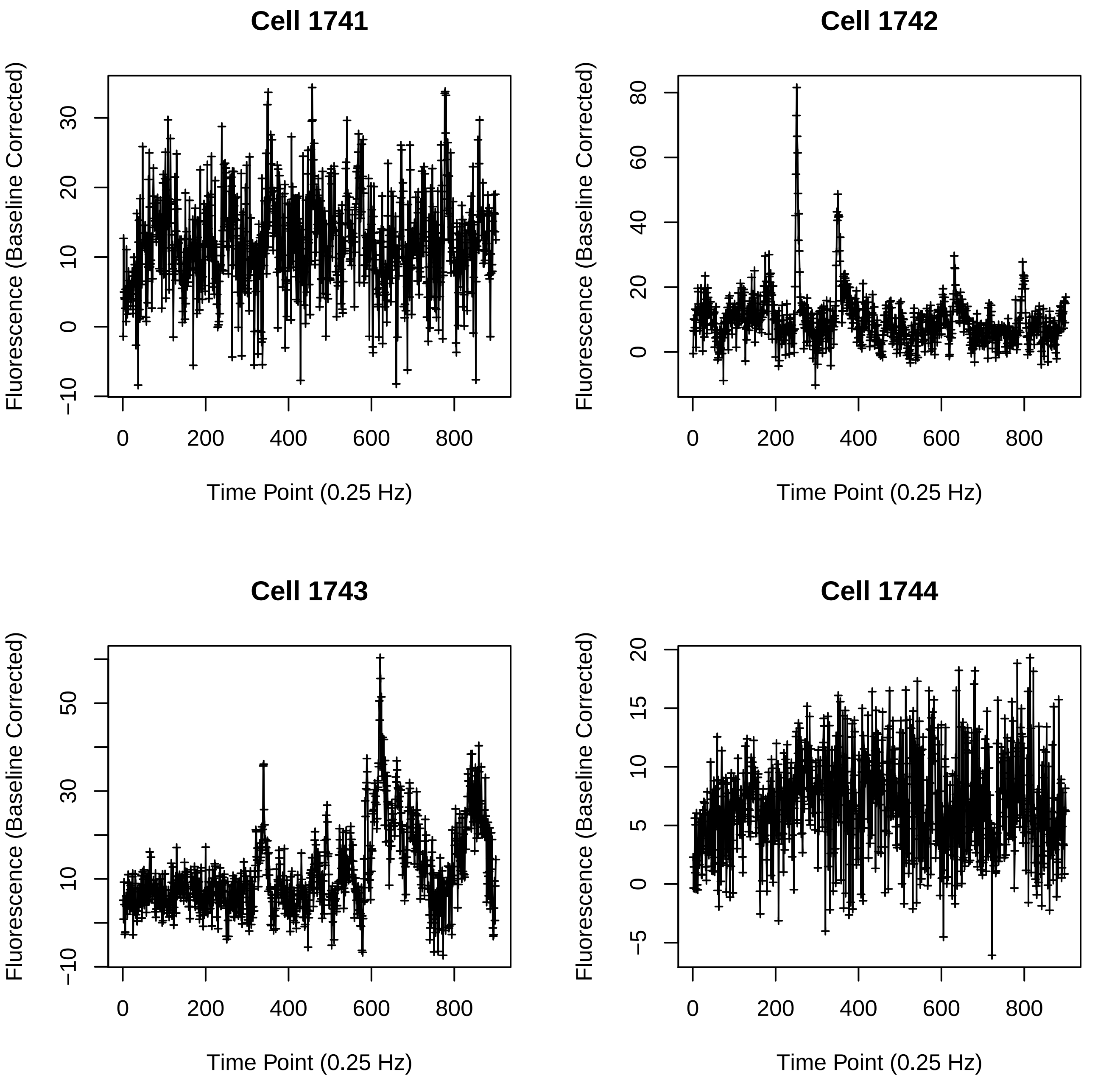
Cell 1741
Cell 1742
Cell 1743
Cell 1744
Fluorescence (Baseline Corrected)
Time Point (0.25 Hz)
−10
0
10
20
30
40
50
60
80
−5
5
15
200
400
600
800

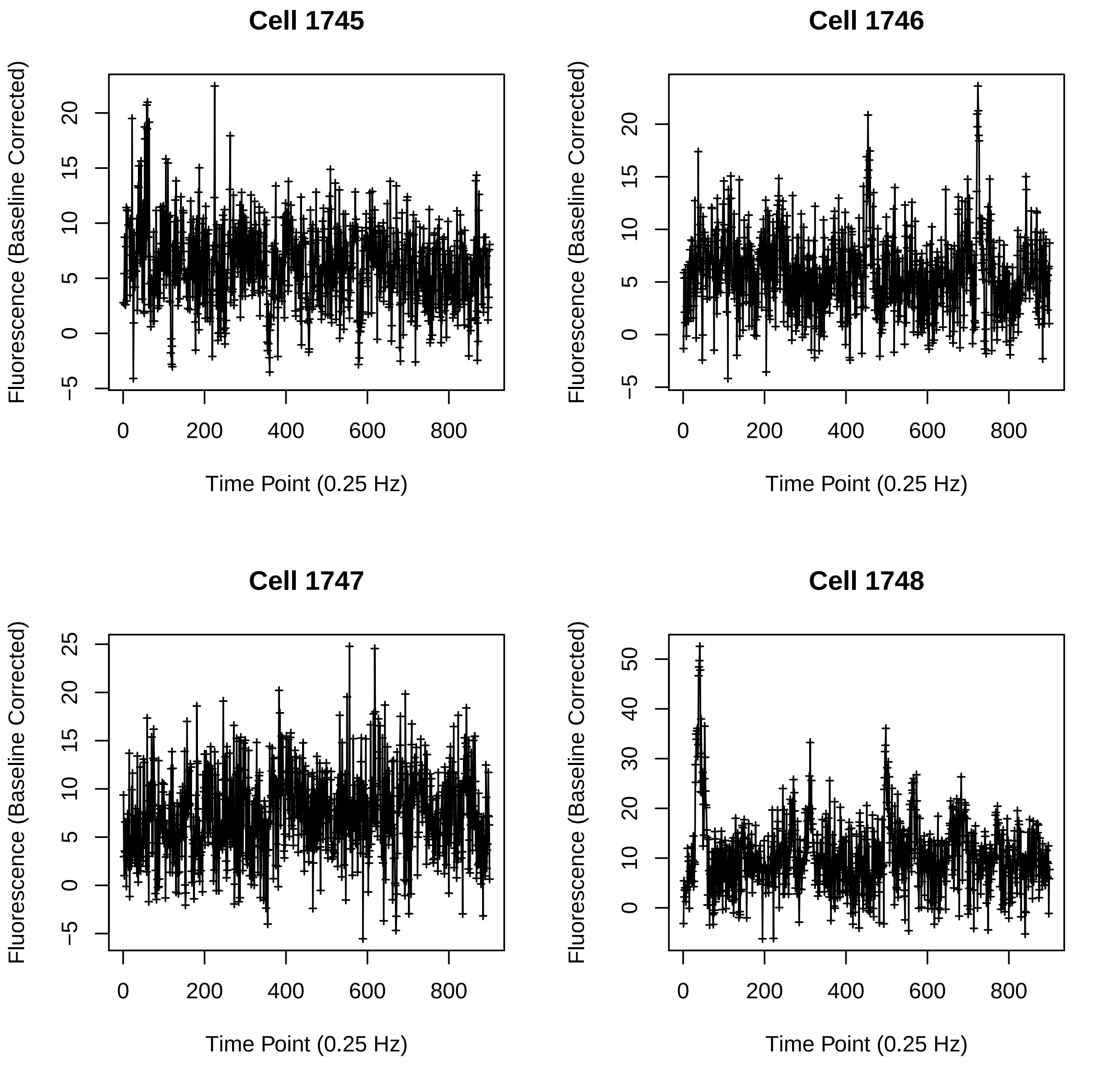
Cell 1745
Cell 1746
Cell 1747
Cell 1748
Fluorescence (Baseline Corrected)
Time Point (0.25 Hz)
0
200
400
600
800
−5
5
10
15
20
25
30
40
50

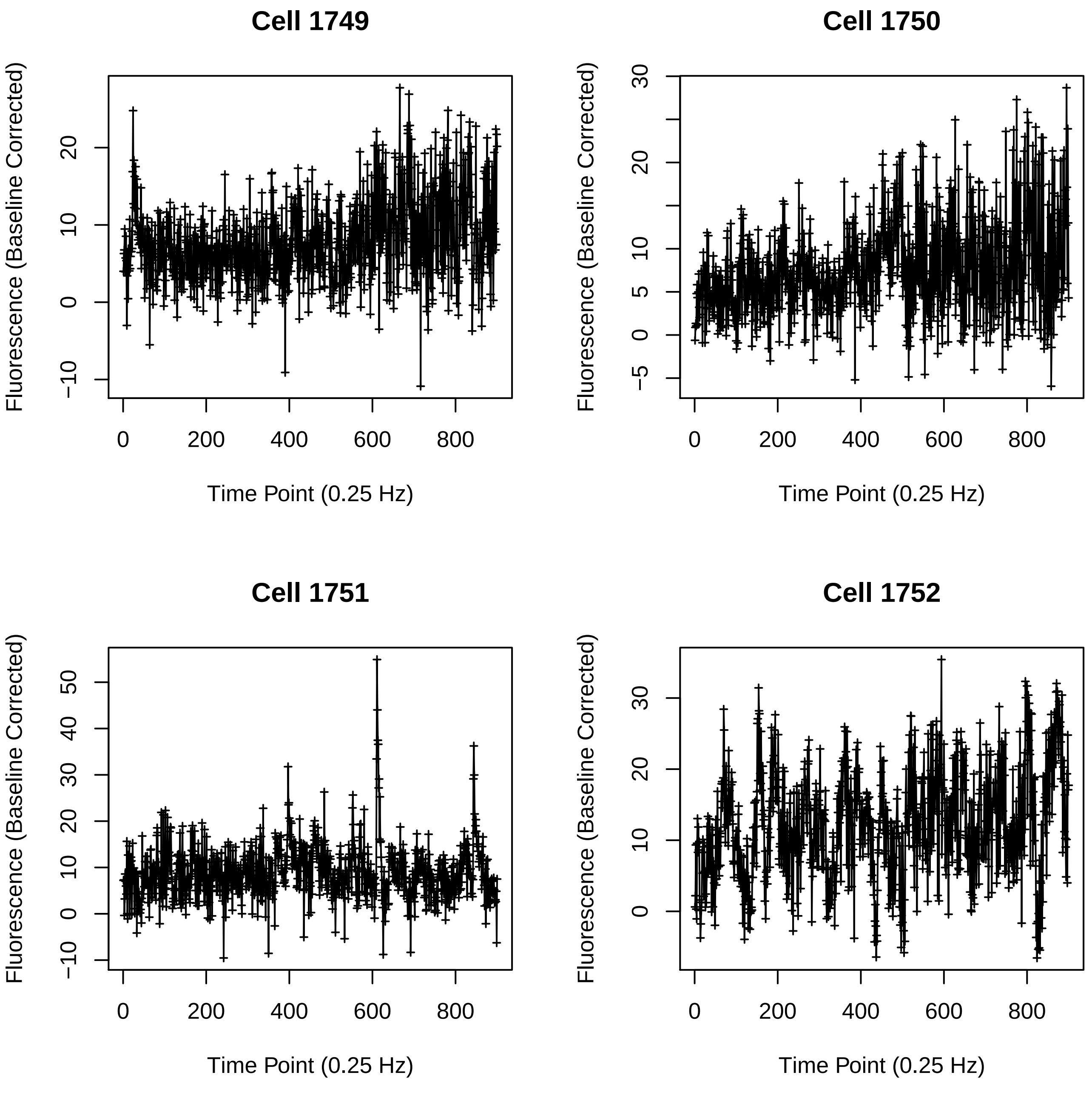
Cell 1749
Fluorescence (Baseline Corrected)
−10
0
10
20
0
200
400
600
800
Time Point (0.25 Hz)
Cell 1750
Fluorescence (Baseline Corrected)
−5
0
5
10
20
30
0
200
400
600
800
Time Point (0.25 Hz)
Cell 1751
Fluorescence (Baseline Corrected)
−10
0
10
20
30
40
50
0
200
400
600
800
Time Point (0.25 Hz)
Cell 1752
Fluorescence (Baseline Corrected)
0
10
20
30
0
200
400
600
800
Time Point (0.25 Hz)

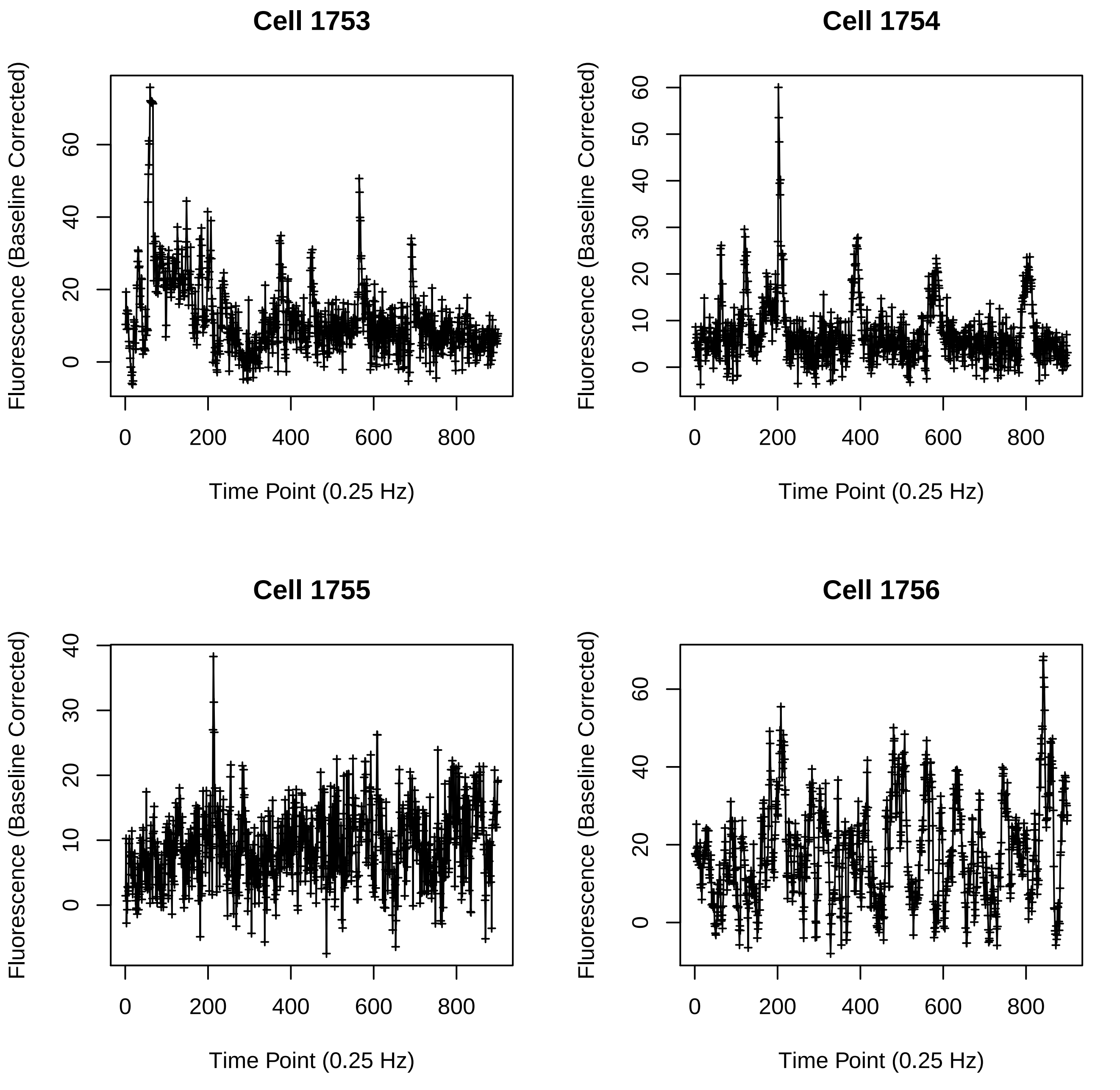
Cell 1753
Cell 1754
Cell 1755
Cell 1756
Fluorescence (Baseline Corrected)
Time Point (0.25 Hz)
0
200
400
600
800
0
10
20
30
40
50
60

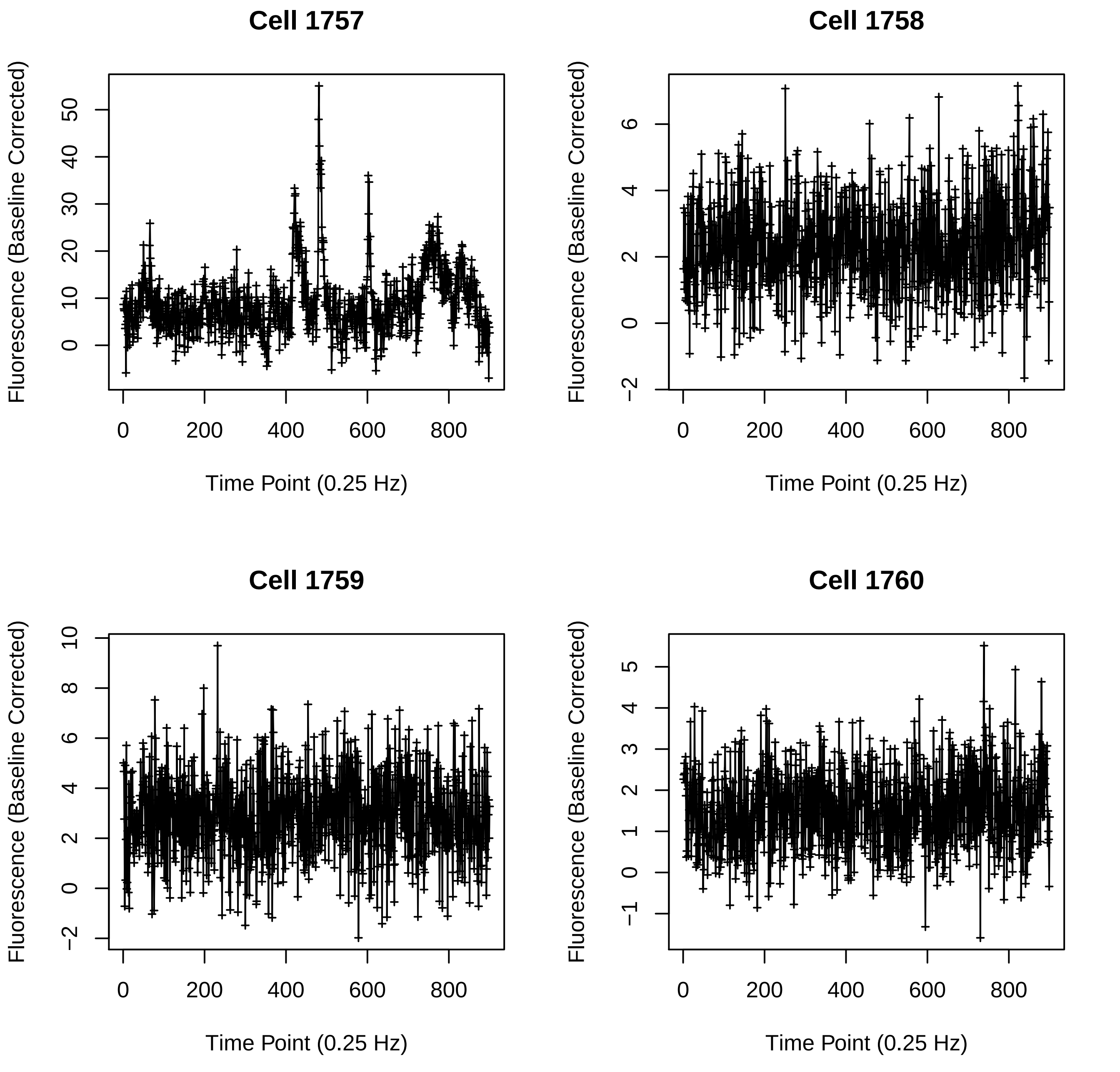

Cell 1757
Cell 1758
Cell 1759
Cell 1760
Fluorescence (Baseline Corrected)
Time Point (0.25 Hz)

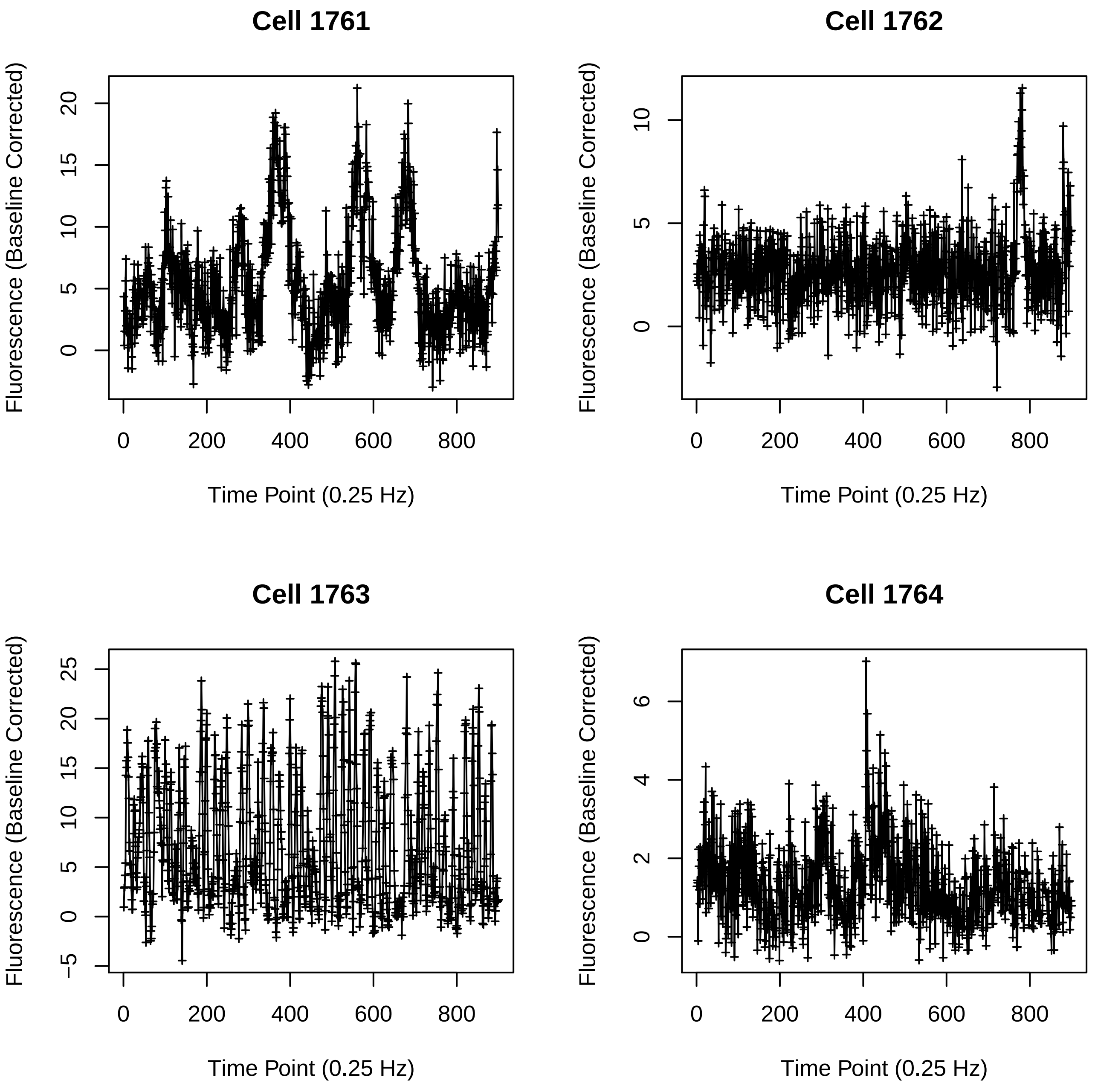
Cell 1761
Fluorescence (Baseline Corrected)
0
5
10
15
20
0
200
400
600
800
Time Point (0.25 Hz)
Cell 1762
Fluorescence (Baseline Corrected)
0
5
10
0
200
400
600
800
Time Point (0.25 Hz)
Cell 1763
Fluorescence (Baseline Corrected)
−5
0
5
10
15
20
25
0
200
400
600
800
Time Point (0.25 Hz)
Cell 1764
Fluorescence (Baseline Corrected)
0
2
4
6
0
200
400
600
800
Time Point (0.25 Hz)

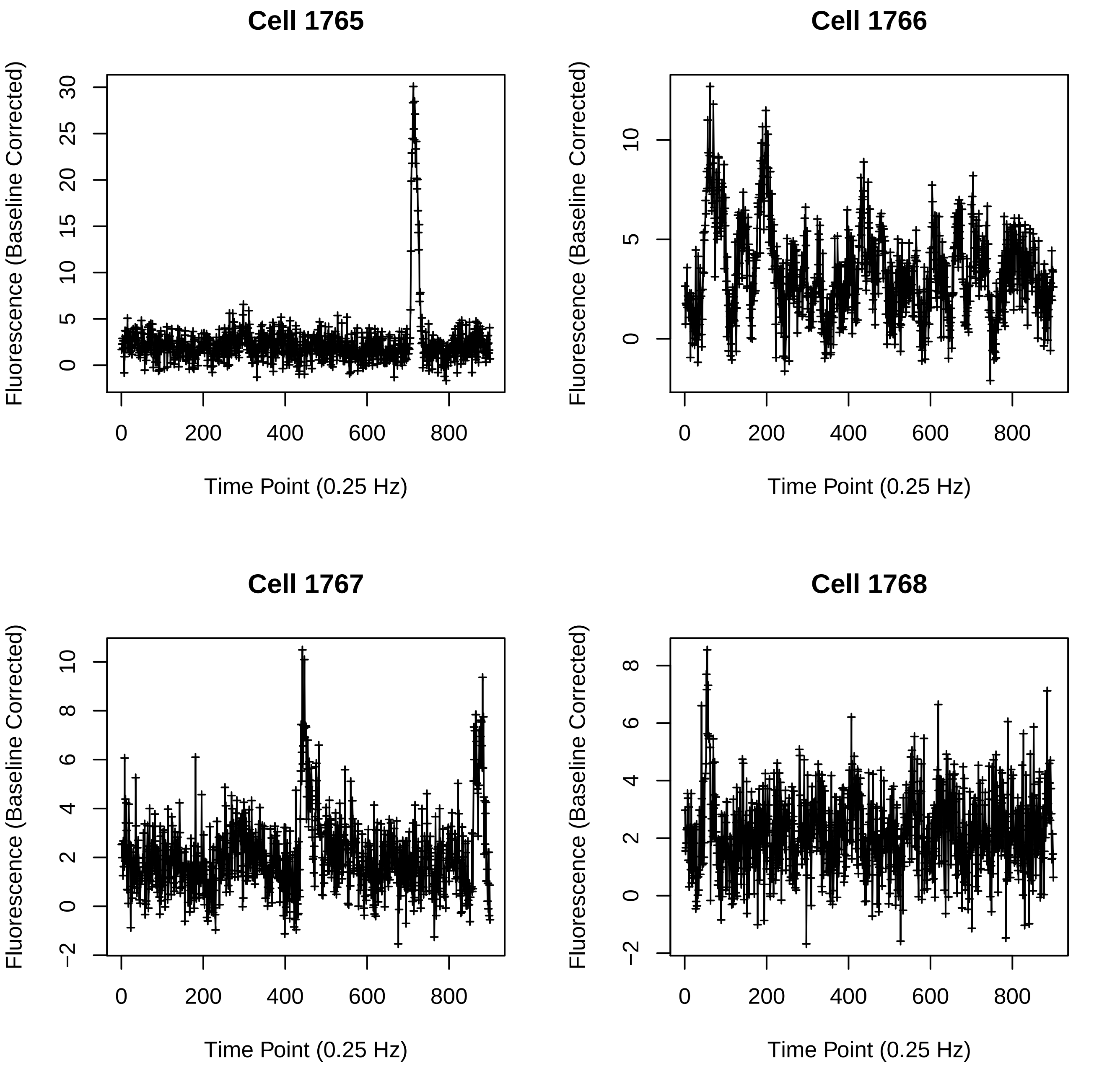
Cell 1765
Fluorescence (Baseline Corrected)
0
5
10
15
20
25
30
0
200
400
600
800
Time Point (0.25 Hz)
Cell 1766
Fluorescence (Baseline Corrected)
0
5
10
0
200
400
600
800
Time Point (0.25 Hz)
Cell 1767
Fluorescence (Baseline Corrected)
−2
0
2
4
6
8
10
0
200
400
600
800
Time Point (0.25 Hz)
Cell 1768
Fluorescence (Baseline Corrected)
−2
0
2
4
6
8
0
200
400
600
800
Time Point (0.25 Hz)

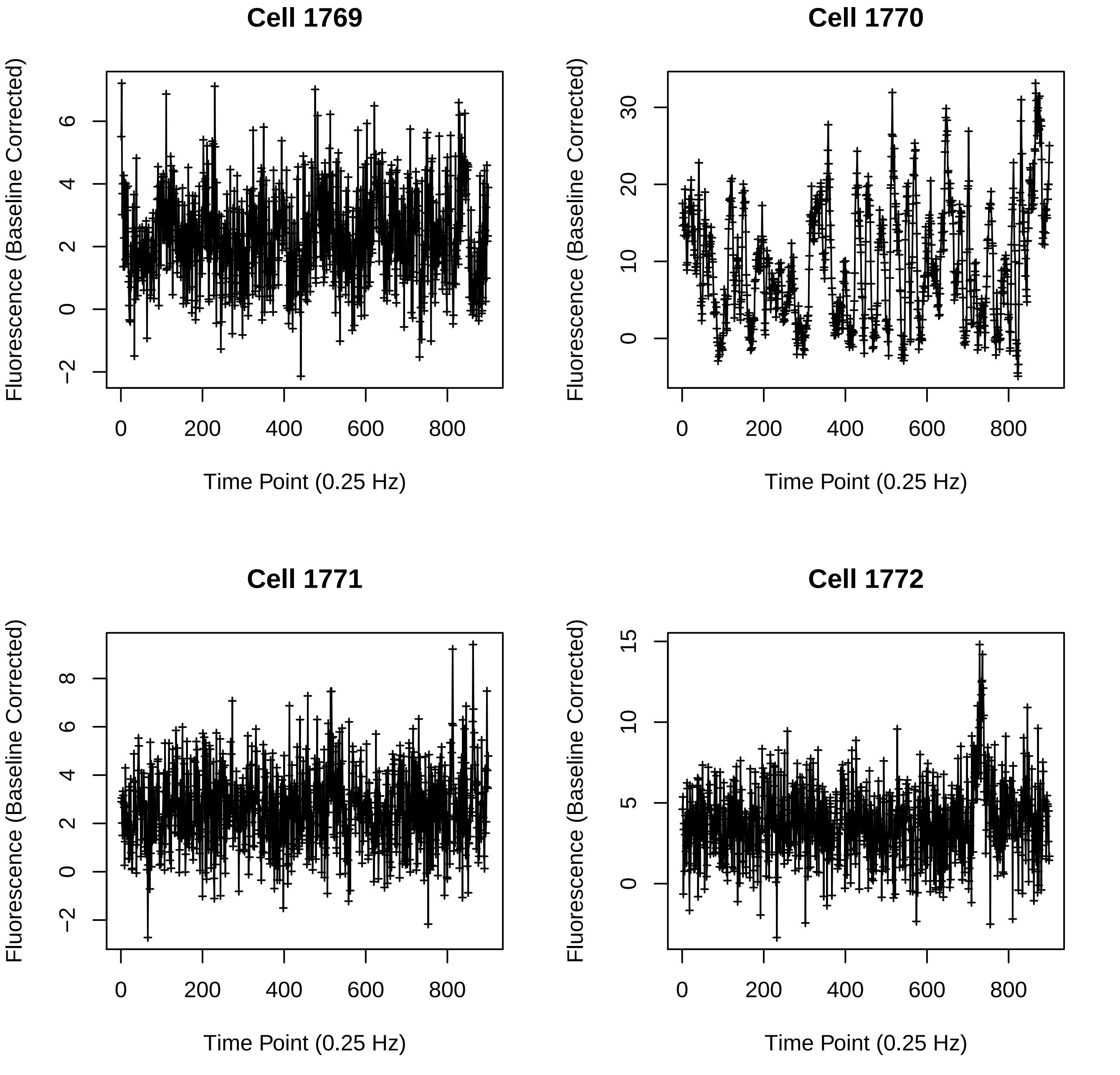
Cell 1769
Cell 1770
Cell 1771
Cell 1772
Fluorescence (Baseline Corrected)
Time Point (0.25 Hz)
-2
0
2
4
6
8
10
15
20
30
200
400
600
800

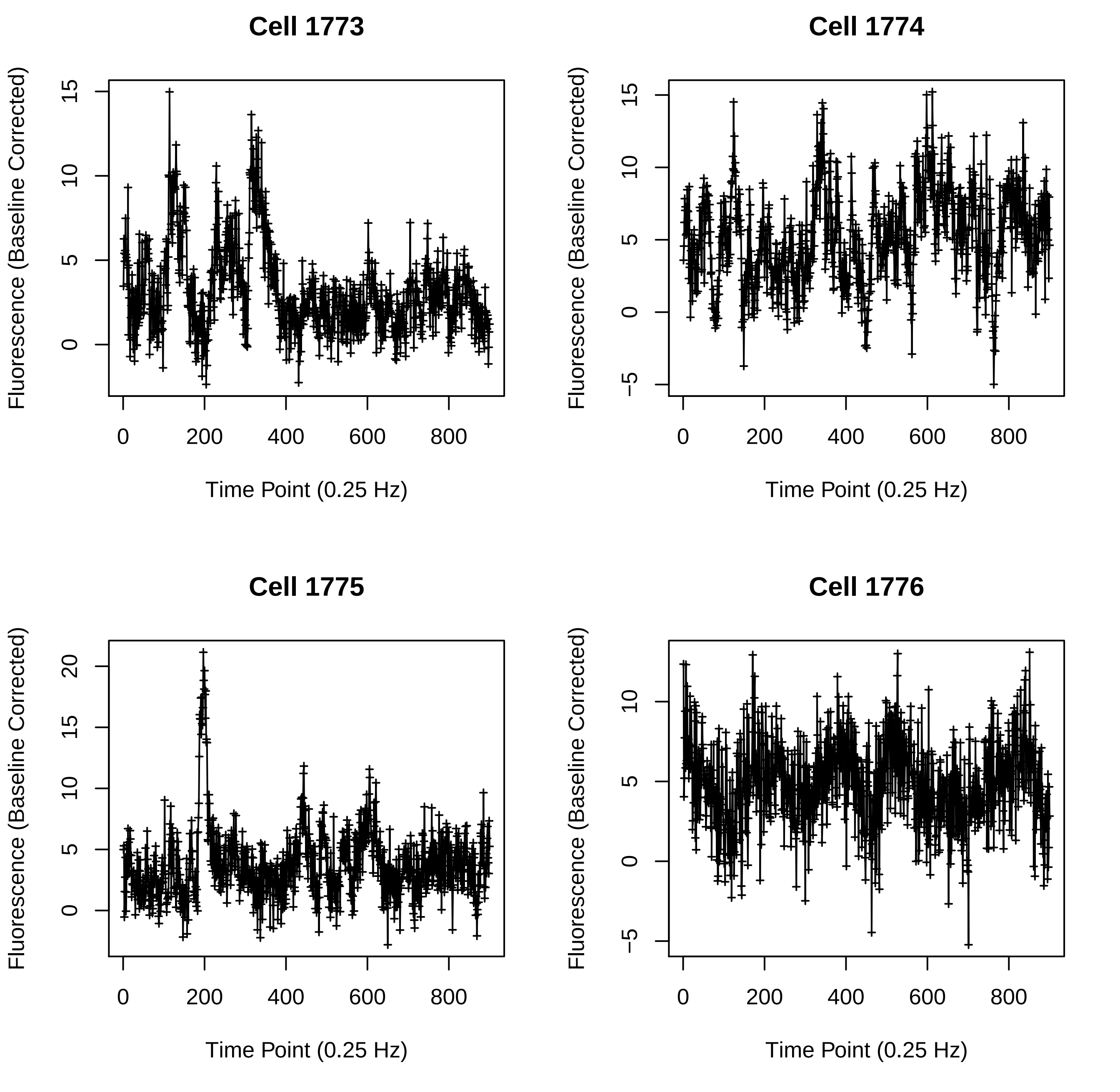
Cell 1773
Cell 1774
Cell 1775
Cell 1776
Fluorescence (Baseline Corrected)
Time Point (0.25 Hz)
0
200
400
600
800
−5
5
10
15
20

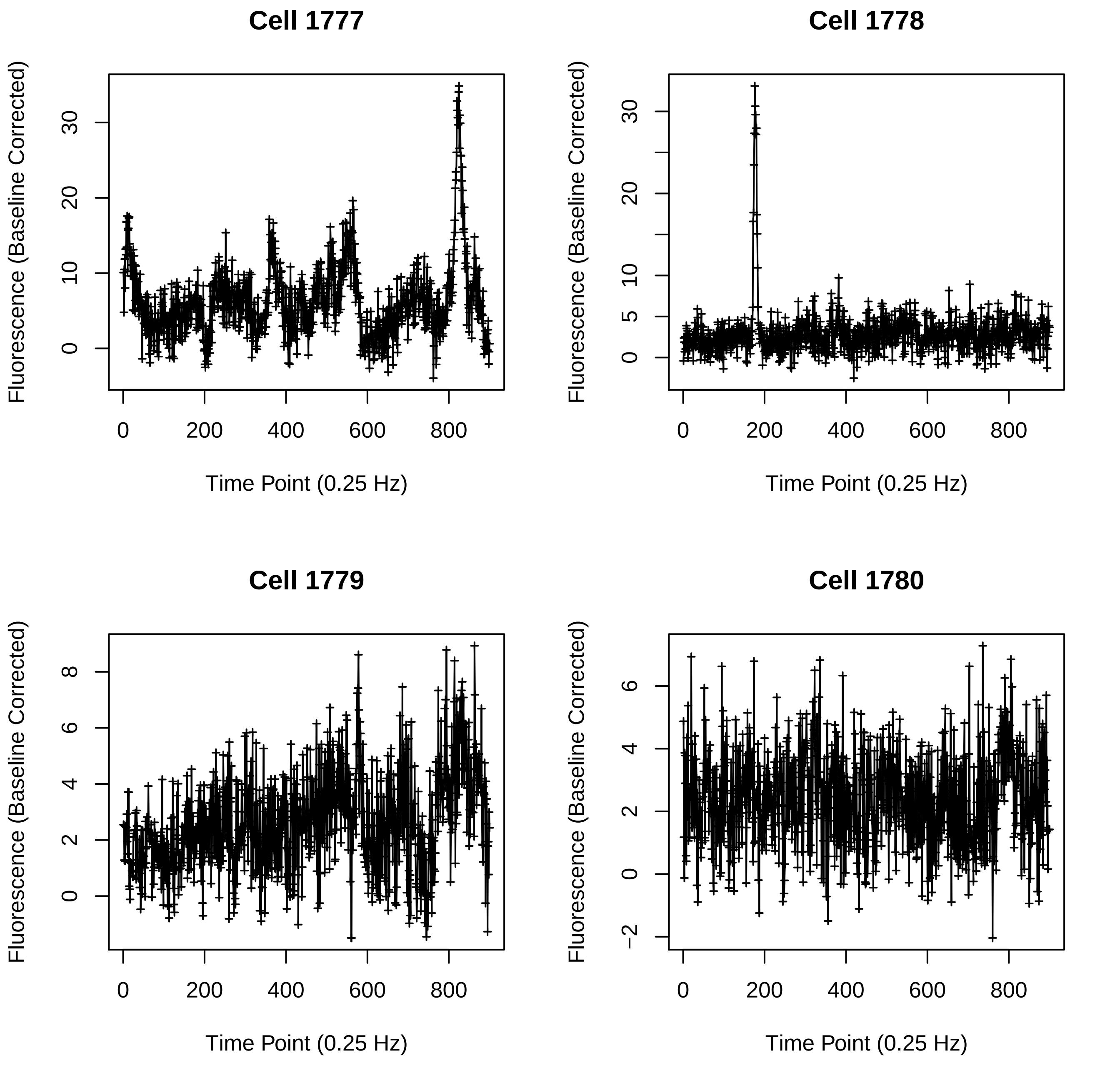
Cell 1777
Cell 1778
Cell 1779
Cell 1780
Fluorescence (Baseline Corrected)
Time Point (0.25 Hz)
0
200
400
600
800
0
10
20
30
5
-2
2
4
6
8

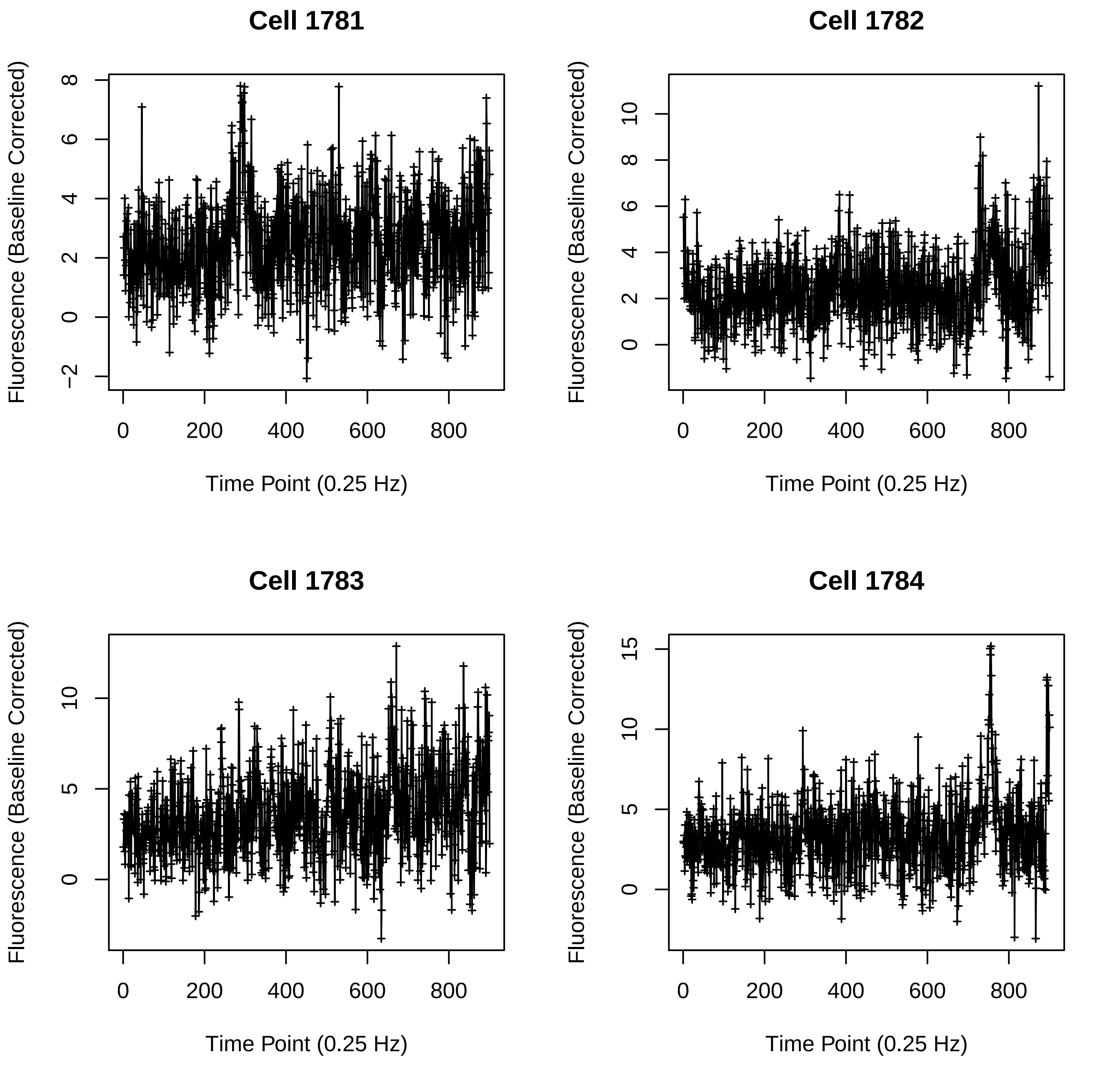
Cell 1781
Cell 1782
Cell 1783
Cell 1784
Fluorescence (Baseline Corrected)
Time Point (0.25 Hz)
0
200
400
600
800
−2
2
4
6
8
10
5
15

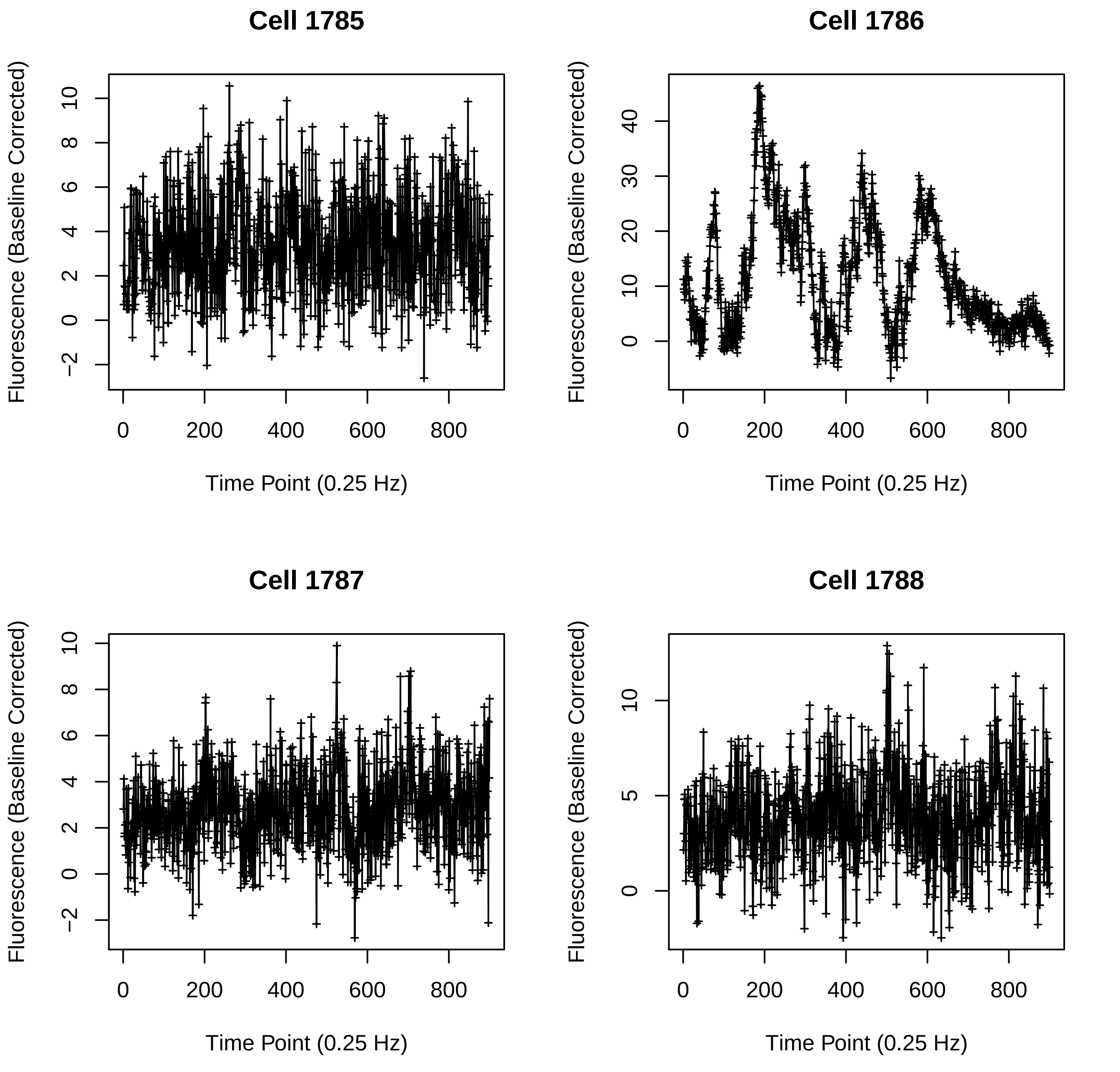
Cell 1785
Cell 1786
Cell 1787
Cell 1788
Fluorescence (Baseline Corrected)
Time Point (0.25 Hz)
−2
0
2
4
6
8
10
20
30
40
5
200
400
600
800

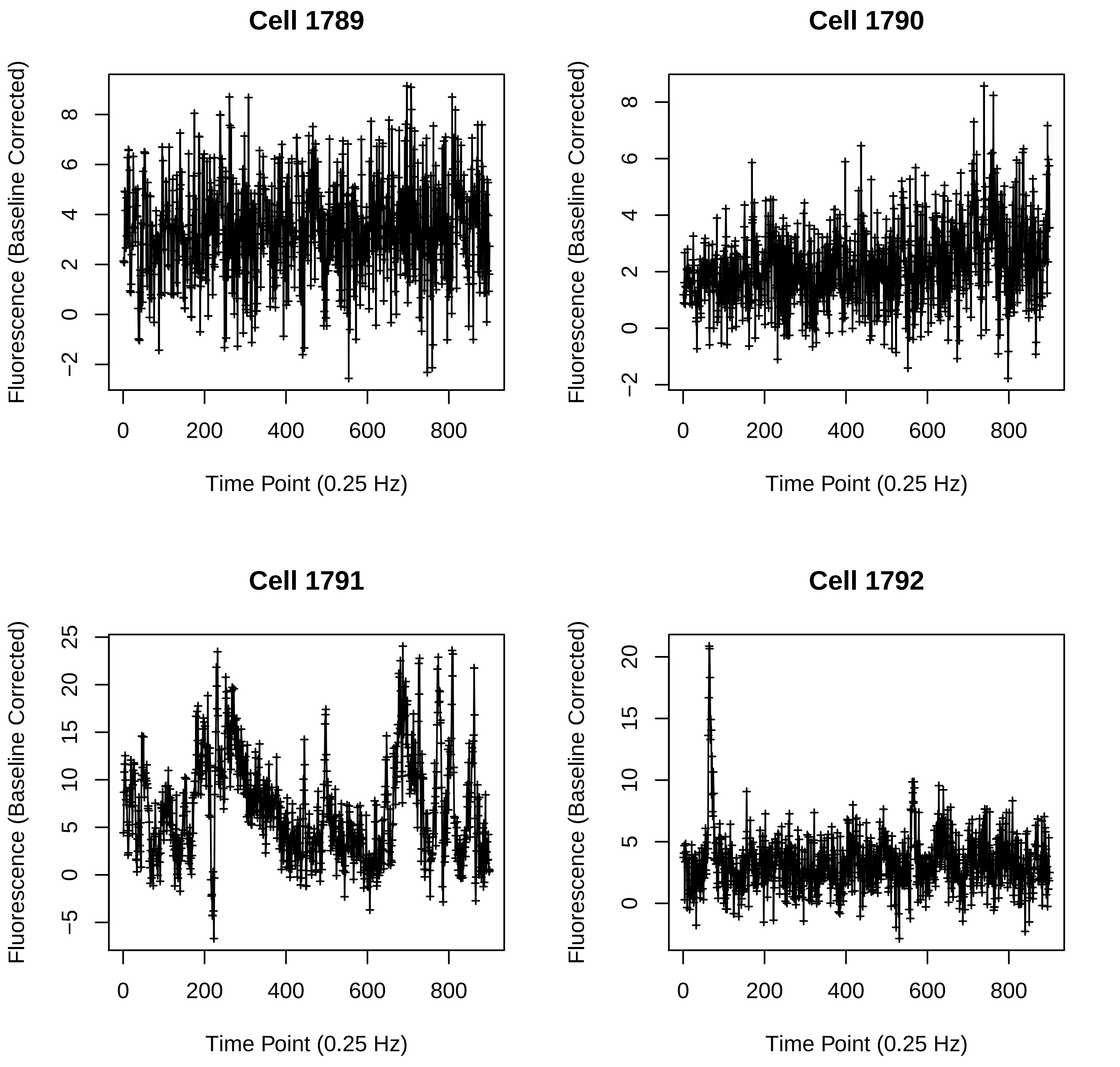
Cell 1789
Cell 1790
Cell 1791
Cell 1792
Fluorescence (Baseline Corrected)
Time Point (0.25 Hz)
0
200
400
600
800
−2
2
4
6
8
−5
5
10
15
20
25

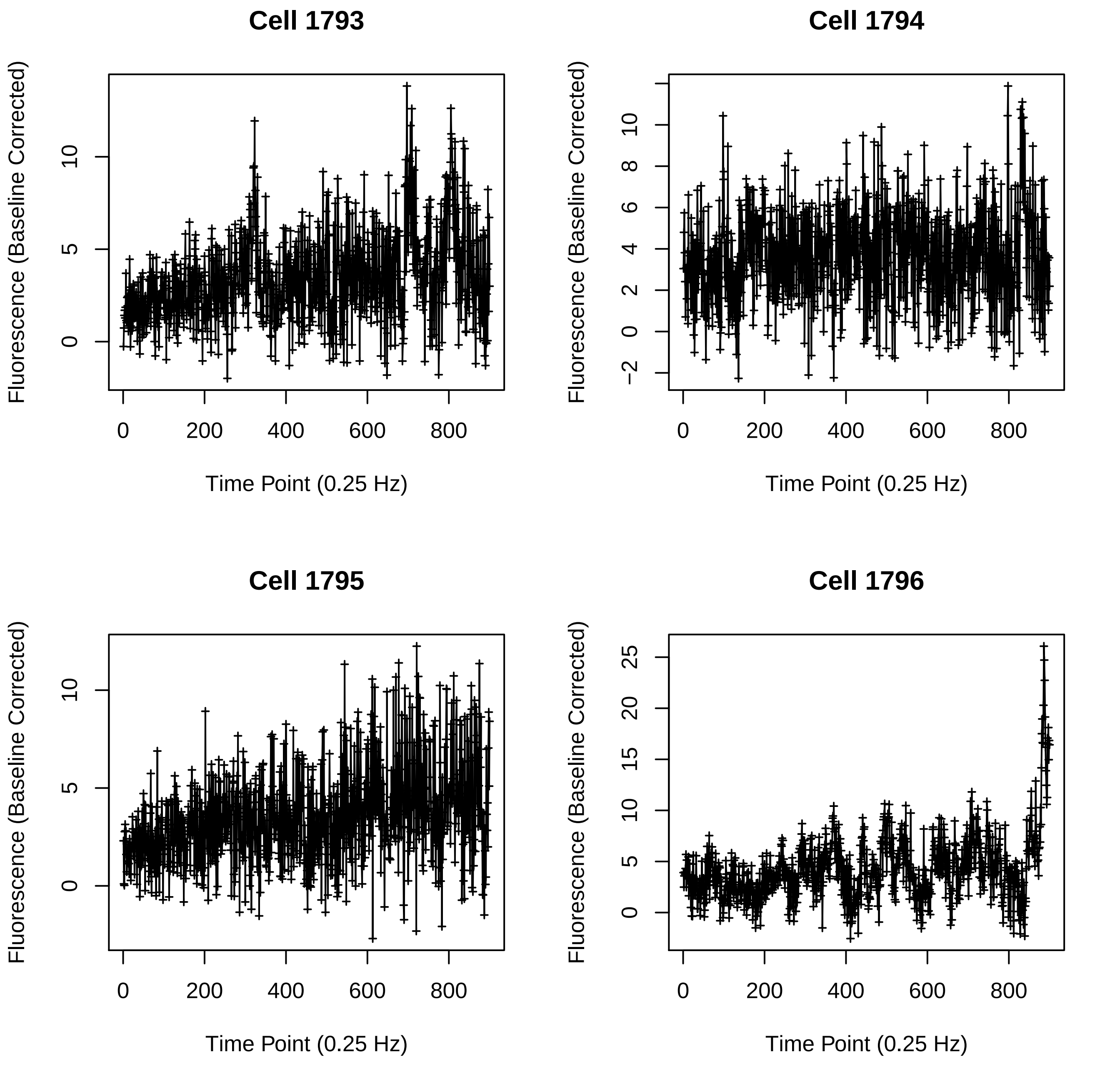
Cell 1793
Cell 1794
Cell 1795
Cell 1796
Fluorescence (Baseline Corrected)
Time Point (0.25 Hz)
0
200
400
600
800
0
5
10
−2
2
4
6
8
15
20
25

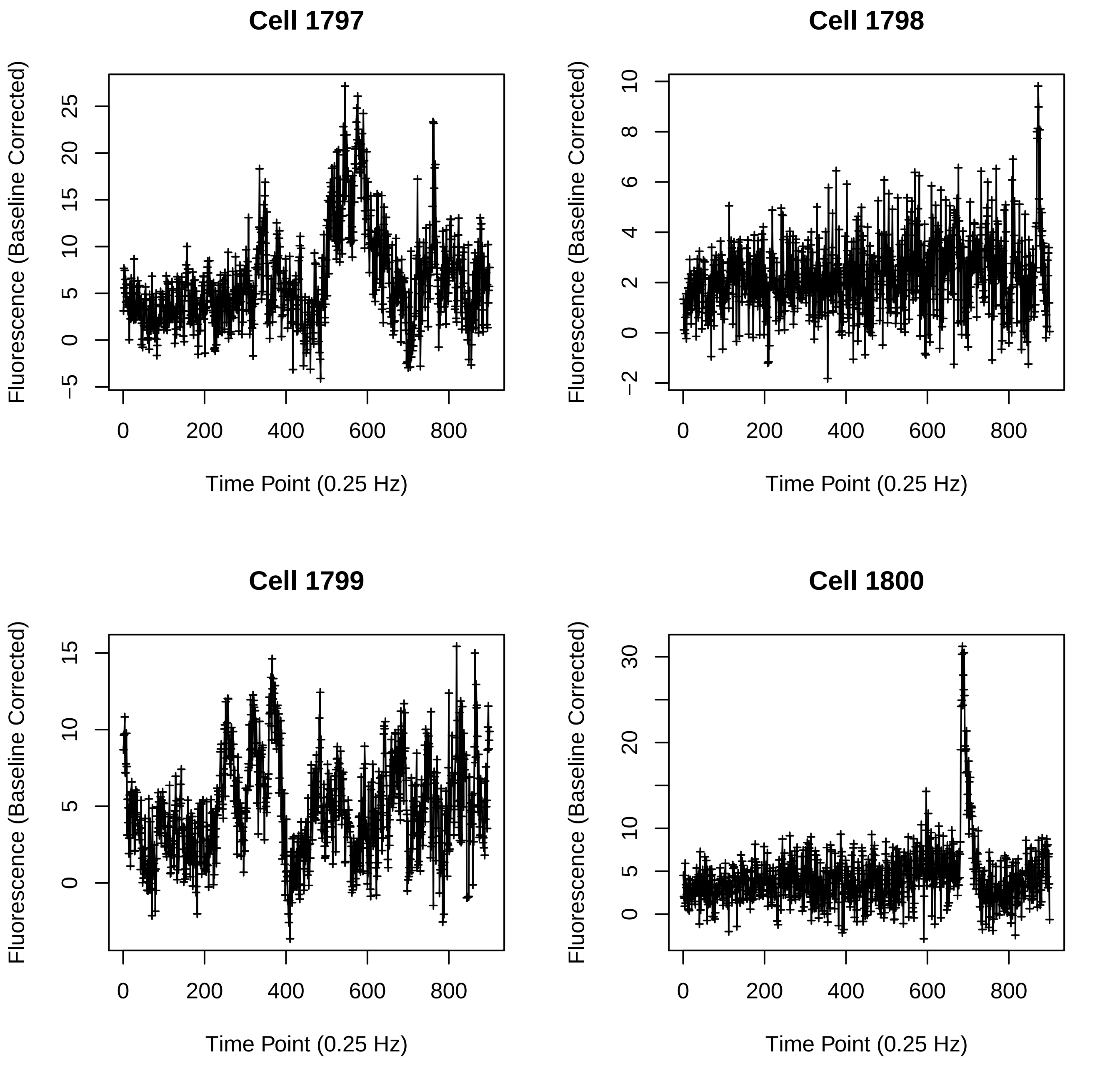

Cell 1797
Cell 1798
Cell 1799
Cell 1800
Fluorescence (Baseline Corrected)
Time Point (0.25 Hz)

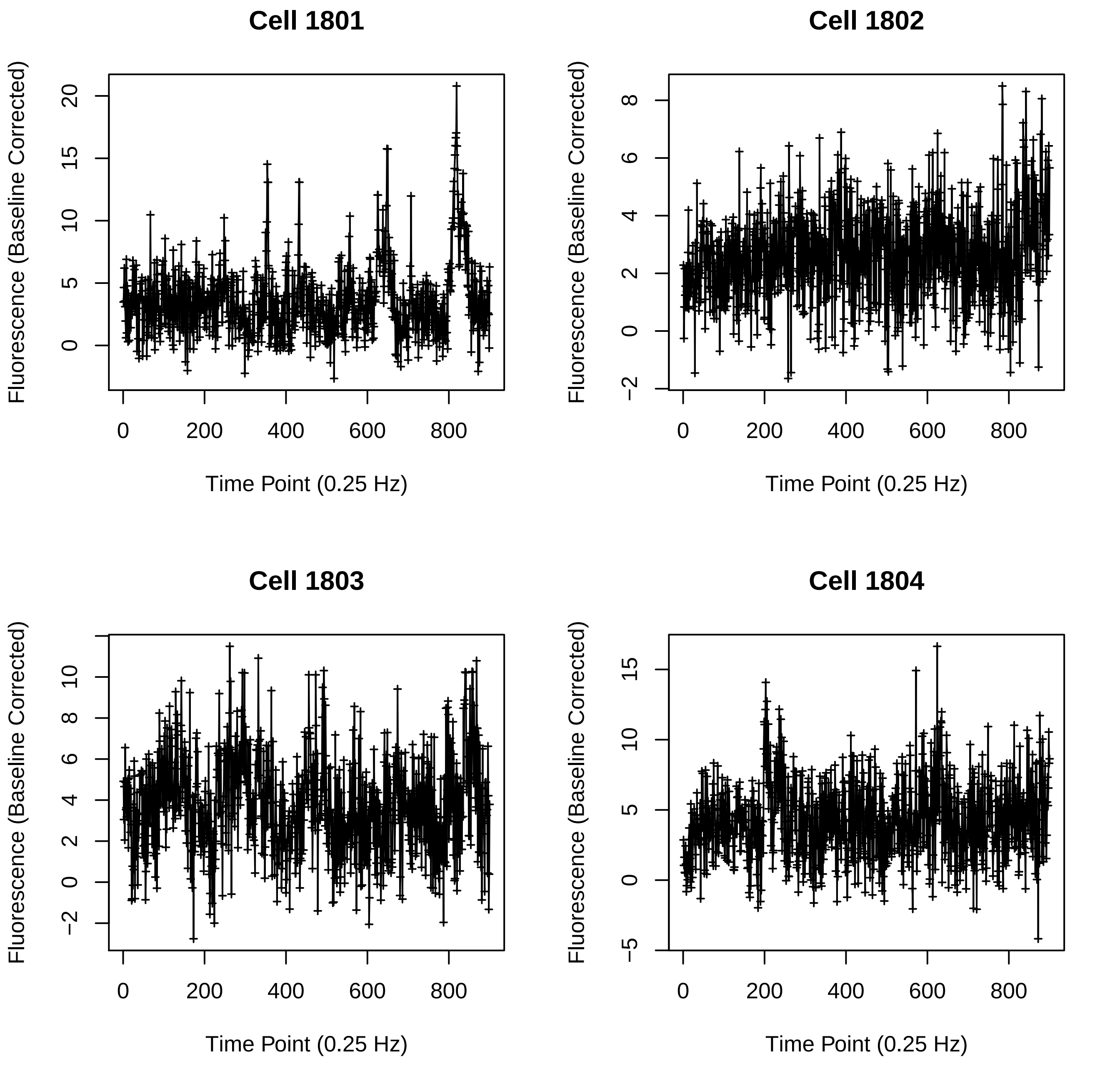

Cell 1801
Cell 1802
Cell 1803
Cell 1804
Fluorescence (Baseline Corrected)
Time Point (0.25 Hz)
0
200
400
600
800

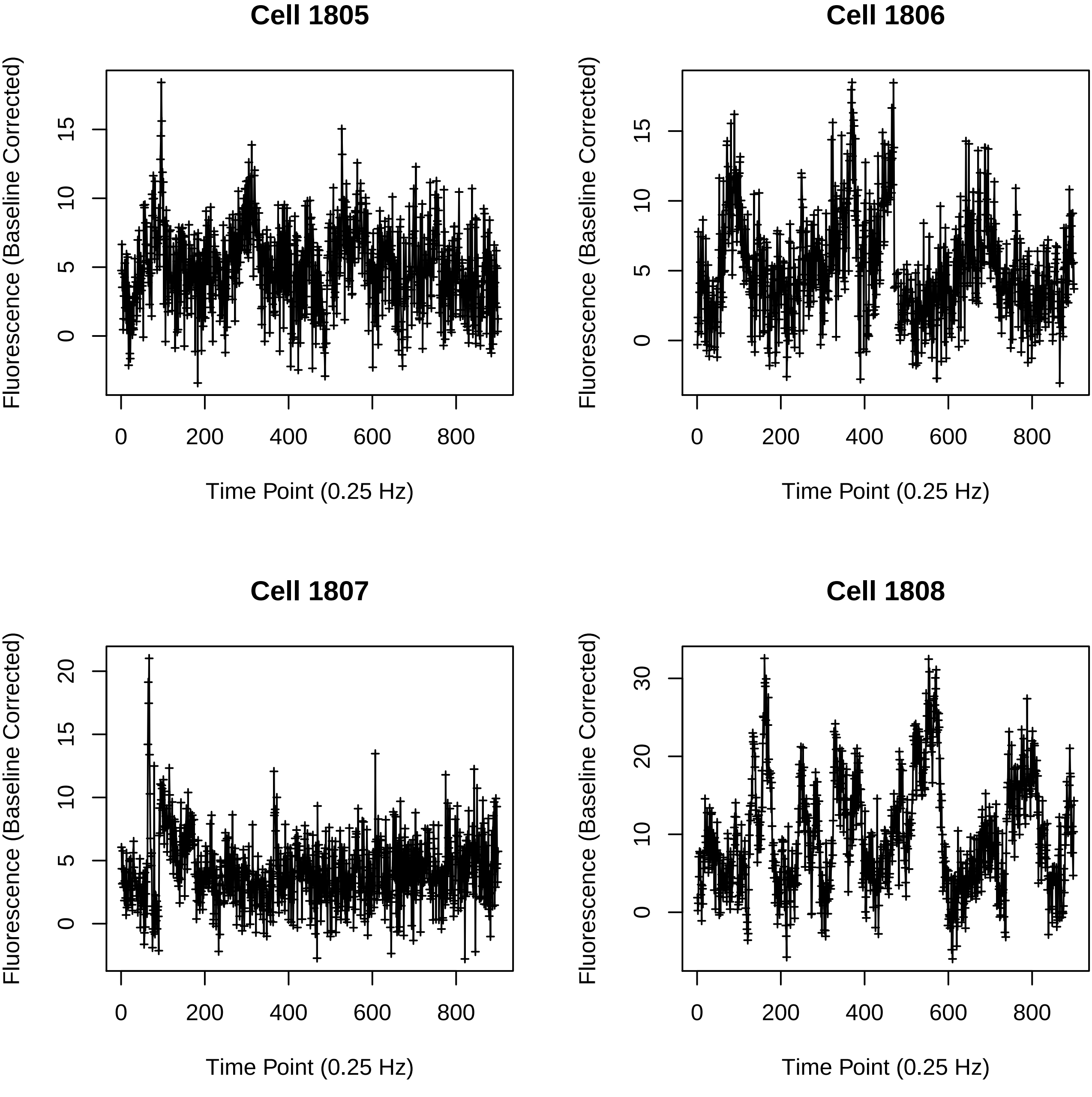
Cell 1805
Cell 1806
Cell 1807
Cell 1808
Fluorescence (Baseline Corrected)
Time Point (0.25 Hz)
0
200
400
600
800
5
10
15
20
30

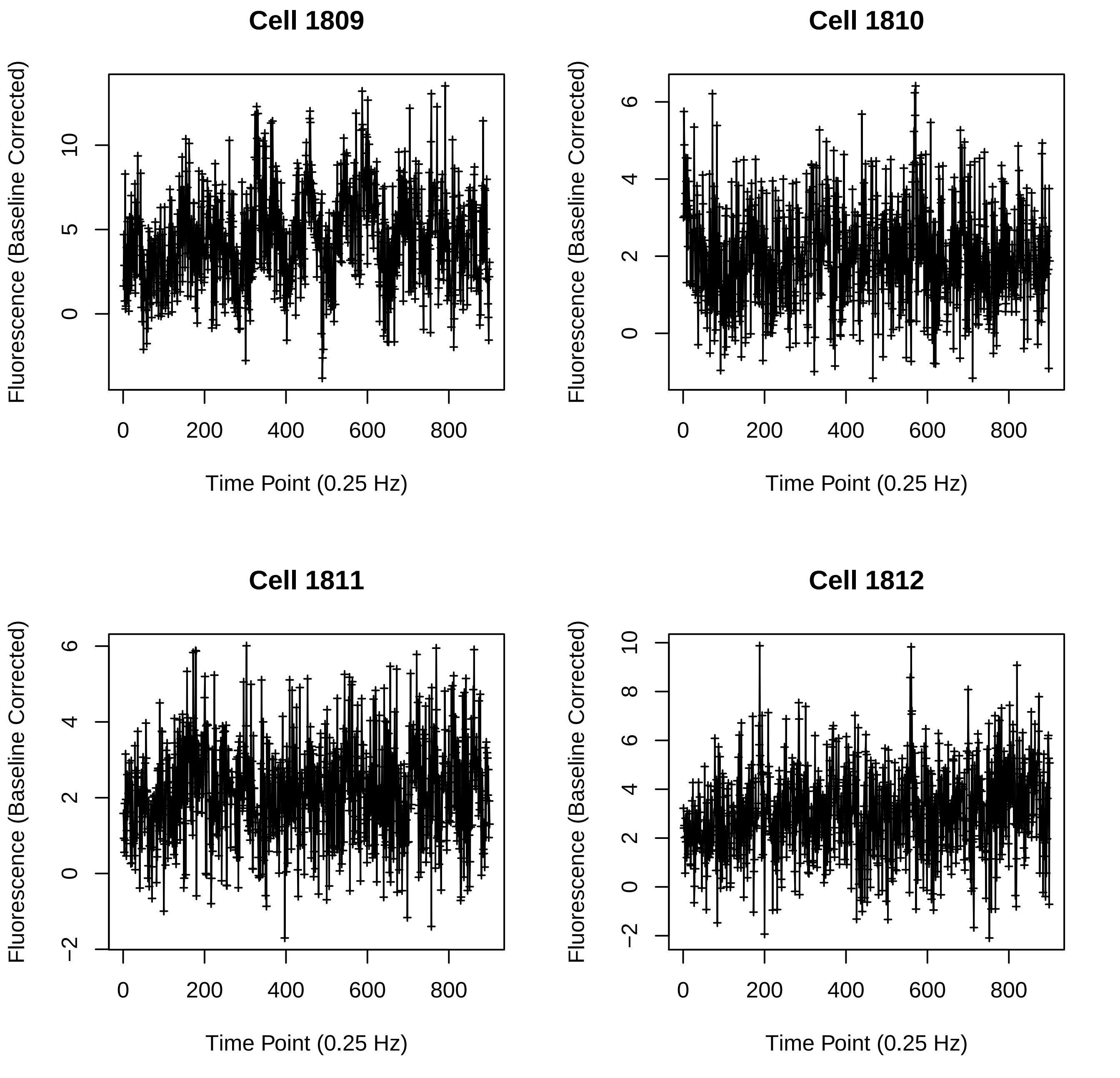
Cell 1809
Cell 1810
Cell 1811
Cell 1812
Fluorescence (Baseline Corrected)
Time Point (0.25 Hz)
0
200
400
600
800
-2
0
2
4
5
6
8
10

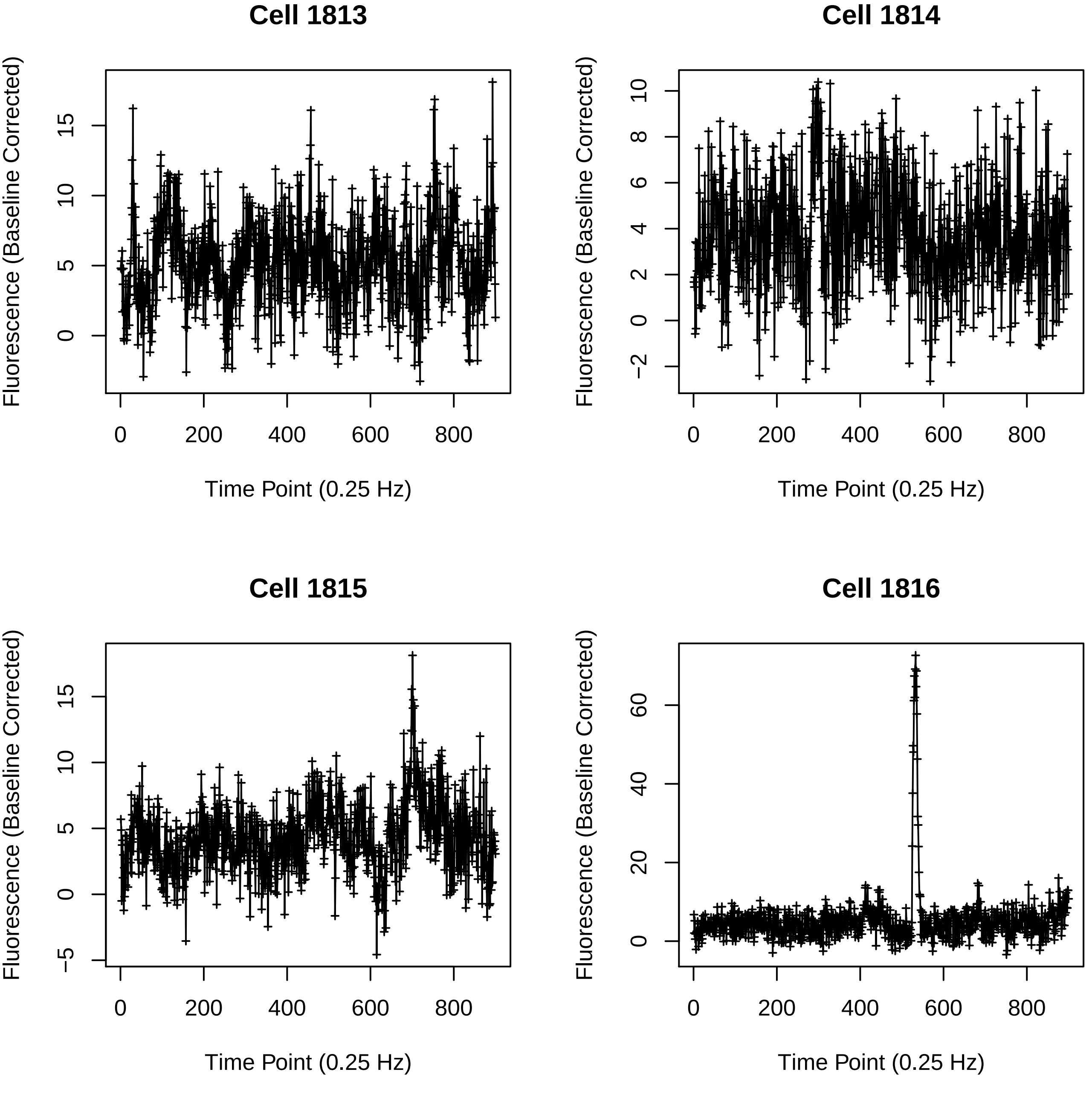
Cell 1813
Fluorescence (Baseline Corrected)
Time Point (0.25 Hz)
Cell 1814
Fluorescence (Baseline Corrected)
Time Point (0.25 Hz)
Cell 1815
Fluorescence (Baseline Corrected)
Time Point (0.25 Hz)
Cell 1816
Fluorescence (Baseline Corrected)
Time Point (0.25 Hz)

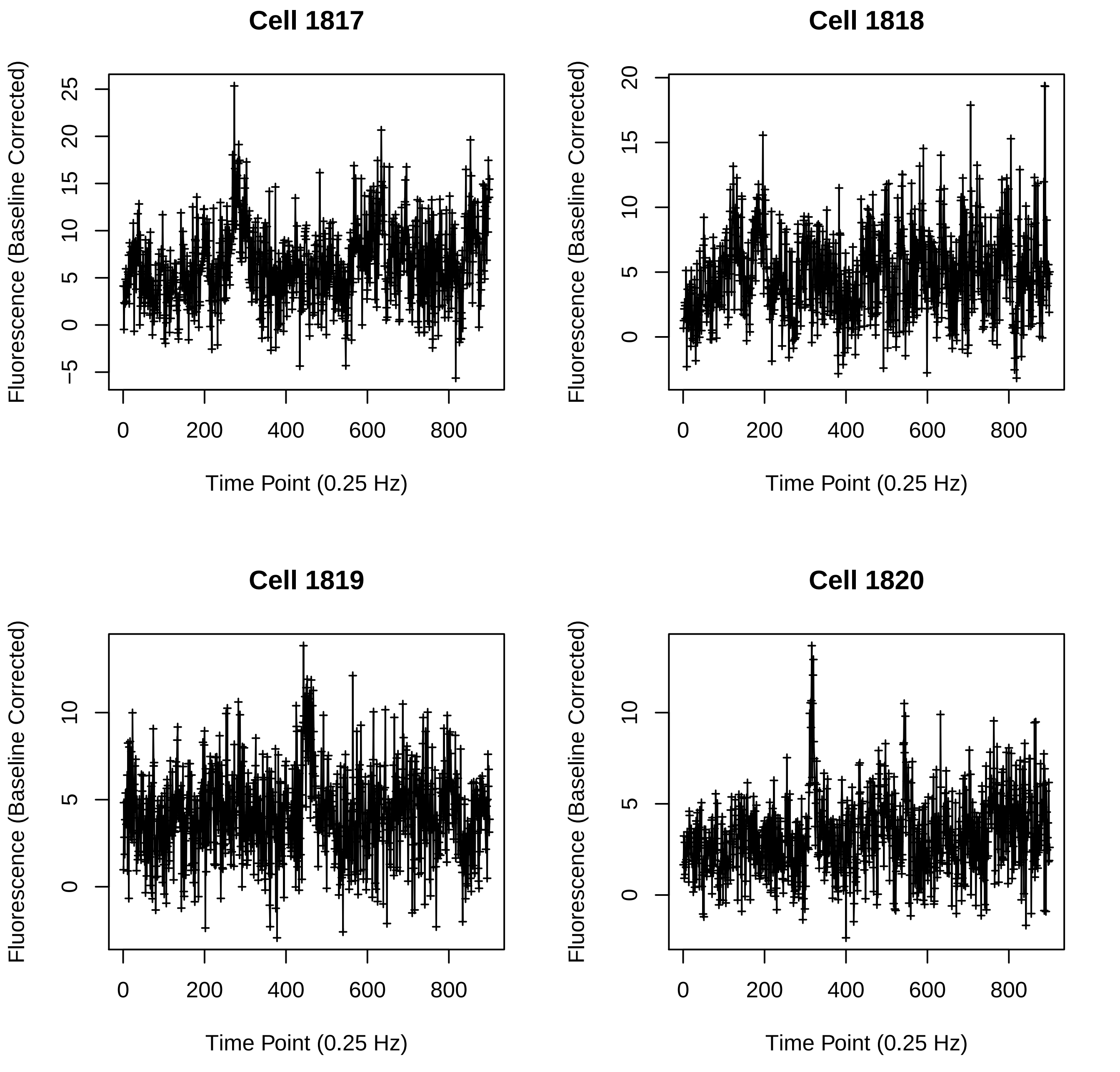
Cell 1817
Cell 1818
Cell 1819
Cell 1820
Fluorescence (Baseline Corrected)
Time Point (0.25 Hz)
0
200
400
600
800
−5
5
10
15
20
25

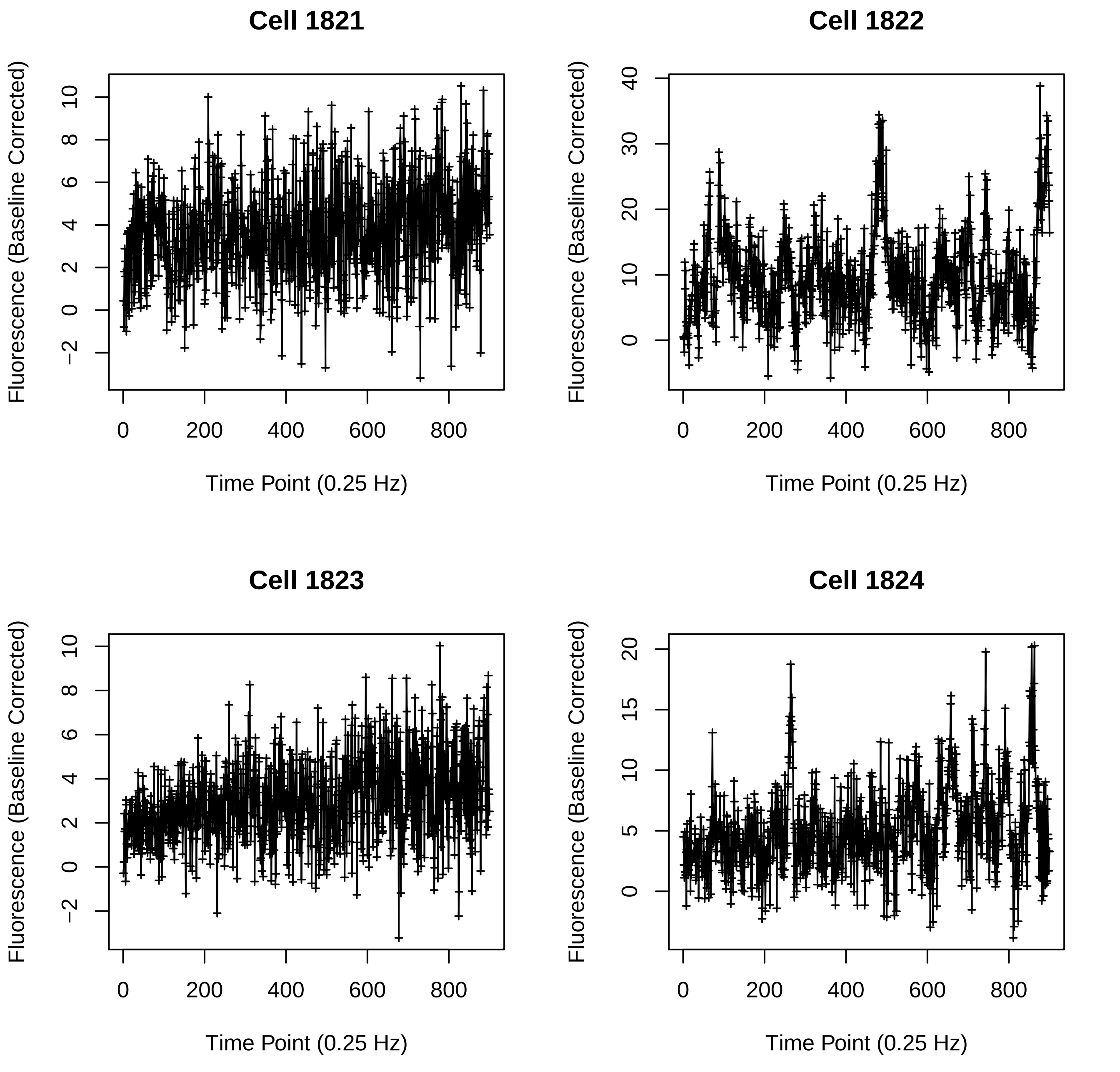

Cell 1821
Cell 1822
Cell 1823
Cell 1824
Fluorescence (Baseline Corrected)
Time Point (0.25 Hz)

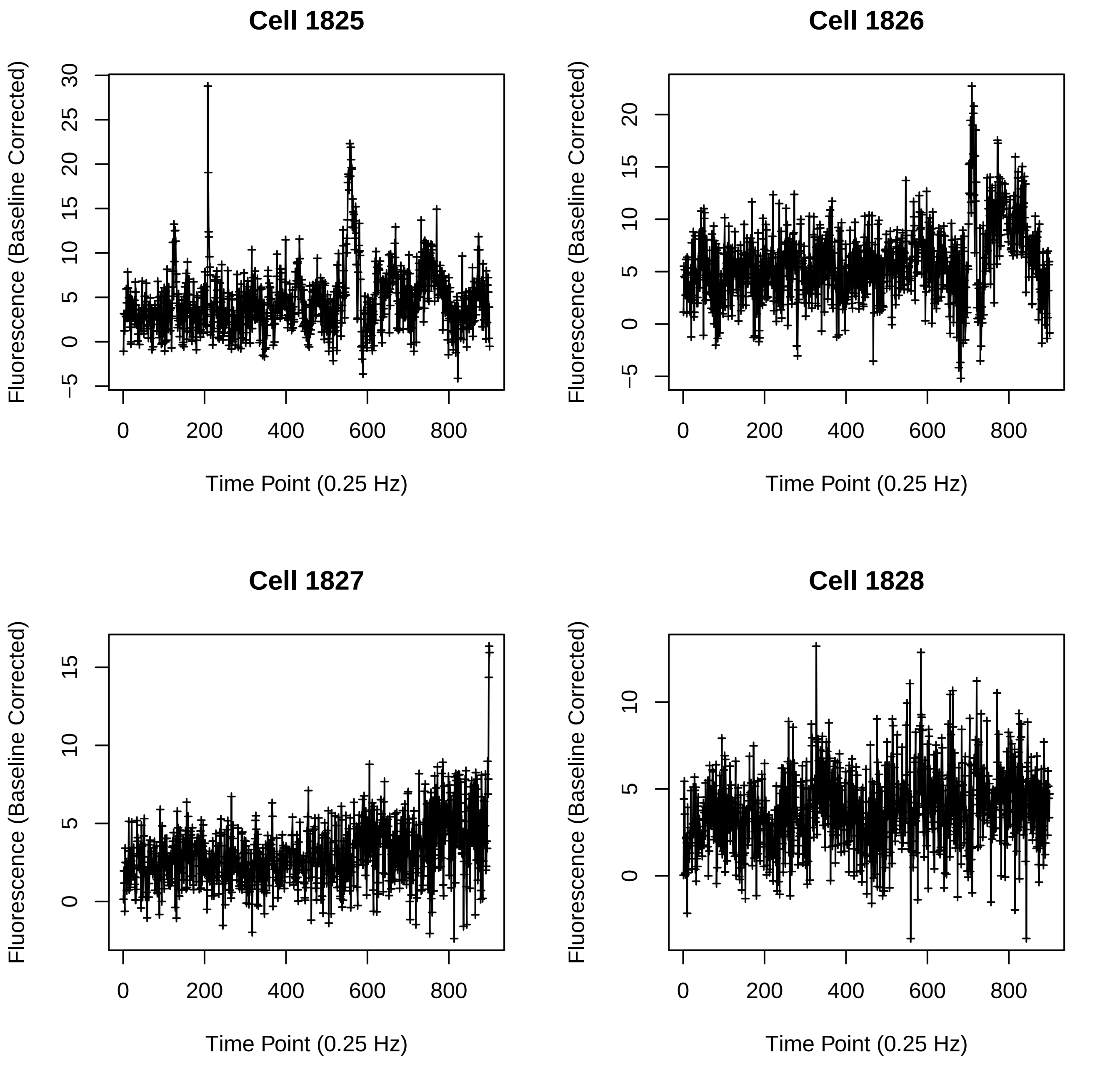
Cell 1825
Cell 1826
Cell 1827
Cell 1828
Fluorescence (Baseline Corrected)
Time Point (0.25 Hz)
0
200
400
600
800
−5
0
5
10
15
20
25
30

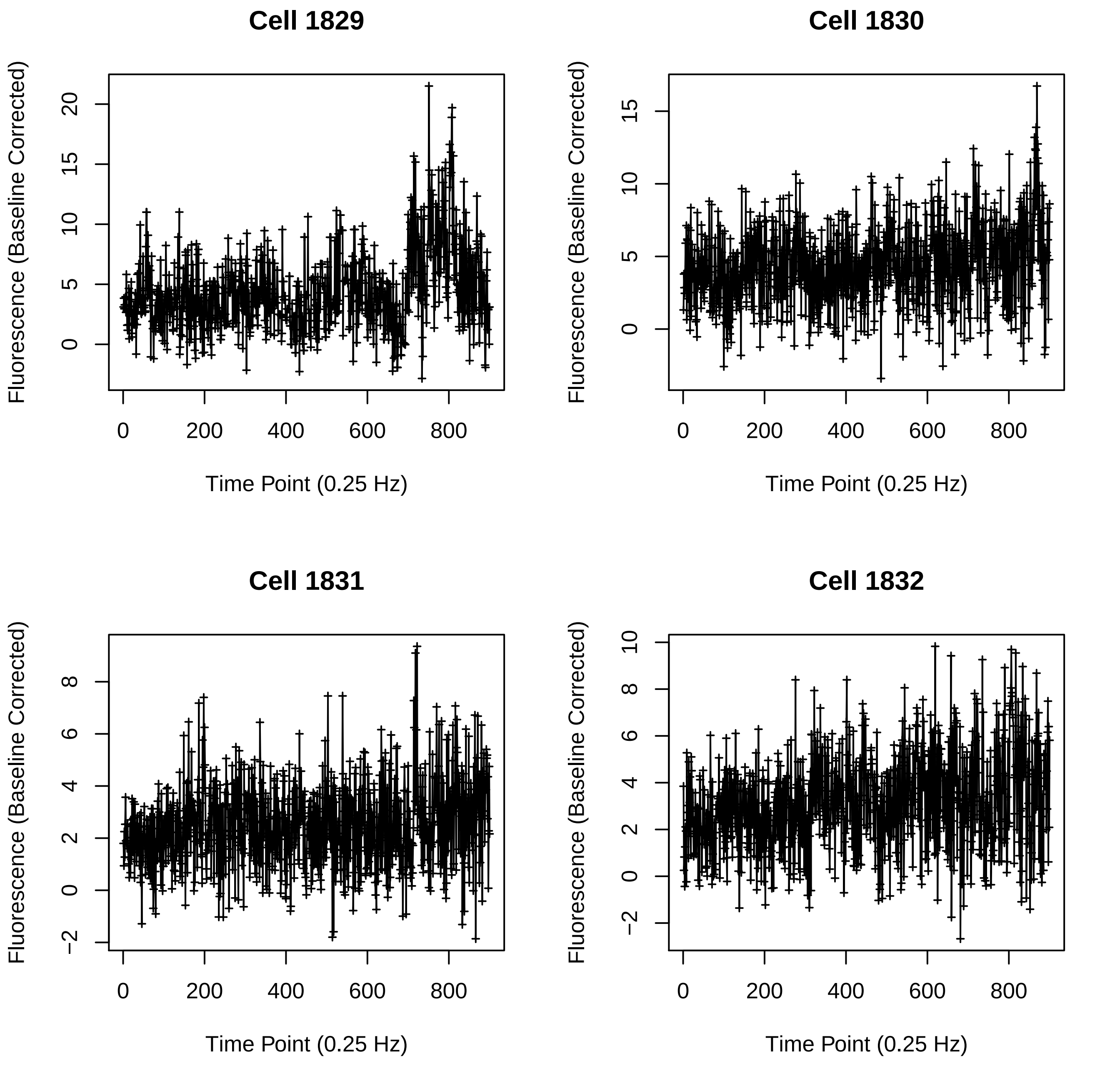
Cell 1829
Cell 1830
Cell 1831
Cell 1832
Fluorescence (Baseline Corrected)
Time Point (0.25 Hz)

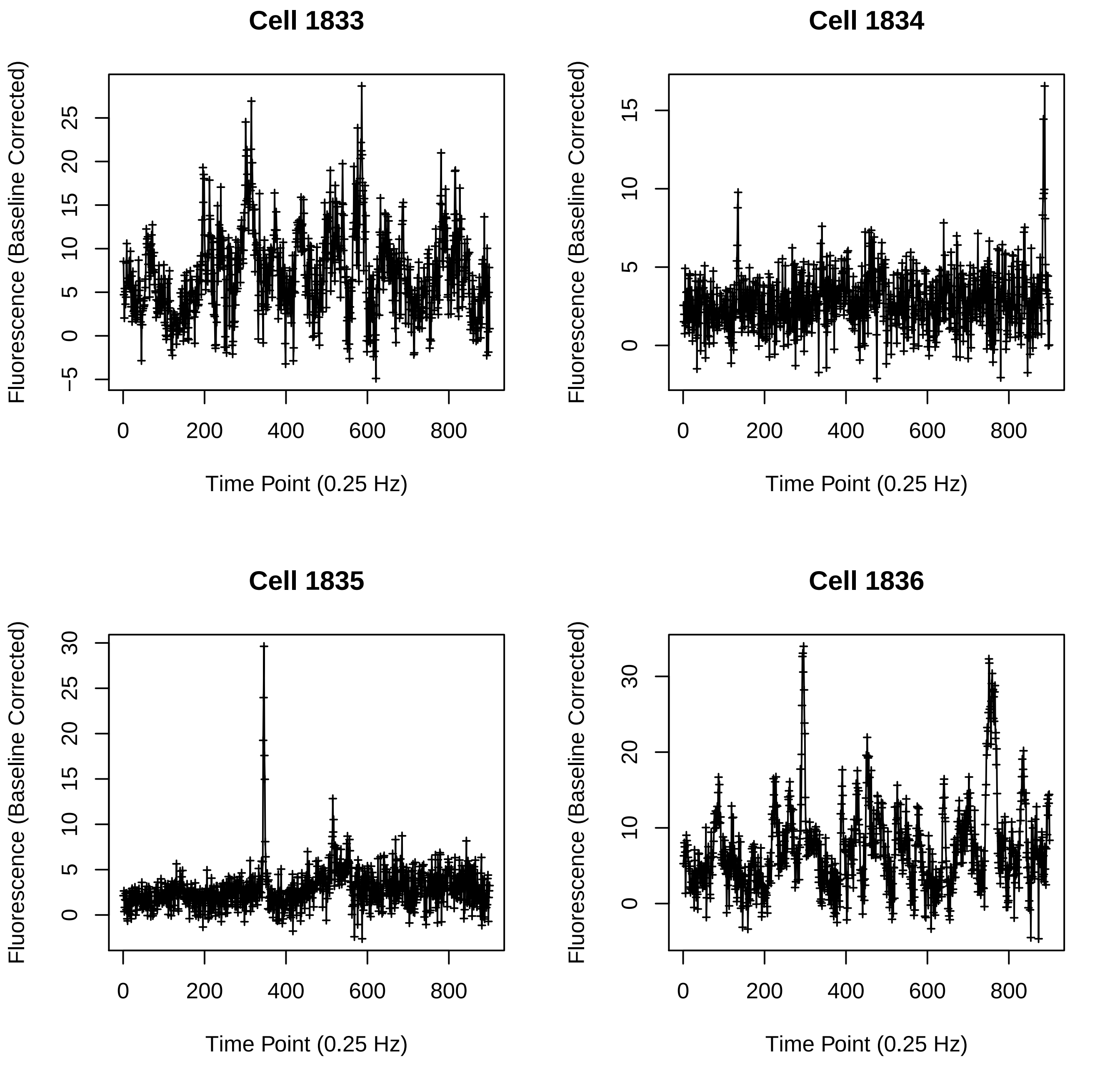
Cell 1833
Cell 1834
Cell 1835
Cell 1836
Fluorescence (Baseline Corrected)
Time Point (0.25 Hz)
-5
0
5
10
15
20
25
30
0
200
400
600
800

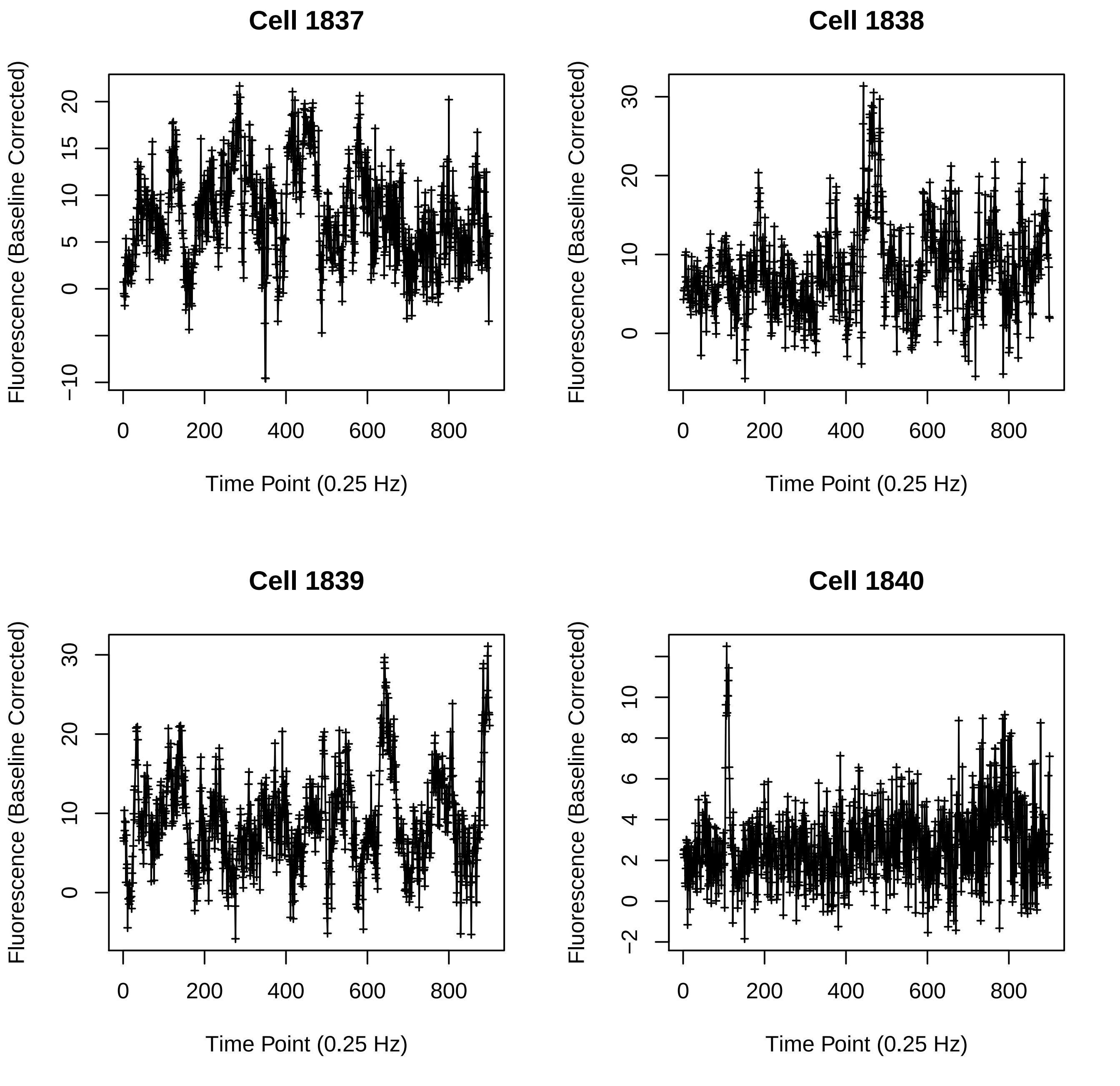
Cell 1837
Cell 1838
Cell 1839
Cell 1840
Fluorescence (Baseline Corrected)
Time Point (0.25 Hz)
0
200
400
600
800
−10
0
5
10
15
20
30
−2
2
4
6
8

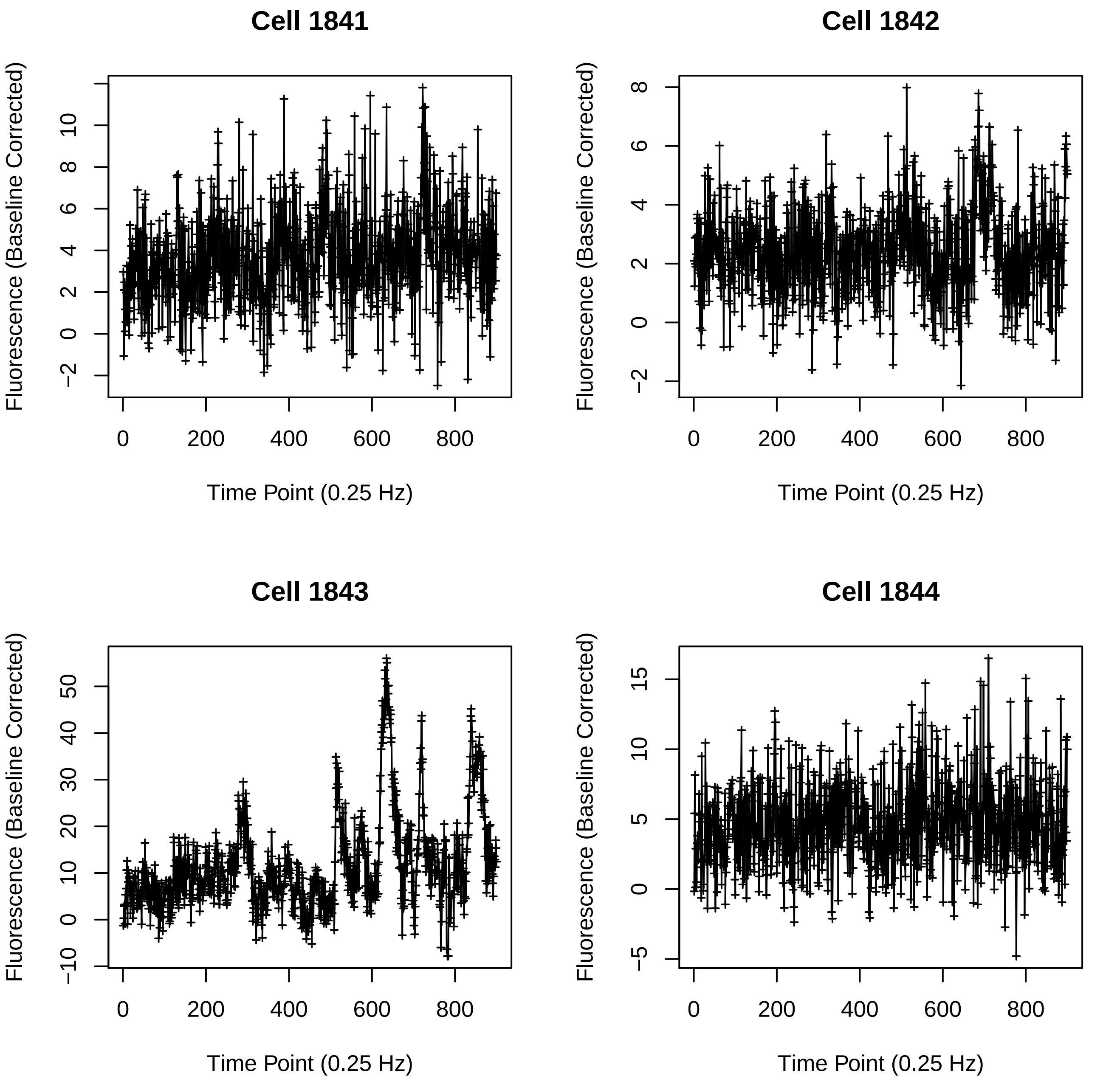
Cell 1841
Fluorescence (Baseline Corrected)
−2 0 2 4 6 8 10
0 200 400 600 800
Time Point (0.25 Hz)
Cell 1842
Fluorescence (Baseline Corrected)
−2 0 2 4 6 8
0 200 400 600 800
Time Point (0.25 Hz)
Cell 1843
Fluorescence (Baseline Corrected)
−10 0 10 20 30 40 50
0 200 400 600 800
Time Point (0.25 Hz)
Cell 1844
Fluorescence (Baseline Corrected)
−5 0 5 10 15
0 200 400 600 800
Time Point (0.25 Hz)

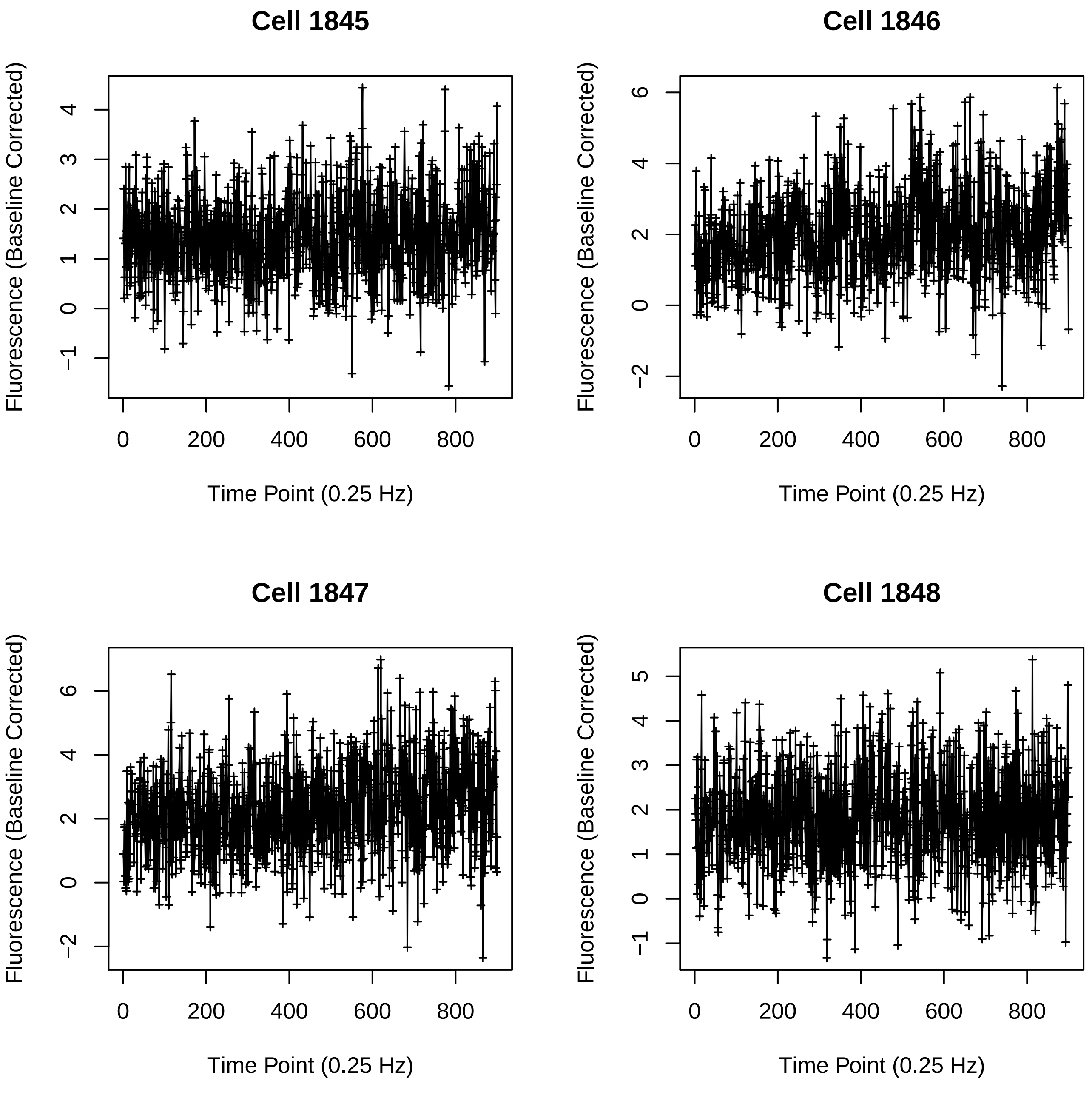
Cell 1845
Cell 1846
Cell 1847
Cell 1848
Fluorescence (Baseline Corrected)
Time Point (0.25 Hz)
0
200
400
600
800

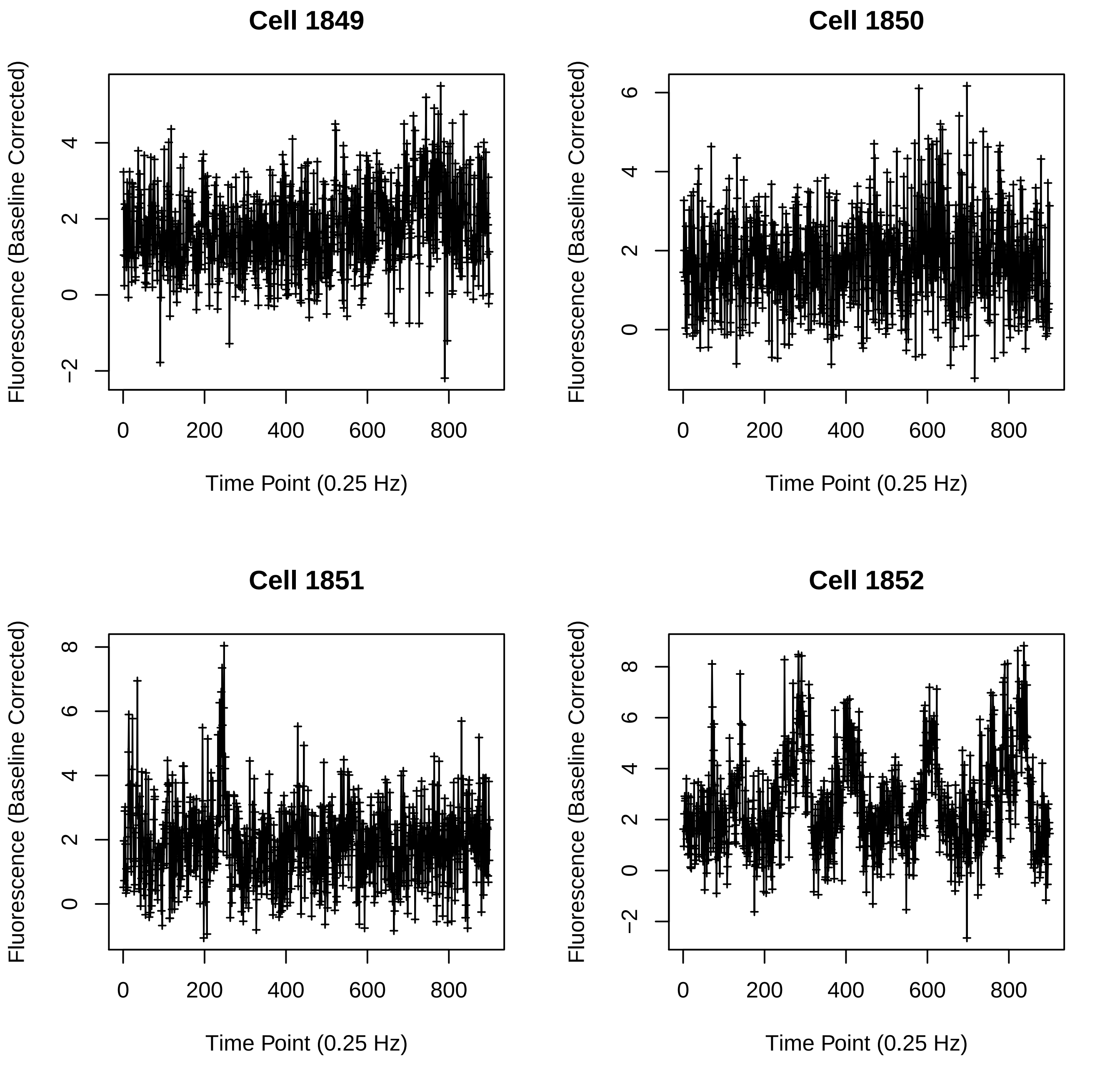
Cell 1849
Cell 1850
Cell 1851
Cell 1852
Fluorescence (Baseline Corrected)
Time Point (0.25 Hz)
0
200
400
600
800
−2
2
4
6
8

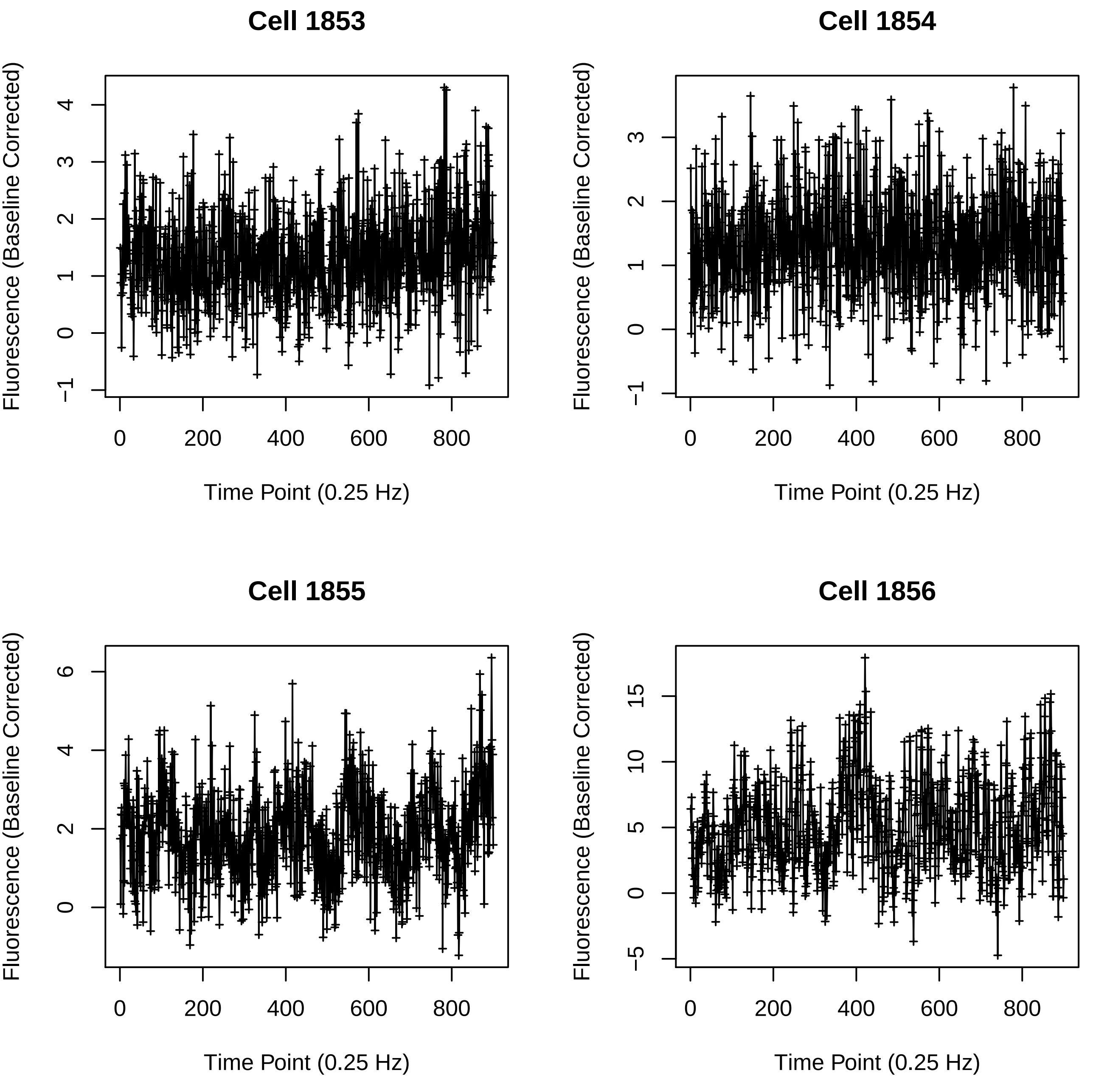

Cell 1853
Cell 1854
Cell 1855
Cell 1856
Fluorescence (Baseline Corrected)
Time Point (0.25 Hz)
−1
0
1
2
3
4
−5
5
10
15
6
0
200
400
600
800

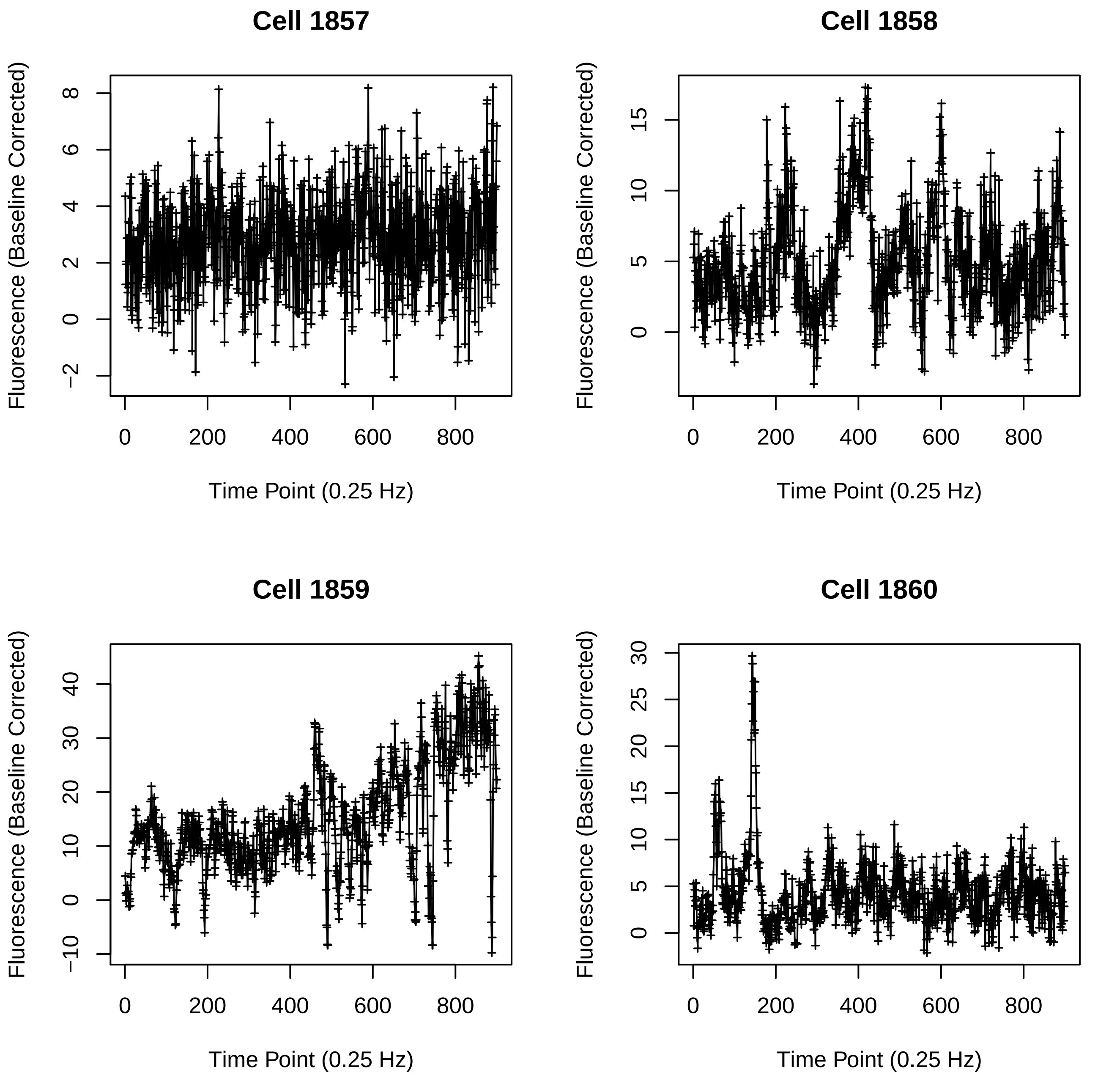

Cell 1857
Fluorescence (Baseline Corrected)
Time Point (0.25 Hz)
Cell 1858
Fluorescence (Baseline Corrected)
Time Point (0.25 Hz)
Cell 1859
Fluorescence (Baseline Corrected)
Time Point (0.25 Hz)
Cell 1860
Fluorescence (Baseline Corrected)
Time Point (0.25 Hz)

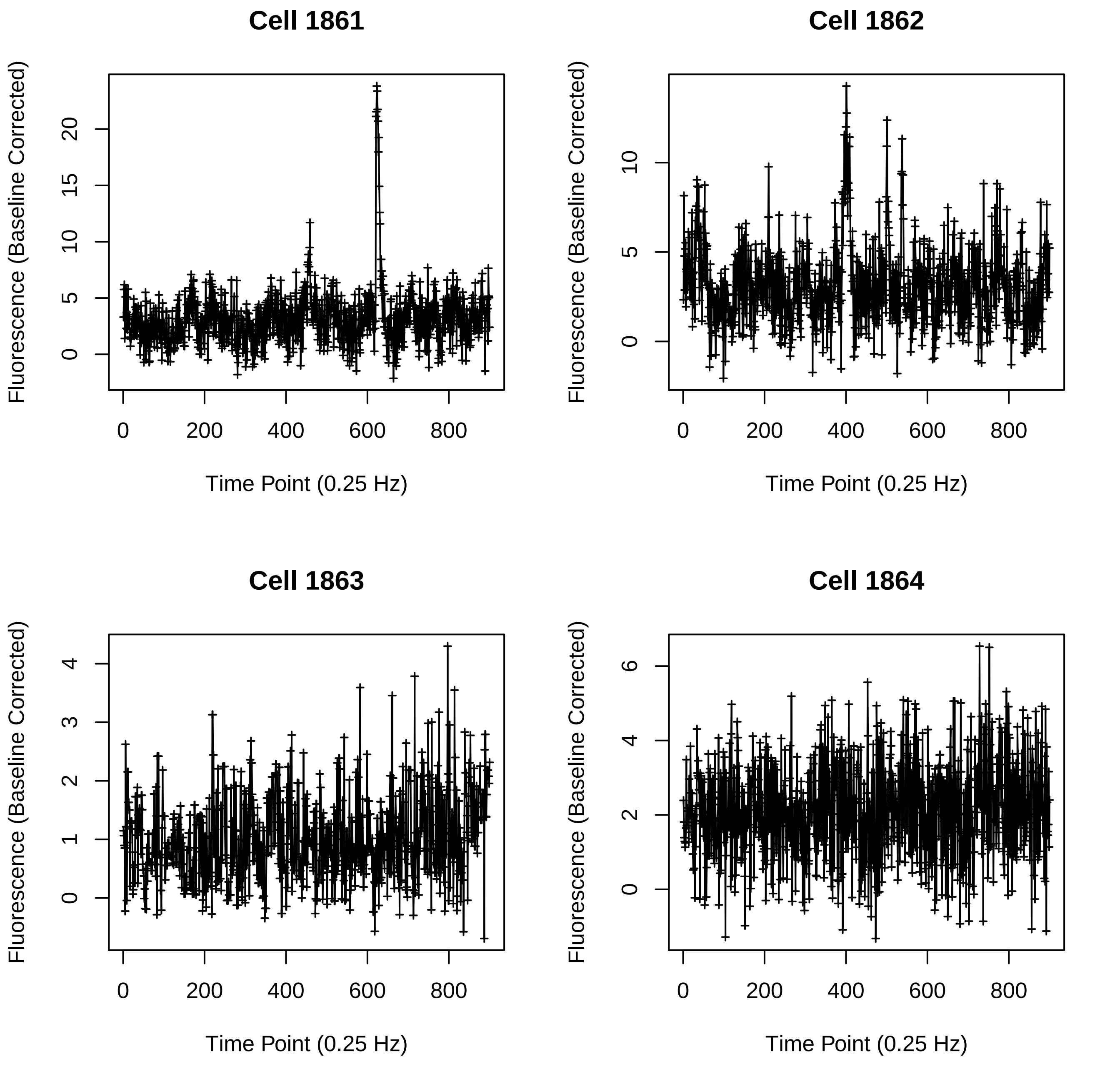
Cell 1861
Cell 1862
Cell 1863
Cell 1864
Fluorescence (Baseline Corrected)
Time Point (0.25 Hz)
0
5
10
15
20
200
400
600
800
1
2
3
4
6

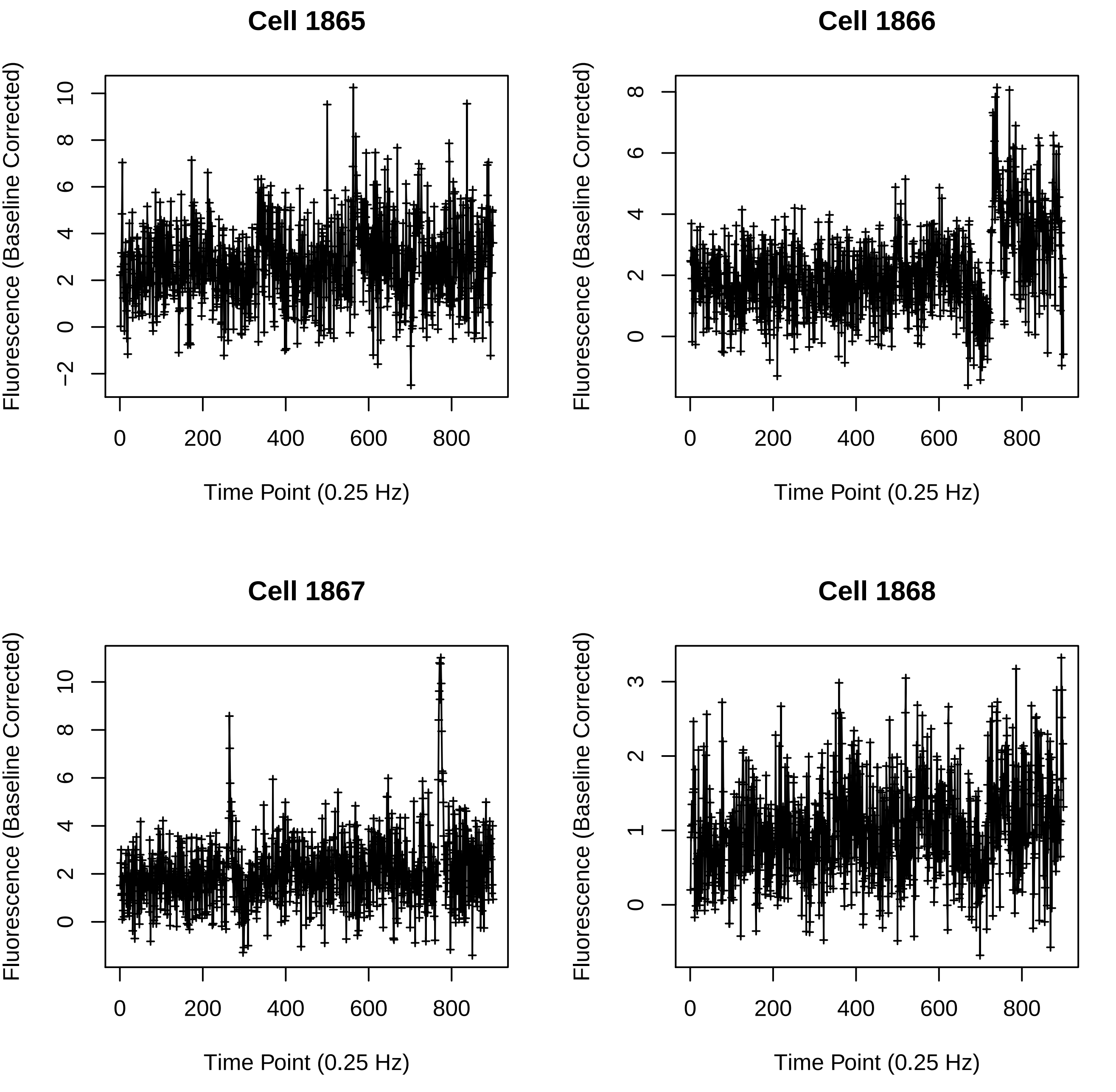
Cell 1865
Cell 1866
Cell 1867
Cell 1868
Fluorescence (Baseline Corrected)
Time Point (0.25 Hz)
0
200
400
600
800
−2
2
4
6
8
10

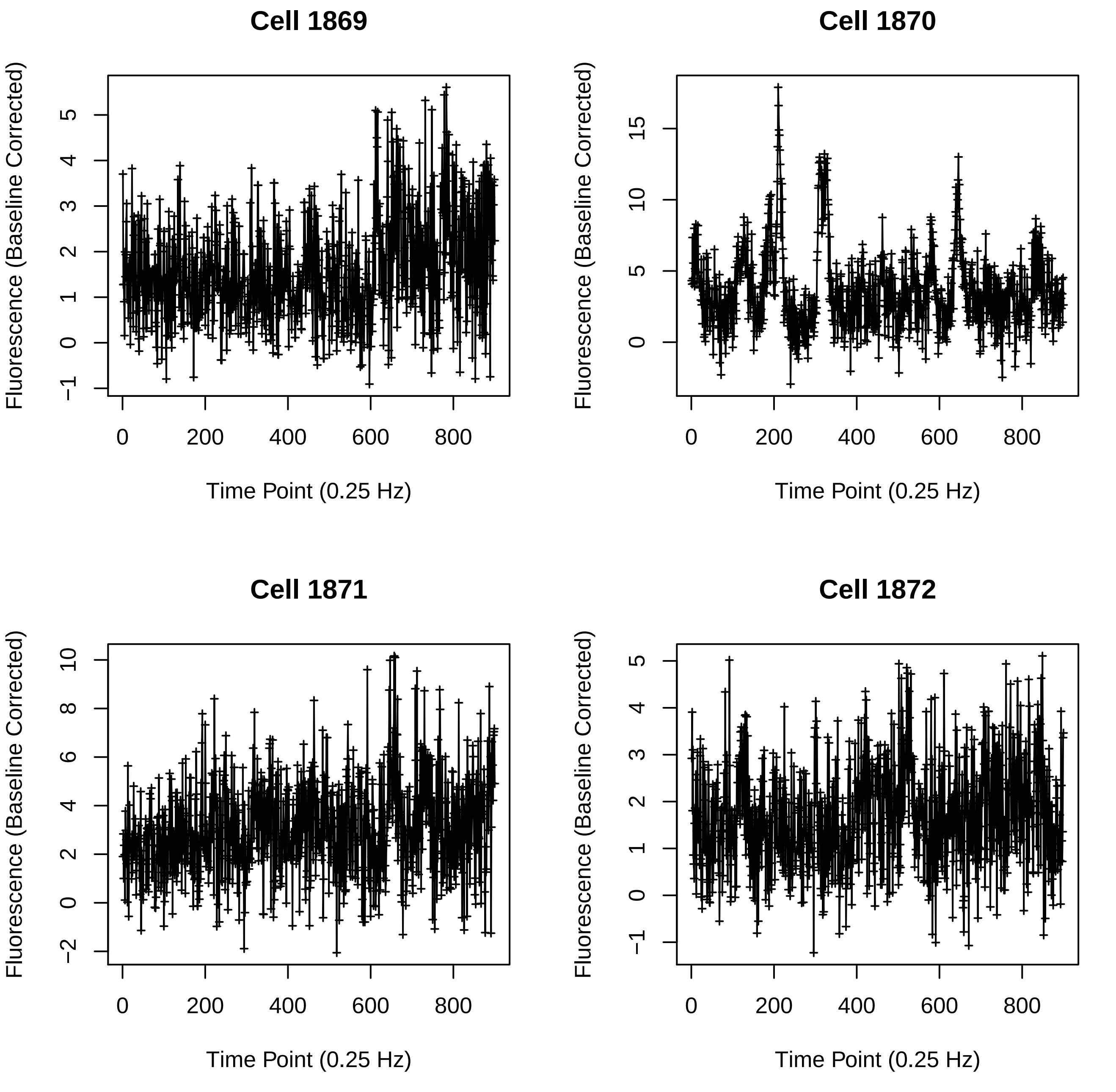
Cell 1869
Cell 1870
Cell 1871
Cell 1872
Fluorescence (Baseline Corrected)
Time Point (0.25 Hz)
0
200
400
600
800
−2
−1
0
1
2
3
4
5
6
8
10
15

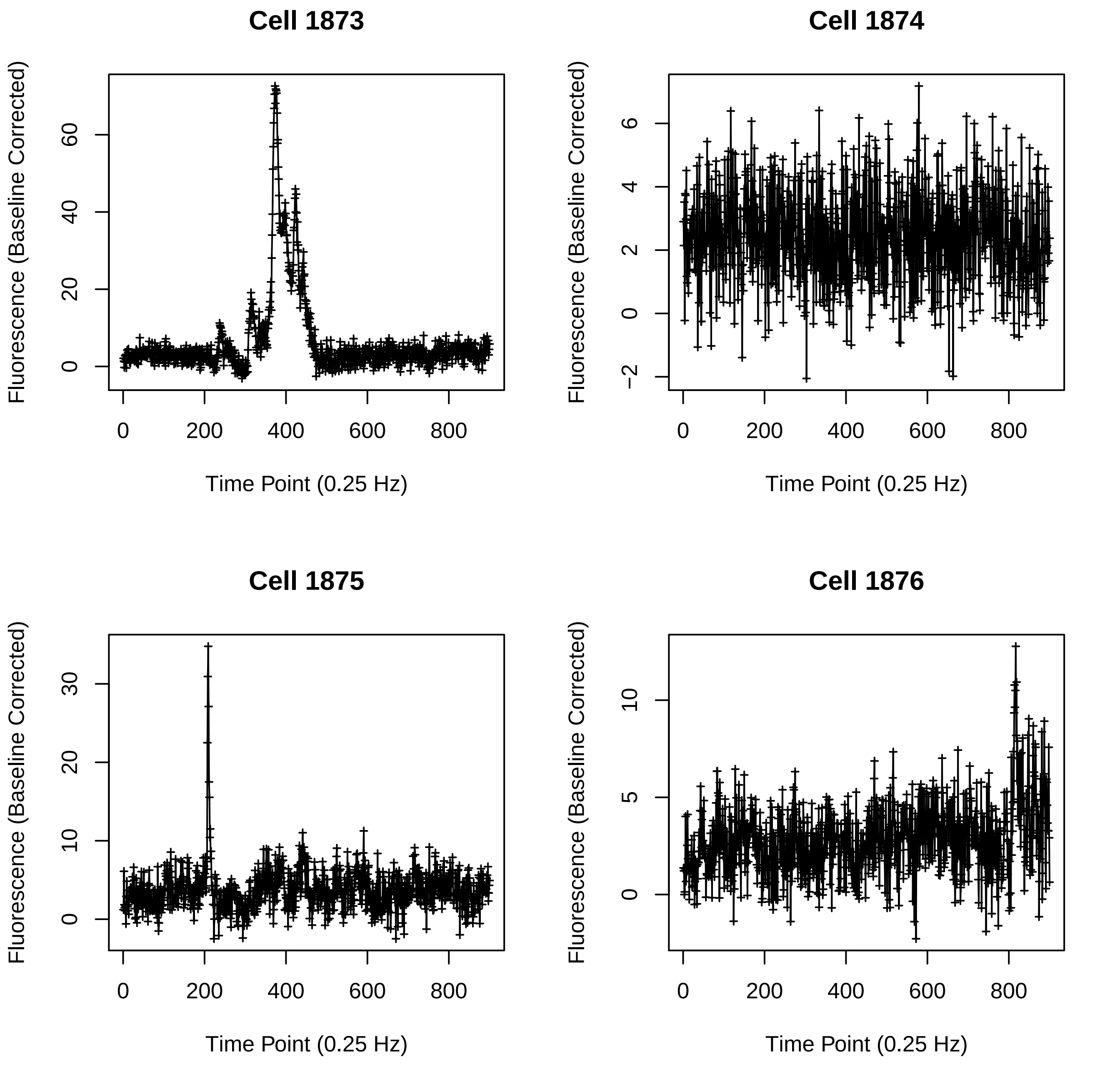

Cell 1873
Cell 1874
Cell 1875
Cell 1876
Fluorescence (Baseline Corrected)
Time Point (0.25 Hz)
0
200
400
600
800
0
20
40
60
−2
2
4
6
10
30
5

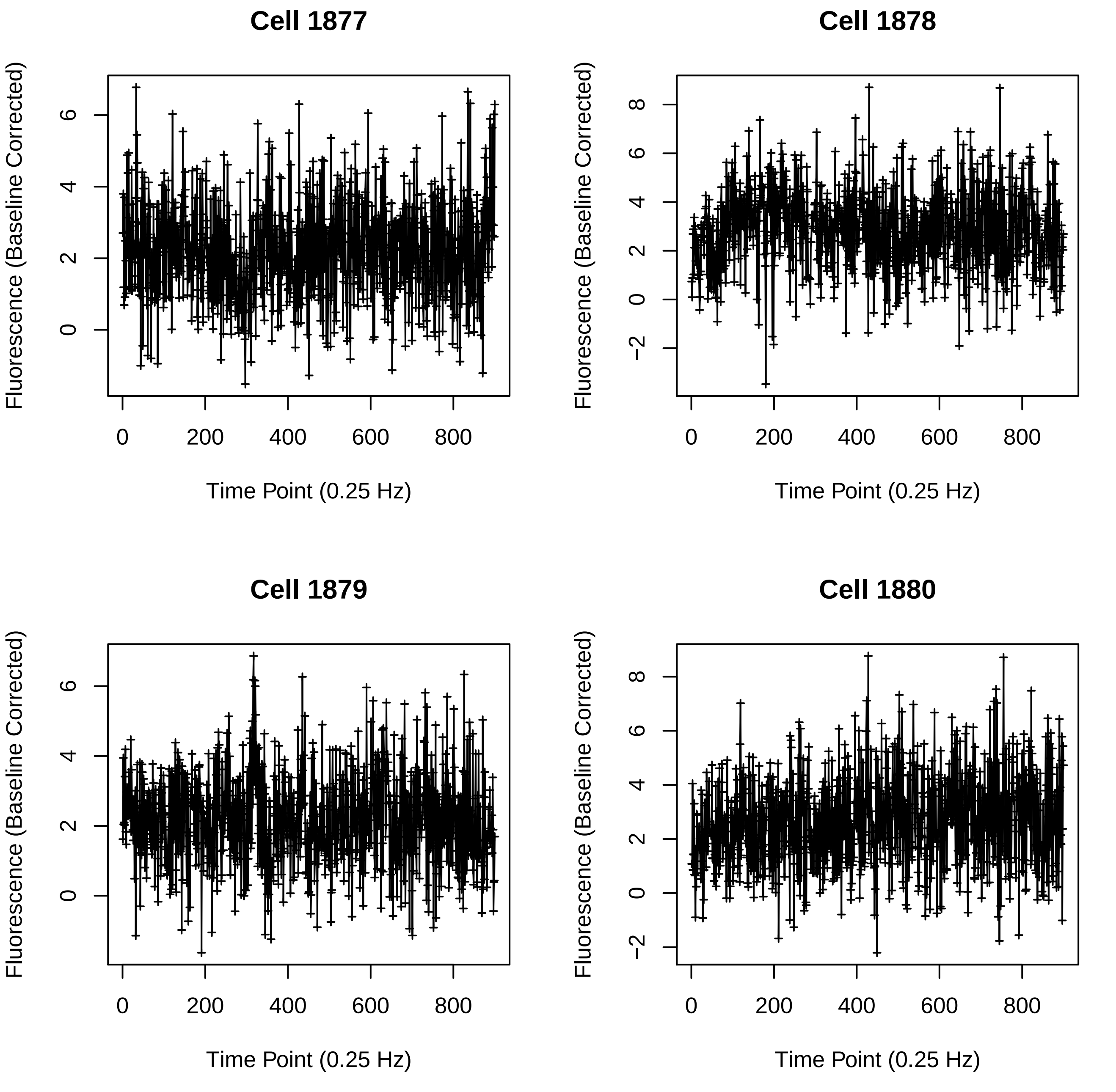
Cell 1877
Cell 1878
Cell 1879
Cell 1880
Fluorescence (Baseline Corrected)
Time Point (0.25 Hz)
0
200
400
600
800
−2
2
4
6
8

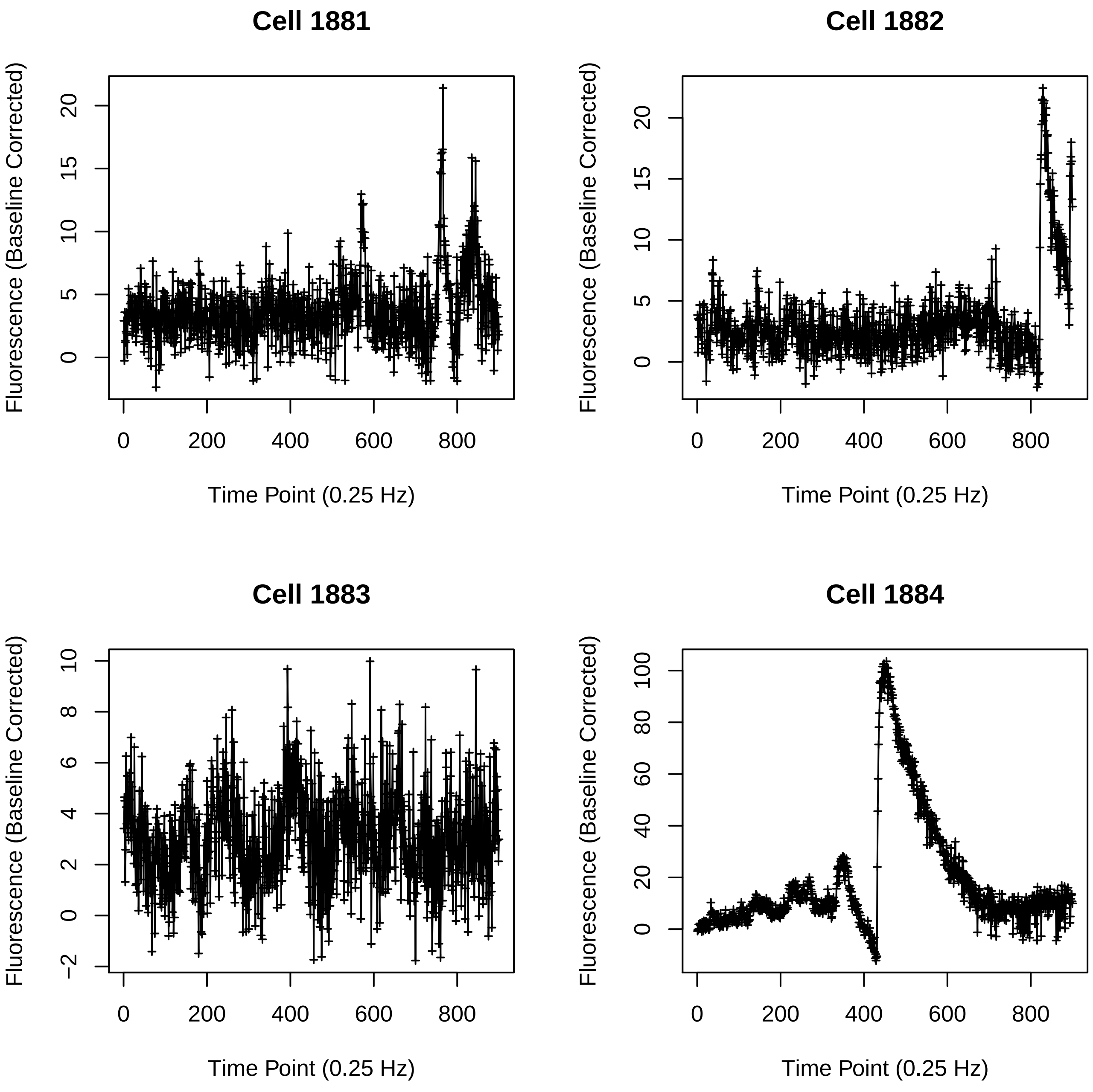

Cell 1881
Cell 1882
Cell 1883
Cell 1884
Fluorescence (Baseline Corrected)
Time Point (0.25 Hz)

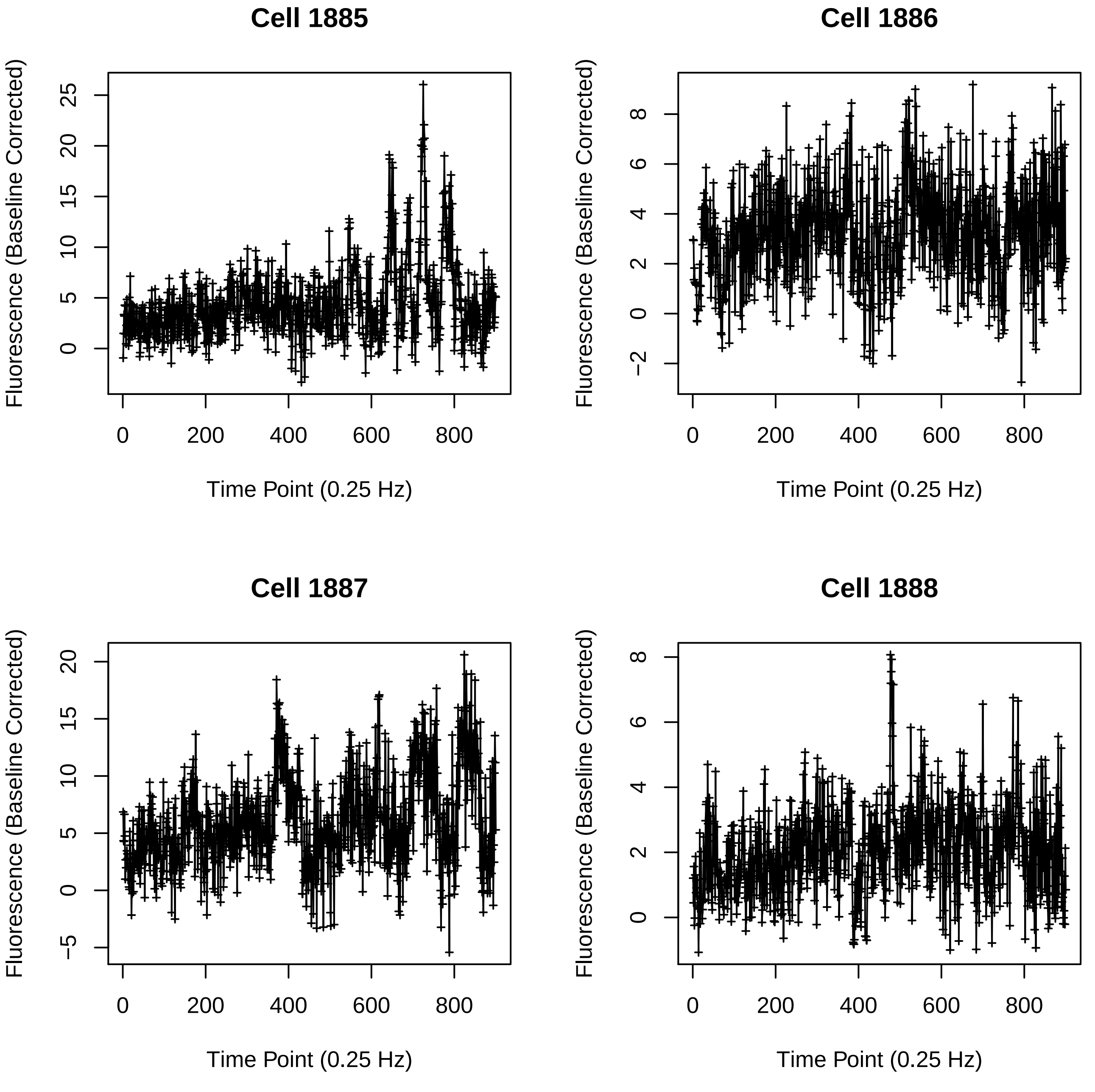
Cell 1885
Cell 1886
Cell 1887
Cell 1888
Fluorescence (Baseline Corrected)
Time Point (0.25 Hz)
0
200
400
600
800
−5
0
5
10
15
20
25
−2
2
4
6
8

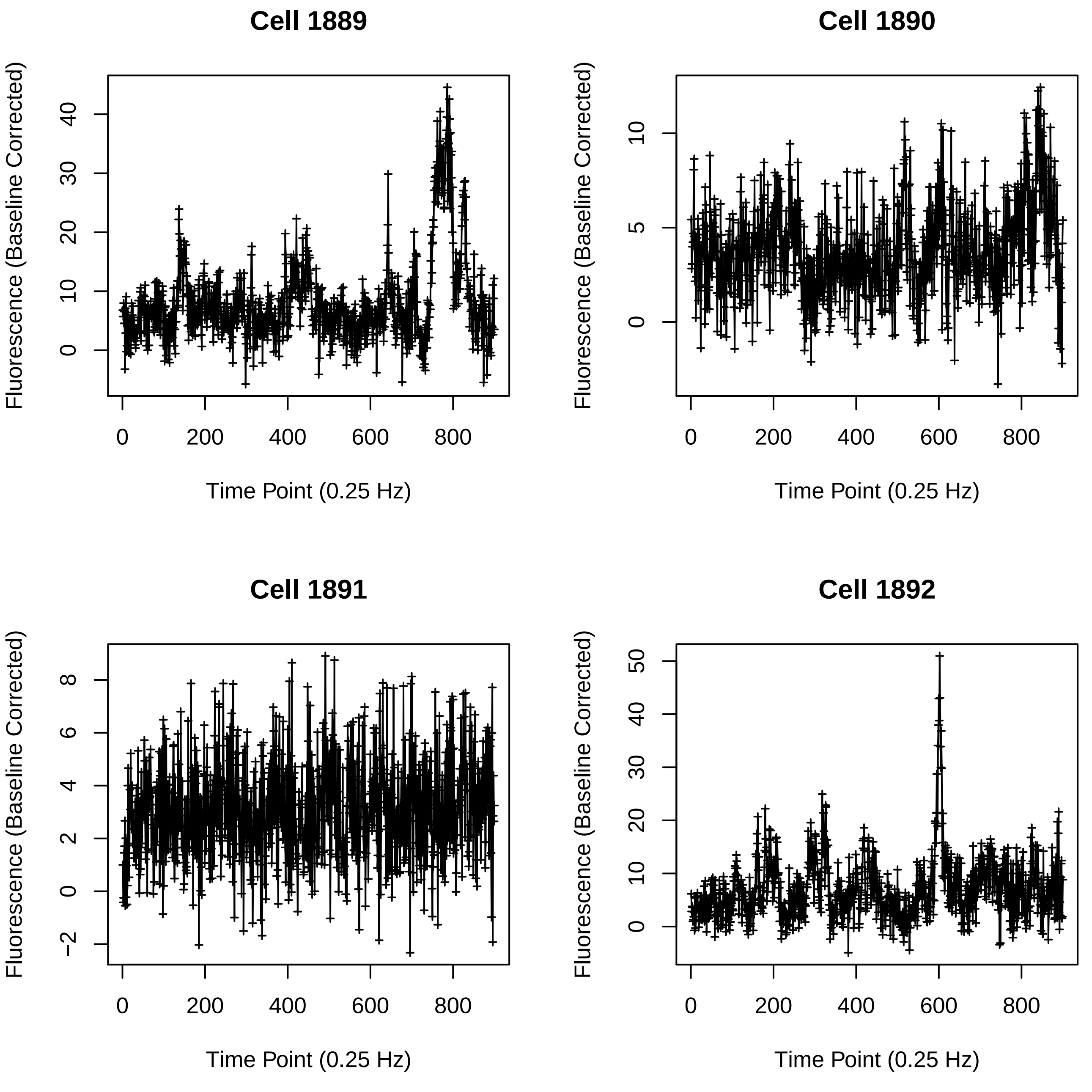
Cell 1889
Cell 1890
Cell 1891
Cell 1892
Fluorescence (Baseline Corrected)
Time Point (0.25 Hz)
0
10
20
30
40
50
5
−2
2
4
6
8
200
400
600
800

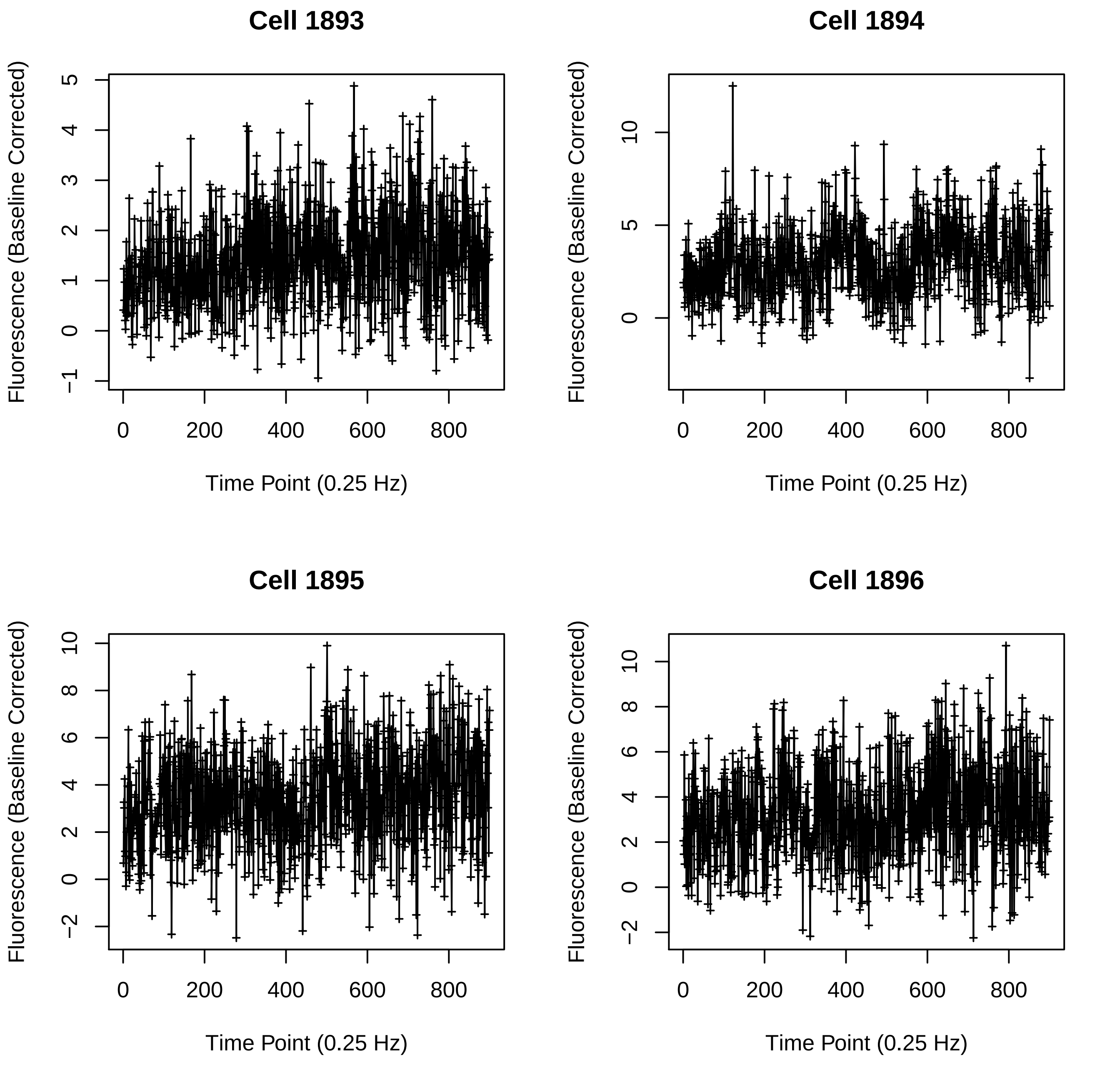
Cell 1893
Cell 1894
Cell 1895
Cell 1896
Fluorescence (Baseline Corrected)
Time Point (0.25 Hz)
0
200
400
600
800
−1
1
2
3
4
5
10
8
6
−2

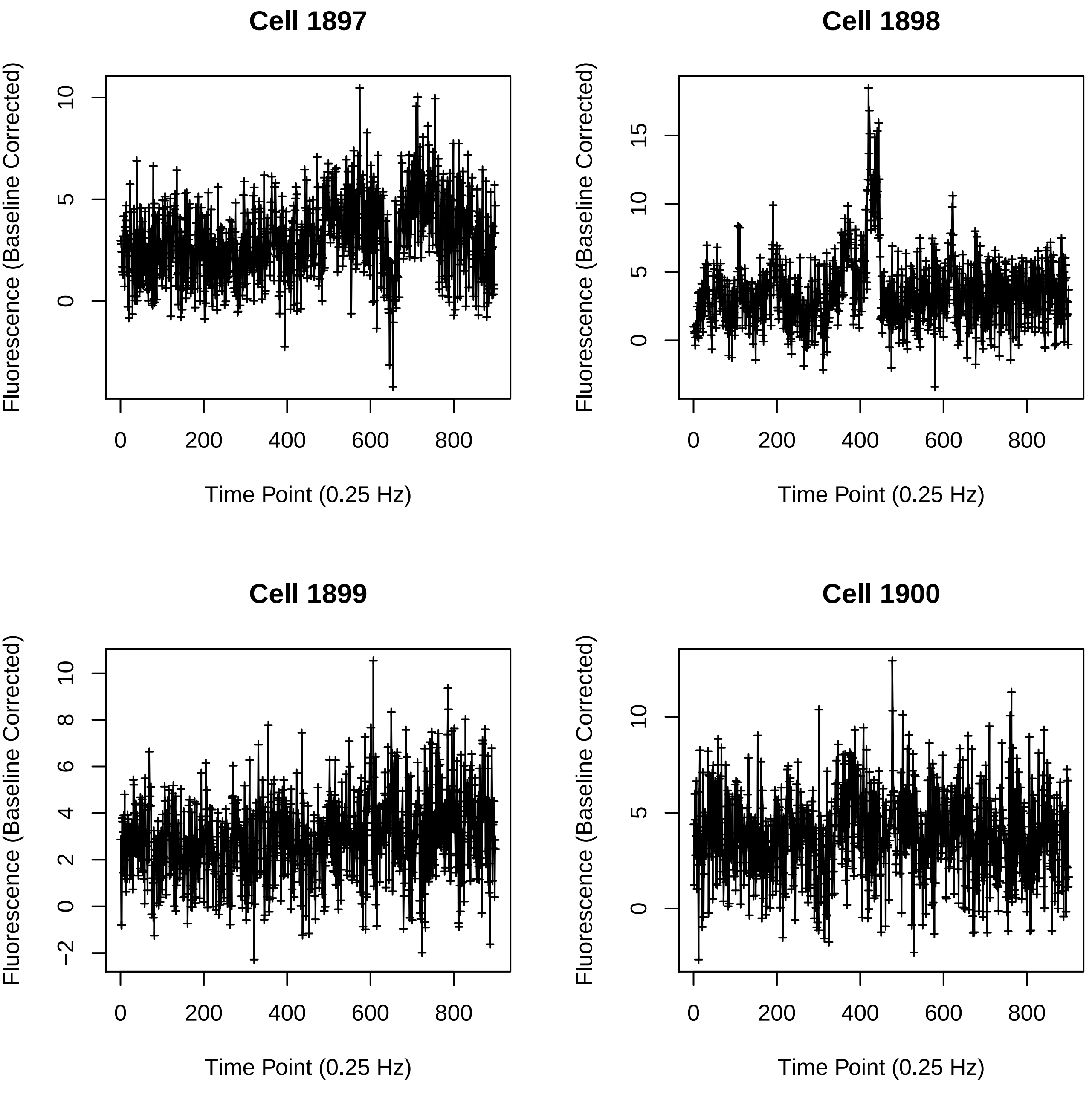
Cell 1897
Cell 1898
Cell 1899
Cell 1900
Fluorescence (Baseline Corrected)
Time Point (0.25 Hz)
0
200
400
600
800
10
5
0
15
8
6
4
2
−2

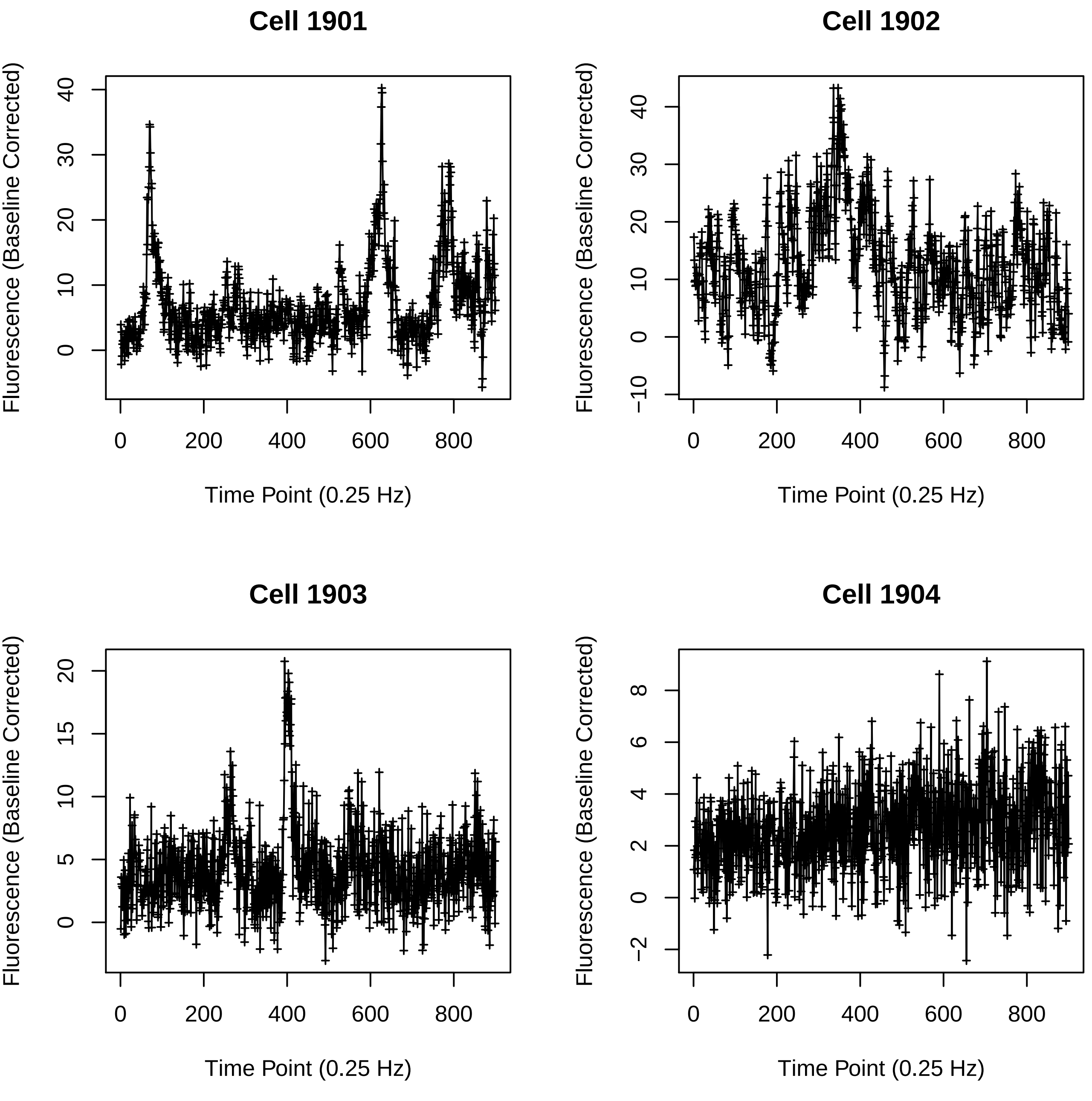

Cell 1901
Cell 1902
Cell 1903
Cell 1904
Fluorescence (Baseline Corrected)
Time Point (0.25 Hz)
0
200
400
600
800
-10
-2
0
2
4
5
6
8
10
15
20
30
40

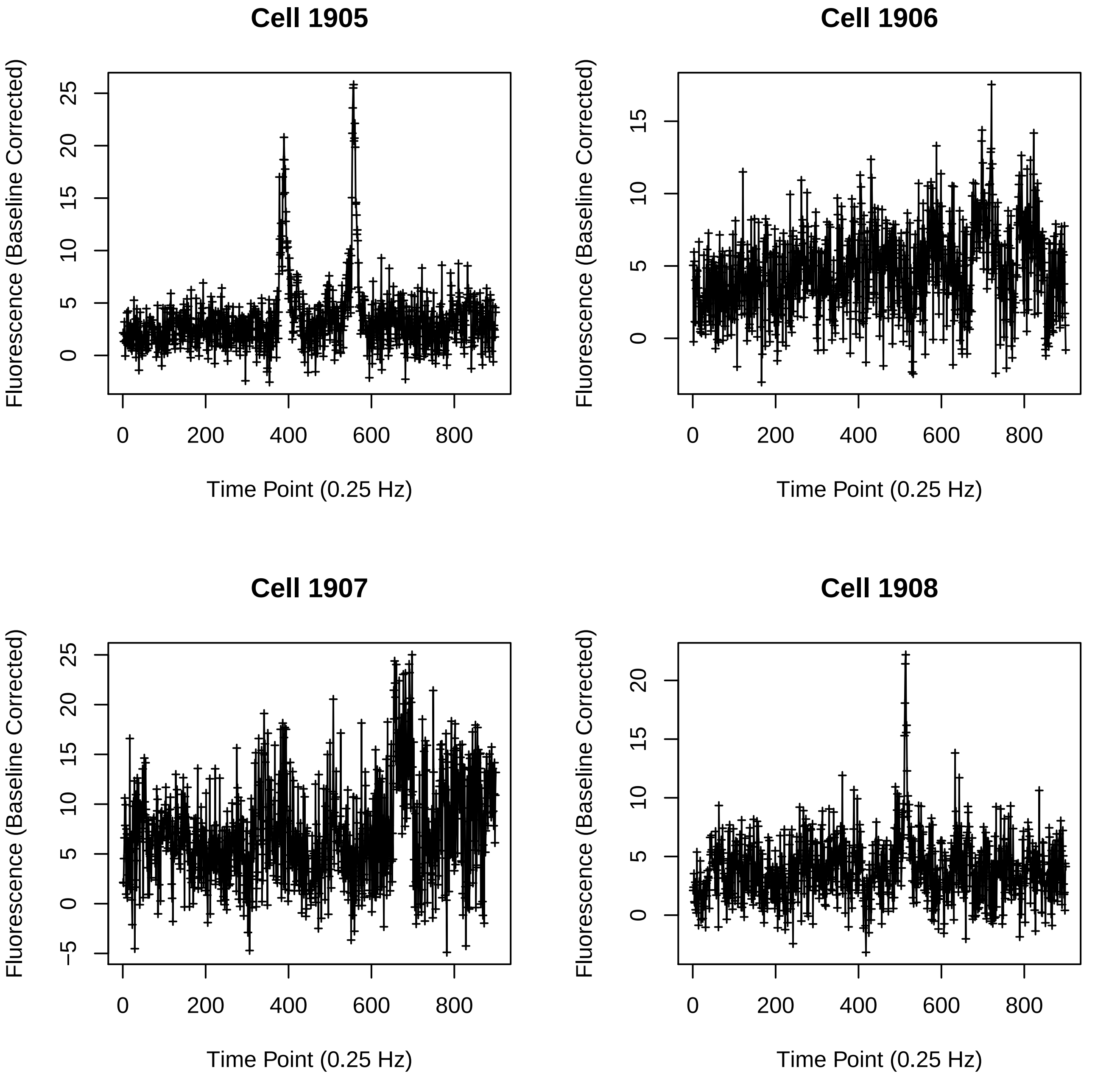

Cell 1905
Fluorescence (Baseline Corrected)
0
5
10
15
20
25
0
200
400
600
800
Time Point (0.25 Hz)
Cell 1906
Fluorescence (Baseline Corrected)
0
5
10
15
0
200
400
600
800
Time Point (0.25 Hz)
Cell 1907
Fluorescence (Baseline Corrected)
−5
0
5
10
15
20
25
0
200
400
600
800
Time Point (0.25 Hz)
Cell 1908
Fluorescence (Baseline Corrected)
0
5
10
15
20
0
200
400
600
800
Time Point (0.25 Hz)

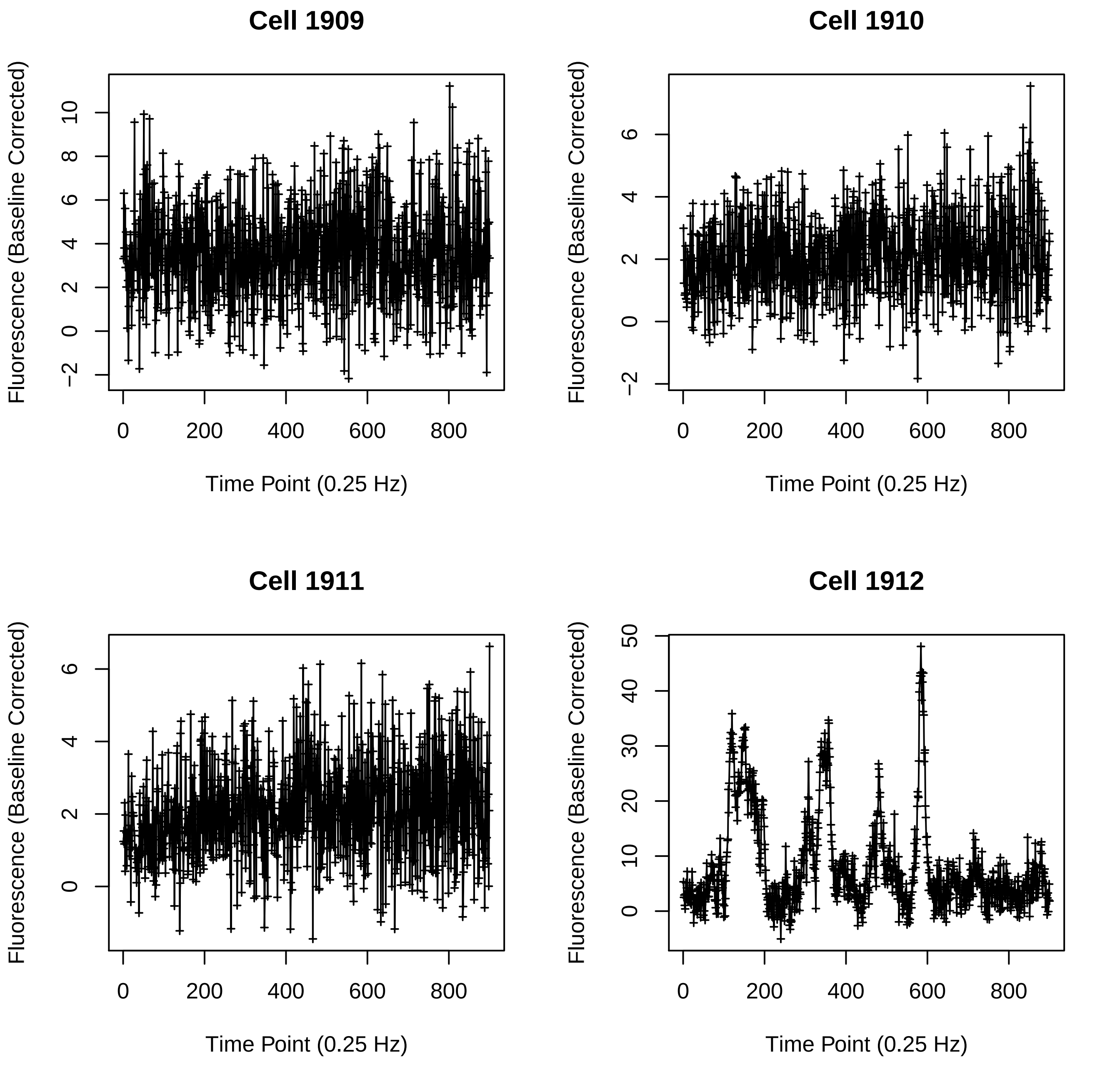
Cell 1909
Cell 1910
Cell 1911
Cell 1912
Fluorescence (Baseline Corrected)
Time Point (0.25 Hz)
0
200
400
600
800
−2
2
4
6
8
10
10
20
30
40
50

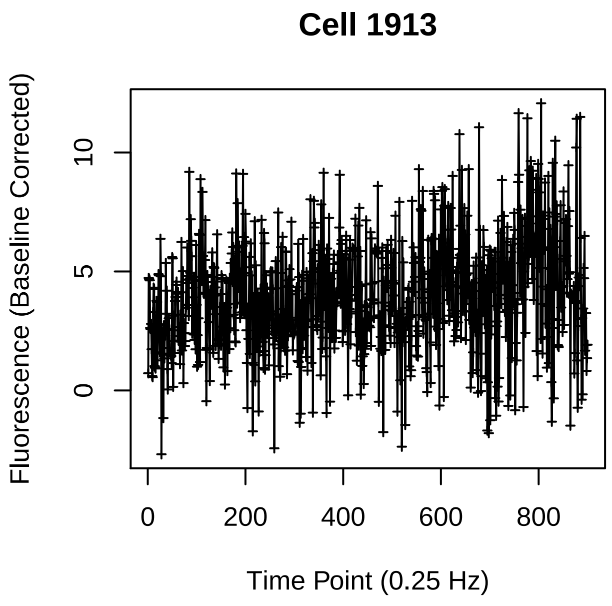
Cell 1913
Fluorescence (Baseline Corrected)
0
5
10
0
200
400
600
800
Time Point (0.25 Hz)

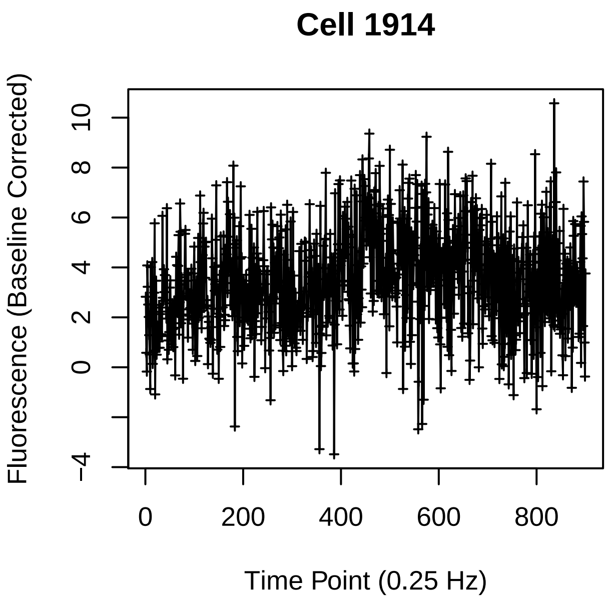
Cell 1914
Fluorescence (Baseline Corrected)
−4
0
2
4
6
8
10
0
200
400
600
800
Time Point (0.25 Hz)

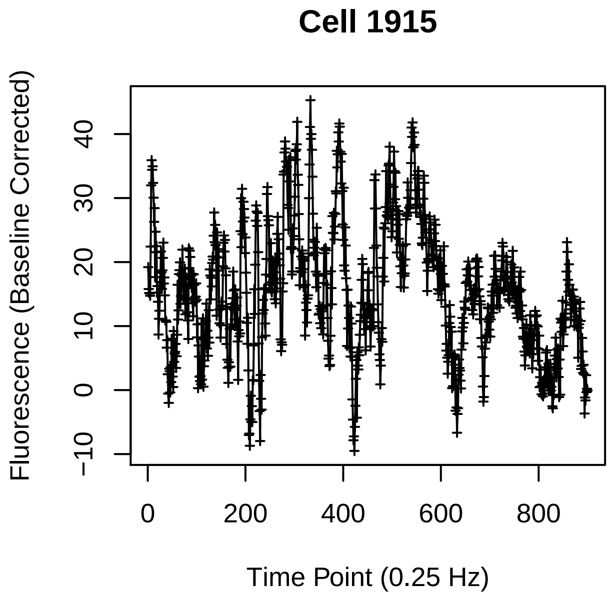
Cell 1915
Fluorescence (Baseline Corrected)
−10
0
10
20
30
40
0
200
400
600
800
Time Point (0.25 Hz)

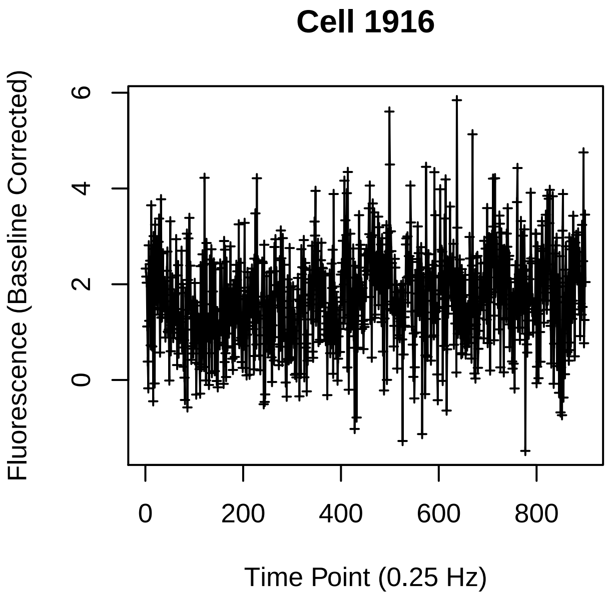
Cell 1916
Fluorescence (Baseline Corrected)
0
2
4
6
0
200
400
600
800
Time Point (0.25 Hz)

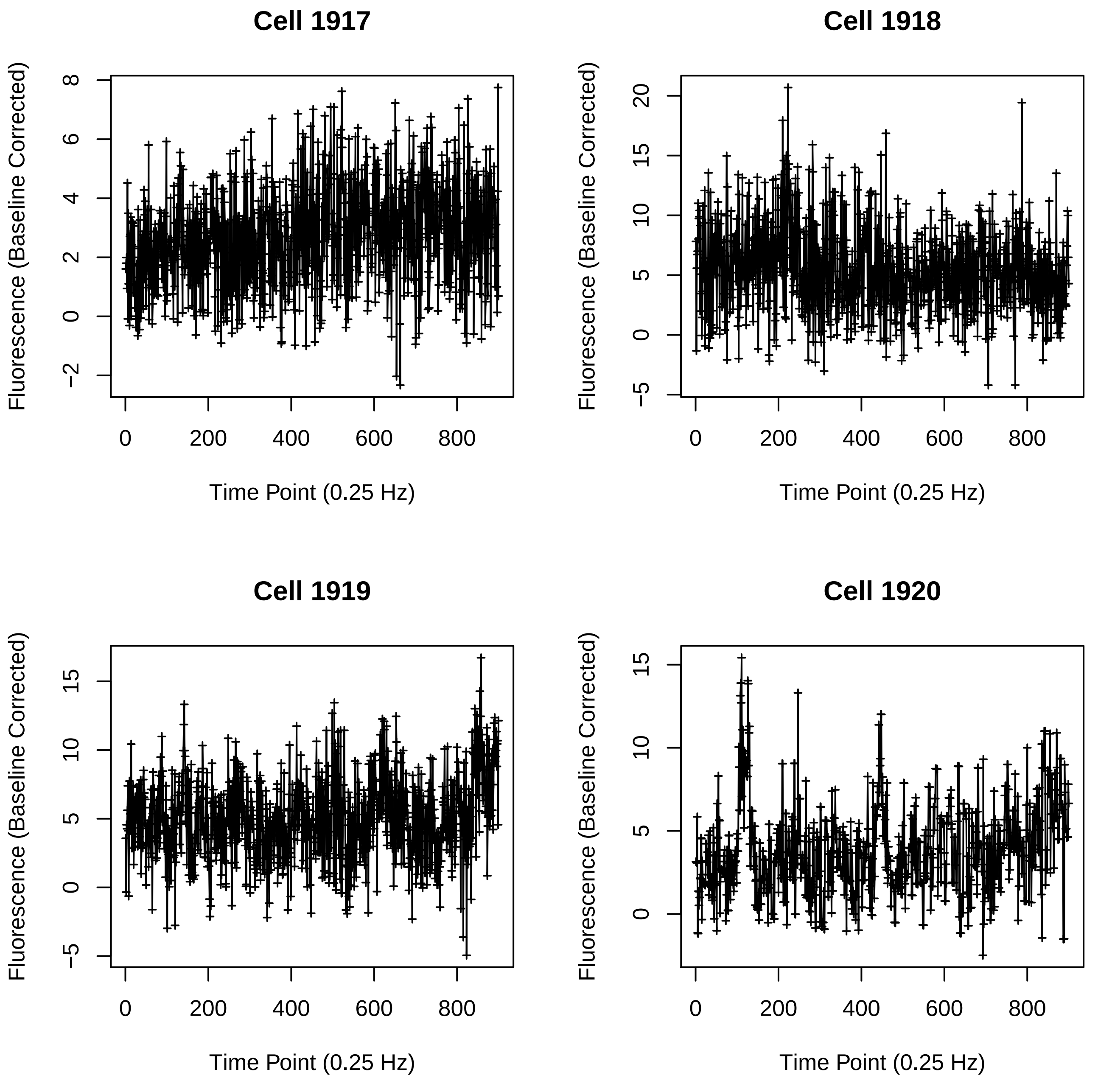
Cell 1917
Fluorescence (Baseline Corrected)
−2
0
2
4
6
8
0
200
400
600
800
Time Point (0.25 Hz)
Cell 1918
Fluorescence (Baseline Corrected)
−5
0
5
10
15
20
0
200
400
600
800
Time Point (0.25 Hz)
Cell 1919
Fluorescence (Baseline Corrected)
−5
0
5
10
15
0
200
400
600
800
Time Point (0.25 Hz)
Cell 1920
Fluorescence (Baseline Corrected)
0
5
10
15
0
200
400
600
800
Time Point (0.25 Hz)

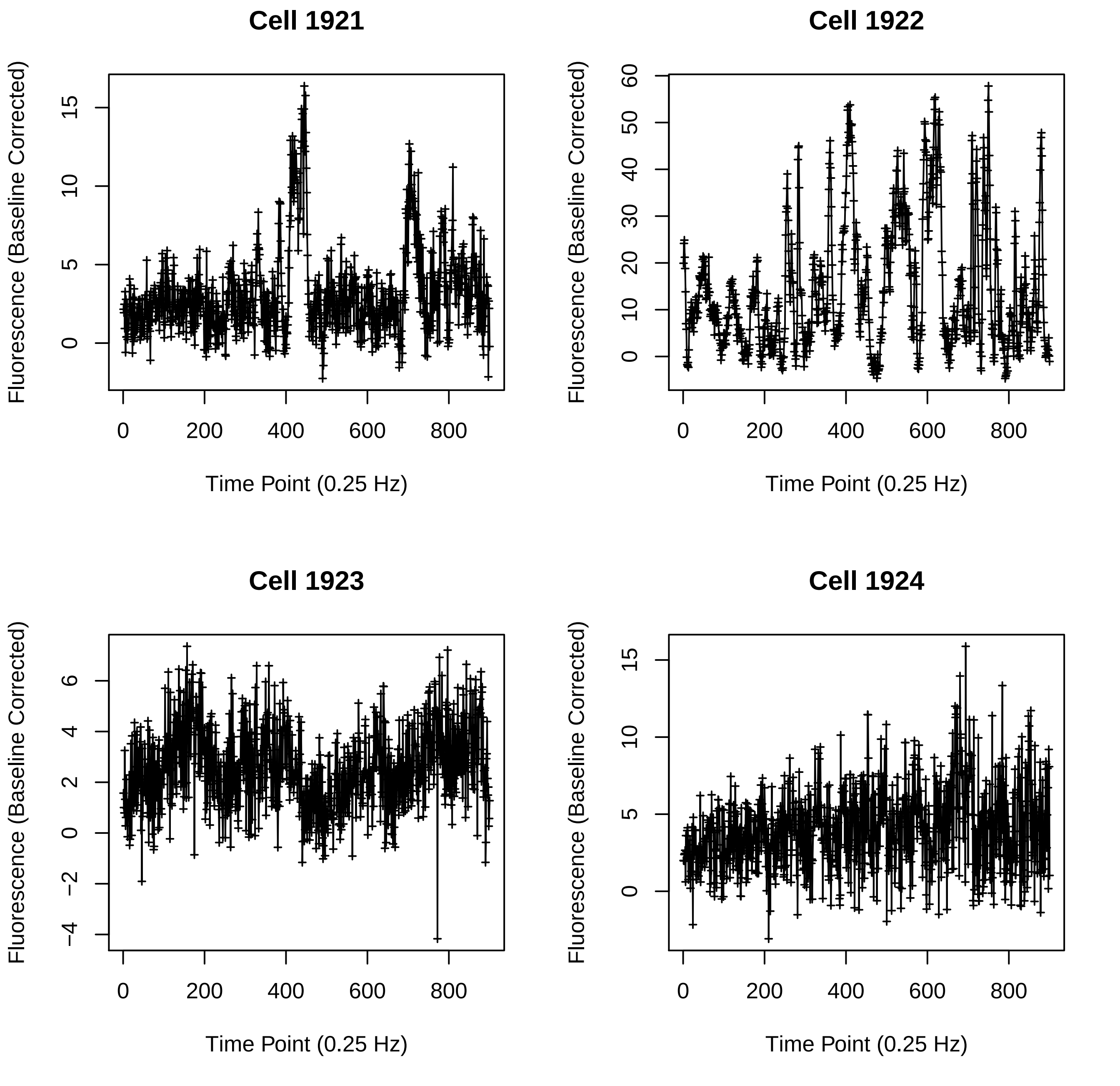
Cell 1921
Cell 1922
Cell 1923
Cell 1924
Fluorescence (Baseline Corrected)
Time Point (0.25 Hz)
0
5
10
15
10
20
30
40
50
60
−4
−2
2
4
6
200
400
600
800

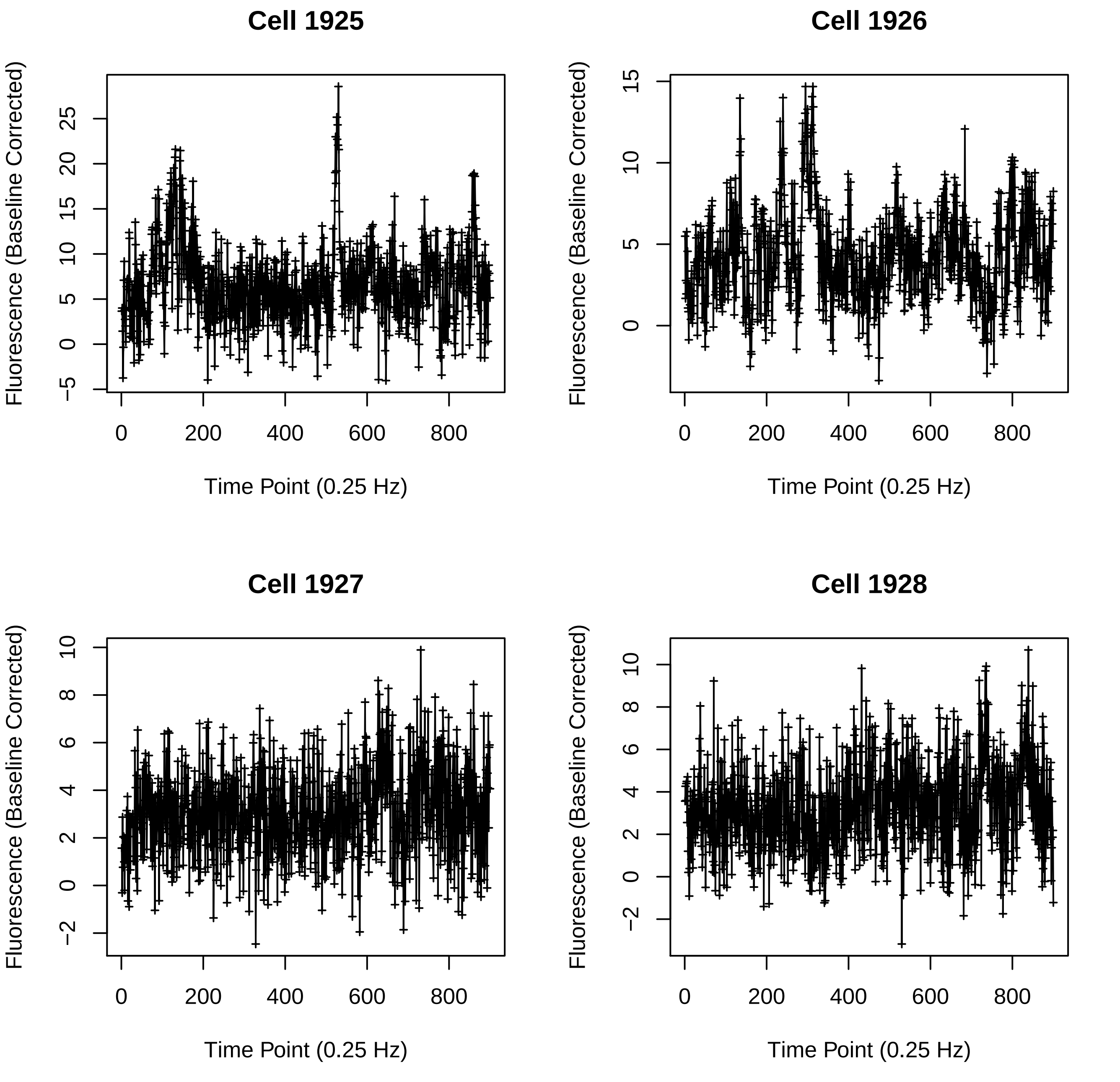
Cell 1925
Cell 1926
Cell 1927
Cell 1928
Fluorescence (Baseline Corrected)
Time Point (0.25 Hz)
0
200
400
600
800
−5
0
5
10
15
20
25
−2
2
4
6
8

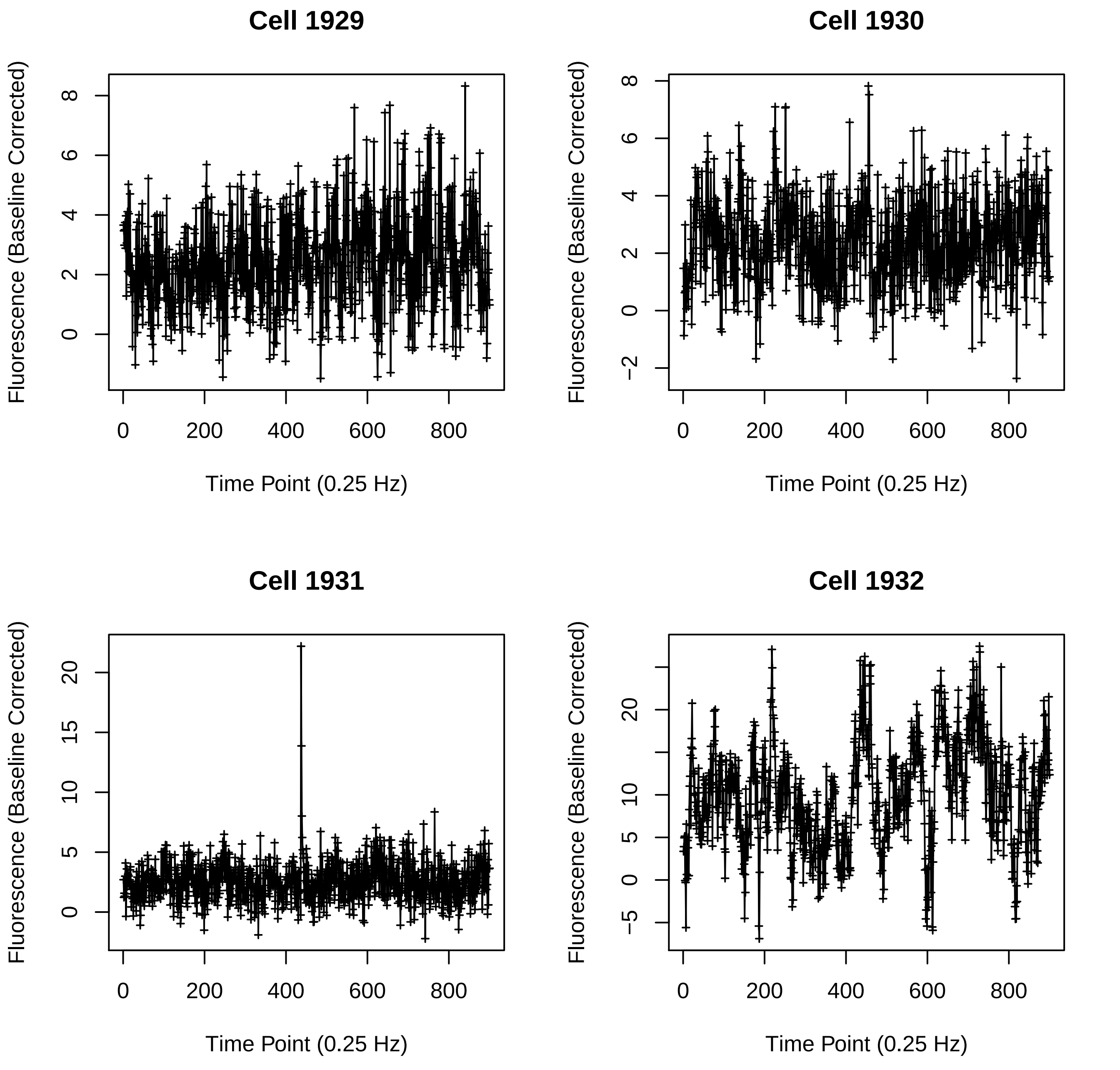

Cell 1929
Cell 1930
Cell 1931
Cell 1932
Fluorescence (Baseline Corrected)
Time Point (0.25 Hz)
0
200
400
600
800
−2
0
2
4
6
8
−5
5
10
15
20

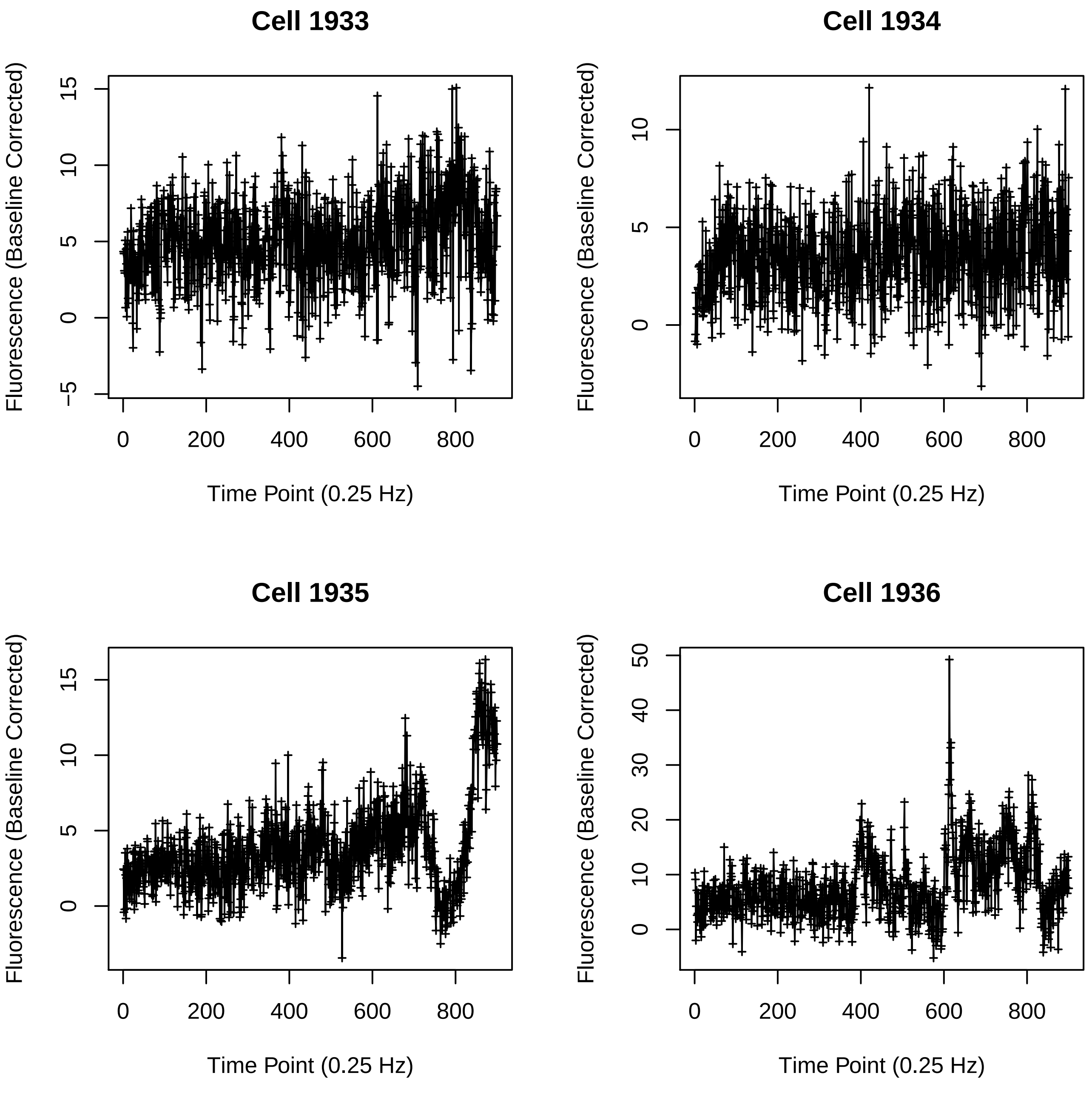

Cell 1933
Cell 1934
Cell 1935
Cell 1936
Fluorescence (Baseline Corrected)
Time Point (0.25 Hz)
0
200
400
600
800
−5
0
5
10
15
20
30
40
50

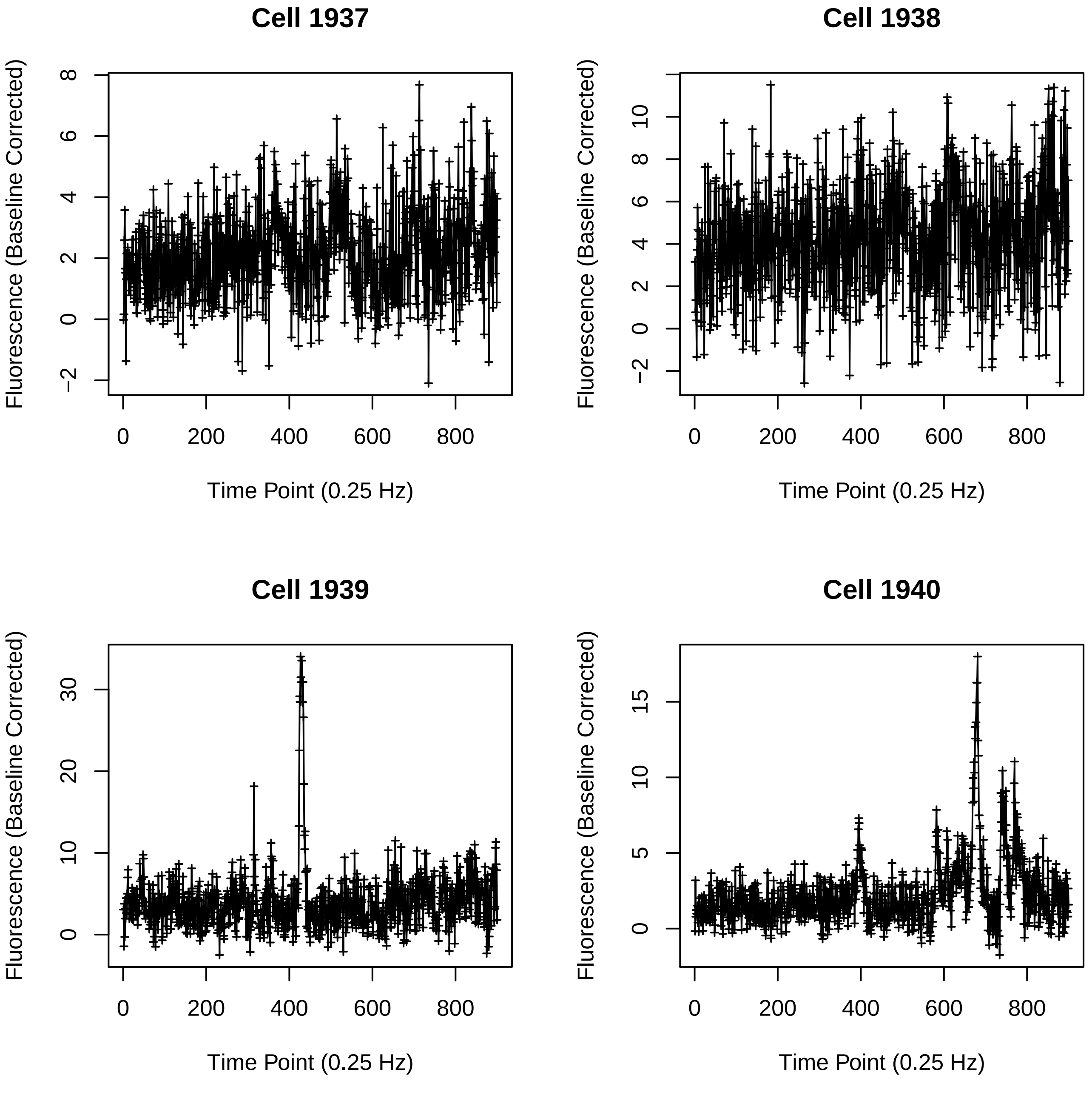
Cell 1937
Cell 1938
Cell 1939
Cell 1940
Fluorescence (Baseline Corrected)
Time Point (0.25 Hz)
0
200
400
600
800
−2
2
4
6
8
10
15
20
30

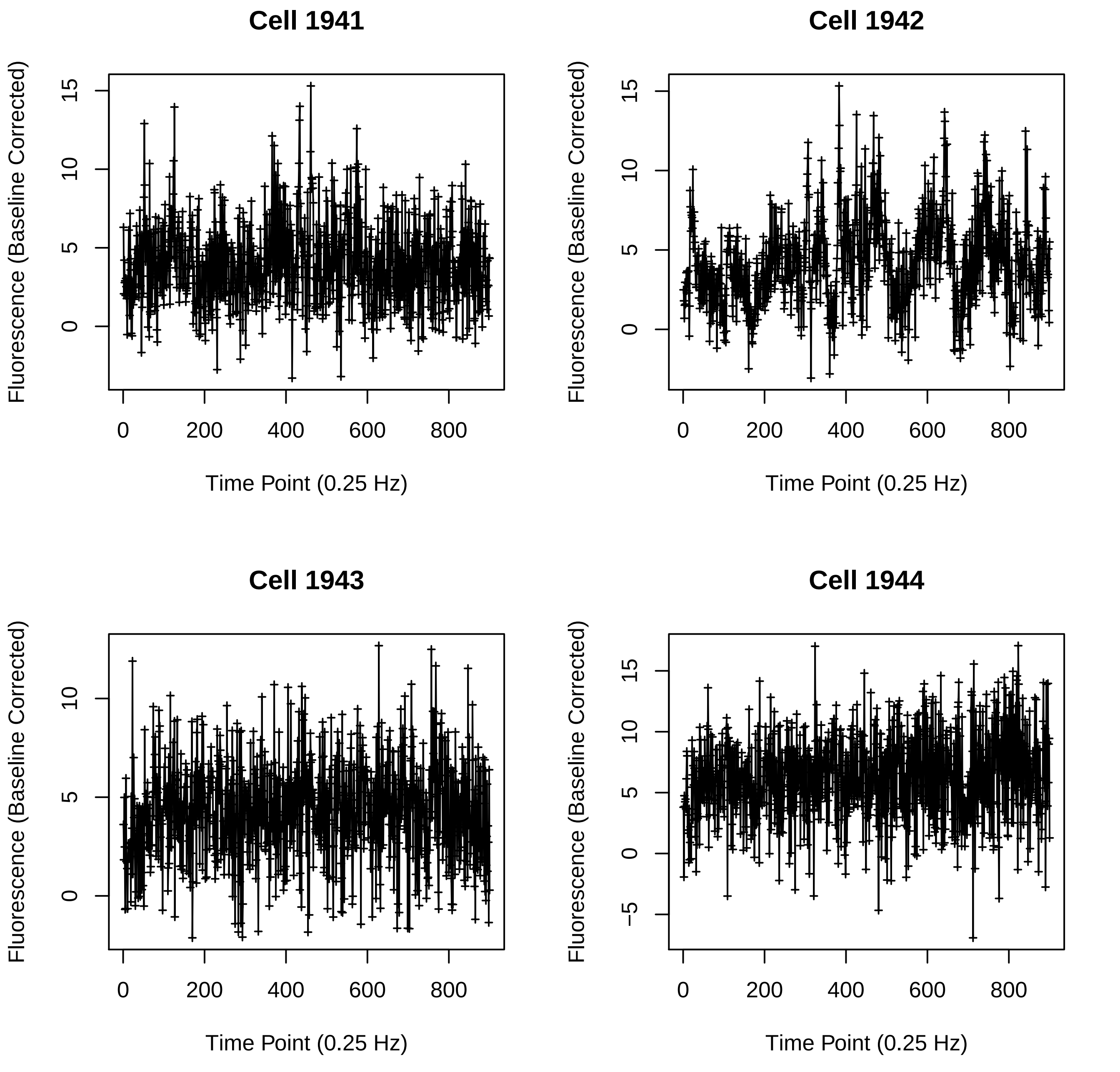
Cell 1941
Cell 1942
Cell 1943
Cell 1944
Fluorescence (Baseline Corrected)
Time Point (0.25 Hz)
0
200
400
600
800
−5
0
5
10
15

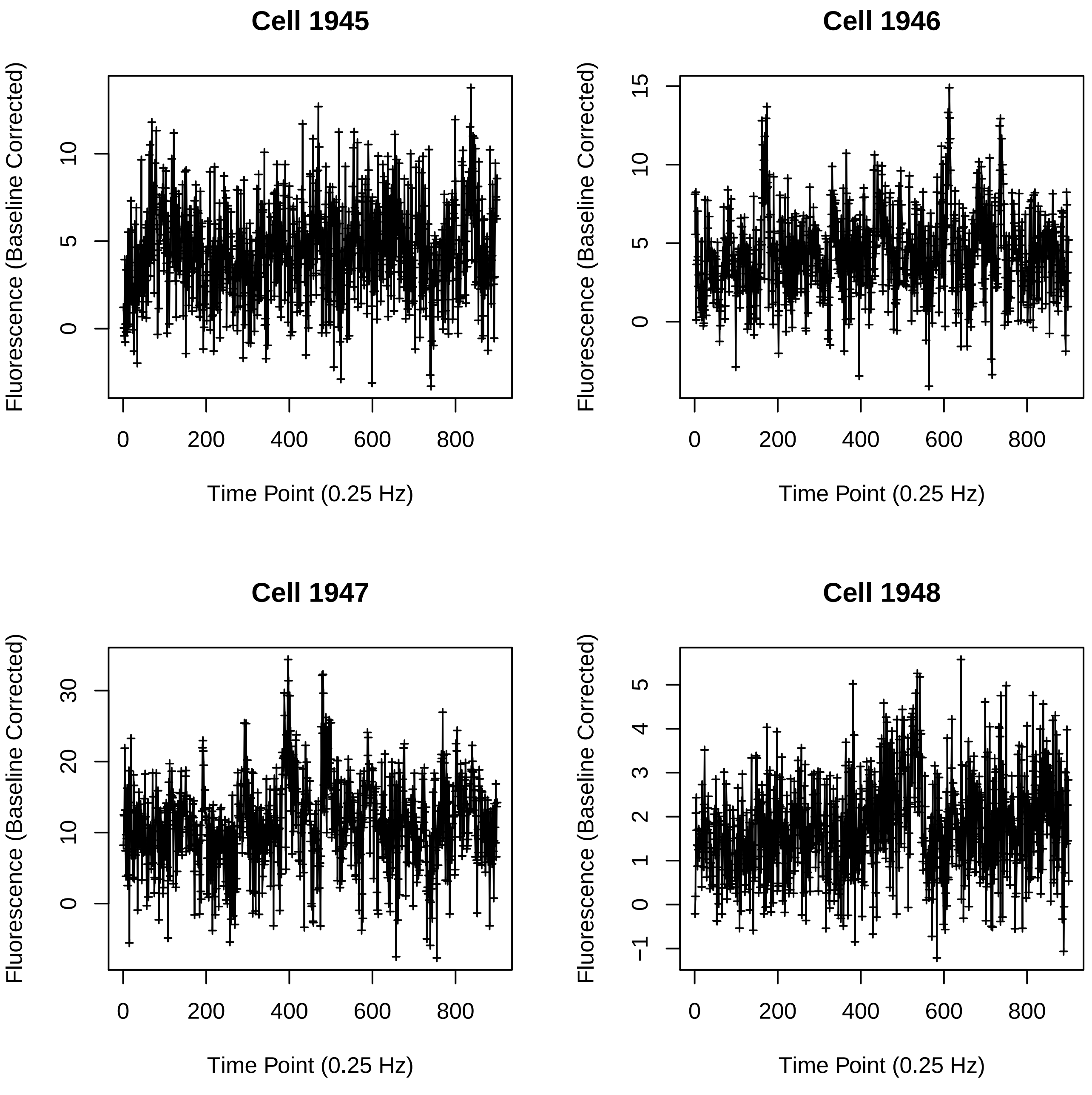
Cell 1945
Cell 1946
Cell 1947
Cell 1948
Fluorescence (Baseline Corrected)
Time Point (0.25 Hz)
0
200
400
600
800
-1
0
1
2
3
4
5
10
15
20
30

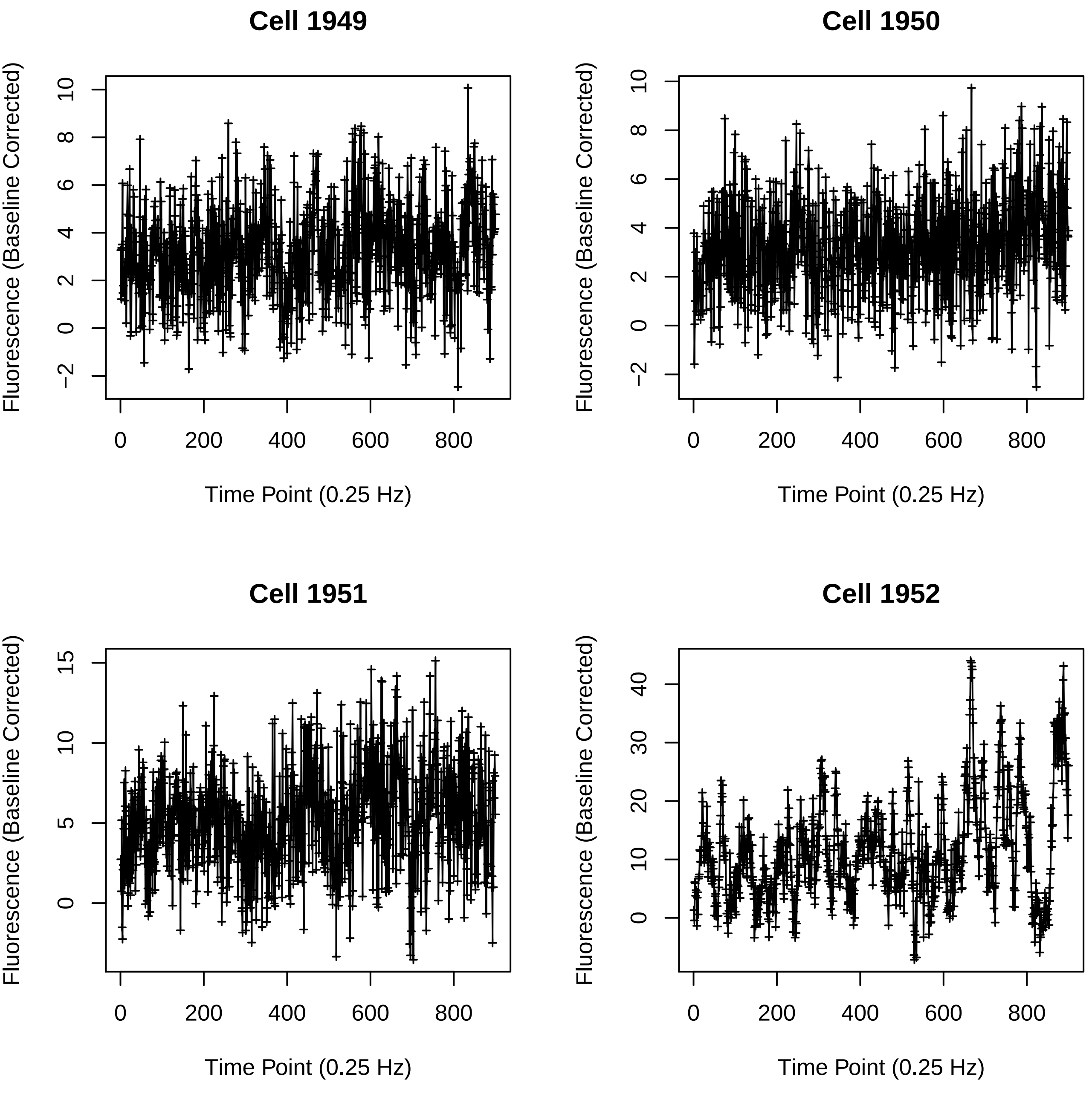
Cell 1949
Cell 1950
Cell 1951
Cell 1952
Fluorescence (Baseline Corrected)
Time Point (0.25 Hz)
0
200
400
600
800
−2
2
4
6
8
10
5
15
20
30
40

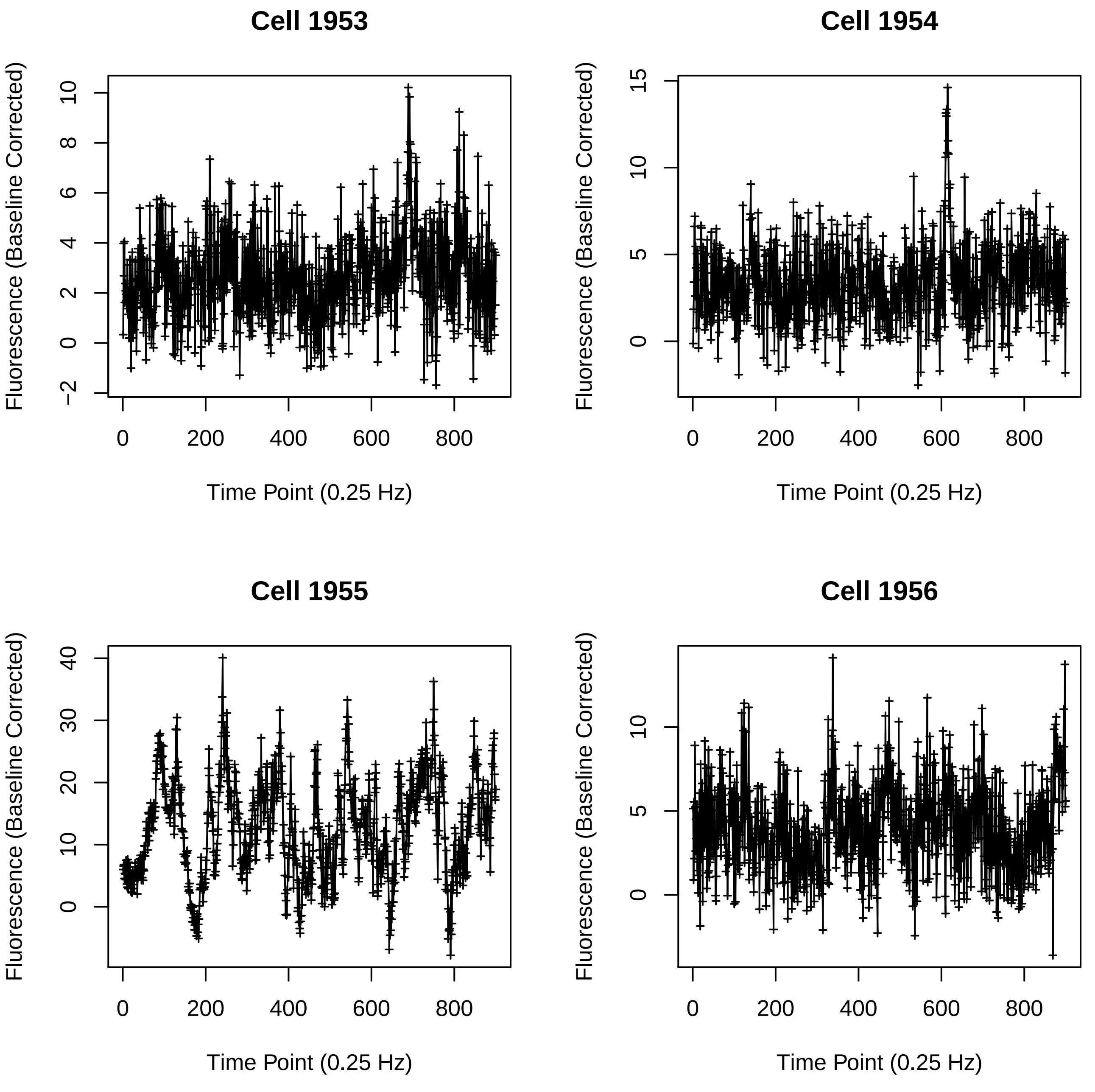
Cell 1953
Fluorescence (Baseline Corrected)
−2
0
2
4
6
8
10
0
200
400
600
800
Time Point (0.25 Hz)
Cell 1954
Fluorescence (Baseline Corrected)
0
5
10
15
0
200
400
600
800
Time Point (0.25 Hz)
Cell 1955
Fluorescence (Baseline Corrected)
0
10
20
30
40
0
200
400
600
800
Time Point (0.25 Hz)
Cell 1956
Fluorescence (Baseline Corrected)
0
5
10
0
200
400
600
800
Time Point (0.25 Hz)

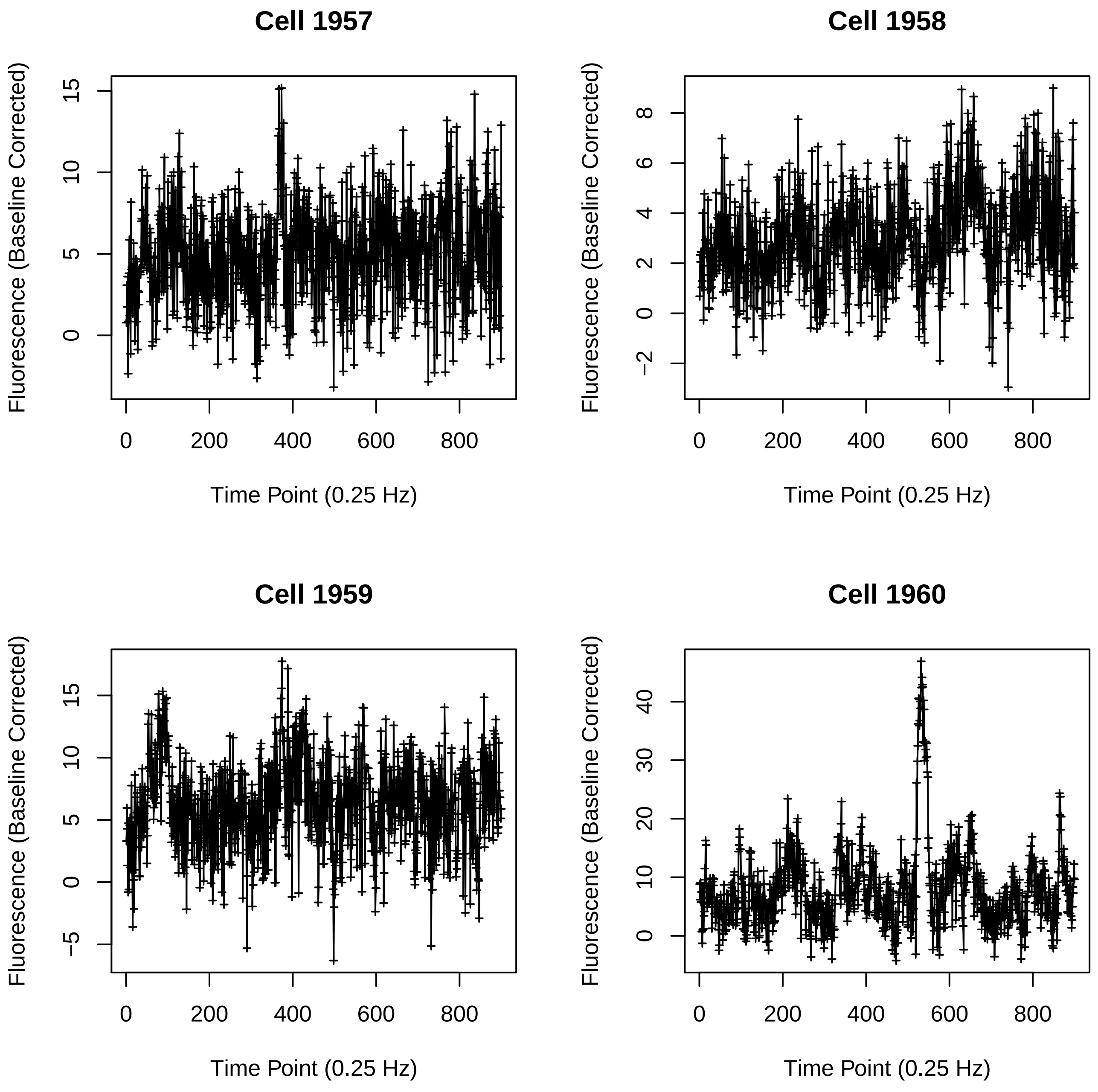
Cell 1957
Fluorescence (Baseline Corrected)
Time Point (0.25 Hz)
Cell 1958
Fluorescence (Baseline Corrected)
Time Point (0.25 Hz)
Cell 1959
Fluorescence (Baseline Corrected)
Time Point (0.25 Hz)
Cell 1960
Fluorescence (Baseline Corrected)
Time Point (0.25 Hz)

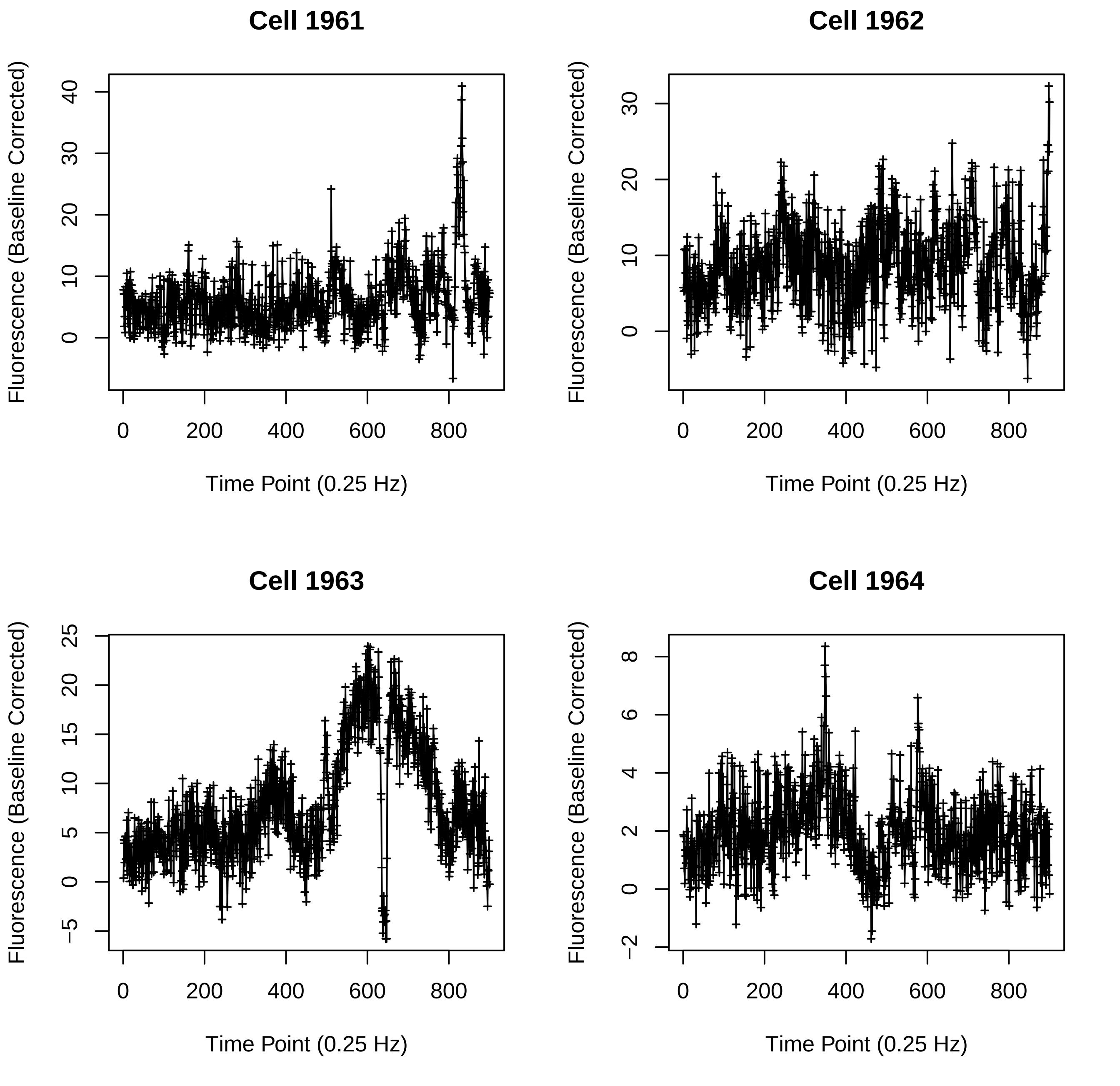

Cell 1961
Cell 1962
Cell 1963
Cell 1964
Fluorescence (Baseline Corrected)
Time Point (0.25 Hz)

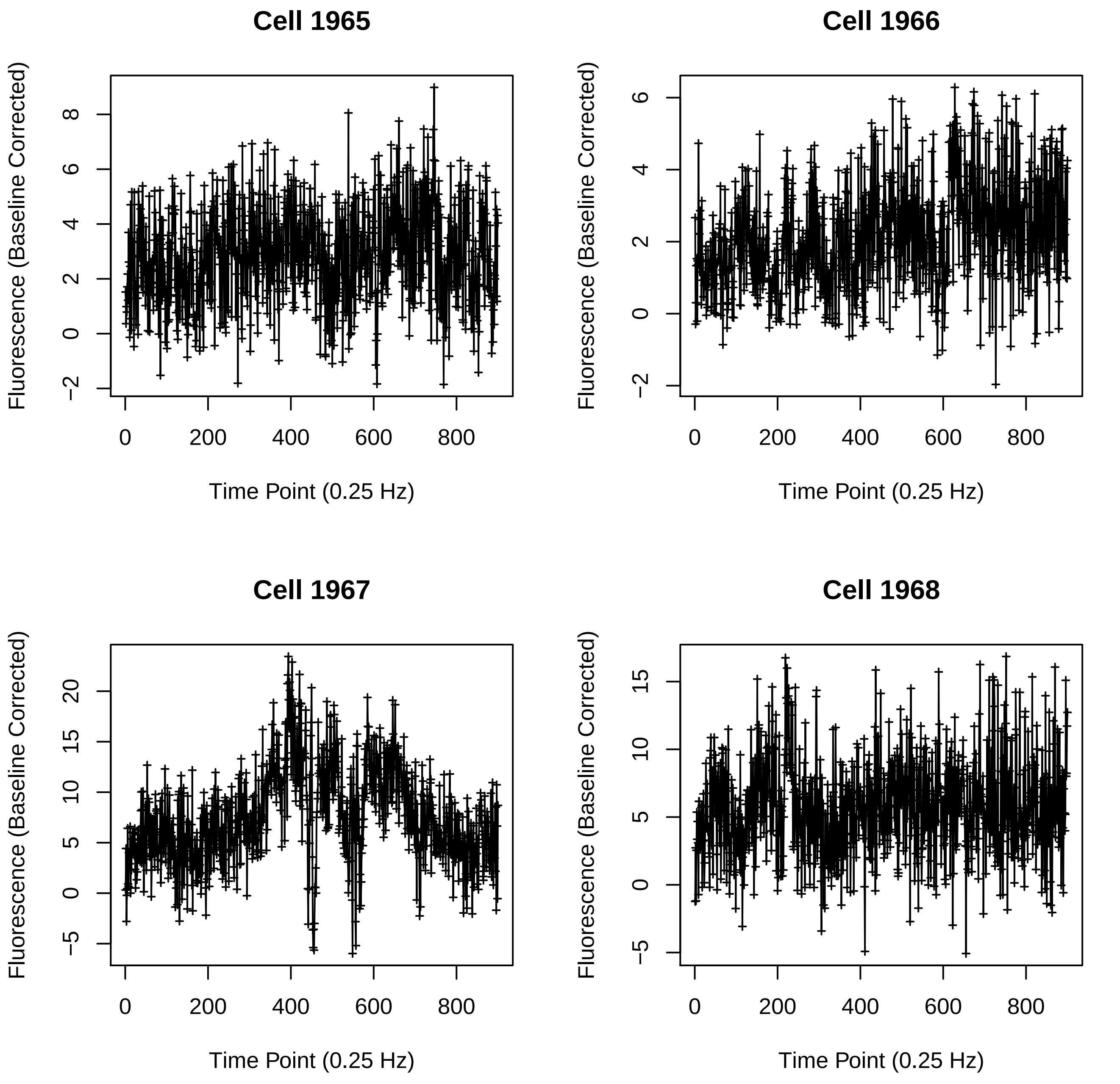

Cell 1965
Cell 1966
Cell 1967
Cell 1968
Fluorescence (Baseline Corrected)
Time Point (0.25 Hz)
0
200
400
600
800
−2
2
4
6
8
−5
5
10
15
20

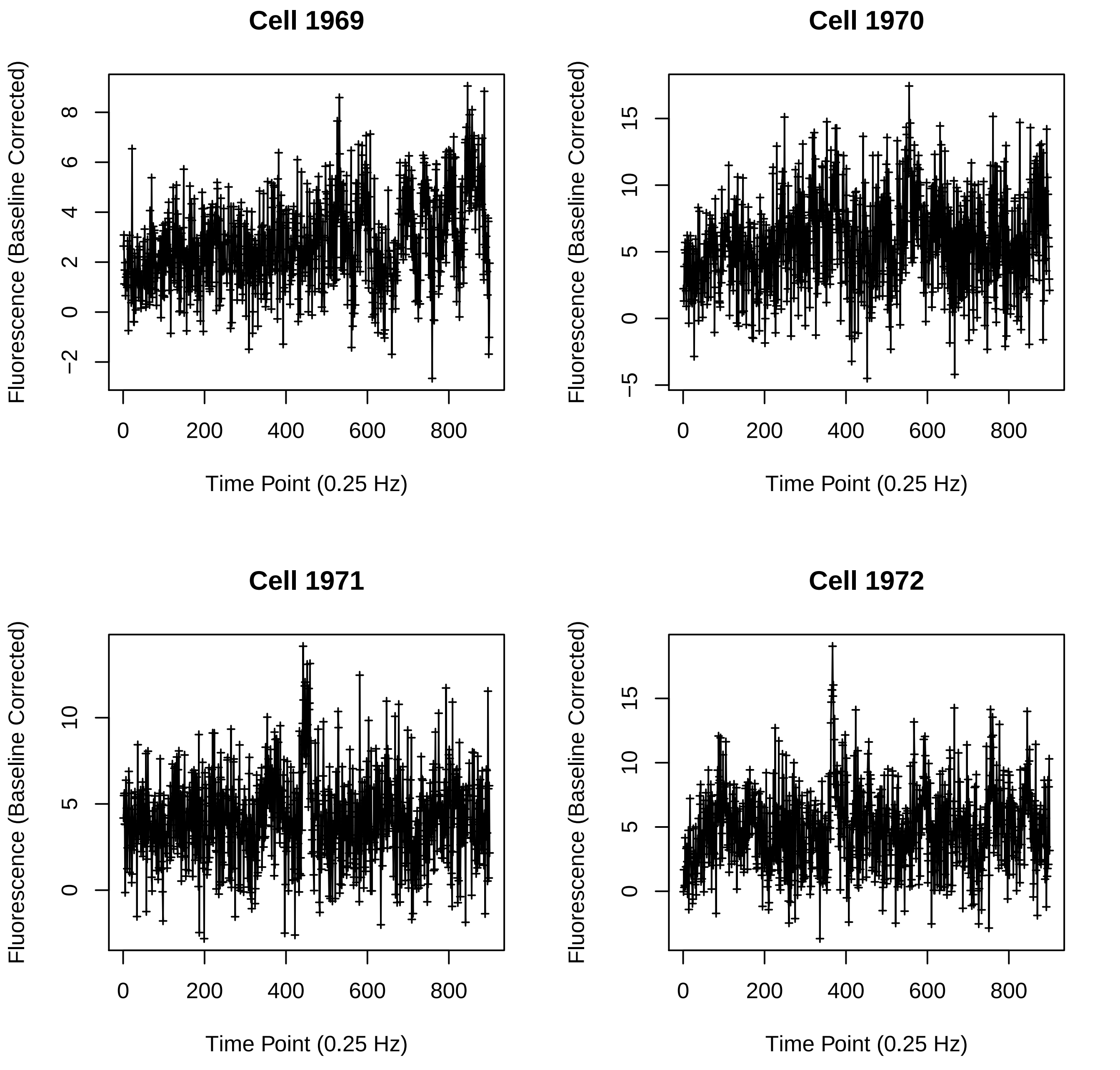
Cell 1969
Fluorescence (Baseline Corrected)
Time Point (0.25 Hz)
−2
0
2
4
6
8
0
200
400
600
800
Cell 1970
Fluorescence (Baseline Corrected)
Time Point (0.25 Hz)
−5
0
5
10
15
0
200
400
600
800
Cell 1971
Fluorescence (Baseline Corrected)
Time Point (0.25 Hz)
0
5
10
0
200
400
600
800
Cell 1972
Fluorescence (Baseline Corrected)
Time Point (0.25 Hz)
0
5
10
15
0
200
400
600
800

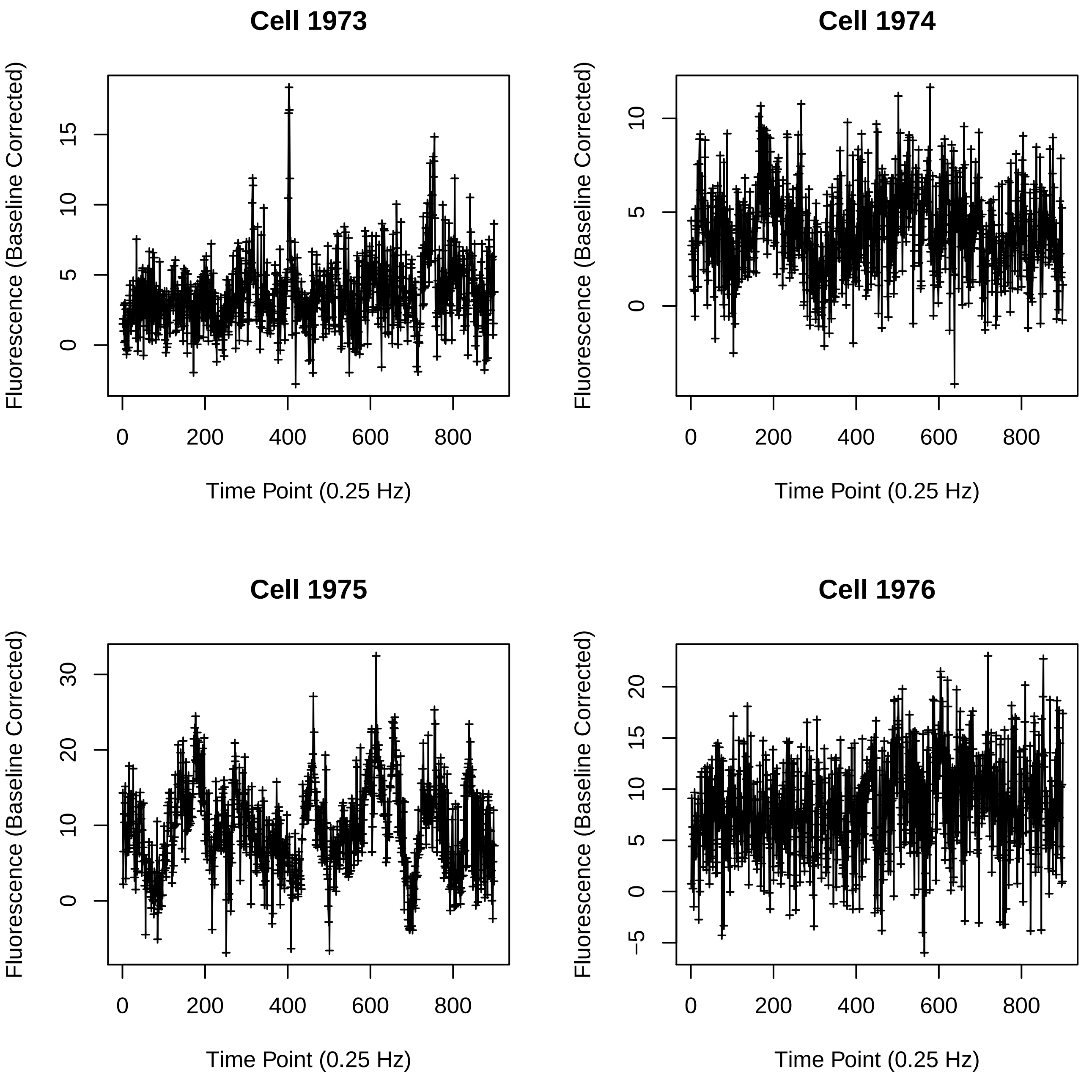
Cell 1973
Cell 1974
Cell 1975
Cell 1976
Fluorescence (Baseline Corrected)
Time Point (0.25 Hz)
0
200
400
600
800
0
5
10
15
20
30
−5

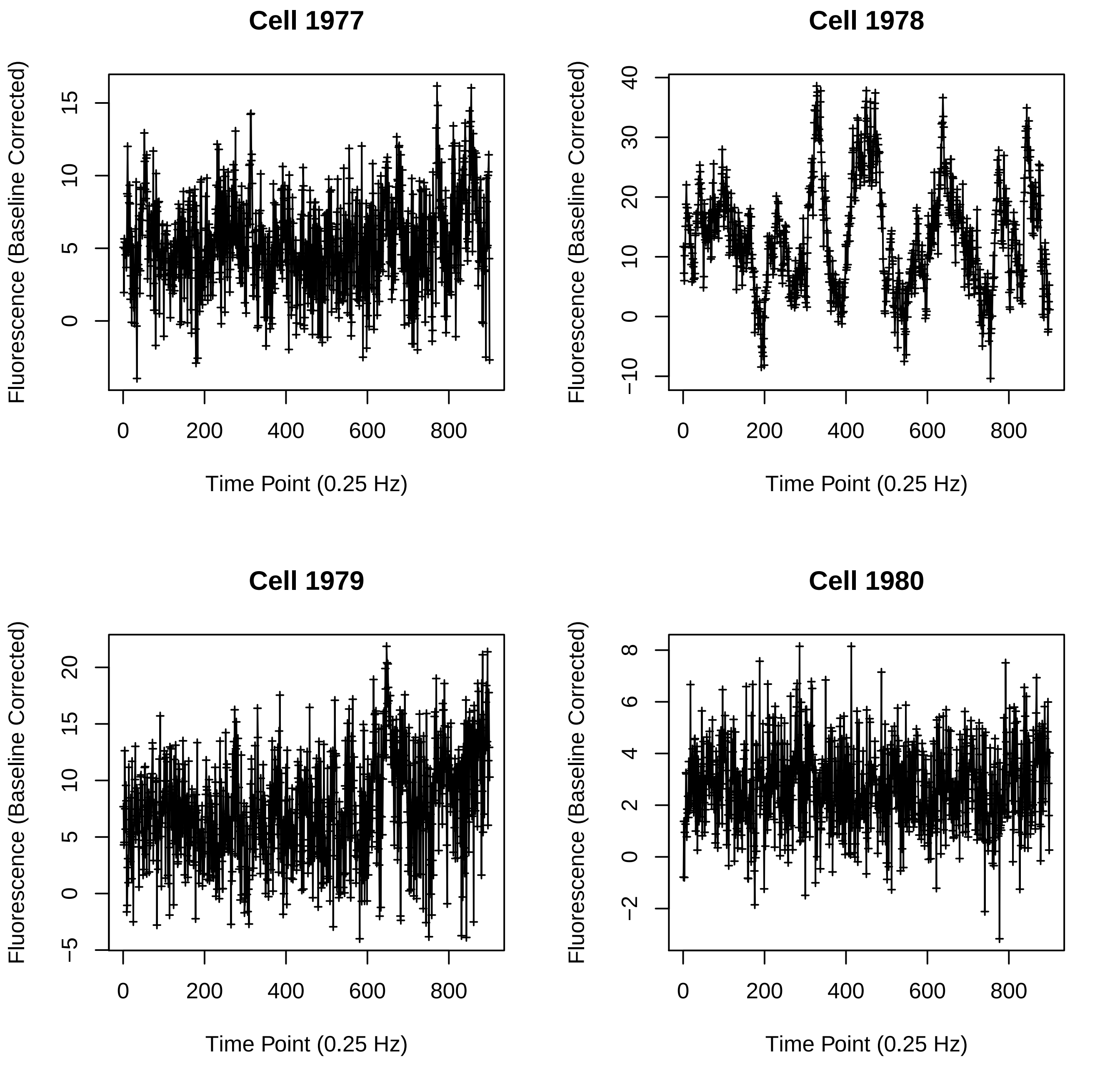

Cell 1977
Cell 1978
Cell 1979
Cell 1980
Fluorescence (Baseline Corrected)
Time Point (0.25 Hz)
0
200
400
600
800

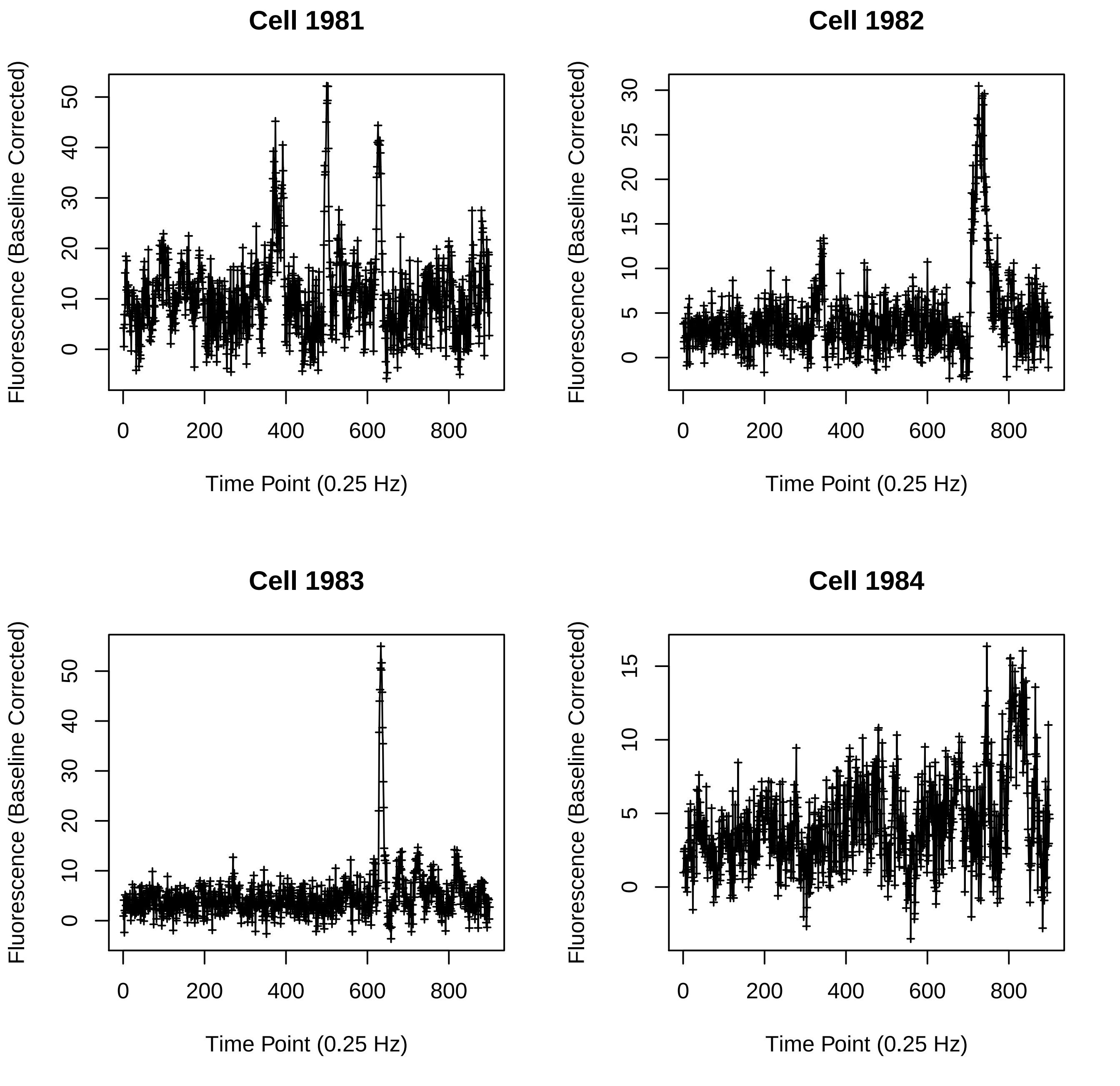
Cell 1981
Cell 1982
Cell 1983
Cell 1984
Fluorescence (Baseline Corrected)
Time Point (0.25 Hz)
0
10
20
30
40
50
0
5
10
15
20
25
30
0
200
400
600
800

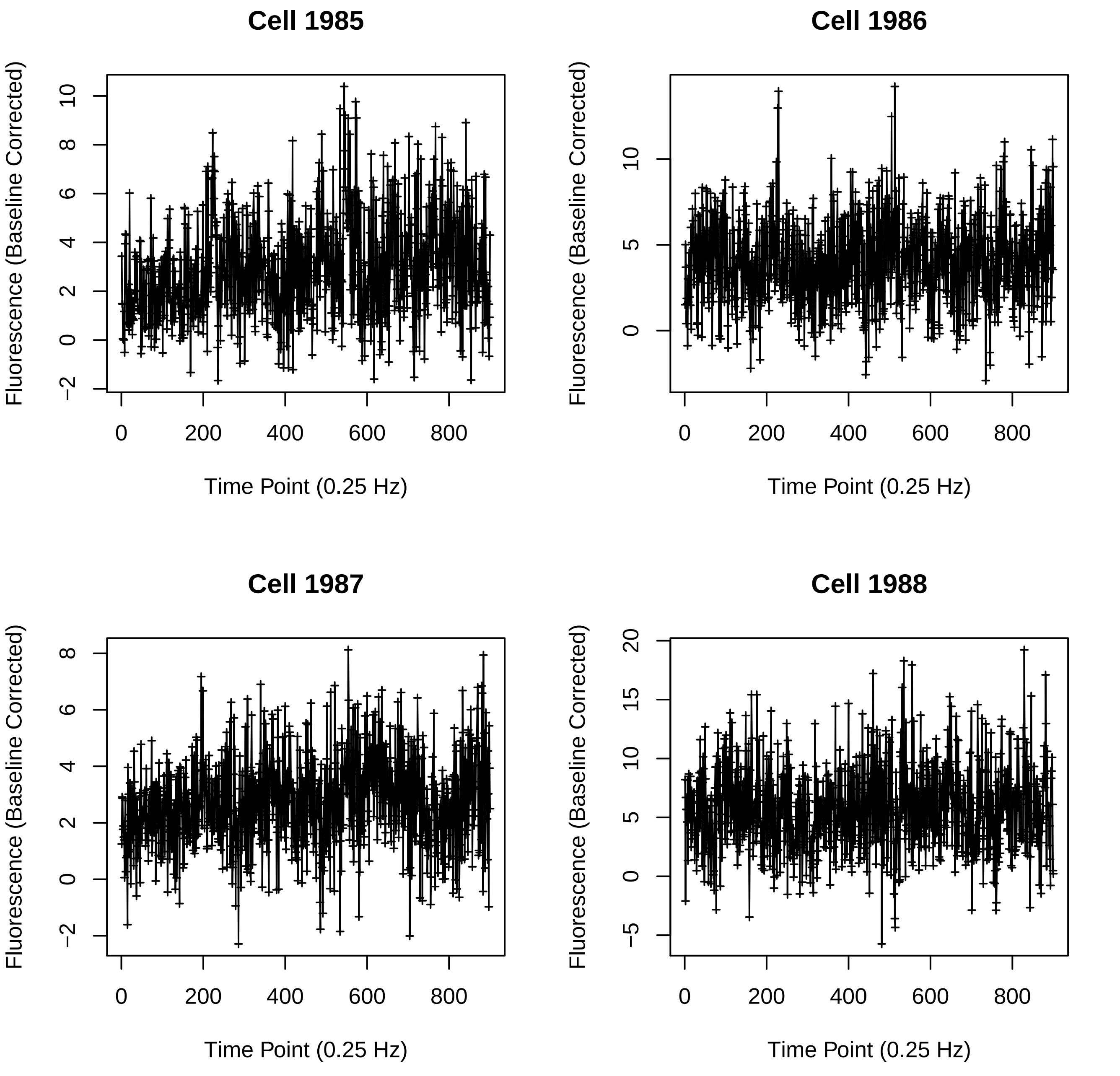
Cell 1985
Fluorescence (Baseline Corrected)
−2
0
2
4
6
8
10
0
200
400
600
800
Time Point (0.25 Hz)
Cell 1986
Fluorescence (Baseline Corrected)
0
5
10
0
200
400
600
800
Time Point (0.25 Hz)
Cell 1987
Fluorescence (Baseline Corrected)
−2
0
2
4
6
8
0
200
400
600
800
Time Point (0.25 Hz)
Cell 1988
Fluorescence (Baseline Corrected)
−5
0
5
10
15
20
0
200
400
600
800
Time Point (0.25 Hz)

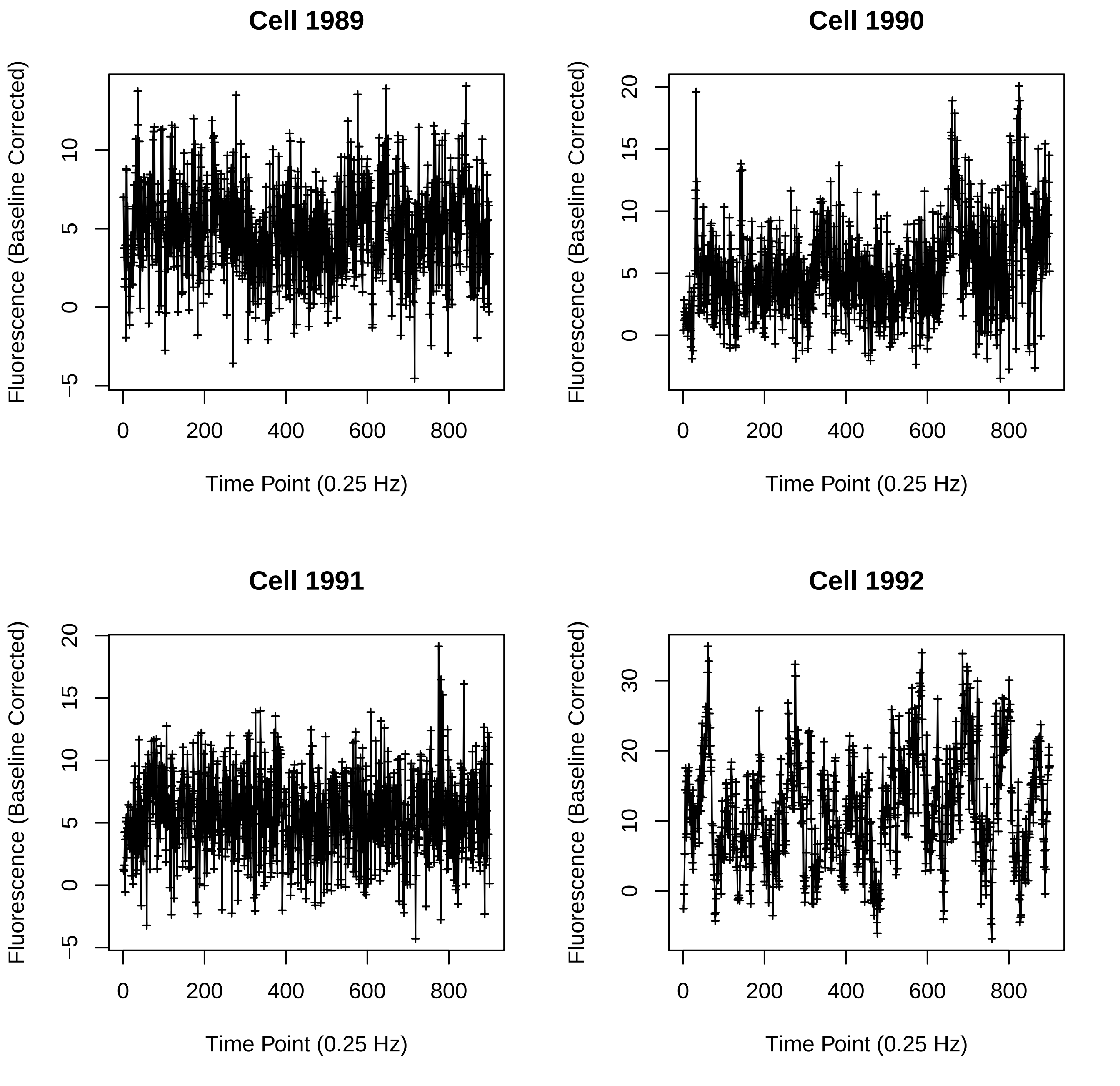

Cell 1989
Cell 1990
Cell 1991
Cell 1992
Fluorescence (Baseline Corrected)
Time Point (0.25 Hz)
0
200
400
600
800
-5
0
5
10
15
20
30

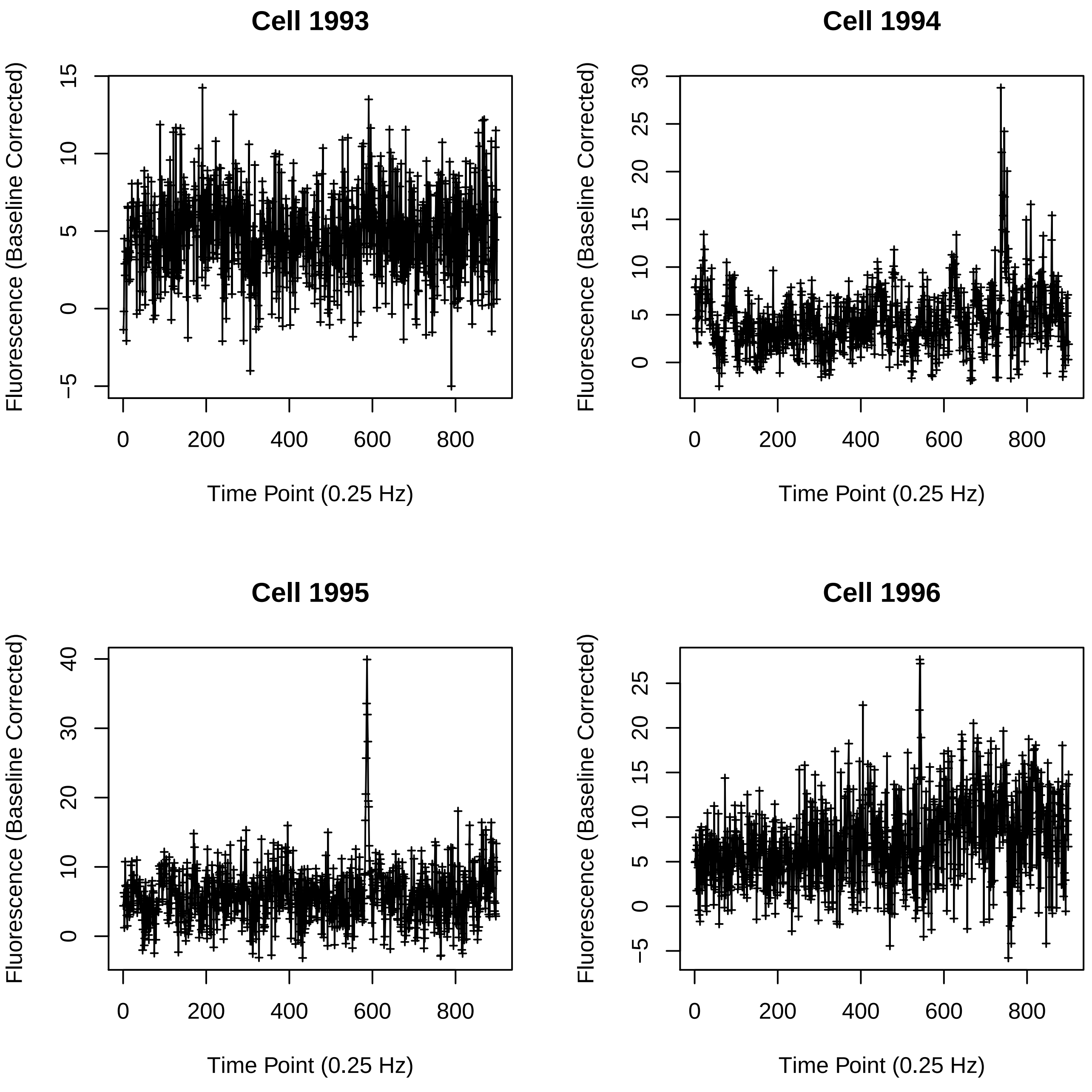

Cell 1993
Cell 1994
Cell 1995
Cell 1996
Fluorescence (Baseline Corrected)
Time Point (0.25 Hz)

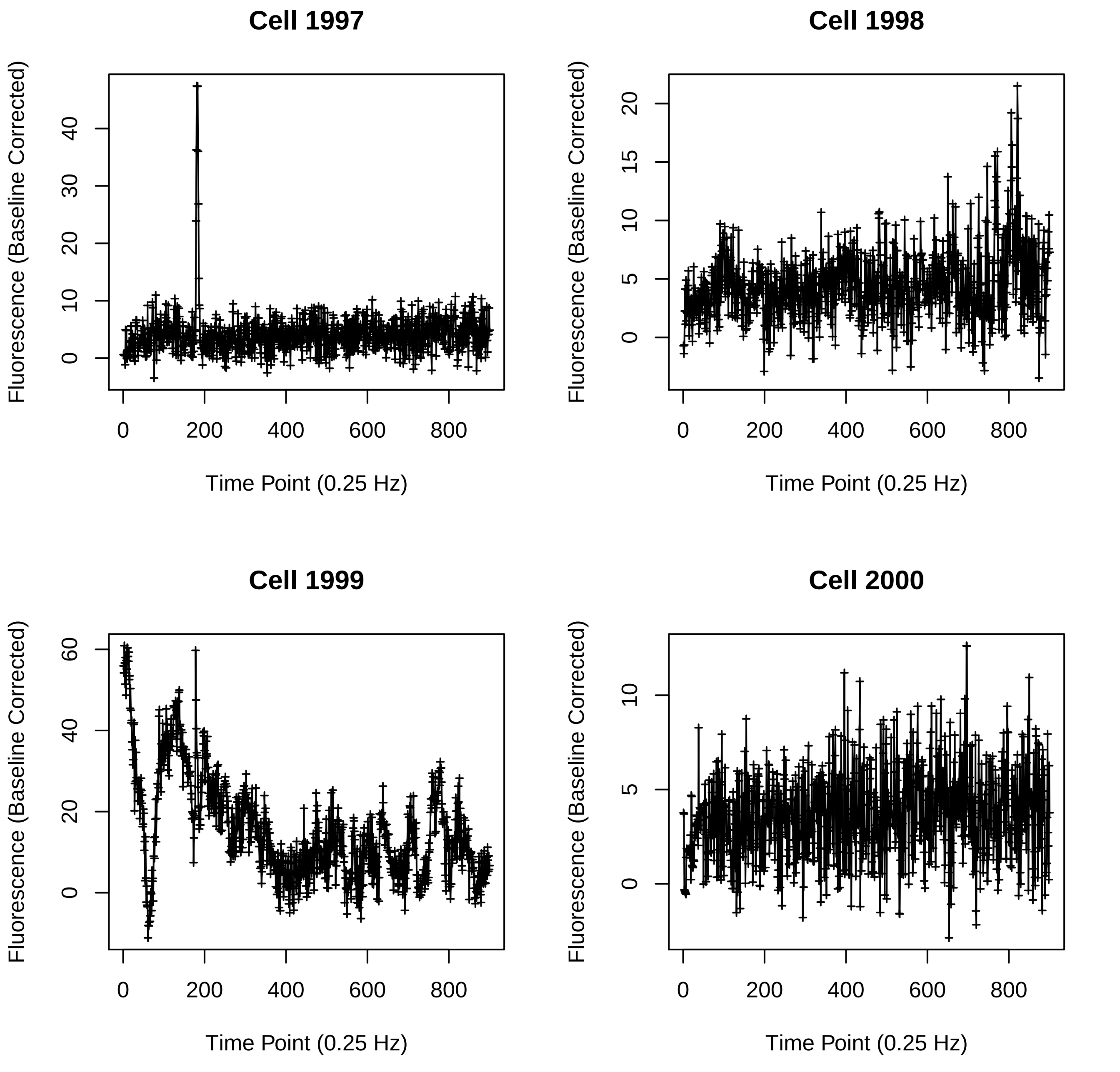
Cell 1997
Fluorescence (Baseline Corrected)
Time Point (0.25 Hz)
Cell 1998
Fluorescence (Baseline Corrected)
Time Point (0.25 Hz)
Cell 1999
Fluorescence (Baseline Corrected)
Time Point (0.25 Hz)
Cell 2000
Fluorescence (Baseline Corrected)
Time Point (0.25 Hz)

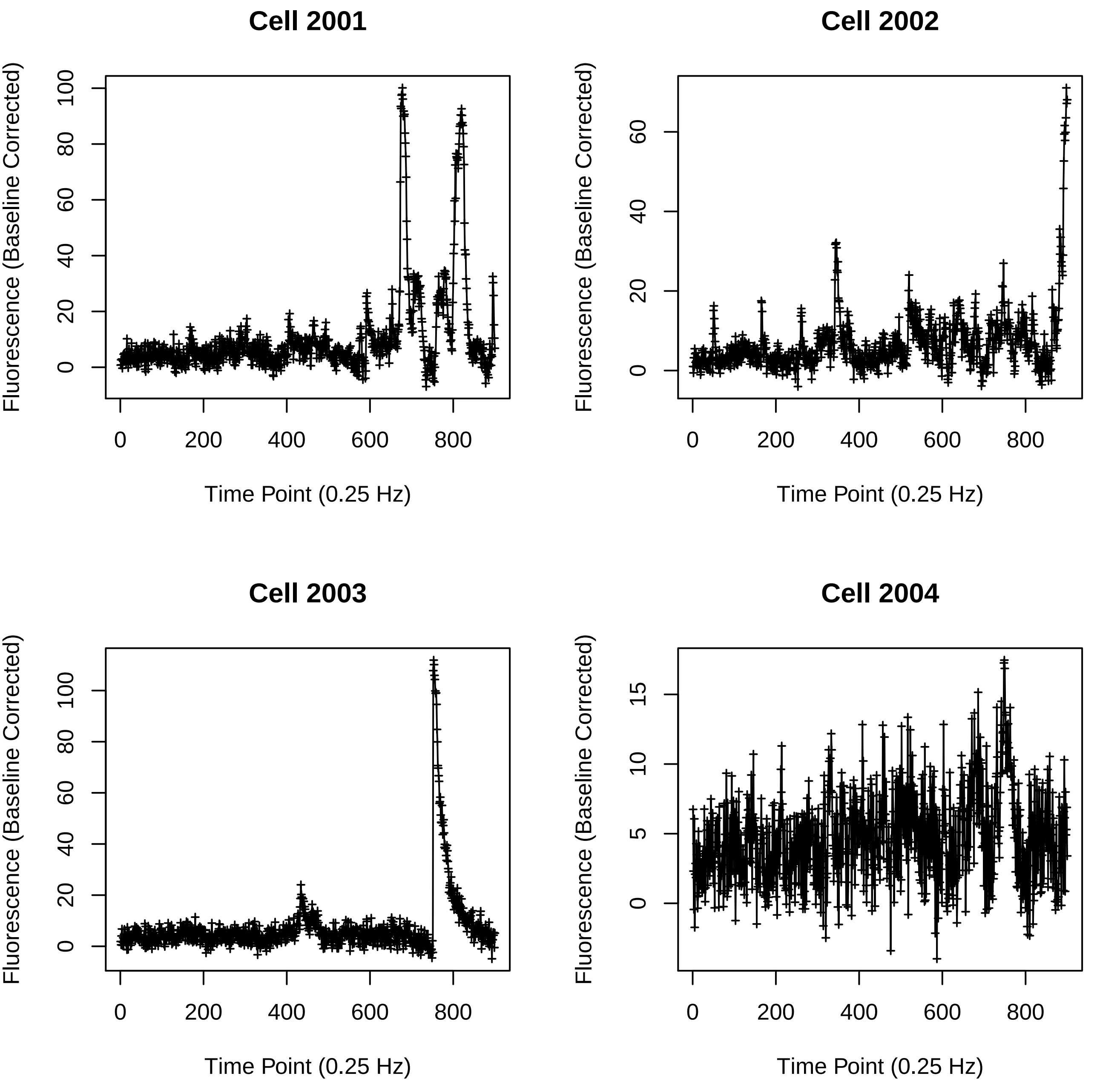

Cell 2001
Cell 2002
Cell 2003
Cell 2004
Fluorescence (Baseline Corrected)
Time Point (0.25 Hz)
0
200
400
600
800
20
40
60
80
100
5
10
15

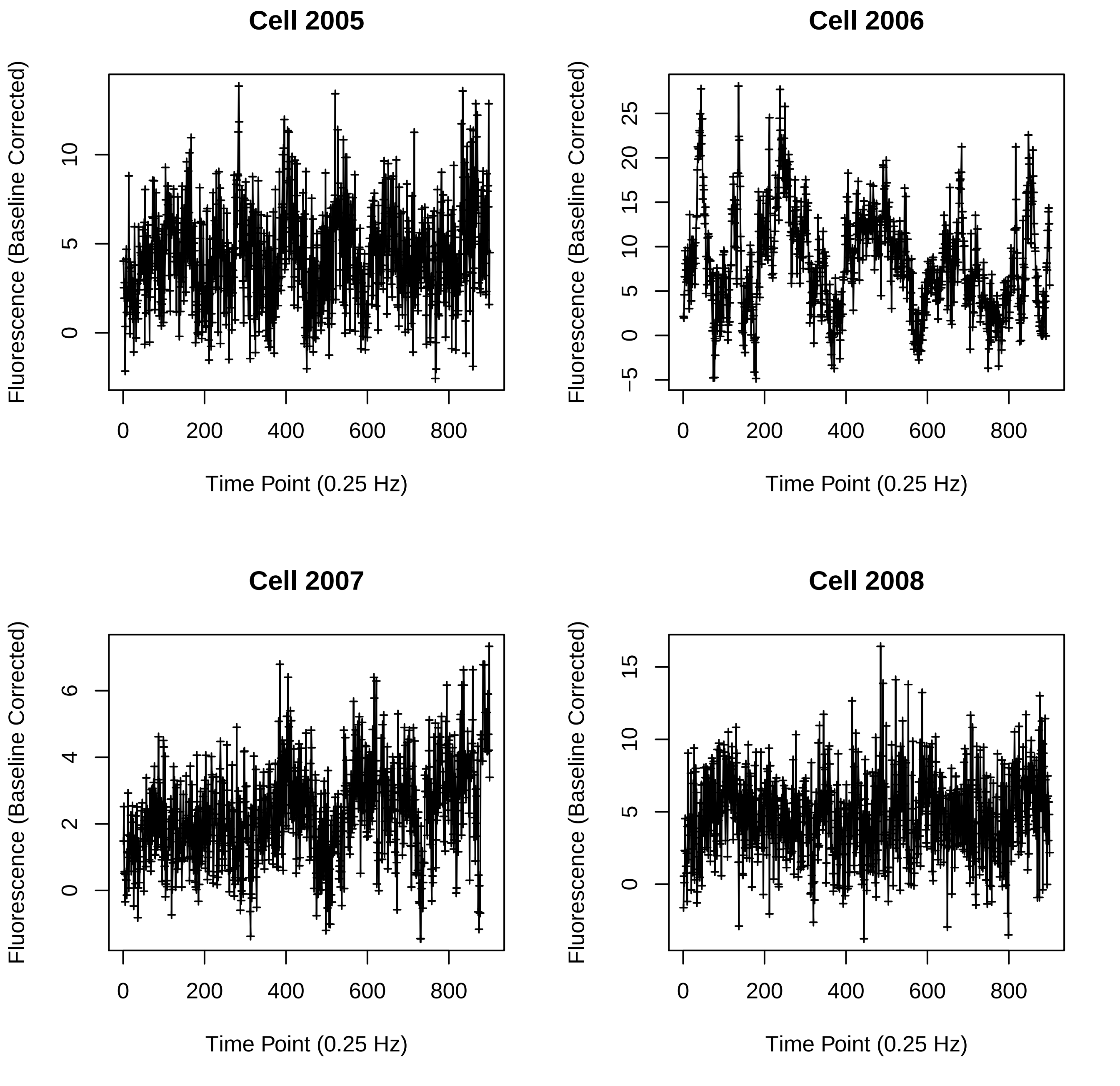
Cell 2005
Cell 2006
Cell 2007
Cell 2008
Fluorescence (Baseline Corrected)
Time Point (0.25 Hz)
0
200
400
600
800
−5
5
10
15
20
25

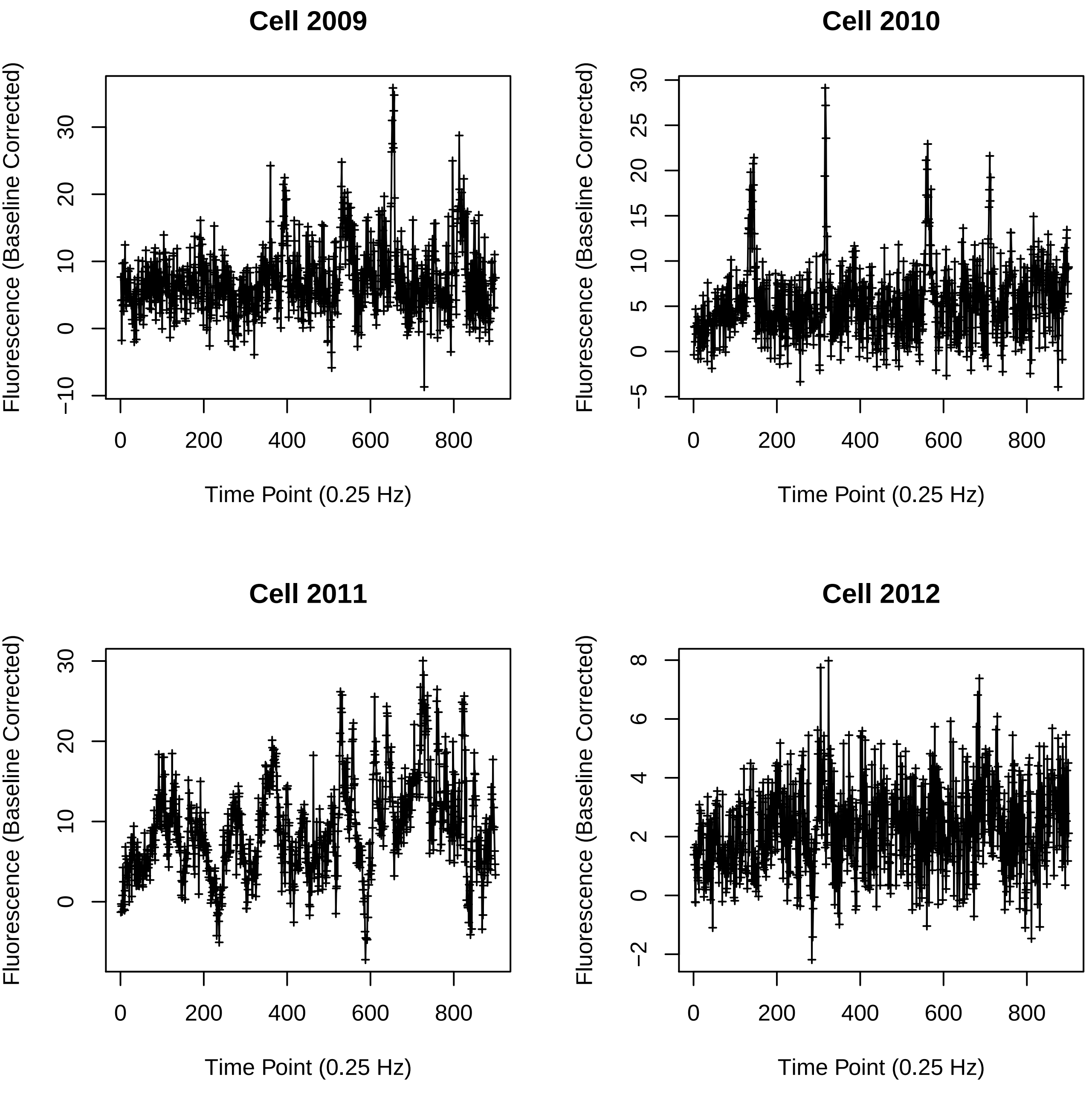
Cell 2009
Cell 2010
Cell 2011
Cell 2012
Fluorescence (Baseline Corrected)
Time Point (0.25 Hz)
−10
0
10
20
30
−5
5
15
25
−2
2
4
6
8
0
200
400
600
800

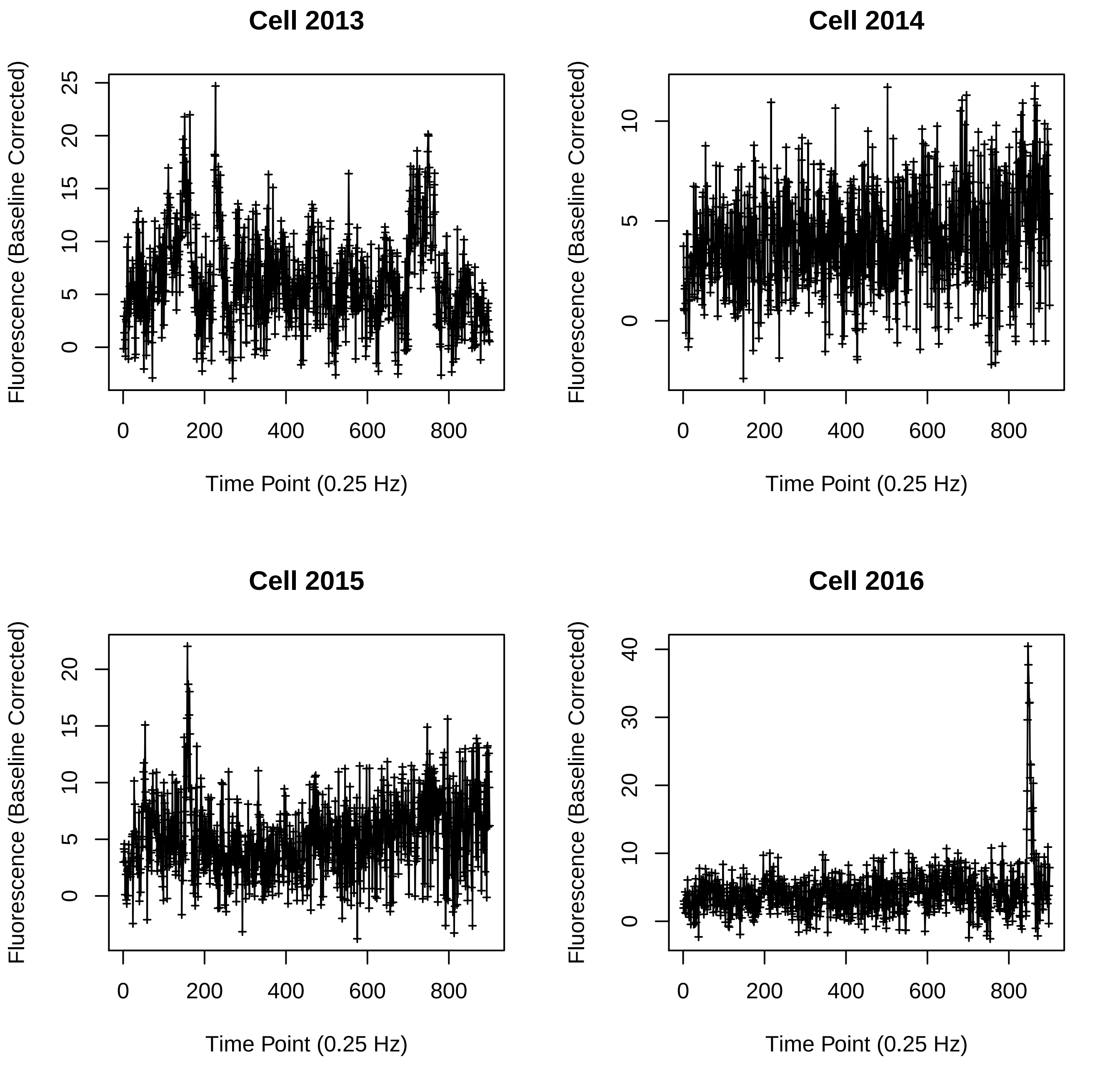

Cell 2013
Cell 2014
Cell 2015
Cell 2016
Fluorescence (Baseline Corrected)
Time Point (0.25 Hz)
0
200
400
600
800
0
5
10
15
20
25
30
40

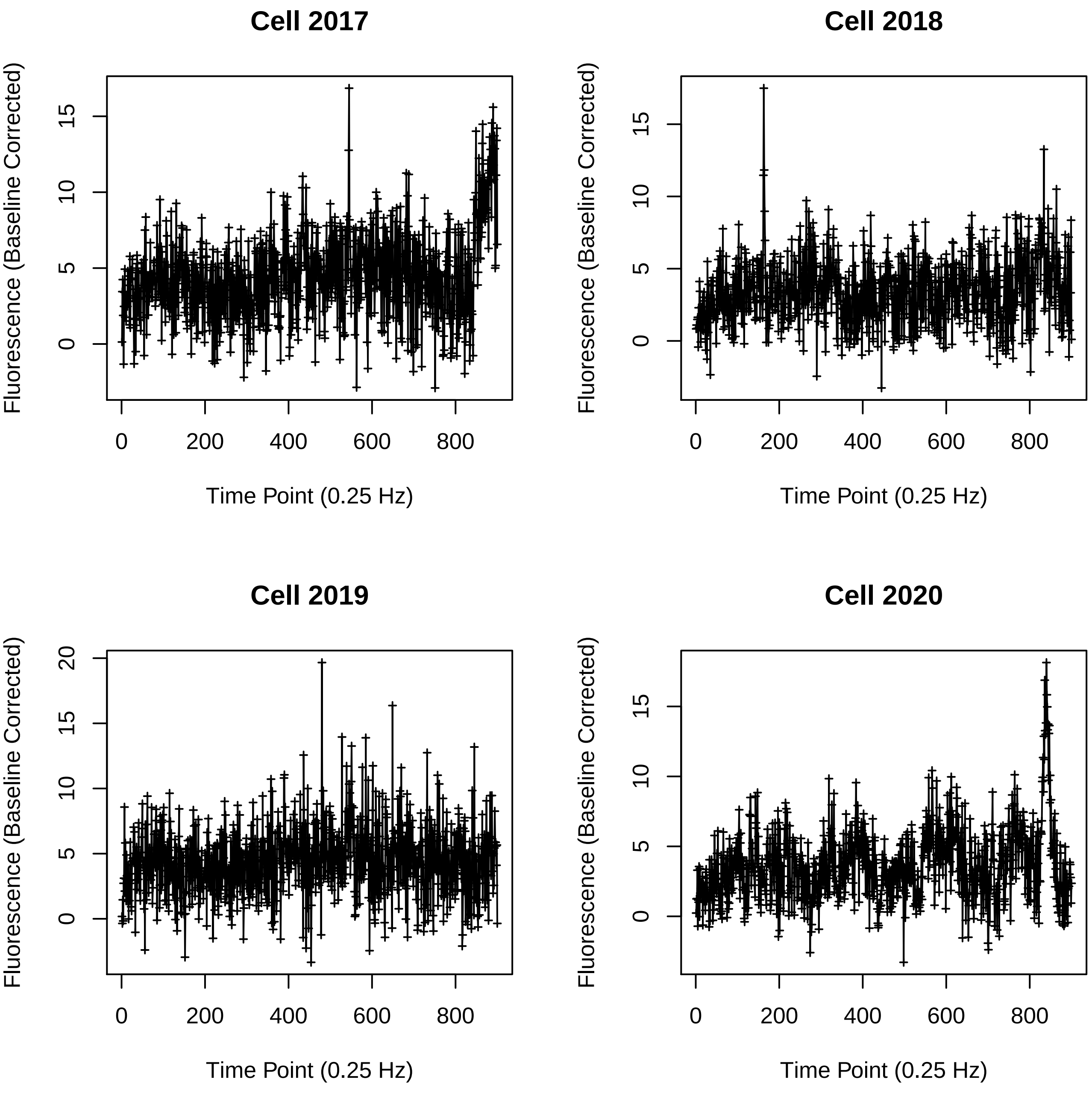
Cell 2017
Cell 2018
Cell 2019
Cell 2020
Fluorescence (Baseline Corrected)
Time Point (0.25 Hz)
0
5
10
15
20
200
400
600
800

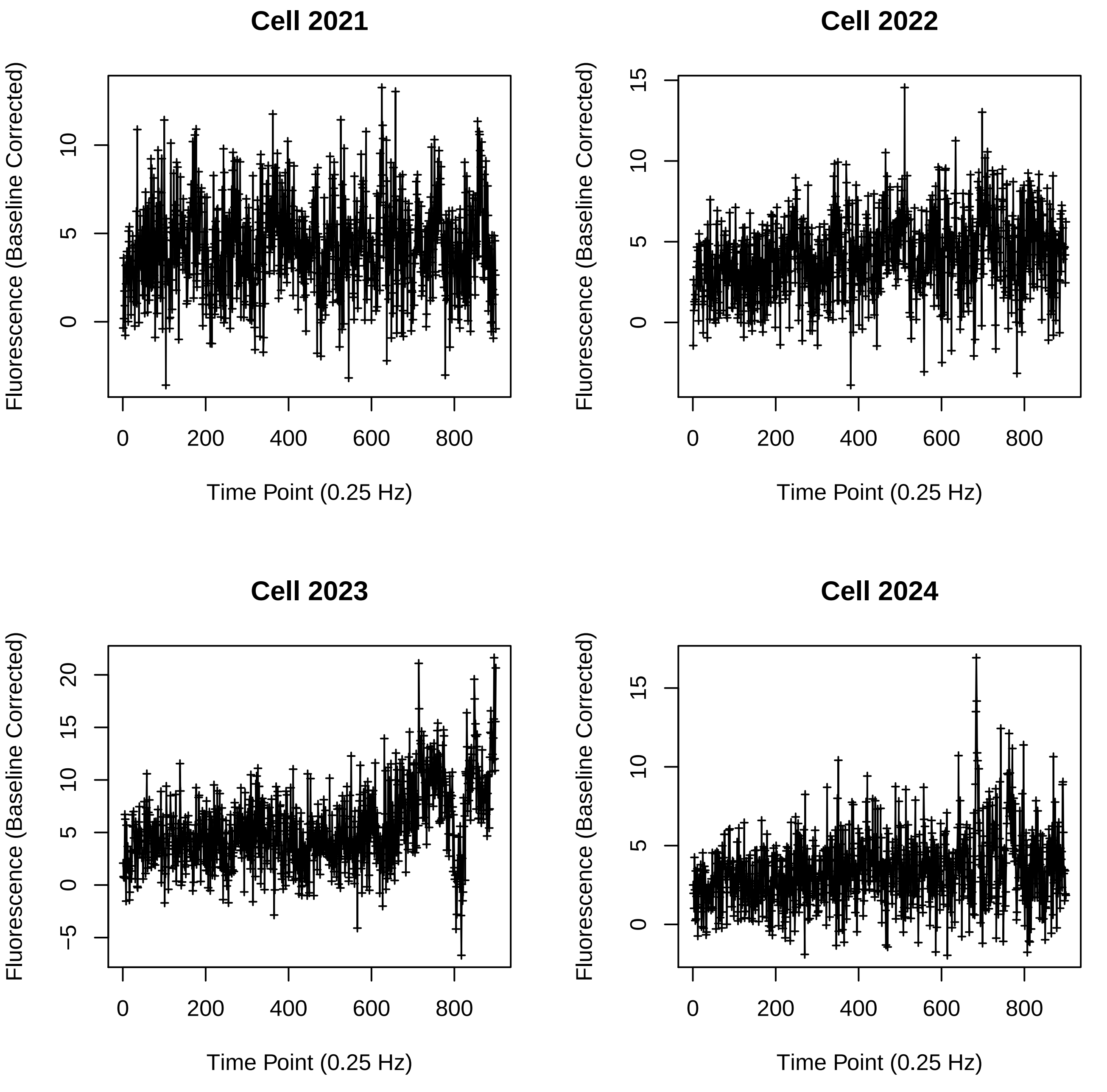
Cell 2021
Cell 2022
Cell 2023
Cell 2024
Fluorescence (Baseline Corrected)
Time Point (0.25 Hz)
0
200
400
600
800
-5
0
5
10
15
20

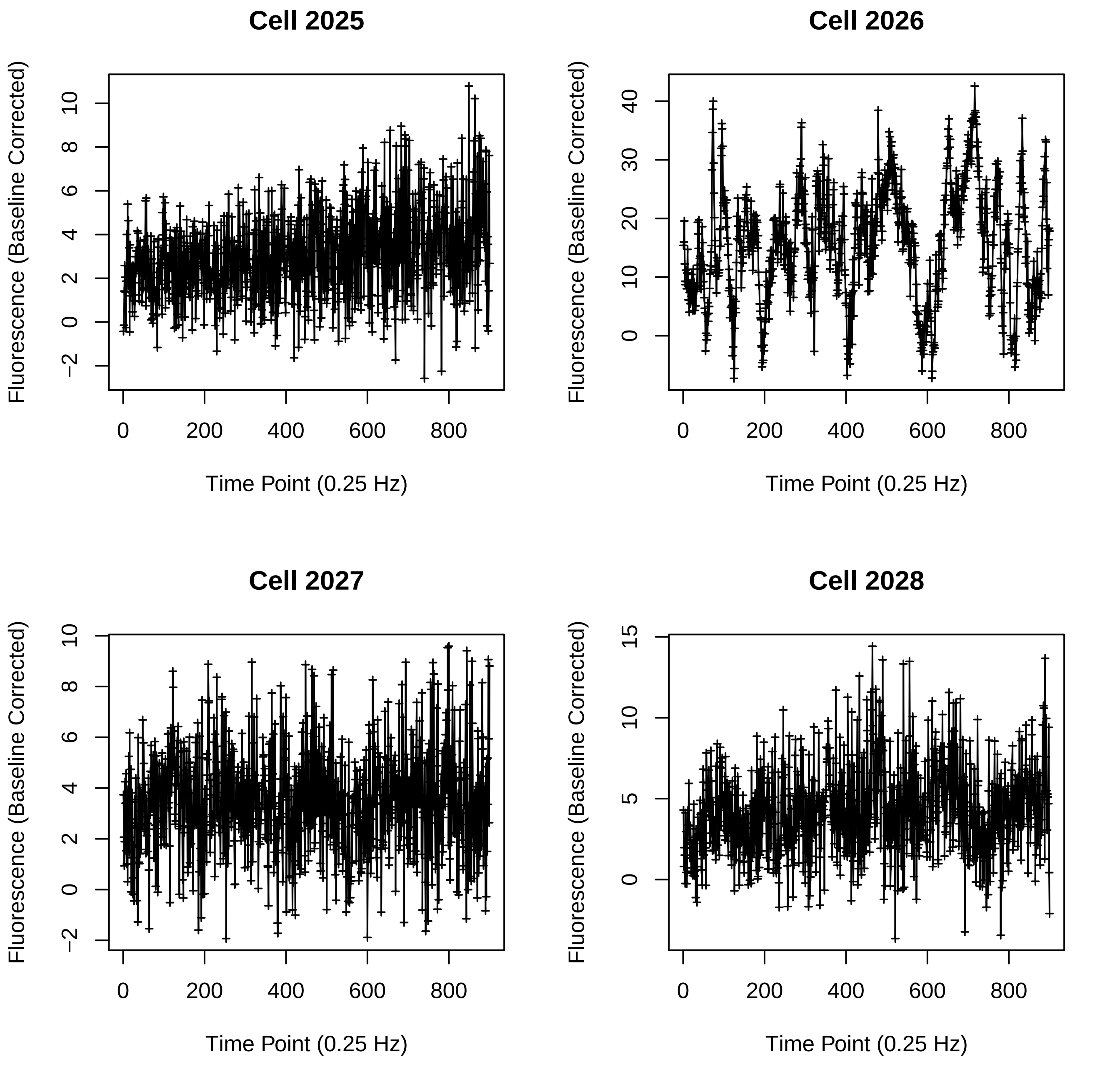

Cell 2025
Cell 2026
Cell 2027
Cell 2028
Fluorescence (Baseline Corrected)
Time Point (0.25 Hz)

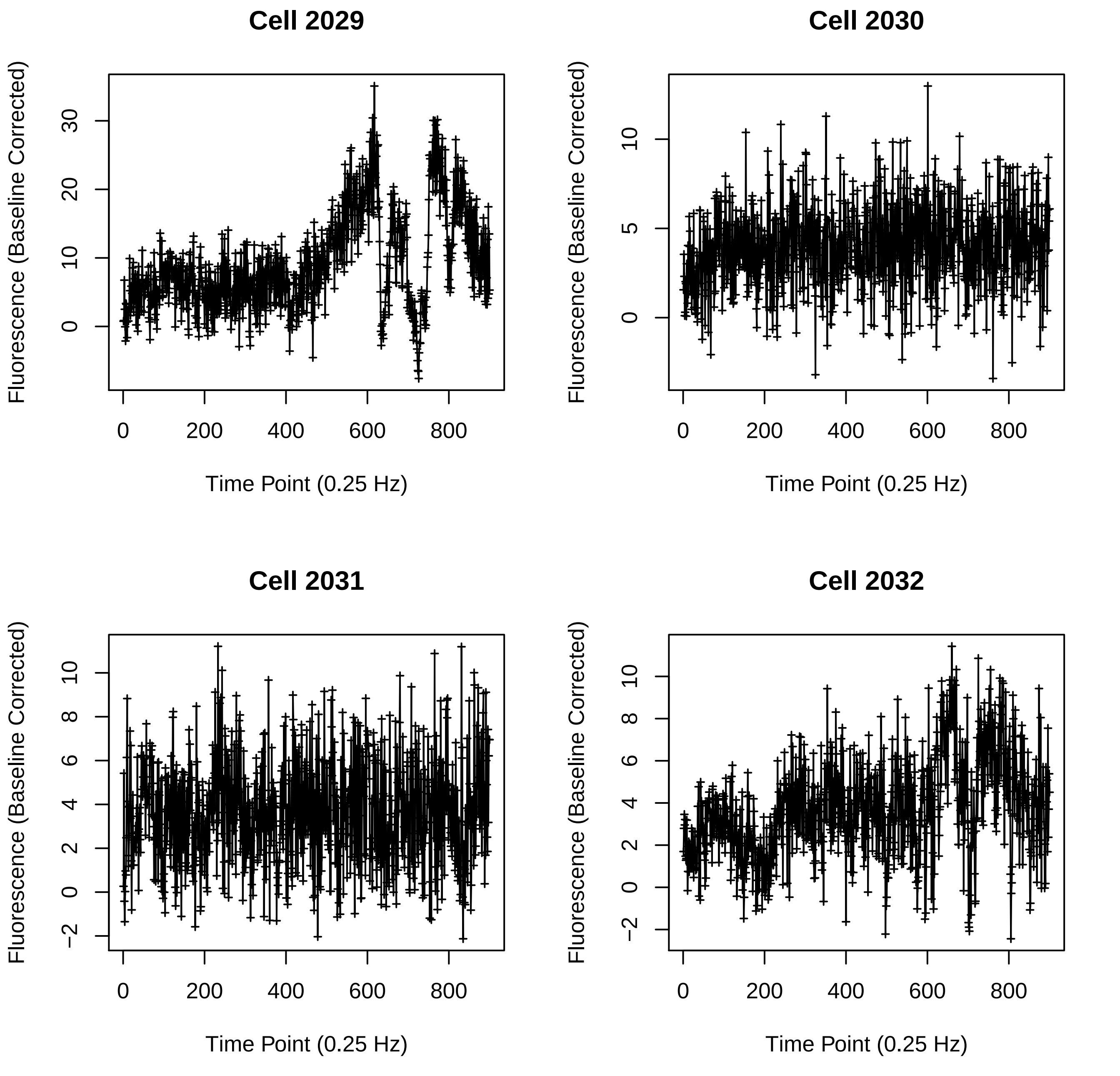
Cell 2029
Cell 2030
Cell 2031
Cell 2032
Fluorescence (Baseline Corrected)
Time Point (0.25 Hz)
0
10
20
30
5
-2
2
4
6
8
200
400
600
800

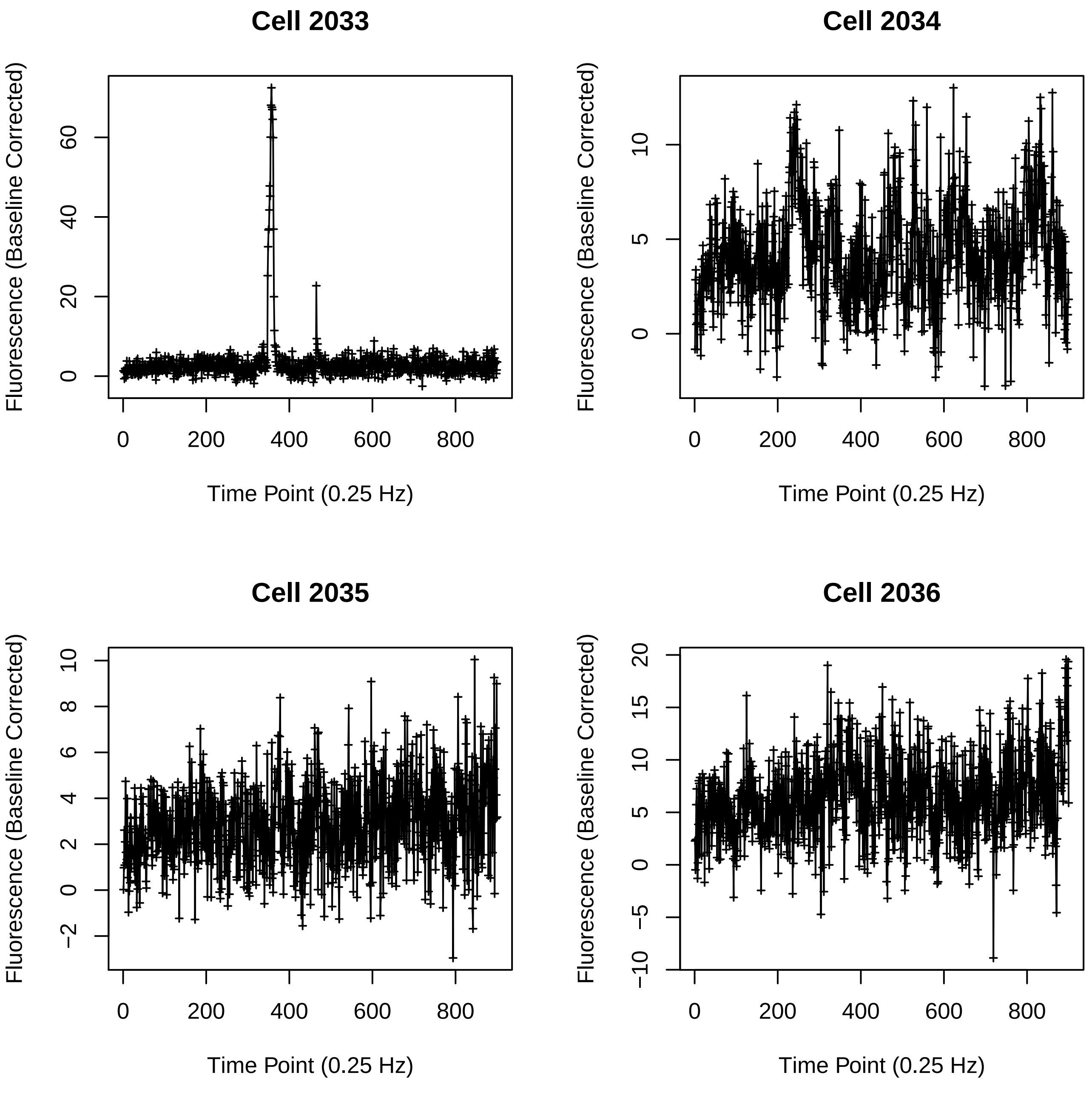
Cell 2033
Fluorescence (Baseline Corrected)
0
20
40
60
0
200
400
600
800
Time Point (0.25 Hz)
Cell 2034
Fluorescence (Baseline Corrected)
0
5
10
0
200
400
600
800
Time Point (0.25 Hz)
Cell 2035
Fluorescence (Baseline Corrected)
−2
0
2
4
6
8
10
0
200
400
600
800
Time Point (0.25 Hz)
Cell 2036
Fluorescence (Baseline Corrected)
−10
−5
0
5
10
15
20
0
200
400
600
800
Time Point (0.25 Hz)

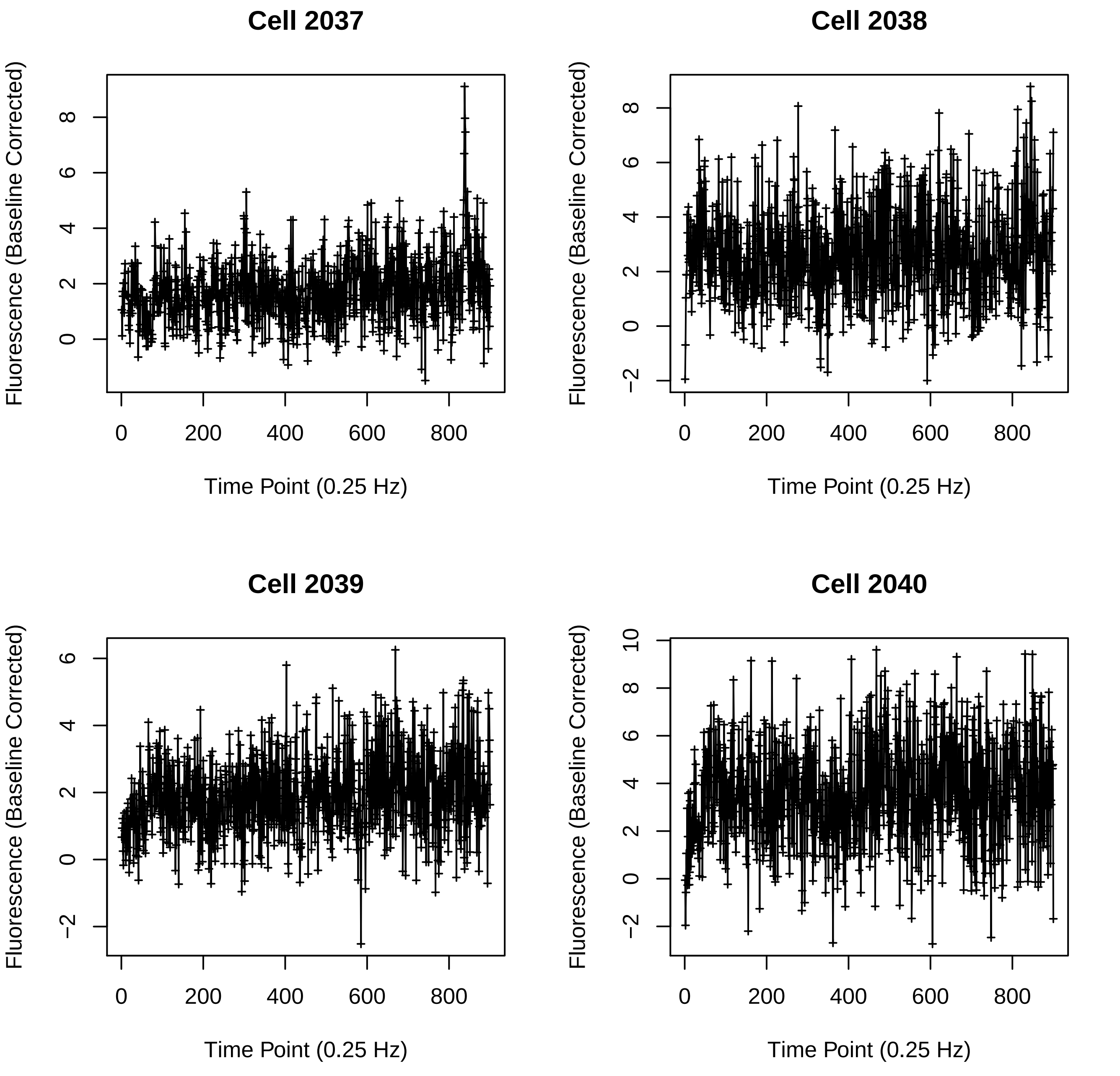
Cell 2037
Cell 2038
Cell 2039
Cell 2040
Fluorescence (Baseline Corrected)
Time Point (0.25 Hz)
0
200
400
600
800
−2
0
2
4
6
8
10

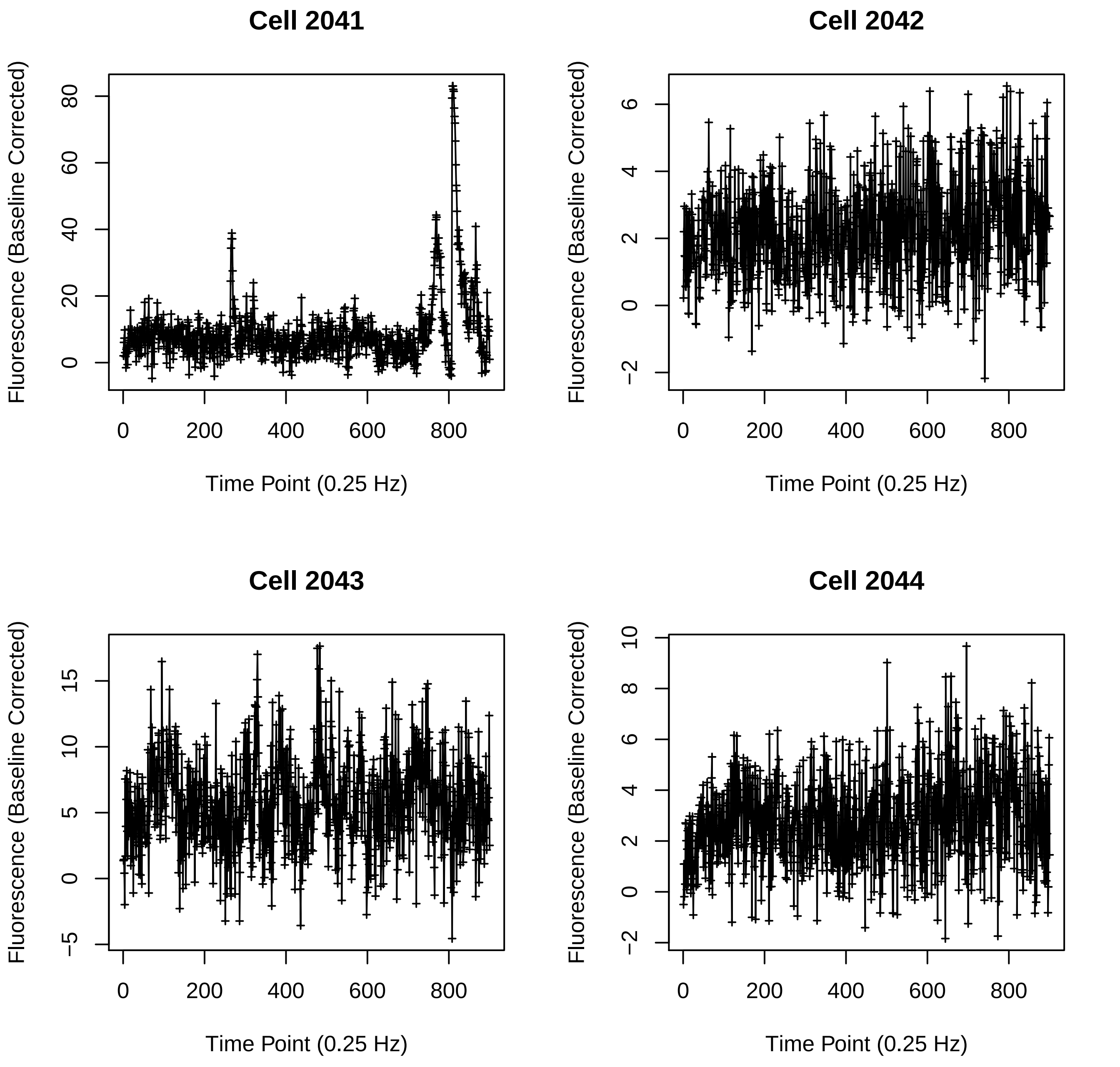
Cell 2041
Cell 2042
Cell 2043
Cell 2044
Fluorescence (Baseline Corrected)
Time Point (0.25 Hz)
0
200
400
600
800
20
40
60
80
−2
2
4
6
8
10
−5
5
15

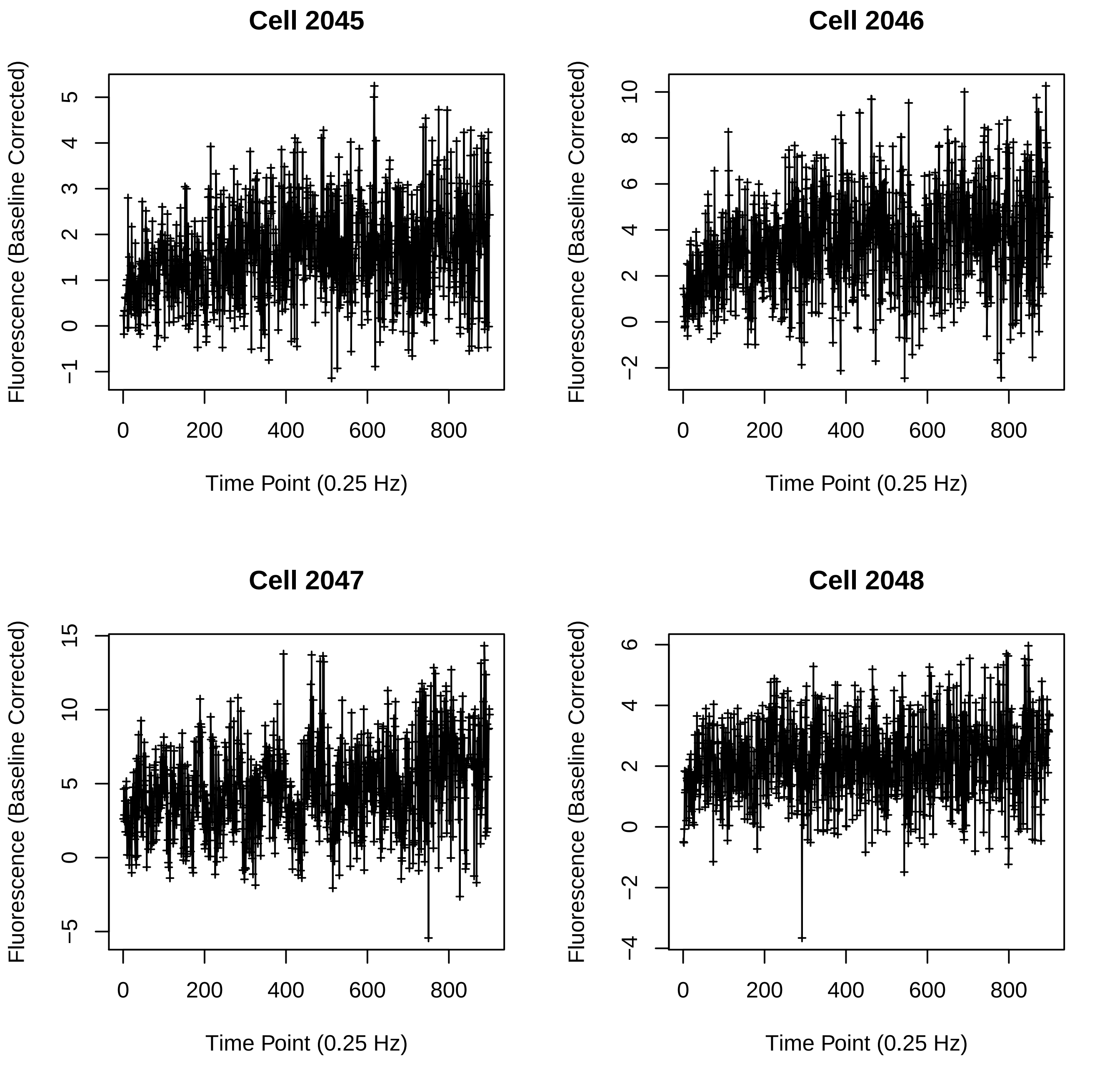
Cell 2045
Cell 2046
Cell 2047
Cell 2048
Fluorescence (Baseline Corrected)
Time Point (0.25 Hz)
0
200
400
600
800
−1
0
1
2
3
4
5
−2
2
4
6
8
10
−5
15
−4

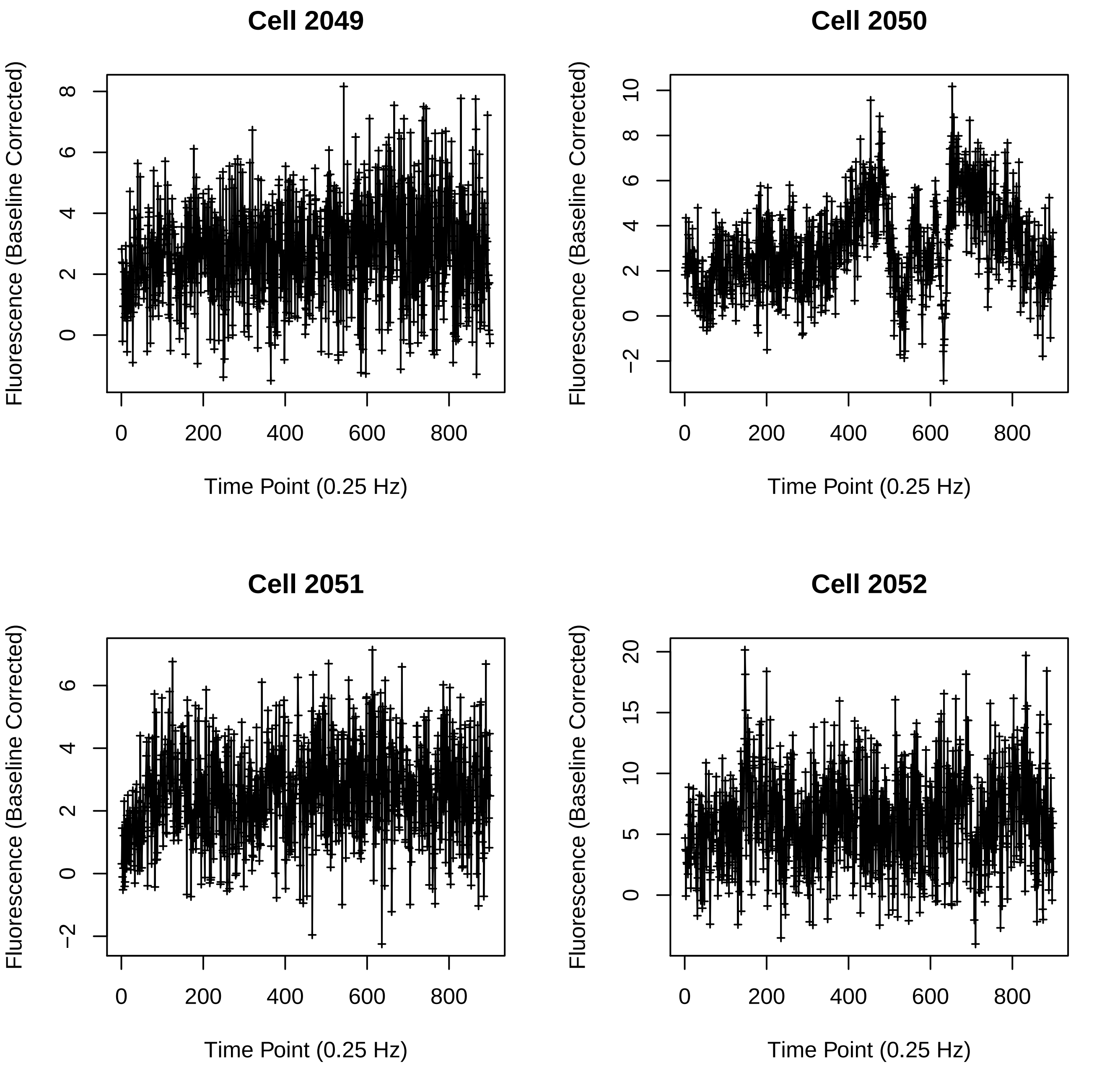
Cell 2049
Fluorescence (Baseline Corrected)
Time Point (0.25 Hz)
0
2
4
6
8
0
200
400
600
800
Cell 2050
Fluorescence (Baseline Corrected)
Time Point (0.25 Hz)
−2
0
2
4
6
8
10
0
200
400
600
800
Cell 2051
Fluorescence (Baseline Corrected)
Time Point (0.25 Hz)
−2
0
2
4
6
0
200
400
600
800
Cell 2052
Fluorescence (Baseline Corrected)
Time Point (0.25 Hz)
0
5
10
15
20
0
200
400
600
800

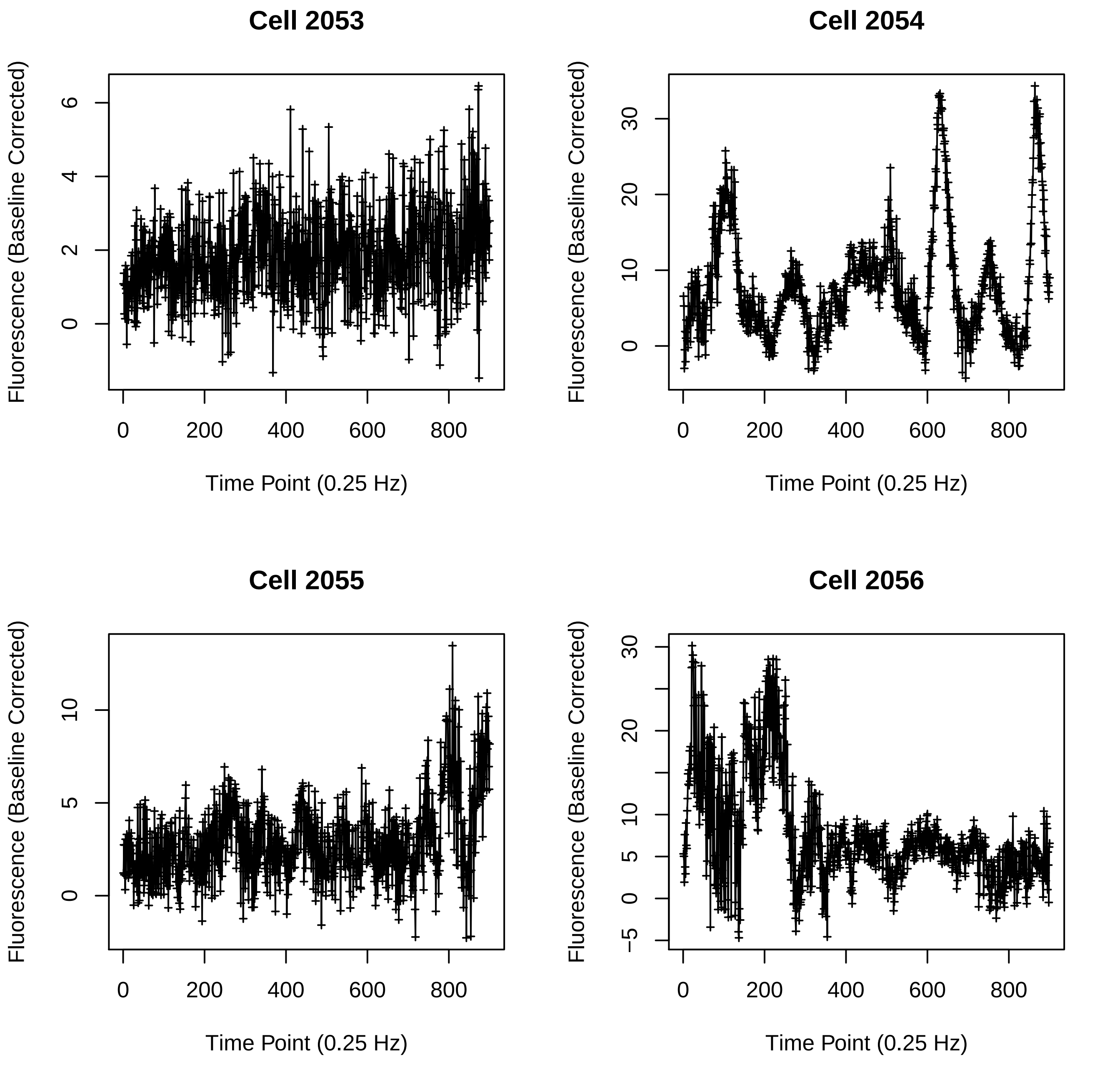

Cell 2053
Cell 2054
Cell 2055
Cell 2056
Fluorescence (Baseline Corrected)
Time Point (0.25 Hz)
0
200
400
600
800

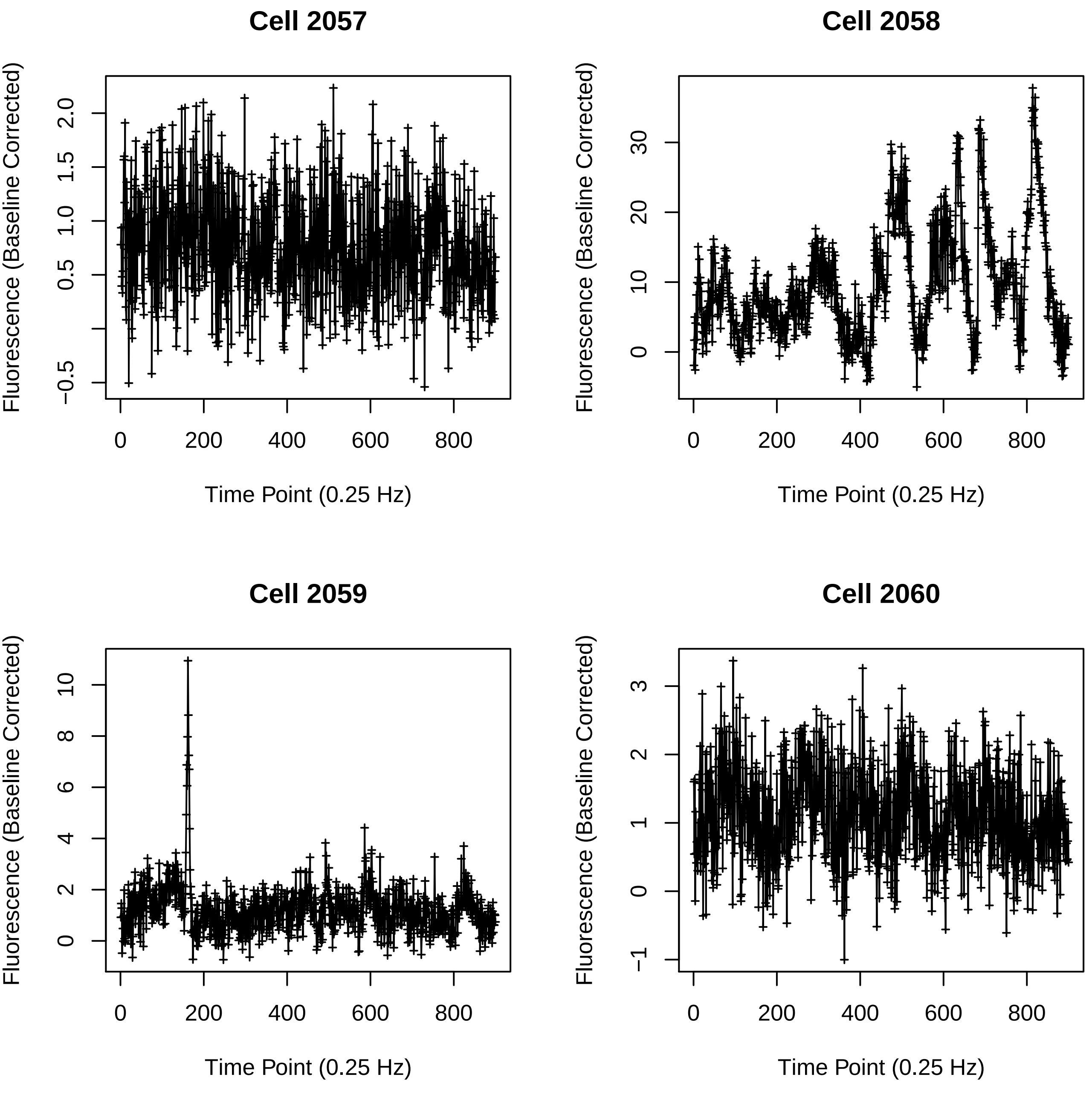
Cell 2057
Cell 2058
Cell 2059
Cell 2060
Fluorescence (Baseline Corrected)
Time Point (0.25 Hz)

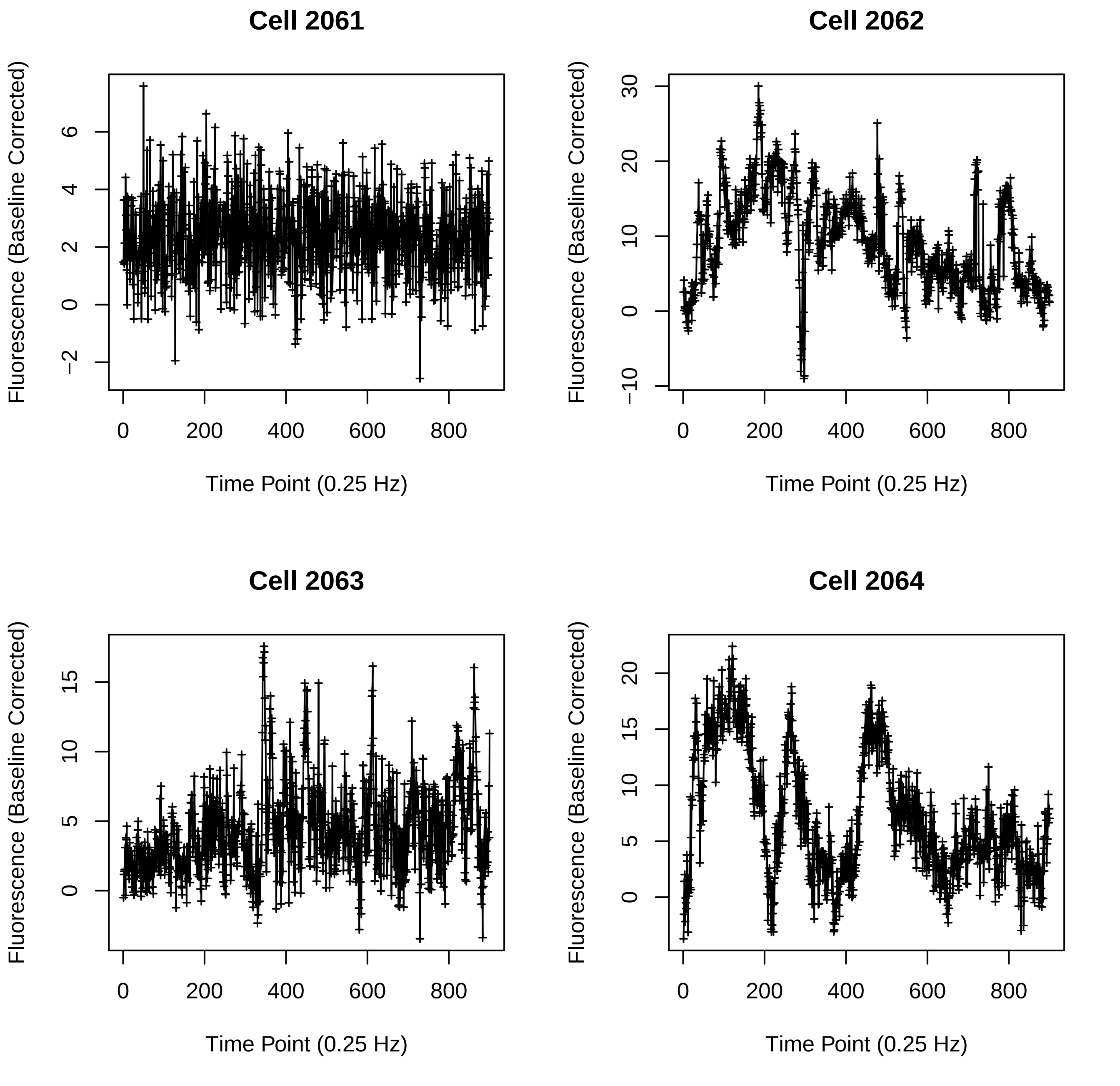
Cell 2061
Cell 2062
Cell 2063
Cell 2064
Fluorescence (Baseline Corrected)
Time Point (0.25 Hz)
0
200
400
600
800

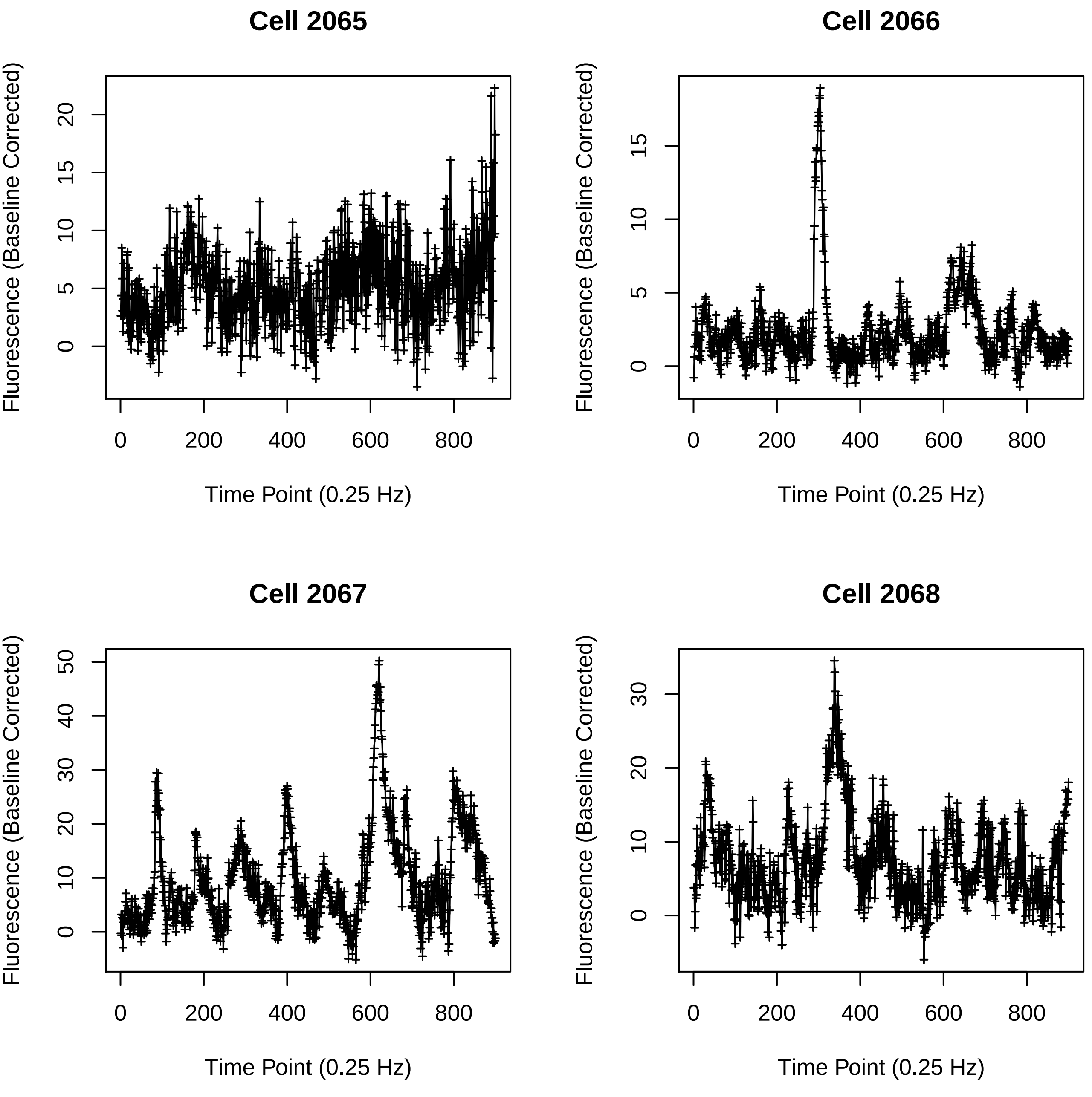
Cell 2065
Cell 2066
Cell 2067
Cell 2068
Fluorescence (Baseline Corrected)
Time Point (0.25 Hz)
0
200
400
600
800
0
5
10
15
20
30
40
50

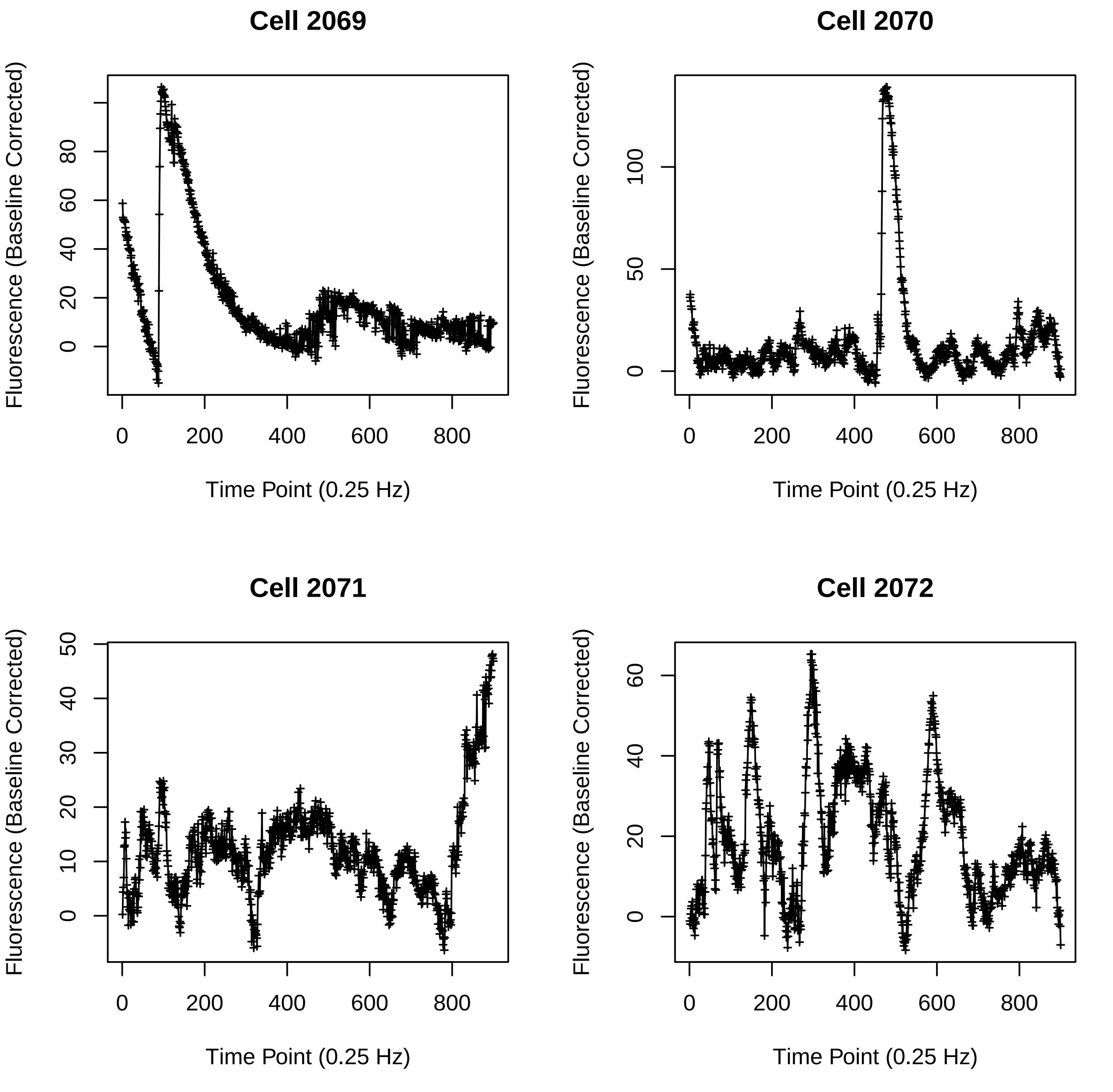
Cell 2069
Cell 2070
Cell 2071
Cell 2072
Fluorescence (Baseline Corrected)
Time Point (0.25 Hz)
0
20
40
60
80
0
50
100
0
10
20
30
40
50
0
20
40
60
0
200
400
600
800

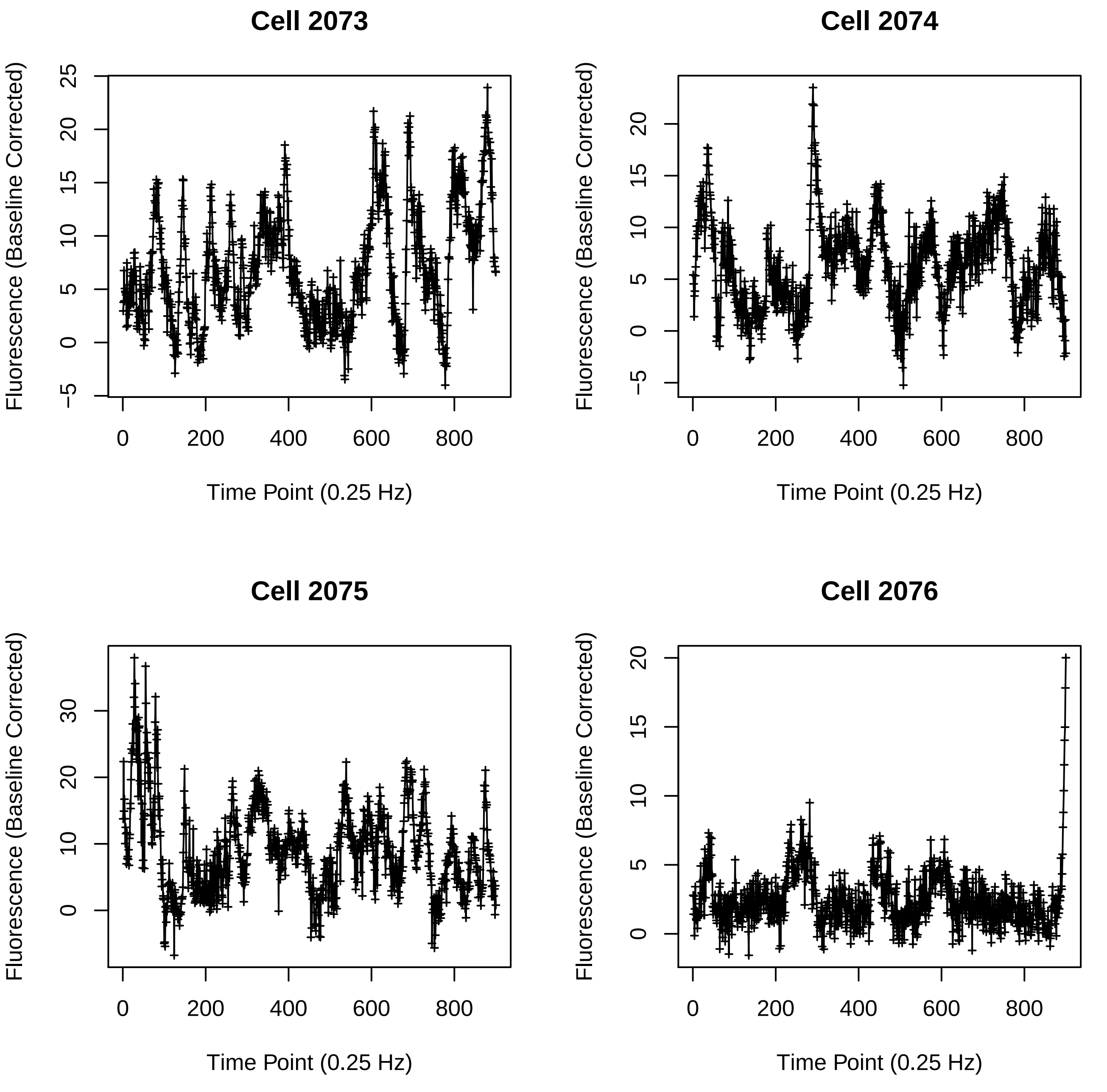
Cell 2073
Fluorescence (Baseline Corrected)
−5
0
5
10
15
20
25
Time Point (0.25 Hz)
0
200
400
600
800
Cell 2074
Fluorescence (Baseline Corrected)
−5
0
5
10
15
20
Time Point (0.25 Hz)
0
200
400
600
800
Cell 2075
Fluorescence (Baseline Corrected)
0
10
20
30
Time Point (0.25 Hz)
0
200
400
600
800
Cell 2076
Fluorescence (Baseline Corrected)
0
5
10
15
20
Time Point (0.25 Hz)
0
200
400
600
800

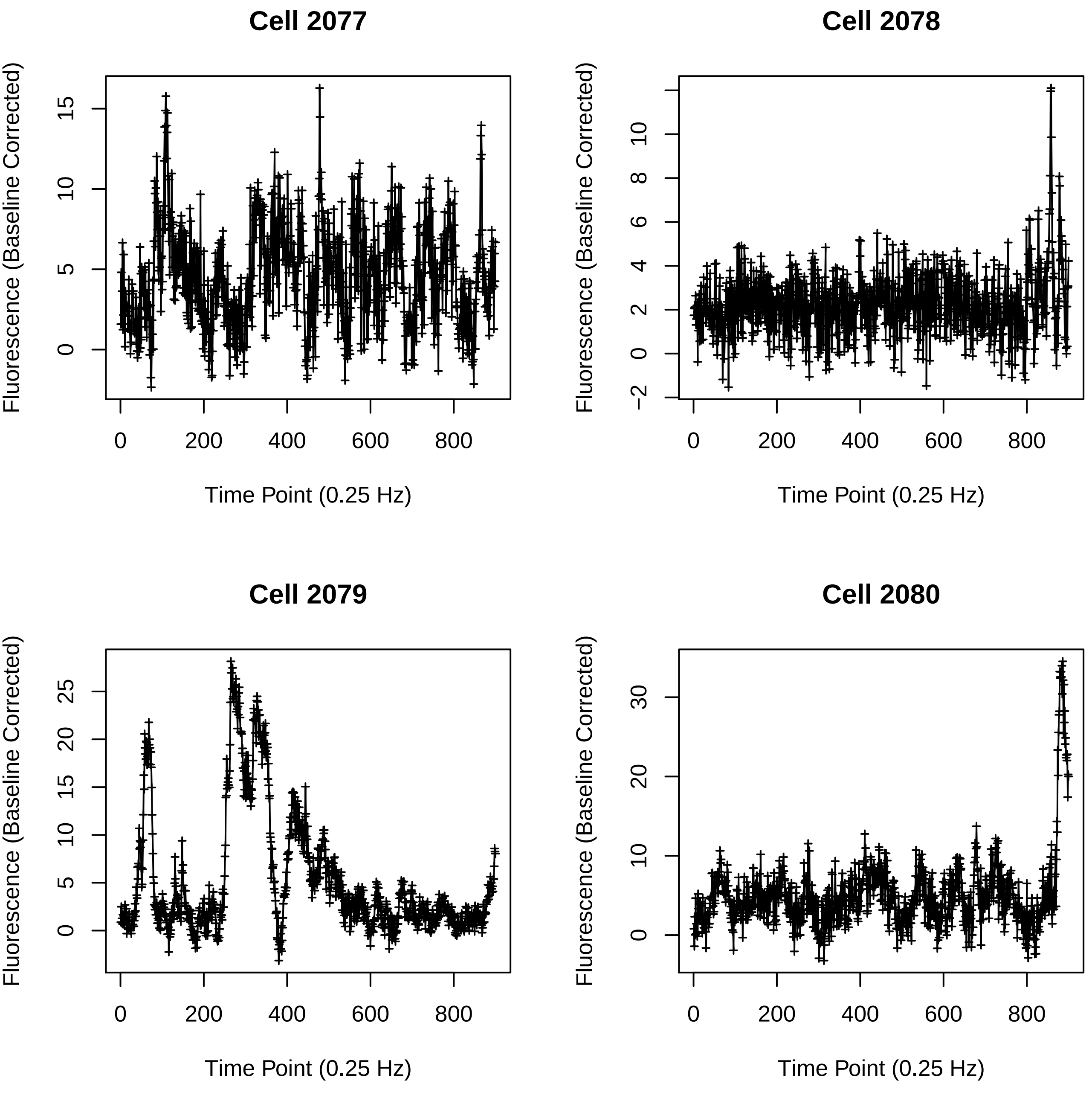

Cell 2077
Cell 2078
Cell 2079
Cell 2080
Fluorescence (Baseline Corrected)
Time Point (0.25 Hz)

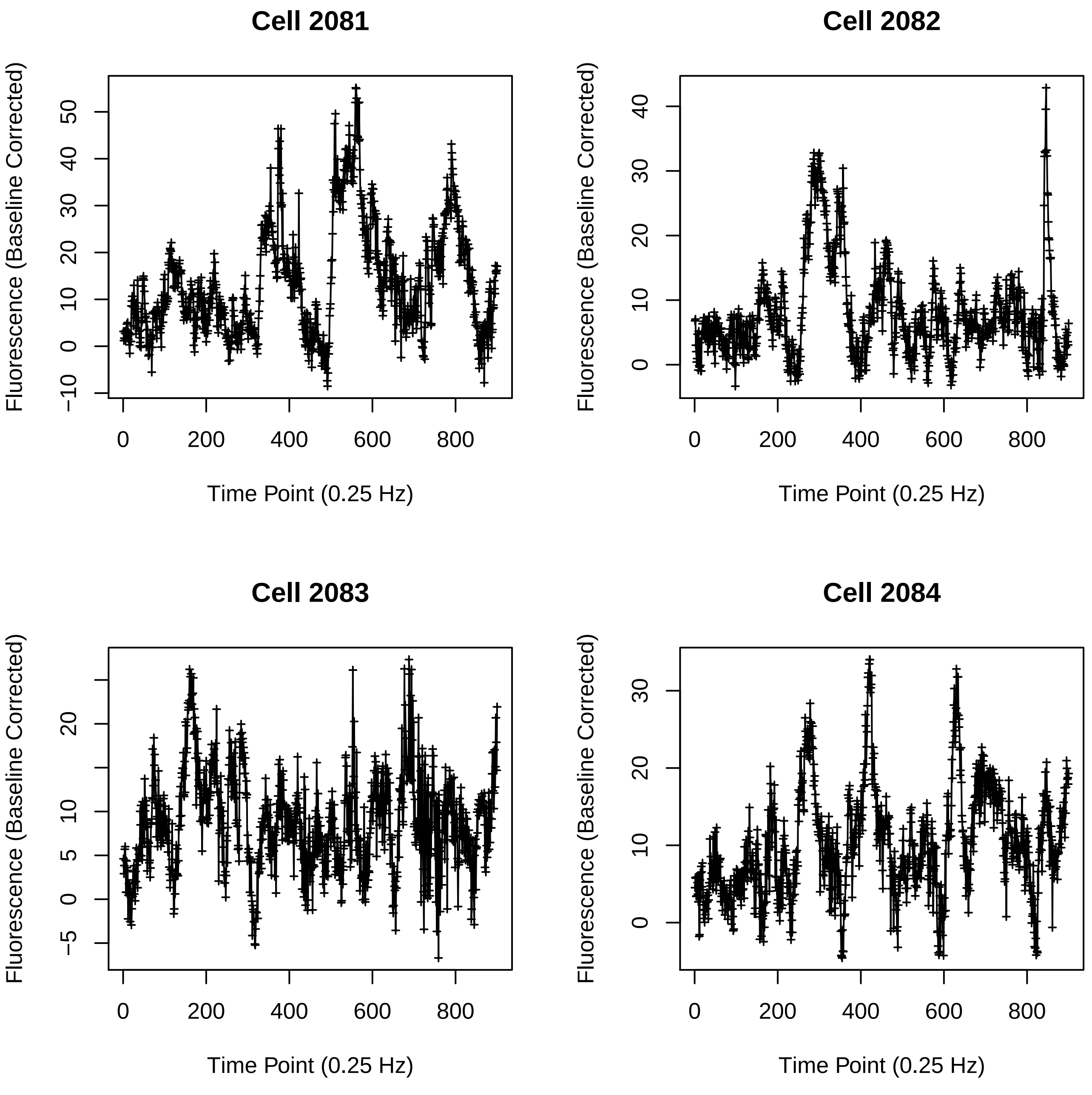
Cell 2081
Fluorescence (Baseline Corrected)
−10
0
10
20
30
40
50
0
200
400
600
800
Time Point (0.25 Hz)
Cell 2082
Fluorescence (Baseline Corrected)
0
10
20
30
40
0
200
400
600
800
Time Point (0.25 Hz)
Cell 2083
Fluorescence (Baseline Corrected)
−5
0
5
10
20
0
200
400
600
800
Time Point (0.25 Hz)
Cell 2084
Fluorescence (Baseline Corrected)
0
10
20
30
0
200
400
600
800
Time Point (0.25 Hz)

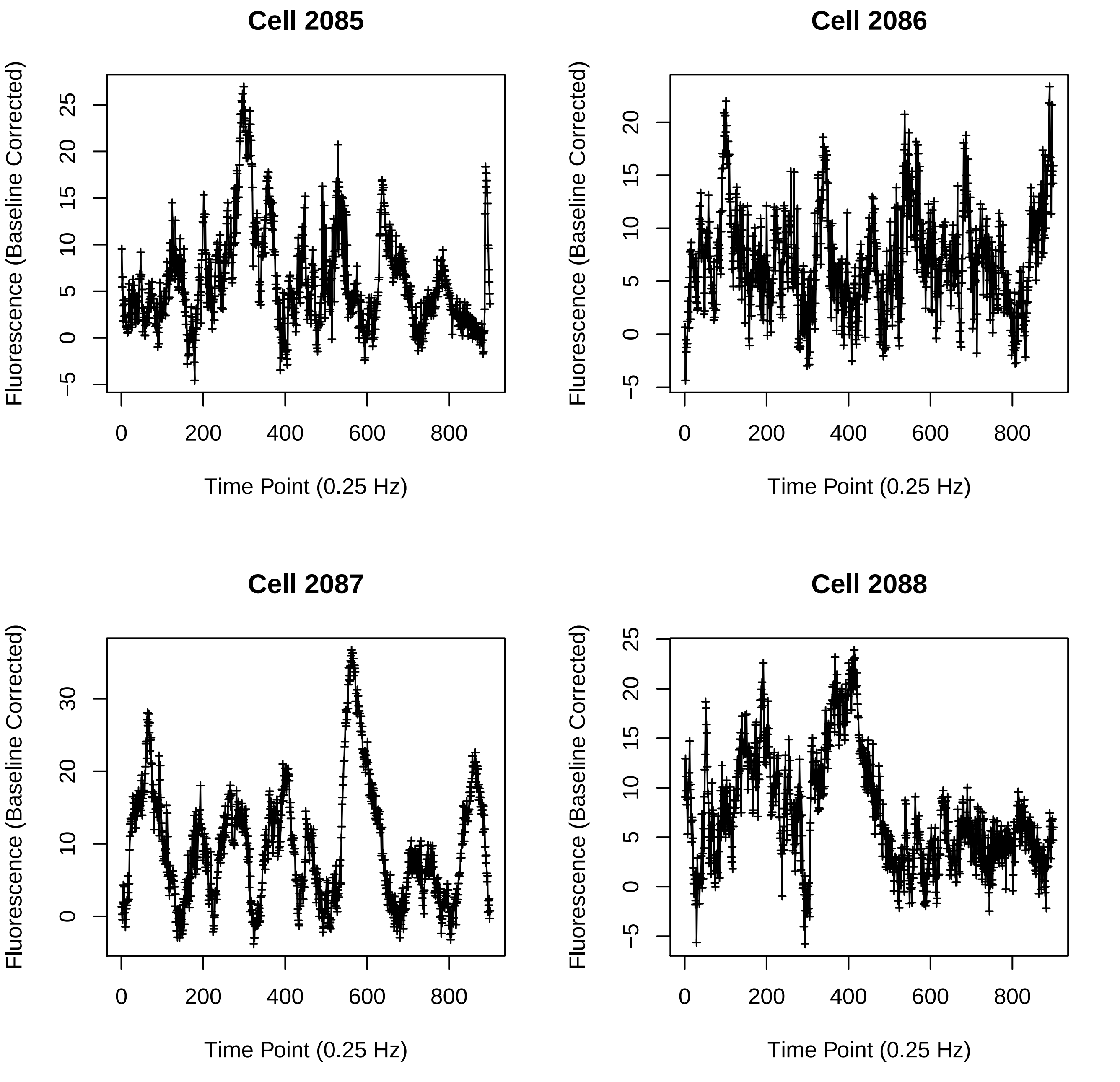

Cell 2085
Cell 2086
Cell 2087
Cell 2088
Fluorescence (Baseline Corrected)
Time Point (0.25 Hz)
0
200
400
600
800
−5
0
5
10
15
20
25
30

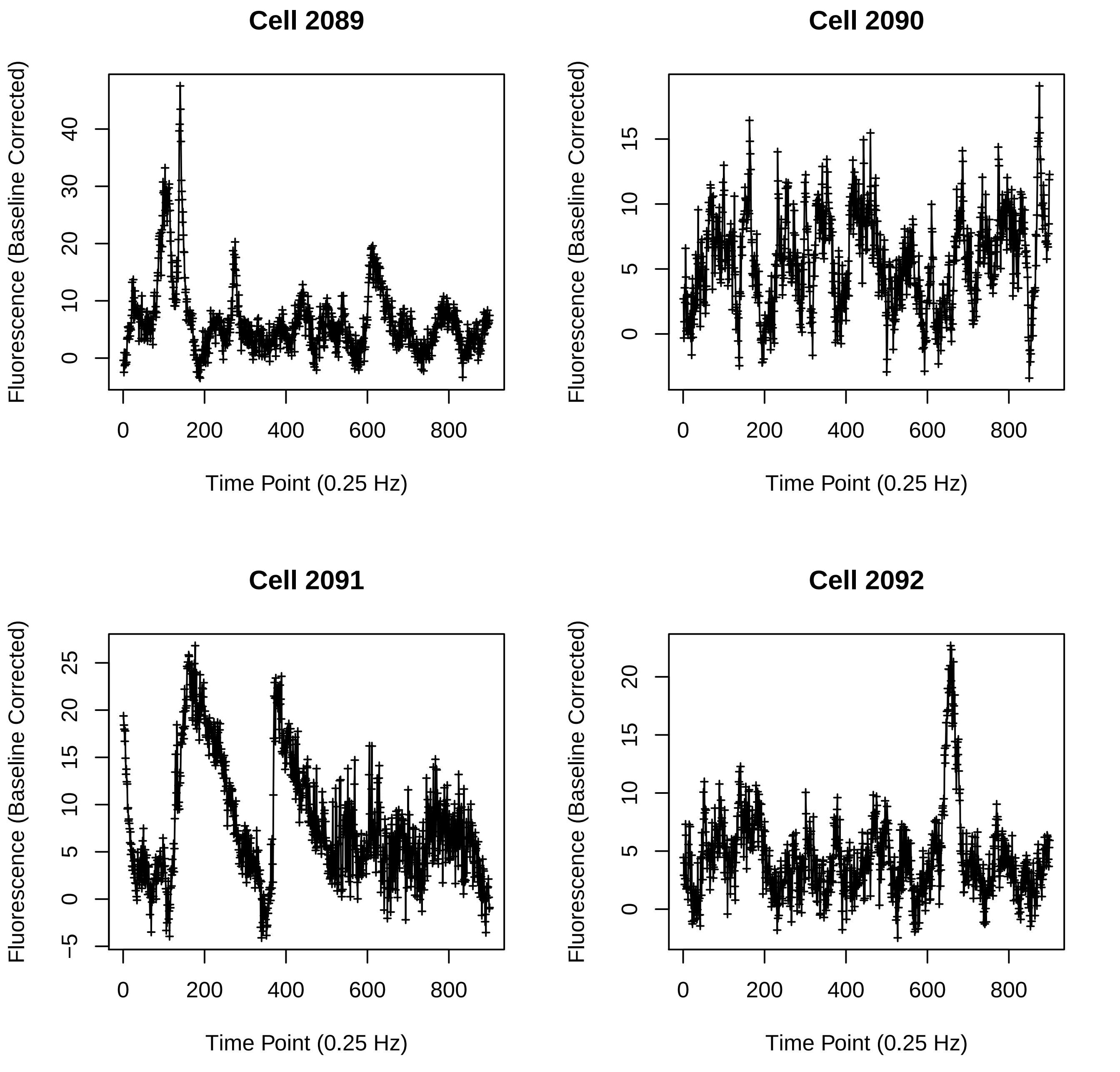

Cell 2089
Cell 2090
Cell 2091
Cell 2092
Fluorescence (Baseline Corrected)
Time Point (0.25 Hz)
0
200
400
600
800
−5
5
10
15
20
25
30
40

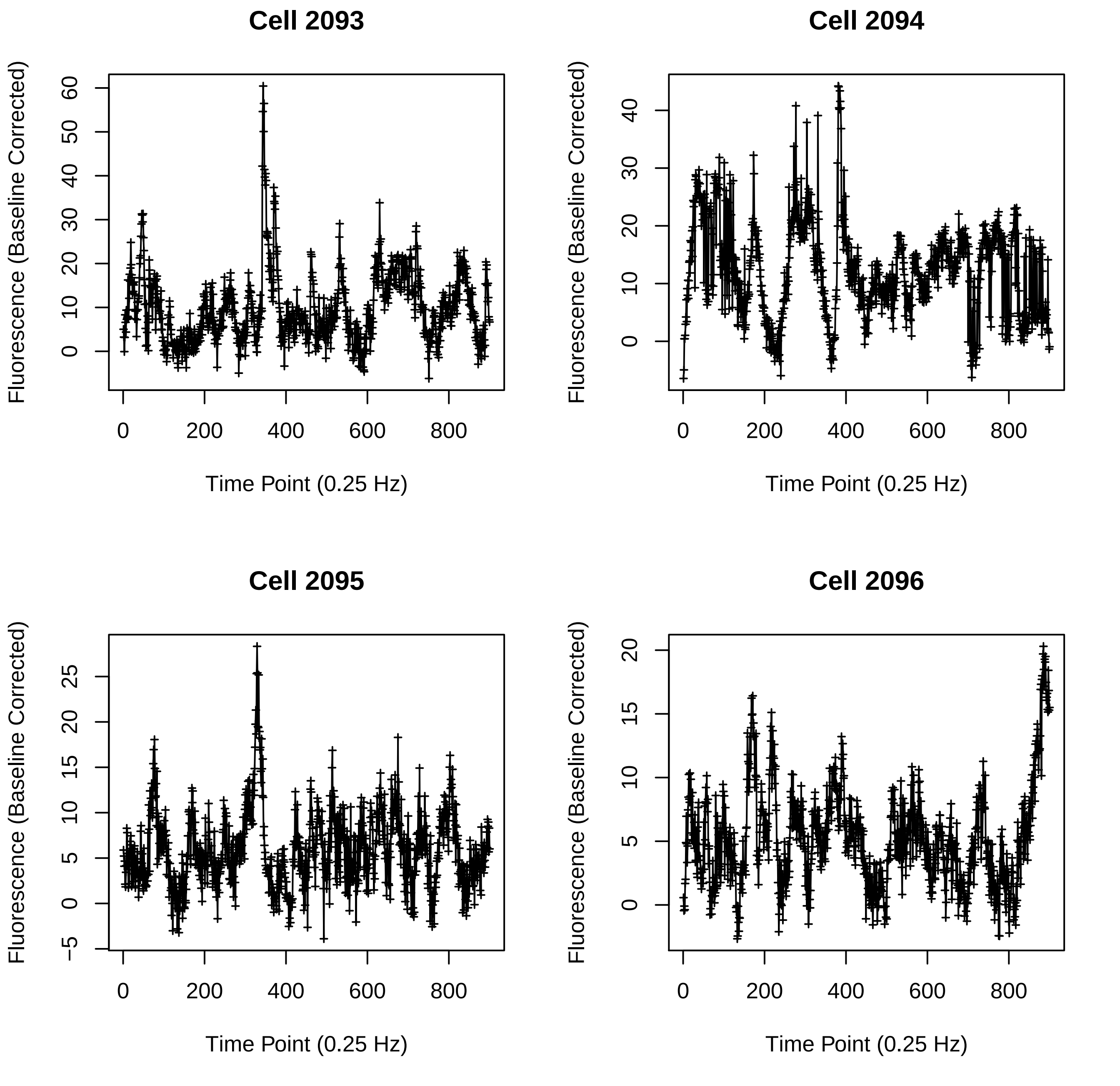
Cell 2093
Cell 2094
Cell 2095
Cell 2096
Fluorescence (Baseline Corrected)
Time Point (0.25 Hz)
0
200
400
600
800
0
10
20
30
40
50
60
−5
5
15
25

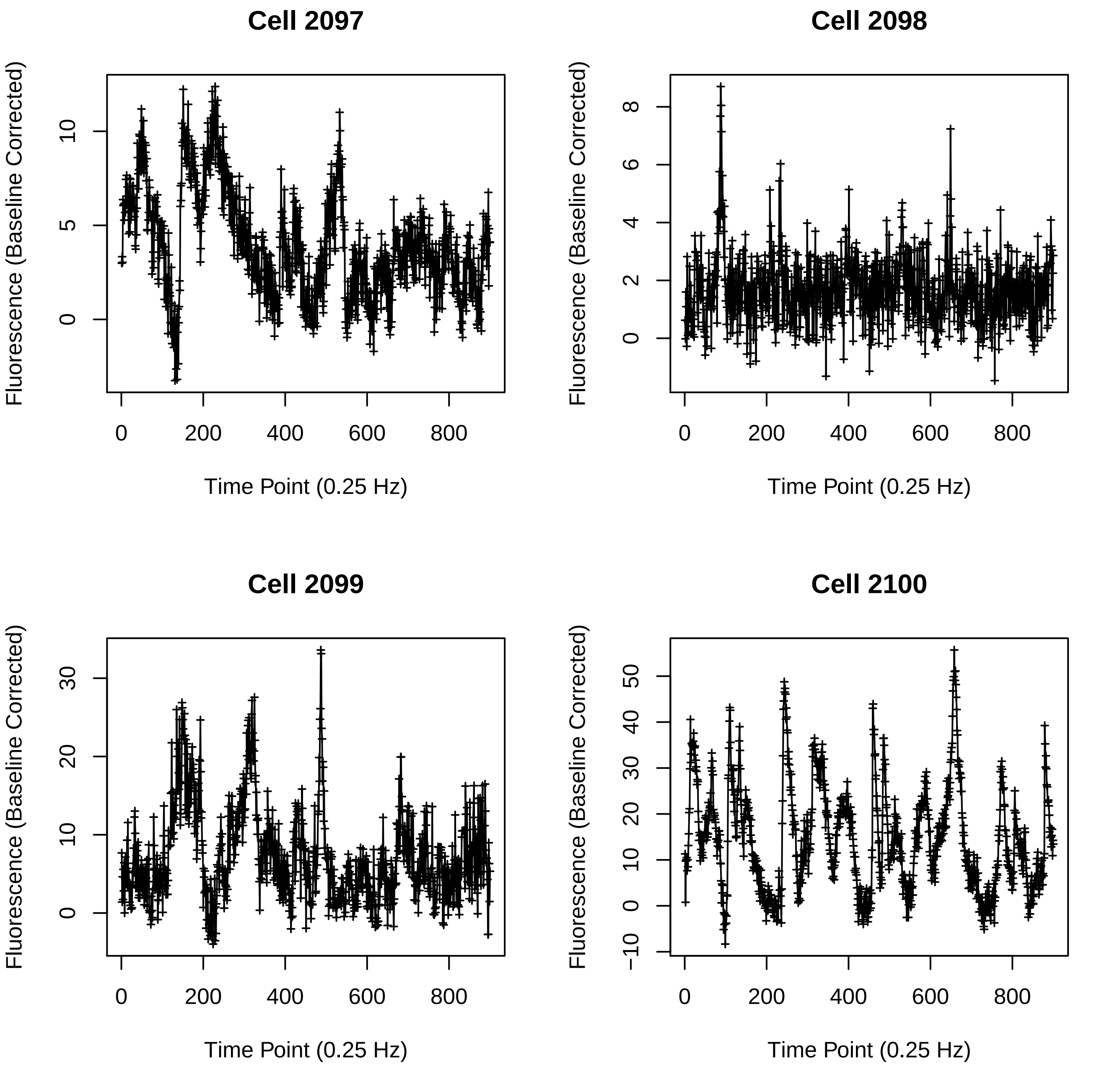
Cell 2097
Fluorescence (Baseline Corrected)
Time Point (0.25 Hz)
0
5
10
0
200
400
600
800
Cell 2098
Fluorescence (Baseline Corrected)
Time Point (0.25 Hz)
0
2
4
6
8
0
200
400
600
800
Cell 2099
Fluorescence (Baseline Corrected)
Time Point (0.25 Hz)
0
10
20
30
0
200
400
600
800
Cell 2100
Fluorescence (Baseline Corrected)
Time Point (0.25 Hz)
−10
0
10
20
30
40
50
0
200
400
600
800

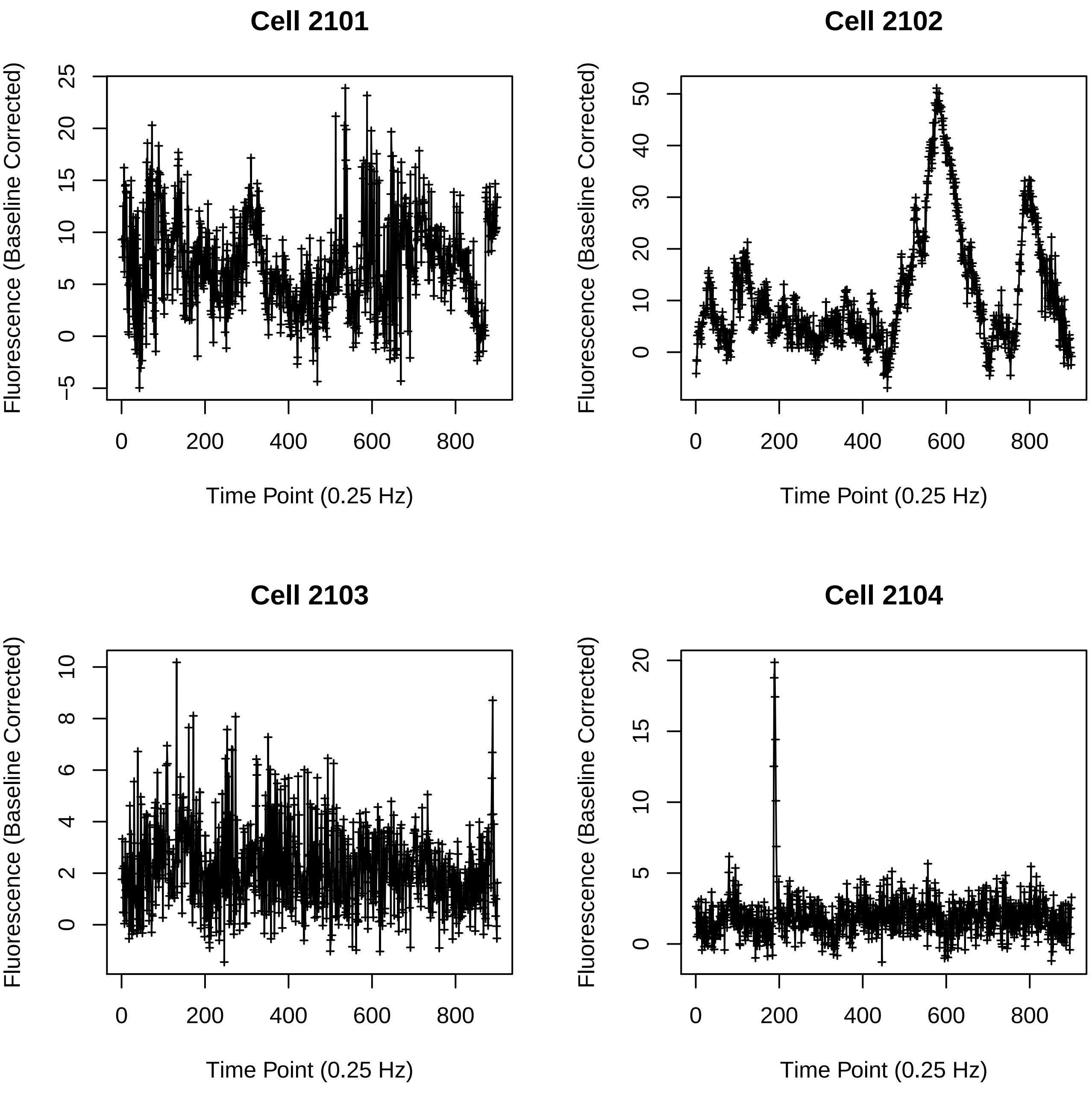
Cell 2101
Cell 2102
Cell 2103
Cell 2104
Fluorescence (Baseline Corrected)
Time Point (0.25 Hz)
0
200
400
600
800
−5
5
10
15
20
25
30
40
50

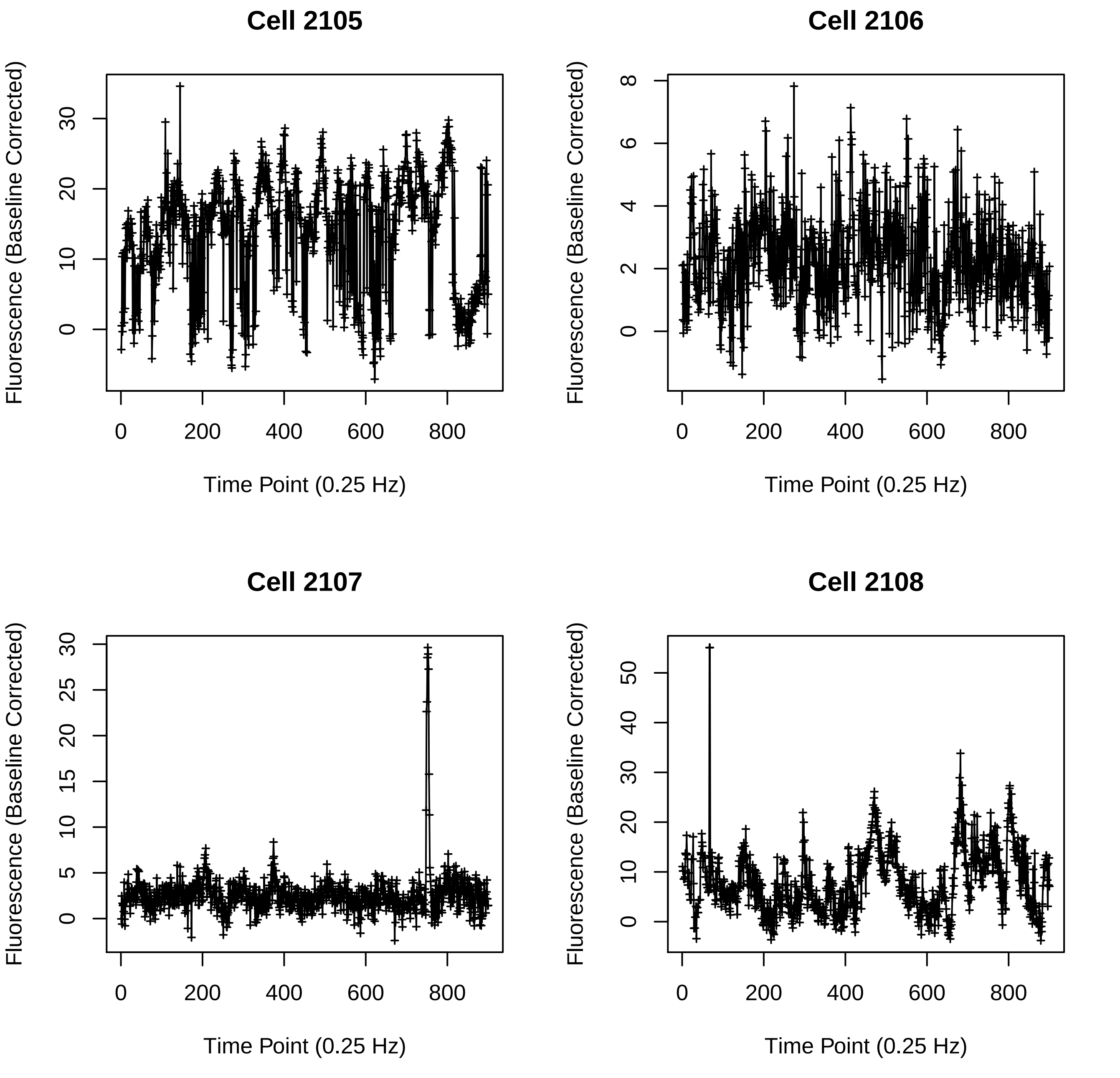
Cell 2105
Cell 2106
Cell 2107
Cell 2108
Fluorescence (Baseline Corrected)
Time Point (0.25 Hz)
0
200
400
600
800
10
20
30
2
4
6
8
5
15
25
40
50

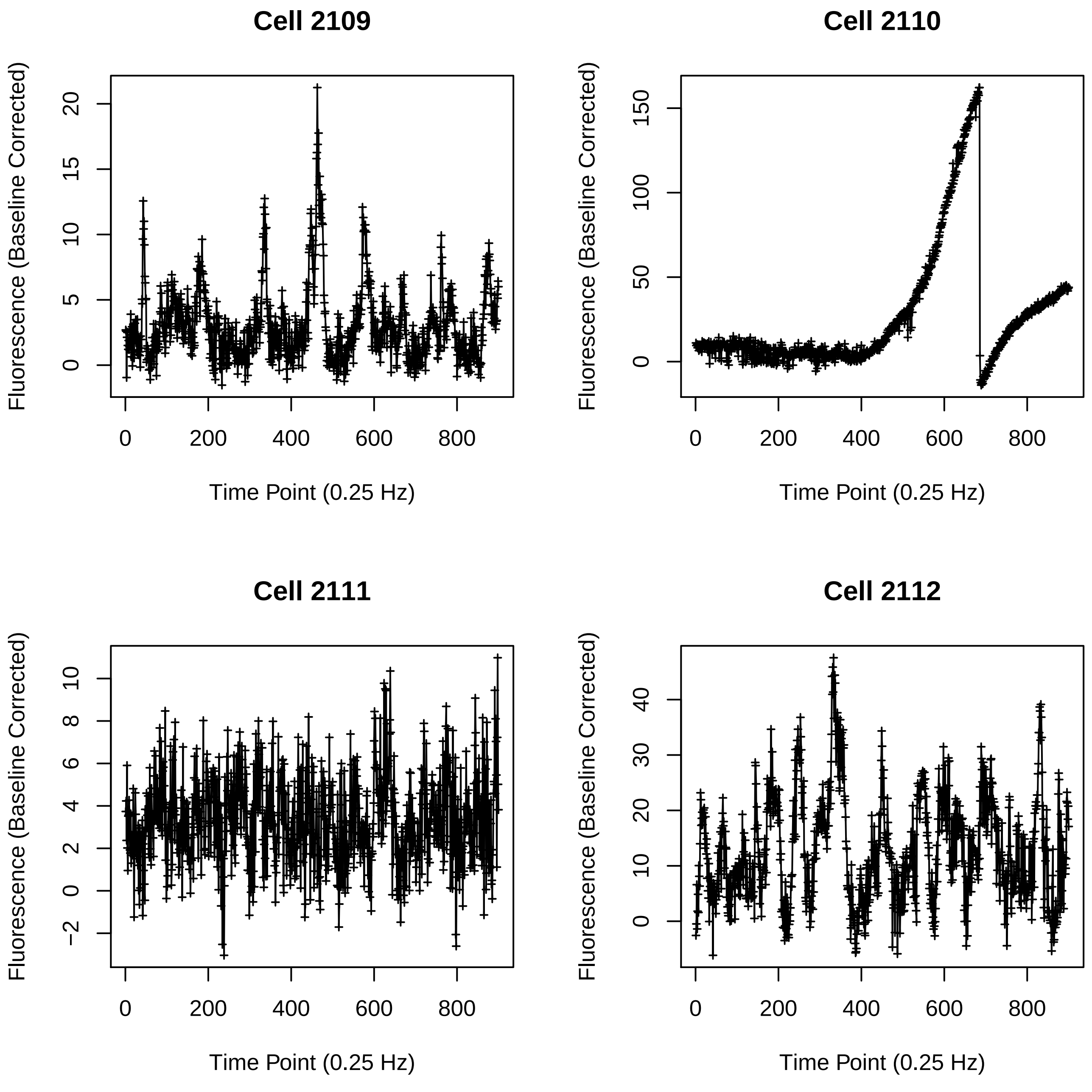
Cell 2109
Cell 2110
Cell 2111
Cell 2112
Fluorescence (Baseline Corrected)
Time Point (0.25 Hz)
0
5
10
15
20
50
100
150
−2
2
4
6
8
30
40
200
400
600
800

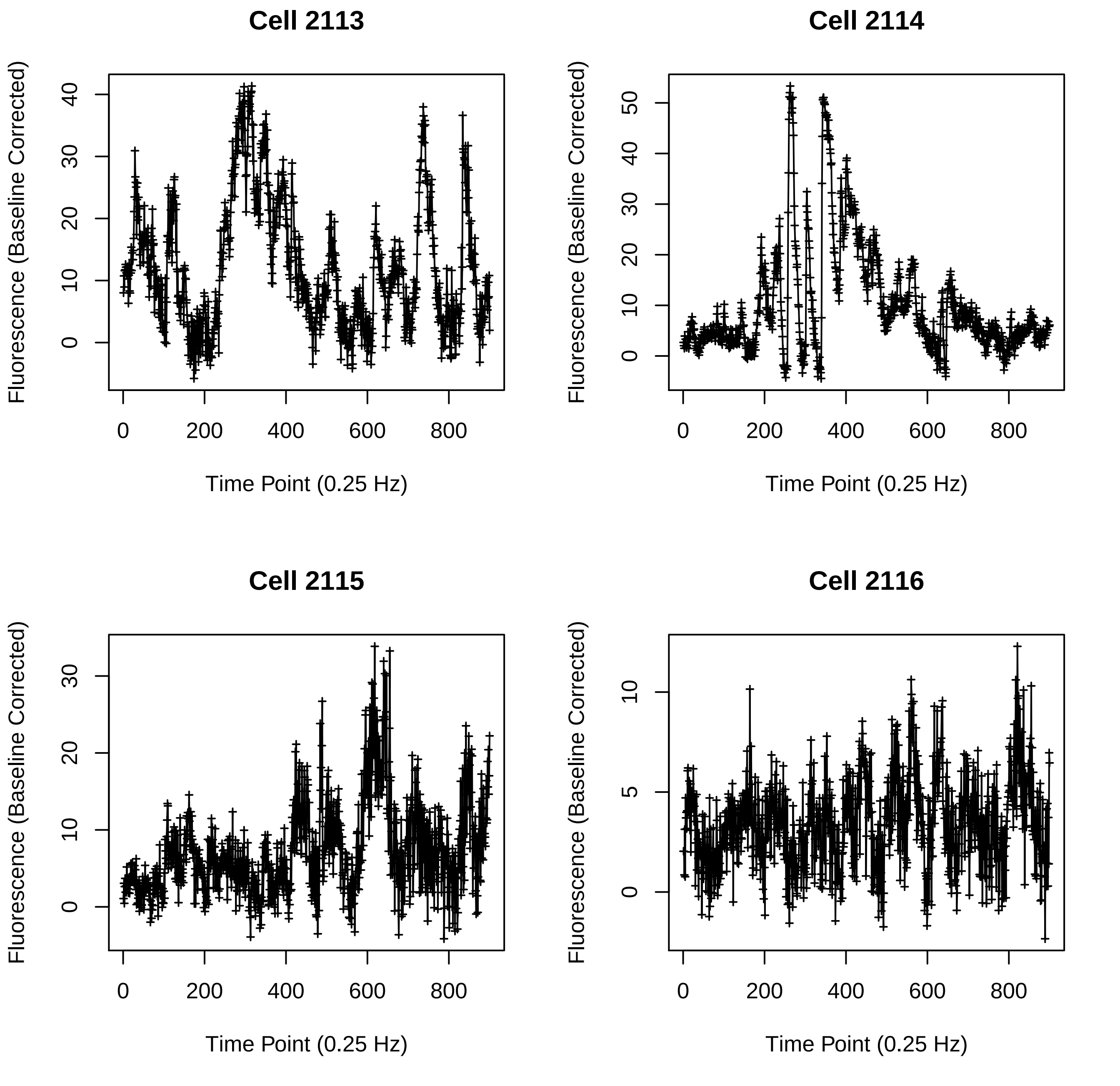
Cell 2113
Cell 2114
Cell 2115
Cell 2116
Fluorescence (Baseline Corrected)
Time Point (0.25 Hz)
0
200
400
600
800
10
20
30
40
50
5

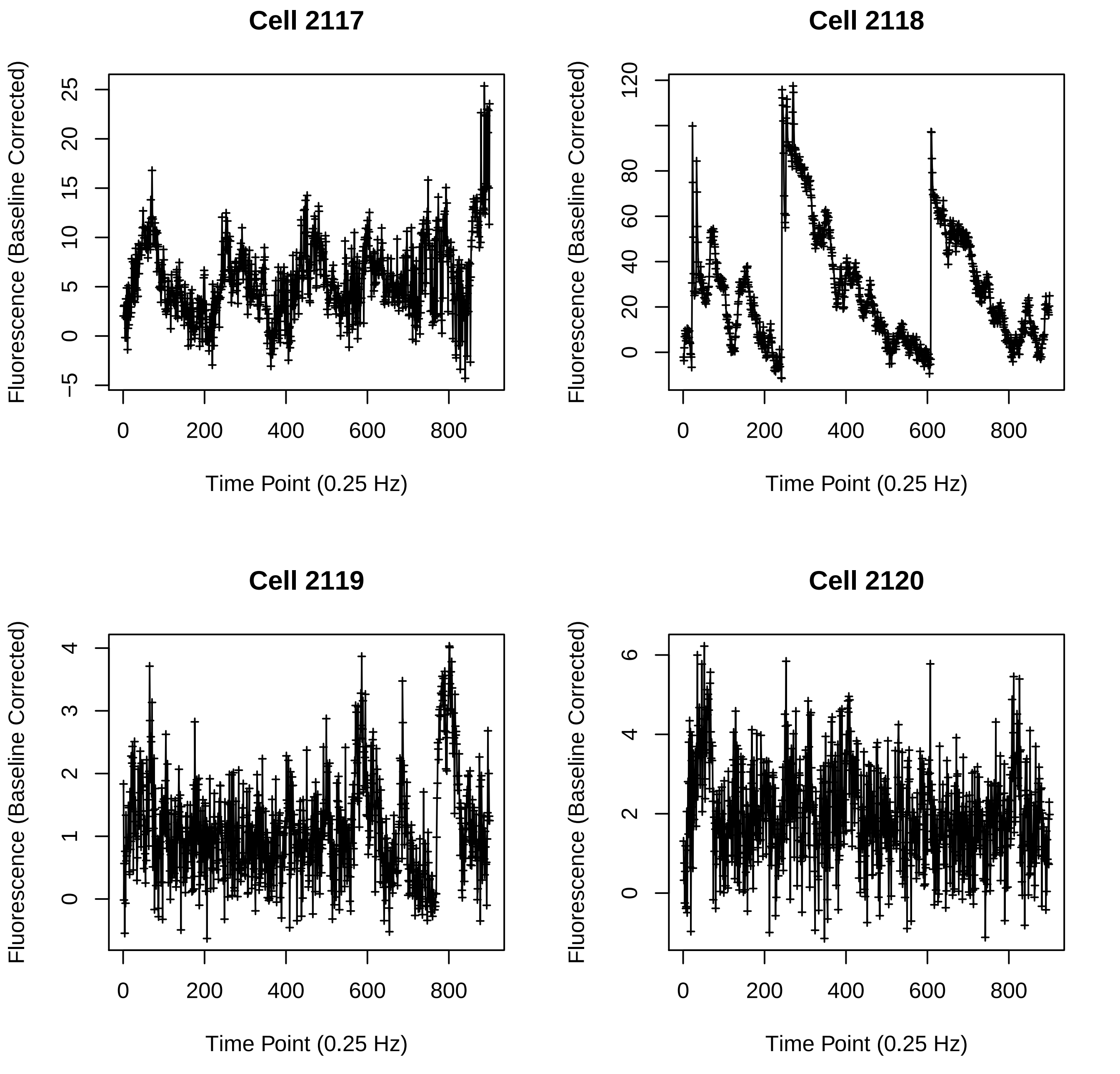
Cell 2117
Cell 2118
Cell 2119
Cell 2120
Fluorescence (Baseline Corrected)
Time Point (0.25 Hz)
-5
0
5
10
15
20
25
0
20
40
60
80
120
1
2
3
4
6
200
400
600
800

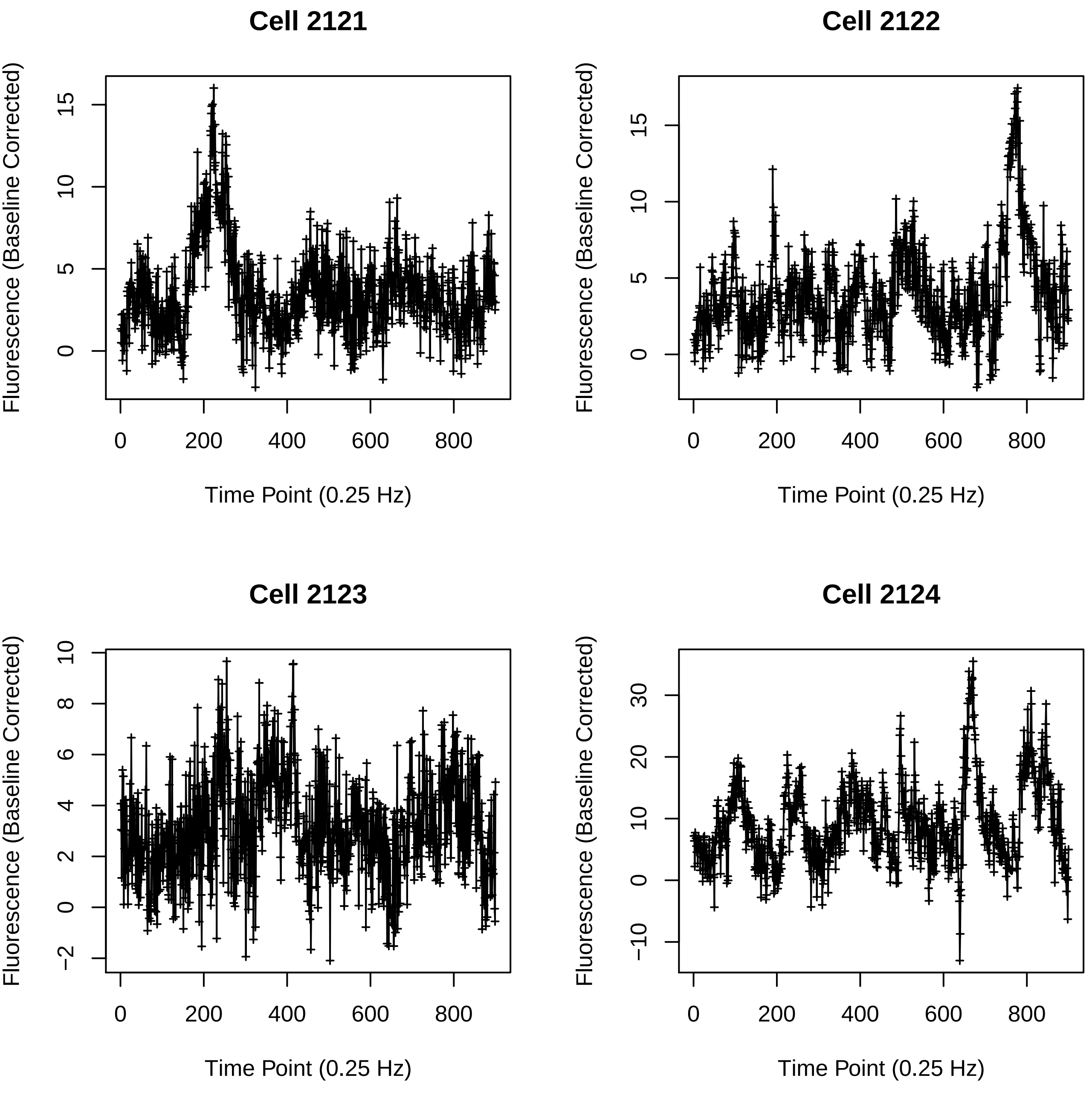
Cell 2121
Cell 2122
Cell 2123
Cell 2124
Fluorescence (Baseline Corrected)
Time Point (0.25 Hz)

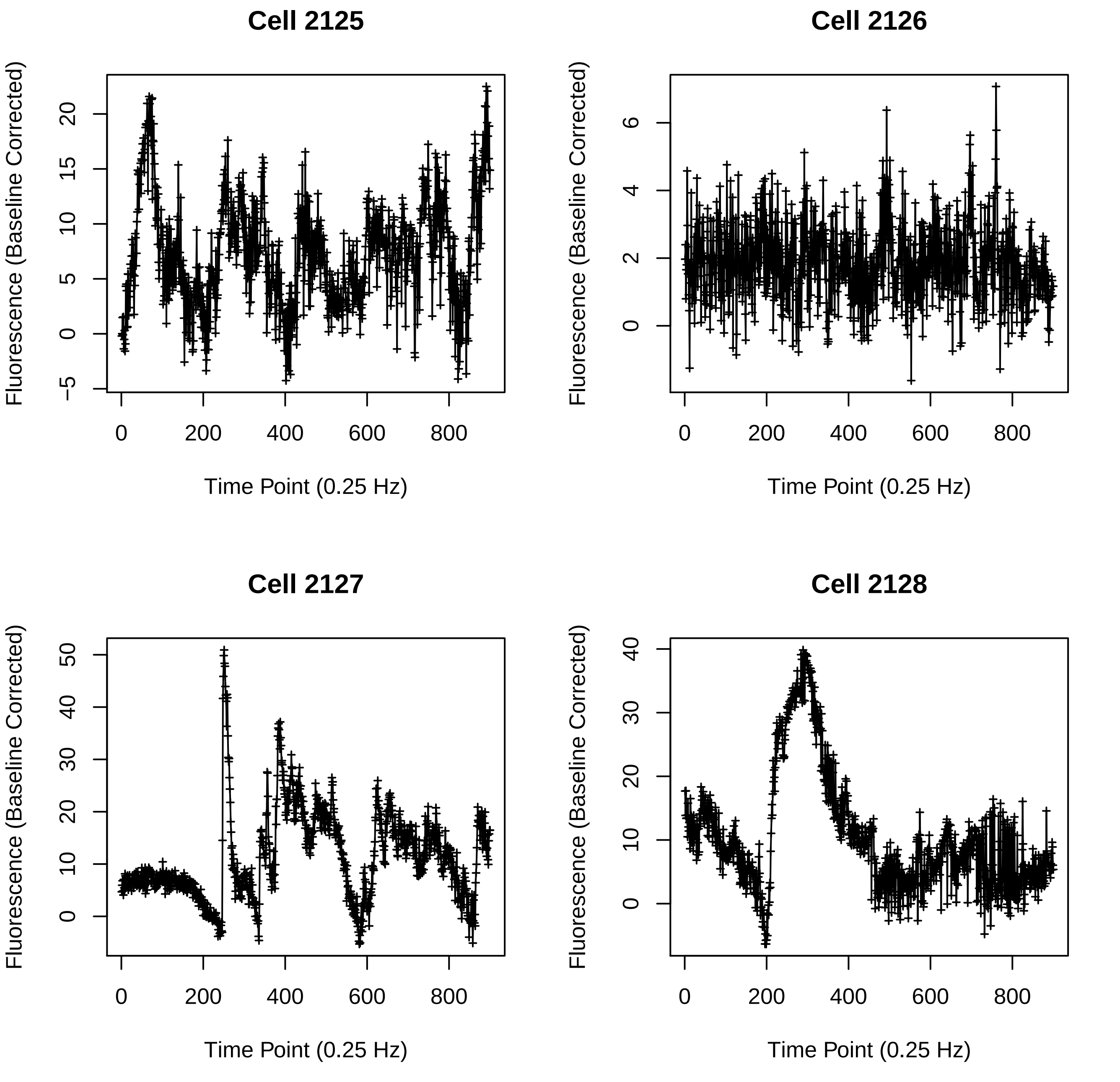
Cell 2125
Fluorescence (Baseline Corrected)
−5
0
5
10
15
20
0
200
400
600
800
Time Point (0.25 Hz)
Cell 2126
Fluorescence (Baseline Corrected)
0
2
4
6
0
200
400
600
800
Time Point (0.25 Hz)
Cell 2127
Fluorescence (Baseline Corrected)
0
10
20
30
40
50
0
200
400
600
800
Time Point (0.25 Hz)
Cell 2128
Fluorescence (Baseline Corrected)
0
10
20
30
40
0
200
400
600
800
Time Point (0.25 Hz)

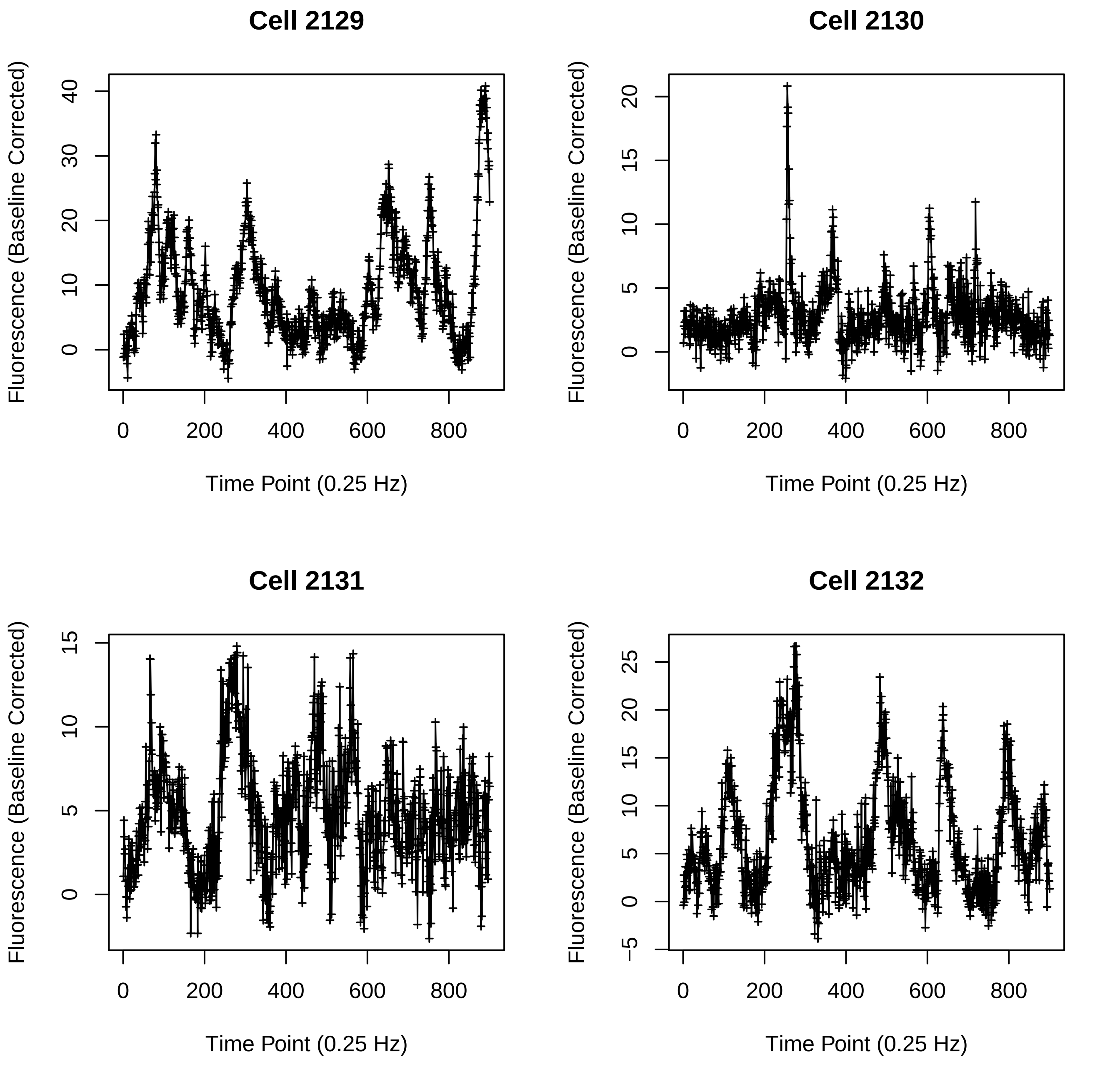
Cell 2129
Cell 2130
Cell 2131
Cell 2132
Fluorescence (Baseline Corrected)
Time Point (0.25 Hz)
0
200
400
600
800
0
10
20
30
40
5
15
25
−5

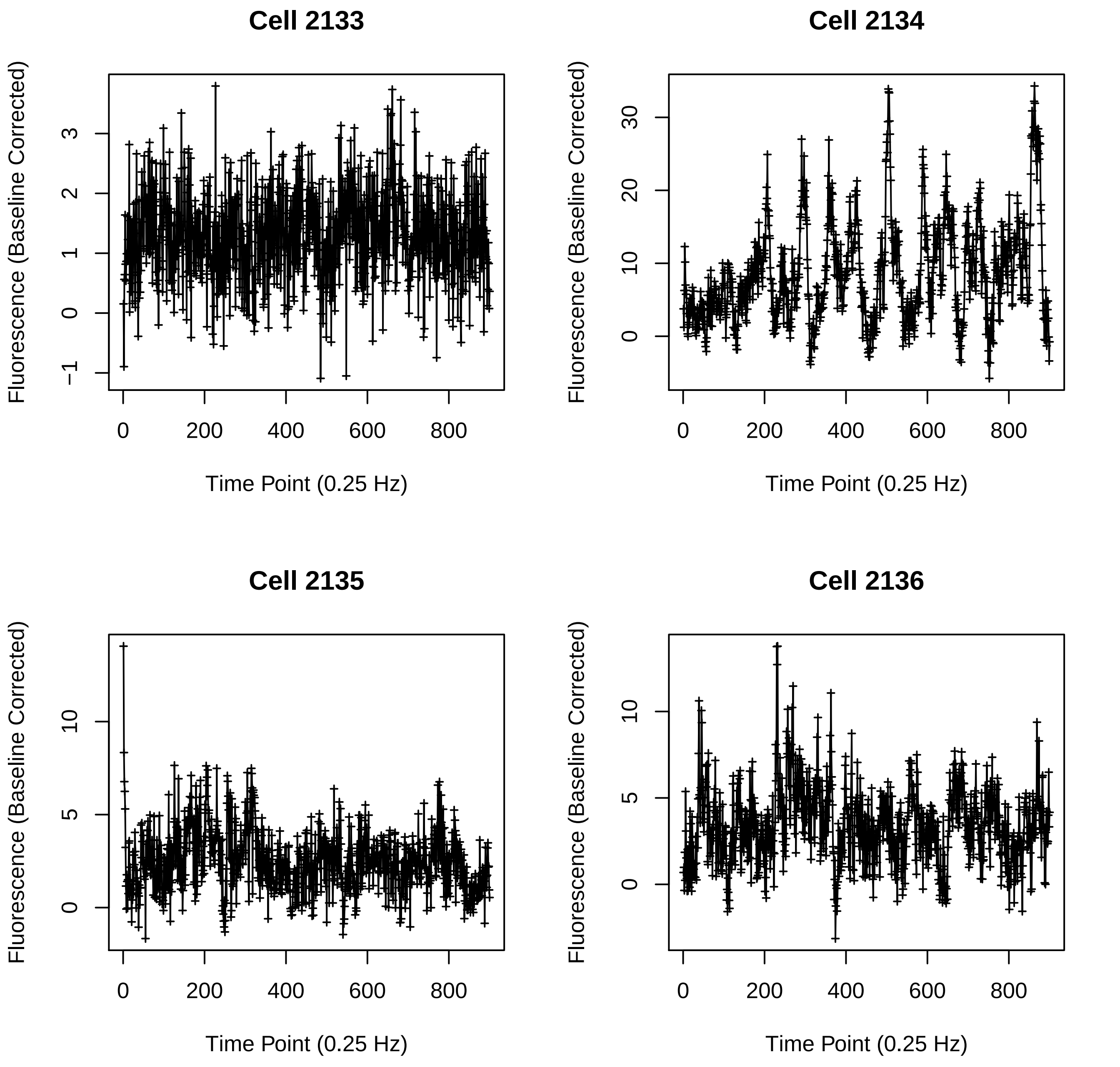
Cell 2133
Fluorescence (Baseline Corrected)
−1
0
1
2
3
0
200
400
600
800
Time Point (0.25 Hz)
Cell 2134
Fluorescence (Baseline Corrected)
0
10
20
30
0
200
400
600
800
Time Point (0.25 Hz)
Cell 2135
Fluorescence (Baseline Corrected)
0
5
10
0
200
400
600
800
Time Point (0.25 Hz)
Cell 2136
Fluorescence (Baseline Corrected)
0
5
10
0
200
400
600
800
Time Point (0.25 Hz)

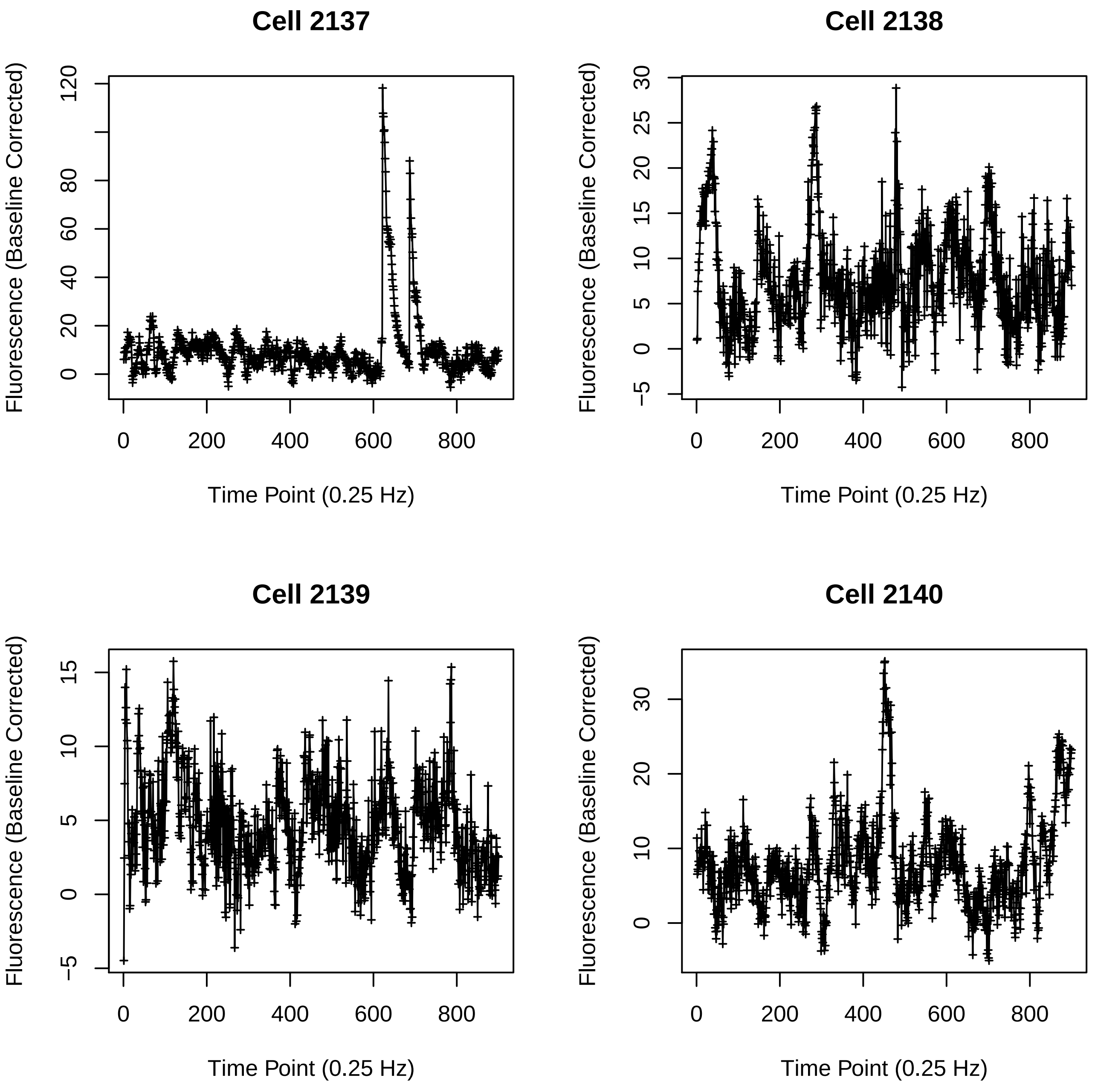

Cell 2137
Cell 2138
Cell 2139
Cell 2140
Fluorescence (Baseline Corrected)
Time Point (0.25 Hz)

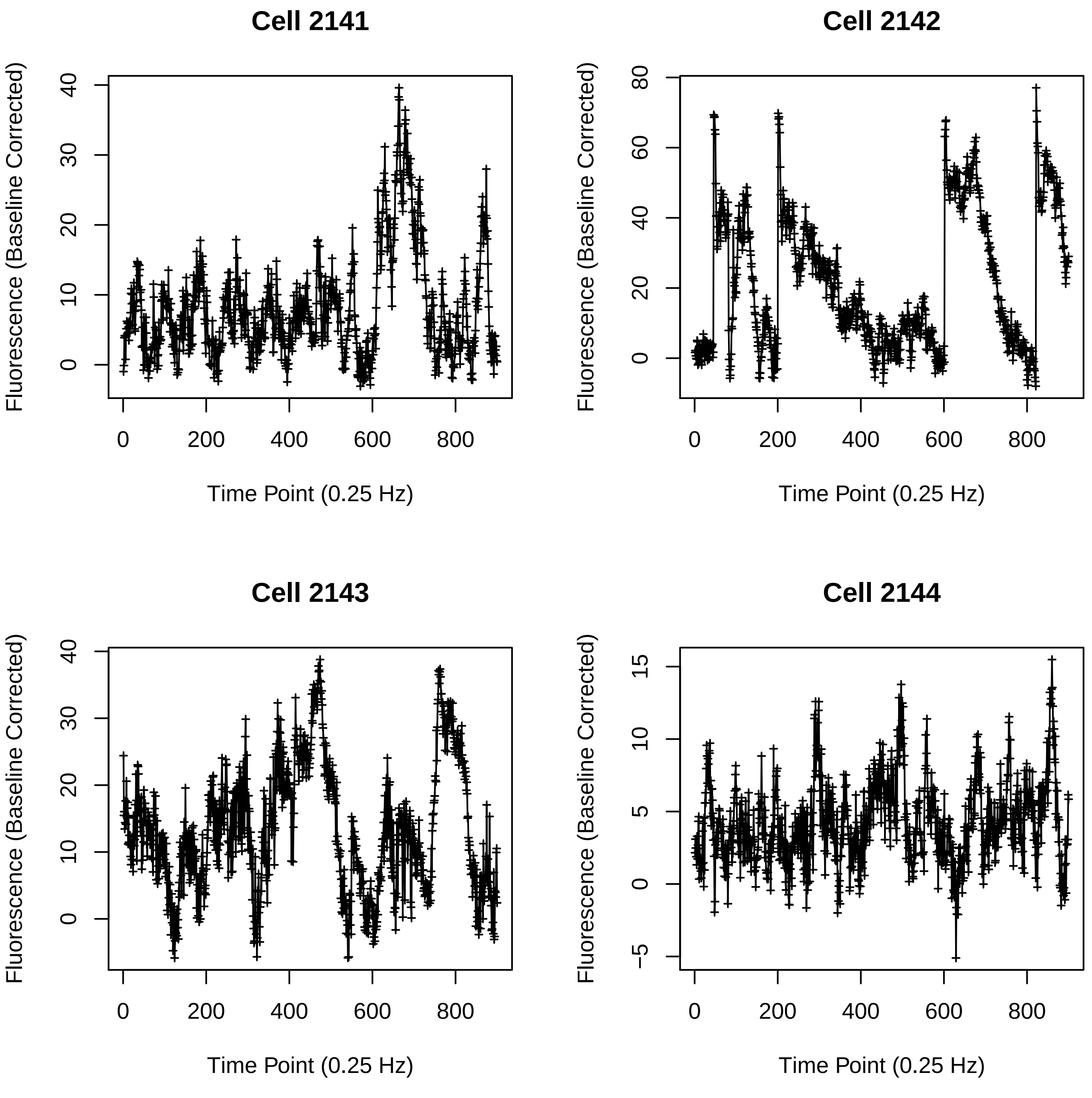

Cell 2141
Fluorescence (Baseline Corrected)
0
10
20
30
40
0
200
400
600
800
Time Point (0.25 Hz)
Cell 2142
Fluorescence (Baseline Corrected)
0
20
40
60
80
0
200
400
600
800
Time Point (0.25 Hz)
Cell 2143
Fluorescence (Baseline Corrected)
0
10
20
30
40
0
200
400
600
800
Time Point (0.25 Hz)
Cell 2144
Fluorescence (Baseline Corrected)
−5
0
5
10
15
0
200
400
600
800
Time Point (0.25 Hz)

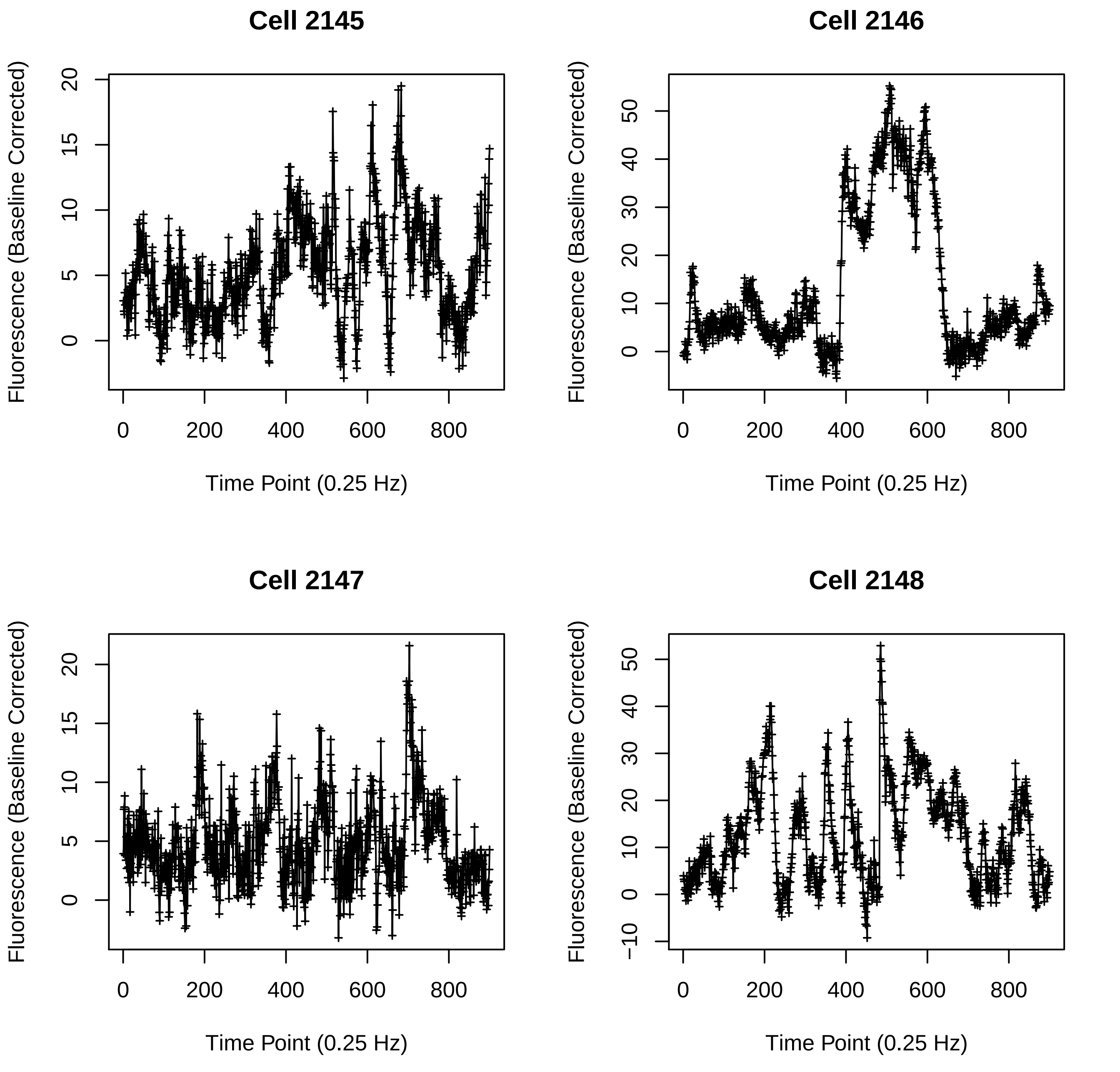
Cell 2145
Cell 2146
Cell 2147
Cell 2148
Fluorescence (Baseline Corrected)
Time Point (0.25 Hz)
0
200
400
600
800
0
5
10
15
20
30
40
50
−10

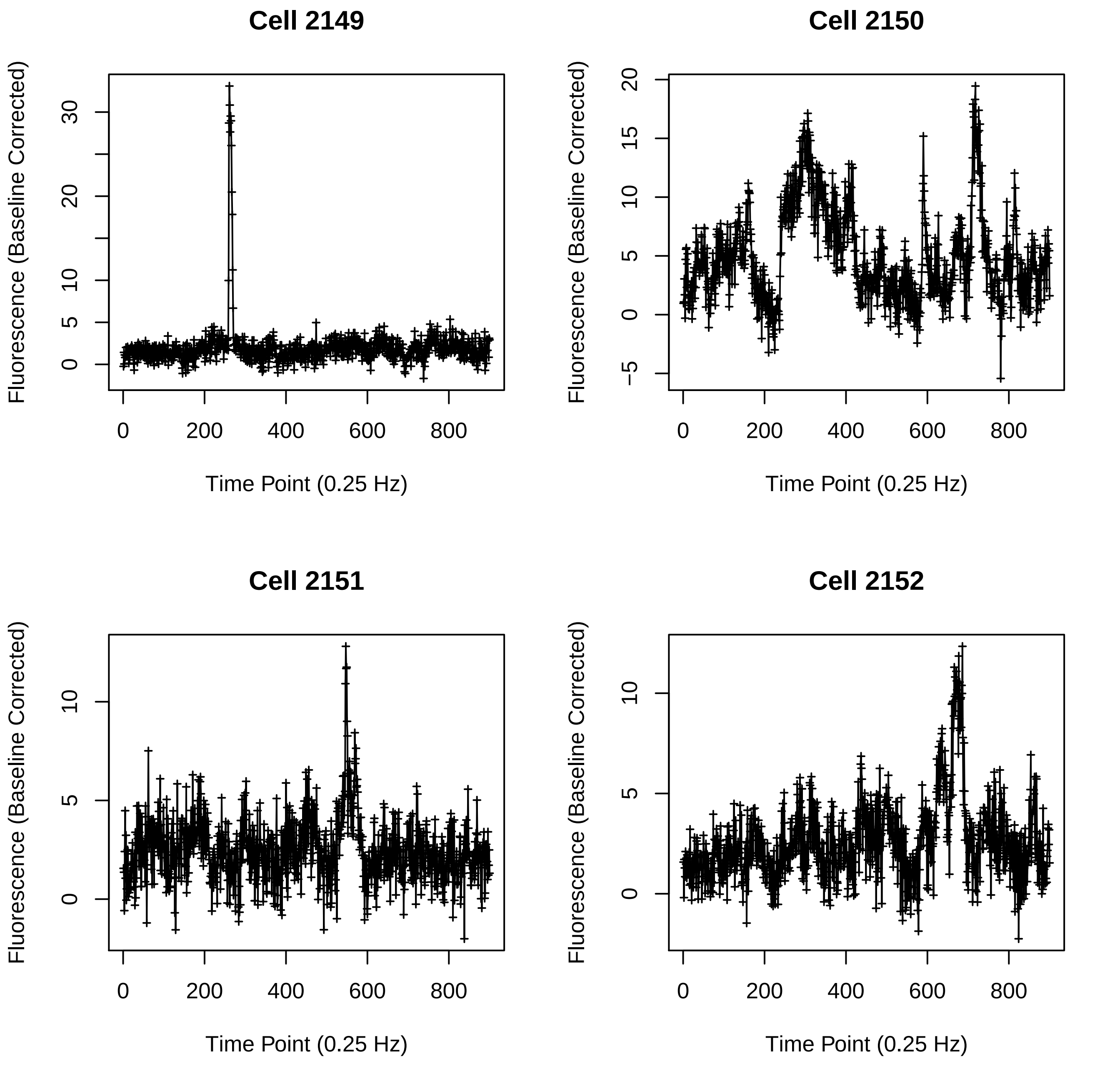
Cell 2149
Cell 2150
Cell 2151
Cell 2152
Fluorescence (Baseline Corrected)
Time Point (0.25 Hz)
0
200
400
600
800
0
5
10
15
20
30
-5

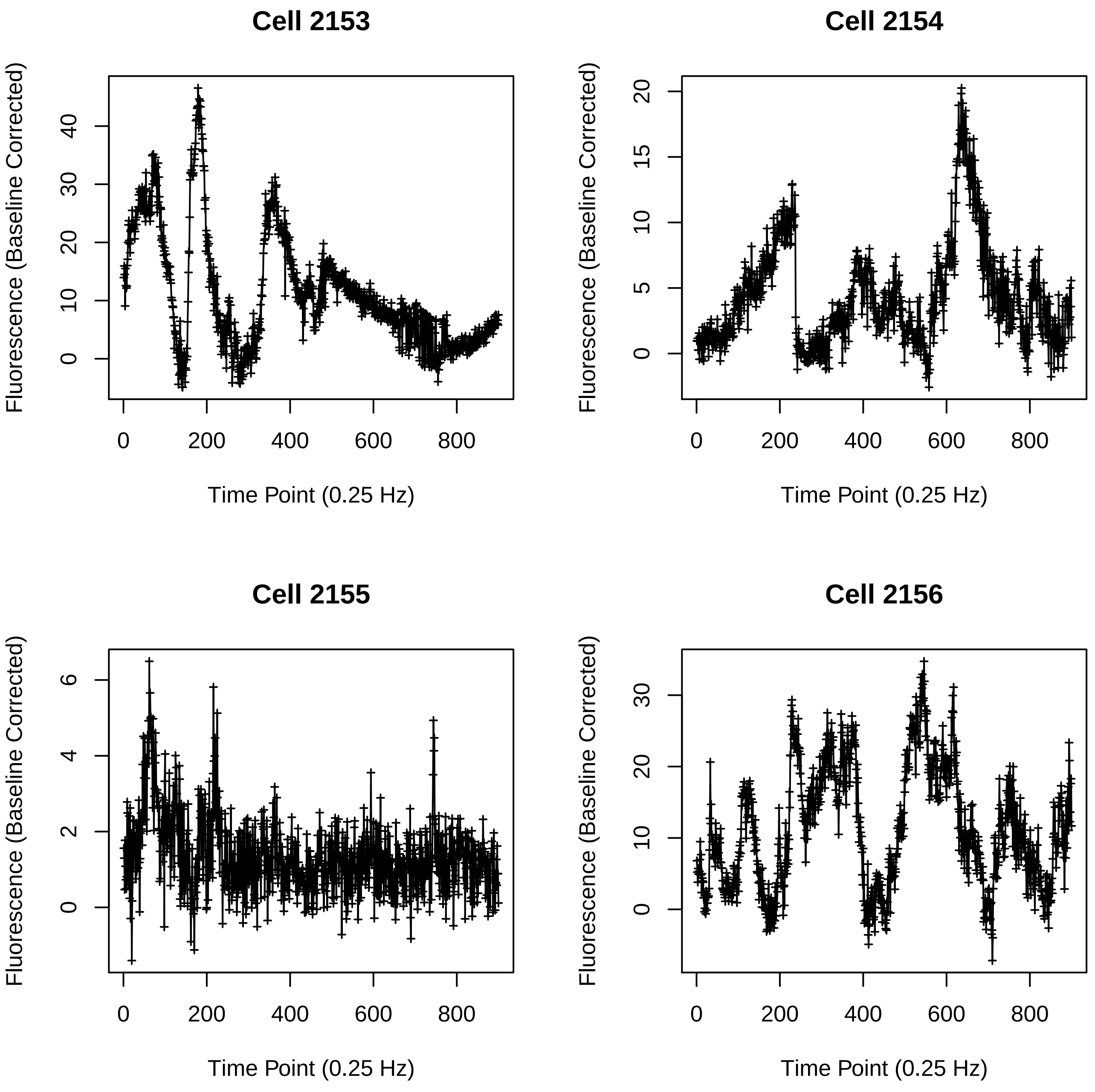
Cell 2153
Cell 2154
Cell 2155
Cell 2156
Fluorescence (Baseline Corrected)
Time Point (0.25 Hz)
0
200
400
600
800
0
10
20
30
40
5
15
2
4
6

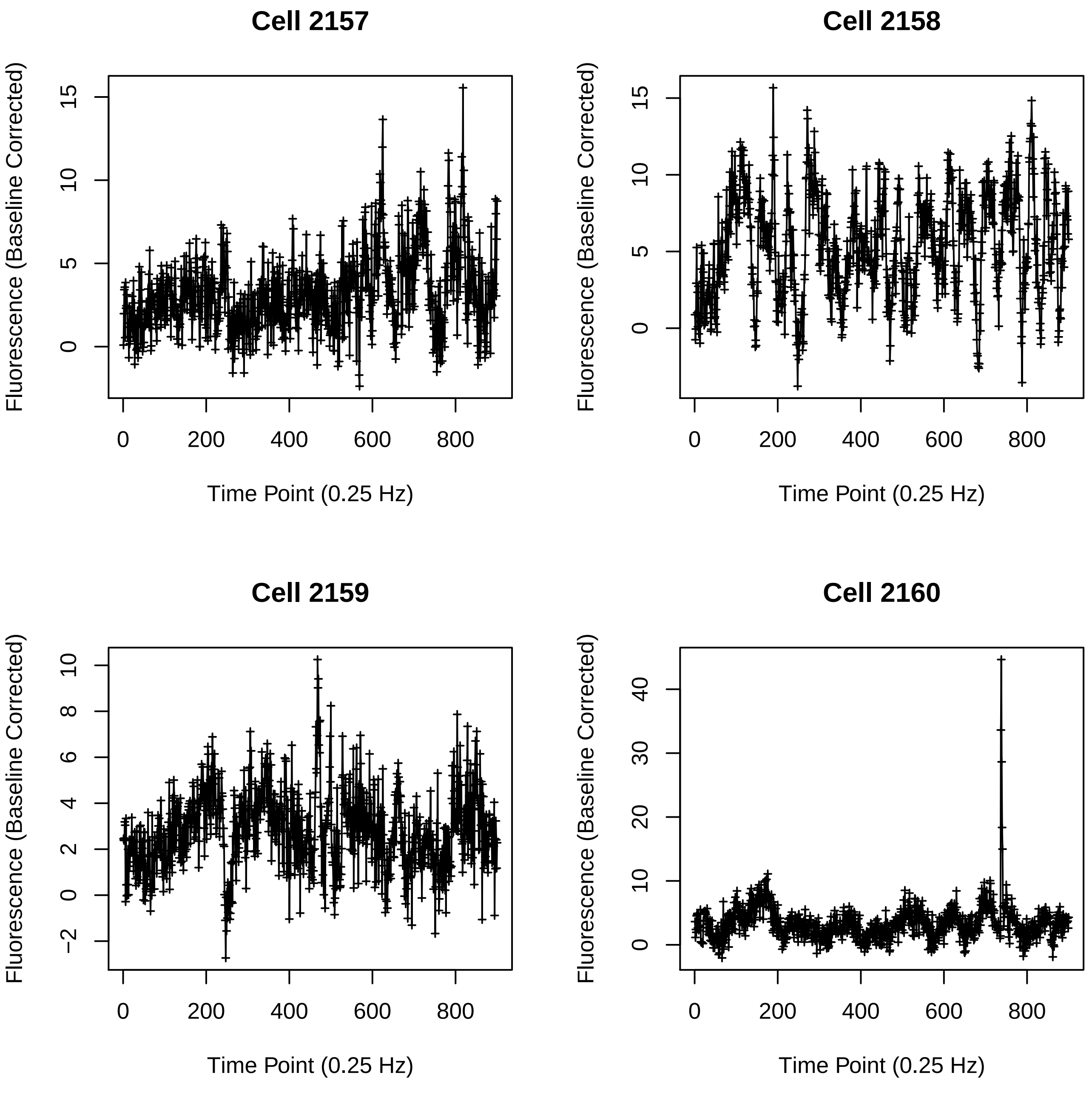
Cell 2157
Cell 2158
Cell 2159
Cell 2160
Fluorescence (Baseline Corrected)
Time Point (0.25 Hz)
0
200
400
600
800
0
5
10
15
−2
2
4
6
8
20
30
40

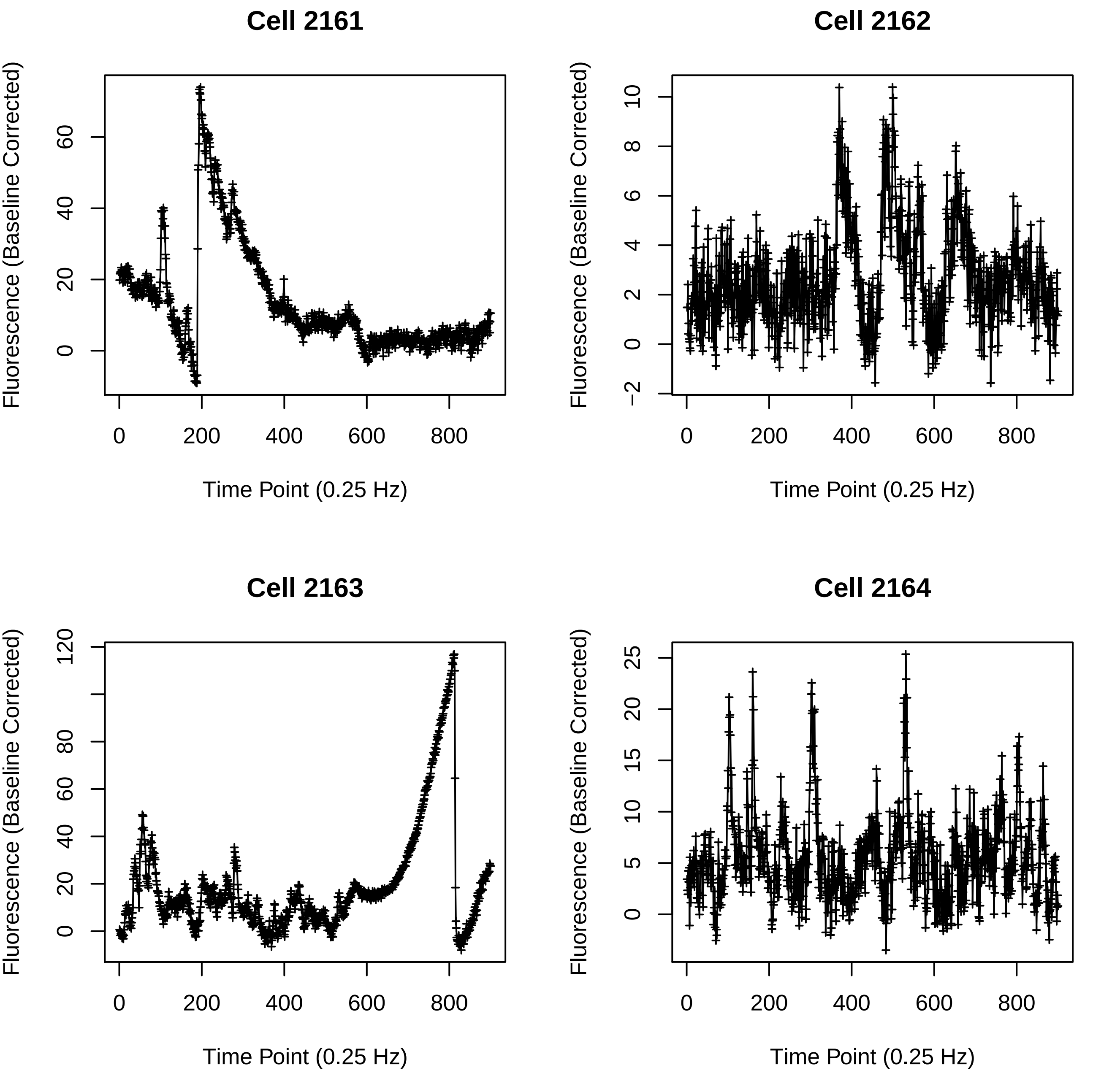

Cell 2161
Cell 2162
Cell 2163
Cell 2164
Fluorescence (Baseline Corrected)
Time Point (0.25 Hz)
0
200
400
600
800

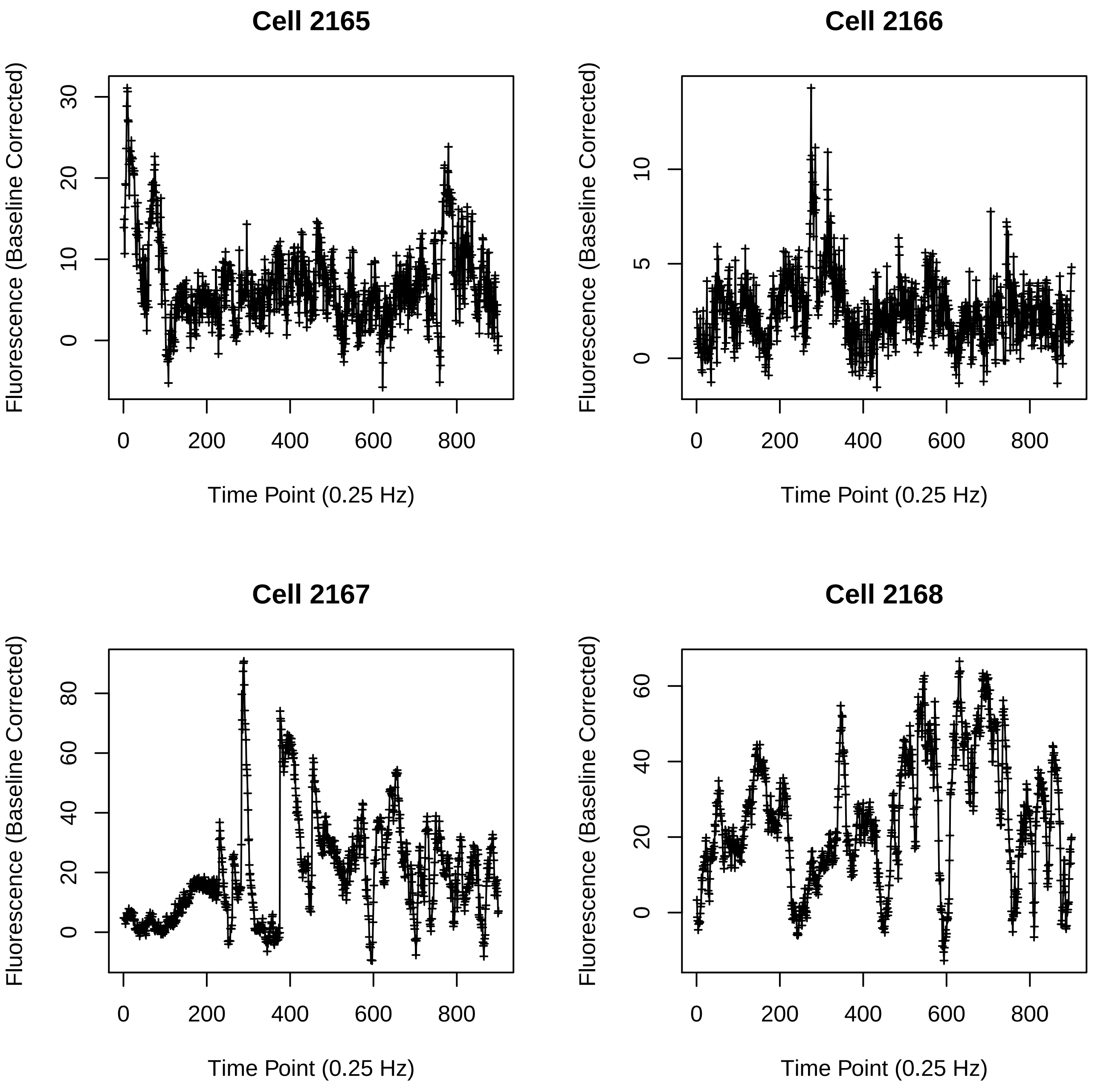
Cell 2165
Cell 2166
Cell 2167
Cell 2168
Fluorescence (Baseline Corrected)
Time Point (0.25 Hz)
0
10
20
30
5
40
60
80
200
400
600
800

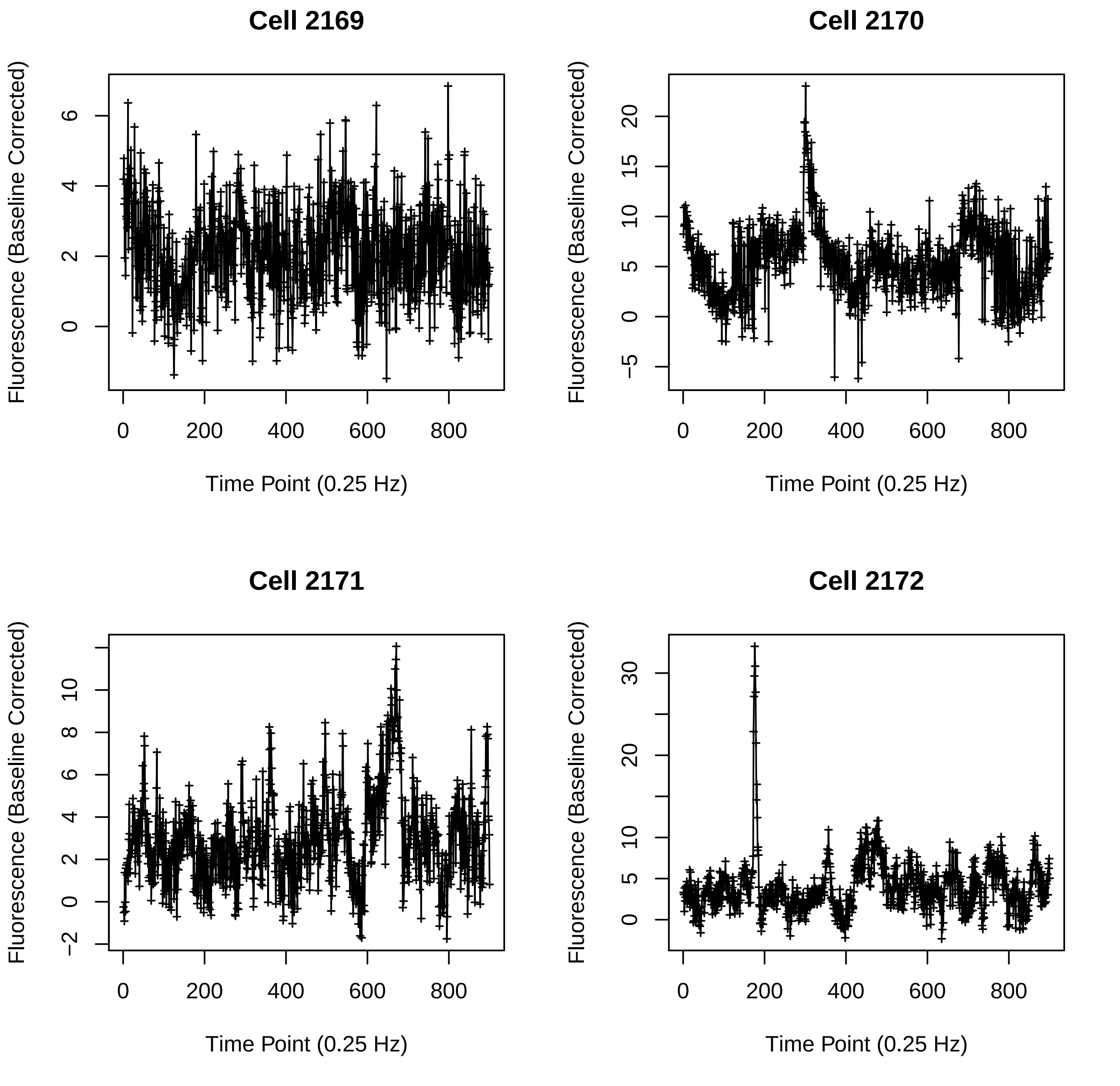

Cell 2169
Cell 2170
Cell 2171
Cell 2172
Fluorescence (Baseline Corrected)
Time Point (0.25 Hz)

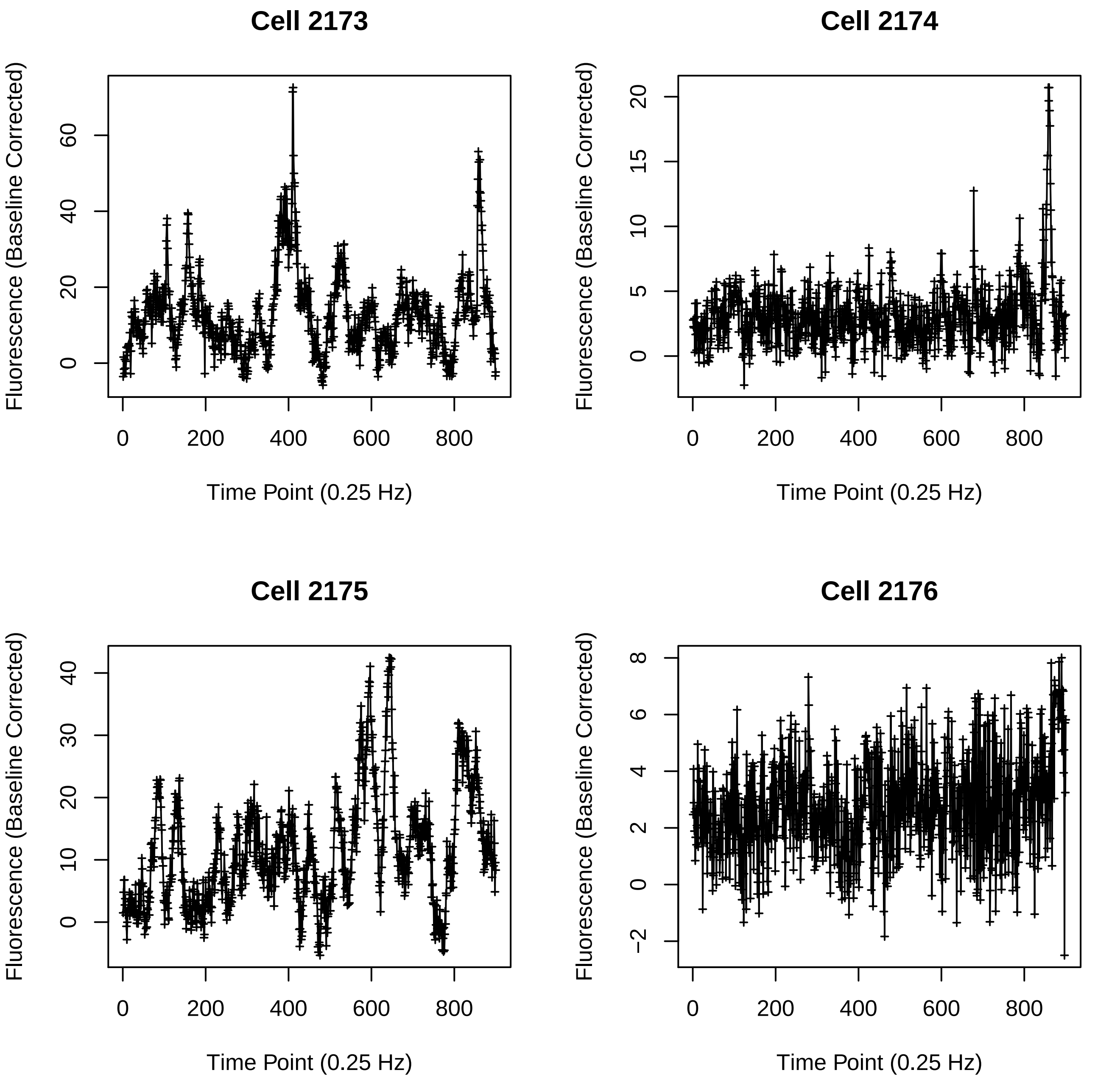
Cell 2173
Cell 2174
Cell 2175
Cell 2176
Fluorescence (Baseline Corrected)
Time Point (0.25 Hz)
0
200
400
600
800
20
40
60
5
10
15
30
−2
2
4
6
8

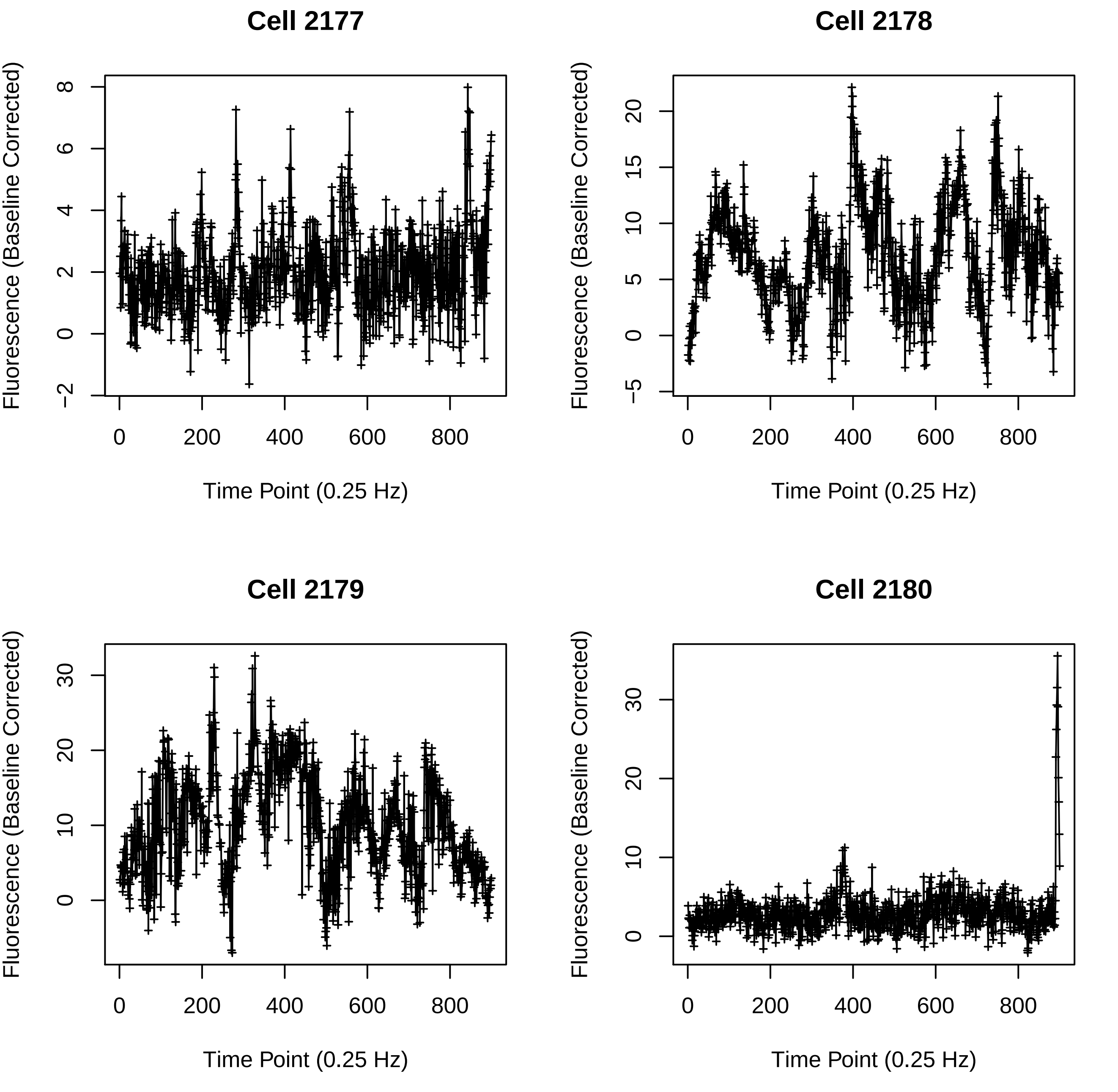
Cell 2177
Cell 2178
Cell 2179
Cell 2180
Fluorescence (Baseline Corrected)
Time Point (0.25 Hz)
0
200
400
600
800
−2
2
4
6
8
−5
5
10
15
20
10
20
30

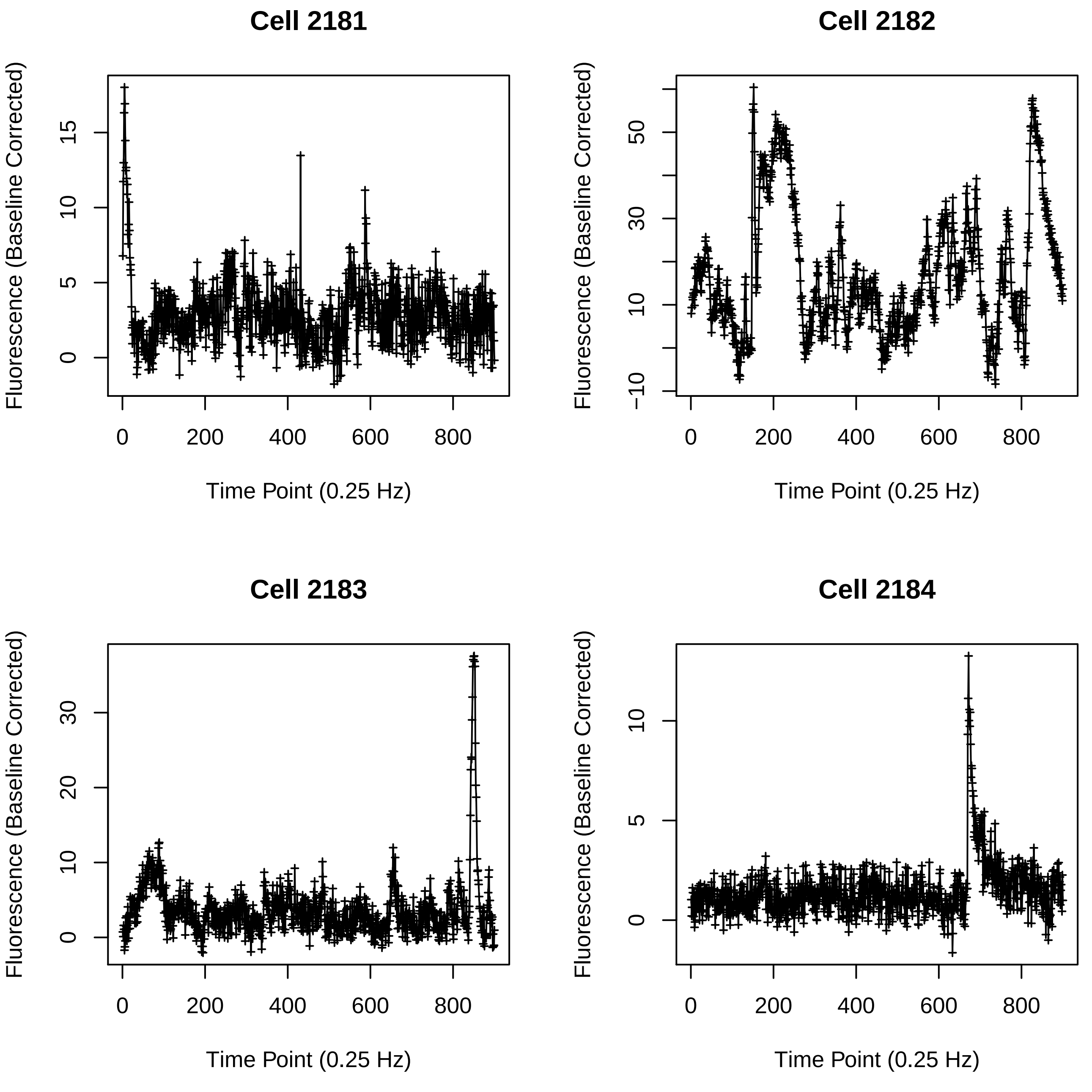
Cell 2181
Cell 2182
Cell 2183
Cell 2184
Fluorescence (Baseline Corrected)
Time Point (0.25 Hz)

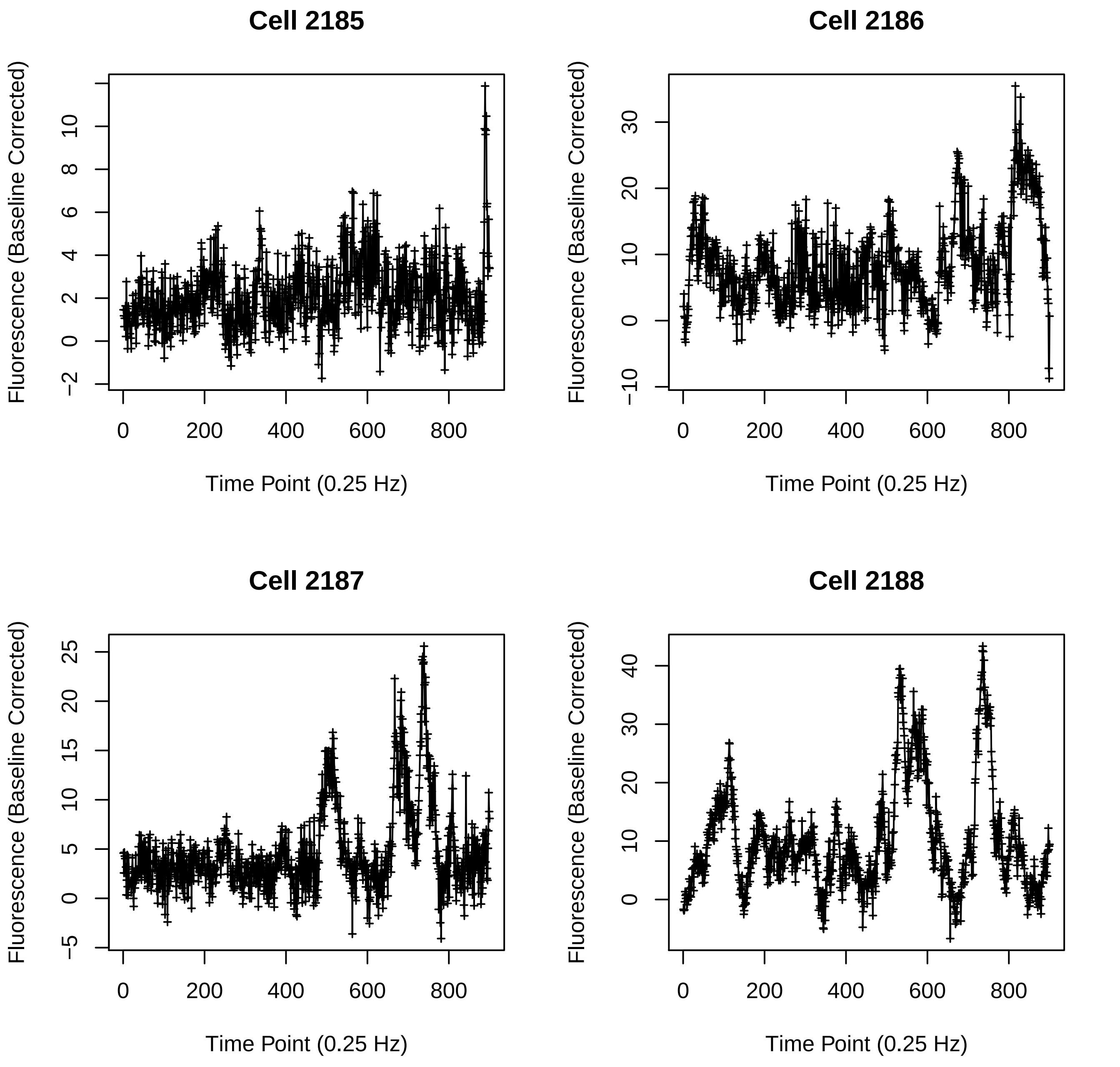
Cell 2185
Cell 2186
Cell 2187
Cell 2188
Fluorescence (Baseline Corrected)
Time Point (0.25 Hz)
−2
0
2
4
6
8
10
−10
0
10
20
30
−5
0
5
10
15
20
25
0
10
20
30
40
0
200
400
600
800

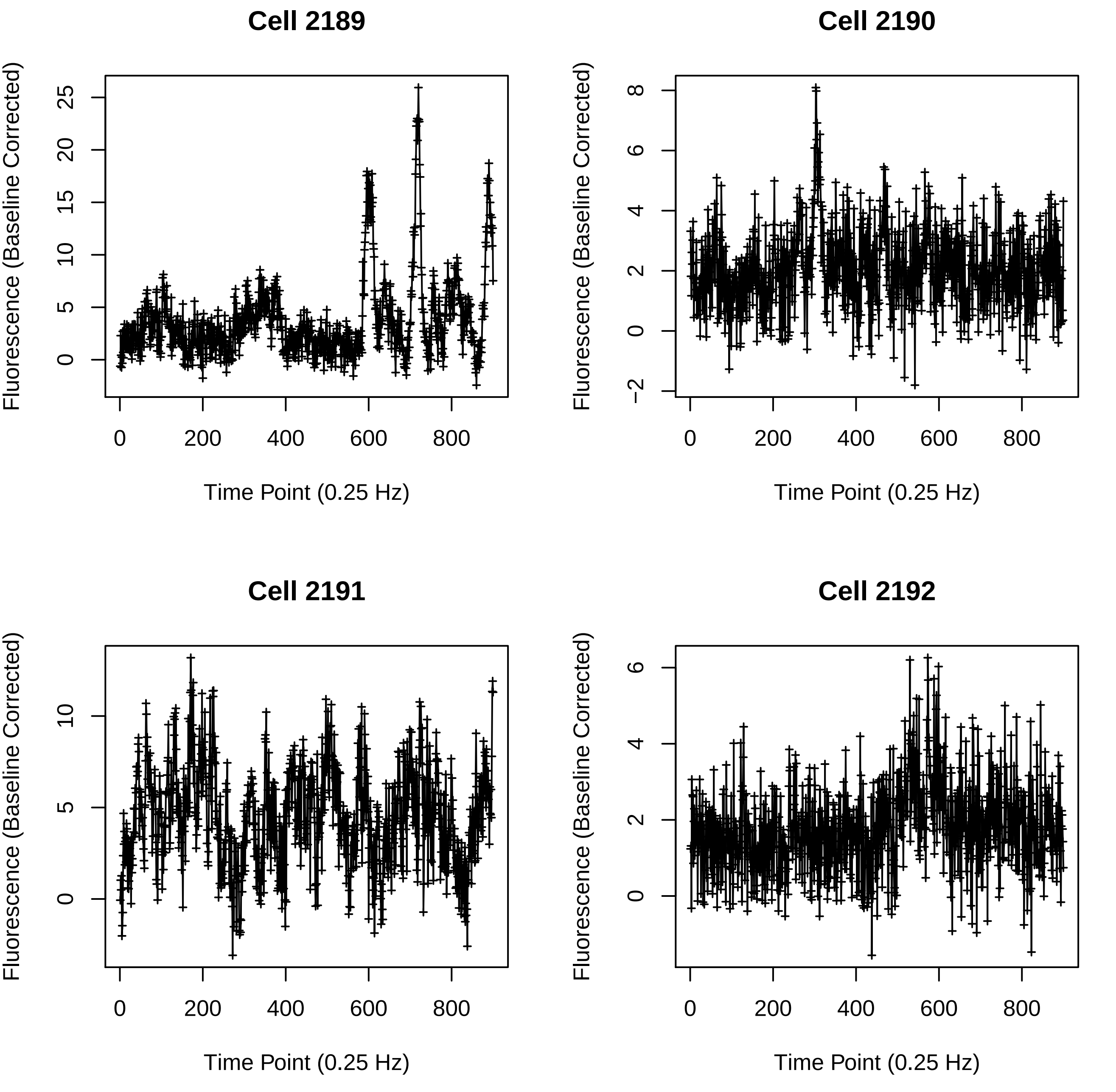
Cell 2189
Cell 2190
Cell 2191
Cell 2192
Fluorescence (Baseline Corrected)
Time Point (0.25 Hz)
0
5
10
15
20
25
−2
2
4
6
8
200
400
600
800

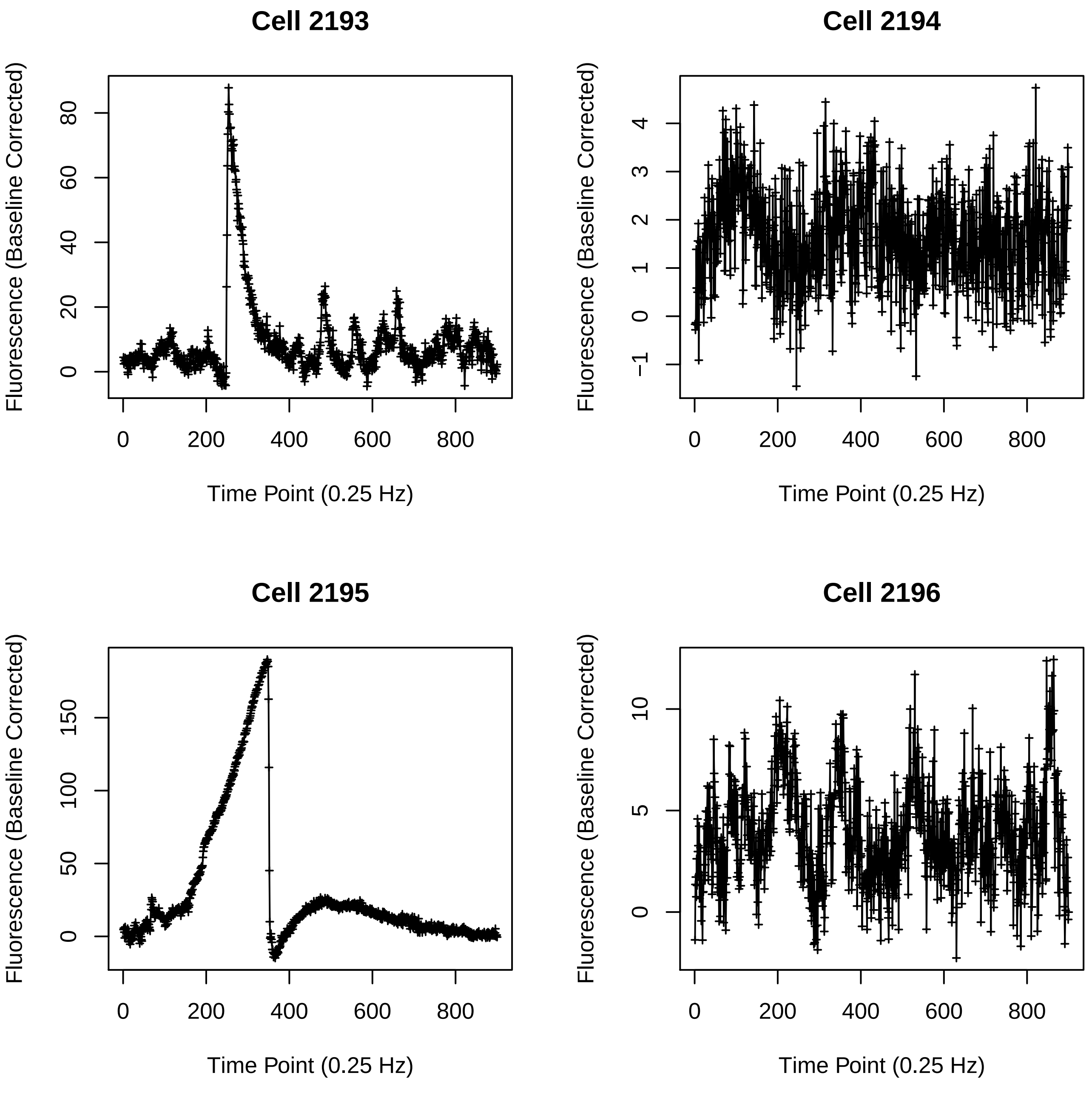
Cell 2193
Fluorescence (Baseline Corrected)
0
20
40
60
80
0
200
400
600
800
Time Point (0.25 Hz)
Cell 2194
Fluorescence (Baseline Corrected)
−1
0
1
2
3
4
0
200
400
600
800
Time Point (0.25 Hz)
Cell 2195
Fluorescence (Baseline Corrected)
0
50
100
150
0
200
400
600
800
Time Point (0.25 Hz)
Cell 2196
Fluorescence (Baseline Corrected)
0
5
10
0
200
400
600
800
Time Point (0.25 Hz)

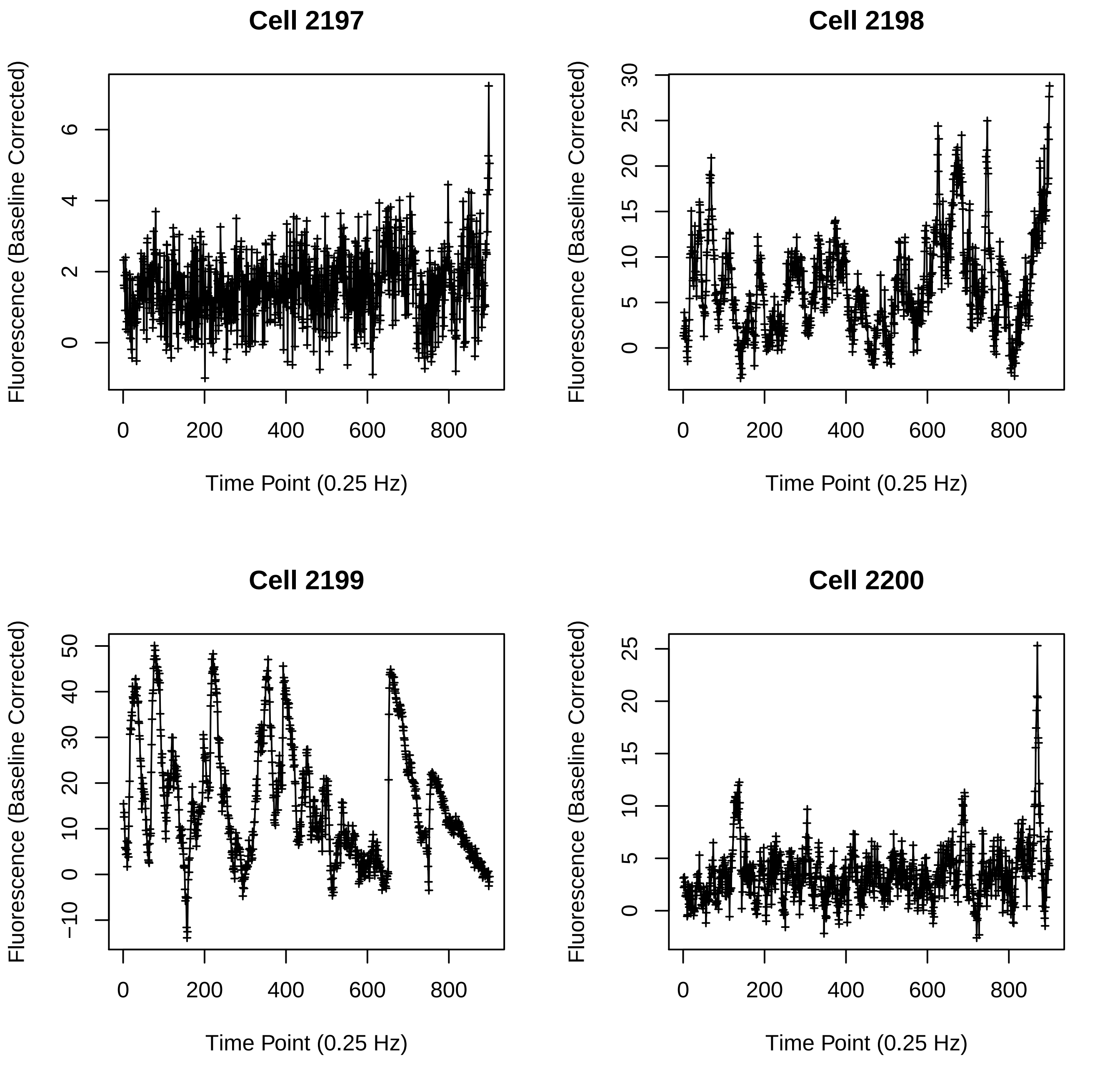

Cell 2197
Cell 2198
Cell 2199
Cell 2200
Fluorescence (Baseline Corrected)
Time Point (0.25 Hz)
0
2
4
6
5
10
15
20
25
30
−10
40
50
200
400
600
800

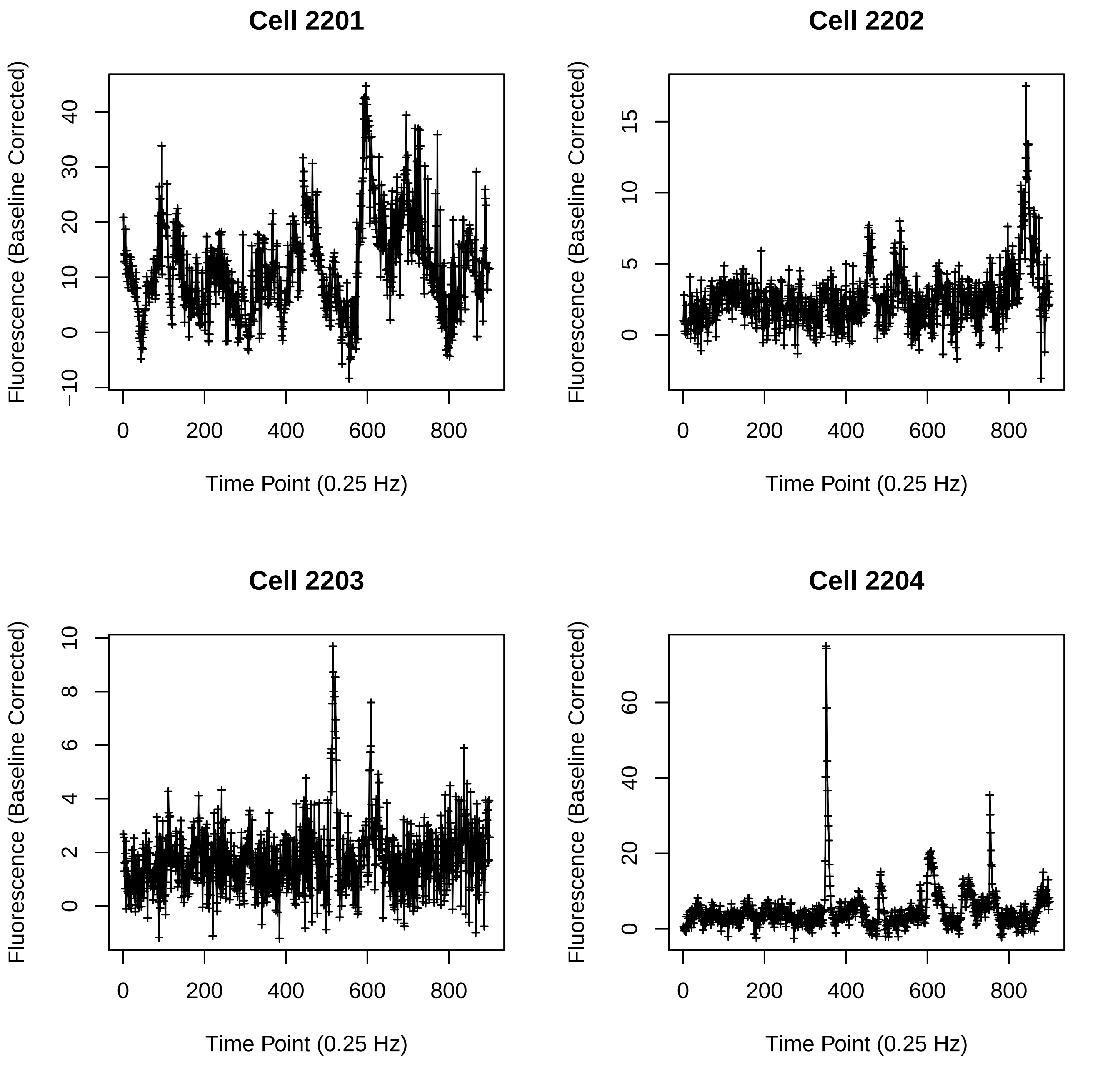

Cell 2201
Cell 2202
Cell 2203
Cell 2204
Fluorescence (Baseline Corrected)
Time Point (0.25 Hz)
0
200
400
600
800
−10
10
20
30
40
5
15
2
4
6
8
60

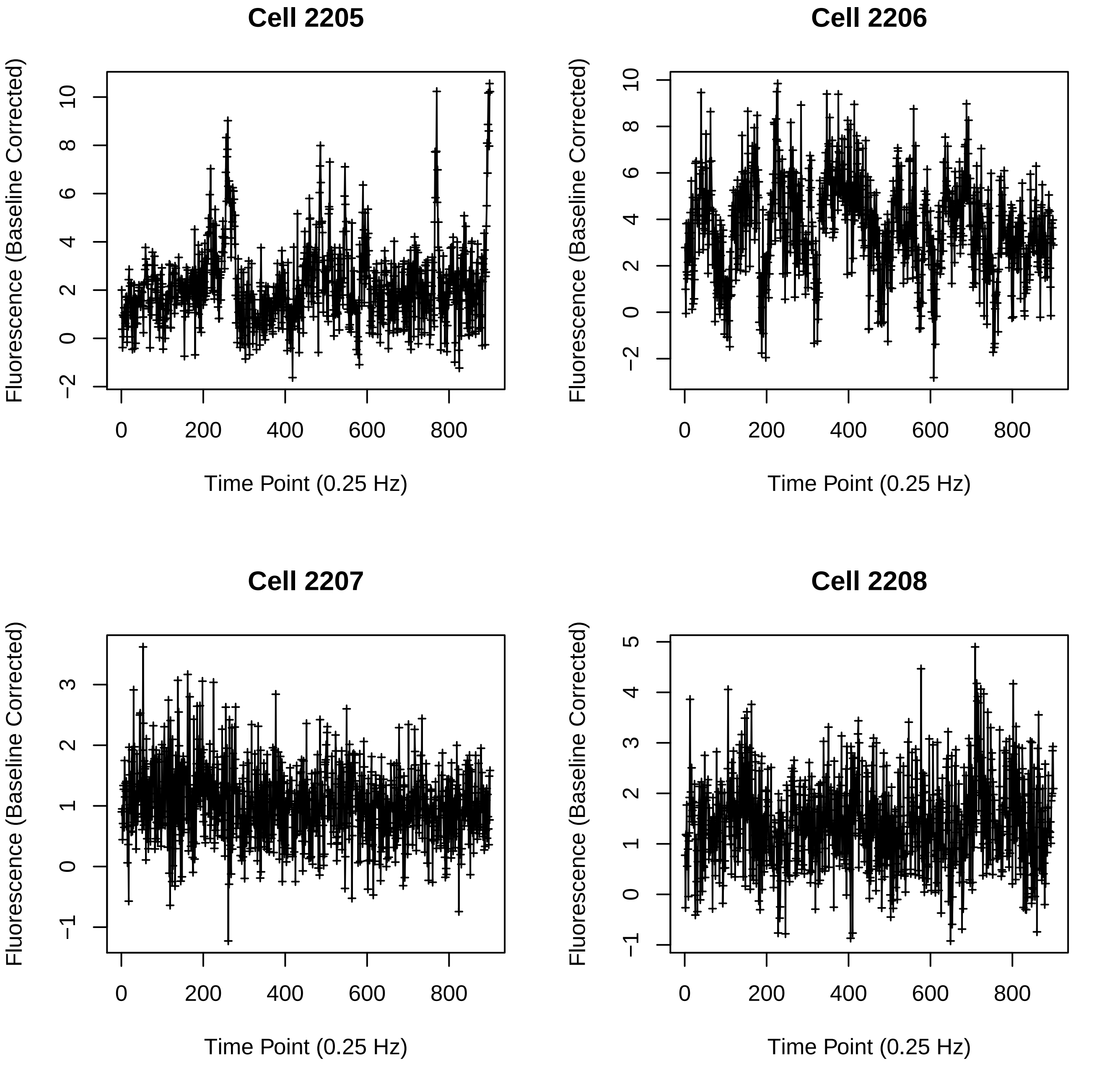
Cell 2205
Cell 2206
Cell 2207
Cell 2208
Fluorescence (Baseline Corrected)
Time Point (0.25 Hz)

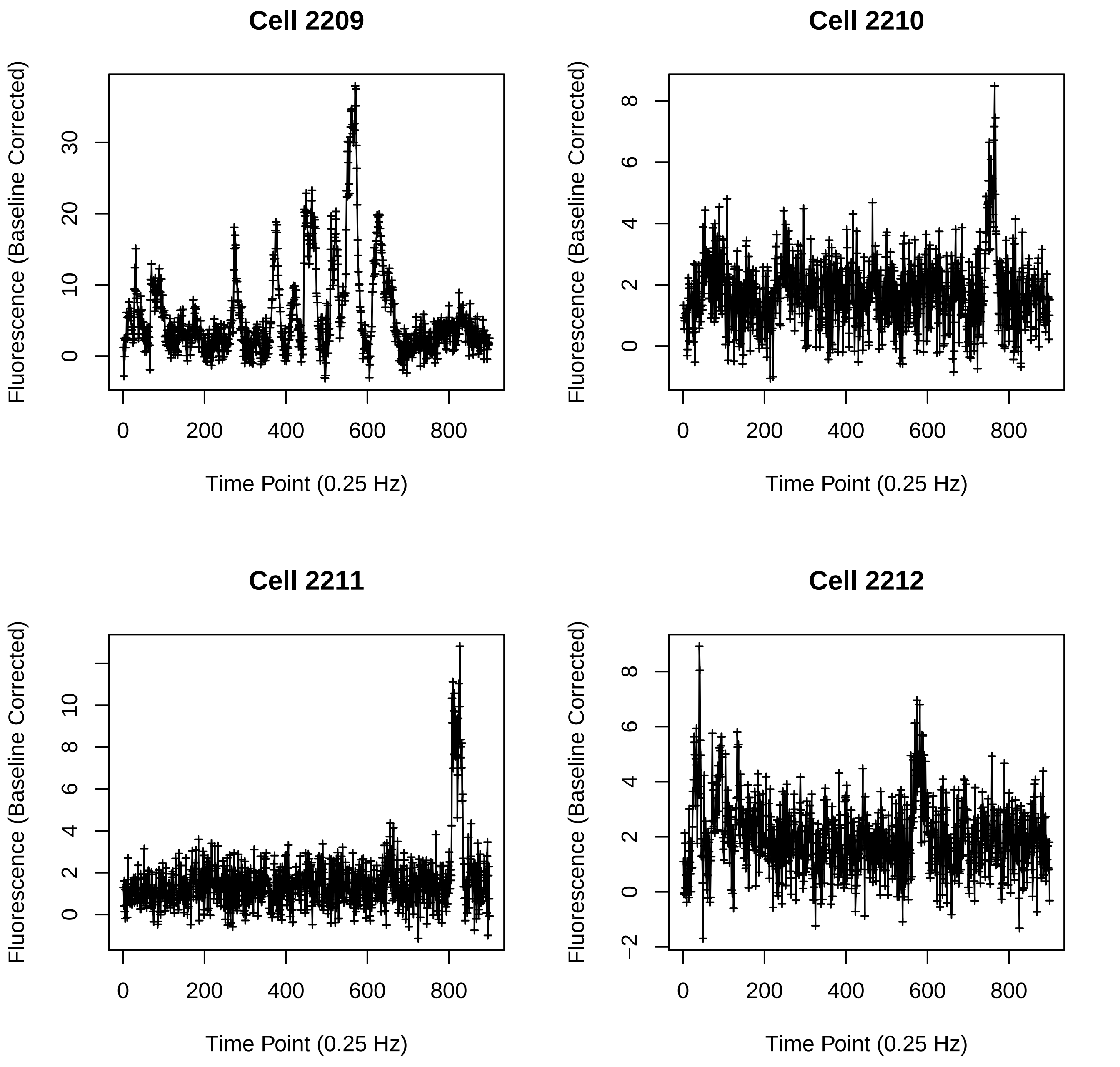

Cell 2209
Cell 2210
Cell 2211
Cell 2212
Fluorescence (Baseline Corrected)
Time Point (0.25 Hz)
0
200
400
600
800
−2
2
4
6
8
10
20
30

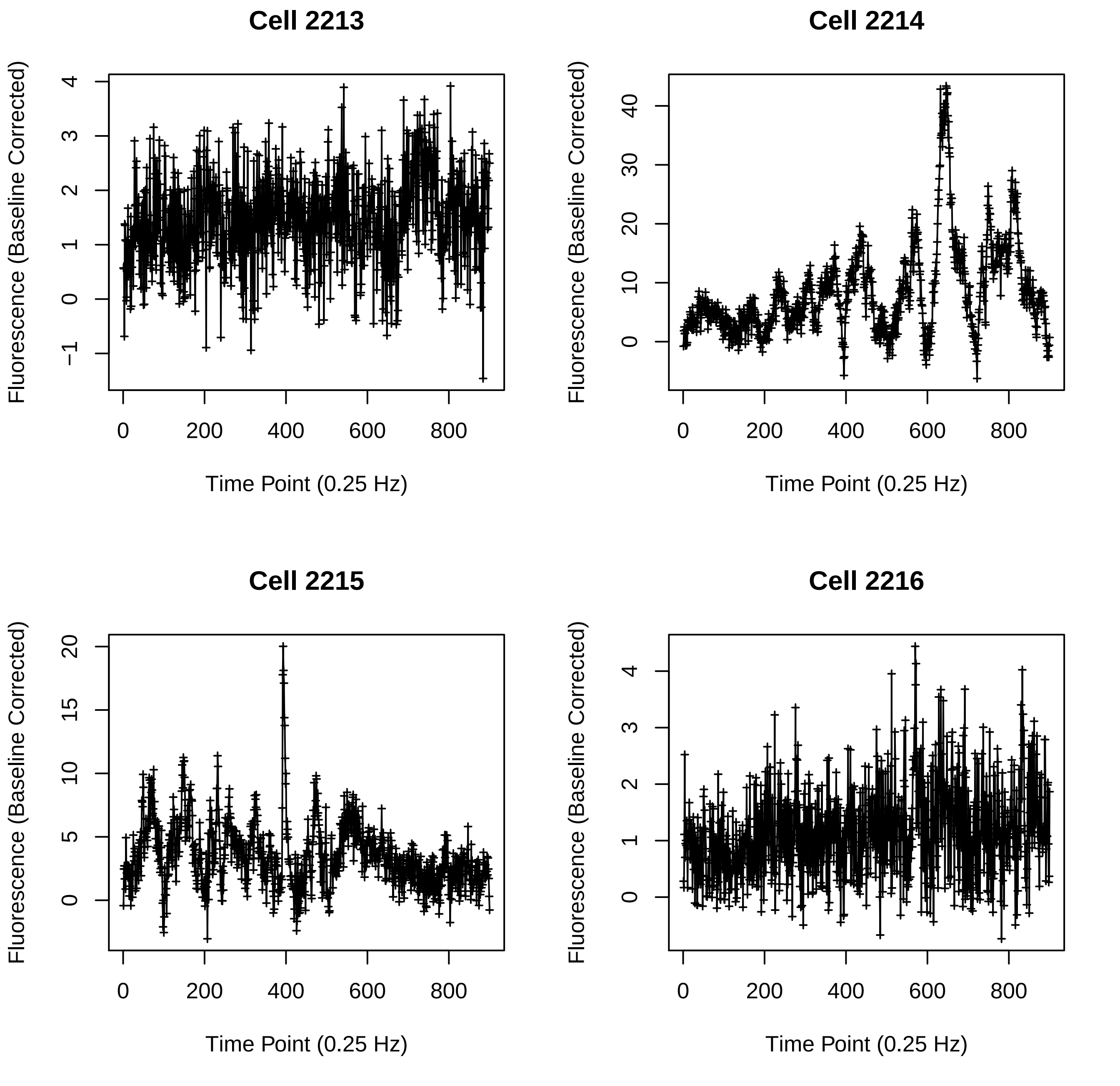
Cell 2213
Cell 2214
Cell 2215
Cell 2216
Fluorescence (Baseline Corrected)
Time Point (0.25 Hz)
0
200
400
600
800
−1
1
2
3
4
10
20
30
40
5
15

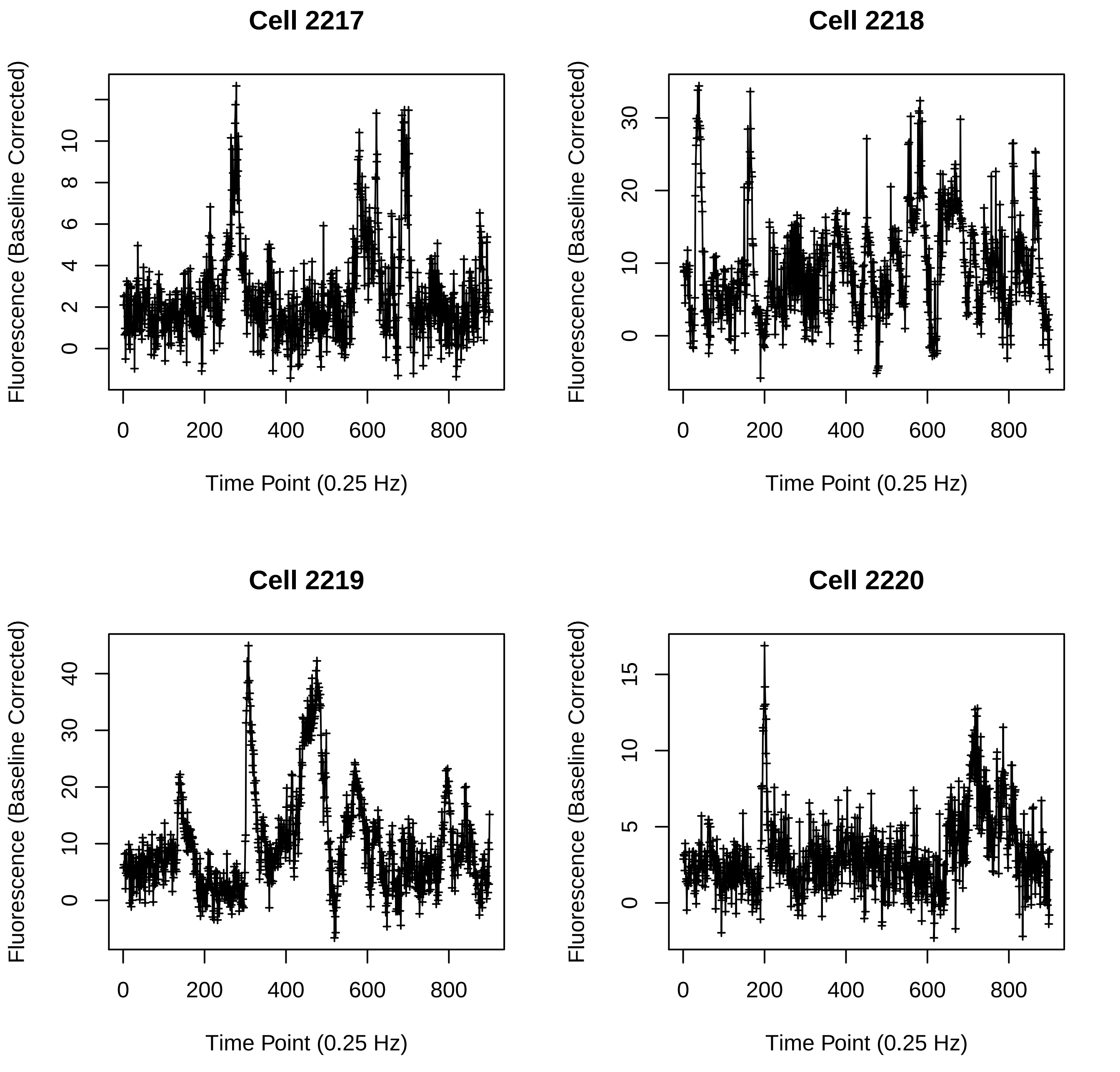

Cell 2217
Cell 2218
Cell 2219
Cell 2220
Fluorescence (Baseline Corrected)
Time Point (0.25 Hz)
0
200
400
600
800

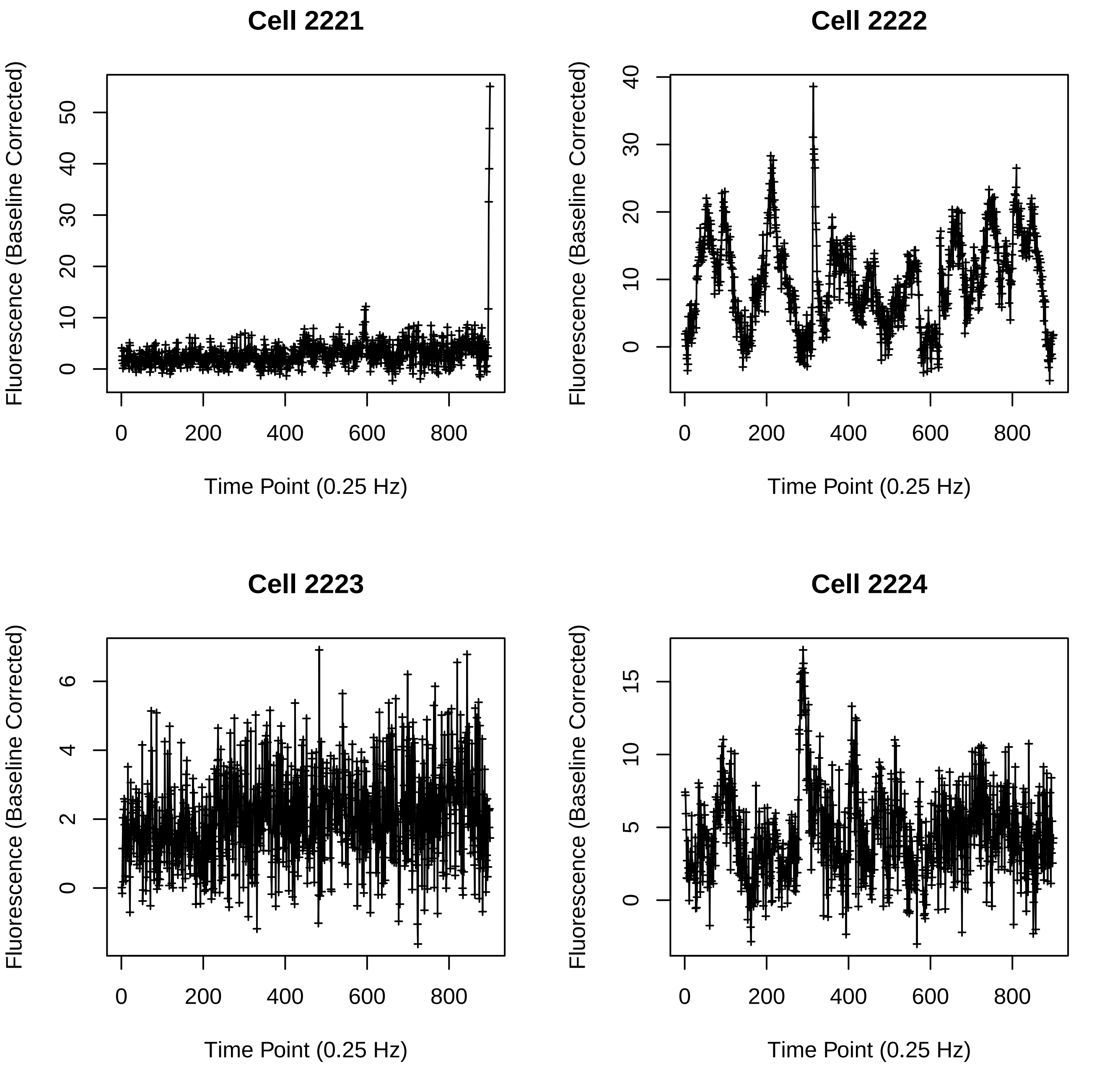
Cell 2221
Cell 2222
Cell 2223
Cell 2224
Fluorescence (Baseline Corrected)
Time Point (0.25 Hz)
0
200
400
600
800
10
20
30
40
50
5
15
2
4
6

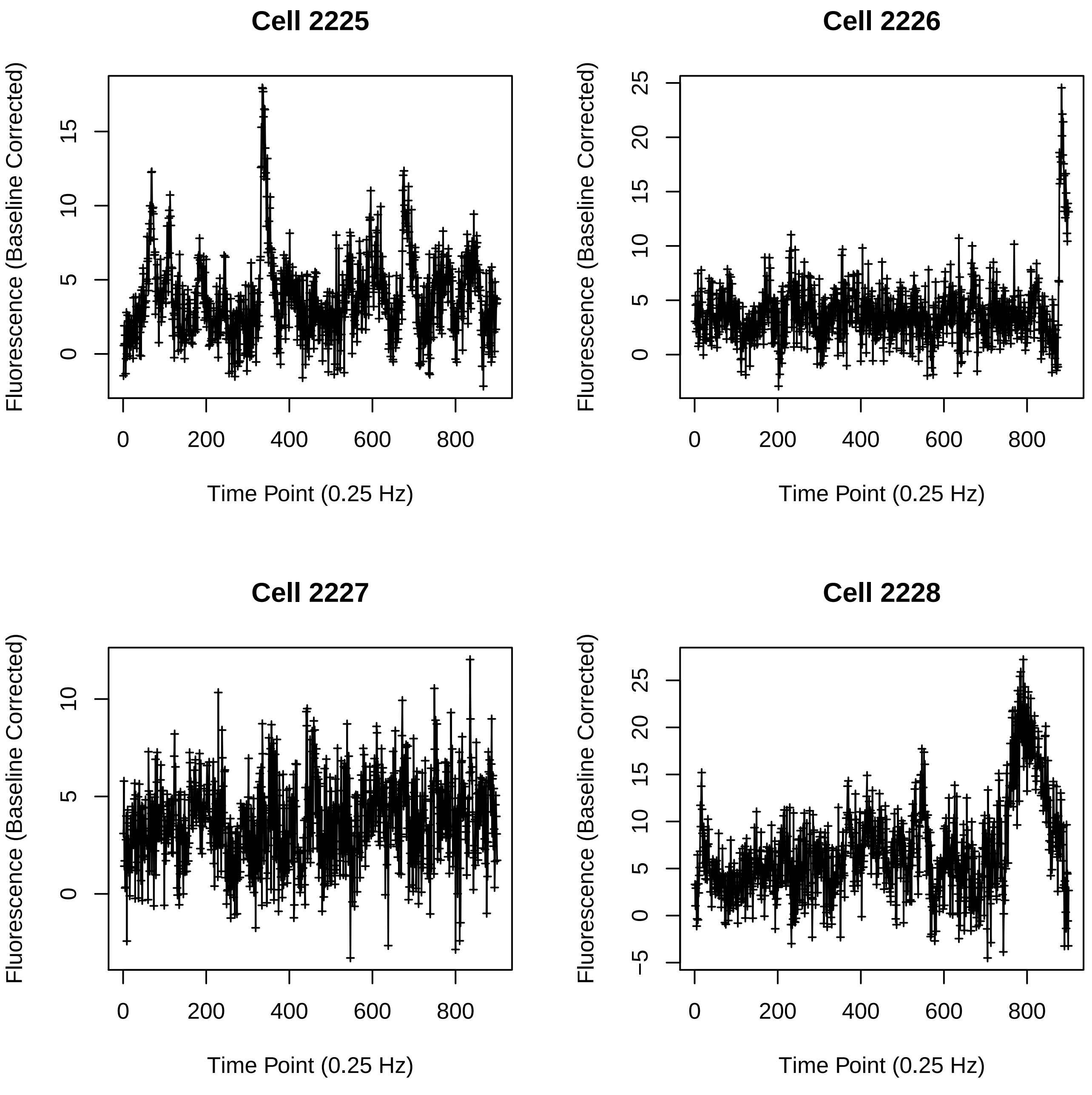
Cell 2225
Cell 2226
Cell 2227
Cell 2228
Fluorescence (Baseline Corrected)
Time Point (0.25 Hz)
0
200
400
600
800
0
5
10
15
20
25
−5

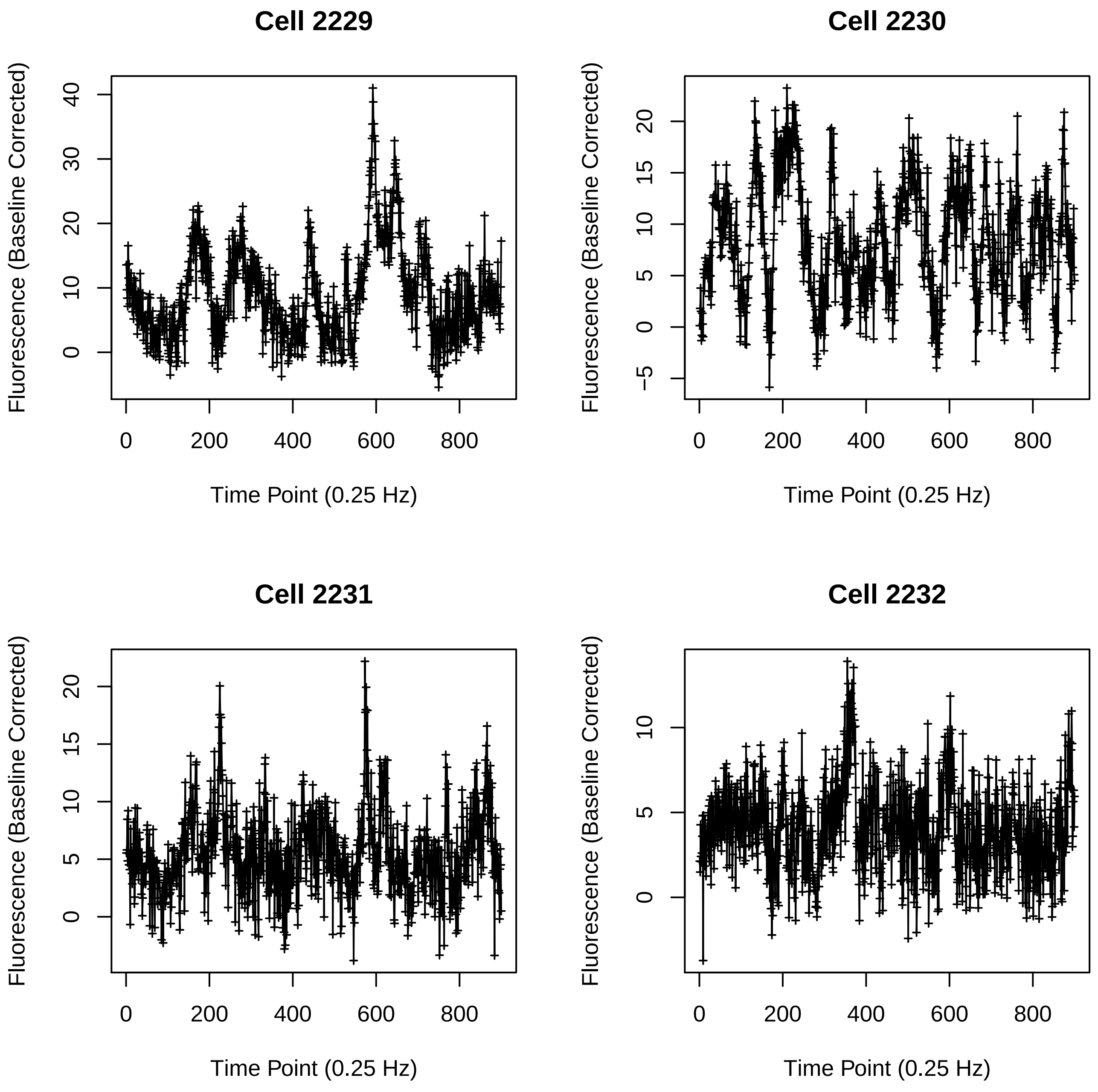

Cell 2229
Cell 2230
Cell 2231
Cell 2232
Fluorescence (Baseline Corrected)
Time Point (0.25 Hz)

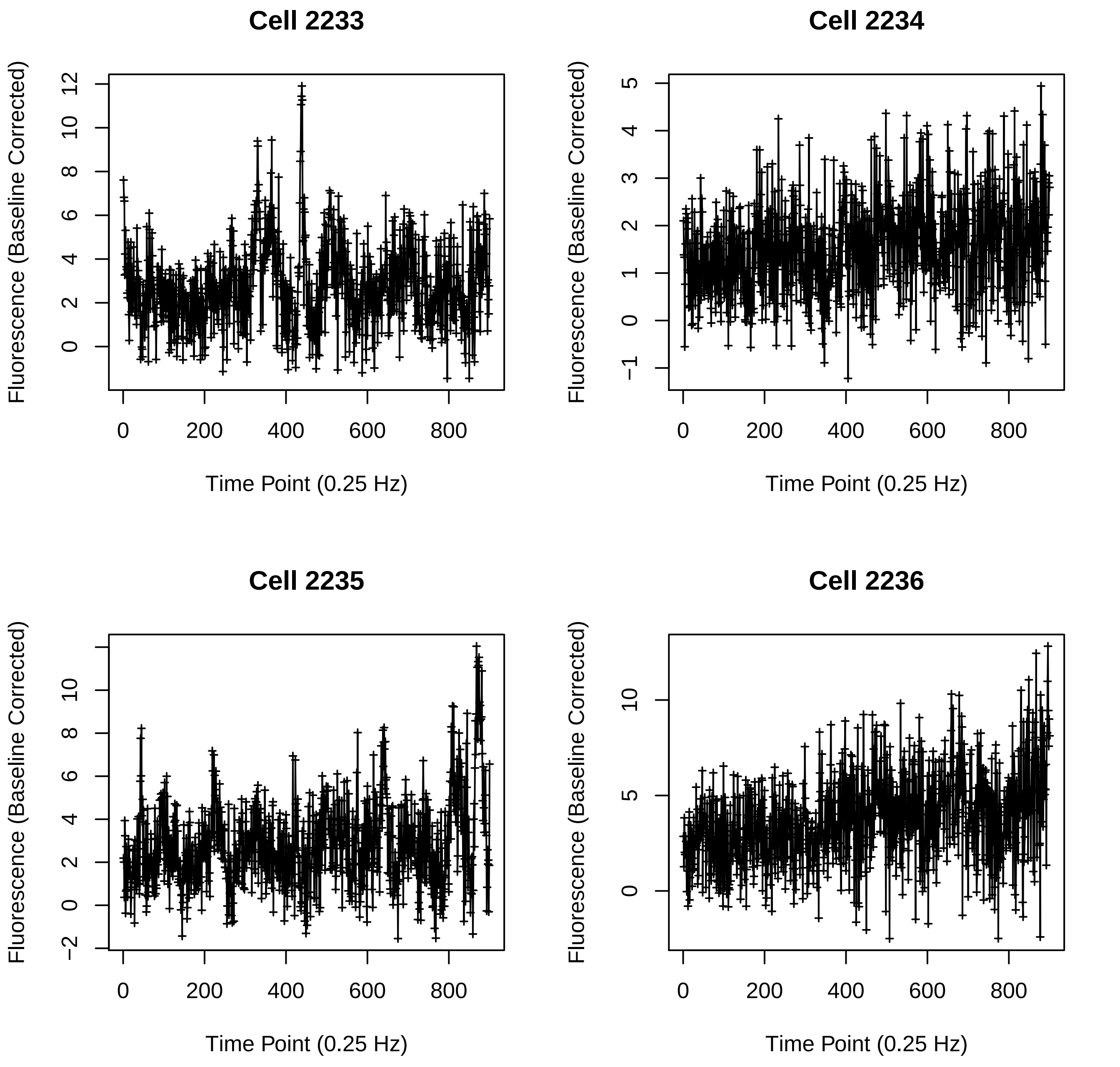
Cell 2233
Cell 2234
Cell 2235
Cell 2236
Fluorescence (Baseline Corrected)
Time Point (0.25 Hz)
0
200
400
600
800
0
2
4
6
8
10
12
−1
1
3
5
−2
0
5
10

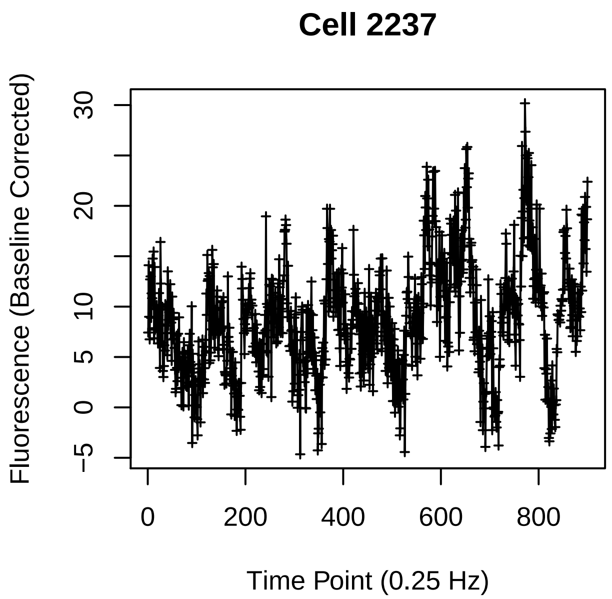
Cell 2237
Fluorescence (Baseline Corrected)
Time Point (0.25 Hz)

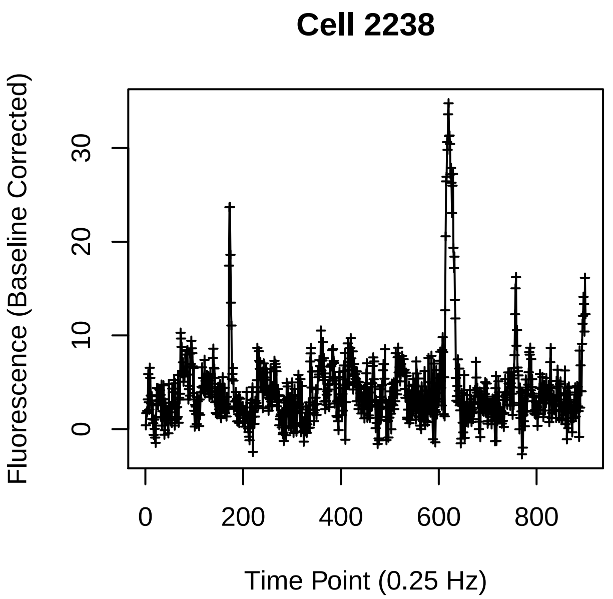
Cell 2238
Fluorescence (Baseline Corrected)
Time Point (0.25 Hz)

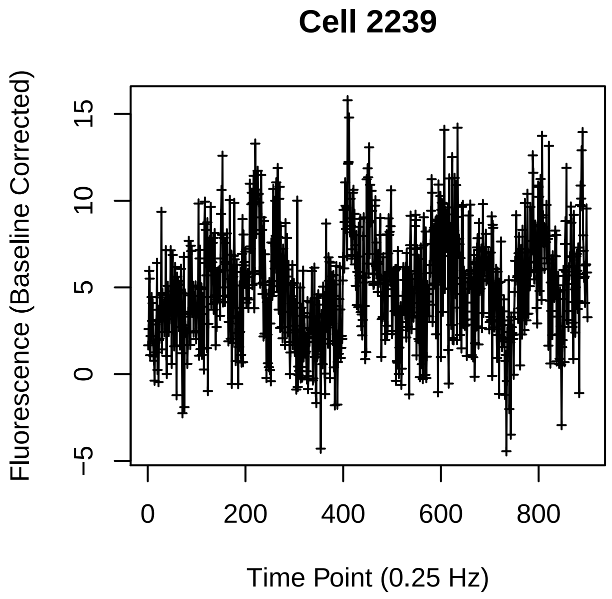
Cell 2239
Fluorescence (Baseline Corrected)
Time Point (0.25 Hz)

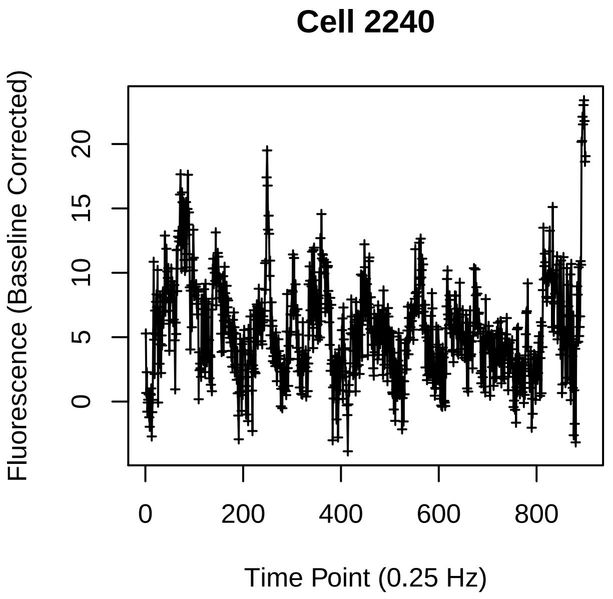
Cell 2240
Fluorescence (Baseline Corrected)
Time Point (0.25 Hz)

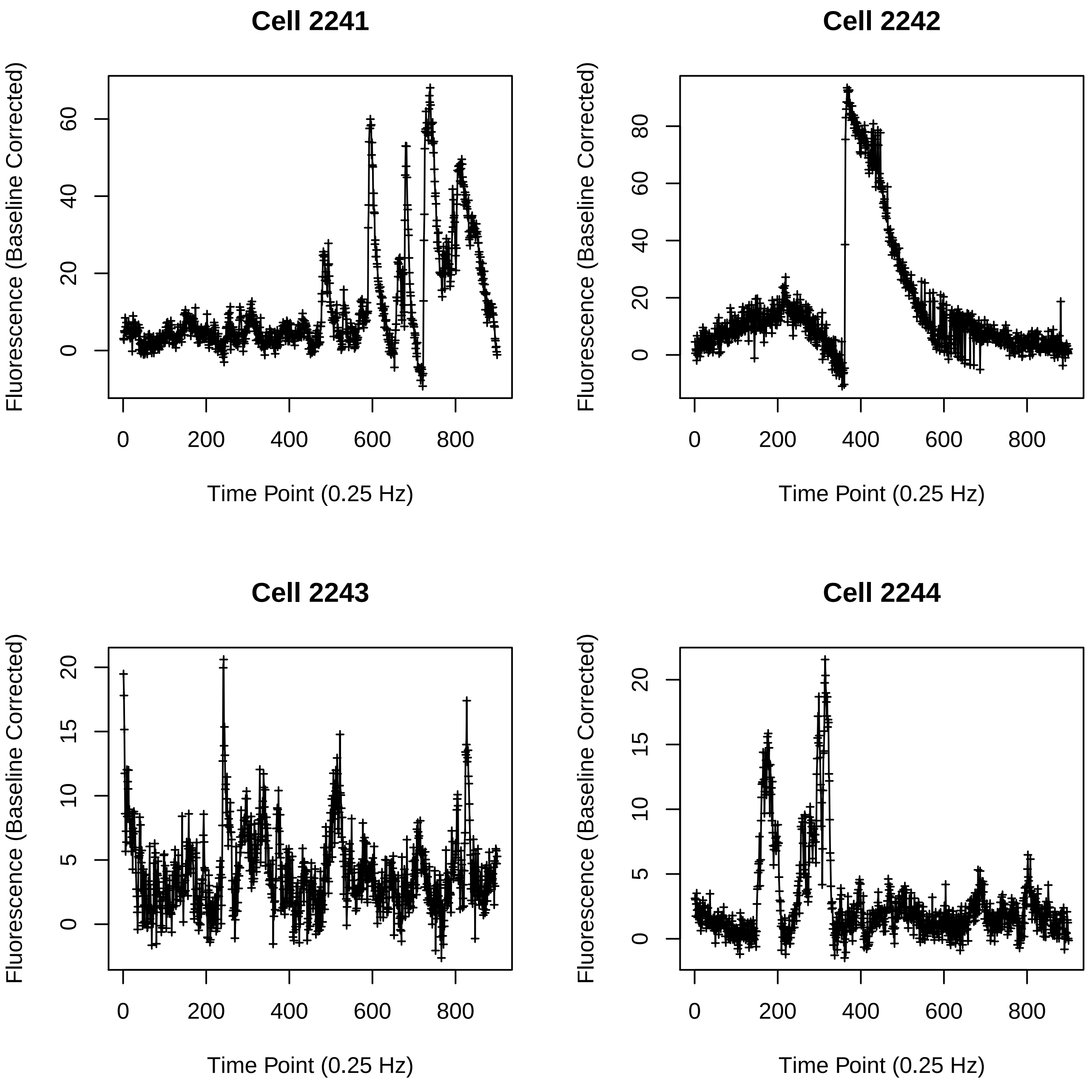
Cell 2241
Cell 2242
Cell 2243
Cell 2244
Fluorescence (Baseline Corrected)
Time Point (0.25 Hz)
0
200
400
600
800
20
40
60
80
5
10
15

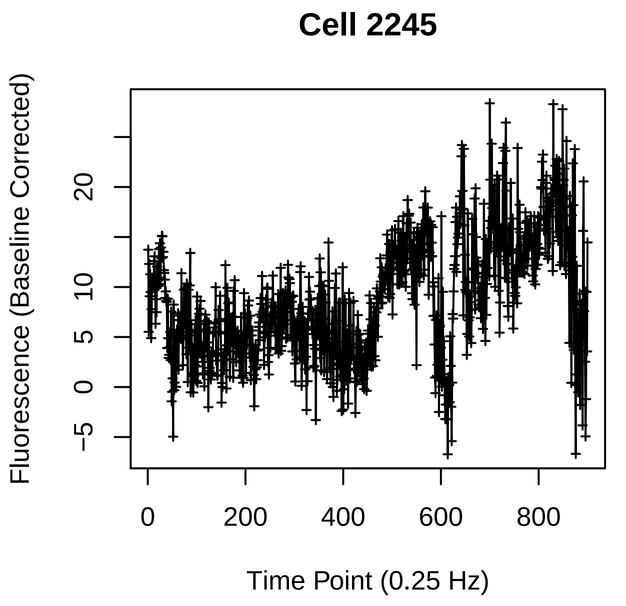

Cell 2245
Fluorescence (Baseline Corrected)
Time Point (0.25 Hz)

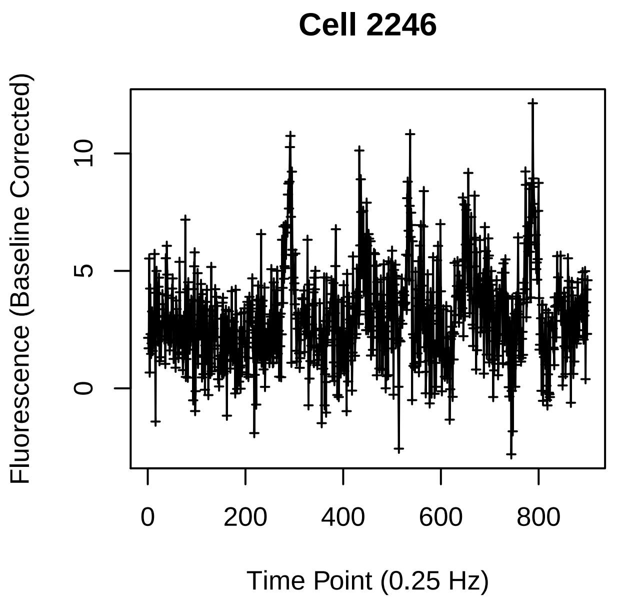

Cell 2246
Fluorescence (Baseline Corrected)
Time Point (0.25 Hz)

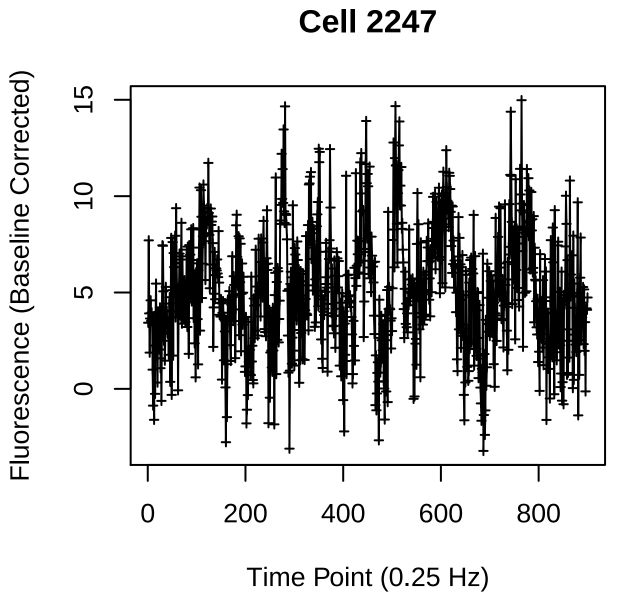

Cell 2247
Fluorescence (Baseline Corrected)
Time Point (0.25 Hz)

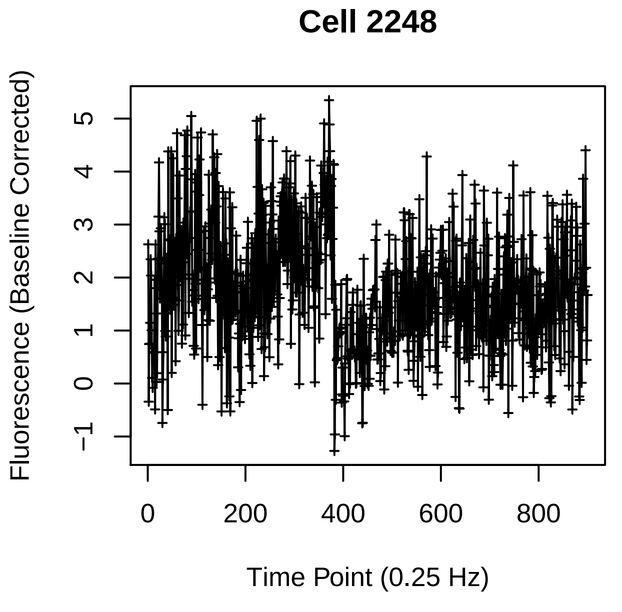

Cell 2248
Fluorescence (Baseline Corrected)
Time Point (0.25 Hz)

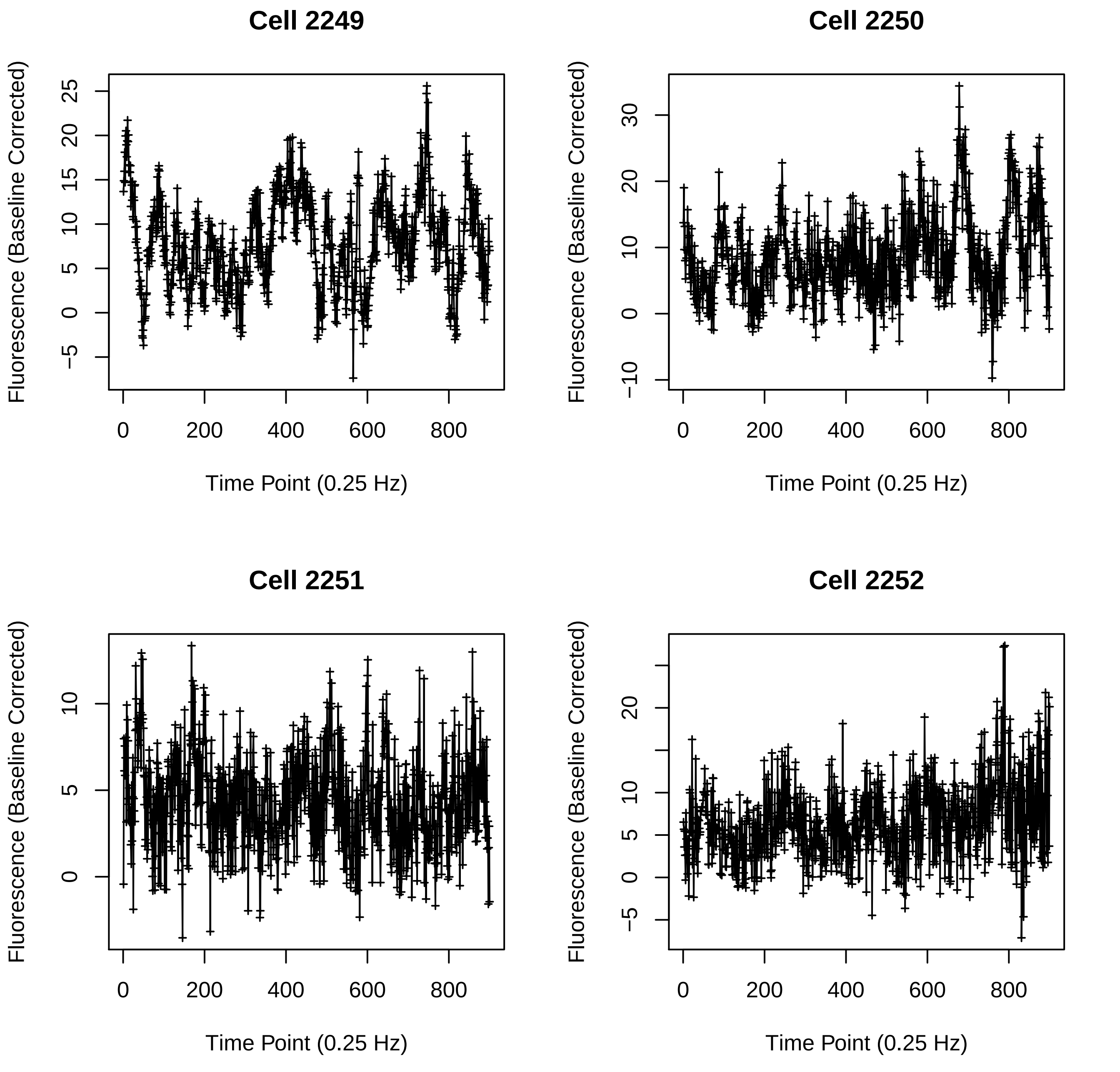
Cell 2249
Cell 2250
Cell 2251
Cell 2252
Fluorescence (Baseline Corrected)
Time Point (0.25 Hz)
0
200
400
600
800
−5
0
5
10
15
20
25
−10
30

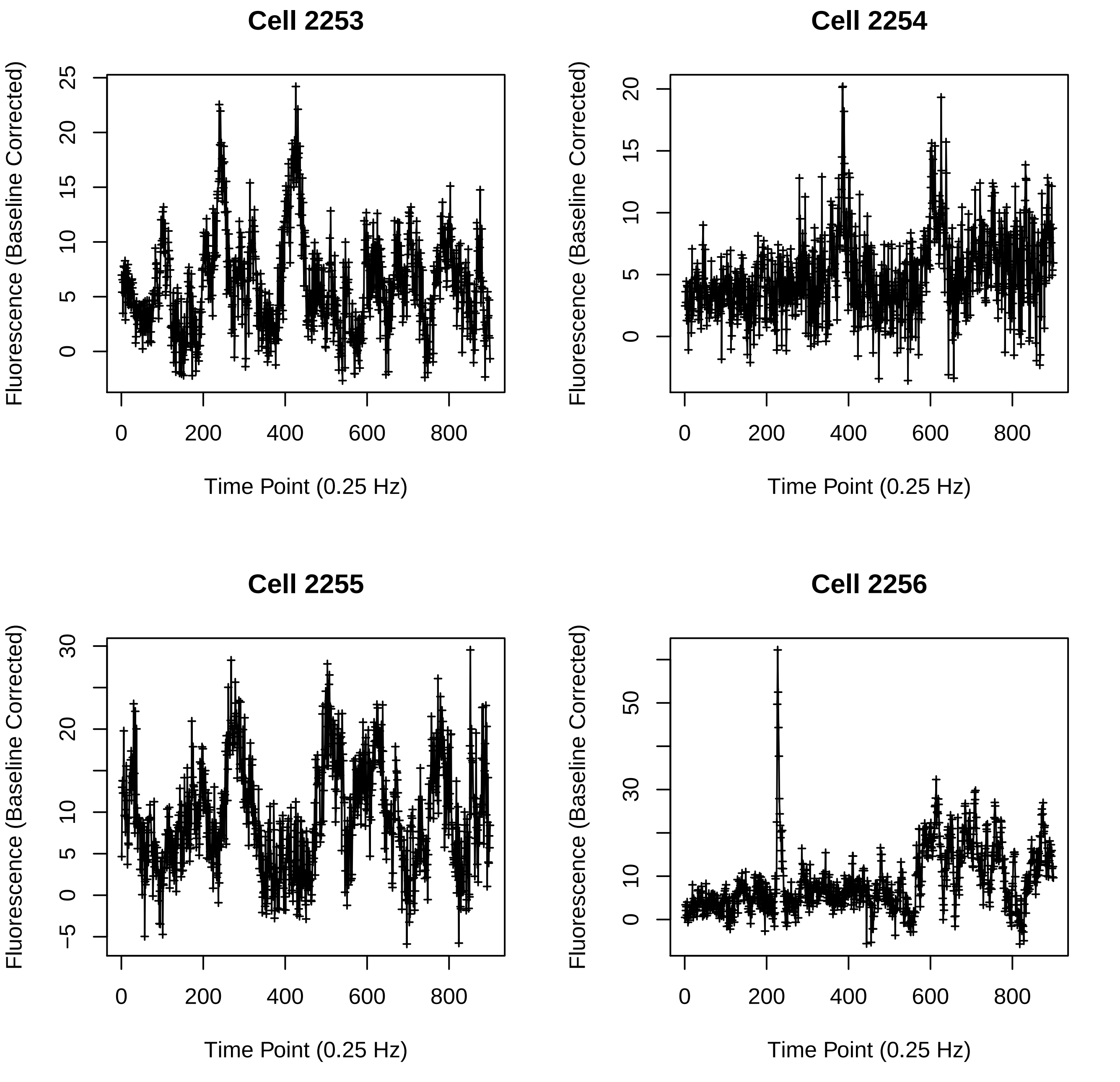
Cell 2253
Cell 2254
Cell 2255
Cell 2256
Fluorescence (Baseline Corrected)
Time Point (0.25 Hz)
0
200
400
600
800
0
5
10
15
20
25
30
−5
50

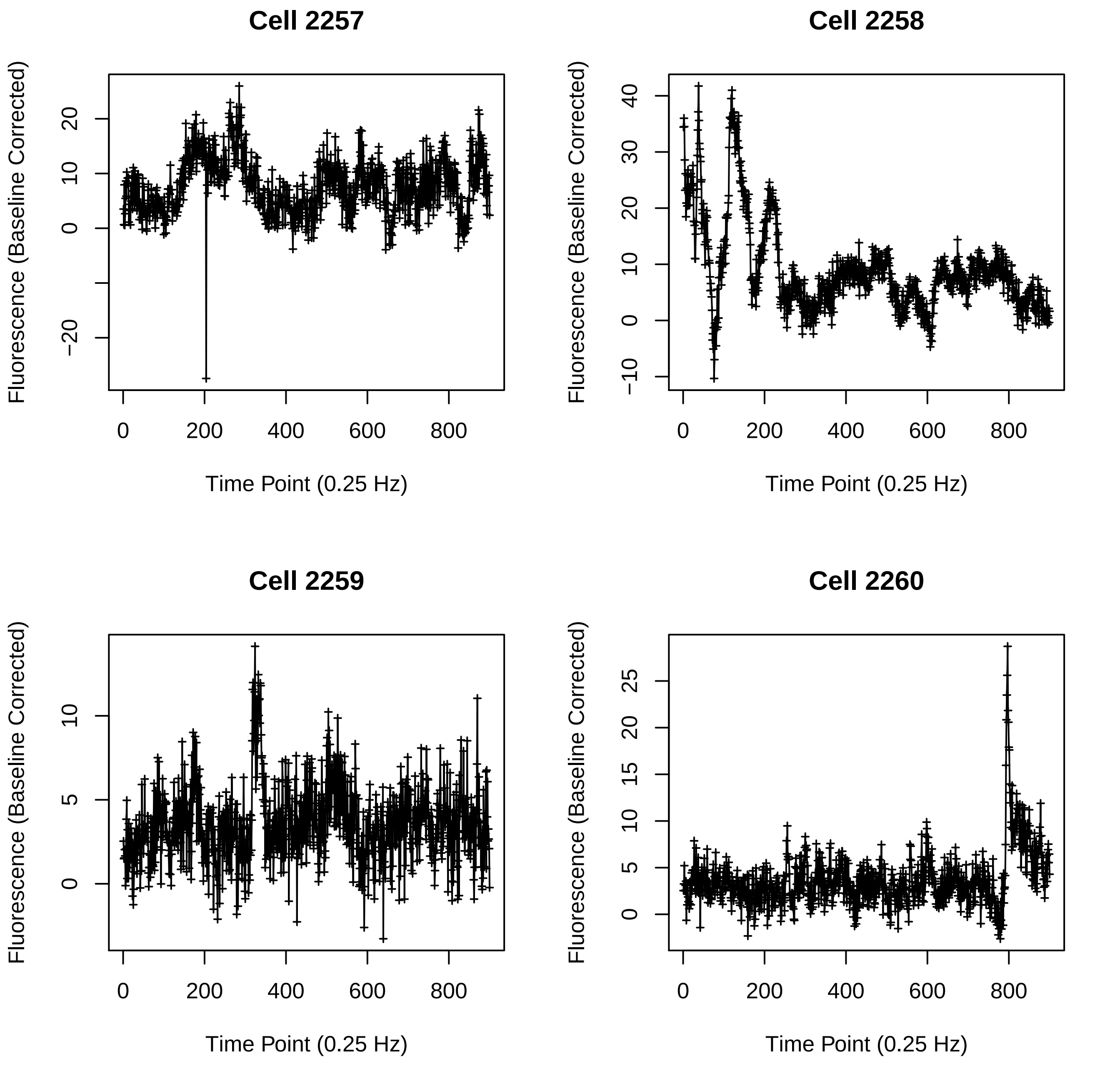

Cell 2257
Cell 2258
Cell 2259
Cell 2260
Fluorescence (Baseline Corrected)
Time Point (0.25 Hz)
0
200
400
600
800
−20
0
10
20
−10
30
40
5
15
25

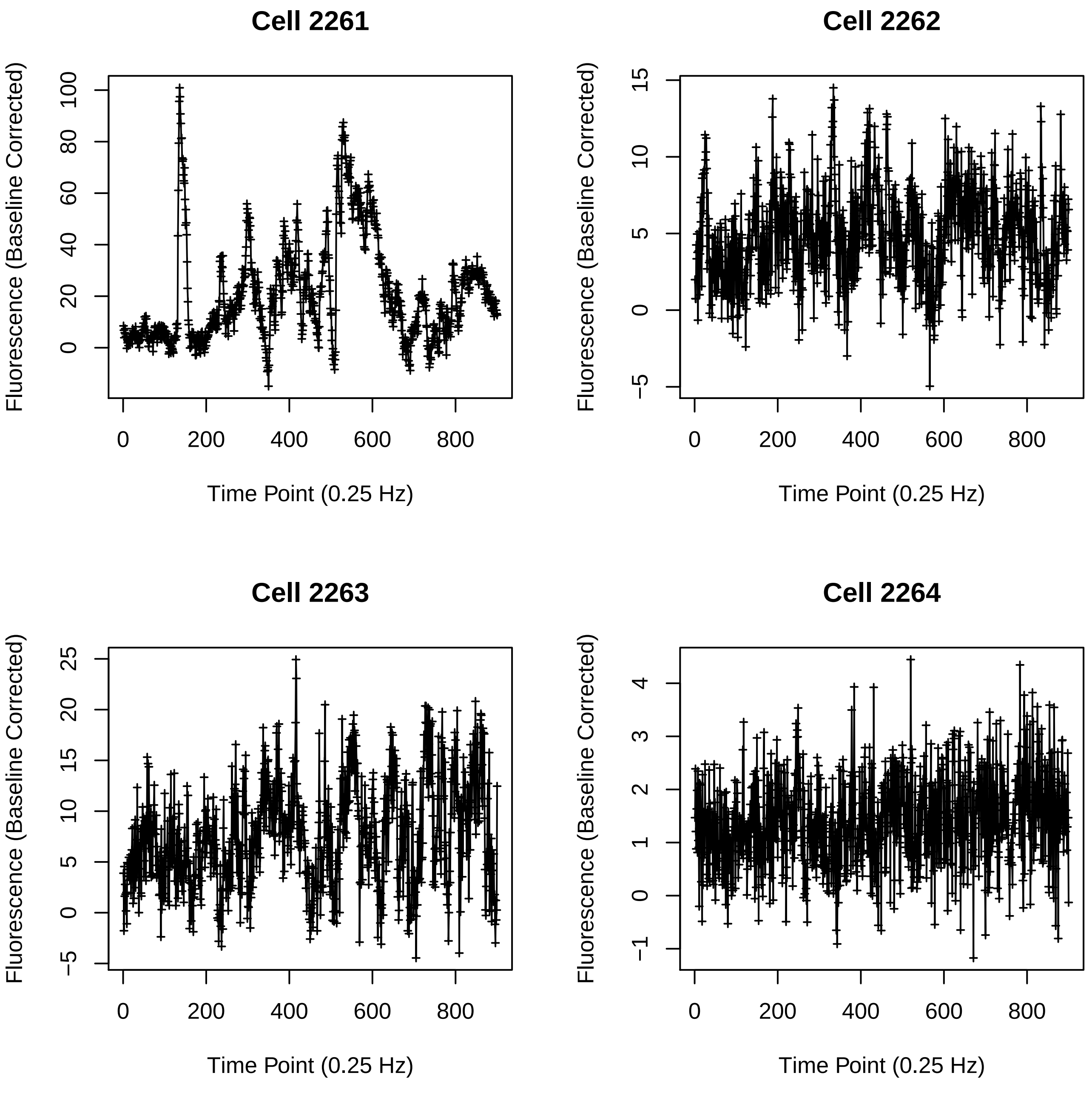
Cell 2261
Fluorescence (Baseline Corrected)
Time Point (0.25 Hz)
0
20
40
60
80
100
0
200
400
600
800
Cell 2262
Fluorescence (Baseline Corrected)
Time Point (0.25 Hz)
−5
0
5
10
15
0
200
400
600
800
Cell 2263
Fluorescence (Baseline Corrected)
Time Point (0.25 Hz)
−5
0
5
10
15
20
25
0
200
400
600
800
Cell 2264
Fluorescence (Baseline Corrected)
Time Point (0.25 Hz)
−1
0
1
2
3
4
0
200
400
600
800

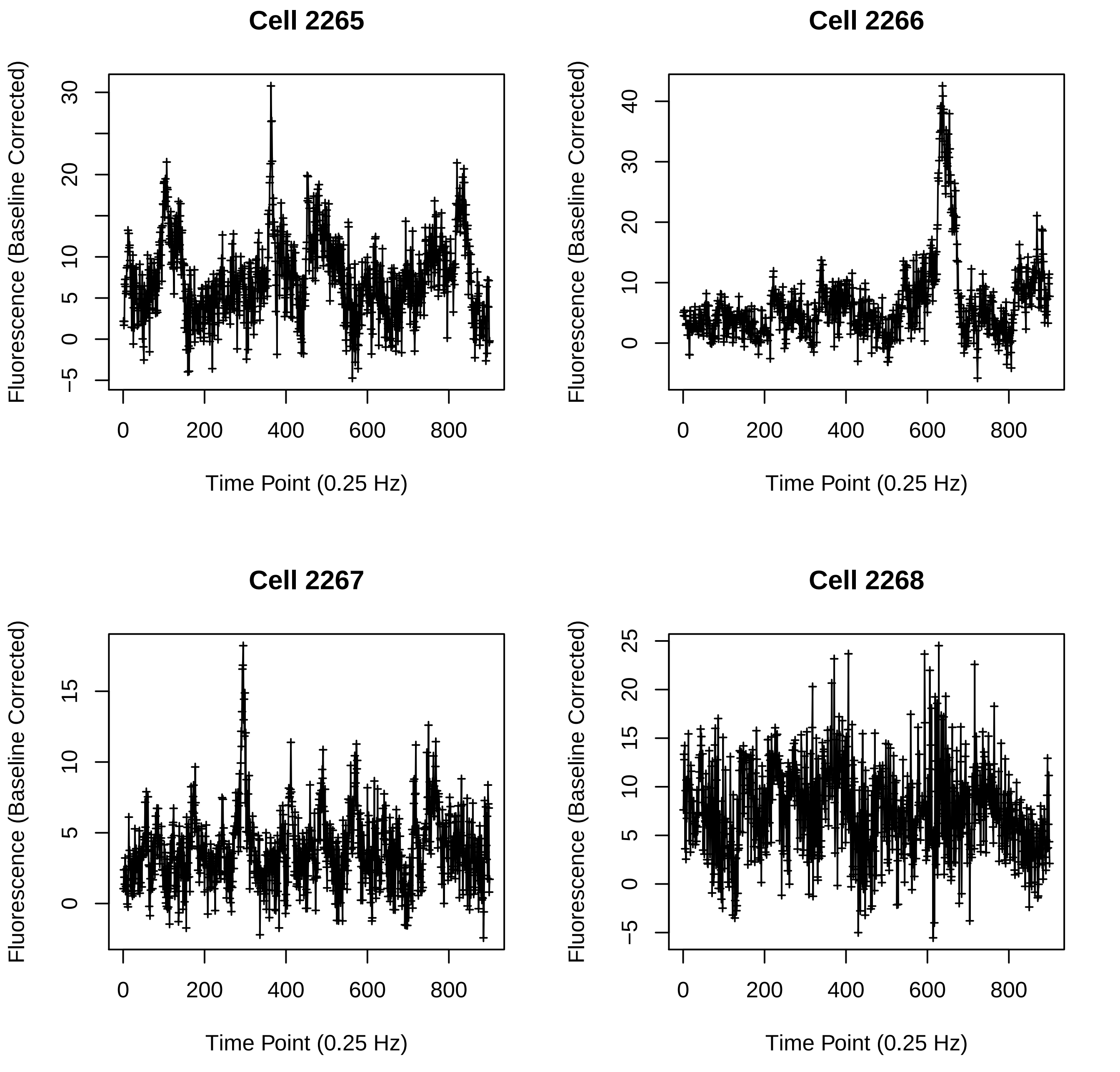

Cell 2265
Cell 2266
Cell 2267
Cell 2268
Fluorescence (Baseline Corrected)
Time Point (0.25 Hz)

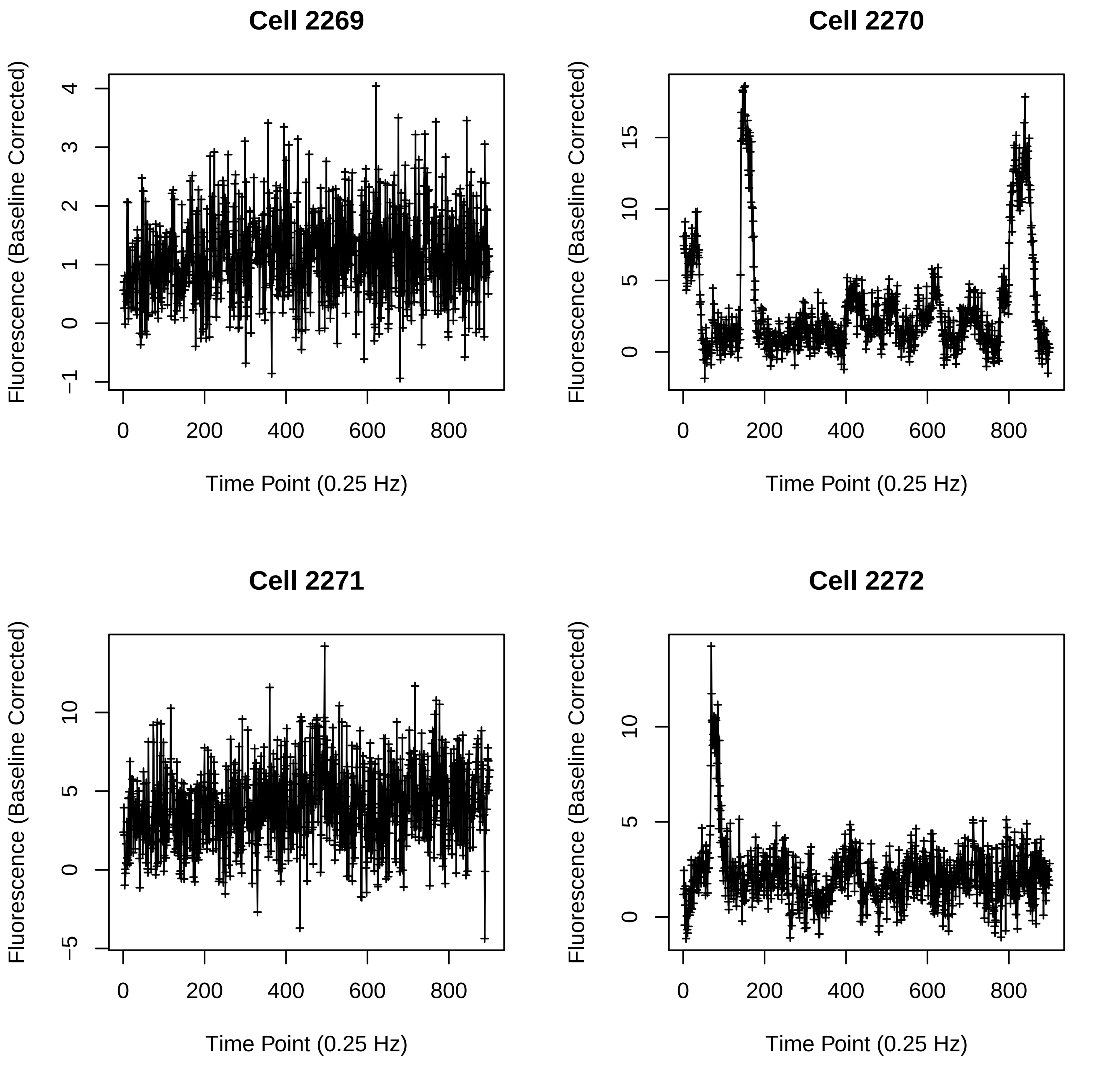
Cell 2269
Cell 2270
Cell 2271
Cell 2272
Fluorescence (Baseline Corrected)
Time Point (0.25 Hz)
0
200
400
600
800
−1
0
1
2
3
4
5
10
15
−5

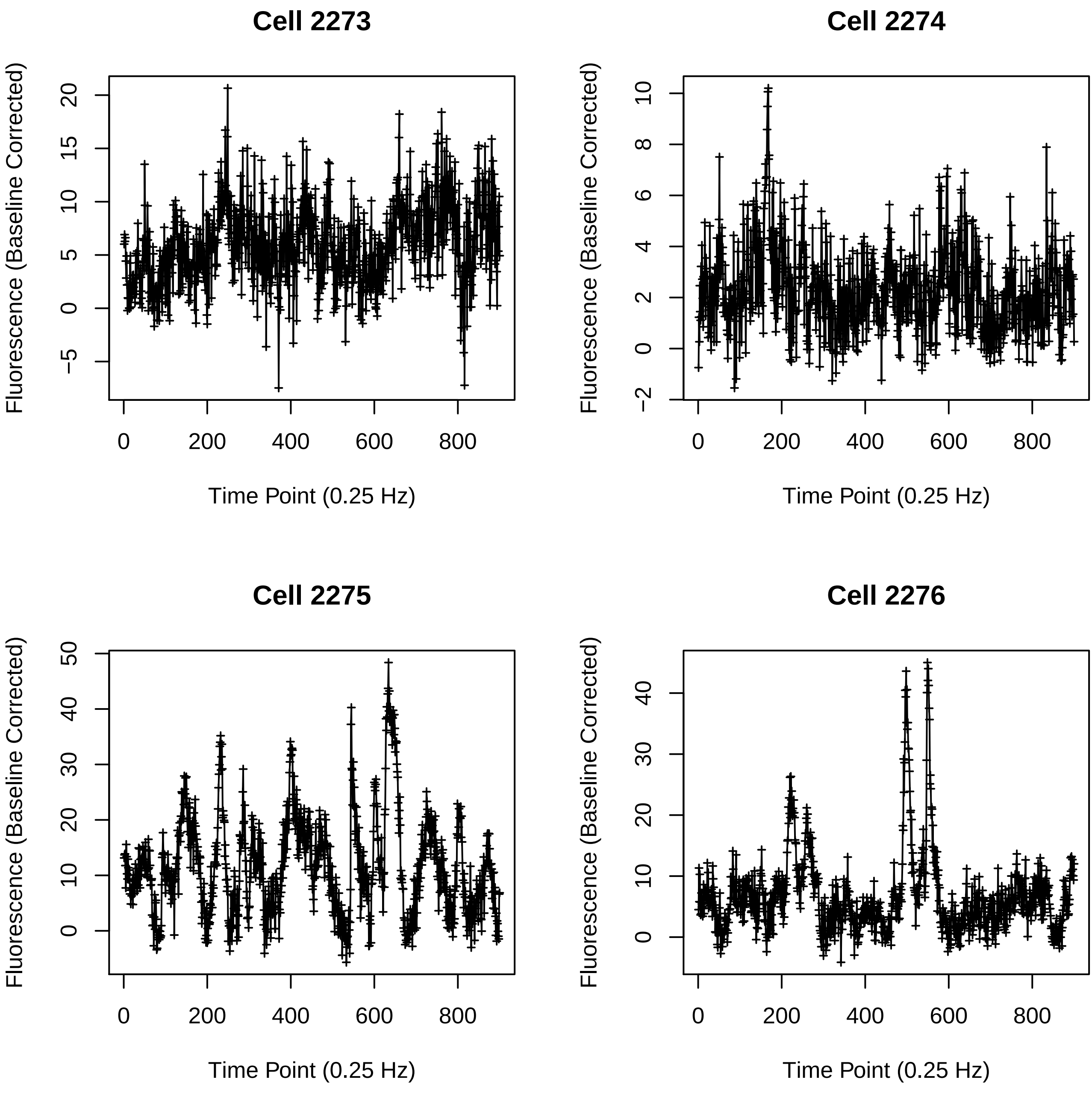
Cell 2273
Cell 2274
Cell 2275
Cell 2276
Fluorescence (Baseline Corrected)
Time Point (0.25 Hz)

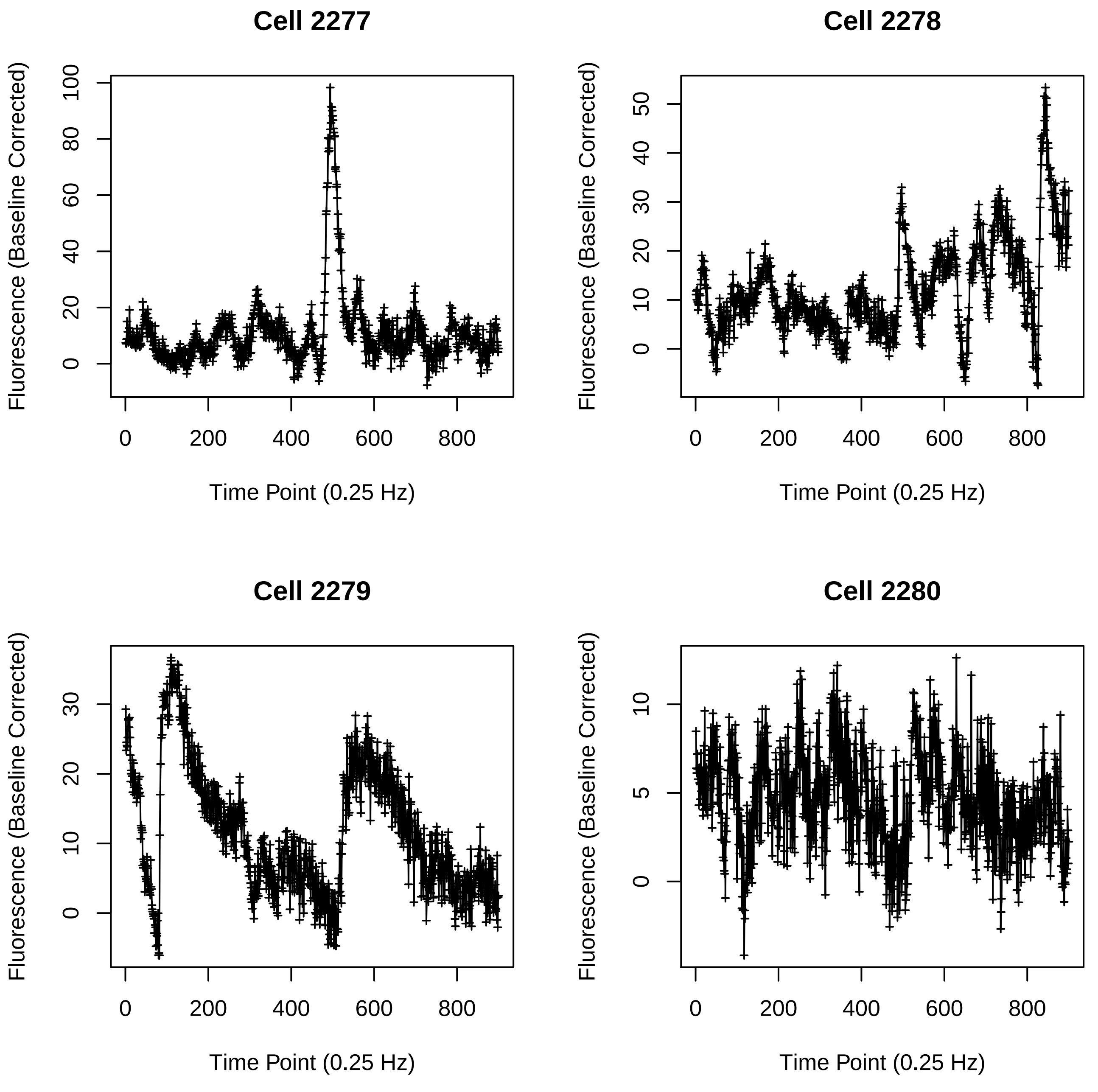
Cell 2277
Cell 2278
Cell 2279
Cell 2280
Fluorescence (Baseline Corrected)
Time Point (0.25 Hz)
0
200
400
600
800
0
20
40
60
80
100
10
30
50
5

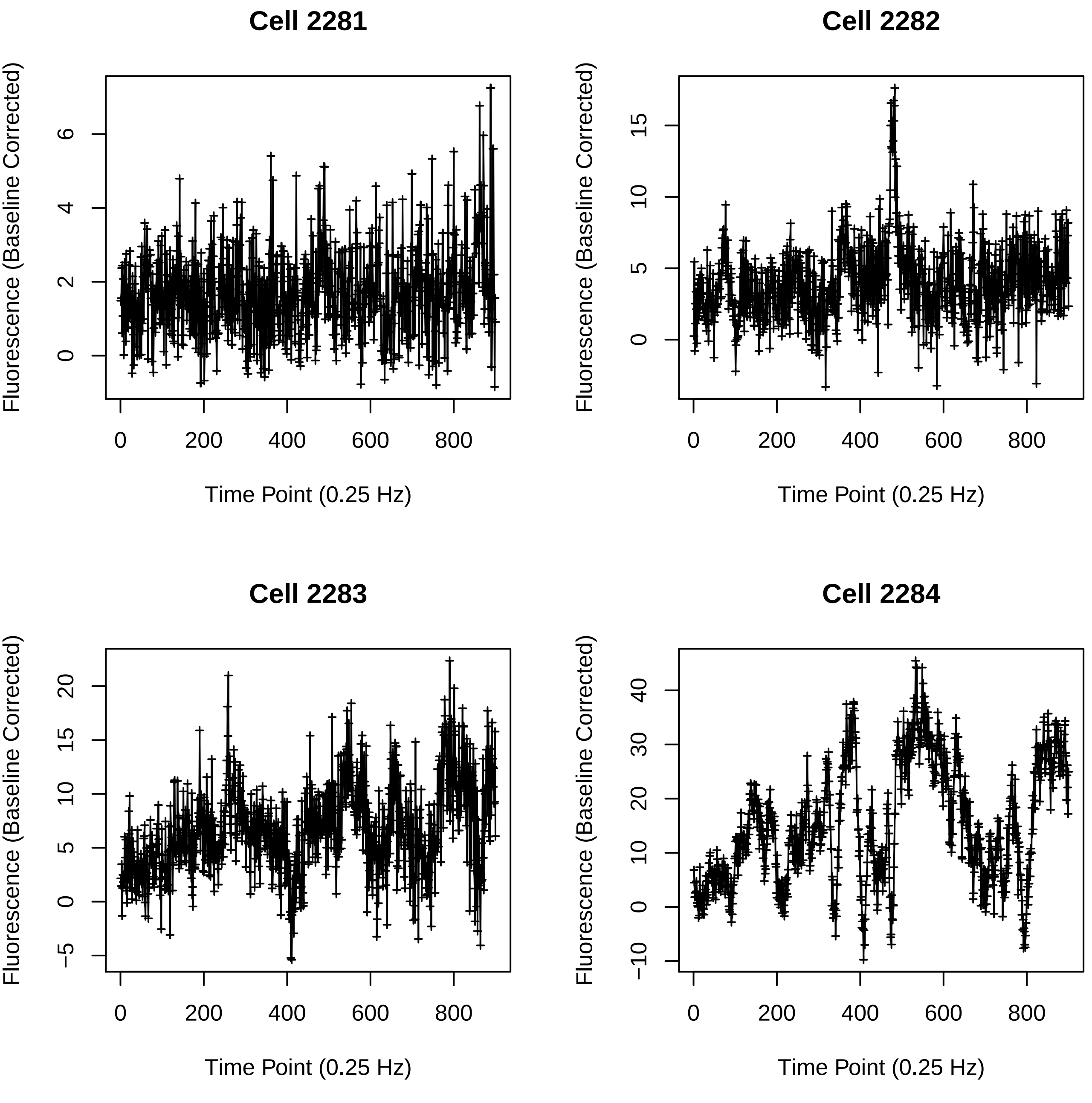
Cell 2281
Cell 2282
Cell 2283
Cell 2284
Fluorescence (Baseline Corrected)
Time Point (0.25 Hz)
0
200
400
600
800
-5
0
2
4
5
6
10
15
20
30
40
-10

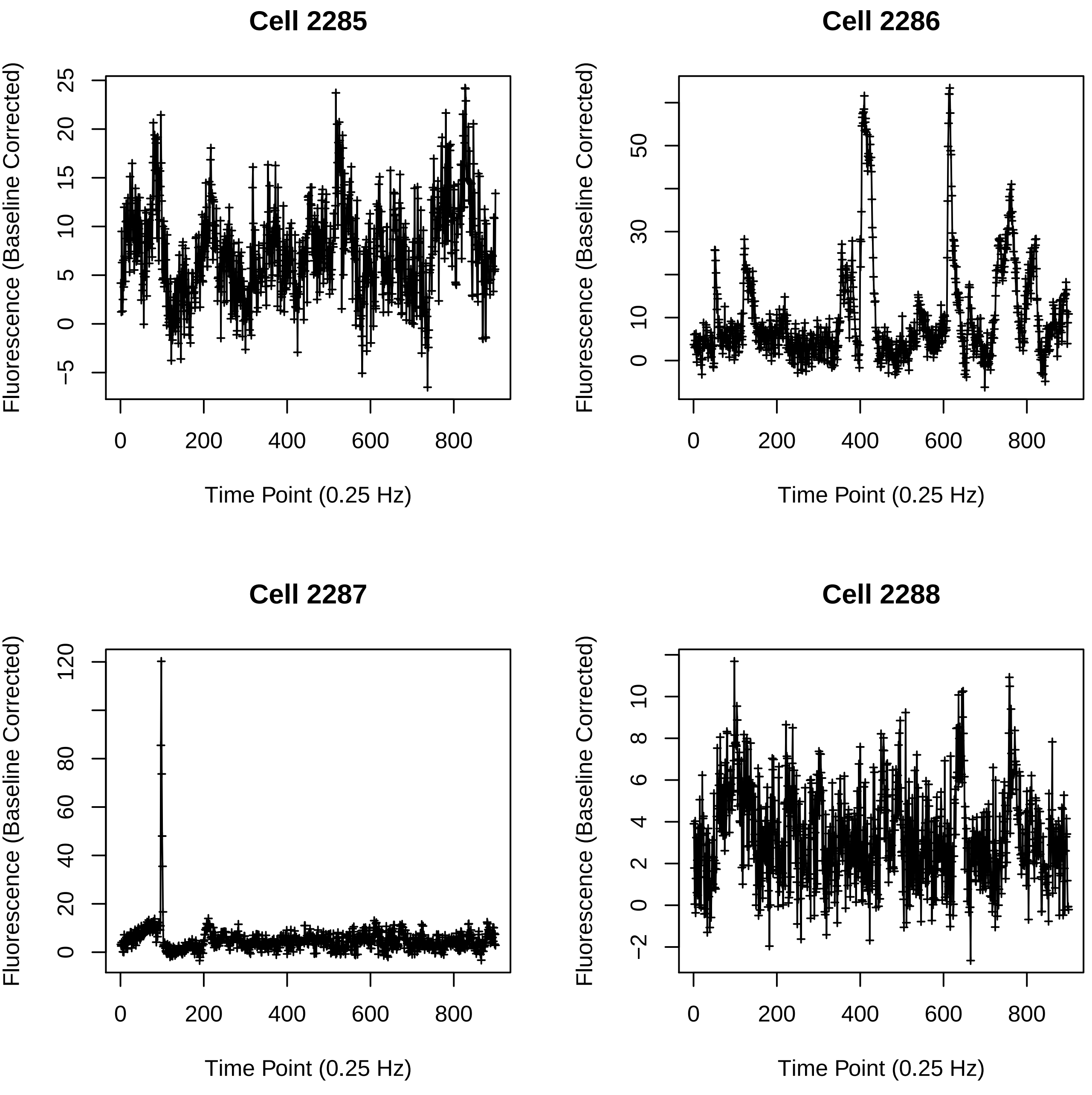
Cell 2285
Cell 2286
Cell 2287
Cell 2288
Fluorescence (Baseline Corrected)
Time Point (0.25 Hz)
−5
0
5
10
15
20
25
0
10
30
50
0
20
40
60
80
120
−2
0
2
4
6
8
10
0
200
400
600
800

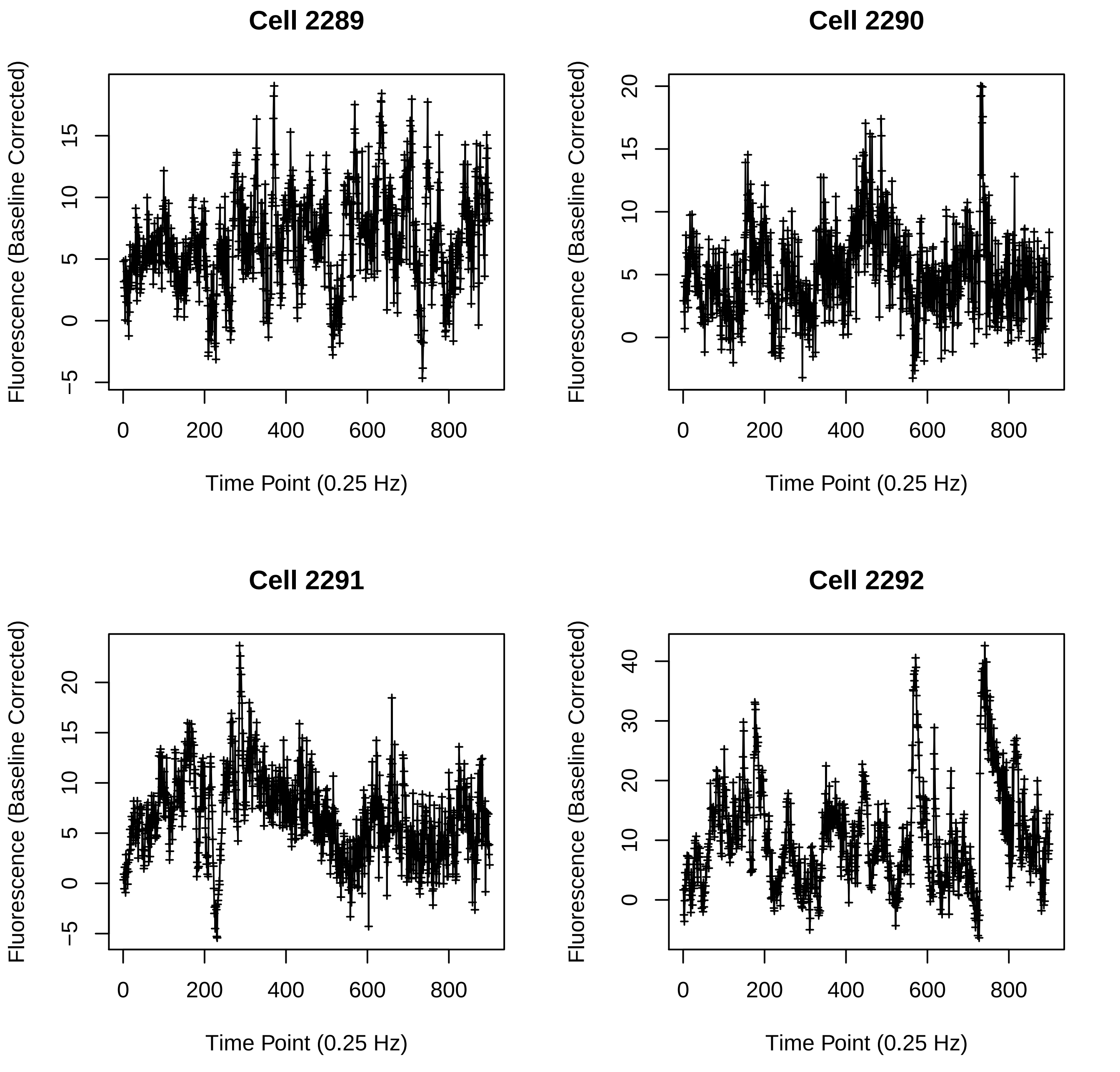

Cell 2289
Cell 2290
Cell 2291
Cell 2292
Fluorescence (Baseline Corrected)
Time Point (0.25 Hz)
0
200
400
600
800
−5
0
5
10
15
20
30
40

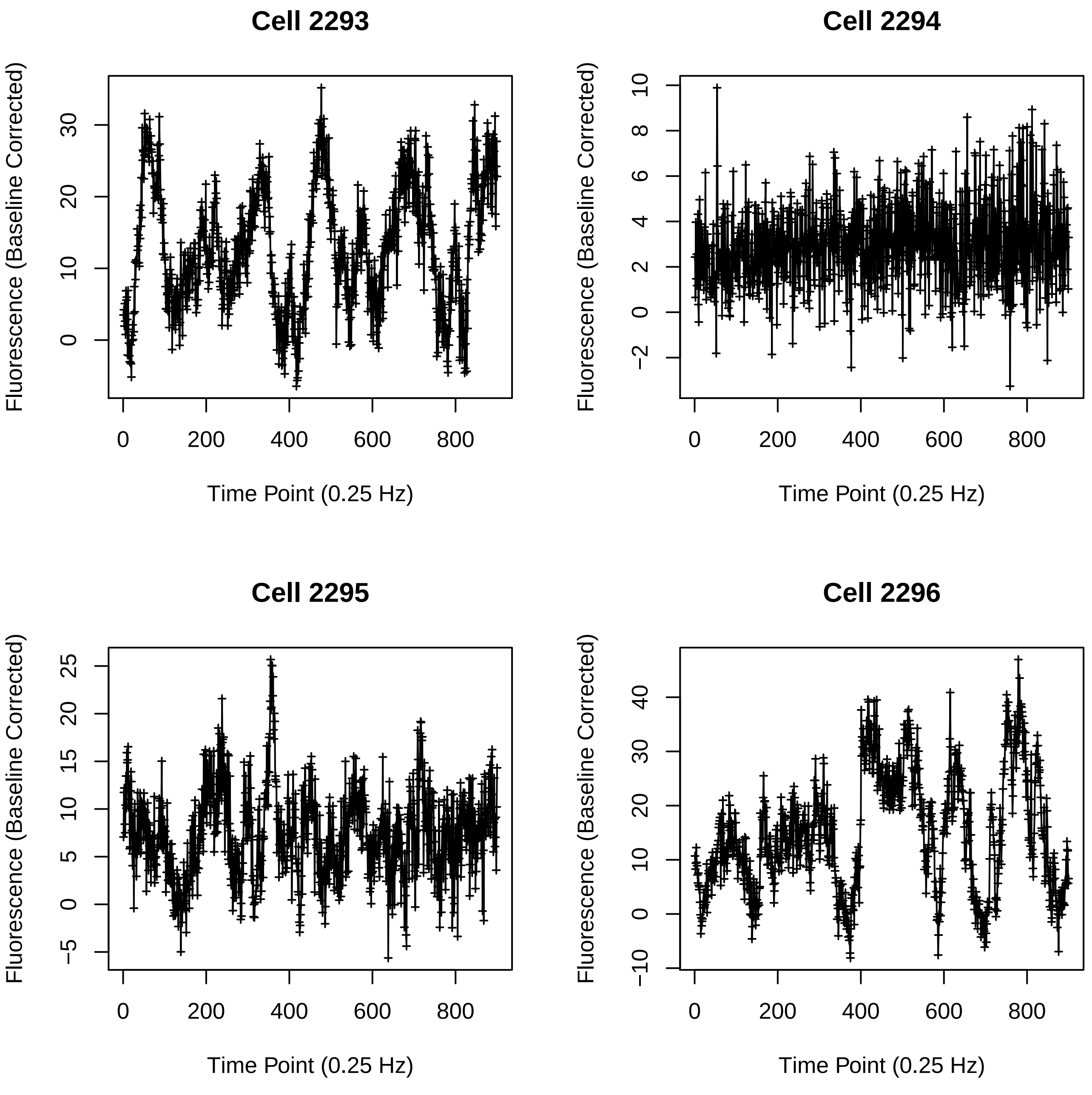

Cell 2293
Cell 2294
Cell 2295
Cell 2296
Fluorescence (Baseline Corrected)
Time Point (0.25 Hz)
0
200
400
600
800

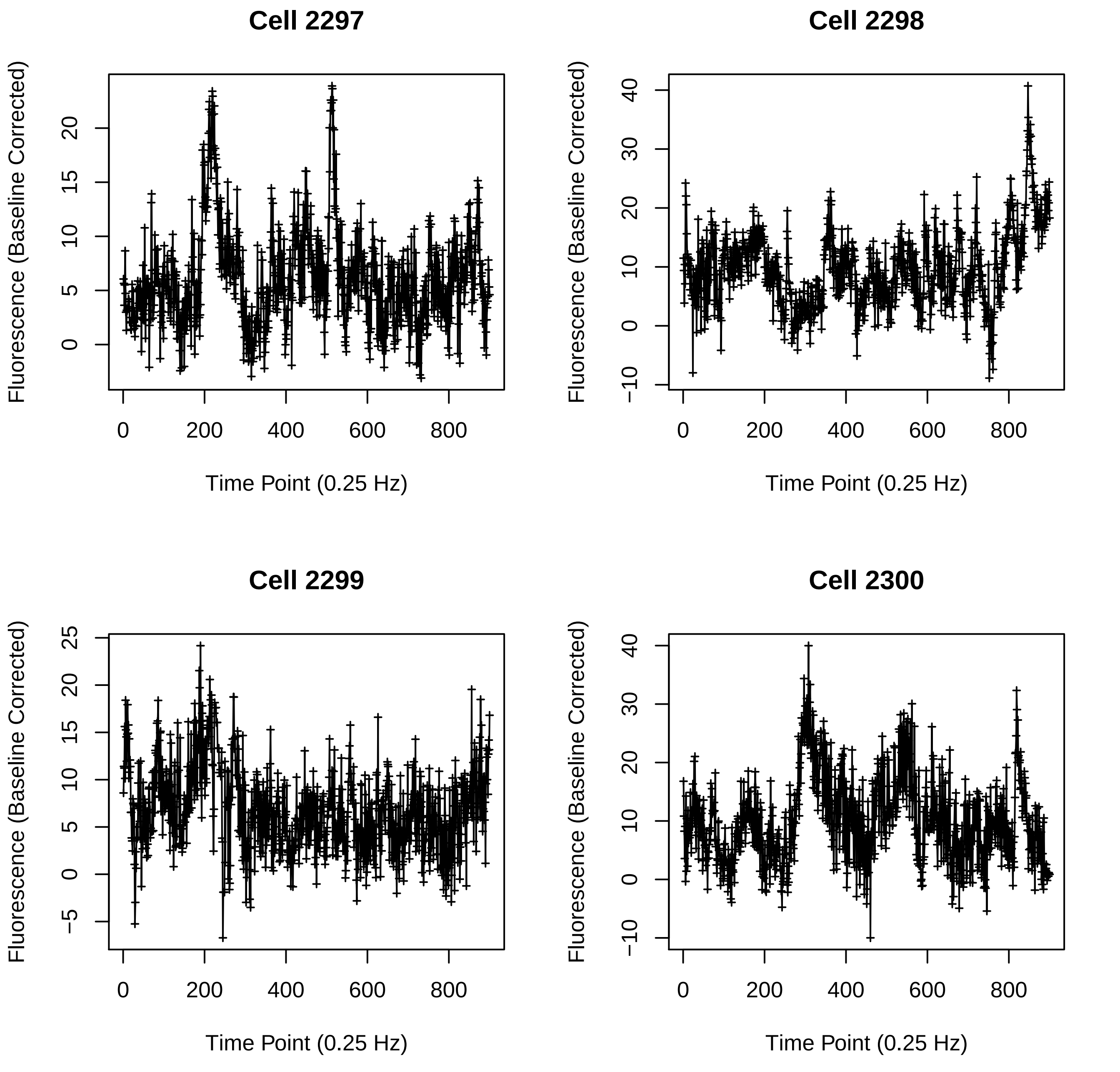
Cell 2297
Cell 2298
Cell 2299
Cell 2300
Fluorescence (Baseline Corrected)
Time Point (0.25 Hz)
0
200
400
600
800
−10
−5
5
10
15
20
25
30
40

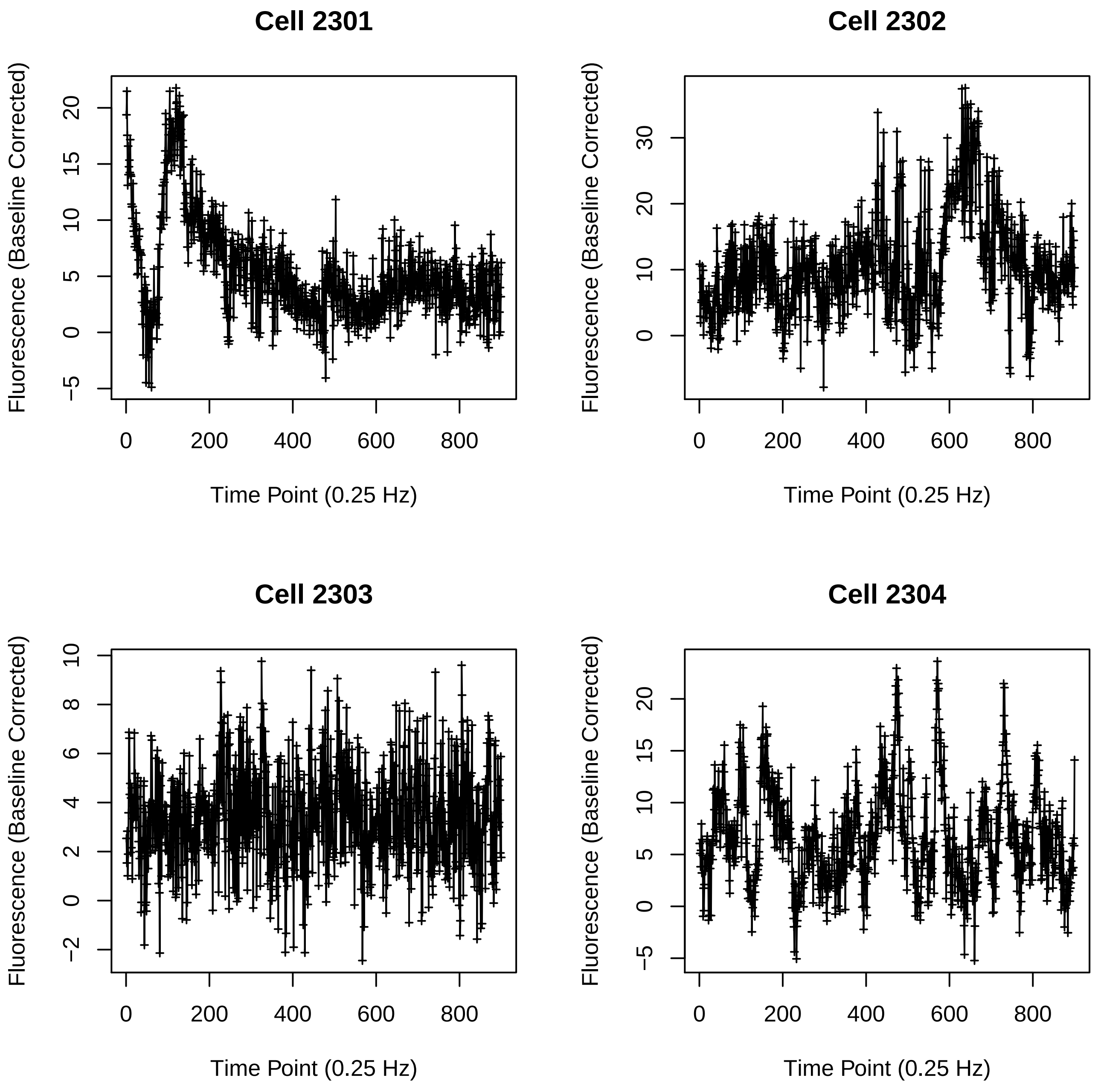
Cell 2301
Cell 2302
Cell 2303
Cell 2304
Fluorescence (Baseline Corrected)
Time Point (0.25 Hz)
0
200
400
600
800
−5
5
10
15
20
30
−2
2
4
6
8

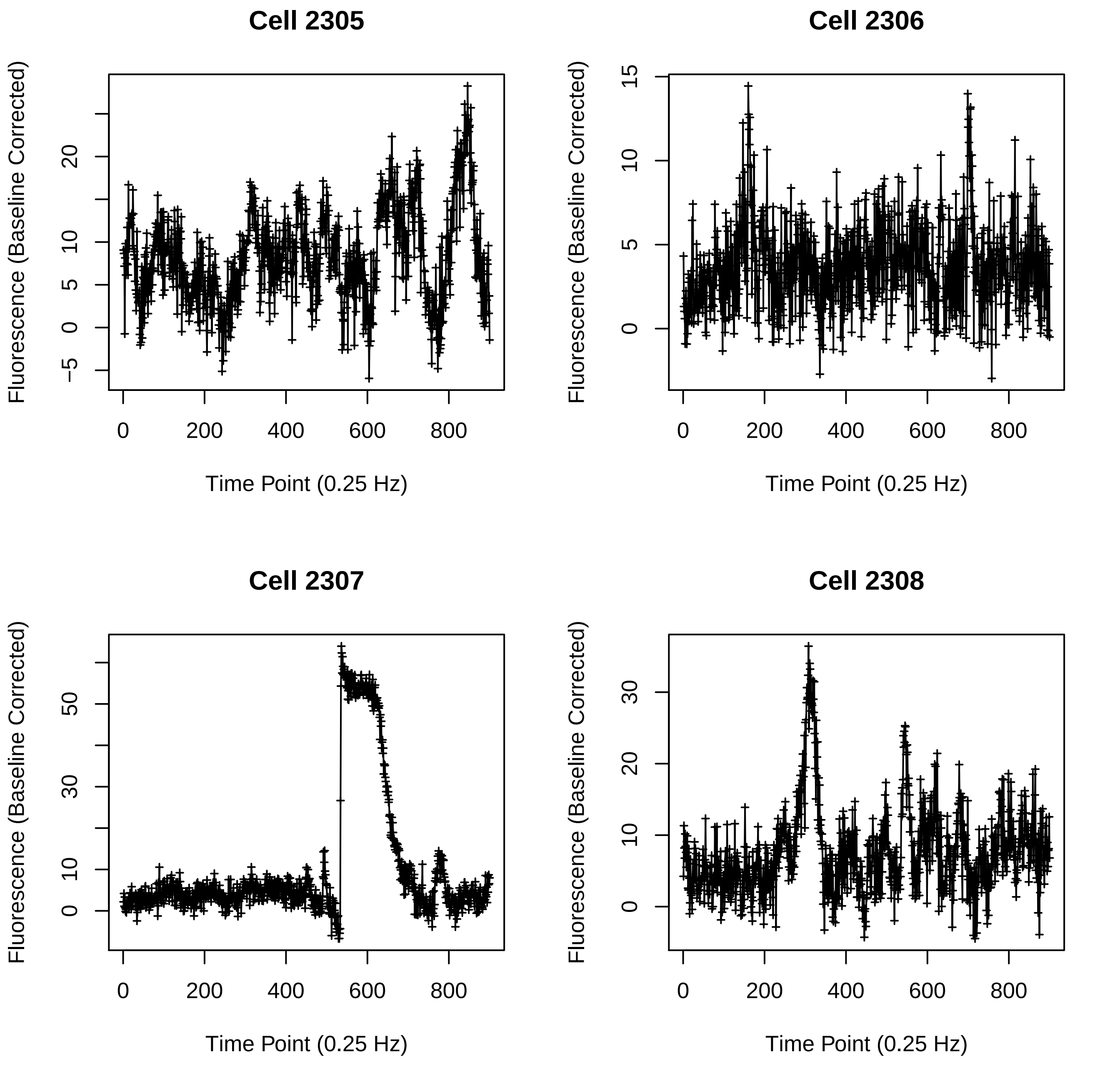
Cell 2305
Cell 2306
Cell 2307
Cell 2308
Fluorescence (Baseline Corrected)
Time Point (0.25 Hz)
0
200
400
600
800
−5
0
5
10
15
20
30
50

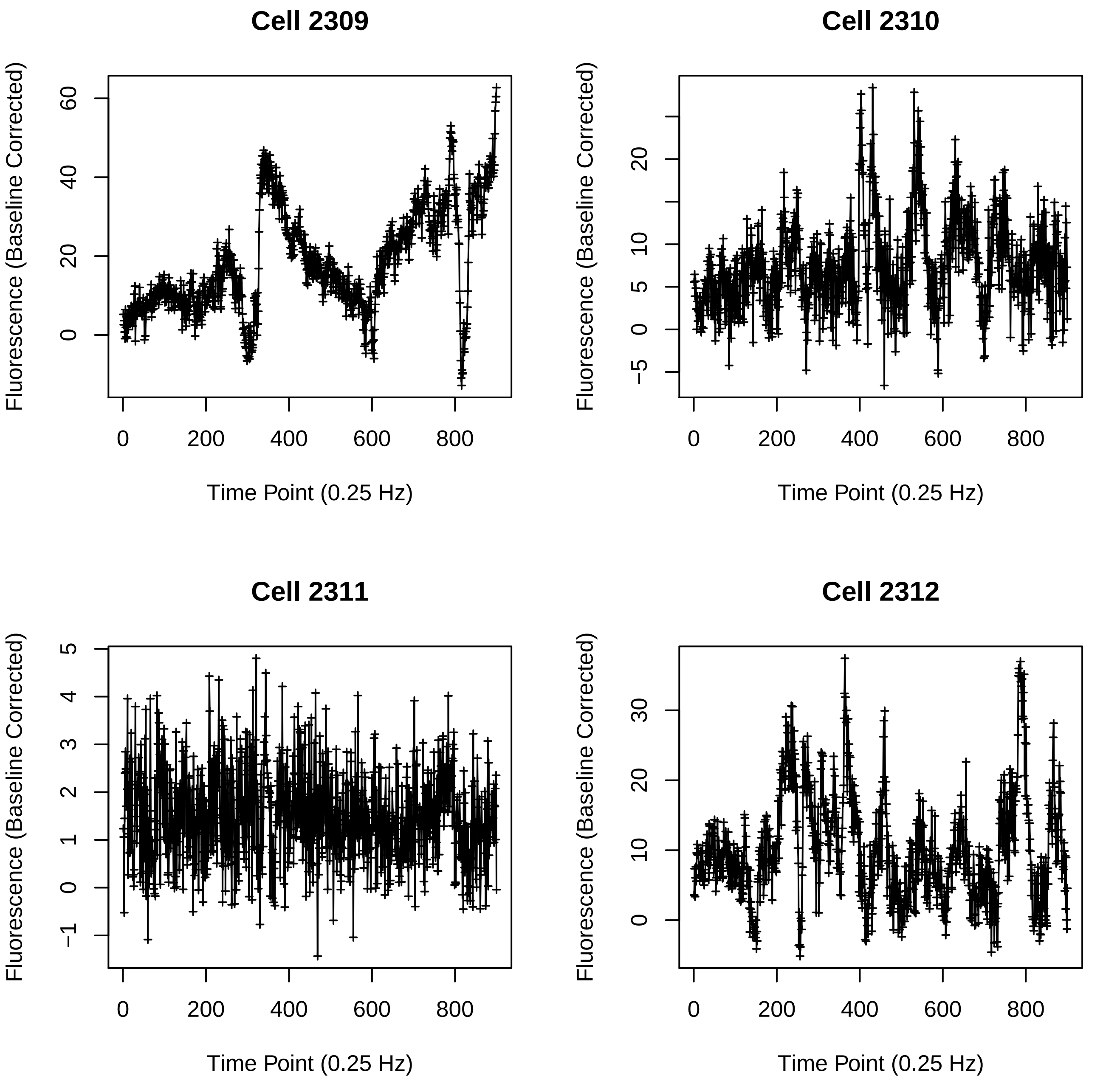

Cell 2309
Fluorescence (Baseline Corrected)
0
20
40
60
0
200
400
600
800
Time Point (0.25 Hz)
Cell 2310
Fluorescence (Baseline Corrected)
−5
0
5
10
20
0
200
400
600
800
Time Point (0.25 Hz)
Cell 2311
Fluorescence (Baseline Corrected)
−1
0
1
2
3
4
5
0
200
400
600
800
Time Point (0.25 Hz)
Cell 2312
Fluorescence (Baseline Corrected)
0
10
20
30
0
200
400
600
800
Time Point (0.25 Hz)

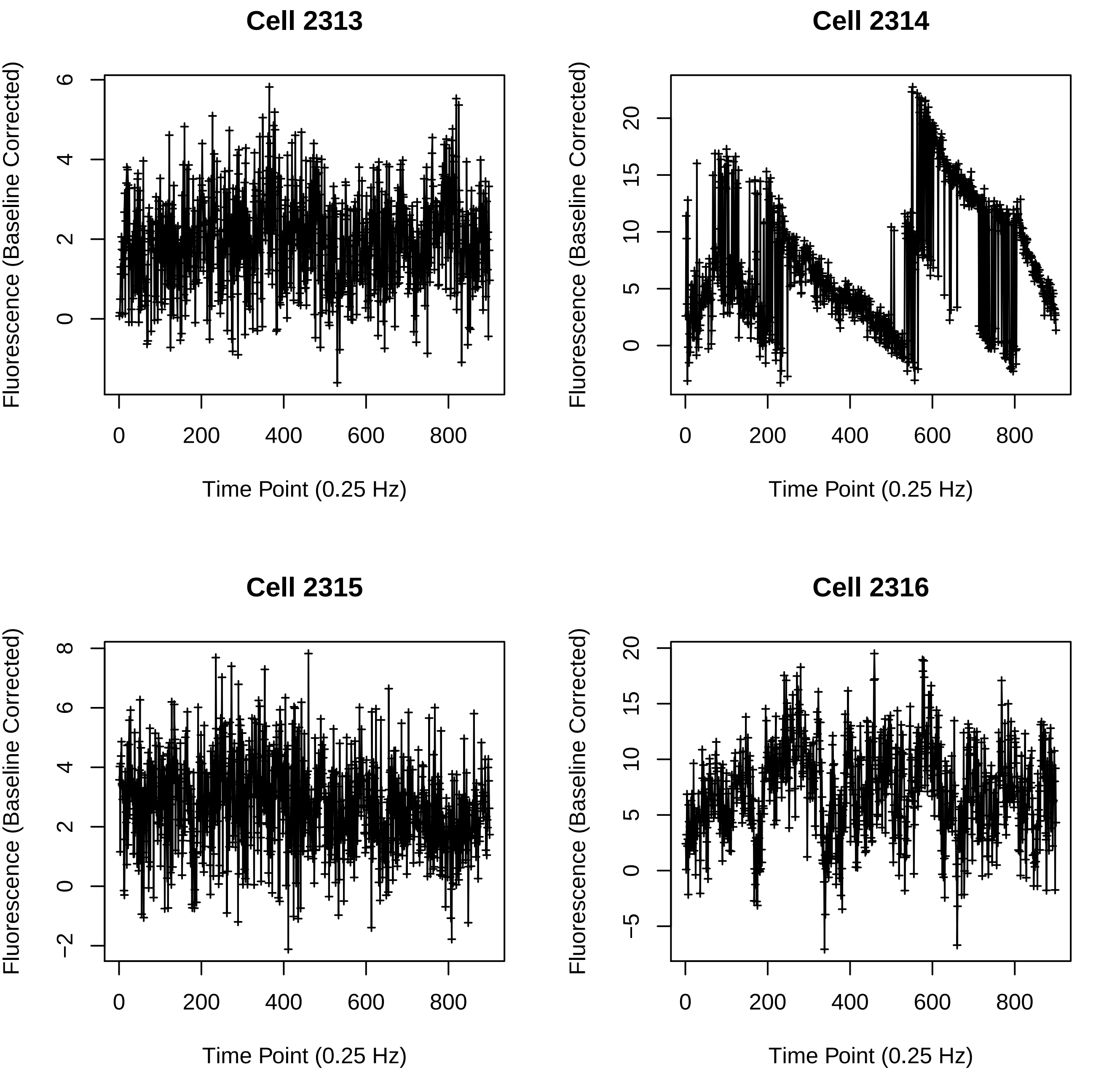

Cell 2313
Fluorescence (Baseline Corrected)
Time Point (0.25 Hz)
0
2
4
6
0
200
400
600
800
Cell 2314
Fluorescence (Baseline Corrected)
Time Point (0.25 Hz)
0
5
10
15
20
0
200
400
600
800
Cell 2315
Fluorescence (Baseline Corrected)
Time Point (0.25 Hz)
−2
0
2
4
6
8
0
200
400
600
800
Cell 2316
Fluorescence (Baseline Corrected)
Time Point (0.25 Hz)
−5
0
5
10
15
20
0
200
400
600
800

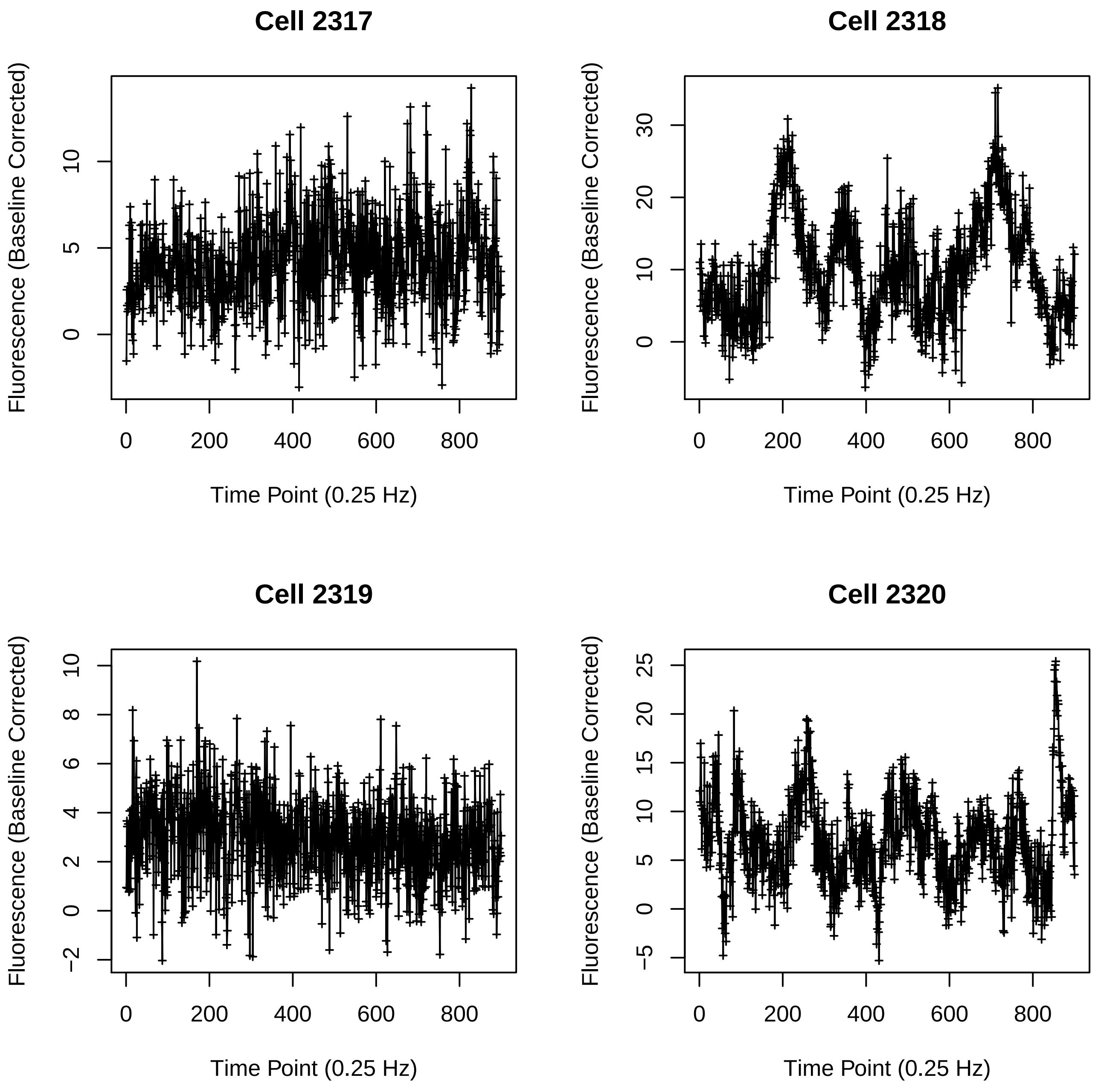
Cell 2317
Cell 2318
Cell 2319
Cell 2320
Fluorescence (Baseline Corrected)
Time Point (0.25 Hz)
0
200
400
600
800
-5
0
5
10
15
20
25
30
-2
2
4
6
8

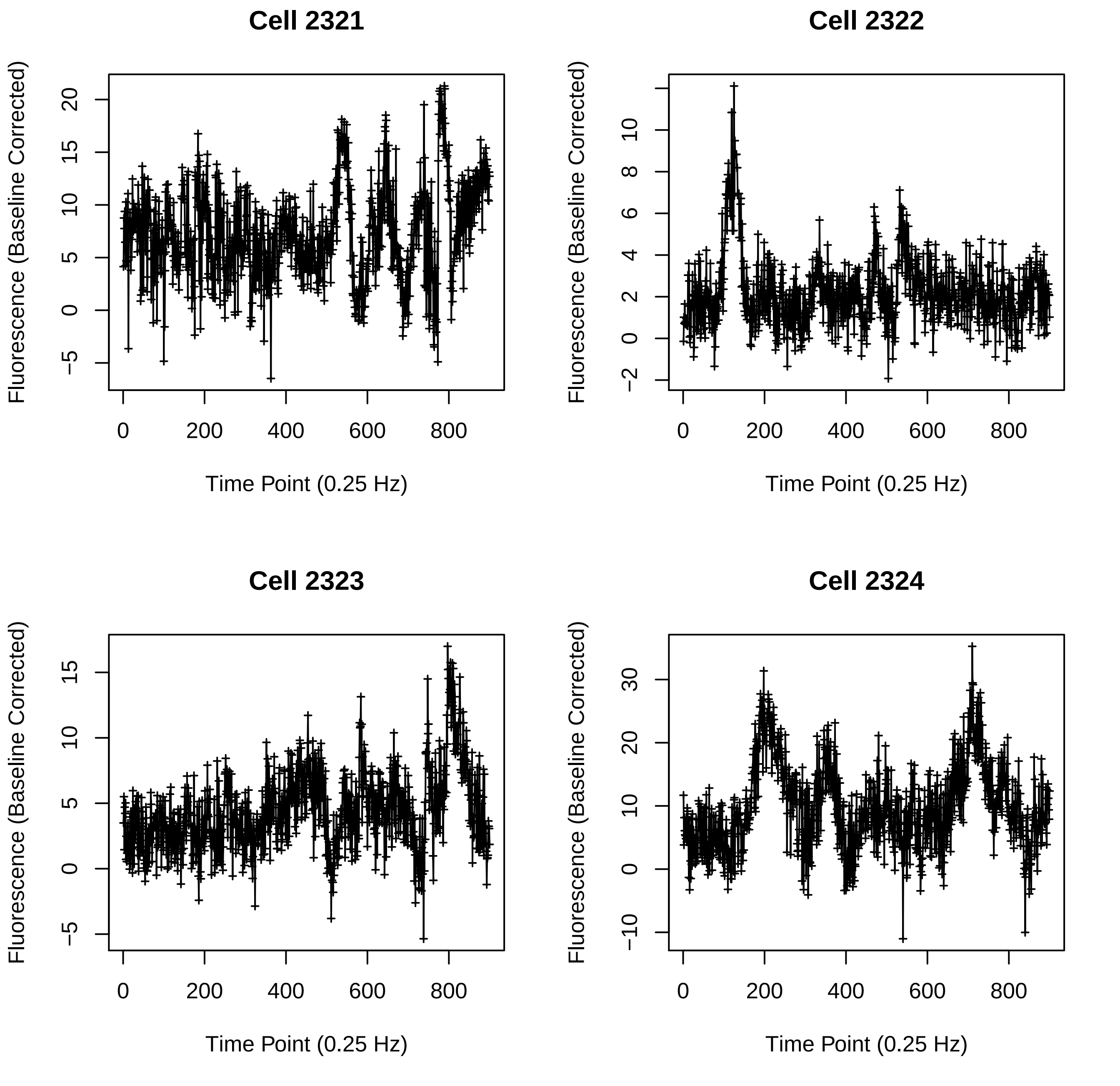
Cell 2321
Cell 2322
Cell 2323
Cell 2324
Fluorescence (Baseline Corrected)
Time Point (0.25 Hz)
0
200
400
600
800
-5
5
10
15
20
-2
2
4
6
8
-10
30

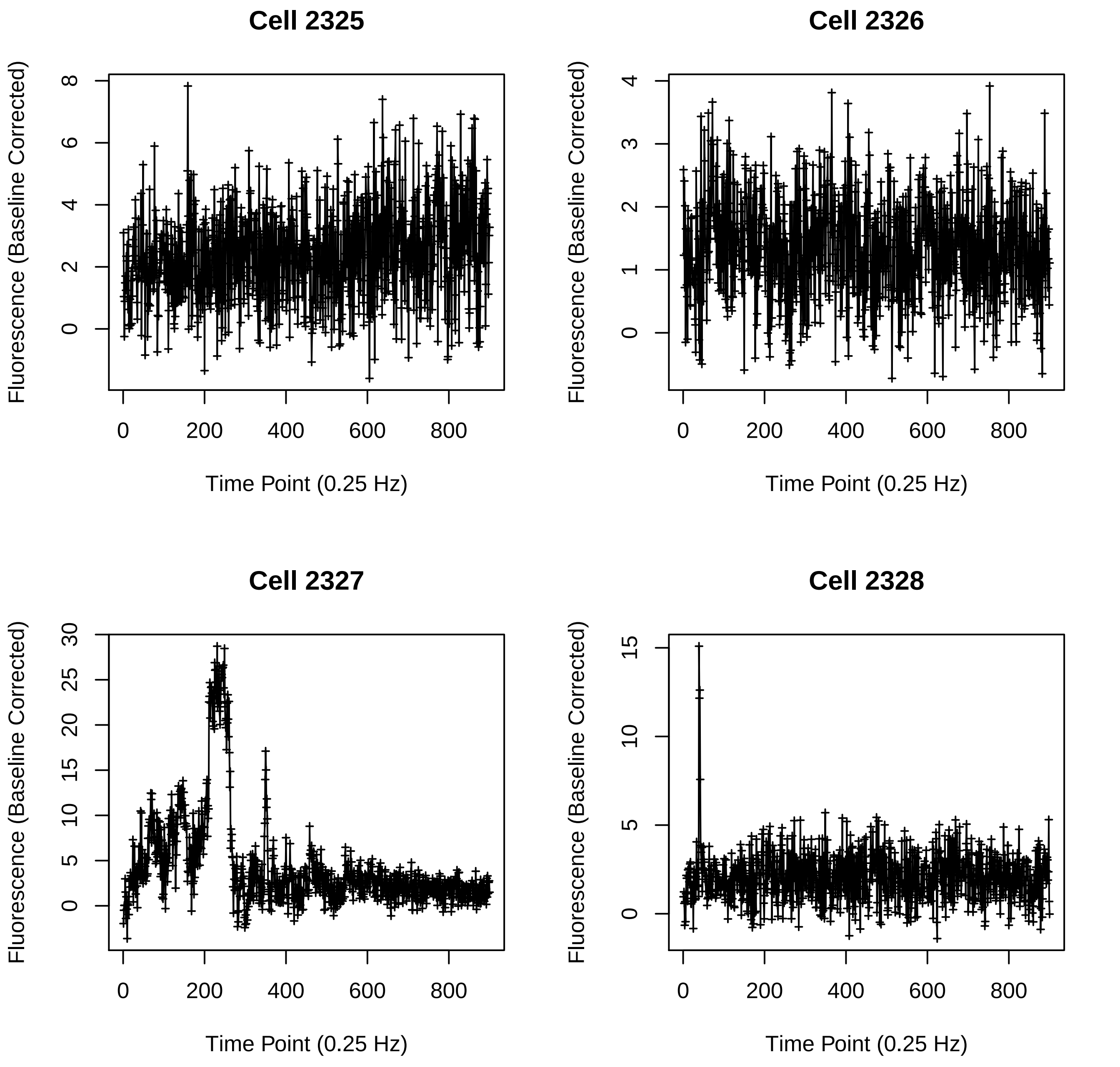
Cell 2325
Cell 2326
Cell 2327
Cell 2328
Fluorescence (Baseline Corrected)
Time Point (0.25 Hz)
0
200
400
600
800
2
4
6
8
1
3
5
10
15
20
25
30

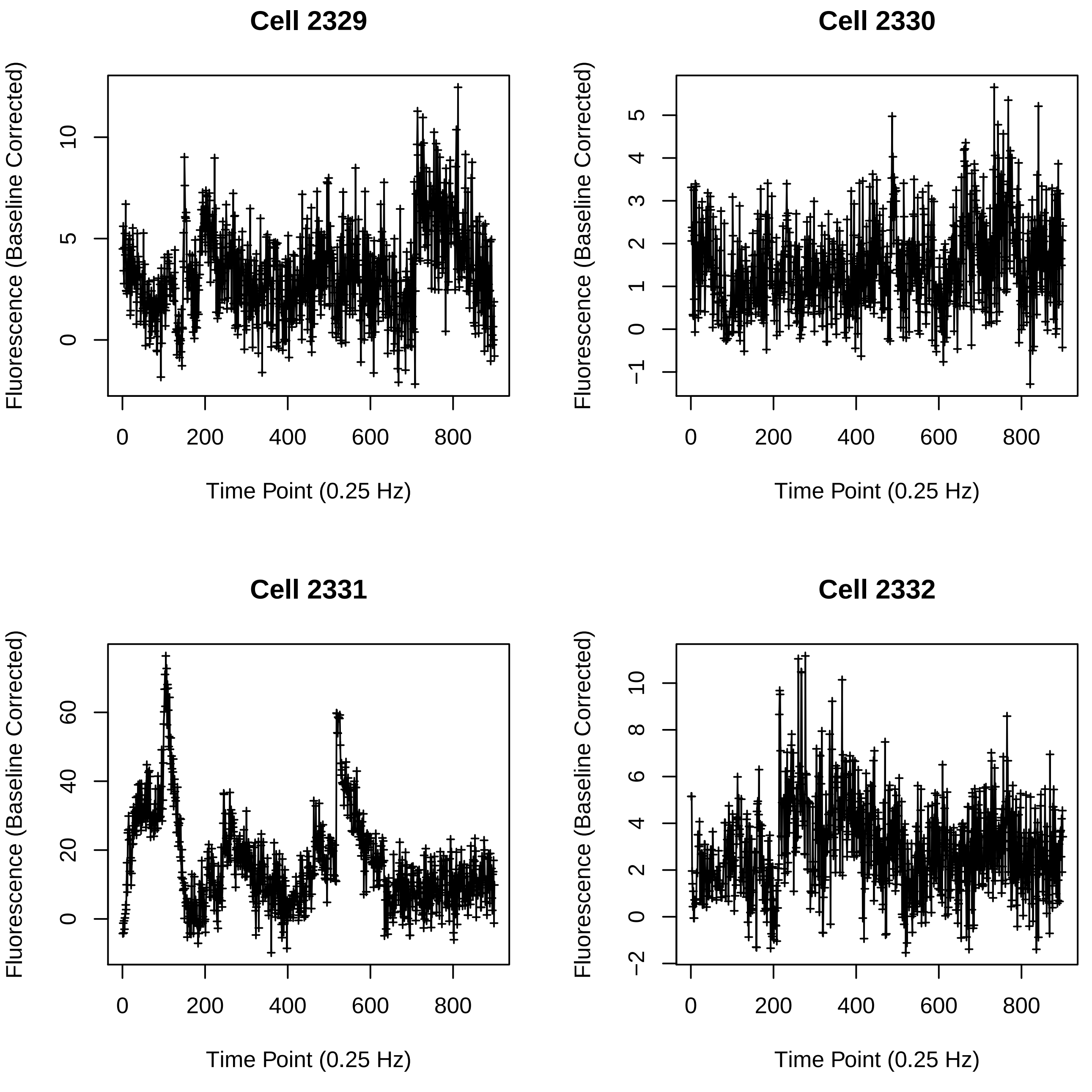
Cell 2329
Cell 2330
Cell 2331
Cell 2332
Fluorescence (Baseline Corrected)
Time Point (0.25 Hz)
0
200
400
600
800
0
5
10
−1
0
1
2
3
4
5
0
20
40
60
−2
0
2
4
6
8
10

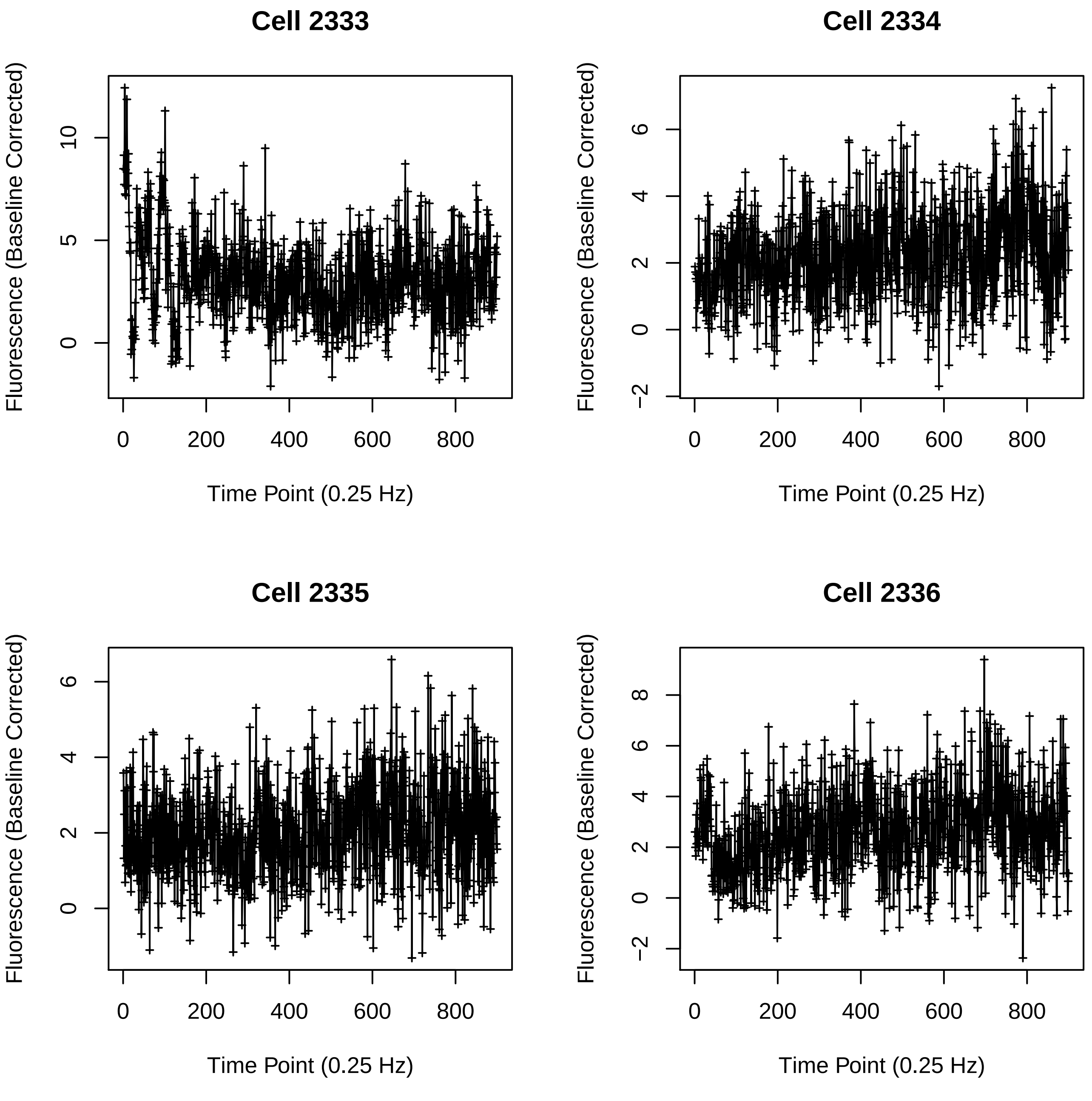
Cell 2333
Fluorescence (Baseline Corrected)
0
5
10
0
200
400
600
800
Time Point (0.25 Hz)
Cell 2334
Fluorescence (Baseline Corrected)
−2
0
2
4
6
0
200
400
600
800
Time Point (0.25 Hz)
Cell 2335
Fluorescence (Baseline Corrected)
0
2
4
6
0
200
400
600
800
Time Point (0.25 Hz)
Cell 2336
Fluorescence (Baseline Corrected)
−2
0
2
4
6
8
0
200
400
600
800
Time Point (0.25 Hz)

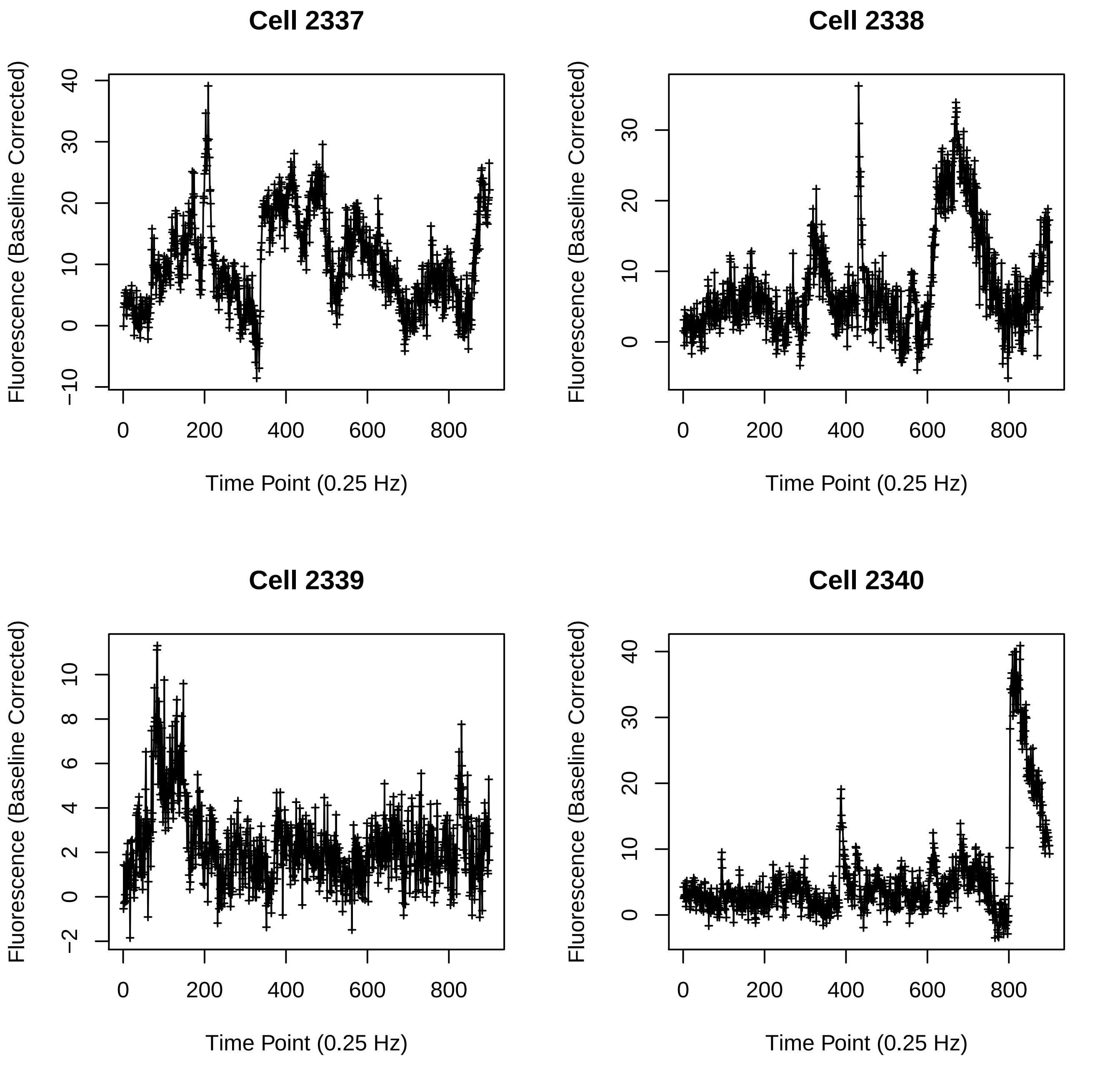
Cell 2337
Cell 2338
Cell 2339
Cell 2340
Fluorescence (Baseline Corrected)
Time Point (0.25 Hz)
−10
0
10
20
30
40
0
200
400
600
800
−2
2
4
6
8
10

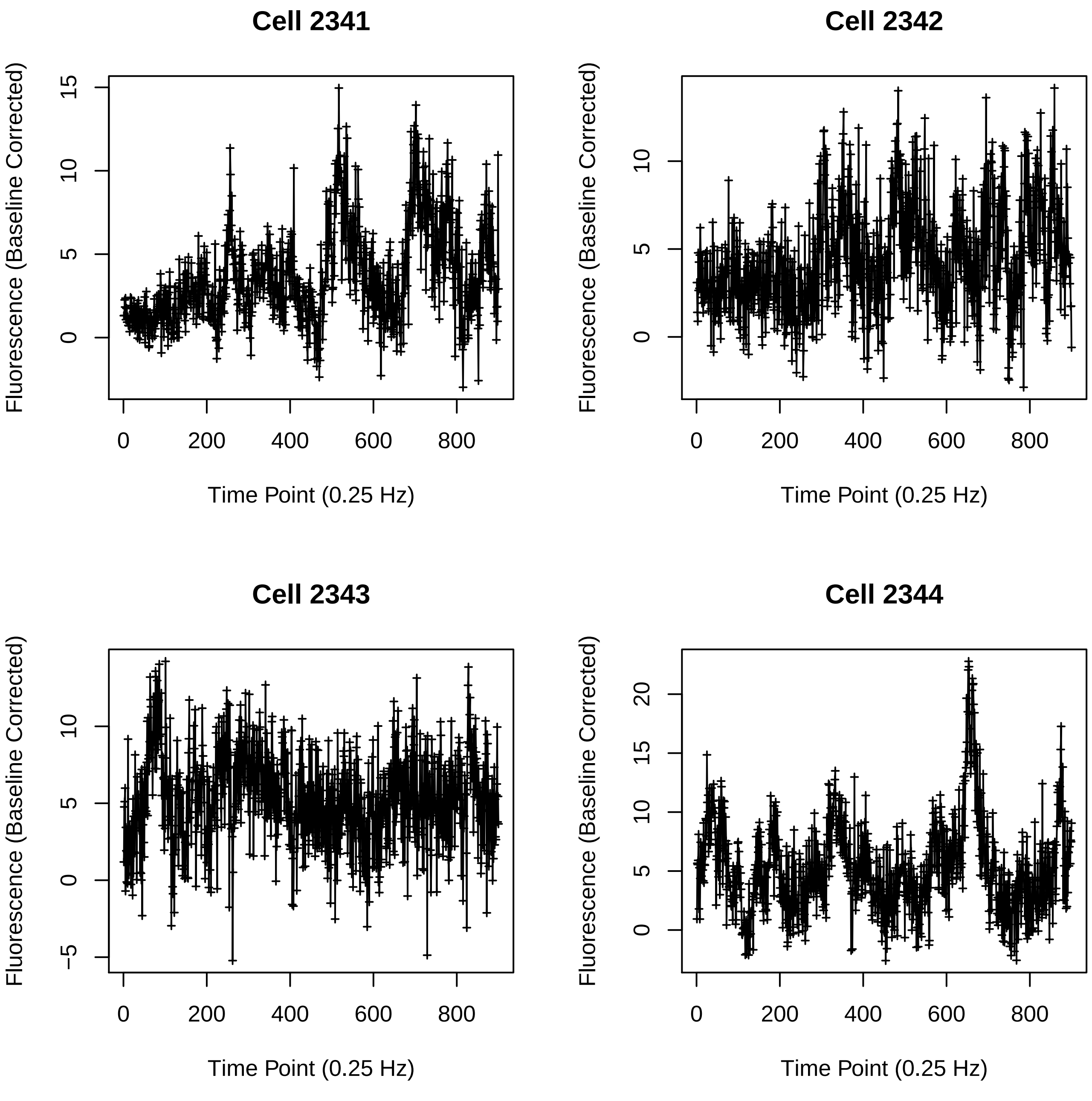
Cell 2341
Cell 2342
Cell 2343
Cell 2344
Fluorescence (Baseline Corrected)
Time Point (0.25 Hz)
0
200
400
600
800
-5
0
5
10
15
20

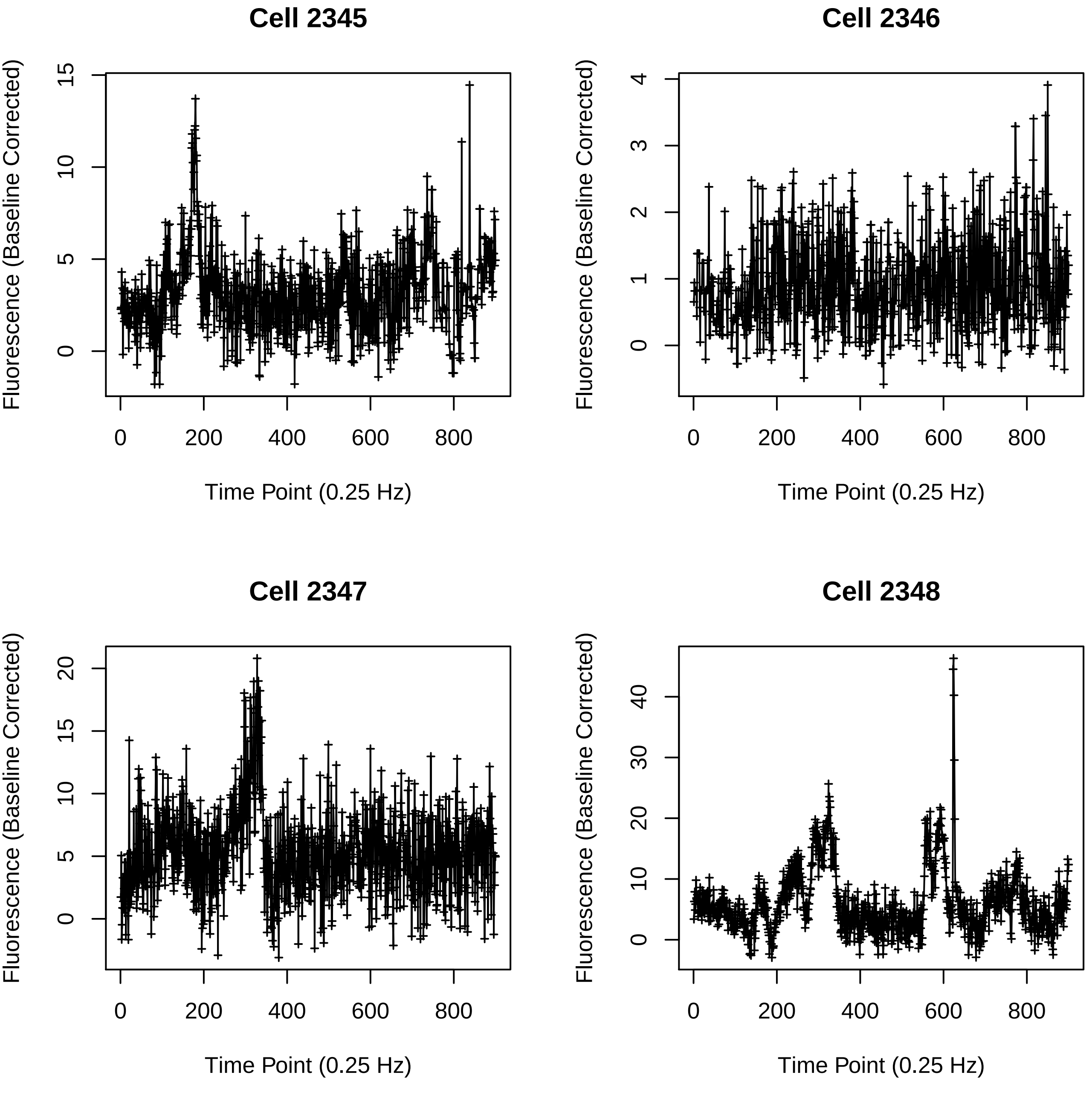
Cell 2345
Cell 2346
Cell 2347
Cell 2348
Fluorescence (Baseline Corrected)
Time Point (0.25 Hz)
0
5
10
15
20
30
40
1
2
3
4
200
400
600
800

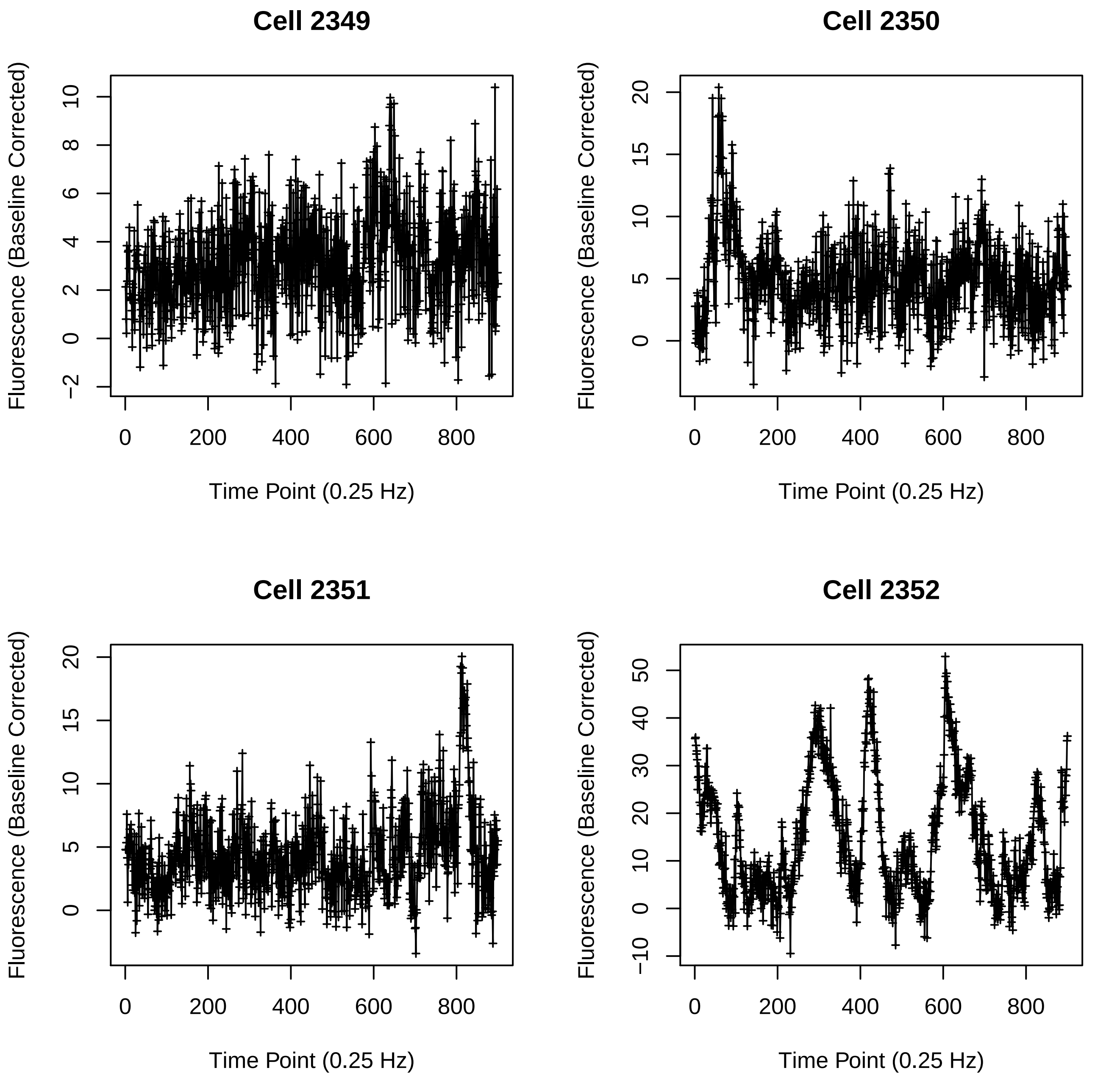
Cell 2349
Cell 2350
Cell 2351
Cell 2352
Fluorescence (Baseline Corrected)
Time Point (0.25 Hz)
0
200
400
600
800
−2
0
2
4
6
8
10
5
15
20
−10
30
40
50

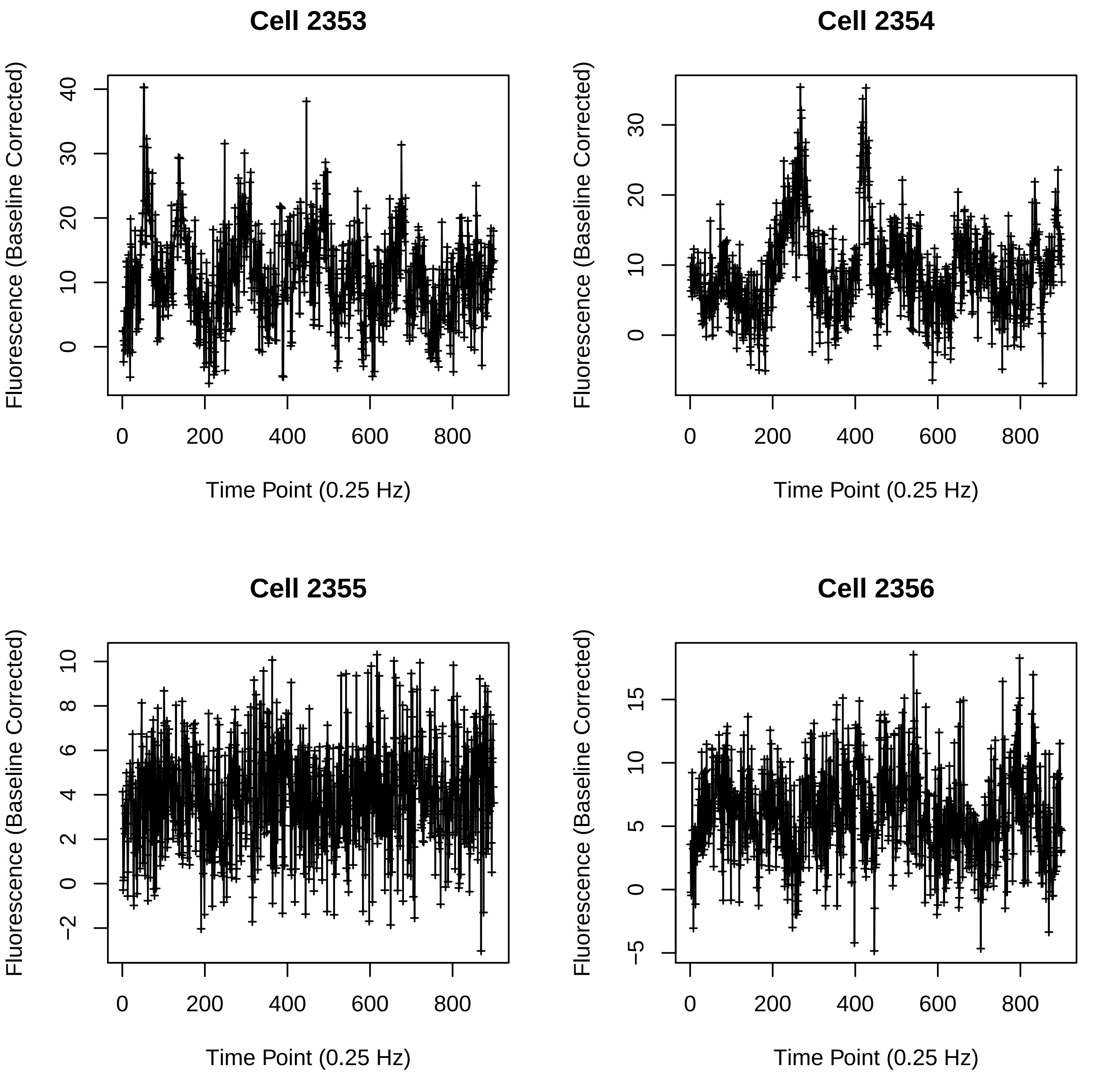
Cell 2353
Fluorescence (Baseline Corrected)
Time Point (0.25 Hz)
0
10
20
30
40
0
200
400
600
800
Cell 2354
Fluorescence (Baseline Corrected)
Time Point (0.25 Hz)
0
10
20
30
0
200
400
600
800
Cell 2355
Fluorescence (Baseline Corrected)
Time Point (0.25 Hz)
−2
0
2
4
6
8
10
0
200
400
600
800
Cell 2356
Fluorescence (Baseline Corrected)
Time Point (0.25 Hz)
−5
0
5
10
15
0
200
400
600
800

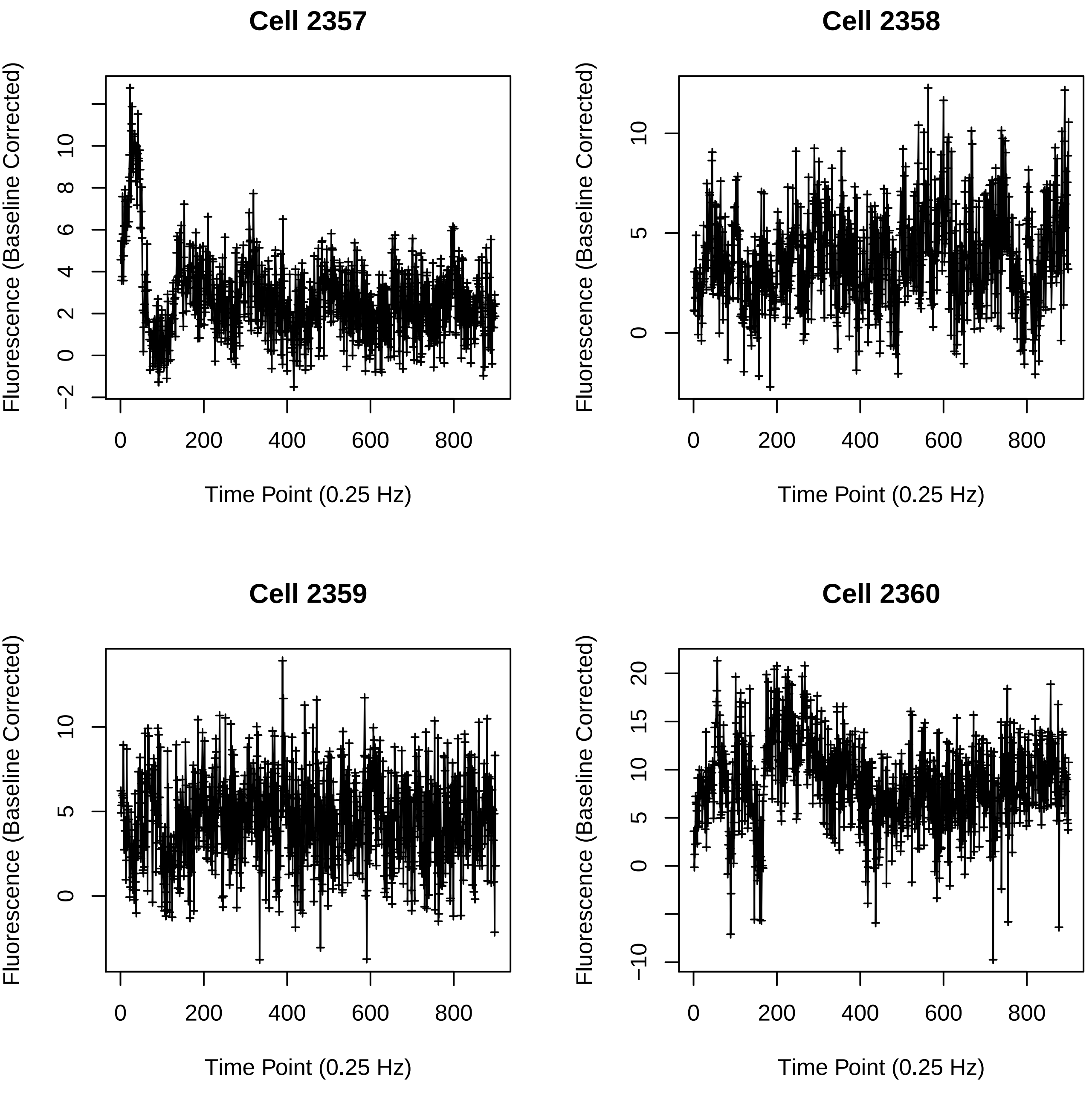

Cell 2357
Cell 2358
Cell 2359
Cell 2360
Fluorescence (Baseline Corrected)
Time Point (0.25 Hz)
0
200
400
600
800

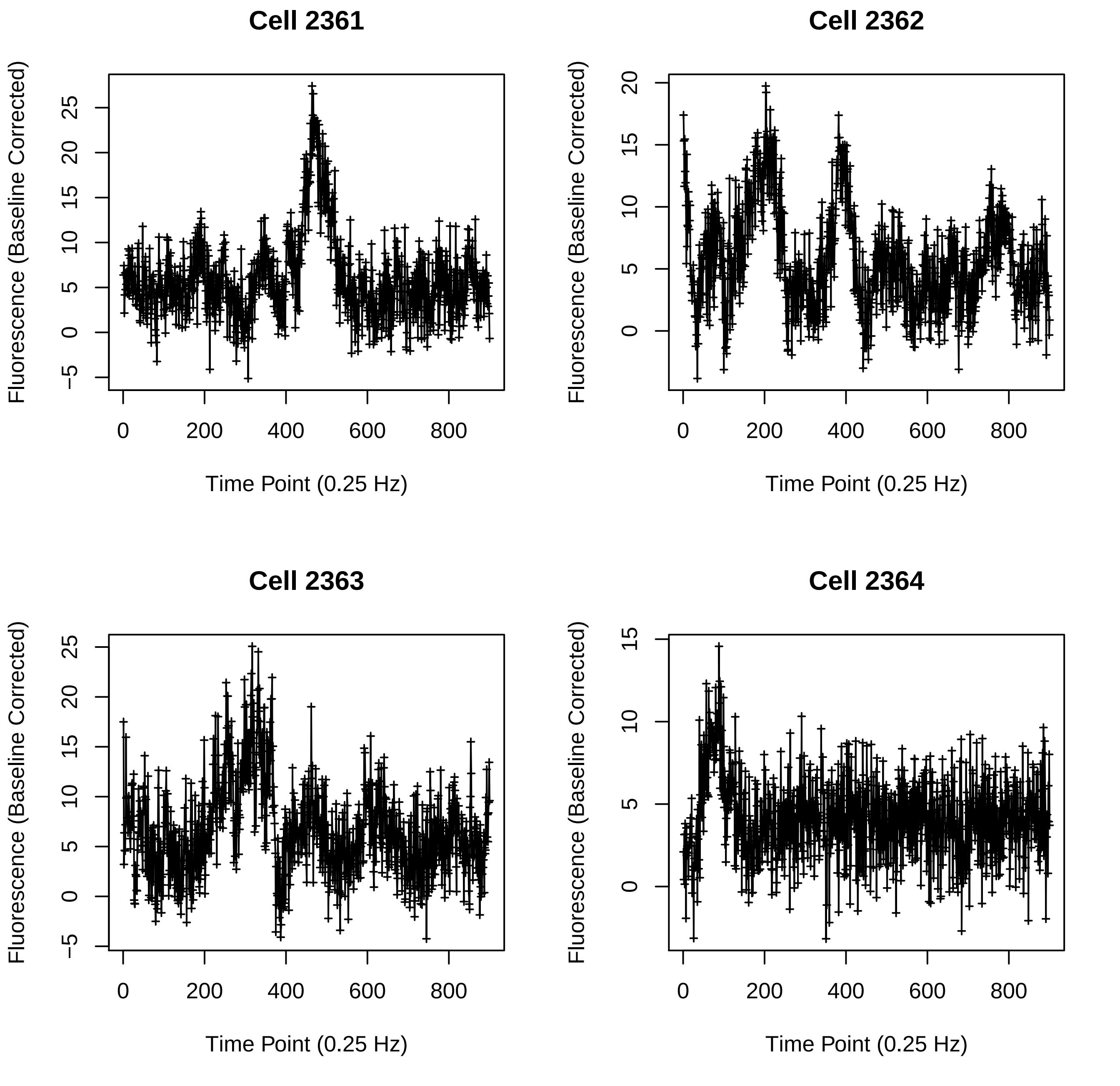
Cell 2361
Cell 2362
Cell 2363
Cell 2364
Fluorescence (Baseline Corrected)
Time Point (0.25 Hz)
0
200
400
600
800
−5
0
5
10
15
20
25

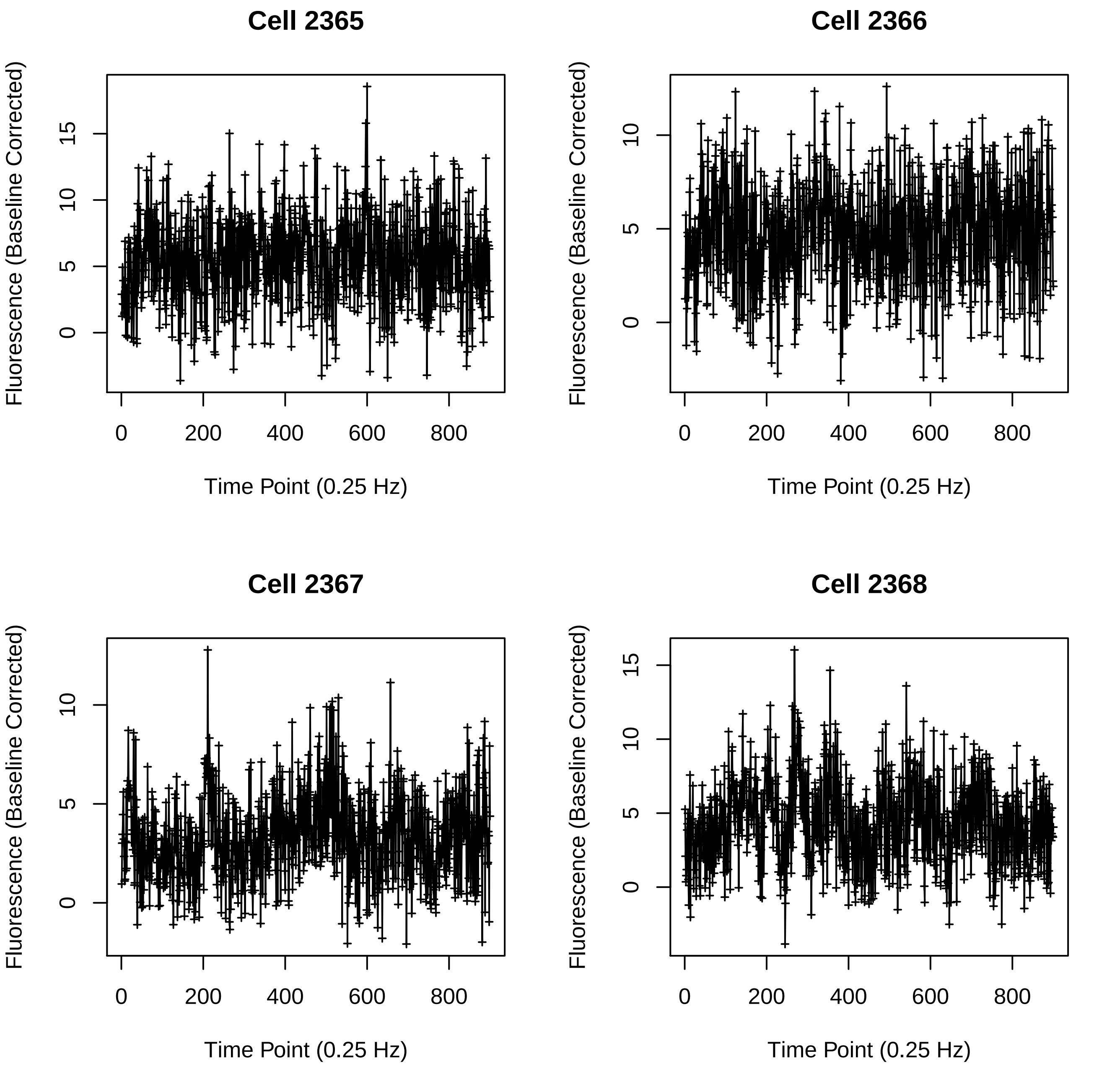
Cell 2365
Cell 2366
Cell 2367
Cell 2368
Fluorescence (Baseline Corrected)
Time Point (0.25 Hz)
0
200
400
600
800
-5
0
5
10
15

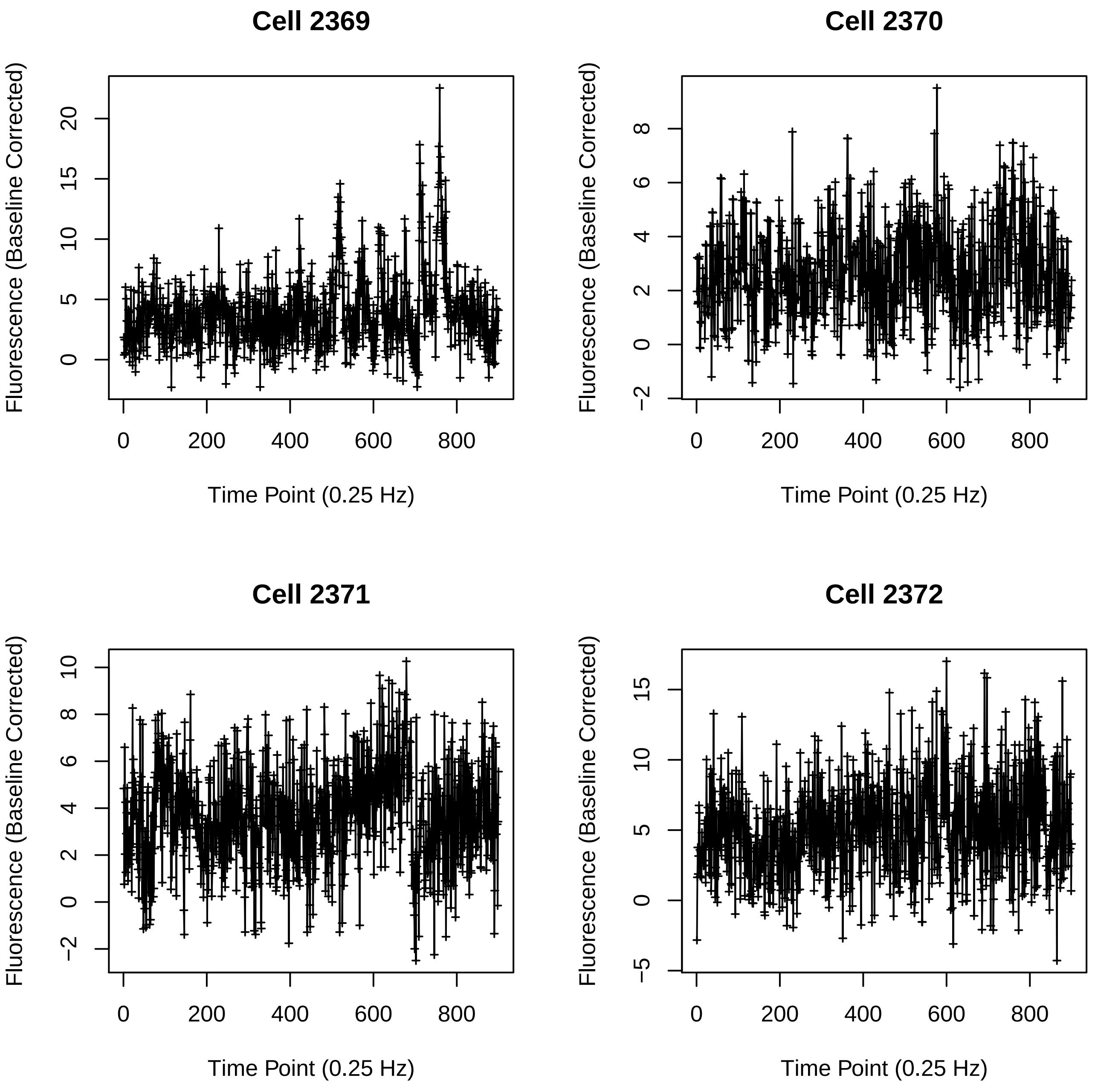
Cell 2369
Cell 2370
Cell 2371
Cell 2372
Fluorescence (Baseline Corrected)
Time Point (0.25 Hz)
0
200
400
600
800
5
10
15
20
−2
2
4
6
8
−5

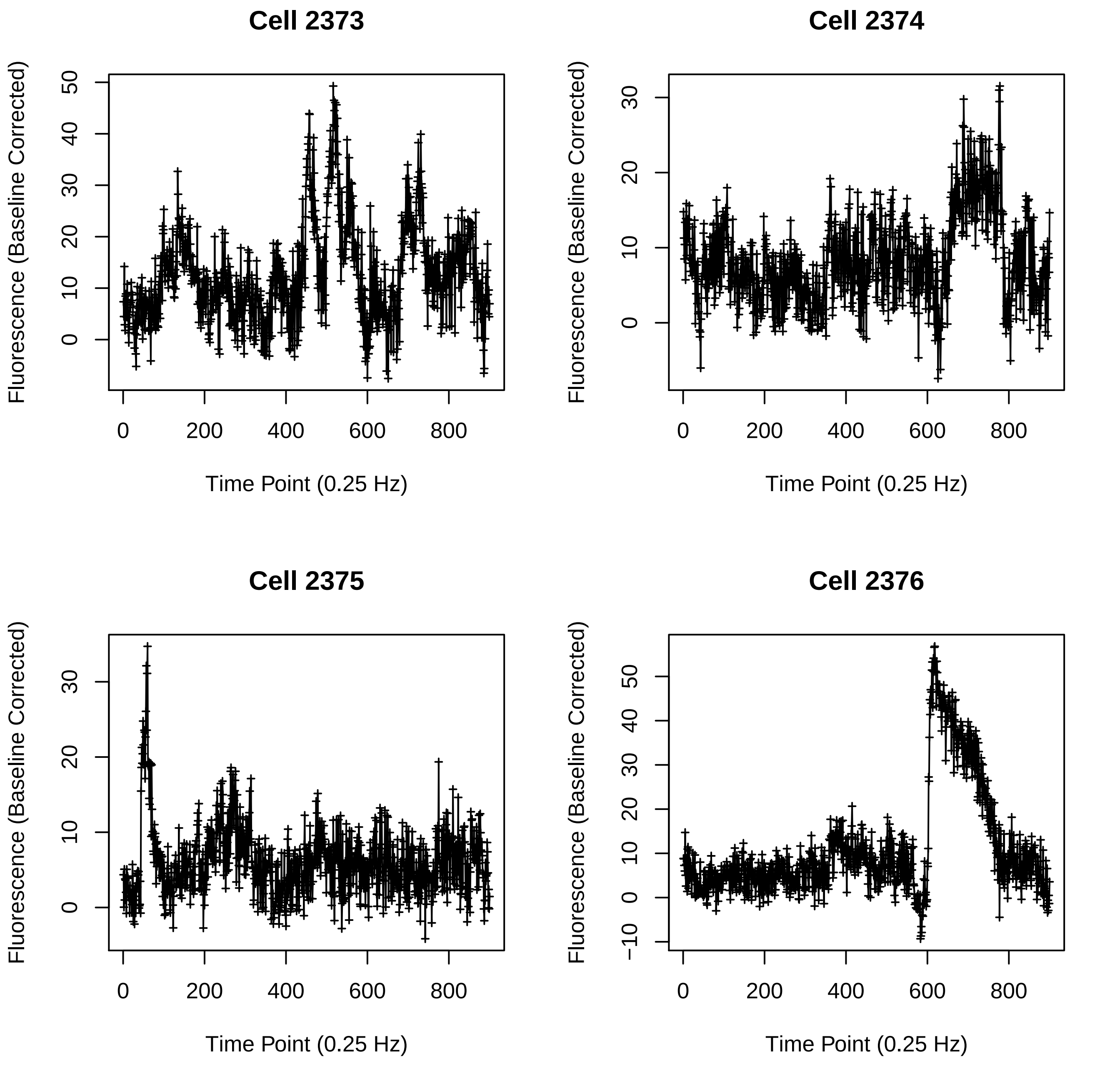
Cell 2373
Cell 2374
Cell 2375
Cell 2376
Fluorescence (Baseline Corrected)
Time Point (0.25 Hz)
0
200
400
600
800
−10
0
10
20
30
40
50

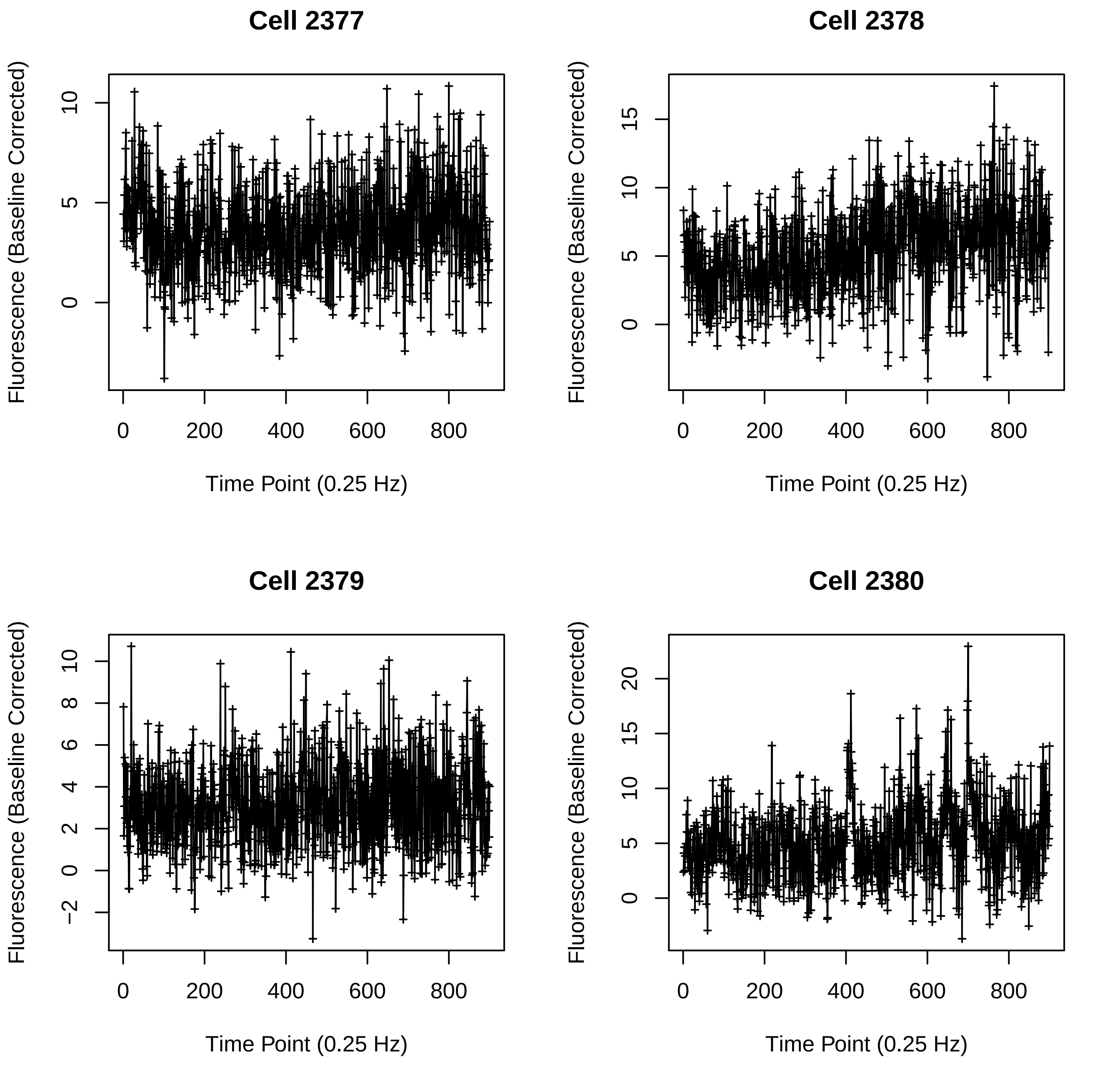

Cell 2377
Cell 2378
Cell 2379
Cell 2380
Fluorescence (Baseline Corrected)
Time Point (0.25 Hz)
0
200
400
600
800

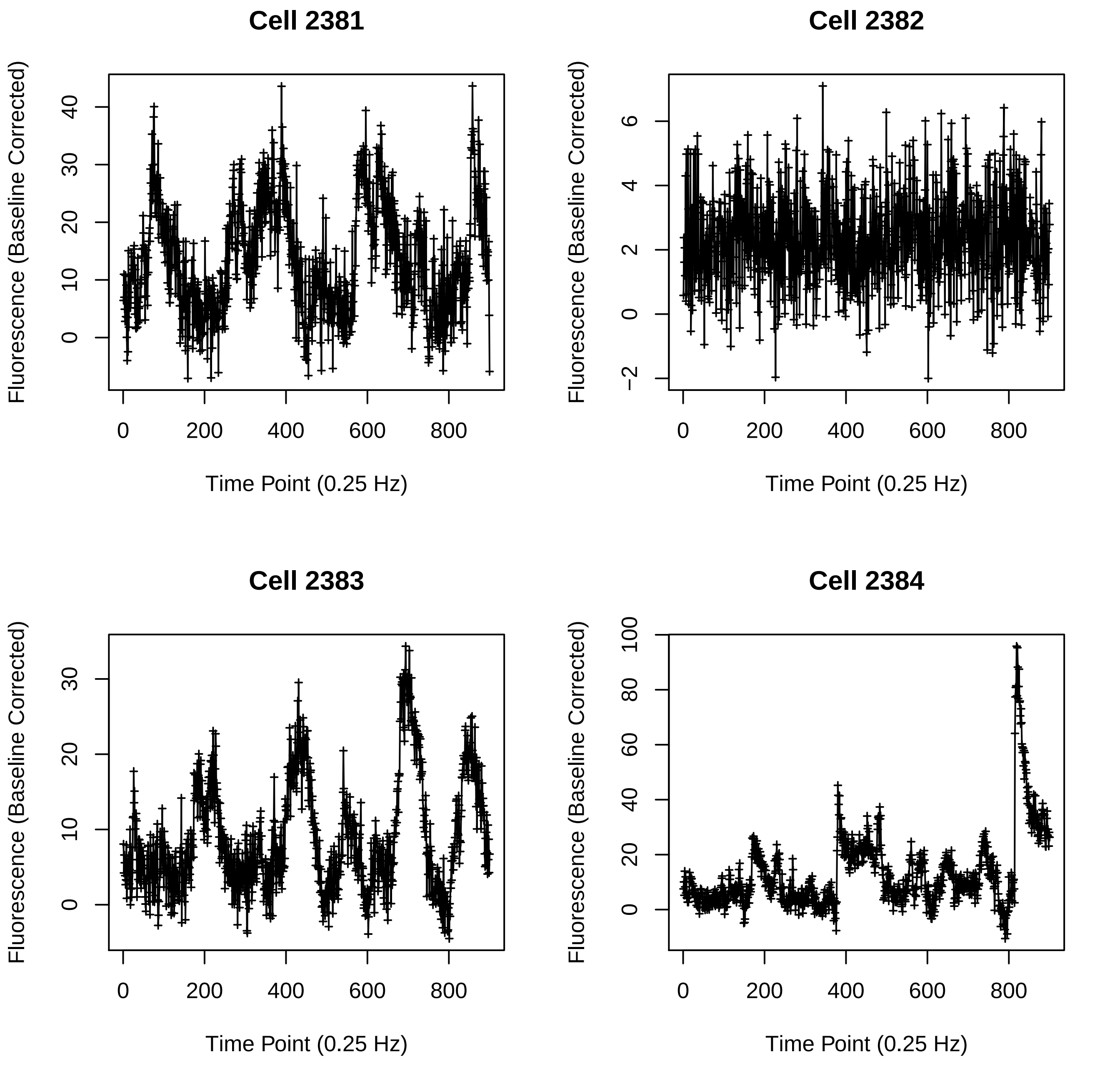

Cell 2381
Cell 2382
Cell 2383
Cell 2384
Fluorescence (Baseline Corrected)
Time Point (0.25 Hz)
0
200
400
600
800

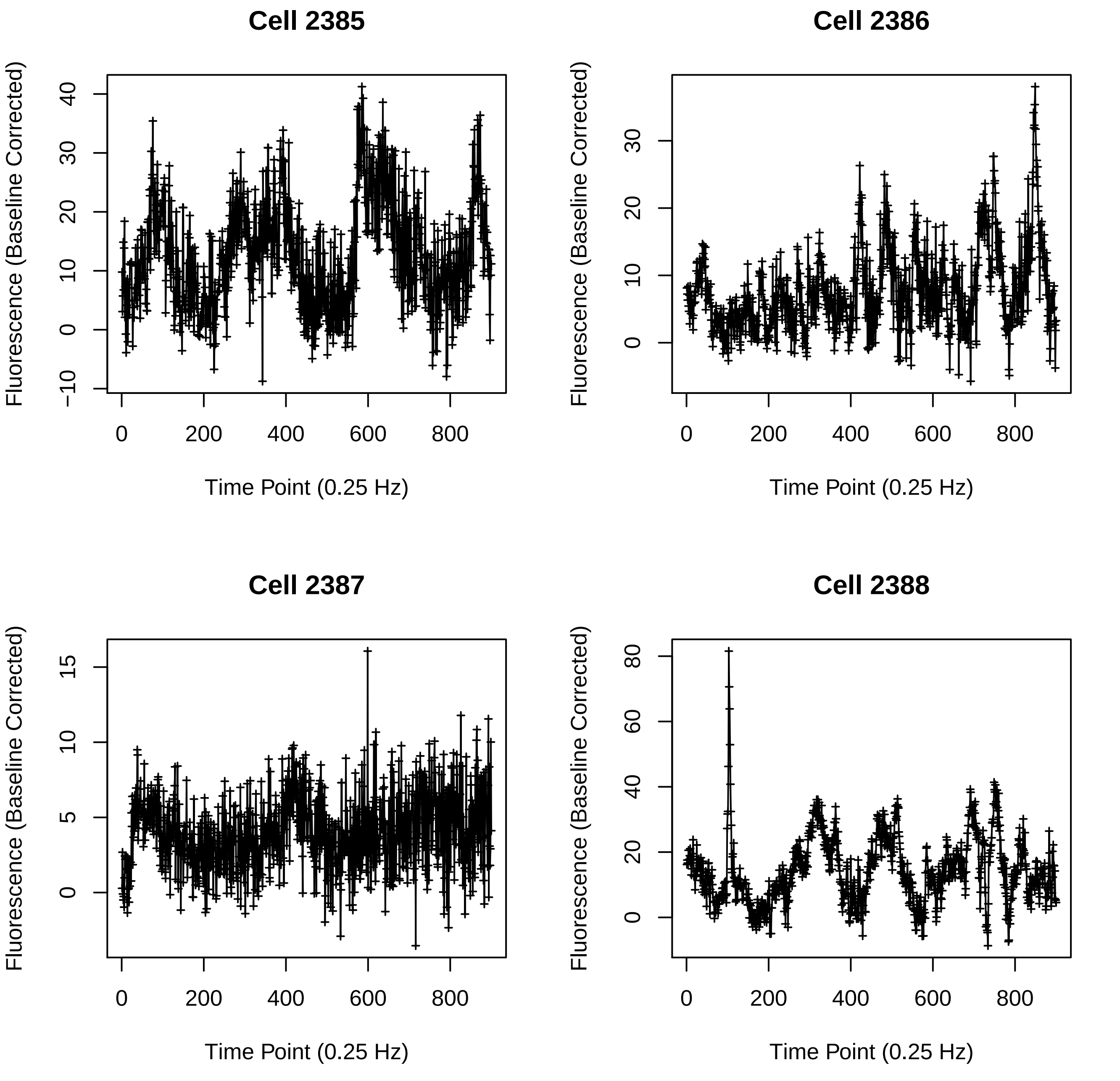
Cell 2385
Fluorescence (Baseline Corrected)
−10
0
10
20
30
40
0
200
400
600
800
Time Point (0.25 Hz)
Cell 2386
Fluorescence (Baseline Corrected)
0
10
20
30
0
200
400
600
800
Time Point (0.25 Hz)
Cell 2387
Fluorescence (Baseline Corrected)
0
5
10
15
0
200
400
600
800
Time Point (0.25 Hz)
Cell 2388
Fluorescence (Baseline Corrected)
0
20
40
60
80
0
200
400
600
800
Time Point (0.25 Hz)

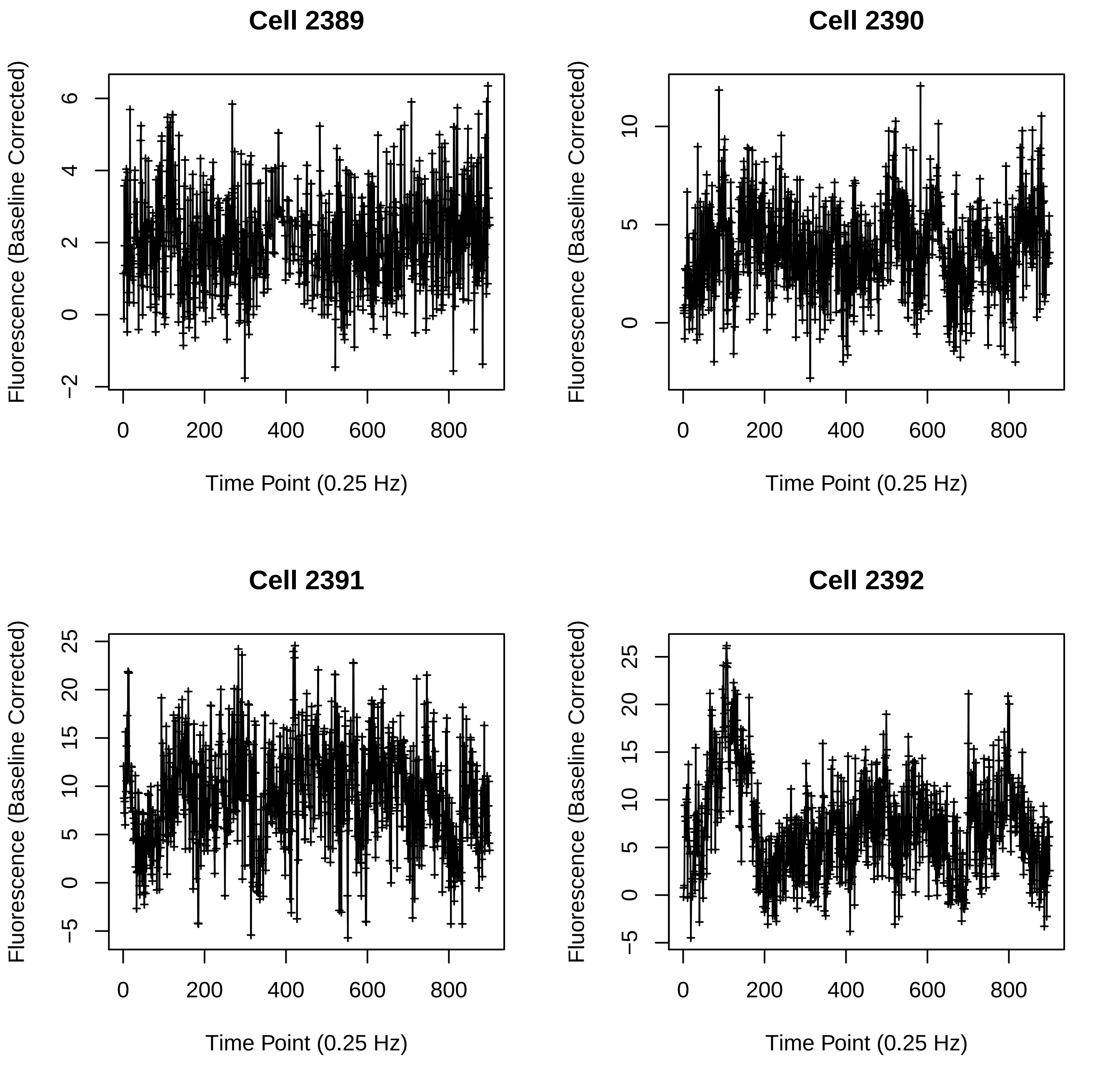
Cell 2389
Fluorescence (Baseline Corrected)
-2
0
2
4
6
0
200
400
600
800
Time Point (0.25 Hz)
Cell 2390
Fluorescence (Baseline Corrected)
0
5
10
0
200
400
600
800
Time Point (0.25 Hz)
Cell 2391
Fluorescence (Baseline Corrected)
-5
0
5
10
15
20
25
0
200
400
600
800
Time Point (0.25 Hz)
Cell 2392
Fluorescence (Baseline Corrected)
-5
0
5
10
15
20
25
0
200
400
600
800
Time Point (0.25 Hz)

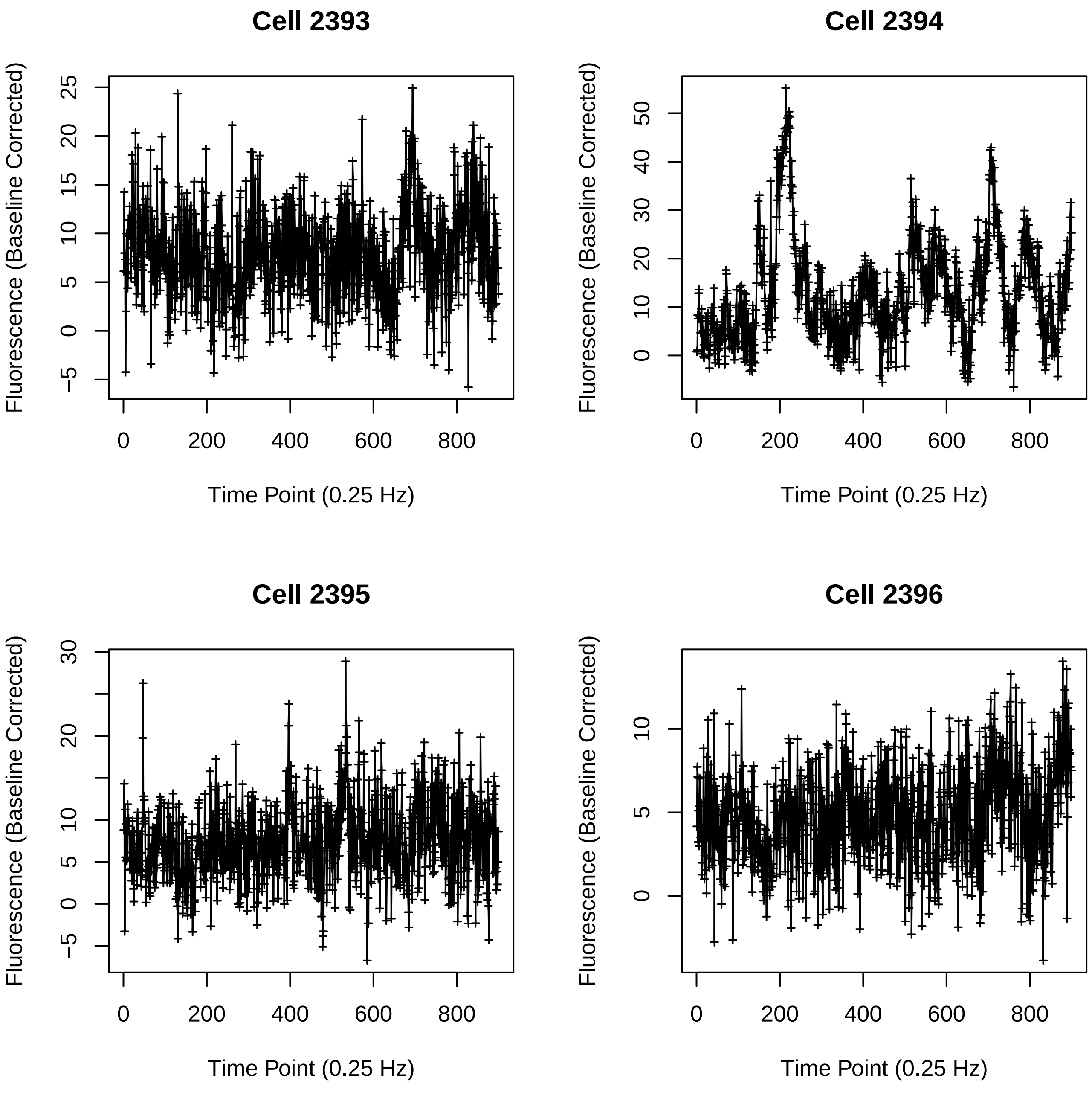
Cell 2393
Cell 2394
Cell 2395
Cell 2396
Fluorescence (Baseline Corrected)
Time Point (0.25 Hz)
−5
0
5
10
15
20
25
30
40
50
0
200
400
600
800

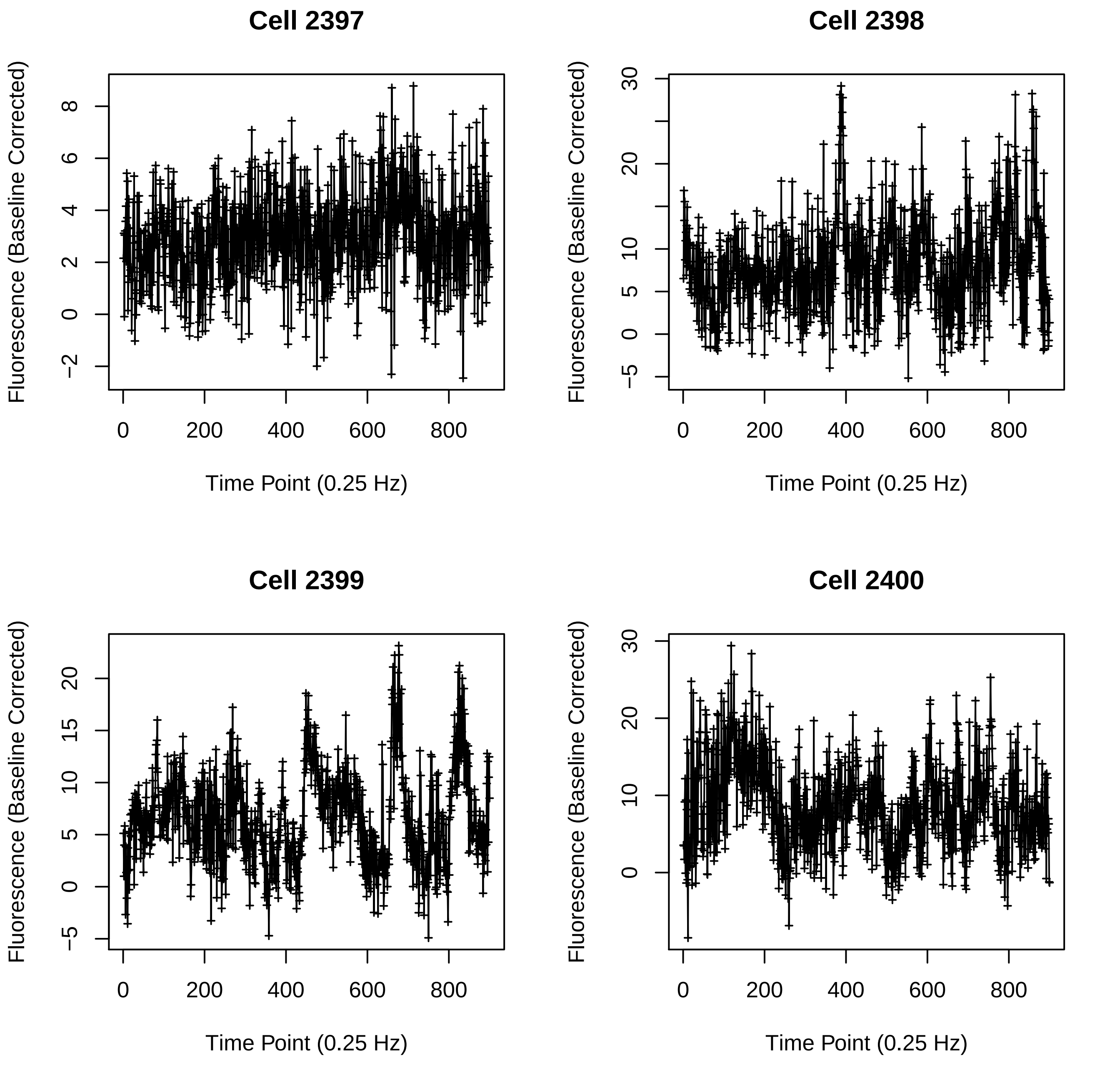
Cell 2397
Cell 2398
Cell 2399
Cell 2400
Fluorescence (Baseline Corrected)
Time Point (0.25 Hz)
−2
0
2
4
6
8
−5
0
5
10
15
20
25
30
200
400
600
800

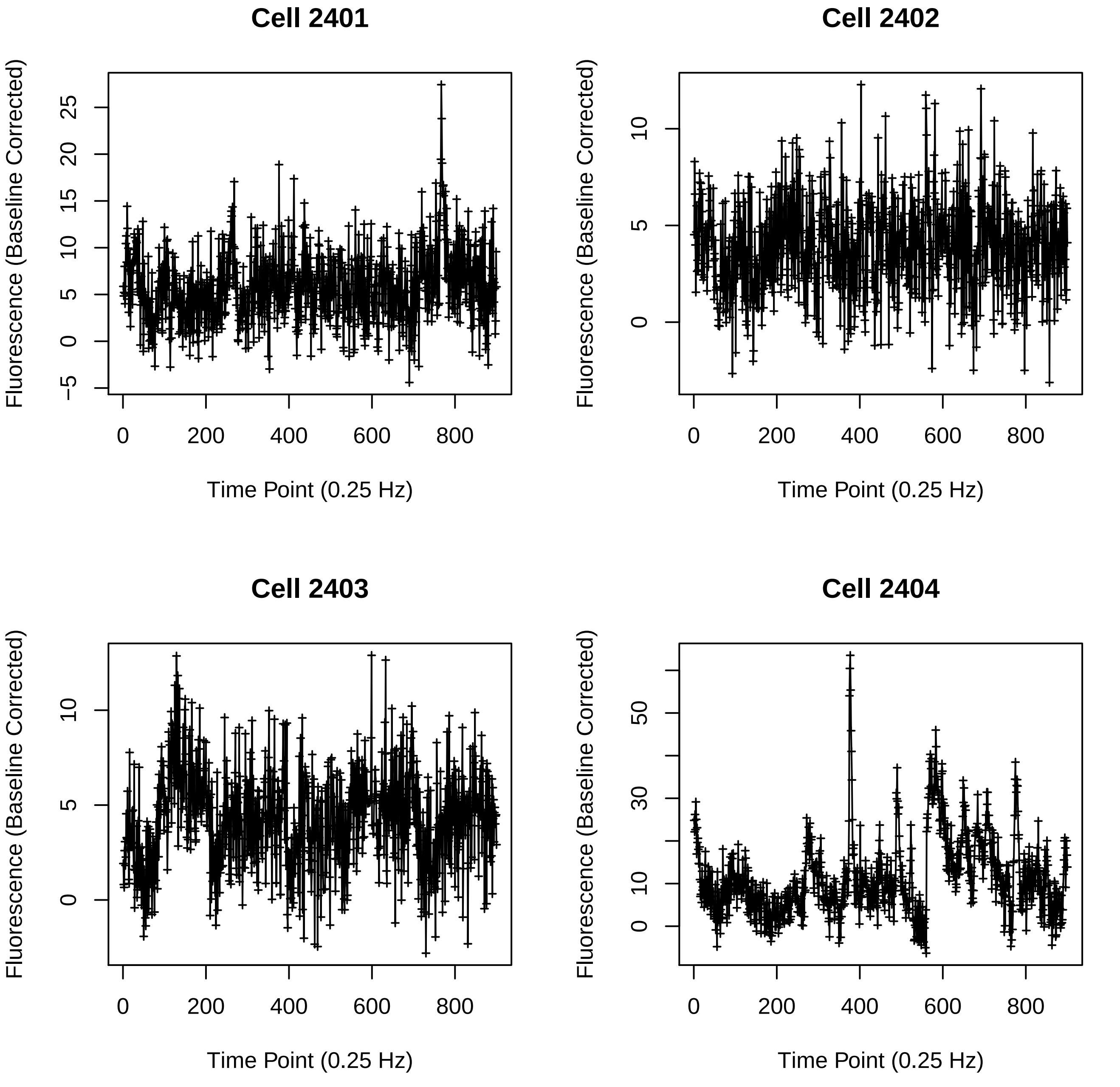
Cell 2401
Cell 2402
Cell 2403
Cell 2404
Fluorescence (Baseline Corrected)
Time Point (0.25 Hz)
−5
0
5
10
15
20
25
30
50
0
200
400
600
800

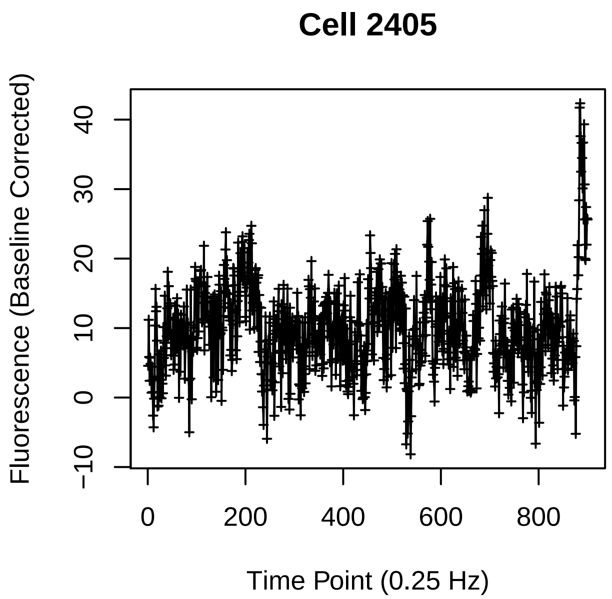
Cell 2405
Fluorescence (Baseline Corrected)
Time Point (0.25 Hz)
−10
0
10
20
30
40
0
200
400
600
800

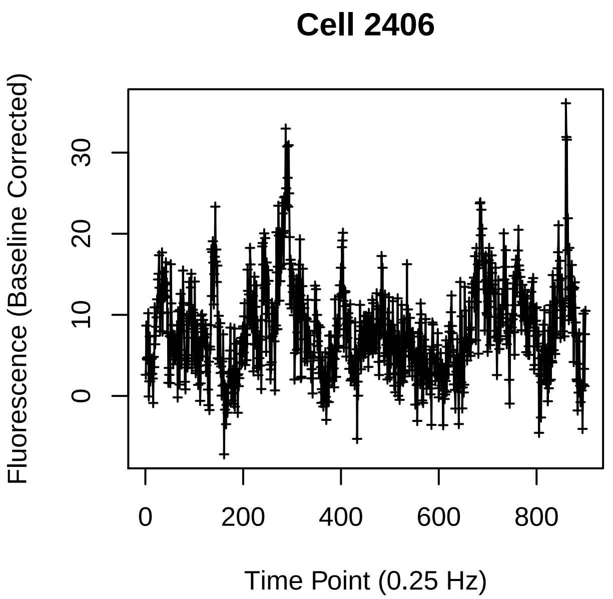
Cell 2406
Fluorescence (Baseline Corrected)
Time Point (0.25 Hz)
0
10
20
30
0
200
400
600
800

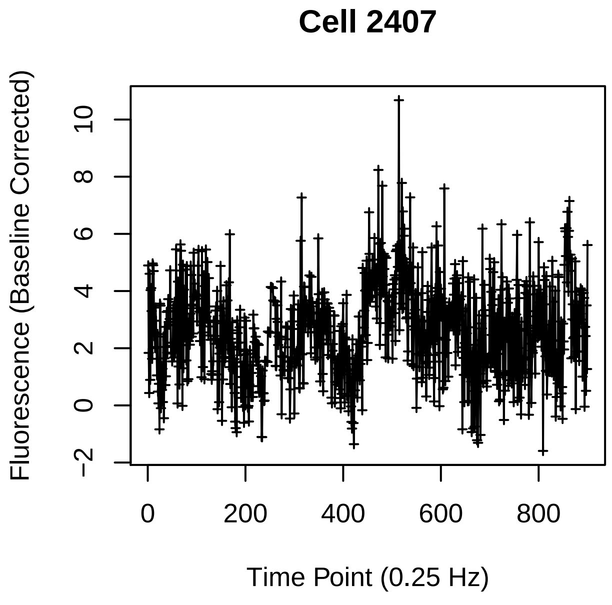
Cell 2407
Fluorescence (Baseline Corrected)
Time Point (0.25 Hz)
−2
0
2
4
6
8
10
0
200
400
600
800

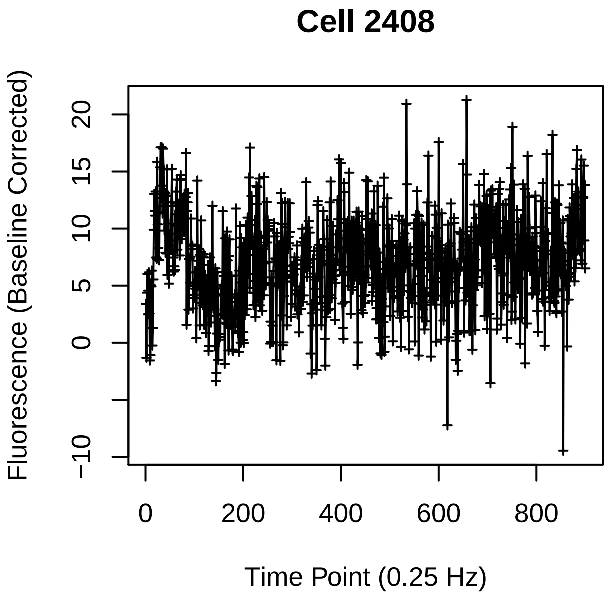
Cell 2408
Fluorescence (Baseline Corrected)
Time Point (0.25 Hz)
−10
0
5
10
15
20
0
200
400
600
800

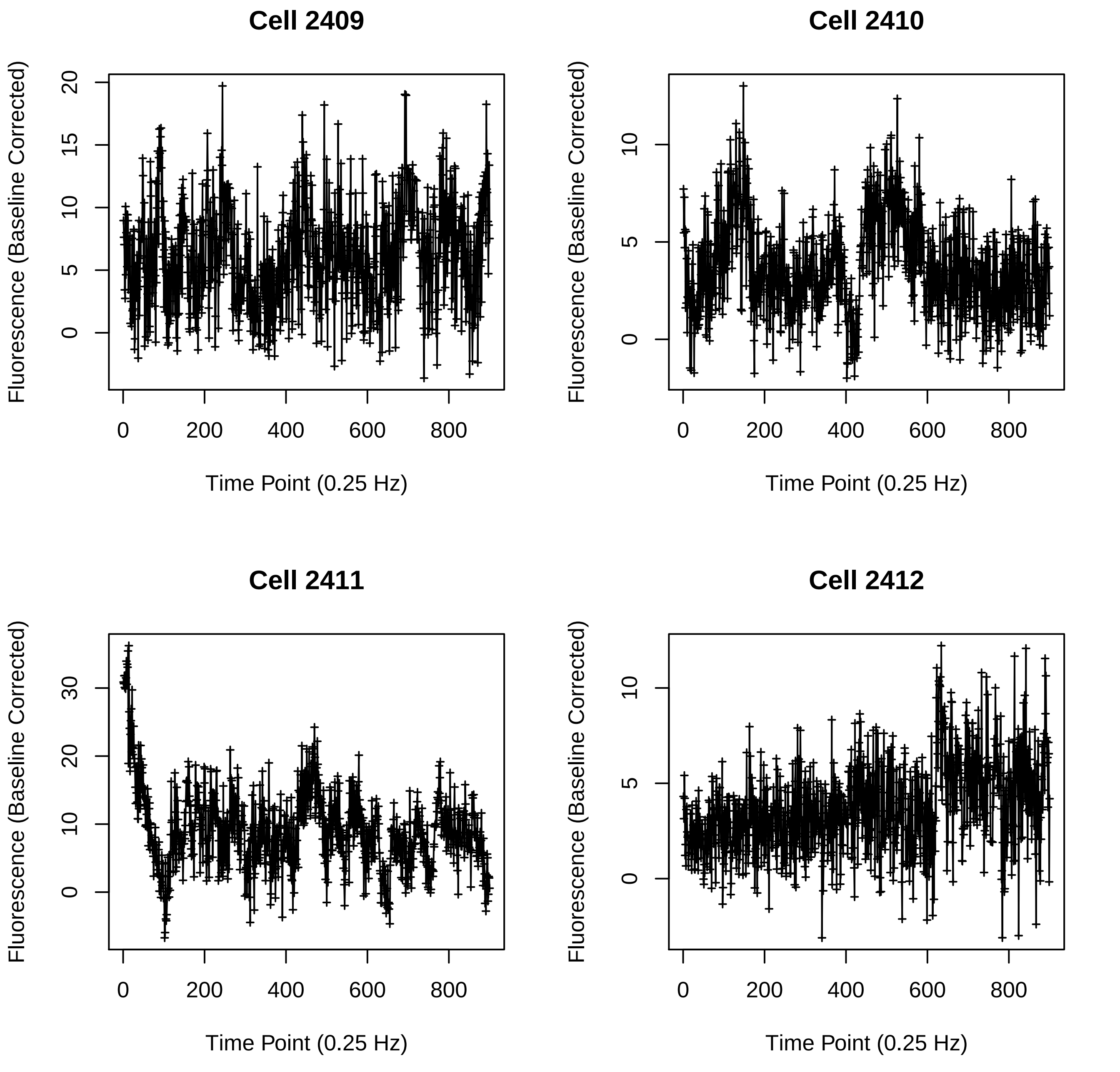
Cell 2409
Cell 2410
Cell 2411
Cell 2412
Fluorescence (Baseline Corrected)
Time Point (0.25 Hz)
0
5
10
15
20
30
200
400
600
800

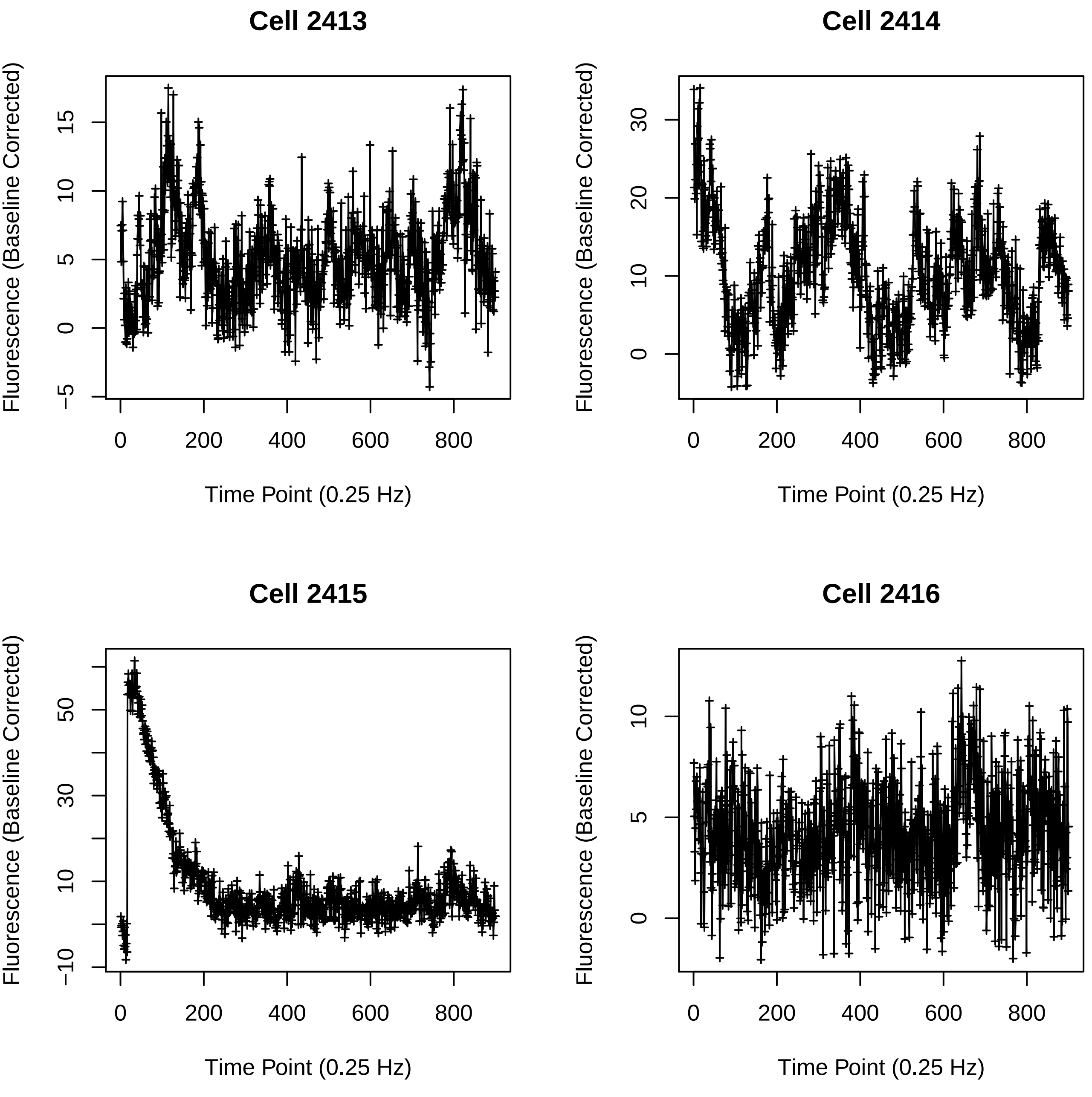

Cell 2413
Cell 2414
Cell 2415
Cell 2416
Fluorescence (Baseline Corrected)
Time Point (0.25 Hz)
0
200
400
600
800
−5
0
5
10
15
20
30
−10
50

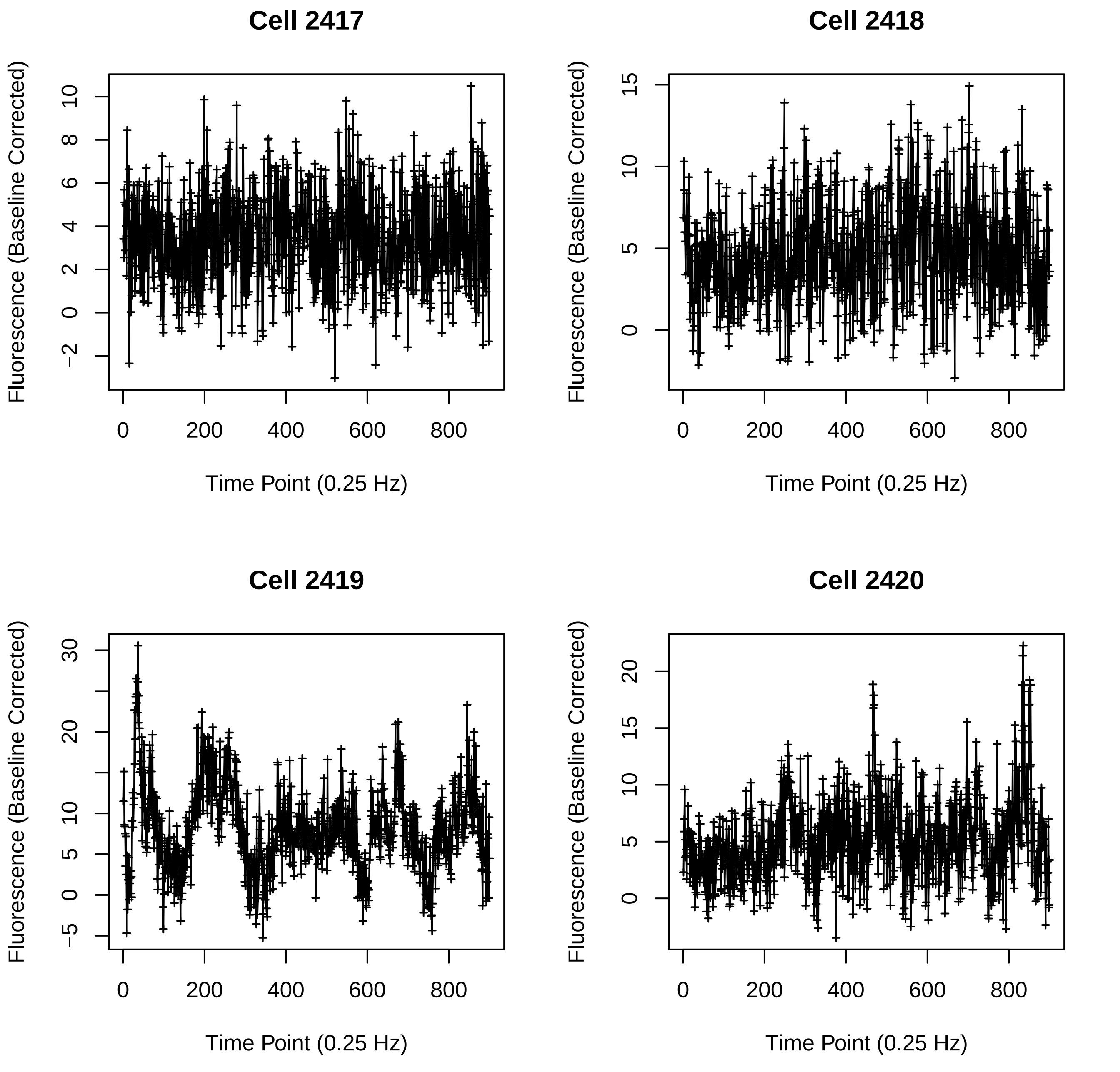
Cell 2417
Cell 2418
Cell 2419
Cell 2420
Fluorescence (Baseline Corrected)
Time Point (0.25 Hz)
−2
0
2
4
6
8
10
0
5
10
15
−5
20
25
30
200
400
600
800

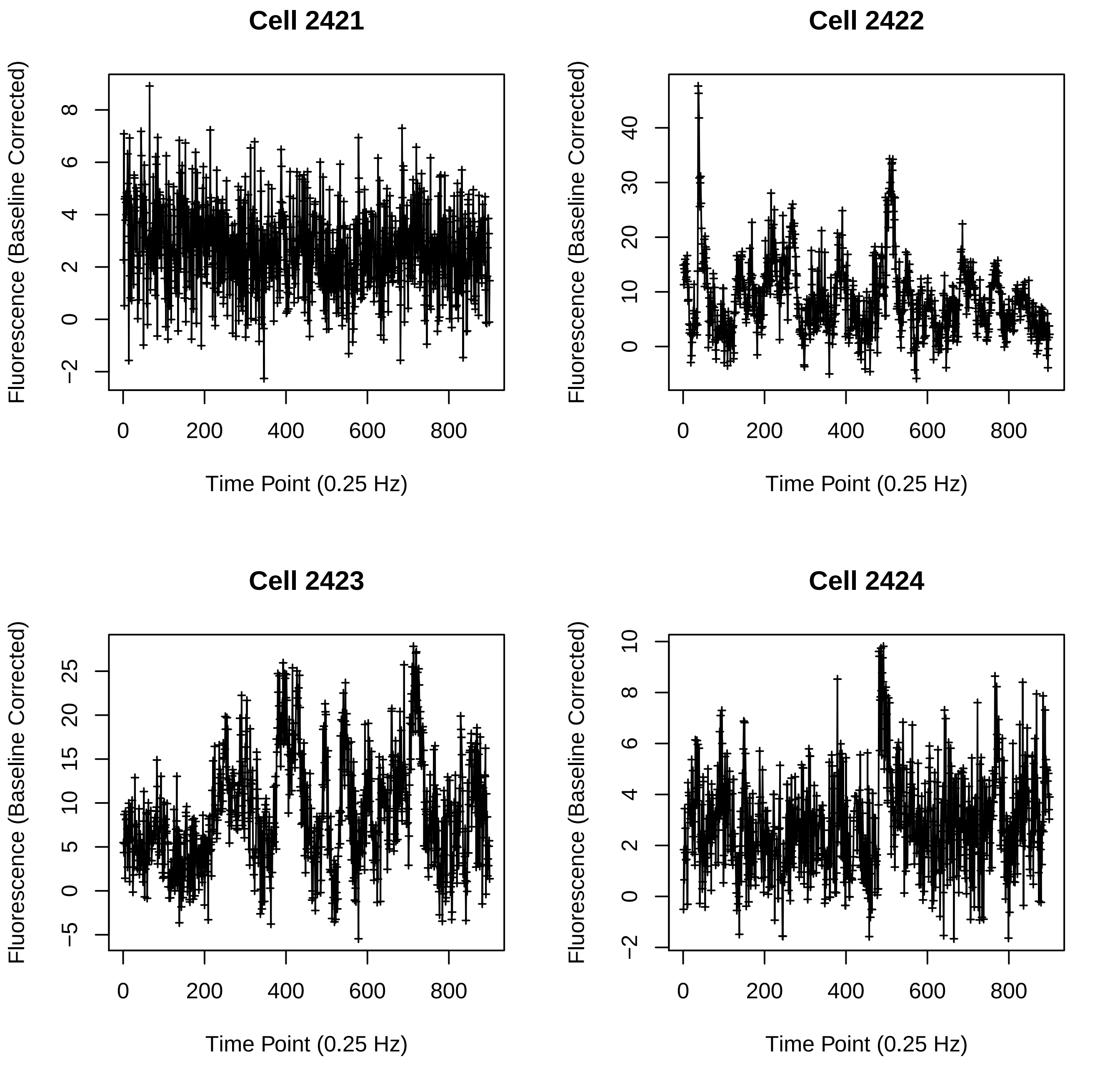
Cell 2421
Cell 2422
Cell 2423
Cell 2424
Fluorescence (Baseline Corrected)
Time Point (0.25 Hz)

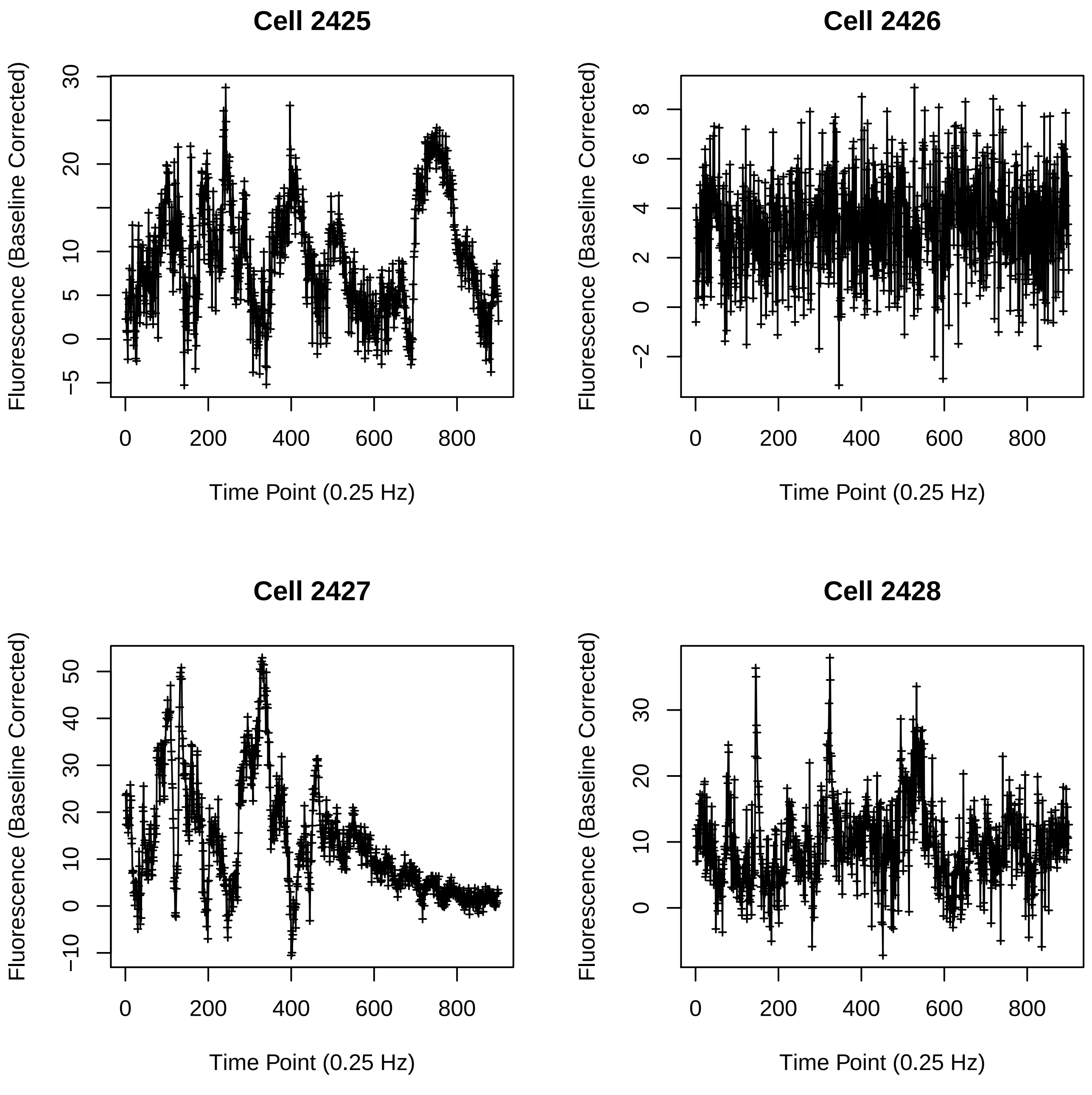
Cell 2425
Cell 2426
Cell 2427
Cell 2428
Fluorescence (Baseline Corrected)
Time Point (0.25 Hz)
0
200
400
600
800
−5
0
5
10
20
30
−2
2
4
6
8
−10
40
50

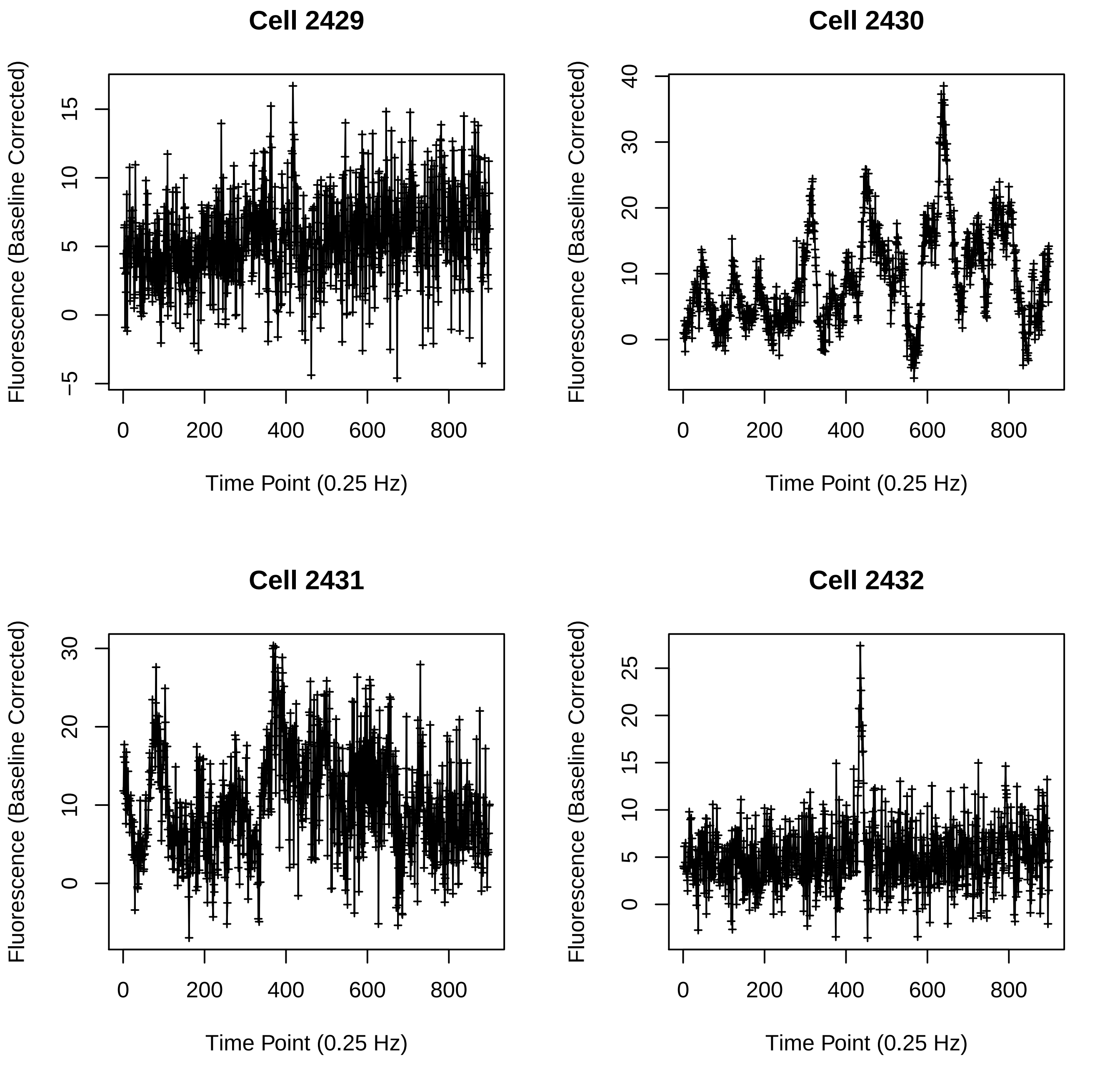
Cell 2429
Cell 2430
Cell 2431
Cell 2432
Fluorescence (Baseline Corrected)
Time Point (0.25 Hz)
0
200
400
600
800
−5
5
10
15
20
25
30
40

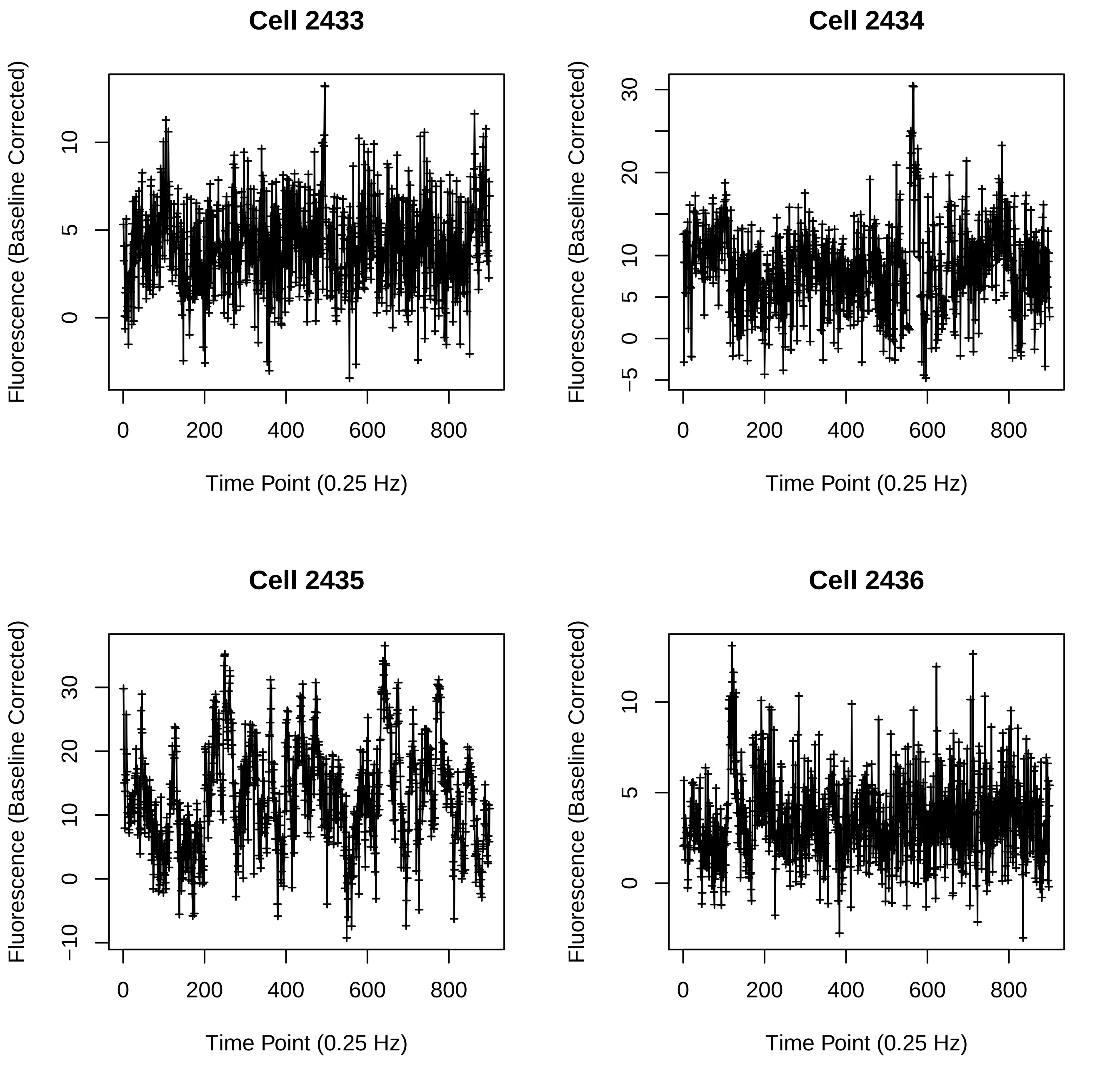
Cell 2433
Cell 2434
Cell 2435
Cell 2436
Fluorescence (Baseline Corrected)
Time Point (0.25 Hz)
0
200
400
600
800
-5
0
5
10
20
25
30
-10

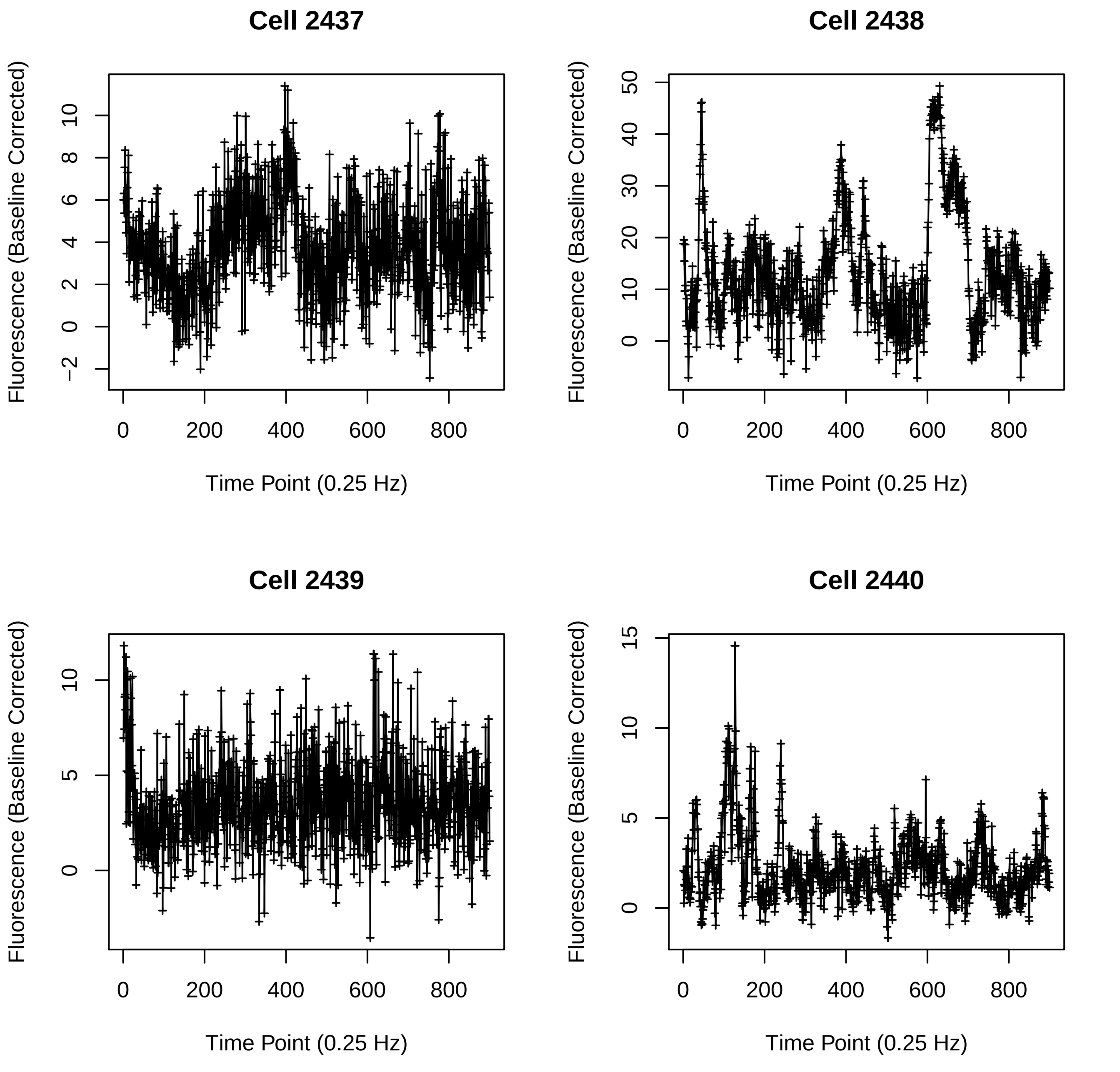

Cell 2437
Fluorescence (Baseline Corrected)
Time Point (0.25 Hz)
Cell 2438
Fluorescence (Baseline Corrected)
Time Point (0.25 Hz)
Cell 2439
Fluorescence (Baseline Corrected)
Time Point (0.25 Hz)
Cell 2440
Fluorescence (Baseline Corrected)
Time Point (0.25 Hz)

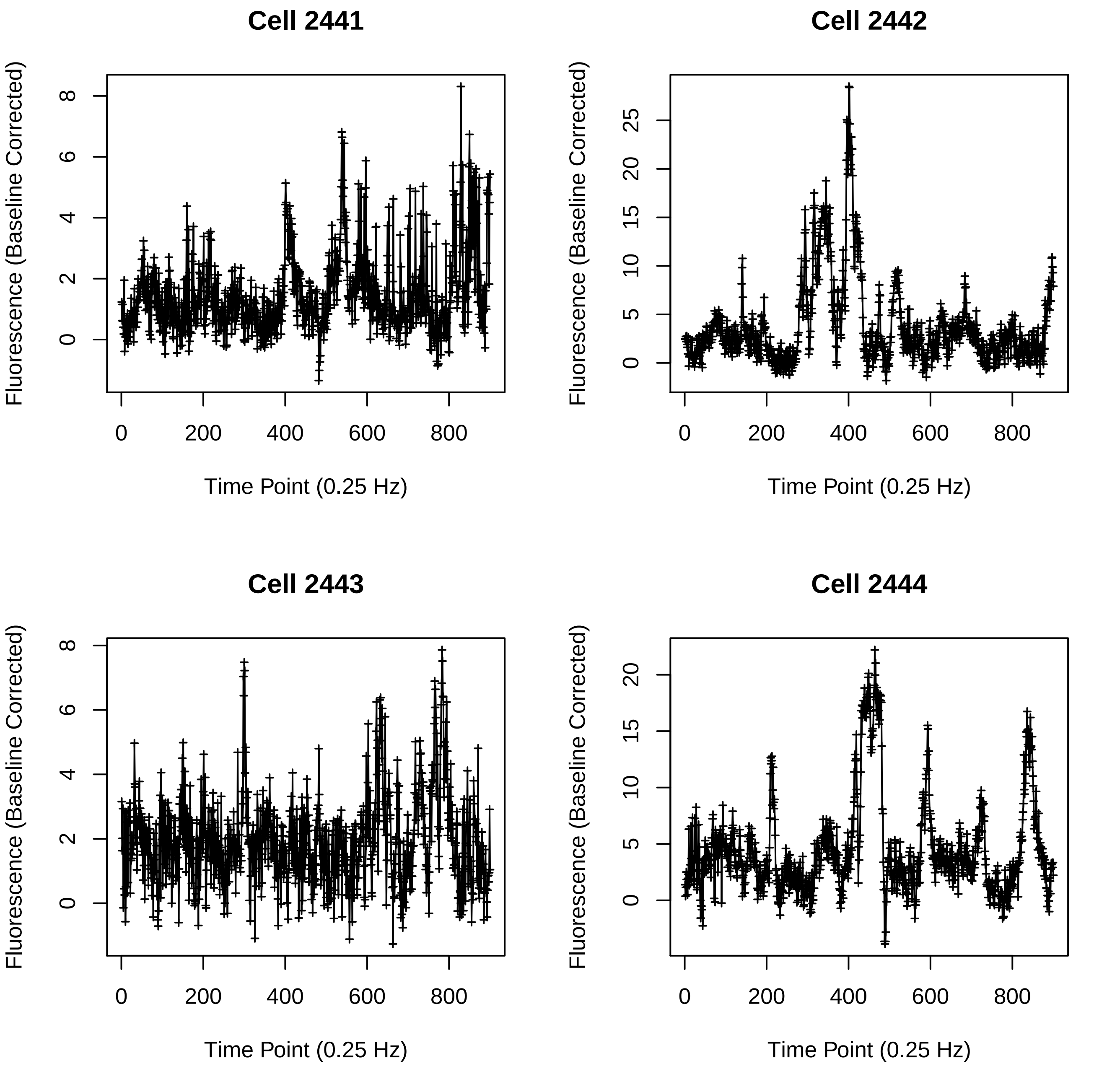

Cell 2441
Fluorescence (Baseline Corrected)
Time Point (0.25 Hz)
Cell 2442
Fluorescence (Baseline Corrected)
Time Point (0.25 Hz)
Cell 2443
Fluorescence (Baseline Corrected)
Time Point (0.25 Hz)
Cell 2444
Fluorescence (Baseline Corrected)
Time Point (0.25 Hz)

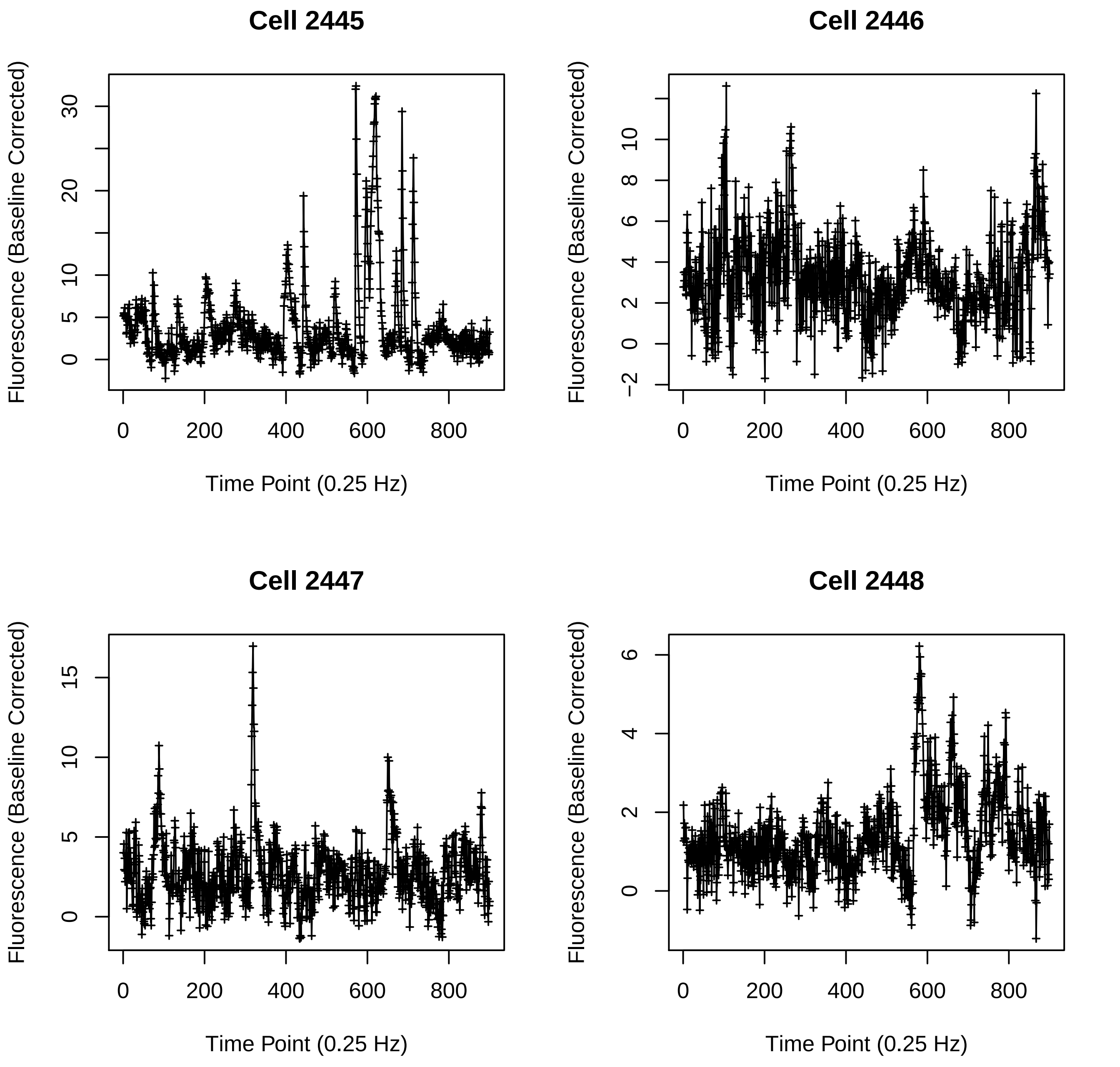
Cell 2445
Cell 2446
Cell 2447
Cell 2448
Fluorescence (Baseline Corrected)
Time Point (0.25 Hz)
0
5
10
20
30
−2
2
4
6
8
15
200
400
600
800

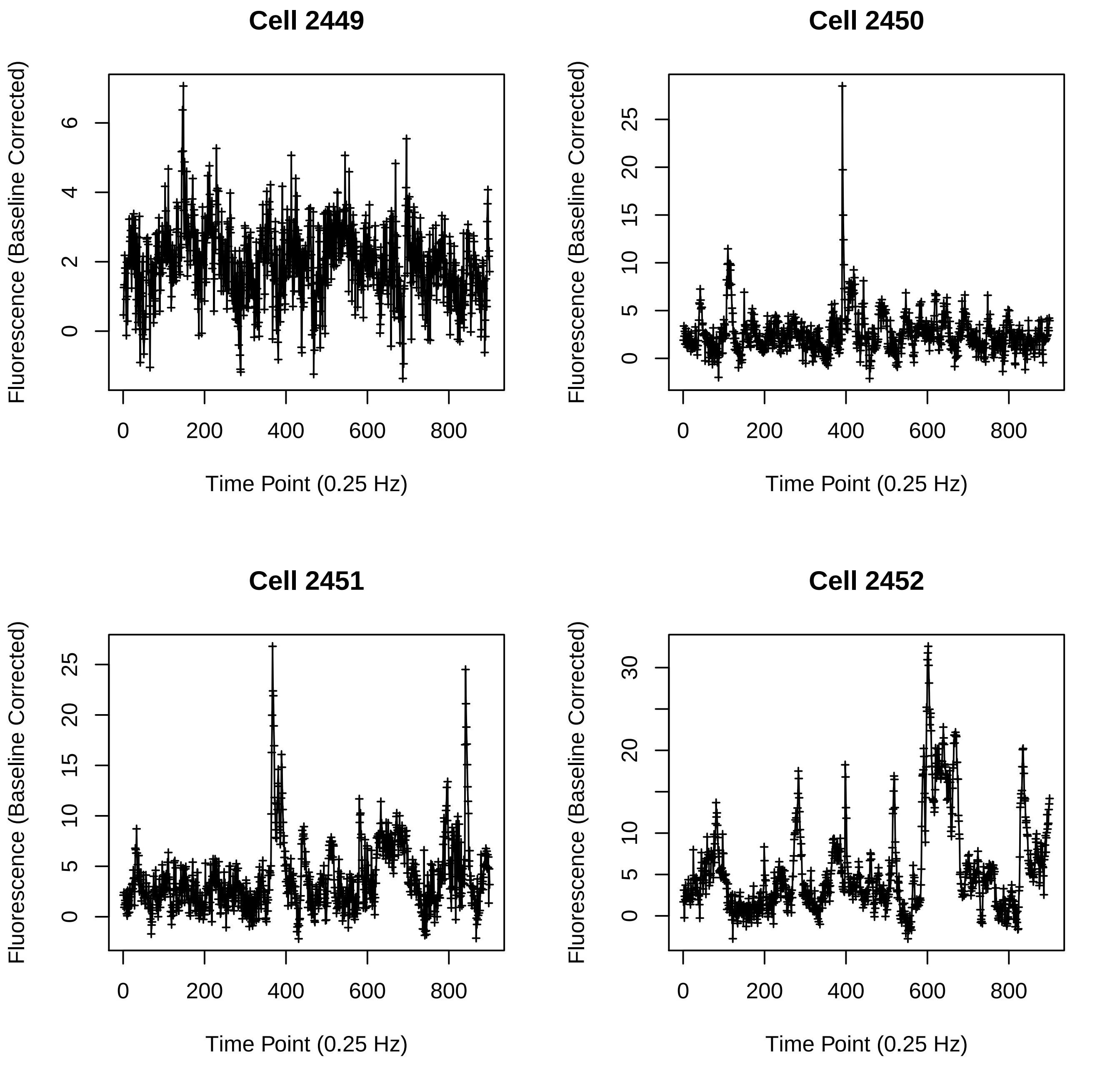
Cell 2449
Cell 2450
Cell 2451
Cell 2452
Fluorescence (Baseline Corrected)
Time Point (0.25 Hz)
0
200
400
600
800
0
2
4
6
5
10
15
20
25
30

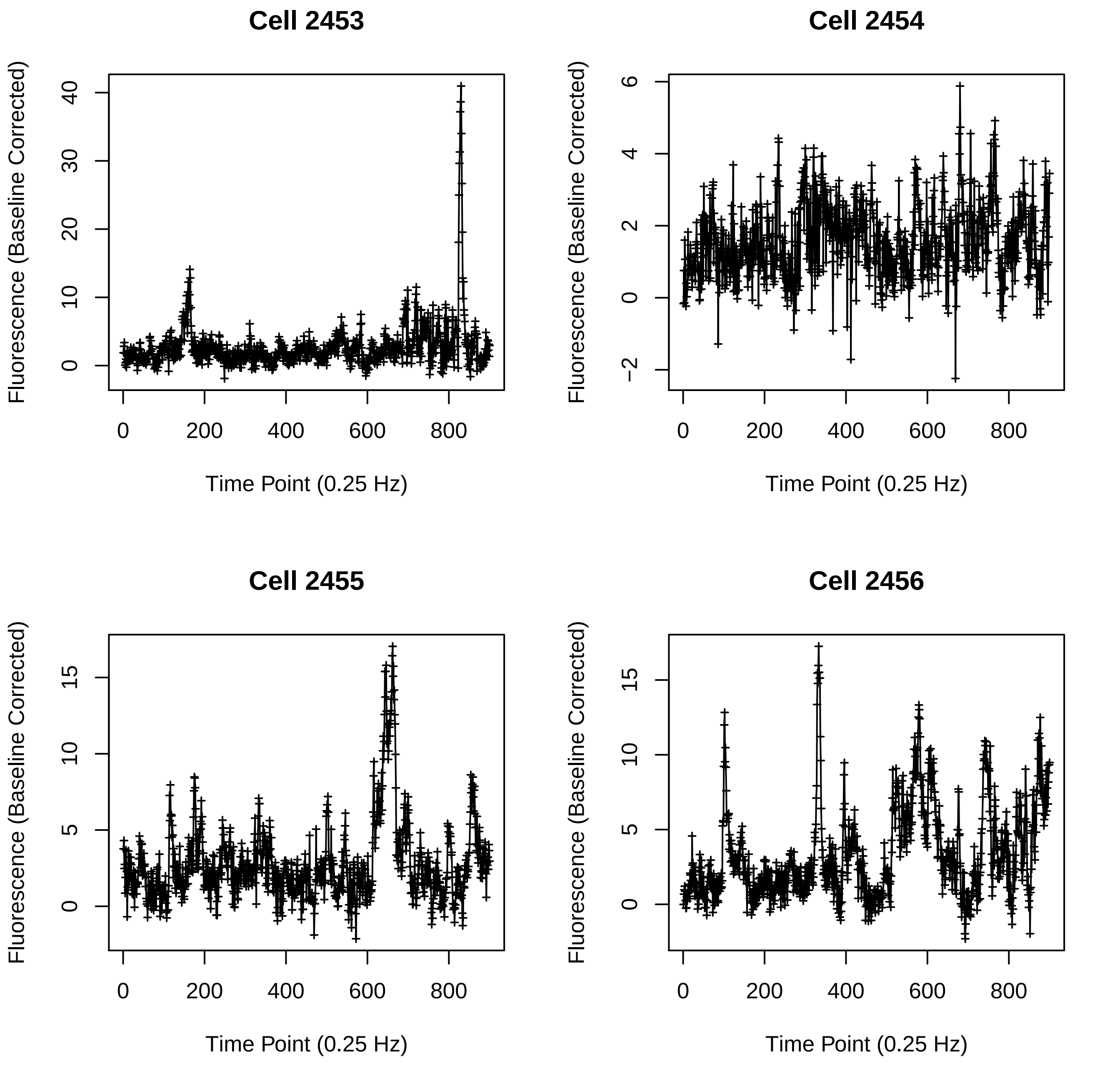

Cell 2453
Fluorescence (Baseline Corrected)
0
10
20
30
40
0
200
400
600
800
Time Point (0.25 Hz)
Cell 2454
Fluorescence (Baseline Corrected)
−2
0
2
4
6
0
200
400
600
800
Time Point (0.25 Hz)
Cell 2455
Fluorescence (Baseline Corrected)
0
5
10
15
0
200
400
600
800
Time Point (0.25 Hz)
Cell 2456
Fluorescence (Baseline Corrected)
0
5
10
15
0
200
400
600
800
Time Point (0.25 Hz)

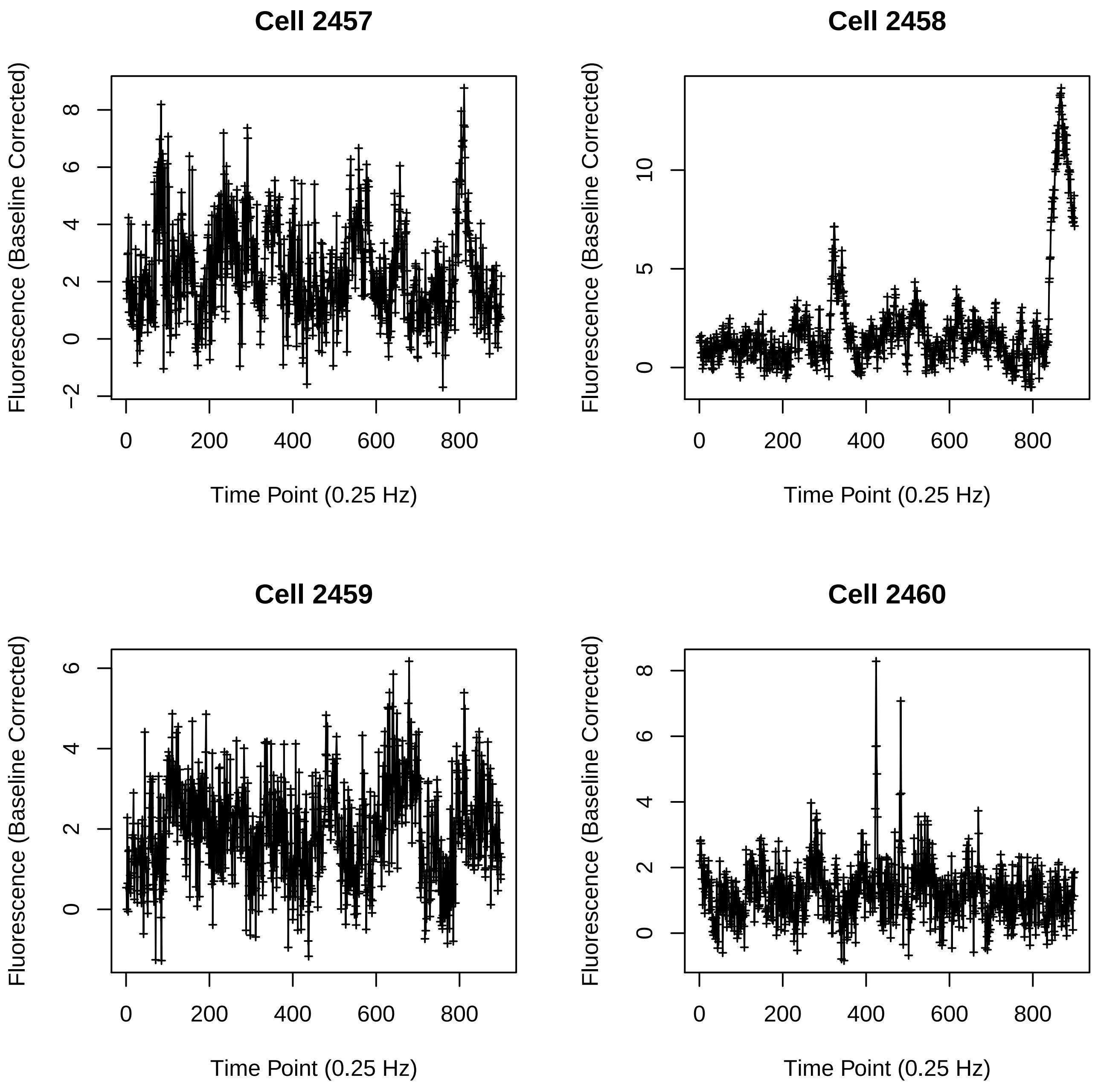
Cell 2457
Cell 2458
Cell 2459
Cell 2460
Fluorescence (Baseline Corrected)
Time Point (0.25 Hz)
−2
0
2
4
6
8
10
5
200
400
600
800

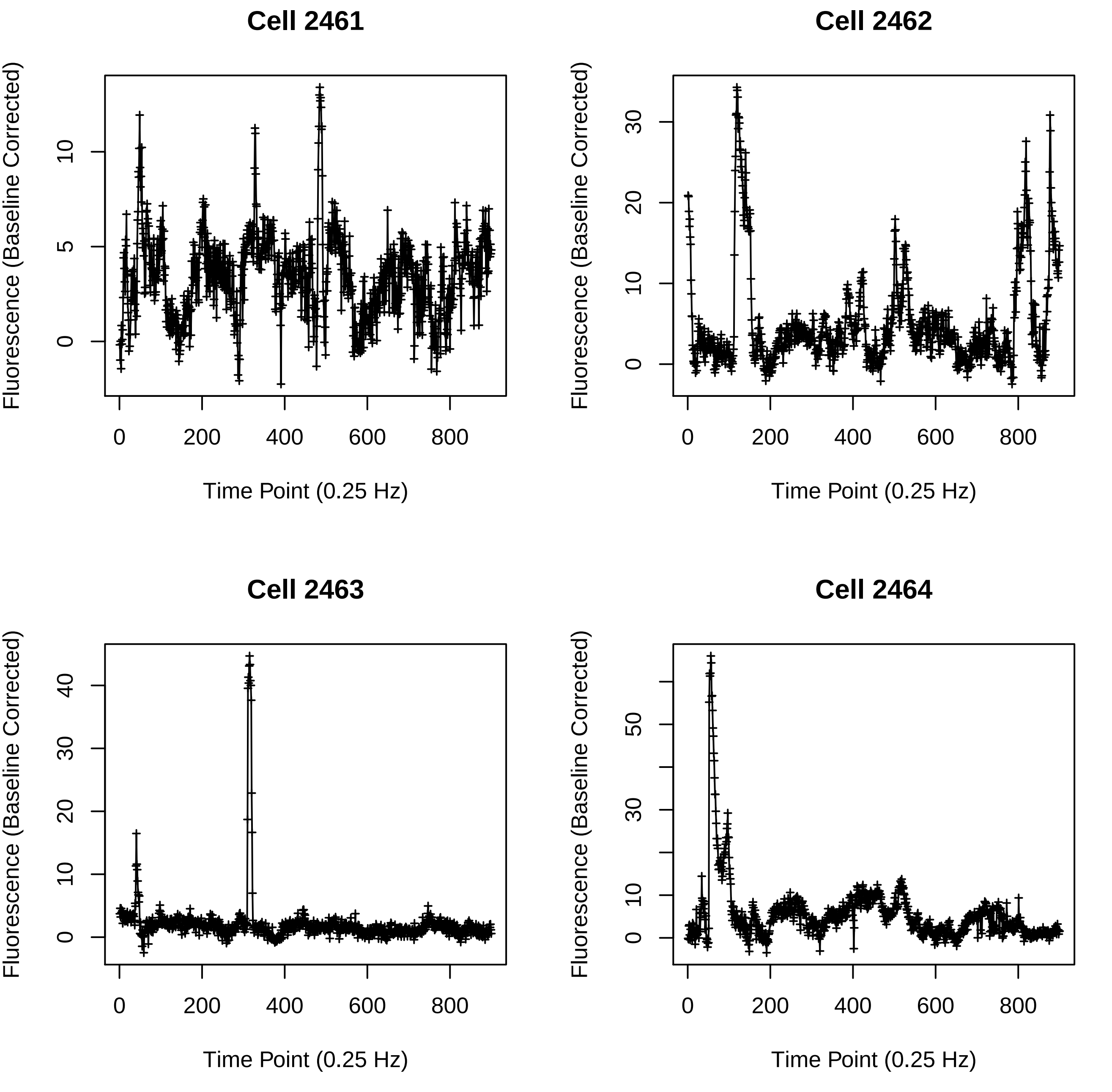
Cell 2461
Cell 2462
Cell 2463
Cell 2464
Fluorescence (Baseline Corrected)
Time Point (0.25 Hz)
0
5
10
10
20
30
40
50
0
200
400
600
800

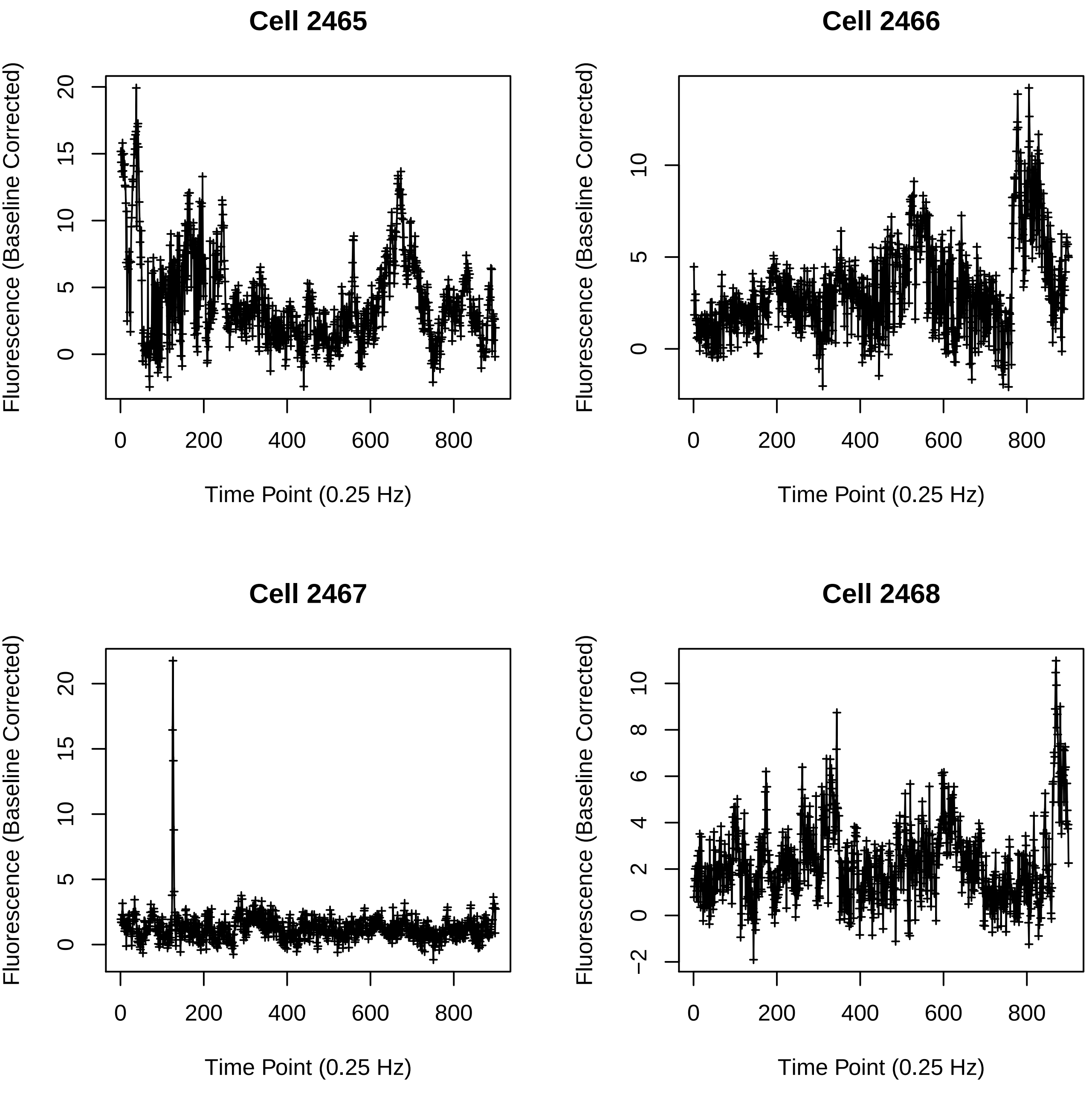
Cell 2465
Cell 2466
Cell 2467
Cell 2468
Fluorescence (Baseline Corrected)
Time Point (0.25 Hz)
0
200
400
600
800
-2
2
4
6
8
10
5
15
20

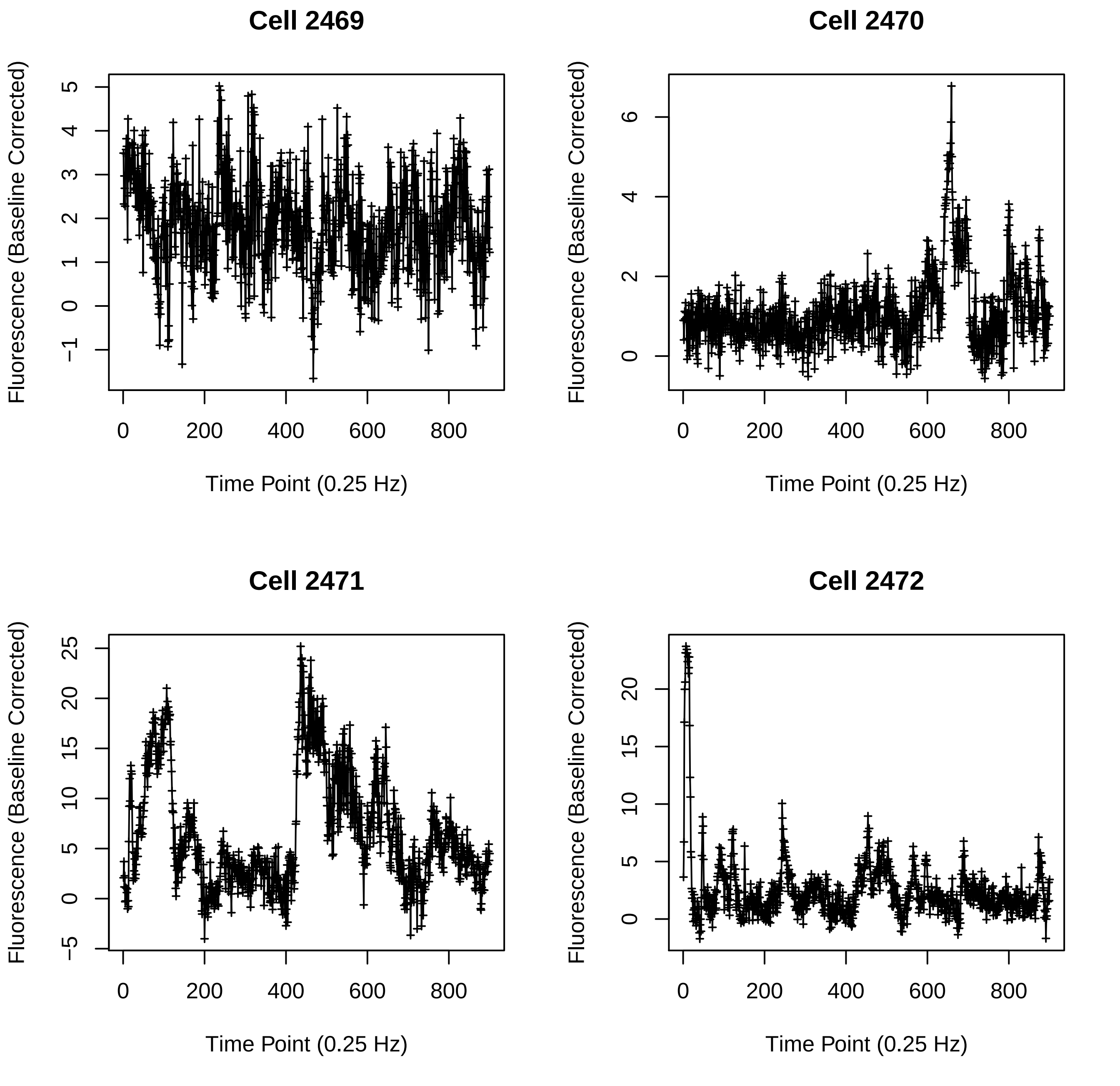
Cell 2469
Cell 2470
Cell 2471
Cell 2472
Fluorescence (Baseline Corrected)
Time Point (0.25 Hz)
0
200
400
600
800
−5
0
5
10
15
20
25
−1
1
2
3
4
6

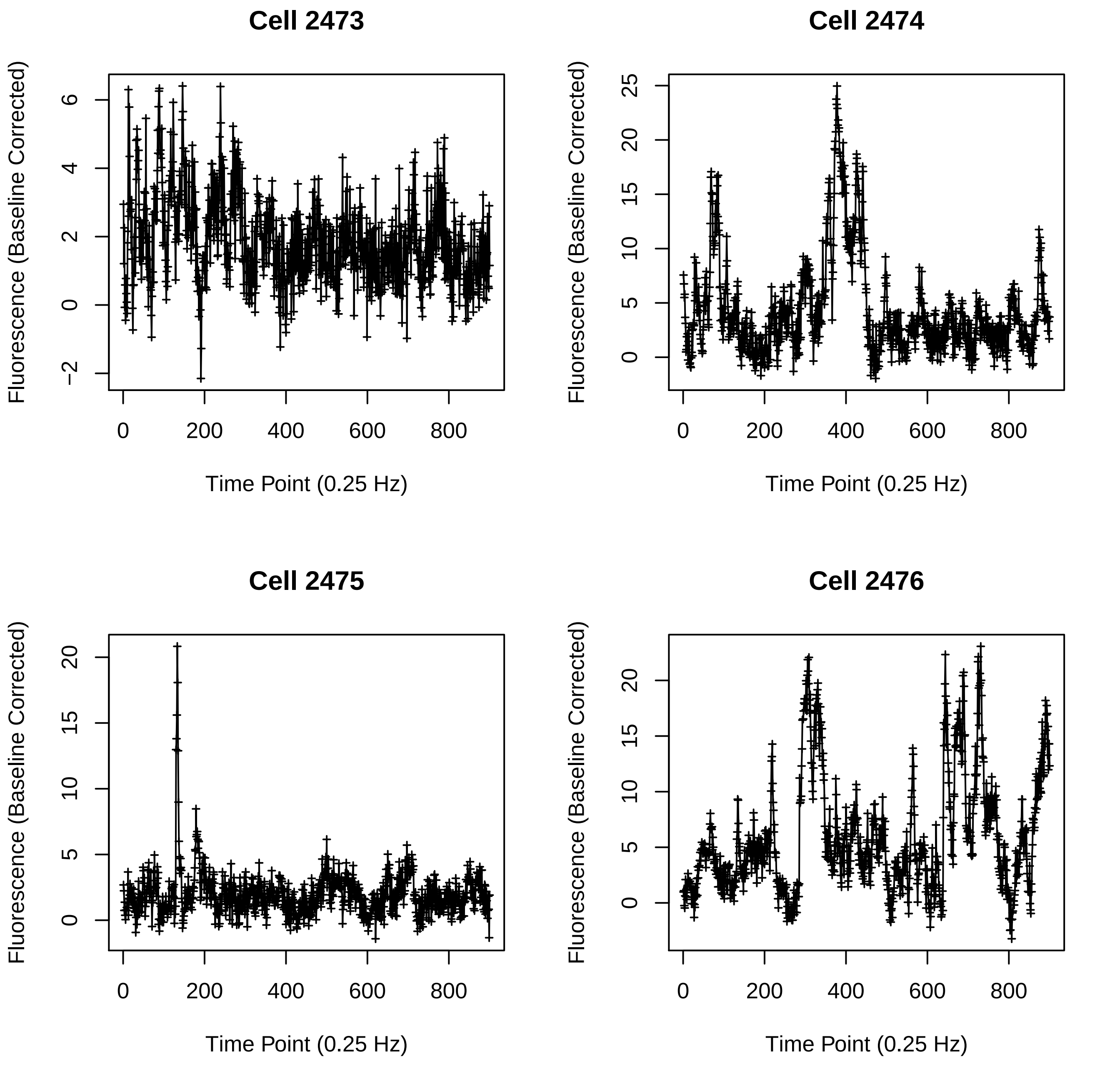
Cell 2473
Cell 2474
Cell 2475
Cell 2476
Fluorescence (Baseline Corrected)
Time Point (0.25 Hz)
0
200
400
600
800
−2
0
2
4
6
5
10
15
20
25

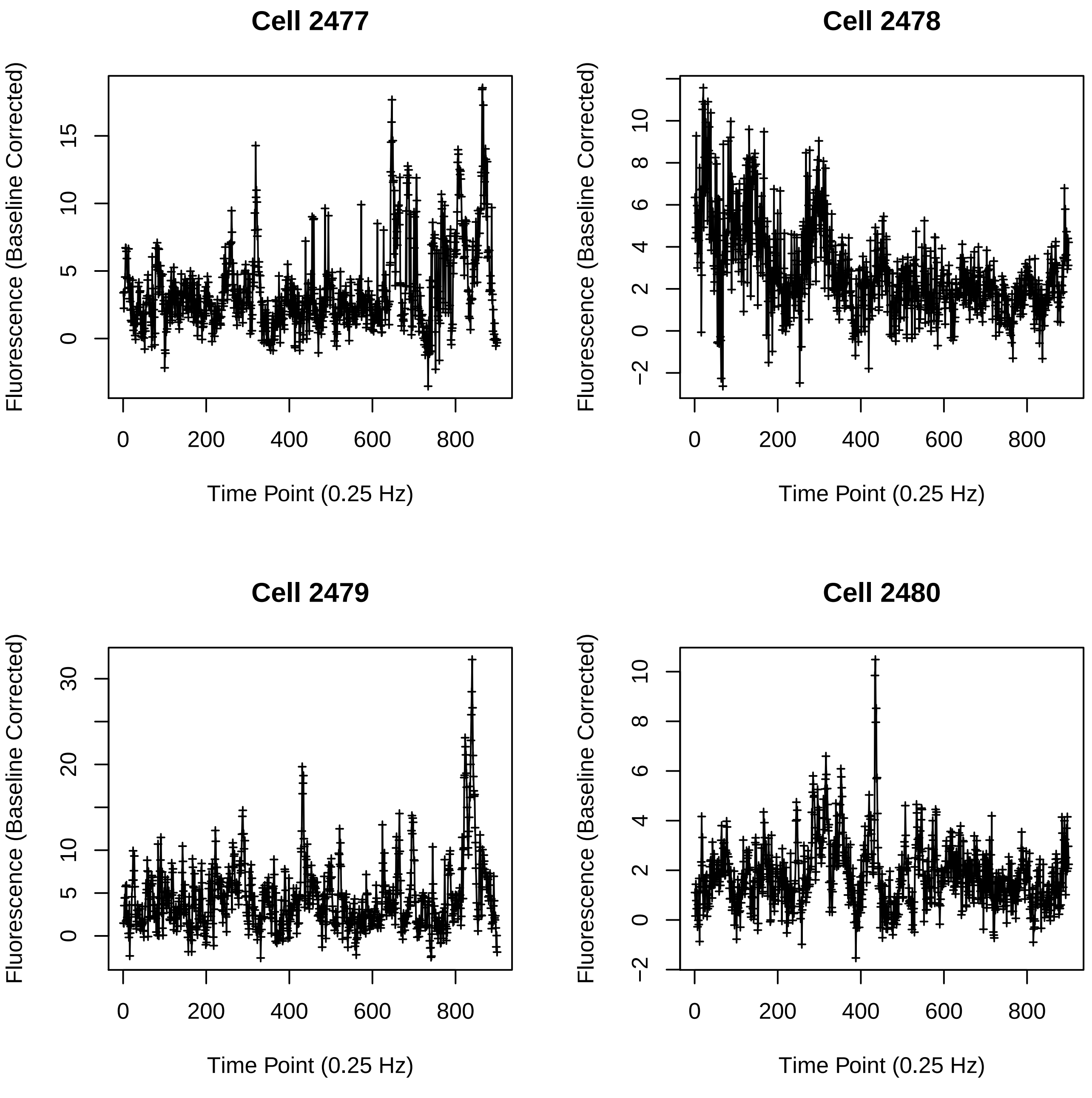
Cell 2477
Fluorescence (Baseline Corrected)
0
5
10
15
0
200
400
600
800
Time Point (0.25 Hz)
Cell 2478
Fluorescence (Baseline Corrected)
−2
0
2
4
6
8
10
0
200
400
600
800
Time Point (0.25 Hz)
Cell 2479
Fluorescence (Baseline Corrected)
0
5
10
20
30
0
200
400
600
800
Time Point (0.25 Hz)
Cell 2480
Fluorescence (Baseline Corrected)
−2
0
2
4
6
8
10
0
200
400
600
800
Time Point (0.25 Hz)

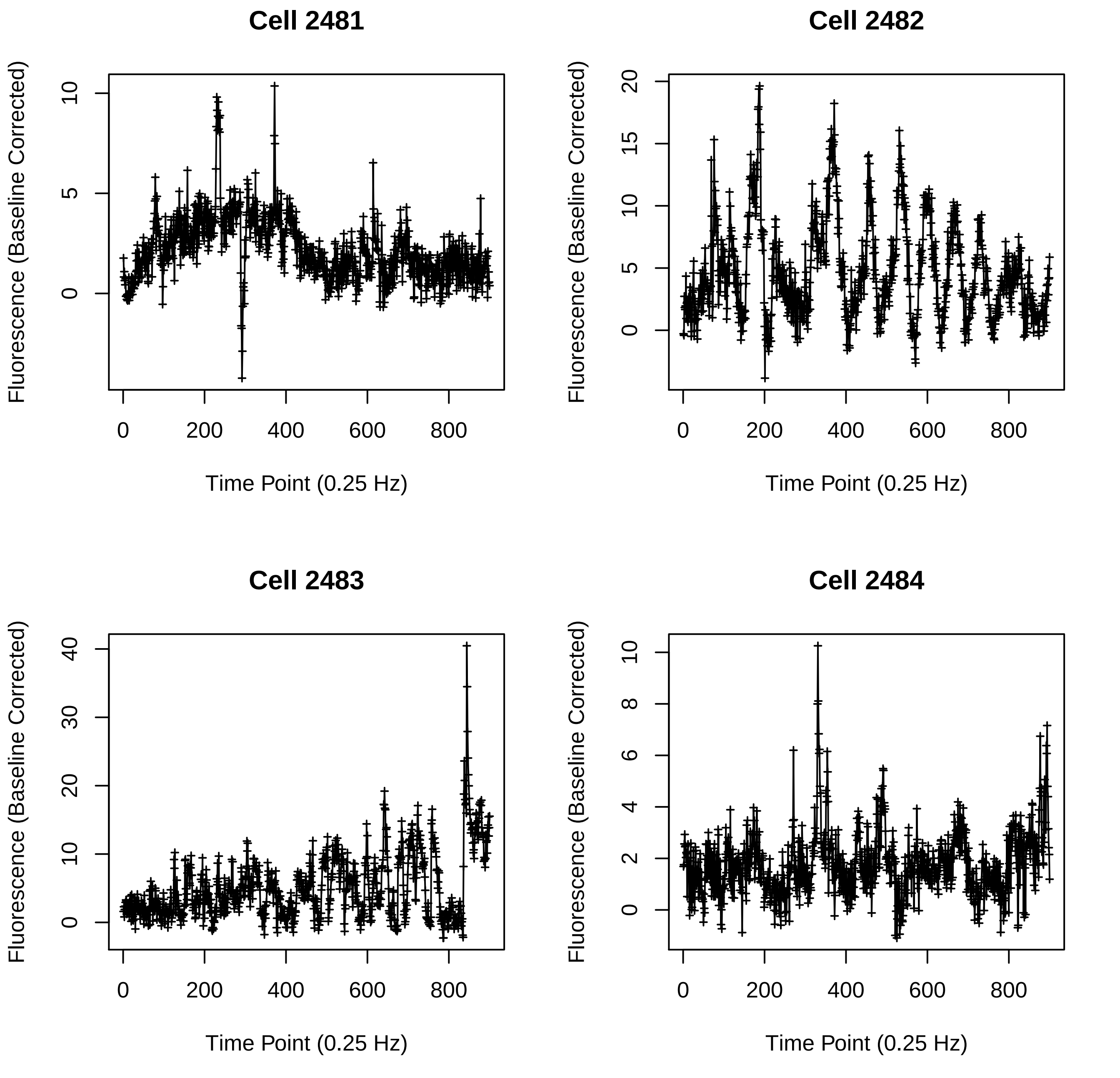
Cell 2481
Fluorescence (Baseline Corrected)
Time Point (0.25 Hz)
0
5
10
0
200
400
600
800
Cell 2482
Fluorescence (Baseline Corrected)
Time Point (0.25 Hz)
0
5
10
15
20
0
200
400
600
800
Cell 2483
Fluorescence (Baseline Corrected)
Time Point (0.25 Hz)
0
10
20
30
40
0
200
400
600
800
Cell 2484
Fluorescence (Baseline Corrected)
Time Point (0.25 Hz)
0
2
4
6
8
10
0
200
400
600
800

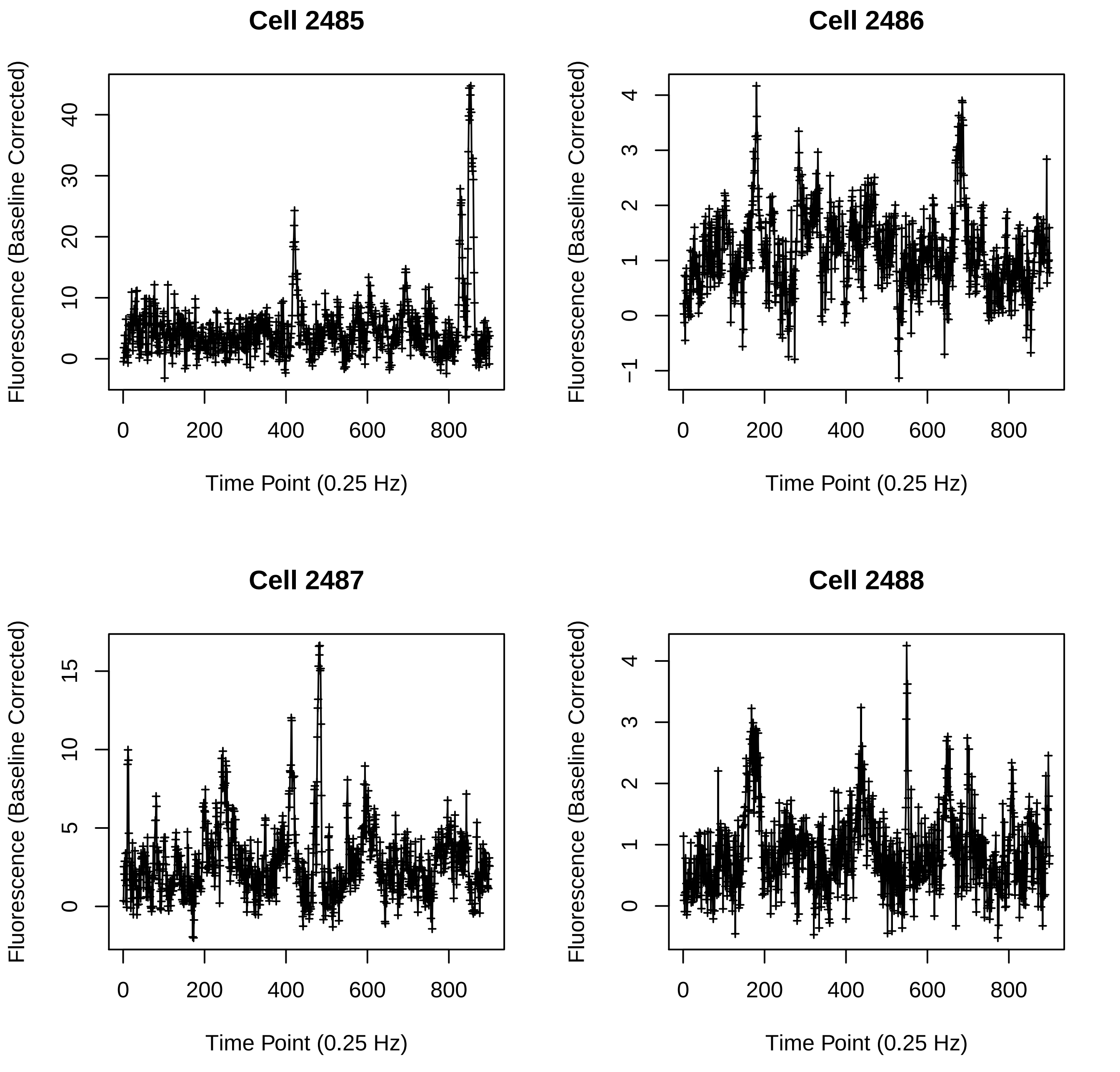
Cell 2485
Cell 2486
Cell 2487
Cell 2488
Fluorescence (Baseline Corrected)
Time Point (0.25 Hz)
0
200
400
600
800
10
20
30
40
−1
1
2
3
4
5
15

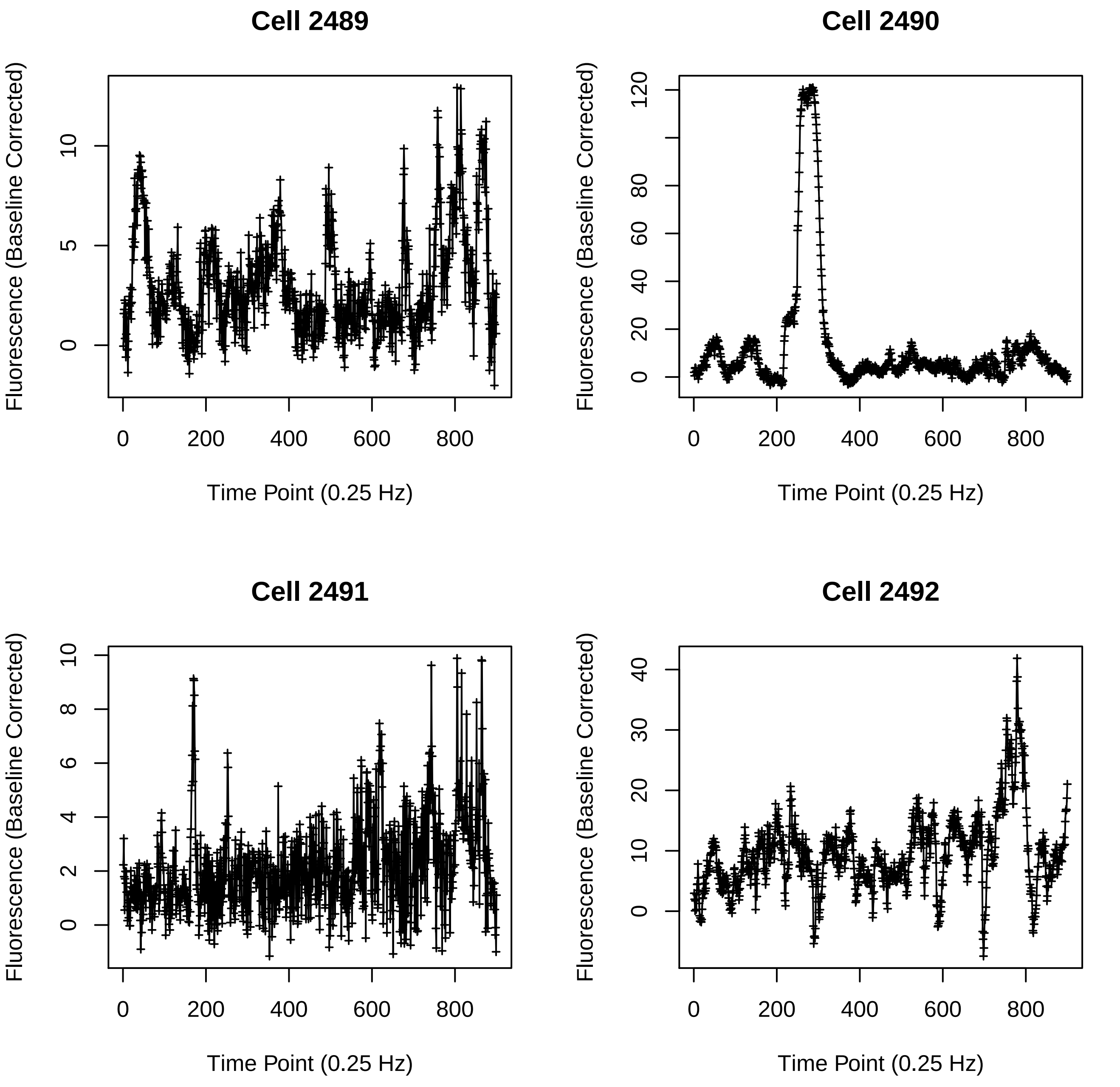
Cell 2489
Cell 2490
Cell 2491
Cell 2492
Fluorescence (Baseline Corrected)
Time Point (0.25 Hz)
0
200
400
600
800
5
10
20
40
60
80
120
2
4
6
8
30

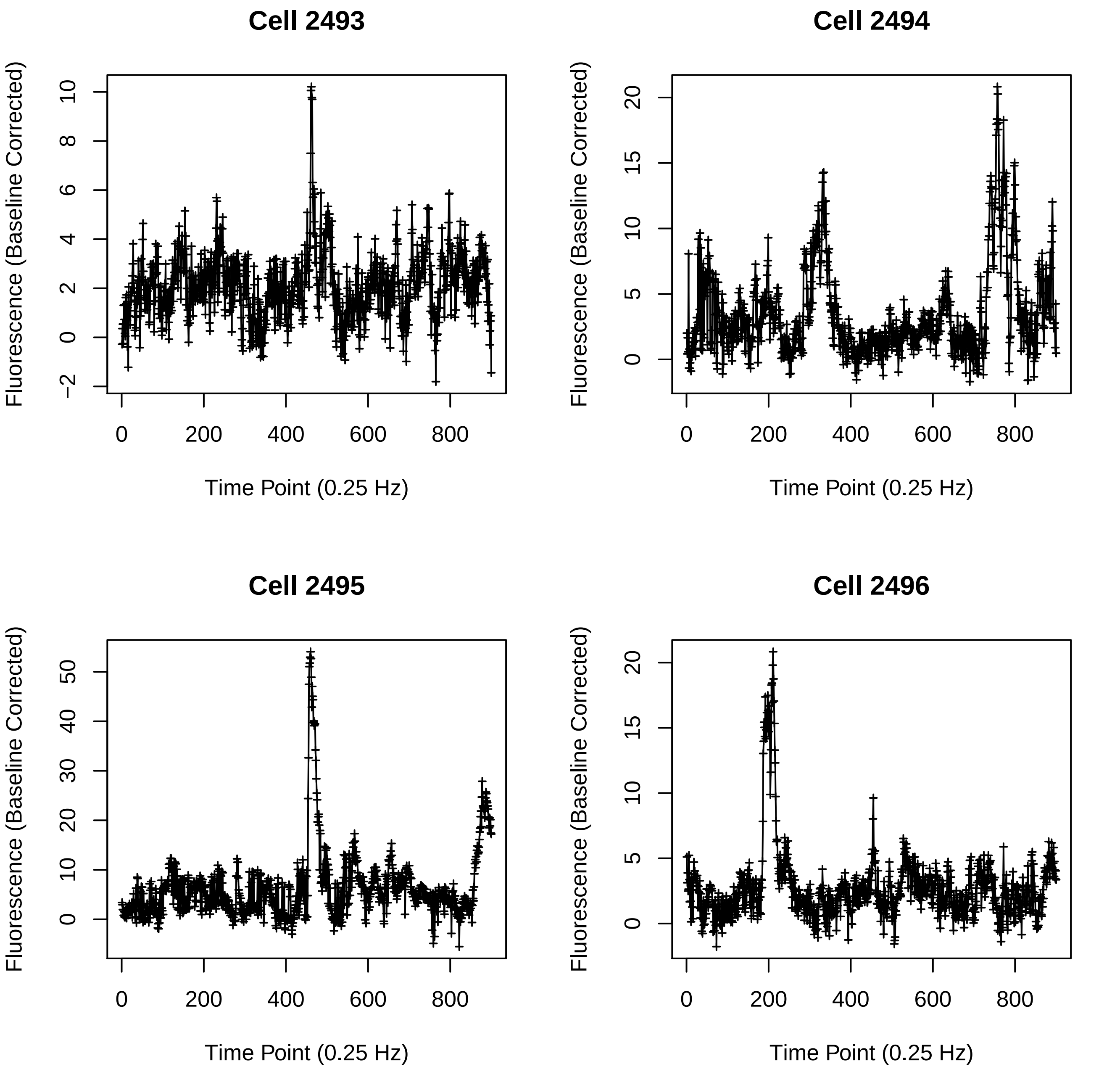
Cell 2493
Cell 2494
Cell 2495
Cell 2496
Fluorescence (Baseline Corrected)
Time Point (0.25 Hz)
0
200
400
600
800
−2
2
4
6
8
10
5
15
20
30
40
50

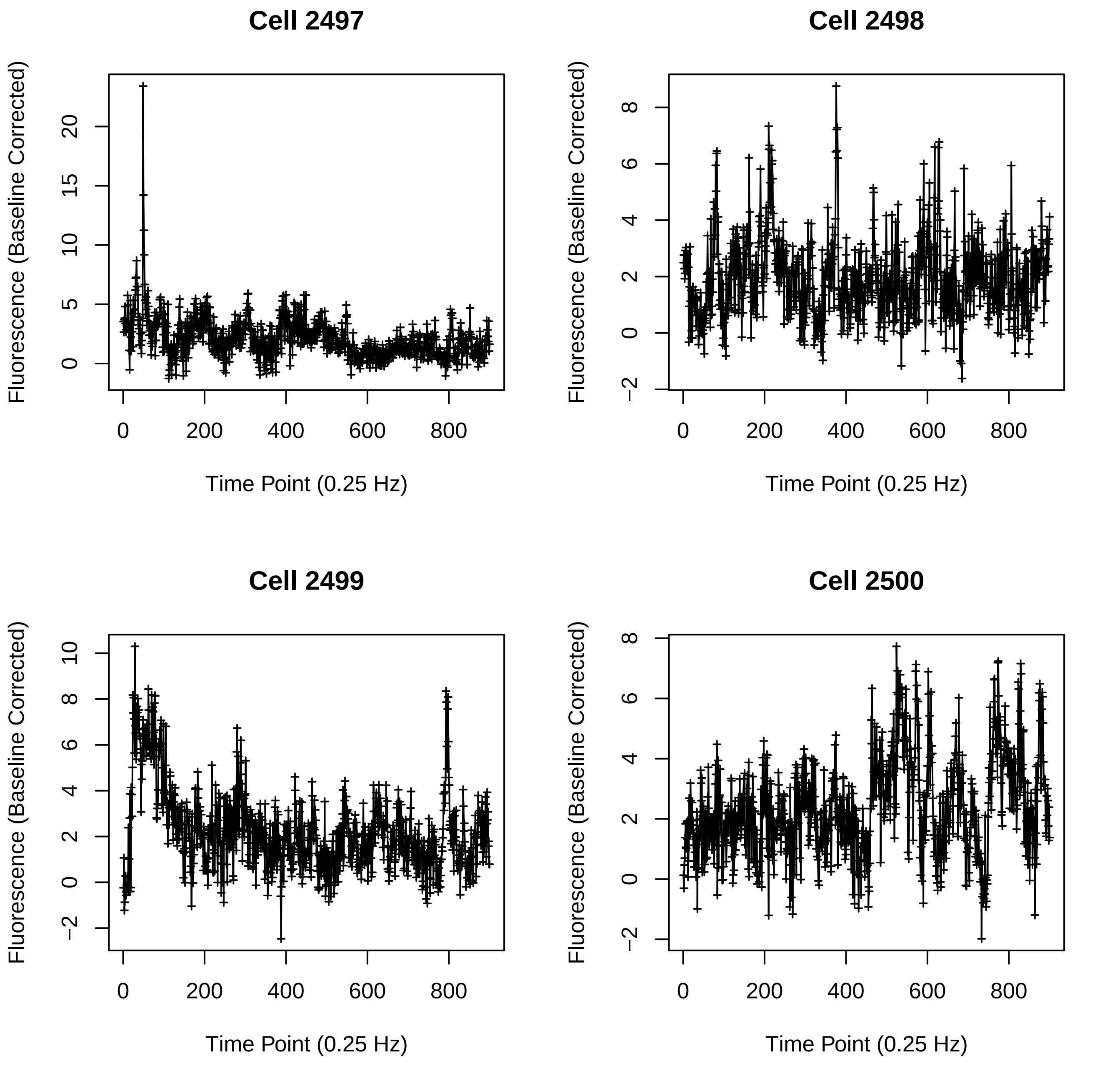

Cell 2497
Cell 2498
Cell 2499
Cell 2500
Fluorescence (Baseline Corrected)
Time Point (0.25 Hz)
0
5
10
15
20
200
400
600
800
−2
2
4
6
8

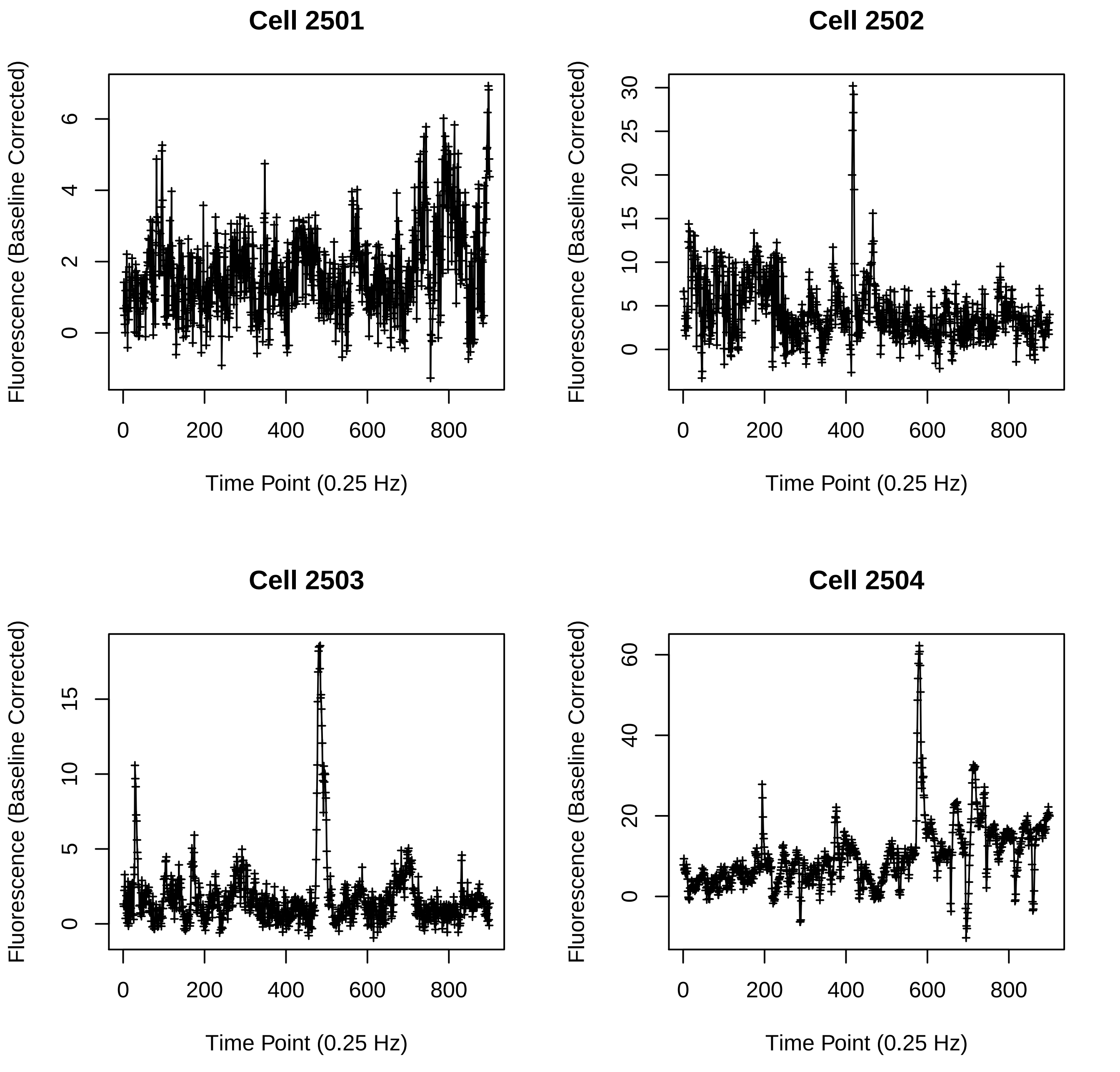

Cell 2501
Cell 2502
Cell 2503
Cell 2504
Fluorescence (Baseline Corrected)
Time Point (0.25 Hz)
0
200
400
600
800

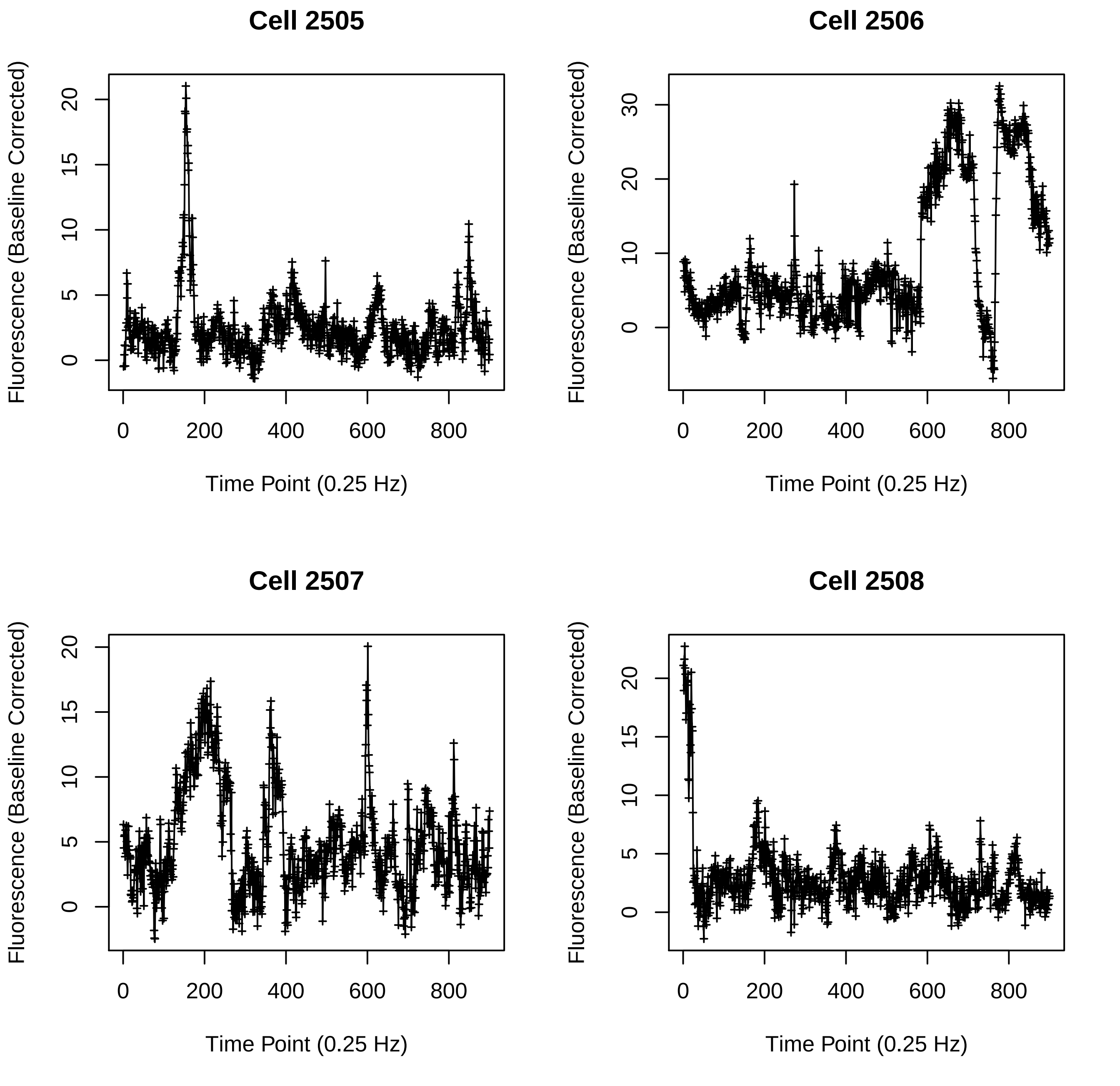

Cell 2505
Cell 2506
Cell 2507
Cell 2508
Fluorescence (Baseline Corrected)
Time Point (0.25 Hz)
0
5
10
15
20
30
200
400
600
800

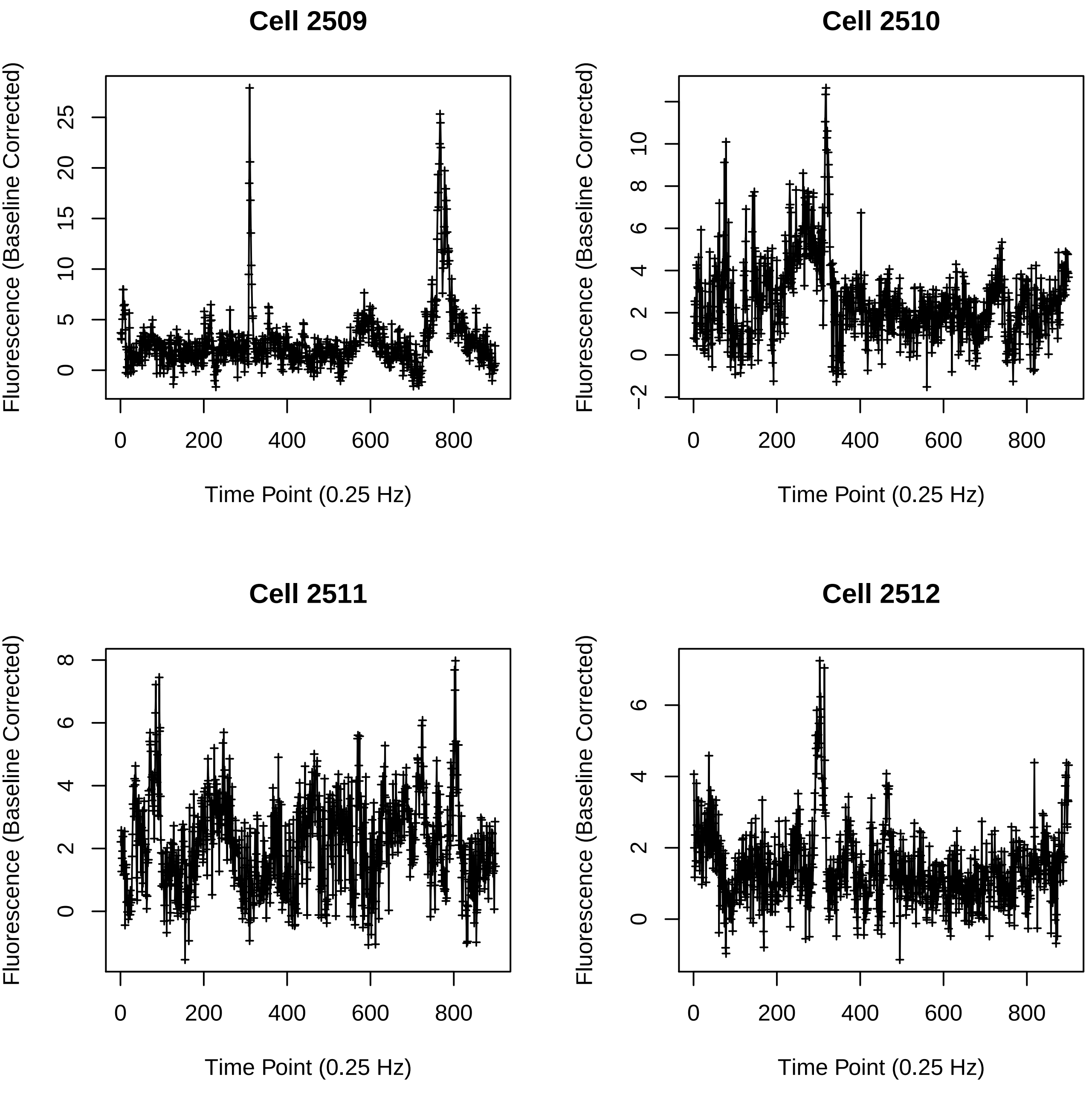

Cell 2509
Cell 2510
Cell 2511
Cell 2512
Fluorescence (Baseline Corrected)
Time Point (0.25 Hz)
0
200
400
600
800
5
10
15
20
25
−2
2
4
6
8

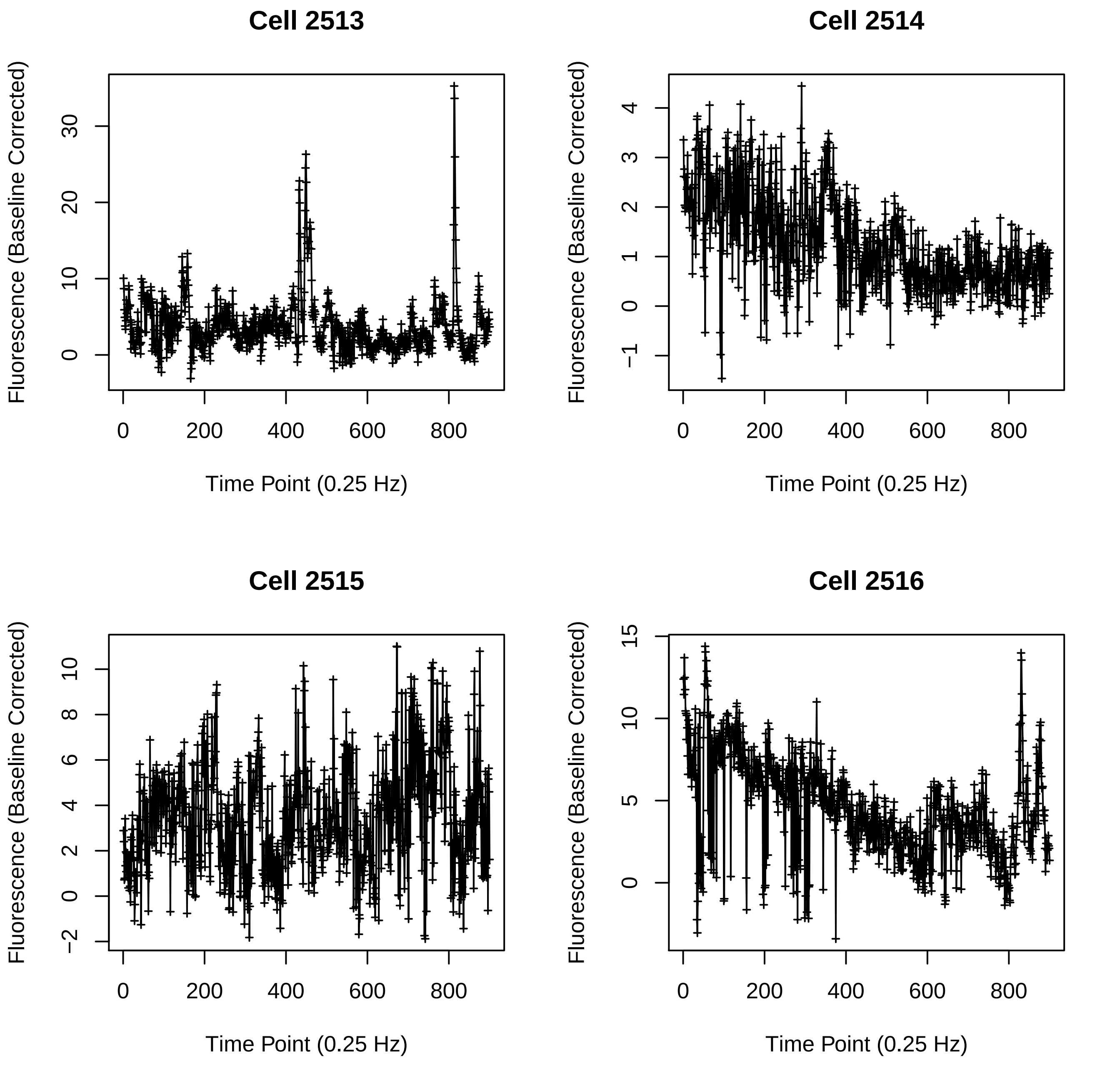
Cell 2513
Cell 2514
Cell 2515
Cell 2516
Fluorescence (Baseline Corrected)
Time Point (0.25 Hz)
0
200
400
600
800
10
20
30
−1
1
2
3
4
−2
6
8
5
15

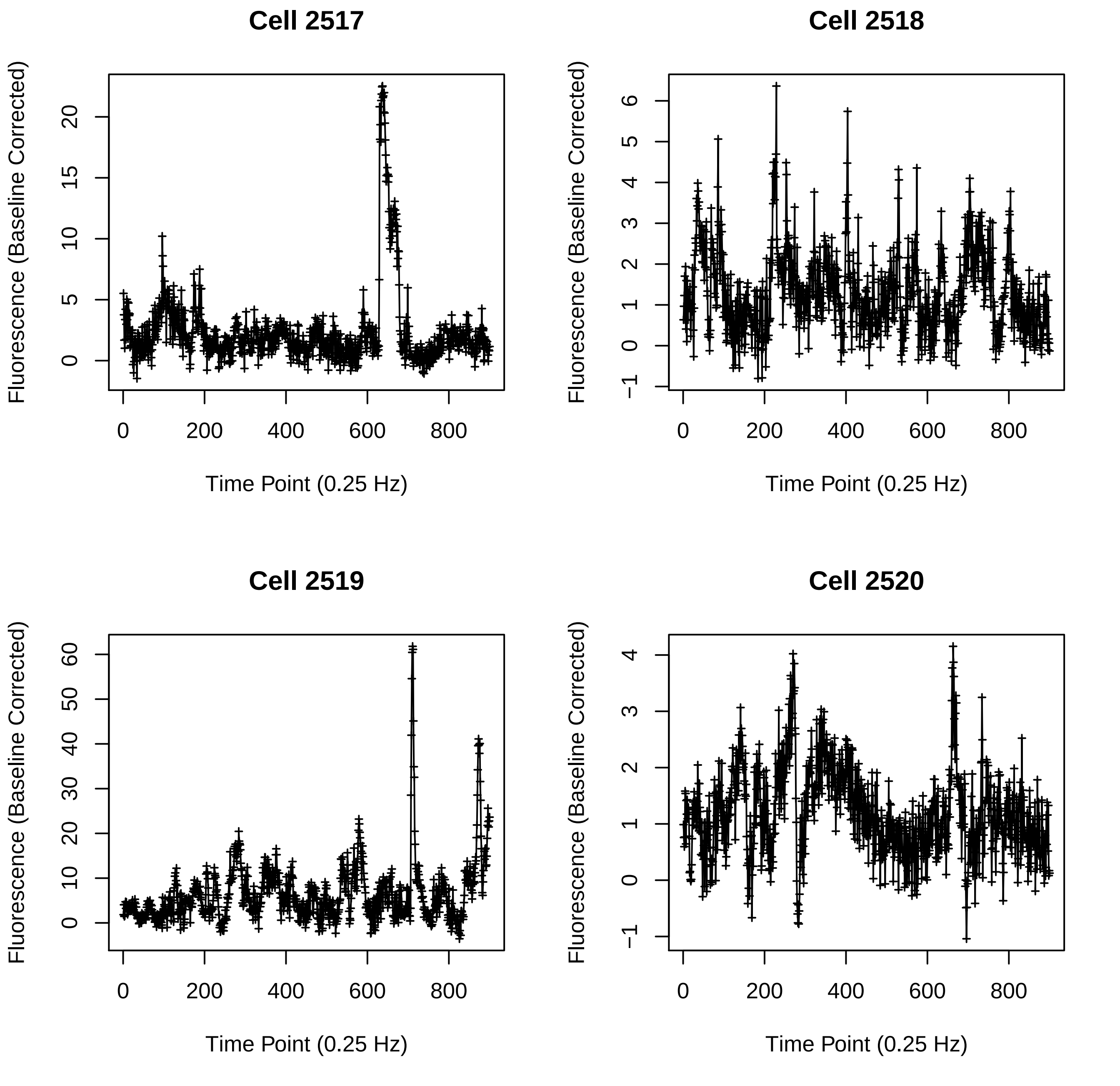

Cell 2517
Cell 2518
Cell 2519
Cell 2520
Fluorescence (Baseline Corrected)
Time Point (0.25 Hz)
0
200
400
600
800

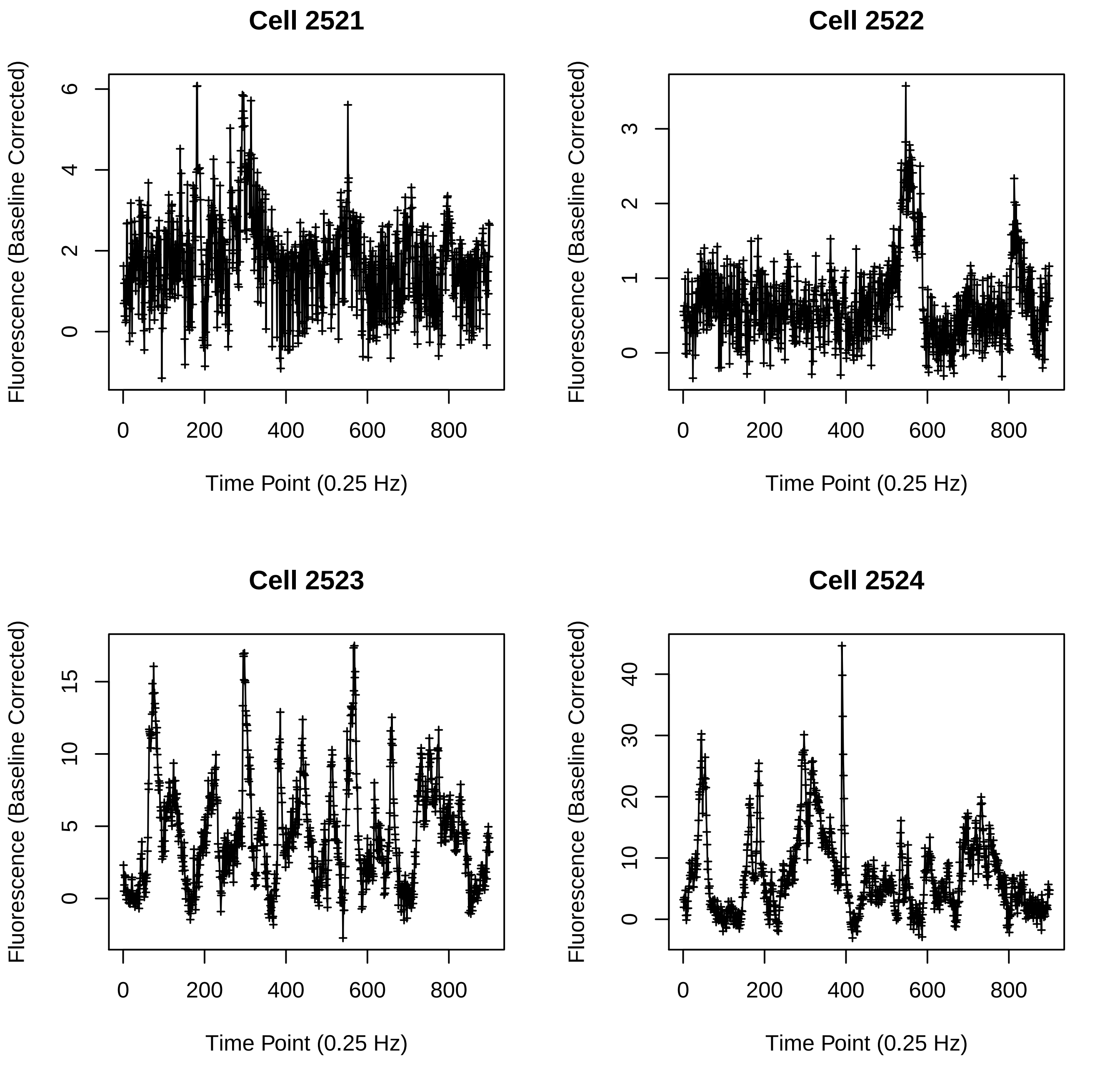

Cell 2521
Cell 2522
Cell 2523
Cell 2524
Fluorescence (Baseline Corrected)
Time Point (0.25 Hz)

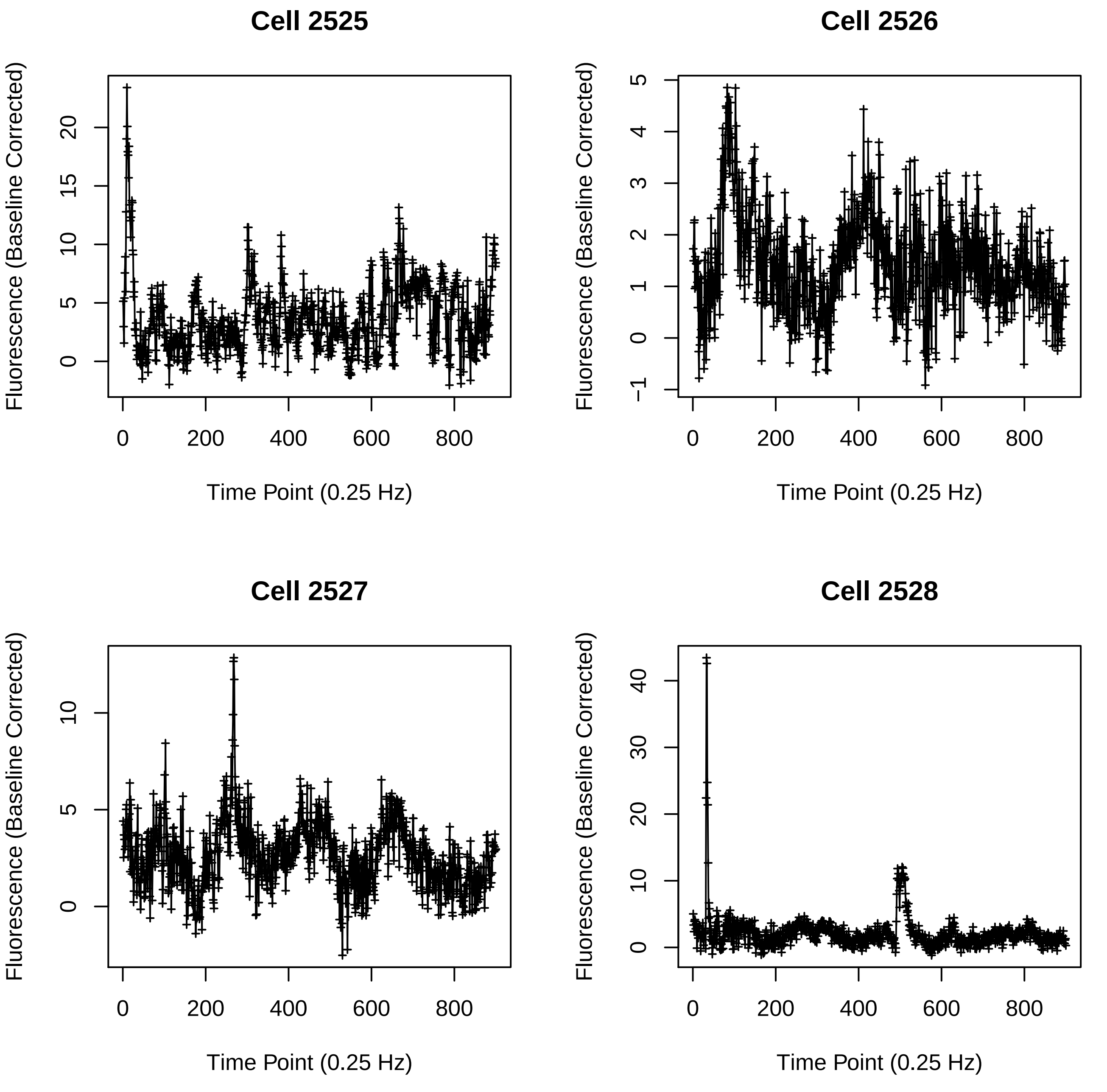
Cell 2525
Cell 2526
Cell 2527
Cell 2528
Fluorescence (Baseline Corrected)
Time Point (0.25 Hz)
0
200
400
600
800
5
10
15
20
−1
1
2
3
4
30
40

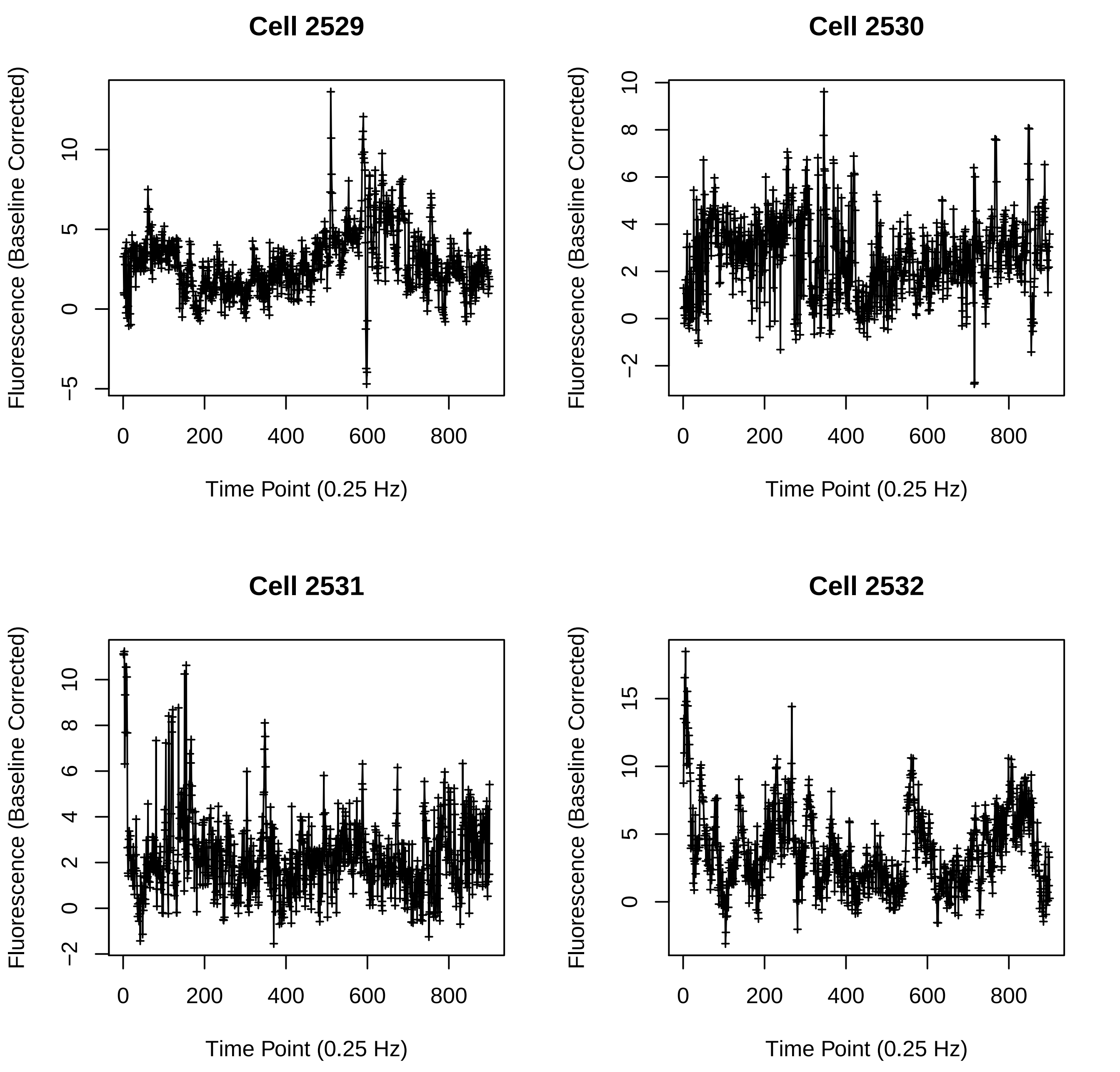
Cell 2529
Cell 2530
Cell 2531
Cell 2532
Fluorescence (Baseline Corrected)
Time Point (0.25 Hz)
0
200
400
600
800
−5
0
5
10
−2
2
4
6
8
15

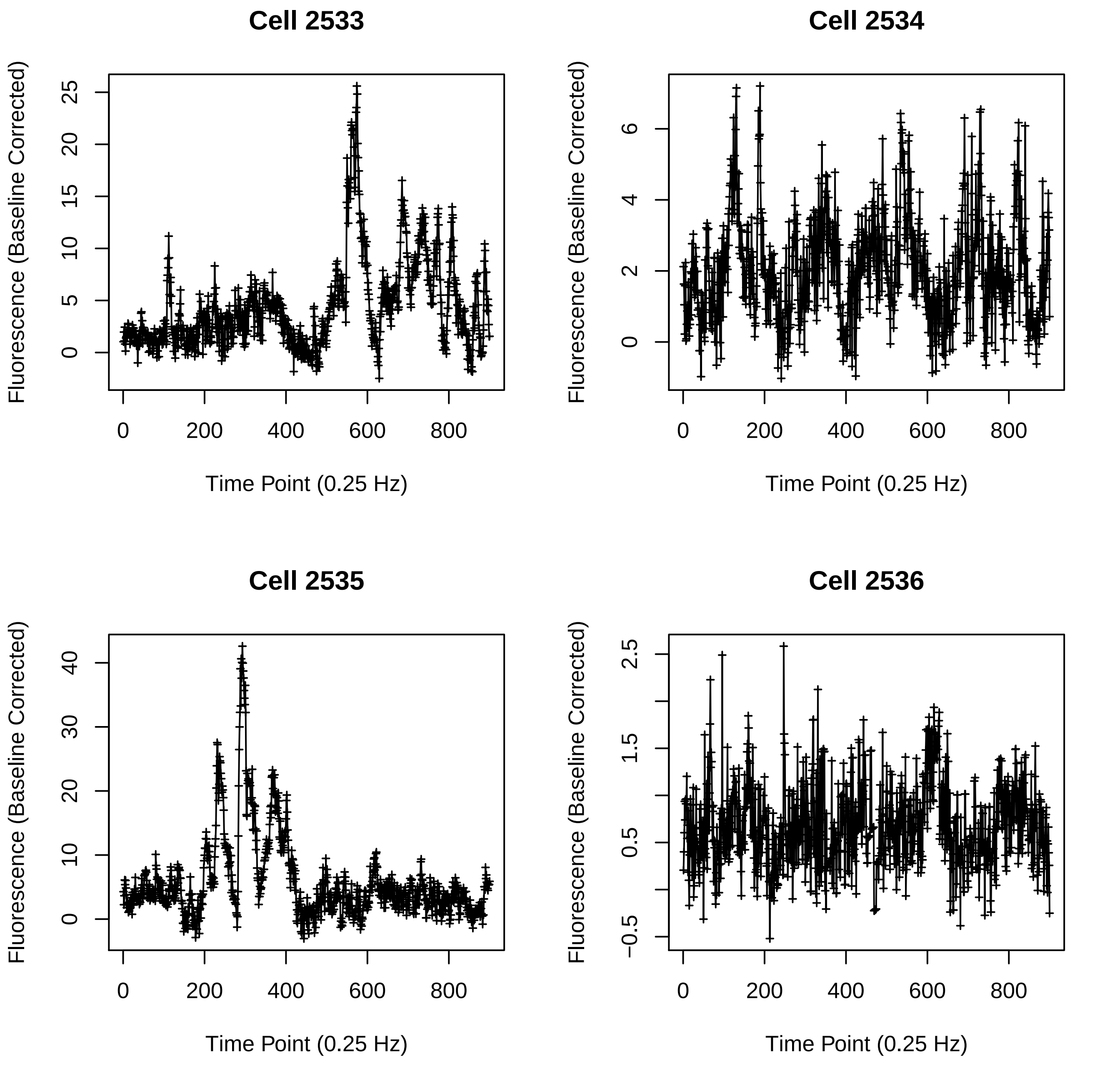

Cell 2533
Cell 2534
Cell 2535
Cell 2536
Fluorescence (Baseline Corrected)
Time Point (0.25 Hz)

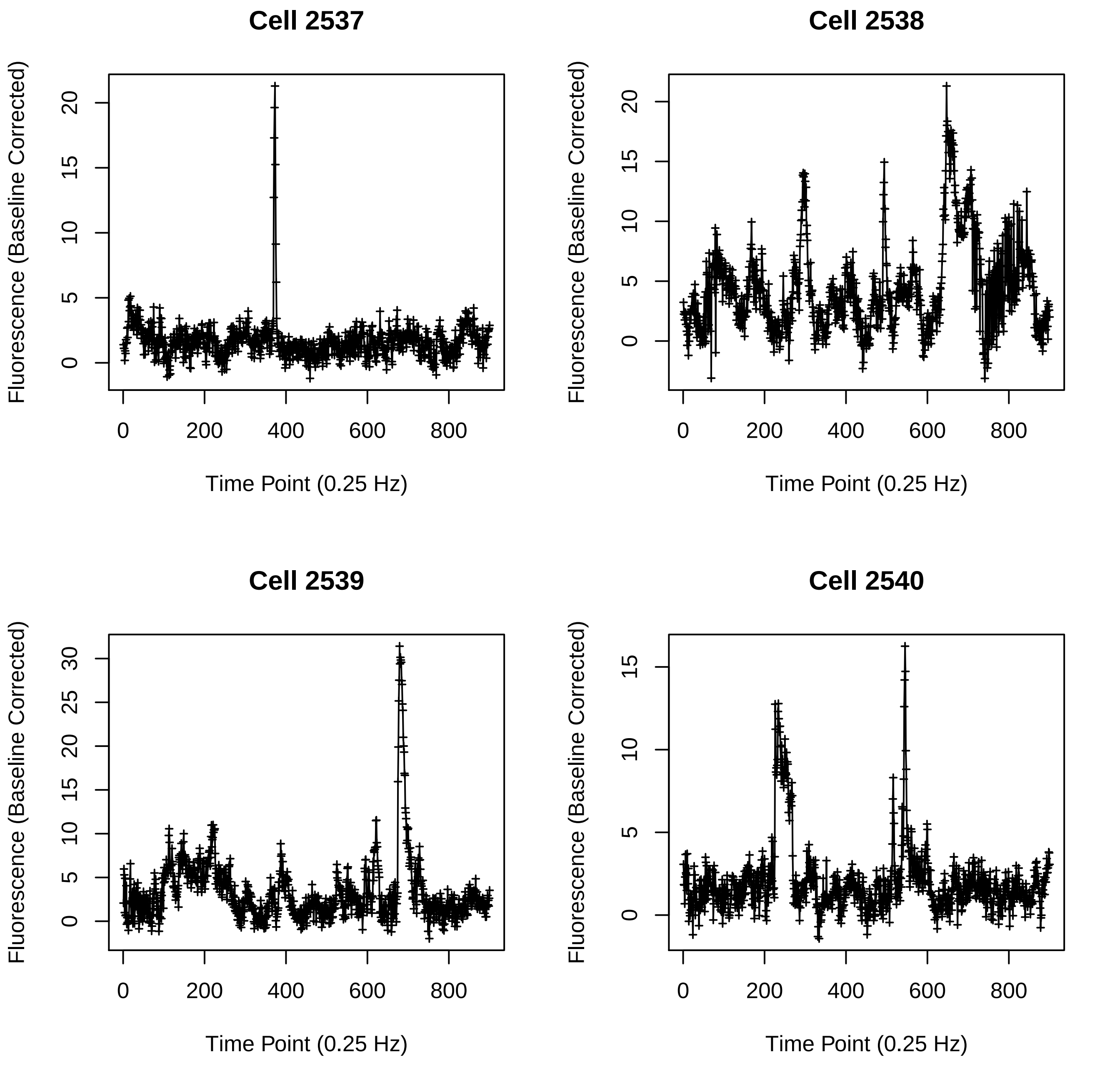
Cell 2537
Cell 2538
Cell 2539
Cell 2540
Fluorescence (Baseline Corrected)
Time Point (0.25 Hz)
0
200
400
600
800
5
10
15
20
25
30

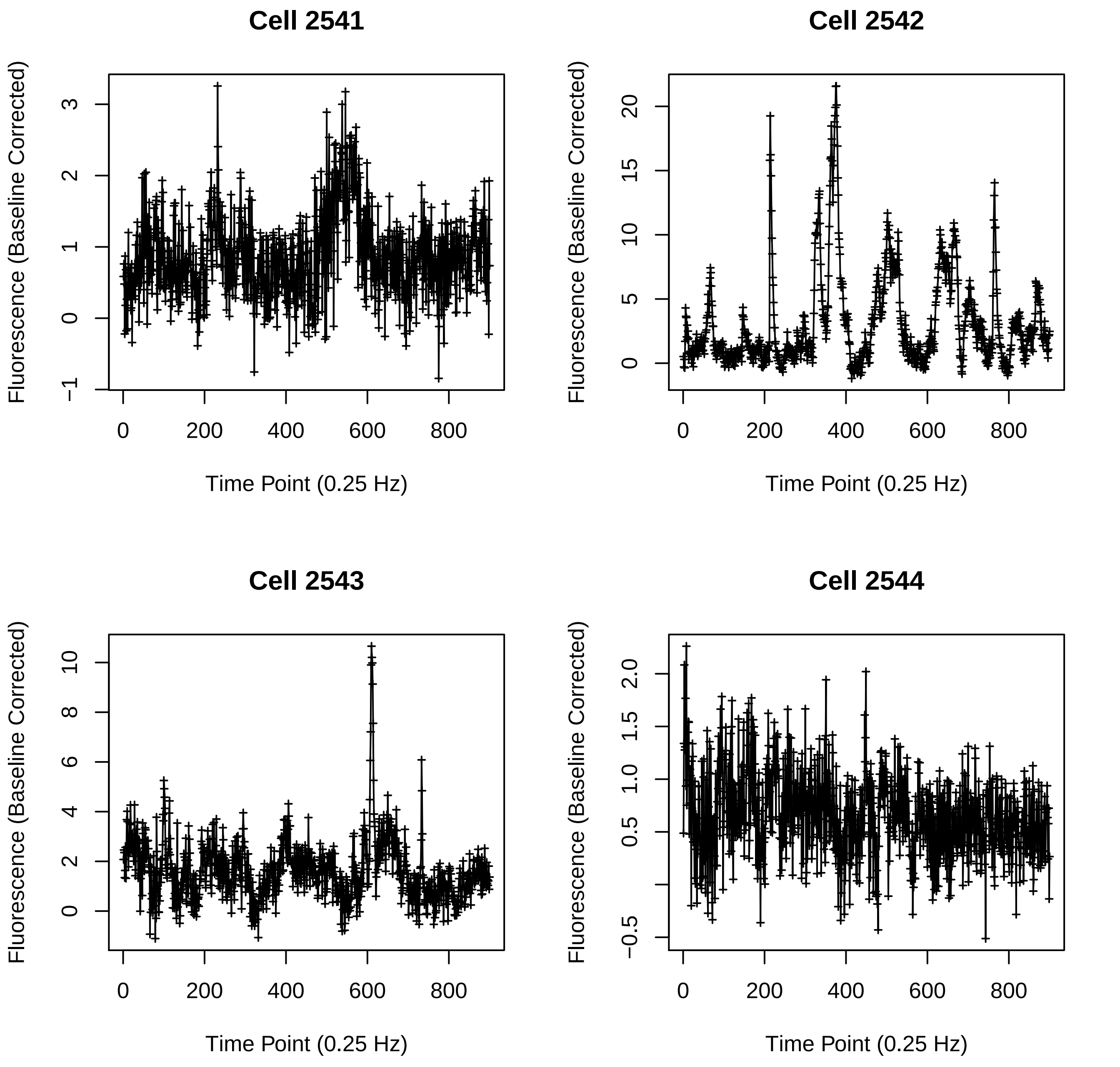
Cell 2541
Cell 2542
Cell 2543
Cell 2544
Fluorescence (Baseline Corrected)
Time Point (0.25 Hz)
−1
0
1
2
3
0
5
10
15
20
0
2
4
6
8
10
−0.5
0.5
1.0
1.5
2.0
0
200
400
600
800

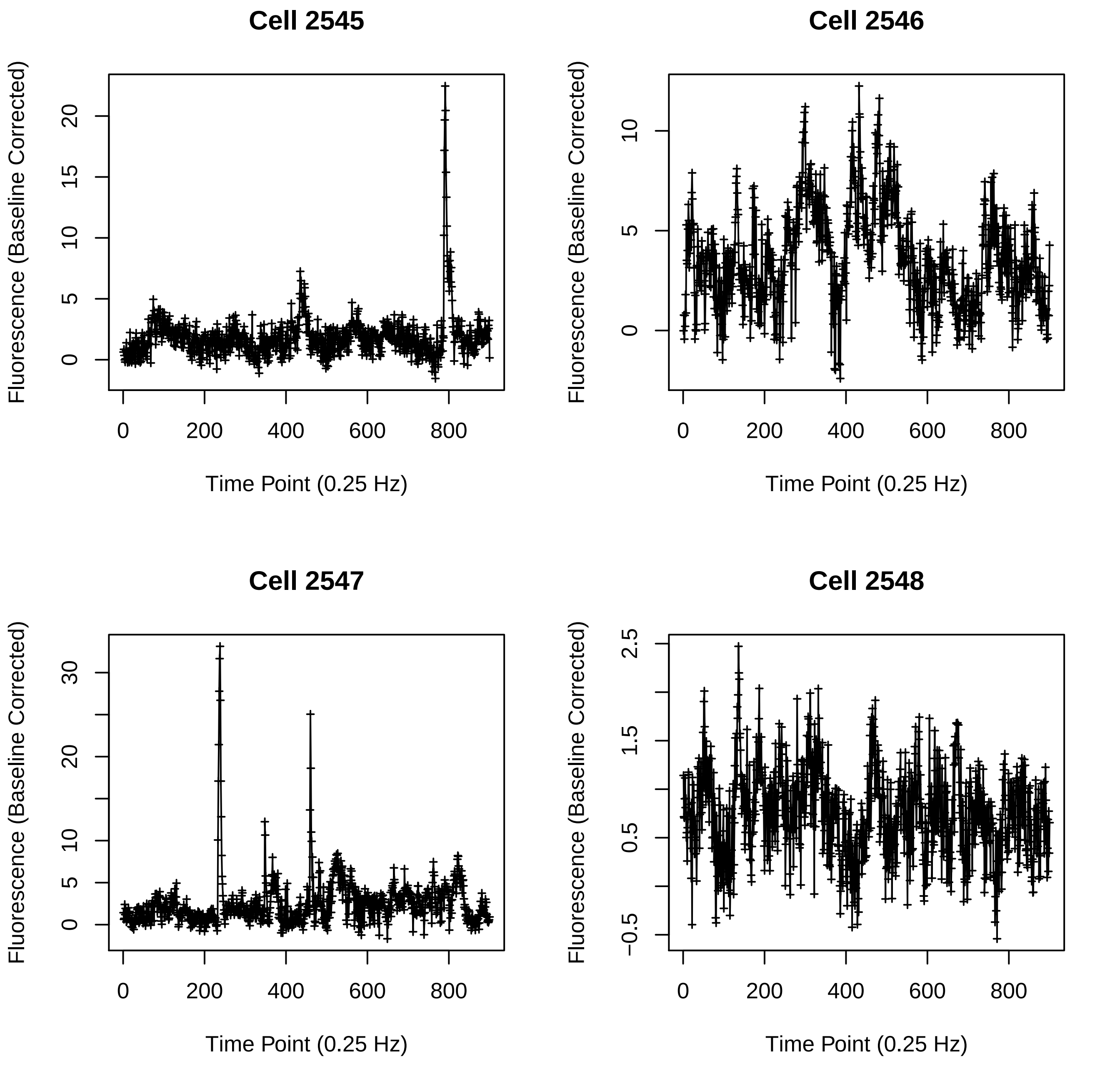
Cell 2545
Cell 2546
Cell 2547
Cell 2548
Fluorescence (Baseline Corrected)
Time Point (0.25 Hz)
0
200
400
600
800
5
10
15
20
30
−0.5
0.5
1.5
2.5

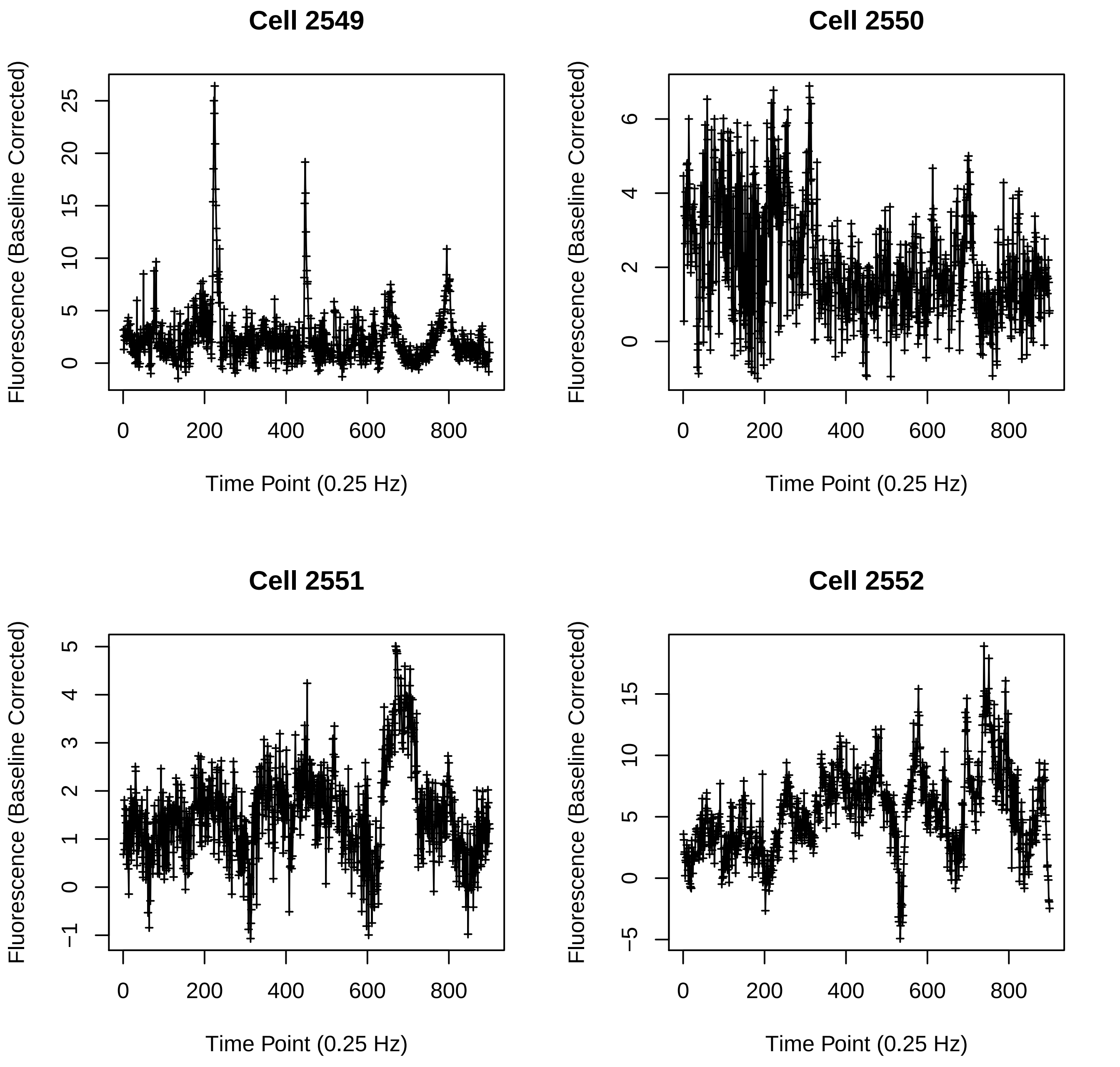

Cell 2549
Cell 2550
Cell 2551
Cell 2552
Fluorescence (Baseline Corrected)
Time Point (0.25 Hz)

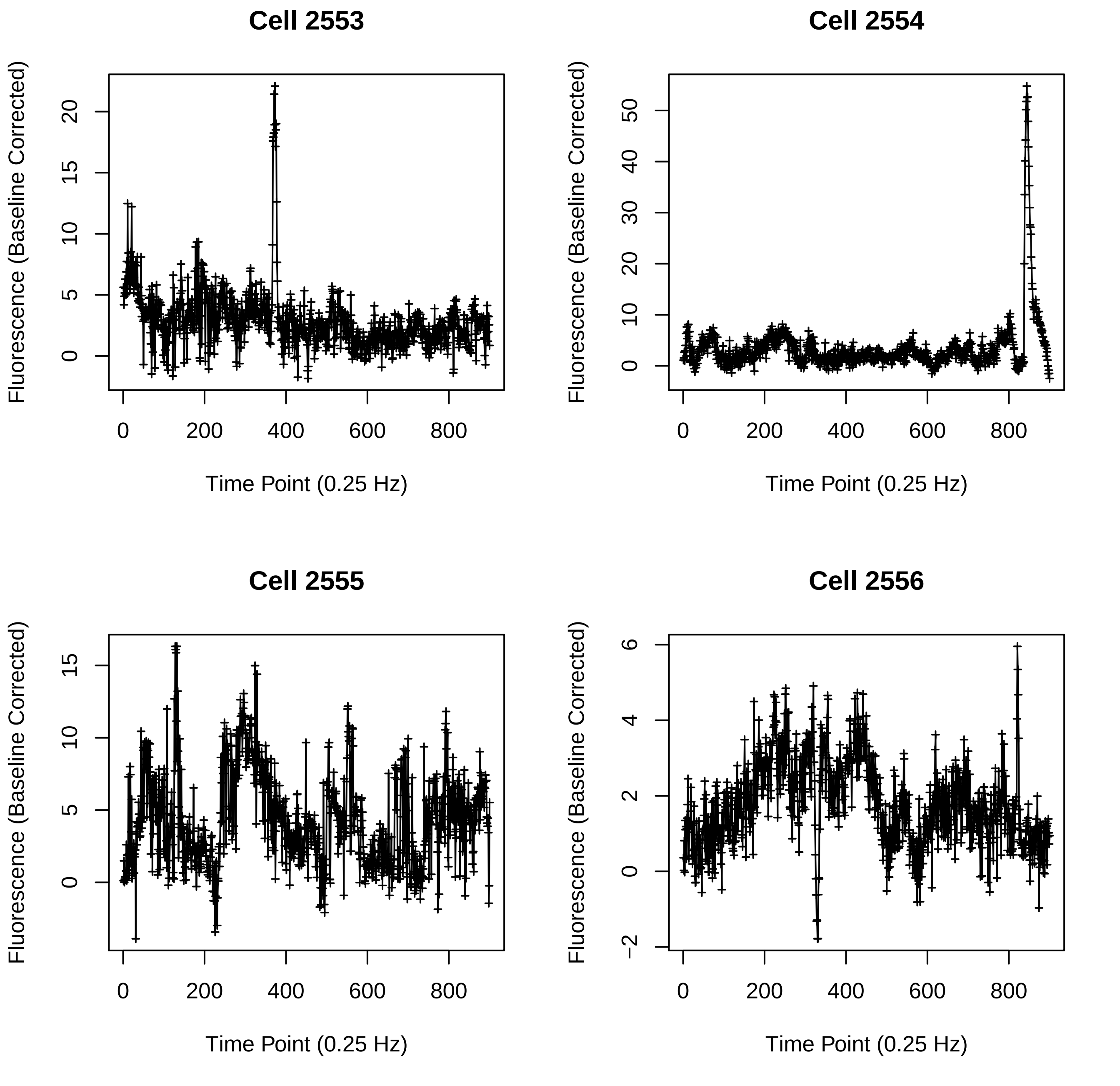

Cell 2553
Cell 2554
Cell 2555
Cell 2556
Fluorescence (Baseline Corrected)
Time Point (0.25 Hz)

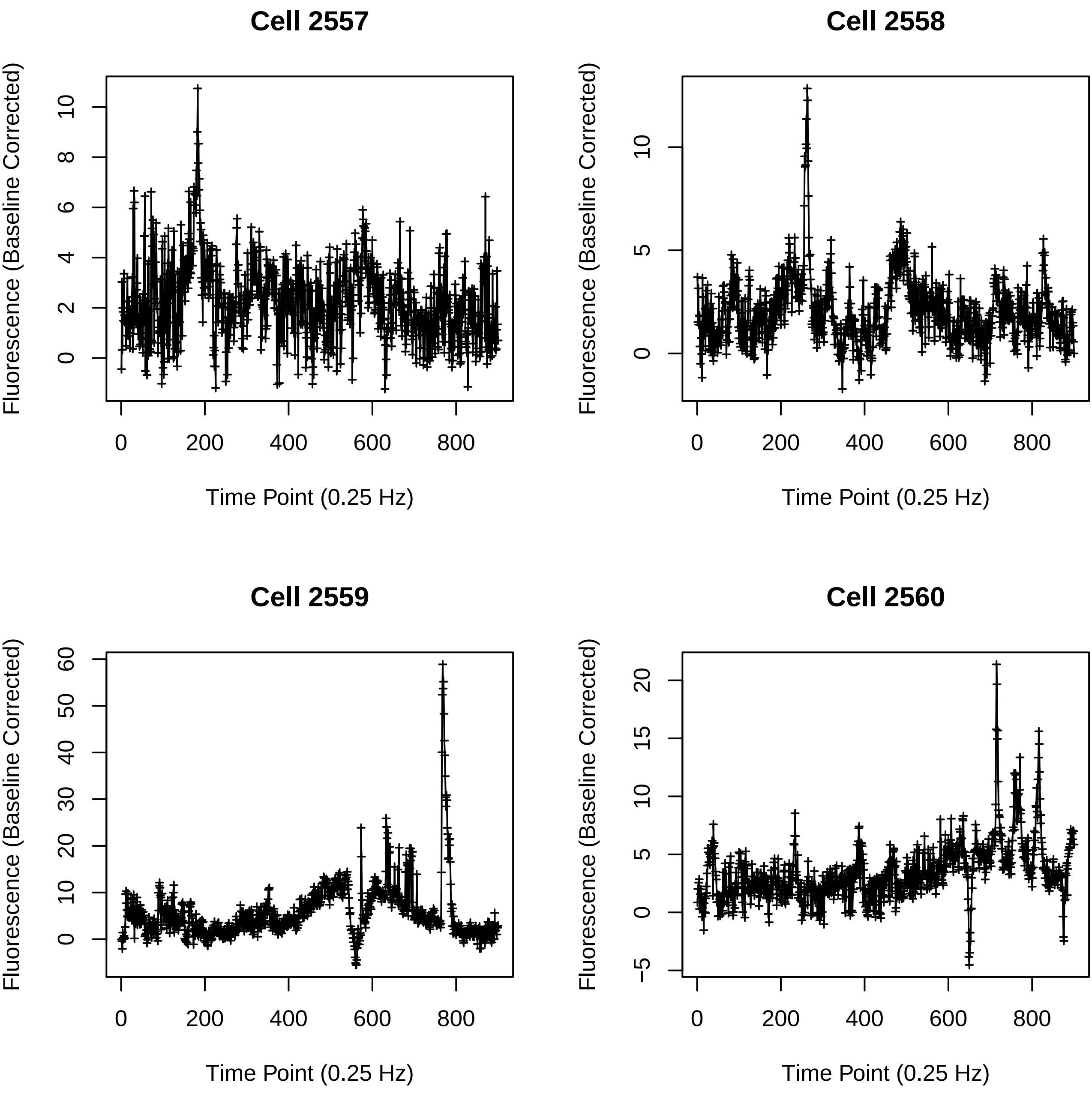
Cell 2557
Cell 2558
Cell 2559
Cell 2560
Fluorescence (Baseline Corrected)
Time Point (0.25 Hz)
0
200
400
600
800
0
2
4
6
8
10
5
10
20
30
40
50
60
−5
15
20

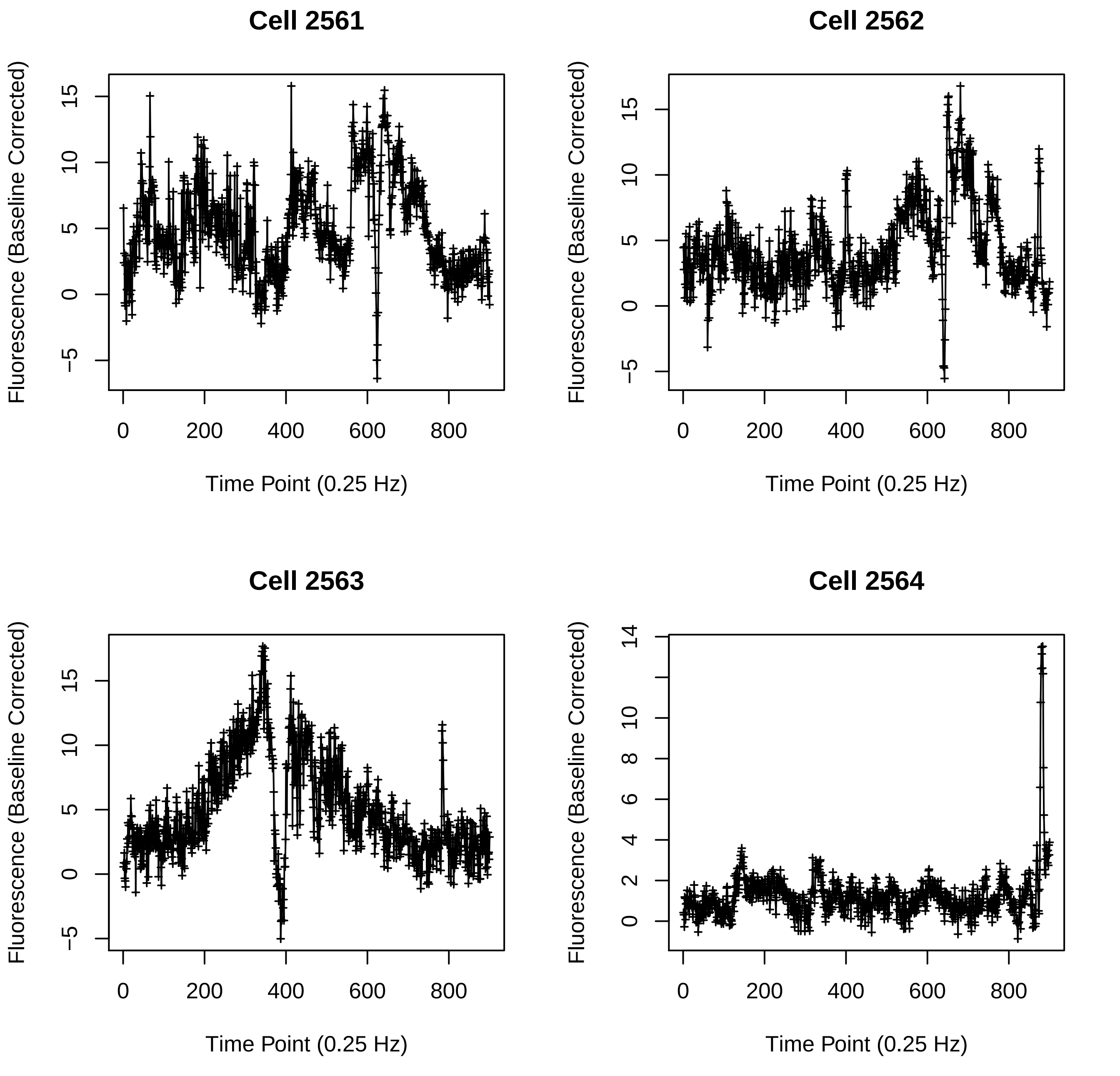
Cell 2561
Cell 2562
Cell 2563
Cell 2564
Fluorescence (Baseline Corrected)
Time Point (0.25 Hz)
−5
0
5
10
15
2
4
6
8
12
14
200
400
600
800

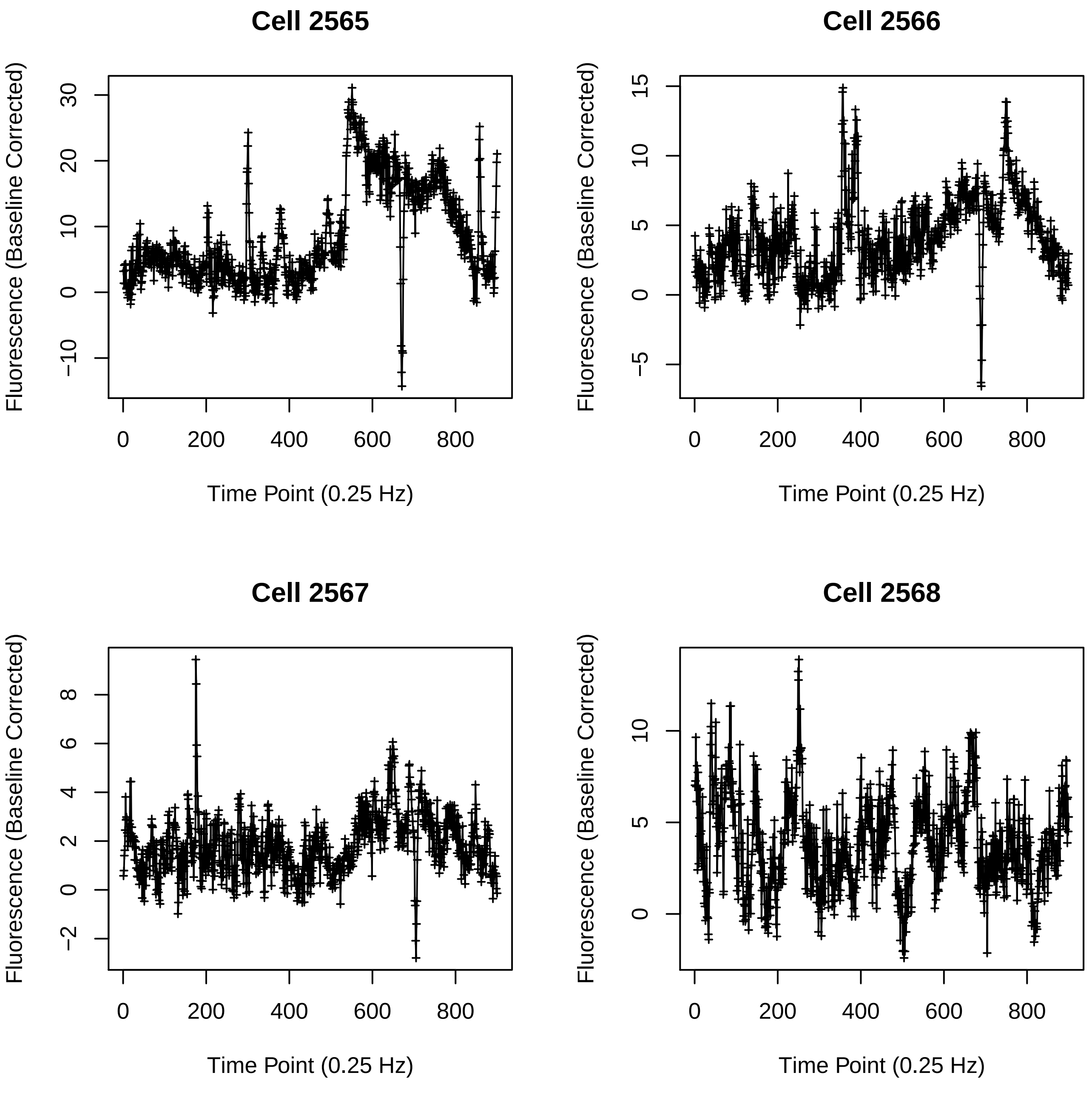
Cell 2565
Cell 2566
Cell 2567
Cell 2568
Fluorescence (Baseline Corrected)
Time Point (0.25 Hz)
−10
0
10
20
30
−5
0
5
10
15
−2
0
2
4
6
8
0
5
10
0
200
400
600
800

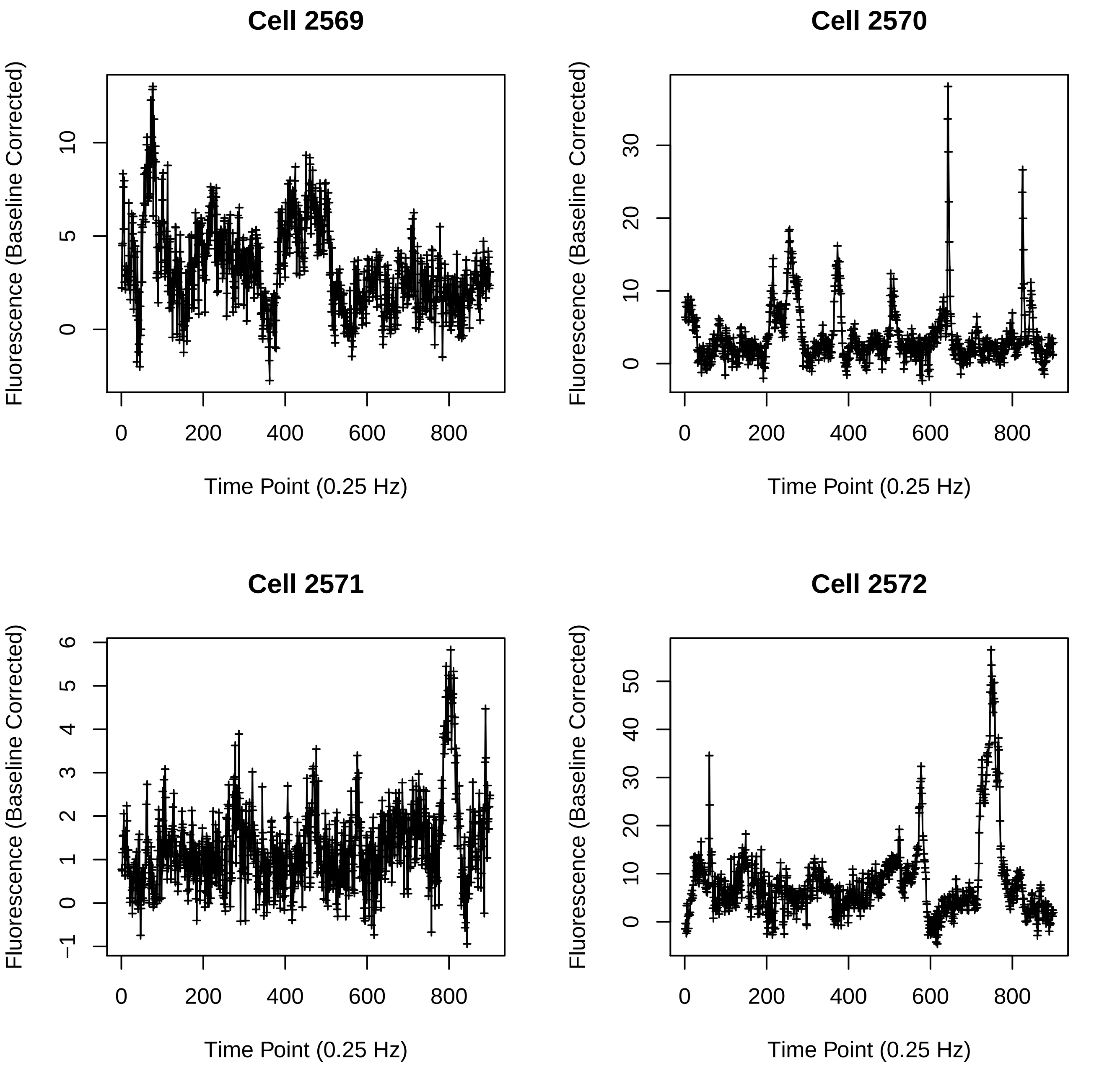
Cell 2569
Cell 2570
Cell 2571
Cell 2572
Fluorescence (Baseline Corrected)
Time Point (0.25 Hz)
0
5
10
0
10
20
30
−1
0
1
2
3
4
5
6
0
10
20
30
40
50
0
200
400
600
800

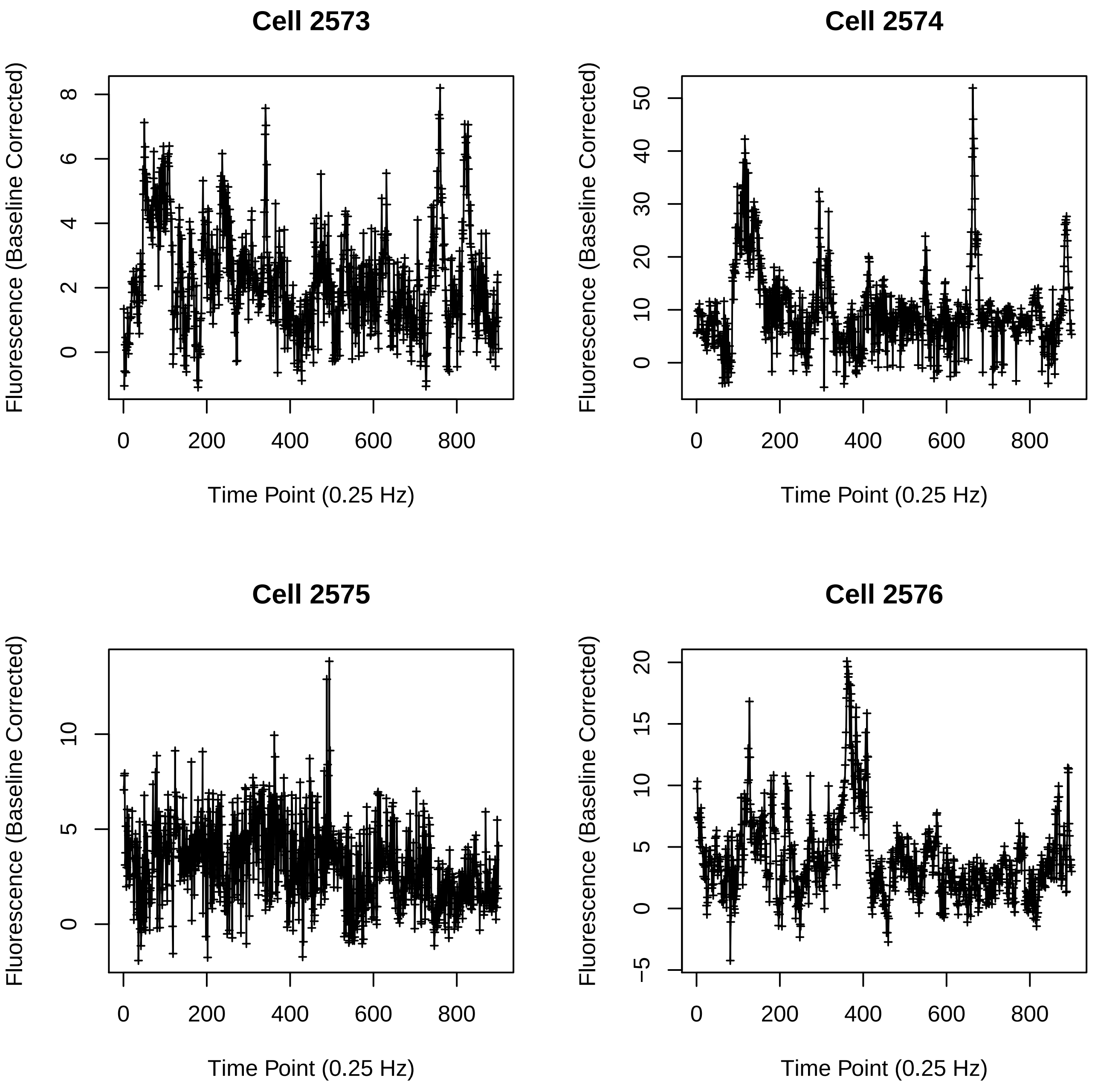
Cell 2573
Cell 2574
Cell 2575
Cell 2576
Fluorescence (Baseline Corrected)
Time Point (0.25 Hz)

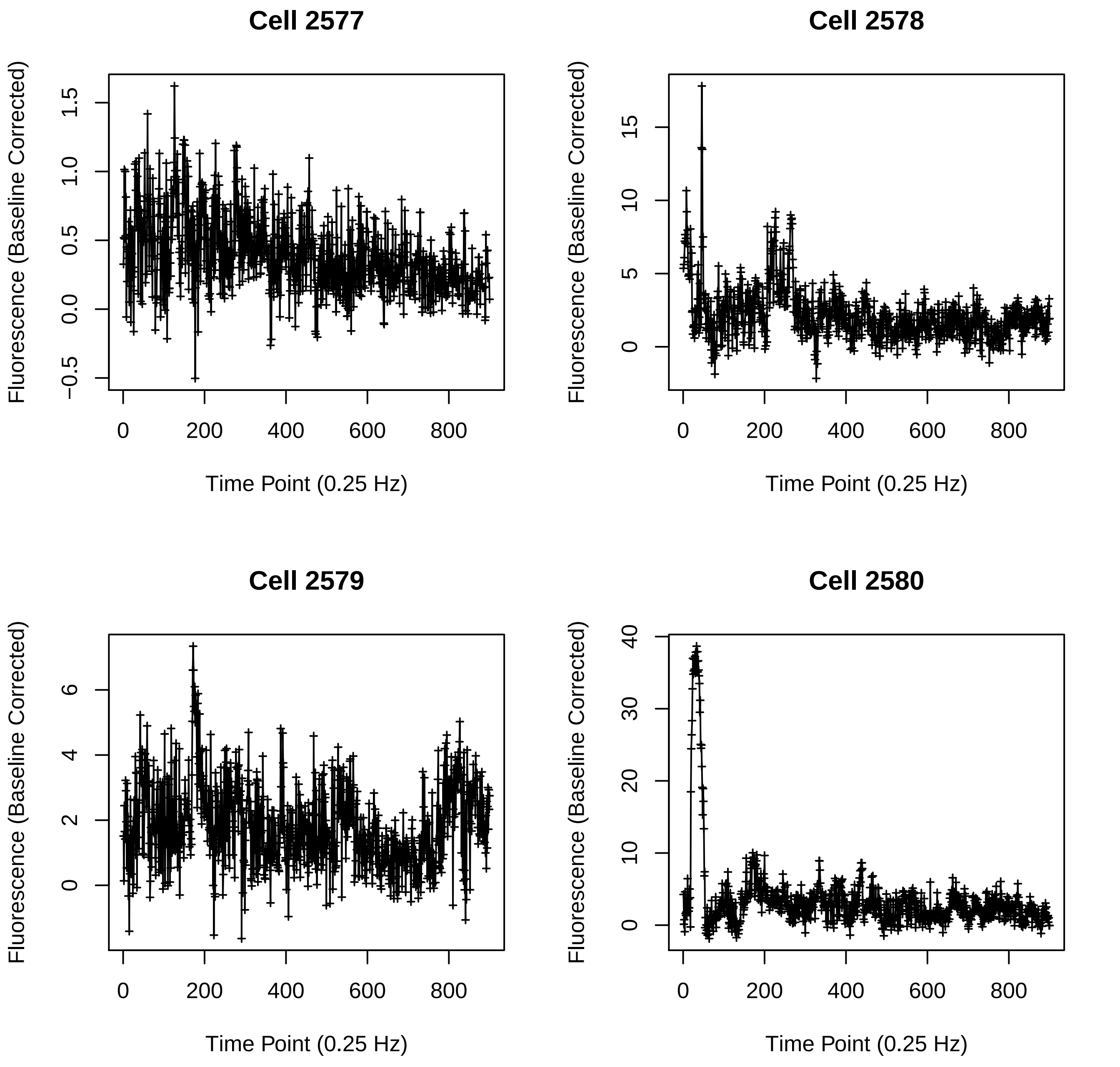
Cell 2577
Cell 2578
Cell 2579
Cell 2580
Fluorescence (Baseline Corrected)
Time Point (0.25 Hz)

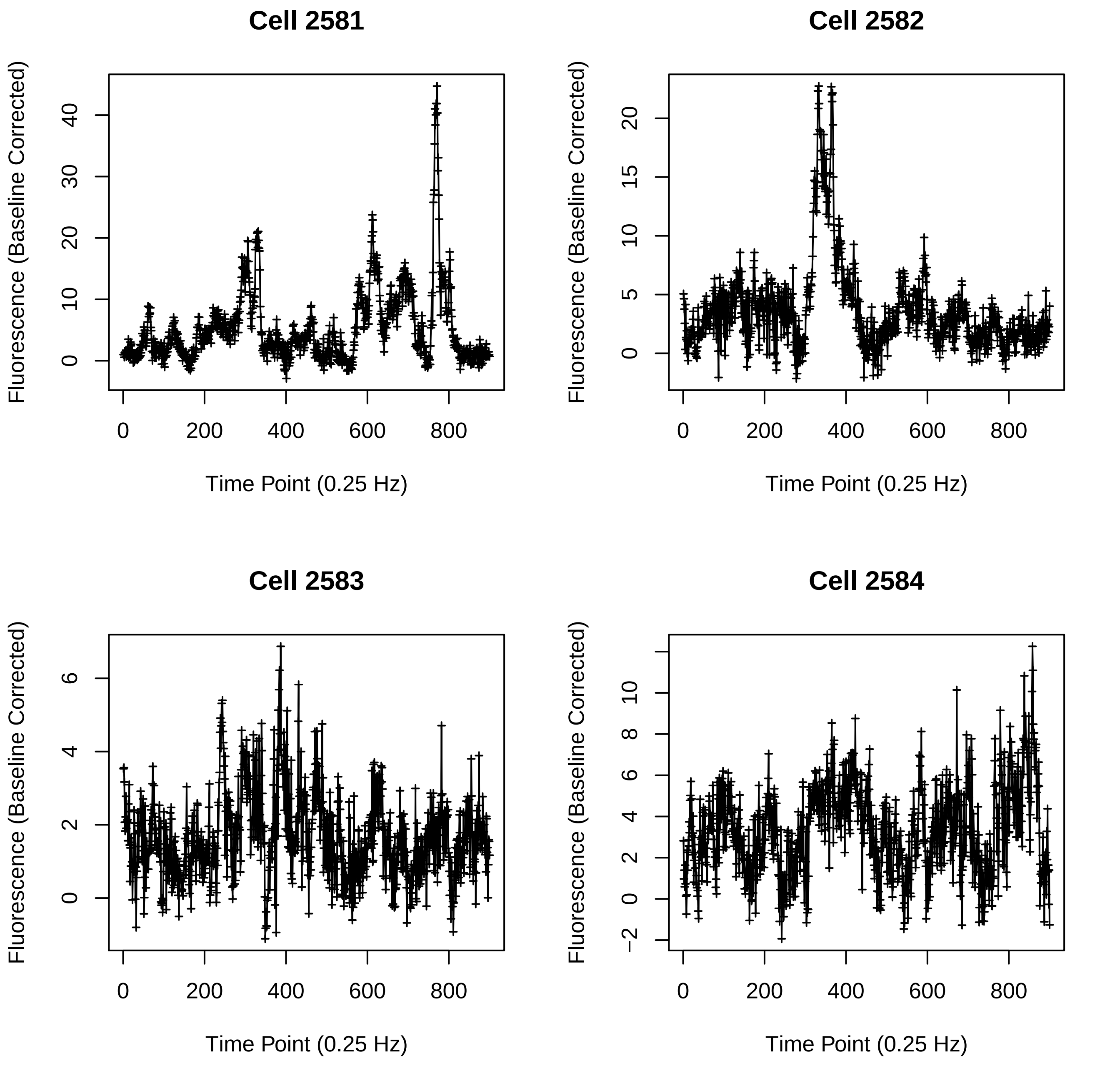

Cell 2581
Cell 2582
Cell 2583
Cell 2584
Fluorescence (Baseline Corrected)
Time Point (0.25 Hz)
0
200
400
600
800
0
10
20
30
40
5
15
2
4
6
8
−2

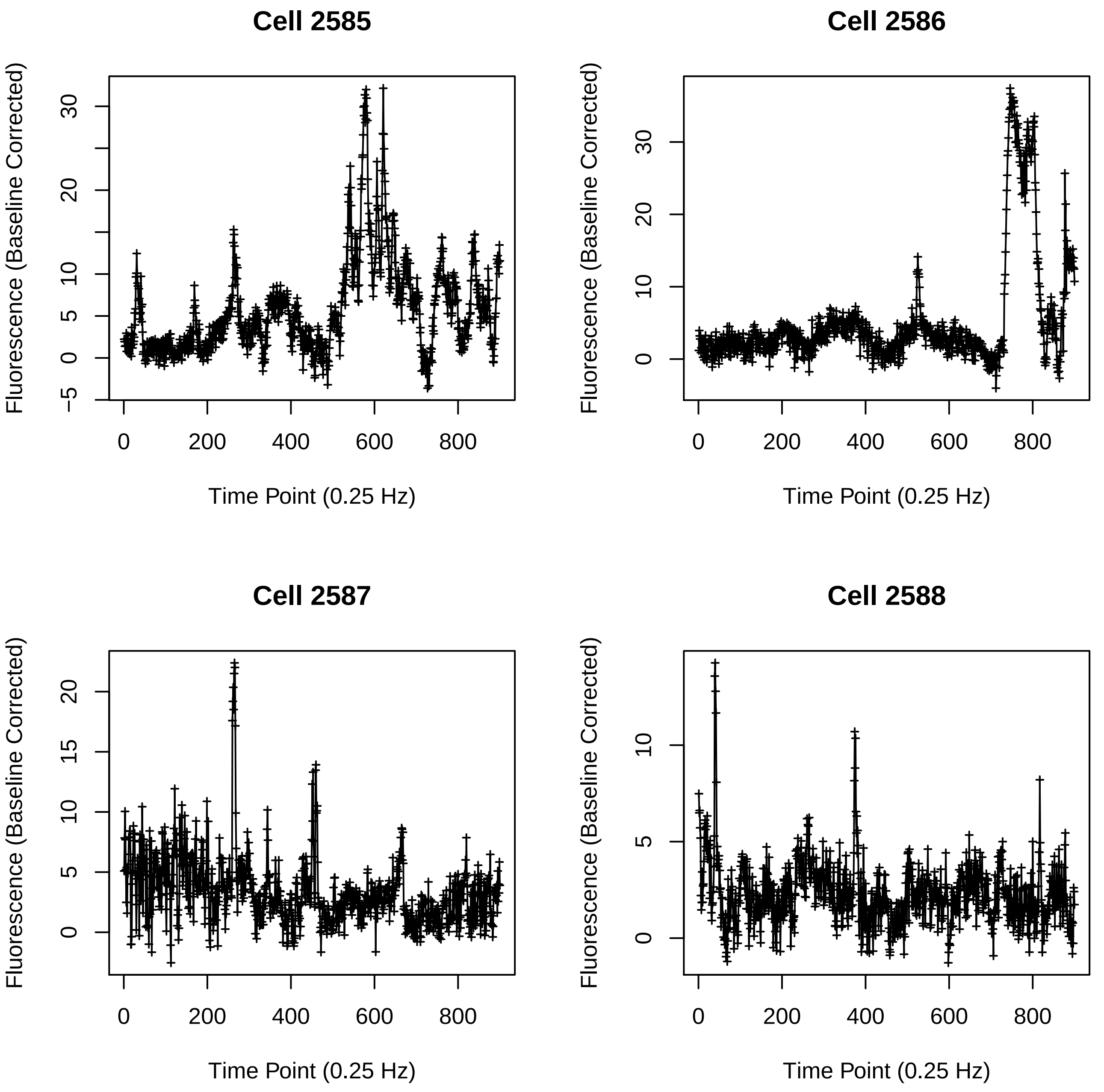
Cell 2585
Cell 2586
Cell 2587
Cell 2588
Fluorescence (Baseline Corrected)
Time Point (0.25 Hz)
0
200
400
600
800
−5
5
10
15
20
30

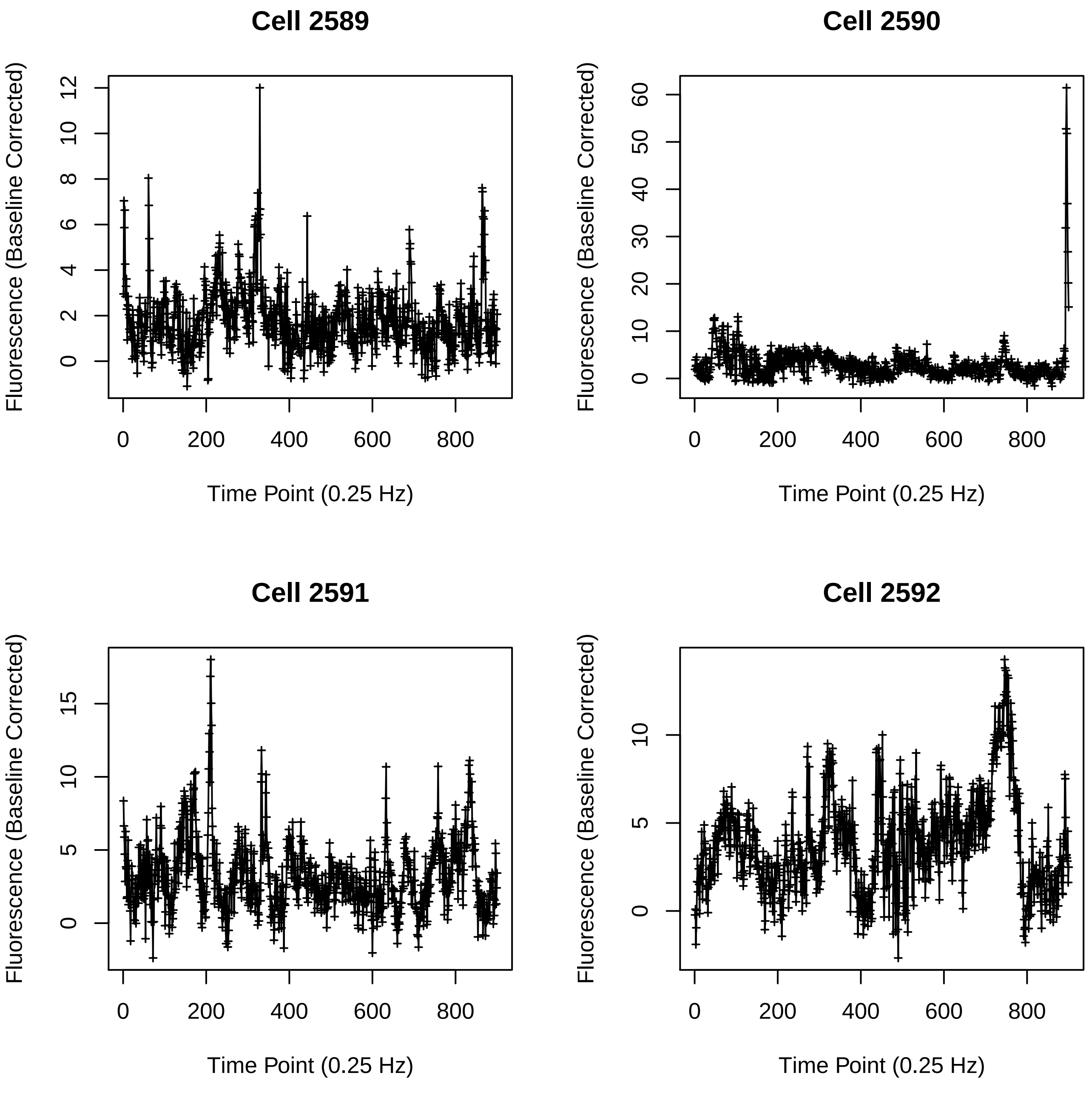
Cell 2589
Cell 2590
Cell 2591
Cell 2592
Fluorescence (Baseline Corrected)
Time Point (0.25 Hz)
0
200
400
600
800
0
2
4
6
8
10
12
10
20
30
40
50
60
5
15

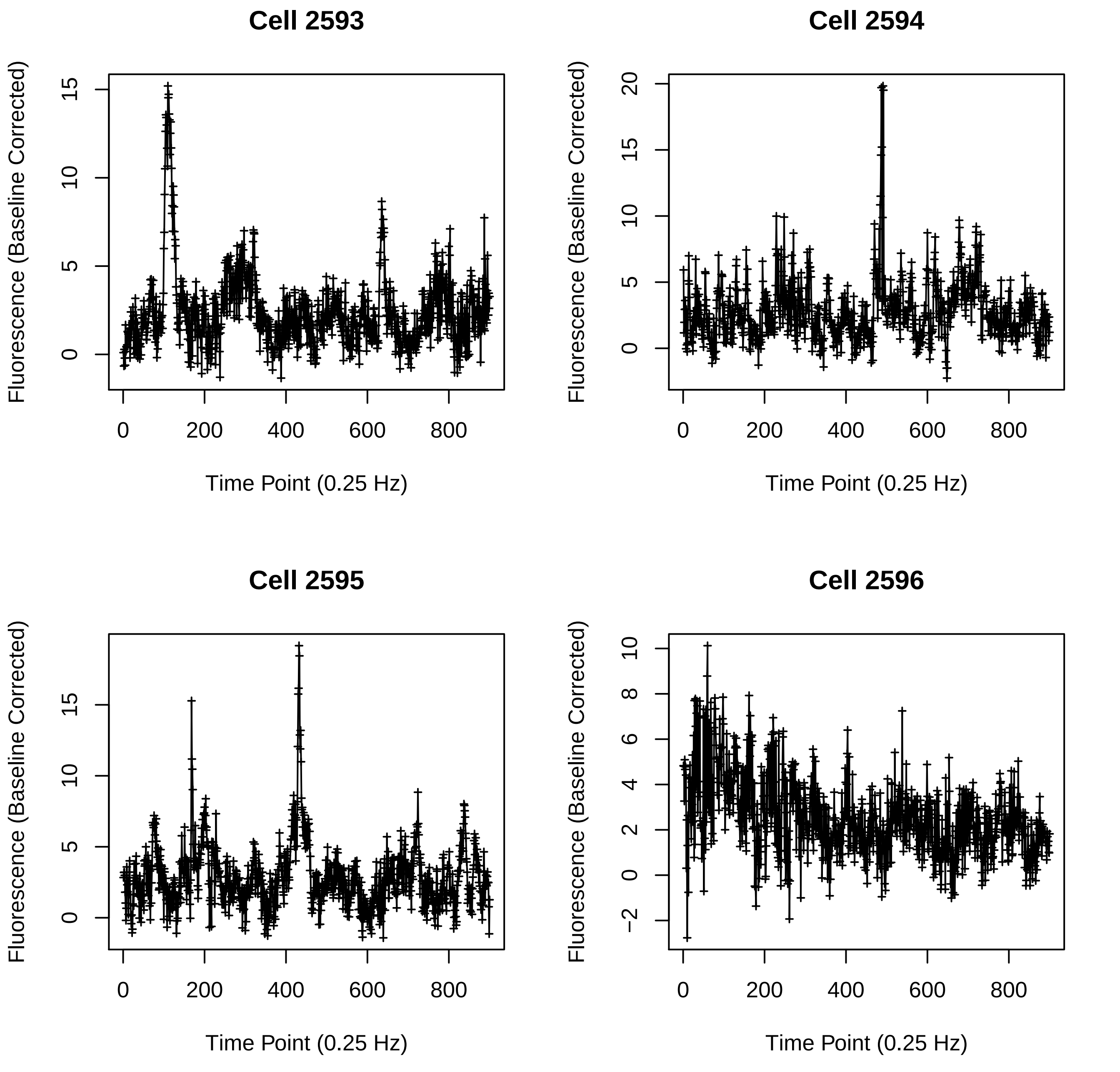
Cell 2593
Cell 2594
Cell 2595
Cell 2596
Fluorescence (Baseline Corrected)
Time Point (0.25 Hz)
0
200
400
600
800
5
10
15
20
-2
2
4
6
8

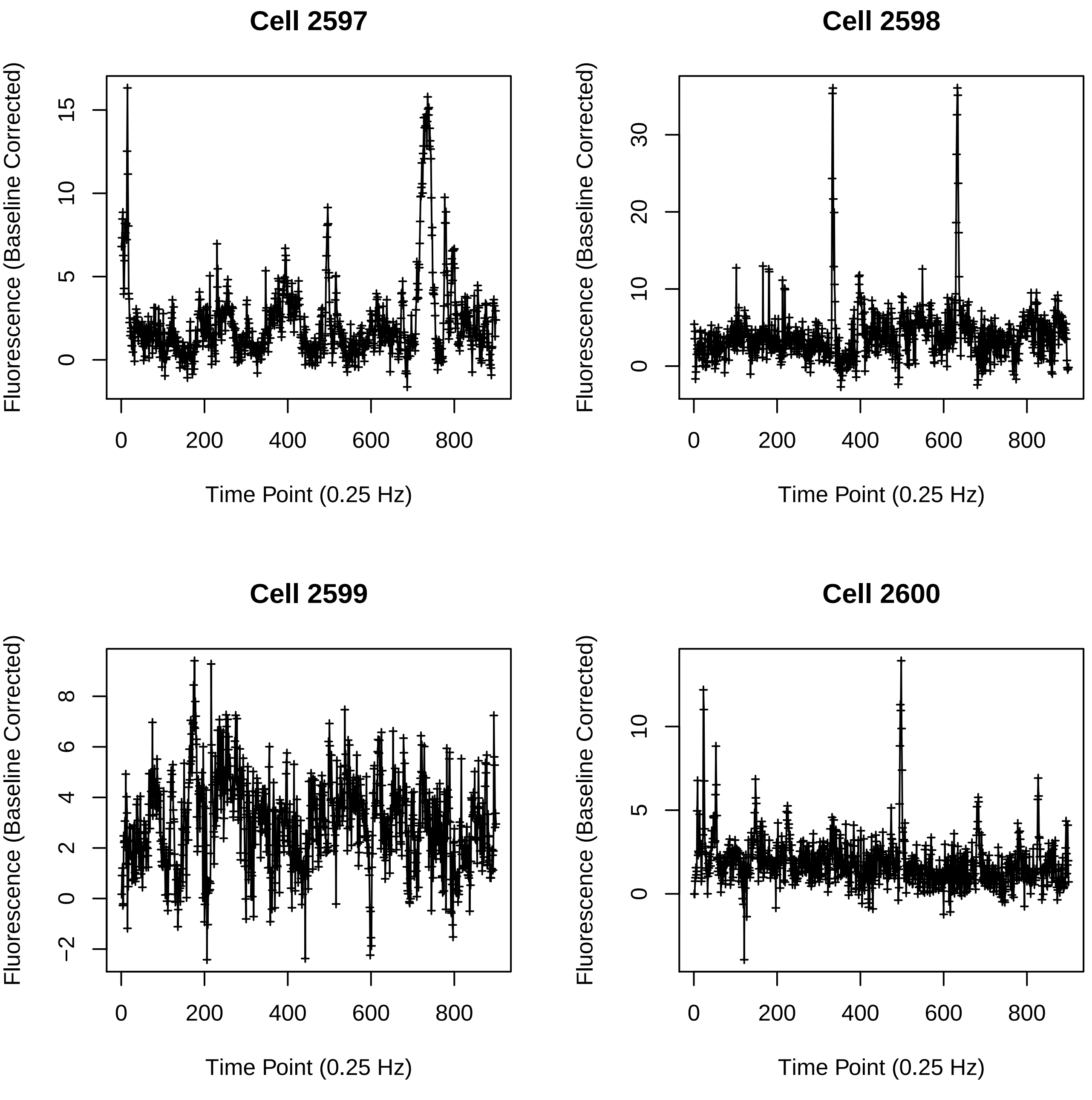
Cell 2597
Cell 2598
Cell 2599
Cell 2600
Fluorescence (Baseline Corrected)
Time Point (0.25 Hz)
0
200
400
600
800
0
5
10
15
20
30
−2
2
4
6
8

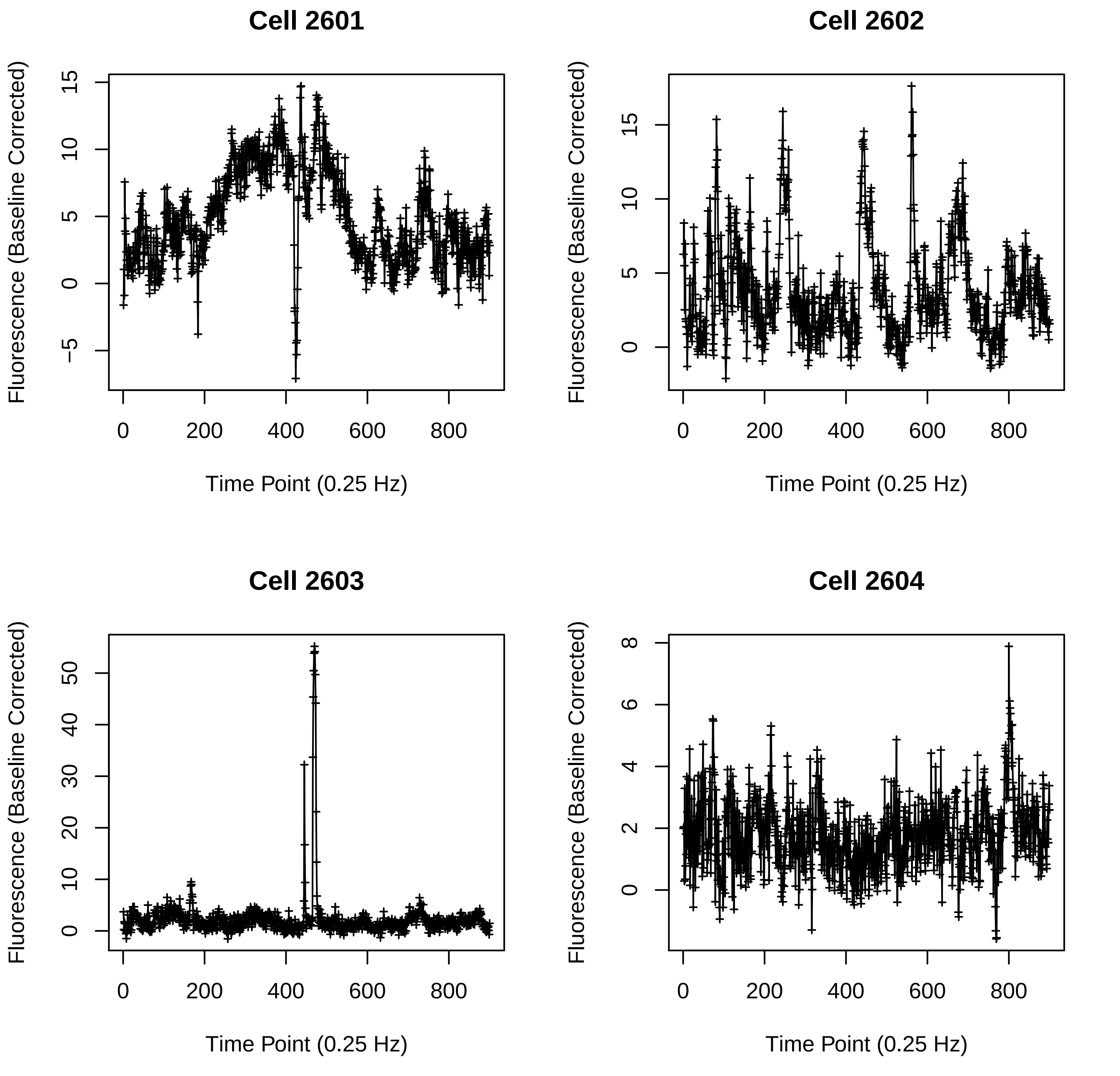
Cell 2601
Cell 2602
Cell 2603
Cell 2604
Fluorescence (Baseline Corrected)
Time Point (0.25 Hz)
0
200
400
600
800
−5
0
5
10
15
20
30
40
50
2
4
6
8

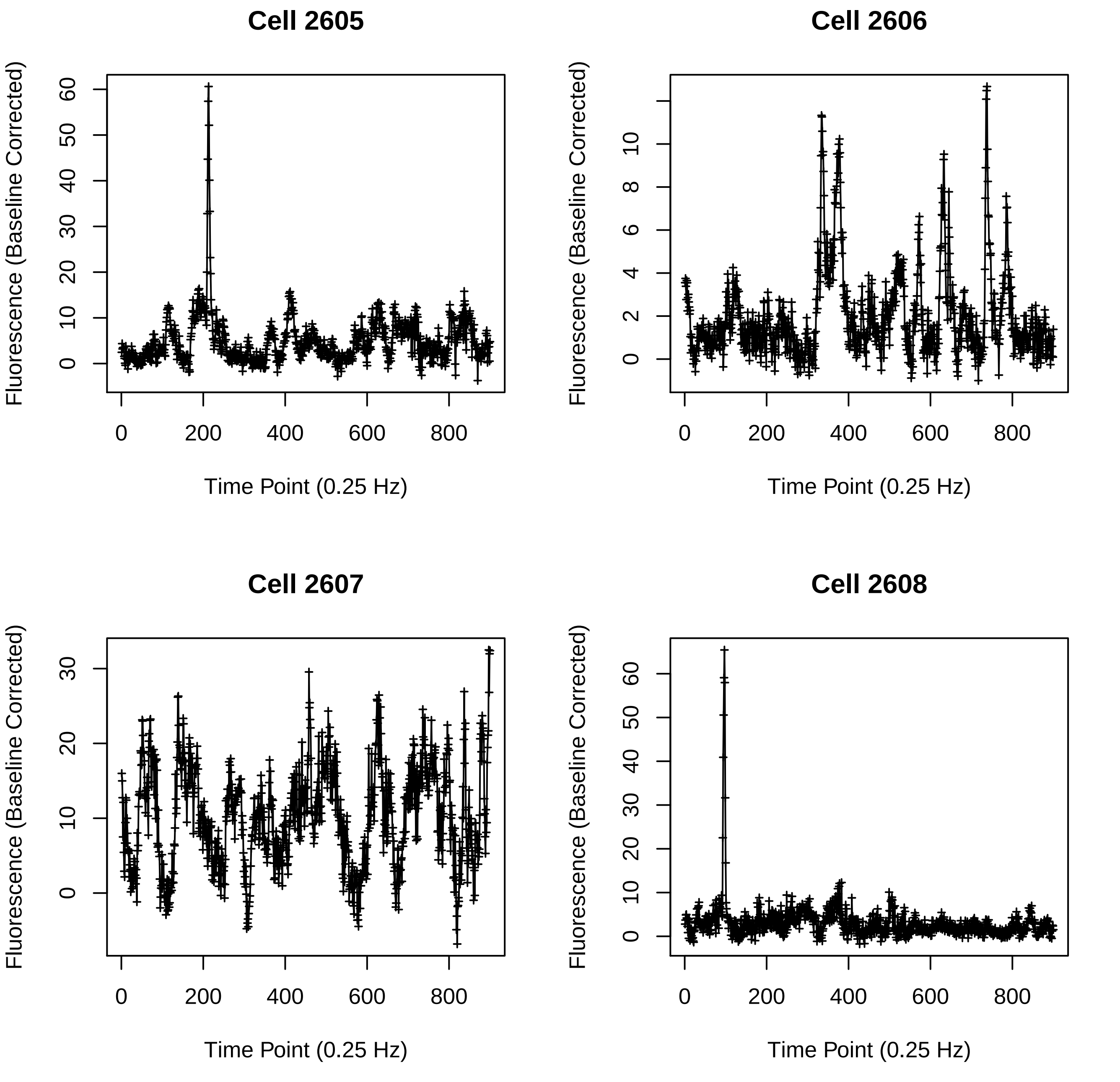
Cell 2605
Fluorescence (Baseline Corrected)
0
10
20
30
40
50
60
0
200
400
600
800
Time Point (0.25 Hz)
Cell 2606
Fluorescence (Baseline Corrected)
0
2
4
6
8
10
0
200
400
600
800
Time Point (0.25 Hz)
Cell 2607
Fluorescence (Baseline Corrected)
0
10
20
30
0
200
400
600
800
Time Point (0.25 Hz)
Cell 2608
Fluorescence (Baseline Corrected)
0
10
20
30
40
50
60
0
200
400
600
800
Time Point (0.25 Hz)

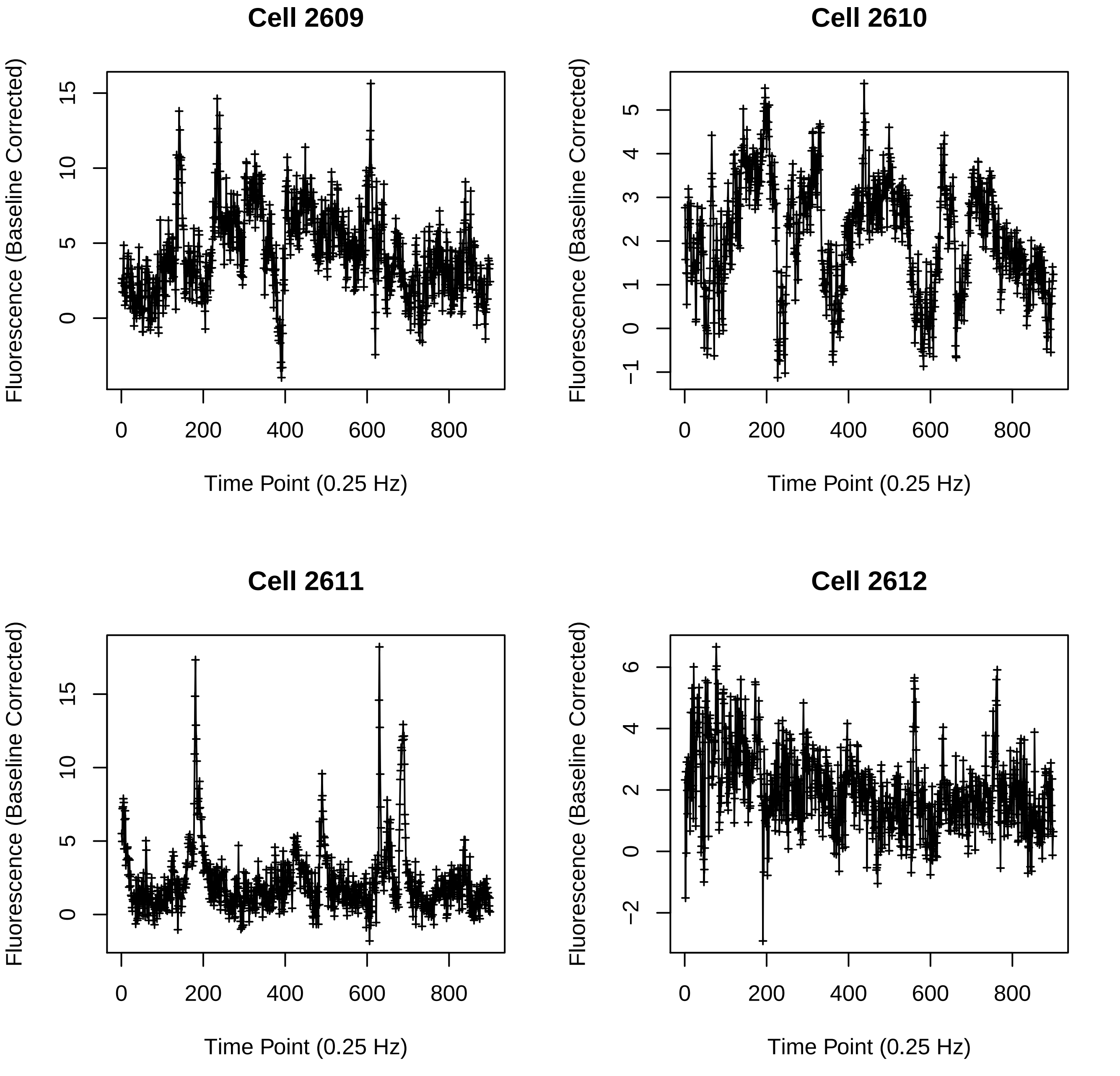
Cell 2609
Cell 2610
Cell 2611
Cell 2612
Fluorescence (Baseline Corrected)
Time Point (0.25 Hz)
0
200
400
600
800
−1
1
2
3
4
5
−2
6
10
15

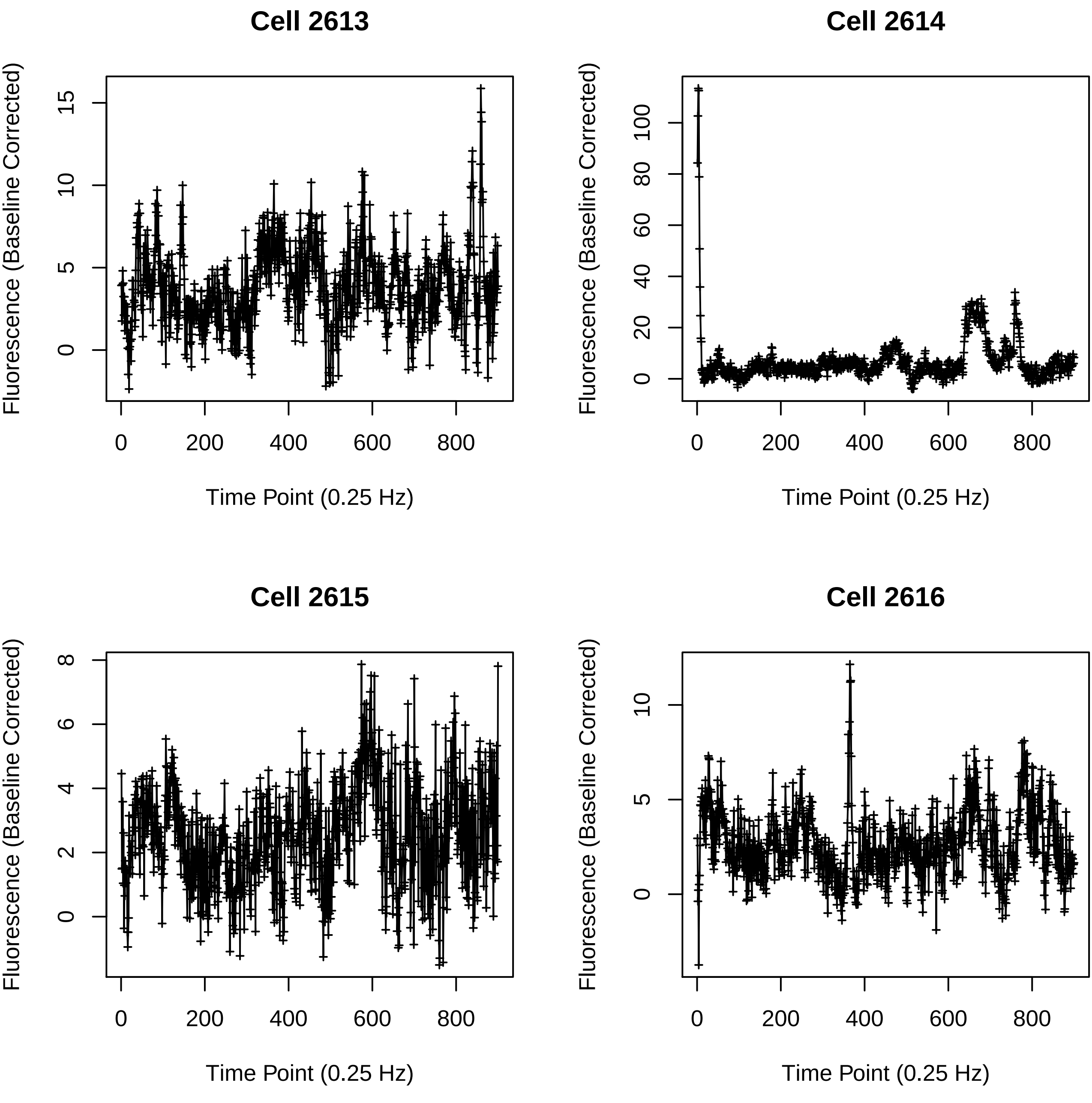
Cell 2613
Cell 2614
Cell 2615
Cell 2616
Fluorescence (Baseline Corrected)
Time Point (0.25 Hz)
0
200
400
600
800

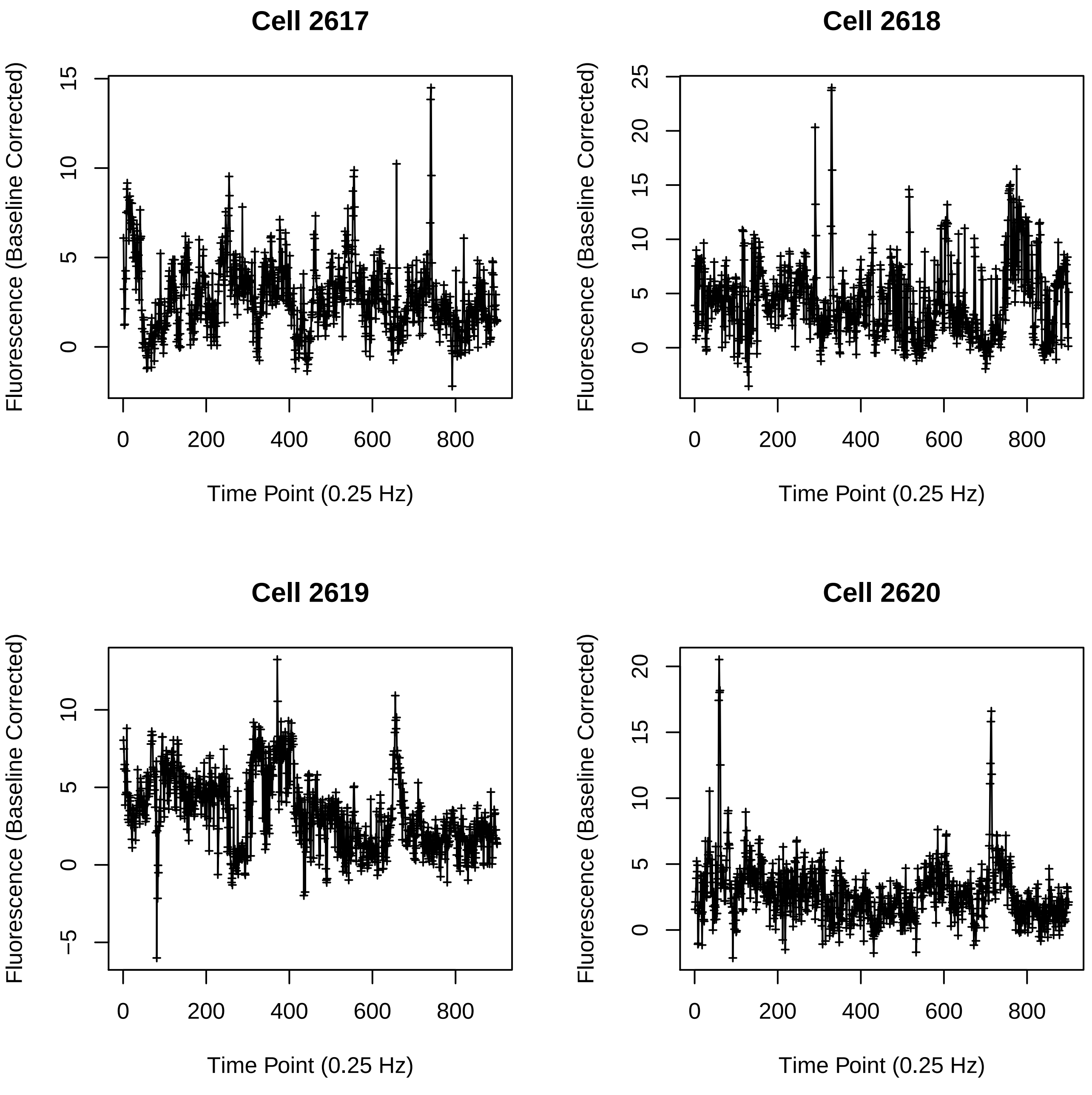
Cell 2617
Cell 2618
Cell 2619
Cell 2620
Fluorescence (Baseline Corrected)
Time Point (0.25 Hz)
0
200
400
600
800
−5
5
10
15
20
25

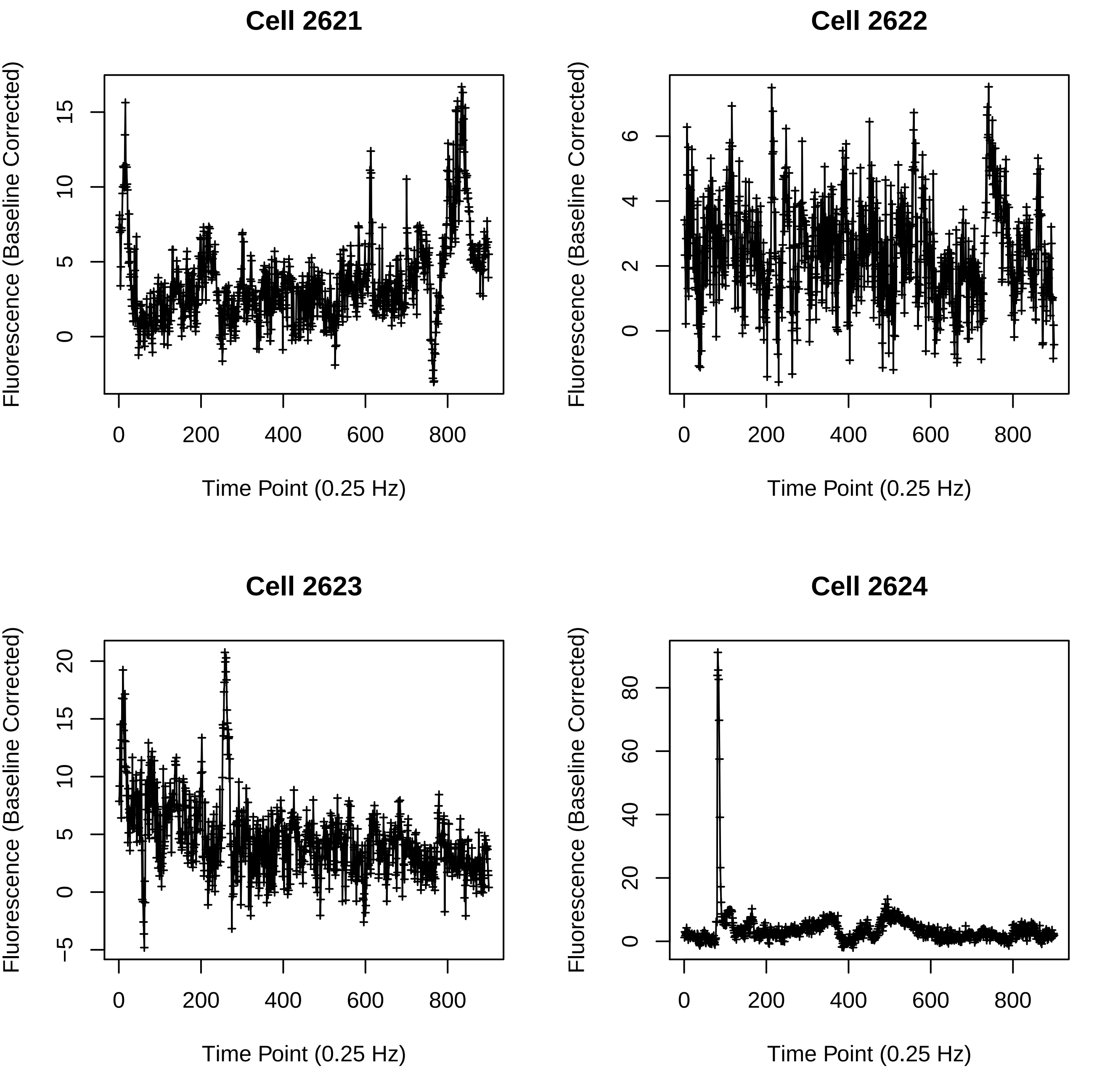
Cell 2621
Cell 2622
Cell 2623
Cell 2624
Fluorescence (Baseline Corrected)
Time Point (0.25 Hz)
0
200
400
600
800
-5
5
10
15
20
2
4
6
40
60
80

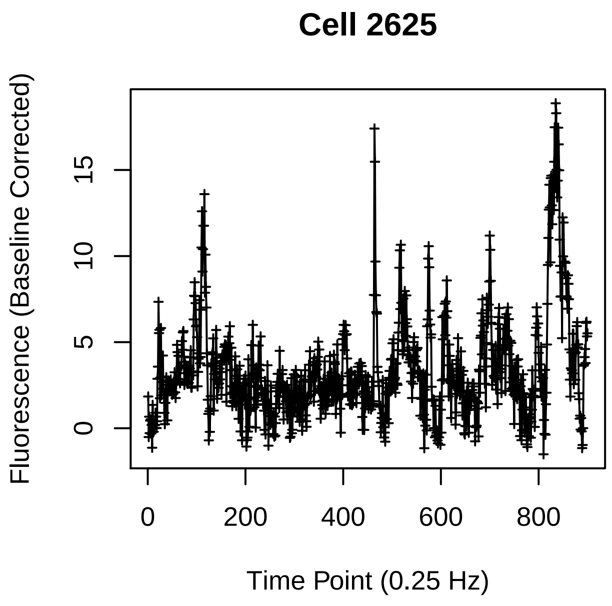
Cell 2625
Fluorescence (Baseline Corrected)
0
5
10
15
0
200
400
600
800
Time Point (0.25 Hz)

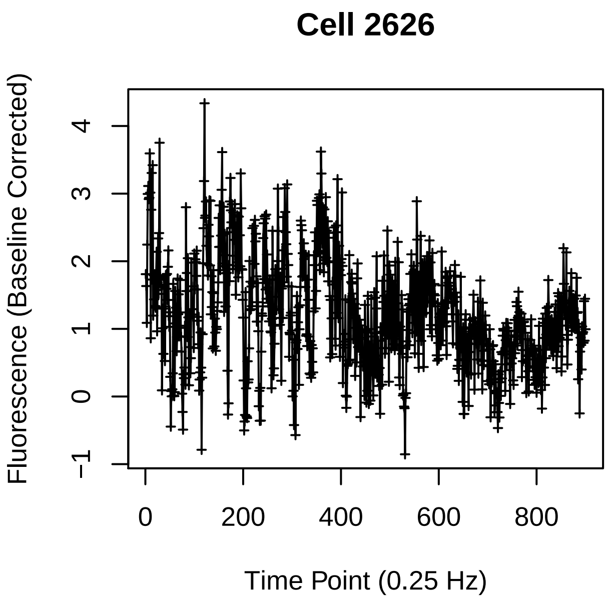
Cell 2626
Fluorescence (Baseline Corrected)
−1
0
1
2
3
4
0
200
400
600
800
Time Point (0.25 Hz)

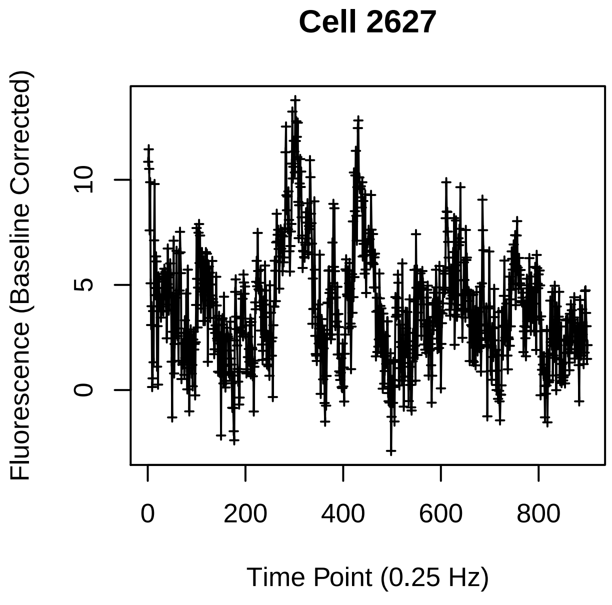
Cell 2627
Fluorescence (Baseline Corrected)
0
5
10
0
200
400
600
800
Time Point (0.25 Hz)

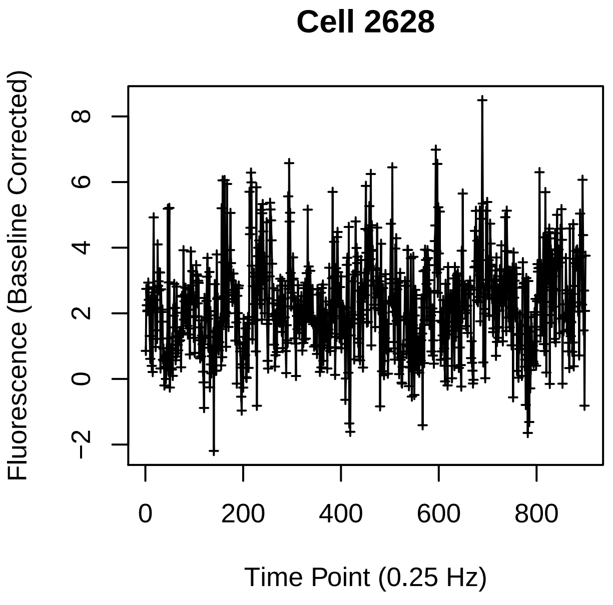
Cell 2628
Fluorescence (Baseline Corrected)
−2
0
2
4
6
8
0
200
400
600
800
Time Point (0.25 Hz)

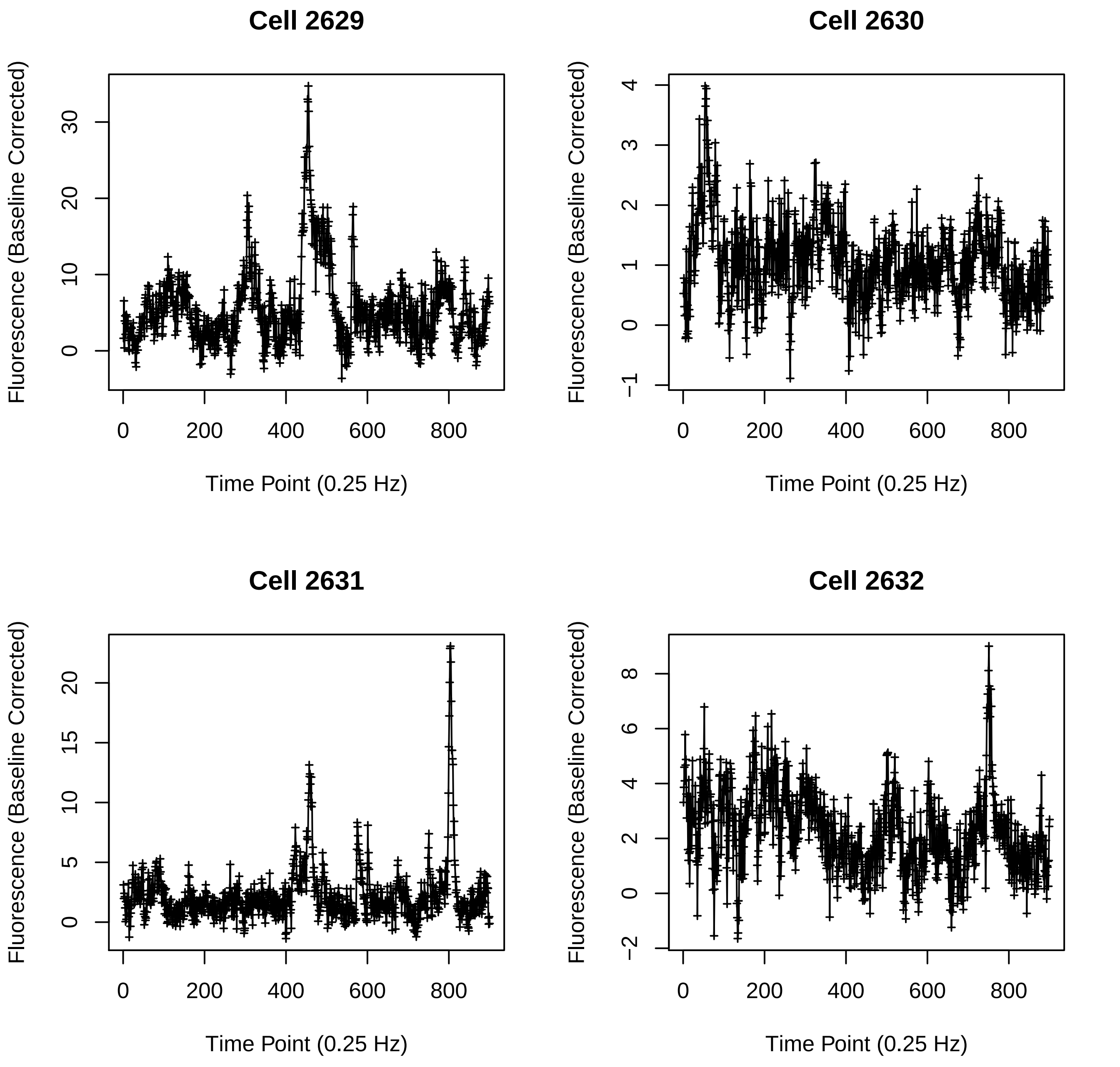
Cell 2629
Cell 2630
Cell 2631
Cell 2632
Fluorescence (Baseline Corrected)
Time Point (0.25 Hz)
0
200
400
600
800
-5
0
10
20
30
-1
1
2
3
4
5
15
-2
6
8

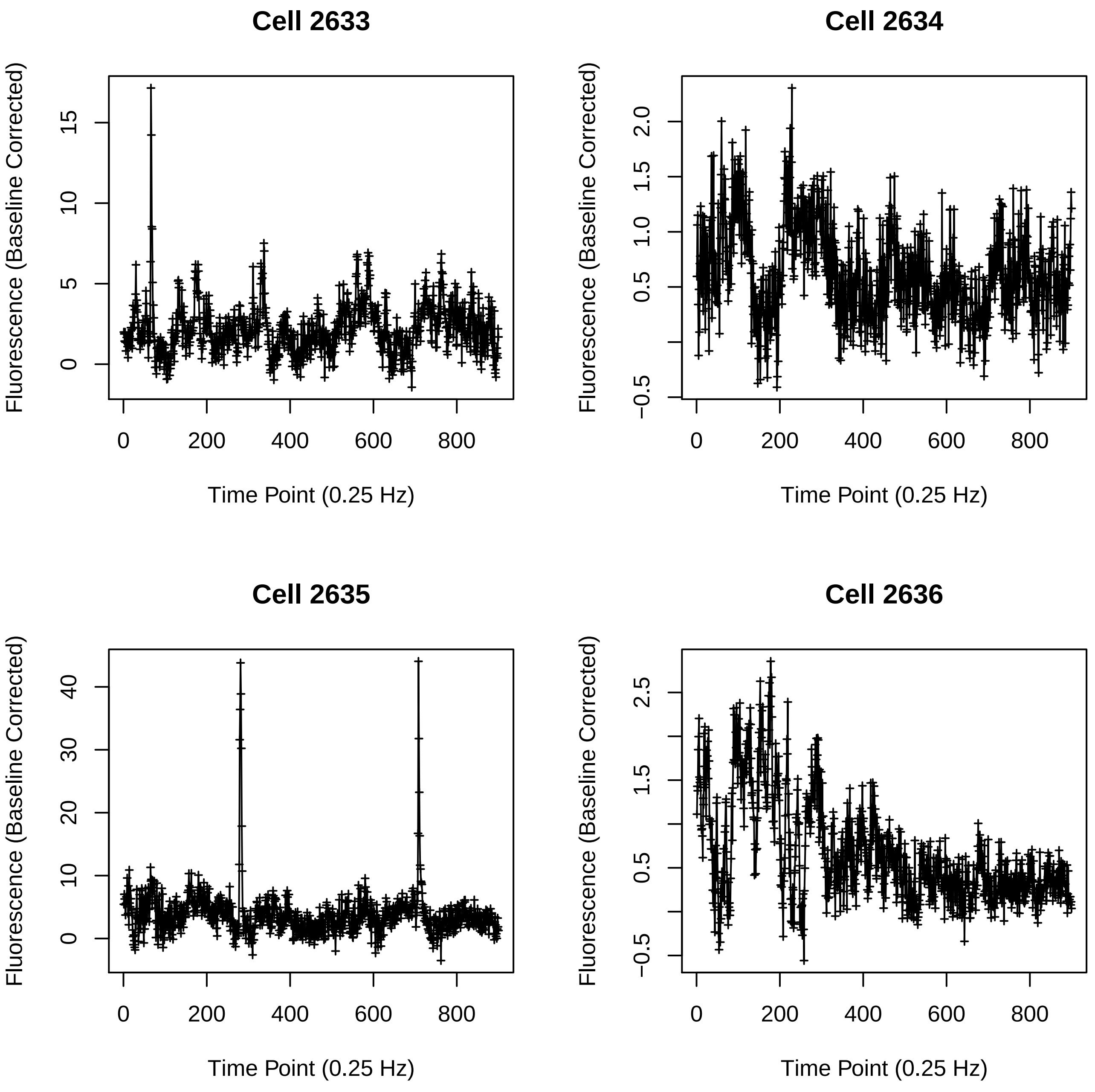
Cell 2633
Fluorescence (Baseline Corrected)
0
5
10
15
0
200
400
600
800
Time Point (0.25 Hz)
Cell 2634
Fluorescence (Baseline Corrected)
−0.5
0.5
1.0
1.5
2.0
0
200
400
600
800
Time Point (0.25 Hz)
Cell 2635
Fluorescence (Baseline Corrected)
0
10
20
30
40
0
200
400
600
800
Time Point (0.25 Hz)
Cell 2636
Fluorescence (Baseline Corrected)
−0.5
0.5
1.5
2.5
0
200
400
600
800
Time Point (0.25 Hz)

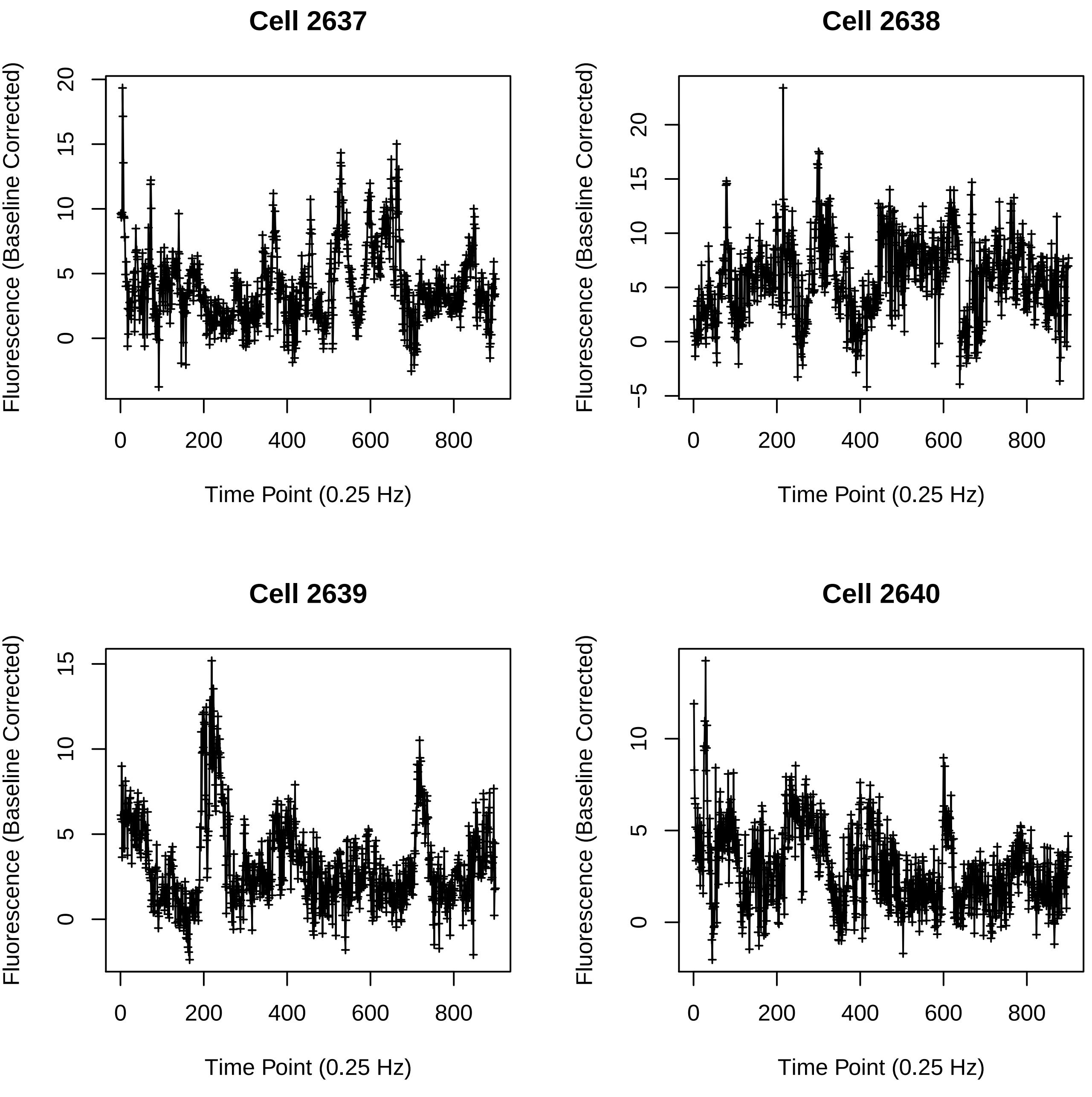

Cell 2637
Cell 2638
Cell 2639
Cell 2640
Fluorescence (Baseline Corrected)
Time Point (0.25 Hz)
0
200
400
600
800
-5
0
5
10
15
20

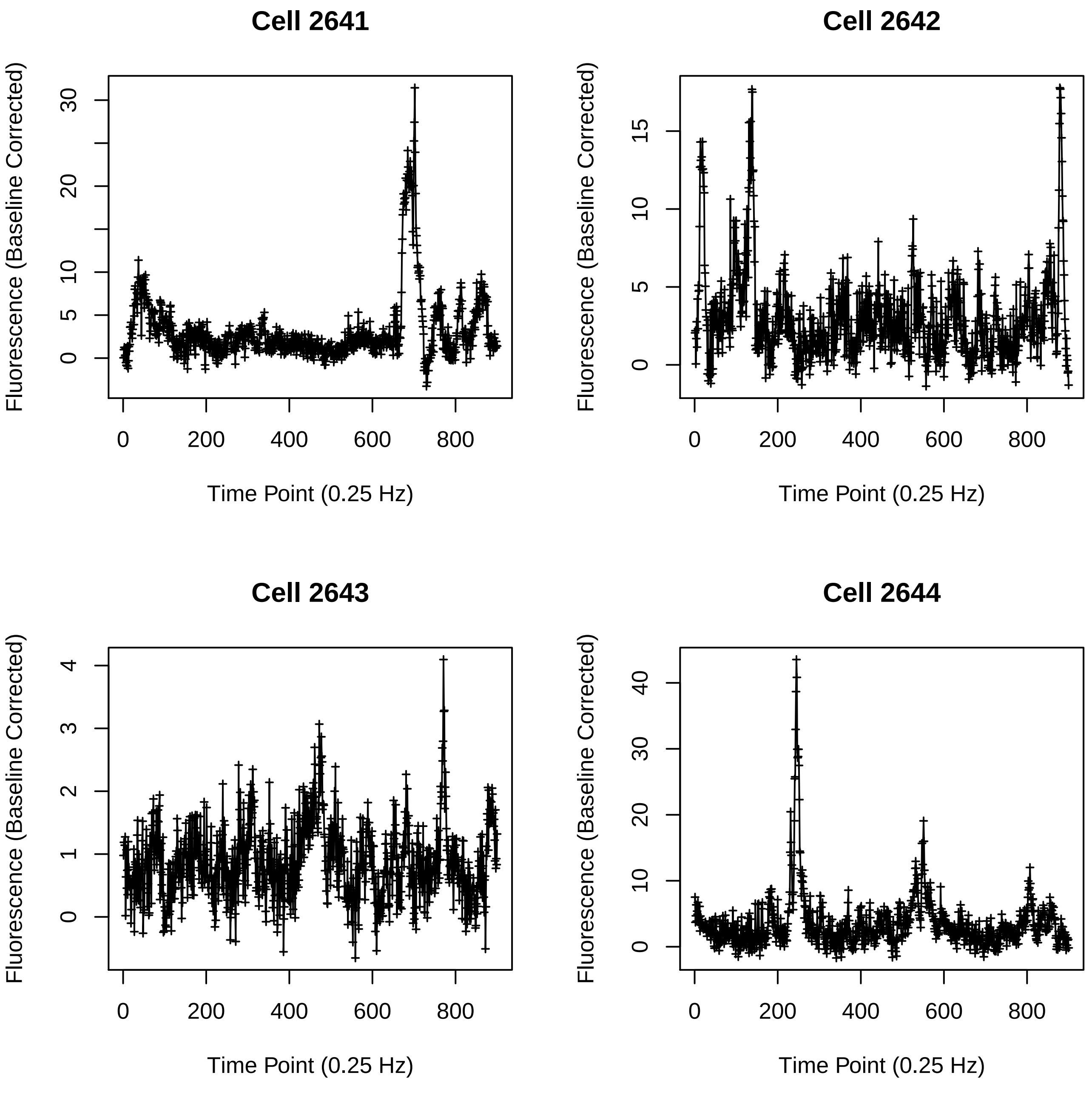
Cell 2641
Cell 2642
Cell 2643
Cell 2644
Fluorescence (Baseline Corrected)
Time Point (0.25 Hz)
0
200
400
600
800
0
5
10
15
20
30
40
1
2
3
4

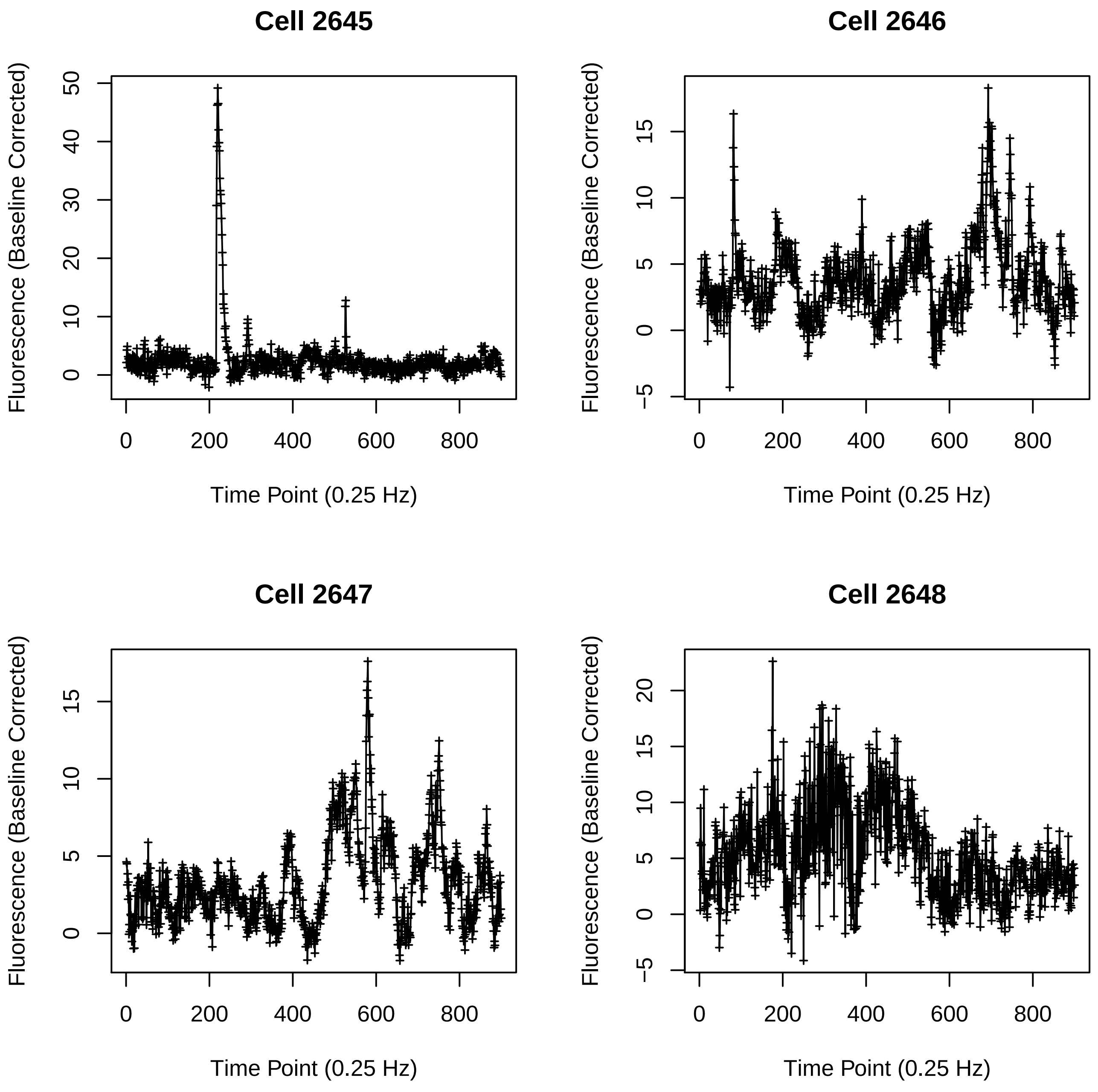
Cell 2645
Cell 2646
Cell 2647
Cell 2648
Fluorescence (Baseline Corrected)
Time Point (0.25 Hz)
0
10
20
30
40
50
5
15
-5
200
400
600
800

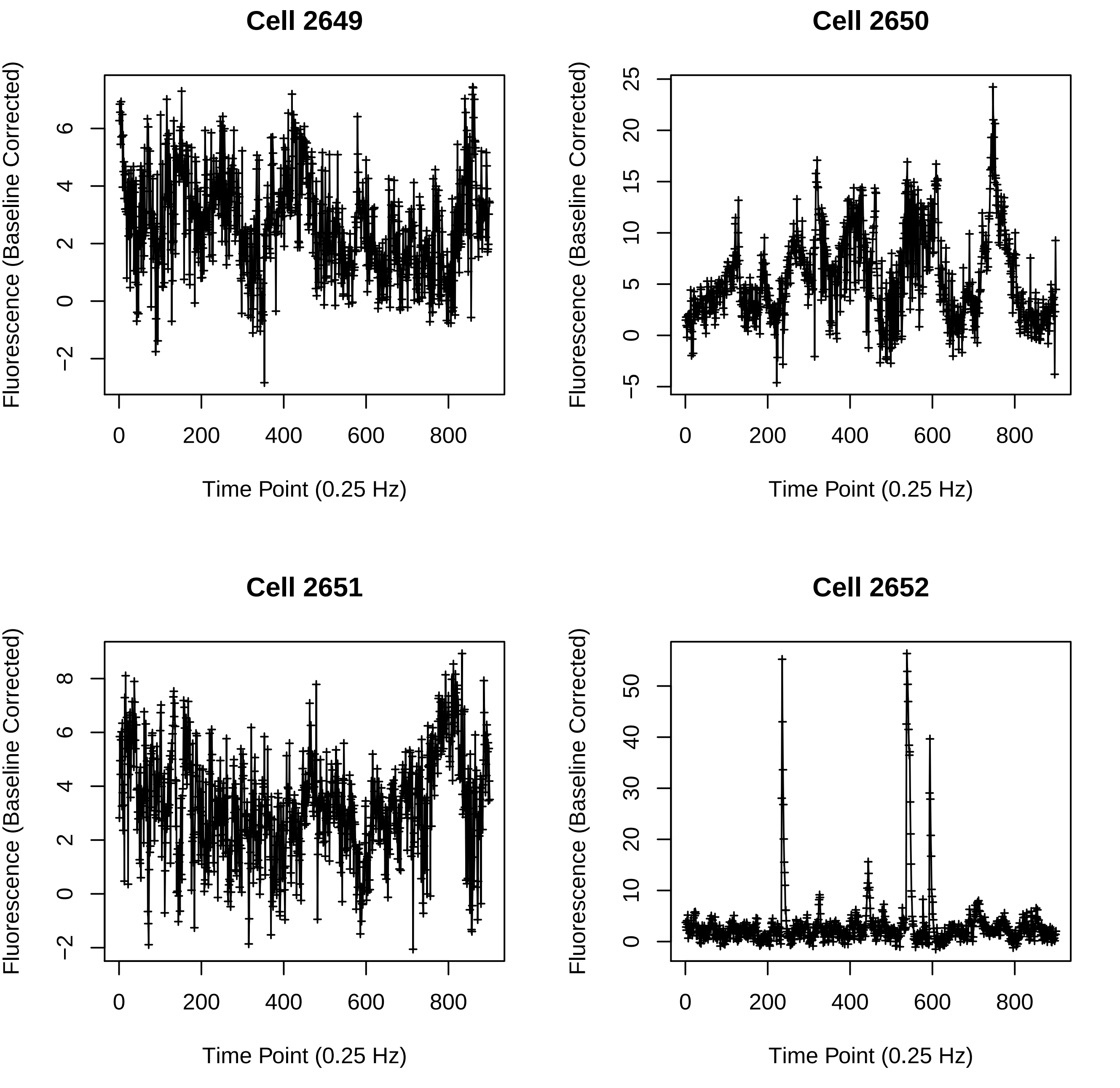

Cell 2649
Cell 2650
Cell 2651
Cell 2652
Fluorescence (Baseline Corrected)
Time Point (0.25 Hz)

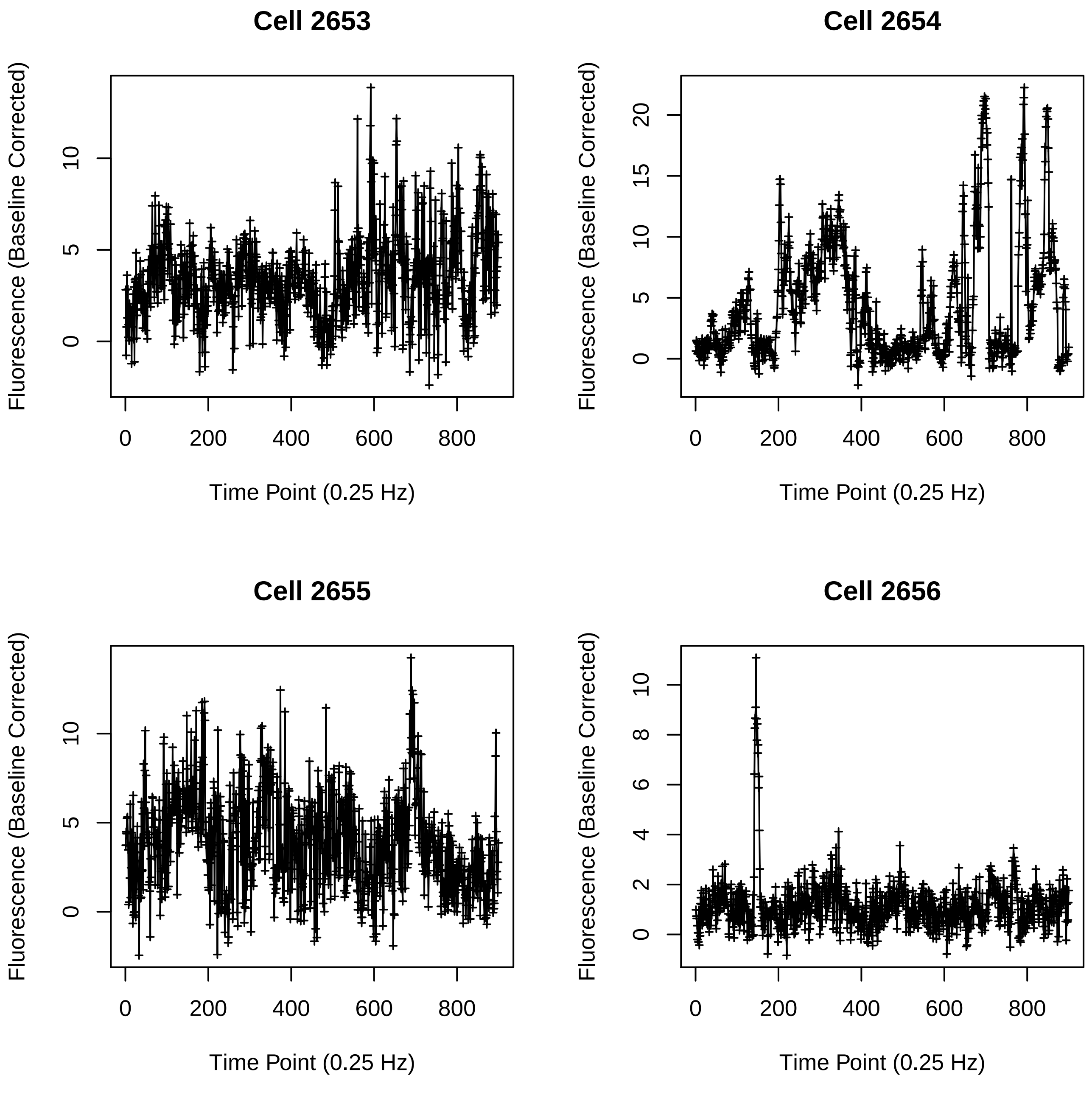
Cell 2653
Cell 2654
Cell 2655
Cell 2656
Fluorescence (Baseline Corrected)
Time Point (0.25 Hz)
0
5
10
15
20
200
400
600
800
2
4
6
8

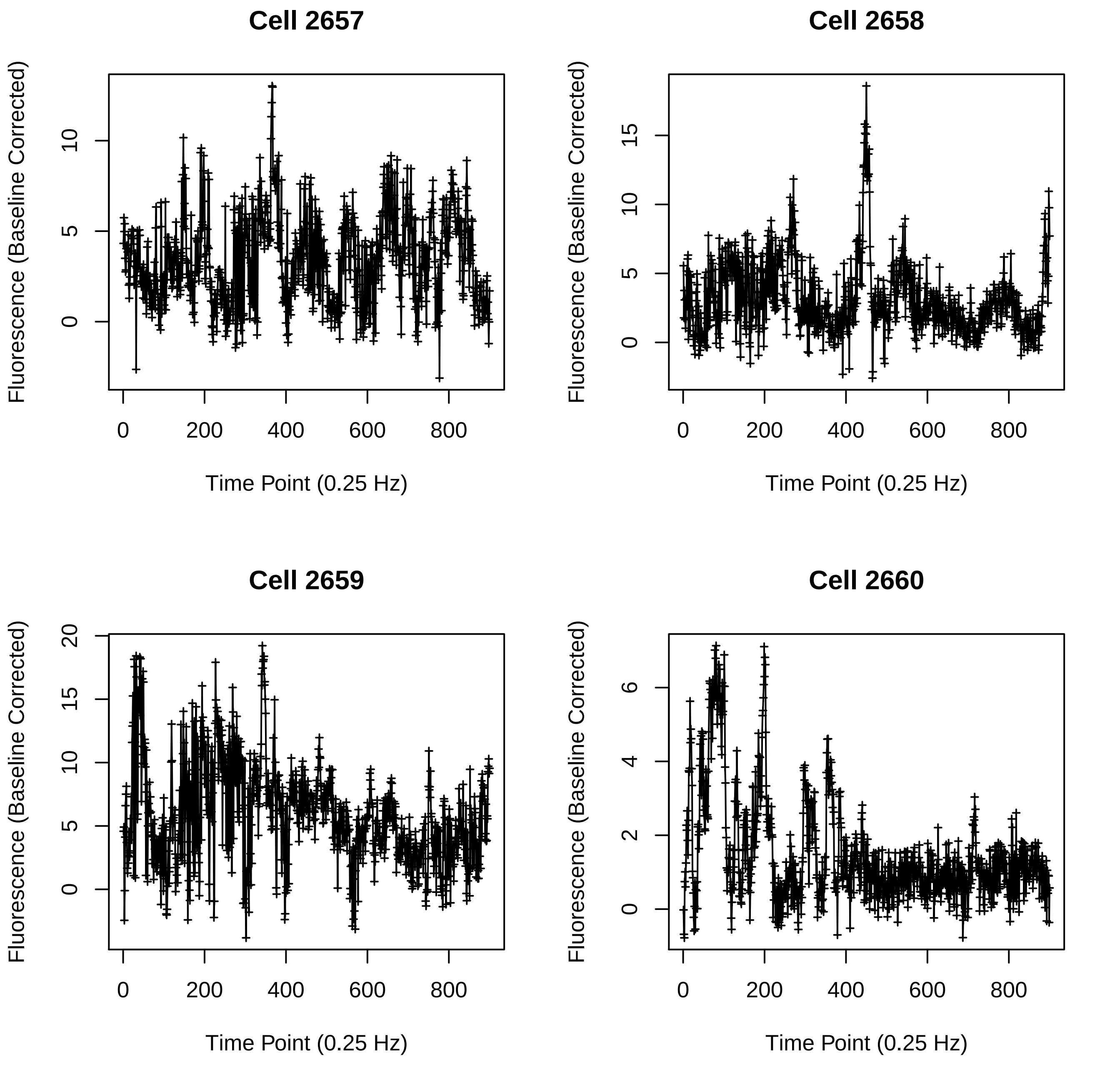
Cell 2657
Cell 2658
Cell 2659
Cell 2660
Fluorescence (Baseline Corrected)
Time Point (0.25 Hz)
0
200
400
600
800
-5
0
5
10
15
20
2
4
6

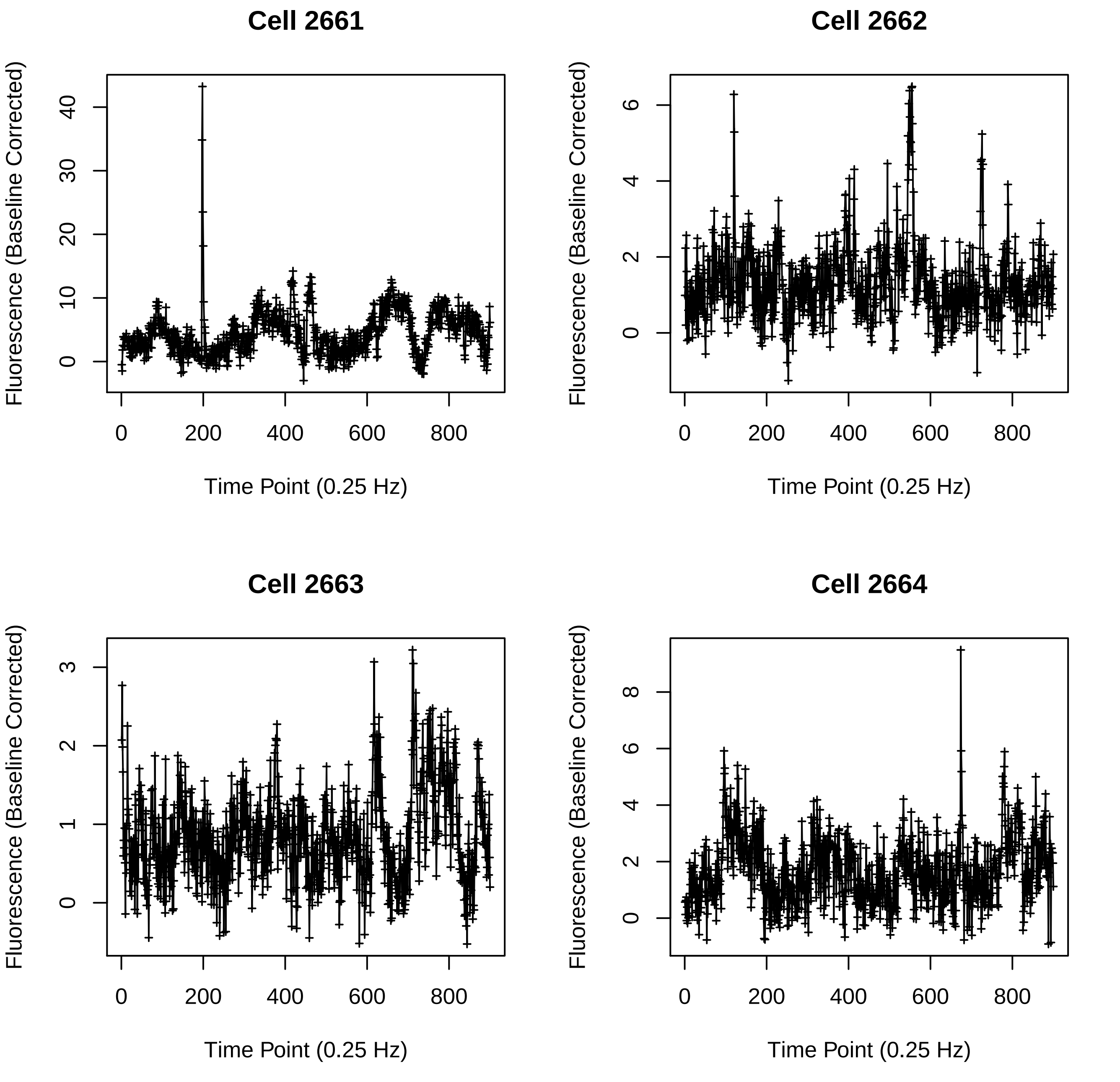
Cell 2661
Fluorescence (Baseline Corrected)
Time Point (0.25 Hz)
Cell 2662
Fluorescence (Baseline Corrected)
Time Point (0.25 Hz)
Cell 2663
Fluorescence (Baseline Corrected)
Time Point (0.25 Hz)
Cell 2664
Fluorescence (Baseline Corrected)
Time Point (0.25 Hz)
