# Supplementary material for: A Markovian Entropy Measure for the Analysis of Calcium Activity Time Series
Source: PLoS One. 2016 Dec 15;11(12):e0168342. doi: 10.1371/journal.pone.0168342 (PMC5158058; doi:10.1371/journal.pone.0168342)
Supplement: S4 File — (PDF) [file pone.0168342.s011.pdf]

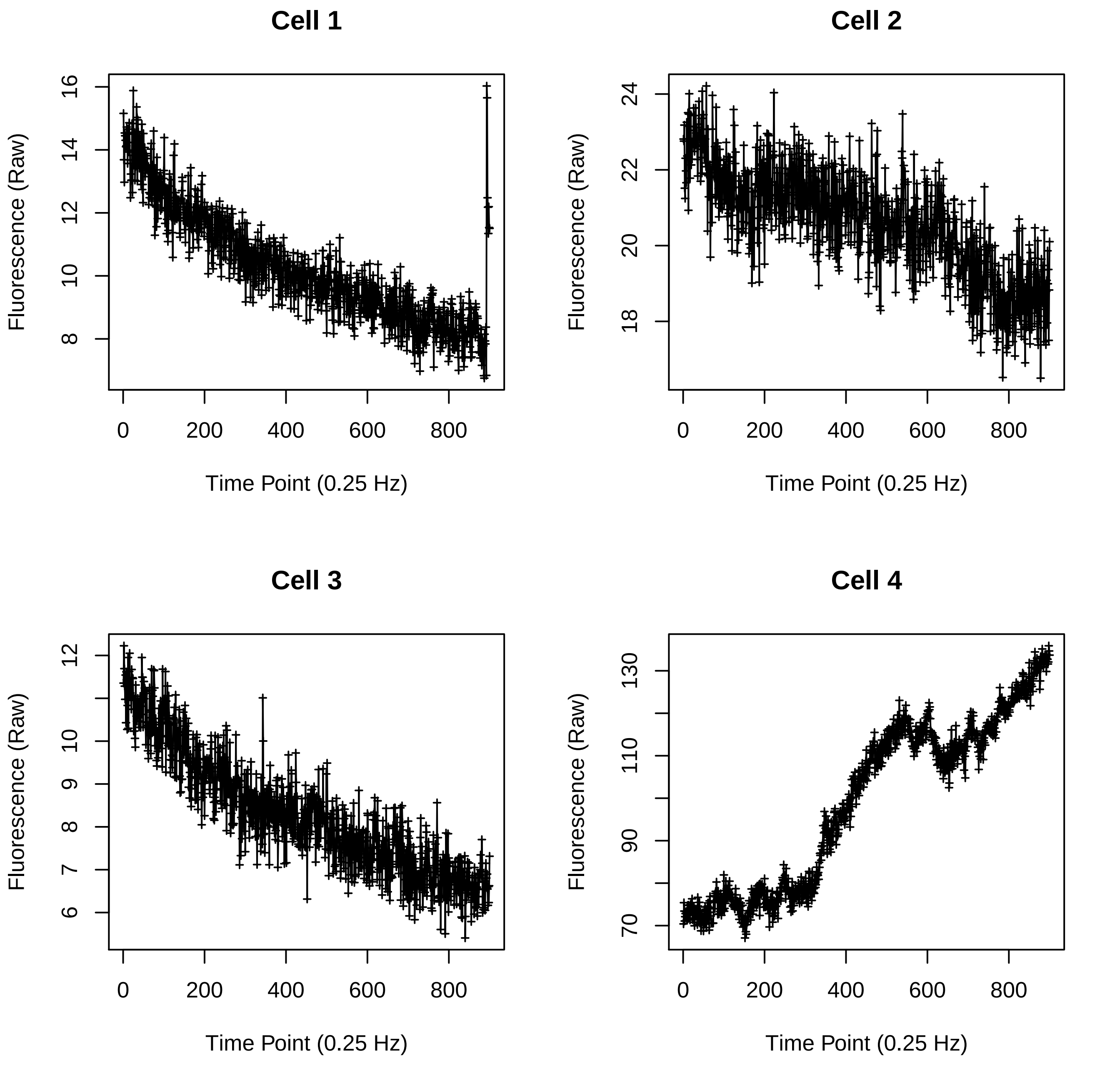
Cell 1
Cell 2
Cell 3
Cell 4
Fluorescence (Raw)
Time Point (0.25 Hz)
0
200
400
600
800
8
10
12
14
16
18
20
22
24
6
7
9
70
90
110
130

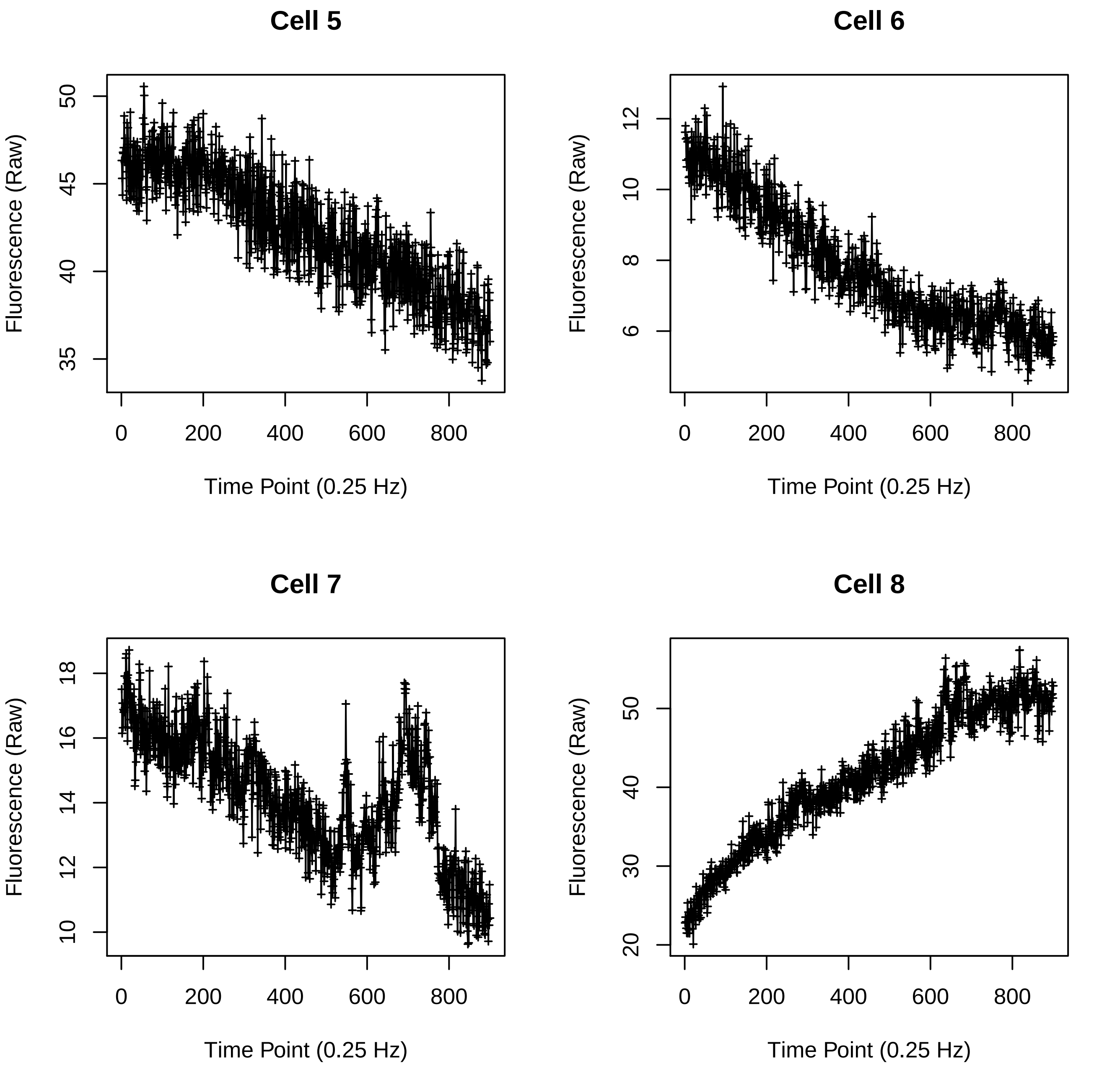
Cell 5
Fluorescence (Raw)
35
40
45
50
0
200
400
600
800
Time Point (0.25 Hz)
Cell 6
Fluorescence (Raw)
6
8
10
12
0
200
400
600
800
Time Point (0.25 Hz)
Cell 7
Fluorescence (Raw)
10
12
14
16
18
0
200
400
600
800
Time Point (0.25 Hz)
Cell 8
Fluorescence (Raw)
20
30
40
50
0
200
400
600
800
Time Point (0.25 Hz)

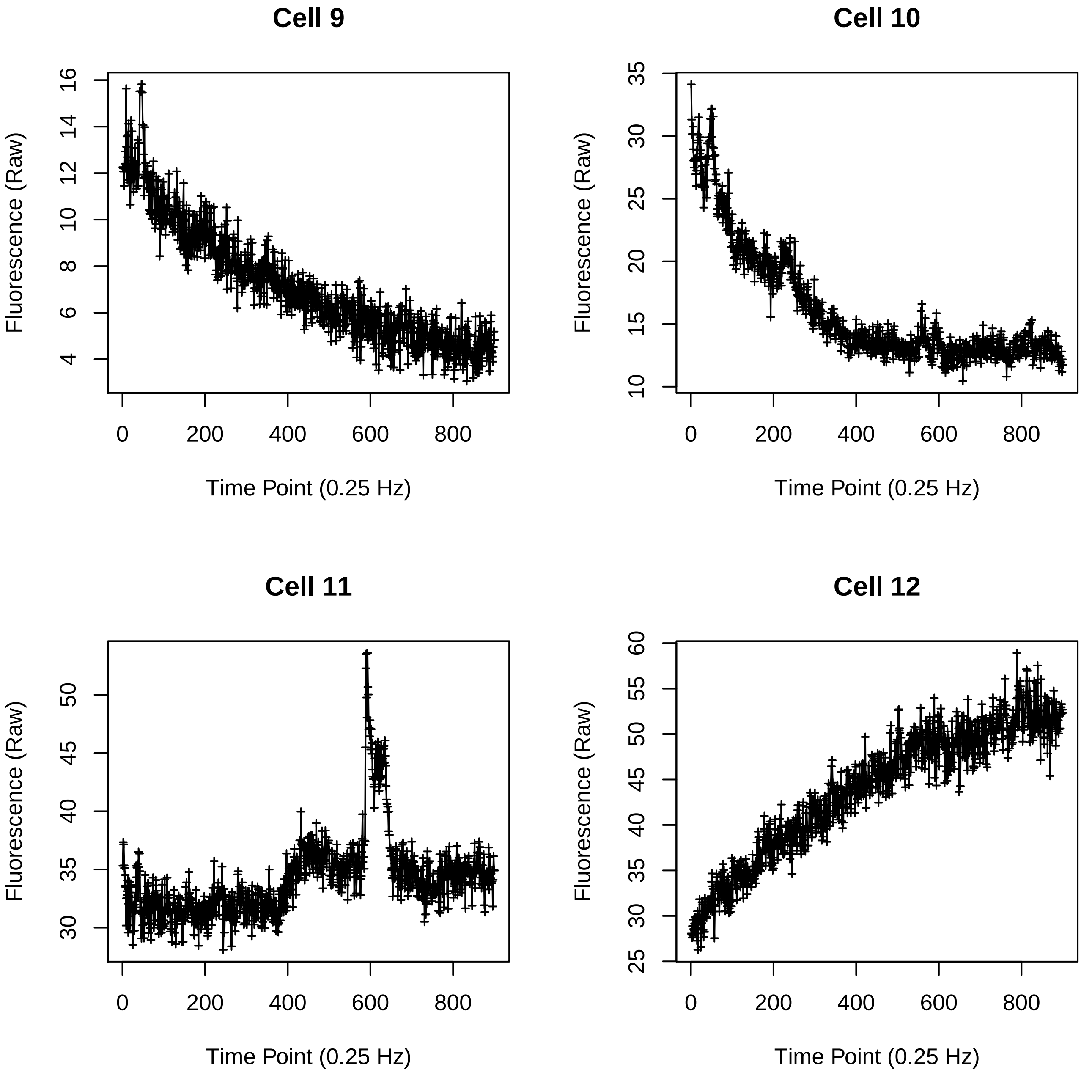

Cell 9
Cell 10
Cell 11
Cell 12
Fluorescence (Raw)
Time Point (0.25 Hz)
0
200
400
600
800
4
6
8
10
12
14
16
10
15
20
25
30
35
40
45
50
25
55
60

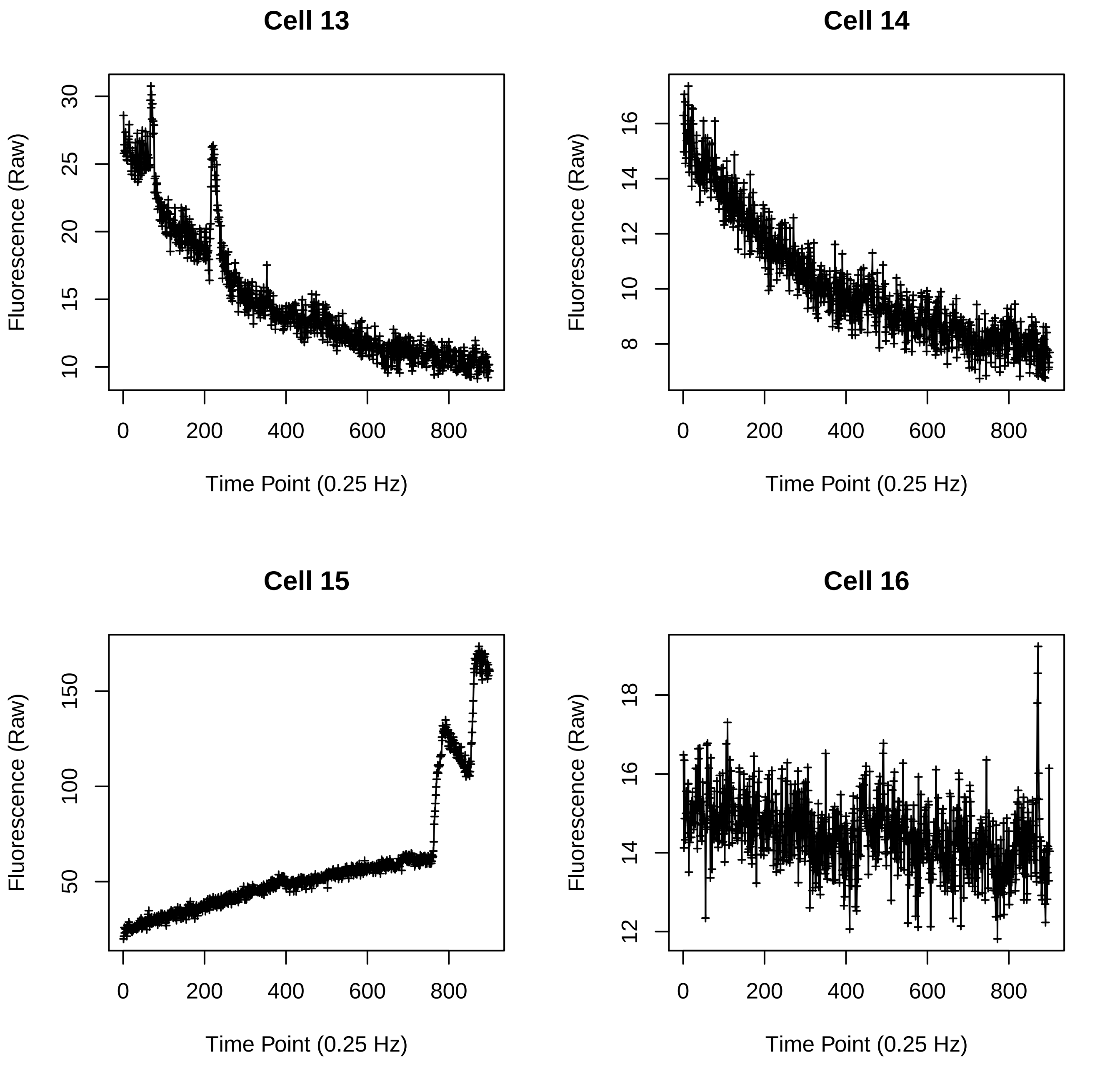
Cell 13
Cell 14
Cell 15
Cell 16
Fluorescence (Raw)
Time Point (0.25 Hz)
0
200
400
600
800
10
15
20
25
30
8
10
12
14
16
50
100
150
12
14
16
18

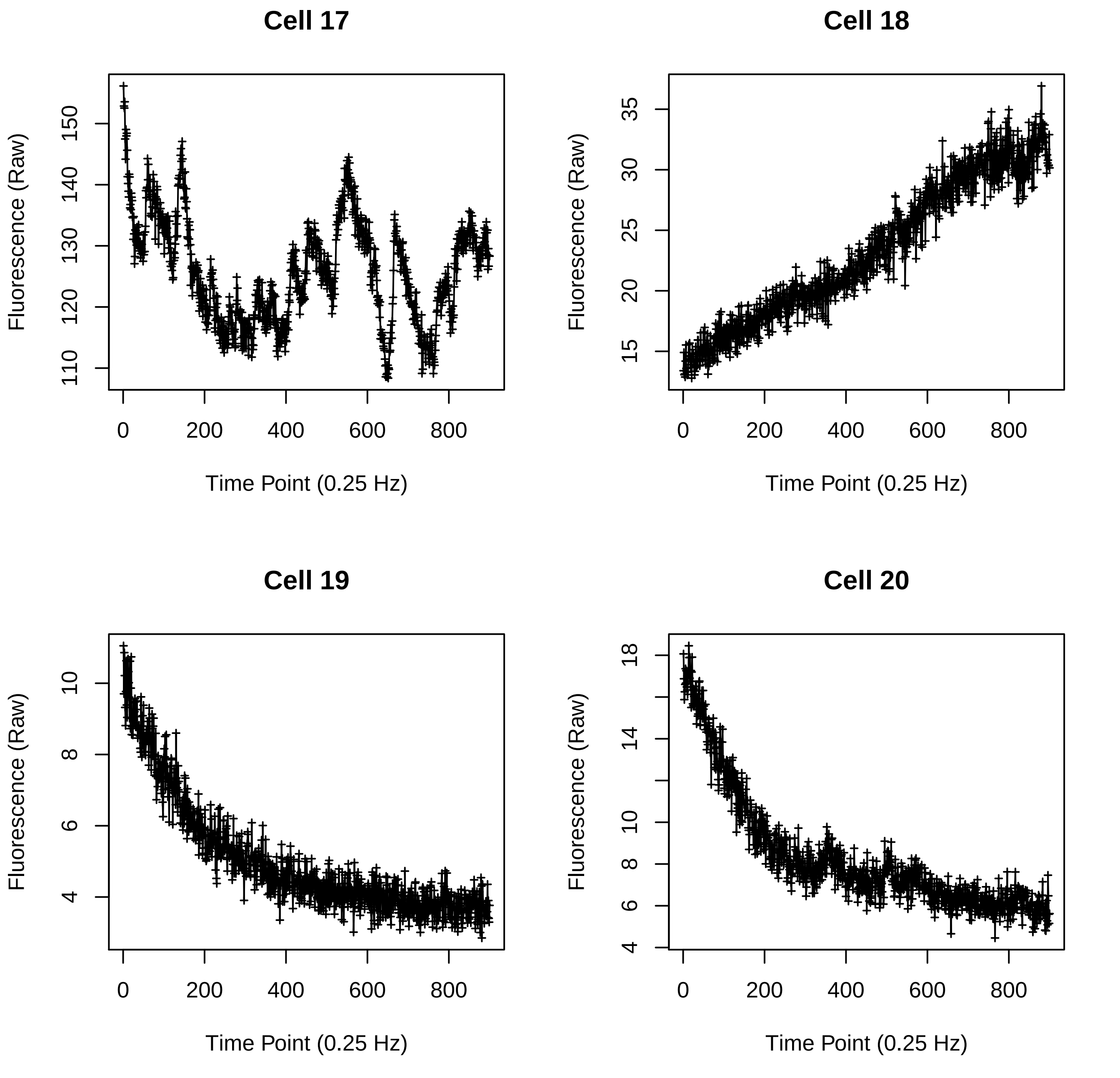
Cell 17
Fluorescence (Raw)
110 120 130 140 150
0 200 400 600 800
Time Point (0.25 Hz)
Cell 18
Fluorescence (Raw)
15 20 25 30 35
0 200 400 600 800
Time Point (0.25 Hz)
Cell 19
Fluorescence (Raw)
4 6 8 10
0 200 400 600 800
Time Point (0.25 Hz)
Cell 20
Fluorescence (Raw)
4 6 8 10 14 18
0 200 400 600 800
Time Point (0.25 Hz)

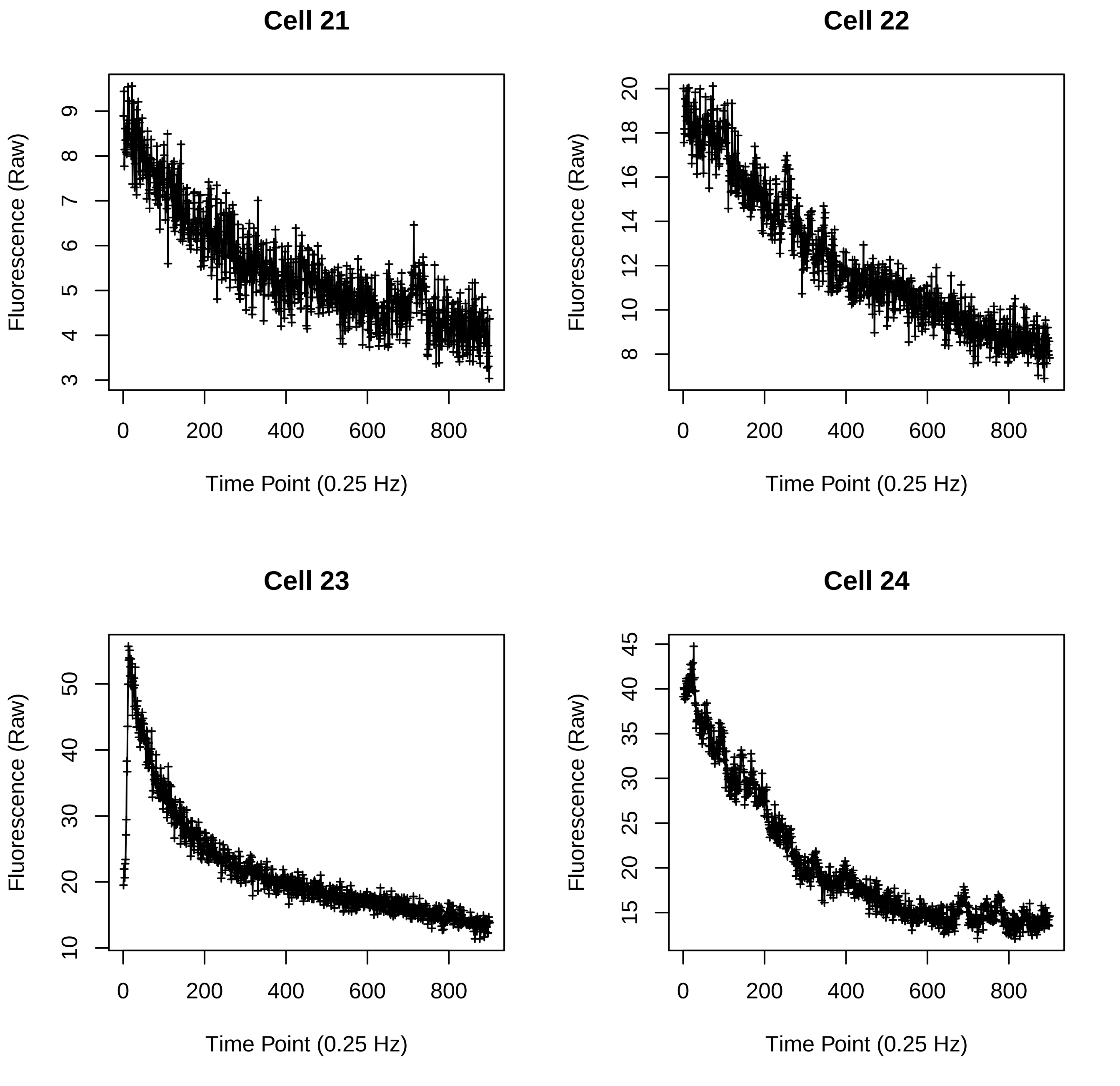
Cell 21
Fluorescence (Raw)
3
4
5
6
7
8
9
0
200
400
600
800
Time Point (0.25 Hz)
Cell 22
Fluorescence (Raw)
8
10
12
14
16
18
20
0
200
400
600
800
Time Point (0.25 Hz)
Cell 23
Fluorescence (Raw)
10
20
30
40
50
0
200
400
600
800
Time Point (0.25 Hz)
Cell 24
Fluorescence (Raw)
15
20
25
30
35
40
45
0
200
400
600
800
Time Point (0.25 Hz)

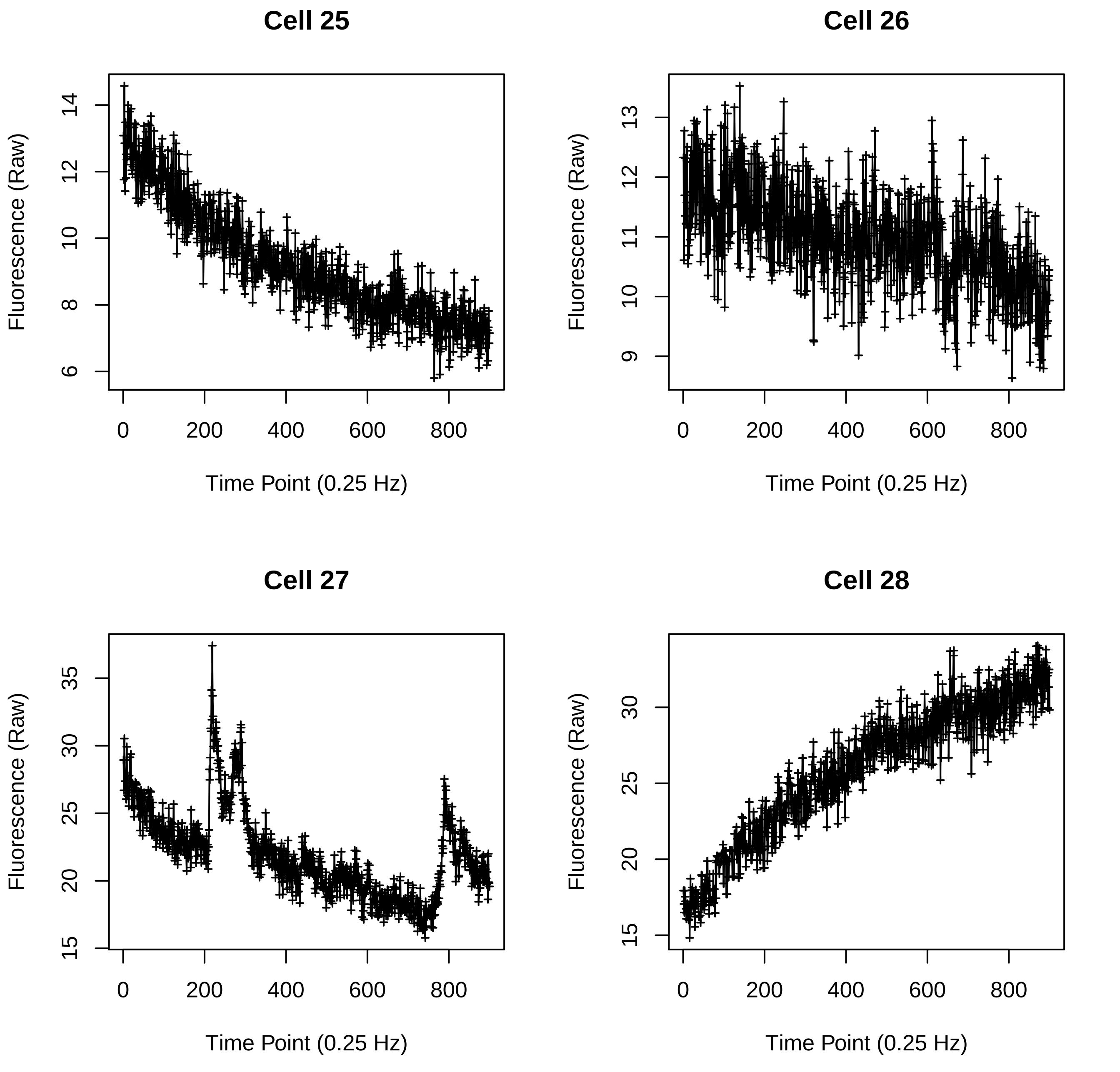
Cell 25
Cell 26
Cell 27
Cell 28
Fluorescence (Raw)
Time Point (0.25 Hz)
0
200
400
600
800
6
8
10
12
14
9
10
11
12
13
15
20
25
30
35

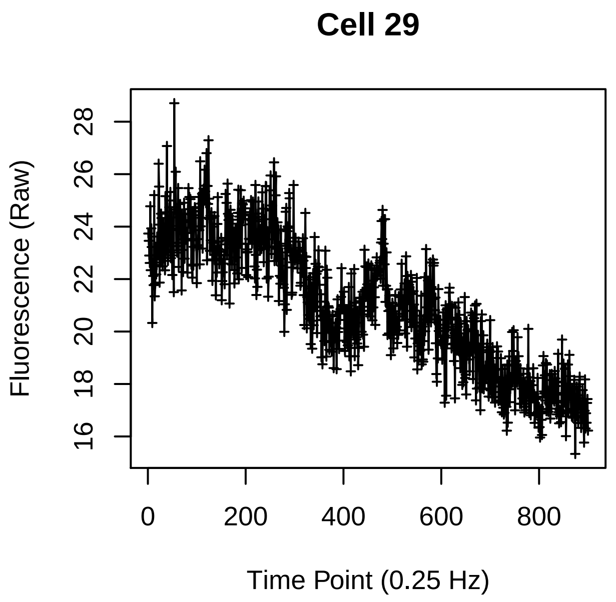
Cell 29
Fluorescence (Raw)
16
18
20
22
24
26
28
0
200
400
600
800
Time Point (0.25 Hz)

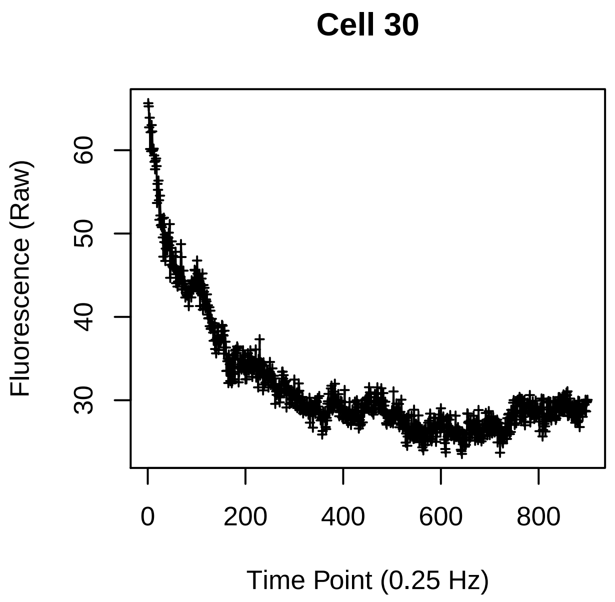
Cell 30
Fluorescence (Raw)
30
40
50
60
0
200
400
600
800
Time Point (0.25 Hz)

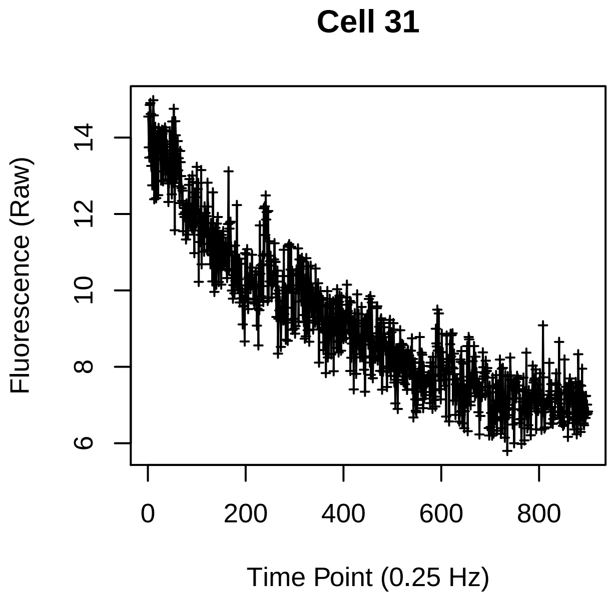
Cell 31
Fluorescence (Raw)
6
8
10
12
14
0
200
400
600
800
Time Point (0.25 Hz)

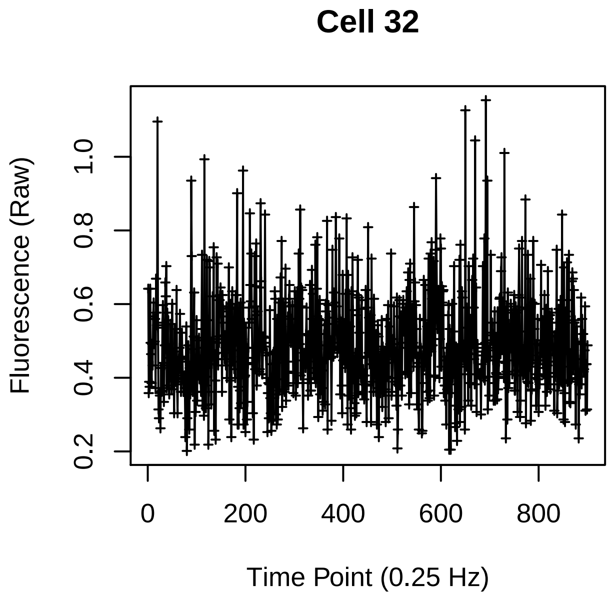
Cell 32
Fluorescence (Raw)
0.2
0.4
0.6
0.8
1.0
0
200
400
600
800
Time Point (0.25 Hz)

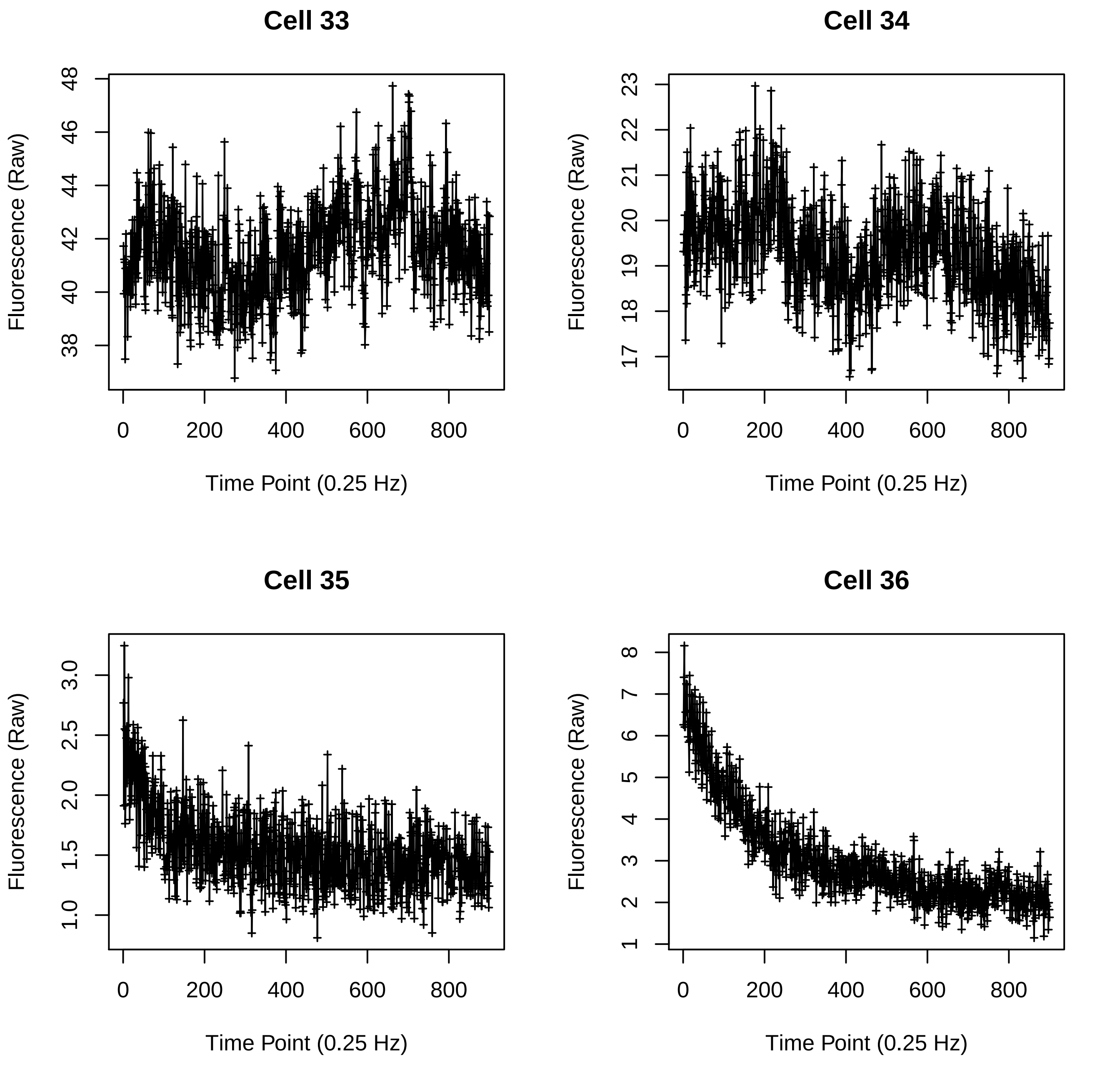
Cell 33
Fluorescence (Raw)
Time Point (0.25 Hz)
Cell 34
Fluorescence (Raw)
Time Point (0.25 Hz)
Cell 35
Fluorescence (Raw)
Time Point (0.25 Hz)
Cell 36
Fluorescence (Raw)
Time Point (0.25 Hz)

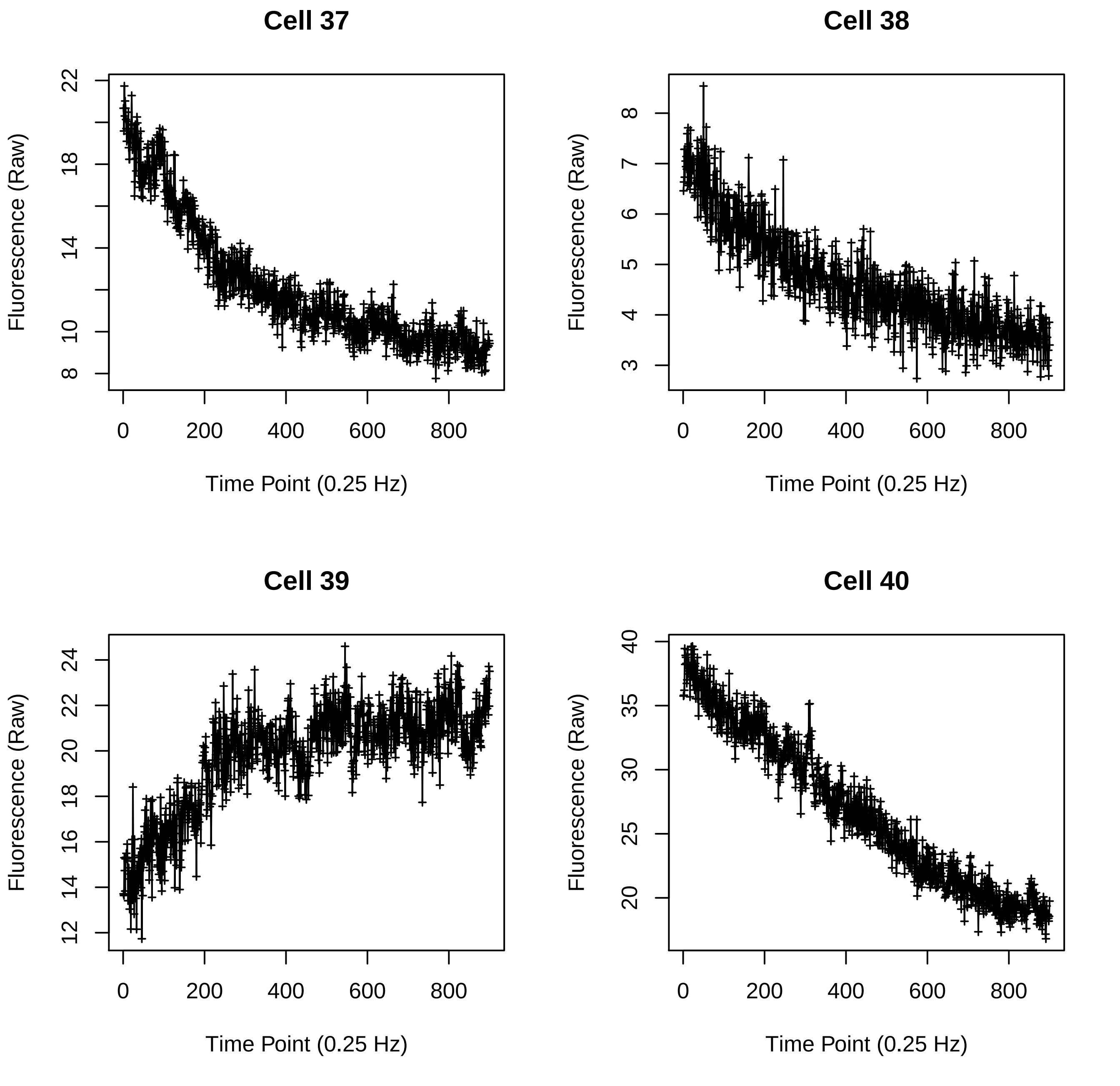
Cell 37
Fluorescence (Raw)
8
10
14
18
22
0
200
400
600
800
Time Point (0.25 Hz)
Cell 38
Fluorescence (Raw)
3
4
5
6
7
8
0
200
400
600
800
Time Point (0.25 Hz)
Cell 39
Fluorescence (Raw)
12
14
16
18
20
22
24
0
200
400
600
800
Time Point (0.25 Hz)
Cell 40
Fluorescence (Raw)
20
25
30
35
40
0
200
400
600
800
Time Point (0.25 Hz)

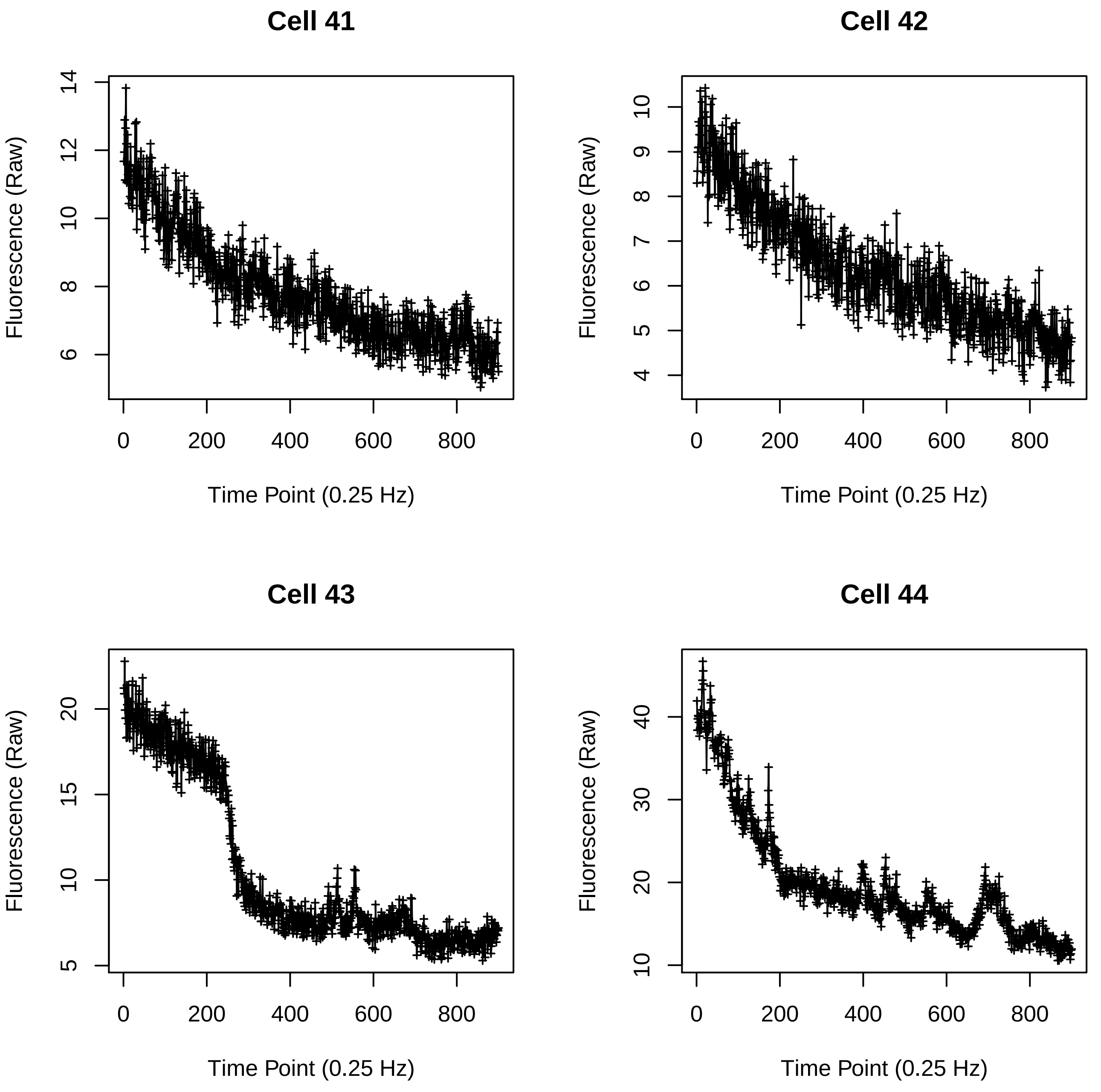
Cell 41
Fluorescence (Raw)
6
8
10
12
14
0
200
400
600
800
Time Point (0.25 Hz)
Cell 42
Fluorescence (Raw)
4
5
6
7
8
9
10
0
200
400
600
800
Time Point (0.25 Hz)
Cell 43
Fluorescence (Raw)
5
10
15
20
0
200
400
600
800
Time Point (0.25 Hz)
Cell 44
Fluorescence (Raw)
10
20
30
40
0
200
400
600
800
Time Point (0.25 Hz)

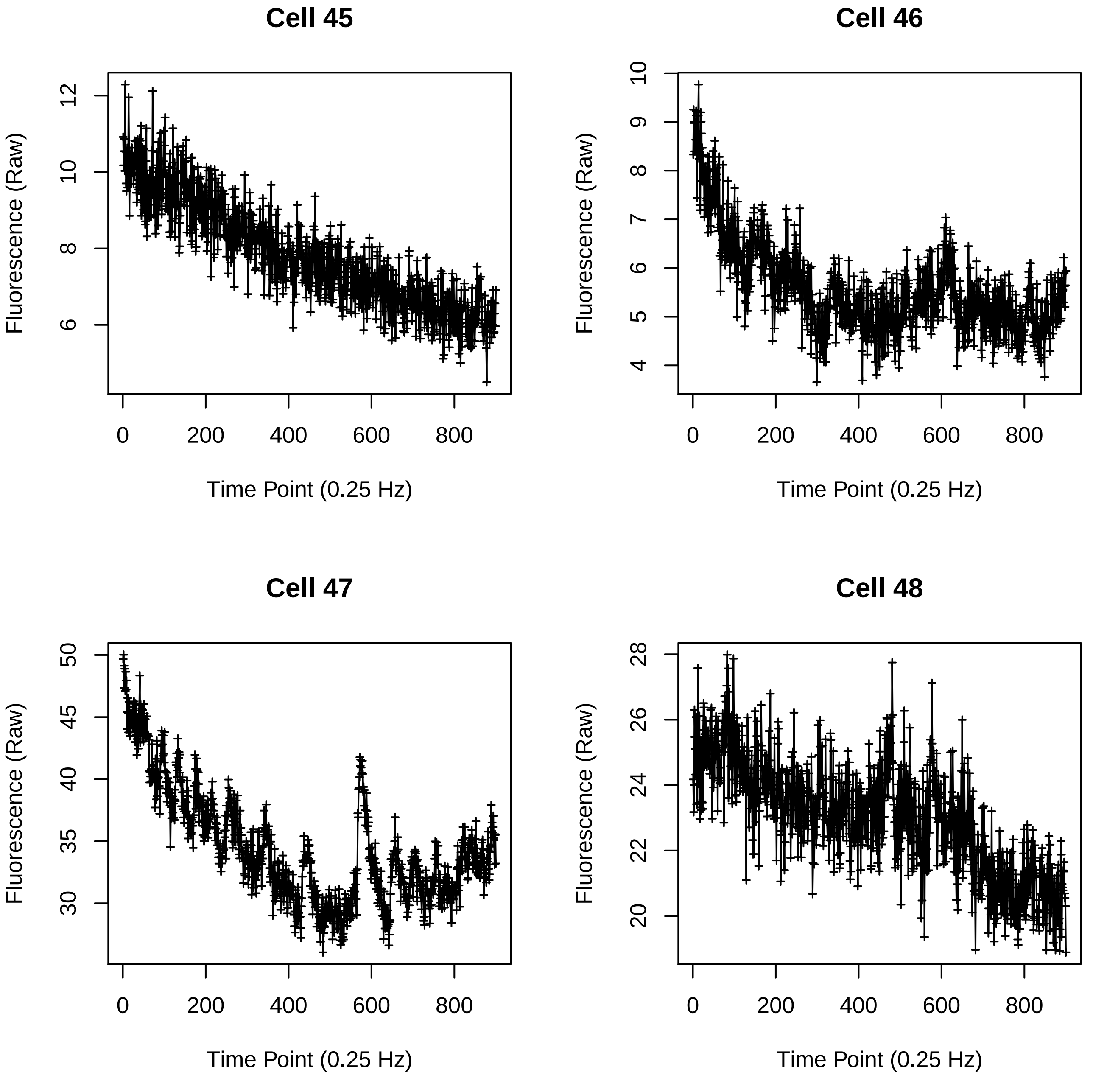

Cell 45
Cell 46
Cell 47
Cell 48
Fluorescence (Raw)
Time Point (0.25 Hz)
0
200
400
600
800
6
8
10
12
4
5
7
9
30
35
40
45
50
20
22
24
26
28

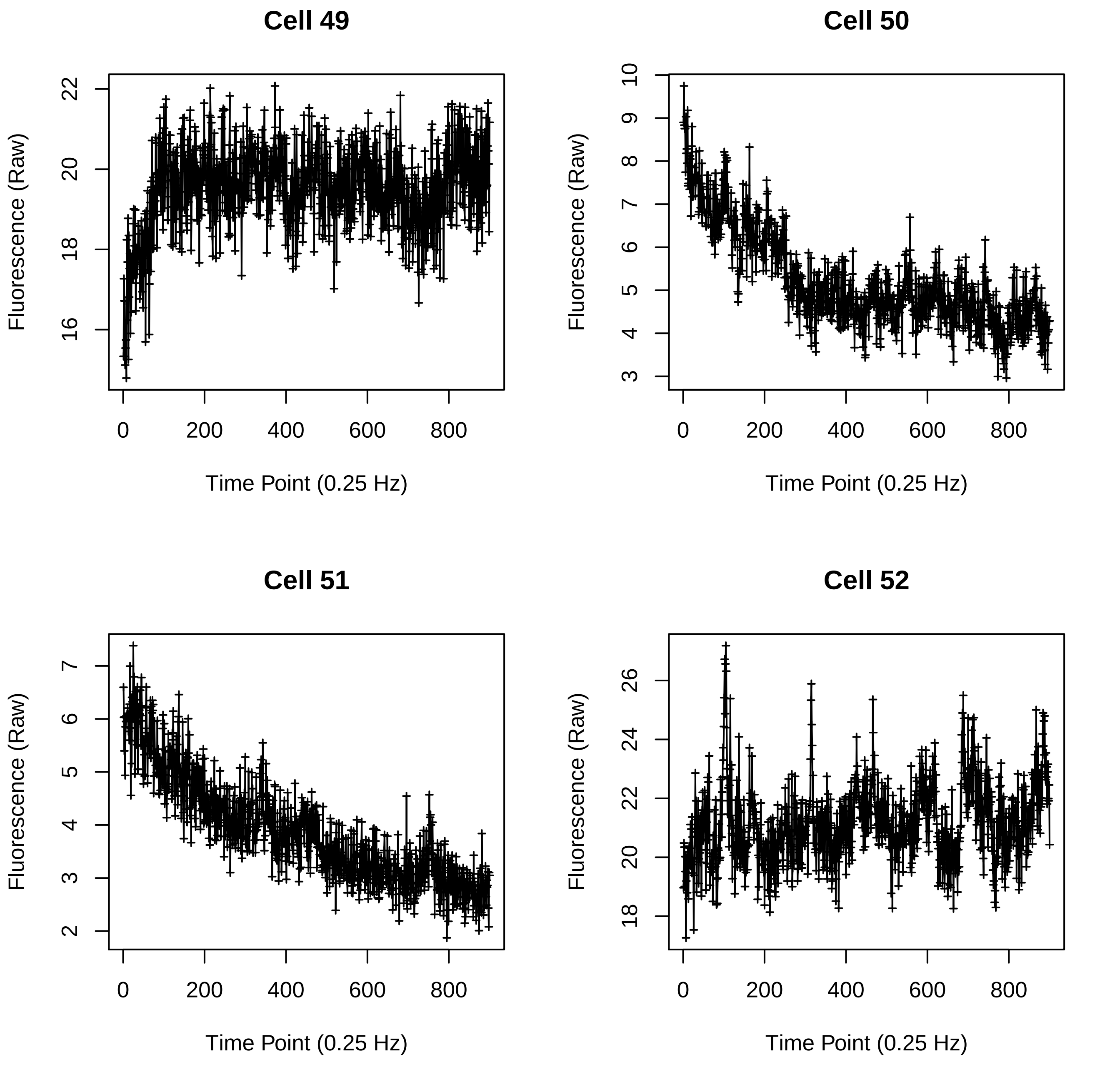
Cell 49
Fluorescence (Raw)
16
18
20
22
0
200
400
600
800
Time Point (0.25 Hz)
Cell 50
Fluorescence (Raw)
3
4
5
6
7
8
9
10
0
200
400
600
800
Time Point (0.25 Hz)
Cell 51
Fluorescence (Raw)
2
3
4
5
6
7
0
200
400
600
800
Time Point (0.25 Hz)
Cell 52
Fluorescence (Raw)
18
20
22
24
26
0
200
400
600
800
Time Point (0.25 Hz)

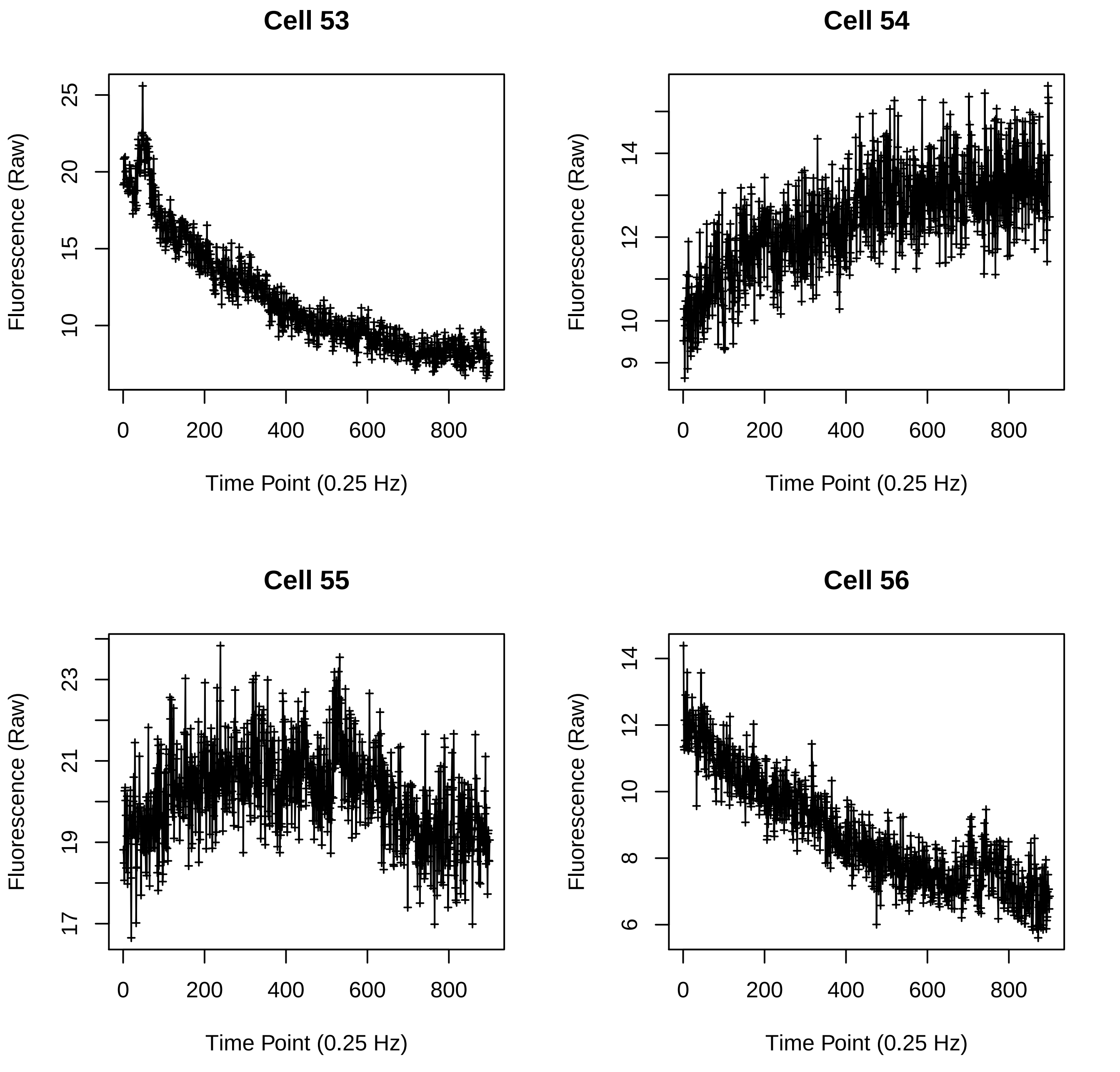
Cell 53
Fluorescence (Raw)
25
20
15
10
0
200
400
600
800
Time Point (0.25 Hz)
Cell 54
Fluorescence (Raw)
14
12
10
9
0
200
400
600
800
Time Point (0.25 Hz)
Cell 55
Fluorescence (Raw)
23
21
19
17
0
200
400
600
800
Time Point (0.25 Hz)
Cell 56
Fluorescence (Raw)
14
12
10
8
6
0
200
400
600
800
Time Point (0.25 Hz)

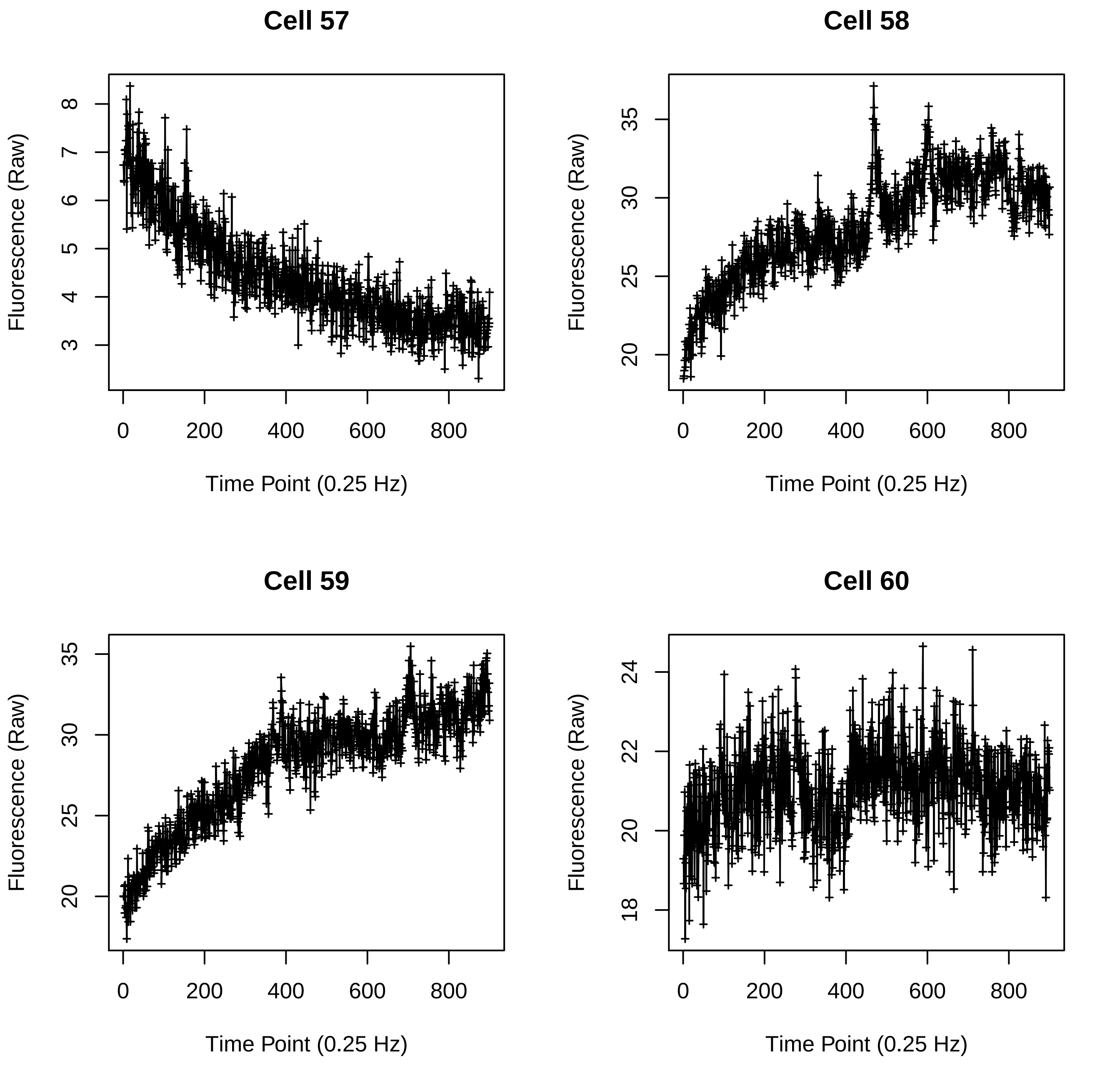
Cell 57
Cell 58
Cell 59
Cell 60
Fluorescence (Raw)
Time Point (0.25 Hz)
0
200
400
600
800
3
4
5
6
7
8
20
25
30
35
18
22
24

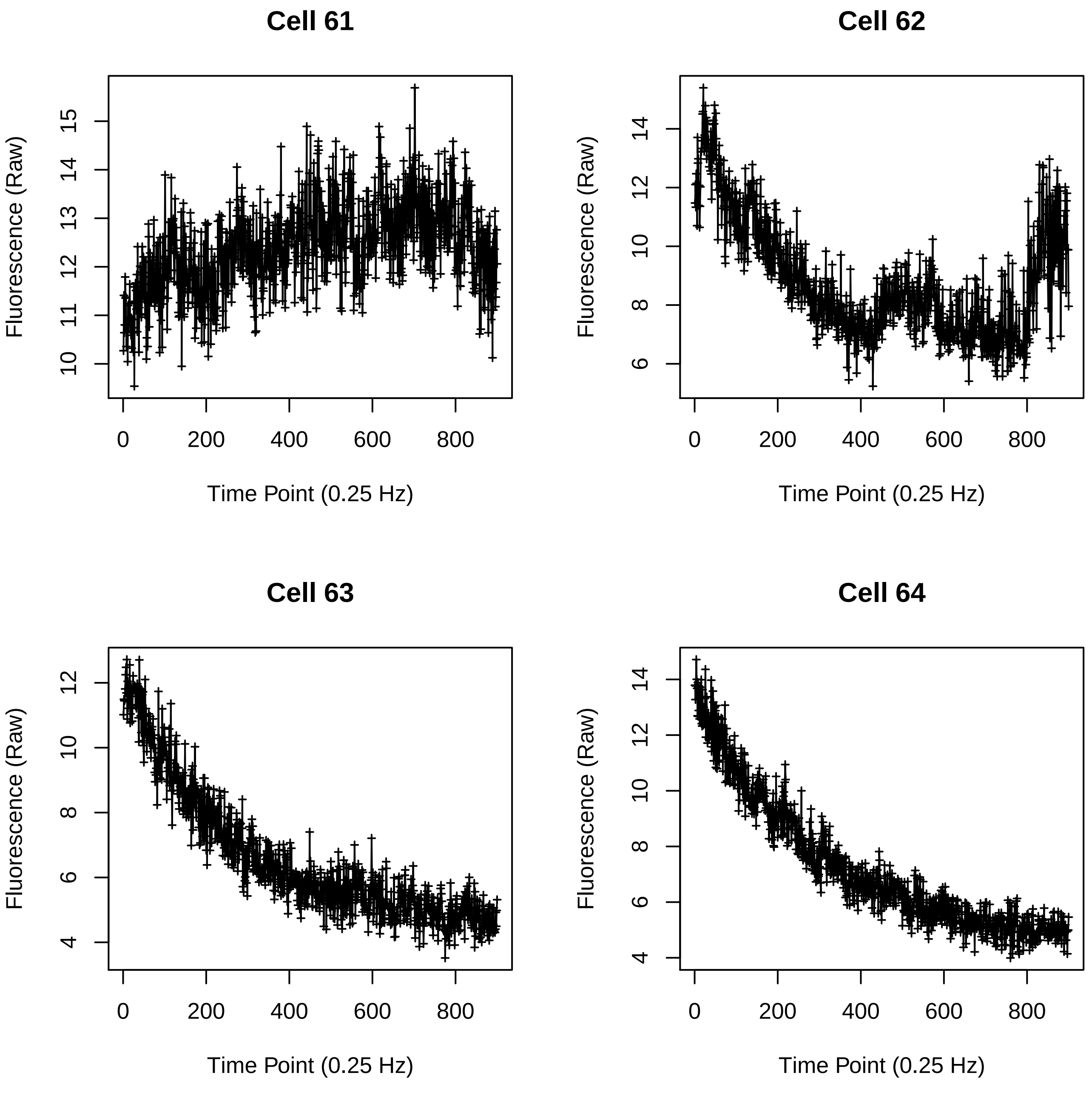

Cell 61
Fluorescence (Raw)
10
11
12
13
14
15
0
200
400
600
800
Time Point (0.25 Hz)
Cell 62
Fluorescence (Raw)
6
8
10
12
14
0
200
400
600
800
Time Point (0.25 Hz)
Cell 63
Fluorescence (Raw)
4
6
8
10
12
0
200
400
600
800
Time Point (0.25 Hz)
Cell 64
Fluorescence (Raw)
4
6
8
10
12
14
0
200
400
600
800
Time Point (0.25 Hz)

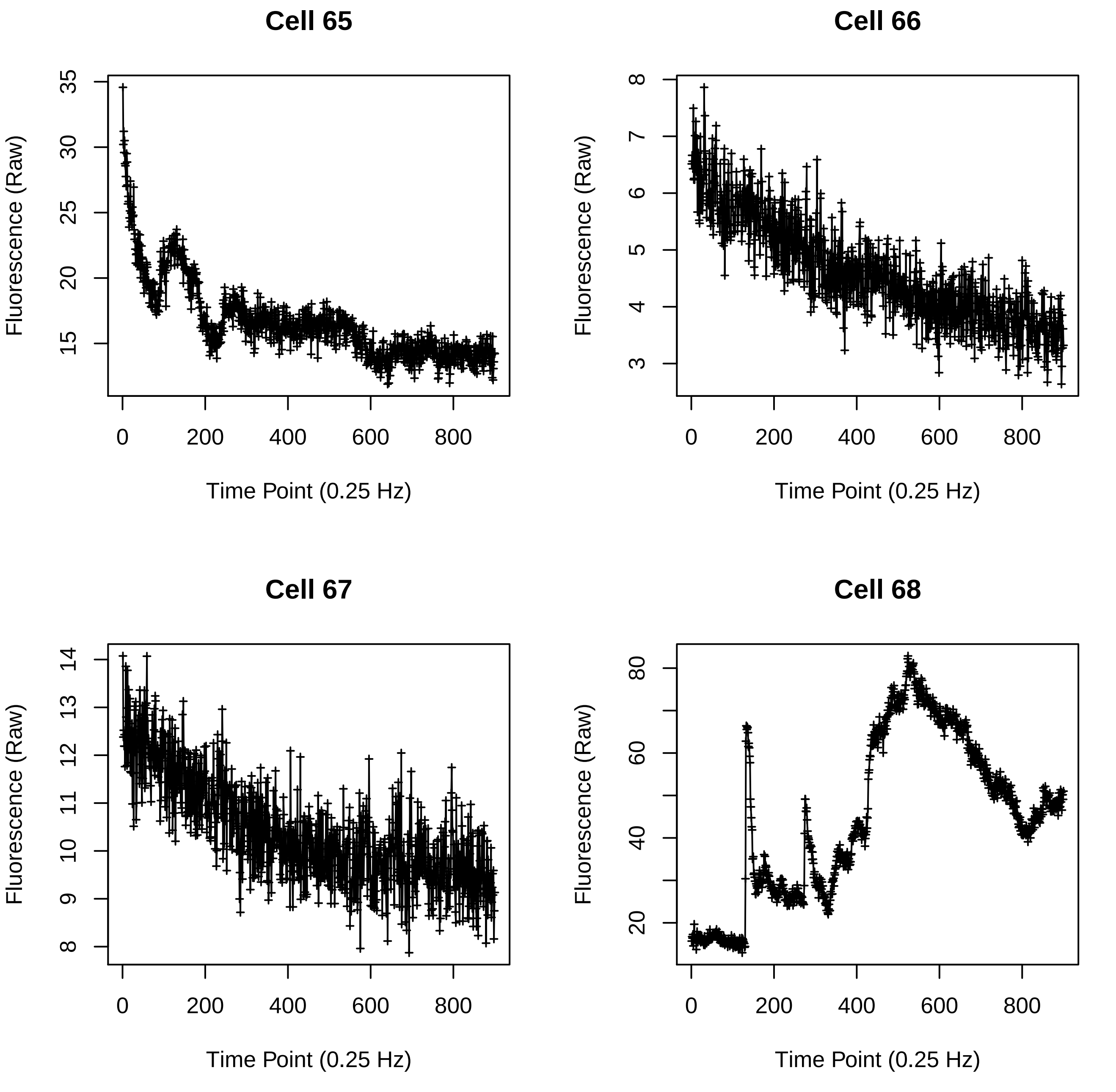

Cell 65
Fluorescence (Raw)
15
20
25
30
35
0
200
400
600
800
Time Point (0.25 Hz)
Cell 66
Fluorescence (Raw)
3
4
5
6
7
8
0
200
400
600
800
Time Point (0.25 Hz)
Cell 67
Fluorescence (Raw)
8
9
10
11
12
13
14
0
200
400
600
800
Time Point (0.25 Hz)
Cell 68
Fluorescence (Raw)
20
40
60
80
0
200
400
600
800
Time Point (0.25 Hz)

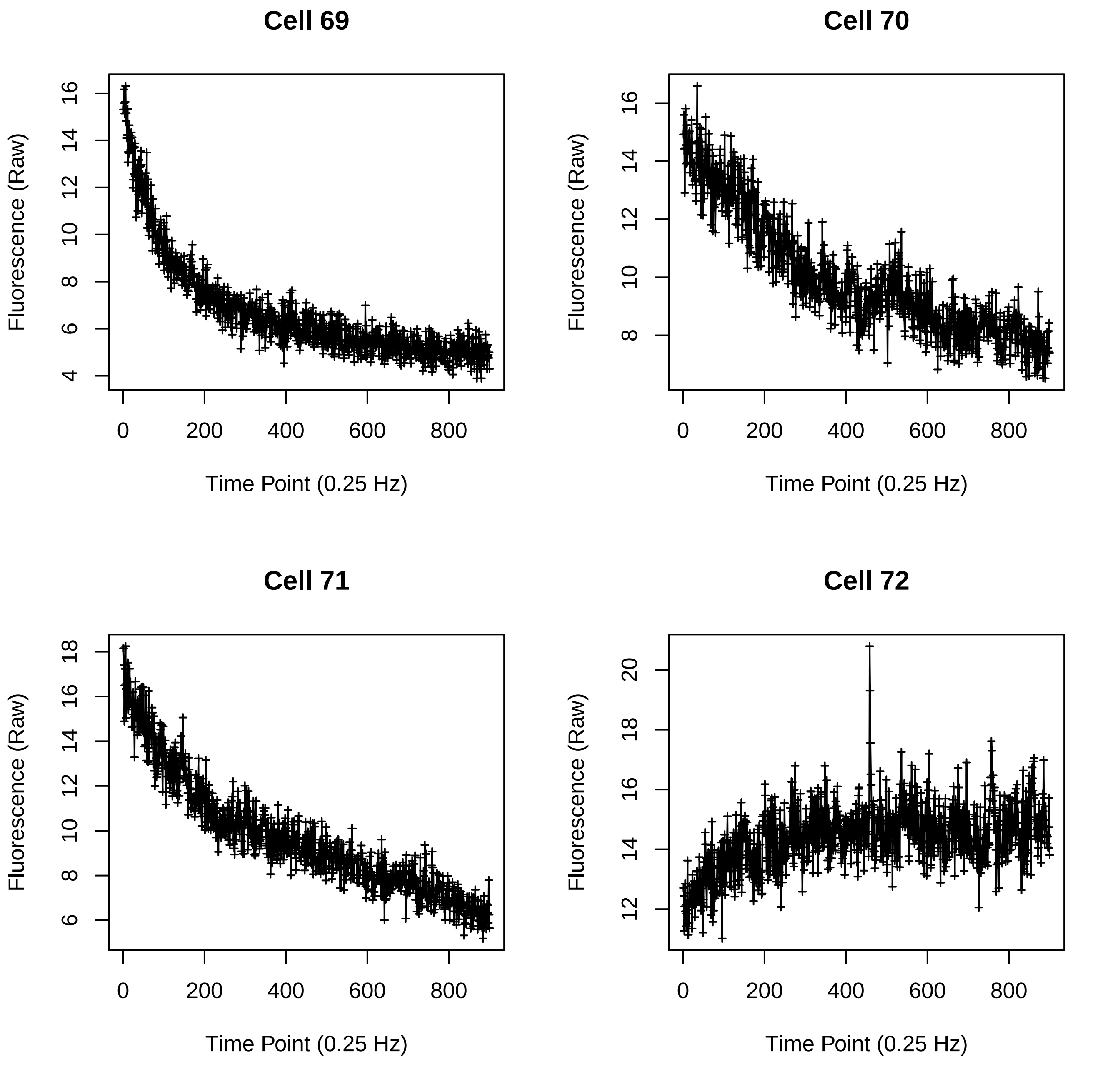
Cell 69
Cell 70
Cell 71
Cell 72
Fluorescence (Raw)
Time Point (0.25 Hz)
0
200
400
600
800
4
6
8
10
12
14
16
18
20

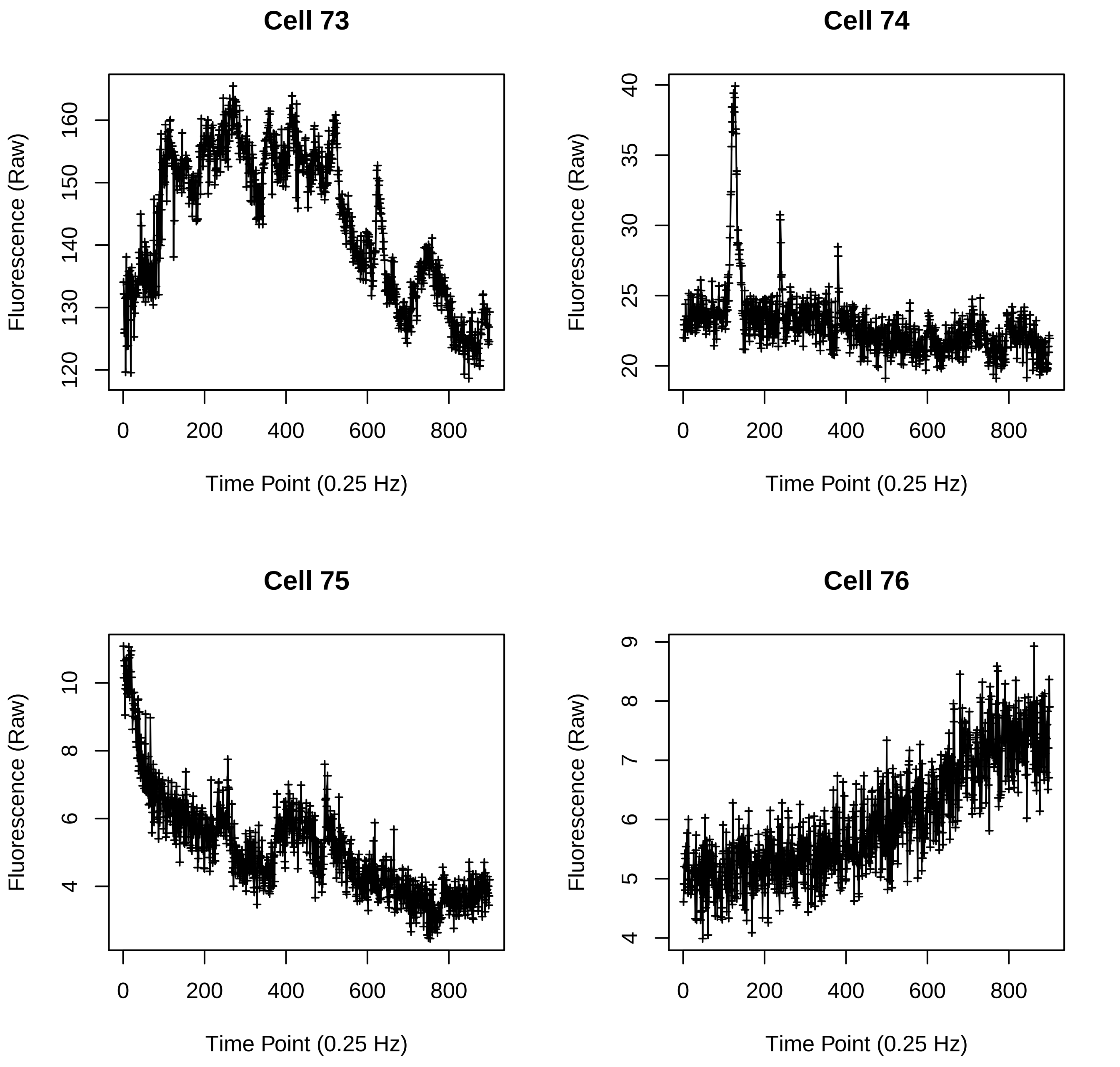
Cell 73
Cell 74
Cell 75
Cell 76
Fluorescence (Raw)
Time Point (0.25 Hz)
120
130
140
150
160
20
25
30
35
40
4
6
8
10
5
7
9
0
200
400
600
800

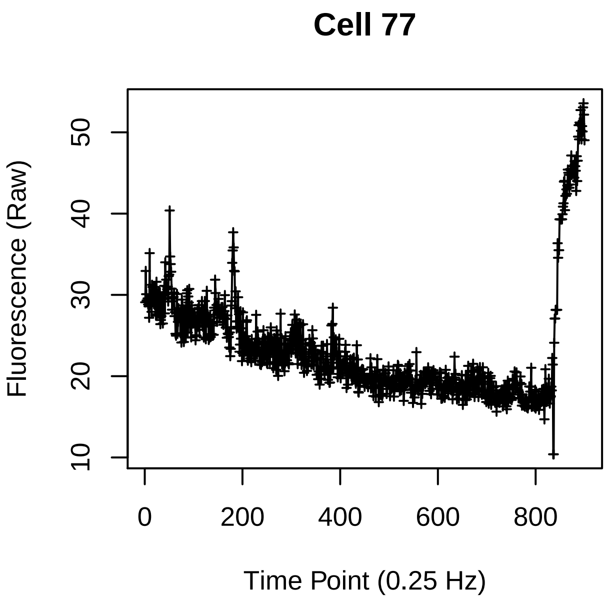
Cell 77
Fluorescence (Raw)
10
20
30
40
50
0
200
400
600
800
Time Point (0.25 Hz)

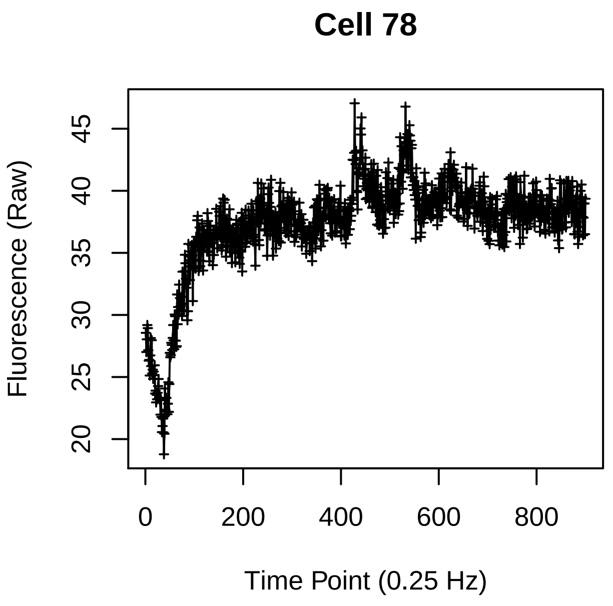
Cell 78
Fluorescence (Raw)
20
25
30
35
40
45
0
200
400
600
800
Time Point (0.25 Hz)

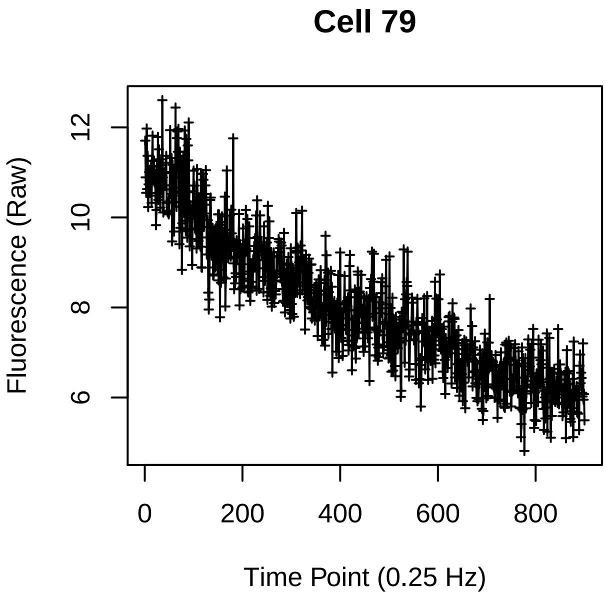
Cell 79
Fluorescence (Raw)
6
8
10
12
0
200
400
600
800
Time Point (0.25 Hz)

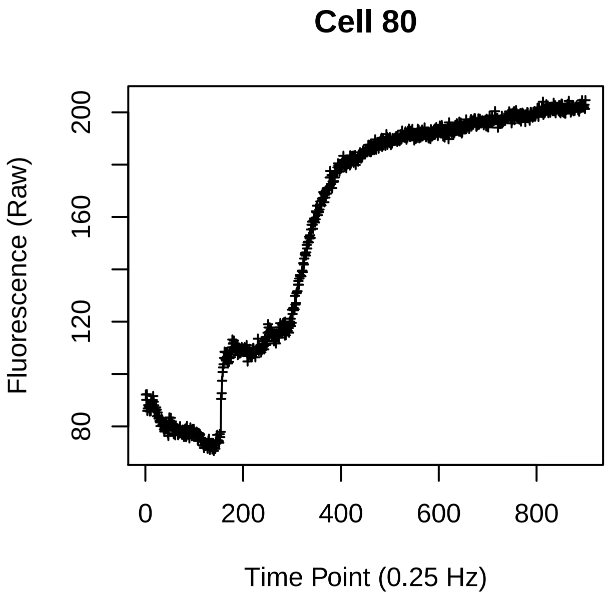
Cell 80
Fluorescence (Raw)
80
120
160
200
0
200
400
600
800
Time Point (0.25 Hz)

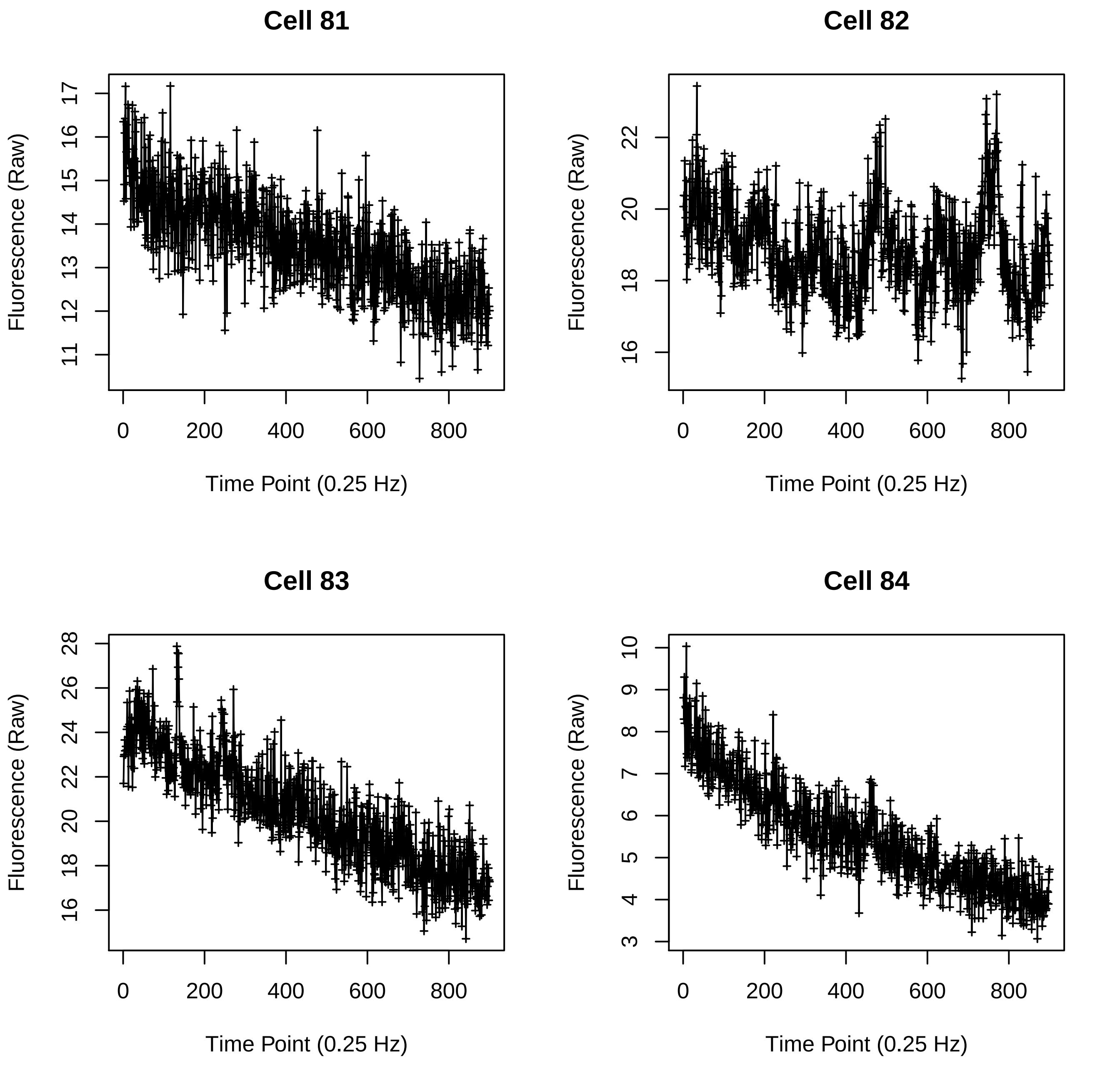
Cell 81
Fluorescence (Raw)
11 12 13 14 15 16 17
0 200 400 600 800
Time Point (0.25 Hz)
Cell 82
Fluorescence (Raw)
16 18 20 22
0 200 400 600 800
Time Point (0.25 Hz)
Cell 83
Fluorescence (Raw)
16 18 20 22 24 26 28
0 200 400 600 800
Time Point (0.25 Hz)
Cell 84
Fluorescence (Raw)
3 4 5 6 7 8 9 10
0 200 400 600 800
Time Point (0.25 Hz)

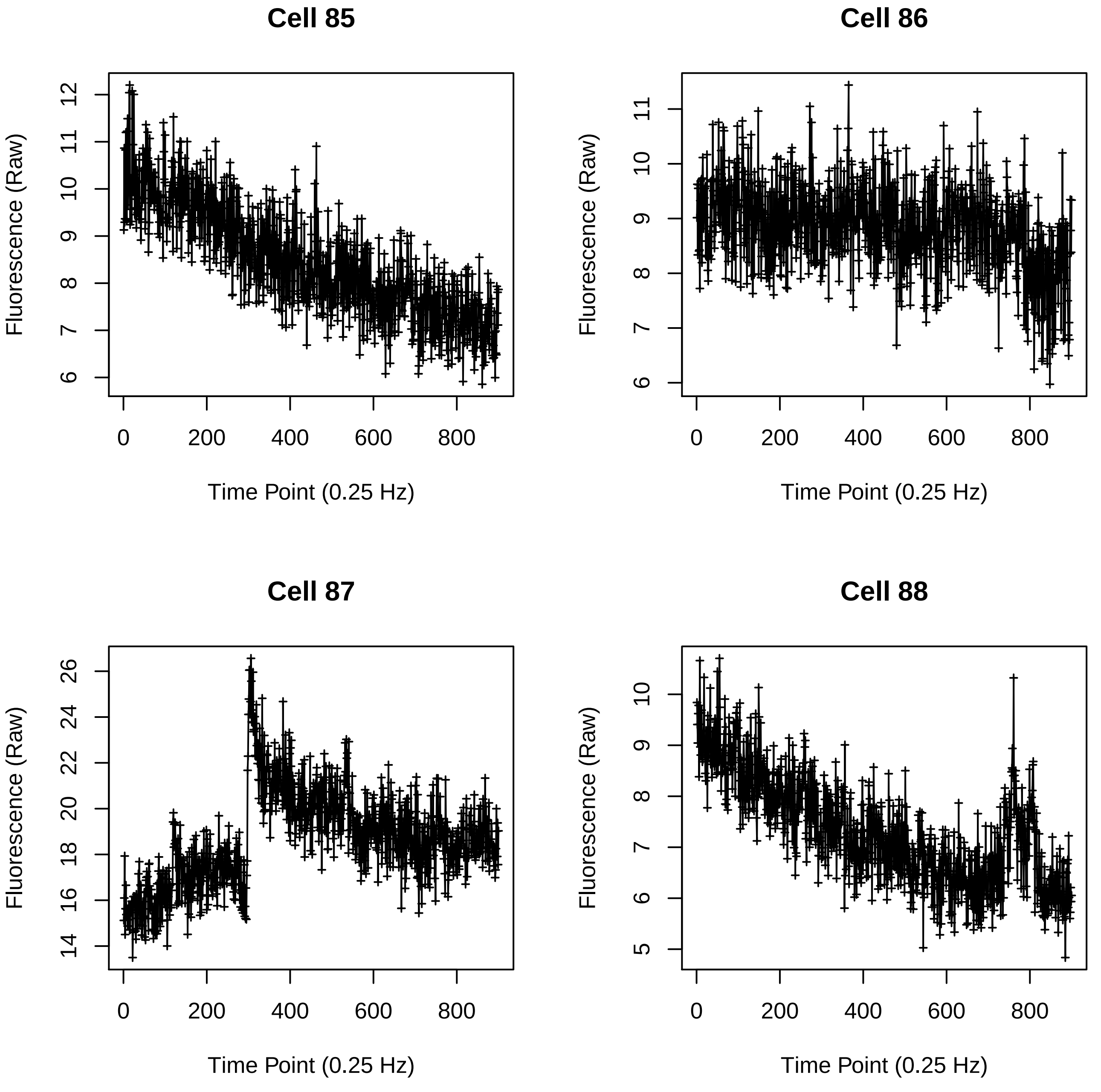
Cell 85
Cell 86
Cell 87
Cell 88
Fluorescence (Raw)
Time Point (0.25 Hz)
0
200
400
600
800
6
7
8
9
10
11
12
14
16
18
20
22
24
26
5

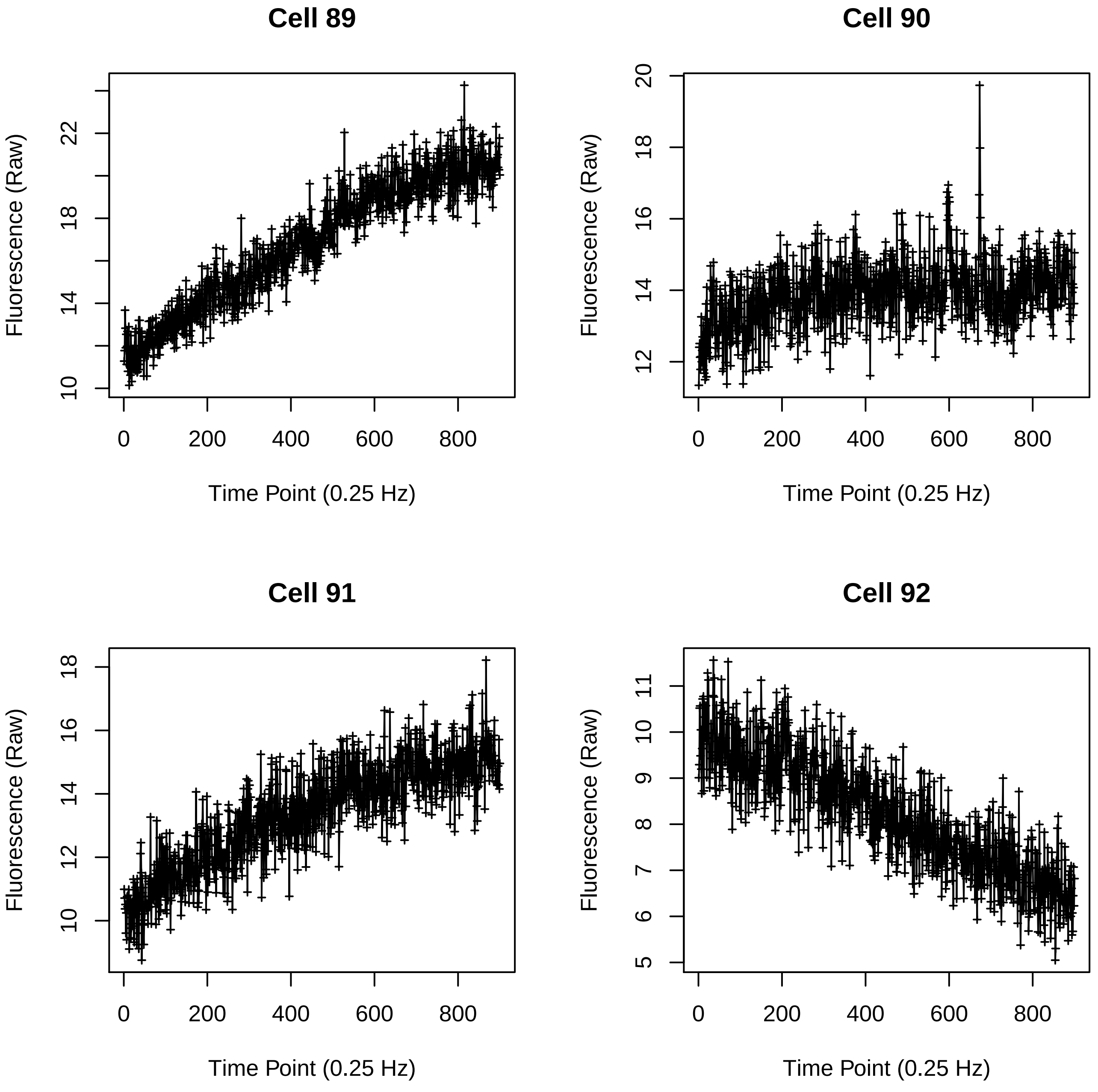
Cell 89
Fluorescence (Raw)
10
14
18
22
0
200
400
600
800
Time Point (0.25 Hz)
Cell 90
Fluorescence (Raw)
12
14
16
18
20
0
200
400
600
800
Time Point (0.25 Hz)
Cell 91
Fluorescence (Raw)
10
12
14
16
18
0
200
400
600
800
Time Point (0.25 Hz)
Cell 92
Fluorescence (Raw)
5
6
7
8
9
10
11
0
200
400
600
800
Time Point (0.25 Hz)

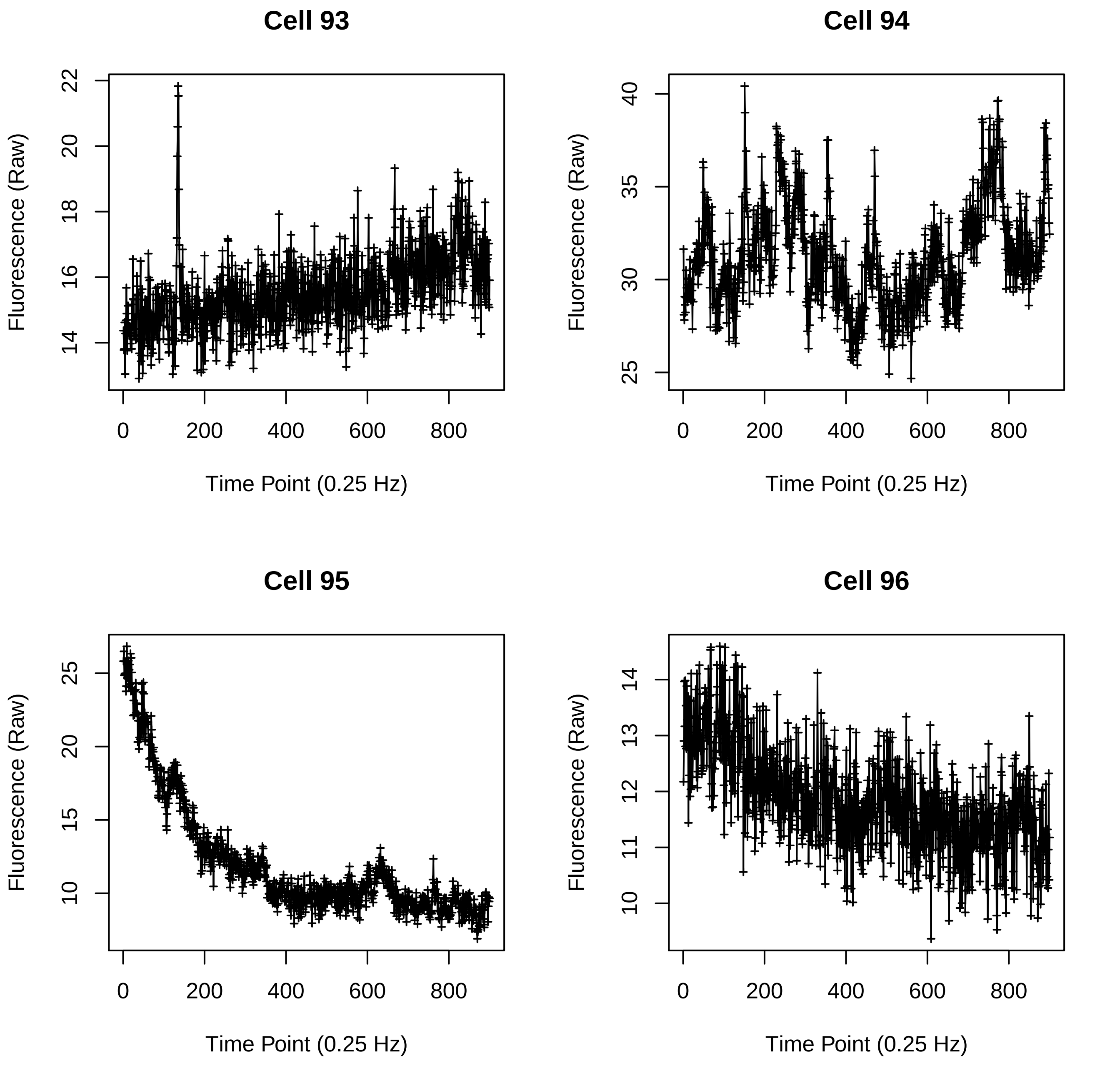

Cell 93
Cell 94
Cell 95
Cell 96
Fluorescence (Raw)
Time Point (0.25 Hz)
0
200
400
600
800
14
16
18
20
22
25
30
35
40
10
15
20
25
10
11
12
13
14

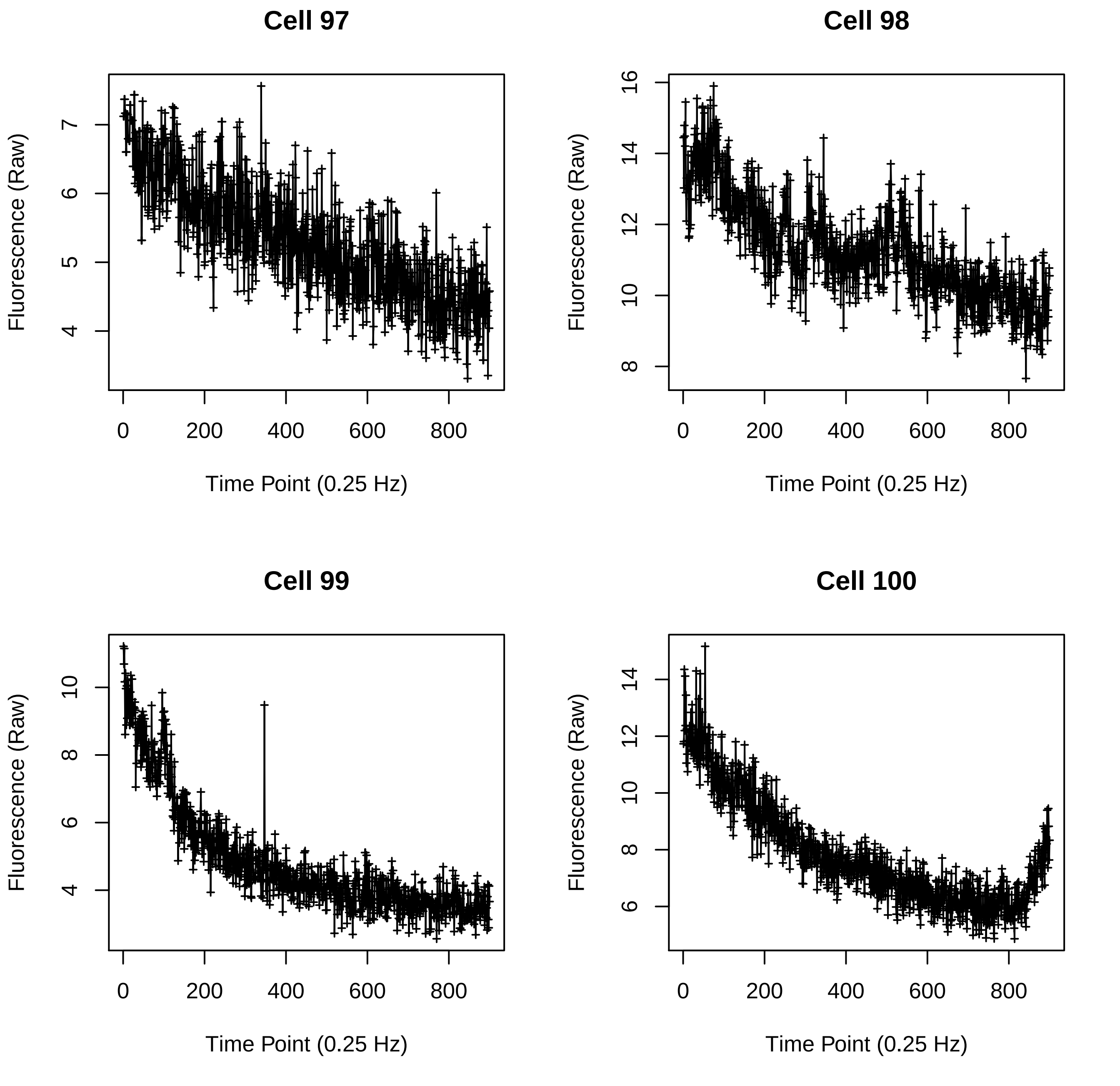
Cell 97
Fluorescence (Raw)
Time Point (0.25 Hz)
Cell 98
Fluorescence (Raw)
Time Point (0.25 Hz)
Cell 99
Fluorescence (Raw)
Time Point (0.25 Hz)
Cell 100
Fluorescence (Raw)
Time Point (0.25 Hz)

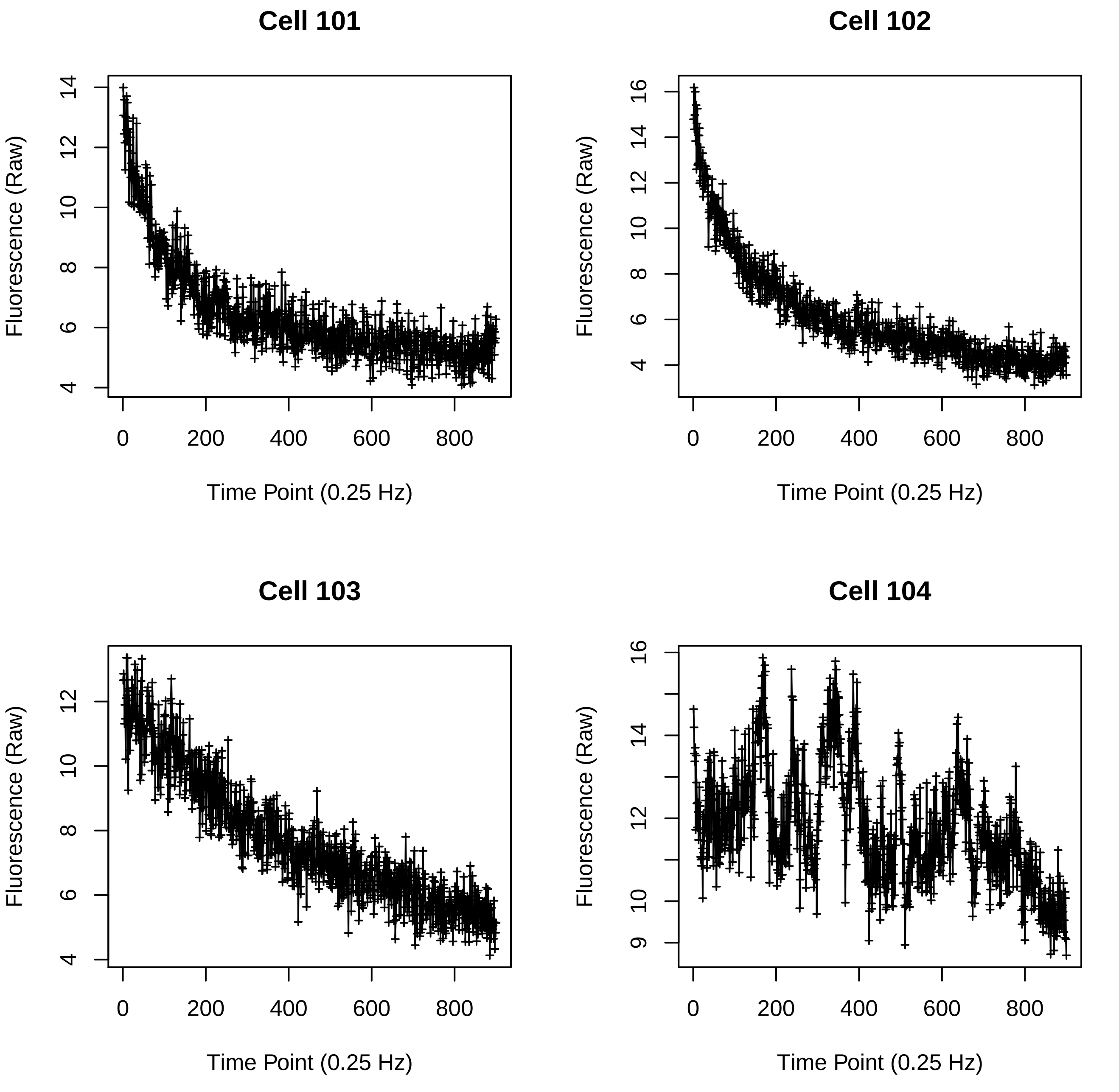
Cell 101
Cell 102
Cell 103
Cell 104
Fluorescence (Raw)
Time Point (0.25 Hz)
0
200
400
600
800
4
6
8
10
12
14
16
9

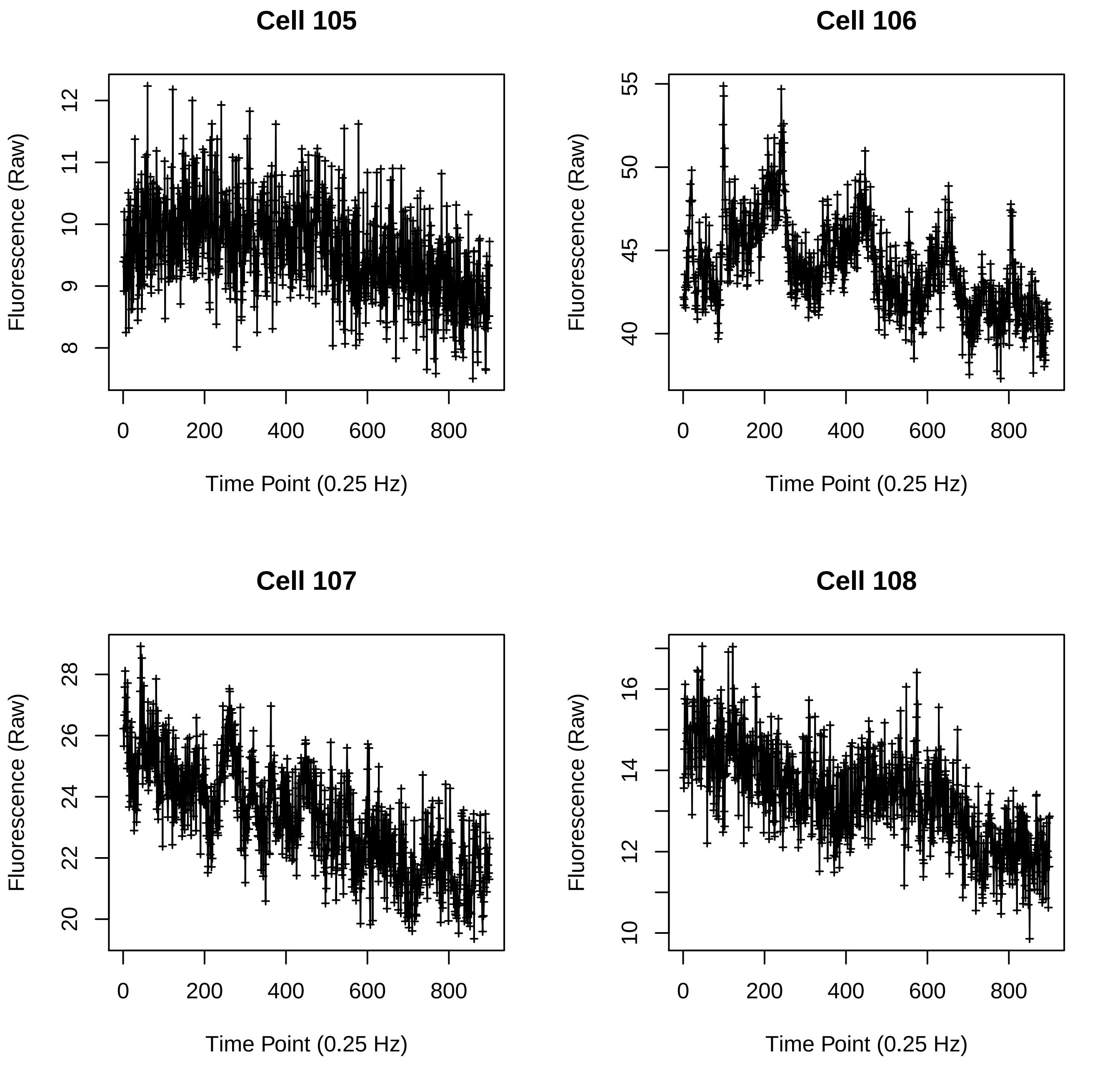

Cell 105
Cell 106
Cell 107
Cell 108
Fluorescence (Raw)
Time Point (0.25 Hz)

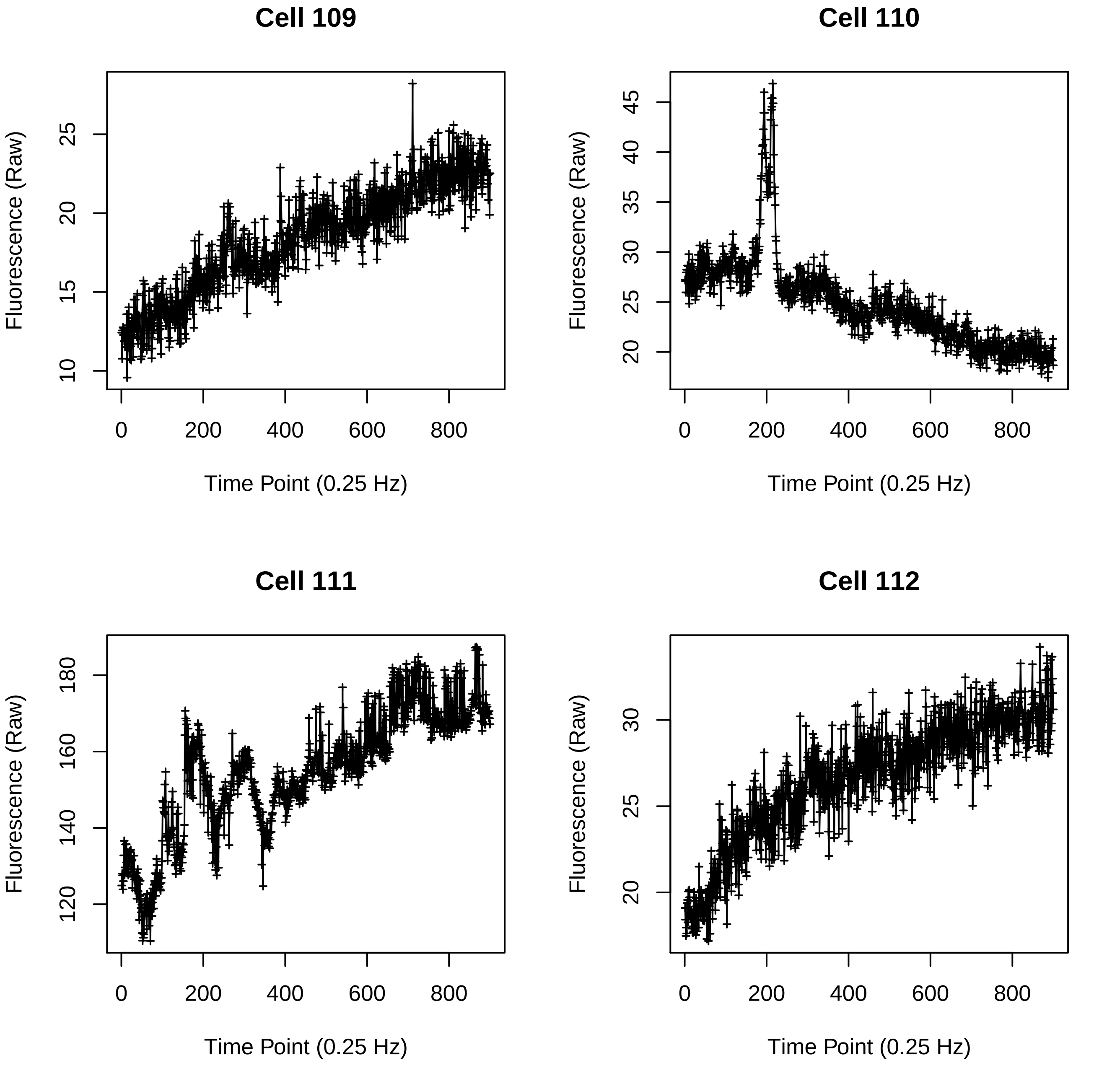
Cell 109
Cell 110
Cell 111
Cell 112
Fluorescence (Raw)
Time Point (0.25 Hz)
10
15
20
25
30
35
40
45
120
140
160
180
0
200
400
600
800

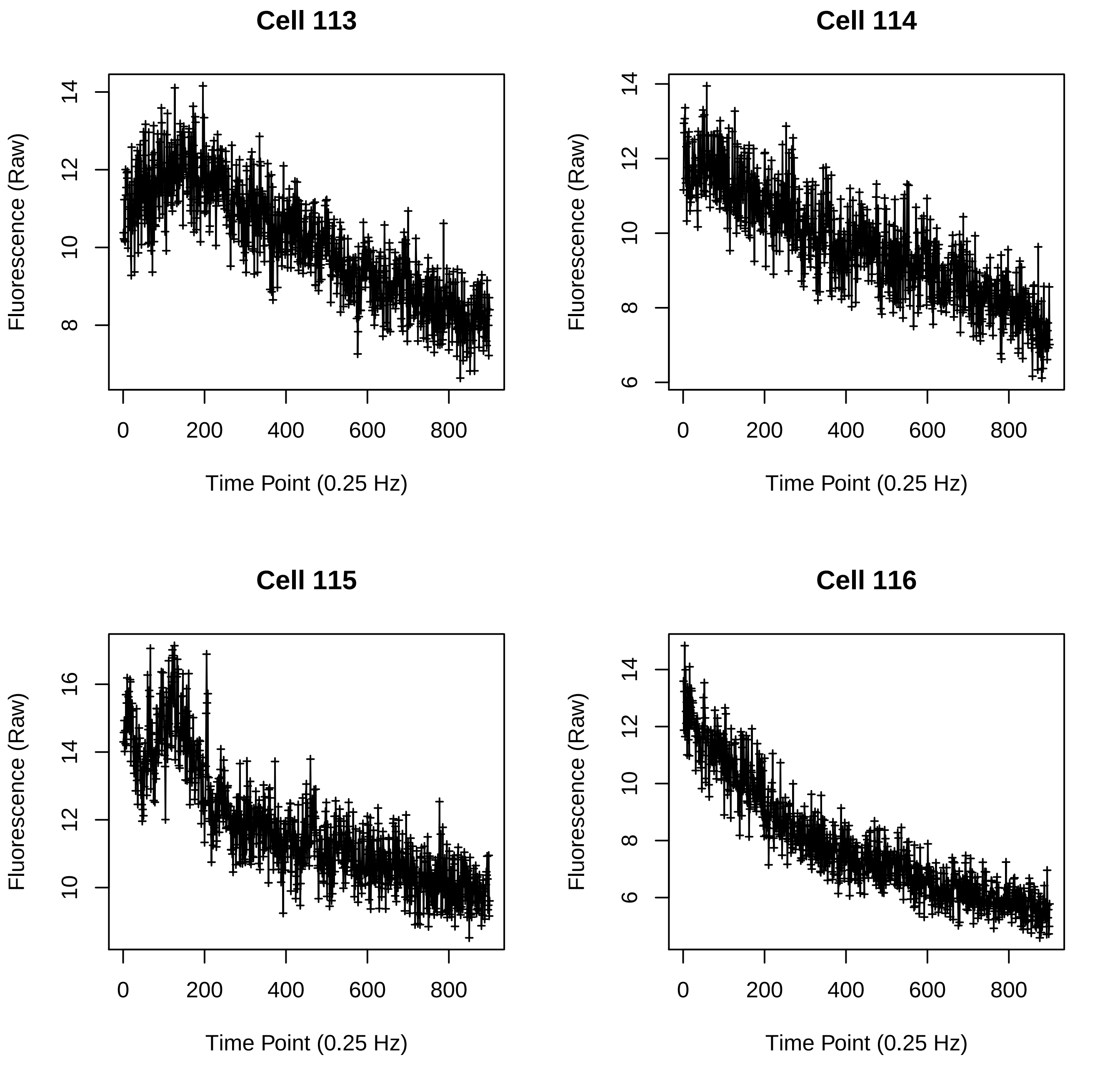

Cell 113
Cell 114
Cell 115
Cell 116
Fluorescence (Raw)
Time Point (0.25 Hz)
0
200
400
600
800

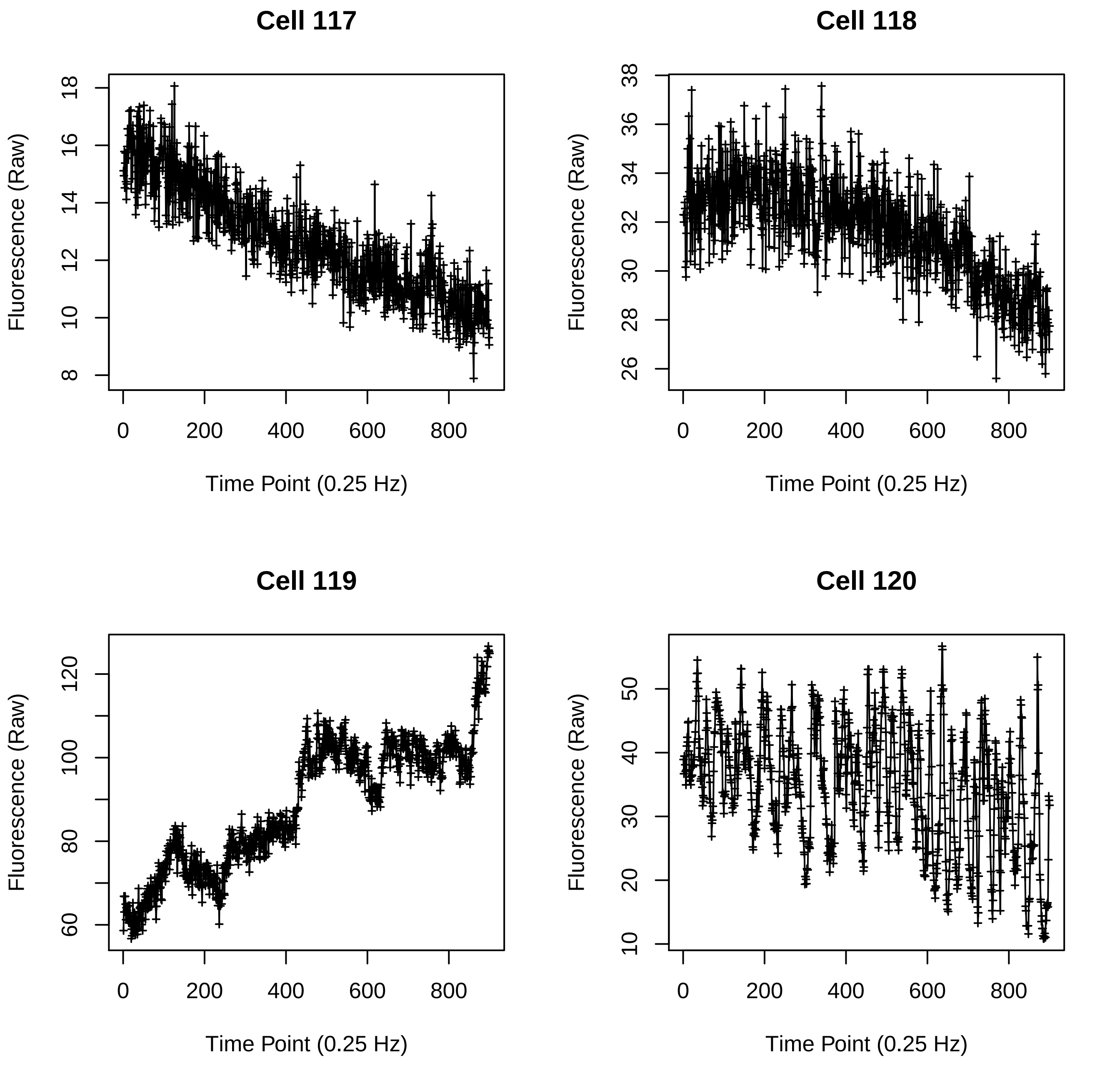
Cell 117
Cell 118
Cell 119
Cell 120
Fluorescence (Raw)
Time Point (0.25 Hz)
0
200
400
600
800
8
10
12
14
16
18
26
28
30
32
34
36
38
60
80
100
120
10
20
30
40
50

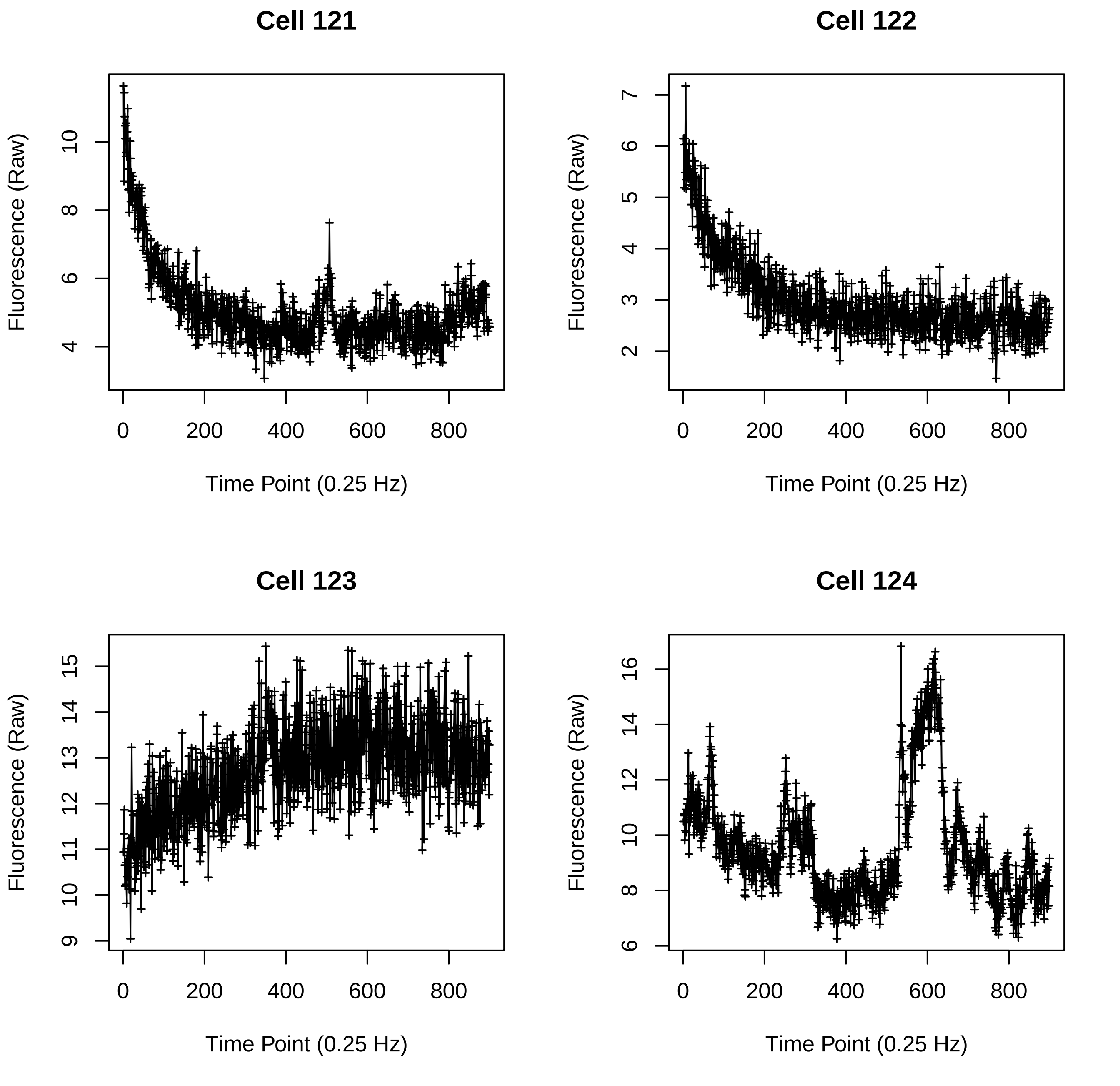

Cell 121
Cell 122
Cell 123
Cell 124
Fluorescence (Raw)
Time Point (0.25 Hz)
0
200
400
600
800

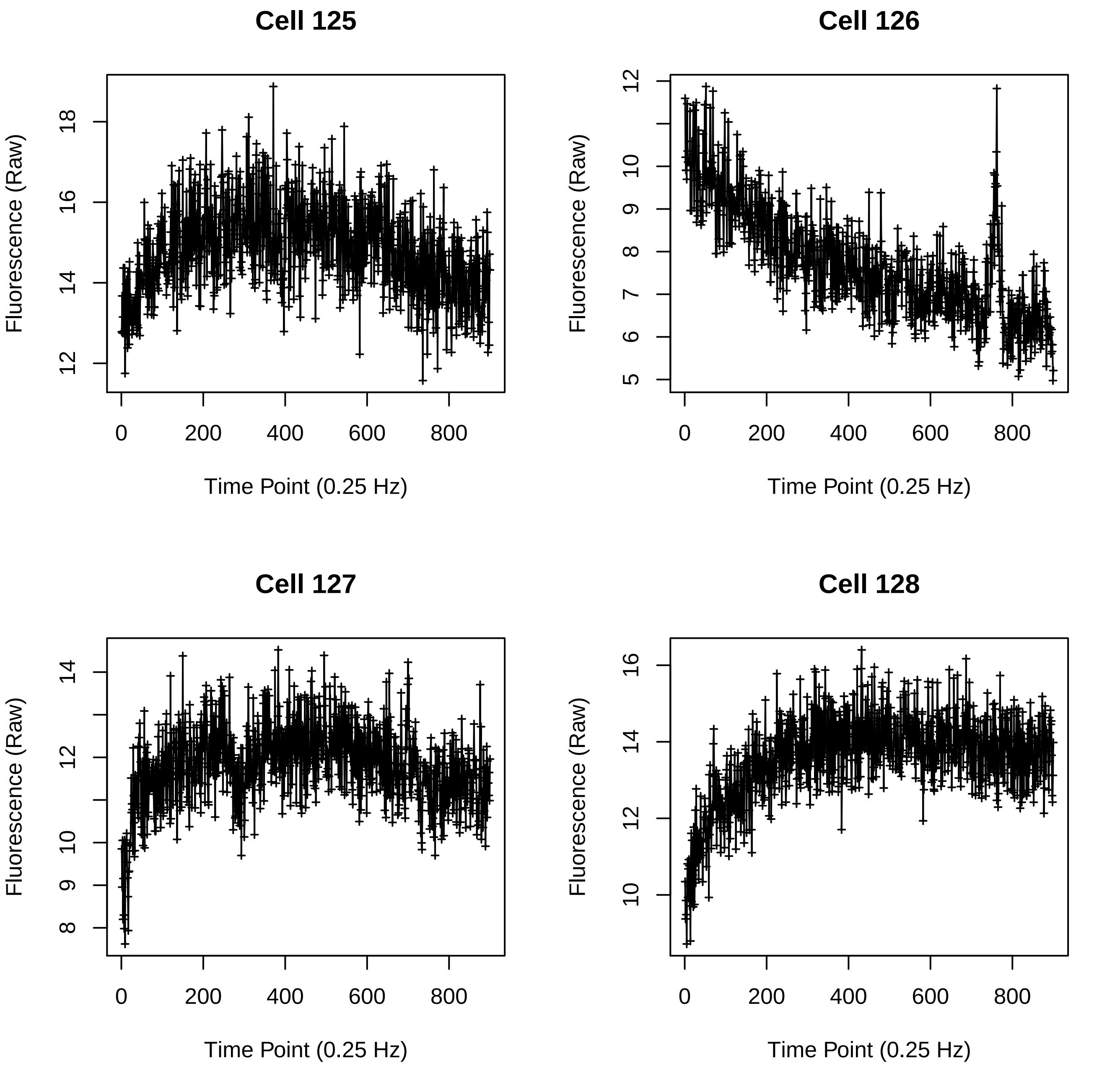
Cell 125
Fluorescence (Raw)
12
14
16
18
0
200
400
600
800
Time Point (0.25 Hz)
Cell 126
Fluorescence (Raw)
5
6
7
8
9
10
12
0
200
400
600
800
Time Point (0.25 Hz)
Cell 127
Fluorescence (Raw)
8
9
10
12
14
0
200
400
600
800
Time Point (0.25 Hz)
Cell 128
Fluorescence (Raw)
10
12
14
16
0
200
400
600
800
Time Point (0.25 Hz)

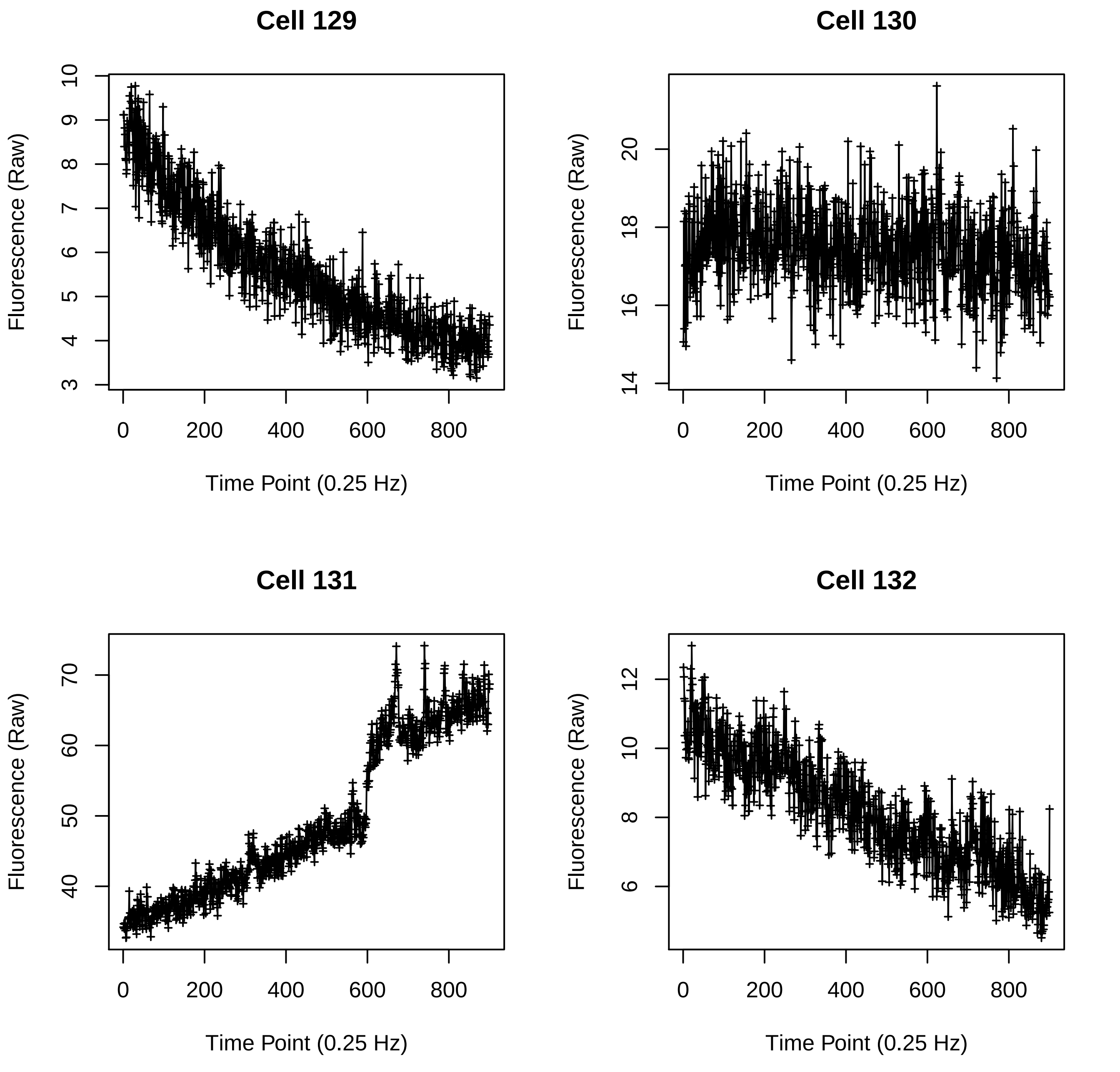
Cell 129
Cell 130
Cell 131
Cell 132
Fluorescence (Raw)
Time Point (0.25 Hz)
0
200
400
600
800
3
4
5
6
7
8
9
10
14
16
18
20
40
50
60
70
6
8
10
12

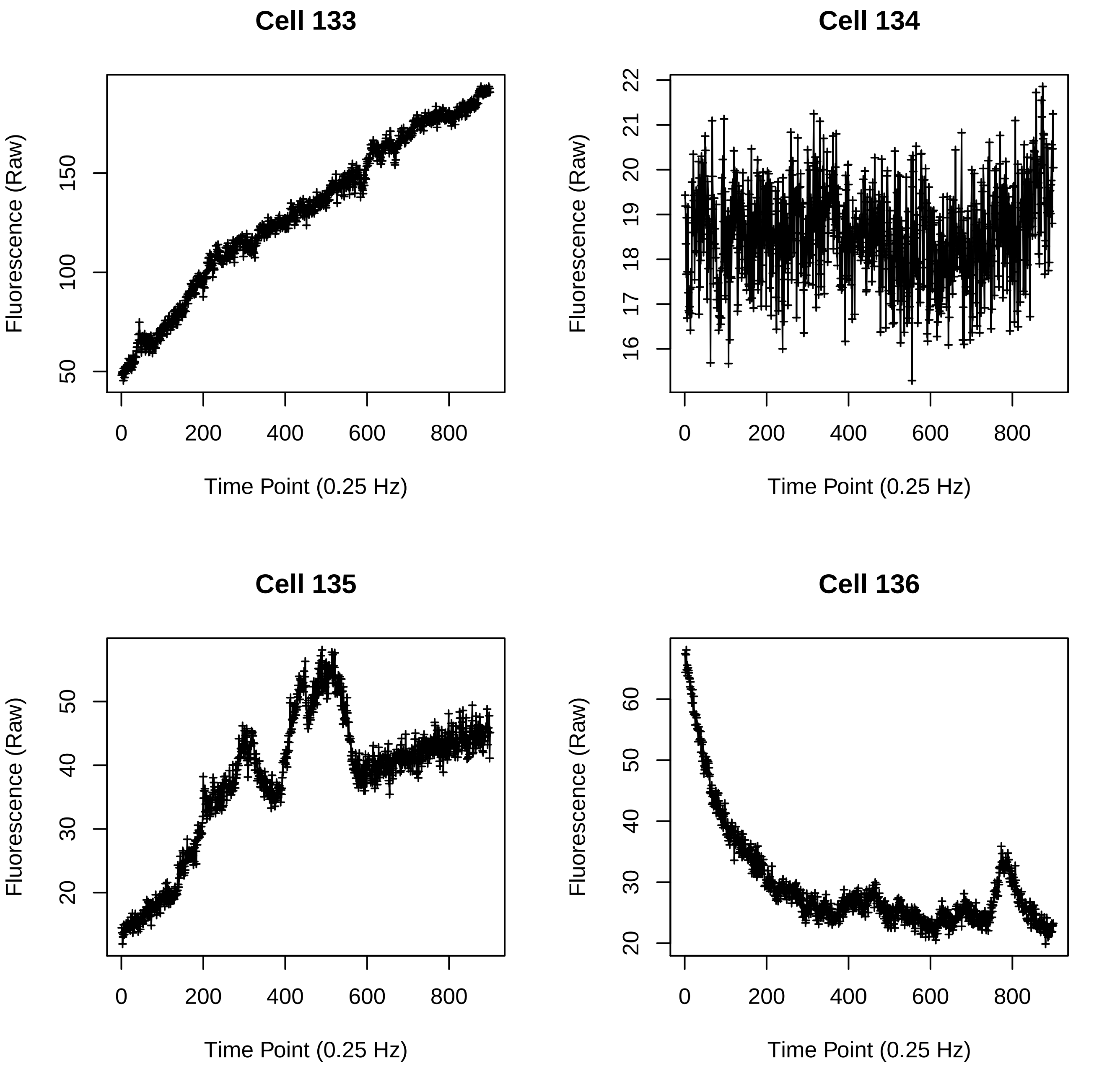
Cell 133
Fluorescence (Raw)
50
100
150
0
200
400
600
800
Time Point (0.25 Hz)
Cell 134
Fluorescence (Raw)
16
17
18
19
20
21
22
0
200
400
600
800
Time Point (0.25 Hz)
Cell 135
Fluorescence (Raw)
20
30
40
50
0
200
400
600
800
Time Point (0.25 Hz)
Cell 136
Fluorescence (Raw)
20
30
40
50
60
0
200
400
600
800
Time Point (0.25 Hz)

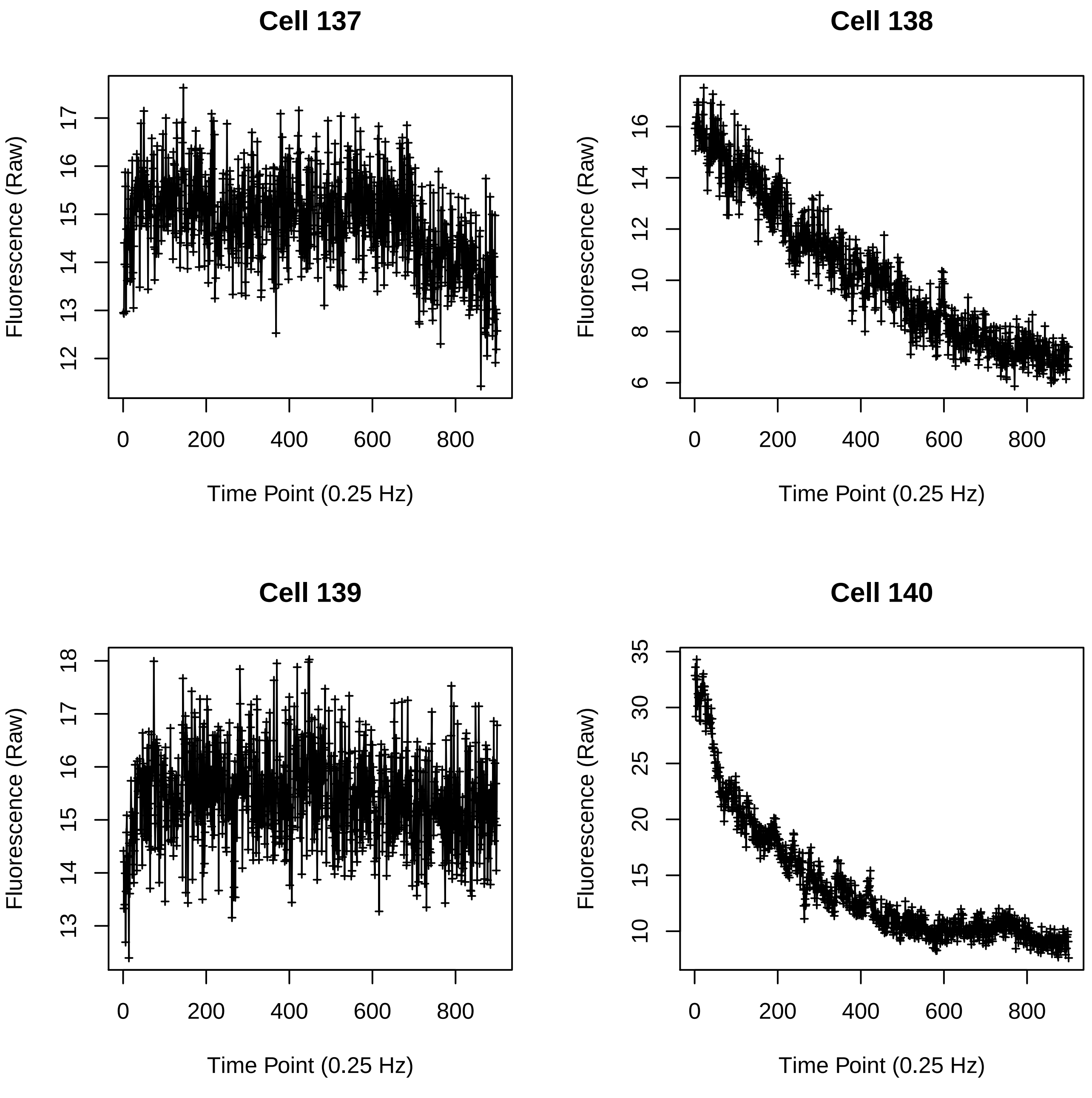

Cell 137
Fluorescence (Raw)
12
13
14
15
16
17
0
200
400
600
800
Time Point (0.25 Hz)
Cell 138
Fluorescence (Raw)
6
8
10
12
14
16
0
200
400
600
800
Time Point (0.25 Hz)
Cell 139
Fluorescence (Raw)
13
14
15
16
17
18
0
200
400
600
800
Time Point (0.25 Hz)
Cell 140
Fluorescence (Raw)
10
15
20
25
30
35
0
200
400
600
800
Time Point (0.25 Hz)

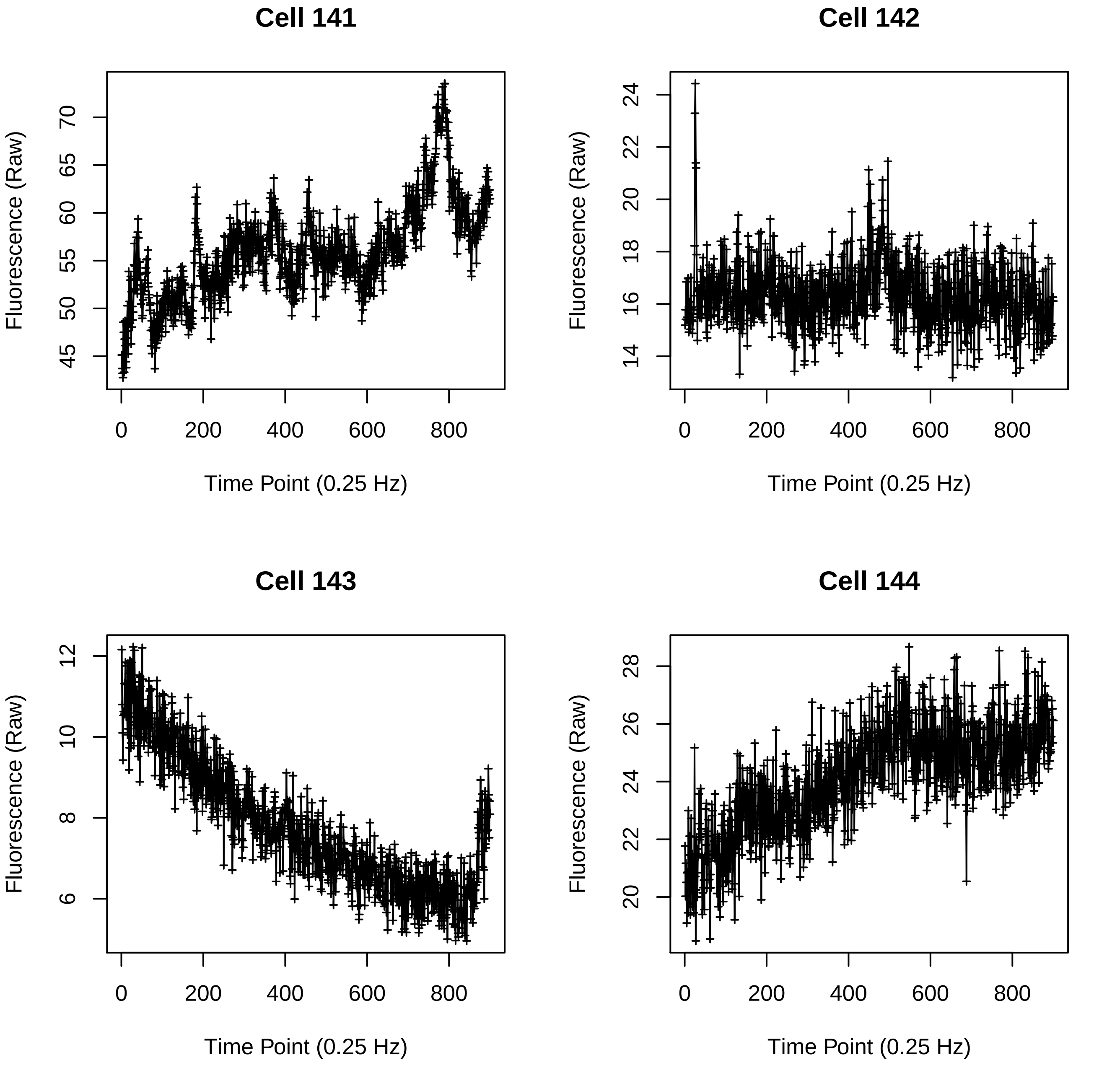
Cell 141
Fluorescence (Raw)
45
50
55
60
65
70
0
200
400
600
800
Time Point (0.25 Hz)
Cell 142
Fluorescence (Raw)
14
16
18
20
22
24
0
200
400
600
800
Time Point (0.25 Hz)
Cell 143
Fluorescence (Raw)
6
8
10
12
0
200
400
600
800
Time Point (0.25 Hz)
Cell 144
Fluorescence (Raw)
20
22
24
26
28
0
200
400
600
800
Time Point (0.25 Hz)

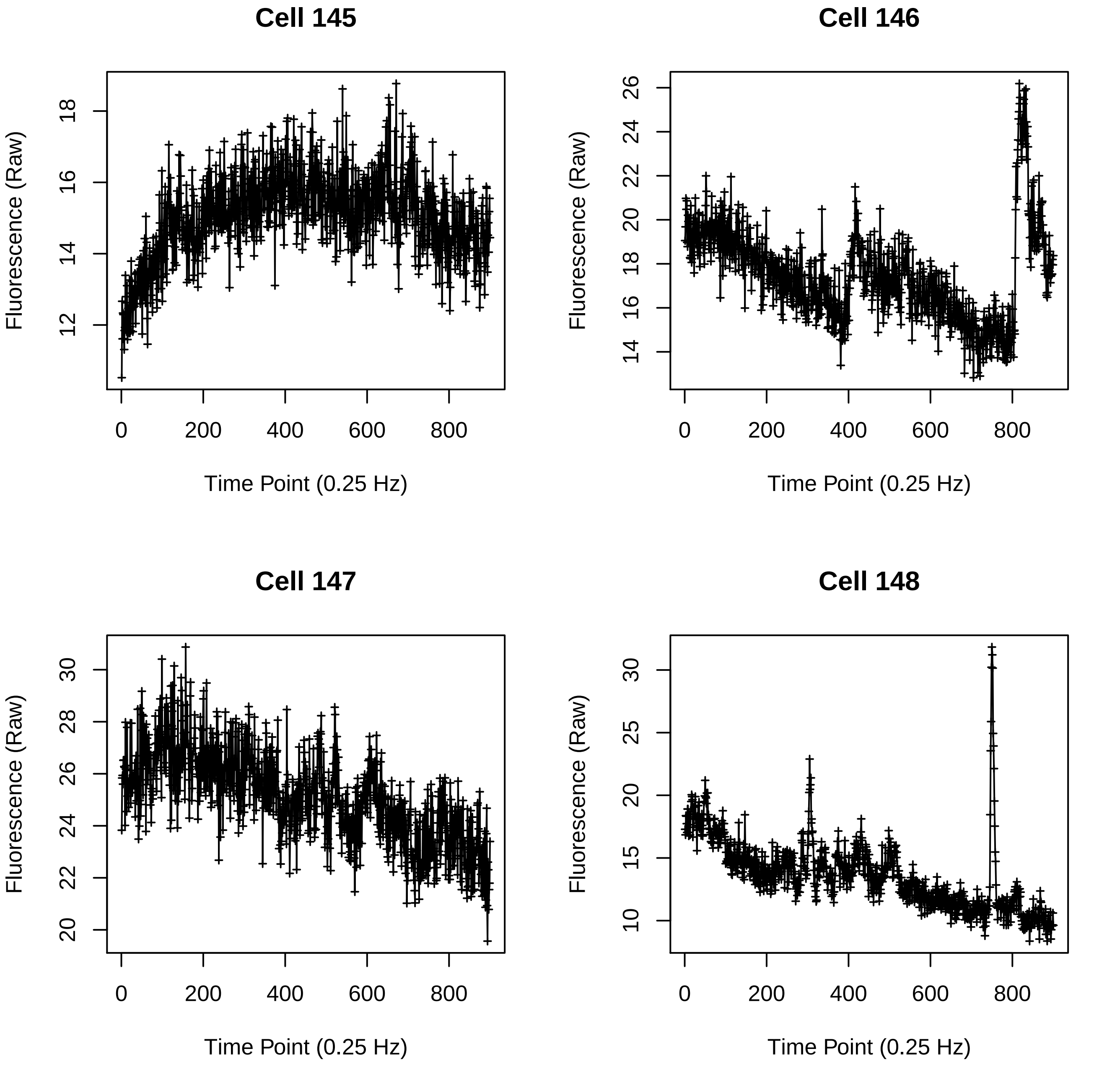
Cell 145
Cell 146
Cell 147
Cell 148
Fluorescence (Raw)
Time Point (0.25 Hz)
0
200
400
600
800
12
14
16
18
20
22
24
26
28
30
10
15
25

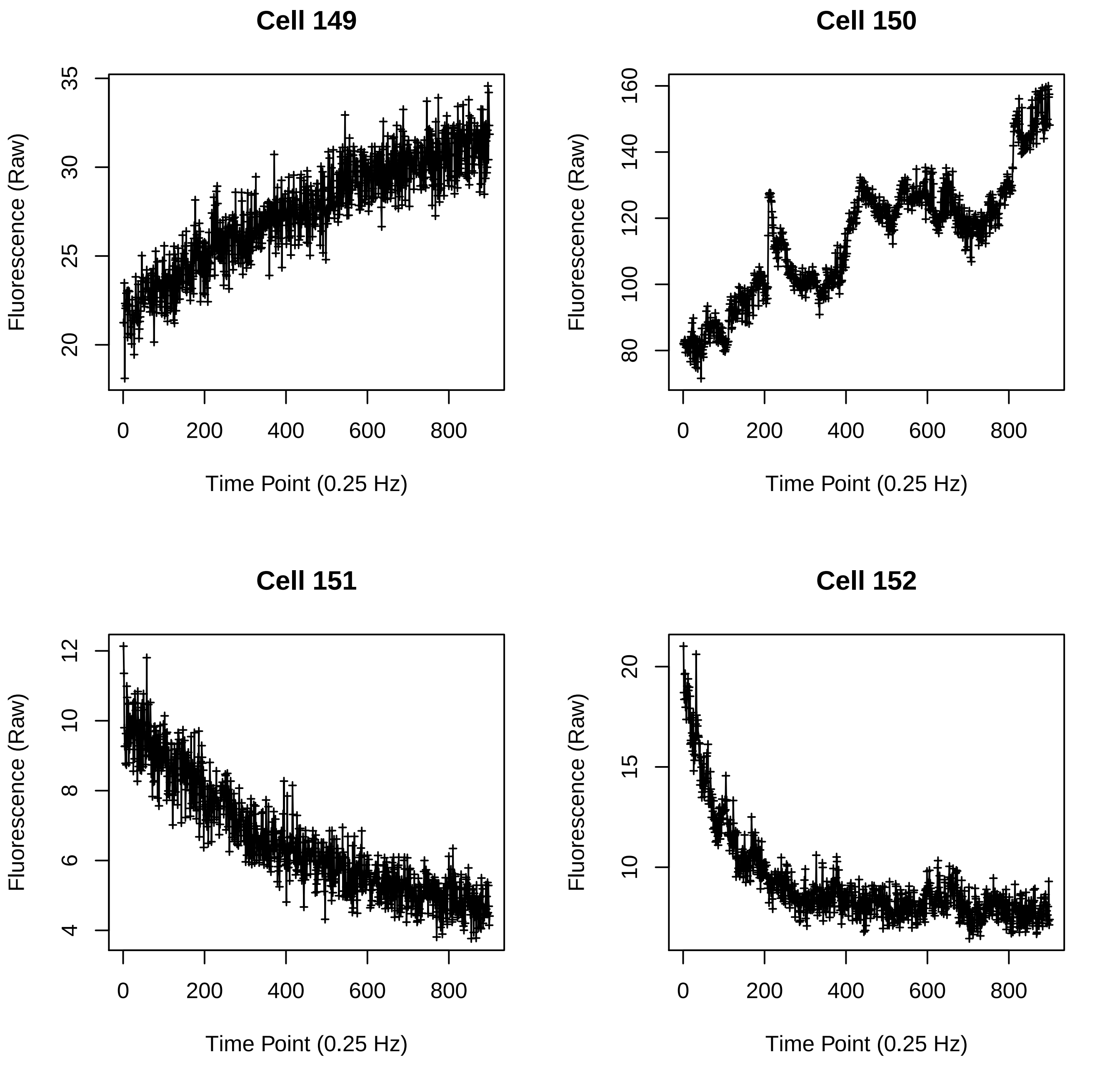
Cell 149
Cell 150
Cell 151
Cell 152
Fluorescence (Raw)
Time Point (0.25 Hz)
0
200
400
600
800
20
25
30
35
80
100
120
140
160
4
6
8
10
12
15

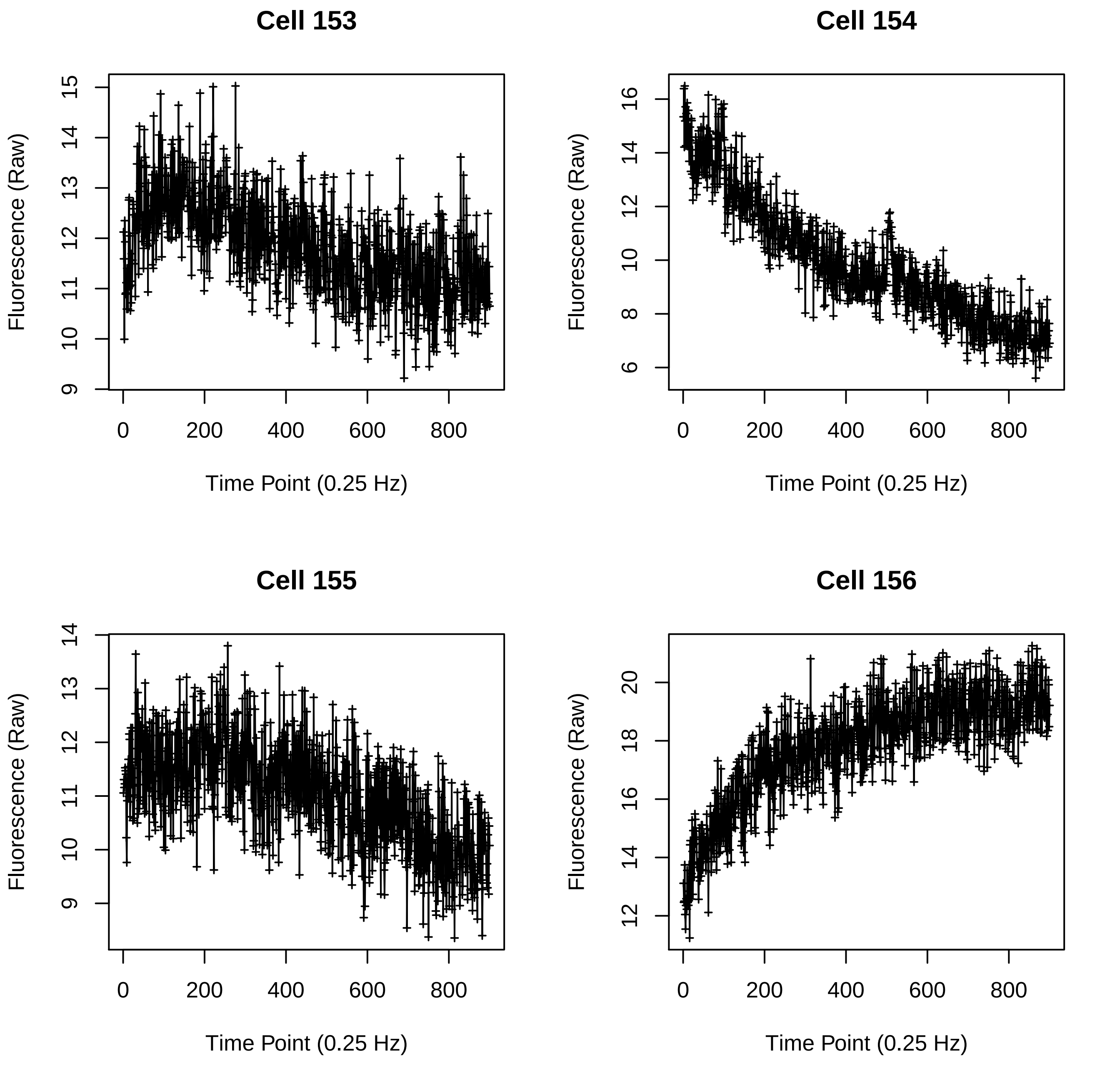
Cell 153
Cell 154
Cell 155
Cell 156
Fluorescence (Raw)
Time Point (0.25 Hz)
0
200
400
600
800
9
10
11
12
13
14
15
6
8
10
12
14
16
20
18

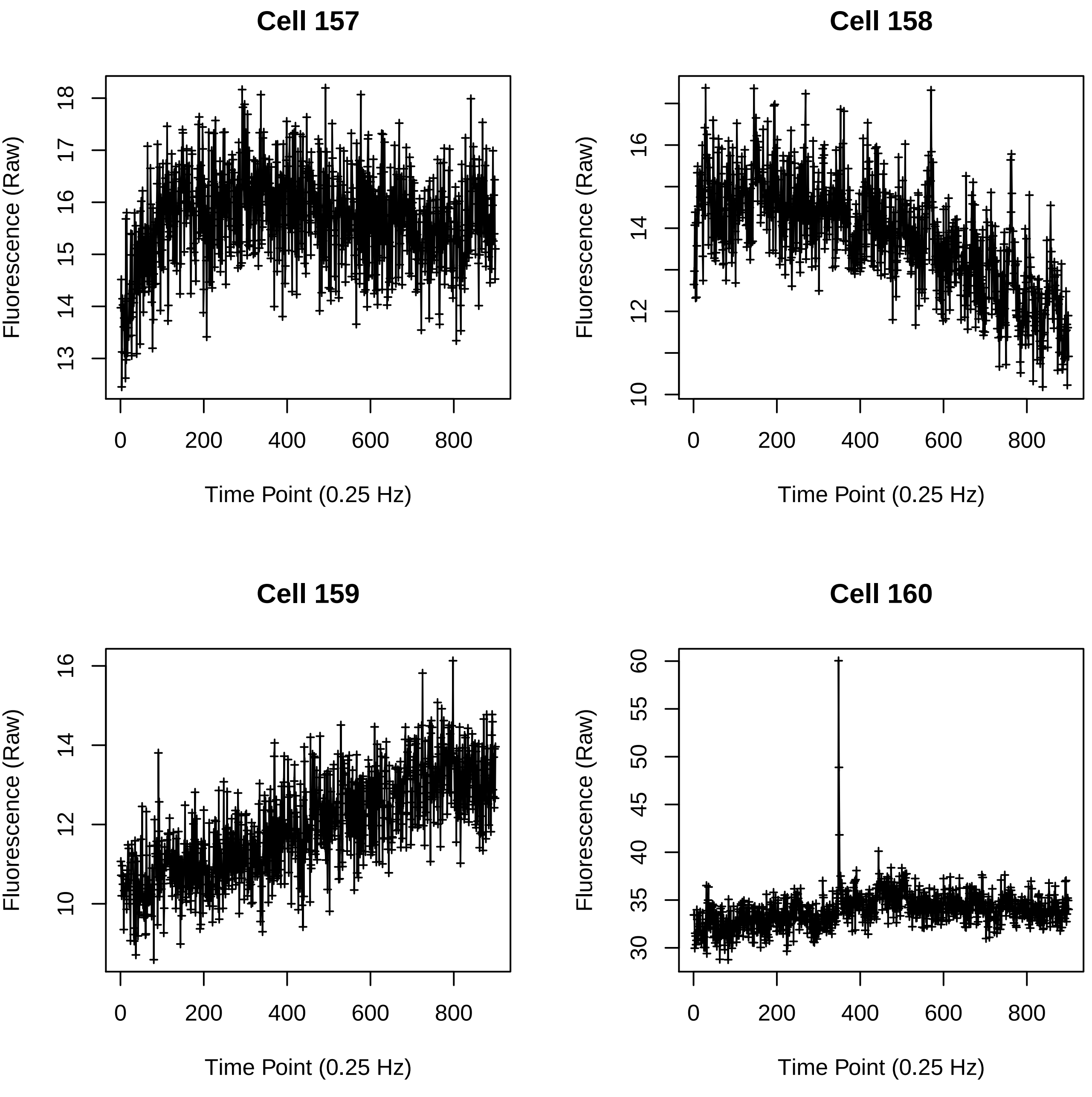
Cell 157
Cell 158
Cell 159
Cell 160
Fluorescence (Raw)
Time Point (0.25 Hz)
0
200
400
600
800
13
14
15
16
17
18
10
12
14
16
30
35
40
45
50
55
60

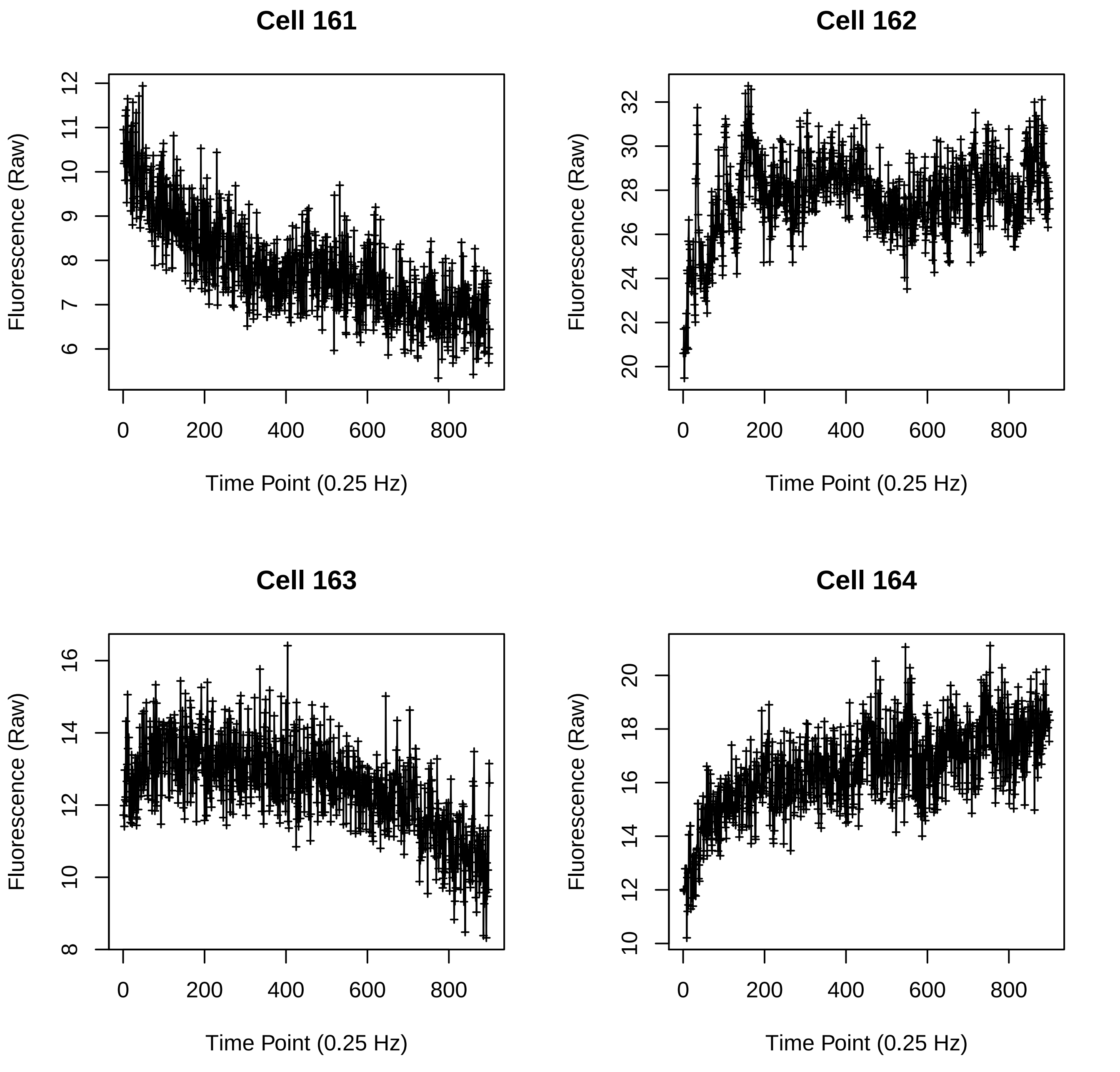
Cell 161
Cell 162
Cell 163
Cell 164
Fluorescence (Raw)
Time Point (0.25 Hz)
0
200
400
600
800
6
7
8
9
10
11
12
20
22
24
26
28
30
32
8
10
12
14
16
10
12
14
16
18
20

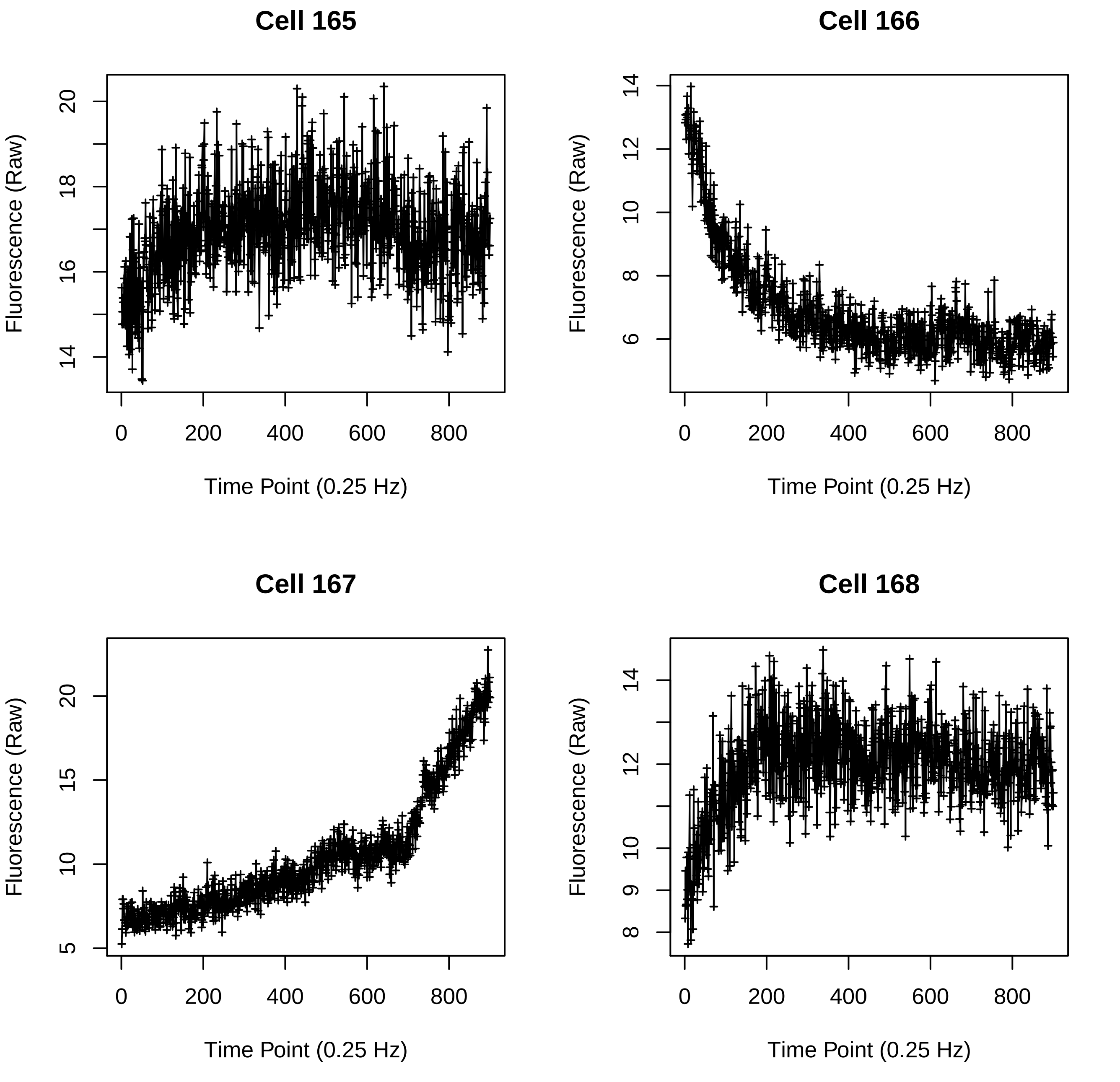

Cell 165
Cell 166
Cell 167
Cell 168
Fluorescence (Raw)
Time Point (0.25 Hz)
0
200
400
600
800
14
16
18
20
6
8
10
12
14
5
10
15
20
8
9
10
12
14

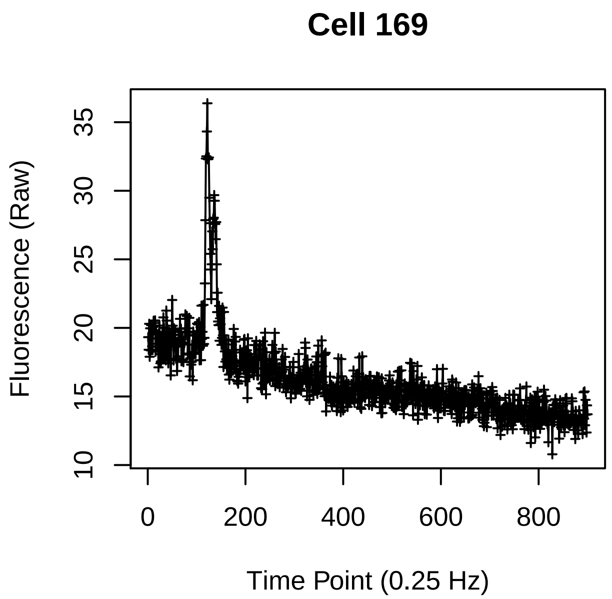
Cell 169
Fluorescence (Raw)
10
15
20
25
30
35
0
200
400
600
800
Time Point (0.25 Hz)

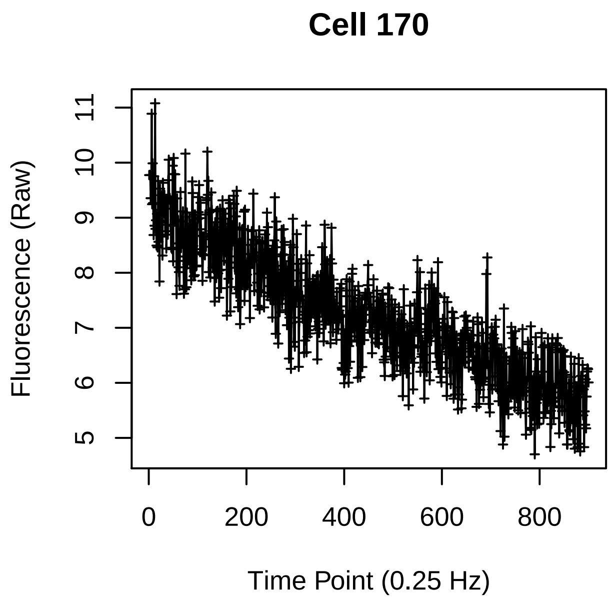
Cell 170
Fluorescence (Raw)
5
6
7
8
9
10
11
0
200
400
600
800
Time Point (0.25 Hz)

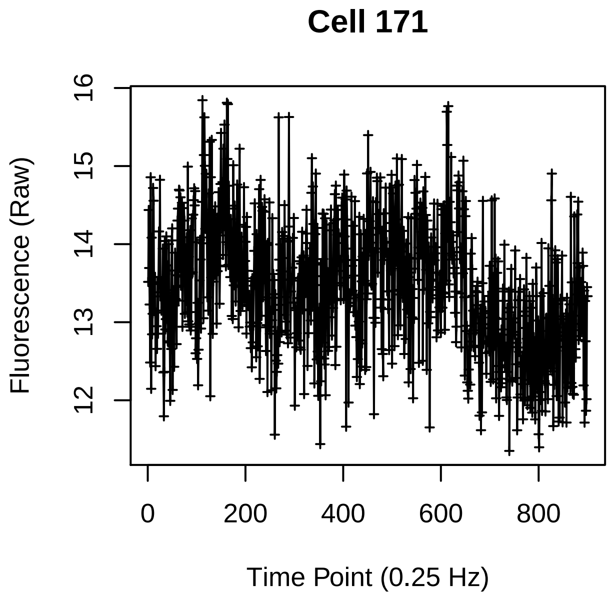
Cell 171
Fluorescence (Raw)
12
13
14
15
16
0
200
400
600
800
Time Point (0.25 Hz)

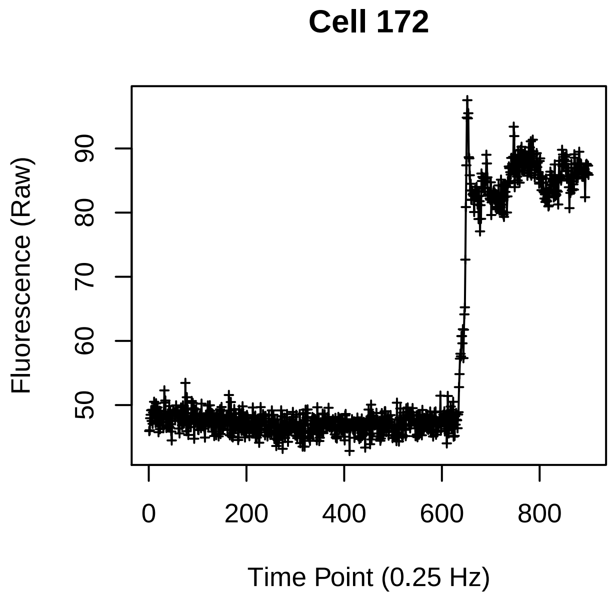
Cell 172
Fluorescence (Raw)
50
60
70
80
90
0
200
400
600
800
Time Point (0.25 Hz)

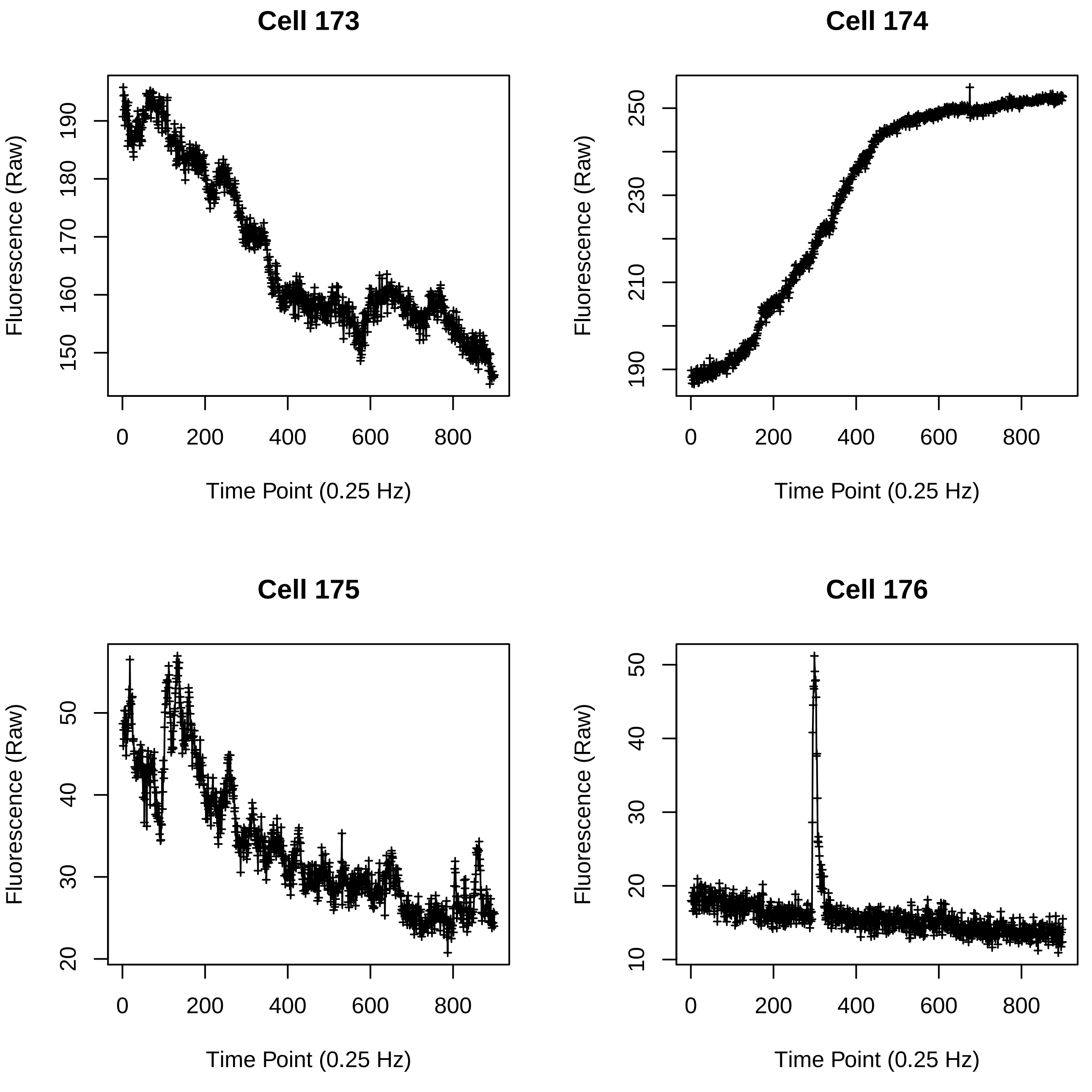
Cell 173
Fluorescence (Raw)
150
160
170
180
190
0
200
400
600
800
Time Point (0.25 Hz)
Cell 174
Fluorescence (Raw)
190
210
230
250
0
200
400
600
800
Time Point (0.25 Hz)
Cell 175
Fluorescence (Raw)
20
30
40
50
0
200
400
600
800
Time Point (0.25 Hz)
Cell 176
Fluorescence (Raw)
10
20
30
40
50
0
200
400
600
800
Time Point (0.25 Hz)

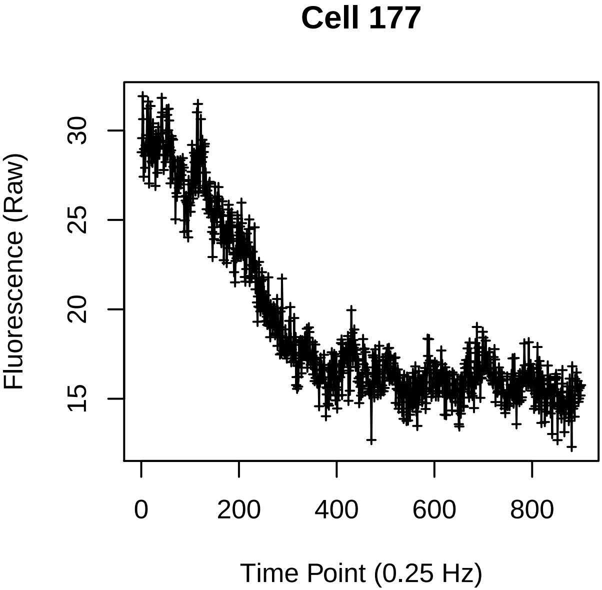
Cell 177
Fluorescence (Raw)
15
20
25
30
0
200
400
600
800
Time Point (0.25 Hz)

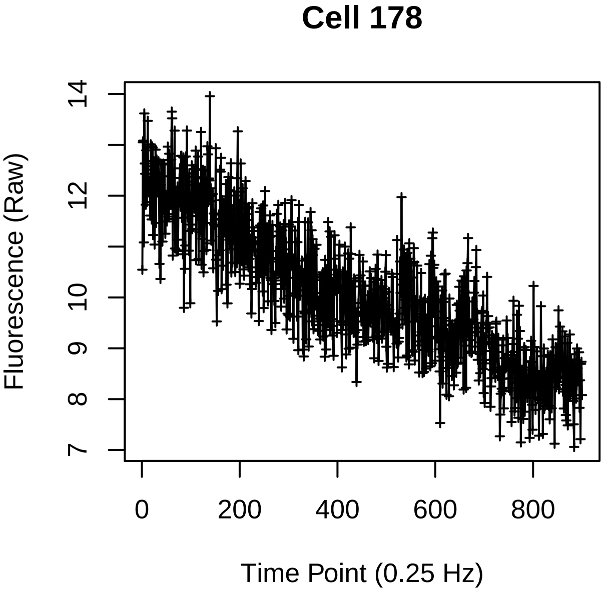
Cell 178
Fluorescence (Raw)
7
8
9
10
12
14
0
200
400
600
800
Time Point (0.25 Hz)

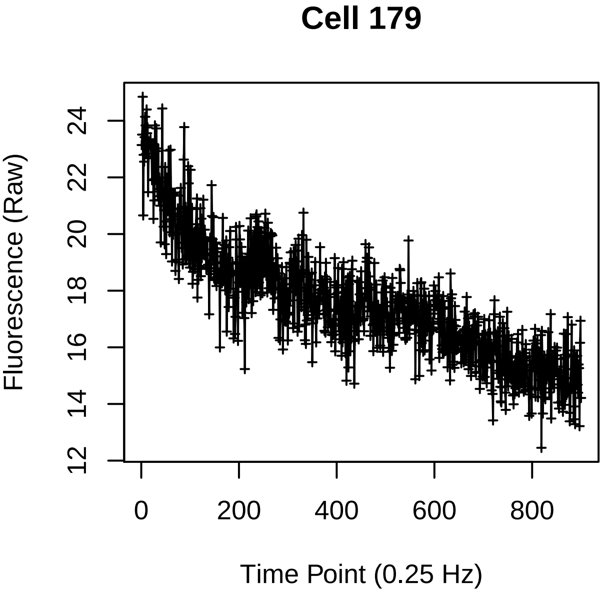
Cell 179
Fluorescence (Raw)
12
14
16
18
20
22
24
0
200
400
600
800
Time Point (0.25 Hz)

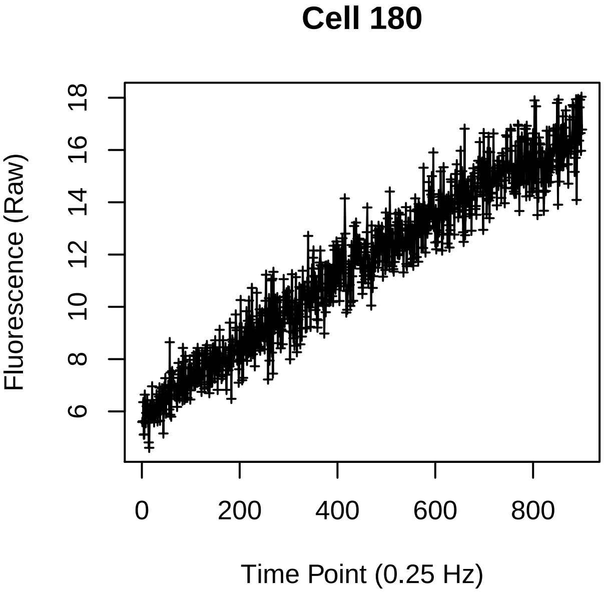
Cell 180
Fluorescence (Raw)
6
8
10
12
14
16
18
0
200
400
600
800
Time Point (0.25 Hz)

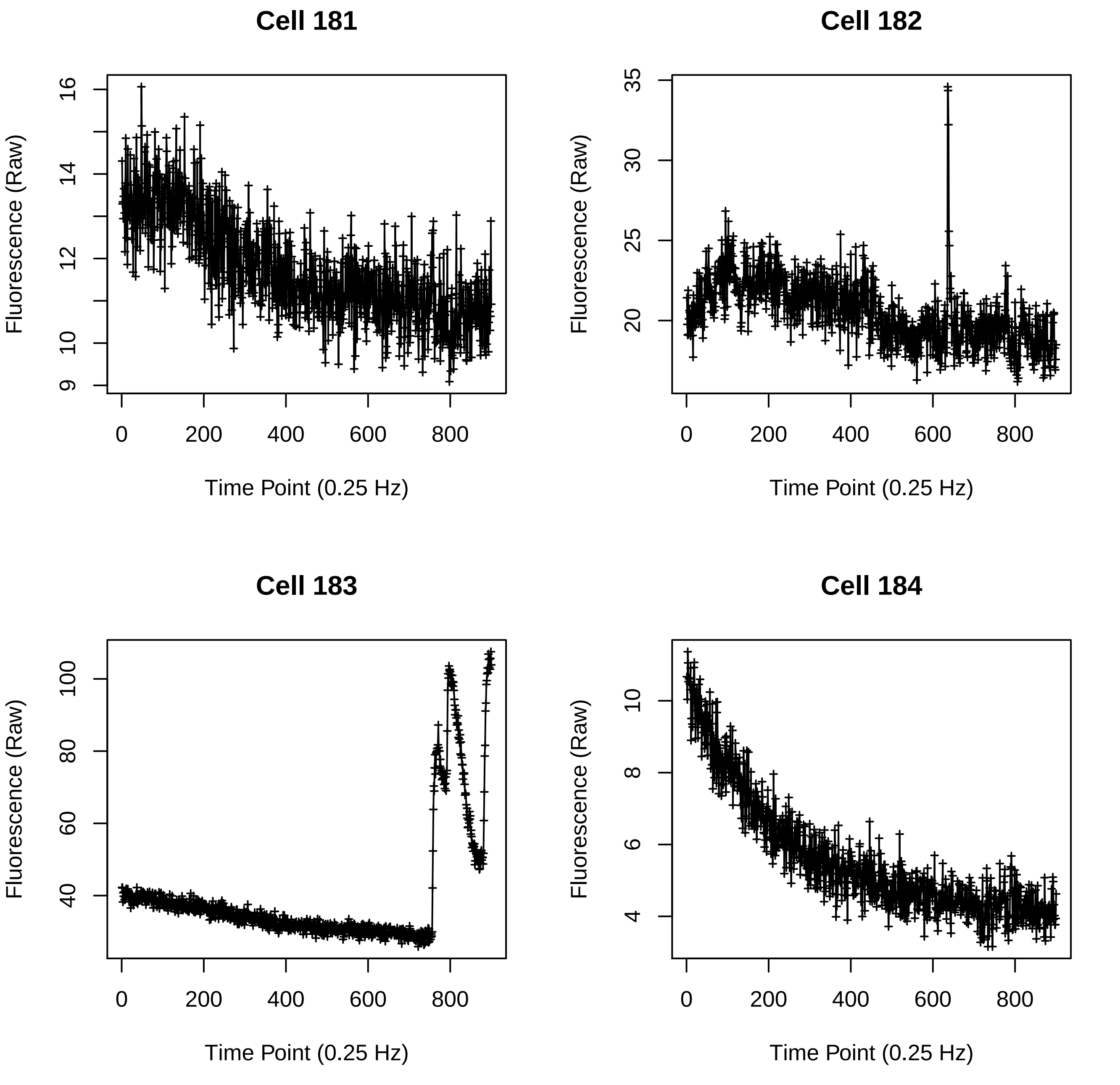
Cell 181
Cell 182
Cell 183
Cell 184
Fluorescence (Raw)
Time Point (0.25 Hz)
0
200
400
600
800
9
10
12
14
16
20
25
30
35
40
60
80
100
4
6
8
10

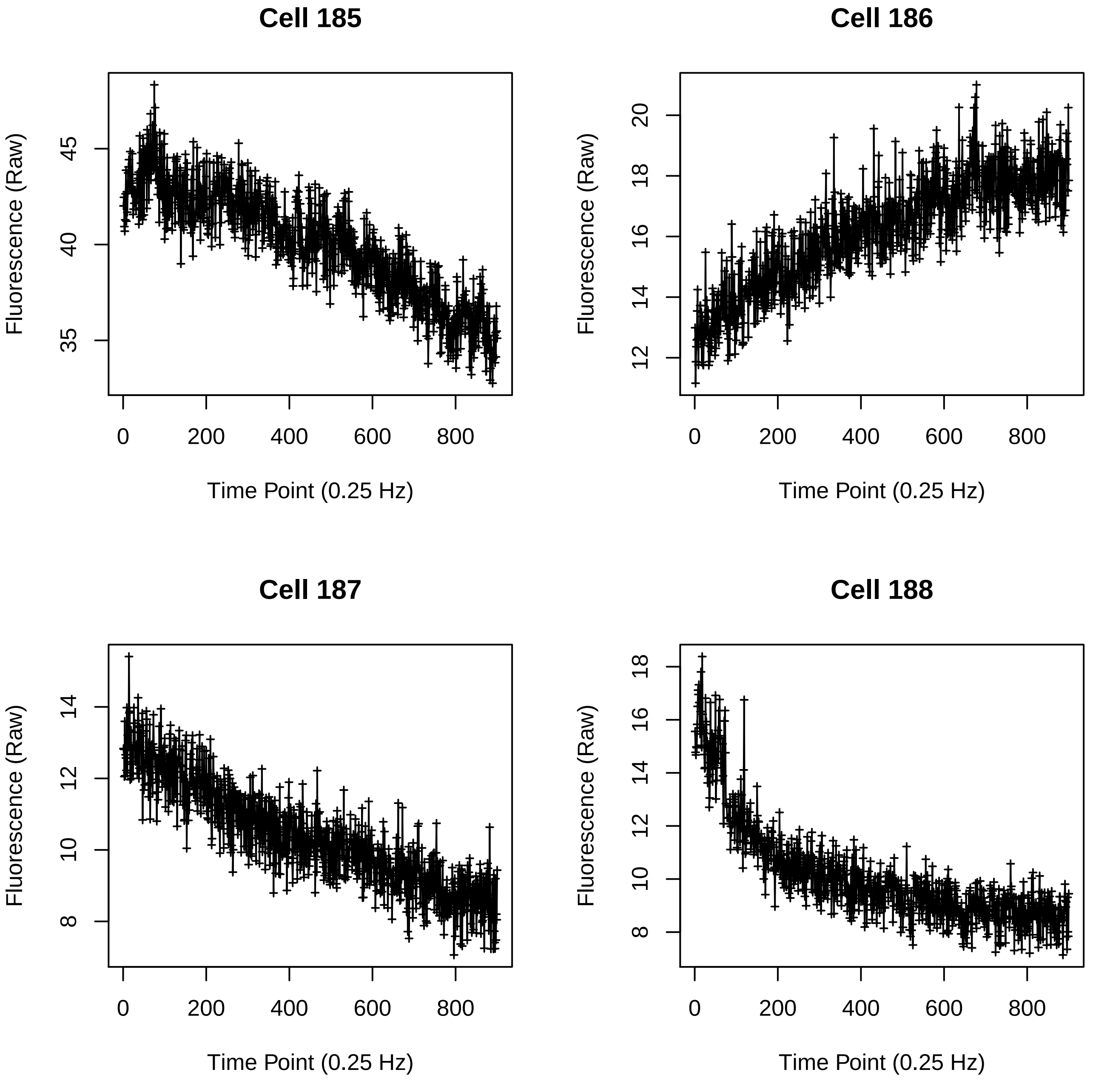

Cell 185
Cell 186
Cell 187
Cell 188
Fluorescence (Raw)
Time Point (0.25 Hz)
0
200
400
600
800
35
40
45
12
14
16
18
20
8
10

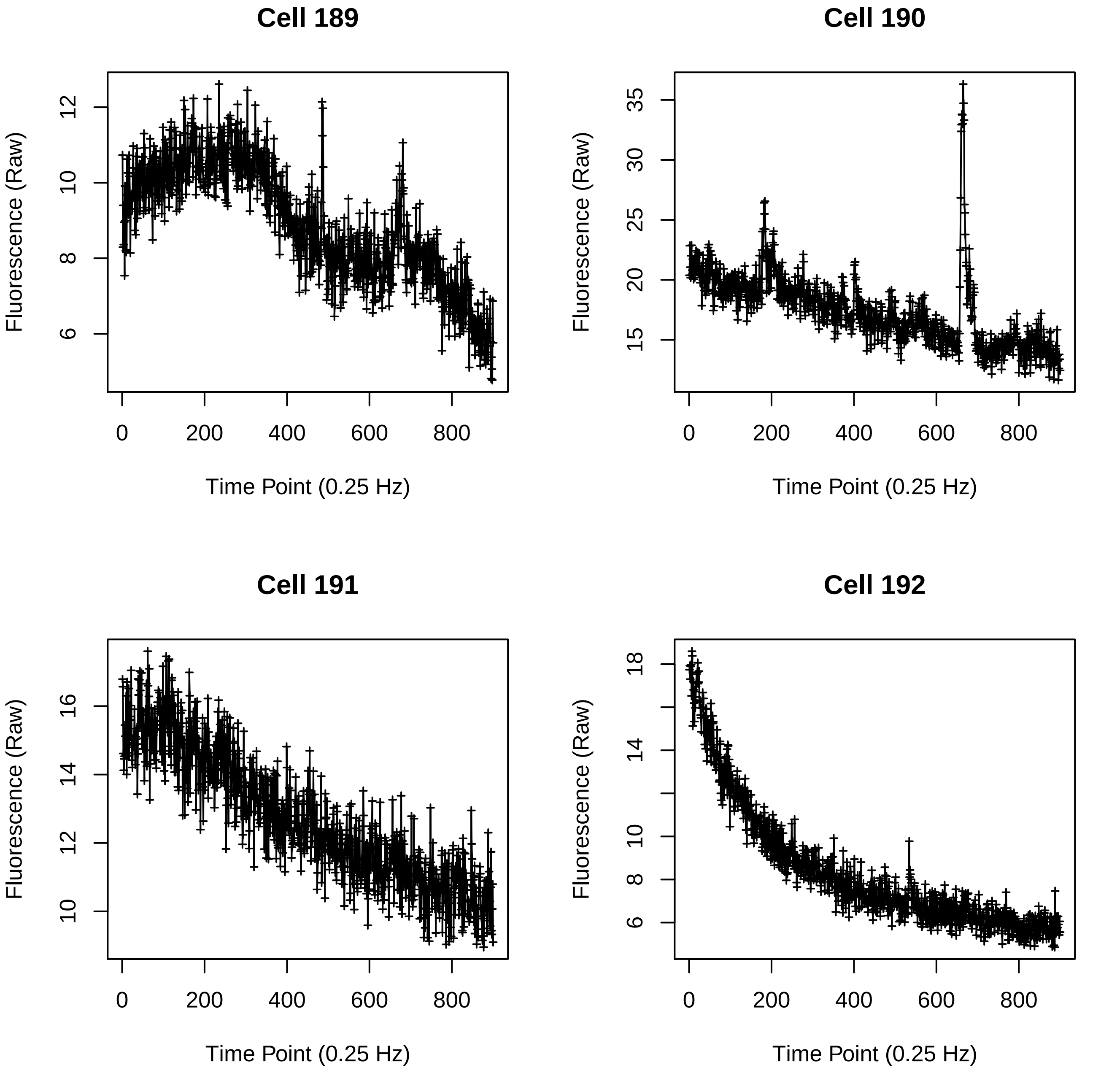

Cell 189
Cell 190
Cell 191
Cell 192
Fluorescence (Raw)
Time Point (0.25 Hz)

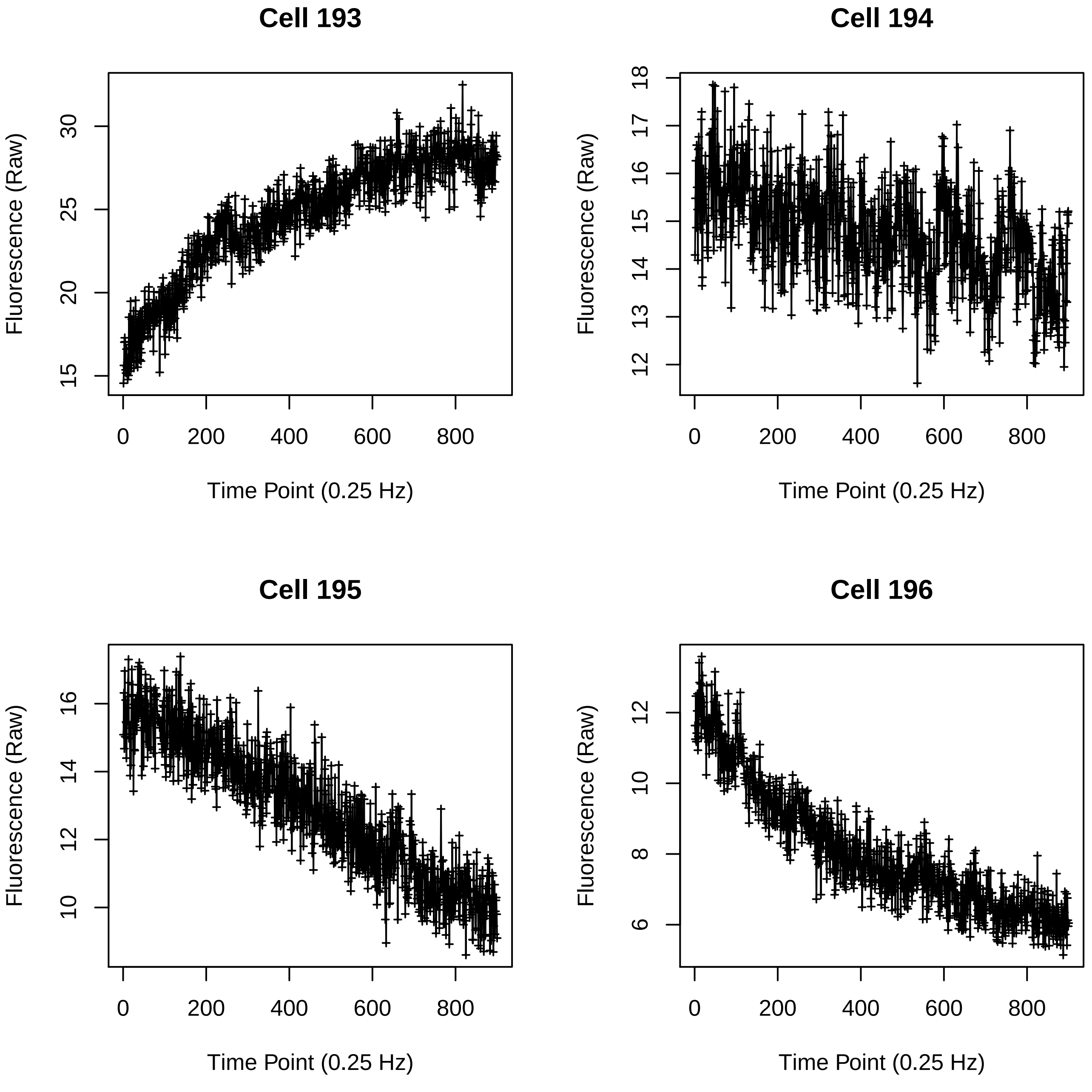
Cell 193
Fluorescence (Raw)
Time Point (0.25 Hz)
15
20
25
30
0
200
400
600
800
Cell 194
Fluorescence (Raw)
Time Point (0.25 Hz)
12
13
14
15
16
17
18
0
200
400
600
800
Cell 195
Fluorescence (Raw)
Time Point (0.25 Hz)
10
12
14
16
0
200
400
600
800
Cell 196
Fluorescence (Raw)
Time Point (0.25 Hz)
6
8
10
12
0
200
400
600
800

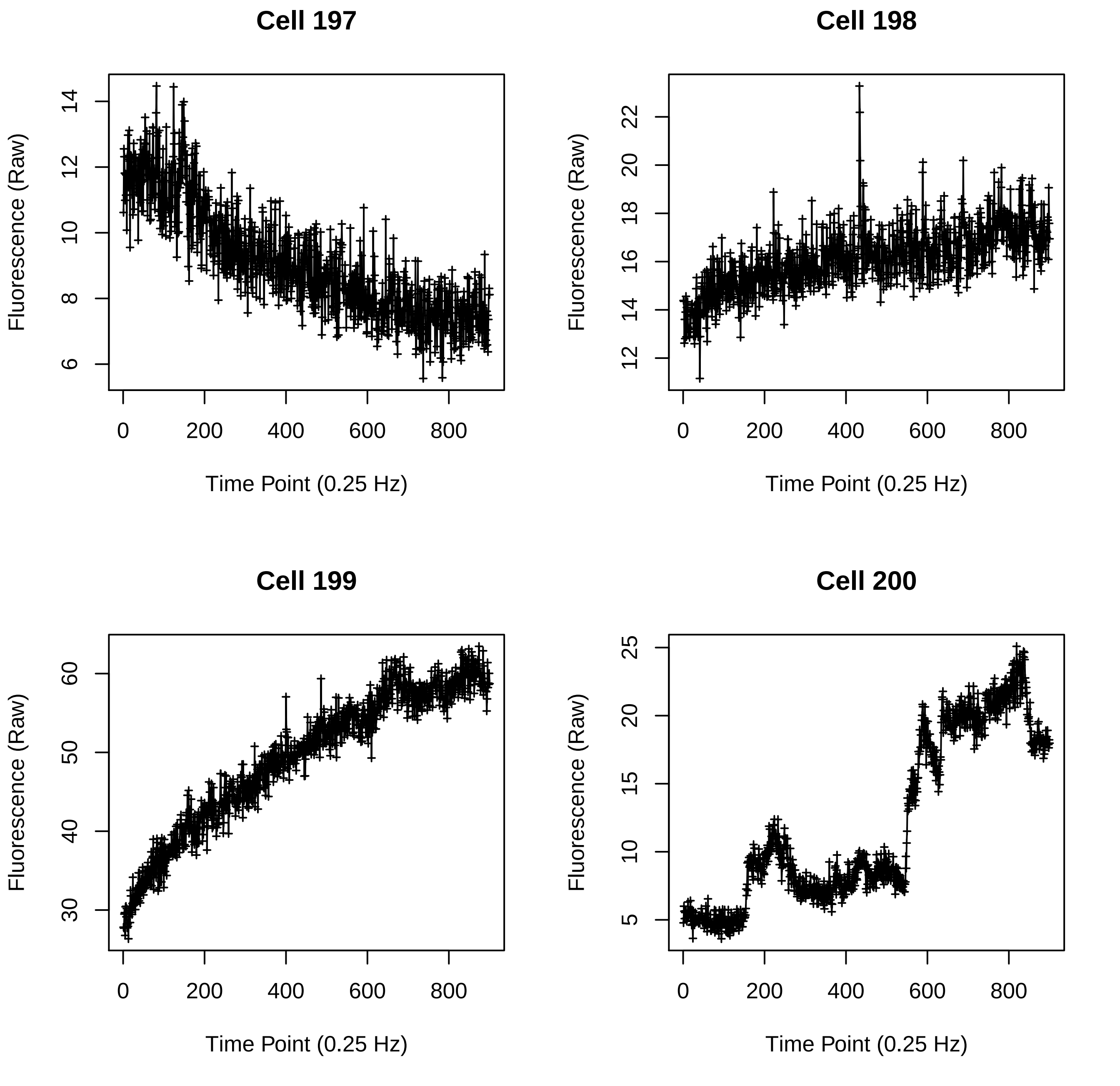

Cell 197
Cell 198
Cell 199
Cell 200
Fluorescence (Raw)
Time Point (0.25 Hz)
0
200
400
600
800
6
8
10
12
14
16
18
20
22
30
40
50
60
5
15
25

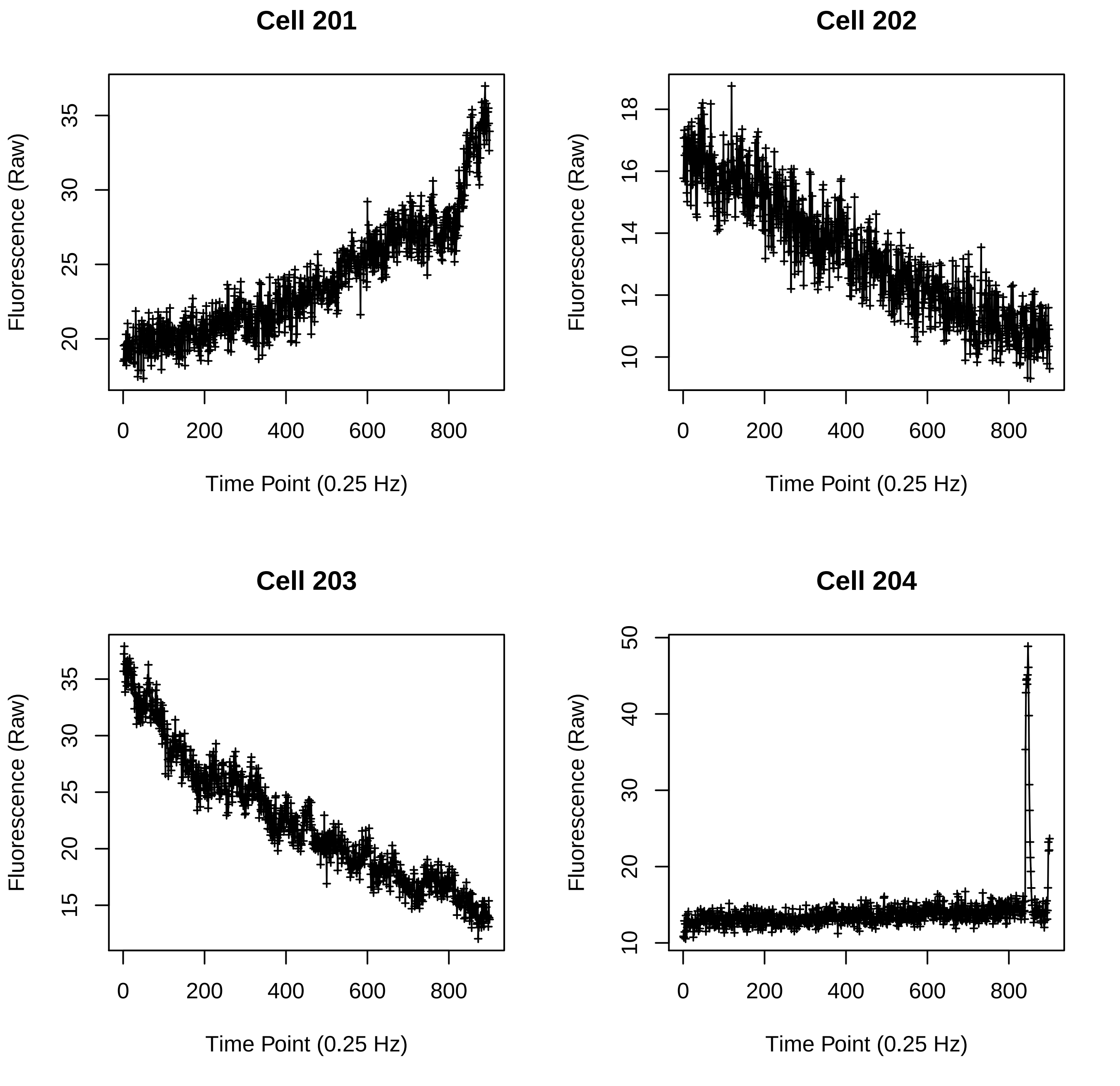
Cell 201
Fluorescence (Raw)
20
25
30
35
0
200
400
600
800
Time Point (0.25 Hz)
Cell 202
Fluorescence (Raw)
10
12
14
16
18
0
200
400
600
800
Time Point (0.25 Hz)
Cell 203
Fluorescence (Raw)
15
20
25
30
35
0
200
400
600
800
Time Point (0.25 Hz)
Cell 204
Fluorescence (Raw)
10
20
30
40
50
0
200
400
600
800
Time Point (0.25 Hz)

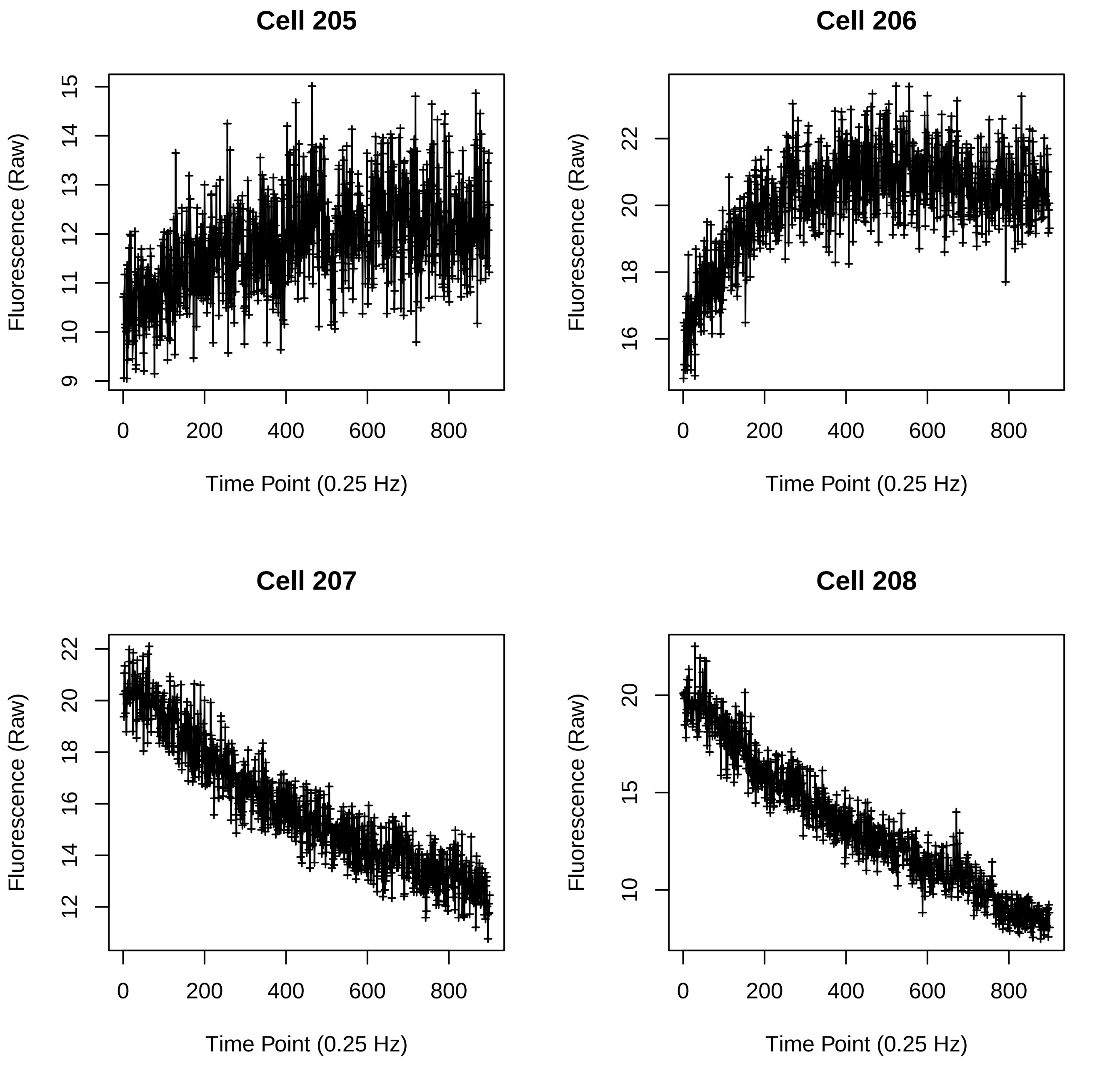

Cell 205
Fluorescence (Raw)
9
10
11
12
13
14
15
0
200
400
600
800
Time Point (0.25 Hz)
Cell 206
Fluorescence (Raw)
16
18
20
22
0
200
400
600
800
Time Point (0.25 Hz)
Cell 207
Fluorescence (Raw)
12
14
16
18
20
22
0
200
400
600
800
Time Point (0.25 Hz)
Cell 208
Fluorescence (Raw)
10
15
20
0
200
400
600
800
Time Point (0.25 Hz)

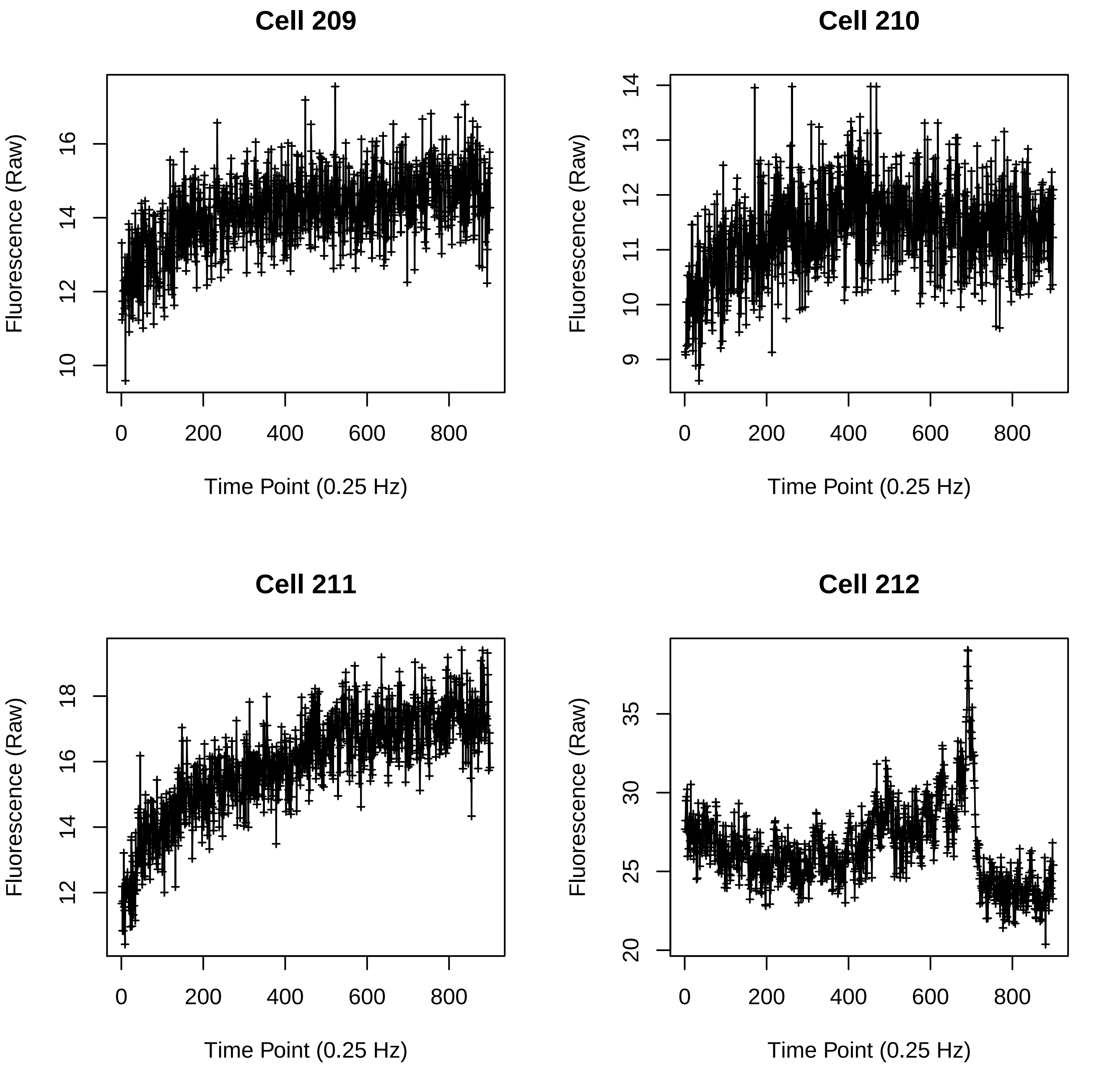
Cell 209
Fluorescence (Raw)
10
12
14
16
0
200
400
600
800
Time Point (0.25 Hz)
Cell 210
Fluorescence (Raw)
9
10
11
12
13
14
0
200
400
600
800
Time Point (0.25 Hz)
Cell 211
Fluorescence (Raw)
12
14
16
18
0
200
400
600
800
Time Point (0.25 Hz)
Cell 212
Fluorescence (Raw)
20
25
30
35
0
200
400
600
800
Time Point (0.25 Hz)

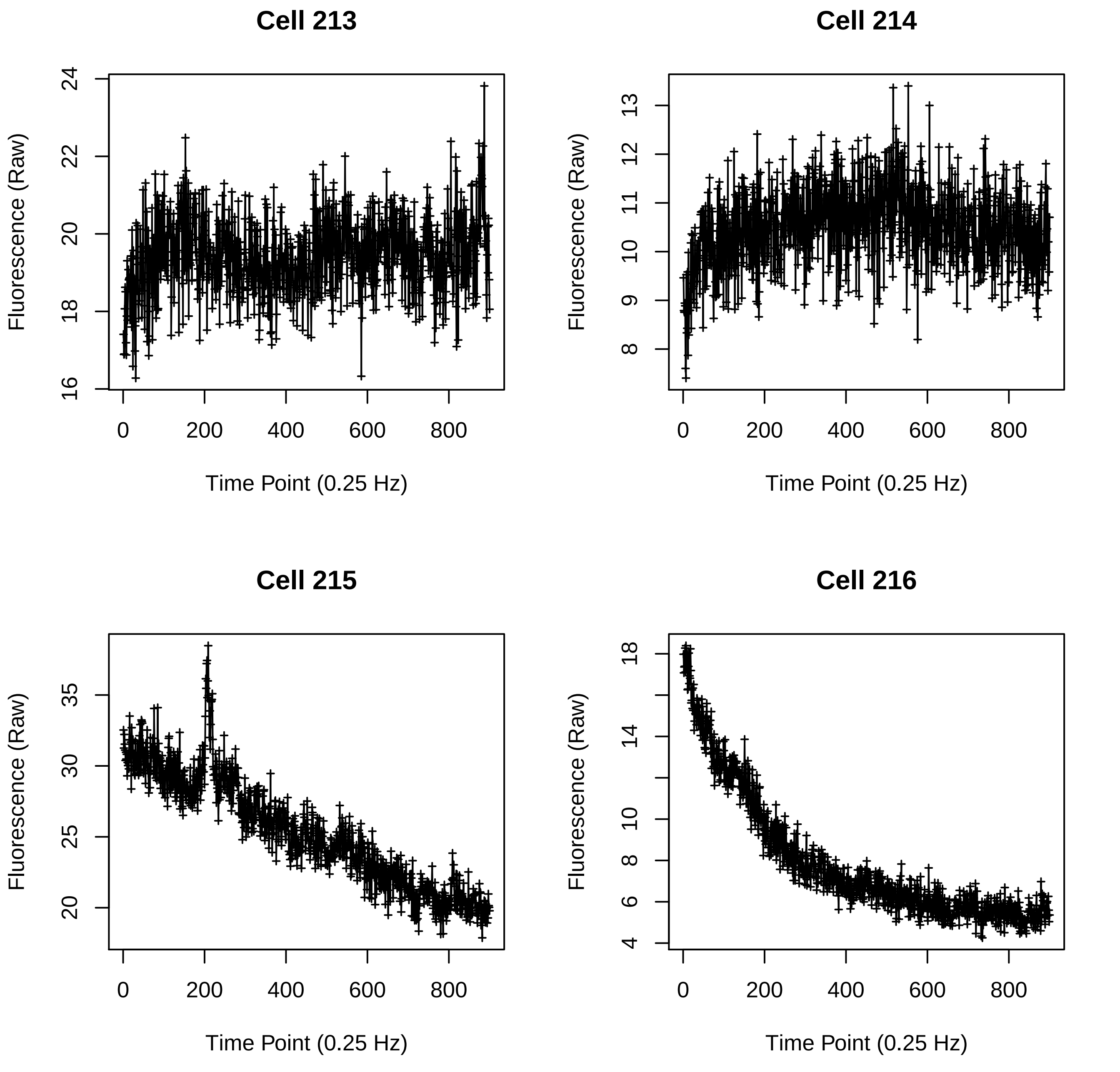
Cell 213
Cell 214
Cell 215
Cell 216
Fluorescence (Raw)
Time Point (0.25 Hz)
0
200
400
600
800
16
18
20
22
24
8
9
10
11
12
13
20
25
30
35
4
6
8
10
14
18

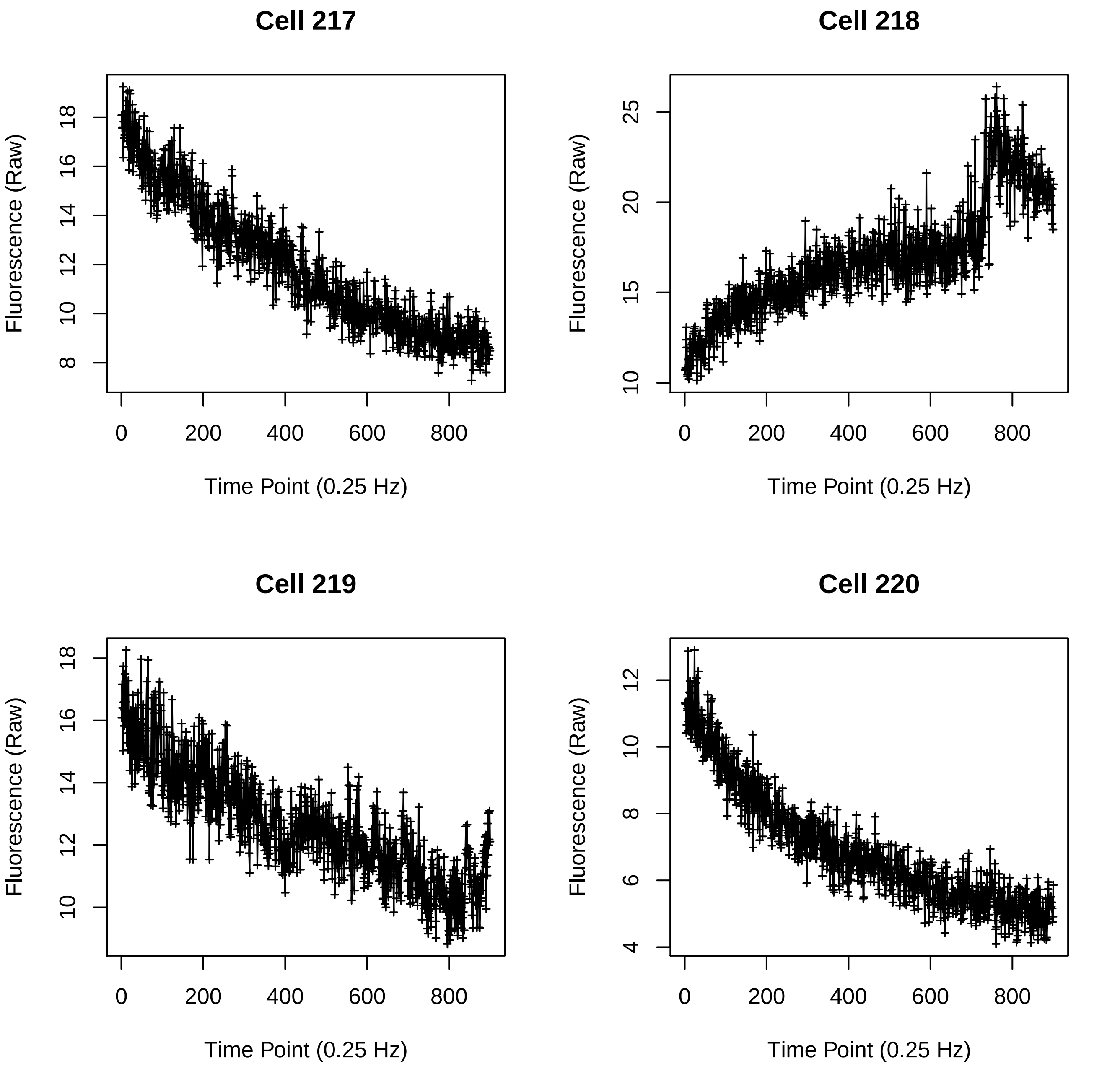
Cell 217
Fluorescence (Raw)
8 10 12 14 16 18
Time Point (0.25 Hz)
0 200 400 600 800
Cell 218
Fluorescence (Raw)
10 15 20 25
Time Point (0.25 Hz)
0 200 400 600 800
Cell 219
Fluorescence (Raw)
10 12 14 16 18
Time Point (0.25 Hz)
0 200 400 600 800
Cell 220
Fluorescence (Raw)
4 6 8 10 12
Time Point (0.25 Hz)
0 200 400 600 800

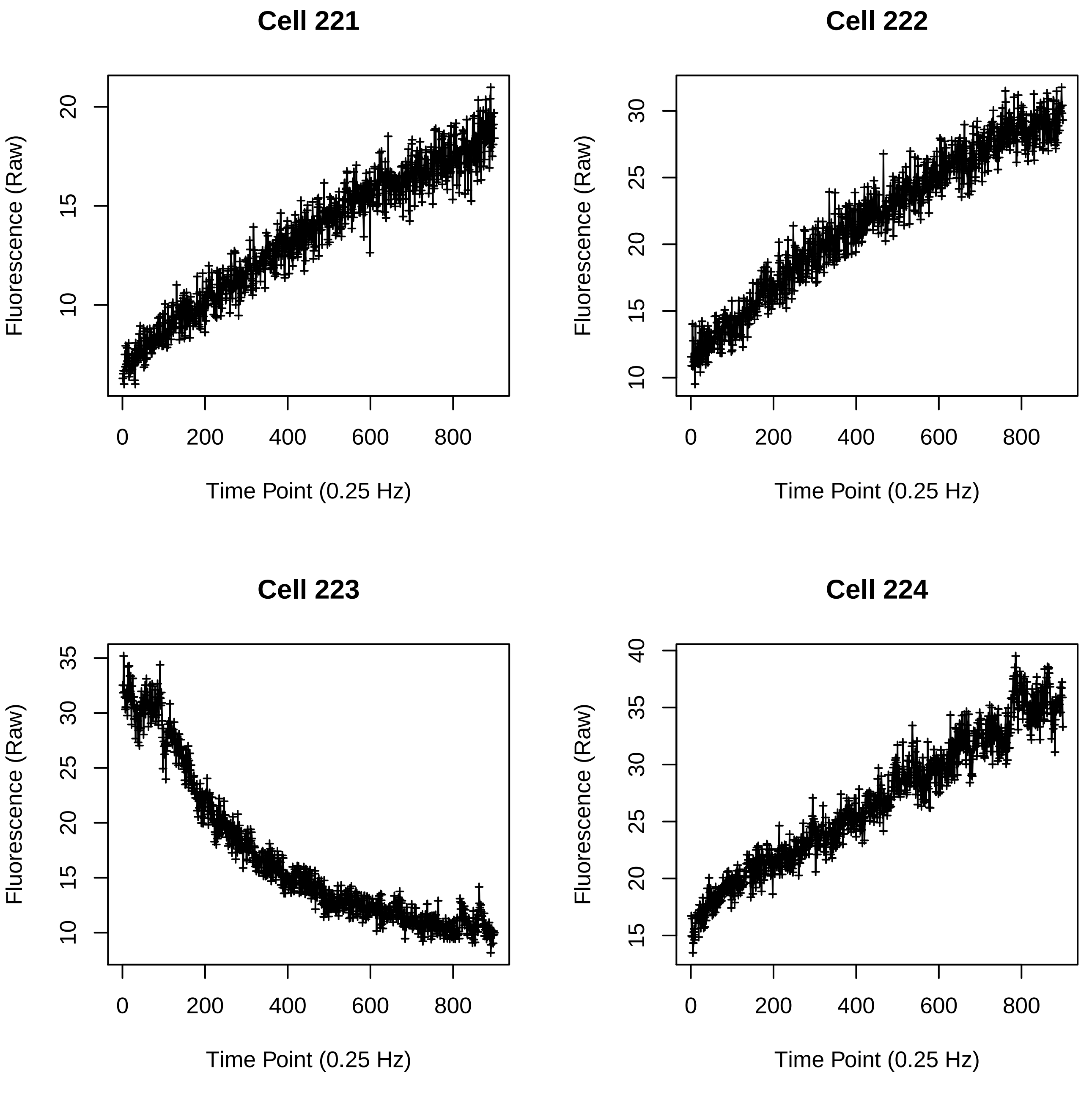
Cell 221
Fluorescence (Raw)
10
15
20
Time Point (0.25 Hz)
0
200
400
600
800
Cell 222
Fluorescence (Raw)
10
15
20
25
30
Time Point (0.25 Hz)
0
200
400
600
800
Cell 223
Fluorescence (Raw)
10
15
20
25
30
35
Time Point (0.25 Hz)
0
200
400
600
800
Cell 224
Fluorescence (Raw)
15
20
25
30
35
40
Time Point (0.25 Hz)
0
200
400
600
800

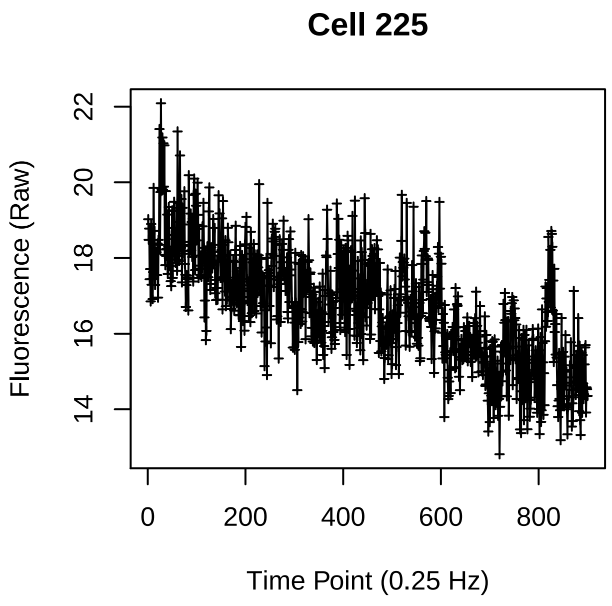
Cell 225
Fluorescence (Raw)
14
16
18
20
22
0
200
400
600
800
Time Point (0.25 Hz)
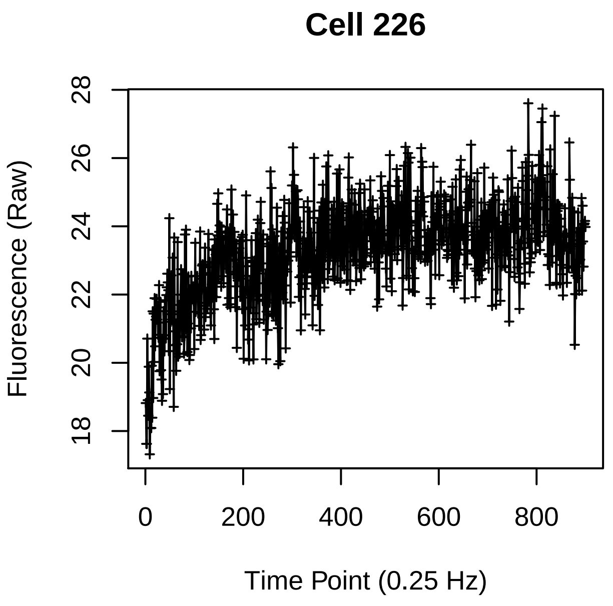
Cell 226
Fluorescence (Raw)
18
20
22
24
26
28
0
200
400
600
800
Time Point (0.25 Hz)
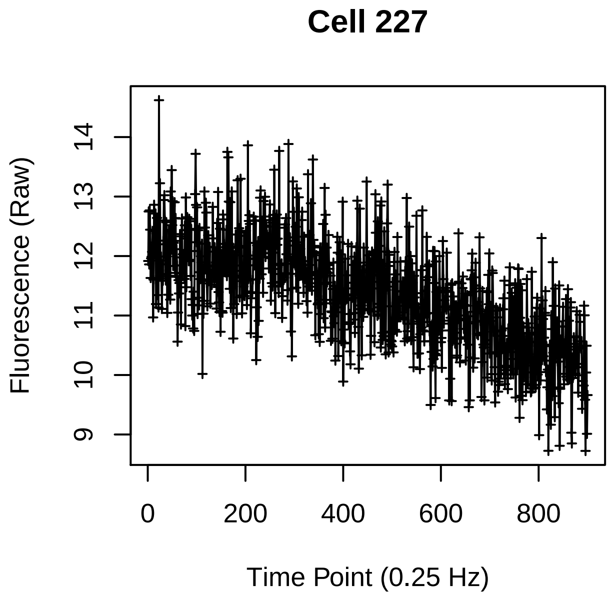
Cell 227
Fluorescence (Raw)
9
10
11
12
13
14
0
200
400
600
800
Time Point (0.25 Hz)
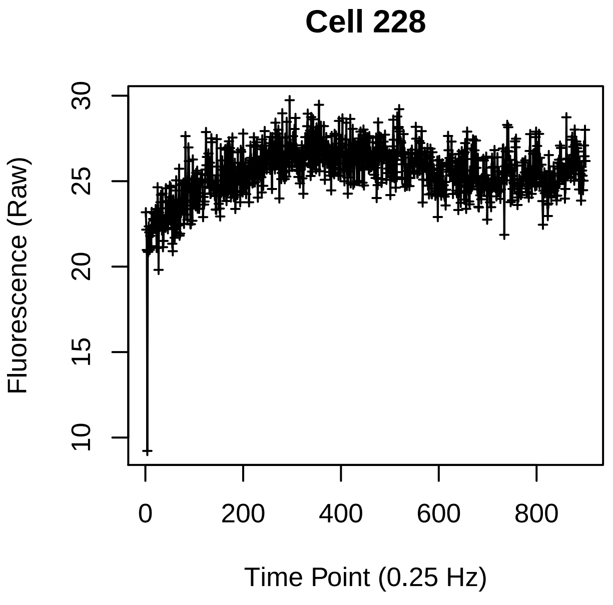
Cell 228
Fluorescence (Raw)
10
15
20
25
30
0
200
400
600
800
Time Point (0.25 Hz)

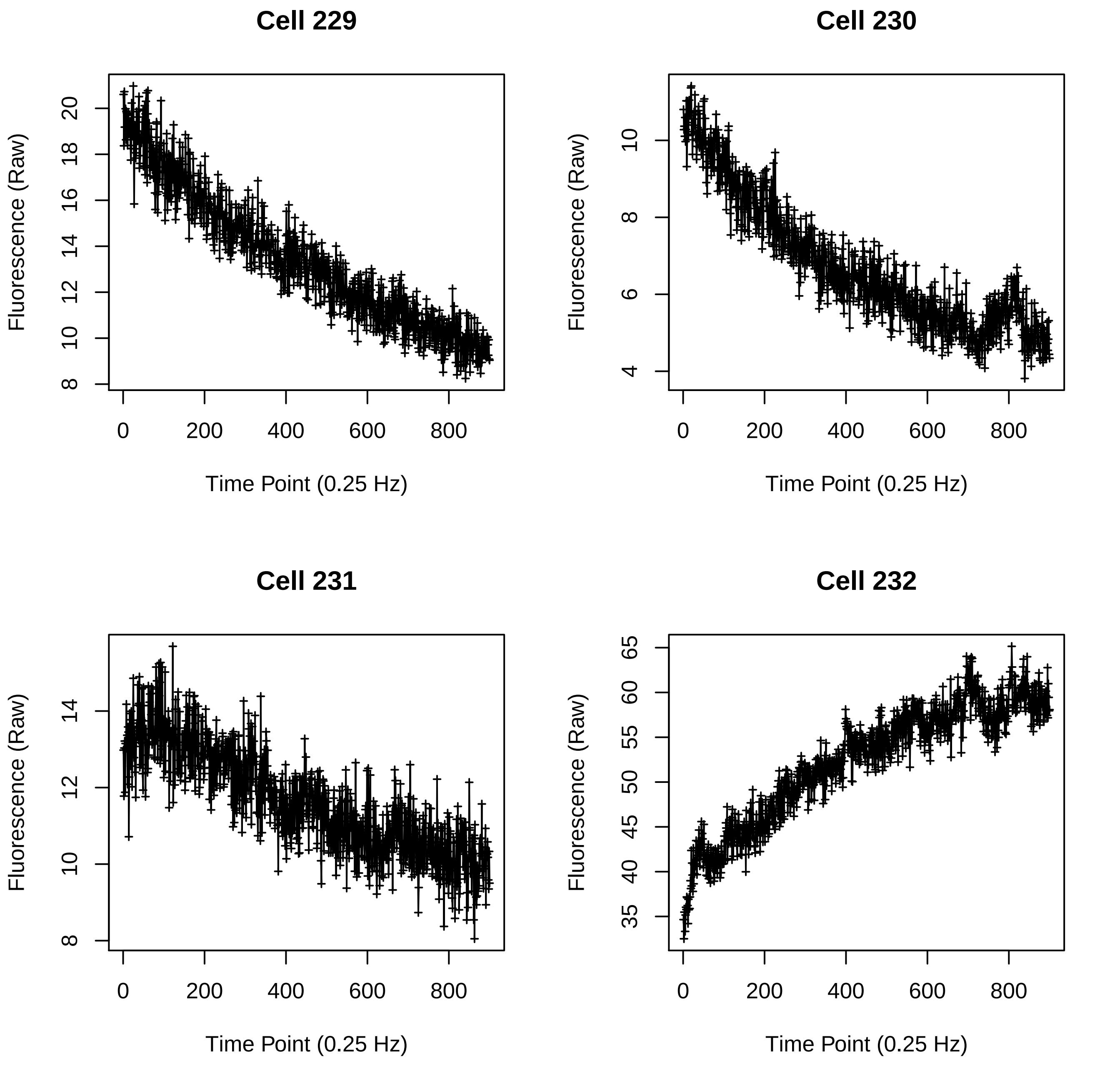
Cell 229
Cell 230
Cell 231
Cell 232
Fluorescence (Raw)
Time Point (0.25 Hz)
0
200
400
600
800

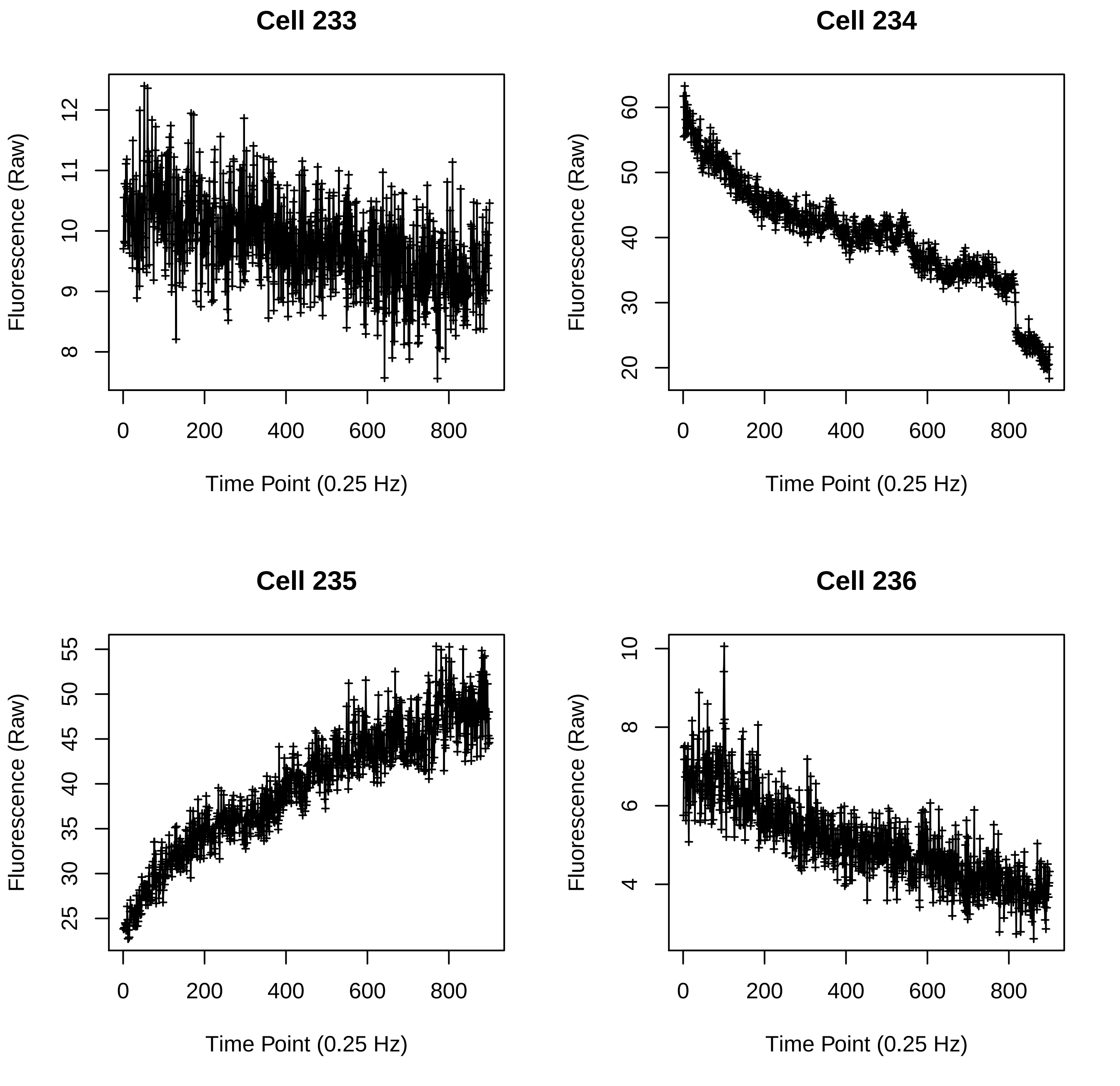
Cell 233
Cell 234
Cell 235
Cell 236
Fluorescence (Raw)
Time Point (0.25 Hz)
0
200
400
600
800

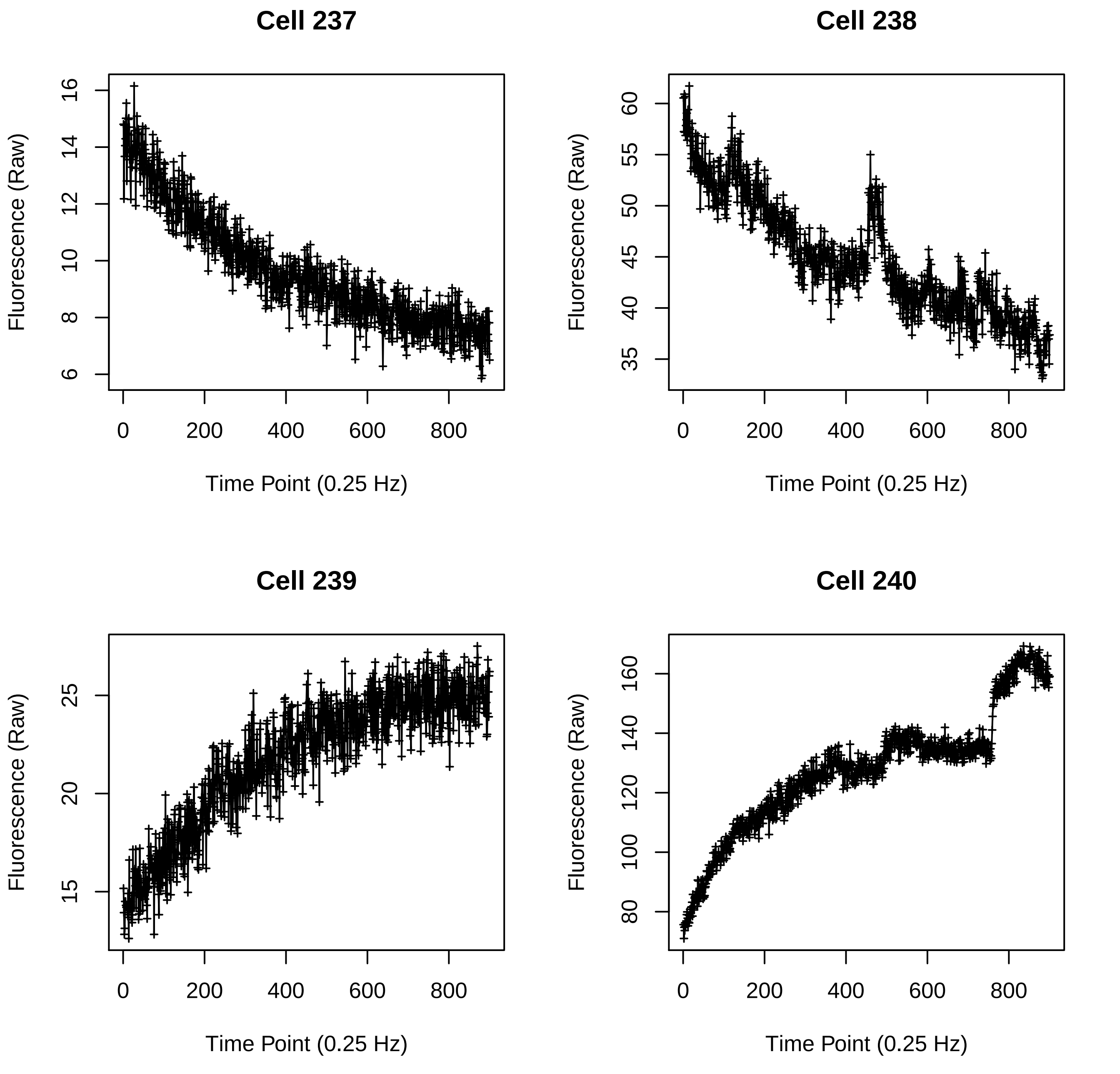
Cell 237
Cell 238
Cell 239
Cell 240
Fluorescence (Raw)
Time Point (0.25 Hz)
0
200
400
600
800
6
8
10
12
14
16
35
40
45
50
55
60
15
20
25
80
100
120
140
160

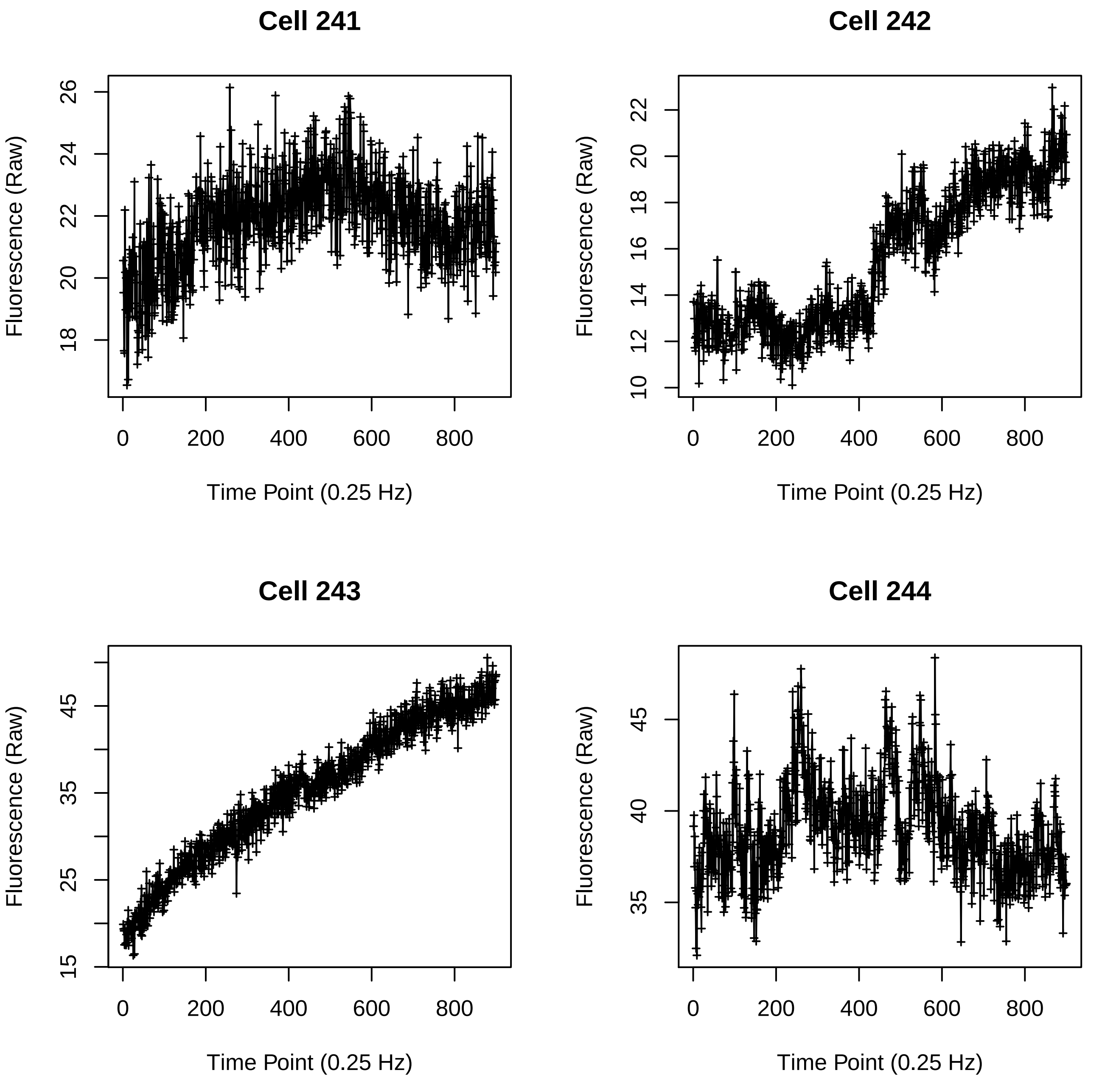
Cell 241
Cell 242
Cell 243
Cell 244
Fluorescence (Raw)
Time Point (0.25 Hz)
0
200
400
600
800
18
20
22
24
26
10
12
14
16
18
20
22
15
25
35
45
35
40
45

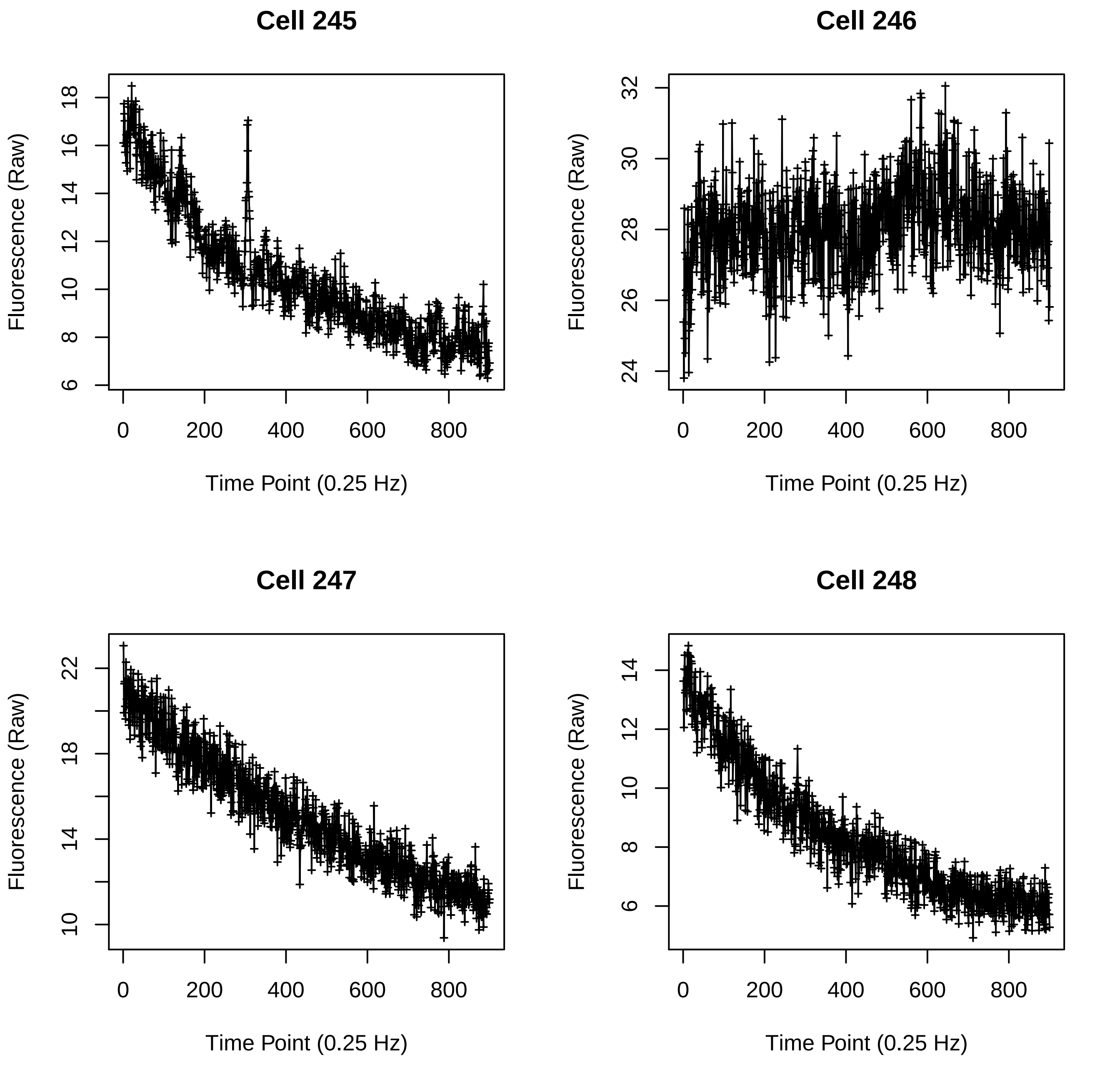
Cell 245
Fluorescence (Raw)
6
8
10
12
14
16
18
0
200
400
600
800
Time Point (0.25 Hz)
Cell 246
Fluorescence (Raw)
24
26
28
30
32
0
200
400
600
800
Time Point (0.25 Hz)
Cell 247
Fluorescence (Raw)
10
14
18
22
0
200
400
600
800
Time Point (0.25 Hz)
Cell 248
Fluorescence (Raw)
6
8
10
12
14
0
200
400
600
800
Time Point (0.25 Hz)

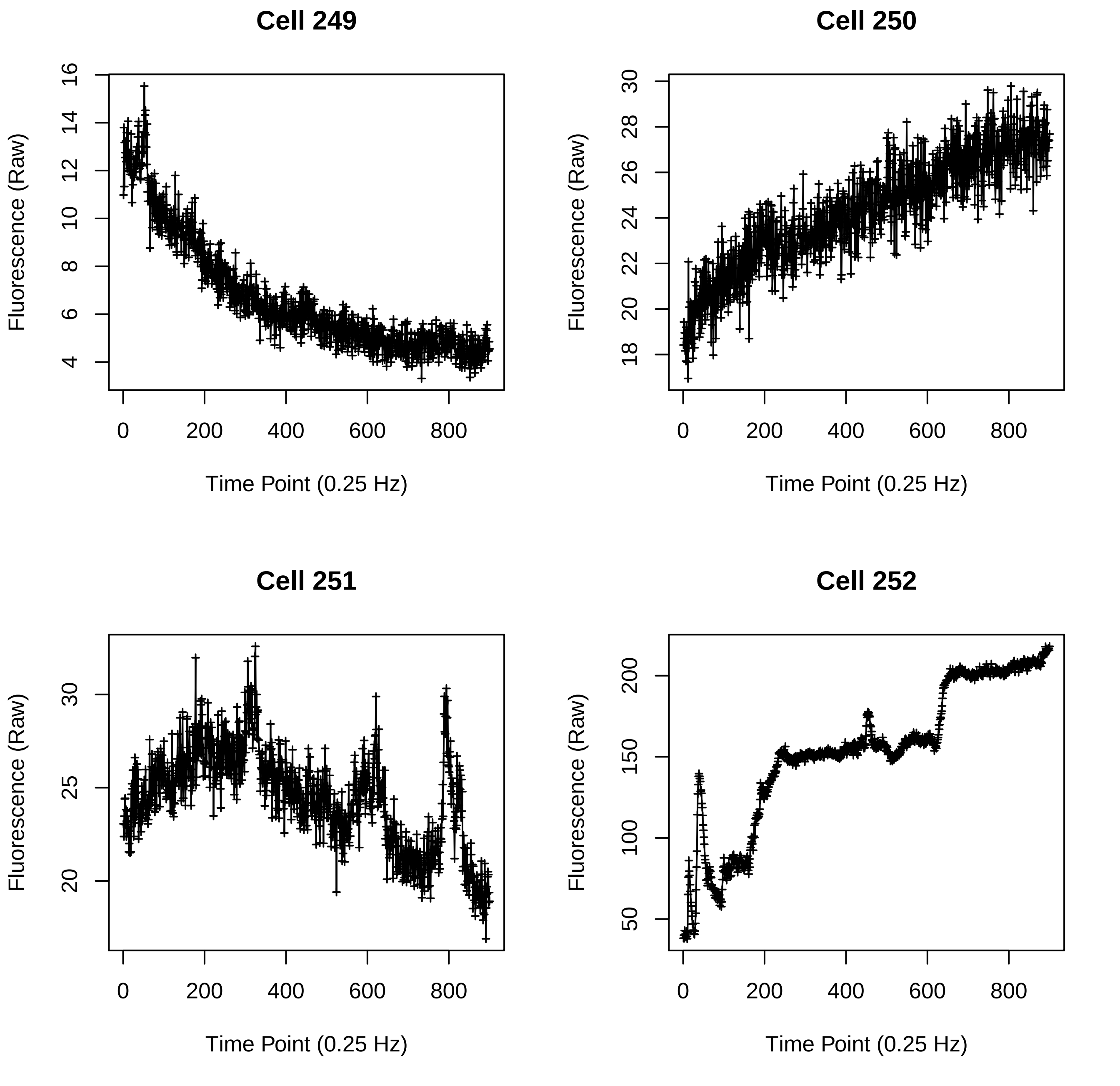
Cell 249
Cell 250
Cell 251
Cell 252
Fluorescence (Raw)
Time Point (0.25 Hz)
0
200
400
600
800

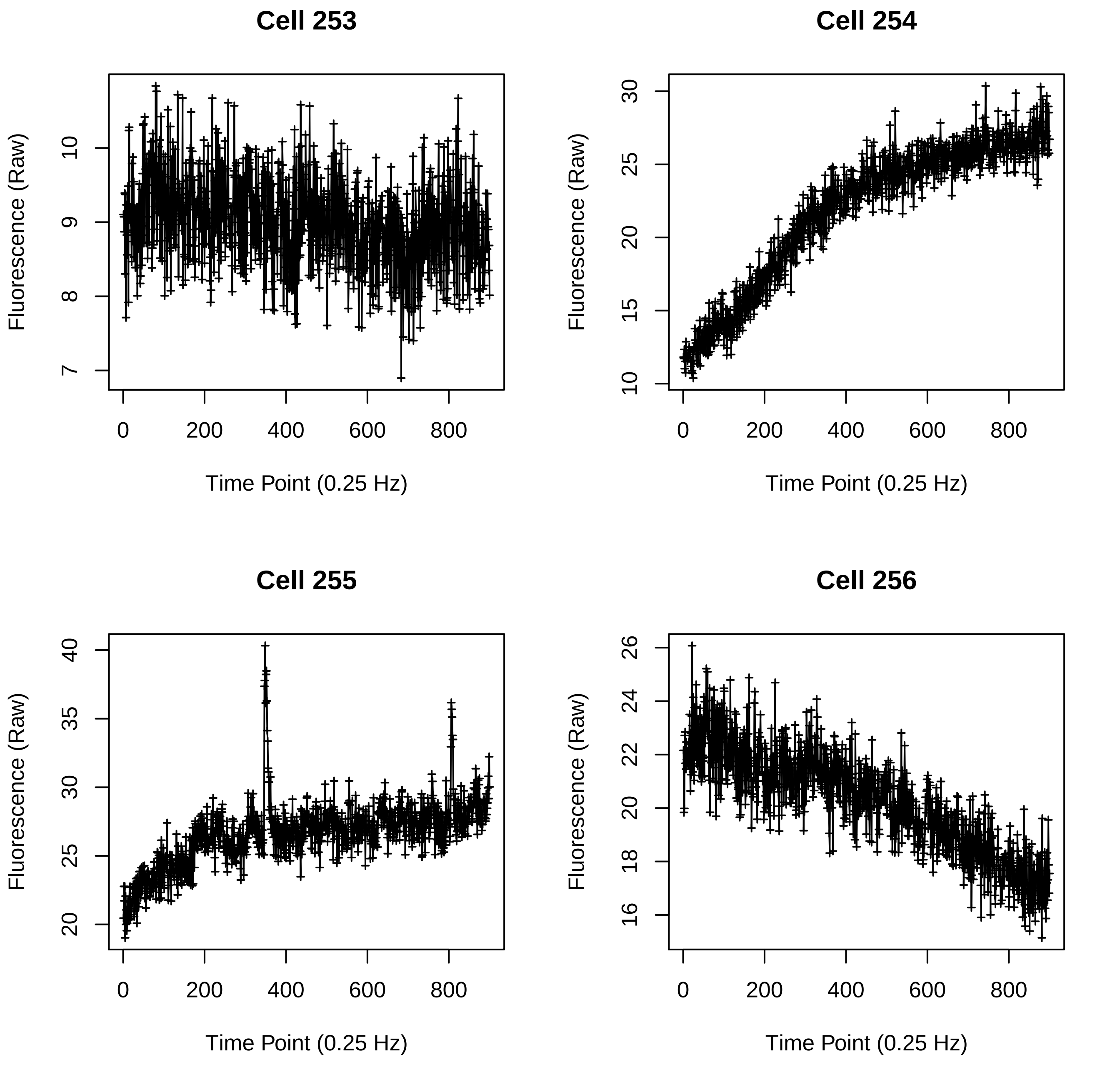
Cell 253
Cell 254
Cell 255
Cell 256
Fluorescence (Raw)
Time Point (0.25 Hz)
0
200
400
600
800
7
8
9
10
10
15
20
25
30
20
25
30
35
40
16
18
20
22
24
26

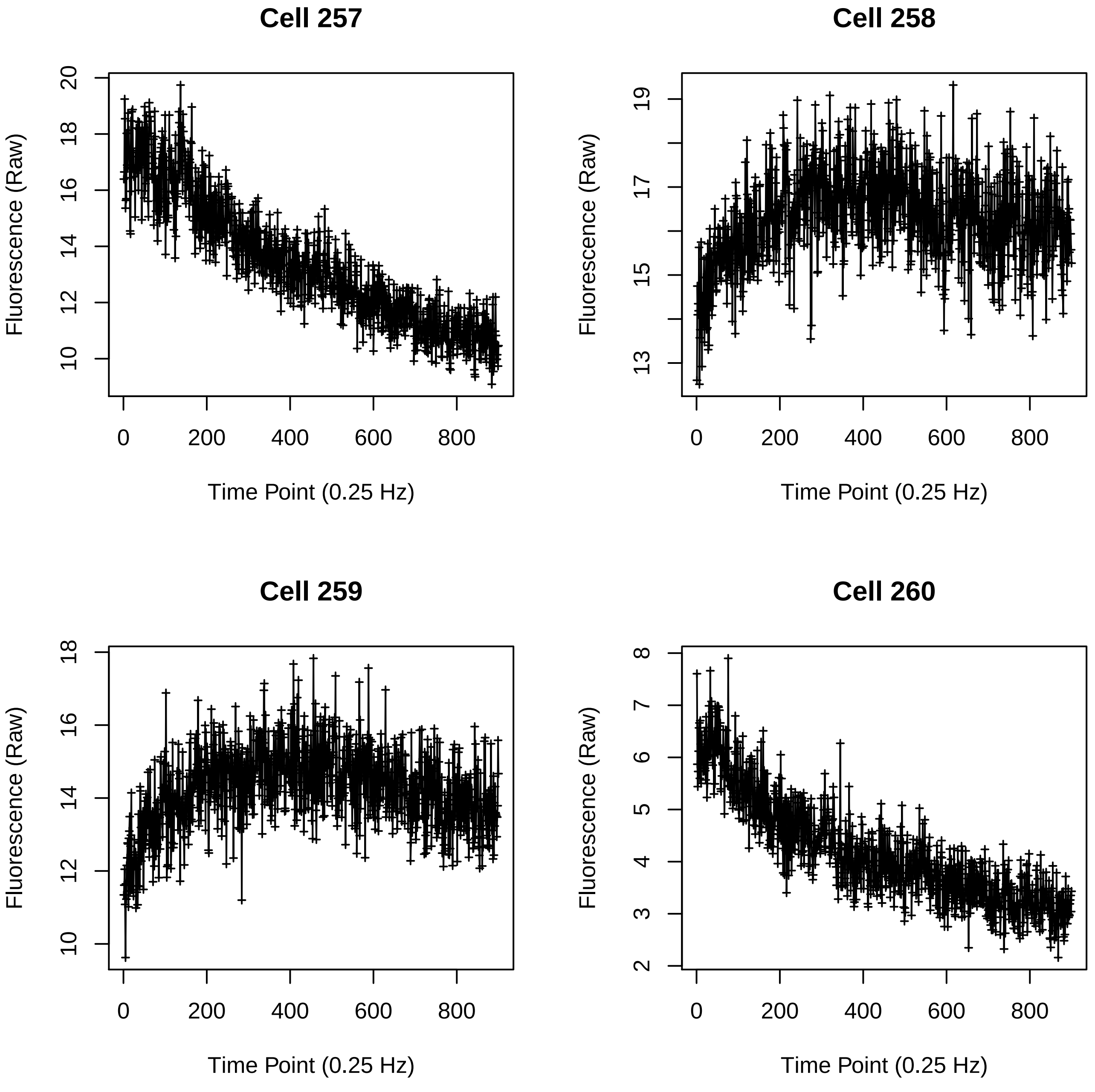

Cell 257
Cell 258
Cell 259
Cell 260
Fluorescence (Raw)
Time Point (0.25 Hz)
0
200
400
600
800
10
12
14
16
18
20
13
15
17
19
2
3
4
5
6
7
8

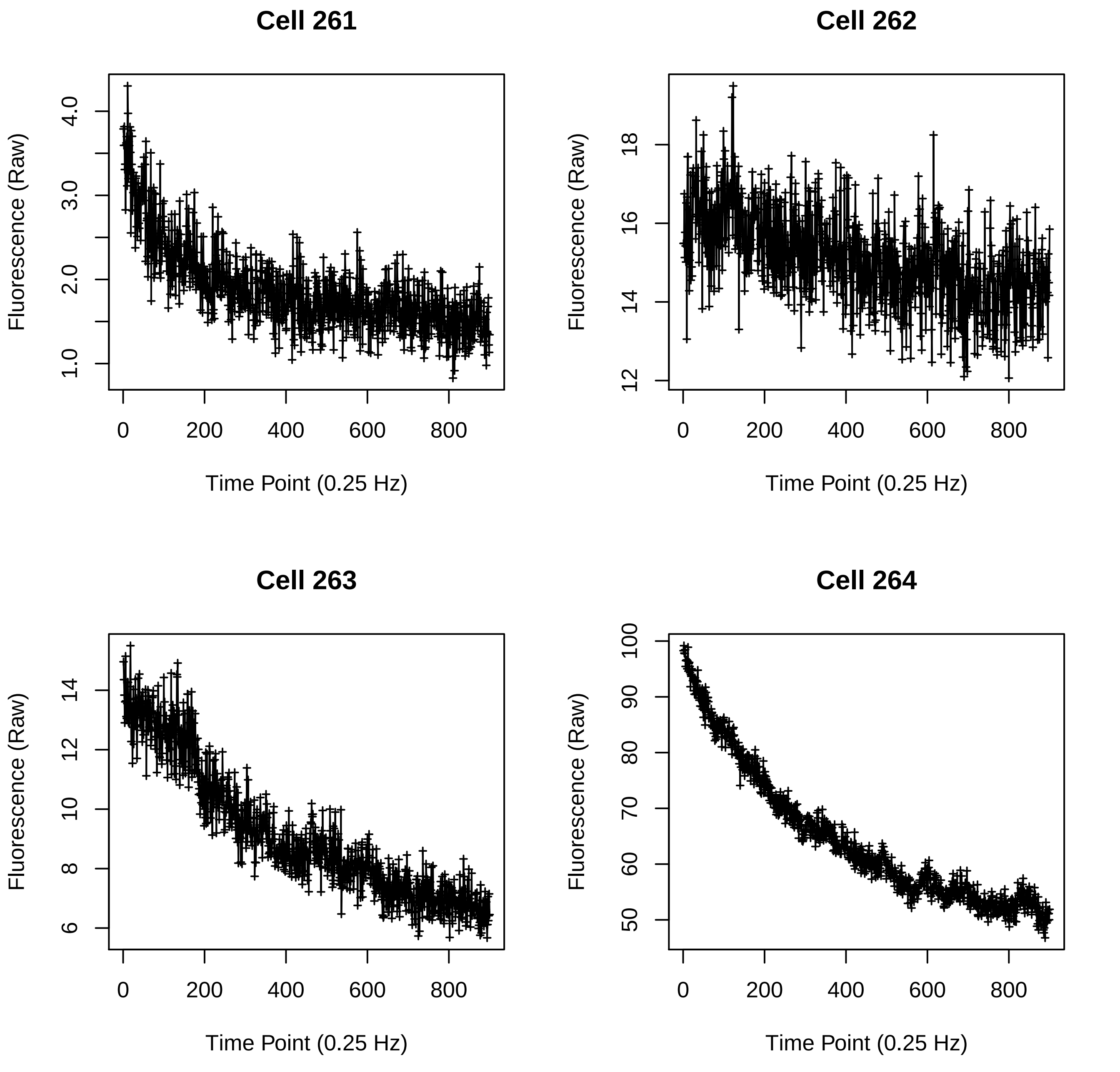
Cell 261
Fluorescence (Raw)
1.0
2.0
3.0
4.0
0
200
400
600
800
Time Point (0.25 Hz)
Cell 262
Fluorescence (Raw)
12
14
16
18
0
200
400
600
800
Time Point (0.25 Hz)
Cell 263
Fluorescence (Raw)
6
8
10
12
14
0
200
400
600
800
Time Point (0.25 Hz)
Cell 264
Fluorescence (Raw)
50
60
70
80
90
100
0
200
400
600
800
Time Point (0.25 Hz)

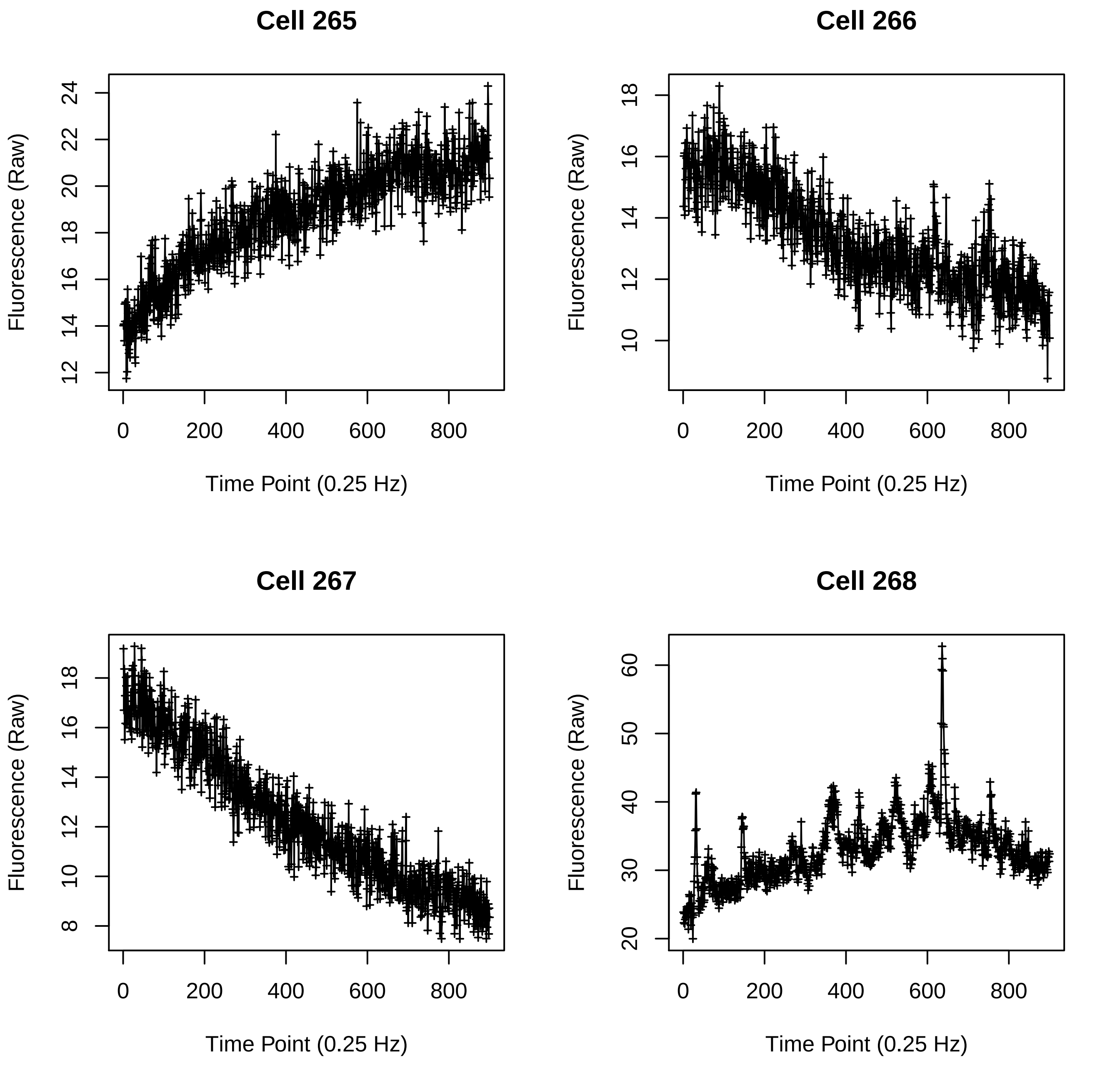

Cell 265
Cell 266
Cell 267
Cell 268
Fluorescence (Raw)
Time Point (0.25 Hz)
0
200
400
600
800
12
14
16
18
20
22
24
10
8
20
30
40
50
60

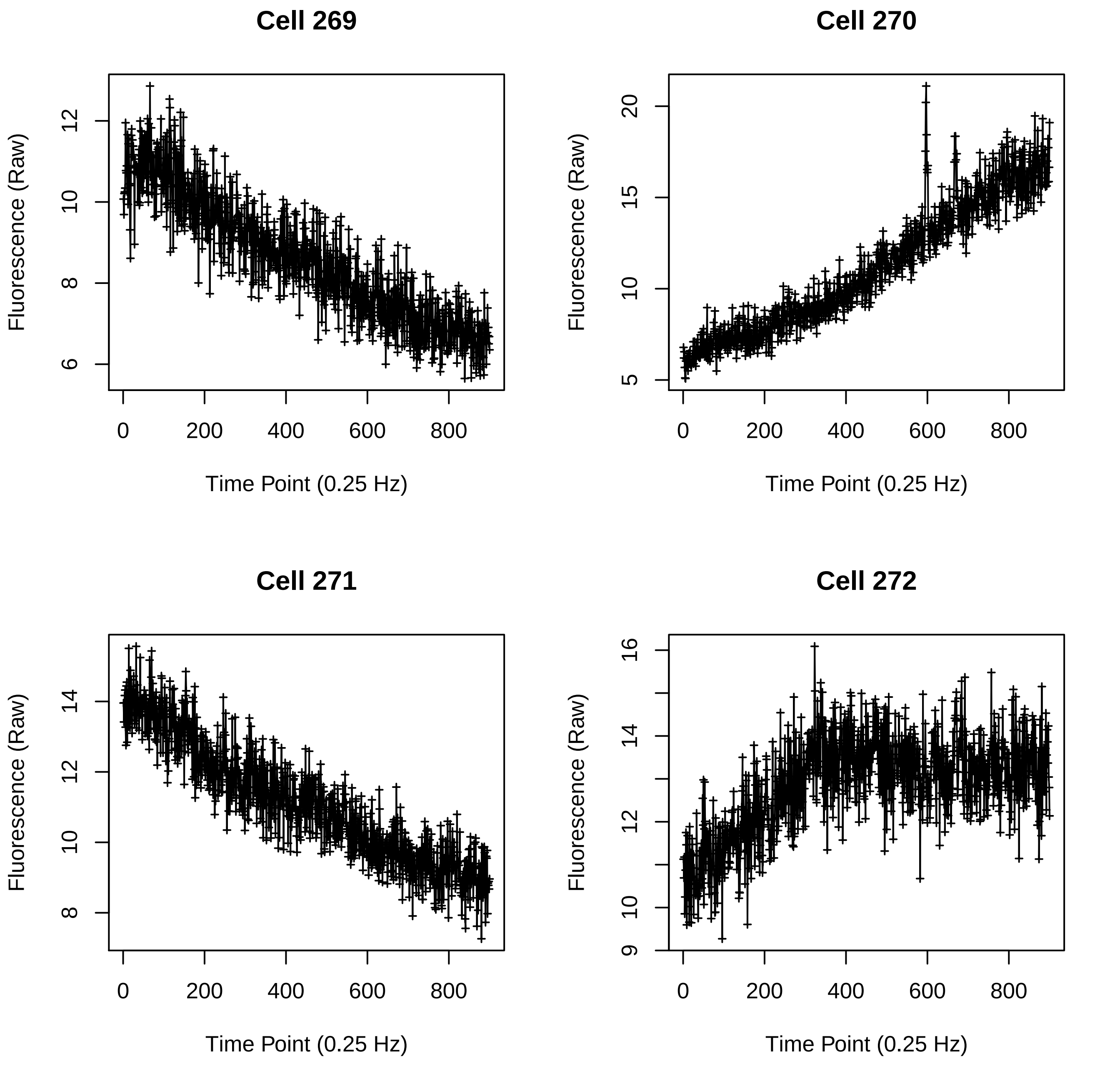
Cell 269
Cell 270
Cell 271
Cell 272
Fluorescence (Raw)
Time Point (0.25 Hz)
0
200
400
600
800
6
8
10
12
5
15
20
14
9
16

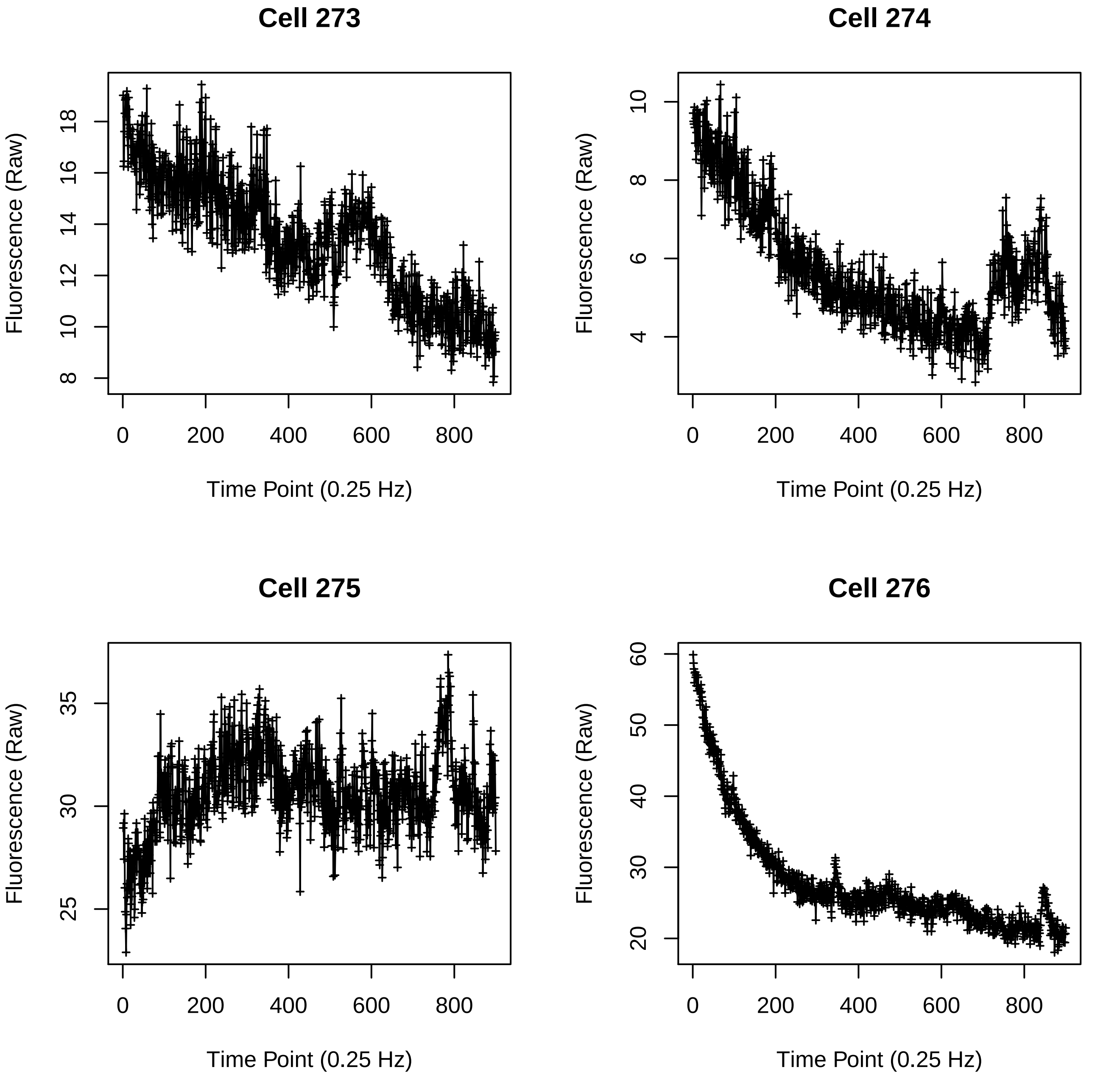
Cell 273
Cell 274
Cell 275
Cell 276
Fluorescence (Raw)
Time Point (0.25 Hz)
0
200
400
600
800
8
10
12
14
16
18
4
6
8
10
25
30
35
20
30
40
50
60

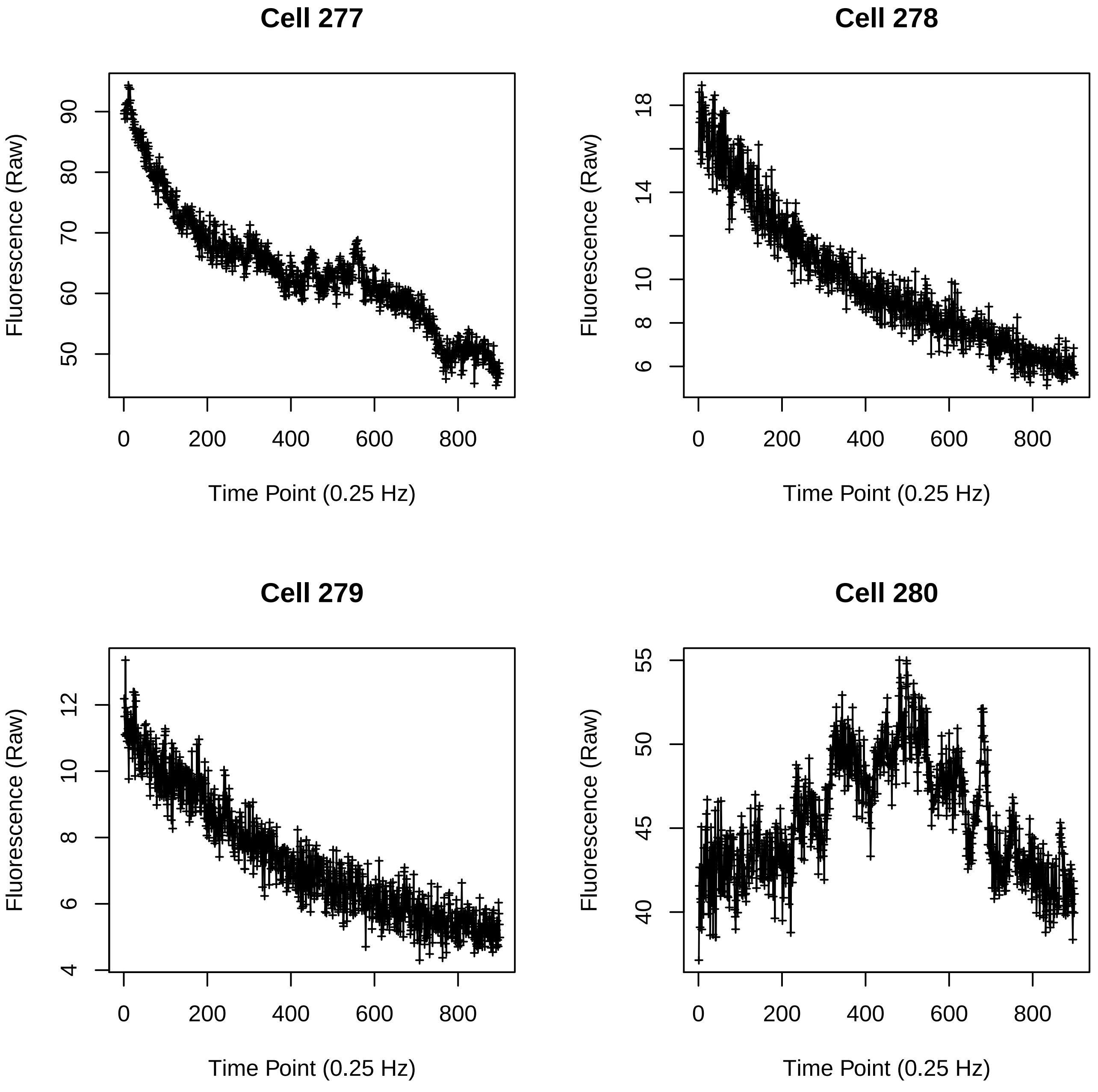
Cell 277
Cell 278
Cell 279
Cell 280
Fluorescence (Raw)
Time Point (0.25 Hz)
0
200
400
600
800
90
80
70
60
50
18
14
10
8
6
12
4
55
45
40

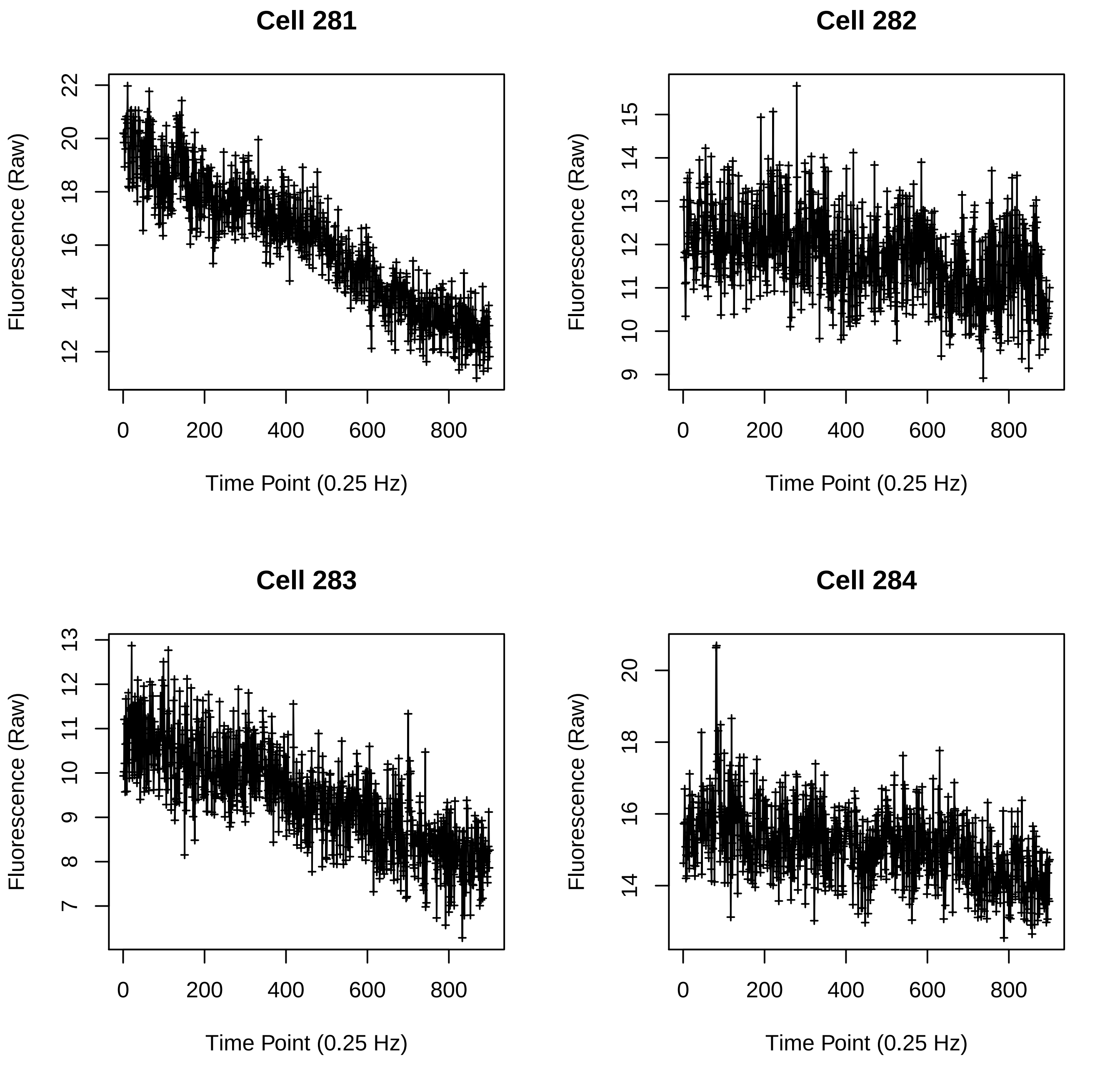

Cell 281
Cell 282
Cell 283
Cell 284
Fluorescence (Raw)
Time Point (0.25 Hz)
0
200
400
600
800
22
20
18
16
14
12
15
13
11
10
9
7
8
20

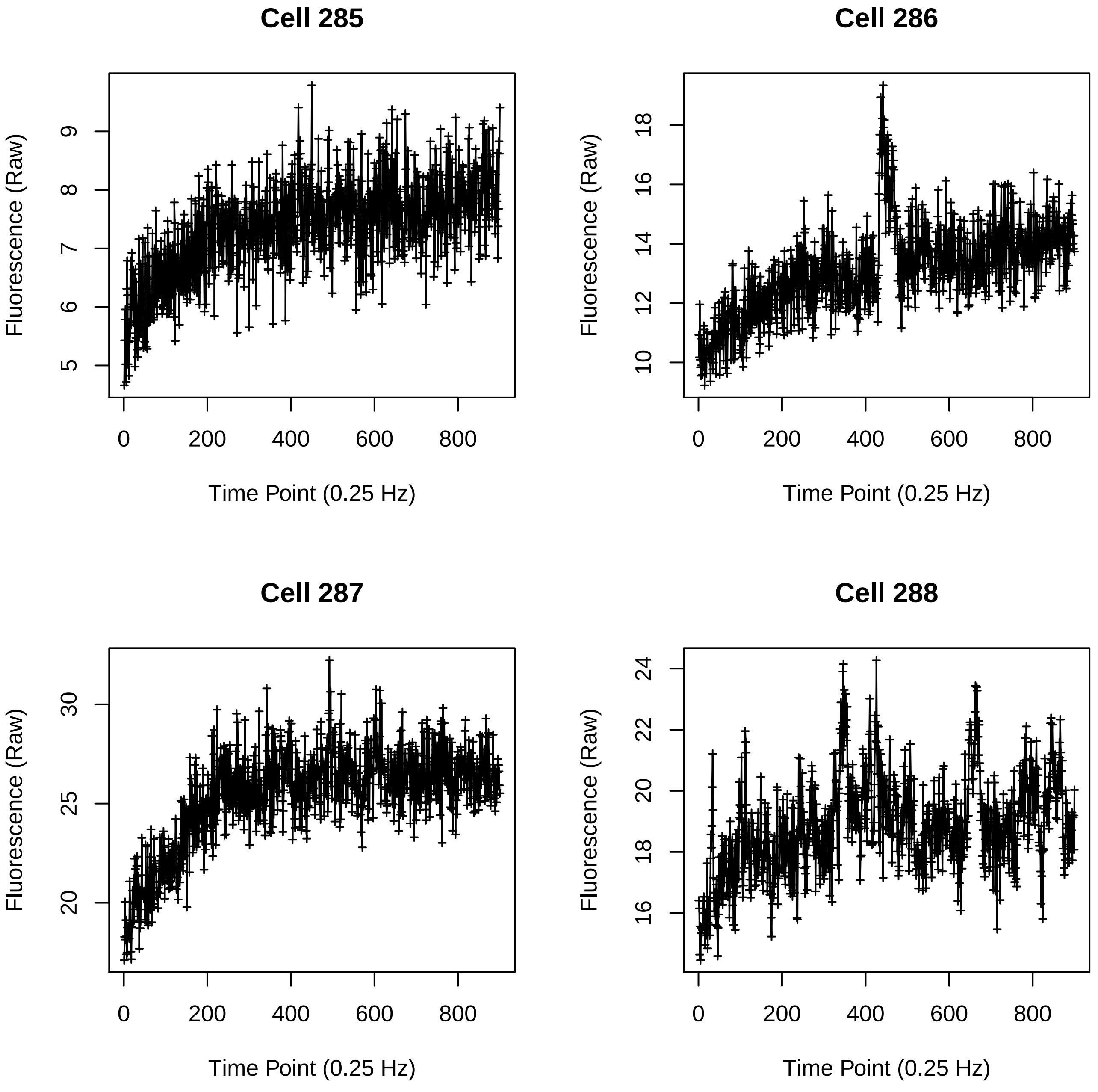
Cell 285
Fluorescence (Raw)
5
6
7
8
9
0
200
400
600
800
Time Point (0.25 Hz)
Cell 286
Fluorescence (Raw)
10
12
14
16
18
0
200
400
600
800
Time Point (0.25 Hz)
Cell 287
Fluorescence (Raw)
20
25
30
0
200
400
600
800
Time Point (0.25 Hz)
Cell 288
Fluorescence (Raw)
16
18
20
22
24
0
200
400
600
800
Time Point (0.25 Hz)

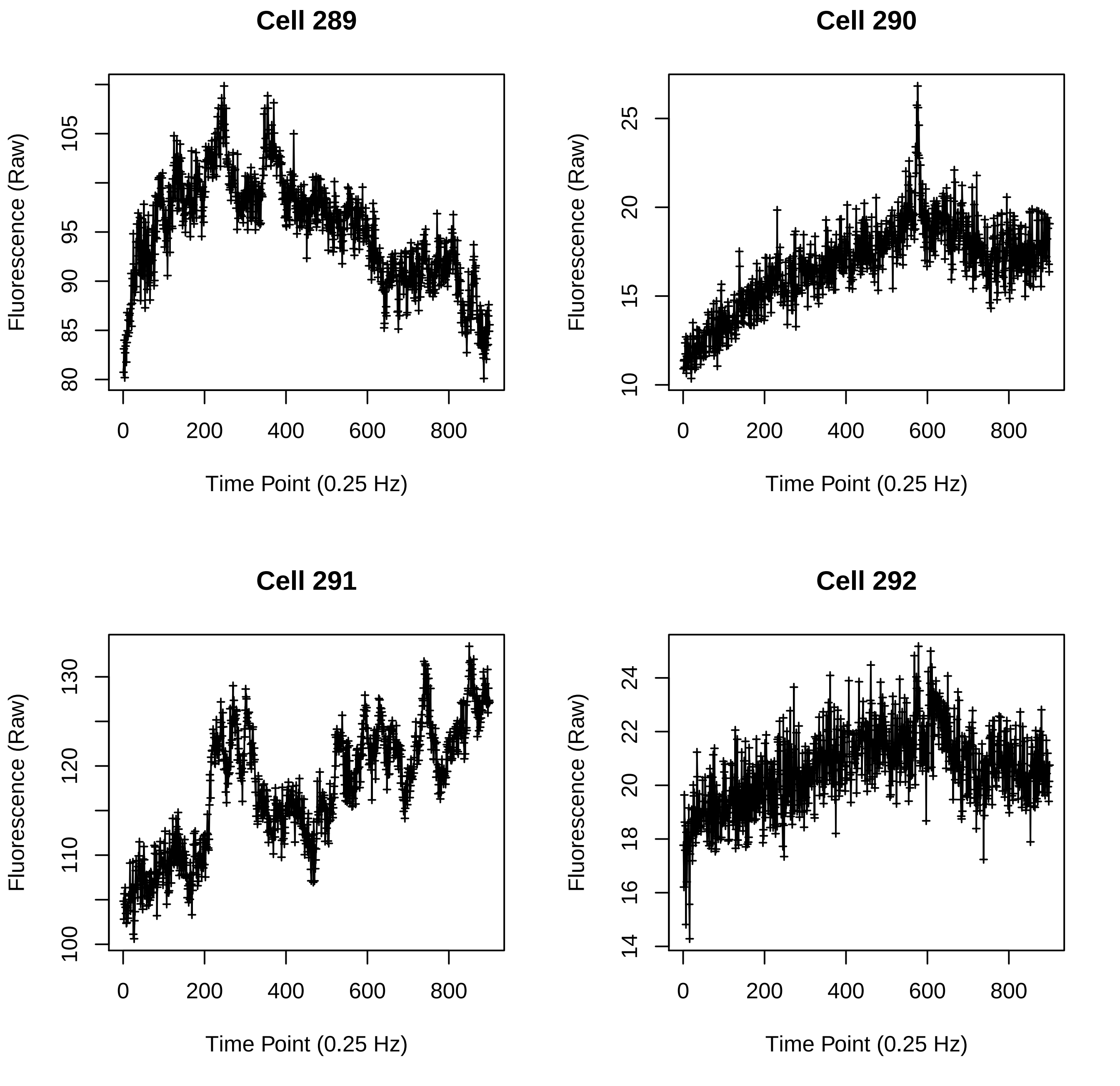
Cell 289
Fluorescence (Raw)
80
85
90
95
105
0
200
400
600
800
Time Point (0.25 Hz)
Cell 290
Fluorescence (Raw)
10
15
20
25
0
200
400
600
800
Time Point (0.25 Hz)
Cell 291
Fluorescence (Raw)
100
110
120
130
0
200
400
600
800
Time Point (0.25 Hz)
Cell 292
Fluorescence (Raw)
14
16
18
20
22
24
0
200
400
600
800
Time Point (0.25 Hz)

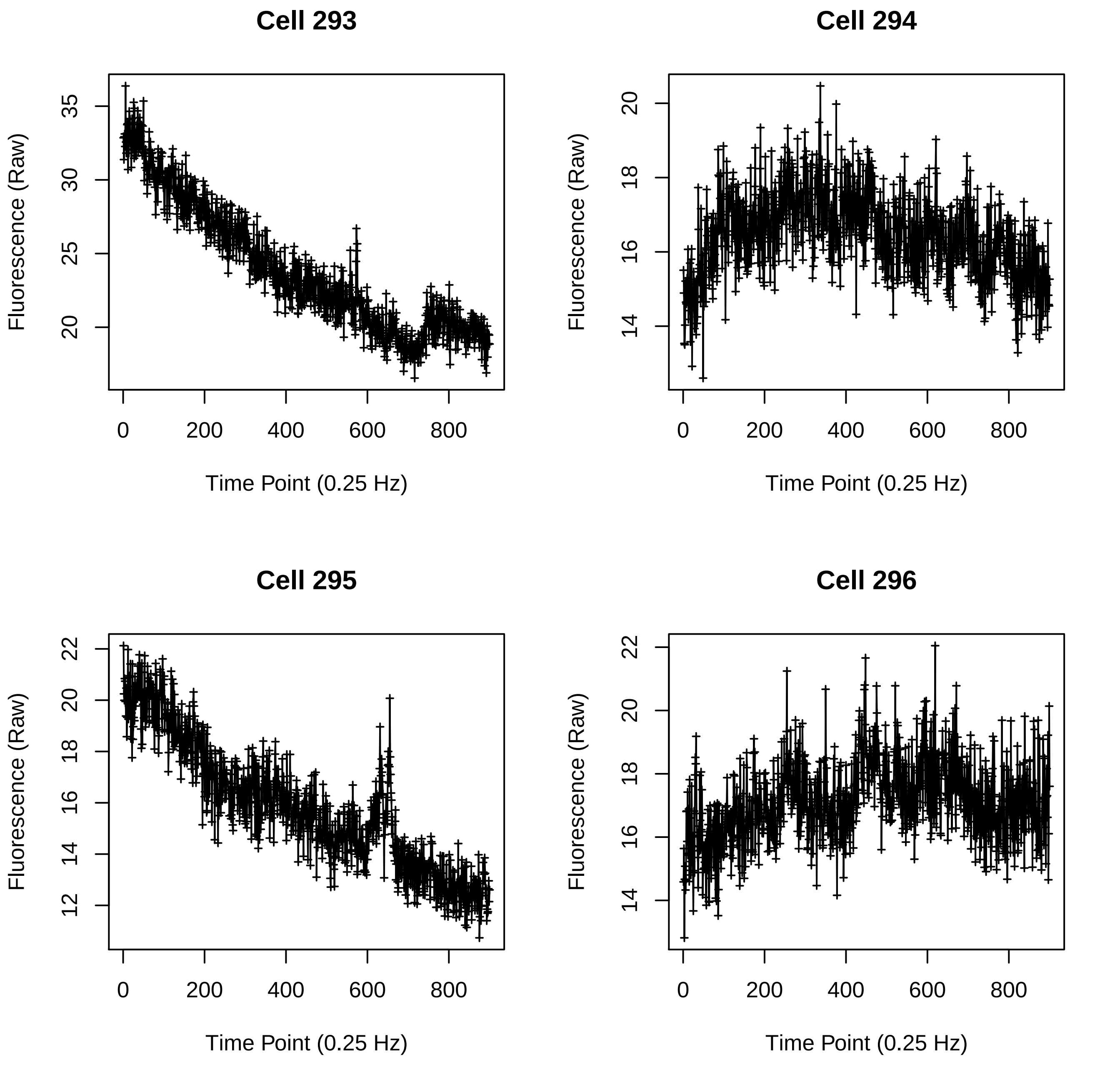
Cell 293
Cell 294
Cell 295
Cell 296
Fluorescence (Raw)
Time Point (0.25 Hz)
0
200
400
600
800
20
25
30
35
14
16
18
20
12
14
16
18
20
22
14
16
18
20
22

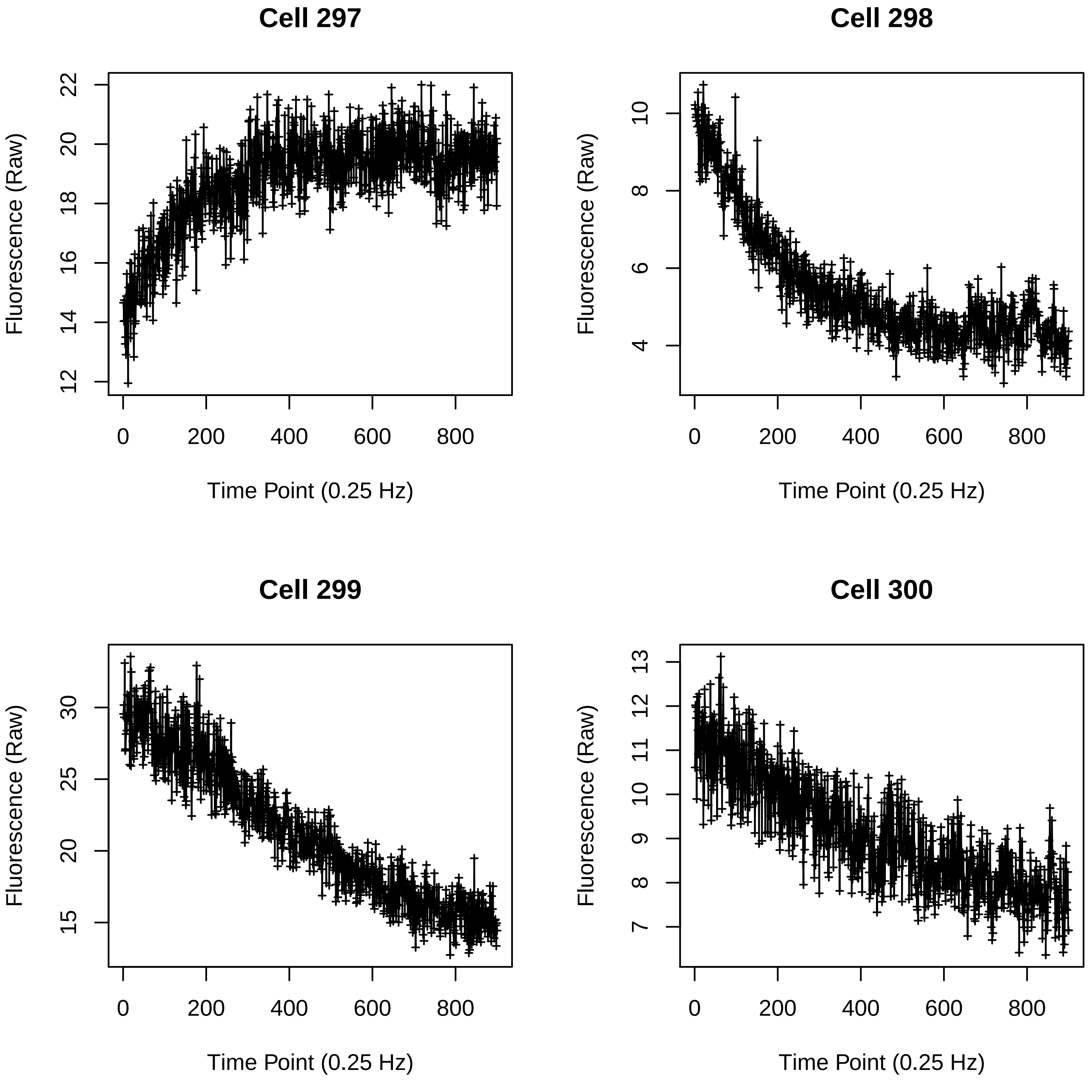
Cell 297
Fluorescence (Raw)
12
14
16
18
20
22
0
200
400
600
800
Time Point (0.25 Hz)
Cell 298
Fluorescence (Raw)
4
6
8
10
0
200
400
600
800
Time Point (0.25 Hz)
Cell 299
Fluorescence (Raw)
15
20
25
30
0
200
400
600
800
Time Point (0.25 Hz)
Cell 300
Fluorescence (Raw)
7
8
9
10
11
12
13
0
200
400
600
800
Time Point (0.25 Hz)

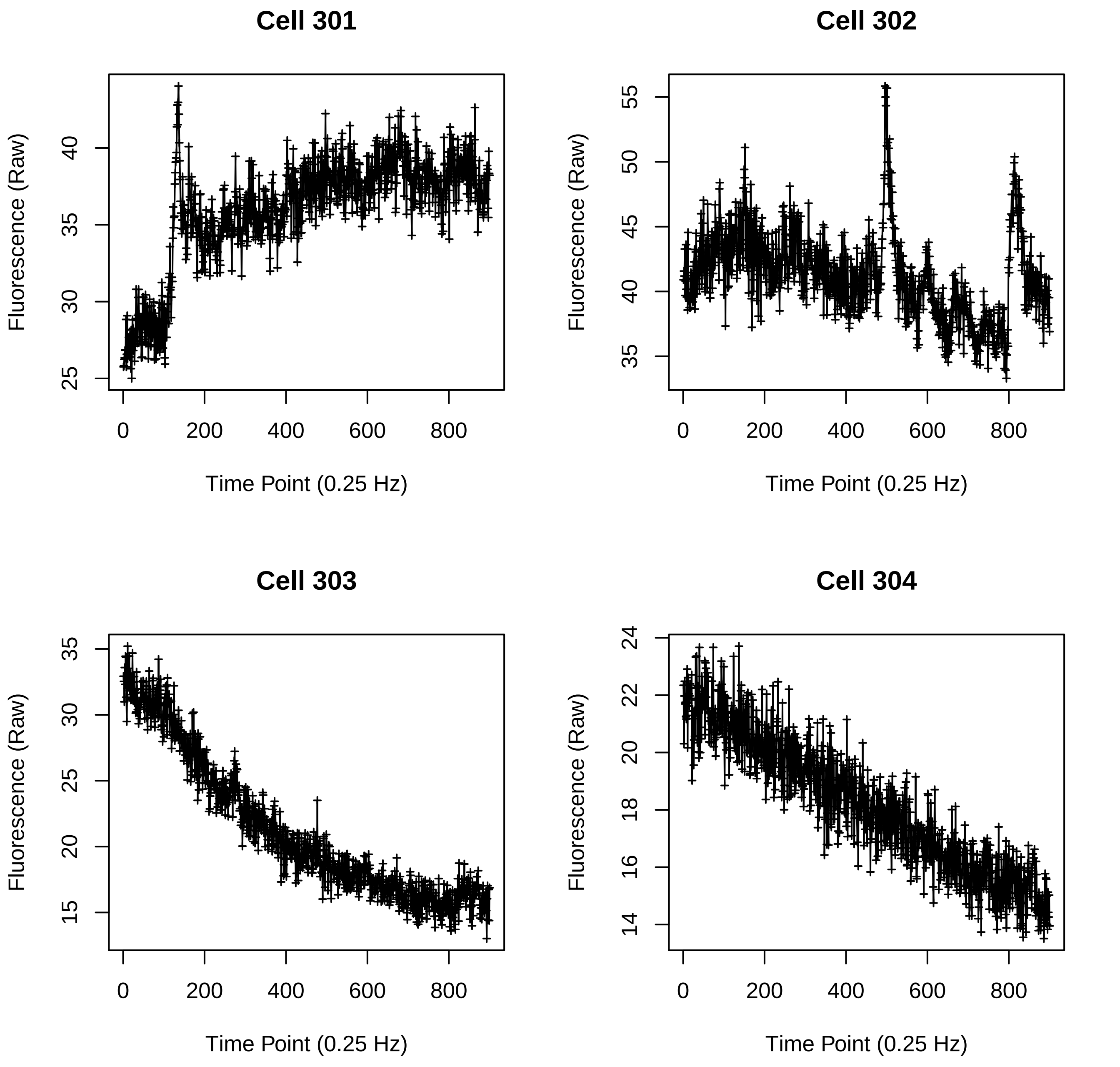

Cell 301
Fluorescence (Raw)
25
30
35
40
0
200
400
600
800
Time Point (0.25 Hz)
Cell 302
Fluorescence (Raw)
35
40
45
50
55
0
200
400
600
800
Time Point (0.25 Hz)
Cell 303
Fluorescence (Raw)
15
20
25
30
35
0
200
400
600
800
Time Point (0.25 Hz)
Cell 304
Fluorescence (Raw)
14
16
18
20
22
24
0
200
400
600
800
Time Point (0.25 Hz)

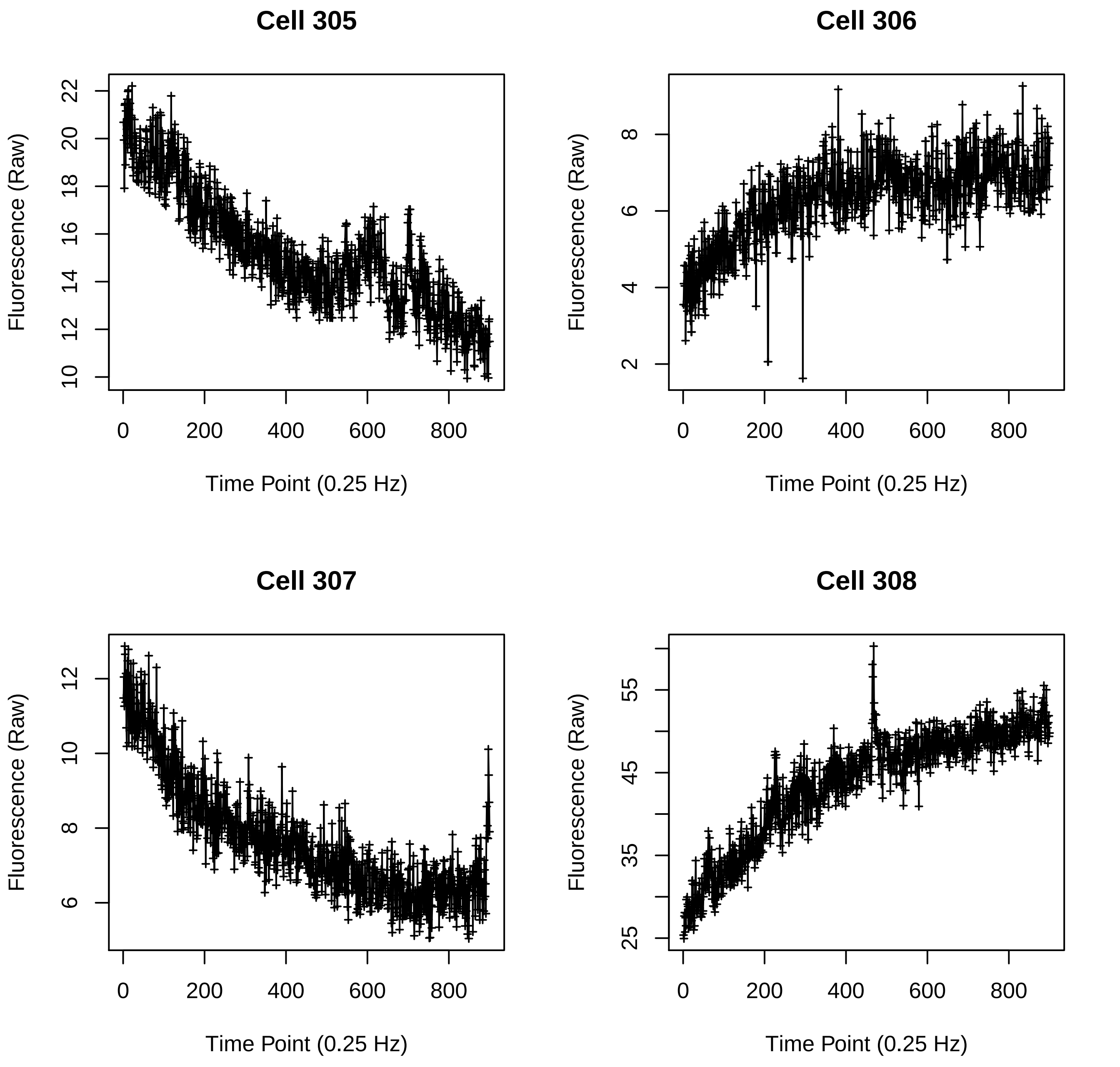
Cell 305
Fluorescence (Raw)
10
12
14
16
18
20
22
Time Point (0.25 Hz)
0
200
400
600
800
Cell 306
Fluorescence (Raw)
2
4
6
8
Time Point (0.25 Hz)
0
200
400
600
800
Cell 307
Fluorescence (Raw)
6
8
10
12
Time Point (0.25 Hz)
0
200
400
600
800
Cell 308
Fluorescence (Raw)
25
35
45
55
Time Point (0.25 Hz)
0
200
400
600
800

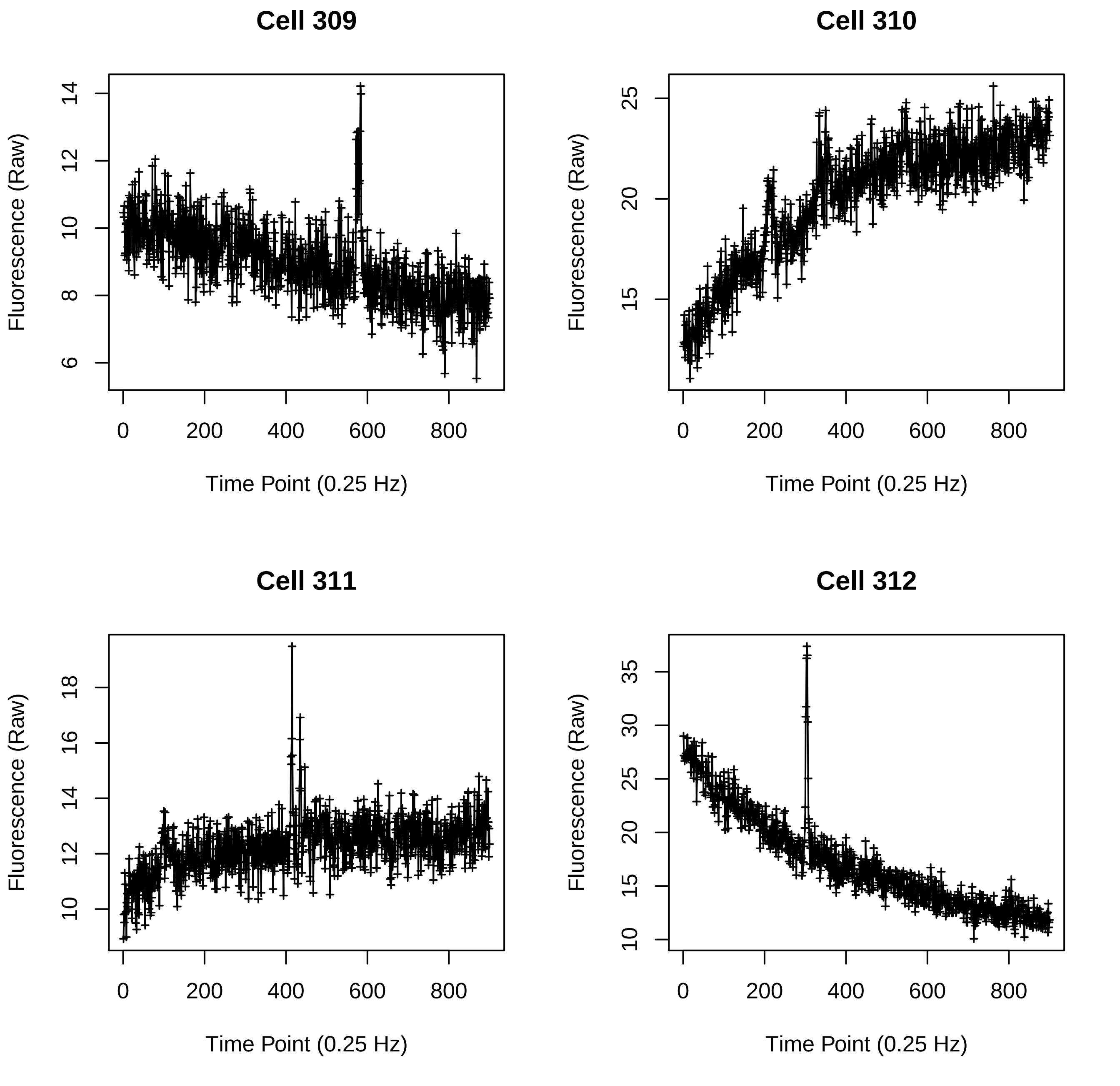
Cell 309
Fluorescence (Raw)
6
8
10
12
14
0
200
400
600
800
Time Point (0.25 Hz)
Cell 310
Fluorescence (Raw)
15
20
25
0
200
400
600
800
Time Point (0.25 Hz)
Cell 311
Fluorescence (Raw)
10
12
14
16
18
0
200
400
600
800
Time Point (0.25 Hz)
Cell 312
Fluorescence (Raw)
10
15
20
25
30
35
0
200
400
600
800
Time Point (0.25 Hz)

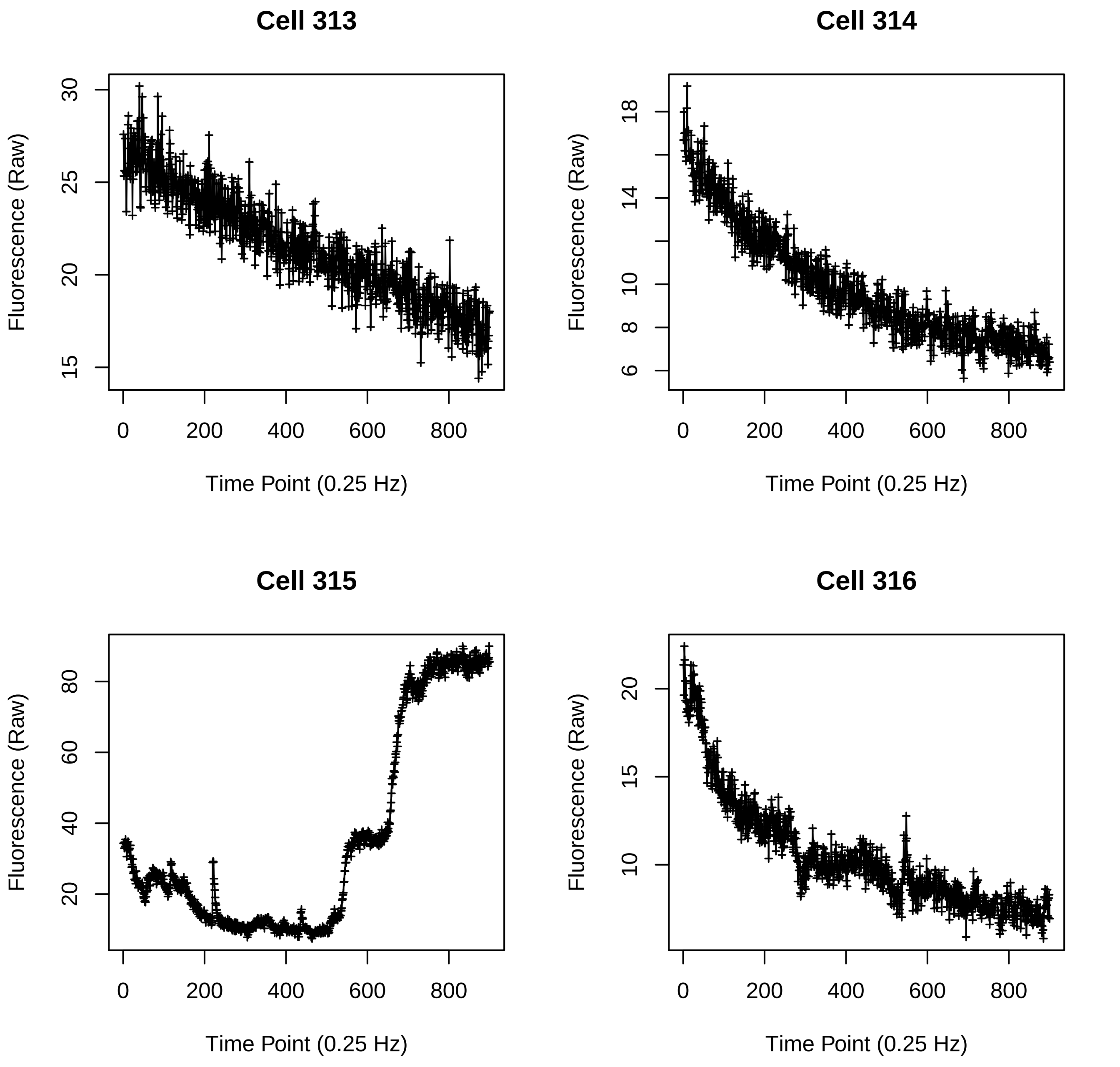
Cell 313
Fluorescence (Raw)
15
20
25
30
0
200
400
600
800
Time Point (0.25 Hz)
Cell 314
Fluorescence (Raw)
6
8
10
14
18
0
200
400
600
800
Time Point (0.25 Hz)
Cell 315
Fluorescence (Raw)
20
40
60
80
0
200
400
600
800
Time Point (0.25 Hz)
Cell 316
Fluorescence (Raw)
10
15
20
0
200
400
600
800
Time Point (0.25 Hz)

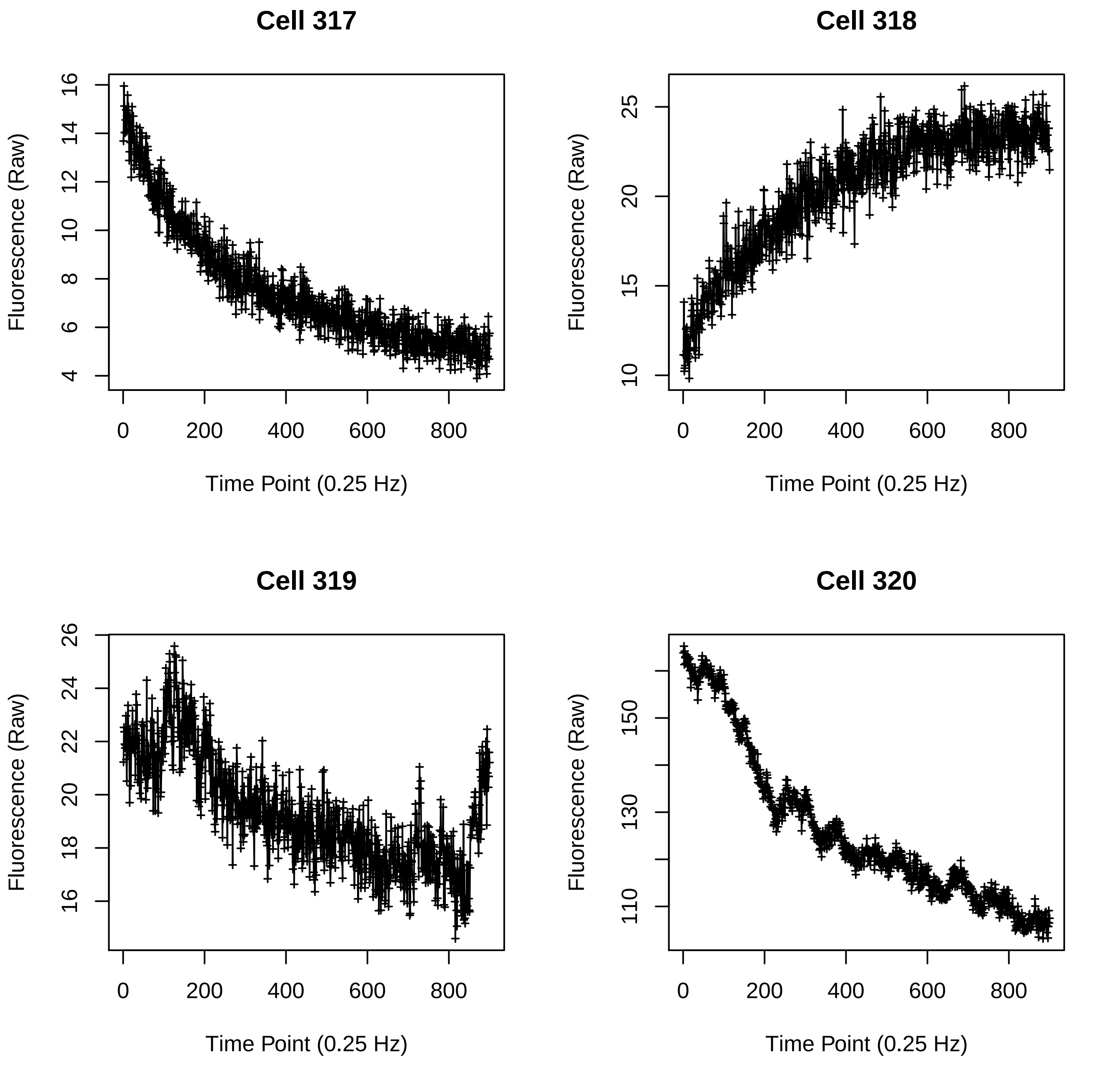
Cell 317
Fluorescence (Raw)
4
6
8
10
12
14
16
0
200
400
600
800
Time Point (0.25 Hz)
Cell 318
Fluorescence (Raw)
10
15
20
25
0
200
400
600
800
Time Point (0.25 Hz)
Cell 319
Fluorescence (Raw)
16
18
20
22
24
26
0
200
400
600
800
Time Point (0.25 Hz)
Cell 320
Fluorescence (Raw)
110
130
150
0
200
400
600
800
Time Point (0.25 Hz)

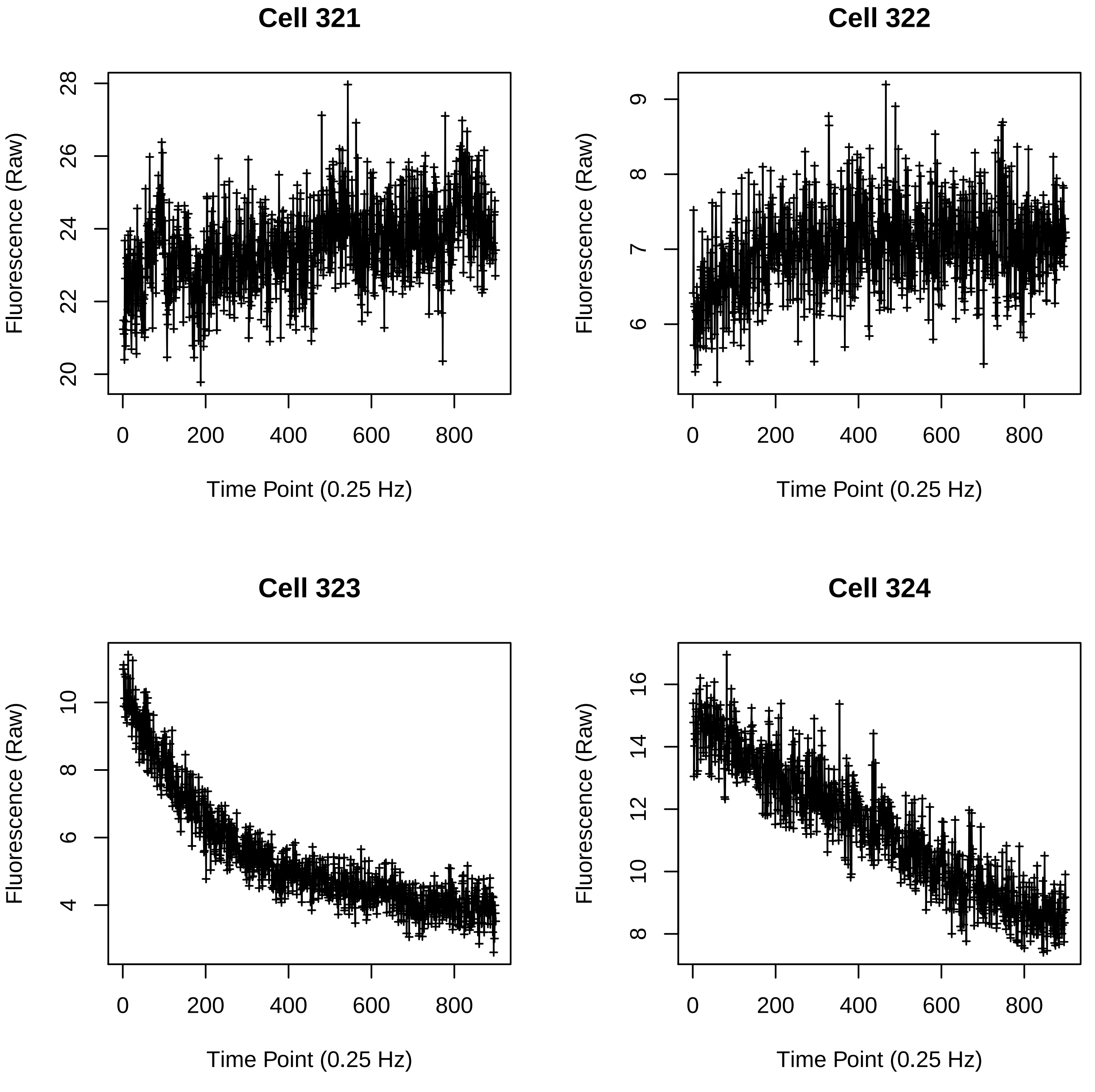
Cell 321
Cell 322
Cell 323
Cell 324
Fluorescence (Raw)
Time Point (0.25 Hz)
20
22
24
26
28
6
7
8
9
4
6
8
10
8
10
12
14
16
0
200
400
600
800

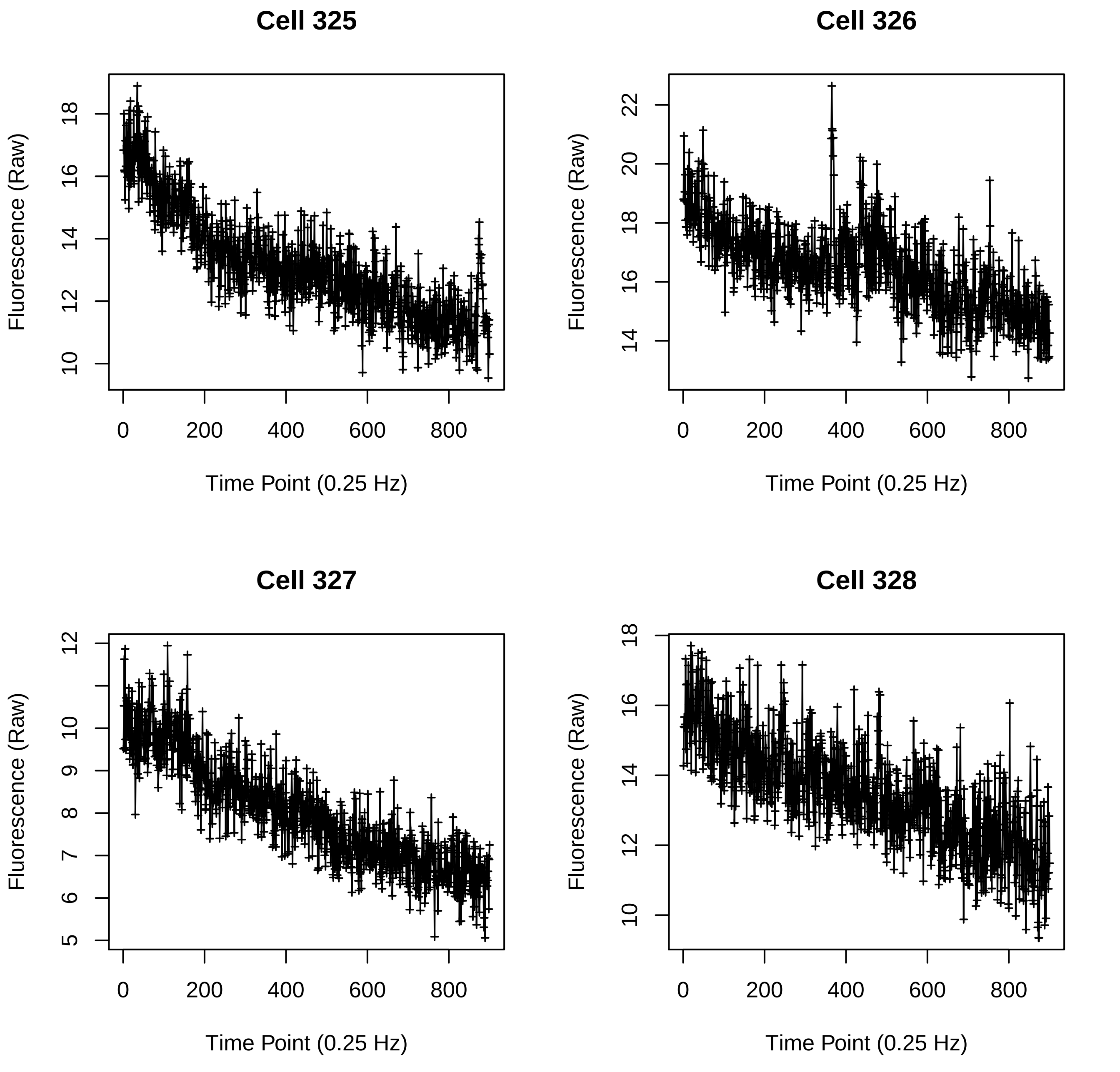
Cell 325
Cell 326
Cell 327
Cell 328
Fluorescence (Raw)
Time Point (0.25 Hz)
0
200
400
600
800
10
12
14
16
18
20
22
5
6
7
8
9

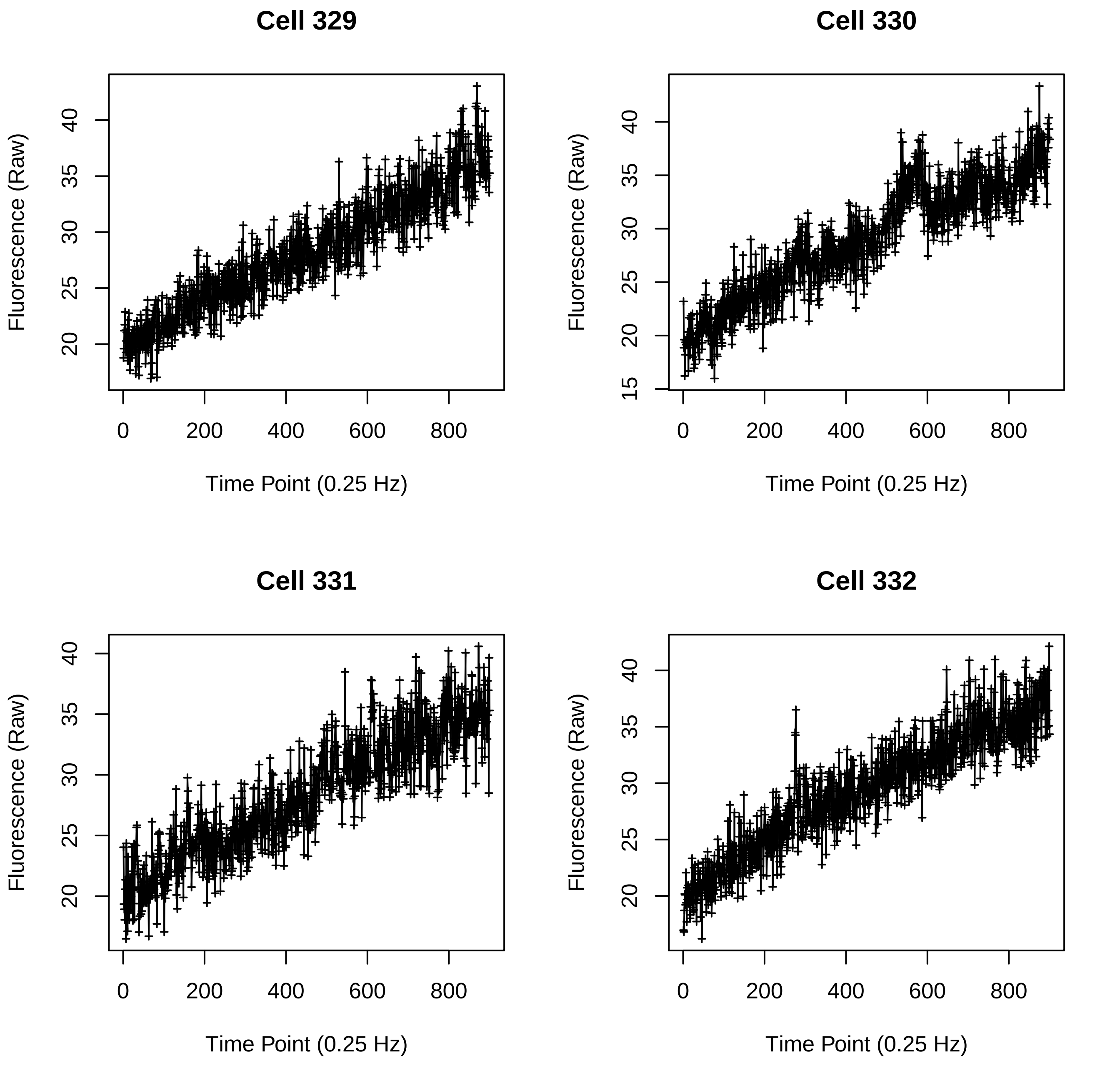
Cell 329
Cell 330
Cell 331
Cell 332
Fluorescence (Raw)
Time Point (0.25 Hz)
0
200
400
600
800
15
20
25
30
35
40

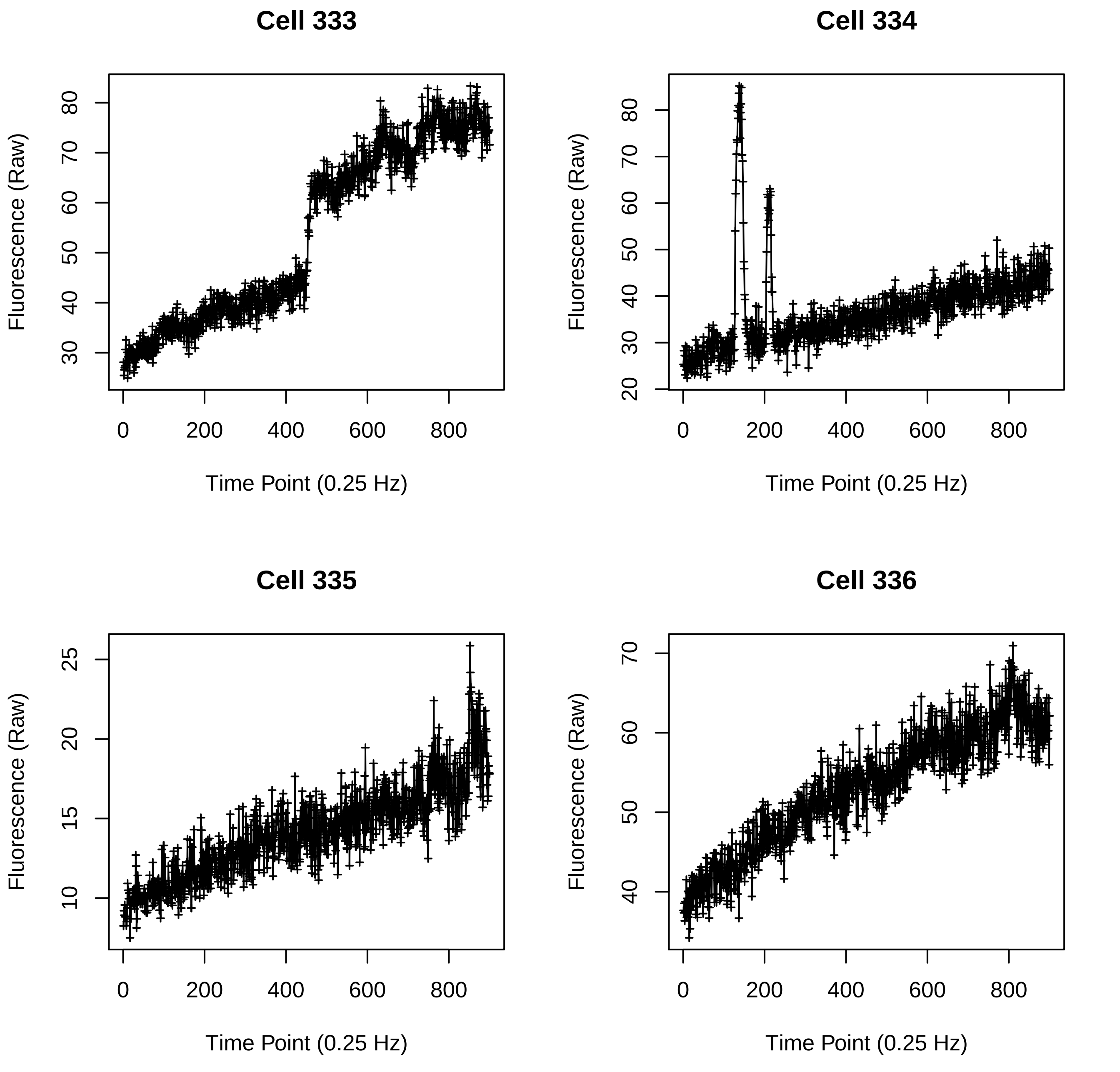
Cell 333
Fluorescence (Raw)
30
40
50
60
70
80
0
200
400
600
800
Time Point (0.25 Hz)
Cell 334
Fluorescence (Raw)
20
30
40
50
60
70
80
0
200
400
600
800
Time Point (0.25 Hz)
Cell 335
Fluorescence (Raw)
10
15
20
25
0
200
400
600
800
Time Point (0.25 Hz)
Cell 336
Fluorescence (Raw)
40
50
60
70
0
200
400
600
800
Time Point (0.25 Hz)

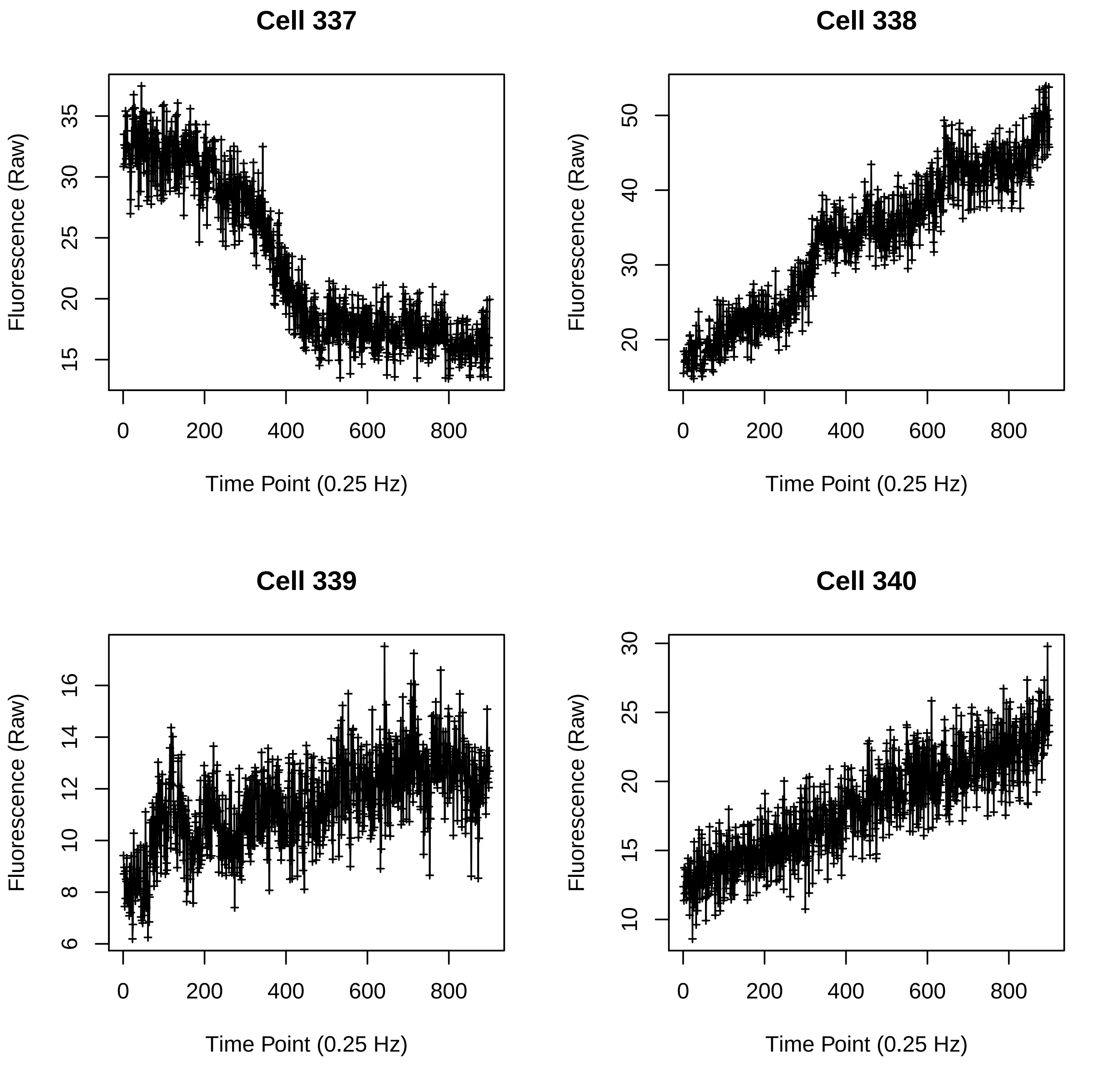
Cell 337
Cell 338
Cell 339
Cell 340
Fluorescence (Raw)
Time Point (0.25 Hz)
0
200
400
600
800
15
20
25
30
35
40
50
6
8
10
12
14
16

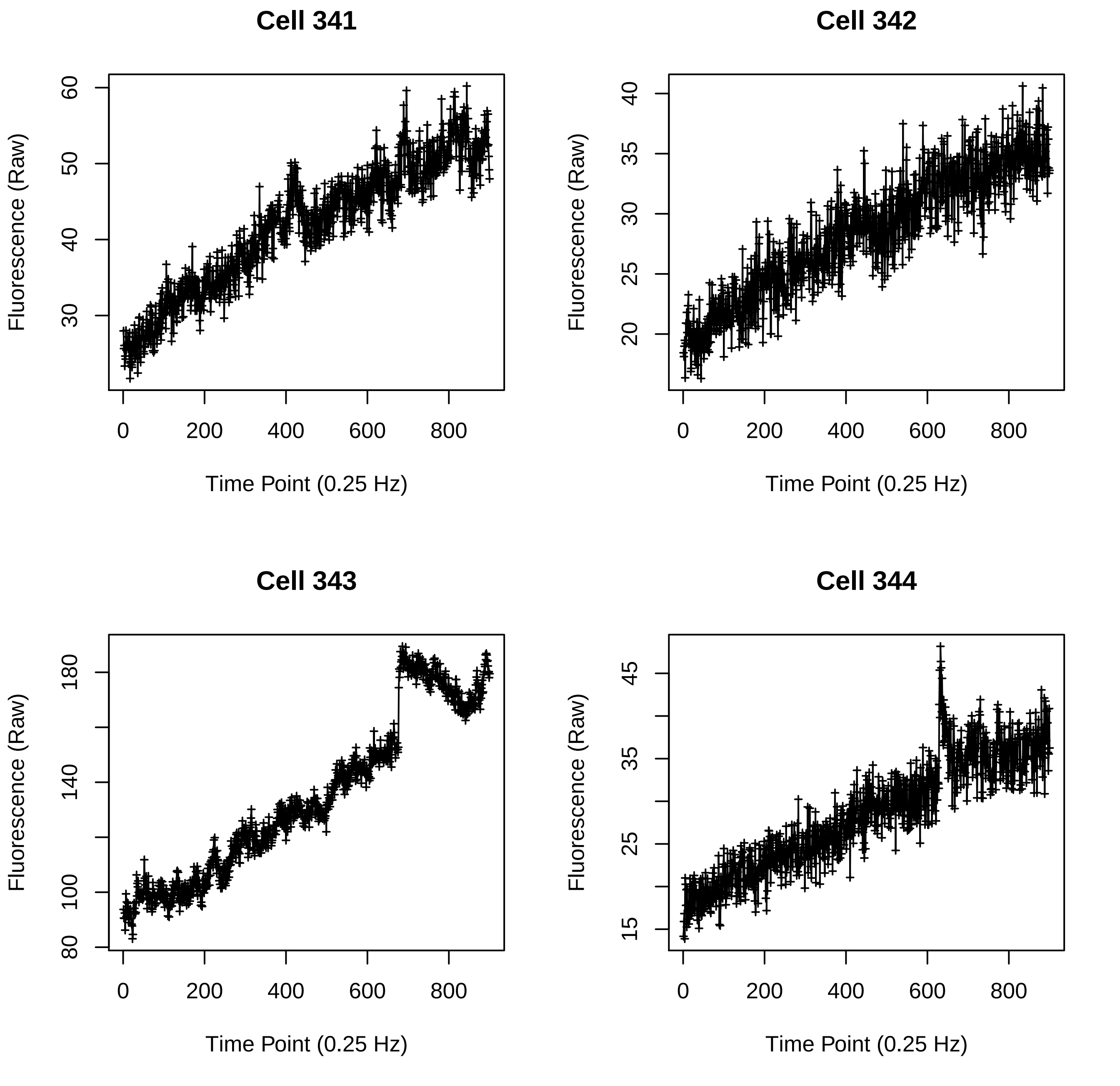
Cell 341
Cell 342
Cell 343
Cell 344
Fluorescence (Raw)
Time Point (0.25 Hz)
0
200
400
600
800
30
40
50
60
20
25
30
35
40
80
100
140
180
15
25
35
45

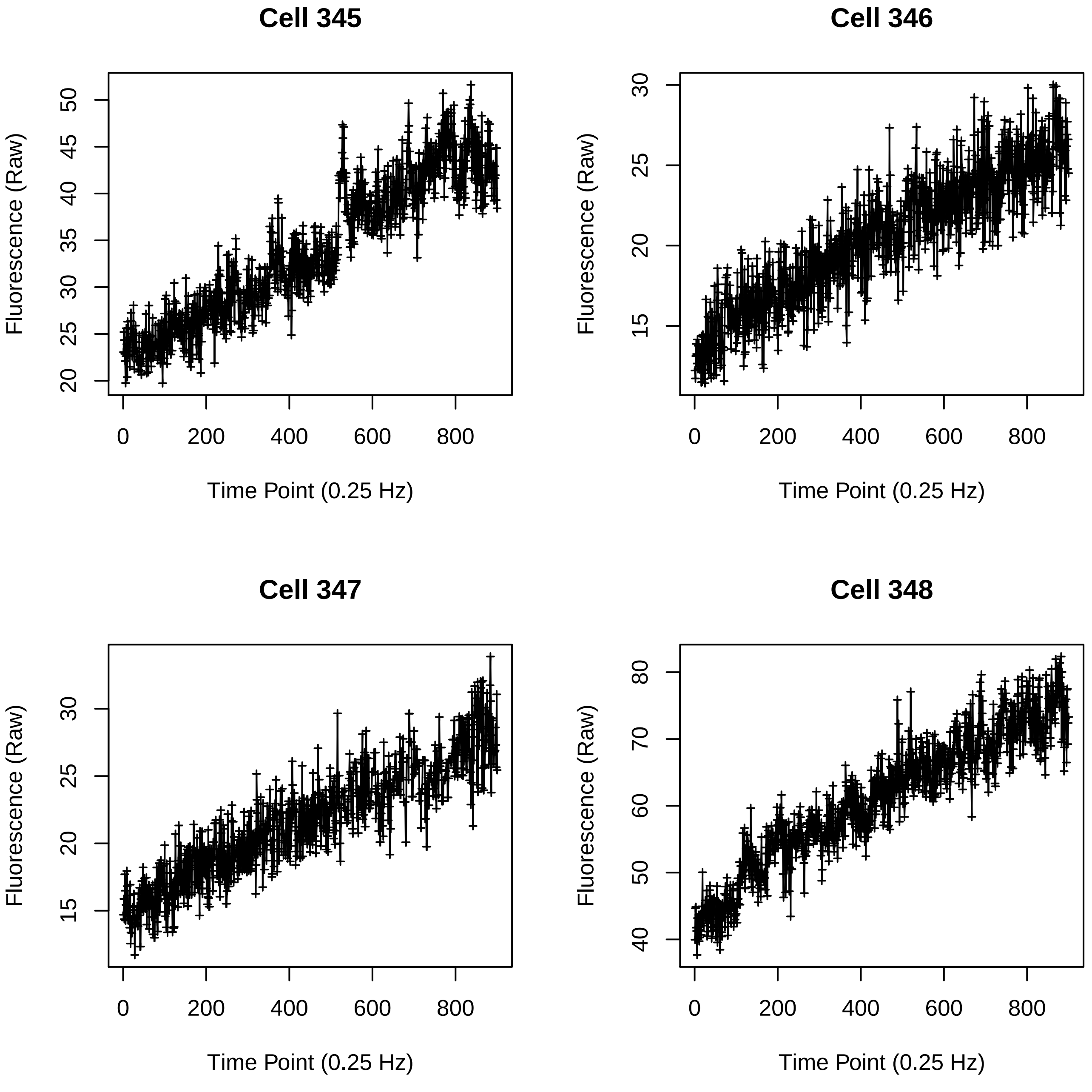
Cell 345
Cell 346
Cell 347
Cell 348
Fluorescence (Raw)
Time Point (0.25 Hz)
0
200
400
600
800
20
25
30
35
40
45
50
15
60
70
80

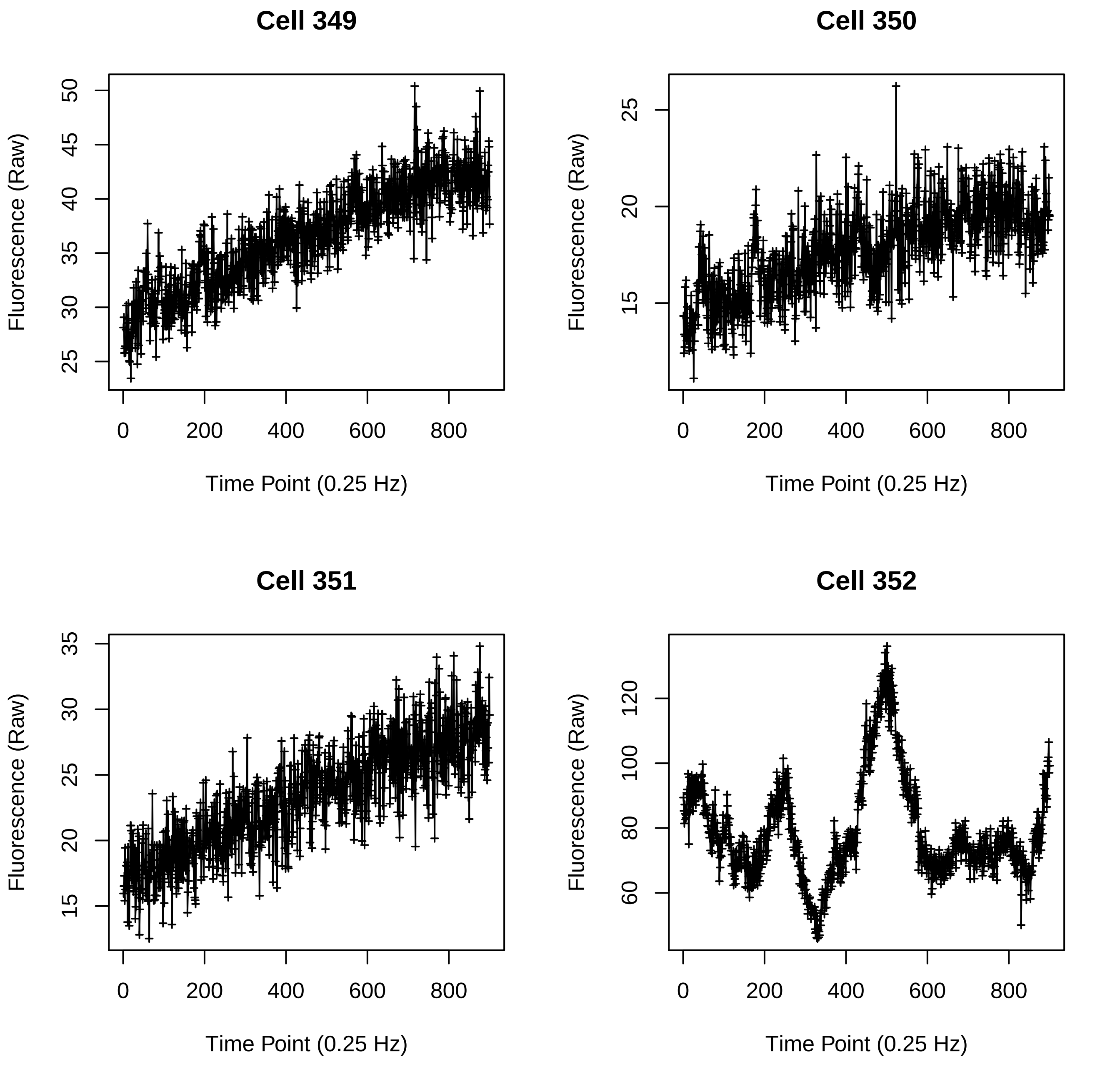
Cell 349
Fluorescence (Raw)
25
30
35
40
45
50
0
200
400
600
800
Time Point (0.25 Hz)
Cell 350
Fluorescence (Raw)
15
20
25
0
200
400
600
800
Time Point (0.25 Hz)
Cell 351
Fluorescence (Raw)
15
20
25
30
35
0
200
400
600
800
Time Point (0.25 Hz)
Cell 352
Fluorescence (Raw)
60
80
100
120
0
200
400
600
800
Time Point (0.25 Hz)

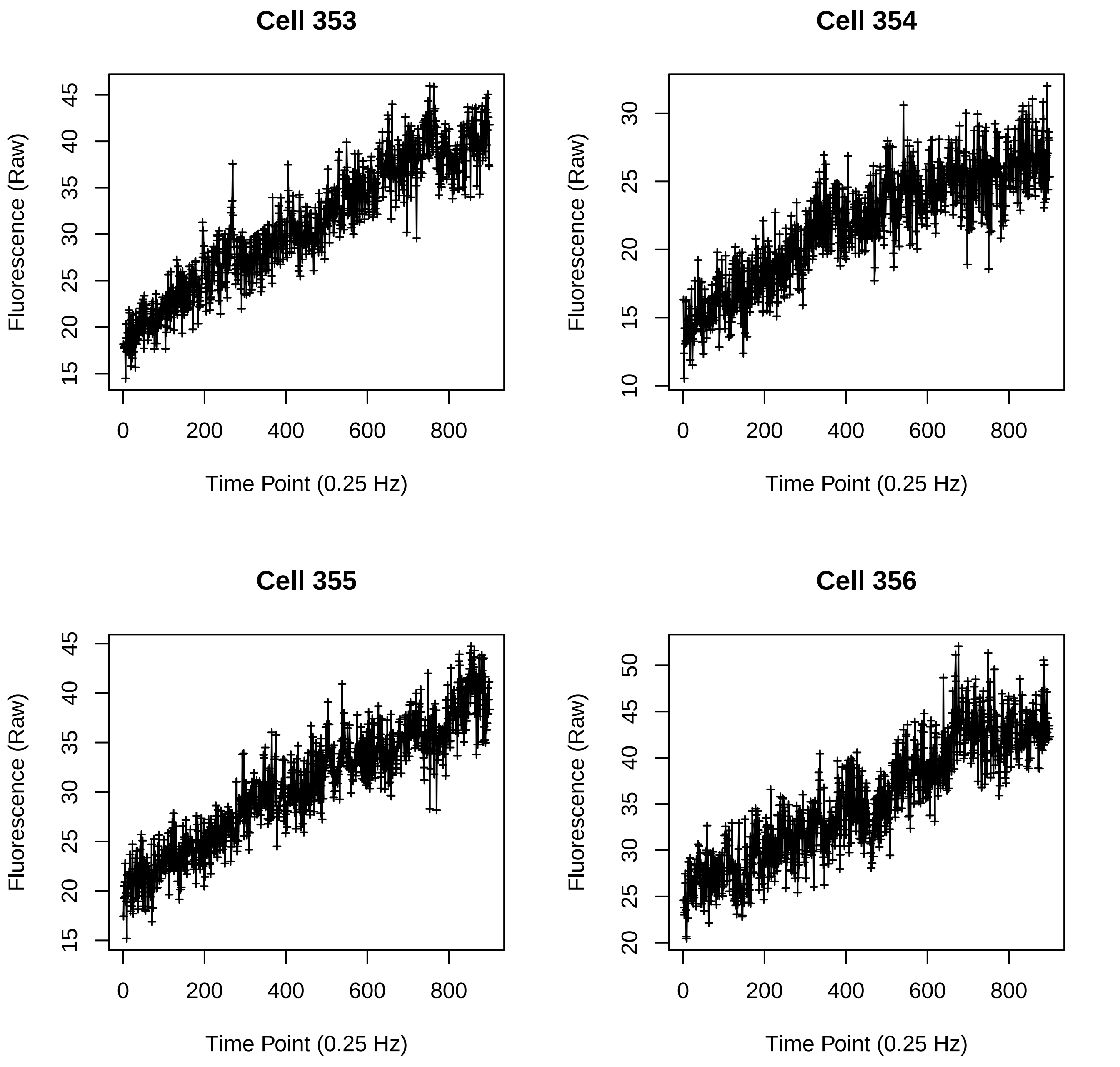

Cell 353
Cell 354
Cell 355
Cell 356
Fluorescence (Raw)
Time Point (0.25 Hz)
0
200
400
600
800
15
20
25
30
35
40
45
10
50

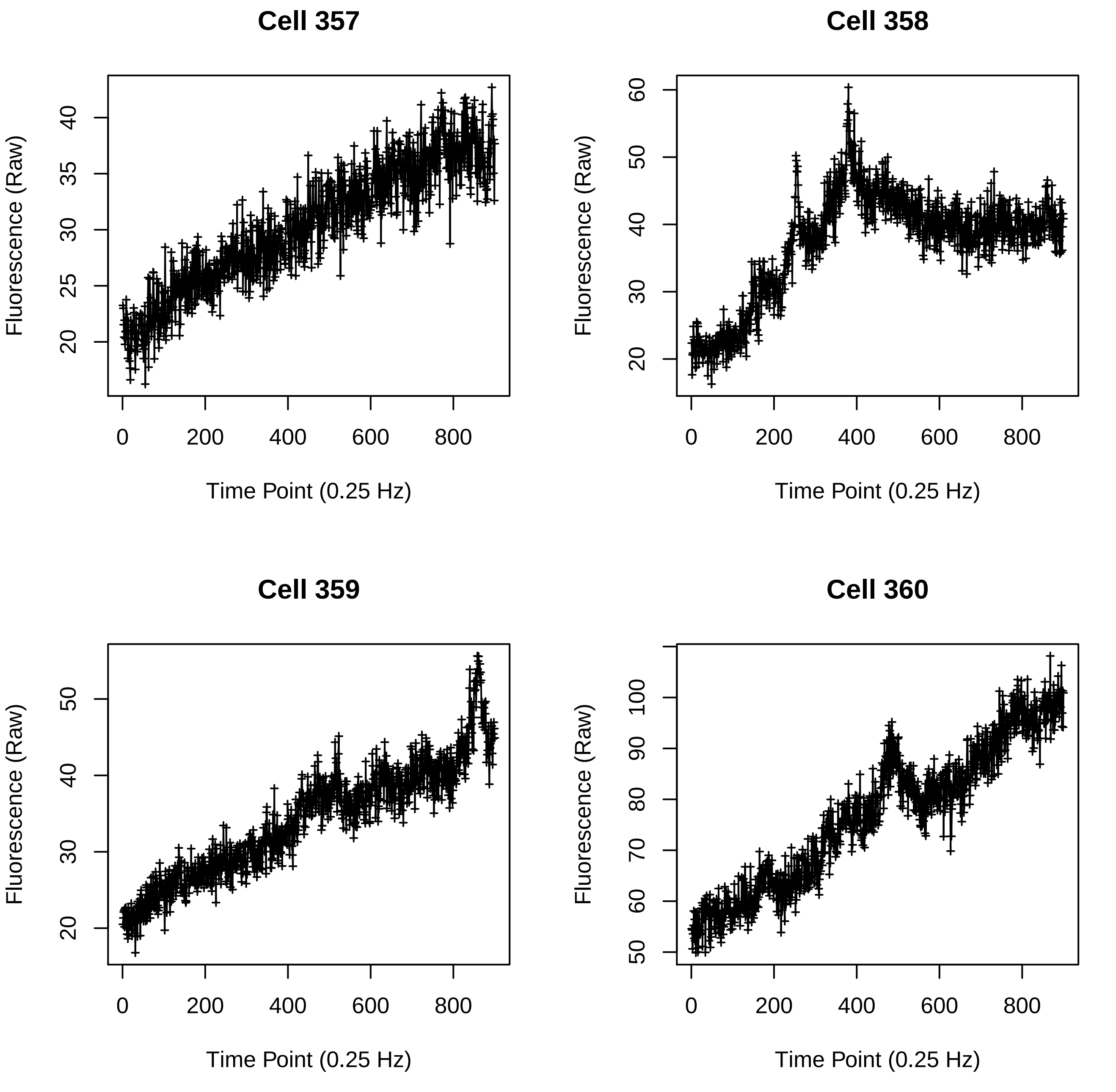

Cell 357
Cell 358
Cell 359
Cell 360
Fluorescence (Raw)
Time Point (0.25 Hz)
0
200
400
600
800
20
25
30
35
40
50
60
70
80
90
100

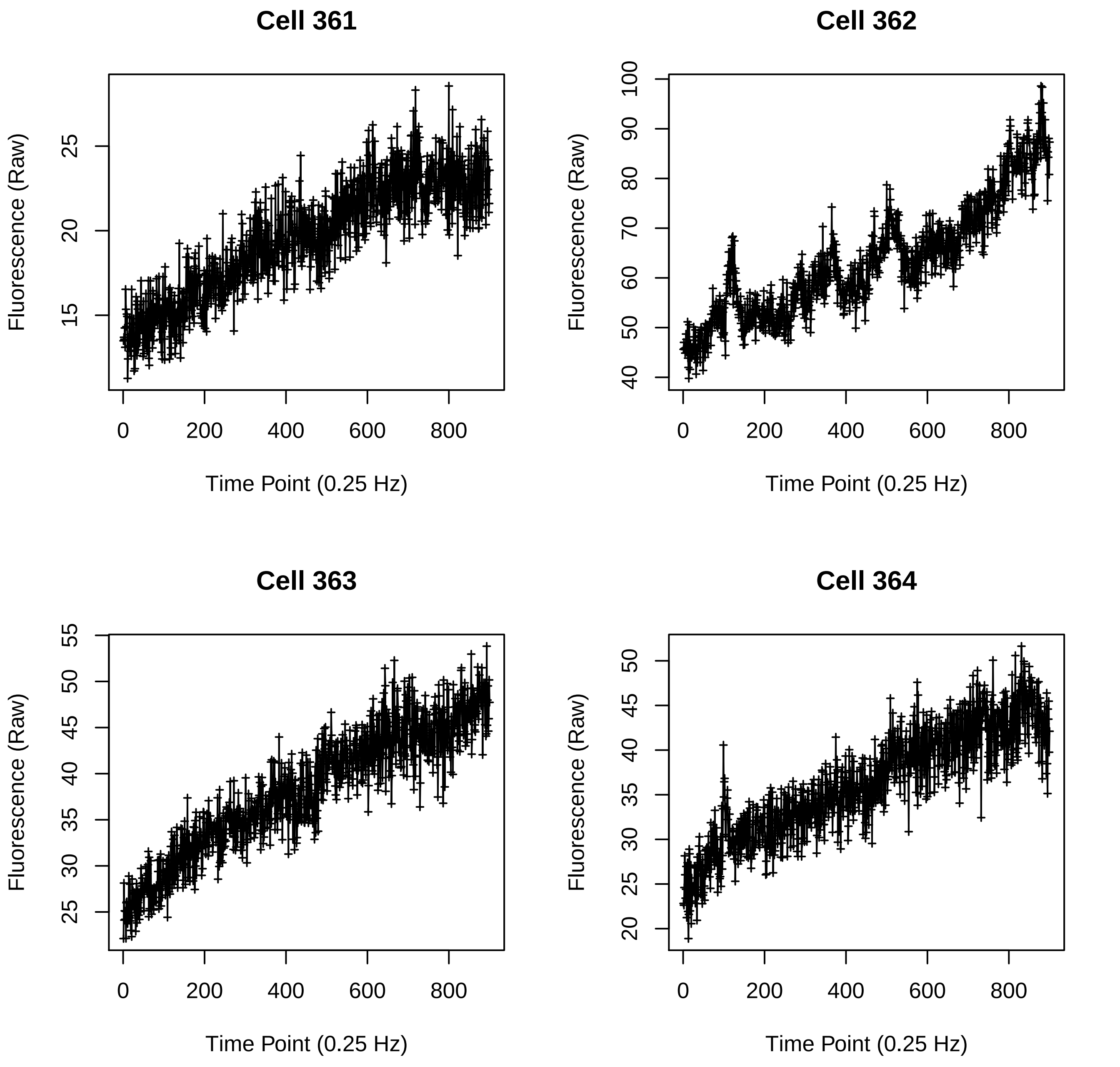
Cell 361
Fluorescence (Raw)
15
20
25
0
200
400
600
800
Time Point (0.25 Hz)
Cell 362
Fluorescence (Raw)
40
50
60
70
80
90
100
0
200
400
600
800
Time Point (0.25 Hz)
Cell 363
Fluorescence (Raw)
25
30
35
40
45
50
55
0
200
400
600
800
Time Point (0.25 Hz)
Cell 364
Fluorescence (Raw)
20
25
30
35
40
45
50
0
200
400
600
800
Time Point (0.25 Hz)

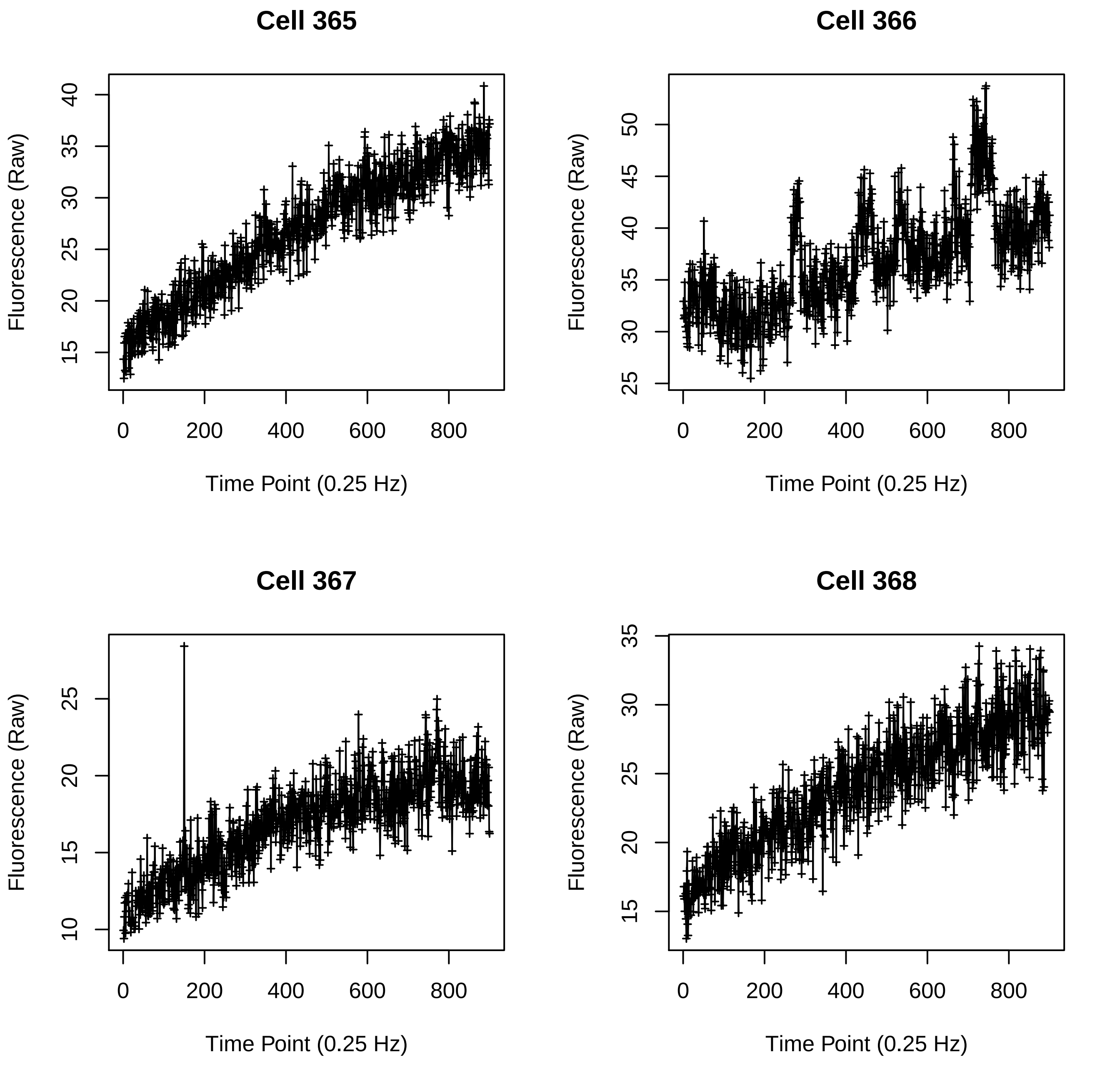

Cell 365
Cell 366
Cell 367
Cell 368
Fluorescence (Raw)
Time Point (0.25 Hz)
0
200
400
600
800
15
20
25
30
35
40
45
50
10

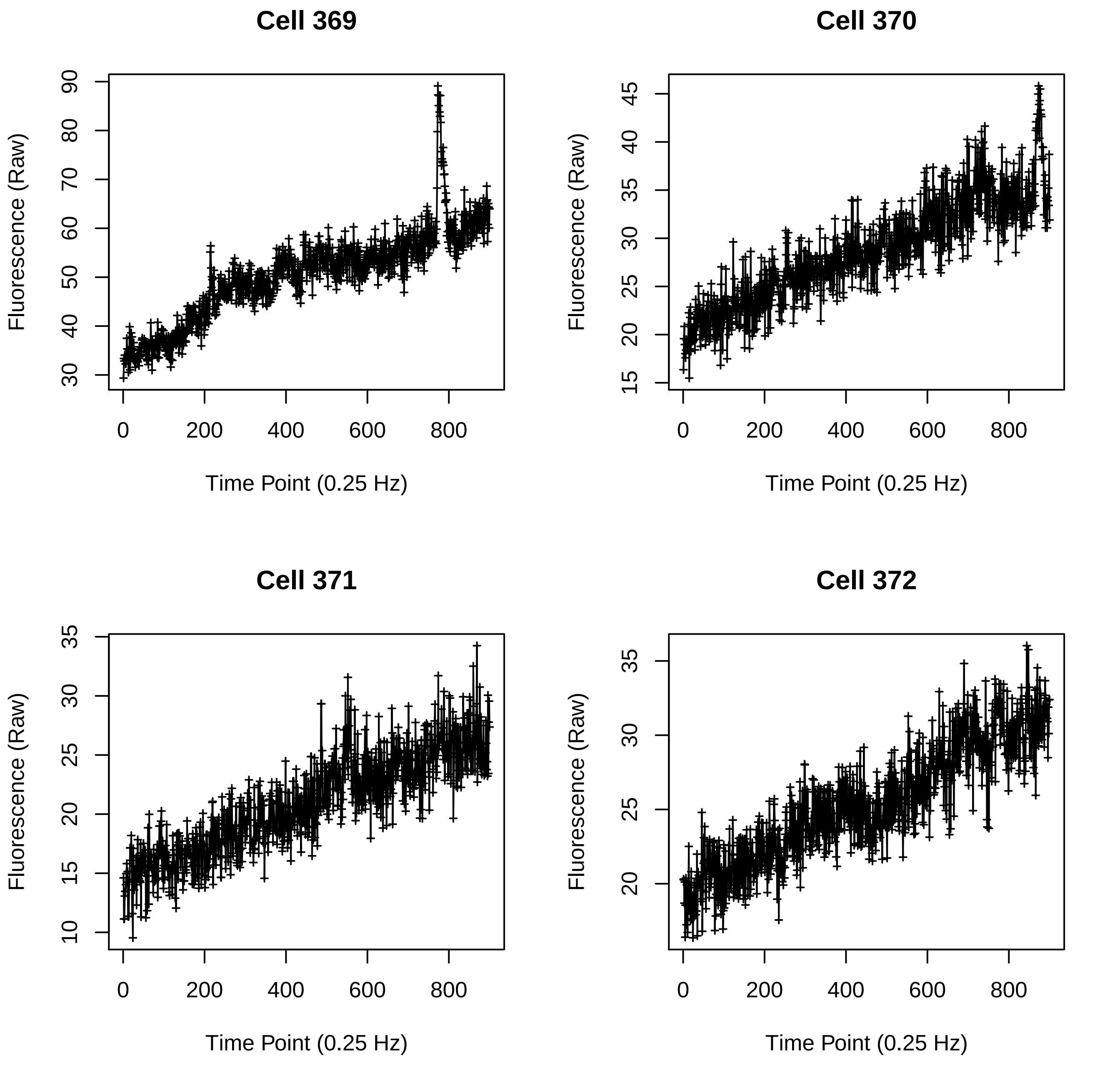
Cell 369
Cell 370
Cell 371
Cell 372
Fluorescence (Raw)
Time Point (0.25 Hz)
0
200
400
600
800
30
40
50
60
70
80
90
15
20
25
30
35
40
45
10

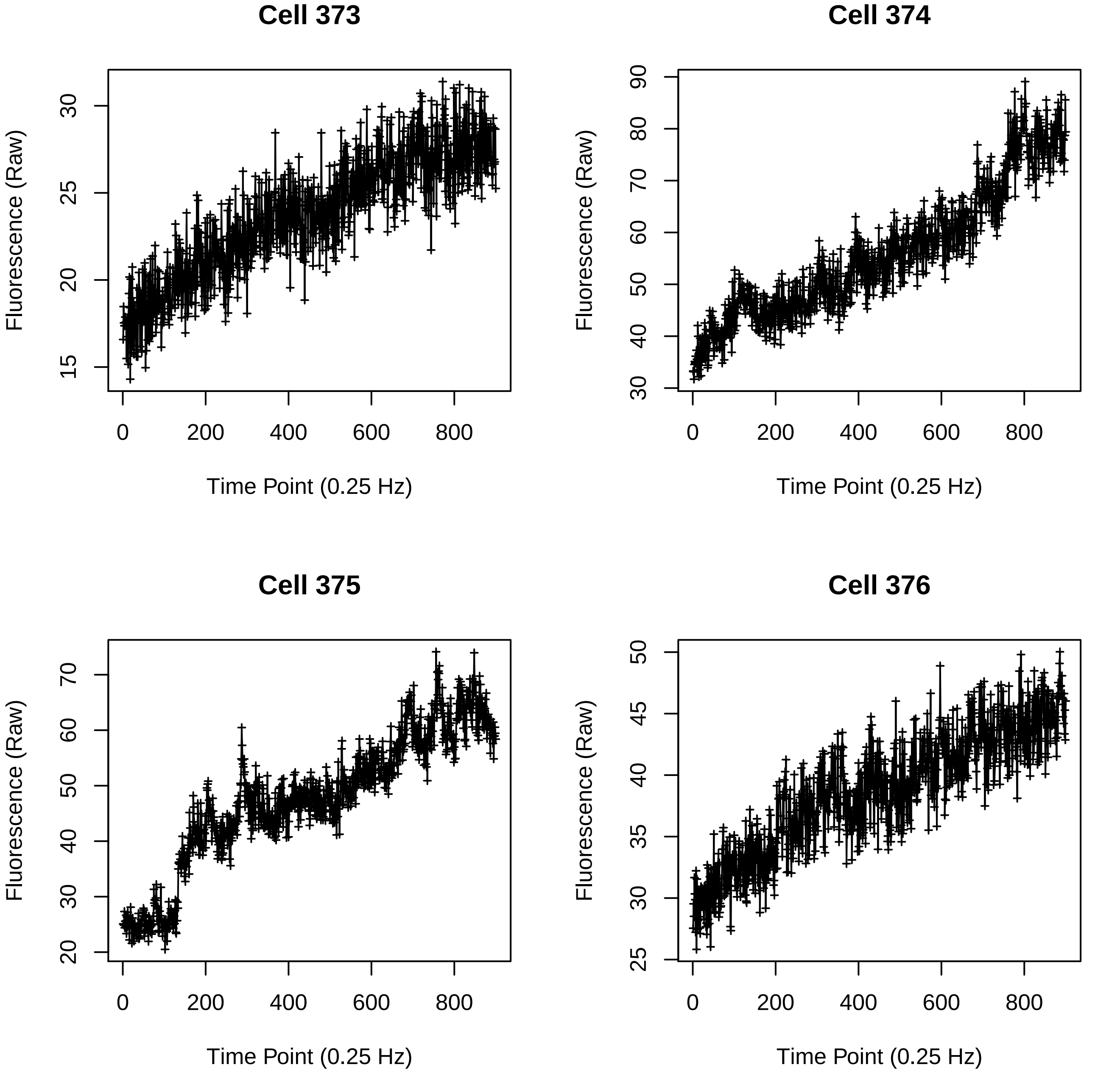
Cell 373
Cell 374
Cell 375
Cell 376
Fluorescence (Raw)
Time Point (0.25 Hz)
0
200
400
600
800
15
20
25
30
40
50
60
70
80
90
35
45

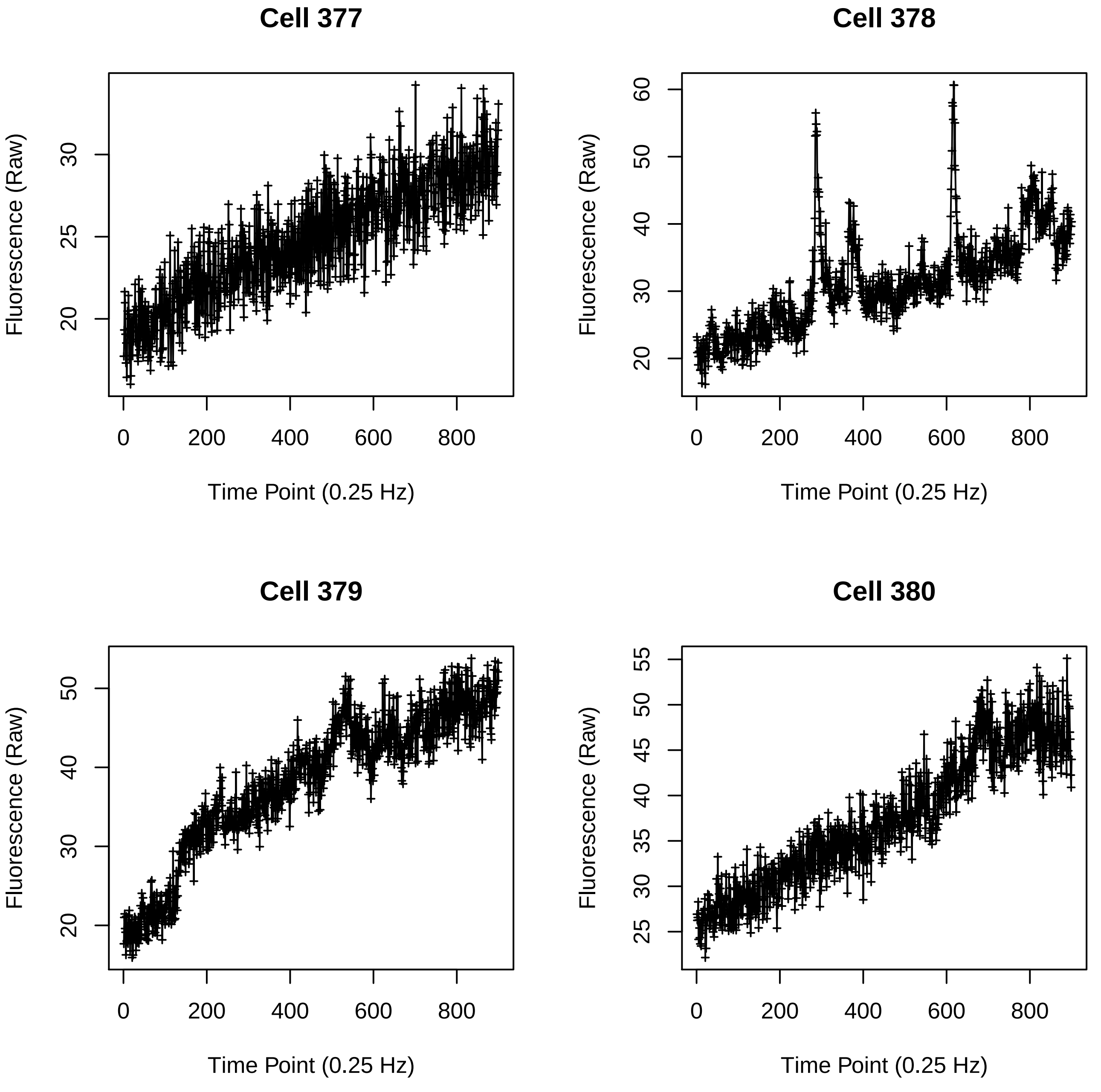
Cell 377
Cell 378
Cell 379
Cell 380
Fluorescence (Raw)
Time Point (0.25 Hz)
0
200
400
600
800
20
25
30
40
50
60
35
45
55

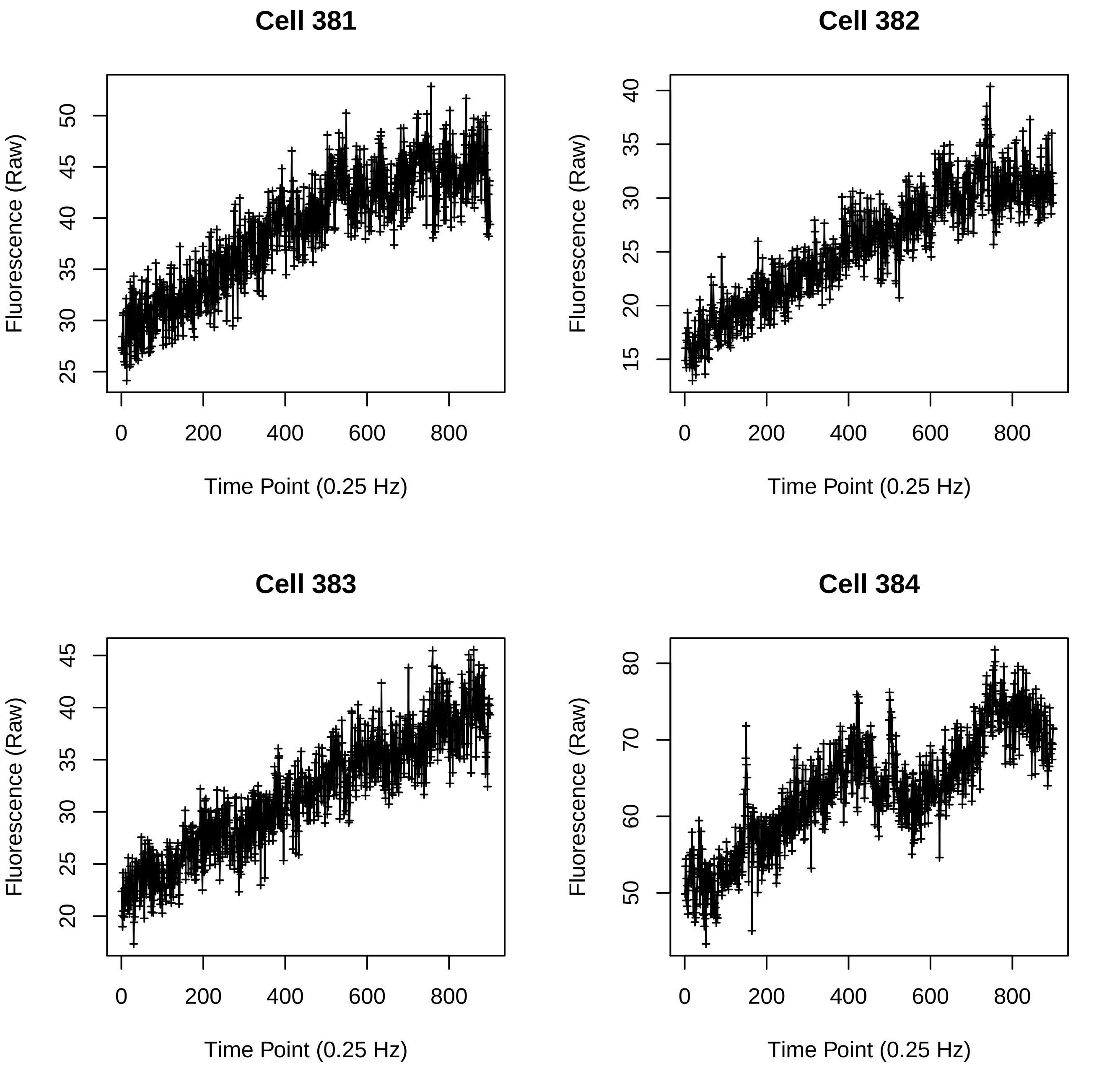
Cell 381
Cell 382
Cell 383
Cell 384
Fluorescence (Raw)
Time Point (0.25 Hz)
0
200
400
600
800
25
30
35
40
45
50
15
20
60
70
80

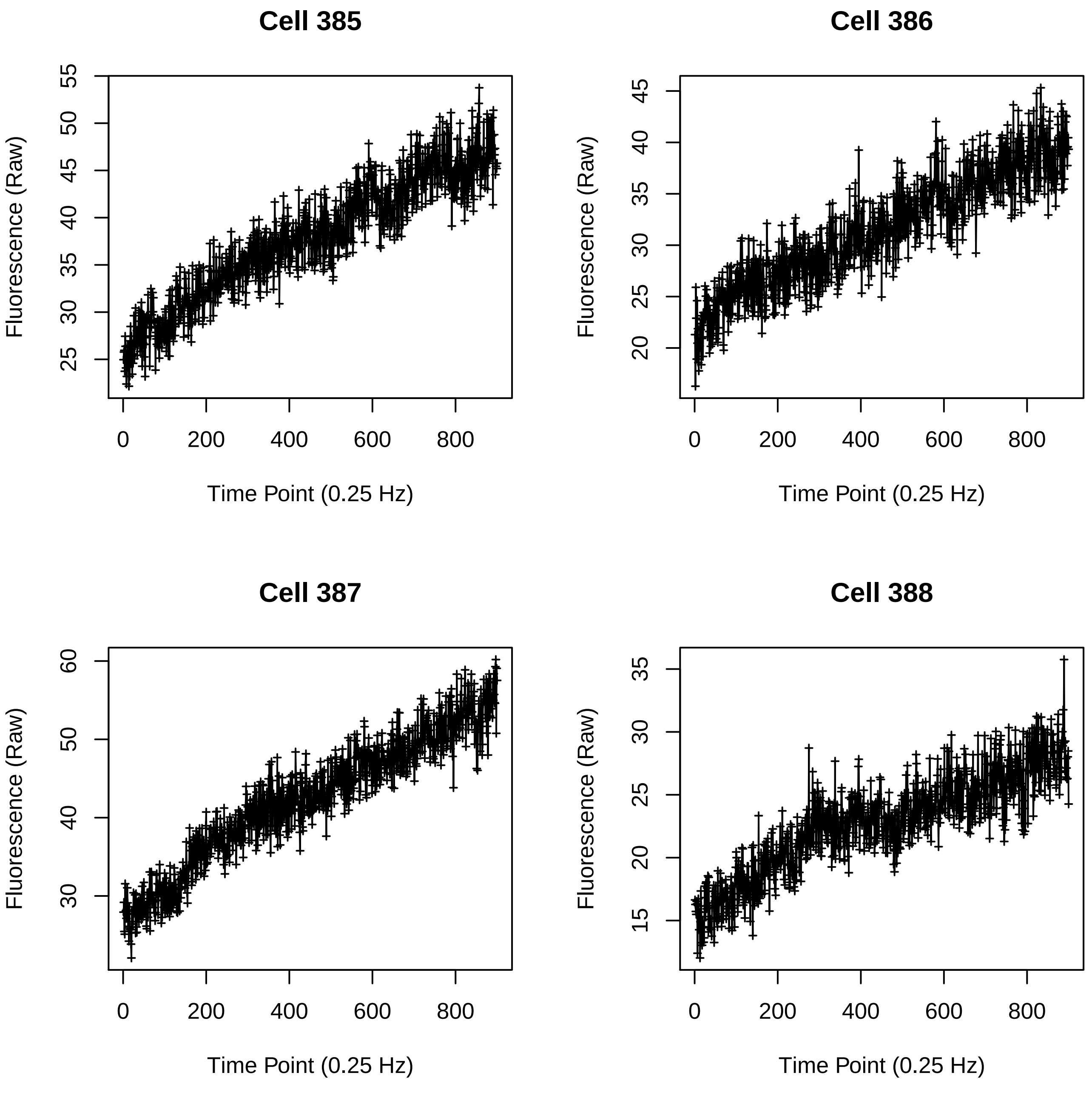
Cell 385
Cell 386
Cell 387
Cell 388
Fluorescence (Raw)
Time Point (0.25 Hz)
0
200
400
600
800
25
30
35
40
45
50
55
20
15
60

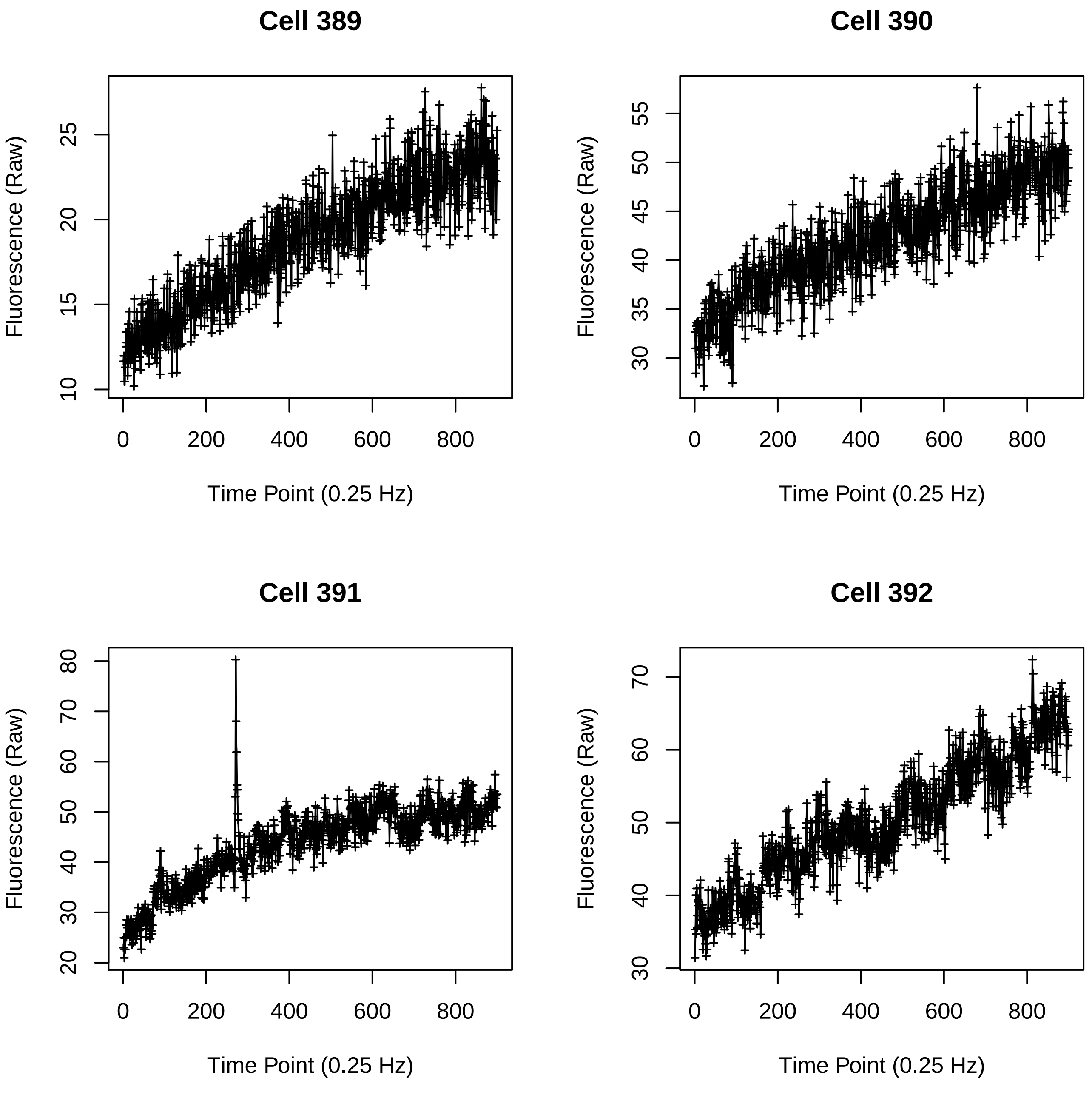
Cell 389
Fluorescence (Raw)
10
15
20
25
0
200
400
600
800
Time Point (0.25 Hz)
Cell 390
Fluorescence (Raw)
30
35
40
45
50
55
0
200
400
600
800
Time Point (0.25 Hz)
Cell 391
Fluorescence (Raw)
20
30
40
50
60
70
80
0
200
400
600
800
Time Point (0.25 Hz)
Cell 392
Fluorescence (Raw)
30
40
50
60
70
0
200
400
600
800
Time Point (0.25 Hz)

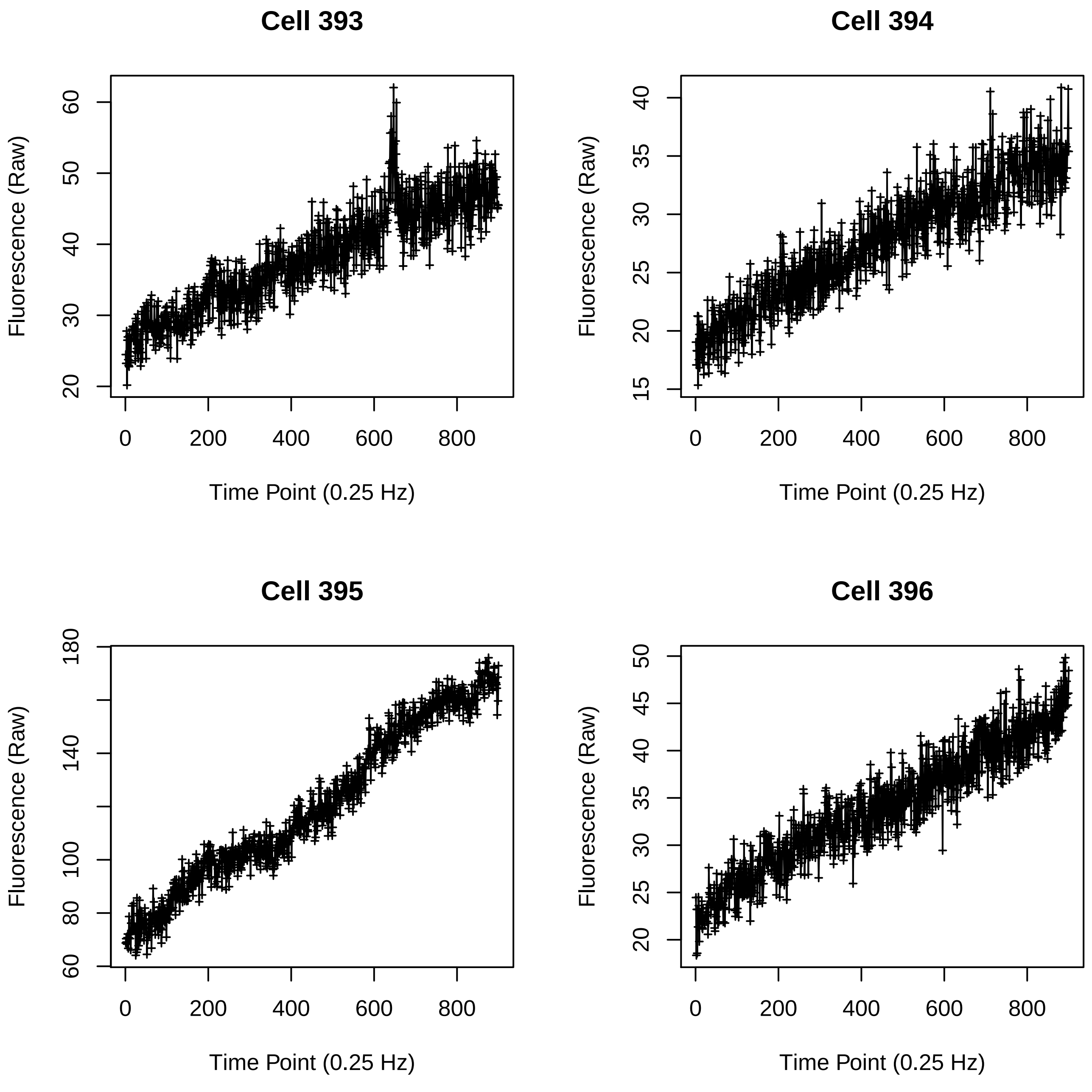
Cell 393
Cell 394
Cell 395
Cell 396
Fluorescence (Raw)
Time Point (0.25 Hz)
0
200
400
600
800
20
30
40
50
60
15
20
25
30
35
40
60
80
100
140
180
20
25
30
35
40
45
50

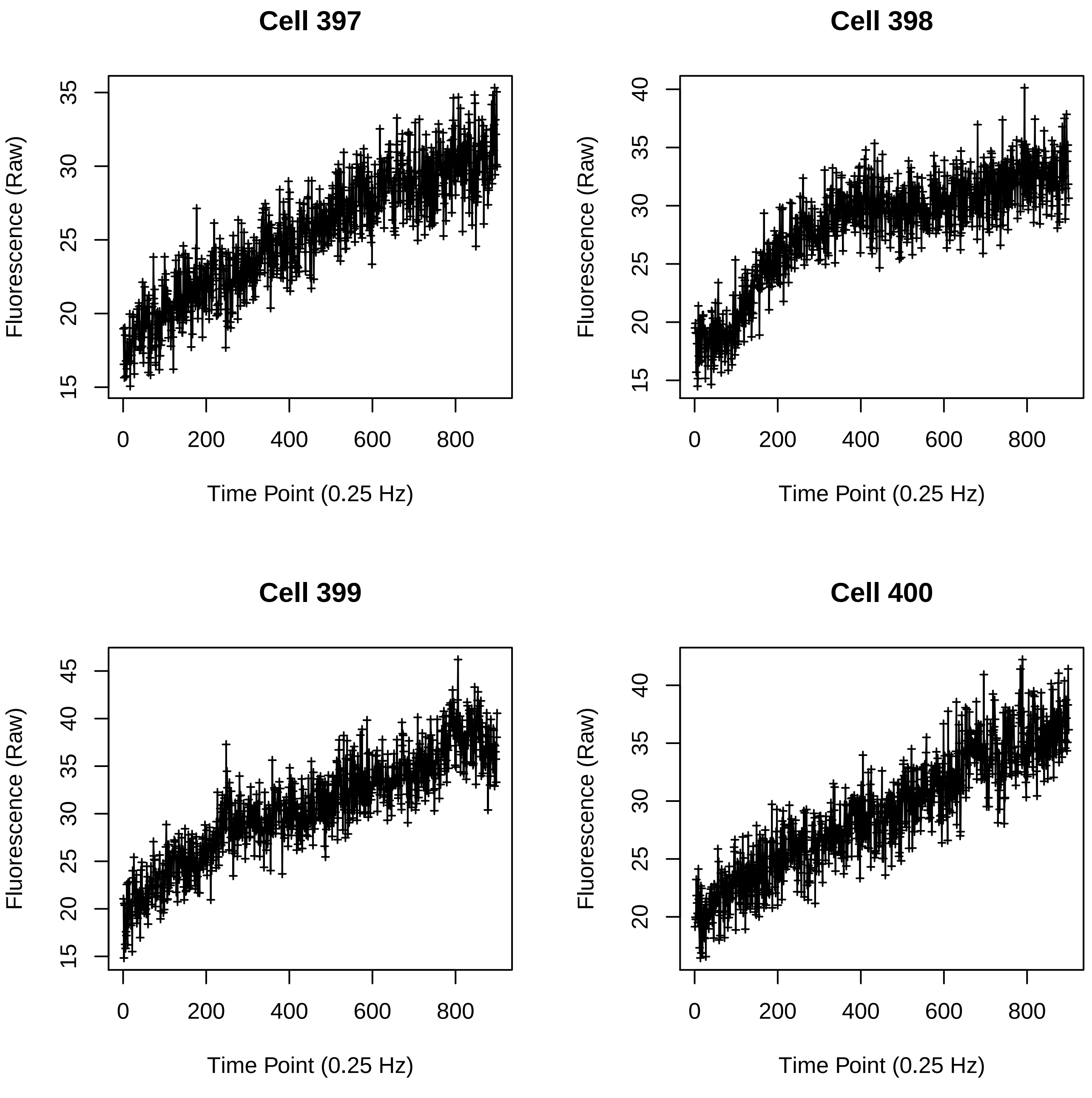
Cell 397
Cell 398
Cell 399
Cell 400
Fluorescence (Raw)
Time Point (0.25 Hz)
15 20 25 30 35
15 20 25 30 35 40
15 20 25 30 35 40 45
20 25 30 35 40
0 200 400 600 800

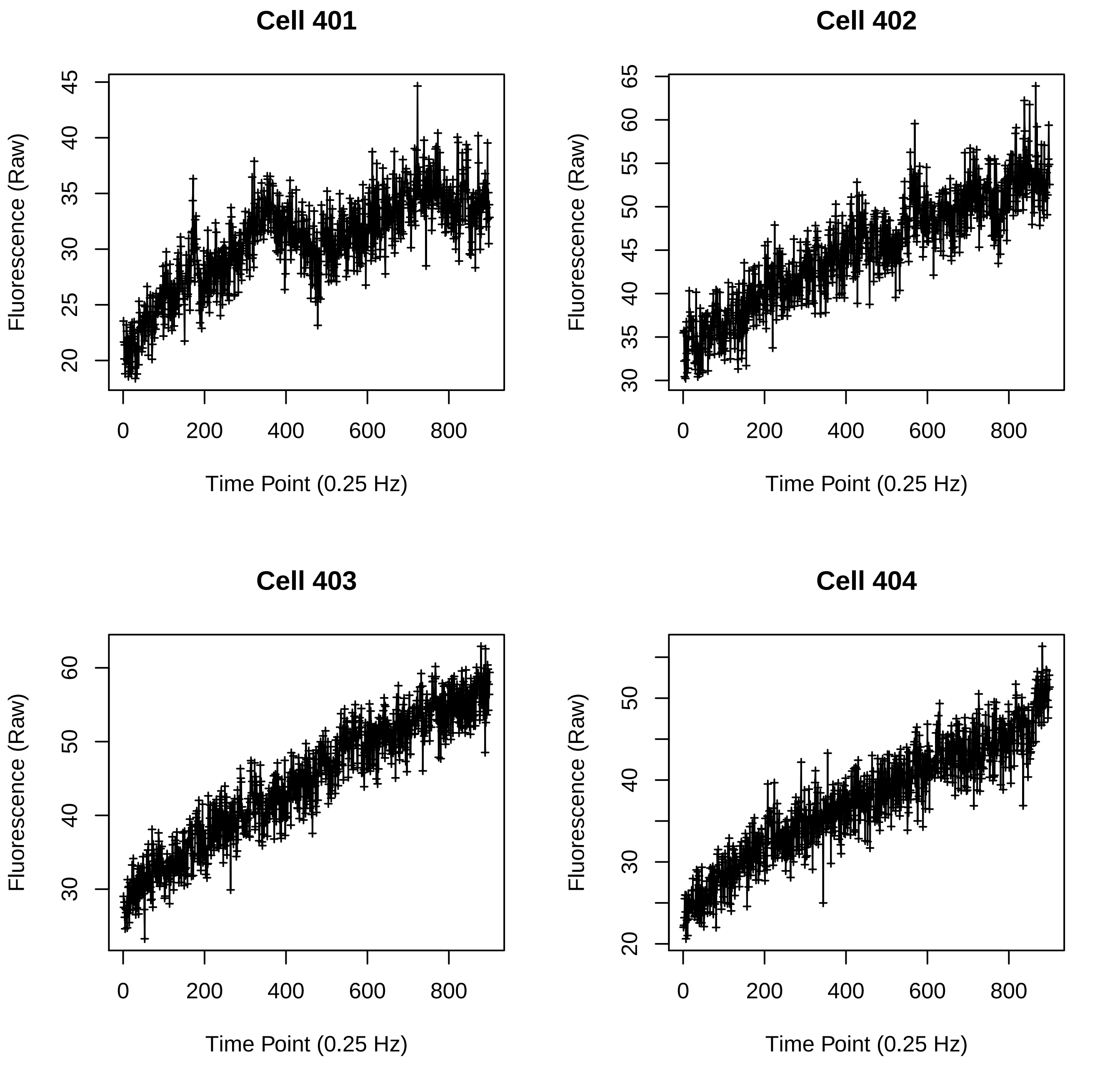
Cell 401
Cell 402
Cell 403
Cell 404
Fluorescence (Raw)
Time Point (0.25 Hz)
0
200
400
600
800
20
25
30
35
40
45
50
55
60
65

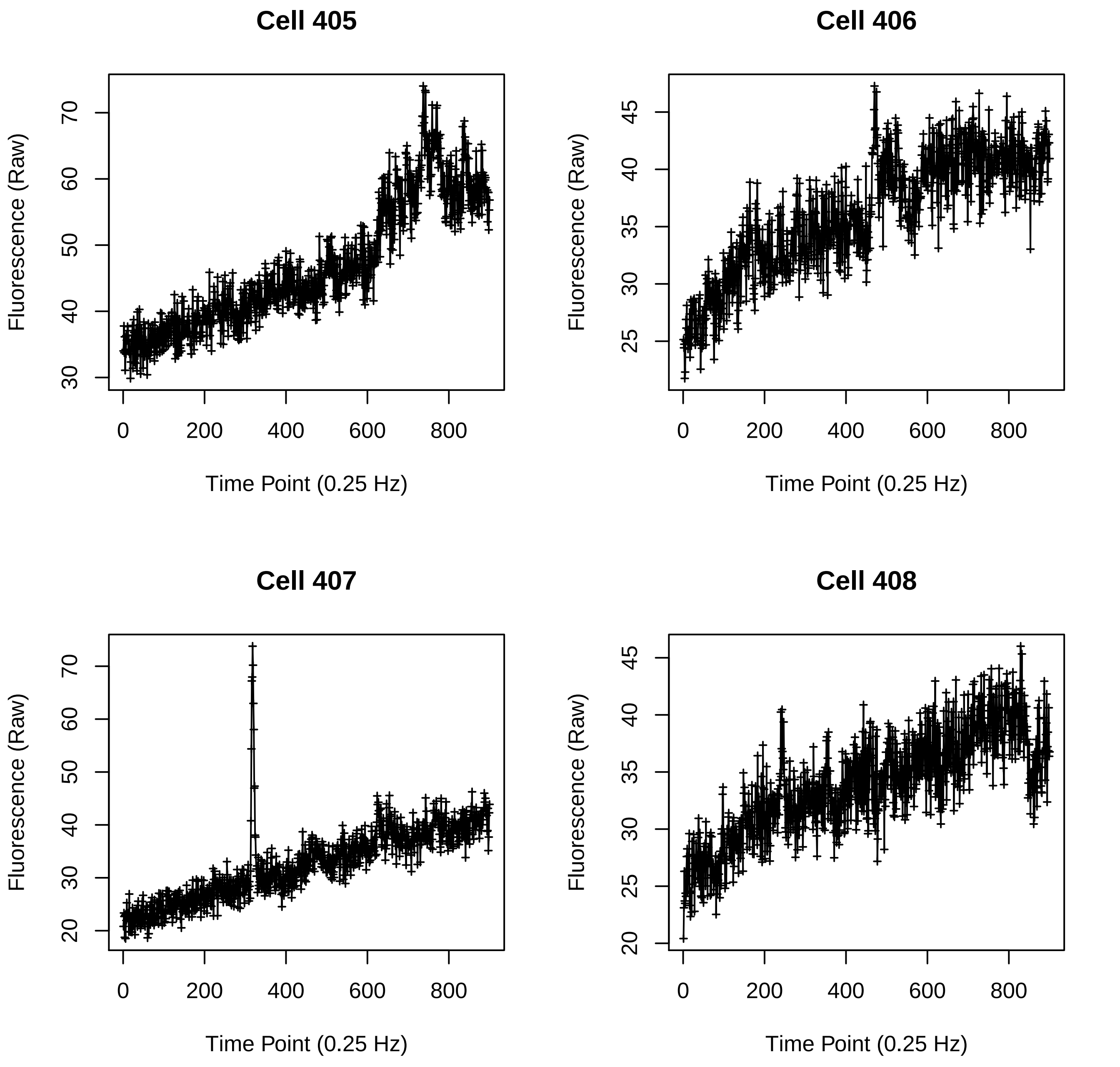
Cell 405
Cell 406
Cell 407
Cell 408
Fluorescence (Raw)
Time Point (0.25 Hz)
0
200
400
600
800
30
40
50
60
70
25
35
45
20

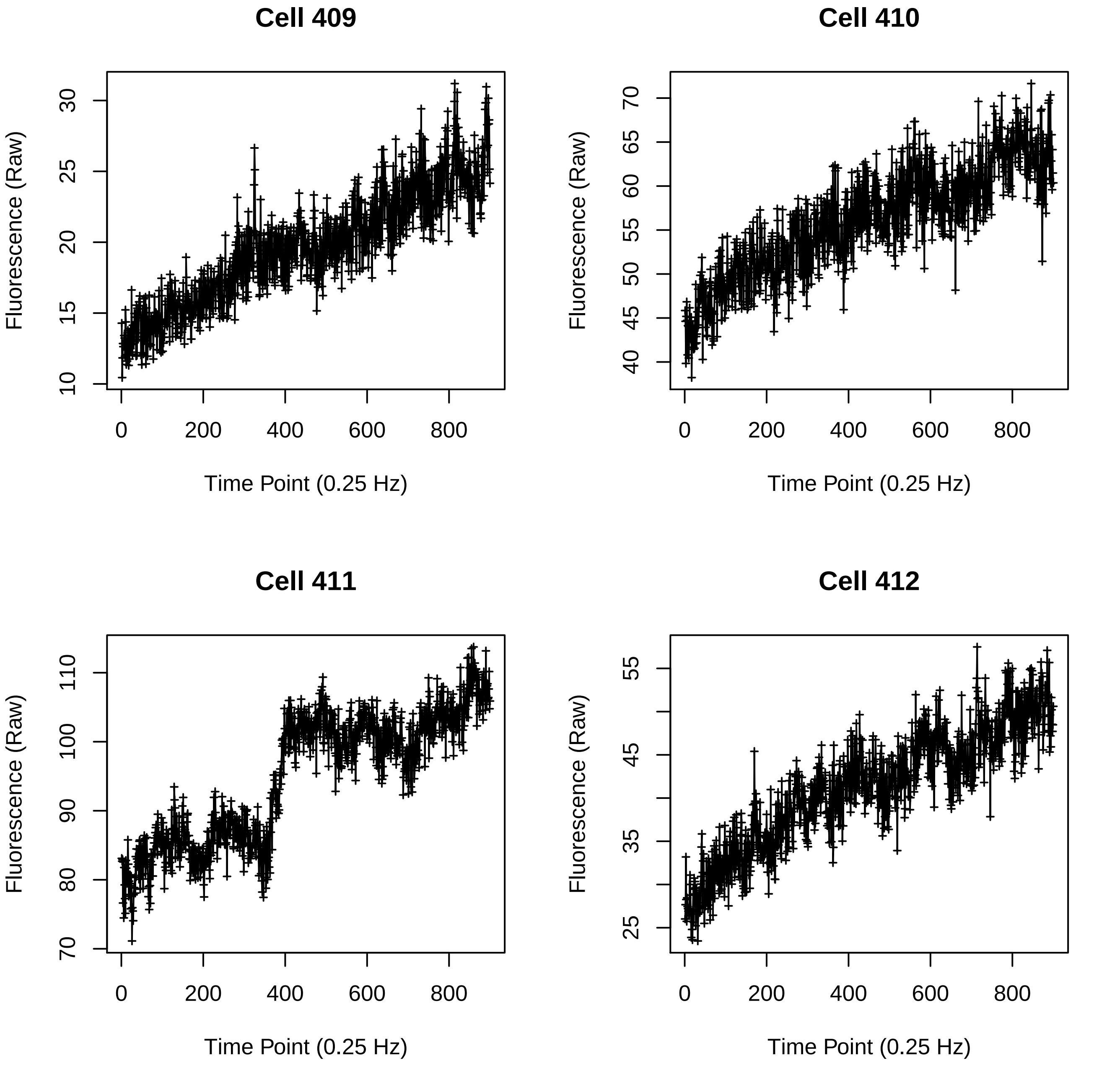
Cell 409
Fluorescence (Raw)
10
15
20
25
30
0
200
400
600
800
Time Point (0.25 Hz)
Cell 410
Fluorescence (Raw)
40
45
50
55
60
65
70
0
200
400
600
800
Time Point (0.25 Hz)
Cell 411
Fluorescence (Raw)
70
80
90
100
110
0
200
400
600
800
Time Point (0.25 Hz)
Cell 412
Fluorescence (Raw)
25
35
45
55
0
200
400
600
800
Time Point (0.25 Hz)

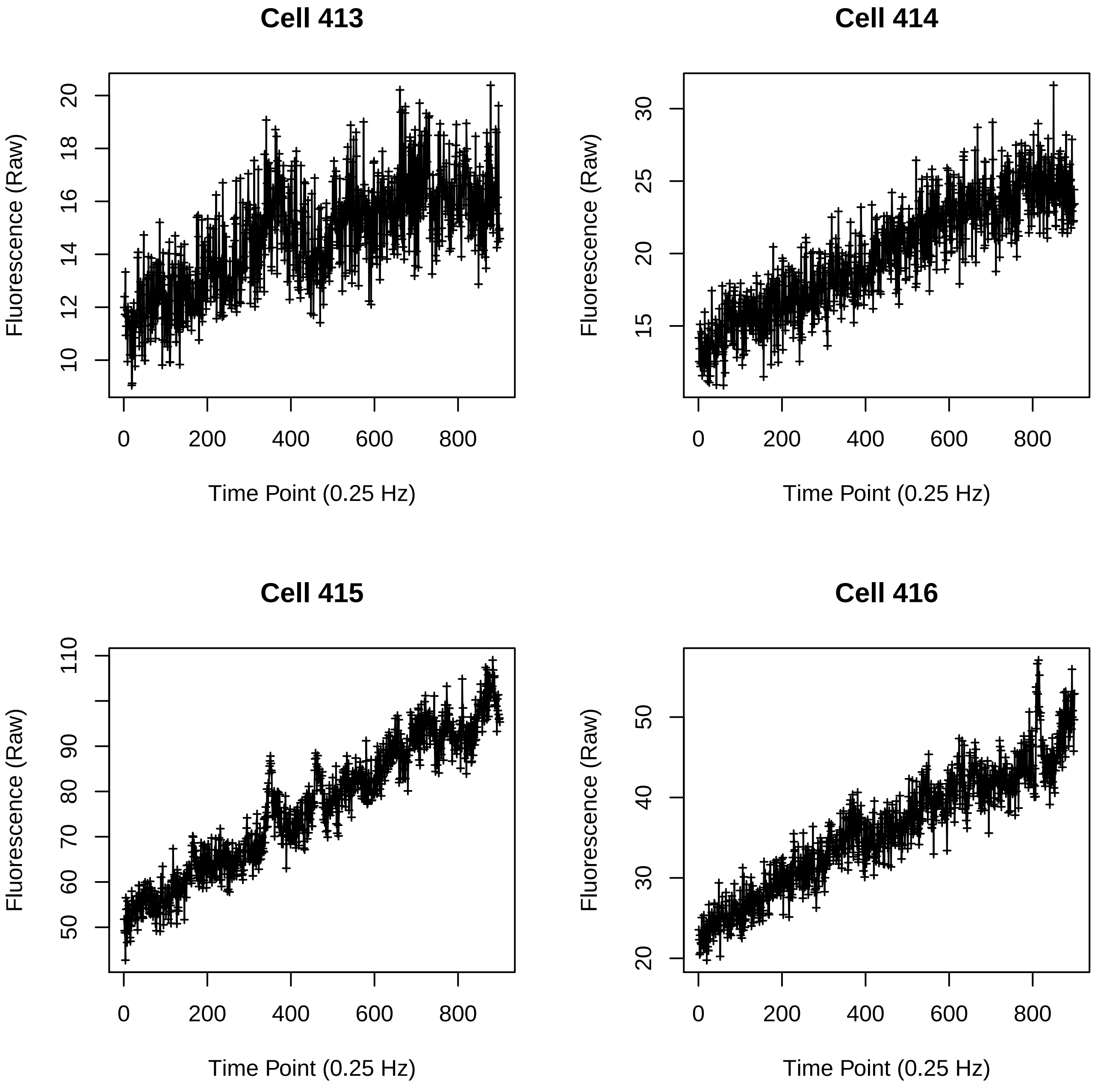
Cell 413
Cell 414
Cell 415
Cell 416
Fluorescence (Raw)
Time Point (0.25 Hz)
0
200
400
600
800
10
12
14
16
18
20
15
20
25
30
50
60
70
80
90
110
40

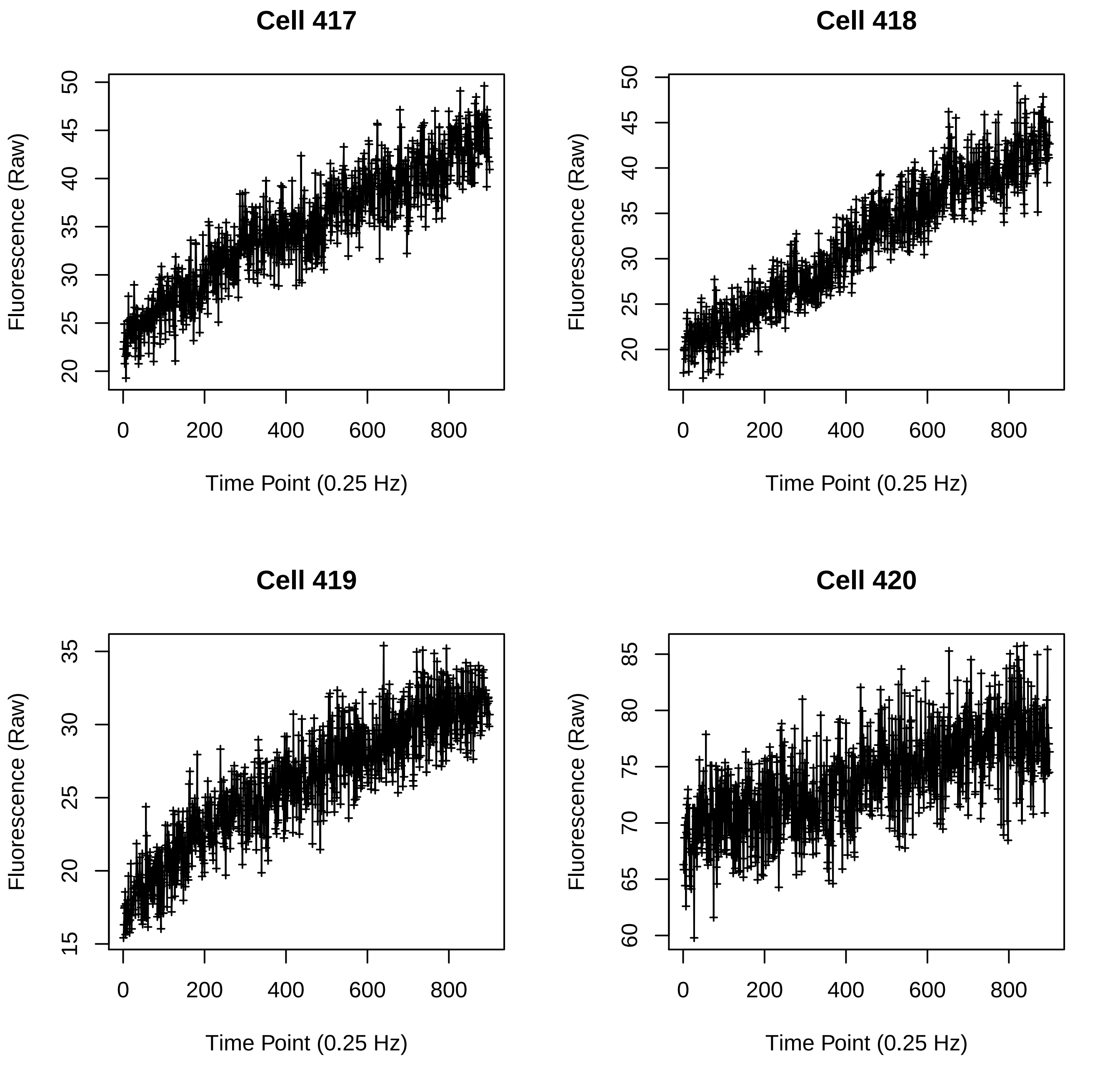

Cell 417
Cell 418
Cell 419
Cell 420
Fluorescence (Raw)
Time Point (0.25 Hz)
0
200
400
600
800
20
25
30
35
40
45
50
15
60
65
70
75
80
85

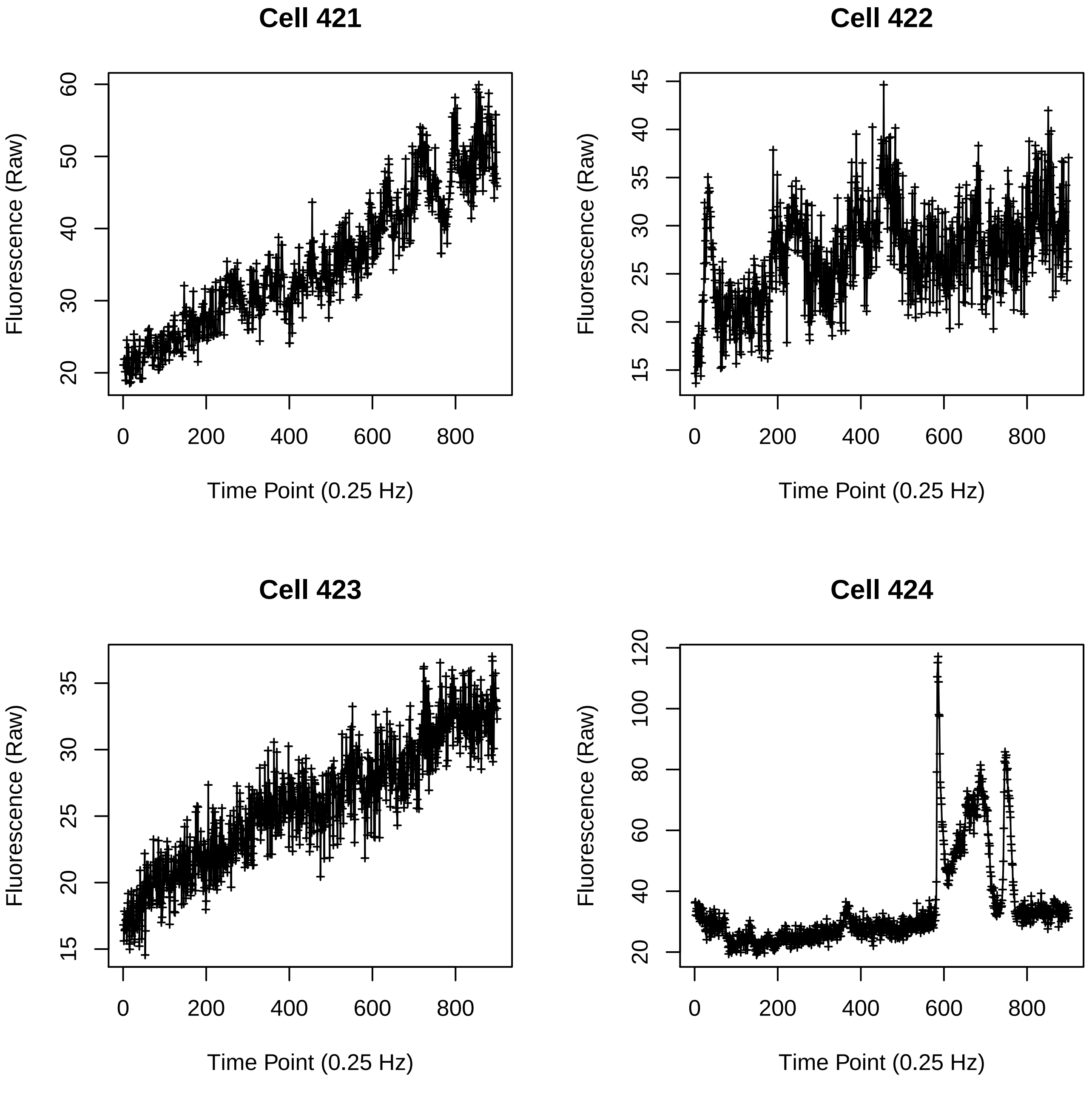
Cell 421
Cell 422
Cell 423
Cell 424
Fluorescence (Raw)
Time Point (0.25 Hz)
0
200
400
600
800
20
30
40
50
60
15
25
35
45
100
120

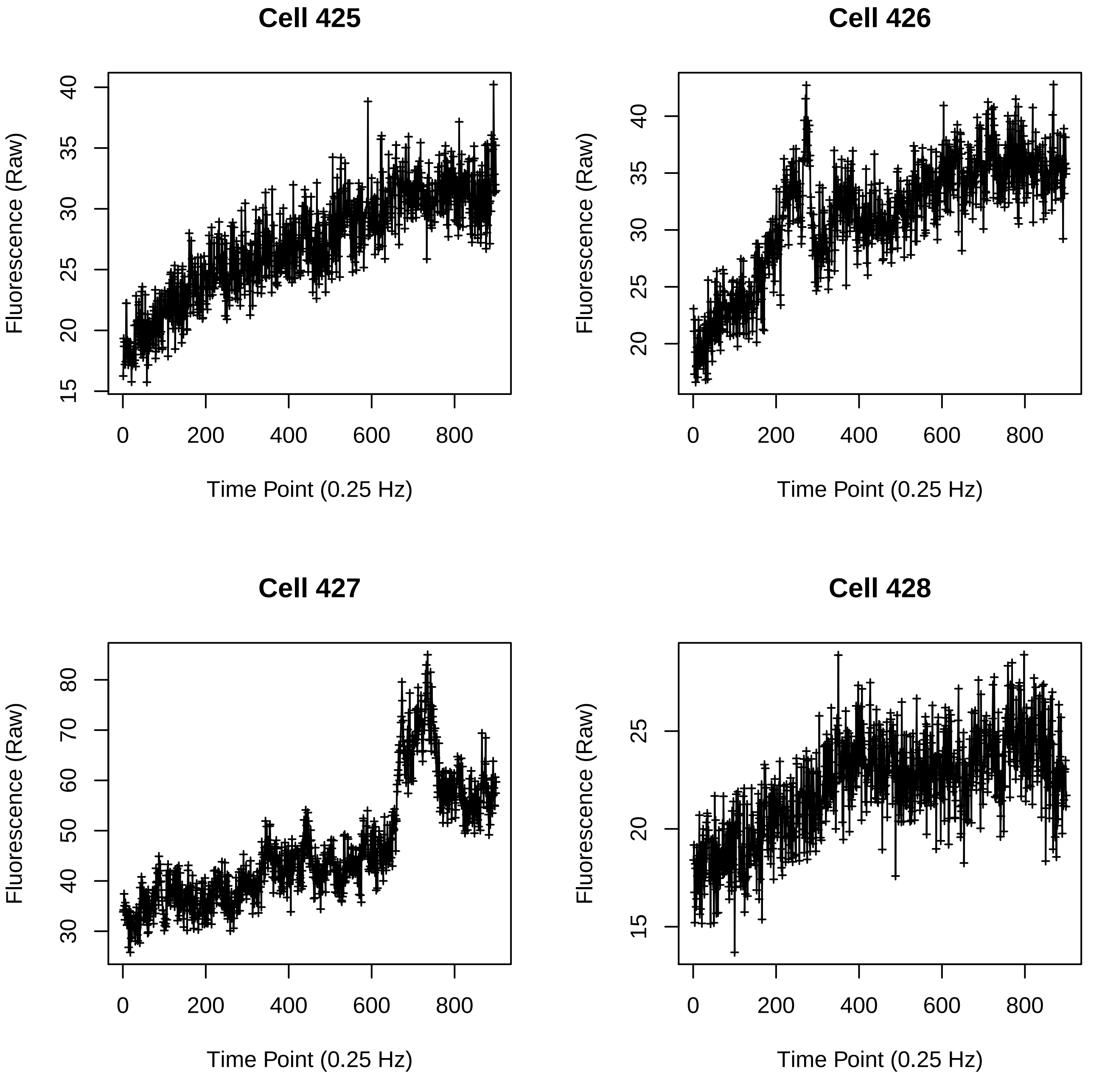
Cell 425
Fluorescence (Raw)
Time Point (0.25 Hz)
Cell 426
Fluorescence (Raw)
Time Point (0.25 Hz)
Cell 427
Fluorescence (Raw)
Time Point (0.25 Hz)
Cell 428
Fluorescence (Raw)
Time Point (0.25 Hz)

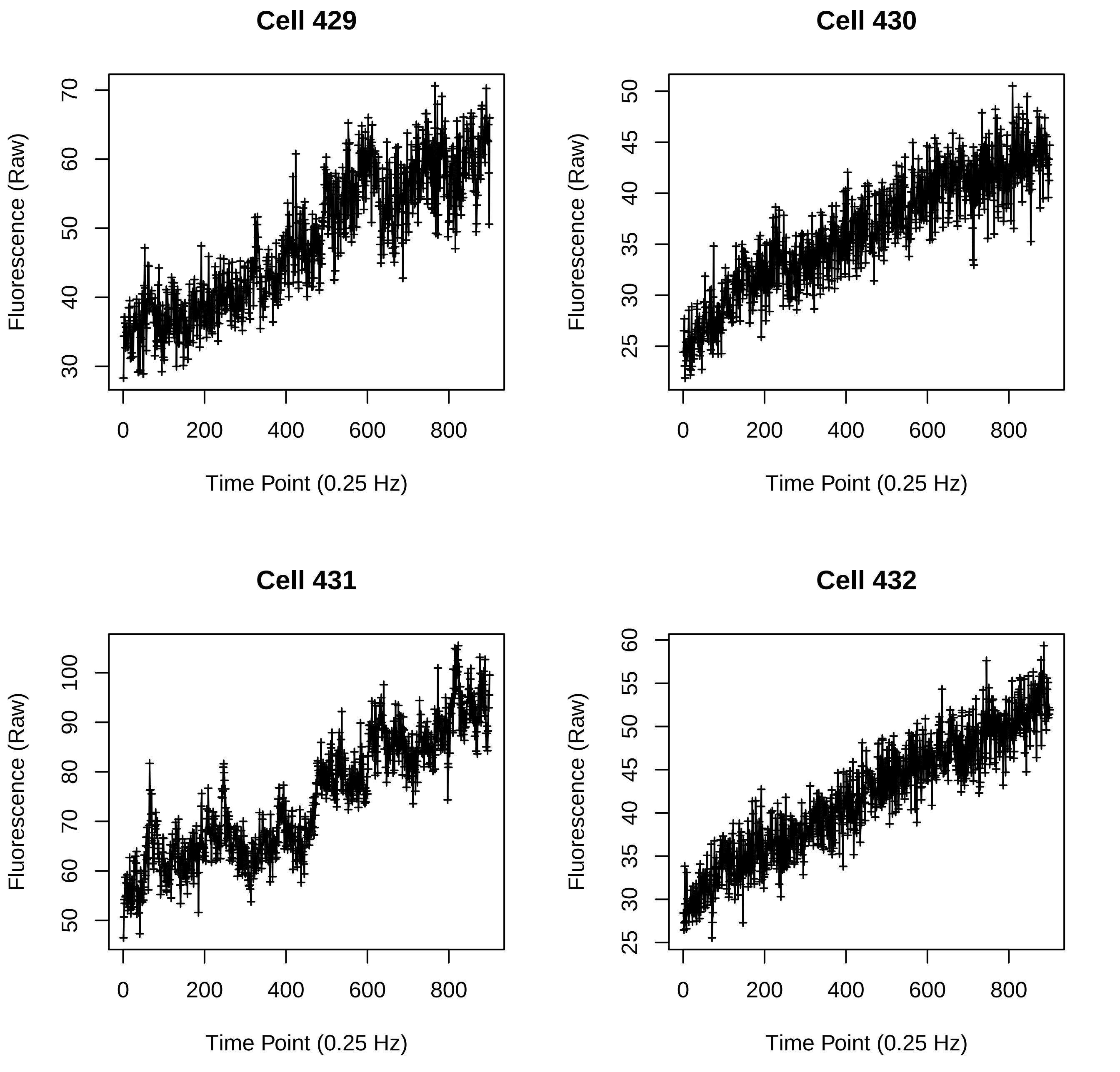
Cell 429
Cell 430
Cell 431
Cell 432
Fluorescence (Raw)
Time Point (0.25 Hz)
30
40
50
60
70
25
35
45
55
80
90
100
0
200
400
600
800

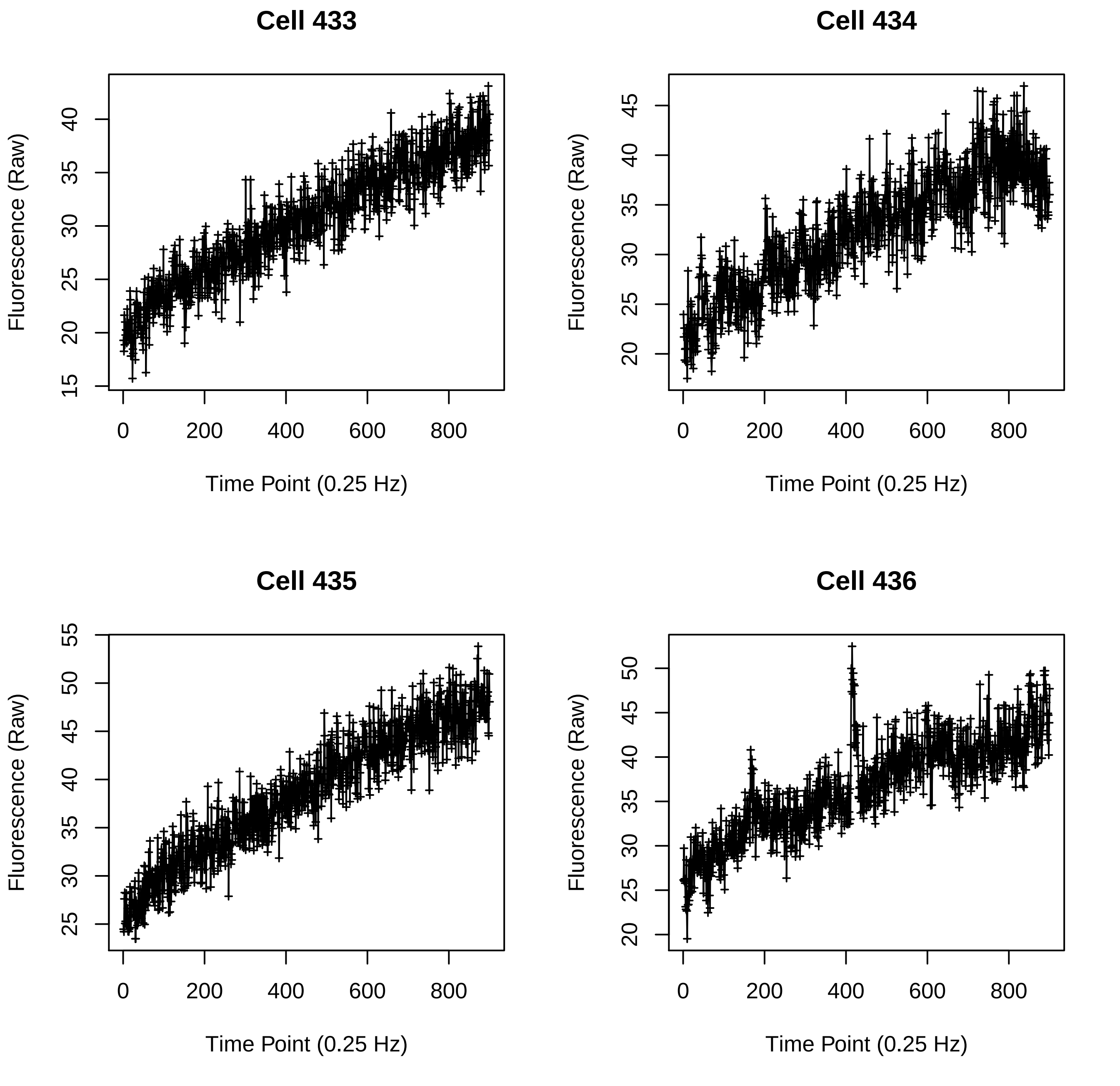
Cell 433
Fluorescence (Raw)
Time Point (0.25 Hz)
Cell 434
Fluorescence (Raw)
Time Point (0.25 Hz)
Cell 435
Fluorescence (Raw)
Time Point (0.25 Hz)
Cell 436
Fluorescence (Raw)
Time Point (0.25 Hz)

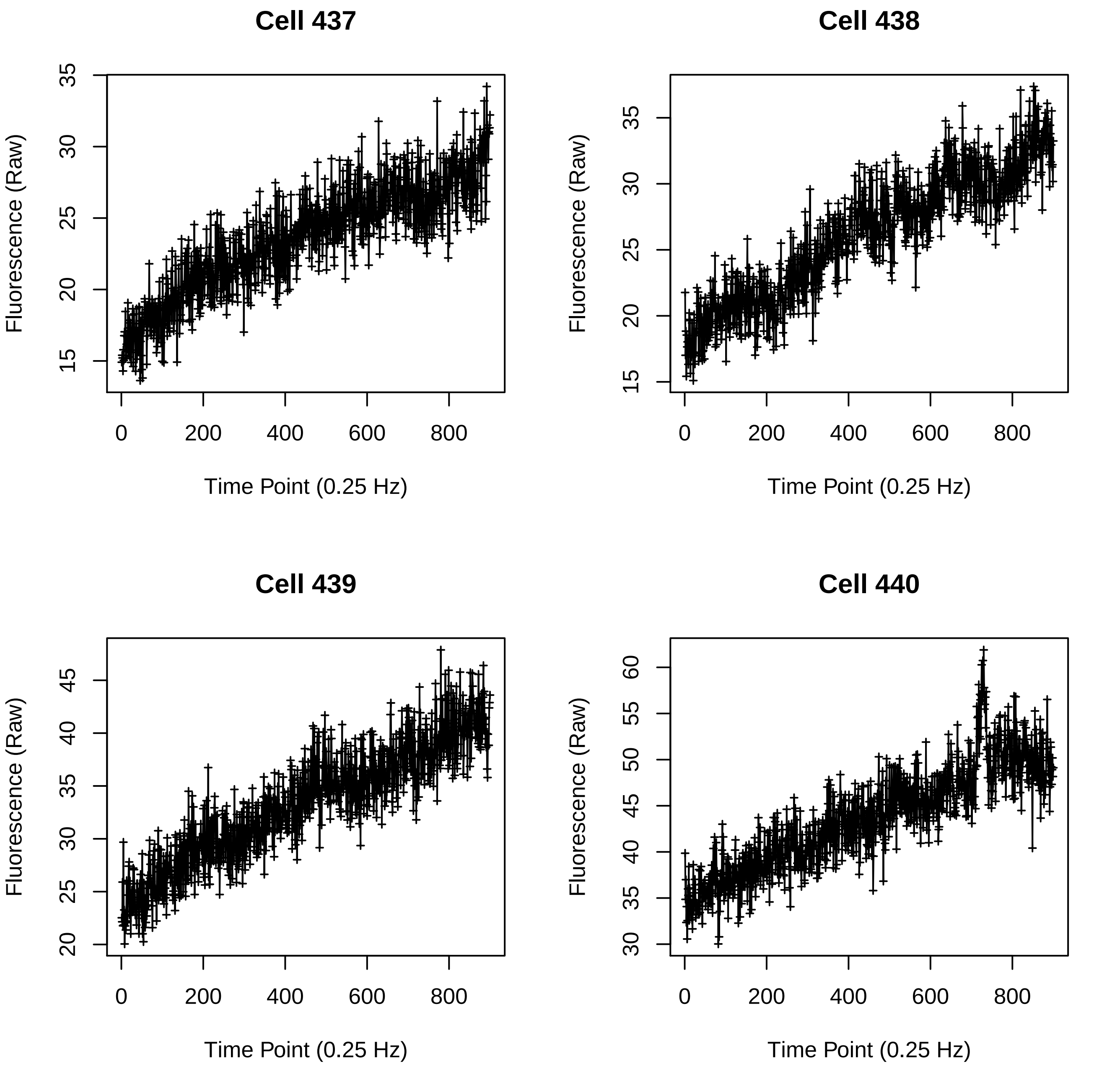
Cell 437
Fluorescence (Raw)
15
20
25
30
35
Time Point (0.25 Hz)
0
200
400
600
800
Cell 438
Fluorescence (Raw)
15
20
25
30
35
Time Point (0.25 Hz)
0
200
400
600
800
Cell 439
Fluorescence (Raw)
20
25
30
35
40
45
Time Point (0.25 Hz)
0
200
400
600
800
Cell 440
Fluorescence (Raw)
30
35
40
45
50
55
60
Time Point (0.25 Hz)
0
200
400
600
800

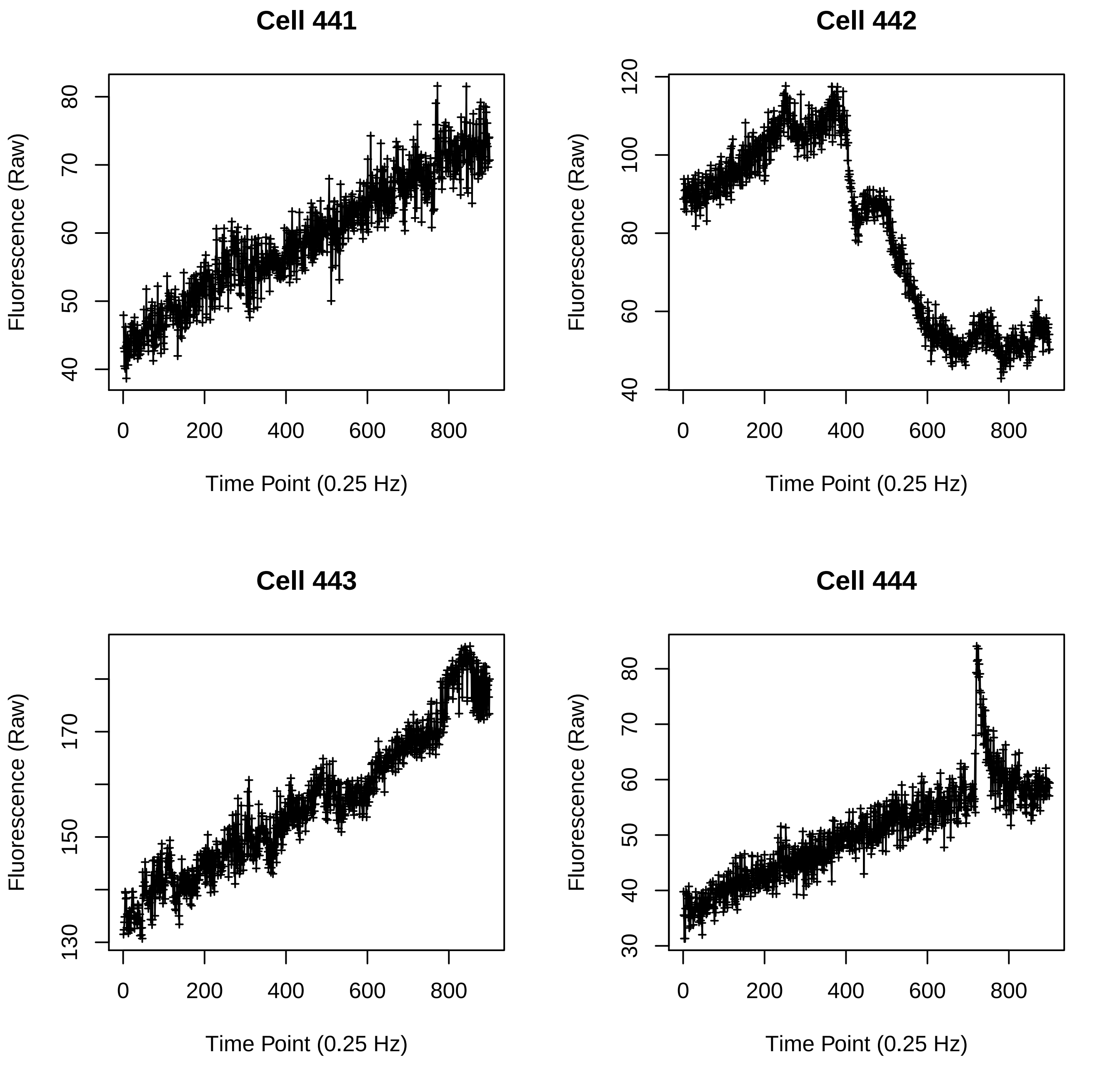
Cell 441
Fluorescence (Raw)
40
50
60
70
80
0
200
400
600
800
Time Point (0.25 Hz)
Cell 442
Fluorescence (Raw)
40
60
80
100
120
0
200
400
600
800
Time Point (0.25 Hz)
Cell 443
Fluorescence (Raw)
130
150
170
0
200
400
600
800
Time Point (0.25 Hz)
Cell 444
Fluorescence (Raw)
30
40
50
60
70
80
0
200
400
600
800
Time Point (0.25 Hz)

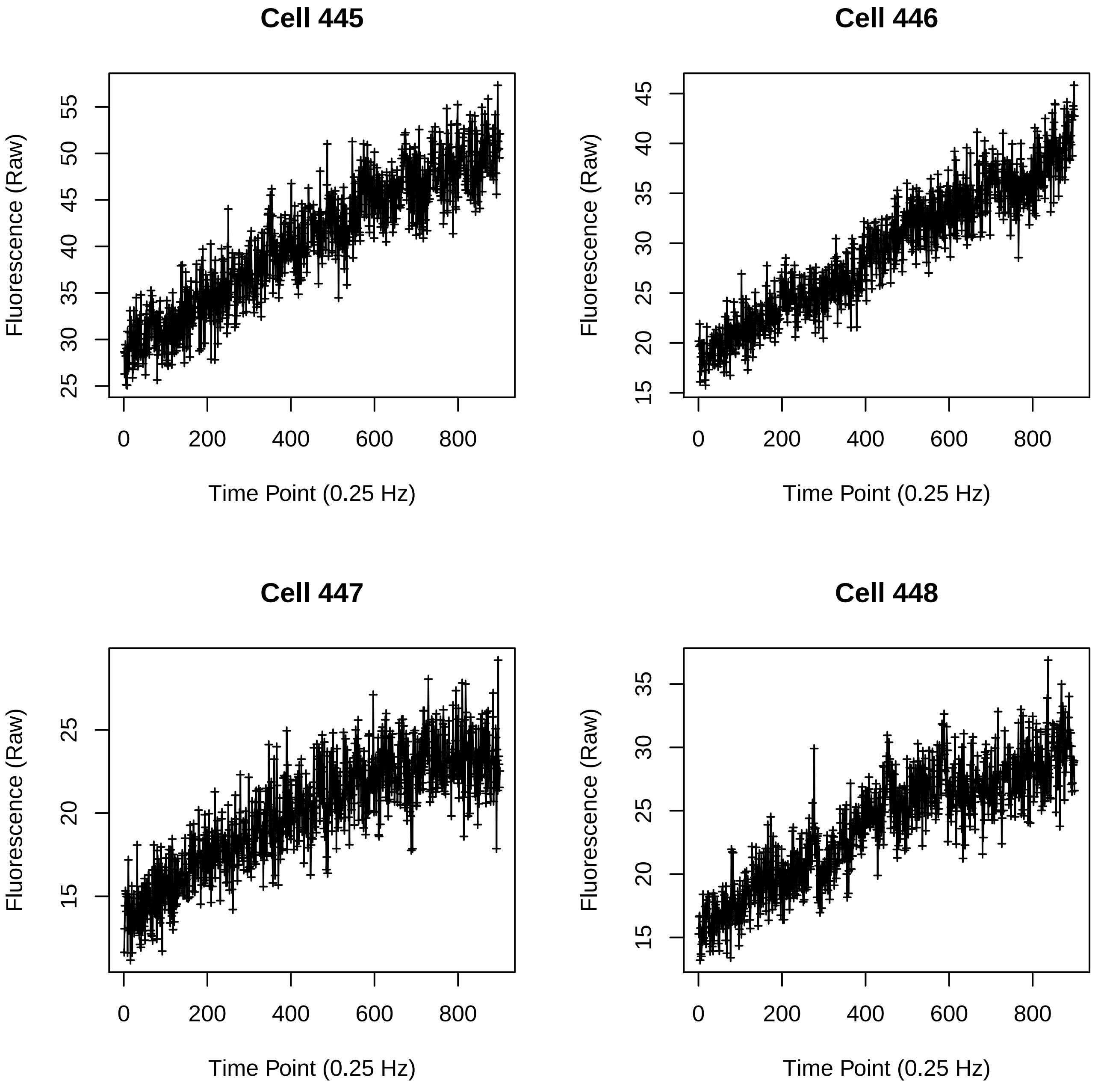
Cell 445
Cell 446
Cell 447
Cell 448
Fluorescence (Raw)
Time Point (0.25 Hz)
25 30 35 40 45 50 55
15 20 25 30 35 40 45
15 20 25
15 20 25 30 35
0 200 400 600 800

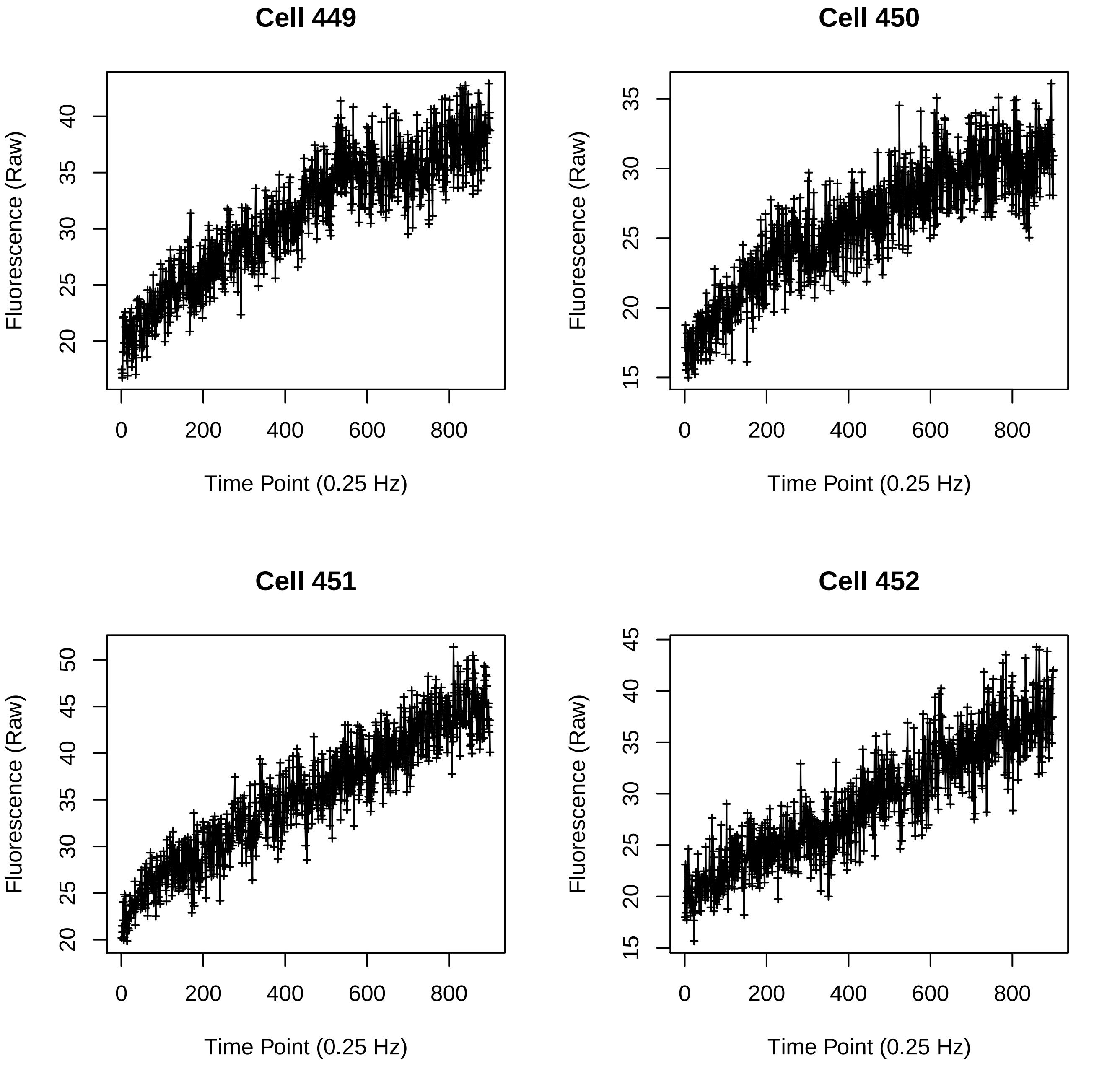
Cell 449
Cell 450
Cell 451
Cell 452
Fluorescence (Raw)
Time Point (0.25 Hz)
0
200
400
600
800
15
20
25
30
35
40
45
50

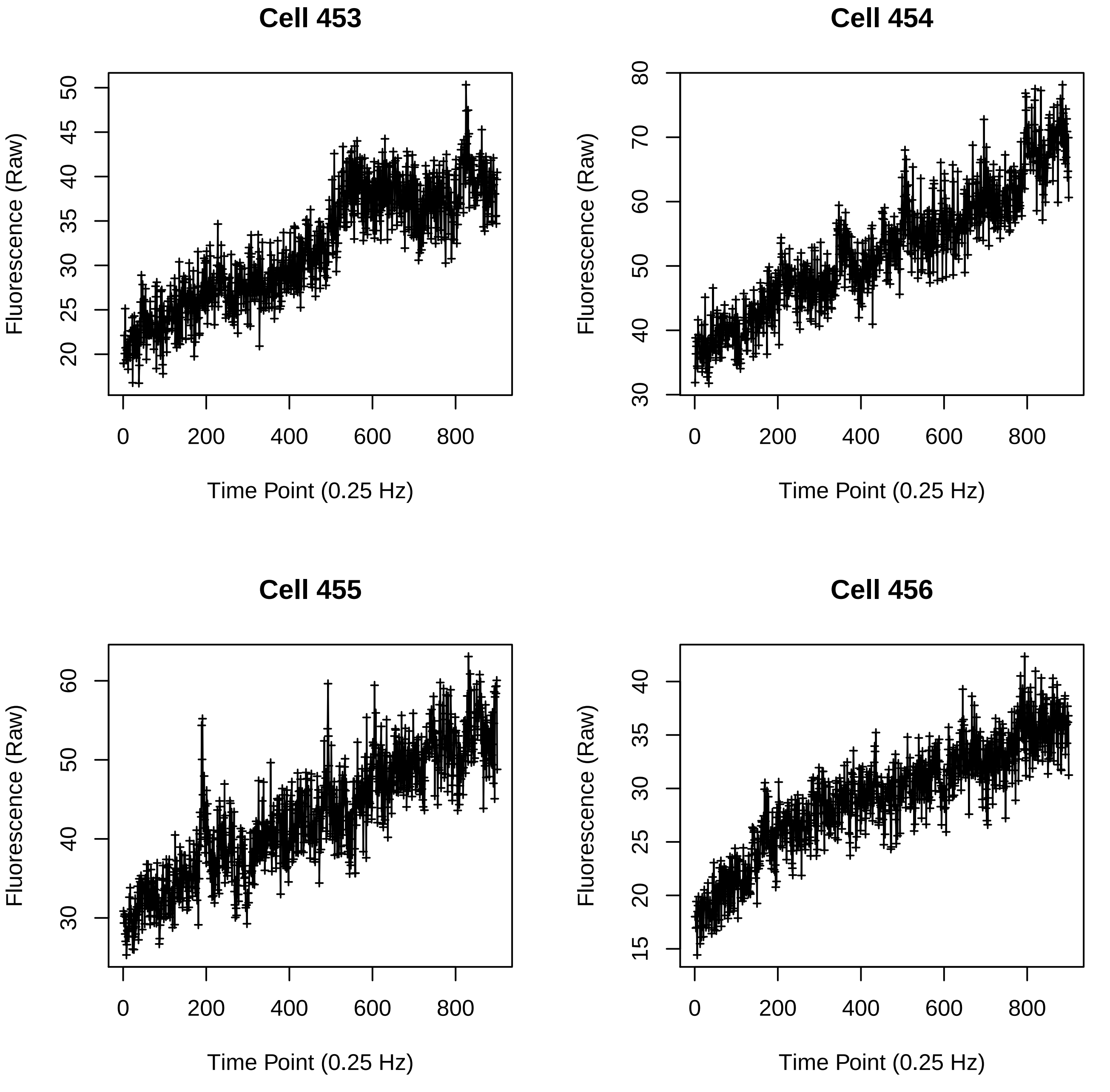
Cell 453
Fluorescence (Raw)
20
25
30
35
40
45
50
0
200
400
600
800
Time Point (0.25 Hz)
Cell 454
Fluorescence (Raw)
30
40
50
60
70
80
0
200
400
600
800
Time Point (0.25 Hz)
Cell 455
Fluorescence (Raw)
30
40
50
60
0
200
400
600
800
Time Point (0.25 Hz)
Cell 456
Fluorescence (Raw)
15
20
25
30
35
40
0
200
400
600
800
Time Point (0.25 Hz)

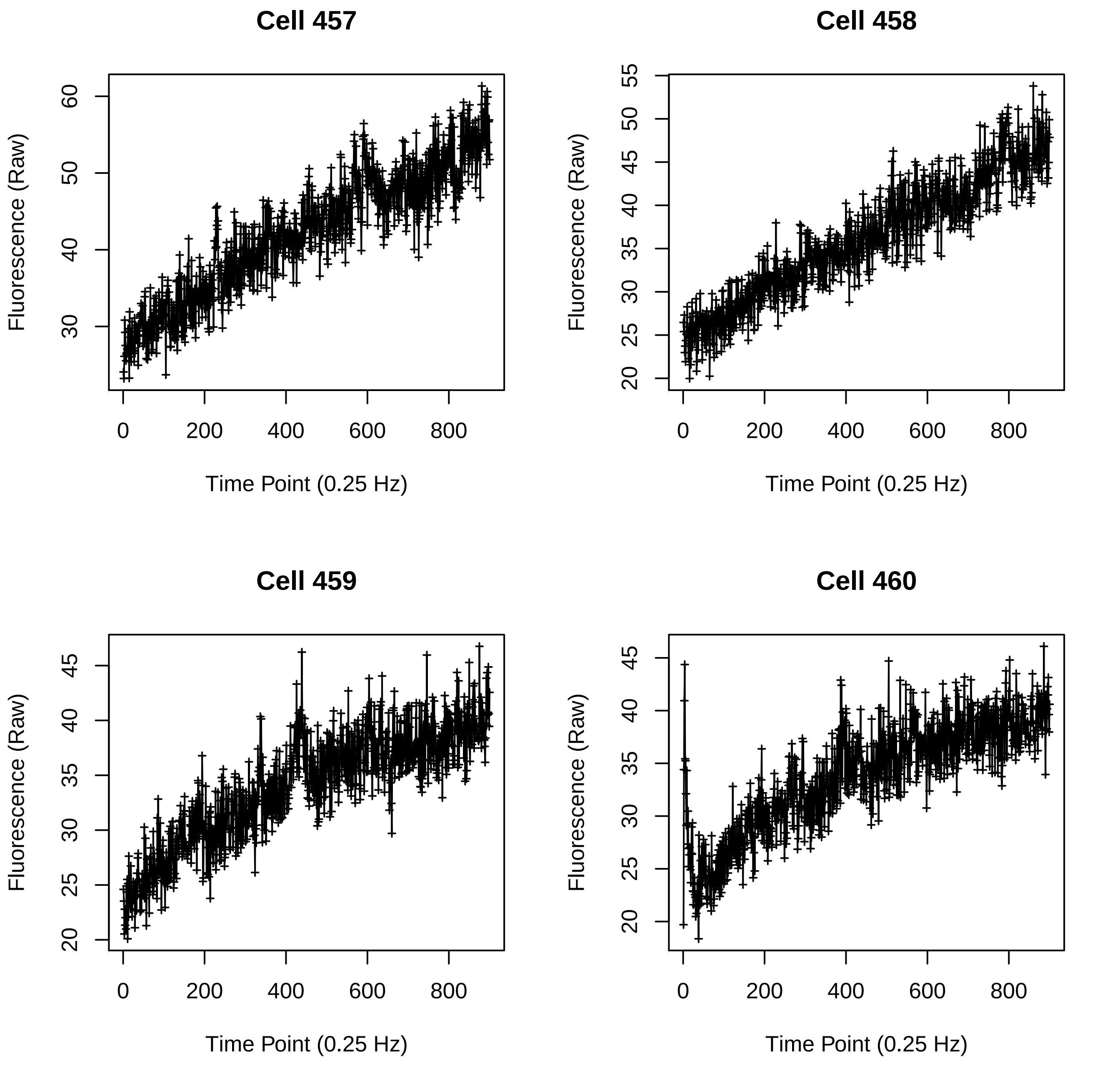
Cell 457
Cell 458
Cell 459
Cell 460
Fluorescence (Raw)
Time Point (0.25 Hz)
0
200
400
600
800
30
40
50
60
20
25
30
35
40
45
50
55

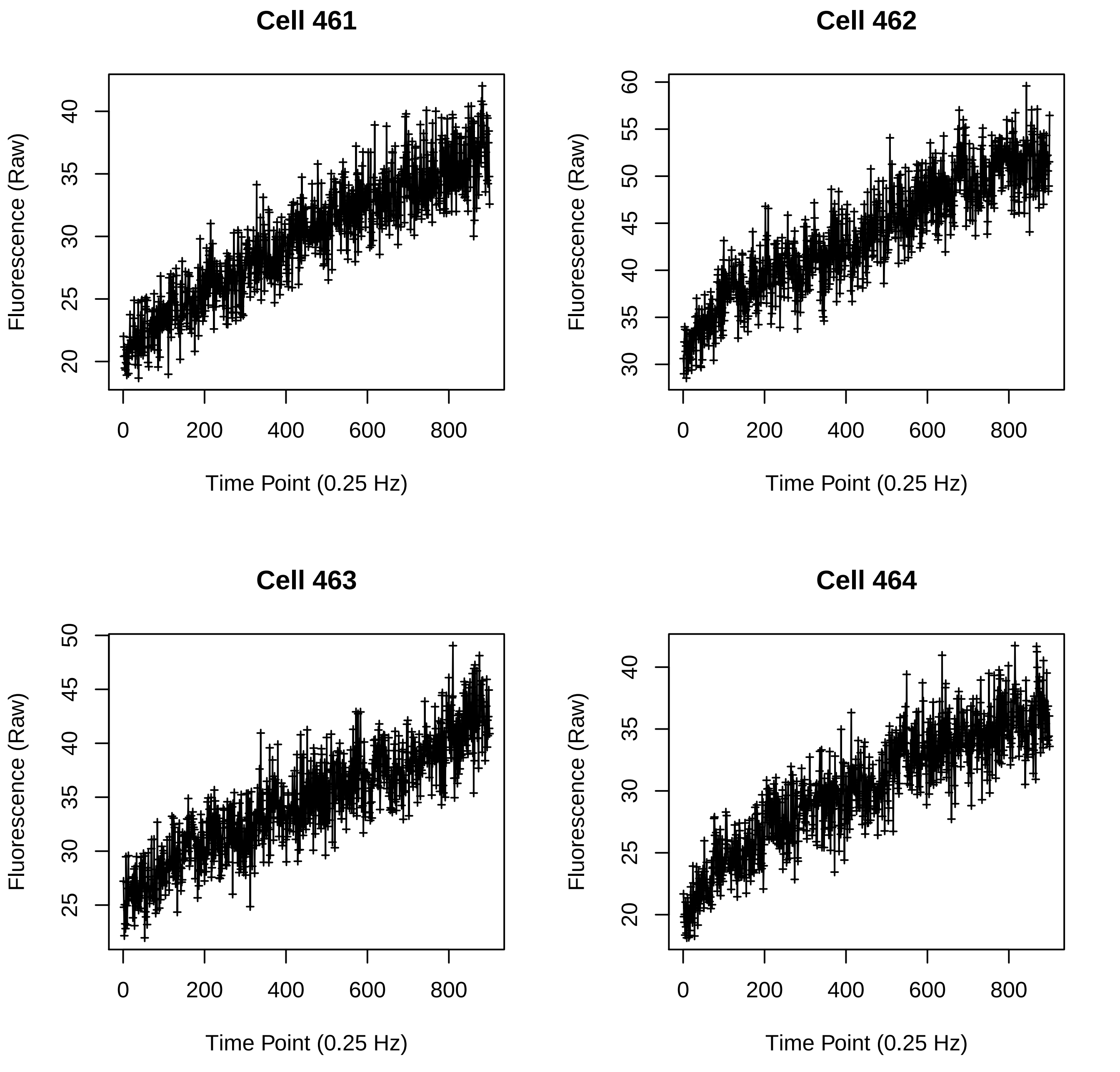
Cell 461
Cell 462
Cell 463
Cell 464
Fluorescence (Raw)
Time Point (0.25 Hz)
0
200
400
600
800
20
25
30
35
40
45
50
55
60

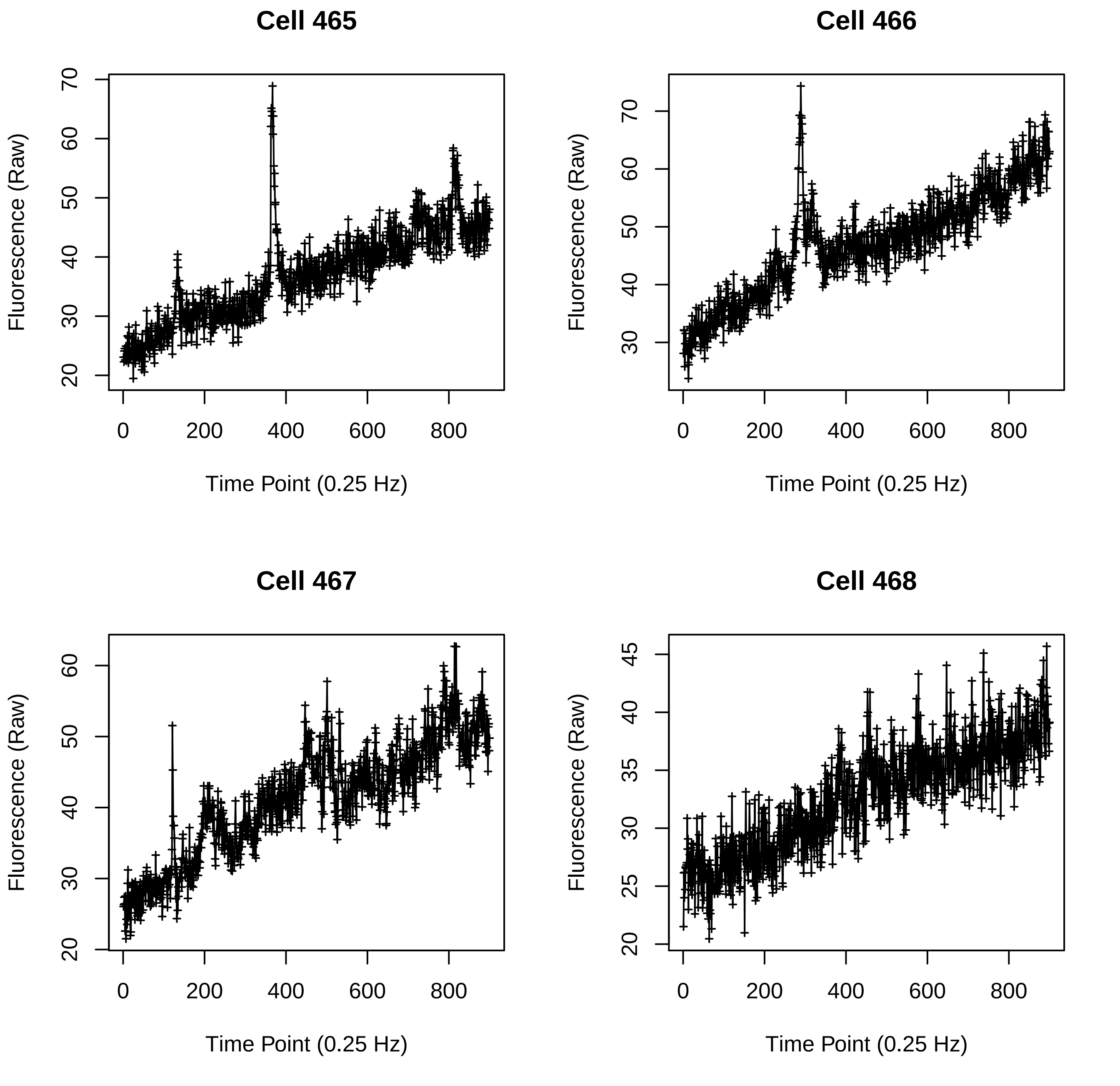
Cell 465
Cell 466
Cell 467
Cell 468
Fluorescence (Raw)
Time Point (0.25 Hz)
0
200
400
600
800
20
30
40
50
60
70
25
35
45

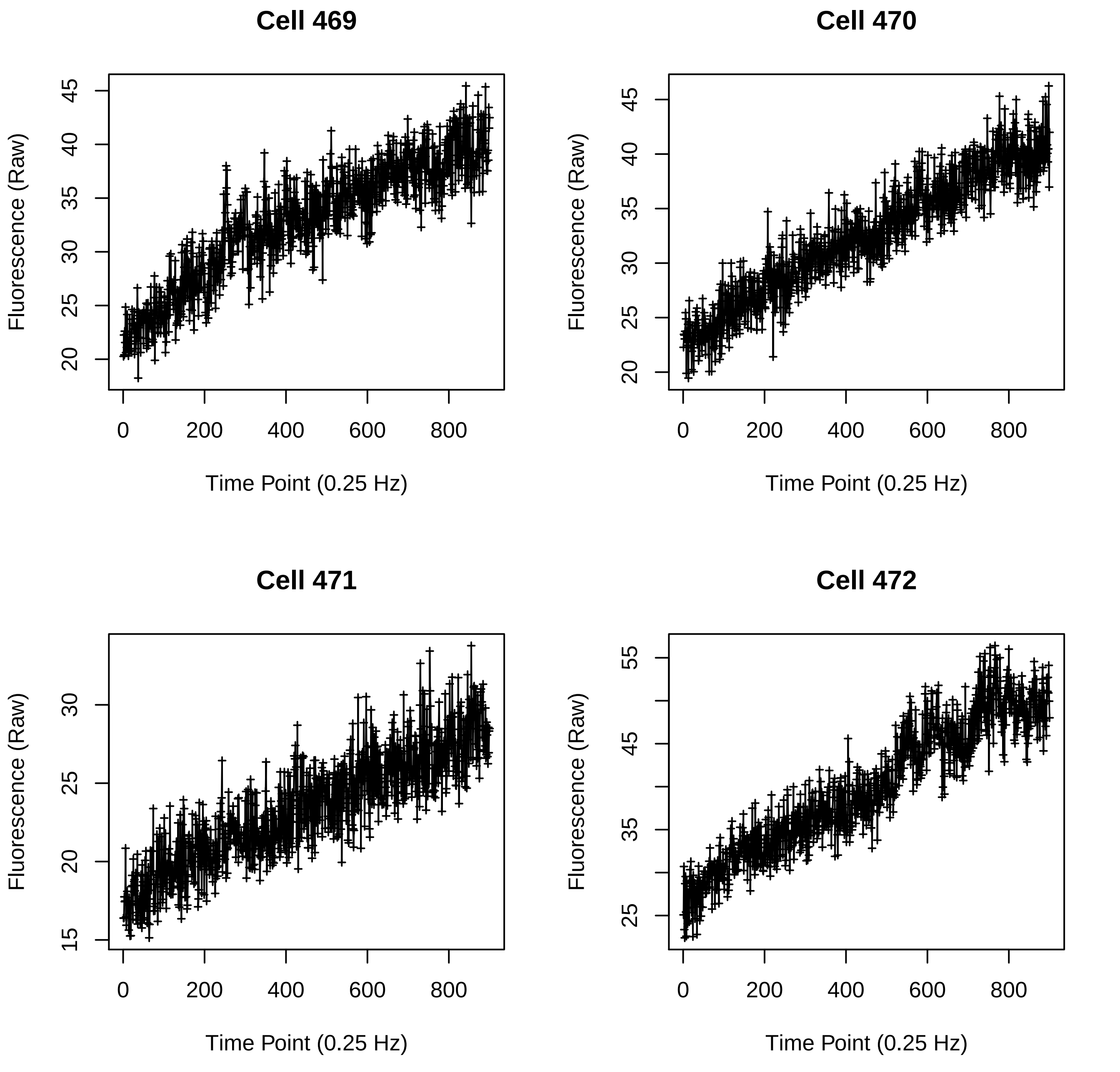
Cell 469
Cell 470
Cell 471
Cell 472
Fluorescence (Raw)
Time Point (0.25 Hz)
0
200
400
600
800
15
20
25
30
35
40
45
50
55

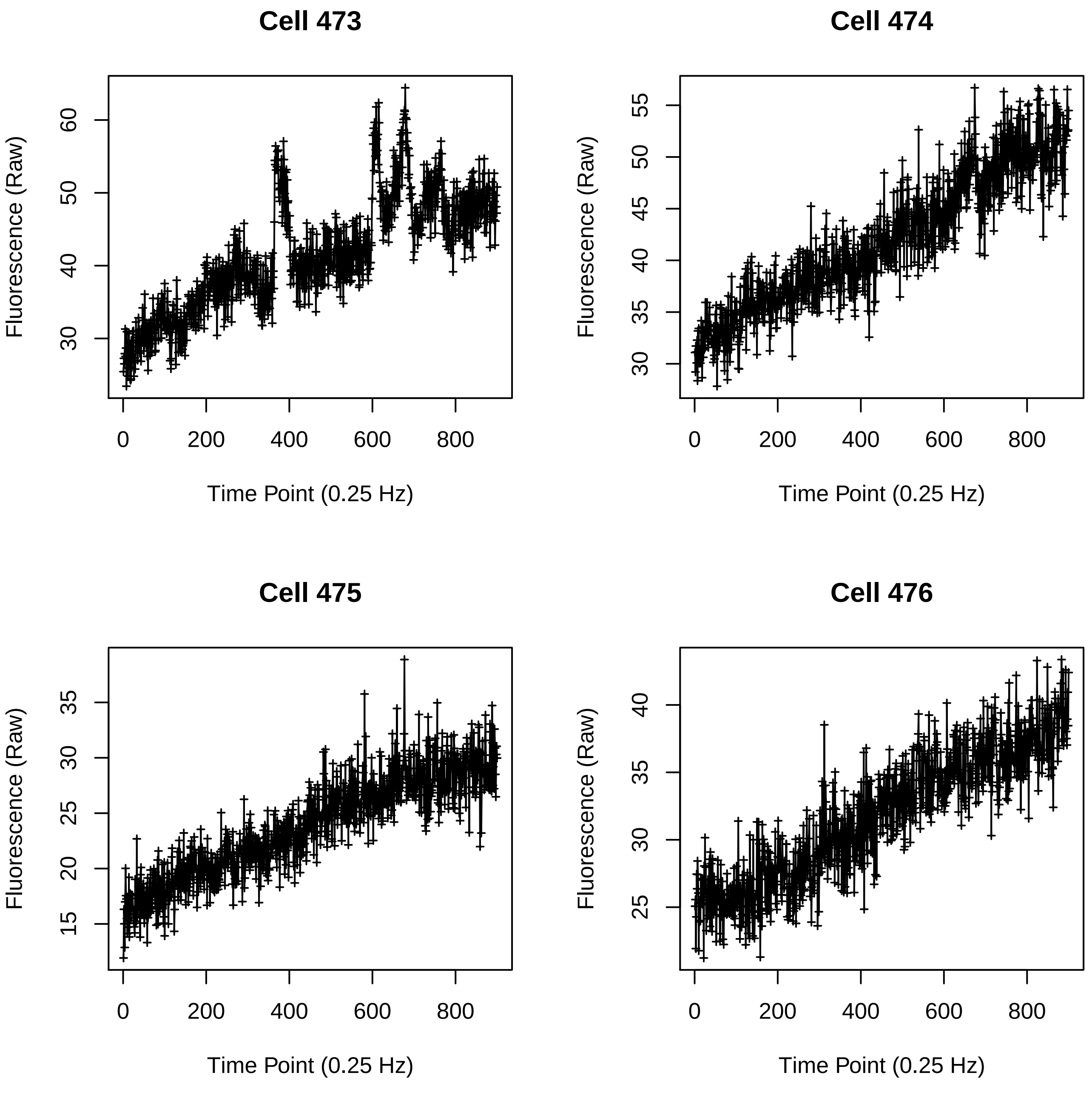
Cell 473
Fluorescence (Raw)
30
40
50
60
0
200
400
600
800
Time Point (0.25 Hz)
Cell 474
Fluorescence (Raw)
30
35
40
45
50
55
0
200
400
600
800
Time Point (0.25 Hz)
Cell 475
Fluorescence (Raw)
15
20
25
30
35
0
200
400
600
800
Time Point (0.25 Hz)
Cell 476
Fluorescence (Raw)
25
30
35
40
0
200
400
600
800
Time Point (0.25 Hz)

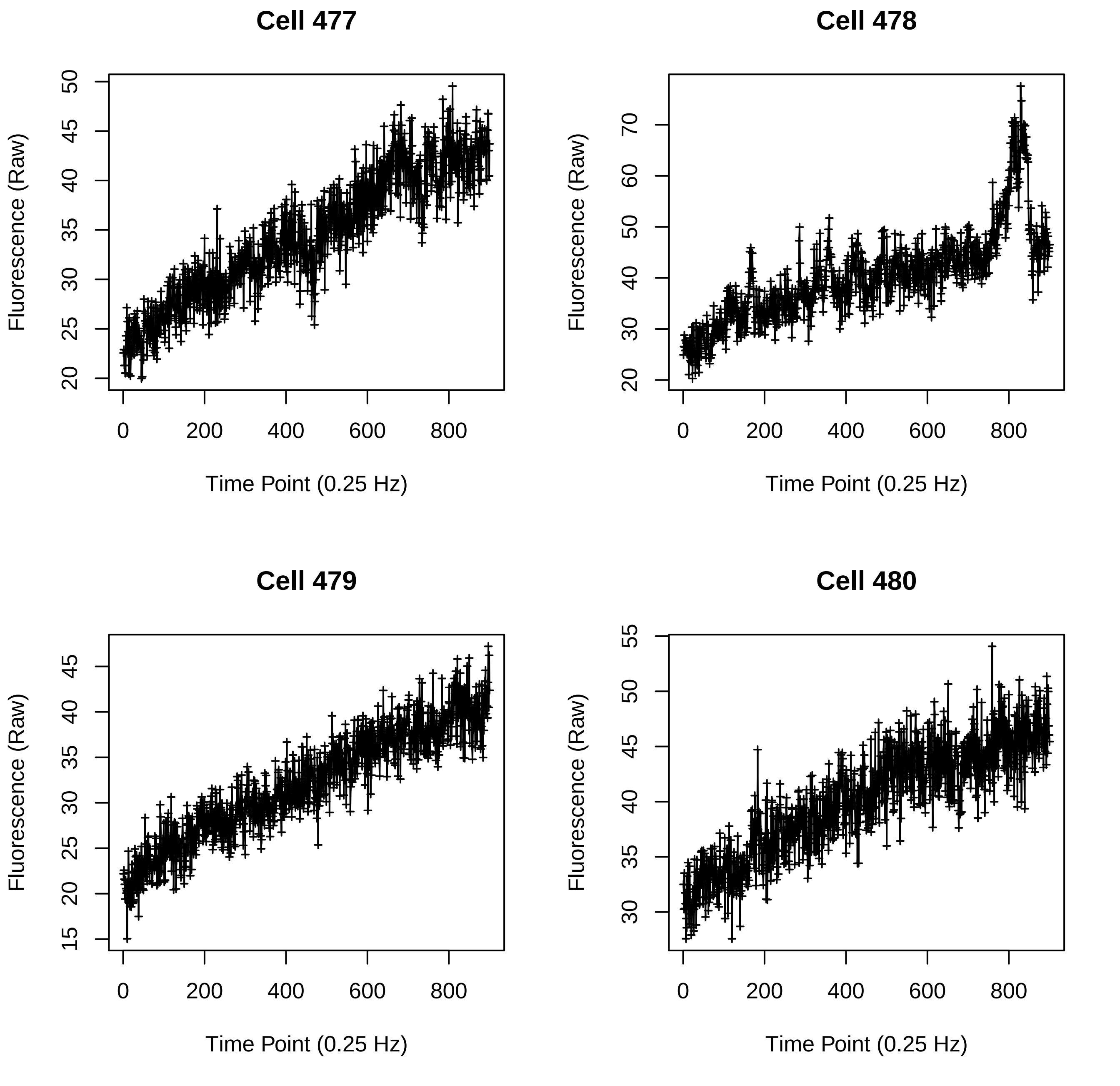

Cell 477
Cell 478
Cell 479
Cell 480
Fluorescence (Raw)
Time Point (0.25 Hz)
0
200
400
600
800
20
25
30
35
40
45
50
60
70
15
55

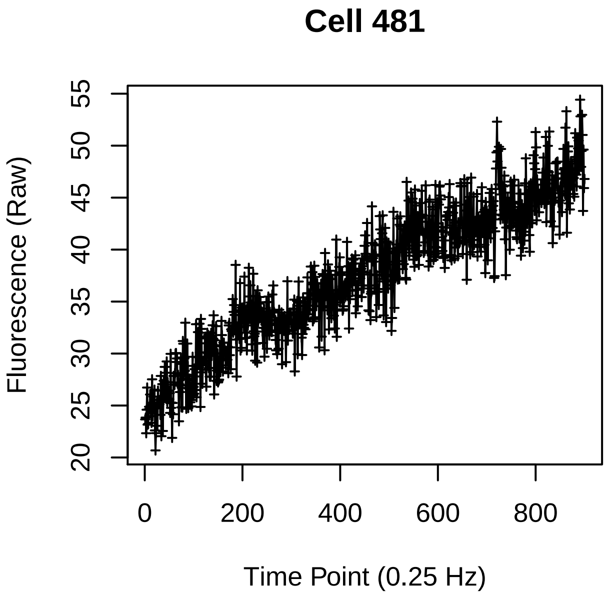
Cell 481
Fluorescence (Raw)
20 25 30 35 40 45 50 55
0 200 400 600 800
Time Point (0.25 Hz)

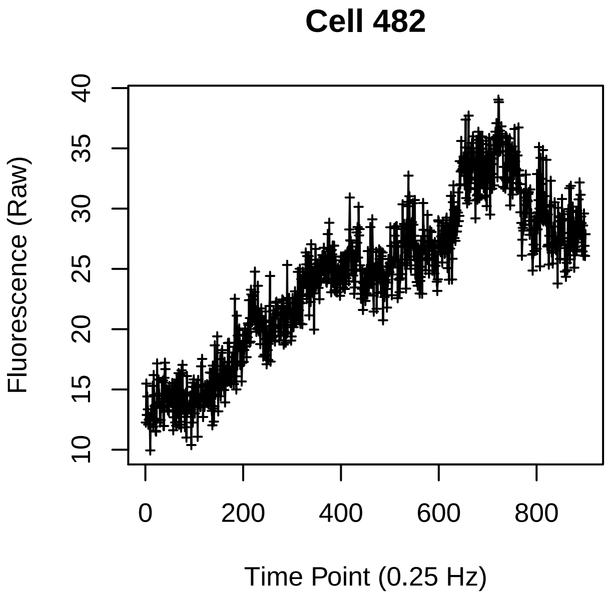
Cell 482
Fluorescence (Raw)
10 15 20 25 30 35 40
0 200 400 600 800
Time Point (0.25 Hz)

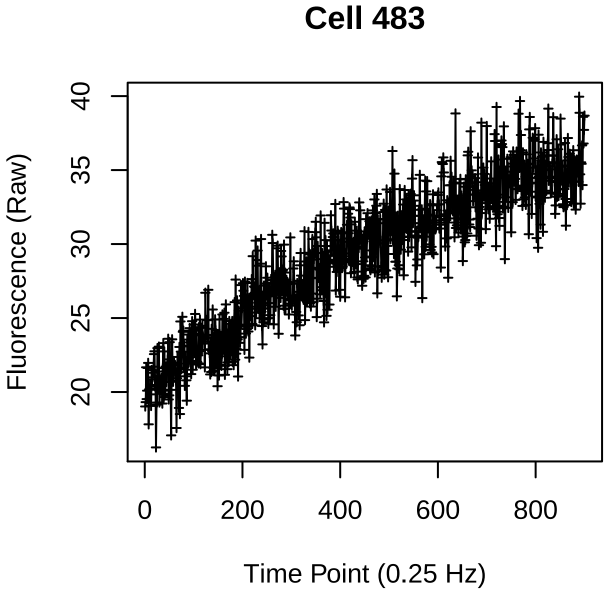
Cell 483
Fluorescence (Raw)
20 25 30 35 40
0 200 400 600 800
Time Point (0.25 Hz)

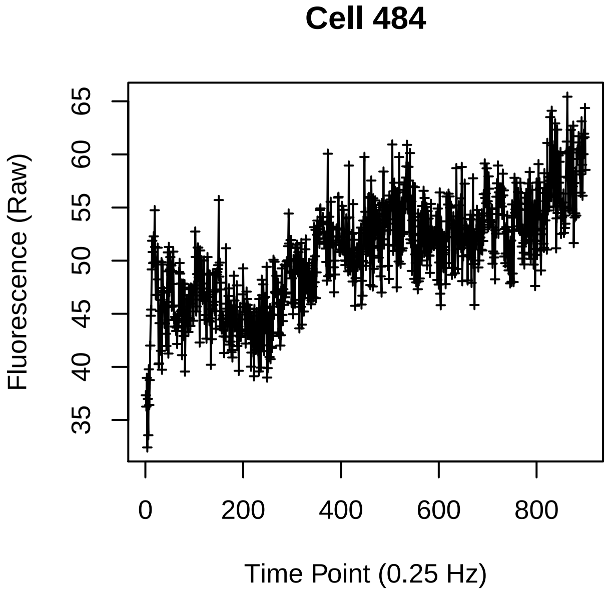
Cell 484
Fluorescence (Raw)
35 40 45 50 55 60 65
0 200 400 600 800
Time Point (0.25 Hz)

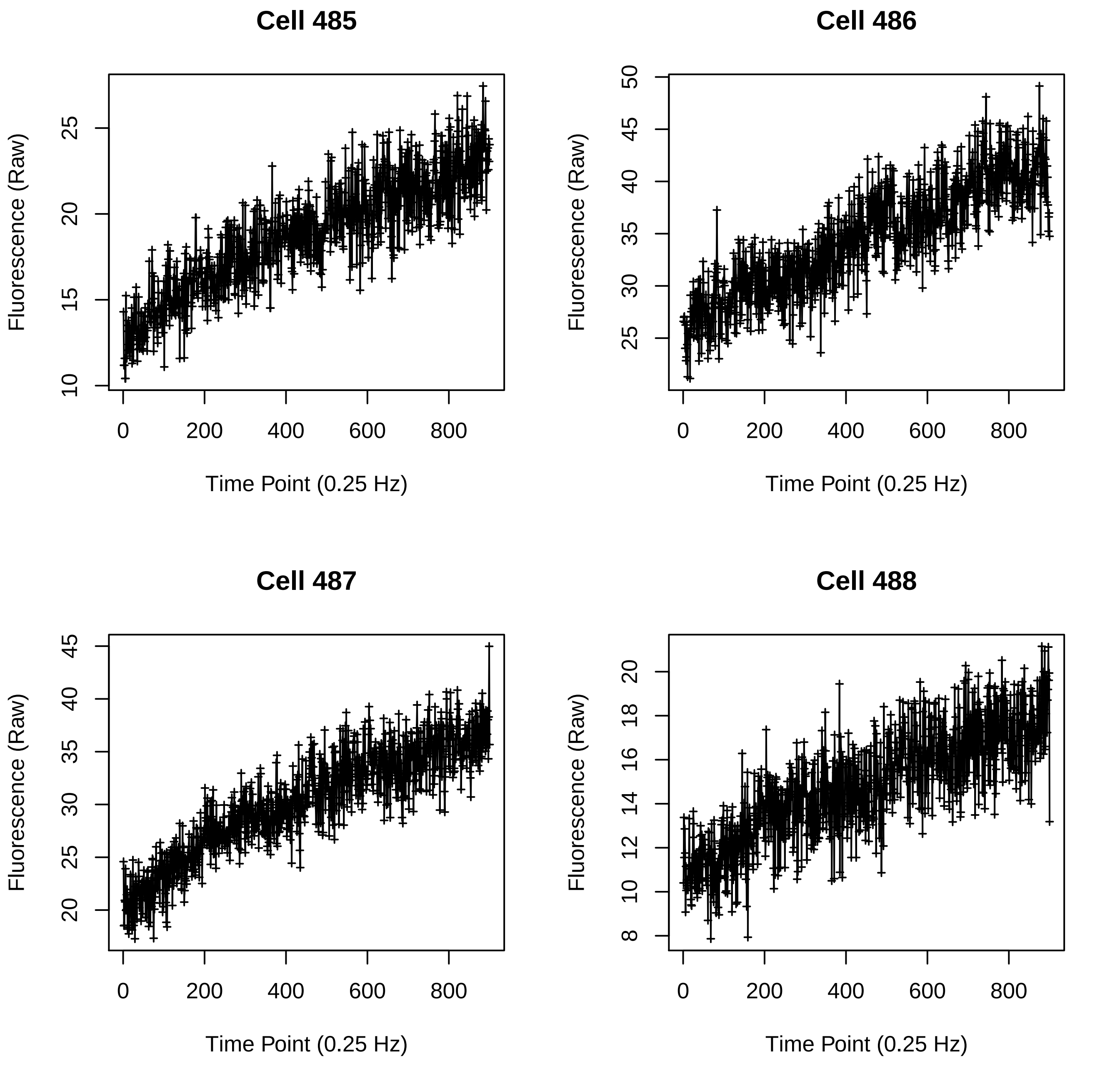

Cell 485
Cell 486
Cell 487
Cell 488
Fluorescence (Raw)
Time Point (0.25 Hz)

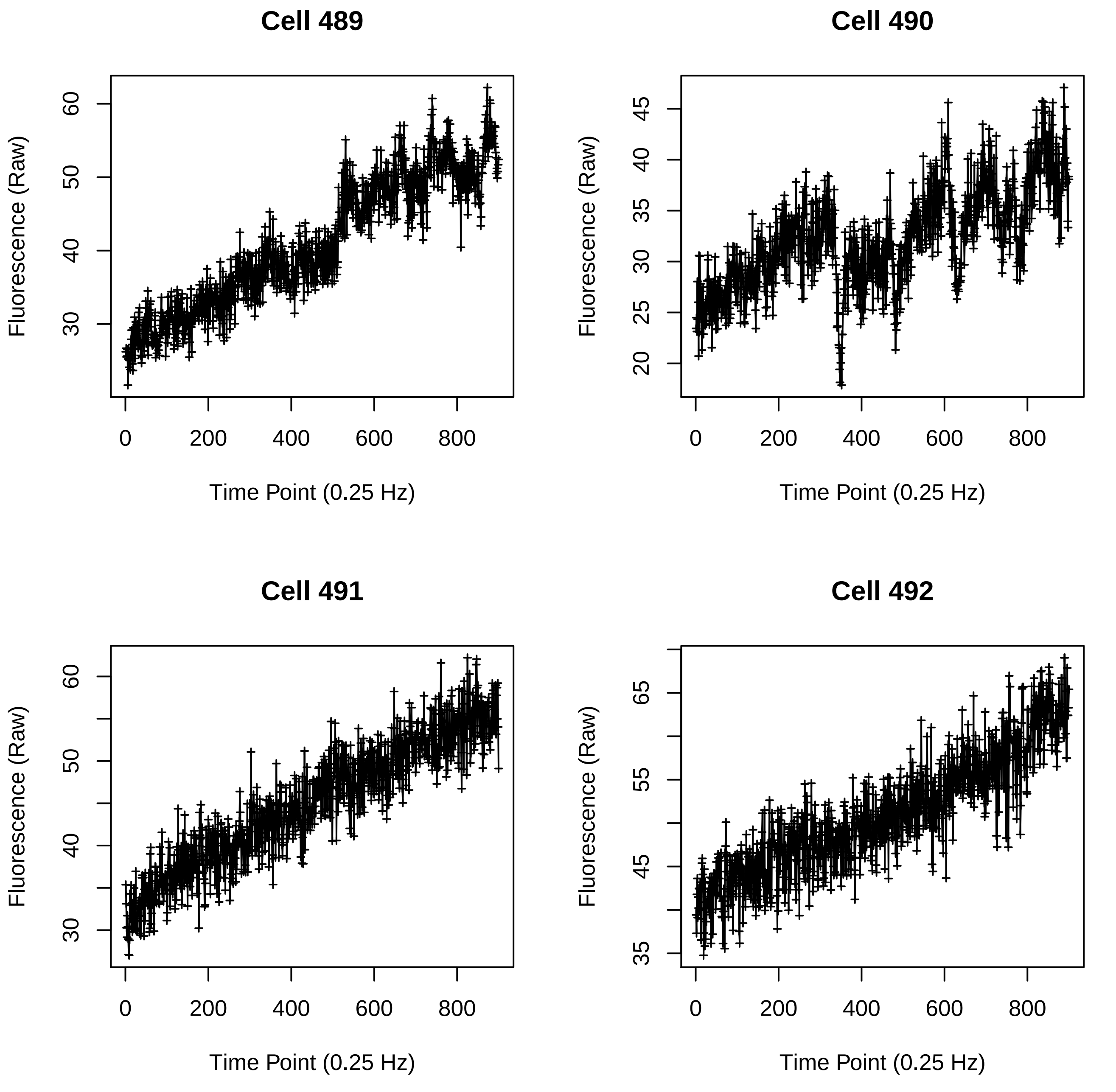
Cell 489
Fluorescence (Raw)
30
40
50
60
0
200
400
600
800
Time Point (0.25 Hz)
Cell 490
Fluorescence (Raw)
20
25
30
35
40
45
0
200
400
600
800
Time Point (0.25 Hz)
Cell 491
Fluorescence (Raw)
30
40
50
60
0
200
400
600
800
Time Point (0.25 Hz)
Cell 492
Fluorescence (Raw)
35
45
55
65
0
200
400
600
800
Time Point (0.25 Hz)

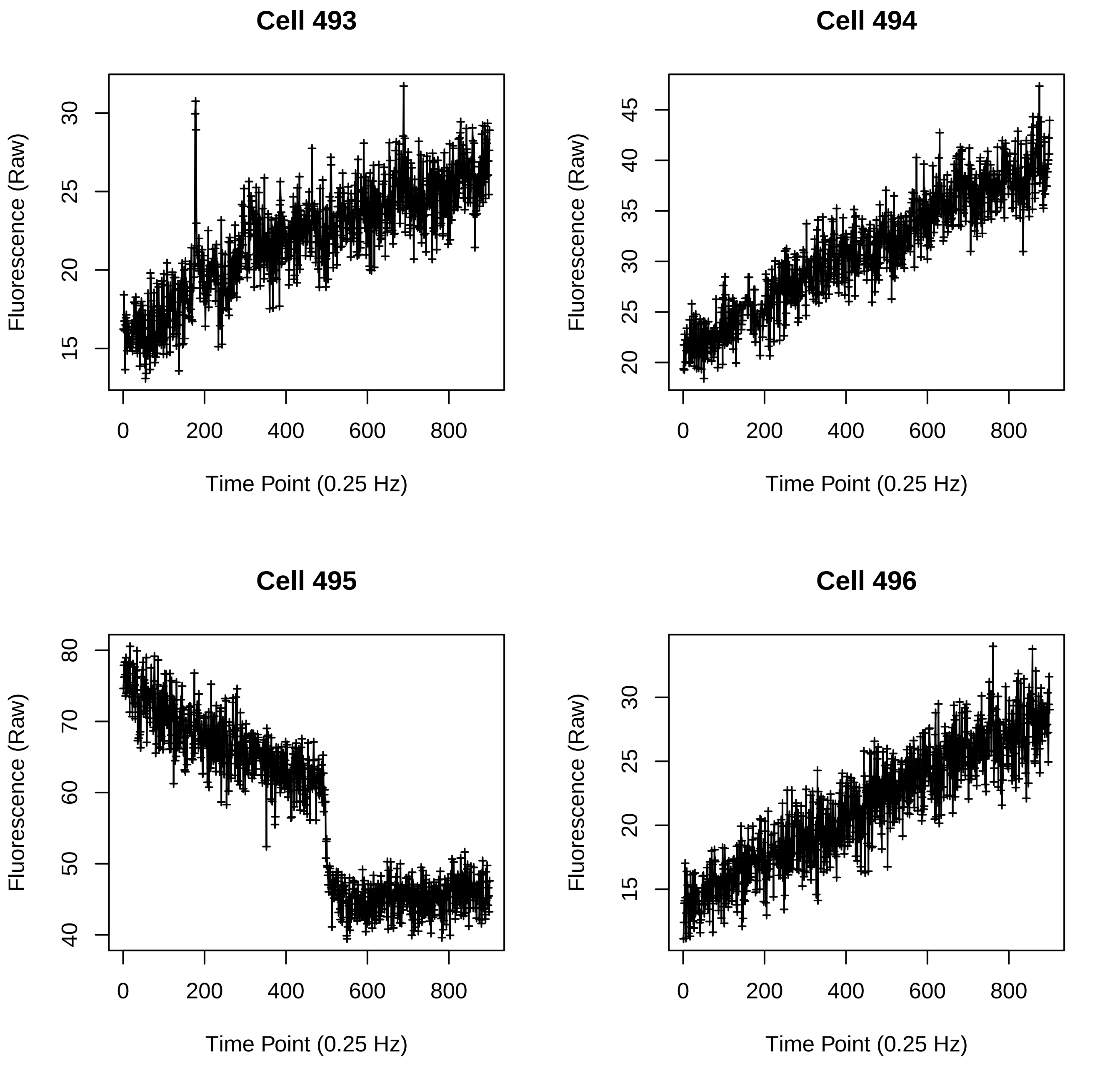
Cell 493
Cell 494
Cell 495
Cell 496
Fluorescence (Raw)
Time Point (0.25 Hz)
15
20
25
30
35
40
45
50
60
70
80
0
200
400
600
800

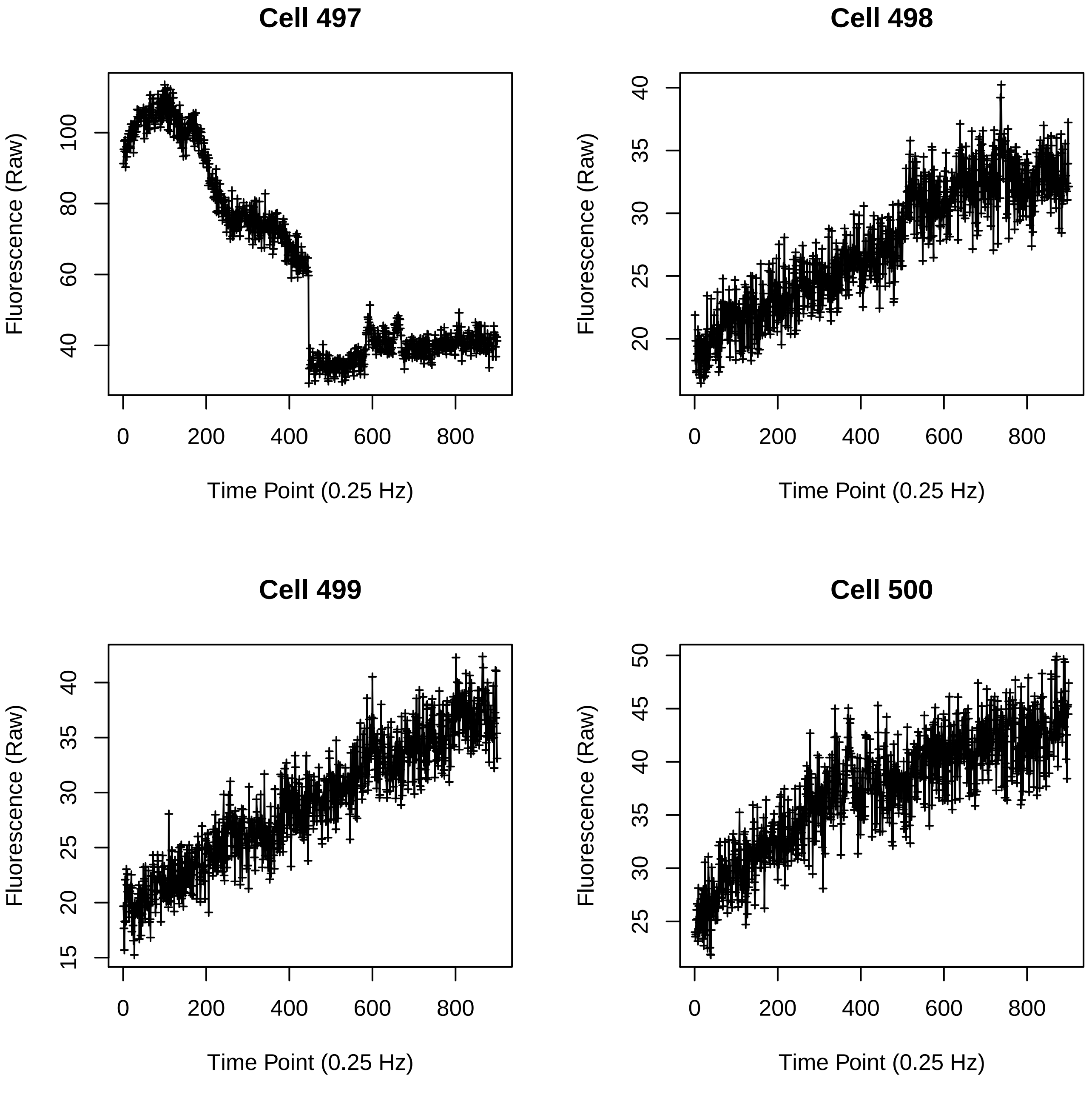
Cell 497
Cell 498
Cell 499
Cell 500
Fluorescence (Raw)
Time Point (0.25 Hz)
0
200
400
600
800
40
60
80
100
20
25
30
35
40
15
20
25
30
35
40
25
30
35
40
45
50

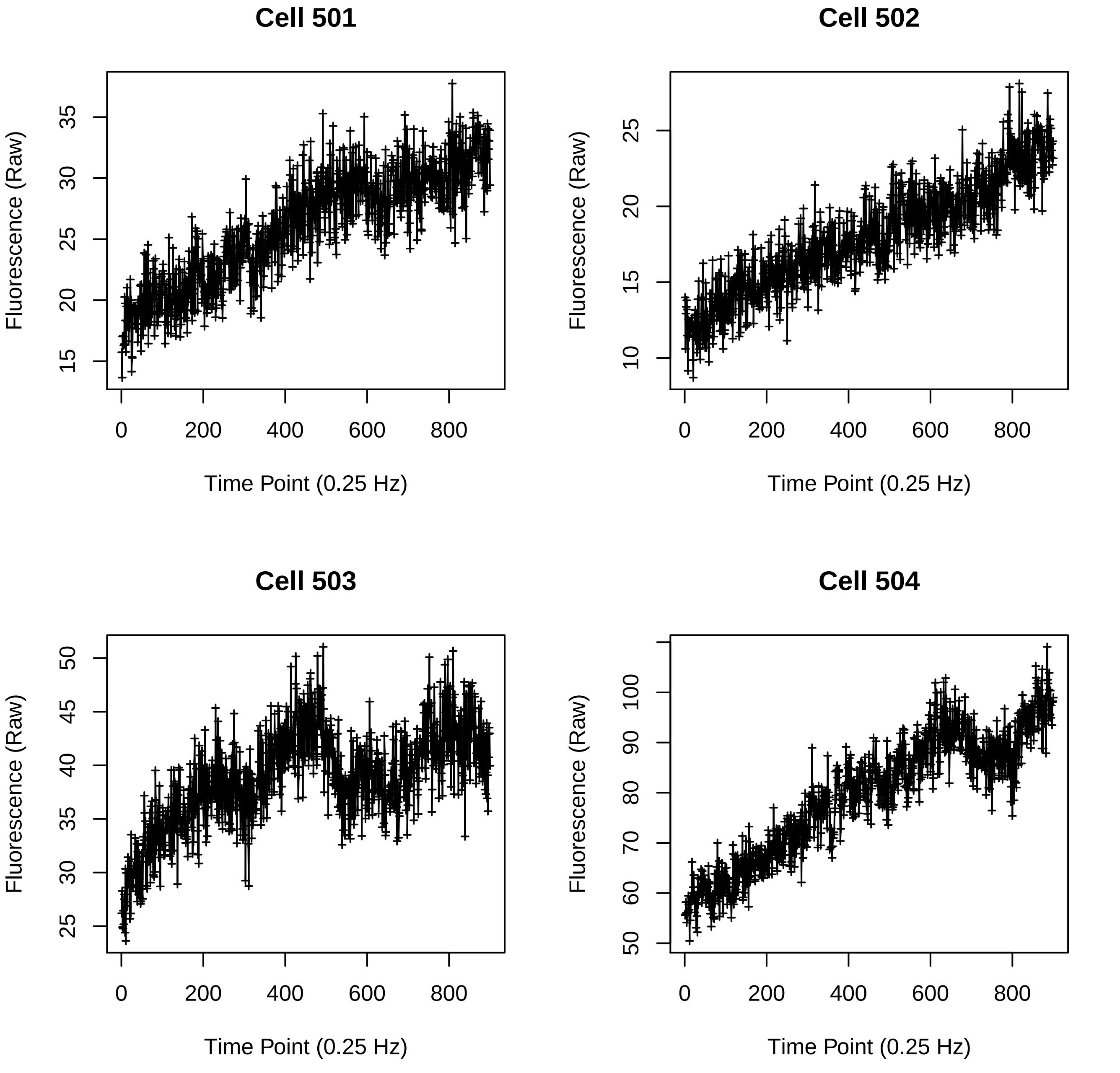
Cell 501
Fluorescence (Raw)
15
20
25
30
35
0
200
400
600
800
Time Point (0.25 Hz)
Cell 502
Fluorescence (Raw)
10
15
20
25
0
200
400
600
800
Time Point (0.25 Hz)
Cell 503
Fluorescence (Raw)
25
30
35
40
45
50
0
200
400
600
800
Time Point (0.25 Hz)
Cell 504
Fluorescence (Raw)
50
60
70
80
90
100
0
200
400
600
800
Time Point (0.25 Hz)

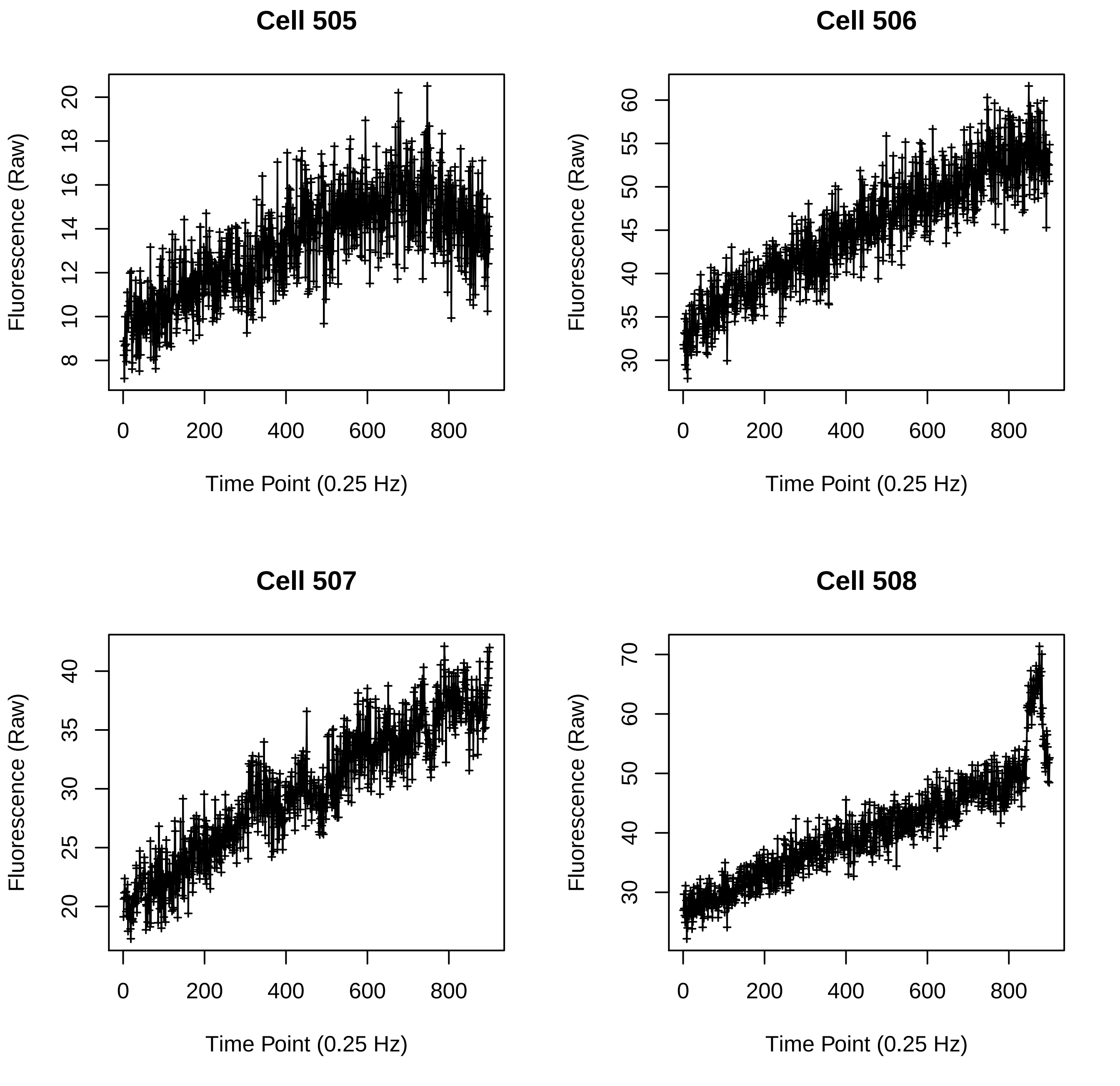

Cell 505
Fluorescence (Raw)
8
10
12
14
16
18
20
0
200
400
600
800
Time Point (0.25 Hz)
Cell 506
Fluorescence (Raw)
30
35
40
45
50
55
60
0
200
400
600
800
Time Point (0.25 Hz)
Cell 507
Fluorescence (Raw)
20
25
30
35
40
0
200
400
600
800
Time Point (0.25 Hz)
Cell 508
Fluorescence (Raw)
30
40
50
60
70
0
200
400
600
800
Time Point (0.25 Hz)

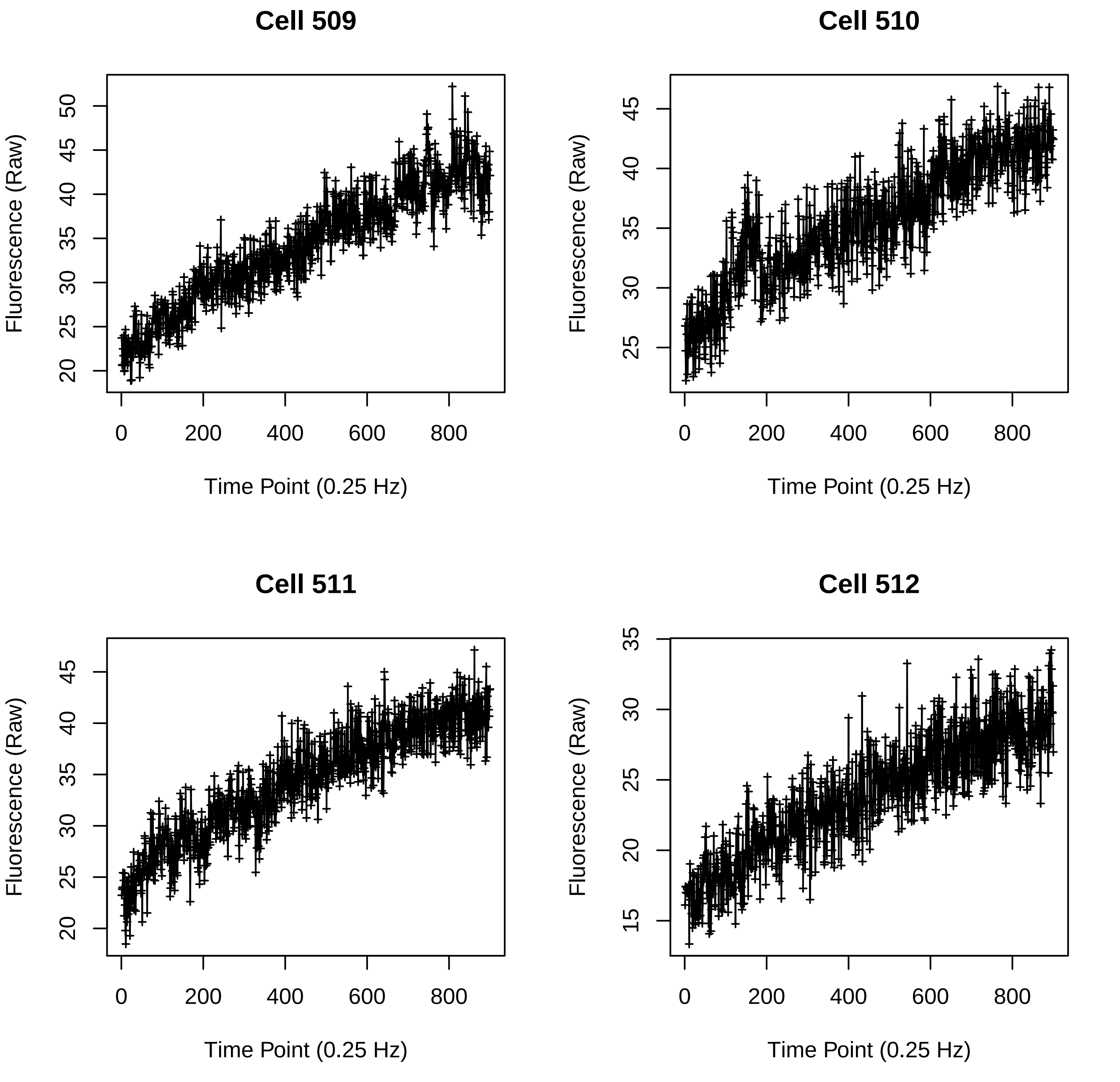

Cell 509
Cell 510
Cell 511
Cell 512
Fluorescence (Raw)
Time Point (0.25 Hz)
0
200
400
600
800
20
25
30
35
40
45
50
15

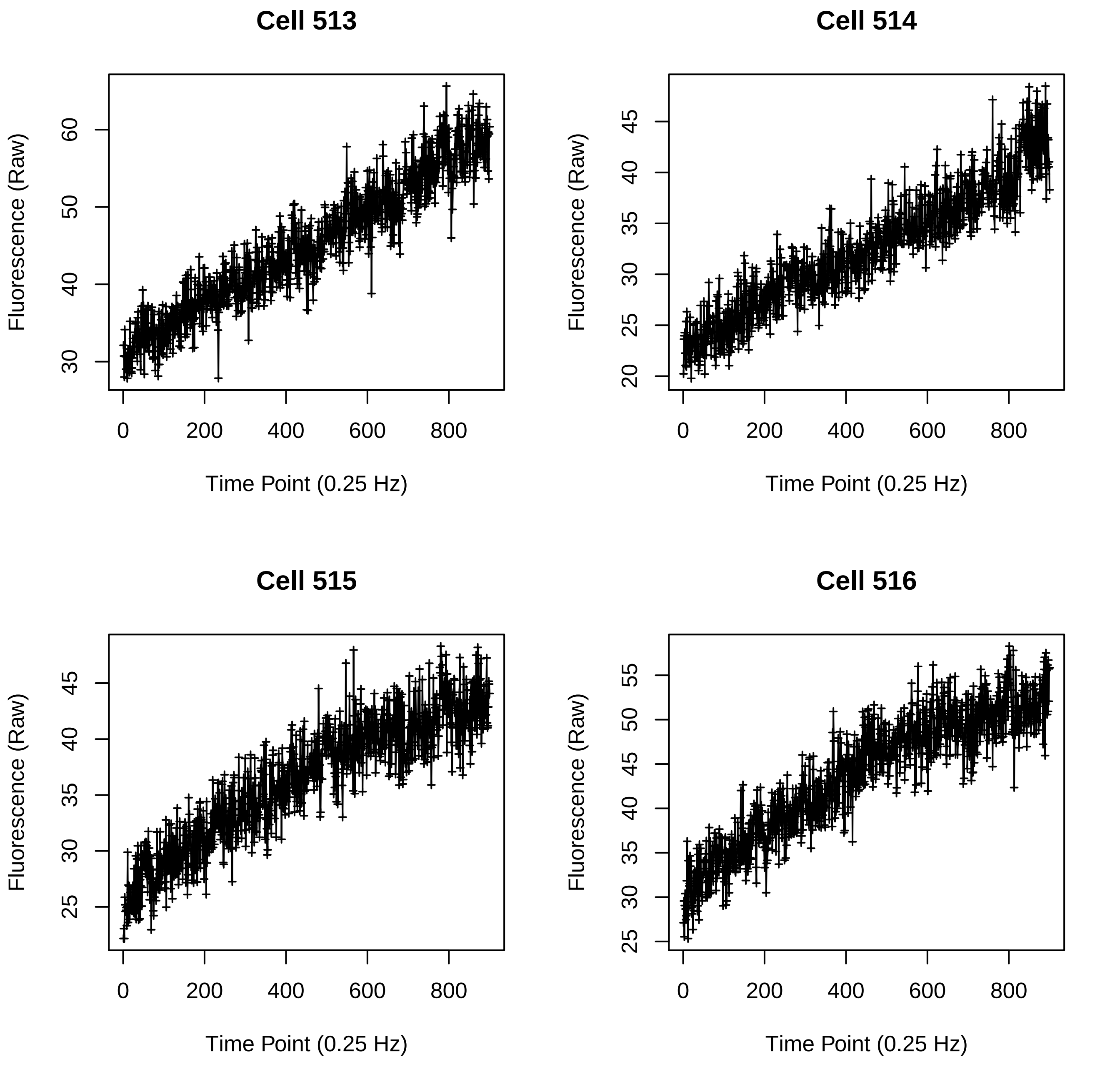
Cell 513
Fluorescence (Raw)
30
40
50
60
0
200
400
600
800
Time Point (0.25 Hz)
Cell 514
Fluorescence (Raw)
20
25
30
35
40
45
0
200
400
600
800
Time Point (0.25 Hz)
Cell 515
Fluorescence (Raw)
25
30
35
40
45
0
200
400
600
800
Time Point (0.25 Hz)
Cell 516
Fluorescence (Raw)
25
30
35
40
45
50
55
0
200
400
600
800
Time Point (0.25 Hz)

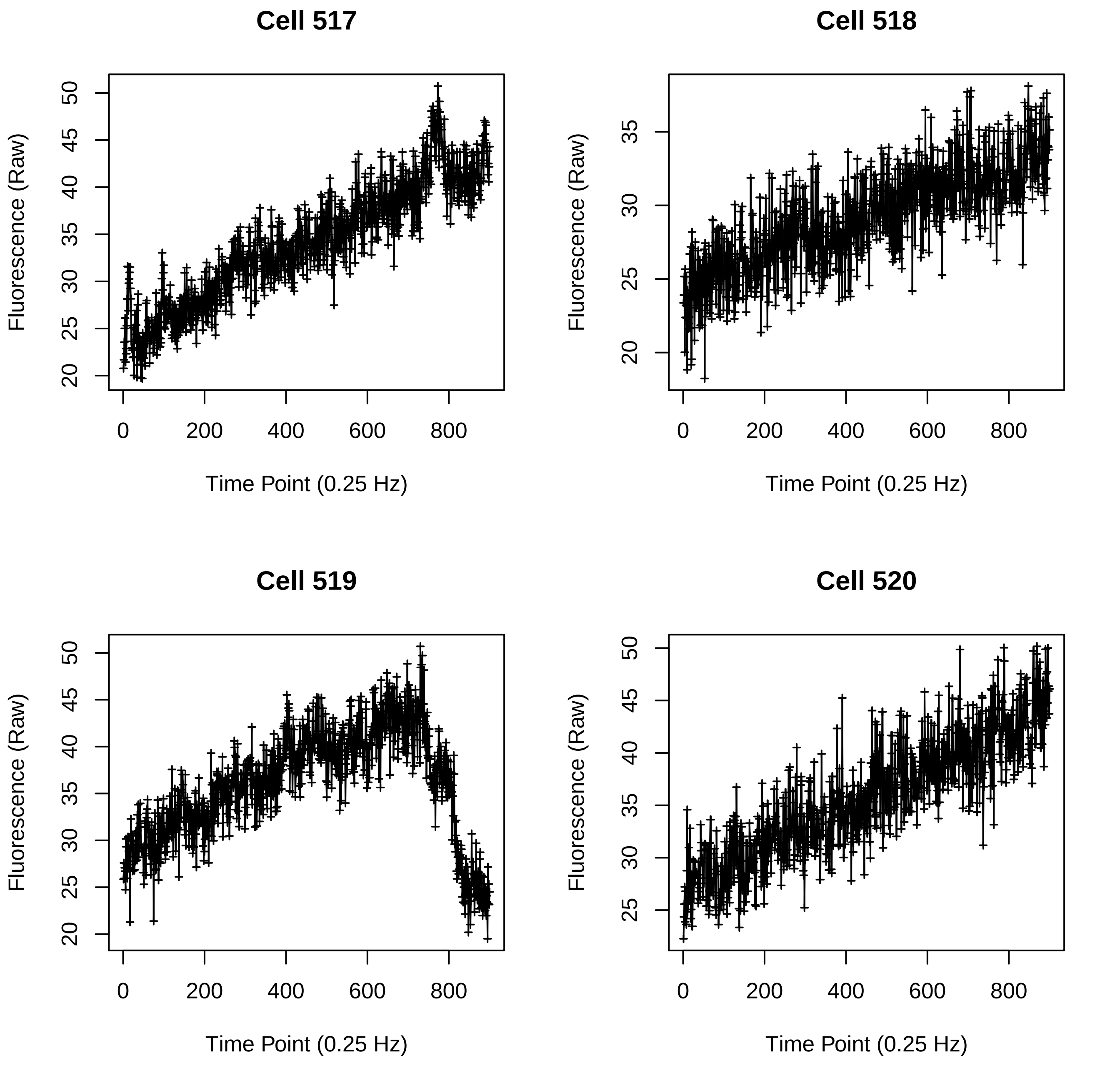
Cell 517
Fluorescence (Raw)
20 25 30 35 40 45 50
0 200 400 600 800
Time Point (0.25 Hz)
Cell 518
Fluorescence (Raw)
20 25 30 35
0 200 400 600 800
Time Point (0.25 Hz)
Cell 519
Fluorescence (Raw)
20 25 30 35 40 45 50
0 200 400 600 800
Time Point (0.25 Hz)
Cell 520
Fluorescence (Raw)
25 30 35 40 45 50
0 200 400 600 800
Time Point (0.25 Hz)

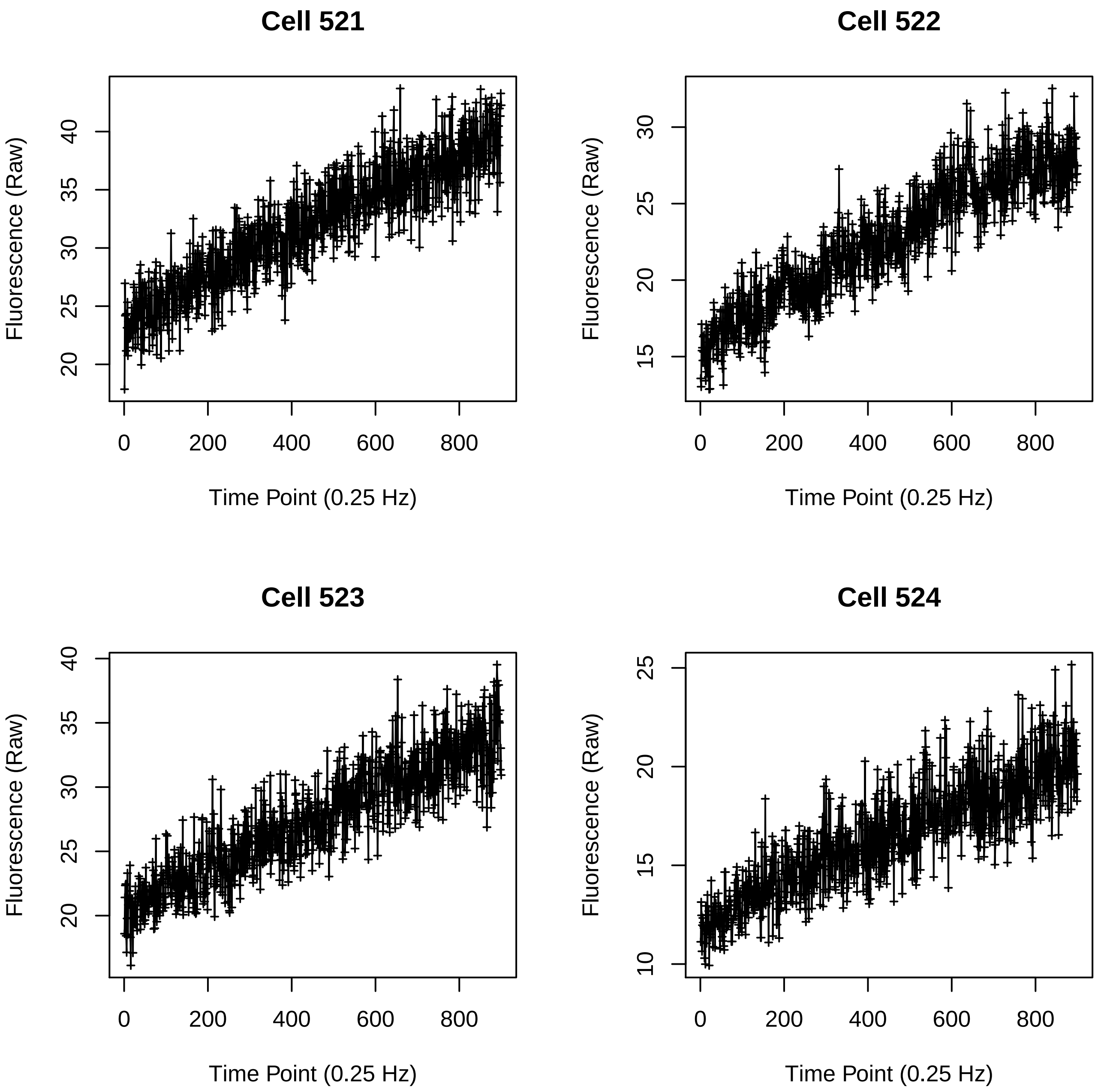
Cell 521
Cell 522
Cell 523
Cell 524
Fluorescence (Raw)
Time Point (0.25 Hz)
0
200
400
600
800
20
25
30
35
40
15
10

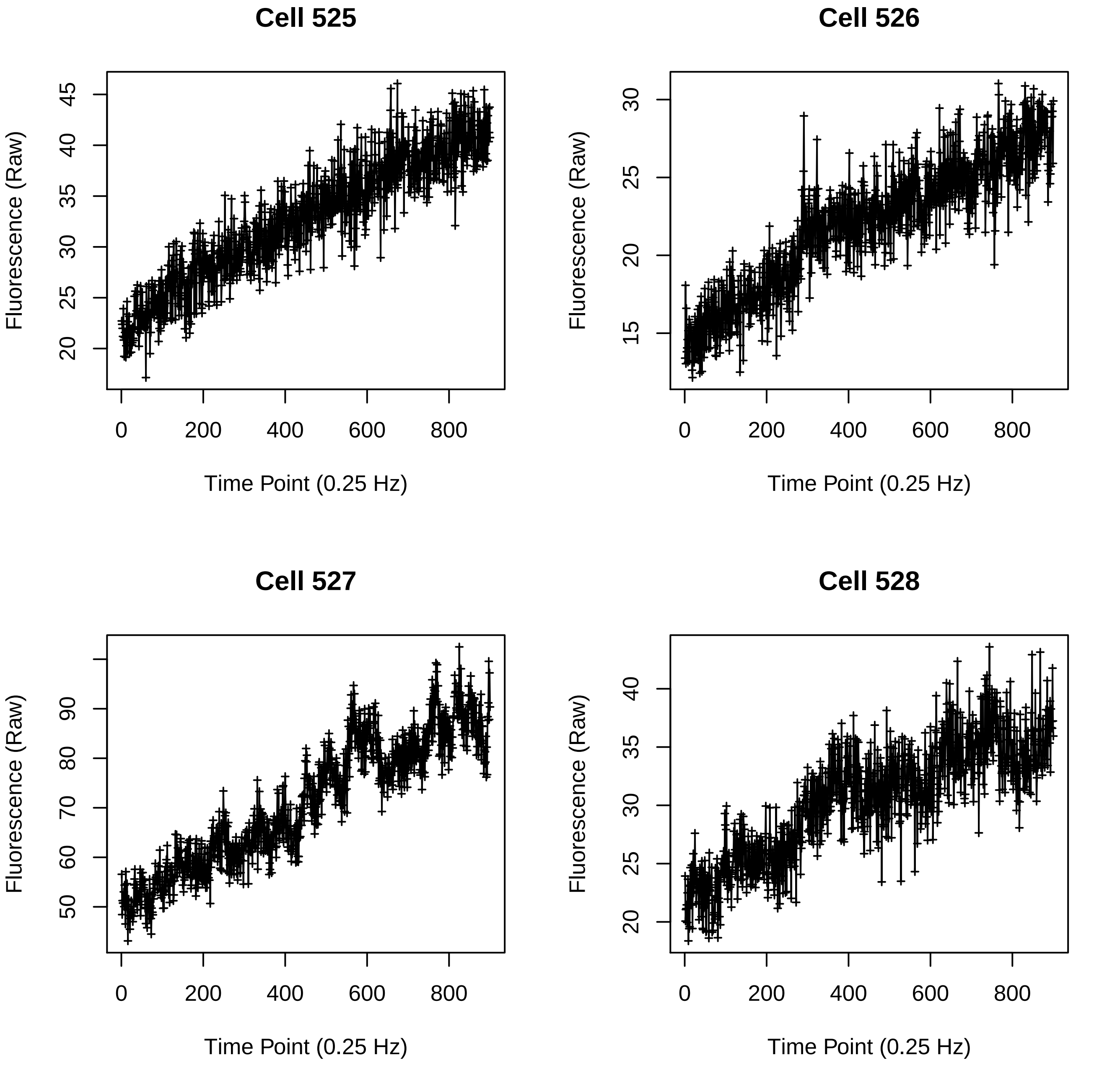
Cell 525
Fluorescence (Raw)
20
25
30
35
40
45
0
200
400
600
800
Time Point (0.25 Hz)
Cell 526
Fluorescence (Raw)
15
20
25
30
0
200
400
600
800
Time Point (0.25 Hz)
Cell 527
Fluorescence (Raw)
50
60
70
80
90
0
200
400
600
800
Time Point (0.25 Hz)
Cell 528
Fluorescence (Raw)
20
25
30
35
40
0
200
400
600
800
Time Point (0.25 Hz)

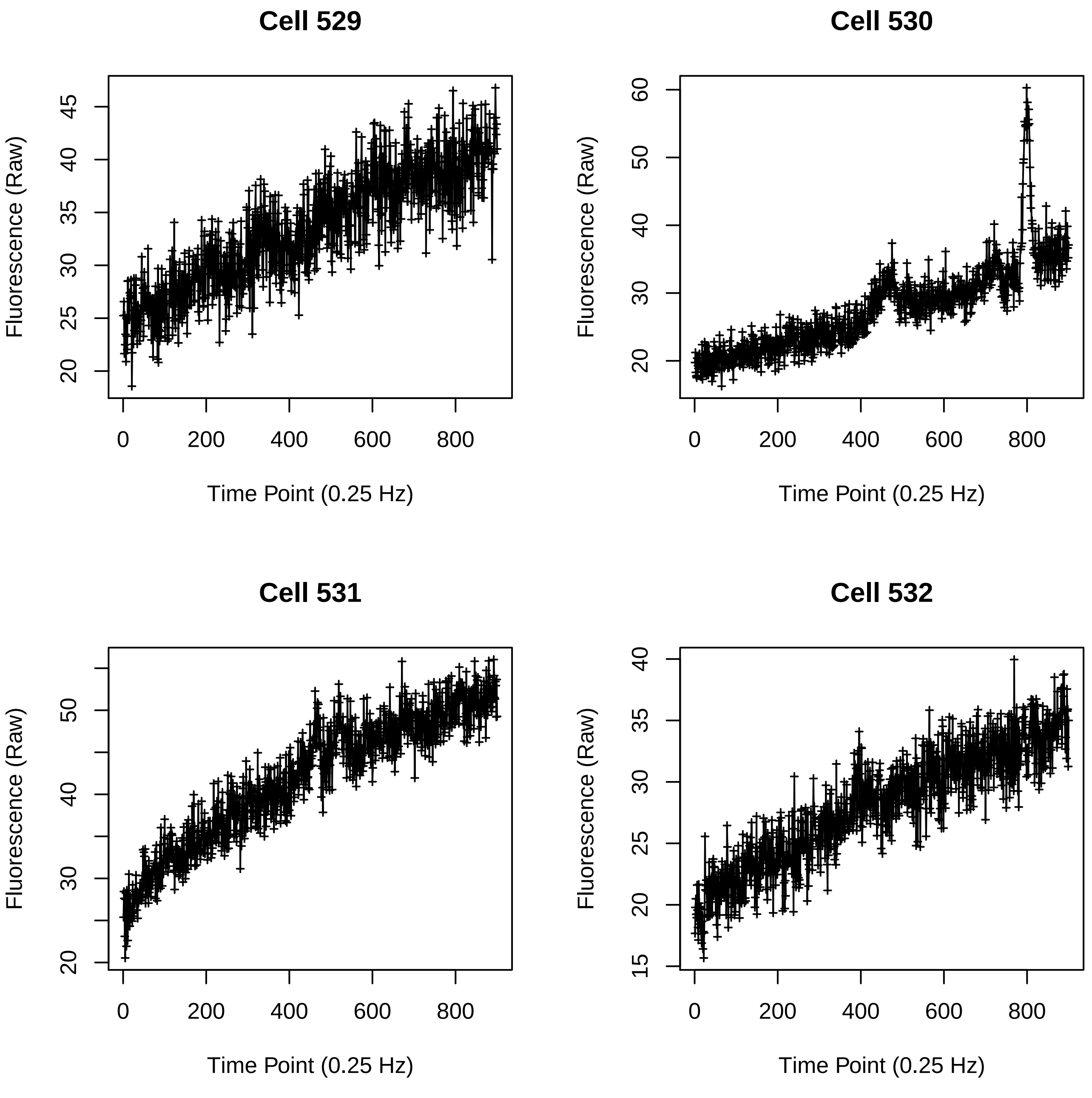
Cell 529
Fluorescence (Raw)
20
25
30
35
40
45
0
200
400
600
800
Time Point (0.25 Hz)
Cell 530
Fluorescence (Raw)
20
30
40
50
60
0
200
400
600
800
Time Point (0.25 Hz)
Cell 531
Fluorescence (Raw)
20
30
40
50
0
200
400
600
800
Time Point (0.25 Hz)
Cell 532
Fluorescence (Raw)
15
20
25
30
35
40
0
200
400
600
800
Time Point (0.25 Hz)

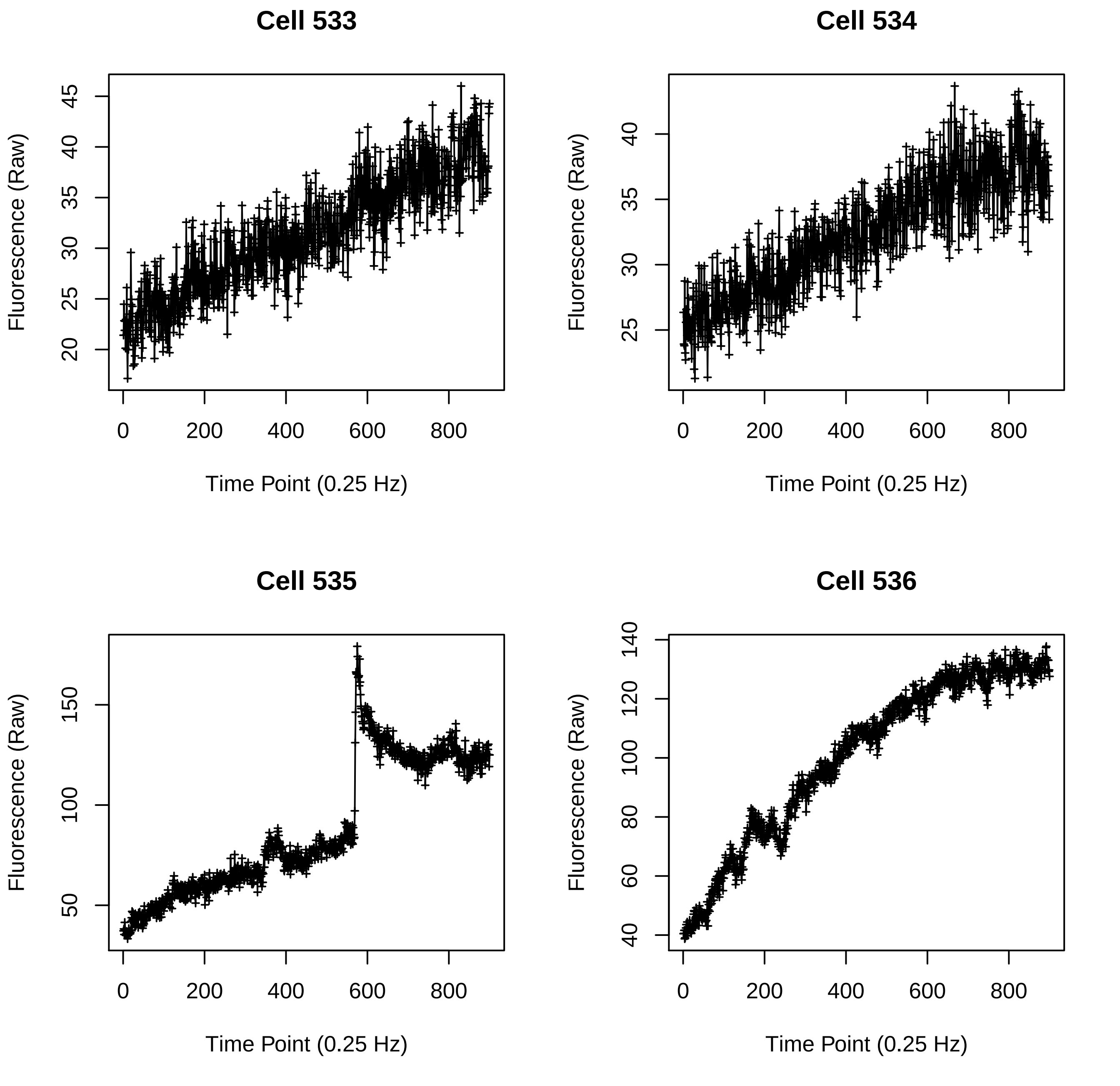
Cell 533
Cell 534
Cell 535
Cell 536
Fluorescence (Raw)
Time Point (0.25 Hz)
0
200
400
600
800
20
25
30
35
40
45
50
100
150
60
80
120
140

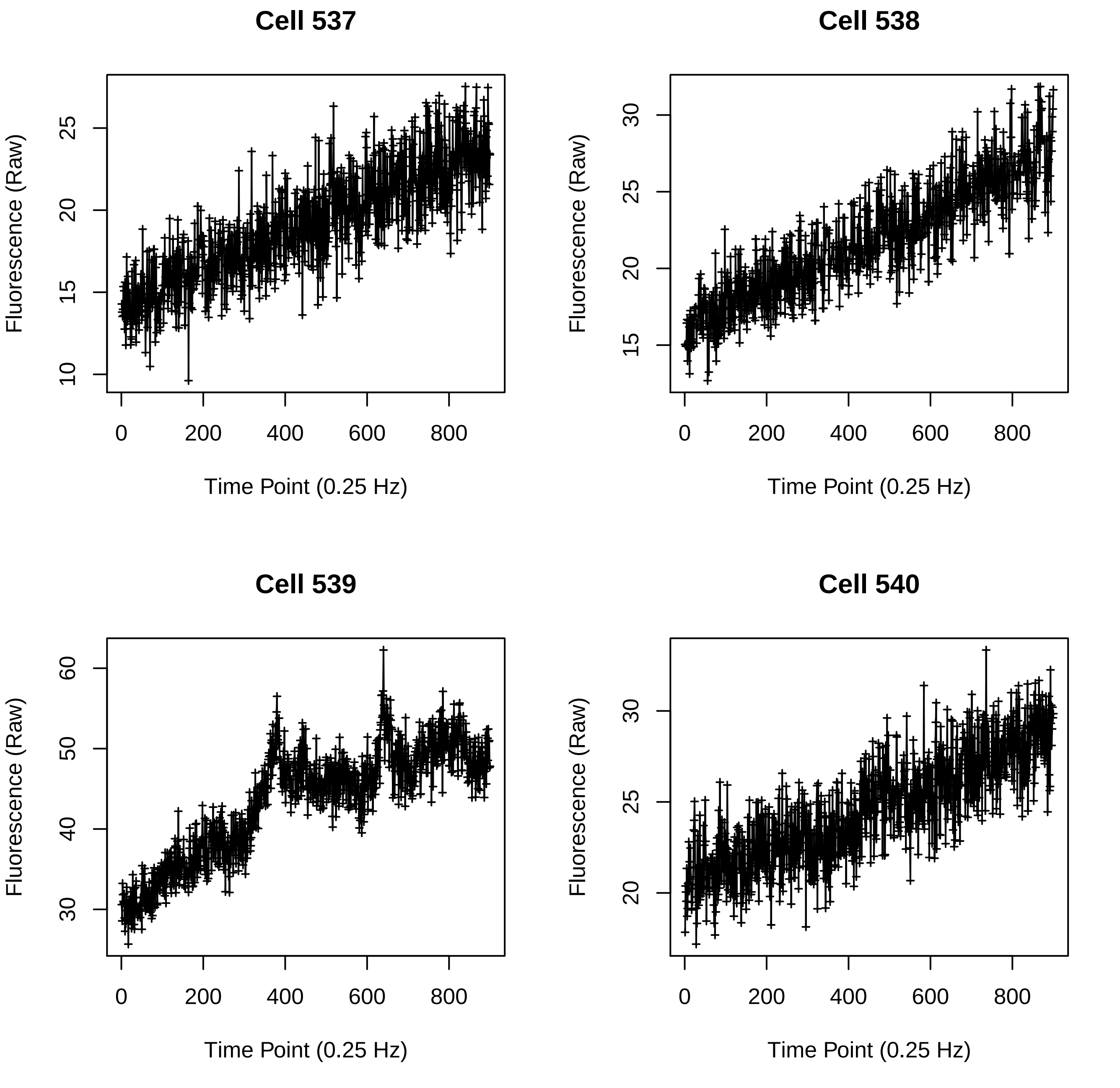
Cell 537
Fluorescence (Raw)
Time Point (0.25 Hz)
Cell 538
Fluorescence (Raw)
Time Point (0.25 Hz)
Cell 539
Fluorescence (Raw)
Time Point (0.25 Hz)
Cell 540
Fluorescence (Raw)
Time Point (0.25 Hz)

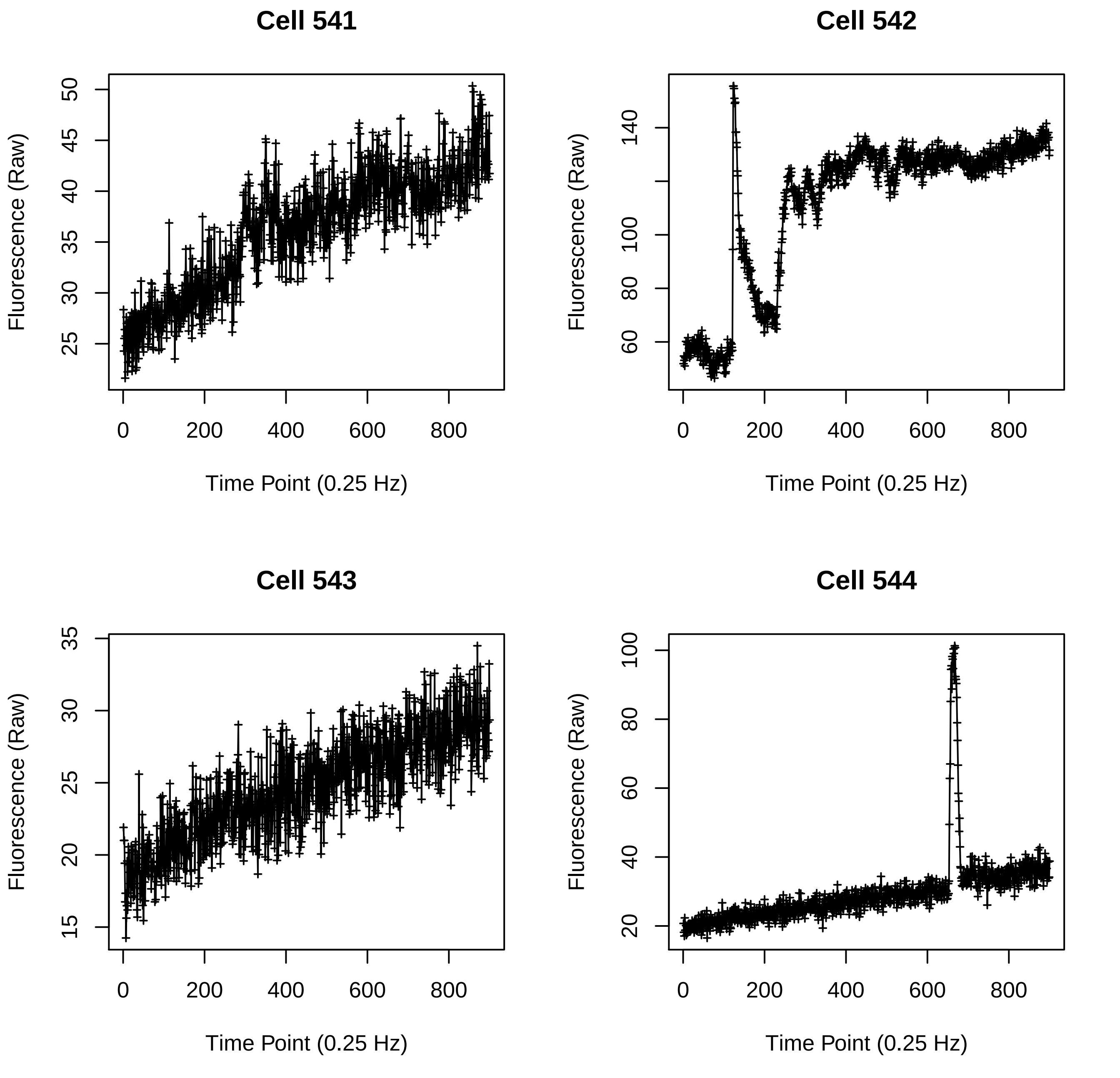
Cell 541
Fluorescence (Raw)
25
30
35
40
45
50
0
200
400
600
800
Time Point (0.25 Hz)
Cell 542
Fluorescence (Raw)
60
80
100
140
0
200
400
600
800
Time Point (0.25 Hz)
Cell 543
Fluorescence (Raw)
15
20
25
30
35
0
200
400
600
800
Time Point (0.25 Hz)
Cell 544
Fluorescence (Raw)
20
40
60
80
100
0
200
400
600
800
Time Point (0.25 Hz)

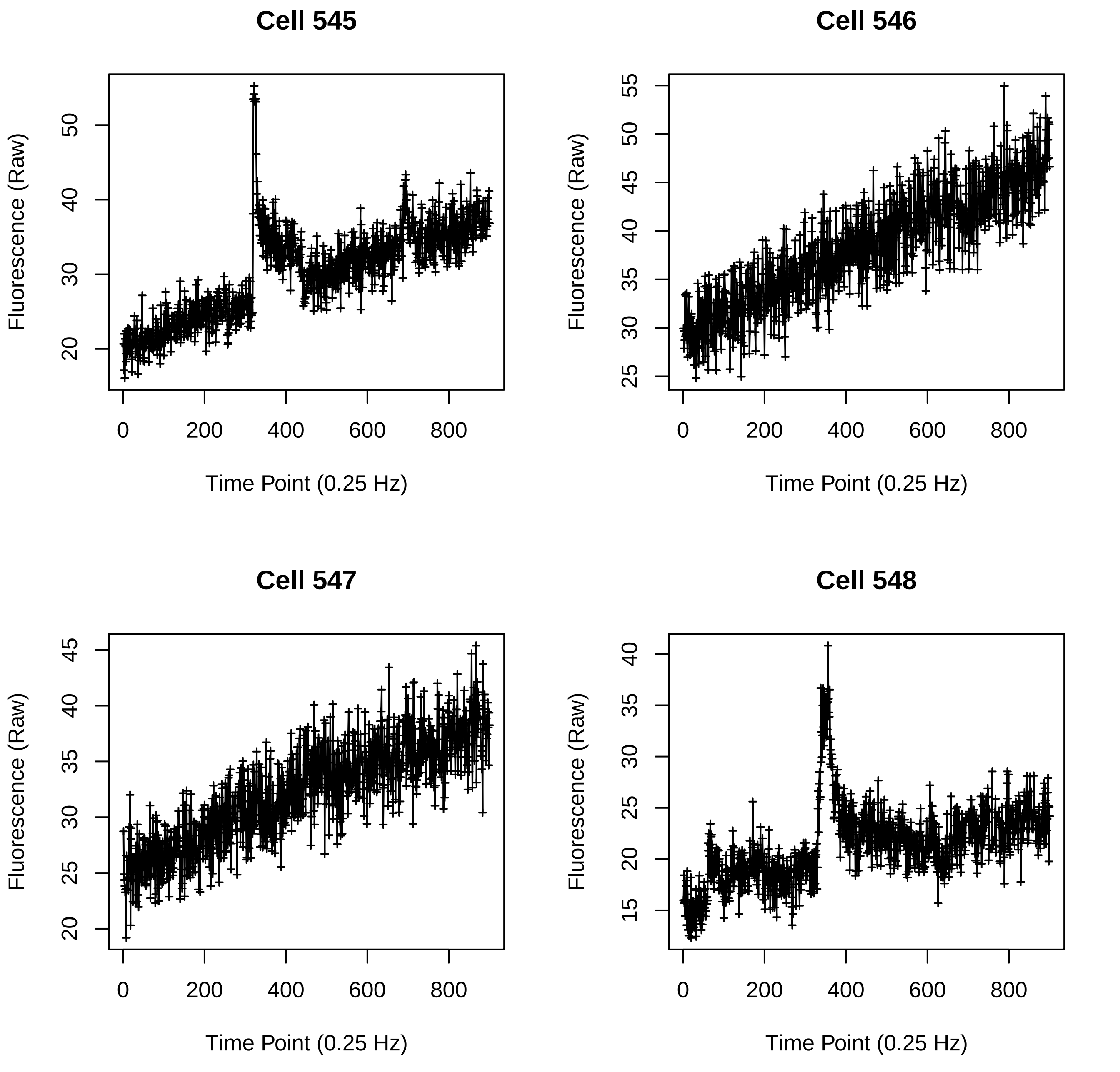
Cell 545
Cell 546
Cell 547
Cell 548
Fluorescence (Raw)
Time Point (0.25 Hz)
0
200
400
600
800
20
30
40
50
25
35
45
55
15

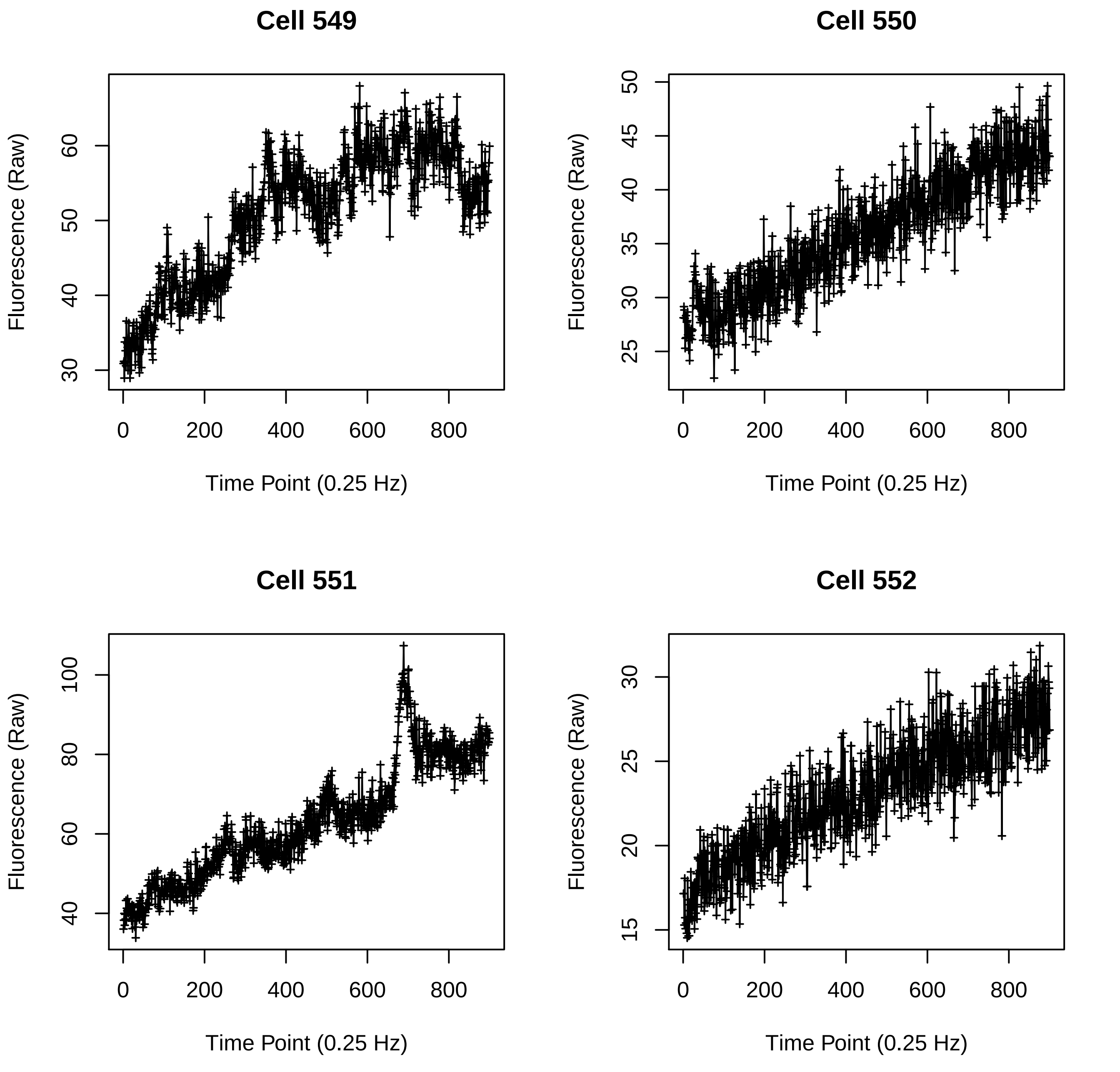
Cell 549
Fluorescence (Raw)
30
40
50
60
0
200
400
600
800
Time Point (0.25 Hz)
Cell 550
Fluorescence (Raw)
25
30
35
40
45
50
0
200
400
600
800
Time Point (0.25 Hz)
Cell 551
Fluorescence (Raw)
40
60
80
100
0
200
400
600
800
Time Point (0.25 Hz)
Cell 552
Fluorescence (Raw)
15
20
25
30
0
200
400
600
800
Time Point (0.25 Hz)

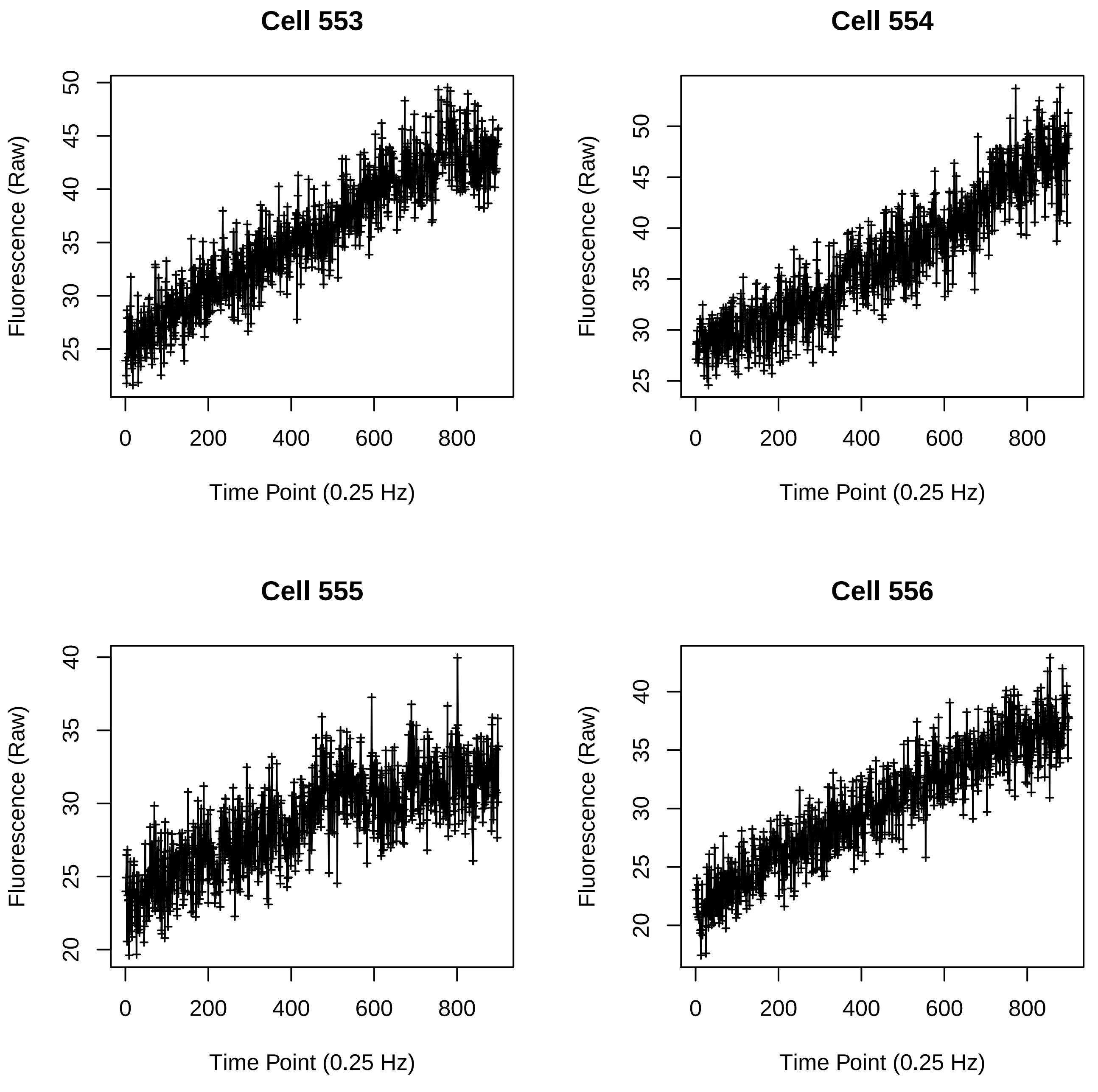
Cell 553
Cell 554
Cell 555
Cell 556
Fluorescence (Raw)
Time Point (0.25 Hz)
0
200
400
600
800
20
25
30
35
40
45
50

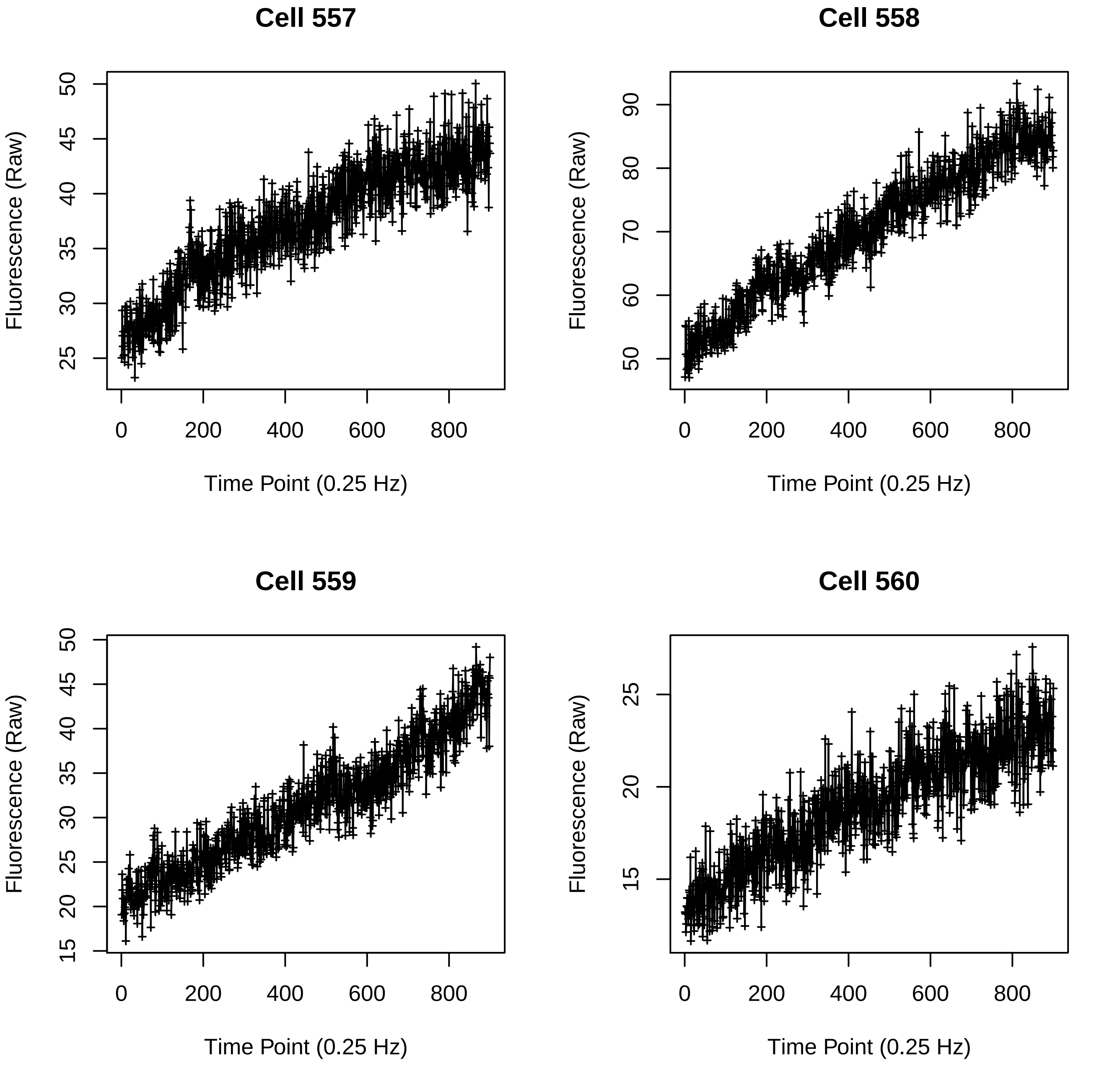
Cell 557
Cell 558
Cell 559
Cell 560
Fluorescence (Raw)
Time Point (0.25 Hz)
0
200
400
600
800
25
30
35
40
45
50
60
70
80
90
15
20

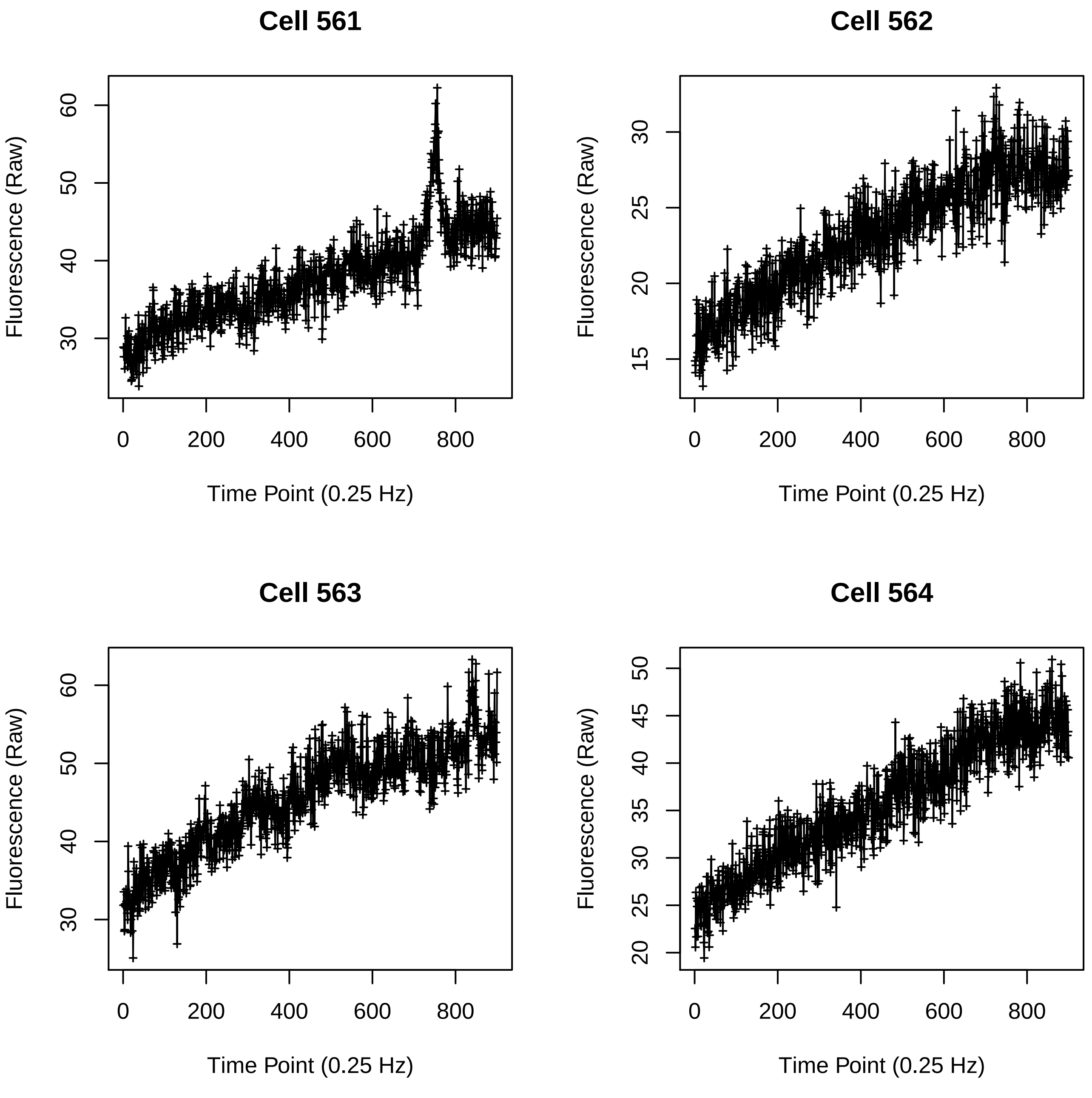
Cell 561
Cell 562
Cell 563
Cell 564
Fluorescence (Raw)
Time Point (0.25 Hz)
0
200
400
600
800
30
40
50
60
15
20
25
30
20
25
30
35
40
45
50

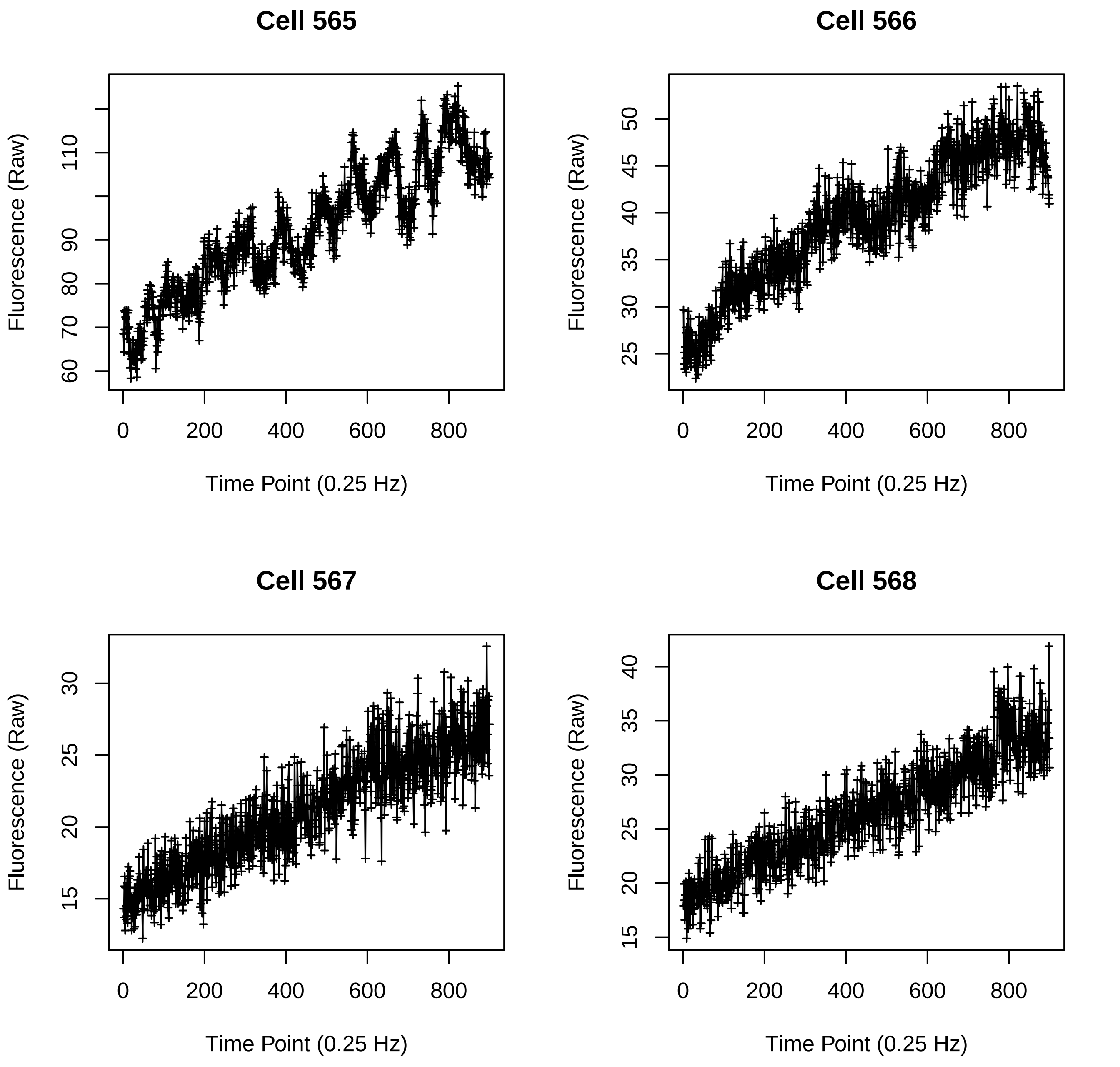
Cell 565
Fluorescence (Raw)
60
70
80
90
110
0
200
400
600
800
Time Point (0.25 Hz)
Cell 566
Fluorescence (Raw)
25
30
35
40
45
50
0
200
400
600
800
Time Point (0.25 Hz)
Cell 567
Fluorescence (Raw)
15
20
25
30
0
200
400
600
800
Time Point (0.25 Hz)
Cell 568
Fluorescence (Raw)
15
20
25
30
35
40
0
200
400
600
800
Time Point (0.25 Hz)

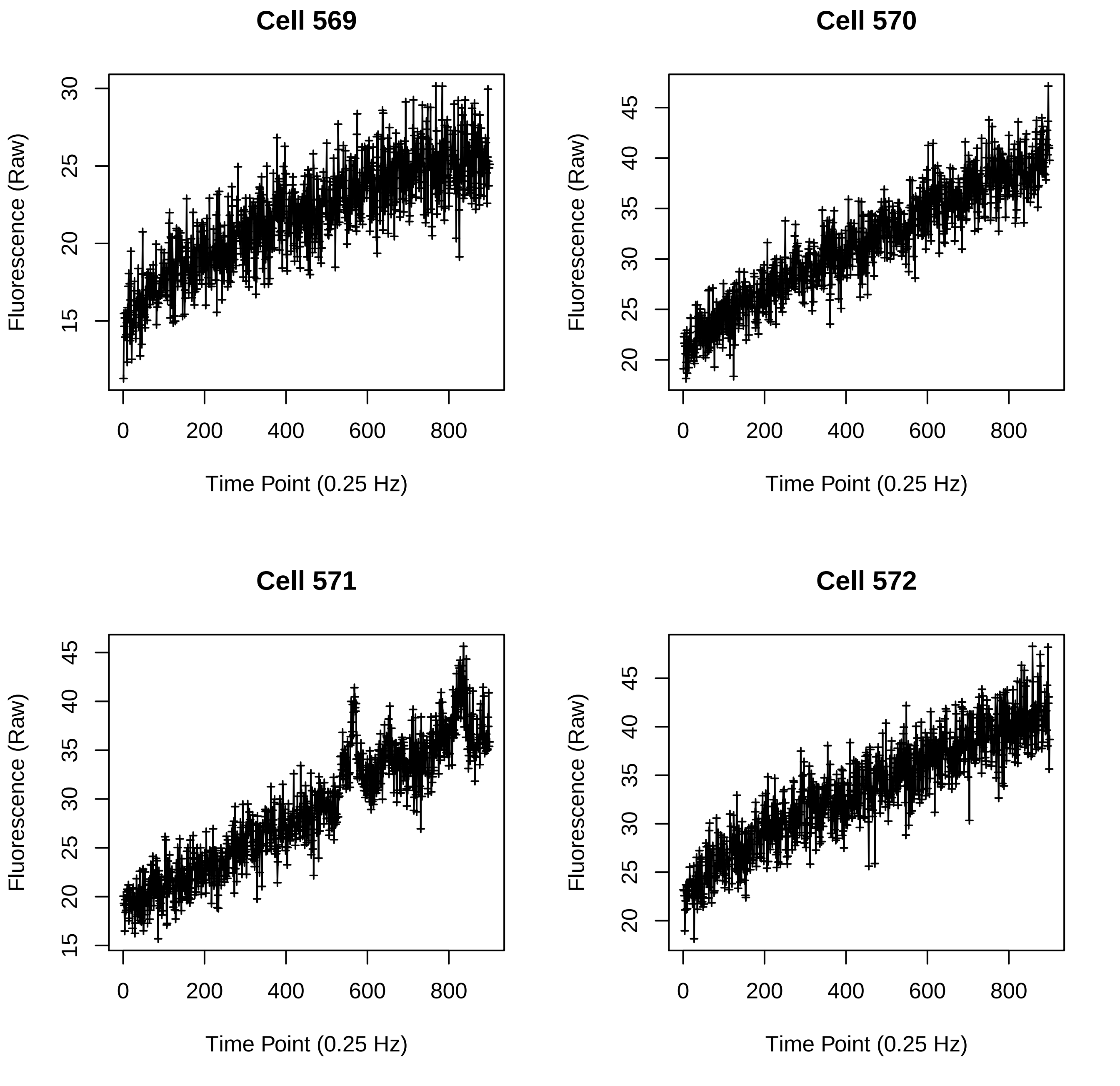

Cell 569
Cell 570
Cell 571
Cell 572
Fluorescence (Raw)
Time Point (0.25 Hz)
0
200
400
600
800
15
20
25
30
35
40
45

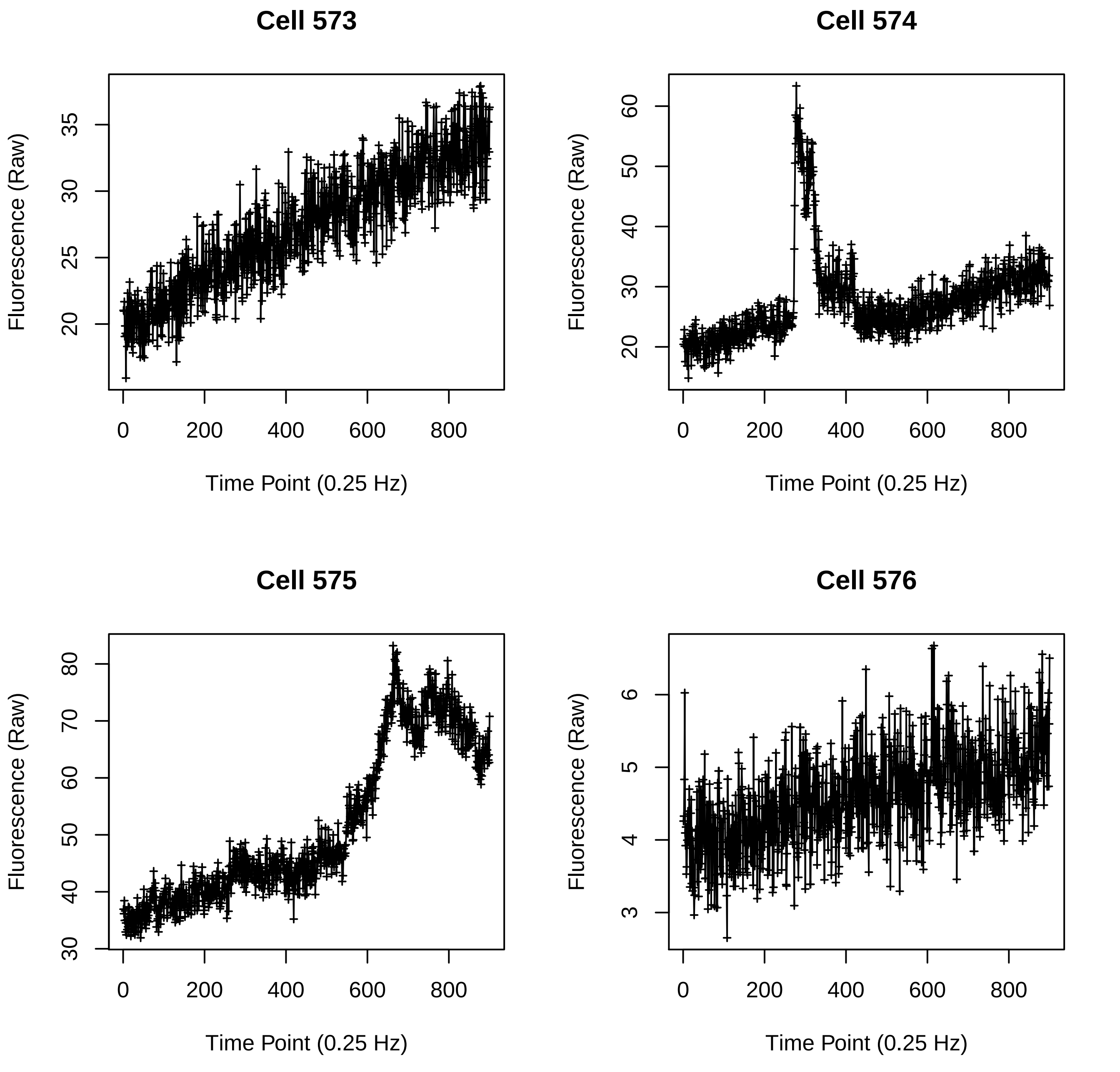
Cell 573
Cell 574
Cell 575
Cell 576
Fluorescence (Raw)
Time Point (0.25 Hz)
20
25
30
35
40
50
60
70
80
3
4
5
6
0
200
400
600
800

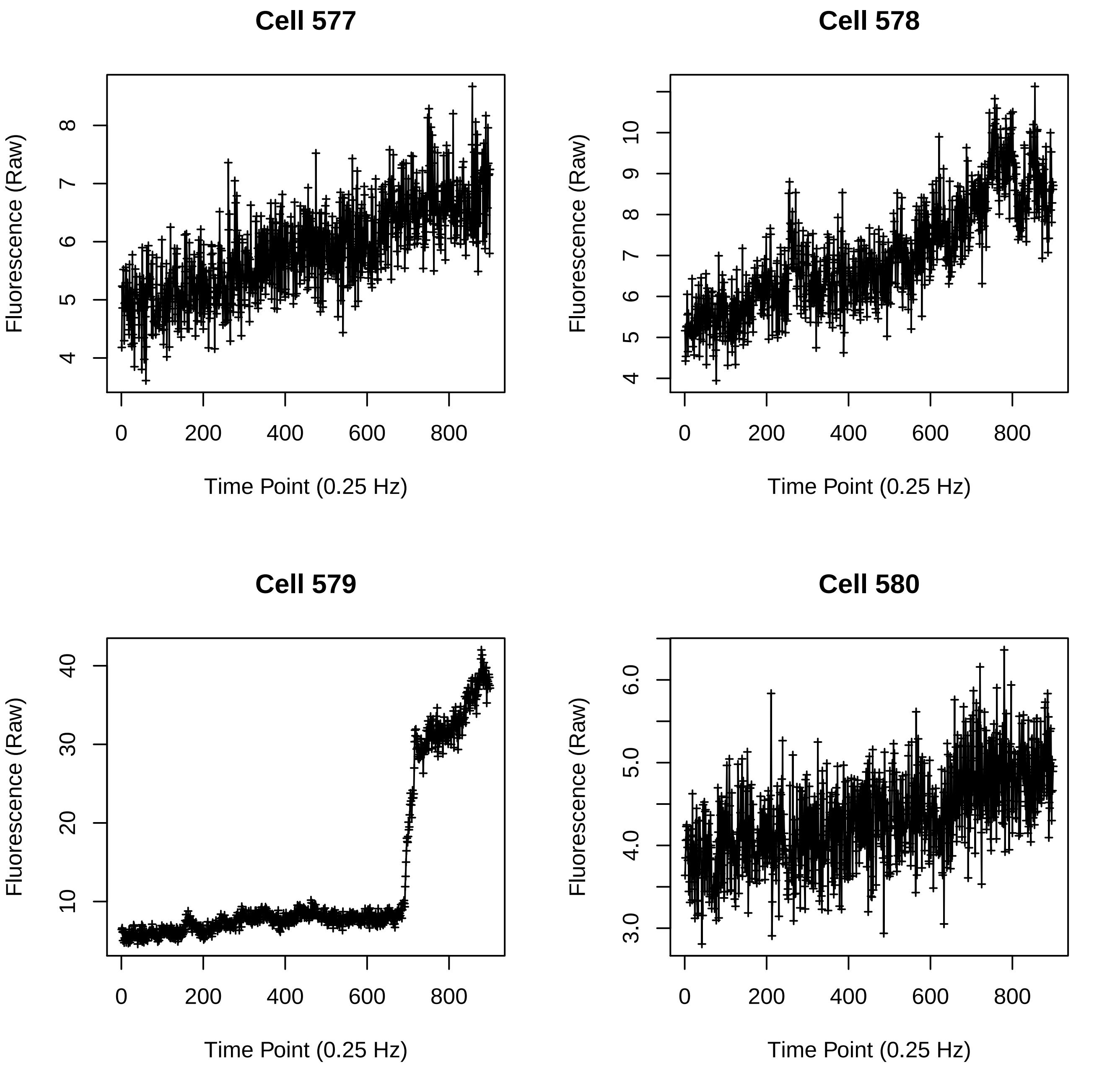
Cell 577
Fluorescence (Raw)
4
5
6
7
8
Time Point (0.25 Hz)
0
200
400
600
800
Cell 578
Fluorescence (Raw)
4
5
6
7
8
9
10
Time Point (0.25 Hz)
0
200
400
600
800
Cell 579
Fluorescence (Raw)
10
20
30
40
Time Point (0.25 Hz)
0
200
400
600
800
Cell 580
Fluorescence (Raw)
3.0
4.0
5.0
6.0
Time Point (0.25 Hz)
0
200
400
600
800

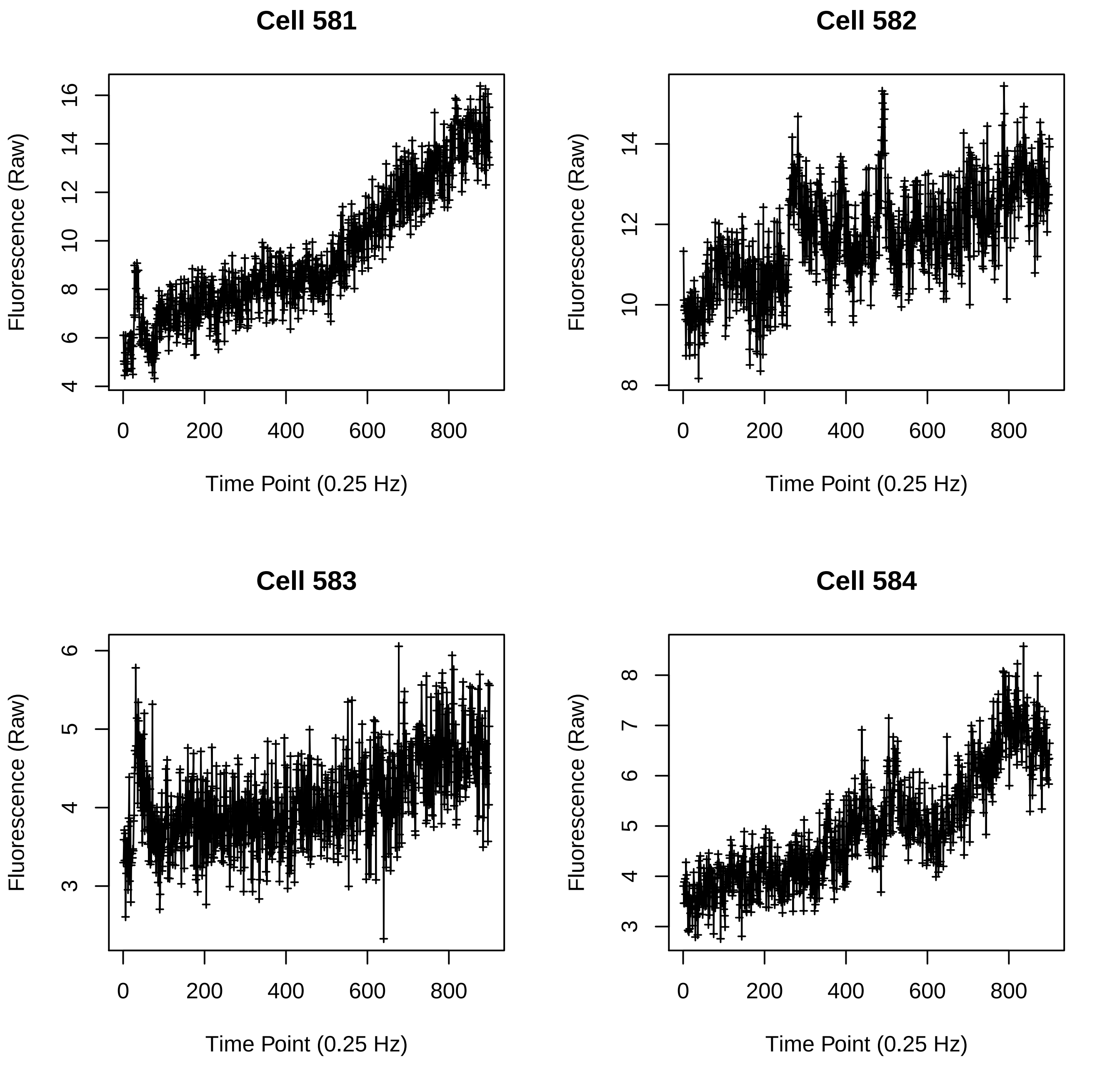
Cell 581
Cell 582
Cell 583
Cell 584
Fluorescence (Raw)
Time Point (0.25 Hz)
0
200
400
600
800
4
6
8
10
12
14
16
8
10
12
14
3
4
5
6
7
8

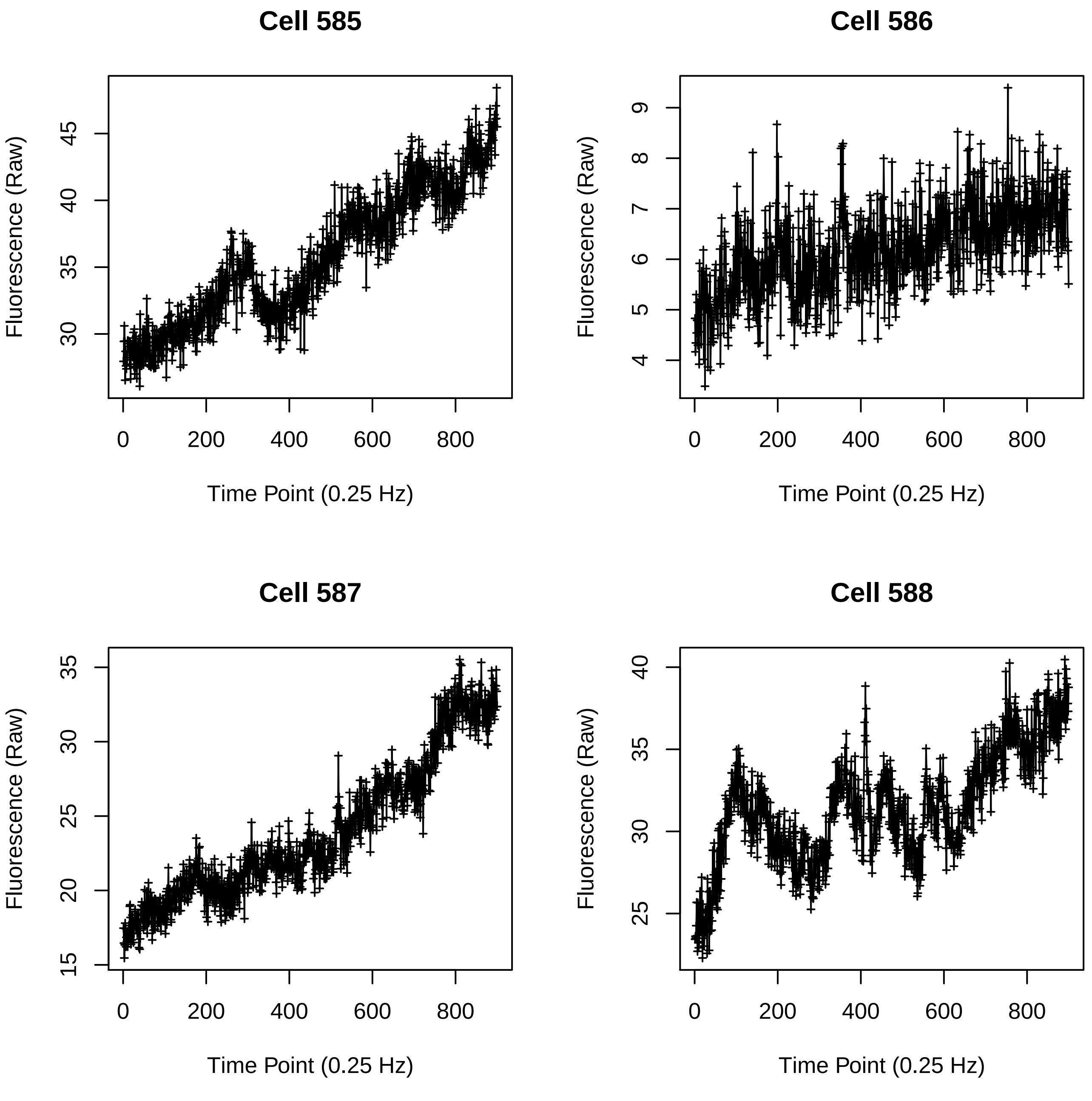

Cell 585
Fluorescence (Raw)
30
35
40
45
0
200
400
600
800
Time Point (0.25 Hz)
Cell 586
Fluorescence (Raw)
4
5
6
7
8
9
0
200
400
600
800
Time Point (0.25 Hz)
Cell 587
Fluorescence (Raw)
15
20
25
30
35
0
200
400
600
800
Time Point (0.25 Hz)
Cell 588
Fluorescence (Raw)
25
30
35
40
0
200
400
600
800
Time Point (0.25 Hz)

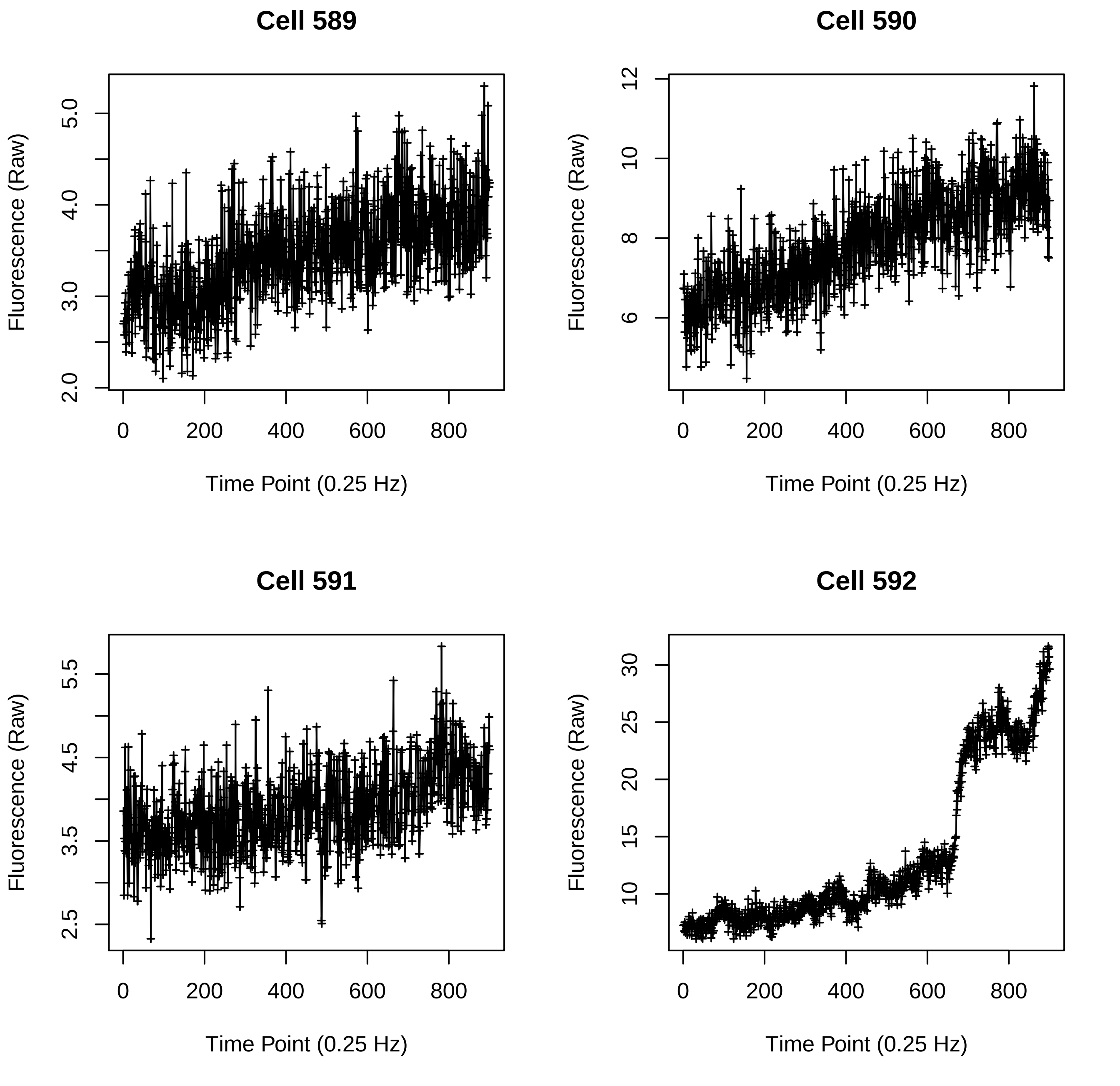
Cell 589
Cell 590
Cell 591
Cell 592
Fluorescence (Raw)
Time Point (0.25 Hz)
0
200
400
600
800
2.0
3.0
4.0
5.0
6
8
10
12
2.5
3.5
4.5
5.5
10
15
20
25
30

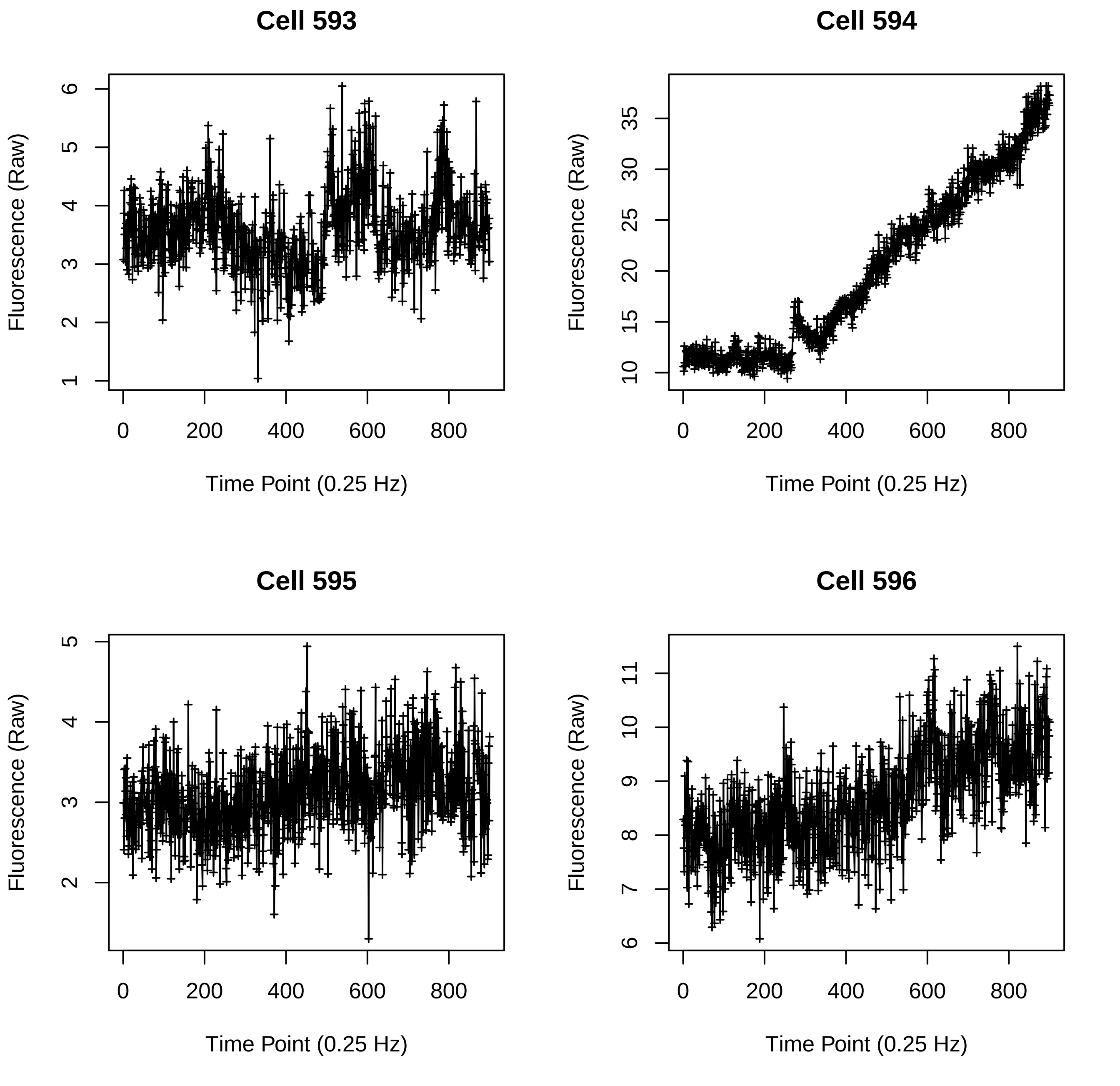
Cell 593
Cell 594
Cell 595
Cell 596
Fluorescence (Raw)
Time Point (0.25 Hz)
0
200
400
600
800
1
2
3
4
5
6
10
15
20
25
30
35
7
8
9
10
11

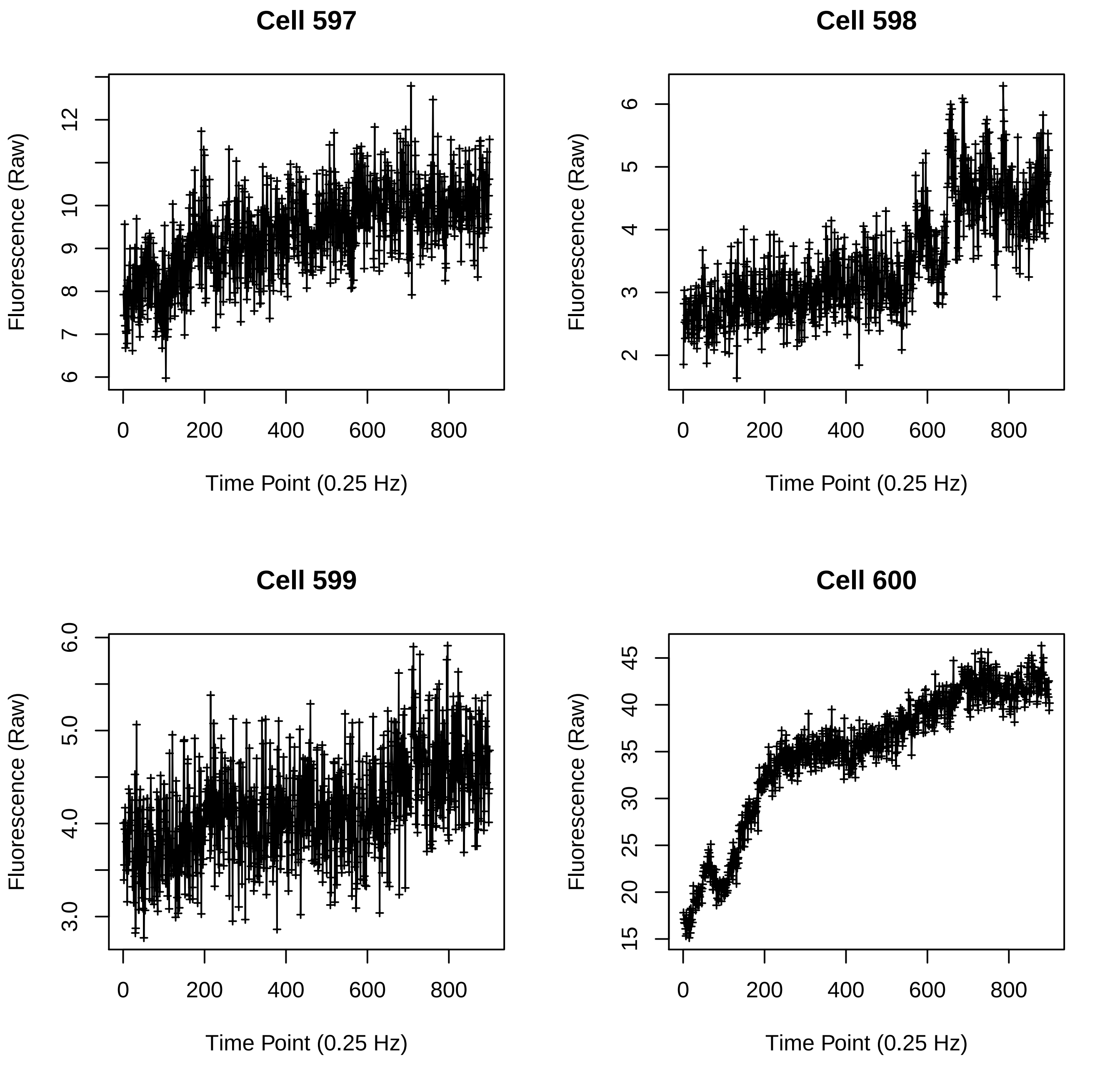

Cell 597
Cell 598
Cell 599
Cell 600
Fluorescence (Raw)
Time Point (0.25 Hz)
0
200
400
600
800
6
7
8
9
10
12
2
3
4
5
6
3.0
4.0
5.0
6.0
15
20
25
30
35
40
45

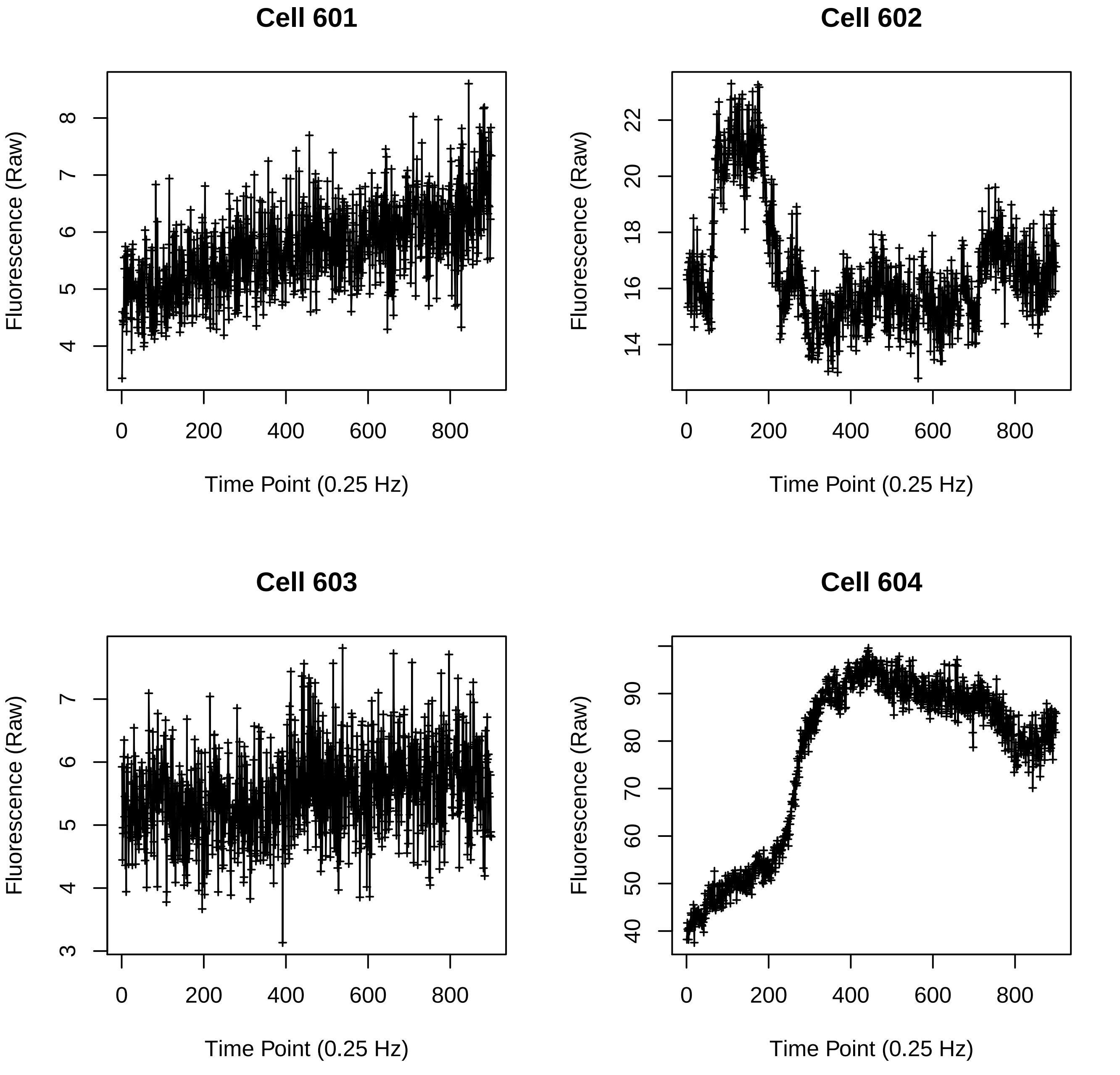
Cell 601
Fluorescence (Raw)
Time Point (0.25 Hz)
Cell 602
Fluorescence (Raw)
Time Point (0.25 Hz)
Cell 603
Fluorescence (Raw)
Time Point (0.25 Hz)
Cell 604
Fluorescence (Raw)
Time Point (0.25 Hz)

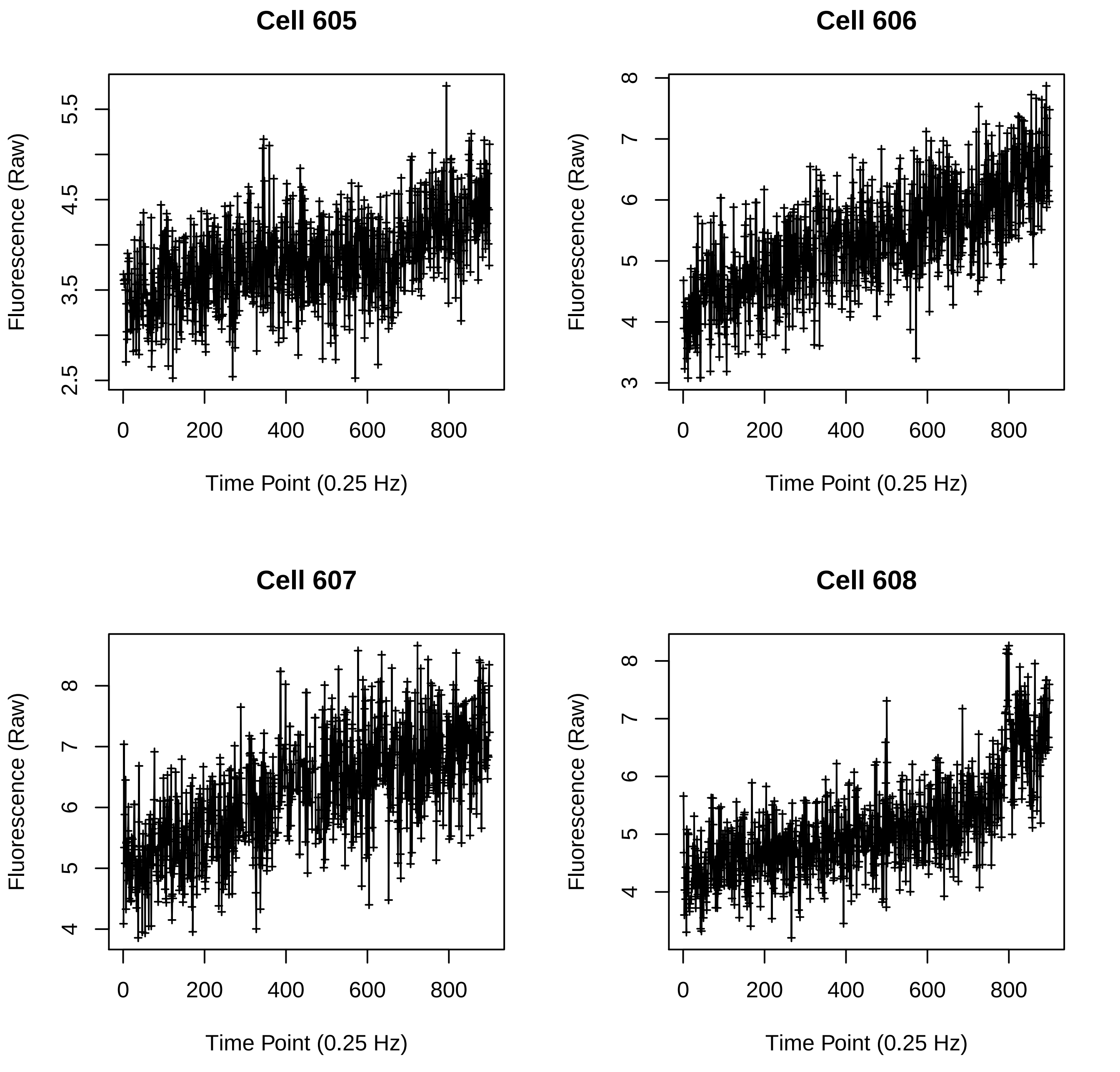
Cell 605
Fluorescence (Raw)
2.5
3.5
4.5
5.5
0
200
400
600
800
Time Point (0.25 Hz)
Cell 606
Fluorescence (Raw)
3
4
5
6
7
8
0
200
400
600
800
Time Point (0.25 Hz)
Cell 607
Fluorescence (Raw)
4
5
6
7
8
0
200
400
600
800
Time Point (0.25 Hz)
Cell 608
Fluorescence (Raw)
4
5
6
7
8
0
200
400
600
800
Time Point (0.25 Hz)

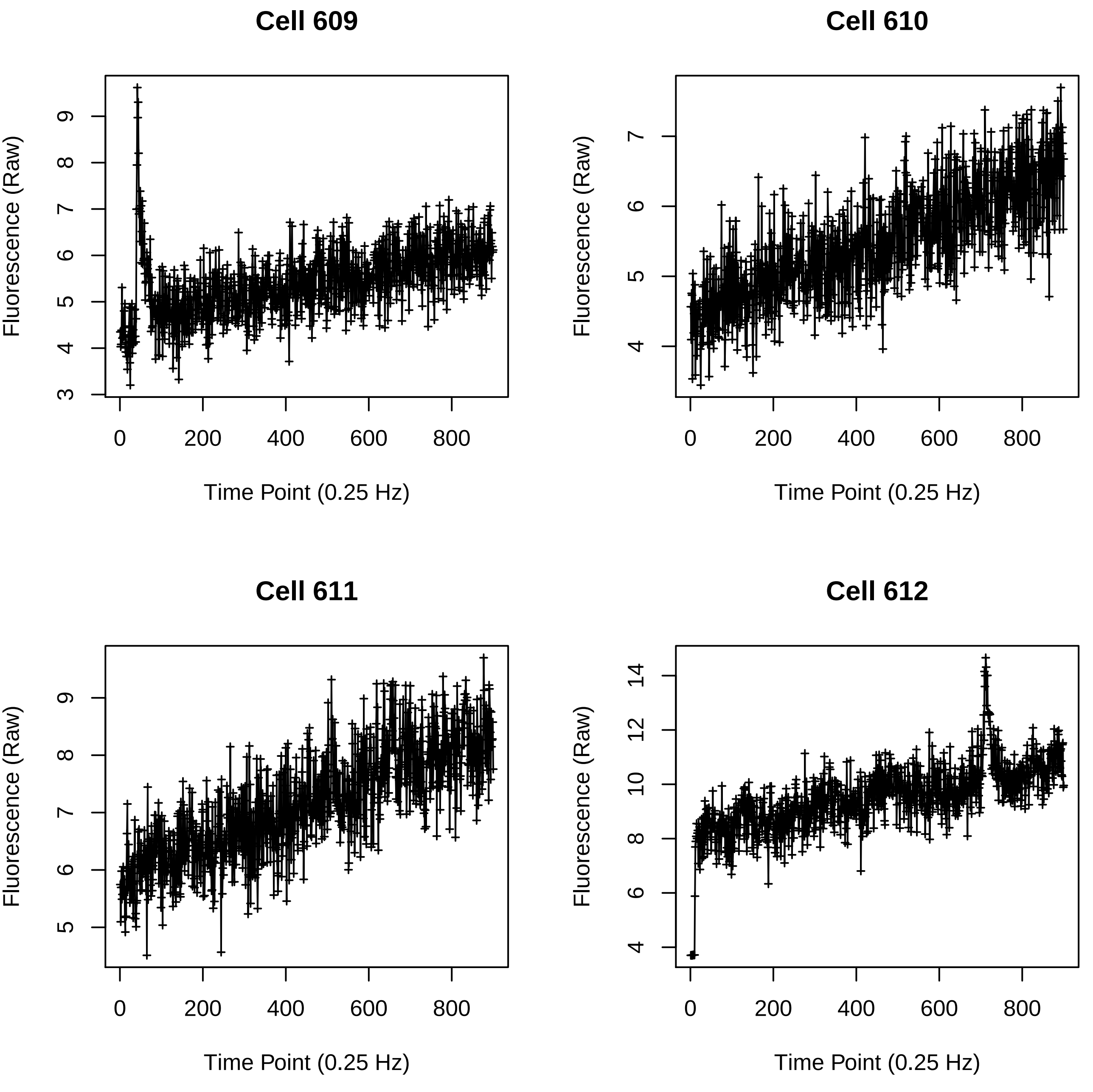
Cell 609
Fluorescence (Raw)
Time Point (0.25 Hz)
Cell 610
Fluorescence (Raw)
Time Point (0.25 Hz)
Cell 611
Fluorescence (Raw)
Time Point (0.25 Hz)
Cell 612
Fluorescence (Raw)
Time Point (0.25 Hz)

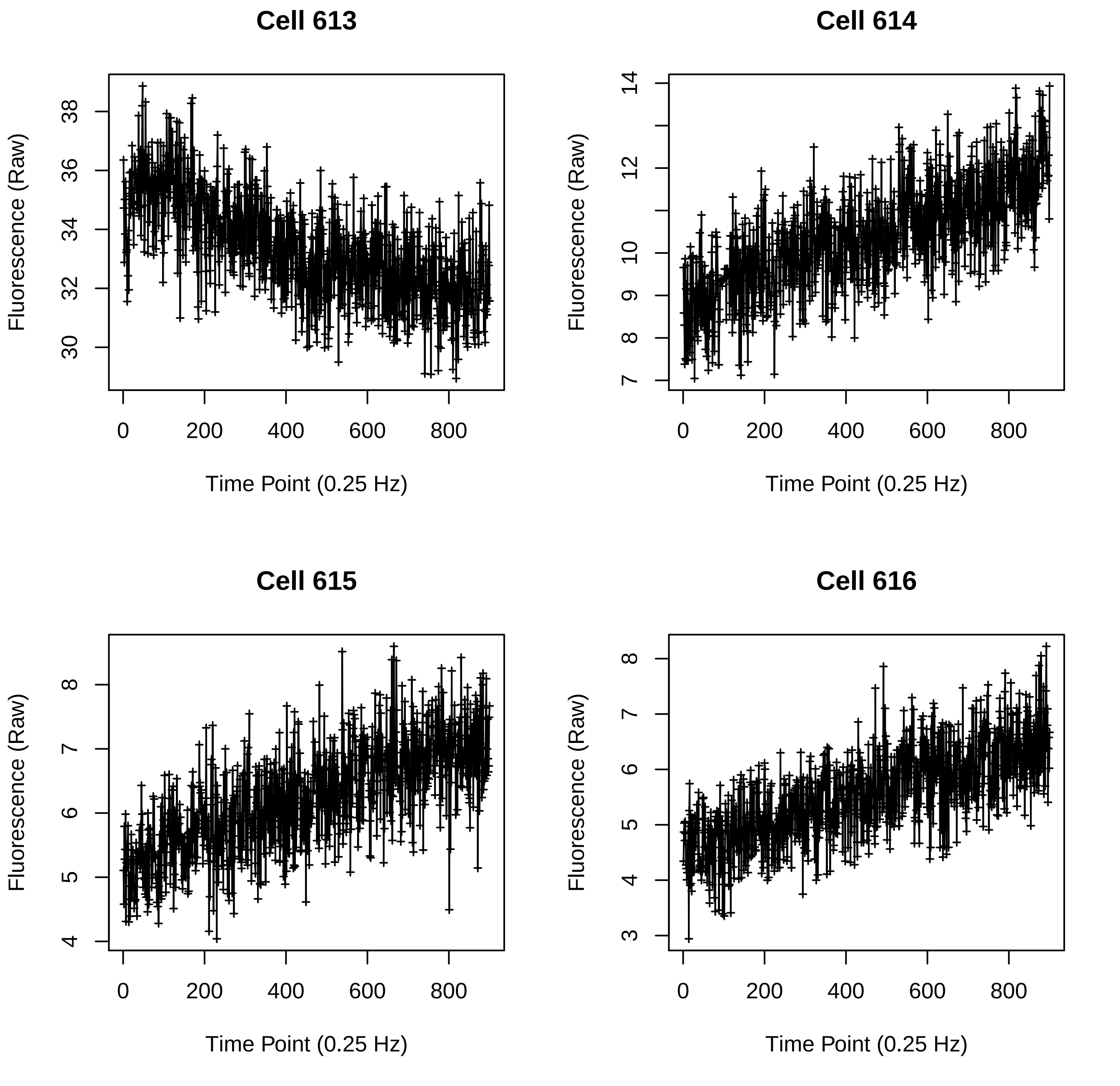

Cell 613
Cell 614
Cell 615
Cell 616
Fluorescence (Raw)
Time Point (0.25 Hz)
0
200
400
600
800
30
32
34
36
38
7
8
9
10
12
14
4
5
6
7
8
3

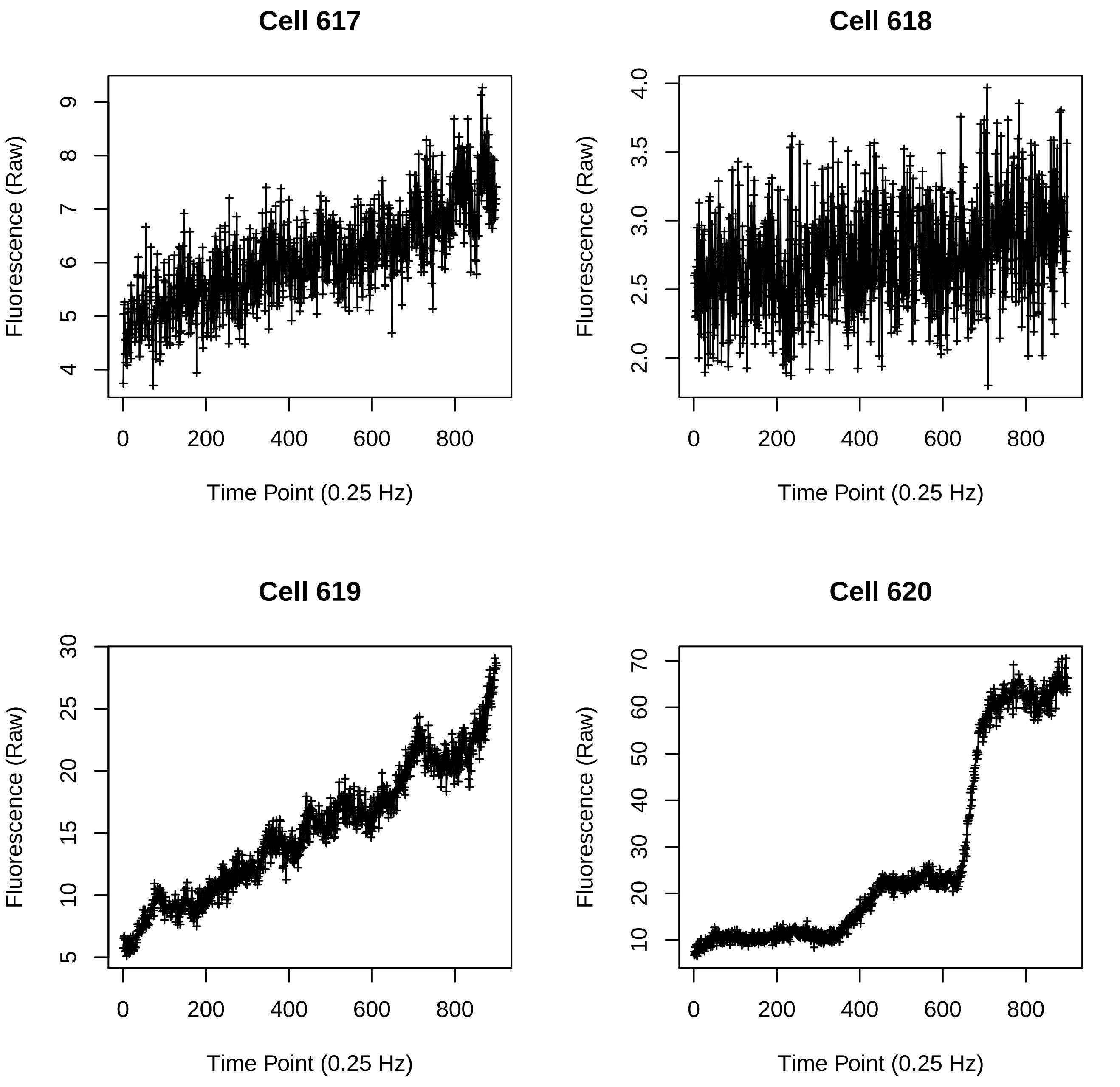
Cell 617
Cell 618
Cell 619
Cell 620
Fluorescence (Raw)
Time Point (0.25 Hz)
4
5
6
7
8
9
2.0
2.5
3.0
3.5
4.0
5
10
15
20
25
30
10
20
30
40
50
60
70
0
200
400
600
800

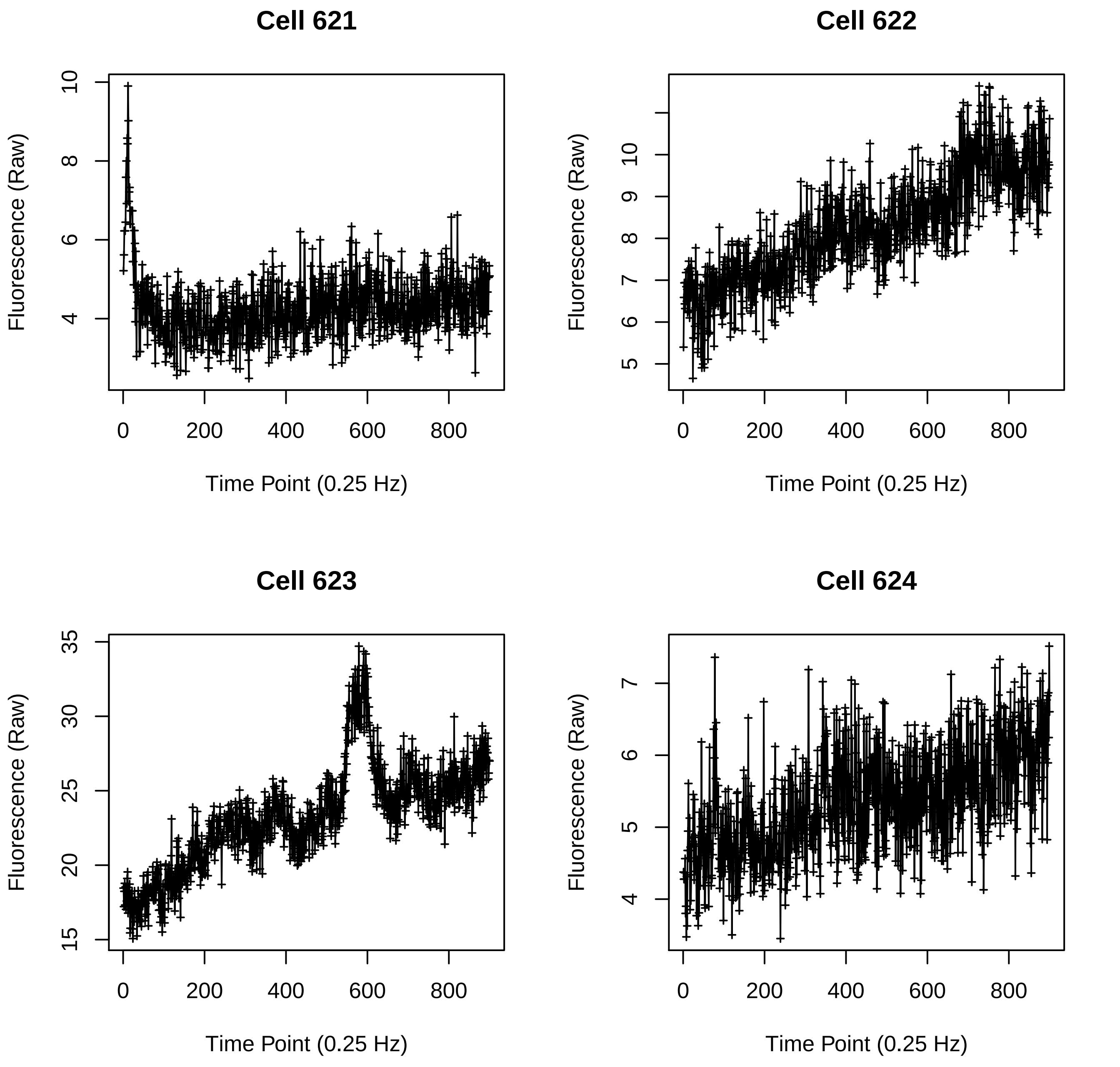
Cell 621
Cell 622
Cell 623
Cell 624
Fluorescence (Raw)
Time Point (0.25 Hz)
0
200
400
600
800
4
6
8
10
5
7
9
15
20
25
30
35

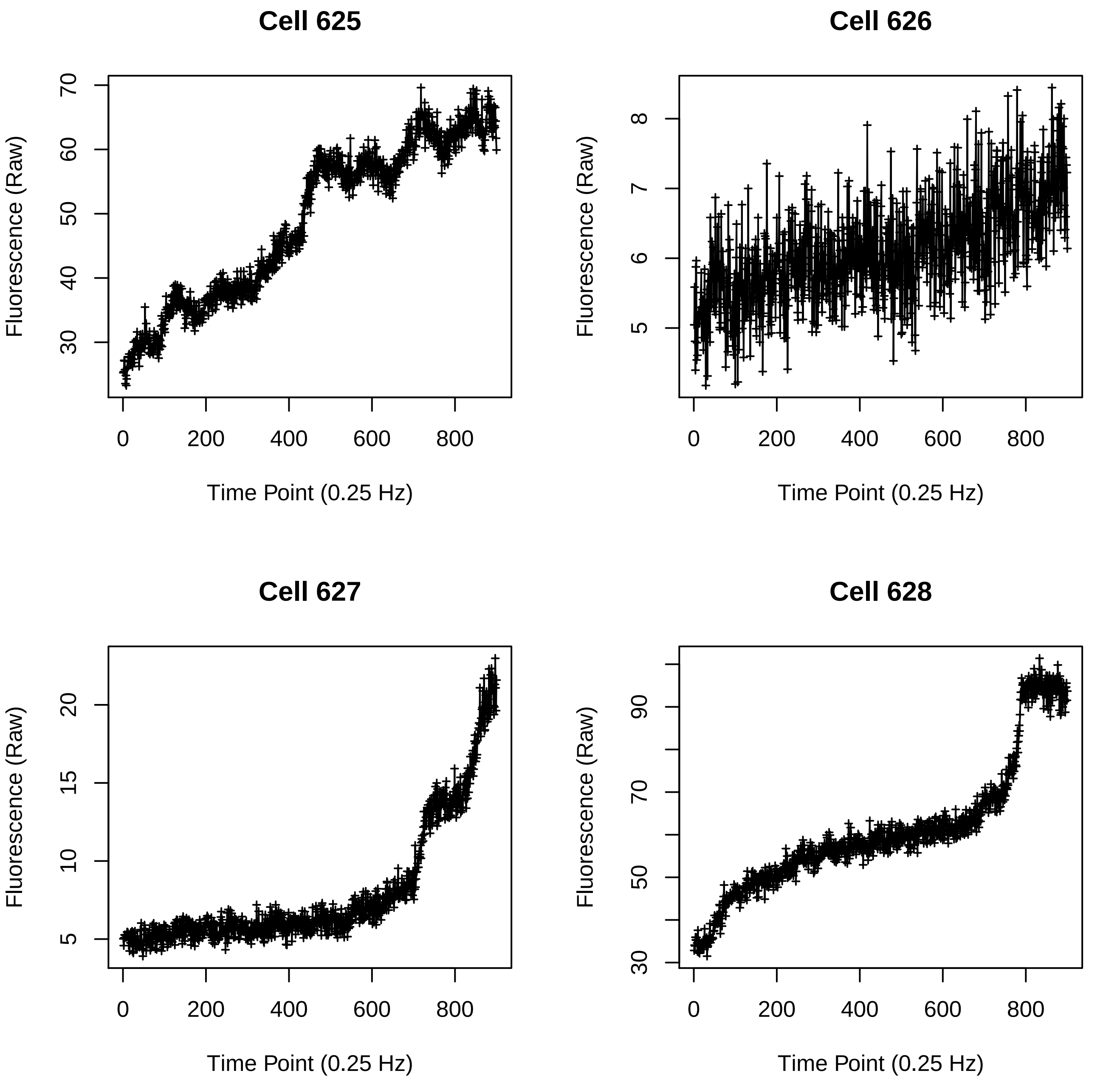
Cell 625
Fluorescence (Raw)
Time Point (0.25 Hz)
Cell 626
Fluorescence (Raw)
Time Point (0.25 Hz)
Cell 627
Fluorescence (Raw)
Time Point (0.25 Hz)
Cell 628
Fluorescence (Raw)
Time Point (0.25 Hz)

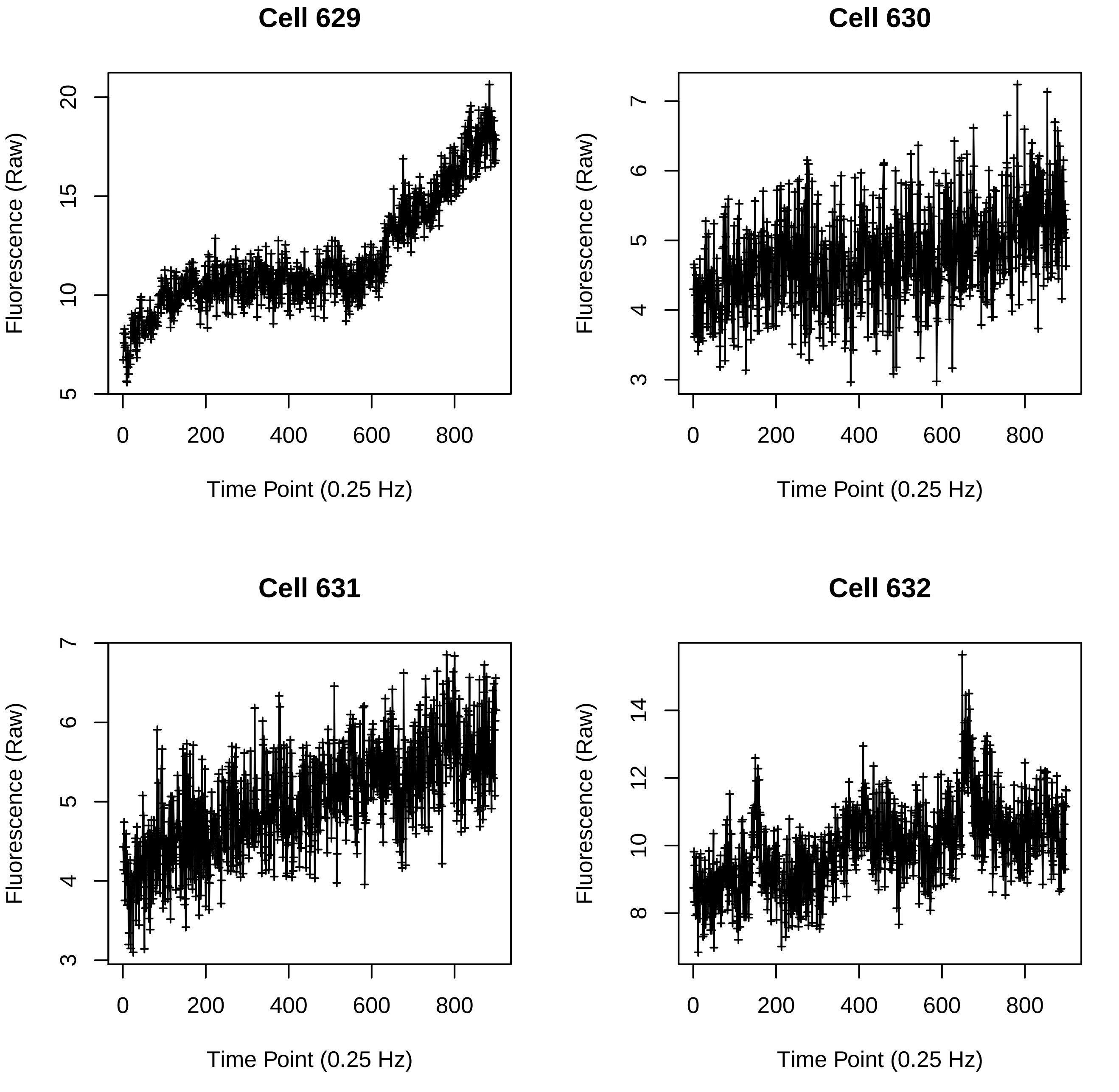
Cell 629
Fluorescence (Raw)
5
10
15
20
0
200
400
600
800
Time Point (0.25 Hz)
Cell 630
Fluorescence (Raw)
3
4
5
6
7
0
200
400
600
800
Time Point (0.25 Hz)
Cell 631
Fluorescence (Raw)
3
4
5
6
7
0
200
400
600
800
Time Point (0.25 Hz)
Cell 632
Fluorescence (Raw)
8
10
12
14
0
200
400
600
800
Time Point (0.25 Hz)

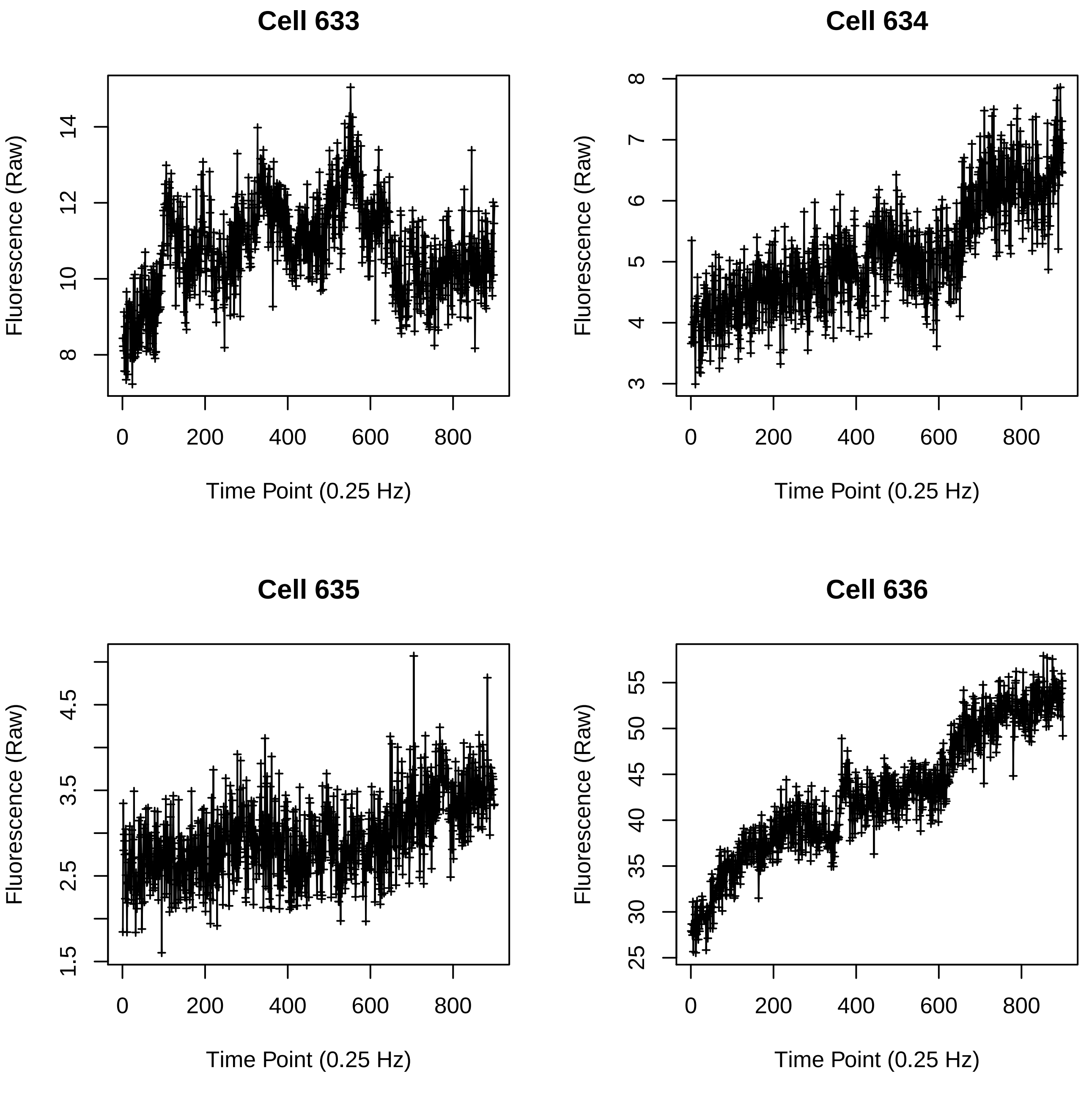
Cell 633
Cell 634
Cell 635
Cell 636
Fluorescence (Raw)
Time Point (0.25 Hz)
0
200
400
600
800
8
10
12
14
3
4
5
6
7
8
1.5
2.5
3.5
4.5
25
30
35
40
45
50
55

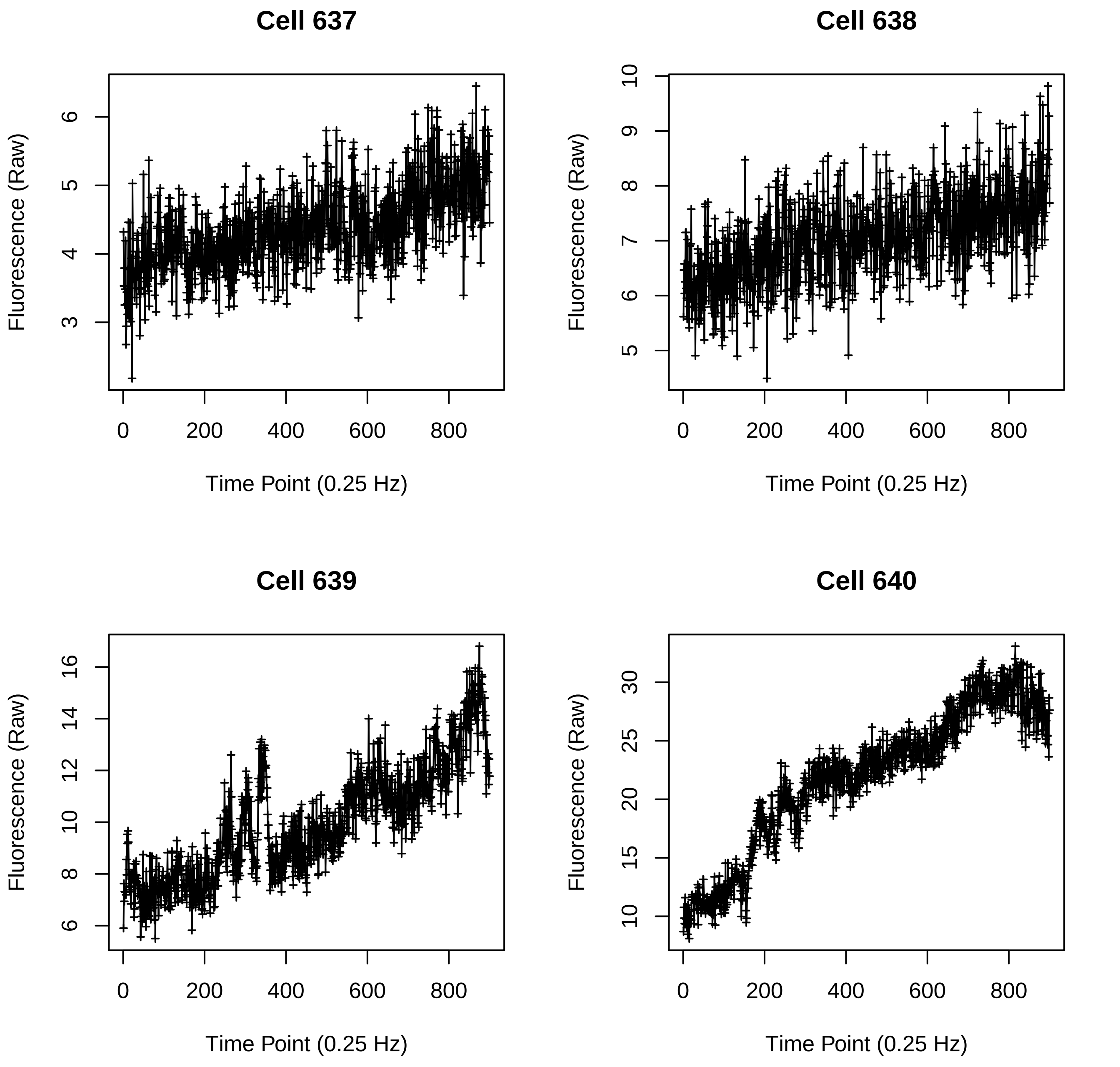
Cell 637
Cell 638
Cell 639
Cell 640
Fluorescence (Raw)
Time Point (0.25 Hz)
0
200
400
600
800

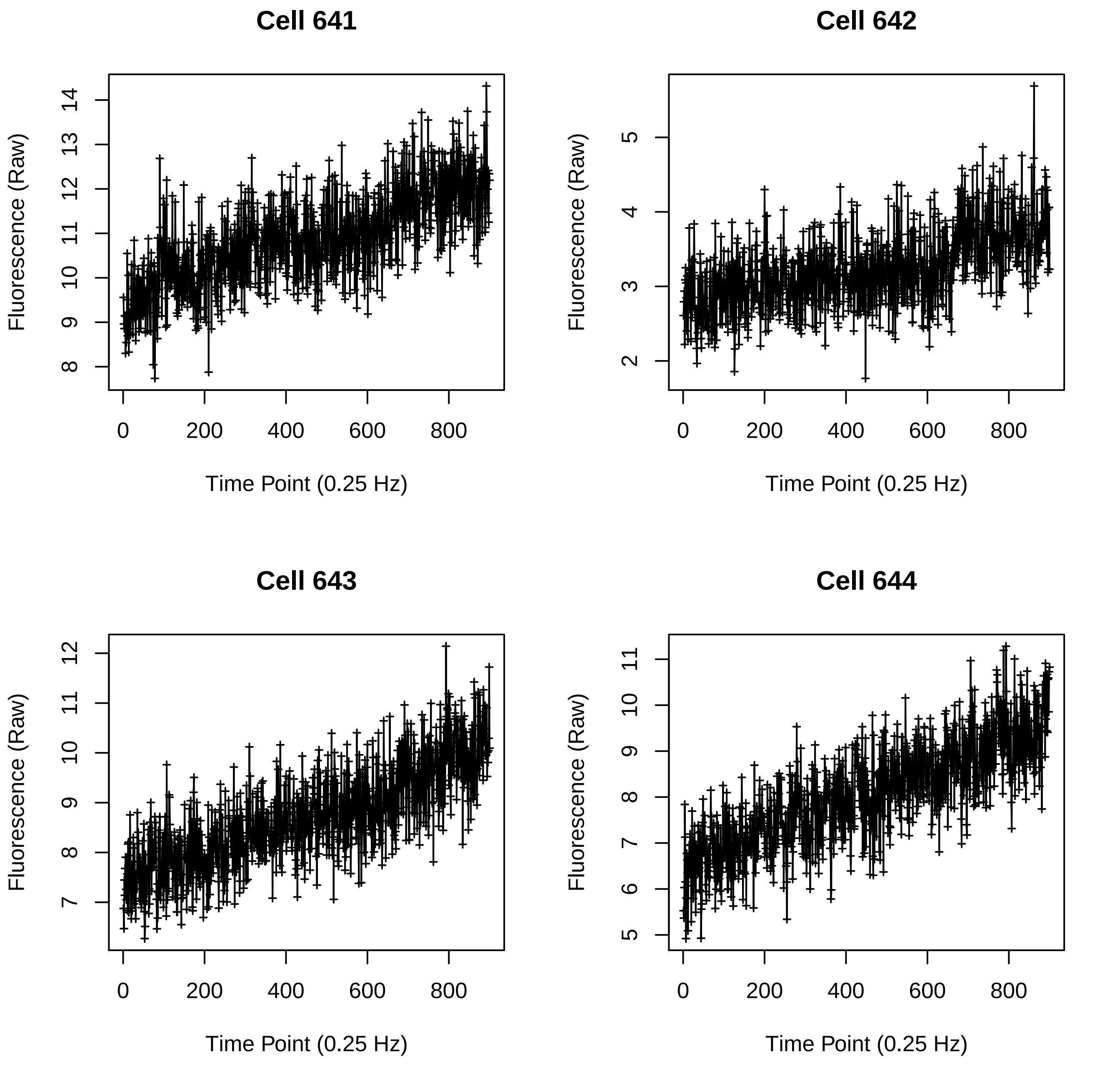

Cell 641
Fluorescence (Raw)
8
9
10
11
12
13
14
0
200
400
600
800
Time Point (0.25 Hz)
Cell 642
Fluorescence (Raw)
2
3
4
5
0
200
400
600
800
Time Point (0.25 Hz)
Cell 643
Fluorescence (Raw)
7
8
9
10
11
12
0
200
400
600
800
Time Point (0.25 Hz)
Cell 644
Fluorescence (Raw)
5
6
7
8
9
10
11
0
200
400
600
800
Time Point (0.25 Hz)

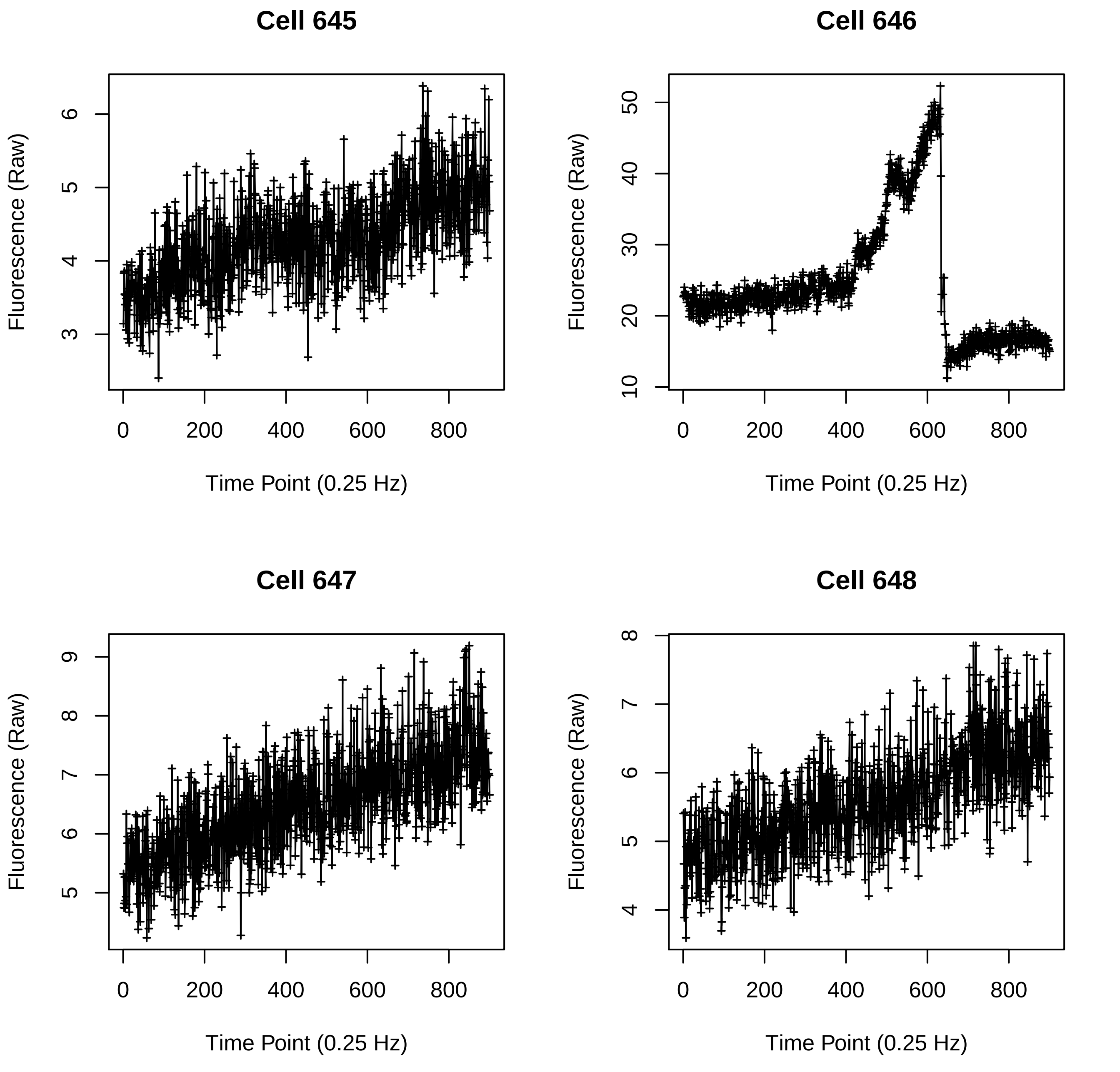
Cell 645
Cell 646
Cell 647
Cell 648
Fluorescence (Raw)
Time Point (0.25 Hz)
0
200
400
600
800
3
4
5
6
10
20
30
40
50
5
6
7
8
9
4
5
6
7
8

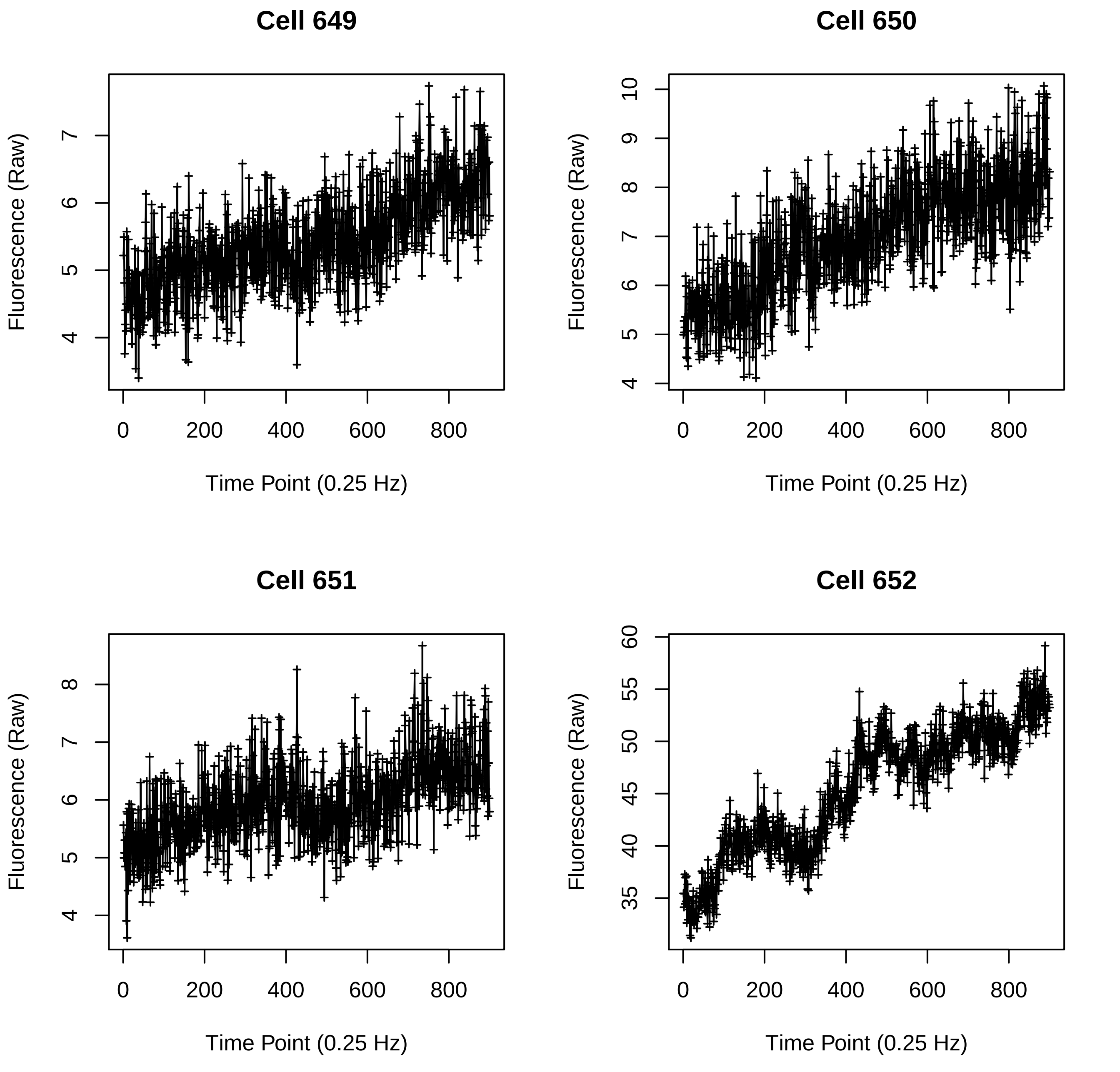
Cell 649
Fluorescence (Raw)
Time Point (0.25 Hz)
Cell 650
Fluorescence (Raw)
Time Point (0.25 Hz)
Cell 651
Fluorescence (Raw)
Time Point (0.25 Hz)
Cell 652
Fluorescence (Raw)
Time Point (0.25 Hz)

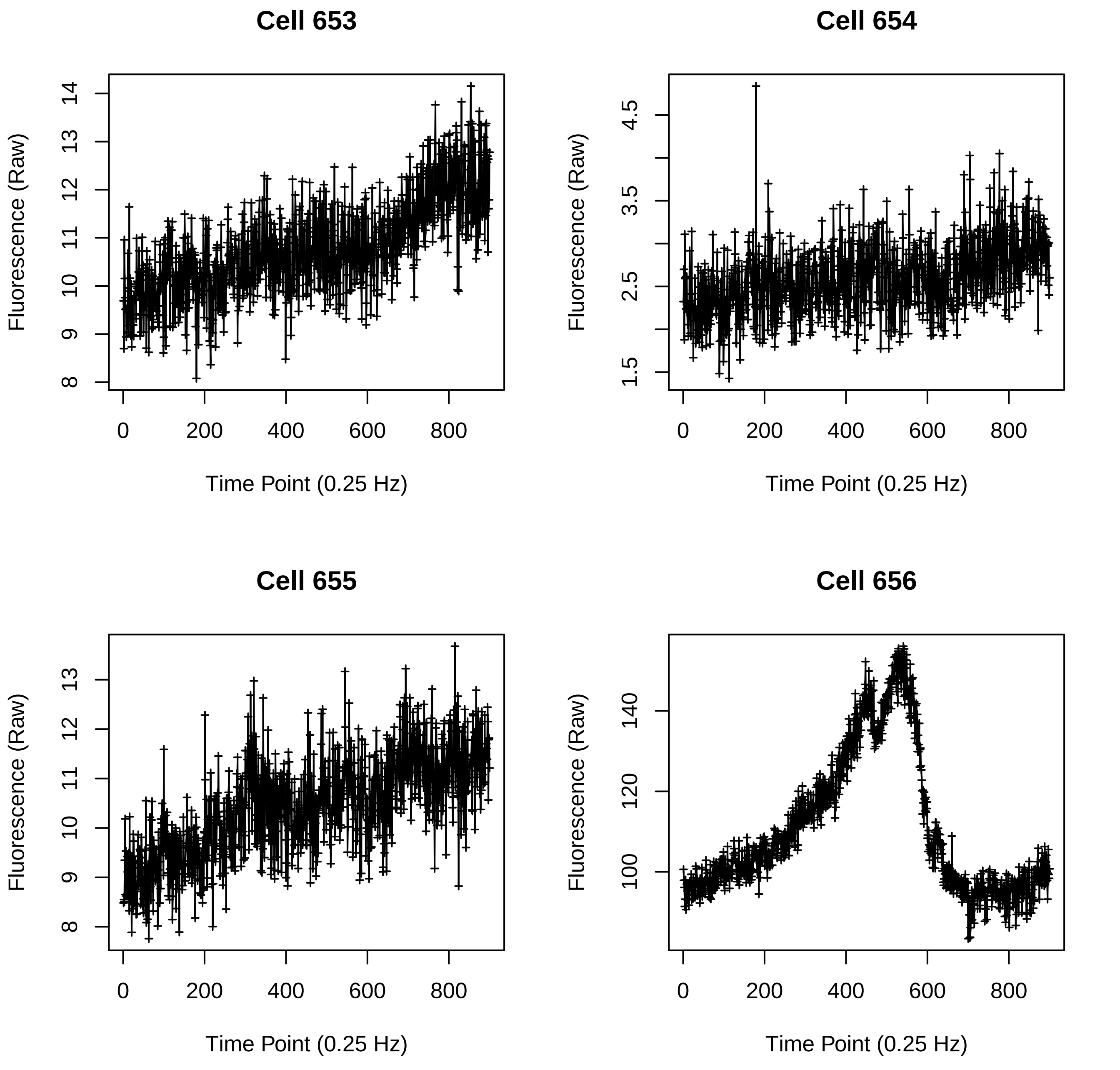
Cell 653
Fluorescence (Raw)
8
9
10
11
12
13
14
0
200
400
600
800
Time Point (0.25 Hz)
Cell 654
Fluorescence (Raw)
1.5
2.5
3.5
4.5
0
200
400
600
800
Time Point (0.25 Hz)
Cell 655
Fluorescence (Raw)
8
9
10
11
12
13
0
200
400
600
800
Time Point (0.25 Hz)
Cell 656
Fluorescence (Raw)
100
120
140
0
200
400
600
800
Time Point (0.25 Hz)

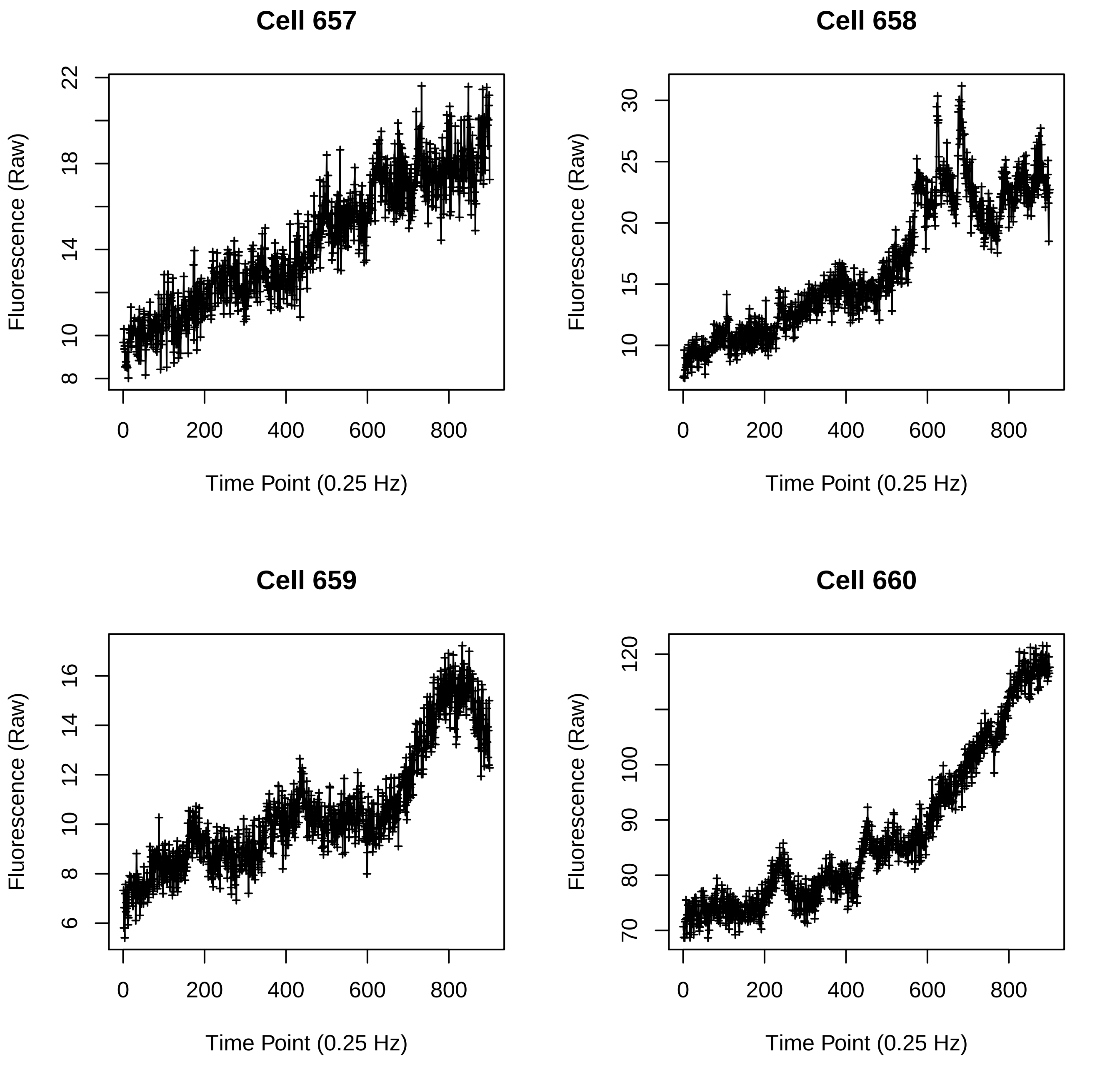
Cell 657
Cell 658
Cell 659
Cell 660
Fluorescence (Raw)
Time Point (0.25 Hz)
0
200
400
600
800
8
10
14
18
22
10
15
20
25
30
6
8
10
12
14
16
70
80
90
100
120

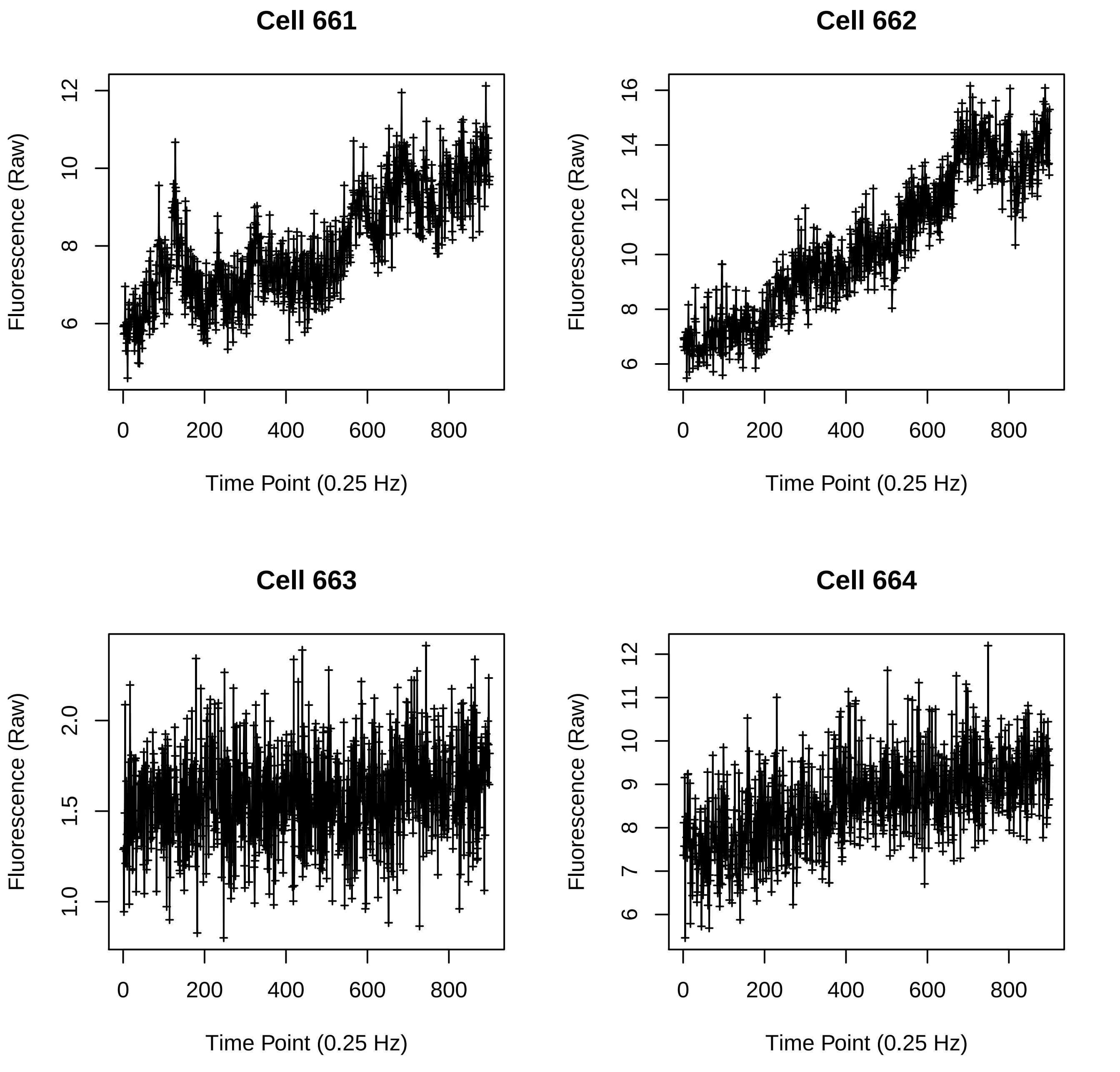
Cell 661
Cell 662
Cell 663
Cell 664
Fluorescence (Raw)
Time Point (0.25 Hz)
0
200
400
600
800
6
8
10
12
14
16
1.0
1.5
2.0
7
9
11

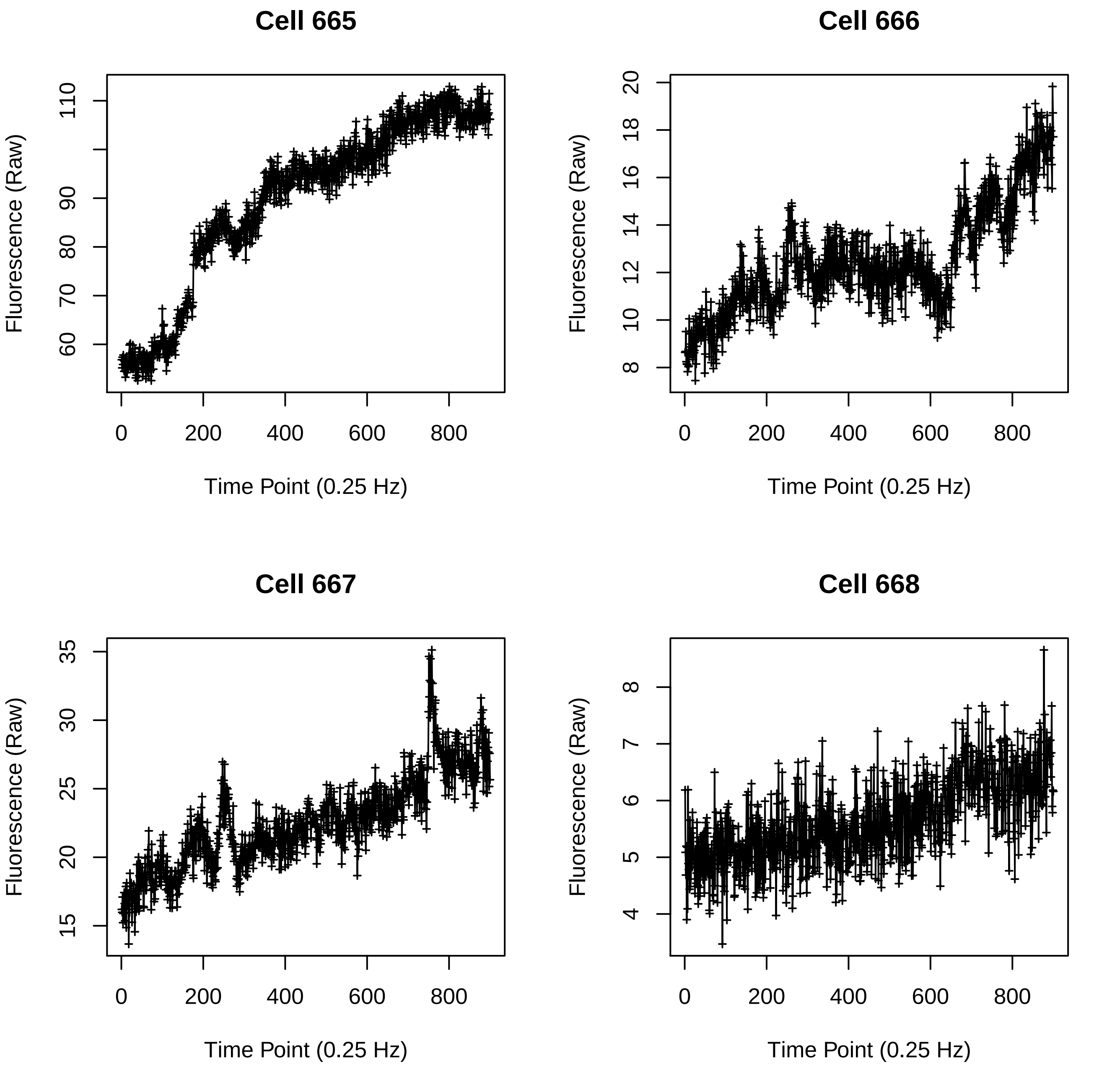
Cell 665
Fluorescence (Raw)
60
70
80
90
110
0
200
400
600
800
Time Point (0.25 Hz)
Cell 666
Fluorescence (Raw)
8
10
12
14
16
18
20
0
200
400
600
800
Time Point (0.25 Hz)
Cell 667
Fluorescence (Raw)
15
20
25
30
35
0
200
400
600
800
Time Point (0.25 Hz)
Cell 668
Fluorescence (Raw)
4
5
6
7
8
0
200
400
600
800
Time Point (0.25 Hz)

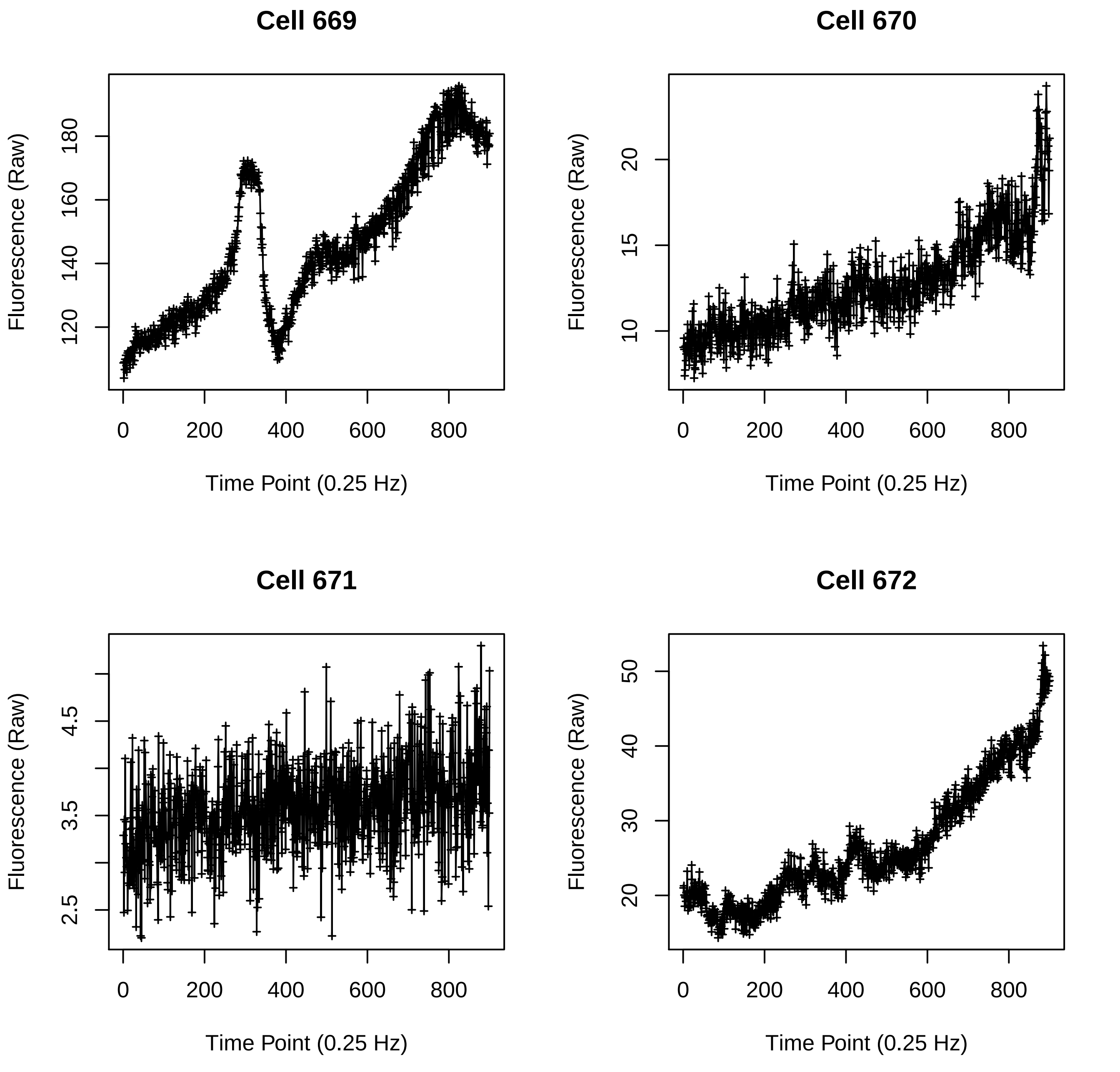
Cell 669
Fluorescence (Raw)
120
140
160
180
0
200
400
600
800
Time Point (0.25 Hz)
Cell 670
Fluorescence (Raw)
10
15
20
0
200
400
600
800
Time Point (0.25 Hz)
Cell 671
Fluorescence (Raw)
2.5
3.5
4.5
0
200
400
600
800
Time Point (0.25 Hz)
Cell 672
Fluorescence (Raw)
20
30
40
50
0
200
400
600
800
Time Point (0.25 Hz)

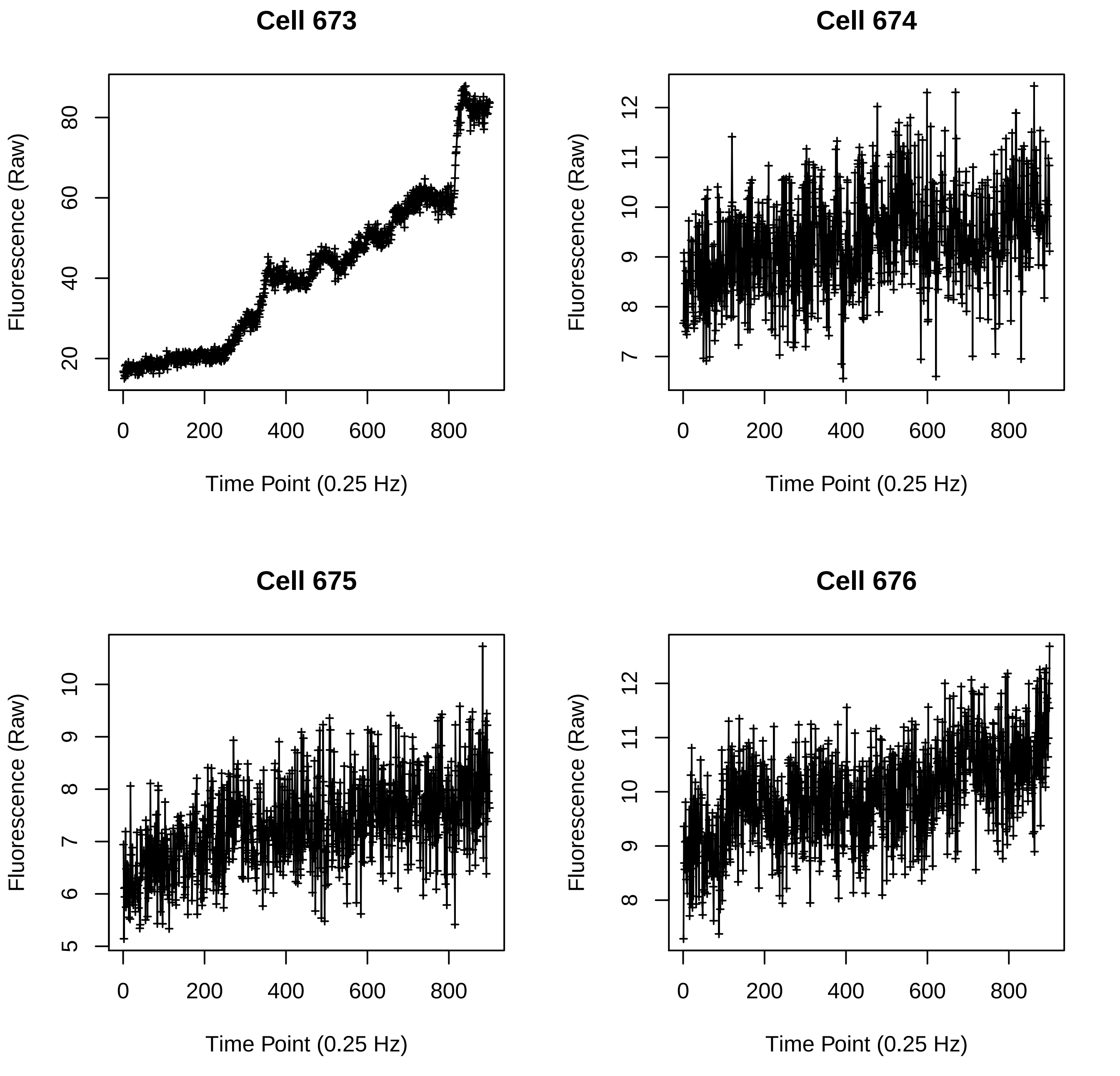
Cell 673
Cell 674
Cell 675
Cell 676
Fluorescence (Raw)
Time Point (0.25 Hz)
0
200
400
600
800
20
40
60
80
7
8
9
10
11
12
5
6

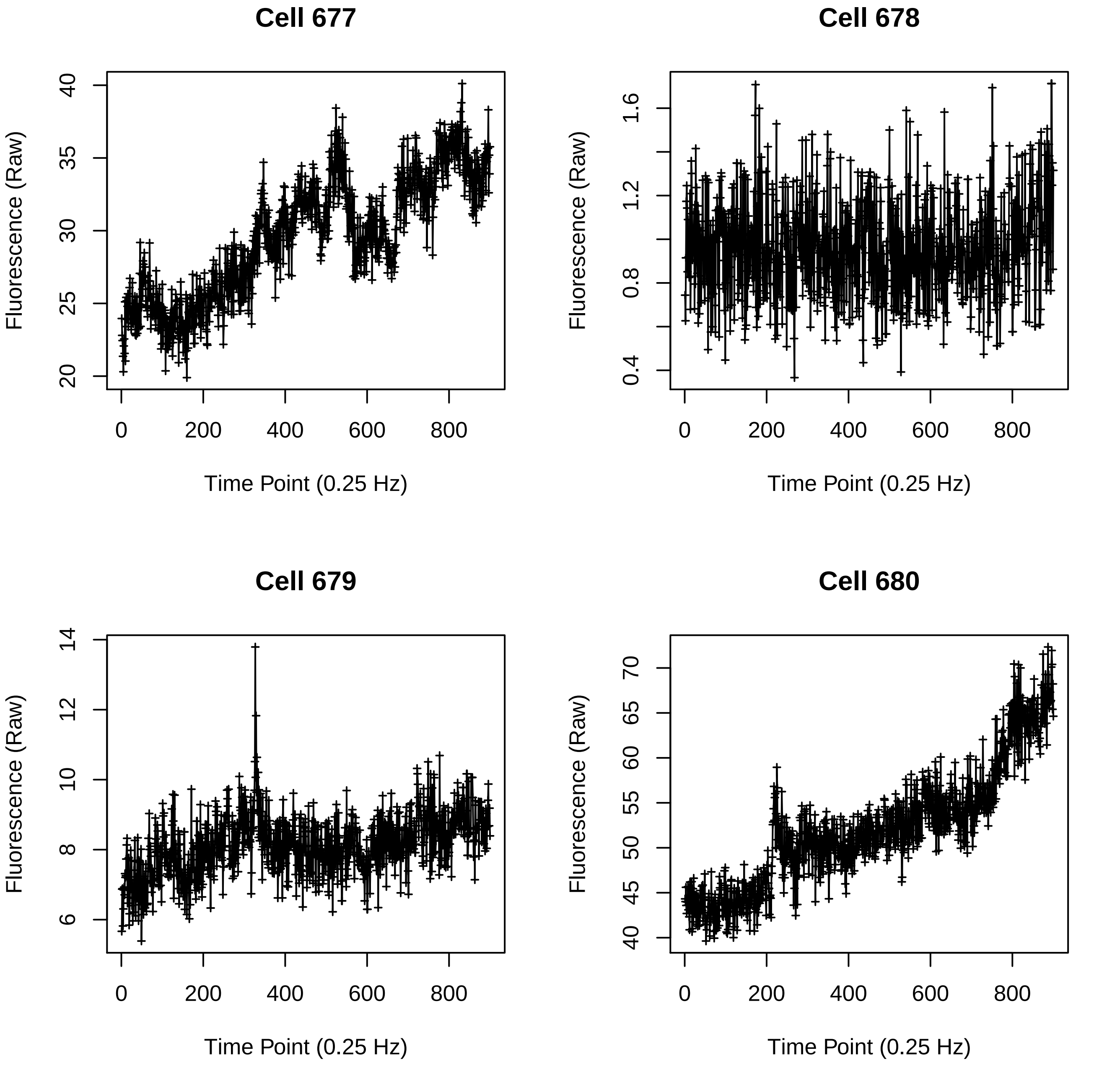
Cell 677
Cell 678
Cell 679
Cell 680
Fluorescence (Raw)
Time Point (0.25 Hz)
20
25
30
35
40
0.4
0.8
1.2
1.6
6
8
10
12
14
40
45
50
55
60
65
70
0
200
400
600
800

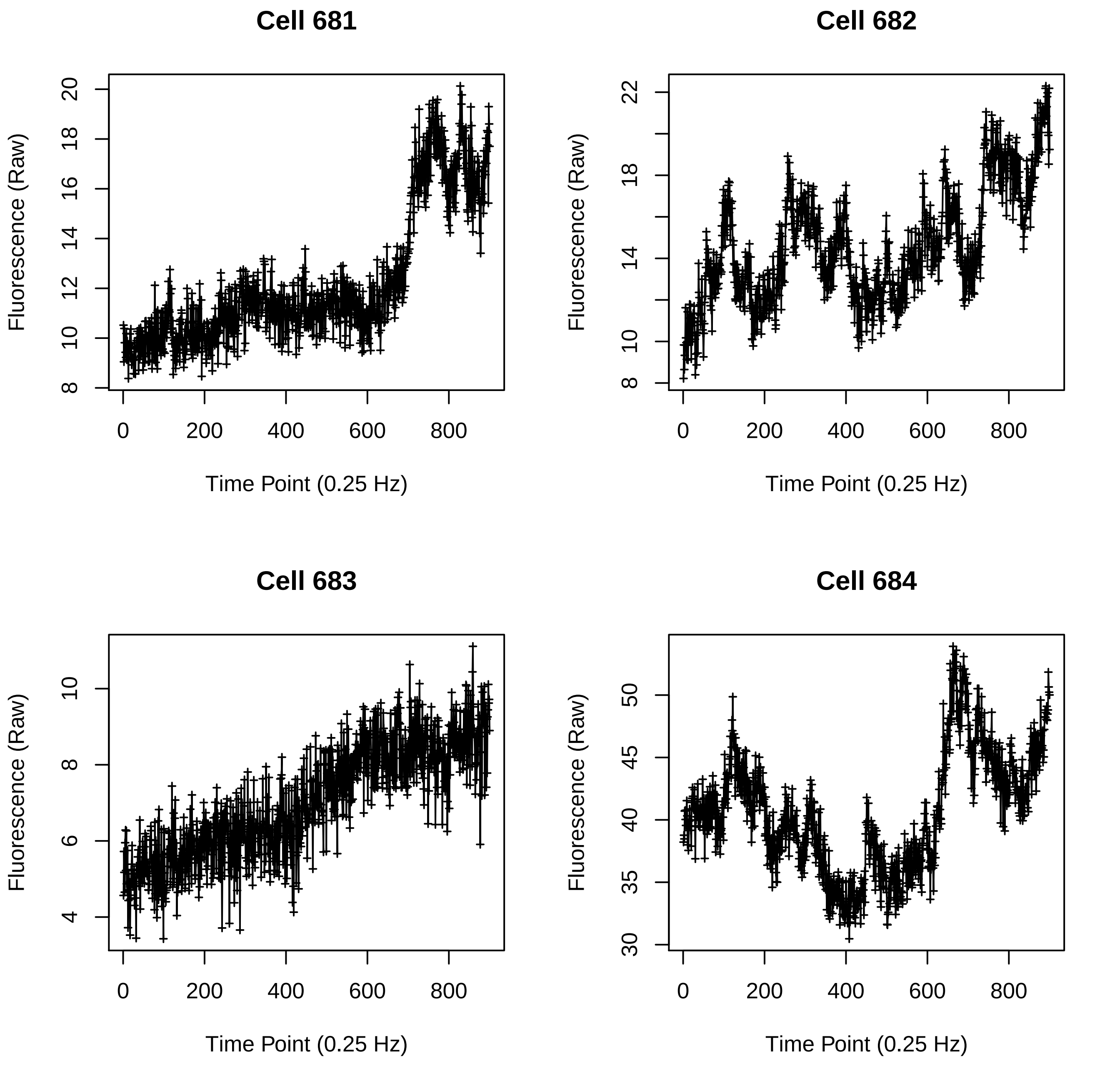
Cell 681
Cell 682
Cell 683
Cell 684
Fluorescence (Raw)
Time Point (0.25 Hz)
0
200
400
600
800
8
10
12
14
16
18
20
22
4
6
30
35
40
45
50

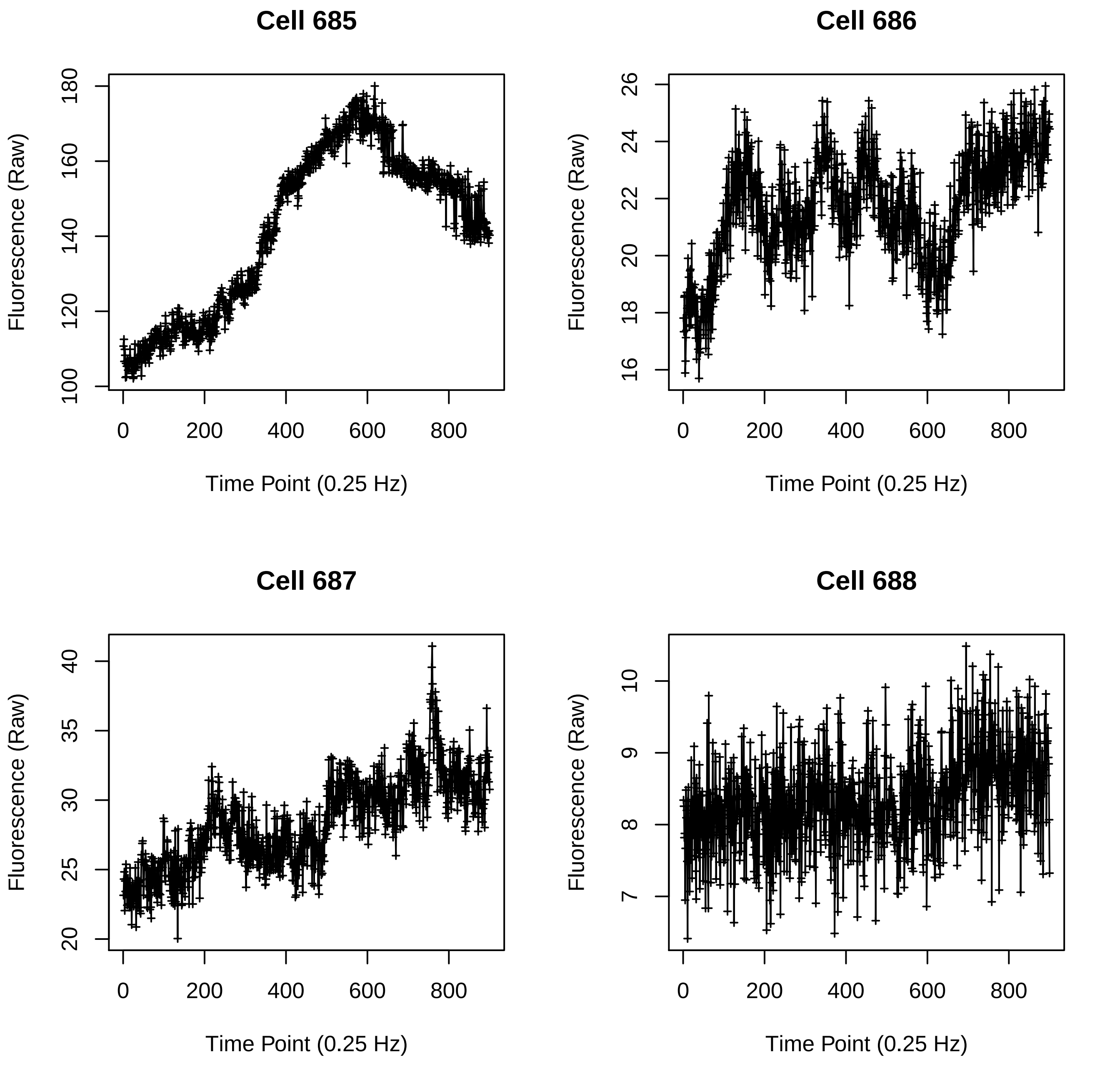
Cell 685
Fluorescence (Raw)
100
120
140
160
180
0
200
400
600
800
Time Point (0.25 Hz)
Cell 686
Fluorescence (Raw)
16
18
20
22
24
26
0
200
400
600
800
Time Point (0.25 Hz)
Cell 687
Fluorescence (Raw)
20
25
30
35
40
0
200
400
600
800
Time Point (0.25 Hz)
Cell 688
Fluorescence (Raw)
7
8
9
10
0
200
400
600
800
Time Point (0.25 Hz)

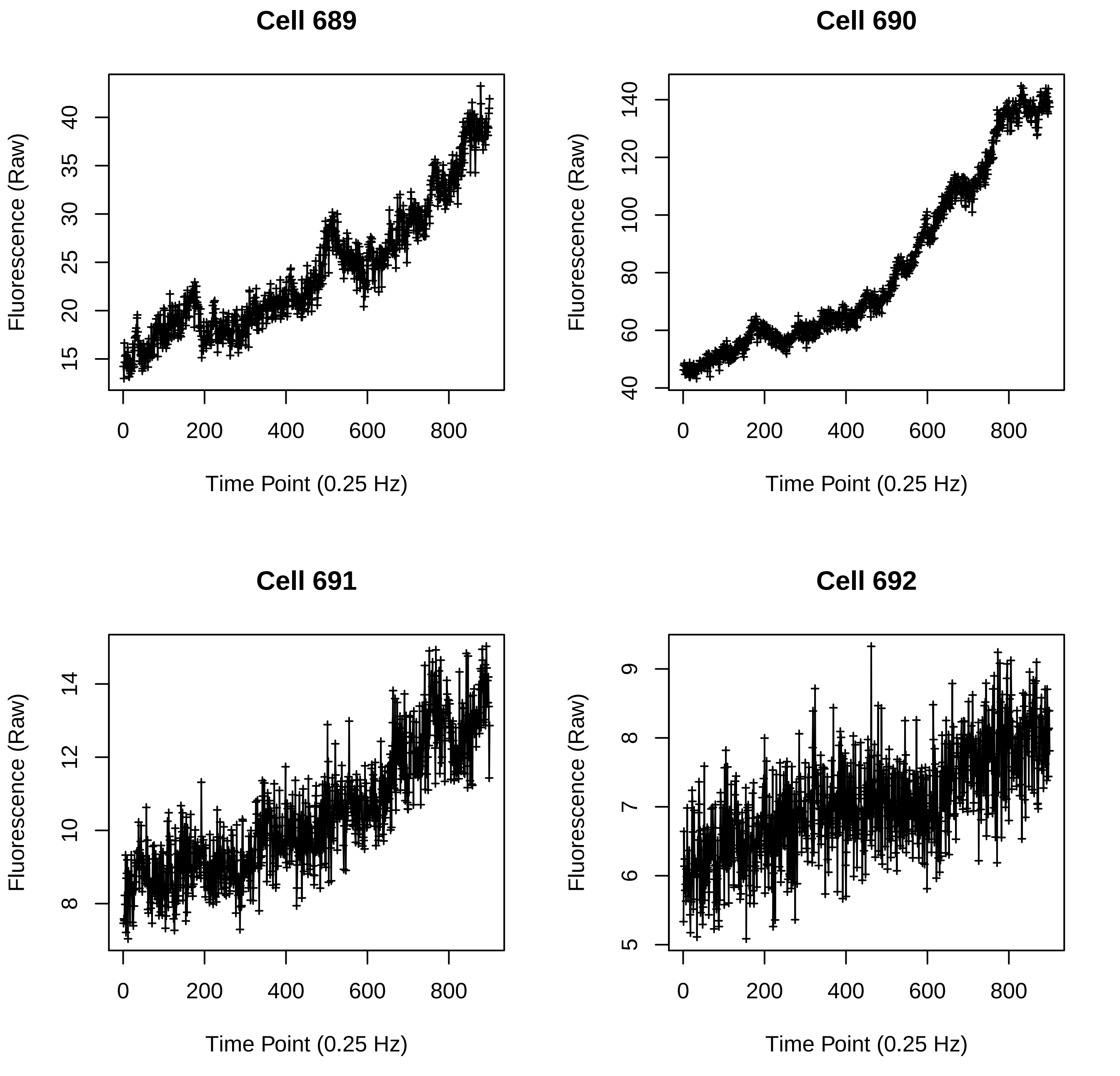
Cell 689
Fluorescence (Raw)
15
20
25
30
35
40
0
200
400
600
800
Time Point (0.25 Hz)
Cell 690
Fluorescence (Raw)
40
60
80
100
120
140
0
200
400
600
800
Time Point (0.25 Hz)
Cell 691
Fluorescence (Raw)
8
10
12
14
0
200
400
600
800
Time Point (0.25 Hz)
Cell 692
Fluorescence (Raw)
5
6
7
8
9
0
200
400
600
800
Time Point (0.25 Hz)

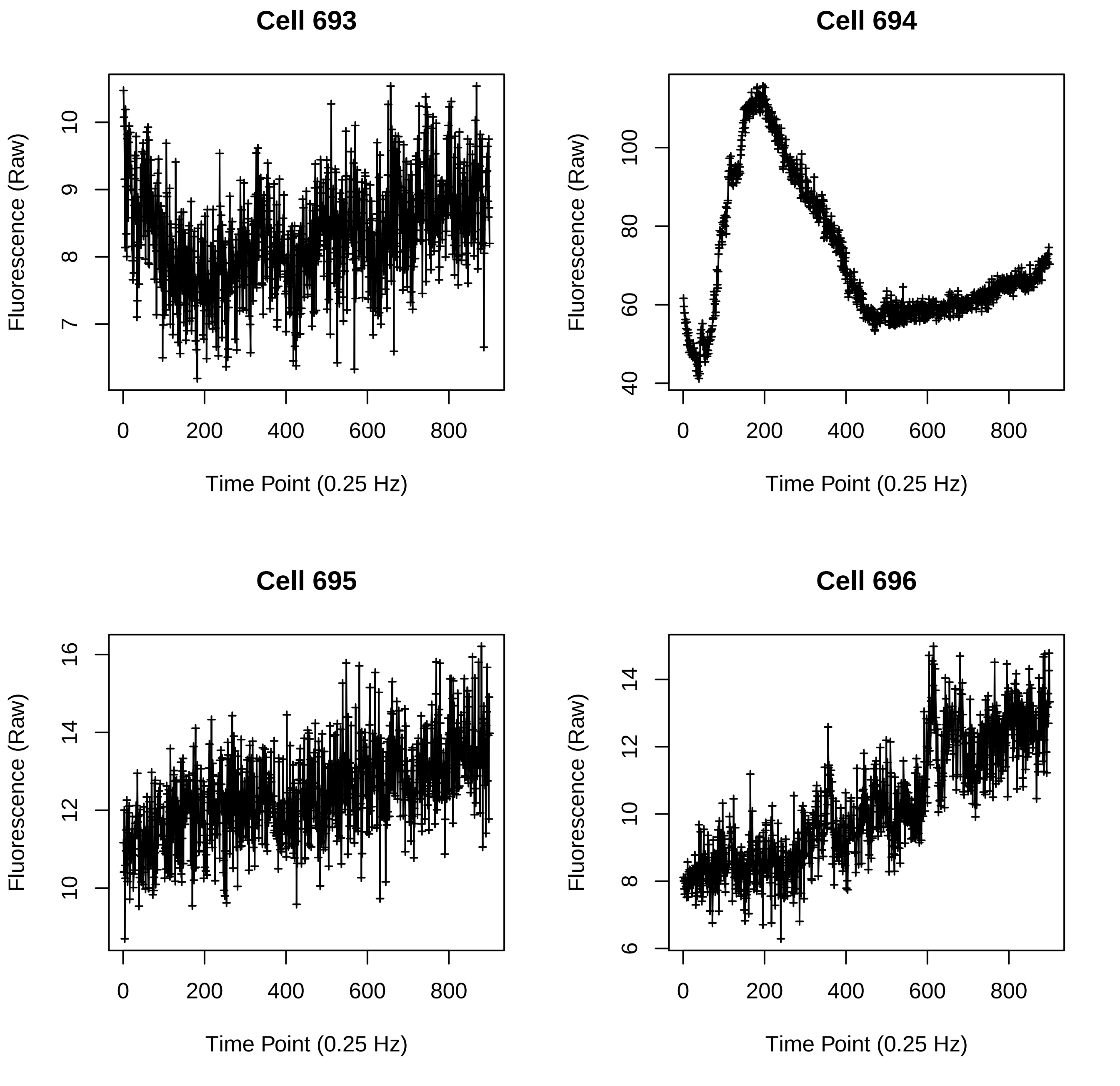

Cell 693
Cell 694
Cell 695
Cell 696
Fluorescence (Raw)
Time Point (0.25 Hz)
0
200
400
600
800

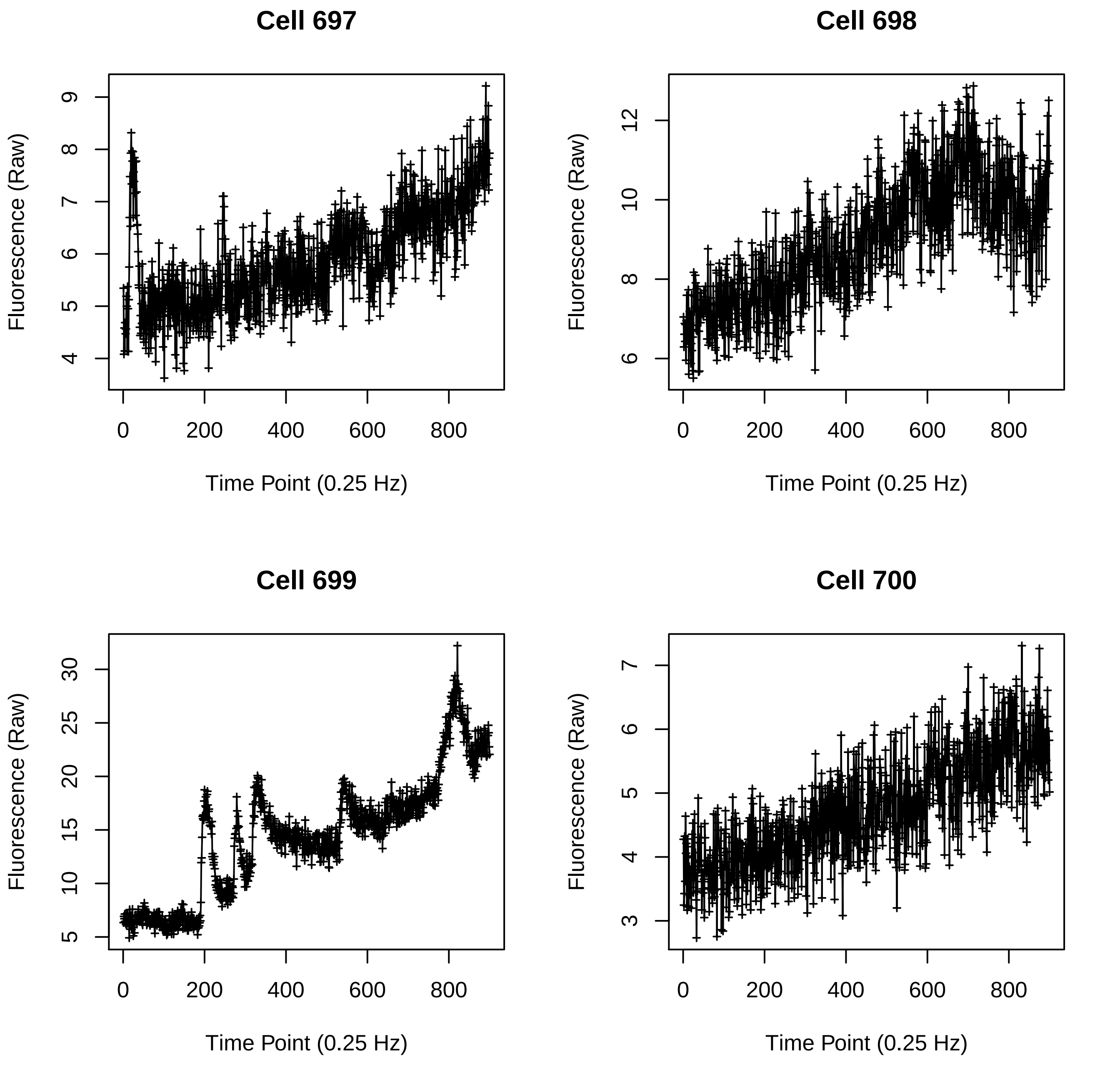
Cell 697
Cell 698
Cell 699
Cell 700
Fluorescence (Raw)
Time Point (0.25 Hz)
0
200
400
600
800

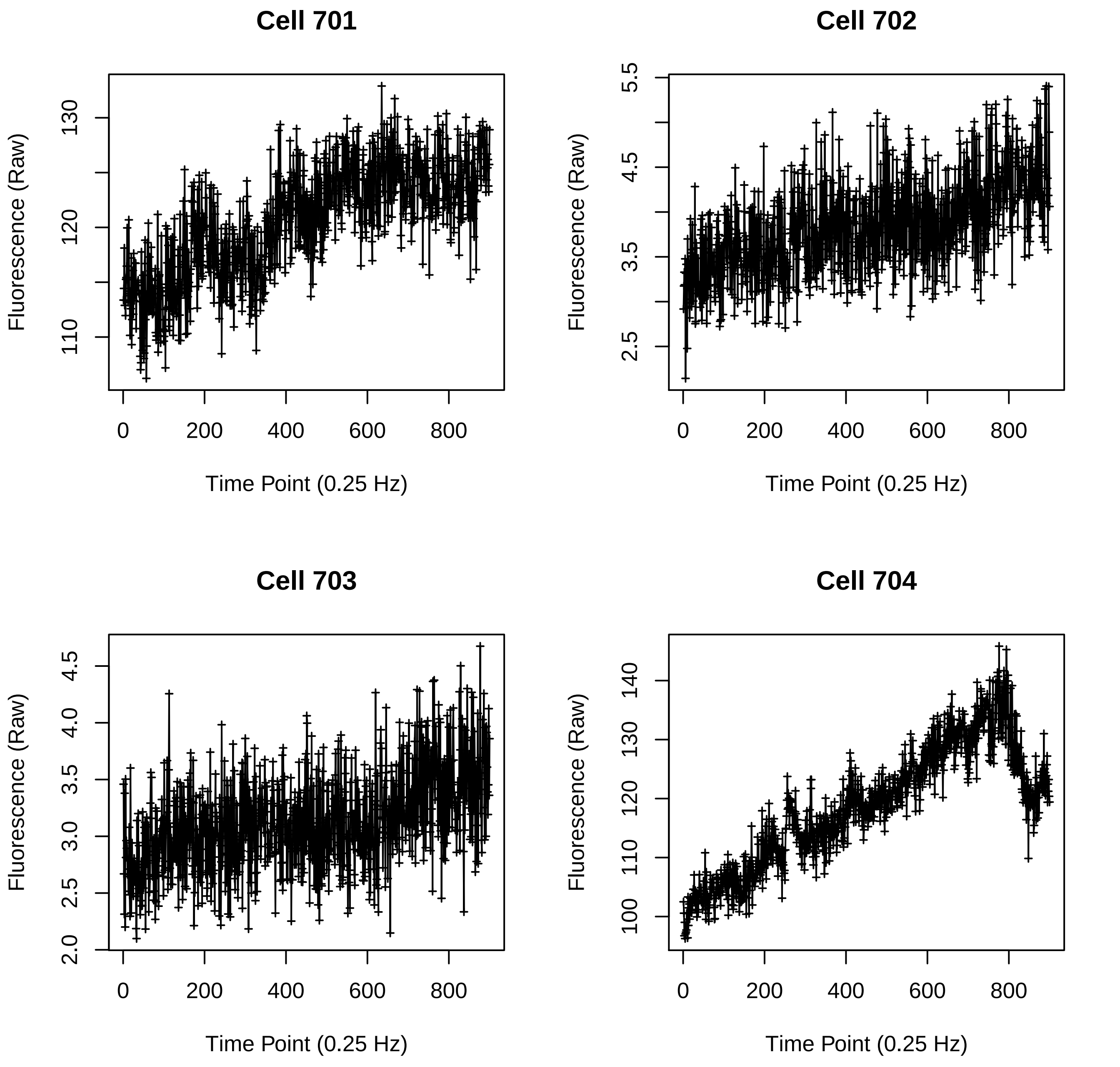
Cell 701
Cell 702
Cell 703
Cell 704
Fluorescence (Raw)
Time Point (0.25 Hz)
0
200
400
600
800
110
120
130
2.5
3.5
4.5
5.5
2.0
3.0
4.0
100
140

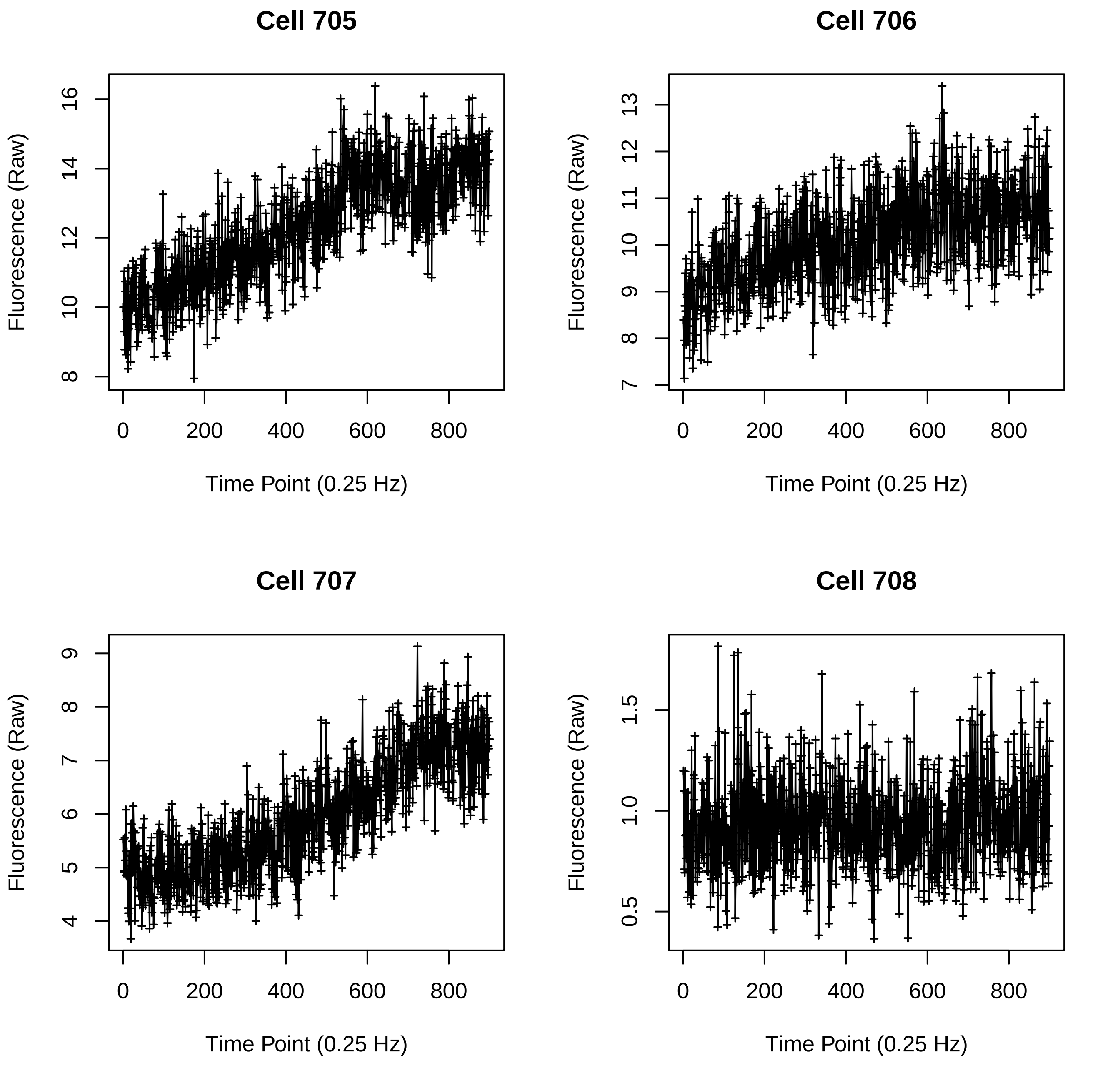
Cell 705
Cell 706
Cell 707
Cell 708
Fluorescence (Raw)
Time Point (0.25 Hz)
0
200
400
600
800
8
10
12
14
16
7
8
9
10
11
12
13
4
5
6
7
8
9
0.5
1.0
1.5

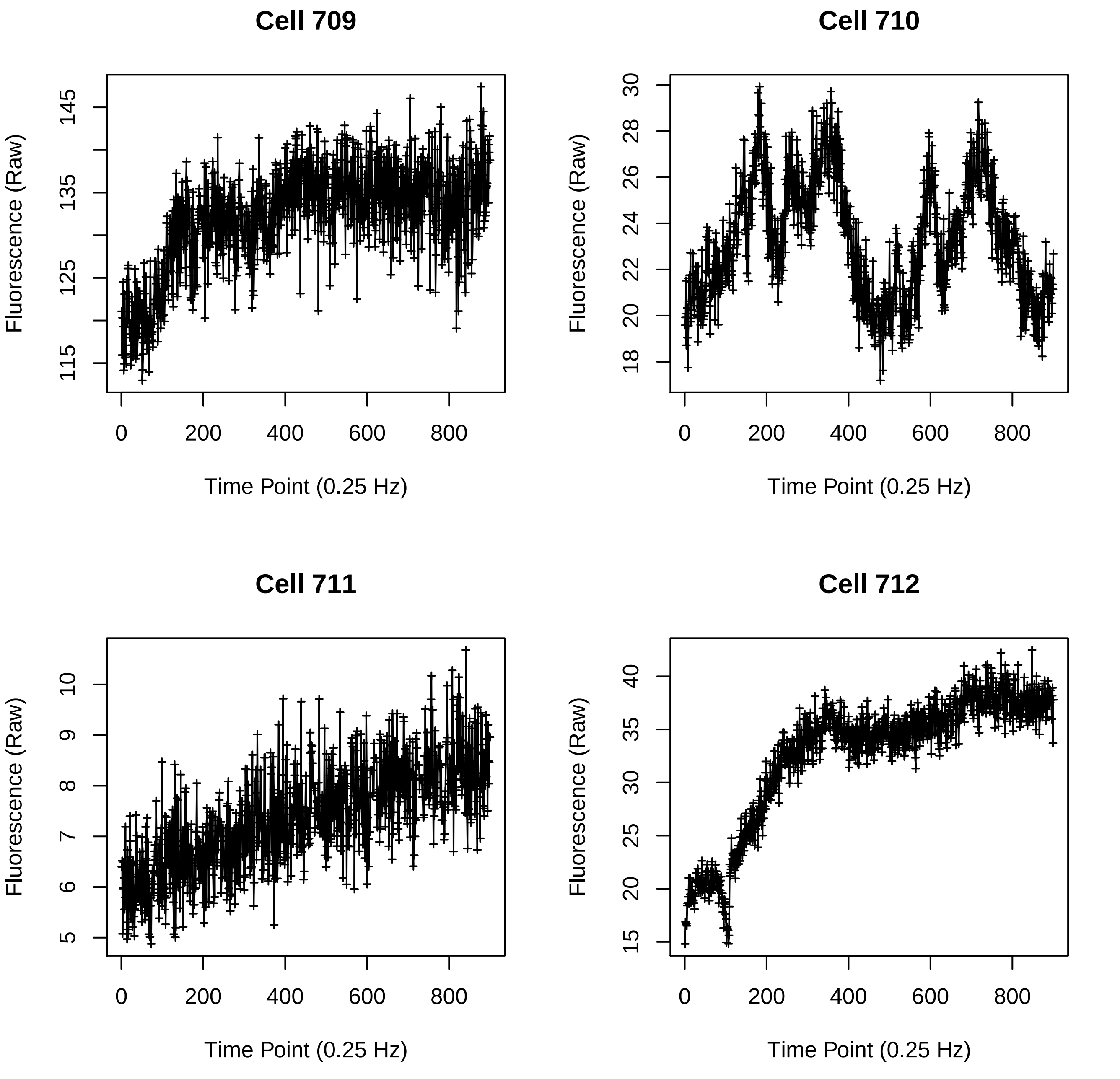

Cell 709
Fluorescence (Raw)
115
125
135
145
0
200
400
600
800
Time Point (0.25 Hz)
Cell 710
Fluorescence (Raw)
18
20
22
24
26
28
30
0
200
400
600
800
Time Point (0.25 Hz)
Cell 711
Fluorescence (Raw)
5
6
7
8
9
10
0
200
400
600
800
Time Point (0.25 Hz)
Cell 712
Fluorescence (Raw)
15
20
25
30
35
40
0
200
400
600
800
Time Point (0.25 Hz)

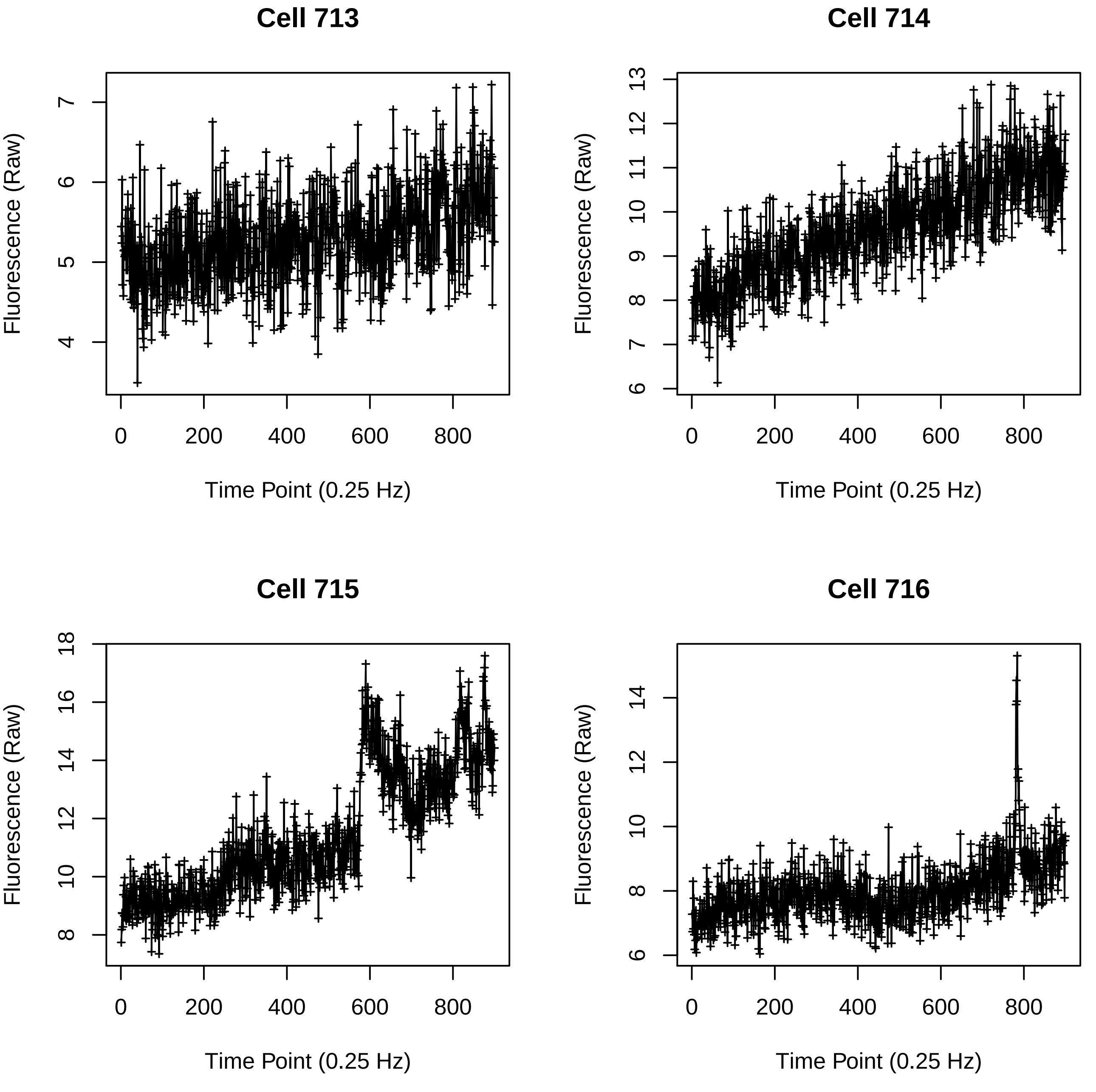
Cell 713
Fluorescence (Raw)
4
5
6
7
0
200
400
600
800
Time Point (0.25 Hz)
Cell 714
Fluorescence (Raw)
6
7
8
9
10
11
12
13
0
200
400
600
800
Time Point (0.25 Hz)
Cell 715
Fluorescence (Raw)
8
10
12
14
16
18
0
200
400
600
800
Time Point (0.25 Hz)
Cell 716
Fluorescence (Raw)
6
8
10
12
14
0
200
400
600
800
Time Point (0.25 Hz)

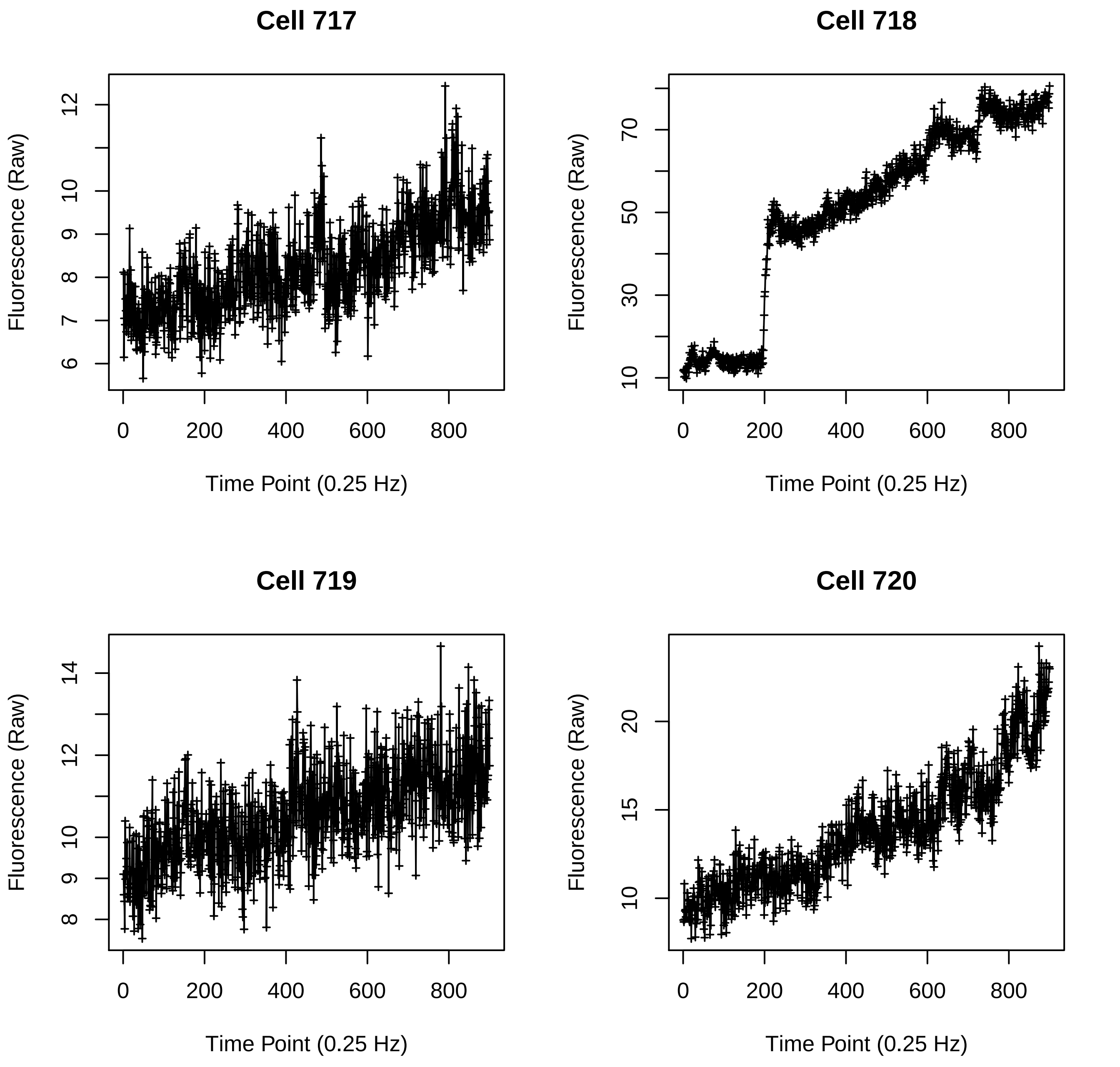
Cell 717
Cell 718
Cell 719
Cell 720
Fluorescence (Raw)
Time Point (0.25 Hz)
0
200
400
600
800
6
7
8
9
10
12
10
30
50
70
8
9
10
12
14
10
15
20

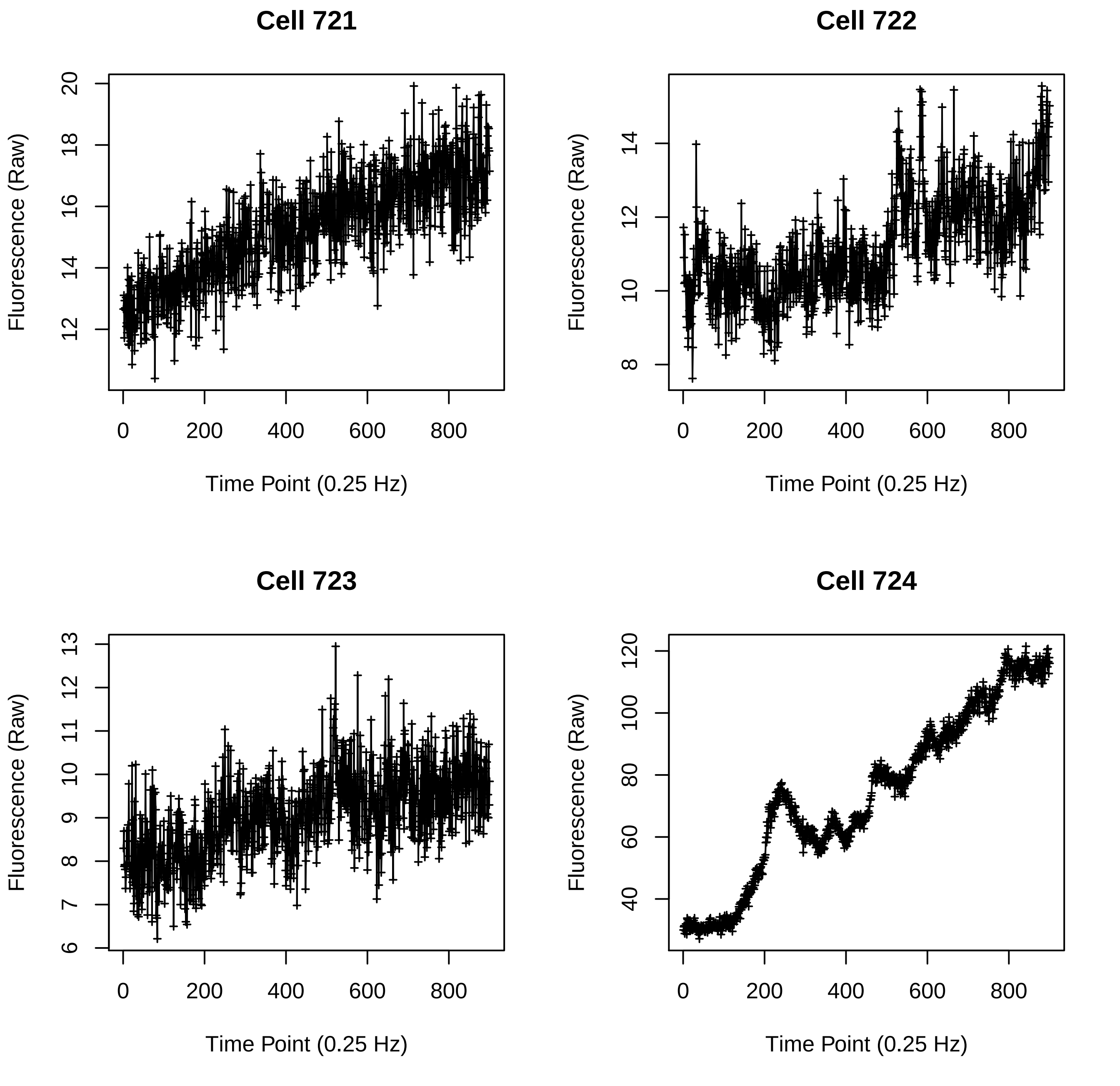
Cell 721
Fluorescence (Raw)
12
14
16
18
20
0
200
400
600
800
Time Point (0.25 Hz)
Cell 722
Fluorescence (Raw)
8
10
12
14
0
200
400
600
800
Time Point (0.25 Hz)
Cell 723
Fluorescence (Raw)
6
7
8
9
10
11
12
13
0
200
400
600
800
Time Point (0.25 Hz)
Cell 724
Fluorescence (Raw)
40
60
80
100
120
0
200
400
600
800
Time Point (0.25 Hz)

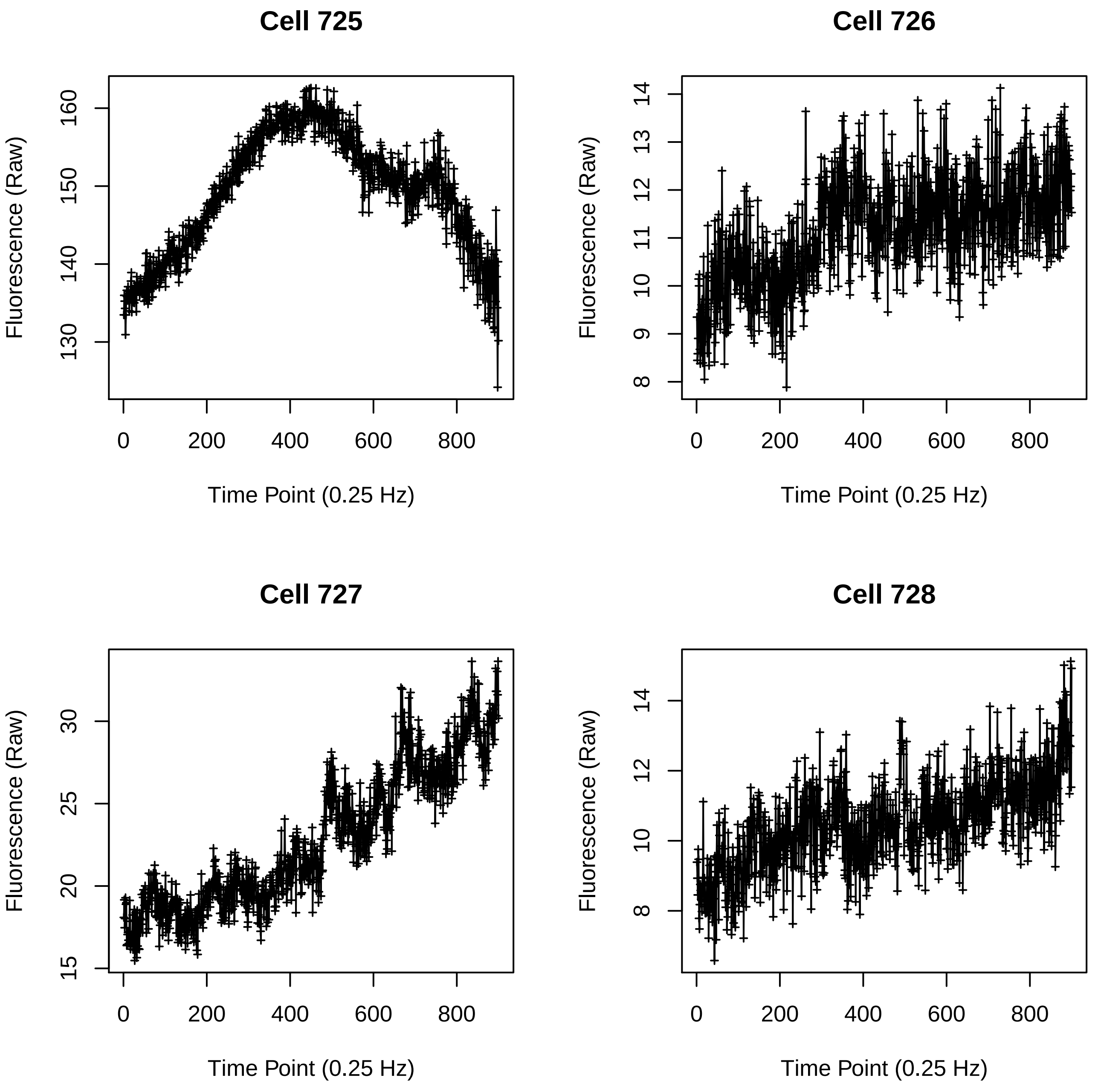

Cell 725
Cell 726
Cell 727
Cell 728
Fluorescence (Raw)
Time Point (0.25 Hz)
0
200
400
600
800
130
140
150
160
8
9
10
11
12
13
14
15
20
25
30

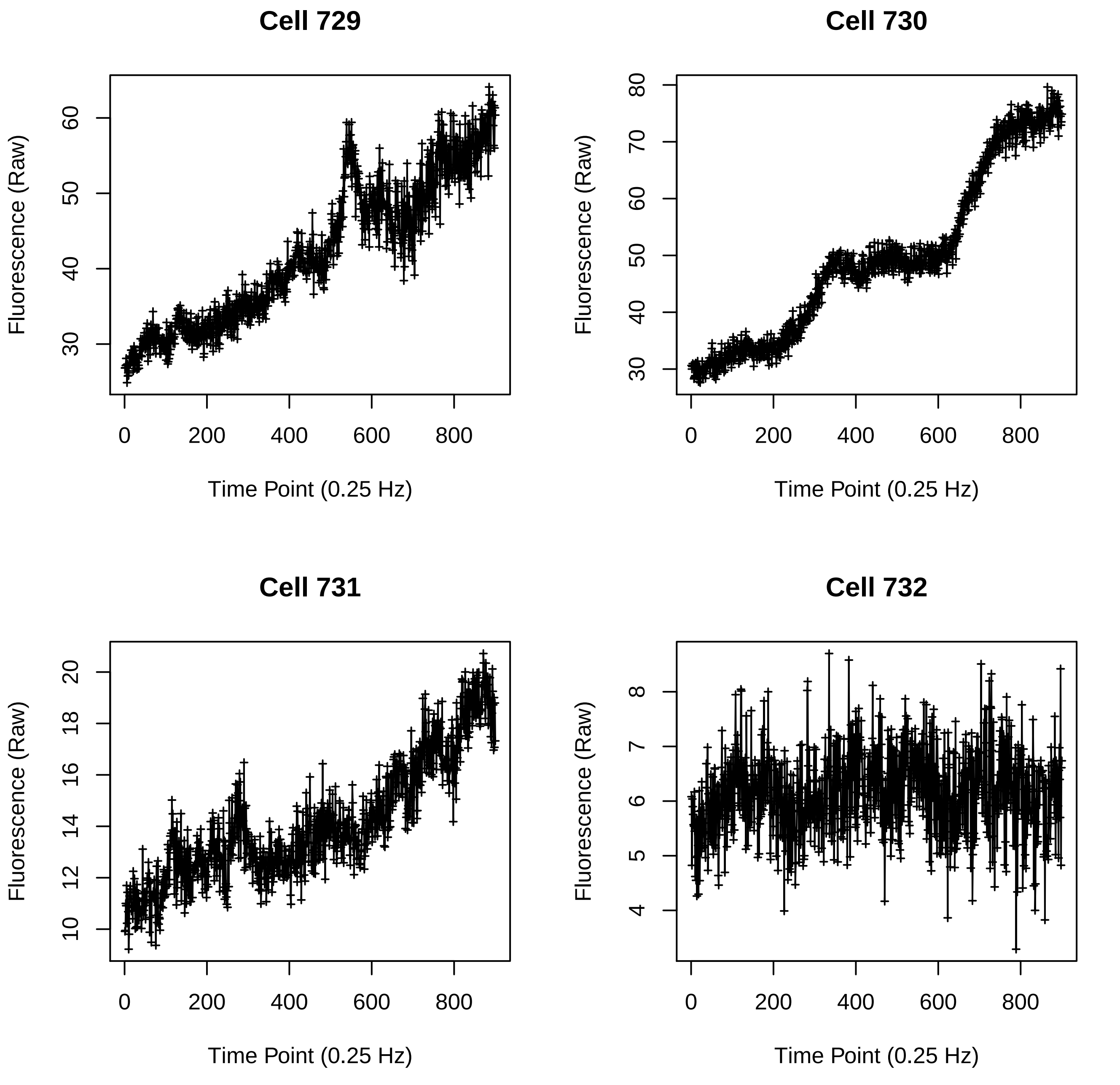
Cell 729
Cell 730
Cell 731
Cell 732
Fluorescence (Raw)
Time Point (0.25 Hz)
0
200
400
600
800
30
40
50
60
70
80
10
12
14
16
18
20
4
5
6
7
8

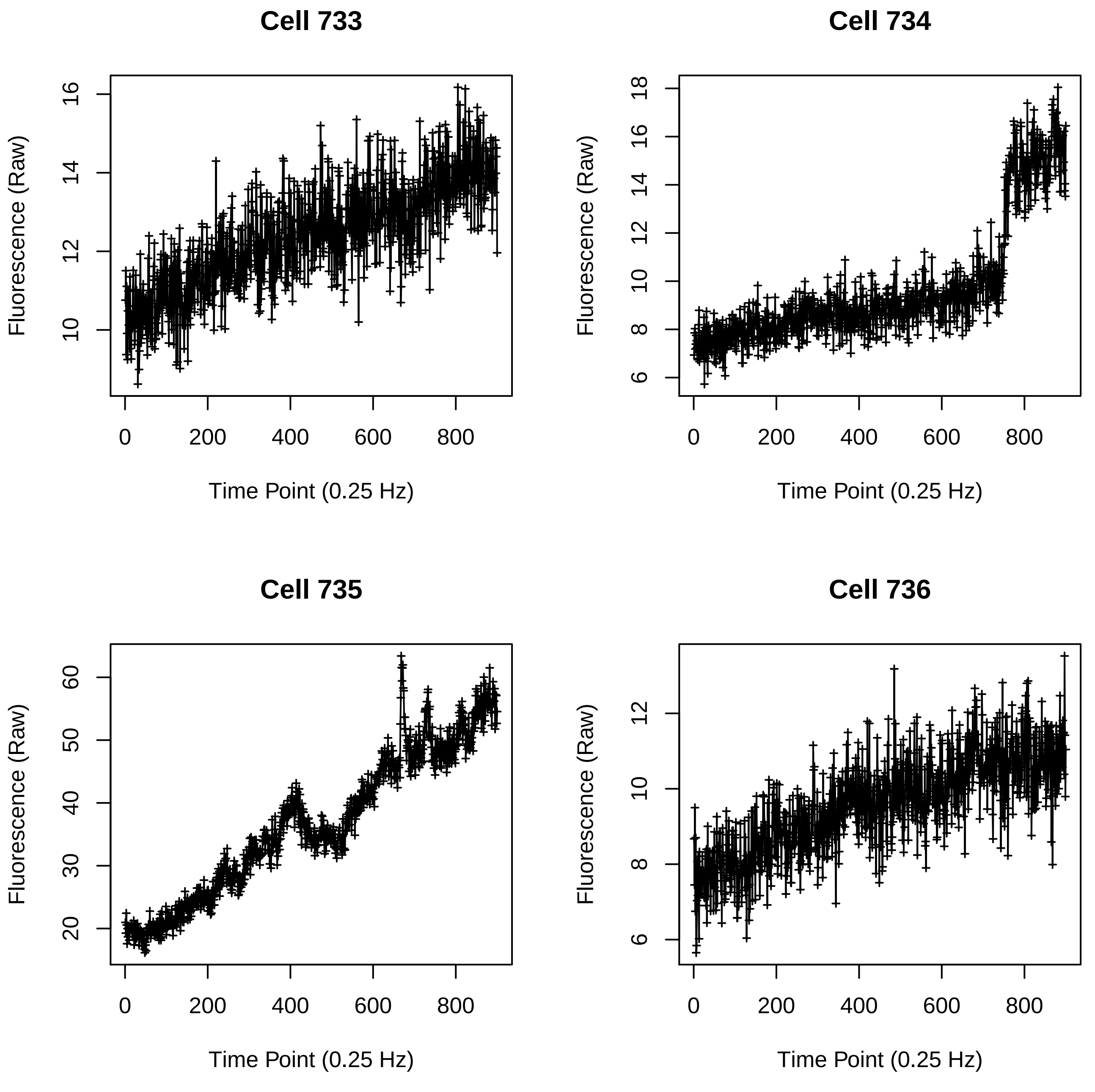

Cell 733
Cell 734
Cell 735
Cell 736
Fluorescence (Raw)
Time Point (0.25 Hz)
0
200
400
600
800
10
12
14
16
6
8
10
12
14
16
18
20
30
40
50
60
6
8
10
12

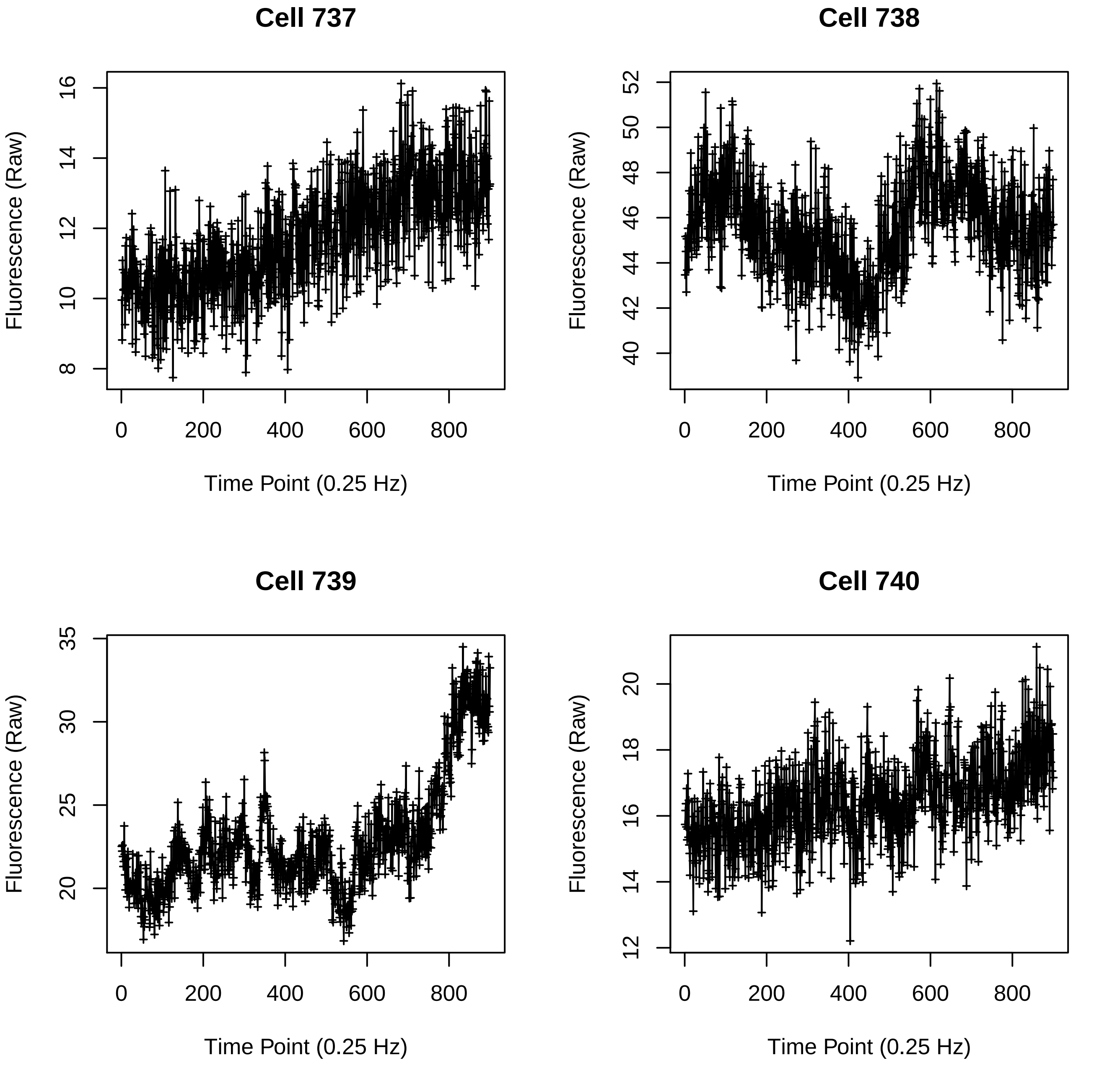
Cell 737
Fluorescence (Raw)
8
10
12
14
16
0
200
400
600
800
Time Point (0.25 Hz)
Cell 738
Fluorescence (Raw)
40
42
44
46
48
50
52
0
200
400
600
800
Time Point (0.25 Hz)
Cell 739
Fluorescence (Raw)
20
25
30
35
0
200
400
600
800
Time Point (0.25 Hz)
Cell 740
Fluorescence (Raw)
12
14
16
18
20
0
200
400
600
800
Time Point (0.25 Hz)

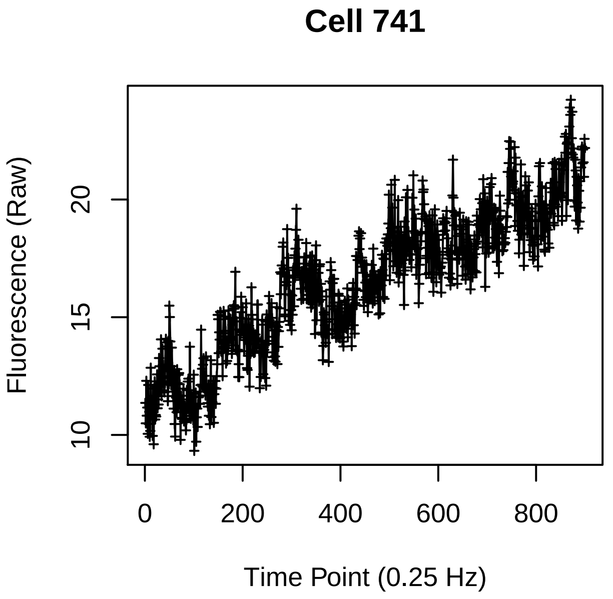
Cell 741
Fluorescence (Raw)
10
15
20
0
200
400
600
800
Time Point (0.25 Hz)

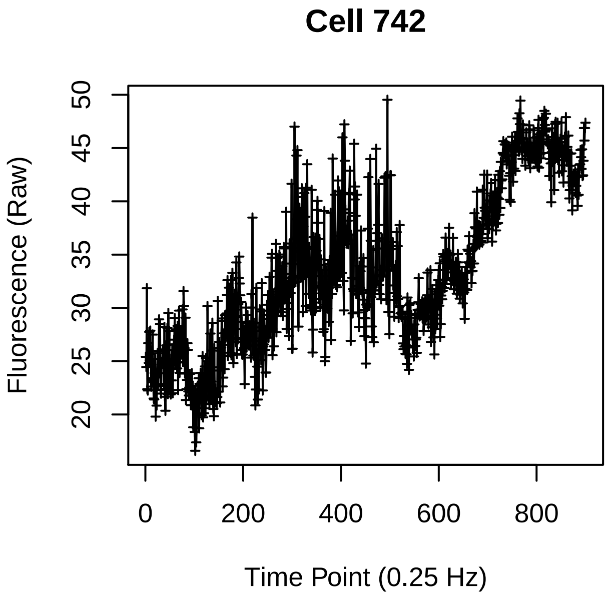
Cell 742
Fluorescence (Raw)
20
25
30
35
40
45
50
0
200
400
600
800
Time Point (0.25 Hz)

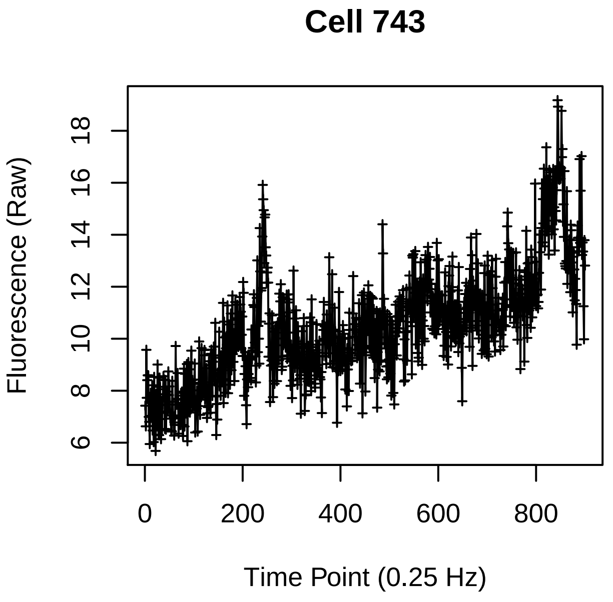
Cell 743
Fluorescence (Raw)
6
8
10
12
14
16
18
0
200
400
600
800
Time Point (0.25 Hz)

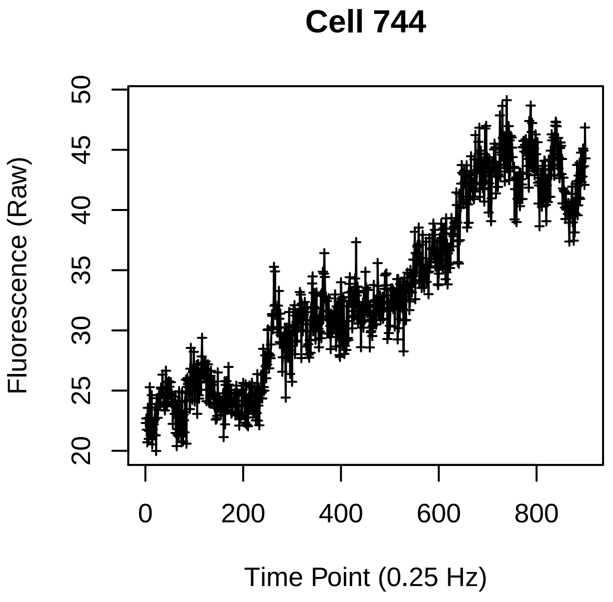
Cell 744
Fluorescence (Raw)
20
25
30
35
40
45
50
0
200
400
600
800
Time Point (0.25 Hz)

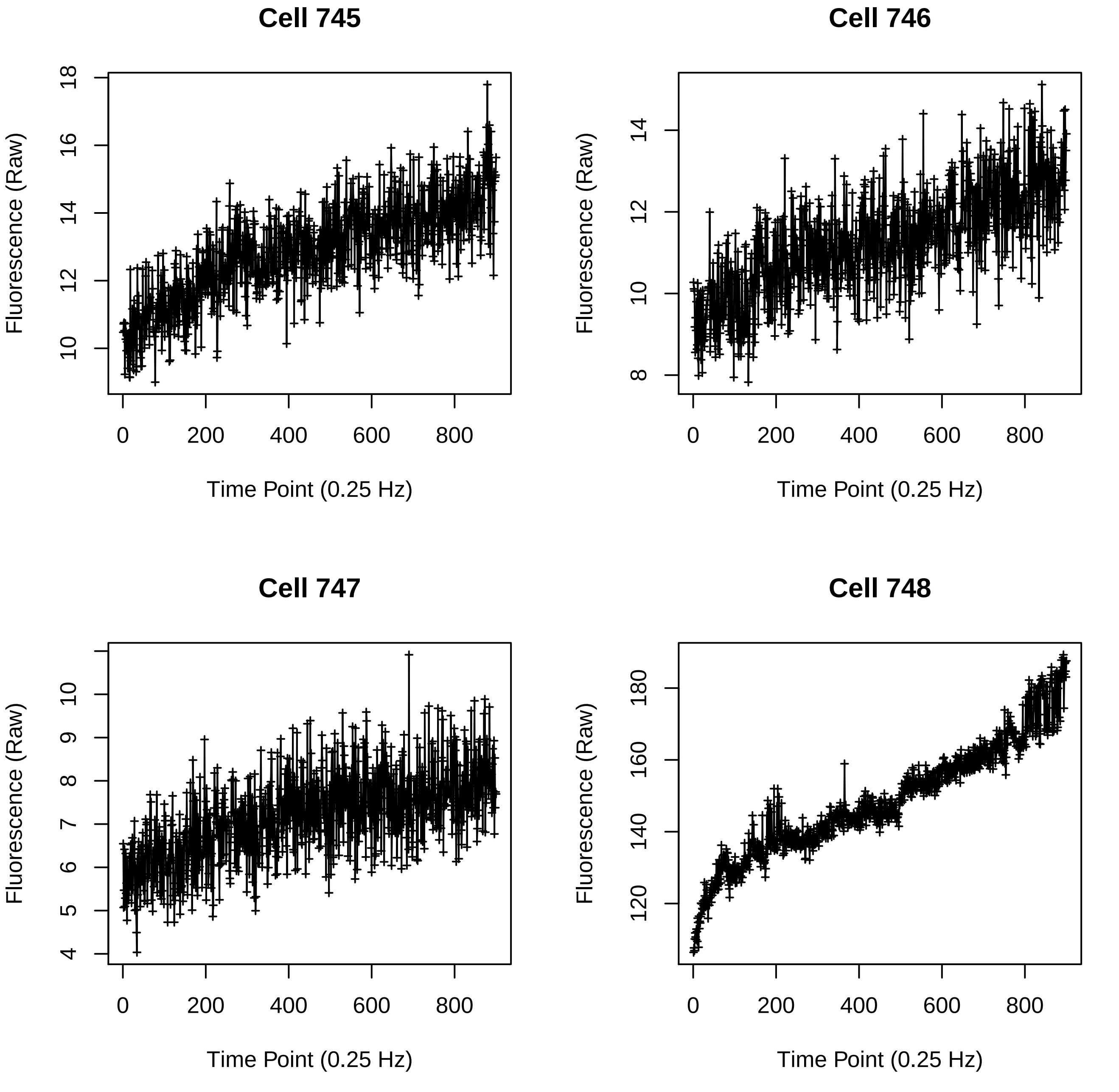

Cell 745
Cell 746
Cell 747
Cell 748
Fluorescence (Raw)
Time Point (0.25 Hz)
10
12
14
16
18
8
0
200
400
600
800
4
5
6
7
9
120
140
160
180

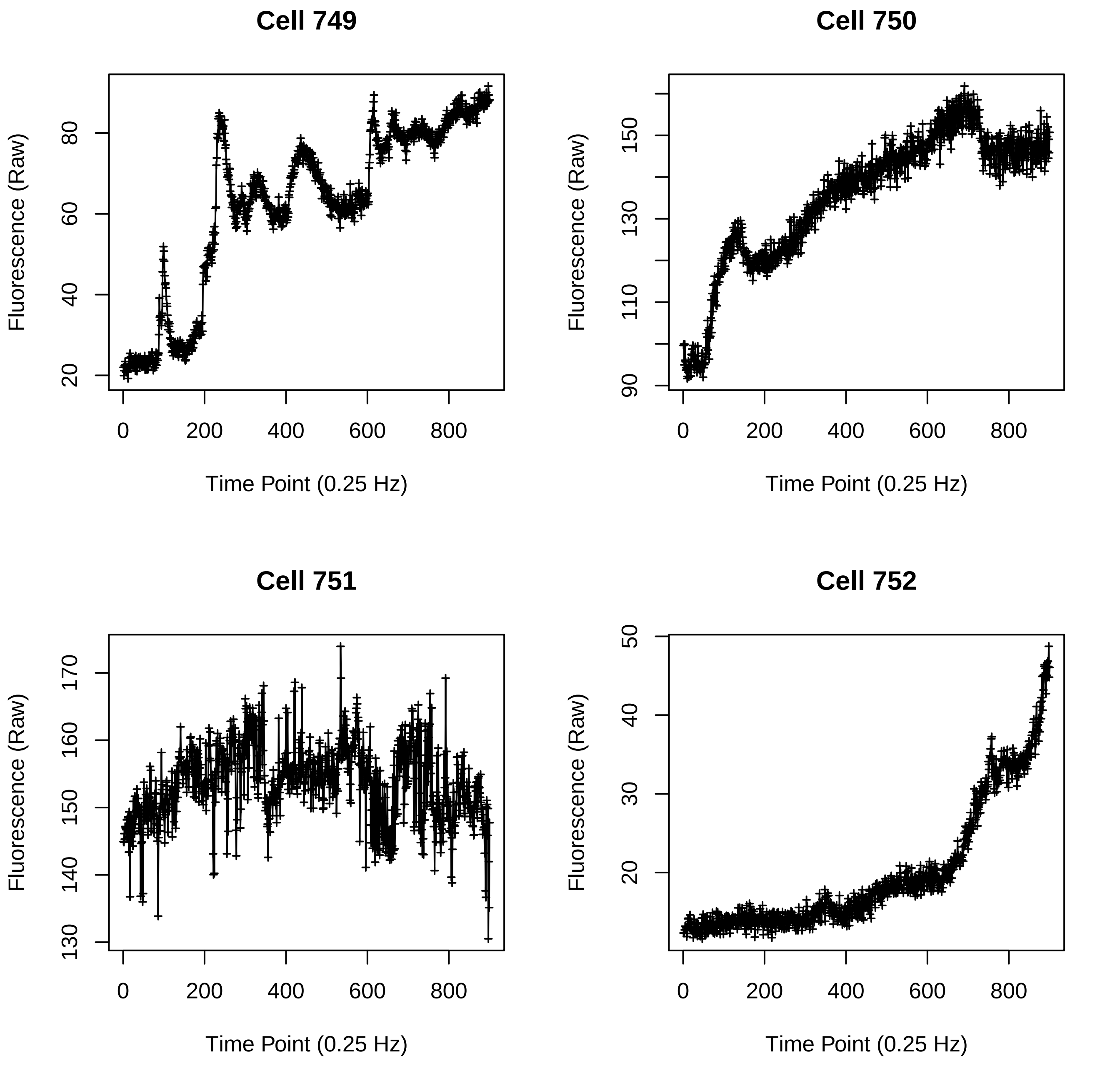
Cell 749
Cell 750
Cell 751
Cell 752
Fluorescence (Raw)
Time Point (0.25 Hz)
0
200
400
600
800
20
40
60
80
90
110
130
150
140
160
170
50
30

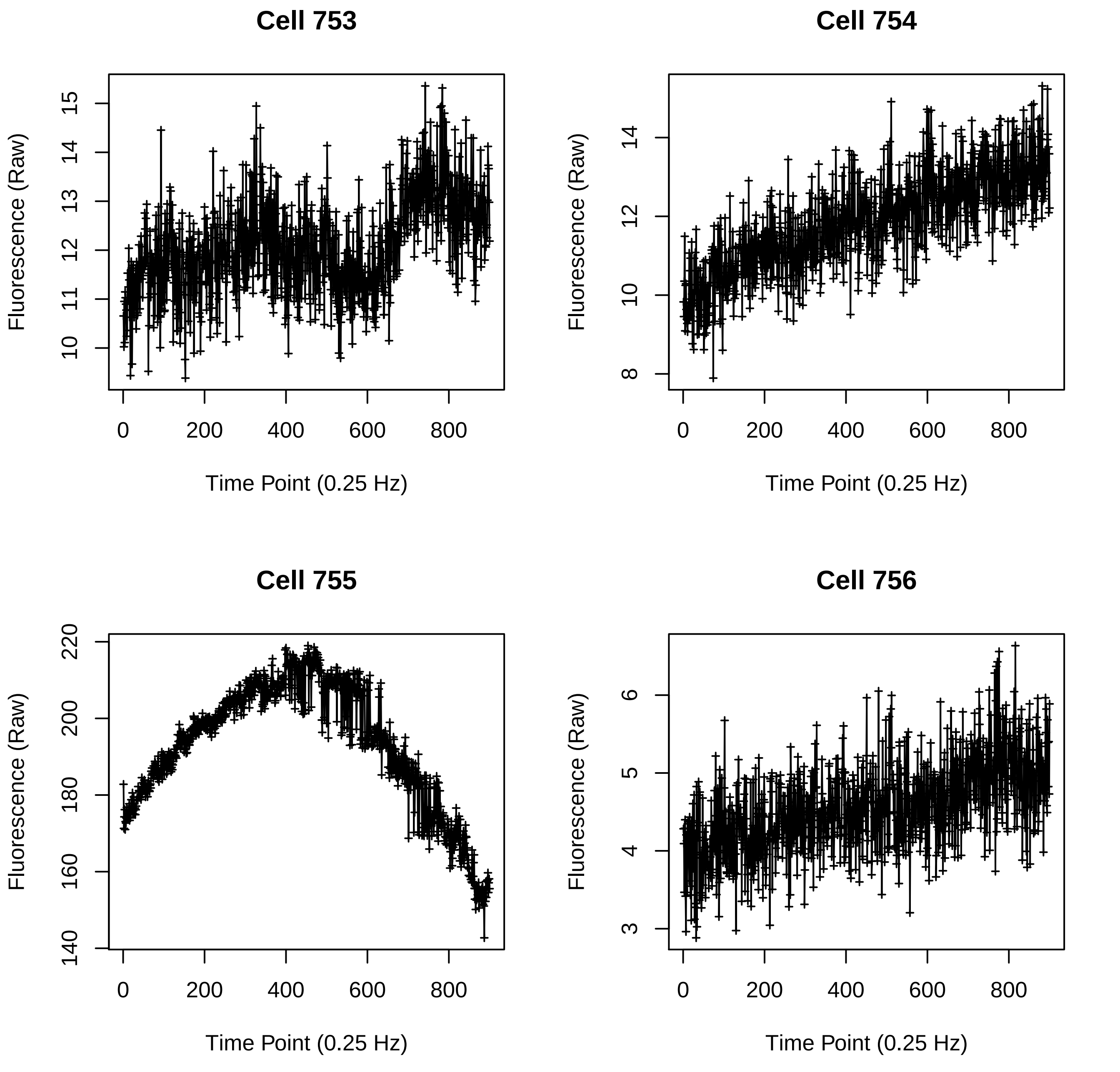
Cell 753
Cell 754
Cell 755
Cell 756
Fluorescence (Raw)
Time Point (0.25 Hz)
0
200
400
600
800
10
11
12
13
14
15
8
10
12
14
140
160
180
200
220
3
4
5
6

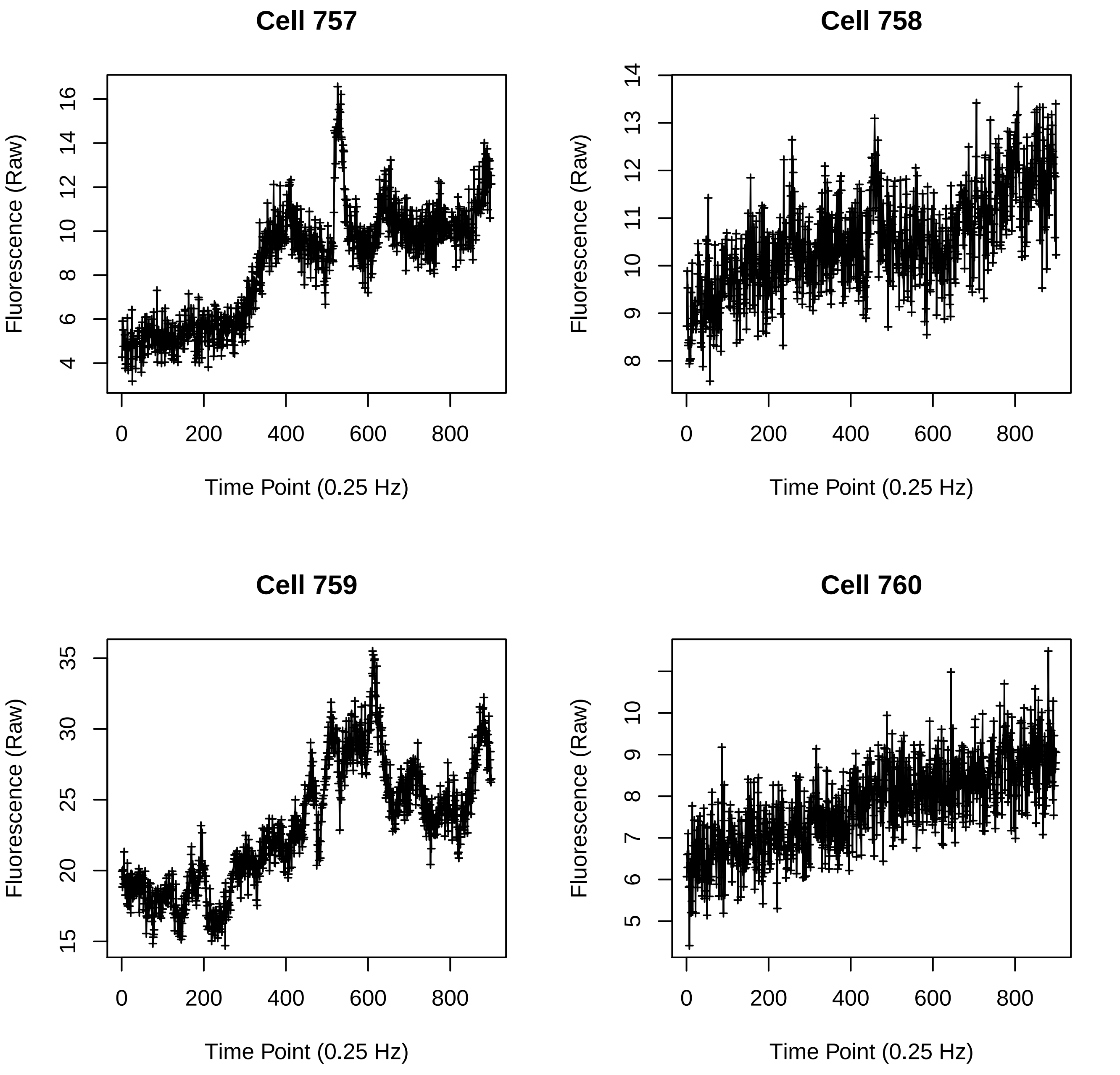
Cell 757
Fluorescence (Raw)
4
6
8
10
12
14
16
0
200
400
600
800
Time Point (0.25 Hz)
Cell 758
Fluorescence (Raw)
8
9
10
11
12
13
14
0
200
400
600
800
Time Point (0.25 Hz)
Cell 759
Fluorescence (Raw)
15
20
25
30
35
0
200
400
600
800
Time Point (0.25 Hz)
Cell 760
Fluorescence (Raw)
5
6
7
8
9
10
0
200
400
600
800
Time Point (0.25 Hz)

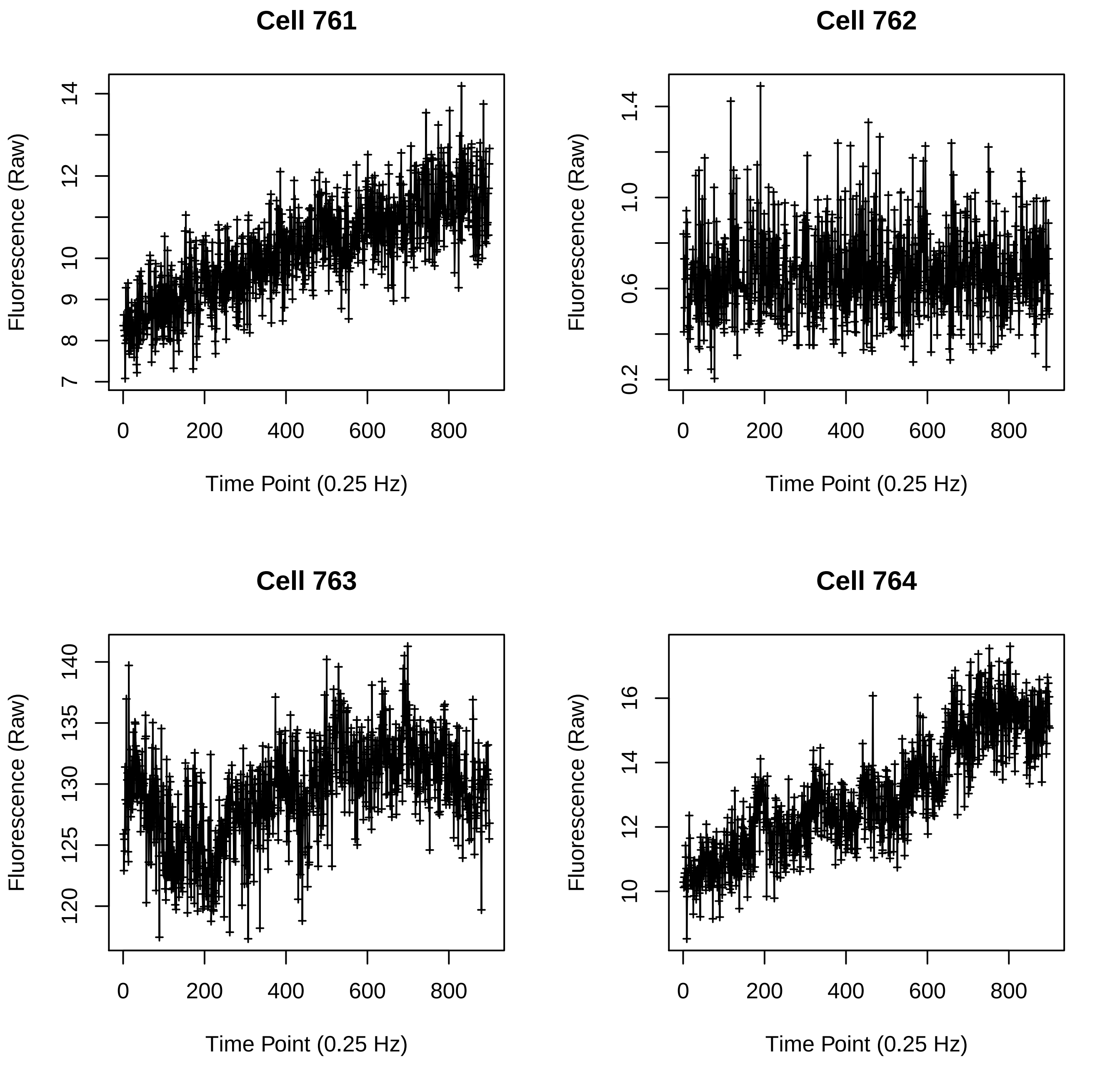
Cell 761
Cell 762
Cell 763
Cell 764
Fluorescence (Raw)
Time Point (0.25 Hz)
0
200
400
600
800
7
8
9
10
12
14
0.2
0.6
1.0
1.4
120
125
130
135
140
10
12
14
16

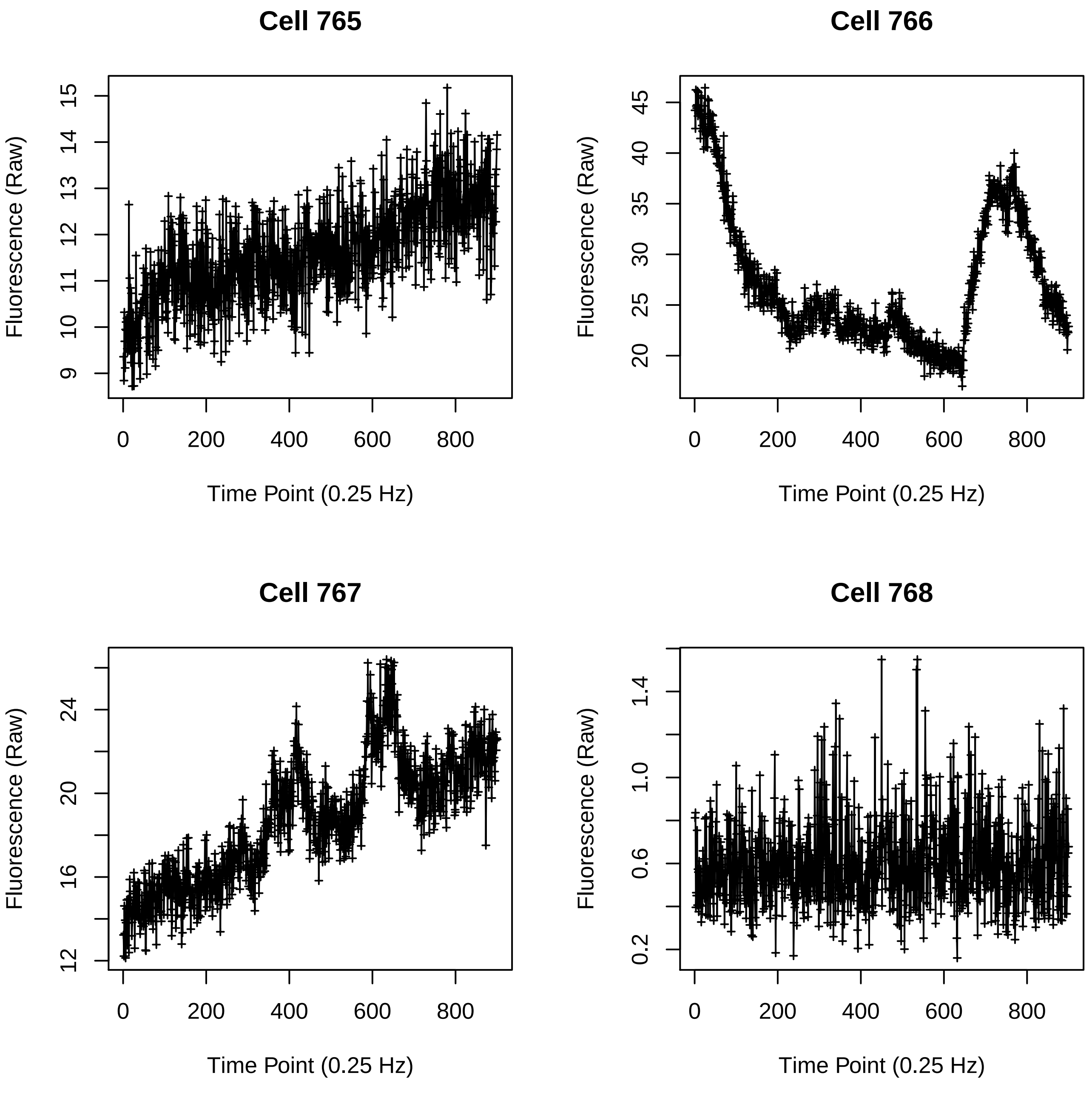

Cell 765
Fluorescence (Raw)
9
10
11
12
13
14
15
0
200
400
600
800
Time Point (0.25 Hz)
Cell 766
Fluorescence (Raw)
20
25
30
35
40
45
0
200
400
600
800
Time Point (0.25 Hz)
Cell 767
Fluorescence (Raw)
12
16
20
24
0
200
400
600
800
Time Point (0.25 Hz)
Cell 768
Fluorescence (Raw)
0.2
0.6
1.0
1.4
0
200
400
600
800
Time Point (0.25 Hz)

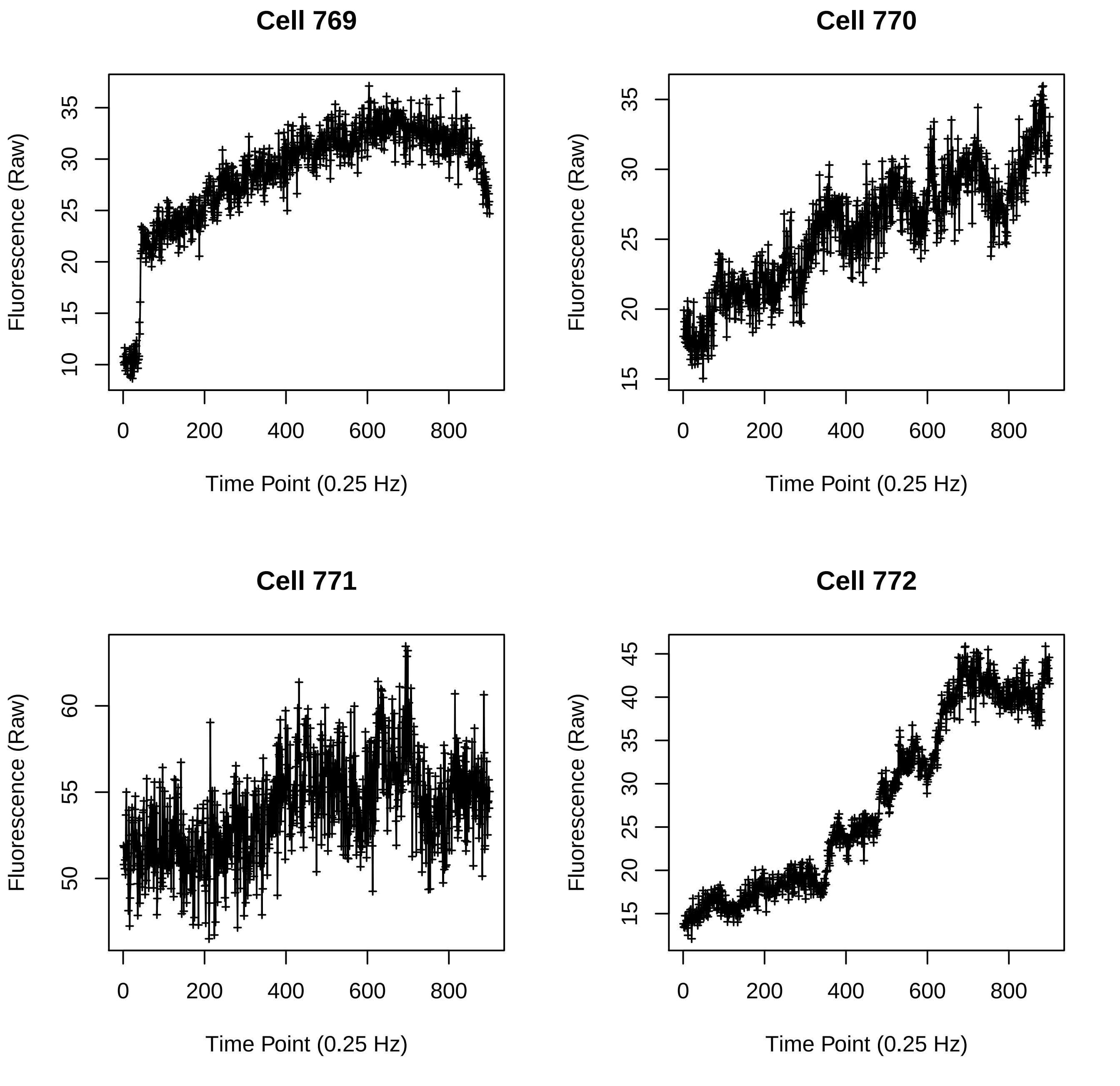
Cell 769
Cell 770
Cell 771
Cell 772
Fluorescence (Raw)
Time Point (0.25 Hz)
0
200
400
600
800
10
15
20
25
30
35
45
40
50
55
60

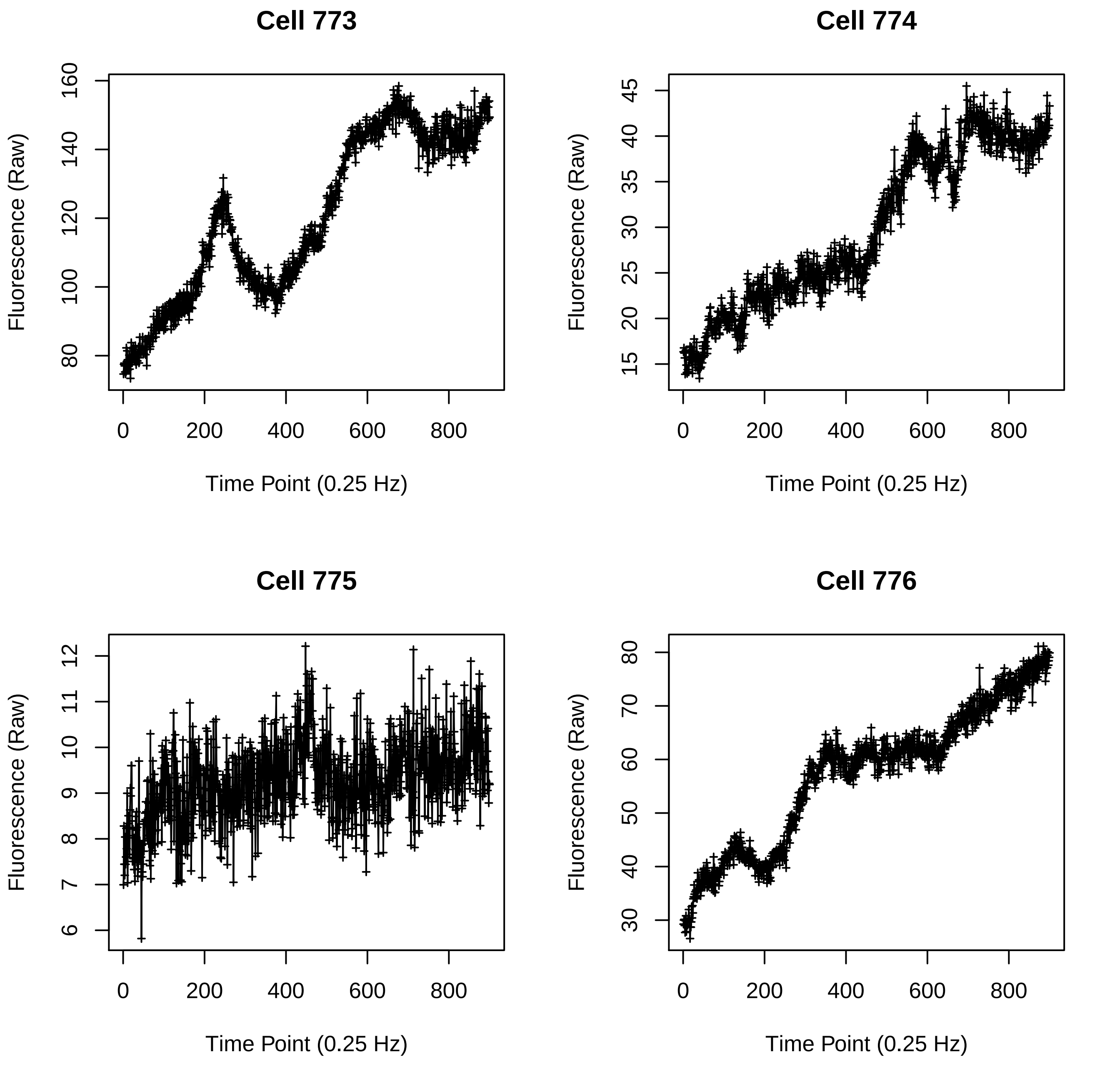

Cell 773
Cell 774
Cell 775
Cell 776
Fluorescence (Raw)
Time Point (0.25 Hz)

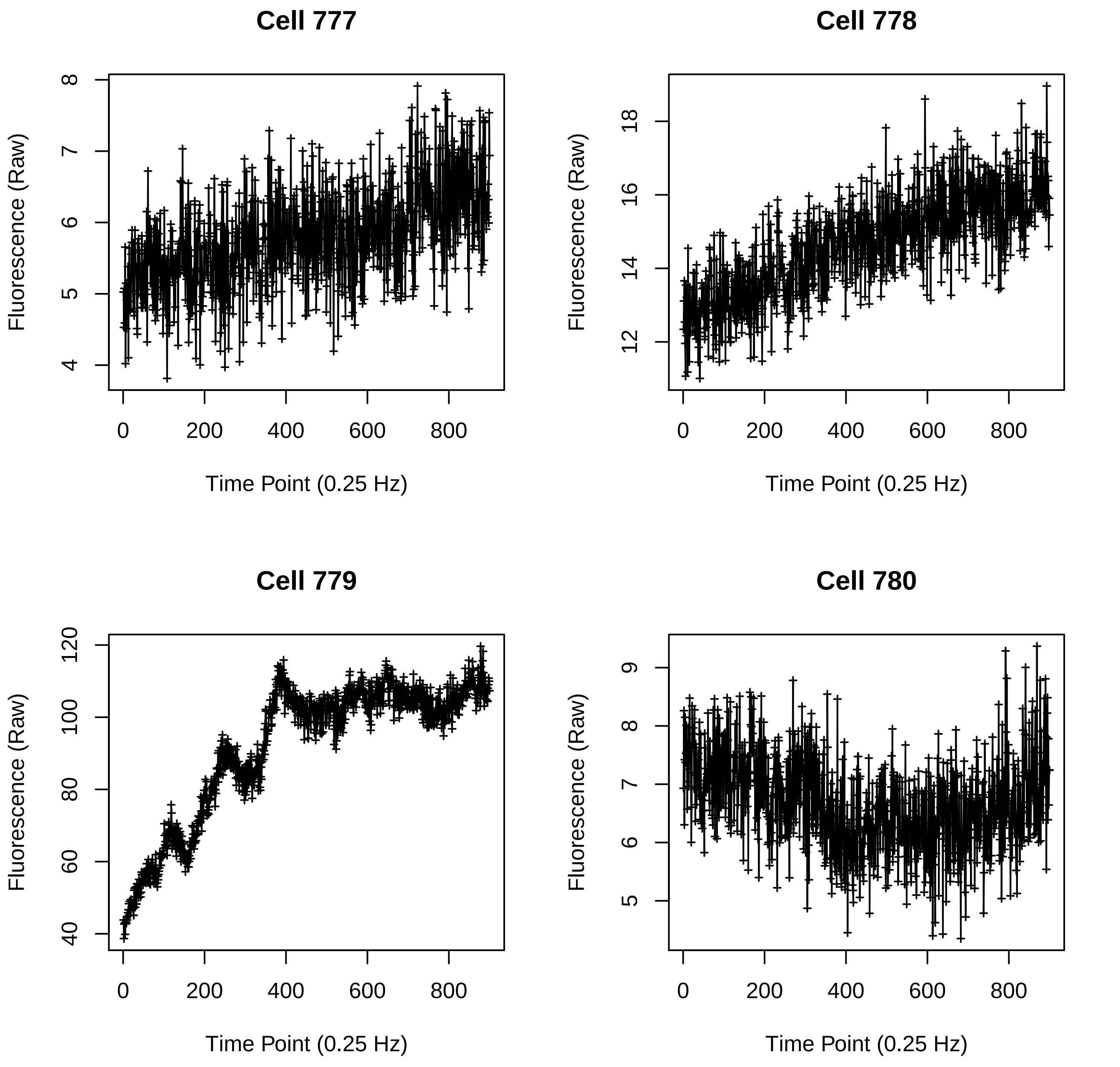
Cell 777
Cell 778
Cell 779
Cell 780
Fluorescence (Raw)
Time Point (0.25 Hz)
0
200
400
600
800
4
5
6
7
8
12
14
16
18
40
60
80
100
120
9

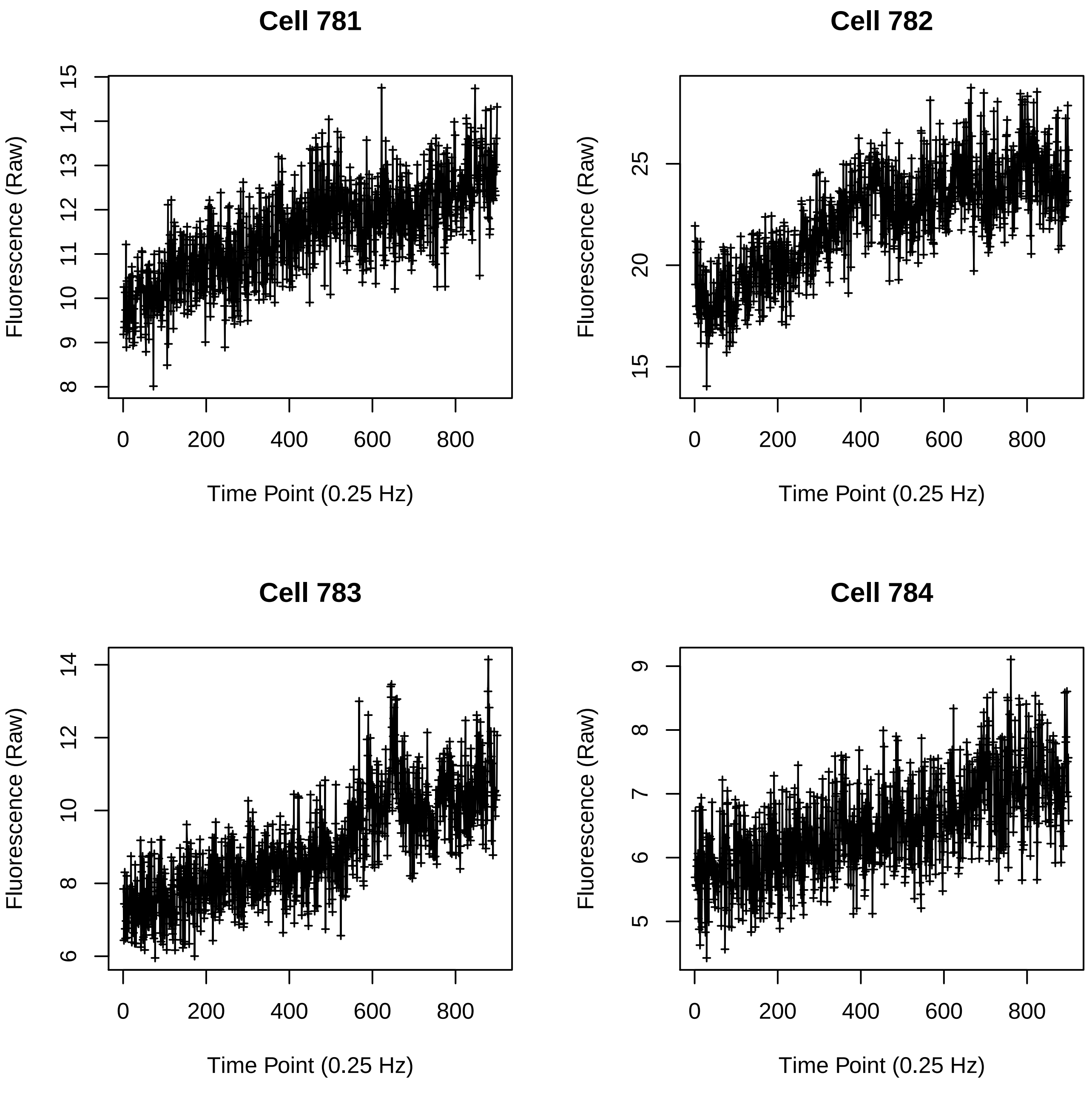
Cell 781
Cell 782
Cell 783
Cell 784
Fluorescence (Raw)
Time Point (0.25 Hz)
0
200
400
600
800
8
9
10
11
12
13
14
15
20
25
6
7

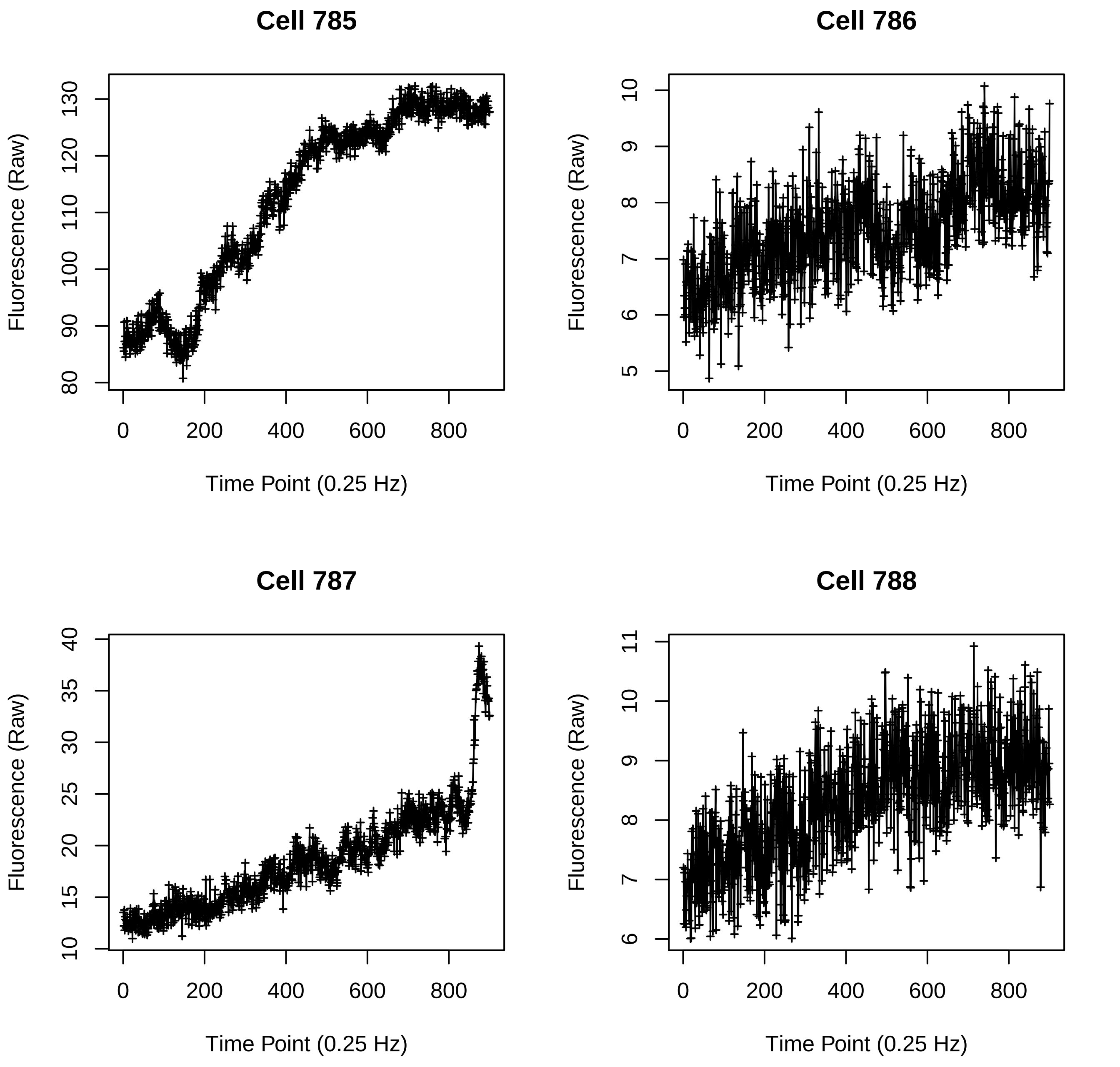

Cell 785
Cell 786
Cell 787
Cell 788
Fluorescence (Raw)
Time Point (0.25 Hz)
0
200
400
600
800
80
90
100
110
120
130
5
6
7
8
9
10
15
20
25
30
35
40
11

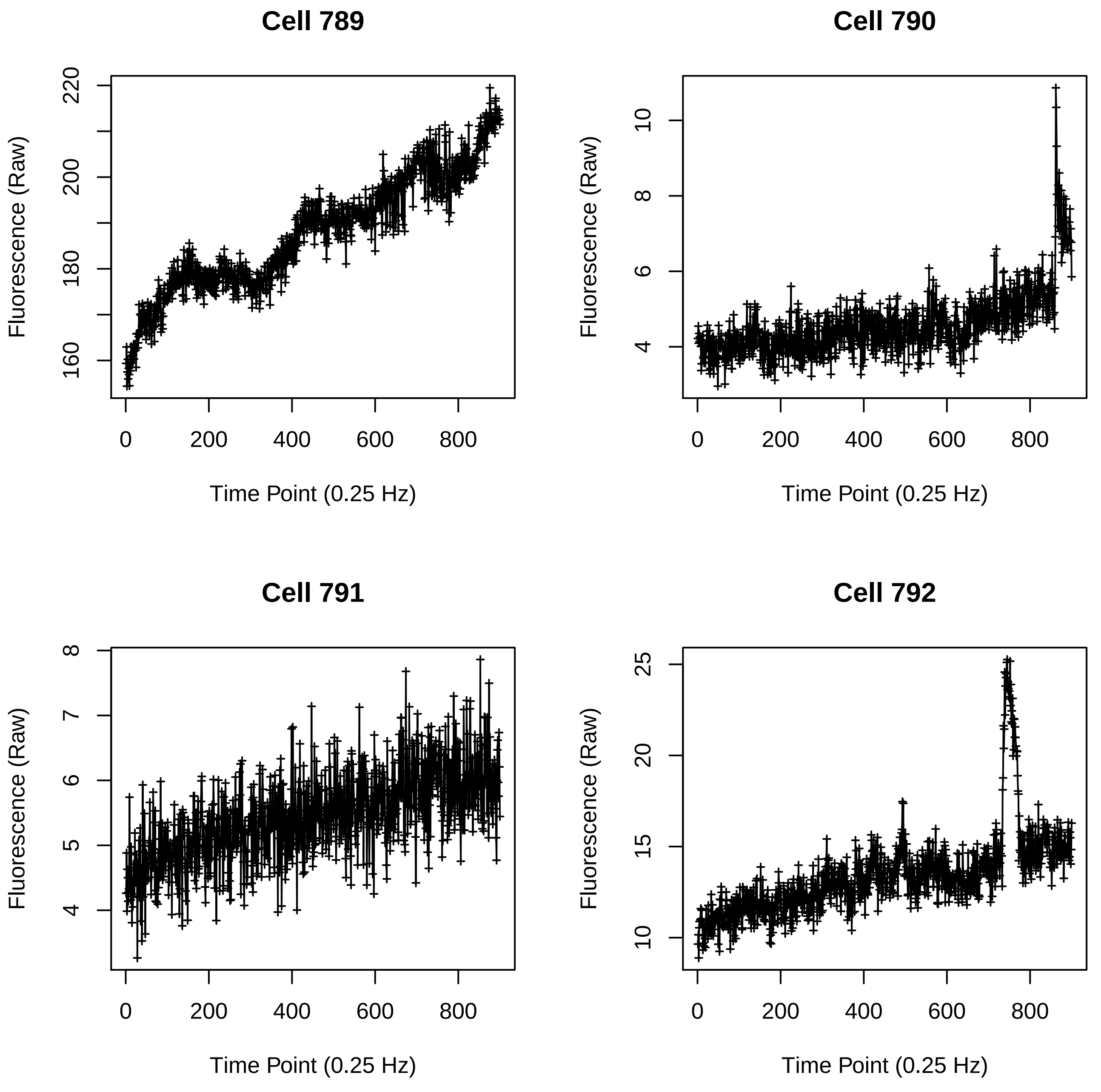
Cell 789
Fluorescence (Raw)
160
180
200
220
0
200
400
600
800
Time Point (0.25 Hz)
Cell 790
Fluorescence (Raw)
4
6
8
10
0
200
400
600
800
Time Point (0.25 Hz)
Cell 791
Fluorescence (Raw)
4
5
6
7
8
0
200
400
600
800
Time Point (0.25 Hz)
Cell 792
Fluorescence (Raw)
10
15
20
25
0
200
400
600
800
Time Point (0.25 Hz)

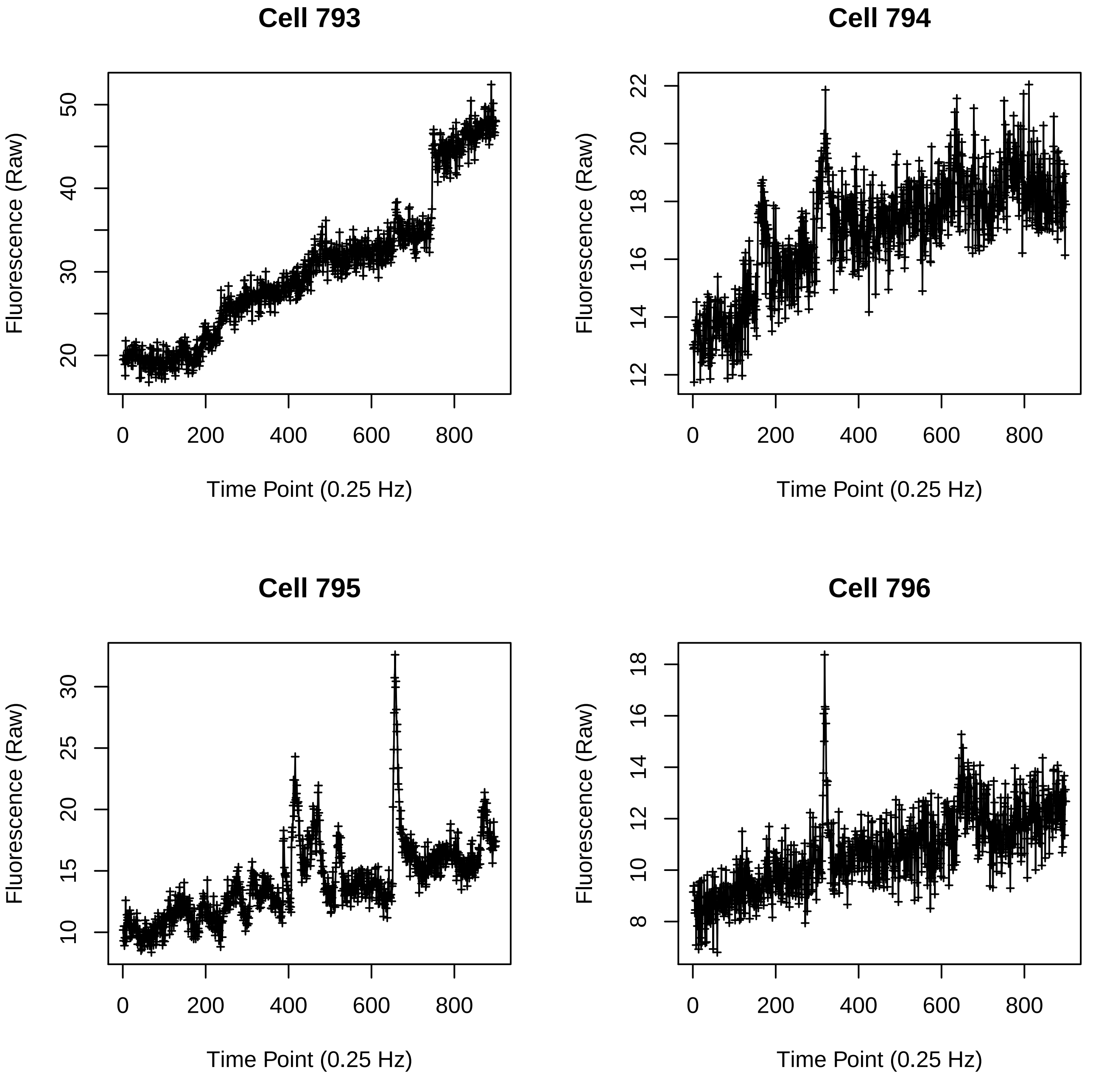
Cell 793
Cell 794
Cell 795
Cell 796
Fluorescence (Raw)
Time Point (0.25 Hz)
0
200
400
600
800
20
30
40
50
12
14
16
18
20
22
10
15
20
25
30
8
10
12
14
16
18

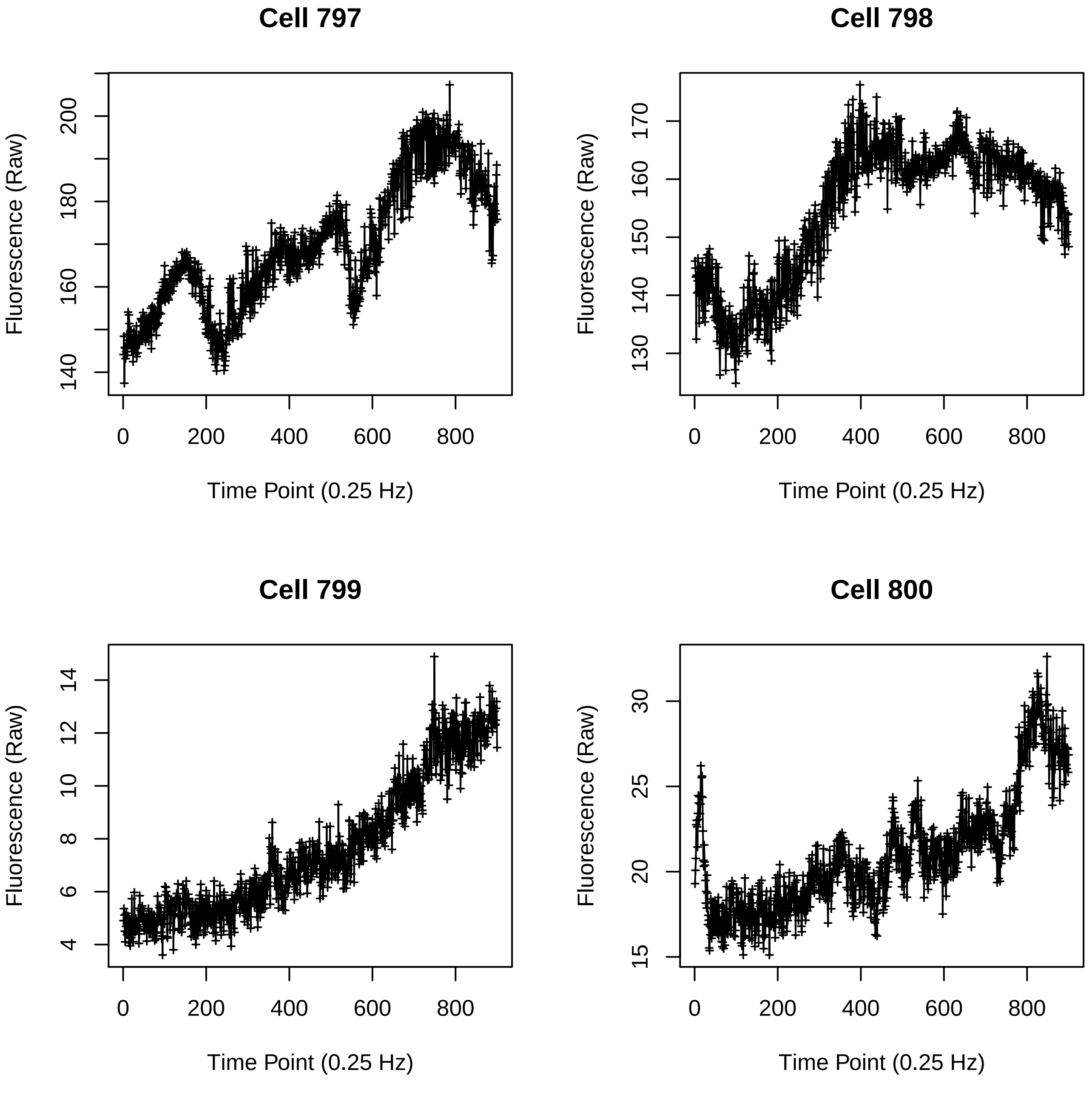
Cell 797
Fluorescence (Raw)
140
160
180
200
0
200
400
600
800
Time Point (0.25 Hz)
Cell 798
Fluorescence (Raw)
130
140
150
160
170
0
200
400
600
800
Time Point (0.25 Hz)
Cell 799
Fluorescence (Raw)
4
6
8
10
12
14
0
200
400
600
800
Time Point (0.25 Hz)
Cell 800
Fluorescence (Raw)
15
20
25
30
0
200
400
600
800
Time Point (0.25 Hz)

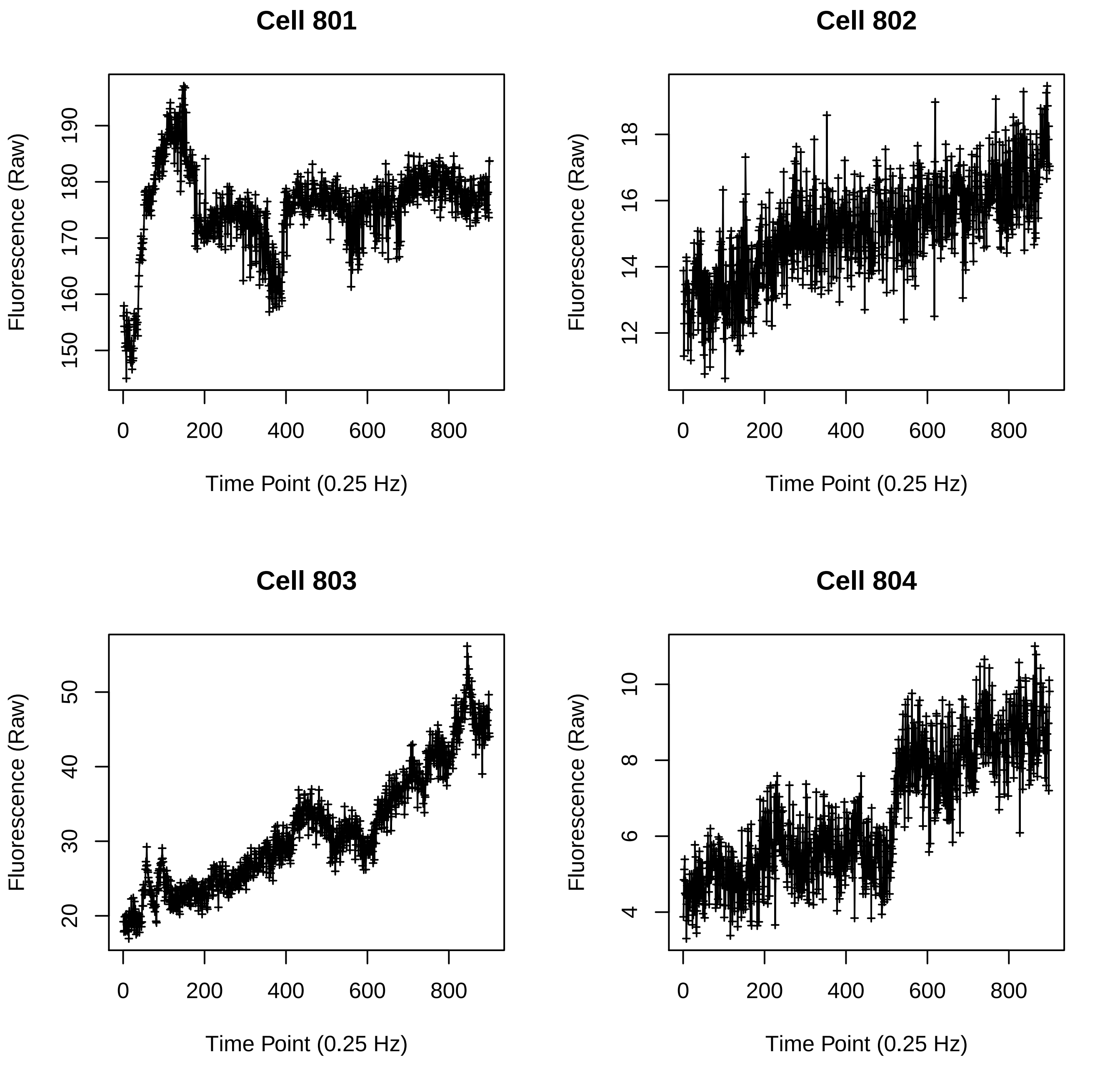
Cell 801
Cell 802
Cell 803
Cell 804
Fluorescence (Raw)
Time Point (0.25 Hz)
150
160
170
180
190
12
14
16
18
20
30
40
50
4
6
8
10
0
200
400
600
800

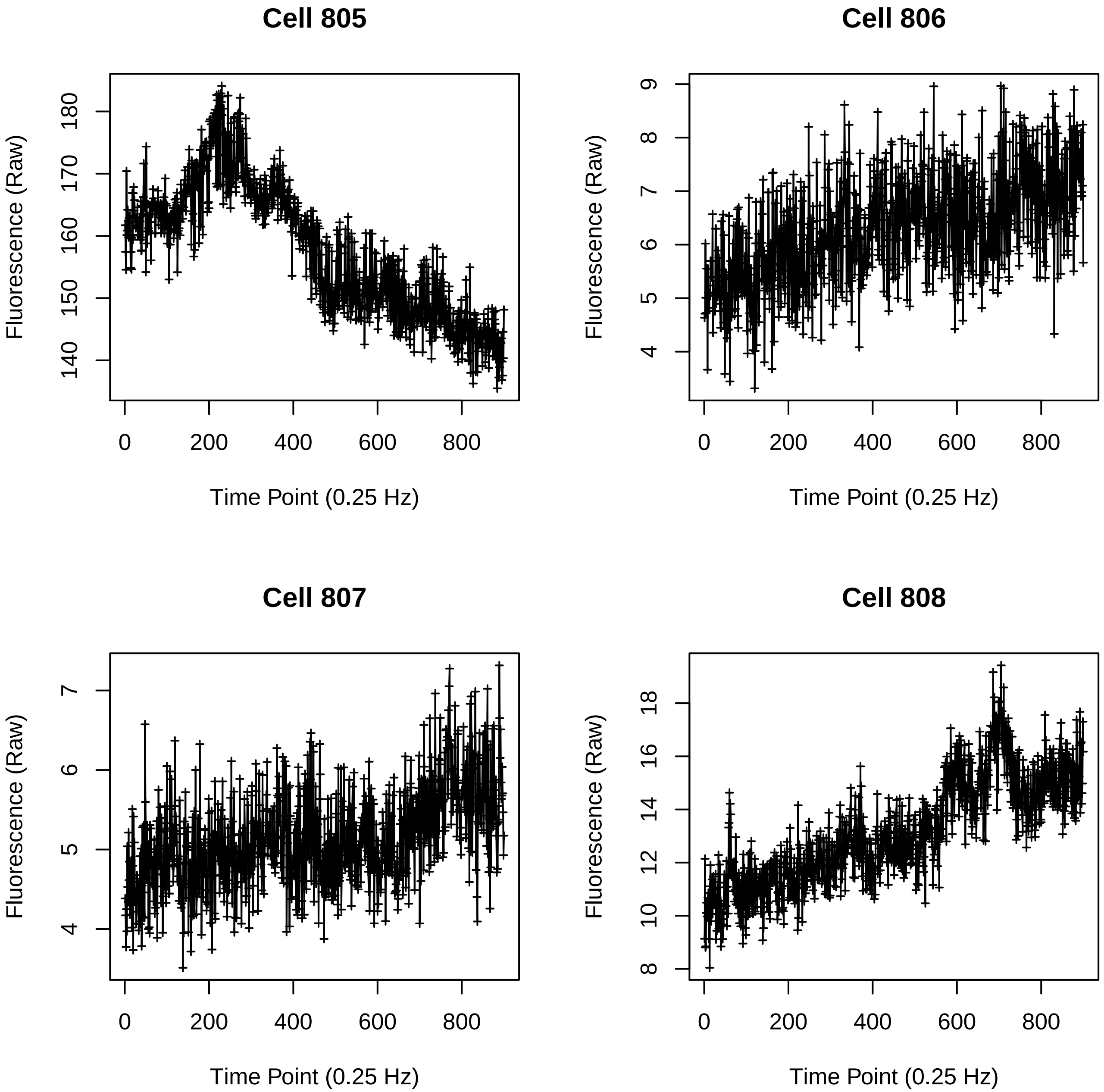
Cell 805
Cell 806
Cell 807
Cell 808
Fluorescence (Raw)
Time Point (0.25 Hz)
0
200
400
600
800
140
150
160
170
180
4
5
6
7
8
9
10
12
14
16
18

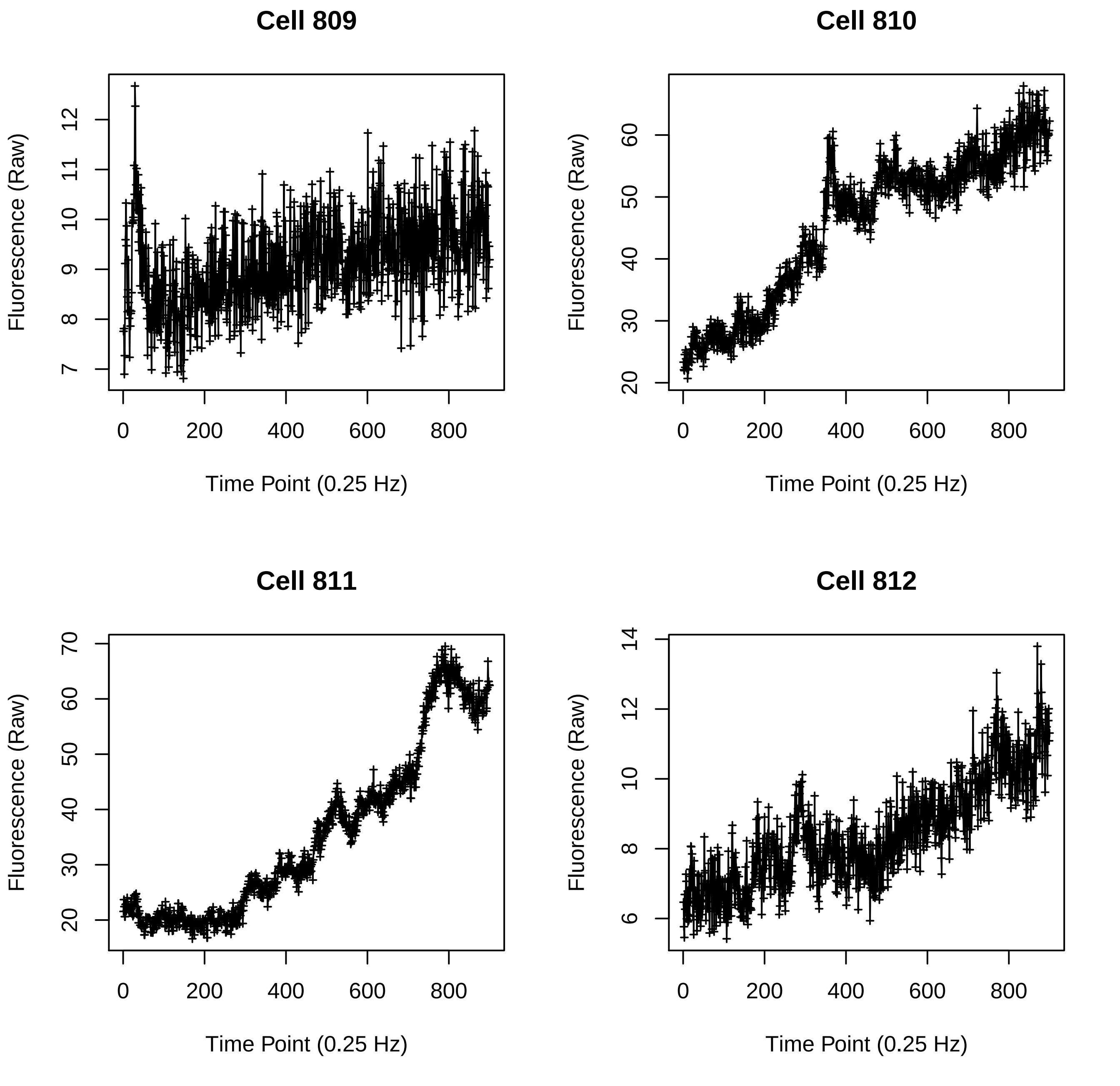

Cell 809
Cell 810
Cell 811
Cell 812
Fluorescence (Raw)
Time Point (0.25 Hz)
0
200
400
600
800

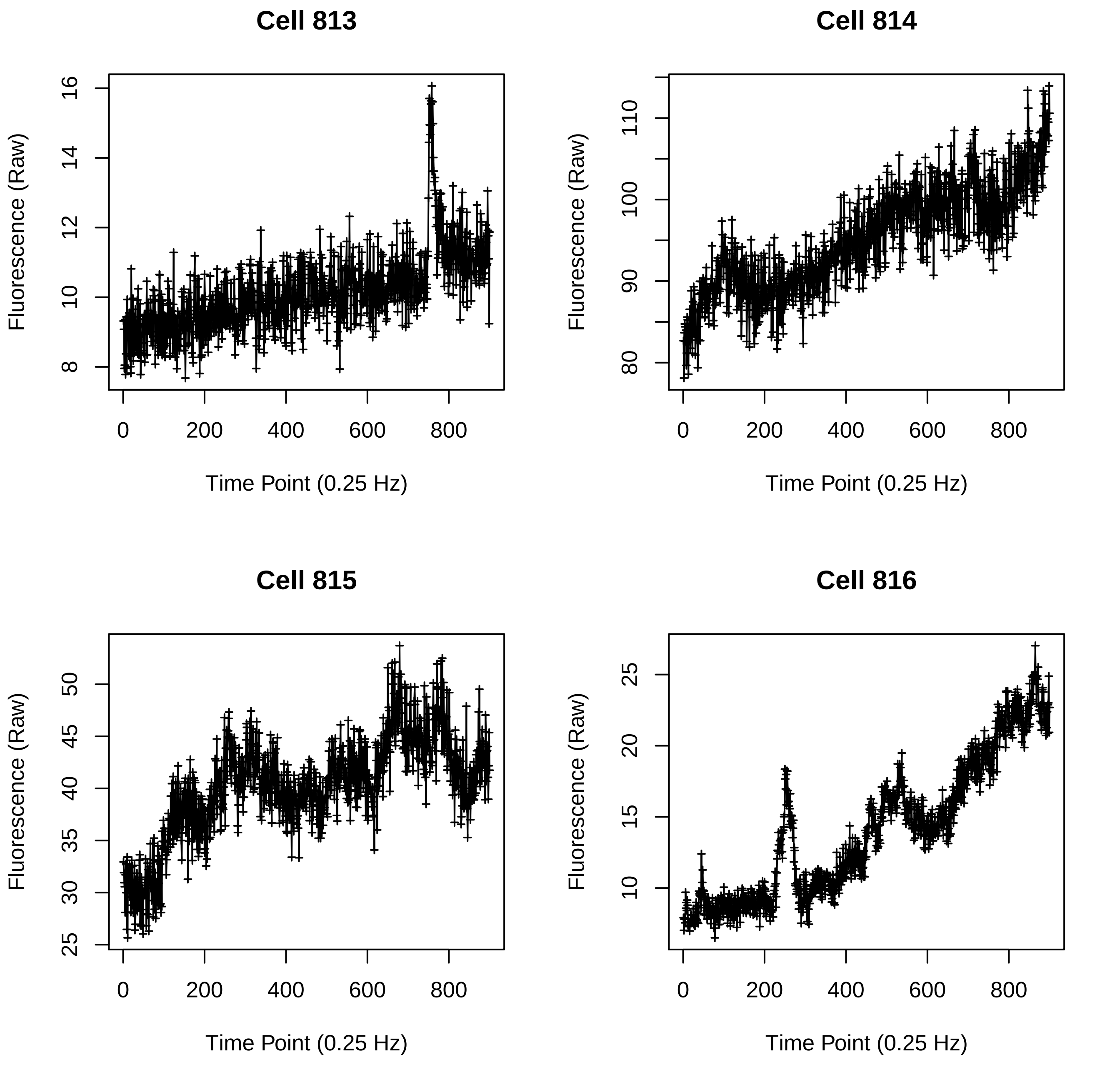
Cell 813
Cell 814
Cell 815
Cell 816
Fluorescence (Raw)
Time Point (0.25 Hz)
8
10
12
14
16
80
90
100
110
25
30
35
40
45
50
15
20
0
200
400
600
800

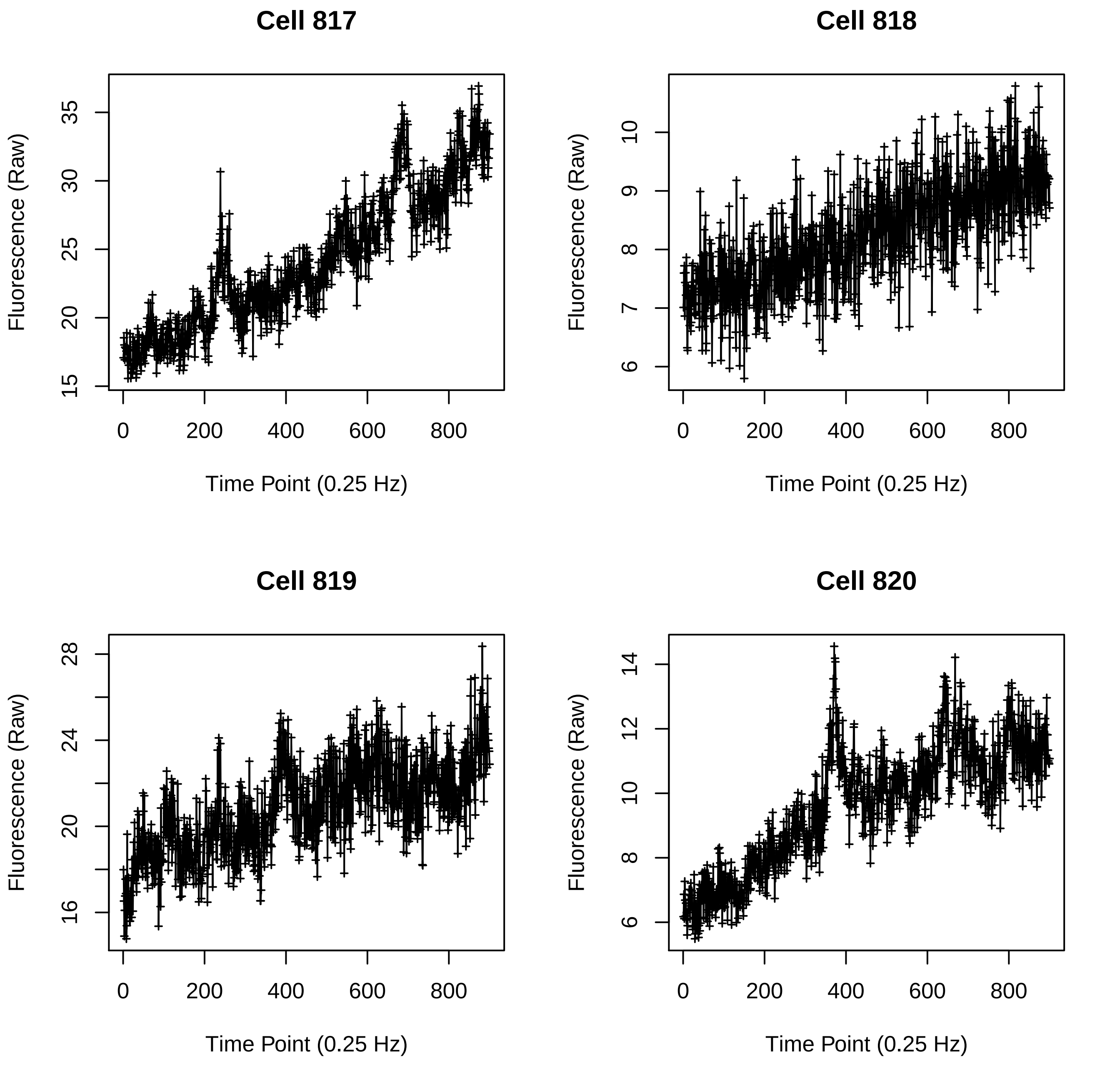
Cell 817
Cell 818
Cell 819
Cell 820
Fluorescence (Raw)
Time Point (0.25 Hz)
0
200
400
600
800
15
20
25
30
35
6
7
8
9
10
16
20
24
28
6
8
10
12
14

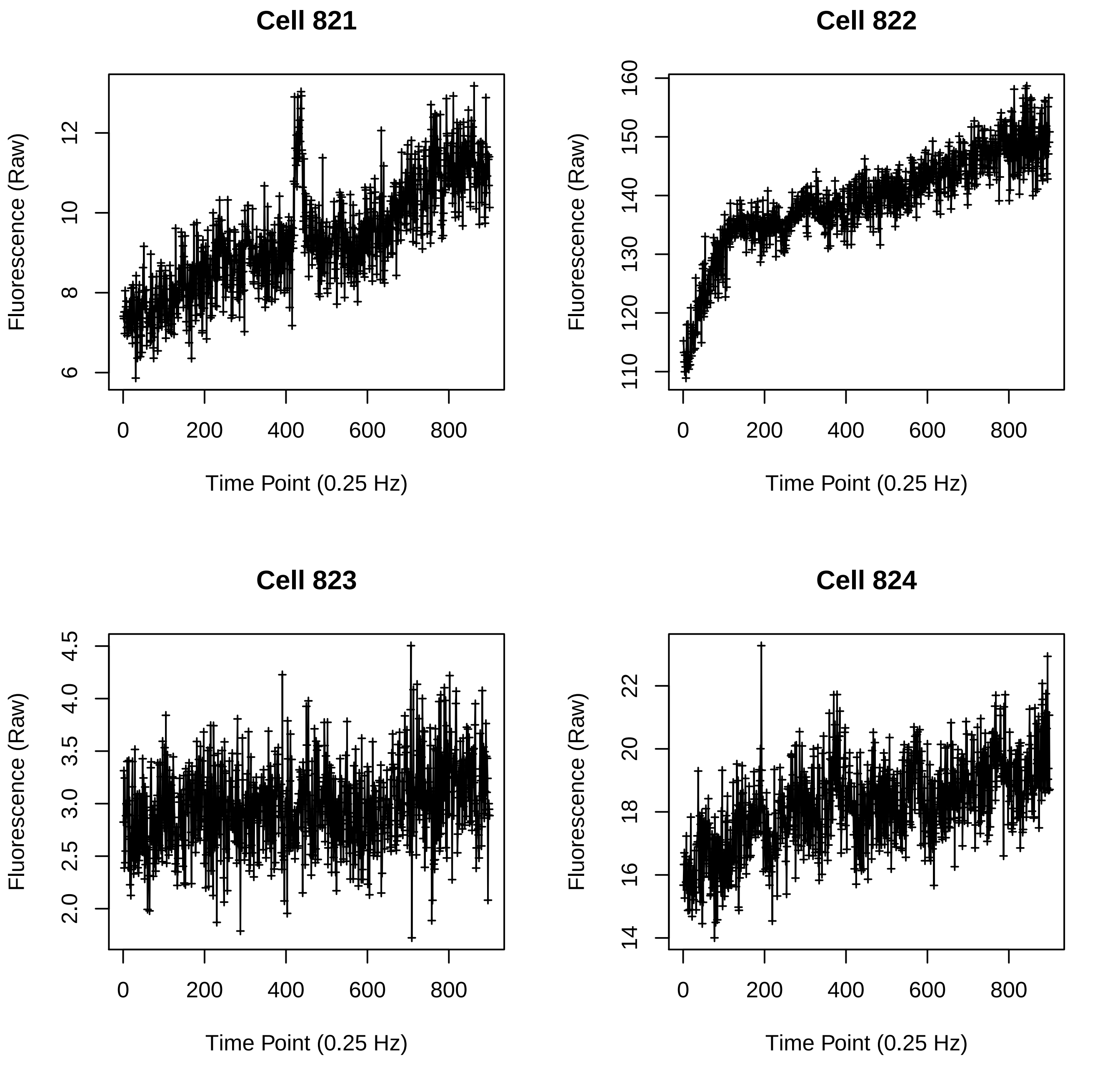
Cell 821
Fluorescence (Raw)
6
8
10
12
0
200
400
600
800
Time Point (0.25 Hz)
Cell 822
Fluorescence (Raw)
110
120
130
140
150
160
0
200
400
600
800
Time Point (0.25 Hz)
Cell 823
Fluorescence (Raw)
2.0
2.5
3.0
3.5
4.0
4.5
0
200
400
600
800
Time Point (0.25 Hz)
Cell 824
Fluorescence (Raw)
14
16
18
20
22
0
200
400
600
800
Time Point (0.25 Hz)

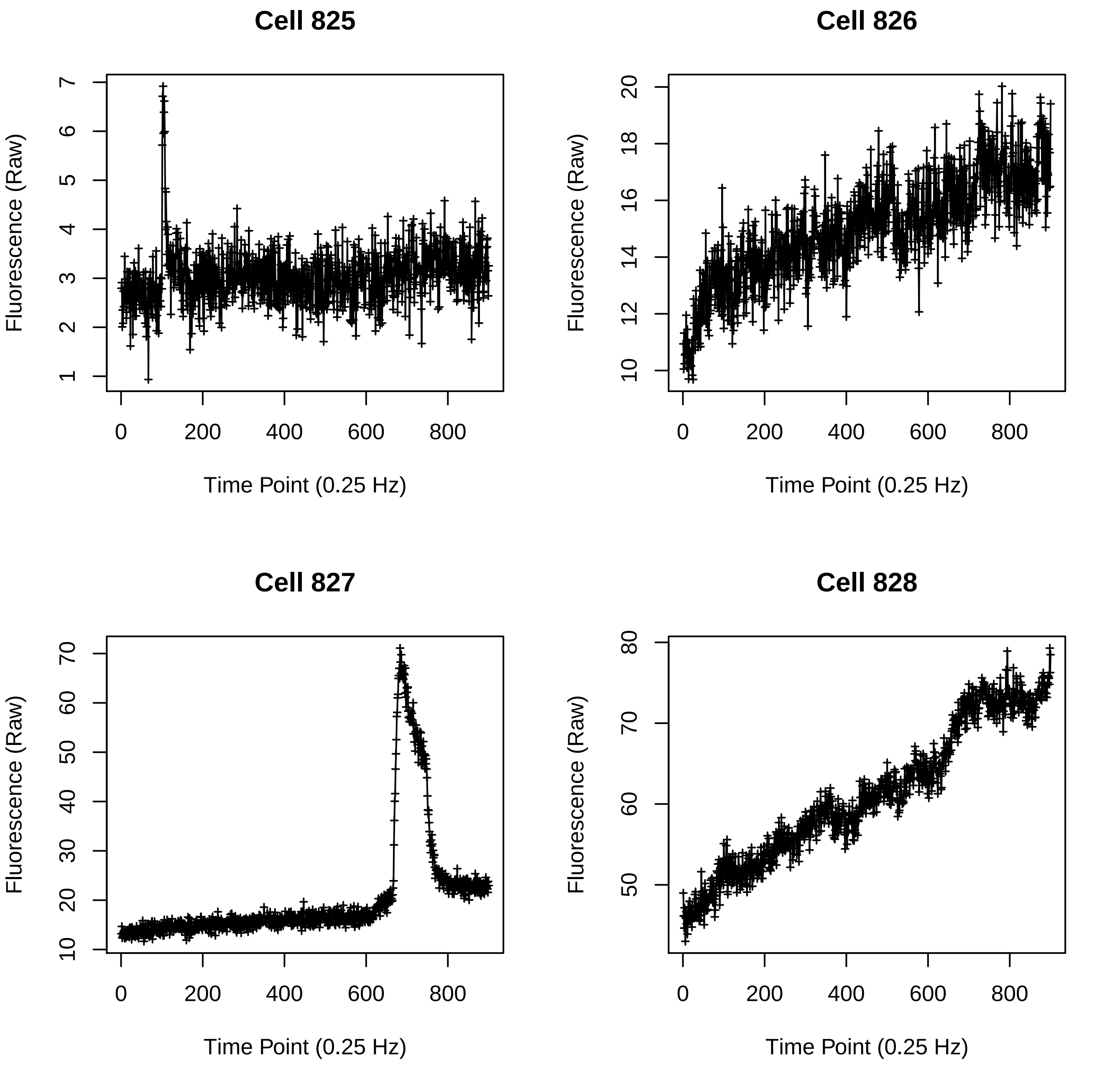
Cell 825
Fluorescence (Raw)
1
2
3
4
5
6
7
0
200
400
600
800
Time Point (0.25 Hz)
Cell 826
Fluorescence (Raw)
10
12
14
16
18
20
0
200
400
600
800
Time Point (0.25 Hz)
Cell 827
Fluorescence (Raw)
10
20
30
40
50
60
70
0
200
400
600
800
Time Point (0.25 Hz)
Cell 828
Fluorescence (Raw)
50
60
70
80
0
200
400
600
800
Time Point (0.25 Hz)

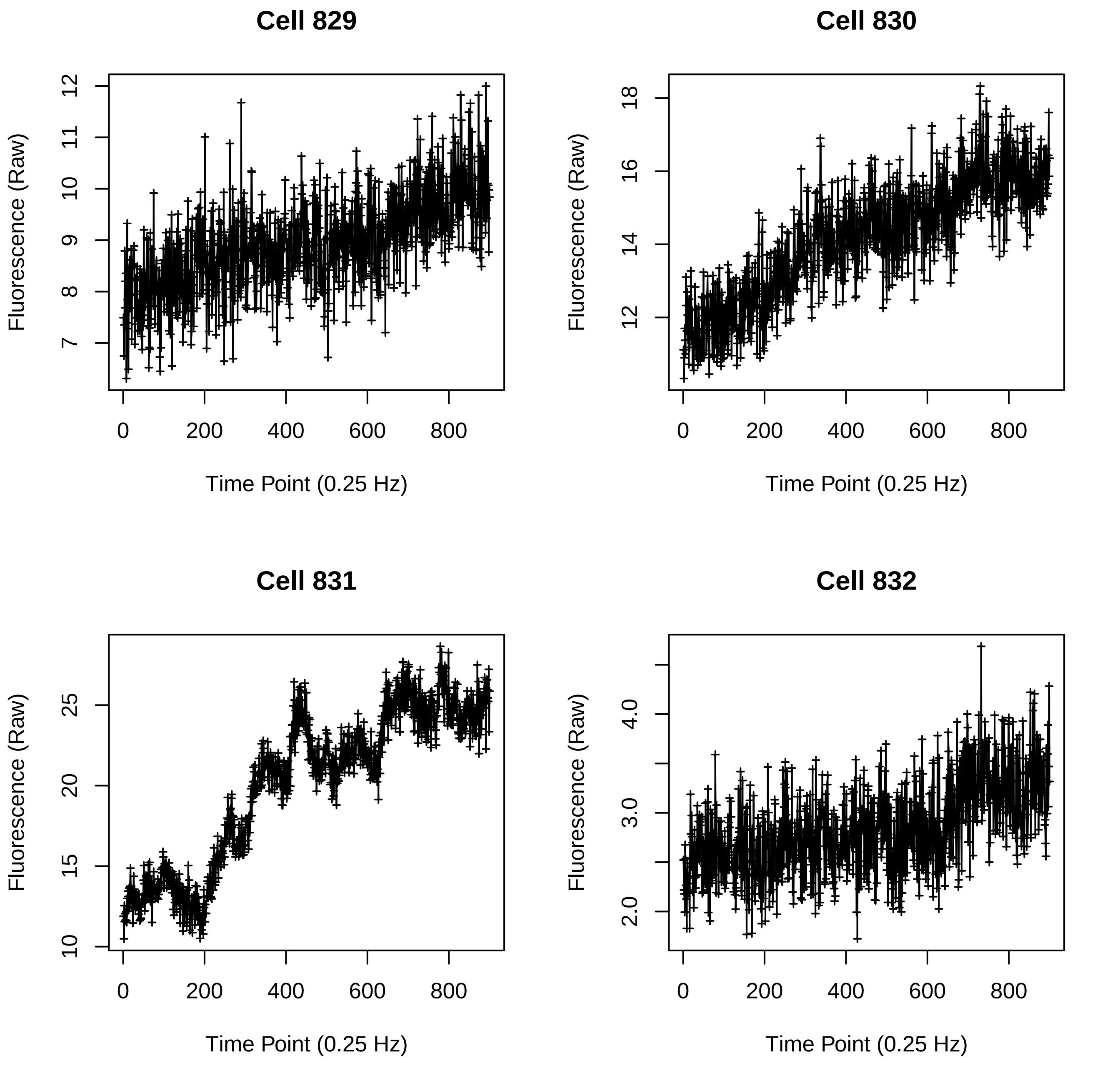
Cell 829
Fluorescence (Raw)
7
8
9
10
11
12
0
200
400
600
800
Time Point (0.25 Hz)
Cell 830
Fluorescence (Raw)
12
14
16
18
0
200
400
600
800
Time Point (0.25 Hz)
Cell 831
Fluorescence (Raw)
10
15
20
25
0
200
400
600
800
Time Point (0.25 Hz)
Cell 832
Fluorescence (Raw)
2.0
3.0
4.0
0
200
400
600
800
Time Point (0.25 Hz)

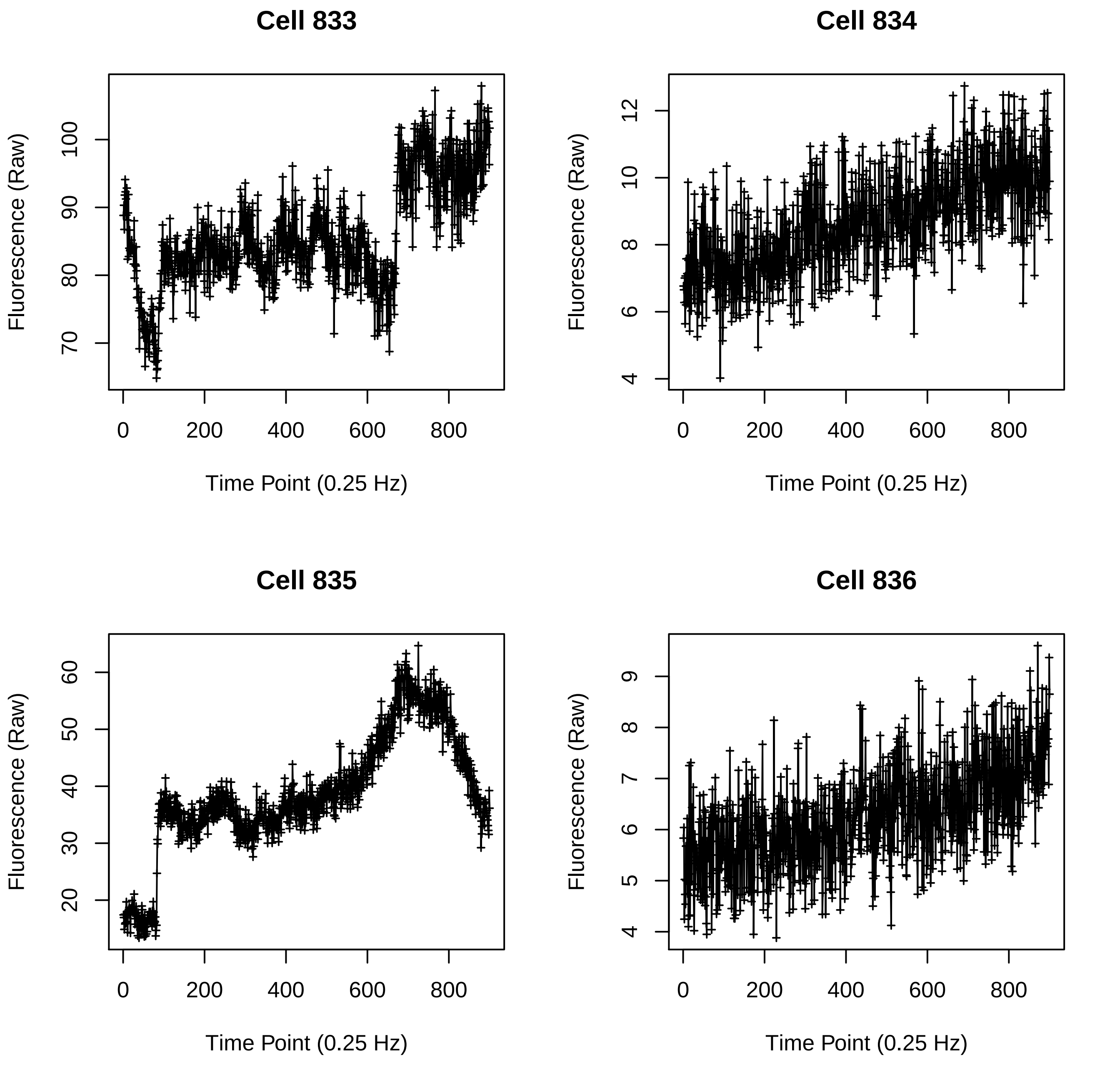

Cell 833
Cell 834
Cell 835
Cell 836
Fluorescence (Raw)
Time Point (0.25 Hz)

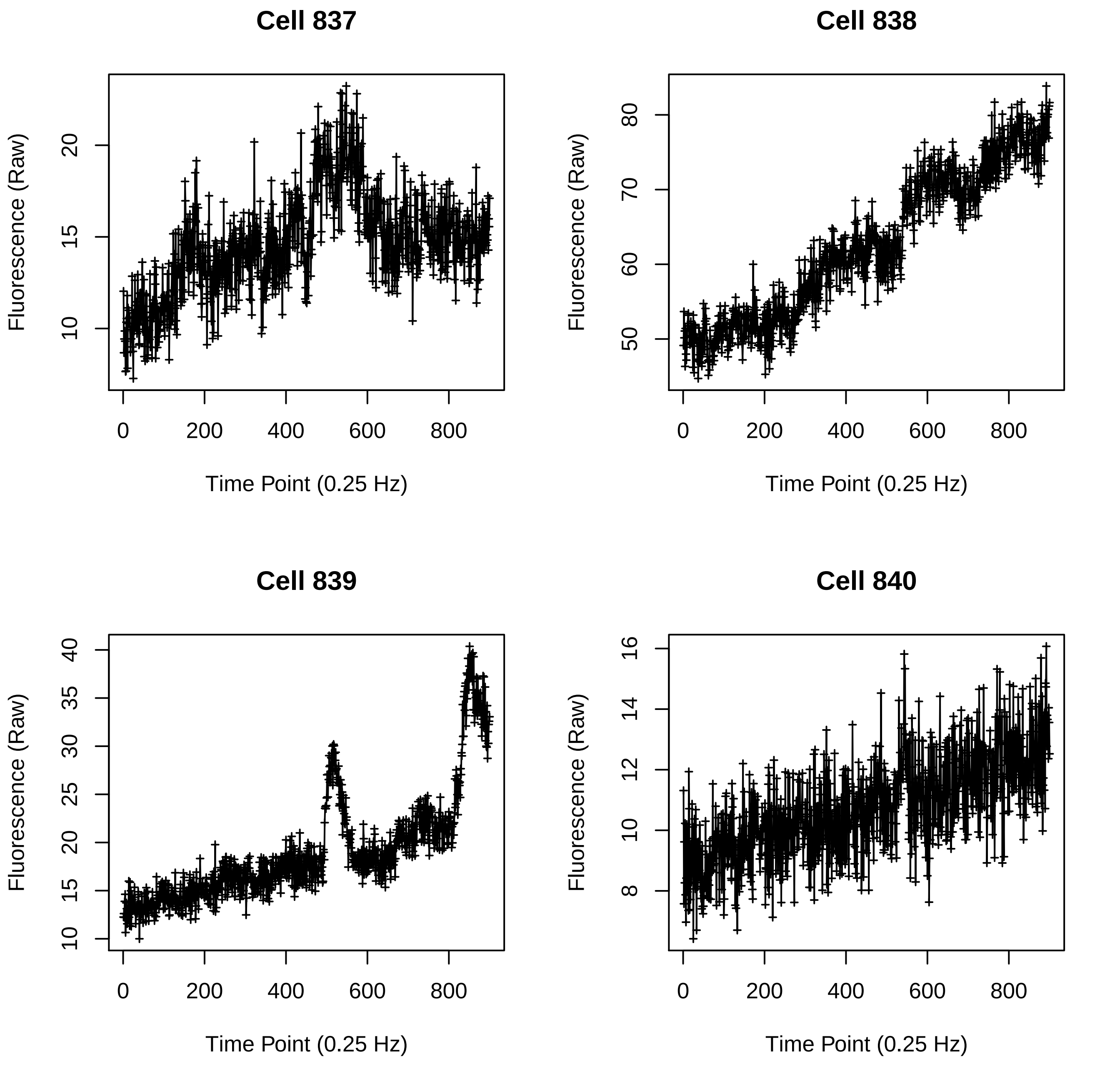
Cell 837
Fluorescence (Raw)
10
15
20
0
200
400
600
800
Time Point (0.25 Hz)
Cell 838
Fluorescence (Raw)
50
60
70
80
0
200
400
600
800
Time Point (0.25 Hz)
Cell 839
Fluorescence (Raw)
10
15
20
25
30
35
40
0
200
400
600
800
Time Point (0.25 Hz)
Cell 840
Fluorescence (Raw)
8
10
12
14
16
0
200
400
600
800
Time Point (0.25 Hz)

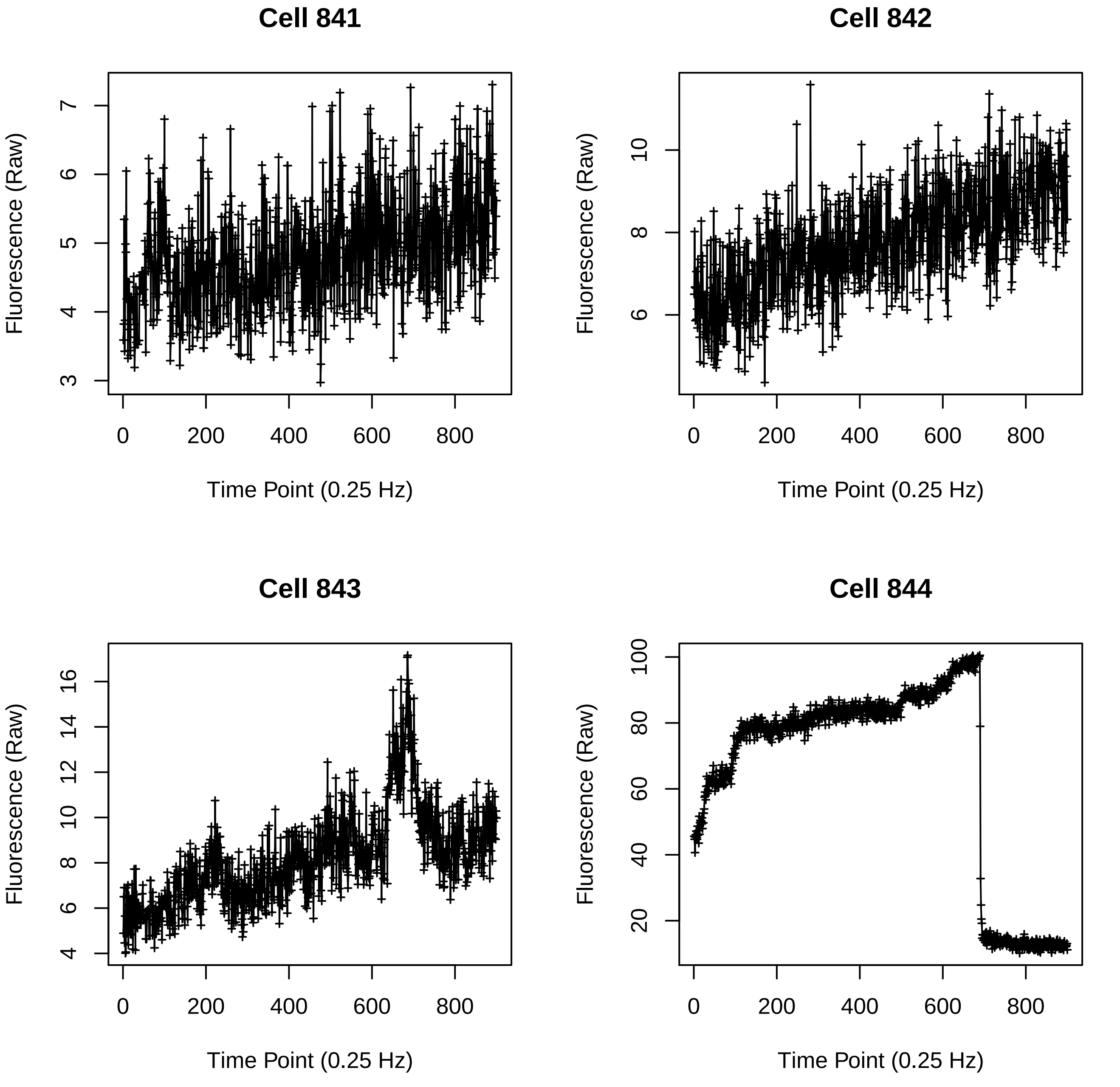
Cell 841
Cell 842
Cell 843
Cell 844
Fluorescence (Raw)
Time Point (0.25 Hz)
0
200
400
600
800
3
4
5
6
7
6
8
10
4
6
8
10
12
14
16
20
40
60
80
100

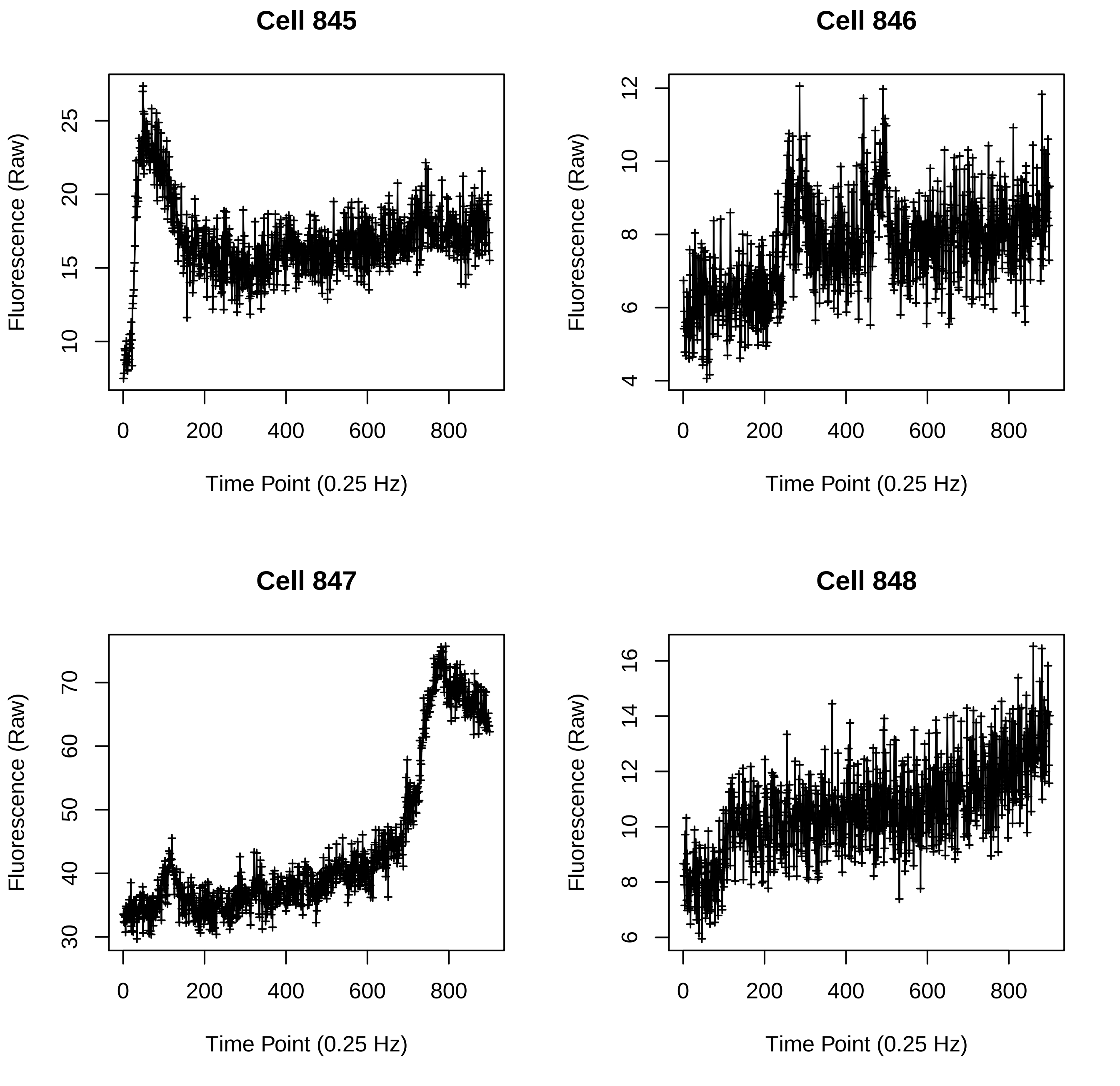
Cell 845
Cell 846
Cell 847
Cell 848
Fluorescence (Raw)
Time Point (0.25 Hz)
0
200
400
600
800

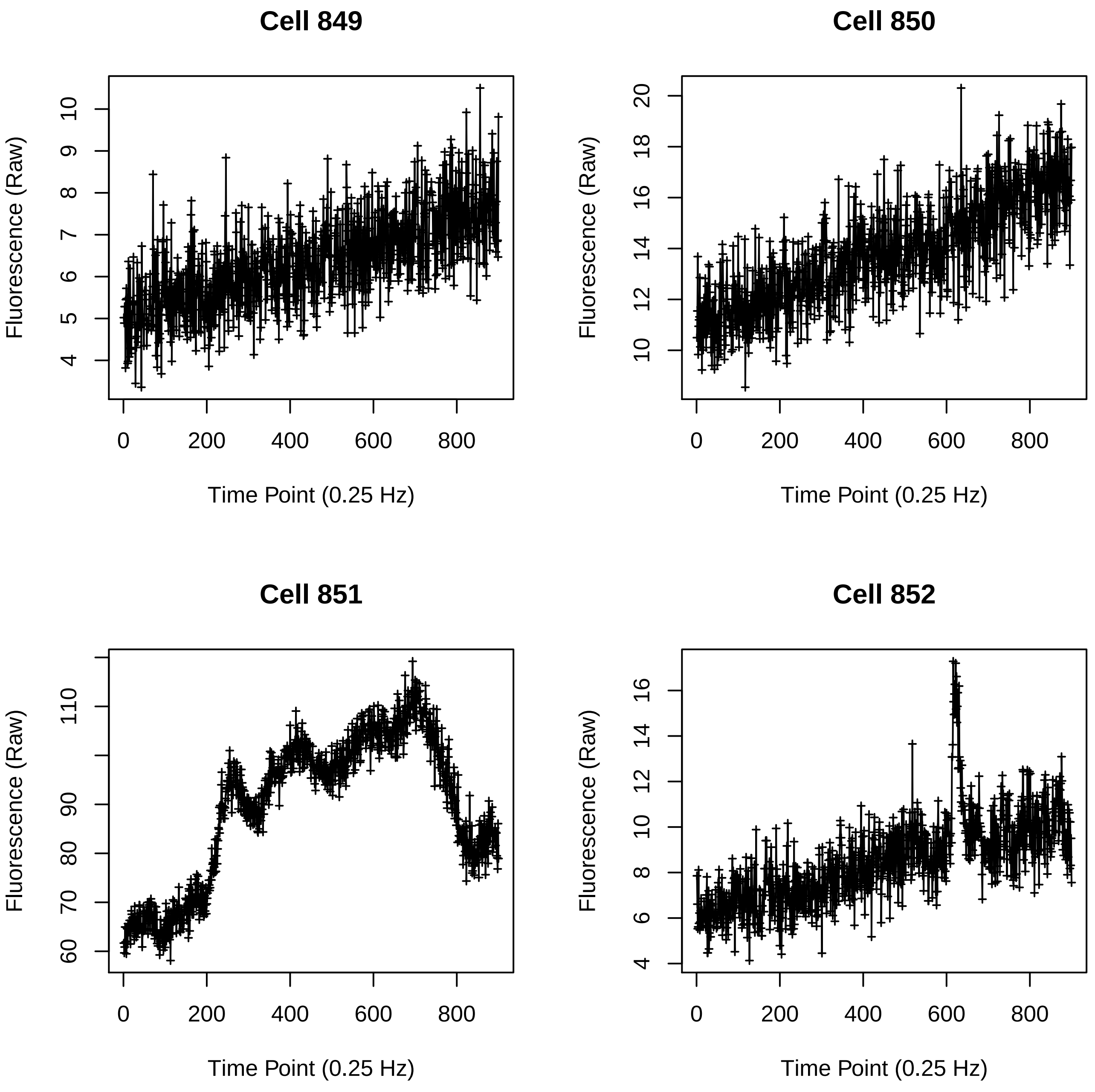

Cell 849
Cell 850
Cell 851
Cell 852
Fluorescence (Raw)
Time Point (0.25 Hz)
0
200
400
600
800
4
5
6
7
8
9
10
12
14
16
18
20
60
70
80
90
110

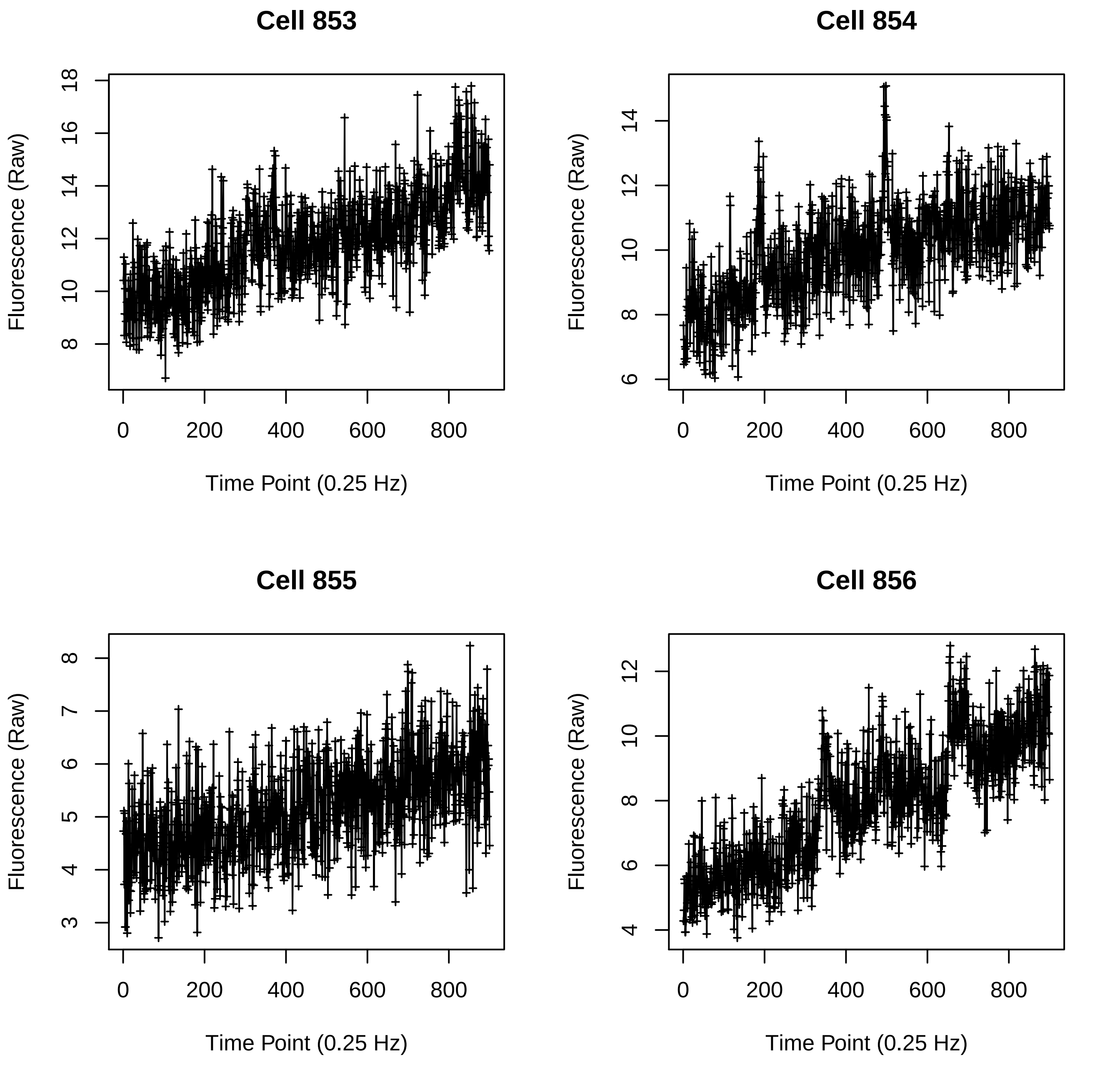

Cell 853
Cell 854
Cell 855
Cell 856
Fluorescence (Raw)
Time Point (0.25 Hz)
0
200
400
600
800

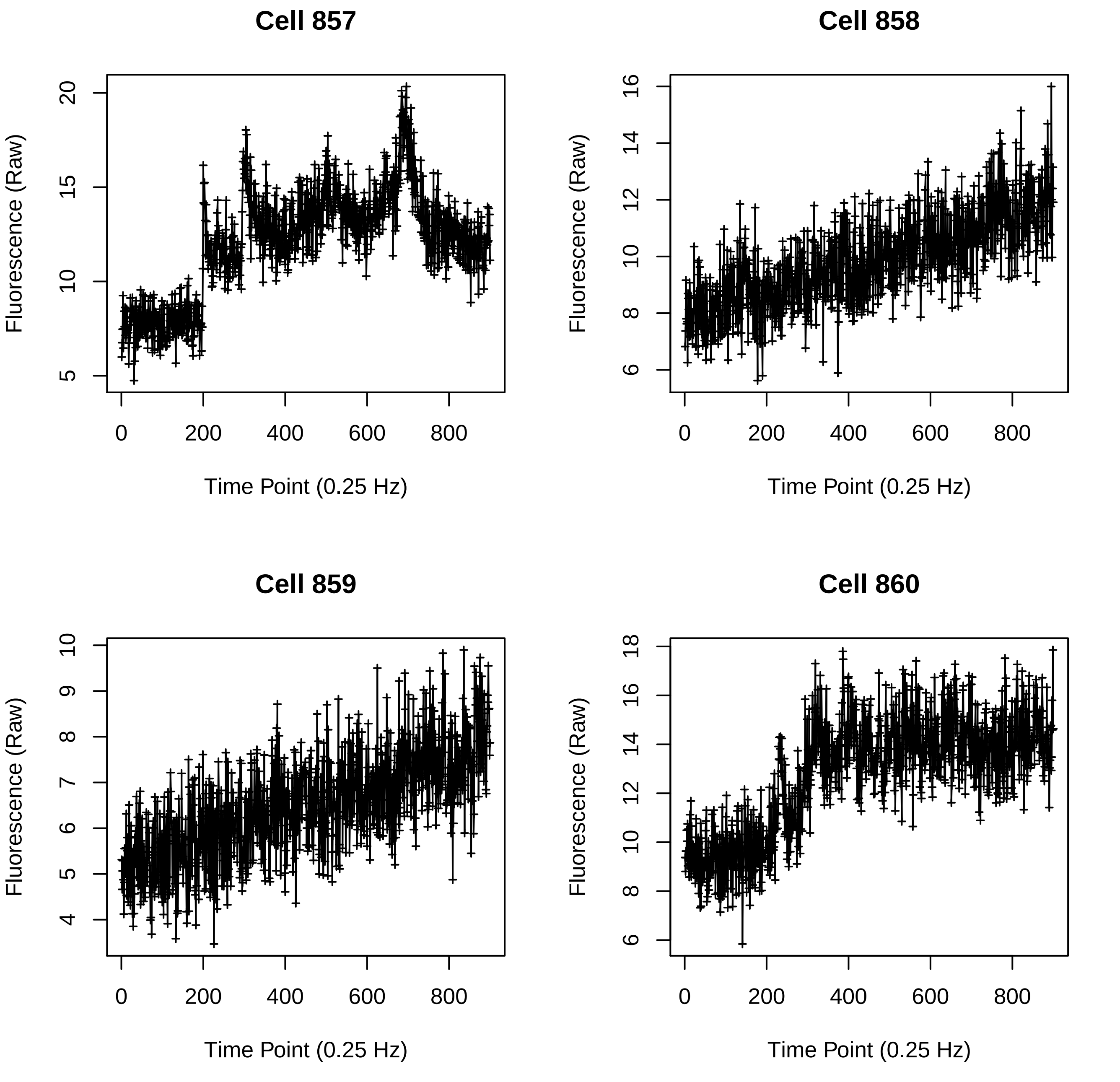

Cell 857
Cell 858
Cell 859
Cell 860
Fluorescence (Raw)
Time Point (0.25 Hz)
0
200
400
600
800
5
10
15
20
6
8
10
12
14
16
4
5
6
7
8
9
10
18

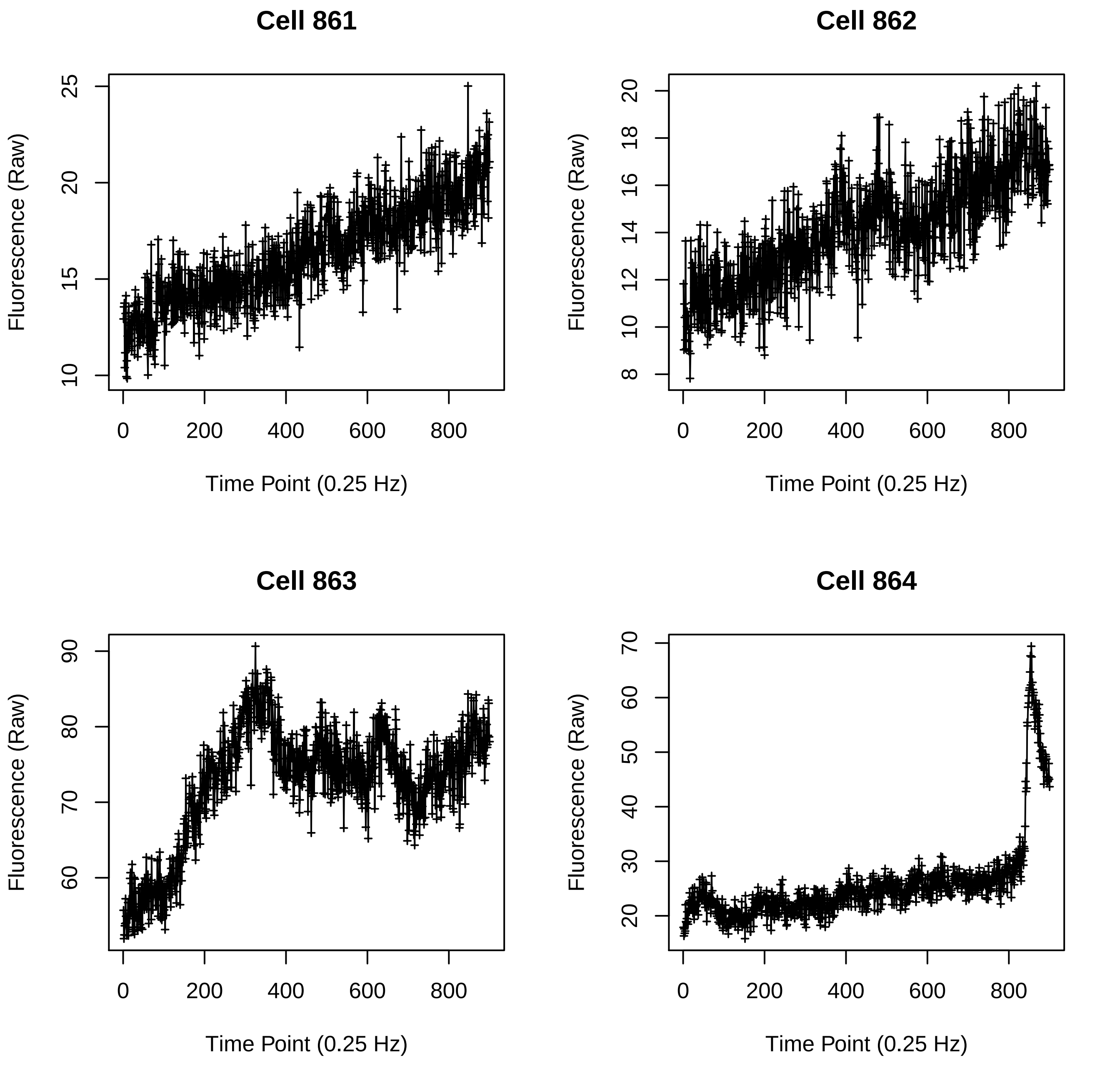
Cell 861
Fluorescence (Raw)
10
15
20
25
0
200
400
600
800
Time Point (0.25 Hz)
Cell 862
Fluorescence (Raw)
8
10
12
14
16
18
20
0
200
400
600
800
Time Point (0.25 Hz)
Cell 863
Fluorescence (Raw)
60
70
80
90
0
200
400
600
800
Time Point (0.25 Hz)
Cell 864
Fluorescence (Raw)
20
30
40
50
60
70
0
200
400
600
800
Time Point (0.25 Hz)

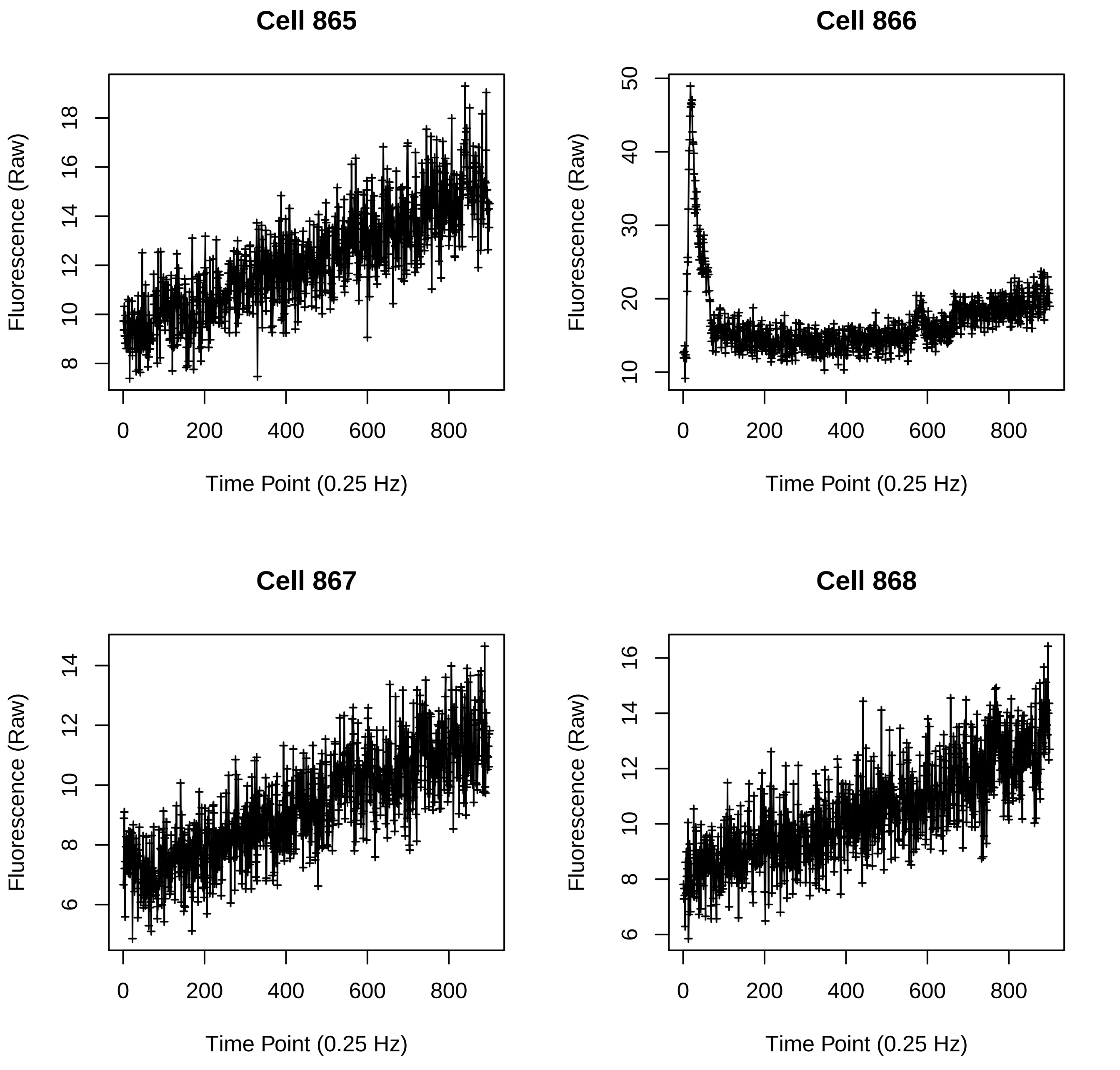
Cell 865
Fluorescence (Raw)
8
10
12
14
16
18
0
200
400
600
800
Time Point (0.25 Hz)
Cell 866
Fluorescence (Raw)
10
20
30
40
50
0
200
400
600
800
Time Point (0.25 Hz)
Cell 867
Fluorescence (Raw)
6
8
10
12
14
0
200
400
600
800
Time Point (0.25 Hz)
Cell 868
Fluorescence (Raw)
6
8
10
12
14
16
0
200
400
600
800
Time Point (0.25 Hz)

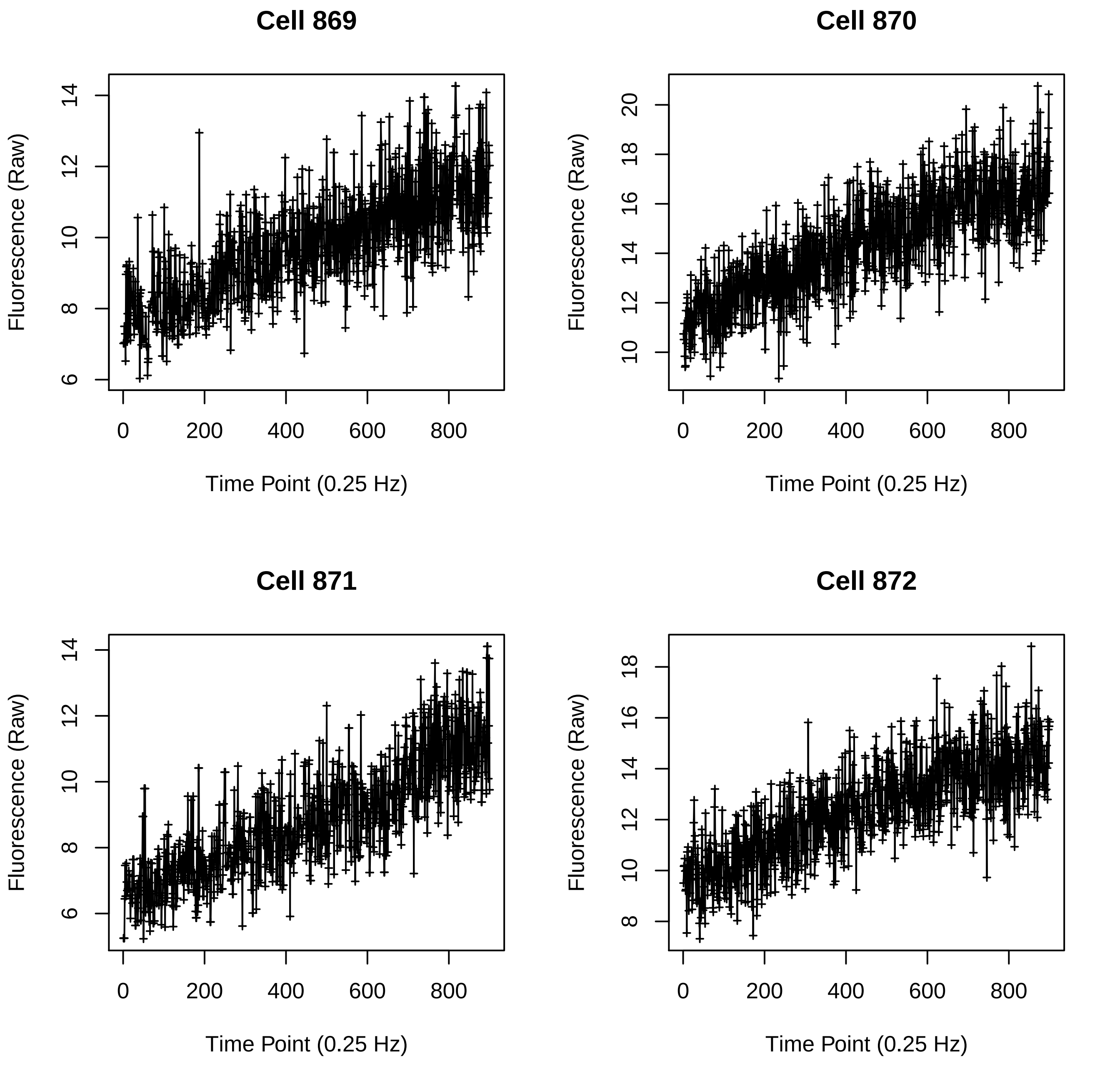
Cell 869
Cell 870
Cell 871
Cell 872
Fluorescence (Raw)
Time Point (0.25 Hz)
0
200
400
600
800
6
8
10
12
14
16
18
20

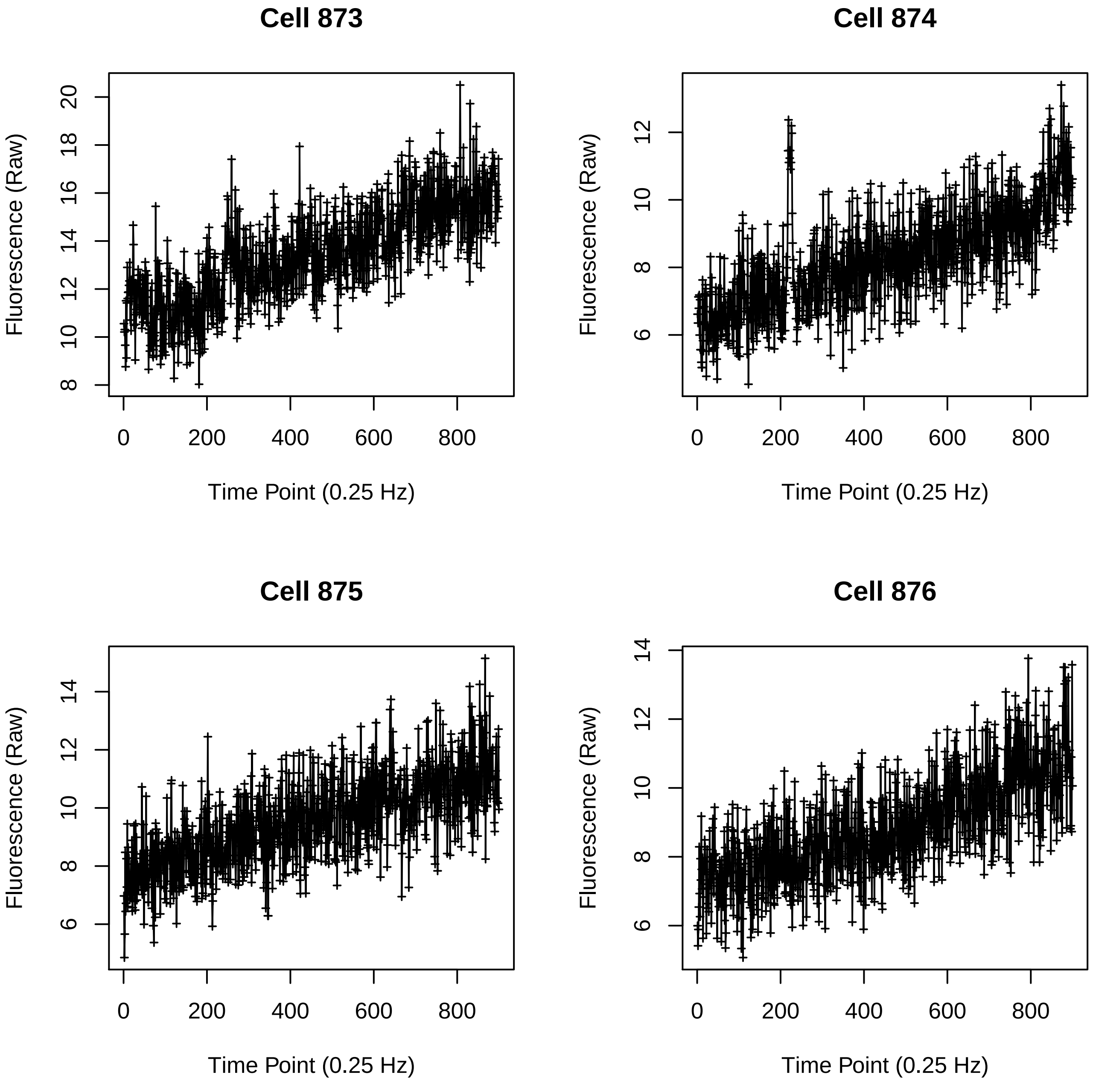
Cell 873
Cell 874
Cell 875
Cell 876
Fluorescence (Raw)
Time Point (0.25 Hz)
0
200
400
600
800

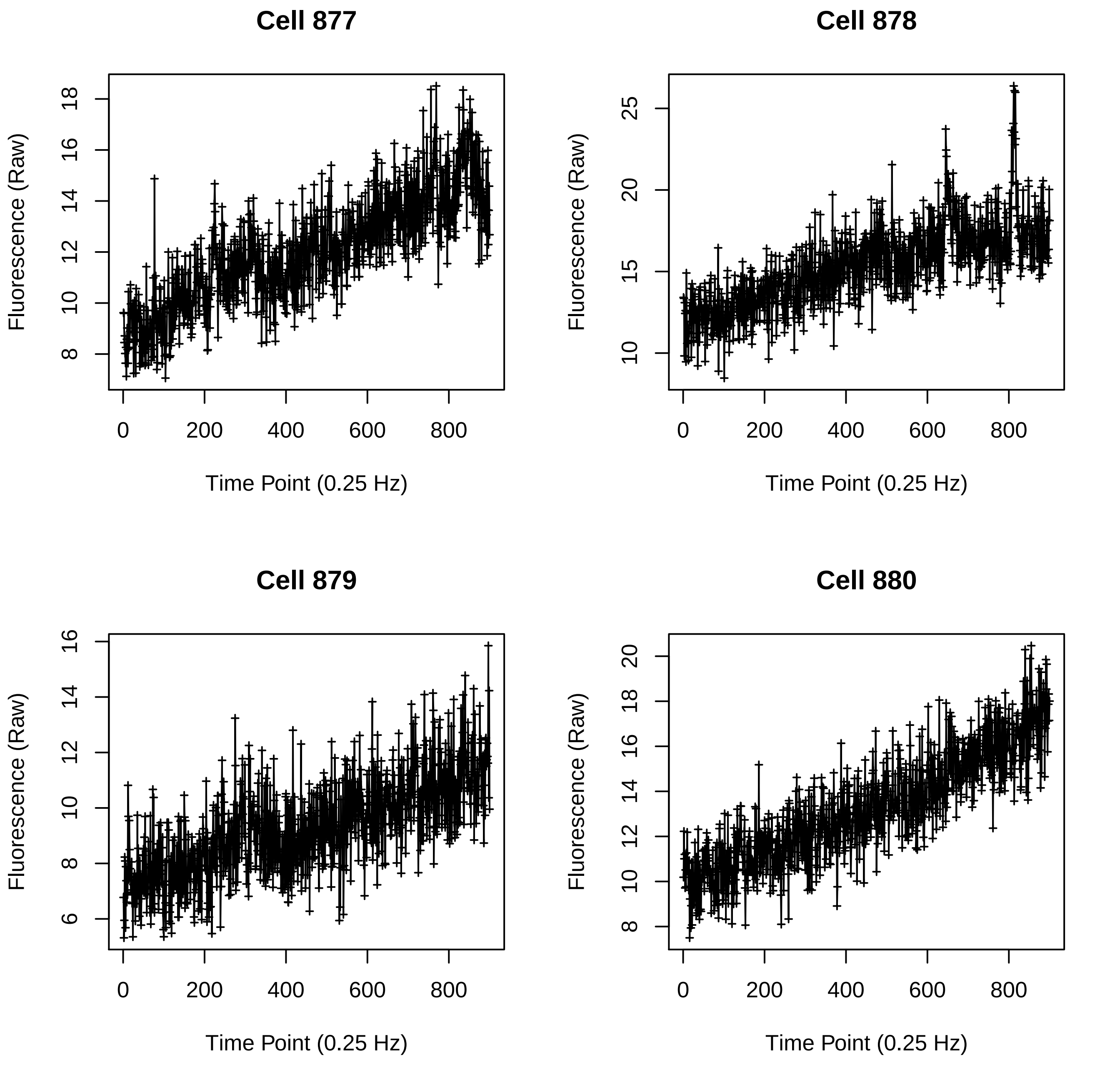

Cell 877
Fluorescence (Raw)
8
10
12
14
16
18
0
200
400
600
800
Time Point (0.25 Hz)
Cell 878
Fluorescence (Raw)
10
15
20
25
0
200
400
600
800
Time Point (0.25 Hz)
Cell 879
Fluorescence (Raw)
6
8
10
12
14
16
0
200
400
600
800
Time Point (0.25 Hz)
Cell 880
Fluorescence (Raw)
8
10
12
14
16
18
20
0
200
400
600
800
Time Point (0.25 Hz)

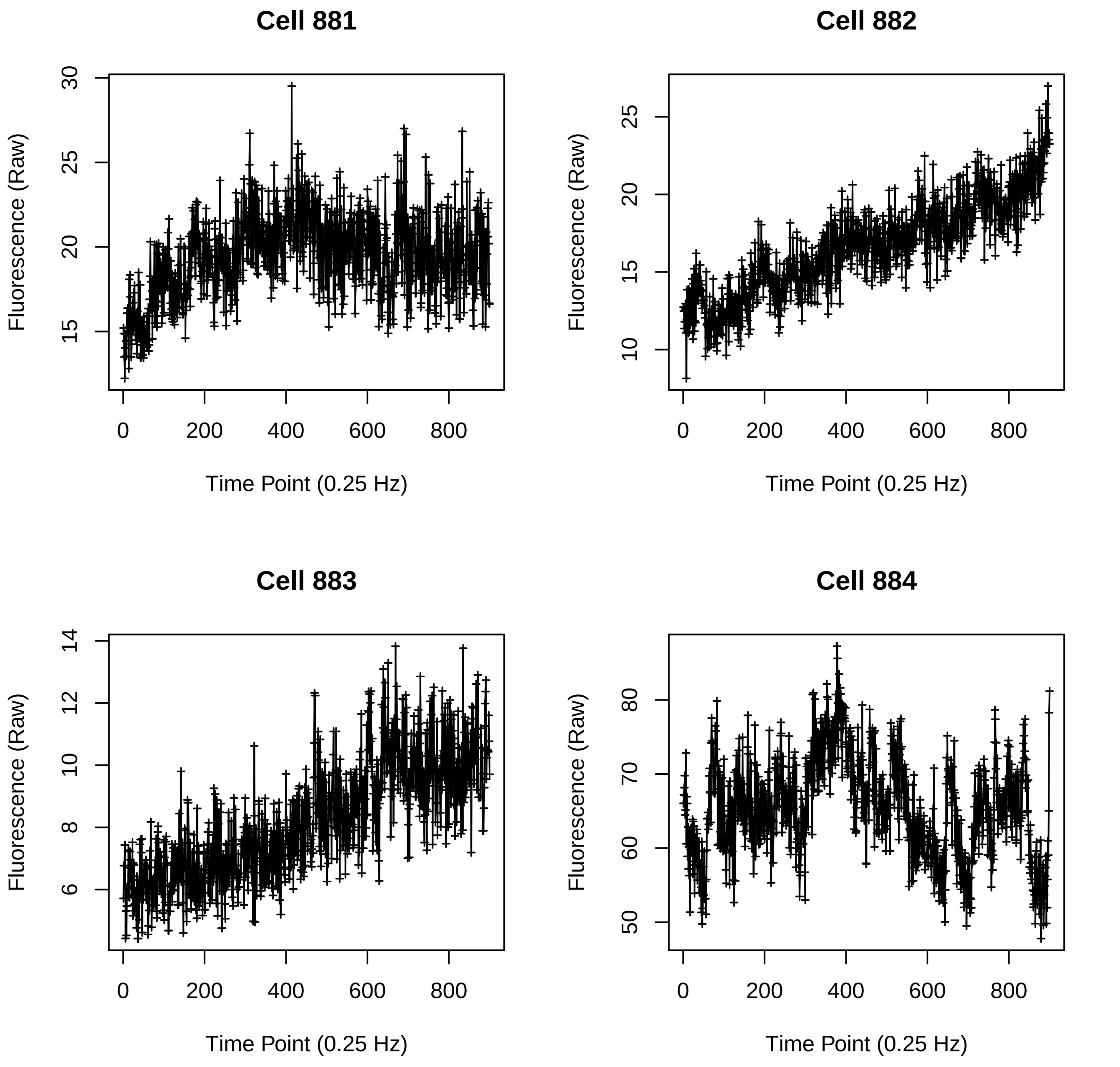
Cell 881
Cell 882
Cell 883
Cell 884
Fluorescence (Raw)
Time Point (0.25 Hz)
0
200
400
600
800
15
20
25
30
10
6
8
12
14
50
60
70
80

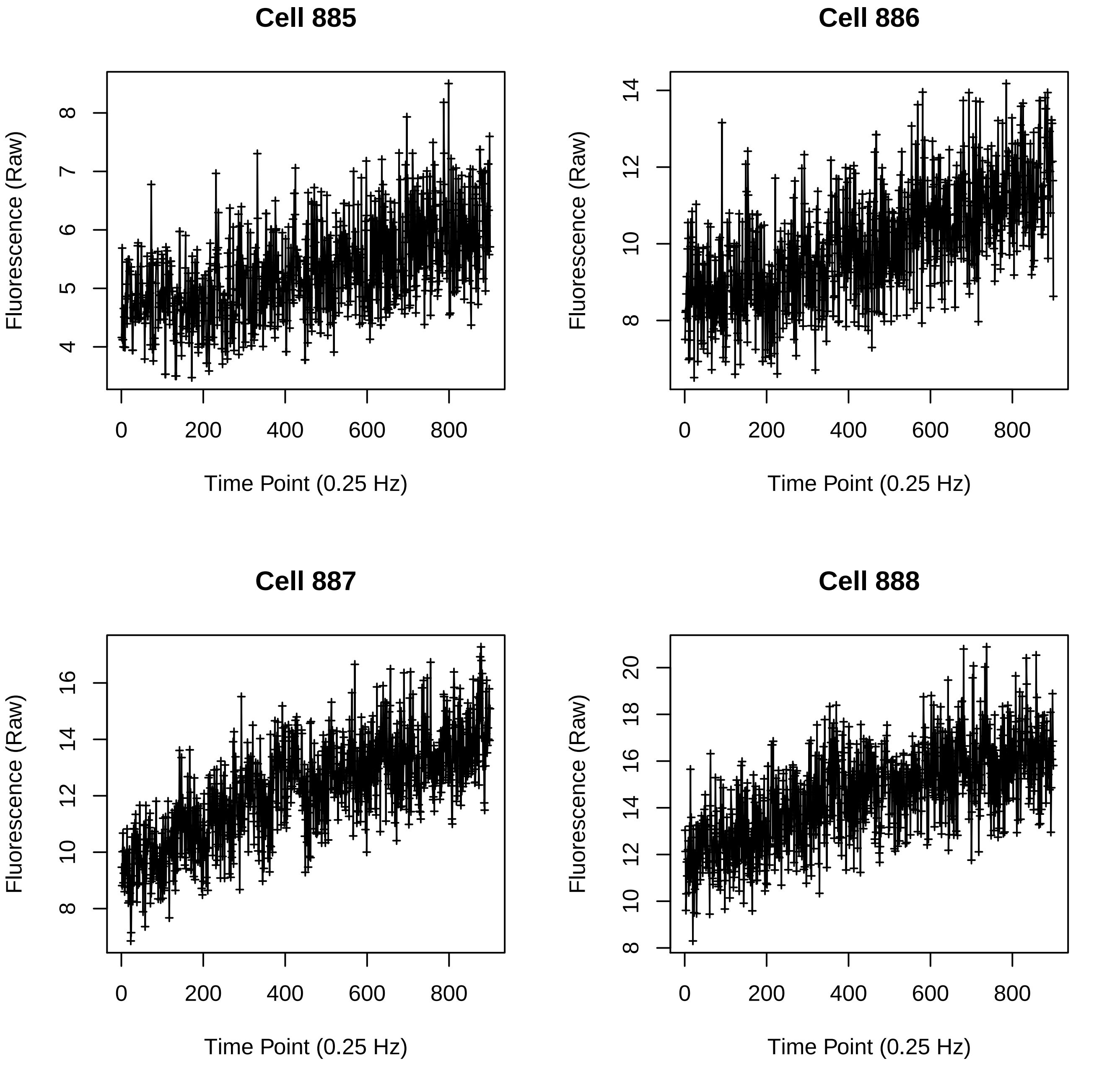
Cell 885
Cell 886
Cell 887
Cell 888
Fluorescence (Raw)
Time Point (0.25 Hz)
0
200
400
600
800
4
5
6
7
8
10
12
14
16
18
20

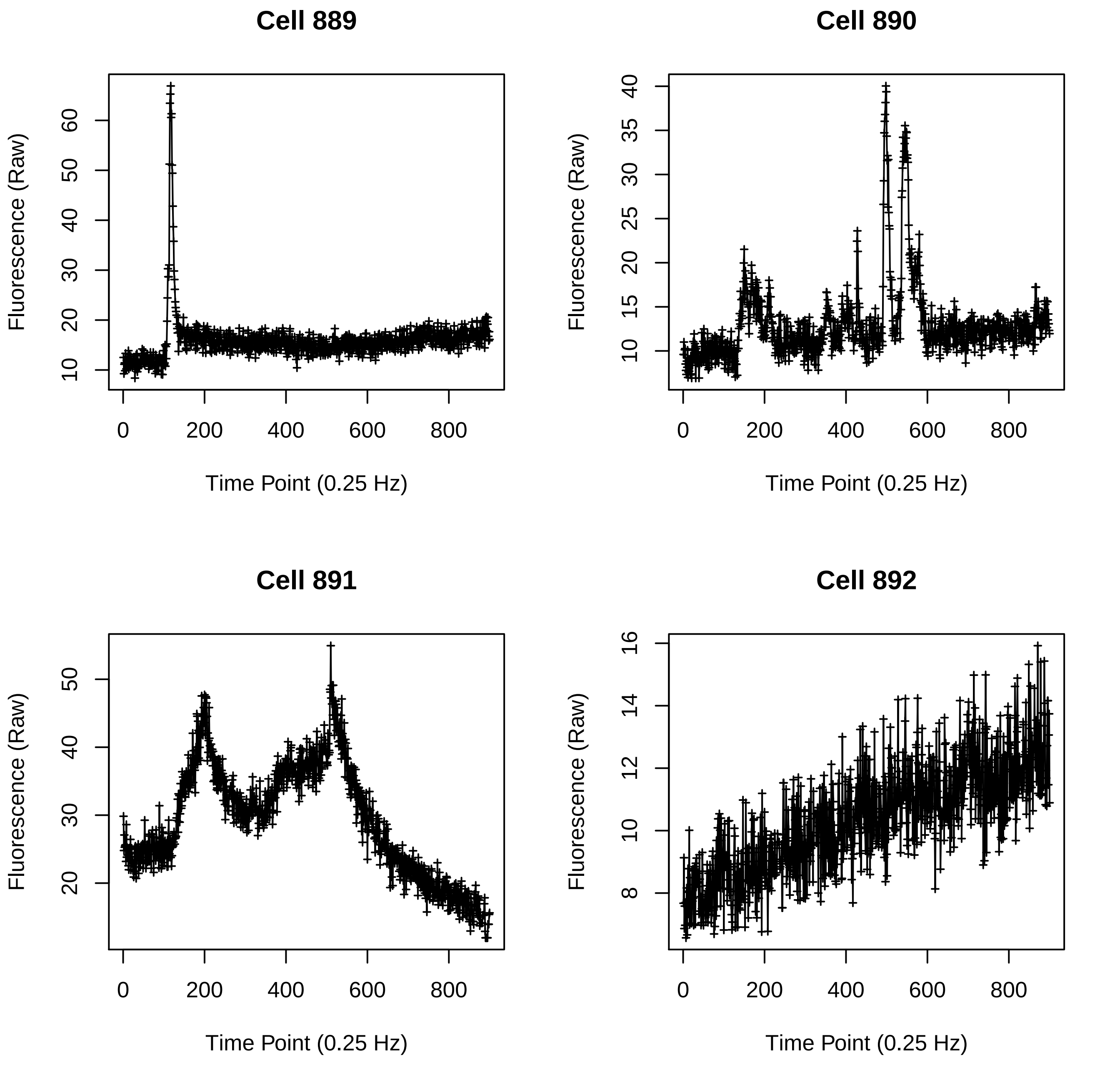
Cell 889
Cell 890
Cell 891
Cell 892
Fluorescence (Raw)
Time Point (0.25 Hz)
0
200
400
600
800
10
20
30
40
50
60
15
25
35
8
12
14
16

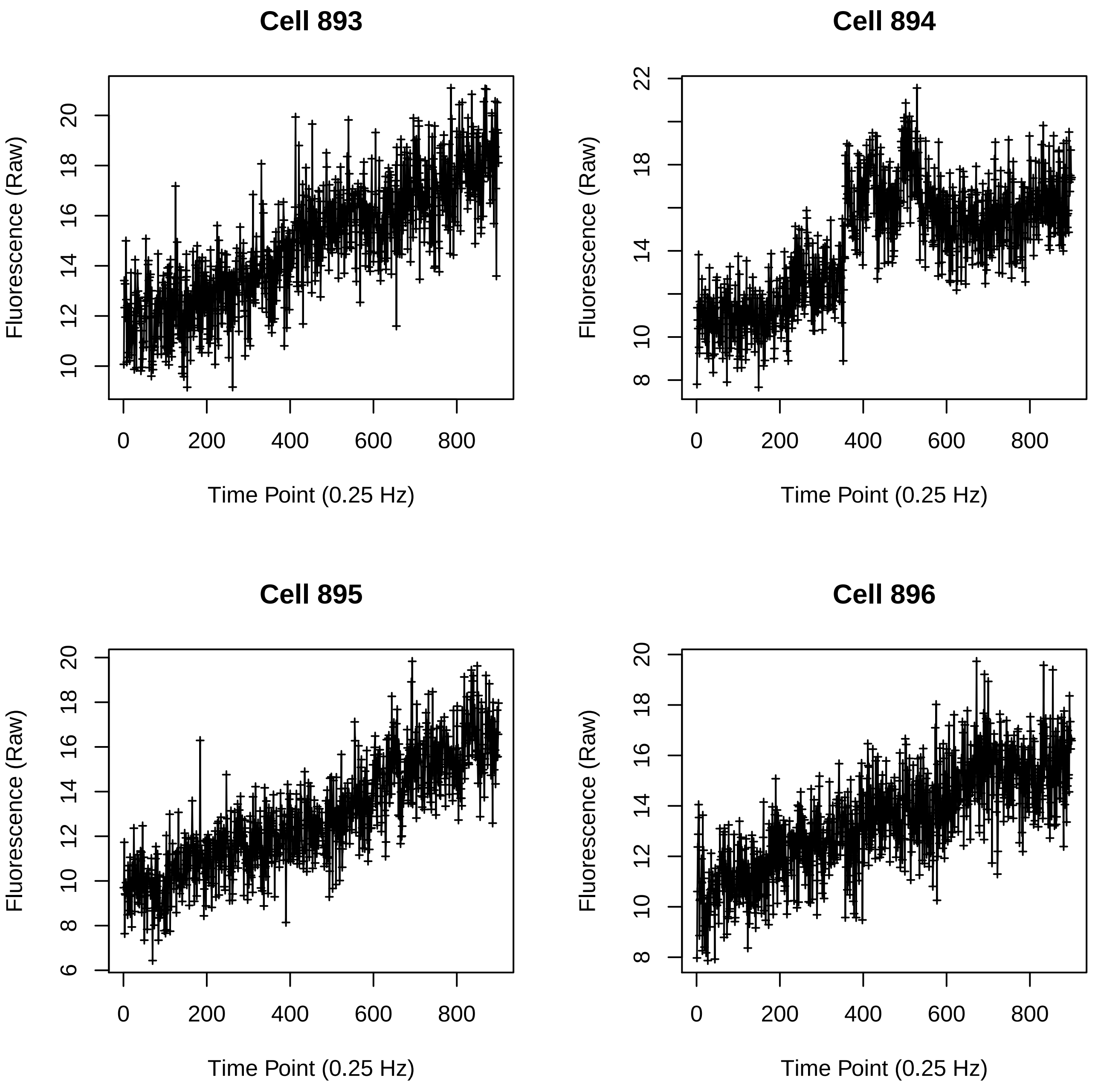
Cell 893
Cell 894
Cell 895
Cell 896
Fluorescence (Raw)
Time Point (0.25 Hz)
0
200
400
600
800
6
8
10
12
14
16
18
20
22

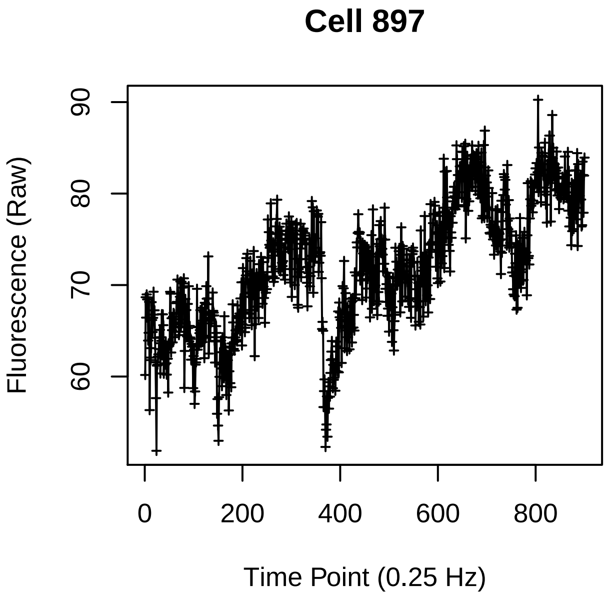
Cell 897
Fluorescence (Raw)
60
70
80
90
0
200
400
600
800
Time Point (0.25 Hz)
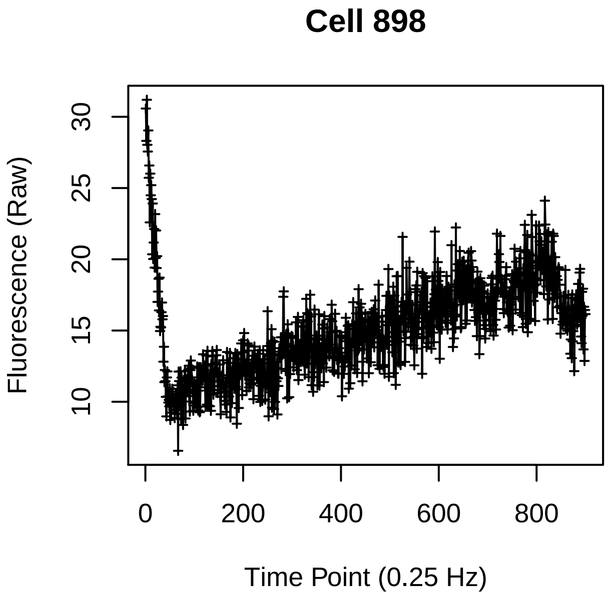
Cell 898
Fluorescence (Raw)
10
15
20
25
30
0
200
400
600
800
Time Point (0.25 Hz)
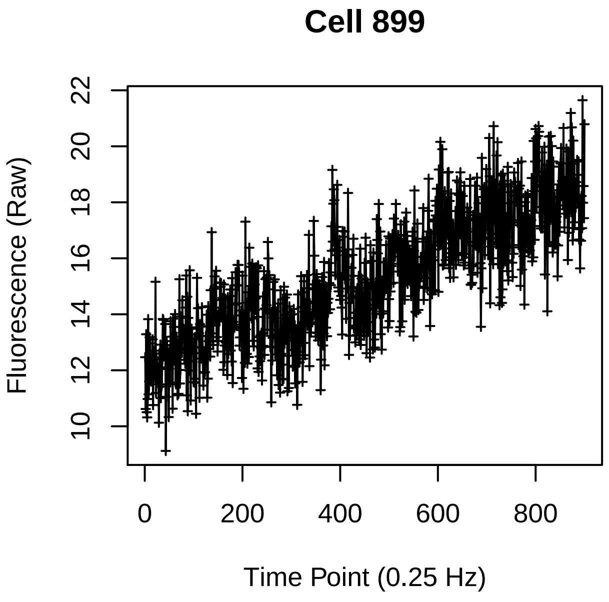
Cell 899
Fluorescence (Raw)
10
12
14
16
18
20
22
0
200
400
600
800
Time Point (0.25 Hz)
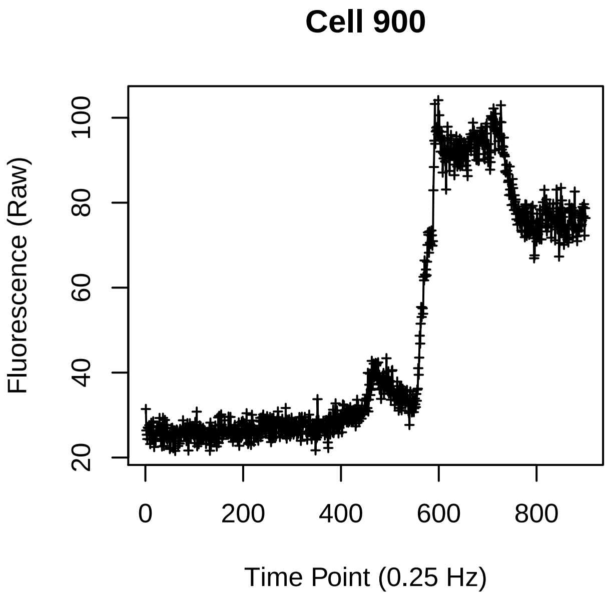
Cell 900
Fluorescence (Raw)
20
40
60
80
100
0
200
400
600
800
Time Point (0.25 Hz)

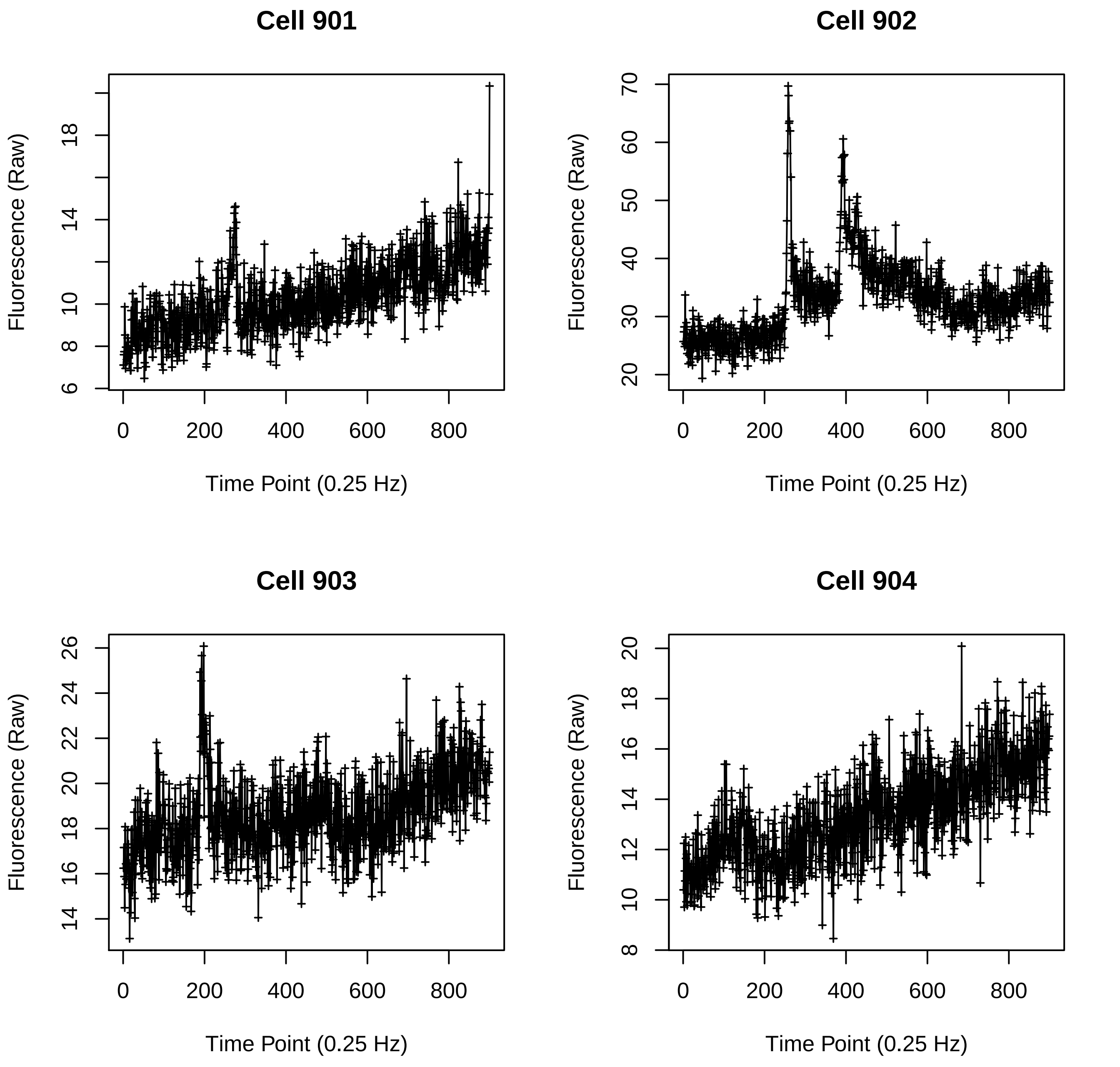
Cell 901
Cell 902
Cell 903
Cell 904
Fluorescence (Raw)
Time Point (0.25 Hz)
0
200
400
600
800
6
8
10
14
18
20
30
40
50
60
70
12
16
22
24
26

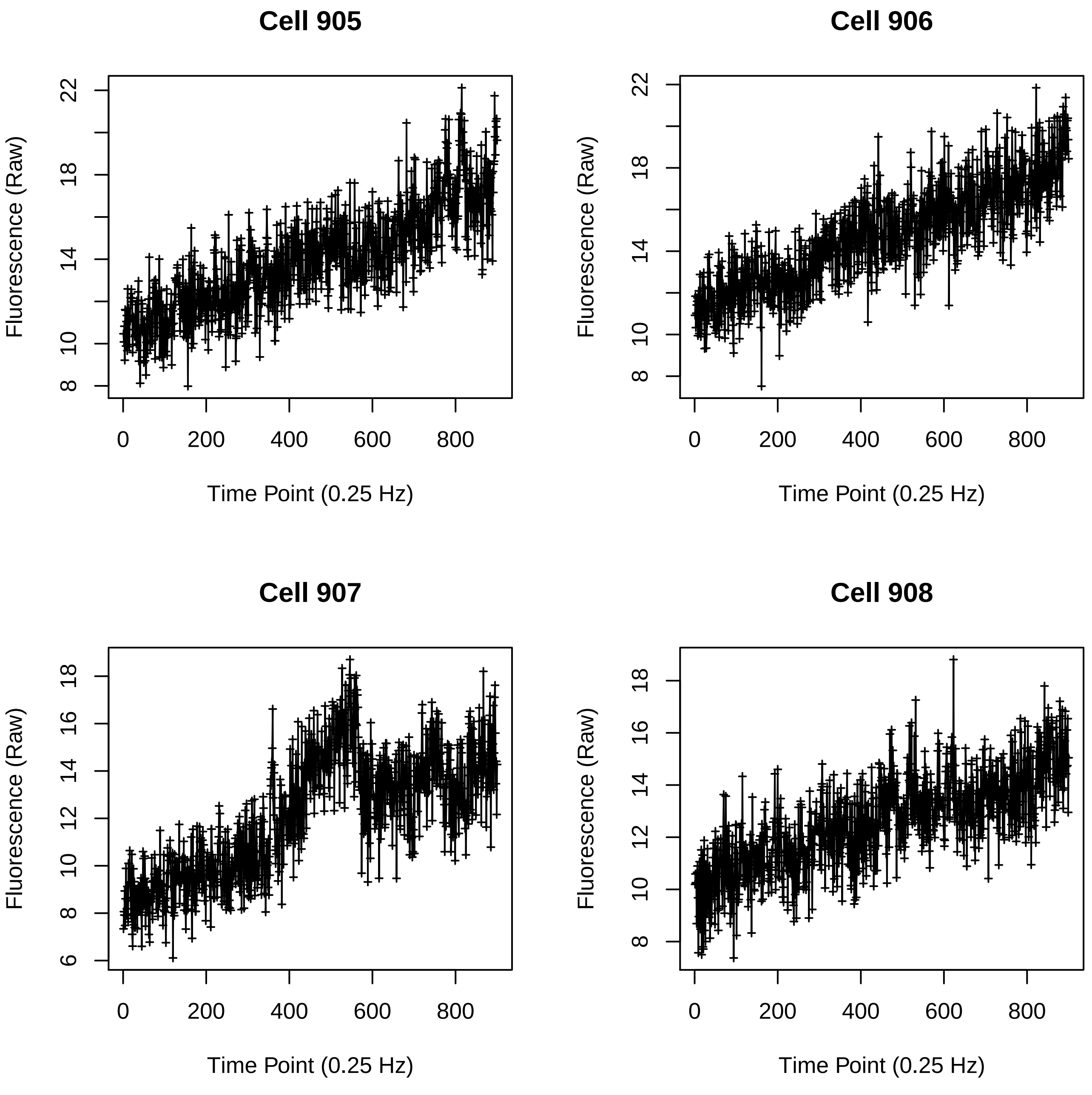
Cell 905
Cell 906
Cell 907
Cell 908
Fluorescence (Raw)
Time Point (0.25 Hz)
8
10
14
18
22
6
12
16
0
200
400
600
800

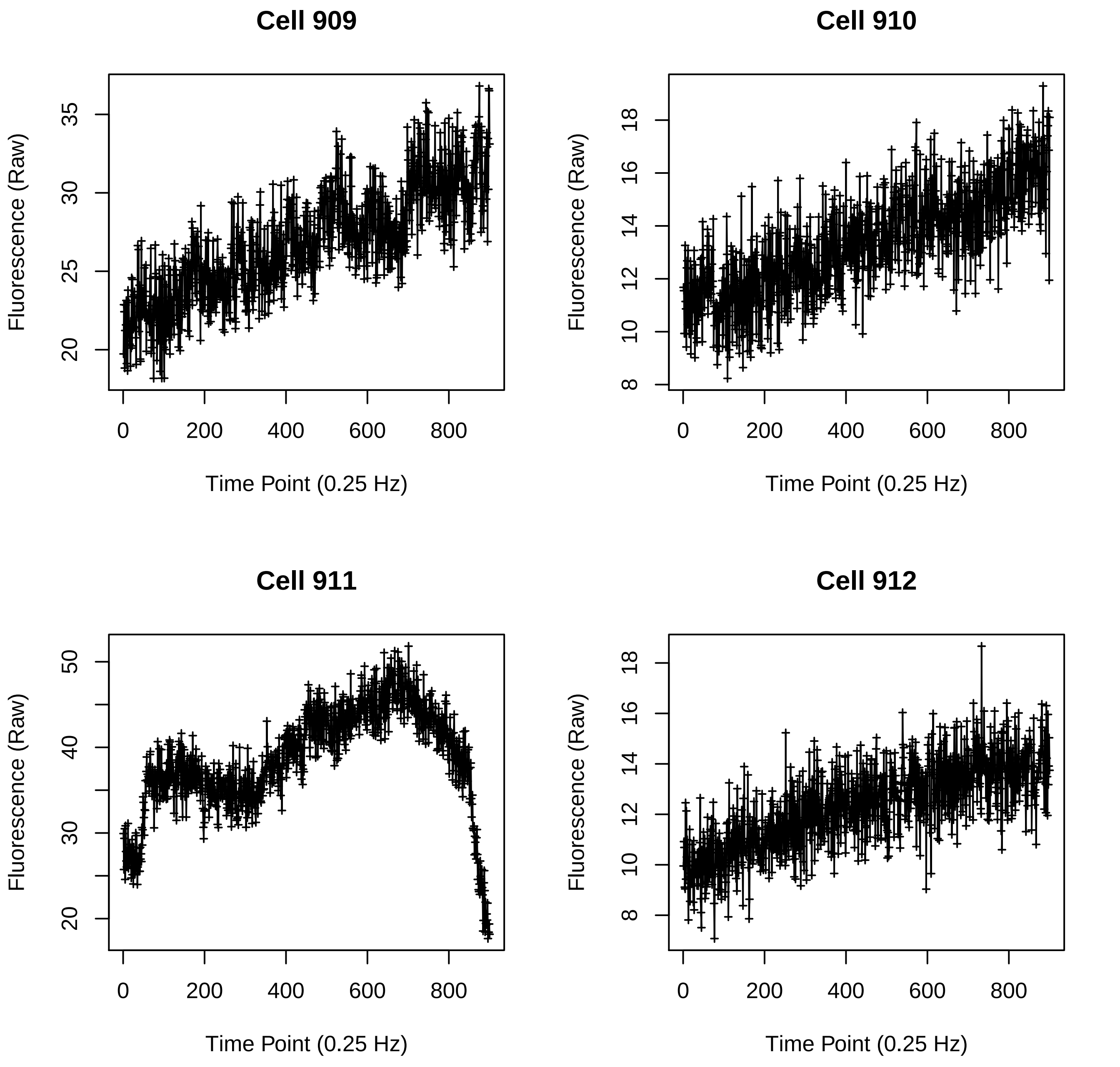

Cell 909
Cell 910
Cell 911
Cell 912
Fluorescence (Raw)
Time Point (0.25 Hz)
0
200
400
600
800
20
25
30
35
8
10
12
14
16
18
20
30
40
50

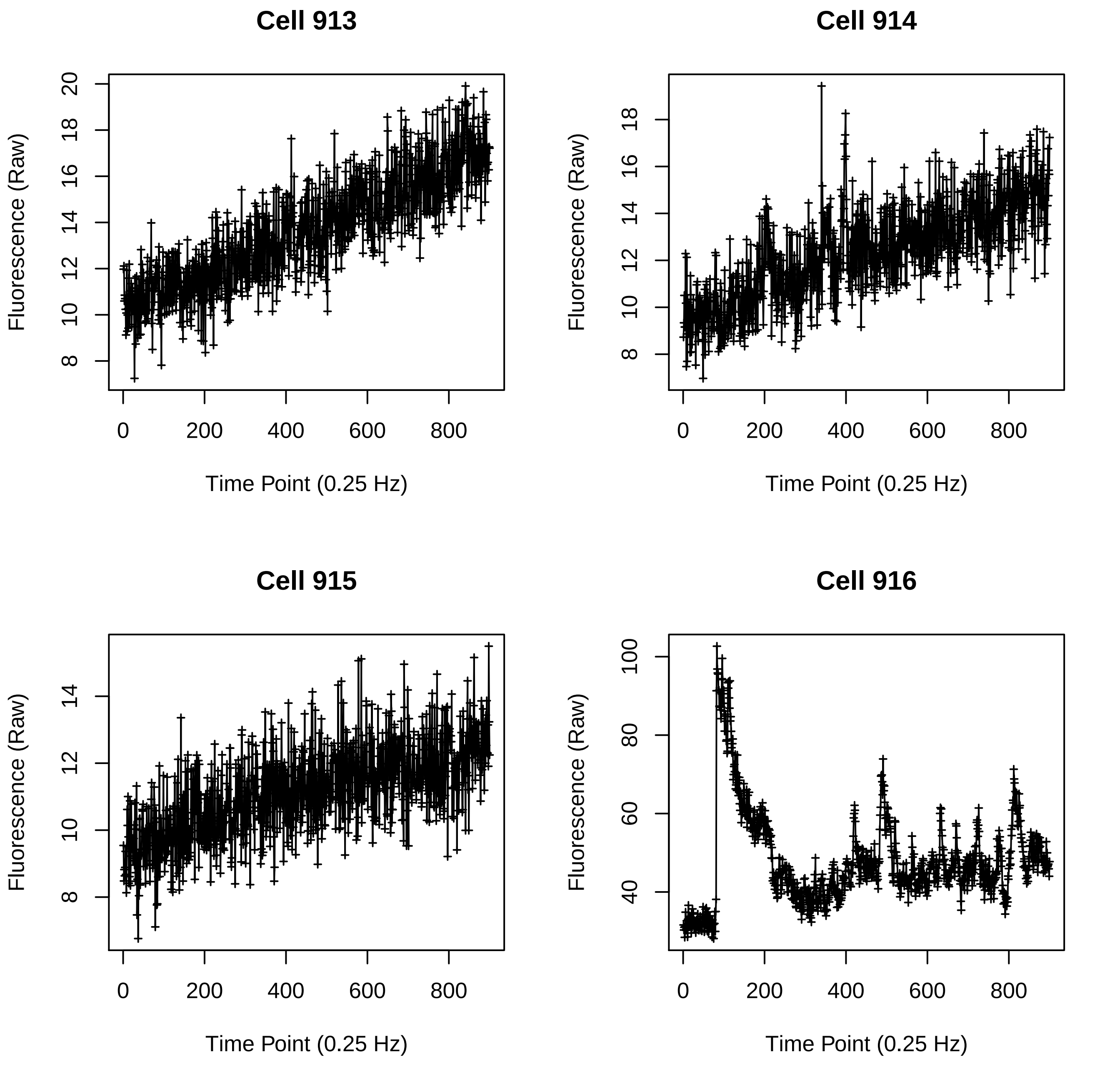
Cell 913
Cell 914
Cell 915
Cell 916
Fluorescence (Raw)
Time Point (0.25 Hz)
0
200
400
600
800
8
10
12
14
16
18
20
40
60
80
100

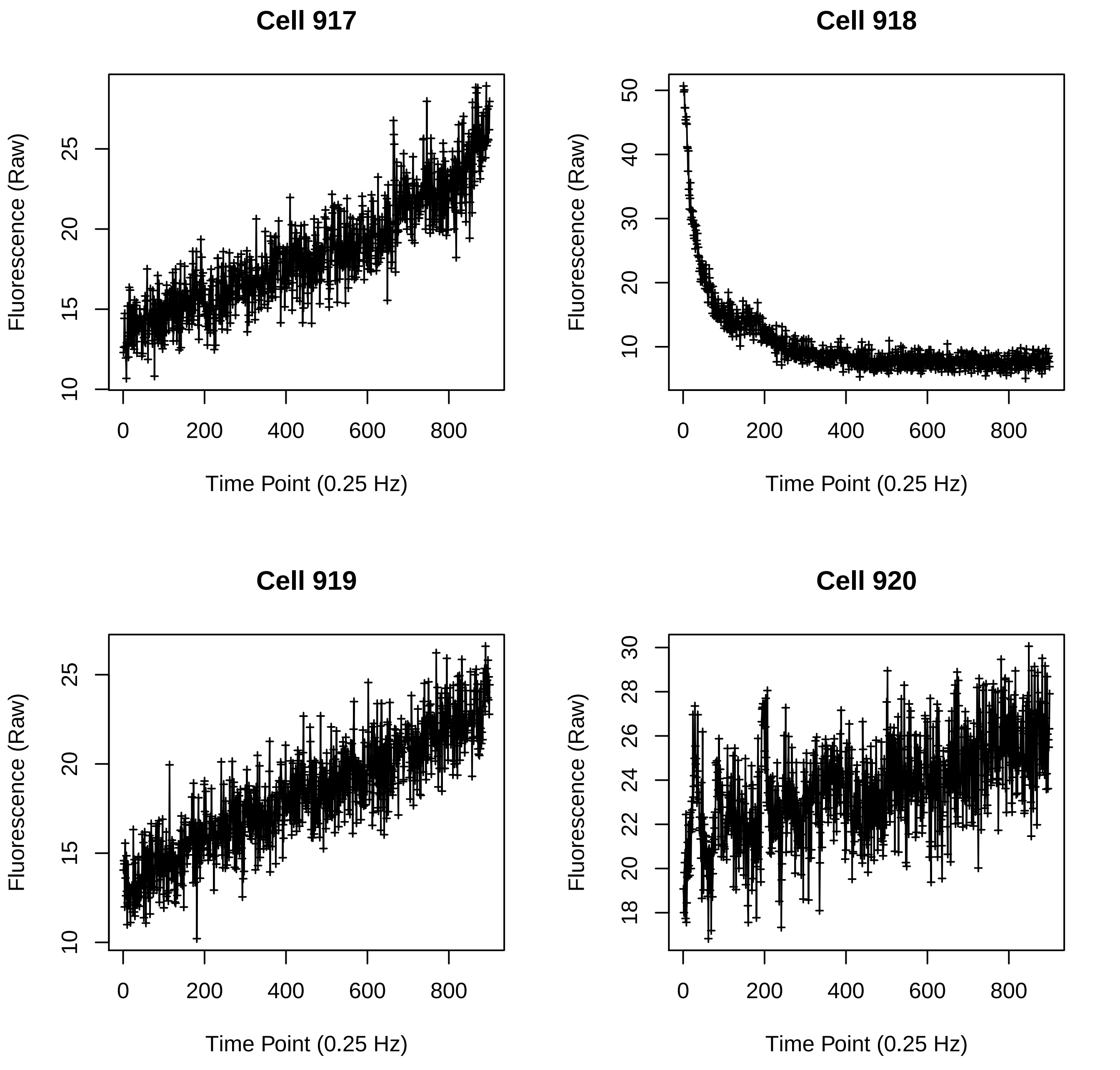
Cell 917
Fluorescence (Raw)
Time Point (0.25 Hz)
10
15
20
25
0
200
400
600
800
Cell 918
Fluorescence (Raw)
Time Point (0.25 Hz)
10
20
30
40
50
0
200
400
600
800
Cell 919
Fluorescence (Raw)
Time Point (0.25 Hz)
10
15
20
25
0
200
400
600
800
Cell 920
Fluorescence (Raw)
Time Point (0.25 Hz)
18
20
22
24
26
28
30
0
200
400
600
800

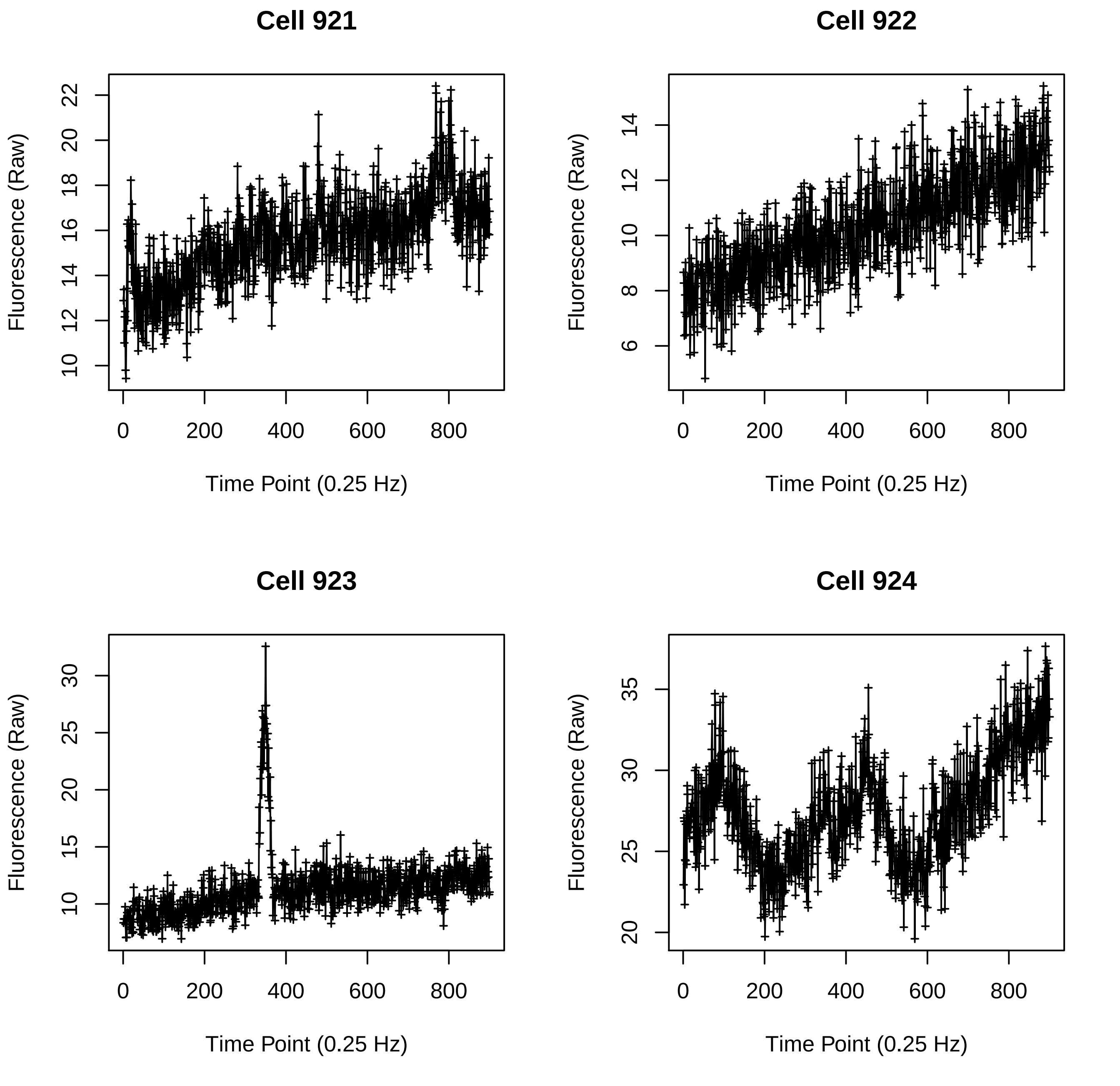

Cell 921
Fluorescence (Raw)
10
12
14
16
18
20
22
0
200
400
600
800
Time Point (0.25 Hz)
Cell 922
Fluorescence (Raw)
6
8
10
12
14
0
200
400
600
800
Time Point (0.25 Hz)
Cell 923
Fluorescence (Raw)
10
15
20
25
30
0
200
400
600
800
Time Point (0.25 Hz)
Cell 924
Fluorescence (Raw)
20
25
30
35
0
200
400
600
800
Time Point (0.25 Hz)

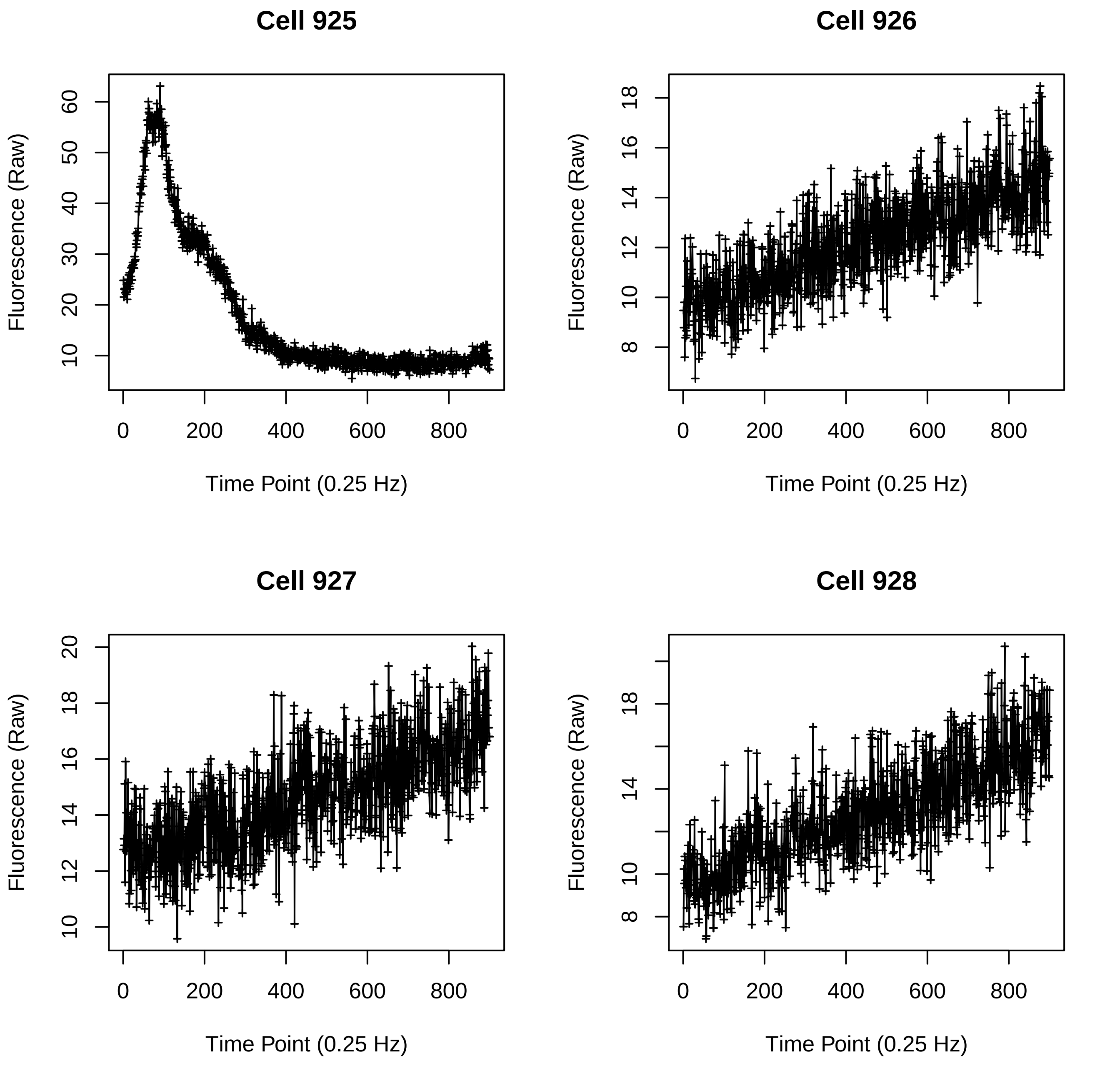

Cell 925
Cell 926
Cell 927
Cell 928
Fluorescence (Raw)
Time Point (0.25 Hz)
0
200
400
600
800
10
20
30
40
50
60
8
12
14
16
18
20

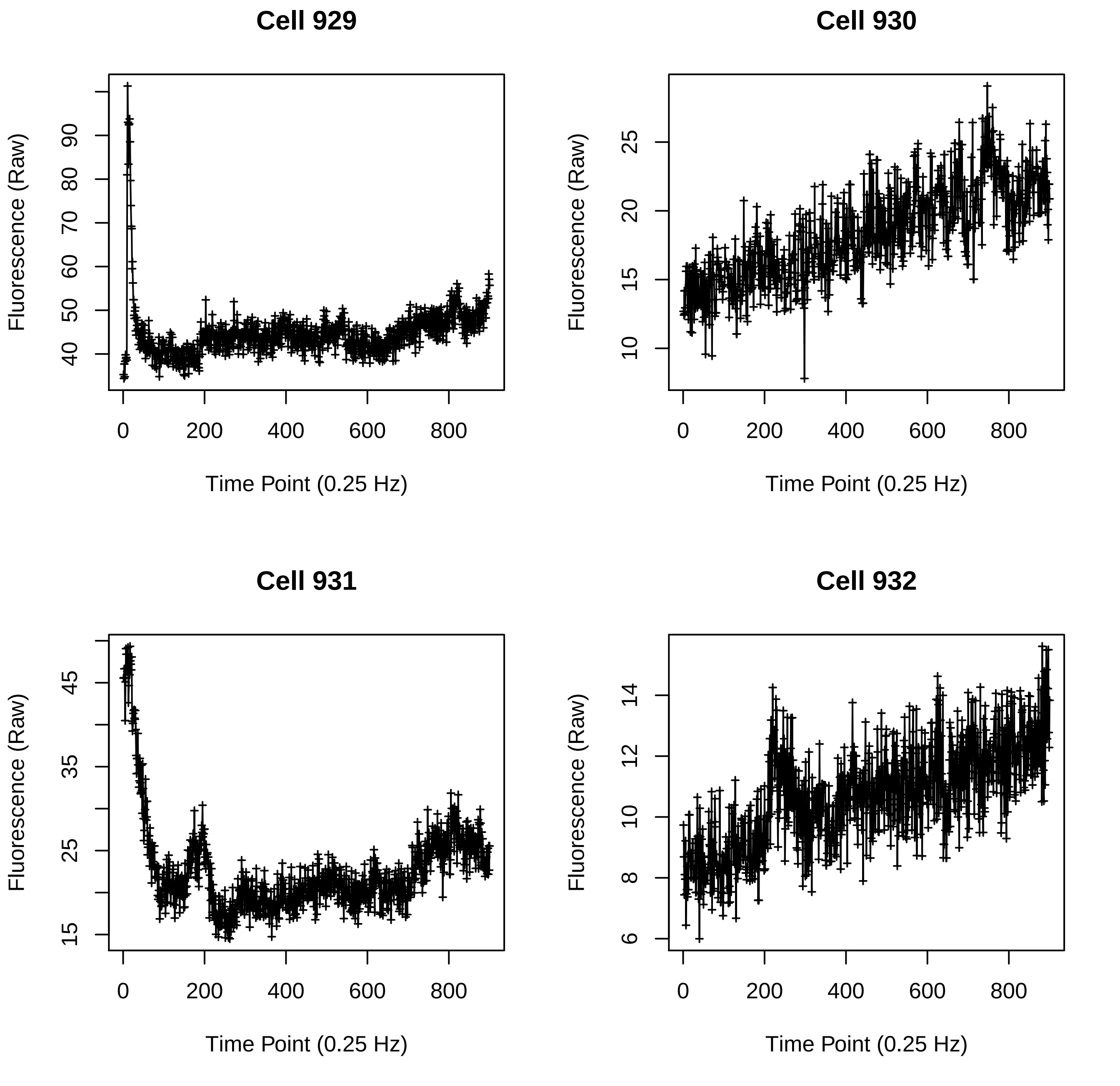
Cell 929
Fluorescence (Raw)
40 50 60 70 80 90
0 200 400 600 800
Time Point (0.25 Hz)
Cell 930
Fluorescence (Raw)
10 15 20 25
0 200 400 600 800
Time Point (0.25 Hz)
Cell 931
Fluorescence (Raw)
15 25 35 45
0 200 400 600 800
Time Point (0.25 Hz)
Cell 932
Fluorescence (Raw)
6 8 10 12 14
0 200 400 600 800
Time Point (0.25 Hz)

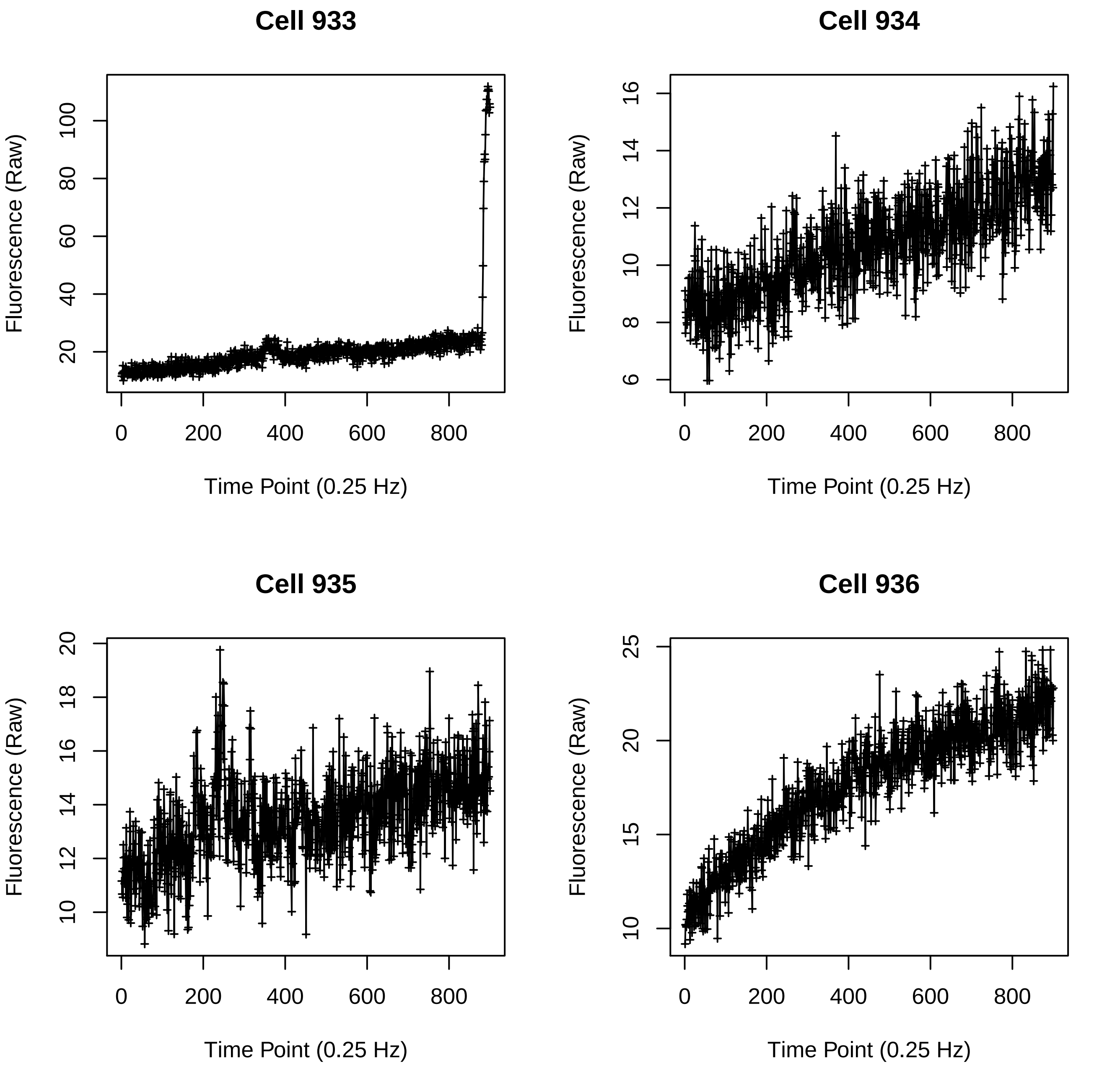
Cell 933
Fluorescence (Raw)
20
40
60
80
100
0
200
400
600
800
Time Point (0.25 Hz)
Cell 934
Fluorescence (Raw)
6
8
10
12
14
16
0
200
400
600
800
Time Point (0.25 Hz)
Cell 935
Fluorescence (Raw)
10
12
14
16
18
20
0
200
400
600
800
Time Point (0.25 Hz)
Cell 936
Fluorescence (Raw)
10
15
20
25
0
200
400
600
800
Time Point (0.25 Hz)

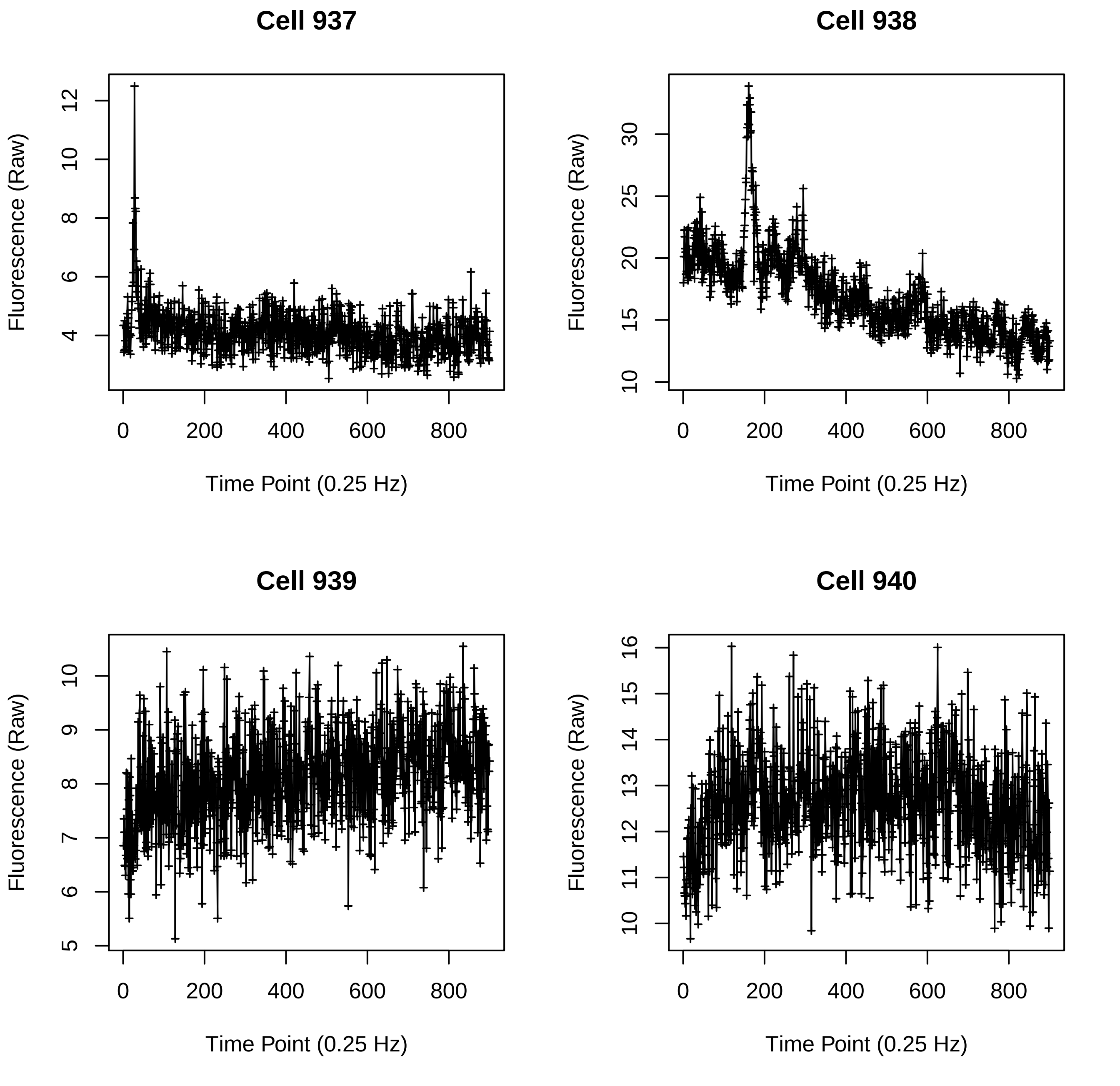
Cell 937
Fluorescence (Raw)
Time Point (0.25 Hz)
Cell 938
Fluorescence (Raw)
Time Point (0.25 Hz)
Cell 939
Fluorescence (Raw)
Time Point (0.25 Hz)
Cell 940
Fluorescence (Raw)
Time Point (0.25 Hz)

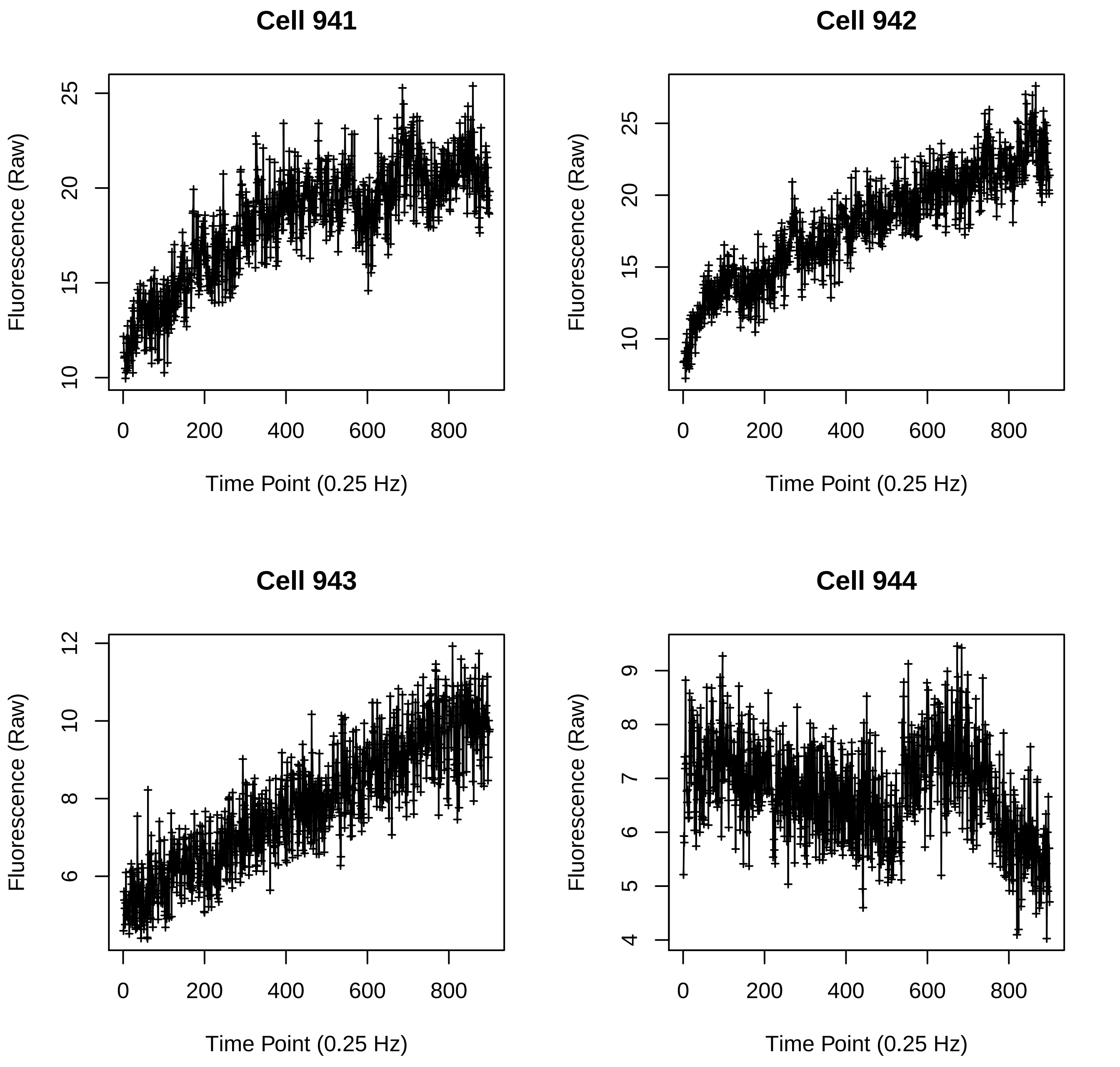
Cell 941
Cell 942
Cell 943
Cell 944
Fluorescence (Raw)
Time Point (0.25 Hz)
0
200
400
600
800
10
15
20
25
6
8
10
12
4
5
6
7
8
9

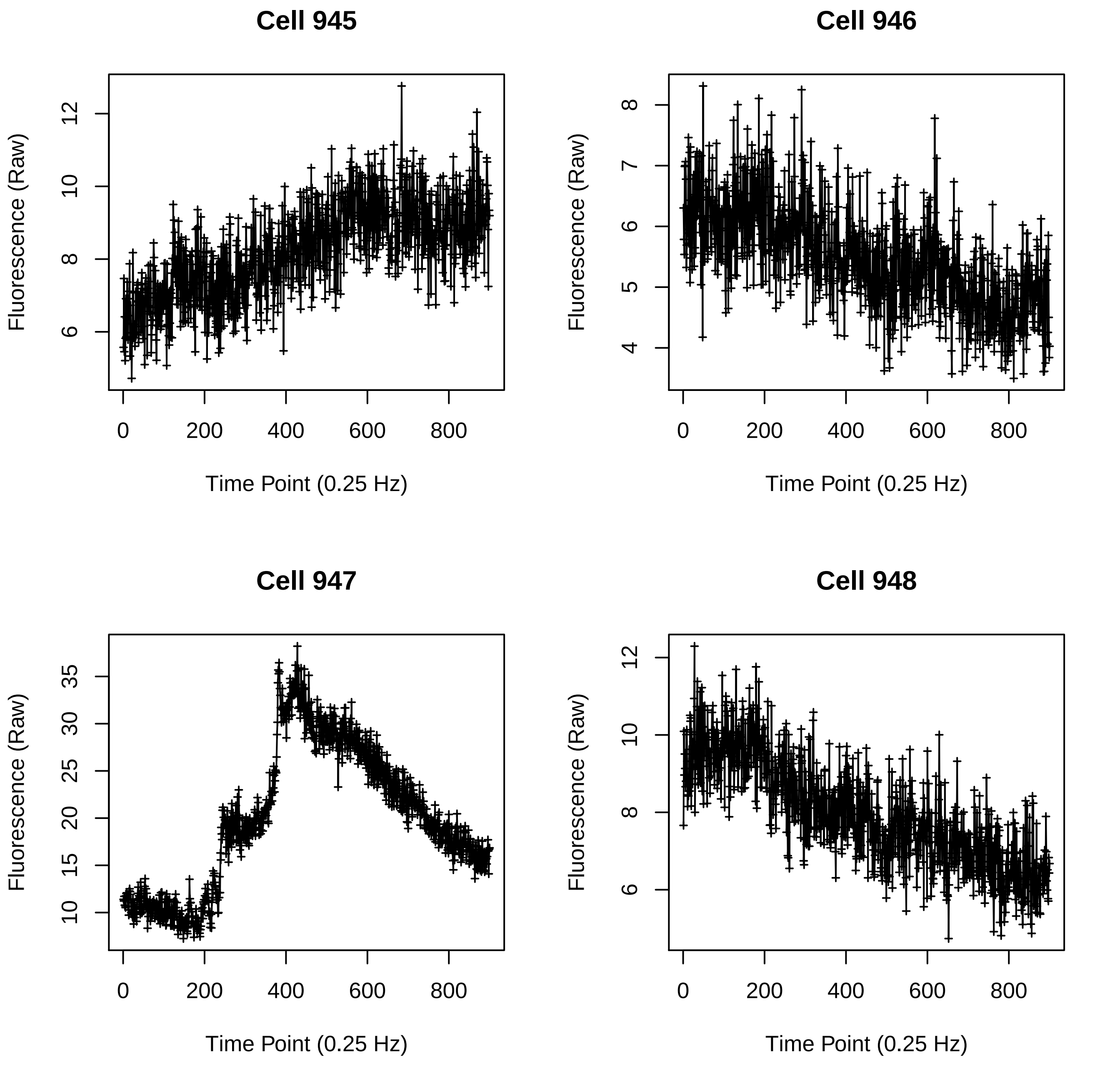
Cell 945
Cell 946
Cell 947
Cell 948
Fluorescence (Raw)
Time Point (0.25 Hz)
0
200
400
600
800
6
8
10
12
4
5
7
15
20
25
30
35

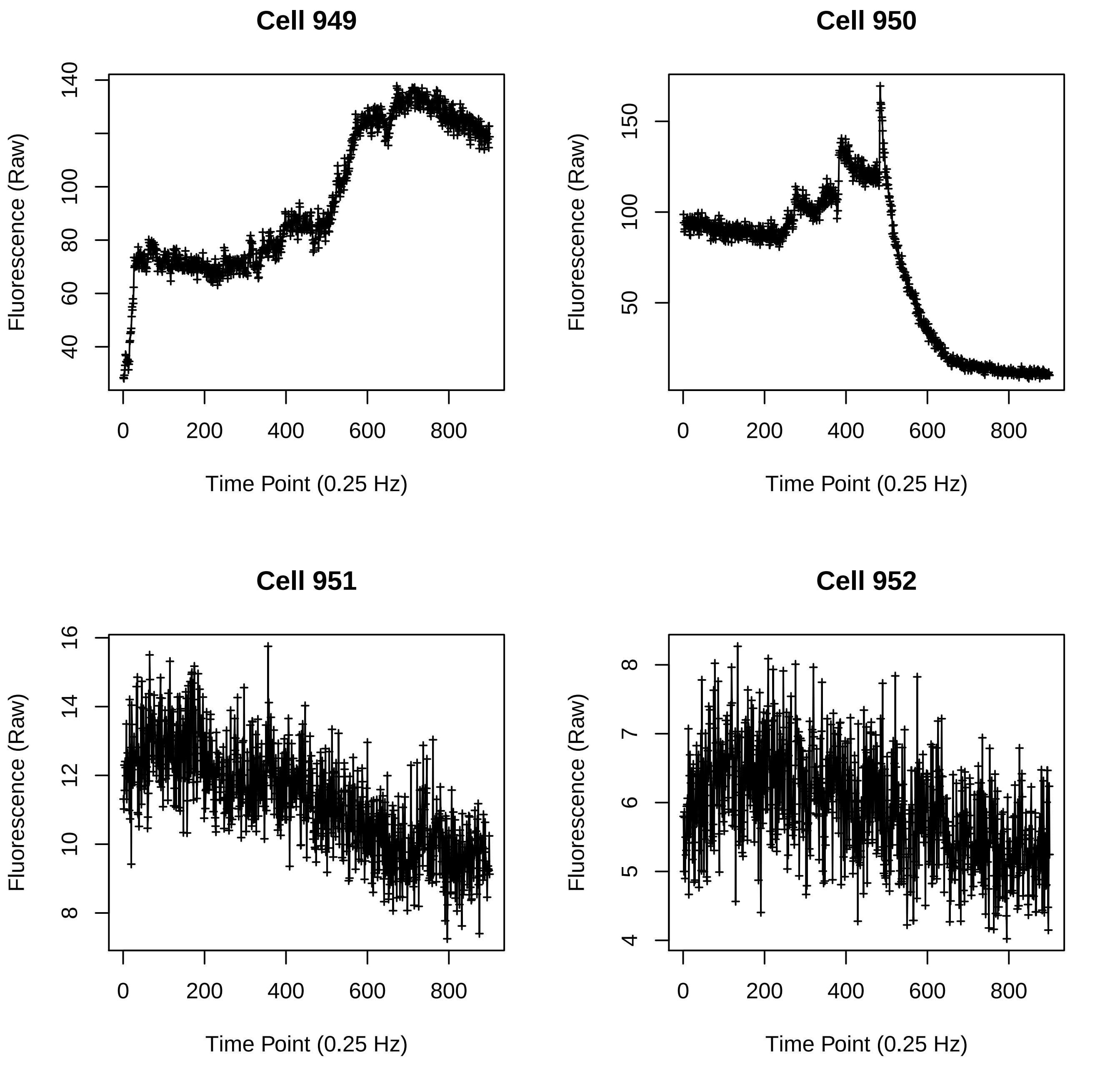
Cell 949
Cell 950
Cell 951
Cell 952
Fluorescence (Raw)
Time Point (0.25 Hz)
0
200
400
600
800
40
60
80
100
140
50
100
150
8
10
12
14
16
4
5
6
7
8

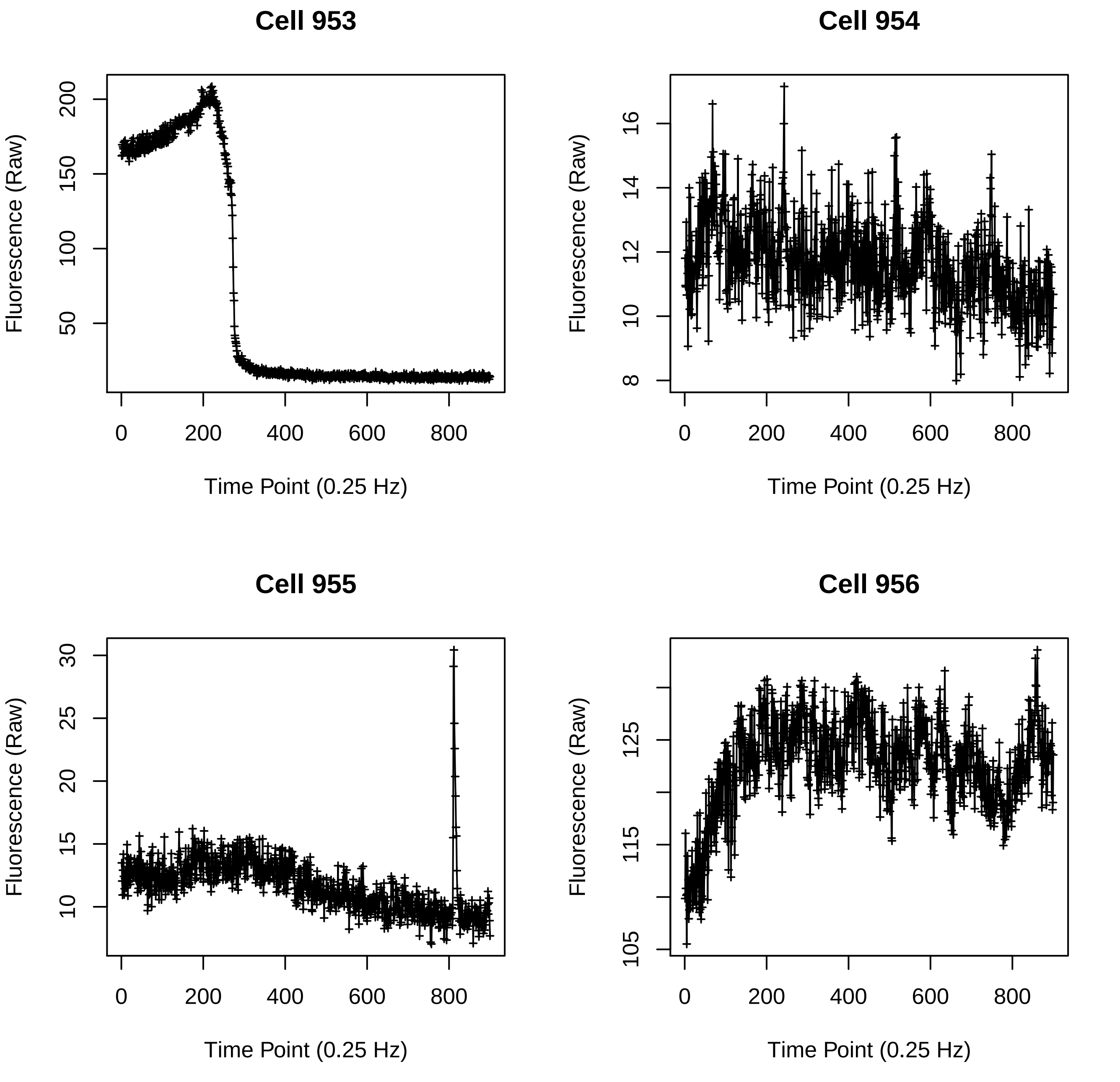
Cell 953
Fluorescence (Raw)
50
100
150
200
0
200
400
600
800
Time Point (0.25 Hz)
Cell 954
Fluorescence (Raw)
8
10
12
14
16
0
200
400
600
800
Time Point (0.25 Hz)
Cell 955
Fluorescence (Raw)
10
15
20
25
30
0
200
400
600
800
Time Point (0.25 Hz)
Cell 956
Fluorescence (Raw)
105
115
125
0
200
400
600
800
Time Point (0.25 Hz)

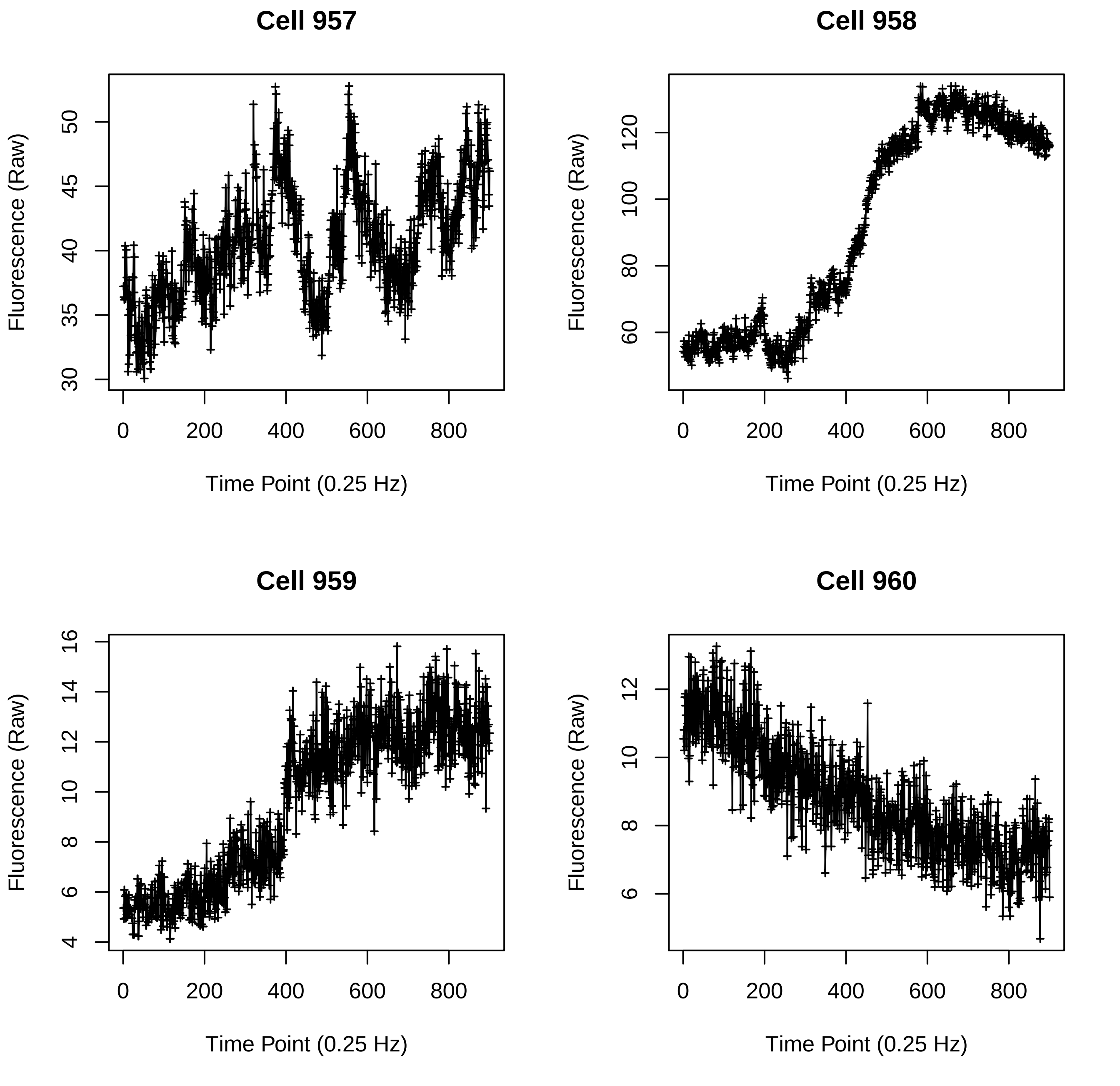

Cell 957
Cell 958
Cell 959
Cell 960
Fluorescence (Raw)
Time Point (0.25 Hz)
0
200
400
600
800
30
35
40
45
50
60
80
100
120
4
6
8
10
12
14
16

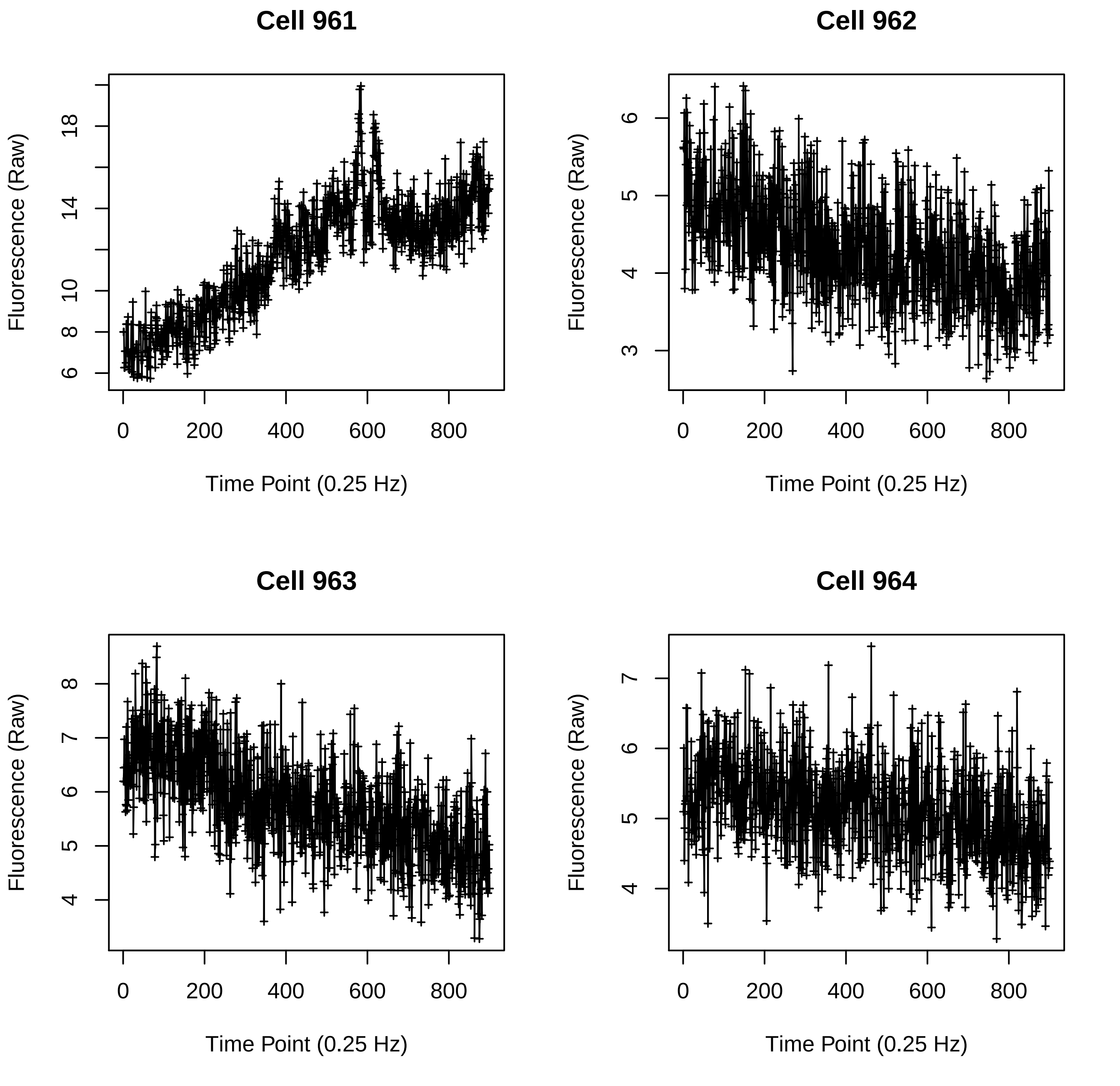
Cell 961
Cell 962
Cell 963
Cell 964
Fluorescence (Raw)
Time Point (0.25 Hz)
0
200
400
600
800
6
8
10
14
18
3
4
5
6
4
5
6
7
8
4
5
6
7

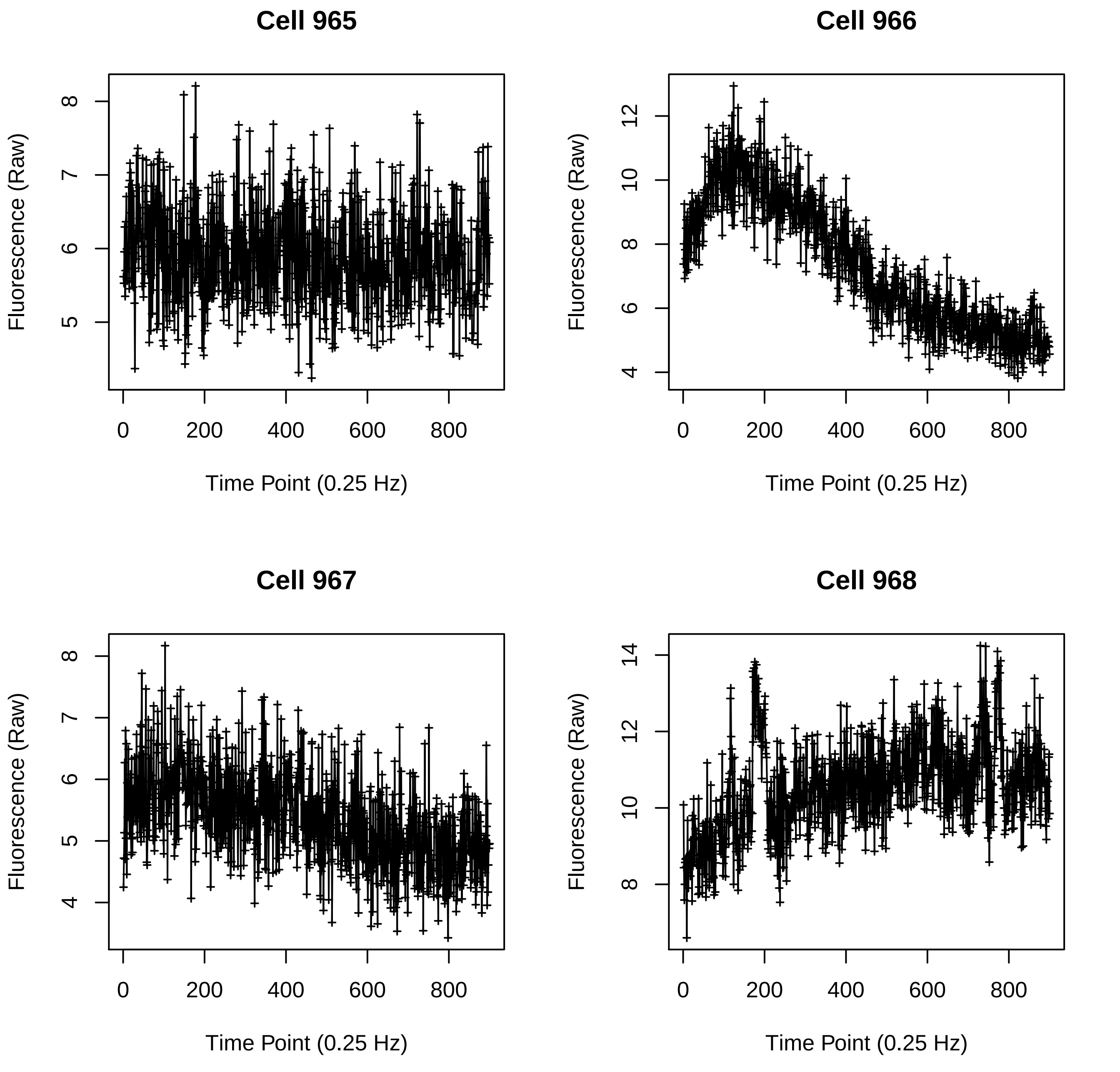
Cell 965
Fluorescence (Raw)
Time Point (0.25 Hz)
Cell 966
Fluorescence (Raw)
Time Point (0.25 Hz)
Cell 967
Fluorescence (Raw)
Time Point (0.25 Hz)
Cell 968
Fluorescence (Raw)
Time Point (0.25 Hz)

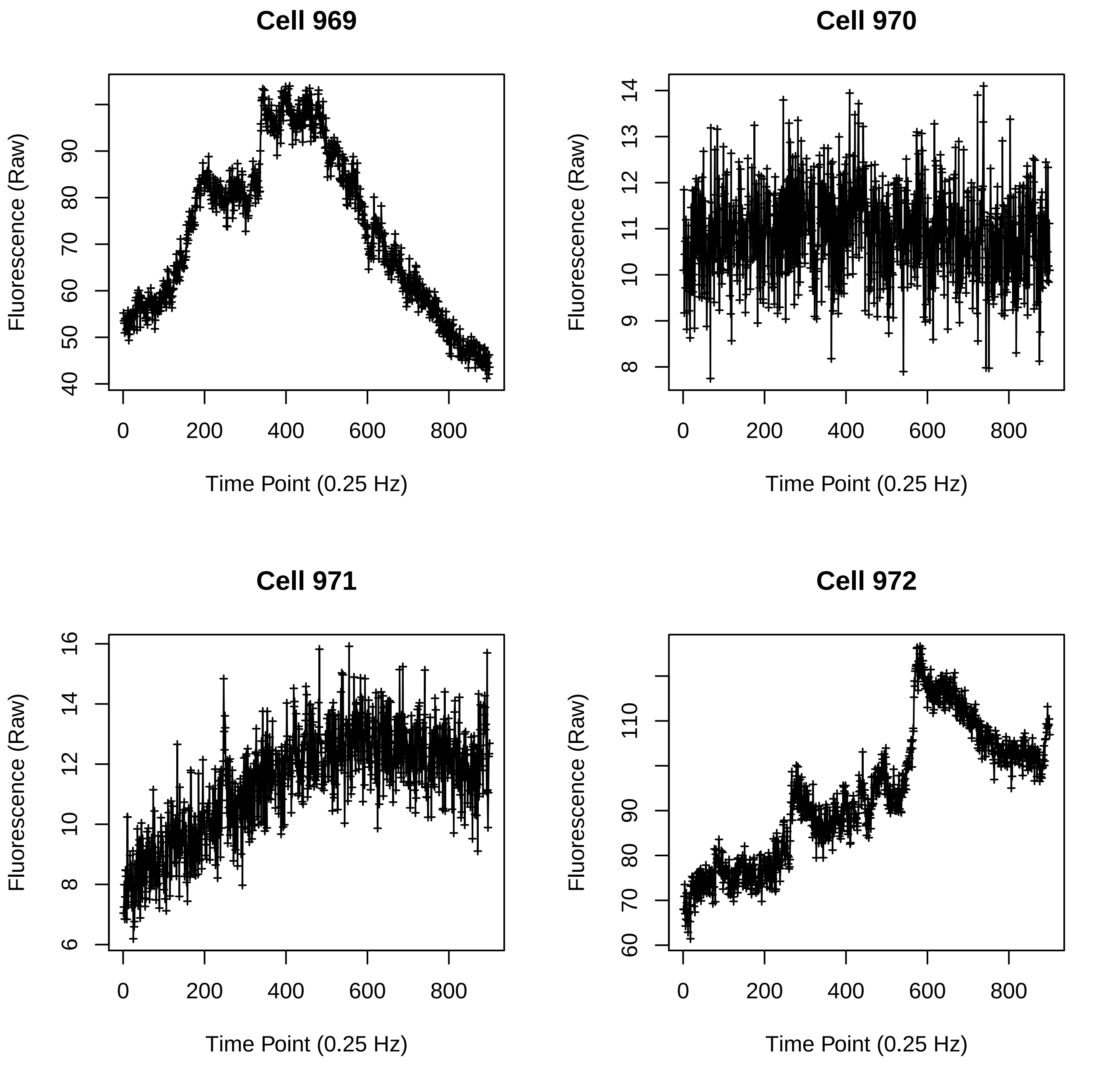
Cell 969
Cell 970
Cell 971
Cell 972
Fluorescence (Raw)
Time Point (0.25 Hz)
0
200
400
600
800
40
50
60
70
80
90
8
9
10
11
12
13
14
6
16
110

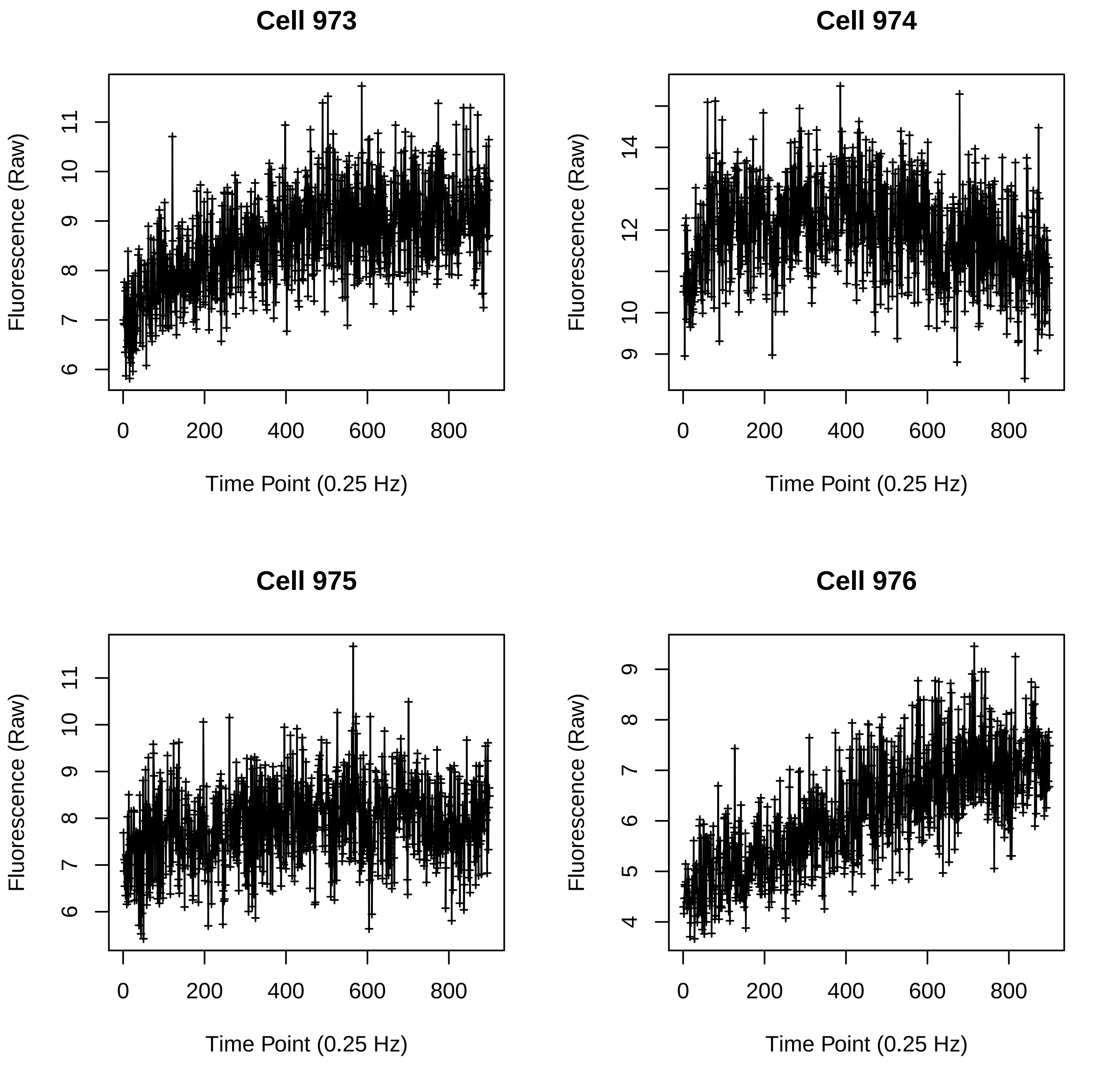
Cell 973
Fluorescence (Raw)
Time Point (0.25 Hz)
Cell 974
Fluorescence (Raw)
Time Point (0.25 Hz)
Cell 975
Fluorescence (Raw)
Time Point (0.25 Hz)
Cell 976
Fluorescence (Raw)
Time Point (0.25 Hz)

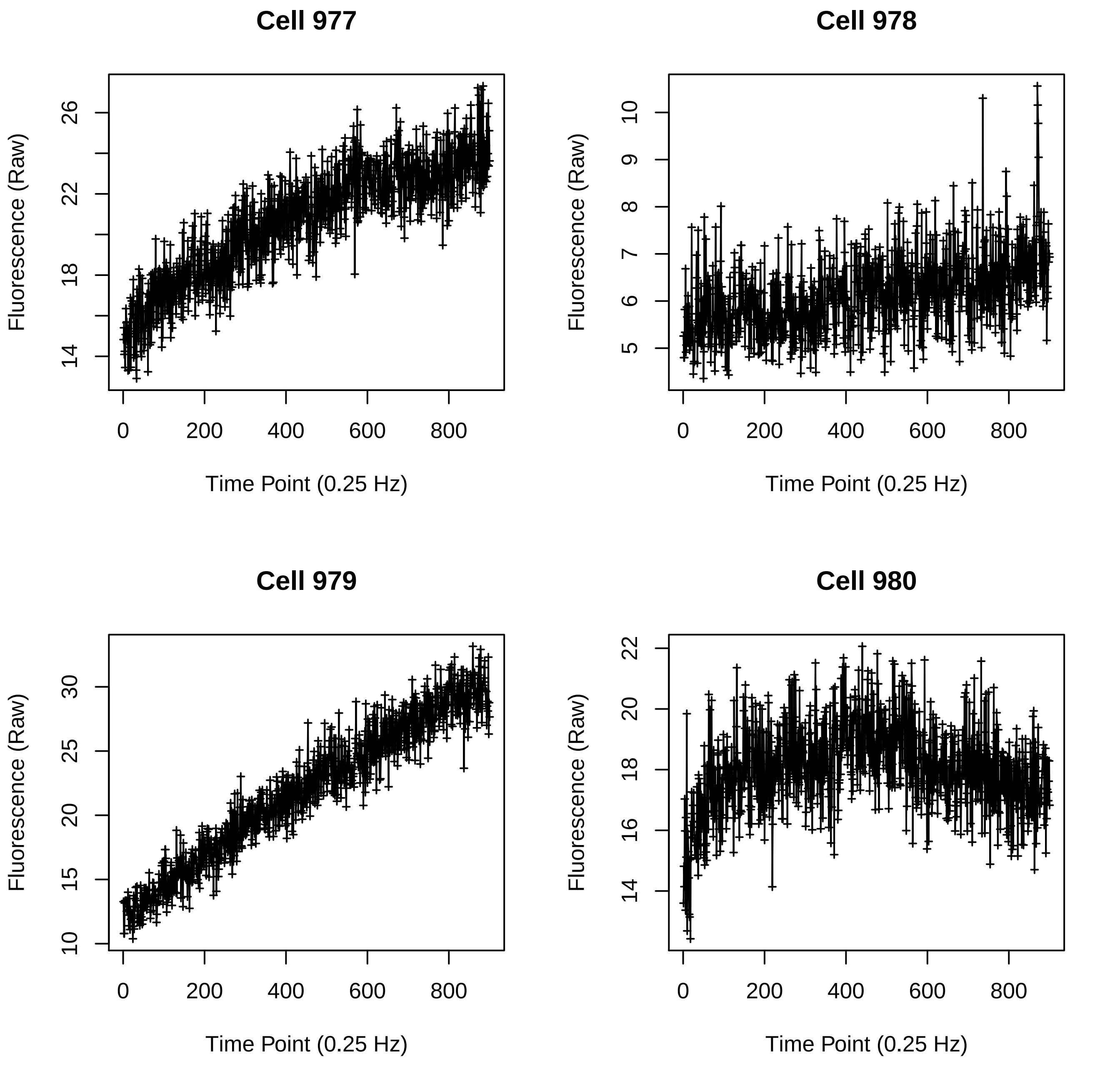
Cell 977
Cell 978
Cell 979
Cell 980
Fluorescence (Raw)
Time Point (0.25 Hz)
0
200
400
600
800
14
18
22
26
5
6
7
8
9
10
15
20
25
30
16

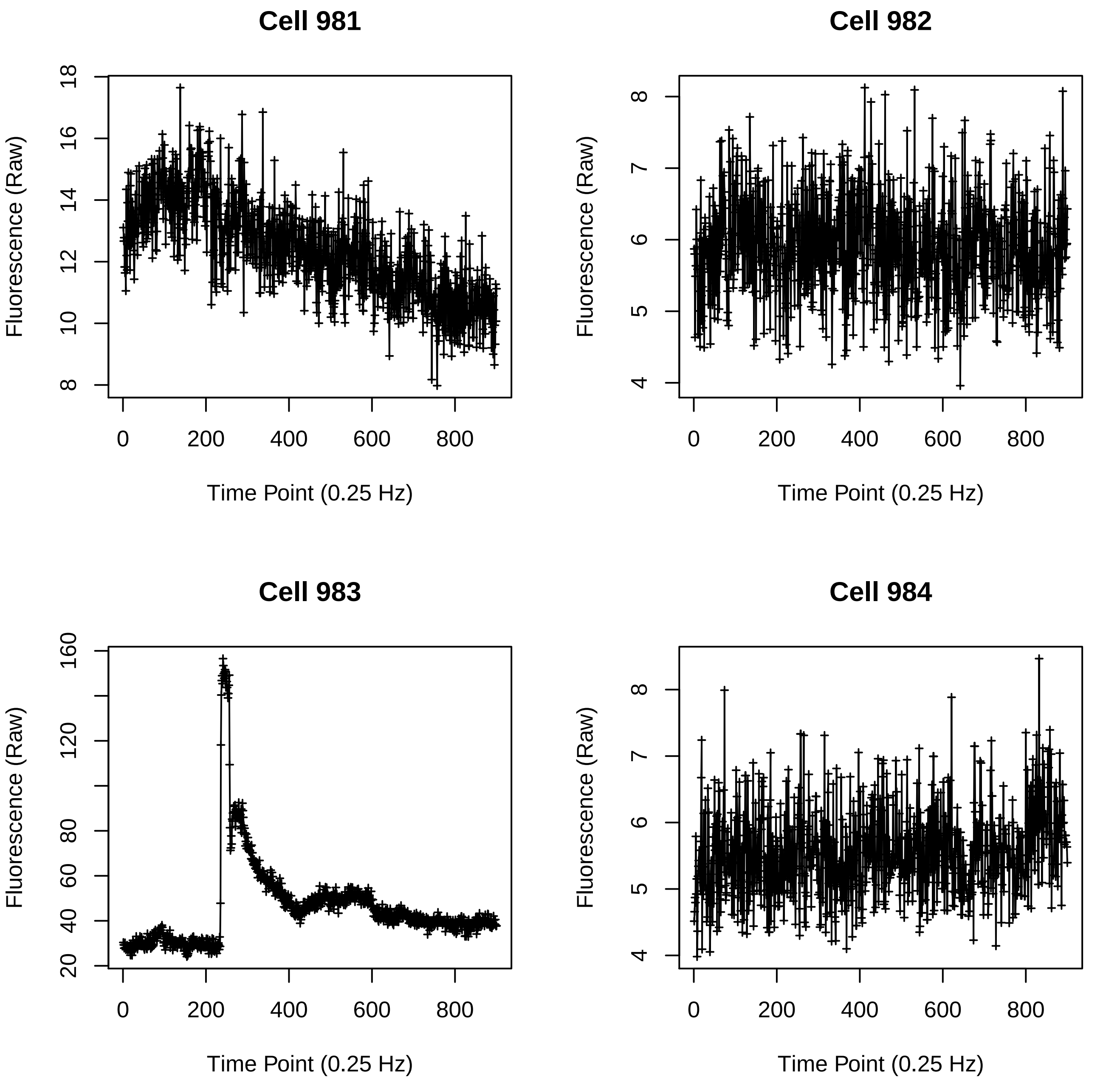
Cell 981
Fluorescence (Raw)
8
10
12
14
16
18
0
200
400
600
800
Time Point (0.25 Hz)
Cell 982
Fluorescence (Raw)
4
5
6
7
8
0
200
400
600
800
Time Point (0.25 Hz)
Cell 983
Fluorescence (Raw)
20
40
60
80
120
160
0
200
400
600
800
Time Point (0.25 Hz)
Cell 984
Fluorescence (Raw)
4
5
6
7
8
0
200
400
600
800
Time Point (0.25 Hz)

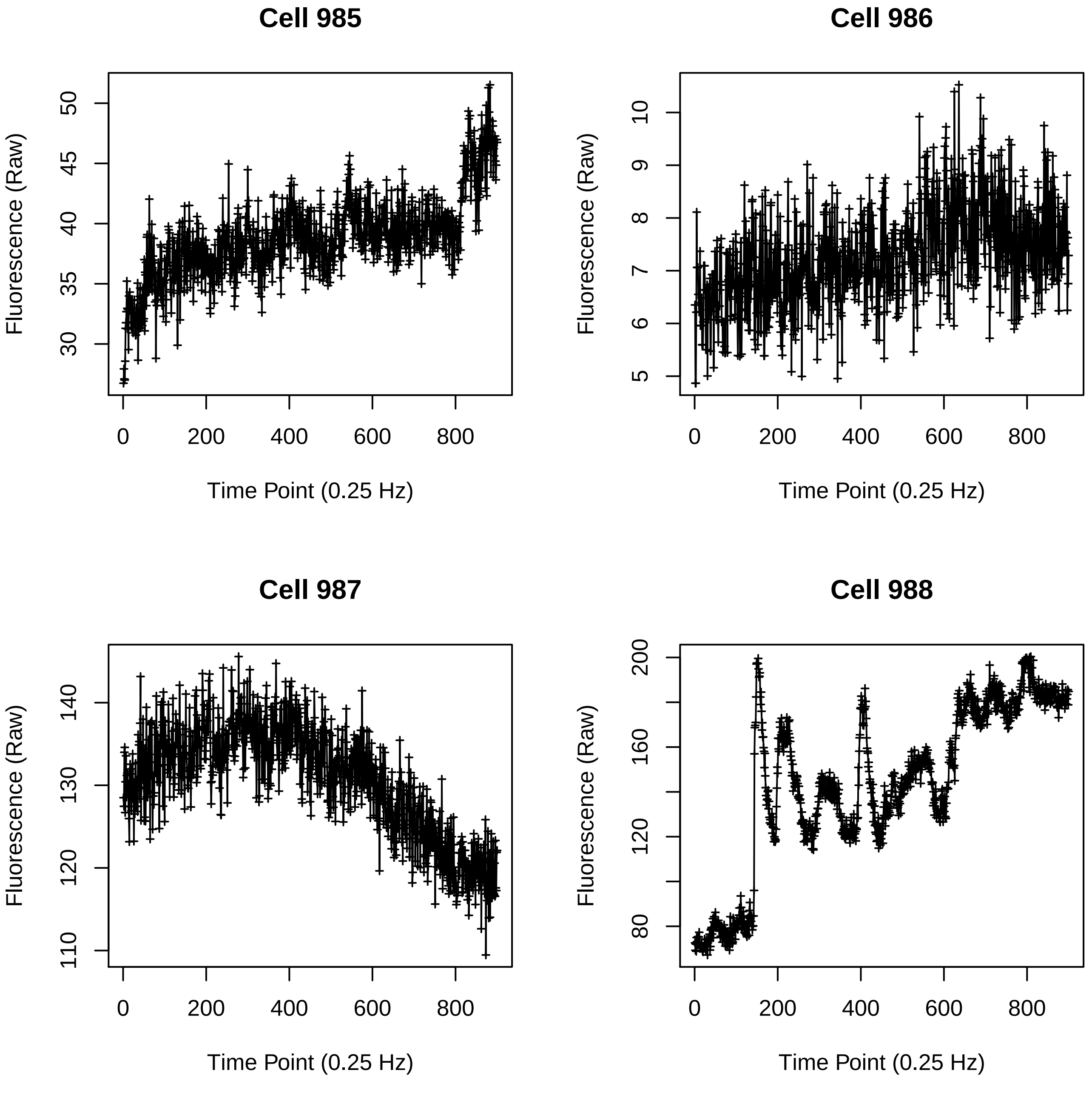
Cell 985
Cell 986
Cell 987
Cell 988
Fluorescence (Raw)
Time Point (0.25 Hz)
0
200
400
600
800
30
35
40
45
50
5
6
7
8
9
10
110
120
130
140
80
120
160
200

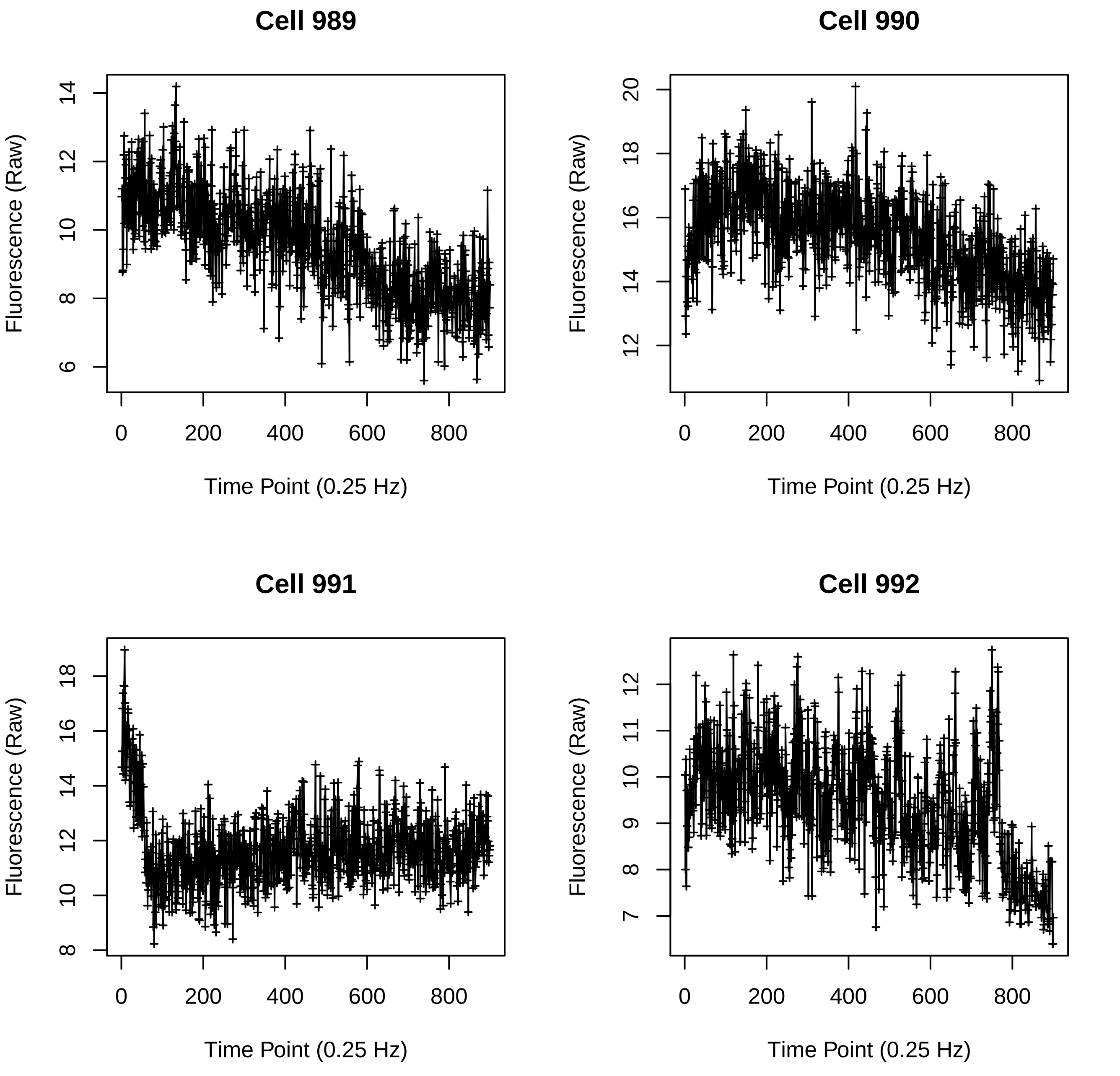
Cell 989
Fluorescence (Raw)
6
8
10
12
14
0
200
400
600
800
Time Point (0.25 Hz)
Cell 990
Fluorescence (Raw)
12
14
16
18
20
0
200
400
600
800
Time Point (0.25 Hz)
Cell 991
Fluorescence (Raw)
8
10
12
14
16
18
0
200
400
600
800
Time Point (0.25 Hz)
Cell 992
Fluorescence (Raw)
7
8
9
10
11
12
0
200
400
600
800
Time Point (0.25 Hz)

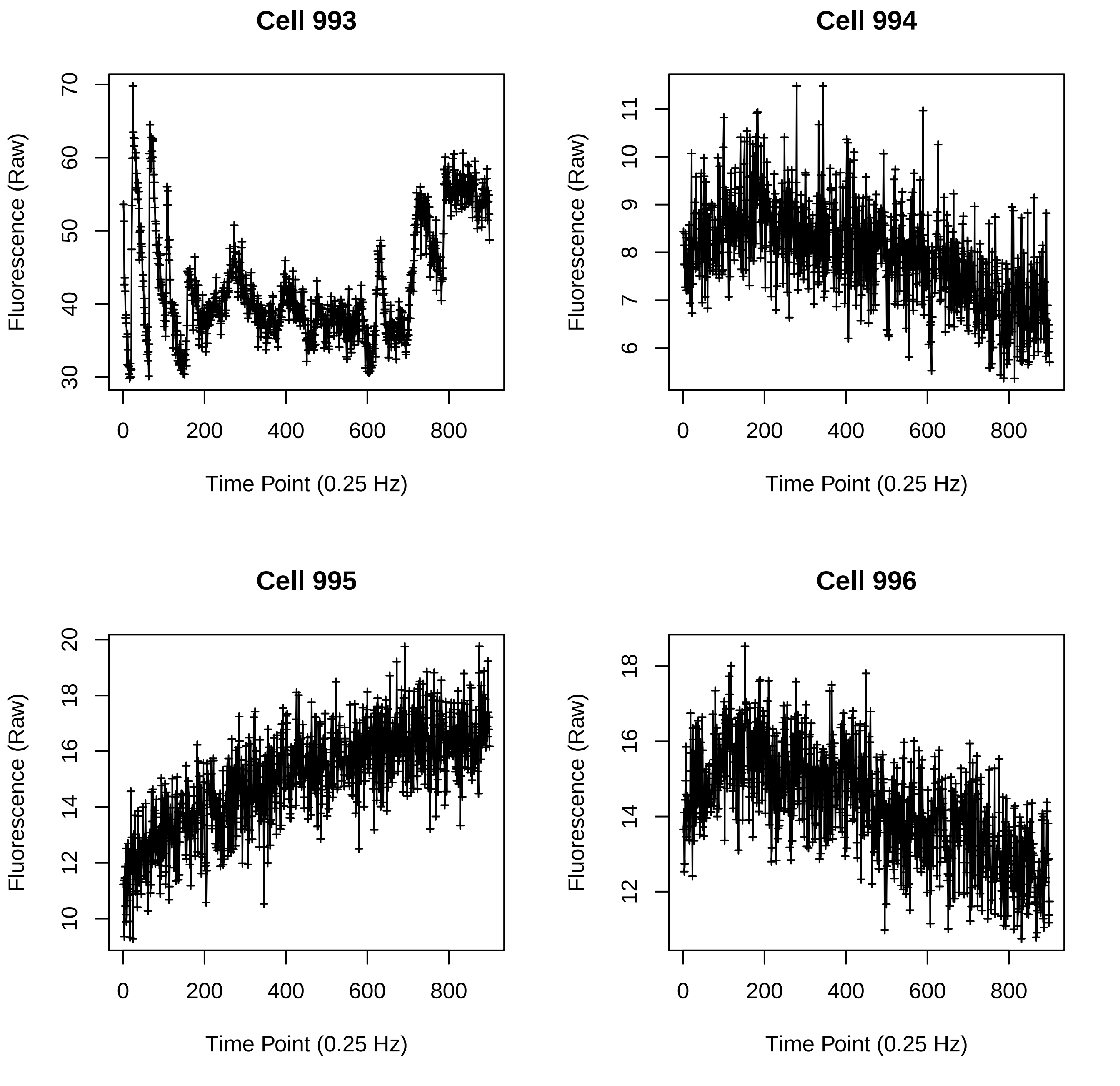
Cell 993
Cell 994
Cell 995
Cell 996
Fluorescence (Raw)
Time Point (0.25 Hz)
30
40
50
60
70
6
7
8
9
10
11
10
12
14
16
18
20
12
14
16
18
0
200
400
600
800

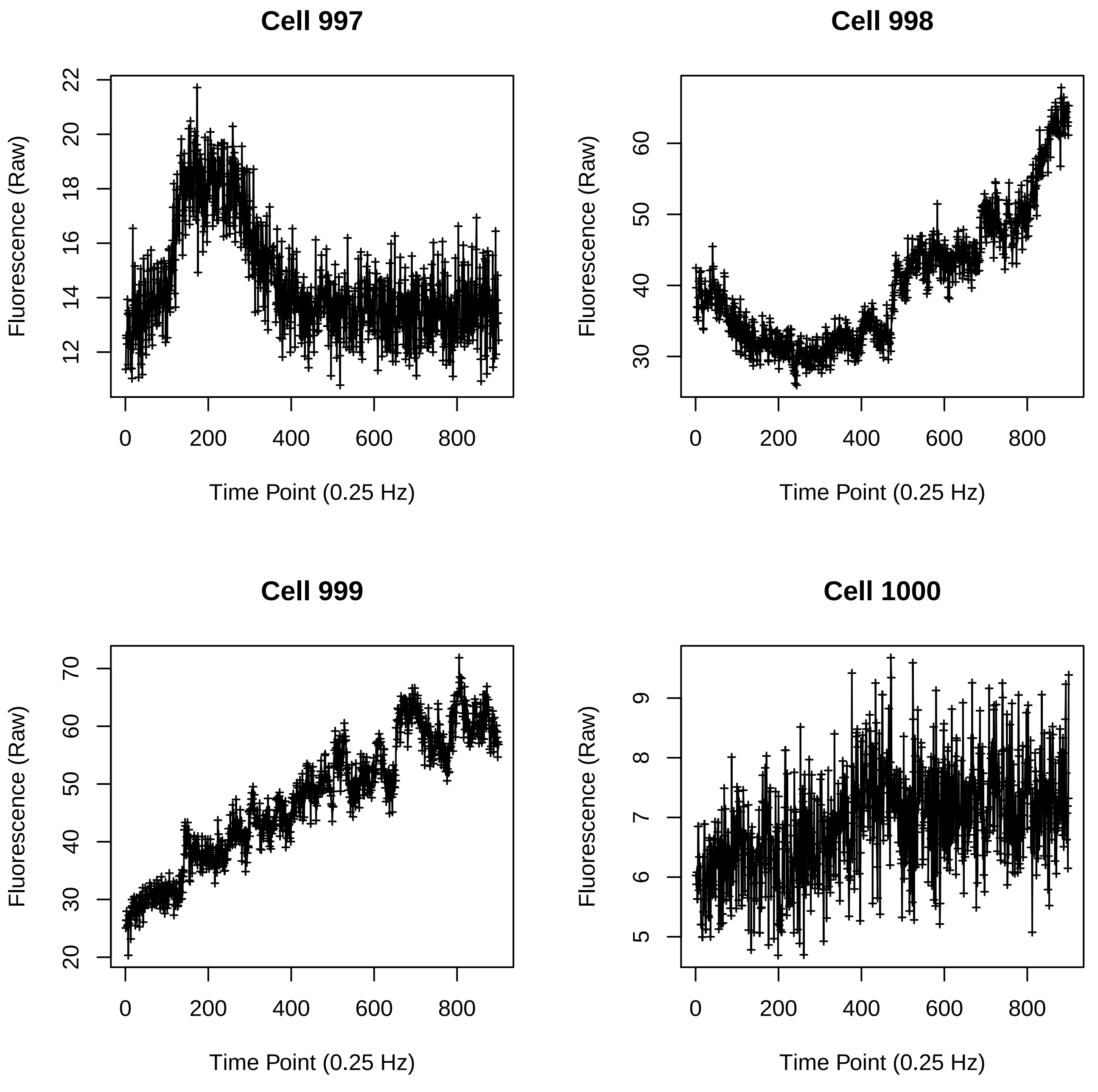
Cell 997
Cell 998
Cell 999
Cell 1000
Fluorescence (Raw)
Time Point (0.25 Hz)
0
200
400
600
800
12
14
16
18
20
22
30
40
50
60
20
30
40
50
60
70
5
6
7
8
9

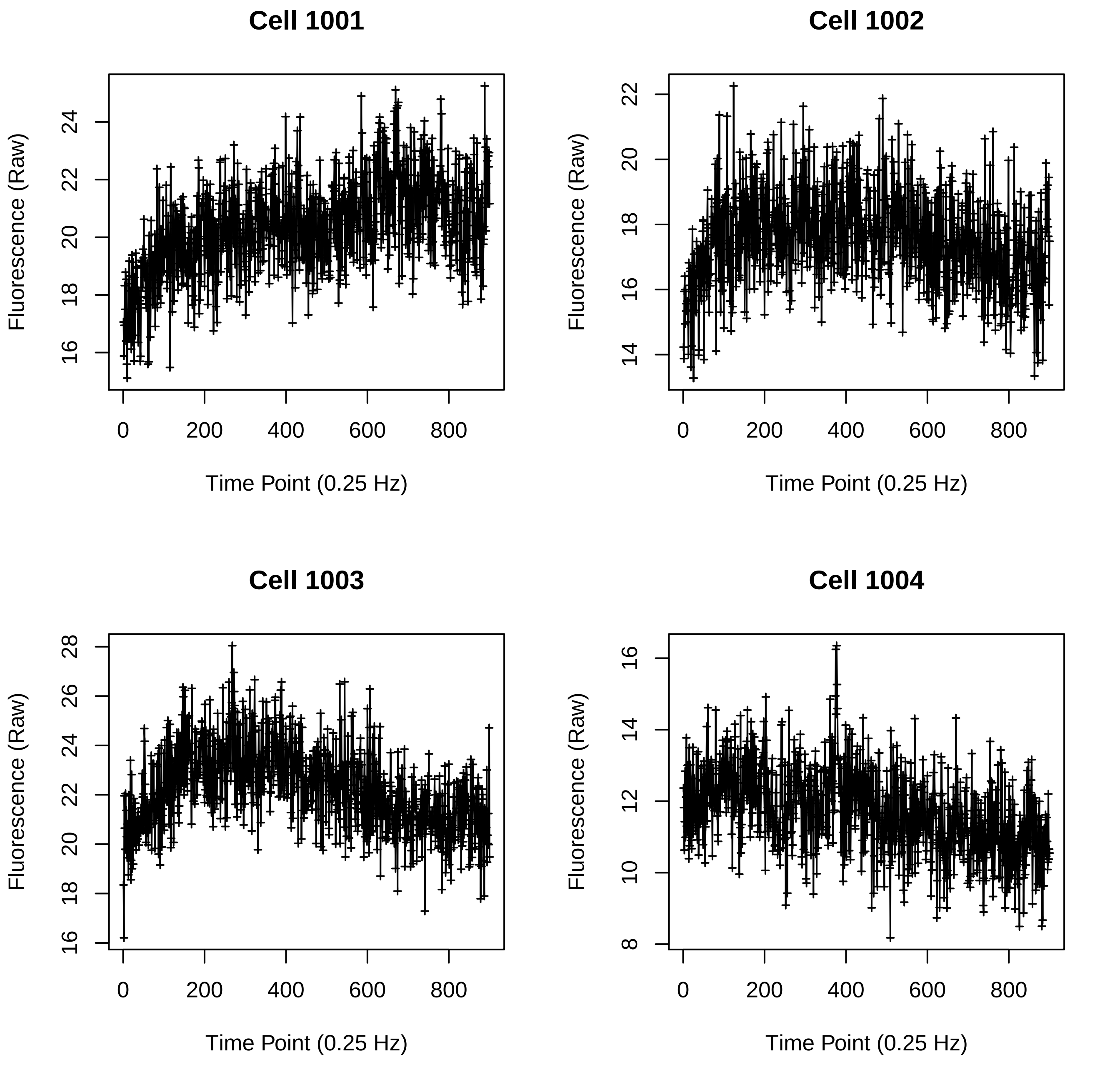

Cell 1001
Cell 1002
Cell 1003
Cell 1004
Fluorescence (Raw)
Time Point (0.25 Hz)
0
200
400
600
800
16
18
20
22
24
14
16
18
20
22
16
18
20
22
24
26
28
8
10
12
14
16

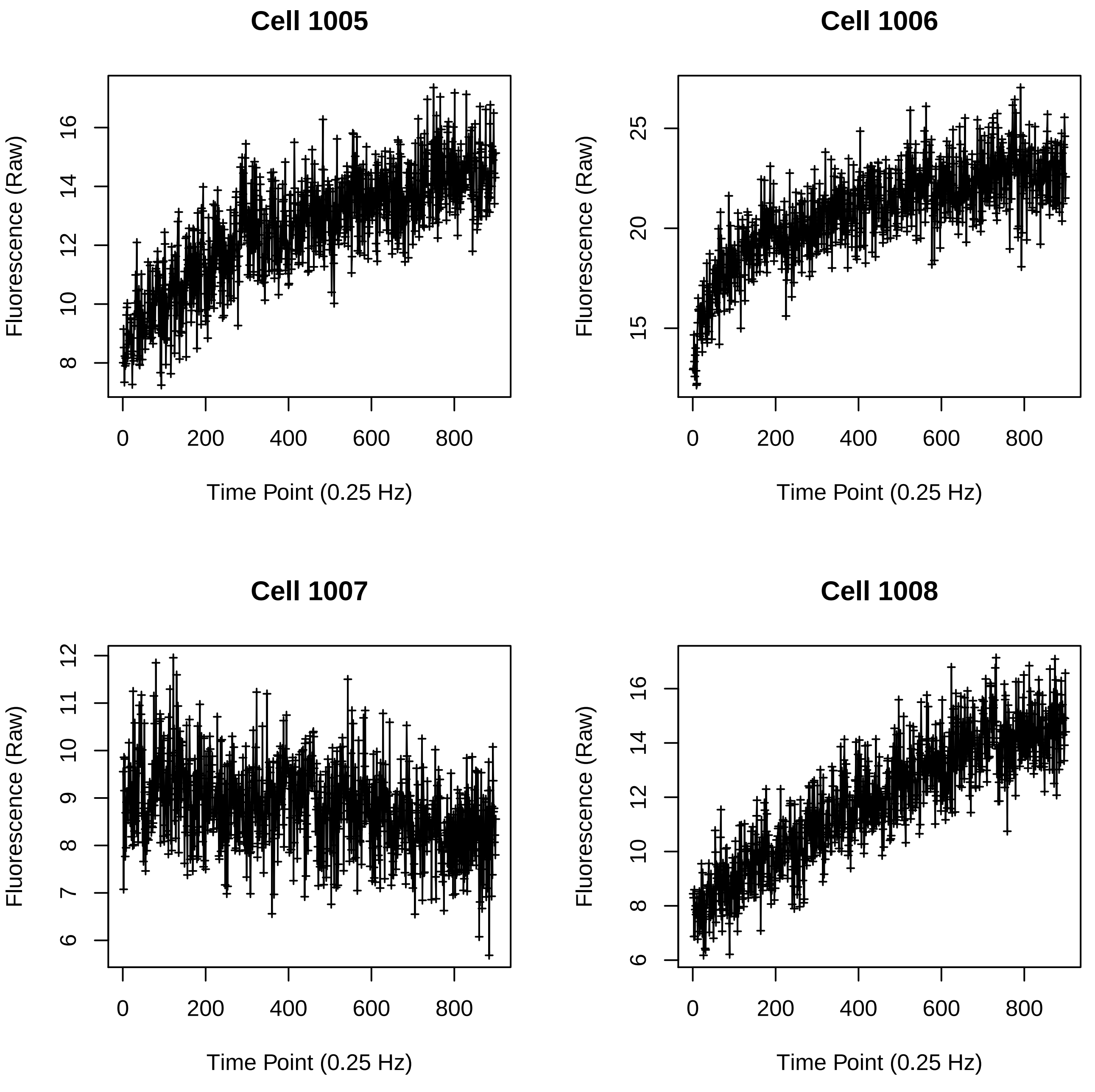
Cell 1005
Fluorescence (Raw)
8
10
12
14
16
0
200
400
600
800
Time Point (0.25 Hz)
Cell 1006
Fluorescence (Raw)
15
20
25
0
200
400
600
800
Time Point (0.25 Hz)
Cell 1007
Fluorescence (Raw)
6
7
8
9
10
11
12
0
200
400
600
800
Time Point (0.25 Hz)
Cell 1008
Fluorescence (Raw)
6
8
10
12
14
16
0
200
400
600
800
Time Point (0.25 Hz)

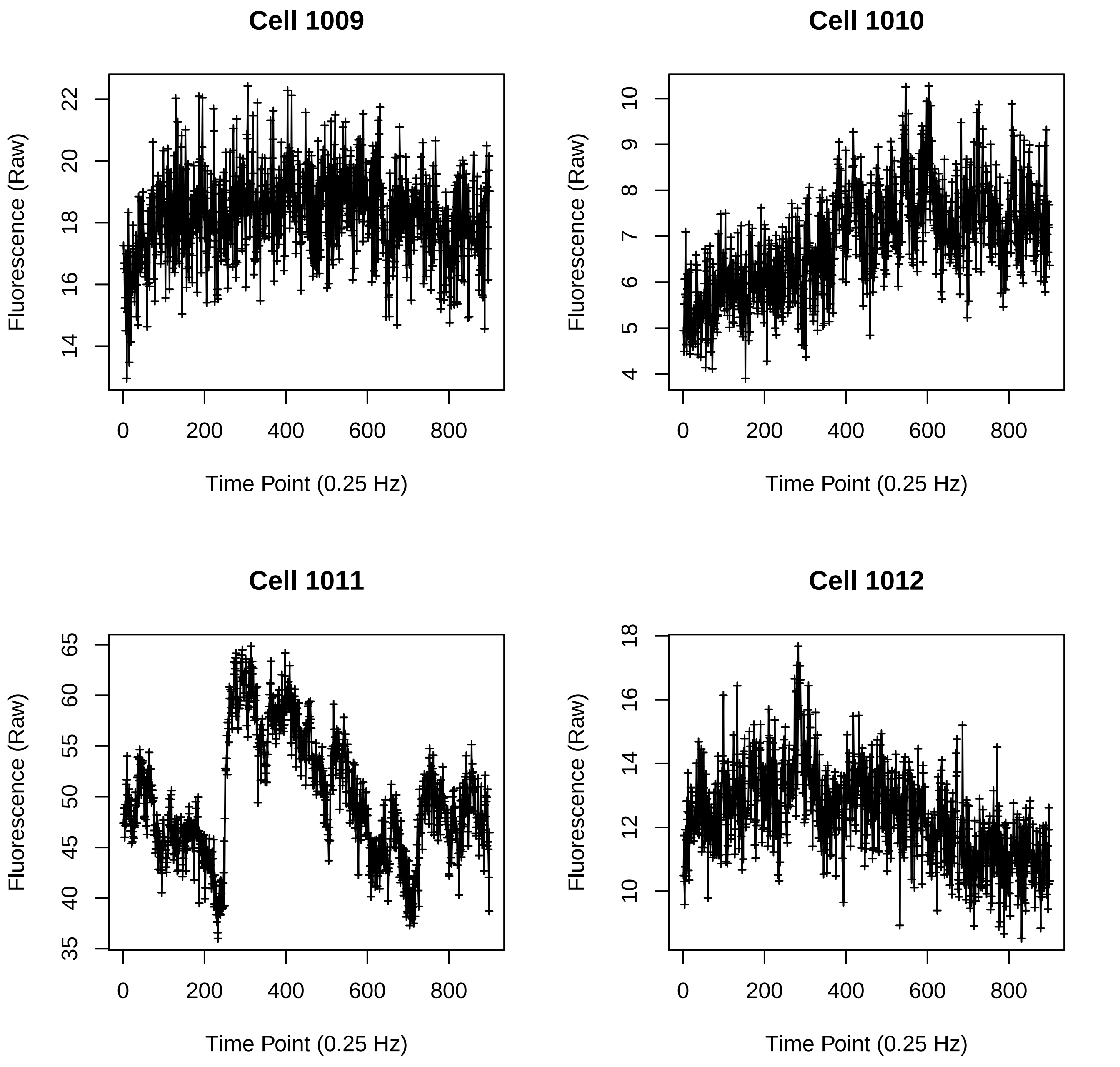

Cell 1009
Cell 1010
Cell 1011
Cell 1012
Fluorescence (Raw)
Time Point (0.25 Hz)
0
200
400
600
800
14
16
18
20
22
4
5
6
7
8
9
10
35
40
45
50
55
60
65
10
12
14
16
18

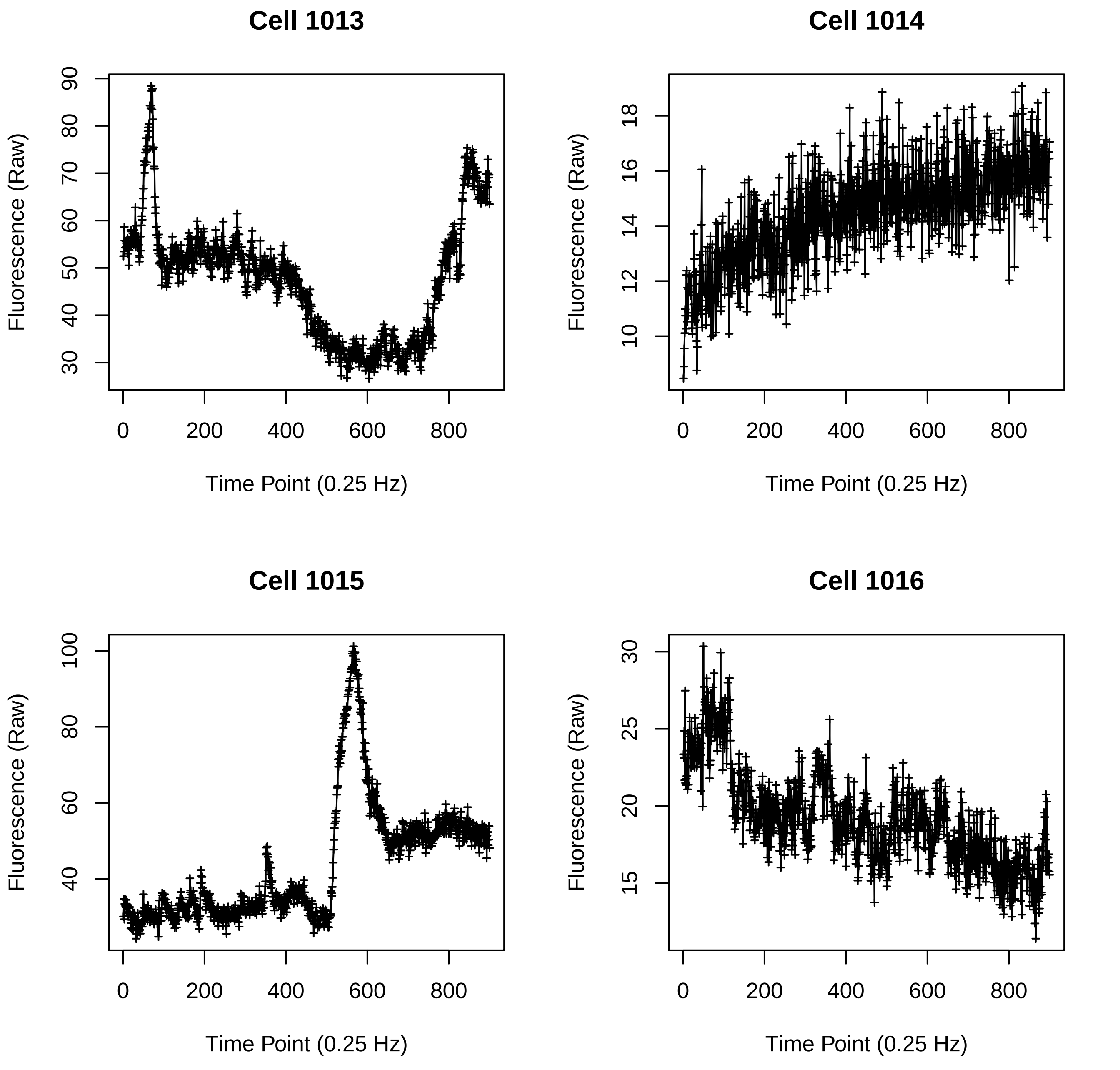
Cell 1013
Cell 1014
Cell 1015
Cell 1016
Fluorescence (Raw)
Time Point (0.25 Hz)
0
200
400
600
800
30
40
50
60
70
80
90
10
12
14
16
18
100
15
20
25

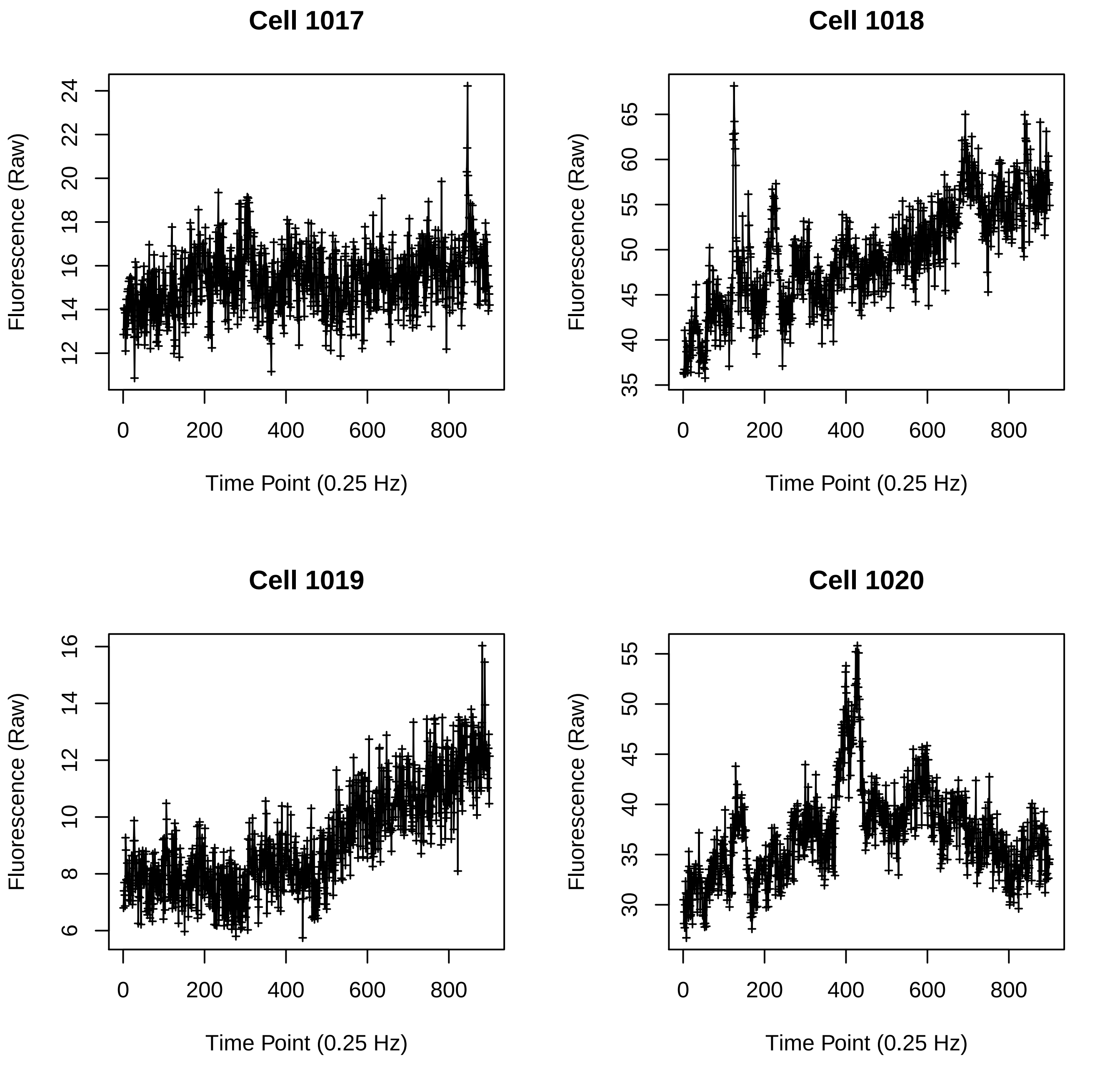
Cell 1017
Cell 1018
Cell 1019
Cell 1020
Fluorescence (Raw)
Time Point (0.25 Hz)
0
200
400
600
800
12
14
16
18
20
22
24
35
40
45
50
55
60
65
6
8
10
12
14
16
30
35
40
45
50
55

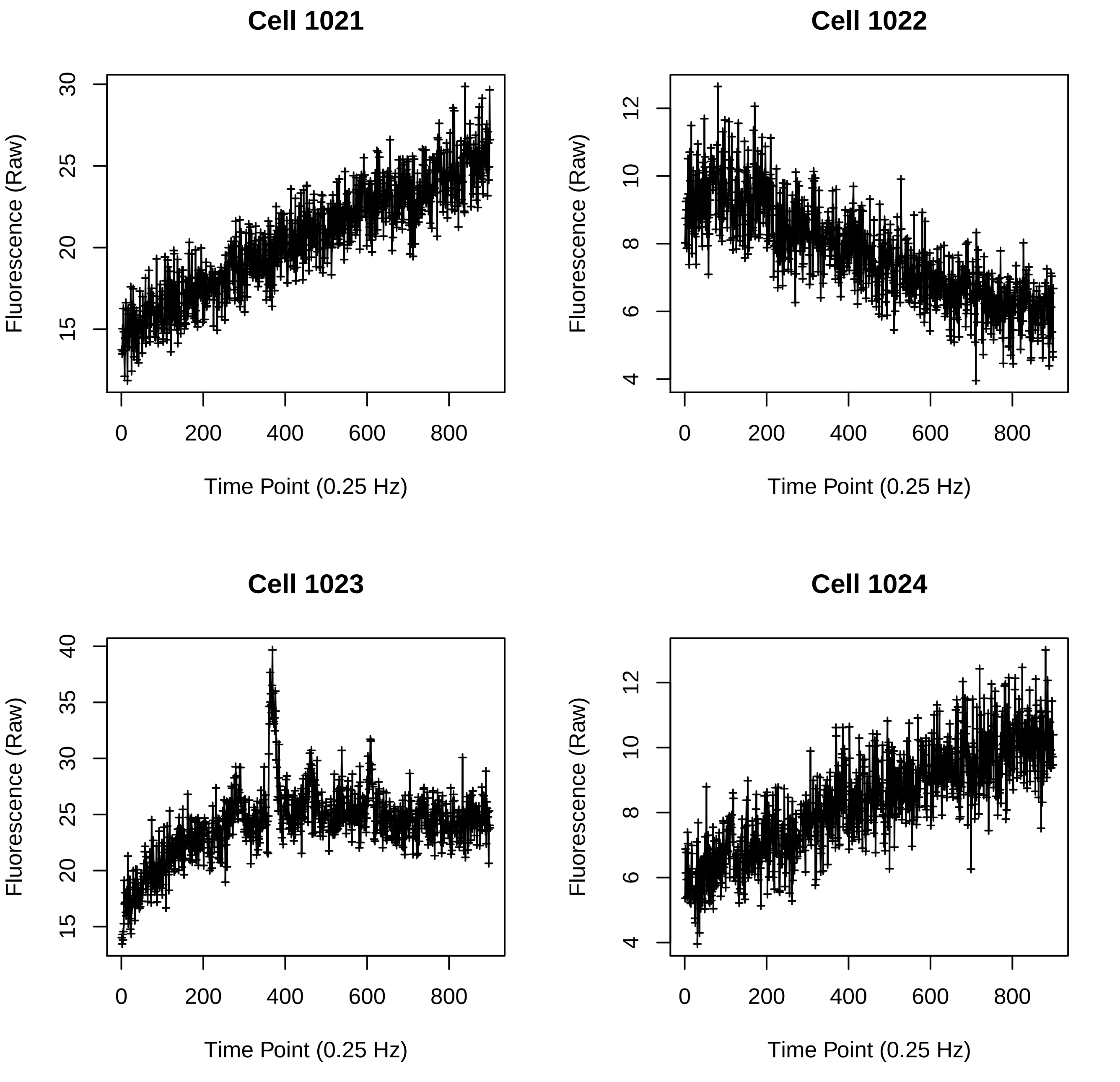
Cell 1021
Fluorescence (Raw)
15
20
25
30
0
200
400
600
800
Time Point (0.25 Hz)
Cell 1022
Fluorescence (Raw)
4
6
8
10
12
0
200
400
600
800
Time Point (0.25 Hz)
Cell 1023
Fluorescence (Raw)
15
20
25
30
35
40
0
200
400
600
800
Time Point (0.25 Hz)
Cell 1024
Fluorescence (Raw)
4
6
8
10
12
0
200
400
600
800
Time Point (0.25 Hz)

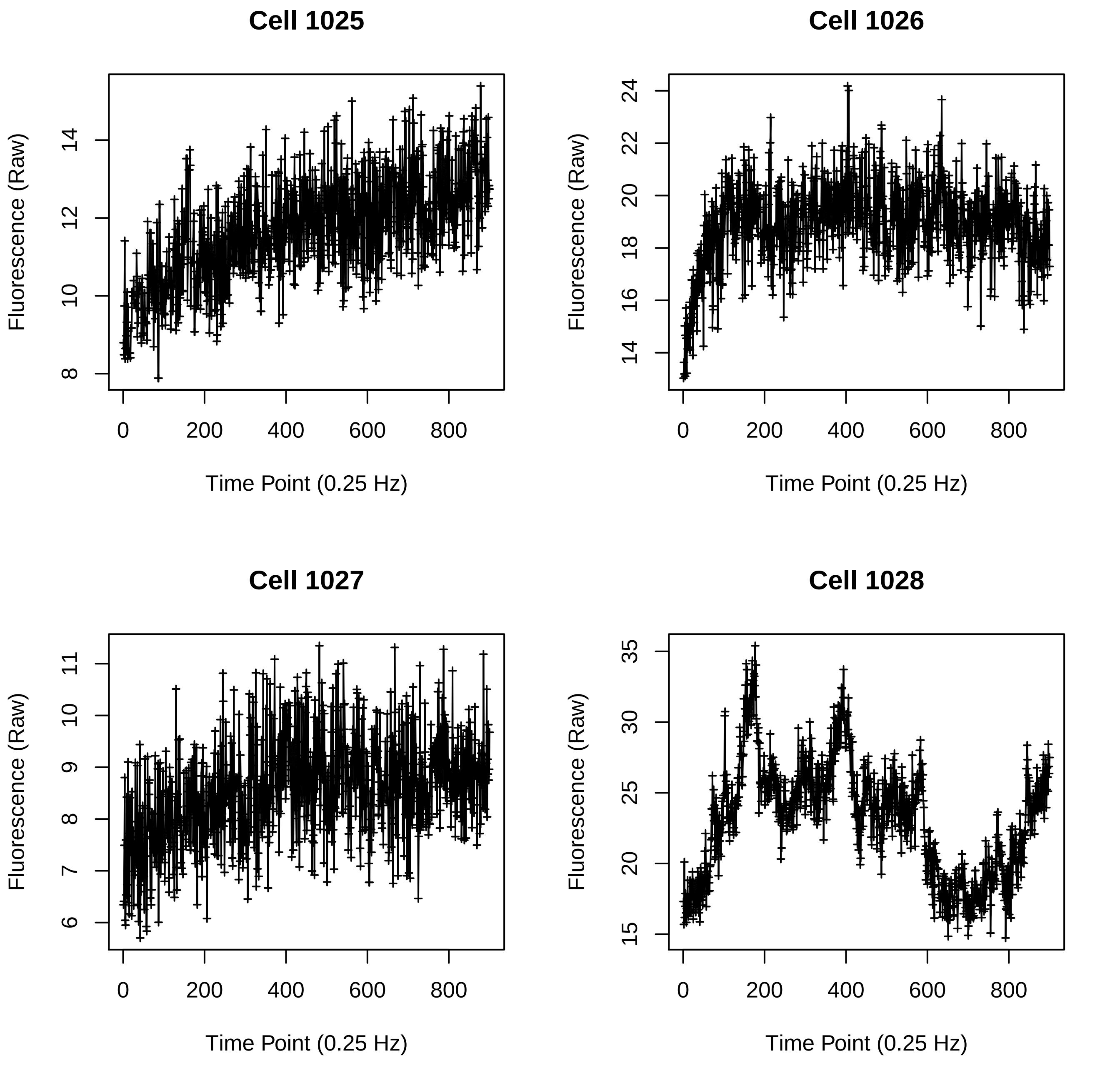
Cell 1025
Cell 1026
Cell 1027
Cell 1028
Fluorescence (Raw)
Time Point (0.25 Hz)
0
200
400
600
800
8
10
12
14
16
18
20
22
24
6
7
9
11
15
25
30
35

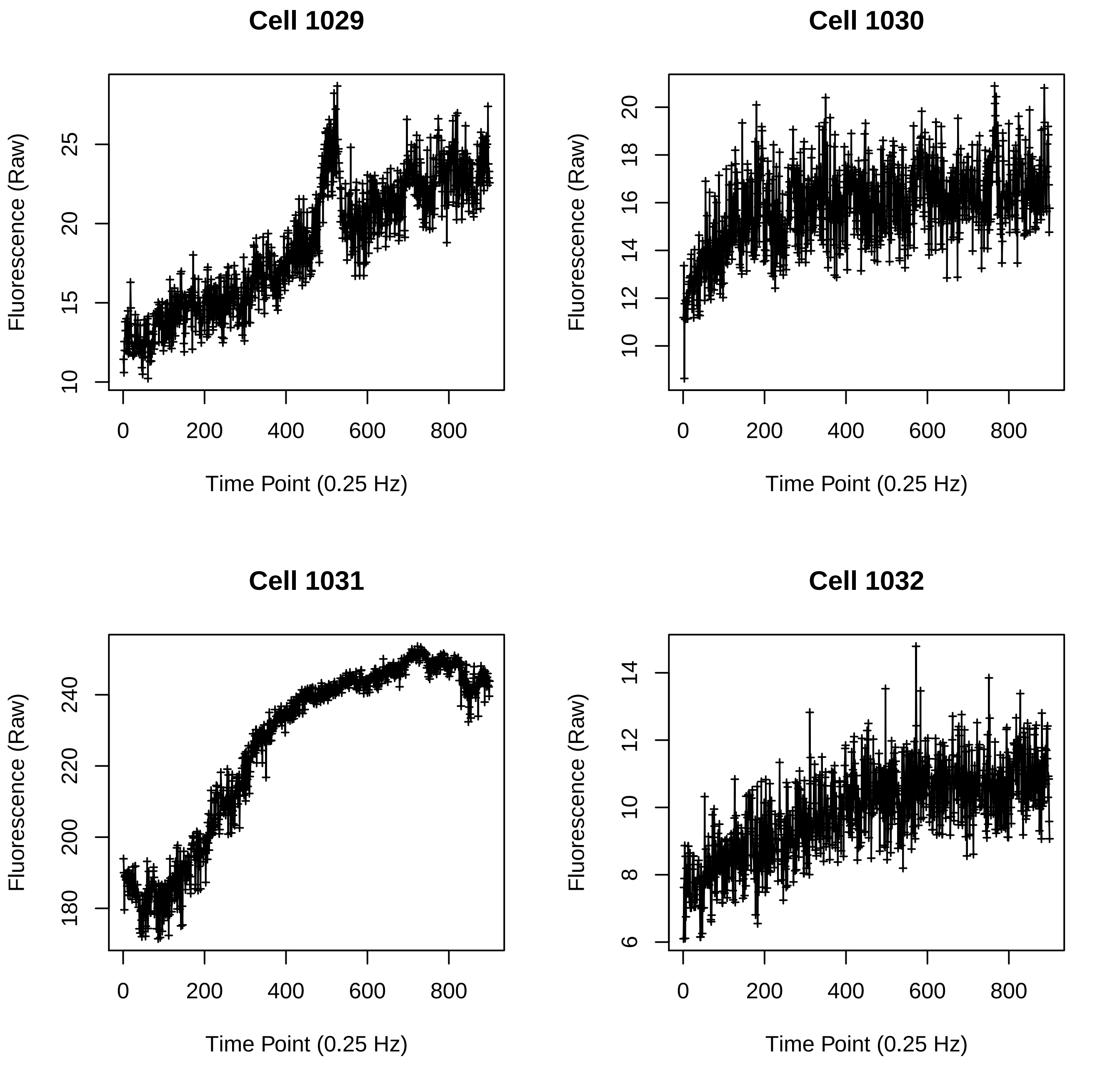
Cell 1029
Cell 1030
Cell 1031
Cell 1032
Fluorescence (Raw)
Time Point (0.25 Hz)
0
200
400
600
800
10
15
20
25
12
14
16
18
20
180
200
220
240
6
8
10
12
14

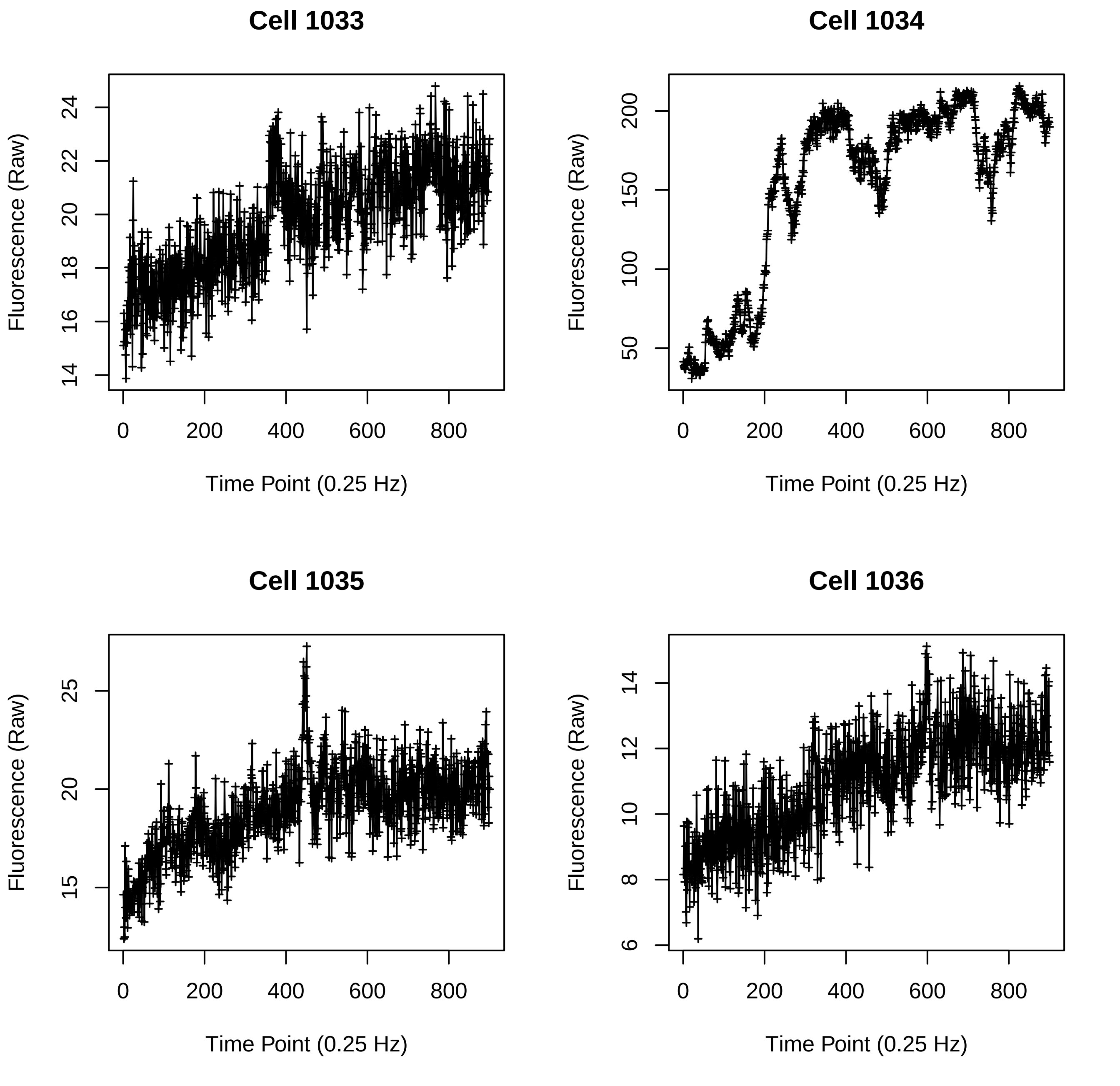
Cell 1033
Fluorescence (Raw)
Time Point (0.25 Hz)
Cell 1034
Fluorescence (Raw)
Time Point (0.25 Hz)
Cell 1035
Fluorescence (Raw)
Time Point (0.25 Hz)
Cell 1036
Fluorescence (Raw)
Time Point (0.25 Hz)

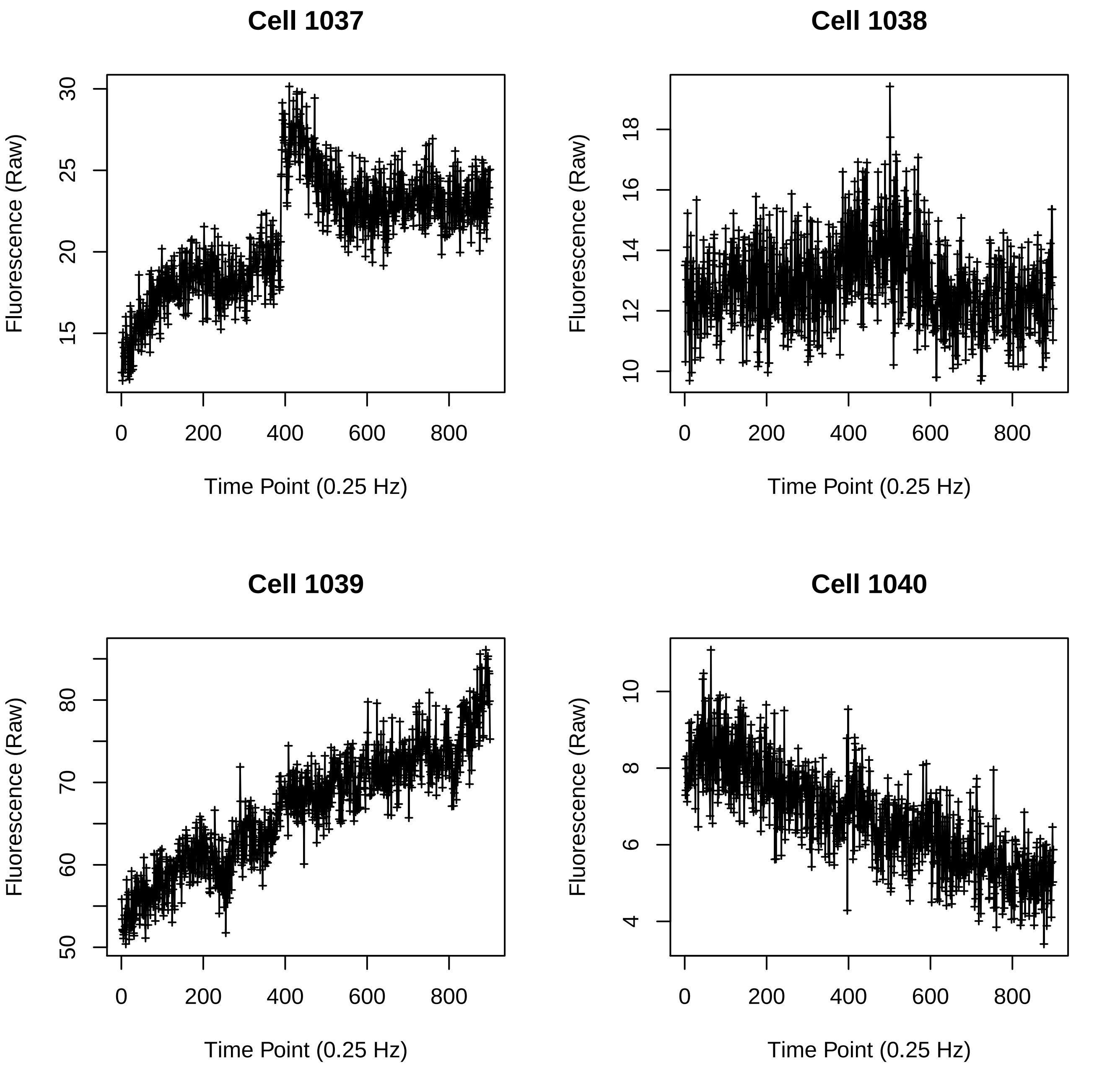
Cell 1037
Cell 1038
Cell 1039
Cell 1040
Fluorescence (Raw)
Time Point (0.25 Hz)
0
200
400
600
800
15
20
25
30
10
12
14
16
18
50
60
70
80
4
6
8
10

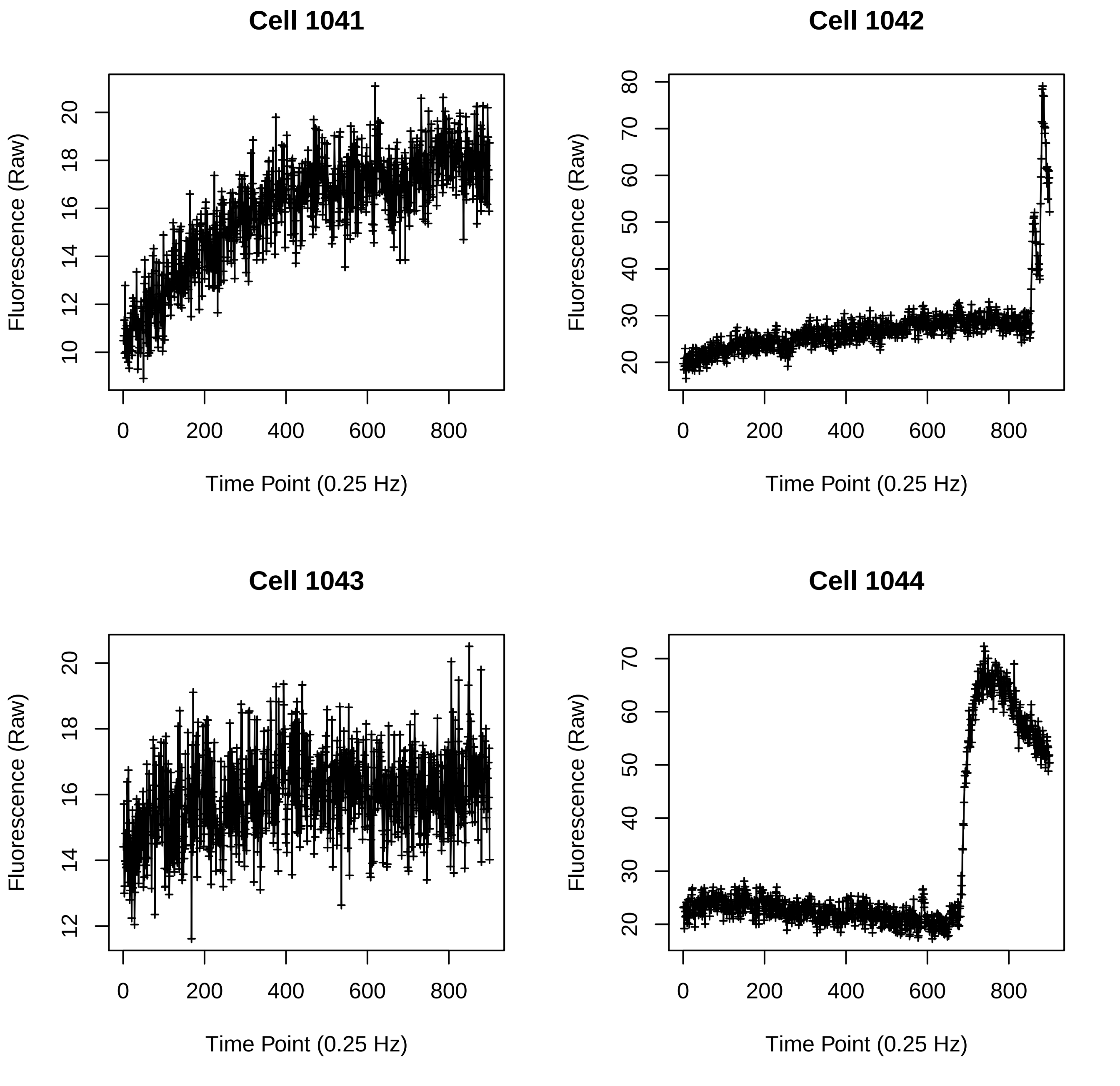

Cell 1041
Cell 1042
Cell 1043
Cell 1044
Fluorescence (Raw)
Time Point (0.25 Hz)
0
200
400
600
800
10
12
14
16
18
20
30
40
50
60
70
80

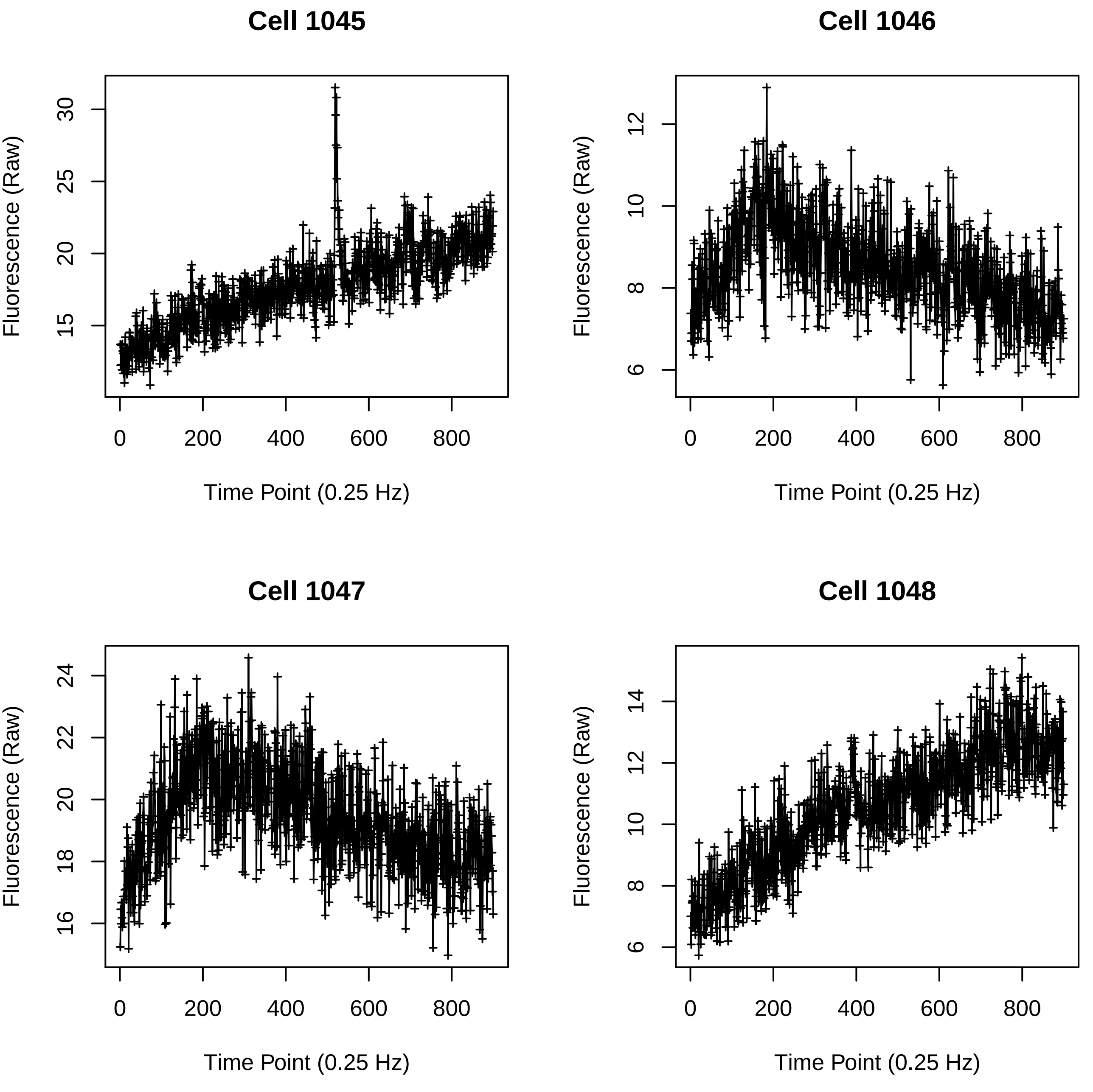

Cell 1045
Cell 1046
Cell 1047
Cell 1048
Fluorescence (Raw)
Time Point (0.25 Hz)
0
200
400
600
800
15
20
25
30
6
8
10
12
16
18
20
22
24
6
8
10
12
14

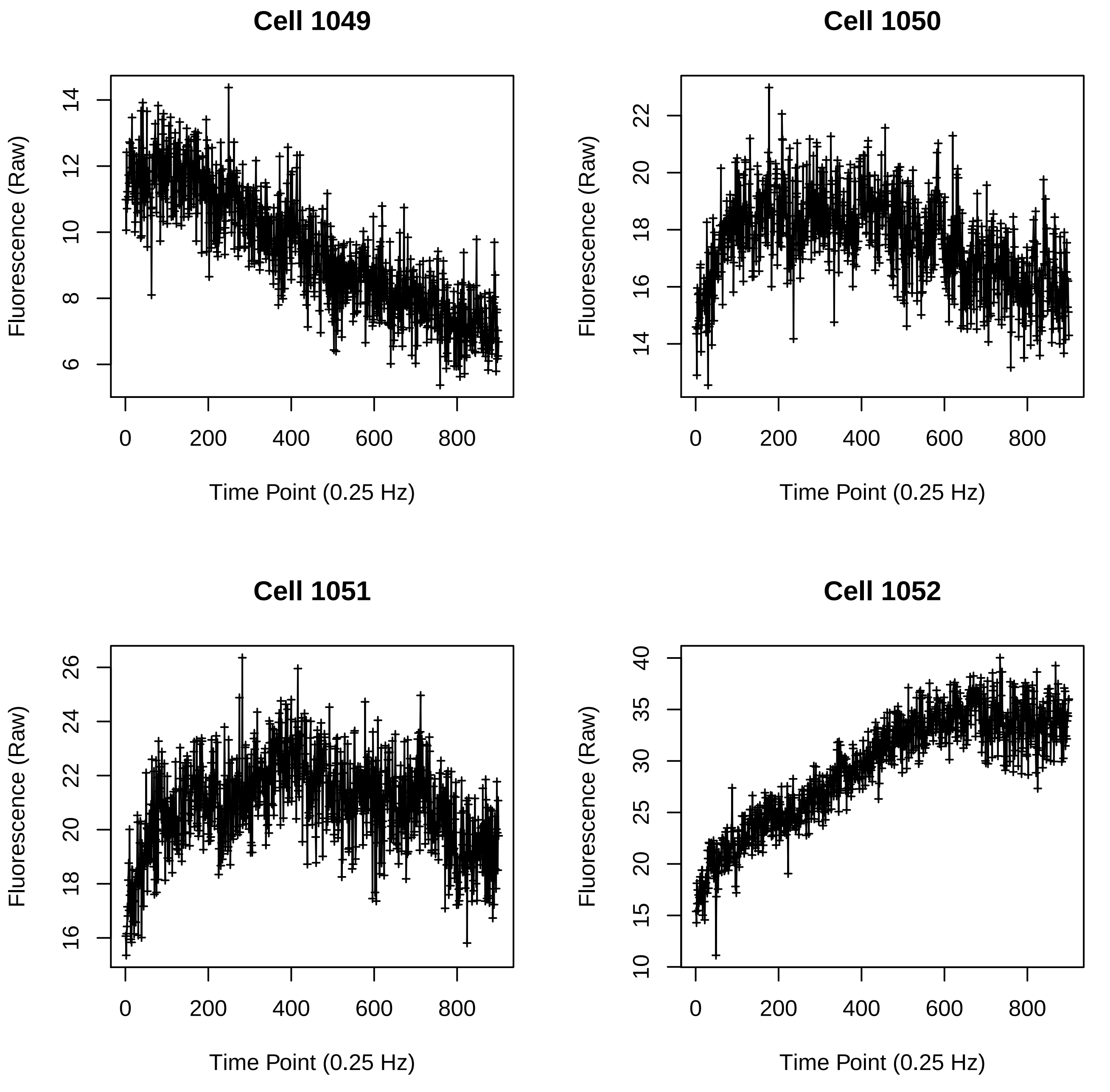
Cell 1049
Cell 1050
Cell 1051
Cell 1052
Fluorescence (Raw)
Time Point (0.25 Hz)
0
200
400
600
800
6
8
10
12
14
16
18
20
22
24
26
15
25
30
35
40

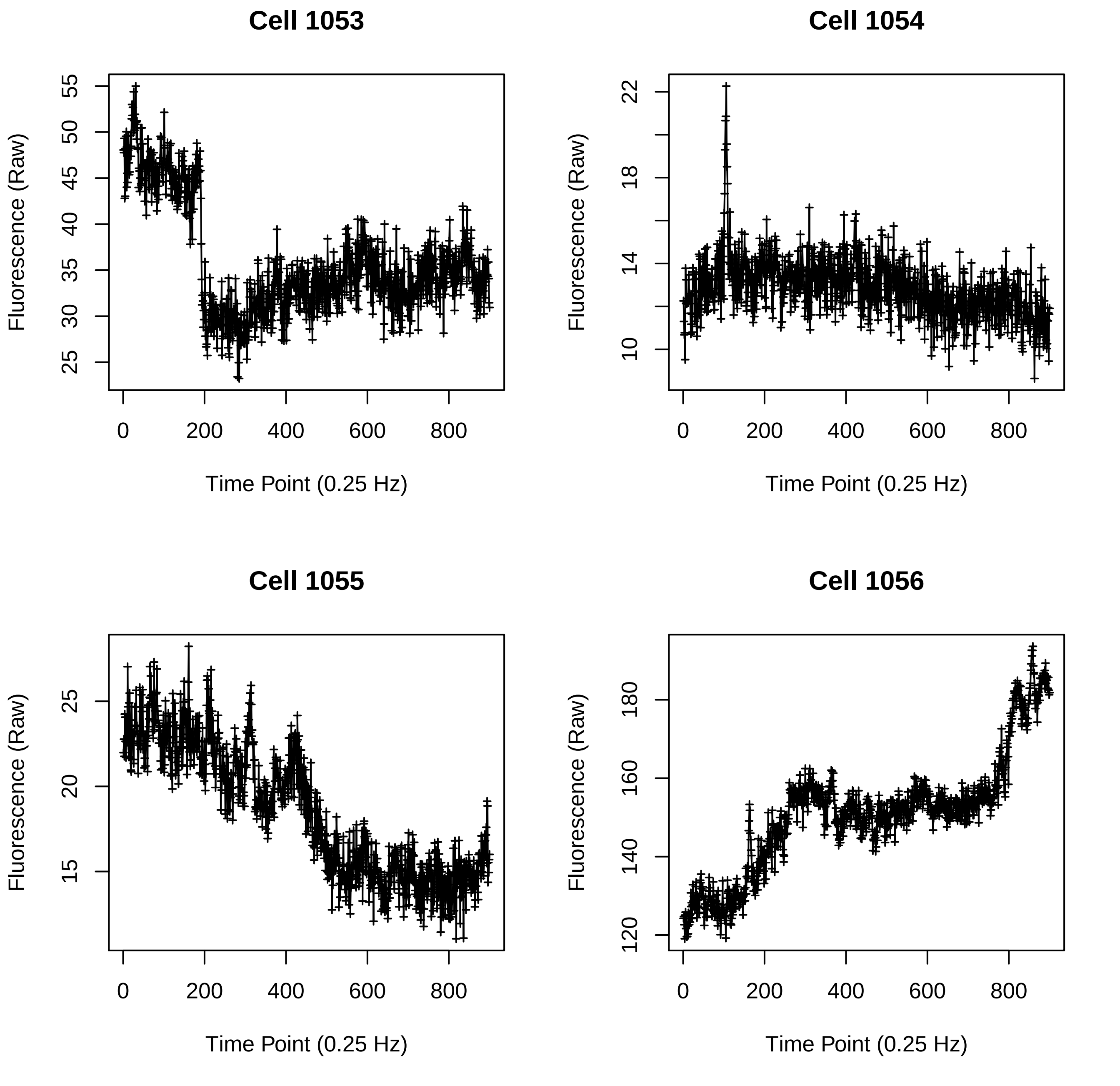

Cell 1053
Cell 1054
Cell 1055
Cell 1056
Fluorescence (Raw)
Time Point (0.25 Hz)
0
200
400
600
800
25
30
35
40
45
50
55
10
14
18
22
15
20
25
120
140
160
180

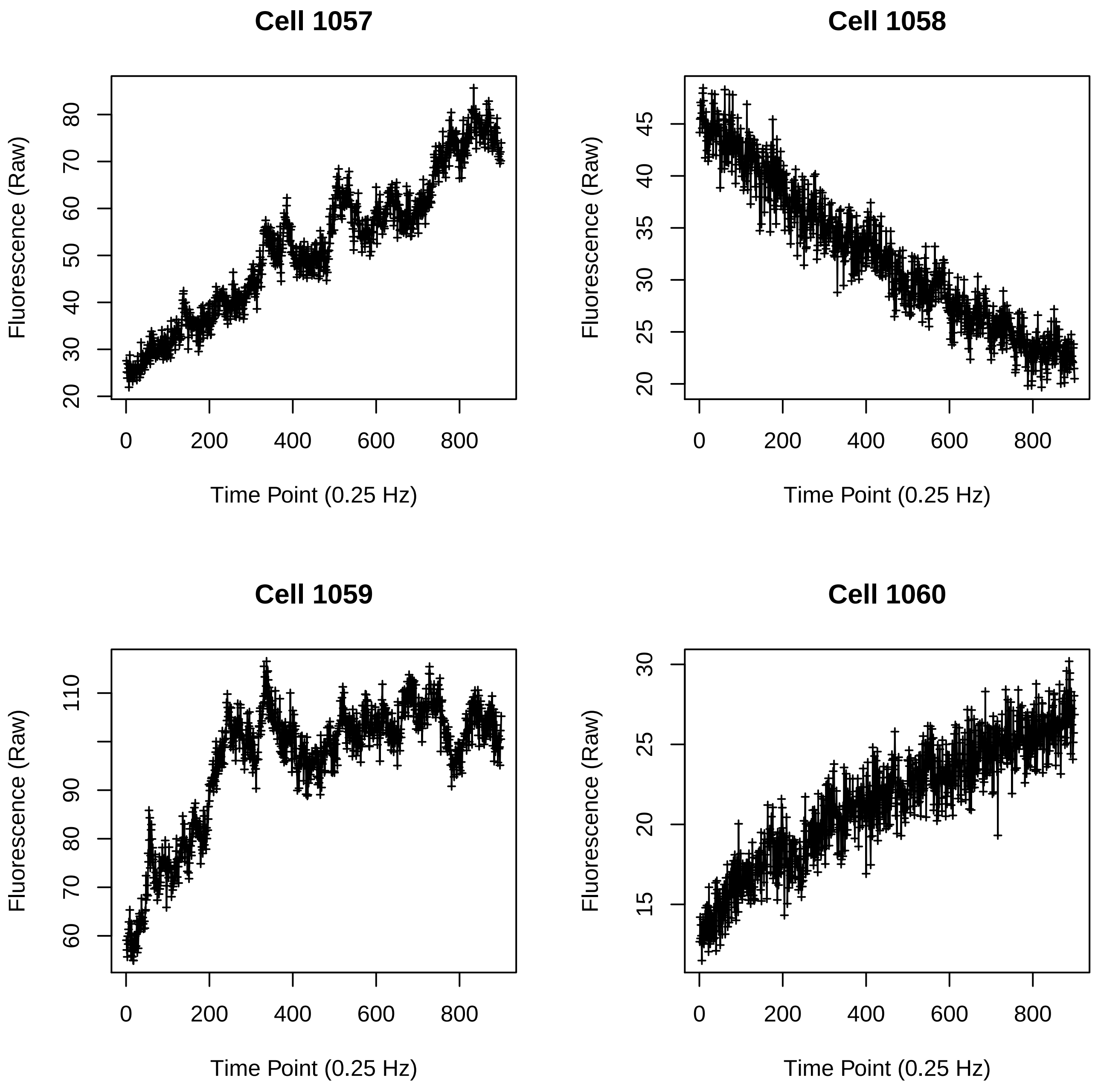
Cell 1057
Fluorescence (Raw)
20
30
40
50
60
70
80
0
200
400
600
800
Time Point (0.25 Hz)
Cell 1058
Fluorescence (Raw)
20
25
30
35
40
45
0
200
400
600
800
Time Point (0.25 Hz)
Cell 1059
Fluorescence (Raw)
60
70
80
90
110
0
200
400
600
800
Time Point (0.25 Hz)
Cell 1060
Fluorescence (Raw)
15
20
25
30
0
200
400
600
800
Time Point (0.25 Hz)

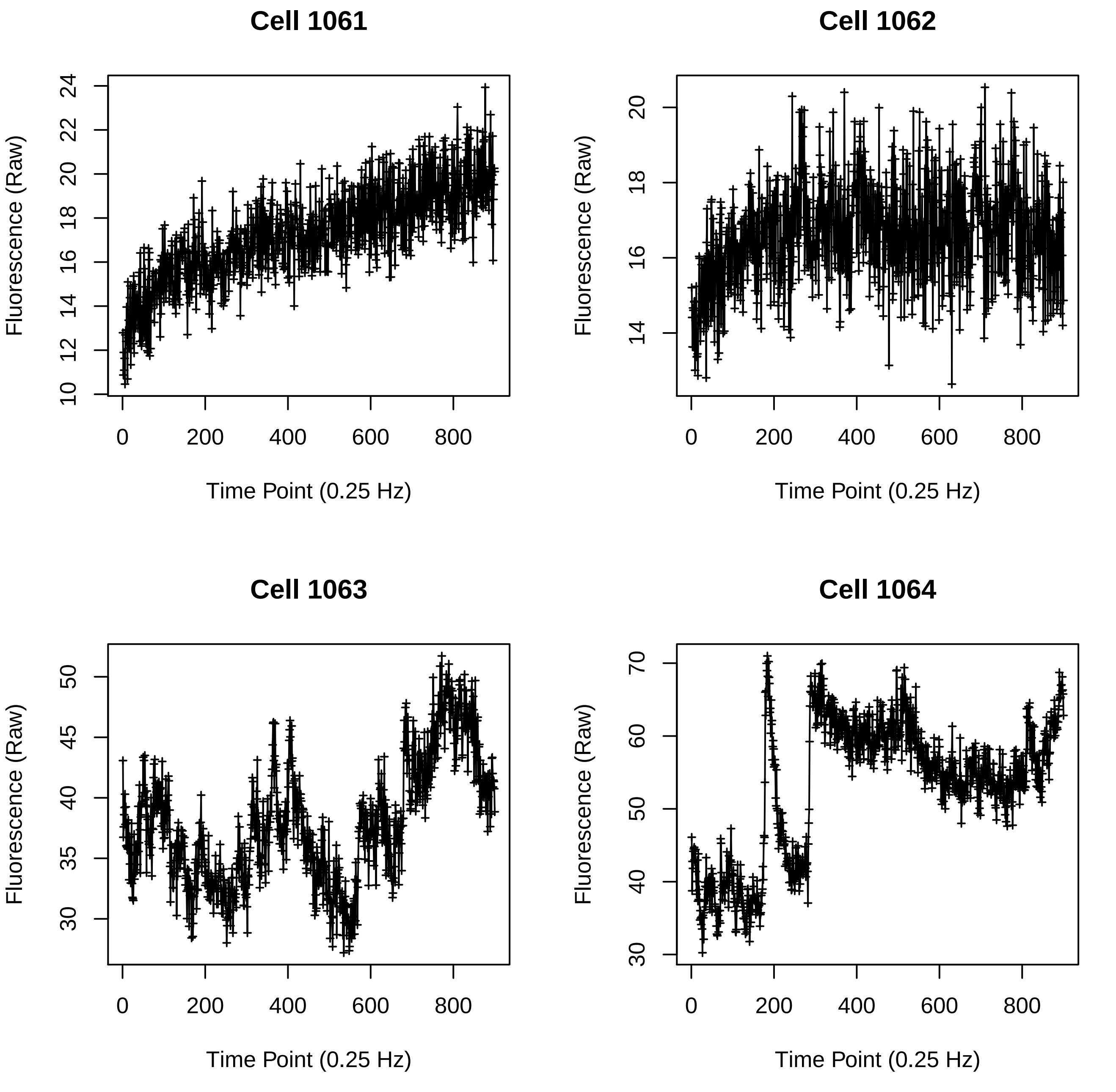
Cell 1061
Fluorescence (Raw)
Time Point (0.25 Hz)
Cell 1062
Fluorescence (Raw)
Time Point (0.25 Hz)
Cell 1063
Fluorescence (Raw)
Time Point (0.25 Hz)
Cell 1064
Fluorescence (Raw)
Time Point (0.25 Hz)

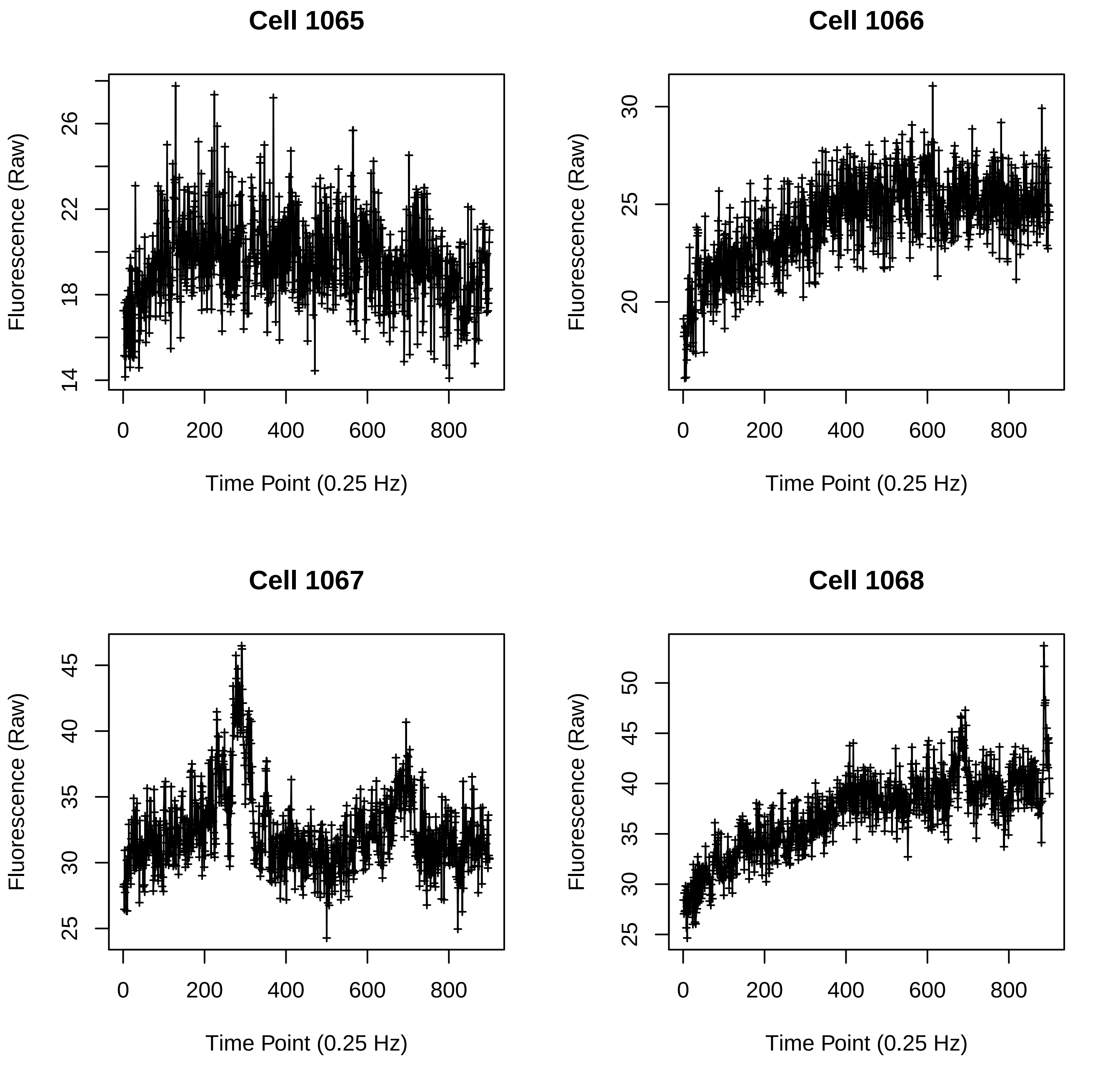

Cell 1065
Fluorescence (Raw)
Time Point (0.25 Hz)
14
18
22
26
0
200
400
600
800
Cell 1066
Fluorescence (Raw)
Time Point (0.25 Hz)
20
25
30
0
200
400
600
800
Cell 1067
Fluorescence (Raw)
Time Point (0.25 Hz)
25
30
35
40
45
0
200
400
600
800
Cell 1068
Fluorescence (Raw)
Time Point (0.25 Hz)
25
30
35
40
45
50
0
200
400
600
800

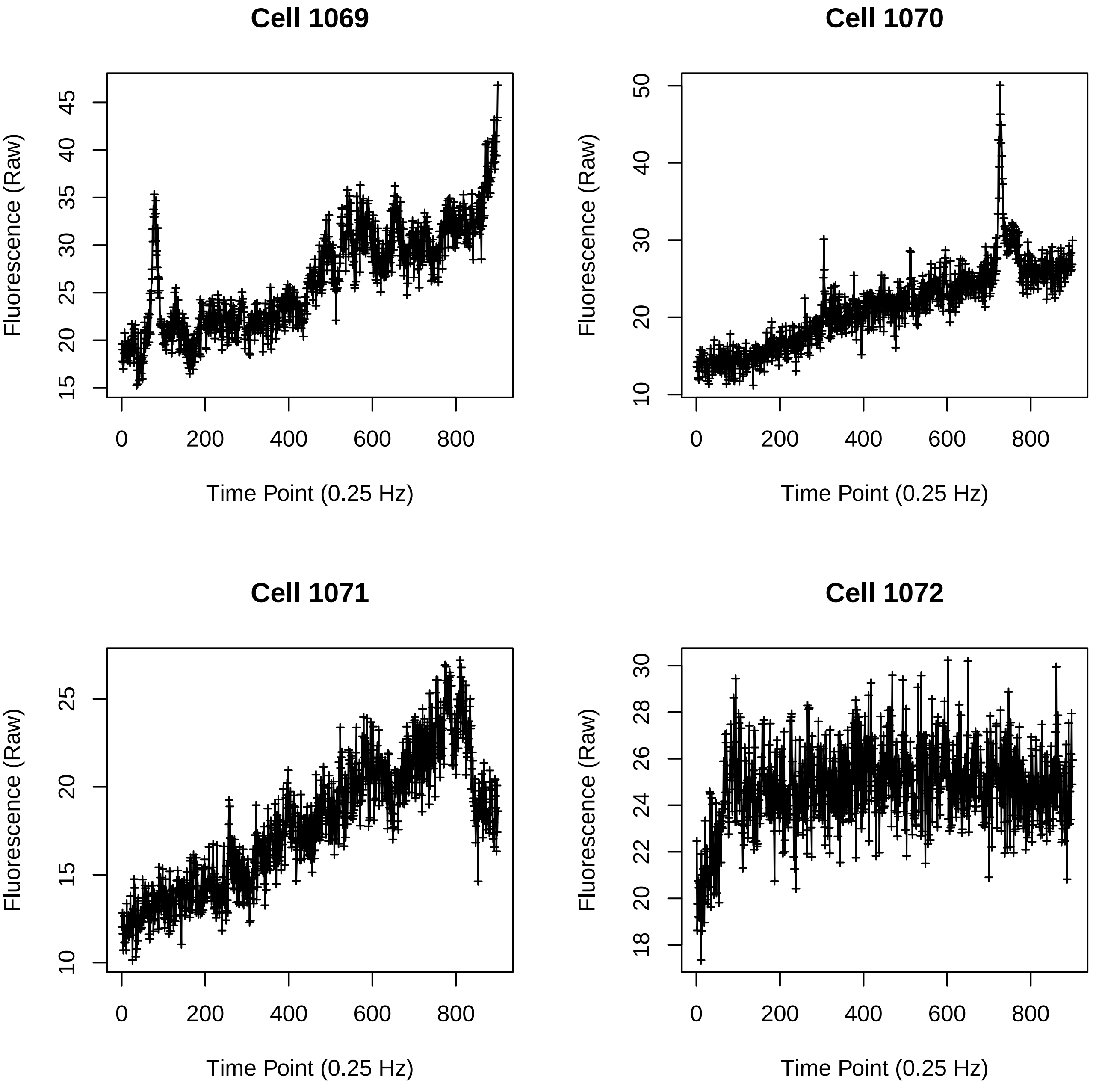

Cell 1069
Cell 1070
Cell 1071
Cell 1072
Fluorescence (Raw)
Time Point (0.25 Hz)
0
200
400
600
800
15
20
25
30
35
40
45
10
50
18
22
24
26
28

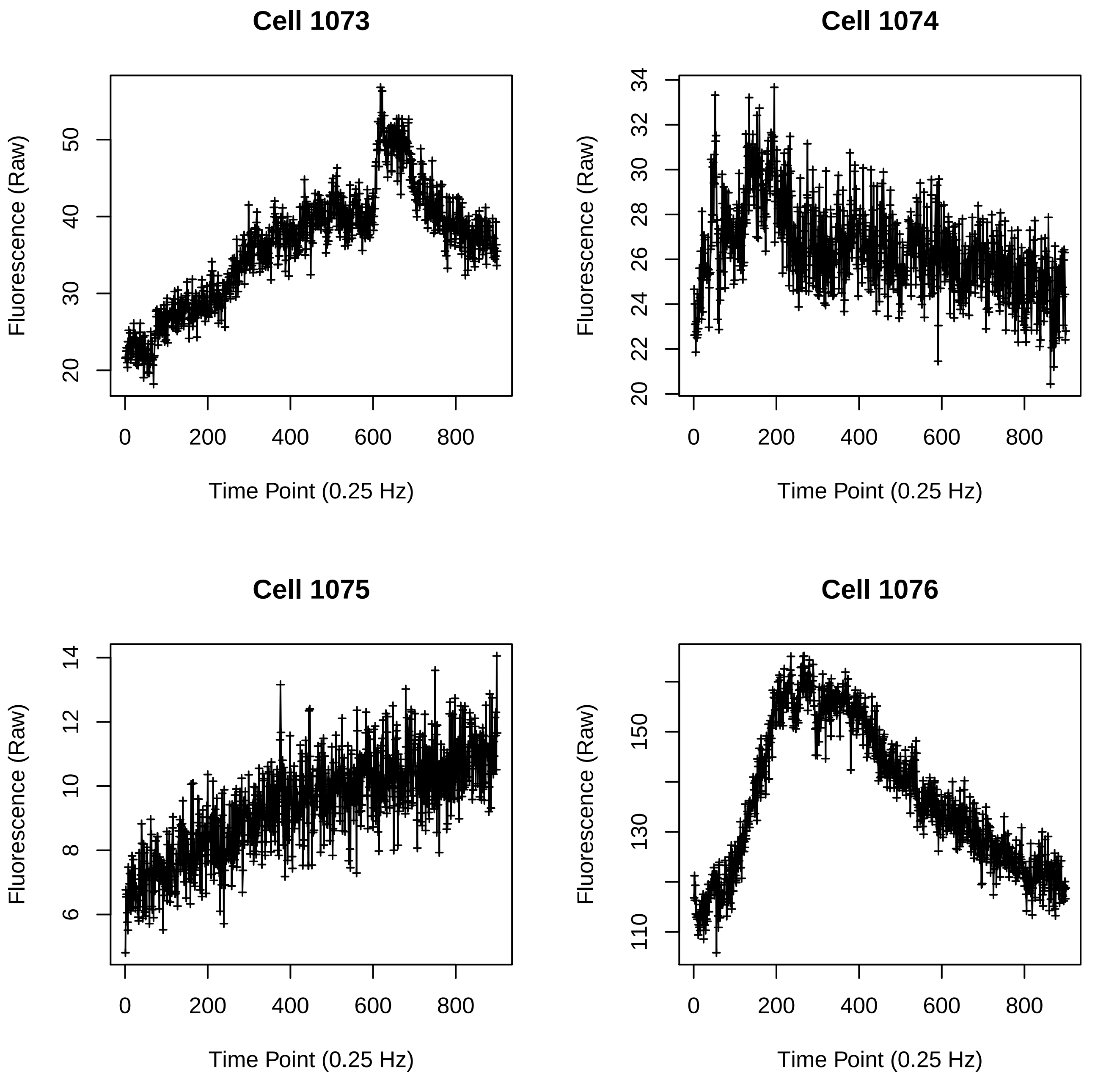
Cell 1073
Fluorescence (Raw)
20
30
40
50
0
200
400
600
800
Time Point (0.25 Hz)
Cell 1074
Fluorescence (Raw)
20
22
24
26
28
30
32
34
0
200
400
600
800
Time Point (0.25 Hz)
Cell 1075
Fluorescence (Raw)
6
8
10
12
14
0
200
400
600
800
Time Point (0.25 Hz)
Cell 1076
Fluorescence (Raw)
110
130
150
0
200
400
600
800
Time Point (0.25 Hz)

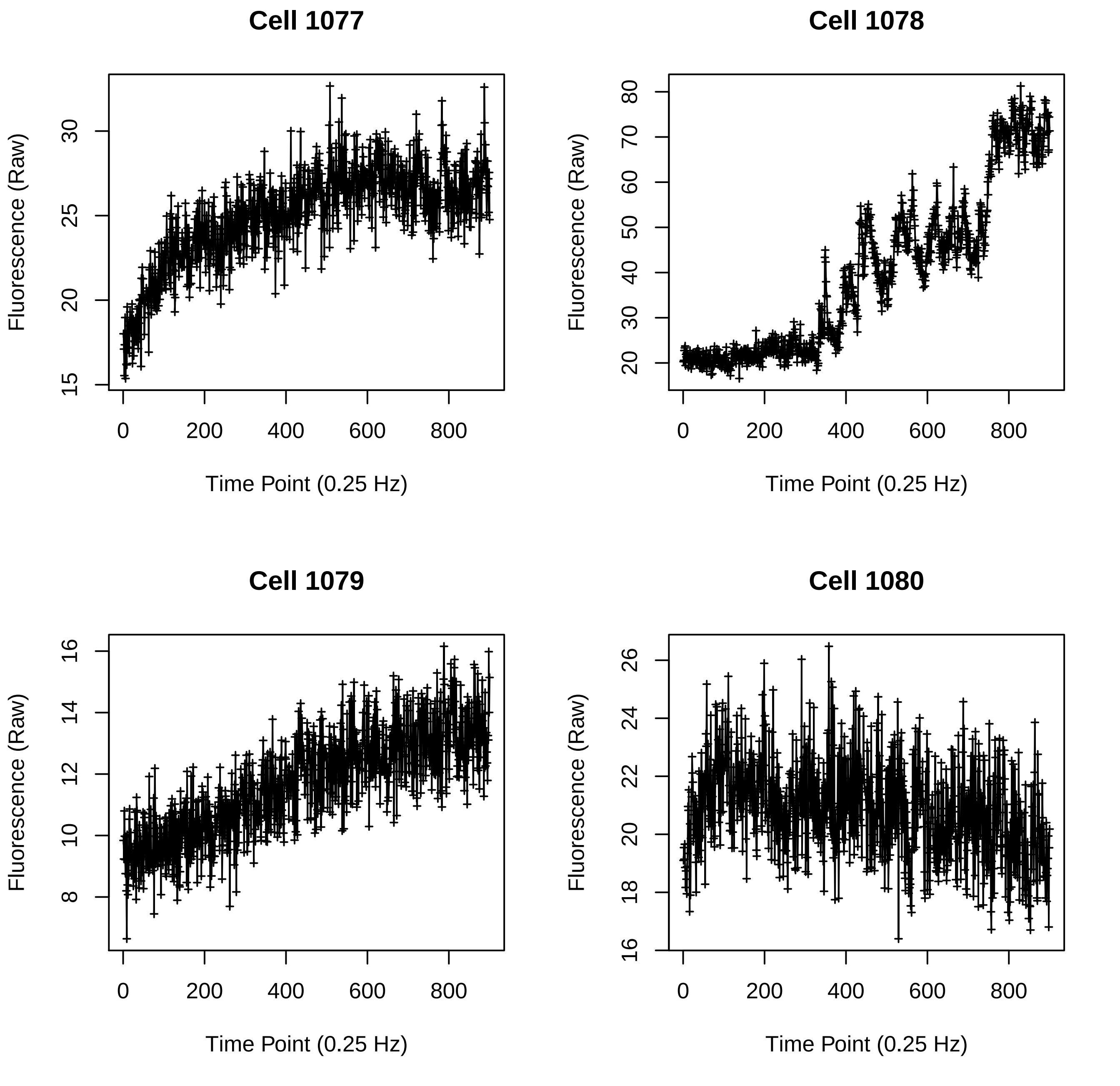
Cell 1077
Cell 1078
Cell 1079
Cell 1080
Fluorescence (Raw)
Time Point (0.25 Hz)
0
200
400
600
800
15
20
25
30
40
50
60
70
80
8
10
12
14
16
18
22
24
26

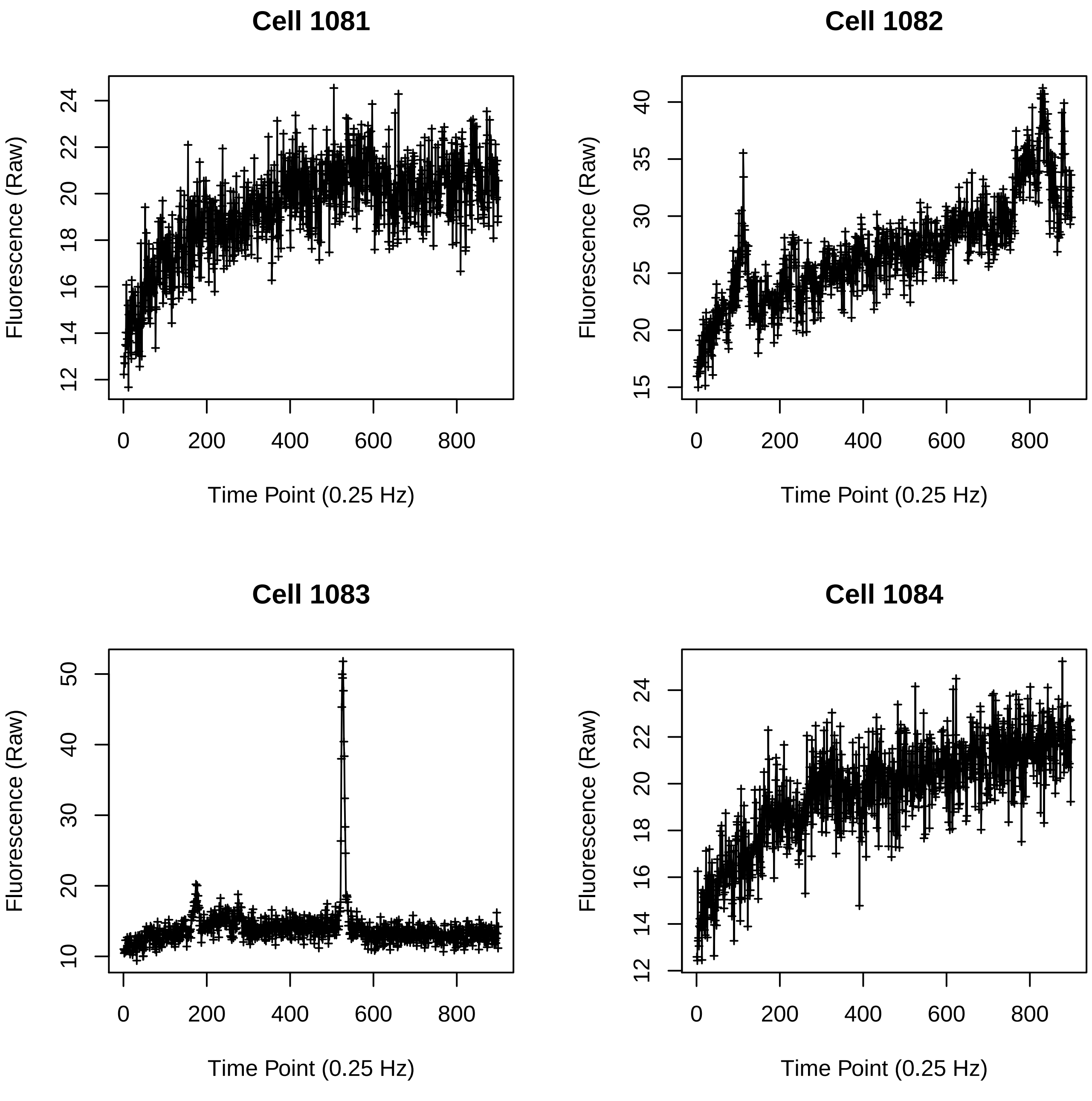
Cell 1081
Cell 1082
Cell 1083
Cell 1084
Fluorescence (Raw)
Time Point (0.25 Hz)
0
200
400
600
800
12
14
16
18
20
22
24
15
25
30
35
40
10
50

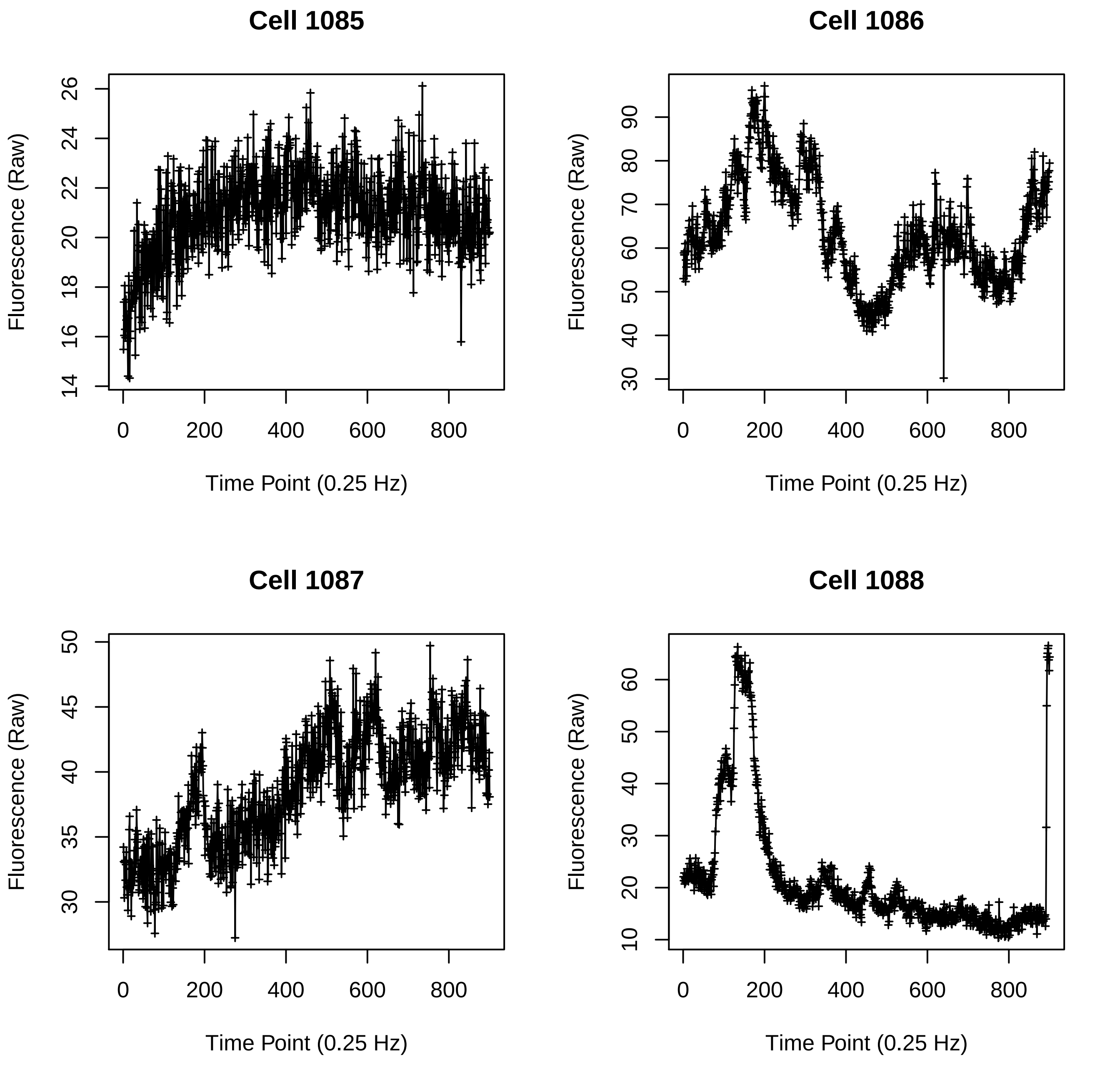
Cell 1085
Cell 1086
Cell 1087
Cell 1088
Fluorescence (Raw)
Time Point (0.25 Hz)
14
16
18
20
22
24
26
30
40
50
60
70
80
90
35
45
10
0
200
400
600
800

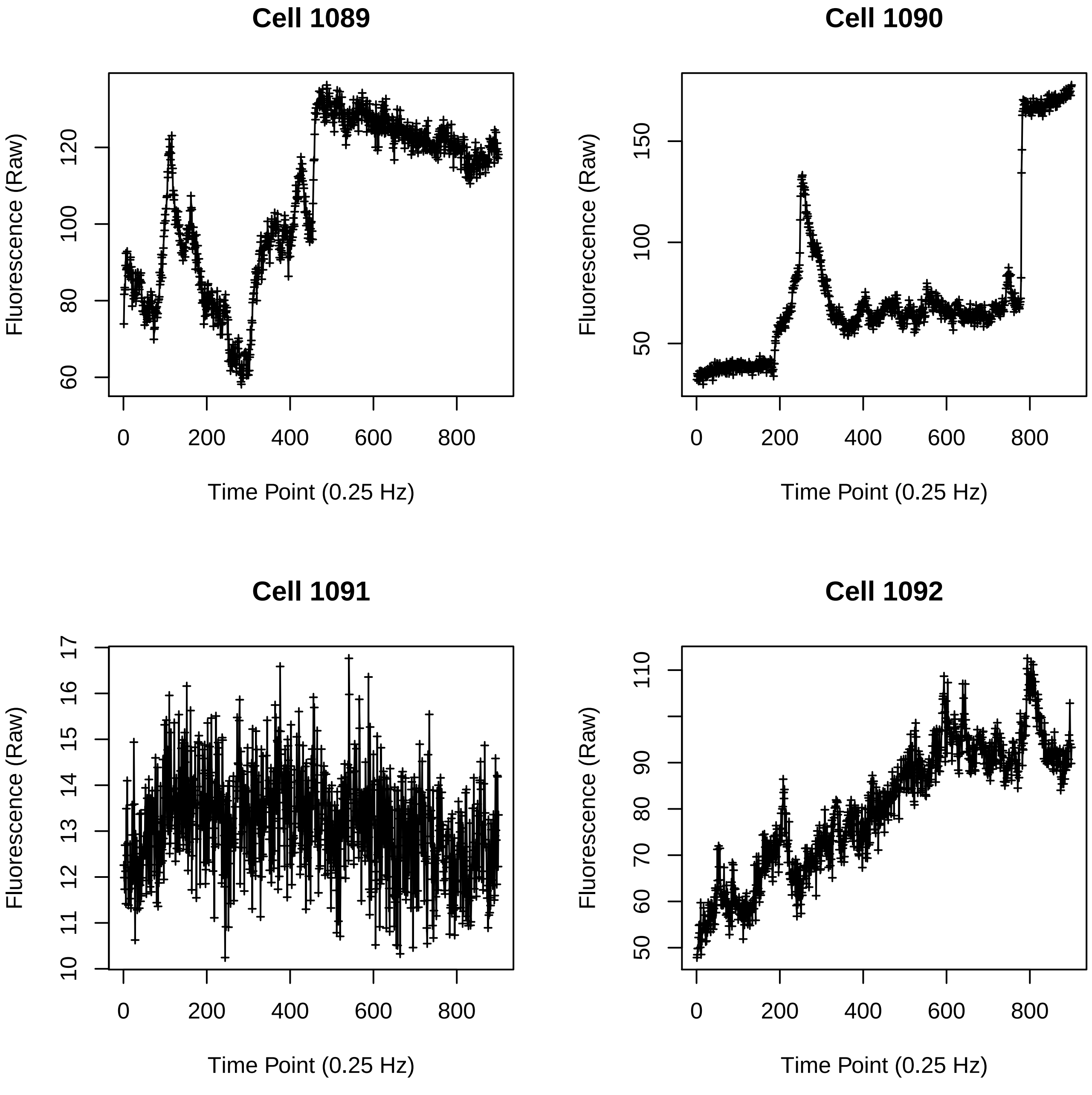
Cell 1089
Fluorescence (Raw)
60
80
100
120
0
200
400
600
800
Time Point (0.25 Hz)
Cell 1090
Fluorescence (Raw)
50
100
150
0
200
400
600
800
Time Point (0.25 Hz)
Cell 1091
Fluorescence (Raw)
10
11
12
13
14
15
16
17
0
200
400
600
800
Time Point (0.25 Hz)
Cell 1092
Fluorescence (Raw)
50
60
70
80
90
110
0
200
400
600
800
Time Point (0.25 Hz)

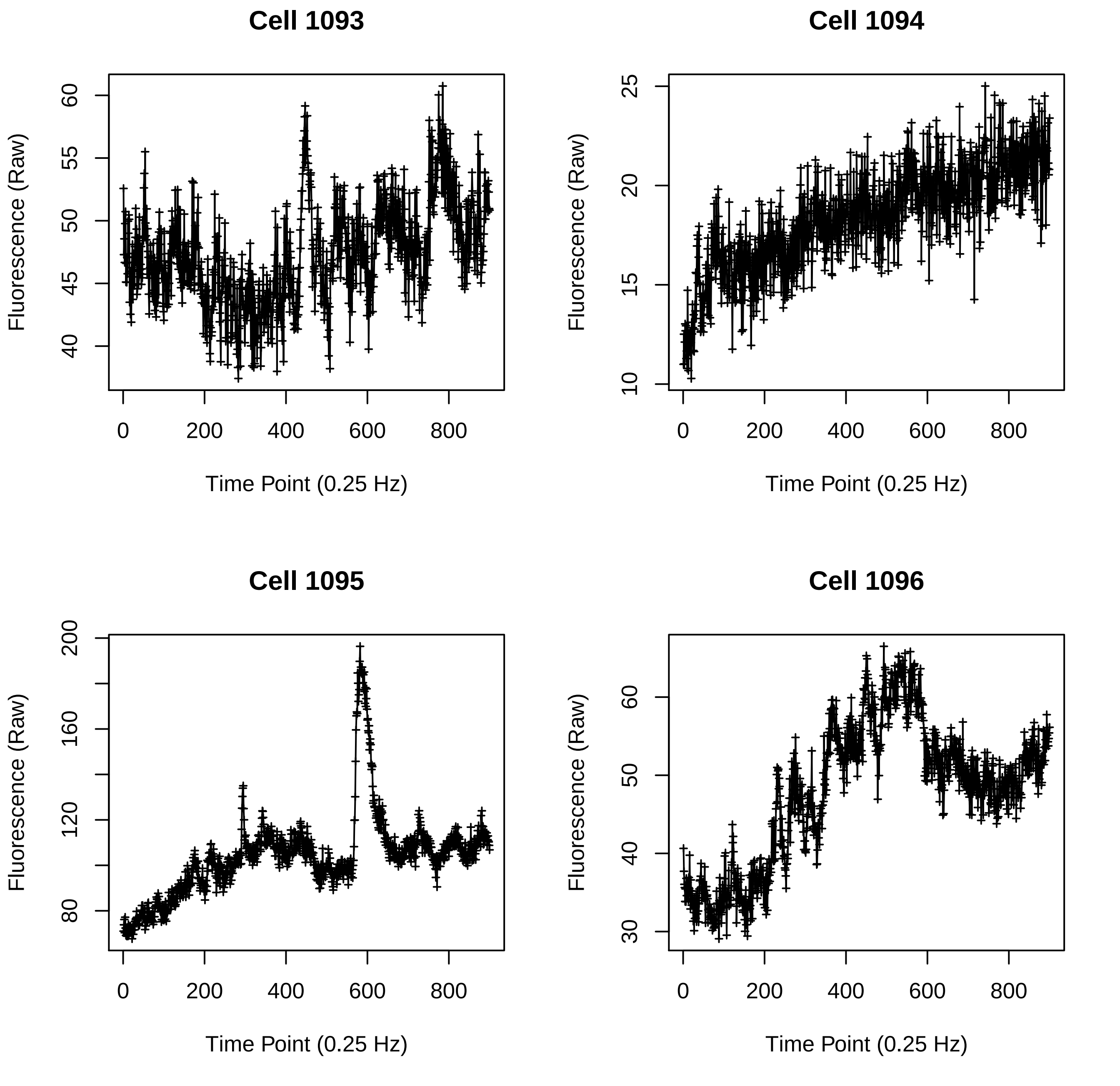
Cell 1093
Cell 1094
Cell 1095
Cell 1096
Fluorescence (Raw)
Time Point (0.25 Hz)
0
200
400
600
800
40
45
50
55
60
10
15
20
25
80
120
160
200
30
40
50
60

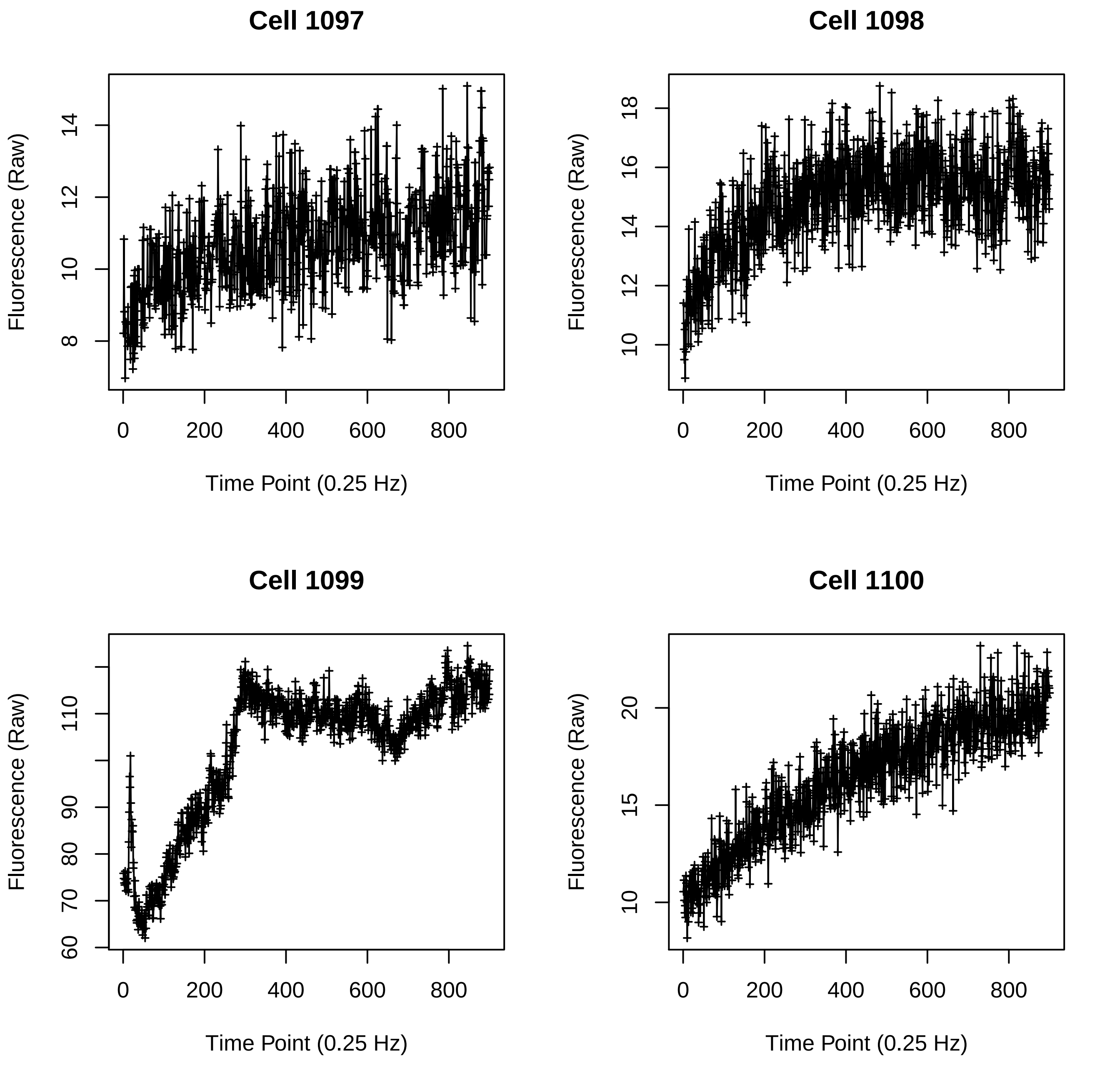

Cell 1097
Cell 1098
Cell 1099
Cell 1100
Fluorescence (Raw)
Time Point (0.25 Hz)
0
200
400
600
800
8
10
12
14
16
18
60
70
80
90
110
15
20

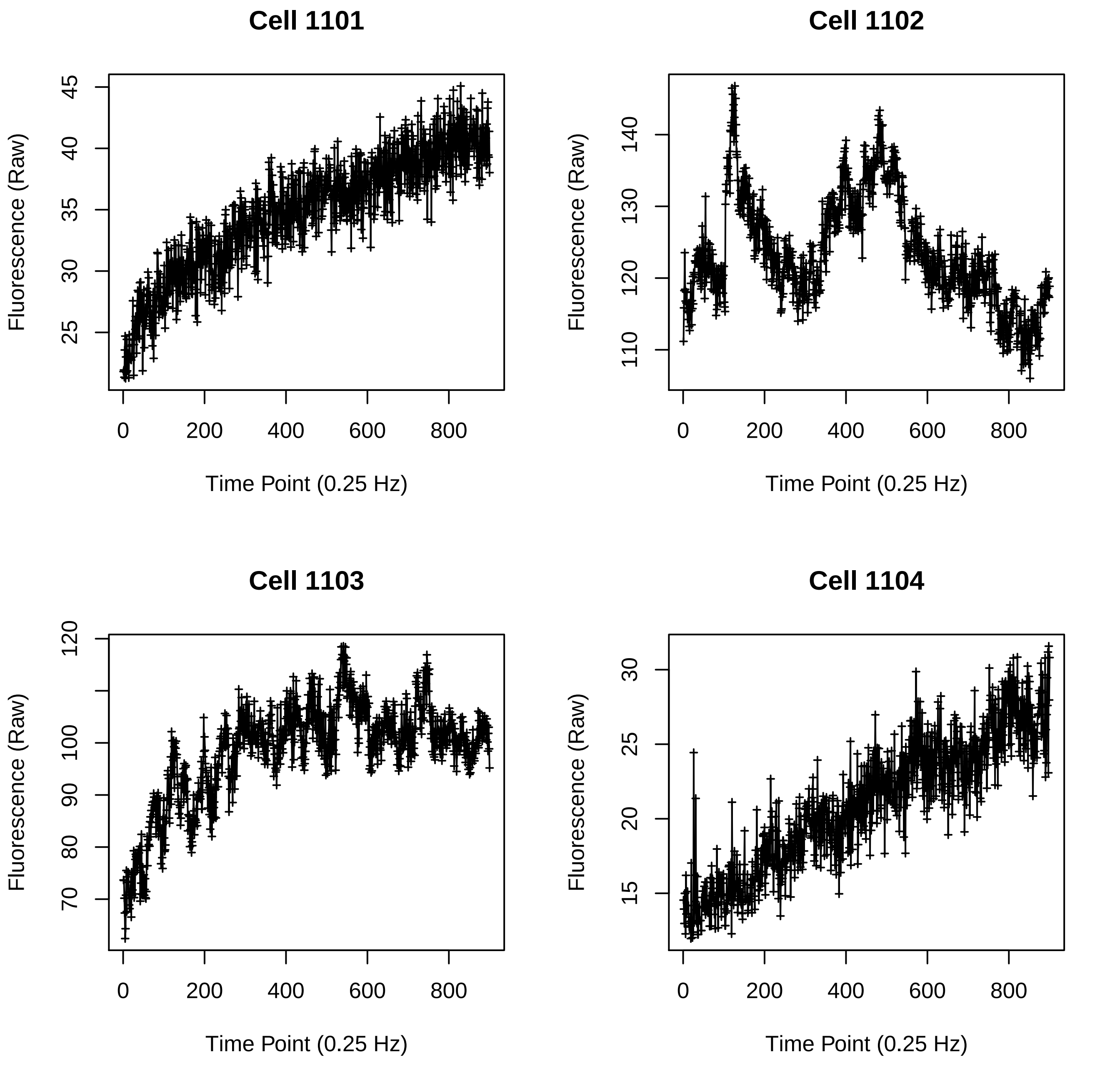

Cell 1101
Cell 1102
Cell 1103
Cell 1104
Fluorescence (Raw)
Time Point (0.25 Hz)
0
200
400
600
800
25
30
35
40
45
110
120
130
140
70
80
90
100
120
15
20
25
30

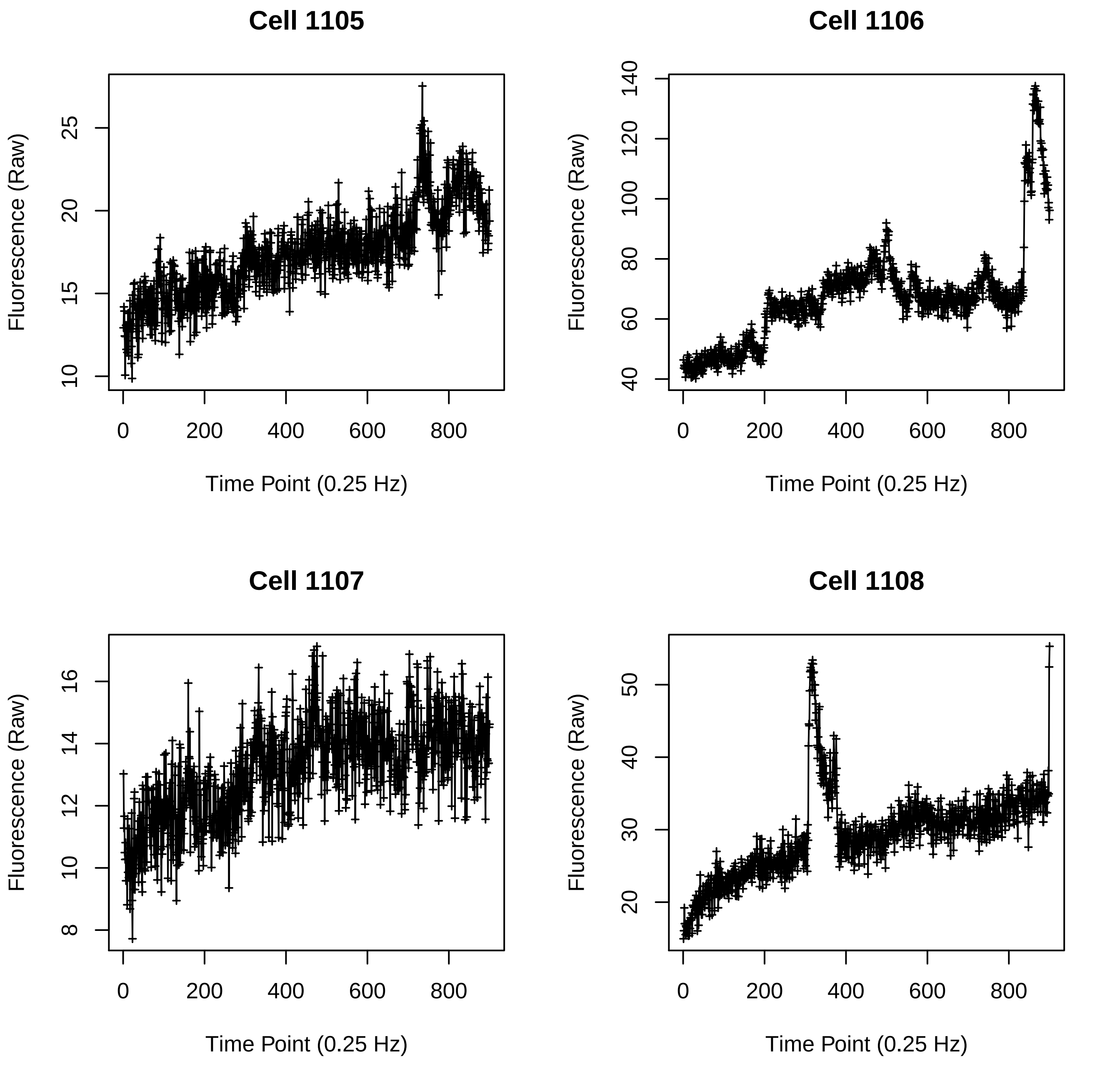

Cell 1105
Cell 1106
Cell 1107
Cell 1108
Fluorescence (Raw)
Time Point (0.25 Hz)
10
15
20
25
40
60
80
100
120
140
8
10
12
14
16
20
30
40
50
0
200
400
600
800

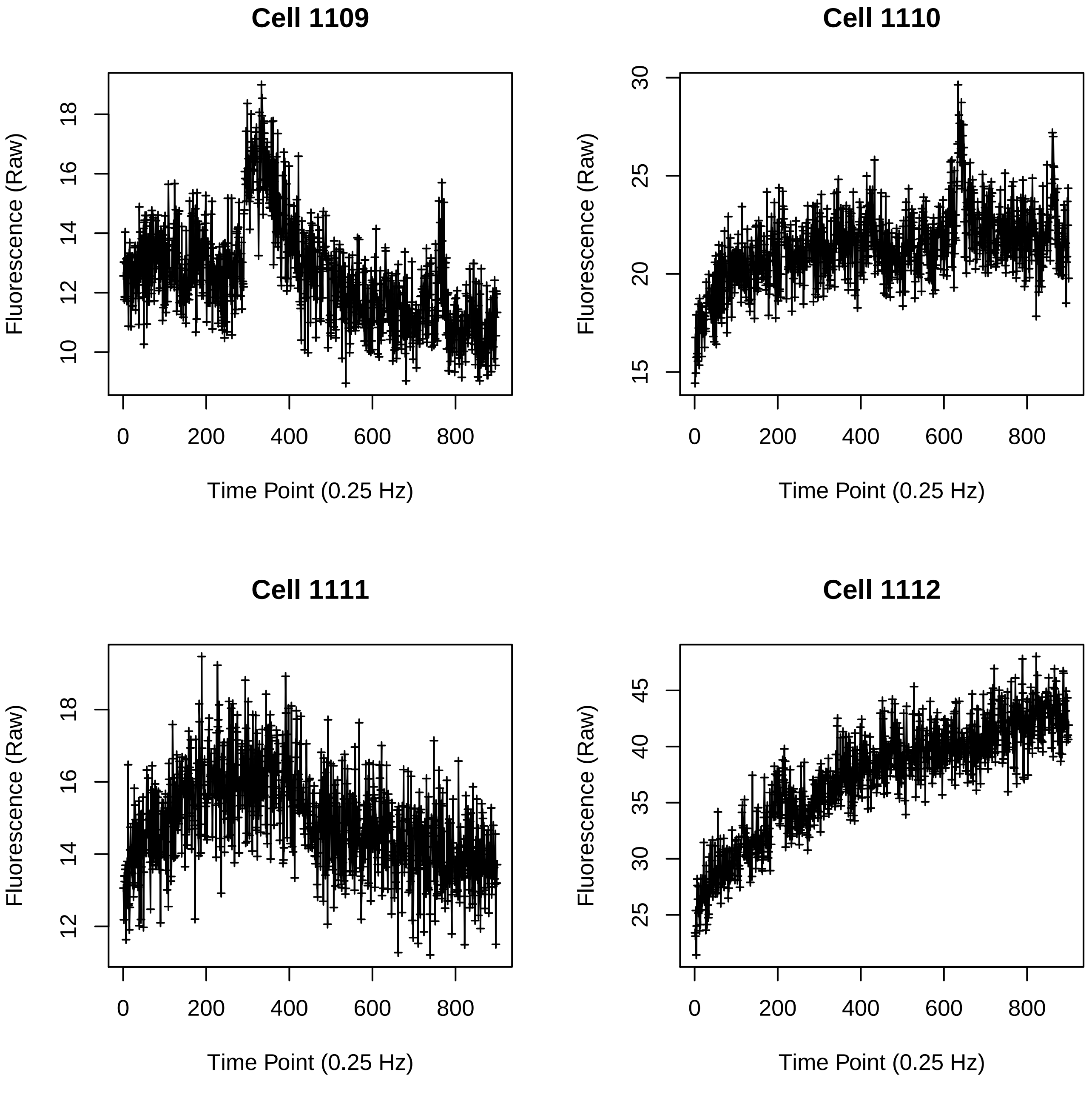

Cell 1109
Fluorescence (Raw)
10
12
14
16
18
0
200
400
600
800
Time Point (0.25 Hz)
Cell 1110
Fluorescence (Raw)
15
20
25
30
0
200
400
600
800
Time Point (0.25 Hz)
Cell 1111
Fluorescence (Raw)
12
14
16
18
0
200
400
600
800
Time Point (0.25 Hz)
Cell 1112
Fluorescence (Raw)
25
30
35
40
45
0
200
400
600
800
Time Point (0.25 Hz)

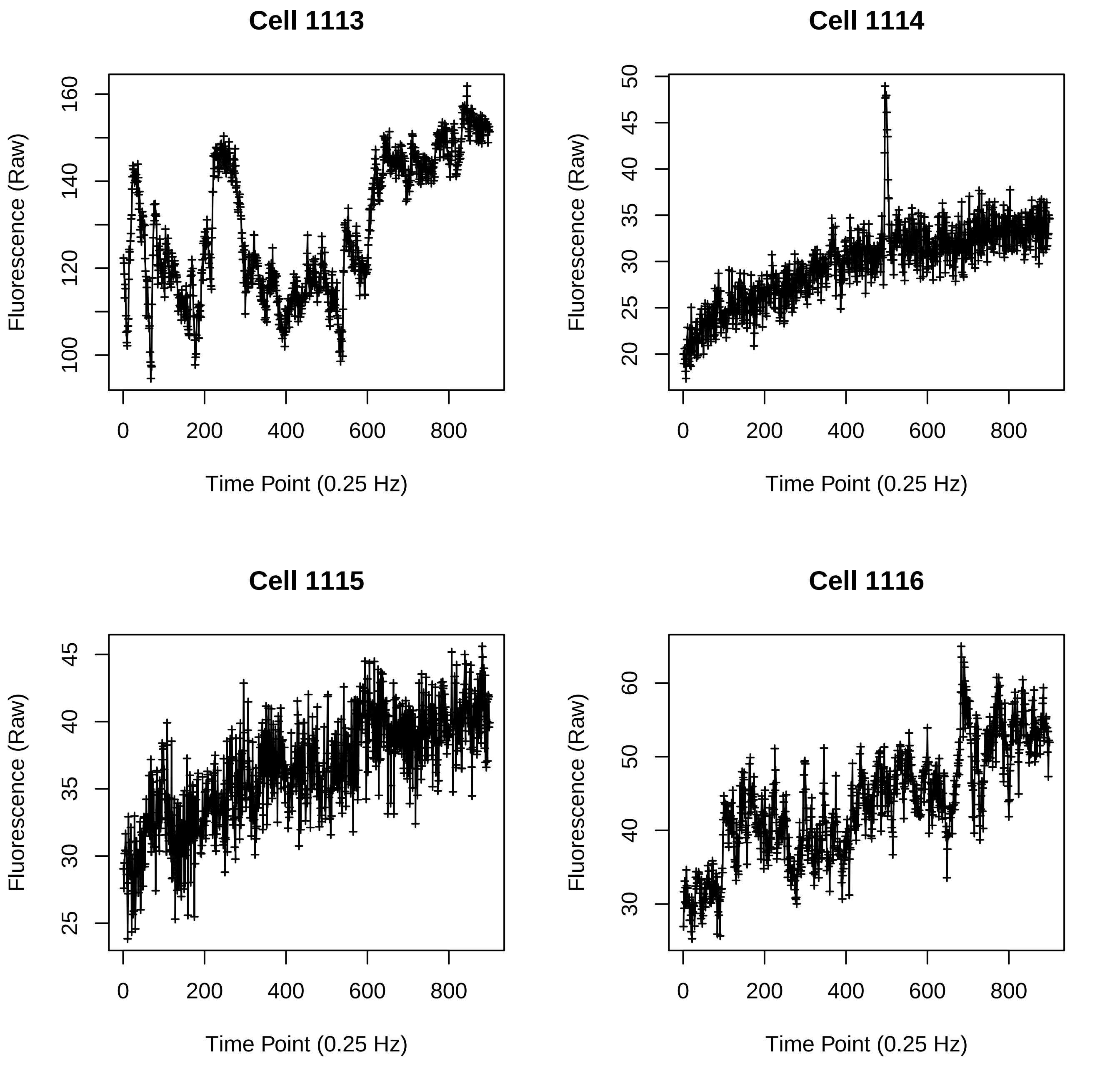

Cell 1113
Fluorescence (Raw)
100
120
140
160
0
200
400
600
800
Time Point (0.25 Hz)
Cell 1114
Fluorescence (Raw)
20
25
30
35
40
45
50
0
200
400
600
800
Time Point (0.25 Hz)
Cell 1115
Fluorescence (Raw)
25
30
35
40
45
0
200
400
600
800
Time Point (0.25 Hz)
Cell 1116
Fluorescence (Raw)
30
40
50
60
0
200
400
600
800
Time Point (0.25 Hz)

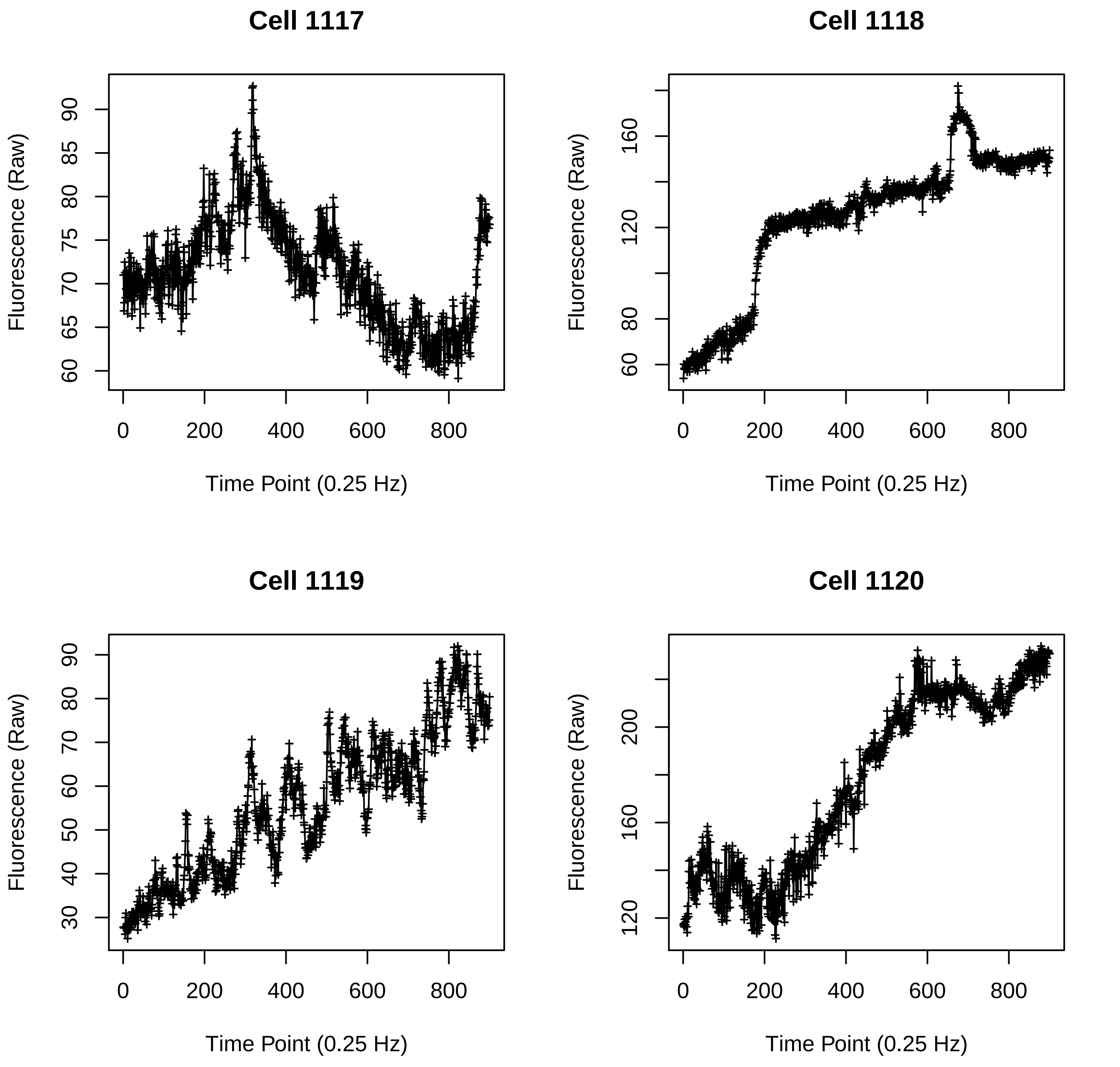
Cell 1117
Cell 1118
Cell 1119
Cell 1120
Fluorescence (Raw)
Time Point (0.25 Hz)
0
200
400
600
800
60
65
70
75
80
85
90
120
160
30
40
50
60
70
80
90
120
160
200

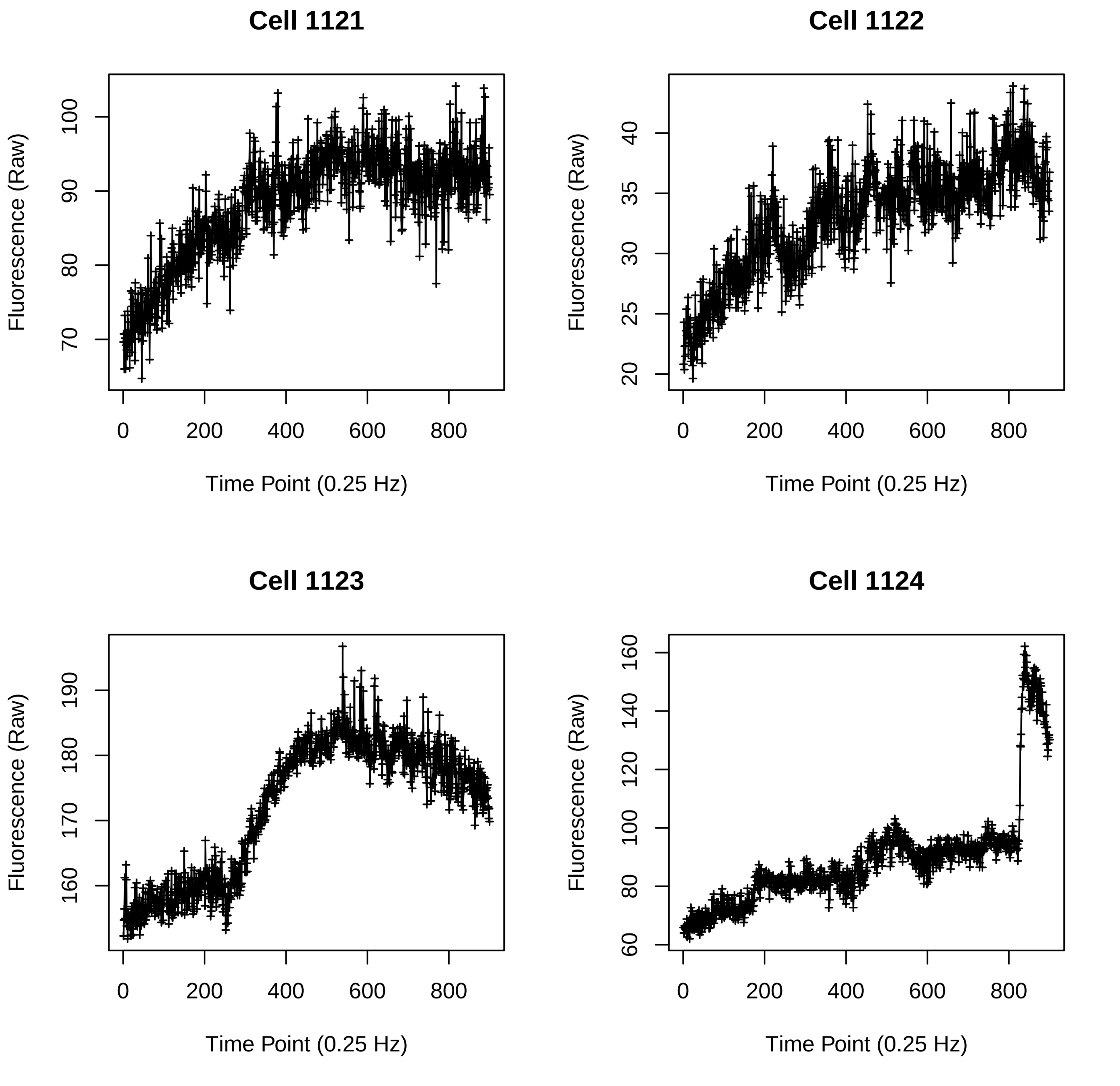
Cell 1121
Cell 1122
Cell 1123
Cell 1124
Fluorescence (Raw)
Time Point (0.25 Hz)
0
200
400
600
800
70
80
90
100
20
25
30
35
40
160
170
180
190
60
80
100
120
140
160

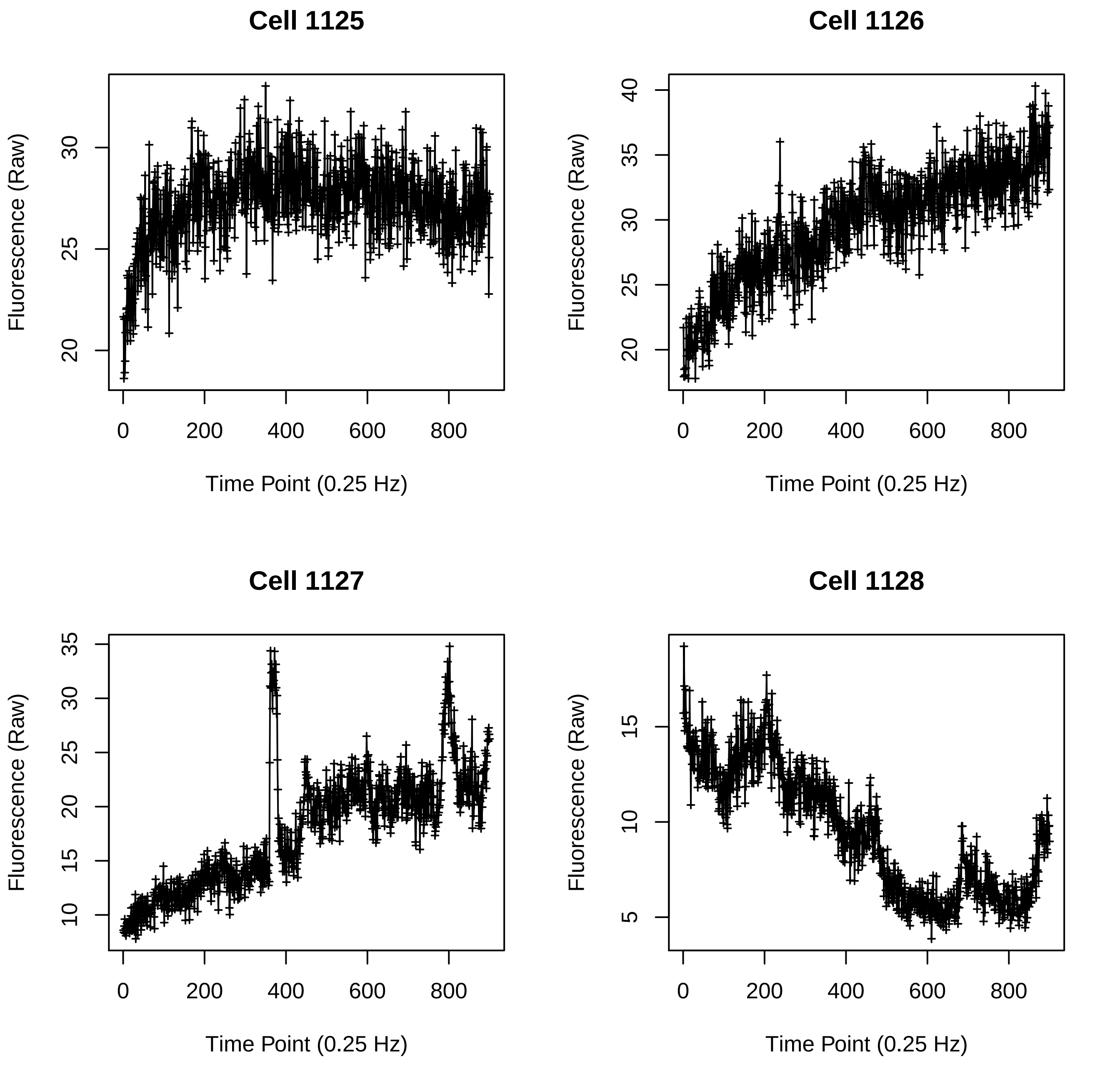
Cell 1125
Cell 1126
Cell 1127
Cell 1128
Fluorescence (Raw)
Time Point (0.25 Hz)
0
200
400
600
800
20
25
30
35
40
10
15
5

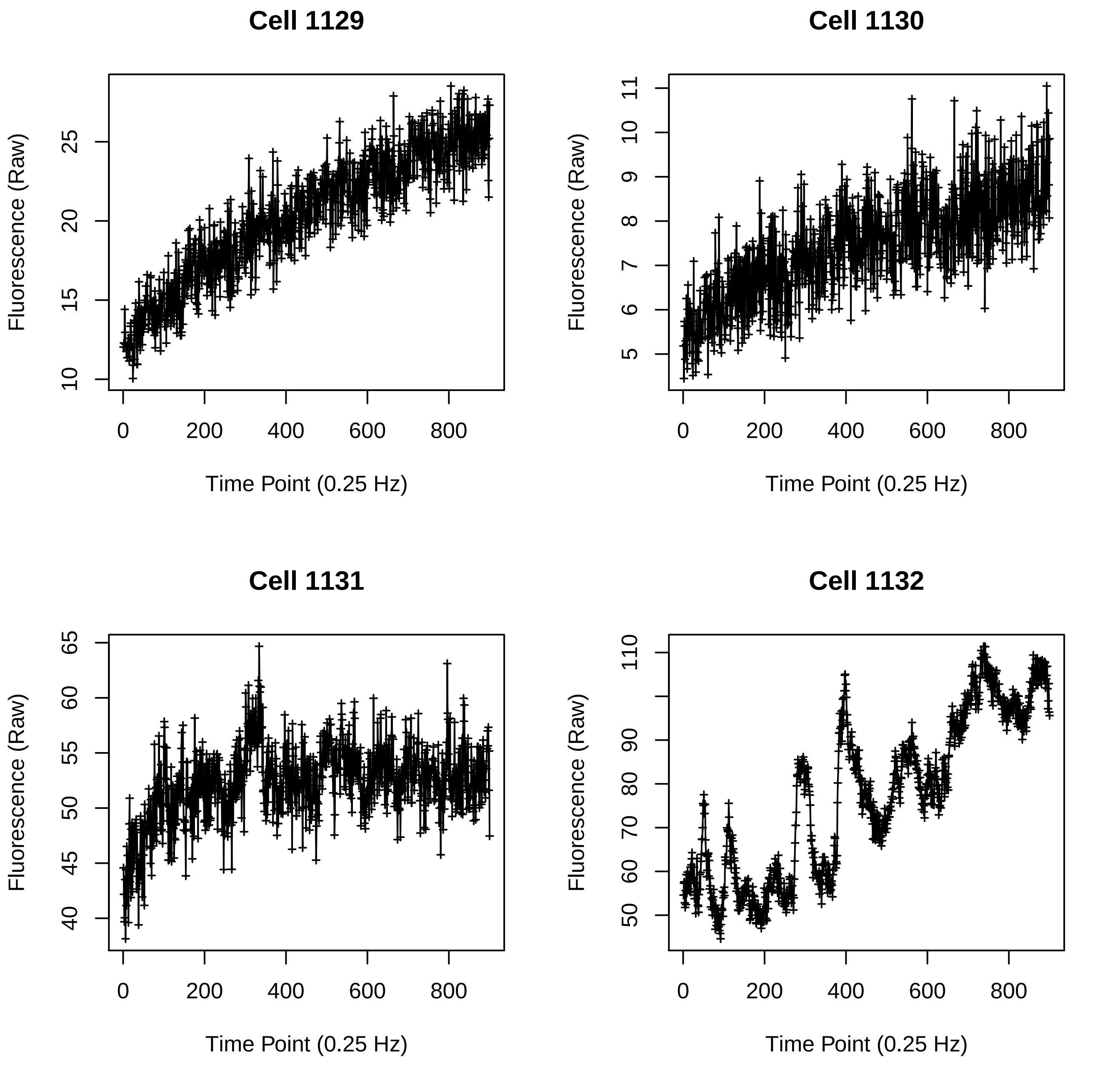
Cell 1129
Cell 1130
Cell 1131
Cell 1132
Fluorescence (Raw)
Time Point (0.25 Hz)
0
200
400
600
800
10
15
20
25
5
6
7
8
9
10
11
40
45
50
55
60
65
50
60
70
80
90
110

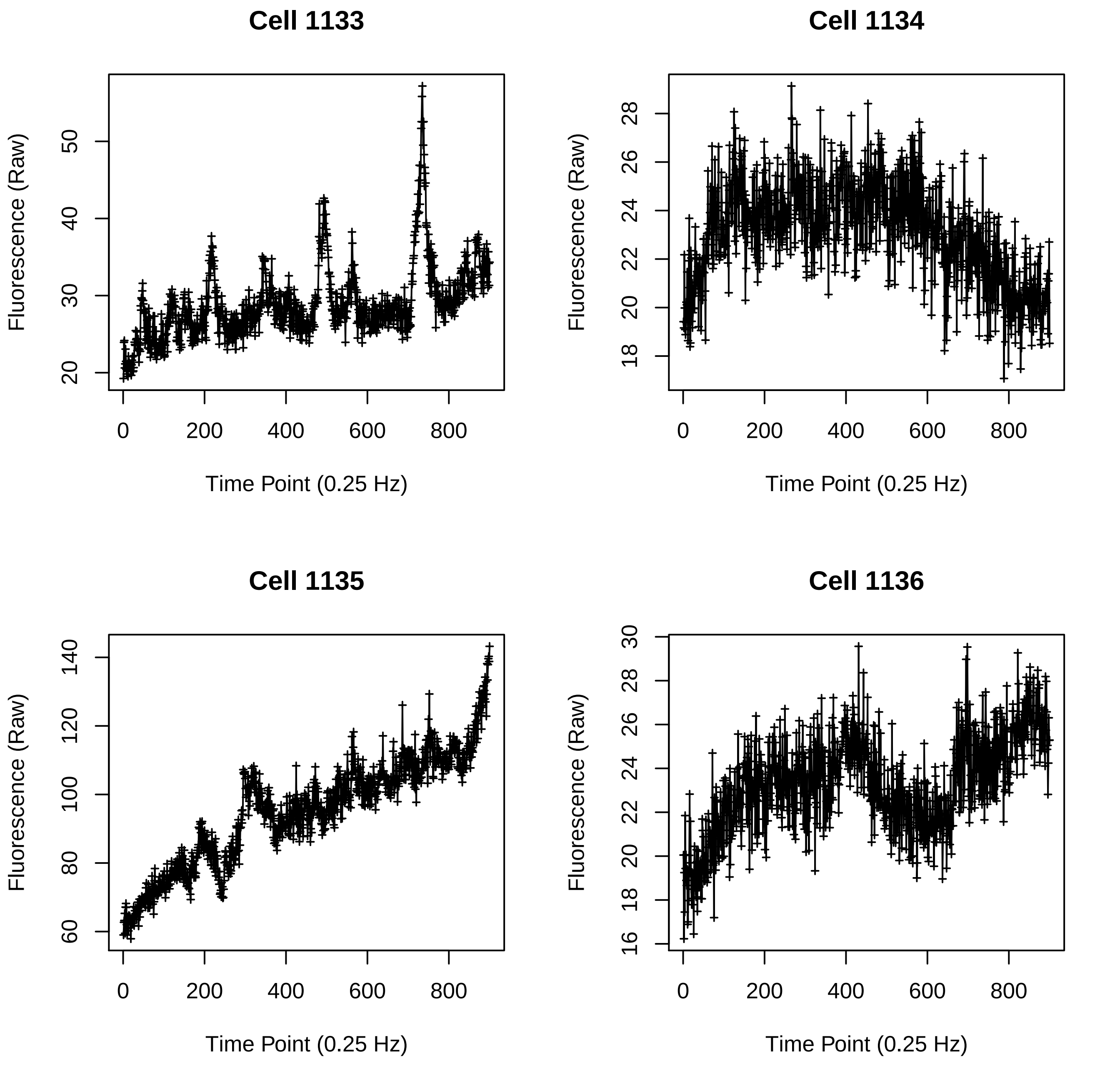
Cell 1133
Cell 1134
Cell 1135
Cell 1136
Fluorescence (Raw)
Time Point (0.25 Hz)
0
200
400
600
800
20
30
40
50
18
22
24
26
28
60
80
100
120
140
16

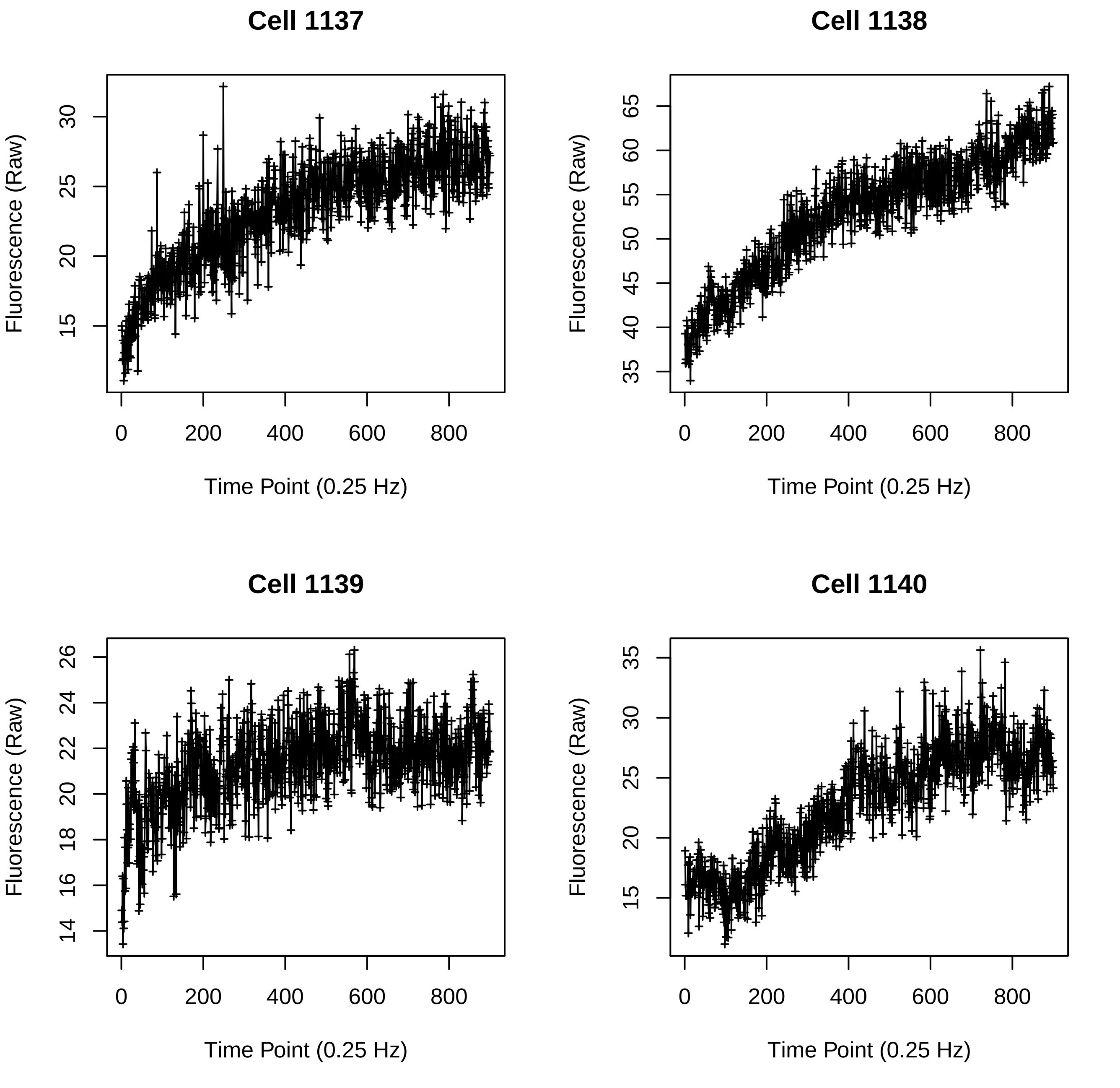
Cell 1137
Fluorescence (Raw)
Time Point (0.25 Hz)
Cell 1138
Fluorescence (Raw)
Time Point (0.25 Hz)
Cell 1139
Fluorescence (Raw)
Time Point (0.25 Hz)
Cell 1140
Fluorescence (Raw)
Time Point (0.25 Hz)

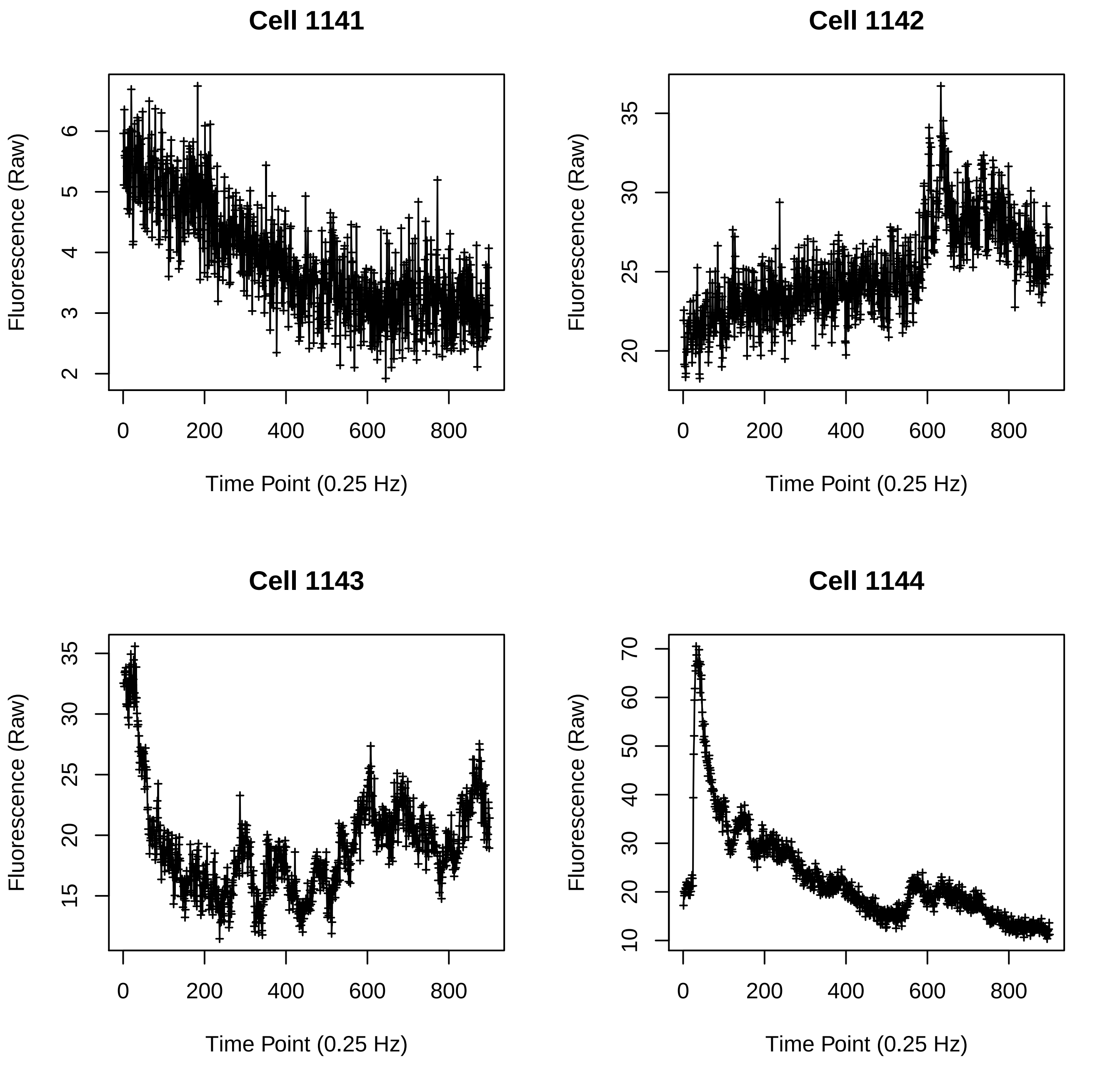
Cell 1141
Cell 1142
Cell 1143
Cell 1144
Fluorescence (Raw)
Time Point (0.25 Hz)
0
200
400
600
800
2
3
4
5
6
20
25
30
35
15
10
40
50
60
70

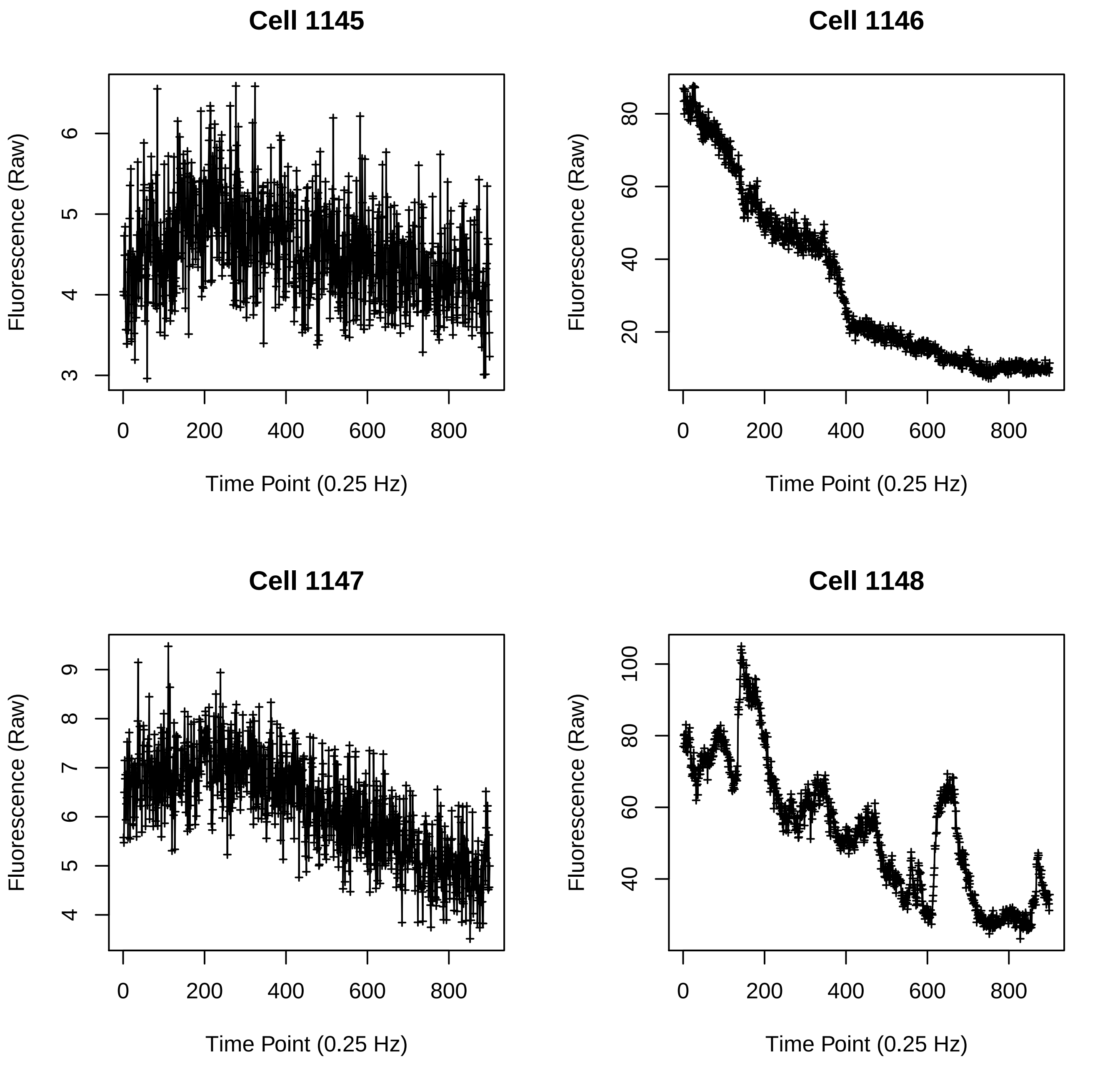
Cell 1145
Cell 1146
Cell 1147
Cell 1148
Fluorescence (Raw)
Time Point (0.25 Hz)
0
200
400
600
800
3
4
5
6
20
40
60
80
7
8
9
100

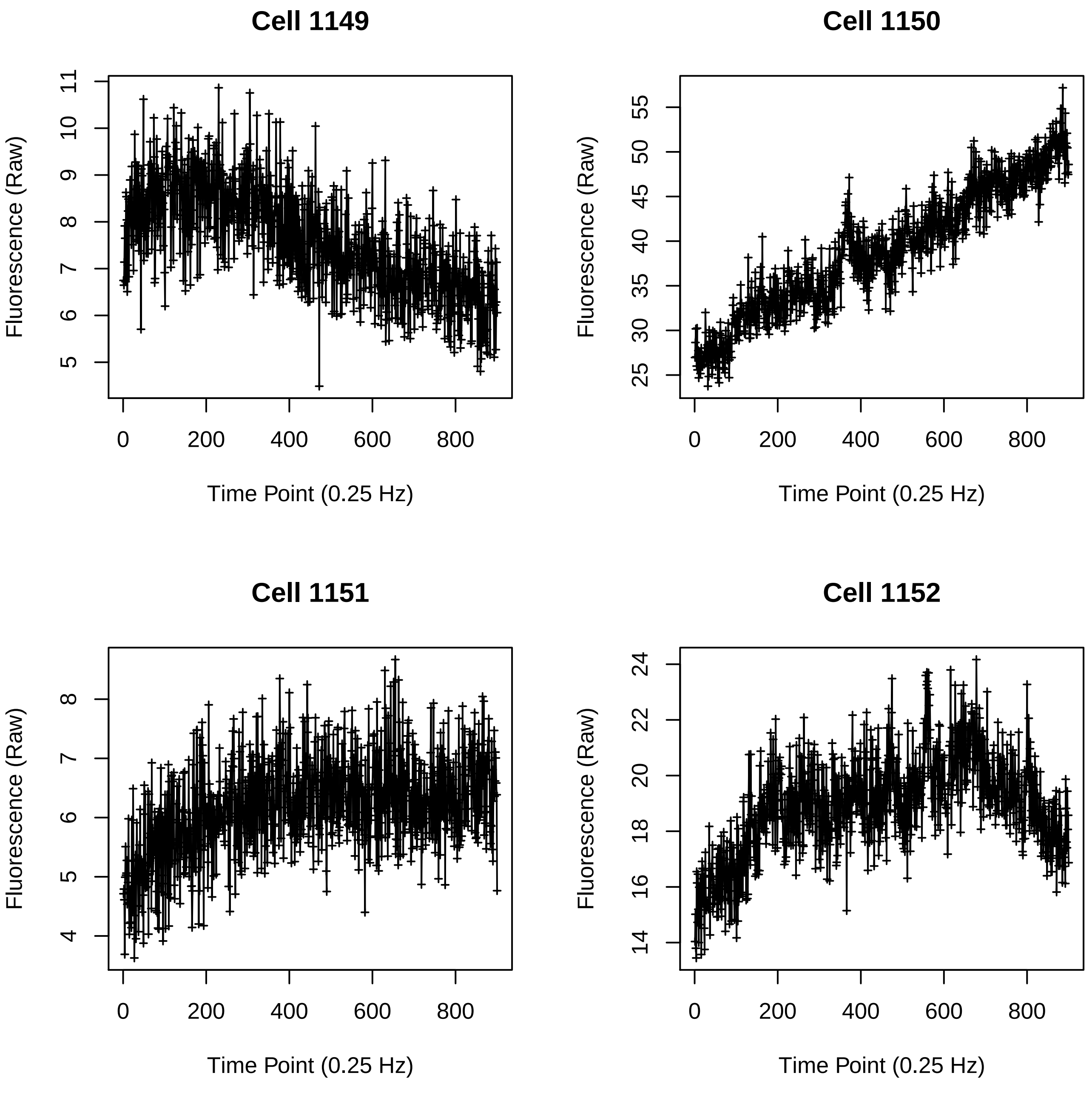
Cell 1149
Fluorescence (Raw)
5
6
7
8
9
10
11
0
200
400
600
800
Time Point (0.25 Hz)
Cell 1150
Fluorescence (Raw)
25
30
35
40
45
50
55
0
200
400
600
800
Time Point (0.25 Hz)
Cell 1151
Fluorescence (Raw)
4
5
6
7
8
0
200
400
600
800
Time Point (0.25 Hz)
Cell 1152
Fluorescence (Raw)
14
16
18
20
22
24
0
200
400
600
800
Time Point (0.25 Hz)

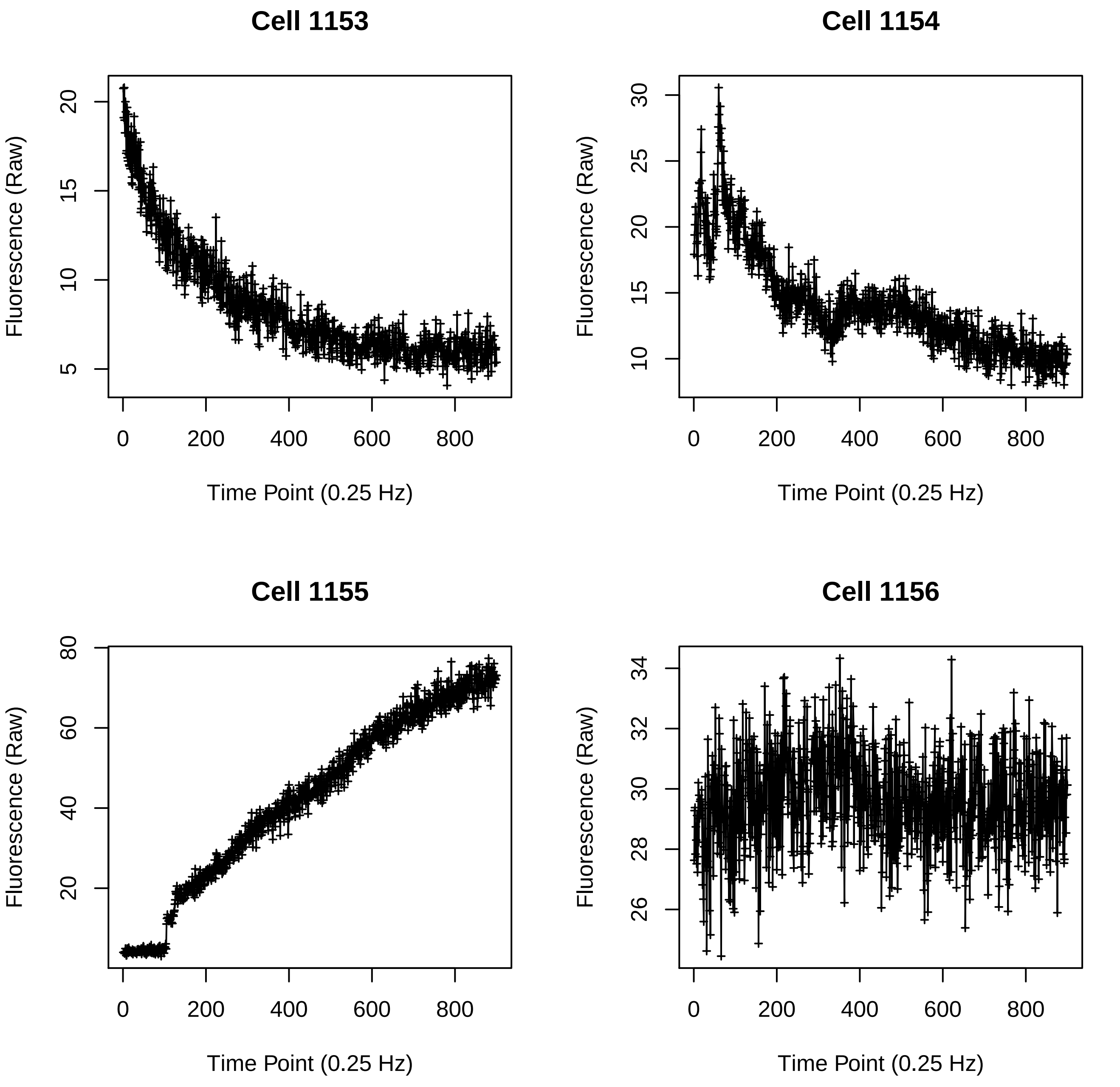
Cell 1153
Fluorescence (Raw)
5
10
15
20
0
200
400
600
800
Time Point (0.25 Hz)
Cell 1154
Fluorescence (Raw)
10
15
20
25
30
0
200
400
600
800
Time Point (0.25 Hz)
Cell 1155
Fluorescence (Raw)
20
40
60
80
0
200
400
600
800
Time Point (0.25 Hz)
Cell 1156
Fluorescence (Raw)
26
28
30
32
34
0
200
400
600
800
Time Point (0.25 Hz)

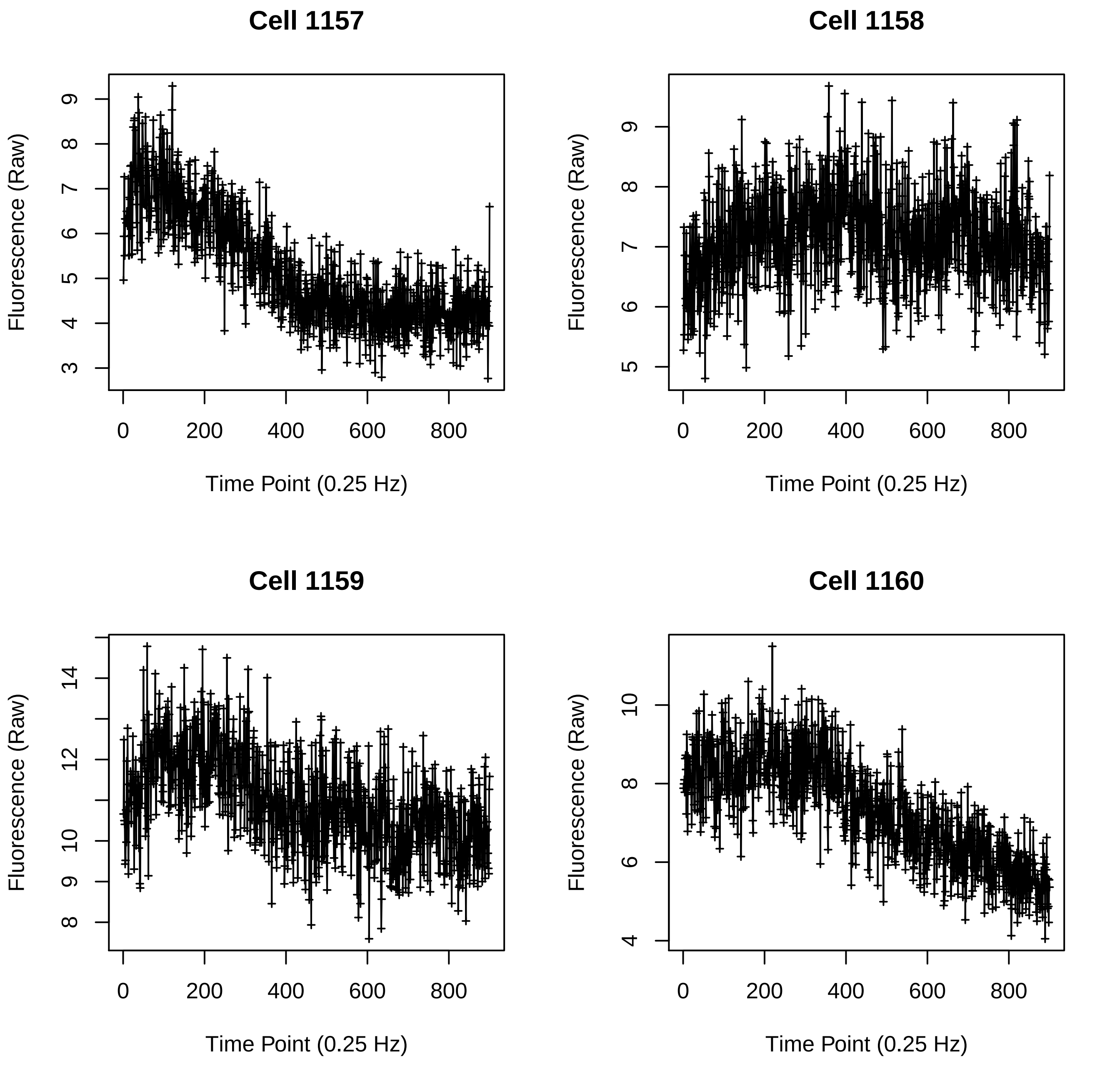

Cell 1157
Fluorescence (Raw)
3
4
5
6
7
8
9
0
200
400
600
800
Time Point (0.25 Hz)
Cell 1158
Fluorescence (Raw)
5
6
7
8
9
0
200
400
600
800
Time Point (0.25 Hz)
Cell 1159
Fluorescence (Raw)
8
9
10
12
14
0
200
400
600
800
Time Point (0.25 Hz)
Cell 1160
Fluorescence (Raw)
4
6
8
10
0
200
400
600
800
Time Point (0.25 Hz)

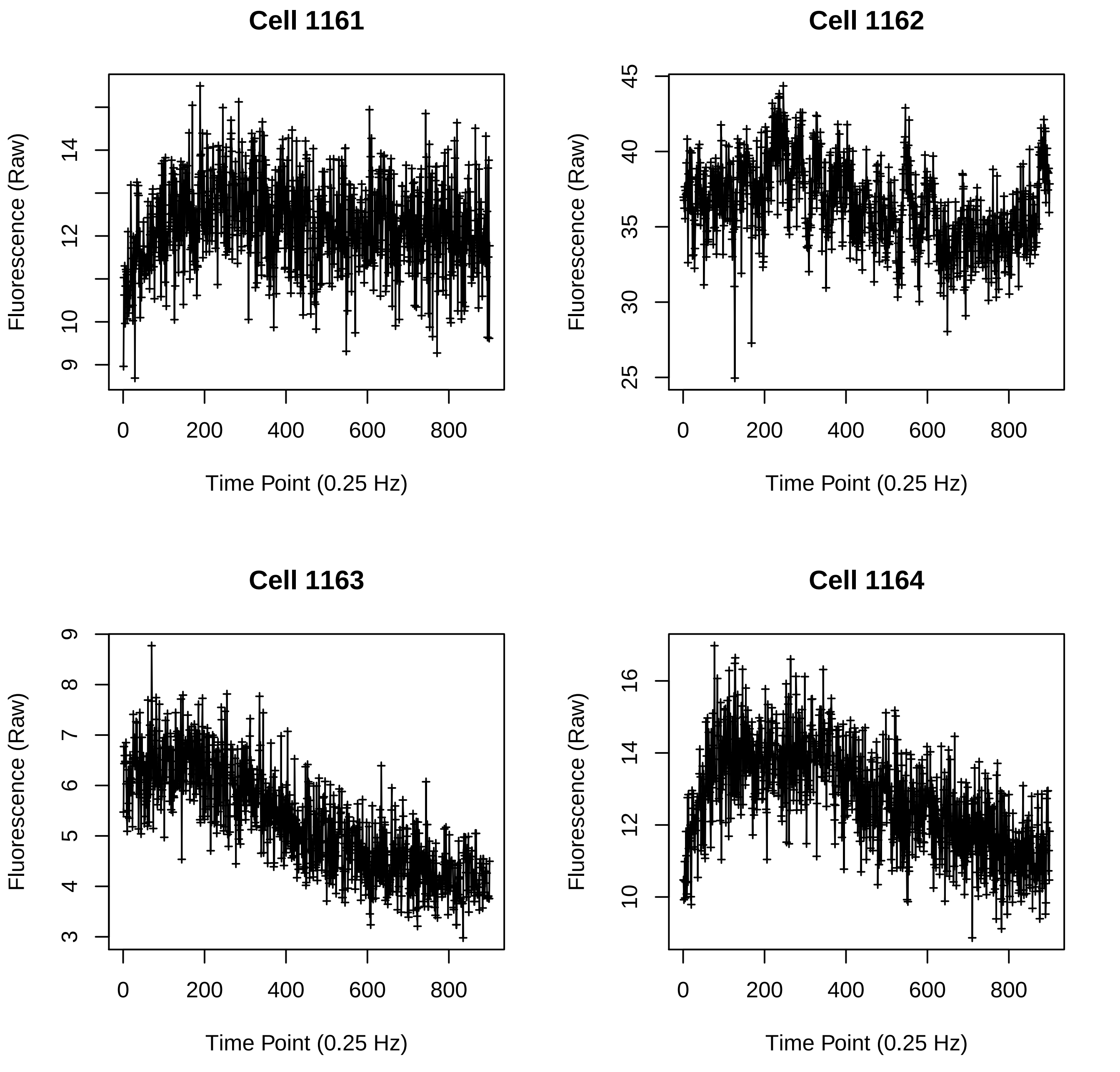

Cell 1161
Fluorescence (Raw)
9
10
12
14
0
200
400
600
800
Time Point (0.25 Hz)
Cell 1162
Fluorescence (Raw)
25
30
35
40
45
0
200
400
600
800
Time Point (0.25 Hz)
Cell 1163
Fluorescence (Raw)
3
4
5
6
7
8
9
0
200
400
600
800
Time Point (0.25 Hz)
Cell 1164
Fluorescence (Raw)
10
12
14
16
0
200
400
600
800
Time Point (0.25 Hz)

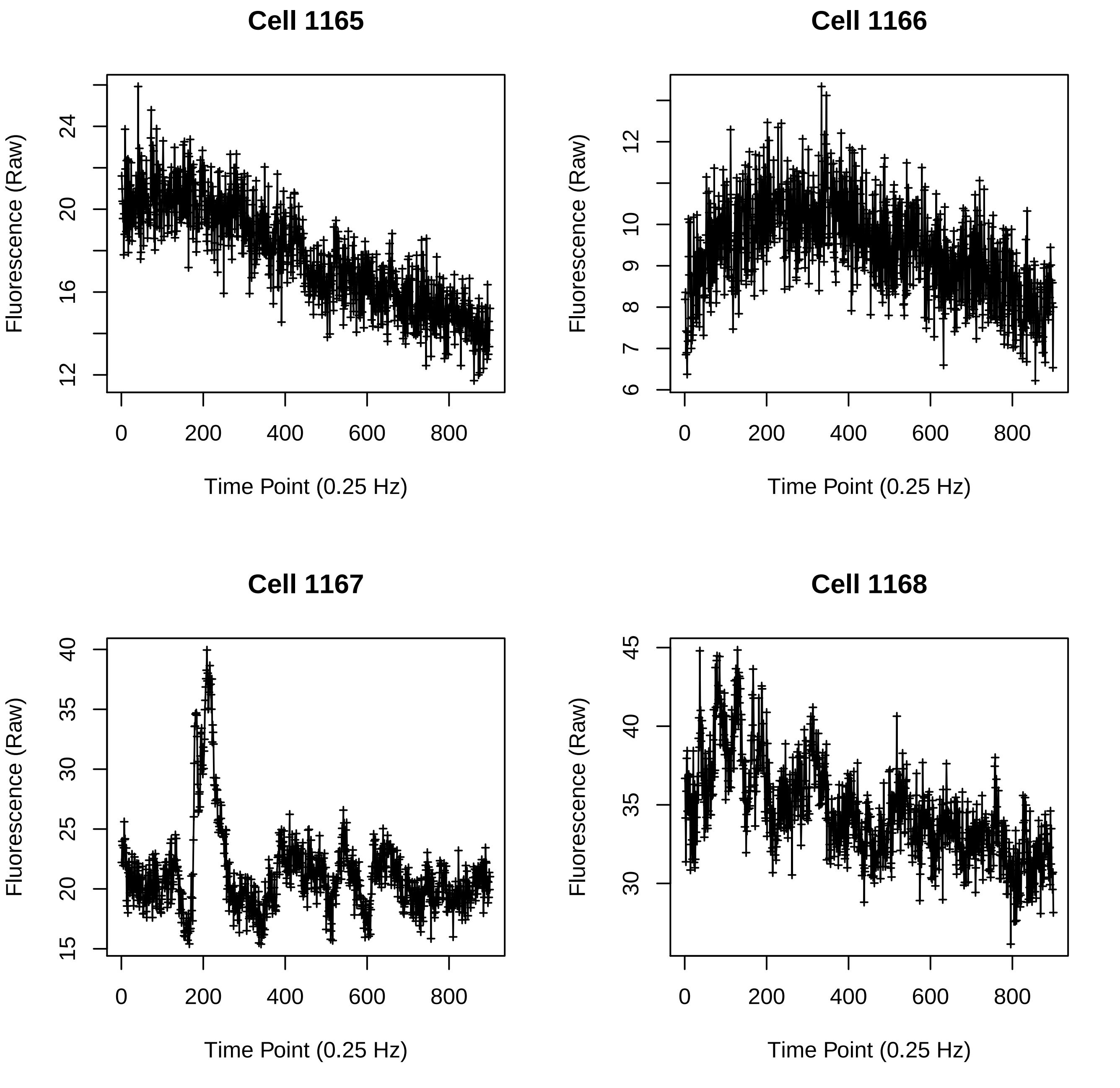
Cell 1165
Cell 1166
Cell 1167
Cell 1168
Fluorescence (Raw)
Time Point (0.25 Hz)
0
200
400
600
800
12
16
20
24
6
7
8
9
10
12
15
25
30
35
40
45

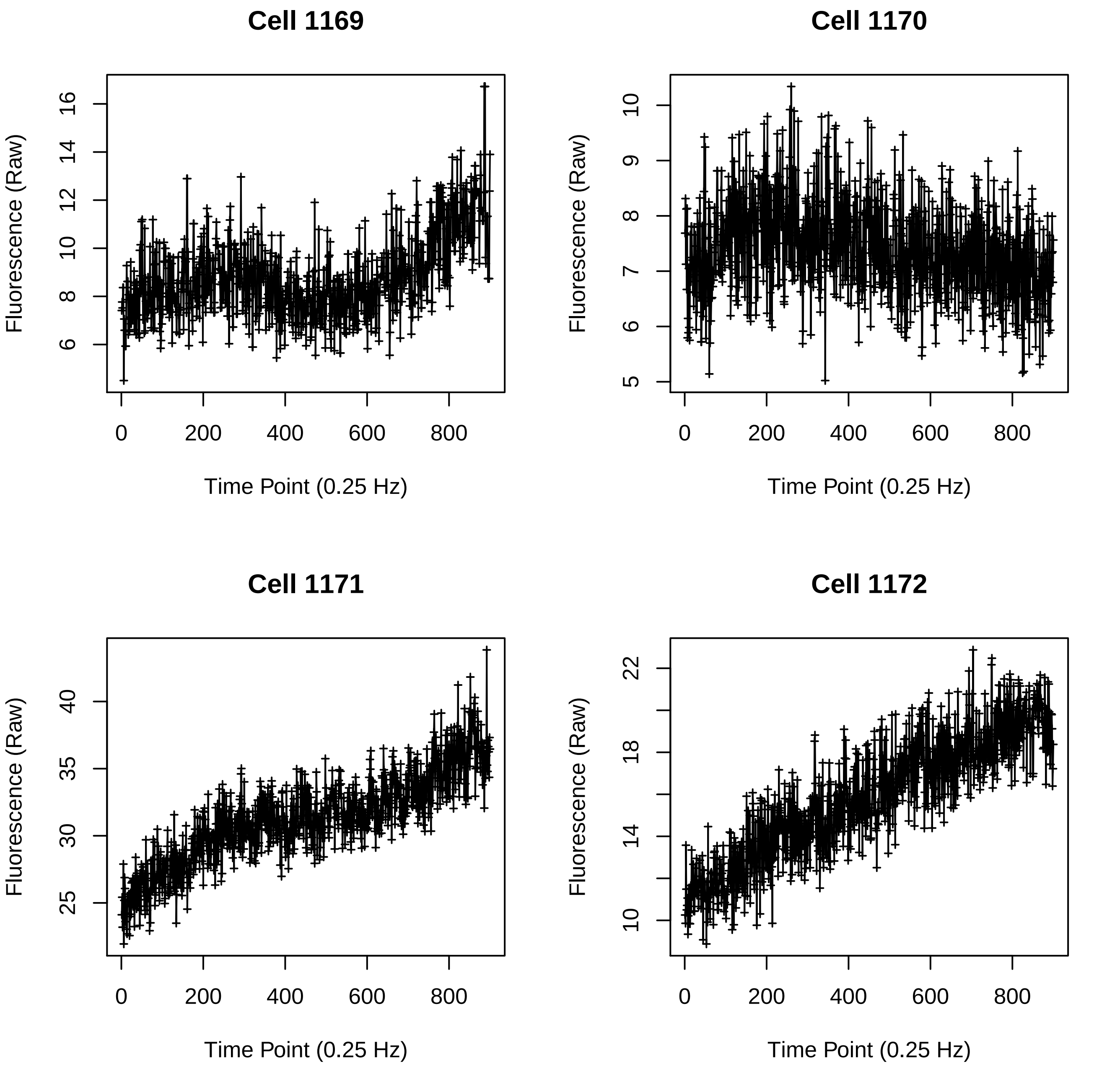
Cell 1169
Cell 1170
Cell 1171
Cell 1172
Fluorescence (Raw)
Time Point (0.25 Hz)
0
200
400
600
800
6
8
10
12
14
16
5
6
7
8
9
10
25
30
35
40
10
14
18
22

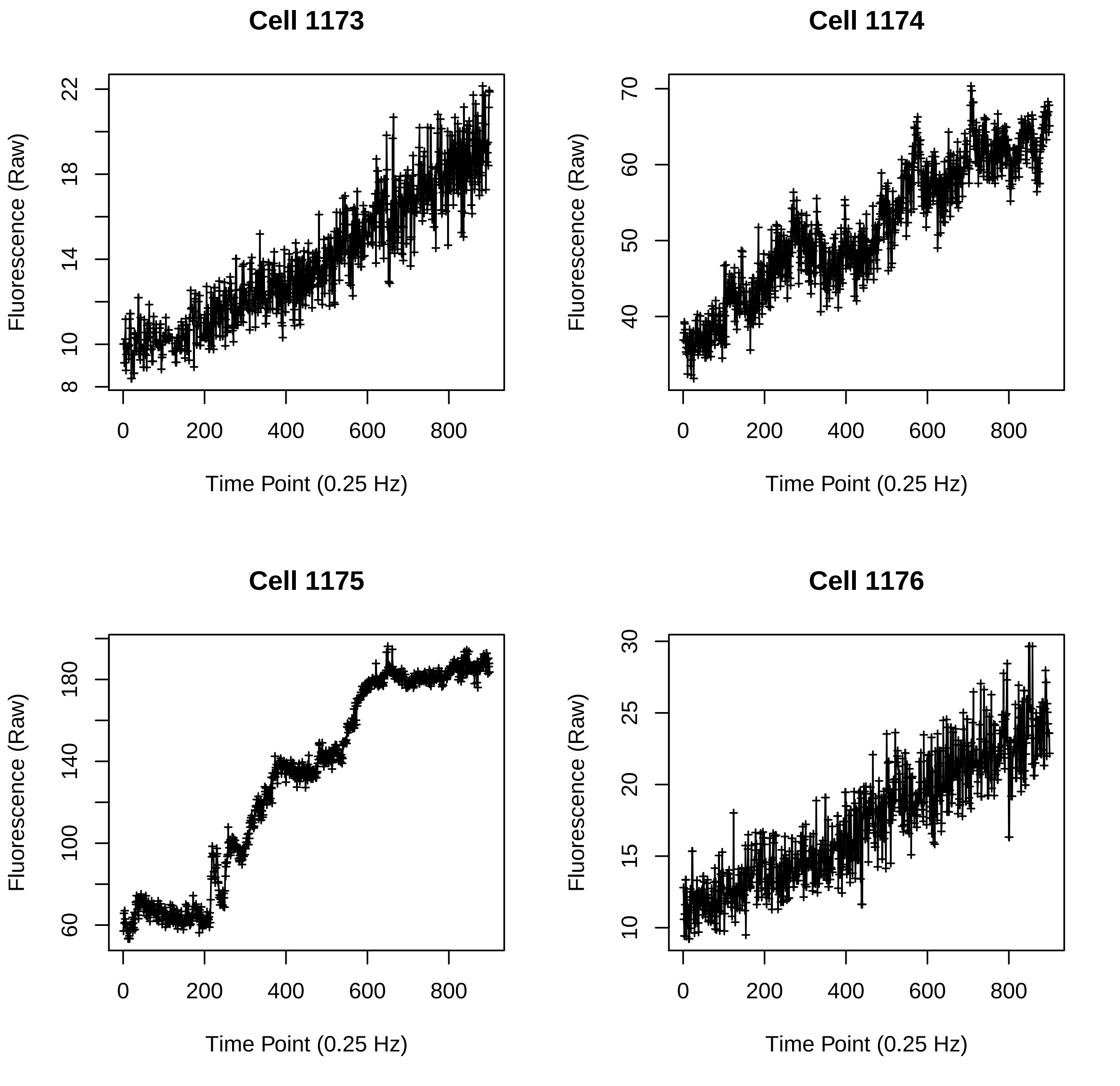

Cell 1173
Fluorescence (Raw)
Time Point (0.25 Hz)
8
10
14
18
22
0
200
400
600
800
Cell 1174
Fluorescence (Raw)
Time Point (0.25 Hz)
40
50
60
70
0
200
400
600
800
Cell 1175
Fluorescence (Raw)
Time Point (0.25 Hz)
60
100
140
180
0
200
400
600
800
Cell 1176
Fluorescence (Raw)
Time Point (0.25 Hz)
10
15
20
25
30
0
200
400
600
800

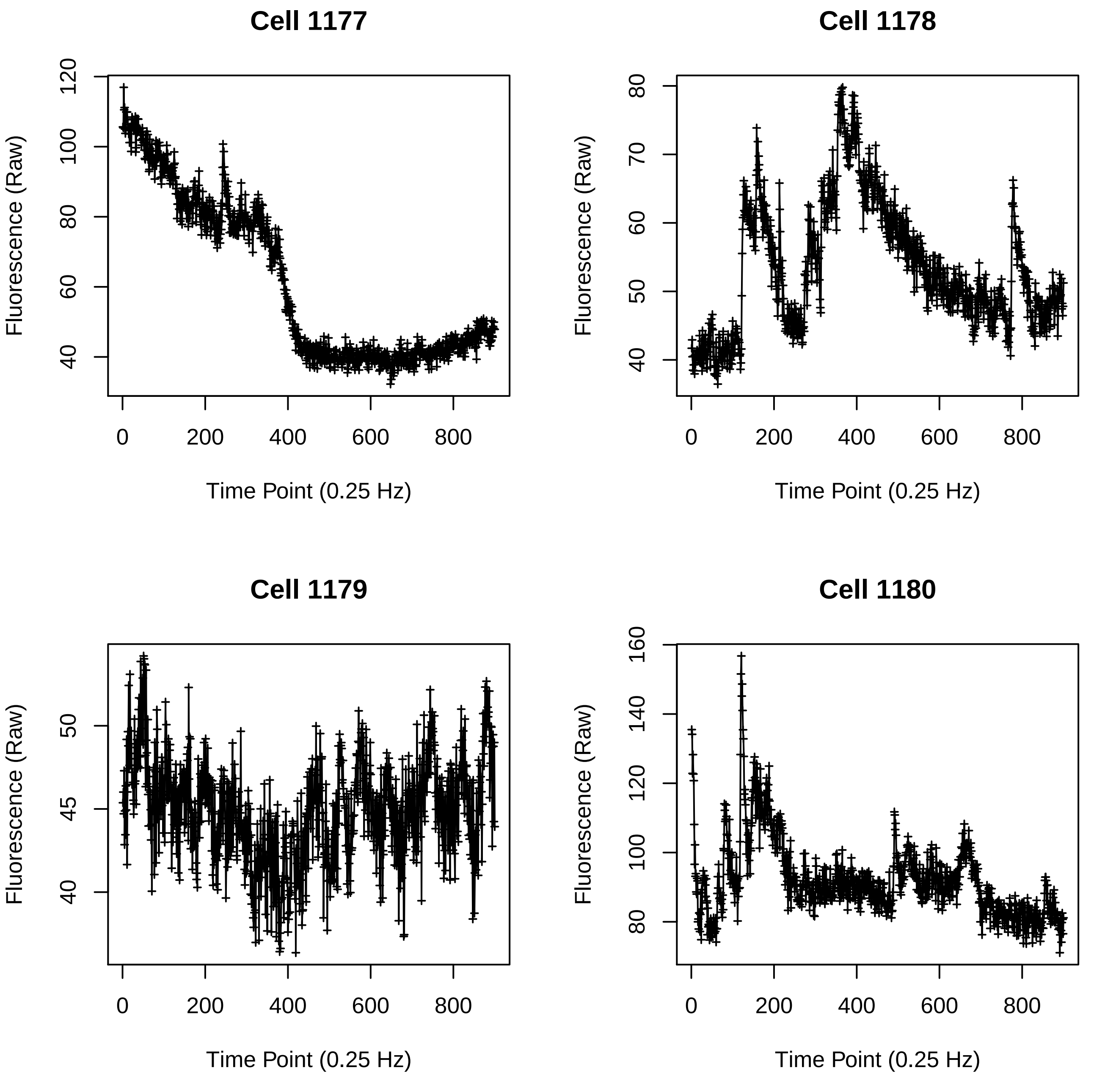
Cell 1177
Cell 1178
Cell 1179
Cell 1180
Fluorescence (Raw)
Time Point (0.25 Hz)
0
200
400
600
800
120
100
80
60
40
70
50
45
160
140

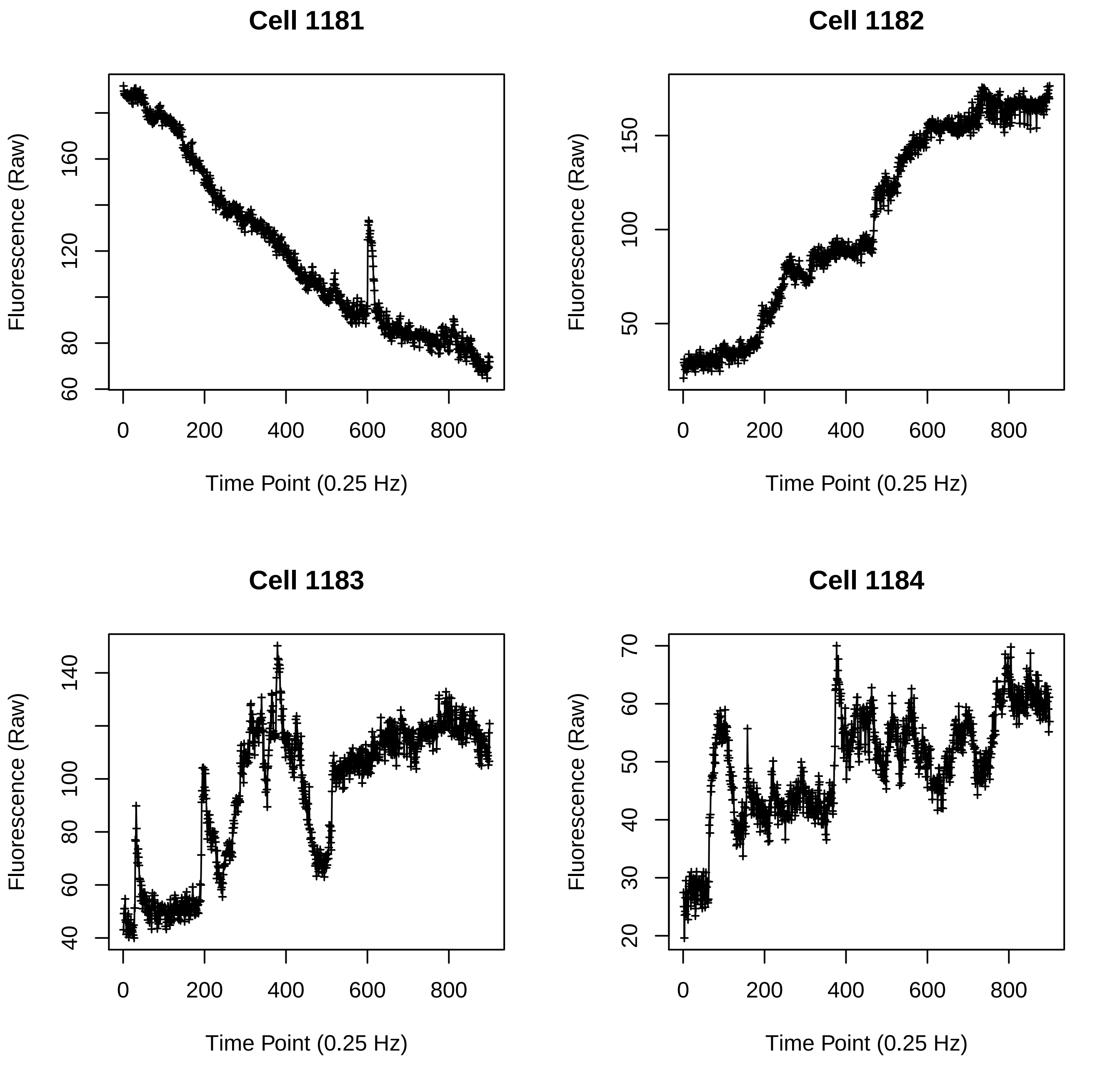
Cell 1181
Fluorescence (Raw)
60
80
120
160
0
200
400
600
800
Time Point (0.25 Hz)
Cell 1182
Fluorescence (Raw)
50
100
150
0
200
400
600
800
Time Point (0.25 Hz)
Cell 1183
Fluorescence (Raw)
40
60
80
100
140
0
200
400
600
800
Time Point (0.25 Hz)
Cell 1184
Fluorescence (Raw)
20
30
40
50
60
70
0
200
400
600
800
Time Point (0.25 Hz)

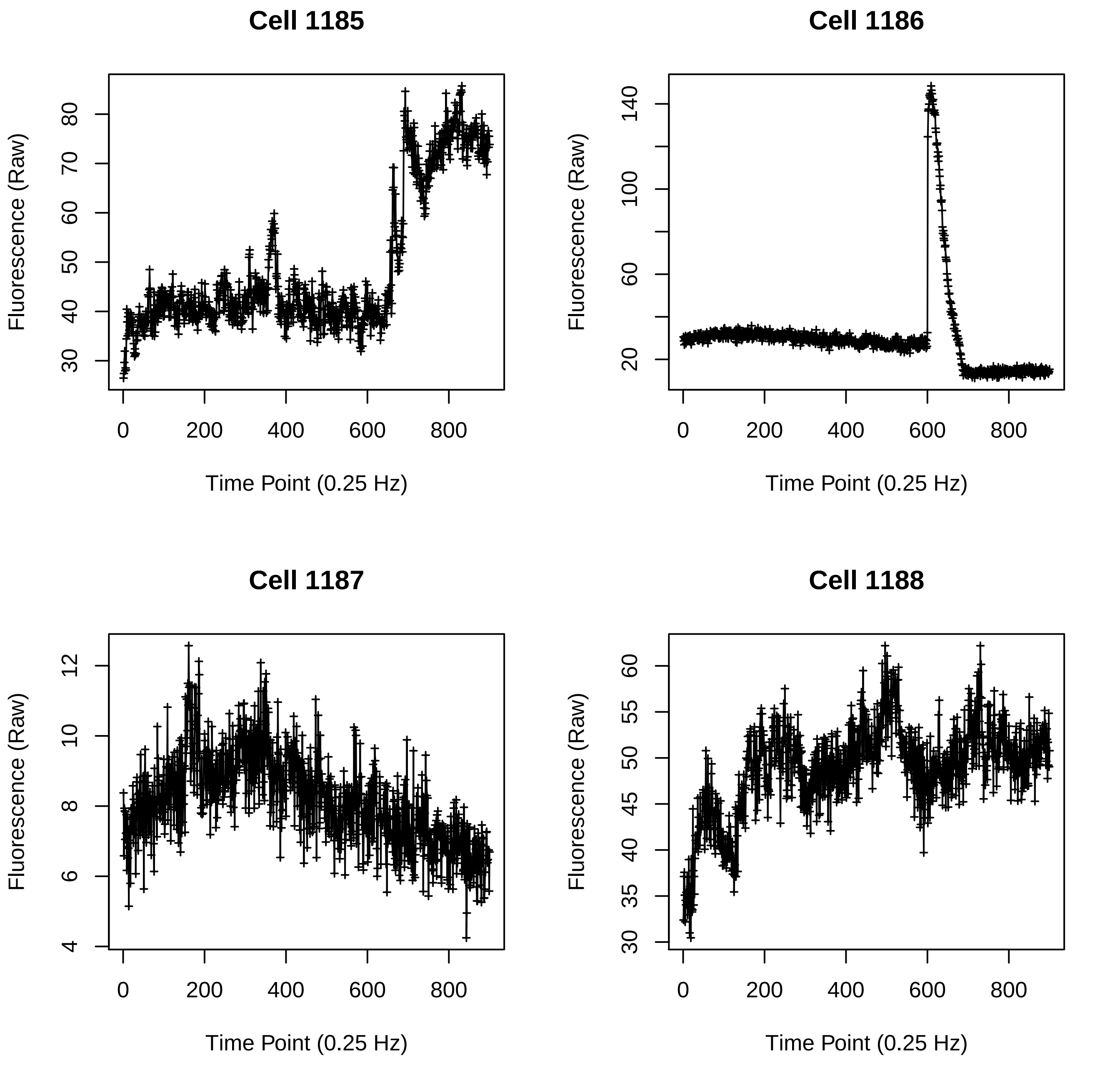

Cell 1185
Fluorescence (Raw)
30
40
50
60
70
80
0
200
400
600
800
Time Point (0.25 Hz)
Cell 1186
Fluorescence (Raw)
20
60
100
140
0
200
400
600
800
Time Point (0.25 Hz)
Cell 1187
Fluorescence (Raw)
4
6
8
10
12
0
200
400
600
800
Time Point (0.25 Hz)
Cell 1188
Fluorescence (Raw)
30
35
40
45
50
55
60
0
200
400
600
800
Time Point (0.25 Hz)

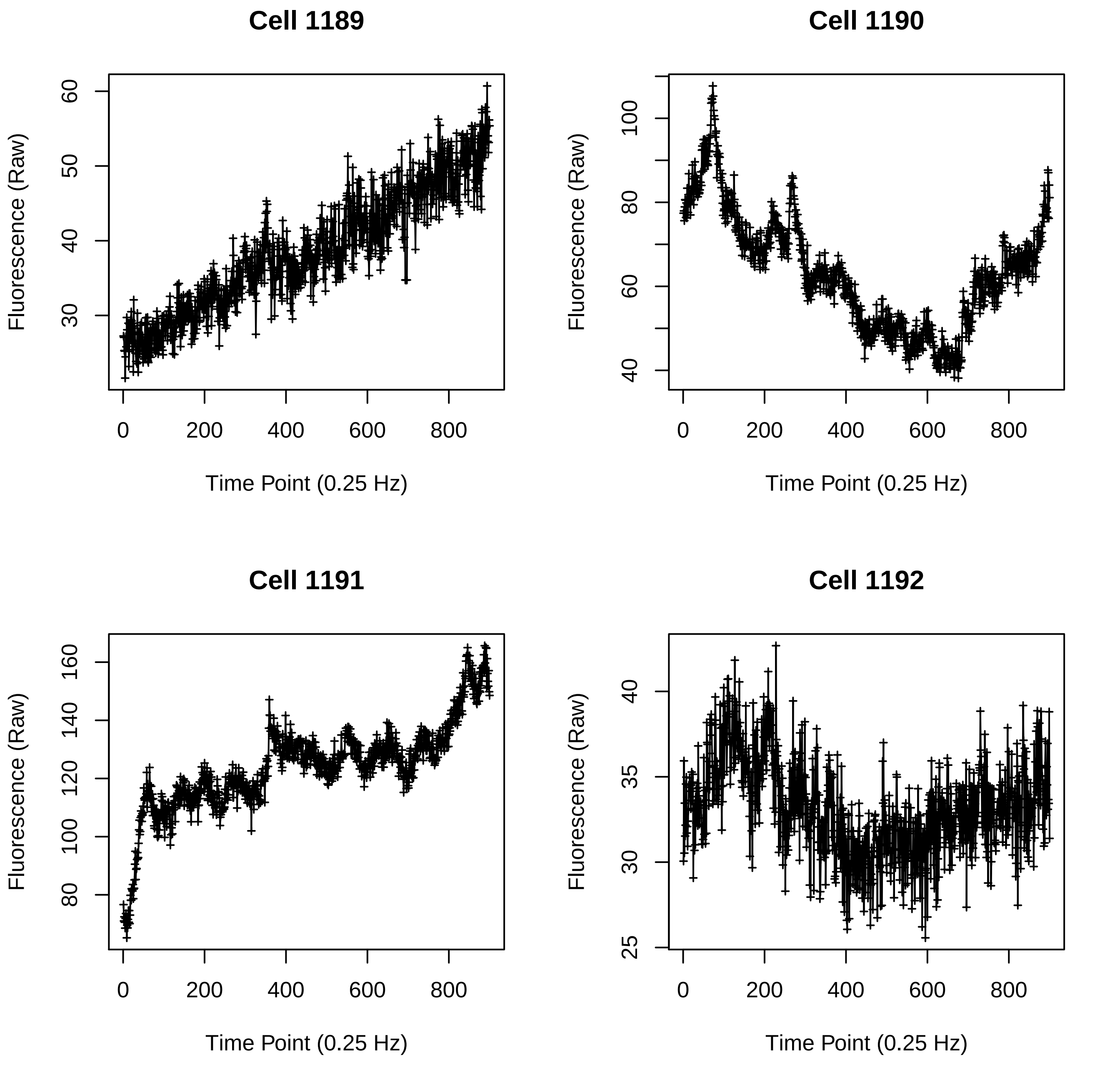

Cell 1189
Fluorescence (Raw)
30
40
50
60
0
200
400
600
800
Time Point (0.25 Hz)
Cell 1190
Fluorescence (Raw)
40
60
80
100
0
200
400
600
800
Time Point (0.25 Hz)
Cell 1191
Fluorescence (Raw)
80
100
120
140
160
0
200
400
600
800
Time Point (0.25 Hz)
Cell 1192
Fluorescence (Raw)
25
30
35
40
0
200
400
600
800
Time Point (0.25 Hz)

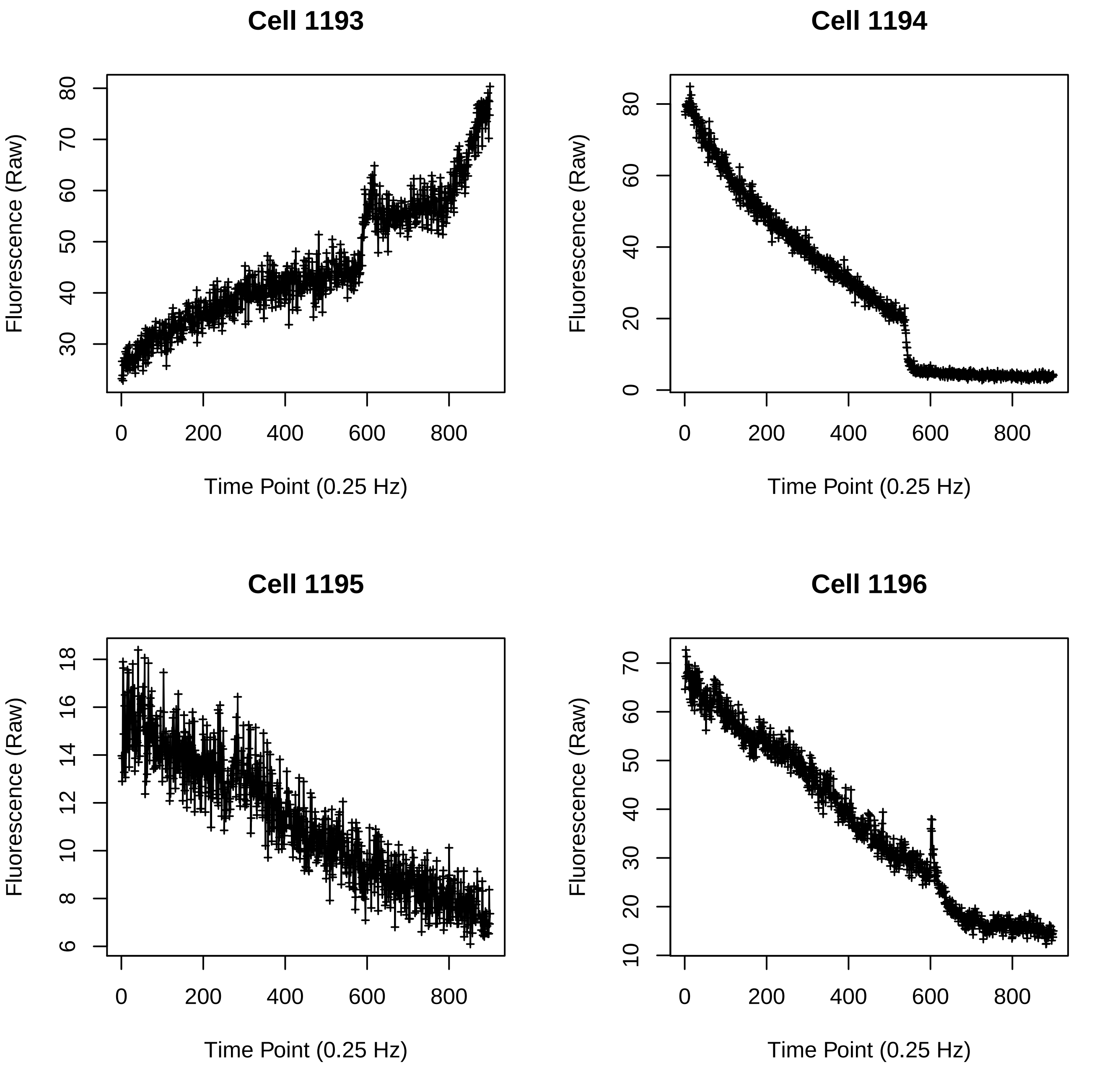
Cell 1193
Cell 1194
Cell 1195
Cell 1196
Fluorescence (Raw)
Time Point (0.25 Hz)
0
200
400
600
800
10
20
30
40
50
60
70
80
0
6
8
10
12
14
16
18

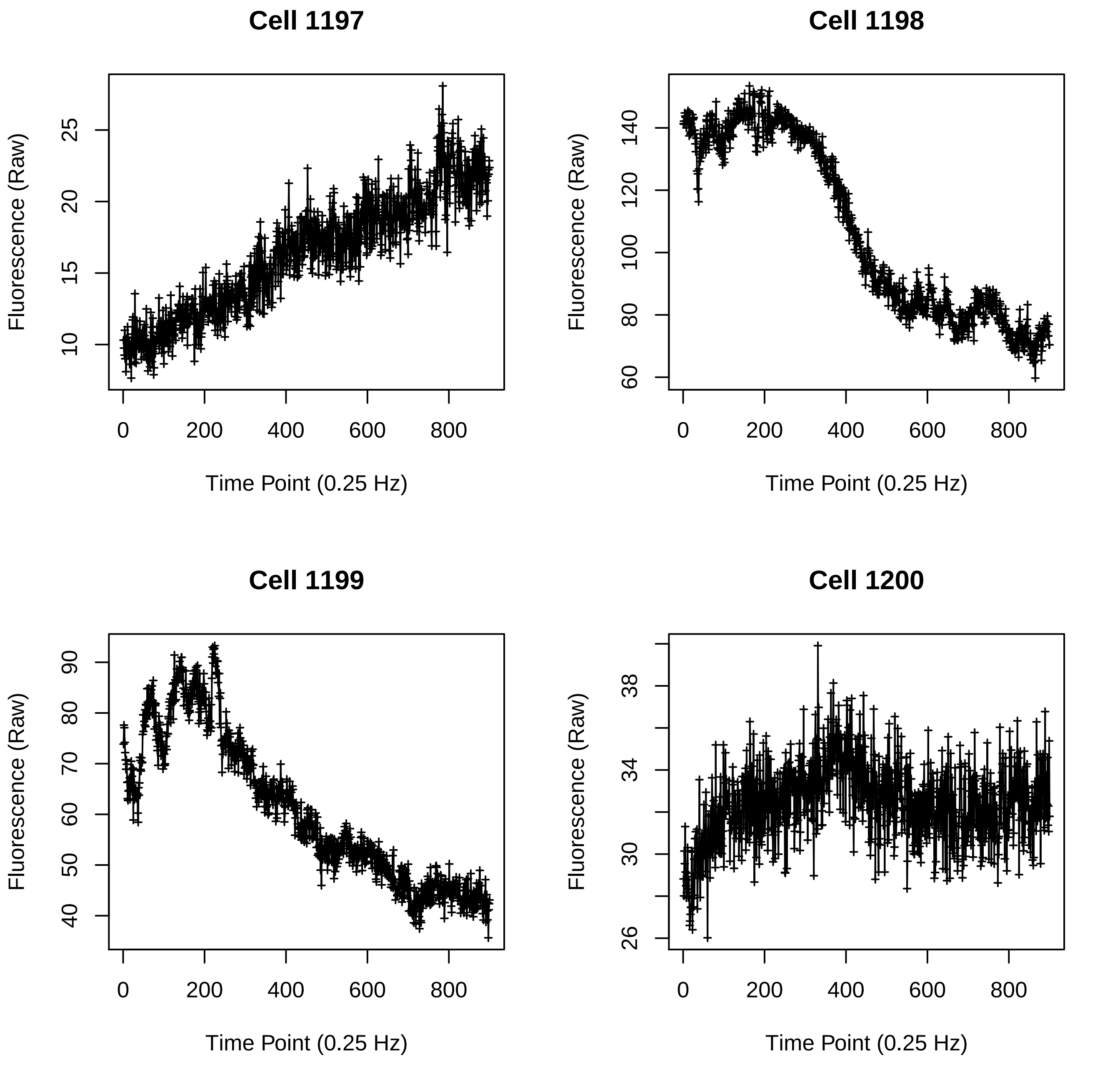
Cell 1197
Cell 1198
Cell 1199
Cell 1200
Fluorescence (Raw)
Time Point (0.25 Hz)
0
200
400
600
800
10
15
20
25
60
80
100
120
140
40
50
60
70
80
90
26
30
34
38

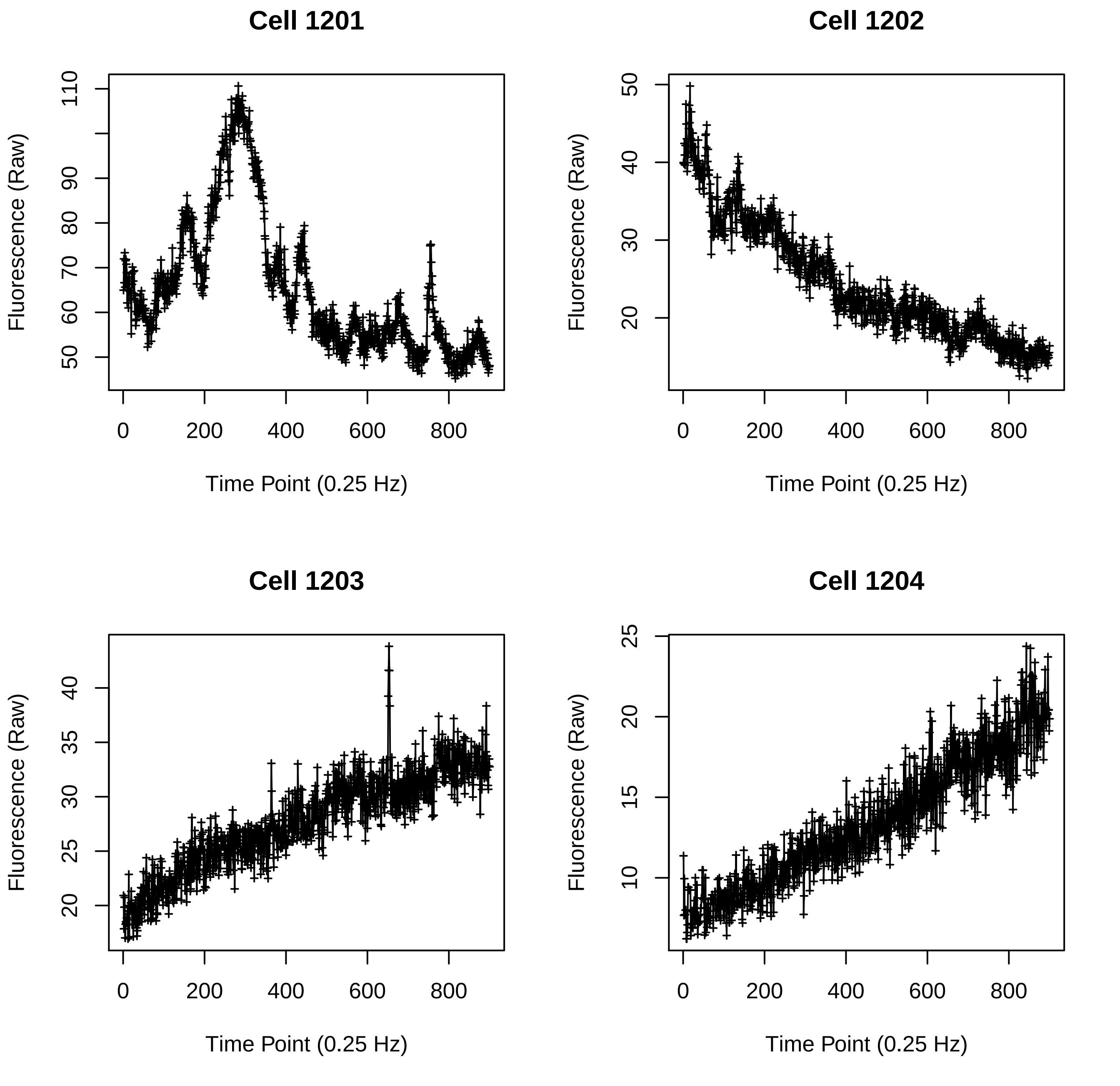
Cell 1201
Cell 1202
Cell 1203
Cell 1204
Fluorescence (Raw)
Time Point (0.25 Hz)
0
200
400
600
800
50
60
70
80
90
110
10
20
30
40
15
25
35

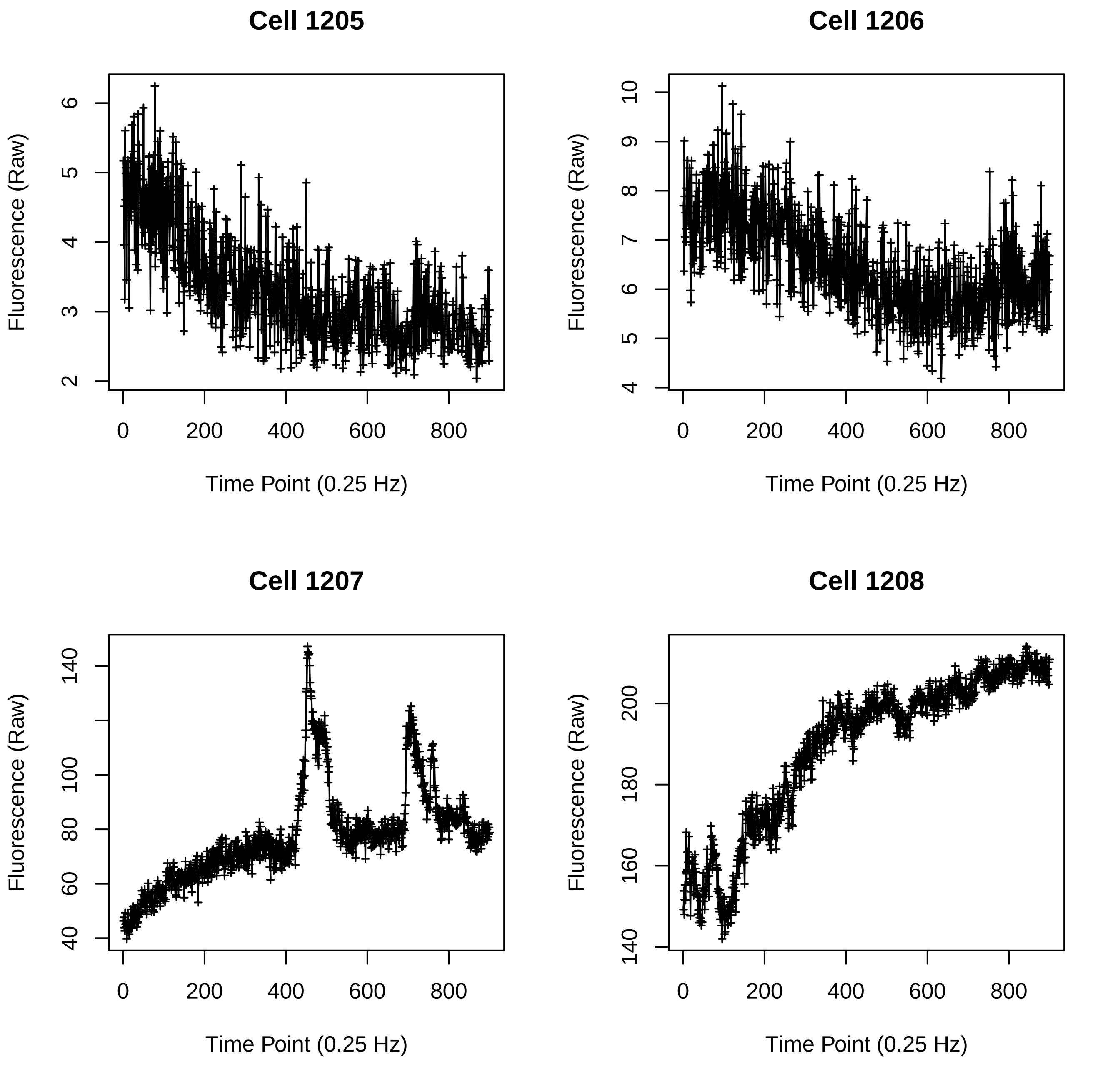
Cell 1205
Cell 1206
Cell 1207
Cell 1208
Fluorescence (Raw)
Time Point (0.25 Hz)
0
200
400
600
800
2
3
4
5
6
7
8
9
10
40
60
80
100
140
160
180
200

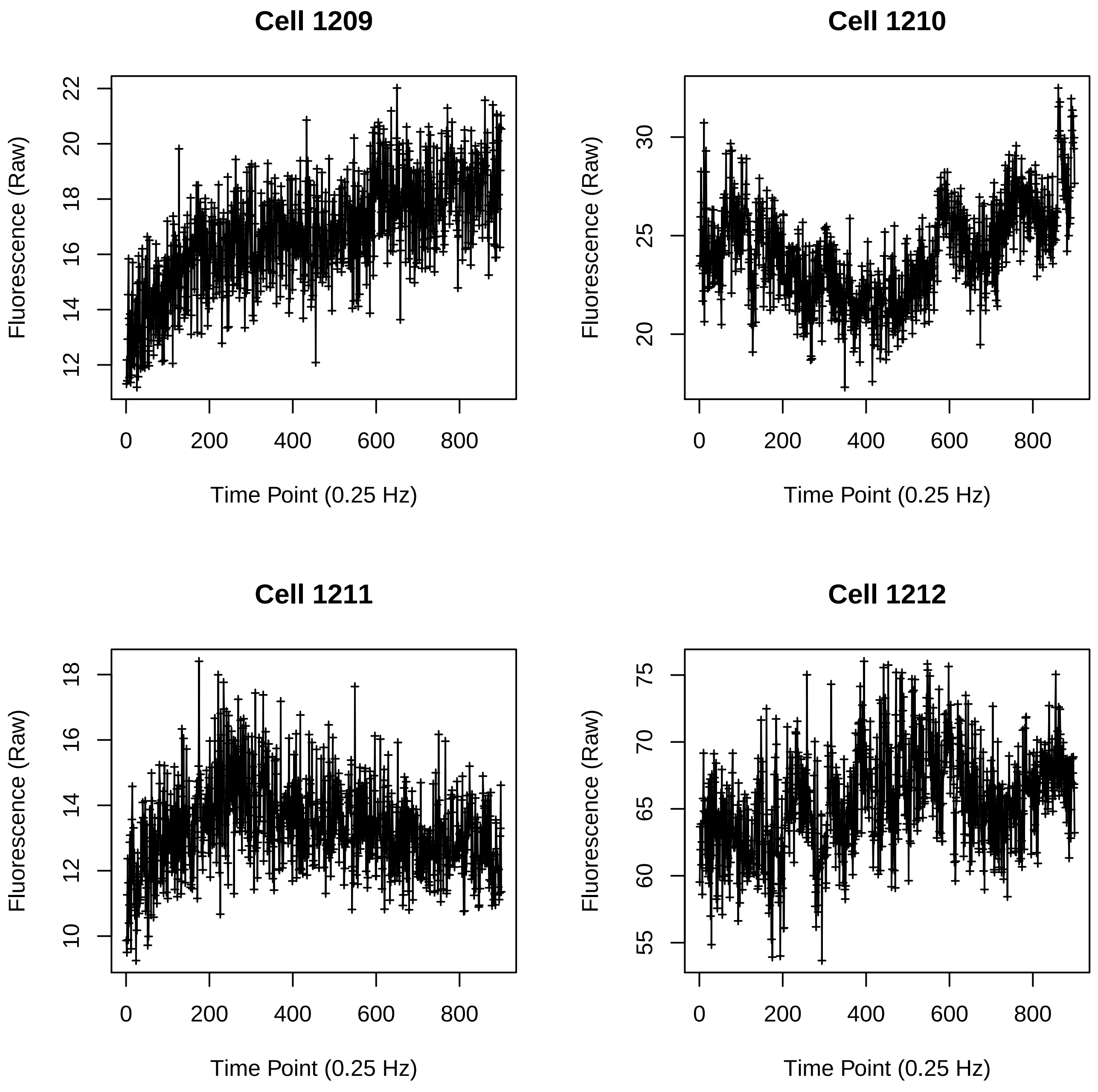
Cell 1209
Cell 1210
Cell 1211
Cell 1212
Fluorescence (Raw)
Time Point (0.25 Hz)
0
200
400
600
800
12
14
16
18
20
22
20
25
30
10
12
14
16
18
55
60
65
70
75

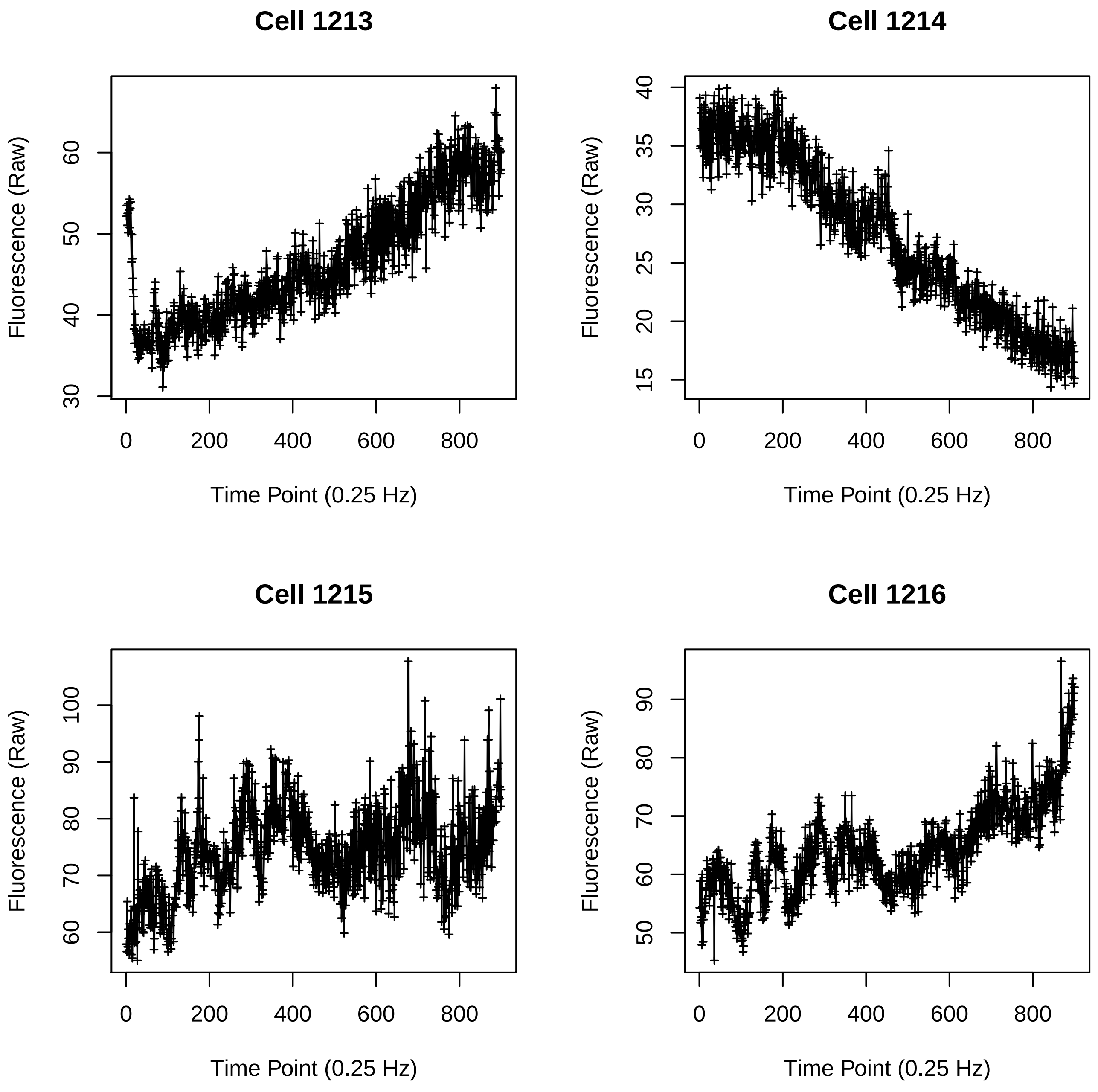

Cell 1213
Cell 1214
Cell 1215
Cell 1216
Fluorescence (Raw)
Time Point (0.25 Hz)
30
40
50
60
15
20
25
30
35
40
60
70
80
90
100
50
60
70
80
90
0
200
400
600
800

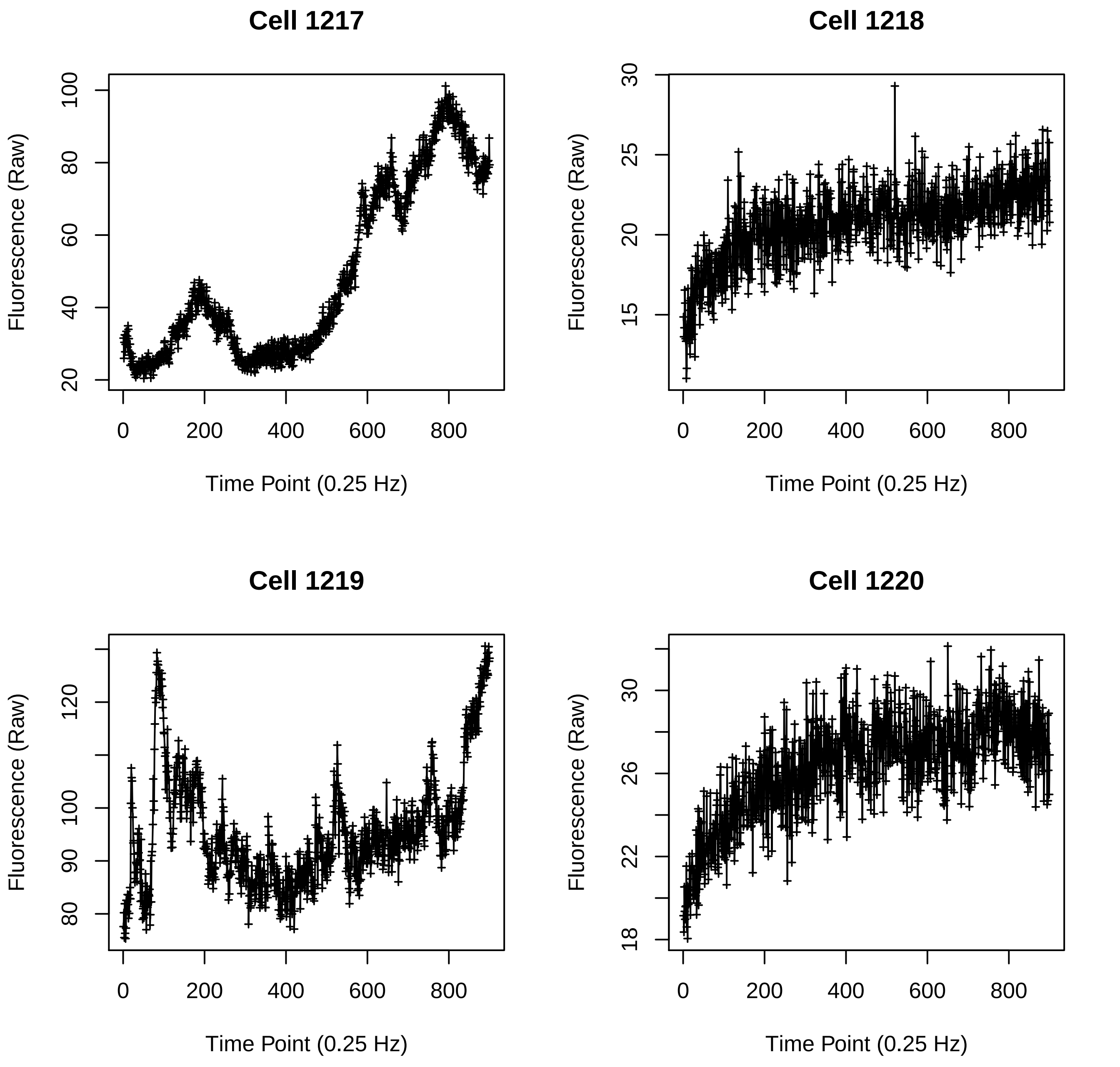

Cell 1217
Cell 1218
Cell 1219
Cell 1220
Fluorescence (Raw)
Time Point (0.25 Hz)
0
200
400
600
800
20
40
60
80
100
15
20
25
30
90
100
120
18
22
26
30

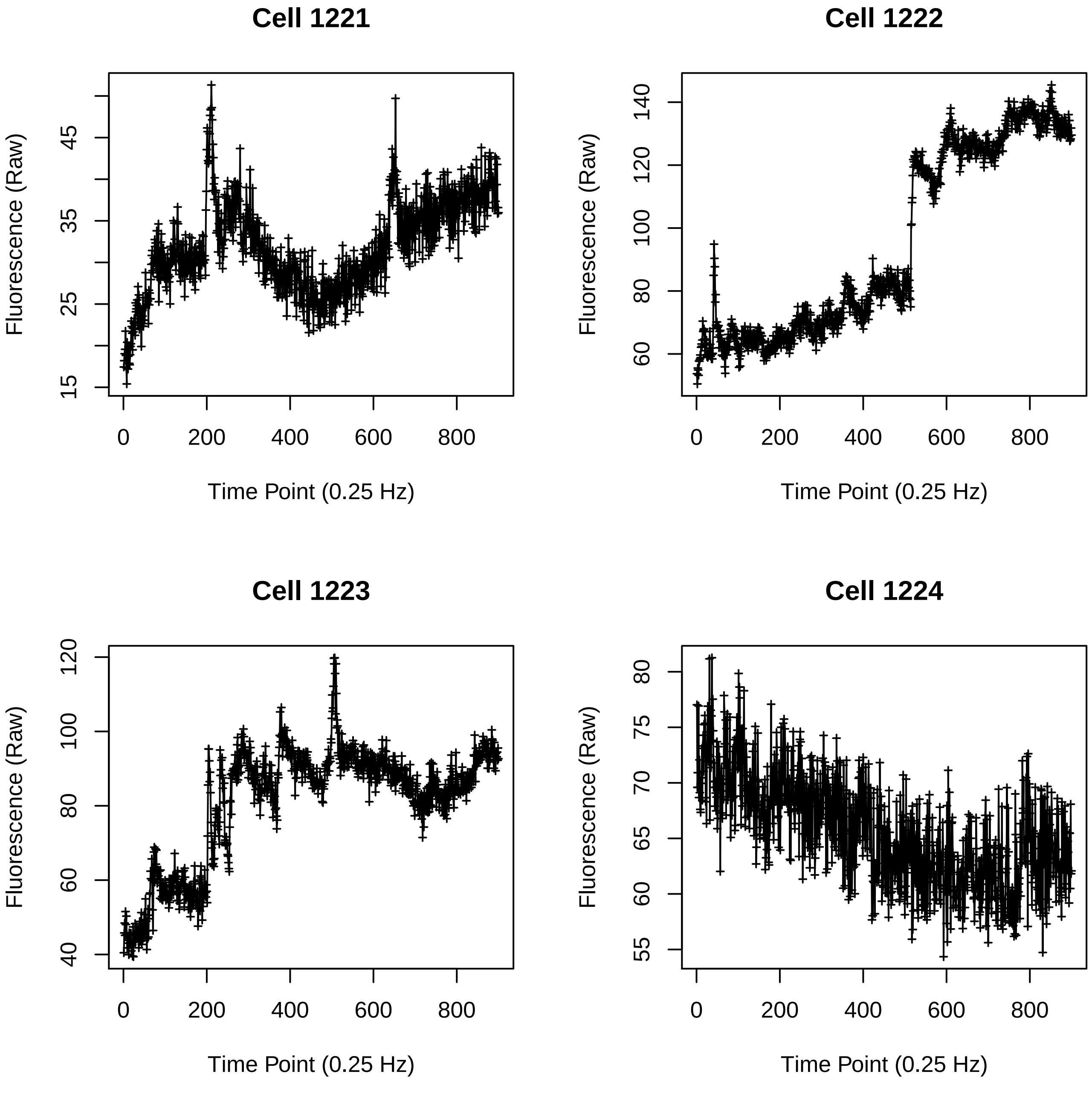
Cell 1221
Cell 1222
Cell 1223
Cell 1224
Fluorescence (Raw)
Time Point (0.25 Hz)
15
25
35
45
60
80
100
120
140
40
55
65
70
75
0
200
400
600
800

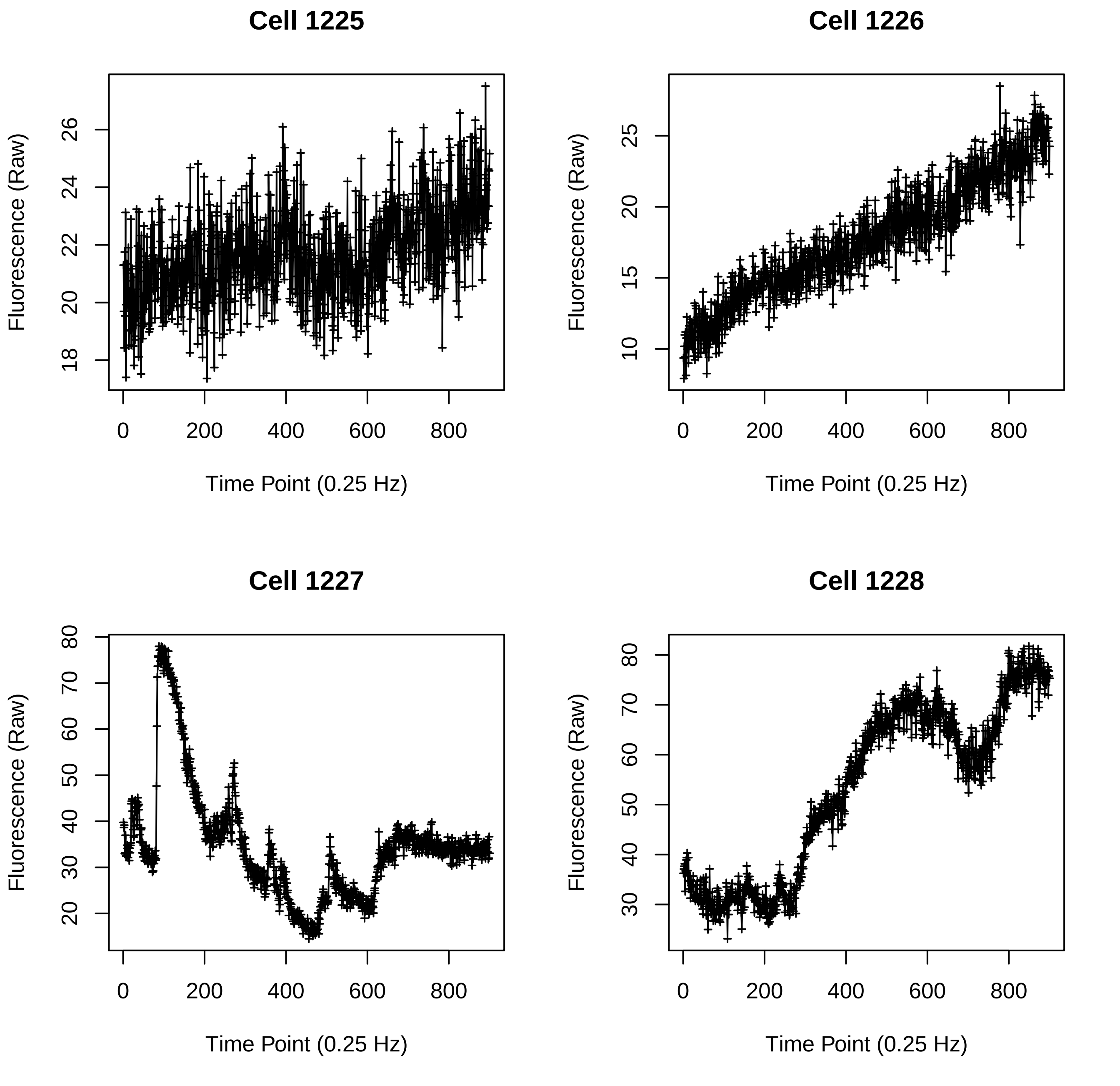

Cell 1225
Cell 1226
Cell 1227
Cell 1228
Fluorescence (Raw)
Time Point (0.25 Hz)
0
200
400
600
800
18
20
22
24
26
10
15
20
25
30
40
50
60
70
80

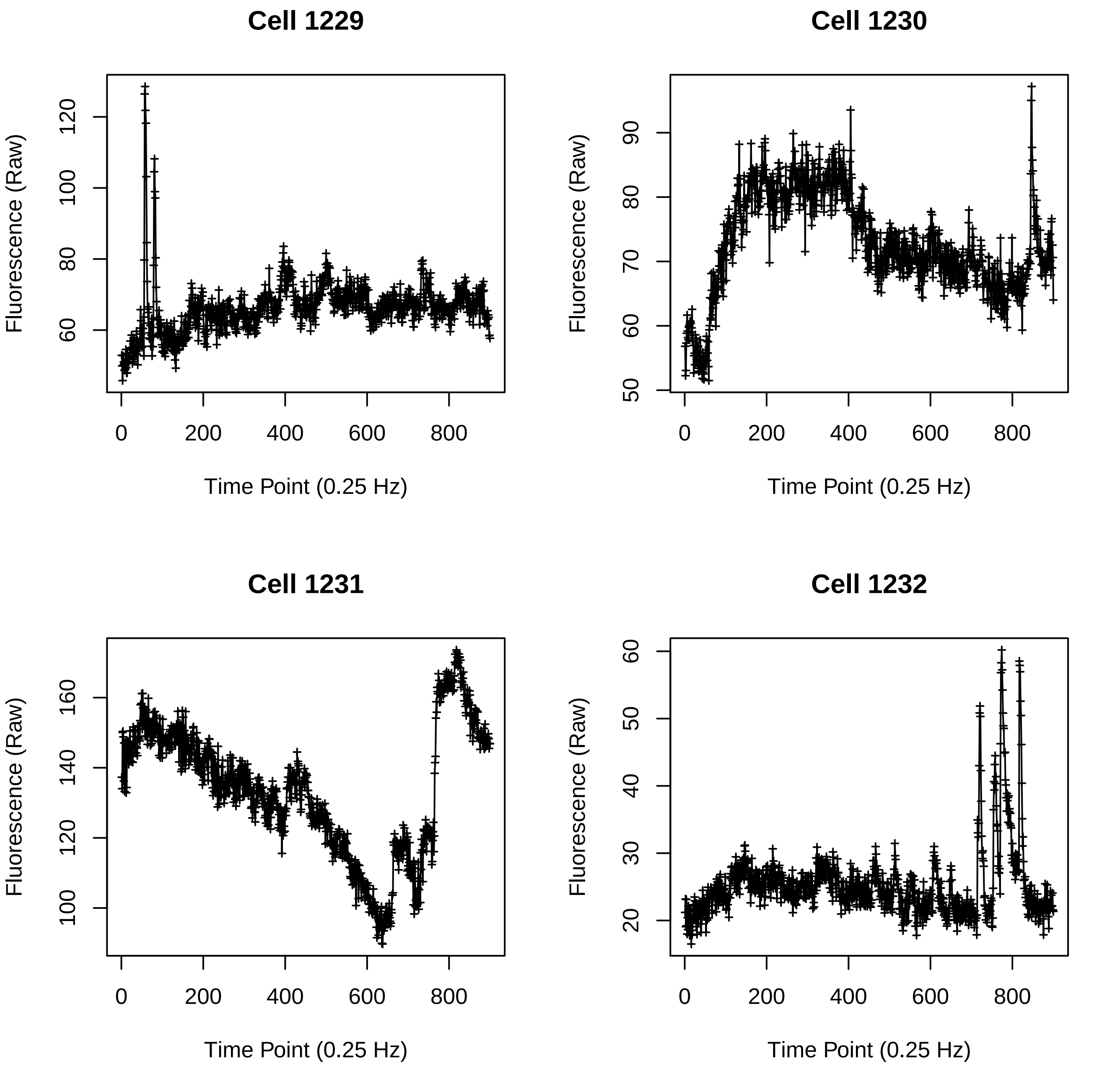

Cell 1229
Cell 1230
Cell 1231
Cell 1232
Fluorescence (Raw)
Time Point (0.25 Hz)

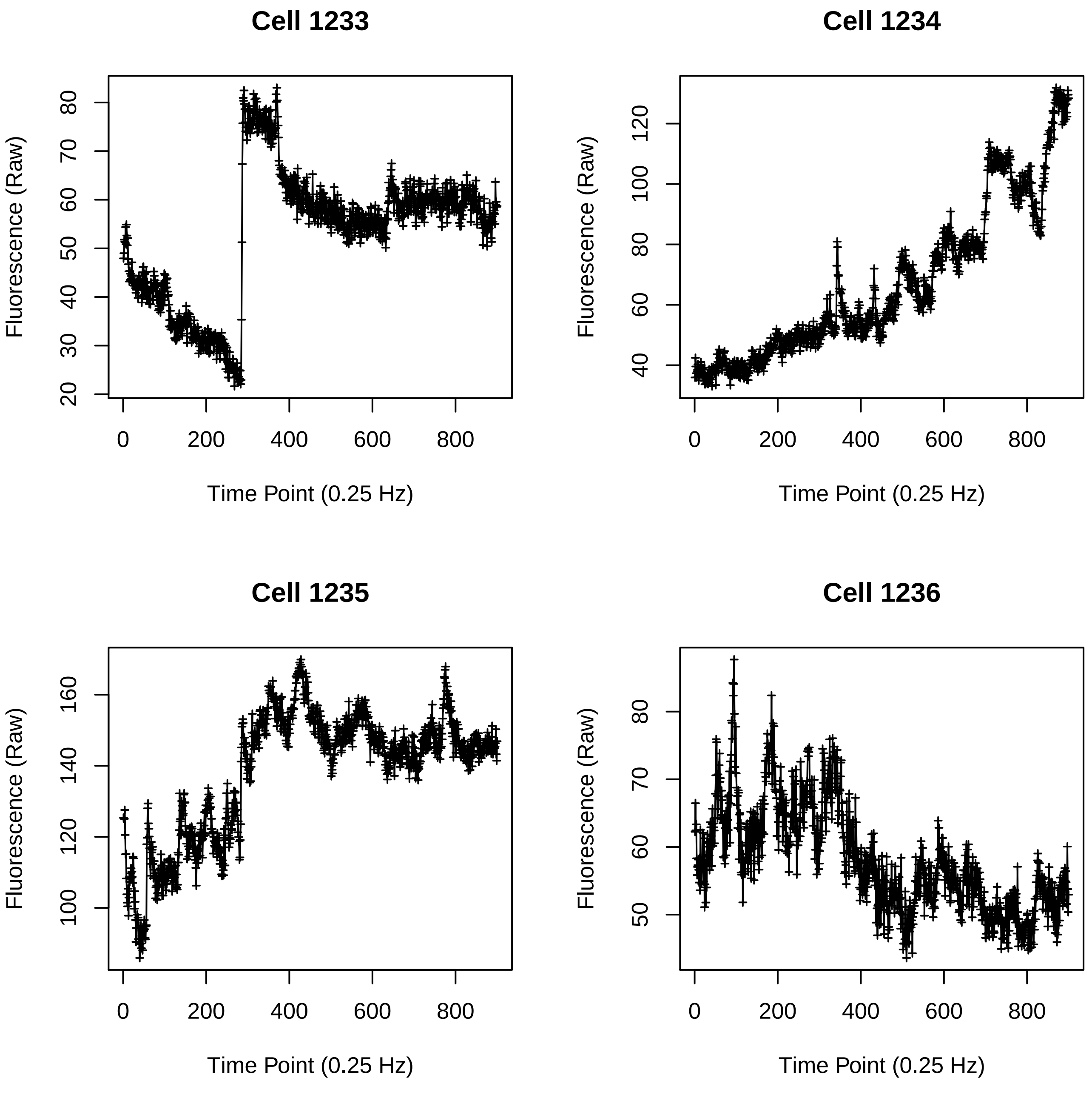
Cell 1233
Cell 1234
Cell 1235
Cell 1236
Fluorescence (Raw)
Time Point (0.25 Hz)
0
200
400
600
800
20
30
40
50
60
70
80
100
120
140
160

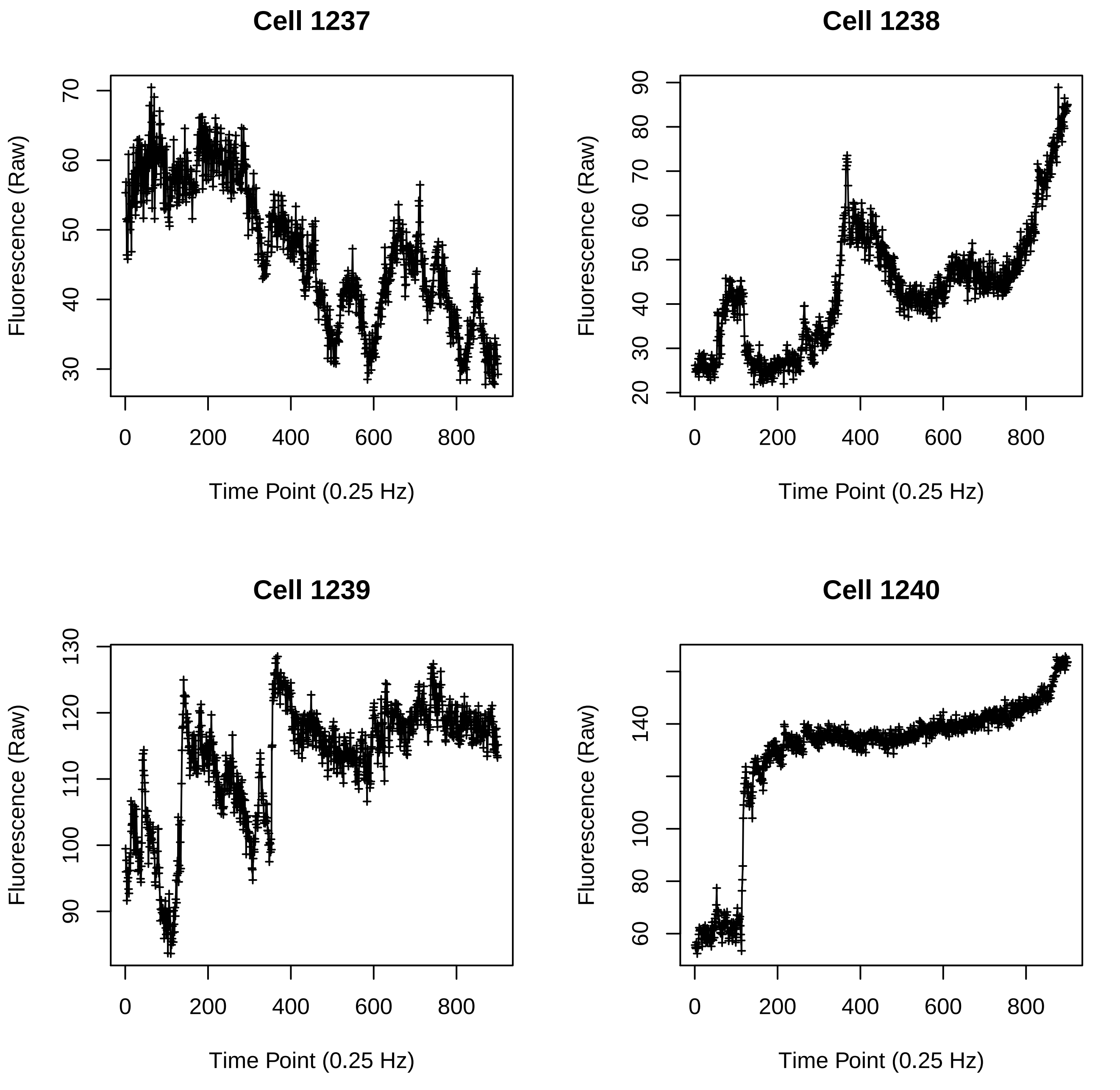
Cell 1237
Fluorescence (Raw)
30
40
50
60
70
0
200
400
600
800
Time Point (0.25 Hz)
Cell 1238
Fluorescence (Raw)
20
30
40
50
60
70
80
90
0
200
400
600
800
Time Point (0.25 Hz)
Cell 1239
Fluorescence (Raw)
90
100
110
120
130
0
200
400
600
800
Time Point (0.25 Hz)
Cell 1240
Fluorescence (Raw)
60
80
100
140
0
200
400
600
800
Time Point (0.25 Hz)

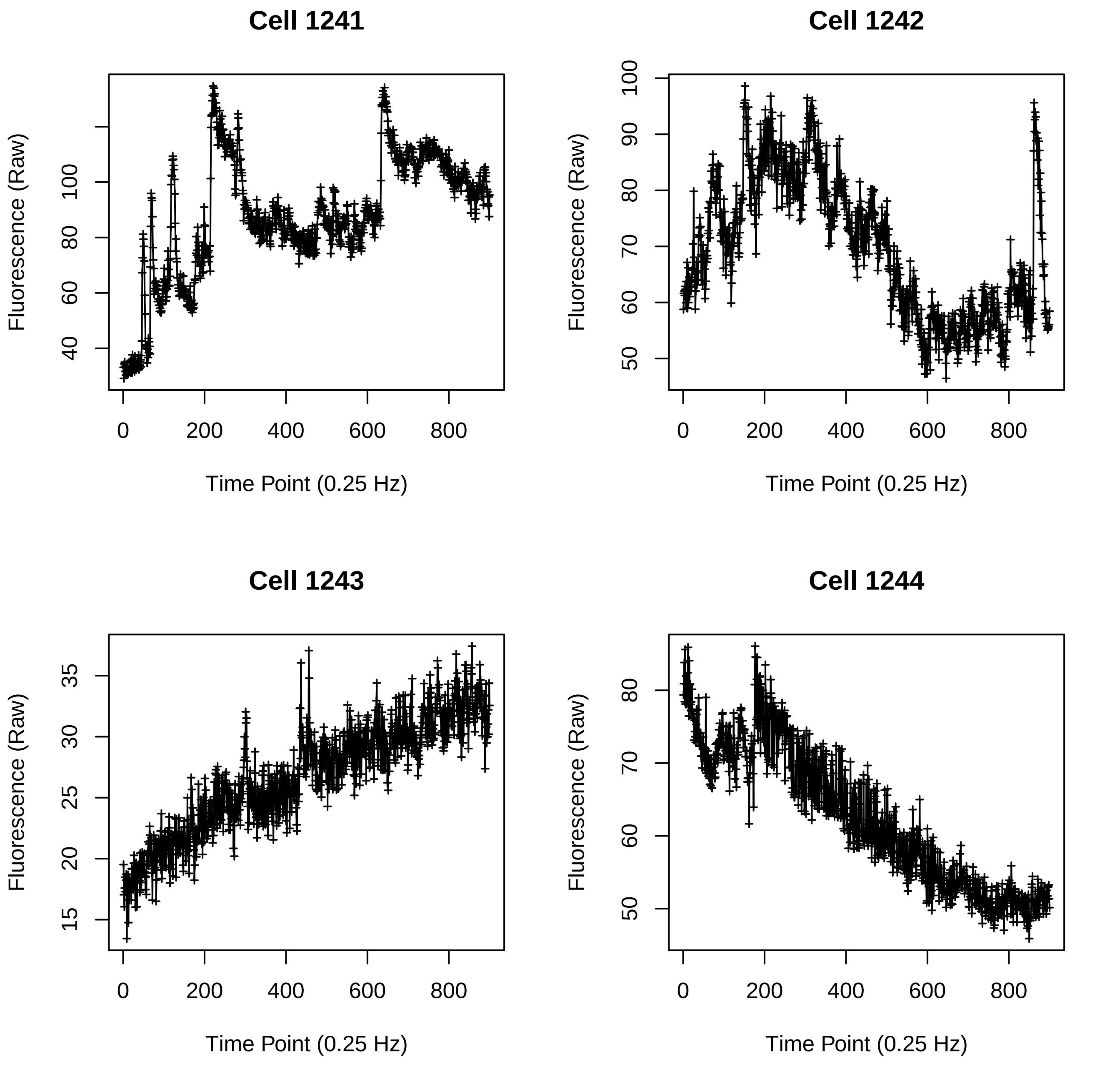
Cell 1241
Cell 1242
Cell 1243
Cell 1244
Fluorescence (Raw)
Time Point (0.25 Hz)
0
200
400
600
800
40
60
80
100
50
70
90
15
20
25
30
35

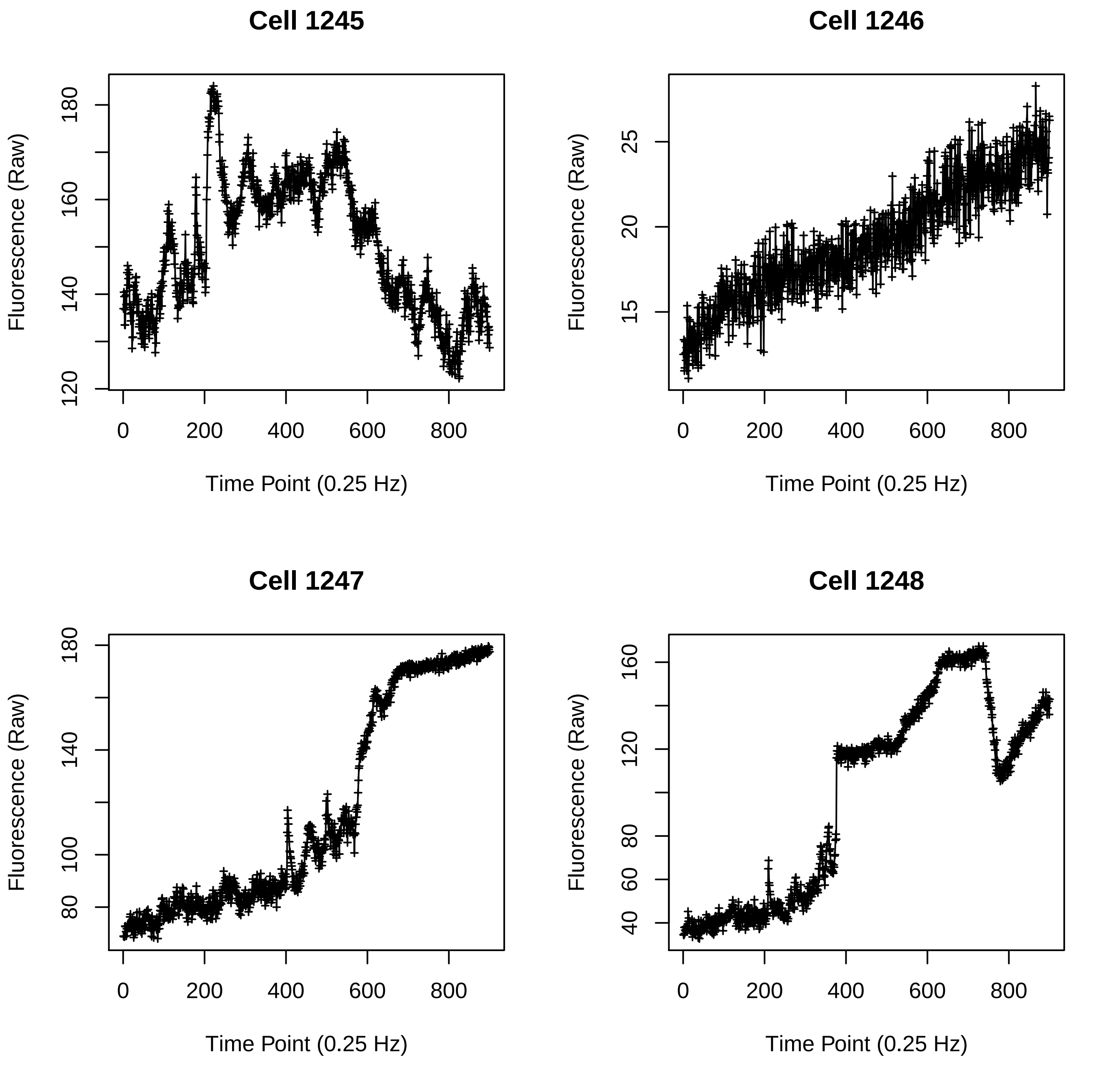
Cell 1245
Fluorescence (Raw)
120
140
160
180
0
200
400
600
800
Time Point (0.25 Hz)
Cell 1246
Fluorescence (Raw)
15
20
25
0
200
400
600
800
Time Point (0.25 Hz)
Cell 1247
Fluorescence (Raw)
80
100
140
180
0
200
400
600
800
Time Point (0.25 Hz)
Cell 1248
Fluorescence (Raw)
40
60
80
120
160
0
200
400
600
800
Time Point (0.25 Hz)

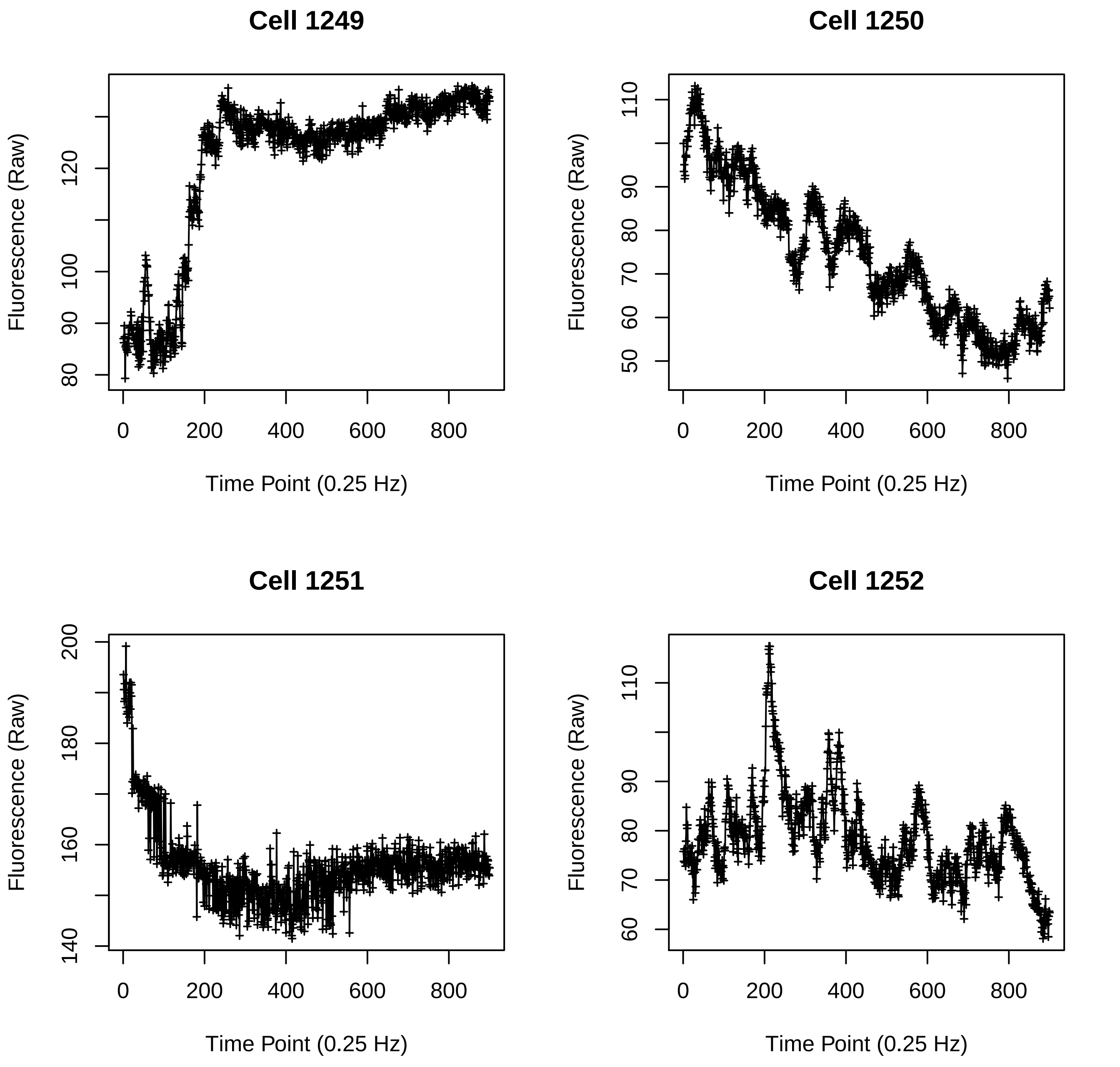

Cell 1249
Cell 1250
Cell 1251
Cell 1252
Fluorescence (Raw)
Time Point (0.25 Hz)
80
90
100
120
50
60
70
80
90
110
140
160
180
200
60
70
80
90
110
0
200
400
600
800

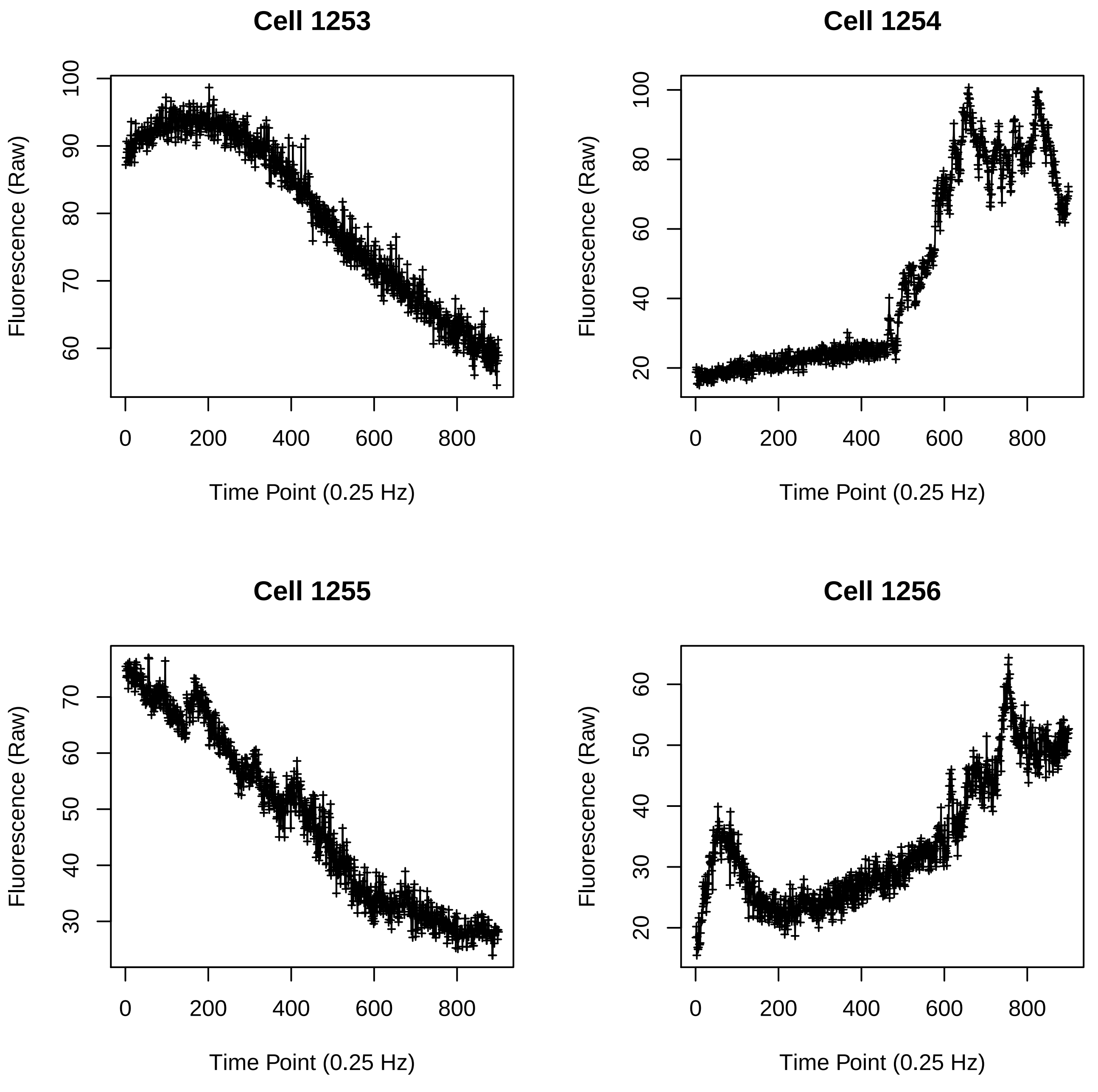
Cell 1253
Cell 1254
Cell 1255
Cell 1256
Fluorescence (Raw)
Time Point (0.25 Hz)
0
200
400
600
800
20
30
40
50
60
70
80
90
100

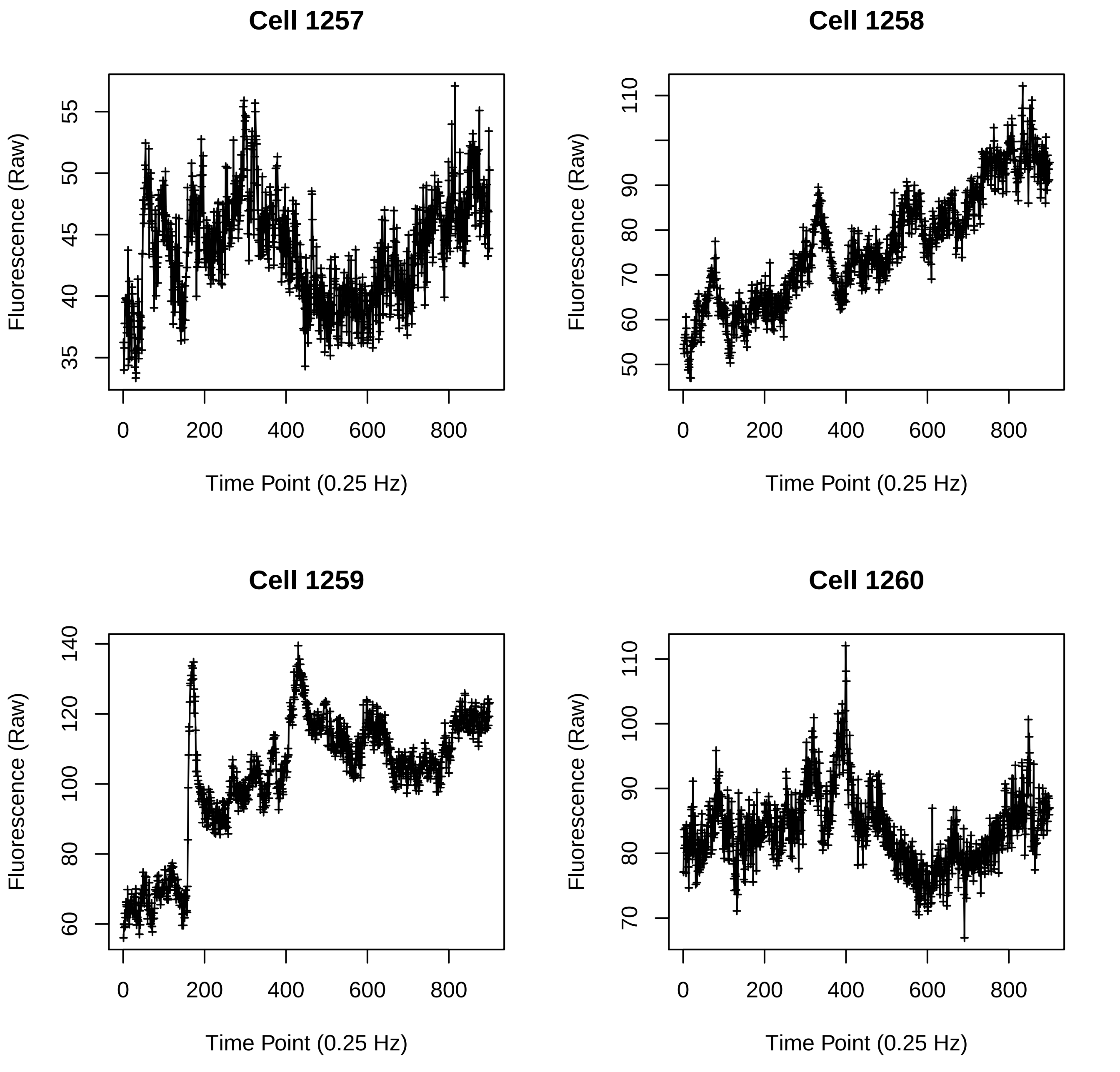
Cell 1257
Cell 1258
Cell 1259
Cell 1260
Fluorescence (Raw)
Time Point (0.25 Hz)
0
200
400
600
800
35
40
45
50
55
50
60
70
80
90
110
60
80
100
120
140
70
80
90
100
110

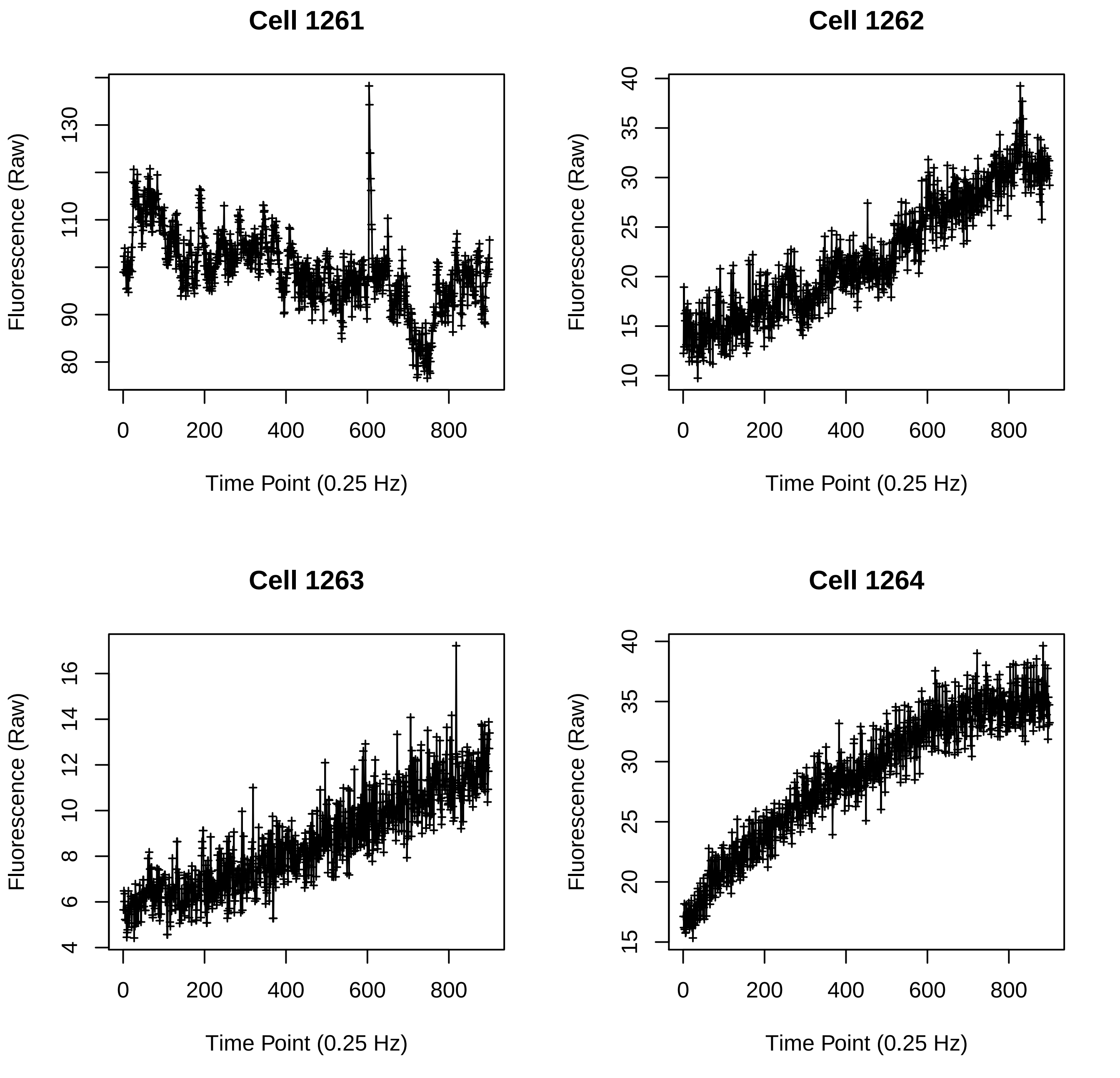
Cell 1261
Cell 1262
Cell 1263
Cell 1264
Fluorescence (Raw)
Time Point (0.25 Hz)
0
200
400
600
800
80
90
110
130
10
15
20
25
30
35
40
4
6
8
10
12
14
16

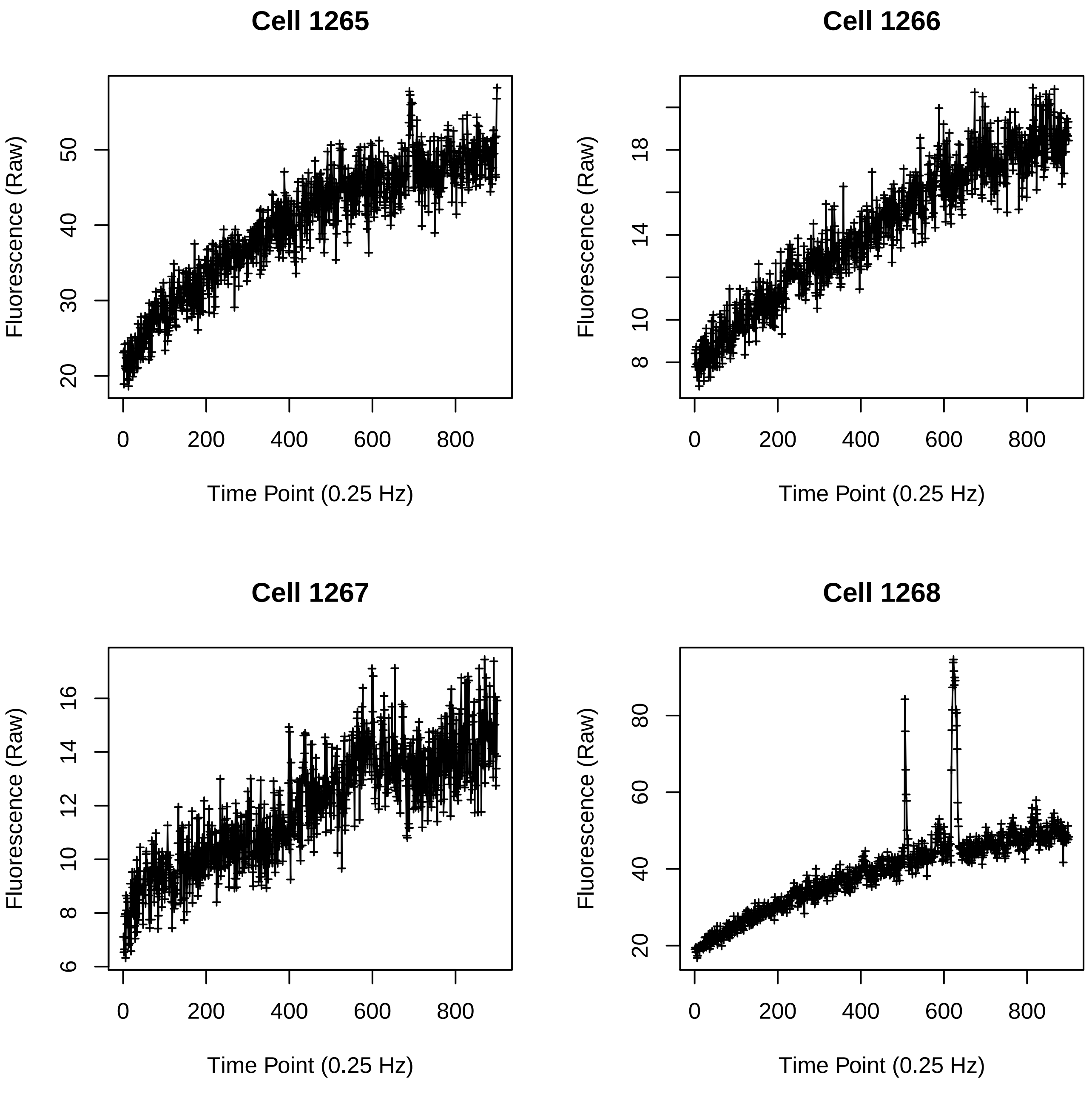
Cell 1265
Fluorescence (Raw)
20
30
40
50
0
200
400
600
800
Time Point (0.25 Hz)
Cell 1266
Fluorescence (Raw)
8
10
14
18
0
200
400
600
800
Time Point (0.25 Hz)
Cell 1267
Fluorescence (Raw)
6
8
10
12
14
16
0
200
400
600
800
Time Point (0.25 Hz)
Cell 1268
Fluorescence (Raw)
20
40
60
80
0
200
400
600
800
Time Point (0.25 Hz)

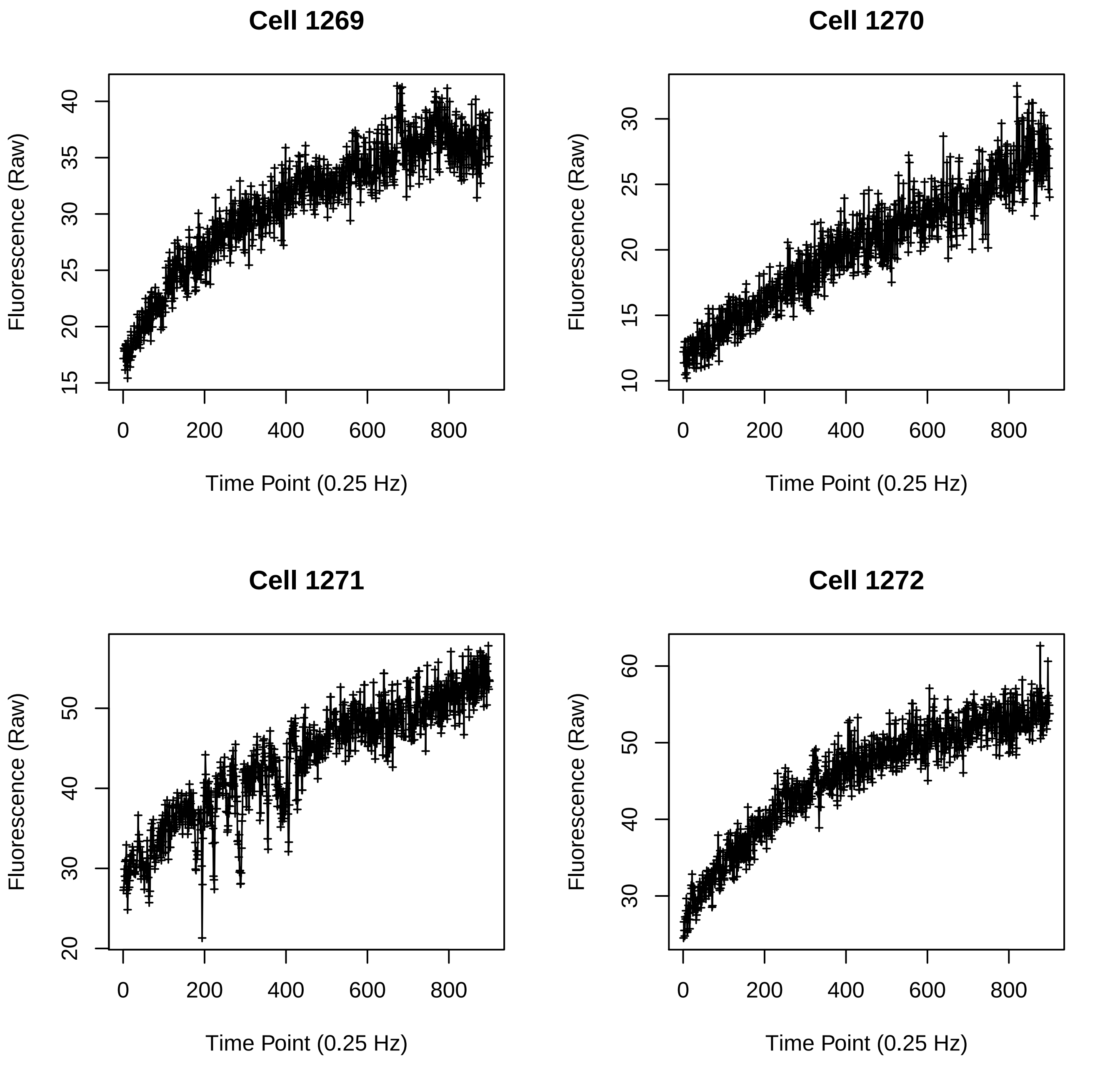
Cell 1269
Fluorescence (Raw)
Time Point (0.25 Hz)
15
20
25
30
35
40
0
200
400
600
800
Cell 1270
Fluorescence (Raw)
Time Point (0.25 Hz)
10
15
20
25
30
0
200
400
600
800
Cell 1271
Fluorescence (Raw)
Time Point (0.25 Hz)
20
30
40
50
0
200
400
600
800
Cell 1272
Fluorescence (Raw)
Time Point (0.25 Hz)
30
40
50
60
0
200
400
600
800

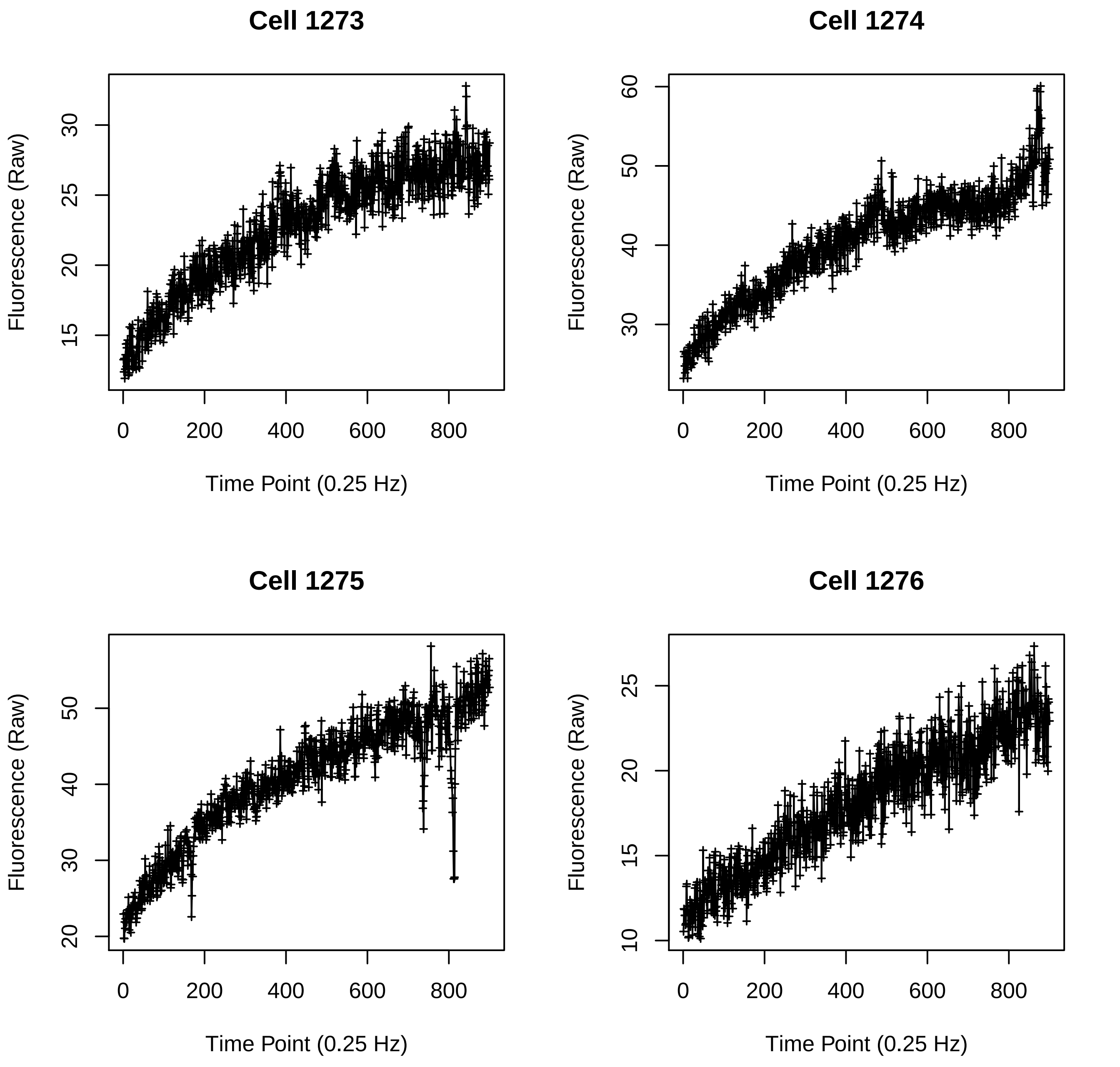
Cell 1273
Cell 1274
Cell 1275
Cell 1276
Fluorescence (Raw)
Time Point (0.25 Hz)
0
200
400
600
800
15
20
25
30
40
50
60
10

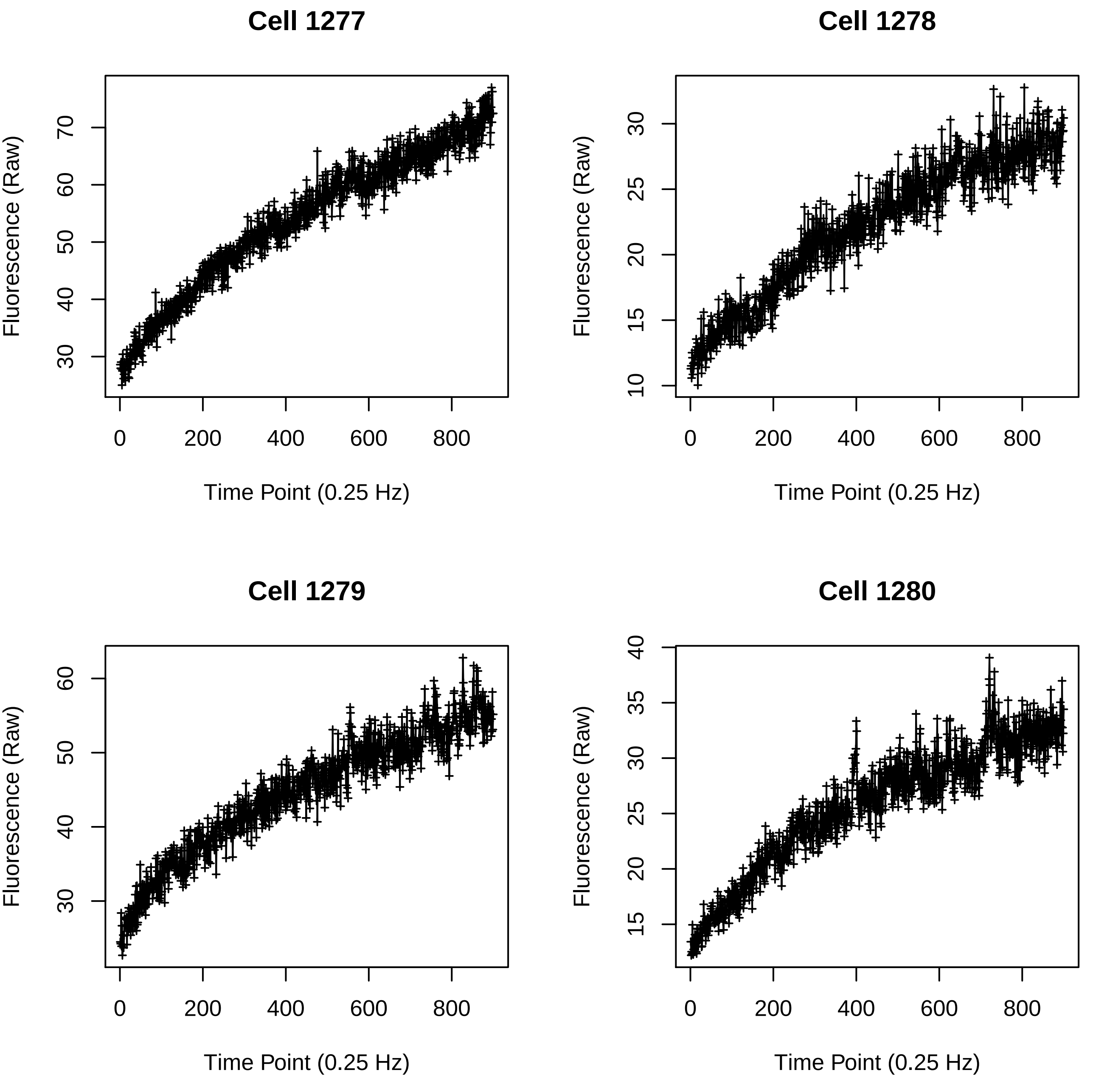

Cell 1277
Cell 1278
Cell 1279
Cell 1280
Fluorescence (Raw)
Time Point (0.25 Hz)
0
200
400
600
800
30
40
50
60
70
10
15
20
25
30
35
40

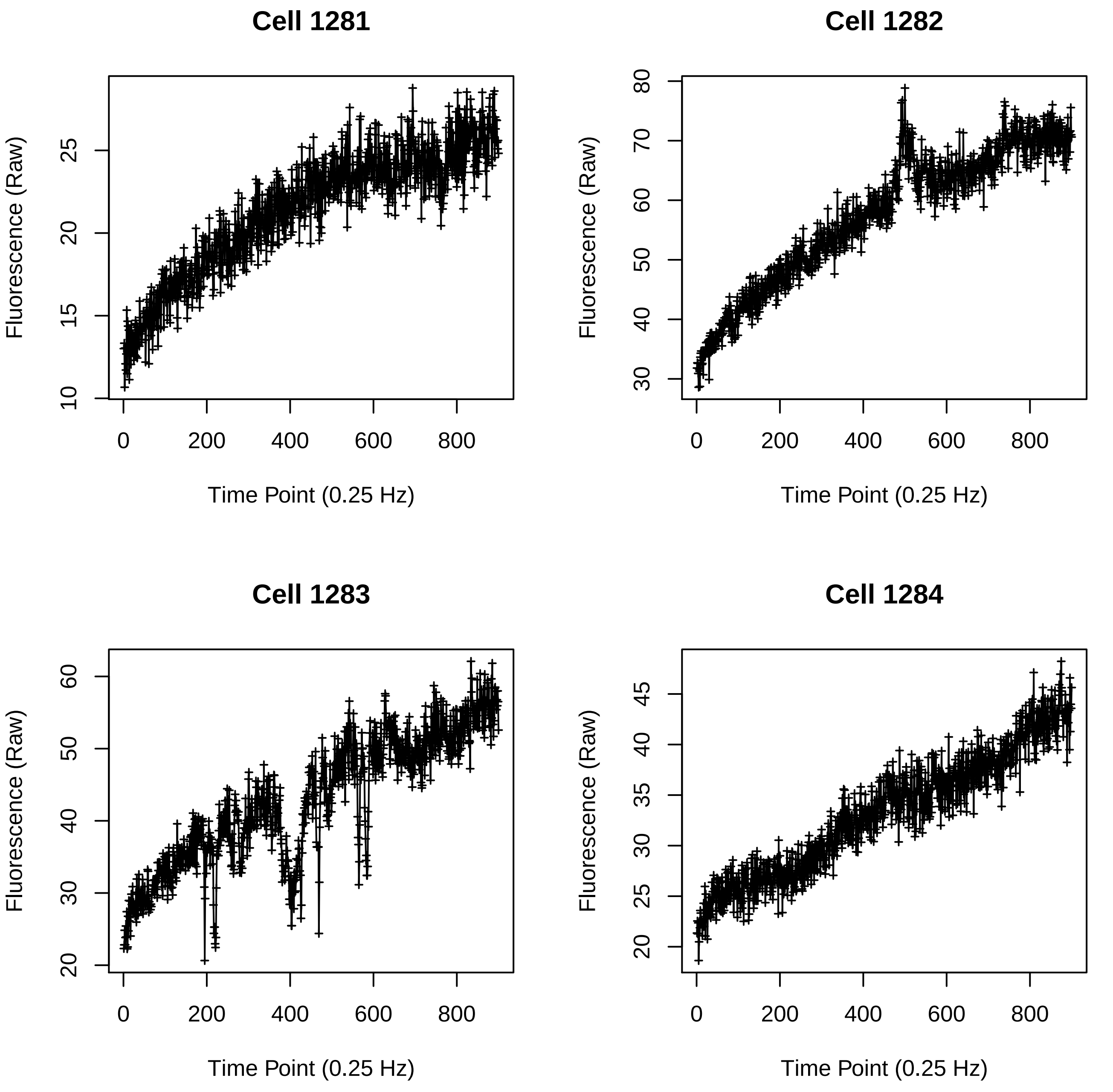
Cell 1281
Fluorescence (Raw)
10
15
20
25
0
200
400
600
800
Time Point (0.25 Hz)
Cell 1282
Fluorescence (Raw)
30
40
50
60
70
80
0
200
400
600
800
Time Point (0.25 Hz)
Cell 1283
Fluorescence (Raw)
20
30
40
50
60
0
200
400
600
800
Time Point (0.25 Hz)
Cell 1284
Fluorescence (Raw)
20
25
30
35
40
45
0
200
400
600
800
Time Point (0.25 Hz)

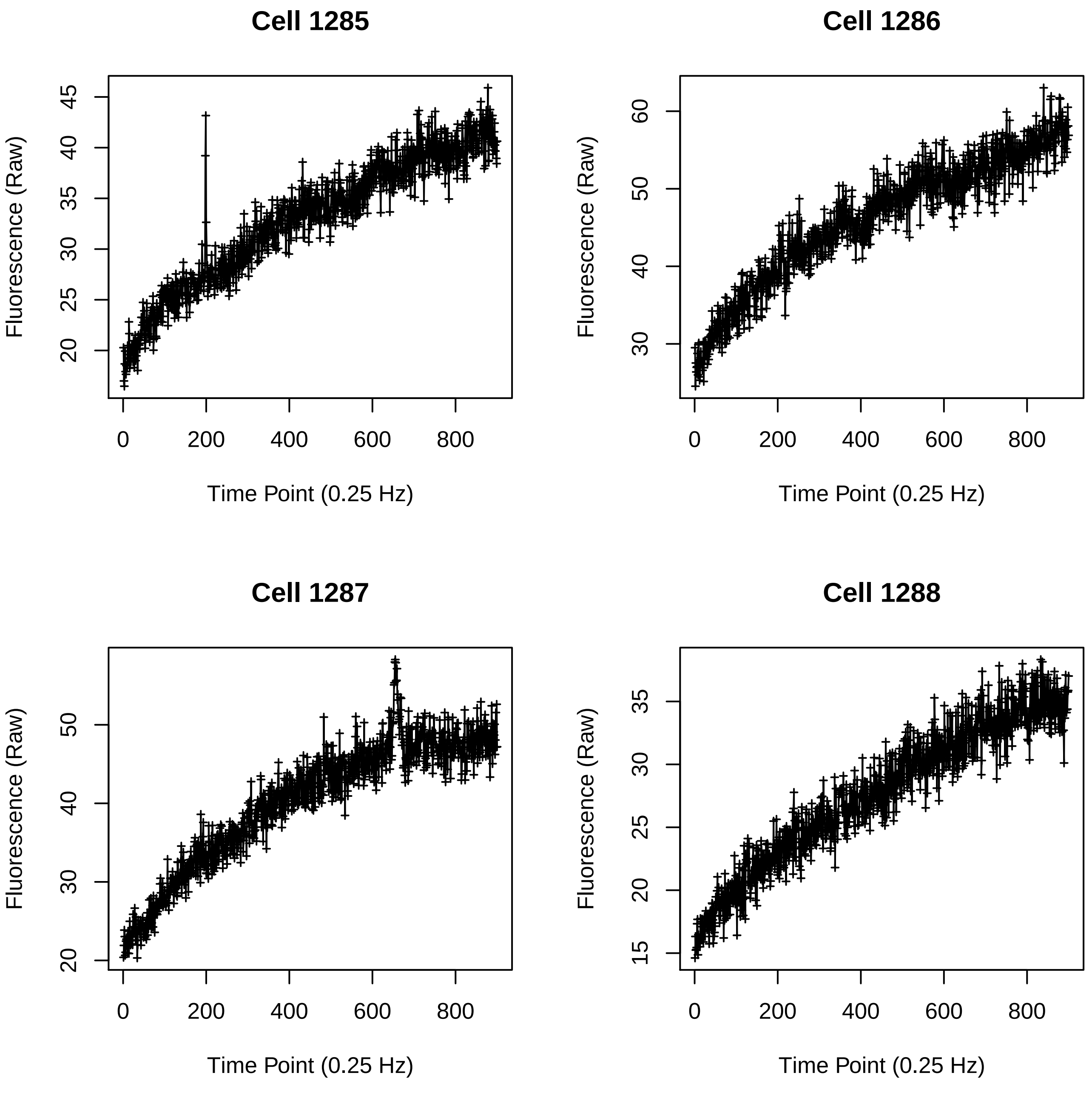
Cell 1285
Cell 1286
Cell 1287
Cell 1288
Fluorescence (Raw)
Time Point (0.25 Hz)
0
200
400
600
800
20
25
30
35
40
45
50
60
15

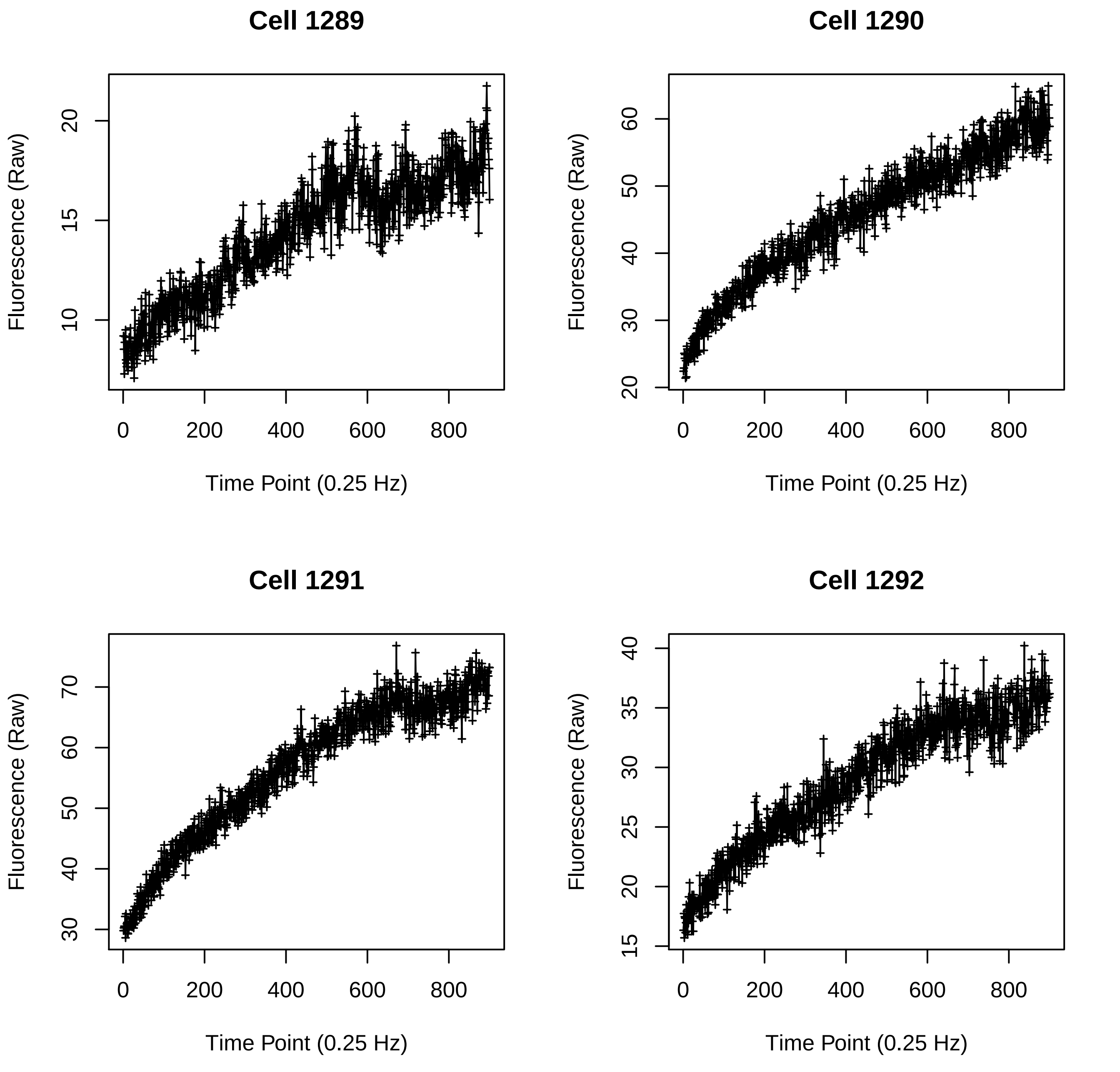
Cell 1289
Cell 1290
Cell 1291
Cell 1292
Fluorescence (Raw)
Time Point (0.25 Hz)
0
200
400
600
800
10
15
20
30
40
50
60
70
25
35

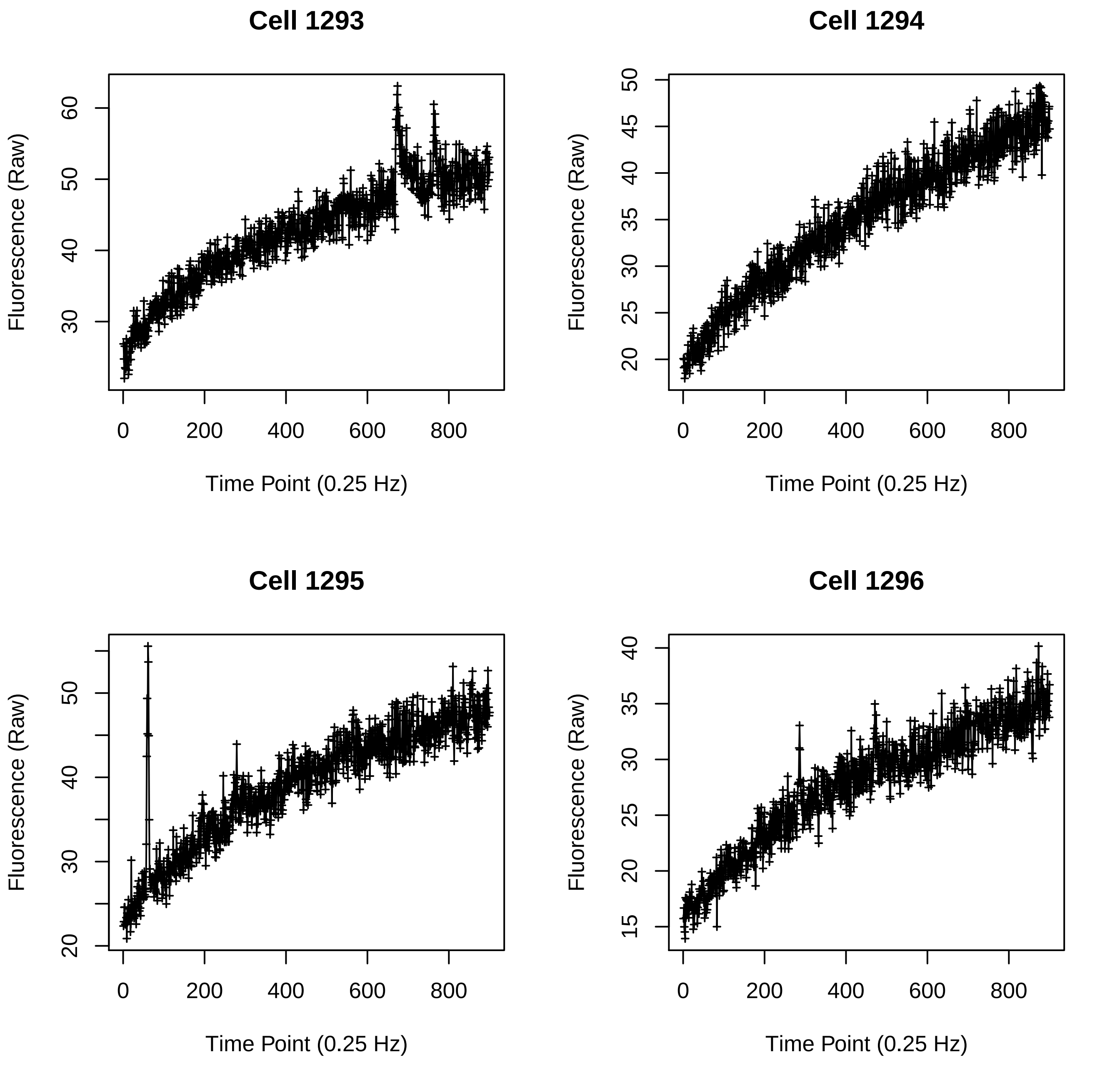
Cell 1293
Cell 1294
Cell 1295
Cell 1296
Fluorescence (Raw)
Time Point (0.25 Hz)
0
200
400
600
800
20
25
30
35
40
45
50
60
15

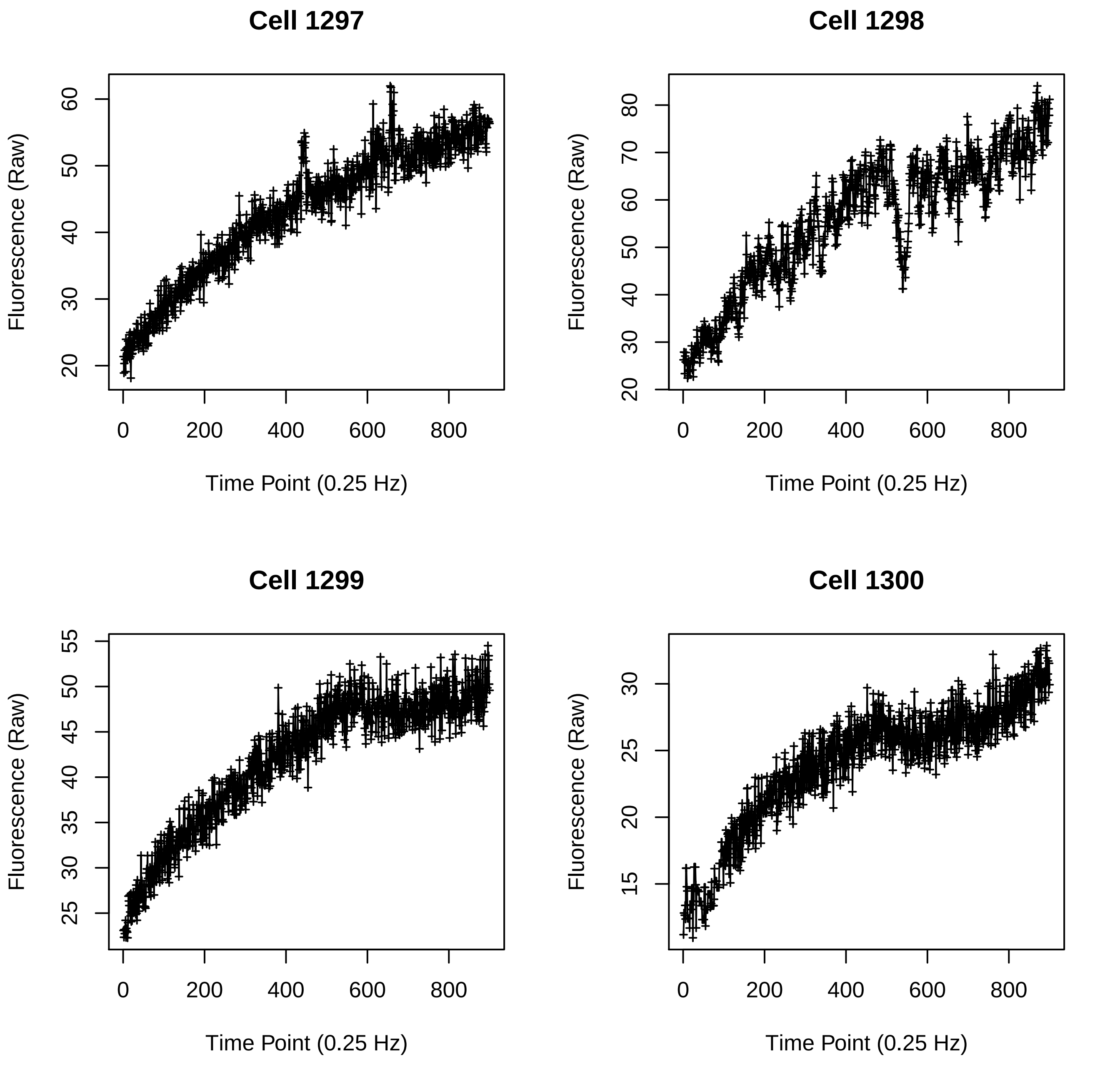

Cell 1297
Fluorescence (Raw)
20
30
40
50
60
0
200
400
600
800
Time Point (0.25 Hz)
Cell 1298
Fluorescence (Raw)
20
30
40
50
60
70
80
0
200
400
600
800
Time Point (0.25 Hz)
Cell 1299
Fluorescence (Raw)
25
30
35
40
45
50
55
0
200
400
600
800
Time Point (0.25 Hz)
Cell 1300
Fluorescence (Raw)
15
20
25
30
0
200
400
600
800
Time Point (0.25 Hz)

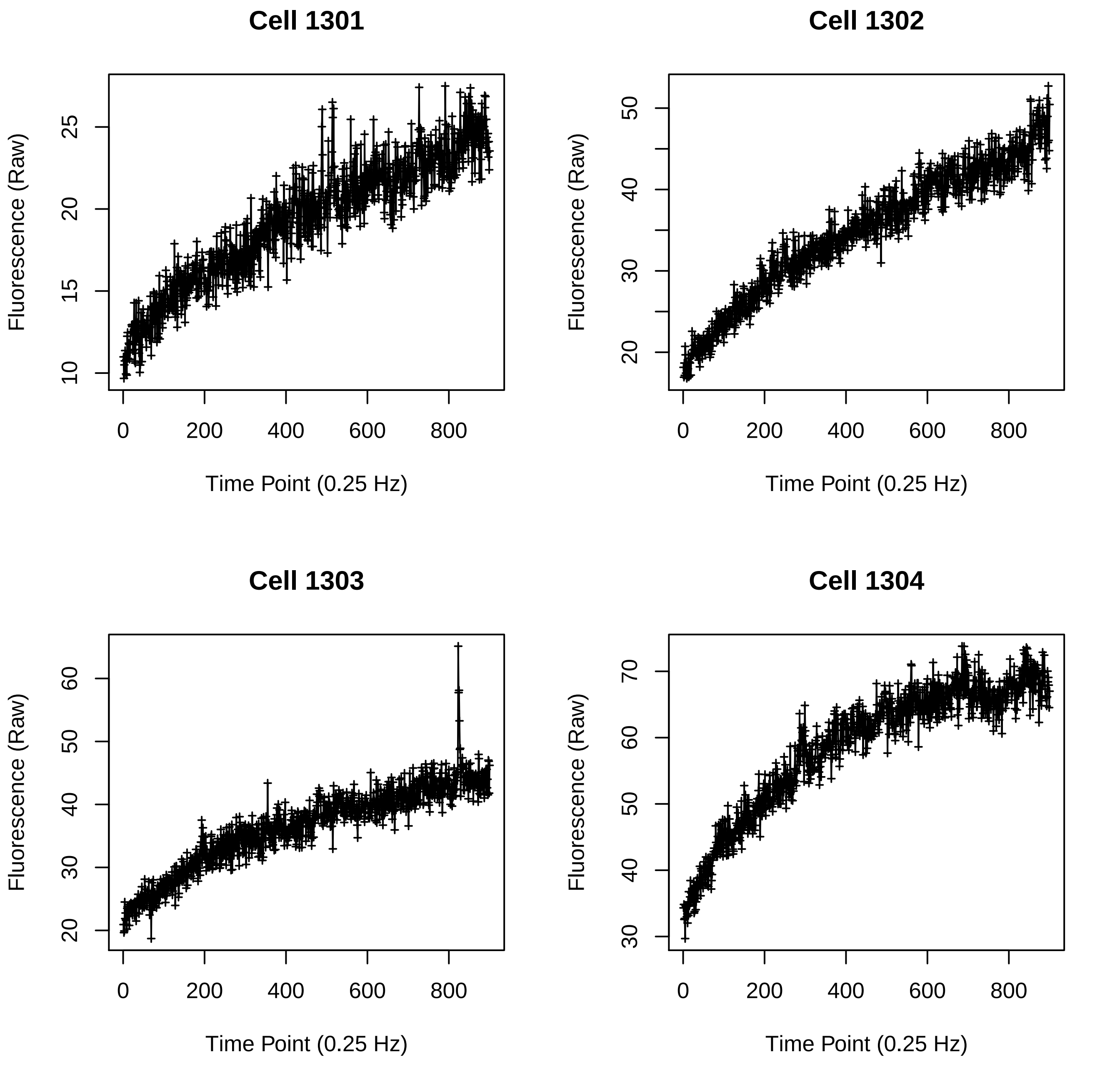
Cell 1301
Cell 1302
Cell 1303
Cell 1304
Fluorescence (Raw)
Time Point (0.25 Hz)
0
200
400
600
800
10
15
20
25
30
40
50
60
70

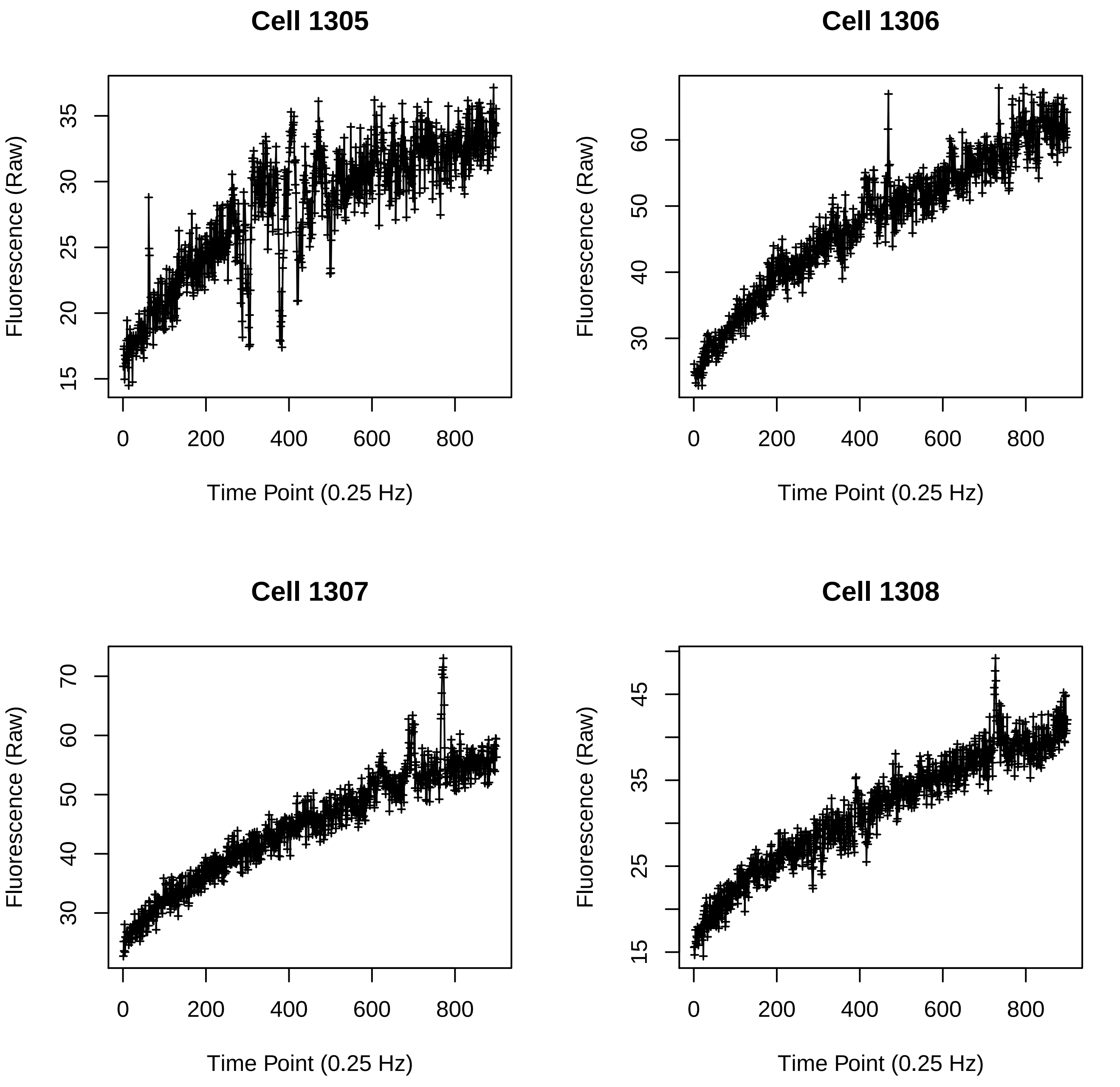
Cell 1305
Cell 1306
Cell 1307
Cell 1308
Fluorescence (Raw)
Time Point (0.25 Hz)
15
20
25
30
35
40
45
50
60
70
0
200
400
600
800

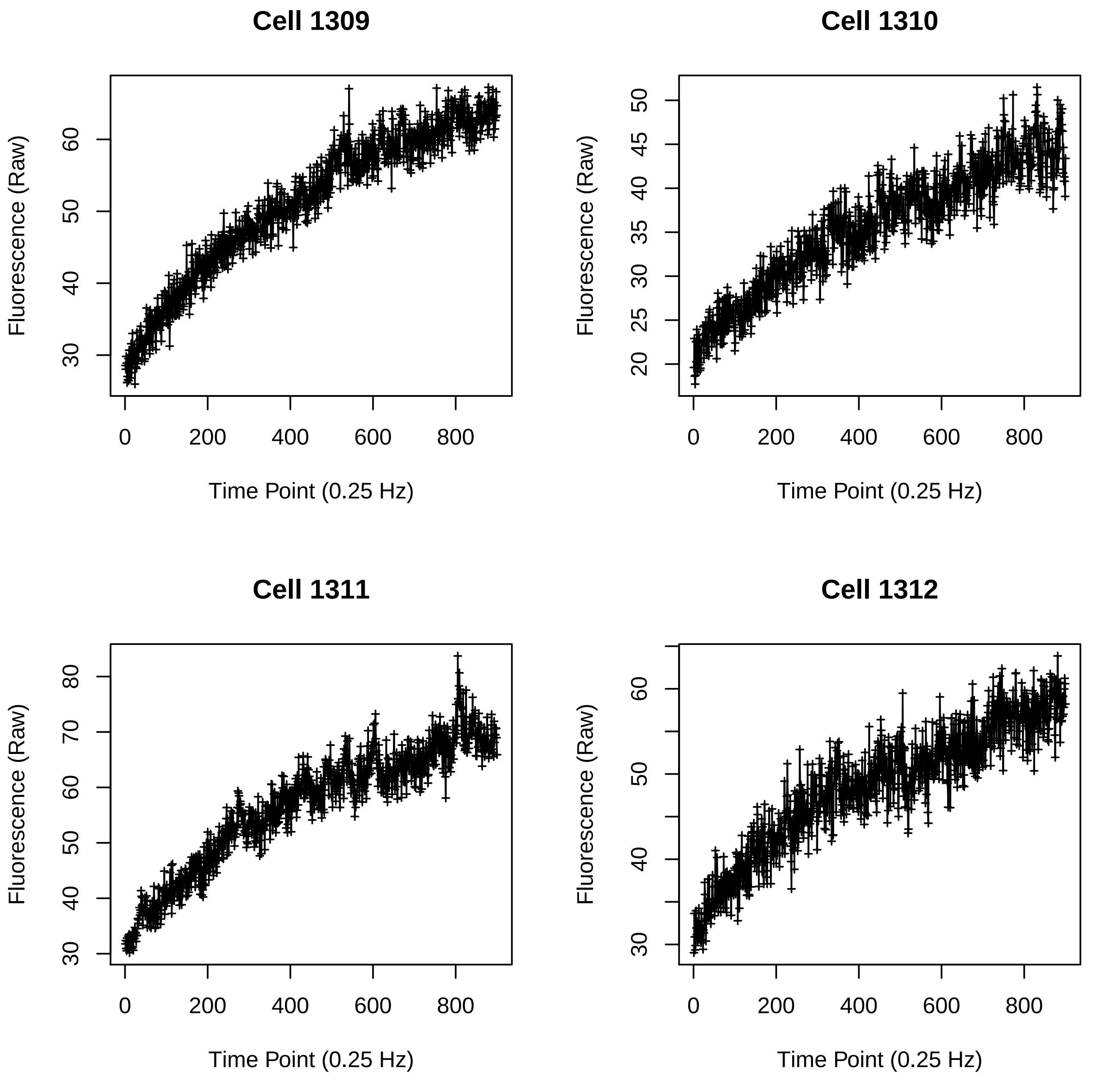
Cell 1309
Fluorescence (Raw)
30
40
50
60
0
200
400
600
800
Time Point (0.25 Hz)
Cell 1310
Fluorescence (Raw)
20
25
30
35
40
45
50
0
200
400
600
800
Time Point (0.25 Hz)
Cell 1311
Fluorescence (Raw)
30
40
50
60
70
80
0
200
400
600
800
Time Point (0.25 Hz)
Cell 1312
Fluorescence (Raw)
30
40
50
60
0
200
400
600
800
Time Point (0.25 Hz)

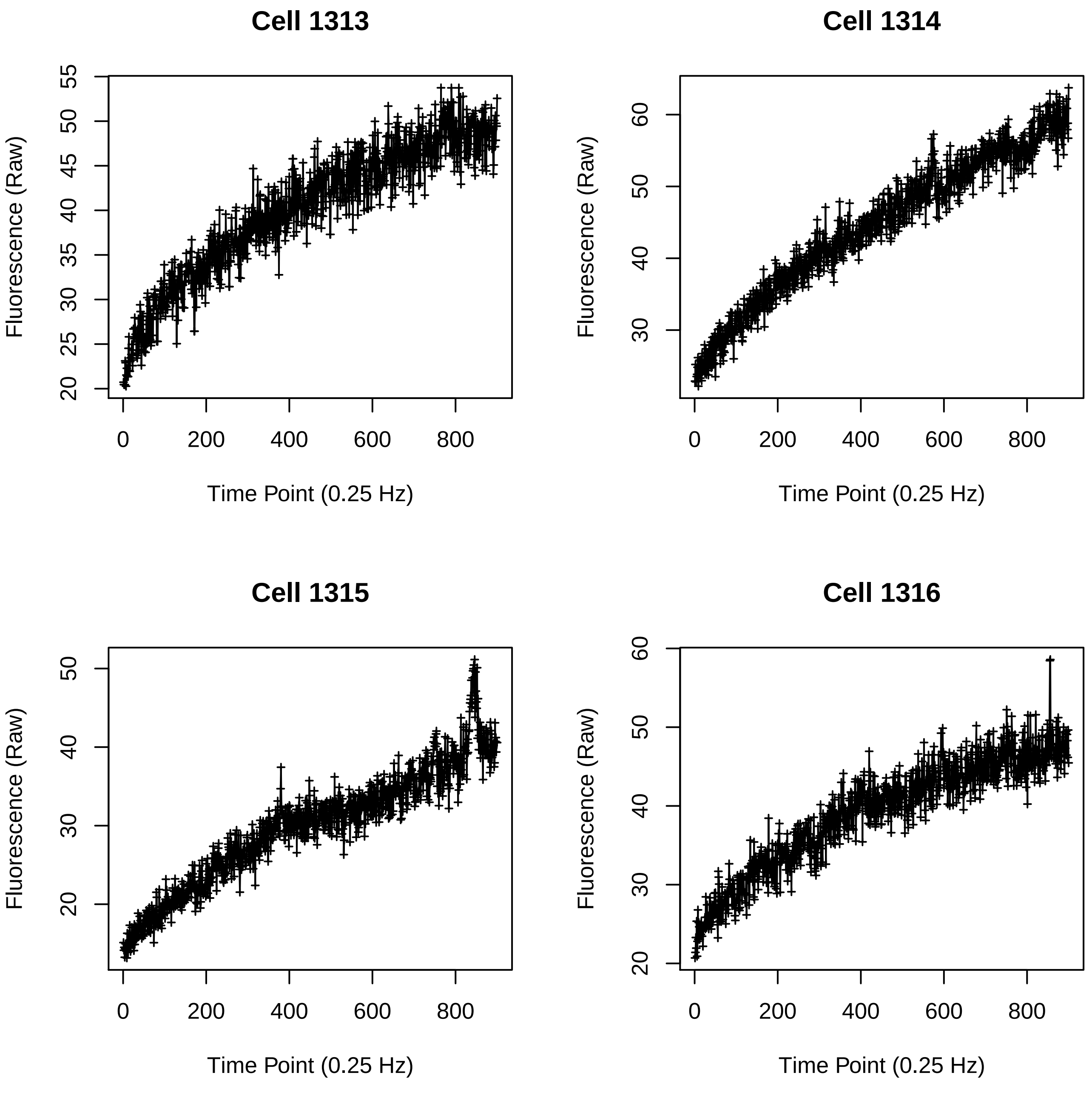
Cell 1313
Cell 1314
Cell 1315
Cell 1316
Fluorescence (Raw)
Time Point (0.25 Hz)
0
200
400
600
800
20
25
30
35
40
45
50
55
60

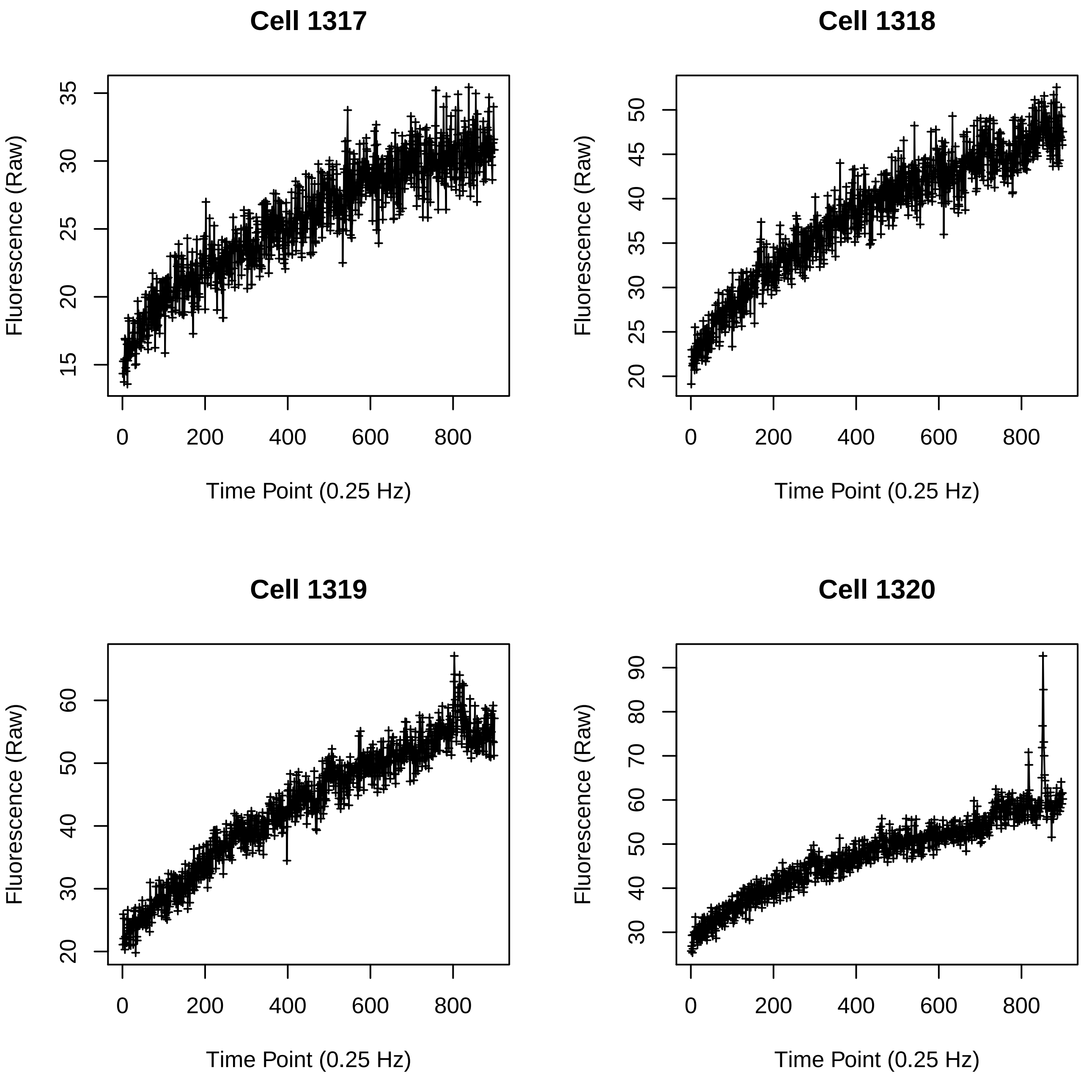
Cell 1317
Cell 1318
Cell 1319
Cell 1320
Fluorescence (Raw)
Time Point (0.25 Hz)
0
200
400
600
800
15
20
25
30
35
40
45
50
60
70
80
90

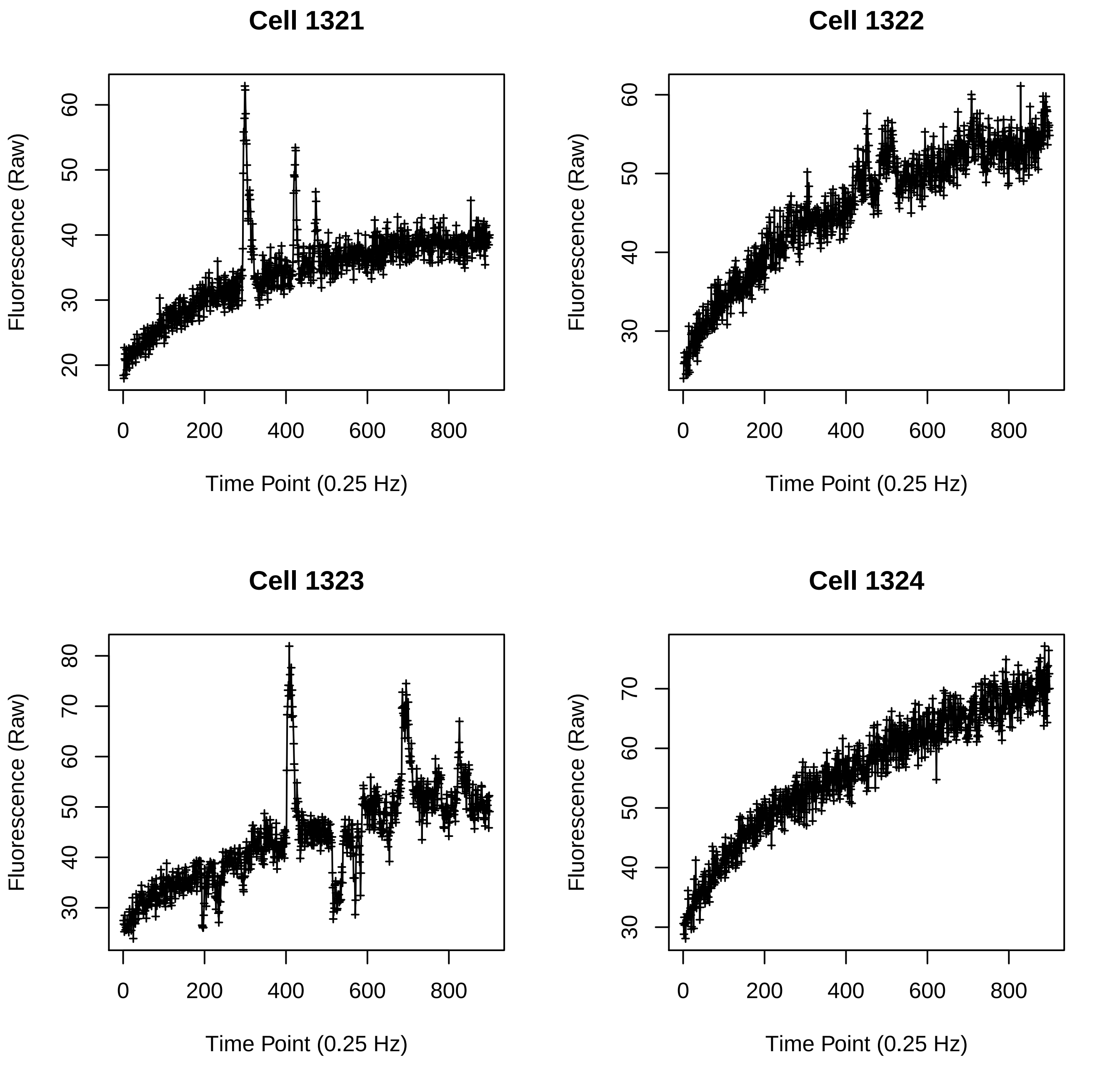
Cell 1321
Cell 1322
Cell 1323
Cell 1324
Fluorescence (Raw)
Time Point (0.25 Hz)
0
200
400
600
800
20
30
40
50
60
70
80

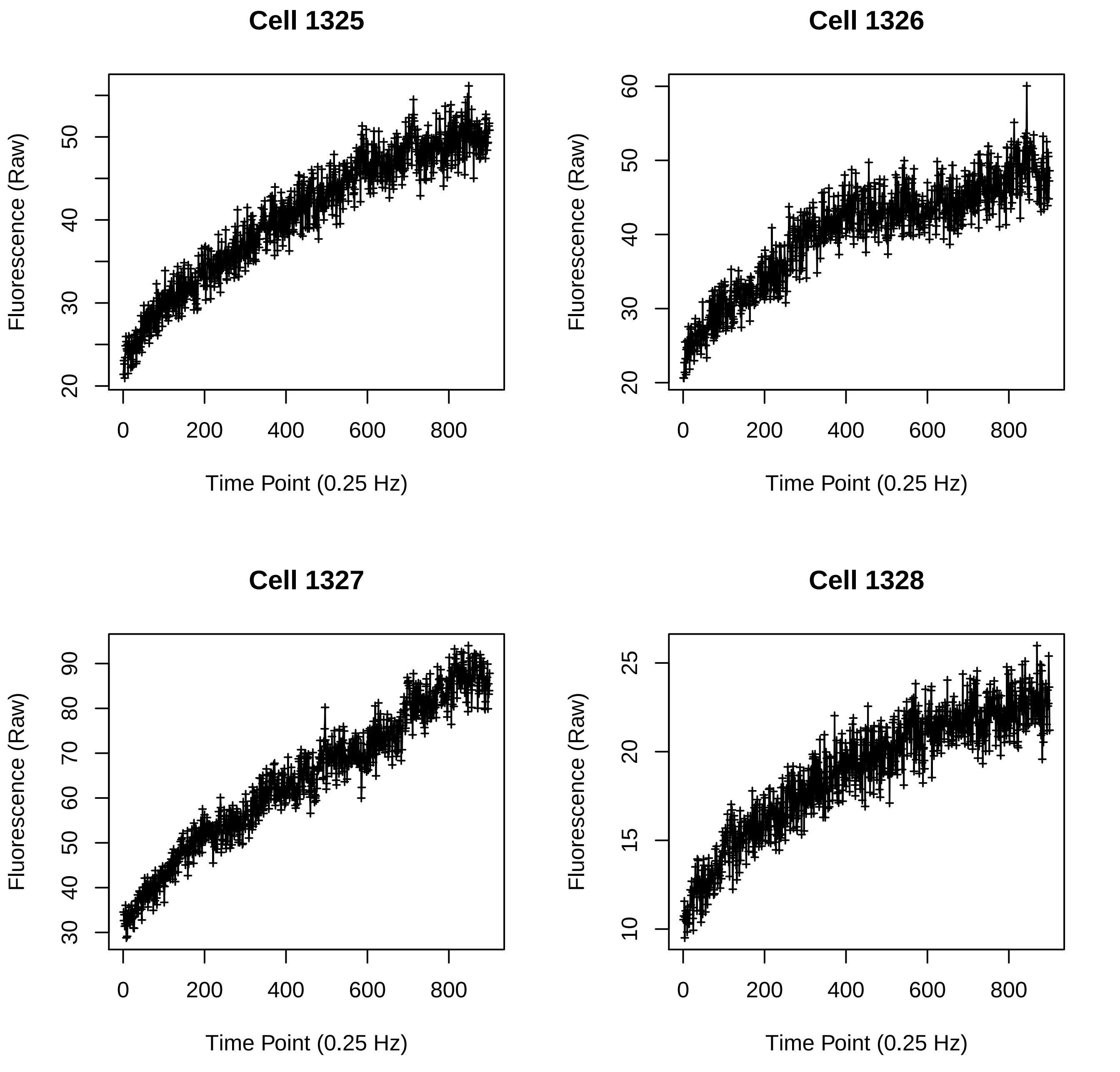
Cell 1325
Cell 1326
Cell 1327
Cell 1328
Fluorescence (Raw)
Time Point (0.25 Hz)
0
200
400
600
800
20
30
40
50
60
70
80
90
10
15
25

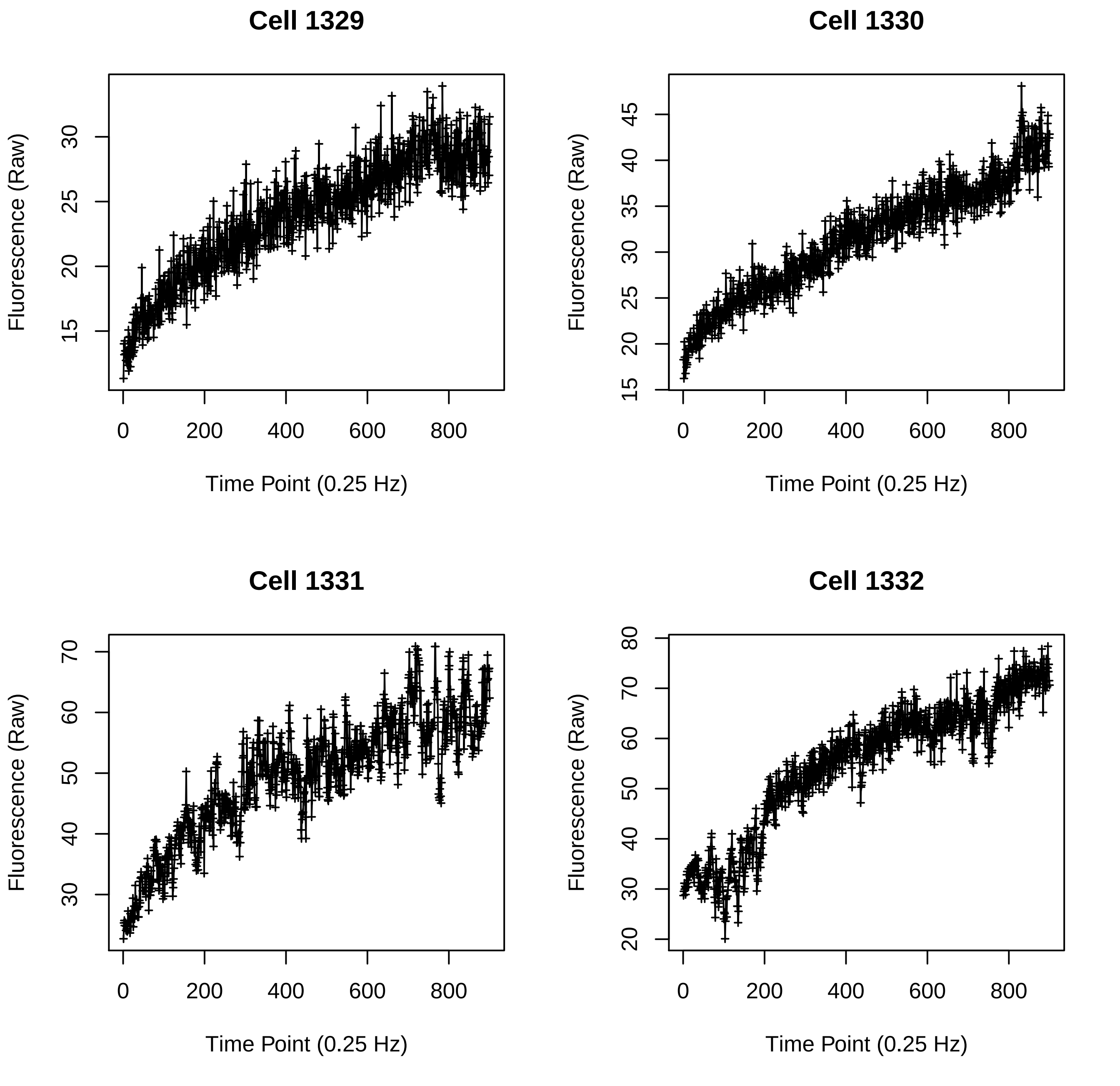
Cell 1329
Cell 1330
Cell 1331
Cell 1332
Fluorescence (Raw)
Time Point (0.25 Hz)
0
200
400
600
800
15
20
25
30
35
40
45
50
60
70
80

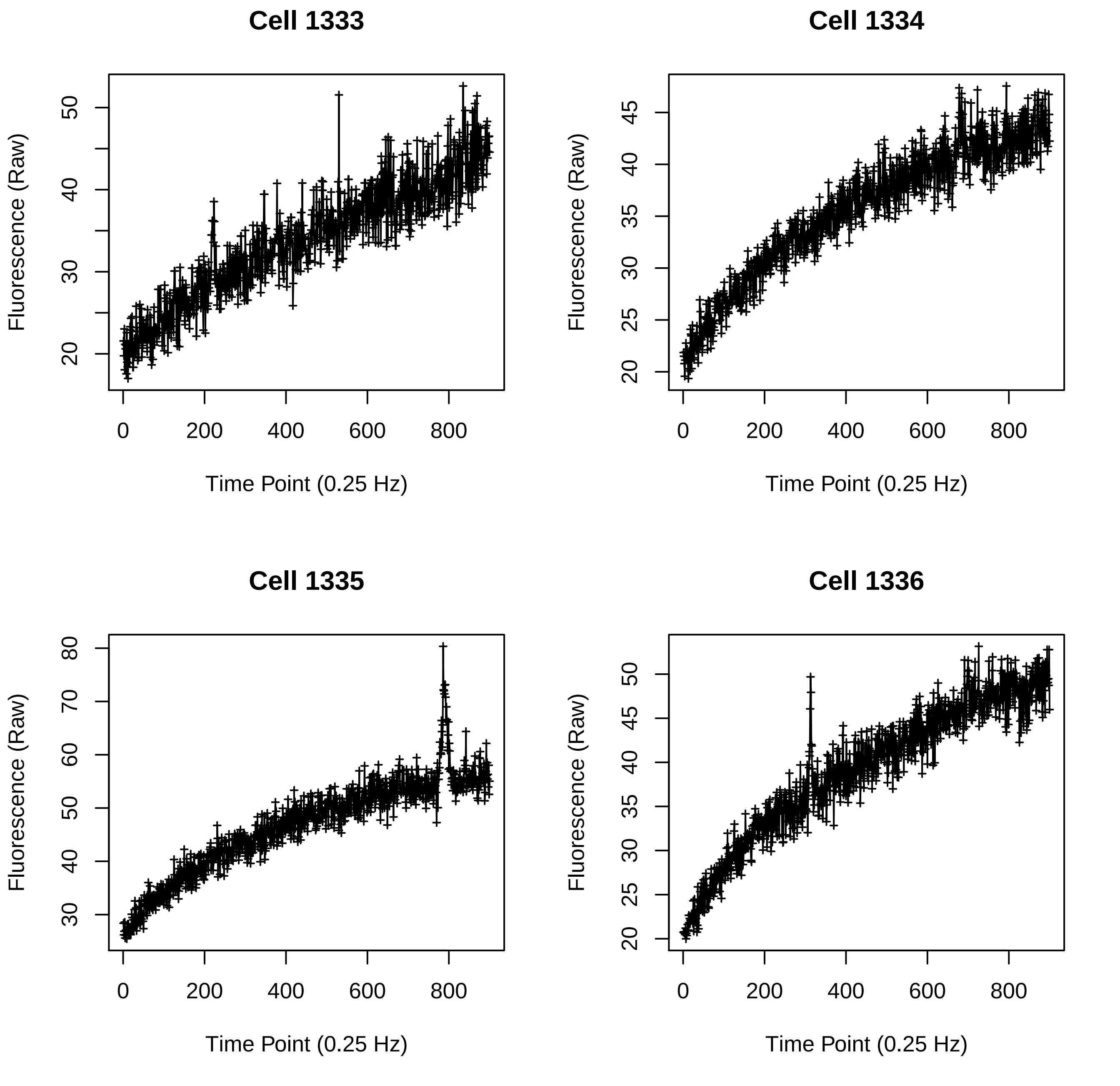
Cell 1333
Cell 1334
Cell 1335
Cell 1336
Fluorescence (Raw)
Time Point (0.25 Hz)
0
200
400
600
800
20
25
30
35
40
45
50
60
70
80

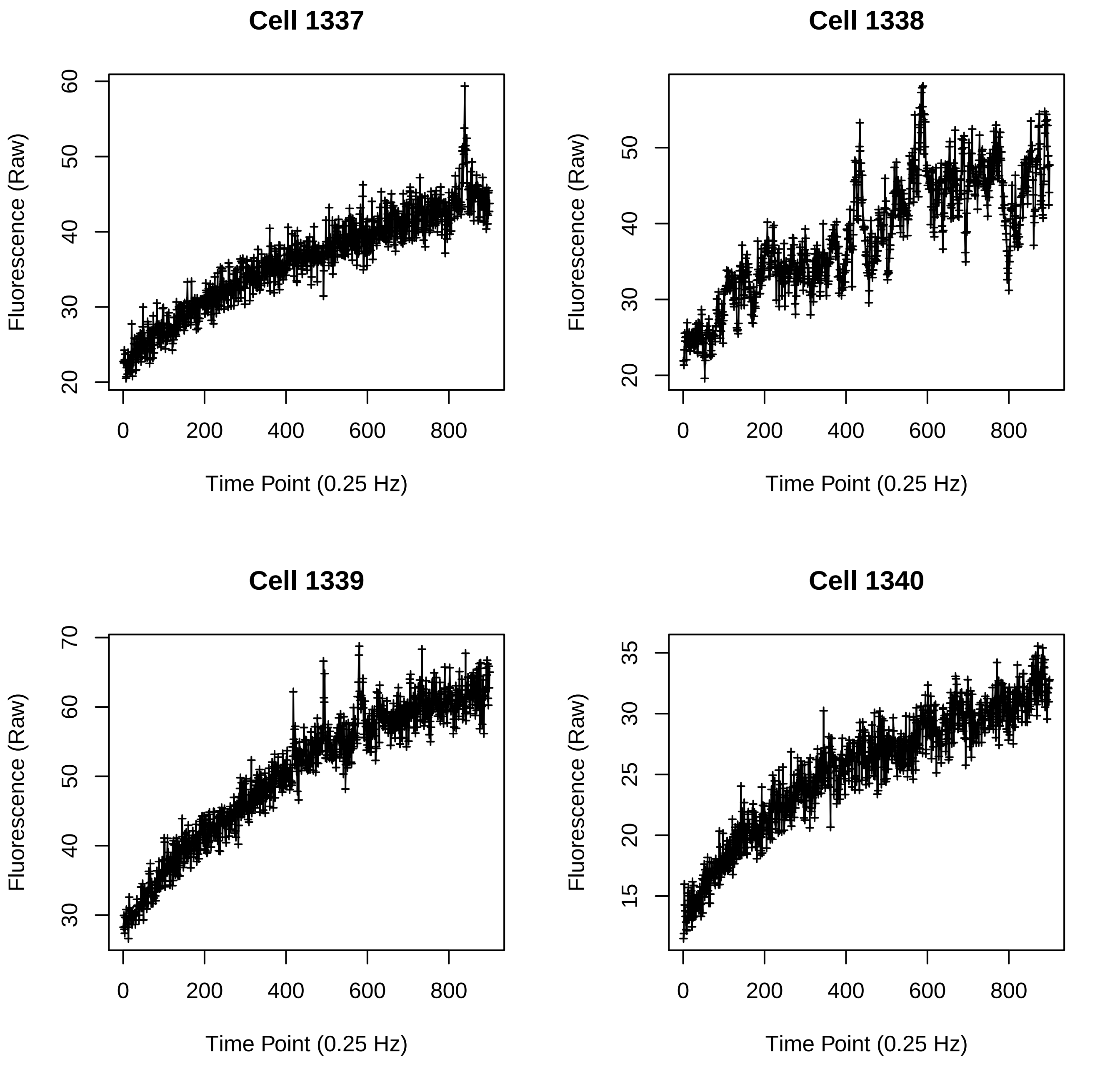
Cell 1337
Cell 1338
Cell 1339
Cell 1340
Fluorescence (Raw)
Time Point (0.25 Hz)
0
200
400
600
800

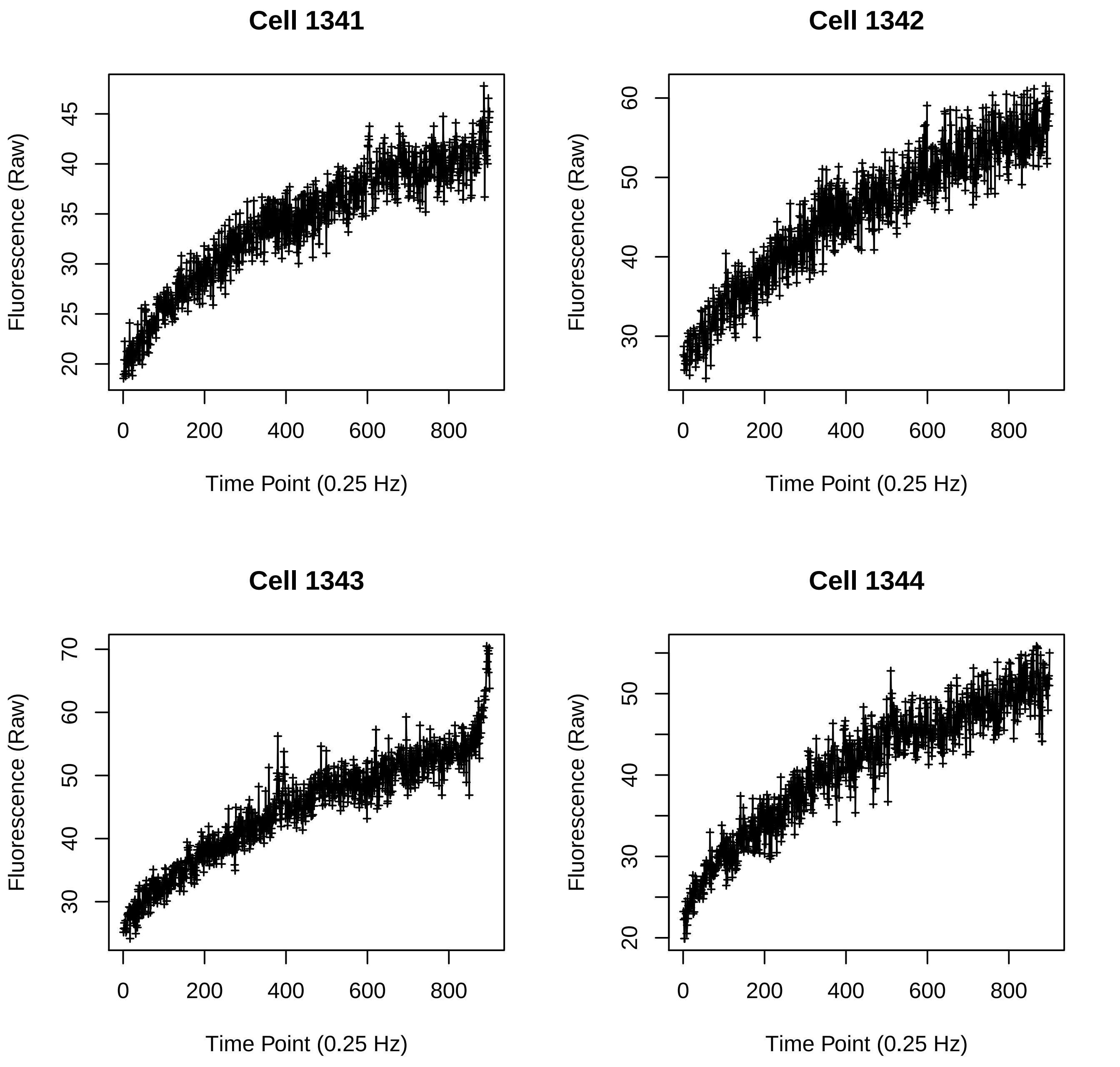
Cell 1341
Cell 1342
Cell 1343
Cell 1344
Fluorescence (Raw)
Time Point (0.25 Hz)
0
200
400
600
800
20
25
30
35
40
45
50
60
70

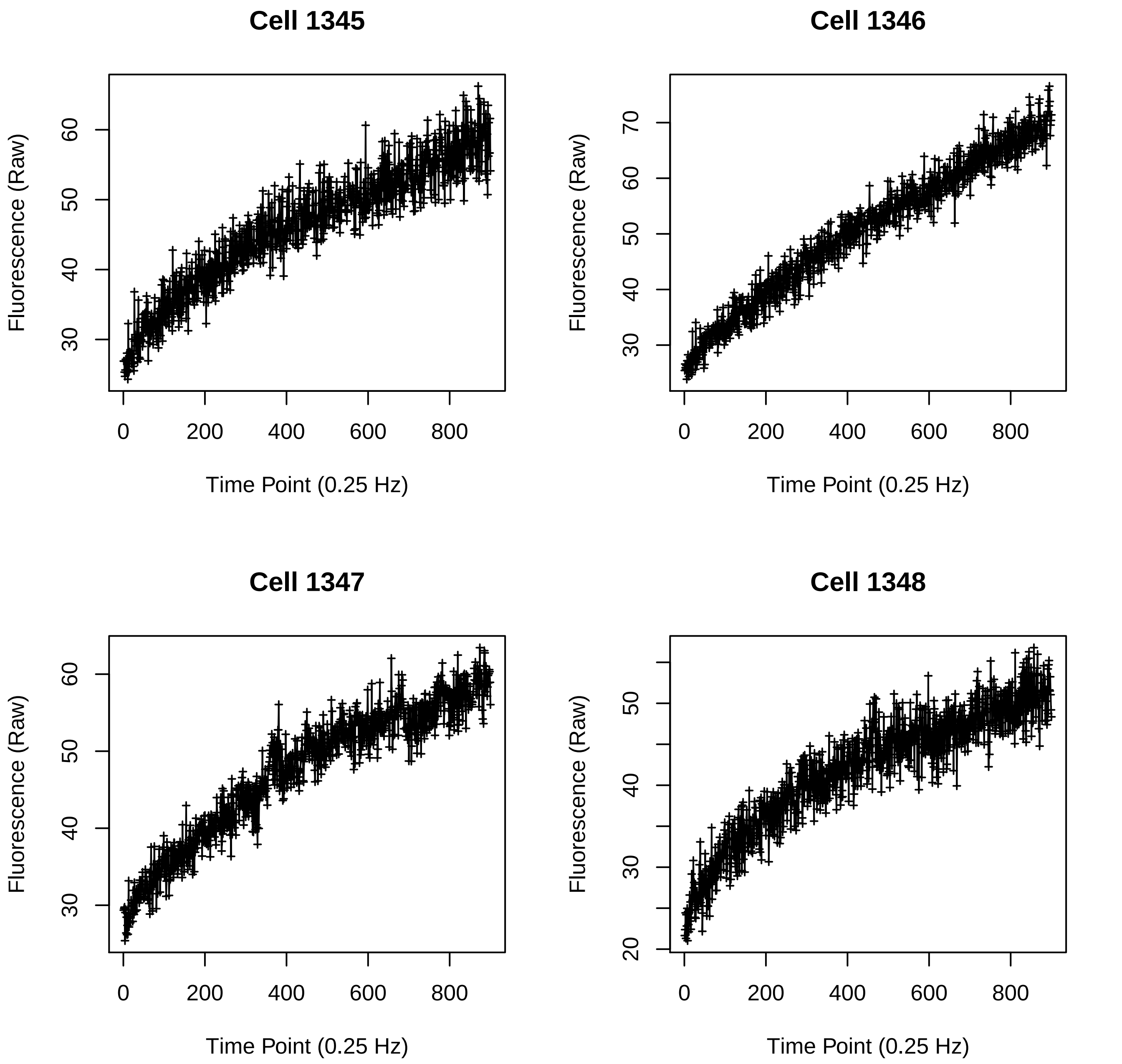

Cell 1345
Fluorescence (Raw)
30
40
50
60
0
200
400
600
800
Time Point (0.25 Hz)
Cell 1346
Fluorescence (Raw)
30
40
50
60
70
0
200
400
600
800
Time Point (0.25 Hz)
Cell 1347
Fluorescence (Raw)
30
40
50
60
0
200
400
600
800
Time Point (0.25 Hz)
Cell 1348
Fluorescence (Raw)
20
30
40
50
0
200
400
600
800
Time Point (0.25 Hz)

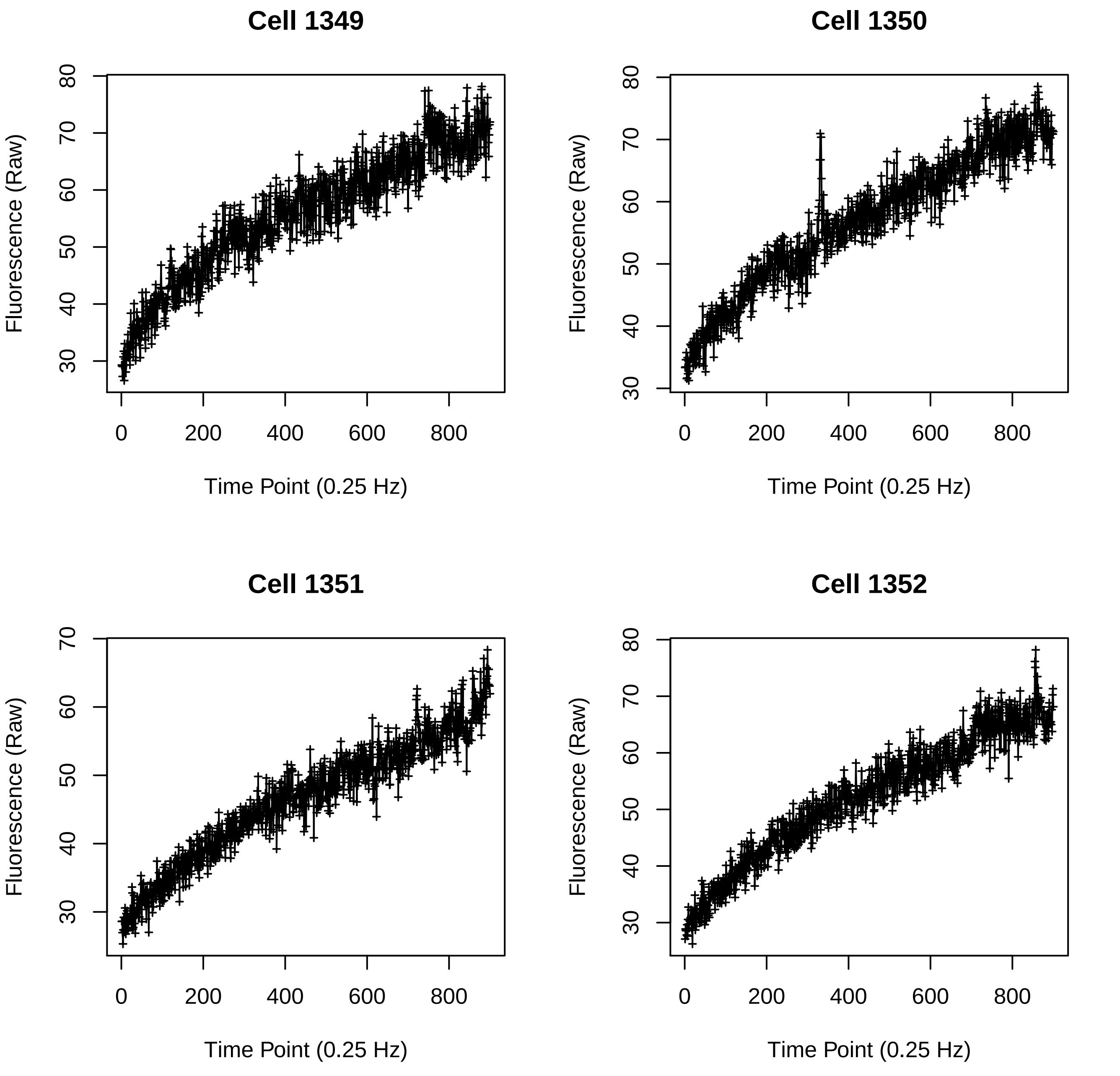

Cell 1349
Cell 1350
Cell 1351
Cell 1352
Fluorescence (Raw)
Time Point (0.25 Hz)
0
200
400
600
800
30
40
50
60
70
80

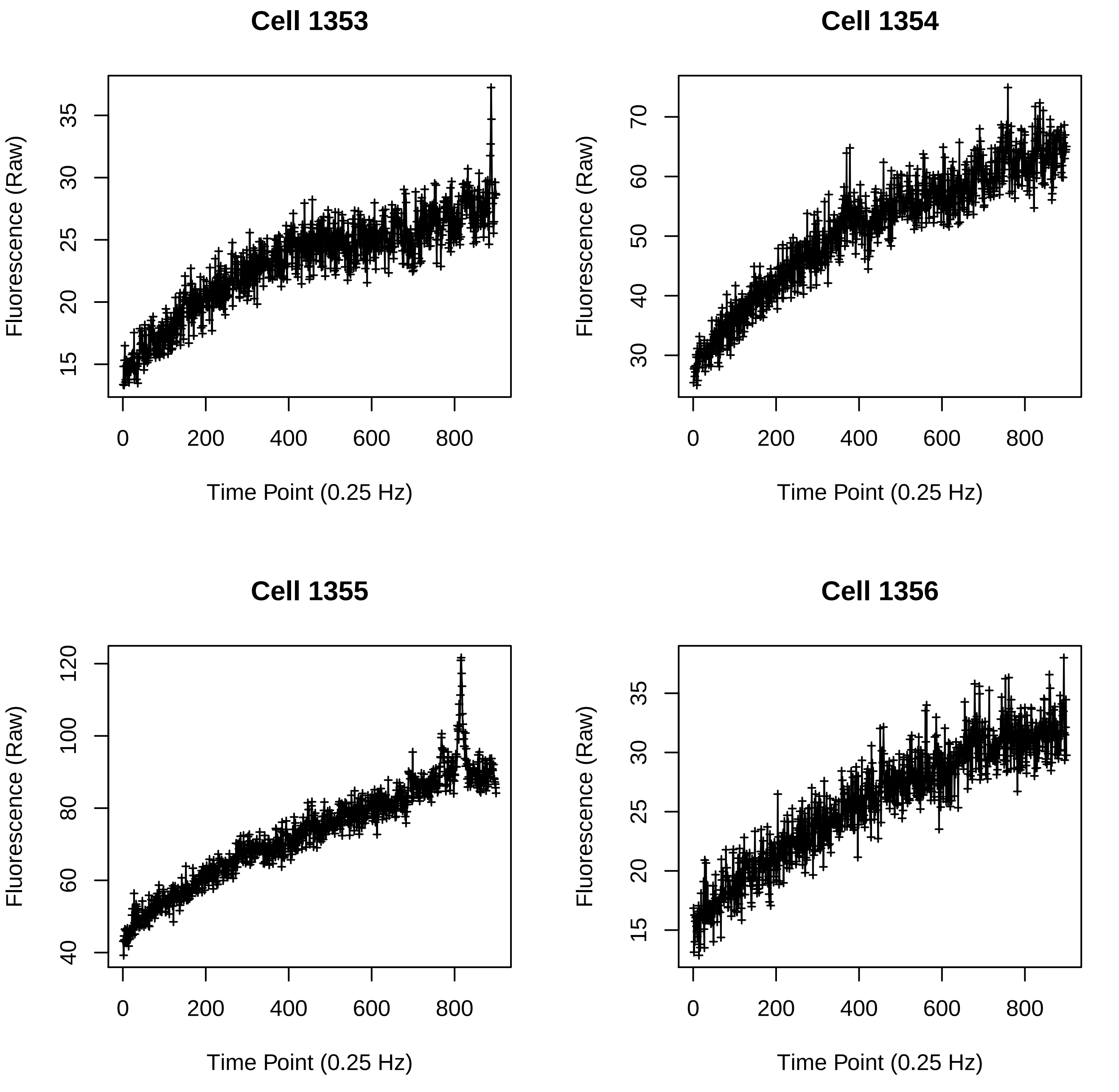
Cell 1353
Fluorescence (Raw)
15
20
25
30
35
0
200
400
600
800
Time Point (0.25 Hz)
Cell 1354
Fluorescence (Raw)
30
40
50
60
70
0
200
400
600
800
Time Point (0.25 Hz)
Cell 1355
Fluorescence (Raw)
40
60
80
100
120
0
200
400
600
800
Time Point (0.25 Hz)
Cell 1356
Fluorescence (Raw)
15
20
25
30
35
0
200
400
600
800
Time Point (0.25 Hz)

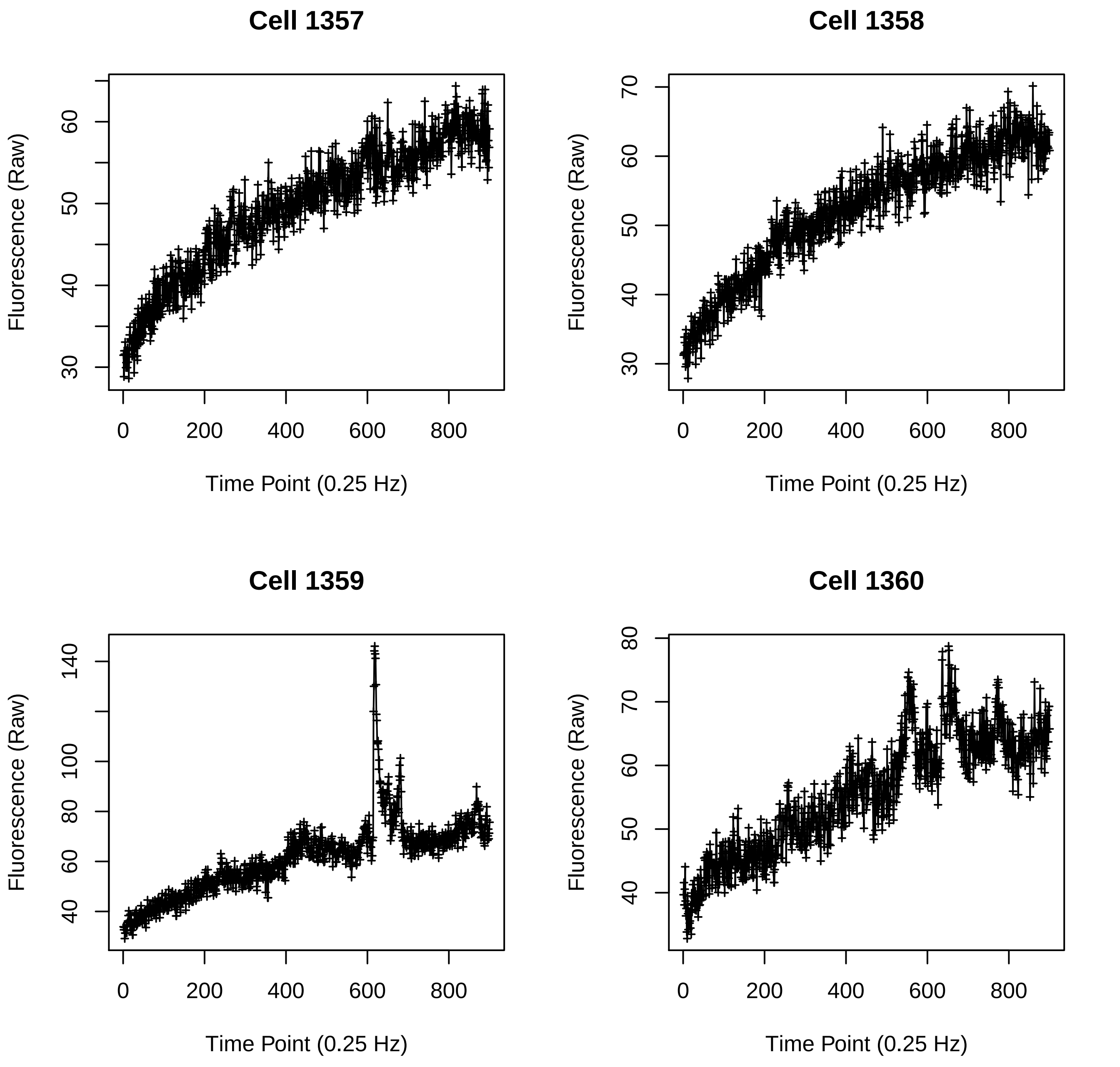
Cell 1357
Cell 1358
Cell 1359
Cell 1360
Fluorescence (Raw)
Time Point (0.25 Hz)
0
200
400
600
800
30
40
50
60
70
80
100
140

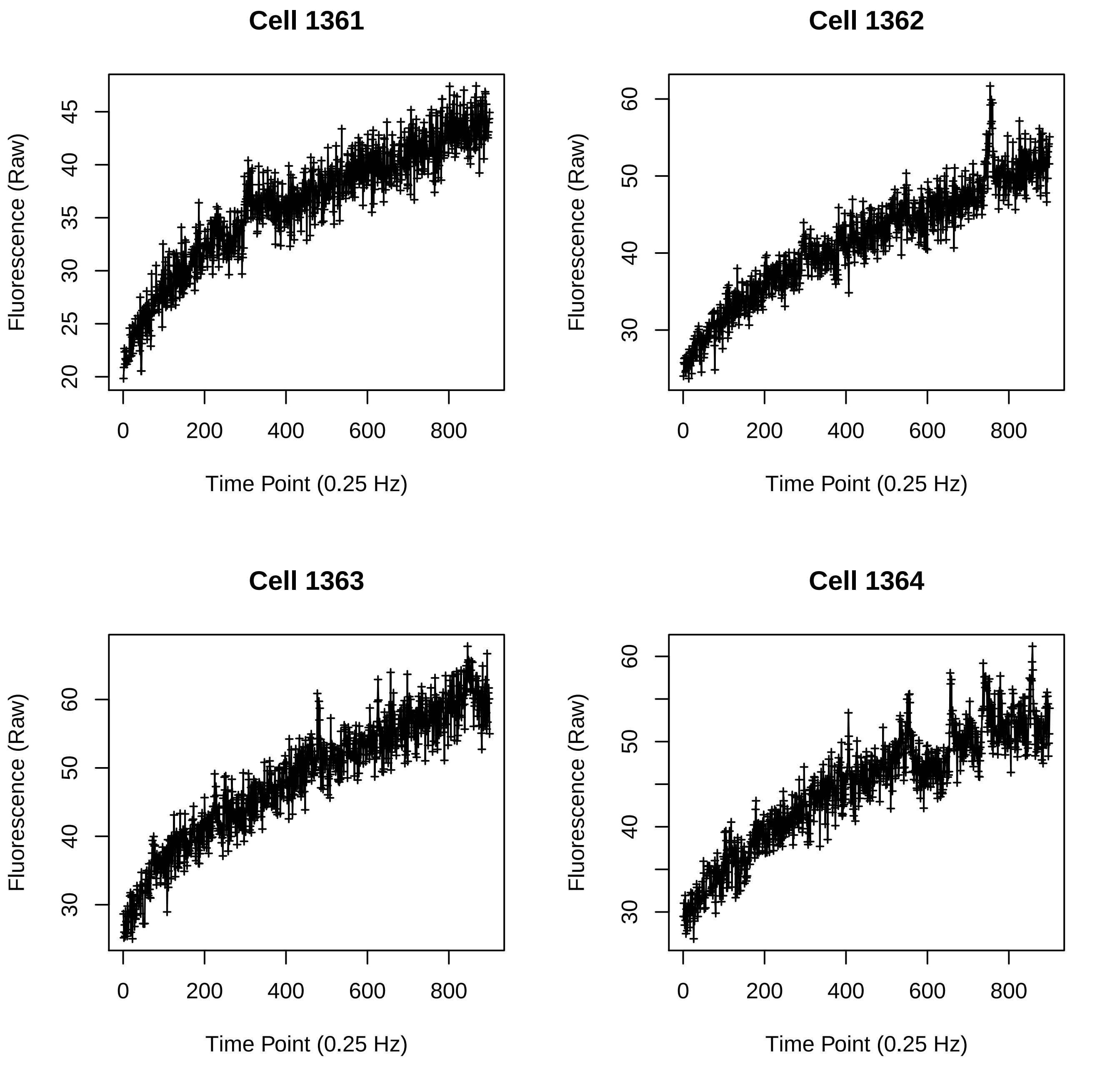

Cell 1361
Fluorescence (Raw)
20
25
30
35
40
45
0
200
400
600
800
Time Point (0.25 Hz)
Cell 1362
Fluorescence (Raw)
30
40
50
60
0
200
400
600
800
Time Point (0.25 Hz)
Cell 1363
Fluorescence (Raw)
30
40
50
60
0
200
400
600
800
Time Point (0.25 Hz)
Cell 1364
Fluorescence (Raw)
30
40
50
60
0
200
400
600
800
Time Point (0.25 Hz)

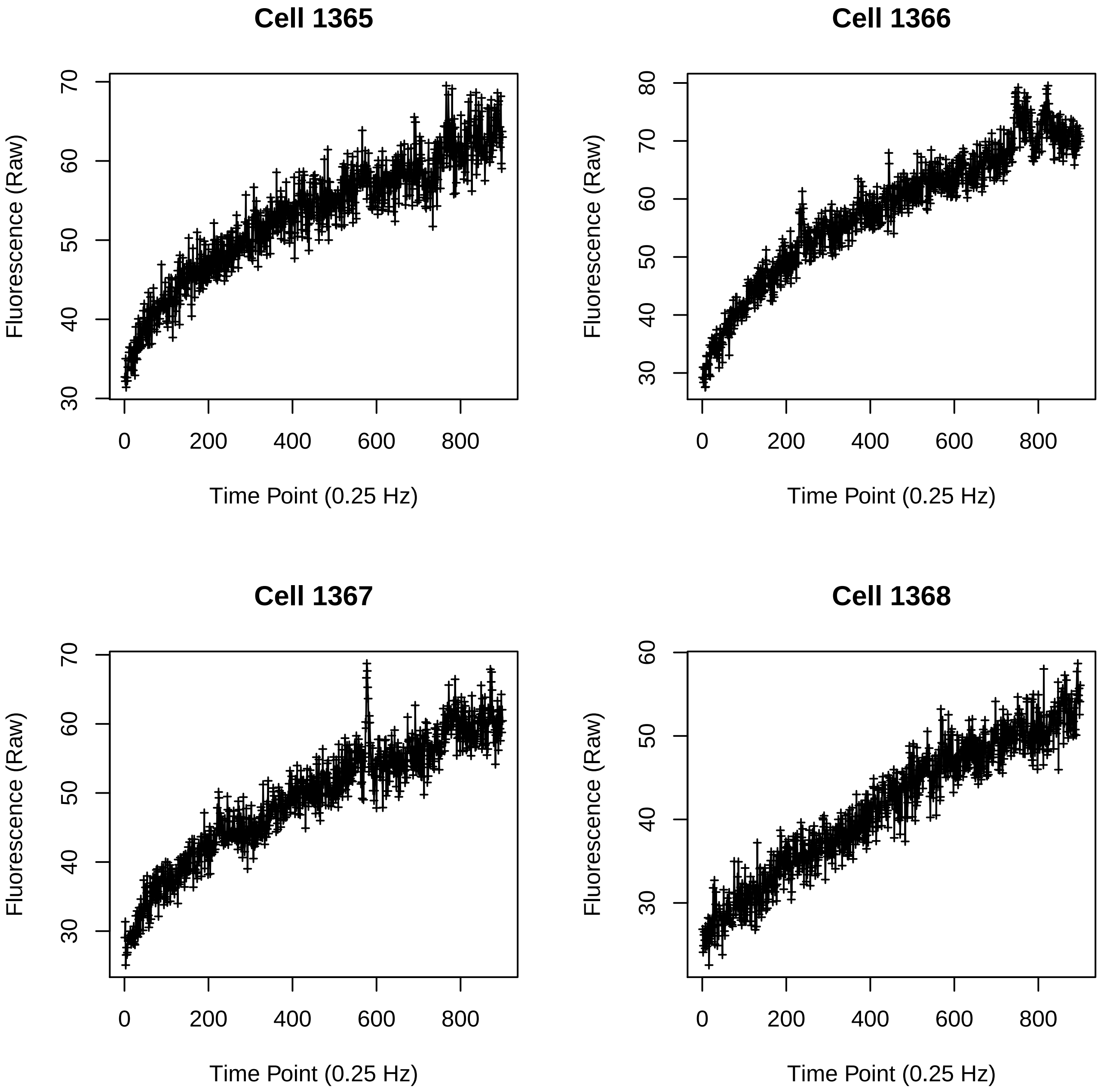
Cell 1365
Fluorescence (Raw)
30
40
50
60
70
0
200
400
600
800
Time Point (0.25 Hz)
Cell 1366
Fluorescence (Raw)
30
40
50
60
70
80
0
200
400
600
800
Time Point (0.25 Hz)
Cell 1367
Fluorescence (Raw)
30
40
50
60
70
0
200
400
600
800
Time Point (0.25 Hz)
Cell 1368
Fluorescence (Raw)
30
40
50
60
0
200
400
600
800
Time Point (0.25 Hz)

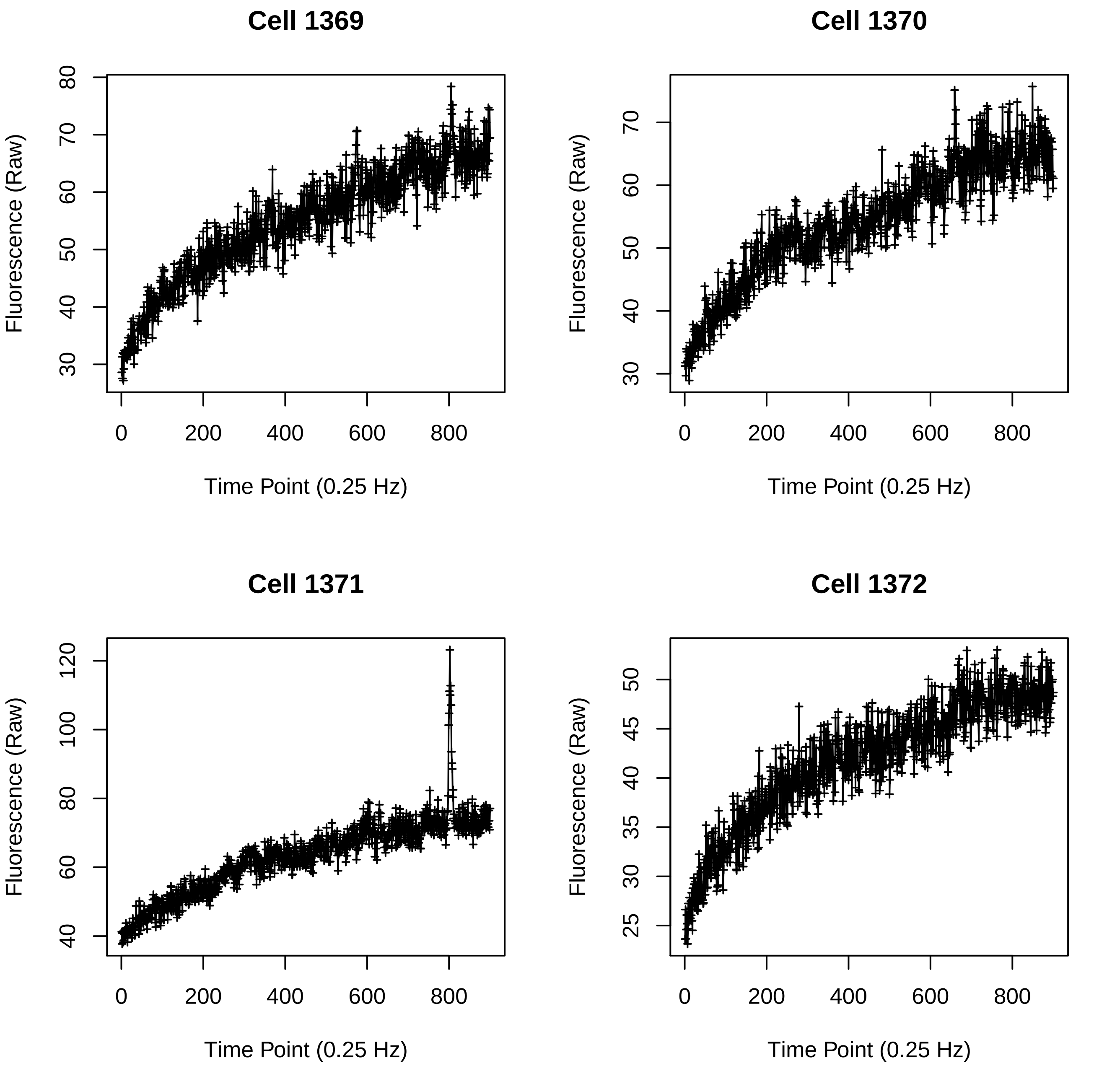

Cell 1369
Fluorescence (Raw)
Time Point (0.25 Hz)
30
40
50
60
70
80
0
200
400
600
800
Cell 1370
Fluorescence (Raw)
Time Point (0.25 Hz)
30
40
50
60
70
0
200
400
600
800
Cell 1371
Fluorescence (Raw)
Time Point (0.25 Hz)
40
60
80
100
120
0
200
400
600
800
Cell 1372
Fluorescence (Raw)
Time Point (0.25 Hz)
25
30
35
40
45
50
0
200
400
600
800

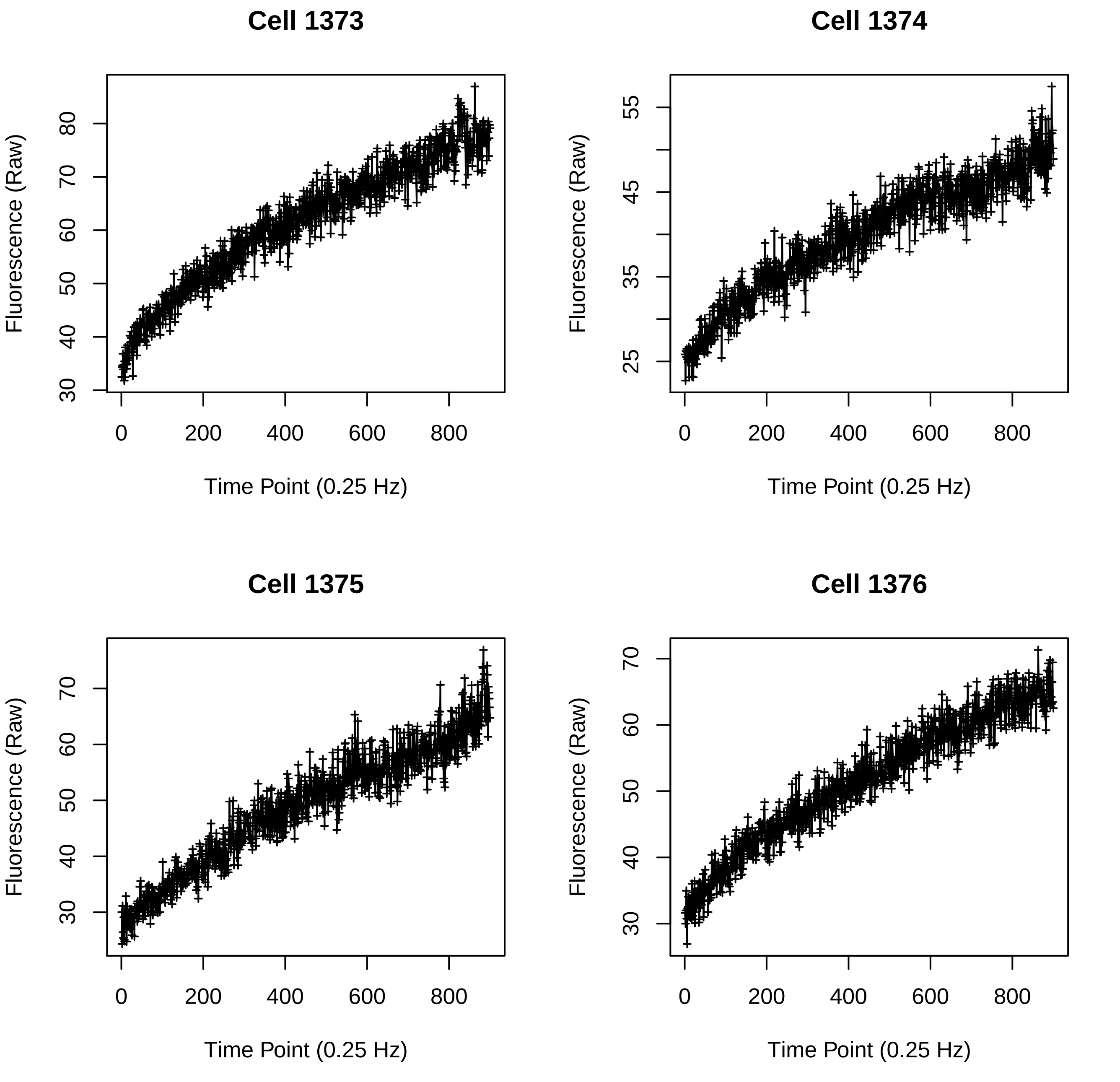
Cell 1373
Fluorescence (Raw)
30
40
50
60
70
80
0
200
400
600
800
Time Point (0.25 Hz)
Cell 1374
Fluorescence (Raw)
25
35
45
55
0
200
400
600
800
Time Point (0.25 Hz)
Cell 1375
Fluorescence (Raw)
30
40
50
60
70
0
200
400
600
800
Time Point (0.25 Hz)
Cell 1376
Fluorescence (Raw)
30
40
50
60
70
0
200
400
600
800
Time Point (0.25 Hz)

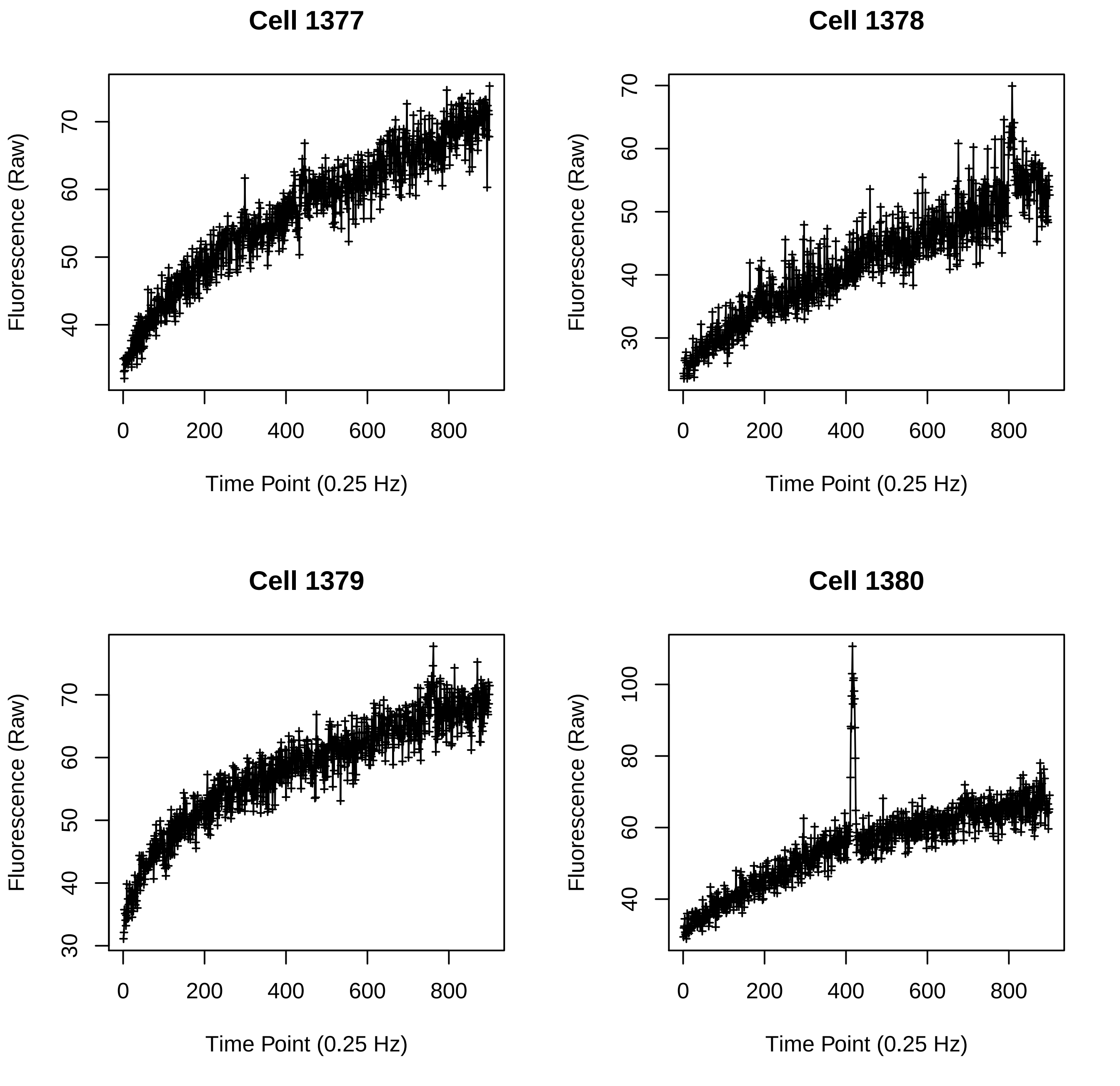
Cell 1377
Cell 1378
Cell 1379
Cell 1380
Fluorescence (Raw)
Time Point (0.25 Hz)
0
200
400
600
800
30
40
50
60
70
80
100

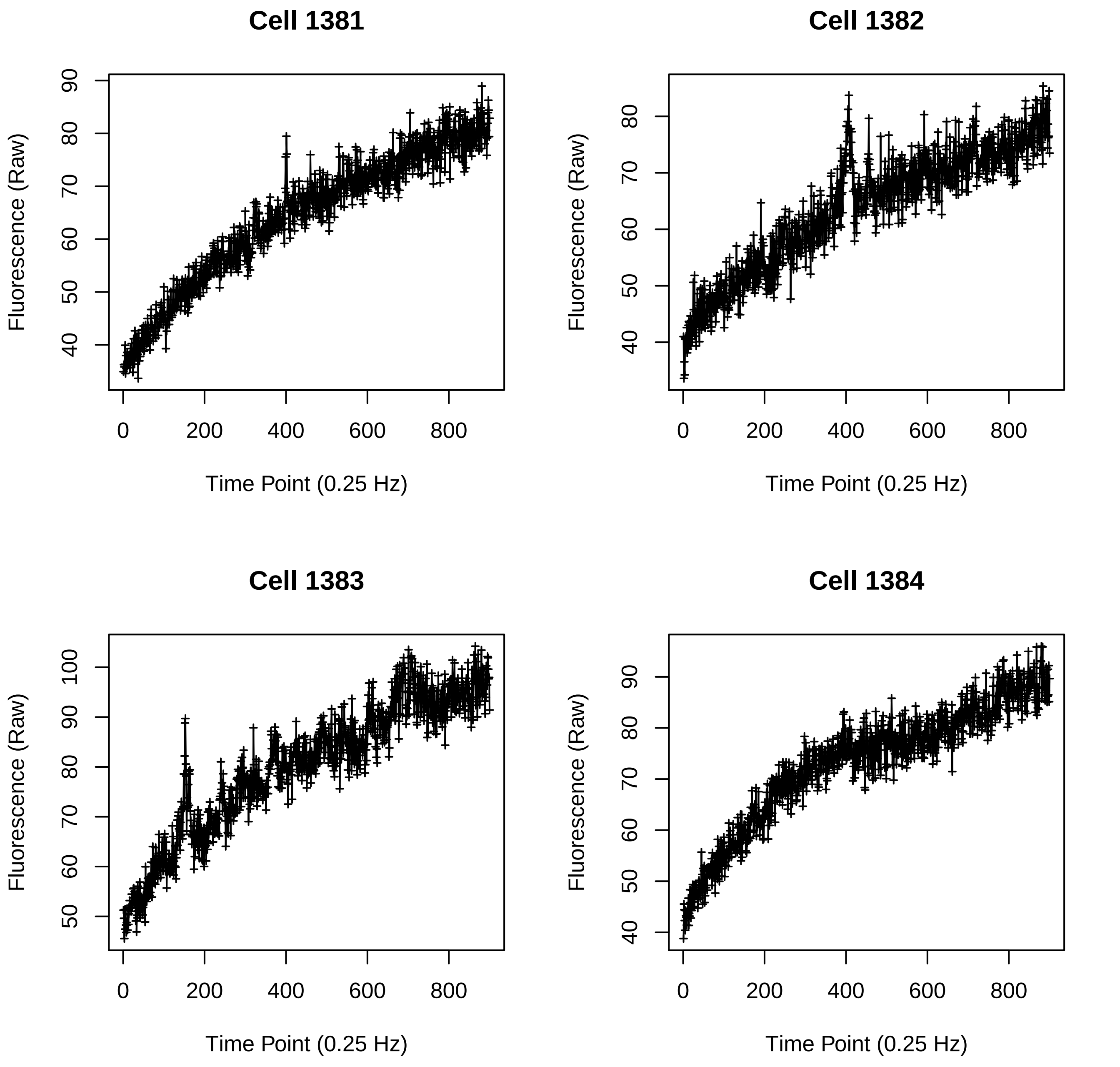
Cell 1381
Cell 1382
Cell 1383
Cell 1384
Fluorescence (Raw)
Time Point (0.25 Hz)
0
200
400
600
800
40
50
60
70
80
90
100

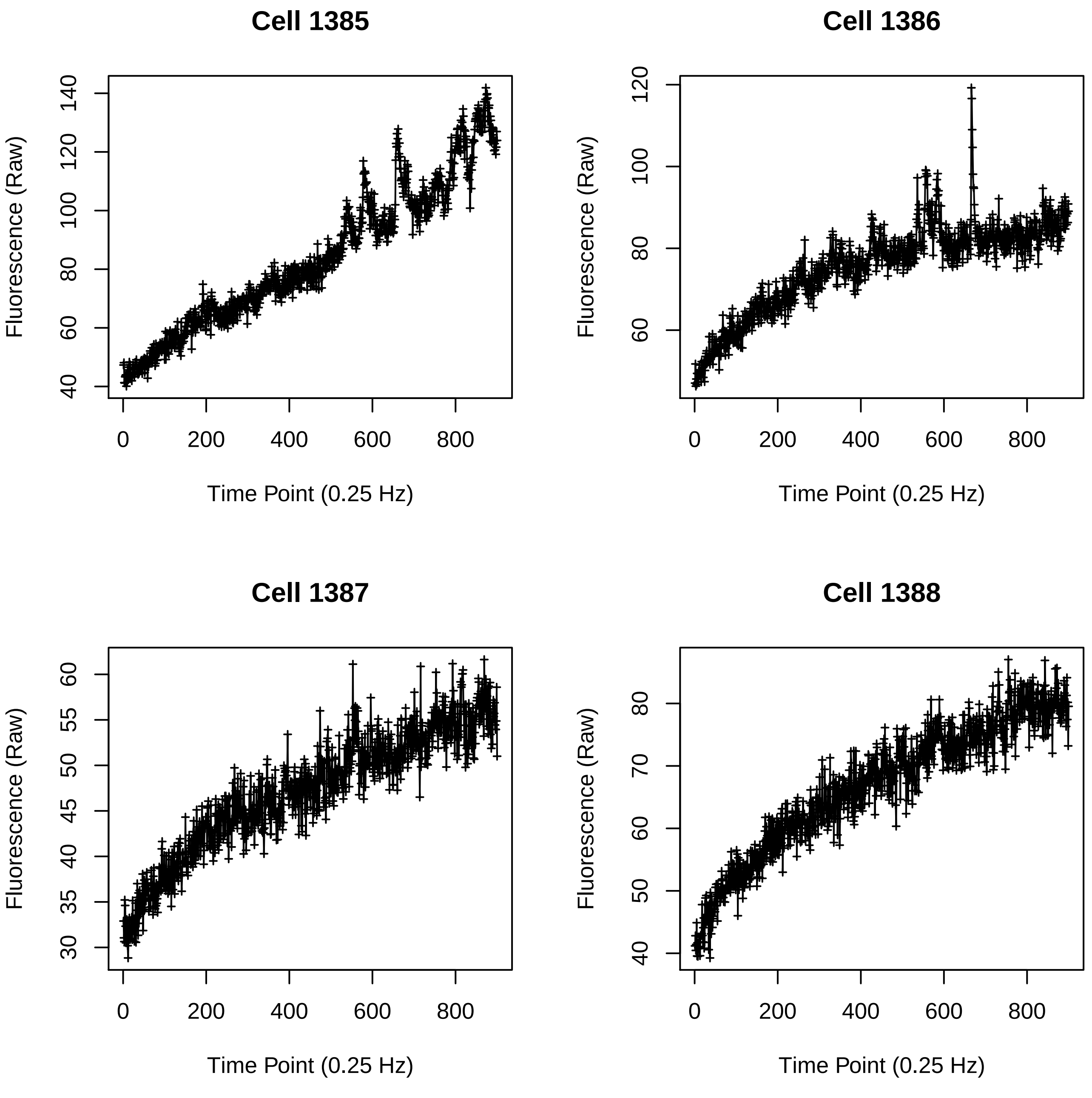
Cell 1385
Fluorescence (Raw)
40
60
80
100
120
140
0
200
400
600
800
Time Point (0.25 Hz)
Cell 1386
Fluorescence (Raw)
60
80
100
120
0
200
400
600
800
Time Point (0.25 Hz)
Cell 1387
Fluorescence (Raw)
30
35
40
45
50
55
60
0
200
400
600
800
Time Point (0.25 Hz)
Cell 1388
Fluorescence (Raw)
40
50
60
70
80
0
200
400
600
800
Time Point (0.25 Hz)

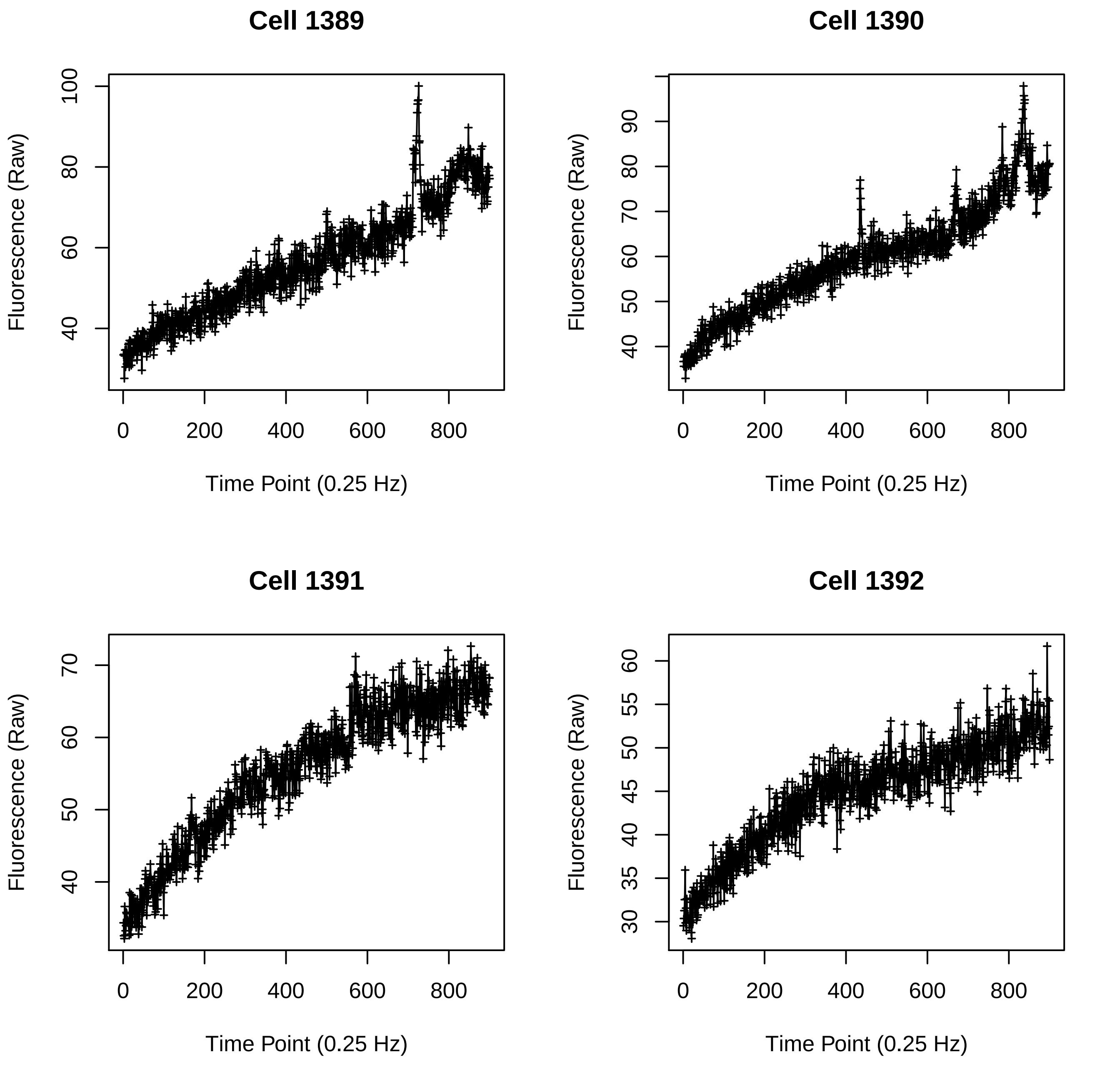
Cell 1389
Fluorescence (Raw)
40
60
80
100
0
200
400
600
800
Time Point (0.25 Hz)
Cell 1390
Fluorescence (Raw)
40
50
60
70
80
90
0
200
400
600
800
Time Point (0.25 Hz)
Cell 1391
Fluorescence (Raw)
40
50
60
70
0
200
400
600
800
Time Point (0.25 Hz)
Cell 1392
Fluorescence (Raw)
30
35
40
45
50
55
60
0
200
400
600
800
Time Point (0.25 Hz)

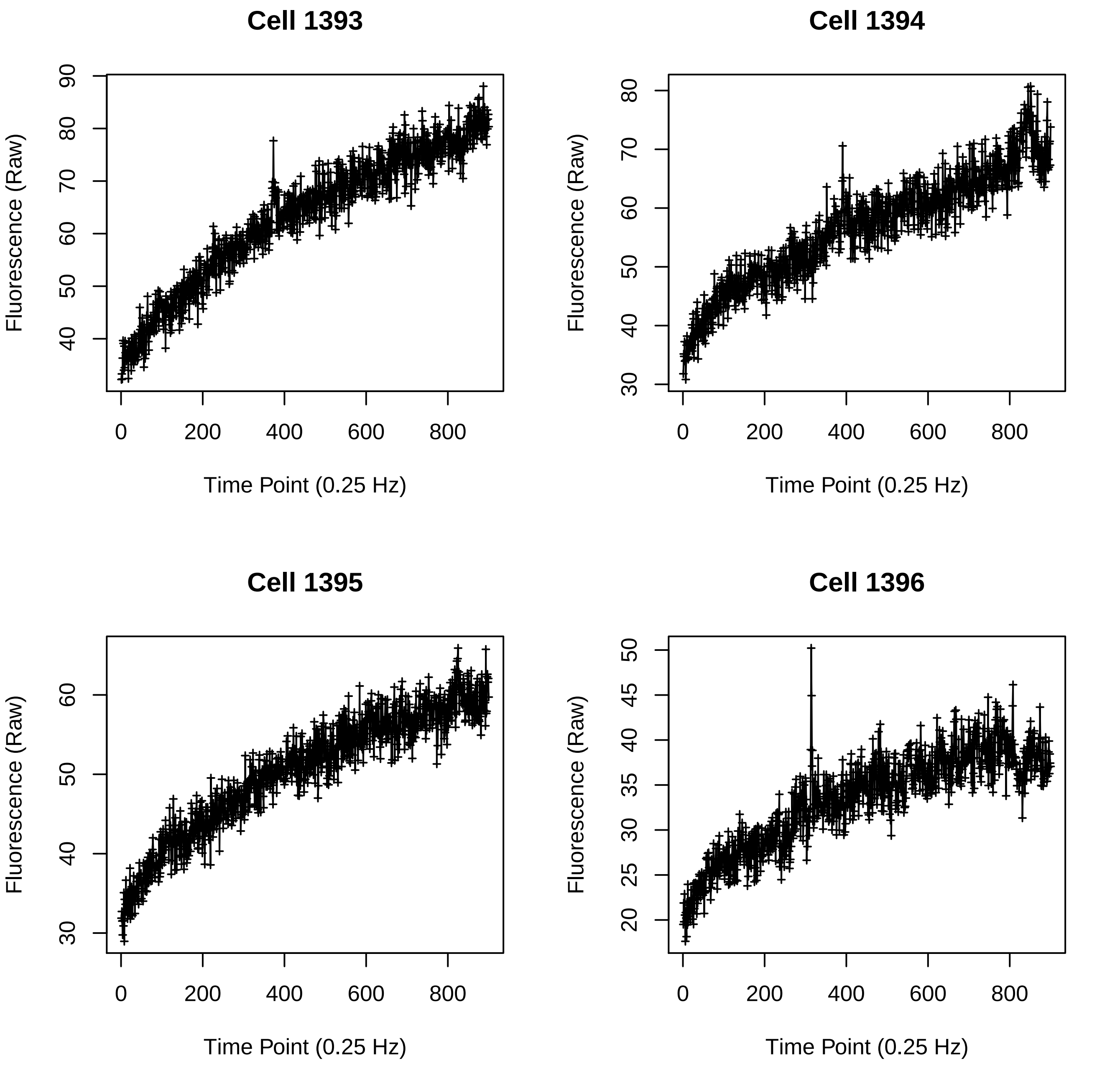
Cell 1393
Cell 1394
Cell 1395
Cell 1396
Fluorescence (Raw)
Time Point (0.25 Hz)
0
200
400
600
800
30
40
50
60
70
80
90
20
25
35
45

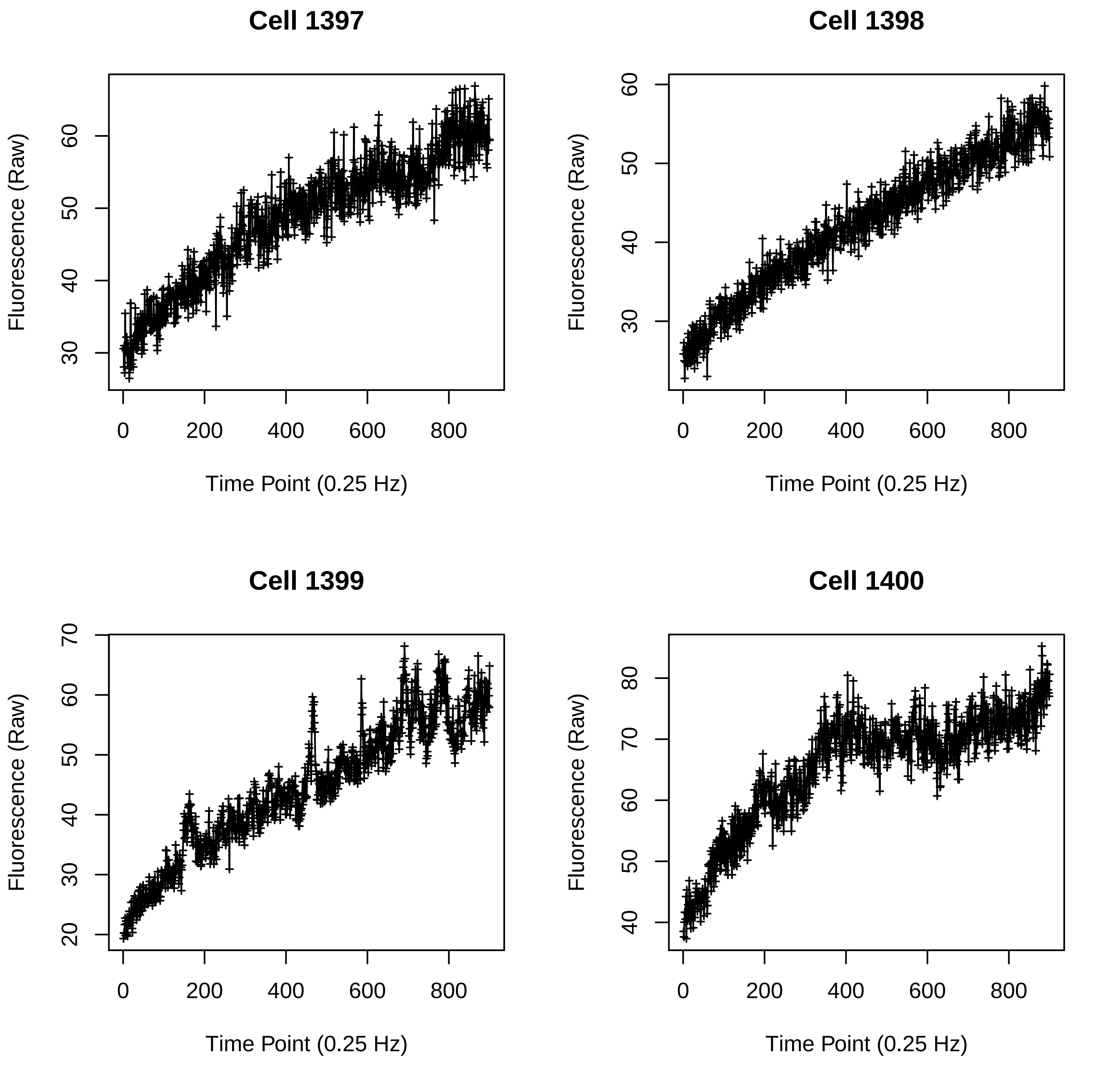

Cell 1397
Cell 1398
Cell 1399
Cell 1400
Fluorescence (Raw)
Time Point (0.25 Hz)
0
200
400
600
800
30
40
50
60
20
70
80

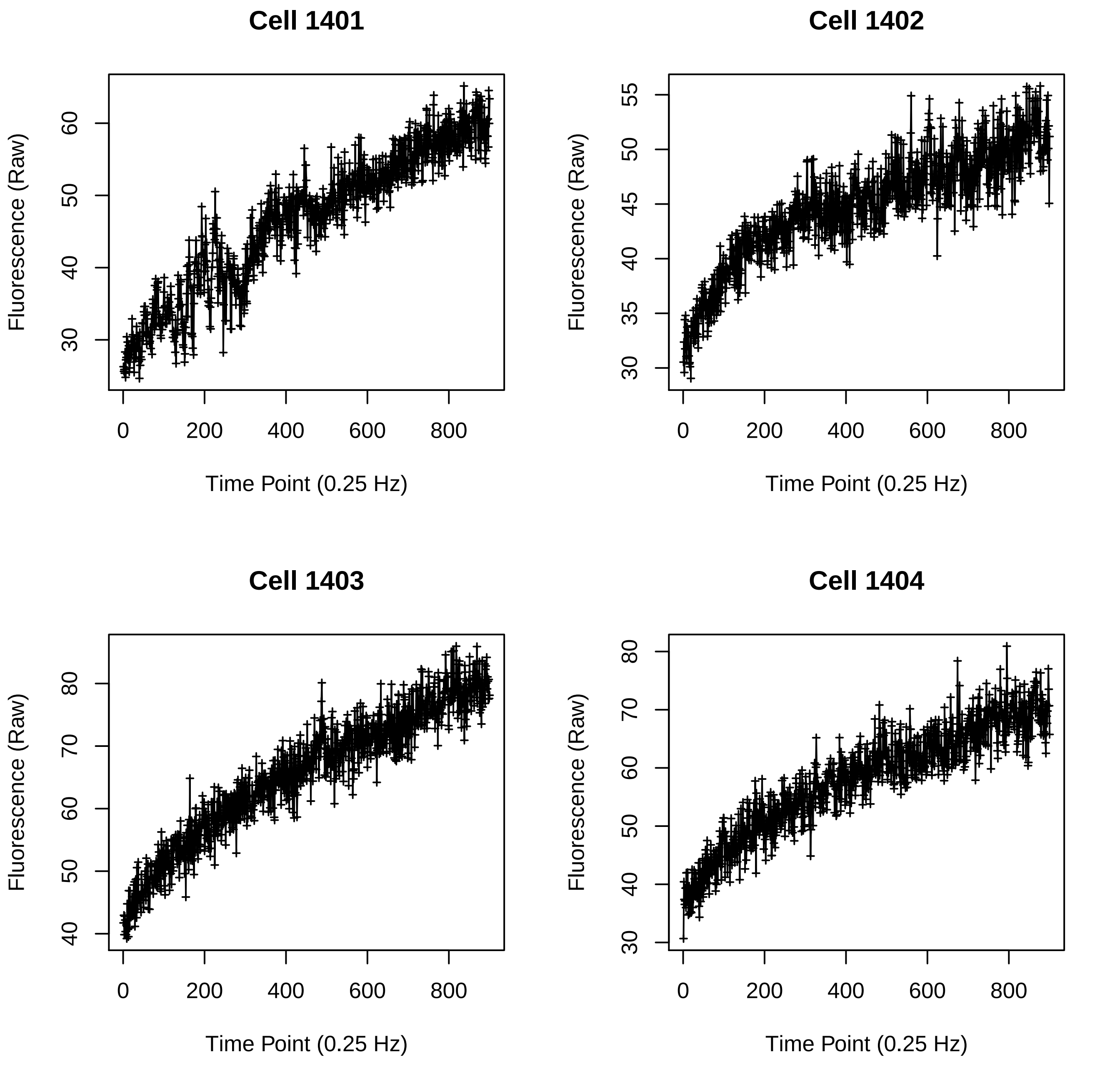
Cell 1401
Cell 1402
Cell 1403
Cell 1404
Fluorescence (Raw)
Time Point (0.25 Hz)
0
200
400
600
800
30
40
50
60
35
45
55
70
80

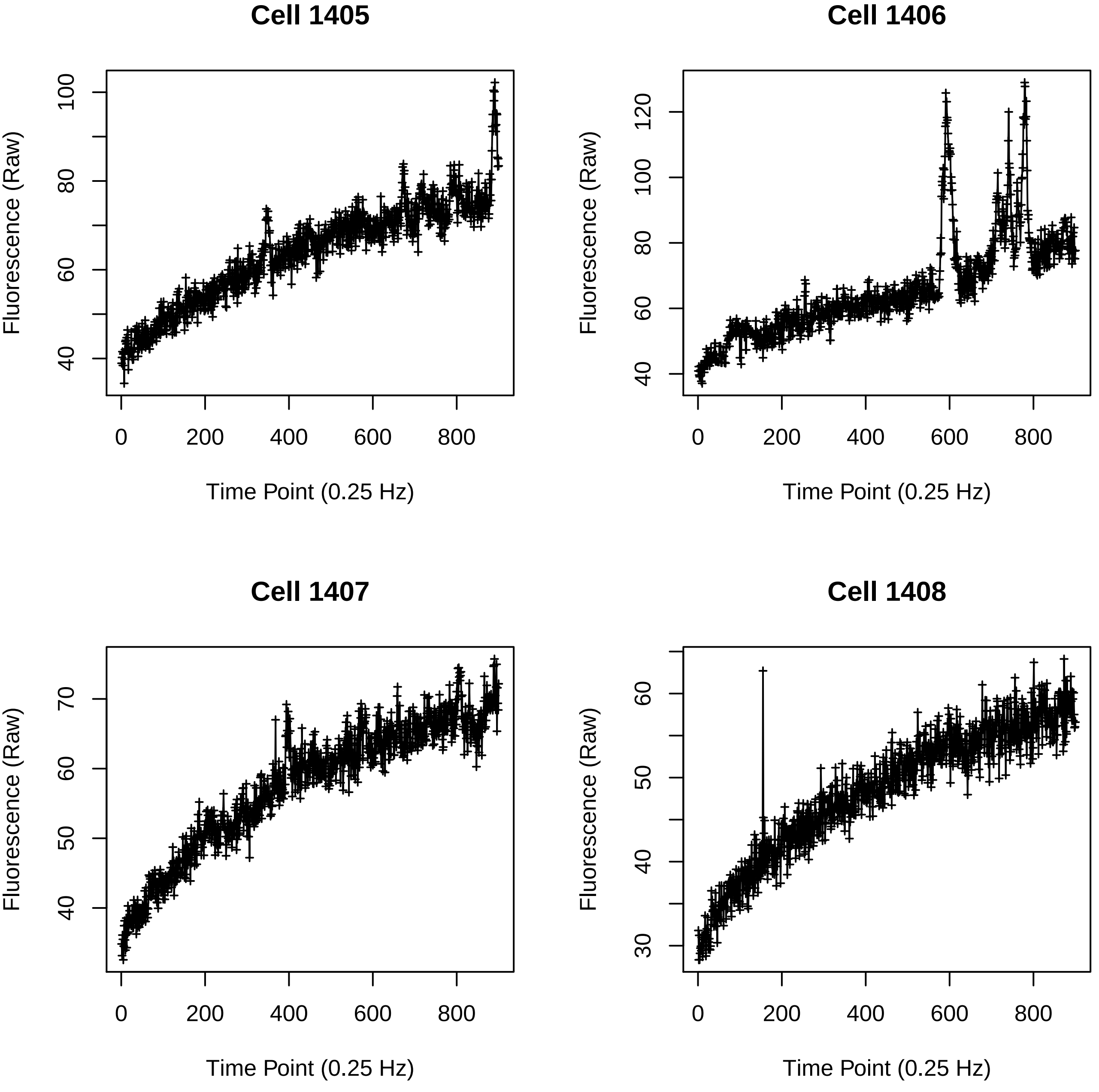

Cell 1405
Cell 1406
Cell 1407
Cell 1408
Fluorescence (Raw)
Time Point (0.25 Hz)
0
200
400
600
800
40
60
80
100
120
30
50
70

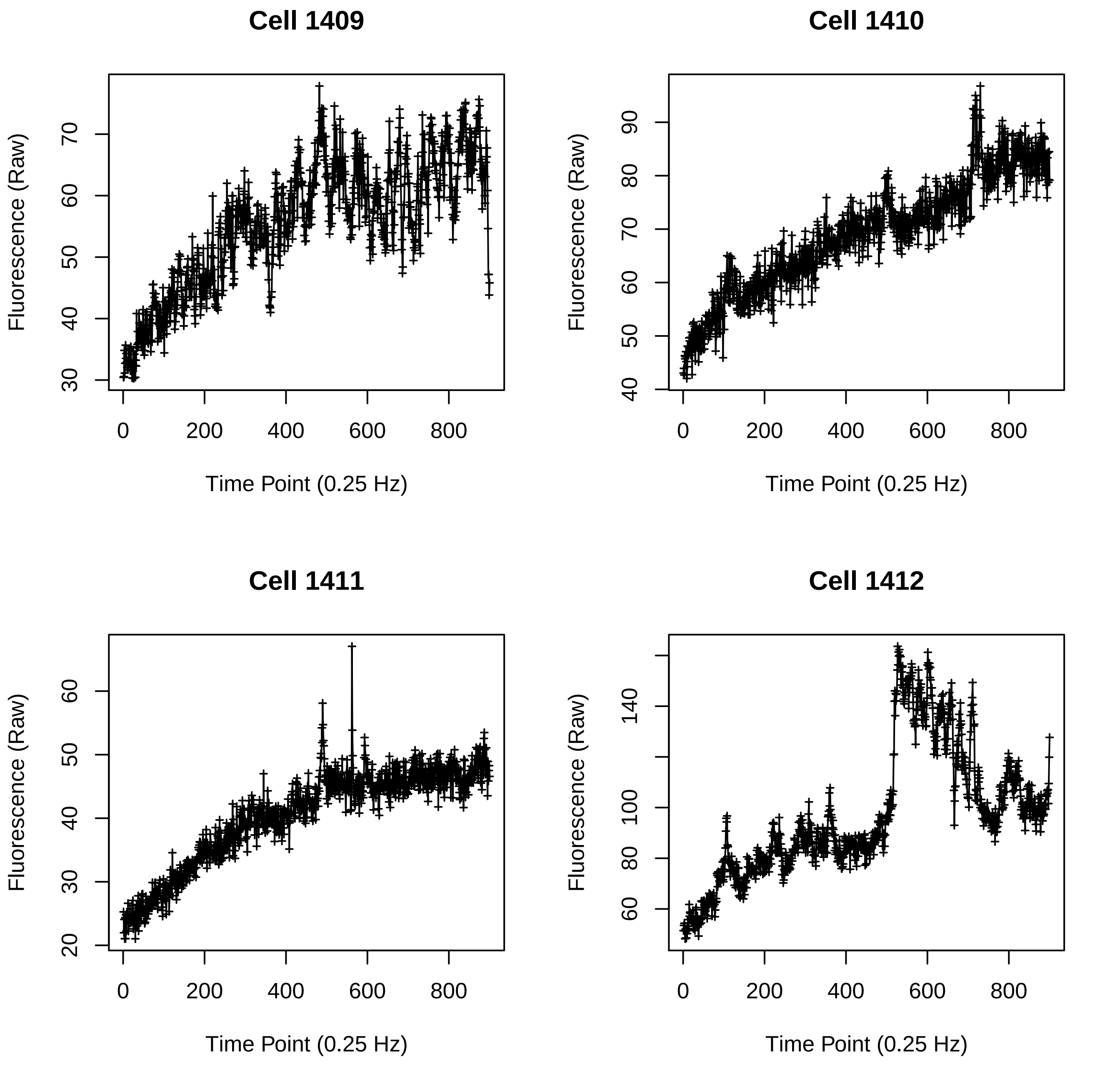

Cell 1409
Fluorescence (Raw)
30
40
50
60
70
0
200
400
600
800
Time Point (0.25 Hz)
Cell 1410
Fluorescence (Raw)
40
50
60
70
80
90
0
200
400
600
800
Time Point (0.25 Hz)
Cell 1411
Fluorescence (Raw)
20
30
40
50
60
0
200
400
600
800
Time Point (0.25 Hz)
Cell 1412
Fluorescence (Raw)
60
80
100
140
0
200
400
600
800
Time Point (0.25 Hz)

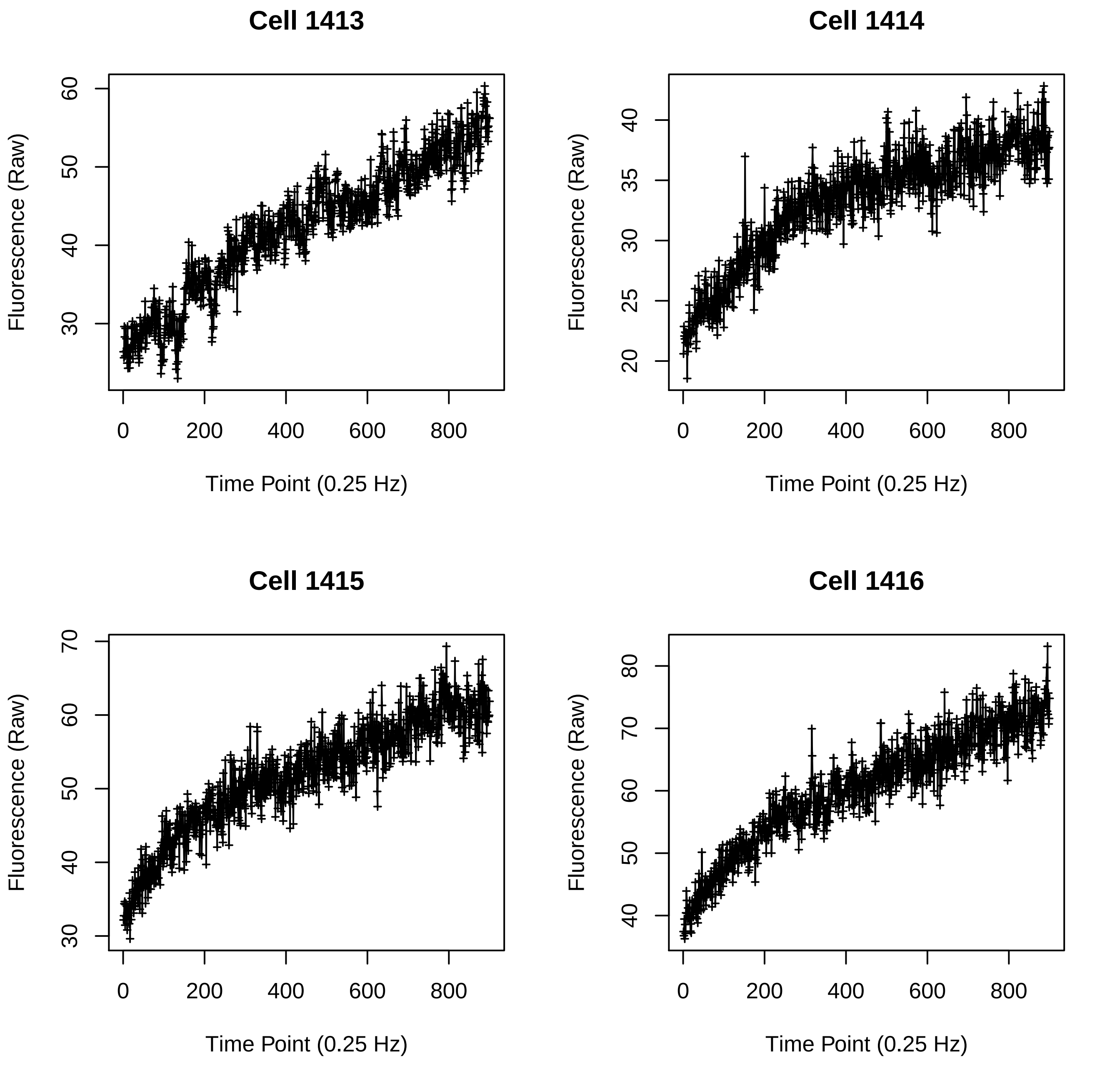

Cell 1413
Fluorescence (Raw)
30
40
50
60
0
200
400
600
800
Time Point (0.25 Hz)
Cell 1414
Fluorescence (Raw)
20
25
30
35
40
0
200
400
600
800
Time Point (0.25 Hz)
Cell 1415
Fluorescence (Raw)
30
40
50
60
70
0
200
400
600
800
Time Point (0.25 Hz)
Cell 1416
Fluorescence (Raw)
40
50
60
70
80
0
200
400
600
800
Time Point (0.25 Hz)

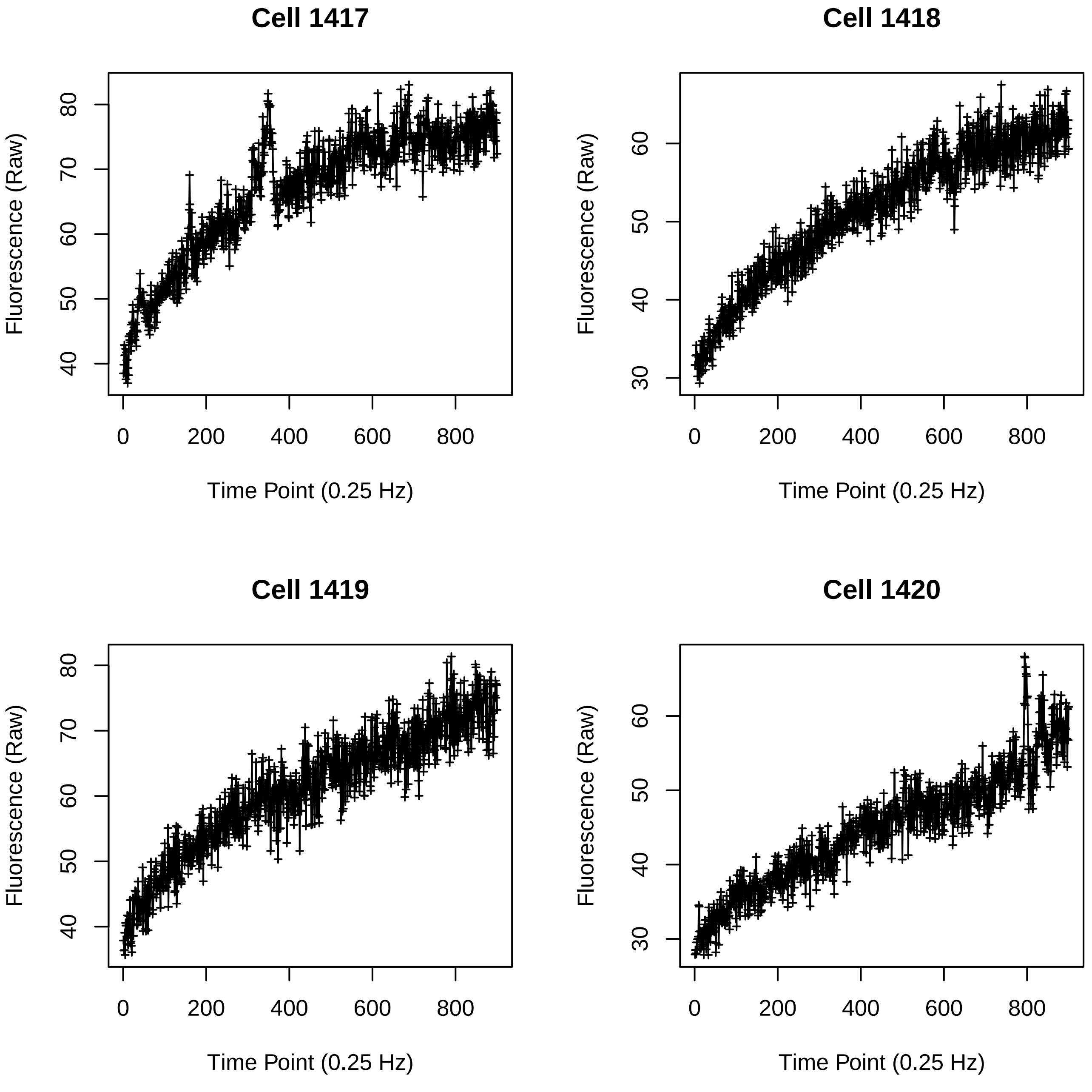
Cell 1417
Fluorescence (Raw)
40
50
60
70
80
0
200
400
600
800
Time Point (0.25 Hz)
Cell 1418
Fluorescence (Raw)
30
40
50
60
0
200
400
600
800
Time Point (0.25 Hz)
Cell 1419
Fluorescence (Raw)
40
50
60
70
80
0
200
400
600
800
Time Point (0.25 Hz)
Cell 1420
Fluorescence (Raw)
30
40
50
60
0
200
400
600
800
Time Point (0.25 Hz)

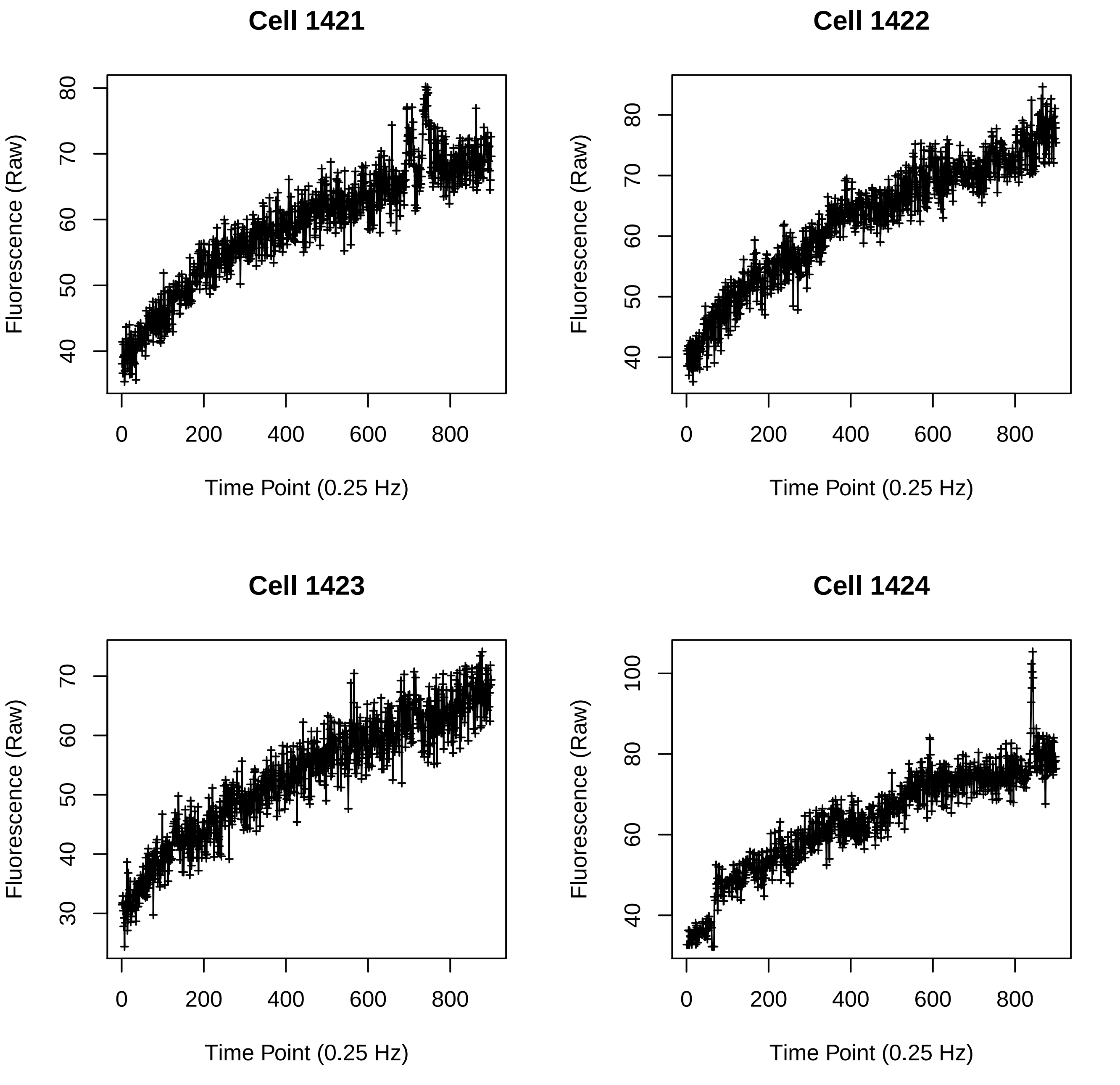
Cell 1421
Fluorescence (Raw)
40
50
60
70
80
0
200
400
600
800
Time Point (0.25 Hz)
Cell 1422
Fluorescence (Raw)
40
50
60
70
80
0
200
400
600
800
Time Point (0.25 Hz)
Cell 1423
Fluorescence (Raw)
30
40
50
60
70
0
200
400
600
800
Time Point (0.25 Hz)
Cell 1424
Fluorescence (Raw)
40
60
80
100
0
200
400
600
800
Time Point (0.25 Hz)

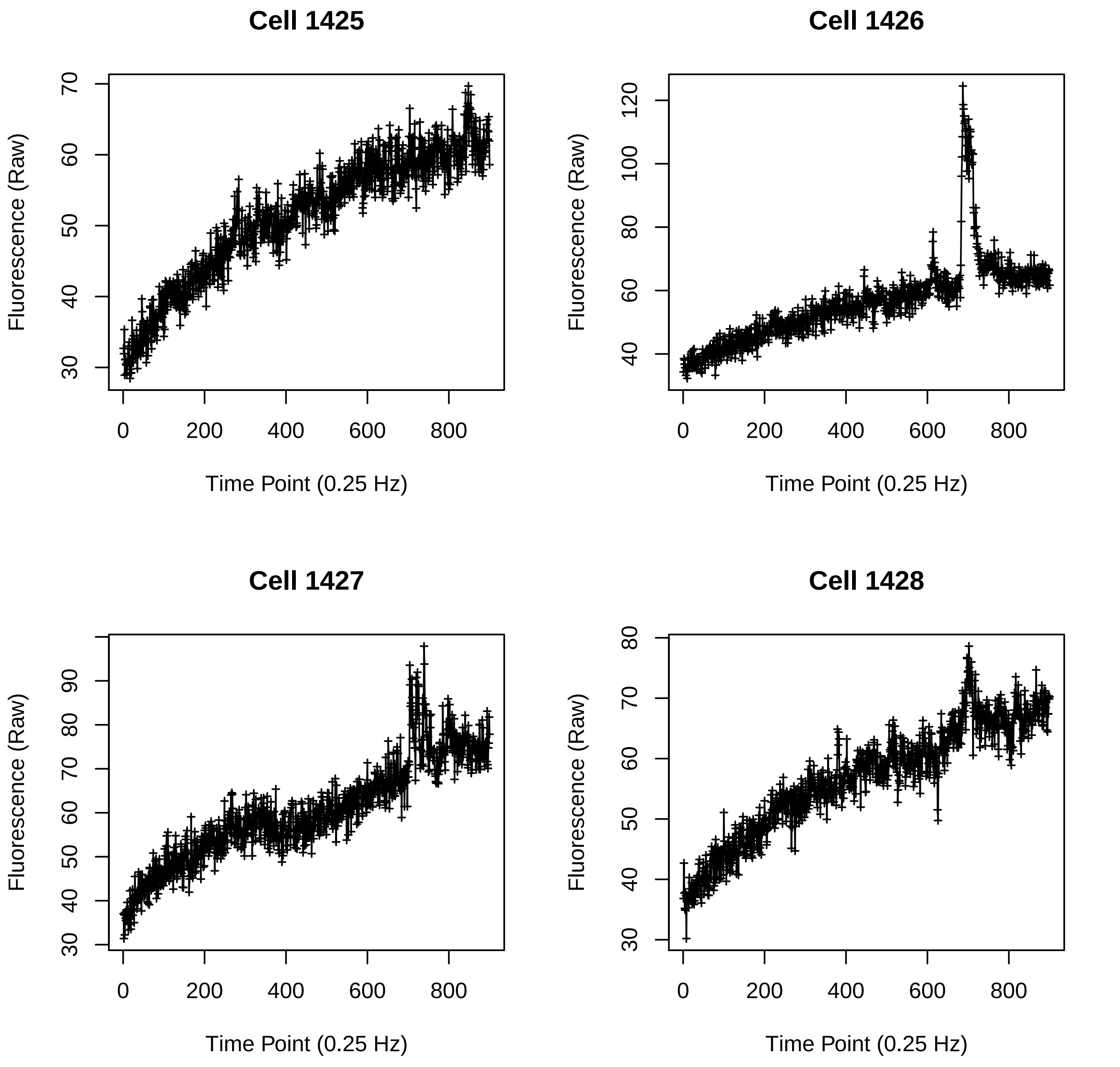
Cell 1425
Fluorescence (Raw)
30
40
50
60
70
0
200
400
600
800
Time Point (0.25 Hz)
Cell 1426
Fluorescence (Raw)
40
60
80
100
120
0
200
400
600
800
Time Point (0.25 Hz)
Cell 1427
Fluorescence (Raw)
30
40
50
60
70
80
90
0
200
400
600
800
Time Point (0.25 Hz)
Cell 1428
Fluorescence (Raw)
30
40
50
60
70
80
0
200
400
600
800
Time Point (0.25 Hz)

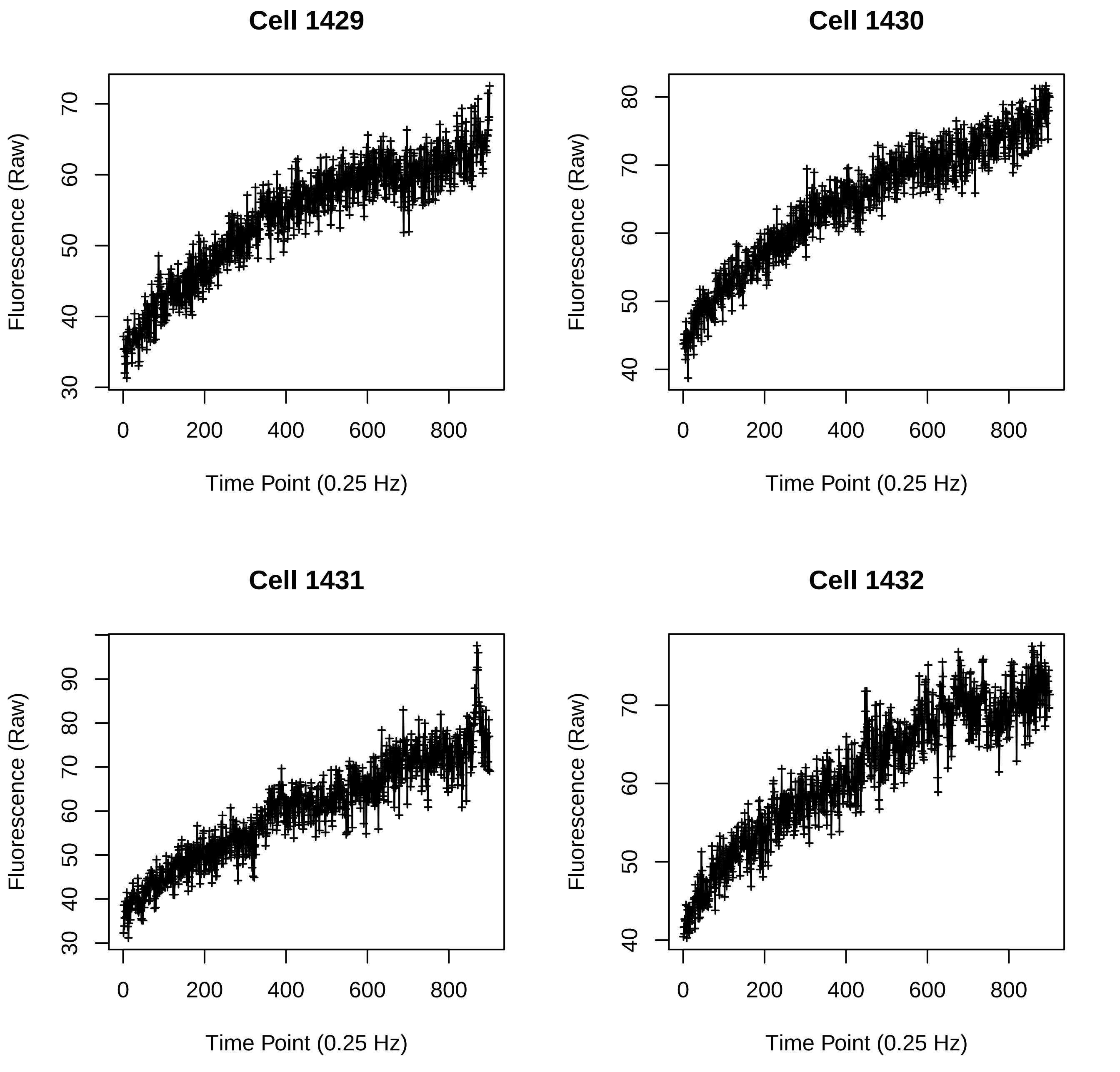
Cell 1429
Fluorescence (Raw)
Time Point (0.25 Hz)
30
40
50
60
70
0
200
400
600
800
Cell 1430
Fluorescence (Raw)
Time Point (0.25 Hz)
40
50
60
70
80
0
200
400
600
800
Cell 1431
Fluorescence (Raw)
Time Point (0.25 Hz)
30
40
50
60
70
80
90
0
200
400
600
800
Cell 1432
Fluorescence (Raw)
Time Point (0.25 Hz)
40
50
60
70
0
200
400
600
800

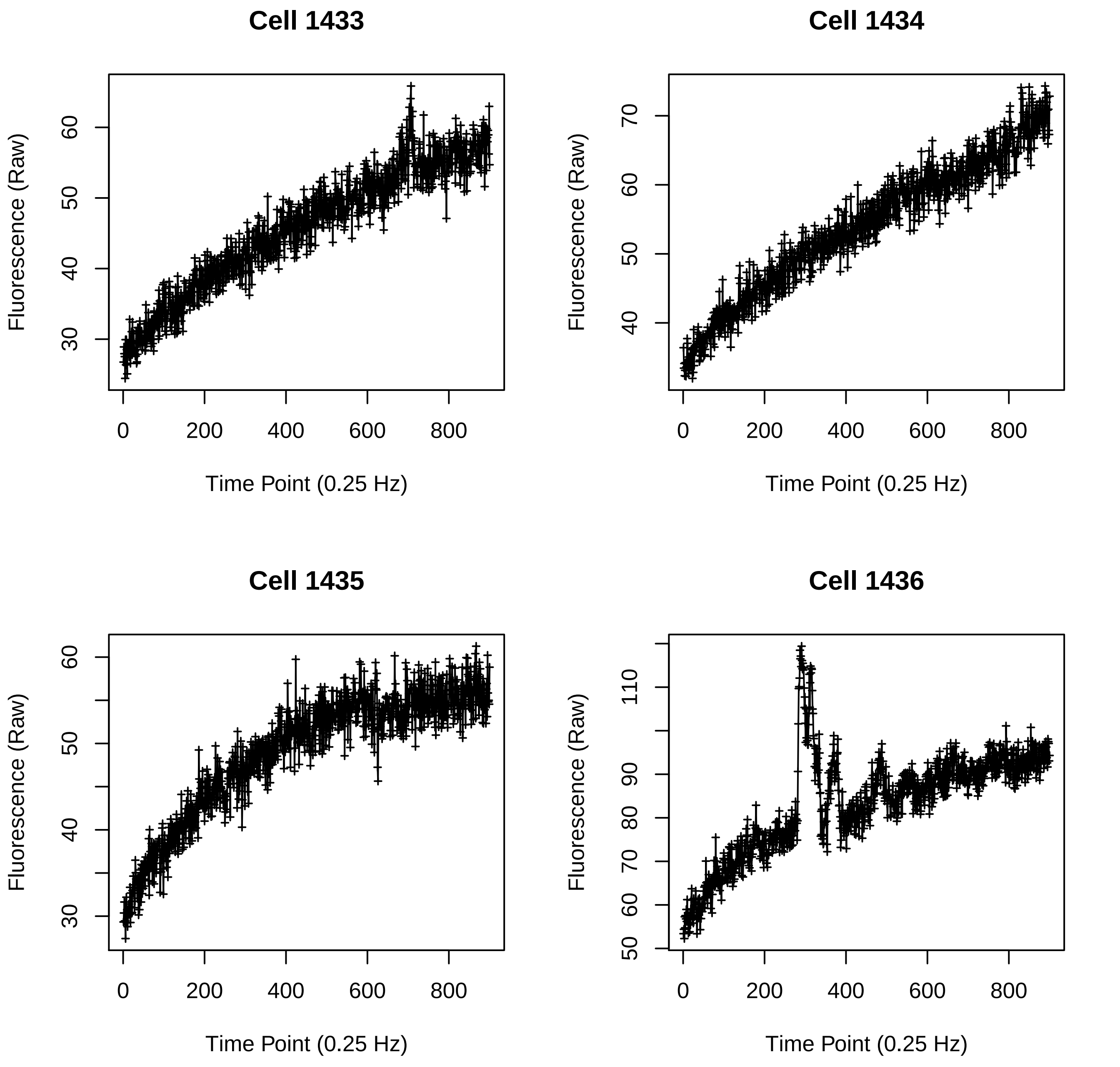

Cell 1433
Fluorescence (Raw)
30
40
50
60
0
200
400
600
800
Time Point (0.25 Hz)
Cell 1434
Fluorescence (Raw)
40
50
60
70
0
200
400
600
800
Time Point (0.25 Hz)
Cell 1435
Fluorescence (Raw)
30
40
50
60
0
200
400
600
800
Time Point (0.25 Hz)
Cell 1436
Fluorescence (Raw)
50
60
70
80
90
110
0
200
400
600
800
Time Point (0.25 Hz)

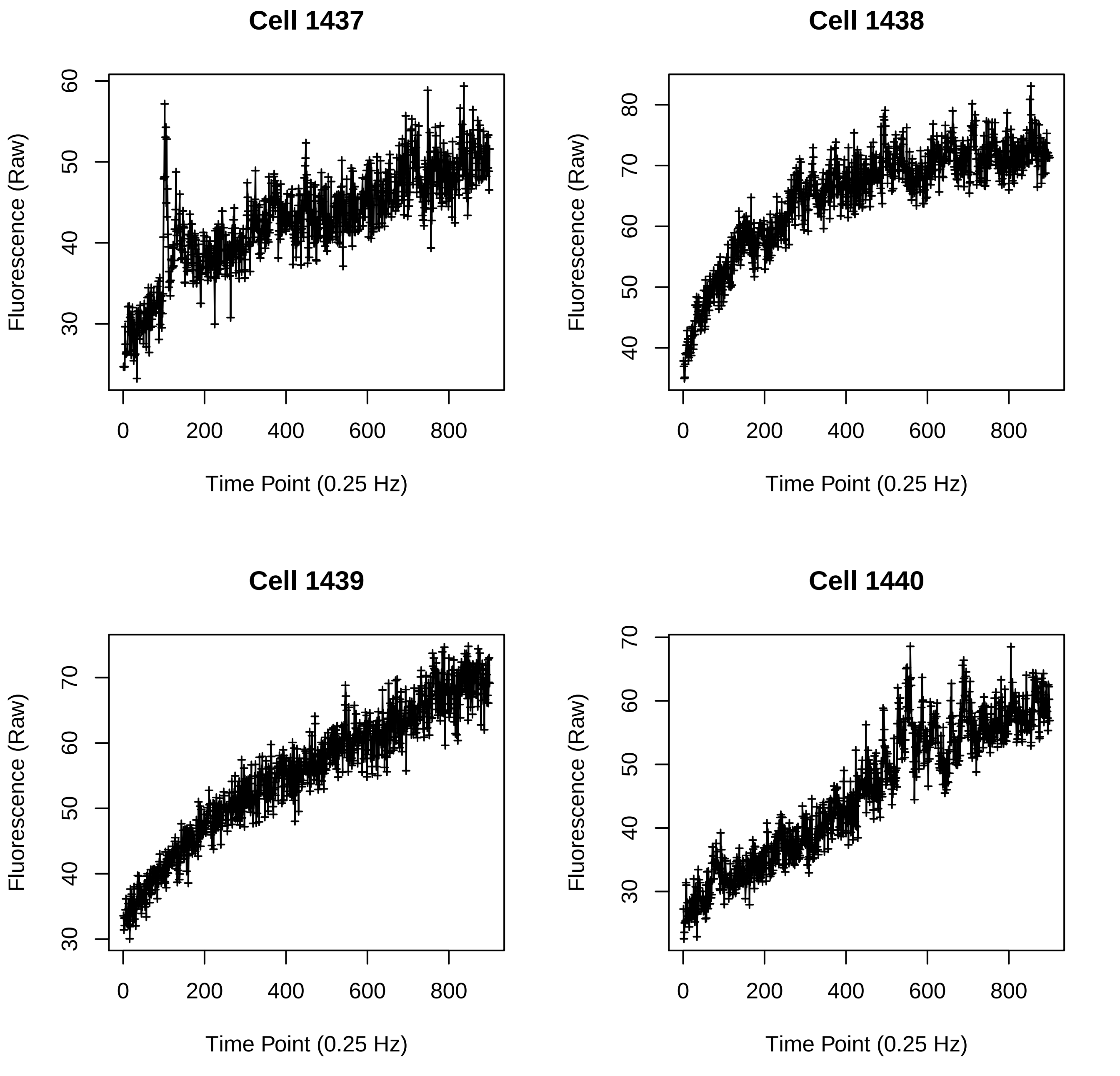
Cell 1437
Fluorescence (Raw)
30
40
50
60
0
200
400
600
800
Time Point (0.25 Hz)
Cell 1438
Fluorescence (Raw)
40
50
60
70
80
0
200
400
600
800
Time Point (0.25 Hz)
Cell 1439
Fluorescence (Raw)
30
40
50
60
70
0
200
400
600
800
Time Point (0.25 Hz)
Cell 1440
Fluorescence (Raw)
30
40
50
60
70
0
200
400
600
800
Time Point (0.25 Hz)

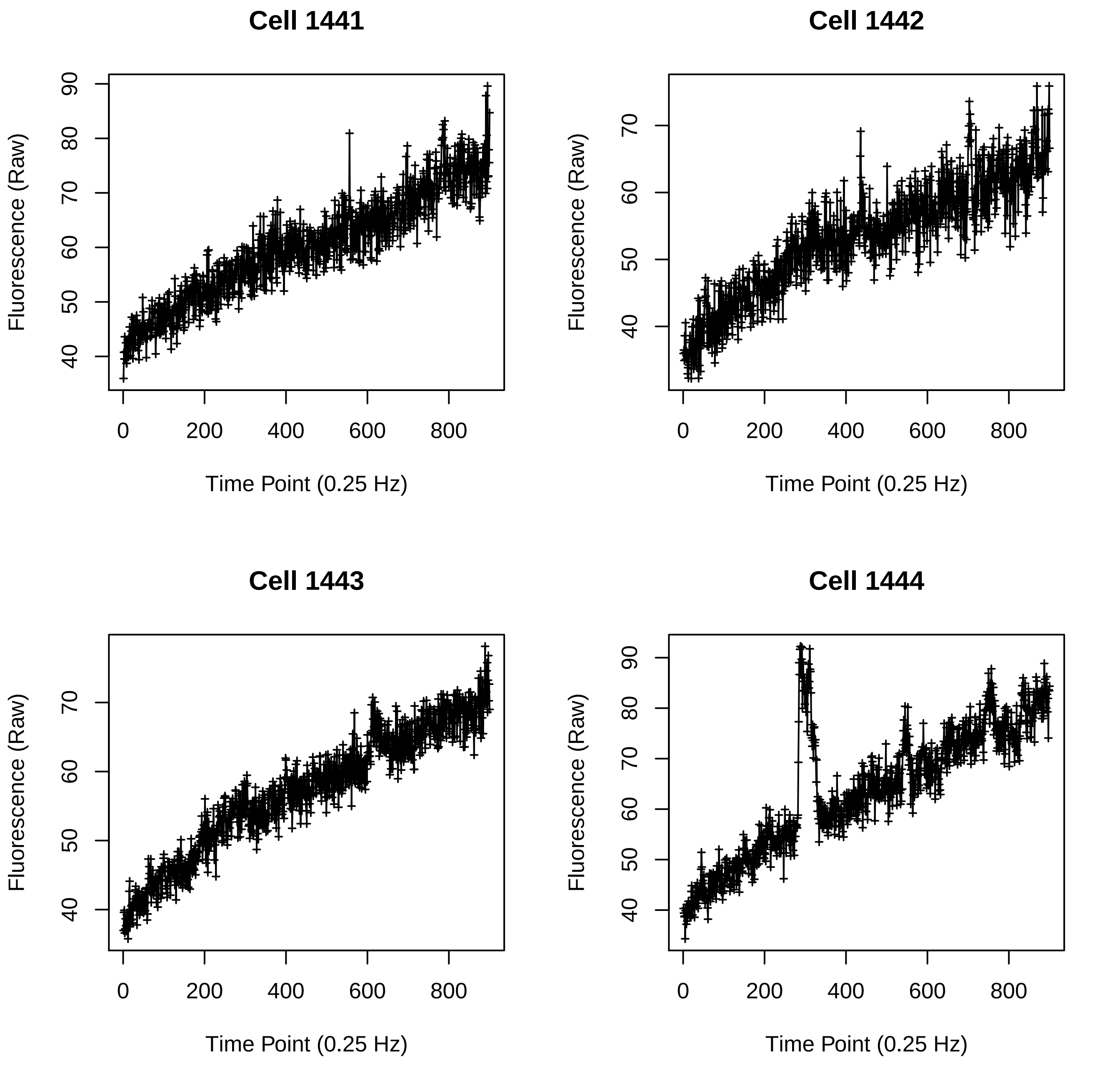

Cell 1441
Cell 1442
Cell 1443
Cell 1444
Fluorescence (Raw)
Time Point (0.25 Hz)
0
200
400
600
800
40
50
60
70
80
90

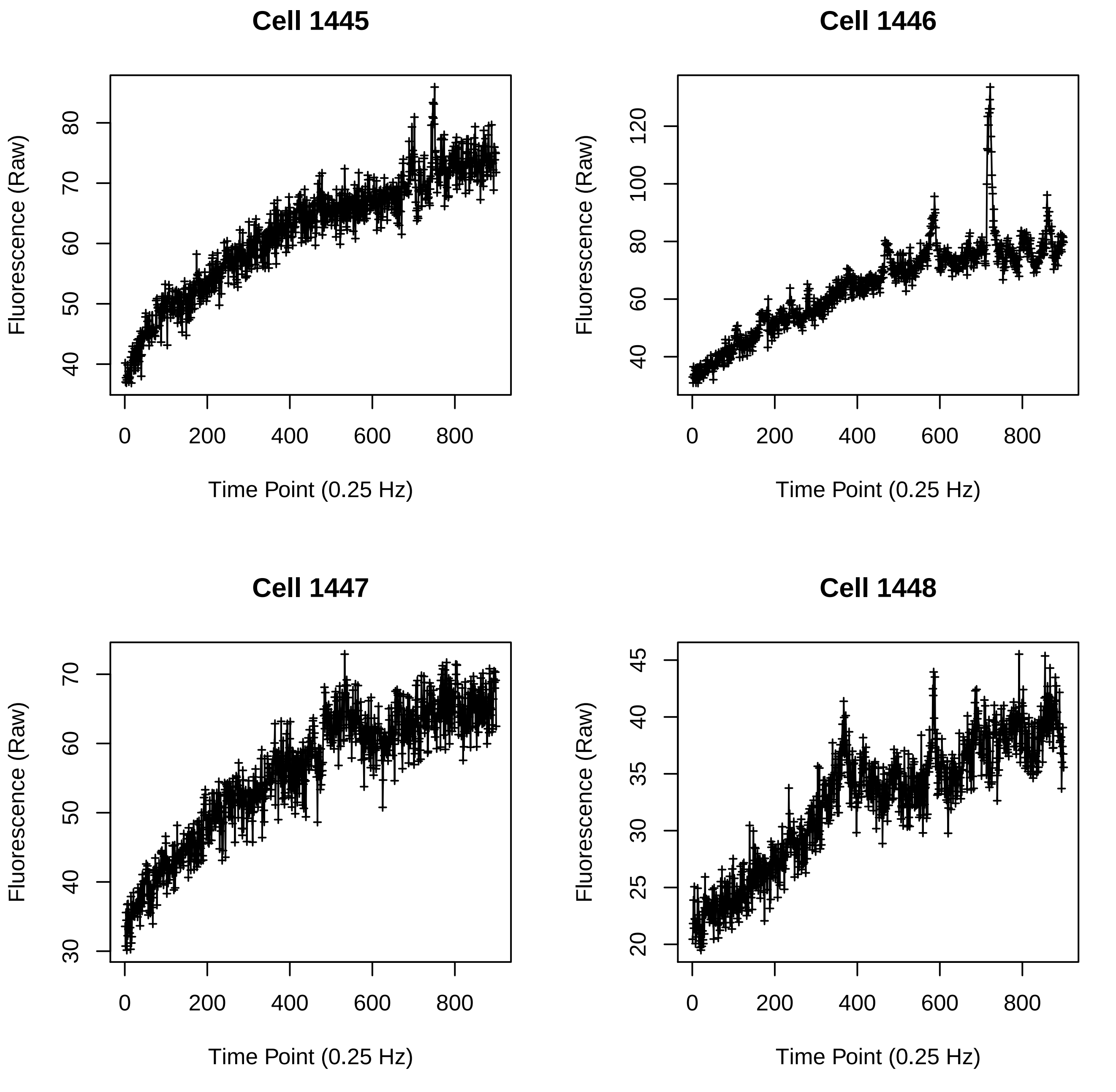
Cell 1445
Cell 1446
Cell 1447
Cell 1448
Fluorescence (Raw)
Time Point (0.25 Hz)
0
200
400
600
800
30
40
50
60
70
80
100
120
20
25
35
45

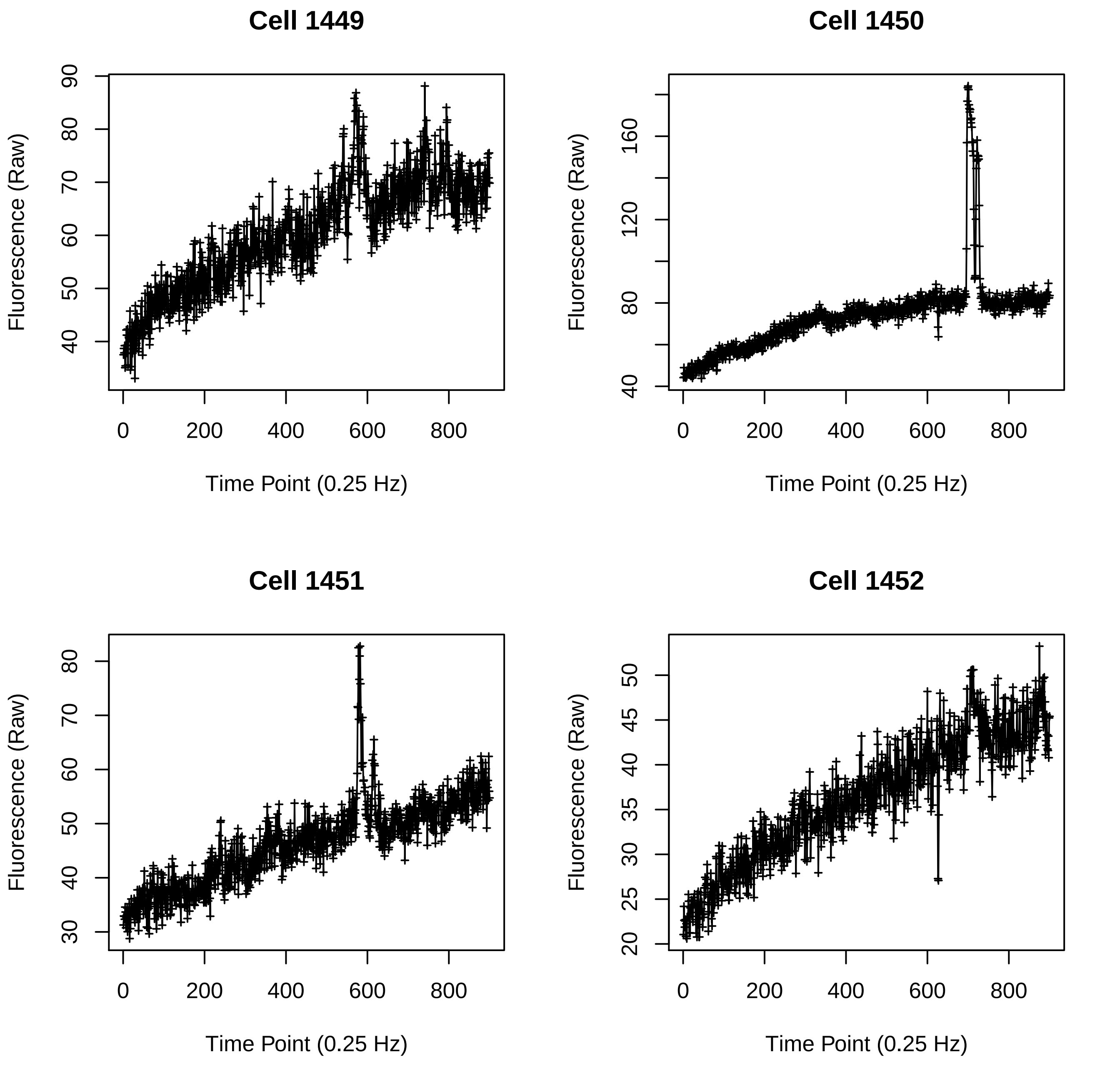

Cell 1449
Cell 1450
Cell 1451
Cell 1452
Fluorescence (Raw)
Time Point (0.25 Hz)
0
200
400
600
800
90
80
70
60
50
40
160
120
80
40
30
45
35
25
20

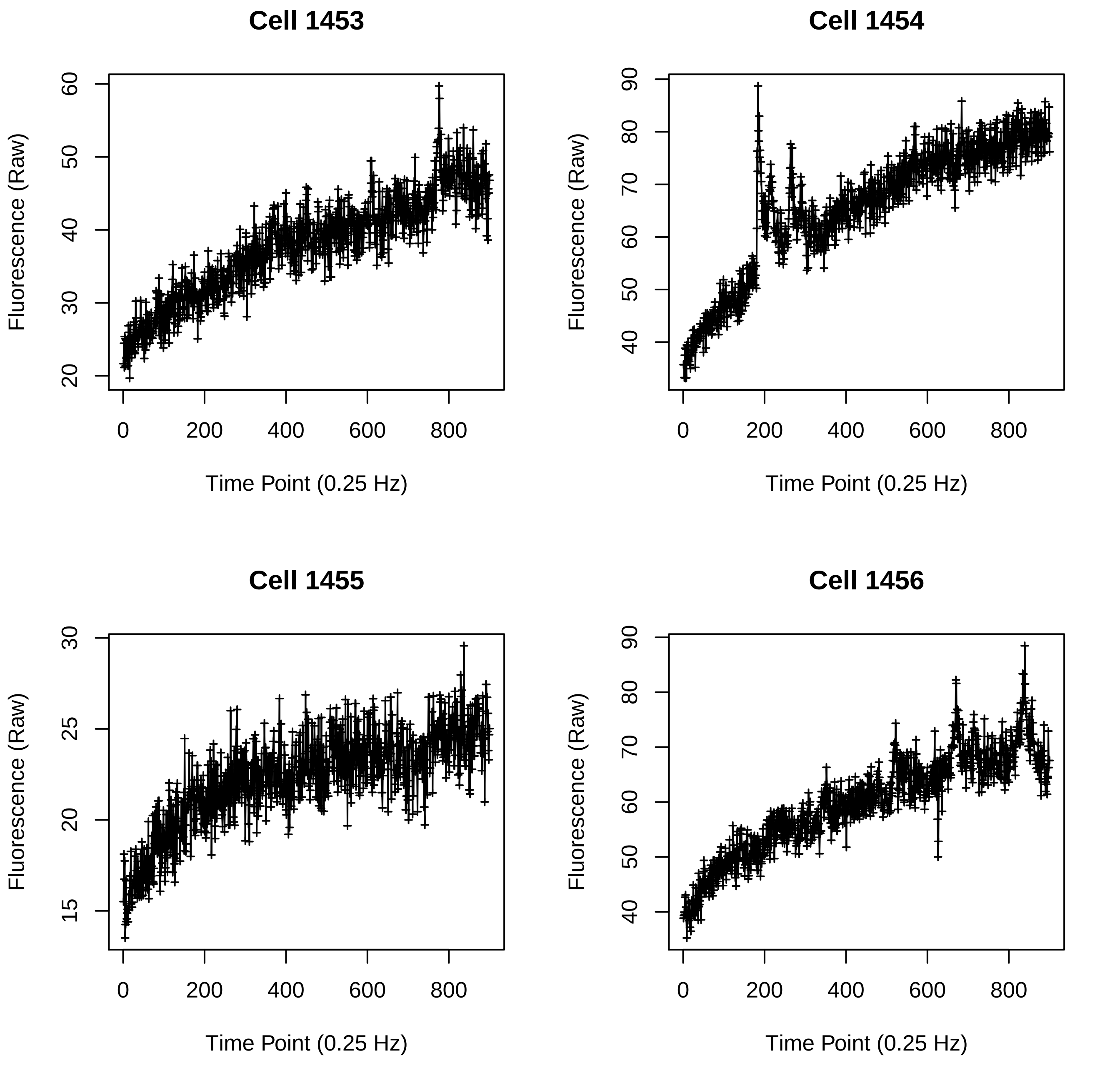

Cell 1453
Cell 1454
Cell 1455
Cell 1456
Fluorescence (Raw)
Time Point (0.25 Hz)
0
200
400
600
800
20
30
40
50
60
70
80
90
15
25

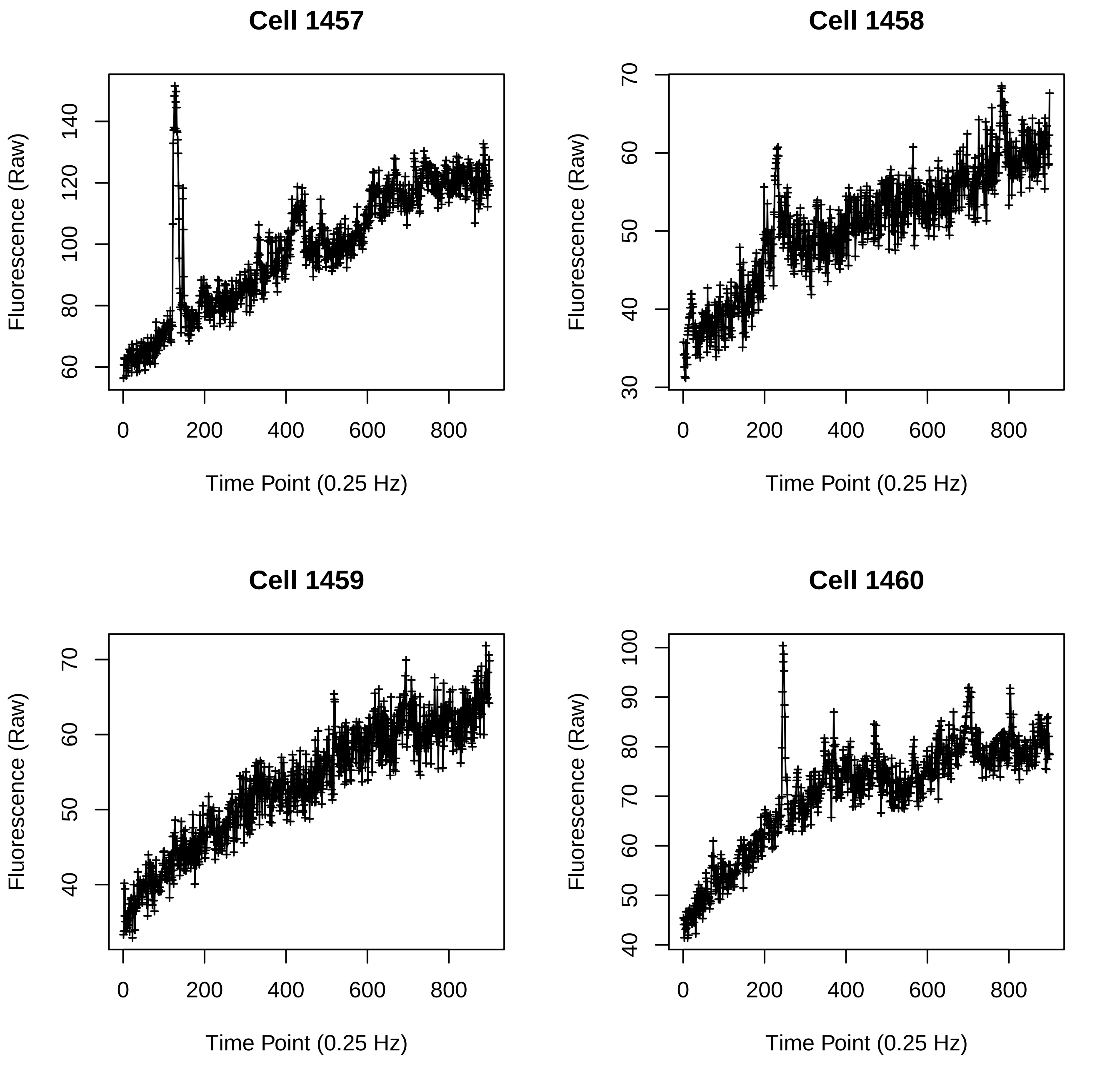

Cell 1457
Fluorescence (Raw)
Time Point (0.25 Hz)
60
80
100
120
140
0
200
400
600
800
Cell 1458
Fluorescence (Raw)
Time Point (0.25 Hz)
30
40
50
60
70
0
200
400
600
800
Cell 1459
Fluorescence (Raw)
Time Point (0.25 Hz)
40
50
60
70
0
200
400
600
800
Cell 1460
Fluorescence (Raw)
Time Point (0.25 Hz)
40
50
60
70
80
90
100
0
200
400
600
800

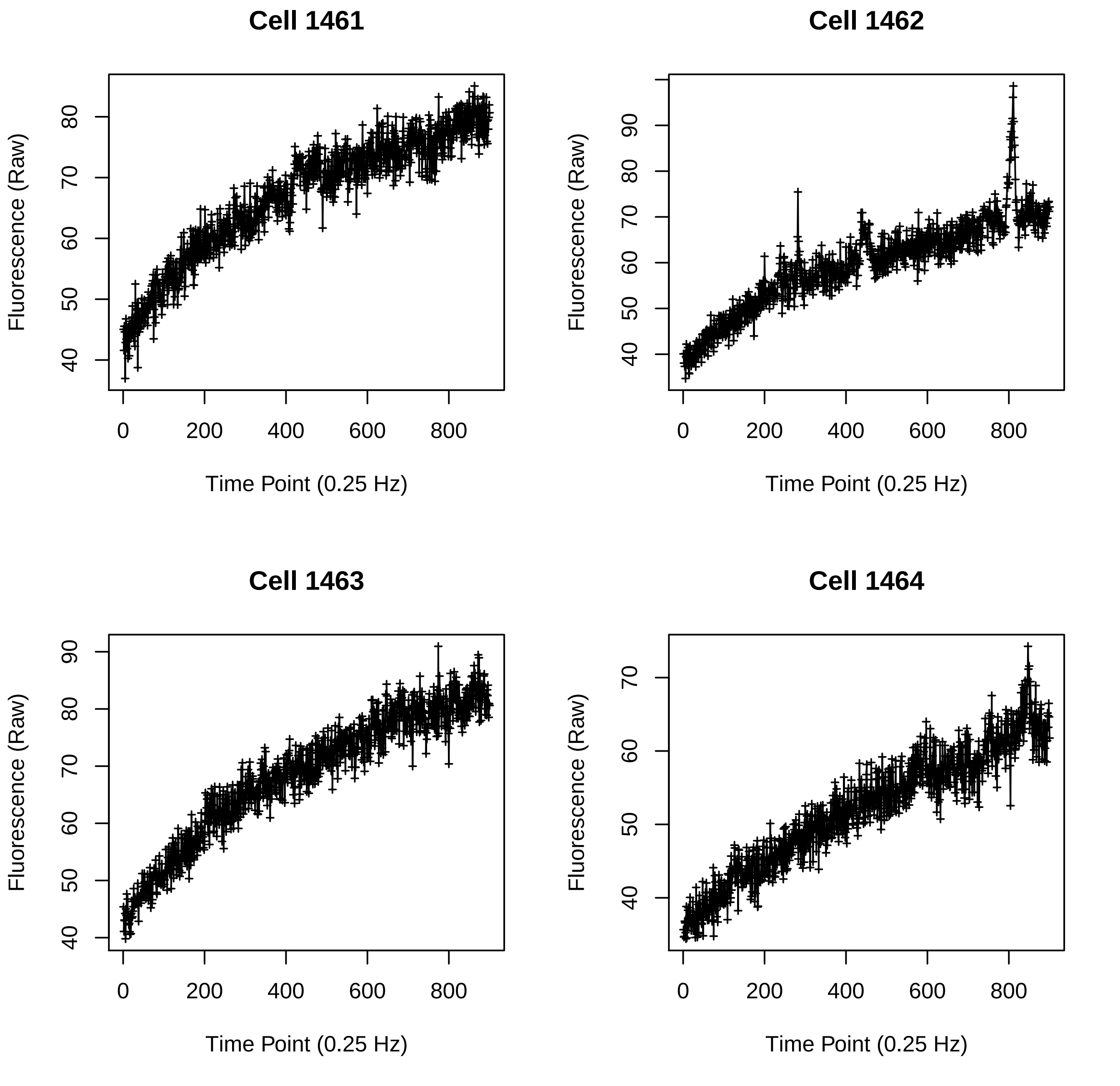
Cell 1461
Cell 1462
Cell 1463
Cell 1464
Fluorescence (Raw)
Time Point (0.25 Hz)
0
200
400
600
800
40
50
60
70
80
90

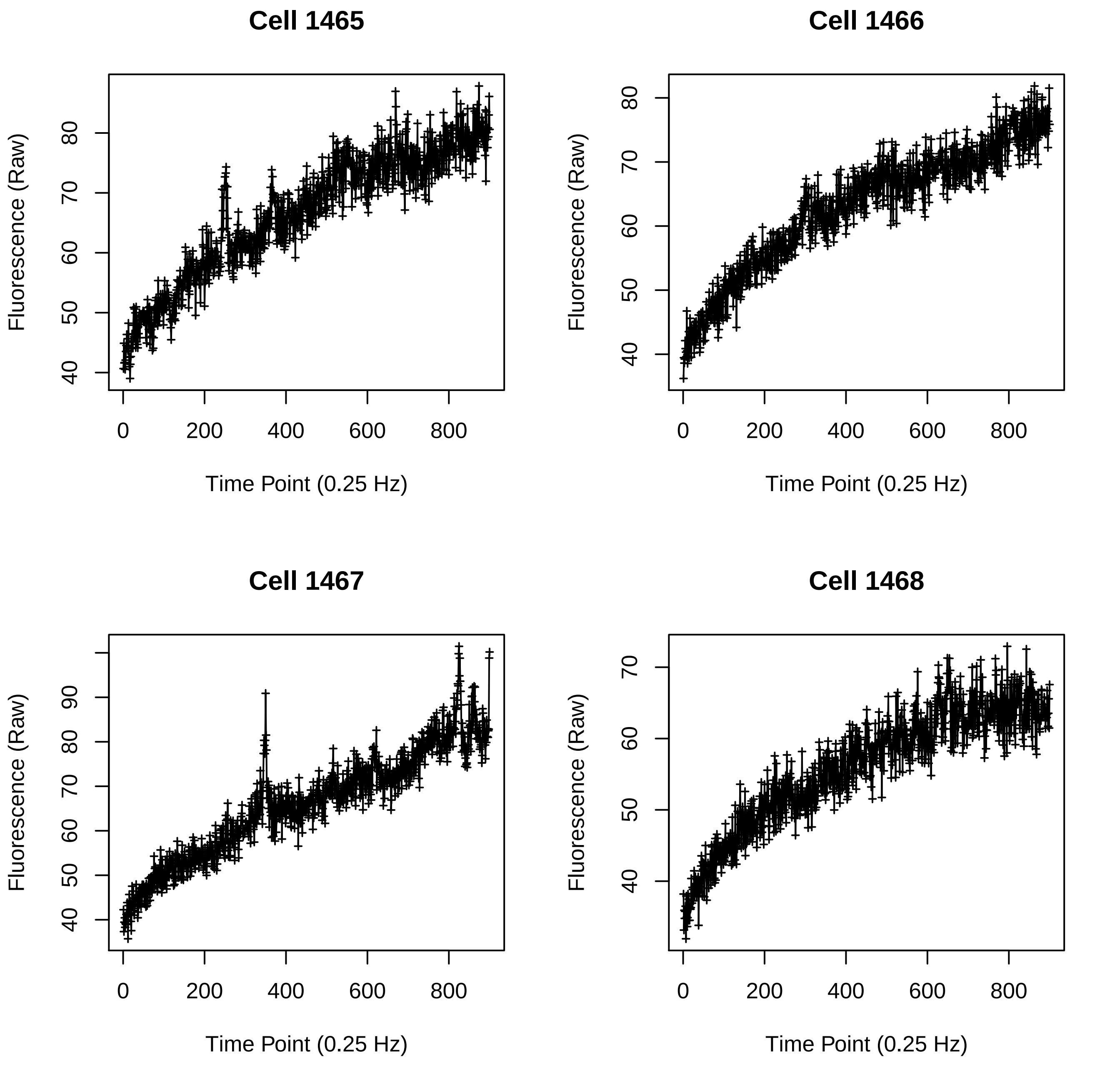
Cell 1465
Fluorescence (Raw)
40
50
60
70
80
0
200
400
600
800
Time Point (0.25 Hz)
Cell 1466
Fluorescence (Raw)
40
50
60
70
80
0
200
400
600
800
Time Point (0.25 Hz)
Cell 1467
Fluorescence (Raw)
40
50
60
70
80
90
0
200
400
600
800
Time Point (0.25 Hz)
Cell 1468
Fluorescence (Raw)
40
50
60
70
0
200
400
600
800
Time Point (0.25 Hz)

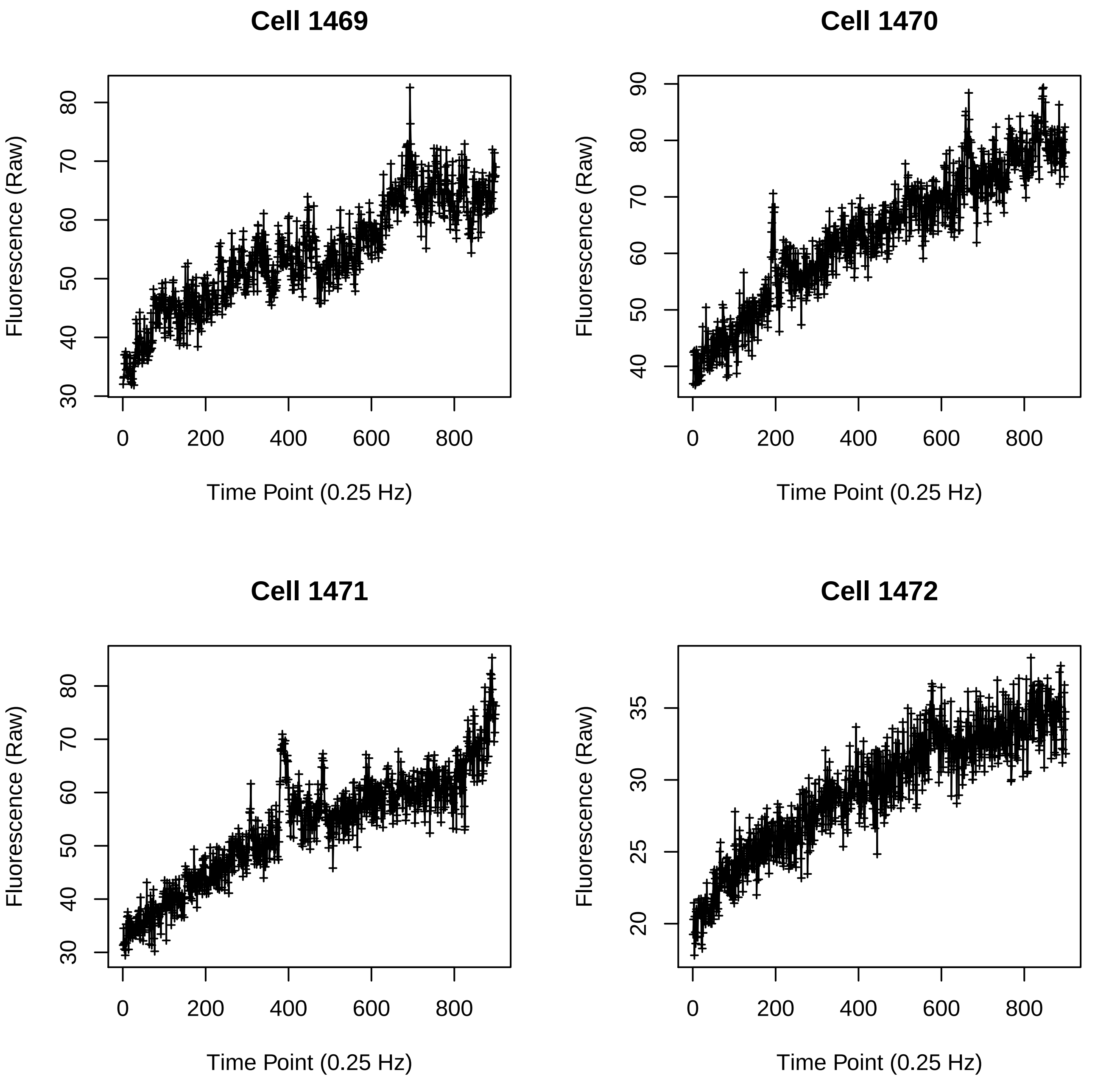

Cell 1469
Fluorescence (Raw)
30
40
50
60
70
80
0
200
400
600
800
Time Point (0.25 Hz)
Cell 1470
Fluorescence (Raw)
40
50
60
70
80
90
0
200
400
600
800
Time Point (0.25 Hz)
Cell 1471
Fluorescence (Raw)
30
40
50
60
70
80
0
200
400
600
800
Time Point (0.25 Hz)
Cell 1472
Fluorescence (Raw)
20
25
30
35
0
200
400
600
800
Time Point (0.25 Hz)

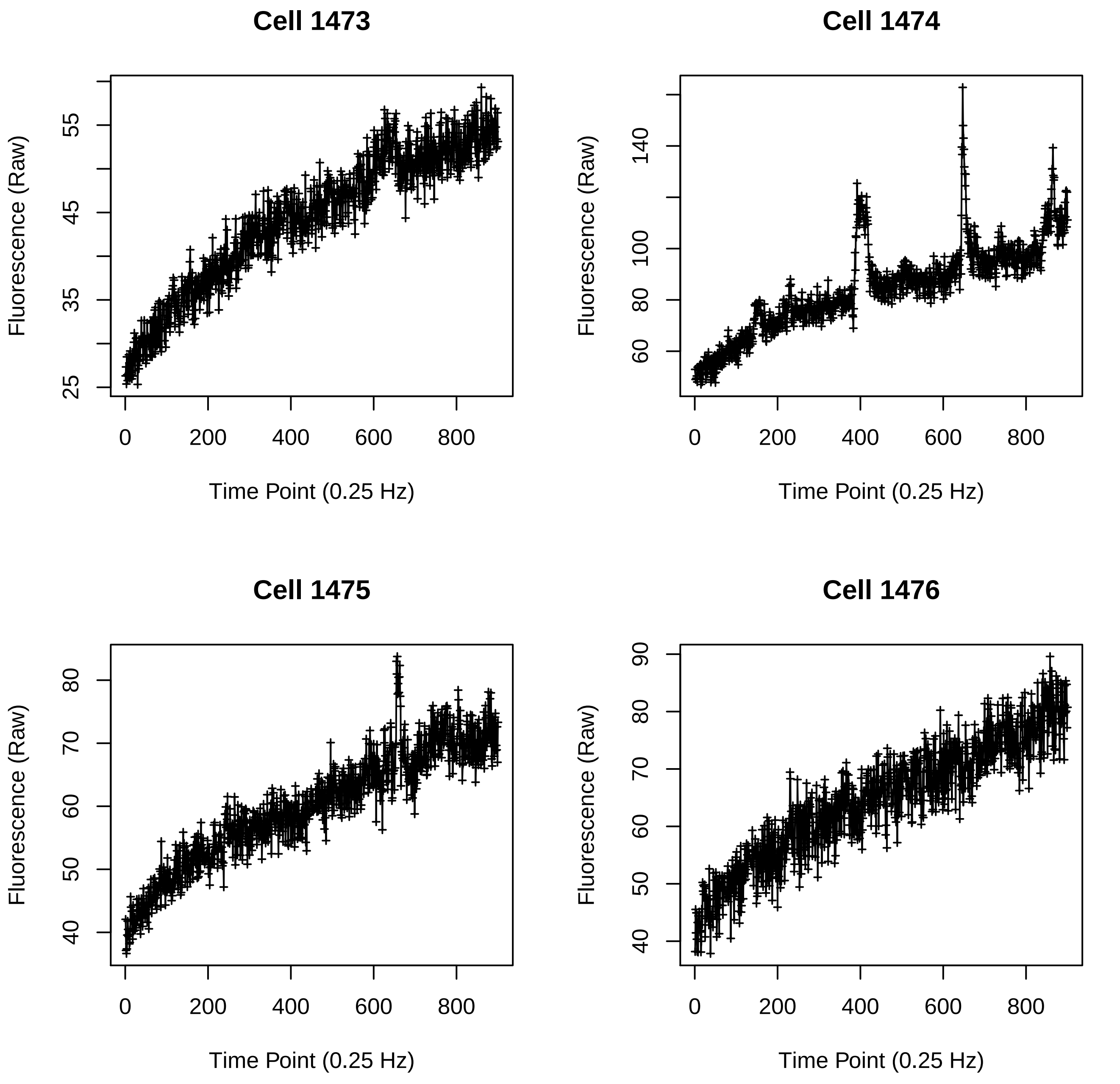
Cell 1473
Cell 1474
Cell 1475
Cell 1476
Fluorescence (Raw)
Time Point (0.25 Hz)
25
35
45
55
60
80
100
140
40
50
60
70
80
90
0
200
400
600
800

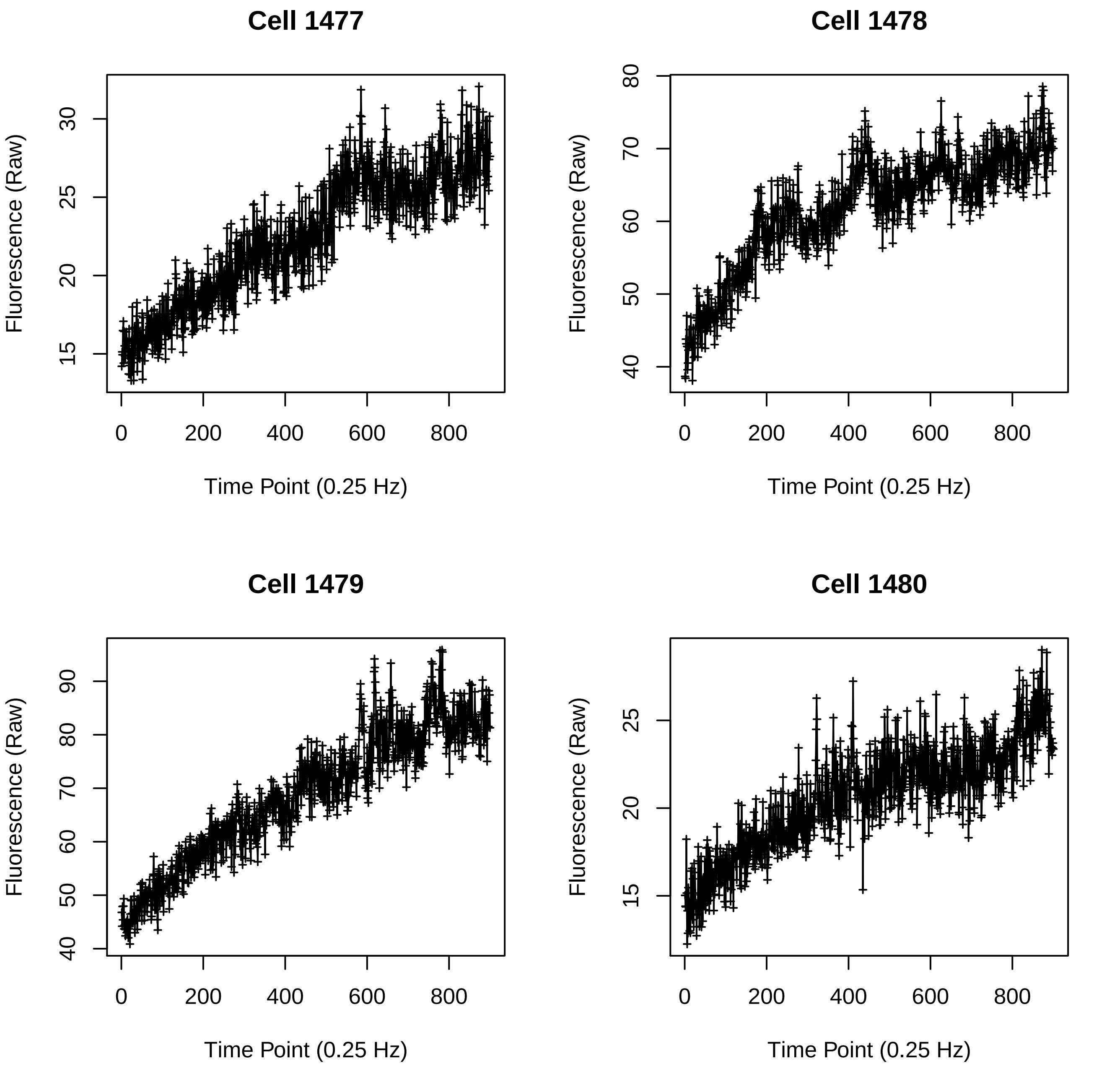
Cell 1477
Fluorescence (Raw)
15
20
25
30
0
200
400
600
800
Time Point (0.25 Hz)
Cell 1478
Fluorescence (Raw)
40
50
60
70
80
0
200
400
600
800
Time Point (0.25 Hz)
Cell 1479
Fluorescence (Raw)
40
50
60
70
80
90
0
200
400
600
800
Time Point (0.25 Hz)
Cell 1480
Fluorescence (Raw)
15
20
25
0
200
400
600
800
Time Point (0.25 Hz)

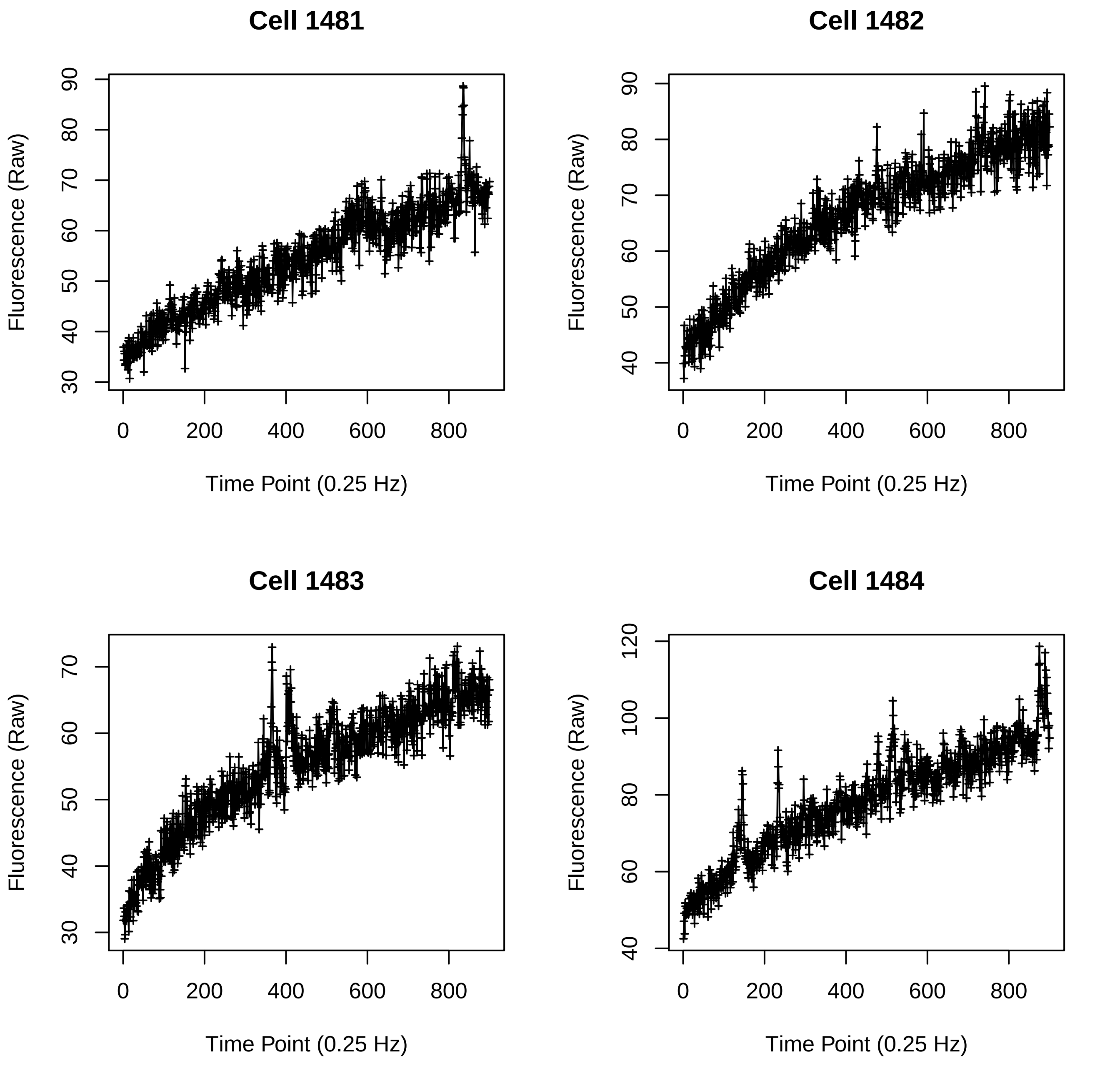
Cell 1481
Cell 1482
Cell 1483
Cell 1484
Fluorescence (Raw)
Time Point (0.25 Hz)
0
200
400
600
800
30
40
50
60
70
80
90
100
120

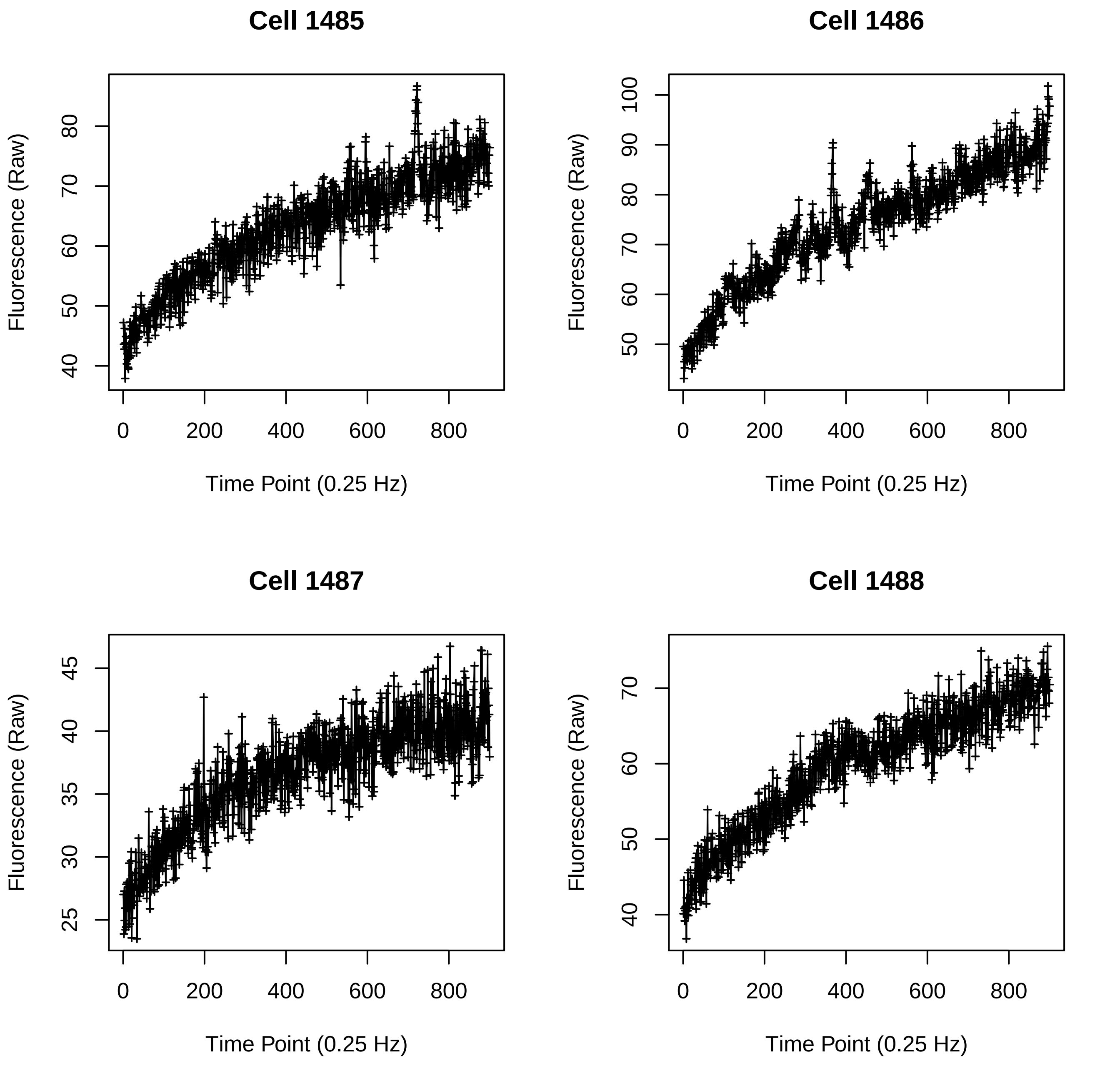

Cell 1485
Cell 1486
Cell 1487
Cell 1488
Fluorescence (Raw)
Time Point (0.25 Hz)
0
200
400
600
800

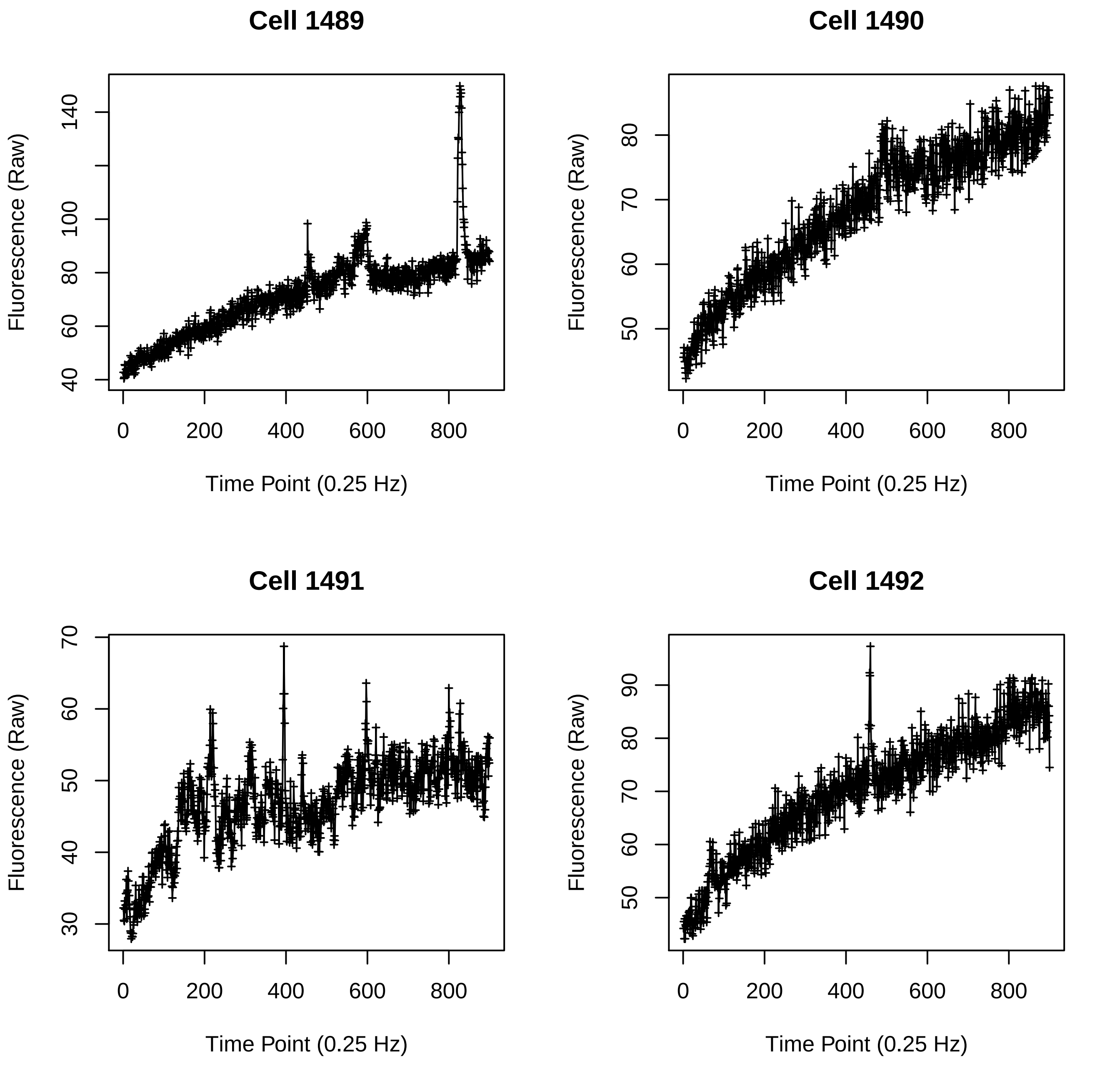
Cell 1489
Cell 1490
Cell 1491
Cell 1492
Fluorescence (Raw)
Time Point (0.25 Hz)
0
200
400
600
800
40
60
80
100
140
50
70
90
30

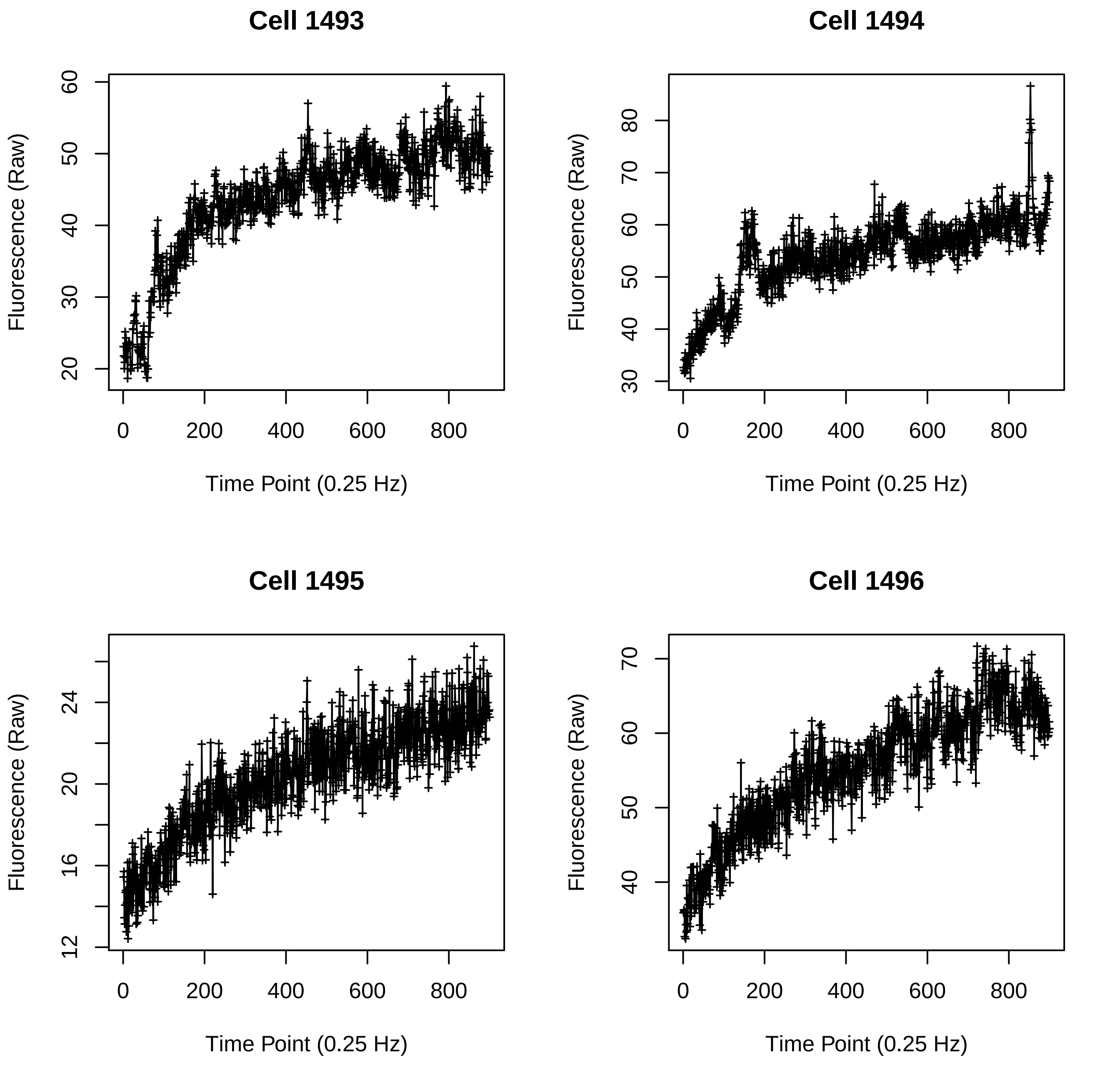
Cell 1493
Cell 1494
Cell 1495
Cell 1496
Fluorescence (Raw)
Time Point (0.25 Hz)
0
200
400
600
800
20
30
40
50
60
70
80
12
16
24

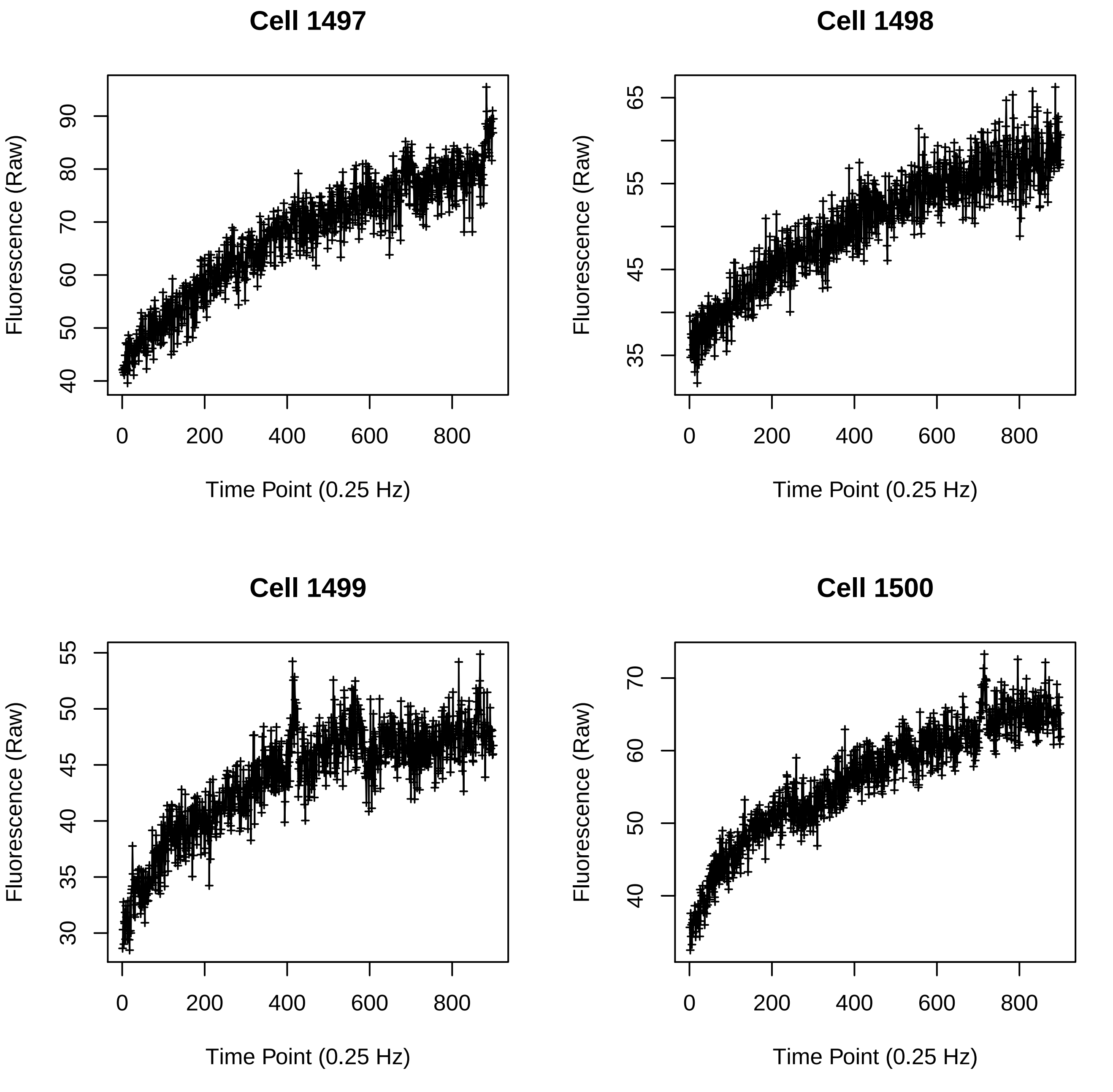
Cell 1497
Fluorescence (Raw)
40
50
60
70
80
90
0
200
400
600
800
Time Point (0.25 Hz)
Cell 1498
Fluorescence (Raw)
35
45
55
65
0
200
400
600
800
Time Point (0.25 Hz)
Cell 1499
Fluorescence (Raw)
30
35
40
45
50
55
0
200
400
600
800
Time Point (0.25 Hz)
Cell 1500
Fluorescence (Raw)
40
50
60
70
0
200
400
600
800
Time Point (0.25 Hz)

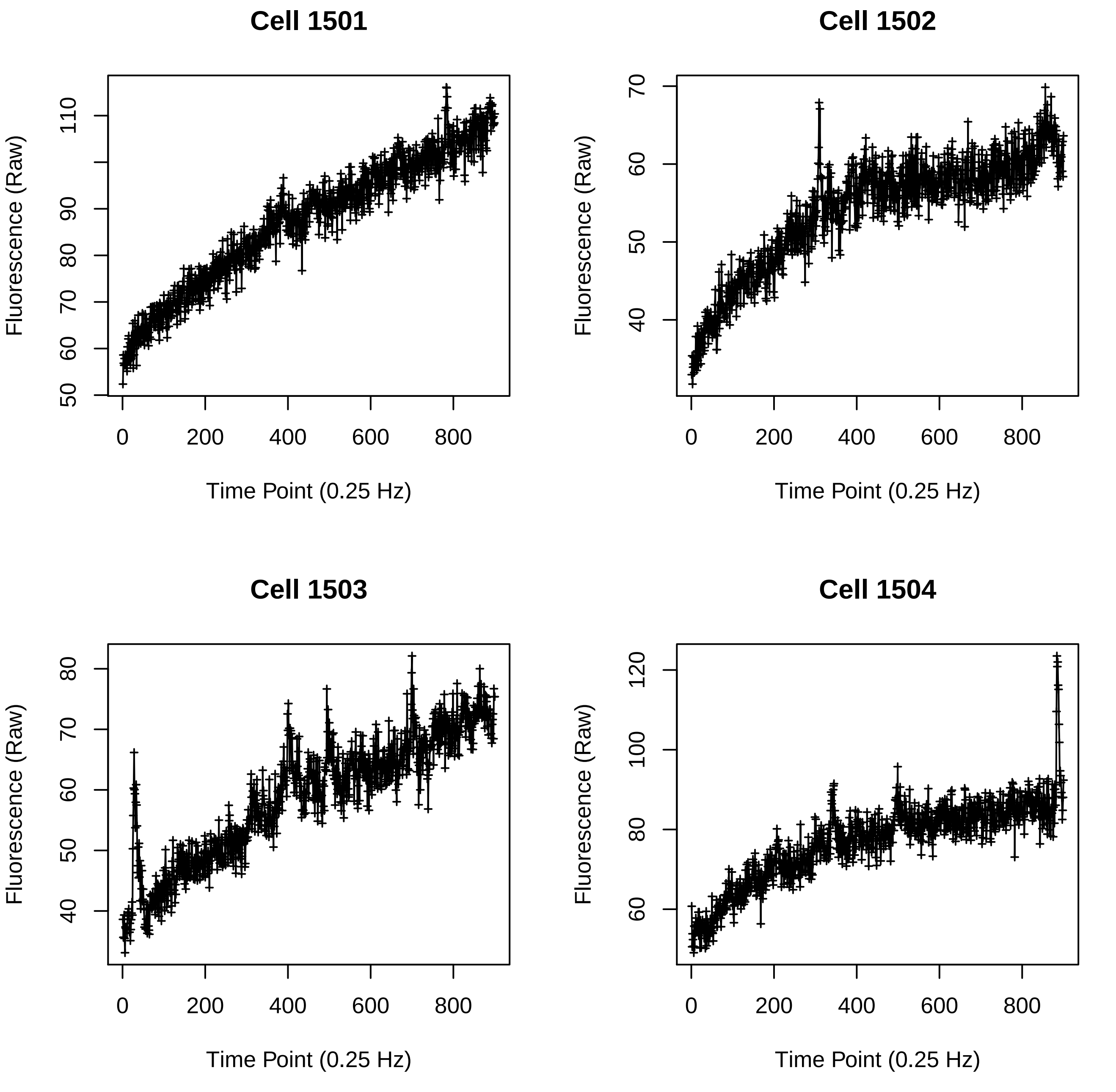

Cell 1501
Cell 1502
Cell 1503
Cell 1504
Fluorescence (Raw)
Time Point (0.25 Hz)
50
60
70
80
90
110
40
120
100
0
200
400
600
800

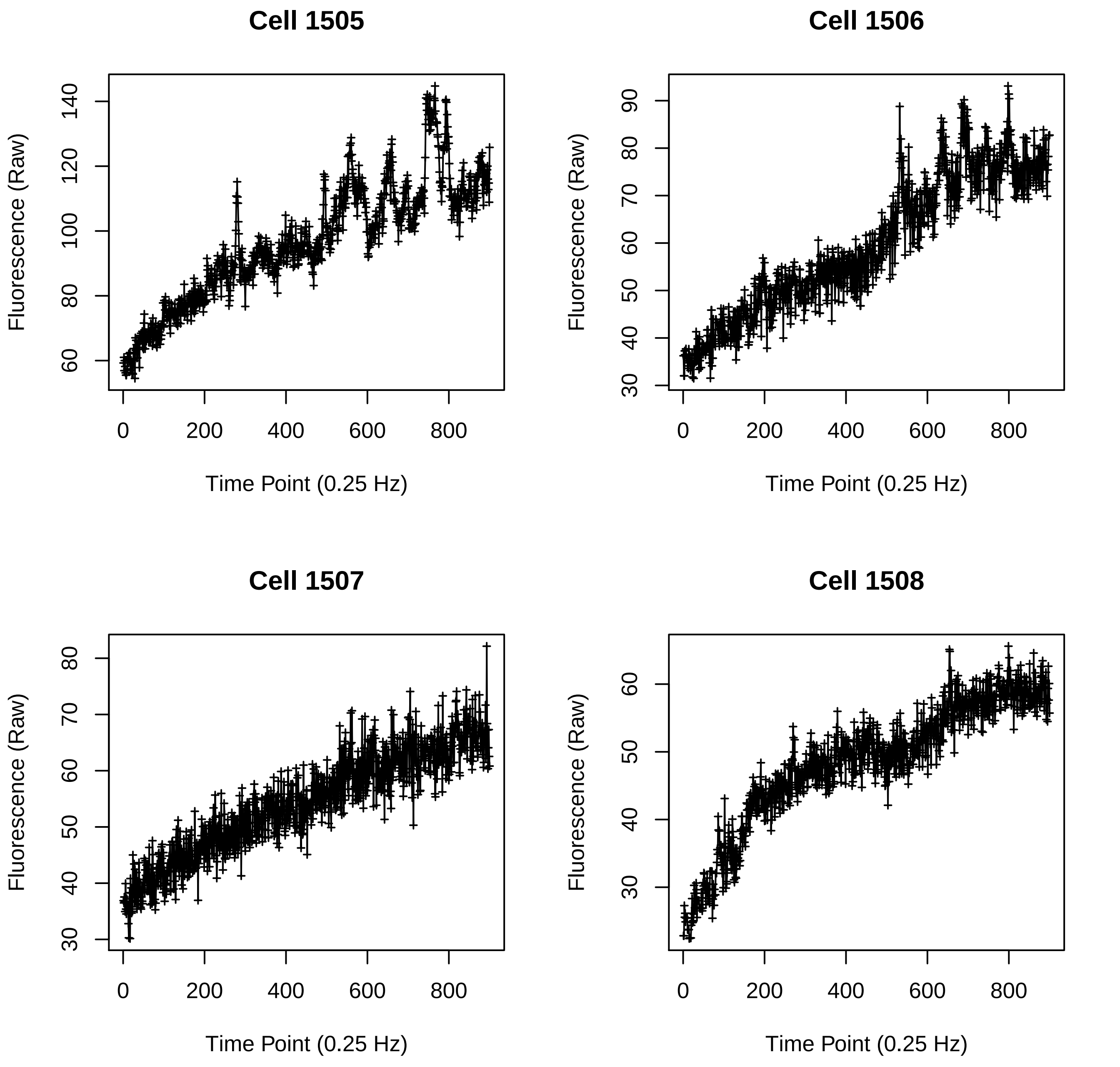
Cell 1505
Cell 1506
Cell 1507
Cell 1508
Fluorescence (Raw)
Time Point (0.25 Hz)
0
200
400
600
800
60
80
100
120
140
30
40
50
60
70
80
90

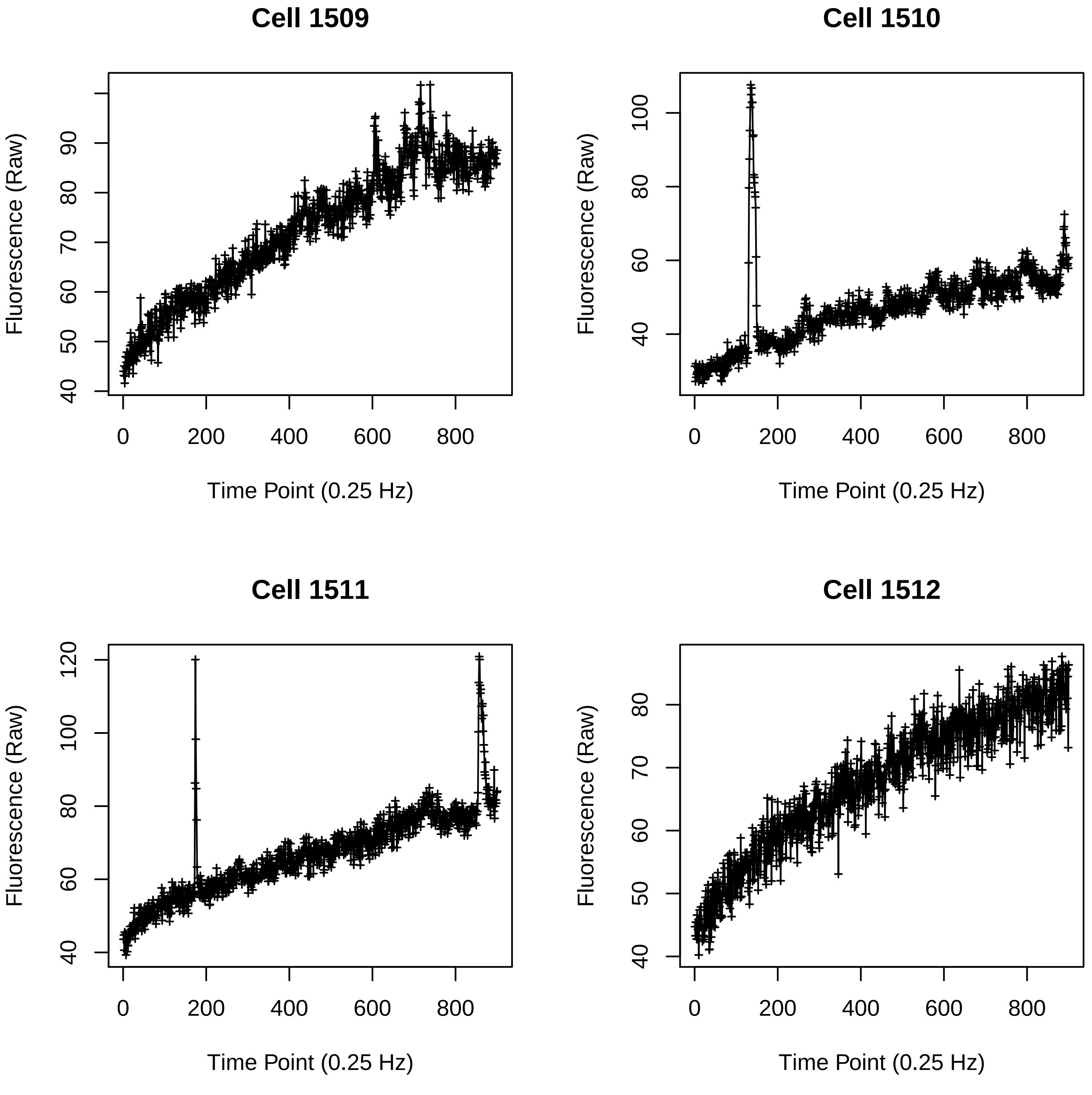
Cell 1509
Cell 1510
Cell 1511
Cell 1512
Fluorescence (Raw)
Time Point (0.25 Hz)
0
200
400
600
800
40
50
60
70
80
90
100
120

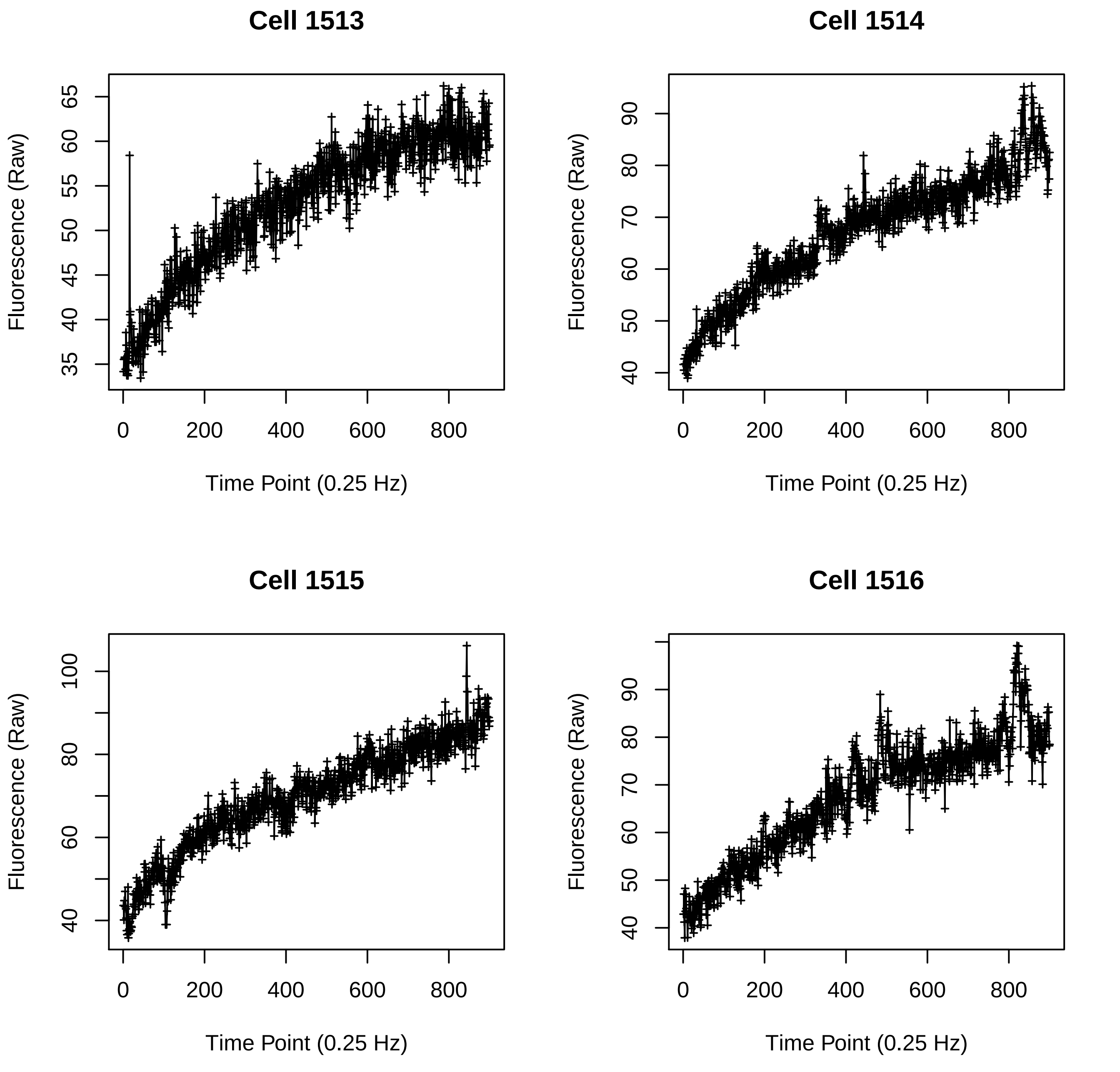
Cell 1513
Cell 1514
Cell 1515
Cell 1516
Fluorescence (Raw)
Time Point (0.25 Hz)
0
200
400
600
800
35
40
45
50
55
60
65
70
80
90
100

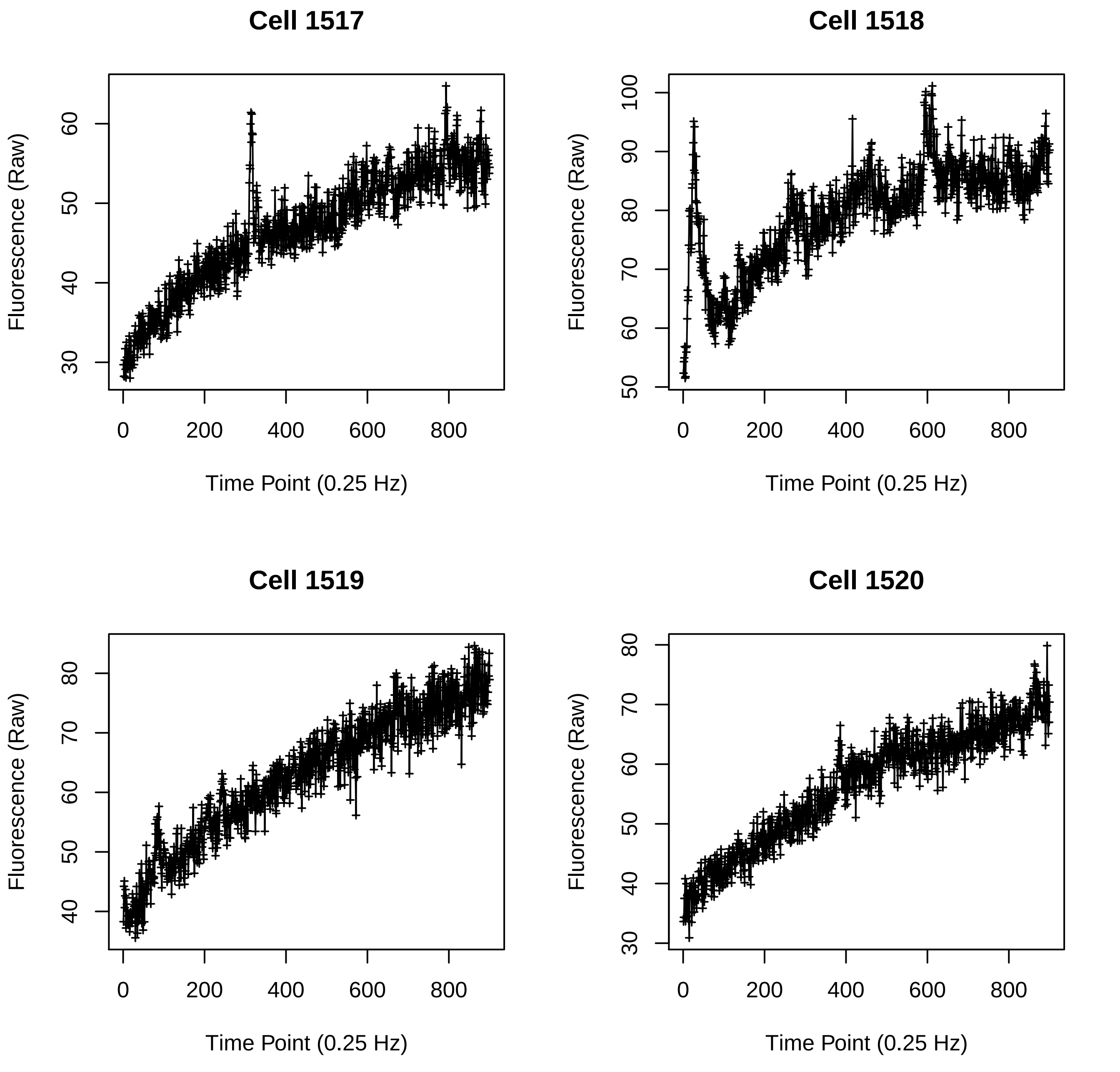
Cell 1517
Fluorescence (Raw)
30
40
50
60
0
200
400
600
800
Time Point (0.25 Hz)
Cell 1518
Fluorescence (Raw)
50
60
70
80
90
100
0
200
400
600
800
Time Point (0.25 Hz)
Cell 1519
Fluorescence (Raw)
40
50
60
70
80
0
200
400
600
800
Time Point (0.25 Hz)
Cell 1520
Fluorescence (Raw)
30
40
50
60
70
80
0
200
400
600
800
Time Point (0.25 Hz)

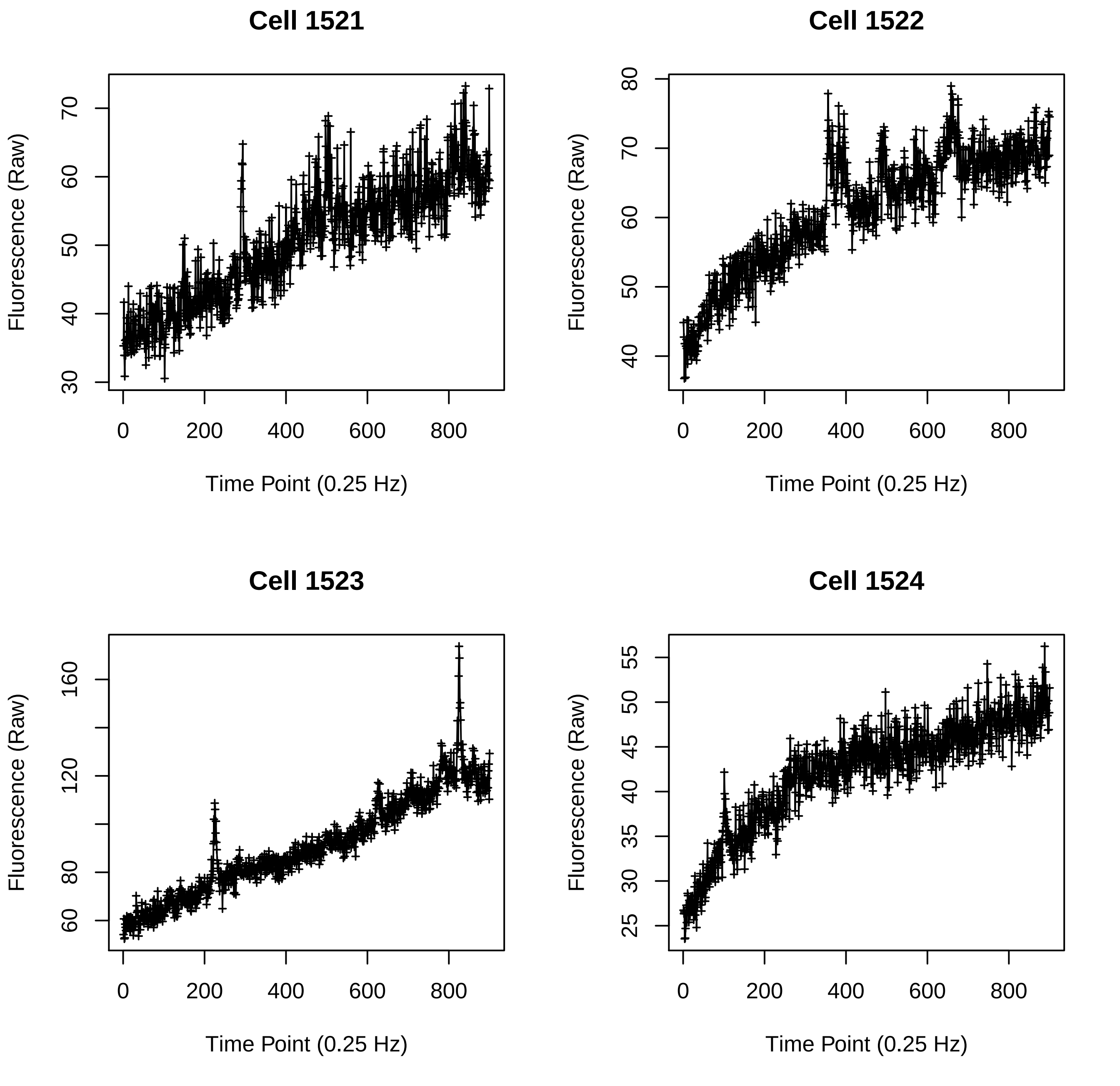
Cell 1521
Fluorescence (Raw)
30
40
50
60
70
0
200
400
600
800
Time Point (0.25 Hz)
Cell 1522
Fluorescence (Raw)
40
50
60
70
80
0
200
400
600
800
Time Point (0.25 Hz)
Cell 1523
Fluorescence (Raw)
60
80
120
160
0
200
400
600
800
Time Point (0.25 Hz)
Cell 1524
Fluorescence (Raw)
25
30
35
40
45
50
55
0
200
400
600
800
Time Point (0.25 Hz)

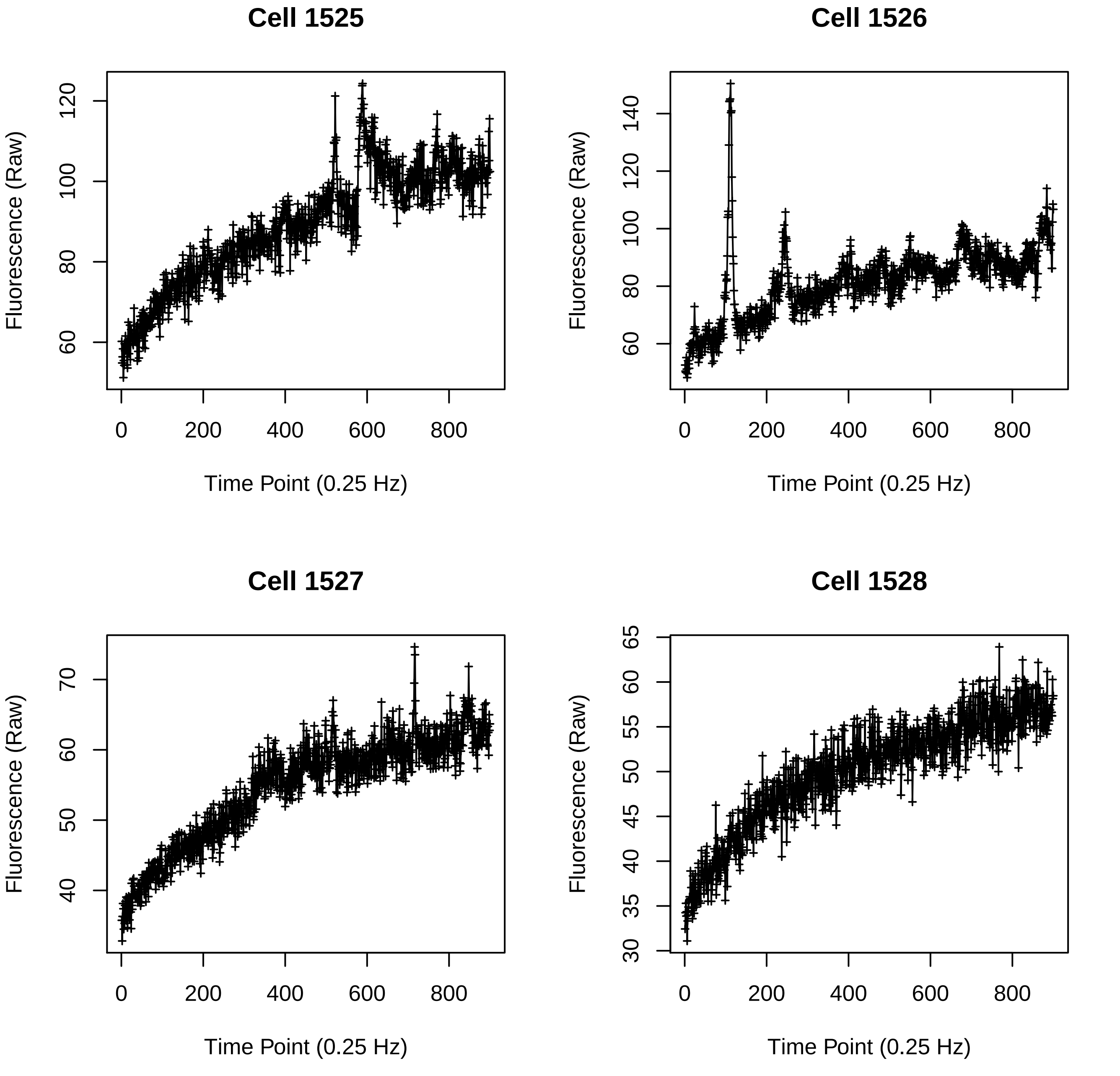
Cell 1525
Fluorescence (Raw)
60
80
100
120
0
200
400
600
800
Time Point (0.25 Hz)
Cell 1526
Fluorescence (Raw)
60
80
100
120
140
0
200
400
600
800
Time Point (0.25 Hz)
Cell 1527
Fluorescence (Raw)
40
50
60
70
0
200
400
600
800
Time Point (0.25 Hz)
Cell 1528
Fluorescence (Raw)
30
35
40
45
50
55
60
65
0
200
400
600
800
Time Point (0.25 Hz)

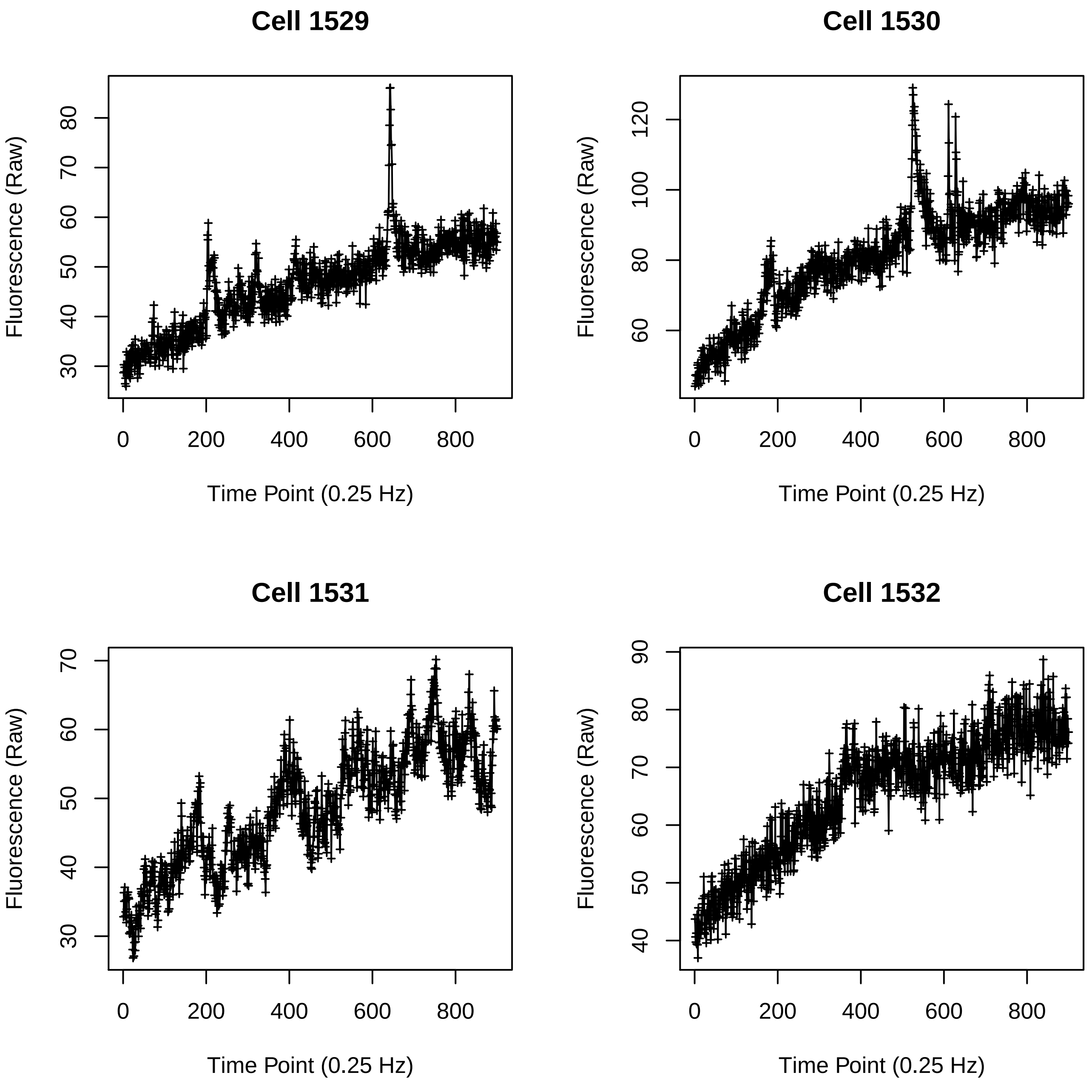
Cell 1529
Fluorescence (Raw)
30
40
50
60
70
80
0
200
400
600
800
Time Point (0.25 Hz)
Cell 1530
Fluorescence (Raw)
60
80
100
120
0
200
400
600
800
Time Point (0.25 Hz)
Cell 1531
Fluorescence (Raw)
30
40
50
60
70
0
200
400
600
800
Time Point (0.25 Hz)
Cell 1532
Fluorescence (Raw)
40
50
60
70
80
90
0
200
400
600
800
Time Point (0.25 Hz)

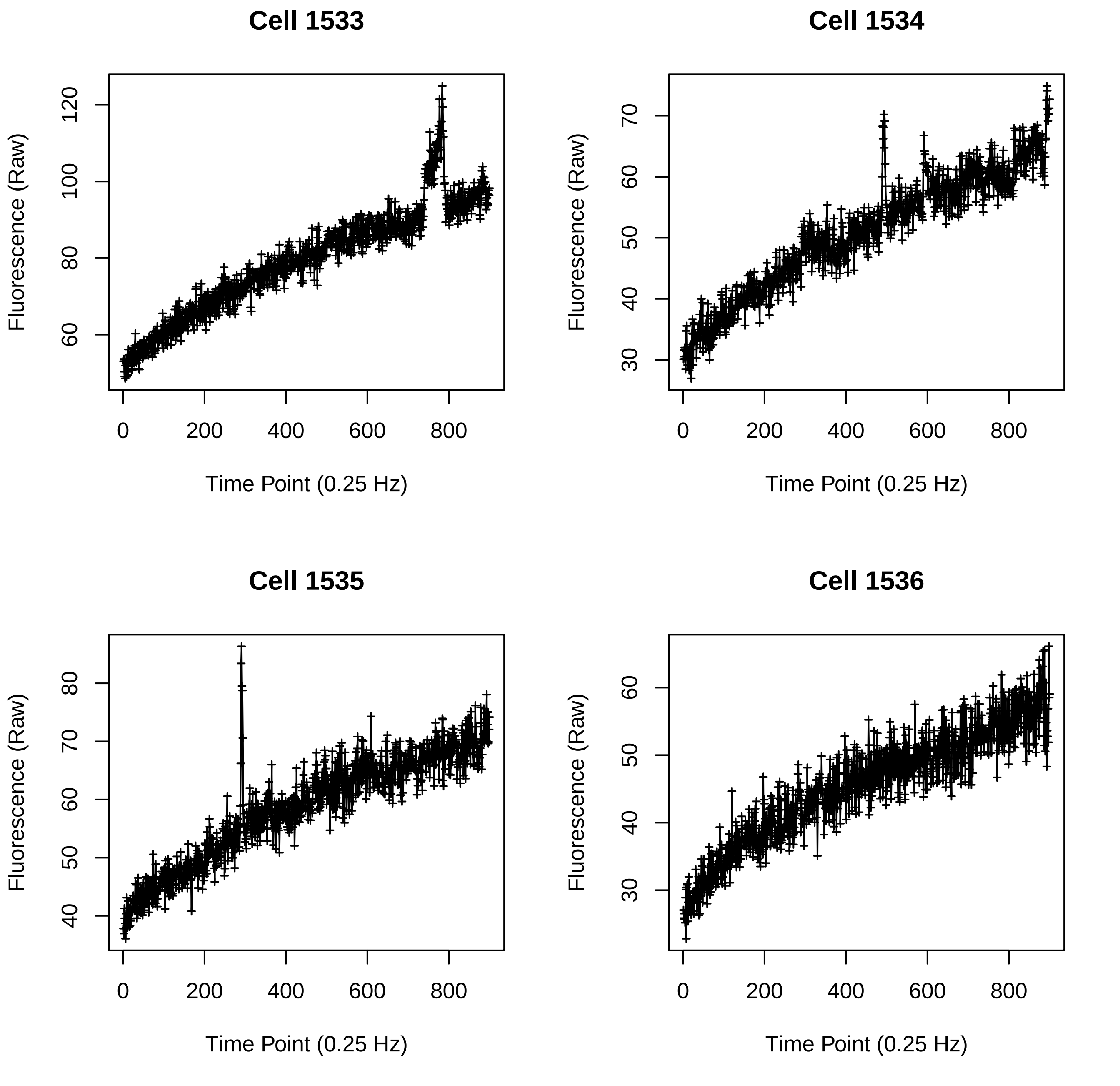

Cell 1533
Cell 1534
Cell 1535
Cell 1536
Fluorescence (Raw)
Time Point (0.25 Hz)
0
200
400
600
800
60
80
100
120
30
40
50
60
70
80

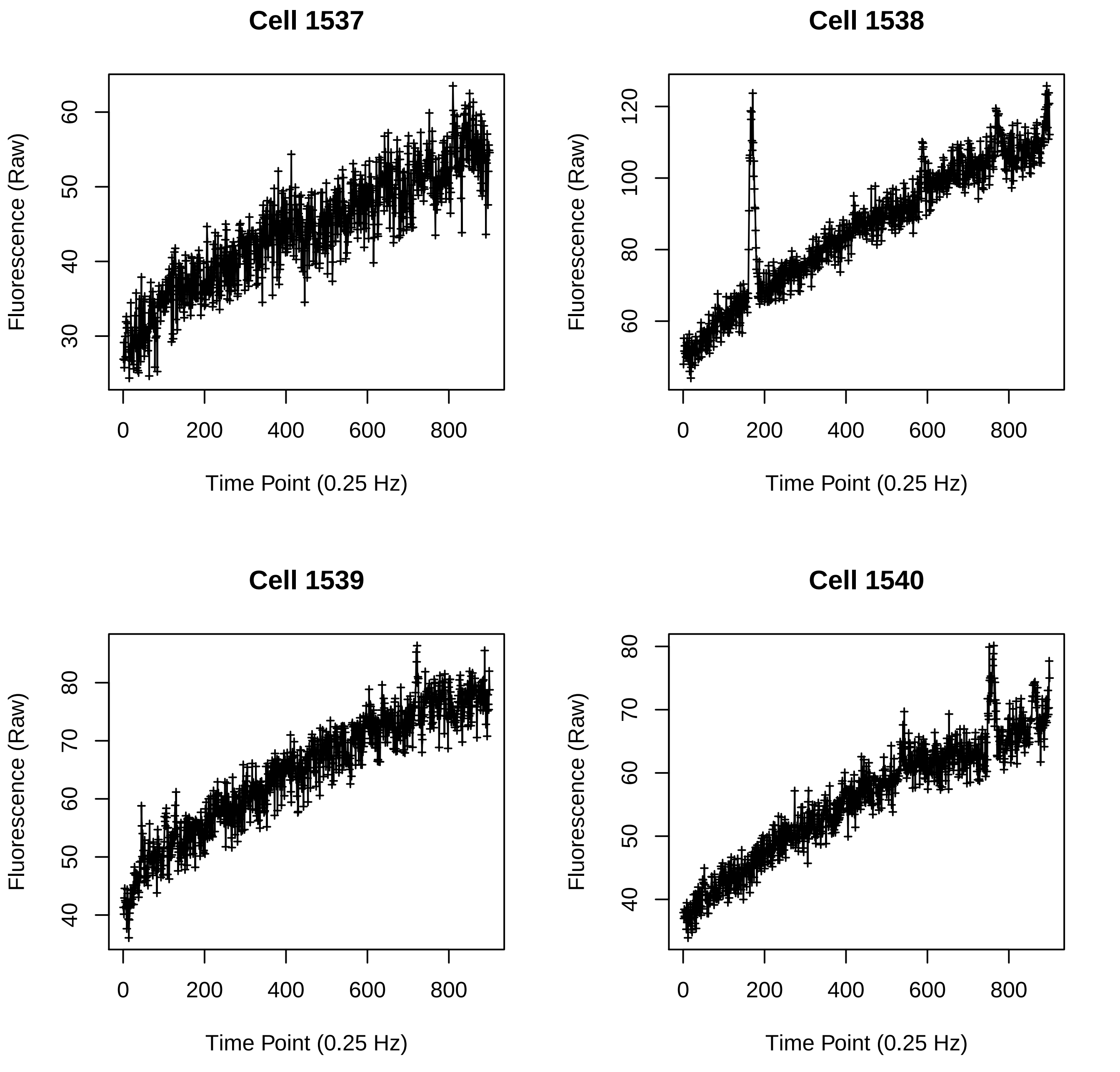
Cell 1537
Fluorescence (Raw)
30
40
50
60
0
200
400
600
800
Time Point (0.25 Hz)
Cell 1538
Fluorescence (Raw)
60
80
100
120
0
200
400
600
800
Time Point (0.25 Hz)
Cell 1539
Fluorescence (Raw)
40
50
60
70
80
0
200
400
600
800
Time Point (0.25 Hz)
Cell 1540
Fluorescence (Raw)
40
50
60
70
80
0
200
400
600
800
Time Point (0.25 Hz)

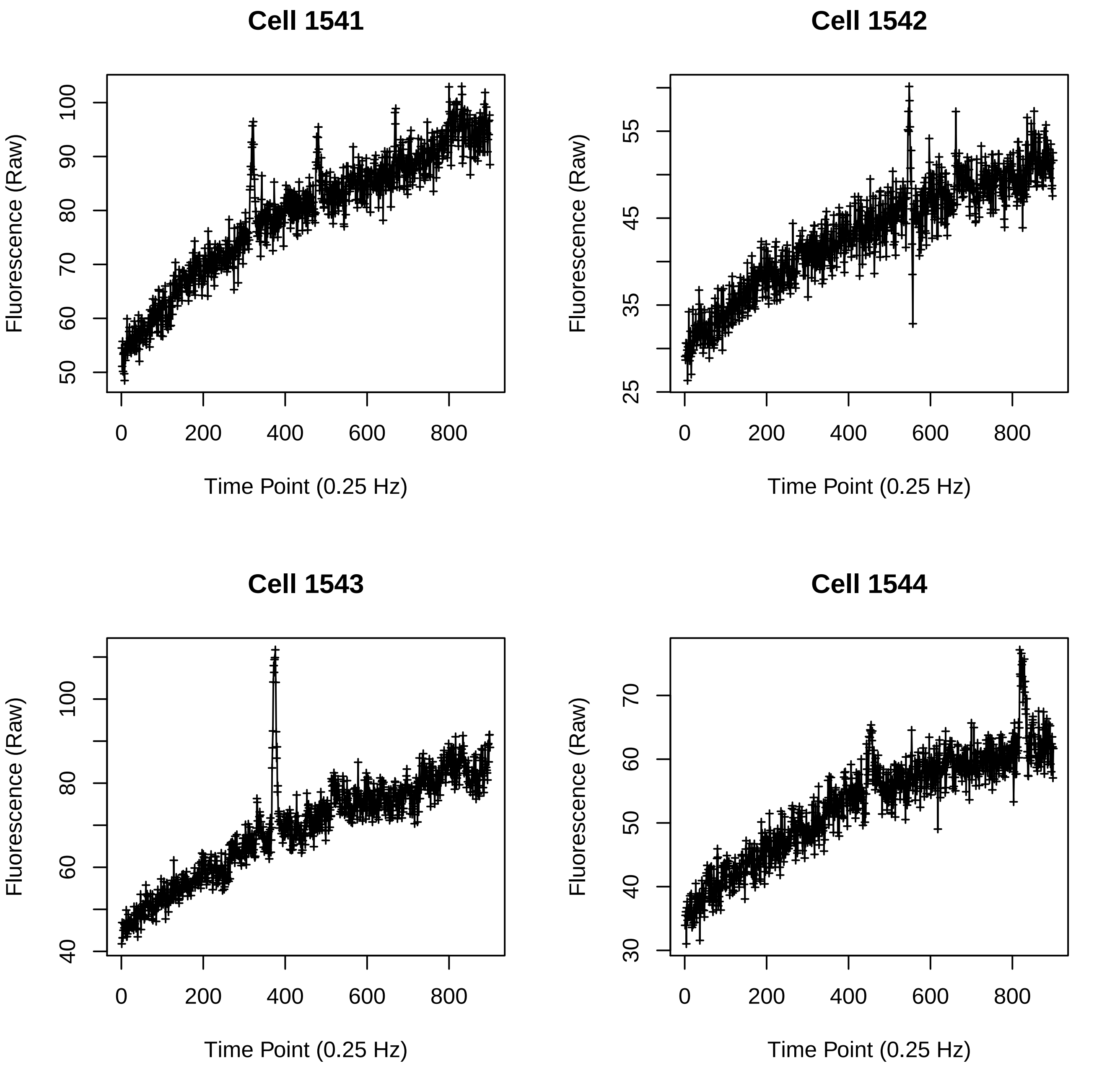
Cell 1541
Fluorescence (Raw)
50
60
70
80
90
100
0
200
400
600
800
Time Point (0.25 Hz)
Cell 1542
Fluorescence (Raw)
25
35
45
55
0
200
400
600
800
Time Point (0.25 Hz)
Cell 1543
Fluorescence (Raw)
40
60
80
100
0
200
400
600
800
Time Point (0.25 Hz)
Cell 1544
Fluorescence (Raw)
30
40
50
60
70
0
200
400
600
800
Time Point (0.25 Hz)

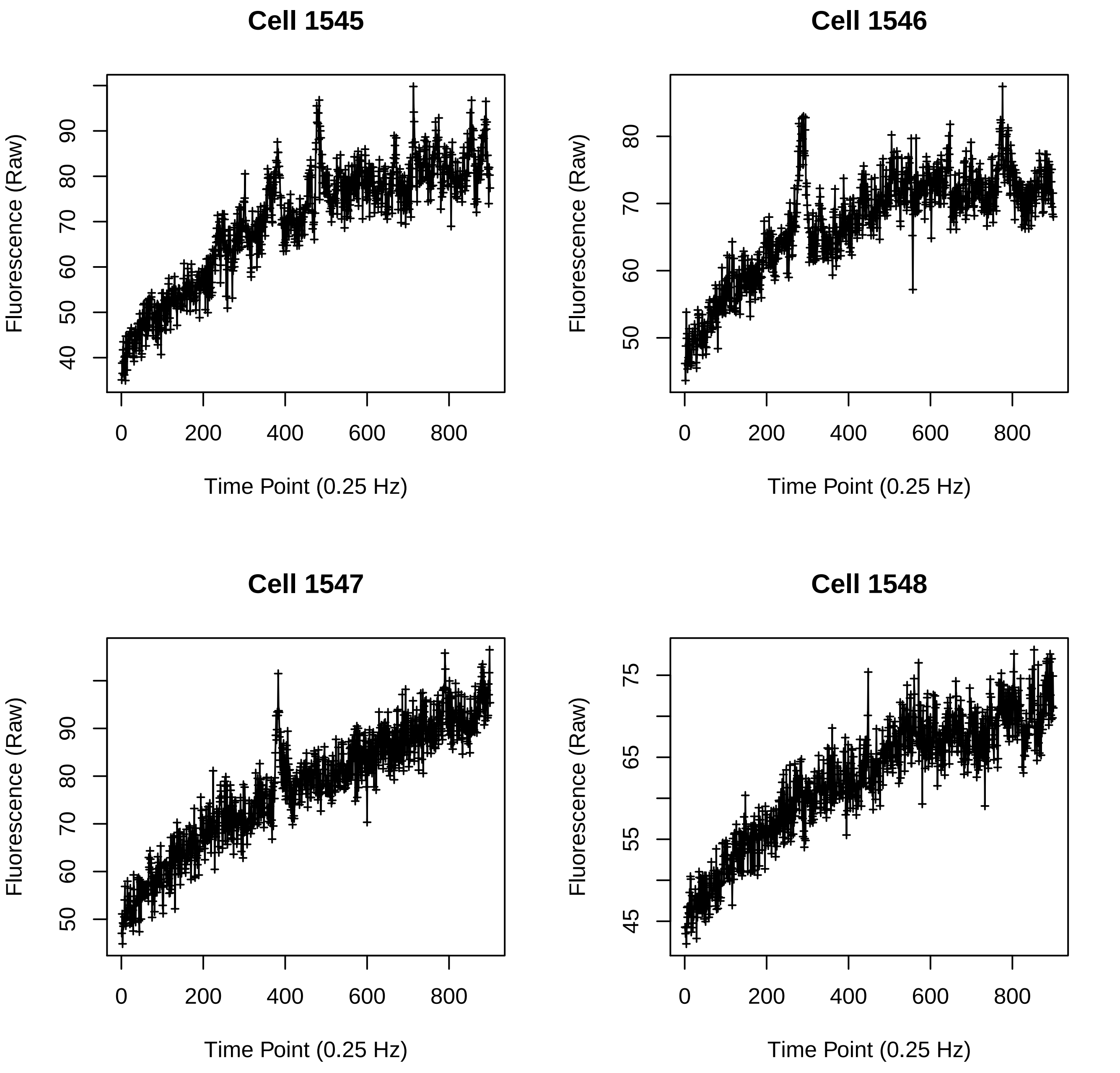

Cell 1545
Cell 1546
Cell 1547
Cell 1548
Fluorescence (Raw)
Time Point (0.25 Hz)
0
200
400
600
800
40
50
60
70
80
90
45
55
65
75

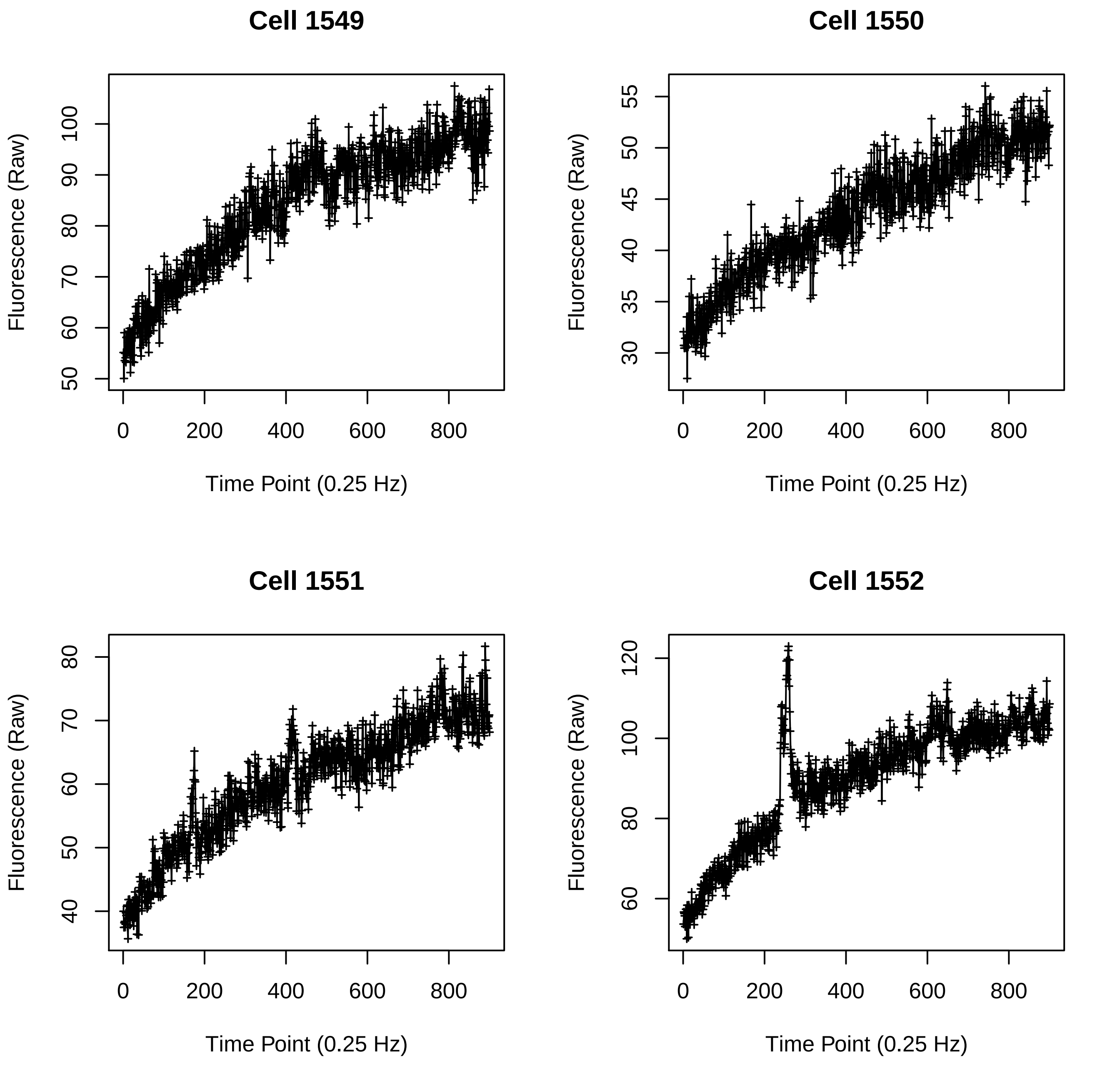
Cell 1549
Cell 1550
Cell 1551
Cell 1552
Fluorescence (Raw)
Time Point (0.25 Hz)
0
200
400
600
800
50
60
70
80
90
100
30
35
40
45
50
55
40
50
60
70
80
60
80
100
120

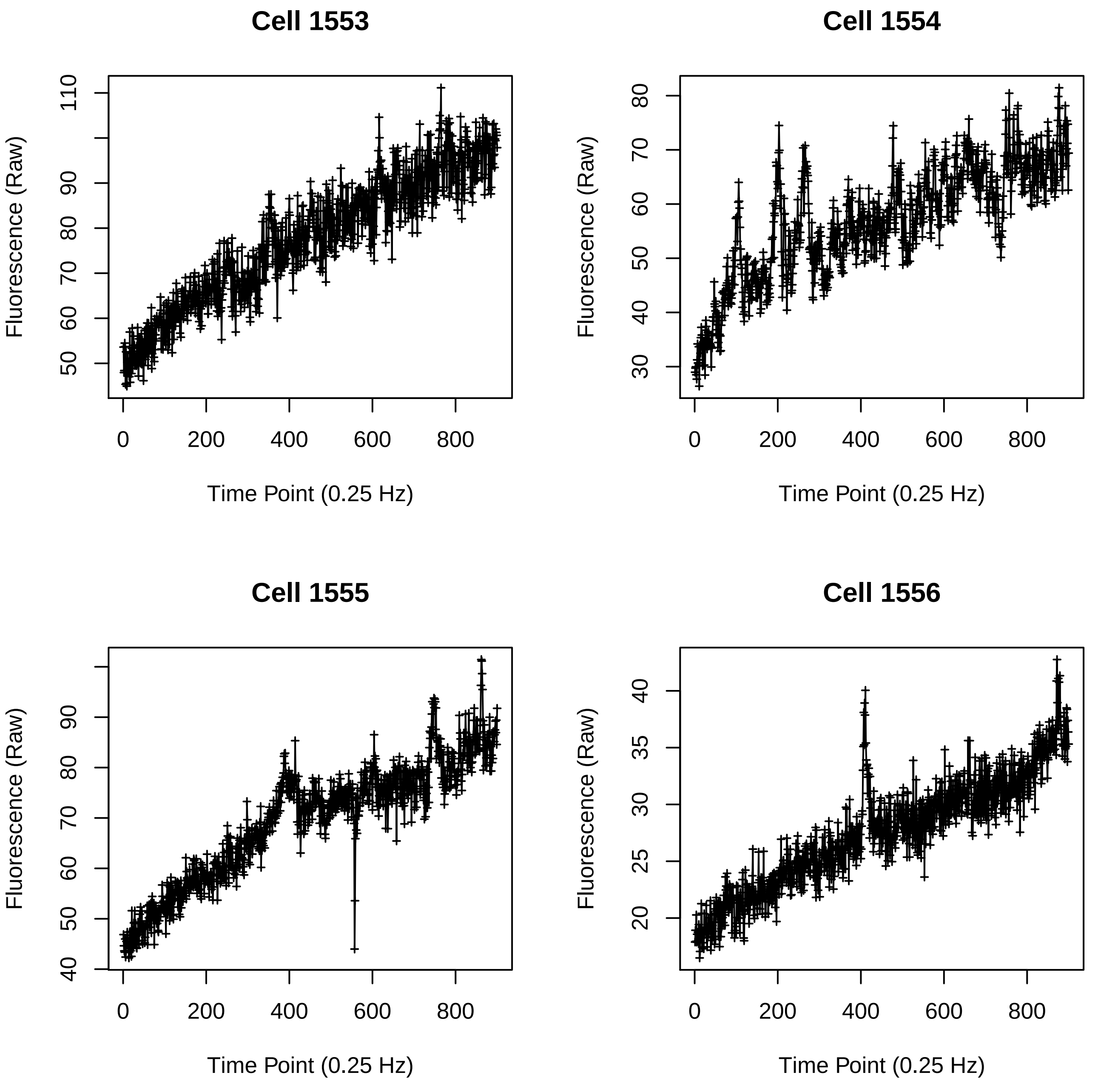

Cell 1553
Fluorescence (Raw)
Time Point (0.25 Hz)
50
60
70
80
90
110
0
200
400
600
800
Cell 1554
Fluorescence (Raw)
Time Point (0.25 Hz)
30
40
50
60
70
80
0
200
400
600
800
Cell 1555
Fluorescence (Raw)
Time Point (0.25 Hz)
40
50
60
70
80
90
0
200
400
600
800
Cell 1556
Fluorescence (Raw)
Time Point (0.25 Hz)
20
25
30
35
40
0
200
400
600
800

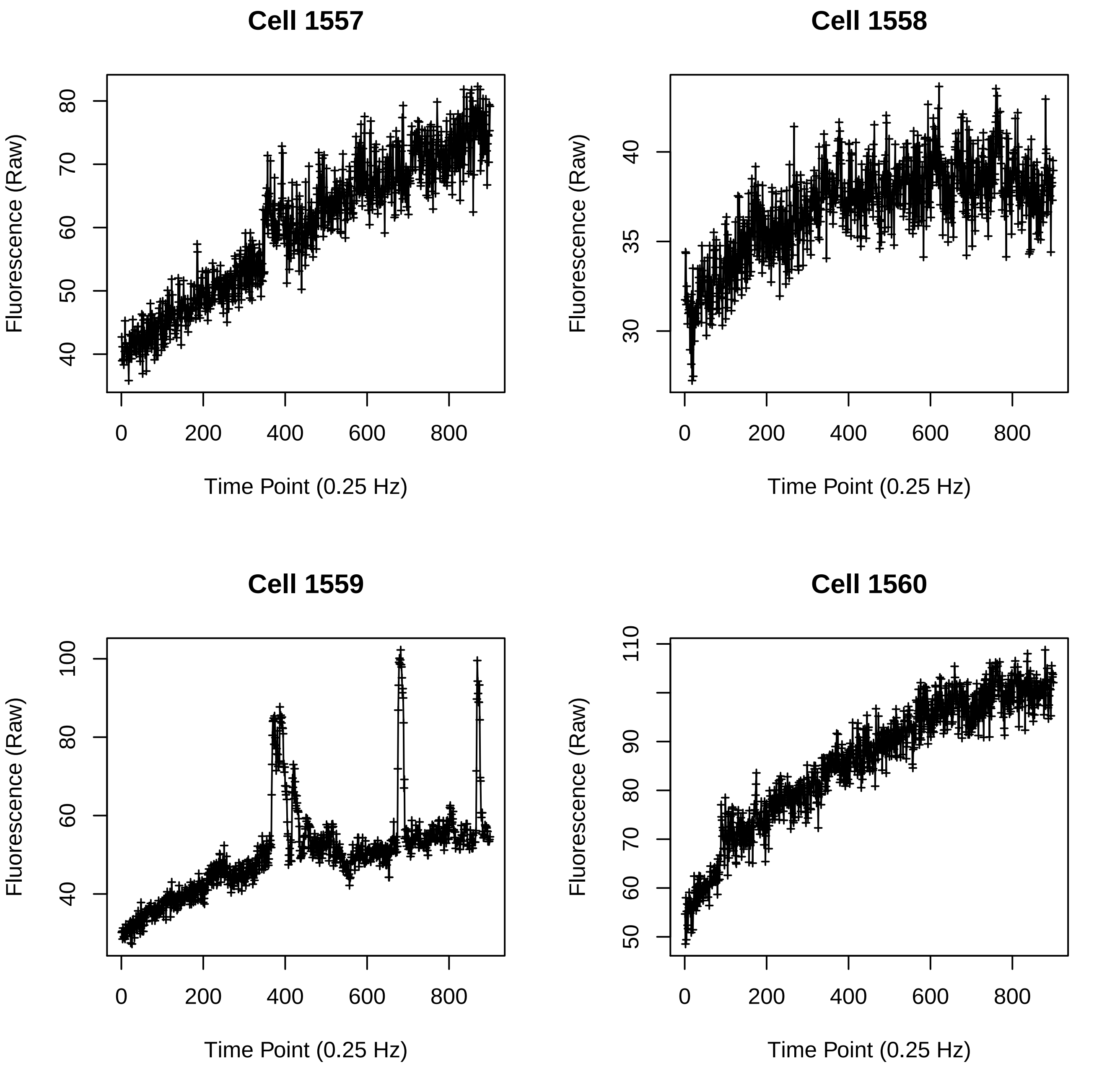
Cell 1557
Fluorescence (Raw)
Time Point (0.25 Hz)
Cell 1558
Fluorescence (Raw)
Time Point (0.25 Hz)
Cell 1559
Fluorescence (Raw)
Time Point (0.25 Hz)
Cell 1560
Fluorescence (Raw)
Time Point (0.25 Hz)

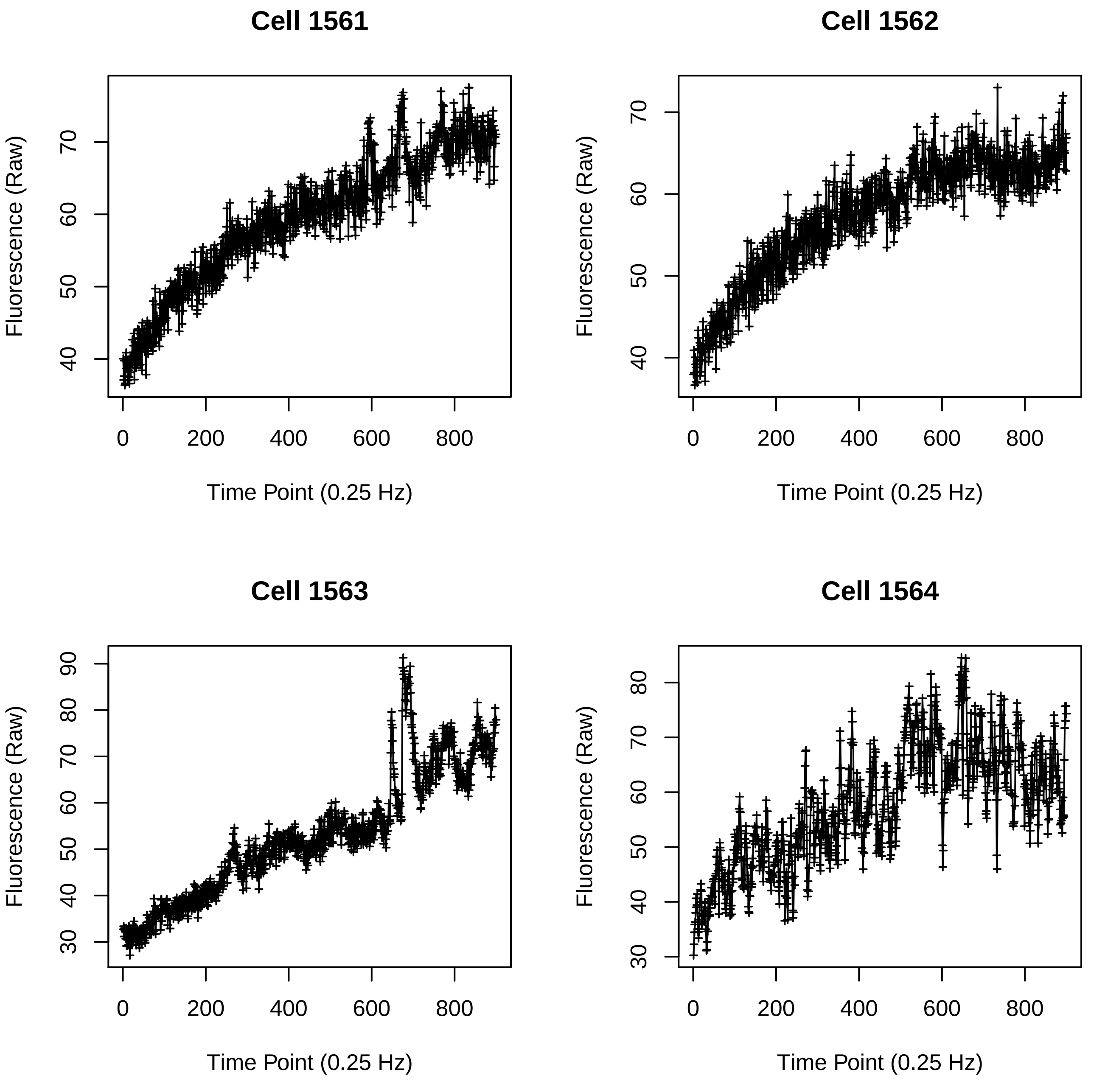
Cell 1561
Fluorescence (Raw)
40
50
60
70
0
200
400
600
800
Time Point (0.25 Hz)
Cell 1562
Fluorescence (Raw)
40
50
60
70
0
200
400
600
800
Time Point (0.25 Hz)
Cell 1563
Fluorescence (Raw)
30
40
50
60
70
80
90
0
200
400
600
800
Time Point (0.25 Hz)
Cell 1564
Fluorescence (Raw)
30
40
50
60
70
80
0
200
400
600
800
Time Point (0.25 Hz)

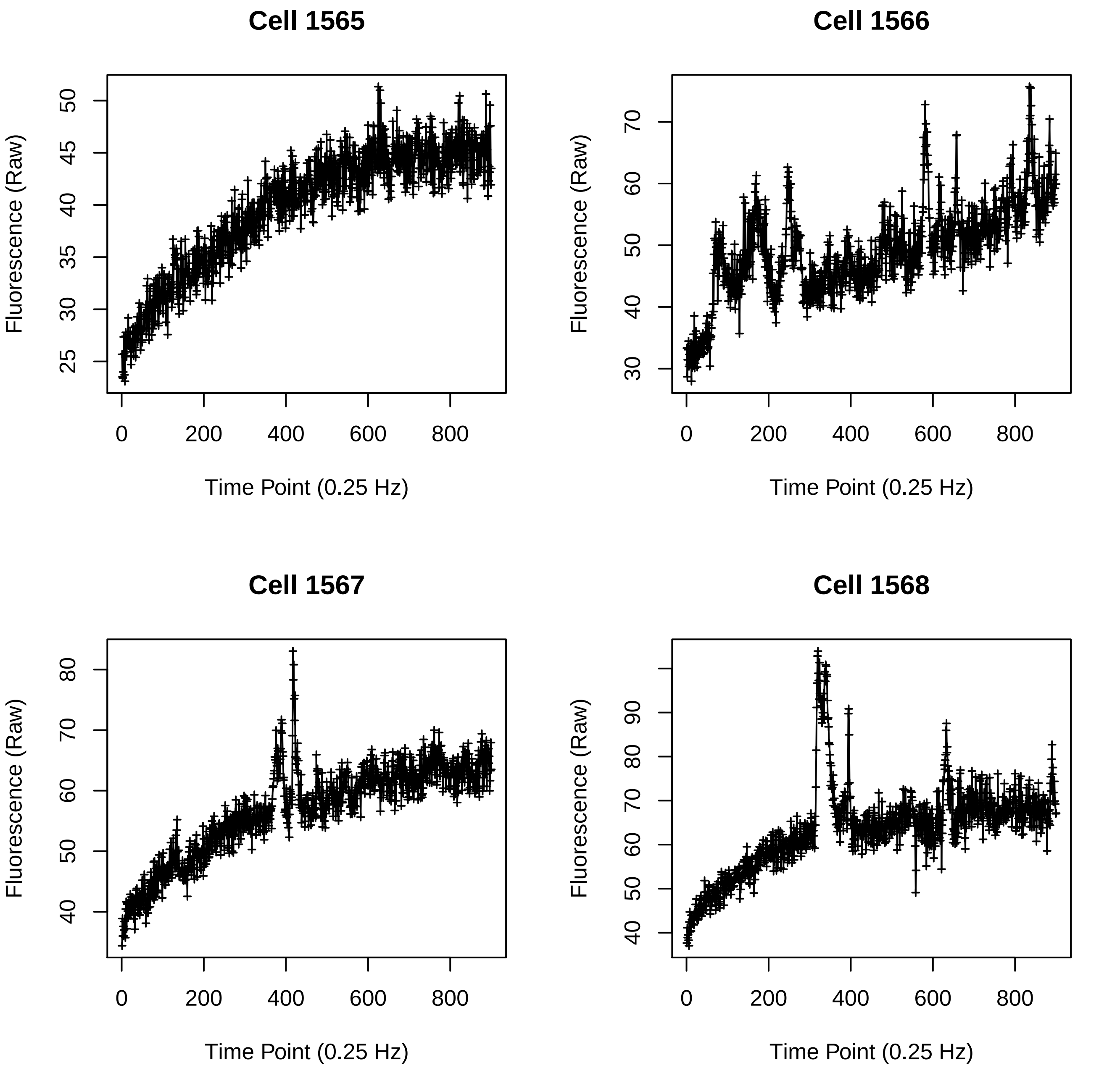

Cell 1565
Cell 1566
Cell 1567
Cell 1568
Fluorescence (Raw)
Time Point (0.25 Hz)
0
200
400
600
800
25
30
35
40
45
50
60
70
80
90

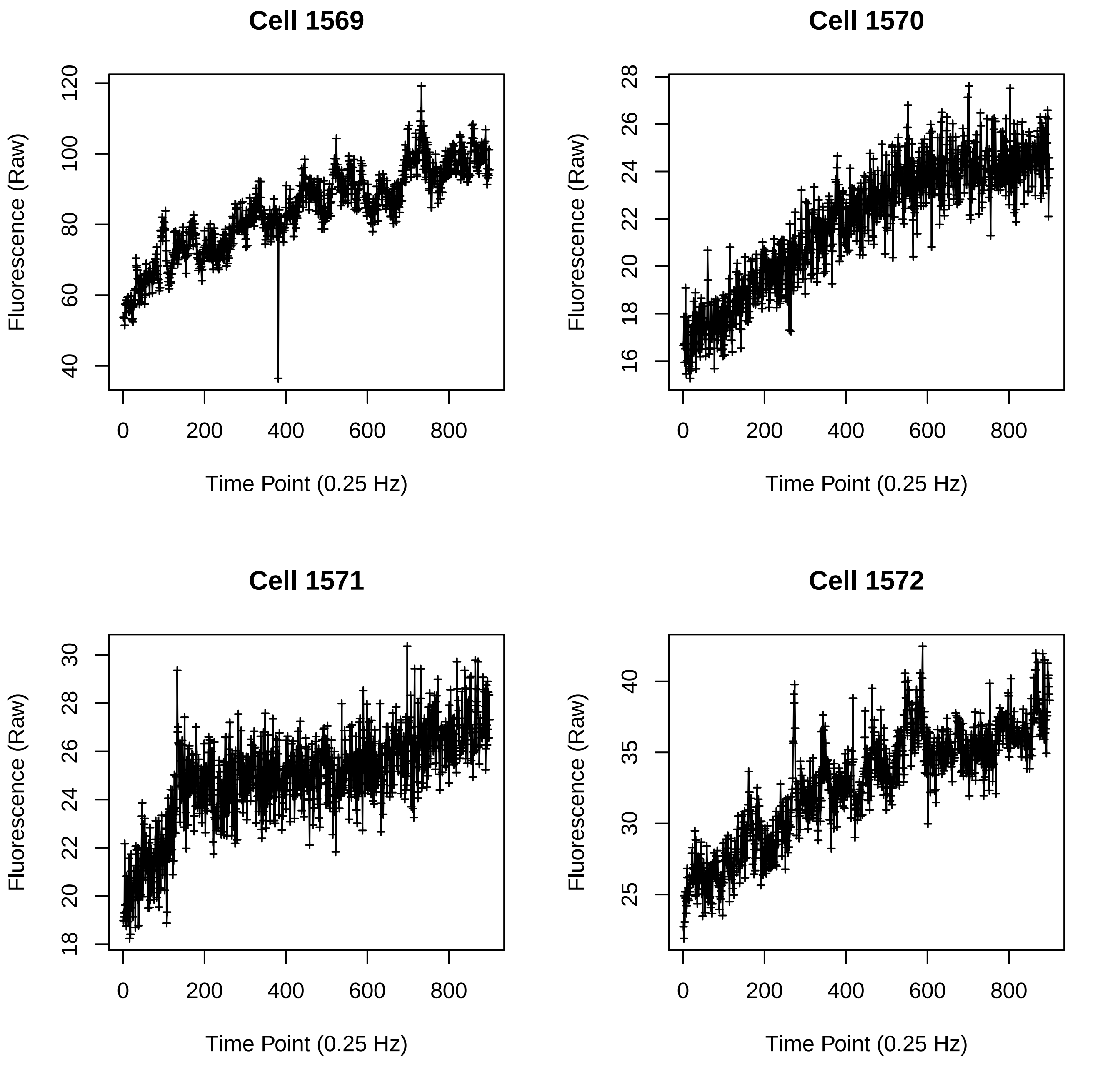
Cell 1569
Fluorescence (Raw)
40
60
80
100
120
0
200
400
600
800
Time Point (0.25 Hz)
Cell 1570
Fluorescence (Raw)
16
18
20
22
24
26
28
0
200
400
600
800
Time Point (0.25 Hz)
Cell 1571
Fluorescence (Raw)
18
20
22
24
26
28
30
0
200
400
600
800
Time Point (0.25 Hz)
Cell 1572
Fluorescence (Raw)
25
30
35
40
0
200
400
600
800
Time Point (0.25 Hz)

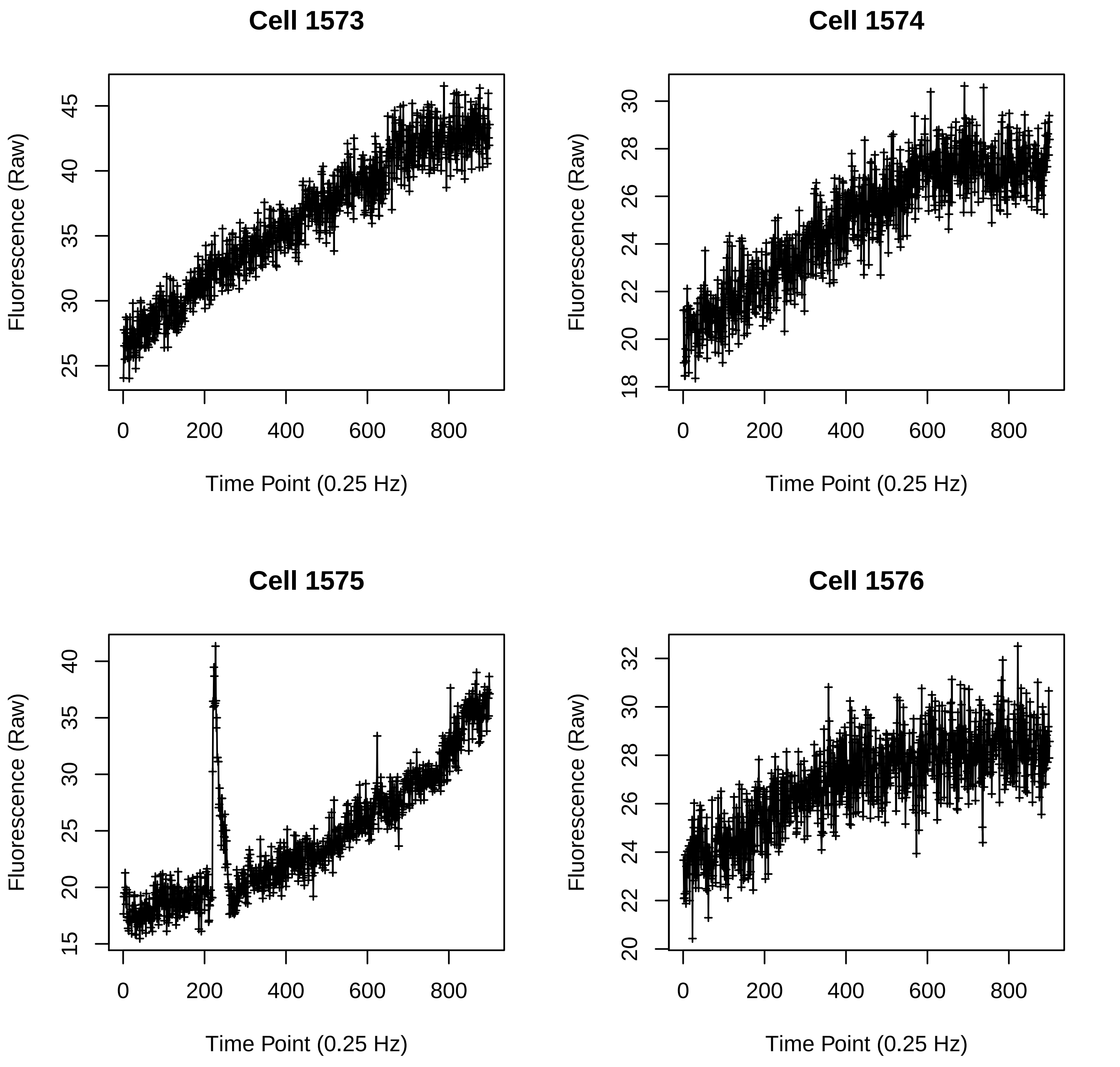
Cell 1573
Cell 1574
Cell 1575
Cell 1576
Fluorescence (Raw)
Time Point (0.25 Hz)
0
200
400
600
800
25
30
35
40
45
18
20
22
24
26
28
30
15
20
25
30
35
40
20
22
24
26
28
30
32

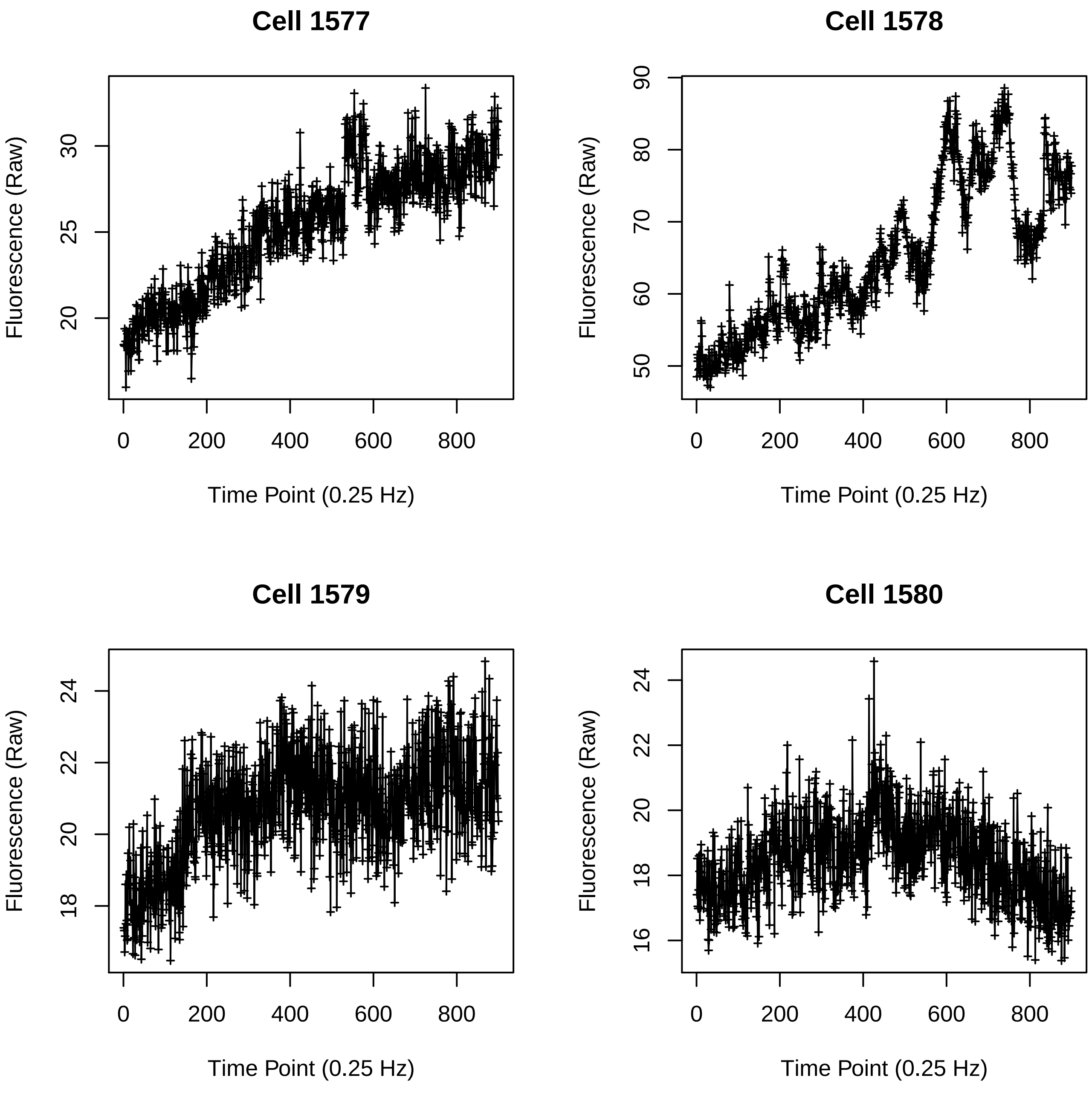
Cell 1577
Fluorescence (Raw)
20
25
30
0
200
400
600
800
Time Point (0.25 Hz)
Cell 1578
Fluorescence (Raw)
50
60
70
80
90
0
200
400
600
800
Time Point (0.25 Hz)
Cell 1579
Fluorescence (Raw)
18
20
22
24
0
200
400
600
800
Time Point (0.25 Hz)
Cell 1580
Fluorescence (Raw)
16
18
20
22
24
0
200
400
600
800
Time Point (0.25 Hz)

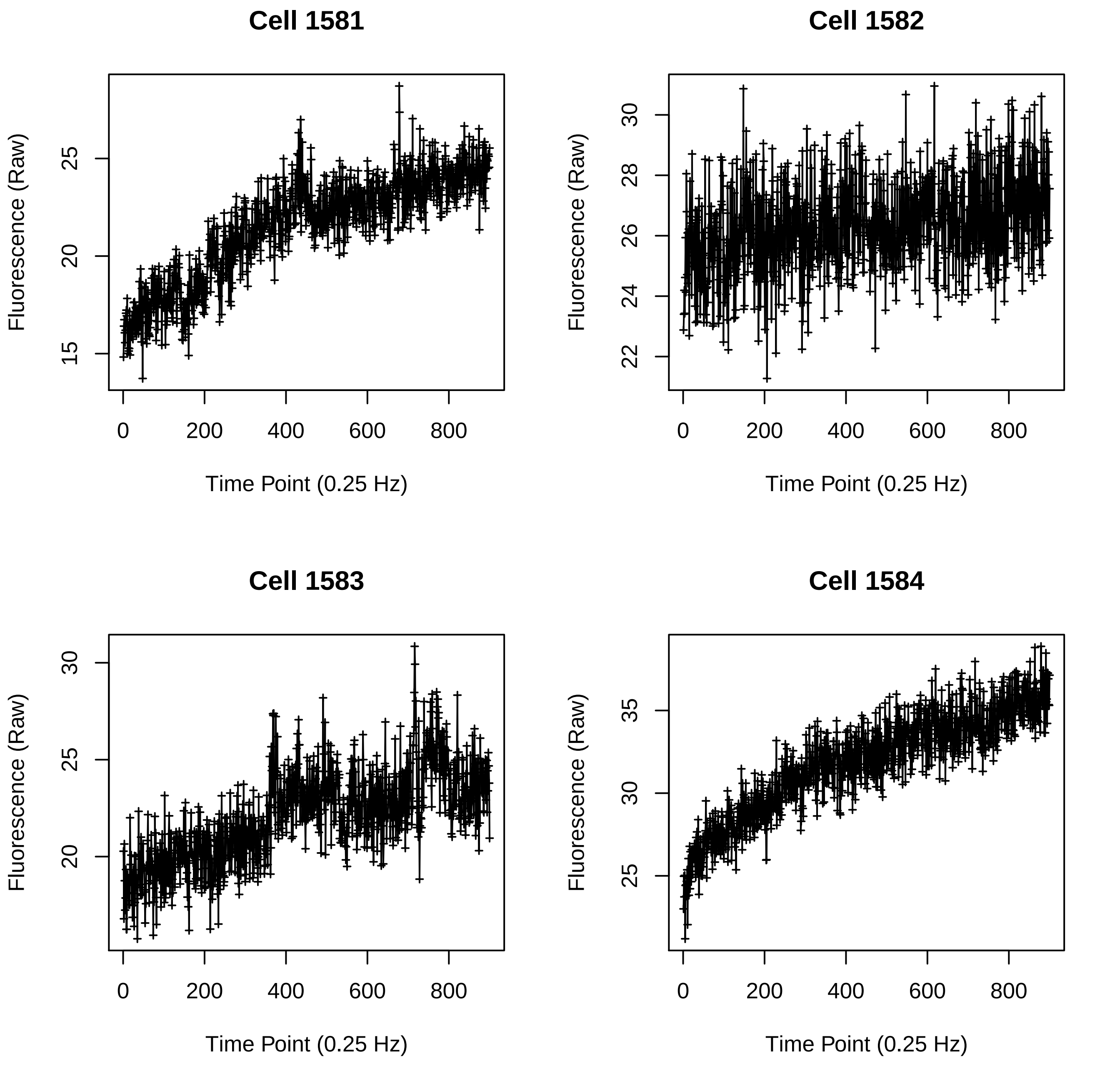

Cell 1581
Cell 1582
Cell 1583
Cell 1584
Fluorescence (Raw)
Time Point (0.25 Hz)
0
200
400
600
800
15
20
25
22
24
26
28
30
35

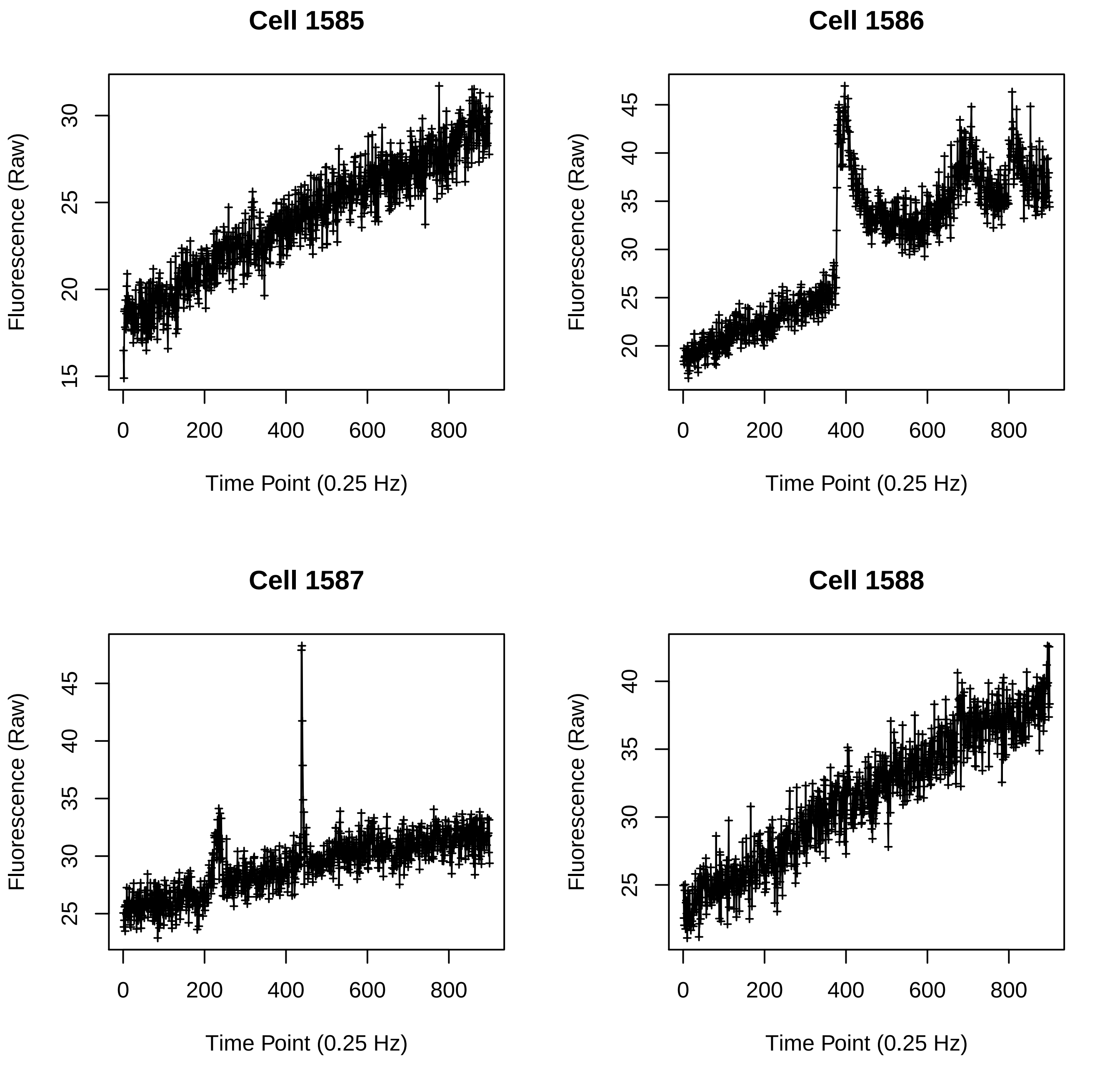

Cell 1585
Cell 1586
Cell 1587
Cell 1588
Fluorescence (Raw)
Time Point (0.25 Hz)
0
200
400
600
800
15
20
25
30
35
40
45

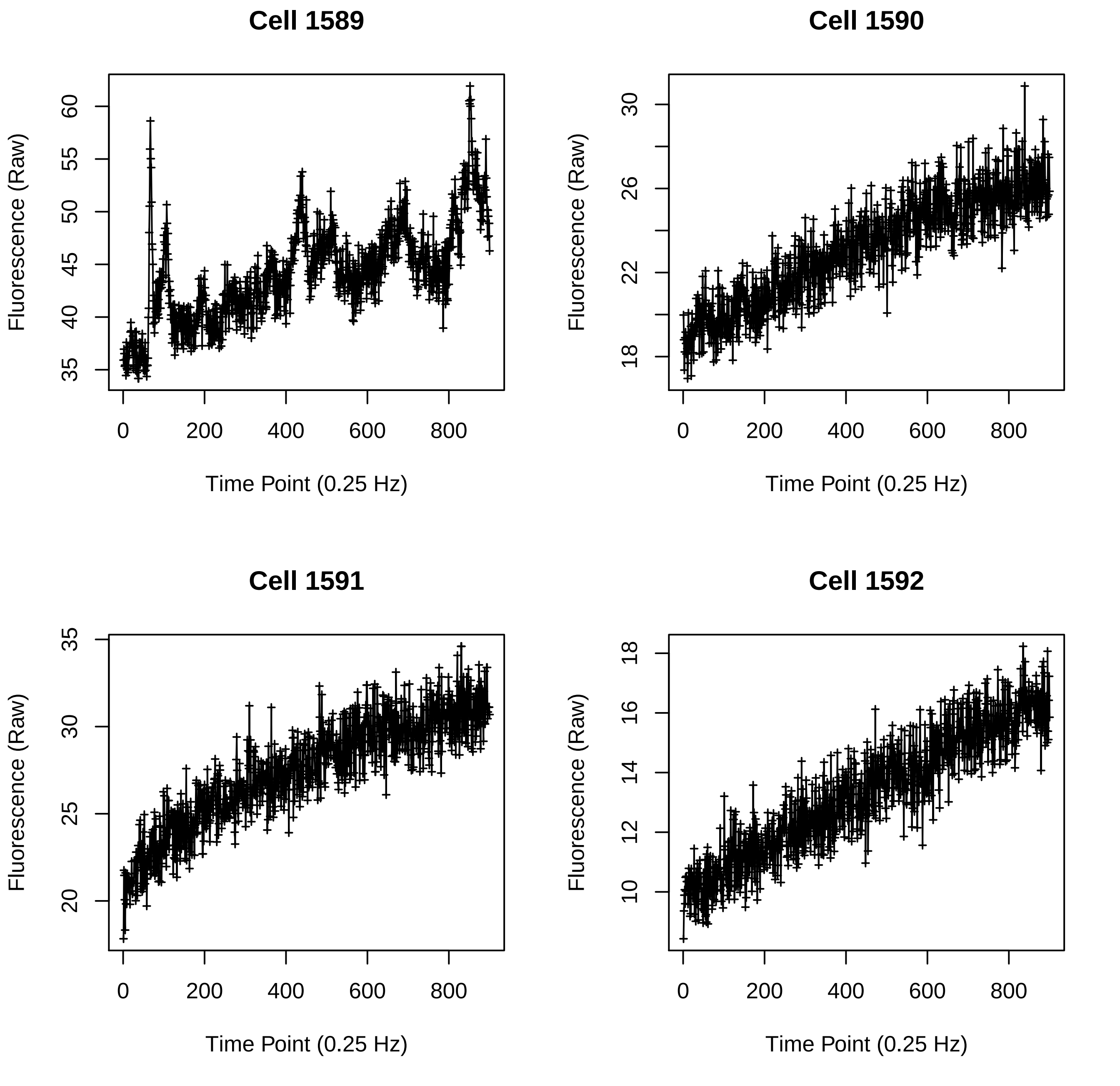
Cell 1589
Fluorescence (Raw)
35
40
45
50
55
60
0
200
400
600
800
Time Point (0.25 Hz)
Cell 1590
Fluorescence (Raw)
18
22
26
30
0
200
400
600
800
Time Point (0.25 Hz)
Cell 1591
Fluorescence (Raw)
20
25
30
35
0
200
400
600
800
Time Point (0.25 Hz)
Cell 1592
Fluorescence (Raw)
10
12
14
16
18
0
200
400
600
800
Time Point (0.25 Hz)

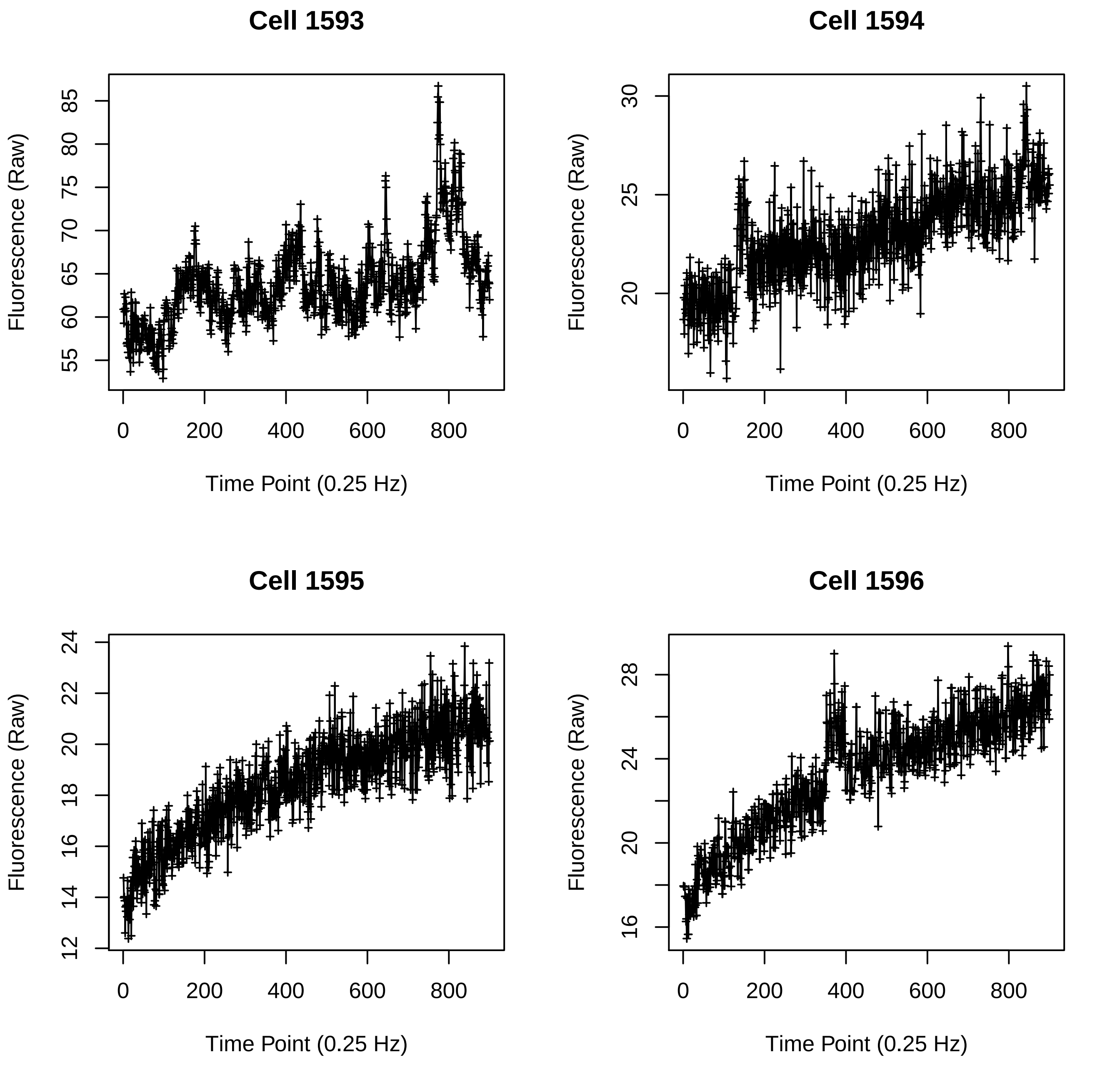

Cell 1593
Cell 1594
Cell 1595
Cell 1596
Fluorescence (Raw)
Time Point (0.25 Hz)
0
200
400
600
800
55
60
65
70
75
80
85
20
25
30
12
14
16
18
20
22
24
16
20
24
28

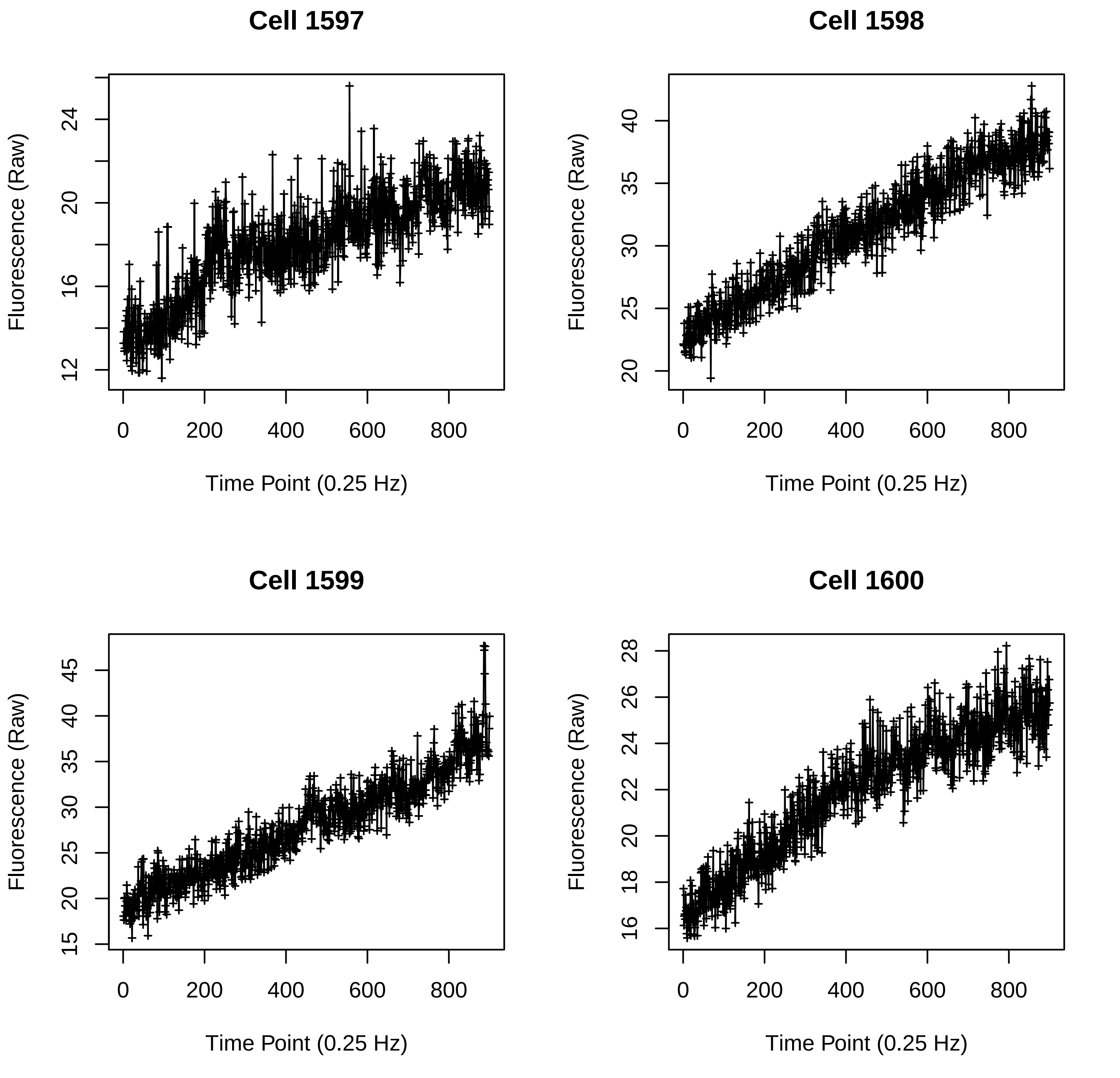
Cell 1597
Cell 1598
Cell 1599
Cell 1600
Fluorescence (Raw)
Time Point (0.25 Hz)
0
200
400
600
800
12
16
20
24
20
25
30
35
40
15
20
25
30
35
40
45
16
18
20
22
24
26
28

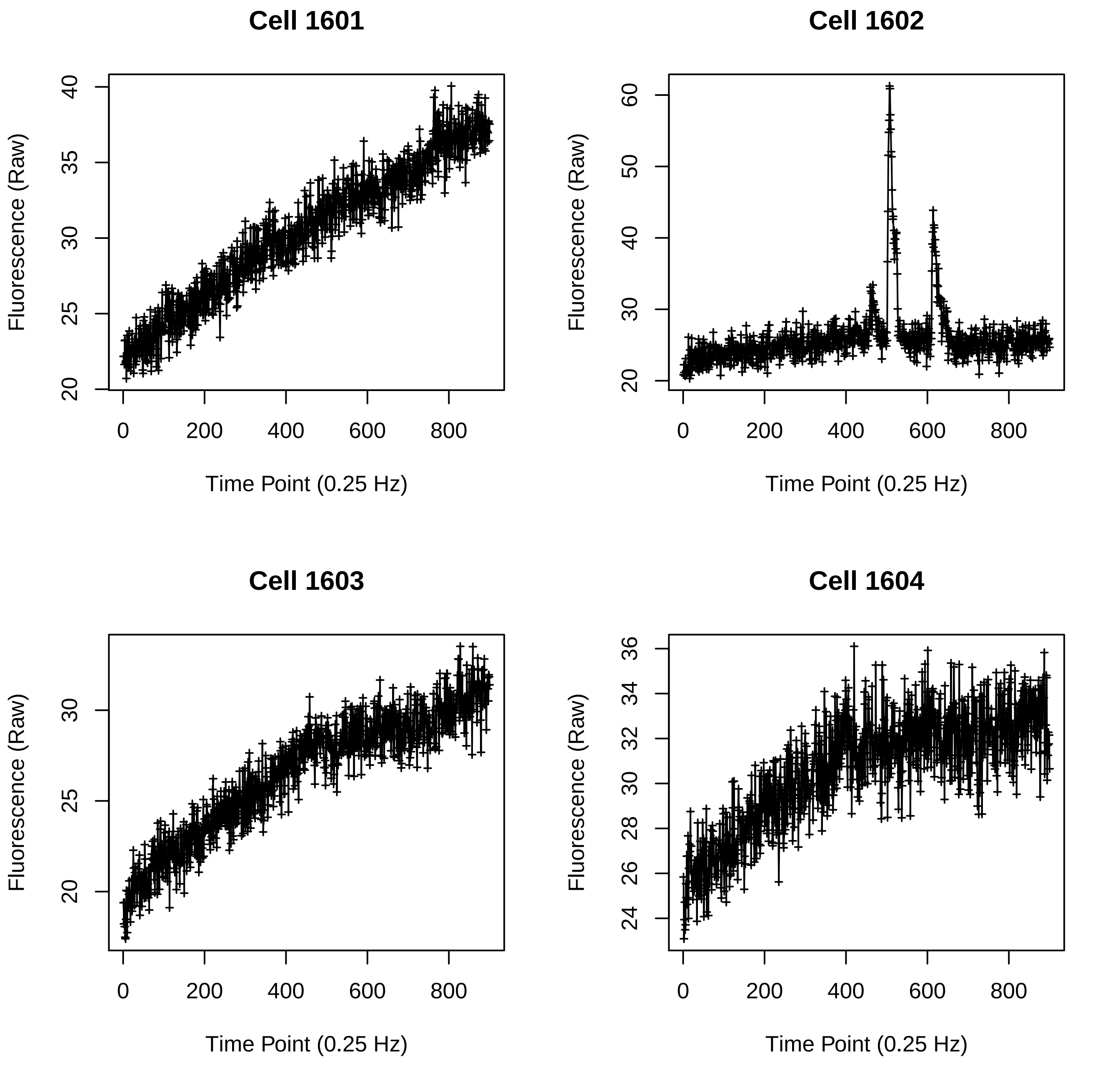
Cell 1601
Fluorescence (Raw)
20
25
30
35
40
0
200
400
600
800
Time Point (0.25 Hz)
Cell 1602
Fluorescence (Raw)
20
30
40
50
60
0
200
400
600
800
Time Point (0.25 Hz)
Cell 1603
Fluorescence (Raw)
20
25
30
0
200
400
600
800
Time Point (0.25 Hz)
Cell 1604
Fluorescence (Raw)
24
26
28
30
32
34
36
0
200
400
600
800
Time Point (0.25 Hz)

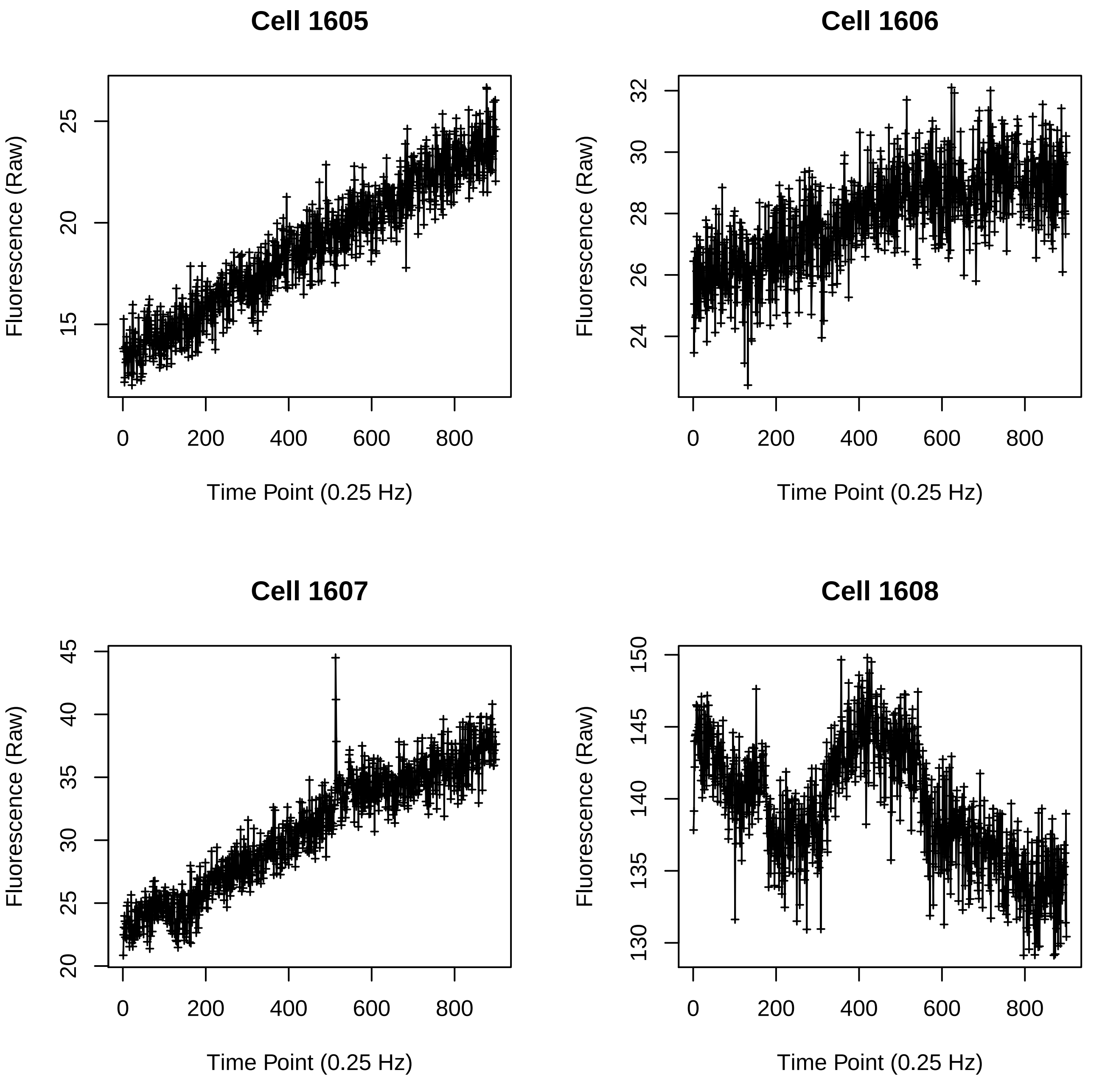
Cell 1605
Cell 1606
Cell 1607
Cell 1608
Fluorescence (Raw)
Time Point (0.25 Hz)
0
200
400
600
800
15
20
25
24
26
28
30
32
20
25
30
35
40
45
130
135
140
145
150

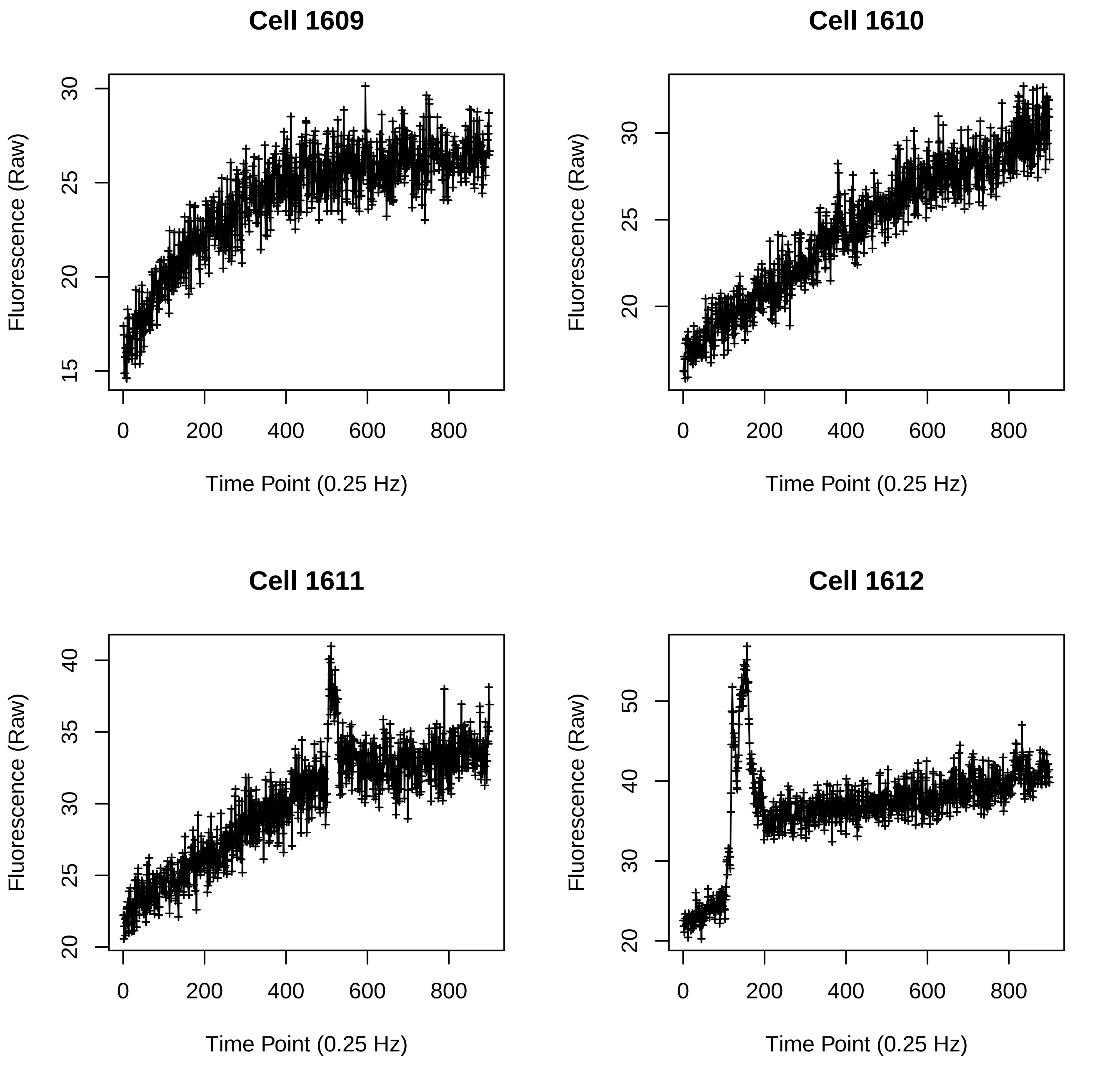

Cell 1609
Cell 1610
Cell 1611
Cell 1612
Fluorescence (Raw)
Time Point (0.25 Hz)
0
200
400
600
800
15
20
25
30
35
40
50

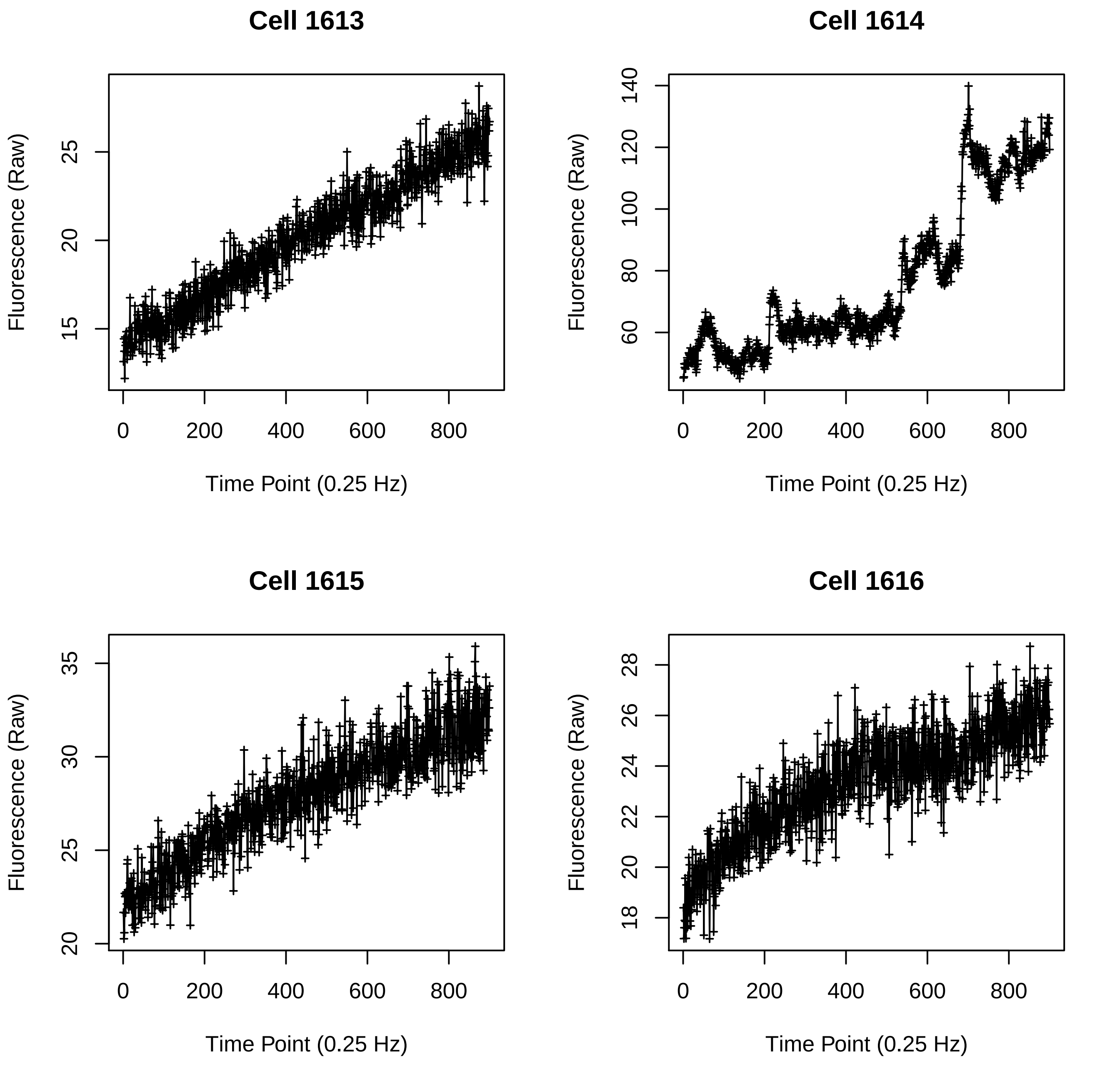
Cell 1613
Fluorescence (Raw)
15
20
25
0
200
400
600
800
Time Point (0.25 Hz)
Cell 1614
Fluorescence (Raw)
60
80
100
120
140
0
200
400
600
800
Time Point (0.25 Hz)
Cell 1615
Fluorescence (Raw)
20
25
30
35
0
200
400
600
800
Time Point (0.25 Hz)
Cell 1616
Fluorescence (Raw)
18
20
22
24
26
28
0
200
400
600
800
Time Point (0.25 Hz)

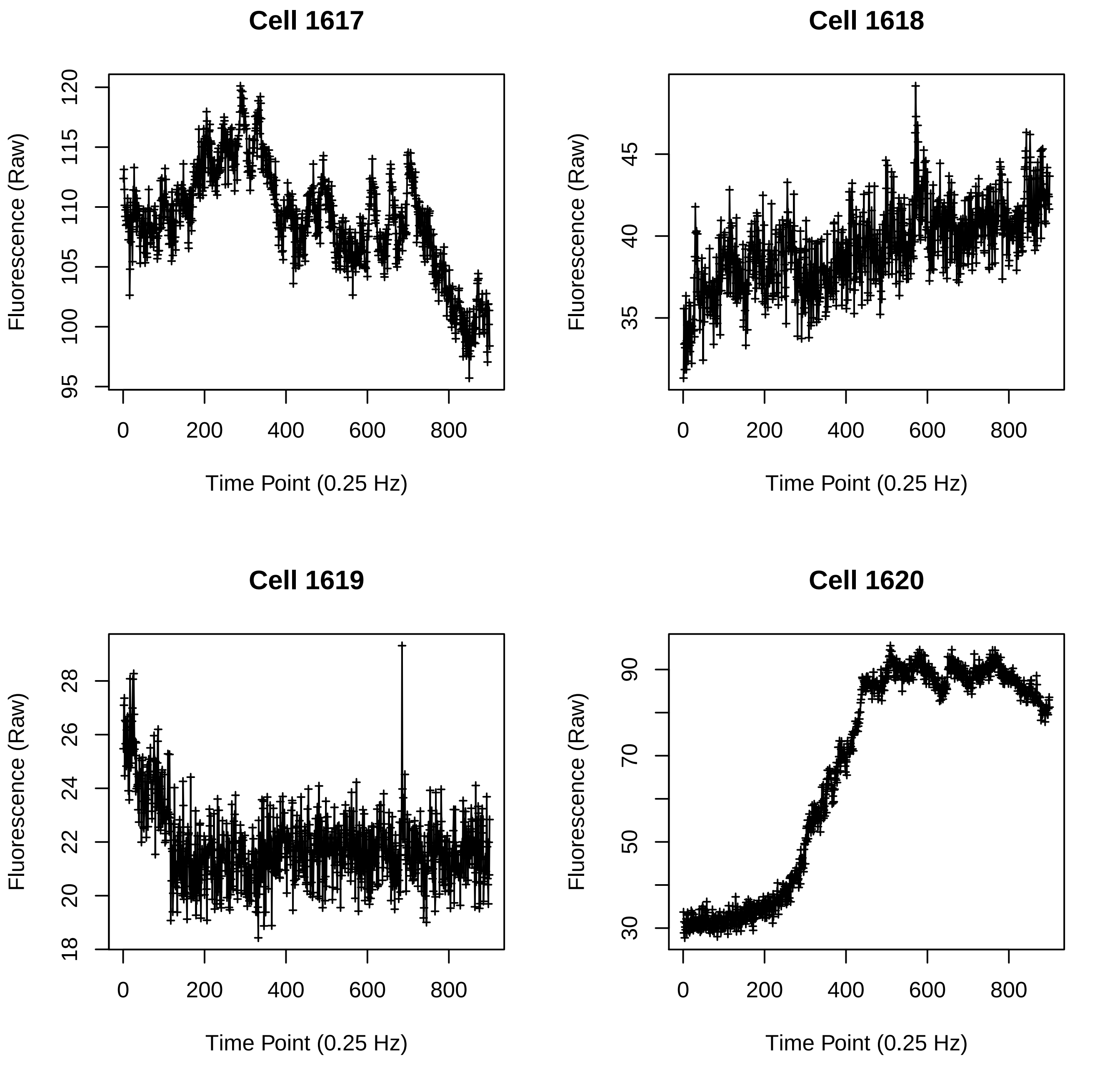
Cell 1617
Fluorescence (Raw)
Time Point (0.25 Hz)
Cell 1618
Fluorescence (Raw)
Time Point (0.25 Hz)
Cell 1619
Fluorescence (Raw)
Time Point (0.25 Hz)
Cell 1620
Fluorescence (Raw)
Time Point (0.25 Hz)

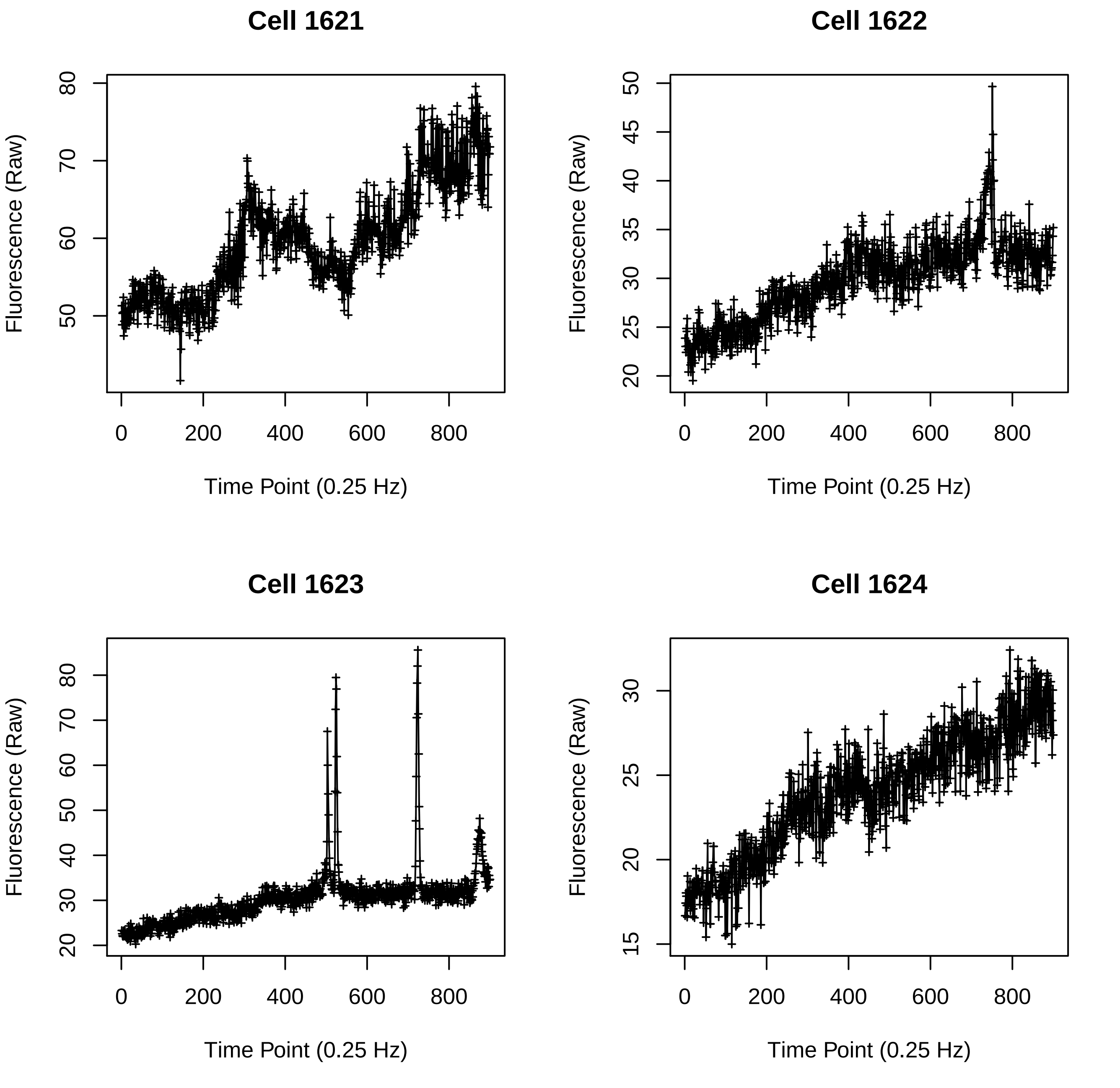
Cell 1621
Fluorescence (Raw)
50
60
70
80
0
200
400
600
800
Time Point (0.25 Hz)
Cell 1622
Fluorescence (Raw)
20
25
30
35
40
45
50
0
200
400
600
800
Time Point (0.25 Hz)
Cell 1623
Fluorescence (Raw)
20
30
40
50
60
70
80
0
200
400
600
800
Time Point (0.25 Hz)
Cell 1624
Fluorescence (Raw)
15
20
25
30
0
200
400
600
800
Time Point (0.25 Hz)

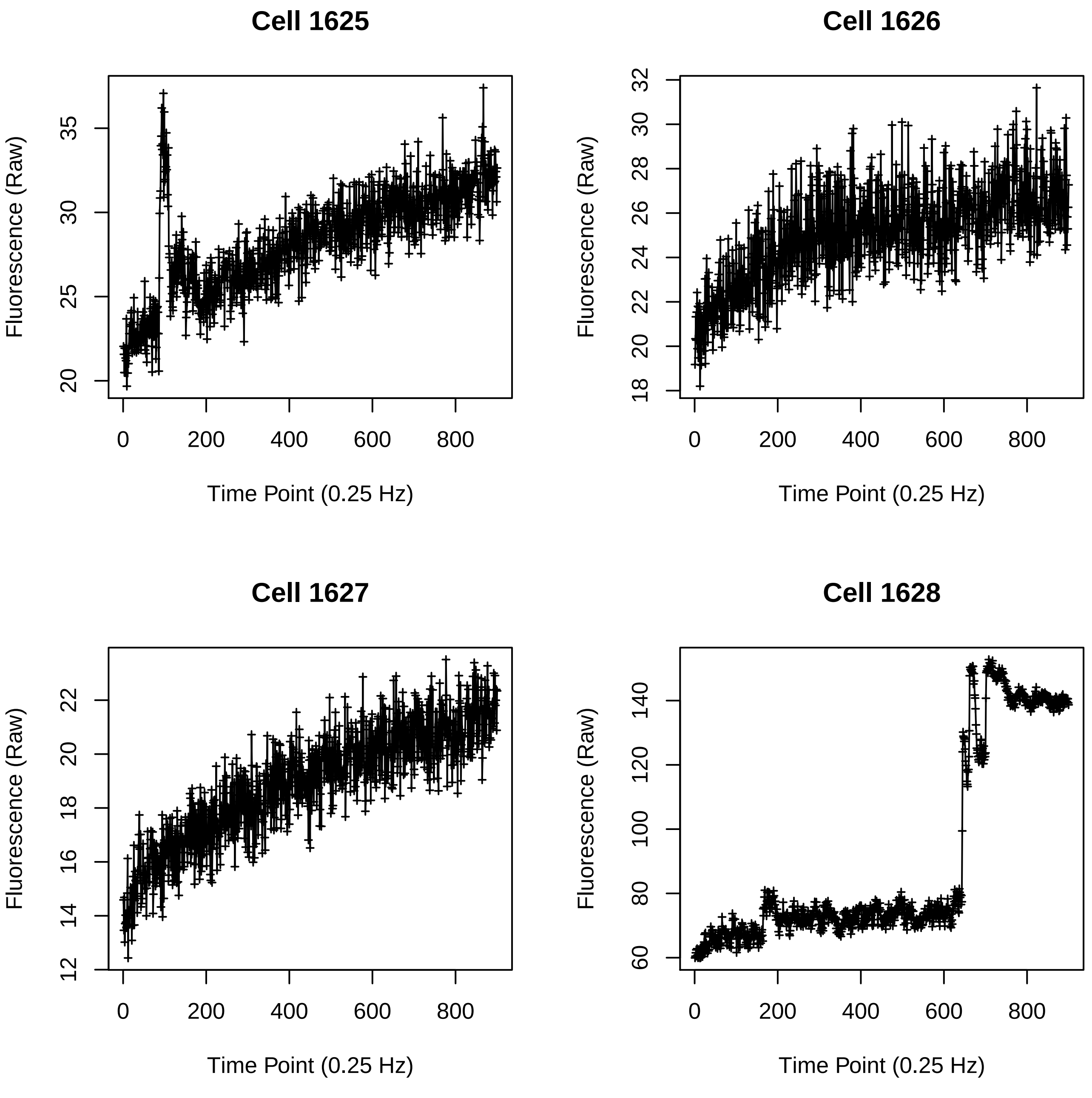
Cell 1625
Cell 1626
Cell 1627
Cell 1628
Fluorescence (Raw)
Time Point (0.25 Hz)
20
25
30
35
18
22
24
26
28
32
12
14
16
60
80
100
120
140
0
200
400
600
800

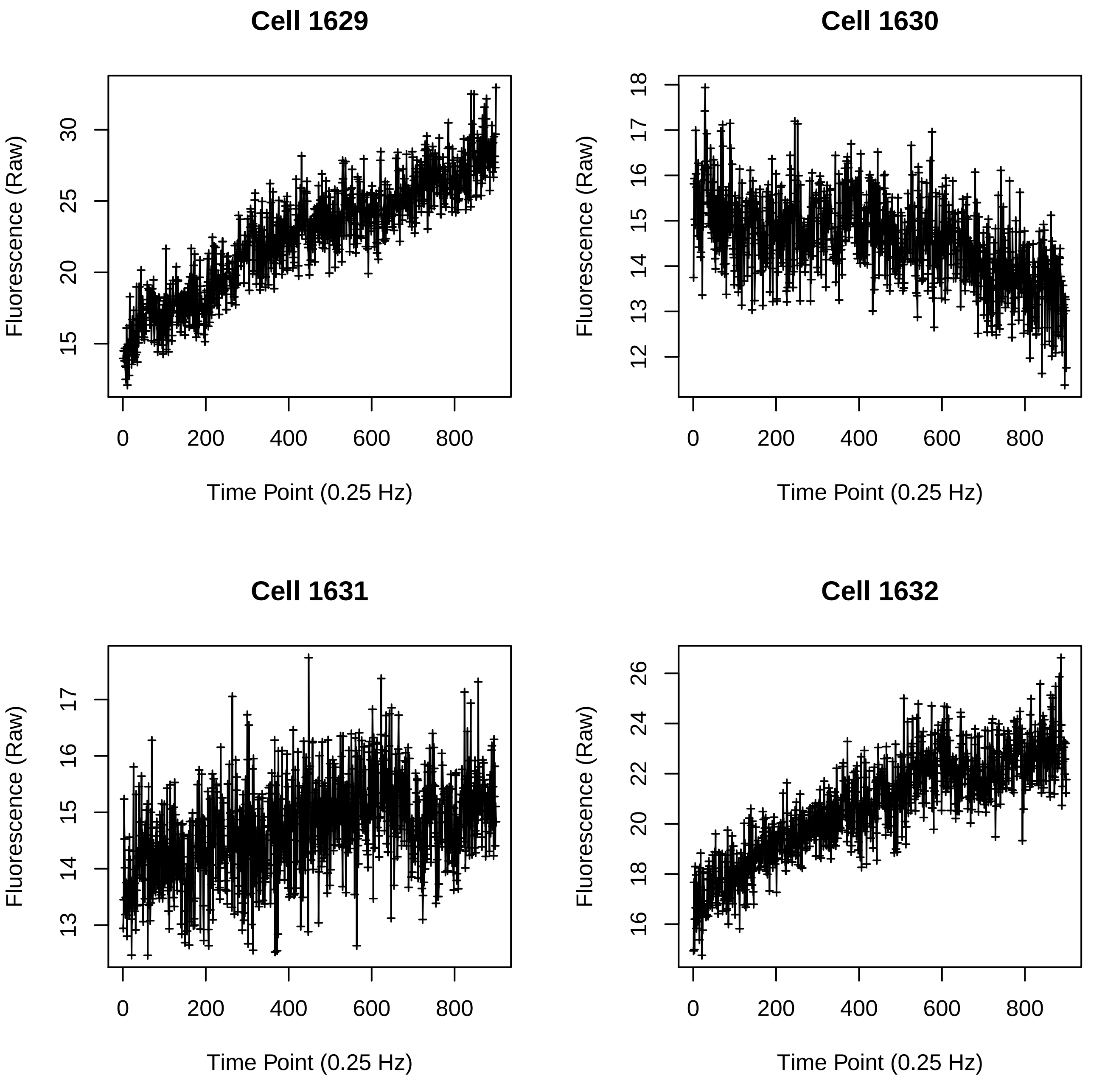
Cell 1629
Fluorescence (Raw)
15
20
25
30
0
200
400
600
800
Time Point (0.25 Hz)
Cell 1630
Fluorescence (Raw)
12
13
14
15
16
17
18
0
200
400
600
800
Time Point (0.25 Hz)
Cell 1631
Fluorescence (Raw)
13
14
15
16
17
0
200
400
600
800
Time Point (0.25 Hz)
Cell 1632
Fluorescence (Raw)
16
18
20
22
24
26
0
200
400
600
800
Time Point (0.25 Hz)

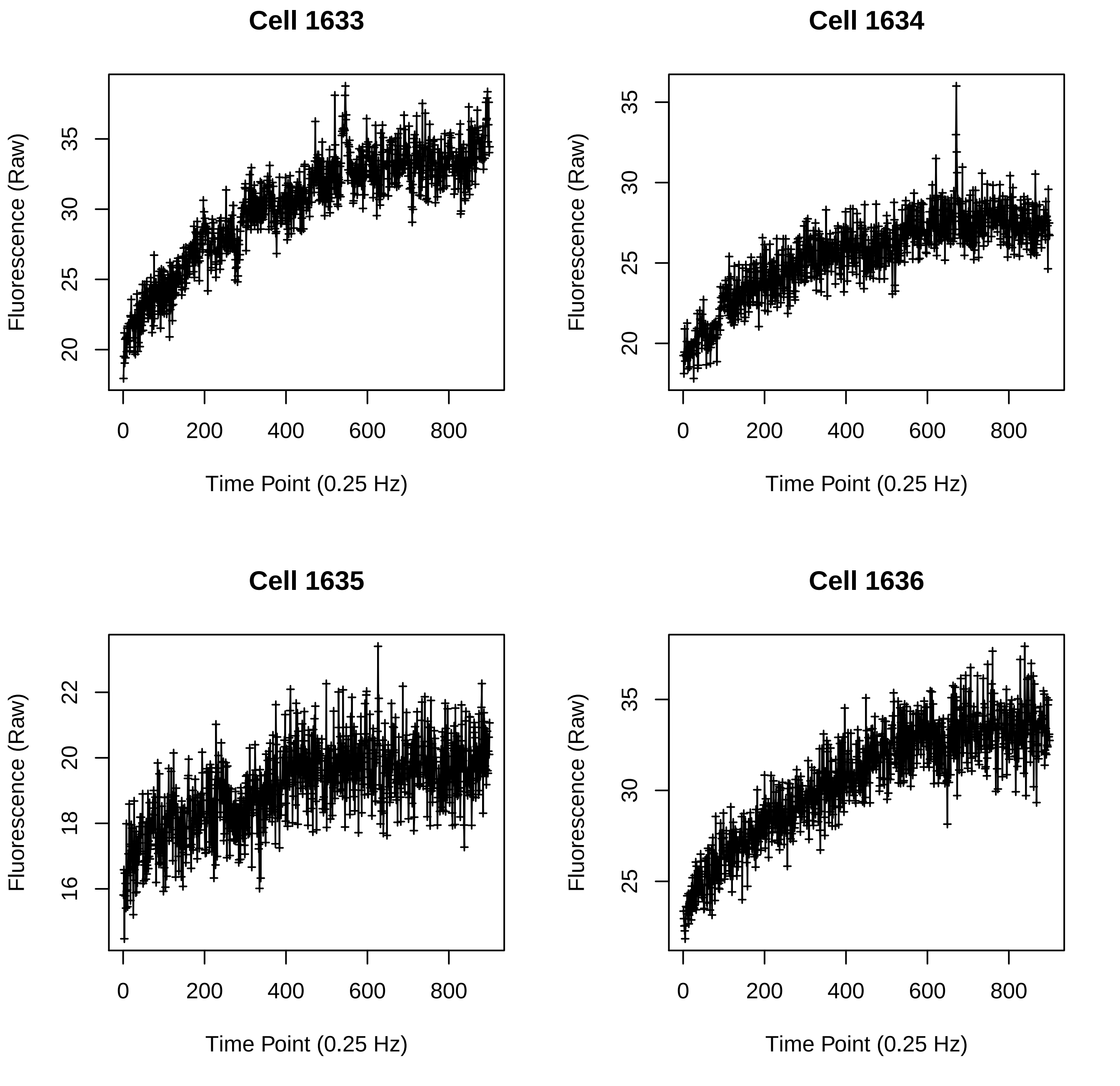

Cell 1633
Cell 1634
Cell 1635
Cell 1636
Fluorescence (Raw)
Time Point (0.25 Hz)
0
200
400
600
800
20
25
30
35
16
18
22

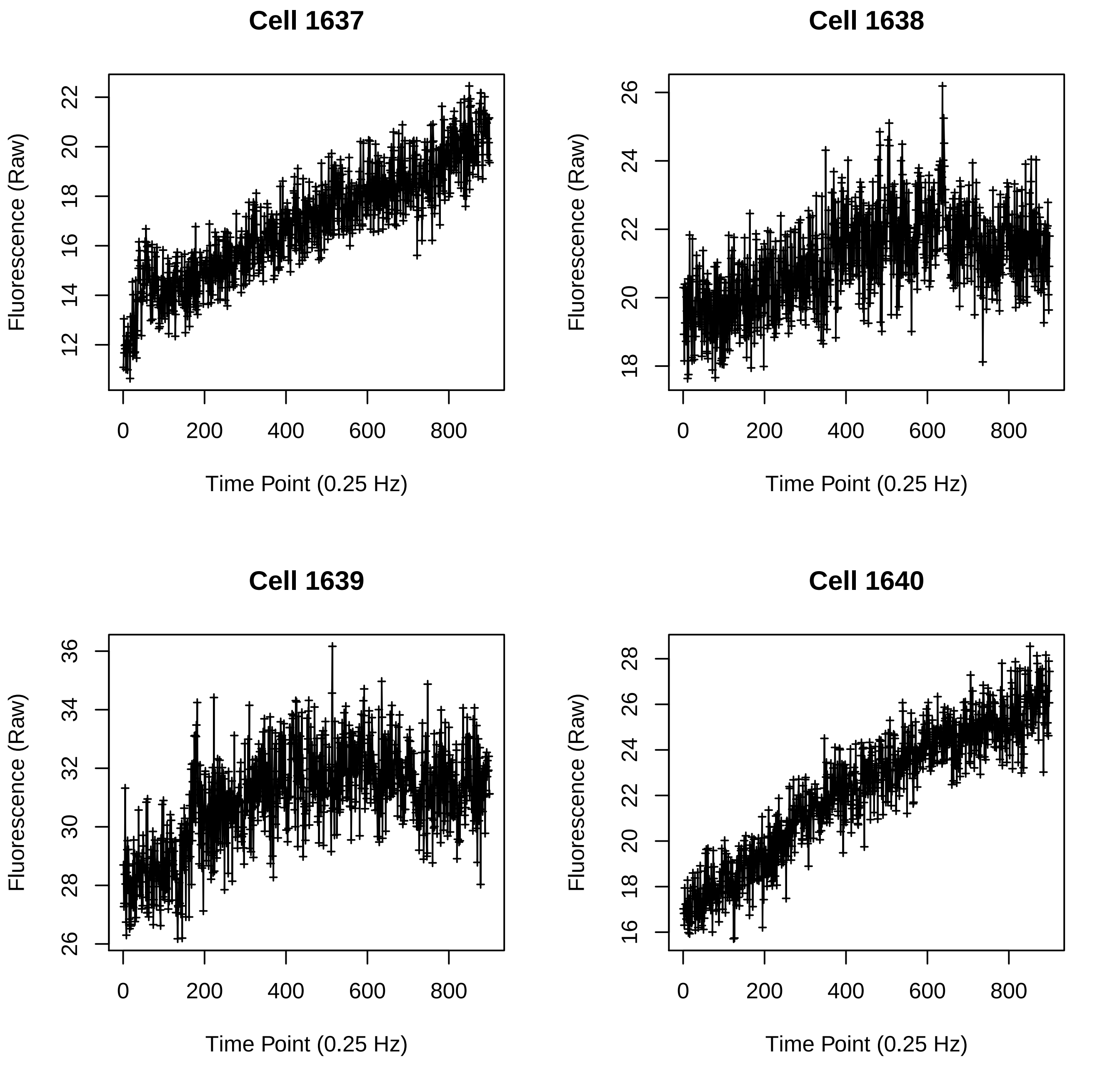
Cell 1637
Cell 1638
Cell 1639
Cell 1640
Fluorescence (Raw)
Time Point (0.25 Hz)
0
200
400
600
800

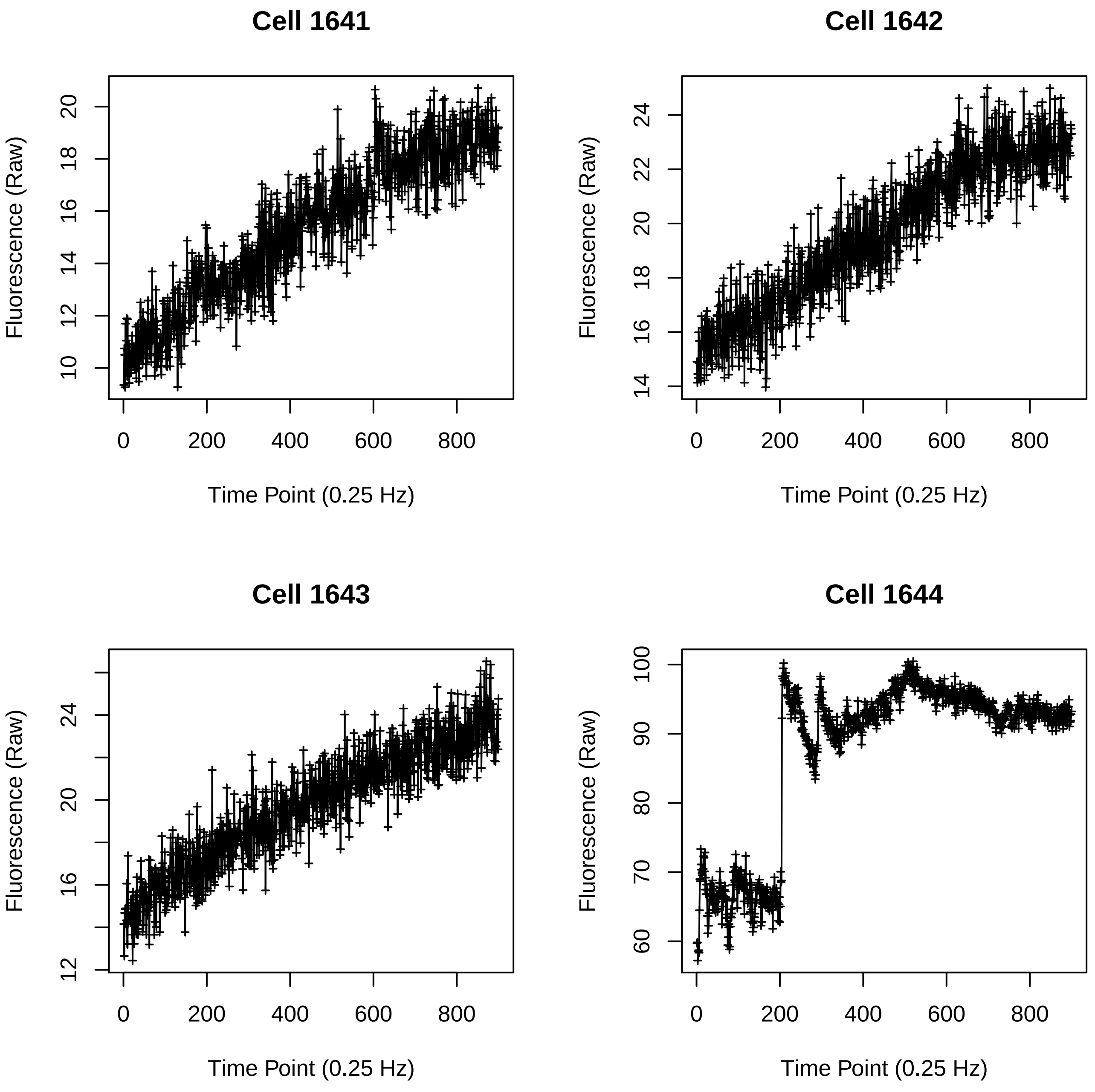
Cell 1641
Cell 1642
Cell 1643
Cell 1644
Fluorescence (Raw)
Time Point (0.25 Hz)
0
200
400
600
800
10
12
14
16
18
20
22
24
60
70
80
90
100

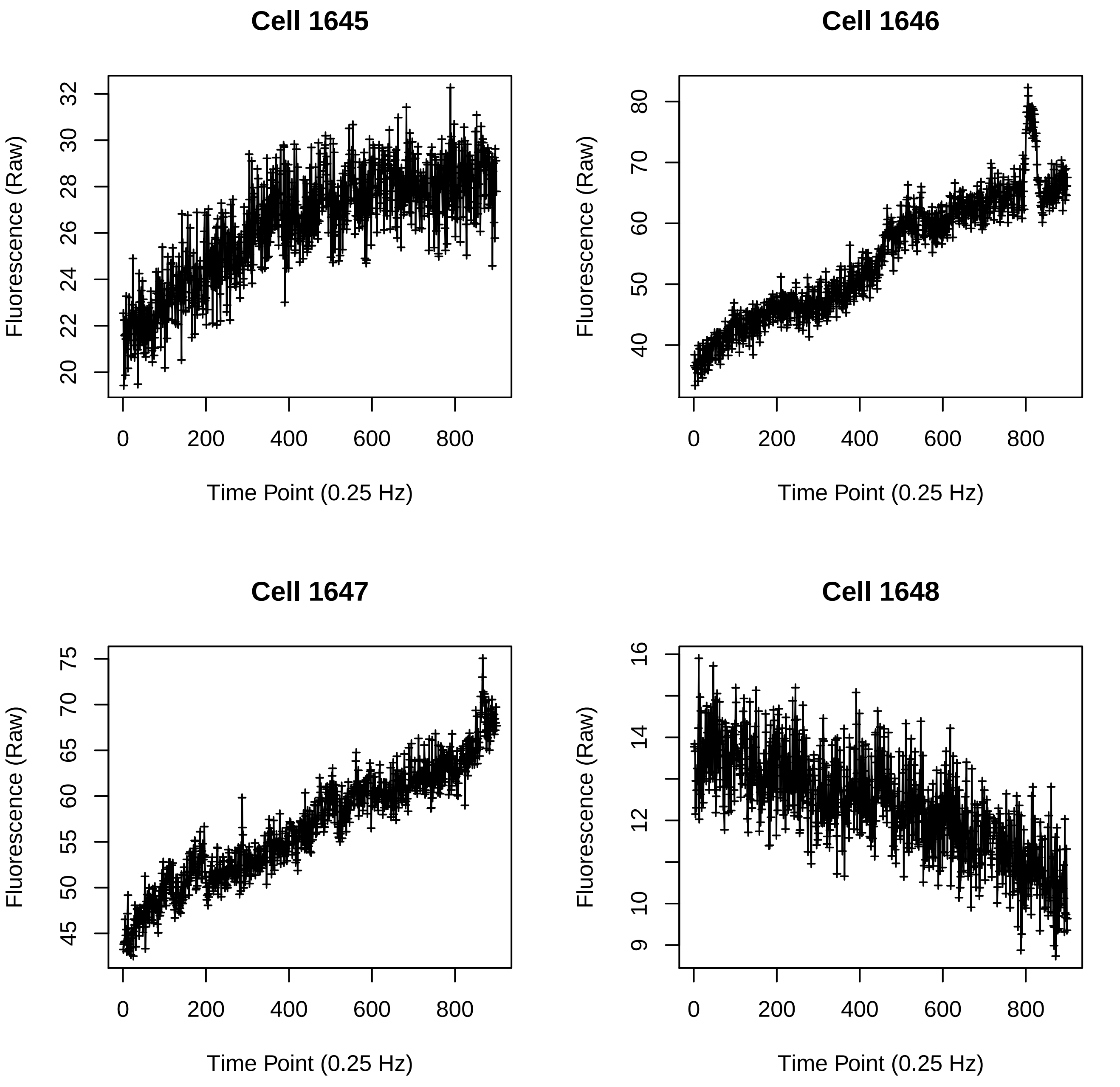
Cell 1645
Fluorescence (Raw)
20
22
24
26
28
30
32
0
200
400
600
800
Time Point (0.25 Hz)
Cell 1646
Fluorescence (Raw)
40
50
60
70
80
0
200
400
600
800
Time Point (0.25 Hz)
Cell 1647
Fluorescence (Raw)
45
50
55
60
65
70
75
0
200
400
600
800
Time Point (0.25 Hz)
Cell 1648
Fluorescence (Raw)
9
10
12
14
16
0
200
400
600
800
Time Point (0.25 Hz)

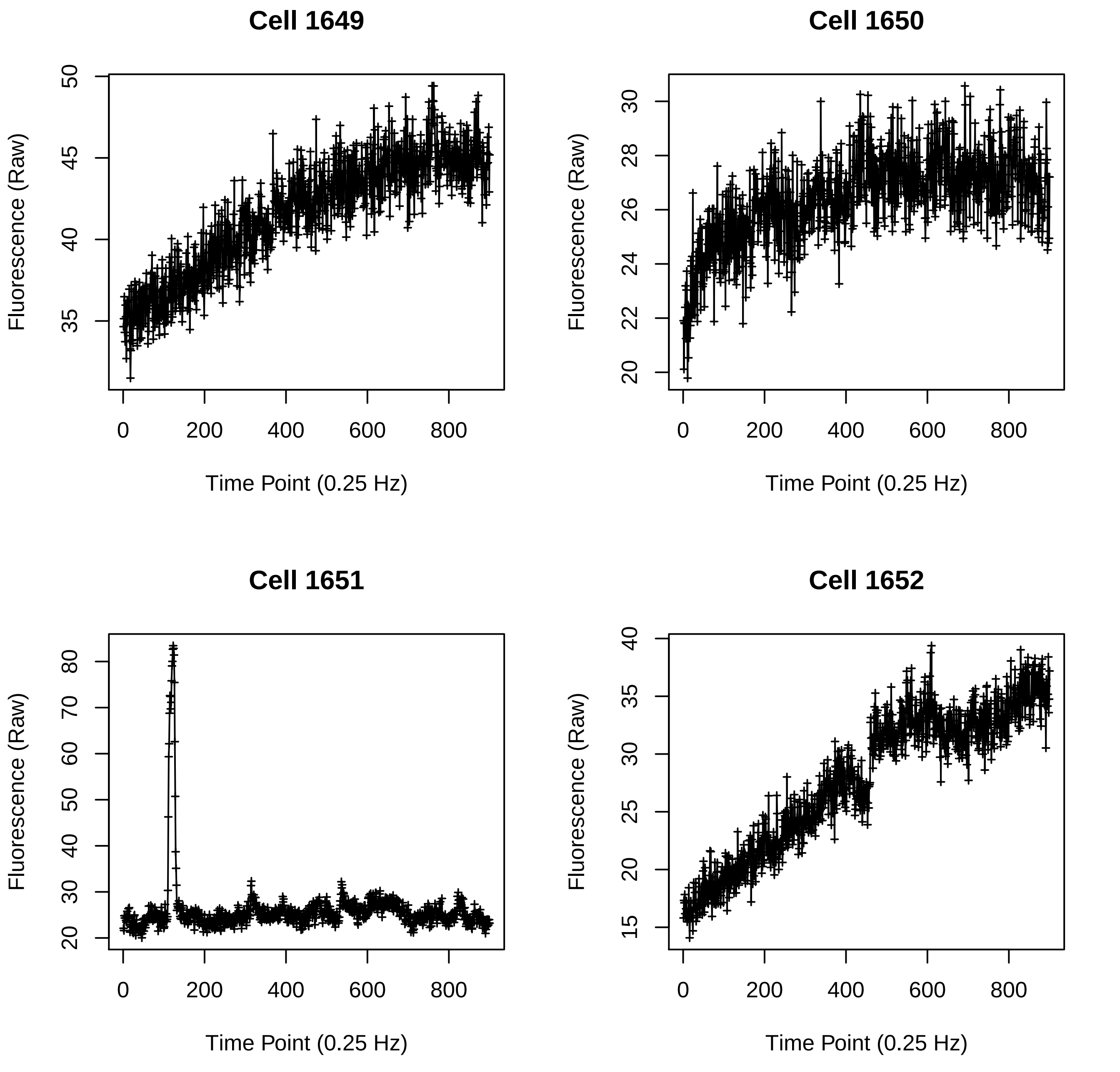

Cell 1649
Cell 1650
Cell 1651
Cell 1652
Fluorescence (Raw)
Time Point (0.25 Hz)
0
200
400
600
800
35
40
45
50
20
22
24
26
28
30
60
70
80
15
25

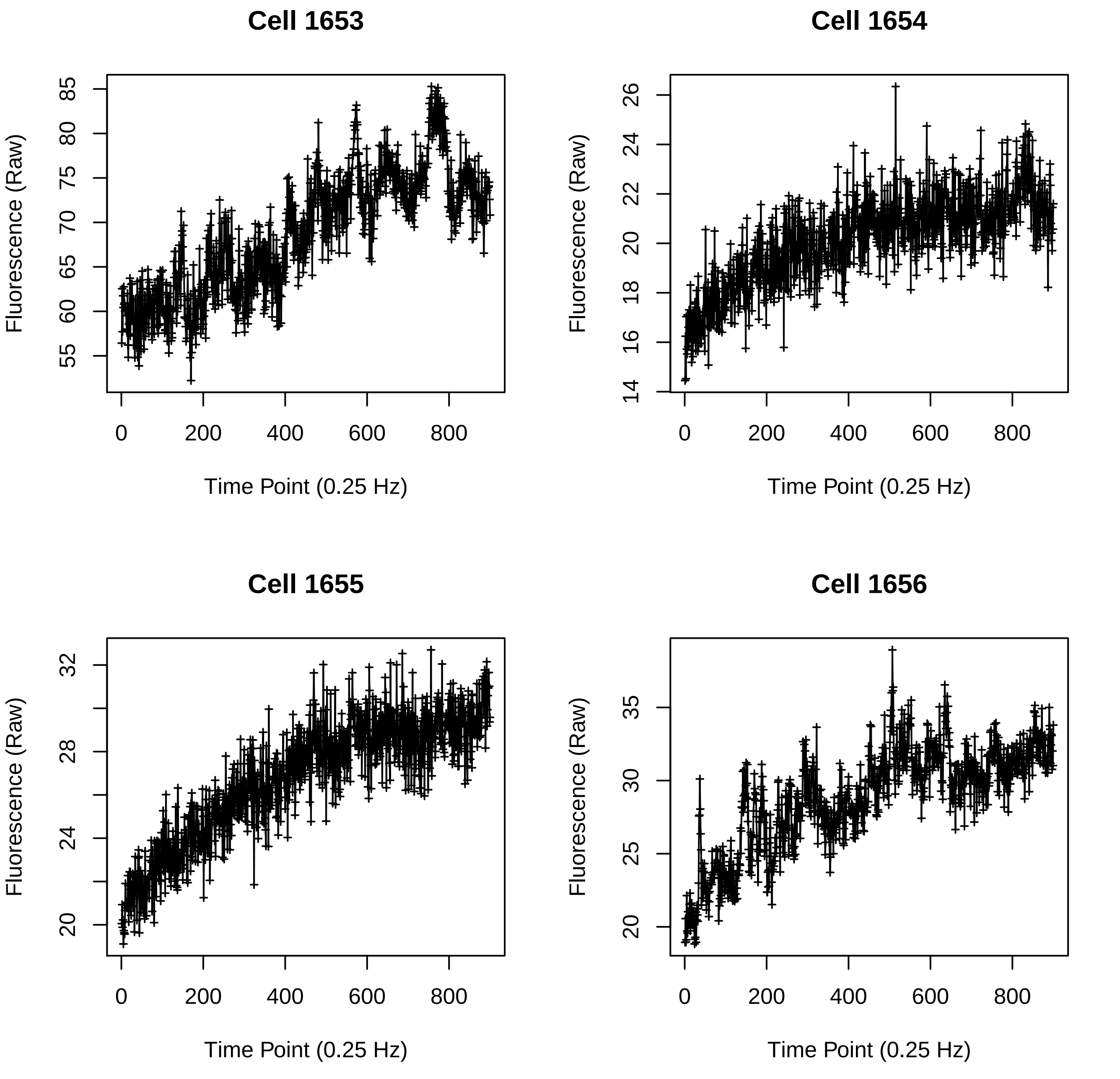
Cell 1653
Cell 1654
Cell 1655
Cell 1656
Fluorescence (Raw)
Time Point (0.25 Hz)
0
200
400
600
800
55
60
65
70
75
80
85
14
16
18
20
22
24
26
20
24
28
32
20
25
30
35

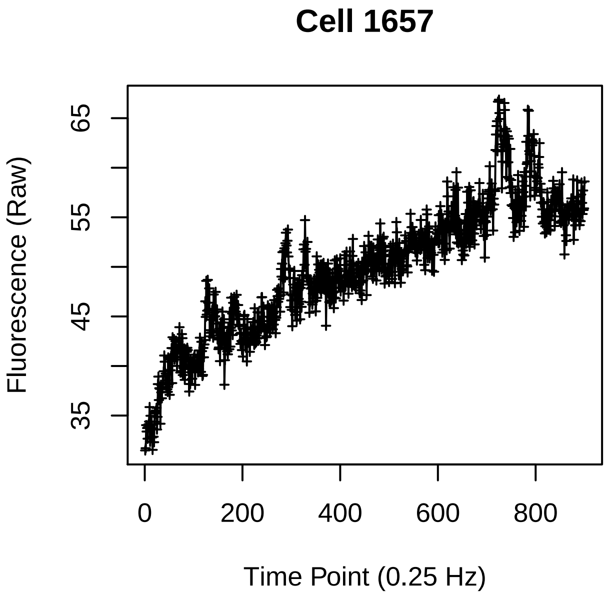
Cell 1657
Fluorescence (Raw)
35
45
55
65
0
200
400
600
800
Time Point (0.25 Hz)

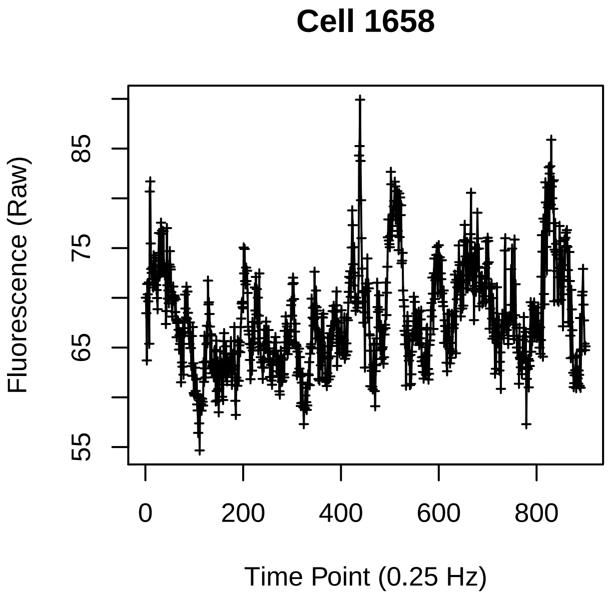
Cell 1658
Fluorescence (Raw)
55
65
75
85
0
200
400
600
800
Time Point (0.25 Hz)

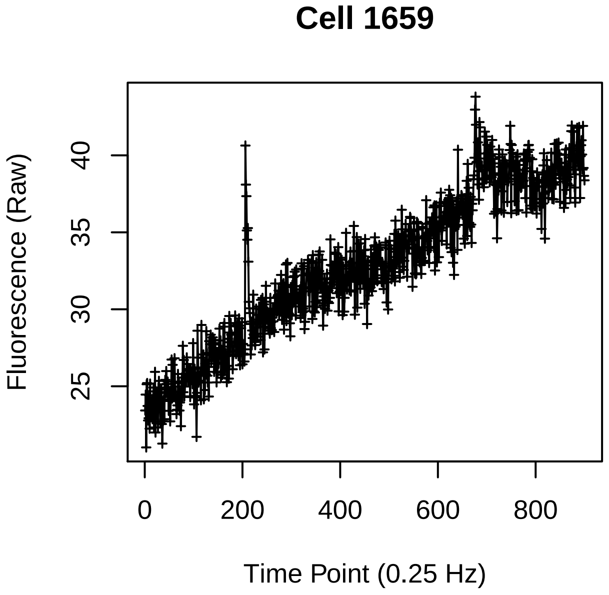
Cell 1659
Fluorescence (Raw)
25
30
35
40
0
200
400
600
800
Time Point (0.25 Hz)

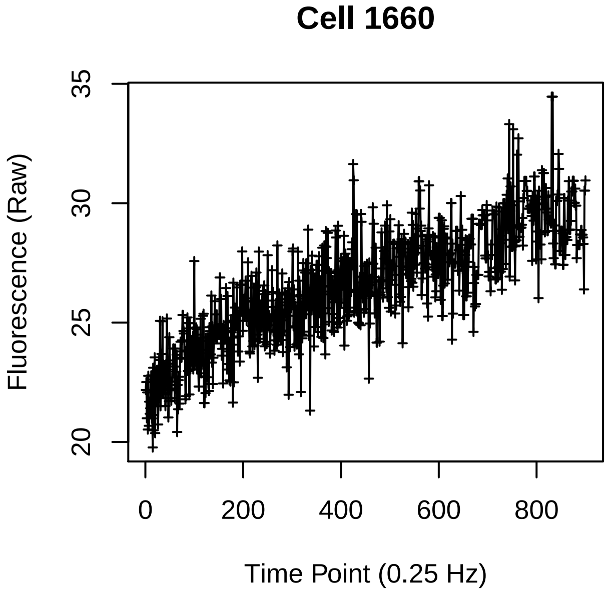
Cell 1660
Fluorescence (Raw)
20
25
30
35
0
200
400
600
800
Time Point (0.25 Hz)

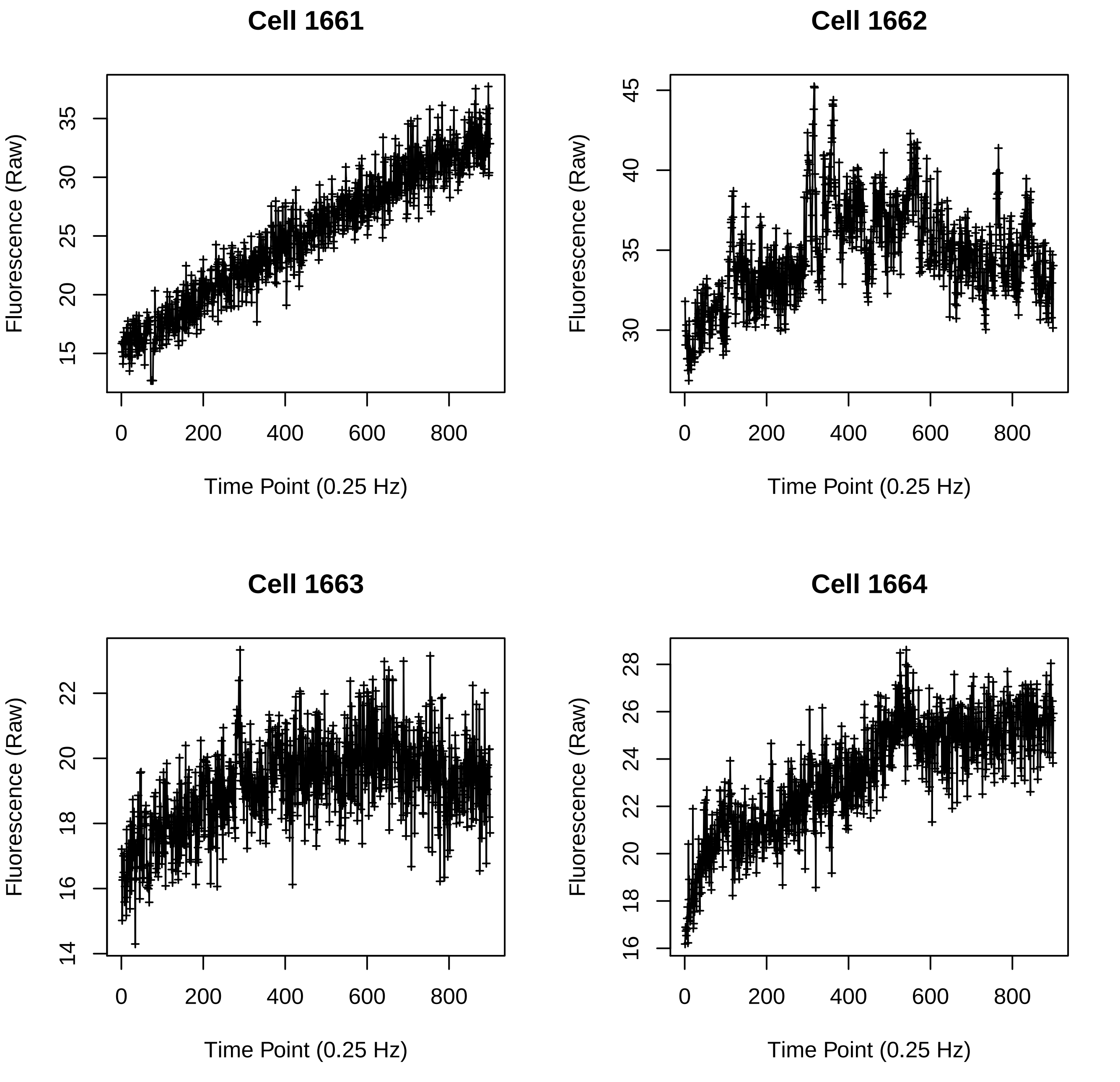
Cell 1661
Cell 1662
Cell 1663
Cell 1664
Fluorescence (Raw)
Time Point (0.25 Hz)
0
200
400
600
800
15
20
25
30
35
40
45
14
16
18
22
24
26
28

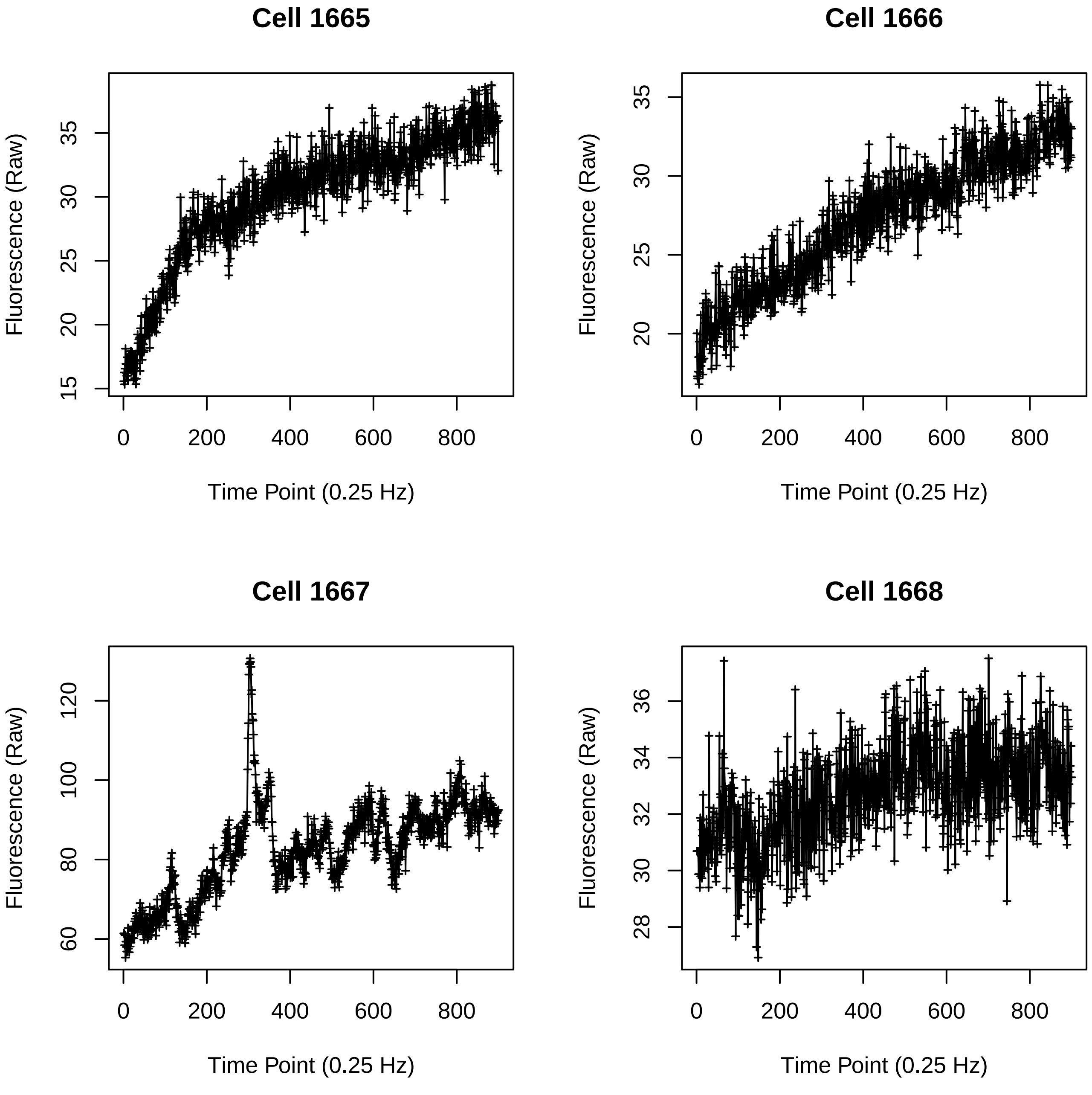
Cell 1665
Fluorescence (Raw)
15
20
25
30
35
0
200
400
600
800
Time Point (0.25 Hz)
Cell 1666
Fluorescence (Raw)
20
25
30
35
0
200
400
600
800
Time Point (0.25 Hz)
Cell 1667
Fluorescence (Raw)
60
80
100
120
0
200
400
600
800
Time Point (0.25 Hz)
Cell 1668
Fluorescence (Raw)
28
30
32
34
36
0
200
400
600
800
Time Point (0.25 Hz)

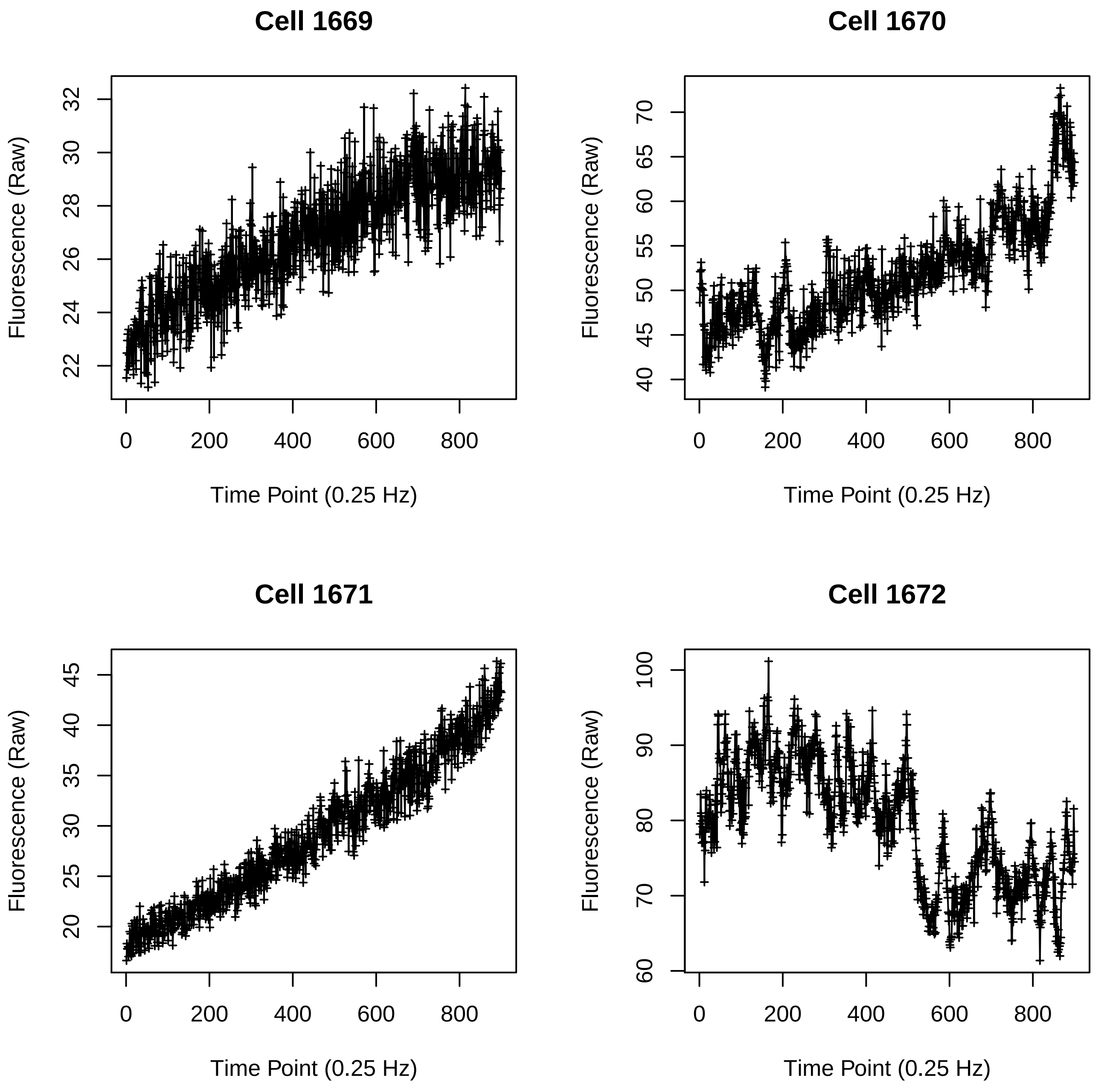
Cell 1669
Fluorescence (Raw)
22
24
26
28
30
32
0
200
400
600
800
Time Point (0.25 Hz)
Cell 1670
Fluorescence (Raw)
40
45
50
55
60
65
70
0
200
400
600
800
Time Point (0.25 Hz)
Cell 1671
Fluorescence (Raw)
20
25
30
35
40
45
0
200
400
600
800
Time Point (0.25 Hz)
Cell 1672
Fluorescence (Raw)
60
70
80
90
100
0
200
400
600
800
Time Point (0.25 Hz)

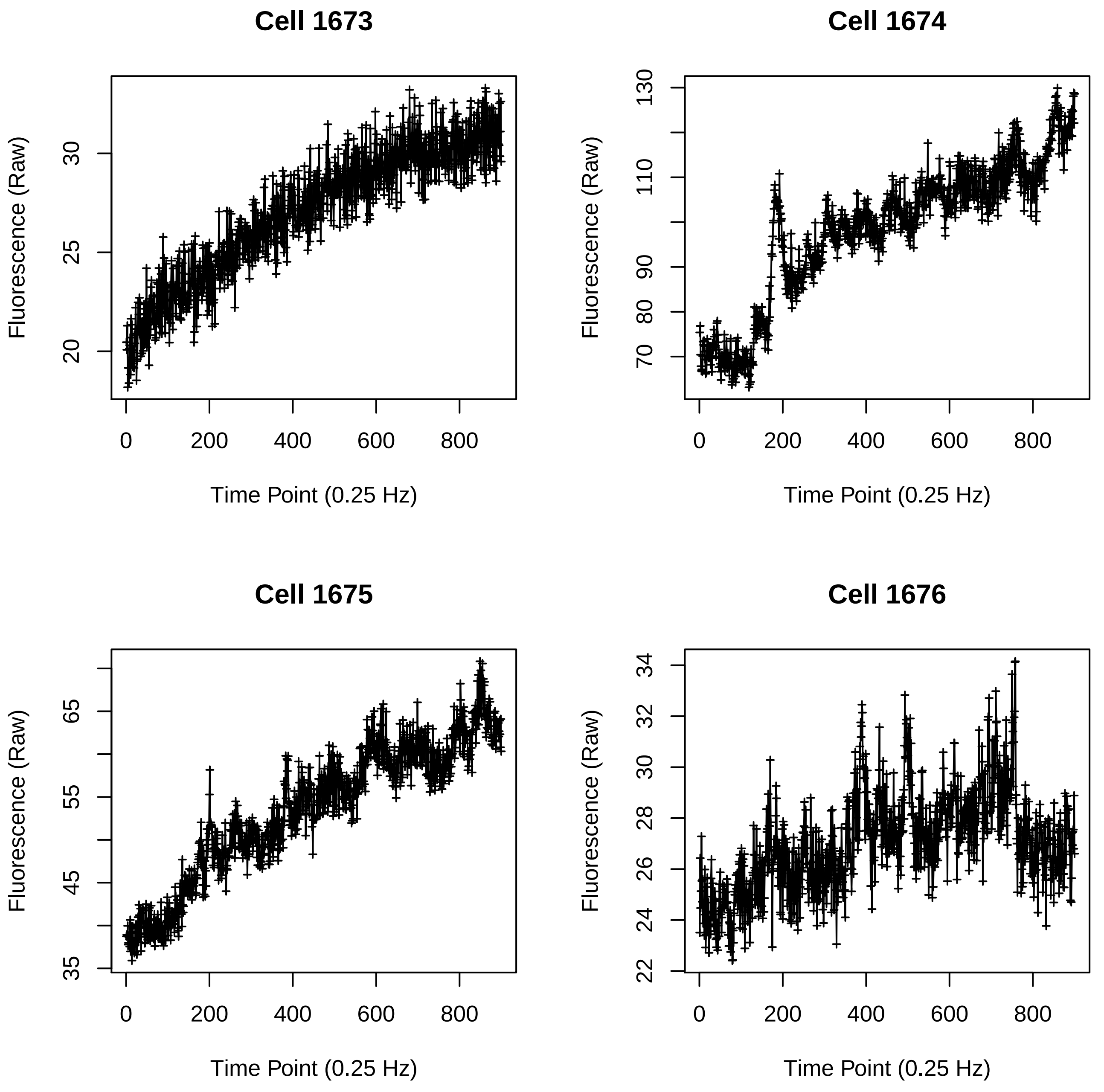

Cell 1673
Cell 1674
Cell 1675
Cell 1676
Fluorescence (Raw)
Time Point (0.25 Hz)
0
200
400
600
800
20
25
30
70
80
90
110
130
35
45
55
65
22
24
26
28
30
32
34

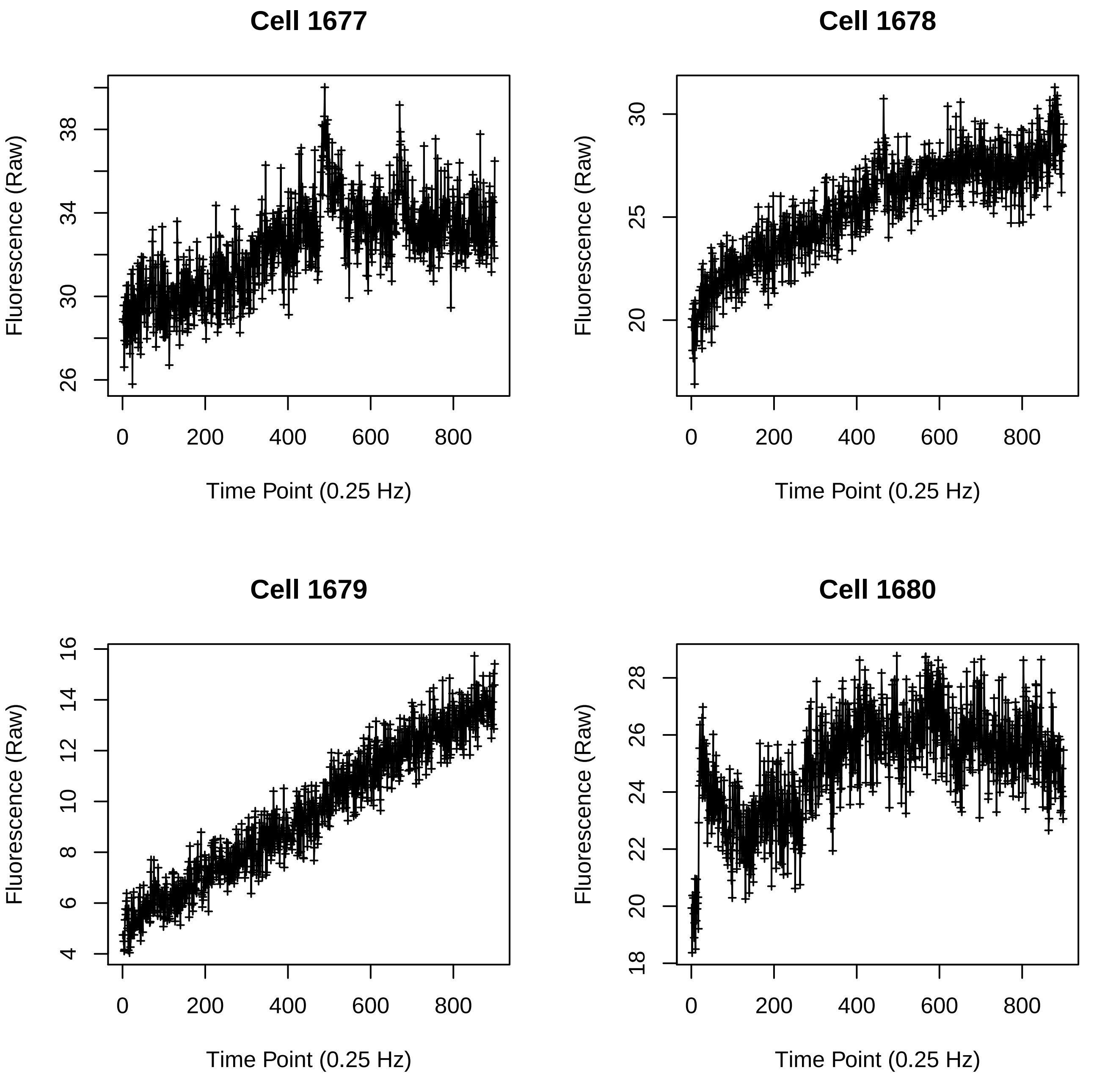

Cell 1677
Fluorescence (Raw)
26
30
34
38
0
200
400
600
800
Time Point (0.25 Hz)
Cell 1678
Fluorescence (Raw)
20
25
30
0
200
400
600
800
Time Point (0.25 Hz)
Cell 1679
Fluorescence (Raw)
4
6
8
10
12
14
16
0
200
400
600
800
Time Point (0.25 Hz)
Cell 1680
Fluorescence (Raw)
18
20
22
24
26
28
0
200
400
600
800
Time Point (0.25 Hz)

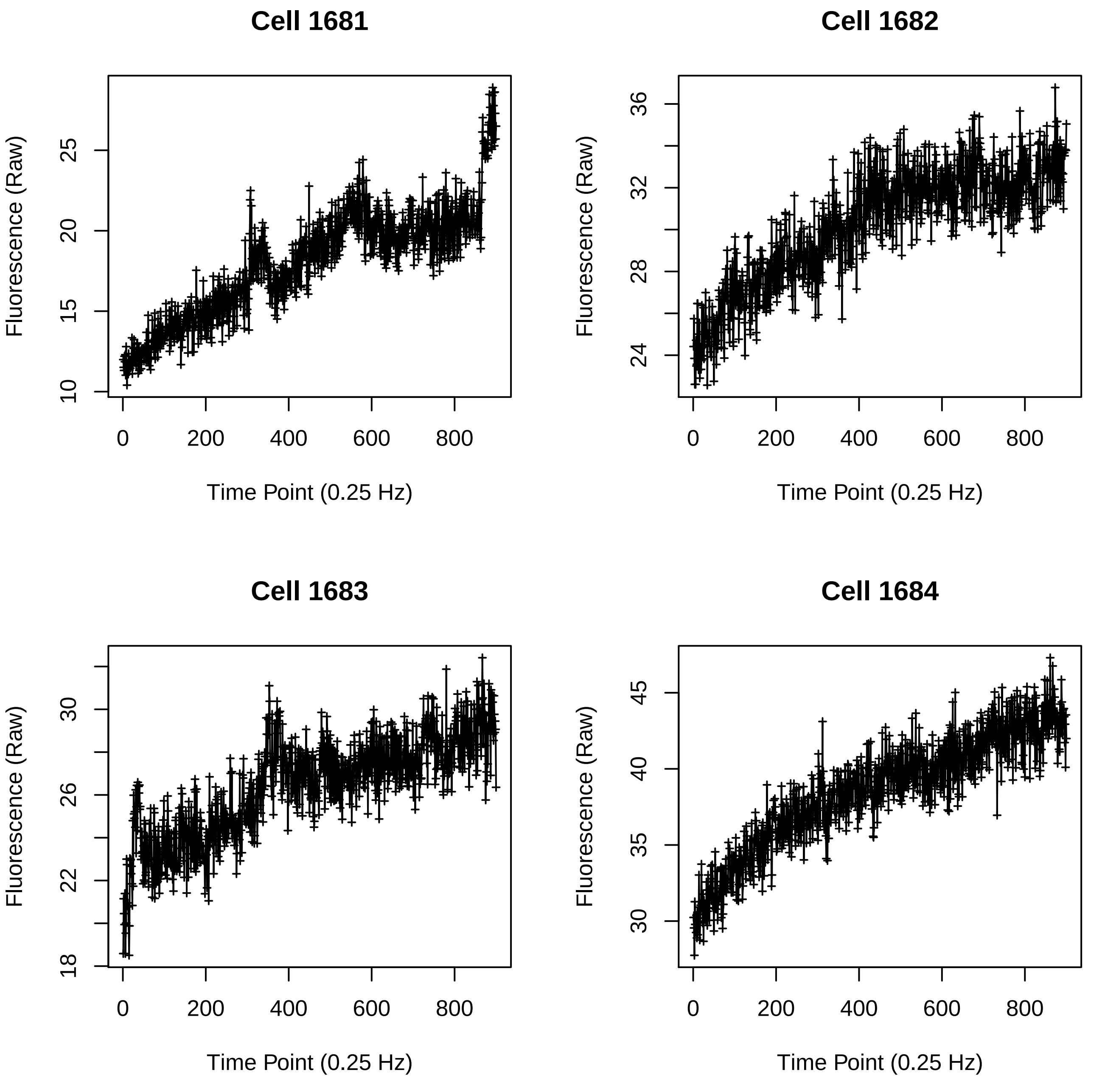
Cell 1681
Cell 1682
Cell 1683
Cell 1684
Fluorescence (Raw)
Time Point (0.25 Hz)
0
200
400
600
800
10
15
20
25
24
28
32
36
18
22
26
30
35
40
45

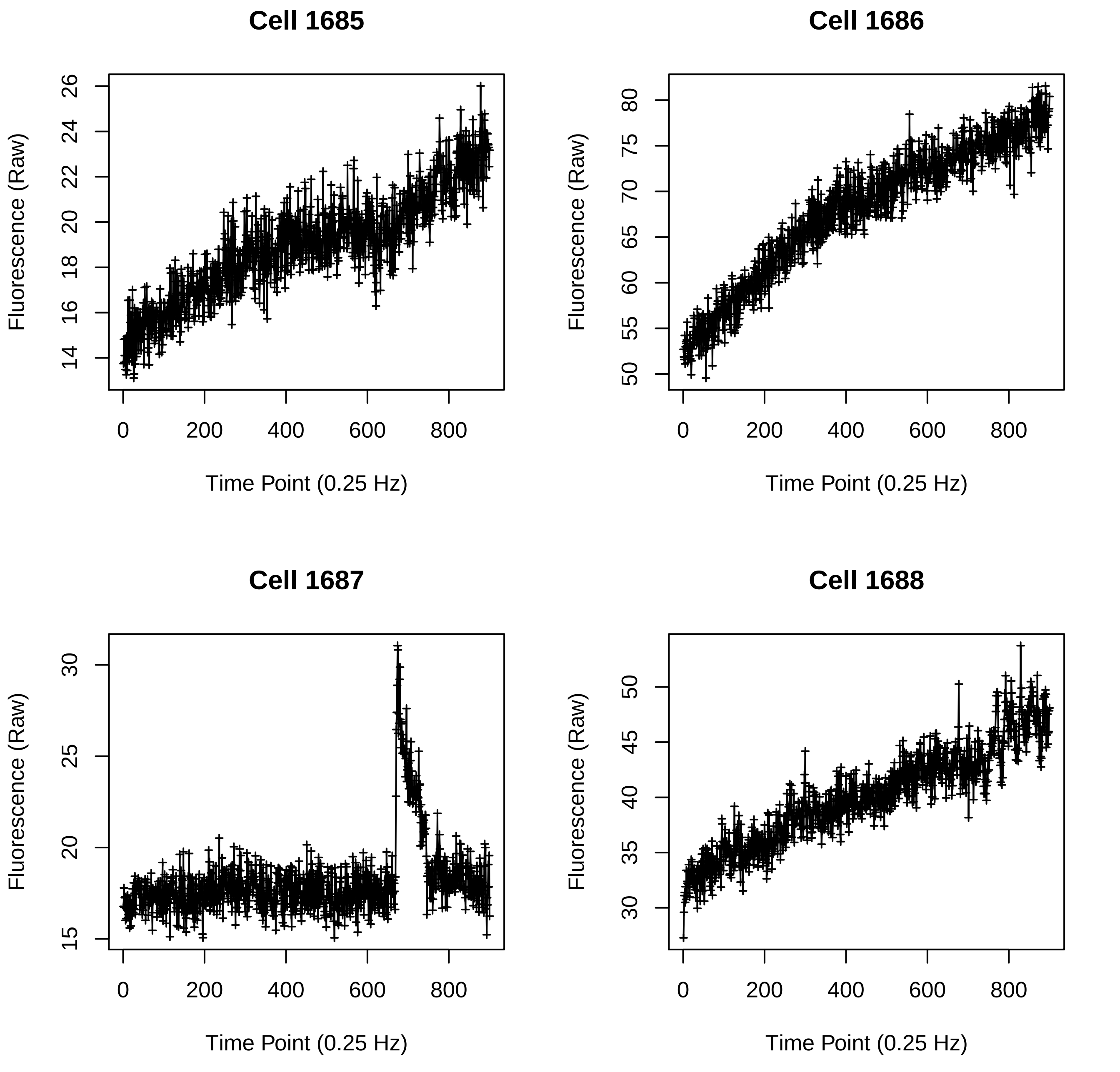
Cell 1685
Cell 1686
Cell 1687
Cell 1688
Fluorescence (Raw)
Time Point (0.25 Hz)
0
200
400
600
800
14
16
18
20
22
24
26
50
55
60
65
70
75
80
15
20
25
30
35
40
45
50

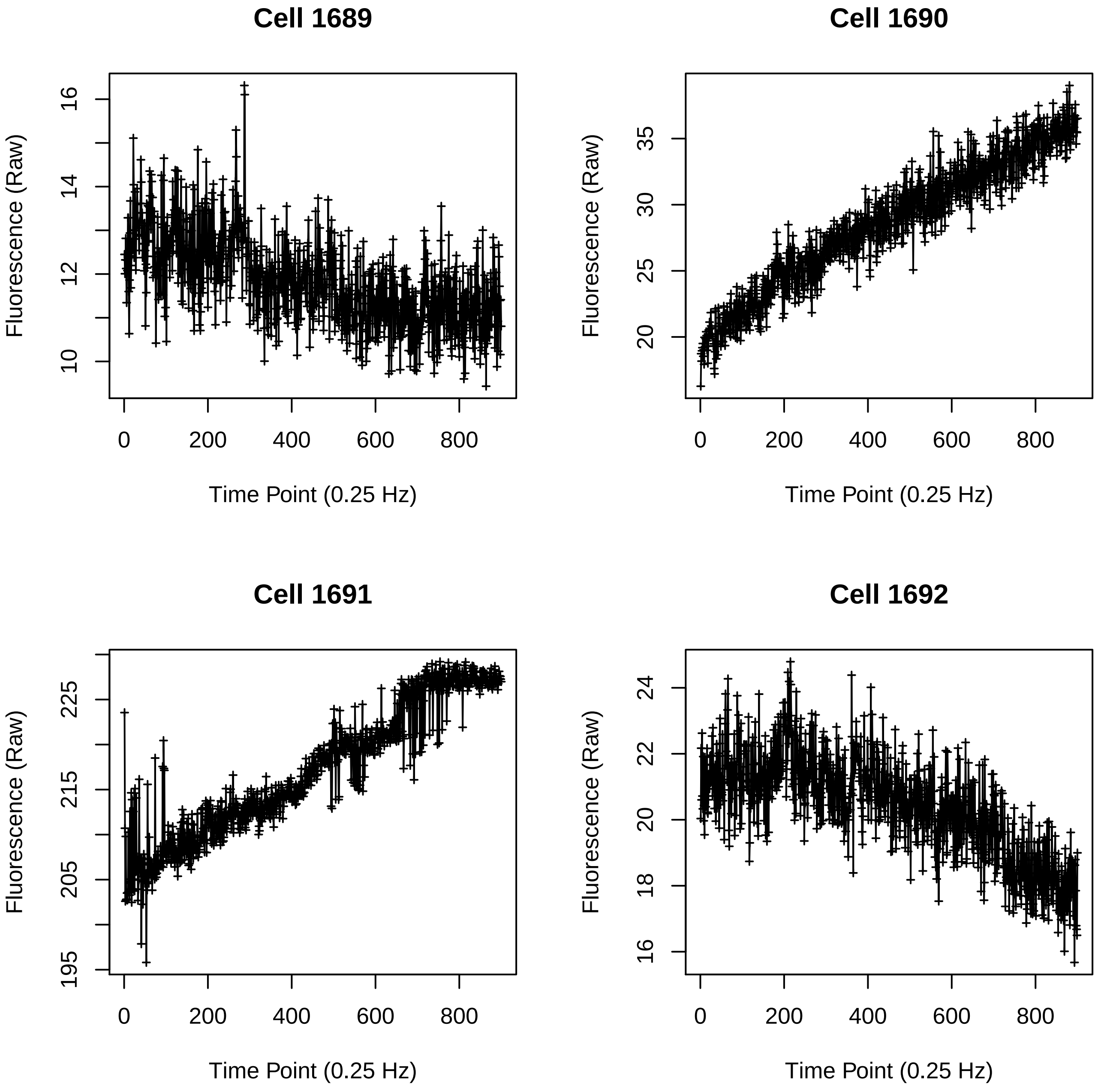
Cell 1689
Cell 1690
Cell 1691
Cell 1692
Fluorescence (Raw)
Time Point (0.25 Hz)
0
200
400
600
800
10
12
14
16
20
25
30
35
195
205
215
225
16
18
20
22
24

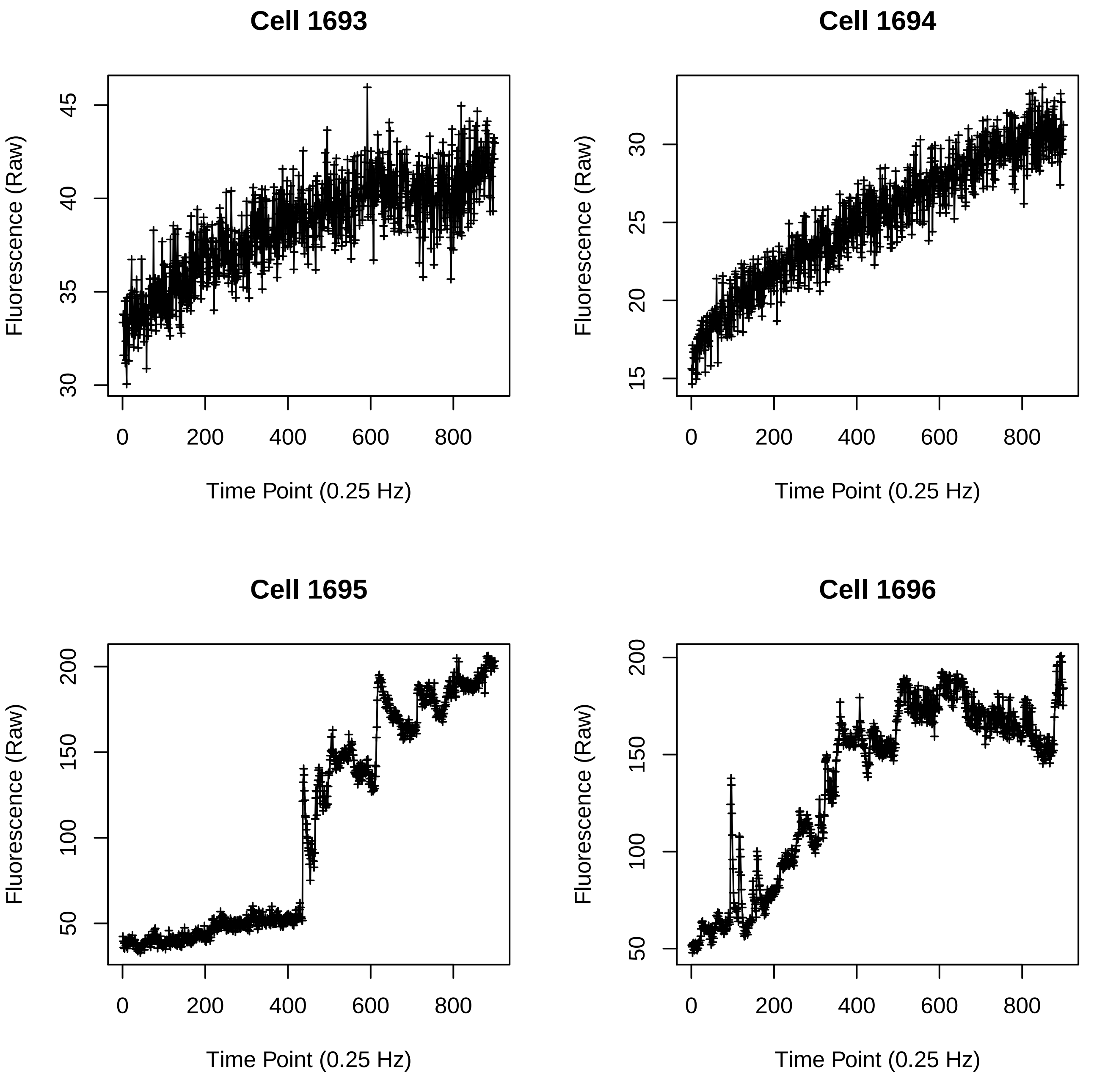
Cell 1693
Cell 1694
Cell 1695
Cell 1696
Fluorescence (Raw)
Time Point (0.25 Hz)
30
35
40
45
15
20
25
30
50
100
150
200
0
200
400
600
800

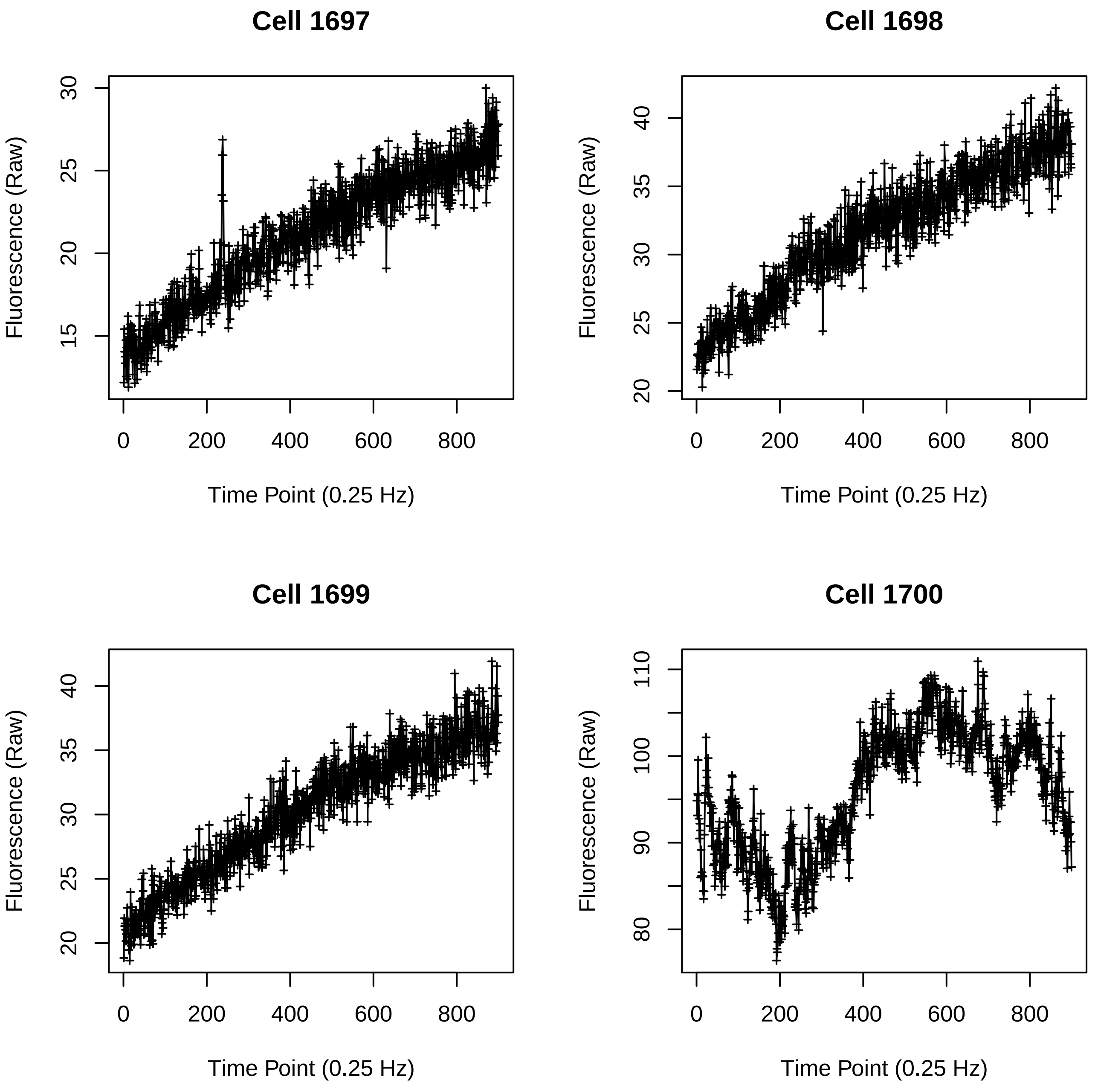
Cell 1697
Cell 1698
Cell 1699
Cell 1700
Fluorescence (Raw)
Time Point (0.25 Hz)
0
200
400
600
800
15
20
25
30
35
40
80
90
100
110

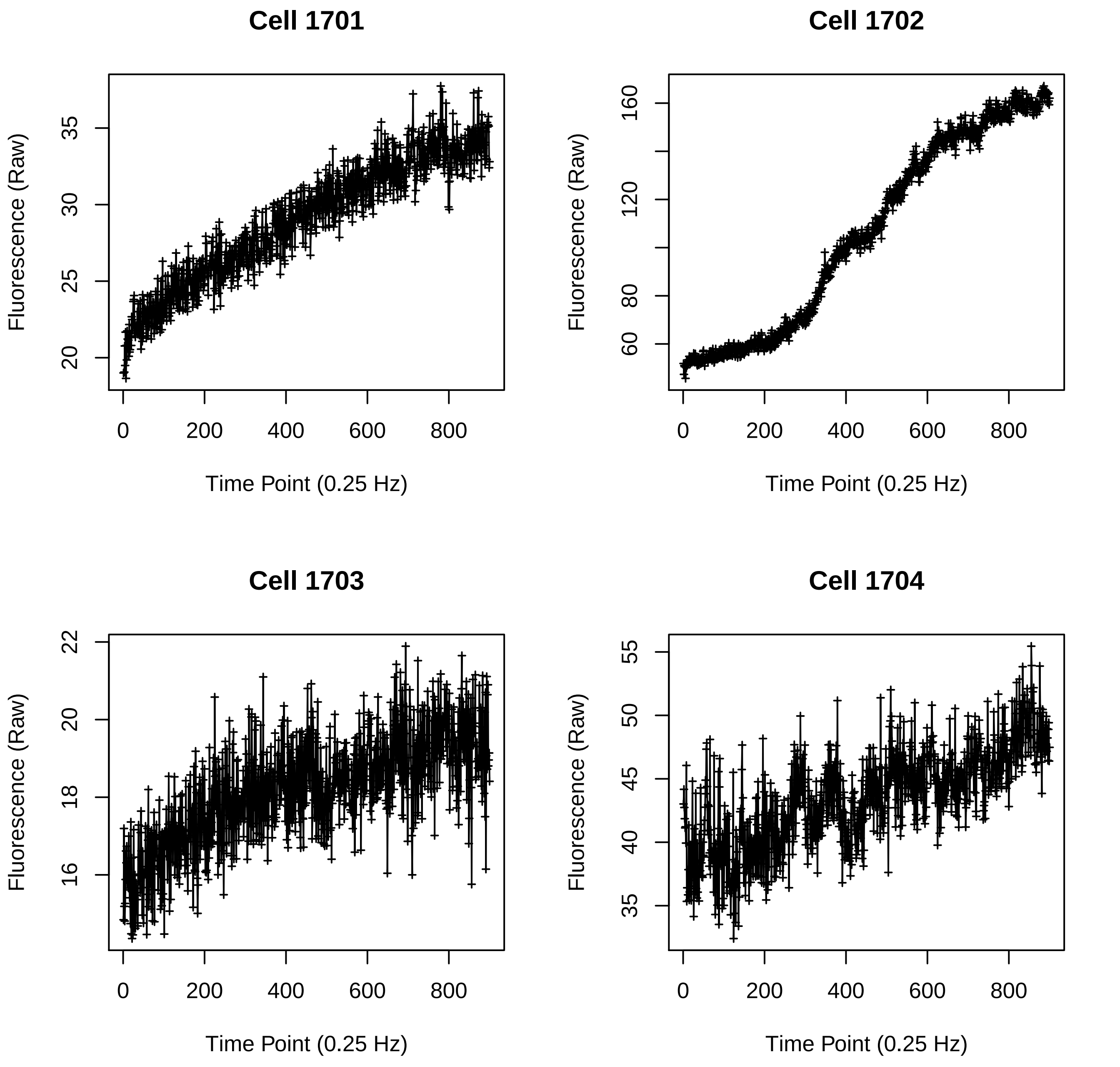
Cell 1701
Cell 1702
Cell 1703
Cell 1704
Fluorescence (Raw)
Time Point (0.25 Hz)
0
200
400
600
800
20
25
30
35
60
80
120
140
160
16
18
20
22
40
45
50
55

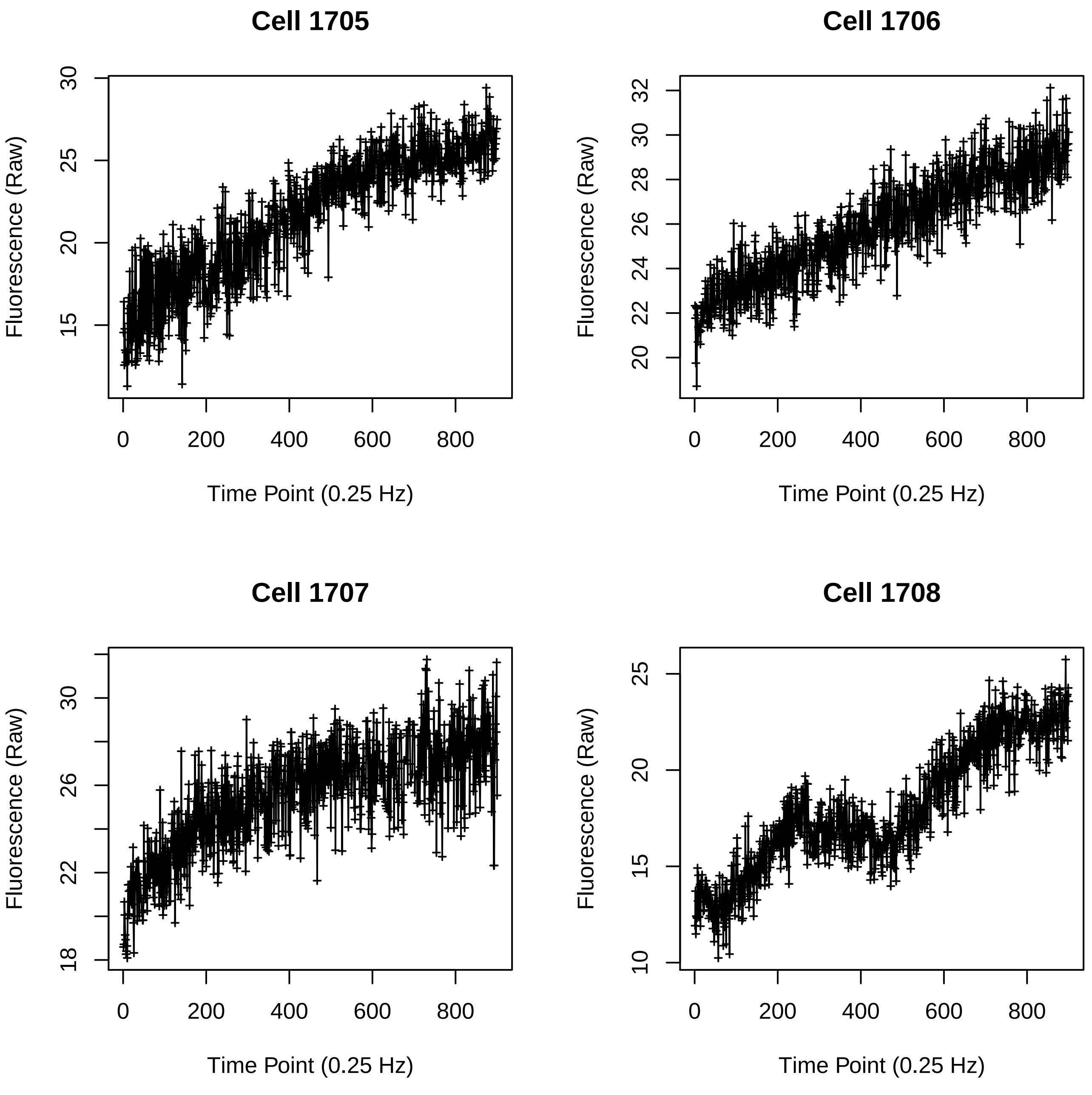
Cell 1705
Cell 1706
Cell 1707
Cell 1708
Fluorescence (Raw)
Time Point (0.25 Hz)
0
200
400
600
800
15
20
25
30
22
24
26
28
32
18
10

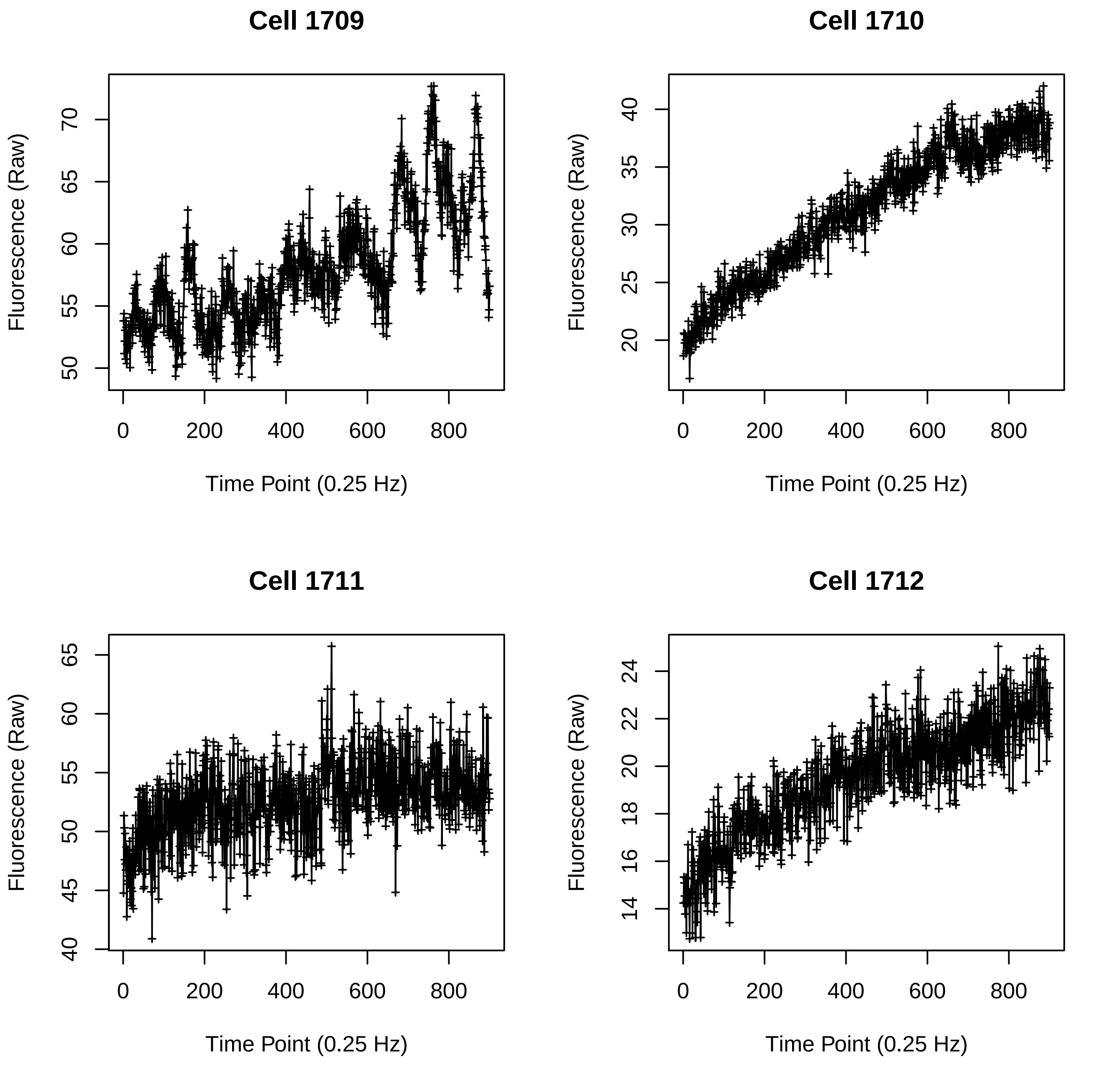
Cell 1709
Fluorescence (Raw)
Time Point (0.25 Hz)
Cell 1710
Fluorescence (Raw)
Time Point (0.25 Hz)
Cell 1711
Fluorescence (Raw)
Time Point (0.25 Hz)
Cell 1712
Fluorescence (Raw)
Time Point (0.25 Hz)

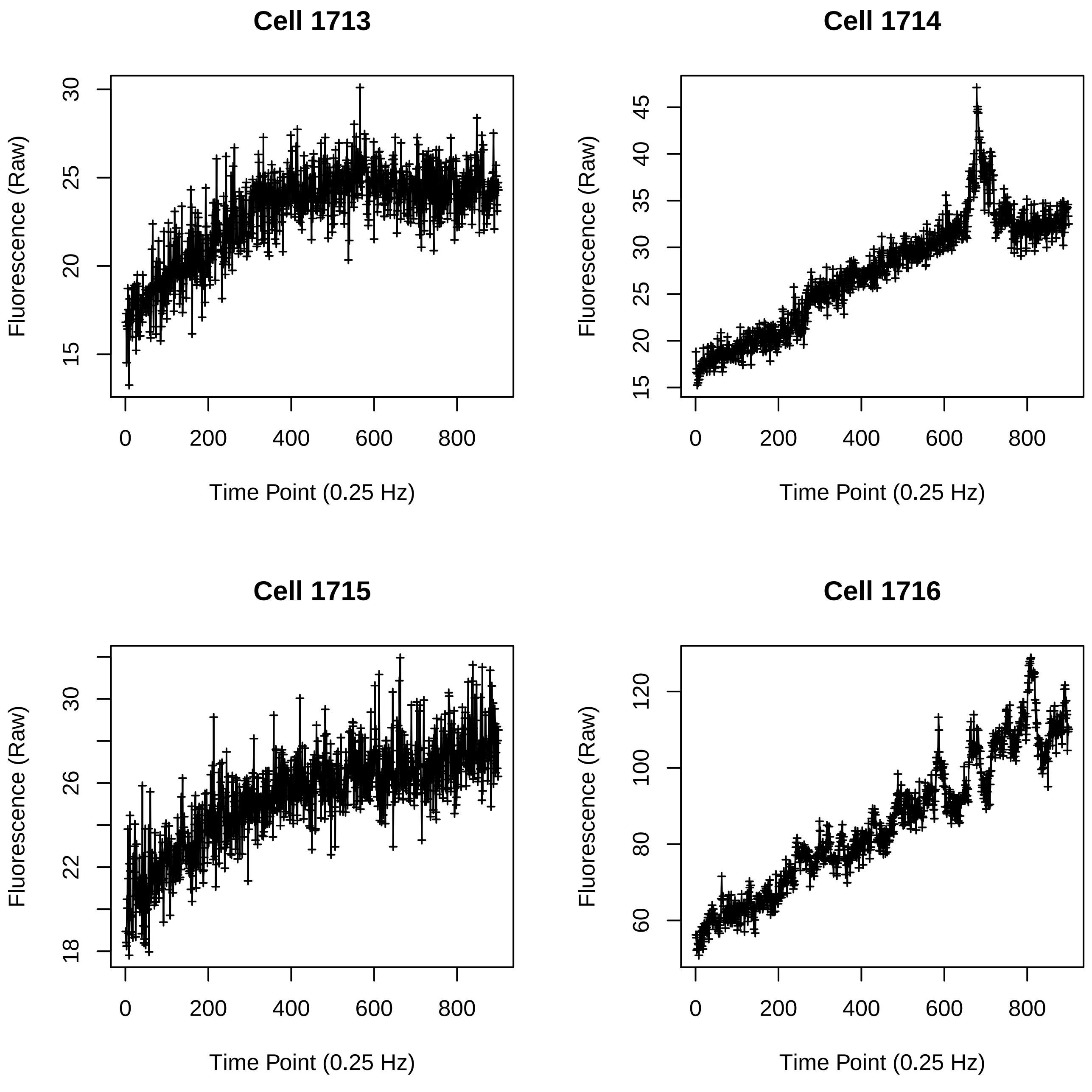
Cell 1713
Cell 1714
Cell 1715
Cell 1716
Fluorescence (Raw)
Time Point (0.25 Hz)

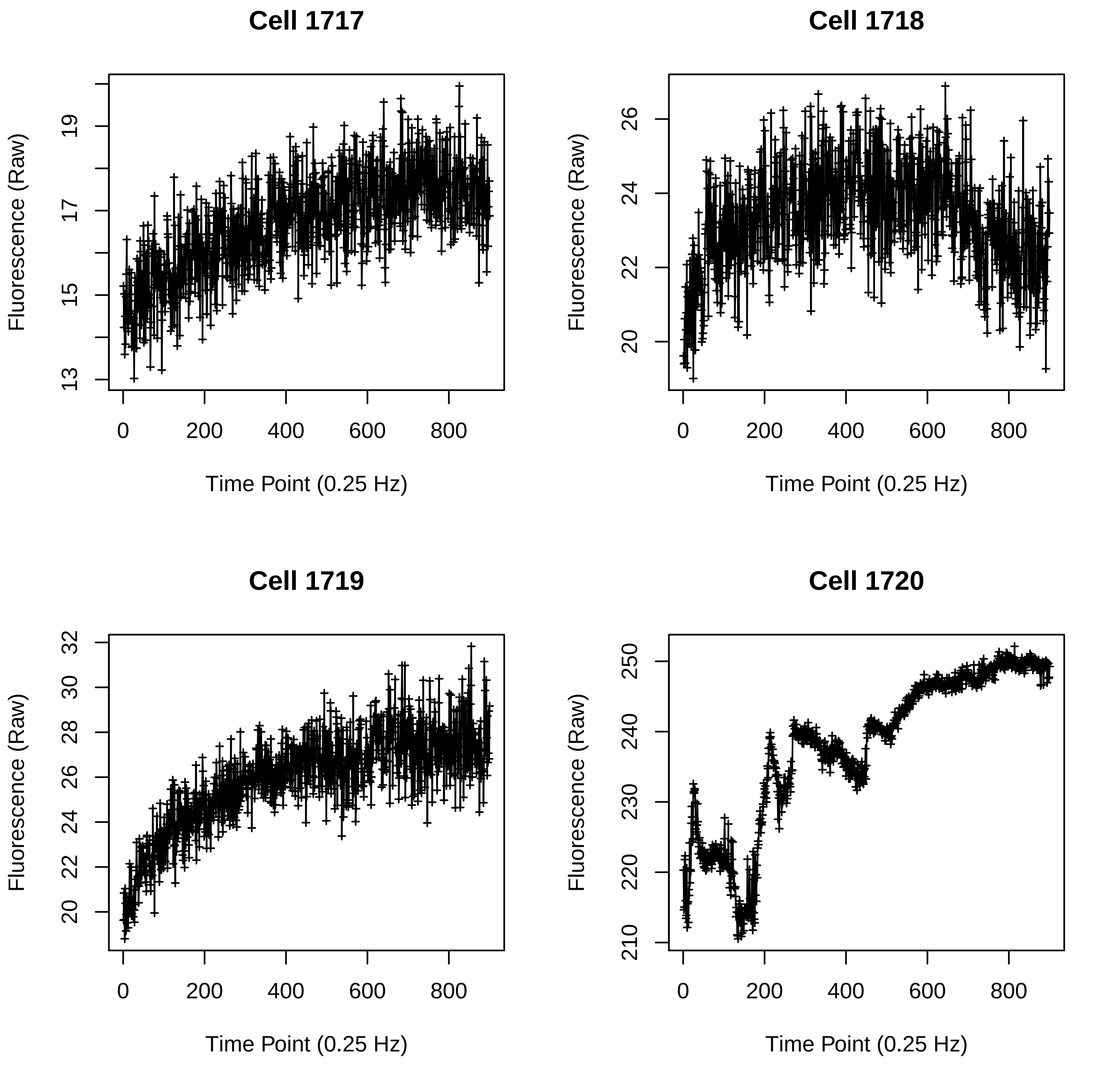
Cell 1717
Cell 1718
Cell 1719
Cell 1720
Fluorescence (Raw)
Time Point (0.25 Hz)
13
15
17
19
20
22
24
26
28
30
32
210
220
230
240
250
0
200
400
600
800

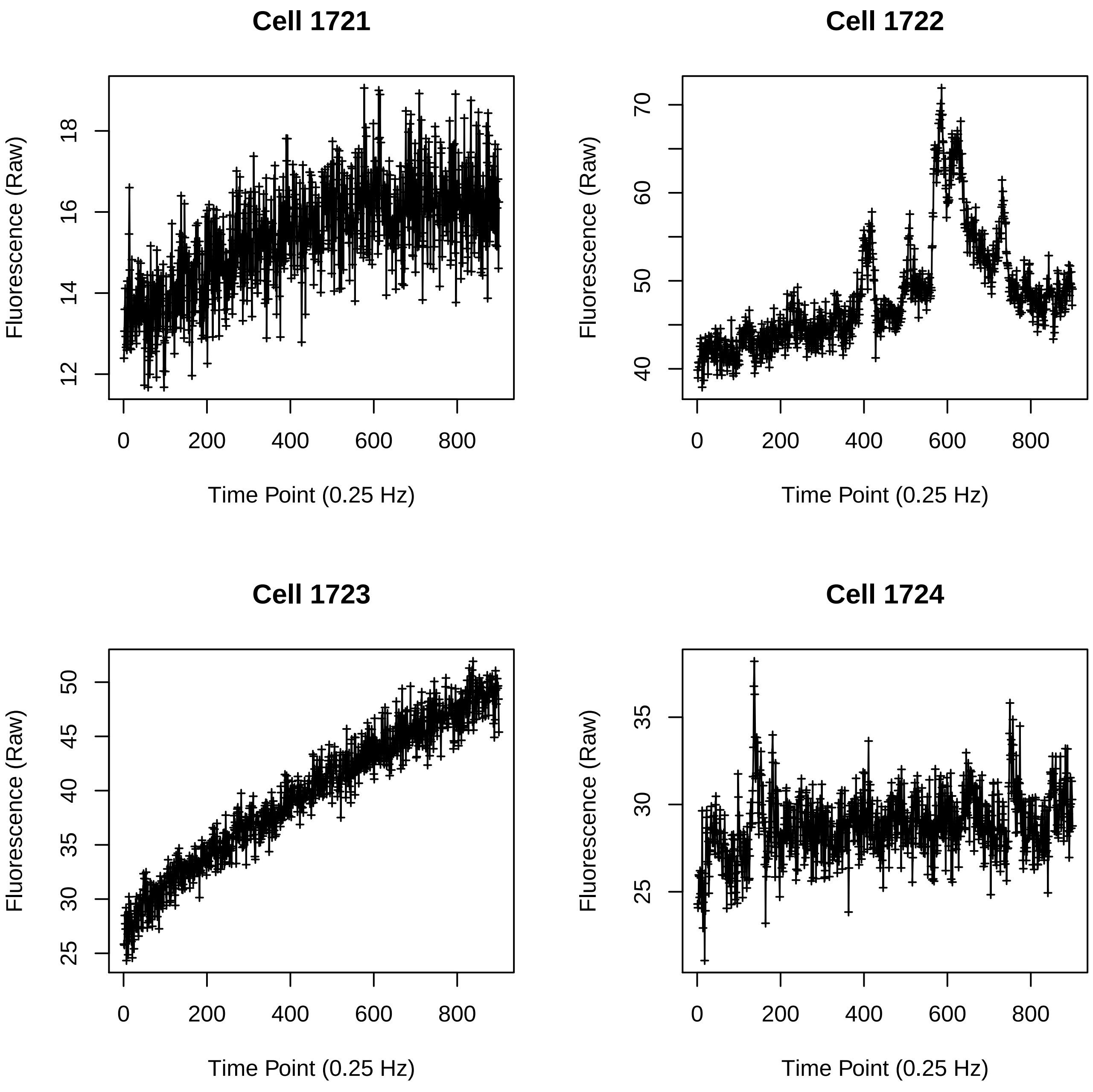
Cell 1721
Fluorescence (Raw)
12
14
16
18
0
200
400
600
800
Time Point (0.25 Hz)
Cell 1722
Fluorescence (Raw)
40
50
60
70
0
200
400
600
800
Time Point (0.25 Hz)
Cell 1723
Fluorescence (Raw)
25
30
35
40
45
50
0
200
400
600
800
Time Point (0.25 Hz)
Cell 1724
Fluorescence (Raw)
25
30
35
0
200
400
600
800
Time Point (0.25 Hz)

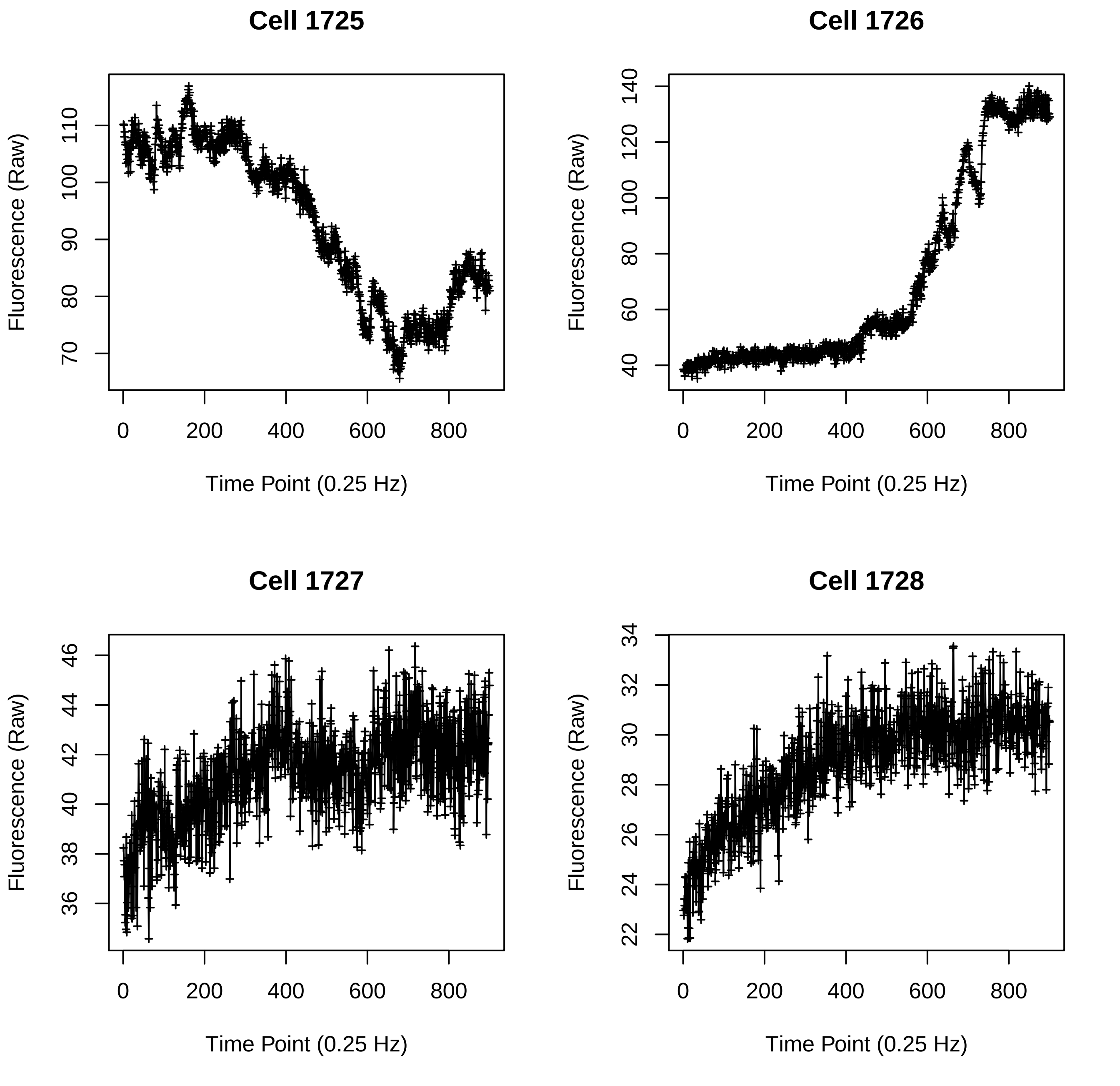

Cell 1725
Fluorescence (Raw)
70
80
90
100
110
0
200
400
600
800
Time Point (0.25 Hz)
Cell 1726
Fluorescence (Raw)
40
60
80
100
120
140
0
200
400
600
800
Time Point (0.25 Hz)
Cell 1727
Fluorescence (Raw)
36
38
40
42
44
46
0
200
400
600
800
Time Point (0.25 Hz)
Cell 1728
Fluorescence (Raw)
22
24
26
28
30
32
34
0
200
400
600
800
Time Point (0.25 Hz)

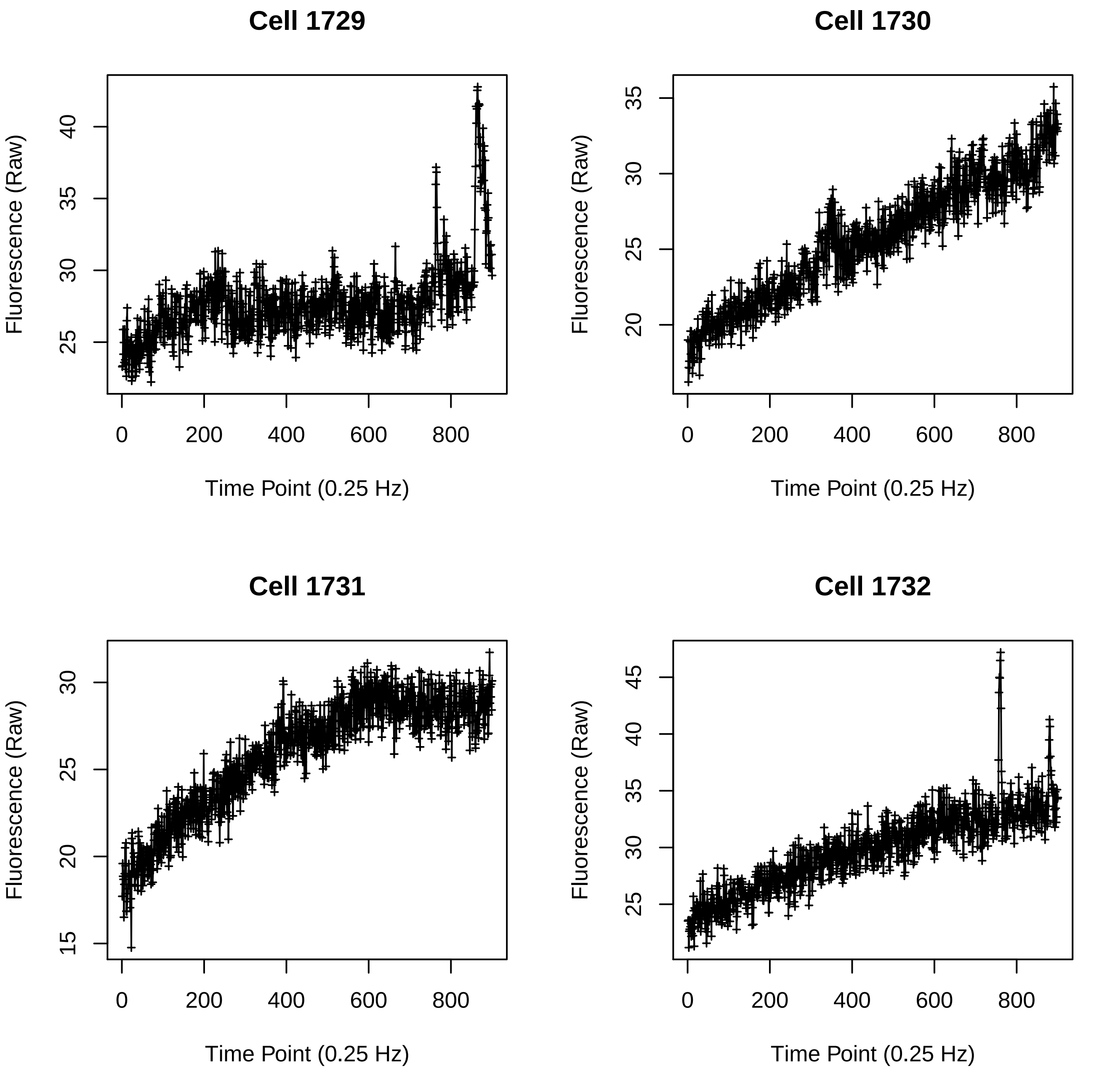
Cell 1729
Cell 1730
Cell 1731
Cell 1732
Fluorescence (Raw)
Time Point (0.25 Hz)
0
200
400
600
800
25
30
35
40
45
20
15

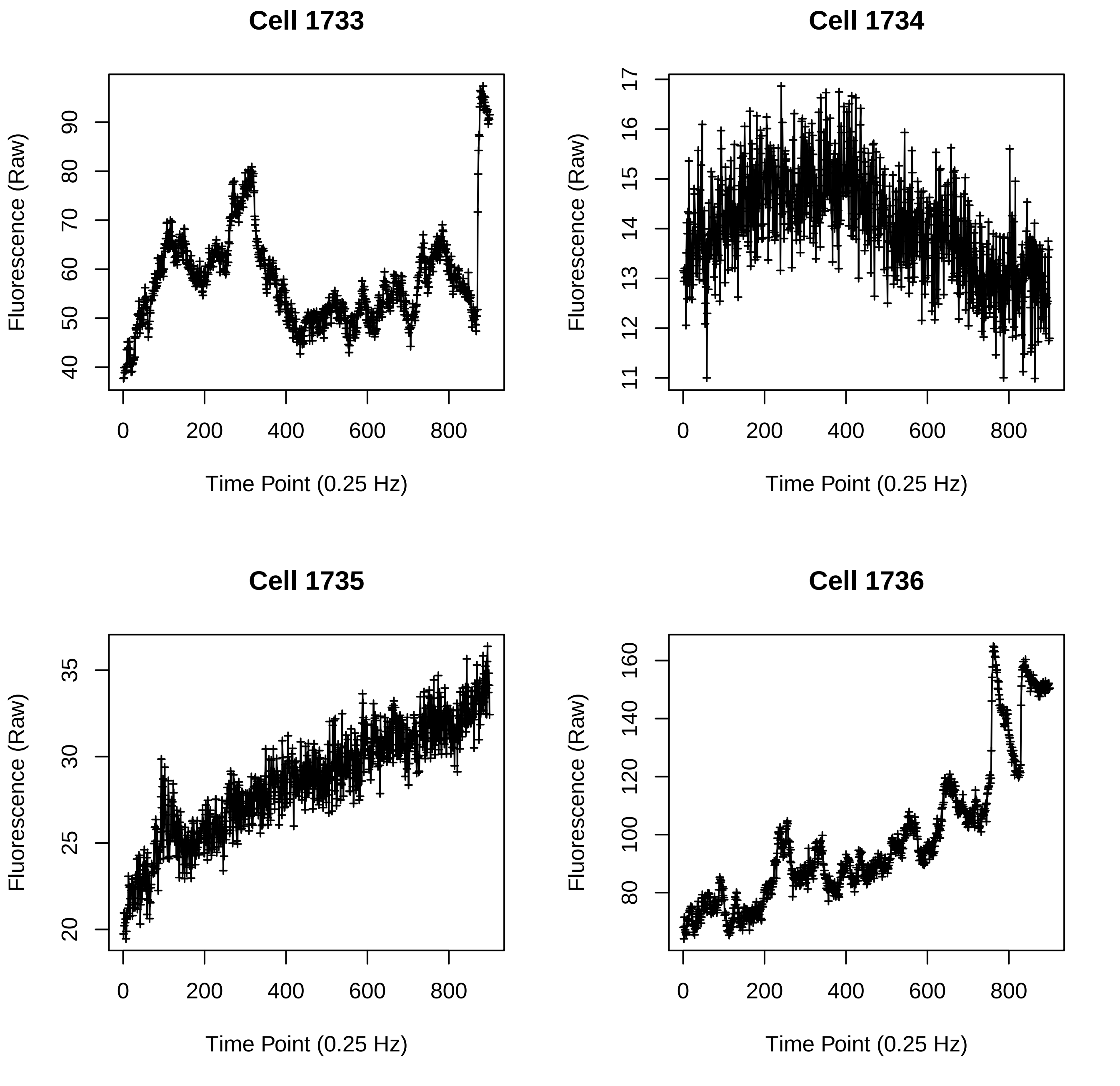

Cell 1733
Cell 1734
Cell 1735
Cell 1736
Fluorescence (Raw)
Time Point (0.25 Hz)
0
200
400
600
800
40
50
60
70
80
90
11
12
13
14
15
16
17
20
25
30
35
80
100
120
140
160

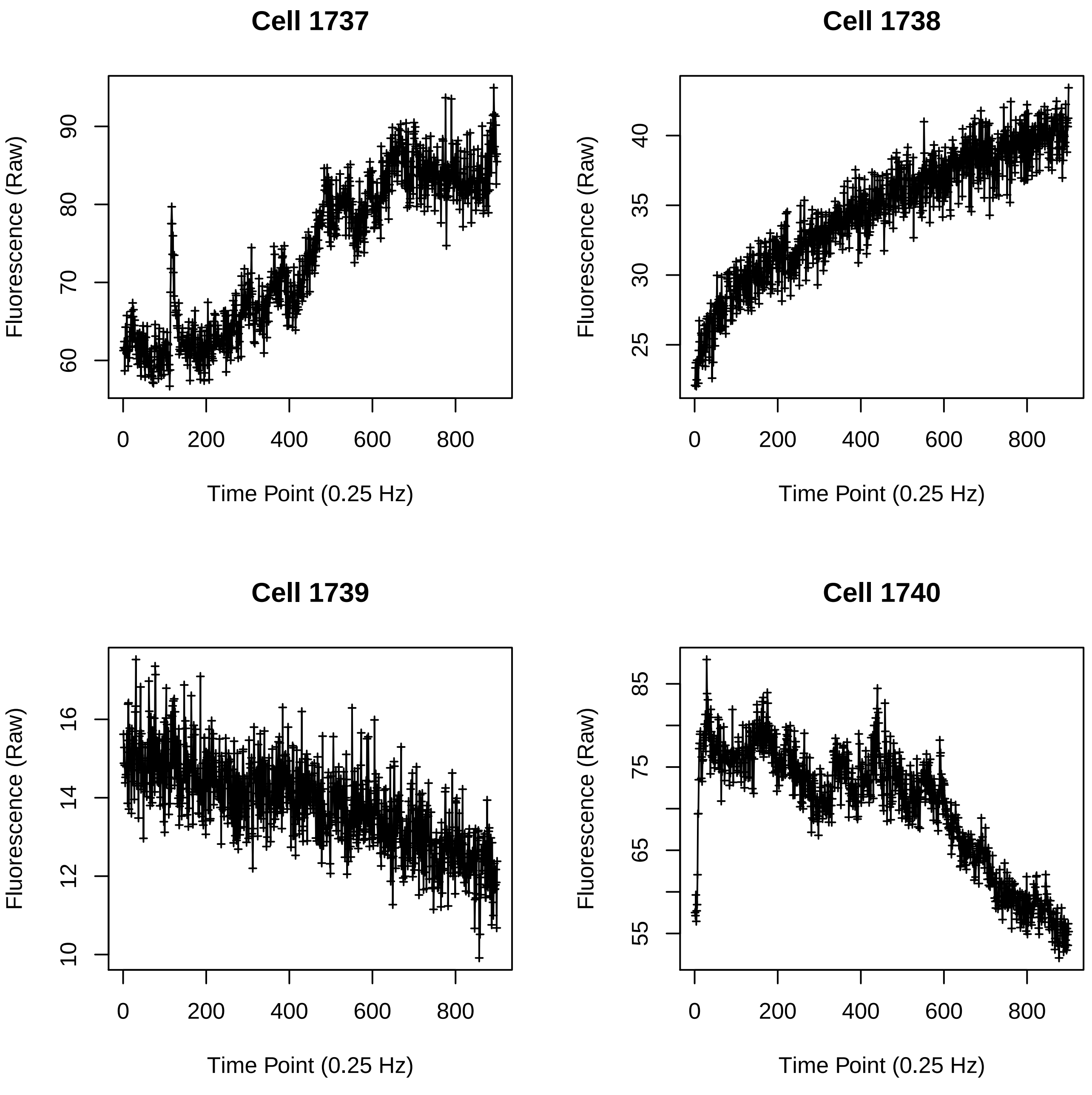
Cell 1737
Cell 1738
Cell 1739
Cell 1740
Fluorescence (Raw)
Time Point (0.25 Hz)
0
200
400
600
800
60
70
80
90
25
30
35
40
10
12
14
16
55
65
75
85

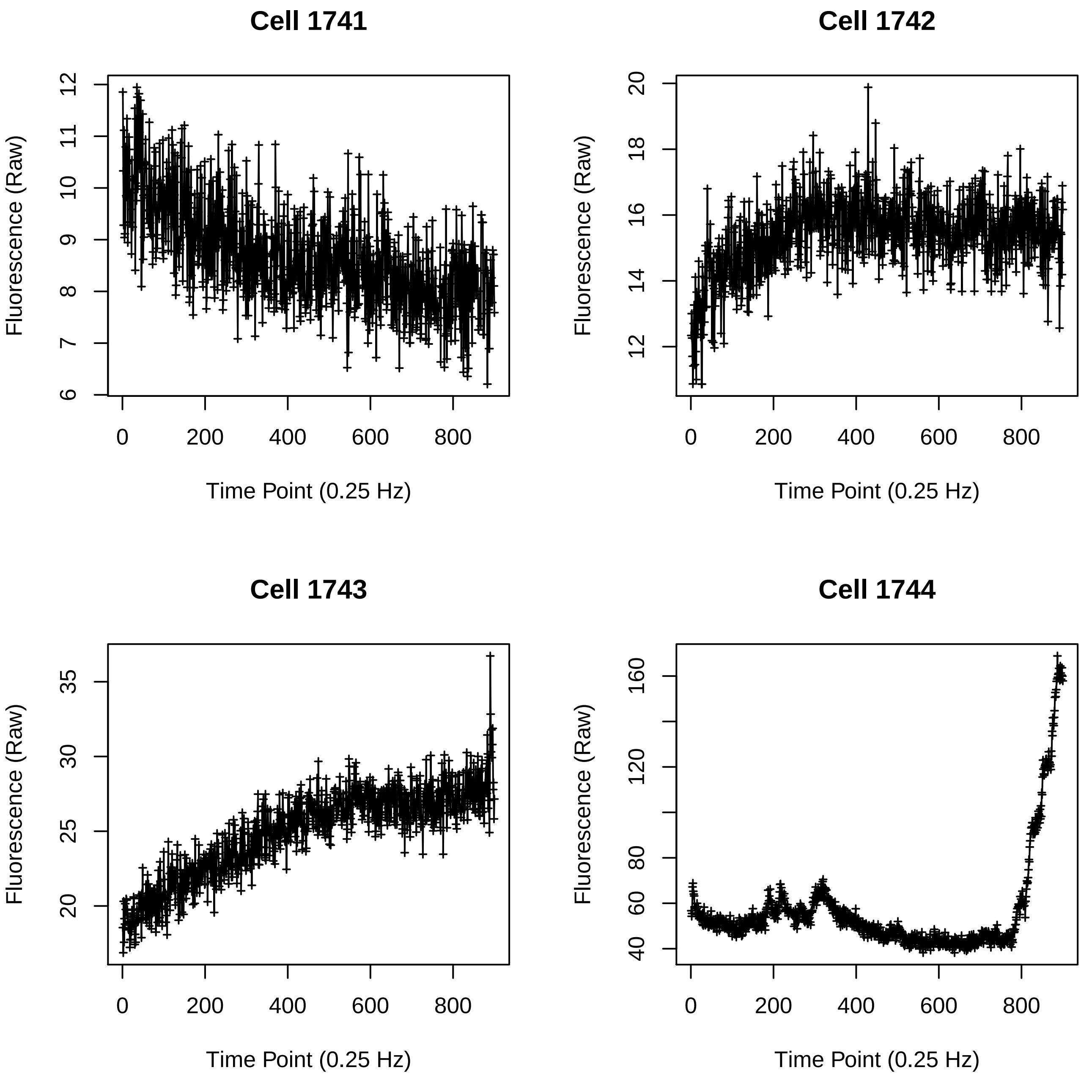
Cell 1741
Fluorescence (Raw)
Time Point (0.25 Hz)
Cell 1742
Fluorescence (Raw)
Time Point (0.25 Hz)
Cell 1743
Fluorescence (Raw)
Time Point (0.25 Hz)
Cell 1744
Fluorescence (Raw)
Time Point (0.25 Hz)

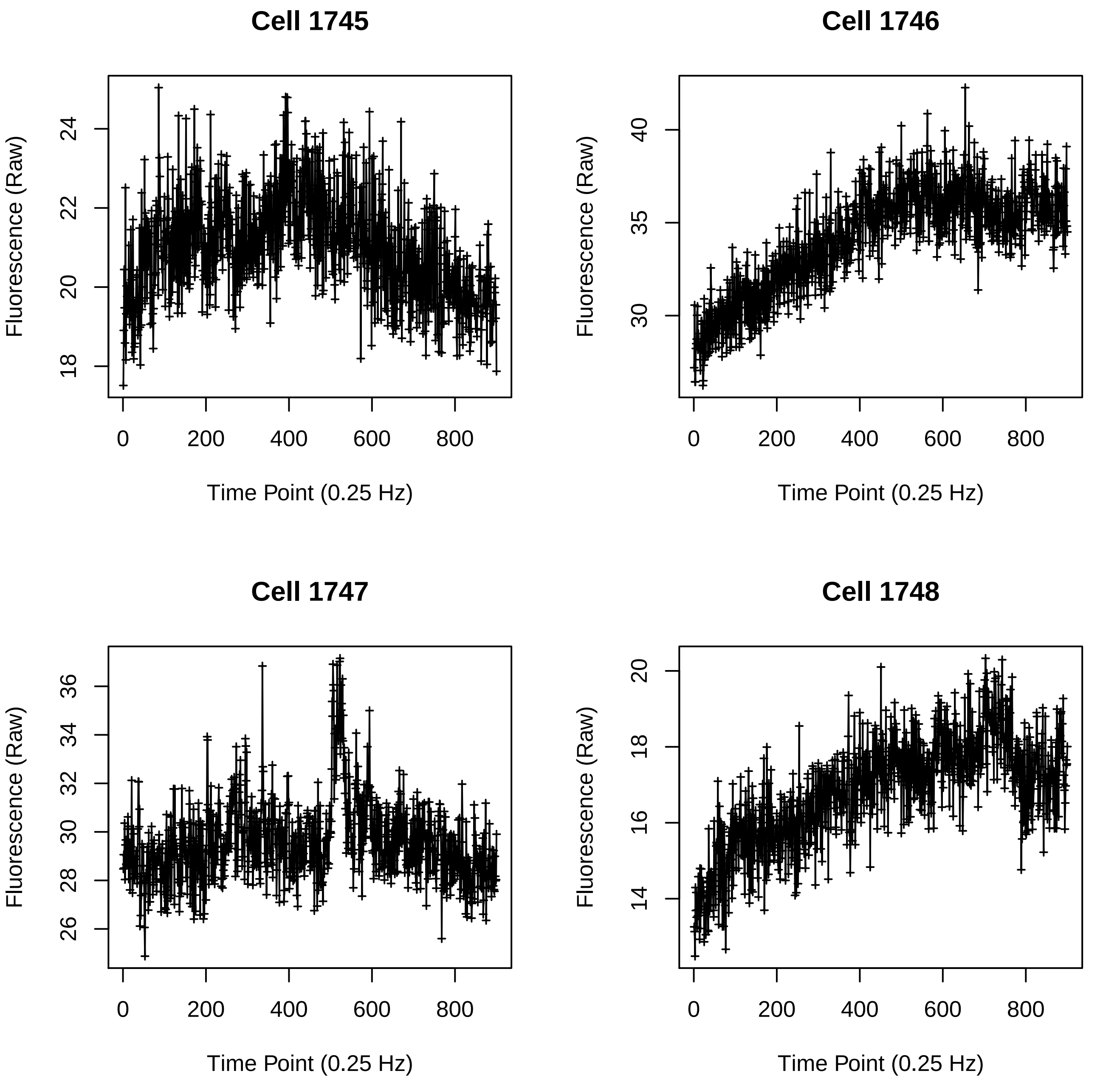
Cell 1745
Fluorescence (Raw)
18
20
22
24
0
200
400
600
800
Time Point (0.25 Hz)
Cell 1746
Fluorescence (Raw)
30
35
40
0
200
400
600
800
Time Point (0.25 Hz)
Cell 1747
Fluorescence (Raw)
26
28
30
32
34
36
0
200
400
600
800
Time Point (0.25 Hz)
Cell 1748
Fluorescence (Raw)
14
16
18
20
0
200
400
600
800
Time Point (0.25 Hz)

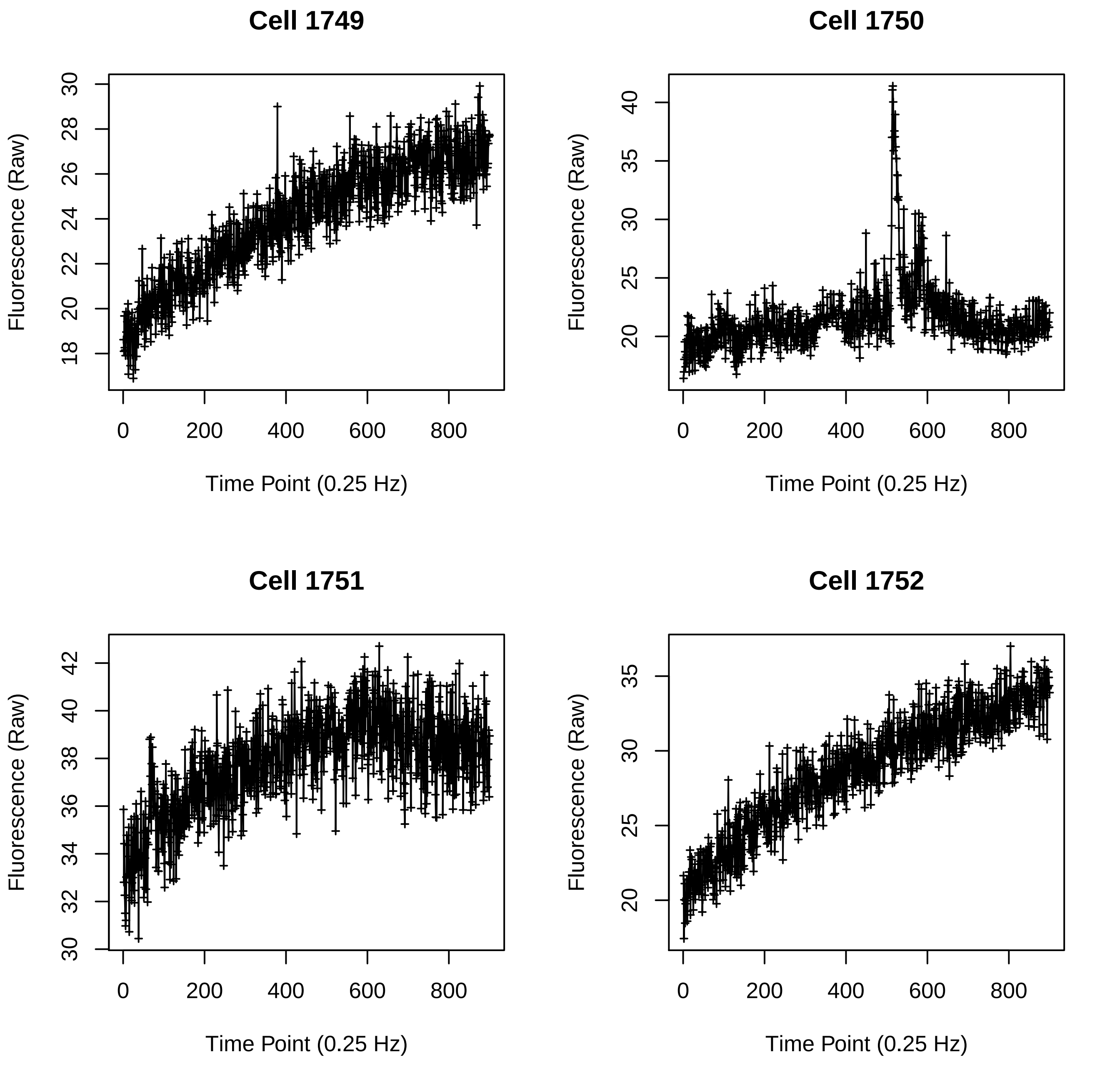

Cell 1749
Cell 1750
Cell 1751
Cell 1752
Fluorescence (Raw)
Time Point (0.25 Hz)
0
200
400
600
800

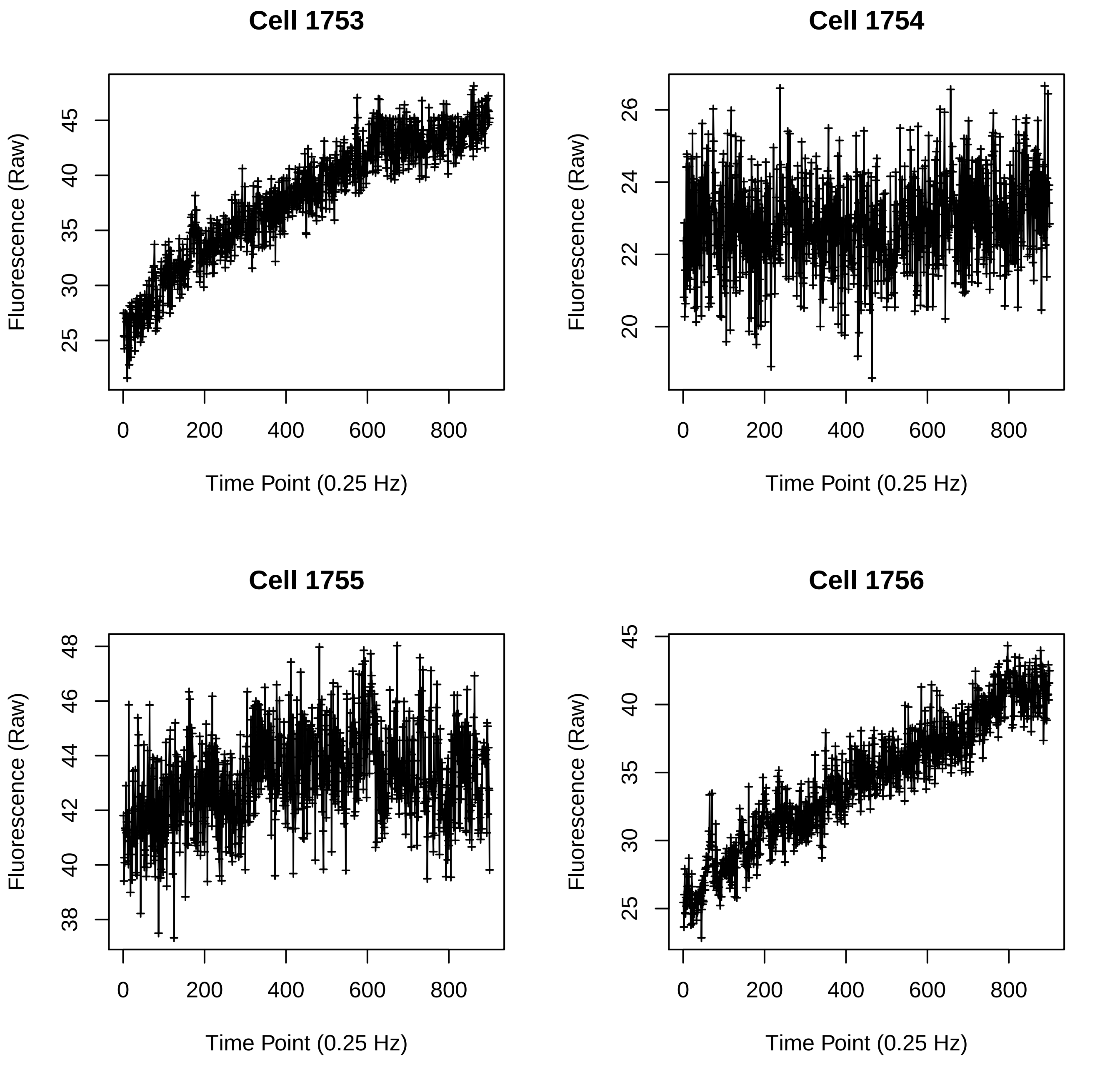
Cell 1753
Fluorescence (Raw)
25
30
35
40
45
0
200
400
600
800
Time Point (0.25 Hz)
Cell 1754
Fluorescence (Raw)
20
22
24
26
0
200
400
600
800
Time Point (0.25 Hz)
Cell 1755
Fluorescence (Raw)
38
40
42
44
46
48
0
200
400
600
800
Time Point (0.25 Hz)
Cell 1756
Fluorescence (Raw)
25
30
35
40
45
0
200
400
600
800
Time Point (0.25 Hz)

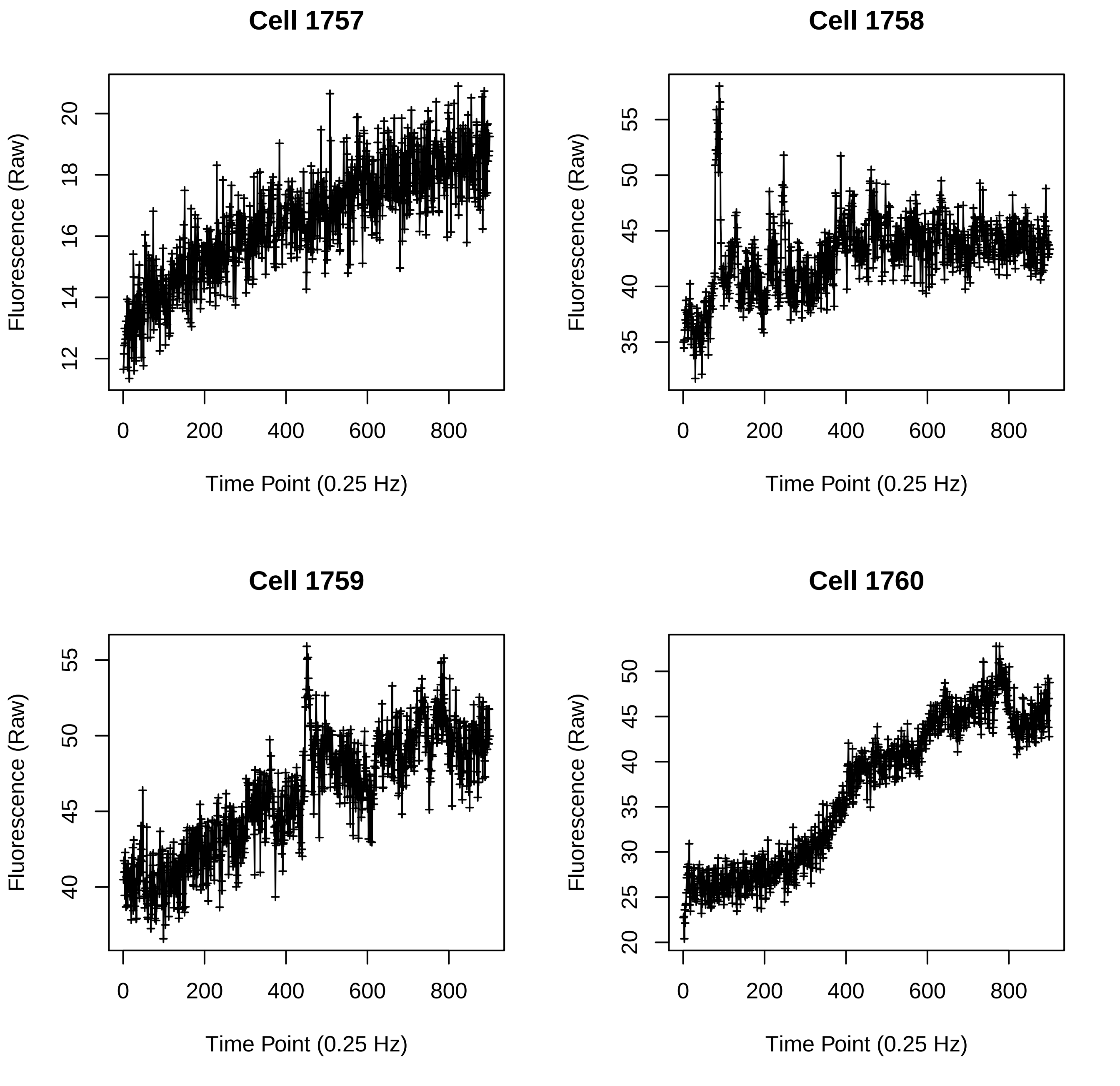

Cell 1757
Cell 1758
Cell 1759
Cell 1760
Fluorescence (Raw)
Time Point (0.25 Hz)
0
200
400
600
800
12
14
16
18
20
35
40
45
50
55
25
30

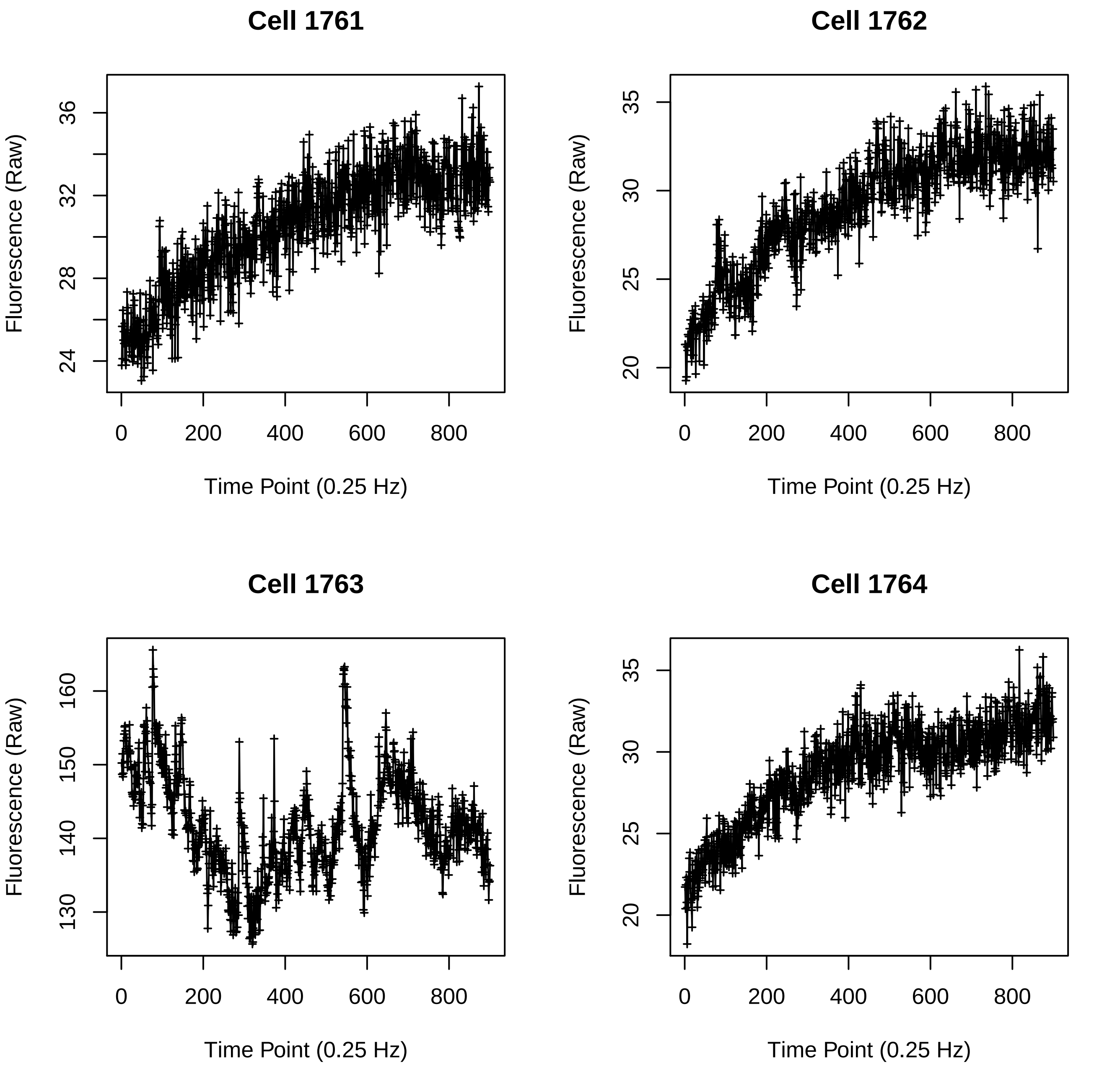
Cell 1761
Fluorescence (Raw)
24
28
32
36
0
200
400
600
800
Time Point (0.25 Hz)
Cell 1762
Fluorescence (Raw)
20
25
30
35
0
200
400
600
800
Time Point (0.25 Hz)
Cell 1763
Fluorescence (Raw)
130
140
150
160
0
200
400
600
800
Time Point (0.25 Hz)
Cell 1764
Fluorescence (Raw)
20
25
30
35
0
200
400
600
800
Time Point (0.25 Hz)

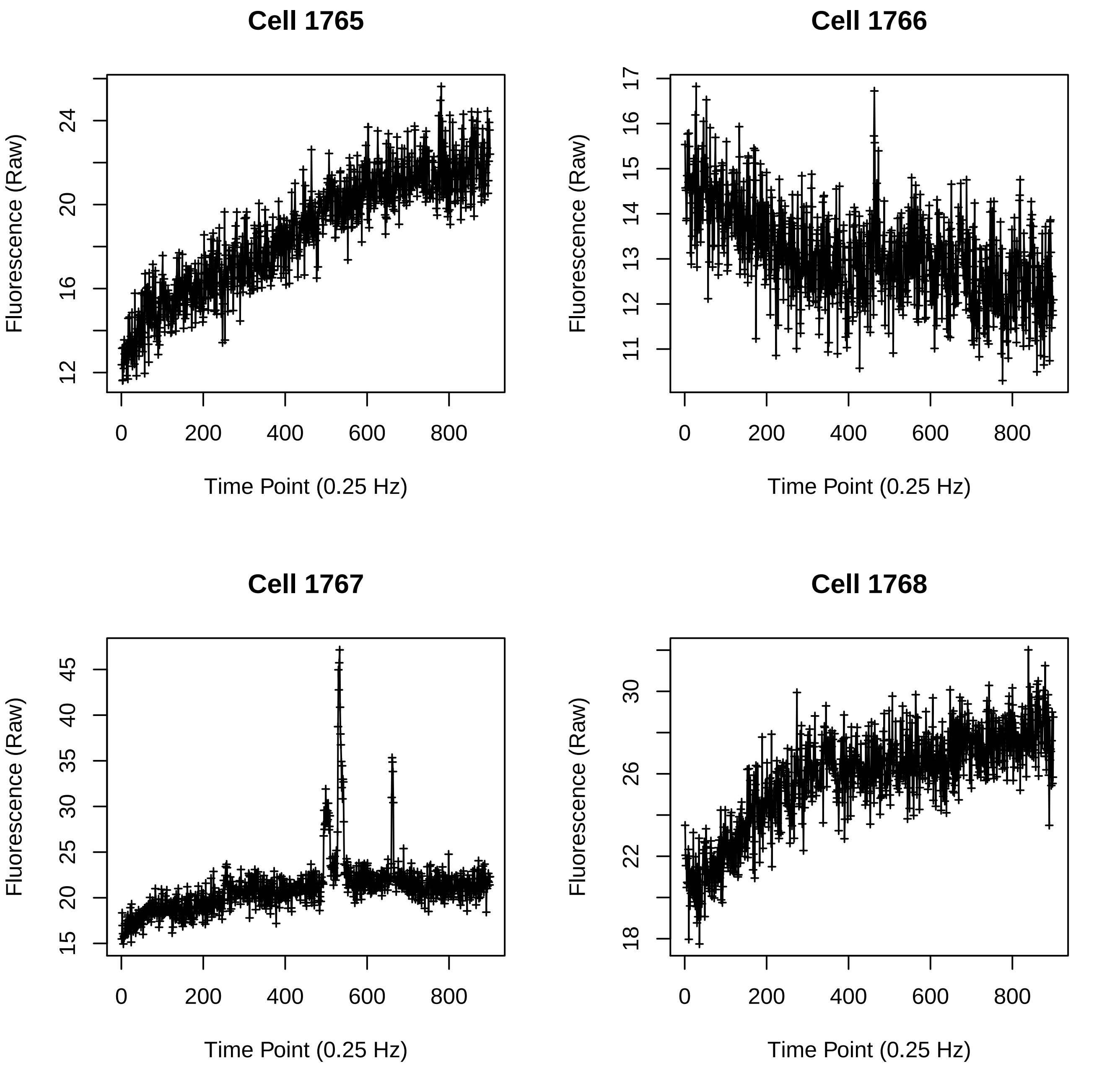
Cell 1765
Cell 1766
Cell 1767
Cell 1768
Fluorescence (Raw)
Time Point (0.25 Hz)
0
200
400
600
800
12
16
20
24
11
12
13
14
15
16
17
15
20
25
30
35
40
45
18
22
26
30

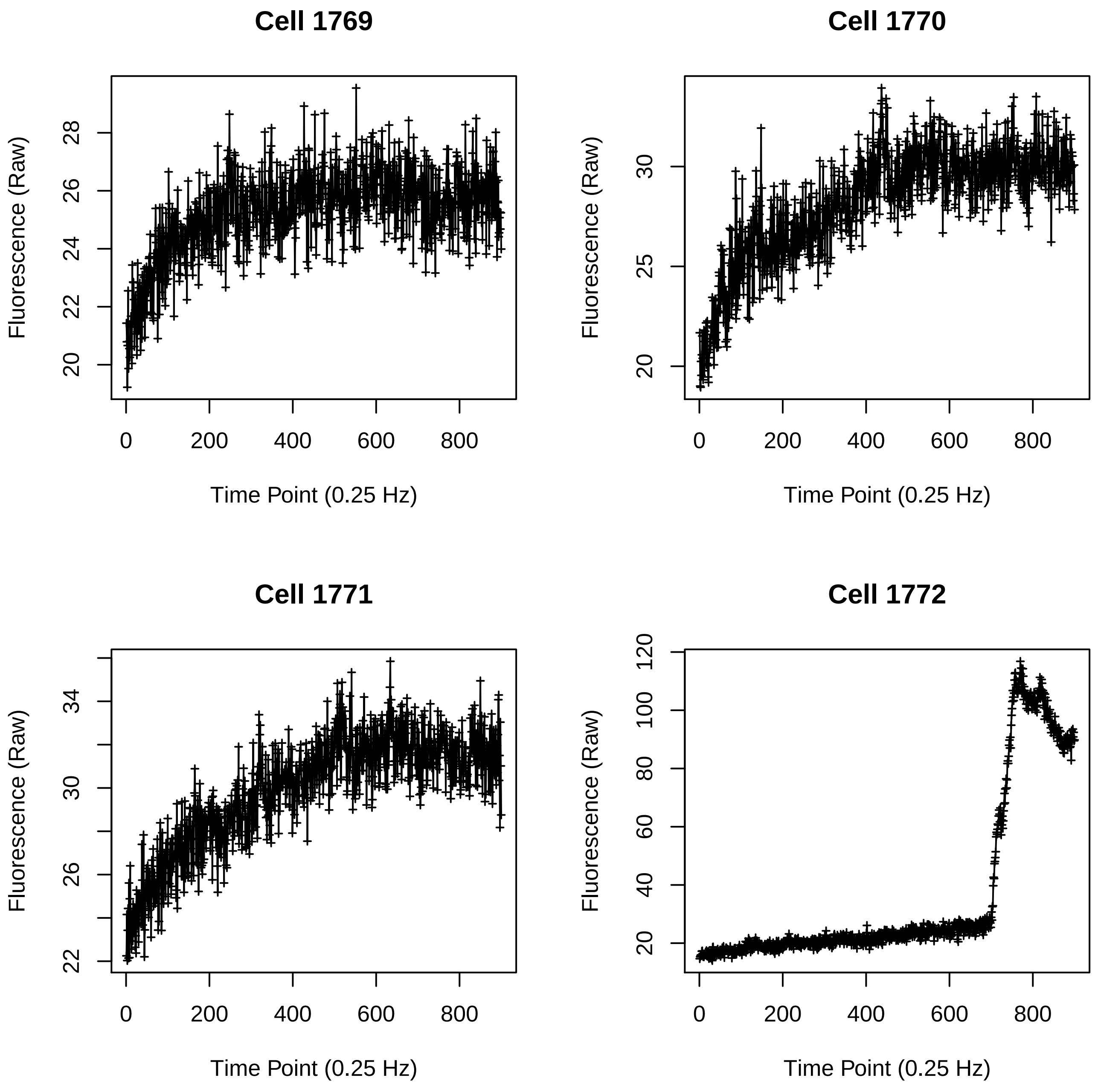
Cell 1769
Fluorescence (Raw)
20
22
24
26
28
0
200
400
600
800
Time Point (0.25 Hz)
Cell 1770
Fluorescence (Raw)
20
25
30
0
200
400
600
800
Time Point (0.25 Hz)
Cell 1771
Fluorescence (Raw)
22
26
30
34
0
200
400
600
800
Time Point (0.25 Hz)
Cell 1772
Fluorescence (Raw)
20
40
60
80
100
120
0
200
400
600
800
Time Point (0.25 Hz)

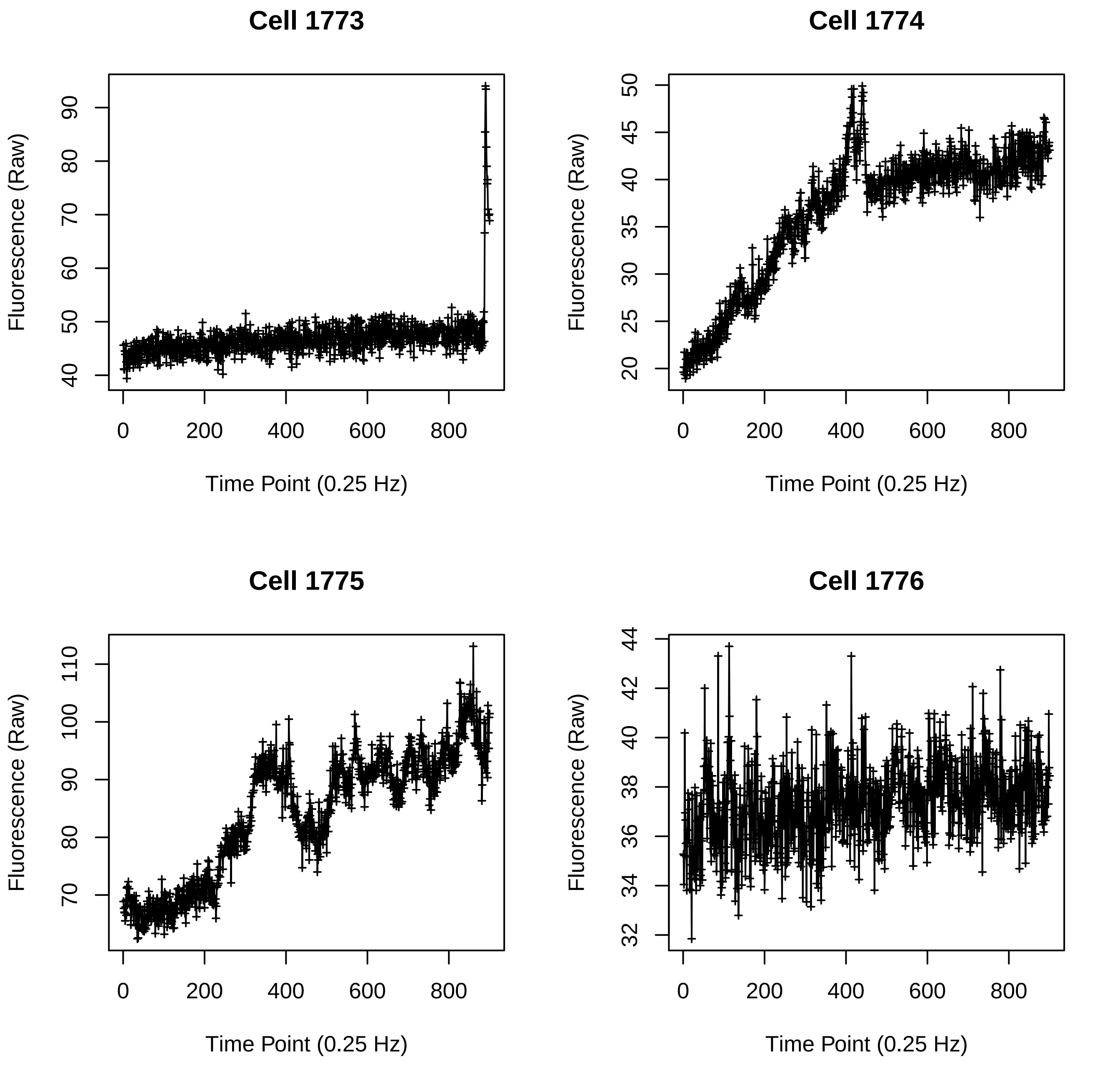

Cell 1773
Fluorescence (Raw)
40
50
60
70
80
90
0
200
400
600
800
Time Point (0.25 Hz)
Cell 1774
Fluorescence (Raw)
20
25
30
35
40
45
50
0
200
400
600
800
Time Point (0.25 Hz)
Cell 1775
Fluorescence (Raw)
70
80
90
100
110
0
200
400
600
800
Time Point (0.25 Hz)
Cell 1776
Fluorescence (Raw)
32
34
36
38
40
42
44
0
200
400
600
800
Time Point (0.25 Hz)

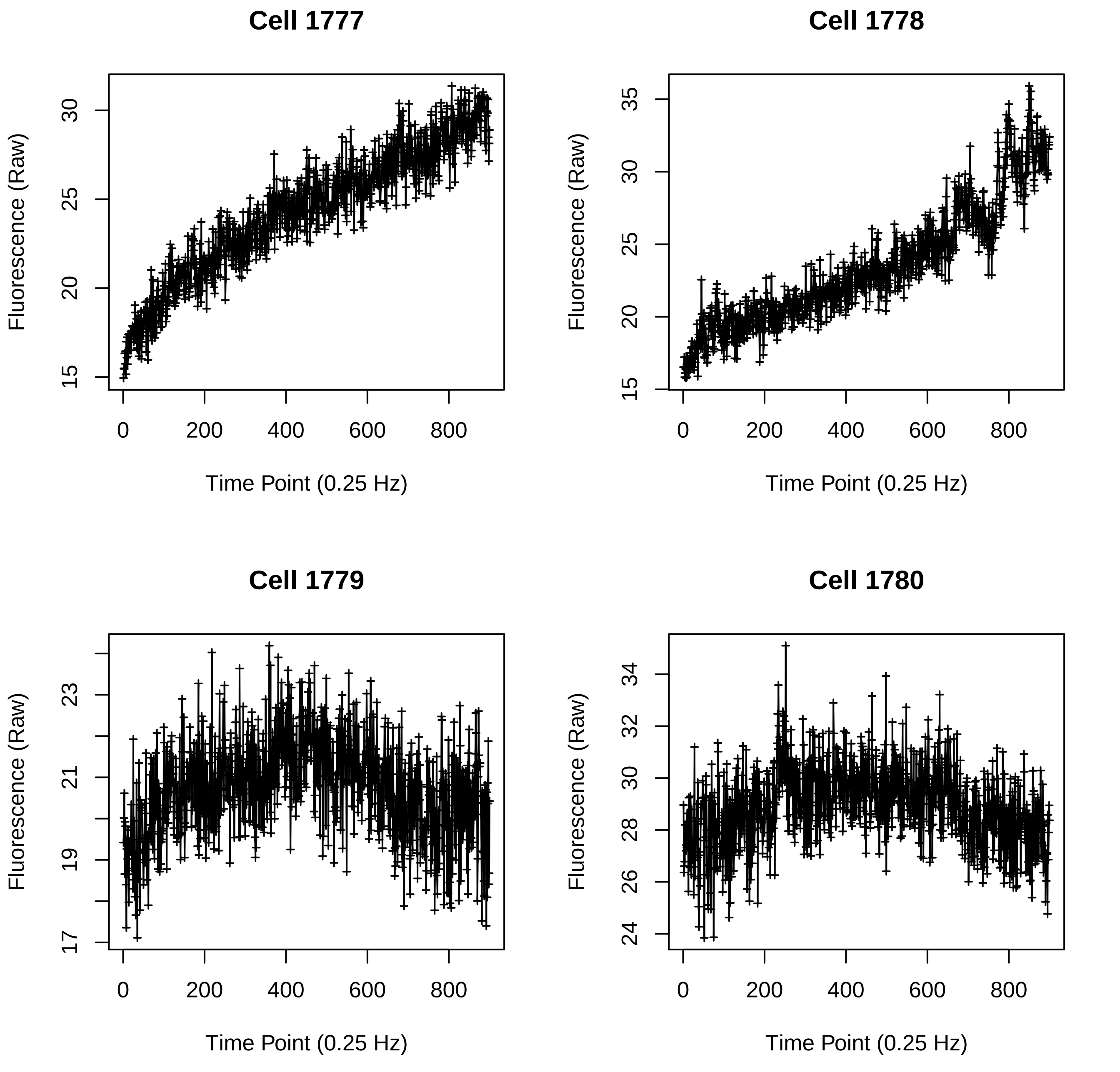

Cell 1777
Fluorescence (Raw)
15
20
25
30
0
200
400
600
800
Time Point (0.25 Hz)
Cell 1778
Fluorescence (Raw)
15
20
25
30
35
0
200
400
600
800
Time Point (0.25 Hz)
Cell 1779
Fluorescence (Raw)
17
19
21
23
0
200
400
600
800
Time Point (0.25 Hz)
Cell 1780
Fluorescence (Raw)
24
26
28
30
32
34
0
200
400
600
800
Time Point (0.25 Hz)

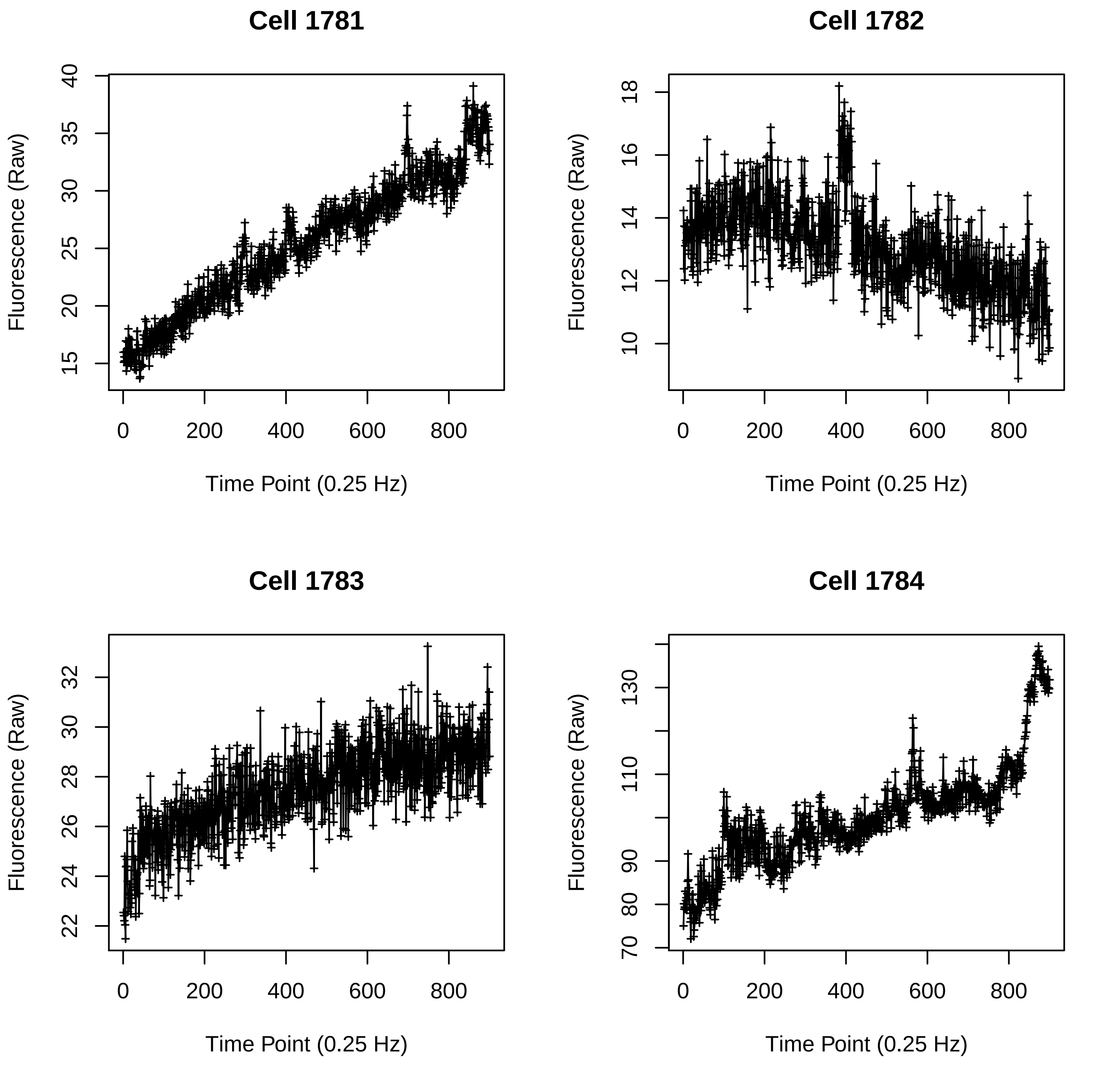

Cell 1781
Cell 1782
Cell 1783
Cell 1784
Fluorescence (Raw)
Time Point (0.25 Hz)
0
200
400
600
800
15
20
25
30
35
40
10
12
14
16
18
22
24
26
28
30
32
70
80
90
110
130

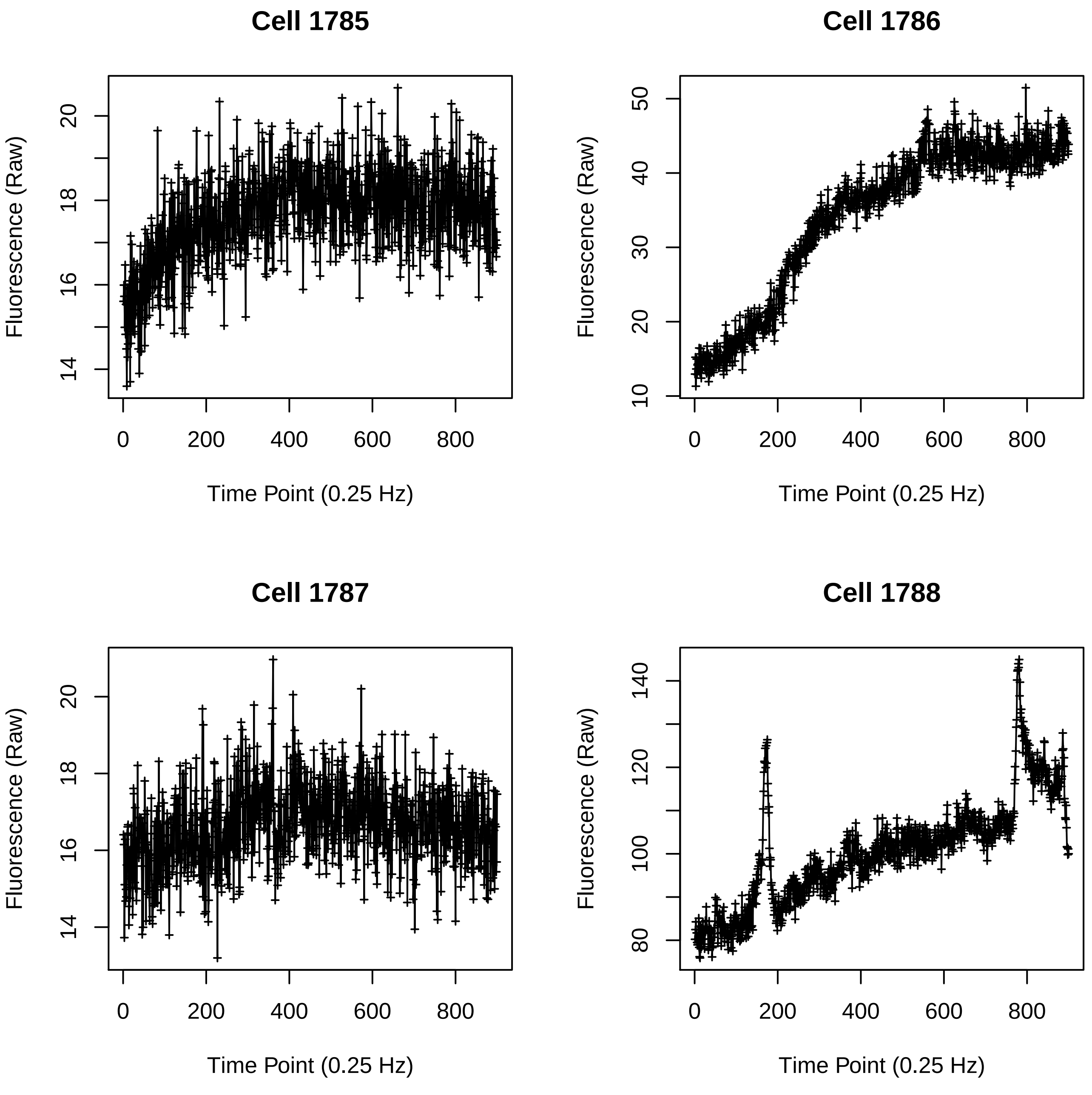
Cell 1785
Cell 1786
Cell 1787
Cell 1788
Fluorescence (Raw)
Time Point (0.25 Hz)
0
200
400
600
800
14
16
18
20
10
20
30
40
50
80
100
120
140

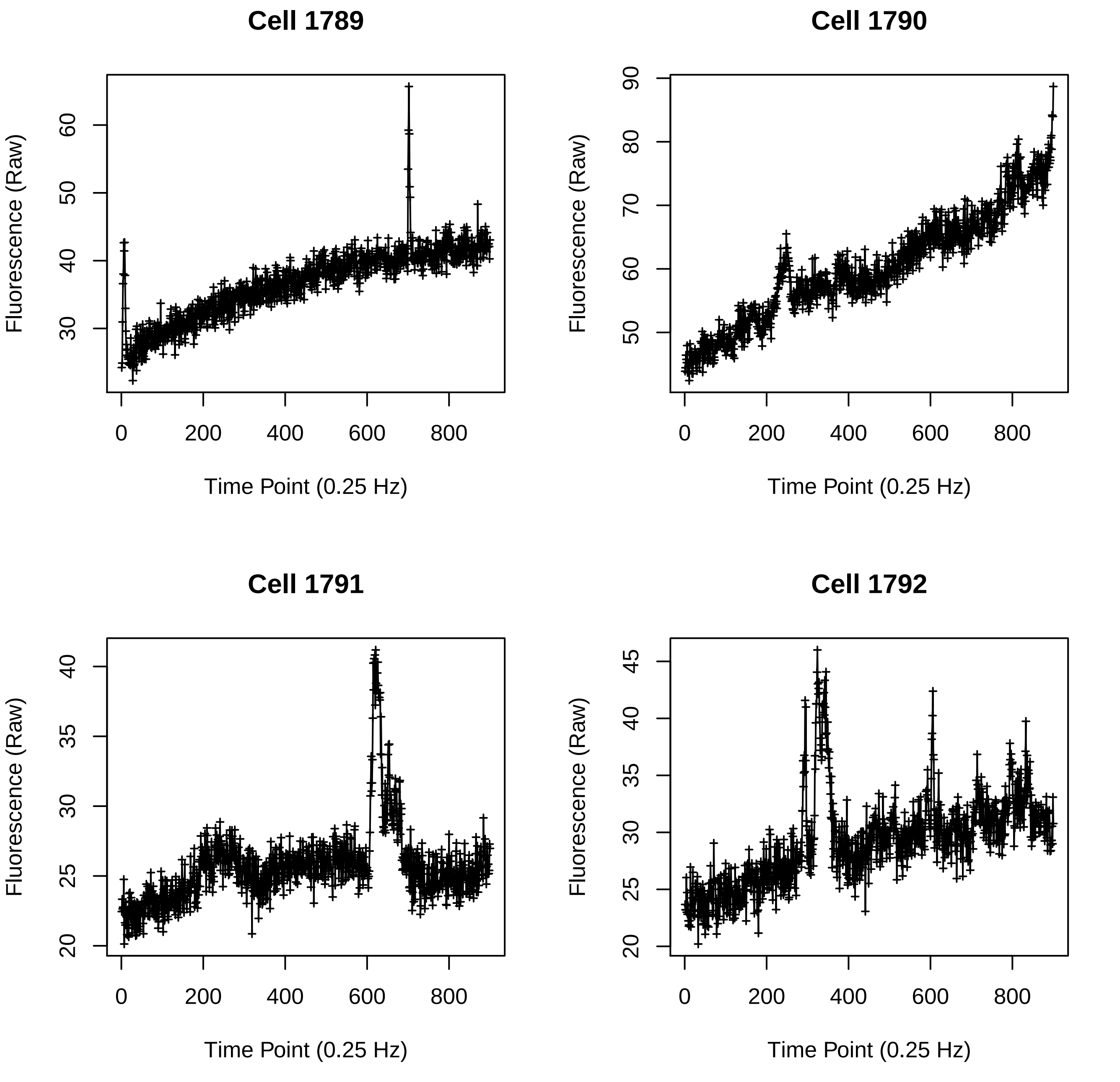
Cell 1789
Fluorescence (Raw)
30
40
50
60
0
200
400
600
800
Time Point (0.25 Hz)
Cell 1790
Fluorescence (Raw)
50
60
70
80
90
0
200
400
600
800
Time Point (0.25 Hz)
Cell 1791
Fluorescence (Raw)
20
25
30
35
40
0
200
400
600
800
Time Point (0.25 Hz)
Cell 1792
Fluorescence (Raw)
20
25
30
35
40
45
0
200
400
600
800
Time Point (0.25 Hz)

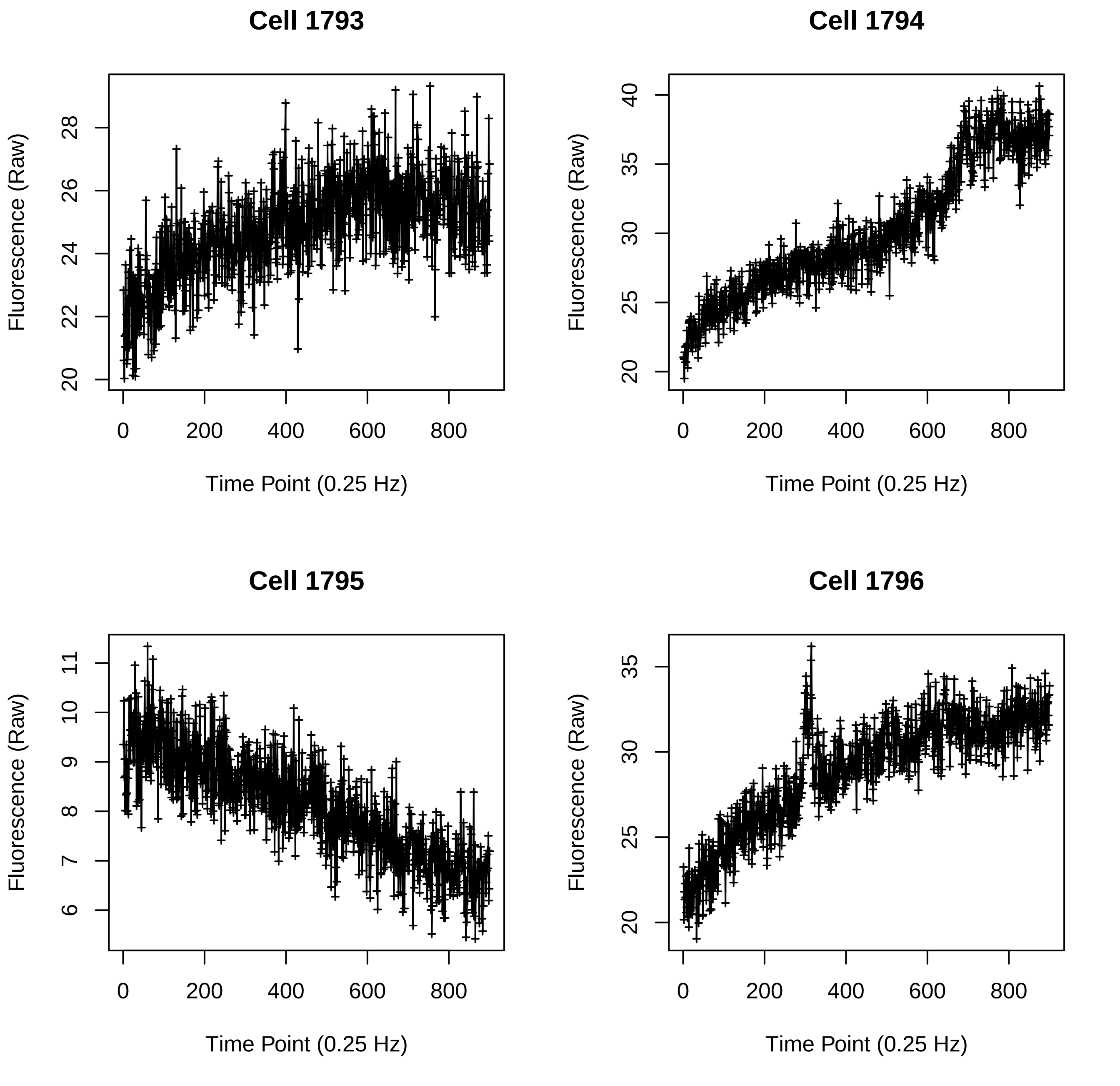

Cell 1793
Cell 1794
Cell 1795
Cell 1796
Fluorescence (Raw)
Time Point (0.25 Hz)

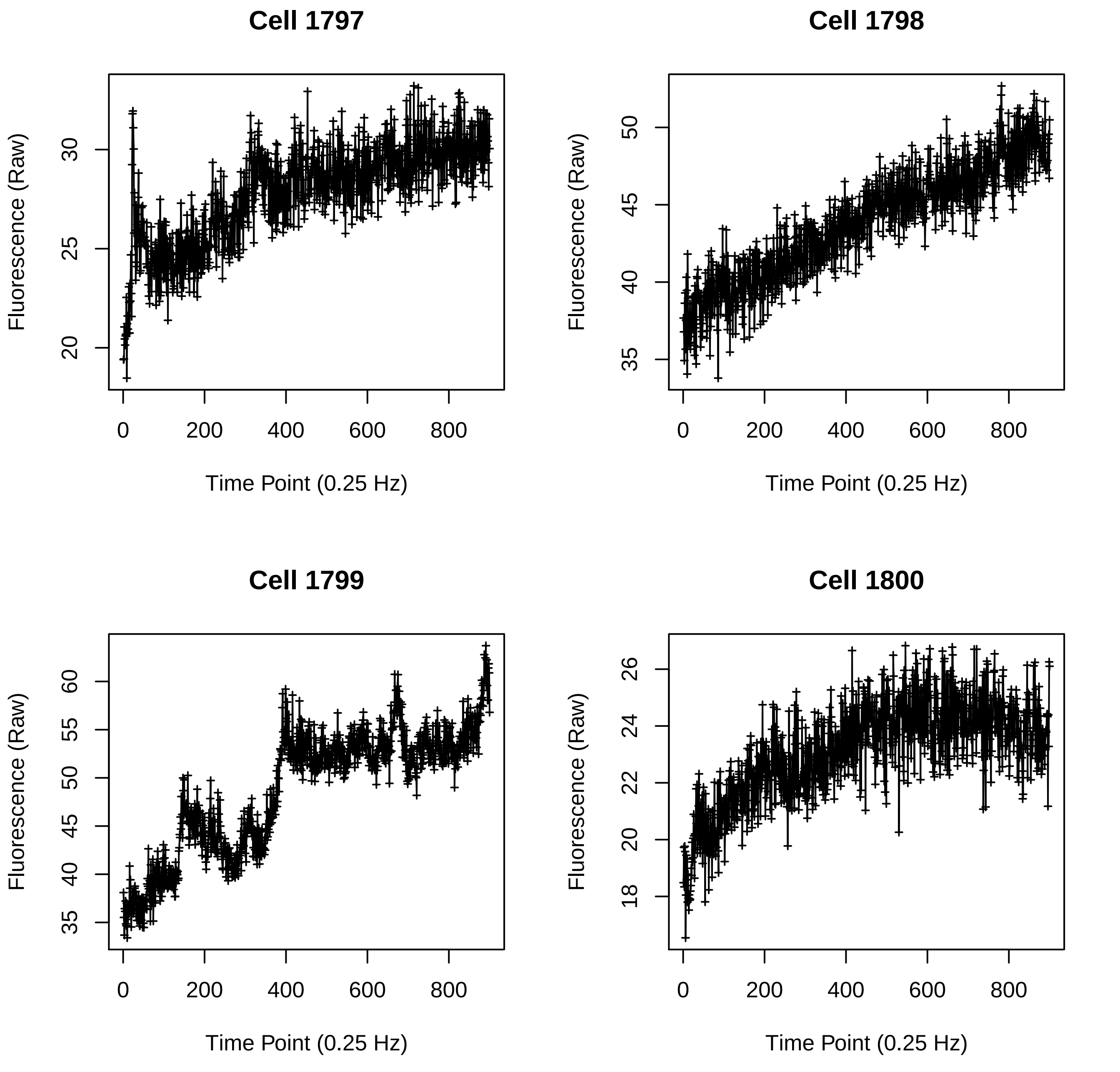
Cell 1797
Cell 1798
Cell 1799
Cell 1800
Fluorescence (Raw)
Time Point (0.25 Hz)
0
200
400
600
800
20
25
30
35
40
45
50
55
60
18
22
24
26

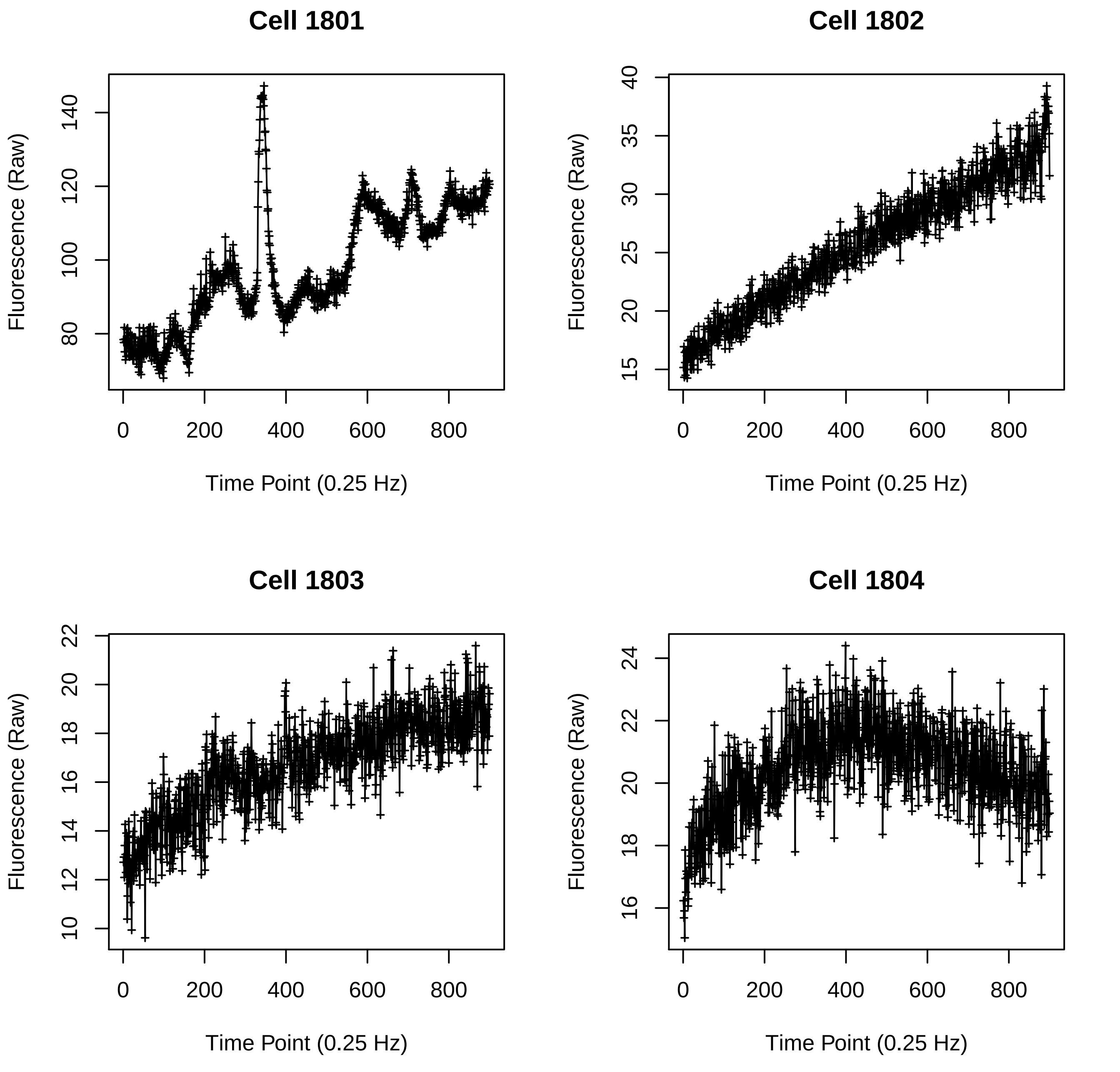
Cell 1801
Fluorescence (Raw)
80
100
120
140
0
200
400
600
800
Time Point (0.25 Hz)
Cell 1802
Fluorescence (Raw)
15
20
25
30
35
40
0
200
400
600
800
Time Point (0.25 Hz)
Cell 1803
Fluorescence (Raw)
10
12
14
16
18
20
22
0
200
400
600
800
Time Point (0.25 Hz)
Cell 1804
Fluorescence (Raw)
16
18
20
22
24
0
200
400
600
800
Time Point (0.25 Hz)

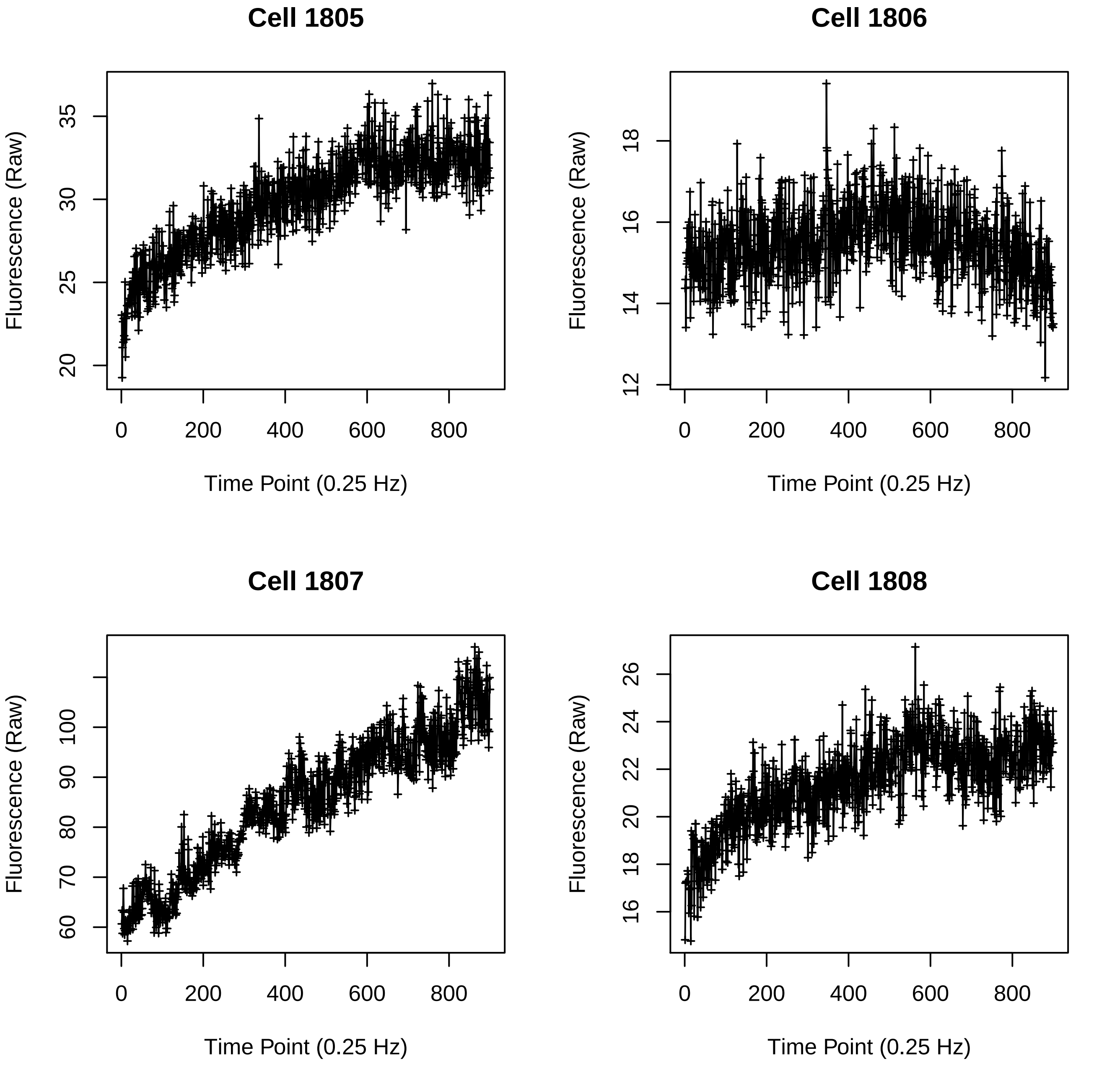
Cell 1805
Fluorescence (Raw)
Time Point (0.25 Hz)
Cell 1806
Fluorescence (Raw)
Time Point (0.25 Hz)
Cell 1807
Fluorescence (Raw)
Time Point (0.25 Hz)
Cell 1808
Fluorescence (Raw)
Time Point (0.25 Hz)

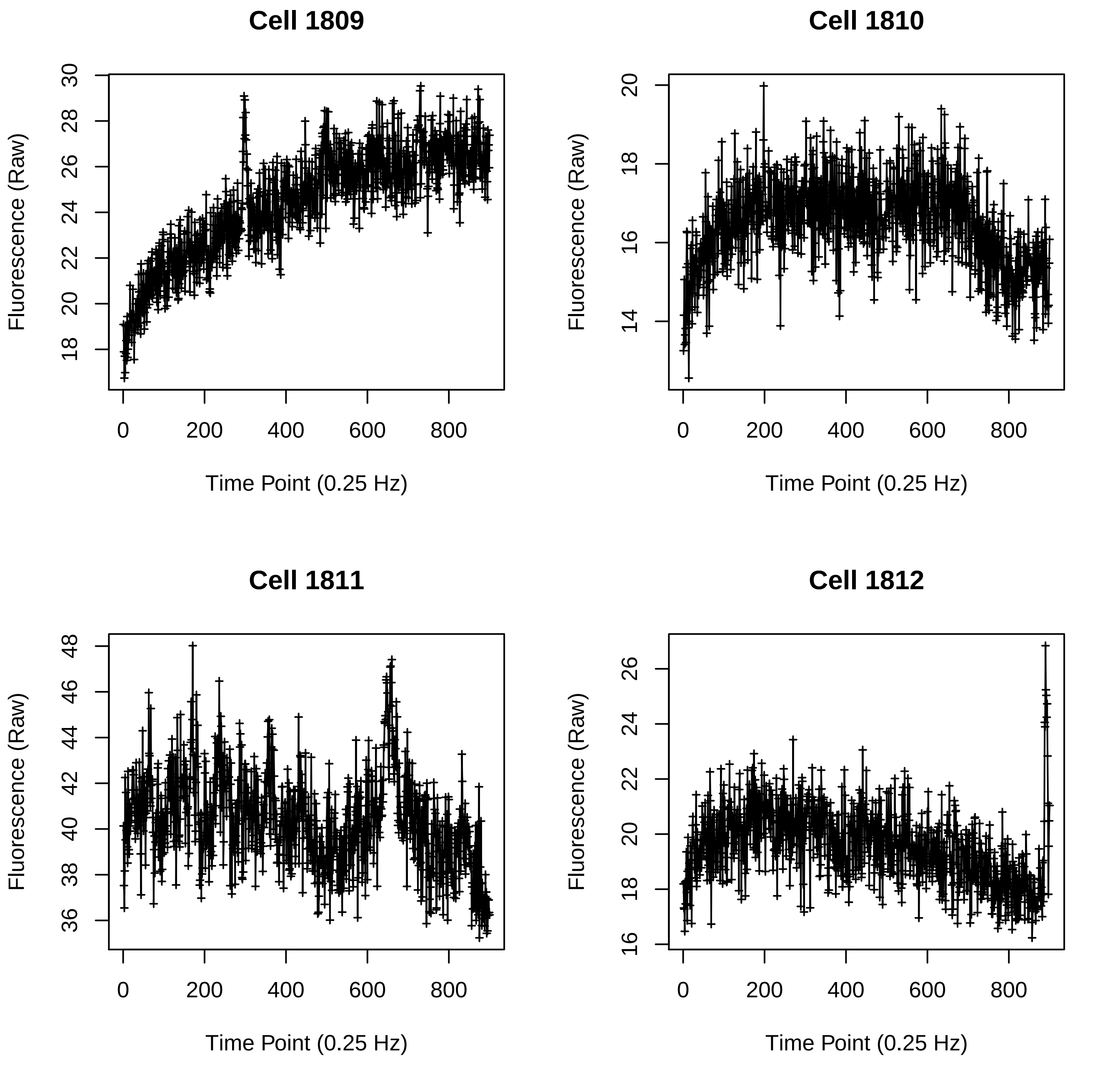
Cell 1809
Cell 1810
Cell 1811
Cell 1812
Fluorescence (Raw)
Time Point (0.25 Hz)
0
200
400
600
800
18
20
22
24
26
28
30
14
16
36
38
40
42
44
46
48

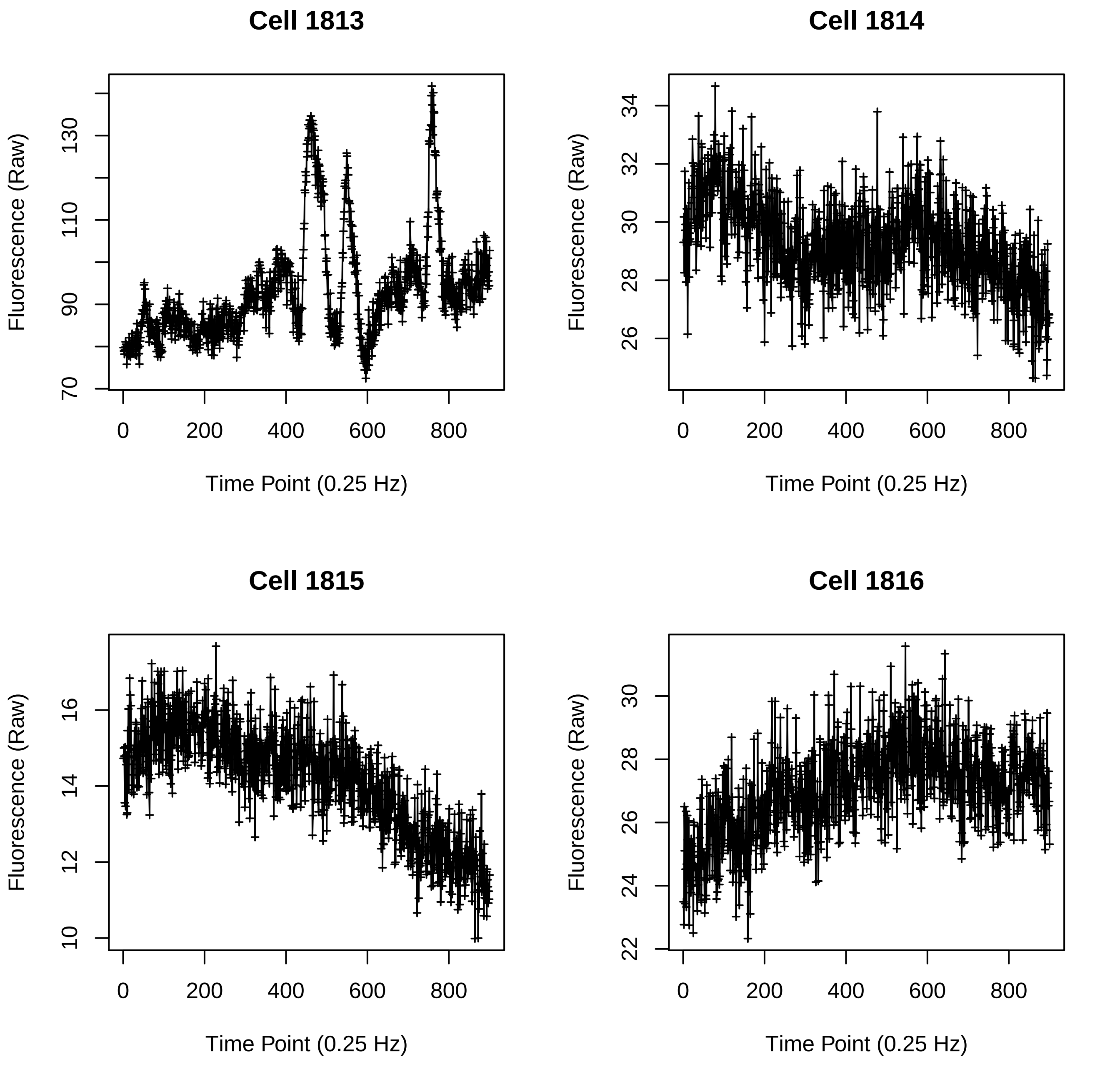
Cell 1813
Cell 1814
Cell 1815
Cell 1816
Fluorescence (Raw)
Time Point (0.25 Hz)
70
90
110
130
34
32
30
28
26
16
14
12
10
22
24
0
200
400
600
800

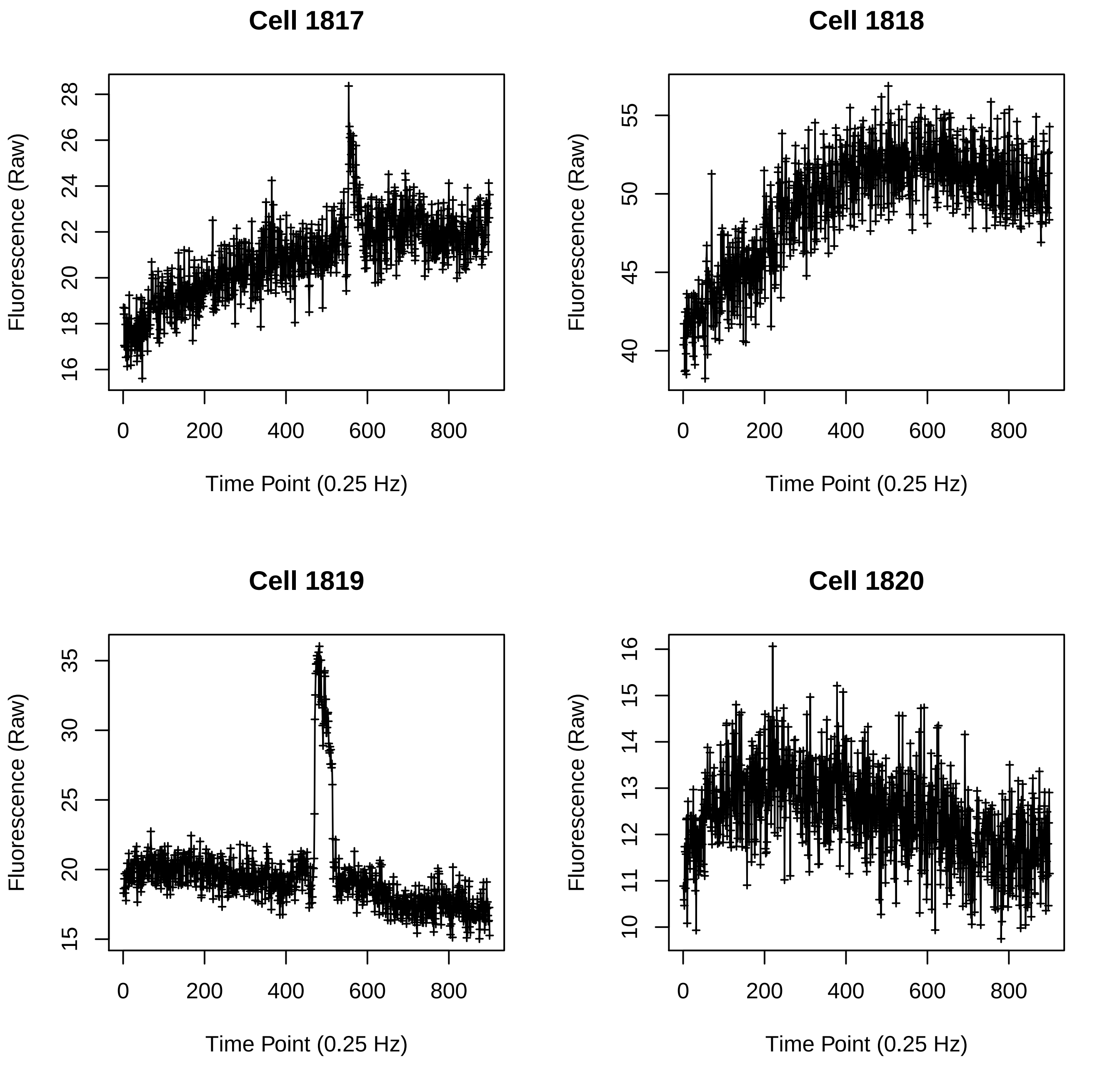

Cell 1817
Cell 1818
Cell 1819
Cell 1820
Fluorescence (Raw)
Time Point (0.25 Hz)
0
200
400
600
800
16
18
20
22
24
26
28
40
45
50
55
15
20
25
30
35
10
11
12
13
14
15
16

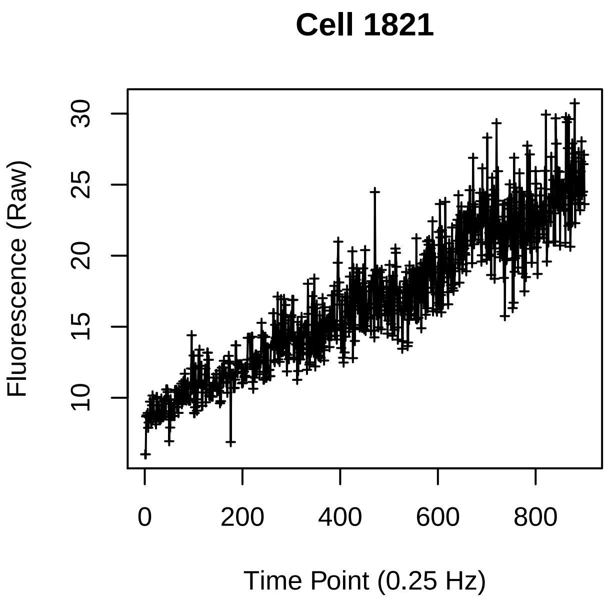
Cell 1821
Fluorescence (Raw)
10
15
20
25
30
0
200
400
600
800
Time Point (0.25 Hz)

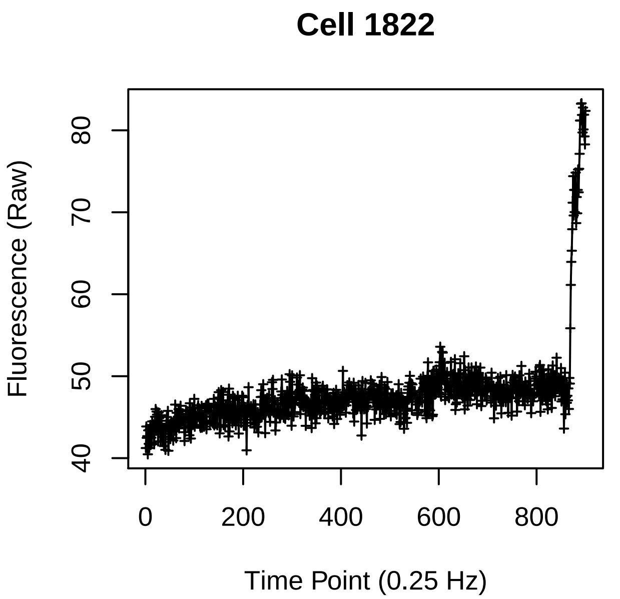
Cell 1822
Fluorescence (Raw)
40
50
60
70
80
0
200
400
600
800
Time Point (0.25 Hz)

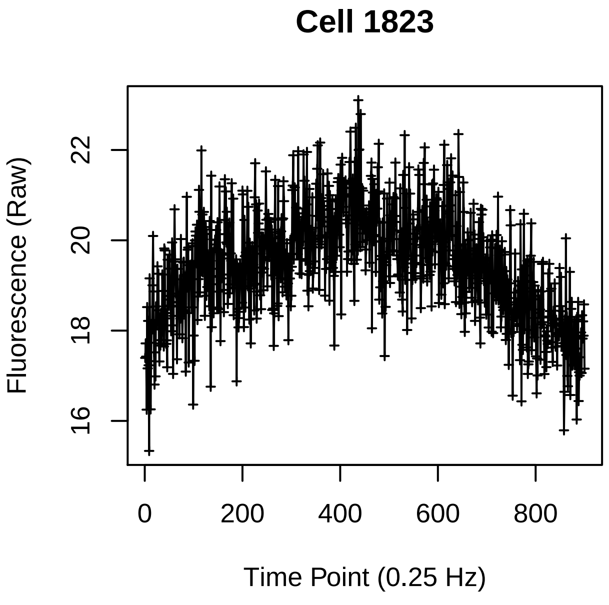
Cell 1823
Fluorescence (Raw)
16
18
20
22
0
200
400
600
800
Time Point (0.25 Hz)

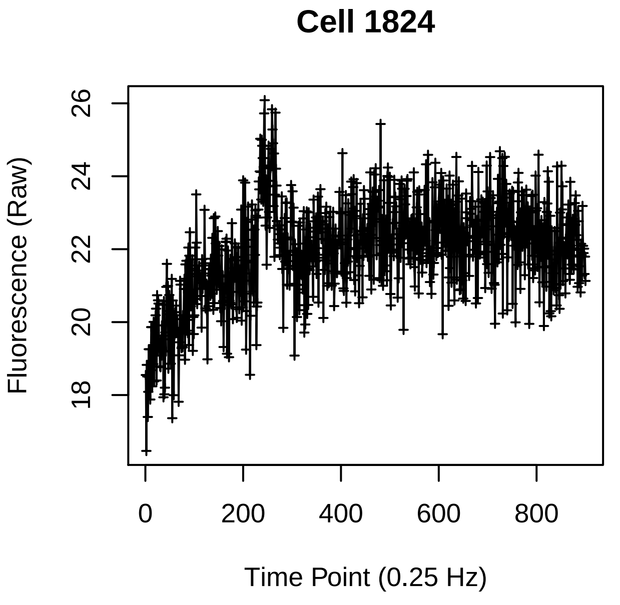
Cell 1824
Fluorescence (Raw)
18
20
22
24
26
0
200
400
600
800
Time Point (0.25 Hz)

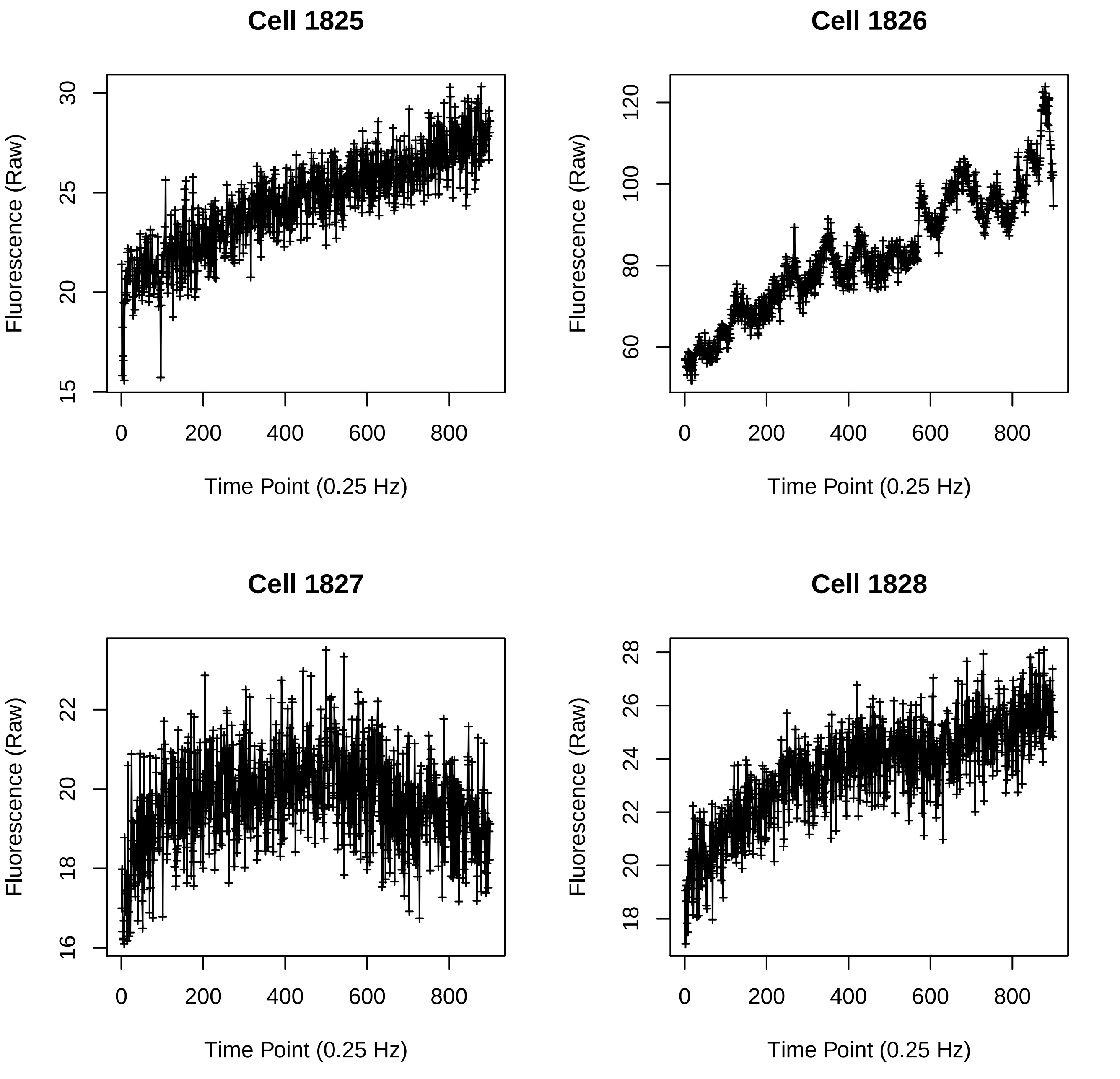
Cell 1825
Fluorescence (Raw)
Time Point (0.25 Hz)
Cell 1826
Fluorescence (Raw)
Time Point (0.25 Hz)
Cell 1827
Fluorescence (Raw)
Time Point (0.25 Hz)
Cell 1828
Fluorescence (Raw)
Time Point (0.25 Hz)

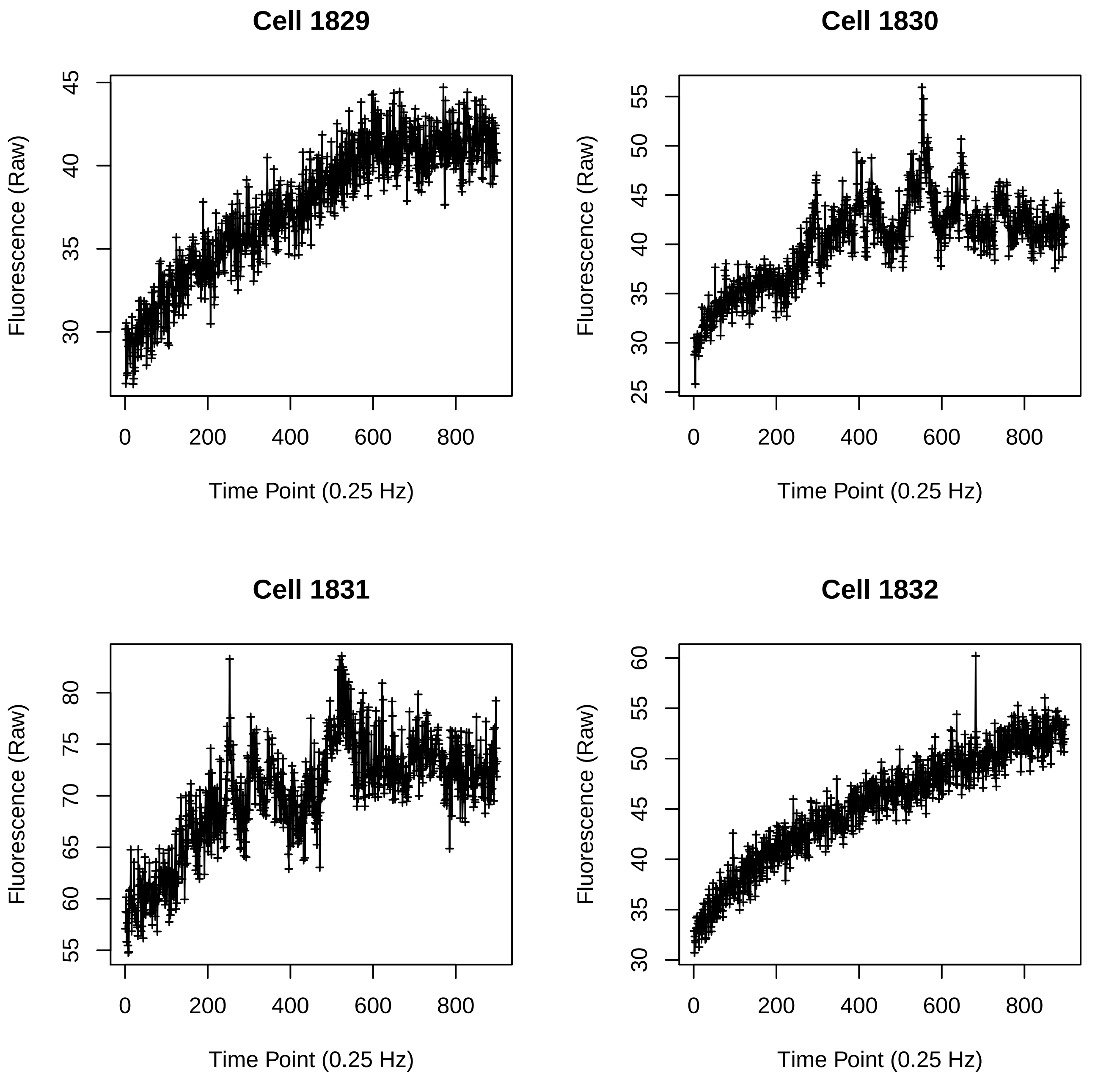
Cell 1829
Fluorescence (Raw)
30
35
40
45
0
200
400
600
800
Time Point (0.25 Hz)
Cell 1830
Fluorescence (Raw)
25
30
35
40
45
50
55
0
200
400
600
800
Time Point (0.25 Hz)
Cell 1831
Fluorescence (Raw)
55
60
65
70
75
80
0
200
400
600
800
Time Point (0.25 Hz)
Cell 1832
Fluorescence (Raw)
30
35
40
45
50
55
60
0
200
400
600
800
Time Point (0.25 Hz)

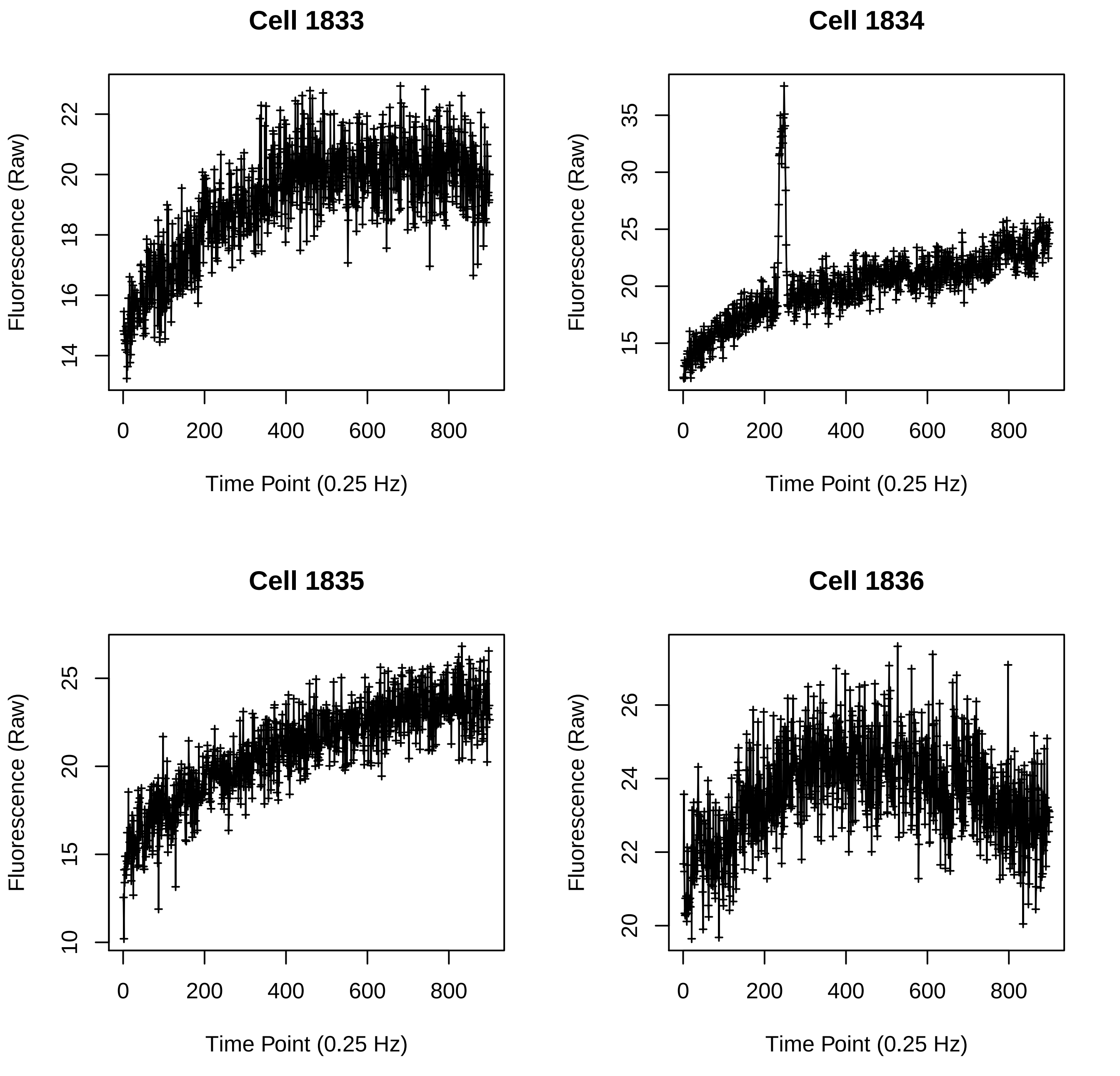
Cell 1833
Fluorescence (Raw)
14
16
18
20
22
0
200
400
600
800
Time Point (0.25 Hz)
Cell 1834
Fluorescence (Raw)
15
20
25
30
35
0
200
400
600
800
Time Point (0.25 Hz)
Cell 1835
Fluorescence (Raw)
10
15
20
25
0
200
400
600
800
Time Point (0.25 Hz)
Cell 1836
Fluorescence (Raw)
20
22
24
26
0
200
400
600
800
Time Point (0.25 Hz)

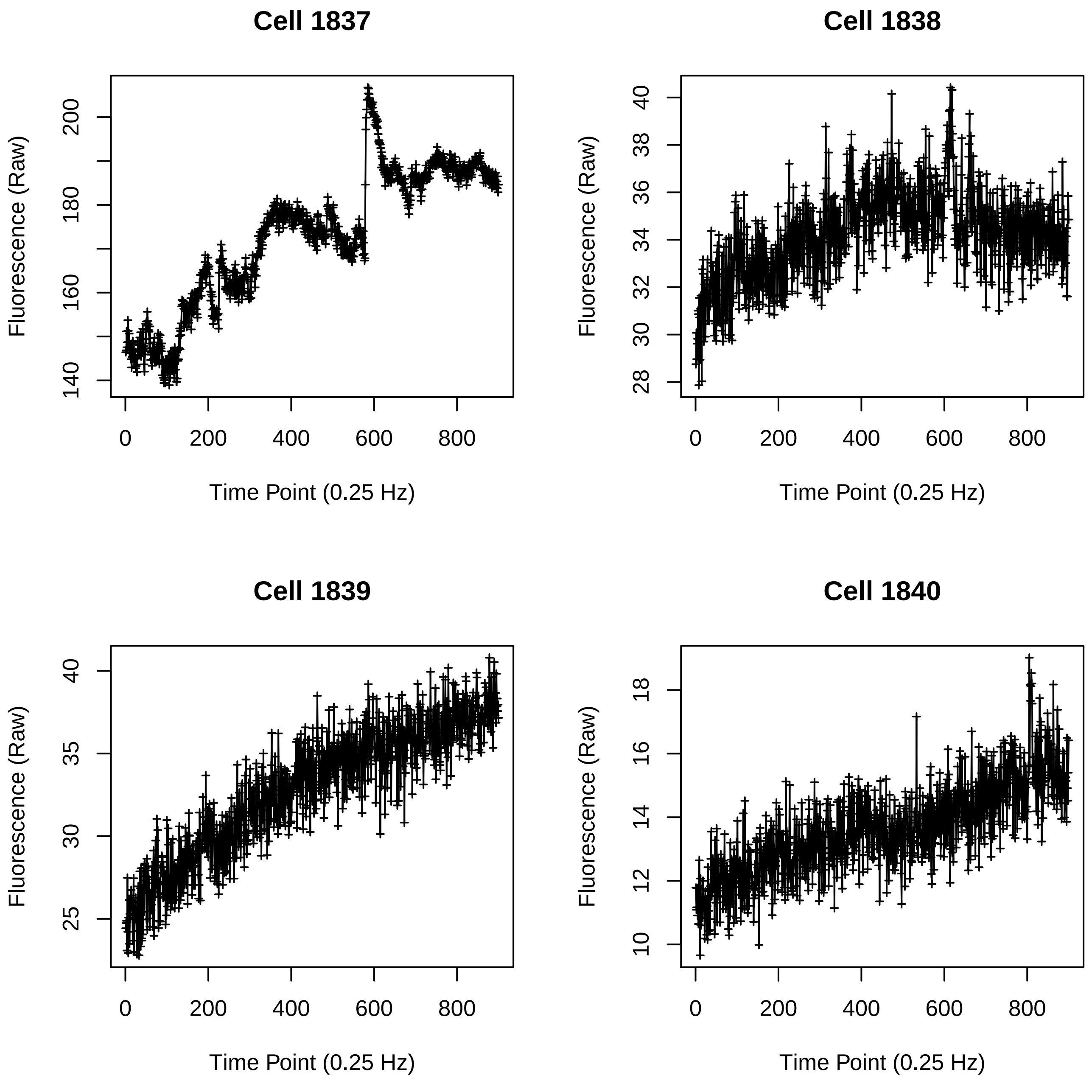
Cell 1837
Cell 1838
Cell 1839
Cell 1840
Fluorescence (Raw)
Time Point (0.25 Hz)
140
160
180
200
28
30
32
34
36
38
40
25
30
35
40
10
12
14
16
18
0
200
400
600
800

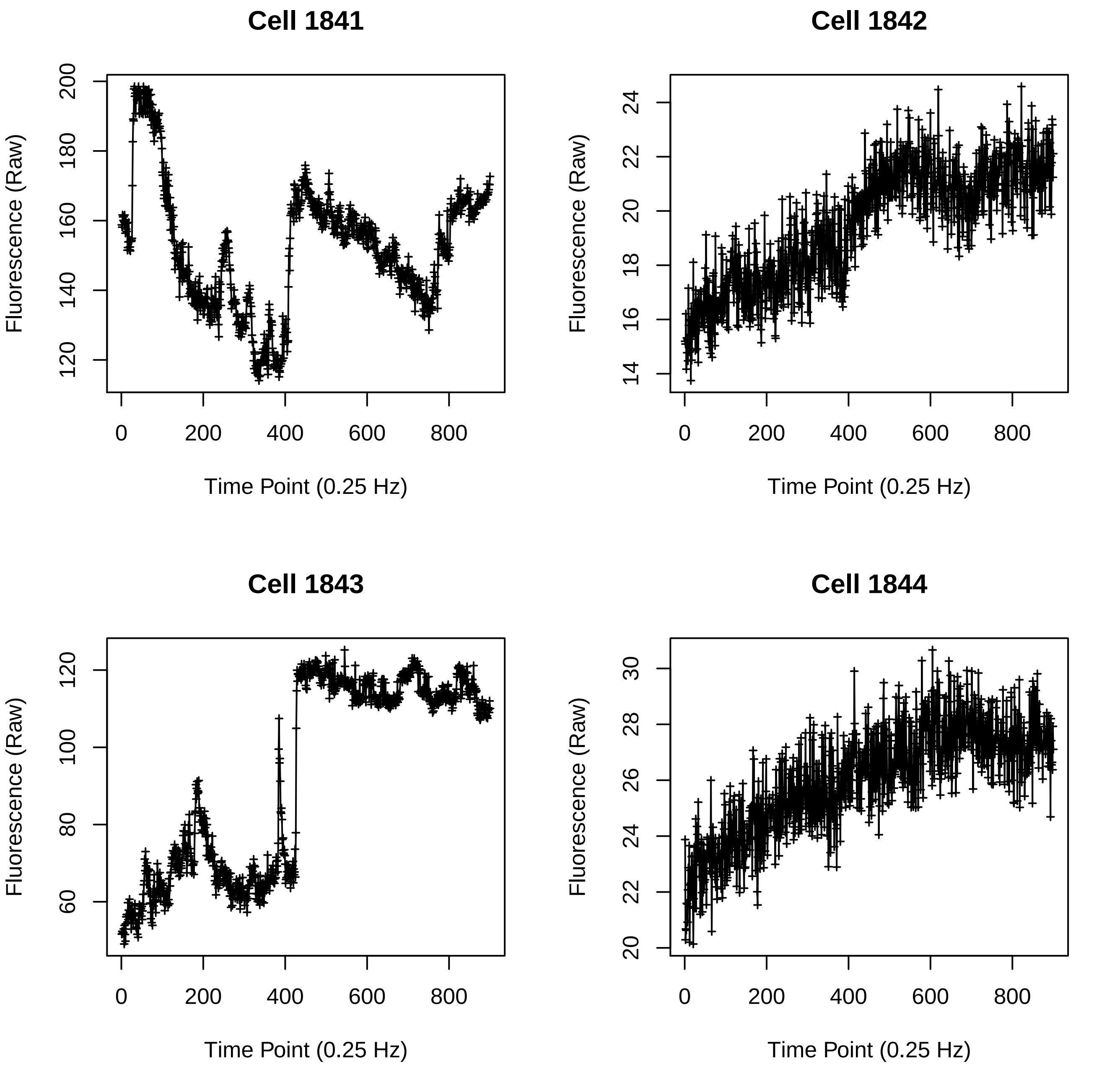
Cell 1841
Fluorescence (Raw)
Time Point (0.25 Hz)
Cell 1842
Fluorescence (Raw)
Time Point (0.25 Hz)
Cell 1843
Fluorescence (Raw)
Time Point (0.25 Hz)
Cell 1844
Fluorescence (Raw)
Time Point (0.25 Hz)

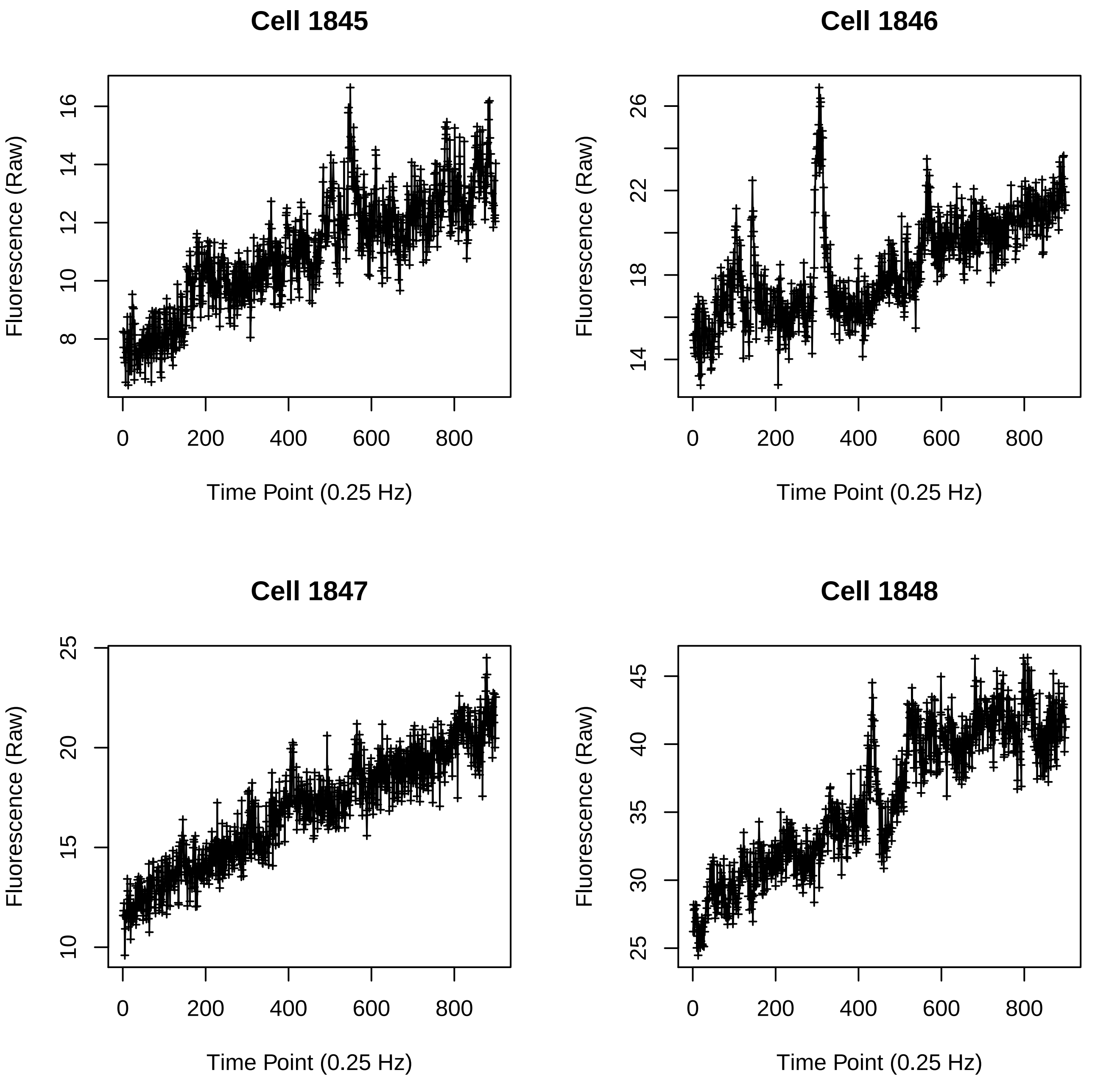
Cell 1845
Fluorescence (Raw)
8
10
12
14
16
0
200
400
600
800
Time Point (0.25 Hz)
Cell 1846
Fluorescence (Raw)
14
18
22
26
0
200
400
600
800
Time Point (0.25 Hz)
Cell 1847
Fluorescence (Raw)
10
15
20
25
0
200
400
600
800
Time Point (0.25 Hz)
Cell 1848
Fluorescence (Raw)
25
30
35
40
45
0
200
400
600
800
Time Point (0.25 Hz)

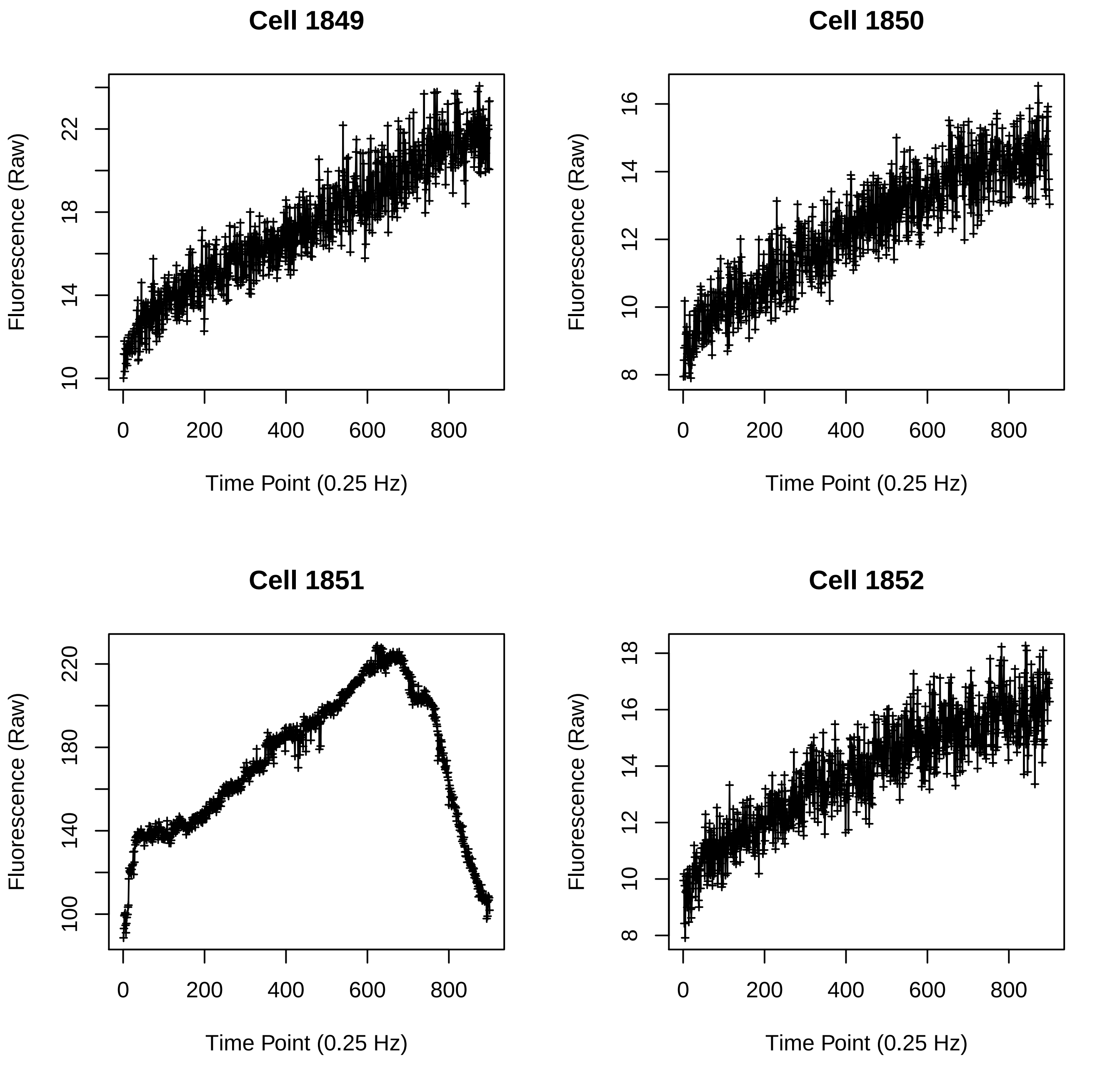
Cell 1849
Cell 1850
Cell 1851
Cell 1852
Fluorescence (Raw)
Time Point (0.25 Hz)
0
200
400
600
800
10
14
18
22
8
10
12
14
16
18
100
140
180
220

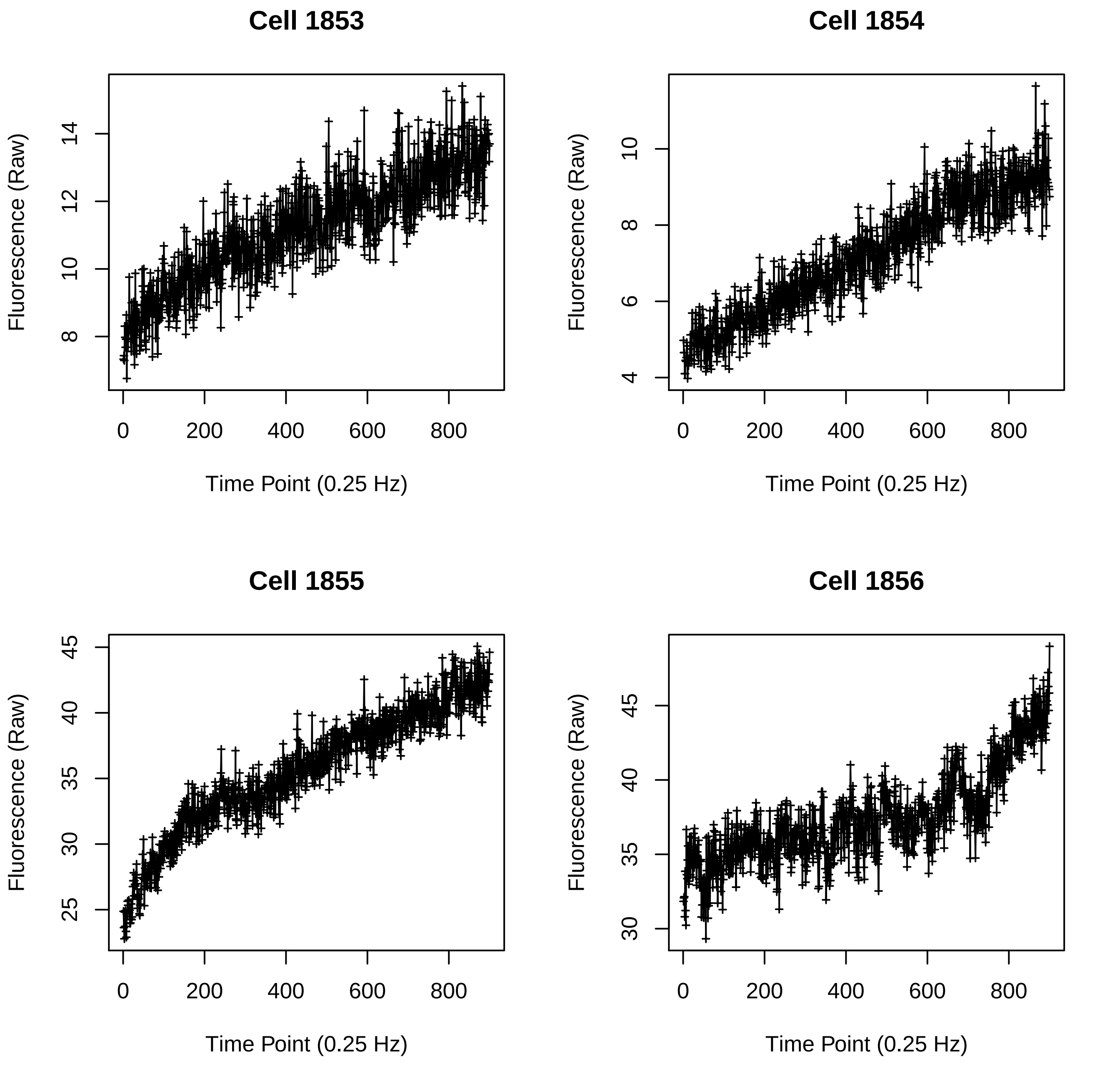

Cell 1853
Cell 1854
Cell 1855
Cell 1856
Fluorescence (Raw)
Time Point (0.25 Hz)
0
200
400
600
800
8
10
12
14
4
6
8
10
25
30
35
40
45
30
35
40
45

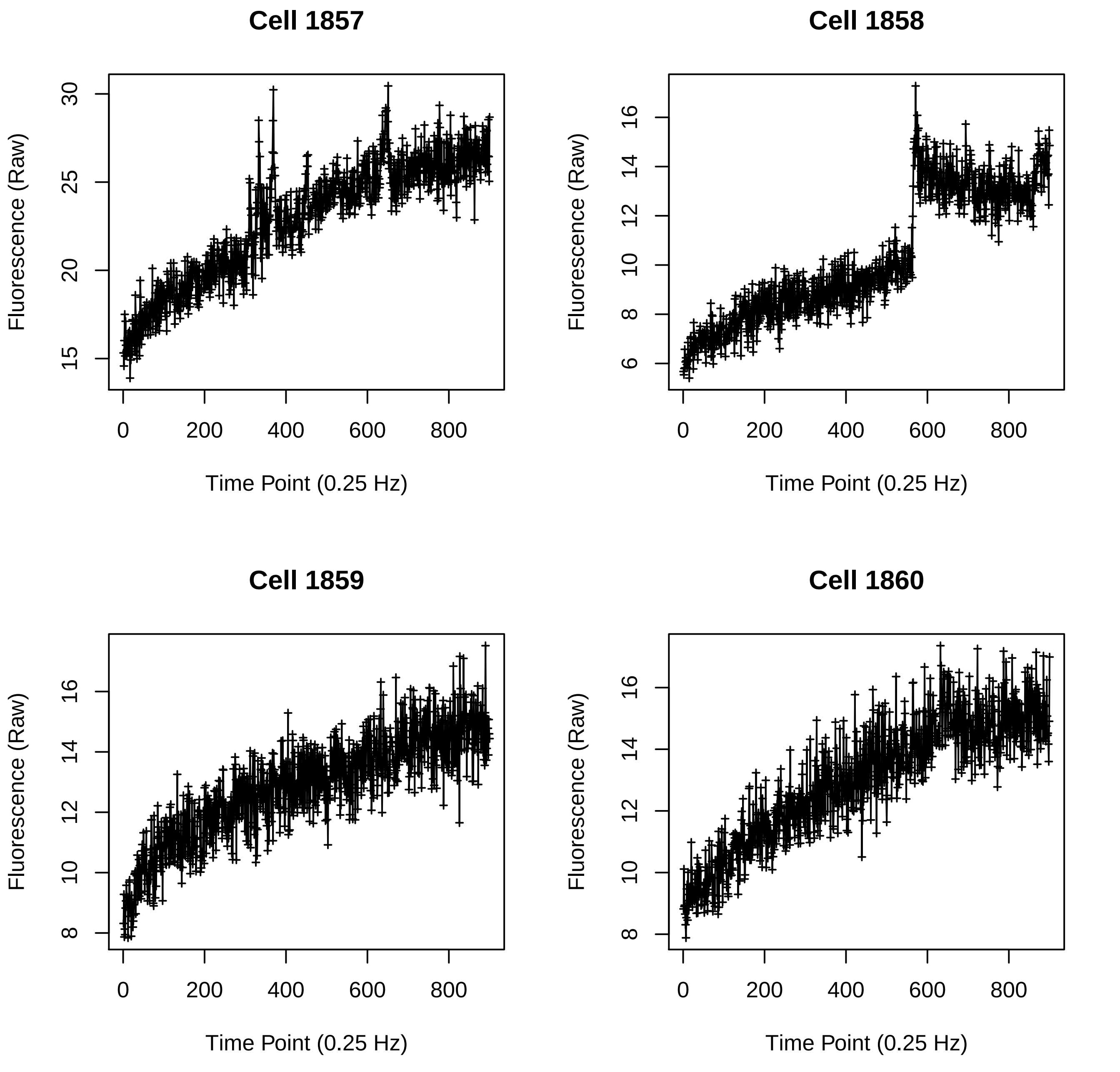
Cell 1857
Cell 1858
Cell 1859
Cell 1860
Fluorescence (Raw)
Time Point (0.25 Hz)
0
200
400
600
800
15
20
25
30
6
8
10
12
14
16

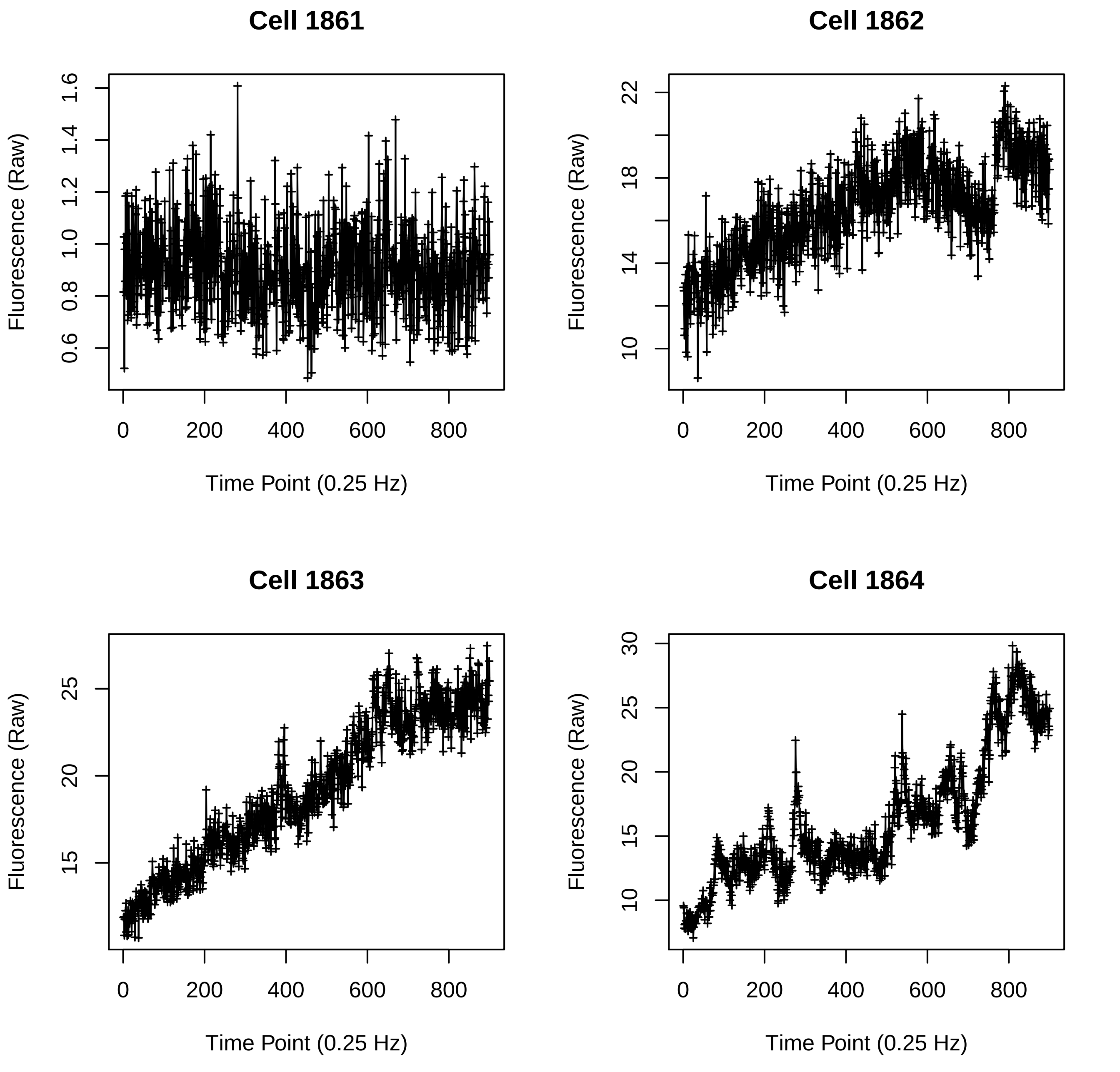

Cell 1861
Cell 1862
Cell 1863
Cell 1864
Fluorescence (Raw)
Time Point (0.25 Hz)
0
200
400
600
800
0.6
0.8
1.0
1.2
1.4
1.6
10
14
18
22
15
20
25
10
15
20
25
30

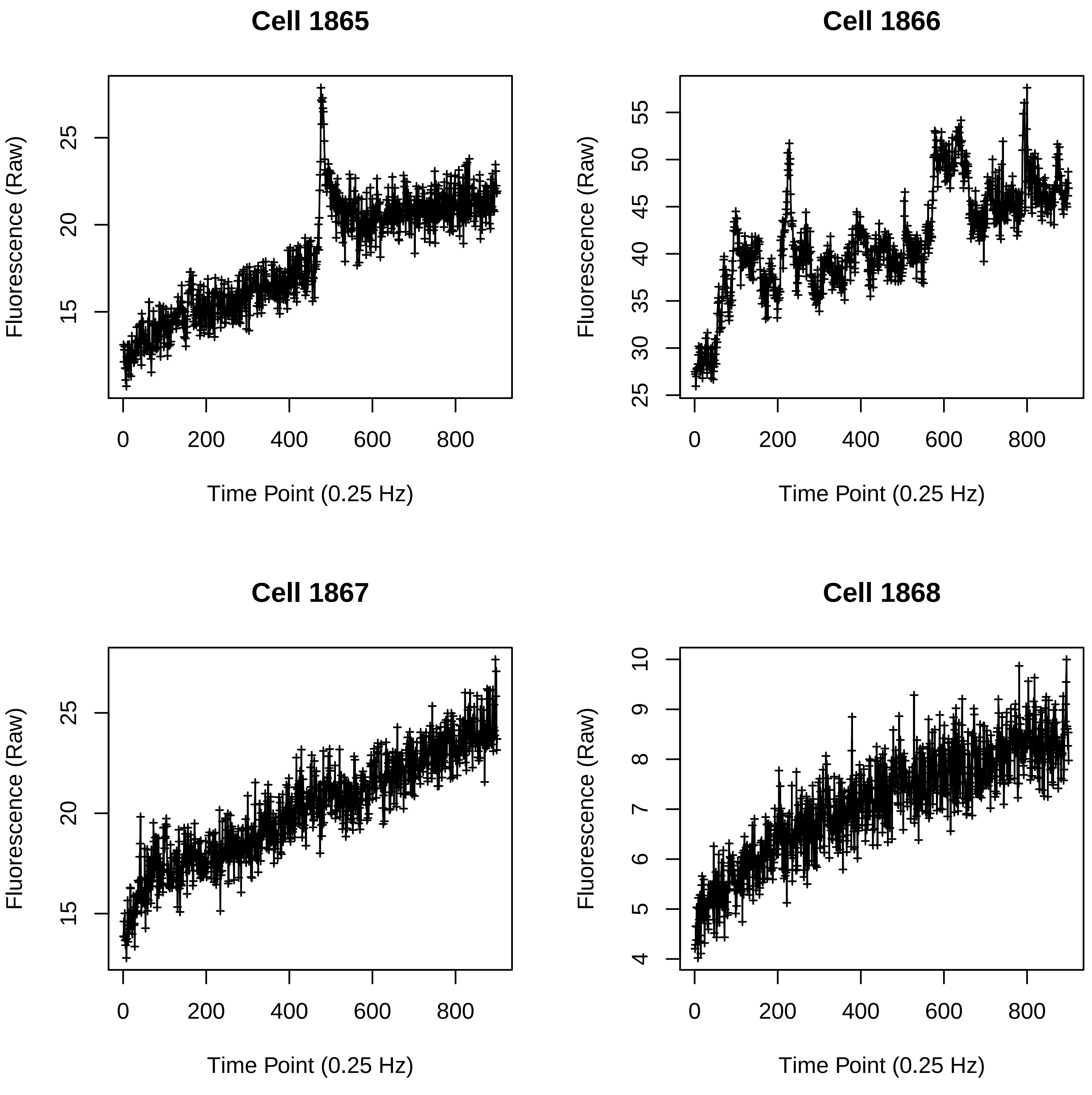
Cell 1865
Fluorescence (Raw)
15
20
25
0
200
400
600
800
Time Point (0.25 Hz)
Cell 1866
Fluorescence (Raw)
25
30
35
40
45
50
55
0
200
400
600
800
Time Point (0.25 Hz)
Cell 1867
Fluorescence (Raw)
15
20
25
0
200
400
600
800
Time Point (0.25 Hz)
Cell 1868
Fluorescence (Raw)
4
5
6
7
8
9
10
0
200
400
600
800
Time Point (0.25 Hz)

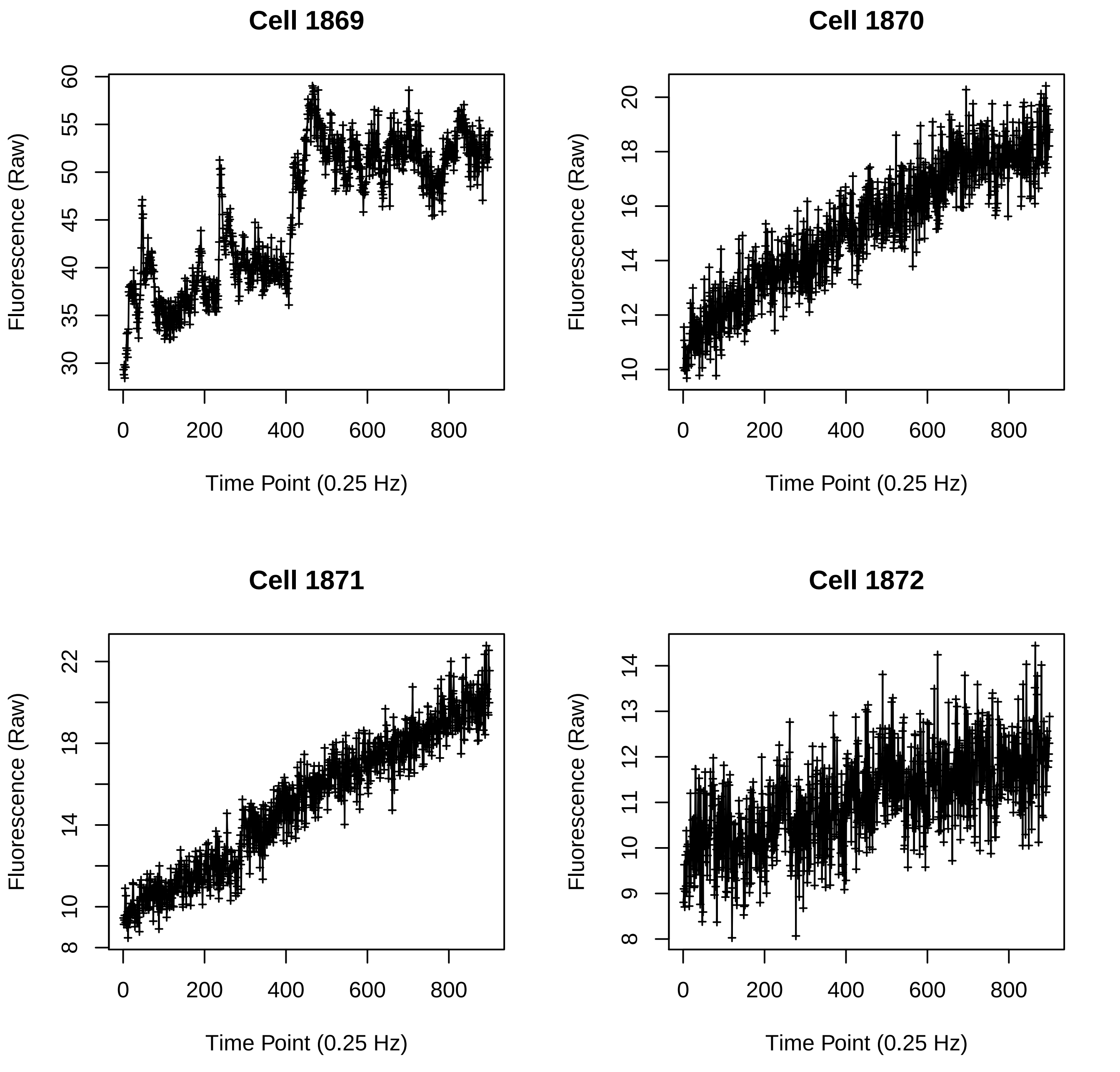
Cell 1869
Fluorescence (Raw)
30
35
40
45
50
55
60
0
200
400
600
800
Time Point (0.25 Hz)
Cell 1870
Fluorescence (Raw)
10
12
14
16
18
20
0
200
400
600
800
Time Point (0.25 Hz)
Cell 1871
Fluorescence (Raw)
8
10
14
18
22
0
200
400
600
800
Time Point (0.25 Hz)
Cell 1872
Fluorescence (Raw)
8
9
10
11
12
13
14
0
200
400
600
800
Time Point (0.25 Hz)

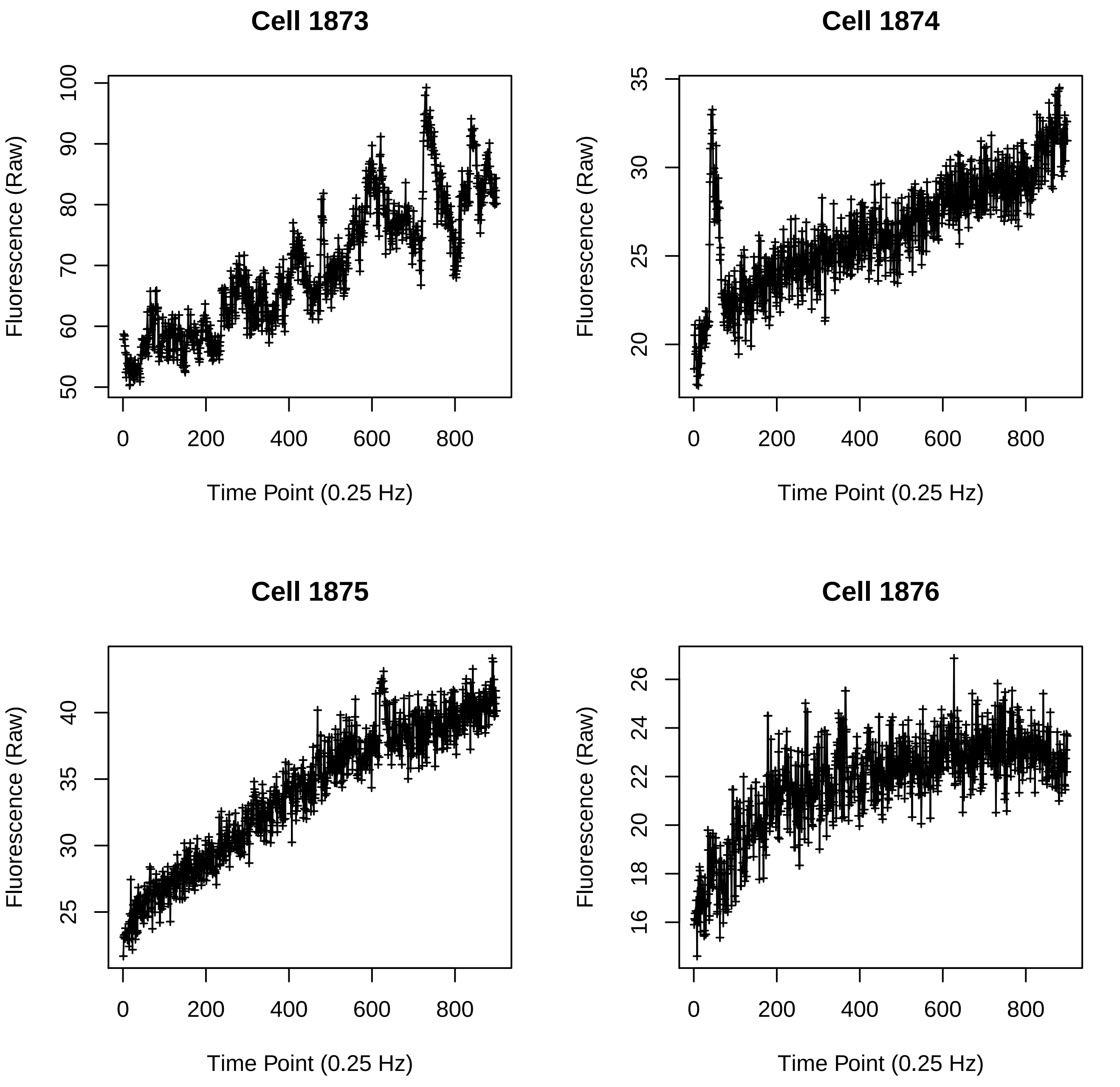
Cell 1873
Fluorescence (Raw)
50
60
70
80
90
100
0
200
400
600
800
Time Point (0.25 Hz)
Cell 1874
Fluorescence (Raw)
20
25
30
35
0
200
400
600
800
Time Point (0.25 Hz)
Cell 1875
Fluorescence (Raw)
25
30
35
40
0
200
400
600
800
Time Point (0.25 Hz)
Cell 1876
Fluorescence (Raw)
16
18
20
22
24
26
0
200
400
600
800
Time Point (0.25 Hz)

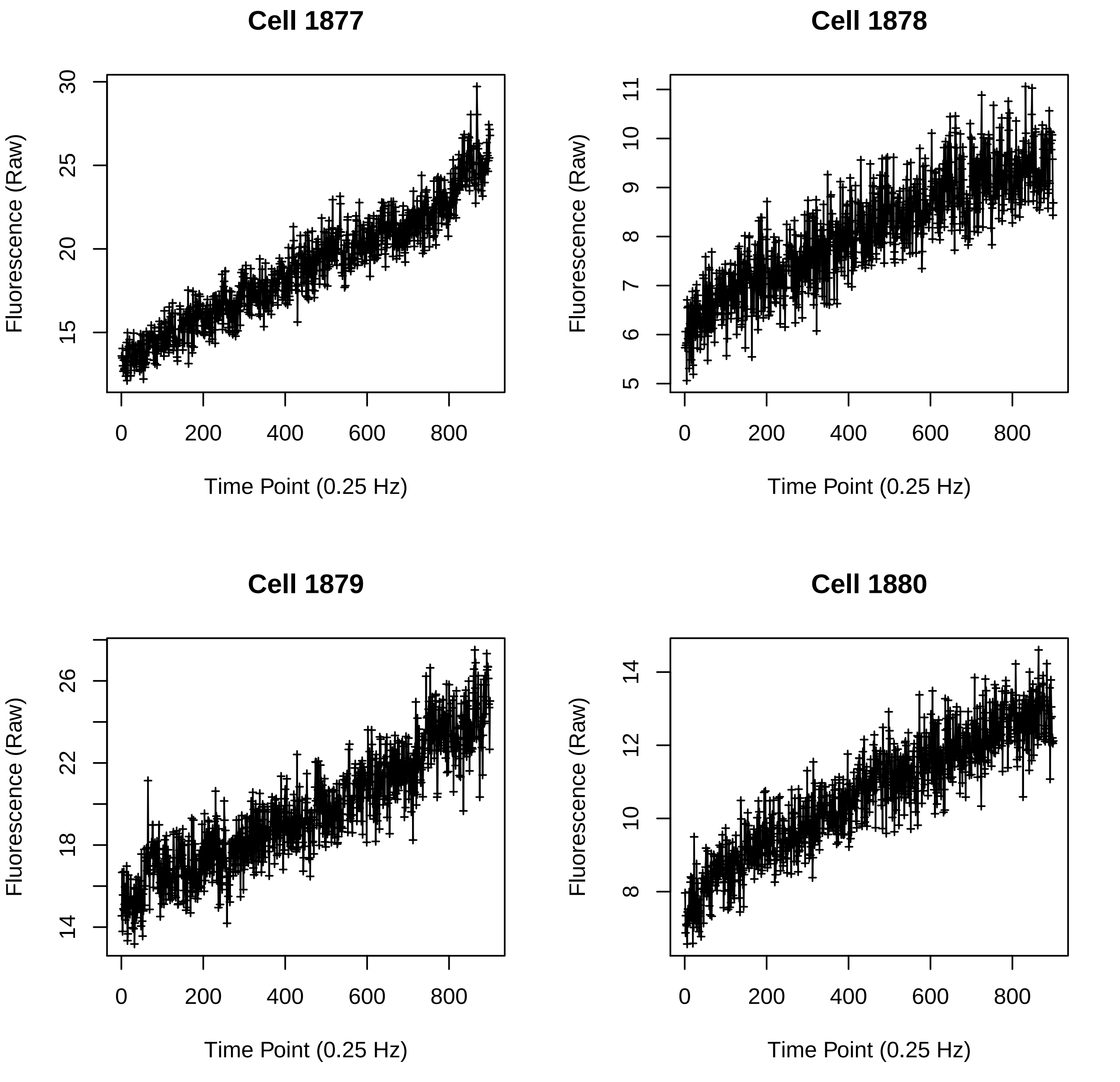
Cell 1877
Fluorescence (Raw)
15
20
25
30
0
200
400
600
800
Time Point (0.25 Hz)
Cell 1878
Fluorescence (Raw)
5
6
7
8
9
10
11
0
200
400
600
800
Time Point (0.25 Hz)
Cell 1879
Fluorescence (Raw)
14
18
22
26
0
200
400
600
800
Time Point (0.25 Hz)
Cell 1880
Fluorescence (Raw)
8
10
12
14
0
200
400
600
800
Time Point (0.25 Hz)

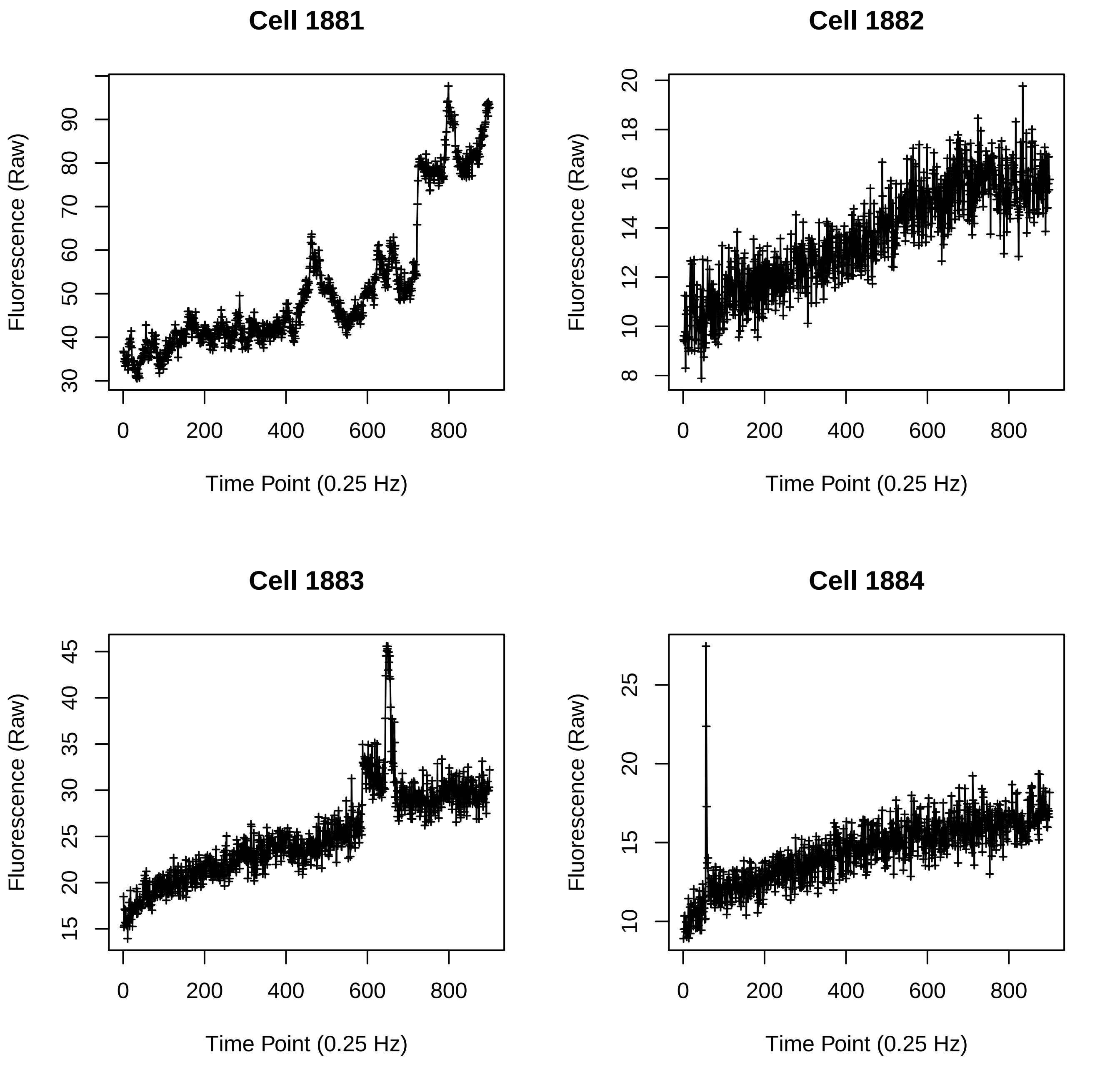
Cell 1881
Cell 1882
Cell 1883
Cell 1884
Fluorescence (Raw)
Time Point (0.25 Hz)
0
200
400
600
800

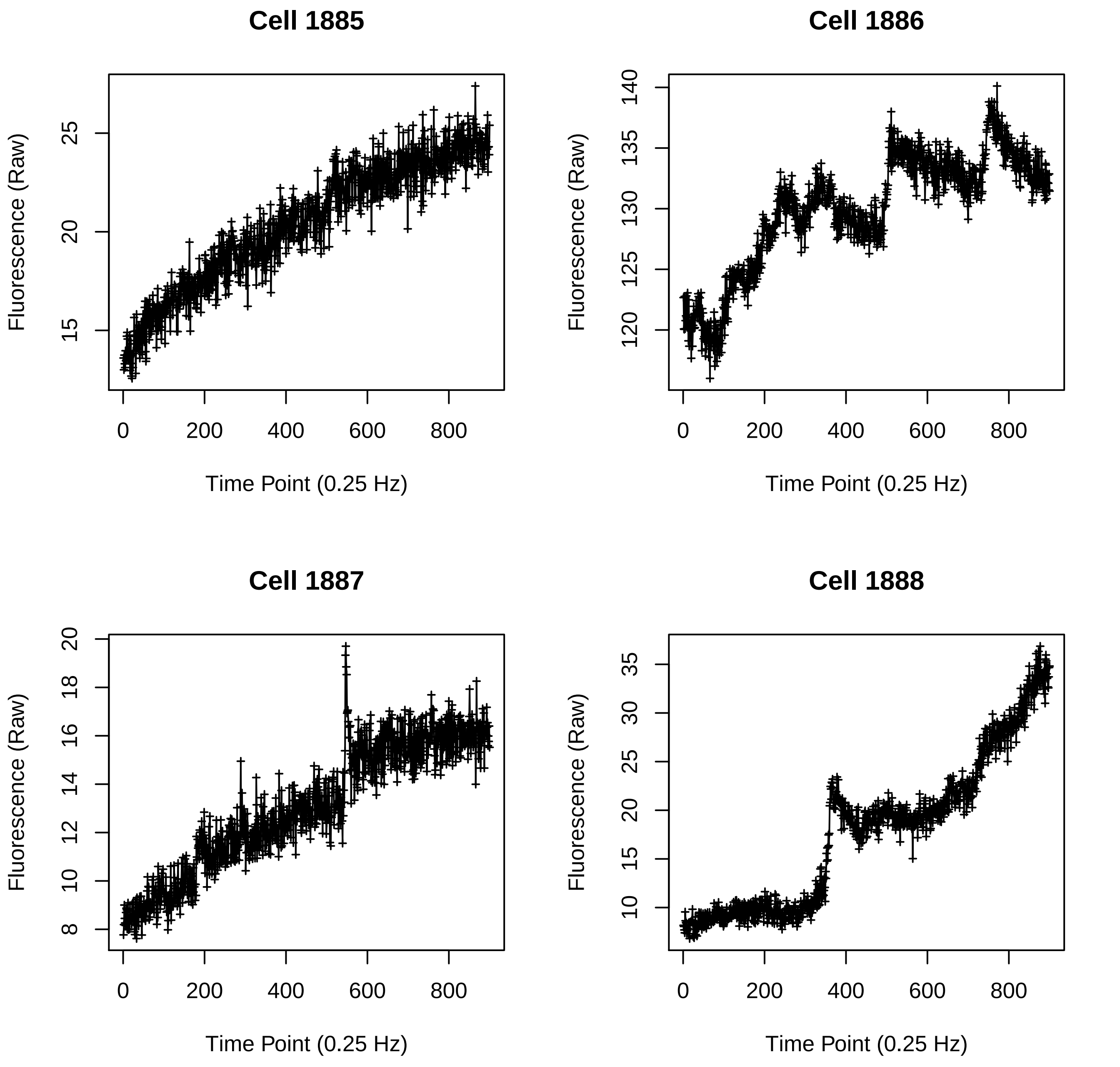
Cell 1885
Cell 1886
Cell 1887
Cell 1888
Fluorescence (Raw)
Time Point (0.25 Hz)
0
200
400
600
800
15
20
25
120
125
130
135
140
8
10
12
14
16
18
20
10
15
20
25
30
35

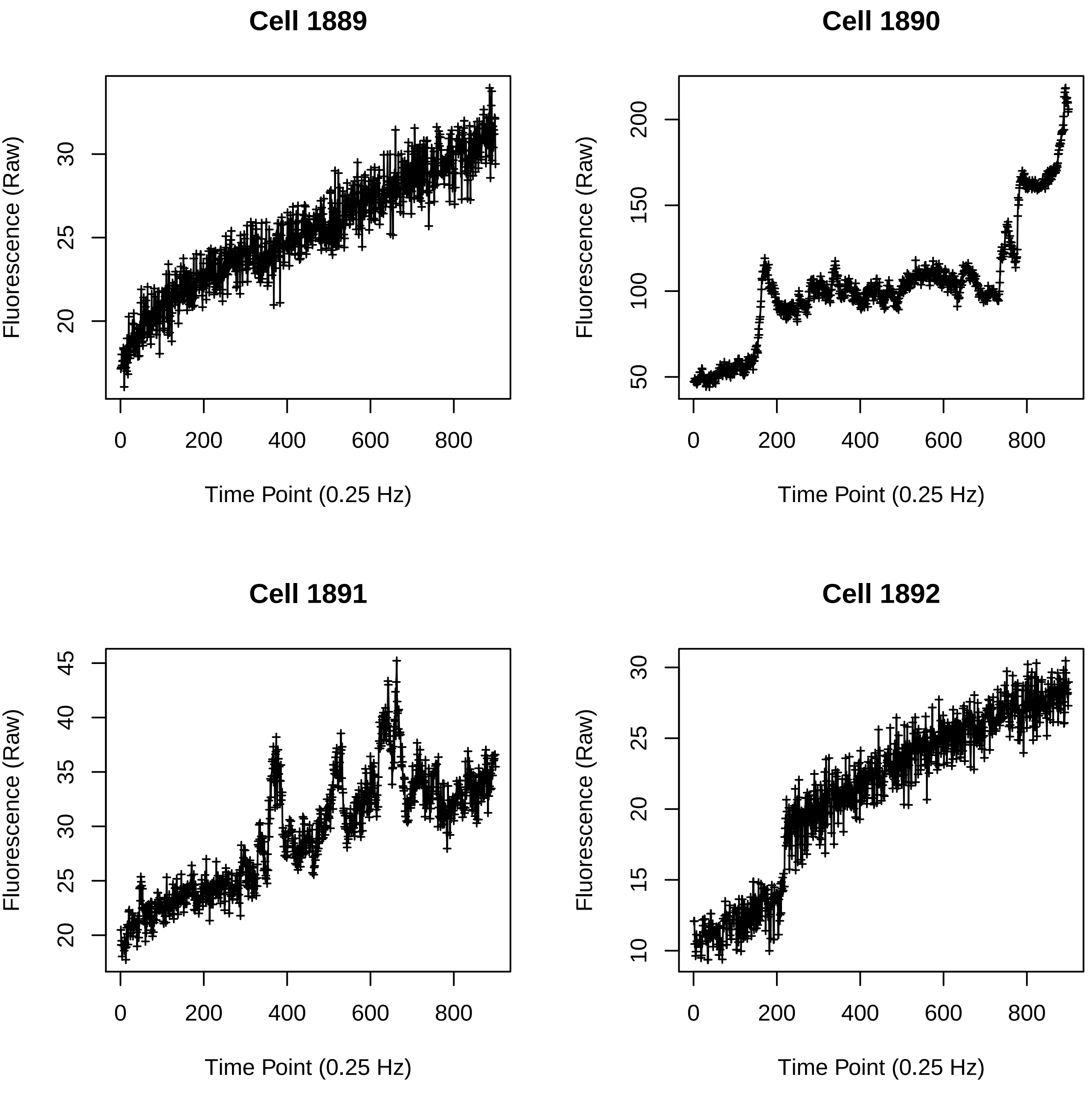

Cell 1889
Fluorescence (Raw)
20
25
30
0
200
400
600
800
Time Point (0.25 Hz)
Cell 1890
Fluorescence (Raw)
50
100
150
200
0
200
400
600
800
Time Point (0.25 Hz)
Cell 1891
Fluorescence (Raw)
20
25
30
35
40
45
0
200
400
600
800
Time Point (0.25 Hz)
Cell 1892
Fluorescence (Raw)
10
15
20
25
30
0
200
400
600
800
Time Point (0.25 Hz)

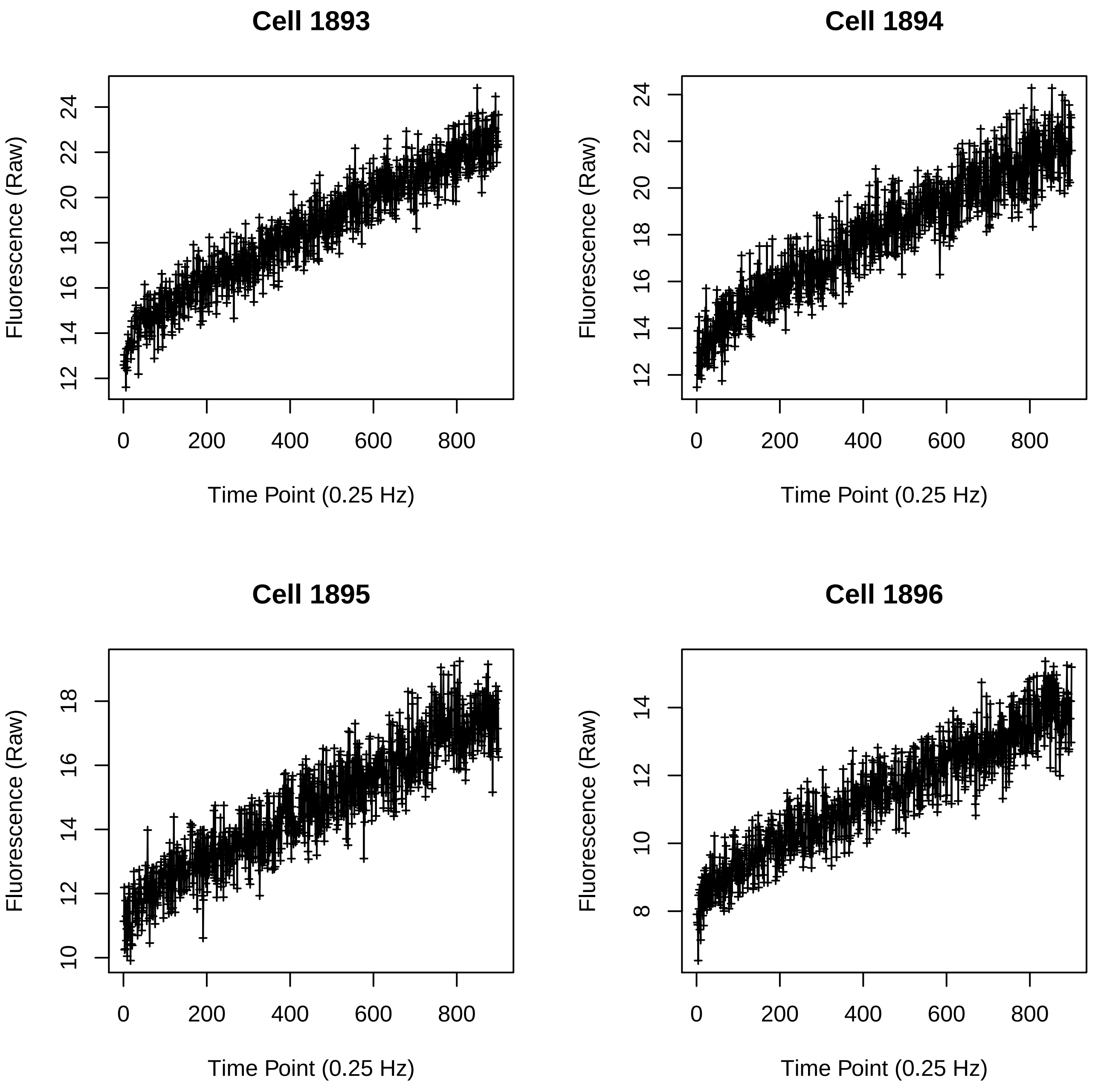
Cell 1893
Cell 1894
Cell 1895
Cell 1896
Fluorescence (Raw)
Time Point (0.25 Hz)
0
200
400
600
800
12
14
16
18
20
22
24
10
8

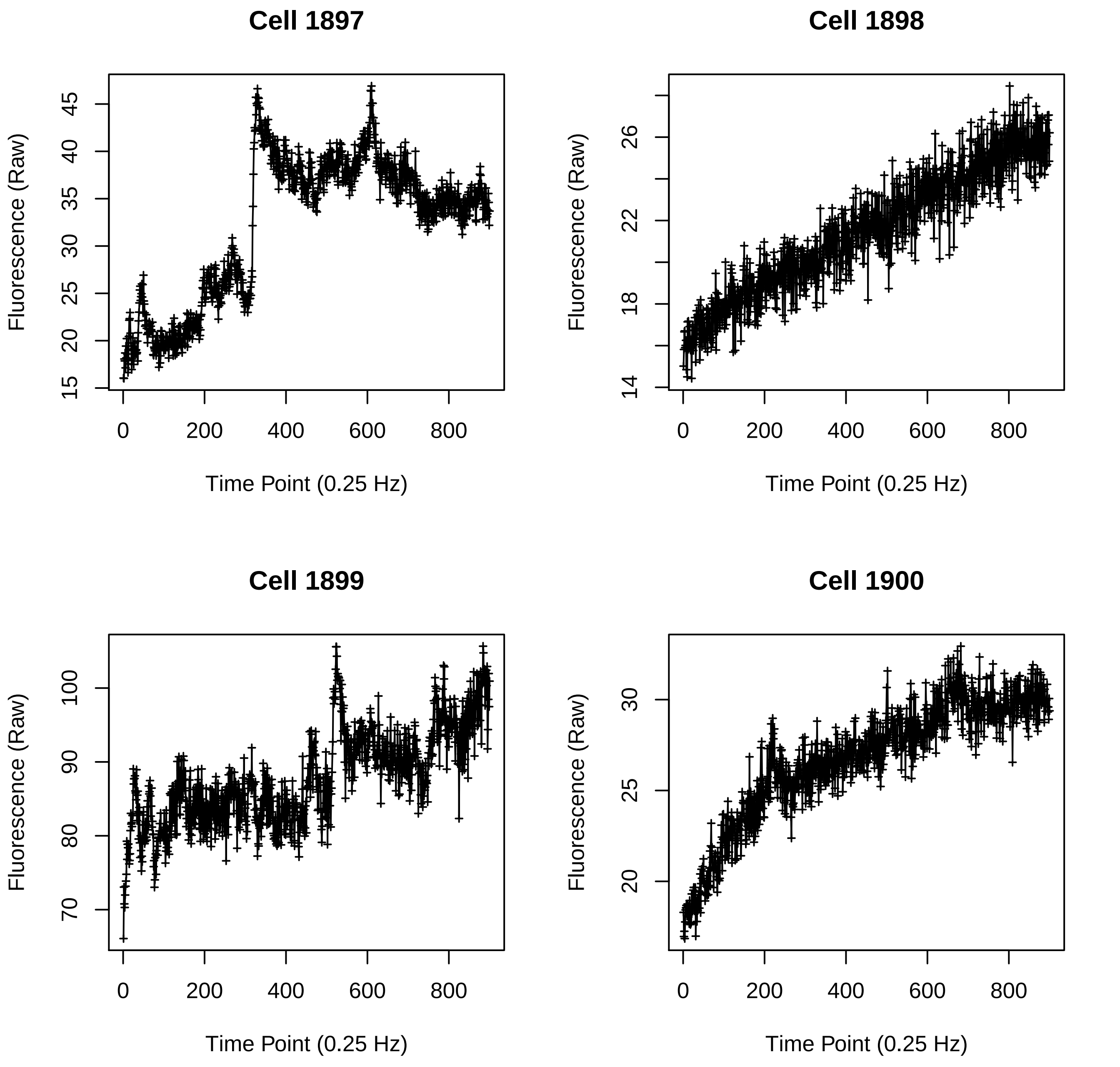
Cell 1897
Fluorescence (Raw)
15
20
25
30
35
40
45
0
200
400
600
800
Time Point (0.25 Hz)
Cell 1898
Fluorescence (Raw)
14
18
22
26
0
200
400
600
800
Time Point (0.25 Hz)
Cell 1899
Fluorescence (Raw)
70
80
90
100
0
200
400
600
800
Time Point (0.25 Hz)
Cell 1900
Fluorescence (Raw)
20
25
30
0
200
400
600
800
Time Point (0.25 Hz)

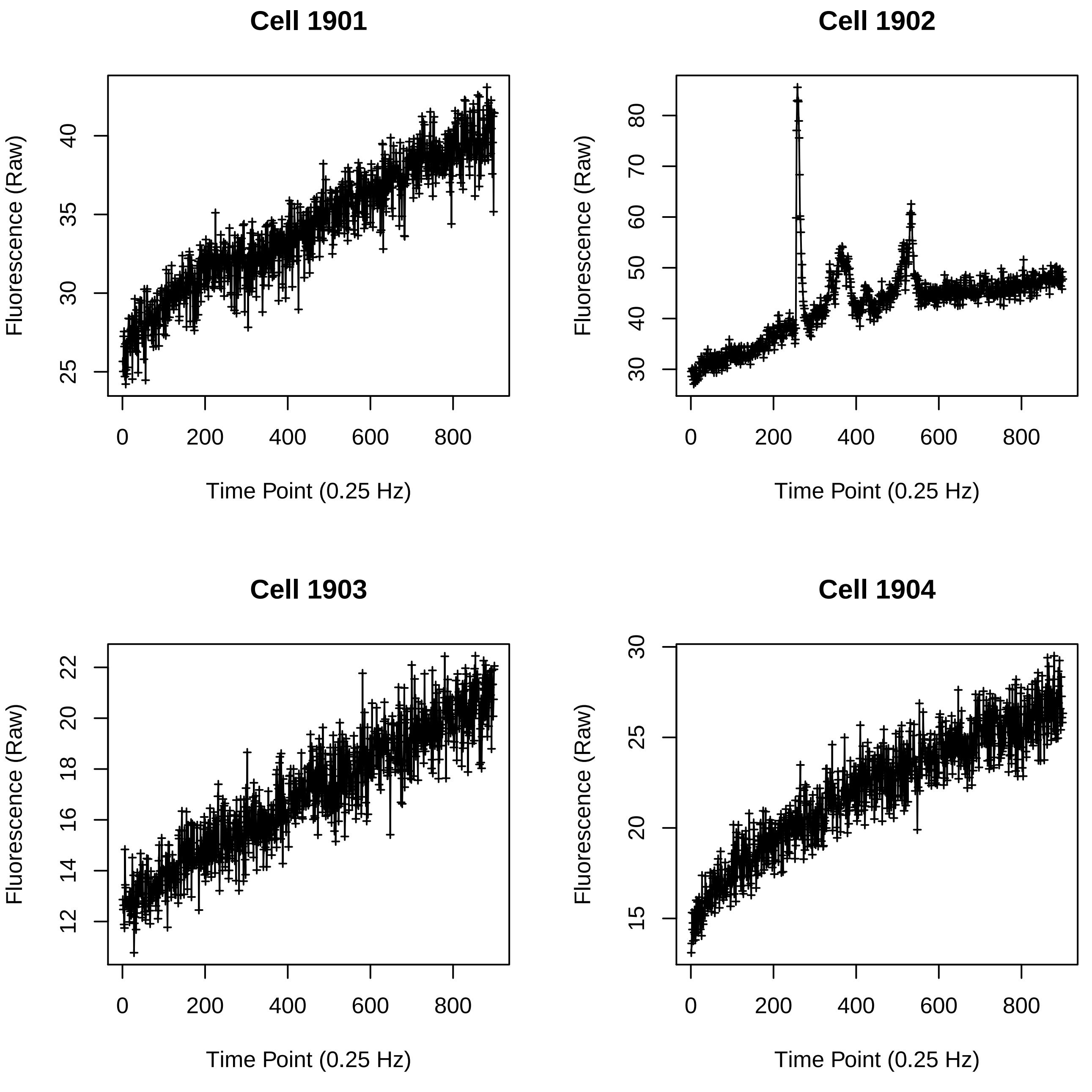
Cell 1901
Cell 1902
Cell 1903
Cell 1904
Fluorescence (Raw)
Time Point (0.25 Hz)
0
200
400
600
800
25
30
35
40
50
60
70
80
12
14
16
18
20
22
15

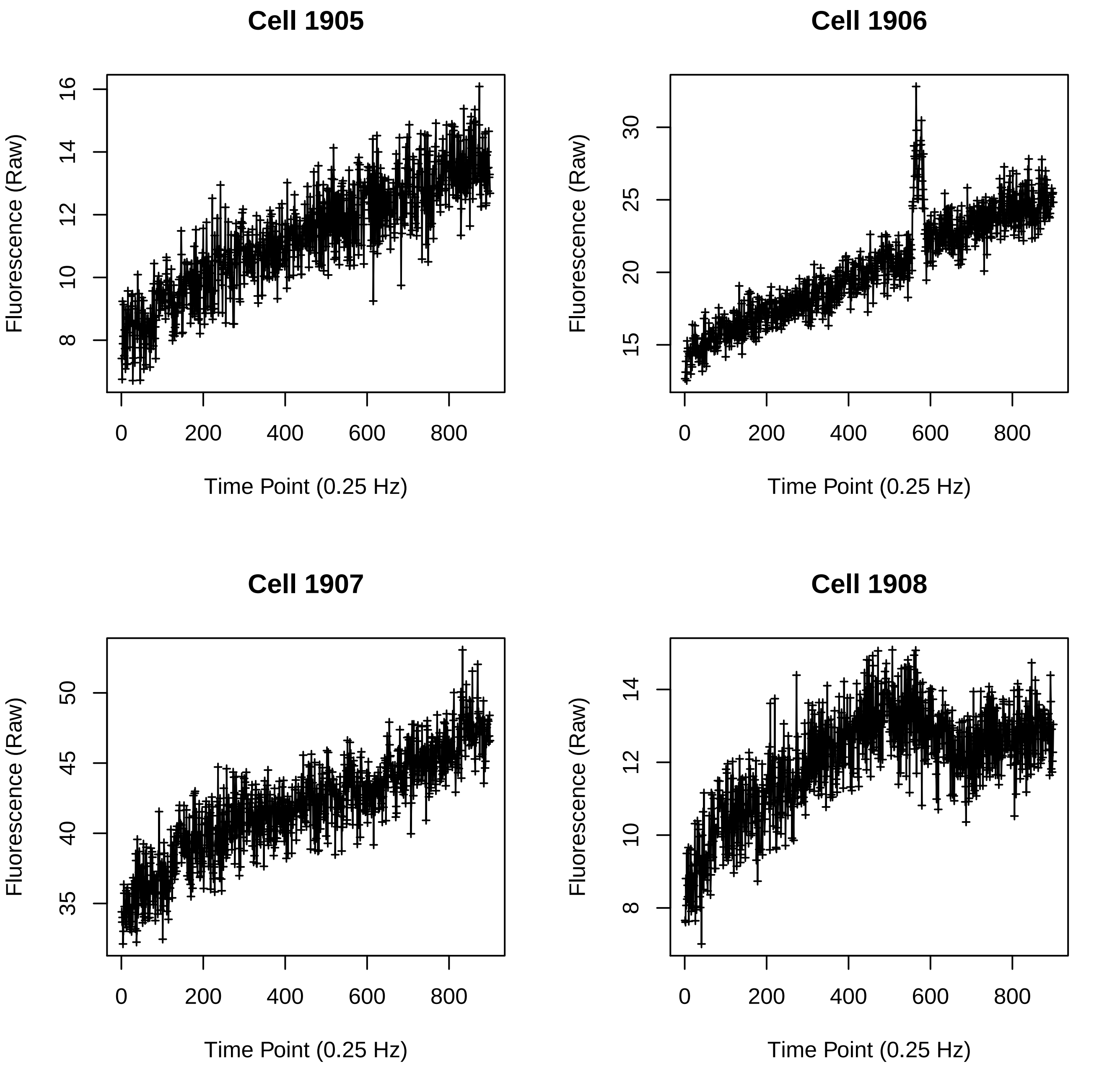
Cell 1905
Fluorescence (Raw)
8
10
12
14
16
0
200
400
600
800
Time Point (0.25 Hz)
Cell 1906
Fluorescence (Raw)
15
20
25
30
0
200
400
600
800
Time Point (0.25 Hz)
Cell 1907
Fluorescence (Raw)
35
40
45
50
0
200
400
600
800
Time Point (0.25 Hz)
Cell 1908
Fluorescence (Raw)
8
10
12
14
0
200
400
600
800
Time Point (0.25 Hz)

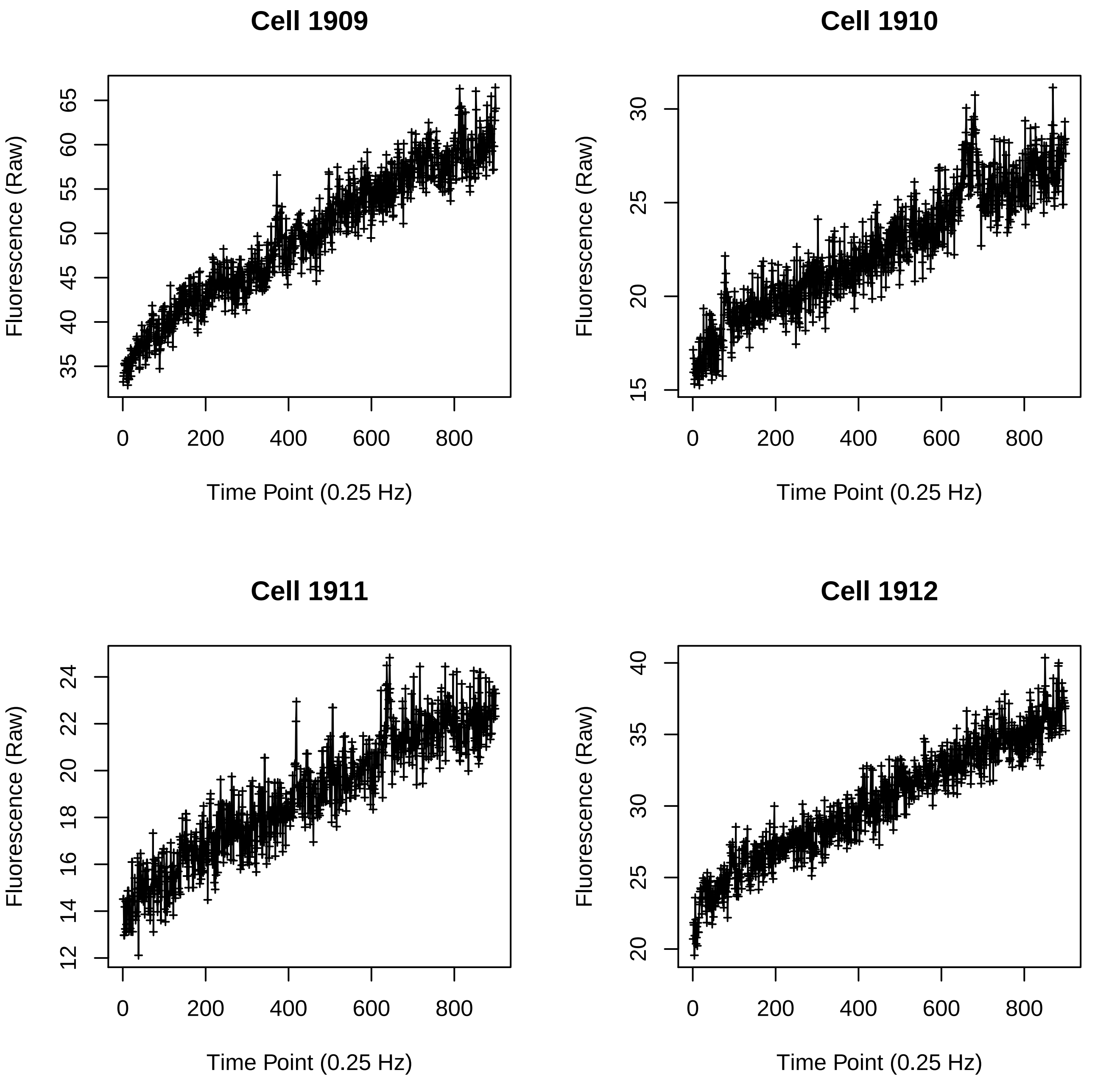
Cell 1909
Cell 1910
Cell 1911
Cell 1912
Fluorescence (Raw)
Time Point (0.25 Hz)
0
200
400
600
800
35
40
45
50
55
60
65
15
20
25
30
12
14
16
18
20
22
24

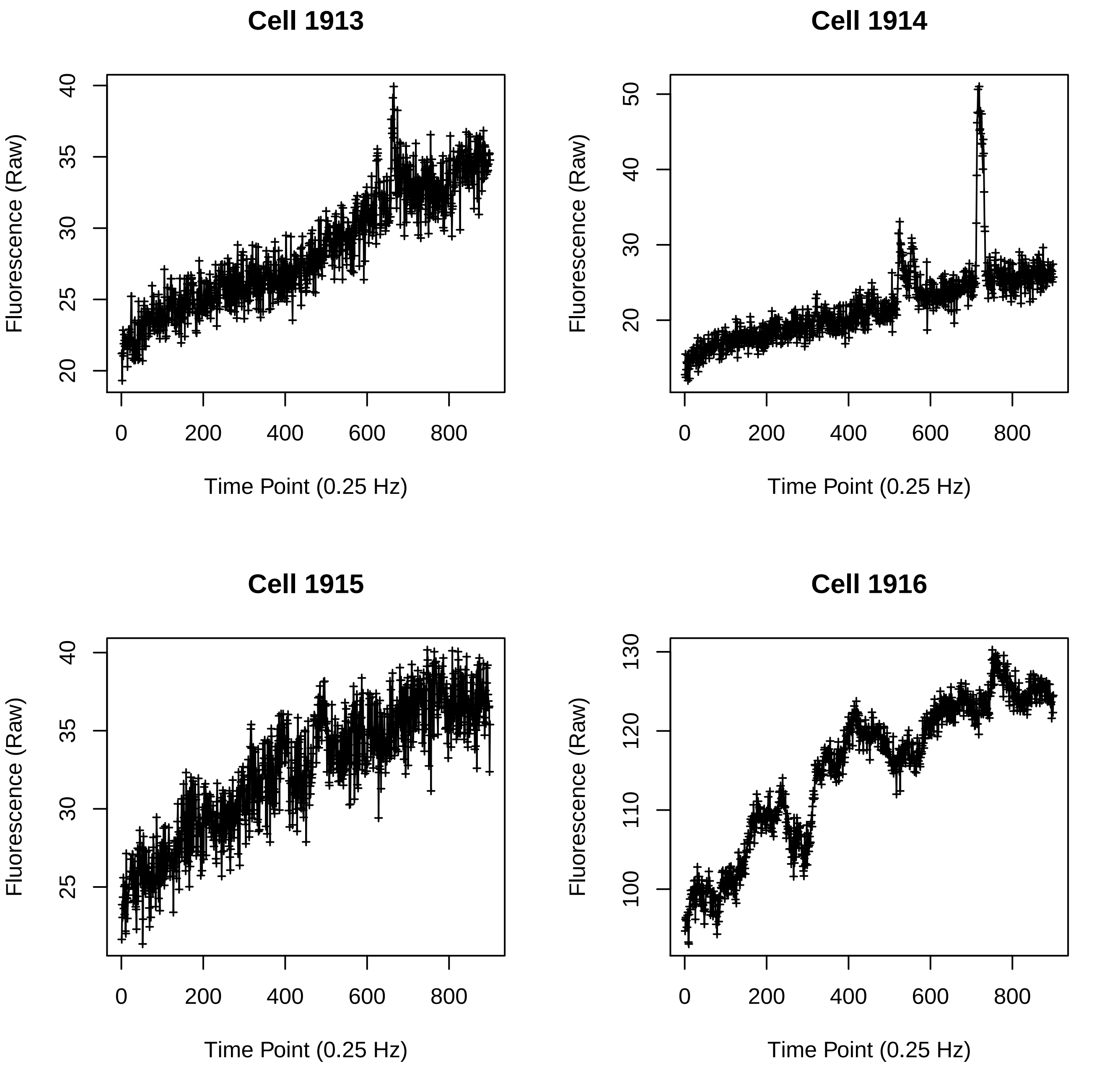

Cell 1913
Fluorescence (Raw)
20
25
30
35
40
0
200
400
600
800
Time Point (0.25 Hz)
Cell 1914
Fluorescence (Raw)
20
30
40
50
0
200
400
600
800
Time Point (0.25 Hz)
Cell 1915
Fluorescence (Raw)
25
30
35
40
0
200
400
600
800
Time Point (0.25 Hz)
Cell 1916
Fluorescence (Raw)
100
110
120
130
0
200
400
600
800
Time Point (0.25 Hz)

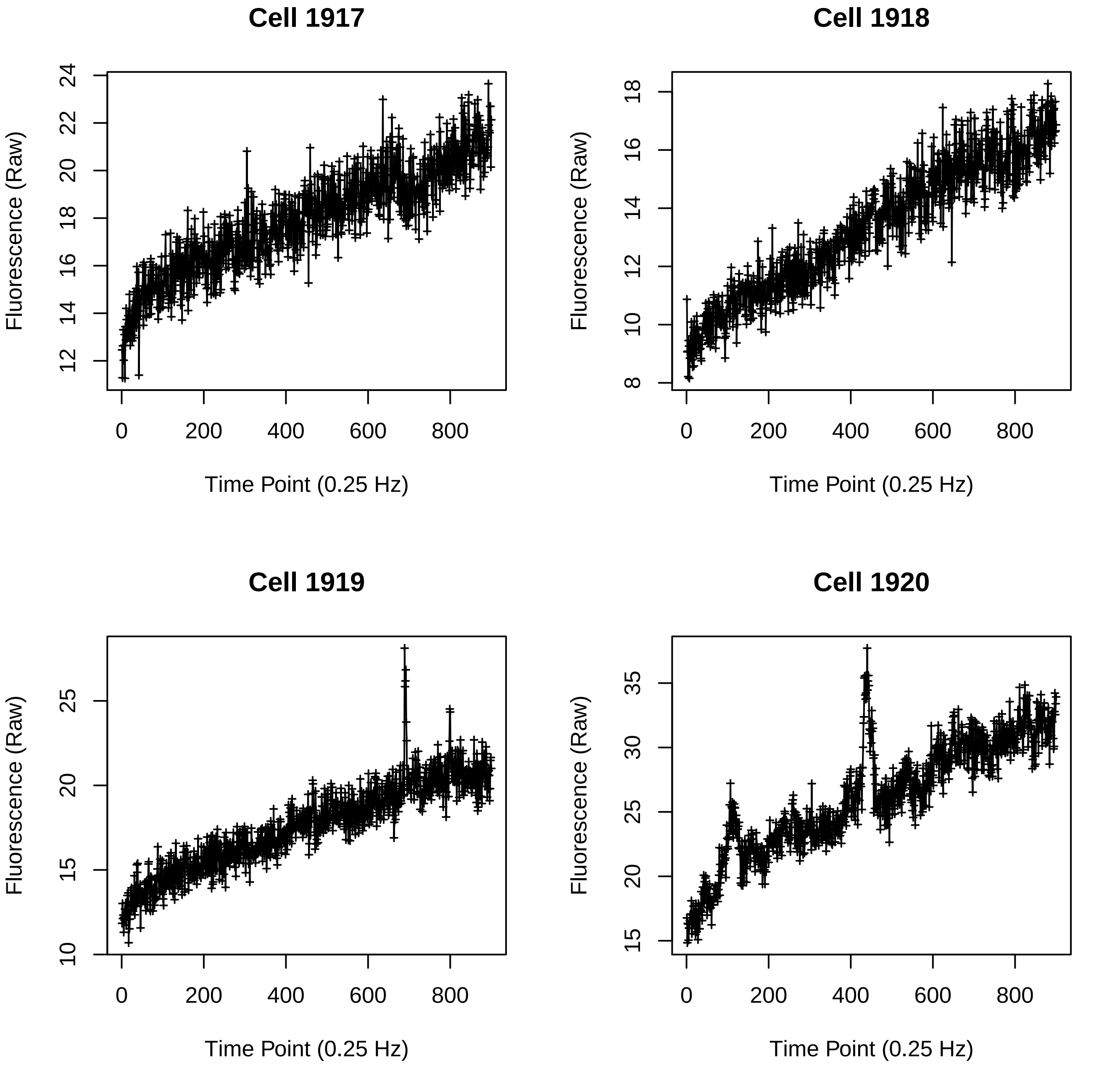
Cell 1917
Fluorescence (Raw)
12
14
16
18
20
22
24
0
200
400
600
800
Time Point (0.25 Hz)
Cell 1918
Fluorescence (Raw)
8
10
12
14
16
18
0
200
400
600
800
Time Point (0.25 Hz)
Cell 1919
Fluorescence (Raw)
10
15
20
25
0
200
400
600
800
Time Point (0.25 Hz)
Cell 1920
Fluorescence (Raw)
15
20
25
30
35
0
200
400
600
800
Time Point (0.25 Hz)

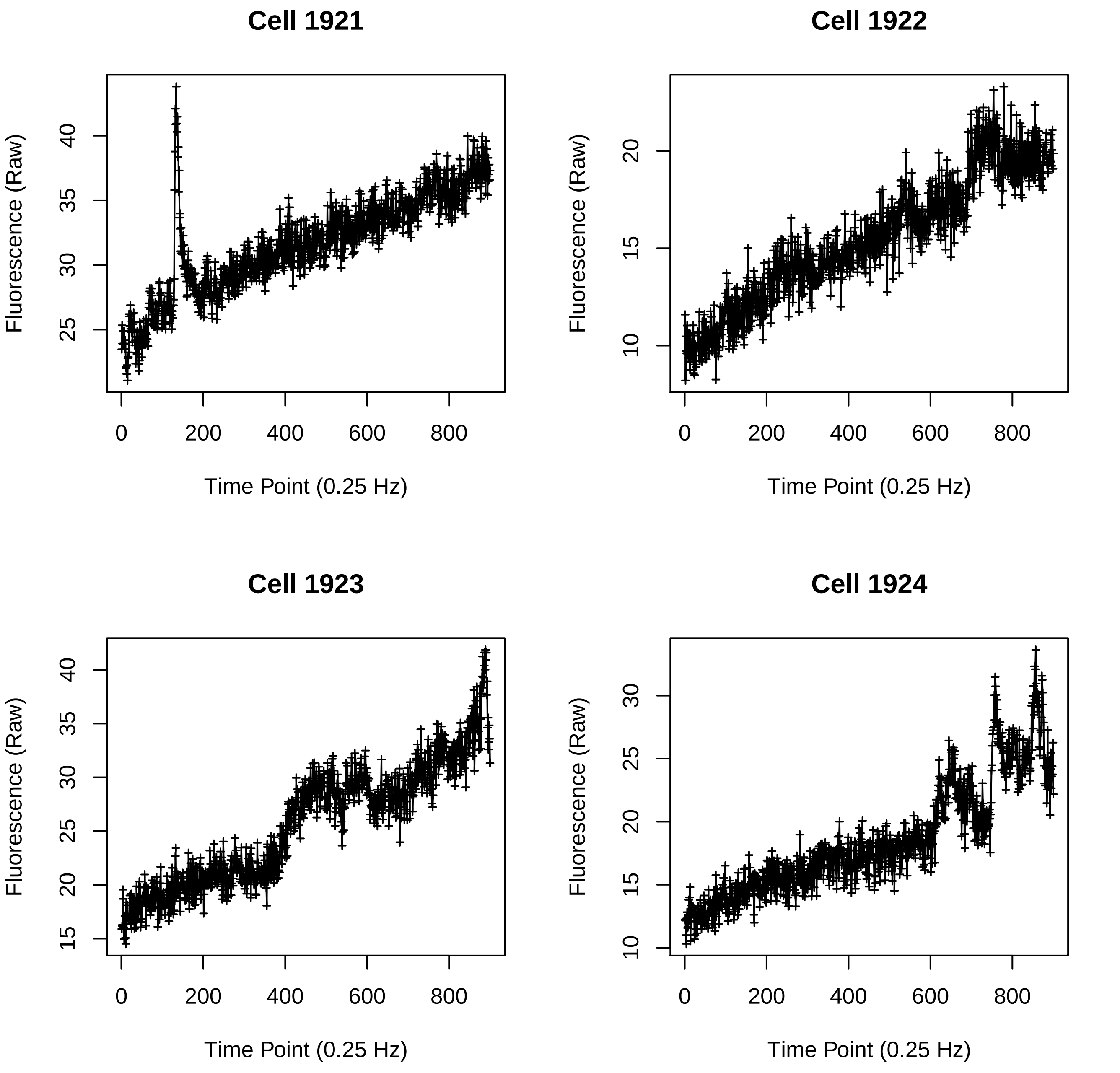
Cell 1921
Cell 1922
Cell 1923
Cell 1924
Fluorescence (Raw)
Time Point (0.25 Hz)
0
200
400
600
800
10
15
20
25
30
35
40

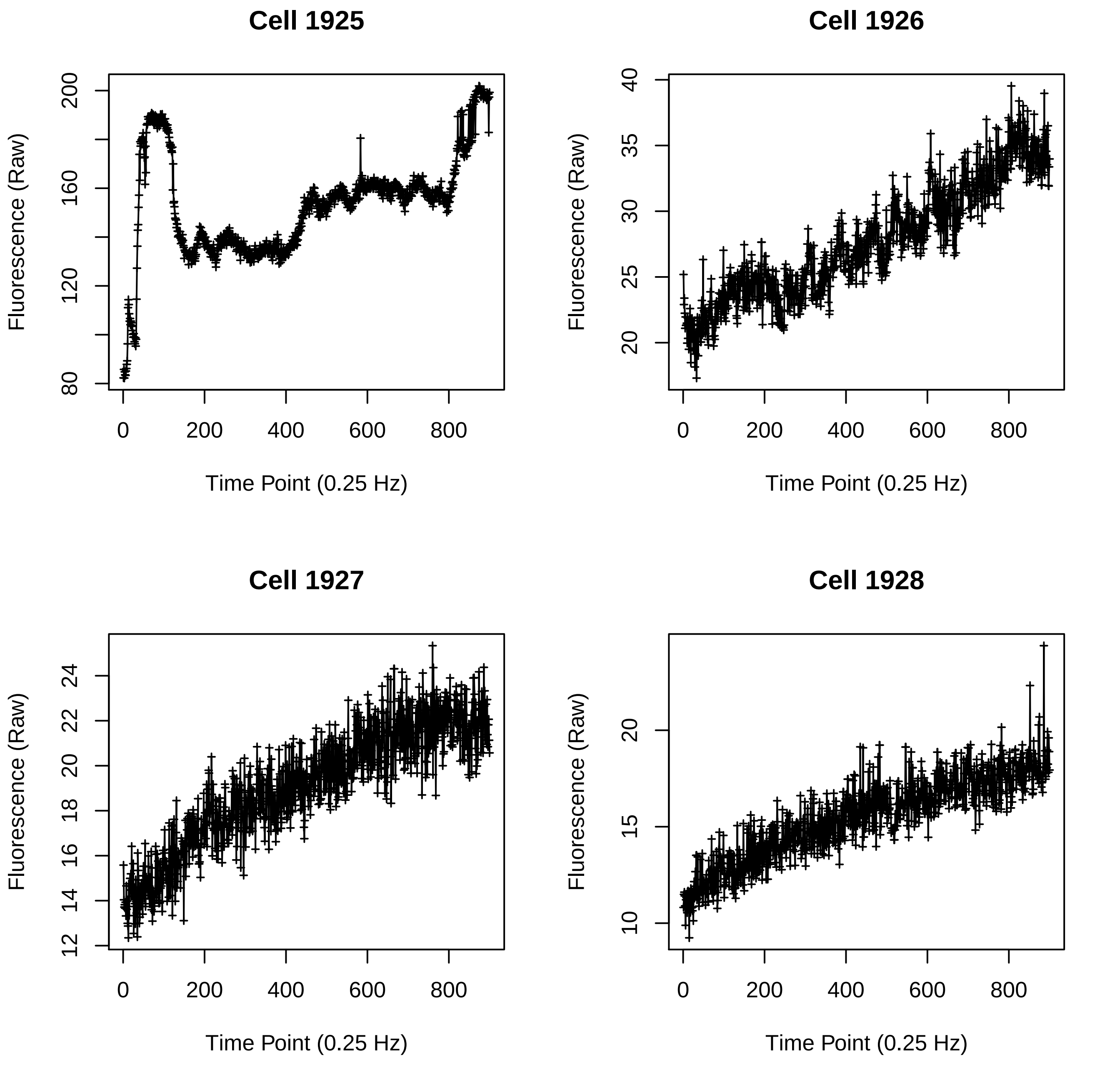
Cell 1925
Cell 1926
Cell 1927
Cell 1928
Fluorescence (Raw)
Time Point (0.25 Hz)
0
200
400
600
800
80
120
160
200
20
25
30
35
40
12
14
16
18
20
22
24
10
15
20

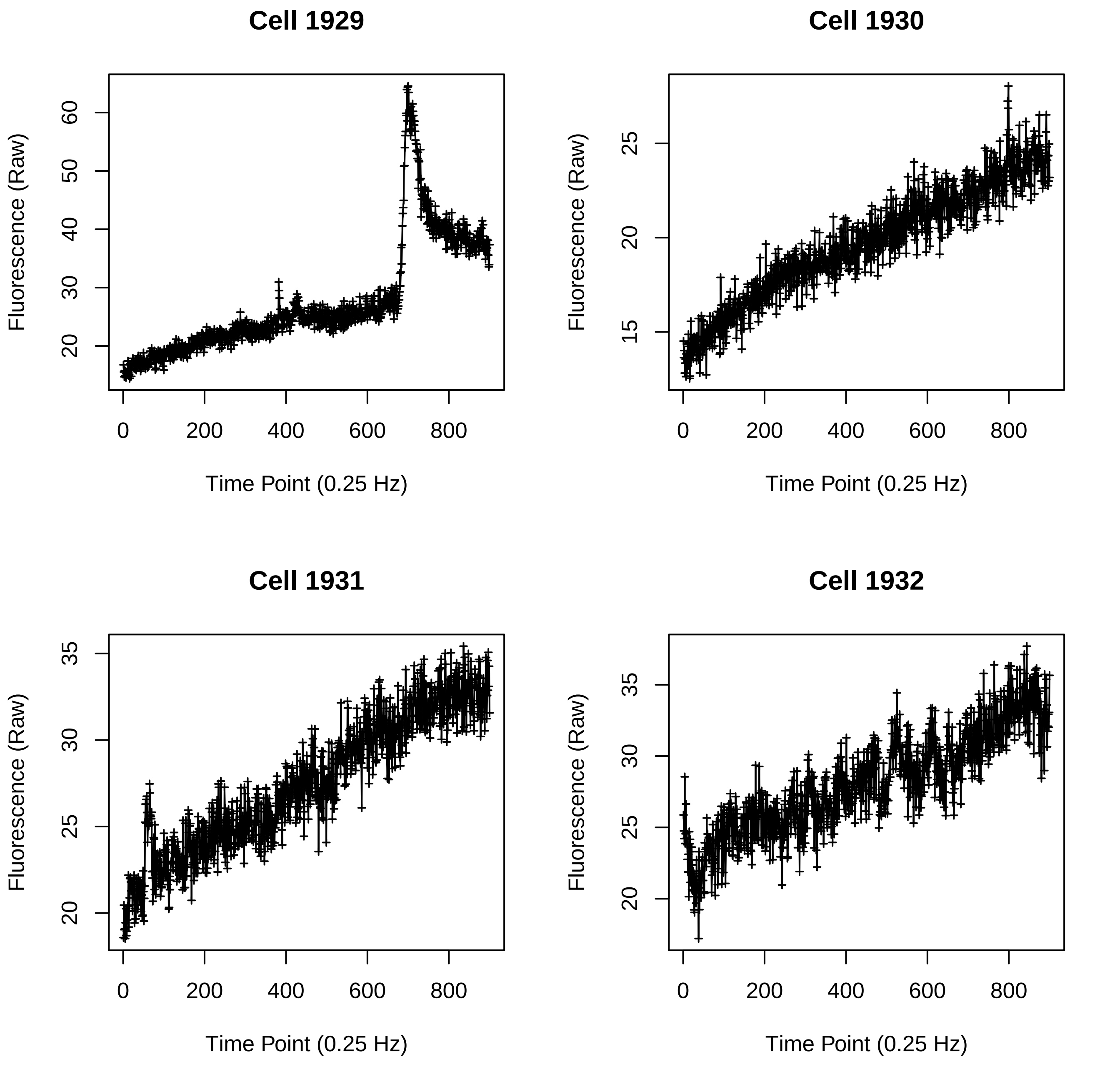
Cell 1929
Cell 1930
Cell 1931
Cell 1932
Fluorescence (Raw)
Time Point (0.25 Hz)
0
200
400
600
800
20
30
40
50
60
15
25
35

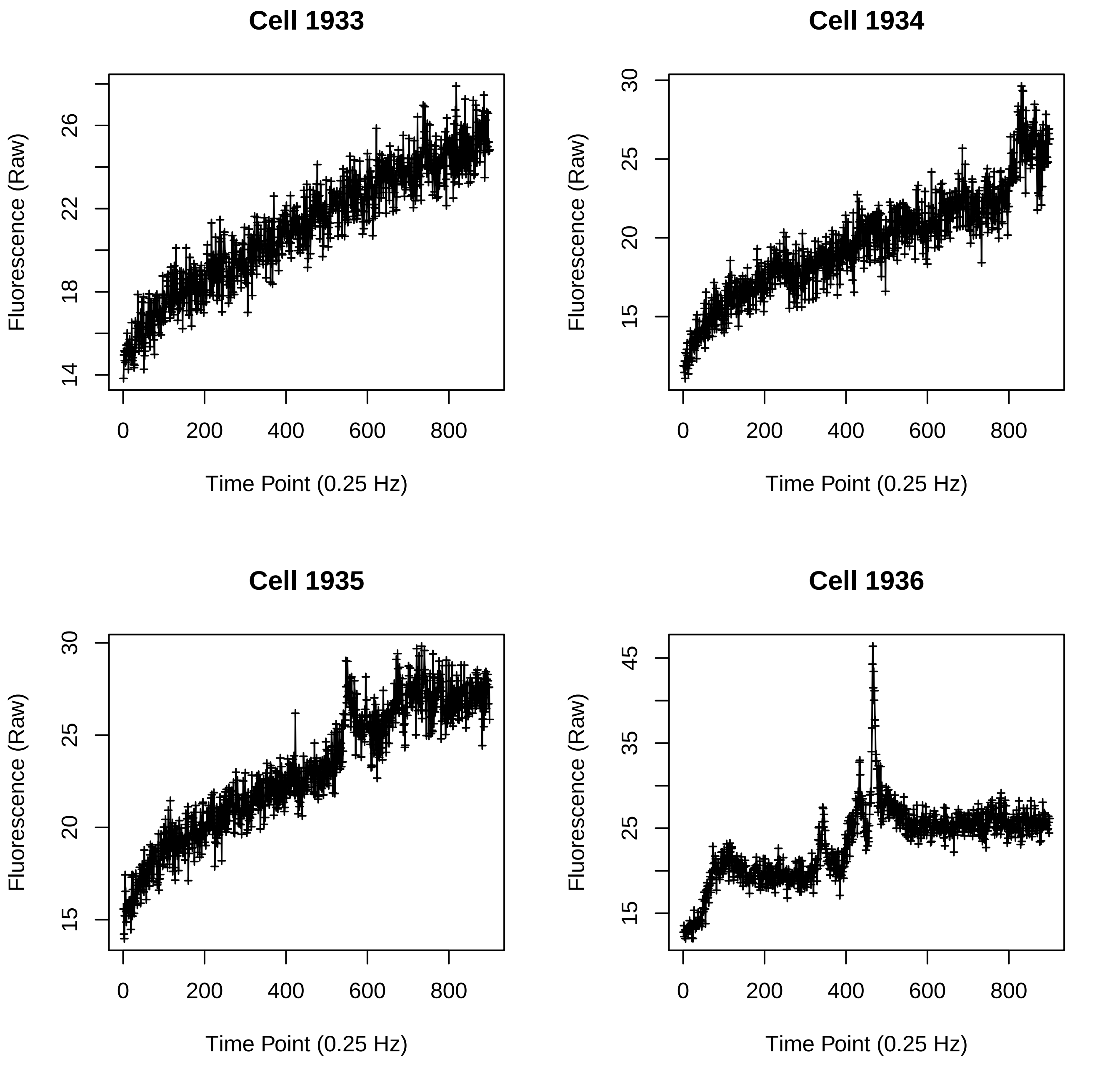
Cell 1933
Cell 1934
Cell 1935
Cell 1936
Fluorescence (Raw)
Time Point (0.25 Hz)
14
18
22
26
15
20
25
30
35
45
0
200
400
600
800

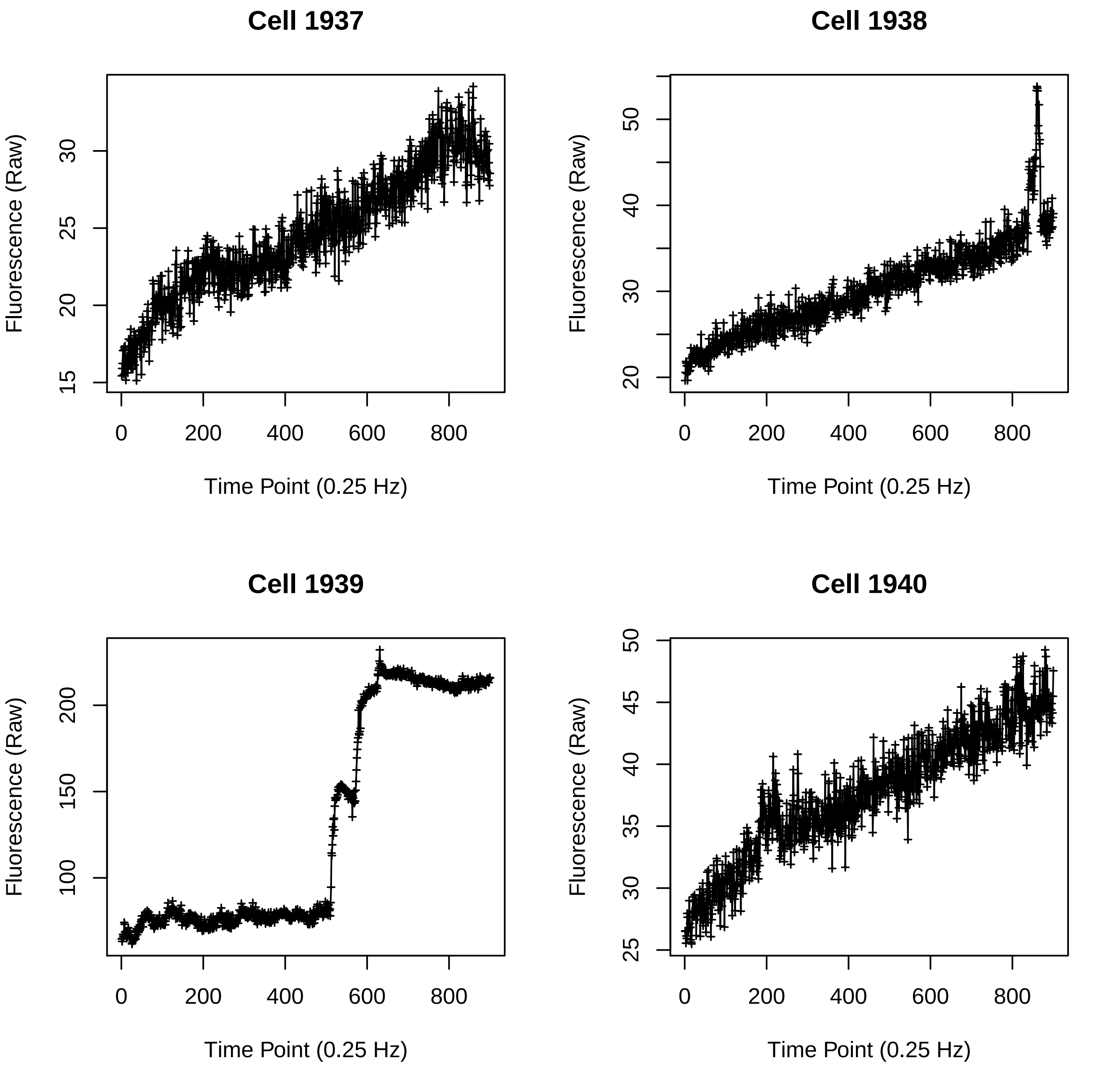
Cell 1937
Fluorescence (Raw)
15
20
25
30
0
200
400
600
800
Time Point (0.25 Hz)
Cell 1938
Fluorescence (Raw)
20
30
40
50
0
200
400
600
800
Time Point (0.25 Hz)
Cell 1939
Fluorescence (Raw)
100
150
200
0
200
400
600
800
Time Point (0.25 Hz)
Cell 1940
Fluorescence (Raw)
25
30
35
40
45
50
0
200
400
600
800
Time Point (0.25 Hz)

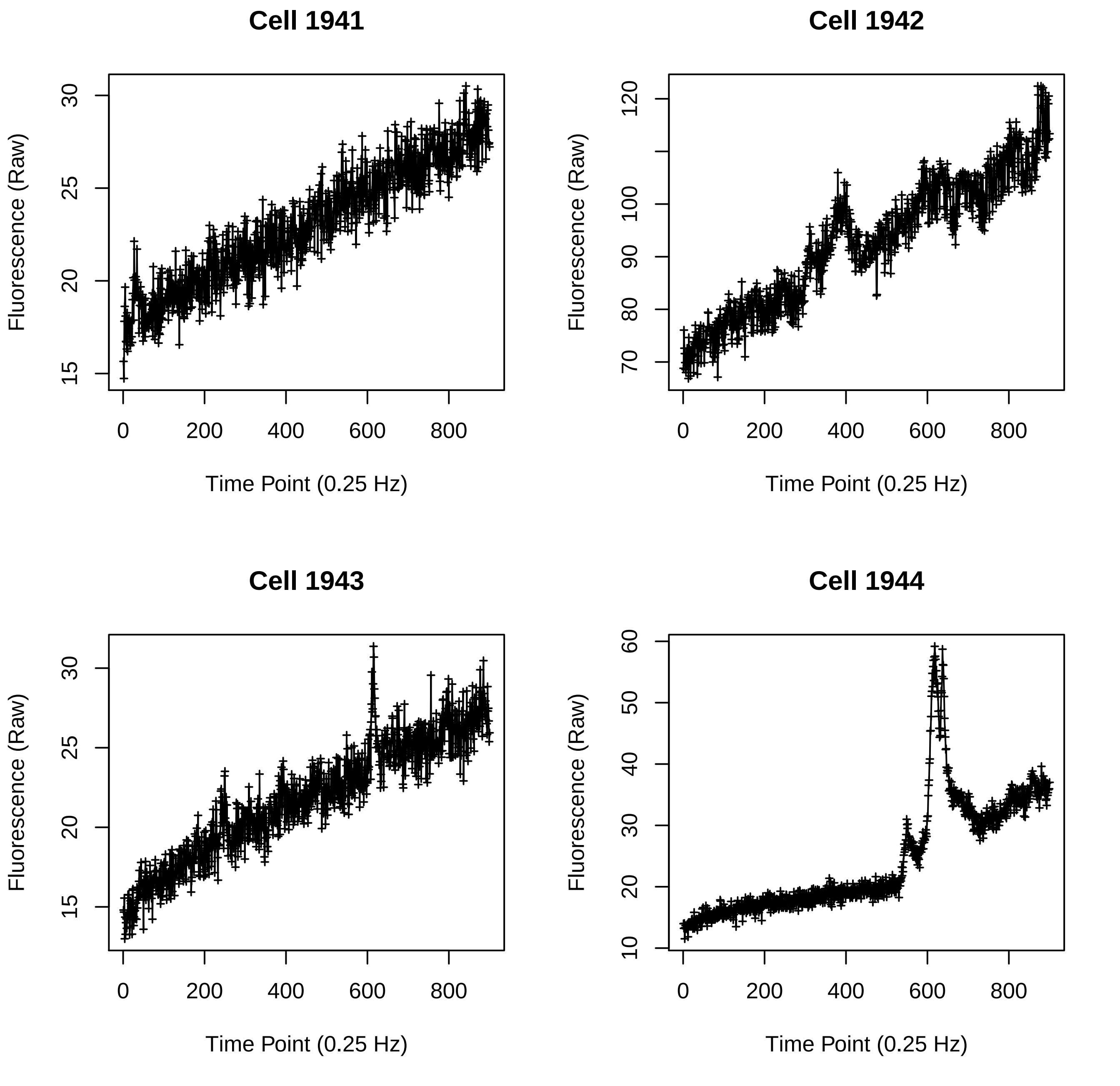

Cell 1941
Cell 1942
Cell 1943
Cell 1944
Fluorescence (Raw)
Time Point (0.25 Hz)
15
20
25
30
70
80
90
100
120
10
40
50
60
0
200
400
600
800

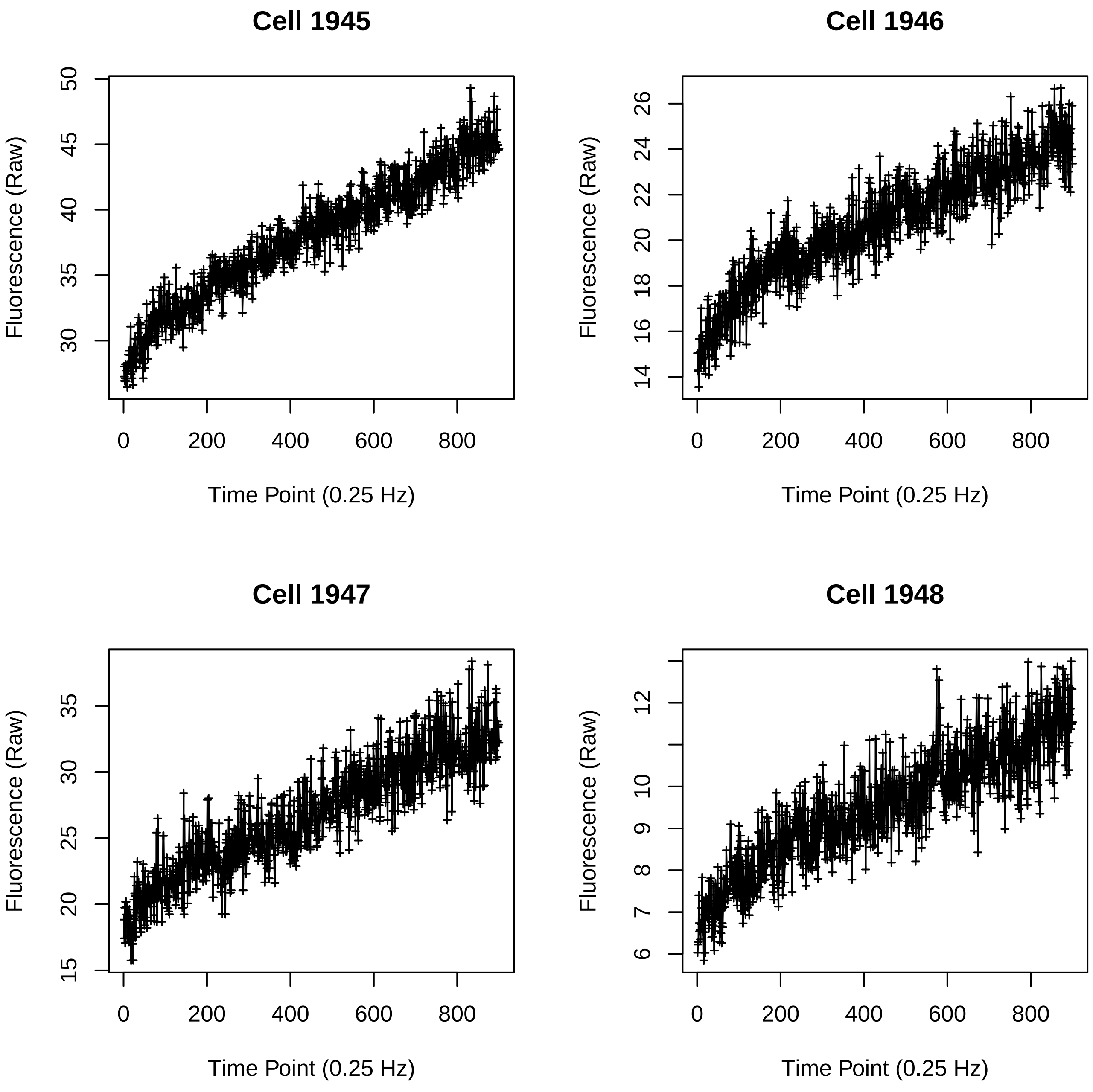
Cell 1945
Cell 1946
Cell 1947
Cell 1948
Fluorescence (Raw)
Time Point (0.25 Hz)
0
200
400
600
800
30
35
40
45
50
14
16
18
20
22
24
26
15
20
25
30
35
6
7
8
9
10
12

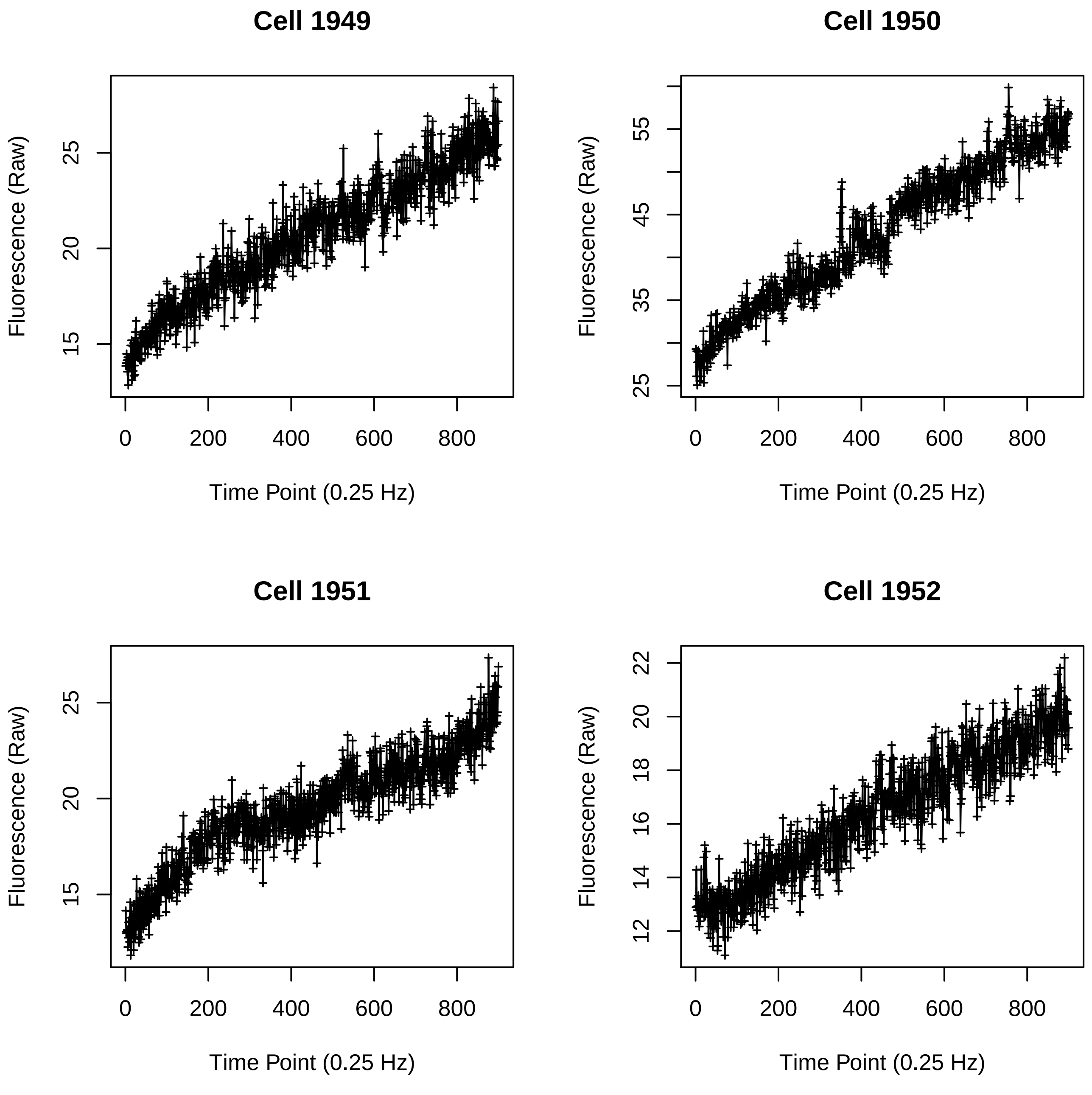
Cell 1949
Cell 1950
Cell 1951
Cell 1952
Fluorescence (Raw)
Time Point (0.25 Hz)
0
200
400
600
800
15
20
25
35
45
55
12
14
16
18
22

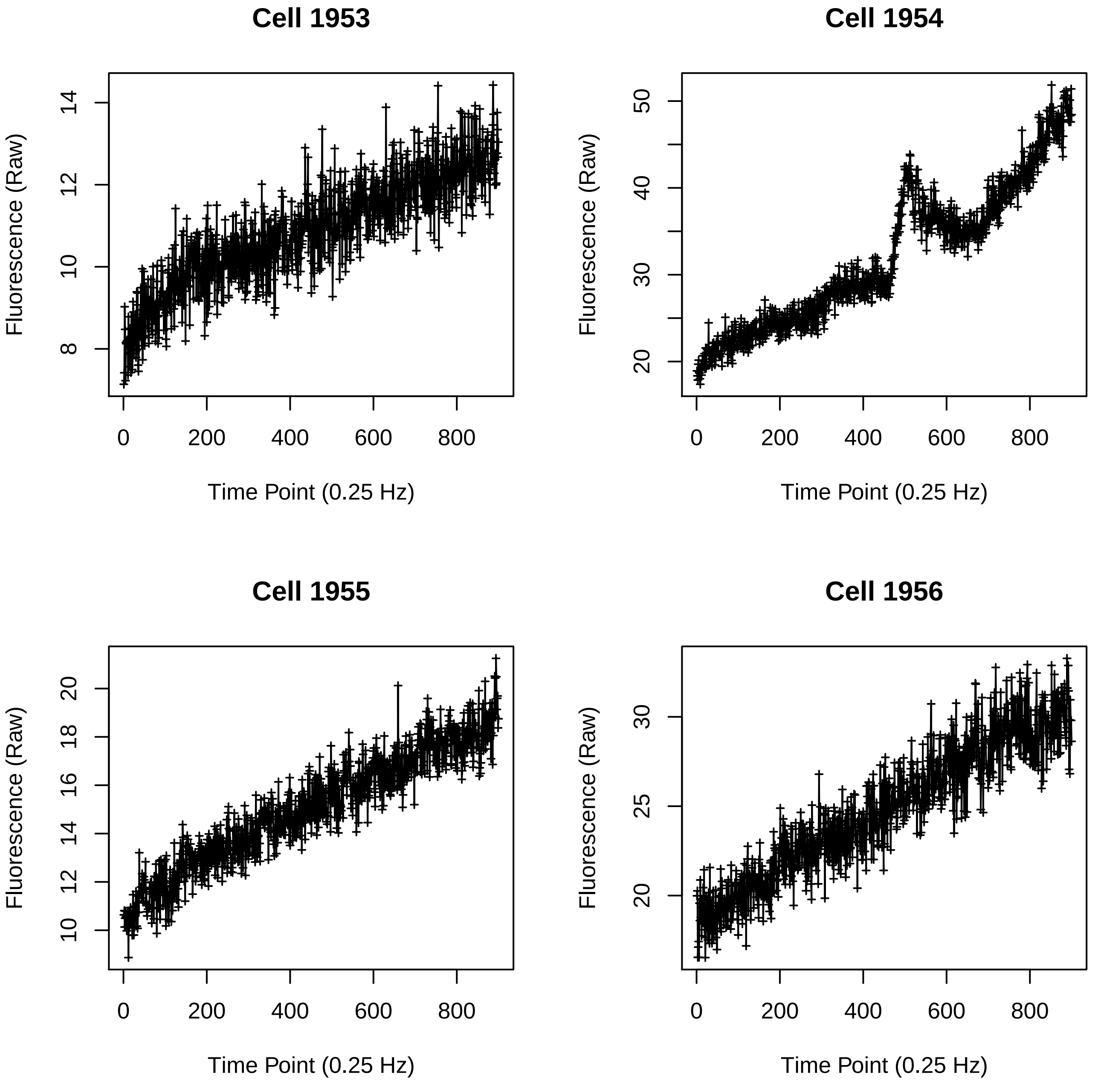
Cell 1953
Cell 1954
Cell 1955
Cell 1956
Fluorescence (Raw)
Time Point (0.25 Hz)
0
200
400
600
800
8
10
12
14
20
30
40
50
16
18
25

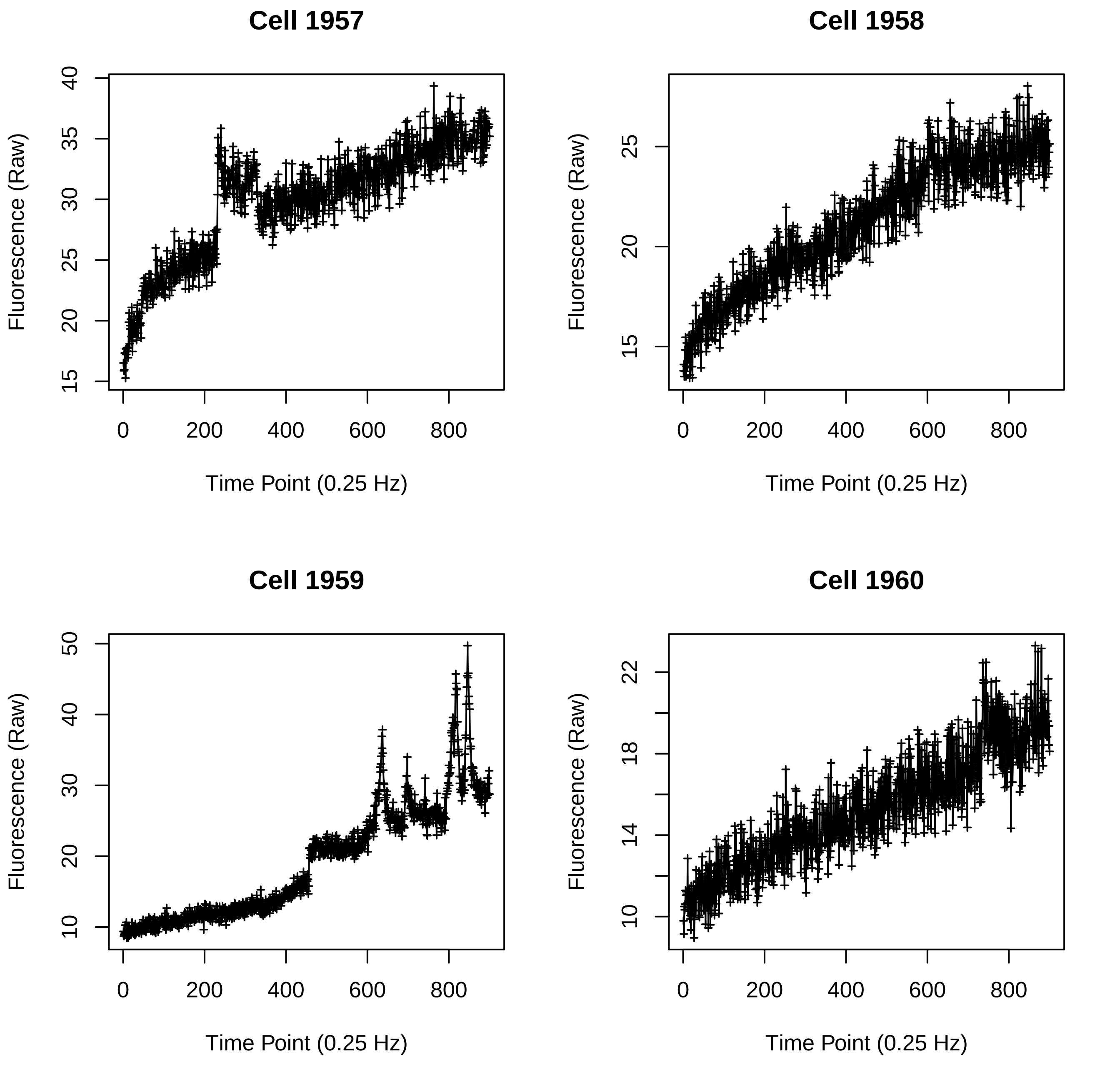
Cell 1957
Cell 1958
Cell 1959
Cell 1960
Fluorescence (Raw)
Time Point (0.25 Hz)
0
200
400
600
800
15
20
25
30
35
40
50
10
14
18
22

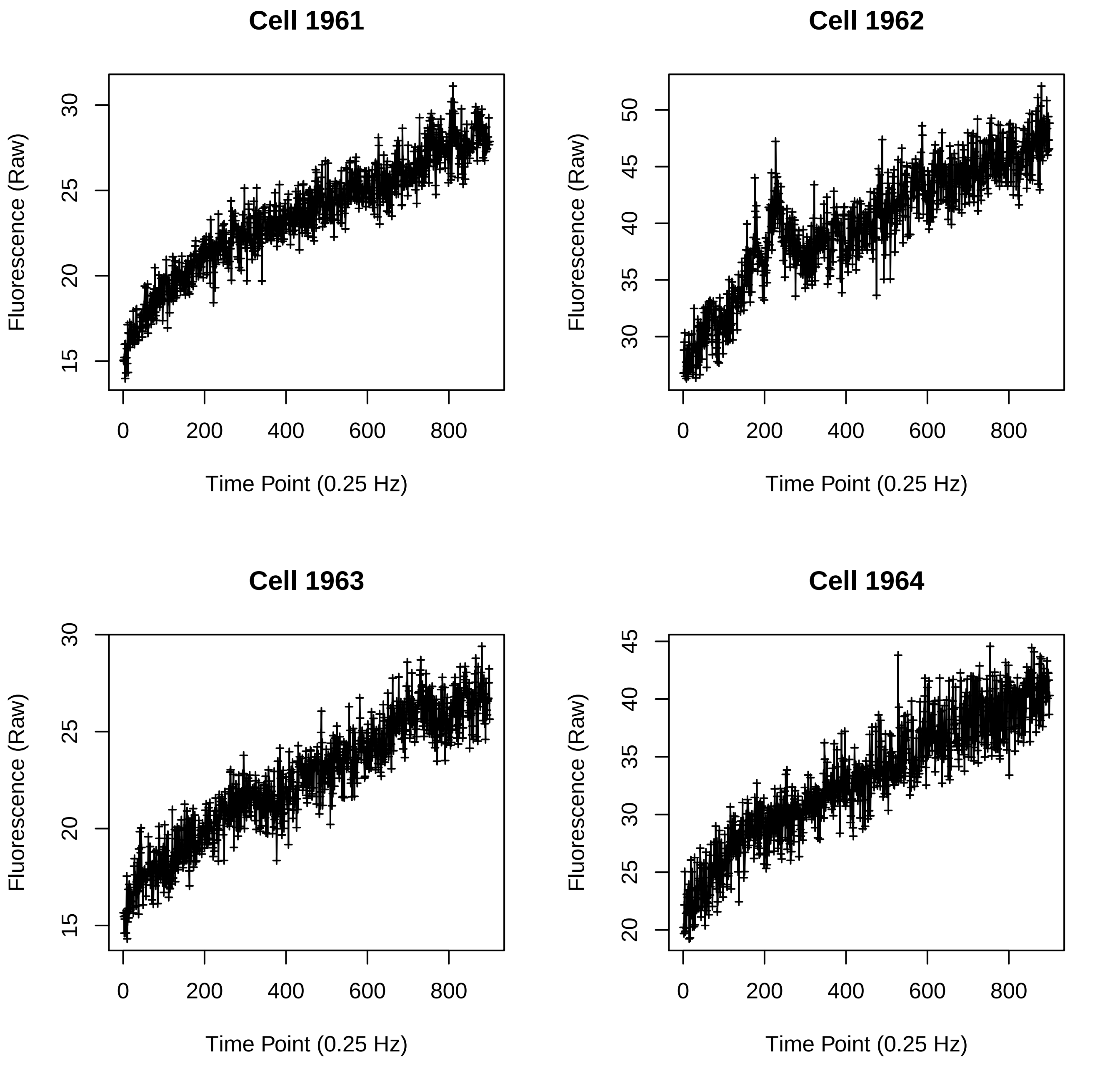

Cell 1961
Fluorescence (Raw)
15
20
25
30
0
200
400
600
800
Time Point (0.25 Hz)
Cell 1962
Fluorescence (Raw)
30
35
40
45
50
0
200
400
600
800
Time Point (0.25 Hz)
Cell 1963
Fluorescence (Raw)
15
20
25
30
0
200
400
600
800
Time Point (0.25 Hz)
Cell 1964
Fluorescence (Raw)
20
25
30
35
40
45
0
200
400
600
800
Time Point (0.25 Hz)

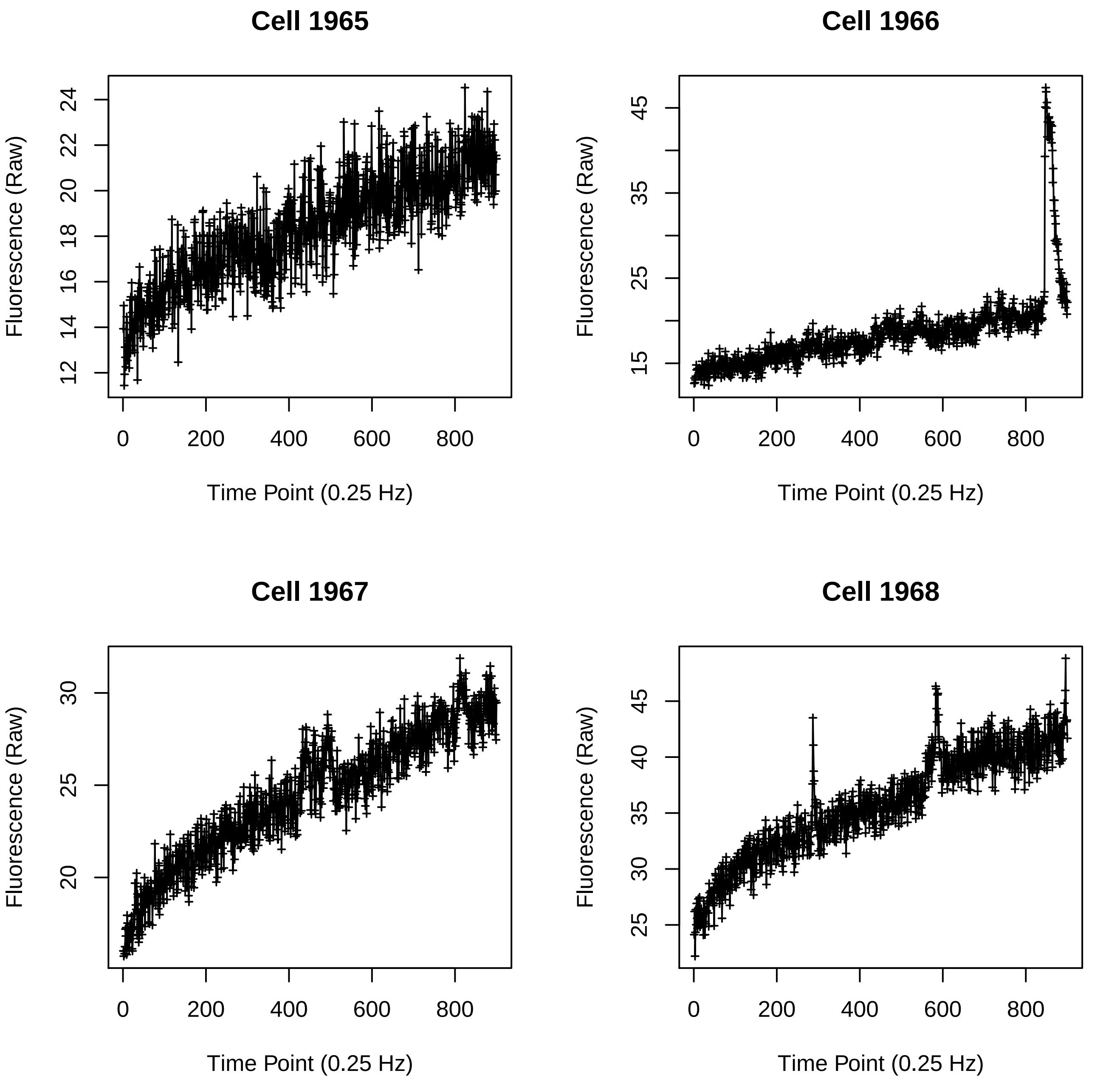
Cell 1965
Fluorescence (Raw)
12
14
16
18
20
22
24
0
200
400
600
800
Time Point (0.25 Hz)
Cell 1966
Fluorescence (Raw)
15
25
35
45
0
200
400
600
800
Time Point (0.25 Hz)
Cell 1967
Fluorescence (Raw)
20
25
30
0
200
400
600
800
Time Point (0.25 Hz)
Cell 1968
Fluorescence (Raw)
25
30
35
40
45
0
200
400
600
800
Time Point (0.25 Hz)

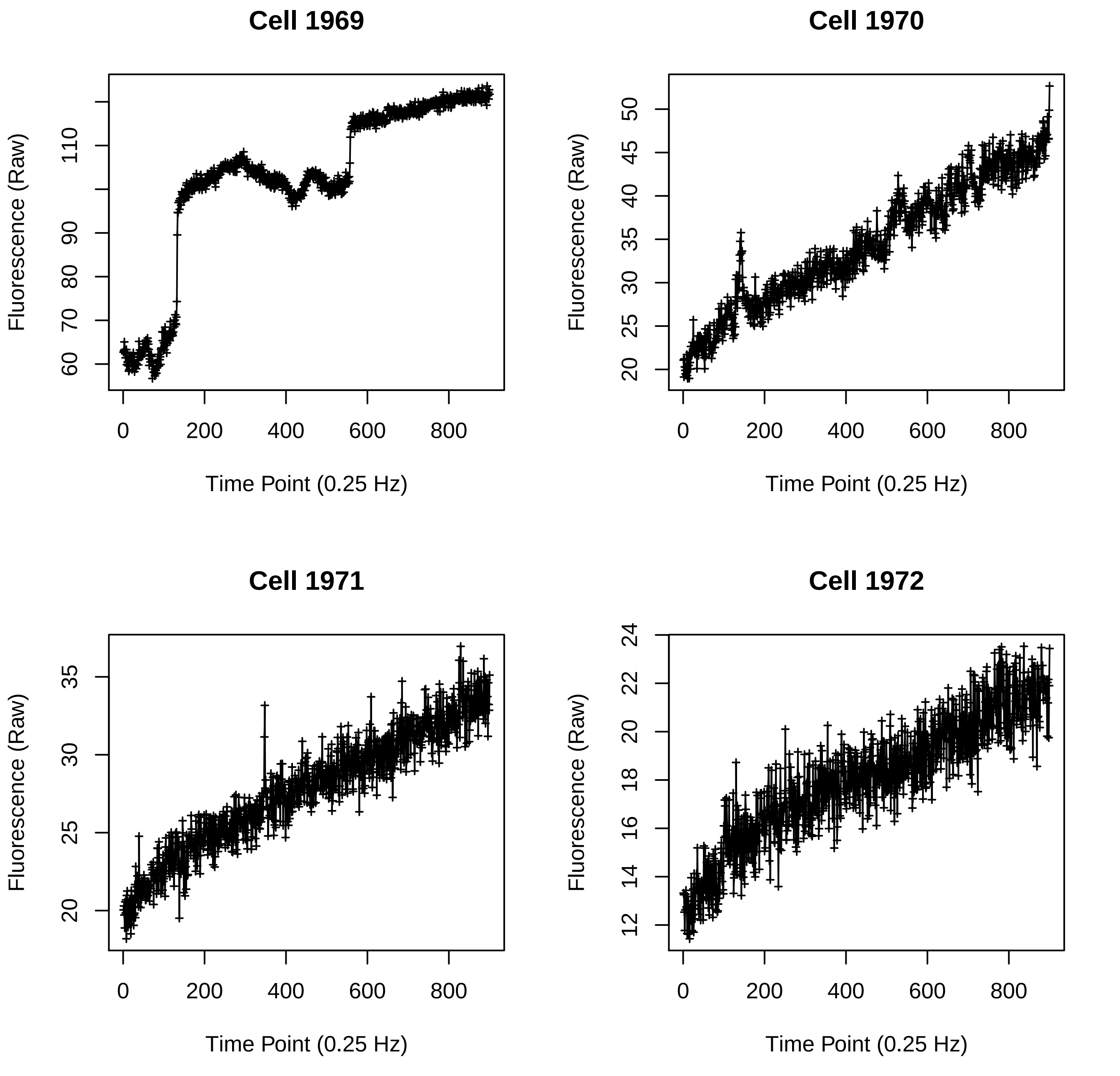
Cell 1969
Fluorescence (Raw)
60
70
80
90
110
Time Point (0.25 Hz)
0
200
400
600
800
Cell 1970
Fluorescence (Raw)
20
25
30
35
40
45
50
Time Point (0.25 Hz)
0
200
400
600
800
Cell 1971
Fluorescence (Raw)
20
25
30
35
Time Point (0.25 Hz)
0
200
400
600
800
Cell 1972
Fluorescence (Raw)
12
14
16
18
20
22
24
Time Point (0.25 Hz)
0
200
400
600
800

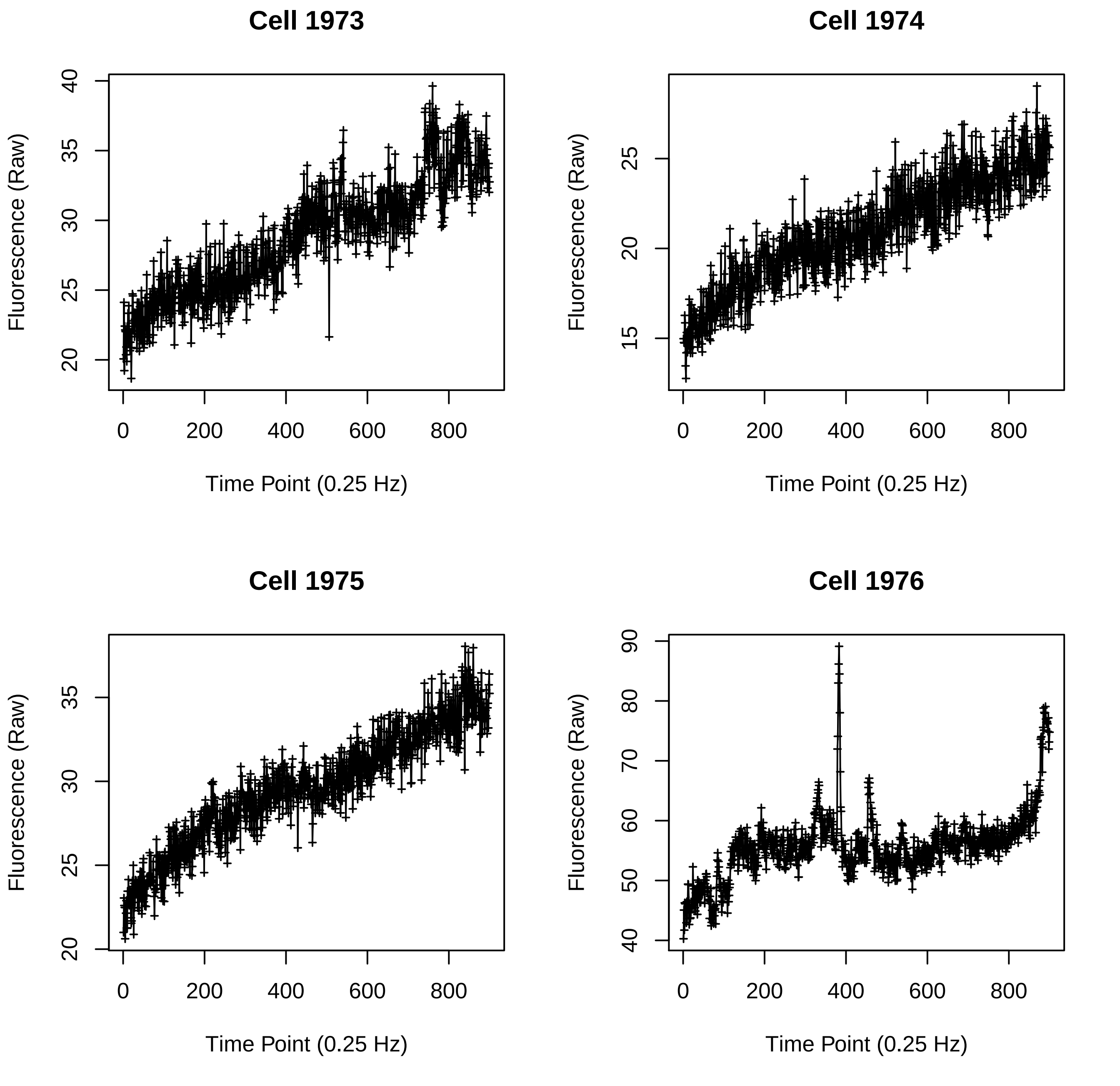
Cell 1973
Cell 1974
Cell 1975
Cell 1976
Fluorescence (Raw)
Time Point (0.25 Hz)
0
200
400
600
800
20
25
30
35
40
15
50
60
70
80
90

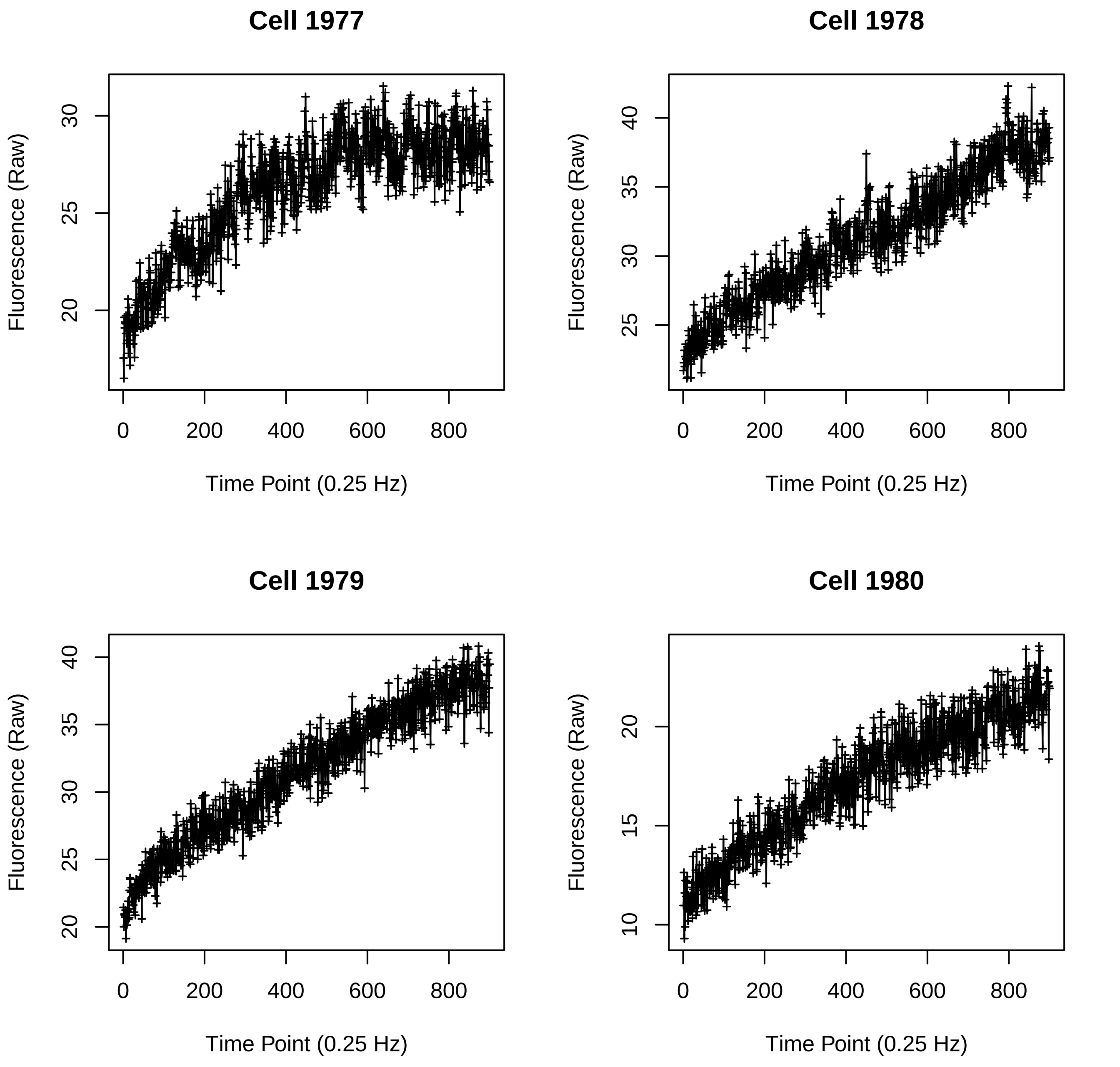
Cell 1977
Cell 1978
Cell 1979
Cell 1980
Fluorescence (Raw)
Time Point (0.25 Hz)
0
200
400
600
800
20
25
30
35
40
10
15

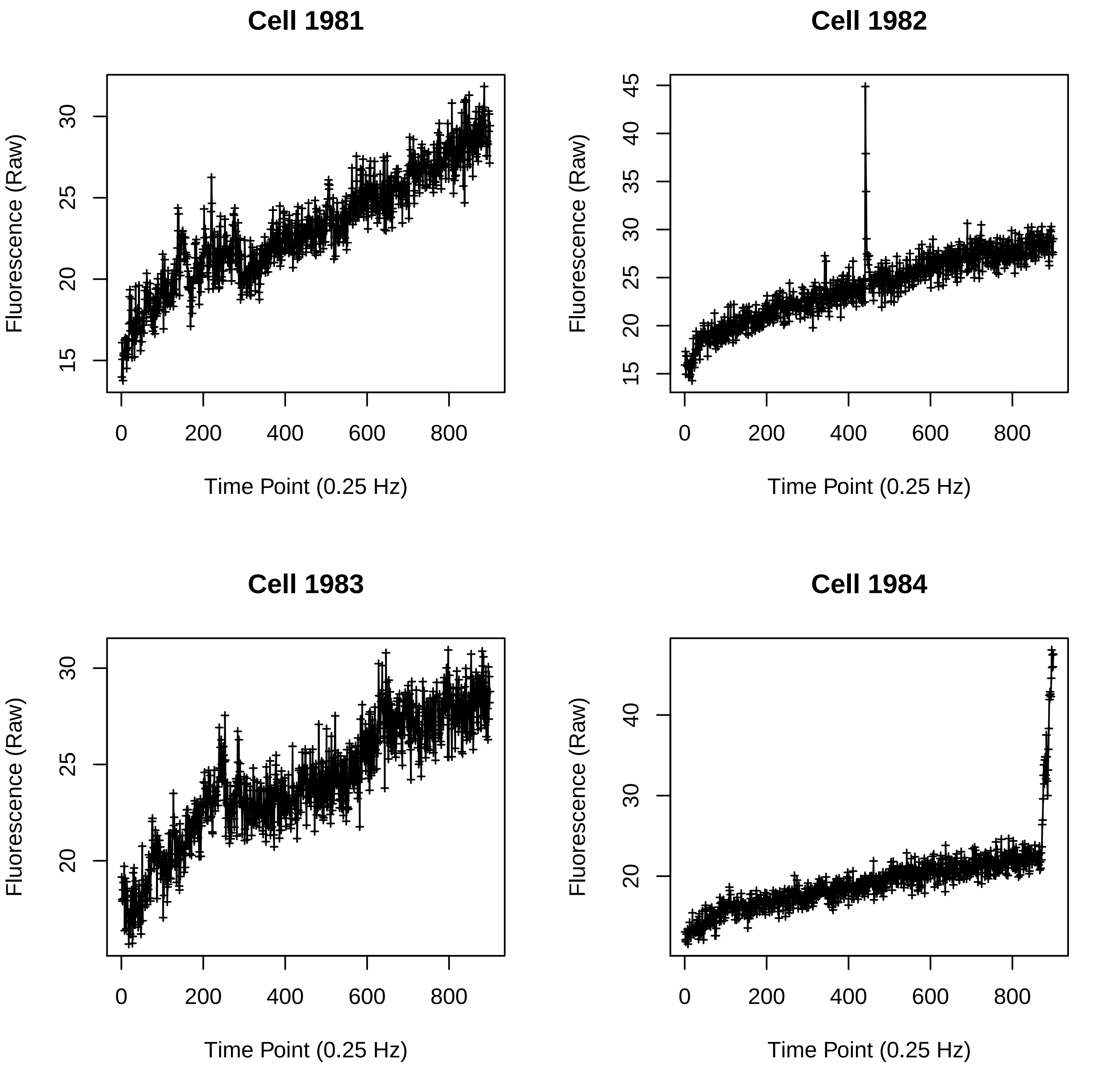
Cell 1981
Fluorescence (Raw)
15
20
25
30
0
200
400
600
800
Time Point (0.25 Hz)
Cell 1982
Fluorescence (Raw)
15
20
25
30
35
40
45
0
200
400
600
800
Time Point (0.25 Hz)
Cell 1983
Fluorescence (Raw)
20
25
30
0
200
400
600
800
Time Point (0.25 Hz)
Cell 1984
Fluorescence (Raw)
20
30
40
0
200
400
600
800
Time Point (0.25 Hz)

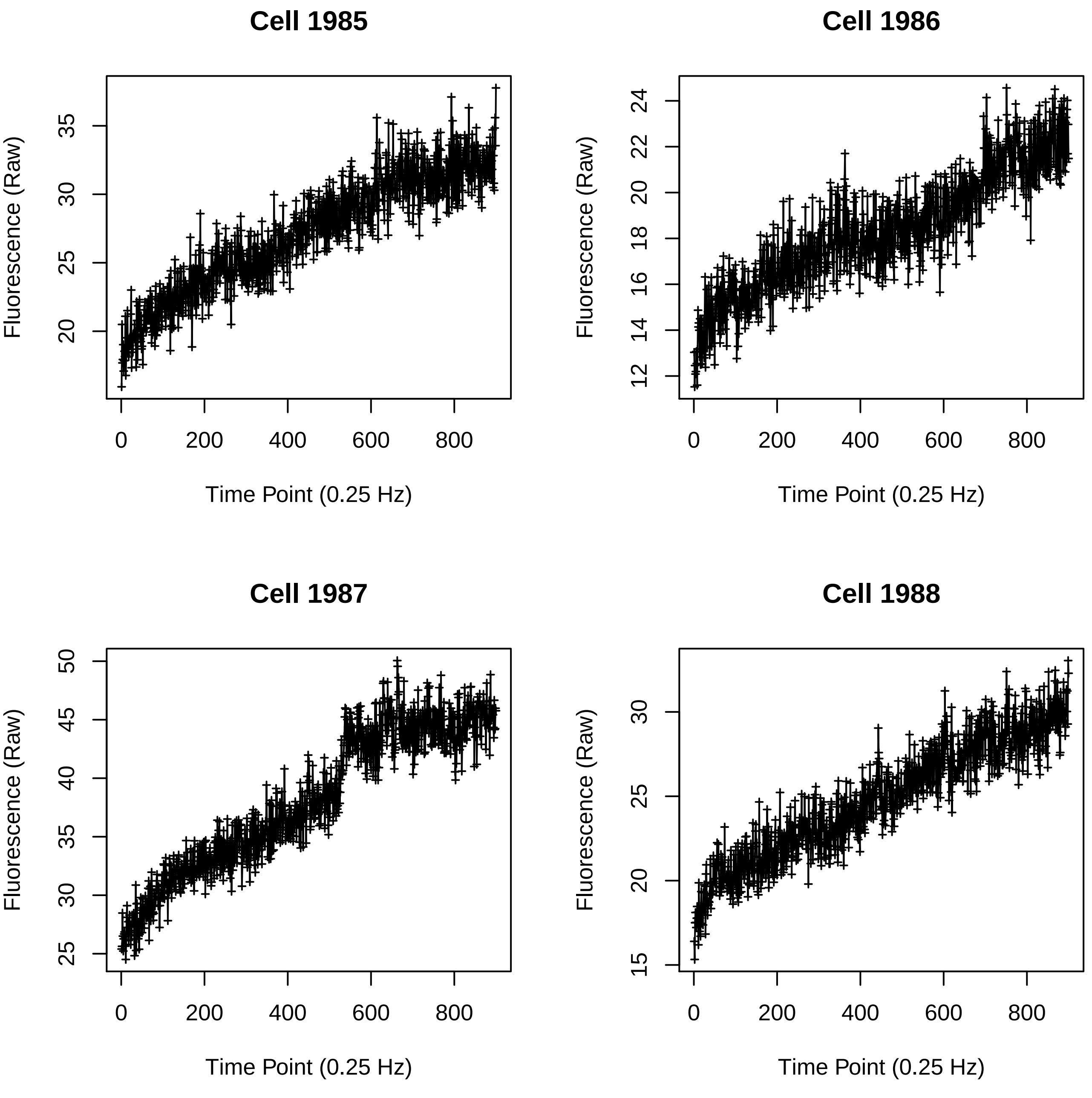

Cell 1985
Cell 1986
Cell 1987
Cell 1988
Fluorescence (Raw)
Time Point (0.25 Hz)
0
200
400
600
800
20
25
30
35
12
14
16
18
20
22
24
25
30
35
40
45
50
15
20
25
30

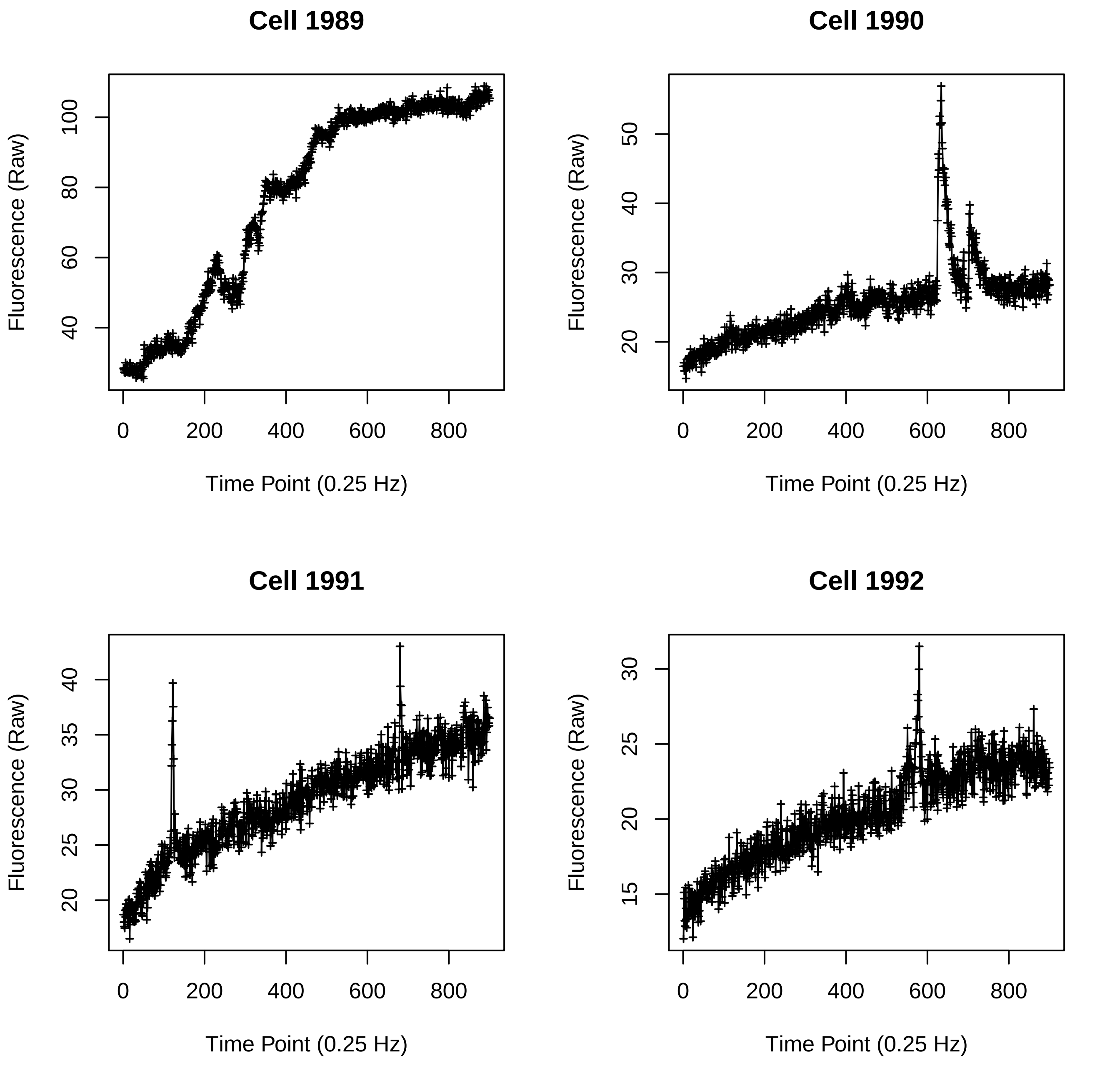
Cell 1989
Fluorescence (Raw)
40
60
80
100
0
200
400
600
800
Time Point (0.25 Hz)
Cell 1990
Fluorescence (Raw)
20
30
40
50
0
200
400
600
800
Time Point (0.25 Hz)
Cell 1991
Fluorescence (Raw)
20
25
30
35
40
0
200
400
600
800
Time Point (0.25 Hz)
Cell 1992
Fluorescence (Raw)
15
20
25
30
0
200
400
600
800
Time Point (0.25 Hz)

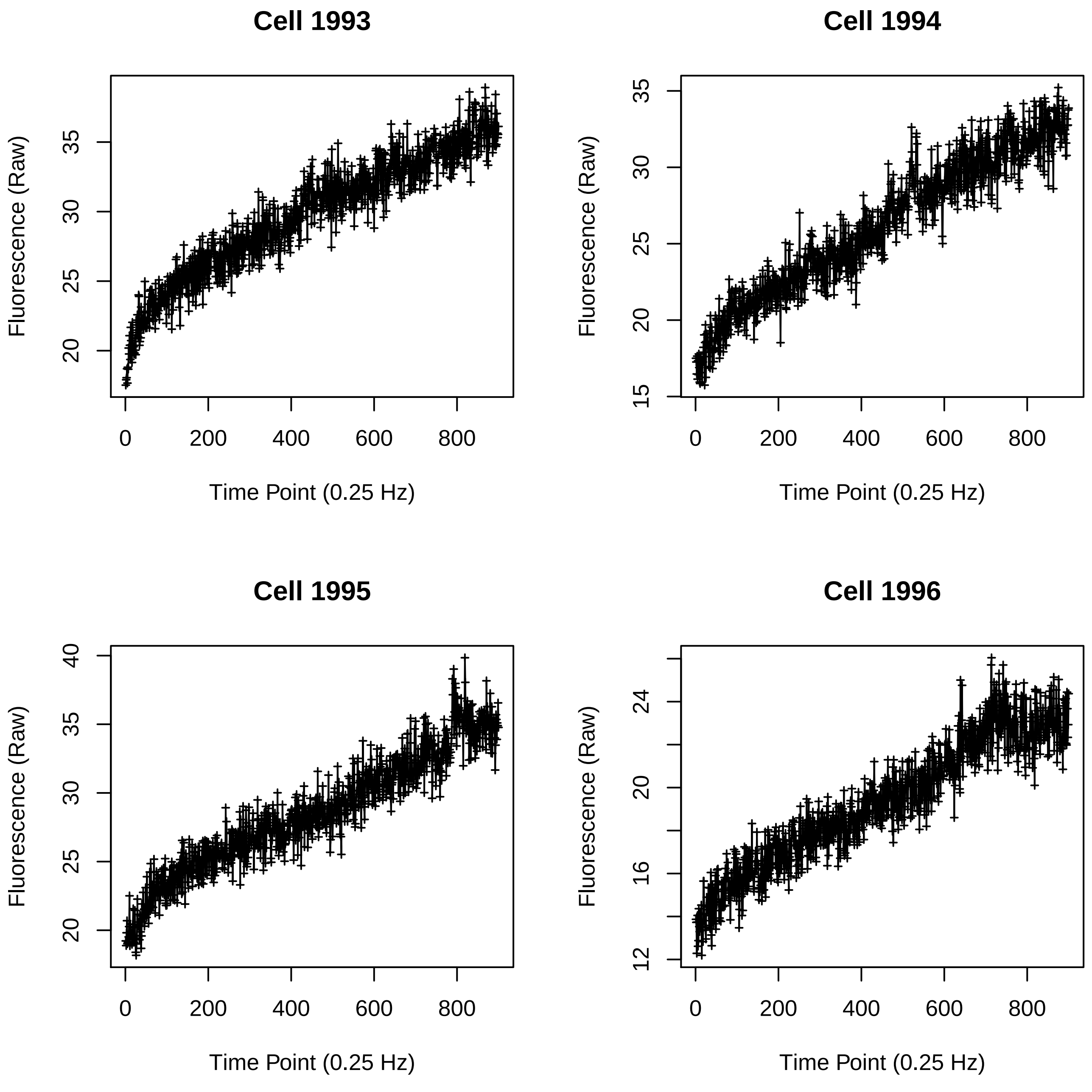
Cell 1993
Cell 1994
Cell 1995
Cell 1996
Fluorescence (Raw)
Time Point (0.25 Hz)
0
200
400
600
800
20
25
30
35
15
40
12
16
20
24

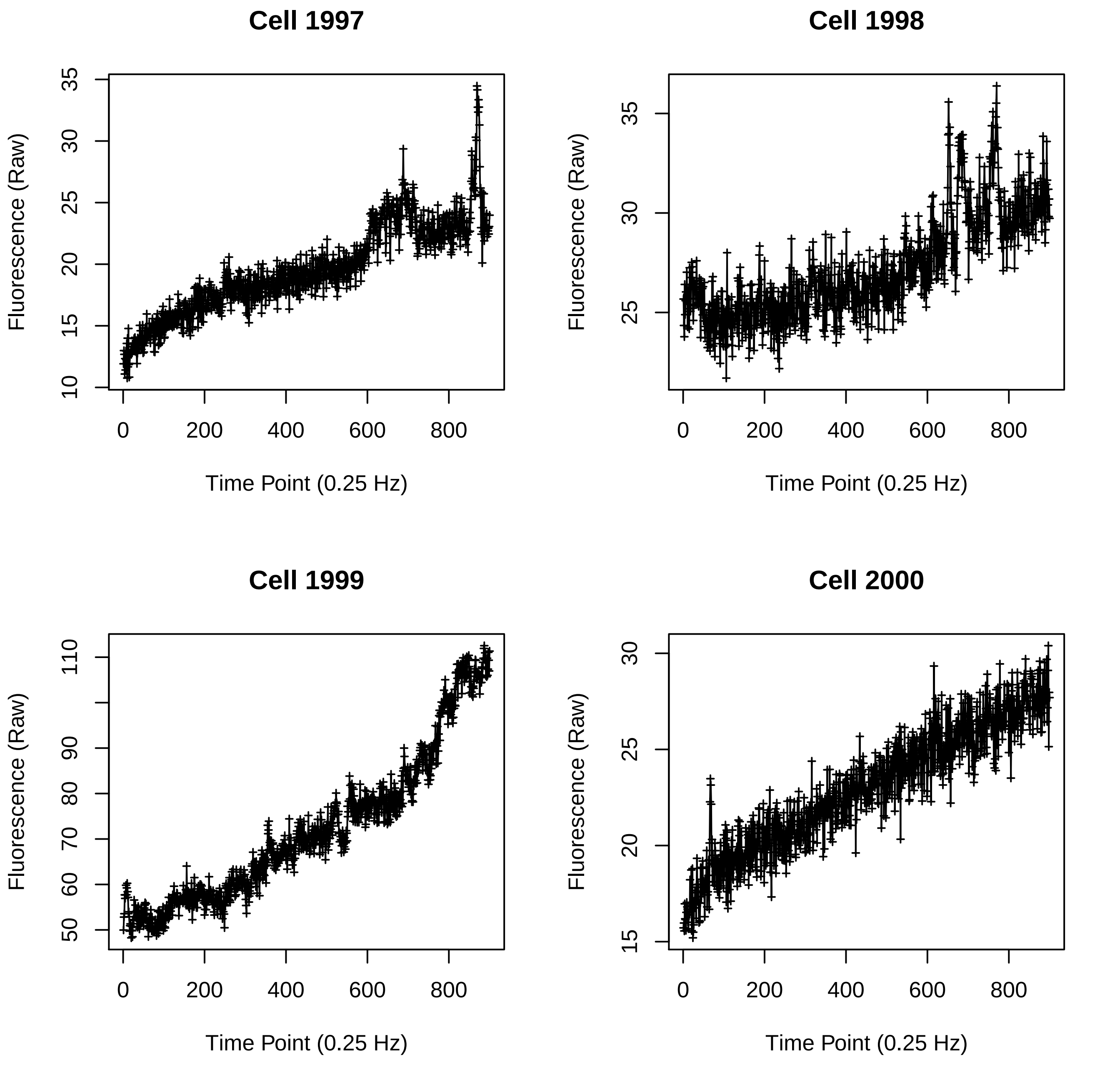

Cell 1997
Fluorescence (Raw)
10
15
20
25
30
35
0
200
400
600
800
Time Point (0.25 Hz)
Cell 1998
Fluorescence (Raw)
25
30
35
0
200
400
600
800
Time Point (0.25 Hz)
Cell 1999
Fluorescence (Raw)
50
60
70
80
90
110
0
200
400
600
800
Time Point (0.25 Hz)
Cell 2000
Fluorescence (Raw)
15
20
25
30
0
200
400
600
800
Time Point (0.25 Hz)

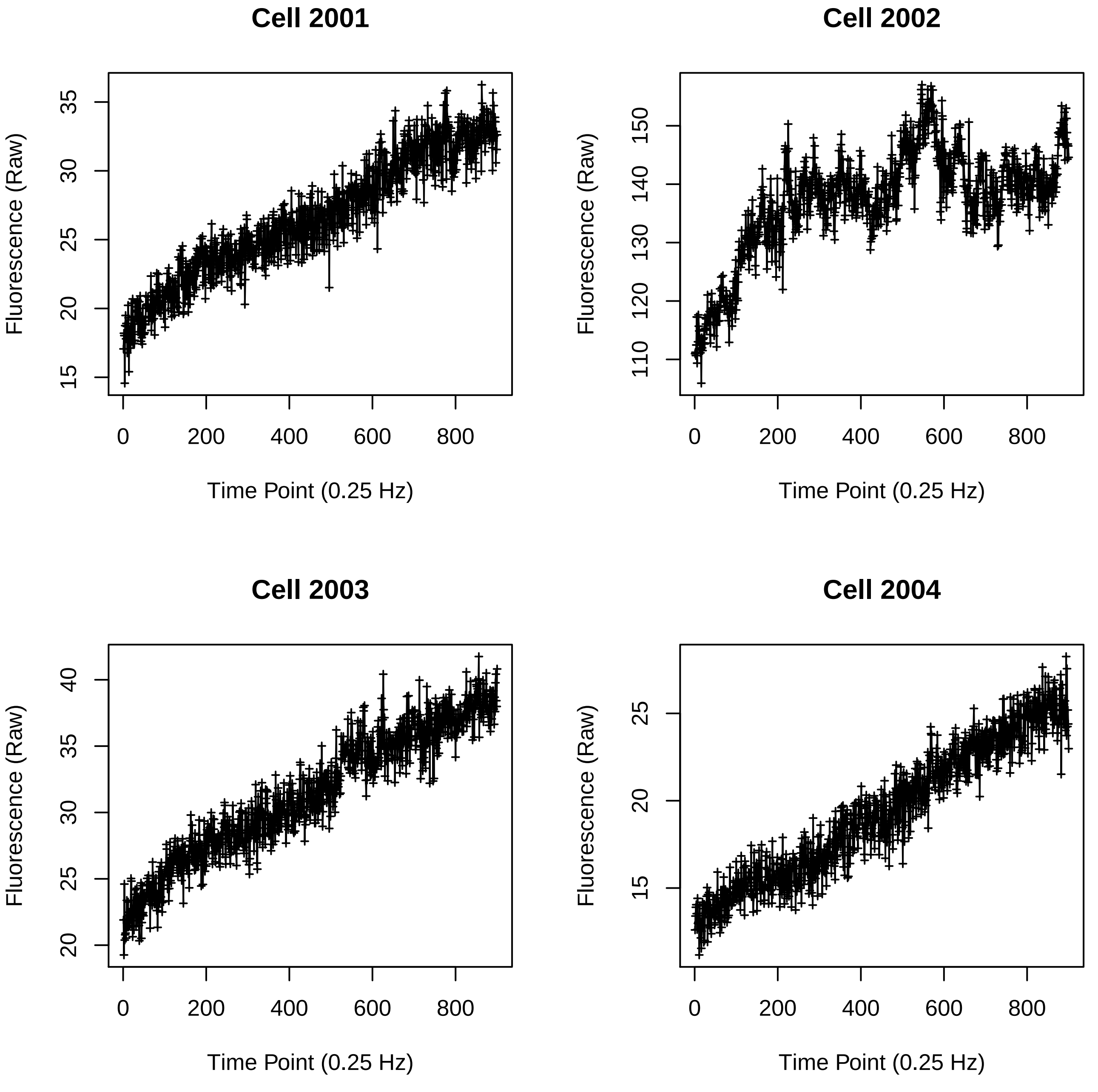
Cell 2001
Cell 2002
Cell 2003
Cell 2004
Fluorescence (Raw)
Time Point (0.25 Hz)
0
200
400
600
800
15
20
25
30
35
40
110
120
130
140
150

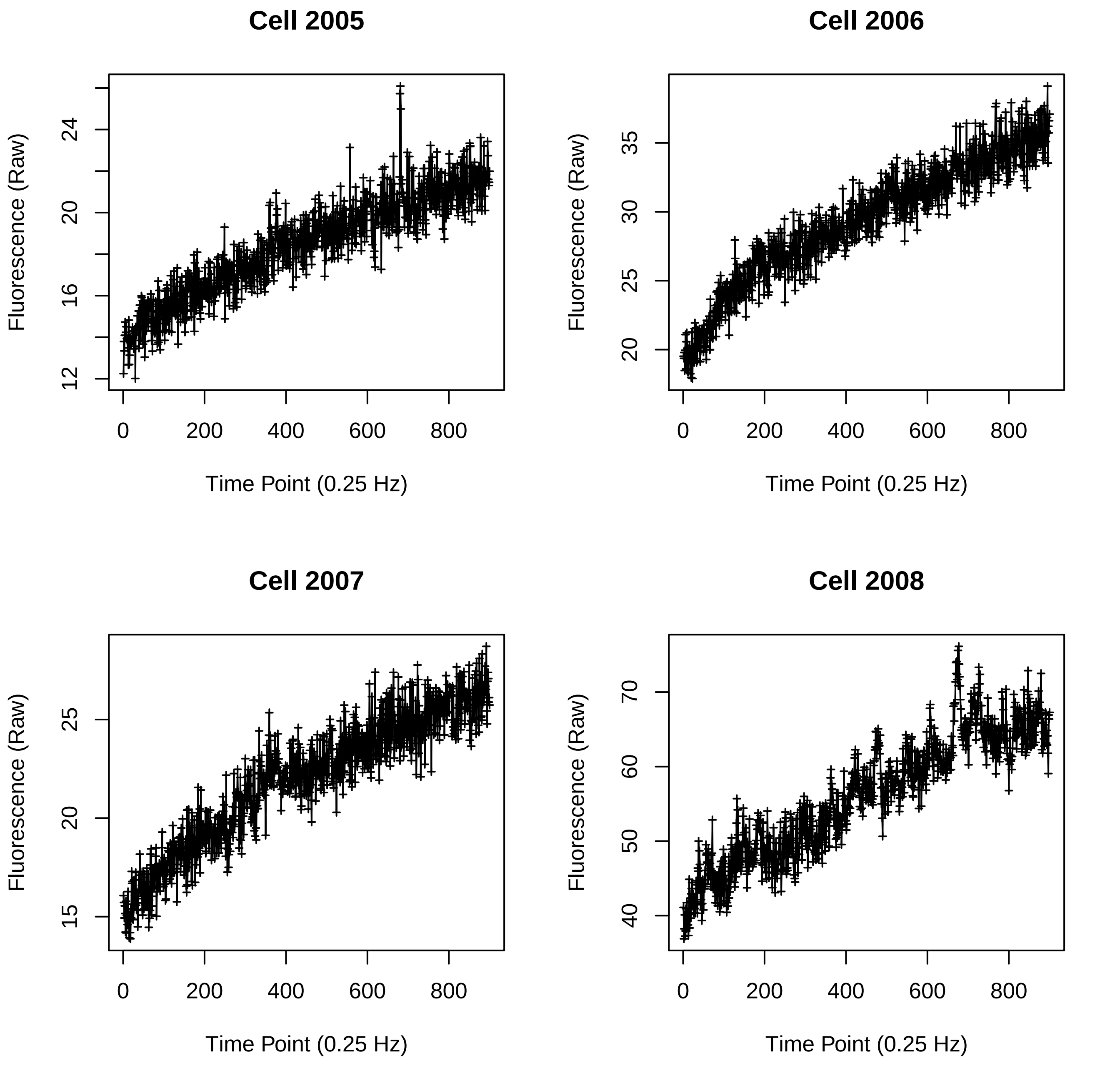
Cell 2005
Cell 2006
Cell 2007
Cell 2008
Fluorescence (Raw)
Time Point (0.25 Hz)
12
16
20
24
20
25
30
35
15
20
25
40
50
60
70
0
200
400
600
800

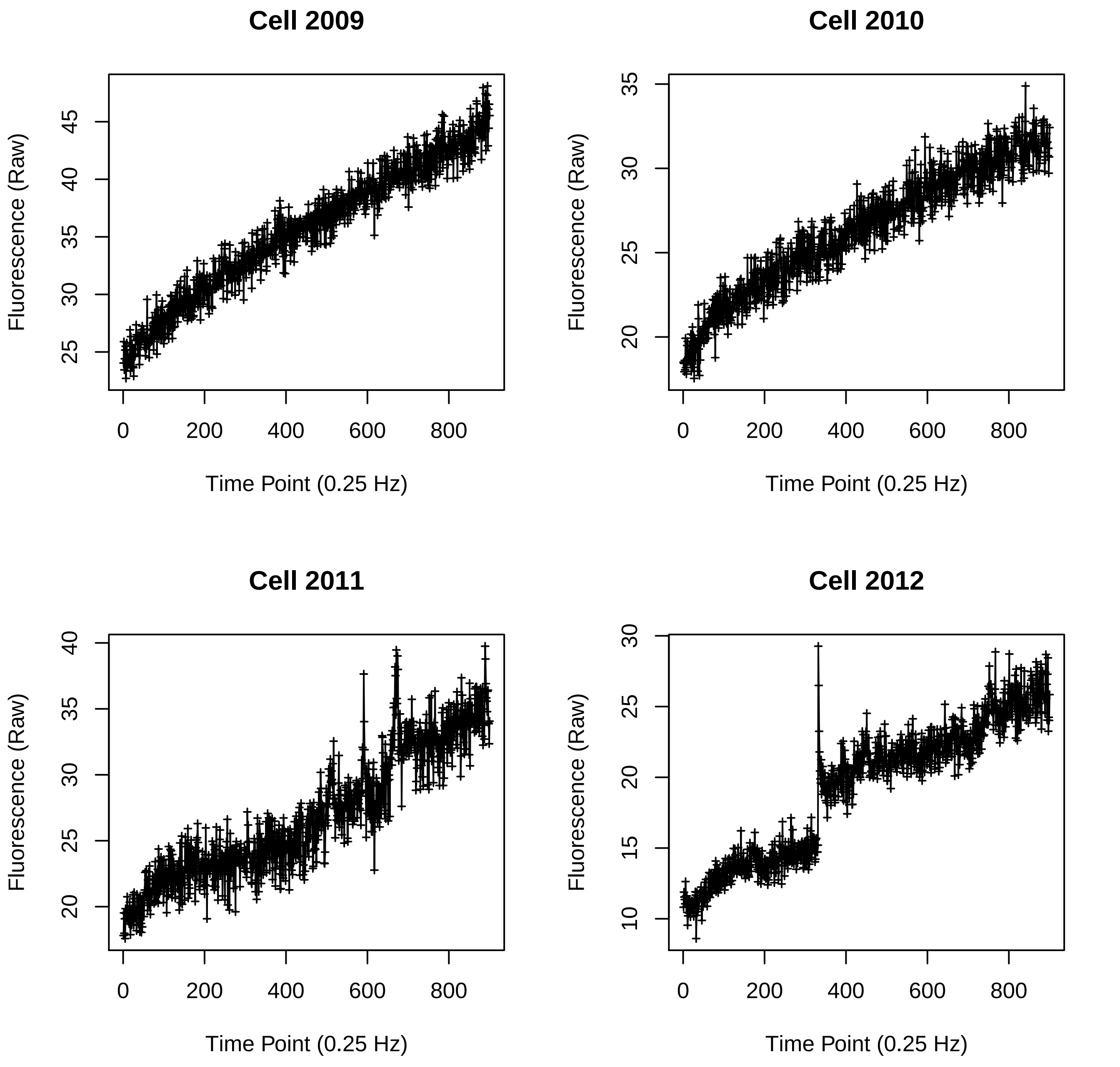

Cell 2009
Cell 2010
Cell 2011
Cell 2012
Fluorescence (Raw)
Time Point (0.25 Hz)
0
200
400
600
800
25
30
35
40
45
20
10
15

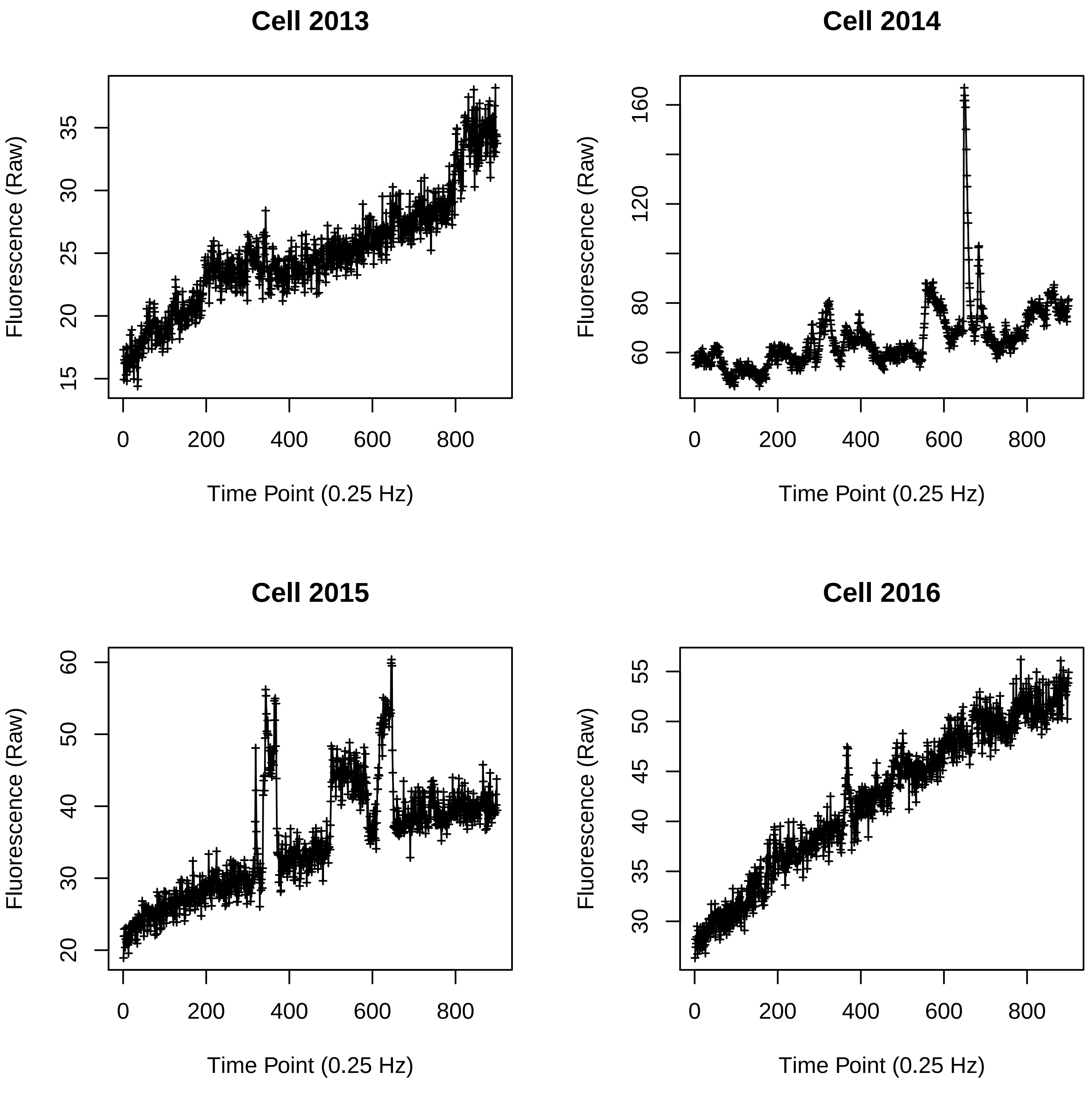
Cell 2013
Cell 2014
Cell 2015
Cell 2016
Fluorescence (Raw)
Time Point (0.25 Hz)
0
200
400
600
800
15
20
25
30
35
60
80
120
140
160
40
50
45
55

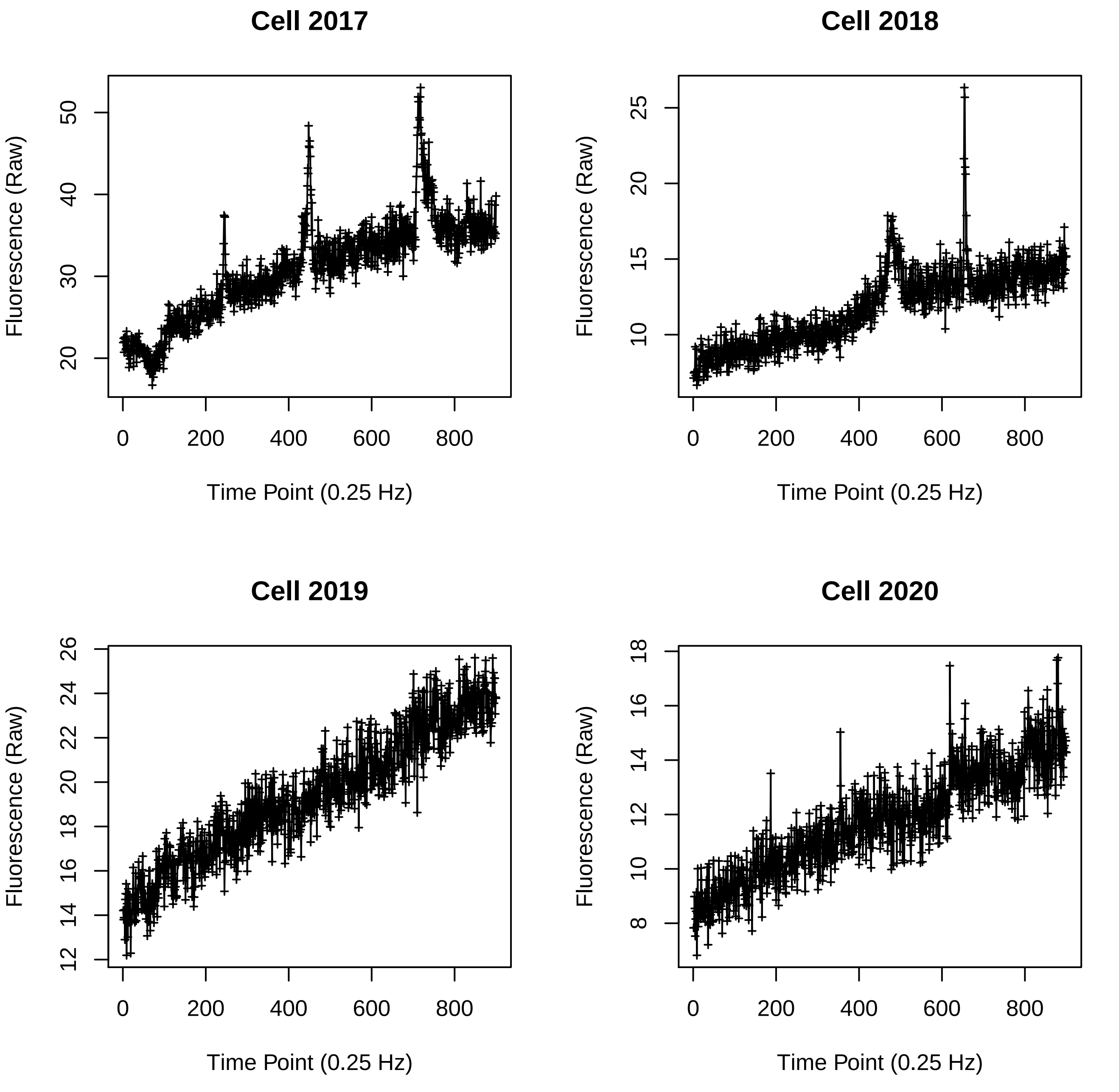

Cell 2017
Cell 2018
Cell 2019
Cell 2020
Fluorescence (Raw)
Time Point (0.25 Hz)
0
200
400
600
800
20
30
40
50
10
15
20
25
12
14
16
18
20
22
24
26
8
10
12
14
16
18

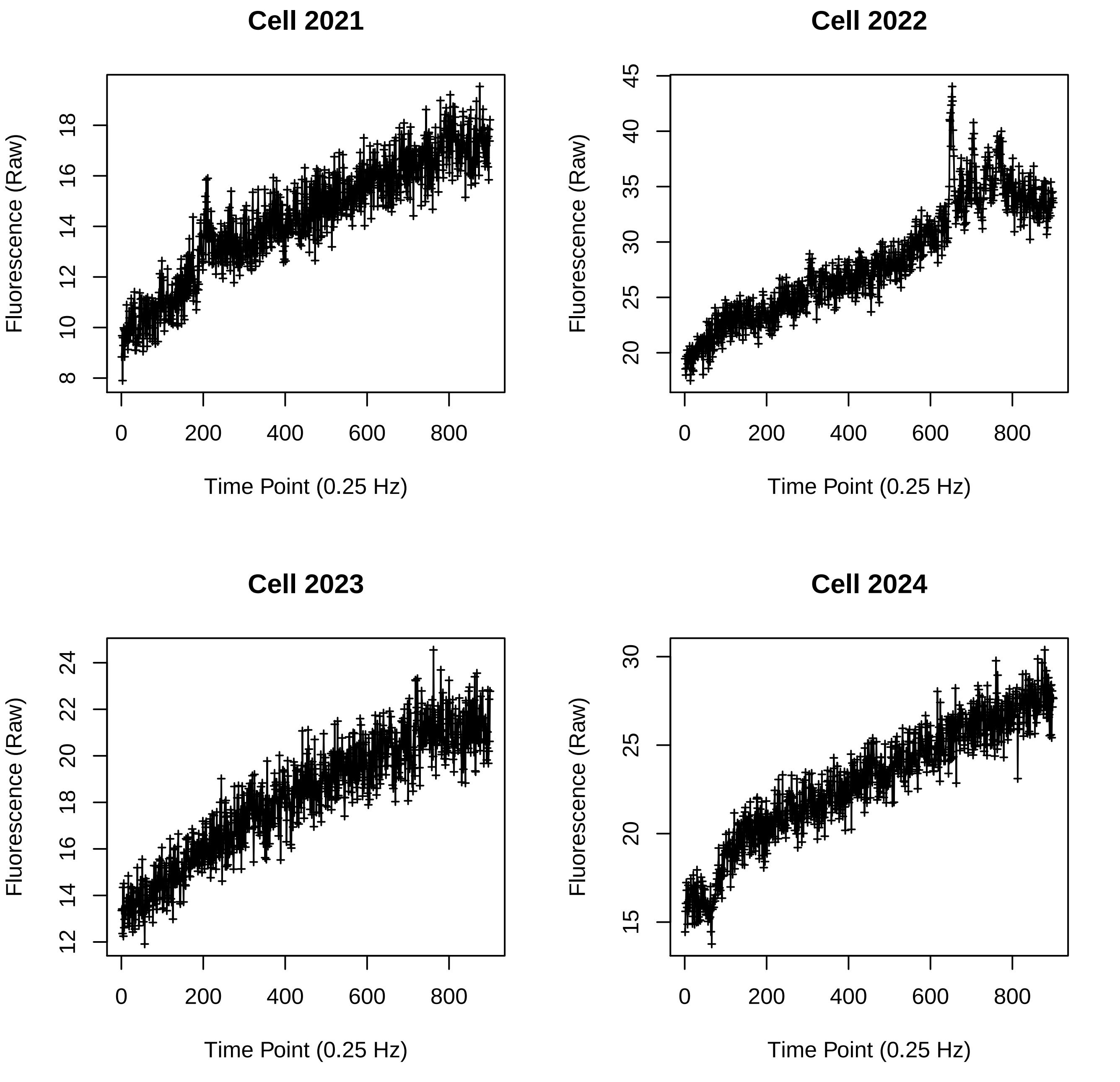
Cell 2021
Fluorescence (Raw)
8
10
12
14
16
18
0
200
400
600
800
Time Point (0.25 Hz)
Cell 2022
Fluorescence (Raw)
20
25
30
35
40
45
0
200
400
600
800
Time Point (0.25 Hz)
Cell 2023
Fluorescence (Raw)
12
14
16
18
20
22
24
0
200
400
600
800
Time Point (0.25 Hz)
Cell 2024
Fluorescence (Raw)
15
20
25
30
0
200
400
600
800
Time Point (0.25 Hz)

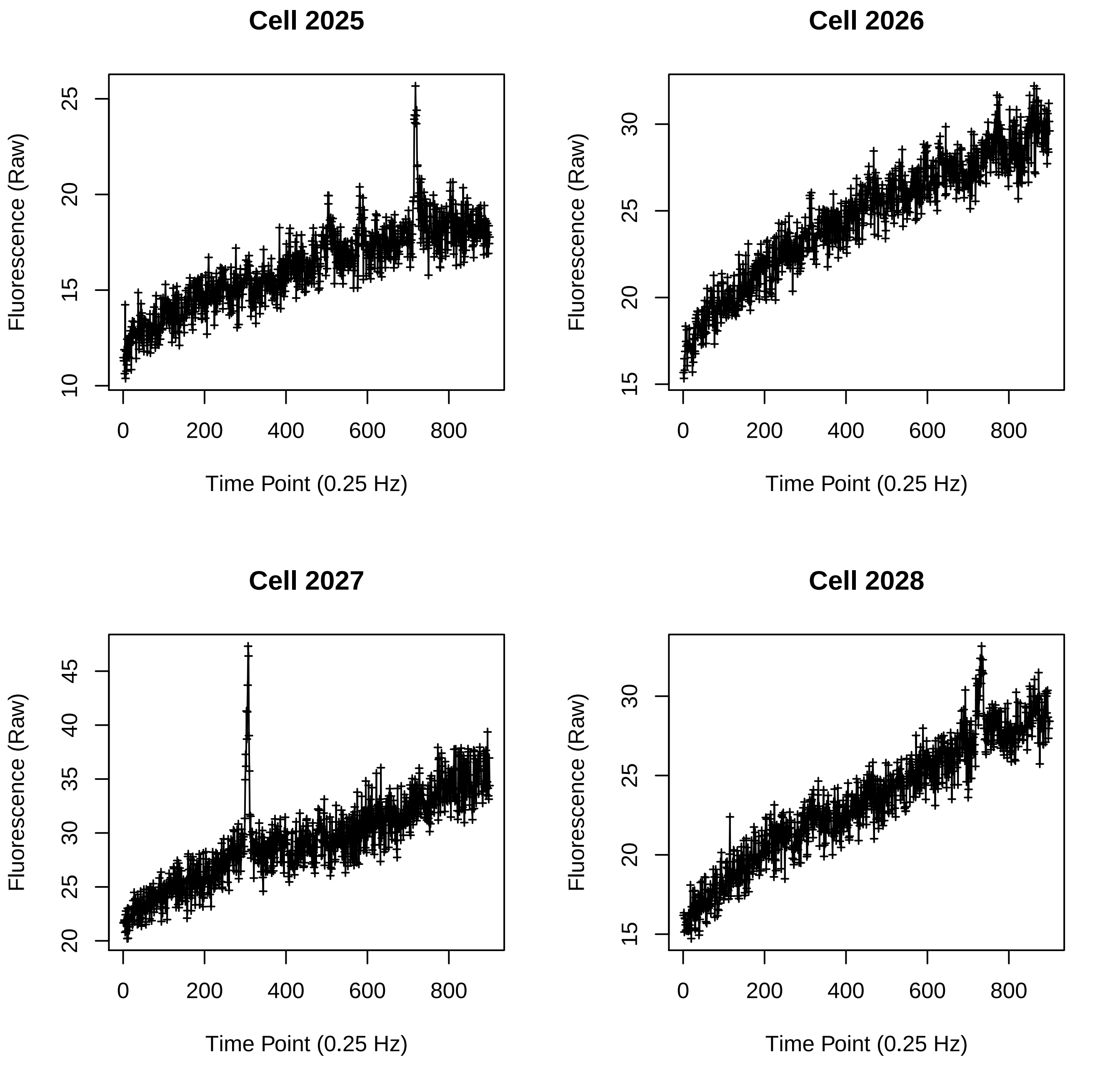
Cell 2025
Cell 2026
Cell 2027
Cell 2028
Fluorescence (Raw)
Time Point (0.25 Hz)
0
200
400
600
800
10
15
20
25
30
35
40
45

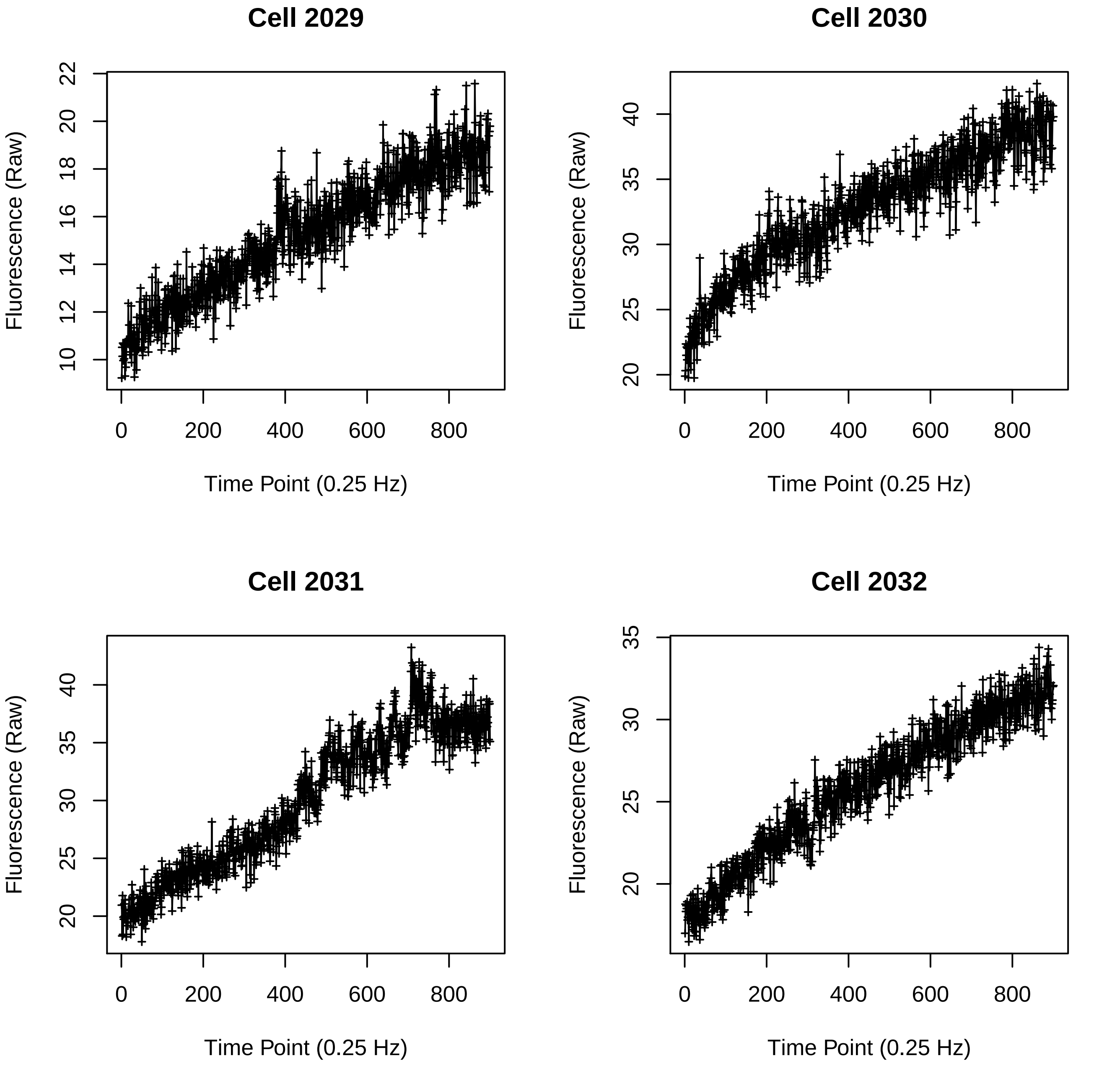
Cell 2029
Cell 2030
Cell 2031
Cell 2032
Fluorescence (Raw)
Time Point (0.25 Hz)
0
200
400
600
800
10
12
14
16
18
20
22
25
30
35
40

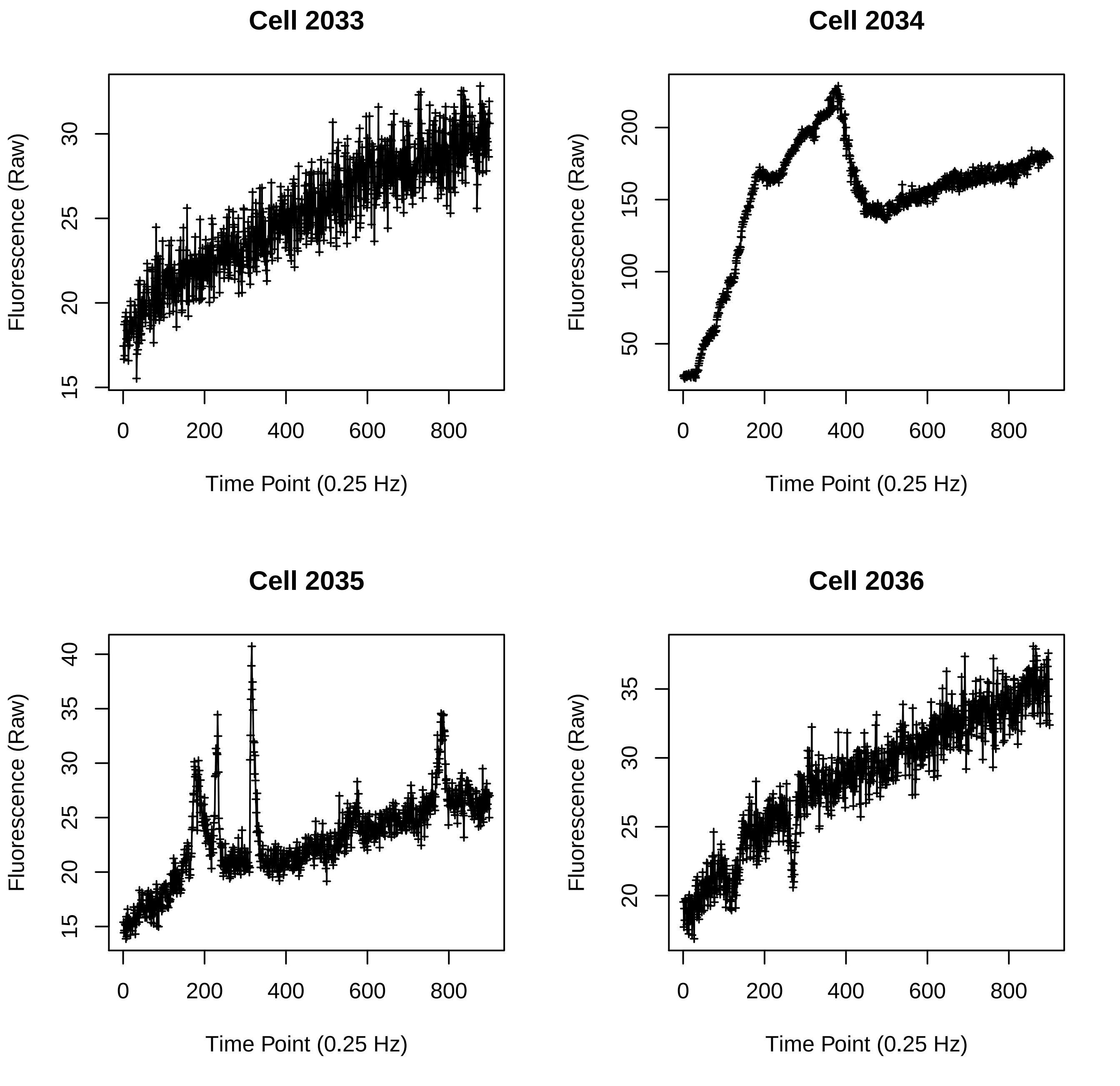

Cell 2033
Cell 2034
Cell 2035
Cell 2036
Fluorescence (Raw)
Time Point (0.25 Hz)
0
200
400
600
800
15
20
25
30
35
40
50
100
150
200

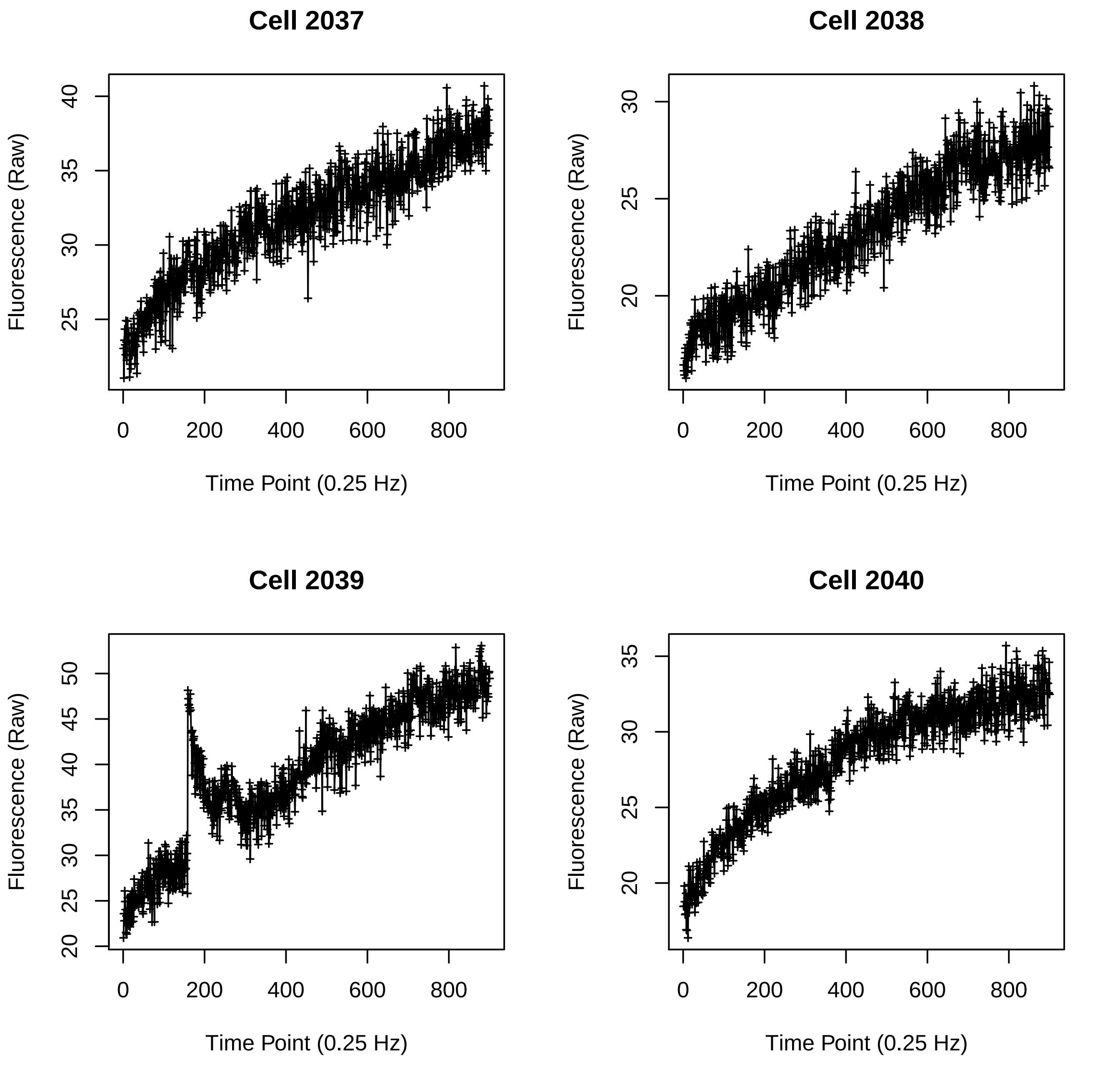
Cell 2037
Cell 2038
Cell 2039
Cell 2040
Fluorescence (Raw)
Time Point (0.25 Hz)
0
200
400
600
800
20
25
30
35
40
45
50

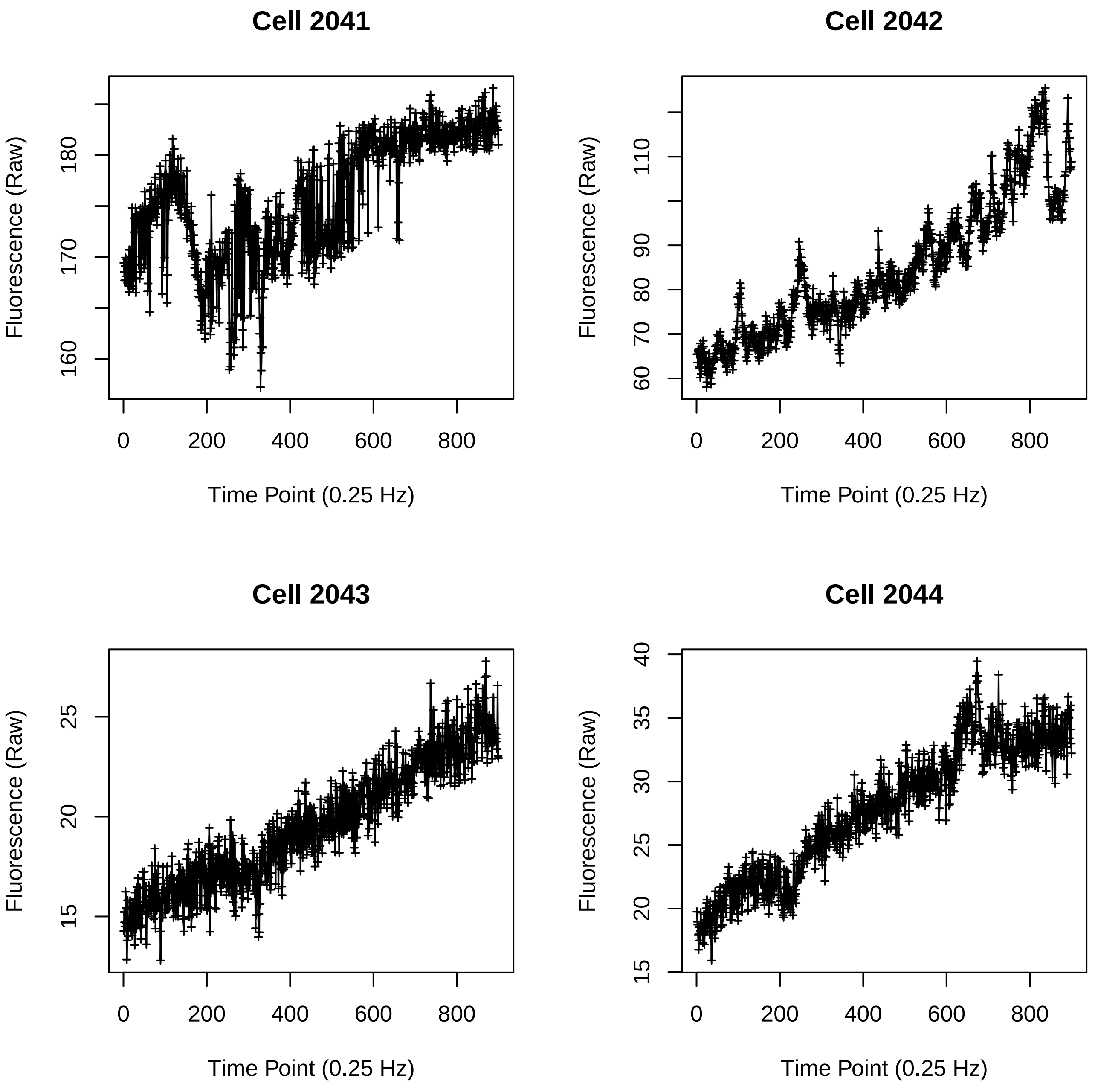

Cell 2041
Cell 2042
Cell 2043
Cell 2044
Fluorescence (Raw)
Time Point (0.25 Hz)
160
170
180
60
70
80
90
110
15
20
25
30
35
40
0
200
400
600
800

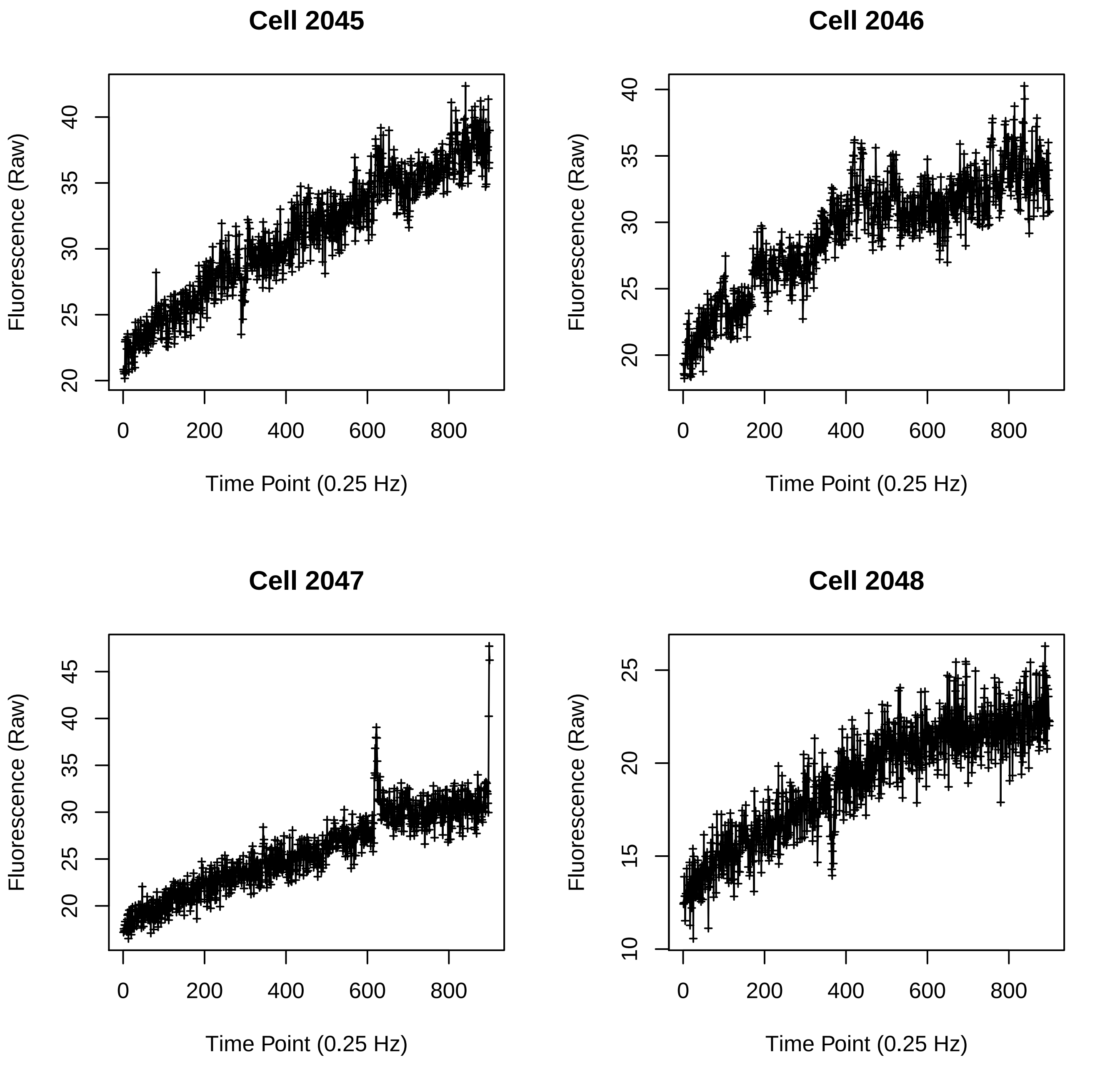
Cell 2045
Cell 2046
Cell 2047
Cell 2048
Fluorescence (Raw)
Time Point (0.25 Hz)
0
200
400
600
800
10
15
20
25
30
35
40
45

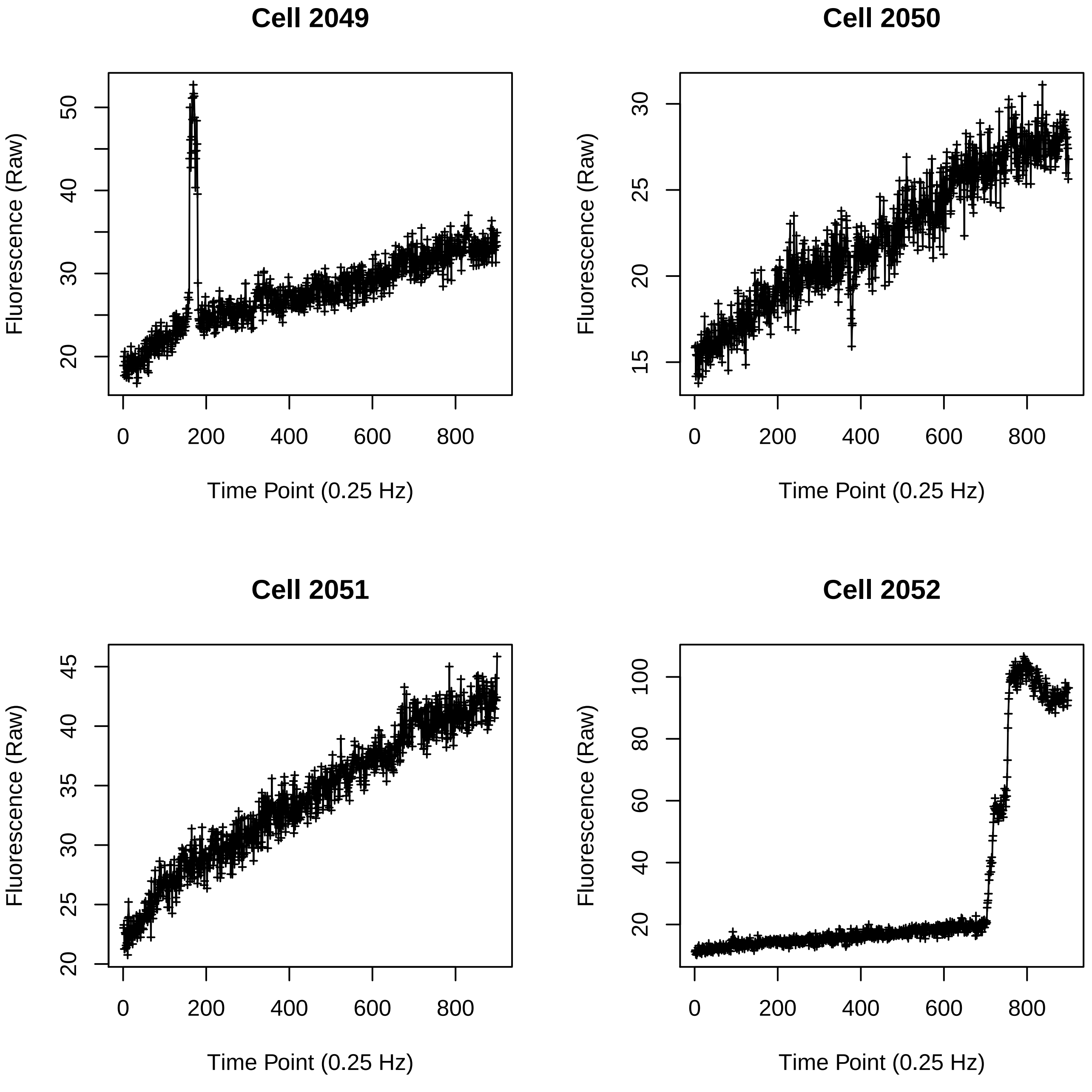
Cell 2049
Cell 2050
Cell 2051
Cell 2052
Fluorescence (Raw)
Time Point (0.25 Hz)
0
200
400
600
800
20
30
40
50
15
20
25
30
25
35
45
60
80
100

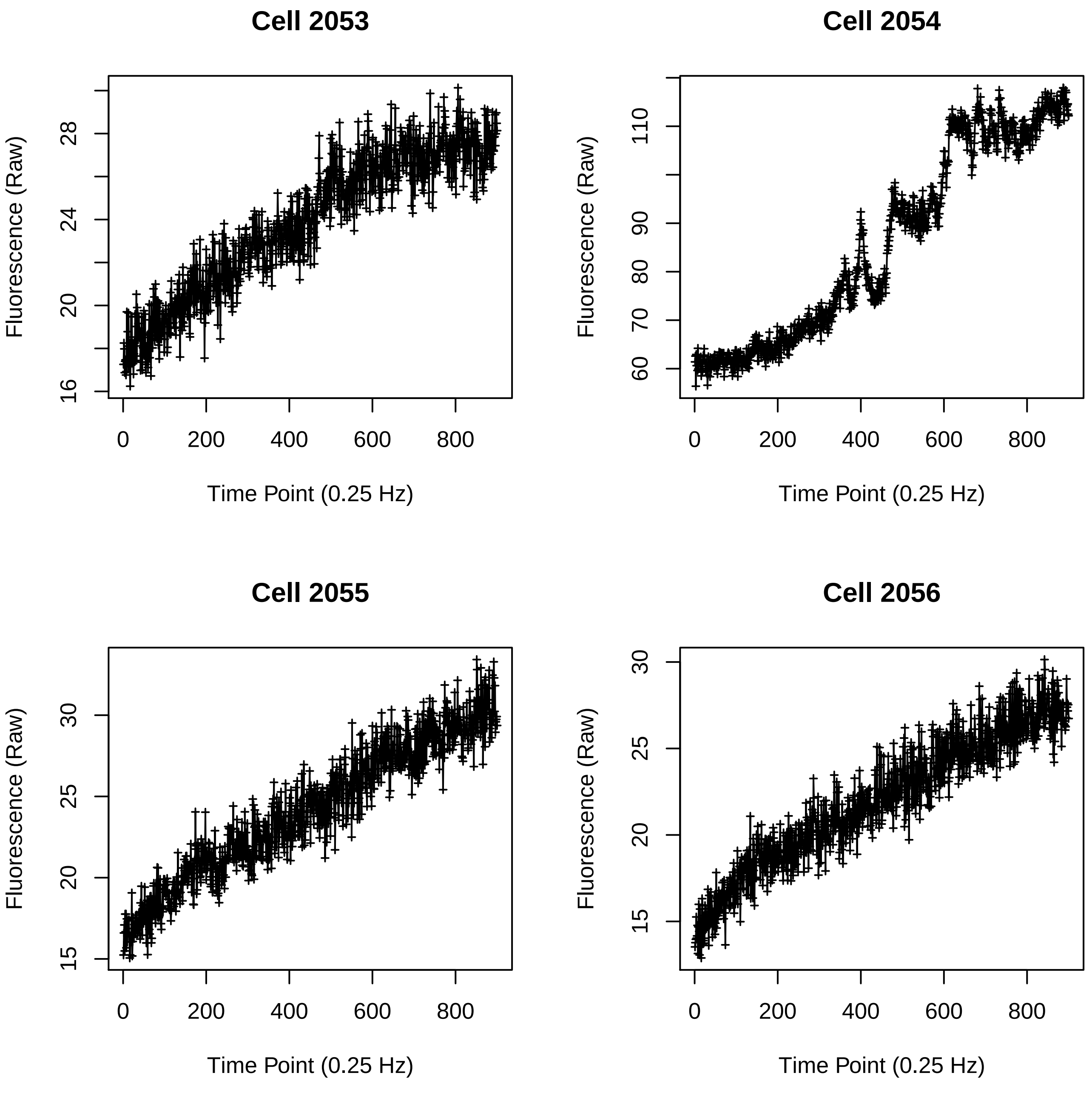

Cell 2053
Fluorescence (Raw)
16
20
24
28
0
200
400
600
800
Time Point (0.25 Hz)
Cell 2054
Fluorescence (Raw)
60
70
80
90
110
0
200
400
600
800
Time Point (0.25 Hz)
Cell 2055
Fluorescence (Raw)
15
20
25
30
0
200
400
600
800
Time Point (0.25 Hz)
Cell 2056
Fluorescence (Raw)
15
20
25
30
0
200
400
600
800
Time Point (0.25 Hz)

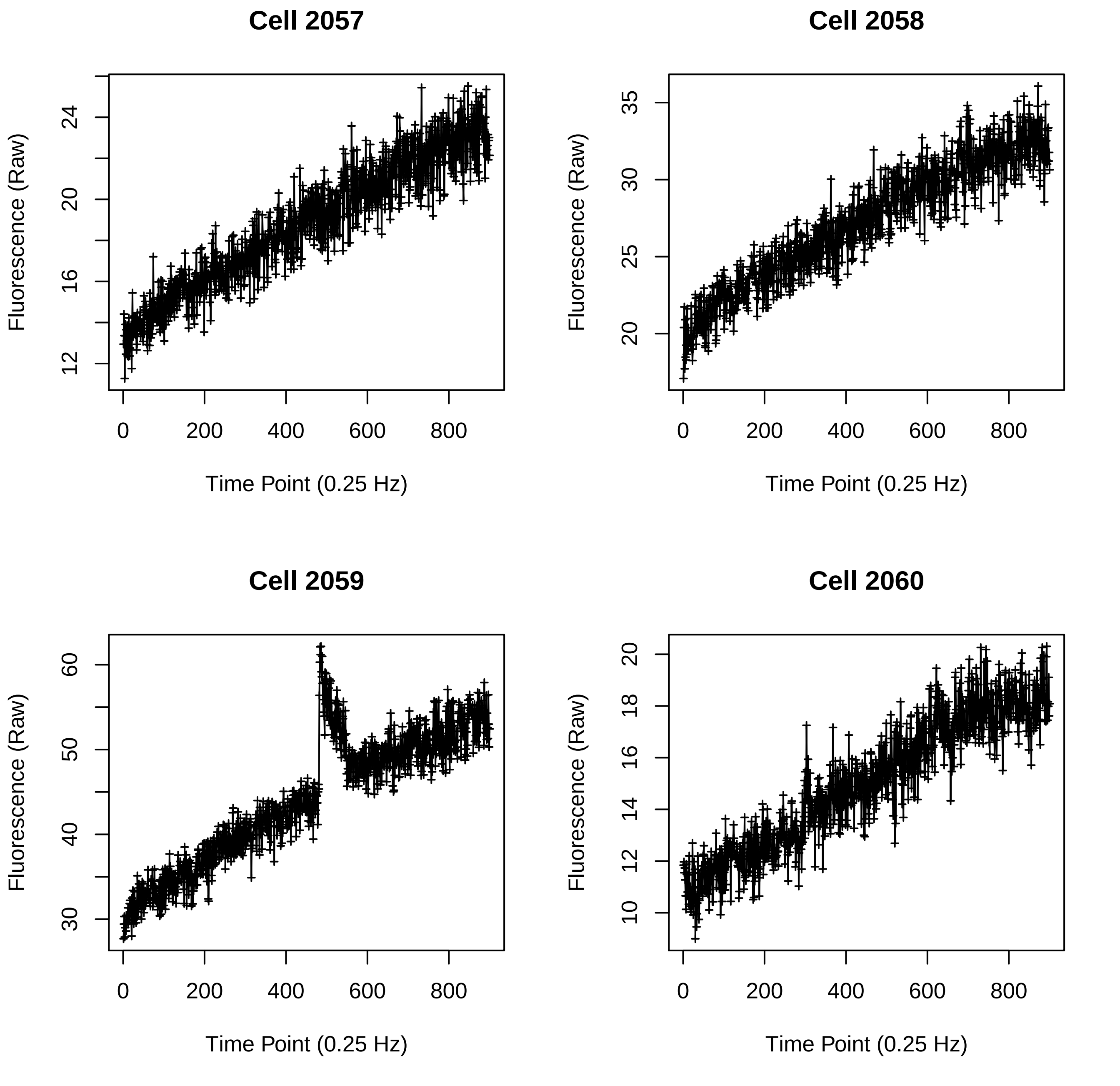

Cell 2057
Cell 2058
Cell 2059
Cell 2060
Fluorescence (Raw)
Time Point (0.25 Hz)
0
200
400
600
800

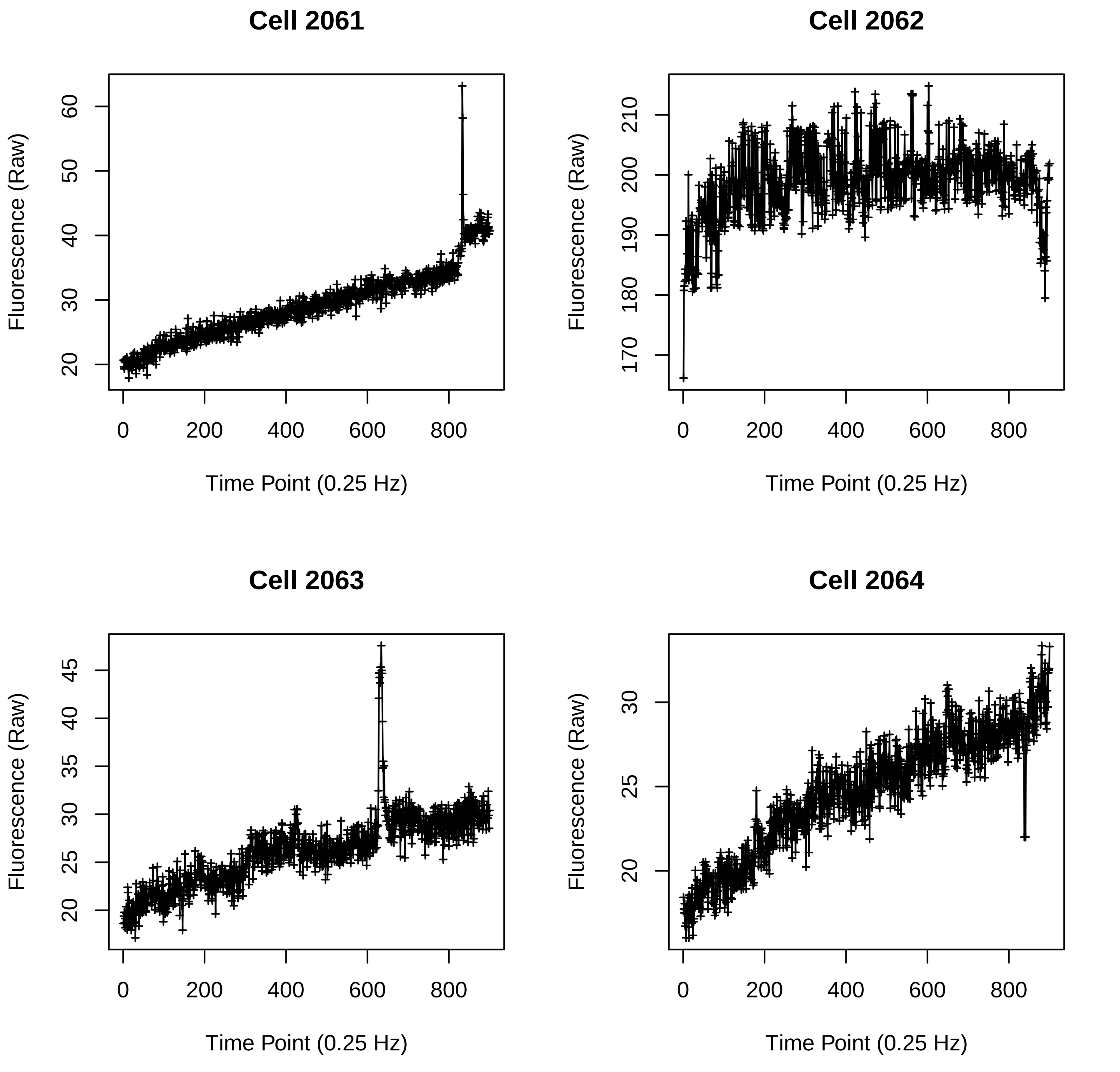

Cell 2061
Cell 2062
Cell 2063
Cell 2064
Fluorescence (Raw)
Time Point (0.25 Hz)
0
200
400
600
800
20
30
40
50
60
170
180
190
200
210
25
35
45

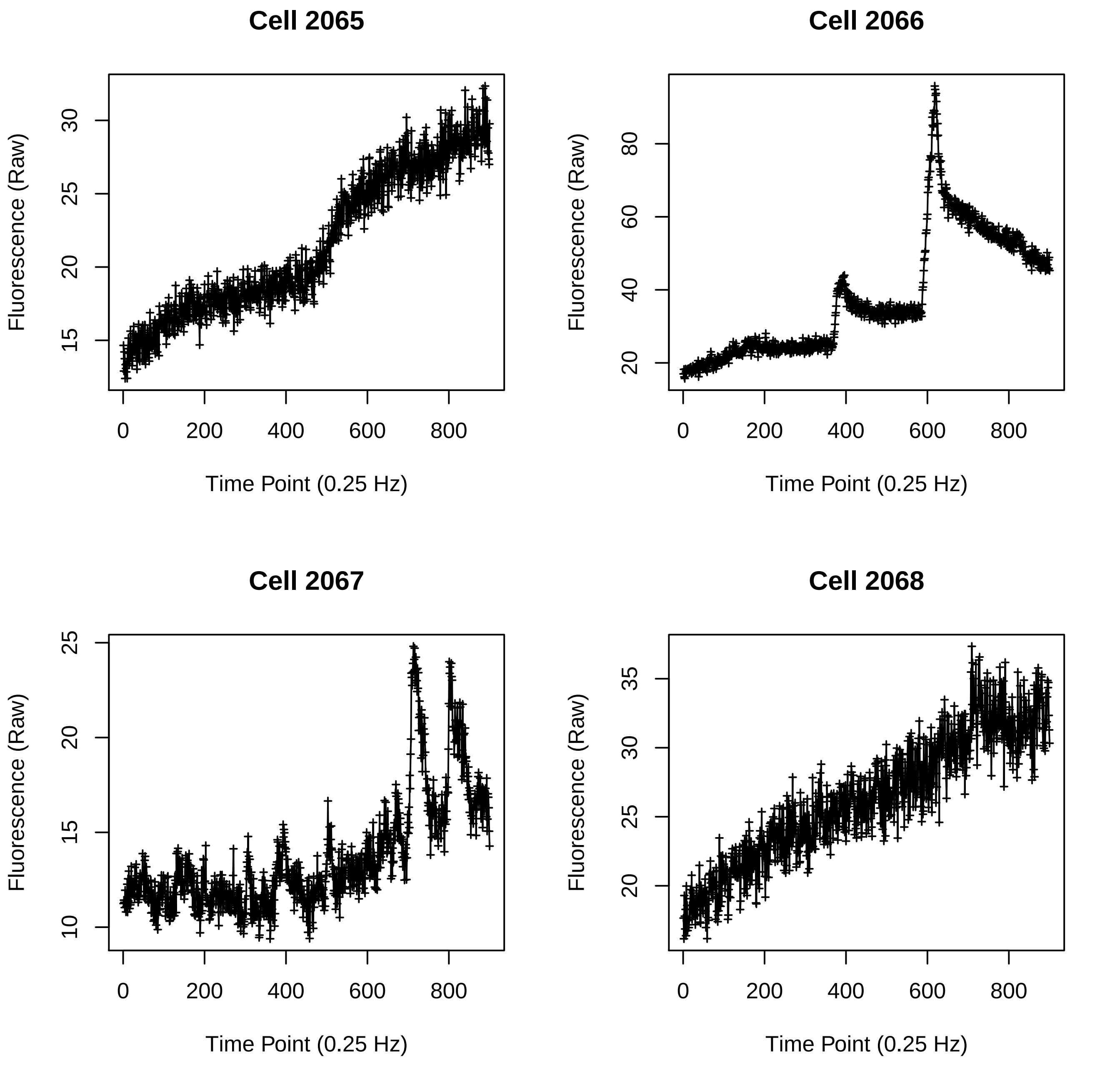

Cell 2065
Cell 2066
Cell 2067
Cell 2068
Fluorescence (Raw)
Time Point (0.25 Hz)
0
200
400
600
800
15
20
25
30
40
60
80
10
35

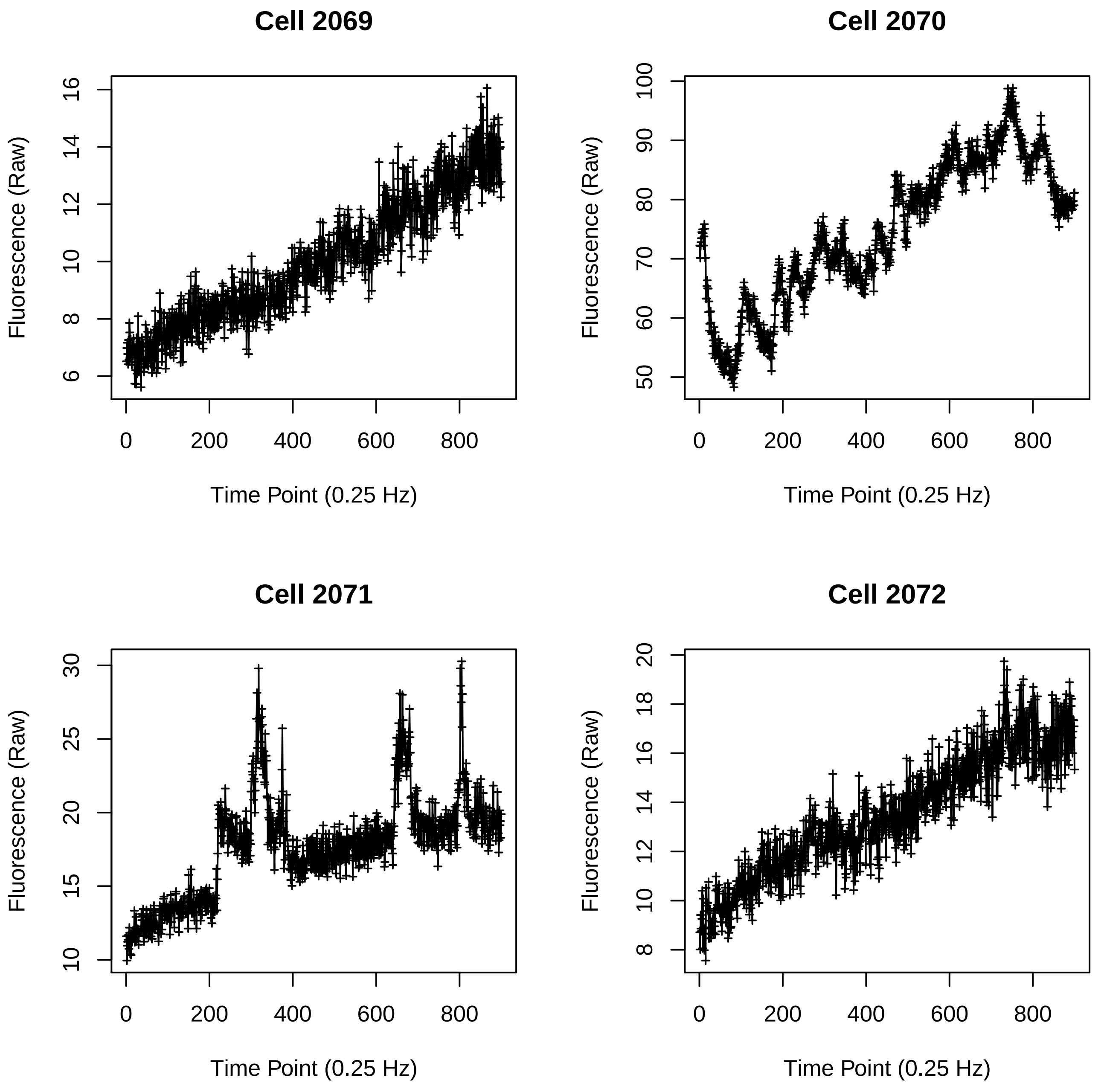
Cell 2069
Cell 2070
Cell 2071
Cell 2072
Fluorescence (Raw)
Time Point (0.25 Hz)
0
200
400
600
800
6
8
10
12
14
16
50
60
70
80
90
100
10
15
20
25
30
8
10
12
14
16
18
20

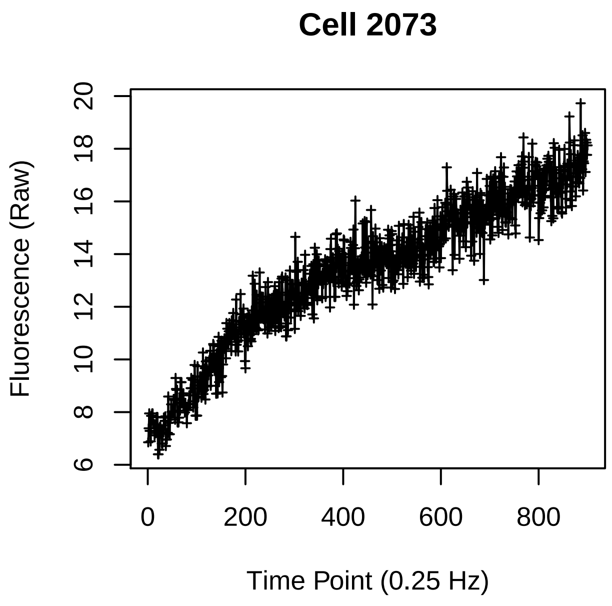
Cell 2073
Fluorescence (Raw)
6
8
10
12
14
16
18
20
0
200
400
600
800
Time Point (0.25 Hz)

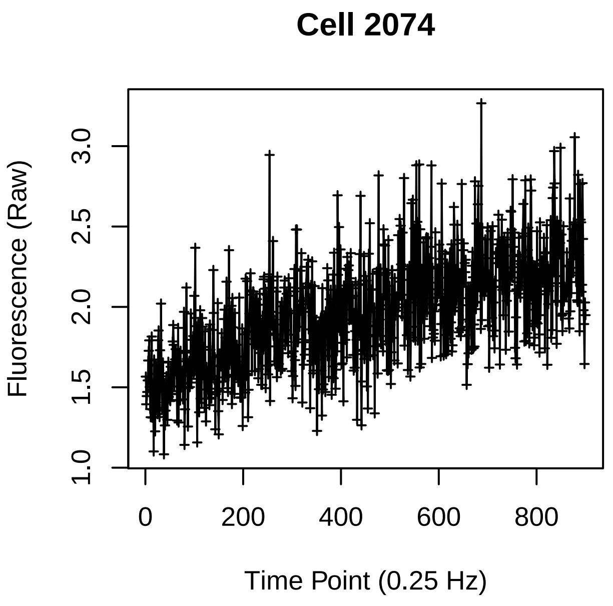
Cell 2074
Fluorescence (Raw)
1.0
1.5
2.0
2.5
3.0
0
200
400
600
800
Time Point (0.25 Hz)

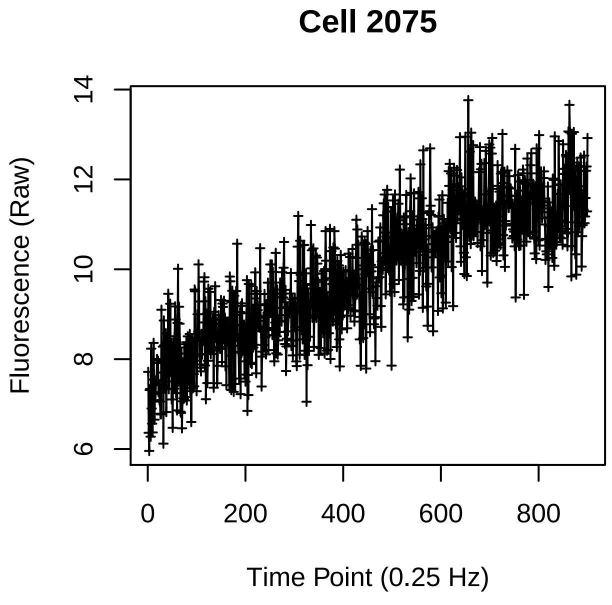
Cell 2075
Fluorescence (Raw)
6
8
10
12
14
0
200
400
600
800
Time Point (0.25 Hz)

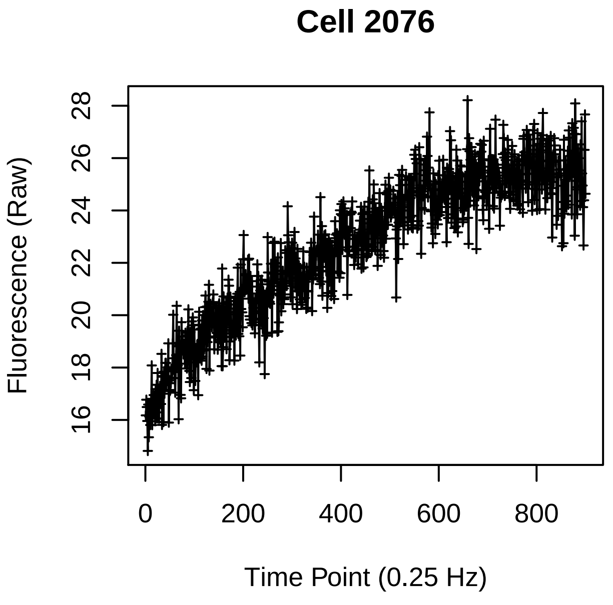
Cell 2076
Fluorescence (Raw)
16
18
20
22
24
26
28
0
200
400
600
800
Time Point (0.25 Hz)

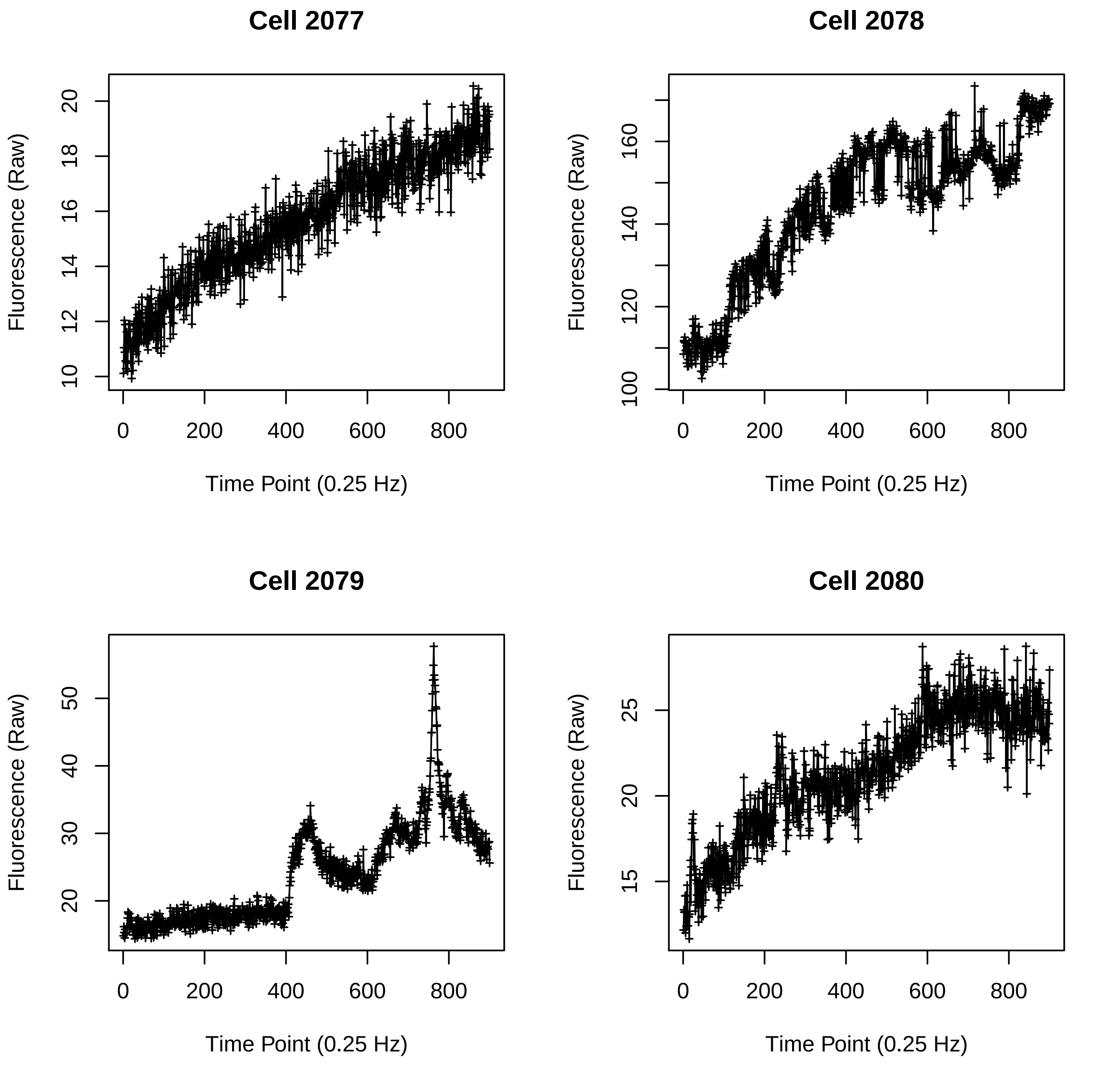

Cell 2077
Cell 2078
Cell 2079
Cell 2080
Fluorescence (Raw)
Time Point (0.25 Hz)
0
200
400
600
800
10
12
14
16
18
20
100
120
140
160
30
40
50
15
25

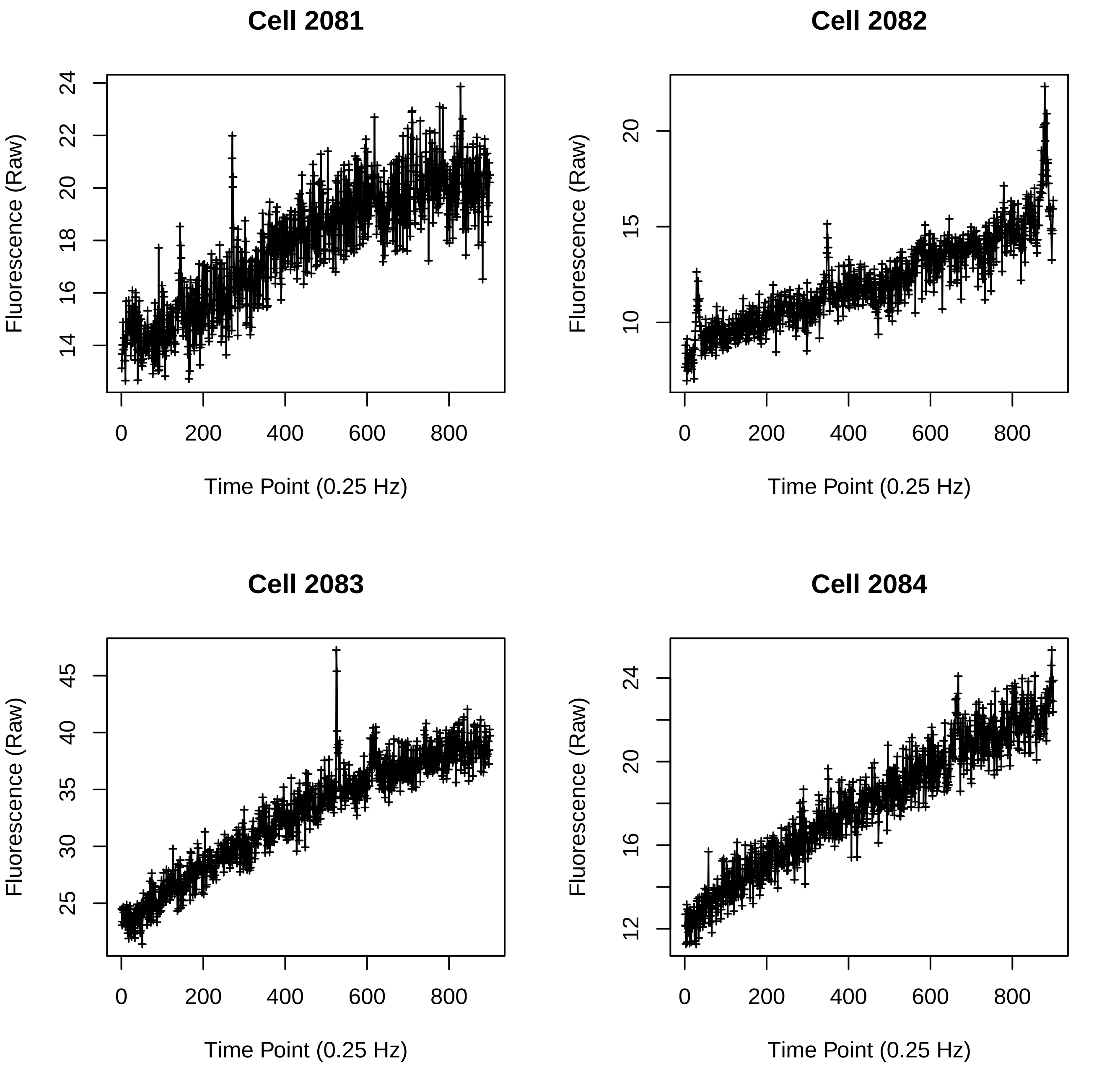
Cell 2081
Fluorescence (Raw)
14
16
18
20
22
24
0
200
400
600
800
Time Point (0.25 Hz)
Cell 2082
Fluorescence (Raw)
10
15
20
0
200
400
600
800
Time Point (0.25 Hz)
Cell 2083
Fluorescence (Raw)
25
30
35
40
45
0
200
400
600
800
Time Point (0.25 Hz)
Cell 2084
Fluorescence (Raw)
12
16
20
24
0
200
400
600
800
Time Point (0.25 Hz)

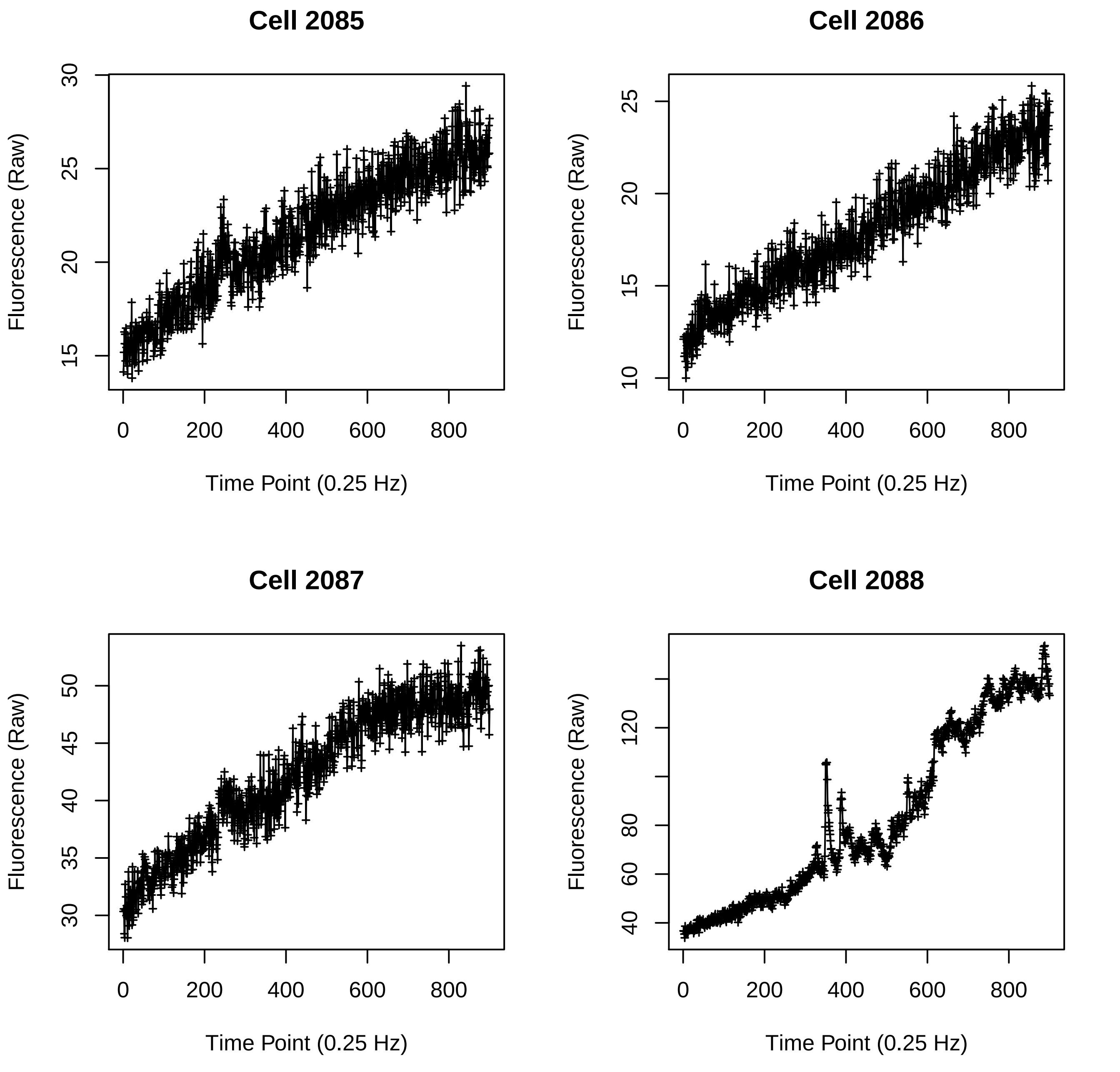
Cell 2085
Cell 2086
Cell 2087
Cell 2088
Fluorescence (Raw)
Time Point (0.25 Hz)
0
200
400
600
800
15
20
25
30
10
35
40
45
50
60
80
120

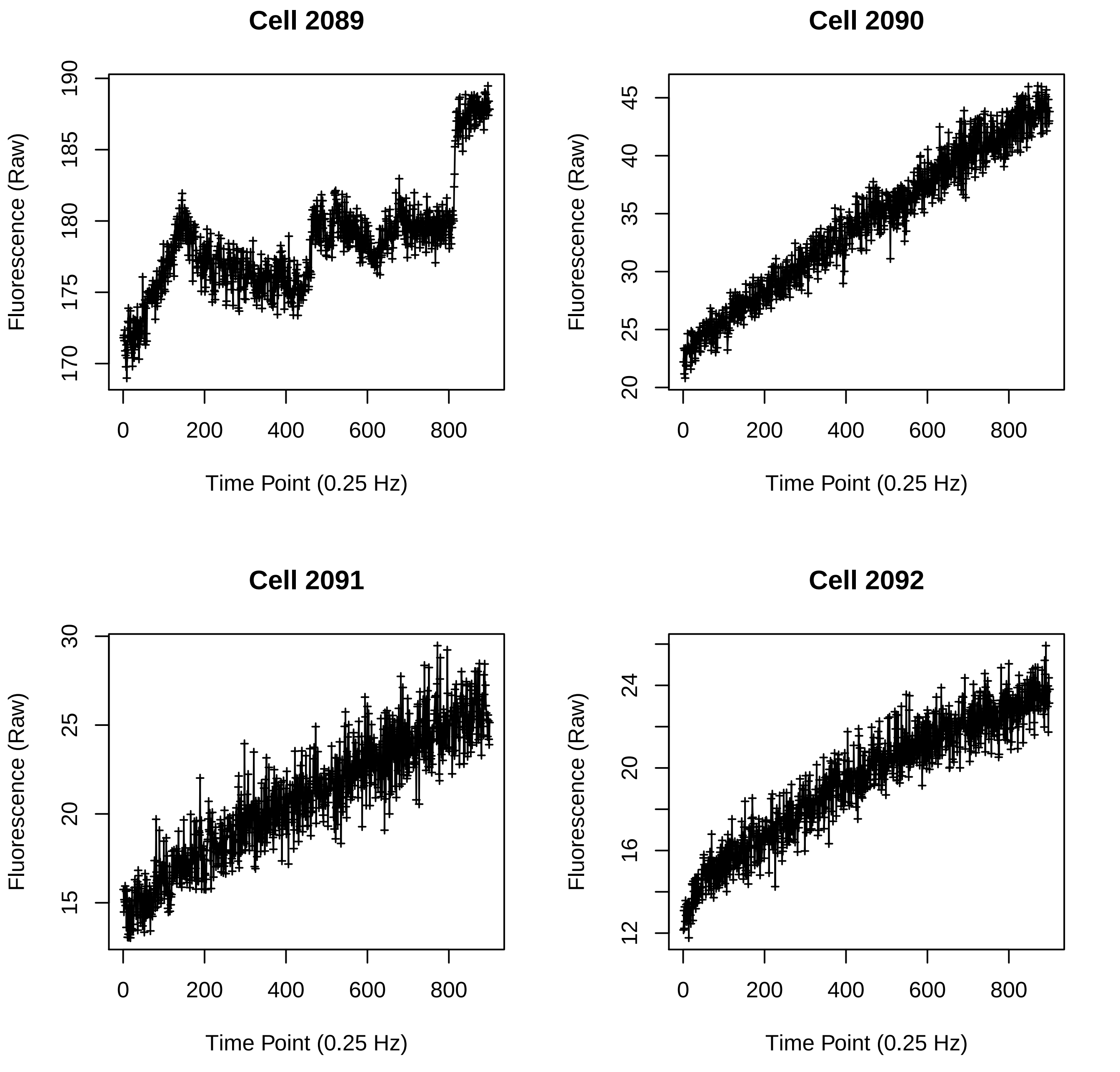
Cell 2089
Cell 2090
Cell 2091
Cell 2092
Fluorescence (Raw)
Time Point (0.25 Hz)
0
200
400
600
800
170
175
180
185
190
20
25
30
35
40
45
15
20
25
30
12
16
20
24

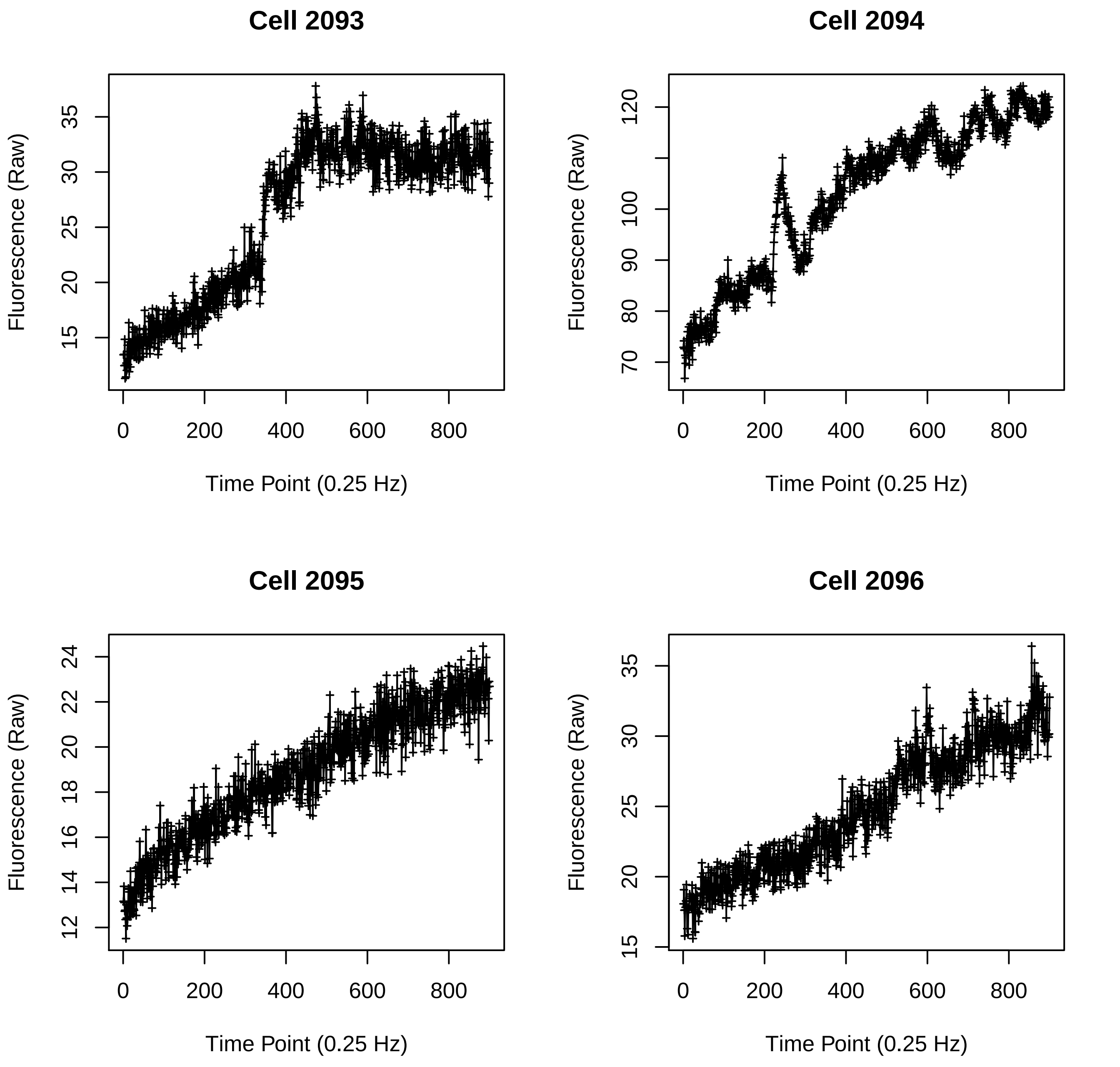
Cell 2093
Fluorescence (Raw)
15 20 25 30 35
0 200 400 600 800
Time Point (0.25 Hz)
Cell 2094
Fluorescence (Raw)
70 80 90 100 120
0 200 400 600 800
Time Point (0.25 Hz)
Cell 2095
Fluorescence (Raw)
12 14 16 18 20 22 24
0 200 400 600 800
Time Point (0.25 Hz)
Cell 2096
Fluorescence (Raw)
15 20 25 30 35
0 200 400 600 800
Time Point (0.25 Hz)

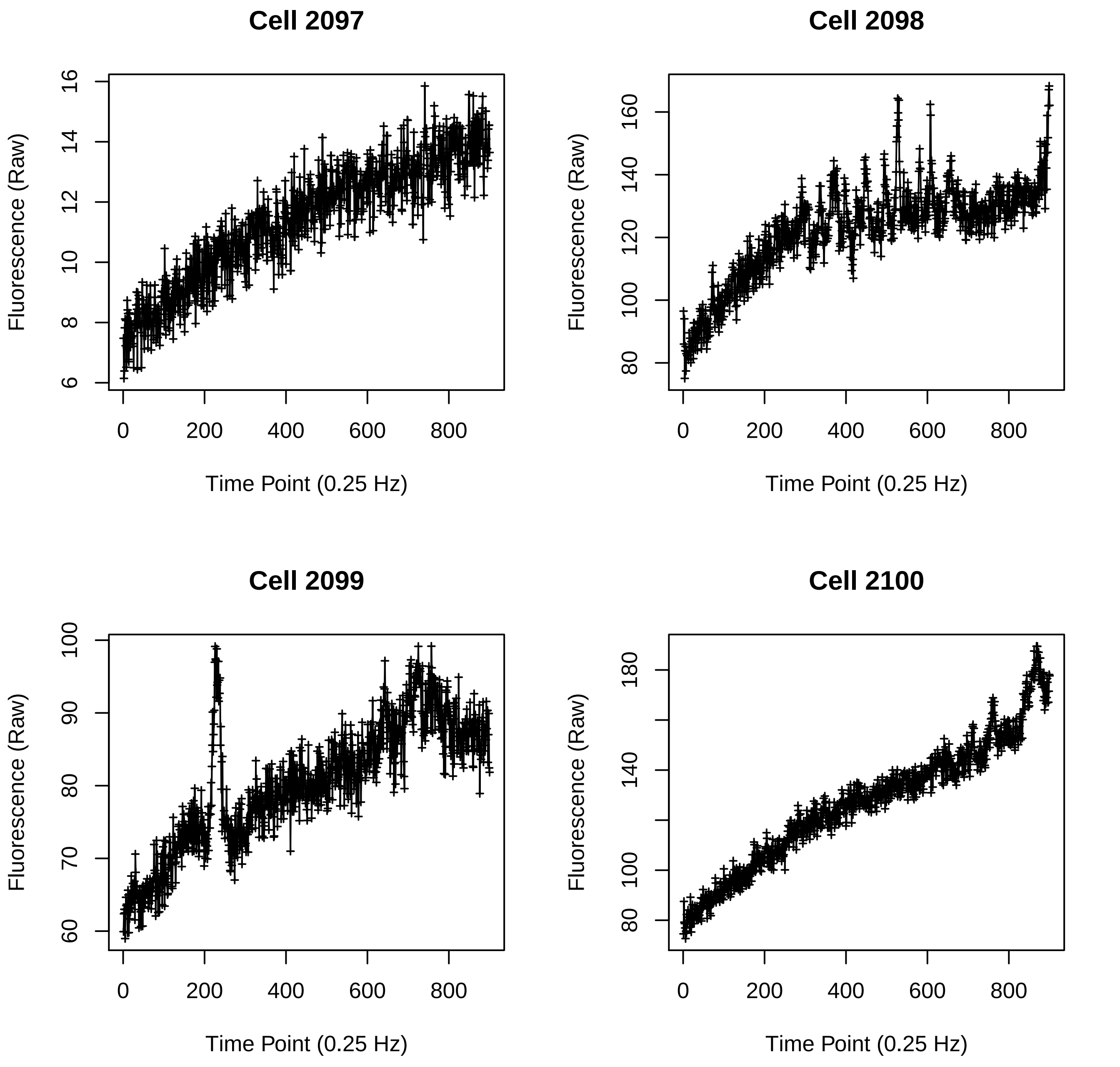
Cell 2097
Cell 2098
Cell 2099
Cell 2100
Fluorescence (Raw)
Time Point (0.25 Hz)
0
200
400
600
800
6
8
10
12
14
16
80
100
120
140
160
60
70
80
90
100
80
100
140
180

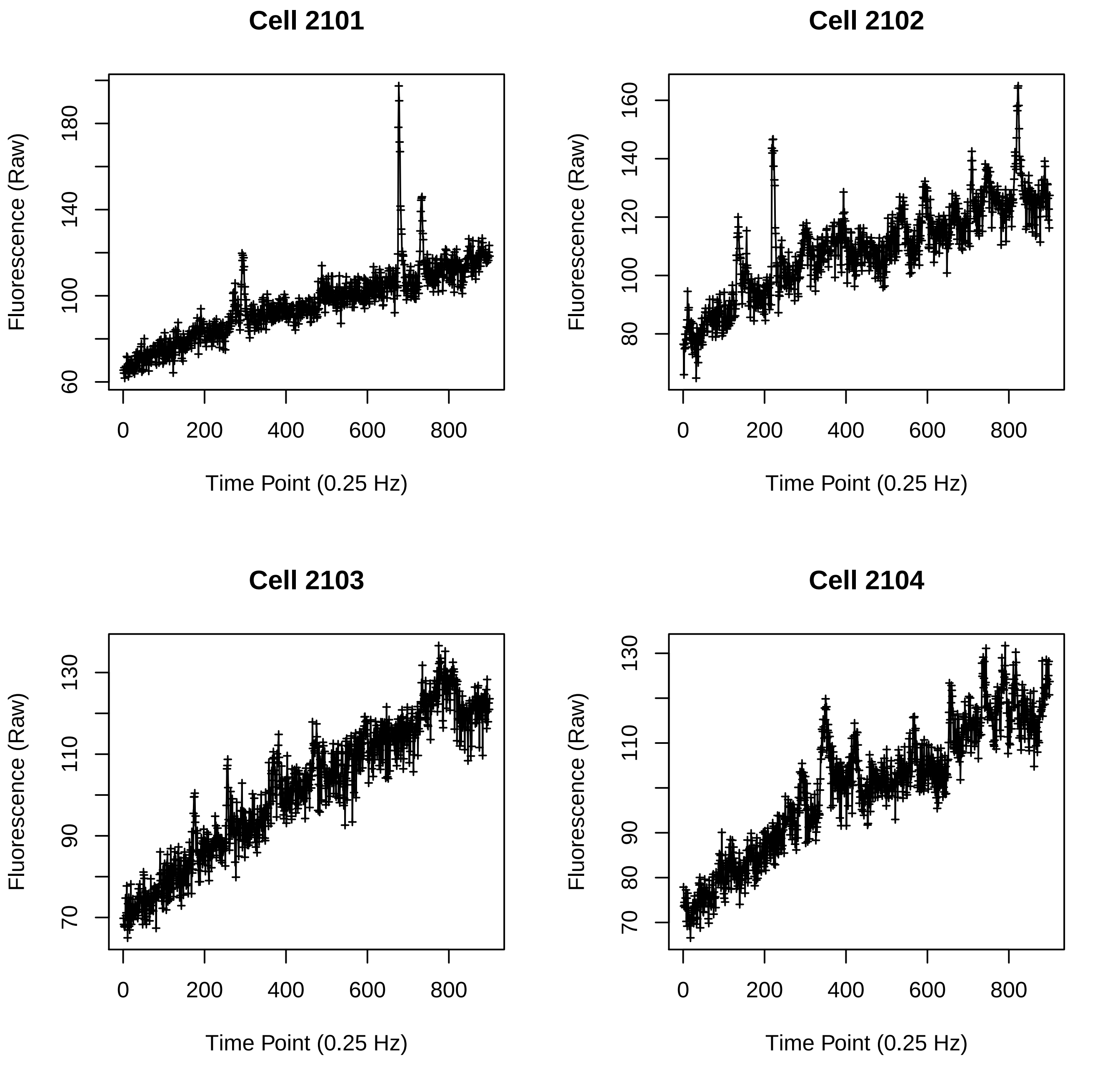
Cell 2101
Cell 2102
Cell 2103
Cell 2104
Fluorescence (Raw)
Time Point (0.25 Hz)
0
200
400
600
800
60
100
140
180
80
120
160
70
90
110
130

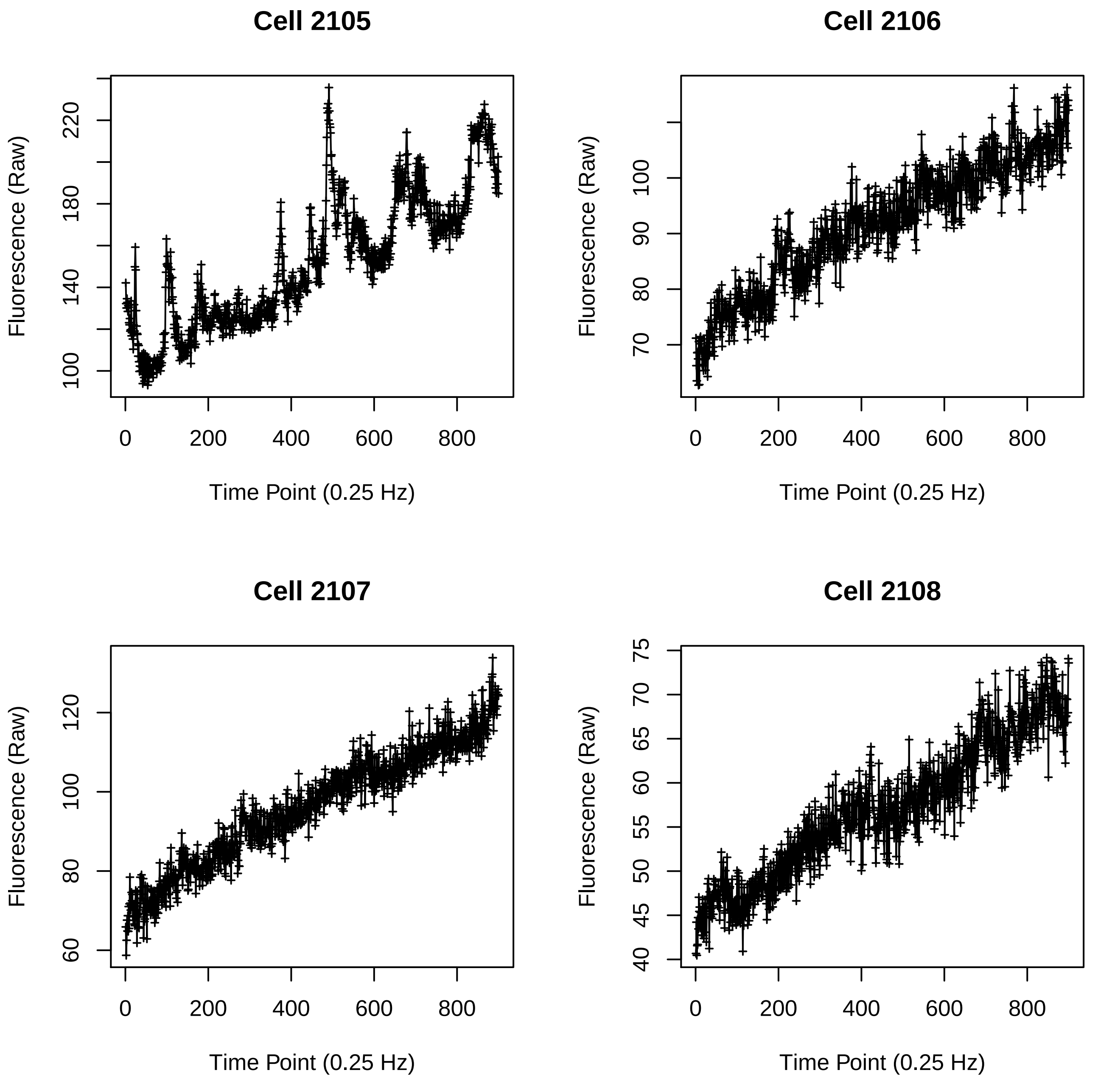
Cell 2105
Cell 2106
Cell 2107
Cell 2108
Fluorescence (Raw)
Time Point (0.25 Hz)
0
200
400
600
800
100
140
180
220
70
80
90
100
60
80
100
120
40
45
50
55
60
65
70
75

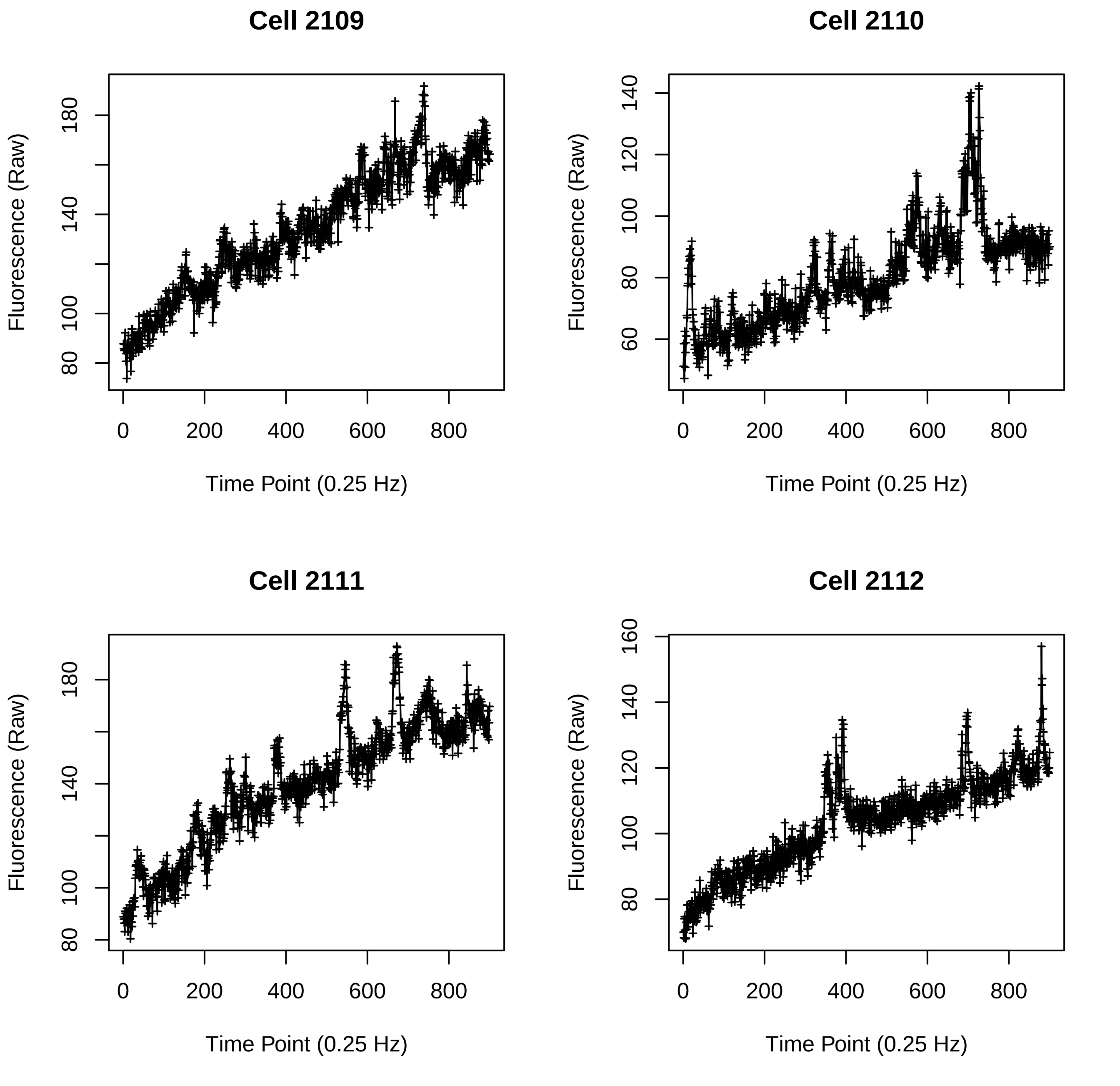
Cell 2109
Cell 2110
Cell 2111
Cell 2112
Fluorescence (Raw)
Time Point (0.25 Hz)
0
200
400
600
800
80
100
140
180
60
120
160

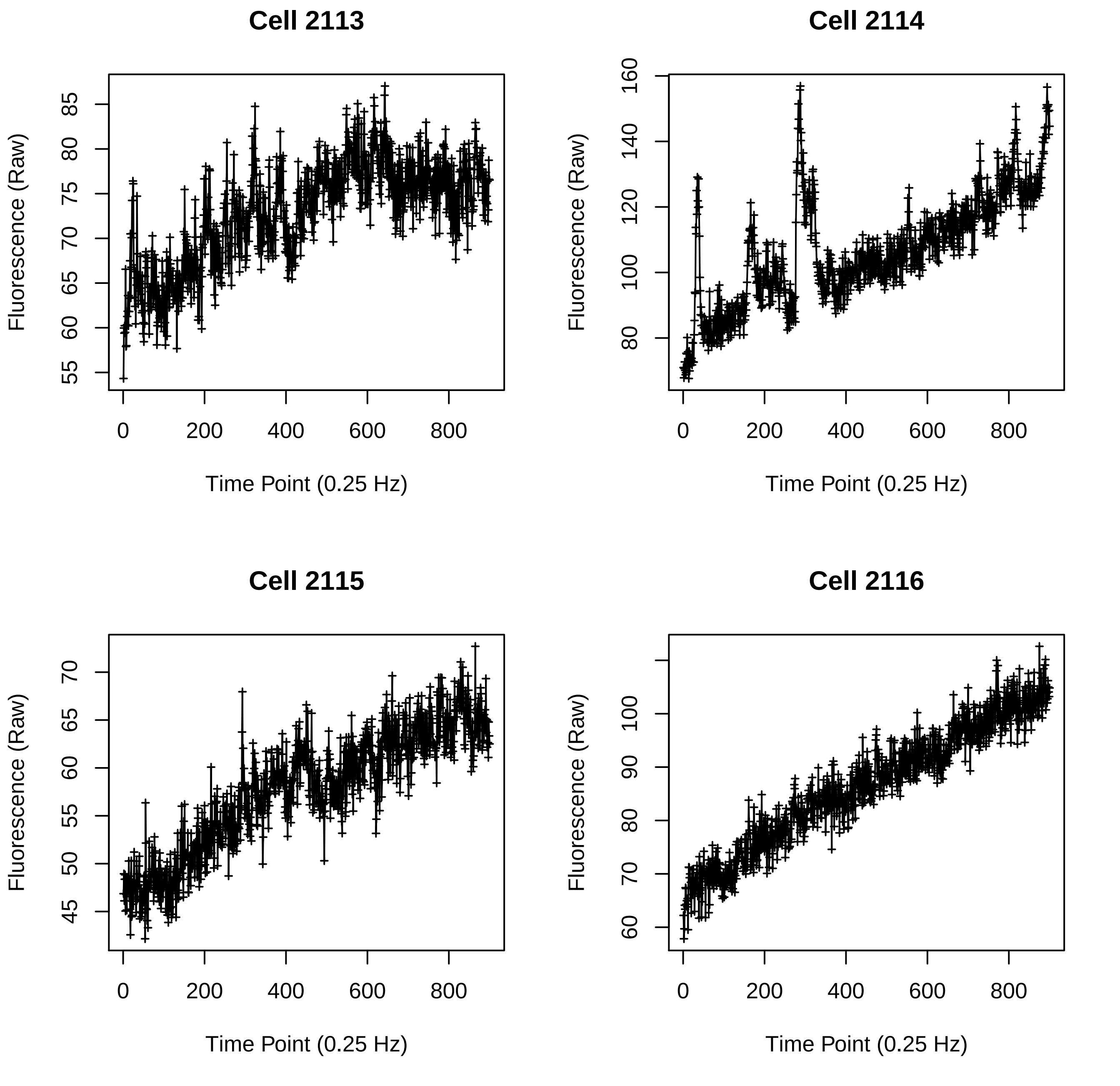
Cell 2113
Fluorescence (Raw)
55
60
65
70
75
80
85
0
200
400
600
800
Time Point (0.25 Hz)
Cell 2114
Fluorescence (Raw)
80
100
120
140
160
0
200
400
600
800
Time Point (0.25 Hz)
Cell 2115
Fluorescence (Raw)
45
50
55
60
65
70
0
200
400
600
800
Time Point (0.25 Hz)
Cell 2116
Fluorescence (Raw)
60
70
80
90
100
0
200
400
600
800
Time Point (0.25 Hz)

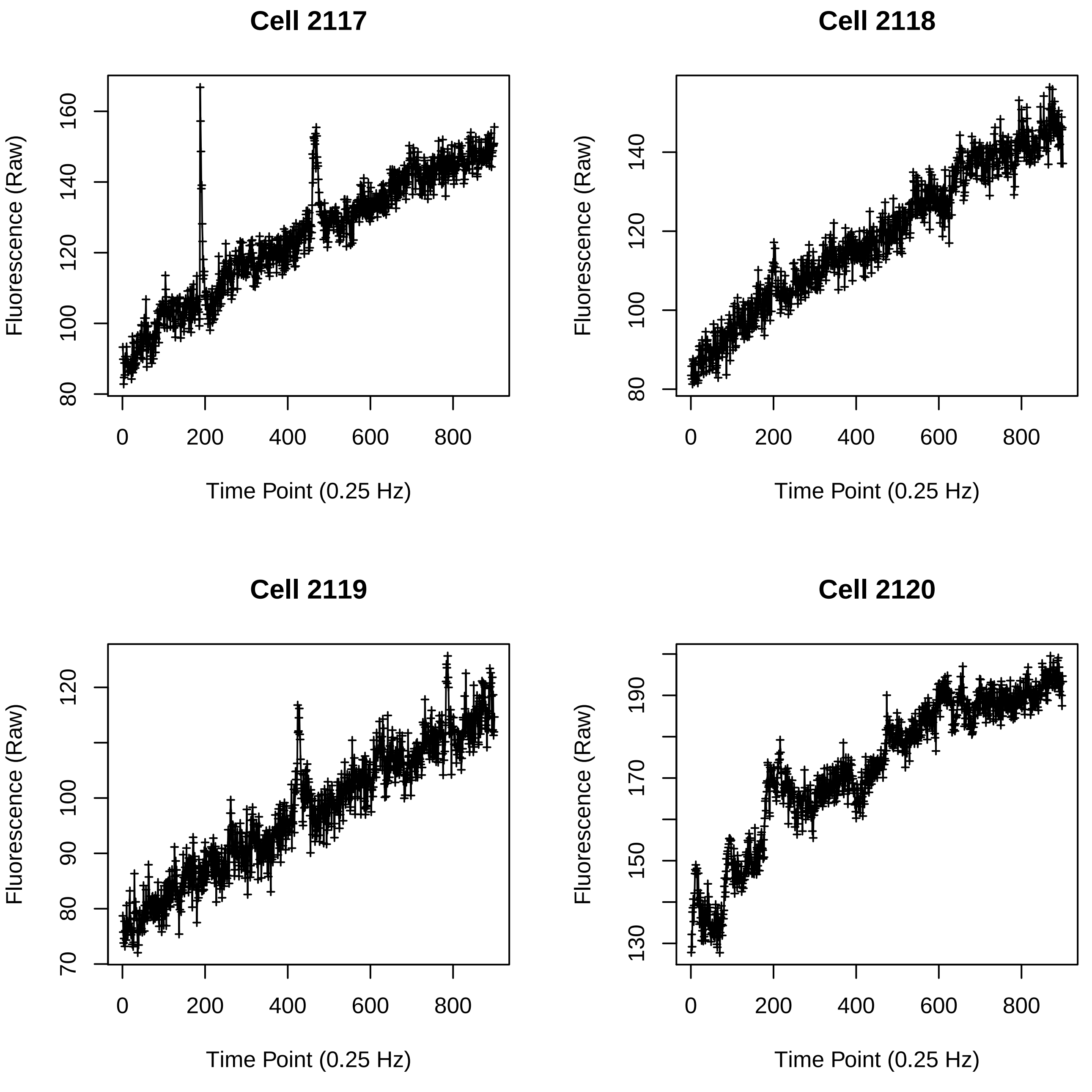
Cell 2117
Cell 2118
Cell 2119
Cell 2120
Fluorescence (Raw)
Time Point (0.25 Hz)
0
200
400
600
800
80
100
120
140
160
70
90
130
150
170
190

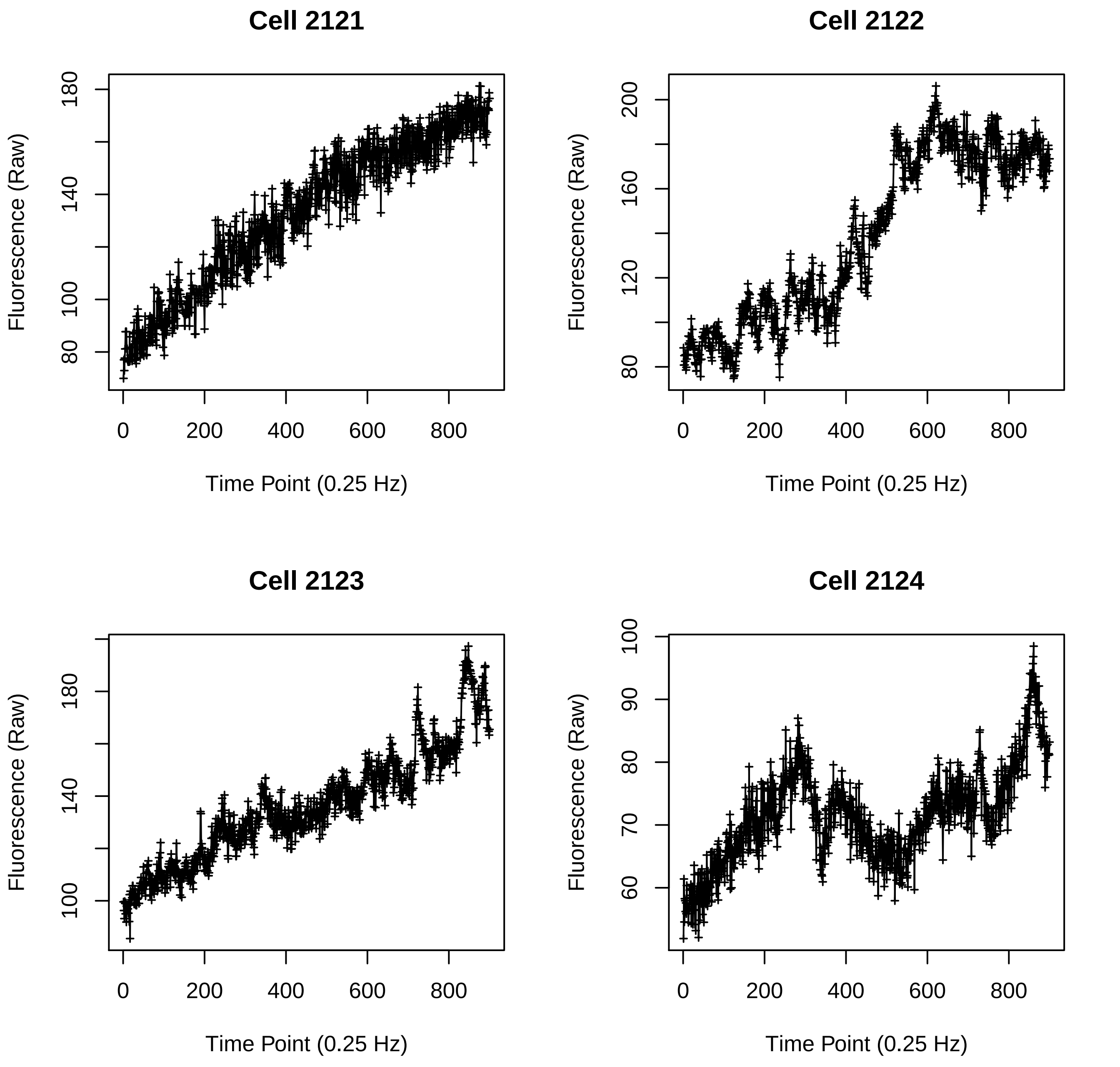
Cell 2121
Fluorescence (Raw)
80
100
140
180
0
200
400
600
800
Time Point (0.25 Hz)
Cell 2122
Fluorescence (Raw)
80
120
160
200
0
200
400
600
800
Time Point (0.25 Hz)
Cell 2123
Fluorescence (Raw)
100
140
180
0
200
400
600
800
Time Point (0.25 Hz)
Cell 2124
Fluorescence (Raw)
60
70
80
90
100
0
200
400
600
800
Time Point (0.25 Hz)

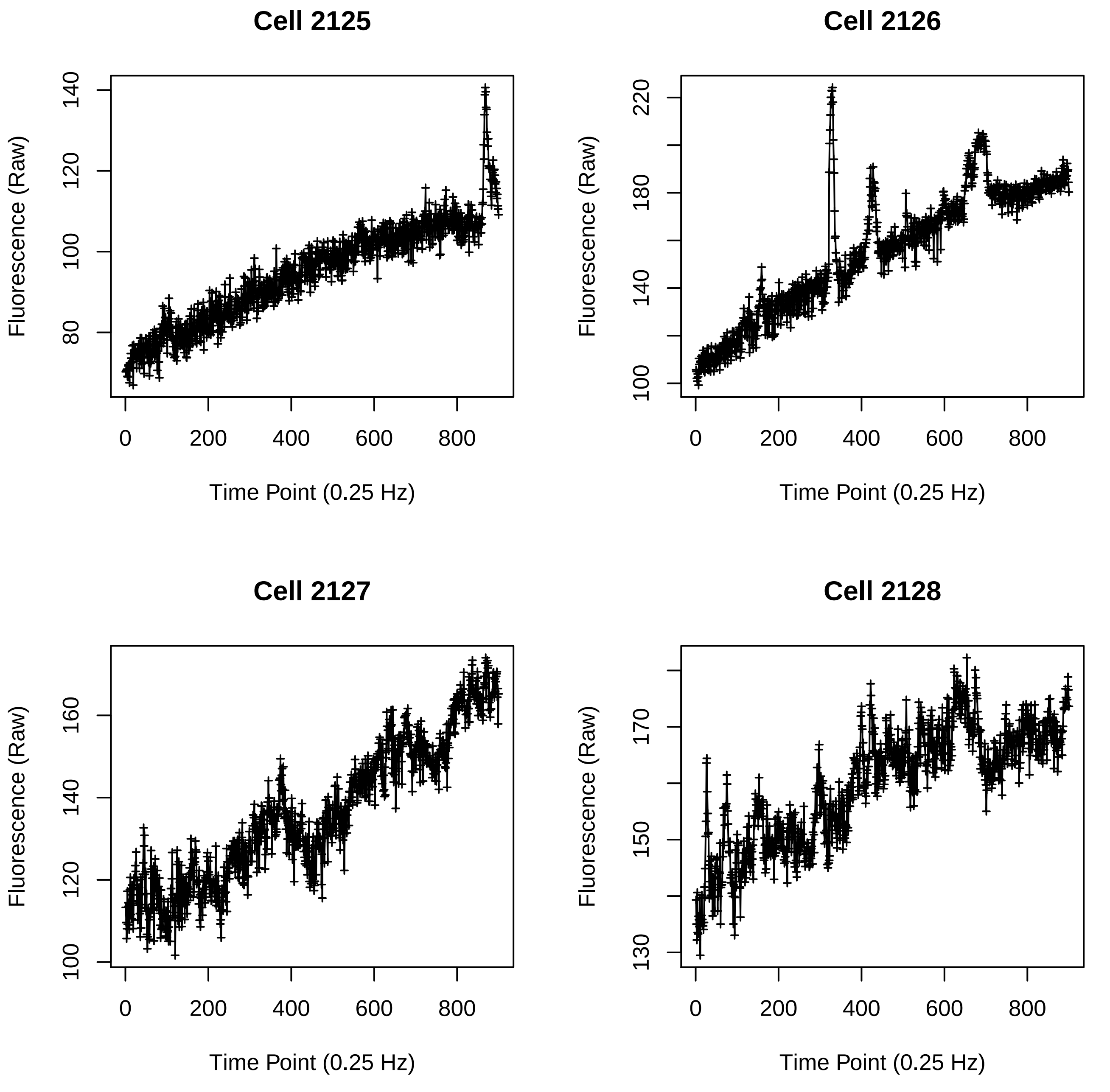
Cell 2125
Fluorescence (Raw)
80
100
120
140
0
200
400
600
800
Time Point (0.25 Hz)
Cell 2126
Fluorescence (Raw)
100
140
180
220
0
200
400
600
800
Time Point (0.25 Hz)
Cell 2127
Fluorescence (Raw)
100
120
140
160
0
200
400
600
800
Time Point (0.25 Hz)
Cell 2128
Fluorescence (Raw)
130
150
170
0
200
400
600
800
Time Point (0.25 Hz)

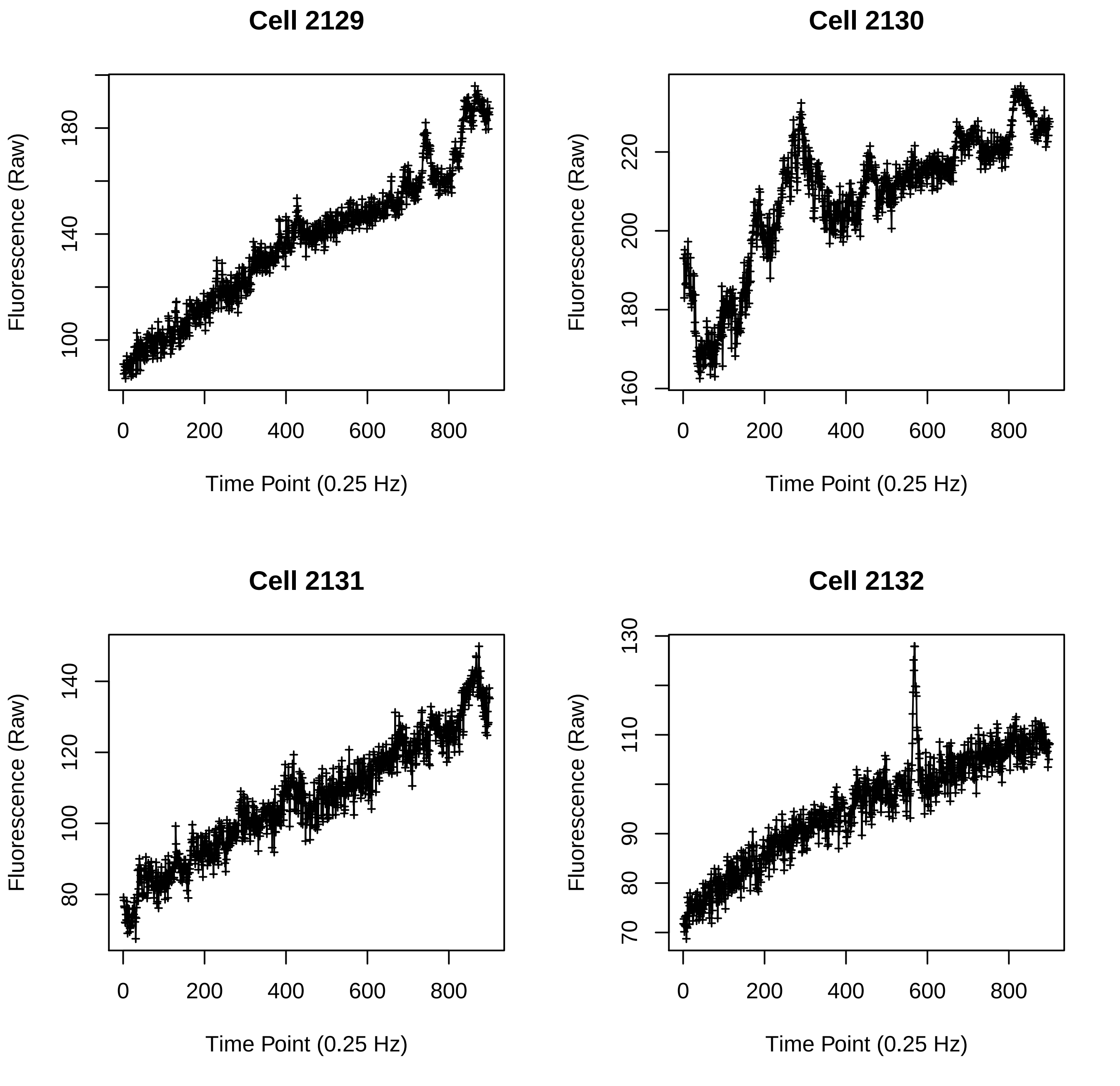
Cell 2129
Fluorescence (Raw)
100
140
180
0
200
400
600
800
Time Point (0.25 Hz)
Cell 2130
Fluorescence (Raw)
160
180
200
220
0
200
400
600
800
Time Point (0.25 Hz)
Cell 2131
Fluorescence (Raw)
80
100
120
140
0
200
400
600
800
Time Point (0.25 Hz)
Cell 2132
Fluorescence (Raw)
70
80
90
110
130
0
200
400
600
800
Time Point (0.25 Hz)

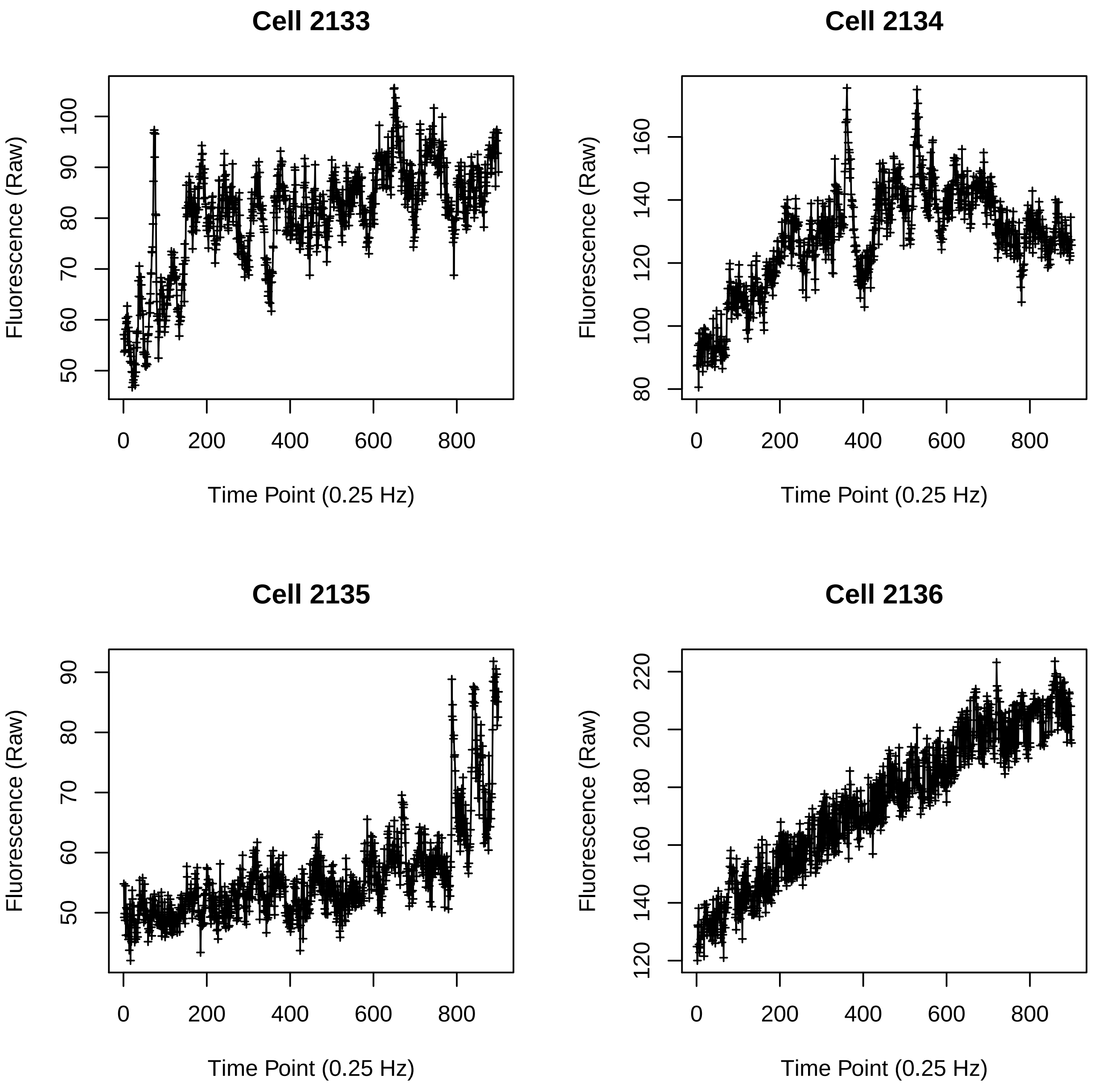
Cell 2133
Cell 2134
Cell 2135
Cell 2136
Fluorescence (Raw)
Time Point (0.25 Hz)
0
200
400
600
800
50
60
70
80
90
100
120
140
160
180
200
220

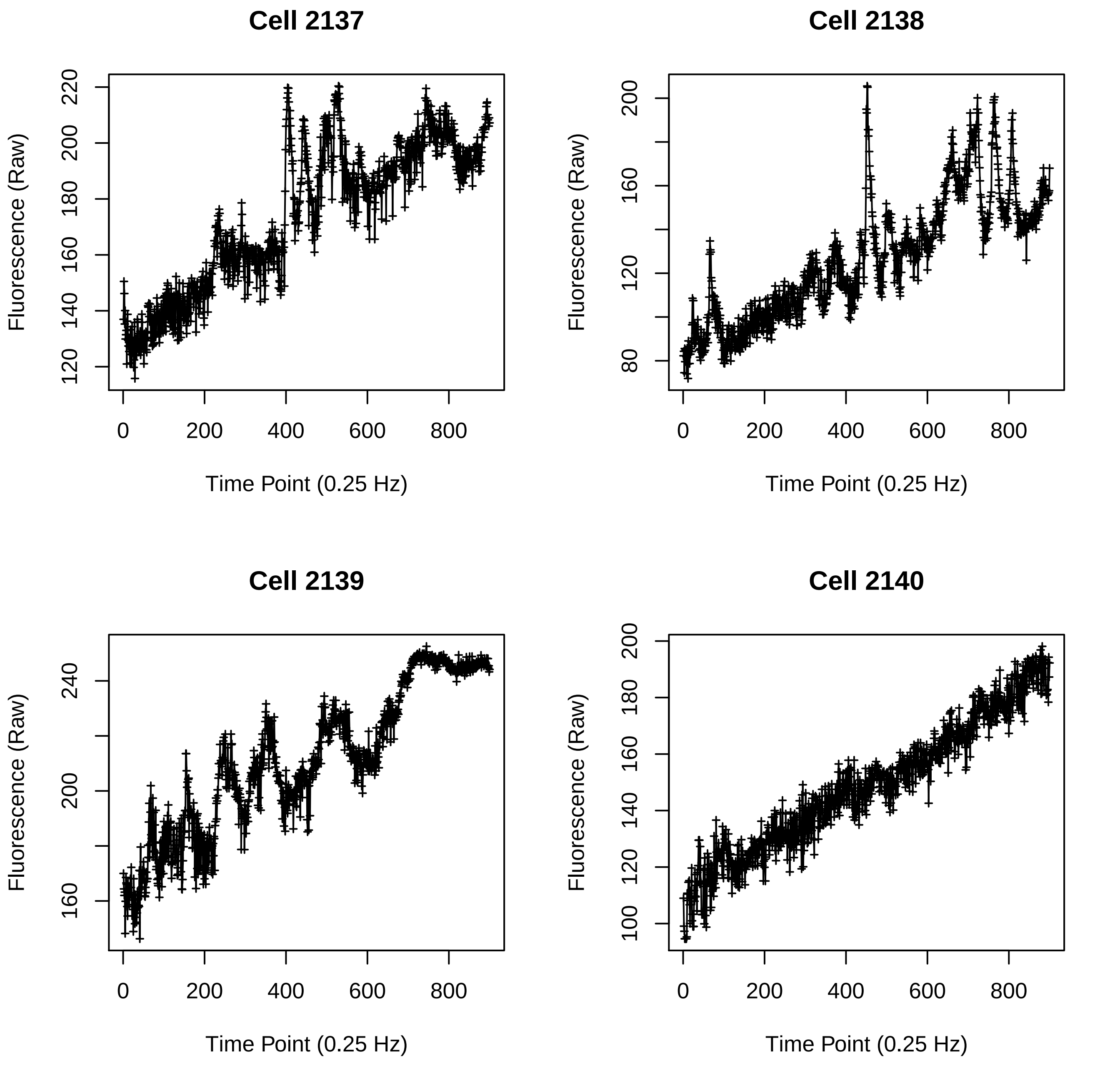
Cell 2137
Cell 2138
Cell 2139
Cell 2140
Fluorescence (Raw)
Time Point (0.25 Hz)
0
200
400
600
800
120
140
160
180
200
220
80
240

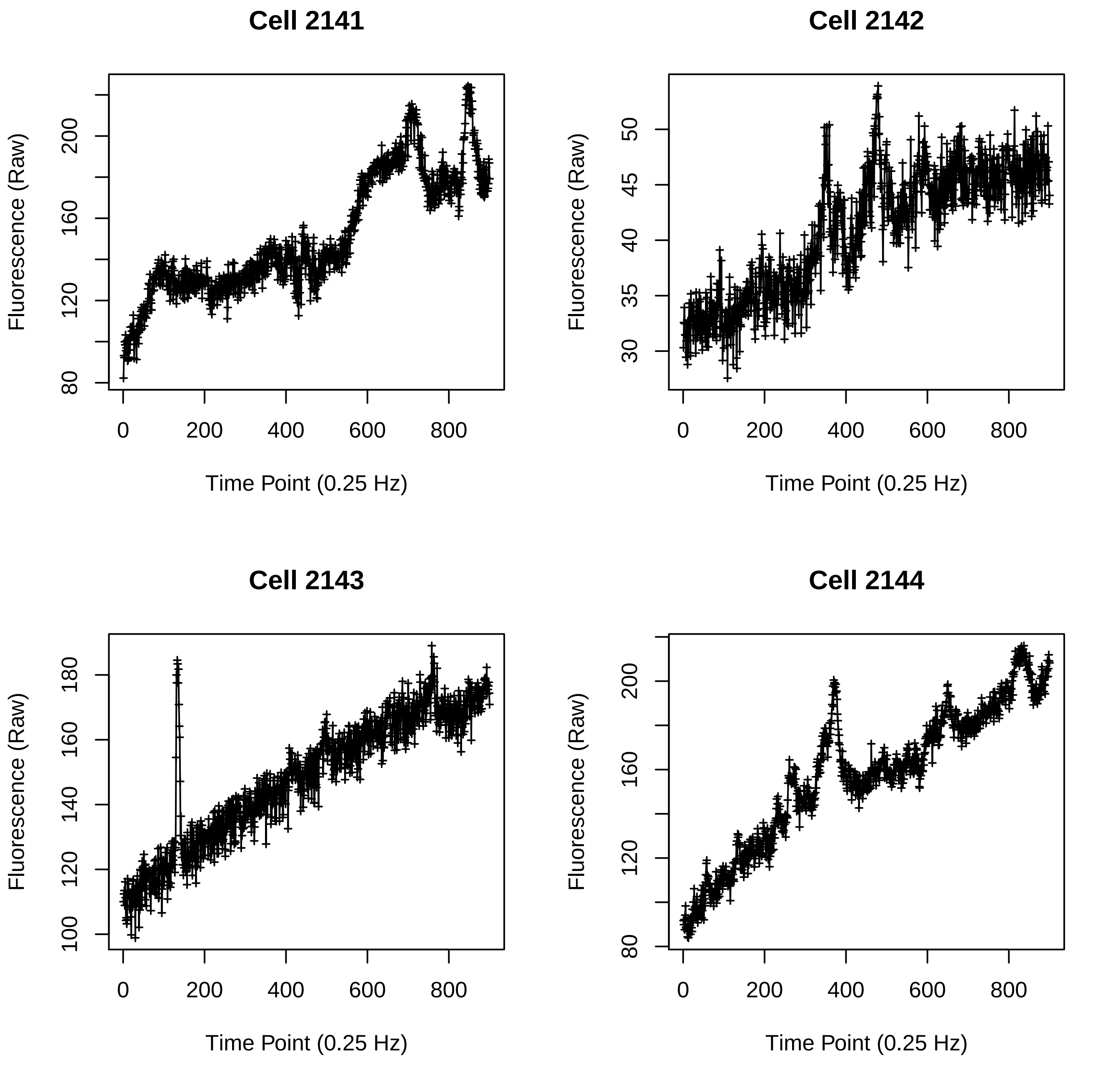
Cell 2141
Fluorescence (Raw)
80
120
160
200
0
200
400
600
800
Time Point (0.25 Hz)
Cell 2142
Fluorescence (Raw)
30
35
40
45
50
0
200
400
600
800
Time Point (0.25 Hz)
Cell 2143
Fluorescence (Raw)
100
120
140
160
180
0
200
400
600
800
Time Point (0.25 Hz)
Cell 2144
Fluorescence (Raw)
80
120
160
200
0
200
400
600
800
Time Point (0.25 Hz)

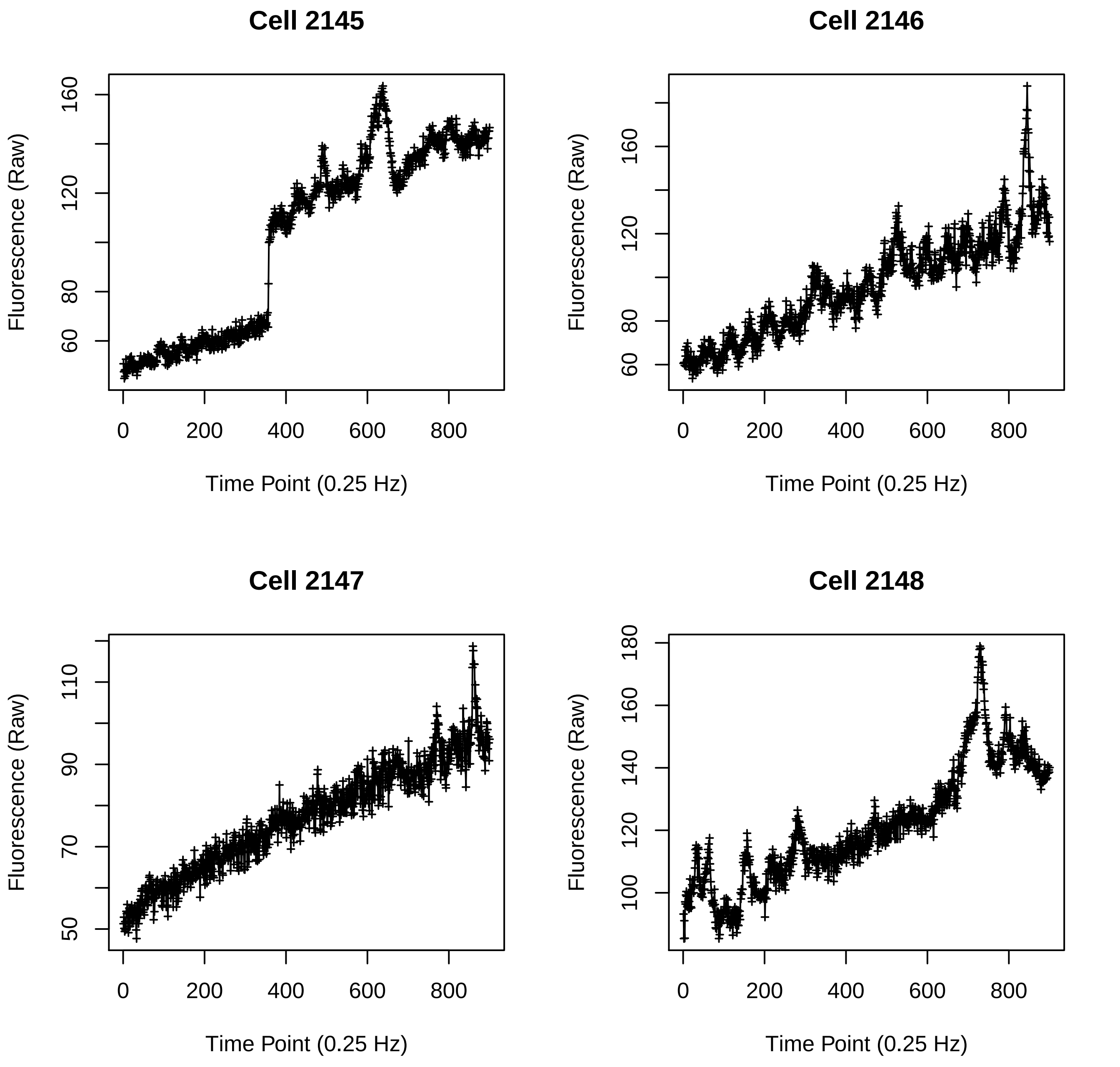

Cell 2145
Cell 2146
Cell 2147
Cell 2148
Fluorescence (Raw)
Time Point (0.25 Hz)

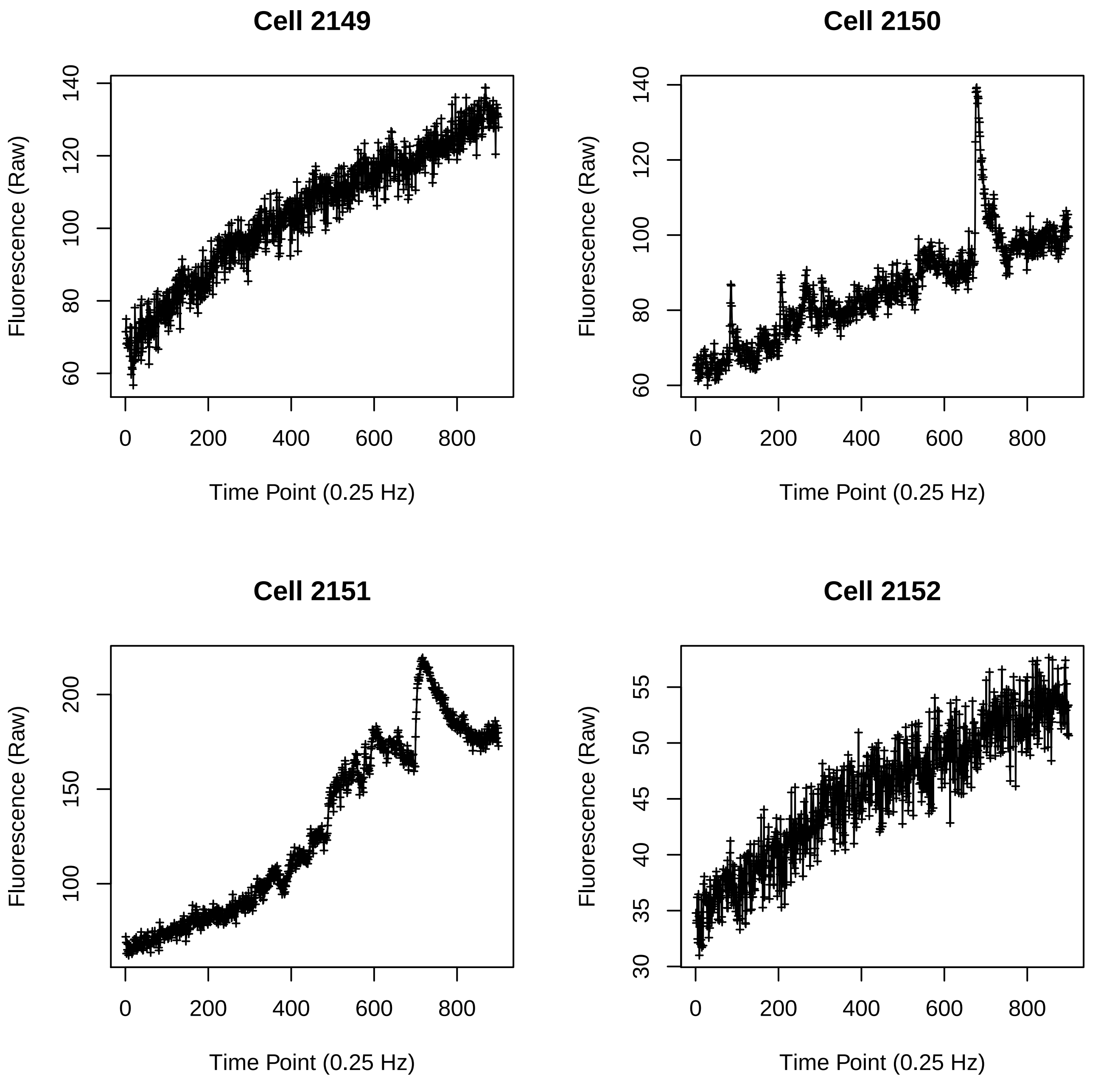
Cell 2149
Fluorescence (Raw)
60
80
100
120
140
0
200
400
600
800
Time Point (0.25 Hz)
Cell 2150
Fluorescence (Raw)
60
80
100
120
140
0
200
400
600
800
Time Point (0.25 Hz)
Cell 2151
Fluorescence (Raw)
100
150
200
0
200
400
600
800
Time Point (0.25 Hz)
Cell 2152
Fluorescence (Raw)
30
35
40
45
50
55
0
200
400
600
800
Time Point (0.25 Hz)

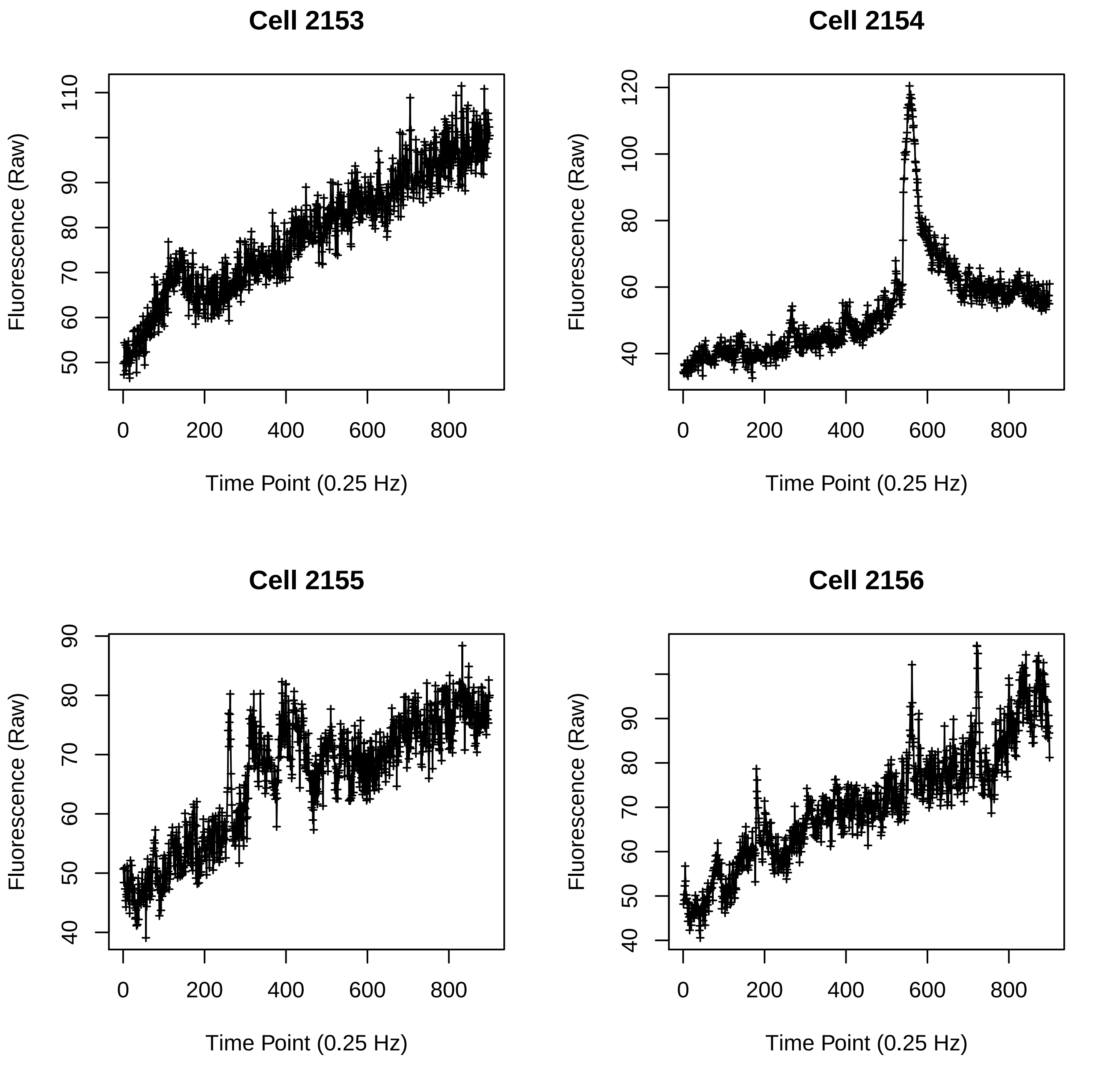
Cell 2153
Fluorescence (Raw)
50
60
70
80
90
110
0
200
400
600
800
Time Point (0.25 Hz)
Cell 2154
Fluorescence (Raw)
40
60
80
100
120
0
200
400
600
800
Time Point (0.25 Hz)
Cell 2155
Fluorescence (Raw)
40
50
60
70
80
90
0
200
400
600
800
Time Point (0.25 Hz)
Cell 2156
Fluorescence (Raw)
40
50
60
70
80
90
0
200
400
600
800
Time Point (0.25 Hz)

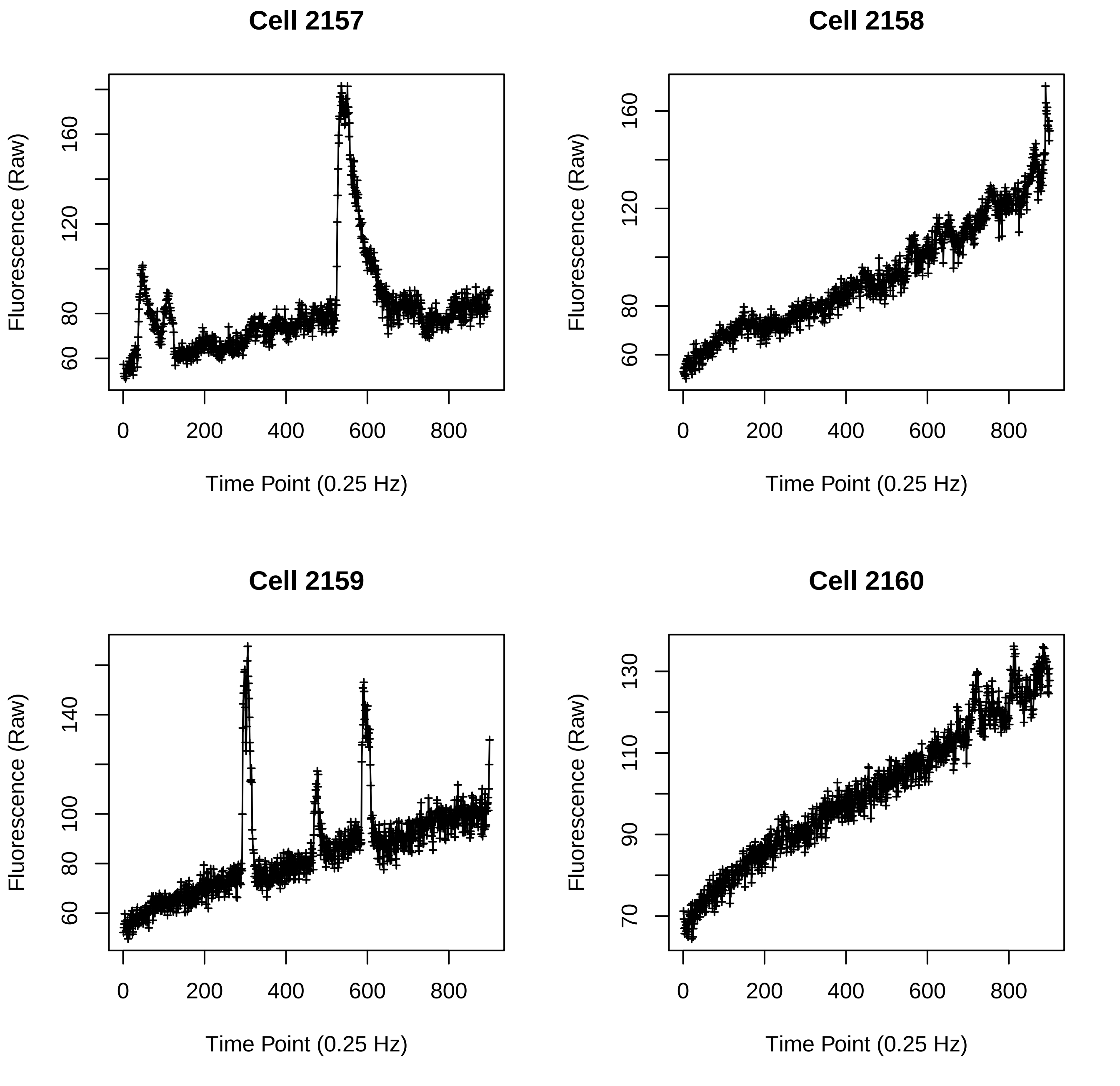

Cell 2157
Cell 2158
Cell 2159
Cell 2160
Fluorescence (Raw)
Time Point (0.25 Hz)
0
200
400
600
800
60
80
120
160
140
100
70
90
110
130

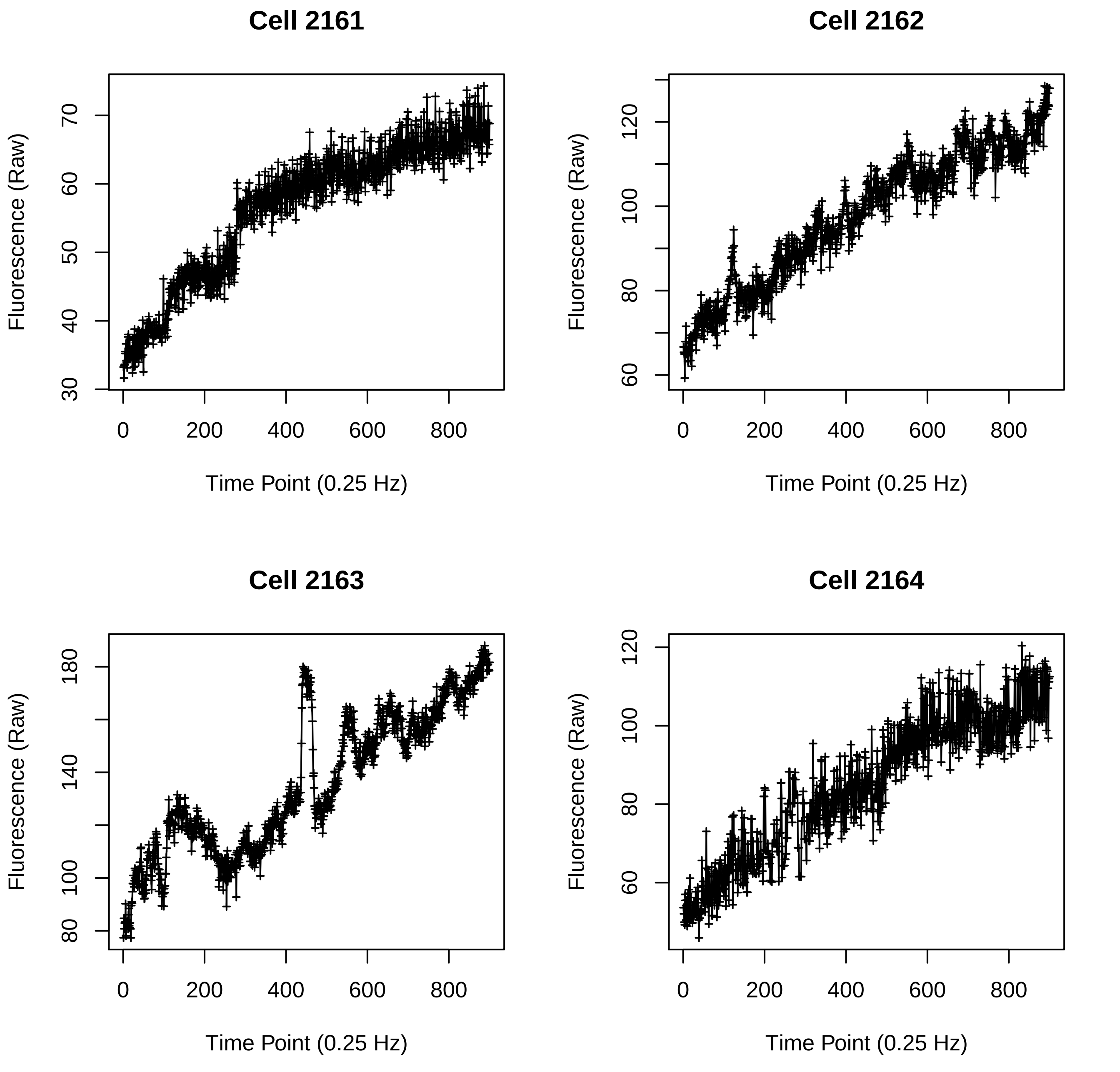
Cell 2161
Cell 2162
Cell 2163
Cell 2164
Fluorescence (Raw)
Time Point (0.25 Hz)
0
200
400
600
800
30
40
50
60
70
80
100
120
140
180

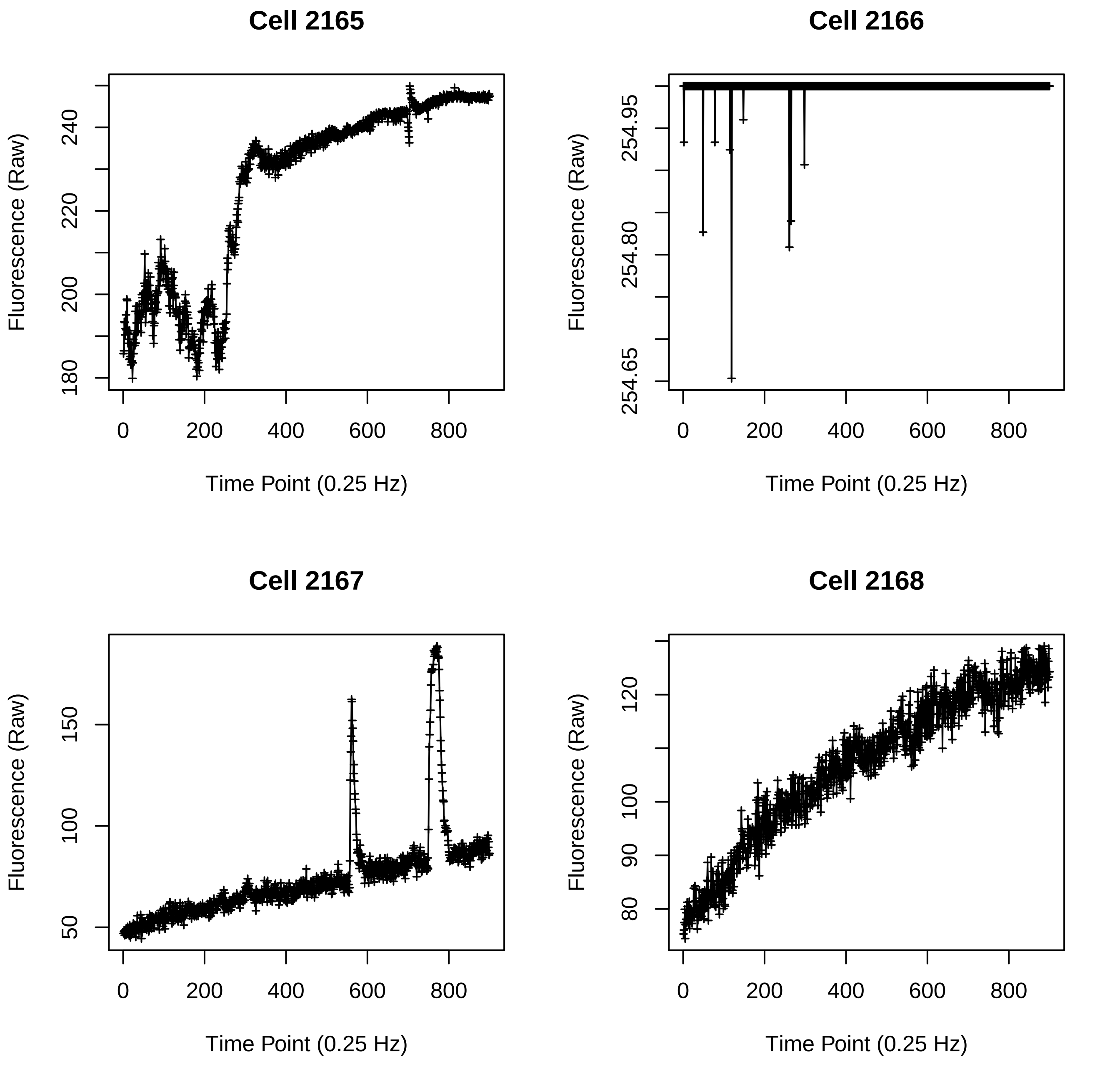

Cell 2165
Cell 2166
Cell 2167
Cell 2168
Fluorescence (Raw)
Time Point (0.25 Hz)
0
200
400
600
800
180
200
220
240
254.65
254.80
254.95
50
100
150
80
90
100
120

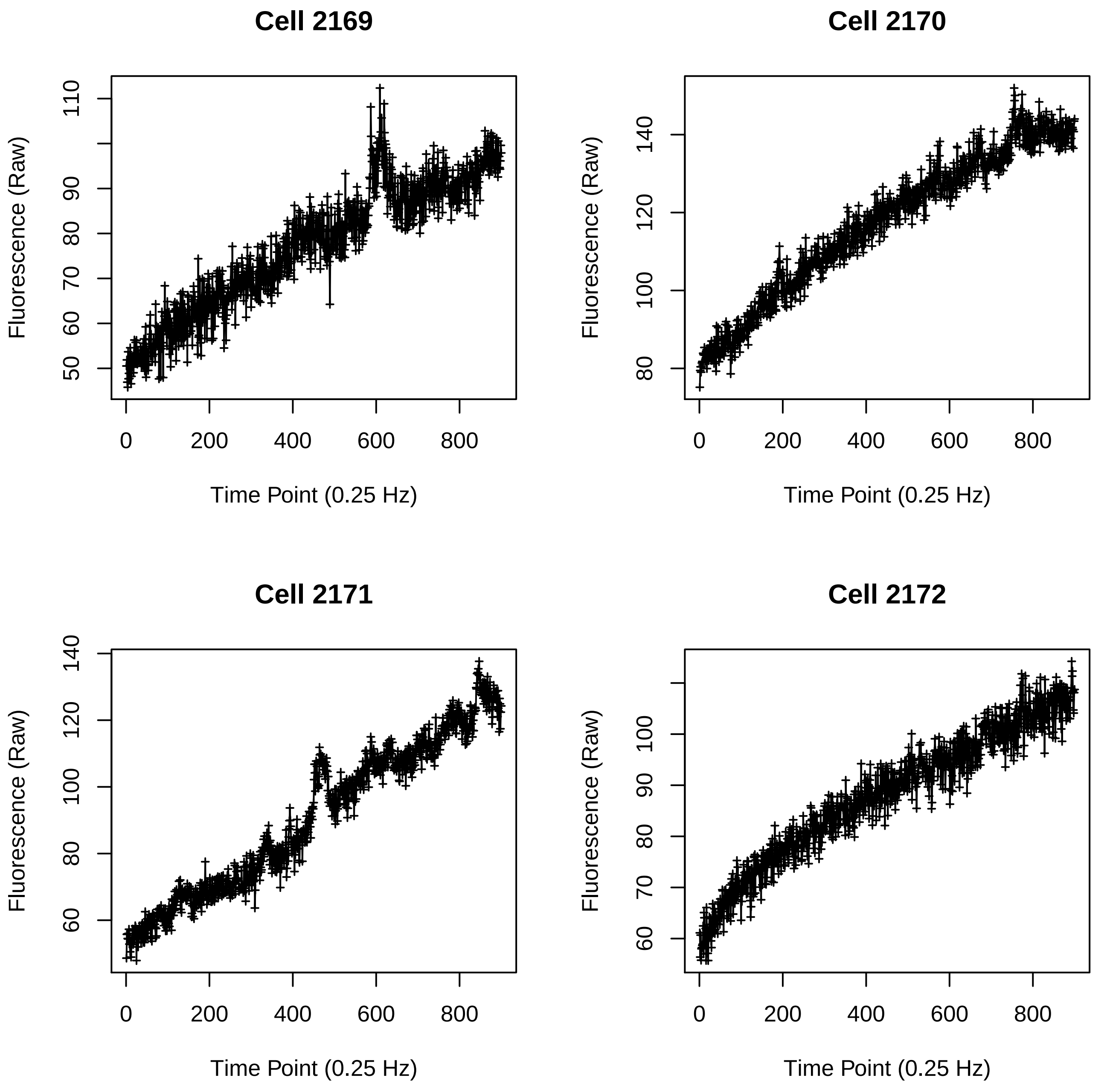
Cell 2169
Fluorescence (Raw)
50
60
70
80
90
110
0
200
400
600
800
Time Point (0.25 Hz)
Cell 2170
Fluorescence (Raw)
80
100
120
140
0
200
400
600
800
Time Point (0.25 Hz)
Cell 2171
Fluorescence (Raw)
60
80
100
120
140
0
200
400
600
800
Time Point (0.25 Hz)
Cell 2172
Fluorescence (Raw)
60
70
80
90
100
0
200
400
600
800
Time Point (0.25 Hz)

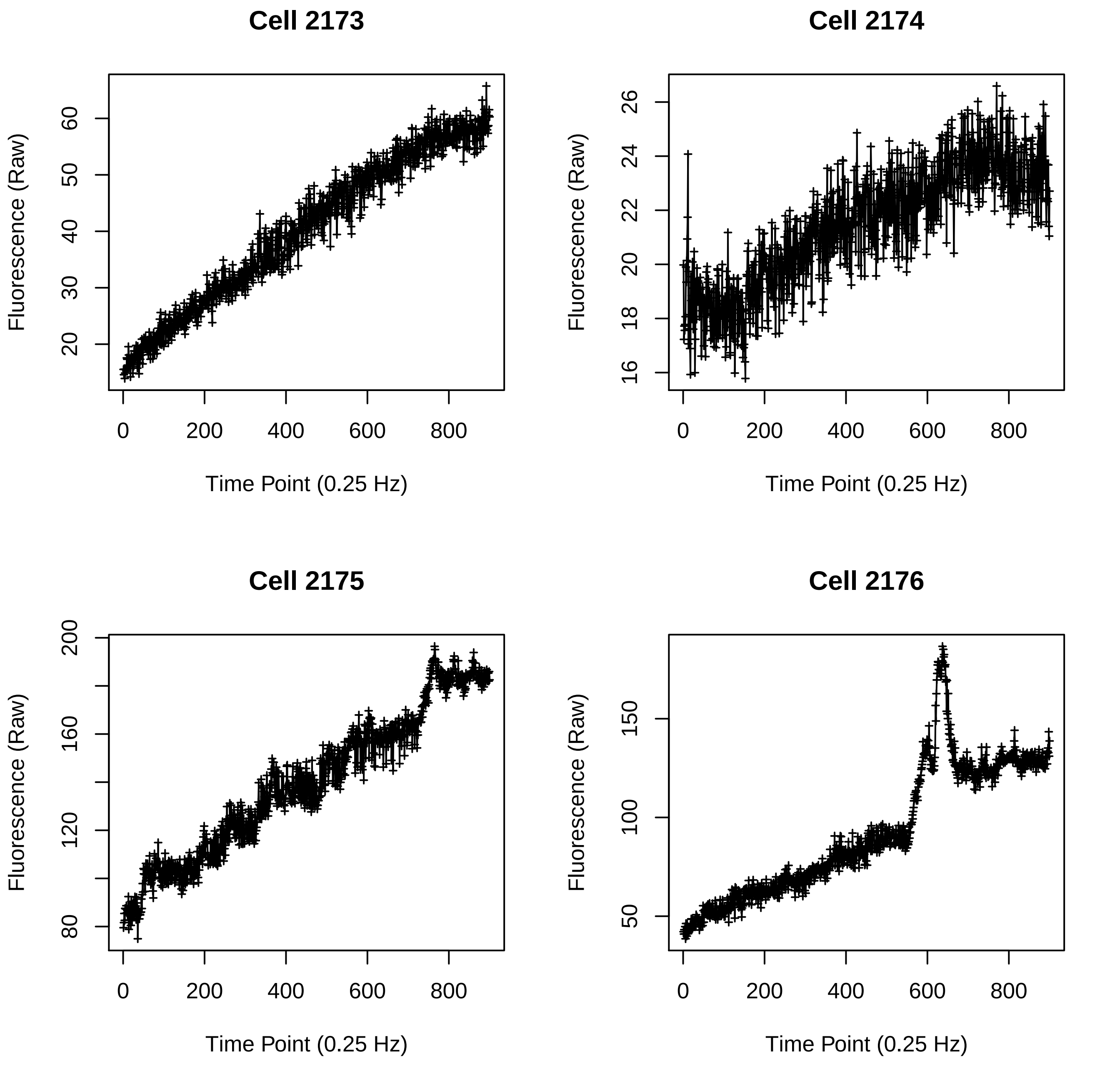
Cell 2173
Fluorescence (Raw)
20
30
40
50
60
0
200
400
600
800
Time Point (0.25 Hz)
Cell 2174
Fluorescence (Raw)
16
18
20
22
24
26
0
200
400
600
800
Time Point (0.25 Hz)
Cell 2175
Fluorescence (Raw)
80
120
160
200
0
200
400
600
800
Time Point (0.25 Hz)
Cell 2176
Fluorescence (Raw)
50
100
150
0
200
400
600
800
Time Point (0.25 Hz)
