# Supplementary material for: A Markovian Entropy Measure for the Analysis of Calcium Activity Time Series
Source: PLoS One. 2016 Dec 15;11(12):e0168342. doi: 10.1371/journal.pone.0168342 (PMC5158058; doi:10.1371/journal.pone.0168342)
Supplement: S5 File — (PDF) [file pone.0168342.s012.pdf]

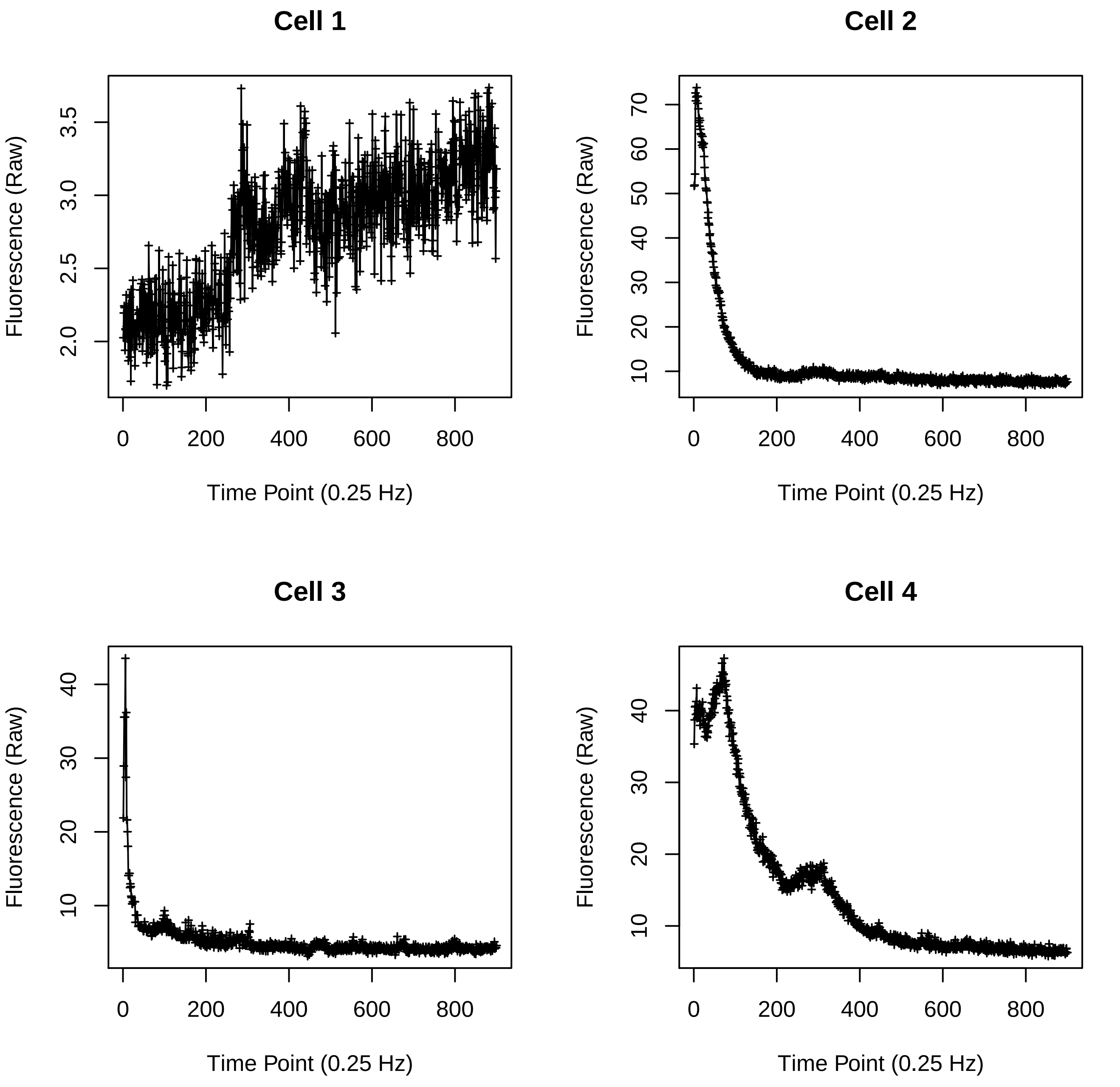
Cell 1
Fluorescence (Raw)
2.0
2.5
3.0
3.5
0
200
400
600
800
Time Point (0.25 Hz)
Cell 2
Fluorescence (Raw)
10
20
30
40
50
60
70
0
200
400
600
800
Time Point (0.25 Hz)
Cell 3
Fluorescence (Raw)
10
20
30
40
0
200
400
600
800
Time Point (0.25 Hz)
Cell 4
Fluorescence (Raw)
10
20
30
40
0
200
400
600
800
Time Point (0.25 Hz)

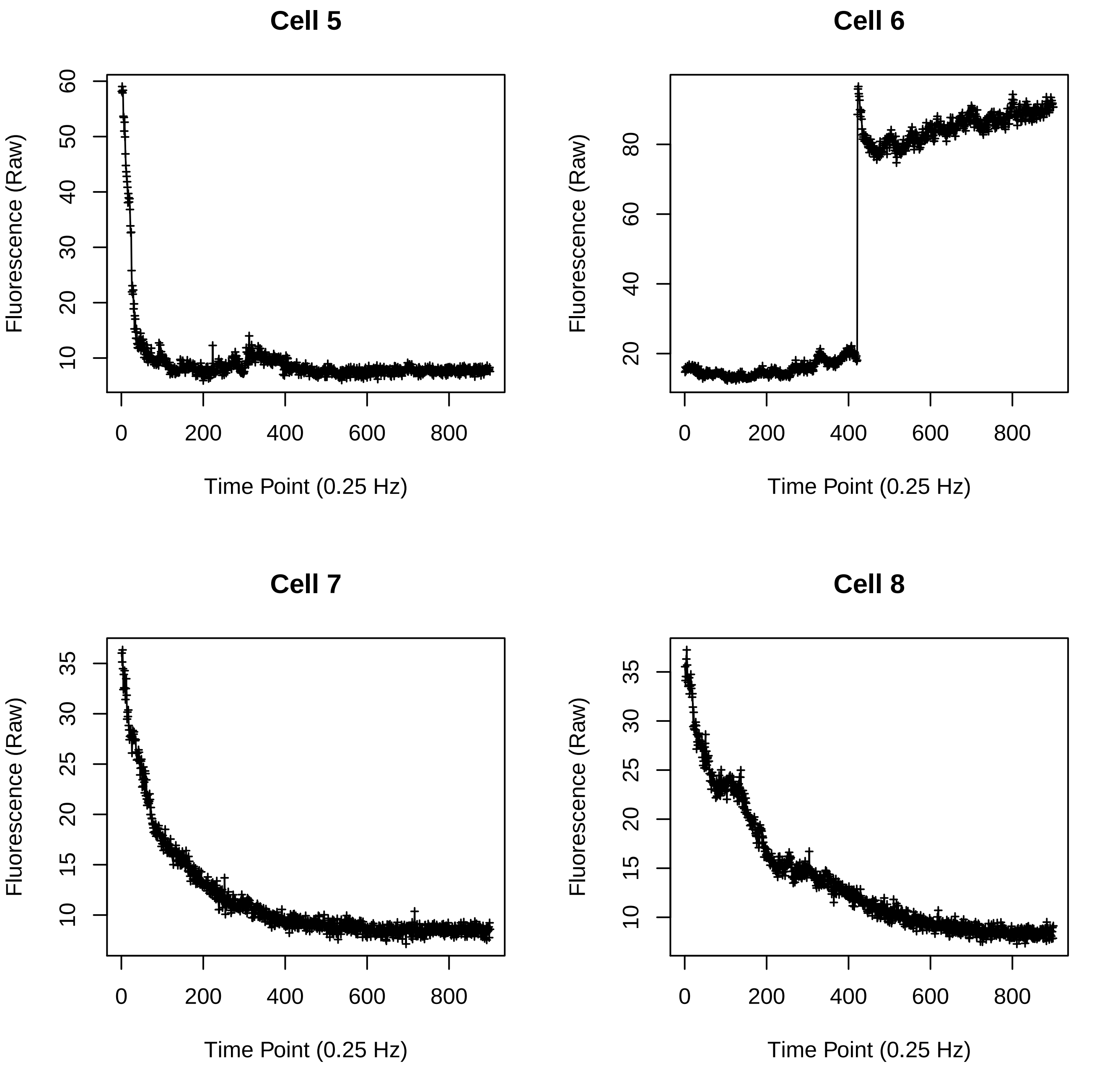

Cell 5
Cell 6
Cell 7
Cell 8
Fluorescence (Raw)
Time Point (0.25 Hz)
0
200
400
600
800
10
20
30
40
50
60
80
15
25
35

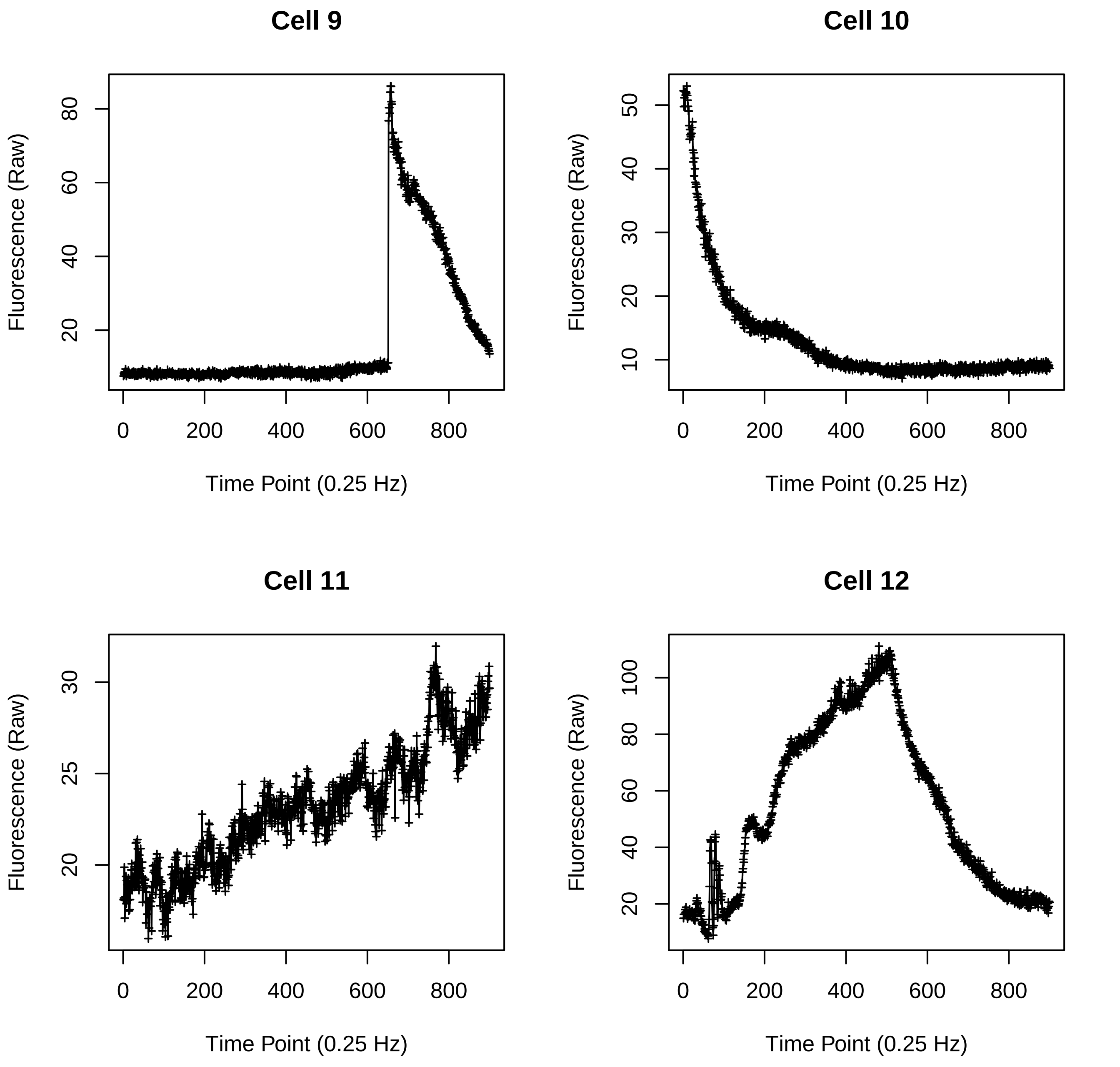

Cell 9
Fluorescence (Raw)
20
40
60
80
0
200
400
600
800
Time Point (0.25 Hz)
Cell 10
Fluorescence (Raw)
10
20
30
40
50
0
200
400
600
800
Time Point (0.25 Hz)
Cell 11
Fluorescence (Raw)
20
25
30
0
200
400
600
800
Time Point (0.25 Hz)
Cell 12
Fluorescence (Raw)
20
40
60
80
100
0
200
400
600
800
Time Point (0.25 Hz)

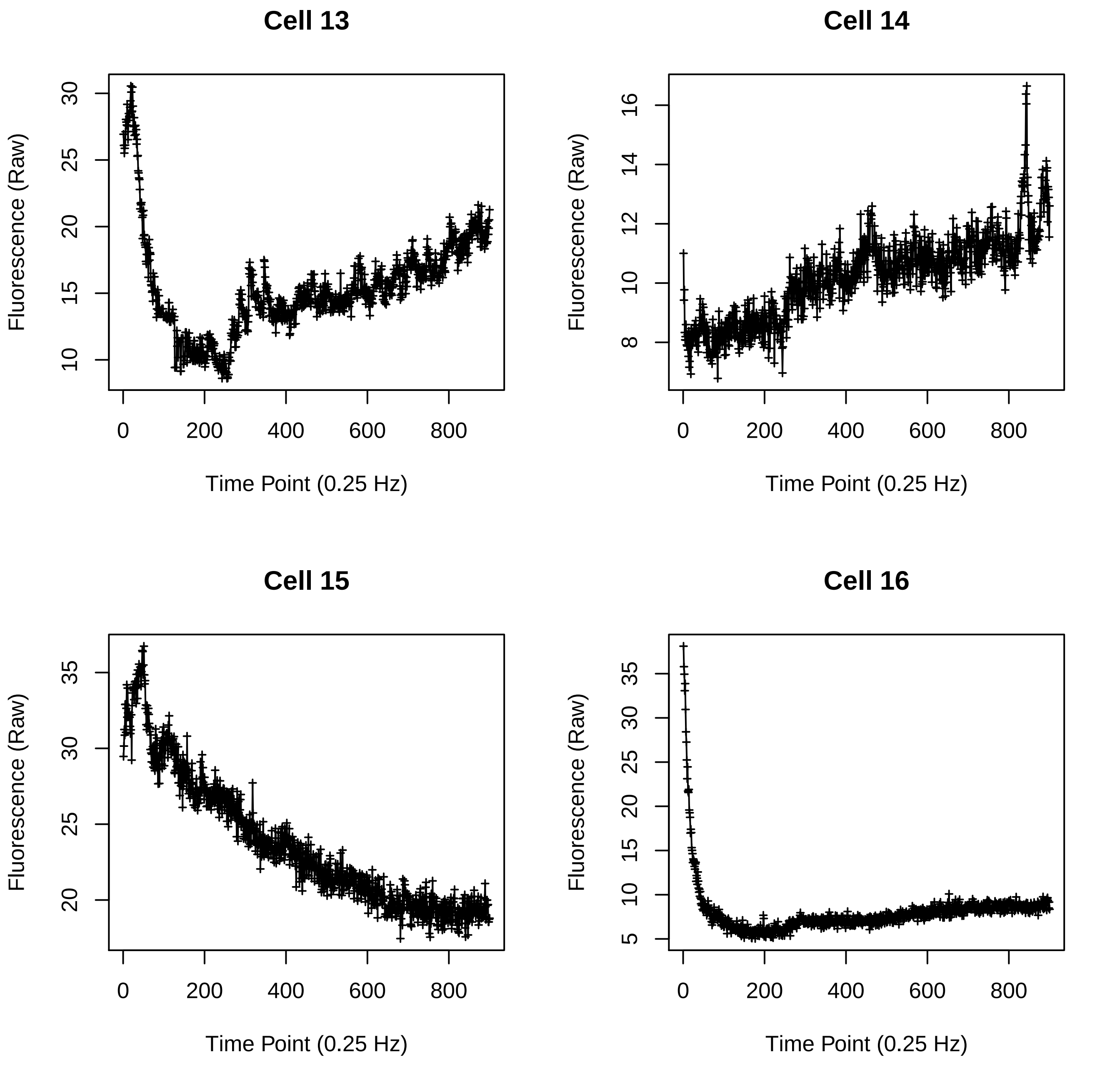

Cell 13
Fluorescence (Raw)
Time Point (0.25 Hz)
Cell 14
Fluorescence (Raw)
Time Point (0.25 Hz)
Cell 15
Fluorescence (Raw)
Time Point (0.25 Hz)
Cell 16
Fluorescence (Raw)
Time Point (0.25 Hz)

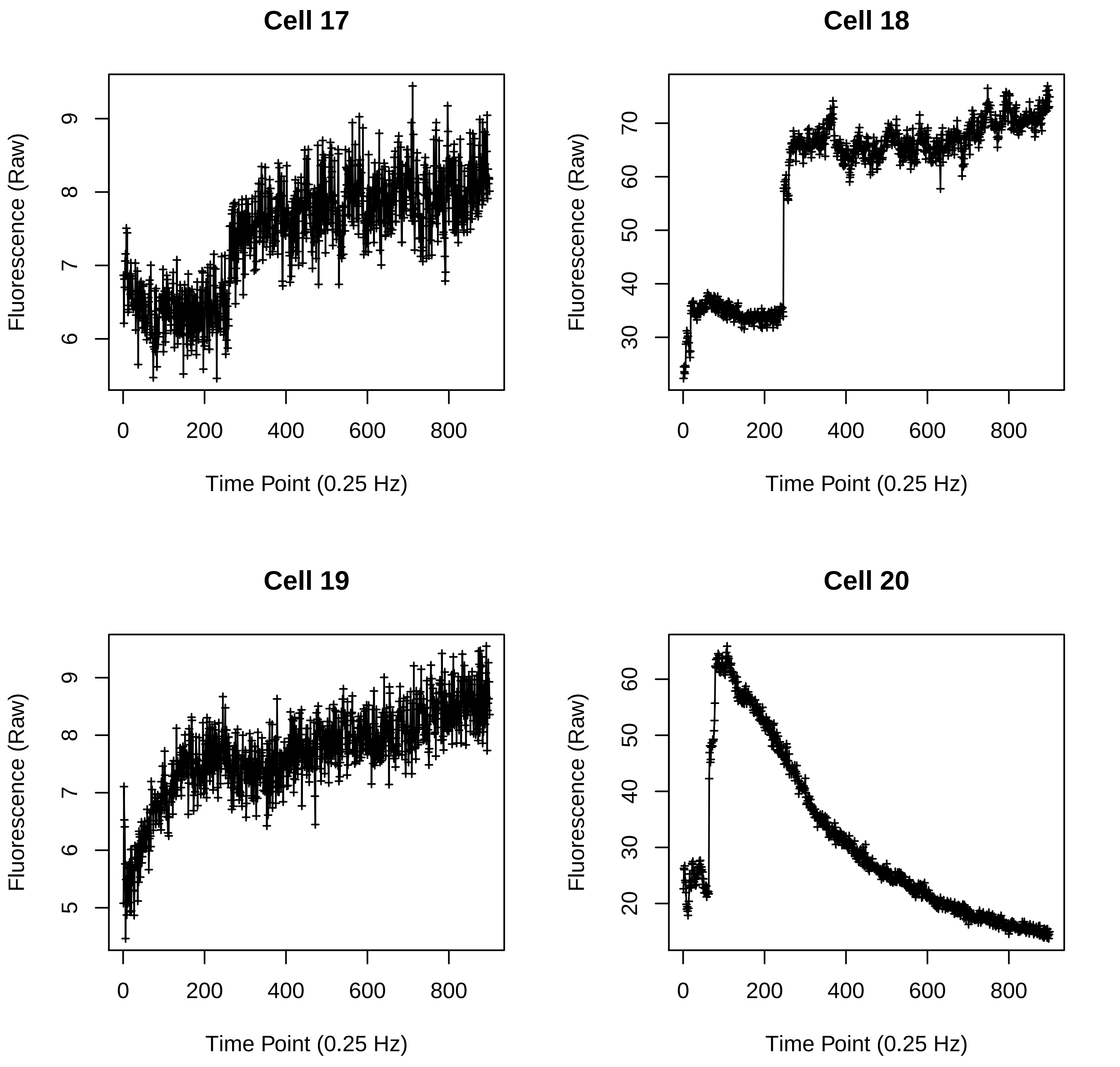
Cell 17
Cell 18
Cell 19
Cell 20
Fluorescence (Raw)
Time Point (0.25 Hz)
0
200
400
600
800
6
7
8
9
30
40
50
60
70
5
20

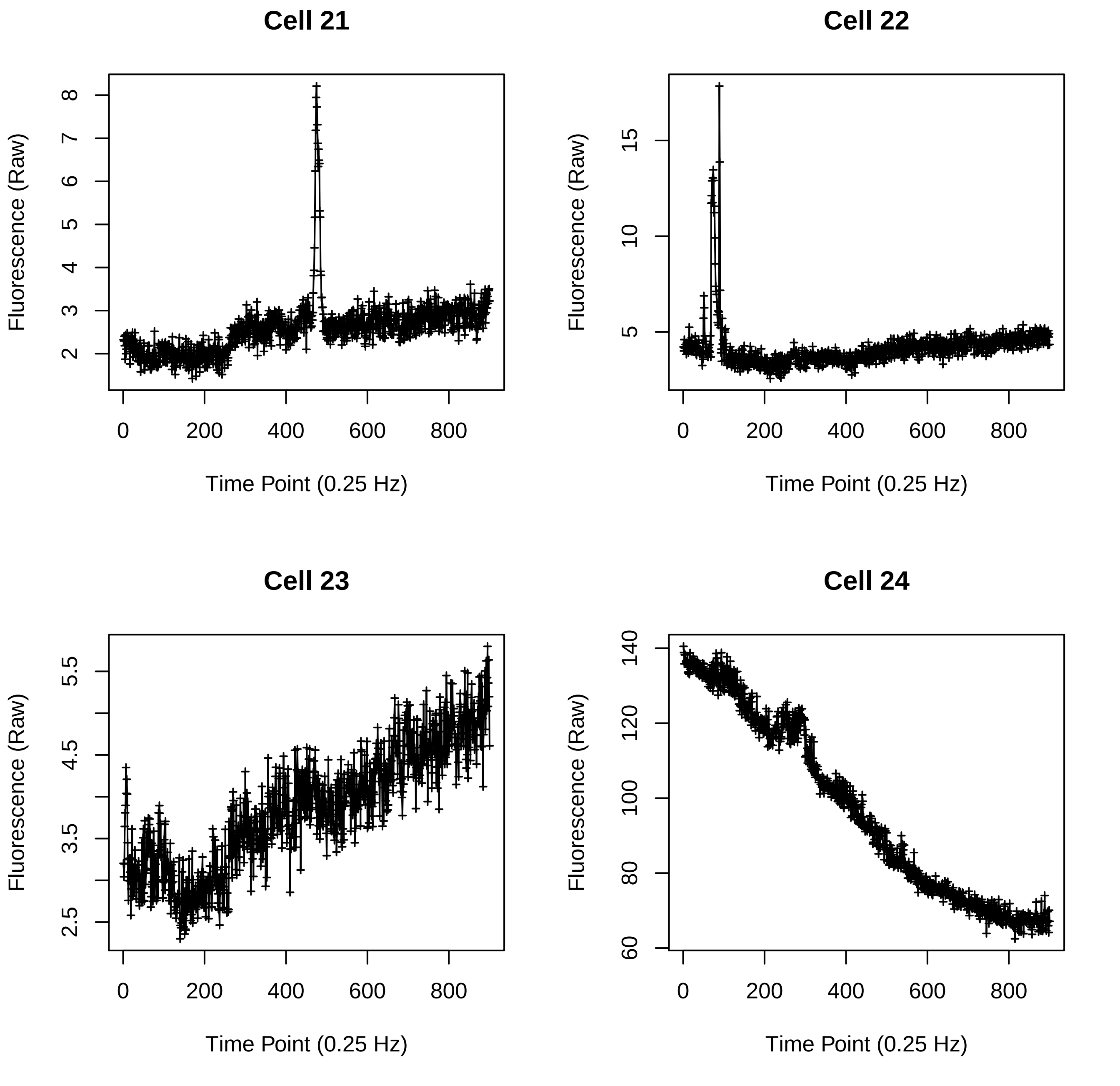

Cell 21
Fluorescence (Raw)
2
3
4
5
6
7
8
0
200
400
600
800
Time Point (0.25 Hz)
Cell 22
Fluorescence (Raw)
5
10
15
0
200
400
600
800
Time Point (0.25 Hz)
Cell 23
Fluorescence (Raw)
2.5
3.5
4.5
5.5
0
200
400
600
800
Time Point (0.25 Hz)
Cell 24
Fluorescence (Raw)
60
80
100
120
140
0
200
400
600
800
Time Point (0.25 Hz)

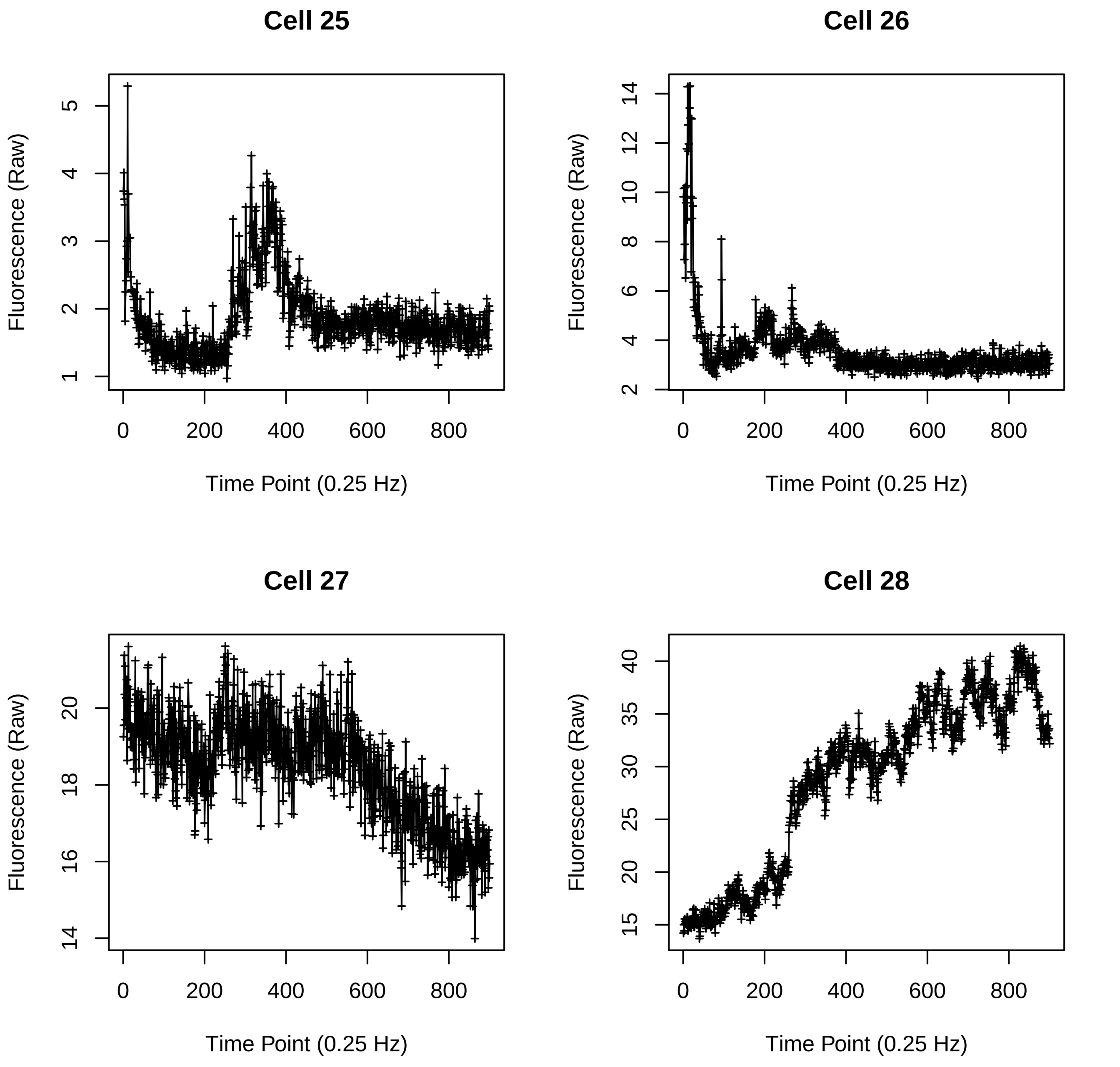

Cell 25
Cell 26
Cell 27
Cell 28
Fluorescence (Raw)
Time Point (0.25 Hz)
0
200
400
600
800
1
2
3
4
5
2
4
6
8
10
12
14
16
18
20
15
25
30
35
40

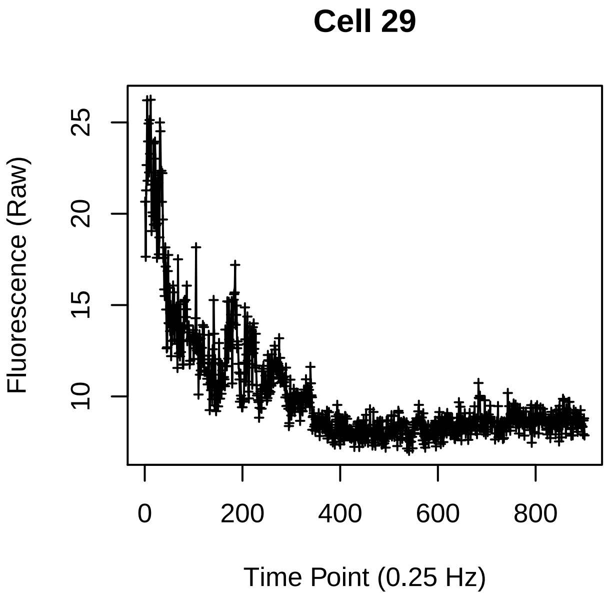
Cell 29
Fluorescence (Raw)
Time Point (0.25 Hz)
10
15
20
25
0
200
400
600
800

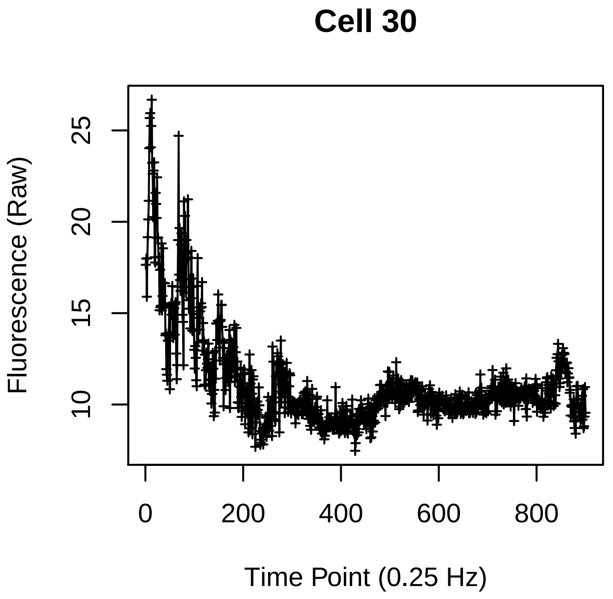
Cell 30
Fluorescence (Raw)
Time Point (0.25 Hz)
10
15
20
25
0
200
400
600
800

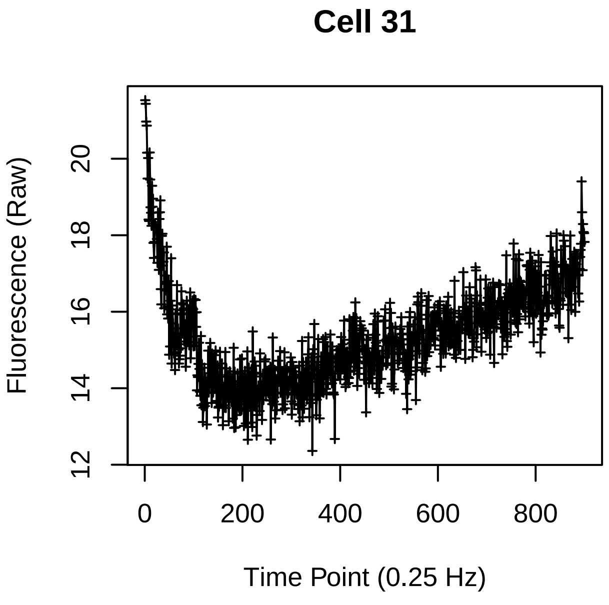
Cell 31
Fluorescence (Raw)
Time Point (0.25 Hz)
12
14
16
18
20
0
200
400
600
800

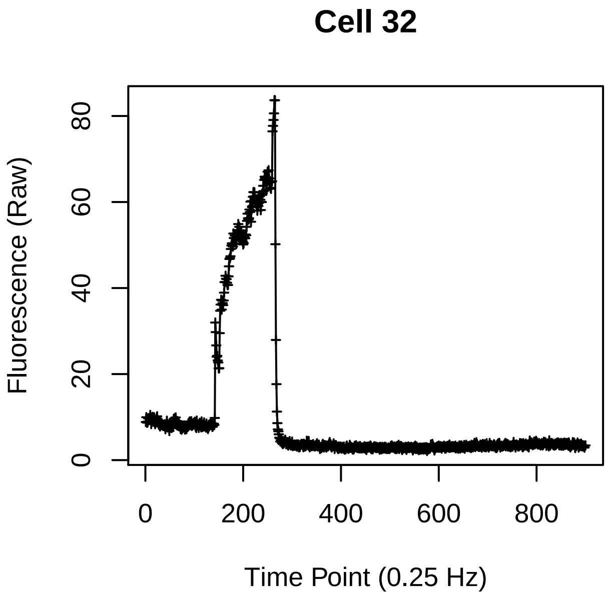
Cell 32
Fluorescence (Raw)
Time Point (0.25 Hz)
0
20
40
60
80
0
200
400
600
800

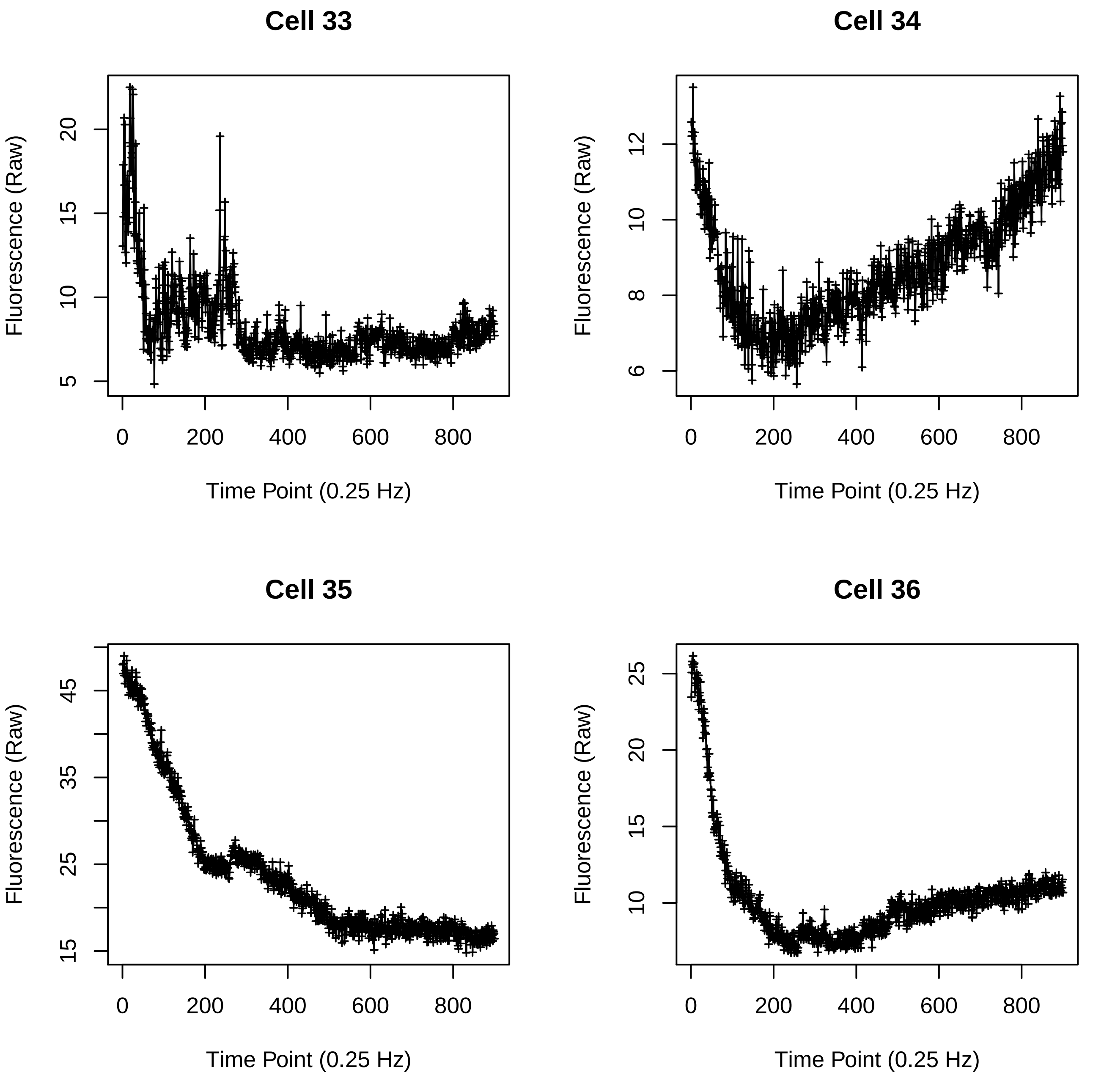
Cell 33
Fluorescence (Raw)
5
10
15
20
0
200
400
600
800
Time Point (0.25 Hz)
Cell 34
Fluorescence (Raw)
6
8
10
12
0
200
400
600
800
Time Point (0.25 Hz)
Cell 35
Fluorescence (Raw)
15
25
35
45
0
200
400
600
800
Time Point (0.25 Hz)
Cell 36
Fluorescence (Raw)
10
15
20
25
0
200
400
600
800
Time Point (0.25 Hz)

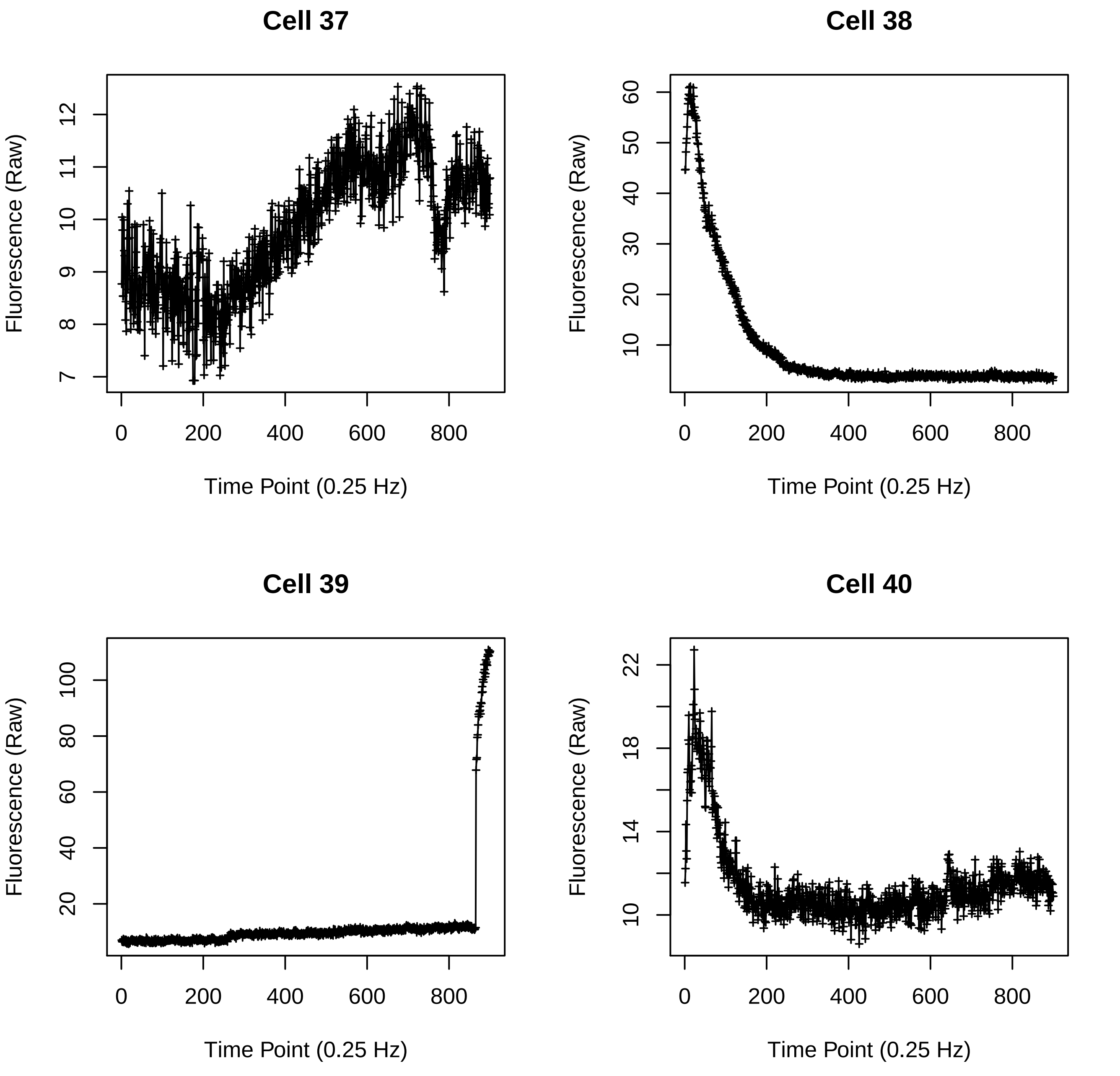
Cell 37
Cell 38
Cell 39
Cell 40
Fluorescence (Raw)
Time Point (0.25 Hz)
0
200
400
600
800
7
8
9
10
11
12
10
20
30
40
50
60
20
40
60
80
100
14
18
22

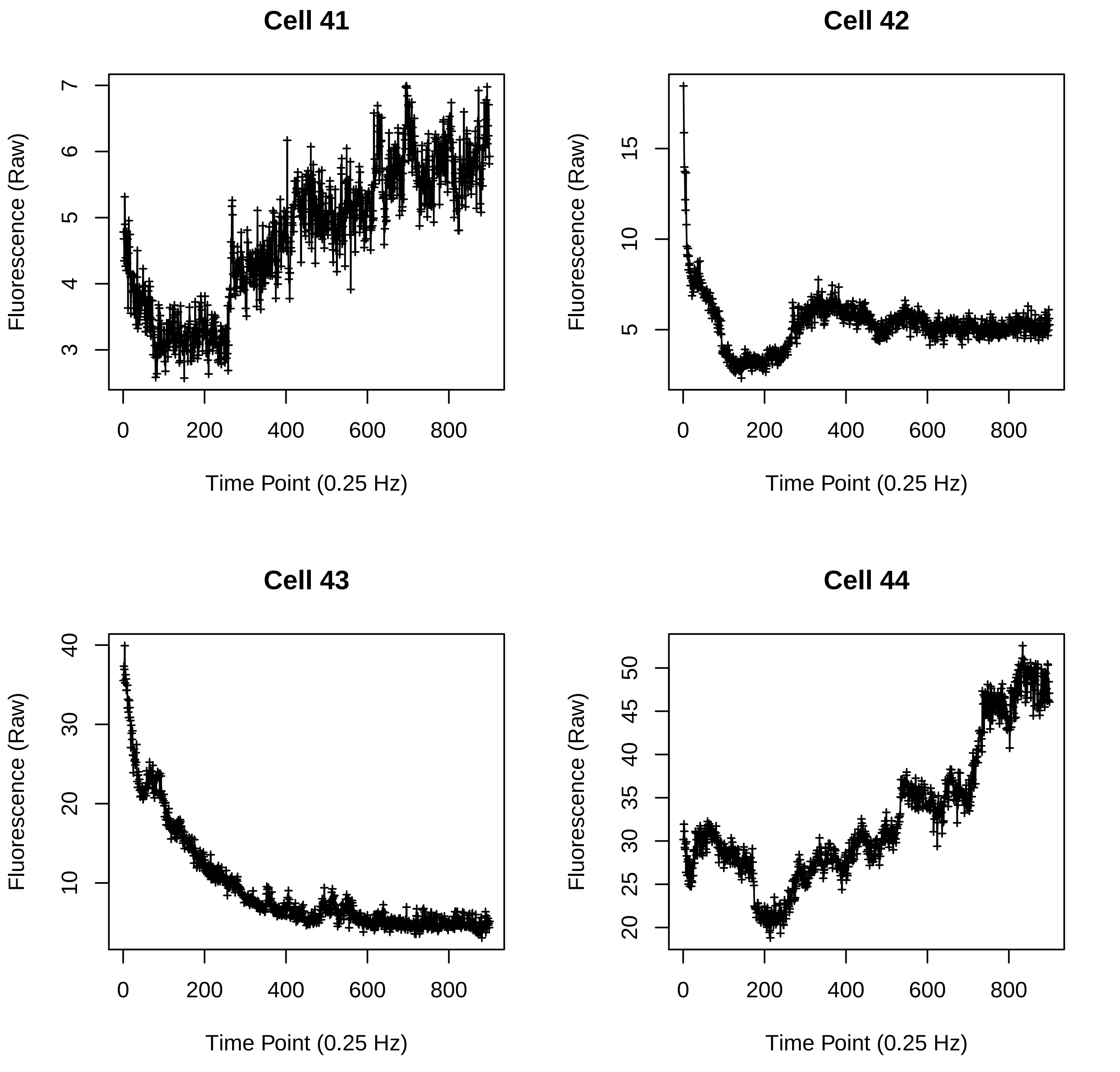

Cell 41
Cell 42
Cell 43
Cell 44
Fluorescence (Raw)
Time Point (0.25 Hz)
0
200
400
600
800
3
4
5
6
7
5
10
15
10
20
30
40
20
25
30
35
40
45
50

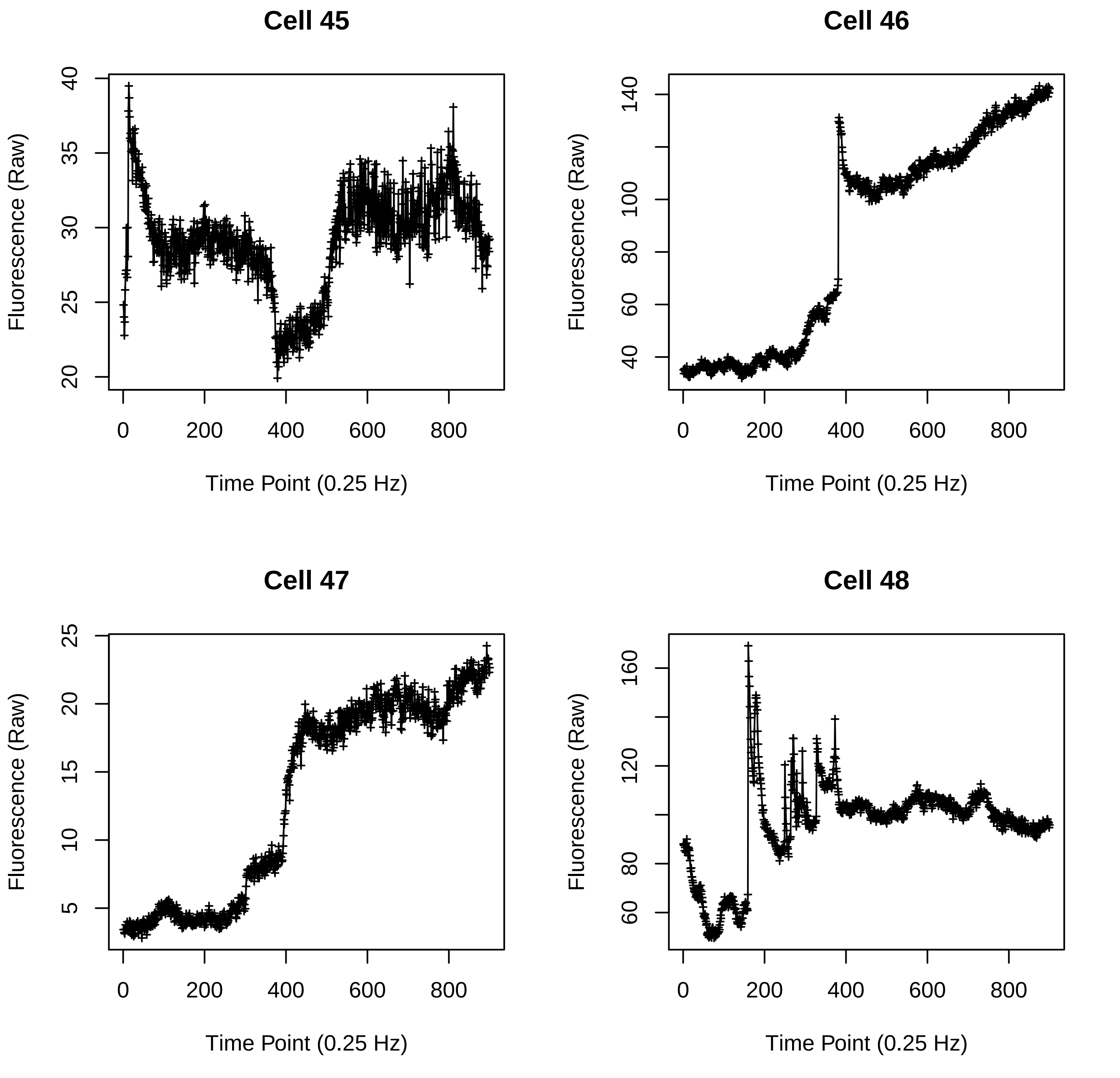
Cell 45
Cell 46
Cell 47
Cell 48
Fluorescence (Raw)
Time Point (0.25 Hz)
0
200
400
600
800
20
25
30
35
40
60
80
100
140
5
10
15
120
160

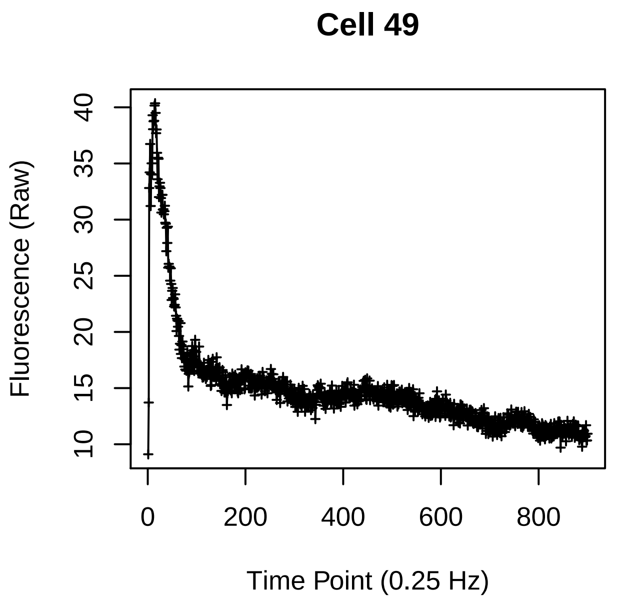

Cell 49
Fluorescence (Raw)
Time Point (0.25 Hz)

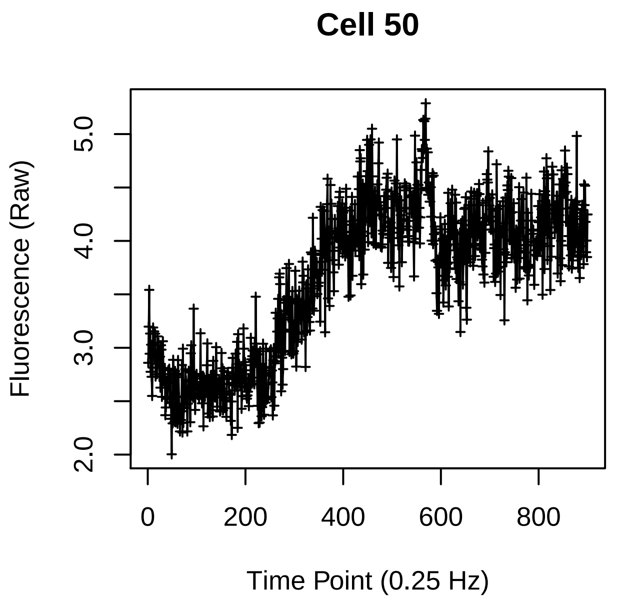

Cell 50
Fluorescence (Raw)
Time Point (0.25 Hz)

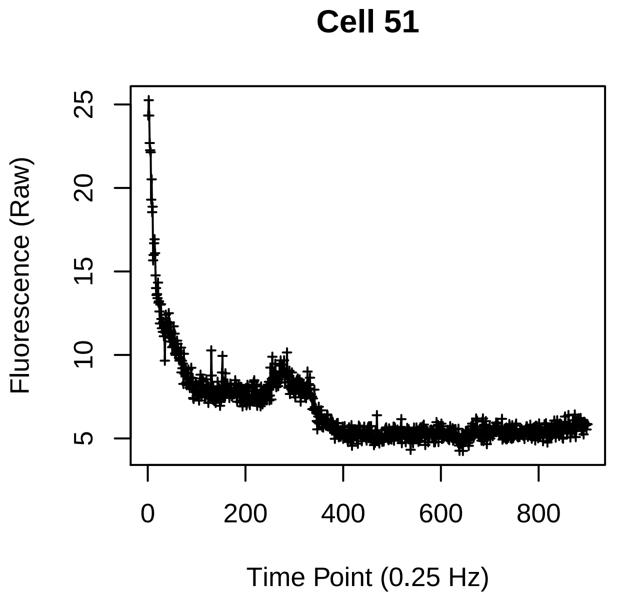

Cell 51
Fluorescence (Raw)
Time Point (0.25 Hz)

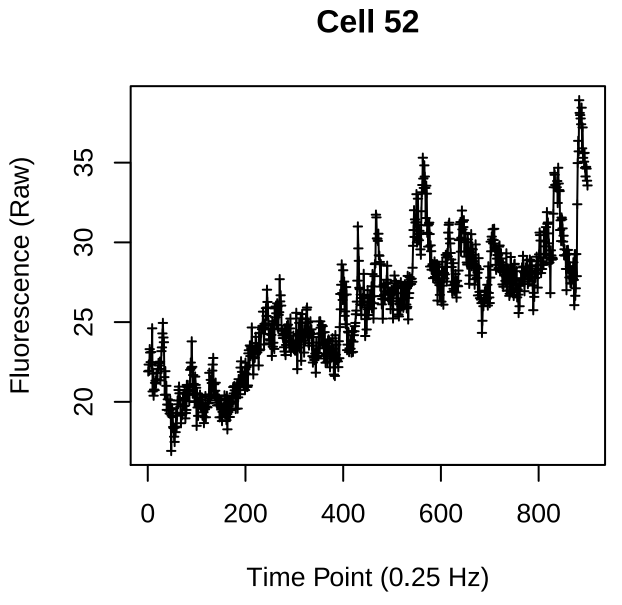

Cell 52
Fluorescence (Raw)
Time Point (0.25 Hz)

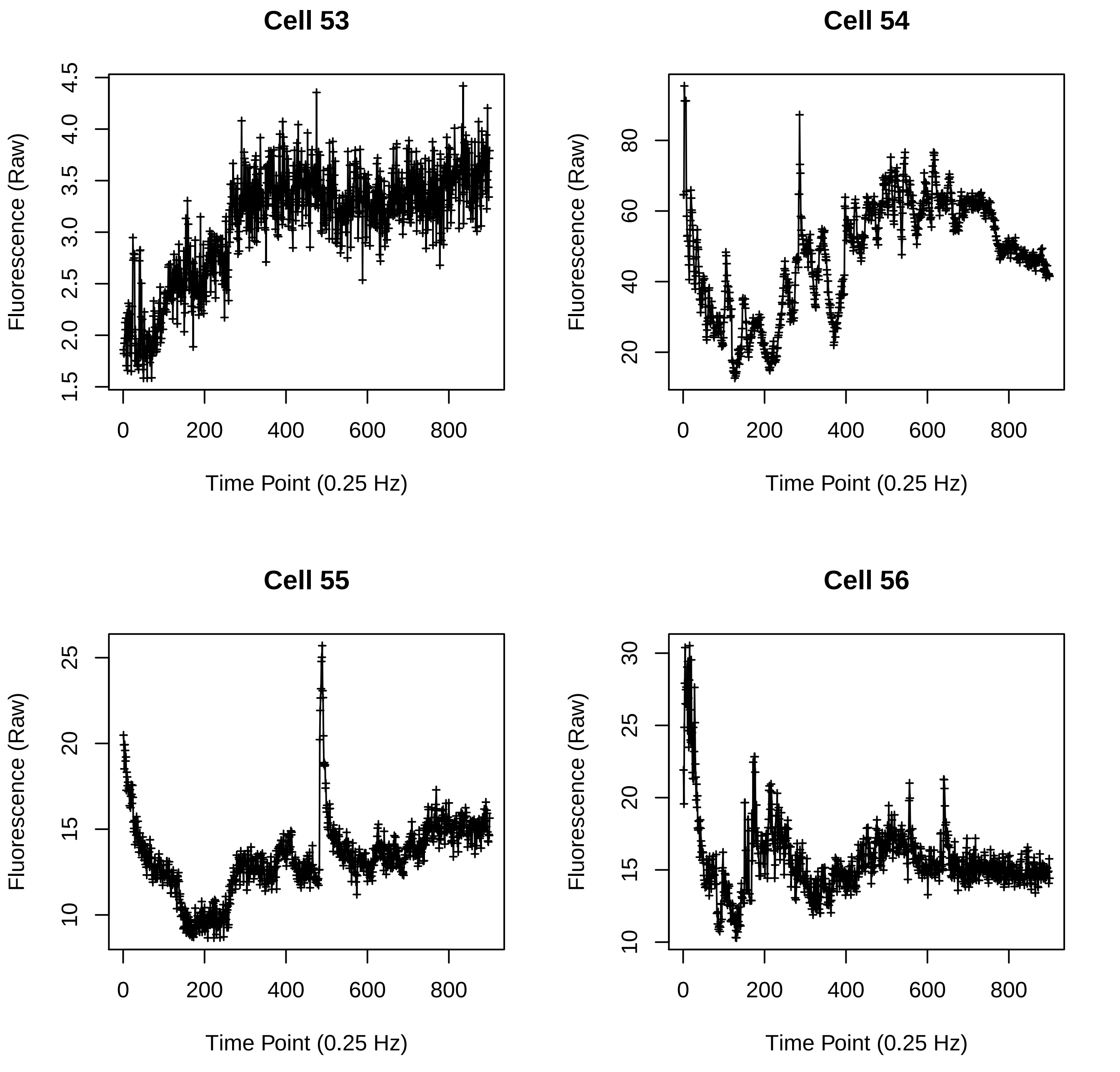
Cell 53
Fluorescence (Raw)
Time Point (0.25 Hz)
Cell 54
Fluorescence (Raw)
Time Point (0.25 Hz)
Cell 55
Fluorescence (Raw)
Time Point (0.25 Hz)
Cell 56
Fluorescence (Raw)
Time Point (0.25 Hz)

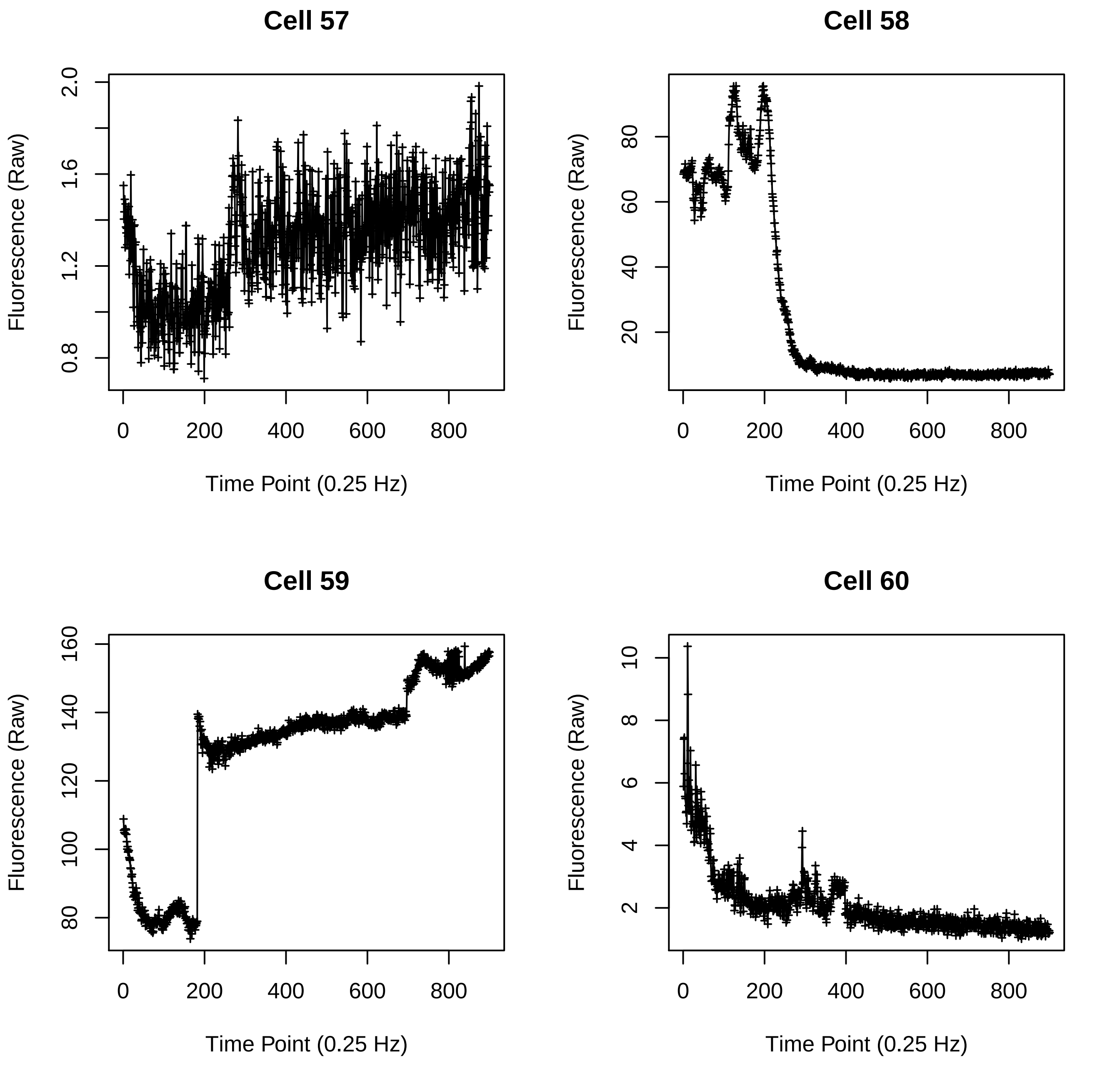

Cell 57
Cell 58
Cell 59
Cell 60
Fluorescence (Raw)
Time Point (0.25 Hz)
0
200
400
600
800
0.8
1.2
1.6
2.0
20
40
60
80
100
120
140
160
2
4
6
8
10

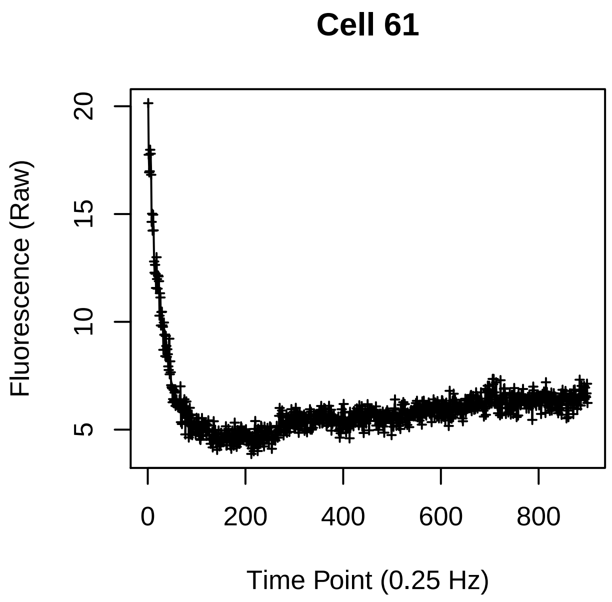
Cell 61
Fluorescence (Raw)
5
10
15
20
0
200
400
600
800
Time Point (0.25 Hz)

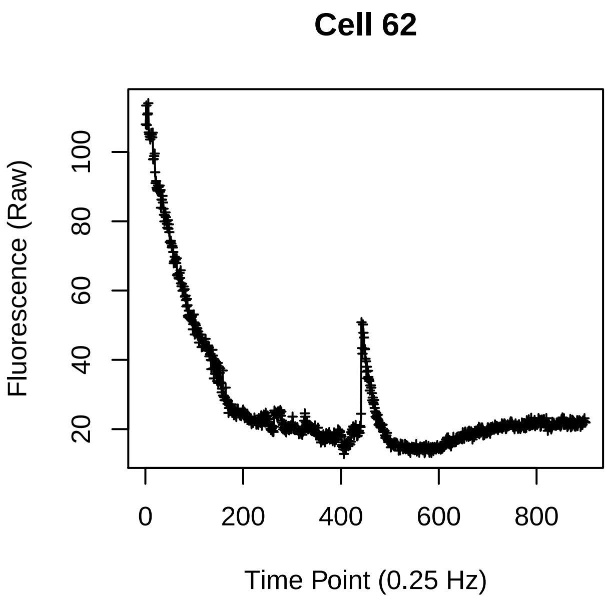
Cell 62
Fluorescence (Raw)
20
40
60
80
100
0
200
400
600
800
Time Point (0.25 Hz)

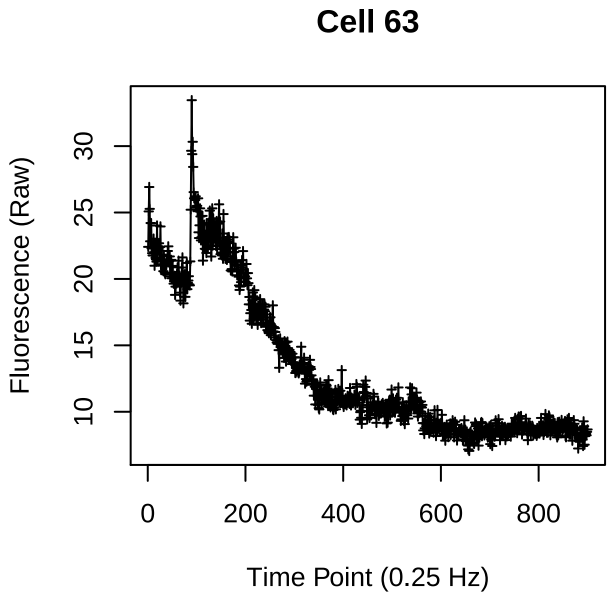
Cell 63
Fluorescence (Raw)
10
15
20
25
30
0
200
400
600
800
Time Point (0.25 Hz)

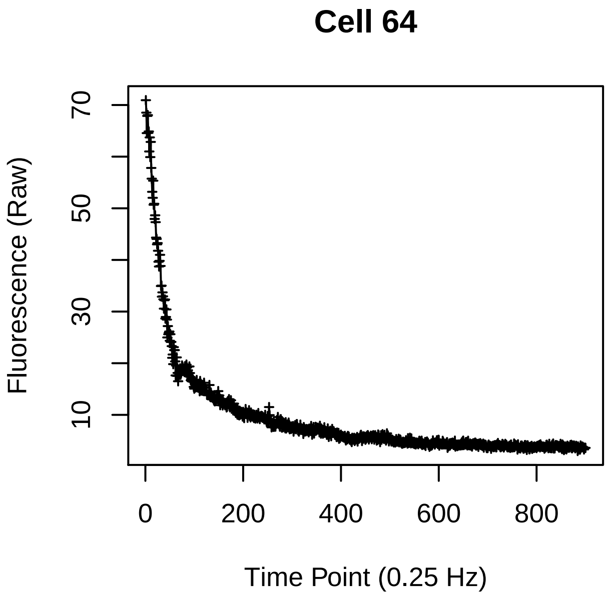
Cell 64
Fluorescence (Raw)
10
30
50
70
0
200
400
600
800
Time Point (0.25 Hz)

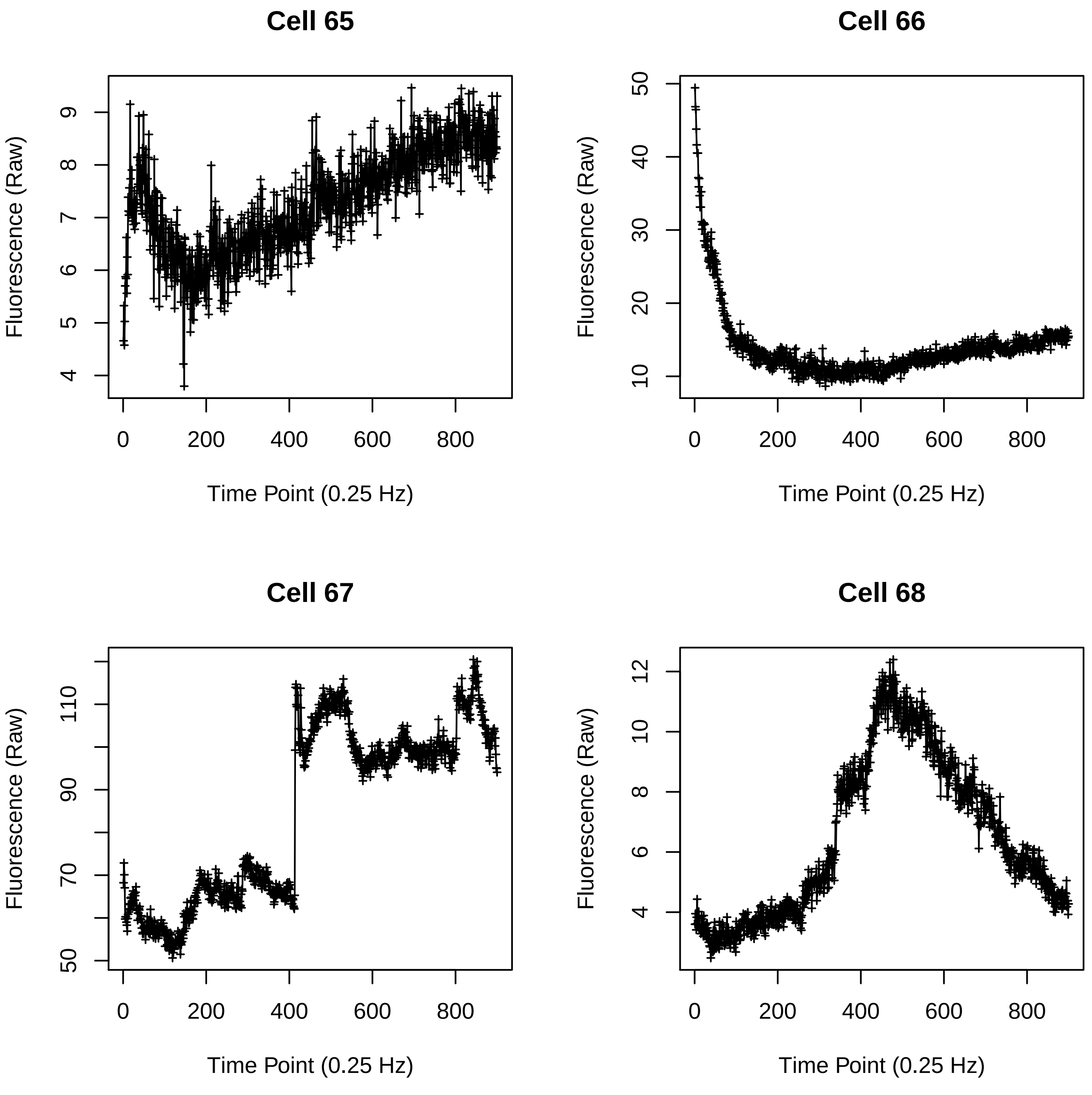

Cell 65
Cell 66
Cell 67
Cell 68
Fluorescence (Raw)
Time Point (0.25 Hz)
0
200
400
600
800
4
5
6
7
8
9
10
20
30
40
50
70
90
110
12

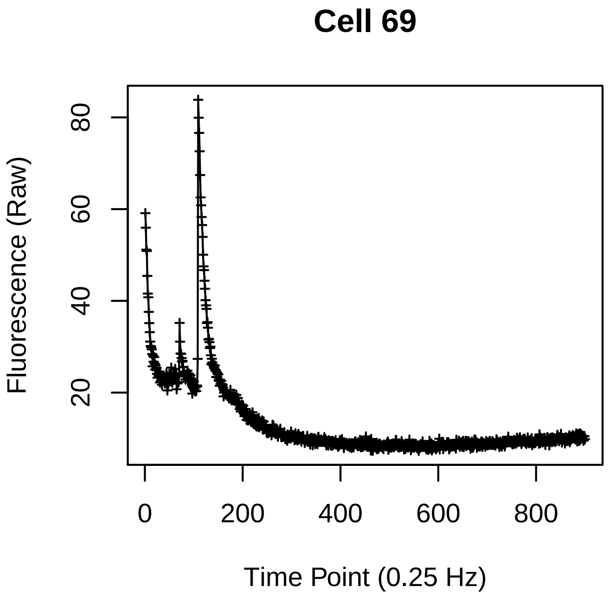
Cell 69
Fluorescence (Raw)
20
40
60
80
0
200
400
600
800
Time Point (0.25 Hz)

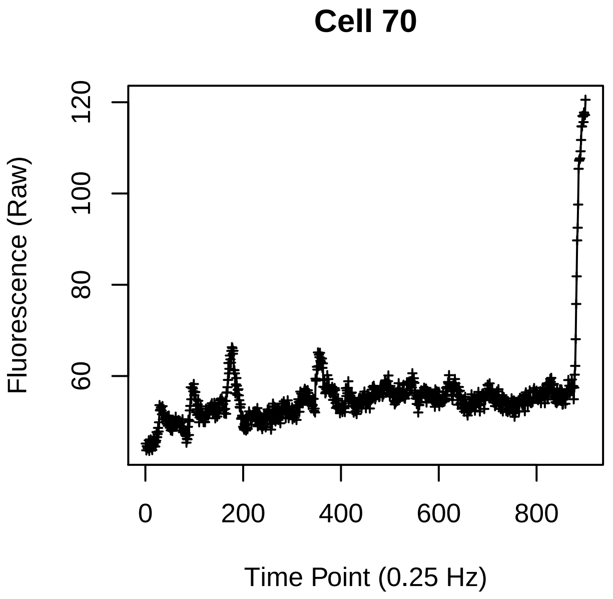
Cell 70
Fluorescence (Raw)
60
80
100
120
0
200
400
600
800
Time Point (0.25 Hz)

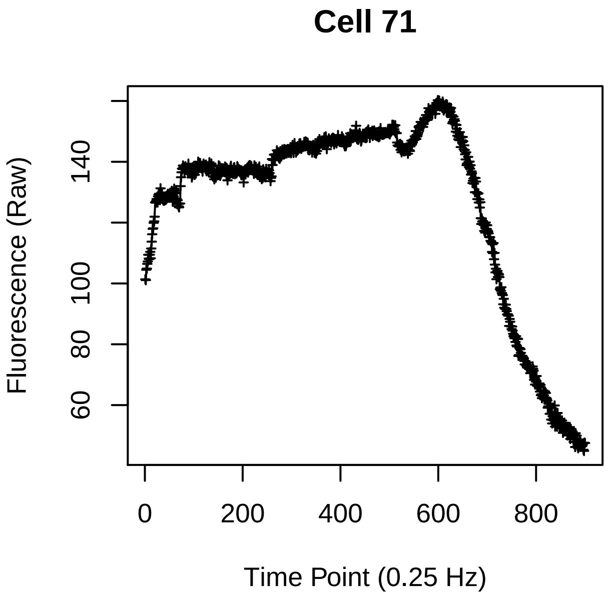
Cell 71
Fluorescence (Raw)
60
80
100
140
0
200
400
600
800
Time Point (0.25 Hz)

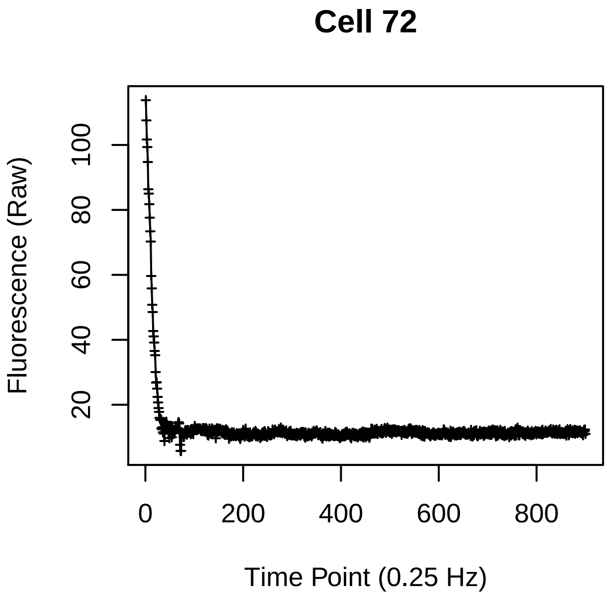
Cell 72
Fluorescence (Raw)
20
40
60
80
100
0
200
400
600
800
Time Point (0.25 Hz)

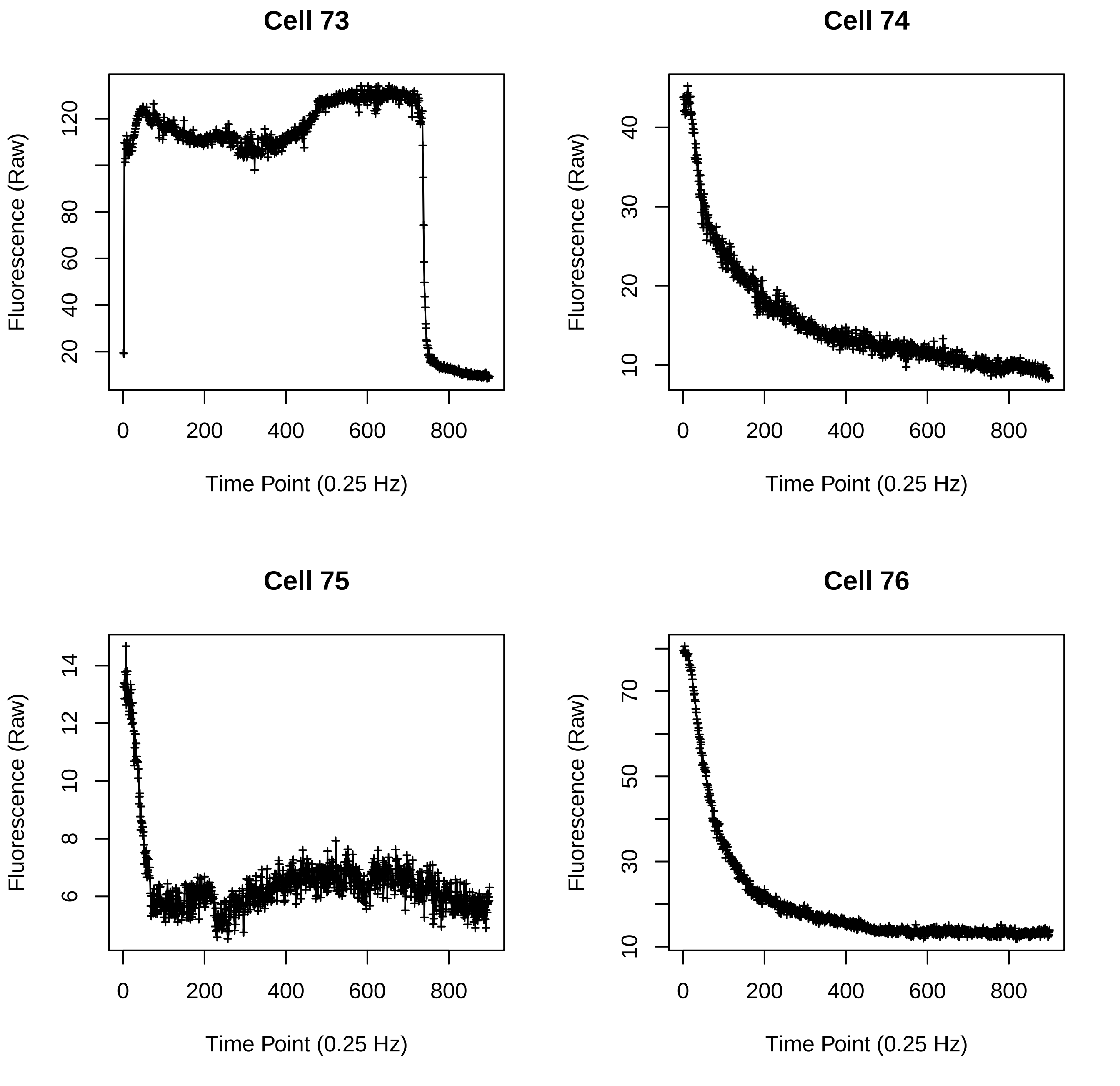

Cell 73
Cell 74
Cell 75
Cell 76
Fluorescence (Raw)
Time Point (0.25 Hz)
0
200
400
600
800
20
40
60
80
120
10
20
30
40
6
8
10
12
14
10
30
50
70

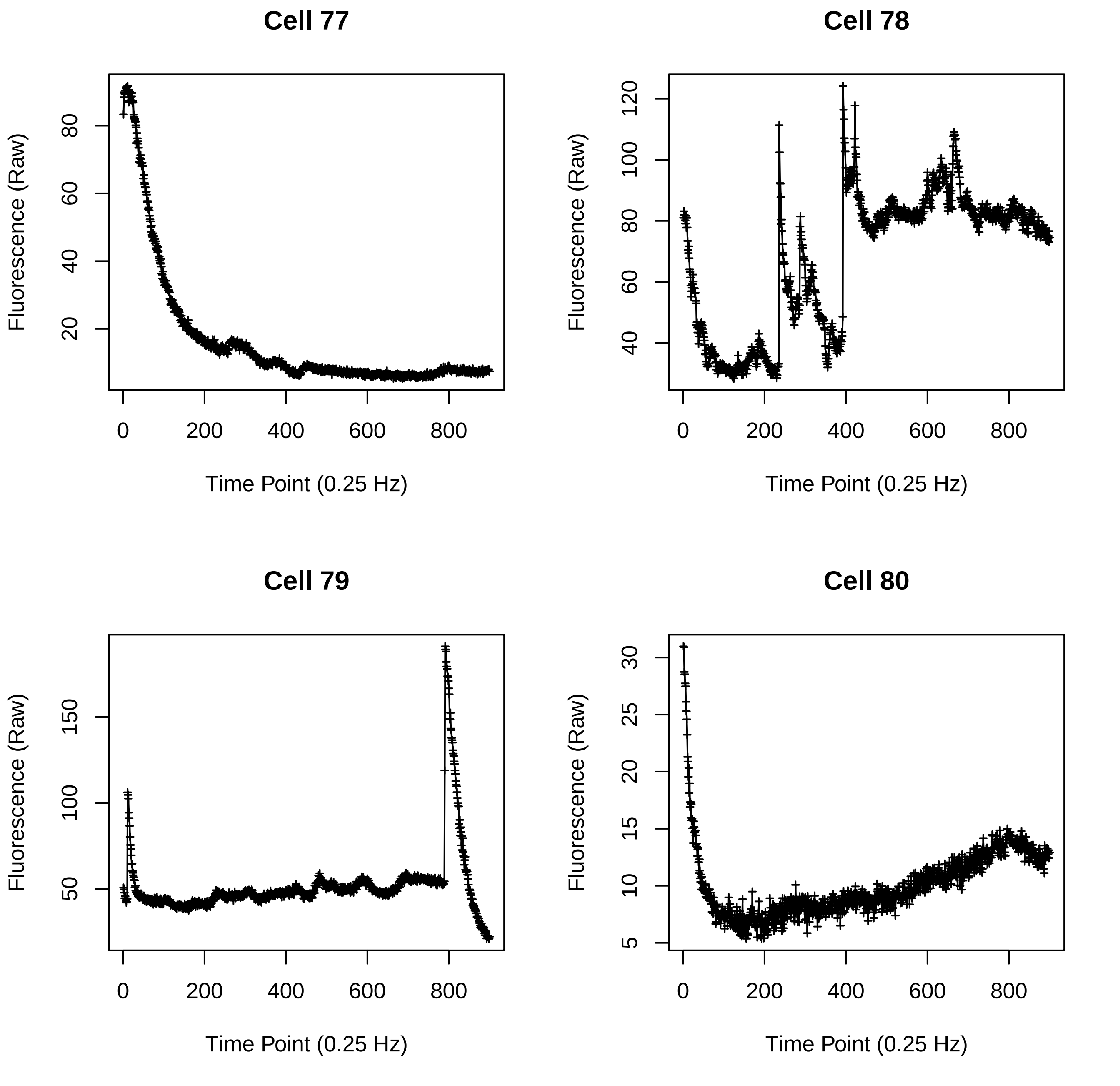

Cell 77
Cell 78
Cell 79
Cell 80
Fluorescence (Raw)
Time Point (0.25 Hz)
0
200
400
600
800
20
40
60
80
100
120
50
150
5
10
15
25
30

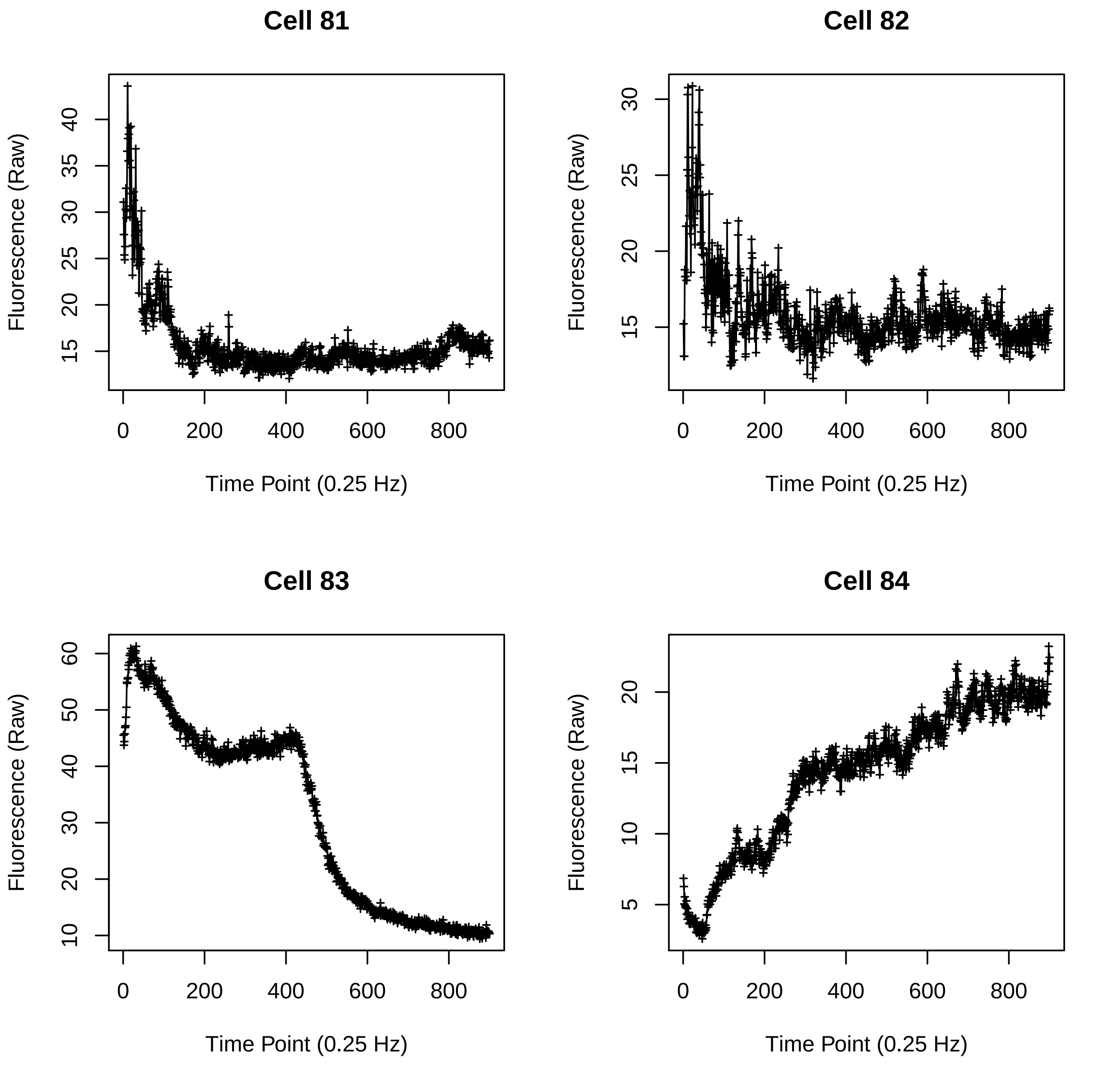

Cell 81
Cell 82
Cell 83
Cell 84
Fluorescence (Raw)
Time Point (0.25 Hz)
0
200
400
600
800
15
20
25
30
35
40
10
50
60
5

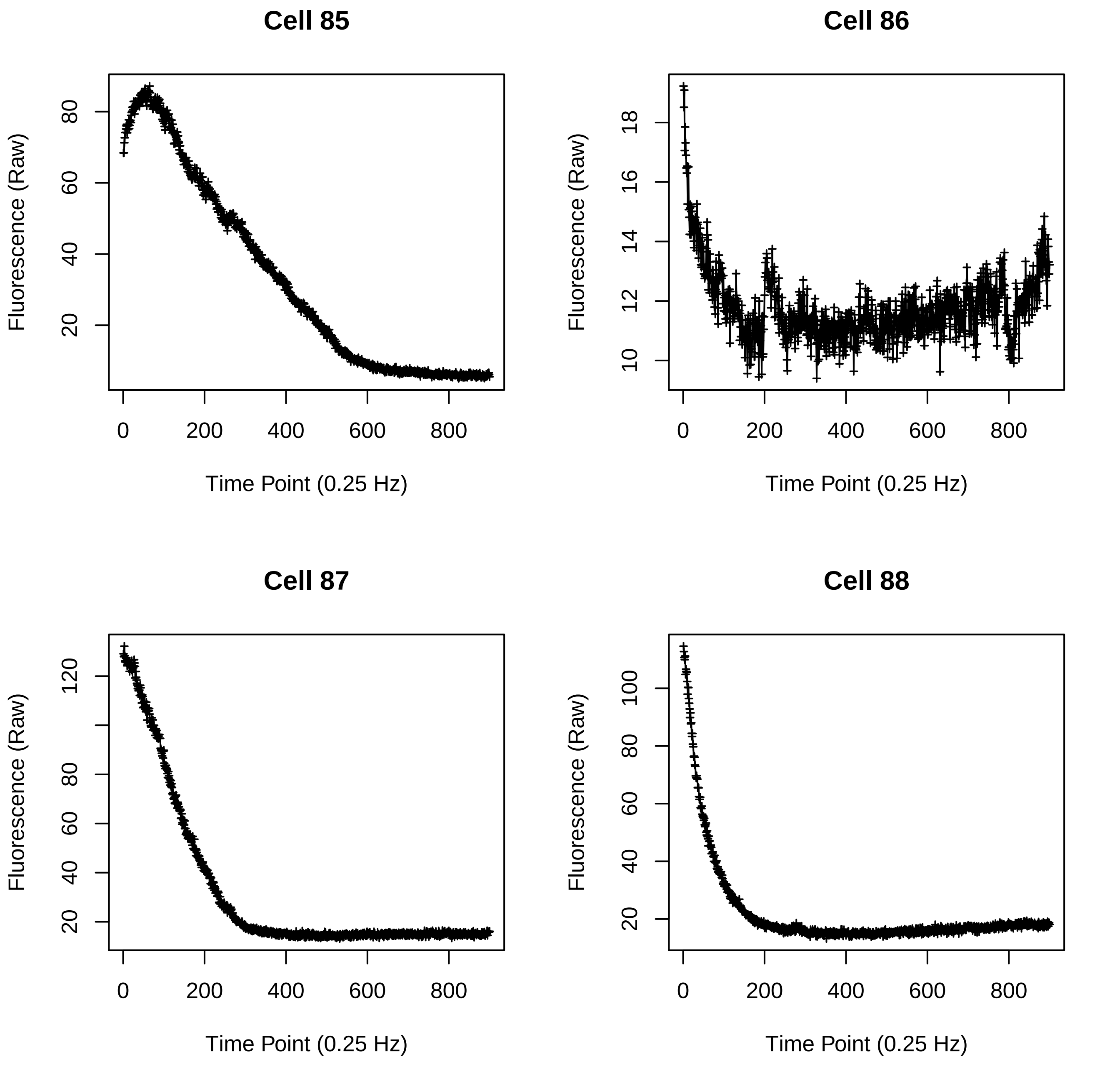
Cell 85
Cell 86
Cell 87
Cell 88
Fluorescence (Raw)
Time Point (0.25 Hz)
0
200
400
600
800
20
40
60
80
10
12
14
16
18
120
100

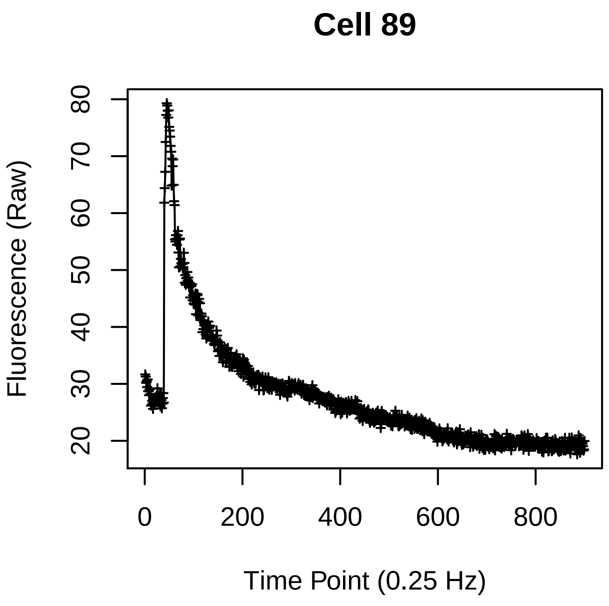
Cell 89
Fluorescence (Raw)
20
30
40
50
60
70
80
0
200
400
600
800
Time Point (0.25 Hz)

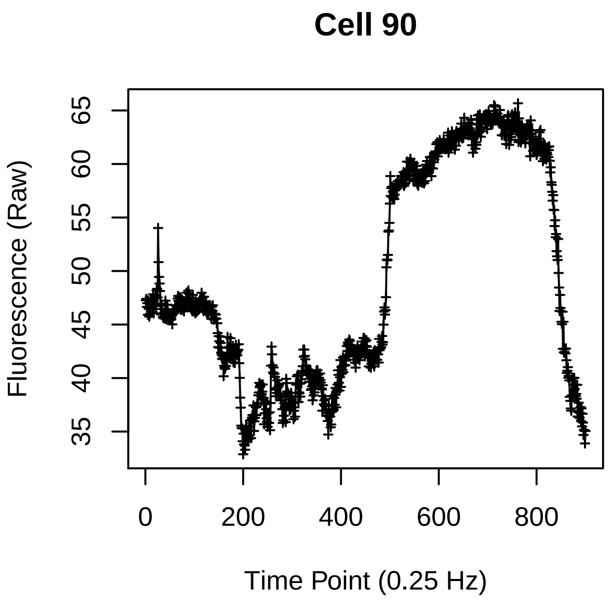
Cell 90
Fluorescence (Raw)
35
40
45
50
55
60
65
0
200
400
600
800
Time Point (0.25 Hz)

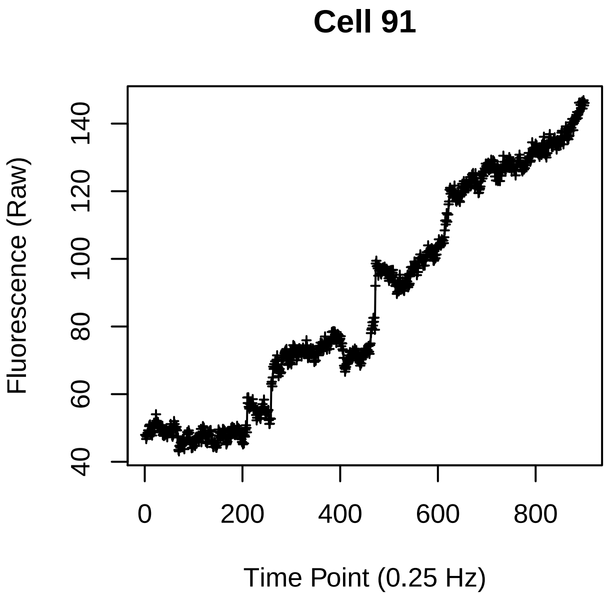
Cell 91
Fluorescence (Raw)
40
60
80
100
120
140
0
200
400
600
800
Time Point (0.25 Hz)

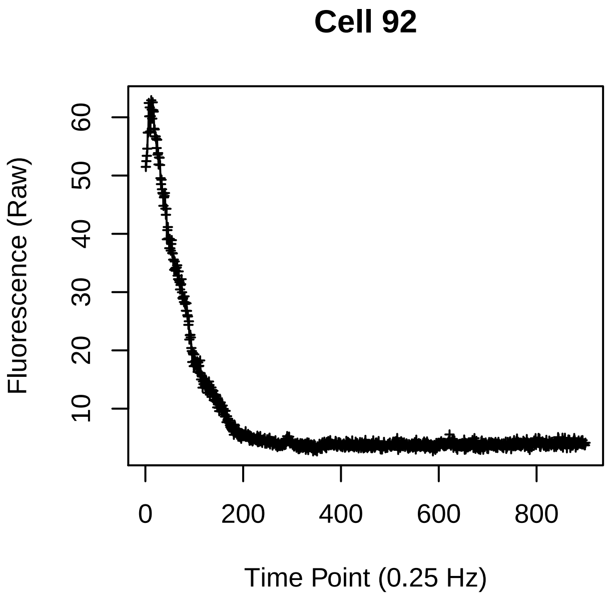
Cell 92
Fluorescence (Raw)
10
20
30
40
50
60
0
200
400
600
800
Time Point (0.25 Hz)

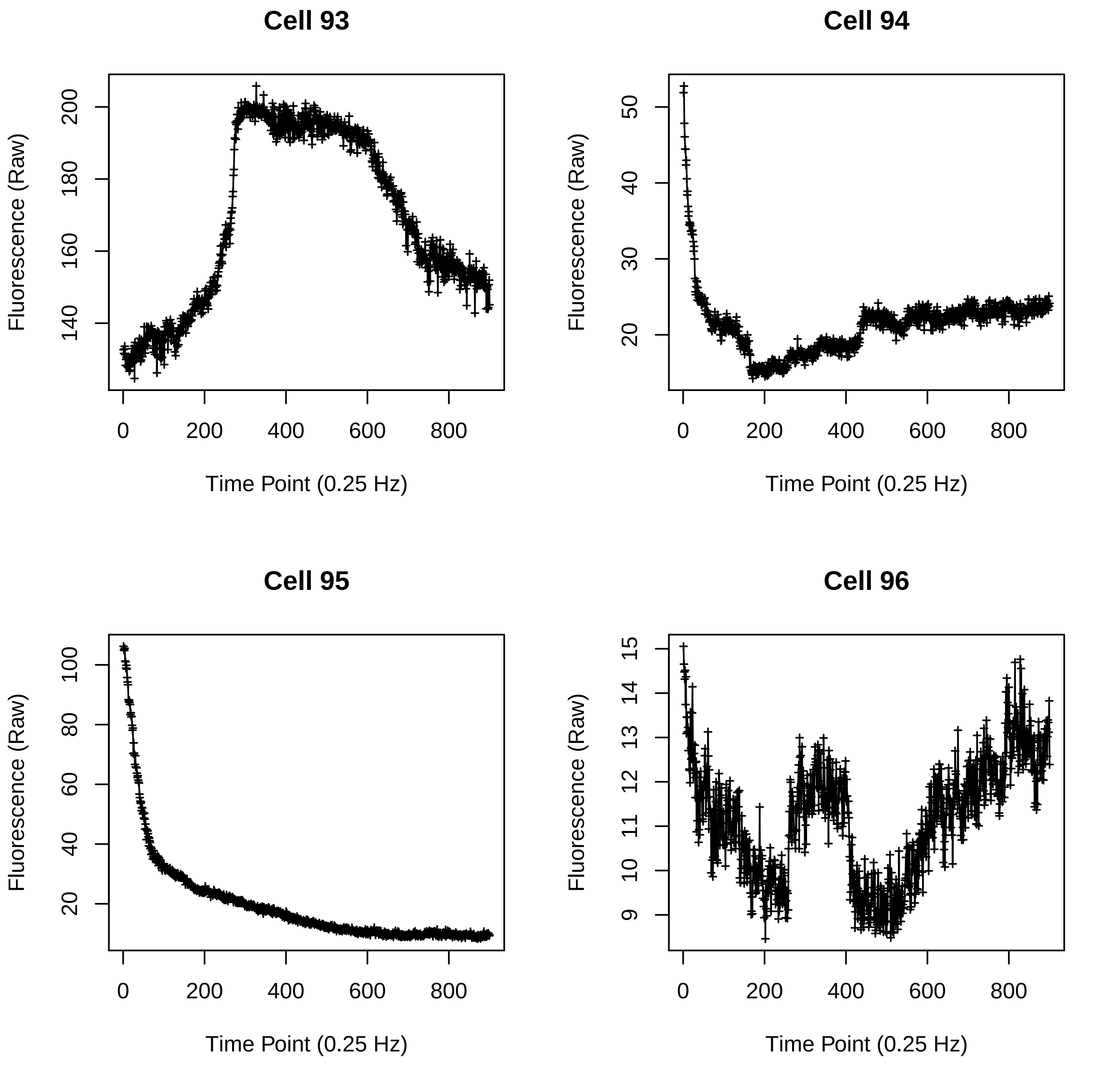

Cell 93
Fluorescence (Raw)
Time Point (0.25 Hz)
Cell 94
Fluorescence (Raw)
Time Point (0.25 Hz)
Cell 95
Fluorescence (Raw)
Time Point (0.25 Hz)
Cell 96
Fluorescence (Raw)
Time Point (0.25 Hz)

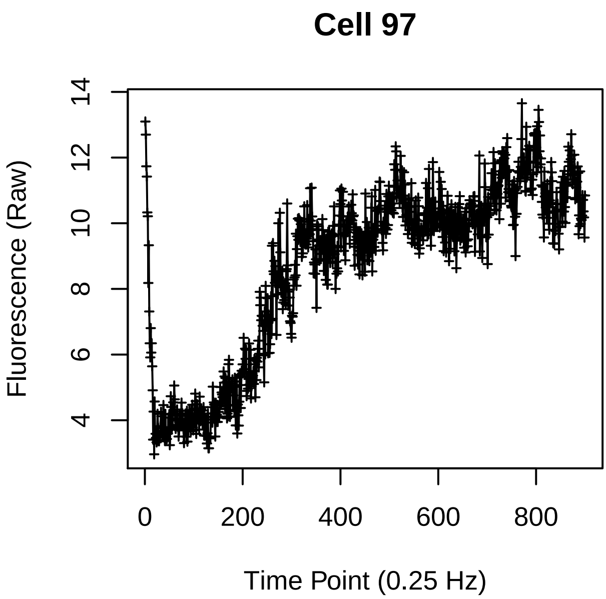
Cell 97
Fluorescence (Raw)
Time Point (0.25 Hz)
4
6
8
10
12
14
0
200
400
600
800

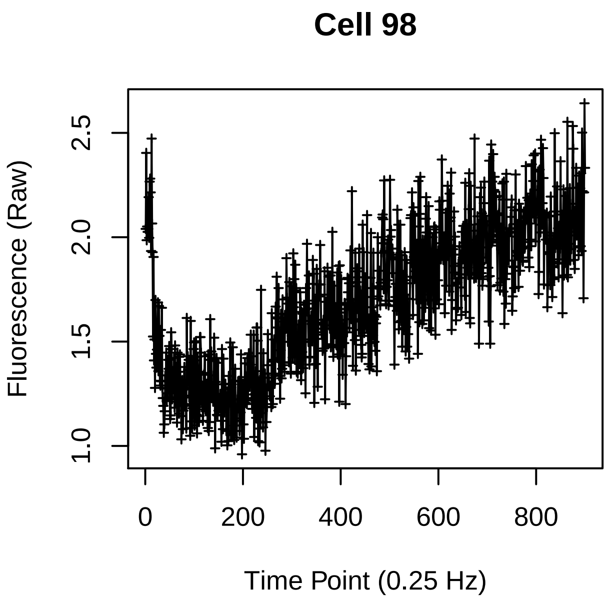
Cell 98
Fluorescence (Raw)
Time Point (0.25 Hz)
1.0
1.5
2.0
2.5
0
200
400
600
800

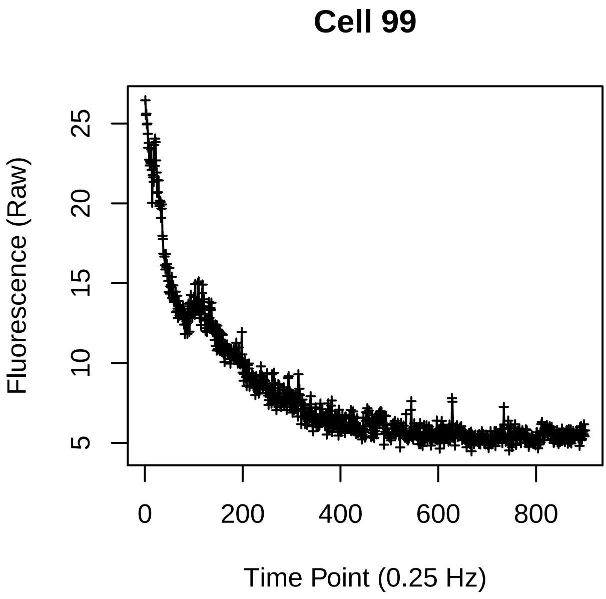
Cell 99
Fluorescence (Raw)
Time Point (0.25 Hz)
5
10
15
20
25
0
200
400
600
800

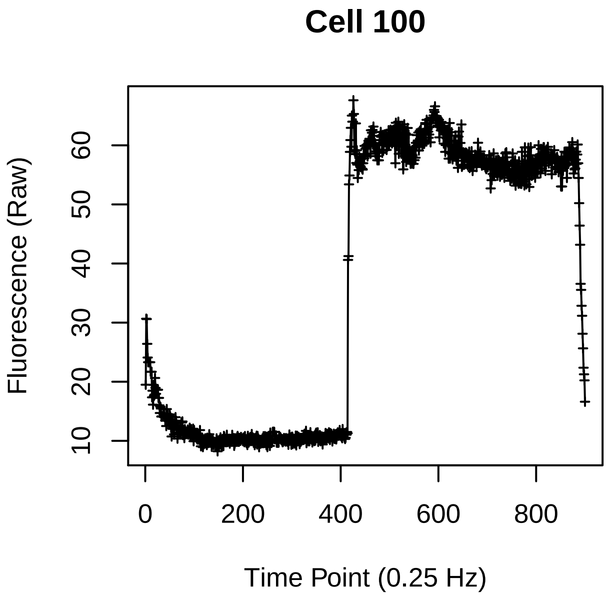
Cell 100
Fluorescence (Raw)
Time Point (0.25 Hz)
10
20
30
40
50
60
0
200
400
600
800

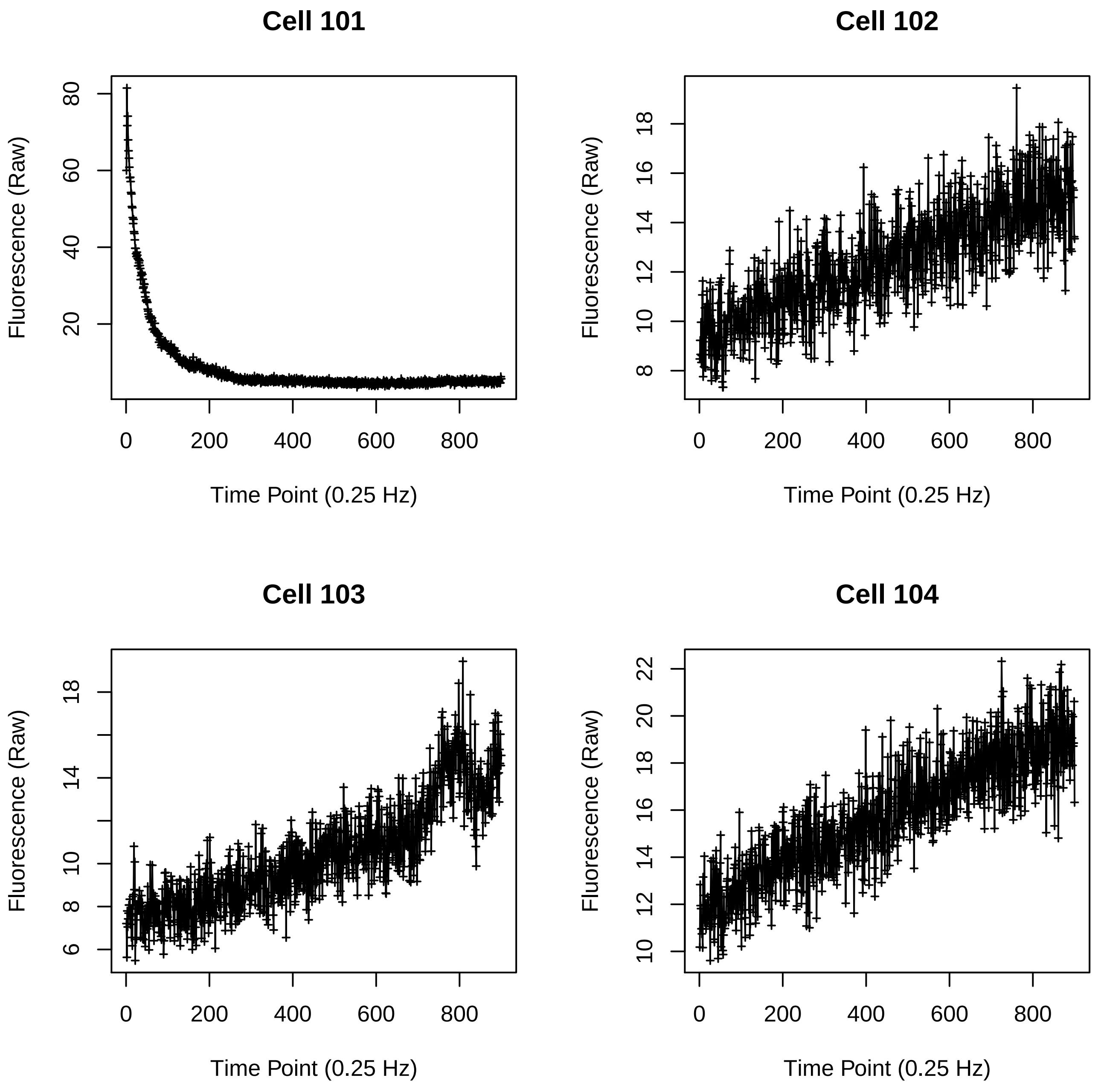
Cell 101
Fluorescence (Raw)
20
40
60
80
0
200
400
600
800
Time Point (0.25 Hz)
Cell 102
Fluorescence (Raw)
8
10
12
14
16
18
0
200
400
600
800
Time Point (0.25 Hz)
Cell 103
Fluorescence (Raw)
6
8
10
14
18
0
200
400
600
800
Time Point (0.25 Hz)
Cell 104
Fluorescence (Raw)
10
12
14
16
18
20
22
0
200
400
600
800
Time Point (0.25 Hz)

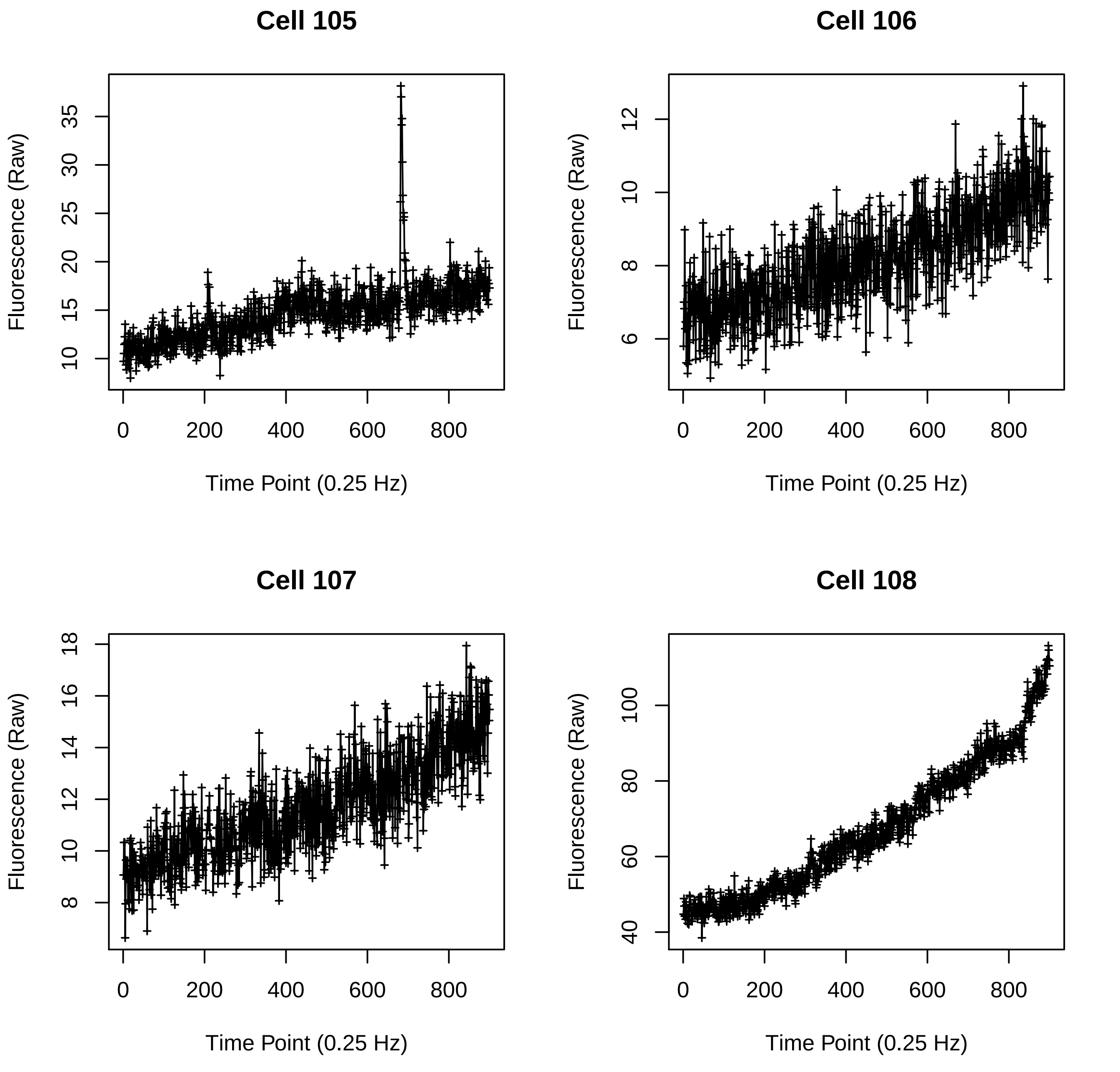
Cell 105
Fluorescence (Raw)
10
15
20
25
30
35
0
200
400
600
800
Time Point (0.25 Hz)
Cell 106
Fluorescence (Raw)
6
8
10
12
0
200
400
600
800
Time Point (0.25 Hz)
Cell 107
Fluorescence (Raw)
8
10
12
14
16
18
0
200
400
600
800
Time Point (0.25 Hz)
Cell 108
Fluorescence (Raw)
40
60
80
100
0
200
400
600
800
Time Point (0.25 Hz)

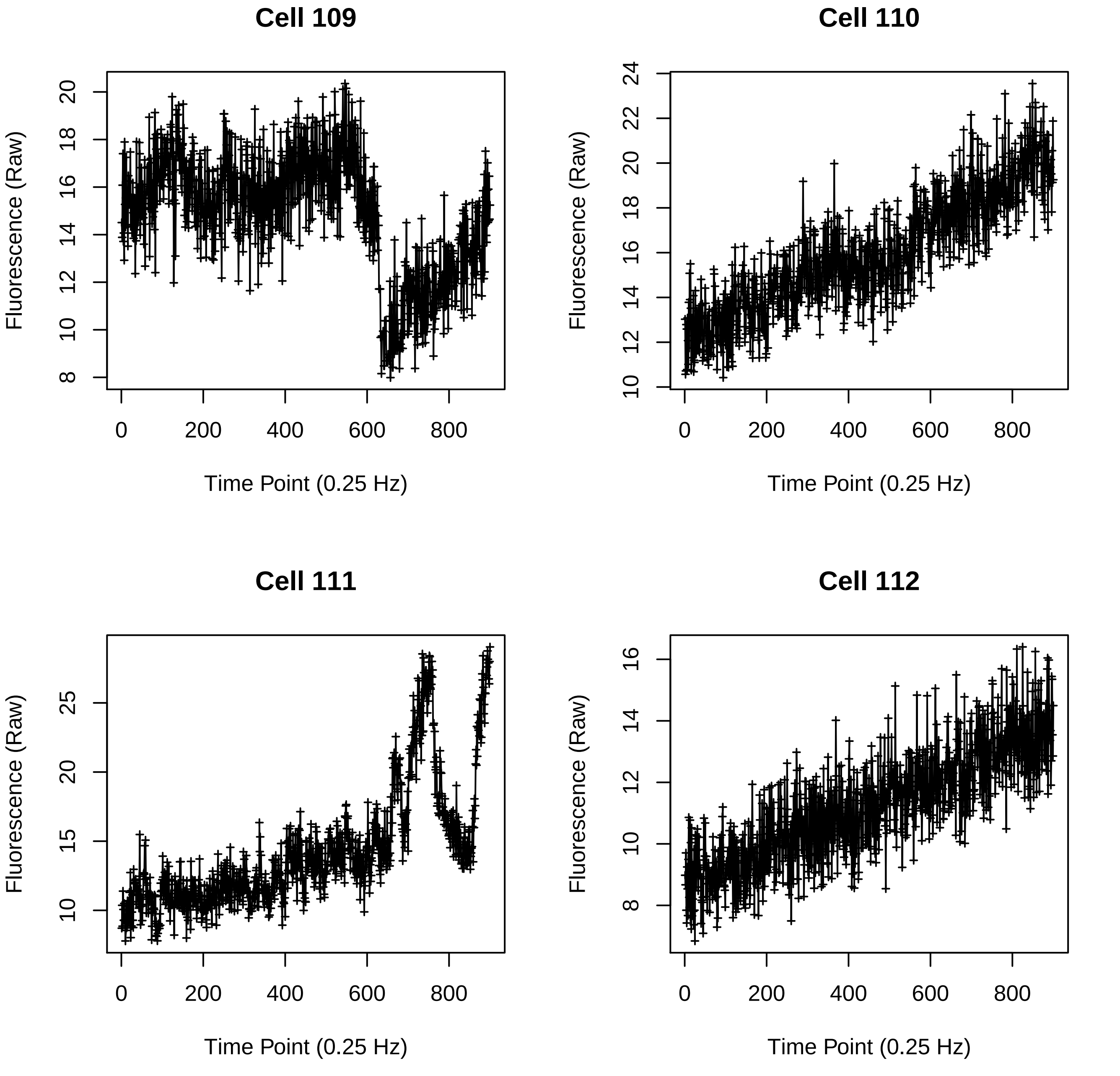
Cell 109
Cell 110
Cell 111
Cell 112
Fluorescence (Raw)
Time Point (0.25 Hz)
0
200
400
600
800
8
10
12
14
16
18
20
22
24
25

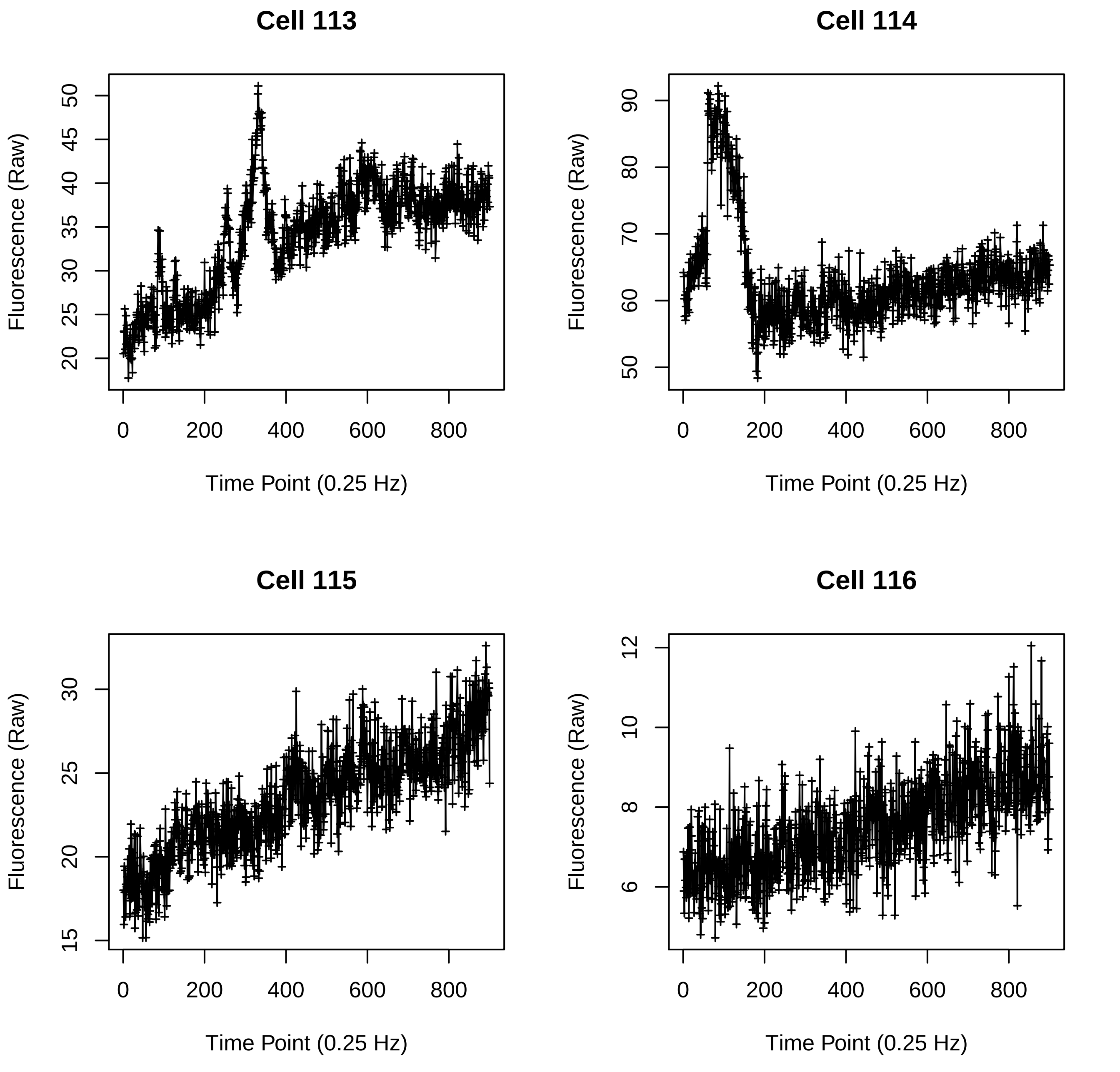
Cell 113
Cell 114
Cell 115
Cell 116
Fluorescence (Raw)
Time Point (0.25 Hz)
0
200
400
600
800
20
25
30
35
40
45
50
60
70
80
90
15
6
8
10
12

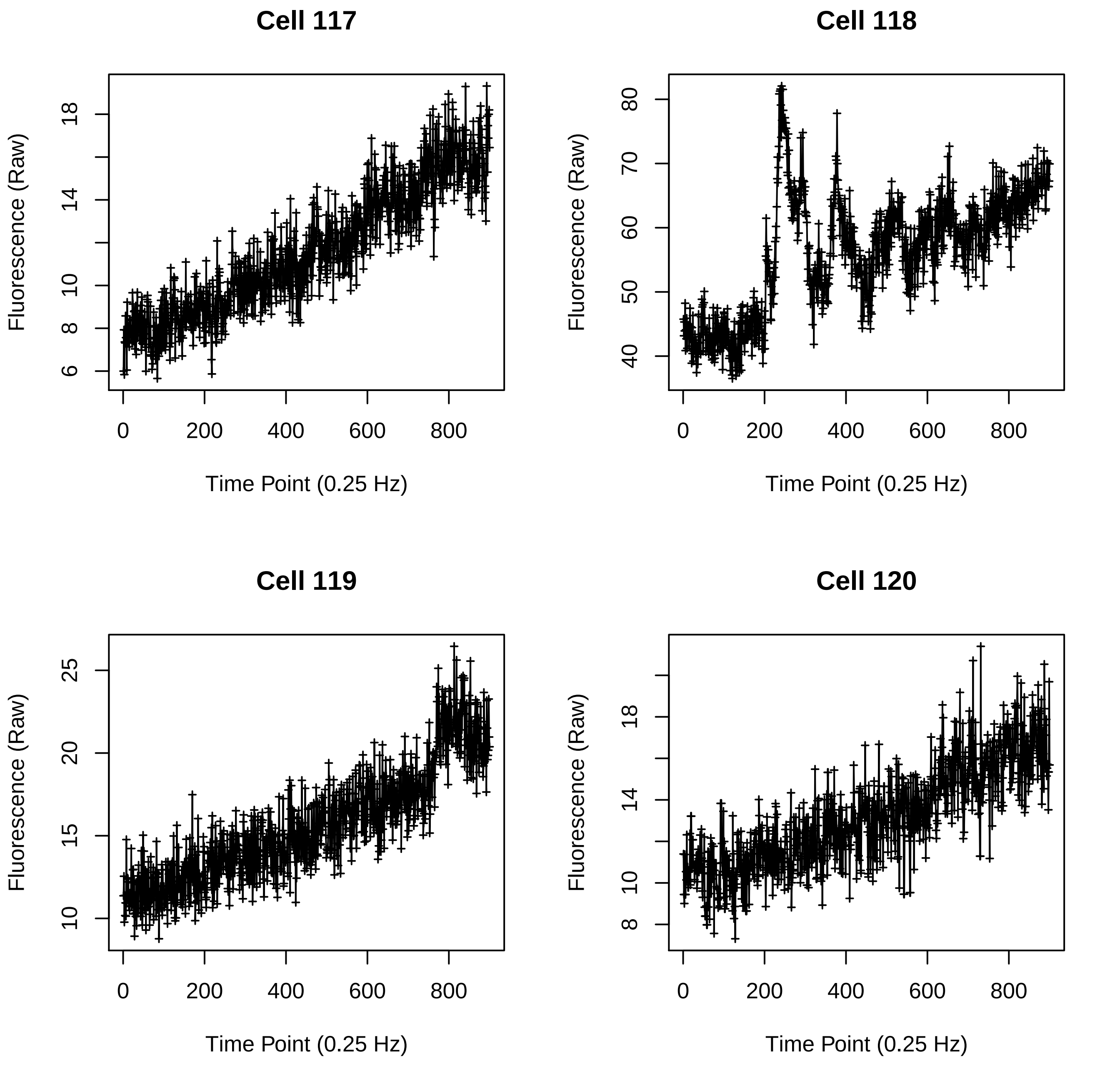

Cell 117
Fluorescence (Raw)
Time Point (0.25 Hz)
Cell 118
Fluorescence (Raw)
Time Point (0.25 Hz)
Cell 119
Fluorescence (Raw)
Time Point (0.25 Hz)
Cell 120
Fluorescence (Raw)
Time Point (0.25 Hz)

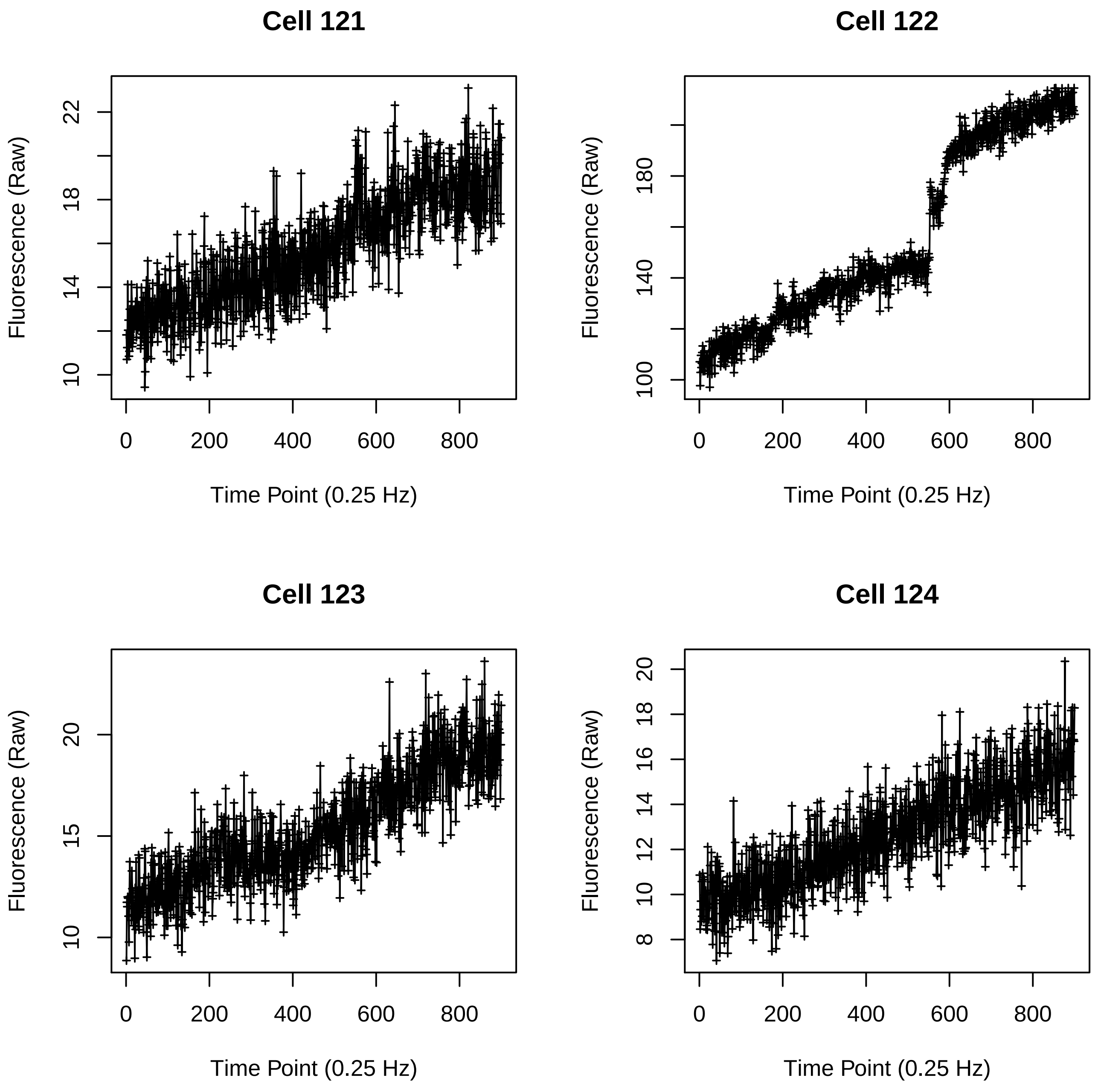
Cell 121
Cell 122
Cell 123
Cell 124
Fluorescence (Raw)
Time Point (0.25 Hz)
10
14
18
22
100
140
180
15
20
8
12
16
0
200
400
600
800

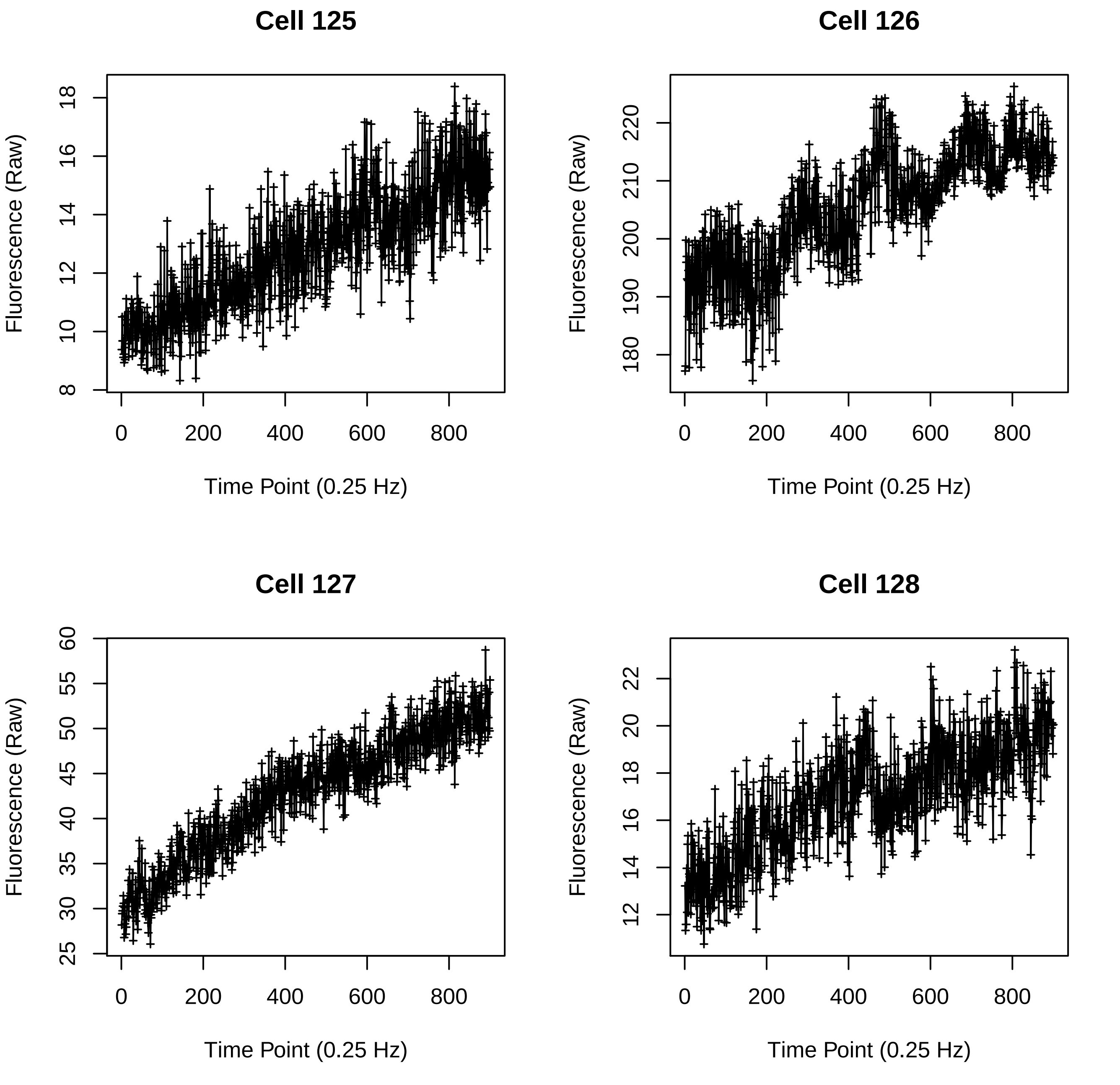

Cell 125
Fluorescence (Raw)
8
10
12
14
16
18
0
200
400
600
800
Time Point (0.25 Hz)
Cell 126
Fluorescence (Raw)
180
190
200
210
220
0
200
400
600
800
Time Point (0.25 Hz)
Cell 127
Fluorescence (Raw)
25
30
35
40
45
50
55
60
0
200
400
600
800
Time Point (0.25 Hz)
Cell 128
Fluorescence (Raw)
12
14
16
18
20
22
0
200
400
600
800
Time Point (0.25 Hz)

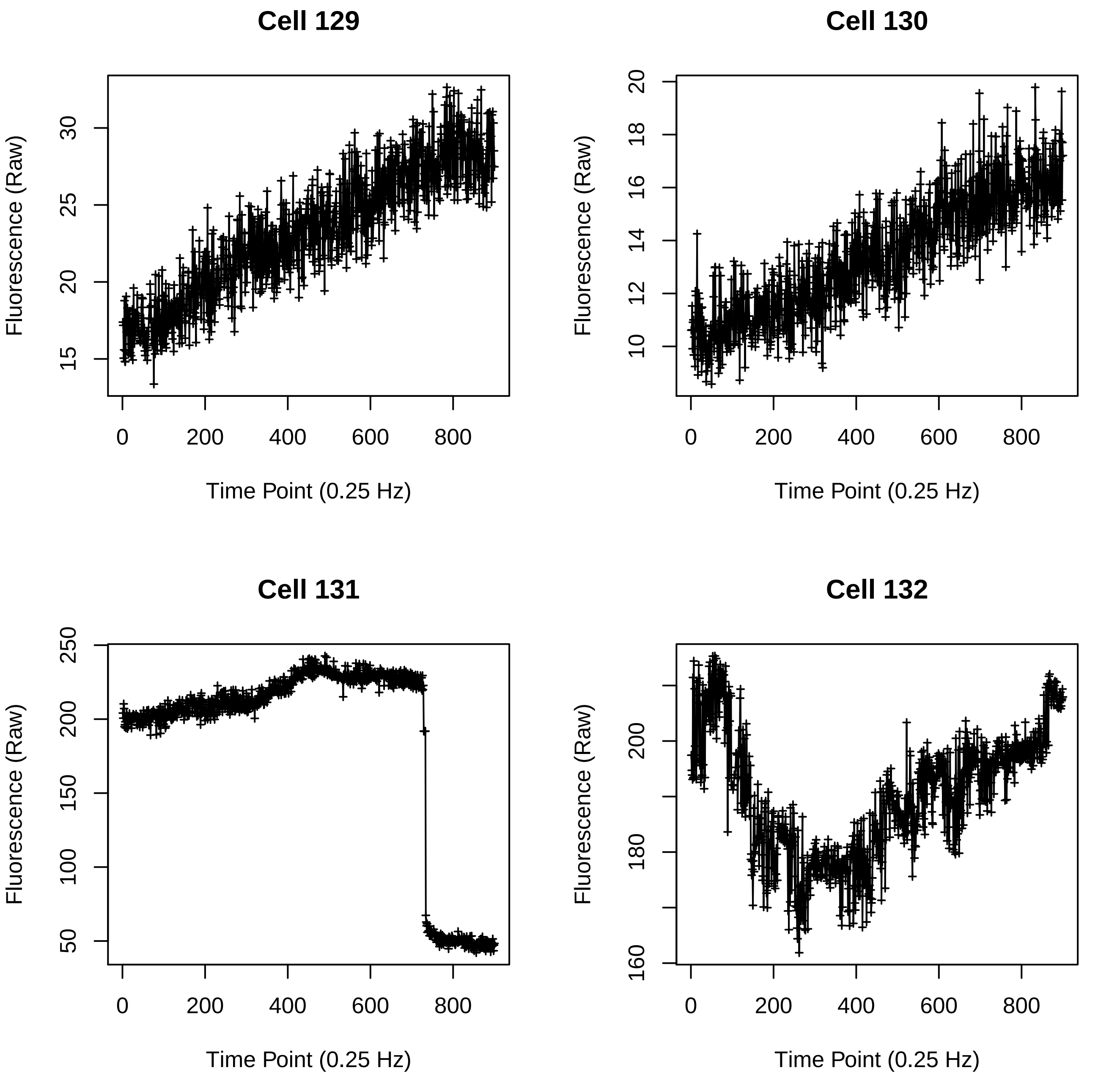
Cell 129
Cell 130
Cell 131
Cell 132
Fluorescence (Raw)
Time Point (0.25 Hz)
0
200
400
600
800
15
20
25
30
10
12
14
16
18
20
50
100
150
200
250
160
180
200

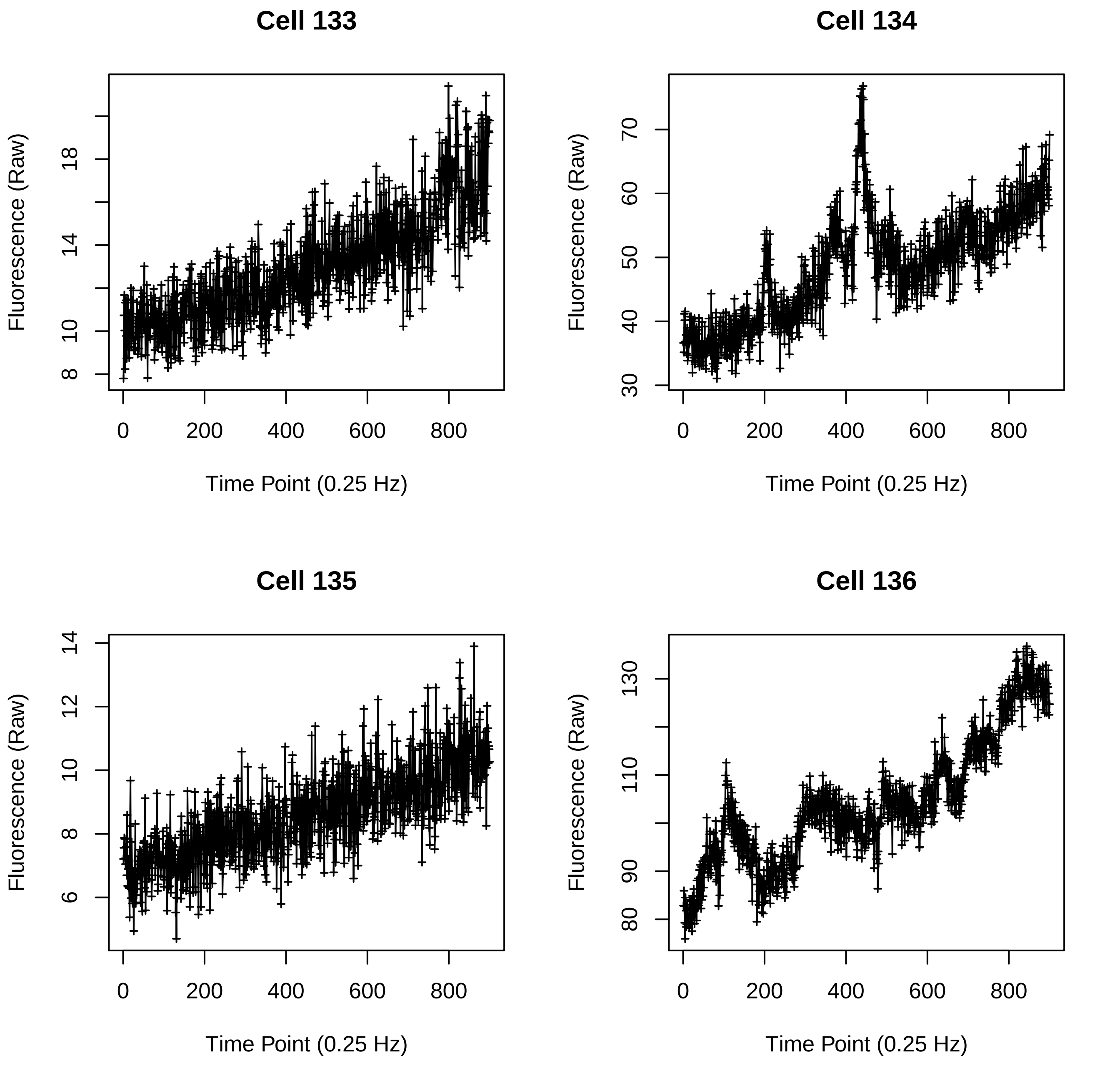
Cell 133
Cell 134
Cell 135
Cell 136
Fluorescence (Raw)
Time Point (0.25 Hz)
0
200
400
600
800
8
10
14
18
30
40
50
60
70
6
8
10
12
14
80
90
110
130

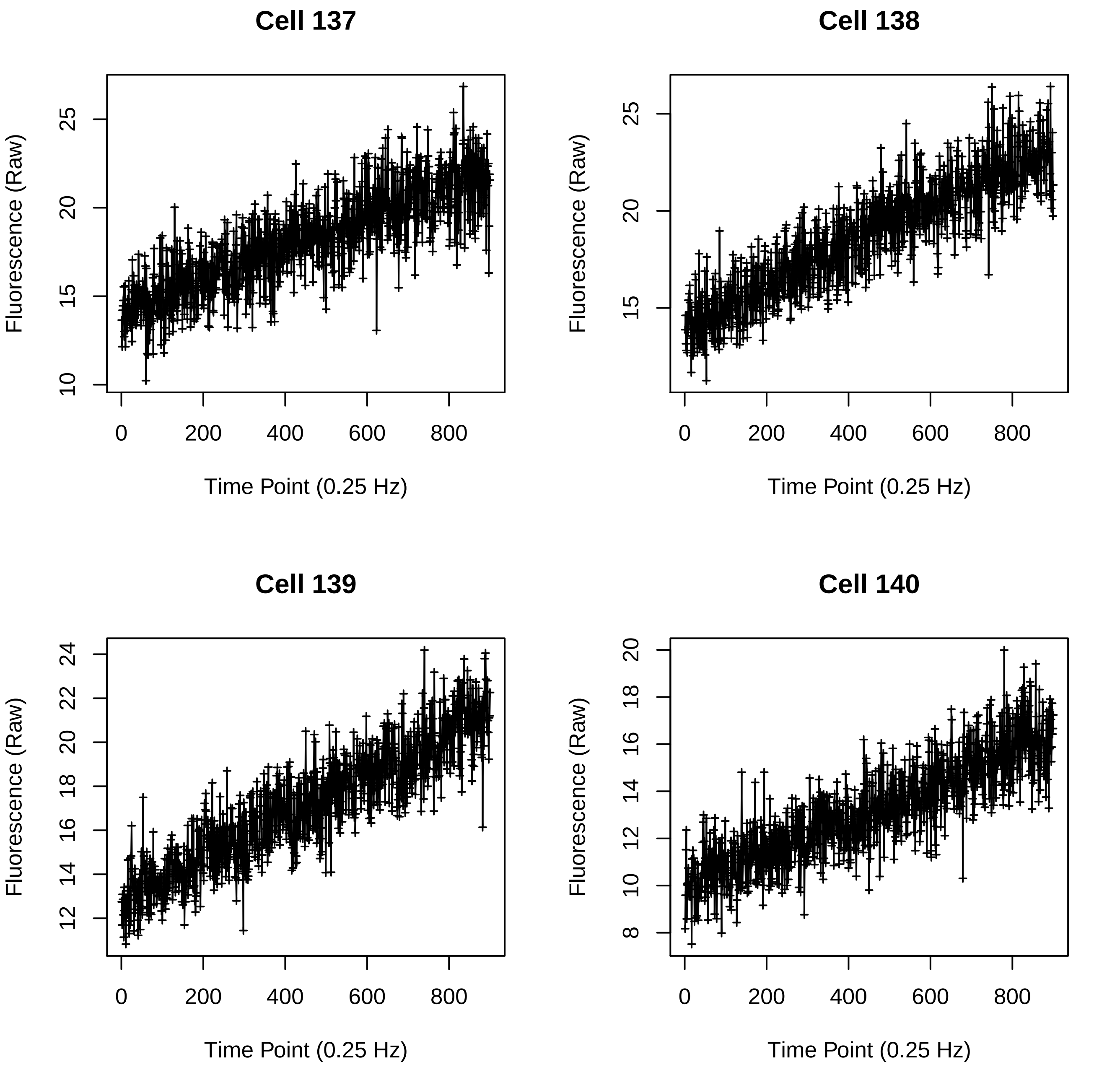

Cell 137
Cell 138
Cell 139
Cell 140
Fluorescence (Raw)
Time Point (0.25 Hz)
0
200
400
600
800
10
15
20
25
8
12
14
16
18
22
24

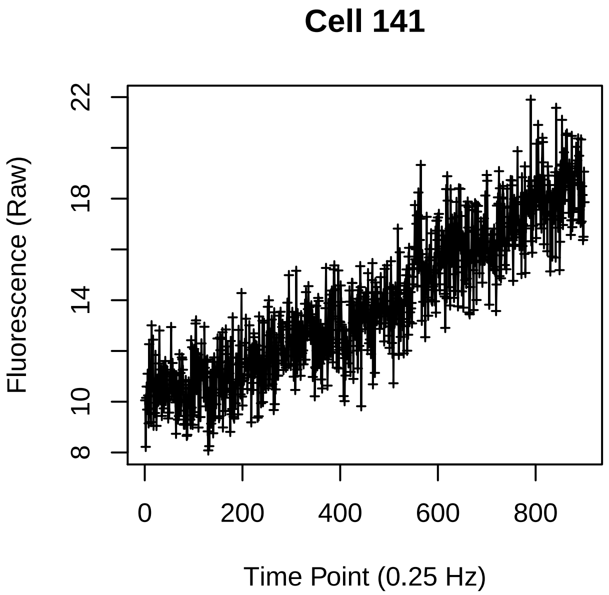
Cell 141
Fluorescence (Raw)
8
10
14
18
22
0
200
400
600
800
Time Point (0.25 Hz)

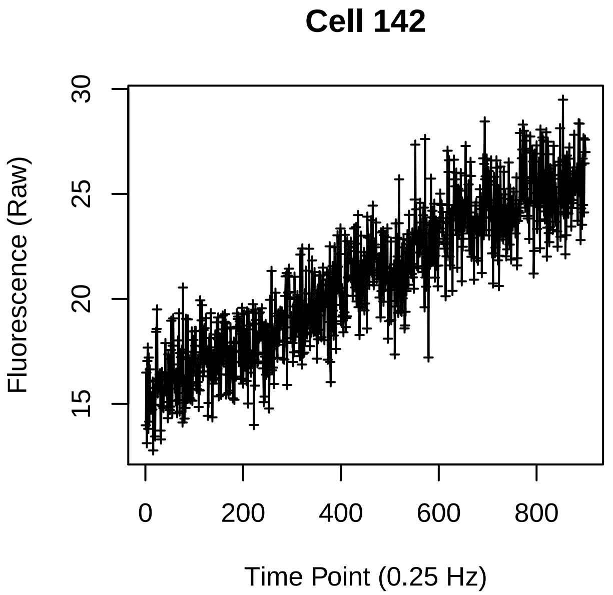
Cell 142
Fluorescence (Raw)
15
20
25
30
0
200
400
600
800
Time Point (0.25 Hz)

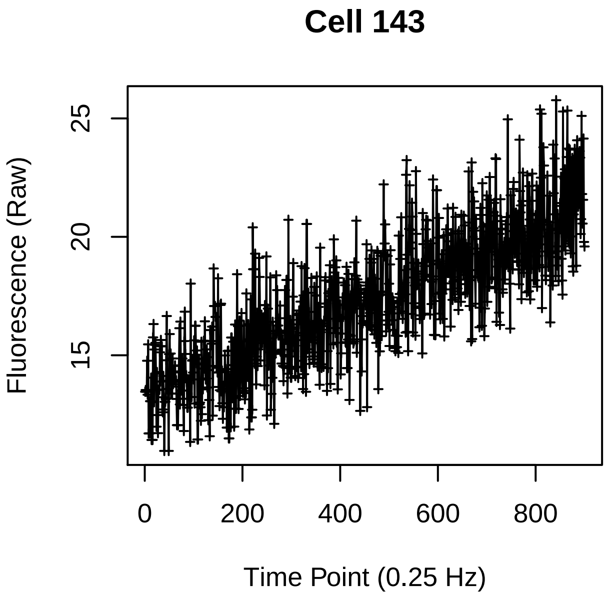
Cell 143
Fluorescence (Raw)
15
20
25
0
200
400
600
800
Time Point (0.25 Hz)

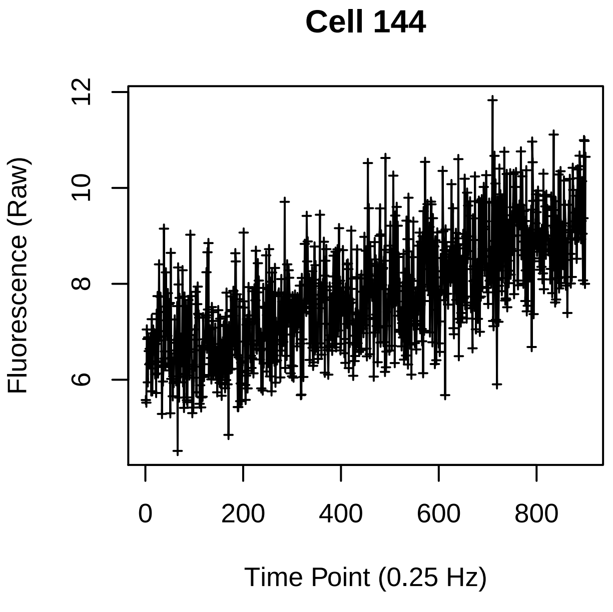
Cell 144
Fluorescence (Raw)
6
8
10
12
0
200
400
600
800
Time Point (0.25 Hz)

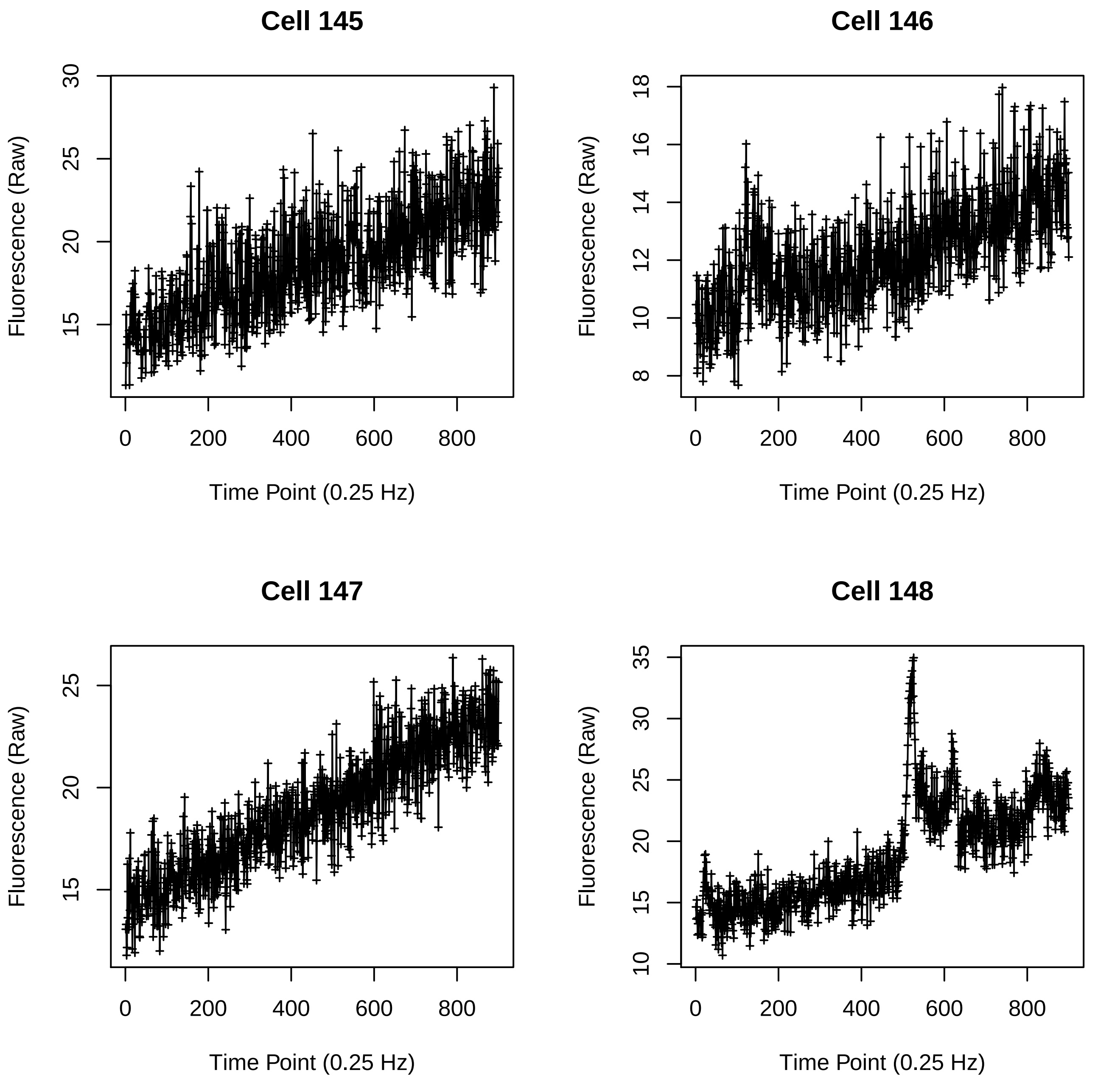

Cell 145
Fluorescence (Raw)
Time Point (0.25 Hz)
15
20
25
30
0
200
400
600
800
Cell 146
Fluorescence (Raw)
Time Point (0.25 Hz)
8
10
12
14
16
18
0
200
400
600
800
Cell 147
Fluorescence (Raw)
Time Point (0.25 Hz)
15
20
25
0
200
400
600
800
Cell 148
Fluorescence (Raw)
Time Point (0.25 Hz)
10
15
20
25
30
35
0
200
400
600
800

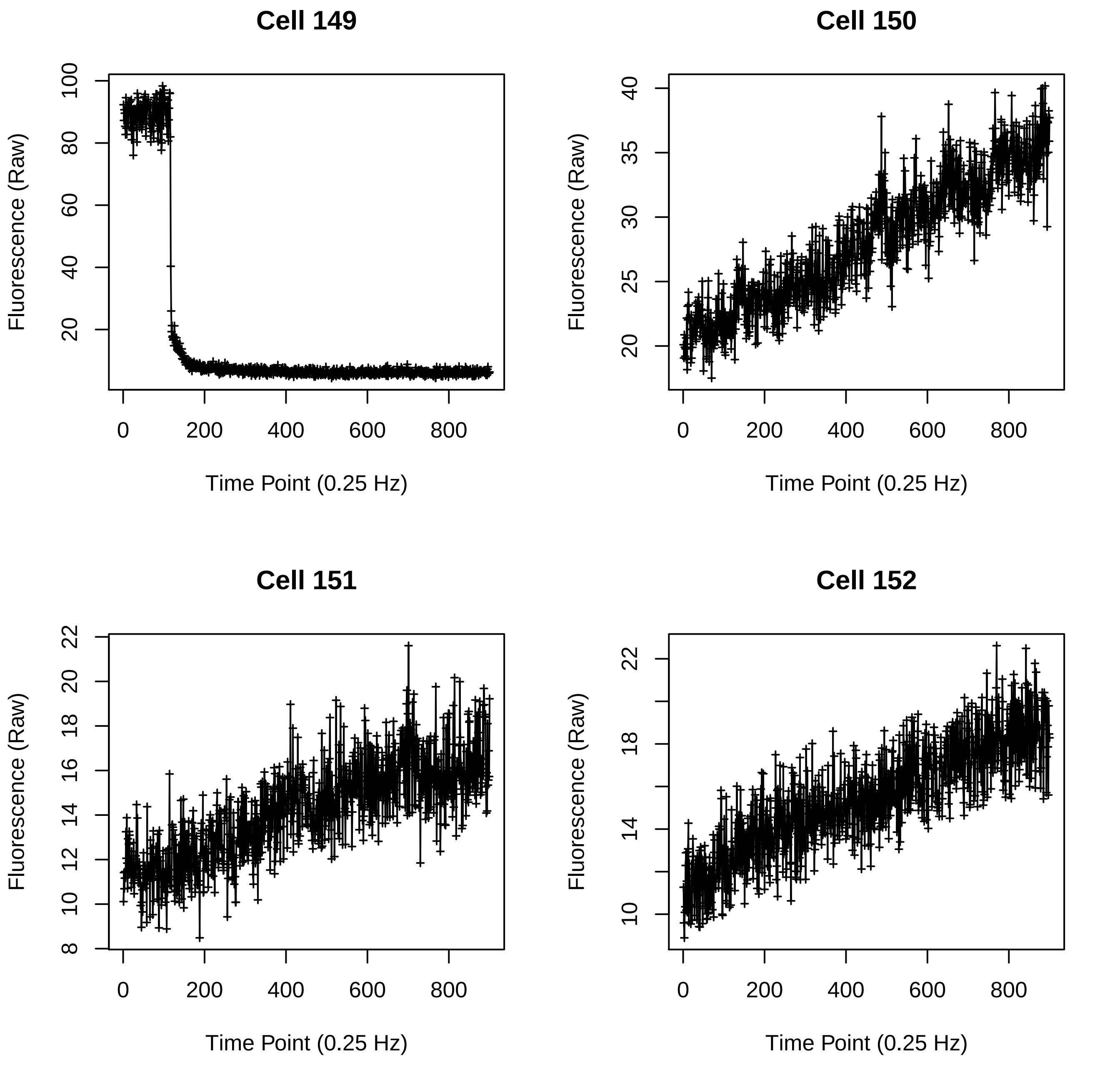
Cell 149
Fluorescence (Raw)
20
40
60
80
100
0
200
400
600
800
Time Point (0.25 Hz)
Cell 150
Fluorescence (Raw)
20
25
30
35
40
0
200
400
600
800
Time Point (0.25 Hz)
Cell 151
Fluorescence (Raw)
8
10
12
14
16
18
20
22
0
200
400
600
800
Time Point (0.25 Hz)
Cell 152
Fluorescence (Raw)
10
14
18
22
0
200
400
600
800
Time Point (0.25 Hz)

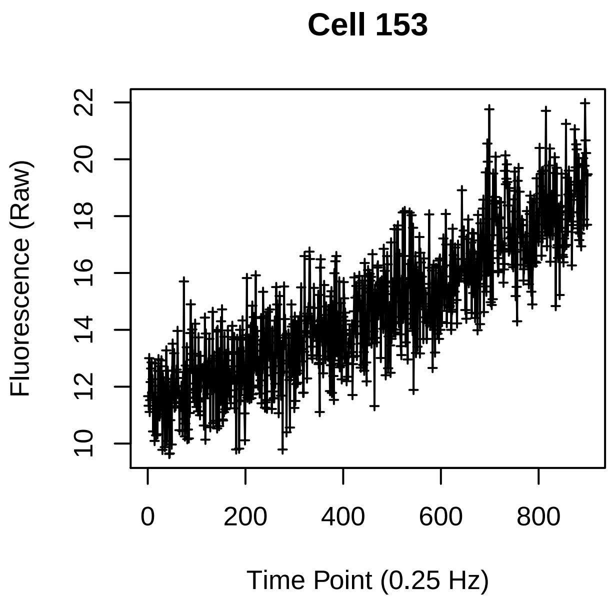
Cell 153
Fluorescence (Raw)
10
12
14
16
18
20
22
0
200
400
600
800
Time Point (0.25 Hz)

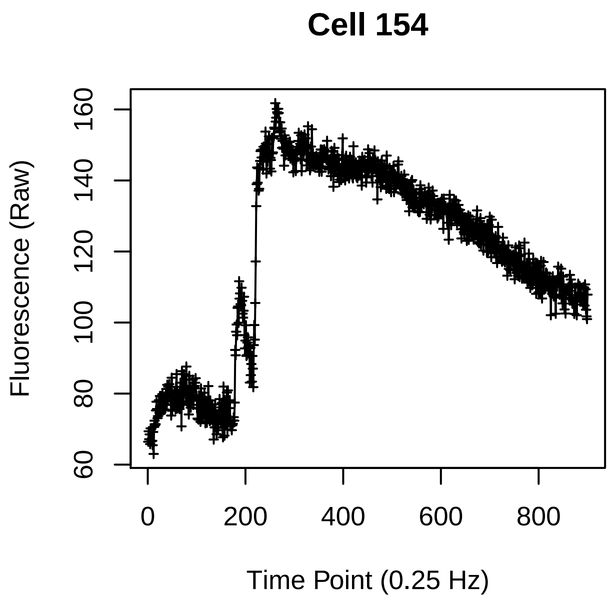
Cell 154
Fluorescence (Raw)
60
80
100
120
140
160
0
200
400
600
800
Time Point (0.25 Hz)

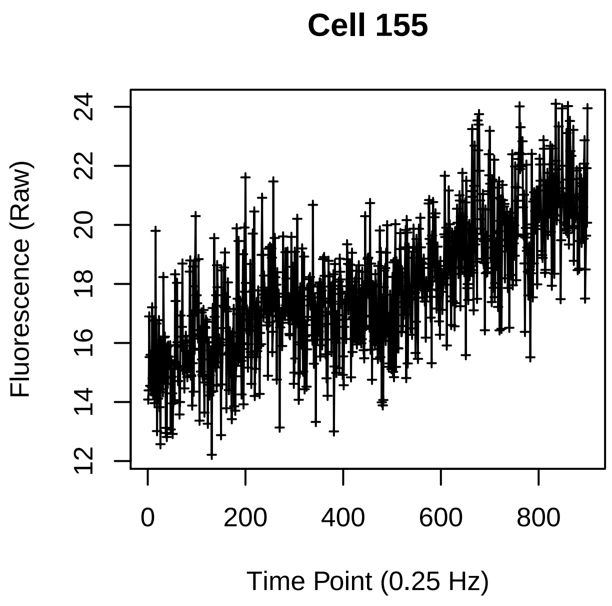
Cell 155
Fluorescence (Raw)
12
14
16
18
20
22
24
0
200
400
600
800
Time Point (0.25 Hz)

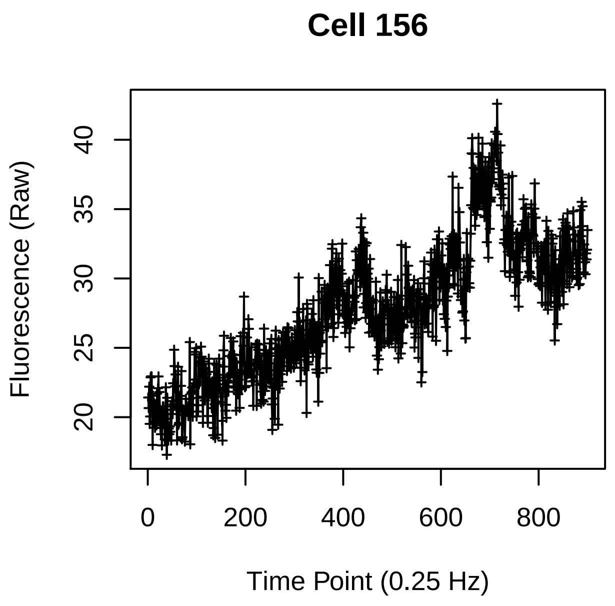
Cell 156
Fluorescence (Raw)
20
25
30
35
40
0
200
400
600
800
Time Point (0.25 Hz)

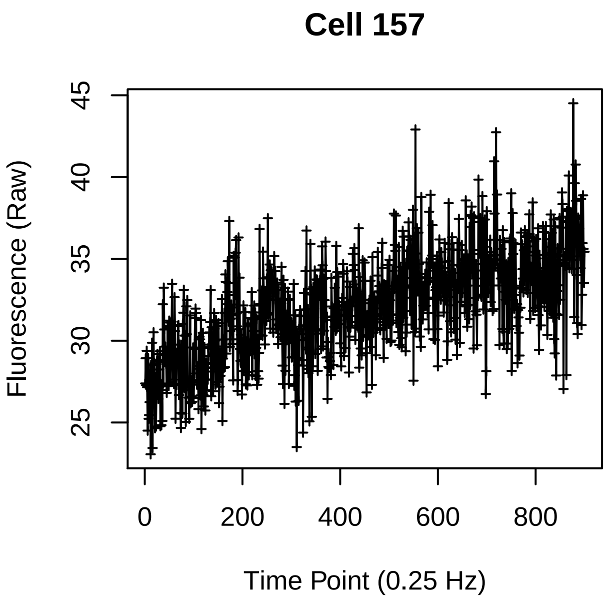
Cell 157
Fluorescence (Raw)
25
30
35
40
45
0
200
400
600
800
Time Point (0.25 Hz)

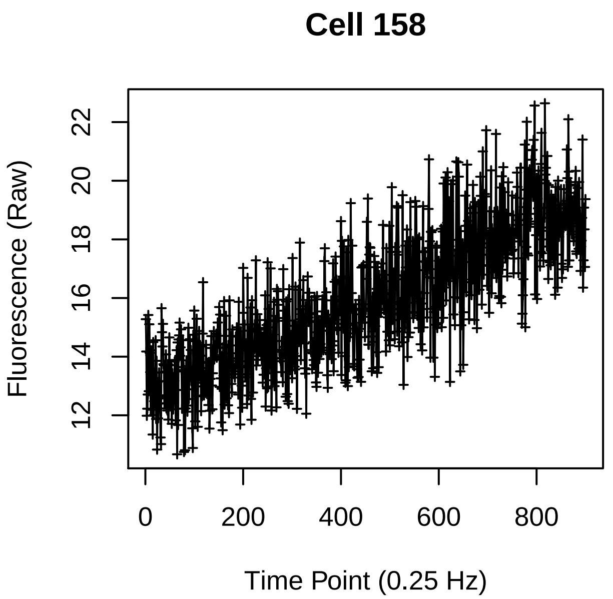
Cell 158
Fluorescence (Raw)
12
14
16
18
20
22
0
200
400
600
800
Time Point (0.25 Hz)

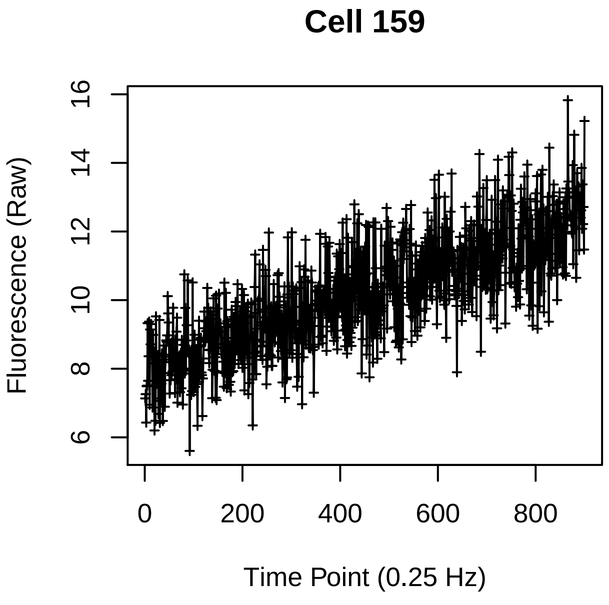
Cell 159
Fluorescence (Raw)
6
8
10
12
14
16
0
200
400
600
800
Time Point (0.25 Hz)

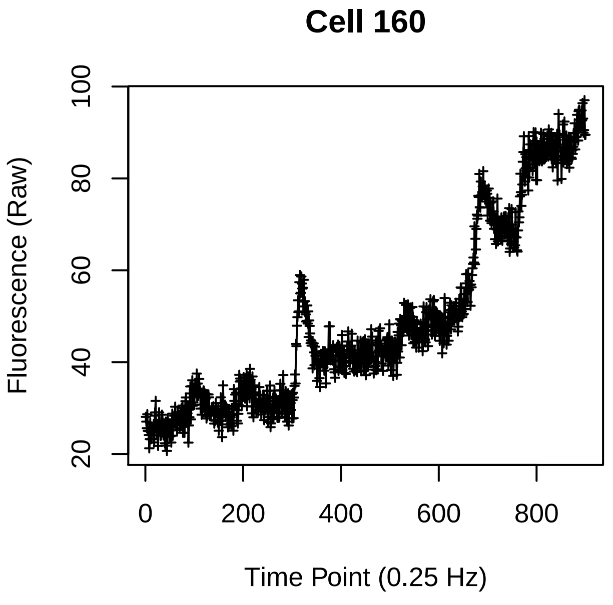
Cell 160
Fluorescence (Raw)
20
40
60
80
100
0
200
400
600
800
Time Point (0.25 Hz)

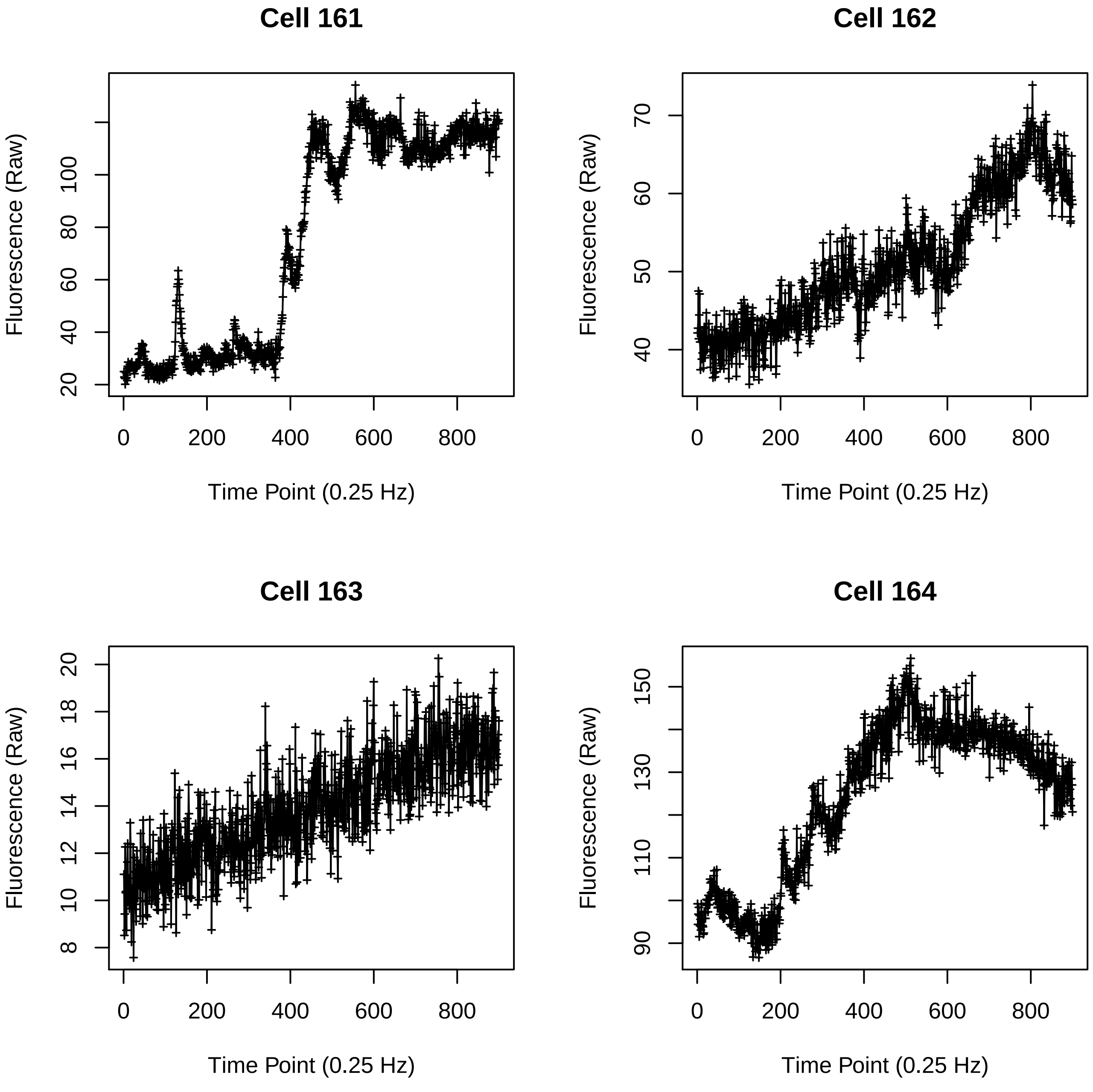
Cell 161
Fluorescence (Raw)
20
40
60
80
100
0
200
400
600
800
Time Point (0.25 Hz)
Cell 162
Fluorescence (Raw)
40
50
60
70
0
200
400
600
800
Time Point (0.25 Hz)
Cell 163
Fluorescence (Raw)
8
10
12
14
16
18
20
0
200
400
600
800
Time Point (0.25 Hz)
Cell 164
Fluorescence (Raw)
90
110
130
150
0
200
400
600
800
Time Point (0.25 Hz)

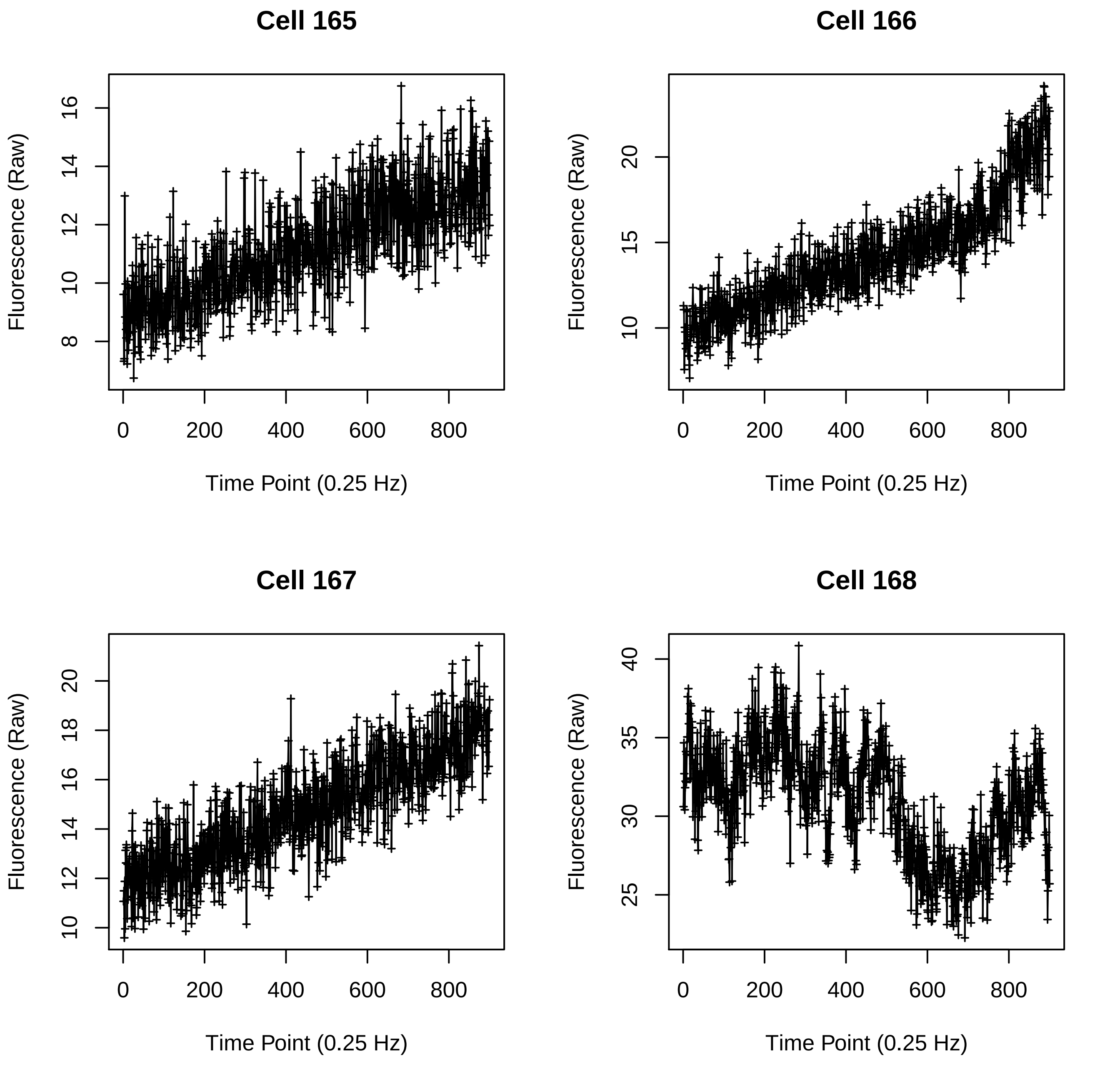
Cell 165
Fluorescence (Raw)
8
10
12
14
16
0
200
400
600
800
Time Point (0.25 Hz)
Cell 166
Fluorescence (Raw)
10
15
20
0
200
400
600
800
Time Point (0.25 Hz)
Cell 167
Fluorescence (Raw)
10
12
14
16
18
20
0
200
400
600
800
Time Point (0.25 Hz)
Cell 168
Fluorescence (Raw)
25
30
35
40
0
200
400
600
800
Time Point (0.25 Hz)

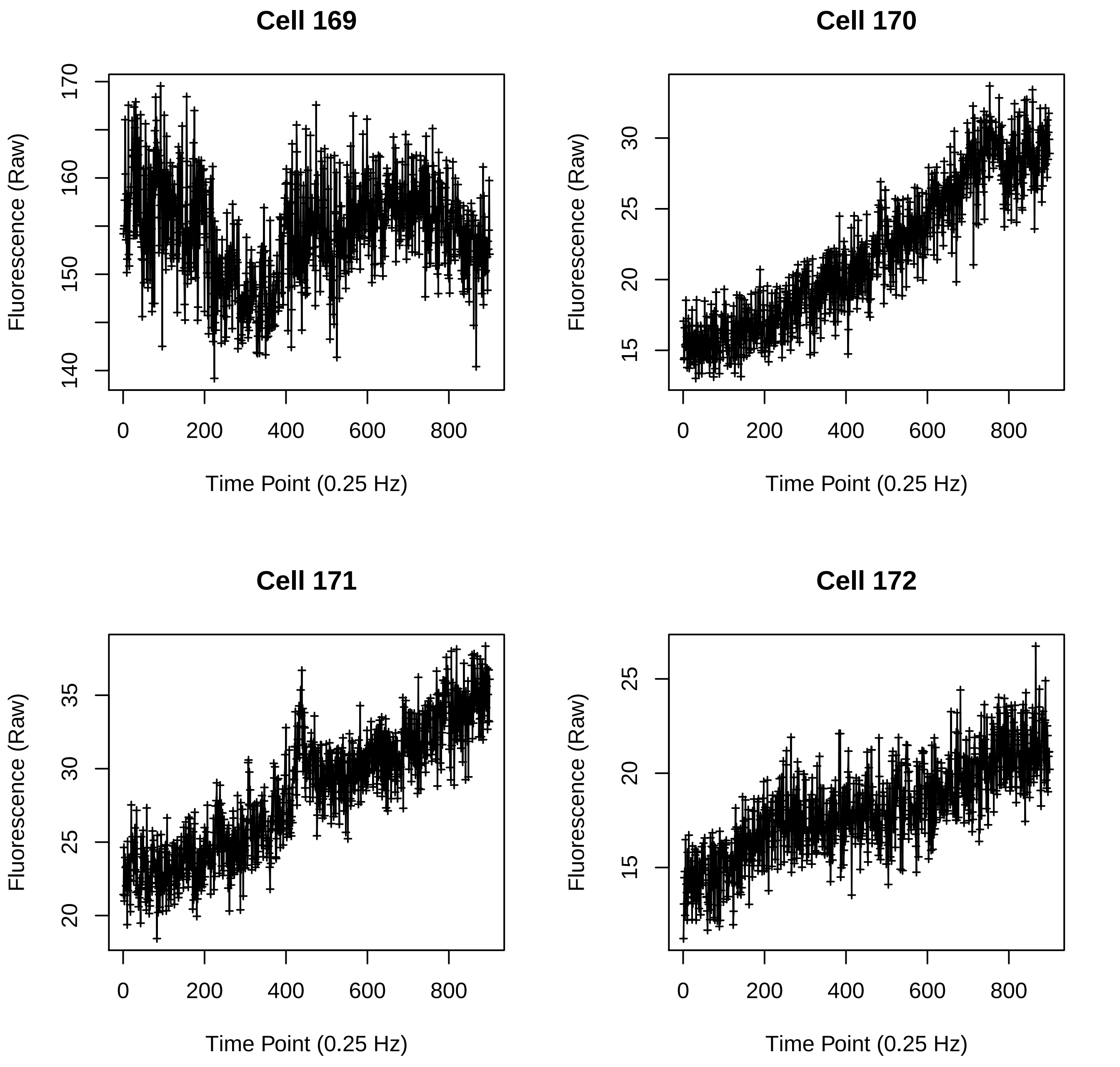
Cell 169
Cell 170
Cell 171
Cell 172
Fluorescence (Raw)
Time Point (0.25 Hz)
140
150
160
170
15
20
25
30
35
0
200
400
600
800

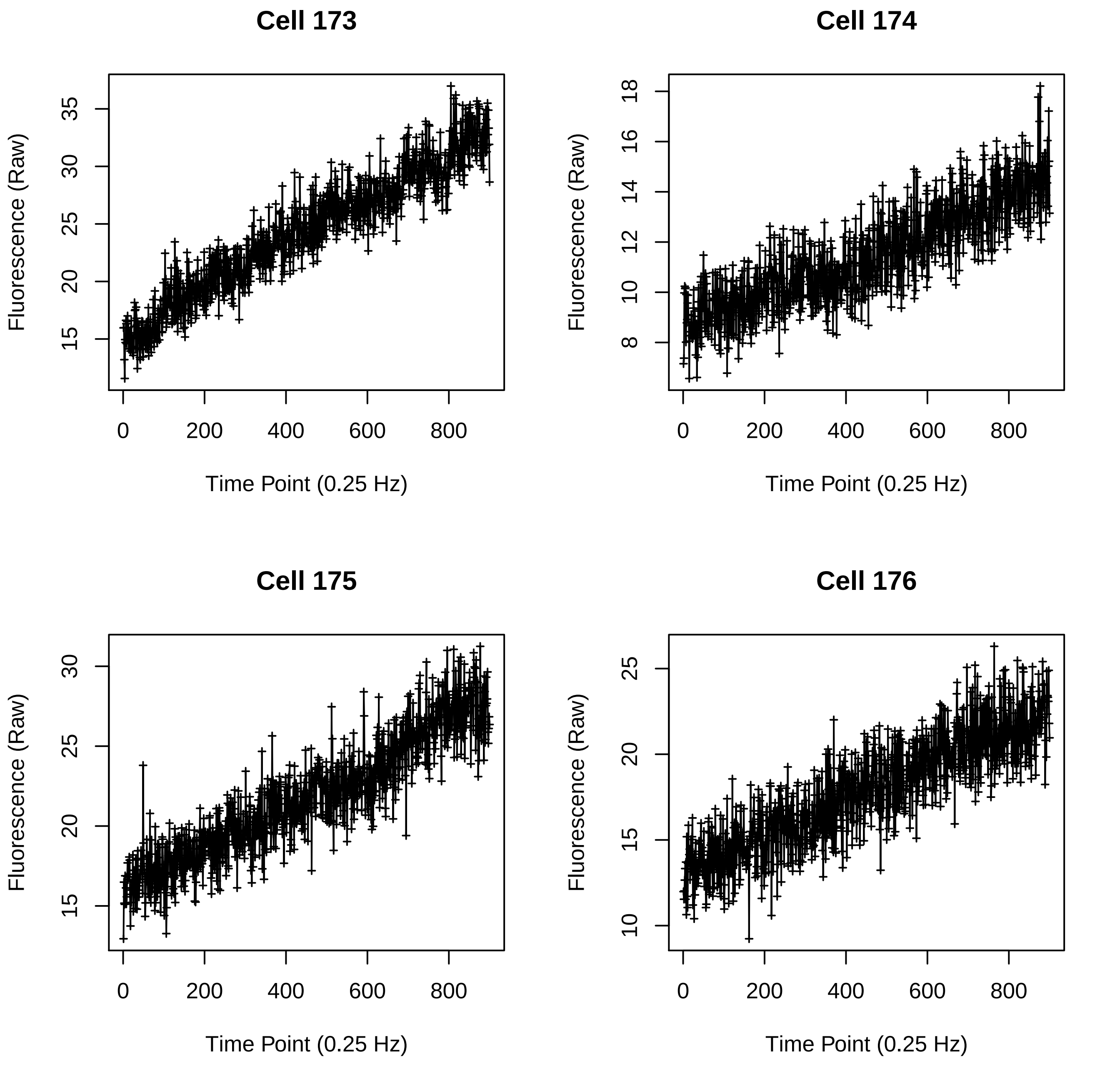

Cell 173
Cell 174
Cell 175
Cell 176
Fluorescence (Raw)
Time Point (0.25 Hz)
0
200
400
600
800
15
20
25
30
35
8
10
12
14
16
18
10

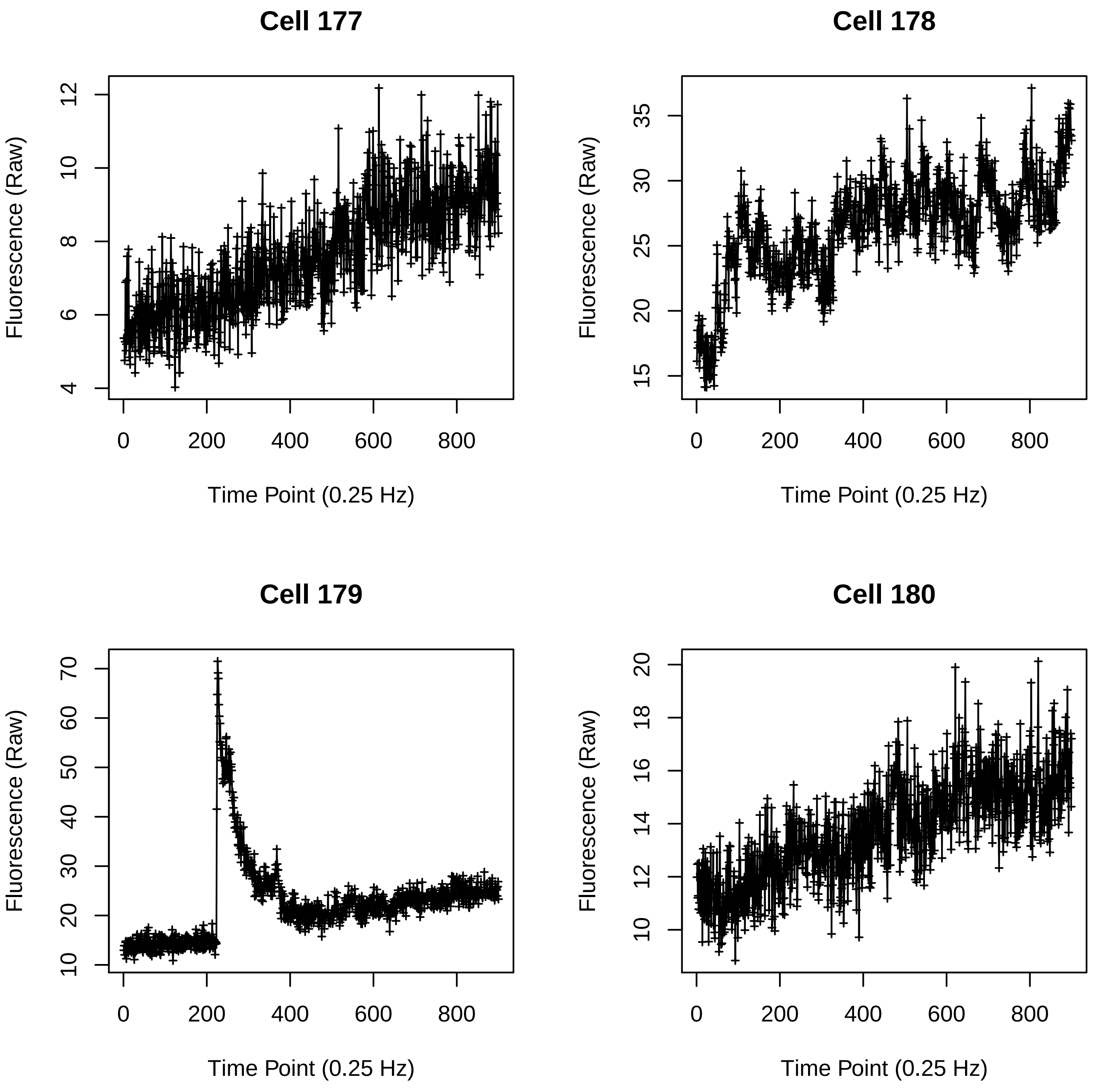

Cell 177
Fluorescence (Raw)
4
6
8
10
12
0
200
400
600
800
Time Point (0.25 Hz)
Cell 178
Fluorescence (Raw)
15
20
25
30
35
0
200
400
600
800
Time Point (0.25 Hz)
Cell 179
Fluorescence (Raw)
10
20
30
40
50
60
70
0
200
400
600
800
Time Point (0.25 Hz)
Cell 180
Fluorescence (Raw)
10
12
14
16
18
20
0
200
400
600
800
Time Point (0.25 Hz)

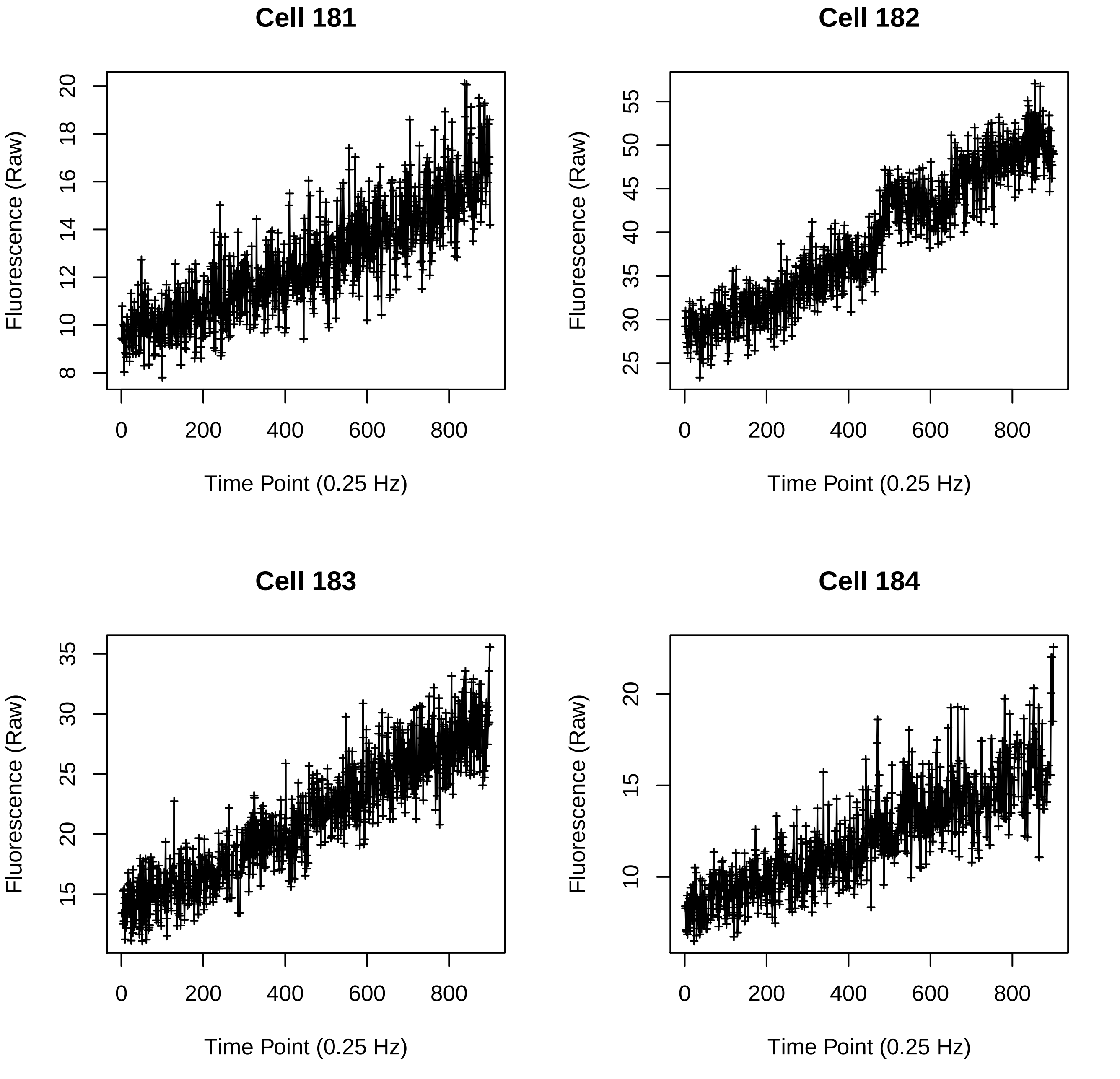

Cell 181
Fluorescence (Raw)
8
10
12
14
16
18
20
0
200
400
600
800
Time Point (0.25 Hz)
Cell 182
Fluorescence (Raw)
25
30
35
40
45
50
55
0
200
400
600
800
Time Point (0.25 Hz)
Cell 183
Fluorescence (Raw)
15
20
25
30
35
0
200
400
600
800
Time Point (0.25 Hz)
Cell 184
Fluorescence (Raw)
10
15
20
0
200
400
600
800
Time Point (0.25 Hz)

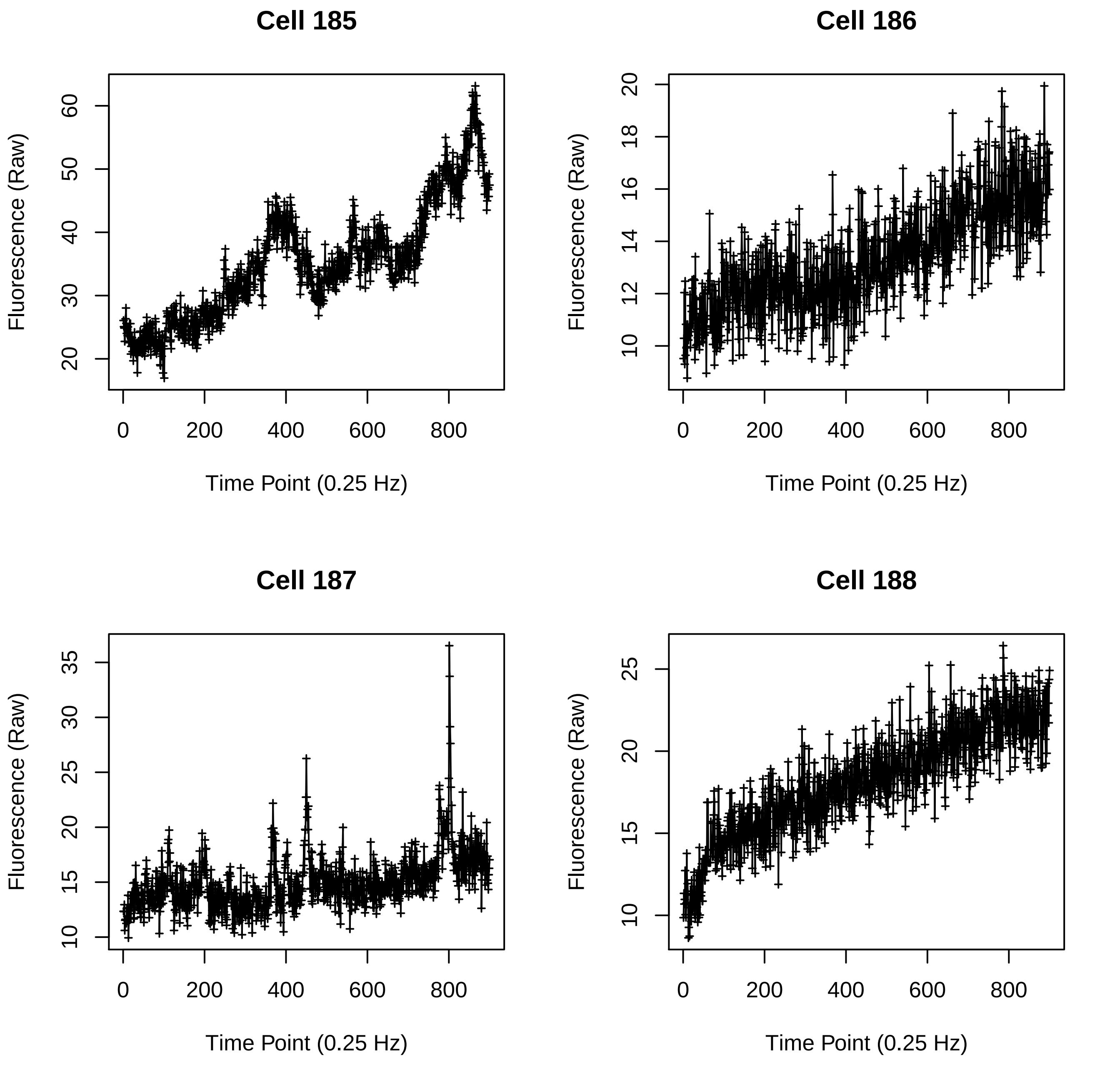
Cell 185
Fluorescence (Raw)
20
30
40
50
60
0
200
400
600
800
Time Point (0.25 Hz)
Cell 186
Fluorescence (Raw)
10
12
14
16
18
20
0
200
400
600
800
Time Point (0.25 Hz)
Cell 187
Fluorescence (Raw)
10
15
20
25
30
35
0
200
400
600
800
Time Point (0.25 Hz)
Cell 188
Fluorescence (Raw)
10
15
20
25
0
200
400
600
800
Time Point (0.25 Hz)

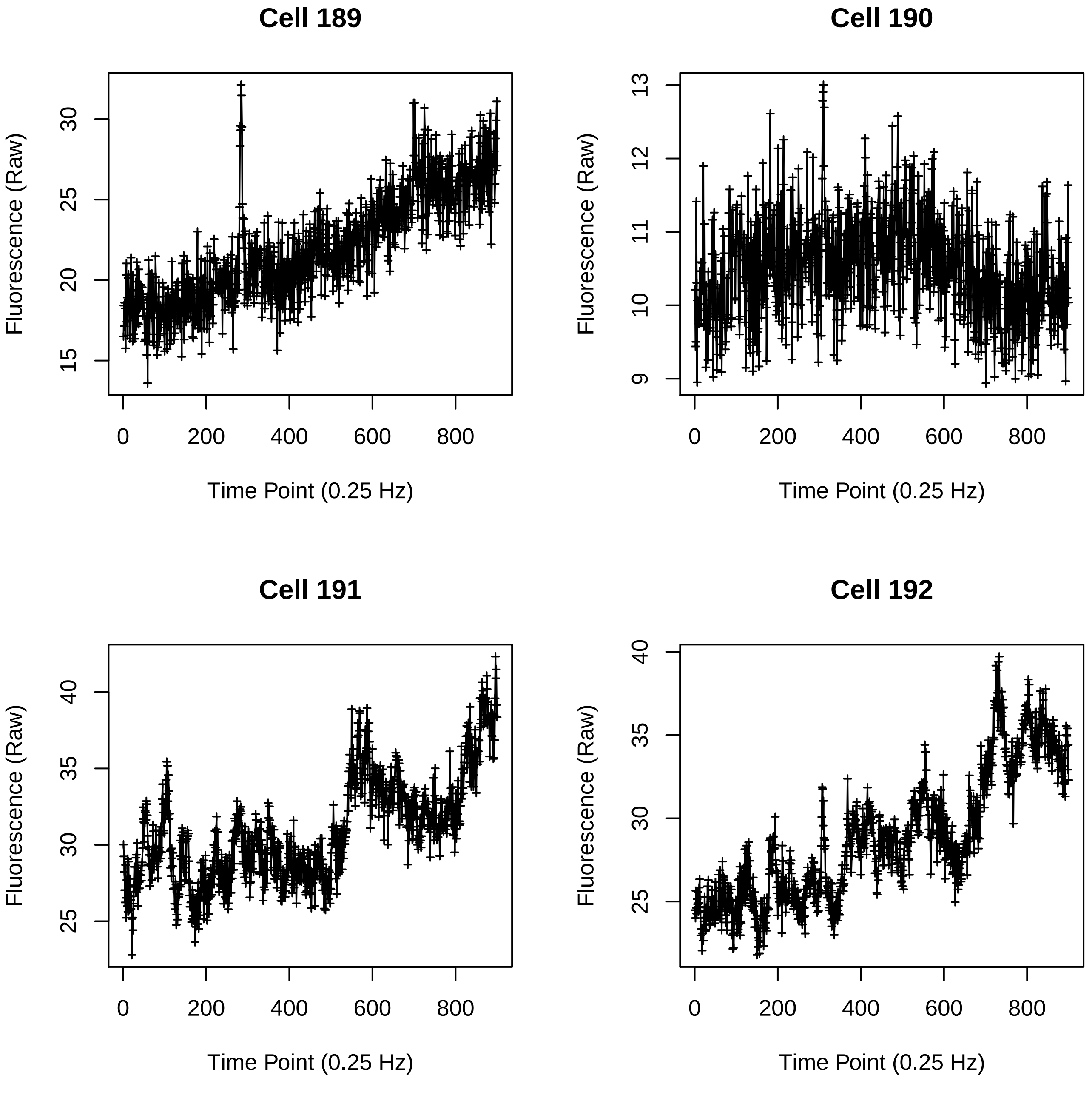
Cell 189
Fluorescence (Raw)
15
20
25
30
0
200
400
600
800
Time Point (0.25 Hz)
Cell 190
Fluorescence (Raw)
9
10
11
12
13
0
200
400
600
800
Time Point (0.25 Hz)
Cell 191
Fluorescence (Raw)
25
30
35
40
0
200
400
600
800
Time Point (0.25 Hz)
Cell 192
Fluorescence (Raw)
25
30
35
40
0
200
400
600
800
Time Point (0.25 Hz)

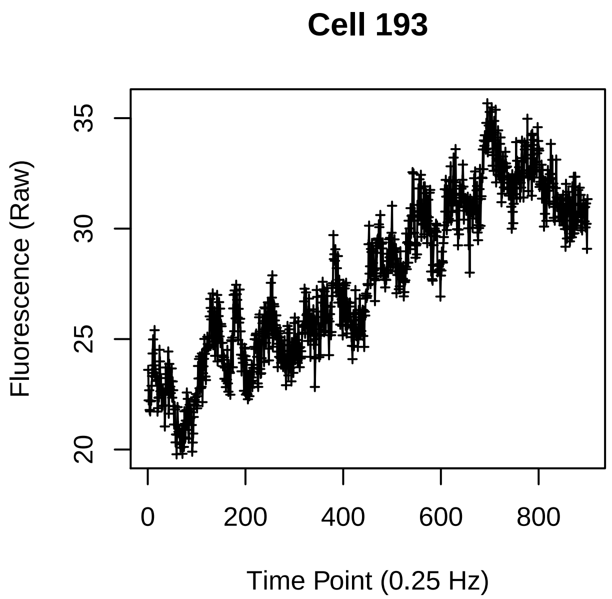
Cell 193
Fluorescence (Raw)
Time Point (0.25 Hz)
20
25
30
35
0
200
400
600
800

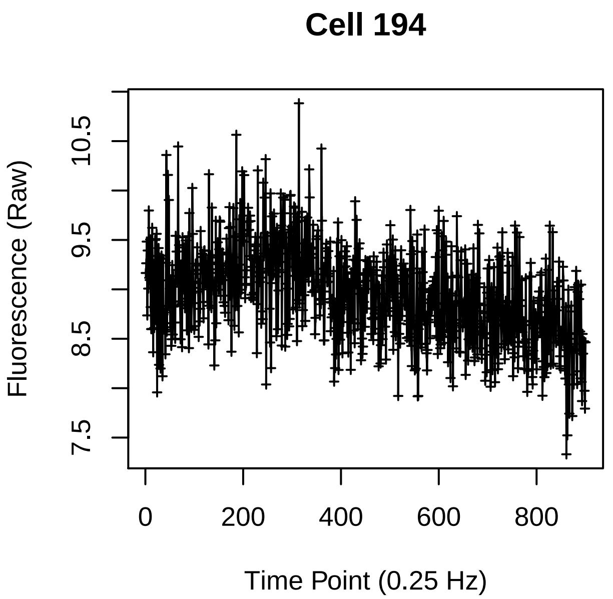
Cell 194
Fluorescence (Raw)
Time Point (0.25 Hz)
7.5
8.5
9.5
10.5
0
200
400
600
800

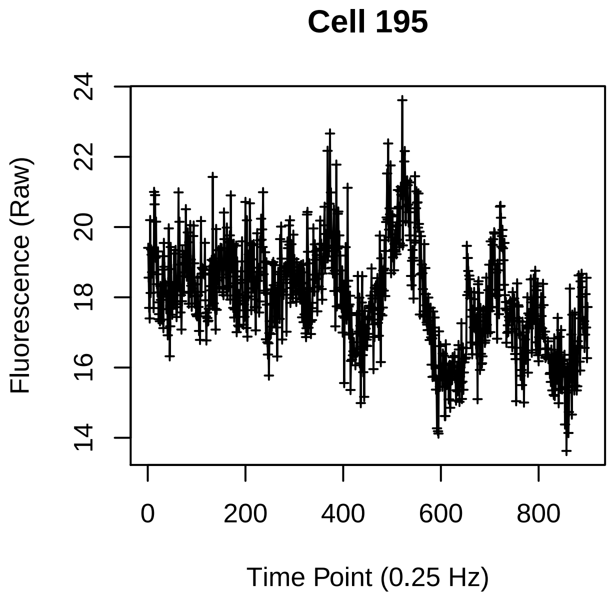
Cell 195
Fluorescence (Raw)
Time Point (0.25 Hz)
14
16
18
20
22
24
0
200
400
600
800

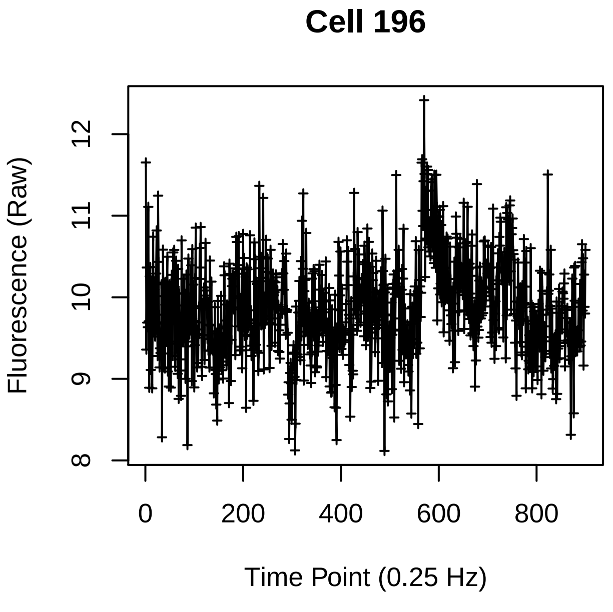
Cell 196
Fluorescence (Raw)
Time Point (0.25 Hz)
8
9
10
11
12
0
200
400
600
800

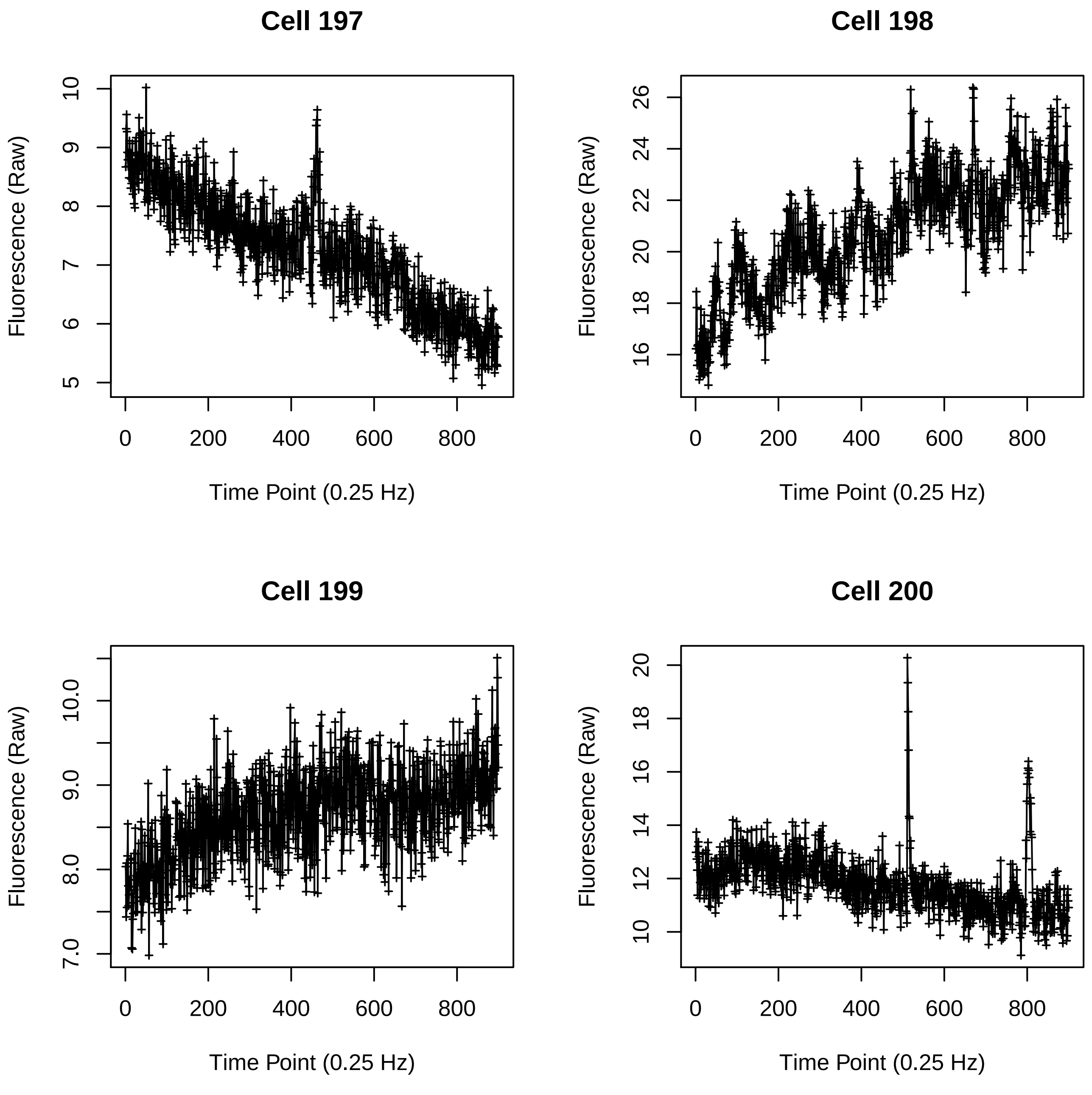
Cell 197
Cell 198
Cell 199
Cell 200
Fluorescence (Raw)
Time Point (0.25 Hz)
5
6
7
8
9
10
16
18
20
22
24
26
7.0
8.0
9.0
10.0
10
12
14
16
18
20
0
200
400
600
800

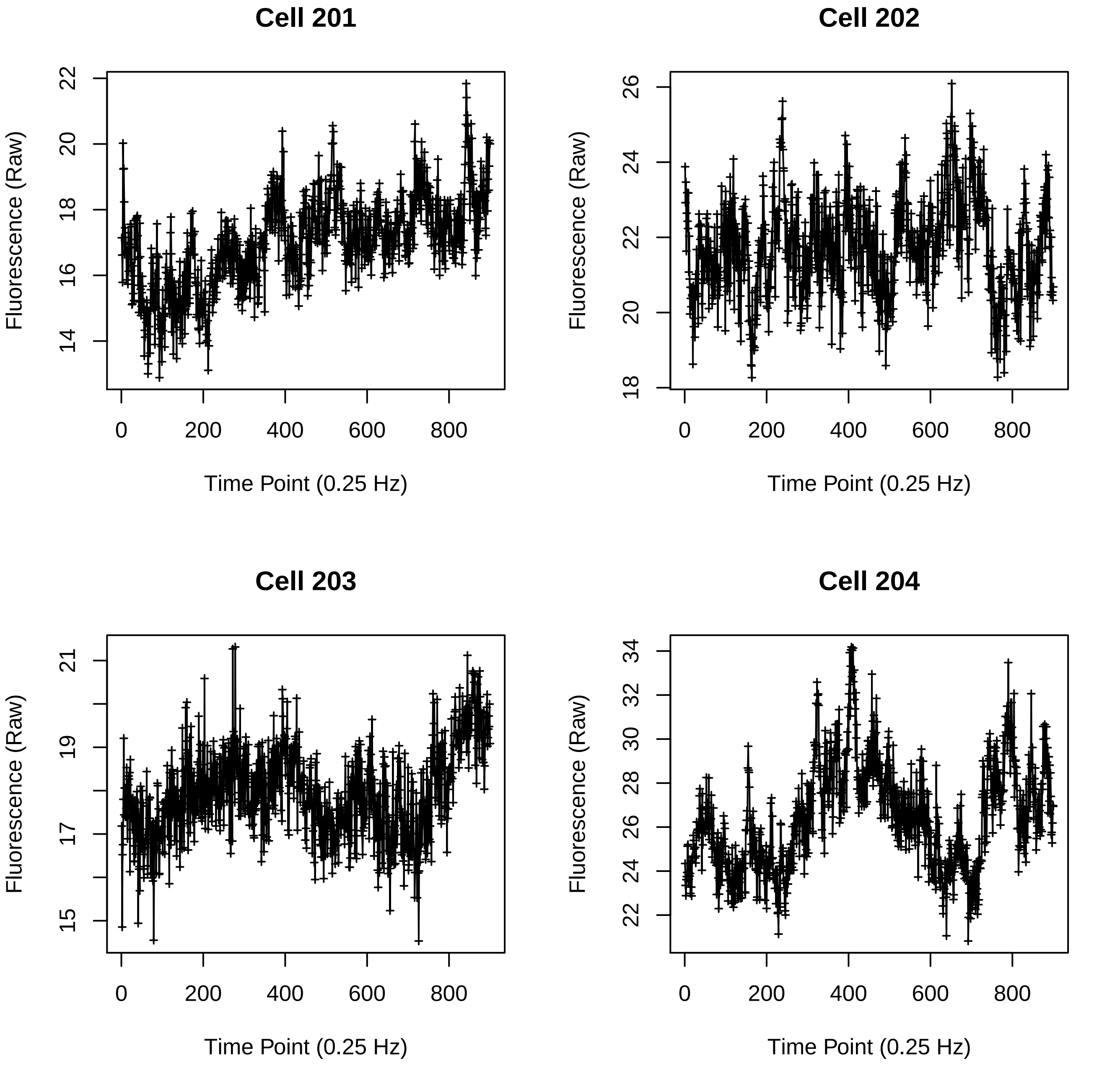
Cell 201
Fluorescence (Raw)
14
16
18
20
22
0
200
400
600
800
Time Point (0.25 Hz)
Cell 202
Fluorescence (Raw)
18
20
22
24
26
0
200
400
600
800
Time Point (0.25 Hz)
Cell 203
Fluorescence (Raw)
15
17
19
21
0
200
400
600
800
Time Point (0.25 Hz)
Cell 204
Fluorescence (Raw)
22
24
26
28
30
32
34
0
200
400
600
800
Time Point (0.25 Hz)

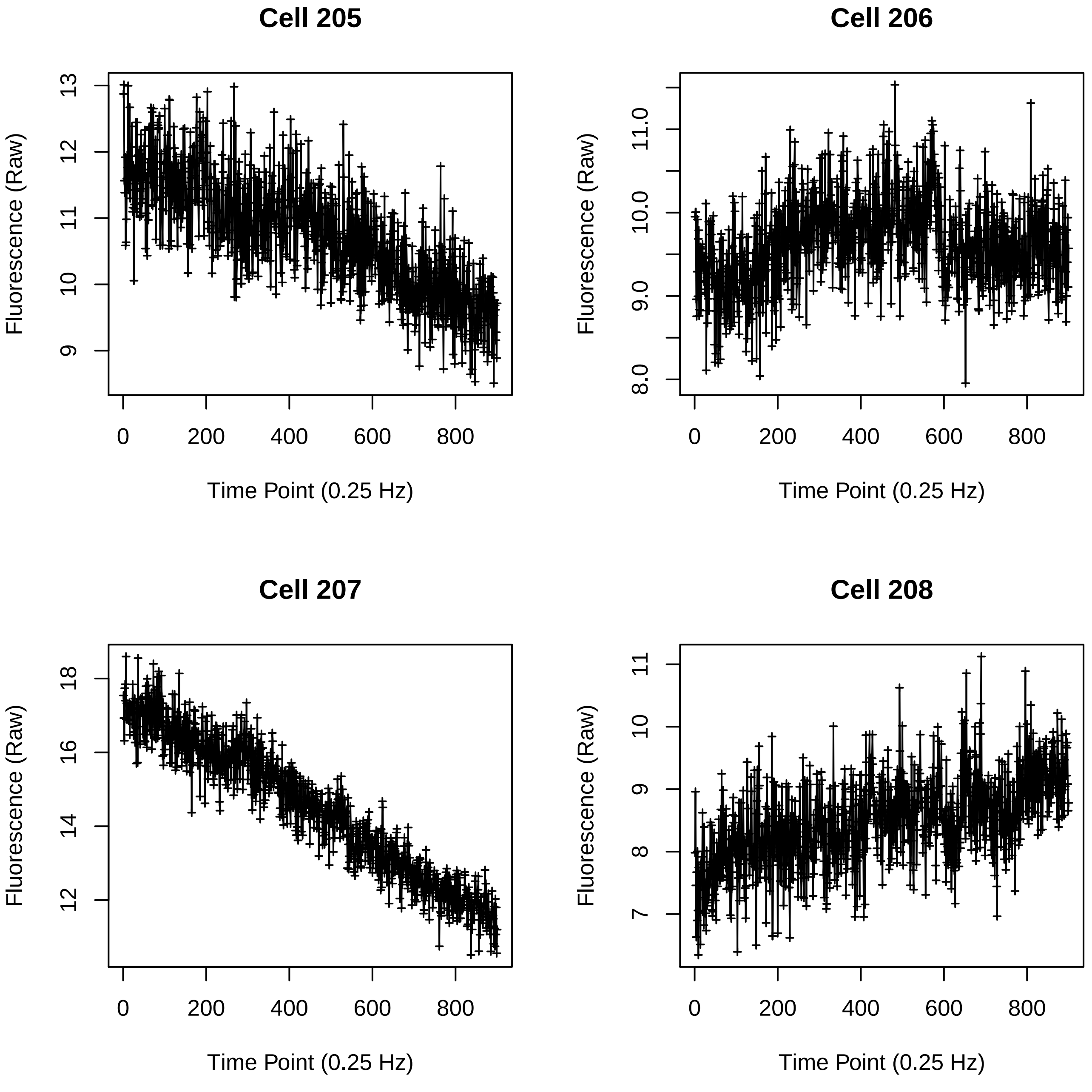

Cell 205
Cell 206
Cell 207
Cell 208
Fluorescence (Raw)
Time Point (0.25 Hz)
0
200
400
600
800
9
10
11
12
13
8.0
9.0
10.0
11.0
12
14
16
18
7
8
9
10
11

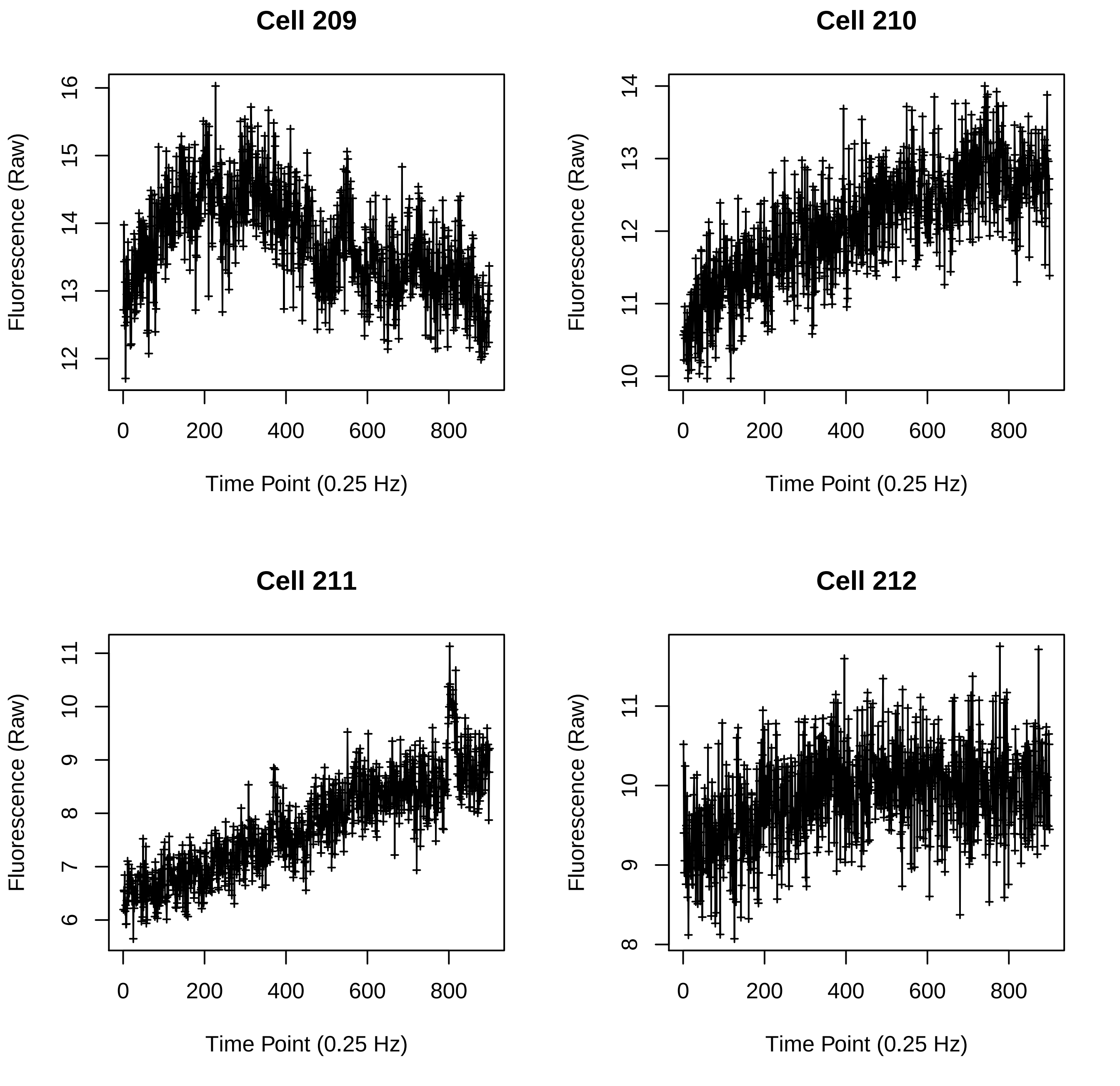

Cell 209
Cell 210
Cell 211
Cell 212
Fluorescence (Raw)
Time Point (0.25 Hz)
0
200
400
600
800
12
13
14
15
16
10
11
12
13
14
6
7
8
9
10
11
8
9
10
11

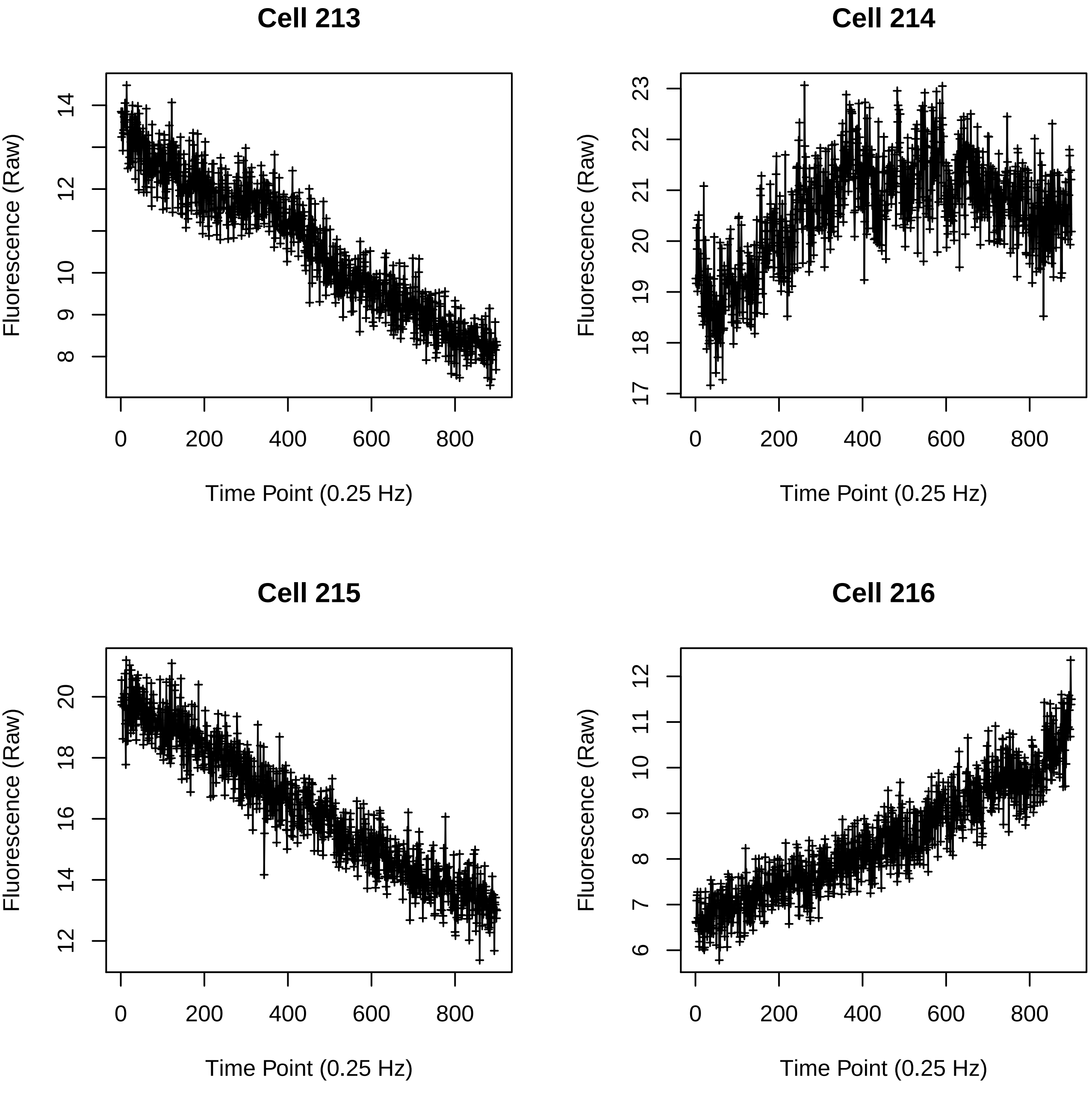
Cell 213
Fluorescence (Raw)
14
12
10
9
8
0
200
400
600
800
Time Point (0.25 Hz)
Cell 214
Fluorescence (Raw)
23
22
21
20
19
18
17
0
200
400
600
800
Time Point (0.25 Hz)
Cell 215
Fluorescence (Raw)
20
18
16
14
12
0
200
400
600
800
Time Point (0.25 Hz)
Cell 216
Fluorescence (Raw)
12
11
10
9
8
7
6
0
200
400
600
800
Time Point (0.25 Hz)

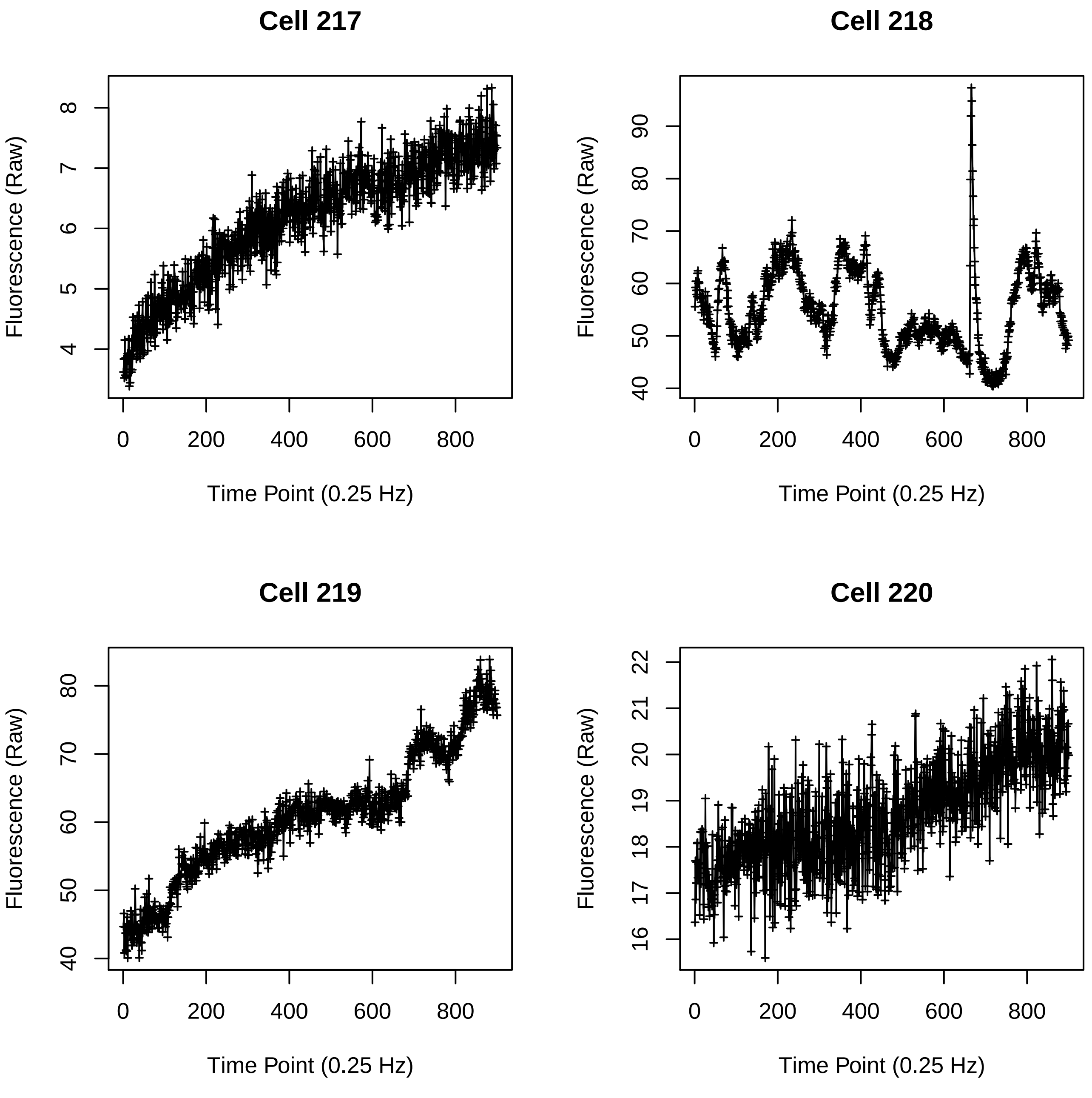

Cell 217
Fluorescence (Raw)
4
5
6
7
8
0
200
400
600
800
Time Point (0.25 Hz)
Cell 218
Fluorescence (Raw)
40
50
60
70
80
90
0
200
400
600
800
Time Point (0.25 Hz)
Cell 219
Fluorescence (Raw)
40
50
60
70
80
0
200
400
600
800
Time Point (0.25 Hz)
Cell 220
Fluorescence (Raw)
16
17
18
19
20
21
22
0
200
400
600
800
Time Point (0.25 Hz)

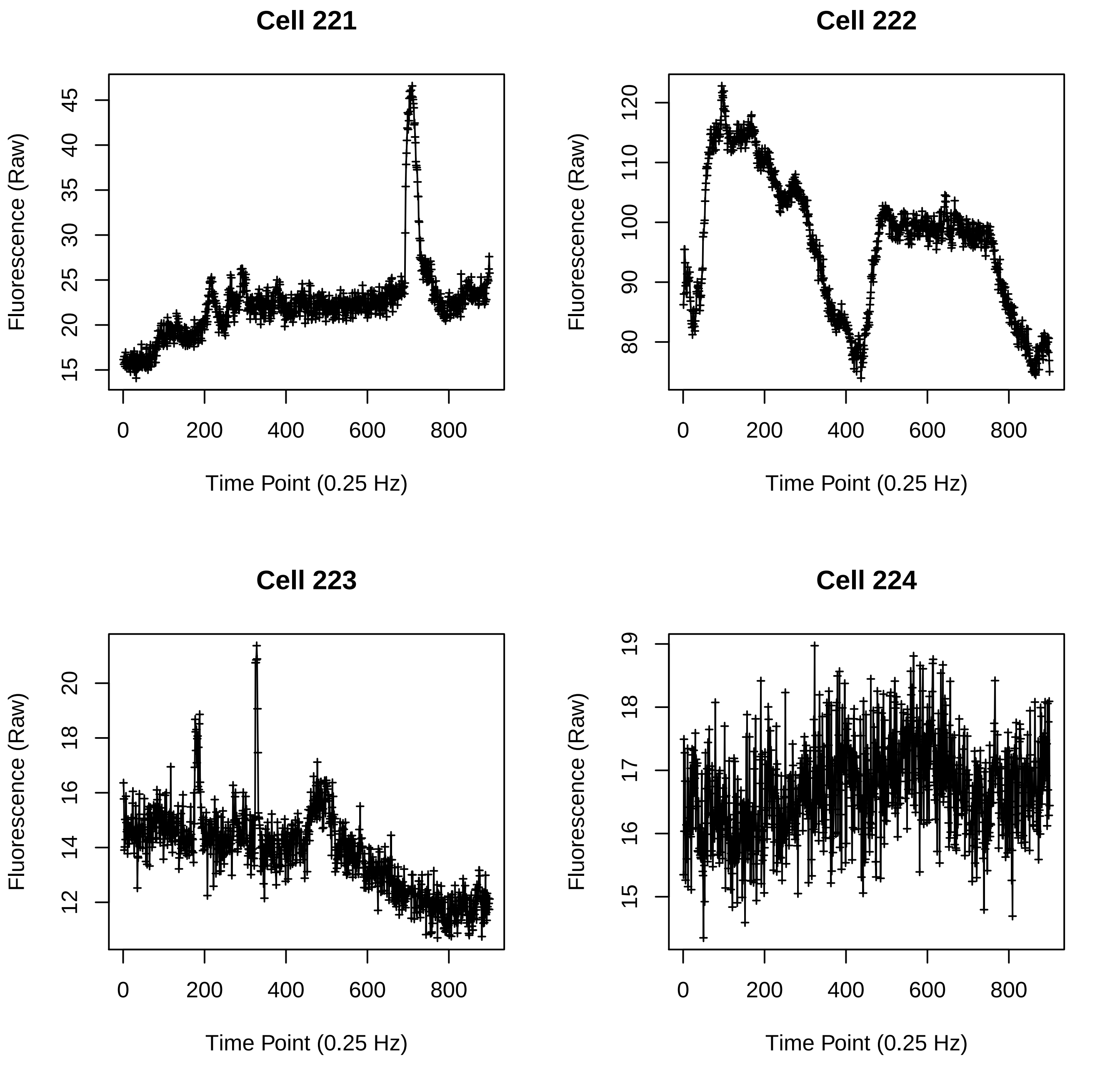
Cell 221
Cell 222
Cell 223
Cell 224
Fluorescence (Raw)
Time Point (0.25 Hz)

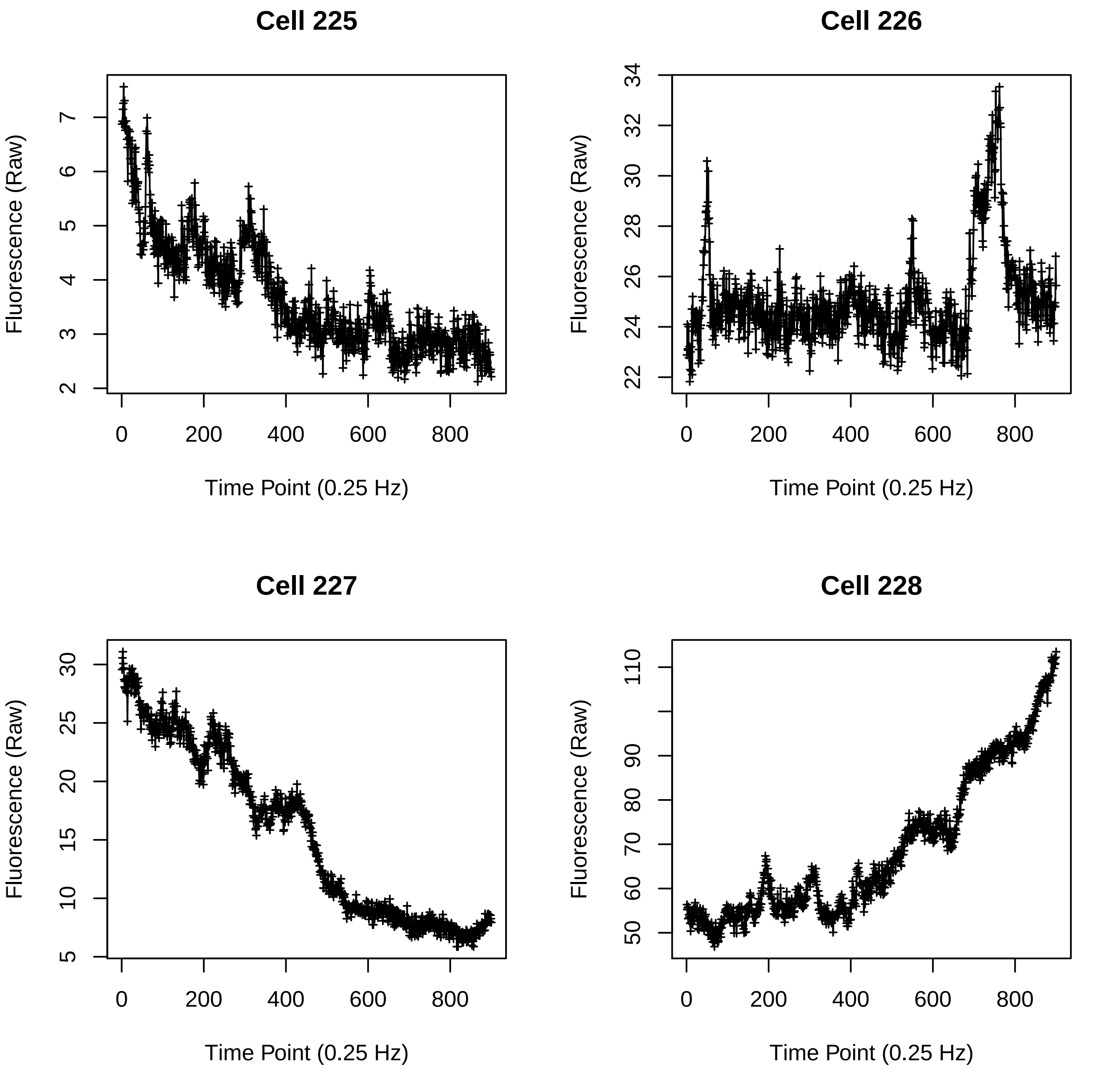
Cell 225
Fluorescence (Raw)
2
3
4
5
6
7
0
200
400
600
800
Time Point (0.25 Hz)
Cell 226
Fluorescence (Raw)
22
24
26
28
30
32
34
0
200
400
600
800
Time Point (0.25 Hz)
Cell 227
Fluorescence (Raw)
5
10
15
20
25
30
0
200
400
600
800
Time Point (0.25 Hz)
Cell 228
Fluorescence (Raw)
50
60
70
80
90
110
0
200
400
600
800
Time Point (0.25 Hz)

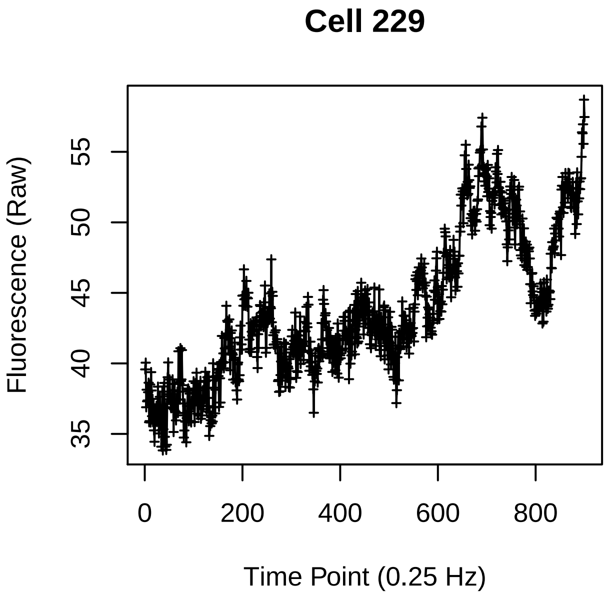
Cell 229
Fluorescence (Raw)
35
40
45
50
55
0
200
400
600
800
Time Point (0.25 Hz)

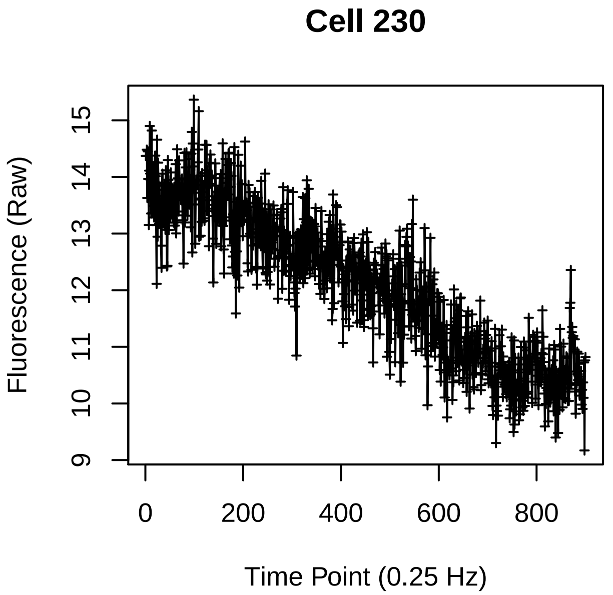
Cell 230
Fluorescence (Raw)
9
10
11
12
13
14
15
0
200
400
600
800
Time Point (0.25 Hz)

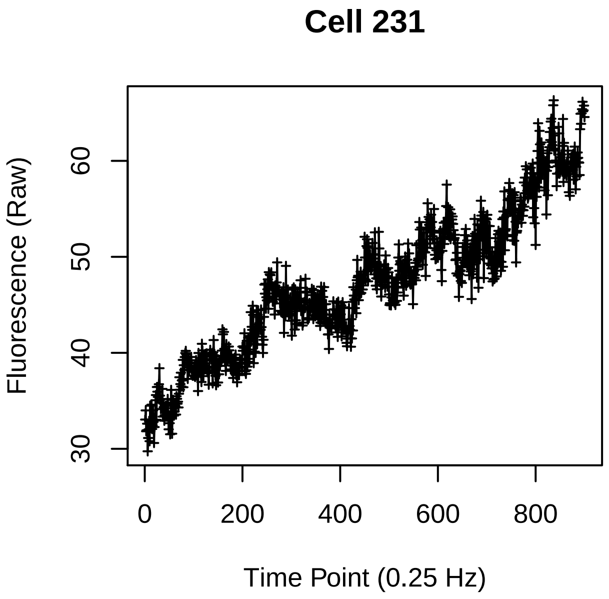
Cell 231
Fluorescence (Raw)
30
40
50
60
0
200
400
600
800
Time Point (0.25 Hz)

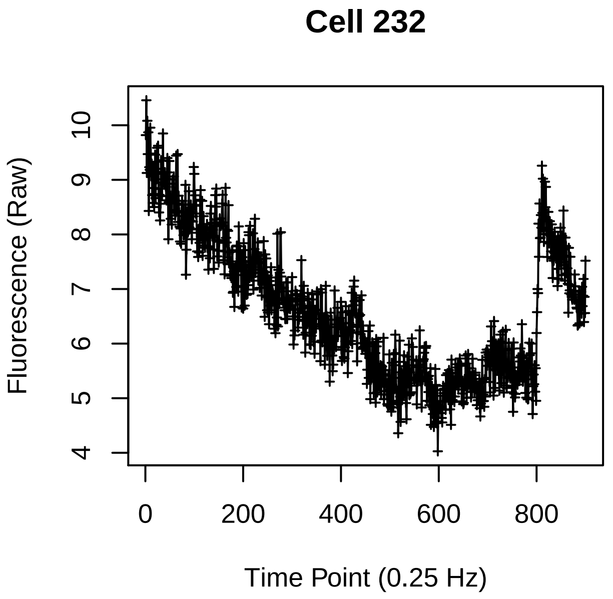
Cell 232
Fluorescence (Raw)
4
5
6
7
8
9
10
0
200
400
600
800
Time Point (0.25 Hz)

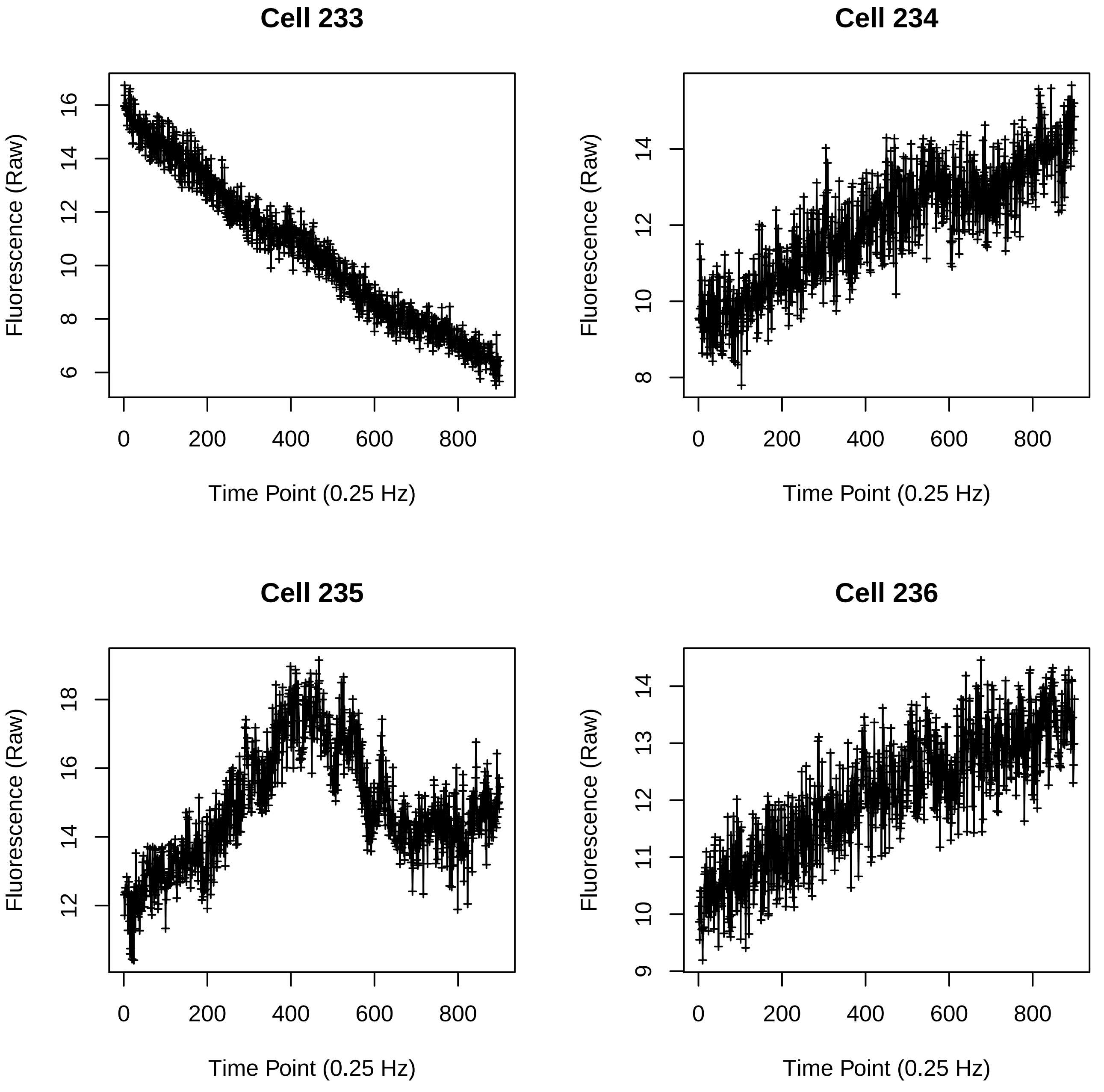
Cell 233
Fluorescence (Raw)
Time Point (0.25 Hz)
6
8
10
12
14
16
0
200
400
600
800
Cell 234
Fluorescence (Raw)
Time Point (0.25 Hz)
8
10
12
14
0
200
400
600
800
Cell 235
Fluorescence (Raw)
Time Point (0.25 Hz)
12
14
16
18
0
200
400
600
800
Cell 236
Fluorescence (Raw)
Time Point (0.25 Hz)
9
10
11
12
13
14
0
200
400
600
800

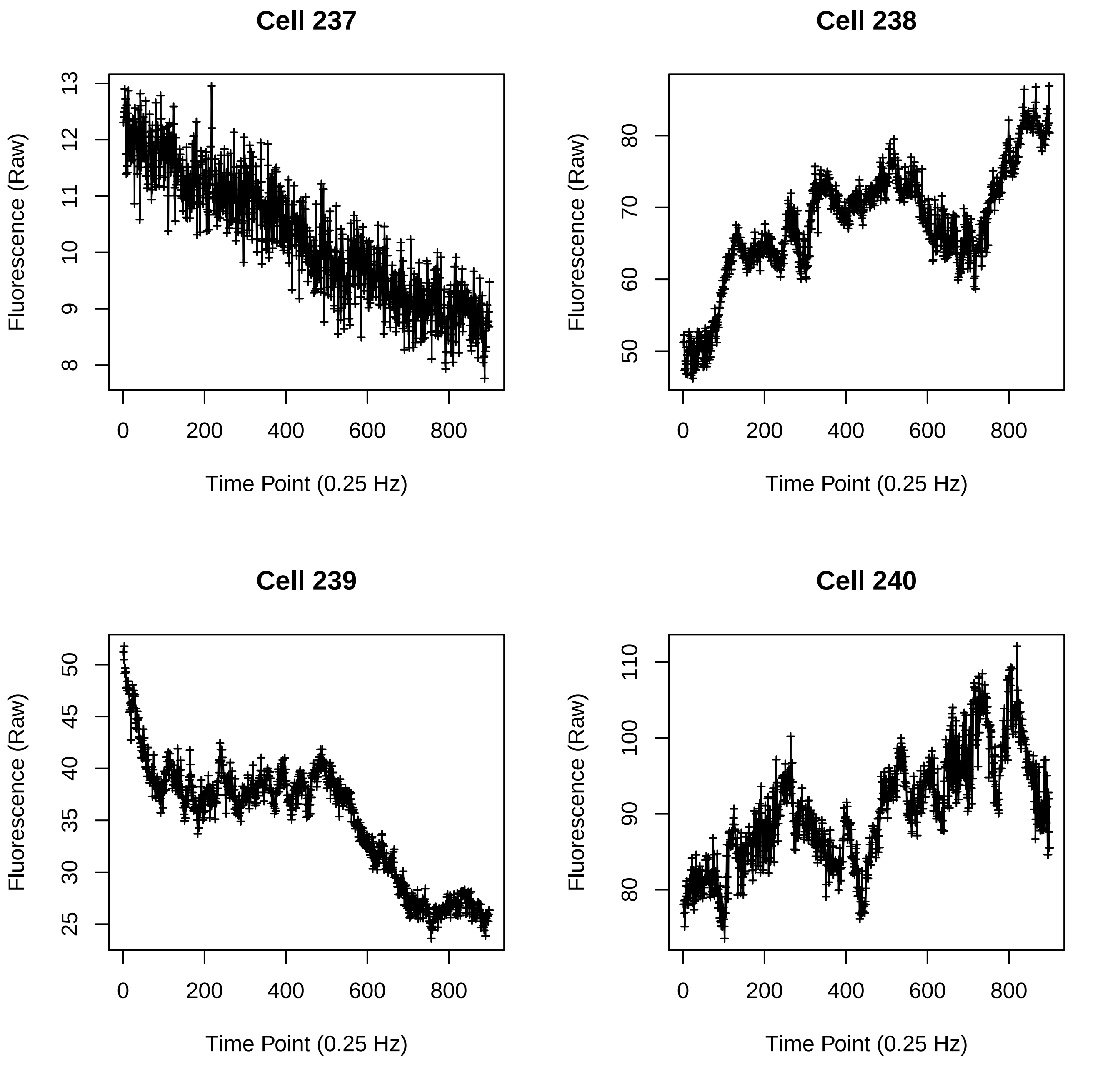

Cell 237
Cell 238
Cell 239
Cell 240
Fluorescence (Raw)
Time Point (0.25 Hz)
0
200
400
600
800
8
9
10
11
12
13
50
60
70
80
25
30
35
40
45
50
80
90
100
110

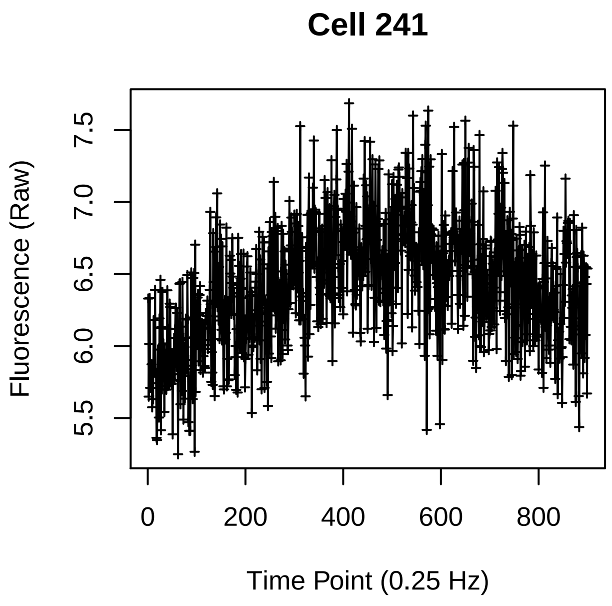
Cell 241
Fluorescence (Raw)
Time Point (0.25 Hz)
5.5
6.0
6.5
7.0
7.5
0
200
400
600
800

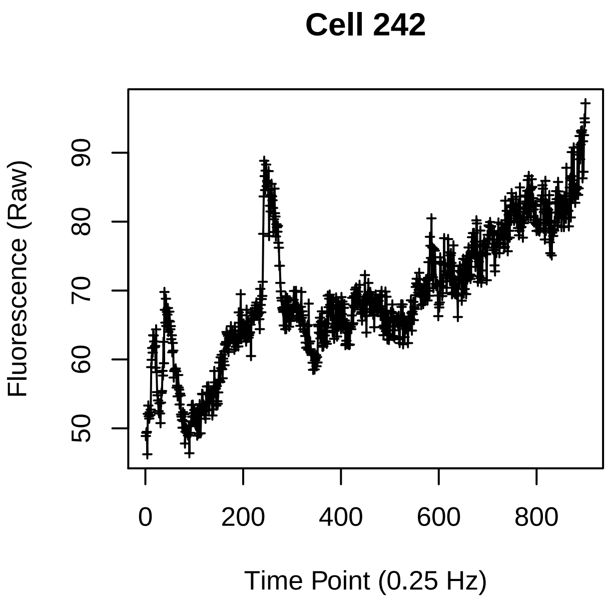
Cell 242
Fluorescence (Raw)
Time Point (0.25 Hz)
50
60
70
80
90
0
200
400
600
800

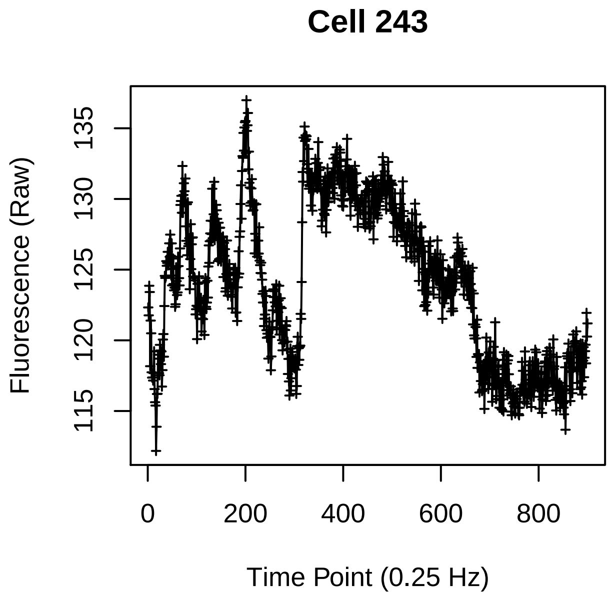
Cell 243
Fluorescence (Raw)
Time Point (0.25 Hz)
115
120
125
130
135
0
200
400
600
800

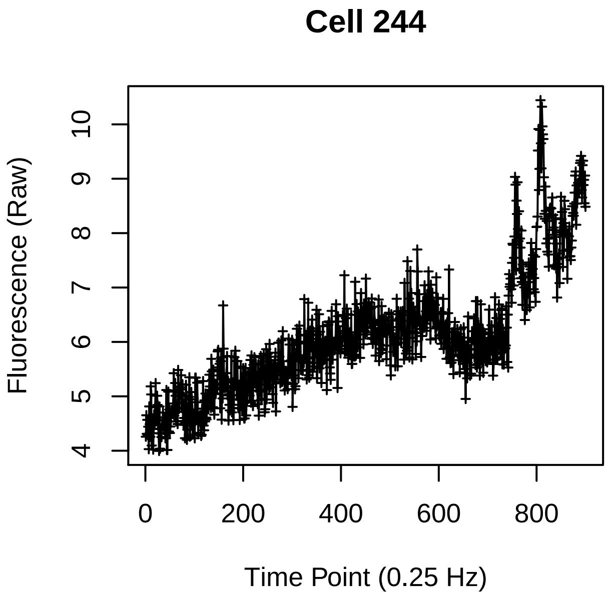
Cell 244
Fluorescence (Raw)
Time Point (0.25 Hz)
4
5
6
7
8
9
10
0
200
400
600
800

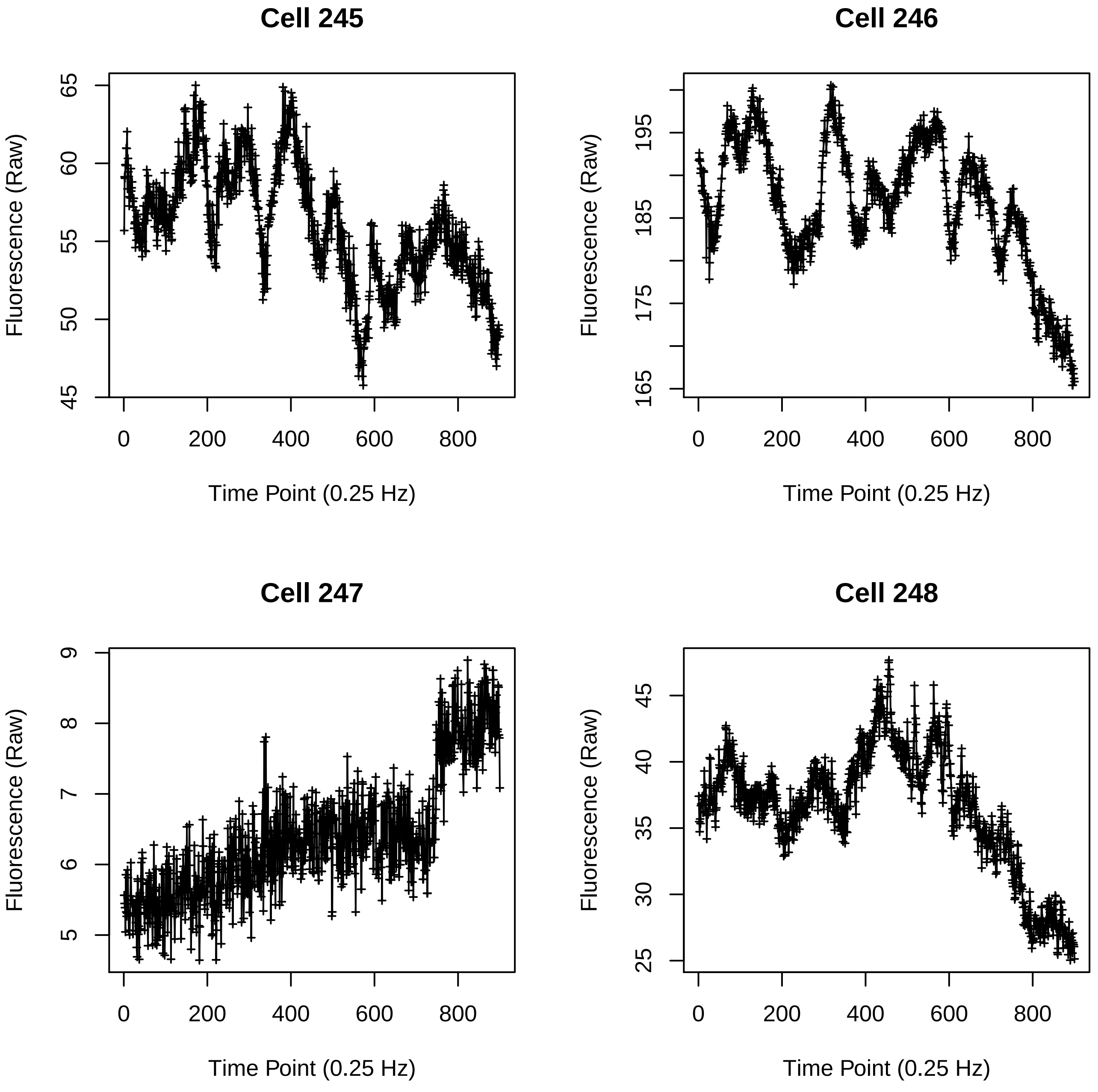
Cell 245
Fluorescence (Raw)
45
50
55
60
65
0
200
400
600
800
Time Point (0.25 Hz)
Cell 246
Fluorescence (Raw)
165
175
185
195
0
200
400
600
800
Time Point (0.25 Hz)
Cell 247
Fluorescence (Raw)
5
6
7
8
9
0
200
400
600
800
Time Point (0.25 Hz)
Cell 248
Fluorescence (Raw)
25
30
35
40
45
0
200
400
600
800
Time Point (0.25 Hz)

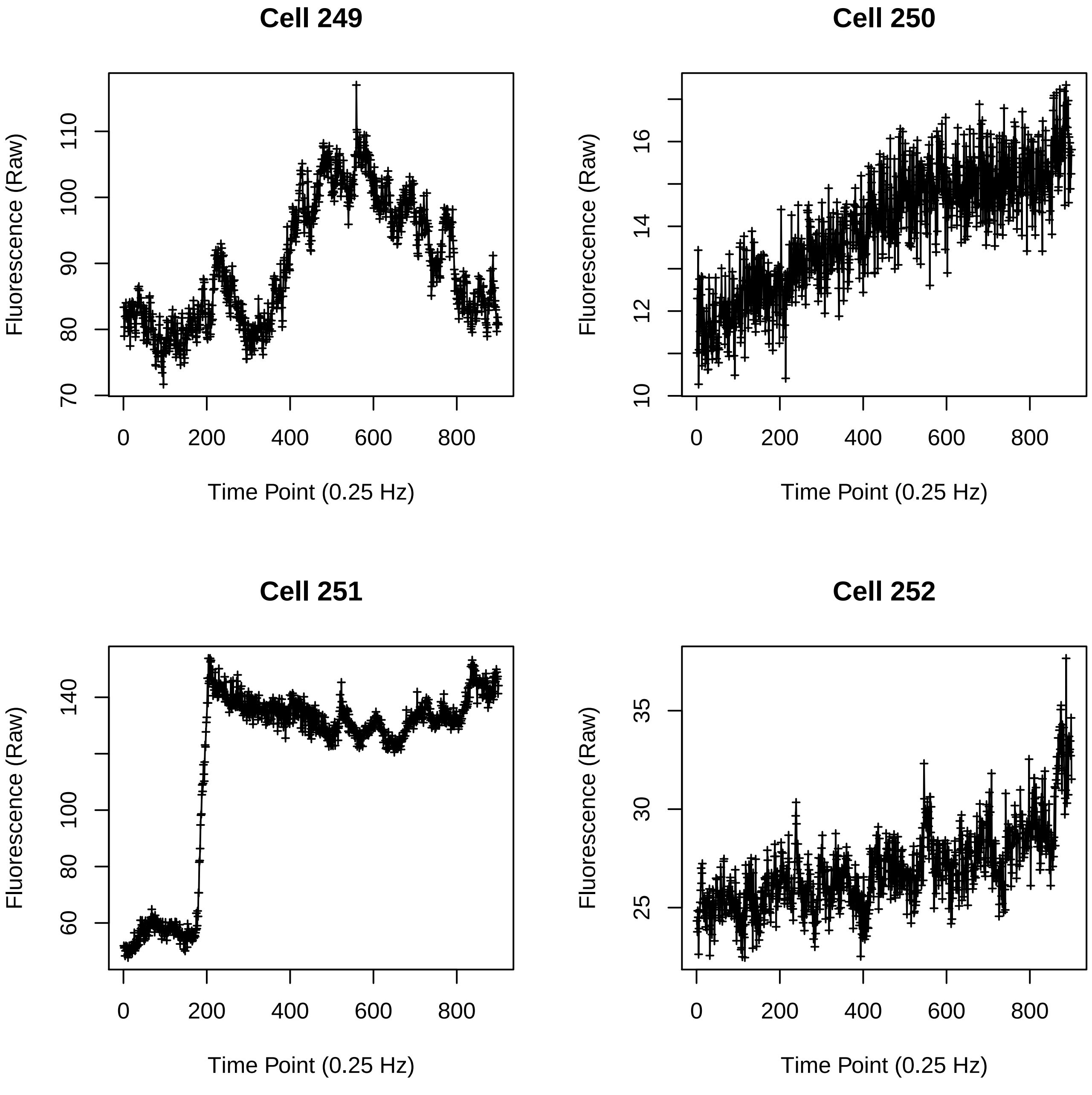
Cell 249
Fluorescence (Raw)
70
80
90
100
110
0
200
400
600
800
Time Point (0.25 Hz)
Cell 250
Fluorescence (Raw)
10
12
14
16
0
200
400
600
800
Time Point (0.25 Hz)
Cell 251
Fluorescence (Raw)
60
80
100
140
0
200
400
600
800
Time Point (0.25 Hz)
Cell 252
Fluorescence (Raw)
25
30
35
0
200
400
600
800
Time Point (0.25 Hz)

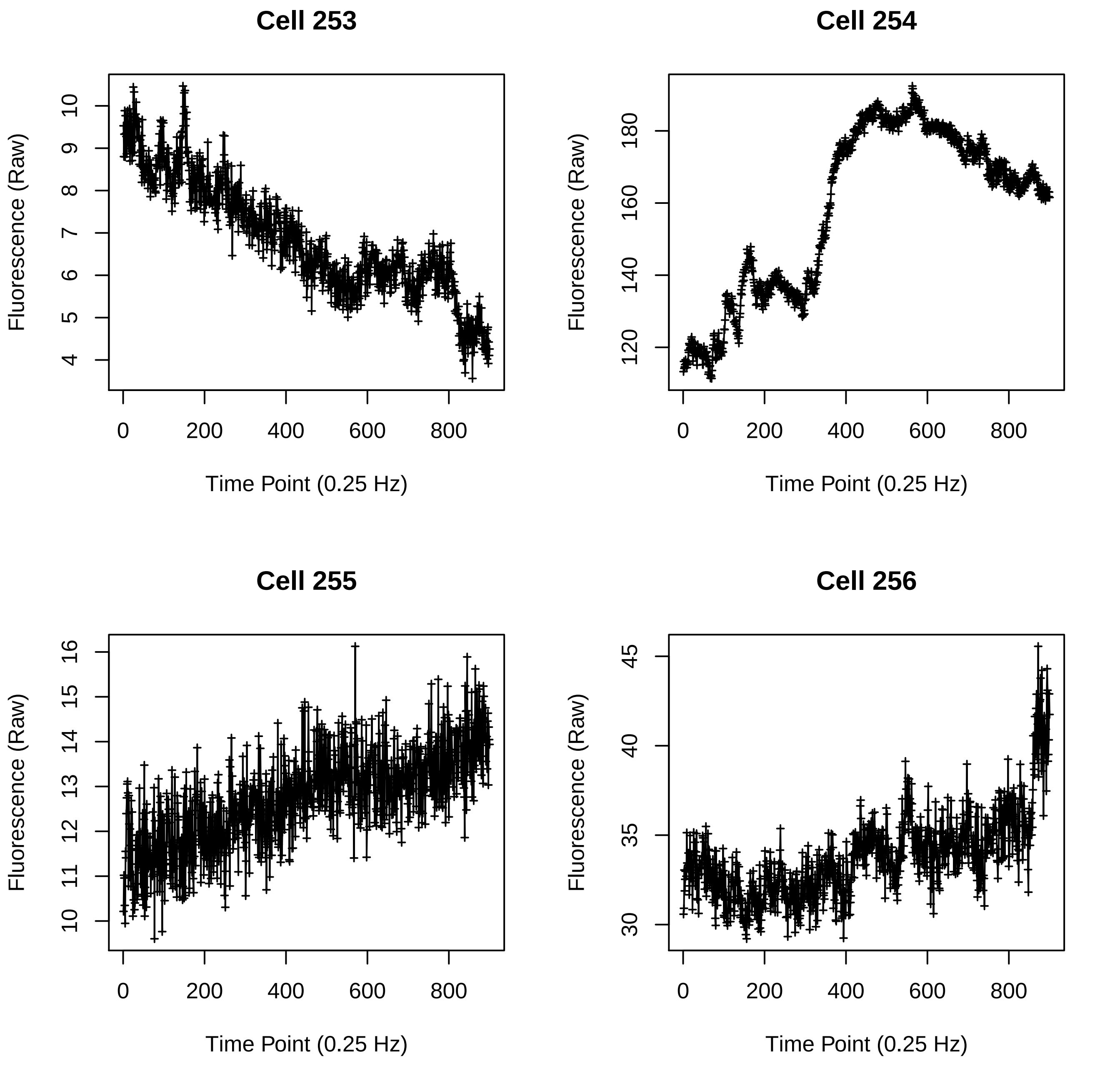

Cell 253
Fluorescence (Raw)
Time Point (0.25 Hz)
Cell 254
Fluorescence (Raw)
Time Point (0.25 Hz)
Cell 255
Fluorescence (Raw)
Time Point (0.25 Hz)
Cell 256
Fluorescence (Raw)
Time Point (0.25 Hz)

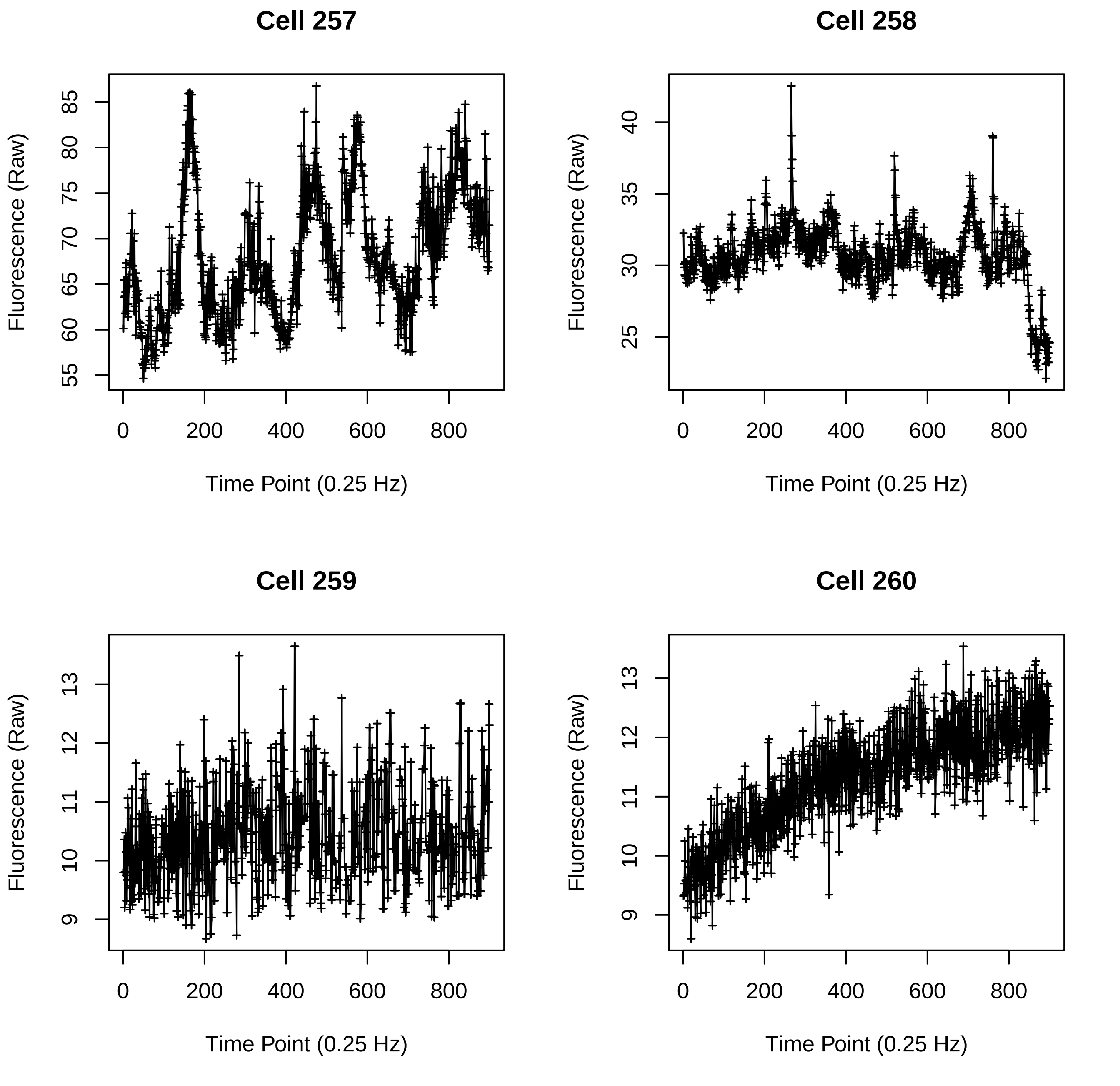
Cell 257
Fluorescence (Raw)
55
60
65
70
75
80
85
0
200
400
600
800
Time Point (0.25 Hz)
Cell 258
Fluorescence (Raw)
25
30
35
40
0
200
400
600
800
Time Point (0.25 Hz)
Cell 259
Fluorescence (Raw)
9
10
11
12
13
0
200
400
600
800
Time Point (0.25 Hz)
Cell 260
Fluorescence (Raw)
9
10
11
12
13
0
200
400
600
800
Time Point (0.25 Hz)

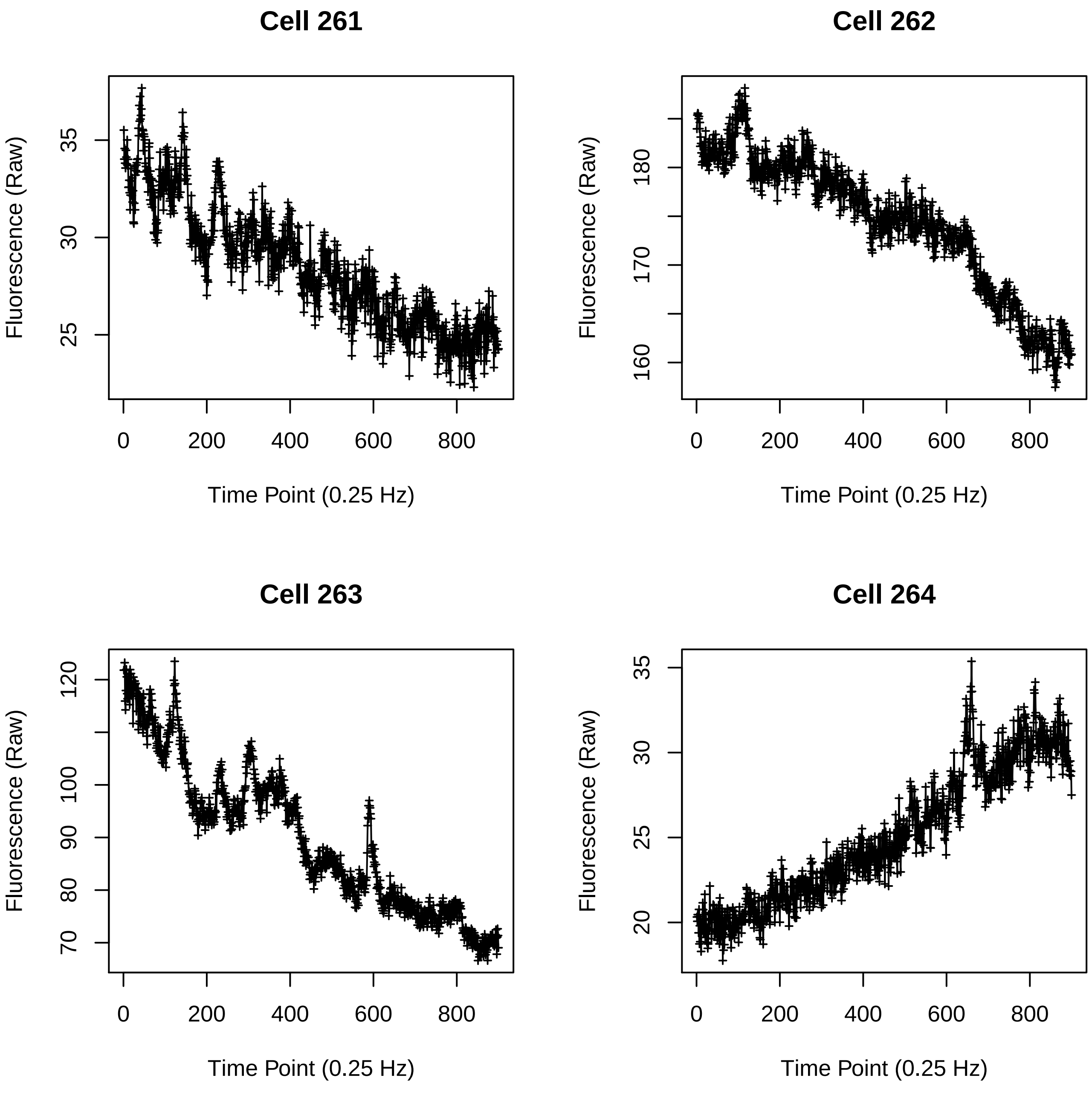
Cell 261
Cell 262
Cell 263
Cell 264
Fluorescence (Raw)
Time Point (0.25 Hz)
0
200
400
600
800
25
30
35
160
170
180
70
80
90
100
120
20

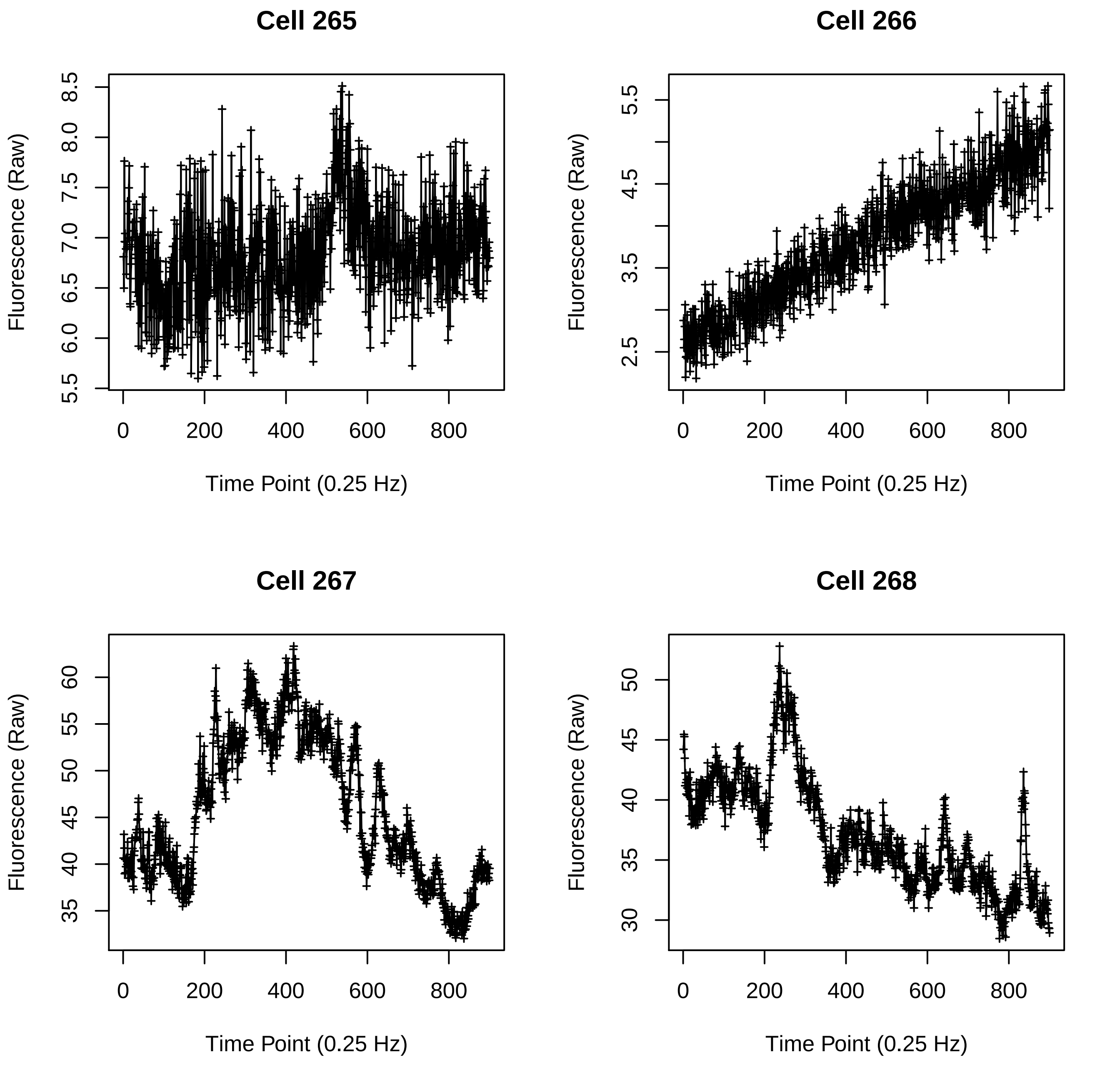

Cell 265
Cell 266
Cell 267
Cell 268
Fluorescence (Raw)
Time Point (0.25 Hz)
5.5
6.0
6.5
7.0
7.5
8.0
8.5
2.5
3.5
4.5
5.5
35
40
45
50
55
60
30
0
200
400
600
800

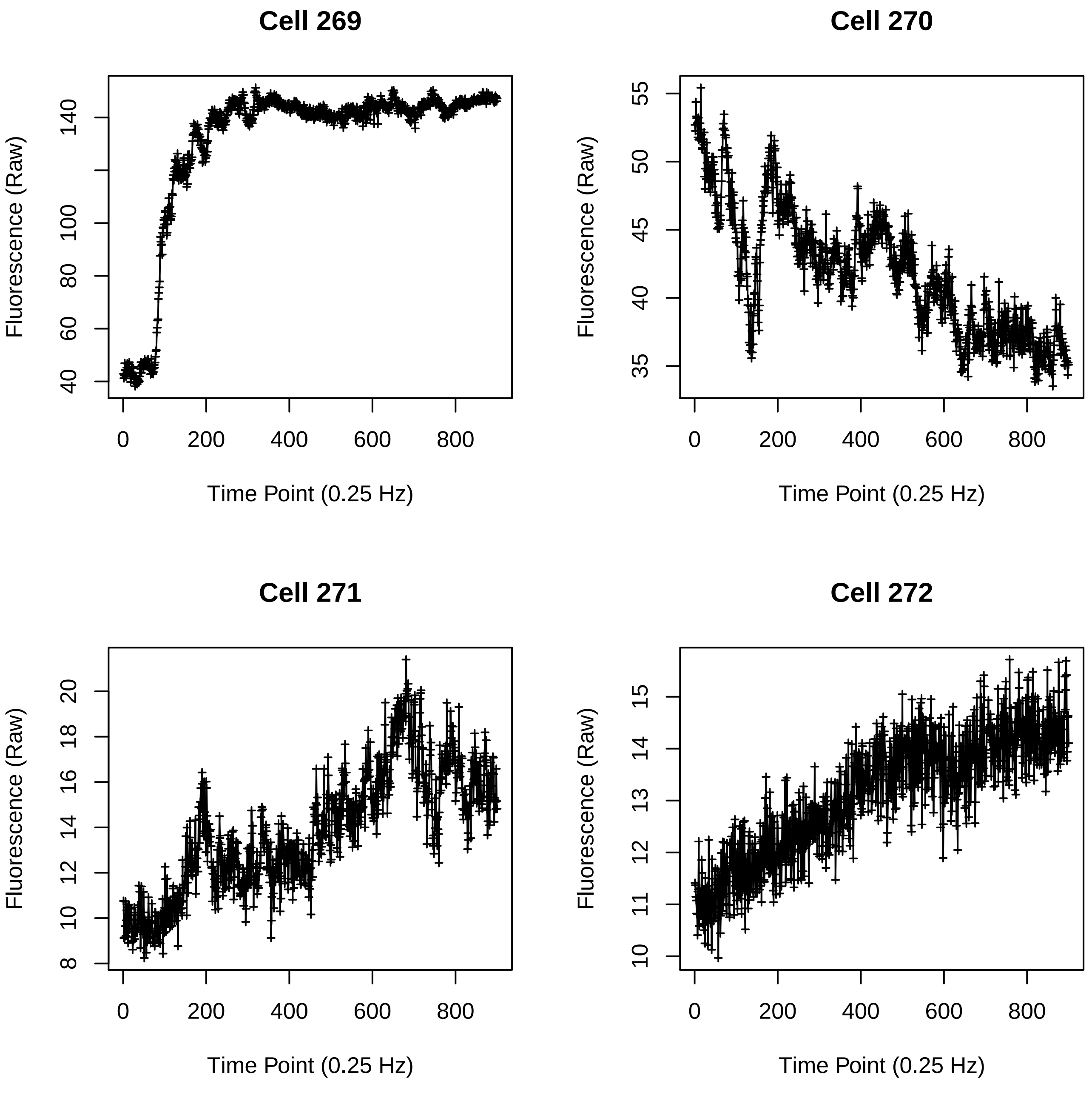
Cell 269
Fluorescence (Raw)
40
60
80
100
140
0
200
400
600
800
Time Point (0.25 Hz)
Cell 270
Fluorescence (Raw)
35
40
45
50
55
0
200
400
600
800
Time Point (0.25 Hz)
Cell 271
Fluorescence (Raw)
8
10
12
14
16
18
20
0
200
400
600
800
Time Point (0.25 Hz)
Cell 272
Fluorescence (Raw)
10
11
12
13
14
15
0
200
400
600
800
Time Point (0.25 Hz)

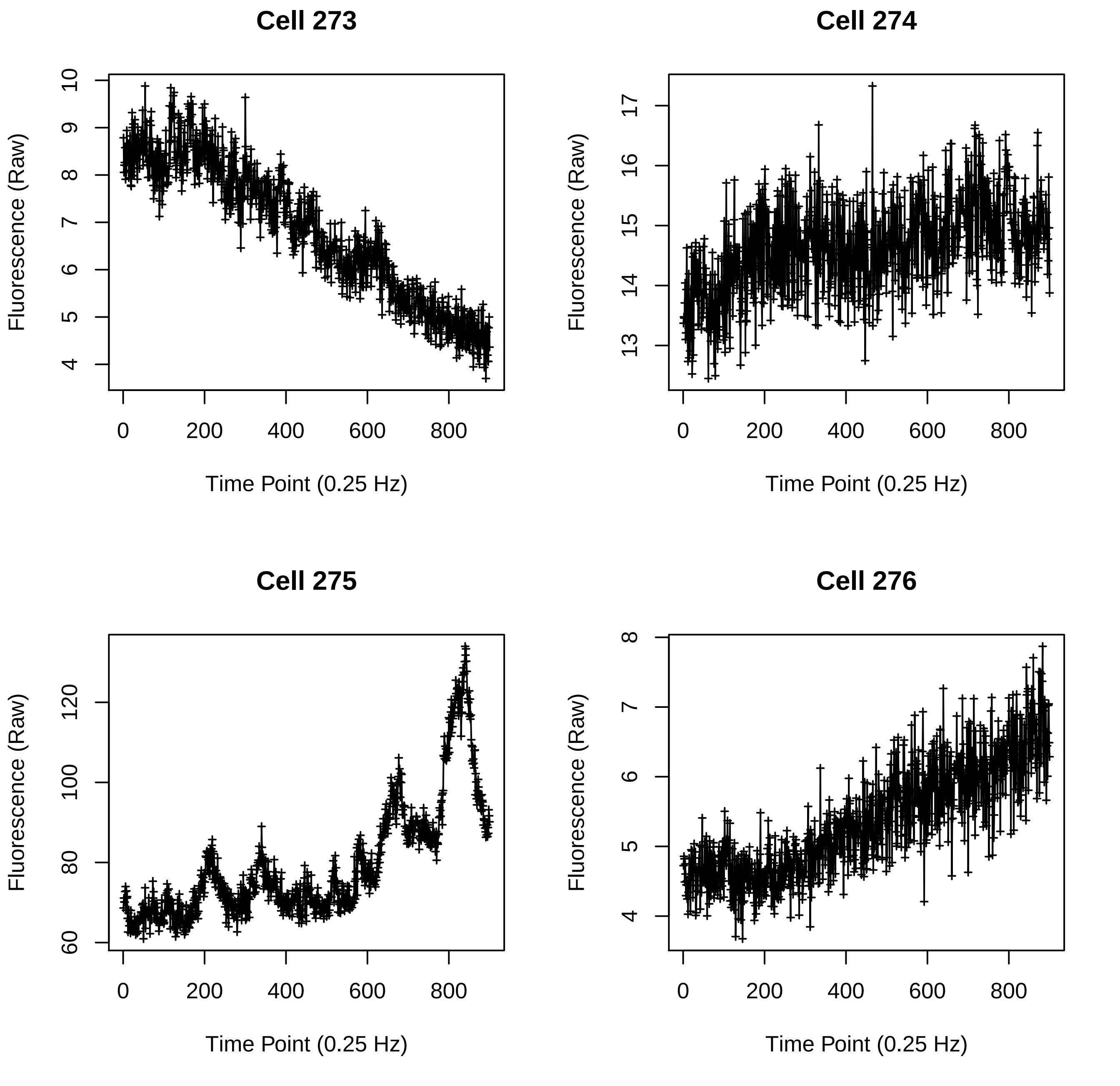
Cell 273
Fluorescence (Raw)
Time Point (0.25 Hz)
Cell 274
Fluorescence (Raw)
Time Point (0.25 Hz)
Cell 275
Fluorescence (Raw)
Time Point (0.25 Hz)
Cell 276
Fluorescence (Raw)
Time Point (0.25 Hz)

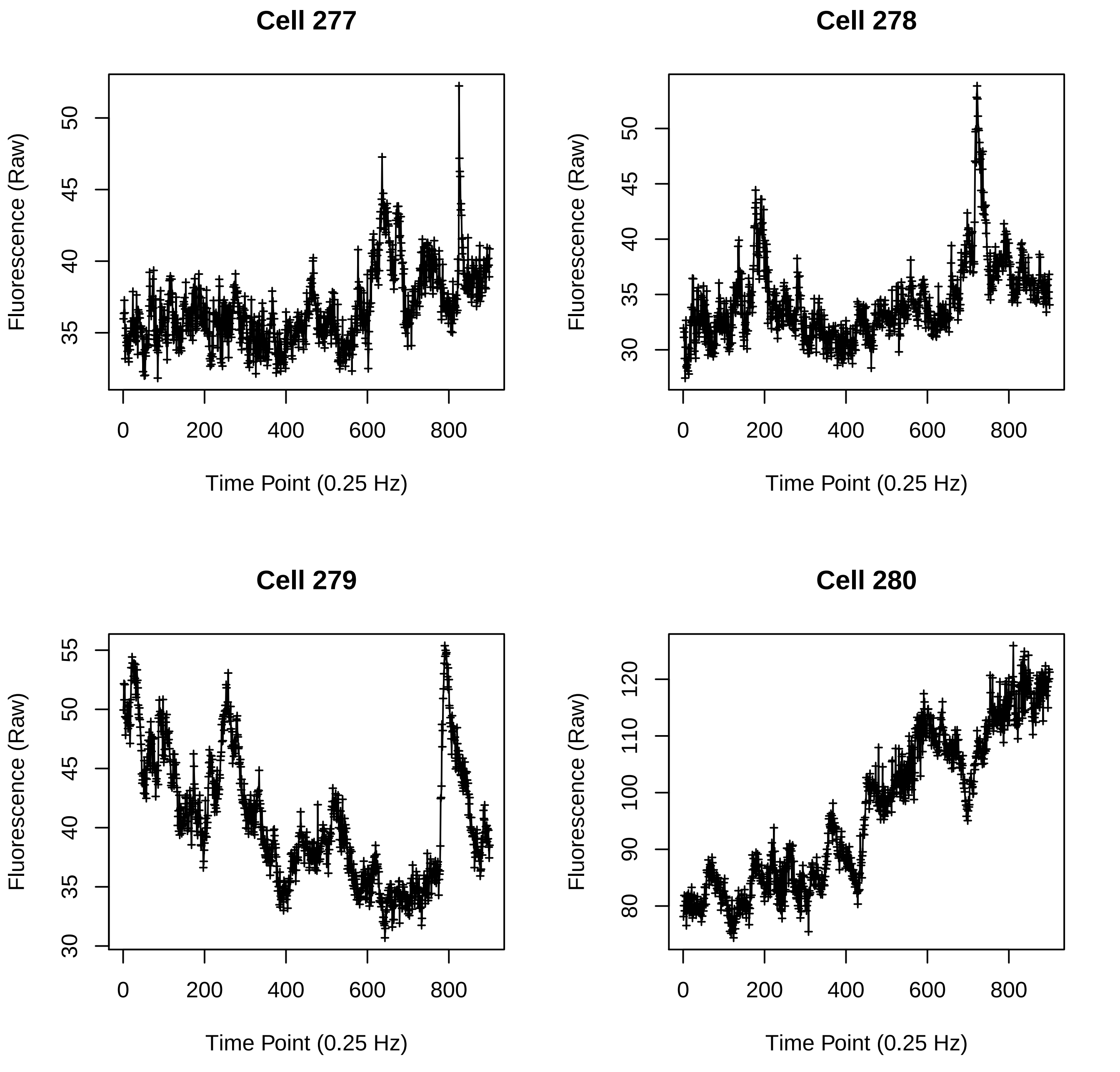

Cell 277
Cell 278
Cell 279
Cell 280
Fluorescence (Raw)
Time Point (0.25 Hz)
0
200
400
600
800
35
40
45
50
30
55
80
90
100
110
120

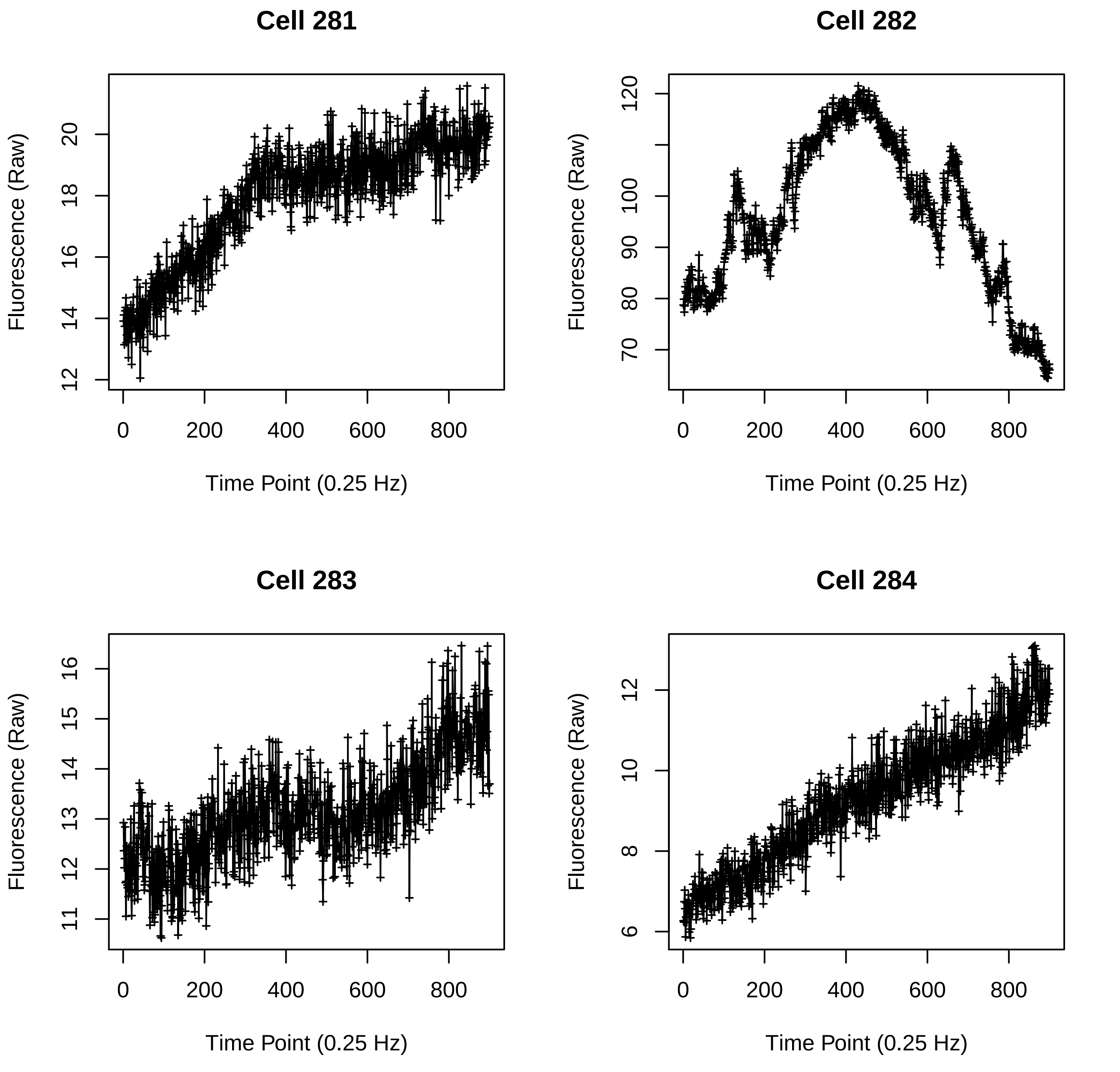
Cell 281
Fluorescence (Raw)
12
14
16
18
20
0
200
400
600
800
Time Point (0.25 Hz)
Cell 282
Fluorescence (Raw)
70
80
90
100
120
0
200
400
600
800
Time Point (0.25 Hz)
Cell 283
Fluorescence (Raw)
11
12
13
14
15
16
0
200
400
600
800
Time Point (0.25 Hz)
Cell 284
Fluorescence (Raw)
6
8
10
12
0
200
400
600
800
Time Point (0.25 Hz)

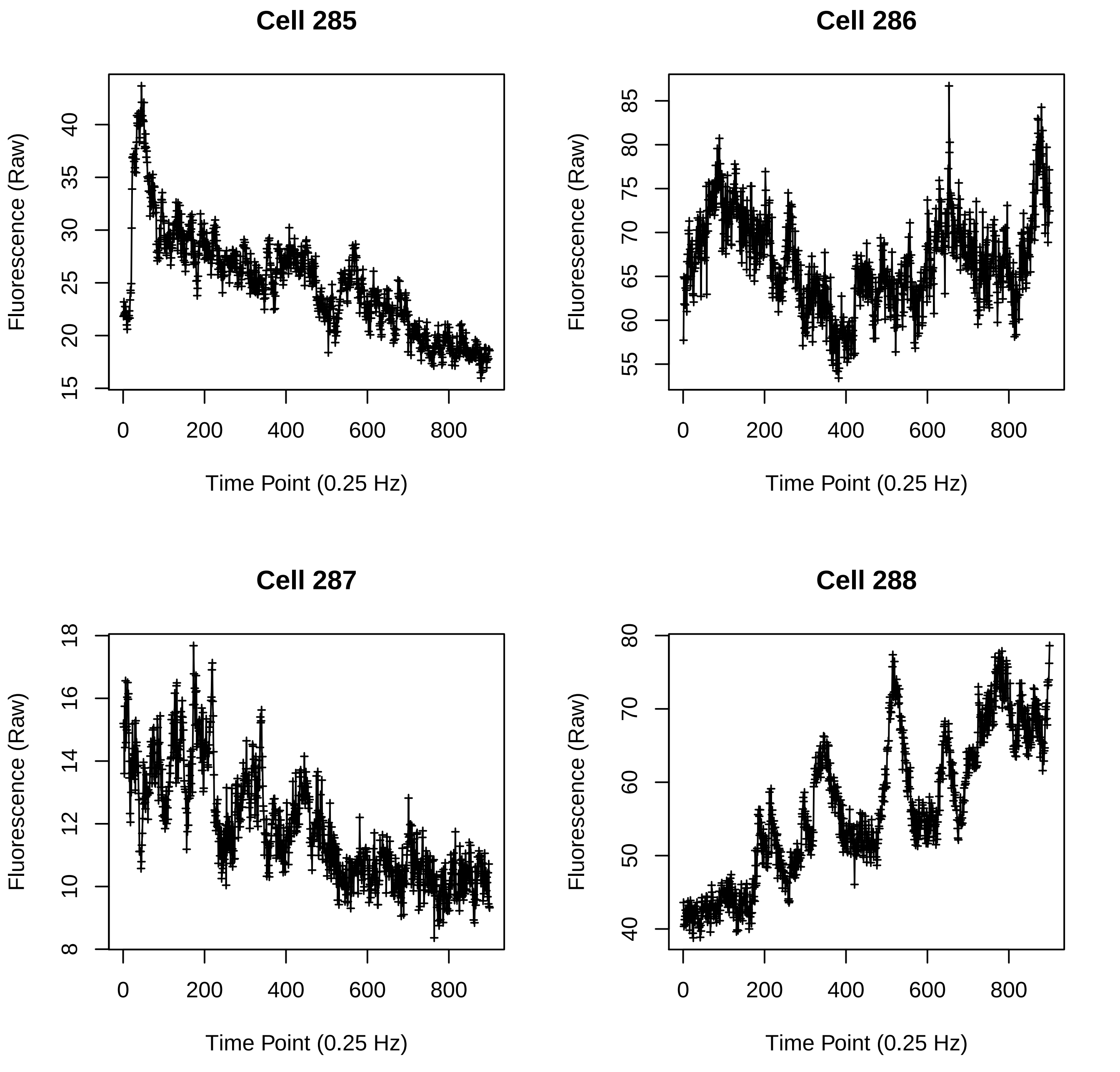
Cell 285
Cell 286
Cell 287
Cell 288
Fluorescence (Raw)
Time Point (0.25 Hz)
15
20
25
30
35
40
55
60
65
70
75
80
85
8
10
12
14
16
18
40
50
60
70
80
0
200
400
600
800

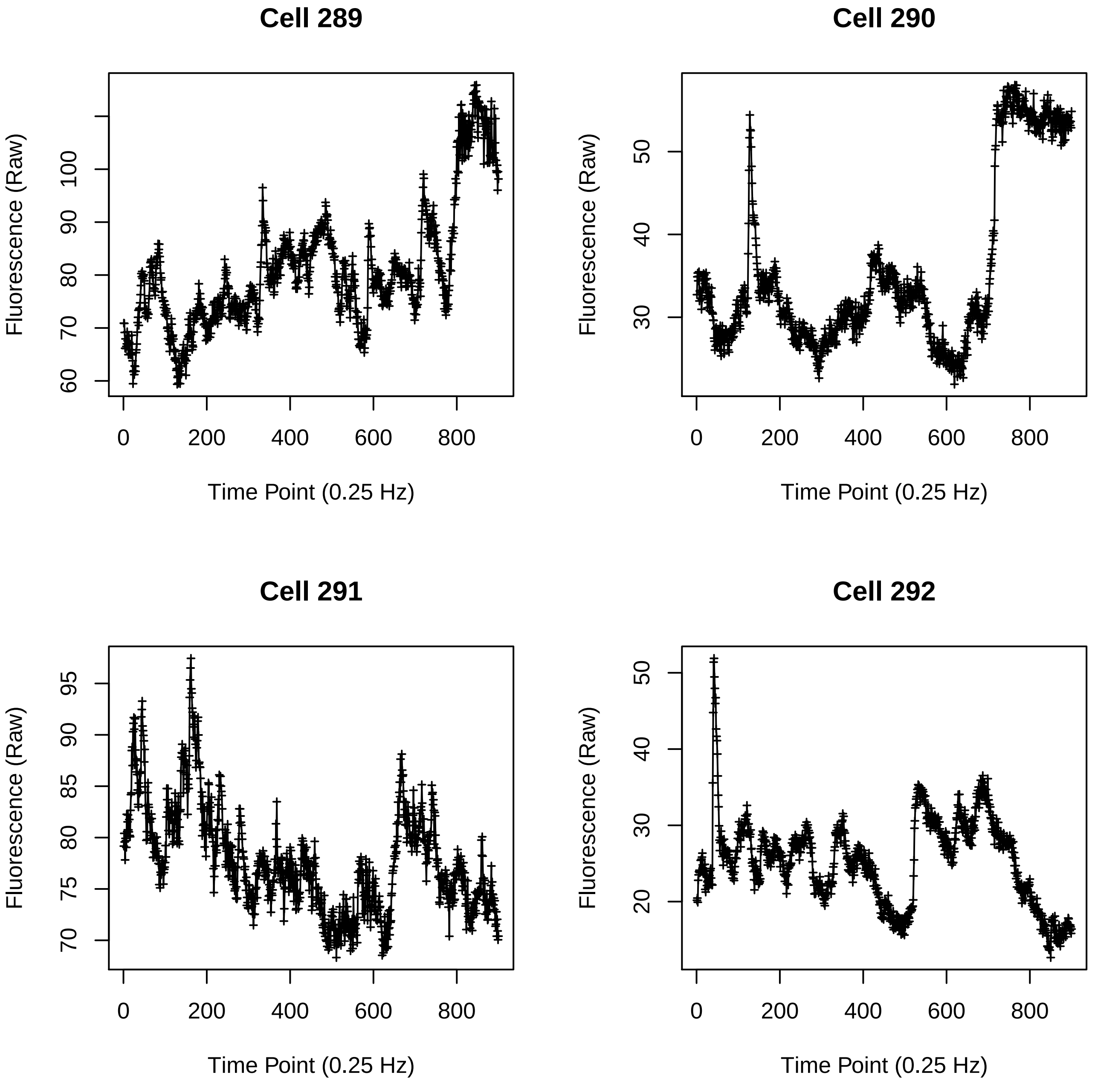

Cell 289
Cell 290
Cell 291
Cell 292
Fluorescence (Raw)
Time Point (0.25 Hz)
0
200
400
600
800
60
70
80
90
100
30
40
50
70
75
80
85
90
95
20
30
40
50

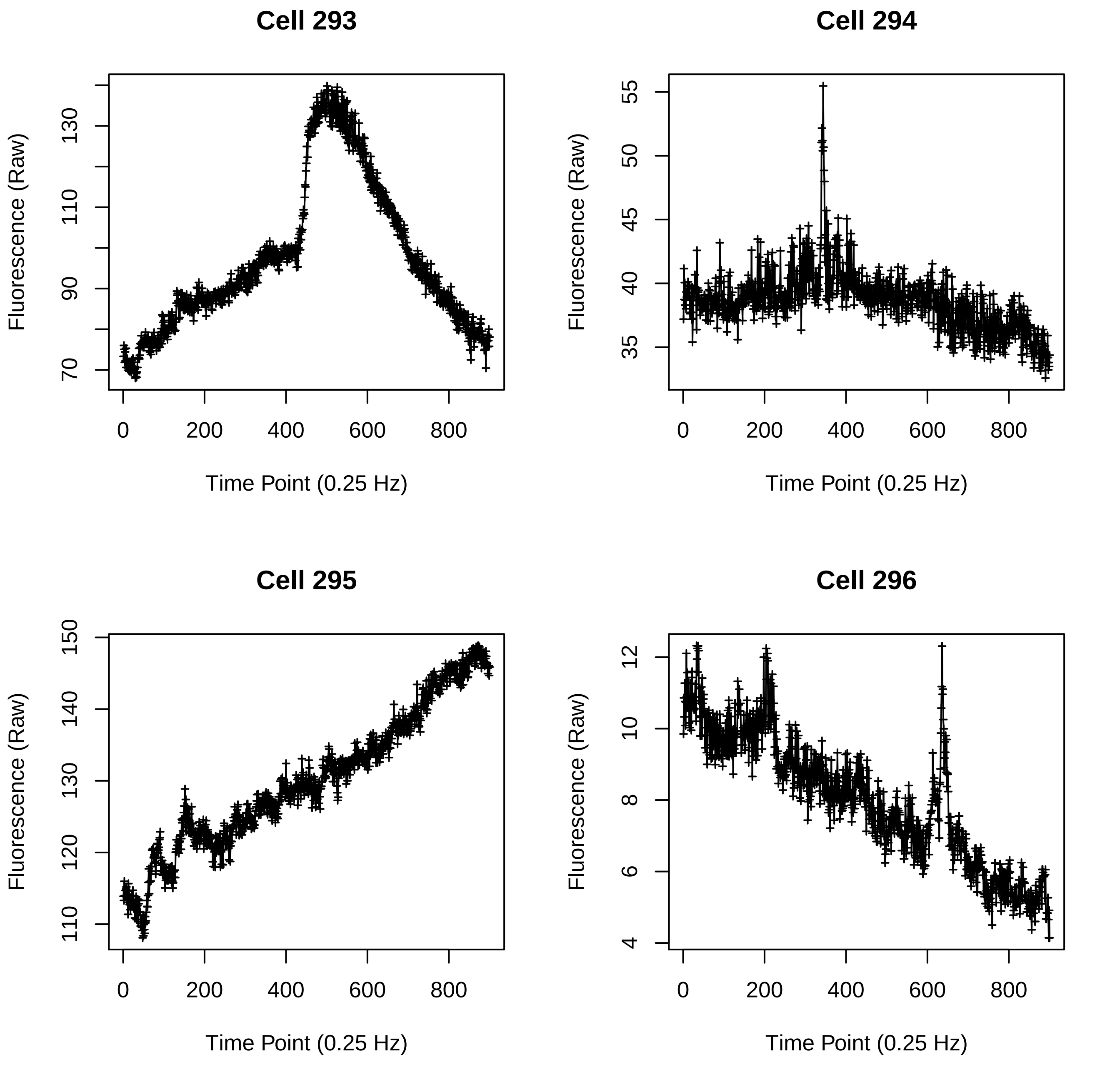
Cell 293
Fluorescence (Raw)
70
90
110
130
0
200
400
600
800
Time Point (0.25 Hz)
Cell 294
Fluorescence (Raw)
35
40
45
50
55
0
200
400
600
800
Time Point (0.25 Hz)
Cell 295
Fluorescence (Raw)
110
120
130
140
150
0
200
400
600
800
Time Point (0.25 Hz)
Cell 296
Fluorescence (Raw)
4
6
8
10
12
0
200
400
600
800
Time Point (0.25 Hz)

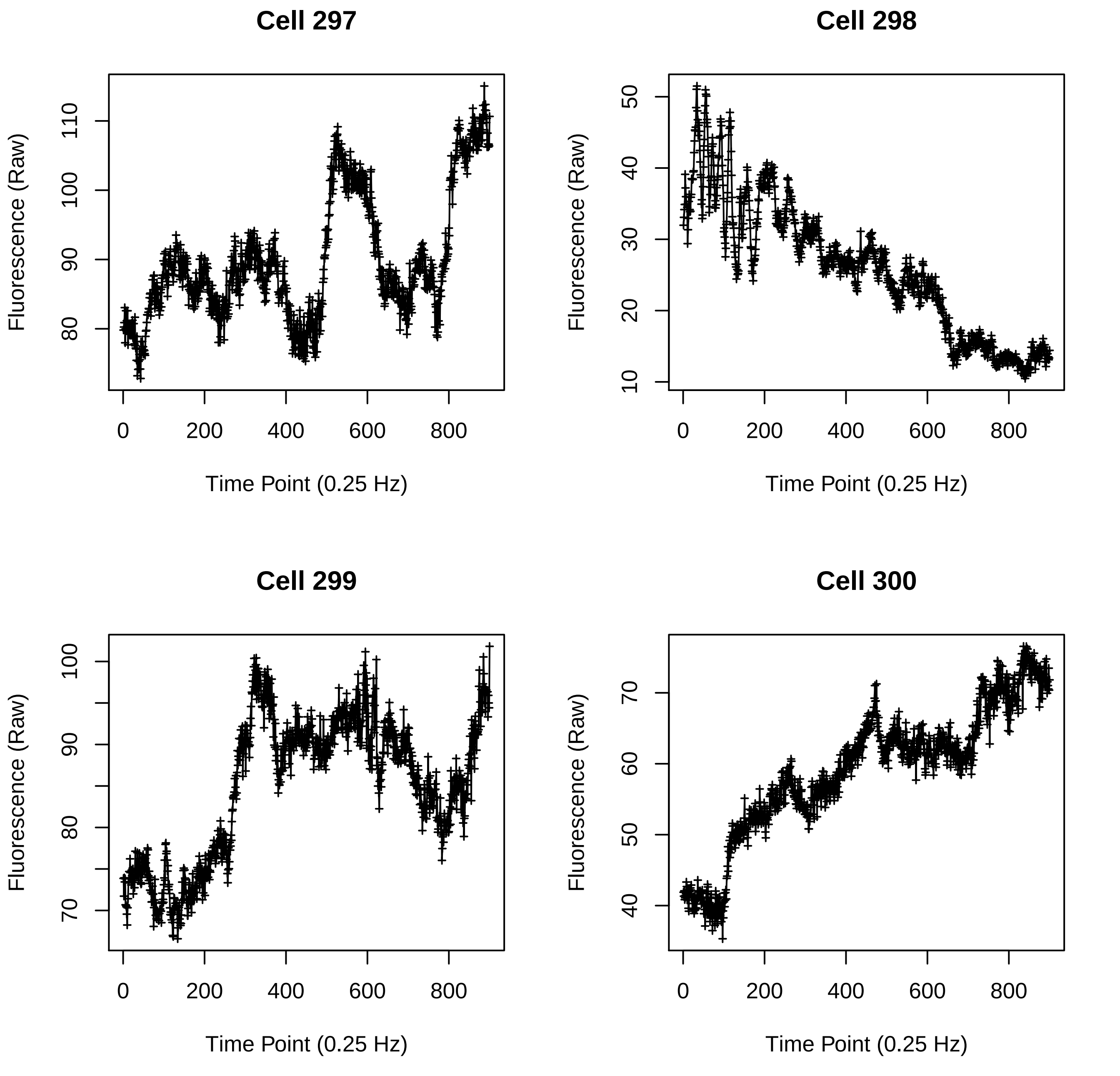
Cell 297
Fluorescence (Raw)
80
90
100
110
0
200
400
600
800
Time Point (0.25 Hz)
Cell 298
Fluorescence (Raw)
10
20
30
40
50
0
200
400
600
800
Time Point (0.25 Hz)
Cell 299
Fluorescence (Raw)
70
80
90
100
0
200
400
600
800
Time Point (0.25 Hz)
Cell 300
Fluorescence (Raw)
40
50
60
70
0
200
400
600
800
Time Point (0.25 Hz)

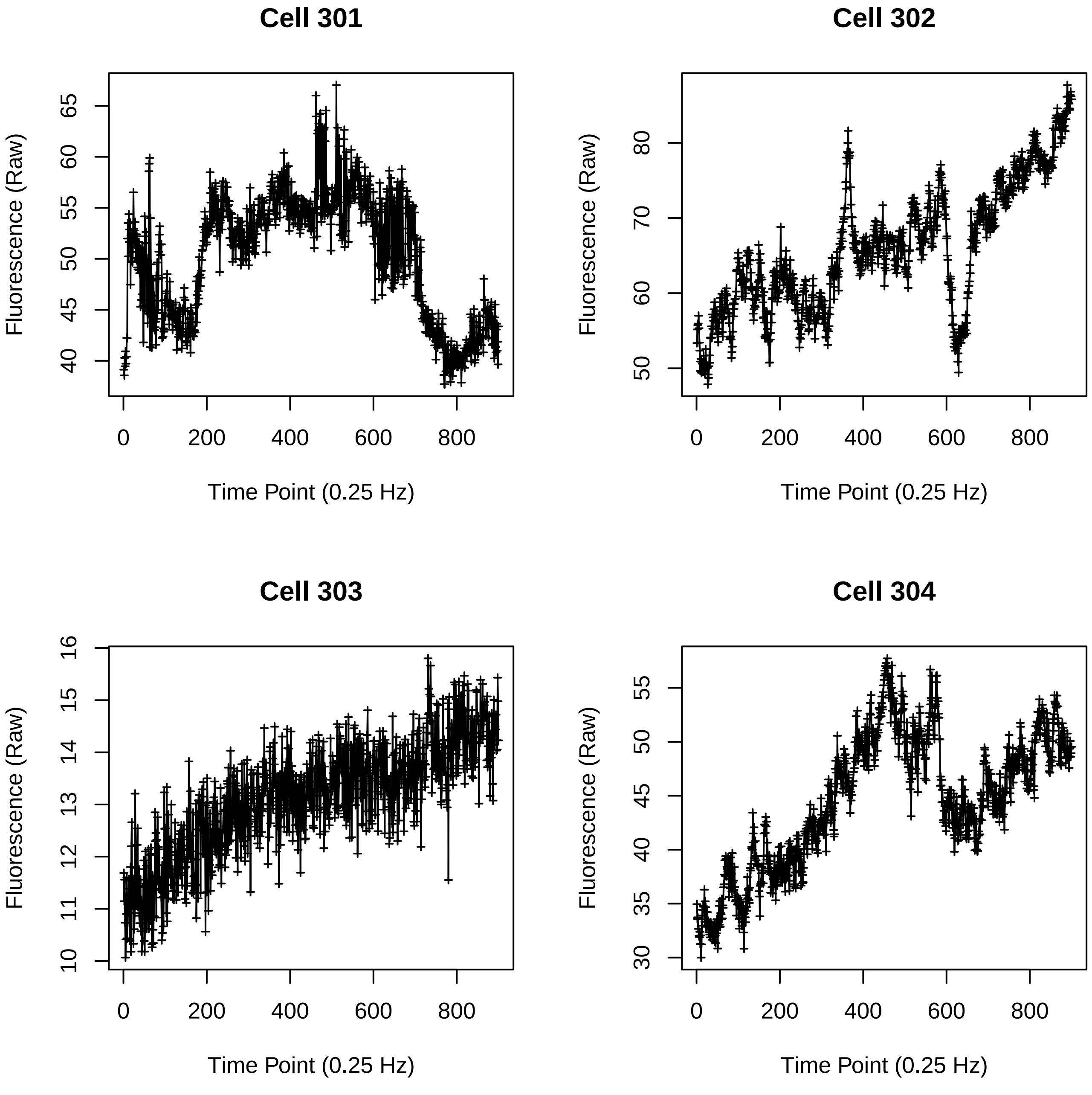
Cell 301
Fluorescence (Raw)
40
45
50
55
60
65
0
200
400
600
800
Time Point (0.25 Hz)
Cell 302
Fluorescence (Raw)
50
60
70
80
0
200
400
600
800
Time Point (0.25 Hz)
Cell 303
Fluorescence (Raw)
10
11
12
13
14
15
16
0
200
400
600
800
Time Point (0.25 Hz)
Cell 304
Fluorescence (Raw)
30
35
40
45
50
55
0
200
400
600
800
Time Point (0.25 Hz)

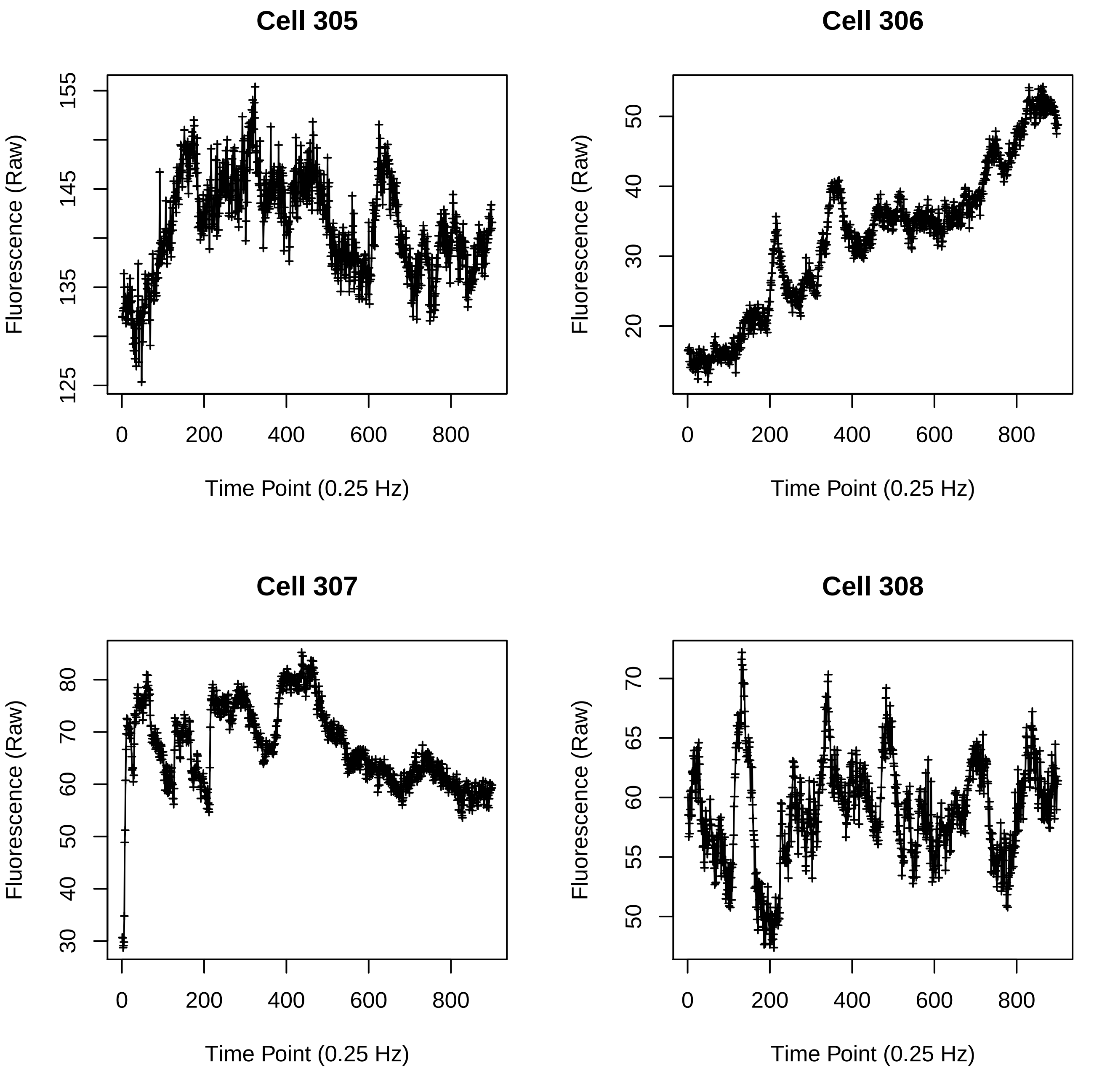
Cell 305
Fluorescence (Raw)
125
135
145
155
0
200
400
600
800
Time Point (0.25 Hz)
Cell 306
Fluorescence (Raw)
20
30
40
50
0
200
400
600
800
Time Point (0.25 Hz)
Cell 307
Fluorescence (Raw)
30
40
50
60
70
80
0
200
400
600
800
Time Point (0.25 Hz)
Cell 308
Fluorescence (Raw)
50
55
60
65
70
0
200
400
600
800
Time Point (0.25 Hz)

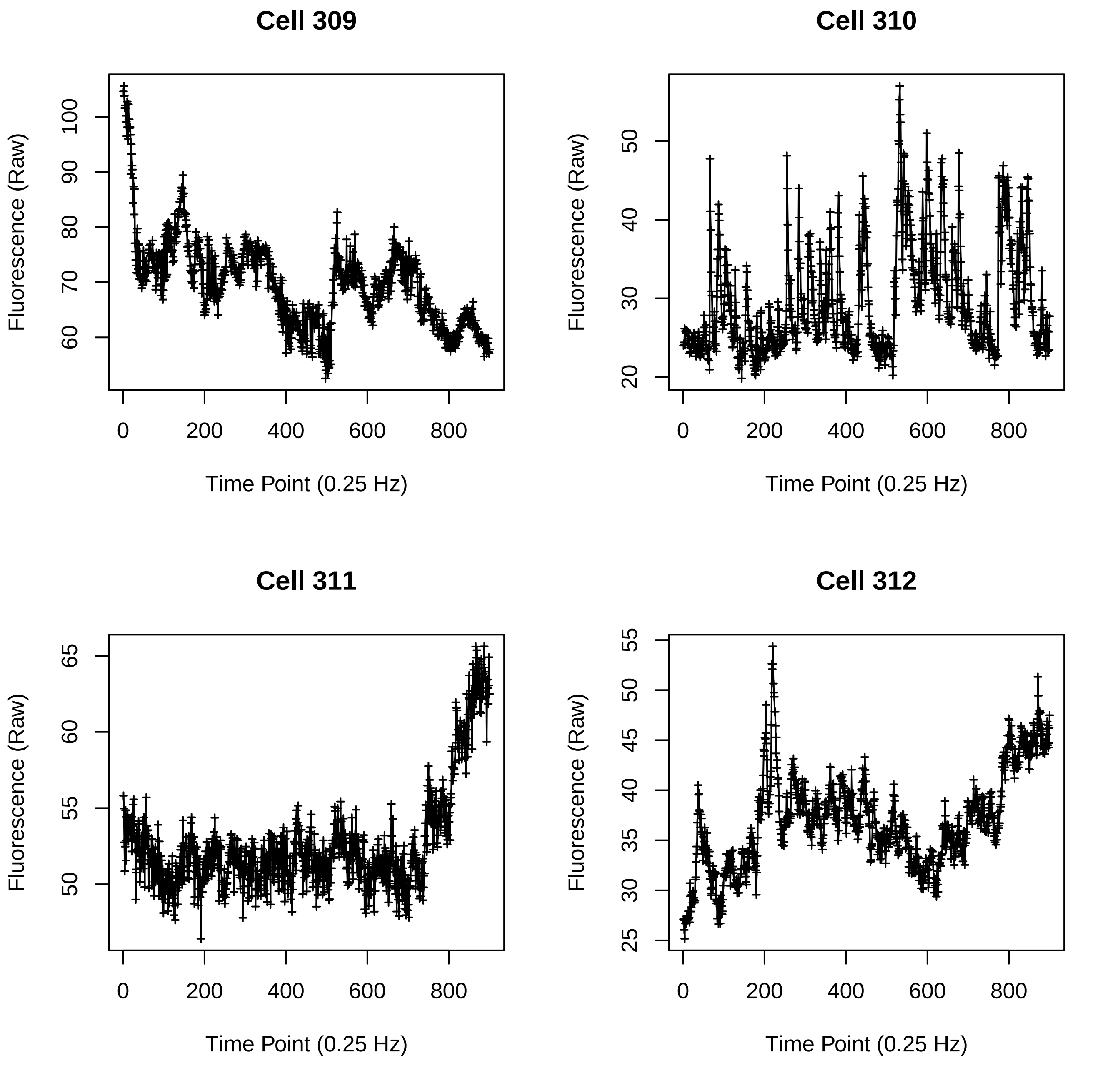

Cell 309
Cell 310
Cell 311
Cell 312
Fluorescence (Raw)
Time Point (0.25 Hz)
0
200
400
600
800
100
90
80
70
60
50
40
30
20
65
55
45
35
25

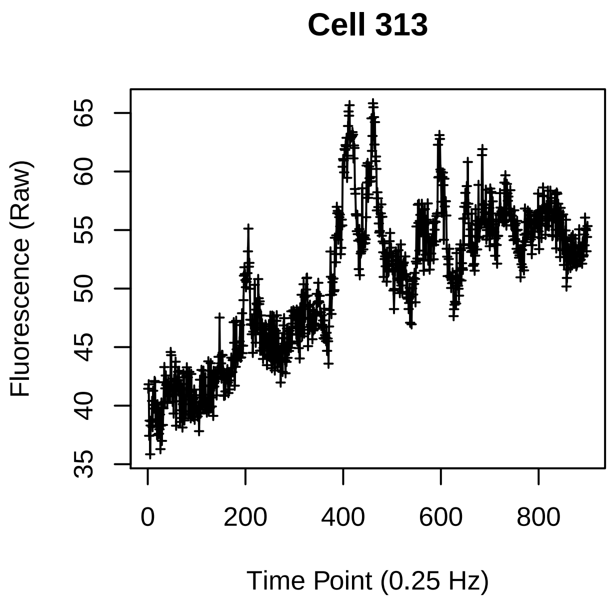
Cell 313
Fluorescence (Raw)
35
40
45
50
55
60
65
0
200
400
600
800
Time Point (0.25 Hz)

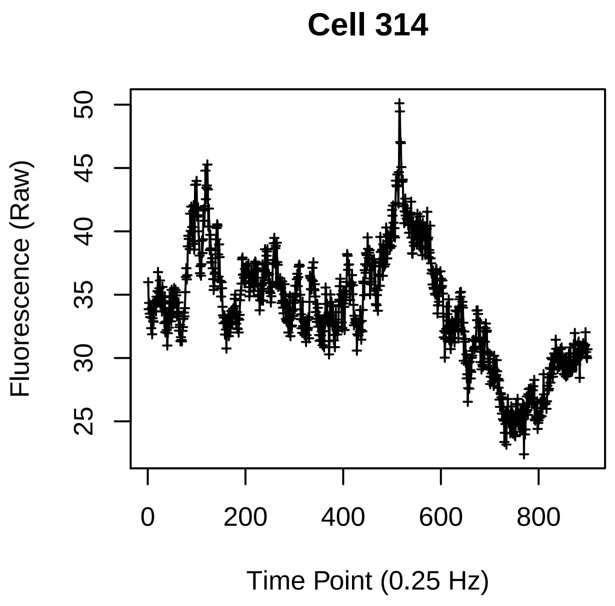
Cell 314
Fluorescence (Raw)
25
30
35
40
45
50
0
200
400
600
800
Time Point (0.25 Hz)

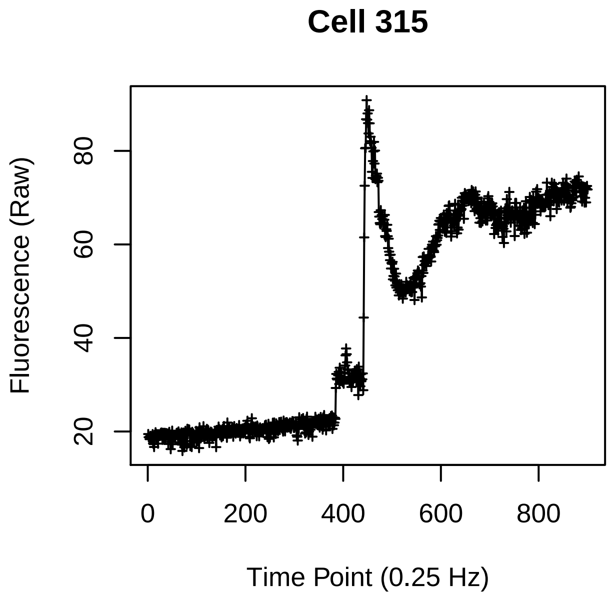
Cell 315
Fluorescence (Raw)
20
40
60
80
0
200
400
600
800
Time Point (0.25 Hz)

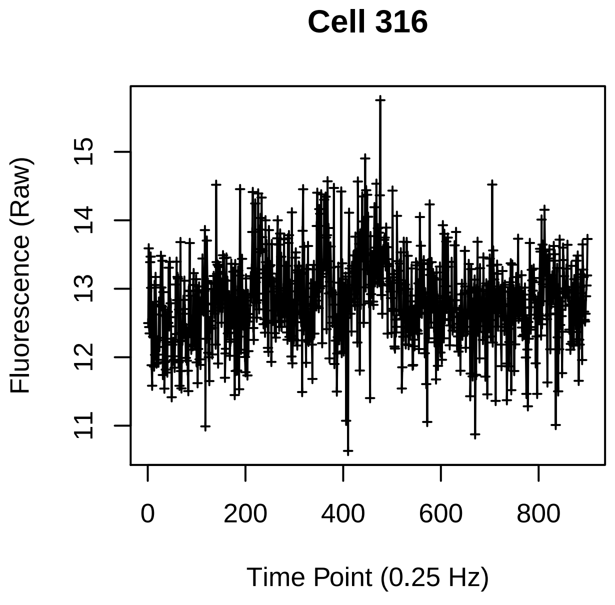
Cell 316
Fluorescence (Raw)
11
12
13
14
15
0
200
400
600
800
Time Point (0.25 Hz)

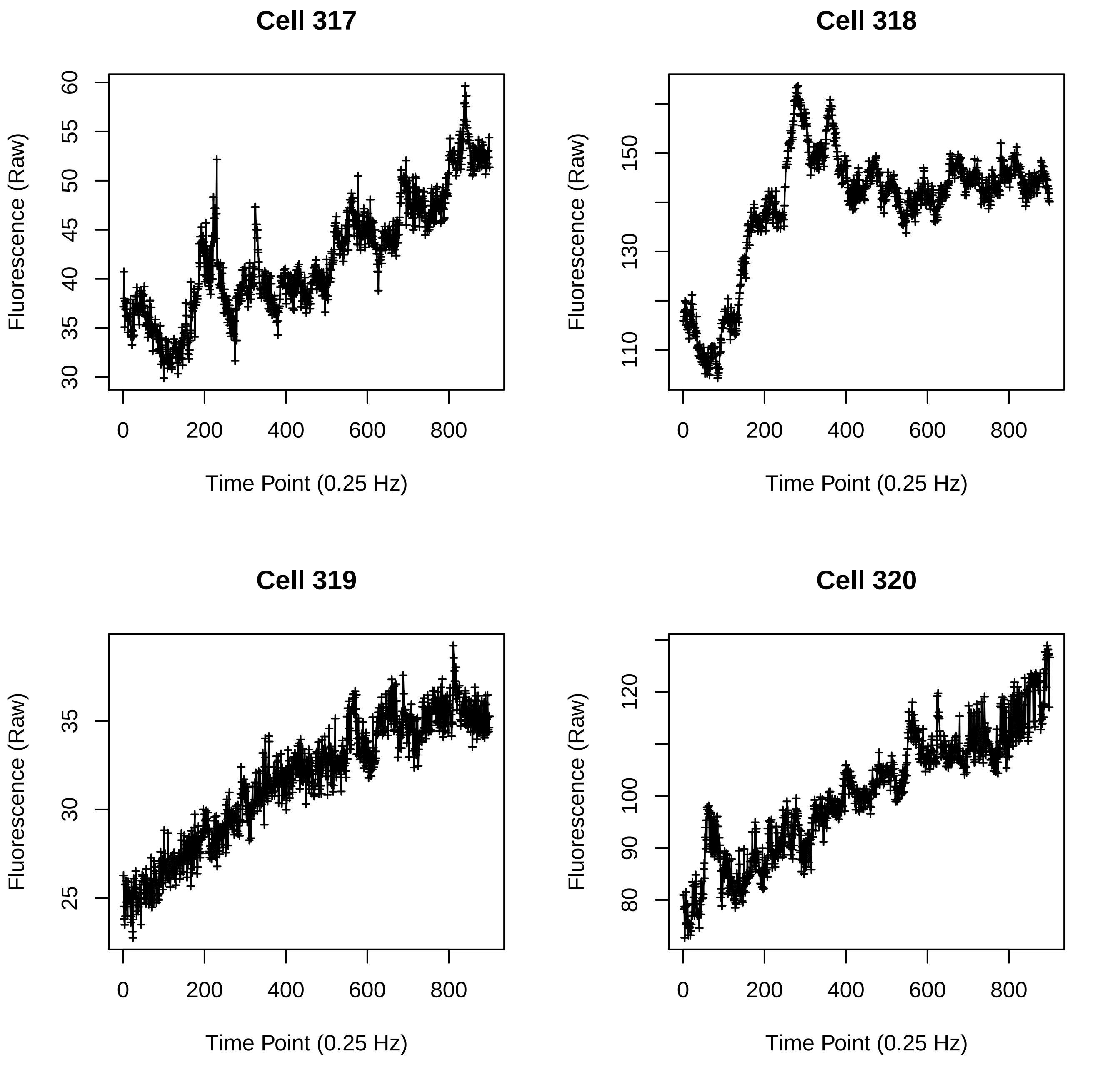

Cell 317
Fluorescence (Raw)
30 35 40 45 50 55 60
0 200 400 600 800
Time Point (0.25 Hz)
Cell 318
Fluorescence (Raw)
110 130 150
0 200 400 600 800
Time Point (0.25 Hz)
Cell 319
Fluorescence (Raw)
25 30 35
0 200 400 600 800
Time Point (0.25 Hz)
Cell 320
Fluorescence (Raw)
80 90 100 120
0 200 400 600 800
Time Point (0.25 Hz)

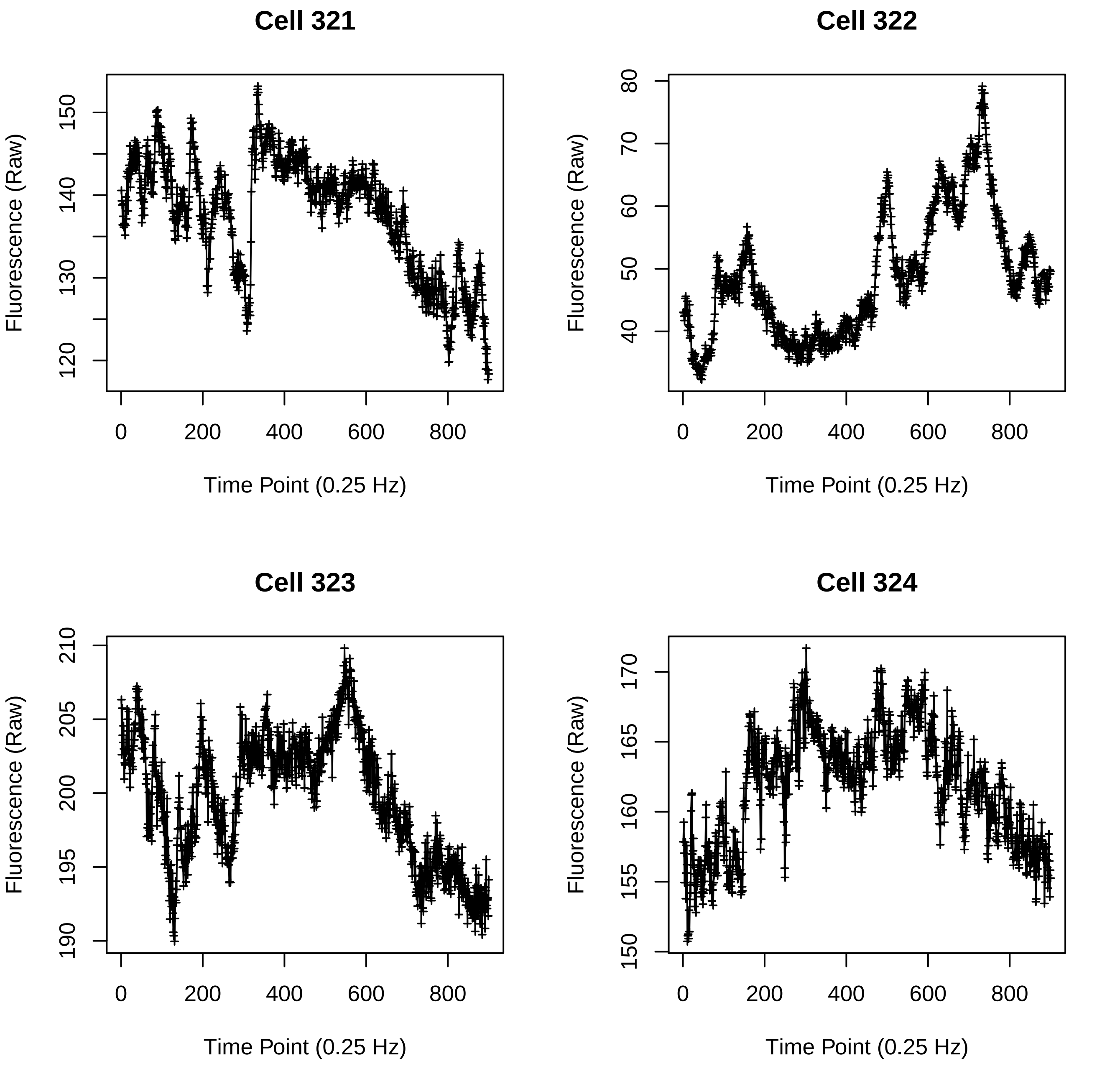
Cell 321
Fluorescence (Raw)
120
130
140
150
0
200
400
600
800
Time Point (0.25 Hz)
Cell 322
Fluorescence (Raw)
40
50
60
70
80
0
200
400
600
800
Time Point (0.25 Hz)
Cell 323
Fluorescence (Raw)
190
195
200
205
210
0
200
400
600
800
Time Point (0.25 Hz)
Cell 324
Fluorescence (Raw)
150
155
160
165
170
0
200
400
600
800
Time Point (0.25 Hz)

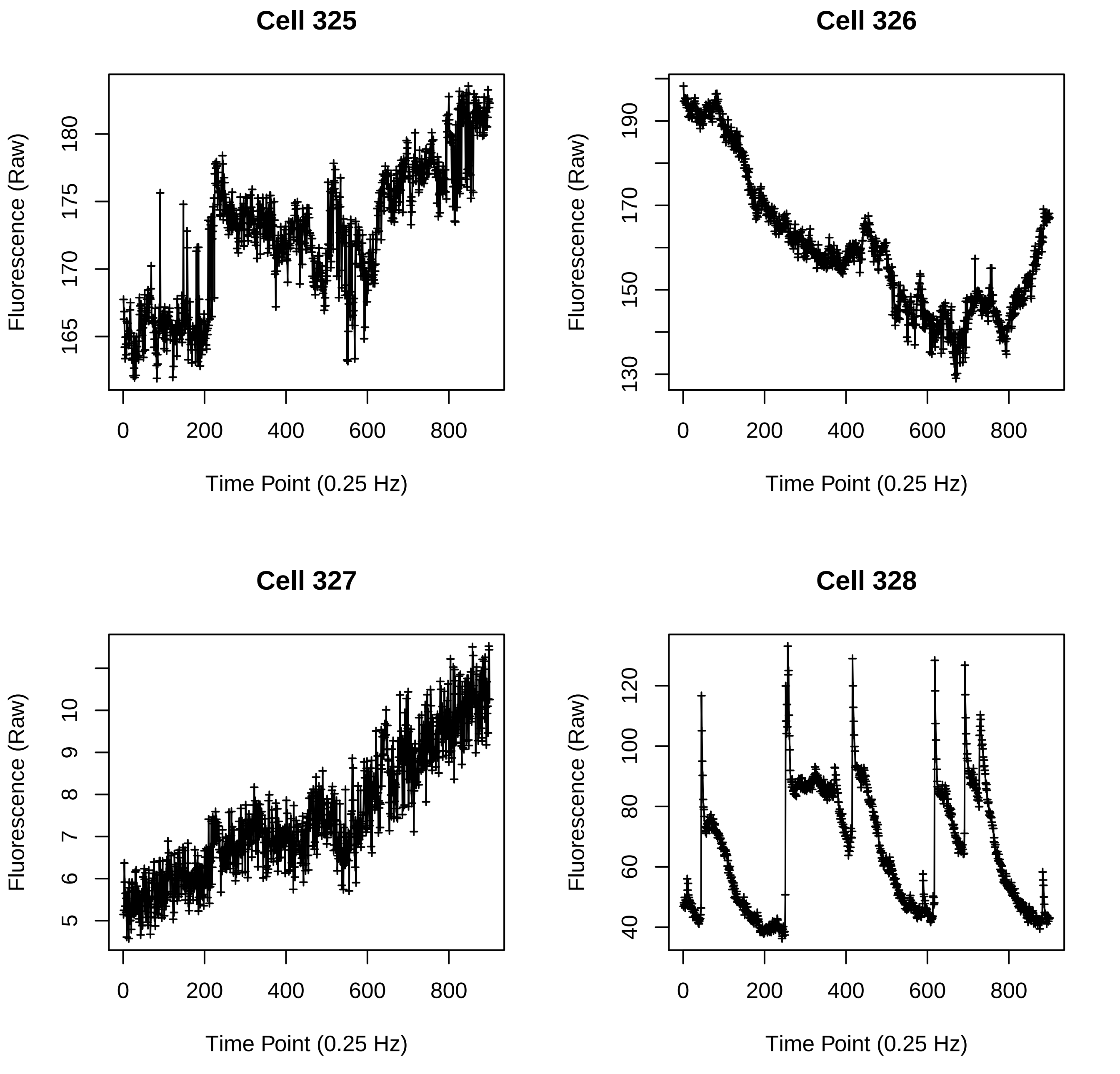
Cell 325
Fluorescence (Raw)
165
170
175
180
0
200
400
600
800
Time Point (0.25 Hz)
Cell 326
Fluorescence (Raw)
130
150
170
190
0
200
400
600
800
Time Point (0.25 Hz)
Cell 327
Fluorescence (Raw)
5
6
7
8
9
10
0
200
400
600
800
Time Point (0.25 Hz)
Cell 328
Fluorescence (Raw)
40
60
80
100
120
0
200
400
600
800
Time Point (0.25 Hz)

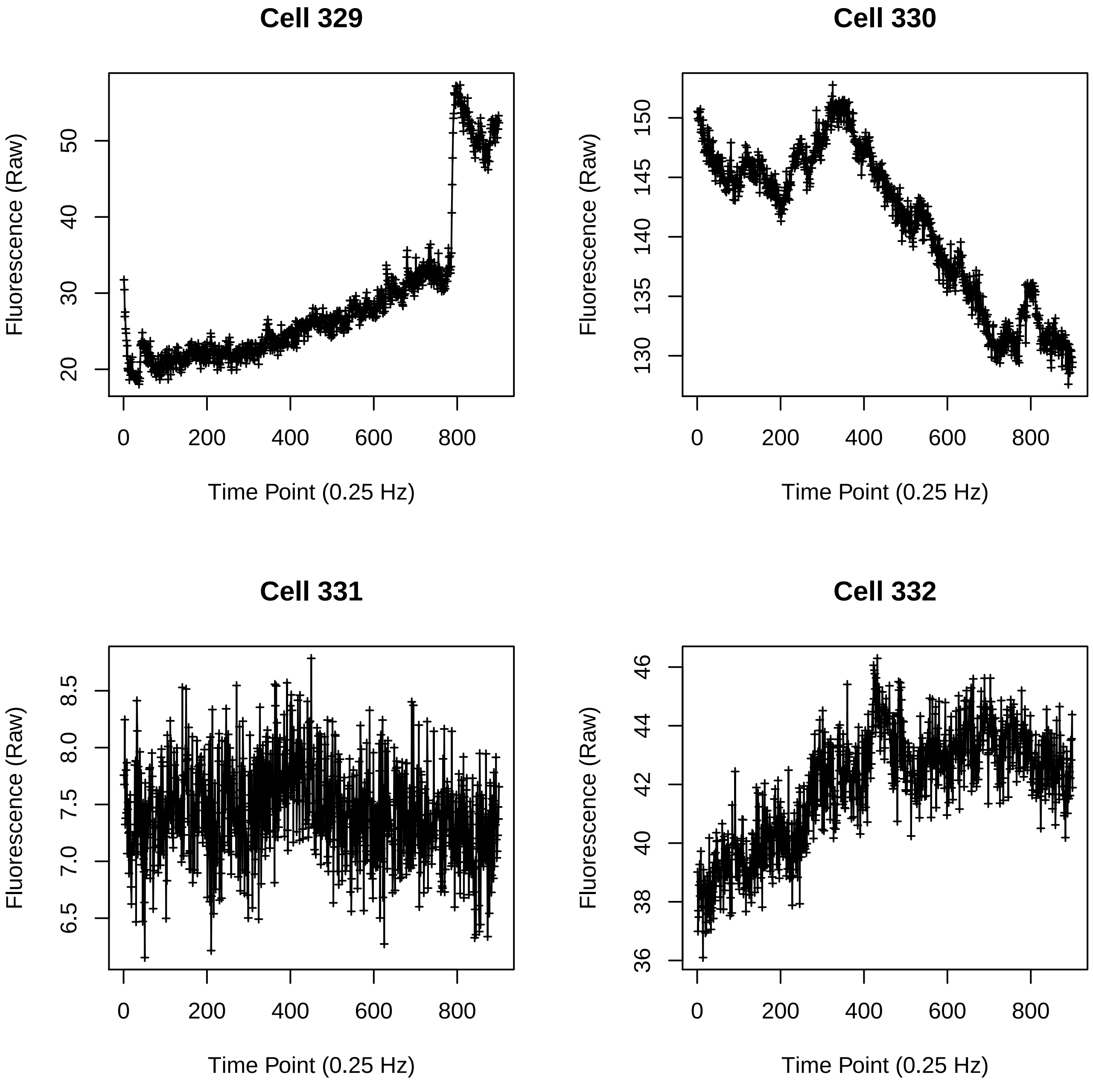

Cell 329
Cell 330
Cell 331
Cell 332
Fluorescence (Raw)
Time Point (0.25 Hz)
20
30
40
50
130
135
140
145
150
6.5
7.0
7.5
8.0
8.5
36
38
40
42
44
46
0
200
400
600
800

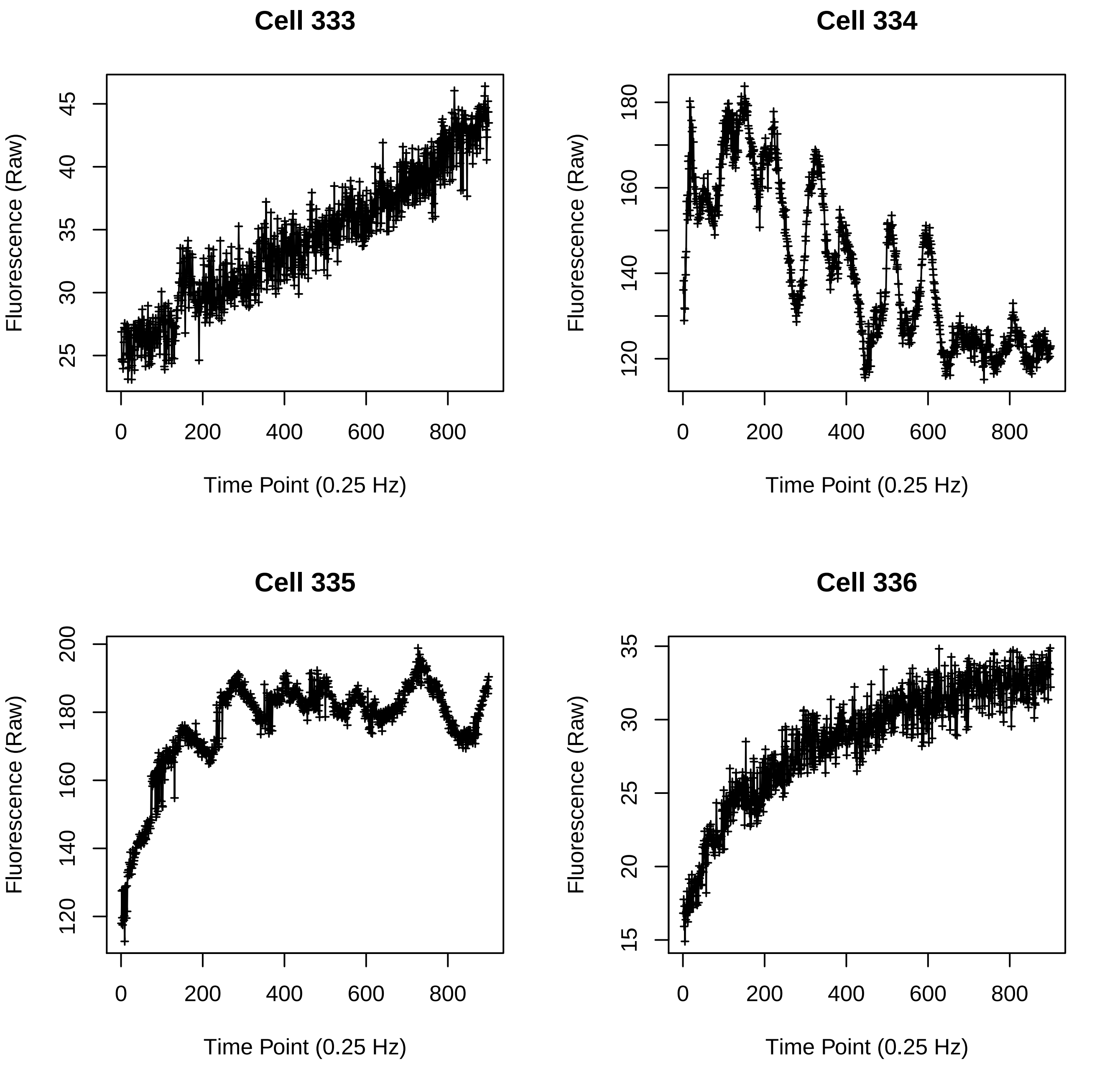

Cell 333
Fluorescence (Raw)
25
30
35
40
45
0
200
400
600
800
Time Point (0.25 Hz)
Cell 334
Fluorescence (Raw)
120
140
160
180
0
200
400
600
800
Time Point (0.25 Hz)
Cell 335
Fluorescence (Raw)
120
140
160
180
200
0
200
400
600
800
Time Point (0.25 Hz)
Cell 336
Fluorescence (Raw)
15
20
25
30
35
0
200
400
600
800
Time Point (0.25 Hz)

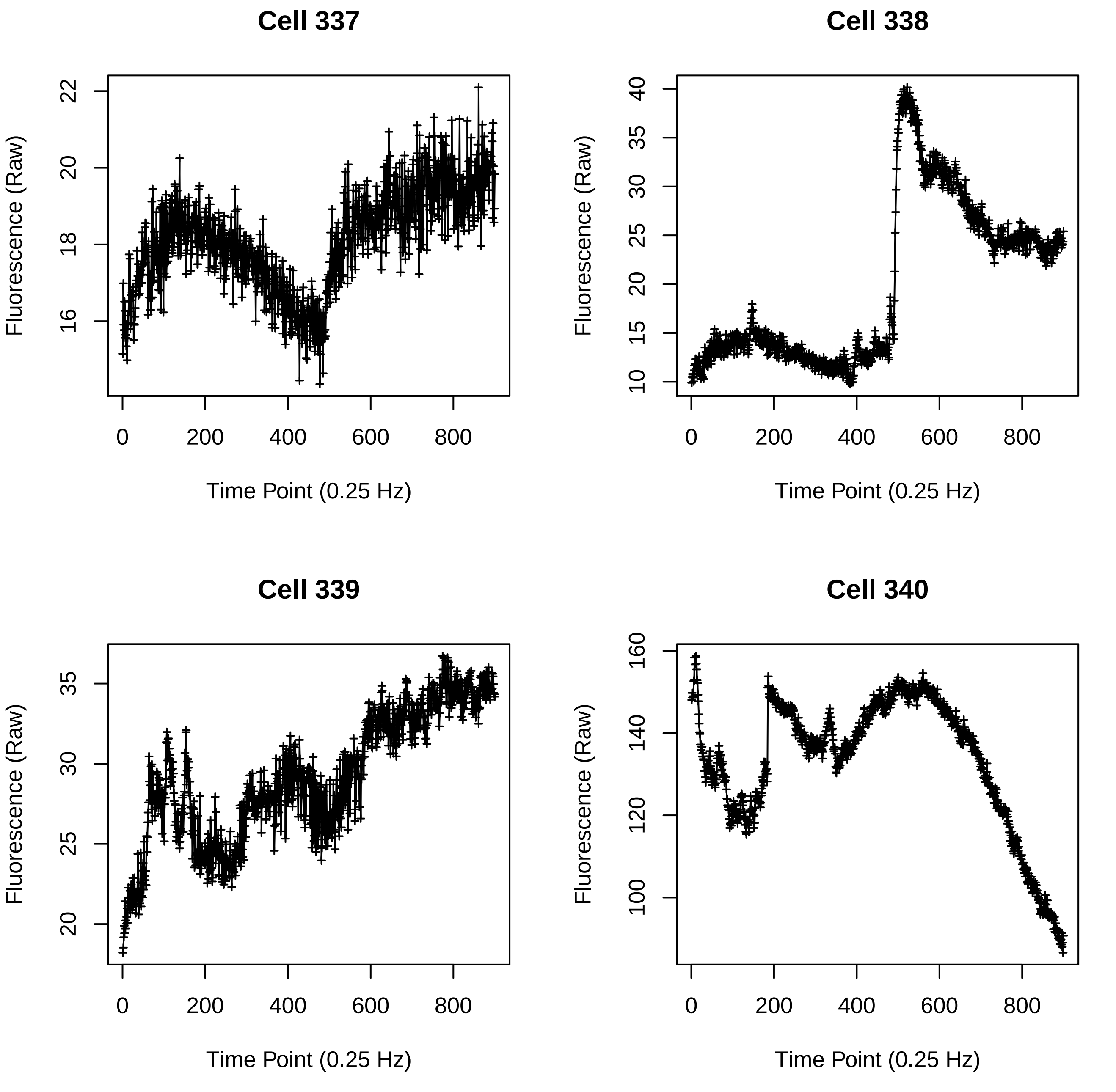
Cell 337
Cell 338
Cell 339
Cell 340
Fluorescence (Raw)
Time Point (0.25 Hz)
0
200
400
600
800
16
18
20
22
10
15
20
25
30
35
40
20
25
30
35
100
120
140
160

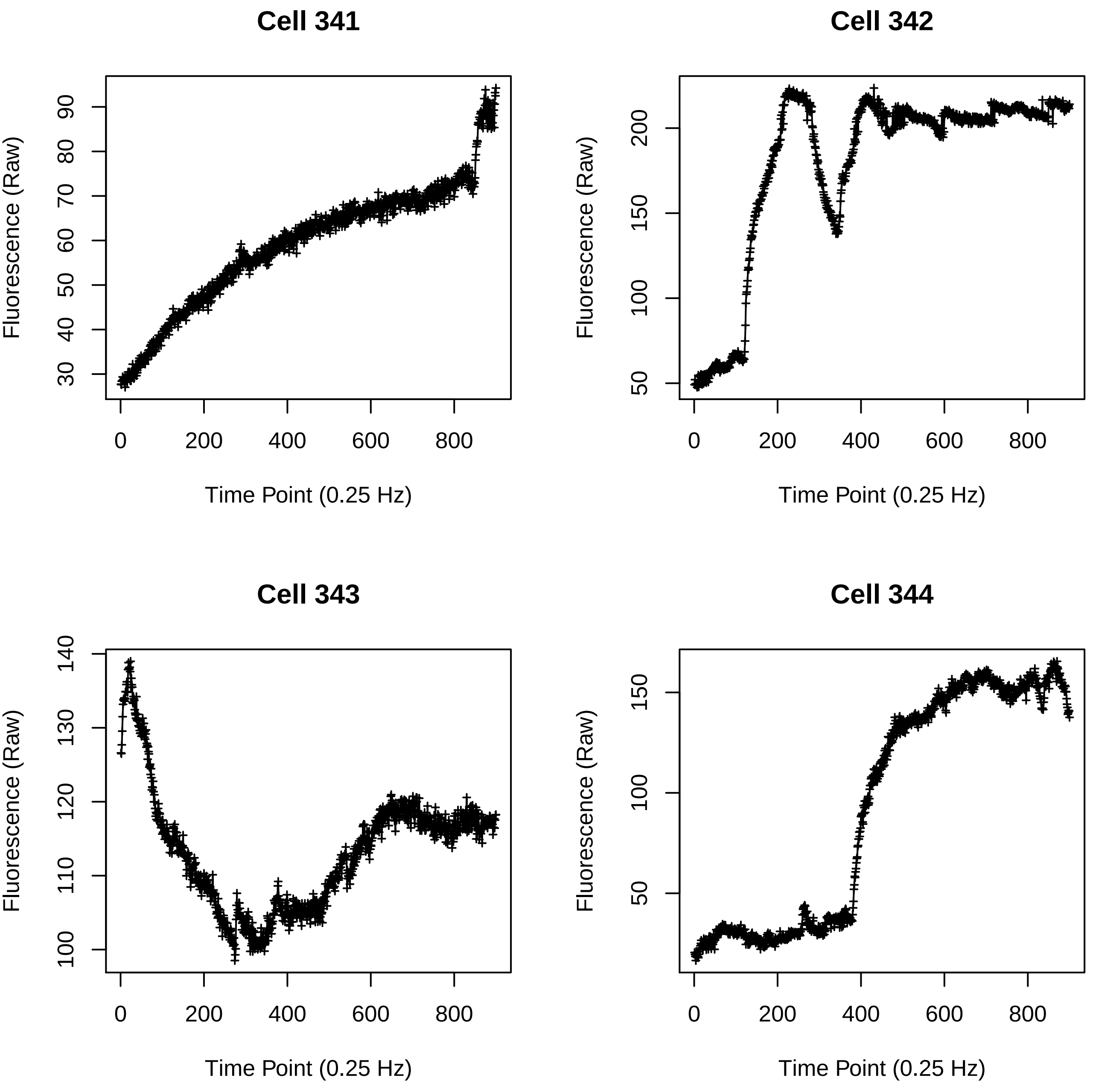
Cell 341
Fluorescence (Raw)
30
40
50
60
70
80
90
0
200
400
600
800
Time Point (0.25 Hz)
Cell 342
Fluorescence (Raw)
50
100
150
200
0
200
400
600
800
Time Point (0.25 Hz)
Cell 343
Fluorescence (Raw)
100
110
120
130
140
0
200
400
600
800
Time Point (0.25 Hz)
Cell 344
Fluorescence (Raw)
50
100
150
0
200
400
600
800
Time Point (0.25 Hz)

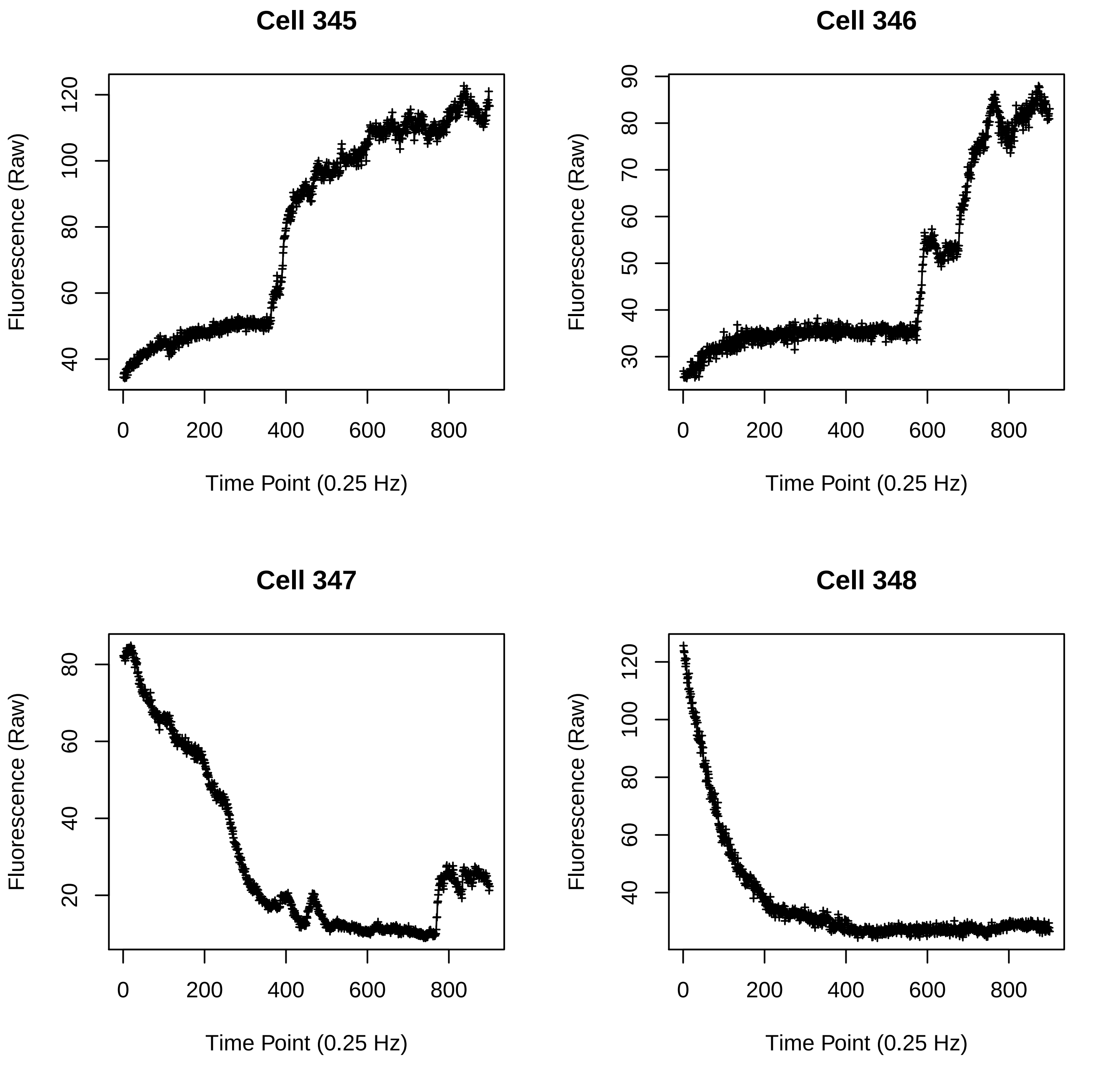
Cell 345
Fluorescence (Raw)
40
60
80
100
120
0
200
400
600
800
Time Point (0.25 Hz)
Cell 346
Fluorescence (Raw)
30
40
50
60
70
80
90
0
200
400
600
800
Time Point (0.25 Hz)
Cell 347
Fluorescence (Raw)
20
40
60
80
0
200
400
600
800
Time Point (0.25 Hz)
Cell 348
Fluorescence (Raw)
40
60
80
100
120
0
200
400
600
800
Time Point (0.25 Hz)

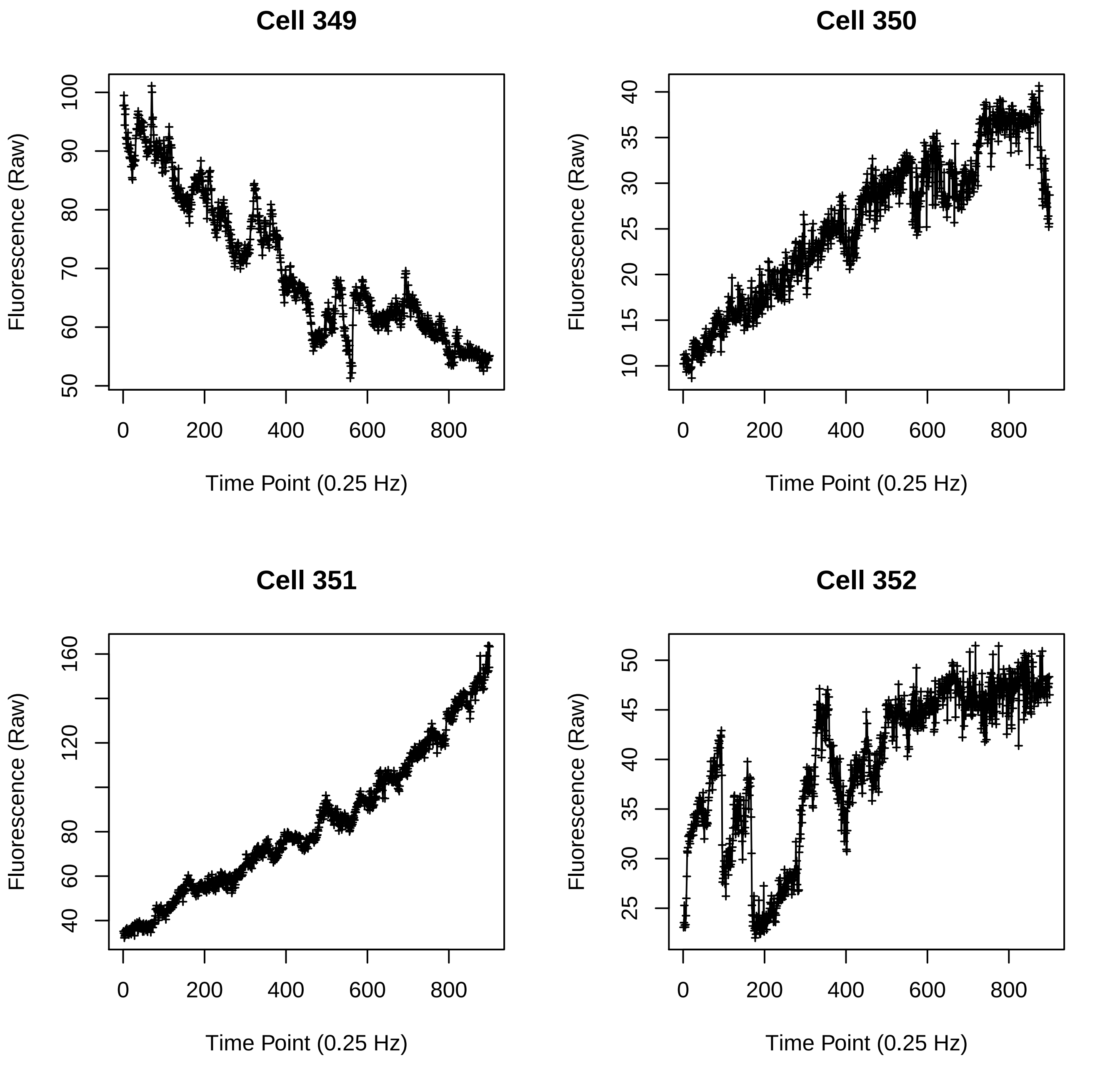
Cell 349
Fluorescence (Raw)
100
90
80
70
60
50
0
200
400
600
800
Time Point (0.25 Hz)
Cell 350
Fluorescence (Raw)
40
35
30
25
20
15
10
0
200
400
600
800
Time Point (0.25 Hz)
Cell 351
Fluorescence (Raw)
160
120
80
60
40
0
200
400
600
800
Time Point (0.25 Hz)
Cell 352
Fluorescence (Raw)
50
45
40
35
30
25
0
200
400
600
800
Time Point (0.25 Hz)

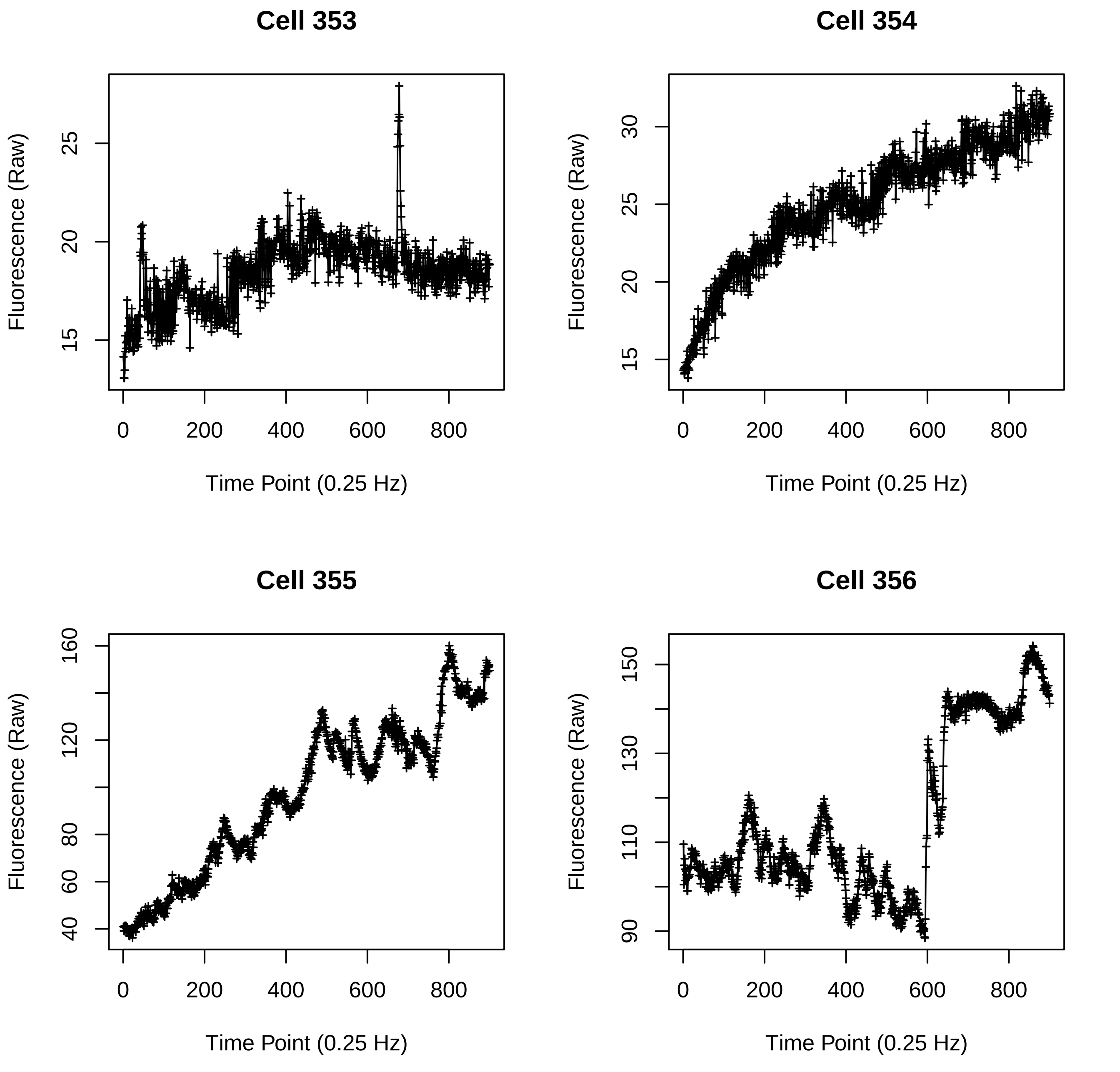
Cell 353
Fluorescence (Raw)
15
20
25
0
200
400
600
800
Time Point (0.25 Hz)
Cell 354
Fluorescence (Raw)
15
20
25
30
0
200
400
600
800
Time Point (0.25 Hz)
Cell 355
Fluorescence (Raw)
40
60
80
120
160
0
200
400
600
800
Time Point (0.25 Hz)
Cell 356
Fluorescence (Raw)
90
110
130
150
0
200
400
600
800
Time Point (0.25 Hz)

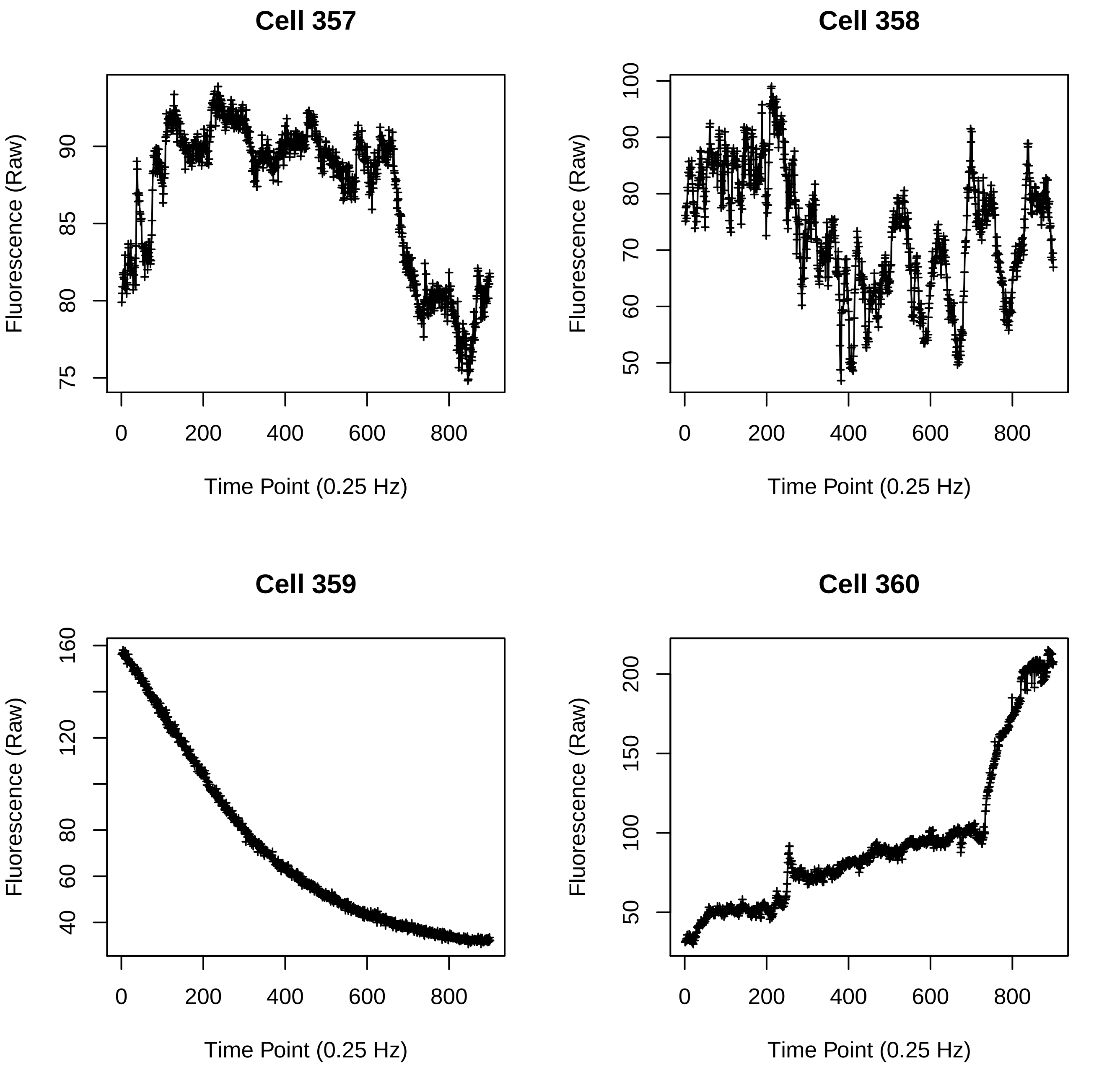

Cell 357
Fluorescence (Raw)
75
80
85
90
0
200
400
600
800
Time Point (0.25 Hz)
Cell 358
Fluorescence (Raw)
50
60
70
80
90
100
0
200
400
600
800
Time Point (0.25 Hz)
Cell 359
Fluorescence (Raw)
40
60
80
120
160
0
200
400
600
800
Time Point (0.25 Hz)
Cell 360
Fluorescence (Raw)
50
100
150
200
0
200
400
600
800
Time Point (0.25 Hz)

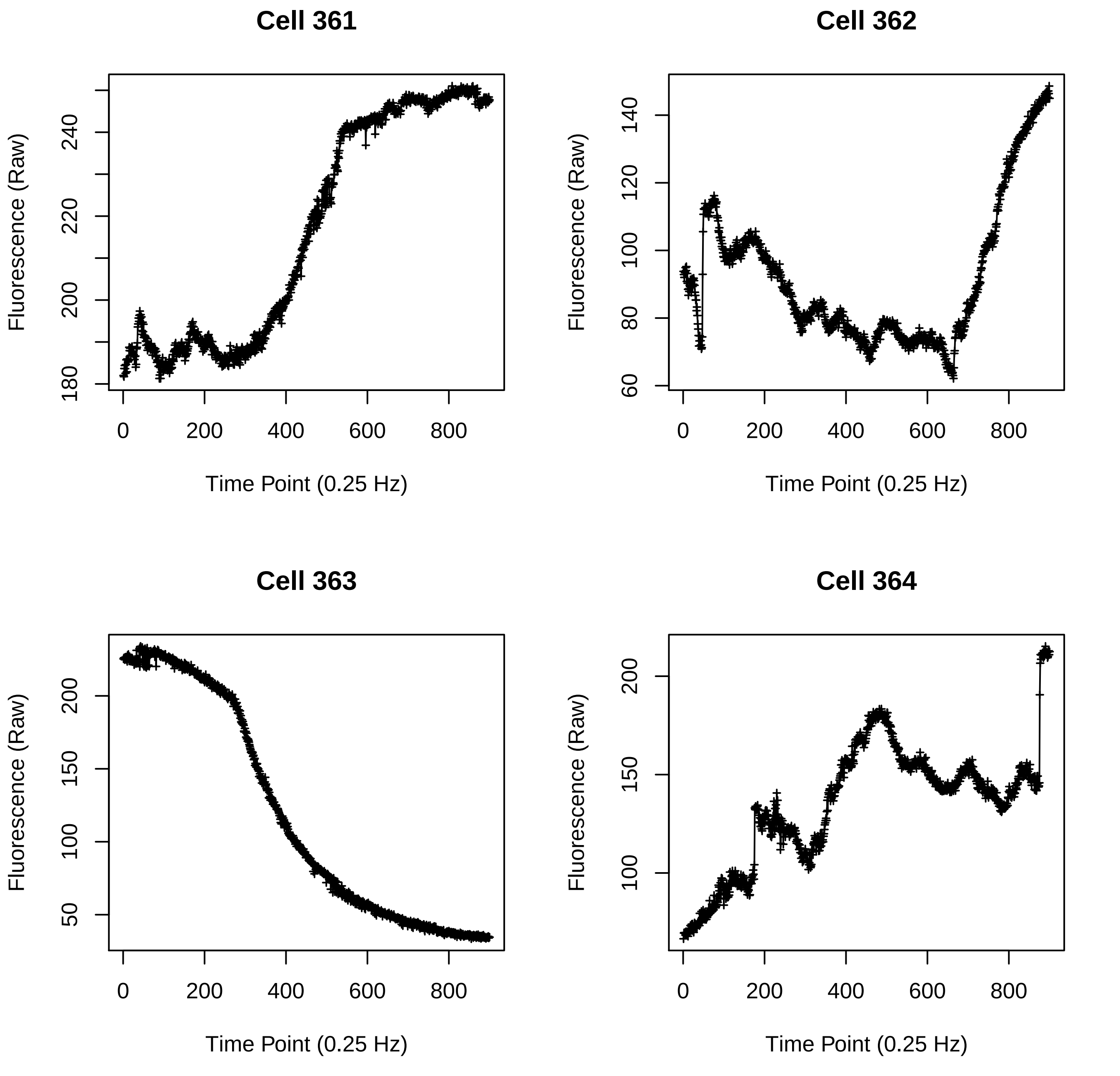
Cell 361
Cell 362
Cell 363
Cell 364
Fluorescence (Raw)
Time Point (0.25 Hz)
180
200
220
240
60
80
100
120
140
50
100
150
200
0
200
400
600
800

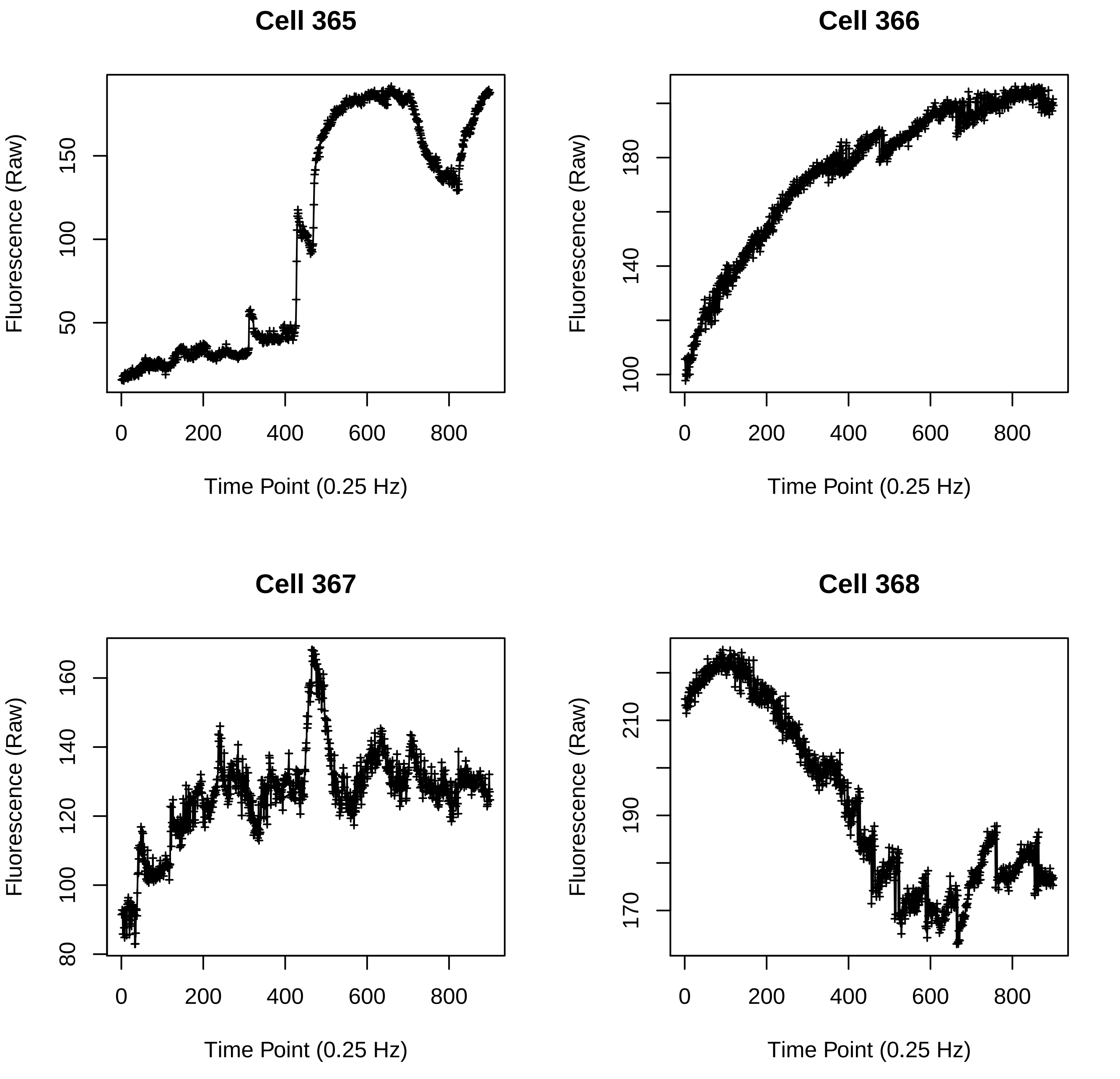

Cell 365
Cell 366
Cell 367
Cell 368
Fluorescence (Raw)
Time Point (0.25 Hz)
0
200
400
600
800
50
100
150
100
140
180
80
100
120
140
160
170
190
210

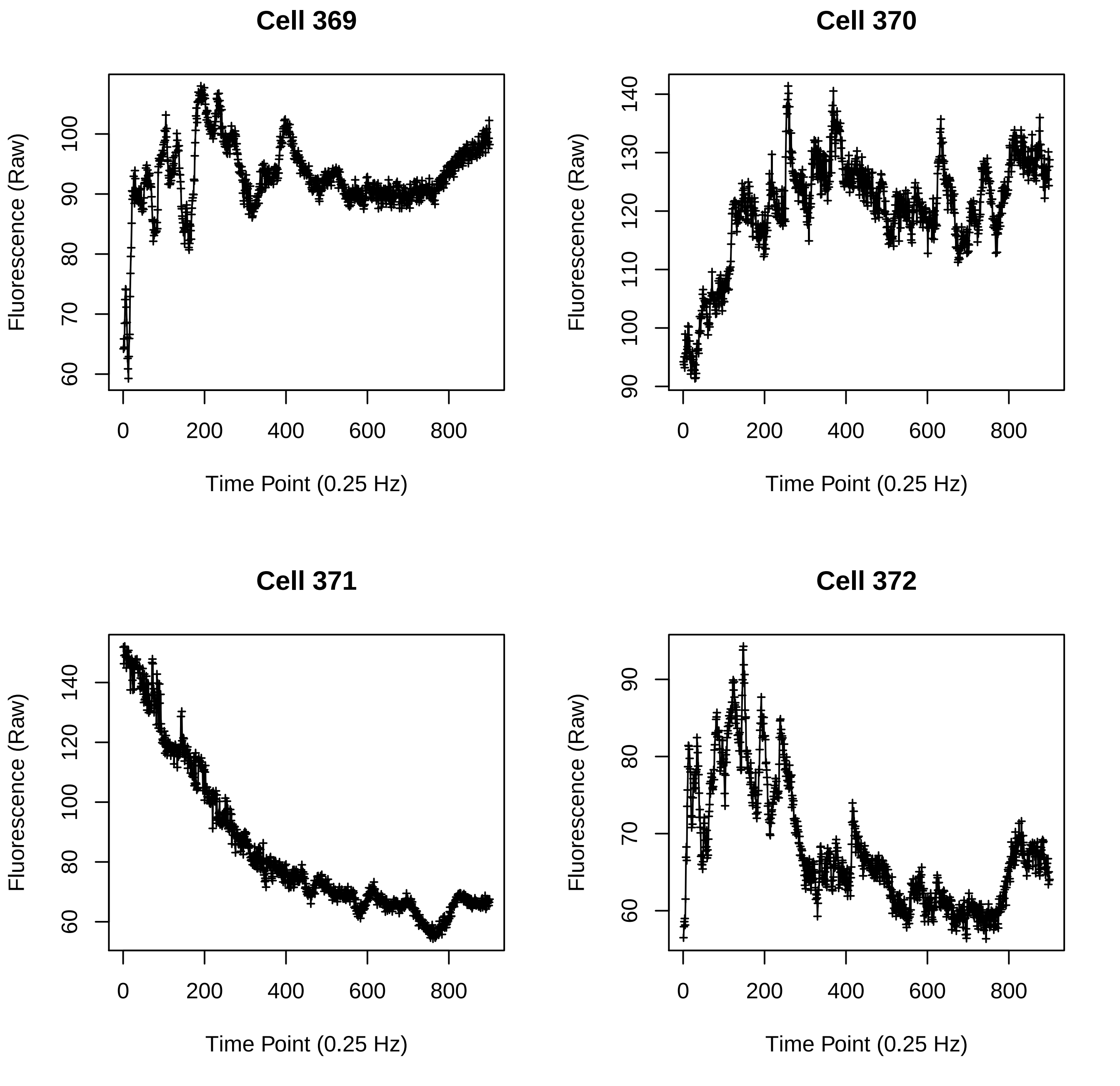

Cell 369
Cell 370
Cell 371
Cell 372
Fluorescence (Raw)
Time Point (0.25 Hz)
0
200
400
600
800
60
70
80
90
100
110
120
130
140

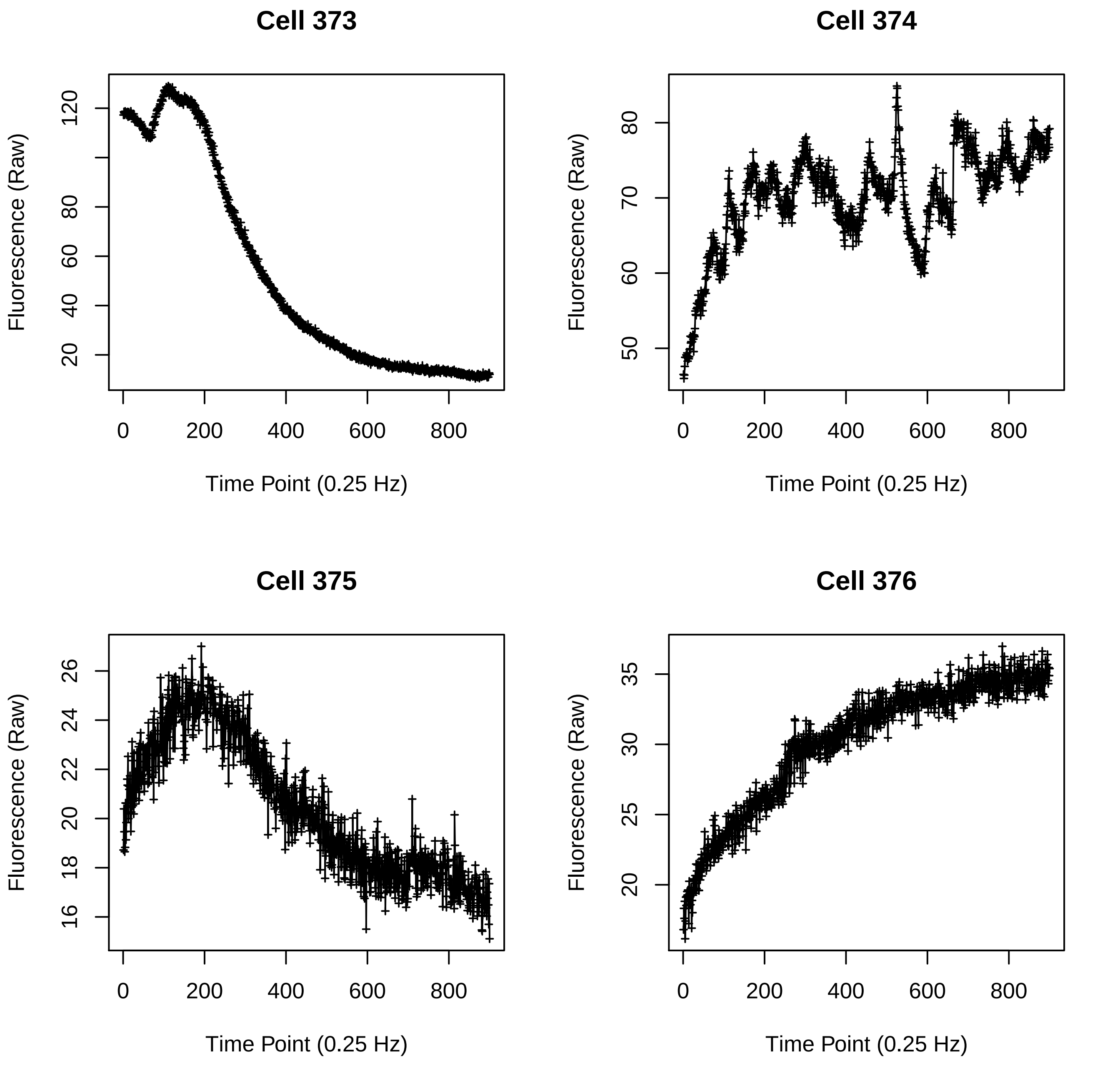
Cell 373
Cell 374
Cell 375
Cell 376
Fluorescence (Raw)
Time Point (0.25 Hz)
0
200
400
600
800
20
40
60
80
120
50
60
70
80
16
18
20
22
24
26
25
30
35

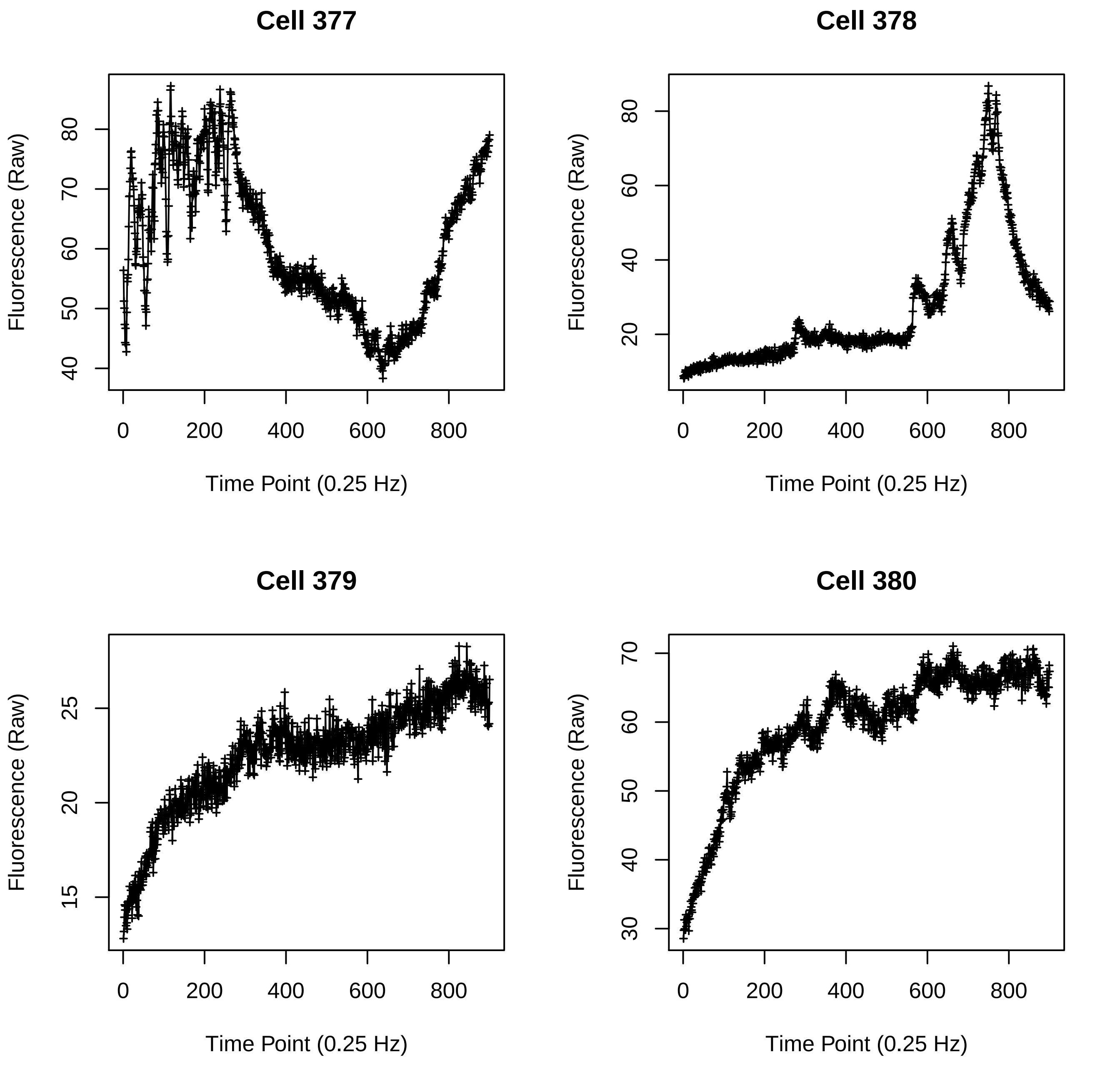
Cell 377
Fluorescence (Raw)
40
50
60
70
80
0
200
400
600
800
Time Point (0.25 Hz)
Cell 378
Fluorescence (Raw)
20
40
60
80
0
200
400
600
800
Time Point (0.25 Hz)
Cell 379
Fluorescence (Raw)
15
20
25
0
200
400
600
800
Time Point (0.25 Hz)
Cell 380
Fluorescence (Raw)
30
40
50
60
70
0
200
400
600
800
Time Point (0.25 Hz)

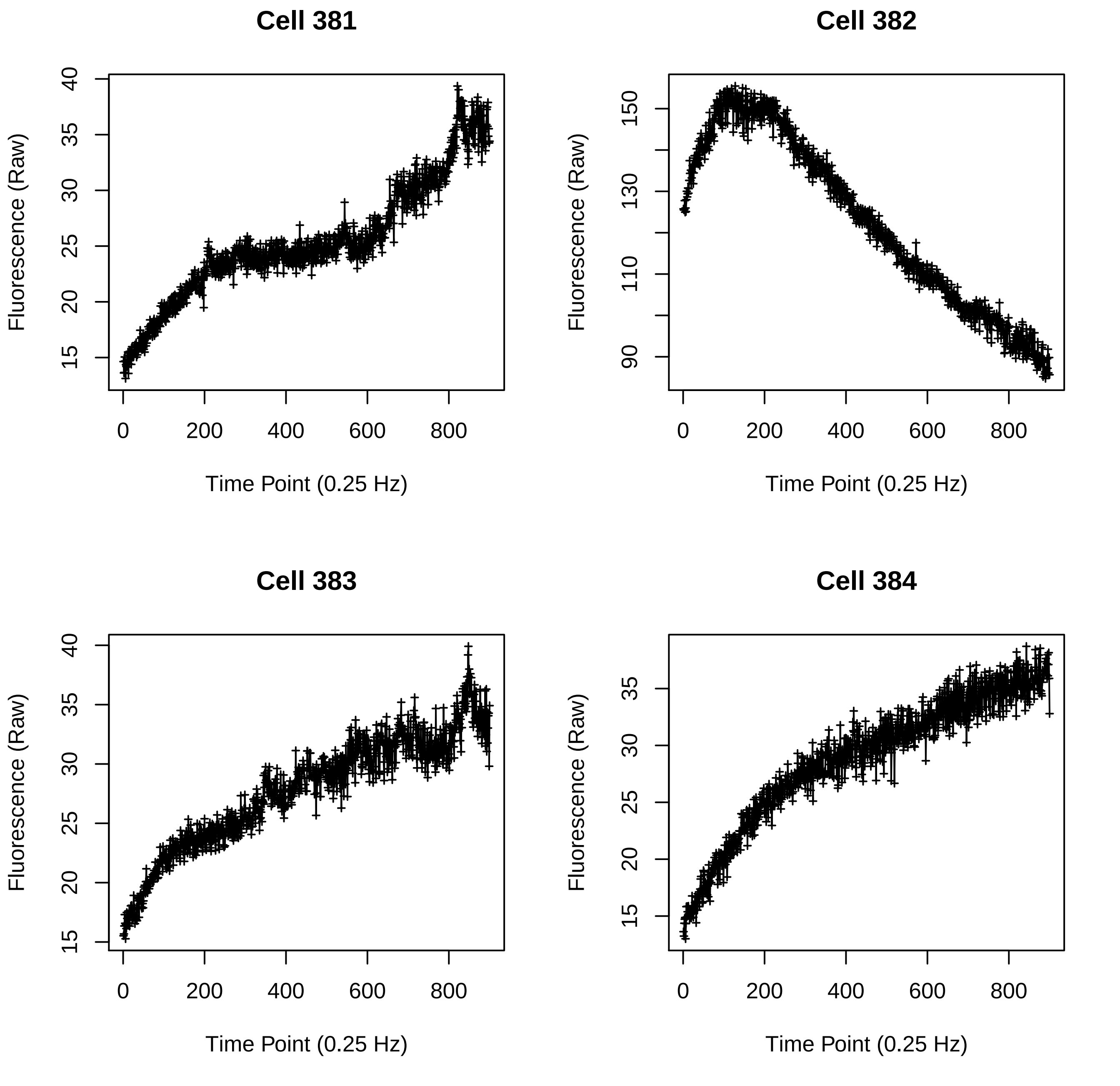

Cell 381
Cell 382
Cell 383
Cell 384
Fluorescence (Raw)
Time Point (0.25 Hz)
0
200
400
600
800
15
20
25
30
35
40
90
110
130
150

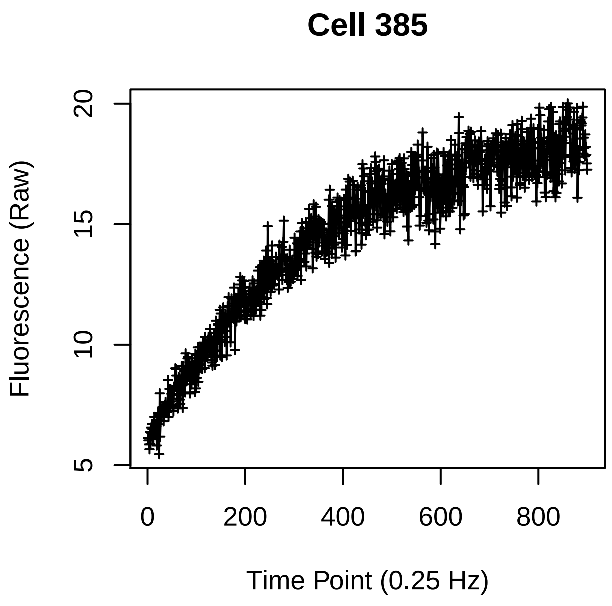
Cell 385
Fluorescence (Raw)
5
10
15
20
0
200
400
600
800
Time Point (0.25 Hz)

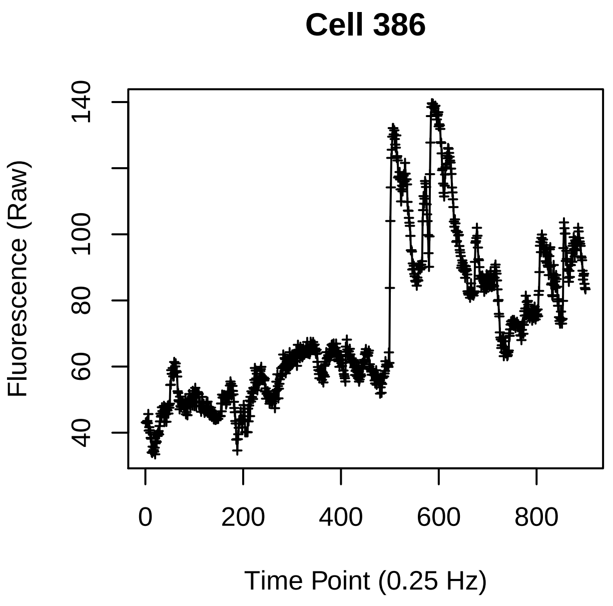
Cell 386
Fluorescence (Raw)
40
60
80
100
140
0
200
400
600
800
Time Point (0.25 Hz)

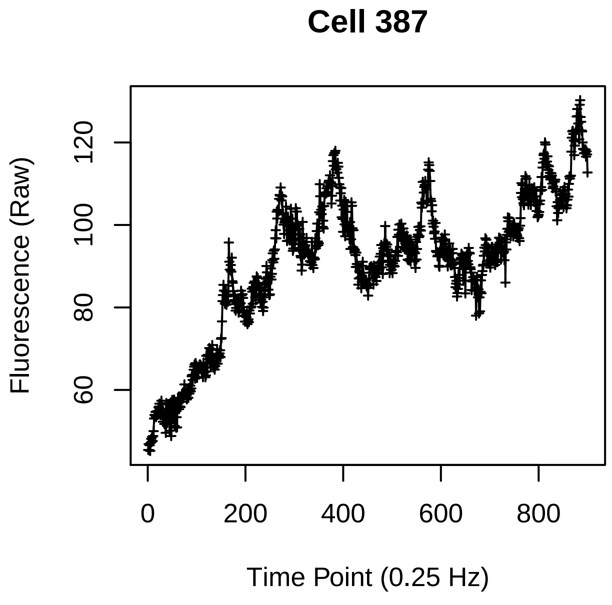
Cell 387
Fluorescence (Raw)
60
80
100
120
0
200
400
600
800
Time Point (0.25 Hz)

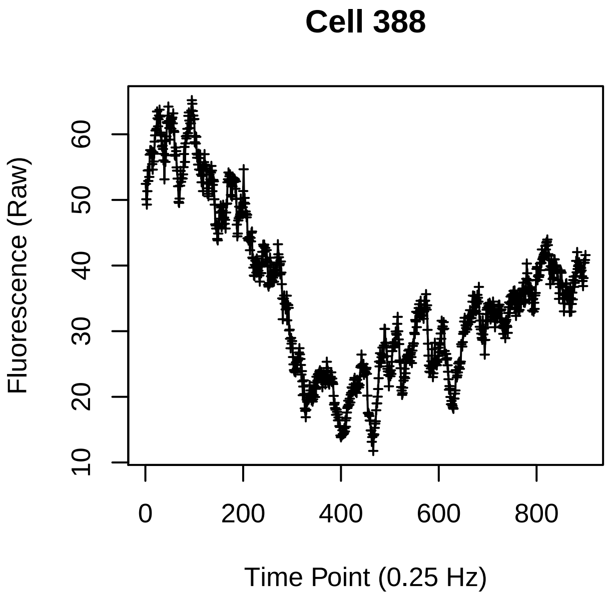
Cell 388
Fluorescence (Raw)
10
20
30
40
50
60
0
200
400
600
800
Time Point (0.25 Hz)

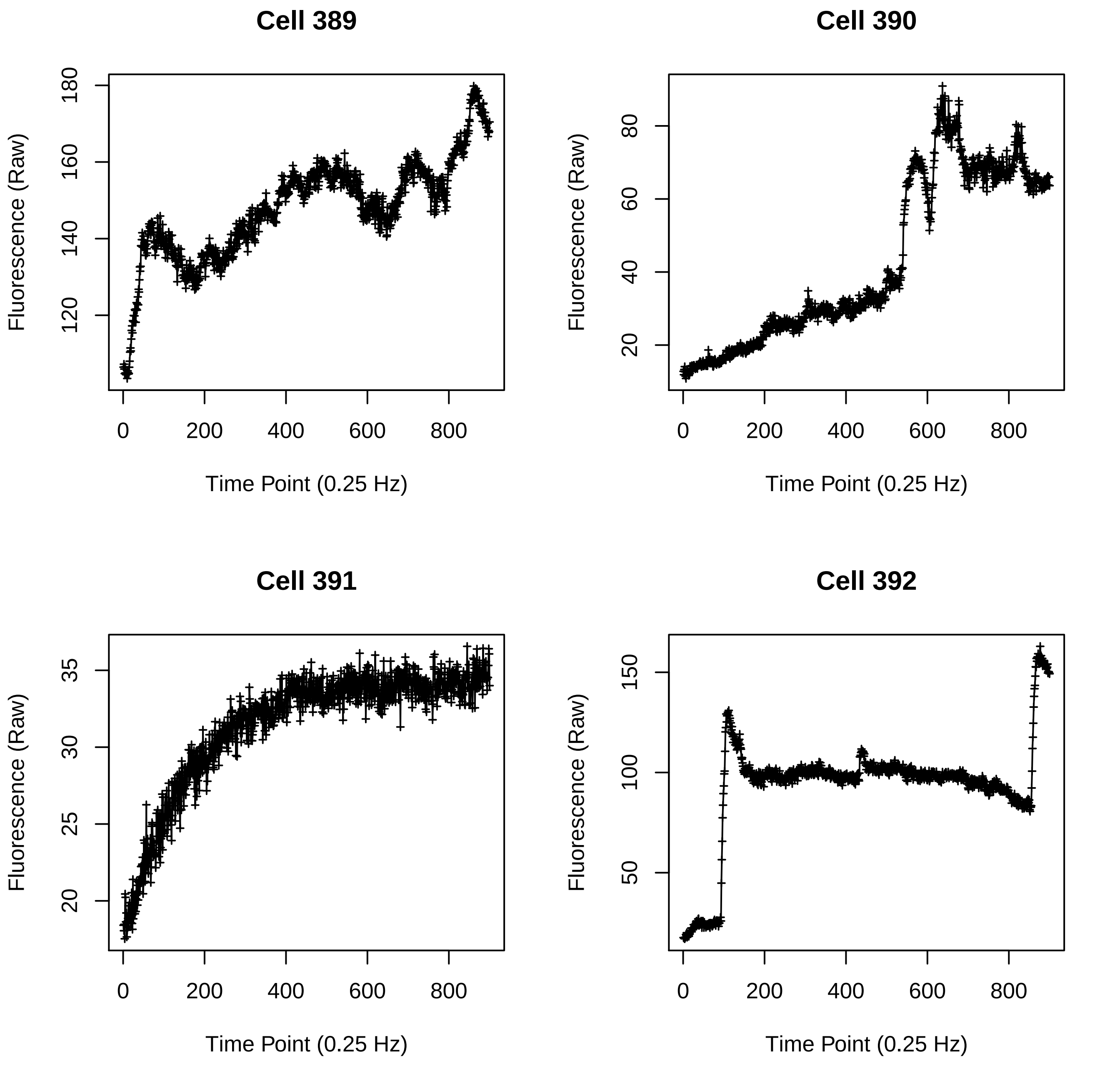

Cell 389
Cell 390
Cell 391
Cell 392
Fluorescence (Raw)
Time Point (0.25 Hz)
0
200
400
600
800
120
140
160
180
20
40
60
80
25
30
35
50
100
150

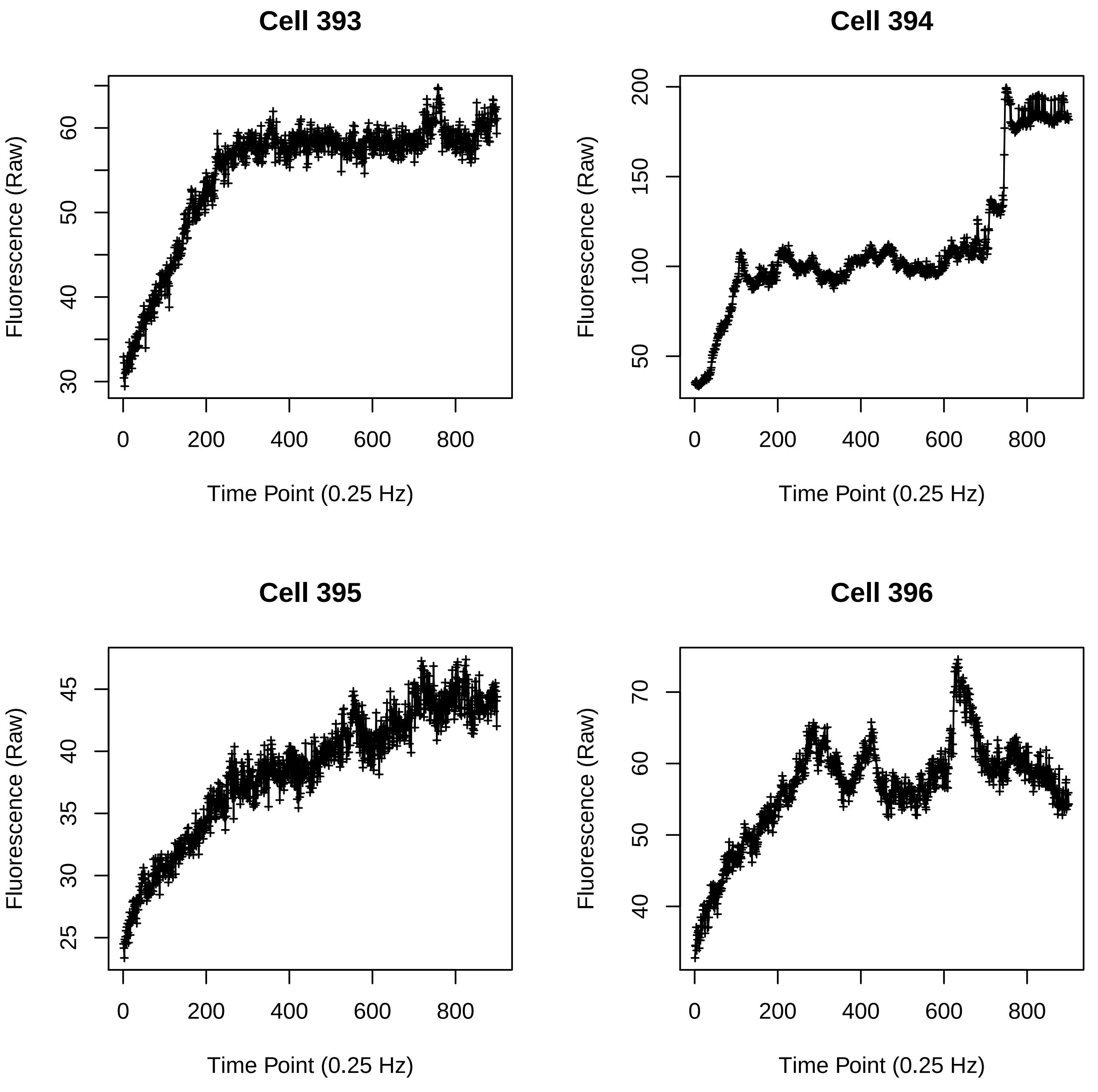
Cell 393
Fluorescence (Raw)
30
40
50
60
0
200
400
600
800
Time Point (0.25 Hz)
Cell 394
Fluorescence (Raw)
50
100
150
200
0
200
400
600
800
Time Point (0.25 Hz)
Cell 395
Fluorescence (Raw)
25
30
35
40
45
0
200
400
600
800
Time Point (0.25 Hz)
Cell 396
Fluorescence (Raw)
40
50
60
70
0
200
400
600
800
Time Point (0.25 Hz)

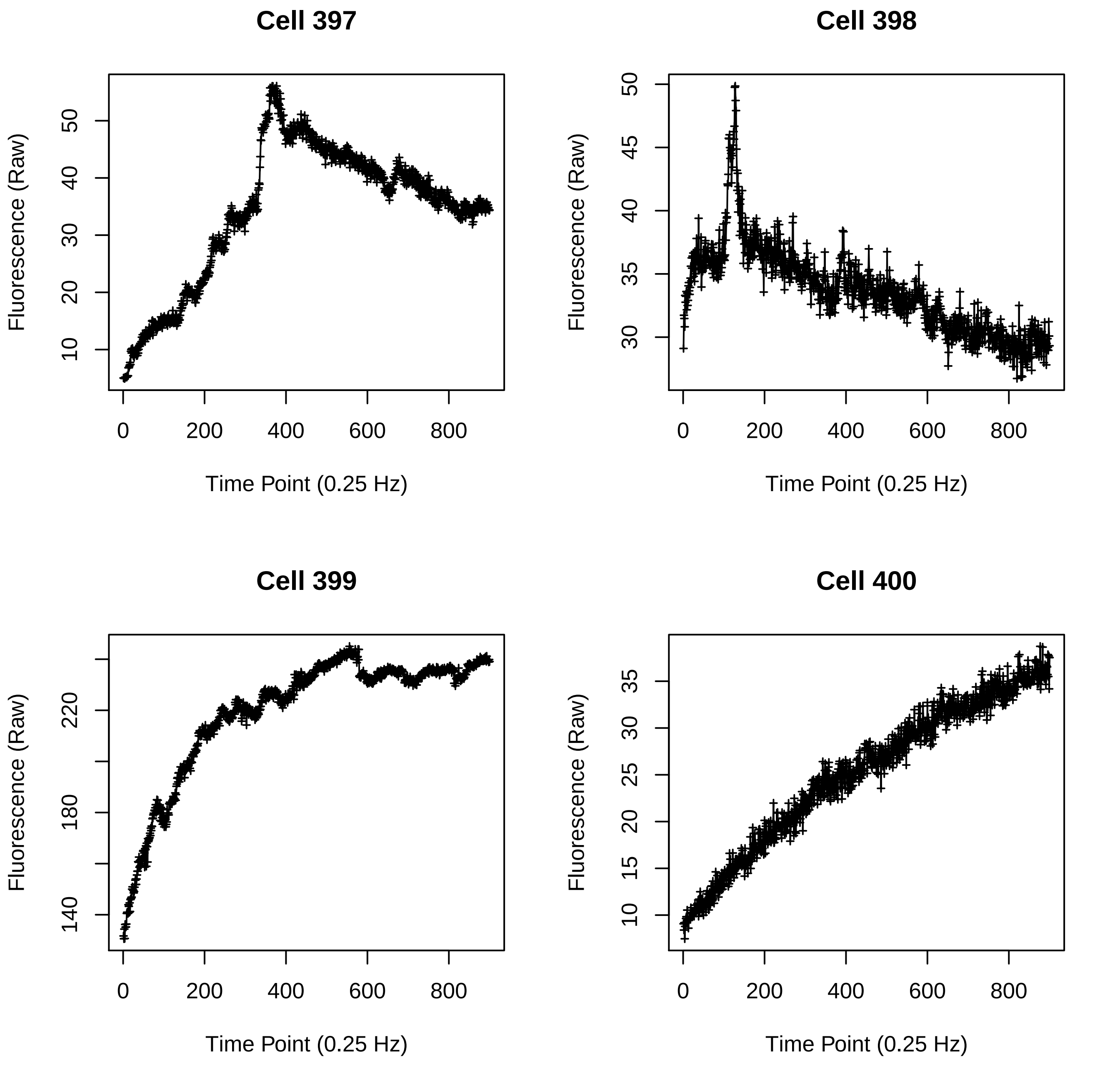

Cell 397
Fluorescence (Raw)
Time Point (0.25 Hz)
0
200
400
600
800
10
20
30
40
50
Cell 398
Fluorescence (Raw)
Time Point (0.25 Hz)
0
200
400
600
800
30
35
40
45
50
Cell 399
Fluorescence (Raw)
Time Point (0.25 Hz)
0
200
400
600
800
140
180
220
Cell 400
Fluorescence (Raw)
Time Point (0.25 Hz)
0
200
400
600
800
10
15
20
25
30
35

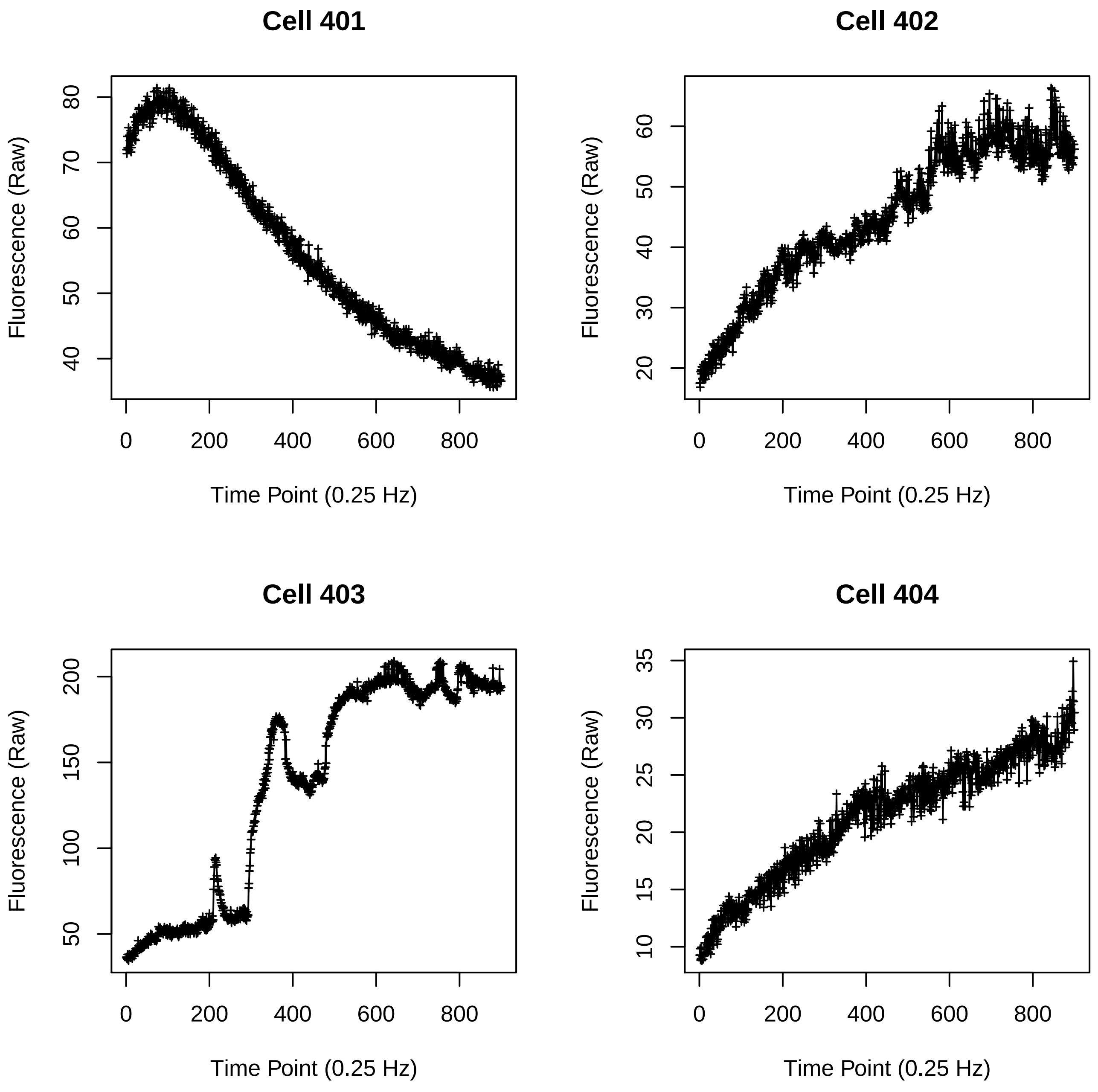

Cell 401
Cell 402
Cell 403
Cell 404
Fluorescence (Raw)
Time Point (0.25 Hz)
40
50
60
70
80
20
30
100
150
200
10
15
25
35
0
200
400
600
800

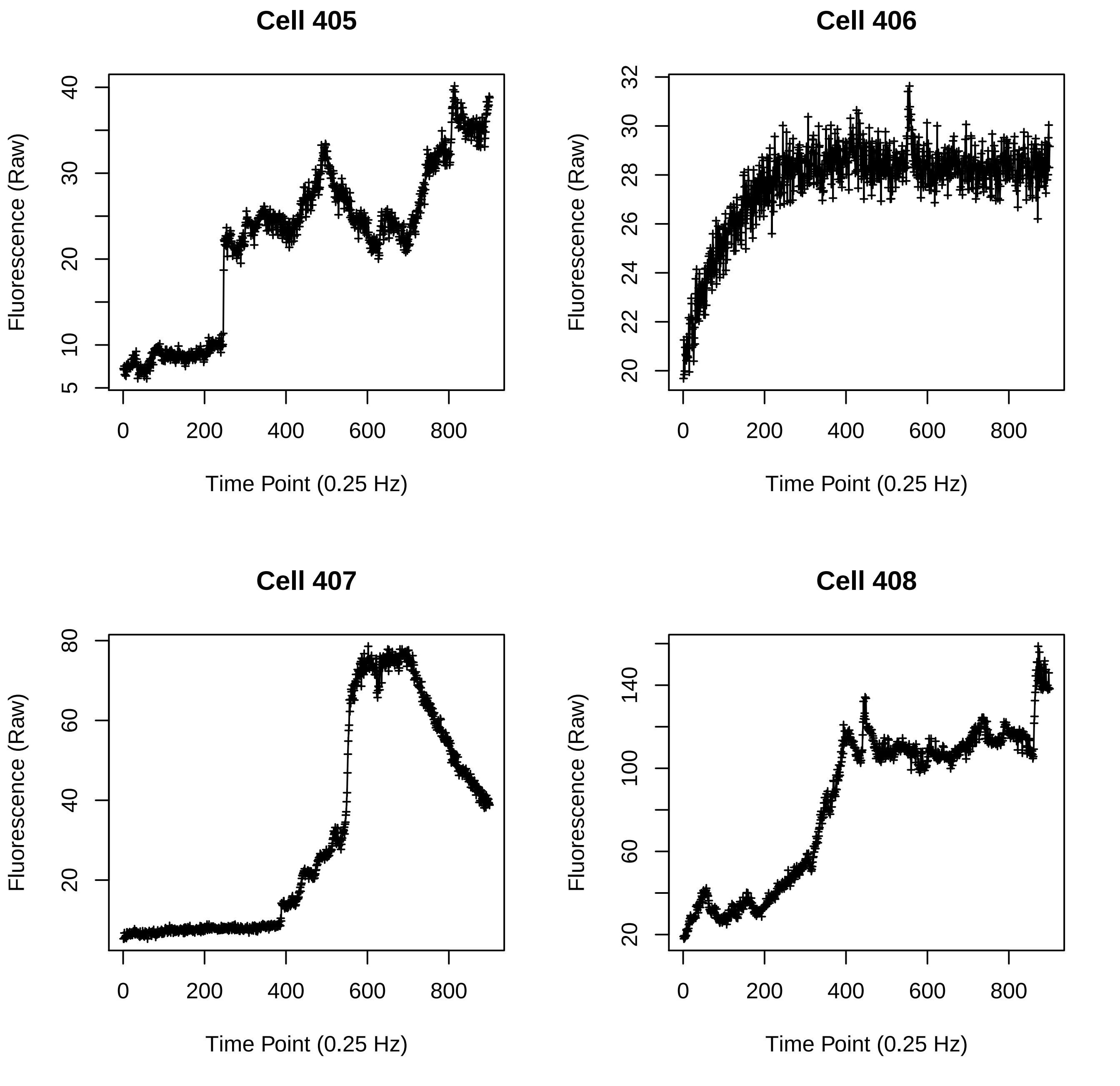

Cell 405
Cell 406
Cell 407
Cell 408
Fluorescence (Raw)
Time Point (0.25 Hz)
5
10
20
30
40
0
200
400
600
800
20
22
24
26
28
30
32
60
80
100
140

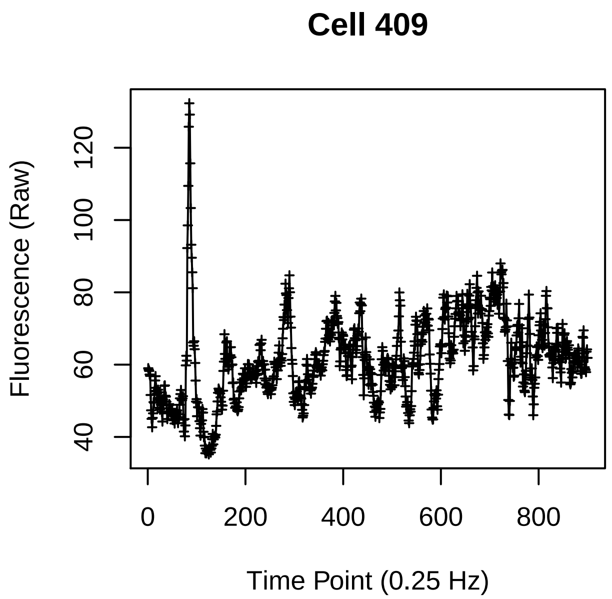
Cell 409
Fluorescence (Raw)
40
60
80
100
120
0
200
400
600
800
Time Point (0.25 Hz)

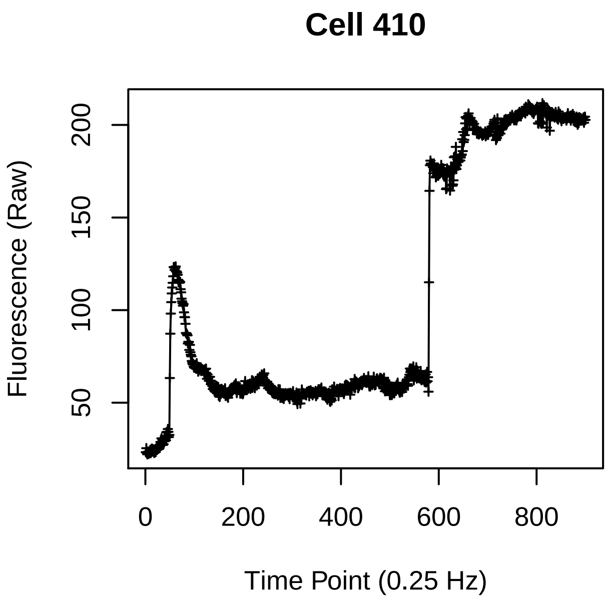
Cell 410
Fluorescence (Raw)
50
100
150
200
0
200
400
600
800
Time Point (0.25 Hz)

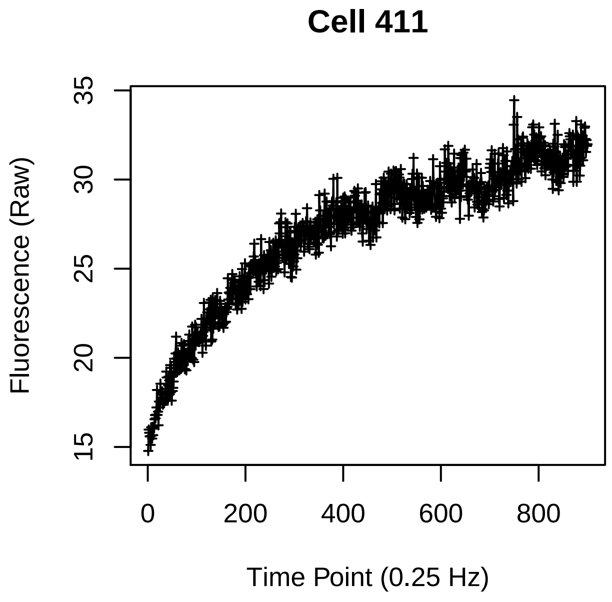
Cell 411
Fluorescence (Raw)
15
20
25
30
35
0
200
400
600
800
Time Point (0.25 Hz)

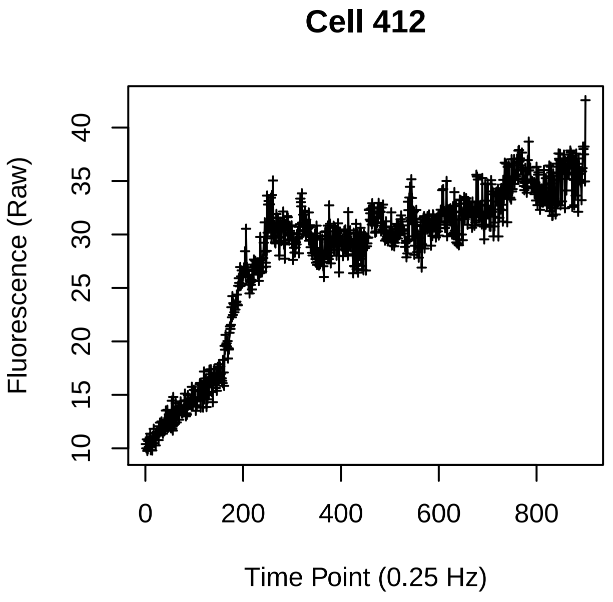
Cell 412
Fluorescence (Raw)
10
15
20
25
30
35
40
0
200
400
600
800
Time Point (0.25 Hz)

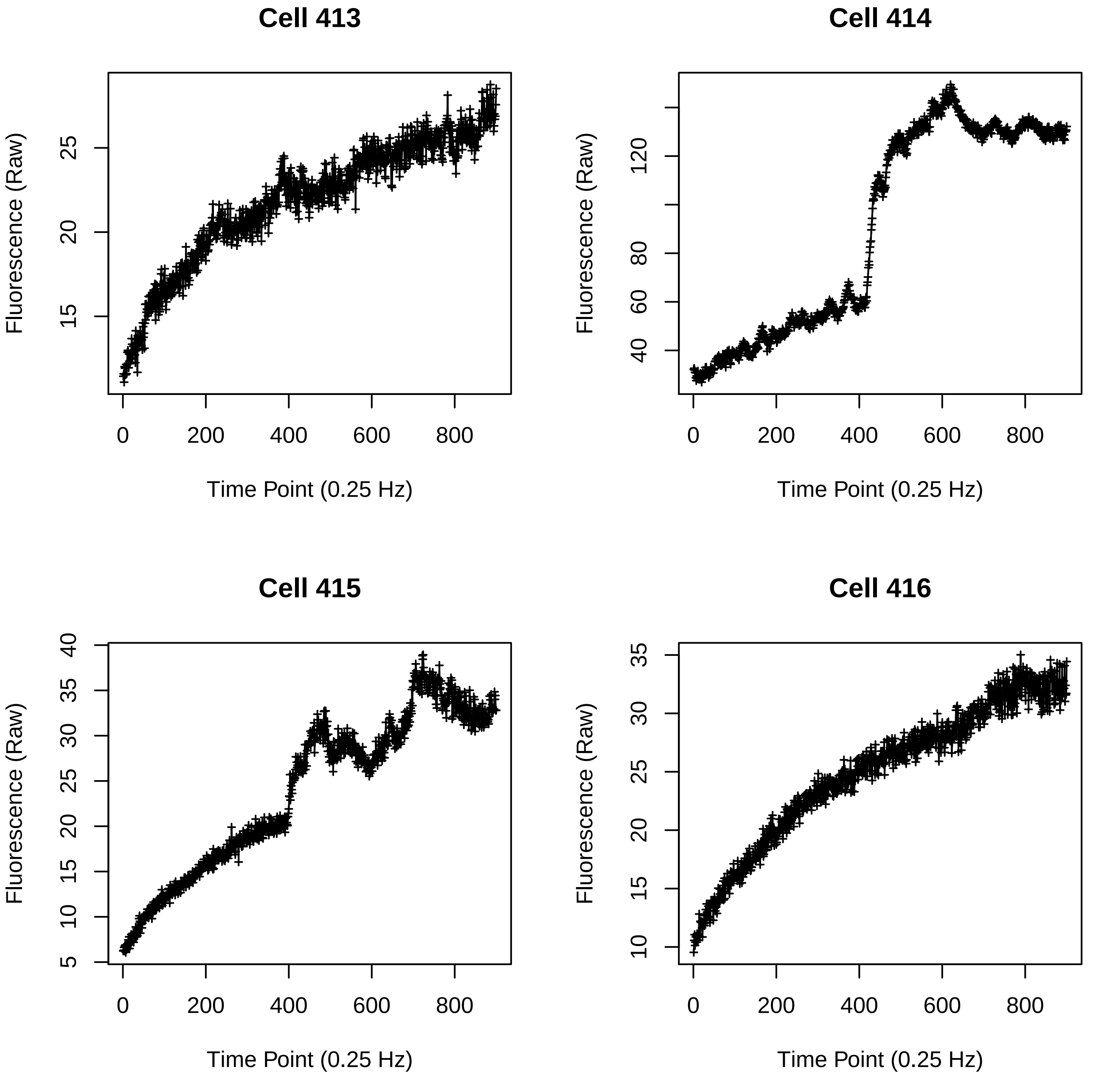
Cell 413
Fluorescence (Raw)
15
20
25
0
200
400
600
800
Time Point (0.25 Hz)
Cell 414
Fluorescence (Raw)
40
60
80
120
0
200
400
600
800
Time Point (0.25 Hz)
Cell 415
Fluorescence (Raw)
5
10
15
20
25
30
35
40
0
200
400
600
800
Time Point (0.25 Hz)
Cell 416
Fluorescence (Raw)
10
15
20
25
30
35
0
200
400
600
800
Time Point (0.25 Hz)

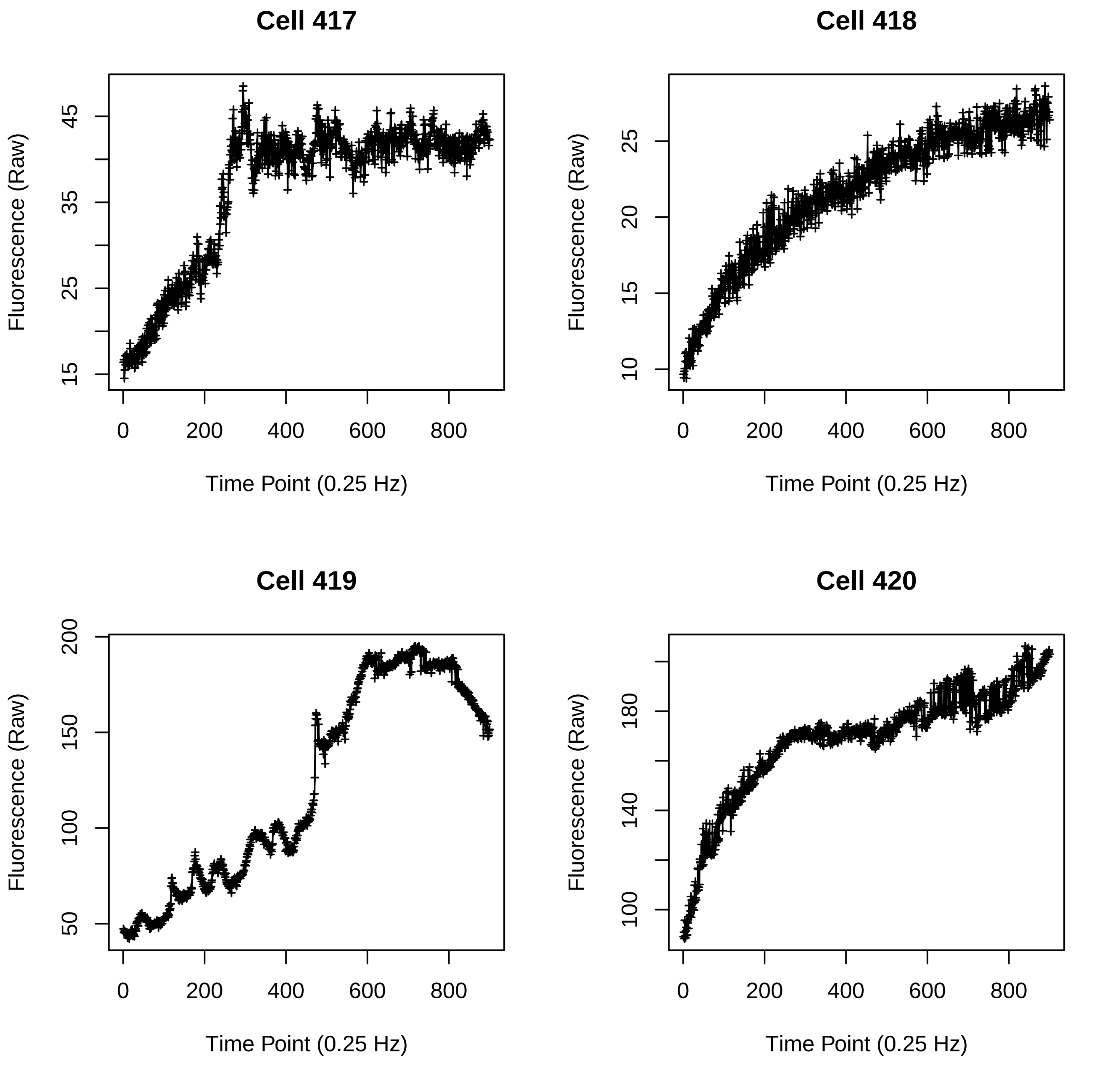

Cell 417
Cell 418
Cell 419
Cell 420
Fluorescence (Raw)
Time Point (0.25 Hz)
15
25
35
45
10
15
20
25
50
100
150
200
100
140
180
0
200
400
600
800

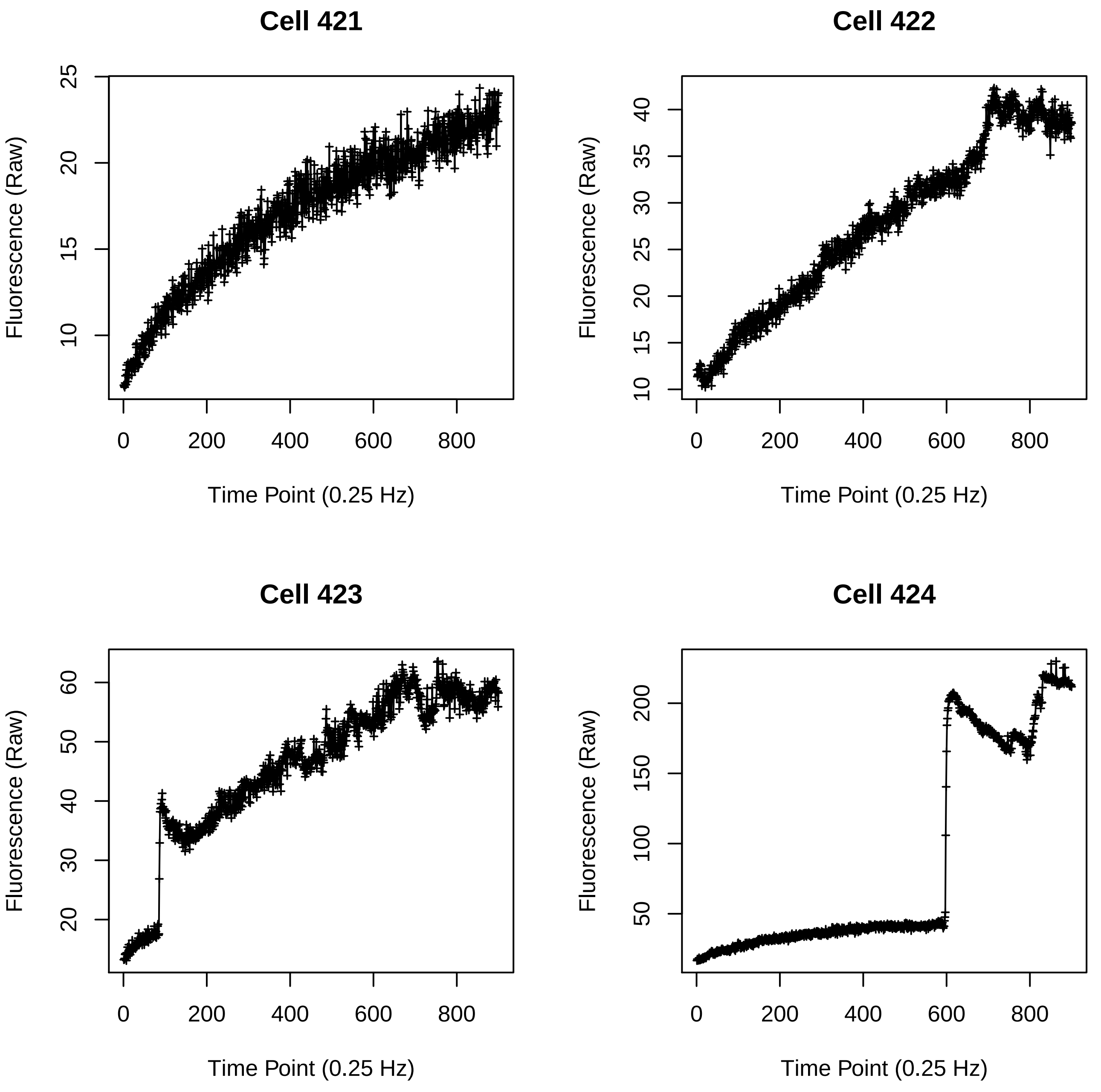

Cell 421
Cell 422
Cell 423
Cell 424
Fluorescence (Raw)
Time Point (0.25 Hz)
0
200
400
600
800
10
15
20
25
30
35
40
50
60
100
150
200

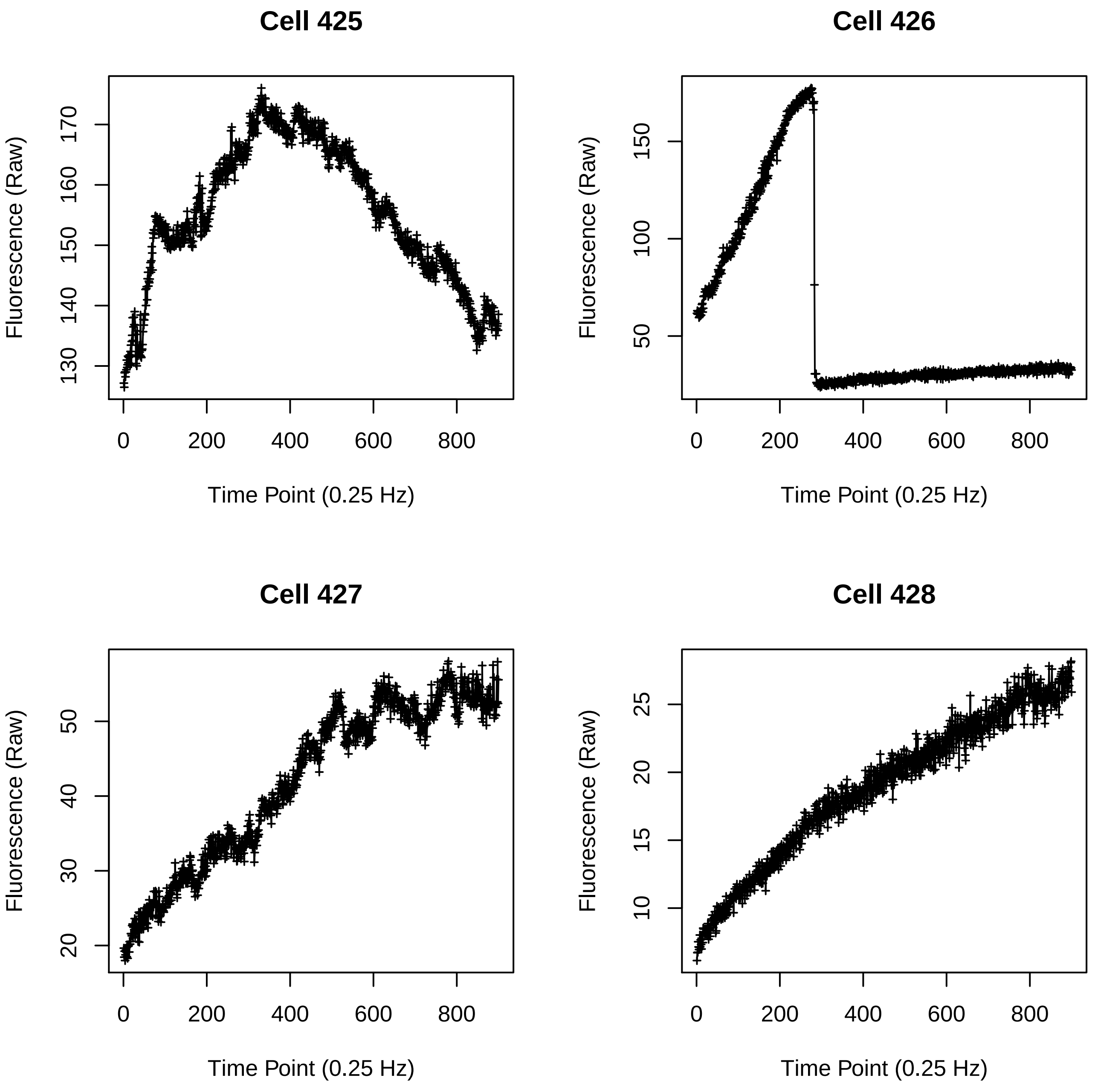
Cell 425
Fluorescence (Raw)
130
140
150
160
170
0
200
400
600
800
Time Point (0.25 Hz)
Cell 426
Fluorescence (Raw)
50
100
150
0
200
400
600
800
Time Point (0.25 Hz)
Cell 427
Fluorescence (Raw)
20
30
40
50
0
200
400
600
800
Time Point (0.25 Hz)
Cell 428
Fluorescence (Raw)
10
15
20
25
0
200
400
600
800
Time Point (0.25 Hz)

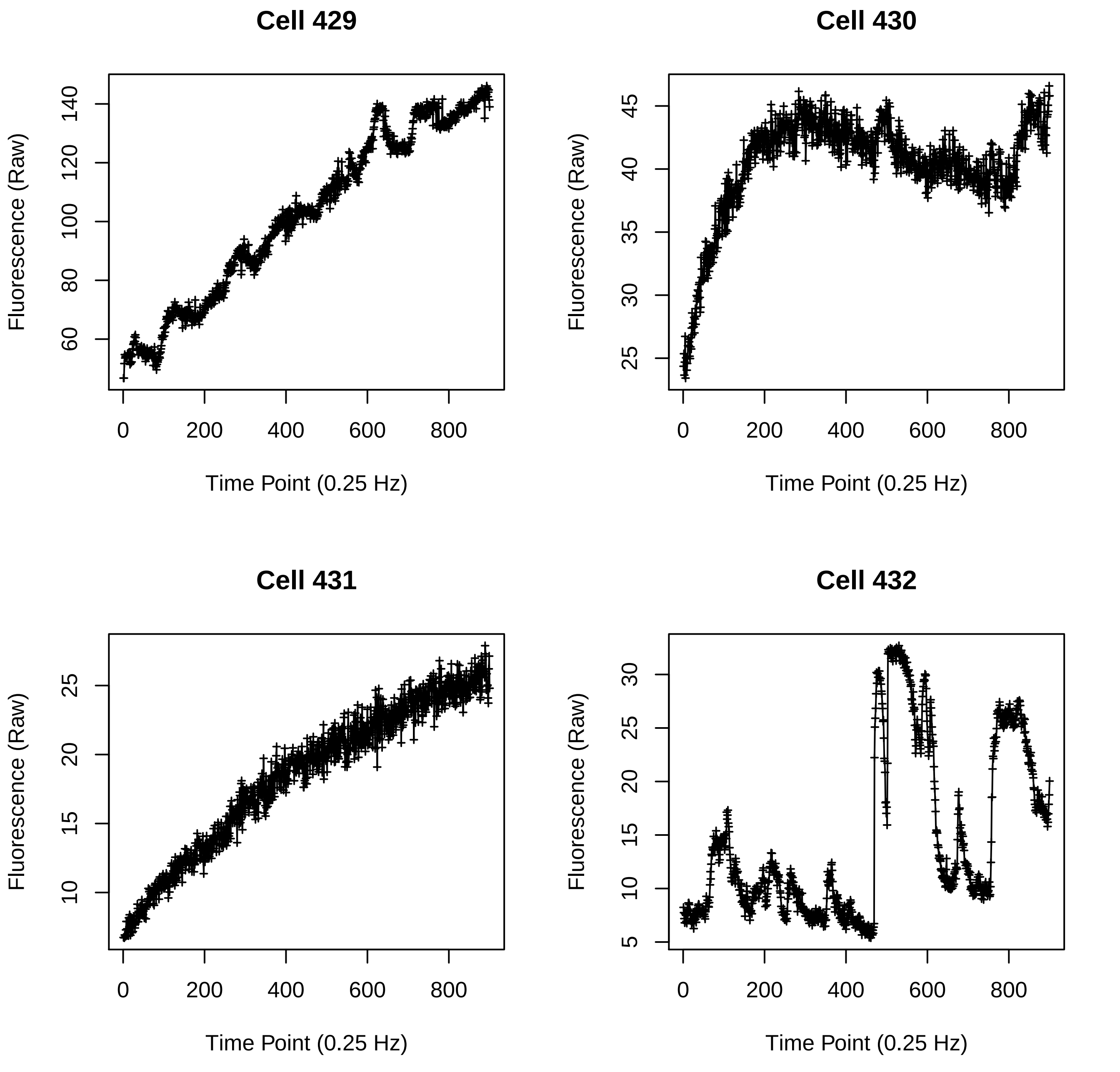
Cell 429
Fluorescence (Raw)
60
80
100
120
140
0
200
400
600
800
Time Point (0.25 Hz)
Cell 430
Fluorescence (Raw)
25
30
35
40
45
0
200
400
600
800
Time Point (0.25 Hz)
Cell 431
Fluorescence (Raw)
10
15
20
25
0
200
400
600
800
Time Point (0.25 Hz)
Cell 432
Fluorescence (Raw)
5
10
15
20
25
30
0
200
400
600
800
Time Point (0.25 Hz)

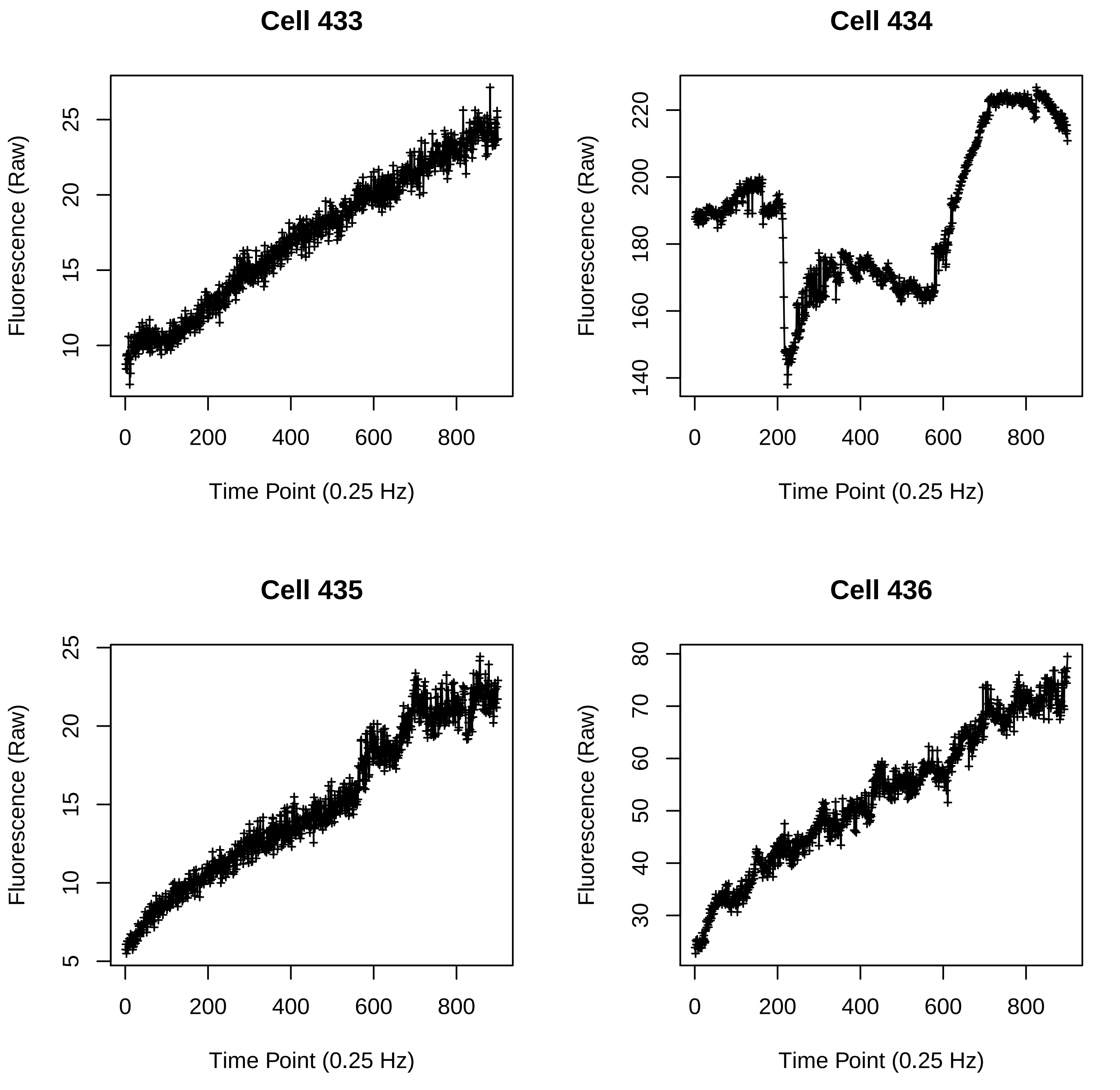

Cell 433
Fluorescence (Raw)
10
15
20
25
0
200
400
600
800
Time Point (0.25 Hz)
Cell 434
Fluorescence (Raw)
140
160
180
200
220
0
200
400
600
800
Time Point (0.25 Hz)
Cell 435
Fluorescence (Raw)
5
10
15
20
25
0
200
400
600
800
Time Point (0.25 Hz)
Cell 436
Fluorescence (Raw)
30
40
50
60
70
80
0
200
400
600
800
Time Point (0.25 Hz)

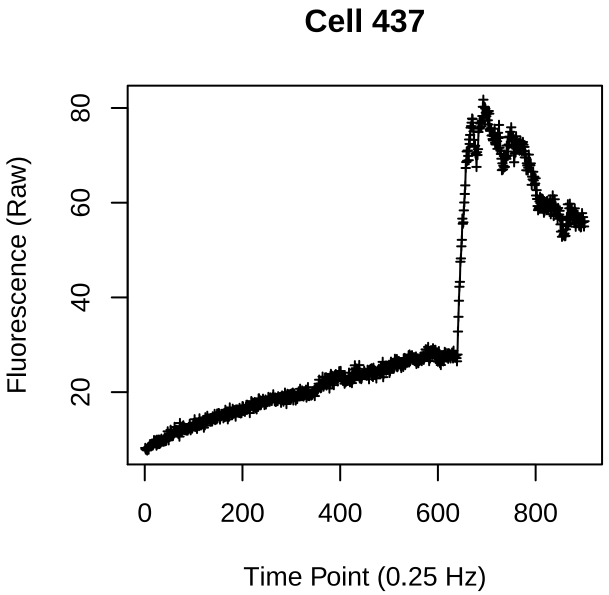
Cell 437
Fluorescence (Raw)
20
40
60
80
0
200
400
600
800
Time Point (0.25 Hz)

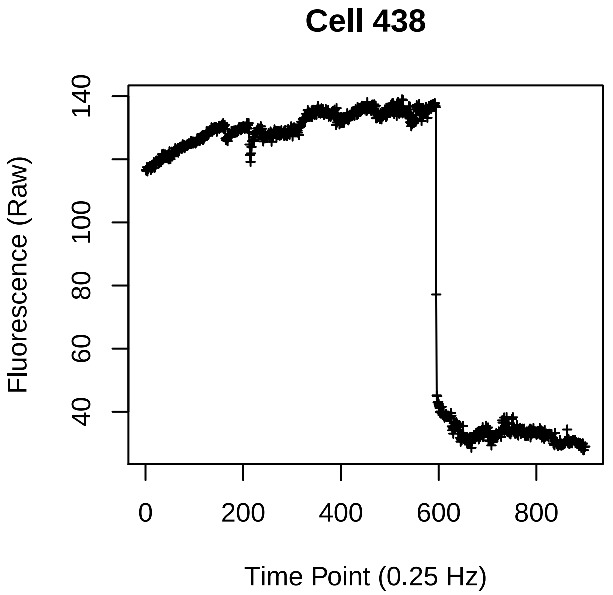
Cell 438
Fluorescence (Raw)
40
60
80
100
140
0
200
400
600
800
Time Point (0.25 Hz)

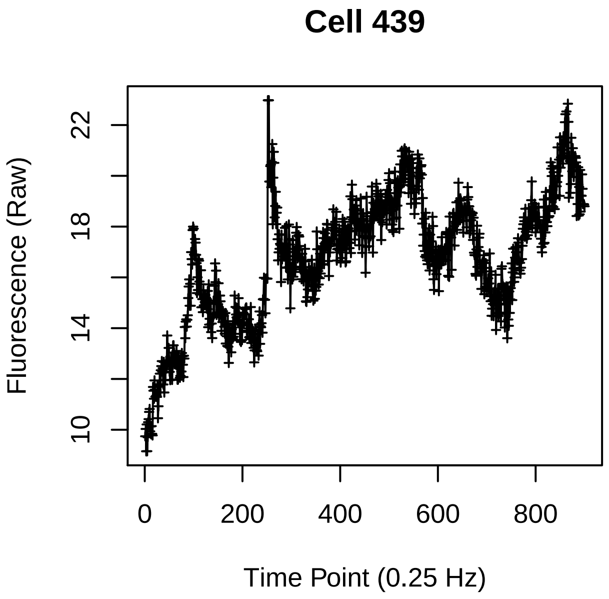
Cell 439
Fluorescence (Raw)
10
14
18
22
0
200
400
600
800
Time Point (0.25 Hz)

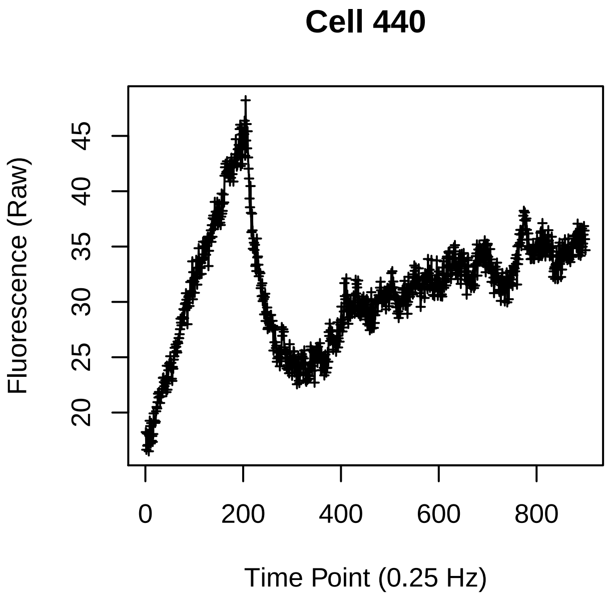
Cell 440
Fluorescence (Raw)
20
25
30
35
40
45
0
200
400
600
800
Time Point (0.25 Hz)

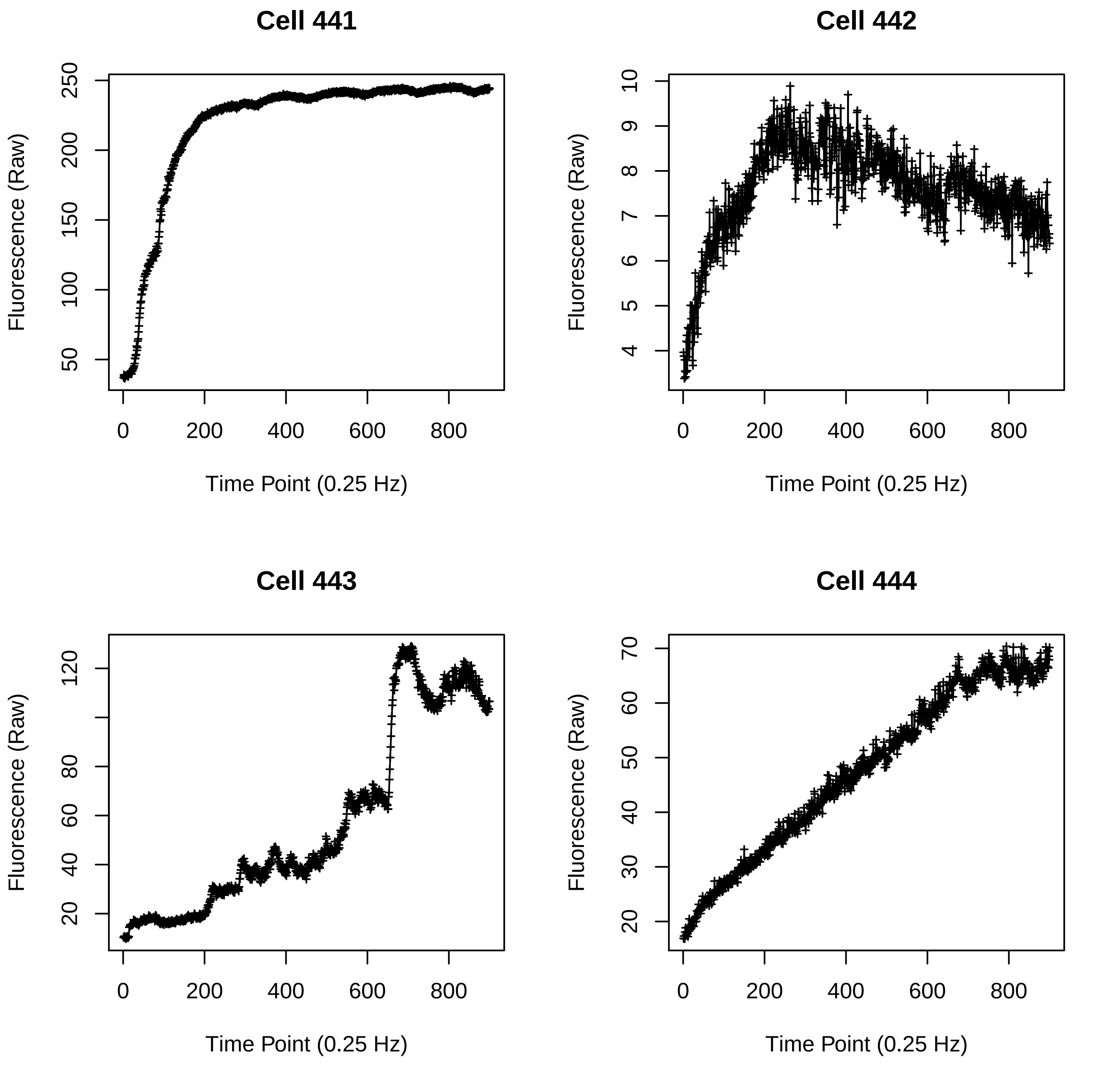
Cell 441
Fluorescence (Raw)
50
100
150
200
250
0
200
400
600
800
Time Point (0.25 Hz)
Cell 442
Fluorescence (Raw)
4
5
6
7
8
9
10
0
200
400
600
800
Time Point (0.25 Hz)
Cell 443
Fluorescence (Raw)
20
40
60
80
120
0
200
400
600
800
Time Point (0.25 Hz)
Cell 444
Fluorescence (Raw)
20
30
40
50
60
70
0
200
400
600
800
Time Point (0.25 Hz)

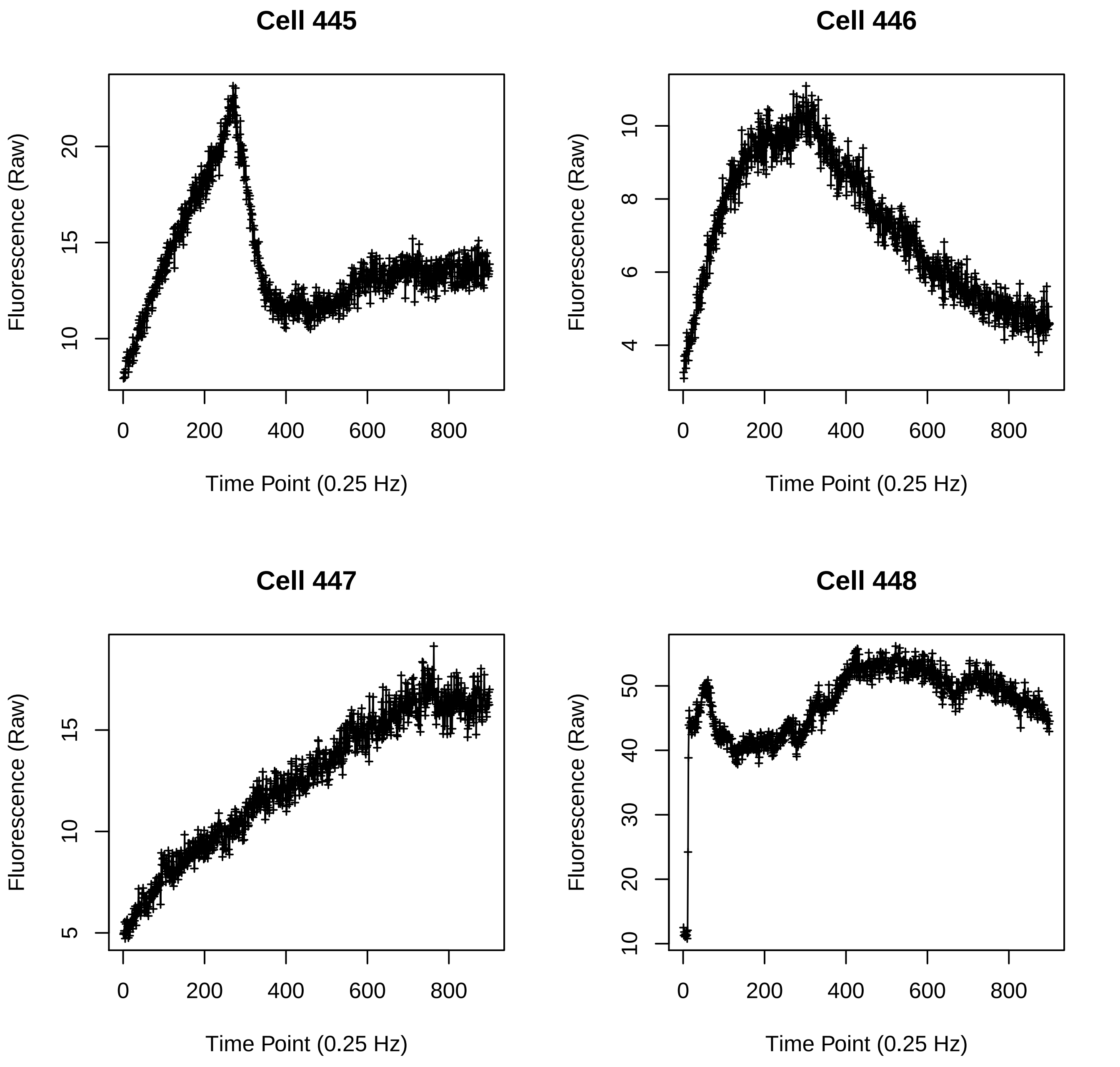

Cell 445
Fluorescence (Raw)
10
15
20
0
200
400
600
800
Time Point (0.25 Hz)
Cell 446
Fluorescence (Raw)
4
6
8
10
0
200
400
600
800
Time Point (0.25 Hz)
Cell 447
Fluorescence (Raw)
5
10
15
0
200
400
600
800
Time Point (0.25 Hz)
Cell 448
Fluorescence (Raw)
10
20
30
40
50
0
200
400
600
800
Time Point (0.25 Hz)

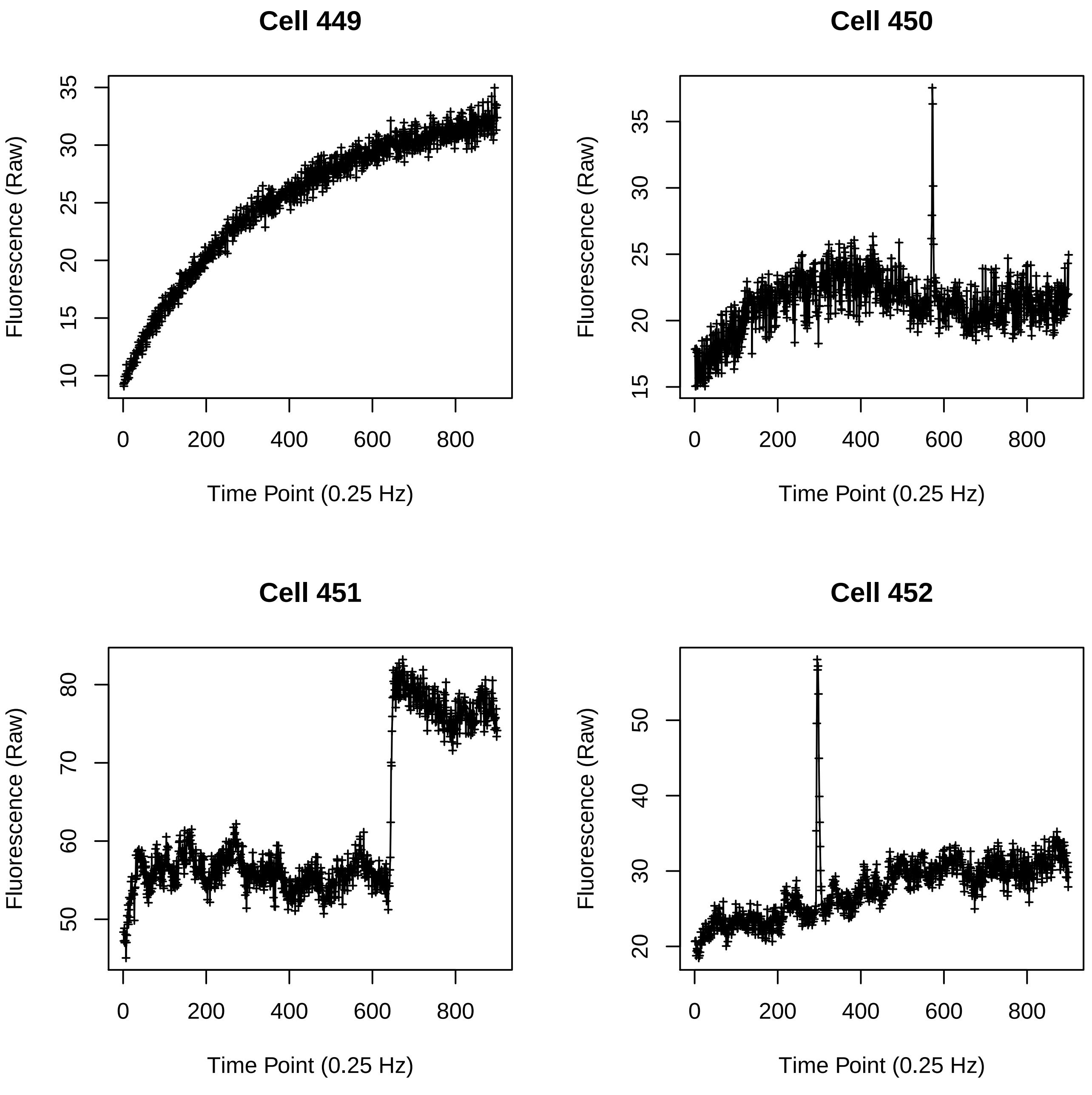
Cell 449
Fluorescence (Raw)
10
15
20
25
30
35
0
200
400
600
800
Time Point (0.25 Hz)
Cell 450
Fluorescence (Raw)
15
20
25
30
35
0
200
400
600
800
Time Point (0.25 Hz)
Cell 451
Fluorescence (Raw)
50
60
70
80
0
200
400
600
800
Time Point (0.25 Hz)
Cell 452
Fluorescence (Raw)
20
30
40
50
0
200
400
600
800
Time Point (0.25 Hz)

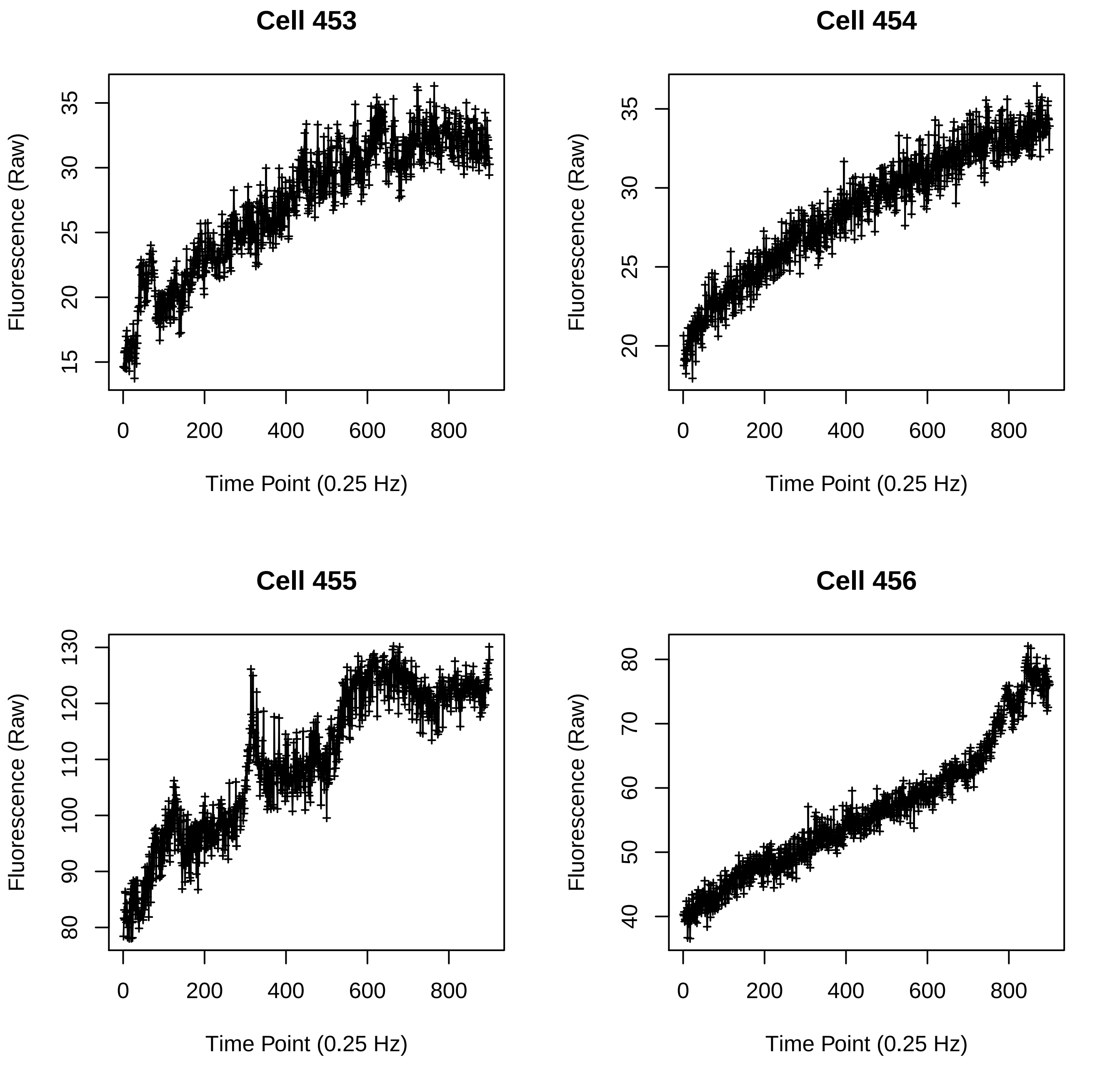

Cell 453
Cell 454
Cell 455
Cell 456
Fluorescence (Raw)
Time Point (0.25 Hz)

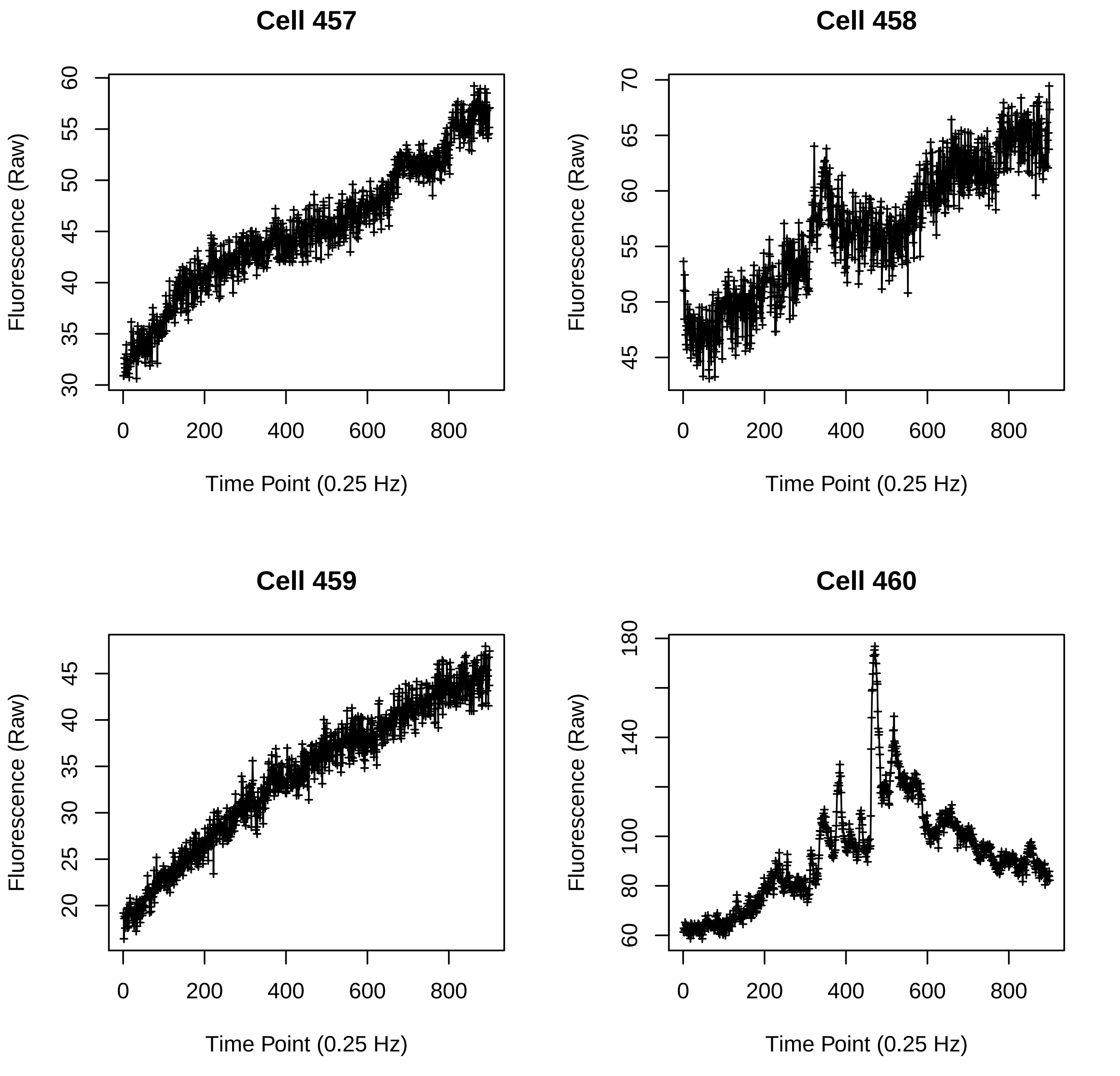

Cell 457
Fluorescence (Raw)
30
35
40
45
50
55
60
0
200
400
600
800
Time Point (0.25 Hz)
Cell 458
Fluorescence (Raw)
45
50
55
60
65
70
0
200
400
600
800
Time Point (0.25 Hz)
Cell 459
Fluorescence (Raw)
20
25
30
35
40
45
0
200
400
600
800
Time Point (0.25 Hz)
Cell 460
Fluorescence (Raw)
60
80
100
140
180
0
200
400
600
800
Time Point (0.25 Hz)

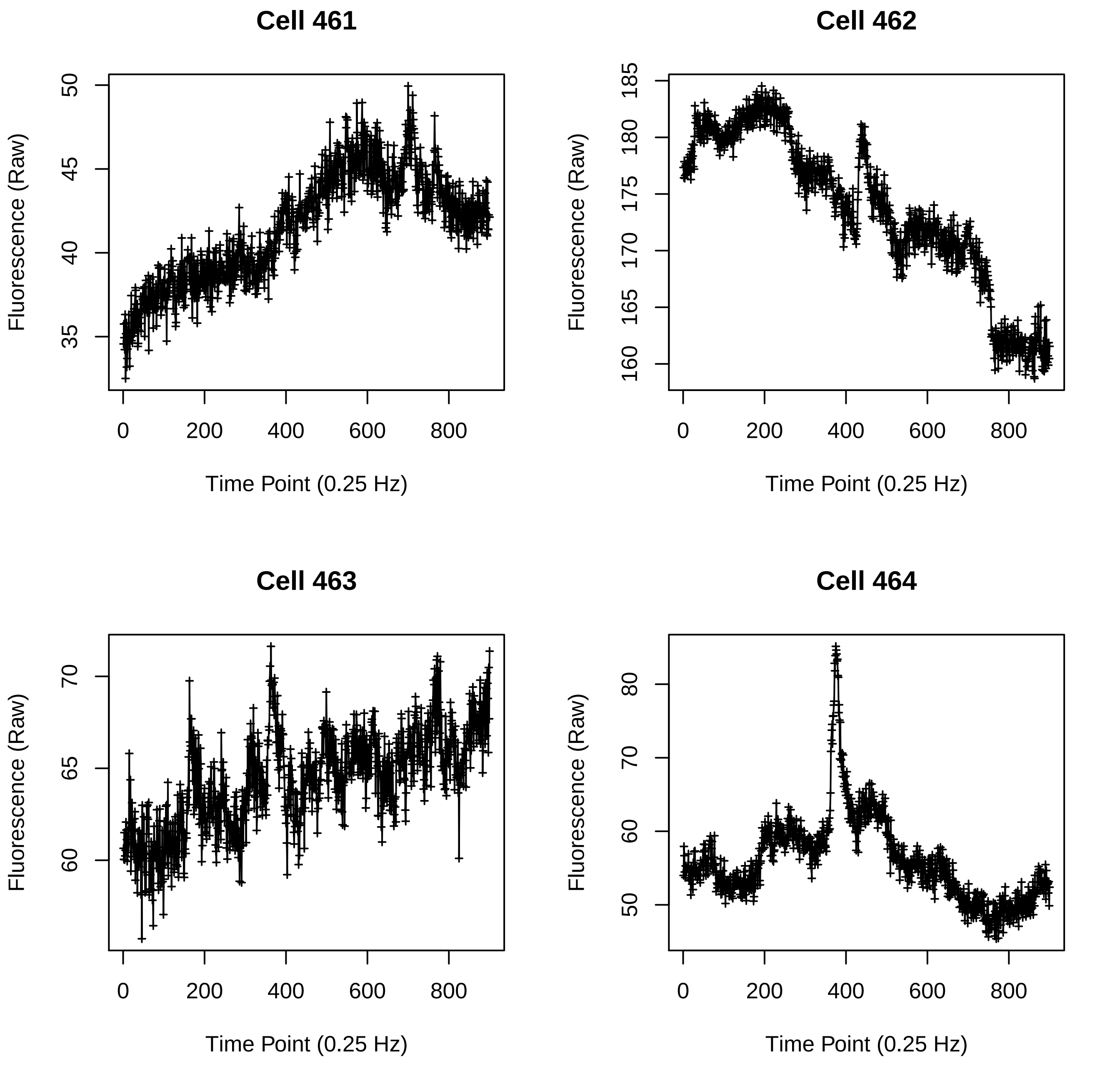

Cell 461
Fluorescence (Raw)
Time Point (0.25 Hz)
35
40
45
50
0
200
400
600
800
Cell 462
Fluorescence (Raw)
Time Point (0.25 Hz)
160
165
170
175
180
185
0
200
400
600
800
Cell 463
Fluorescence (Raw)
Time Point (0.25 Hz)
60
65
70
0
200
400
600
800
Cell 464
Fluorescence (Raw)
Time Point (0.25 Hz)
50
60
70
80
0
200
400
600
800

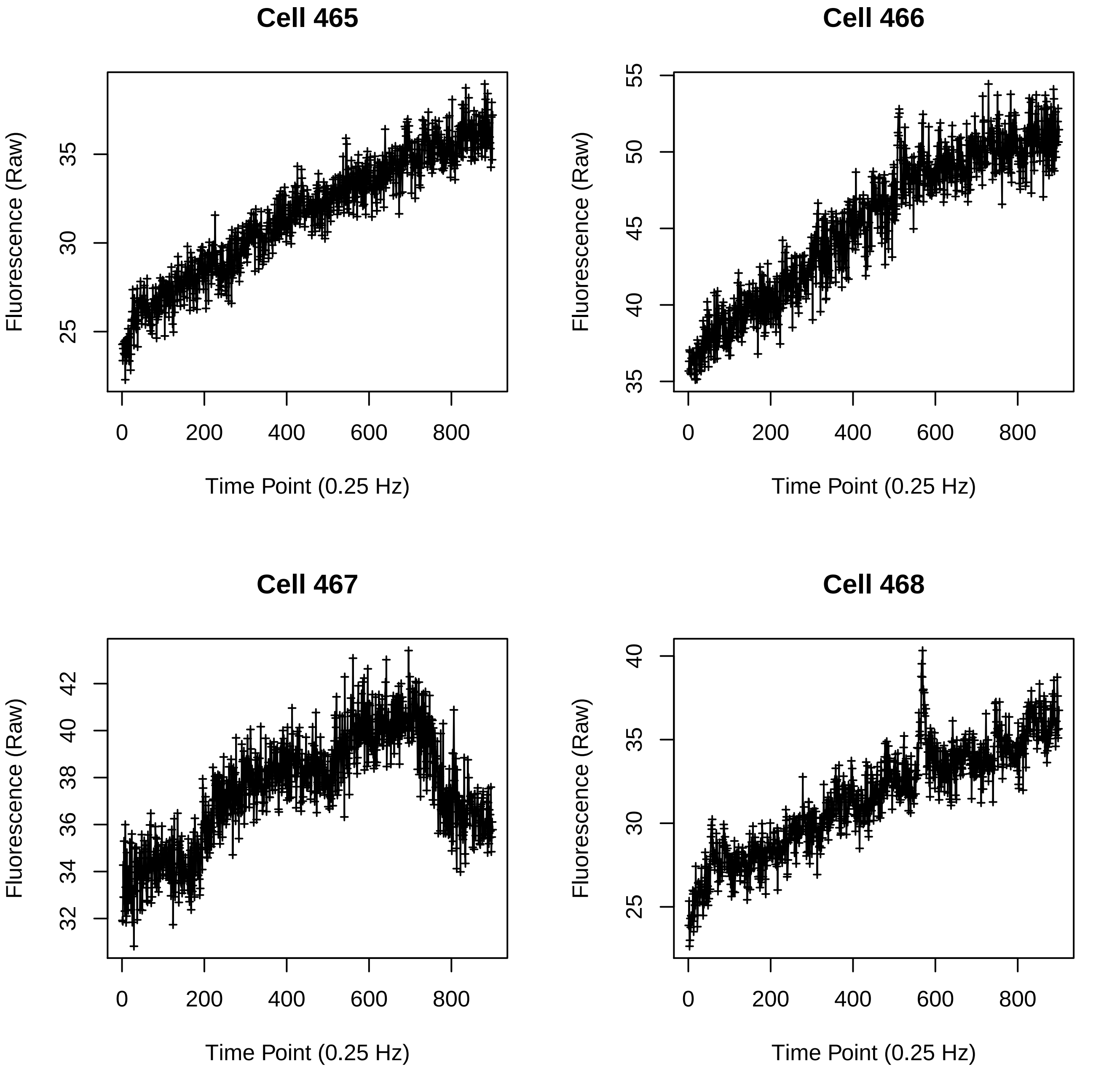
Cell 465
Fluorescence (Raw)
25
30
35
0
200
400
600
800
Time Point (0.25 Hz)
Cell 466
Fluorescence (Raw)
35
40
45
50
55
0
200
400
600
800
Time Point (0.25 Hz)
Cell 467
Fluorescence (Raw)
32
34
36
38
40
42
0
200
400
600
800
Time Point (0.25 Hz)
Cell 468
Fluorescence (Raw)
25
30
35
40
0
200
400
600
800
Time Point (0.25 Hz)

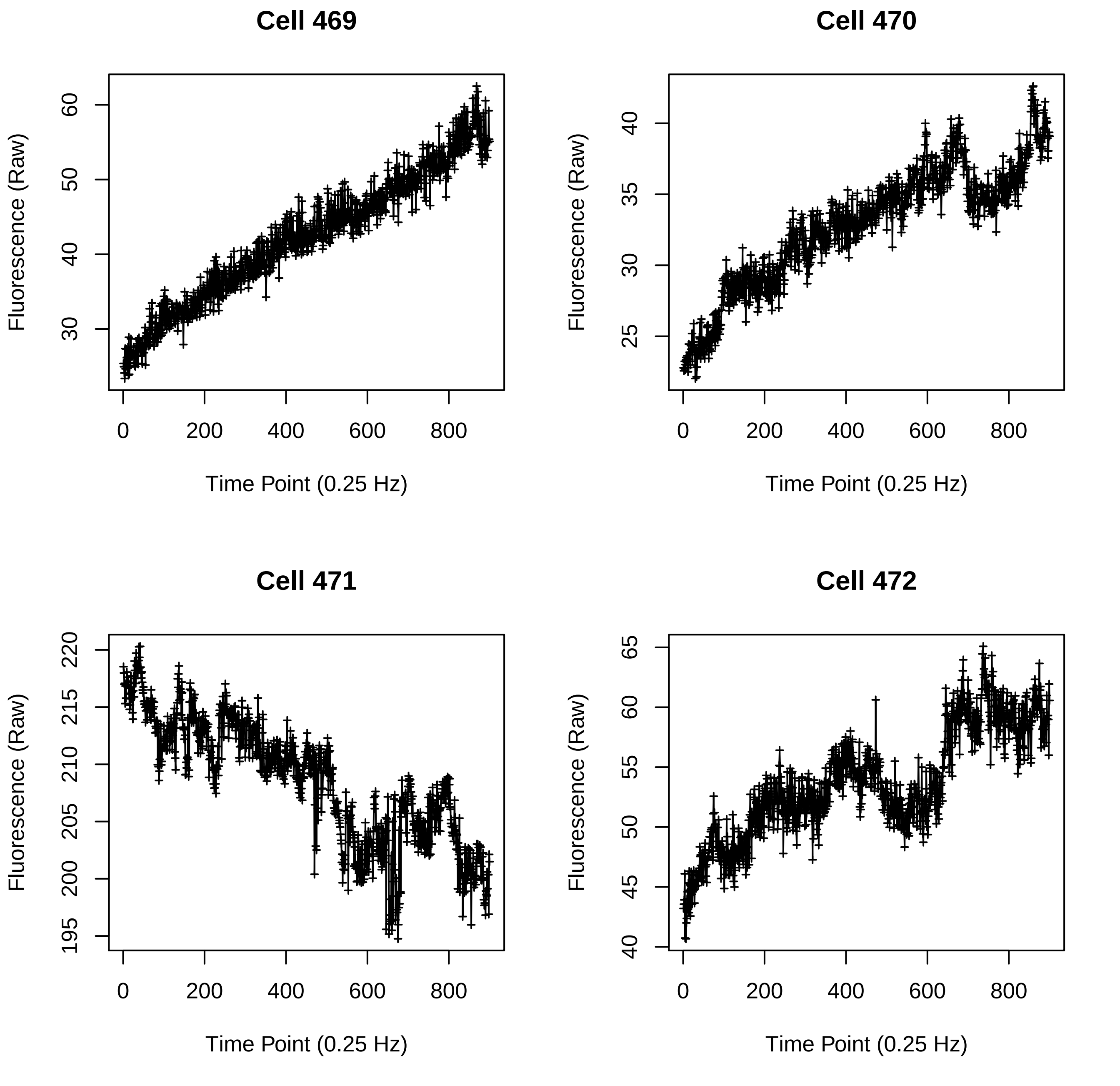

Cell 469
Cell 470
Cell 471
Cell 472
Fluorescence (Raw)
Time Point (0.25 Hz)
0
200
400
600
800
30
40
50
60
25
35
195
200
205
210
215
220
45
55
65

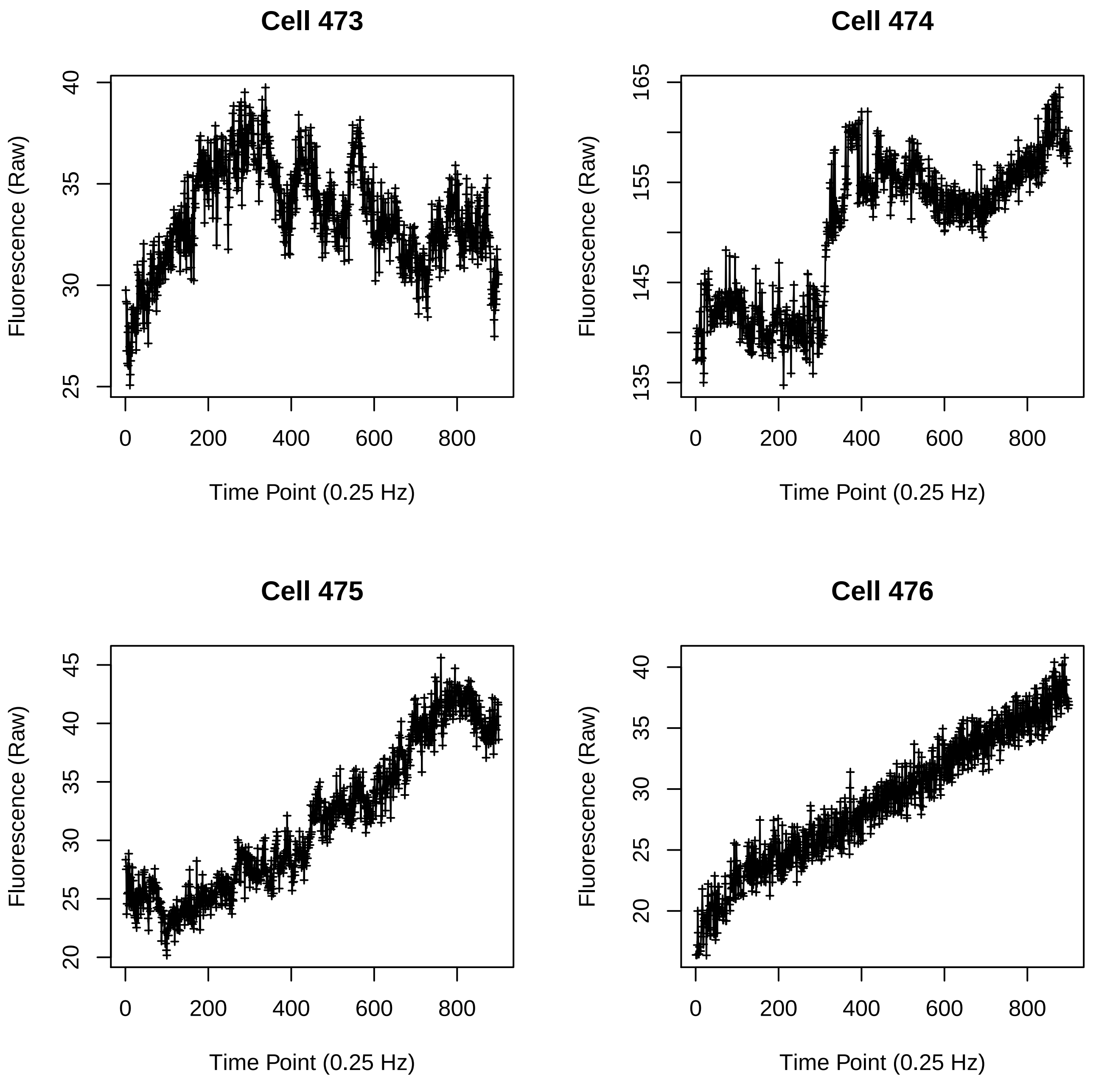
Cell 473
Cell 474
Cell 475
Cell 476
Fluorescence (Raw)
Time Point (0.25 Hz)
25
30
35
40
135
145
155
165
20
45
0
200
400
600
800

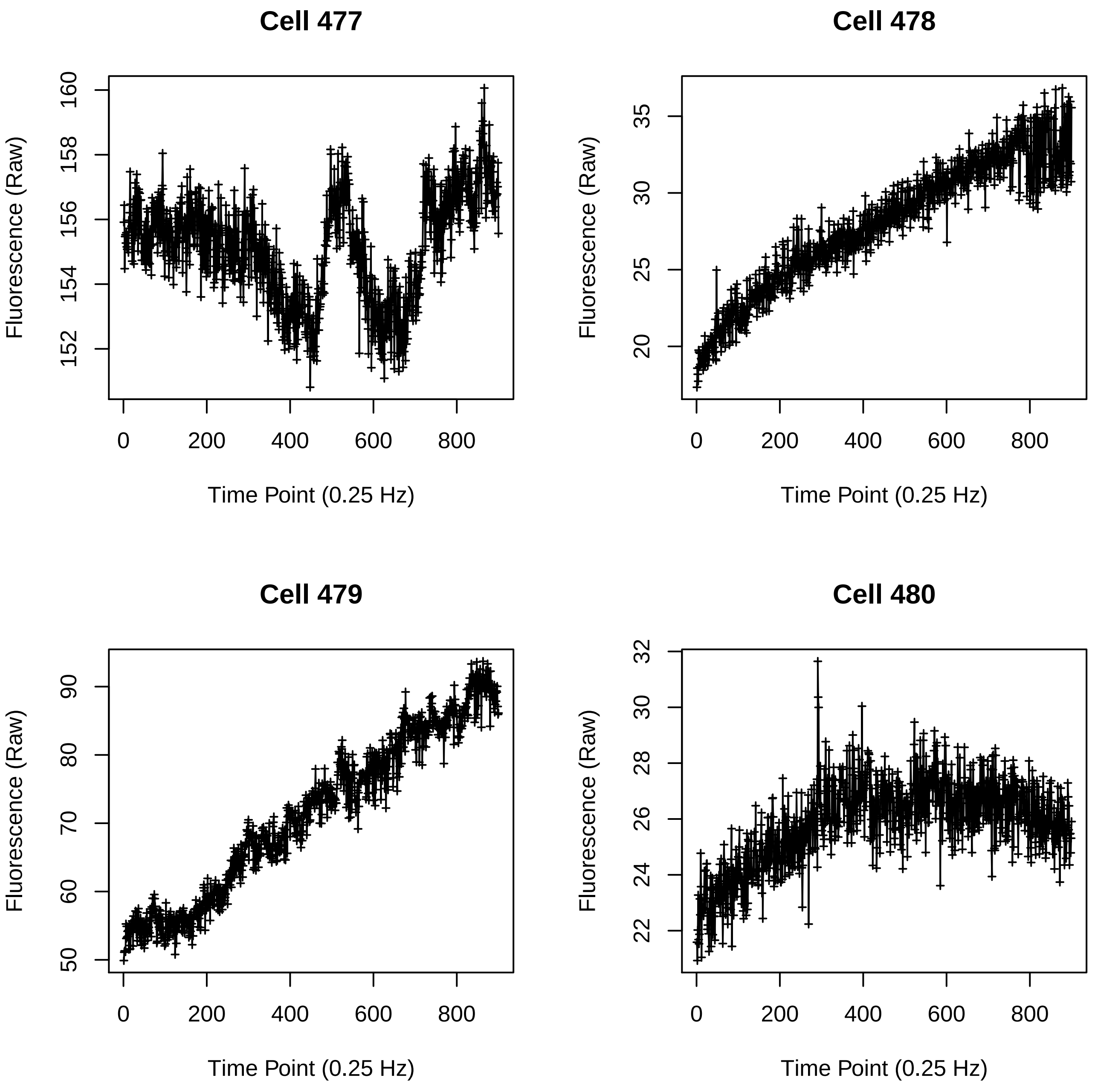
Cell 477
Fluorescence (Raw)
152
154
156
158
160
Time Point (0.25 Hz)
0
200
400
600
800
Cell 478
Fluorescence (Raw)
20
25
30
35
Time Point (0.25 Hz)
0
200
400
600
800
Cell 479
Fluorescence (Raw)
50
60
70
80
90
Time Point (0.25 Hz)
0
200
400
600
800
Cell 480
Fluorescence (Raw)
22
24
26
28
30
32
Time Point (0.25 Hz)
0
200
400
600
800

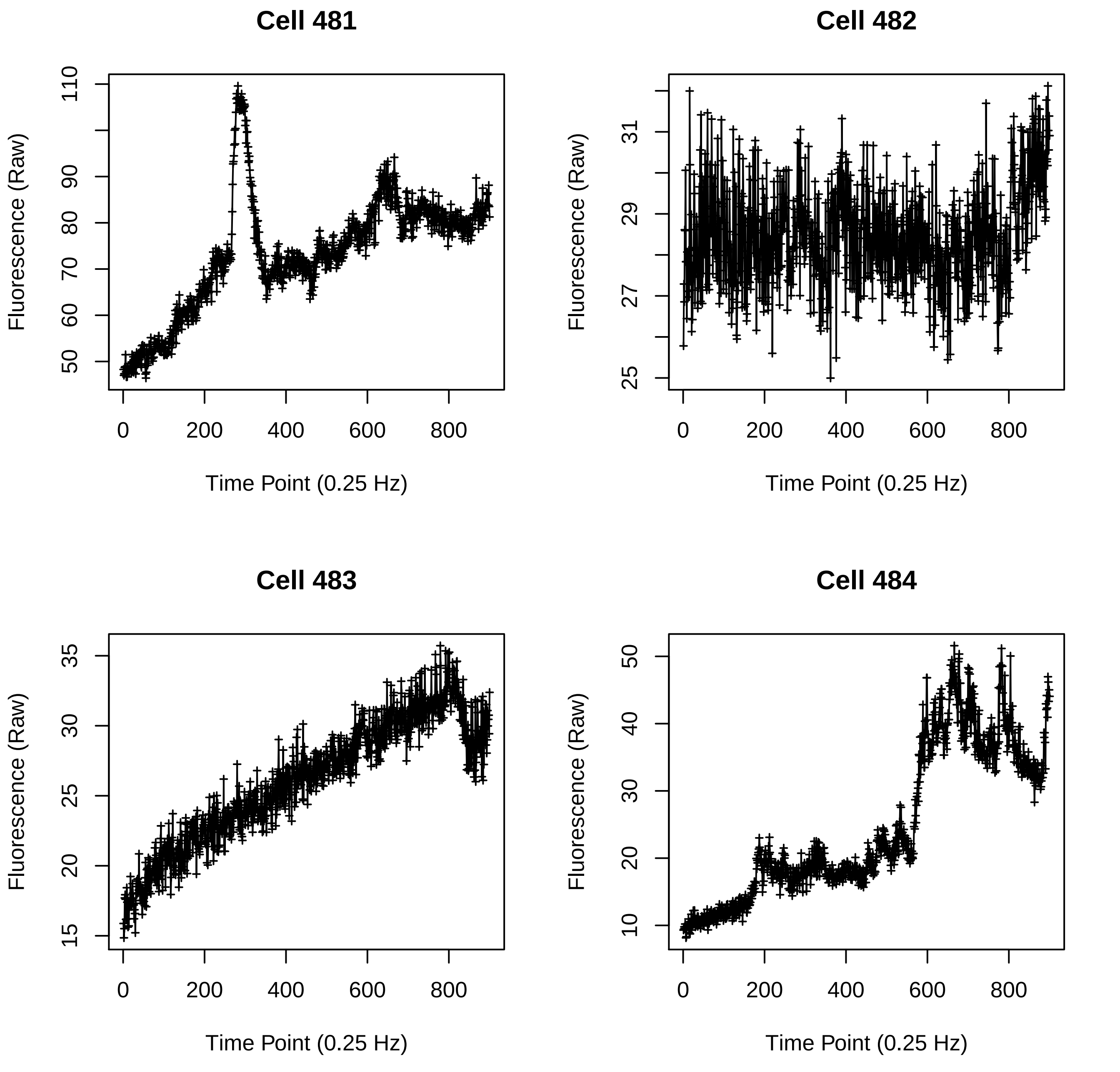
Cell 481
Fluorescence (Raw)
50
60
70
80
90
110
0
200
400
600
800
Time Point (0.25 Hz)
Cell 482
Fluorescence (Raw)
25
27
29
31
0
200
400
600
800
Time Point (0.25 Hz)
Cell 483
Fluorescence (Raw)
15
20
25
30
35
0
200
400
600
800
Time Point (0.25 Hz)
Cell 484
Fluorescence (Raw)
10
20
30
40
50
0
200
400
600
800
Time Point (0.25 Hz)

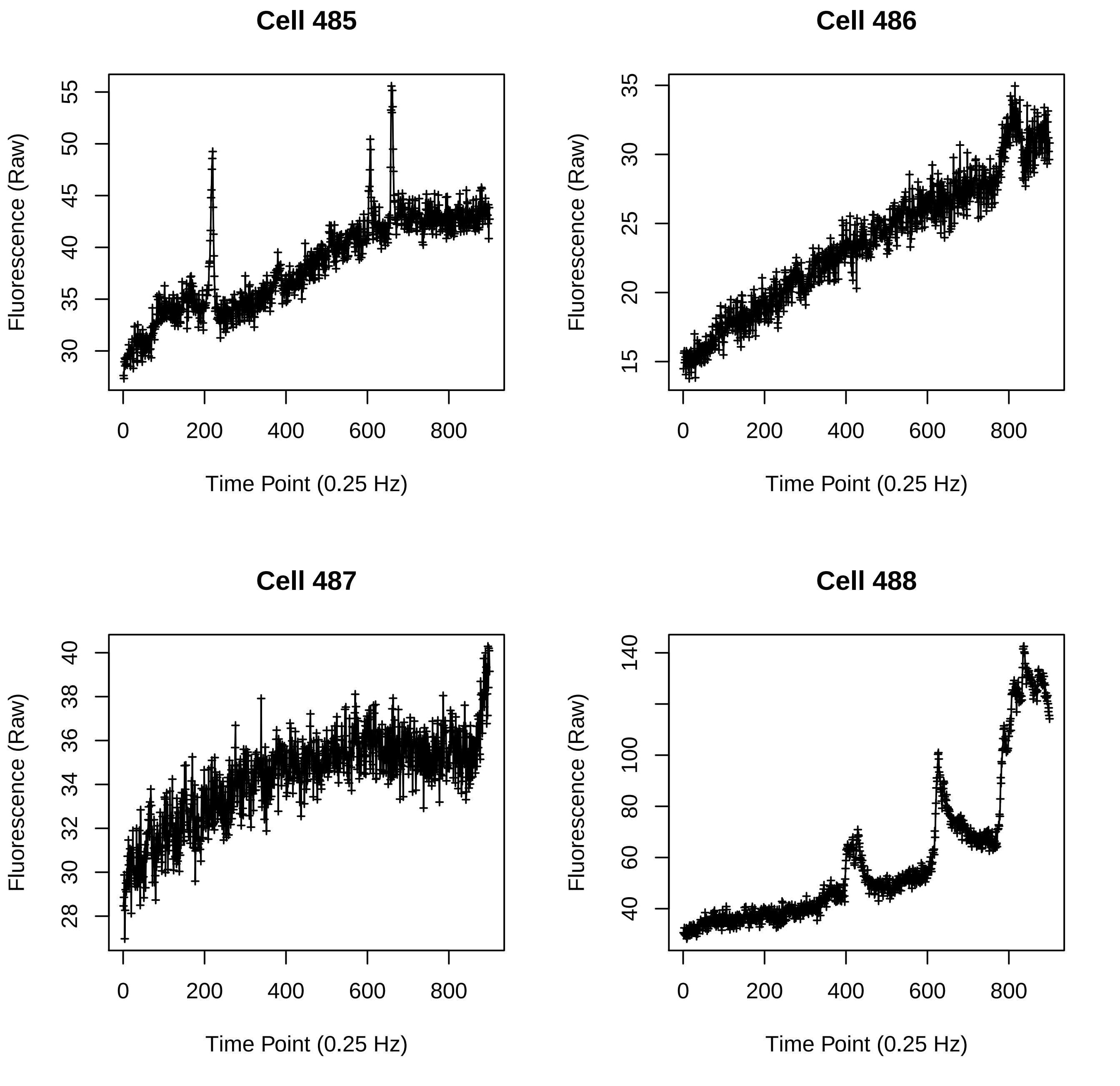

Cell 485
Fluorescence (Raw)
Time Point (0.25 Hz)
0
200
400
600
800
30
35
40
45
50
55
Cell 486
Fluorescence (Raw)
Time Point (0.25 Hz)
0
200
400
600
800
15
20
25
30
35
Cell 487
Fluorescence (Raw)
Time Point (0.25 Hz)
0
200
400
600
800
28
30
32
34
36
38
40
Cell 488
Fluorescence (Raw)
Time Point (0.25 Hz)
0
200
400
600
800
40
60
80
100
140

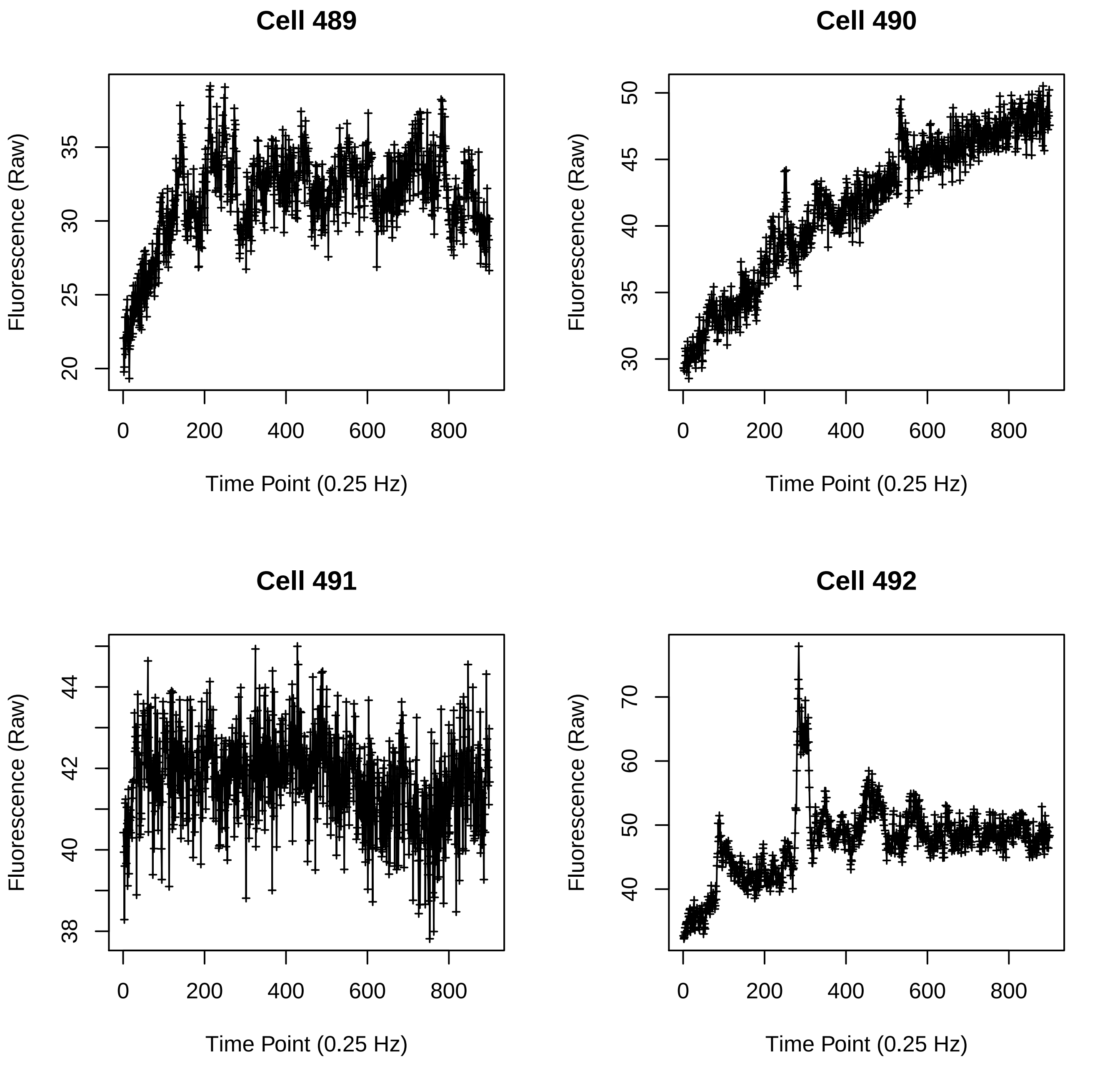
Cell 489
Cell 490
Cell 491
Cell 492
Fluorescence (Raw)
Time Point (0.25 Hz)
0
200
400
600
800
20
25
30
35
40
45
50
38
42
44
60
70

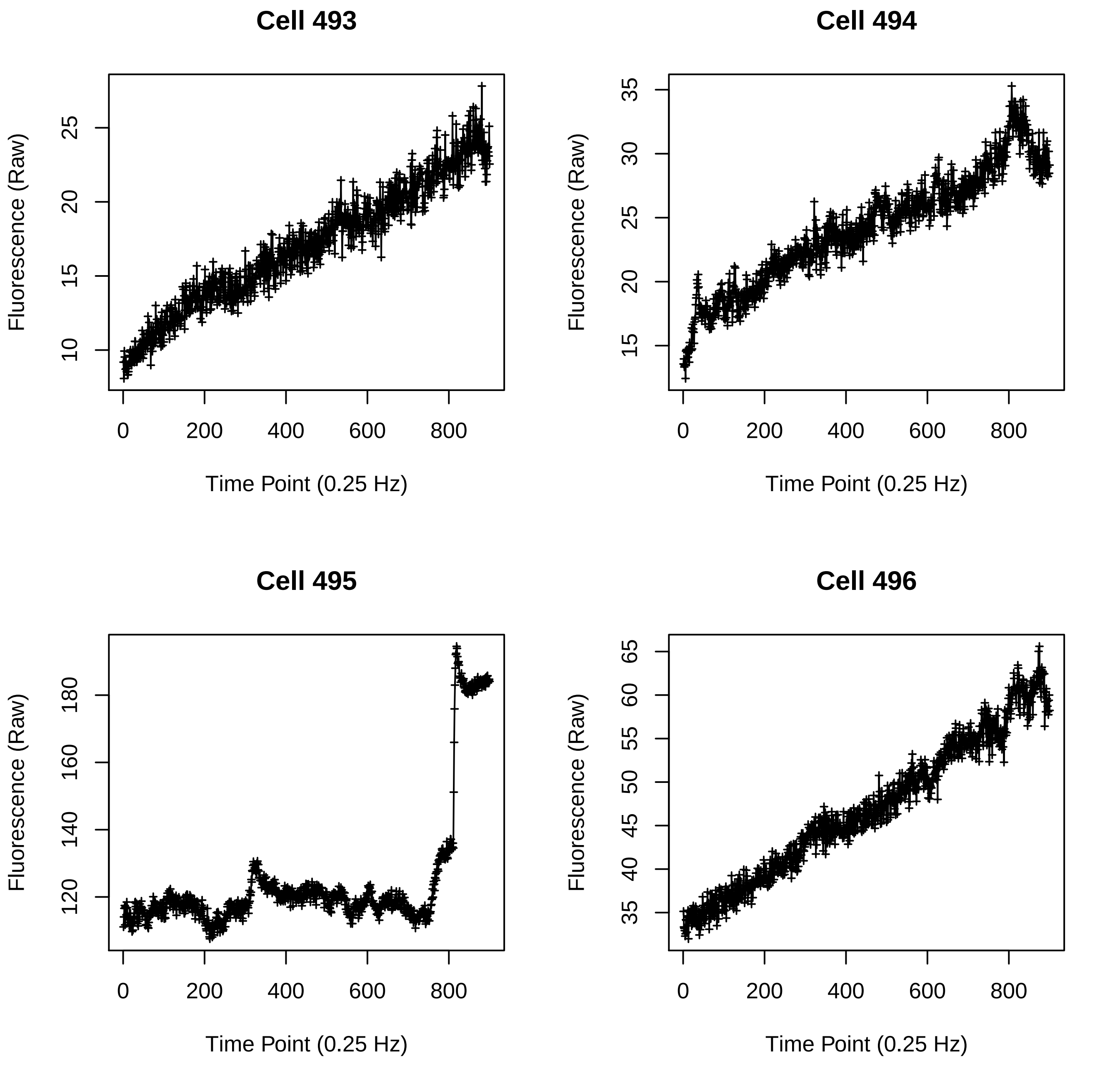
Cell 493
Cell 494
Cell 495
Cell 496
Fluorescence (Raw)
Time Point (0.25 Hz)
0
200
400
600
800
10
15
20
25
30
35
40
45
50
55
60
65
120
140
160
180

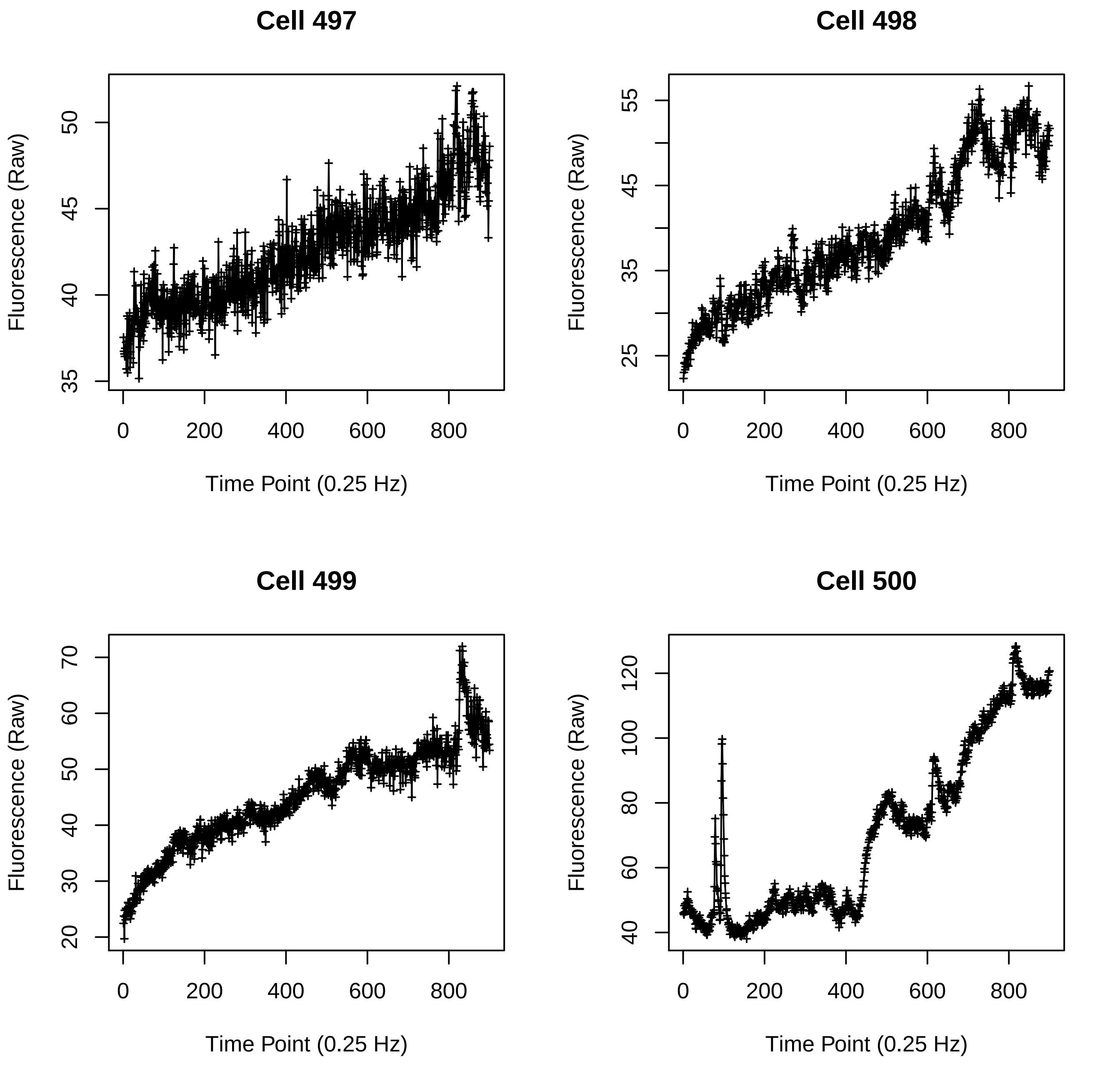
Cell 497
Cell 498
Cell 499
Cell 500
Fluorescence (Raw)
Time Point (0.25 Hz)
0
200
400
600
800
35
40
45
50
25
55
20
30
60
70
80
100
120

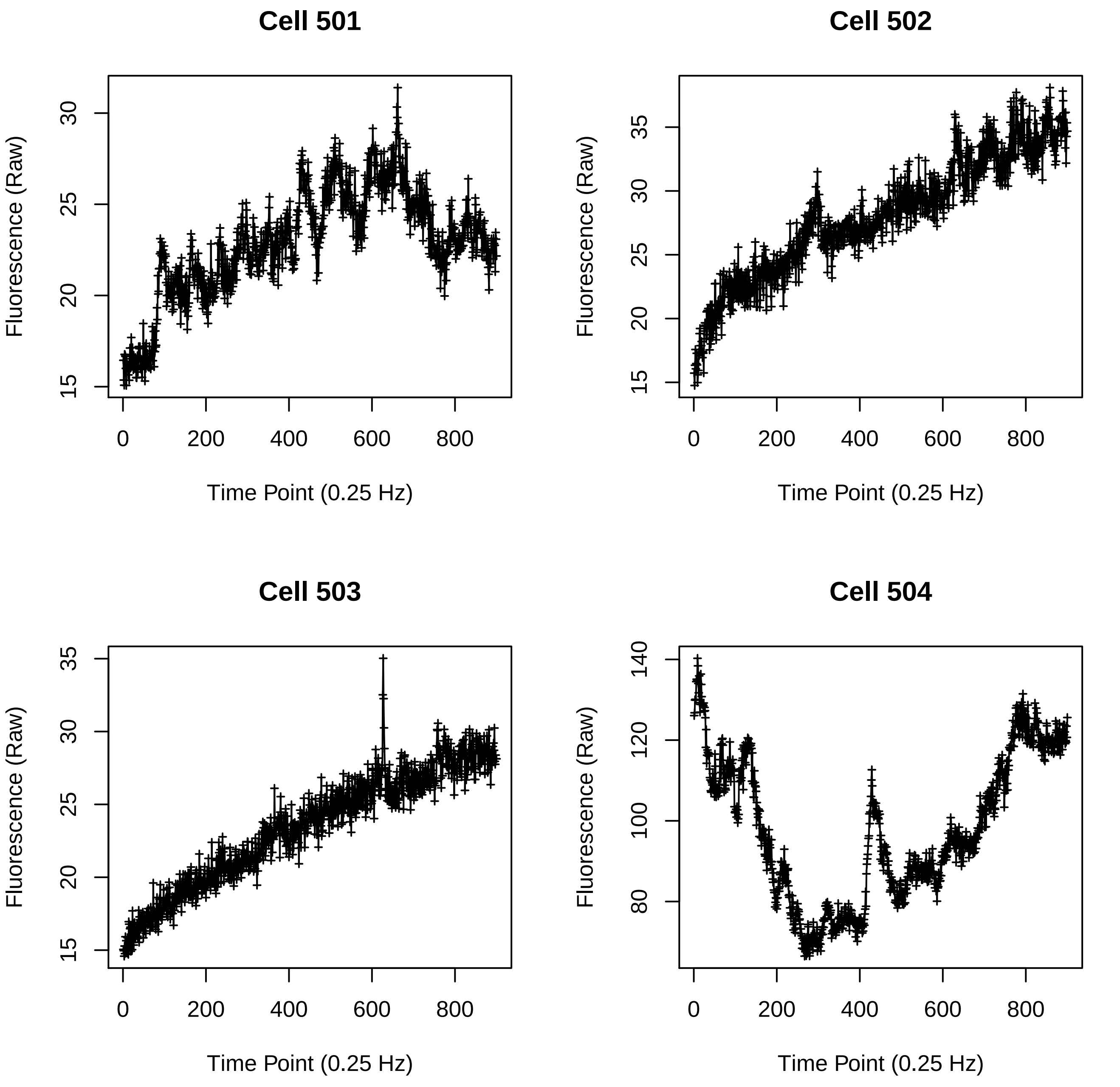

Cell 501
Fluorescence (Raw)
15
20
25
30
0
200
400
600
800
Time Point (0.25 Hz)
Cell 502
Fluorescence (Raw)
15
20
25
30
35
0
200
400
600
800
Time Point (0.25 Hz)
Cell 503
Fluorescence (Raw)
15
20
25
30
35
0
200
400
600
800
Time Point (0.25 Hz)
Cell 504
Fluorescence (Raw)
80
100
120
140
0
200
400
600
800
Time Point (0.25 Hz)

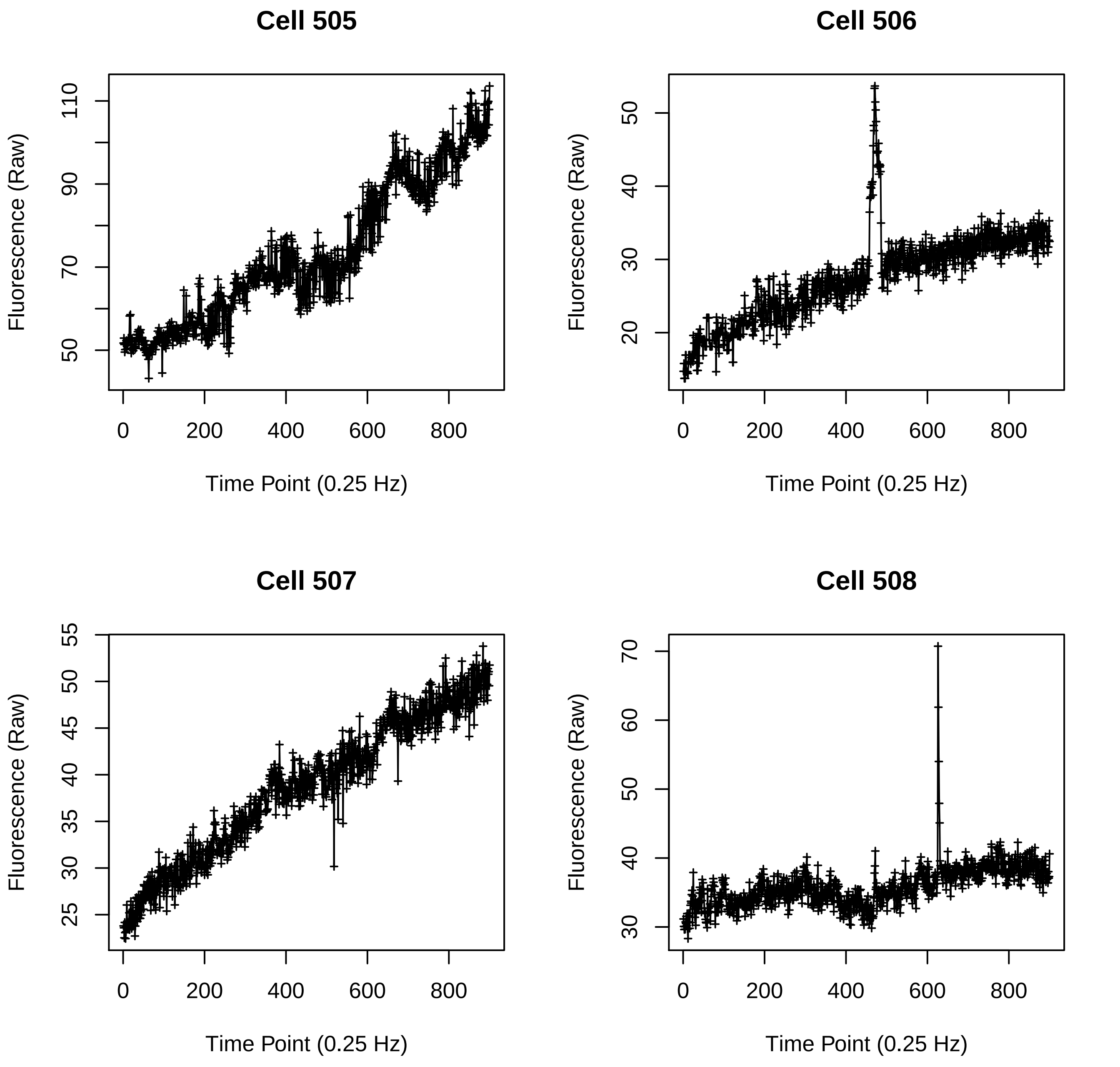
Cell 505
Cell 506
Cell 507
Cell 508
Fluorescence (Raw)
Time Point (0.25 Hz)
0
200
400
600
800
50
70
90
110
20
30
40
50
25
30
35
40
45
55
60

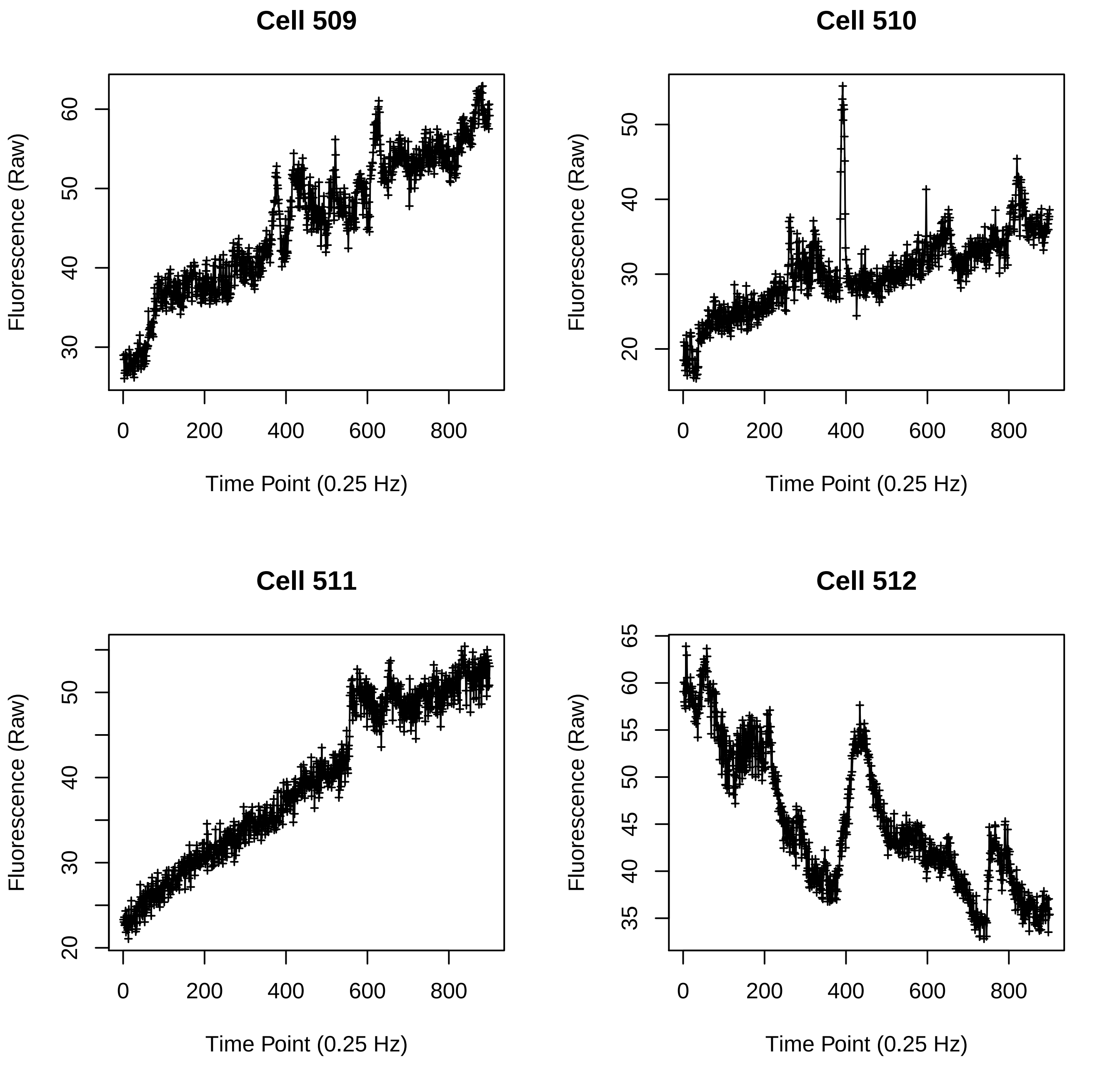

Cell 509
Cell 510
Cell 511
Cell 512
Fluorescence (Raw)
Time Point (0.25 Hz)
0
200
400
600
800

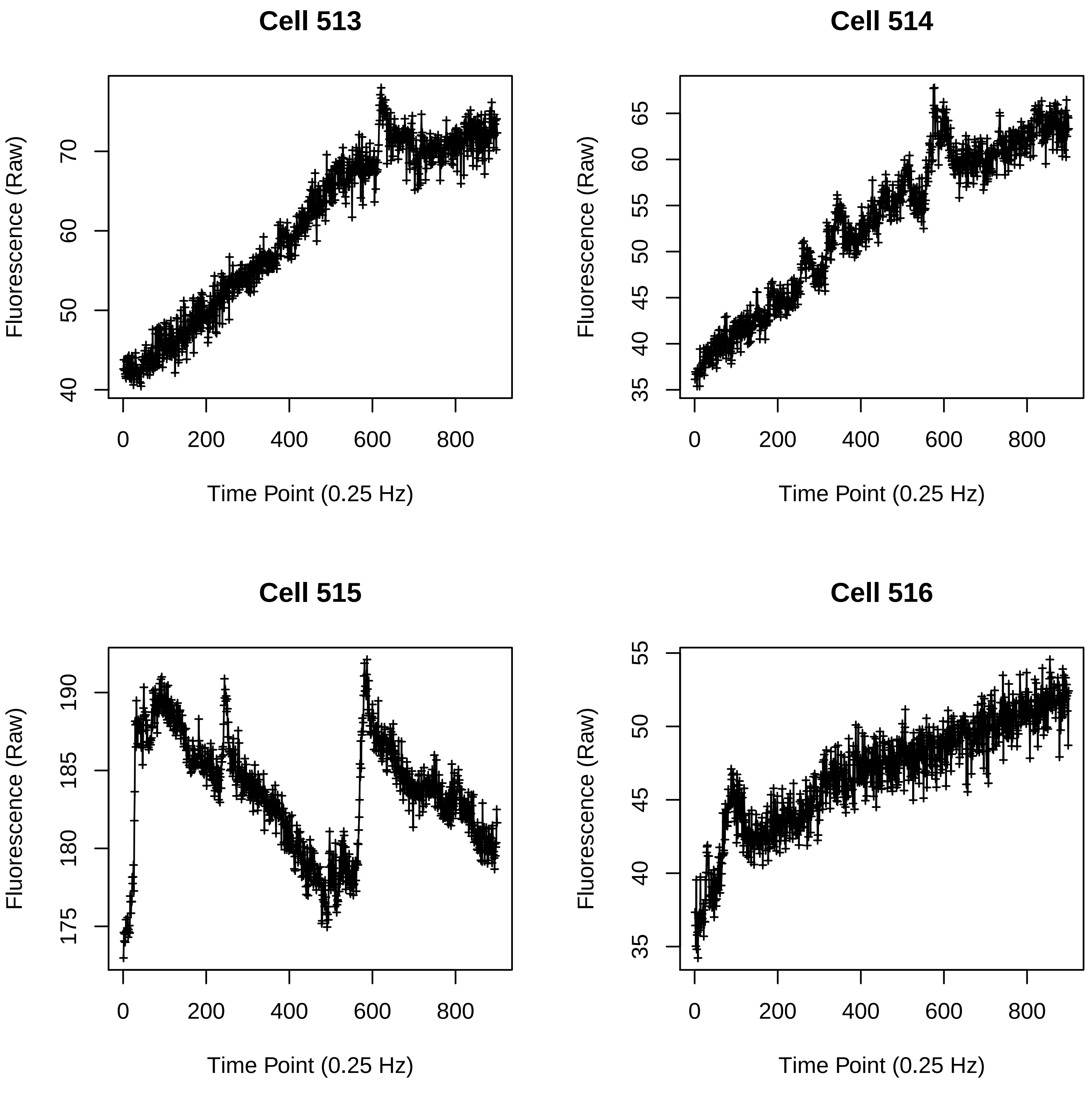

Cell 513
Fluorescence (Raw)
40
50
60
70
0
200
400
600
800
Time Point (0.25 Hz)
Cell 514
Fluorescence (Raw)
35
40
45
50
55
60
65
0
200
400
600
800
Time Point (0.25 Hz)
Cell 515
Fluorescence (Raw)
175
180
185
190
0
200
400
600
800
Time Point (0.25 Hz)
Cell 516
Fluorescence (Raw)
35
40
45
50
55
0
200
400
600
800
Time Point (0.25 Hz)

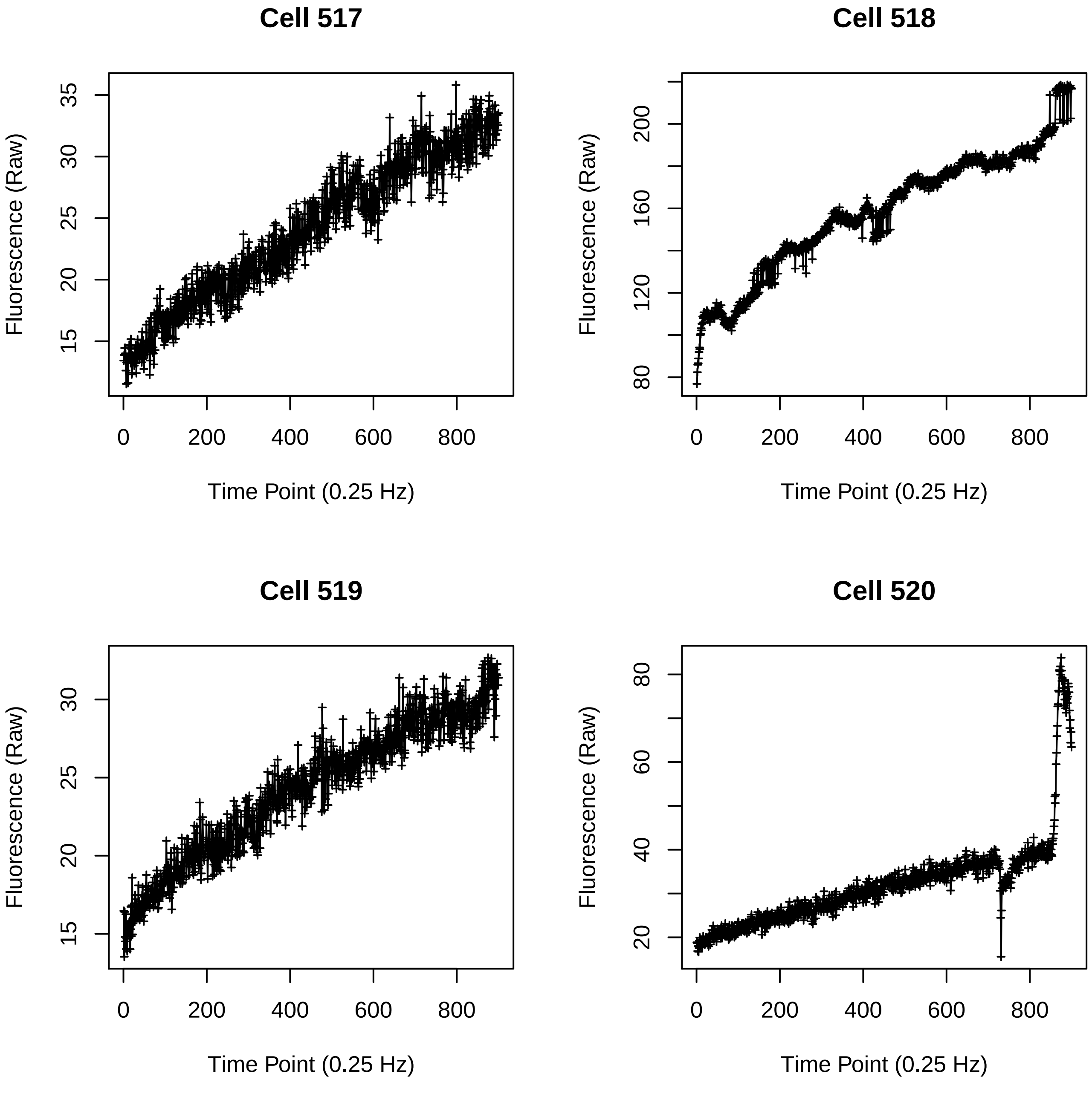

Cell 517
Cell 518
Cell 519
Cell 520
Fluorescence (Raw)
Time Point (0.25 Hz)
15
20
25
30
35
80
120
160
200
40
60
0
200
400
600
800

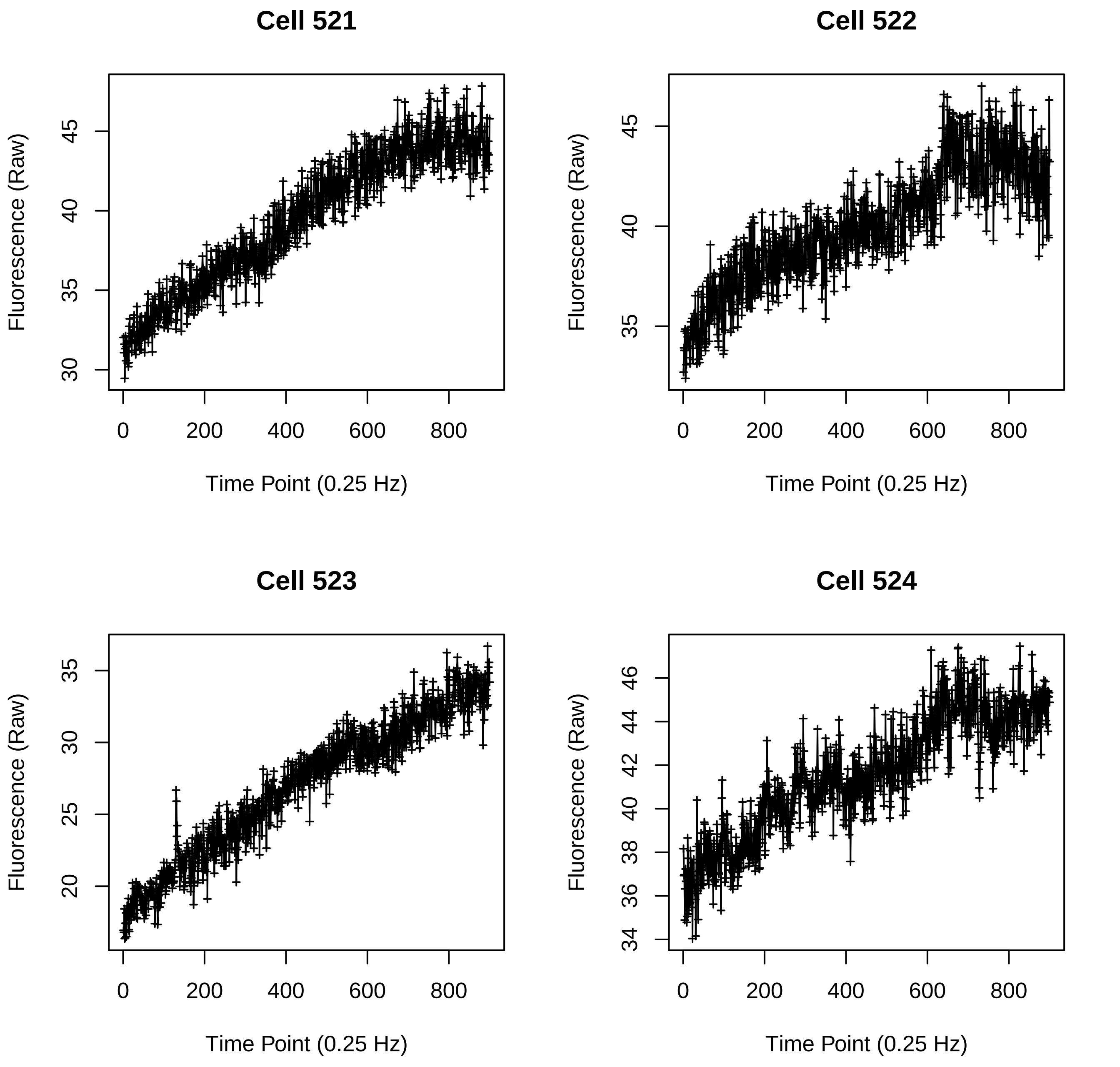

Cell 521
Cell 522
Cell 523
Cell 524
Fluorescence (Raw)
Time Point (0.25 Hz)
0
200
400
600
800
30
35
40
45
20
25
34
36
38
42
44
46

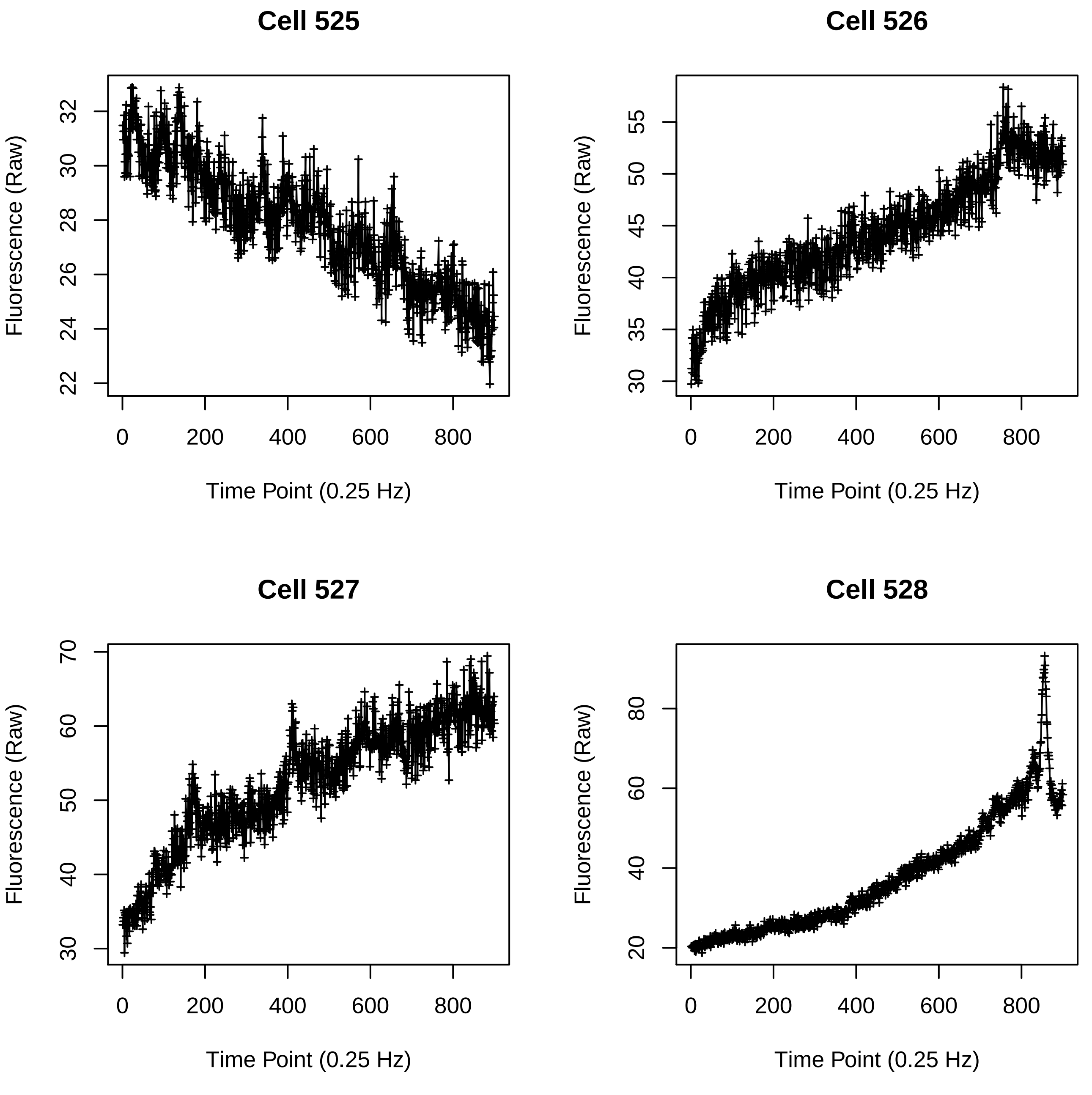
Cell 525
Fluorescence (Raw)
22
24
26
28
30
32
0
200
400
600
800
Time Point (0.25 Hz)
Cell 526
Fluorescence (Raw)
30
35
40
45
50
55
0
200
400
600
800
Time Point (0.25 Hz)
Cell 527
Fluorescence (Raw)
30
40
50
60
70
0
200
400
600
800
Time Point (0.25 Hz)
Cell 528
Fluorescence (Raw)
20
40
60
80
0
200
400
600
800
Time Point (0.25 Hz)

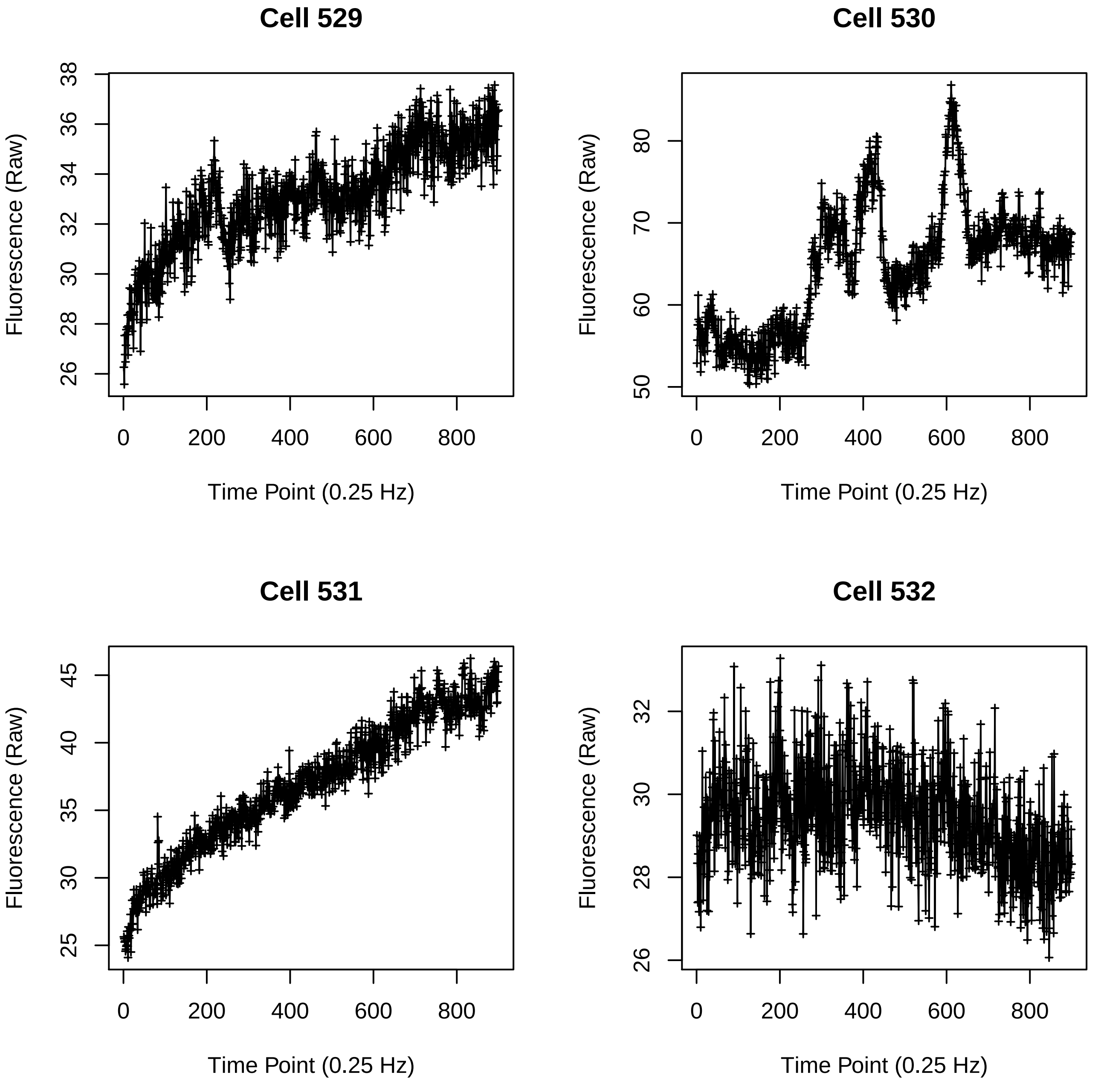
Cell 529
Fluorescence (Raw)
26
28
30
32
34
36
38
0
200
400
600
800
Time Point (0.25 Hz)
Cell 530
Fluorescence (Raw)
50
60
70
80
0
200
400
600
800
Time Point (0.25 Hz)
Cell 531
Fluorescence (Raw)
25
30
35
40
45
0
200
400
600
800
Time Point (0.25 Hz)
Cell 532
Fluorescence (Raw)
26
28
30
32
0
200
400
600
800
Time Point (0.25 Hz)

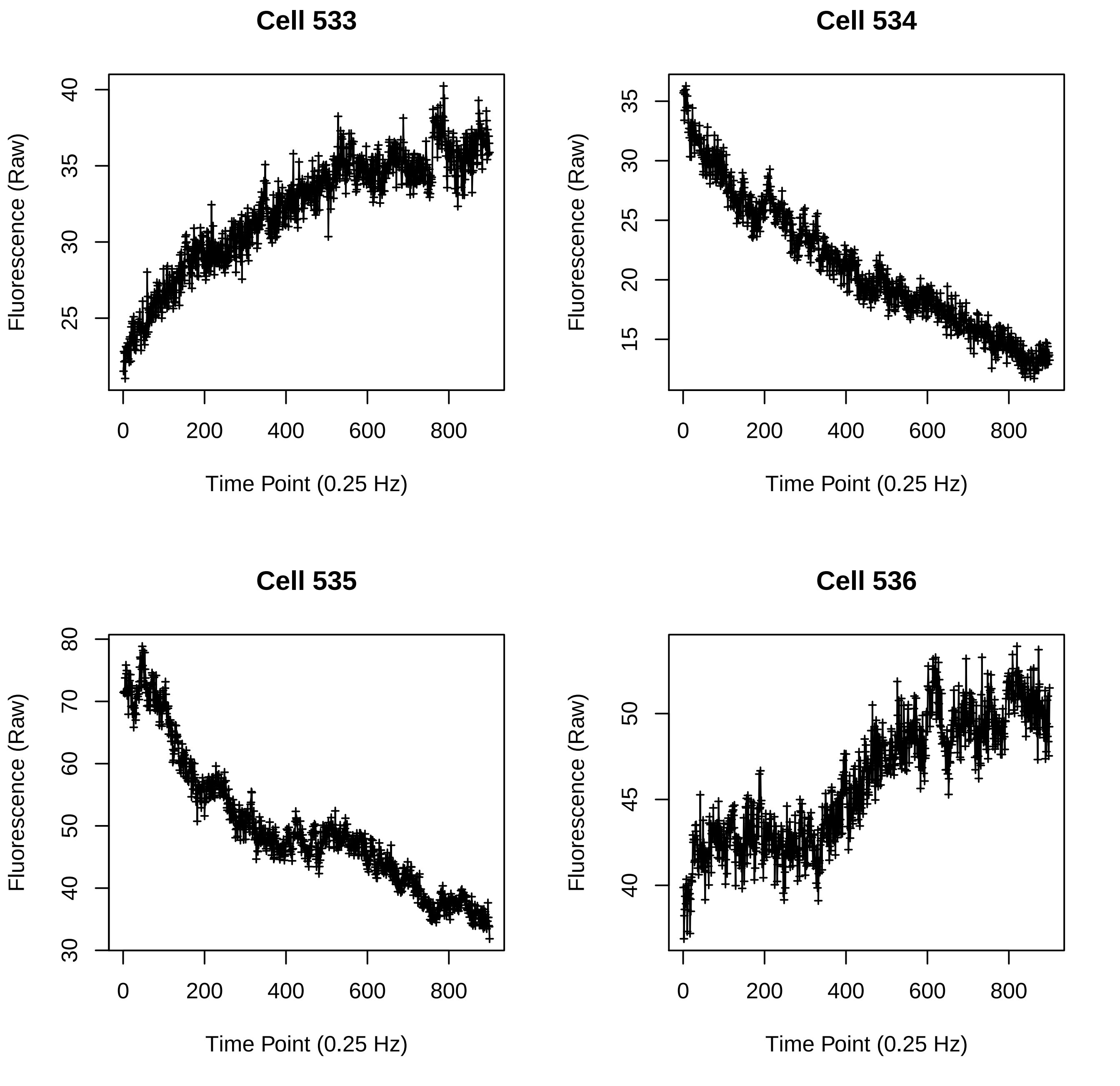
Cell 533
Cell 534
Cell 535
Cell 536
Fluorescence (Raw)
Time Point (0.25 Hz)
0
200
400
600
800
25
30
35
40
15
20
30
40
50
60
70
80
45

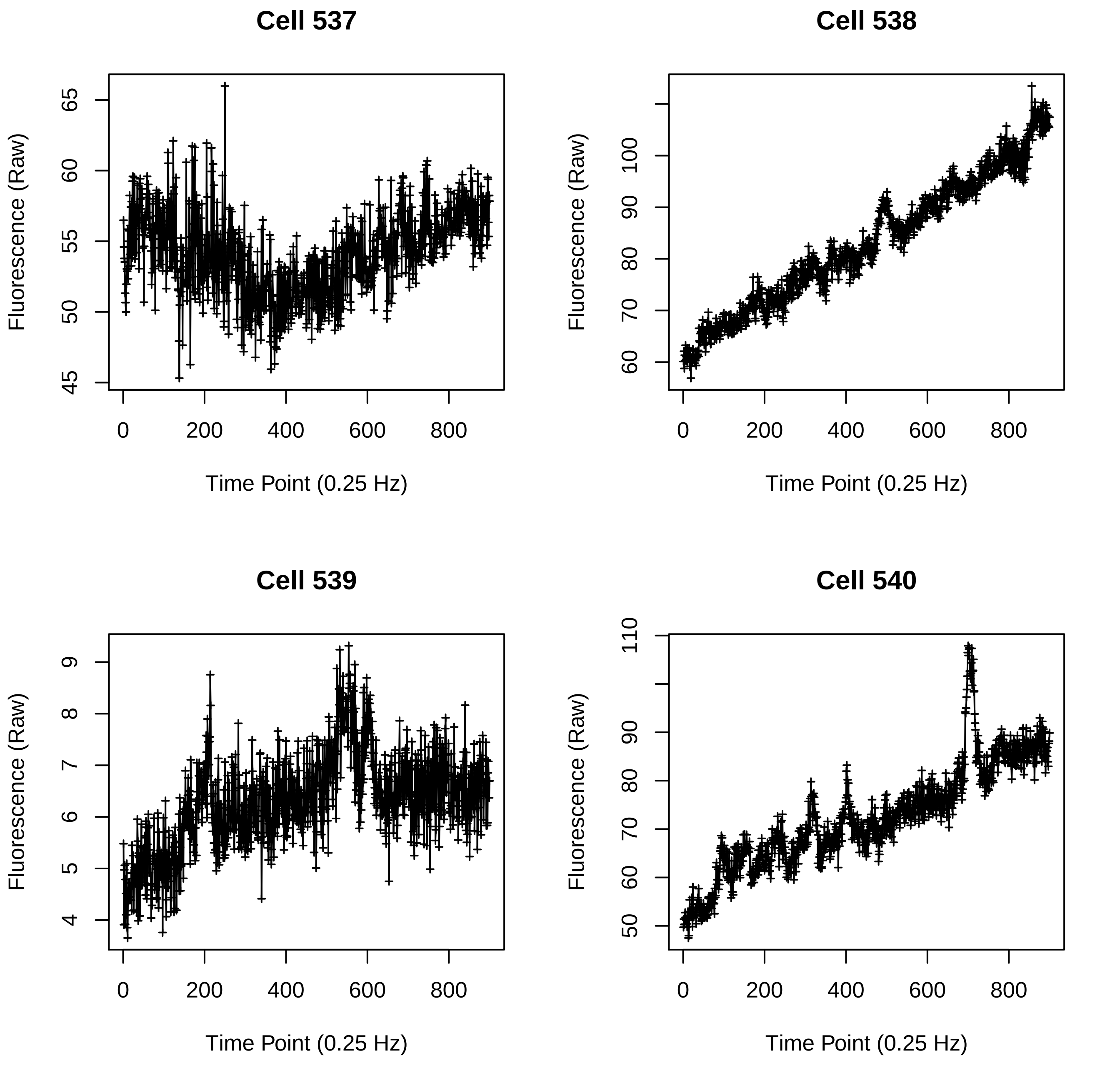

Cell 537
Cell 538
Cell 539
Cell 540
Fluorescence (Raw)
Time Point (0.25 Hz)
45
50
55
60
65
60
70
80
90
100
4
5
6
7
8
9
50
60
70
80
90
110
0
200
400
600
800

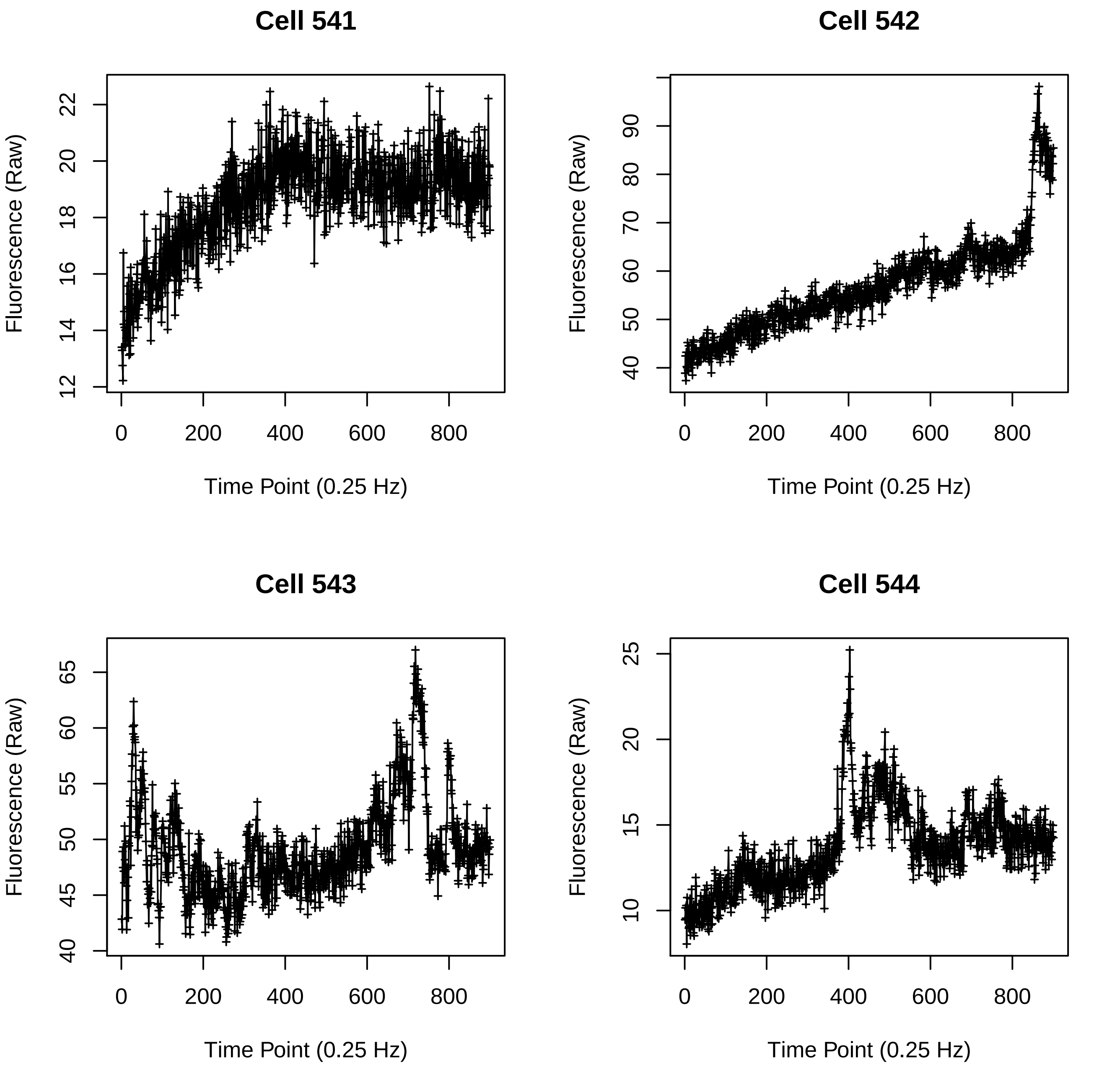
Cell 541
Fluorescence (Raw)
12
14
16
18
20
22
0
200
400
600
800
Time Point (0.25 Hz)
Cell 542
Fluorescence (Raw)
40
50
60
70
80
90
0
200
400
600
800
Time Point (0.25 Hz)
Cell 543
Fluorescence (Raw)
40
45
50
55
60
65
0
200
400
600
800
Time Point (0.25 Hz)
Cell 544
Fluorescence (Raw)
10
15
20
25
0
200
400
600
800
Time Point (0.25 Hz)

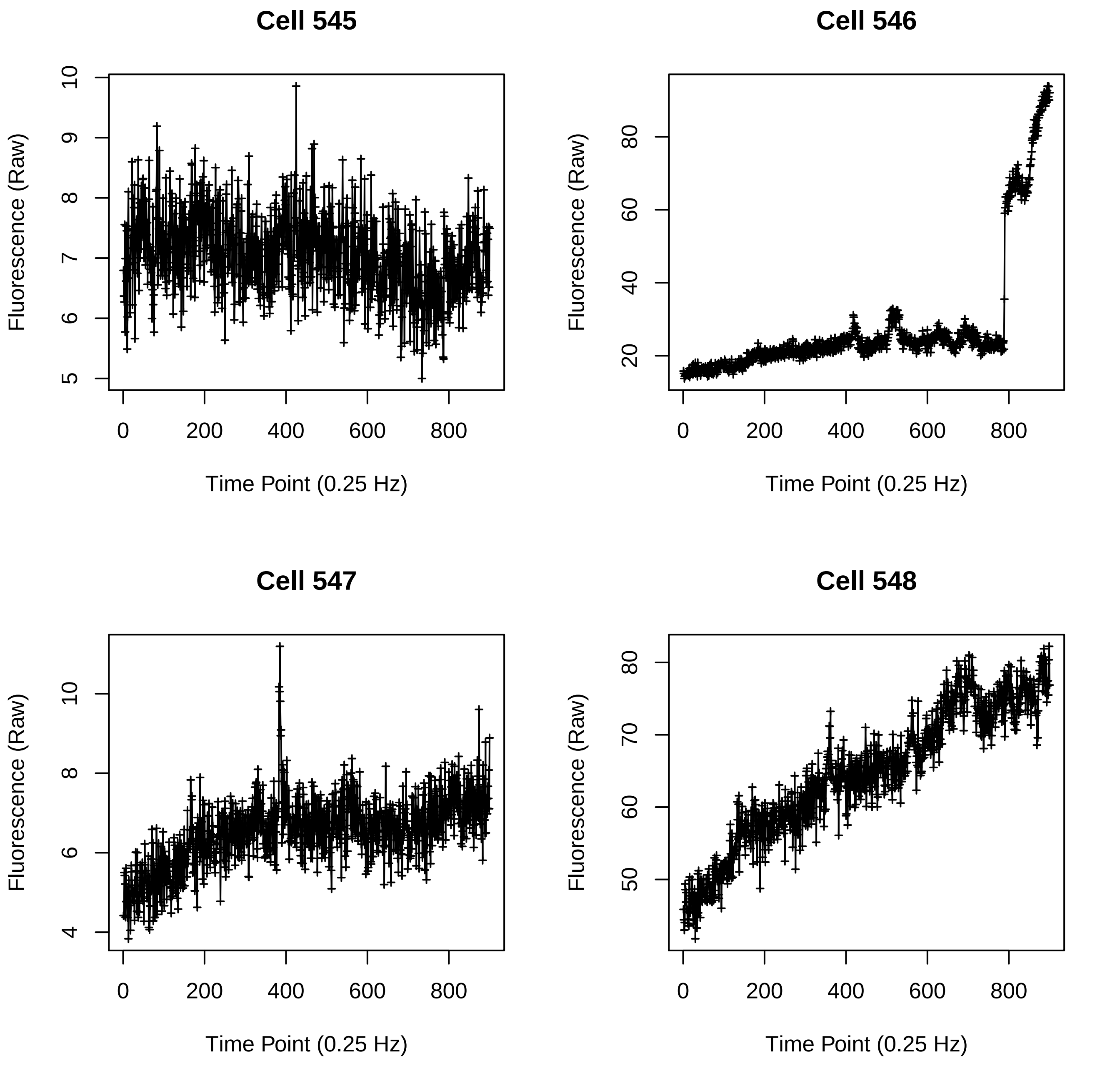
Cell 545
Cell 546
Cell 547
Cell 548
Fluorescence (Raw)
Time Point (0.25 Hz)
0
200
400
600
800
5
6
7
8
9
10
20
40
60
80
4
50
70

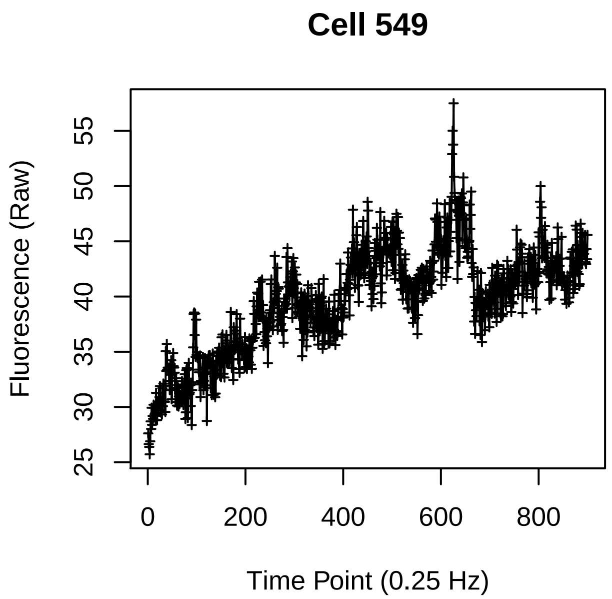
Cell 549
Fluorescence (Raw)
25
30
35
40
45
50
55
0
200
400
600
800
Time Point (0.25 Hz)

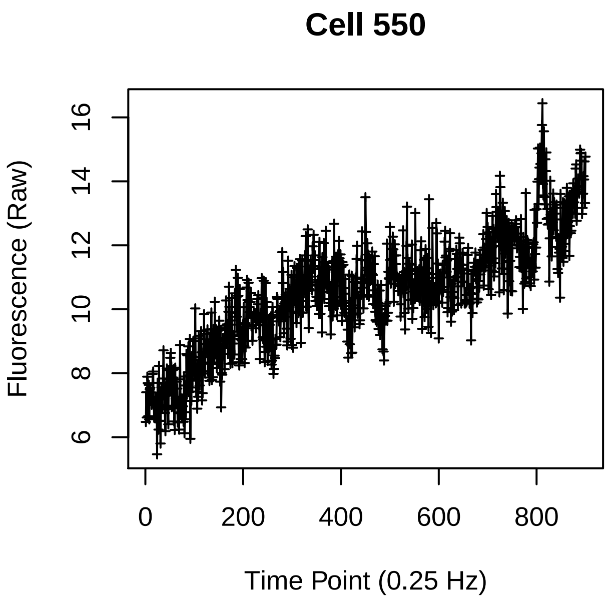
Cell 550
Fluorescence (Raw)
6
8
10
12
14
16
0
200
400
600
800
Time Point (0.25 Hz)

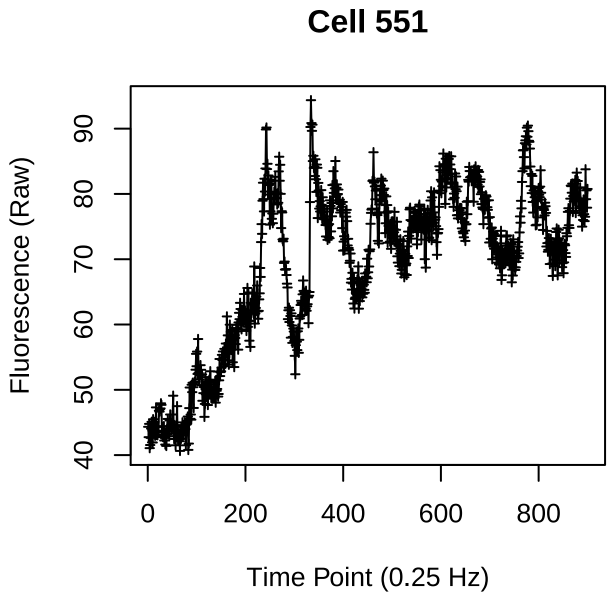
Cell 551
Fluorescence (Raw)
40
50
60
70
80
90
0
200
400
600
800
Time Point (0.25 Hz)

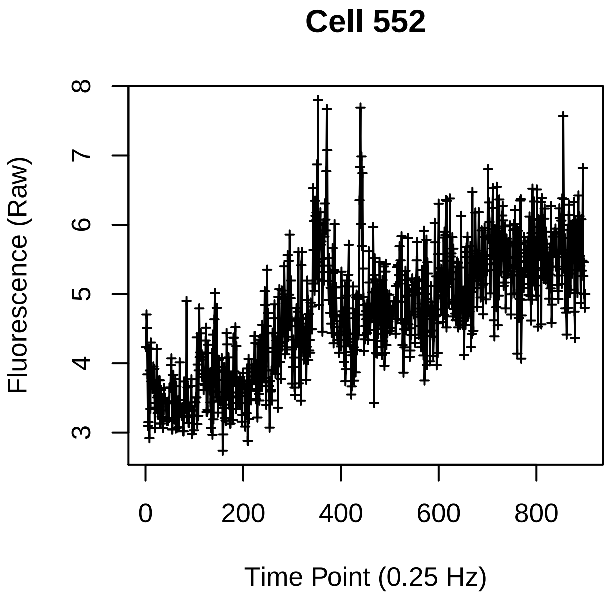
Cell 552
Fluorescence (Raw)
3
4
5
6
7
8
0
200
400
600
800
Time Point (0.25 Hz)

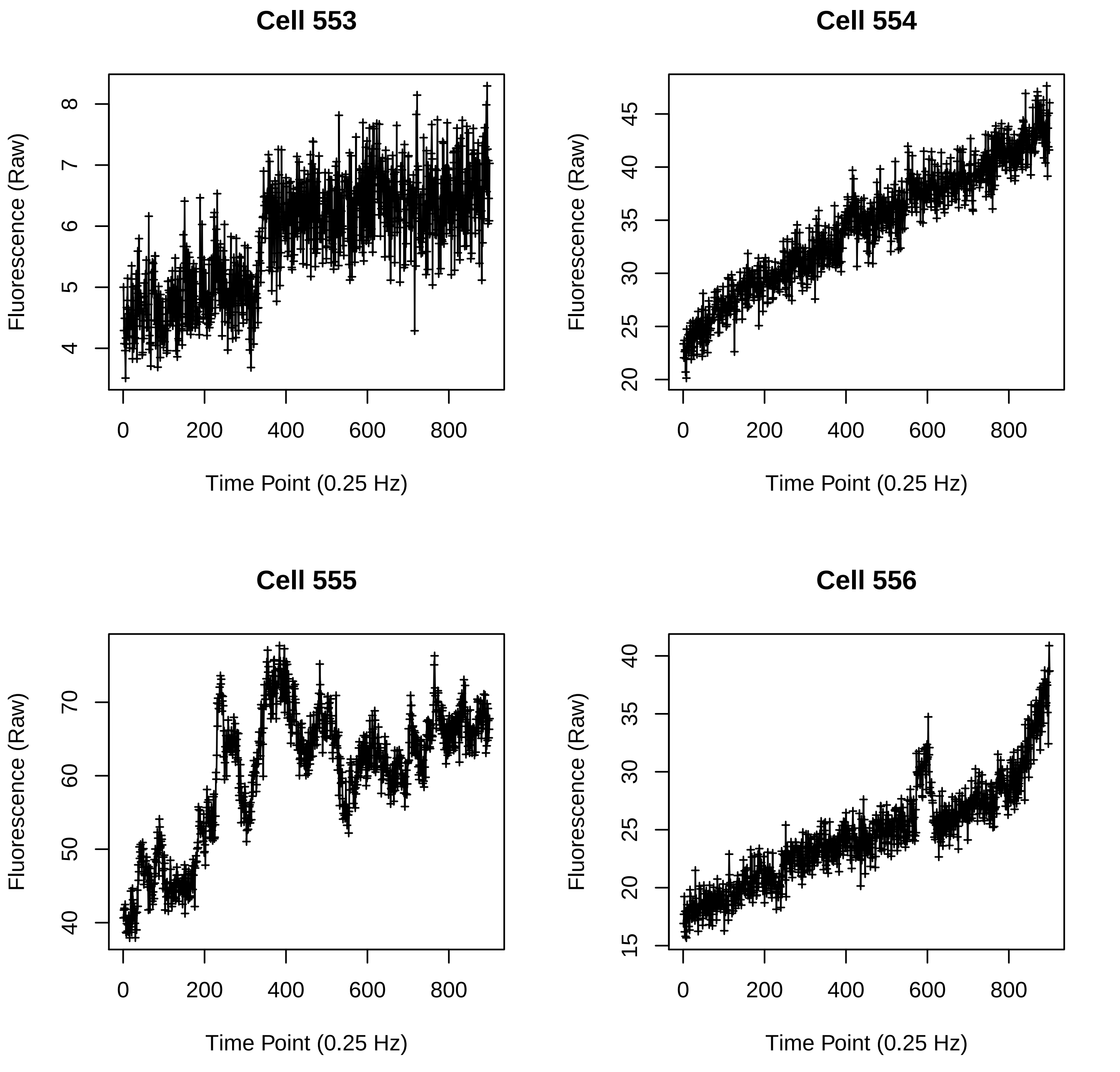

Cell 553
Fluorescence (Raw)
4
5
6
7
8
0
200
400
600
800
Time Point (0.25 Hz)
Cell 554
Fluorescence (Raw)
20
25
30
35
40
45
0
200
400
600
800
Time Point (0.25 Hz)
Cell 555
Fluorescence (Raw)
40
50
60
70
0
200
400
600
800
Time Point (0.25 Hz)
Cell 556
Fluorescence (Raw)
15
20
25
30
35
40
0
200
400
600
800
Time Point (0.25 Hz)

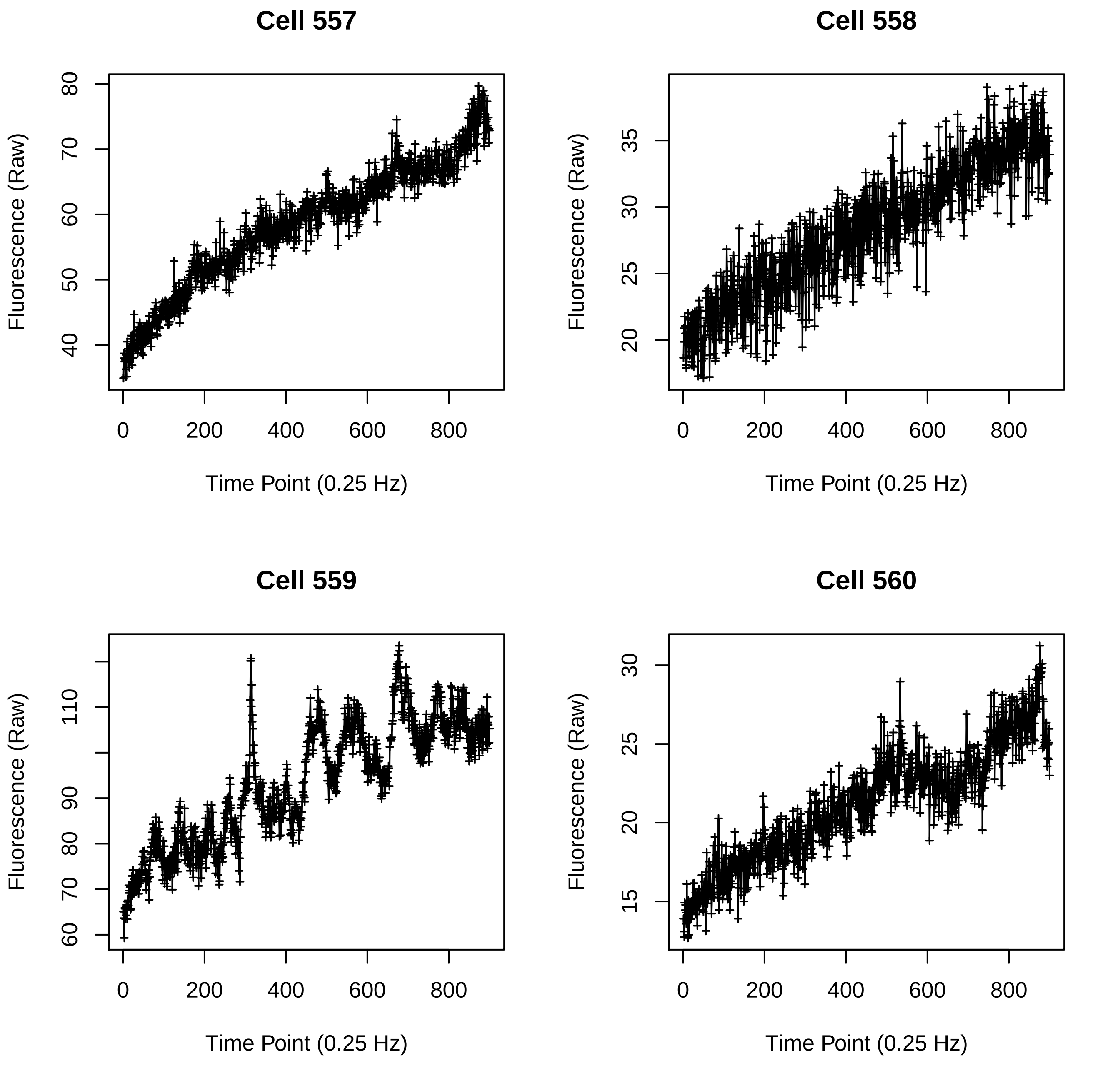
Cell 557
Cell 558
Cell 559
Cell 560
Fluorescence (Raw)
Time Point (0.25 Hz)
0
200
400
600
800
40
50
60
70
80
20
25
30
35
60
70
80
90
110
15

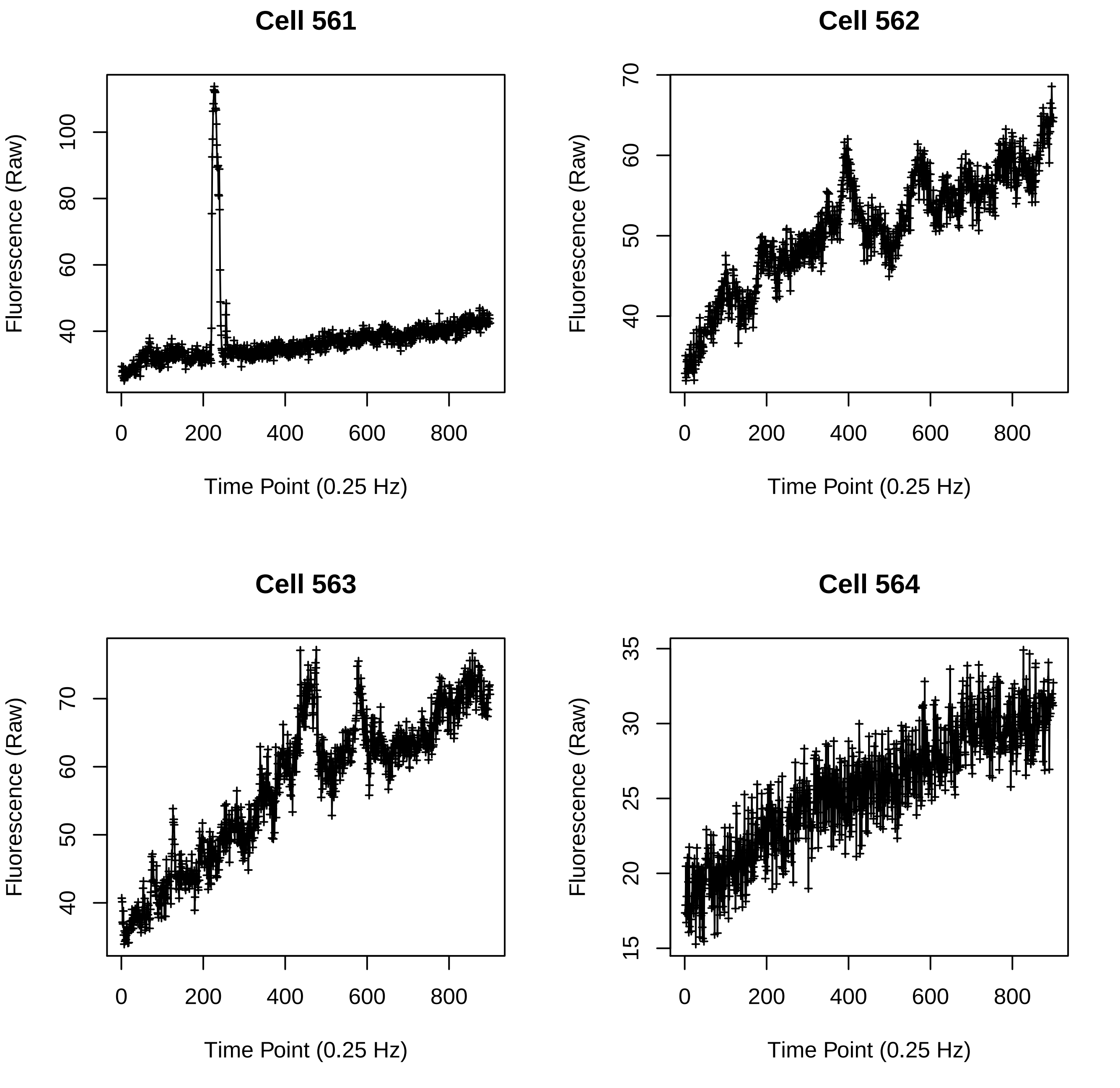
Cell 561
Fluorescence (Raw)
40
60
80
100
0
200
400
600
800
Time Point (0.25 Hz)
Cell 562
Fluorescence (Raw)
40
50
60
70
0
200
400
600
800
Time Point (0.25 Hz)
Cell 563
Fluorescence (Raw)
40
50
60
70
0
200
400
600
800
Time Point (0.25 Hz)
Cell 564
Fluorescence (Raw)
15
20
25
30
35
0
200
400
600
800
Time Point (0.25 Hz)

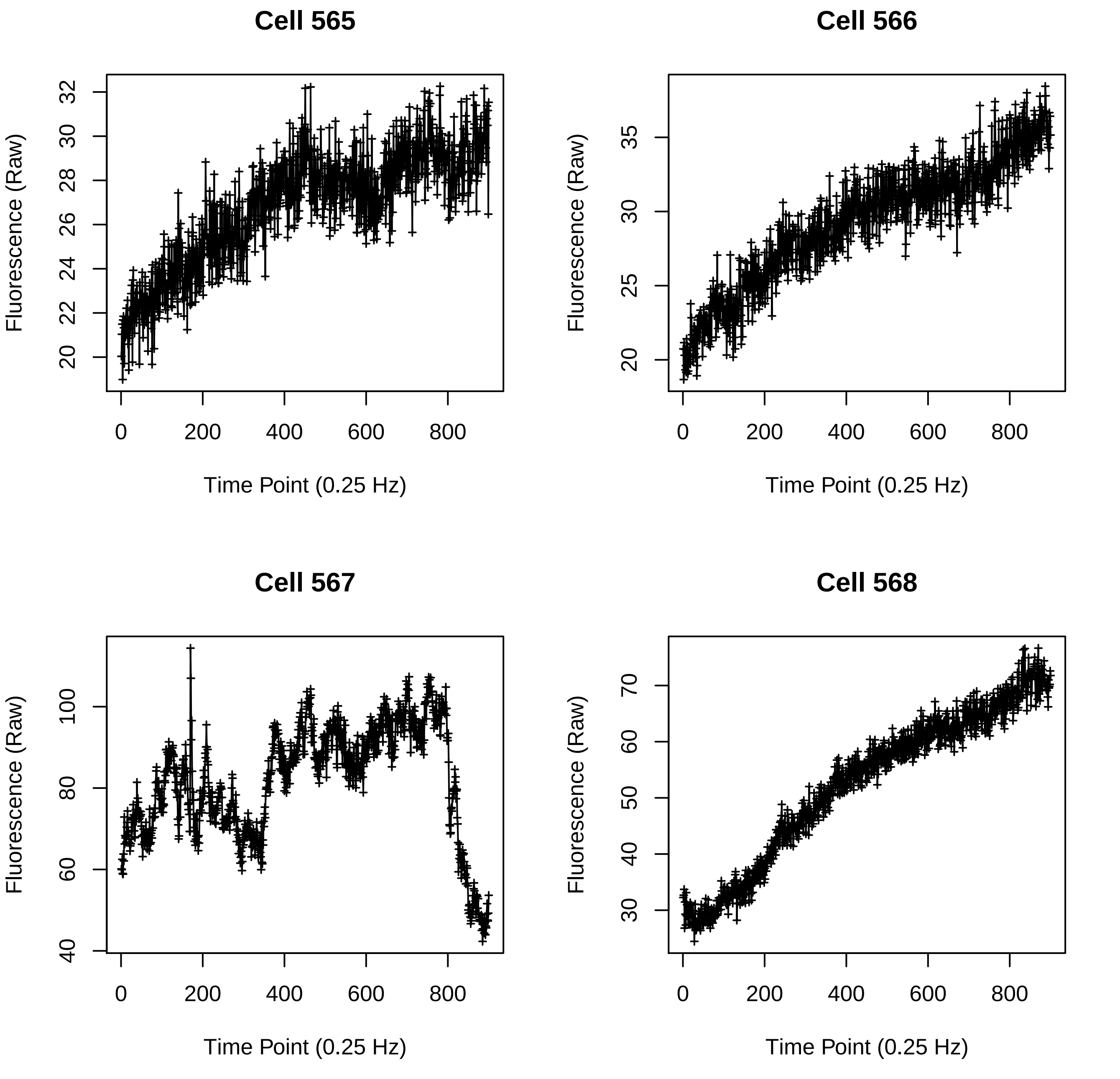

Cell 565
Fluorescence (Raw)
20
22
24
26
28
30
32
Time Point (0.25 Hz)
0
200
400
600
800
Cell 566
Fluorescence (Raw)
20
25
30
35
Time Point (0.25 Hz)
0
200
400
600
800
Cell 567
Fluorescence (Raw)
40
60
80
100
Time Point (0.25 Hz)
0
200
400
600
800
Cell 568
Fluorescence (Raw)
30
40
50
60
70
Time Point (0.25 Hz)
0
200
400
600
800

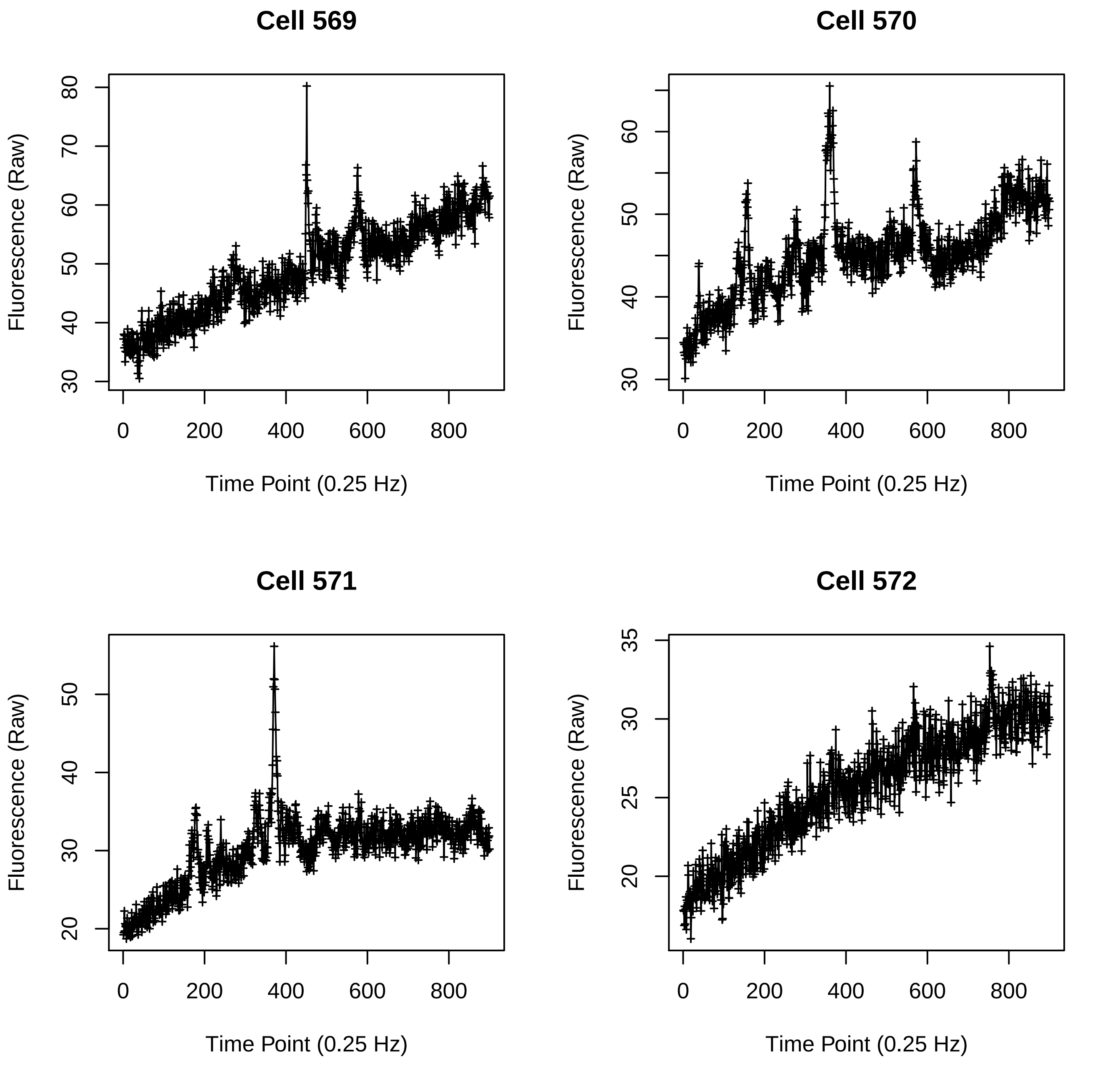

Cell 569
Cell 570
Cell 571
Cell 572
Fluorescence (Raw)
Time Point (0.25 Hz)
0
200
400
600
800
30
40
50
60
70
80
20
25
35

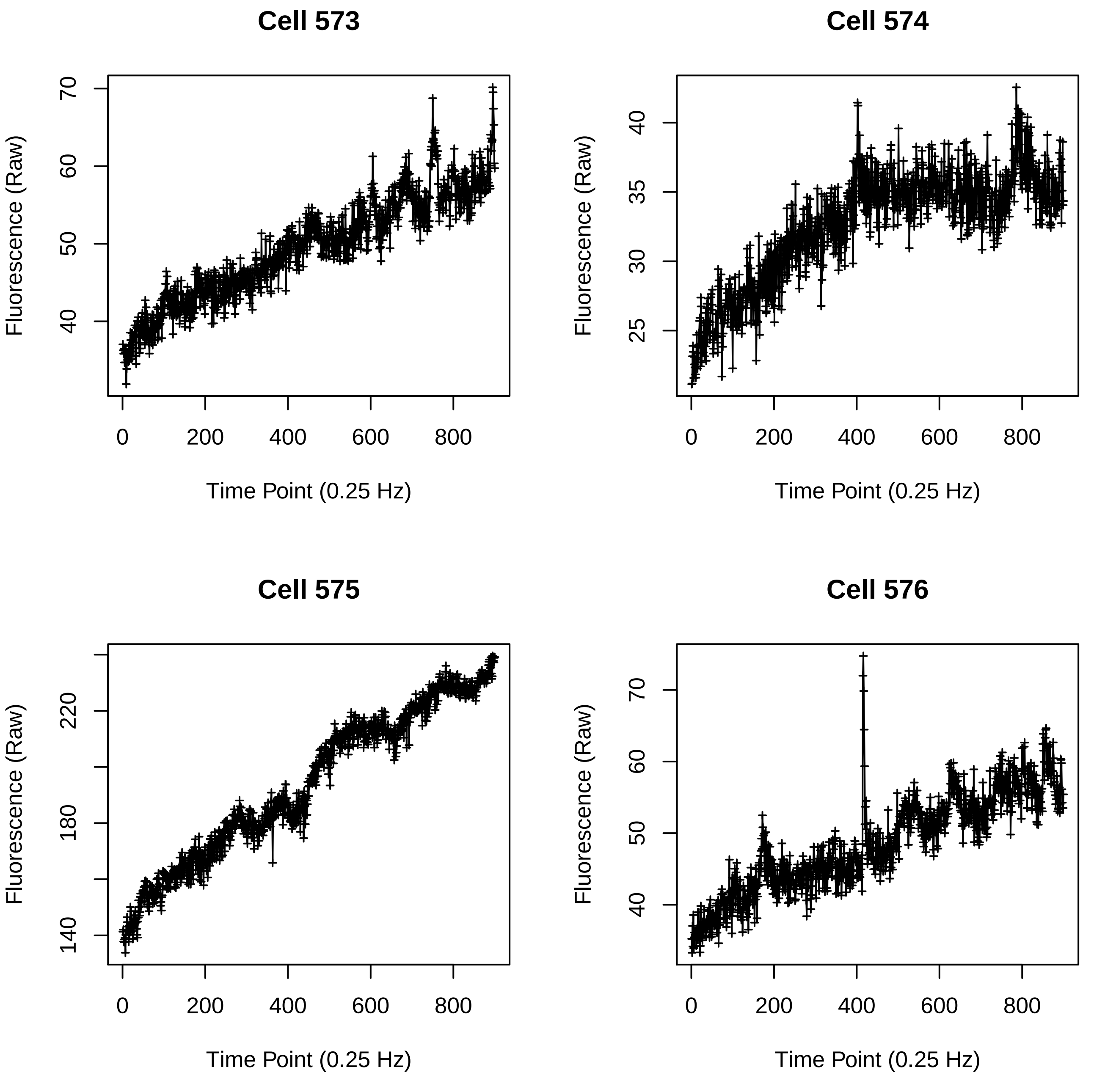

Cell 573
Fluorescence (Raw)
40
50
60
70
0
200
400
600
800
Time Point (0.25 Hz)
Cell 574
Fluorescence (Raw)
25
30
35
40
0
200
400
600
800
Time Point (0.25 Hz)
Cell 575
Fluorescence (Raw)
140
180
220
0
200
400
600
800
Time Point (0.25 Hz)
Cell 576
Fluorescence (Raw)
40
50
60
70
0
200
400
600
800
Time Point (0.25 Hz)

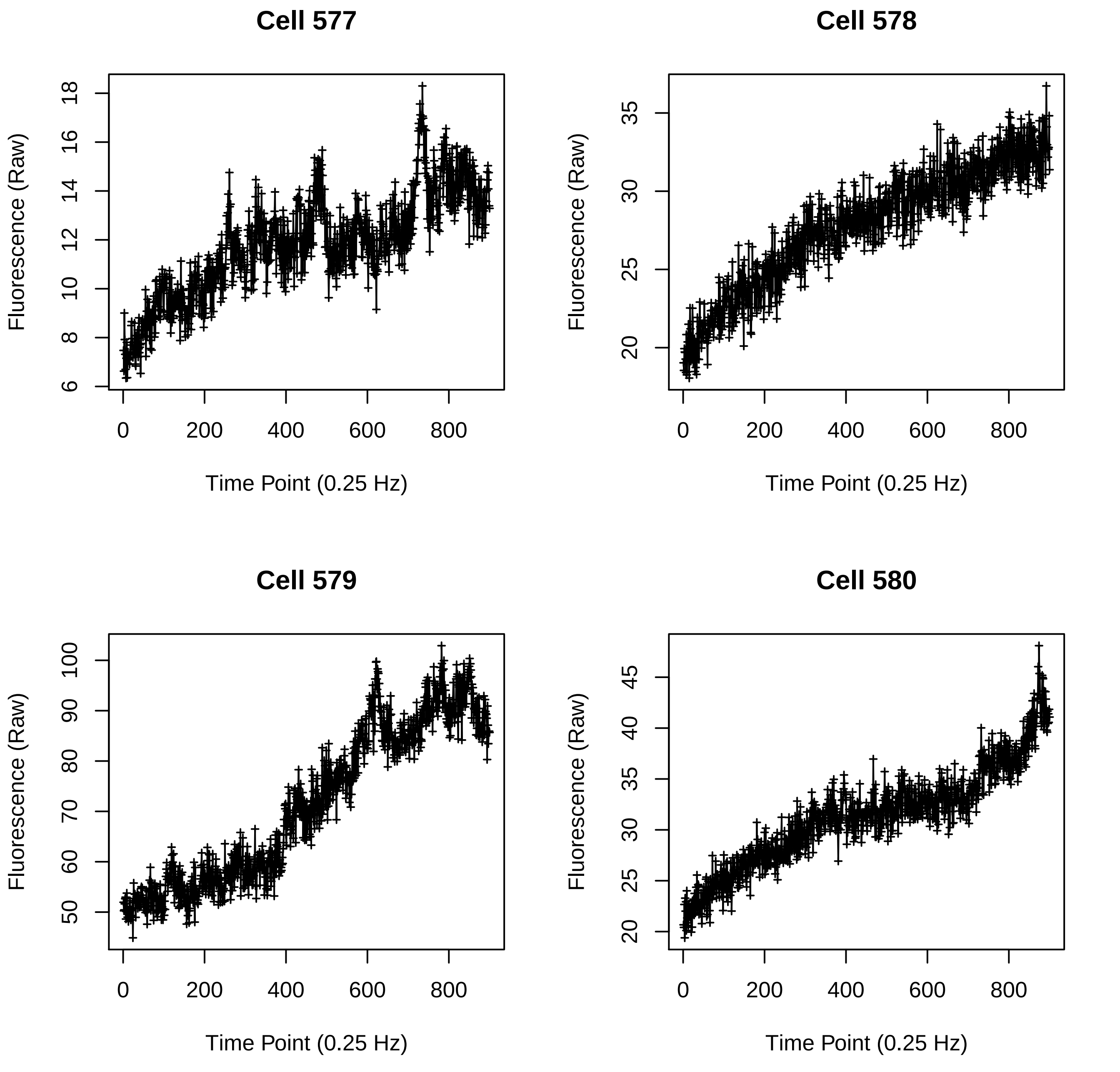
Cell 577
Cell 578
Cell 579
Cell 580
Fluorescence (Raw)
Time Point (0.25 Hz)
6
8
10
12
14
16
18
20
25
30
35
40
45
50
60
70
80
90
100
0
200
400
600
800

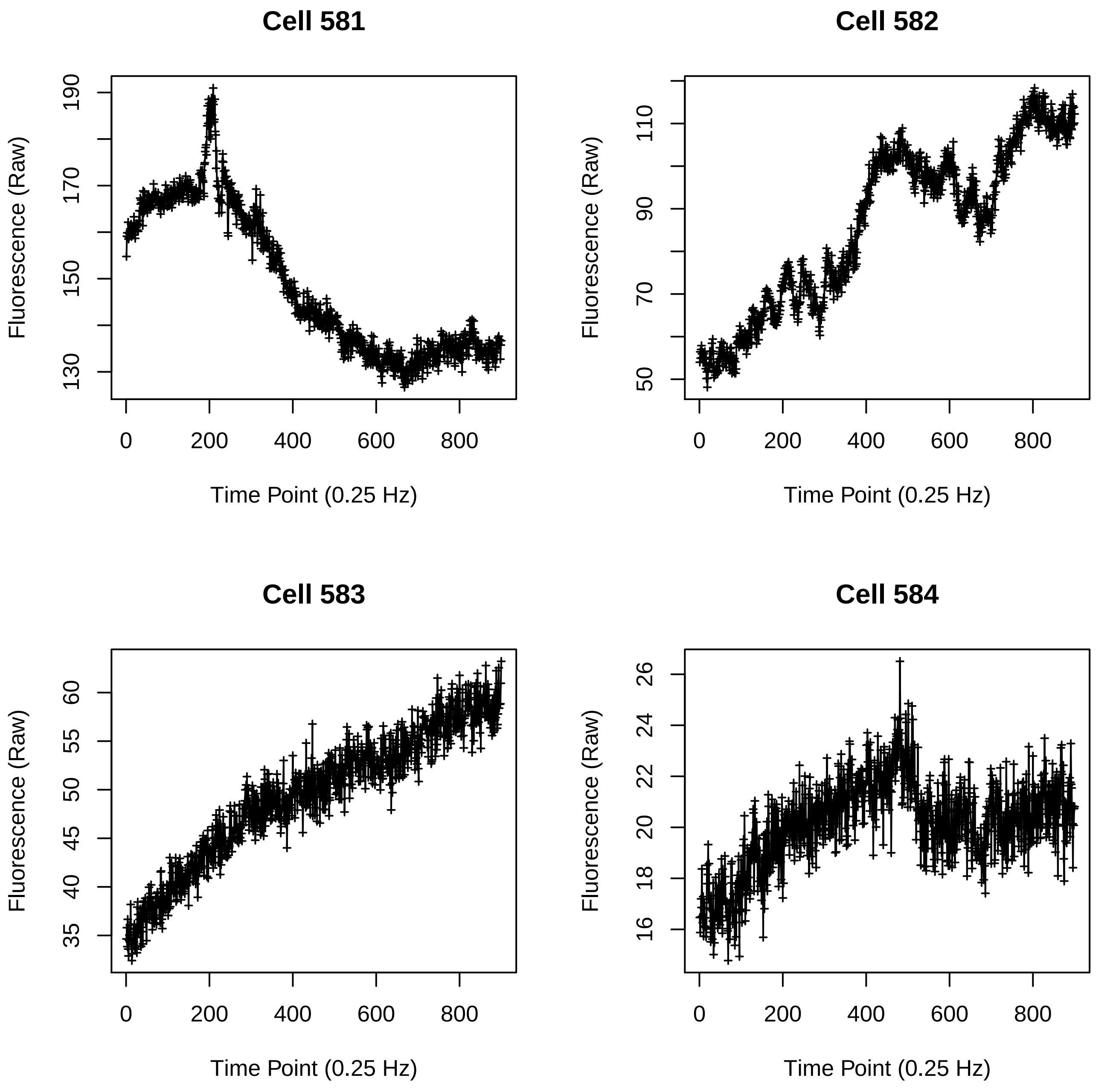
Cell 581
Cell 582
Cell 583
Cell 584
Fluorescence (Raw)
Time Point (0.25 Hz)
130
150
170
190
50
70
90
110
35
40
45
50
55
60
16
18
20
22
24
26
0
200
400
600
800

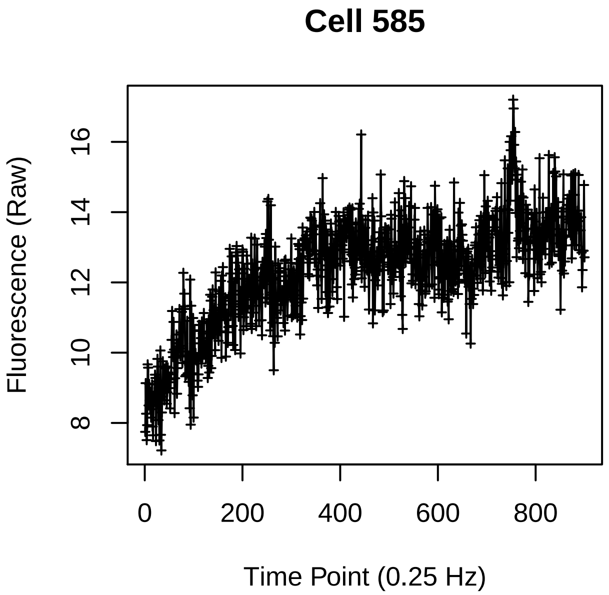
Cell 585
Fluorescence (Raw)
Time Point (0.25 Hz)
8
10
12
14
16
0
200
400
600
800

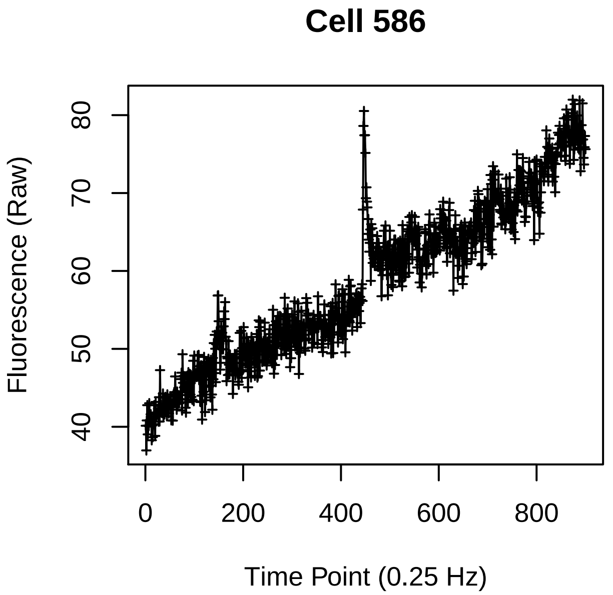
Cell 586
Fluorescence (Raw)
Time Point (0.25 Hz)
40
50
60
70
80
0
200
400
600
800

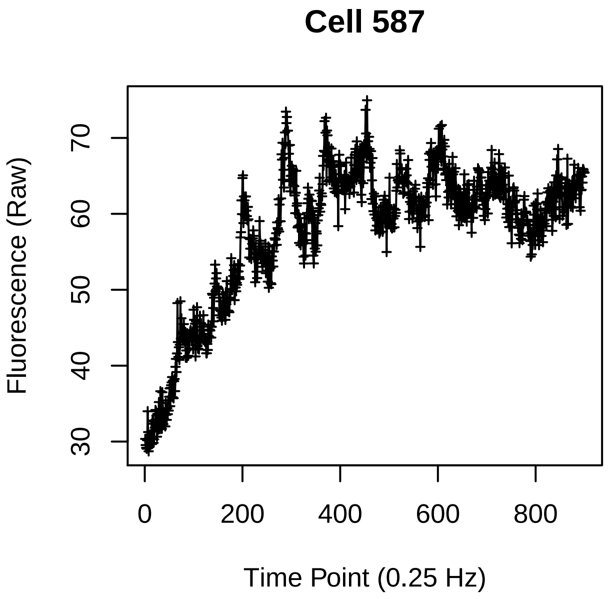
Cell 587
Fluorescence (Raw)
Time Point (0.25 Hz)
30
40
50
60
70
0
200
400
600
800

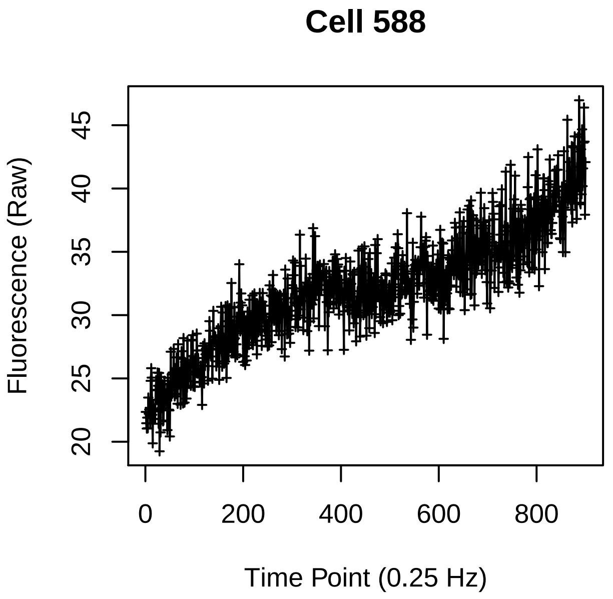
Cell 588
Fluorescence (Raw)
Time Point (0.25 Hz)
20
25
30
35
40
45
0
200
400
600
800

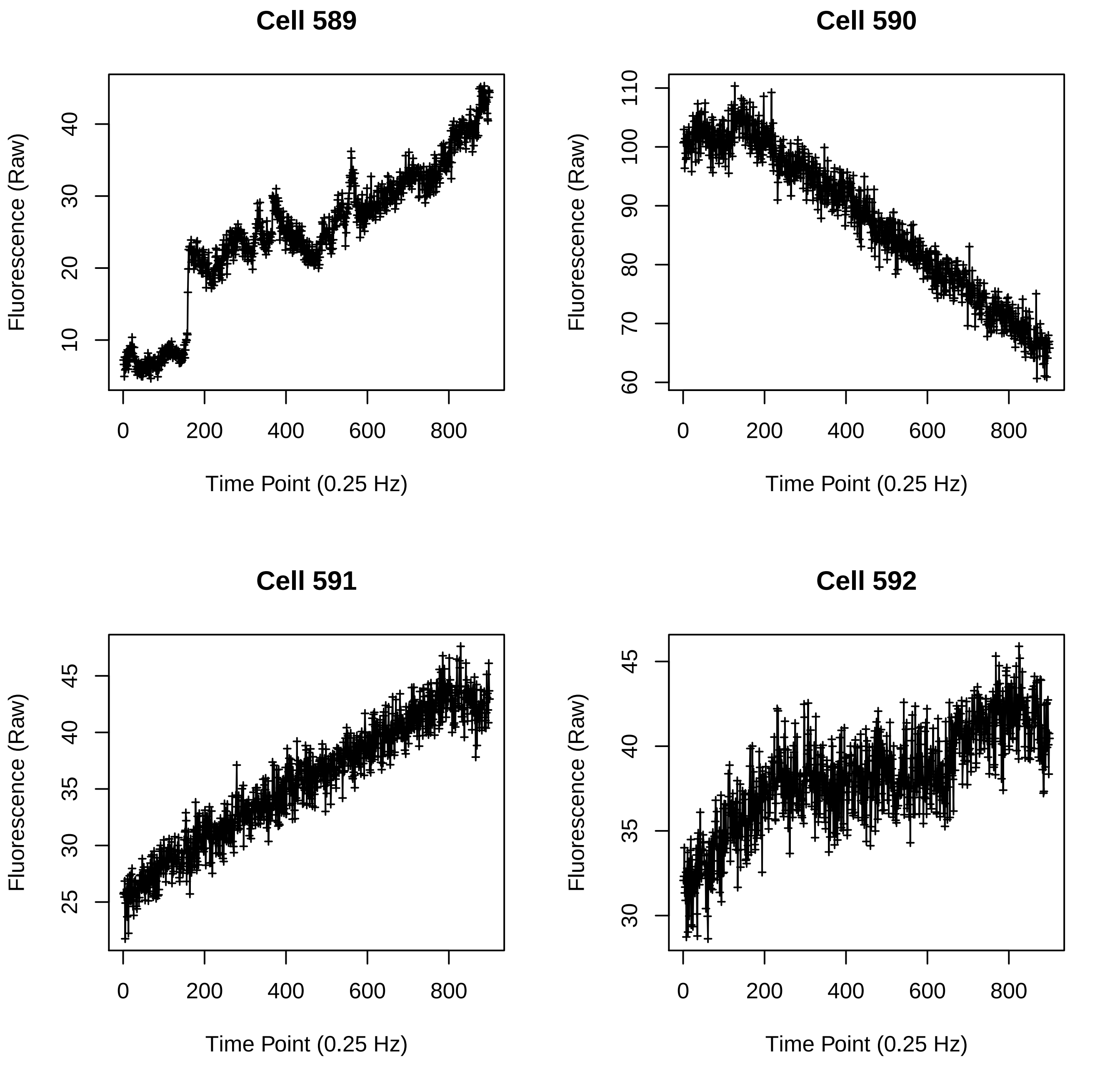
Cell 589
Cell 590
Cell 591
Cell 592
Fluorescence (Raw)
Time Point (0.25 Hz)
0
200
400
600
800
10
20
30
40
60
70
80
90
100
110
25
30
35
40
45

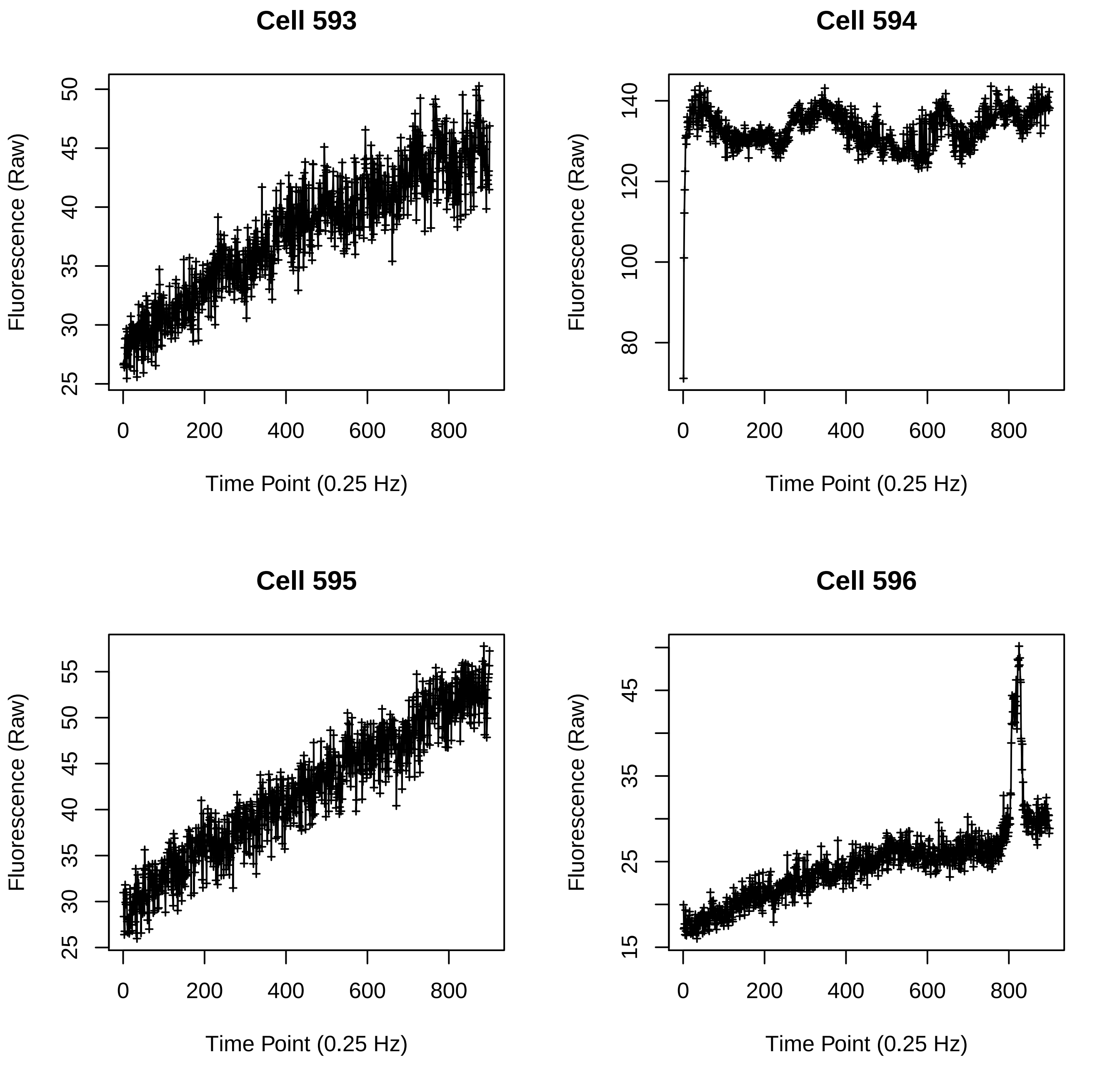
Cell 593
Fluorescence (Raw)
25
30
35
40
45
50
0
200
400
600
800
Time Point (0.25 Hz)
Cell 594
Fluorescence (Raw)
80
100
120
140
0
200
400
600
800
Time Point (0.25 Hz)
Cell 595
Fluorescence (Raw)
25
30
35
40
45
50
55
0
200
400
600
800
Time Point (0.25 Hz)
Cell 596
Fluorescence (Raw)
15
25
35
45
0
200
400
600
800
Time Point (0.25 Hz)

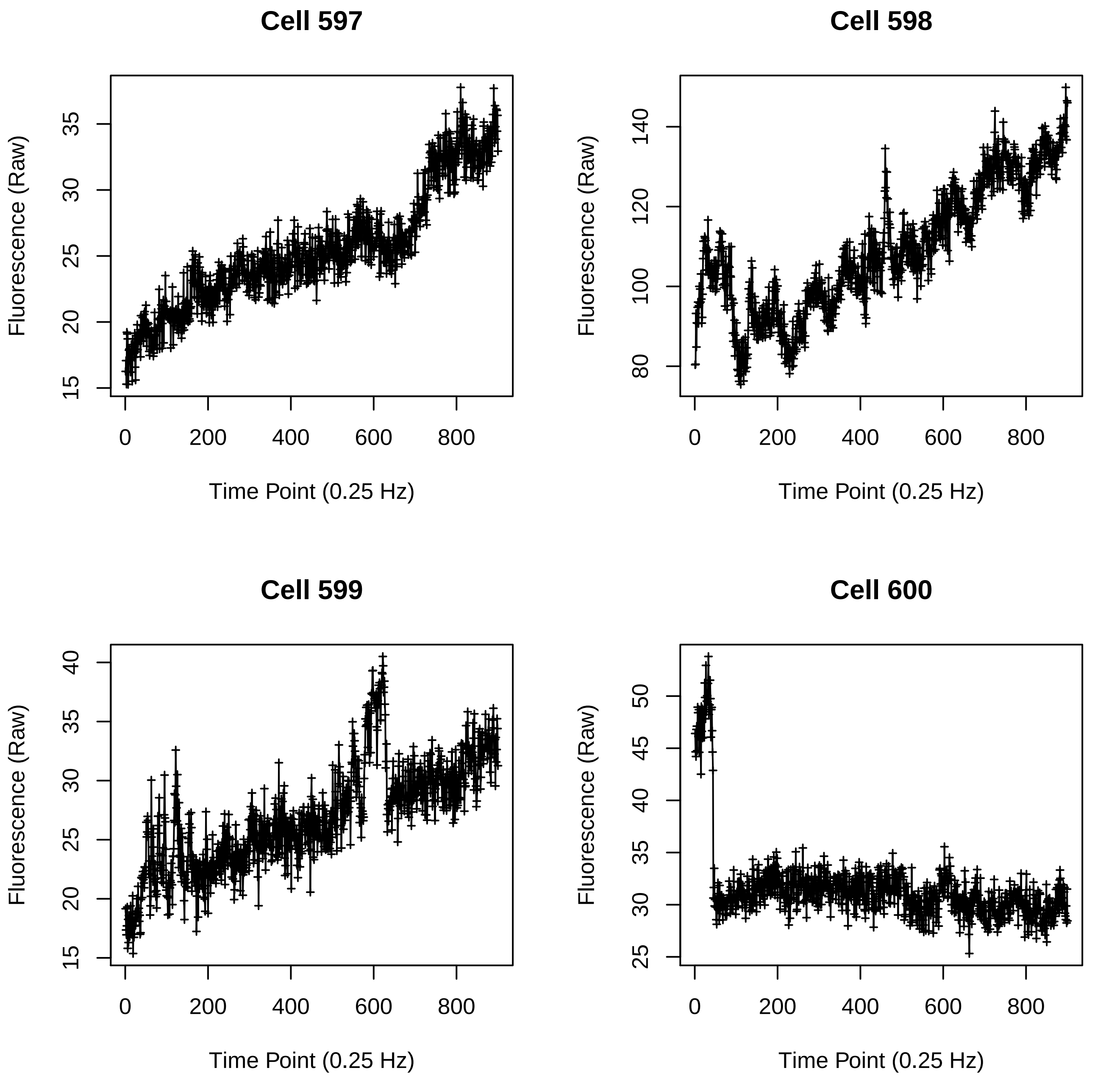

Cell 597
Cell 598
Cell 599
Cell 600
Fluorescence (Raw)
Time Point (0.25 Hz)
0
200
400
600
800
15
20
25
30
35
40
45
50
80
100
120
140

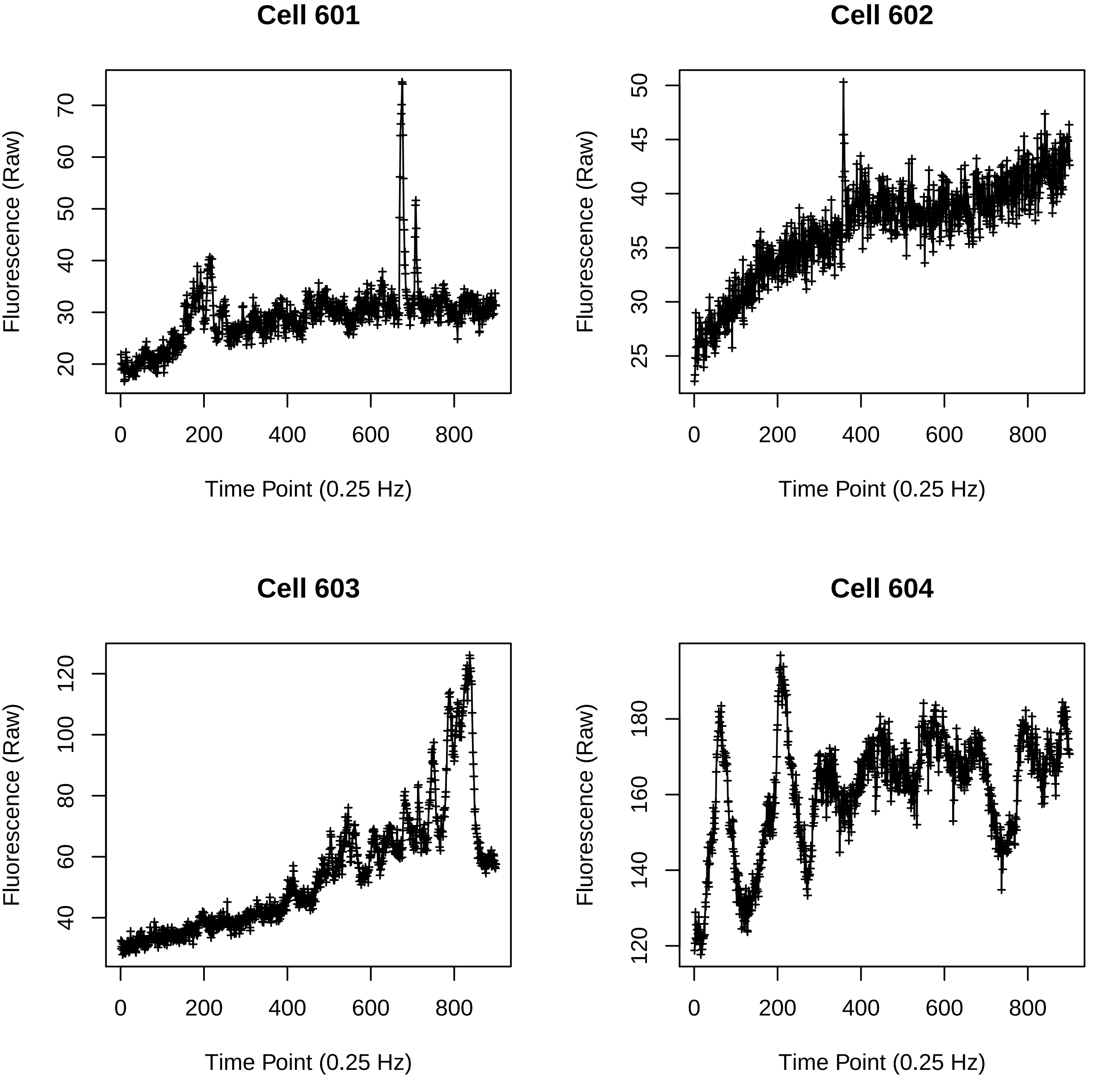
Cell 601
Cell 602
Cell 603
Cell 604
Fluorescence (Raw)
Time Point (0.25 Hz)
0
200
400
600
800
20
30
40
50
60
70
25
35
45
80
100
120
140
160
180

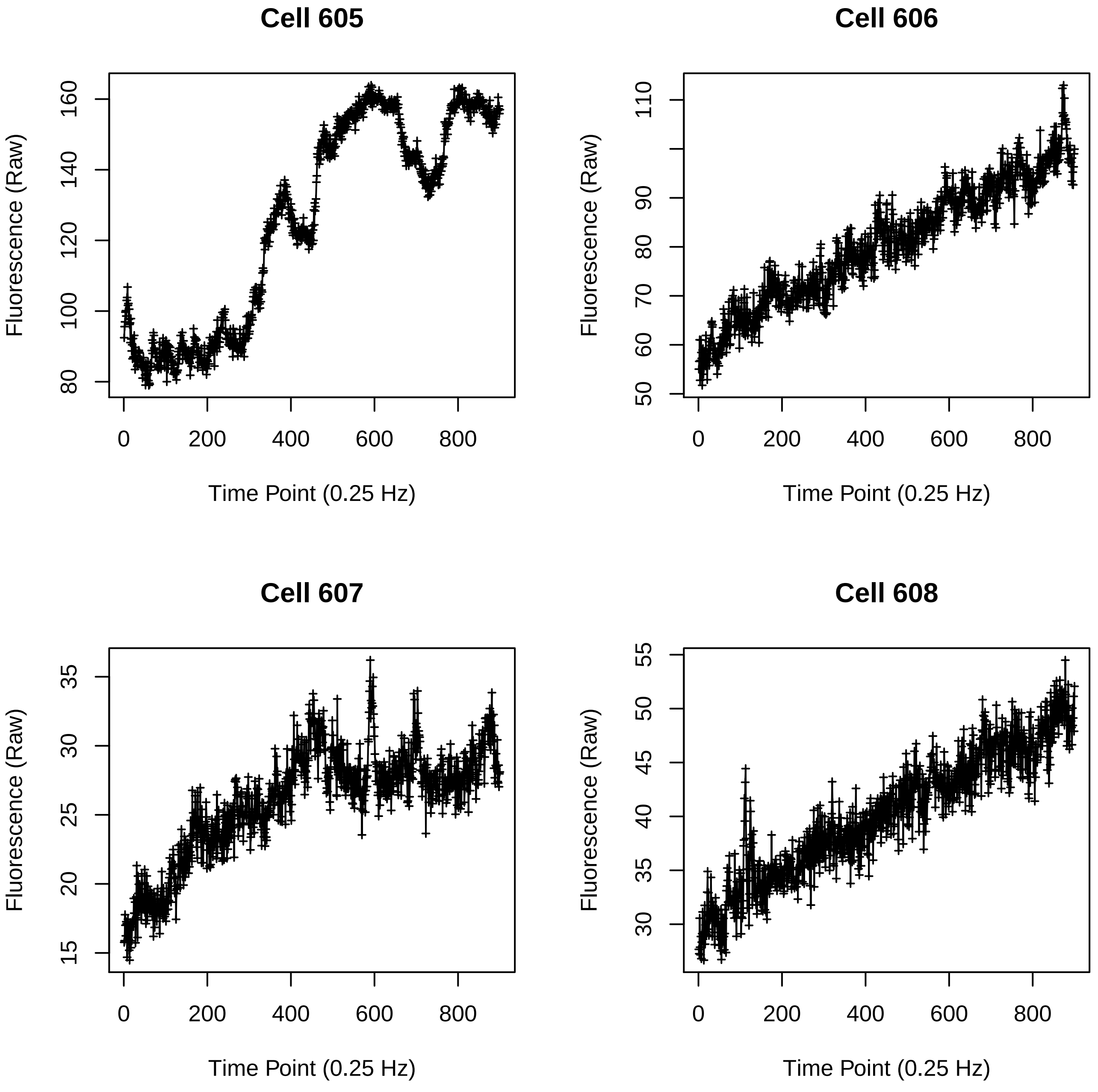

Cell 605
Cell 606
Cell 607
Cell 608
Fluorescence (Raw)
Time Point (0.25 Hz)
0
200
400
600
800
80
100
120
140
160
50
60
70
80
90
110
15
20
25
30
35
30
35
40
45
50
55

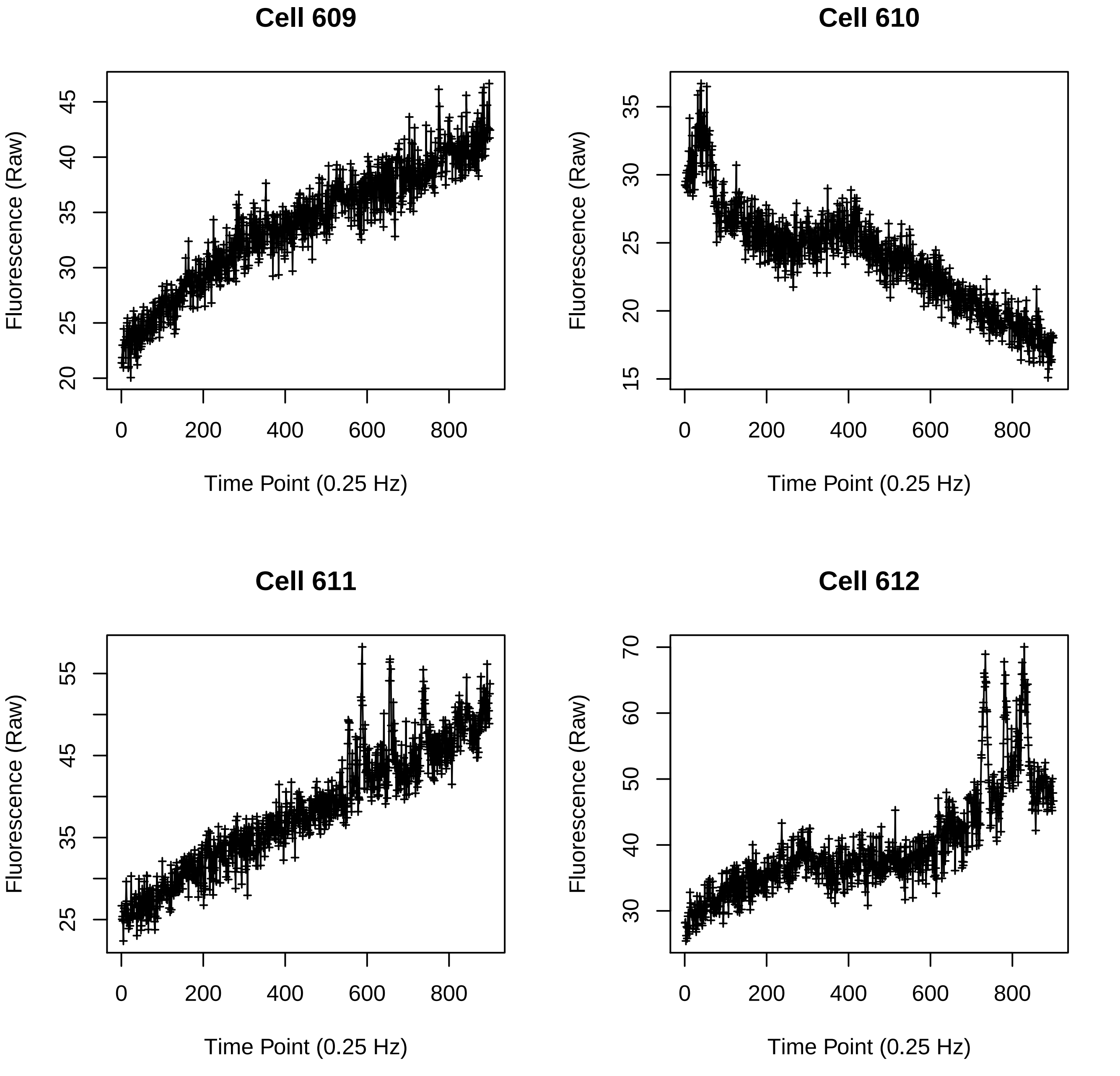
Cell 609
Fluorescence (Raw)
20
25
30
35
40
45
0
200
400
600
800
Time Point (0.25 Hz)
Cell 610
Fluorescence (Raw)
15
20
25
30
35
0
200
400
600
800
Time Point (0.25 Hz)
Cell 611
Fluorescence (Raw)
25
35
45
55
0
200
400
600
800
Time Point (0.25 Hz)
Cell 612
Fluorescence (Raw)
30
40
50
60
70
0
200
400
600
800
Time Point (0.25 Hz)

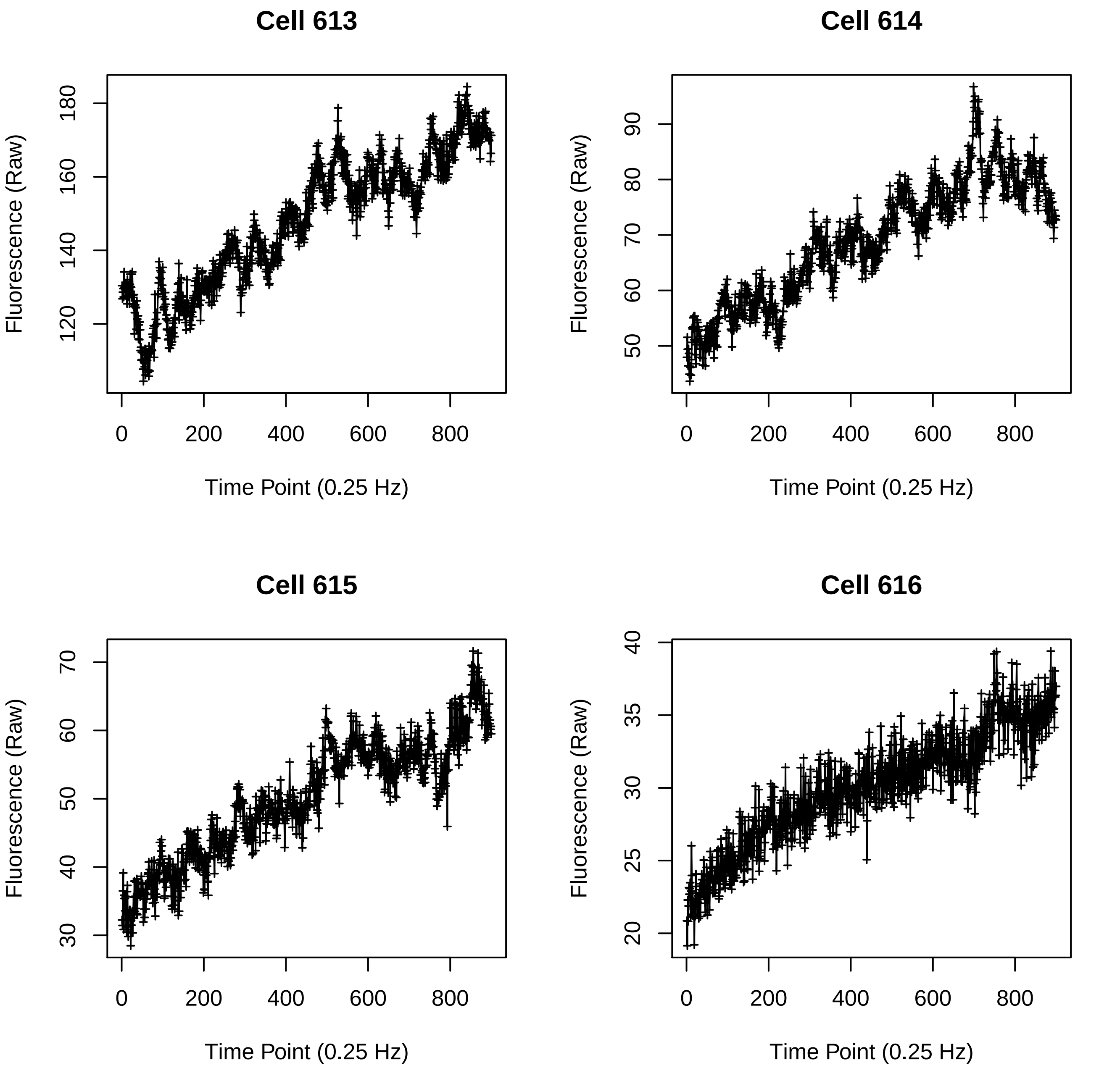

Cell 613
Fluorescence (Raw)
120
140
160
180
0
200
400
600
800
Time Point (0.25 Hz)
Cell 614
Fluorescence (Raw)
50
60
70
80
90
0
200
400
600
800
Time Point (0.25 Hz)
Cell 615
Fluorescence (Raw)
30
40
50
60
70
0
200
400
600
800
Time Point (0.25 Hz)
Cell 616
Fluorescence (Raw)
20
25
30
35
40
0
200
400
600
800
Time Point (0.25 Hz)

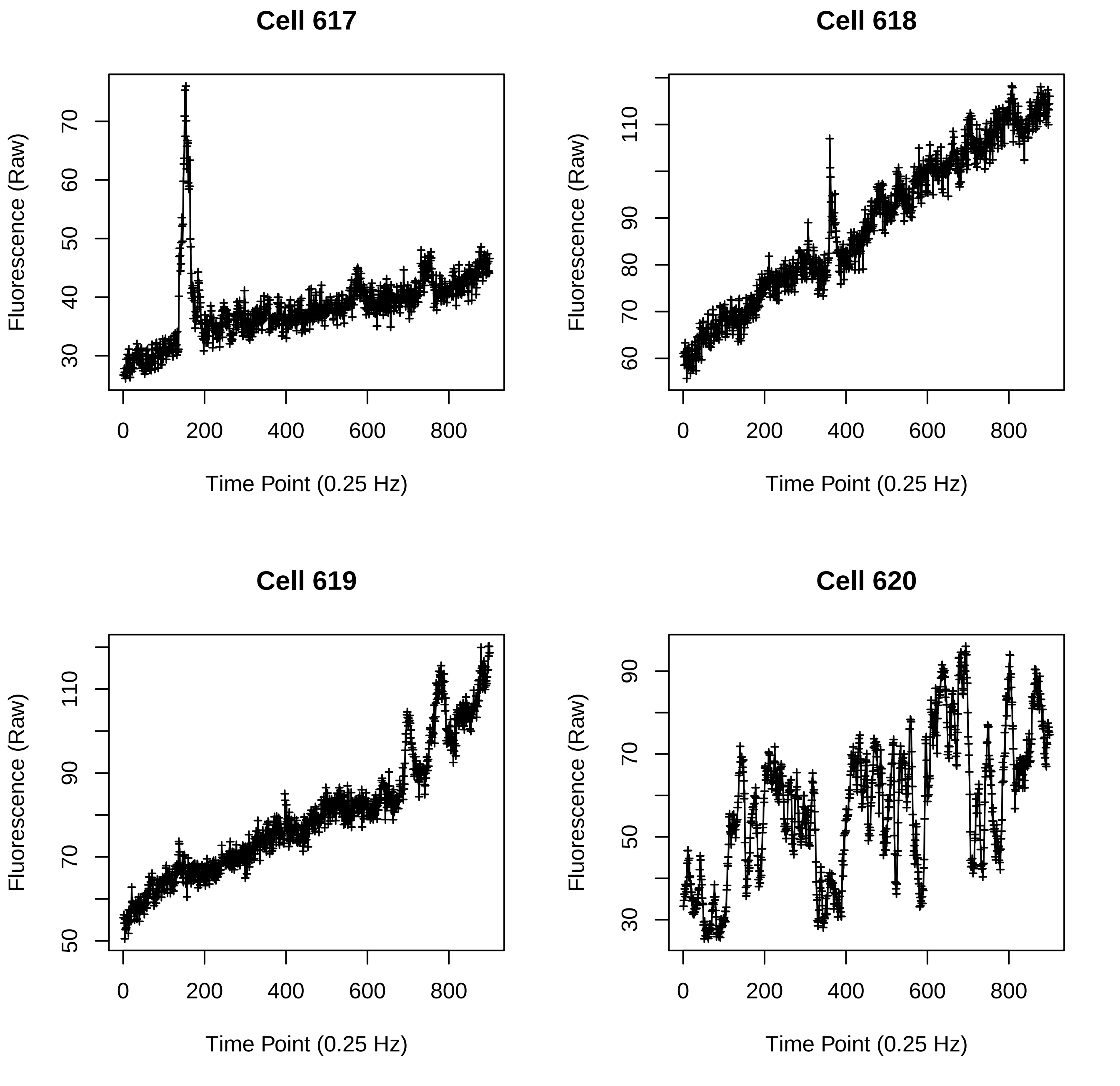

Cell 617
Cell 618
Cell 619
Cell 620
Fluorescence (Raw)
Time Point (0.25 Hz)
0
200
400
600
800
30
40
50
60
70
80
90
110

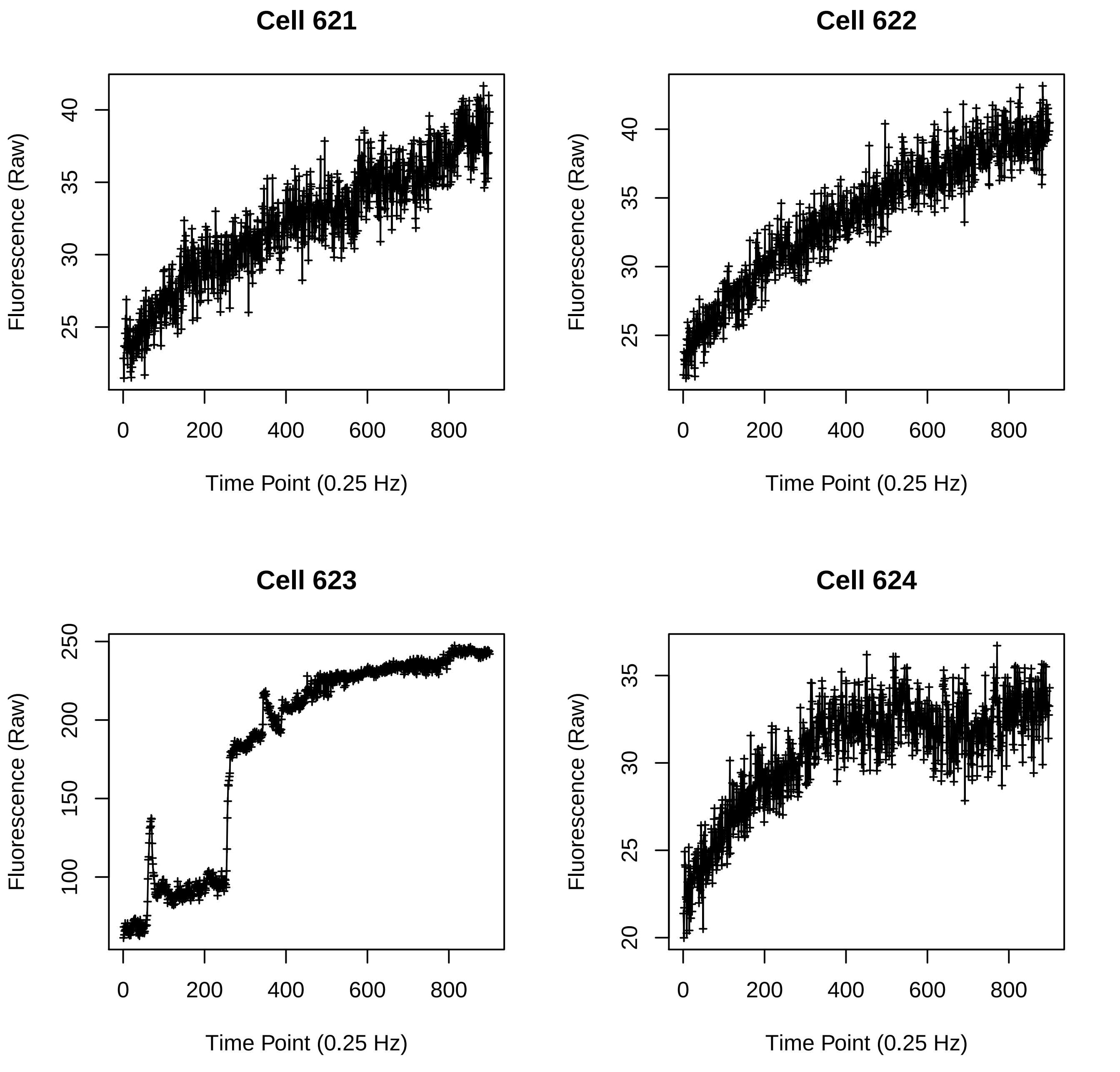

Cell 621
Cell 622
Cell 623
Cell 624
Fluorescence (Raw)
Time Point (0.25 Hz)
0
200
400
600
800
25
30
35
40
100
150
200
250
20

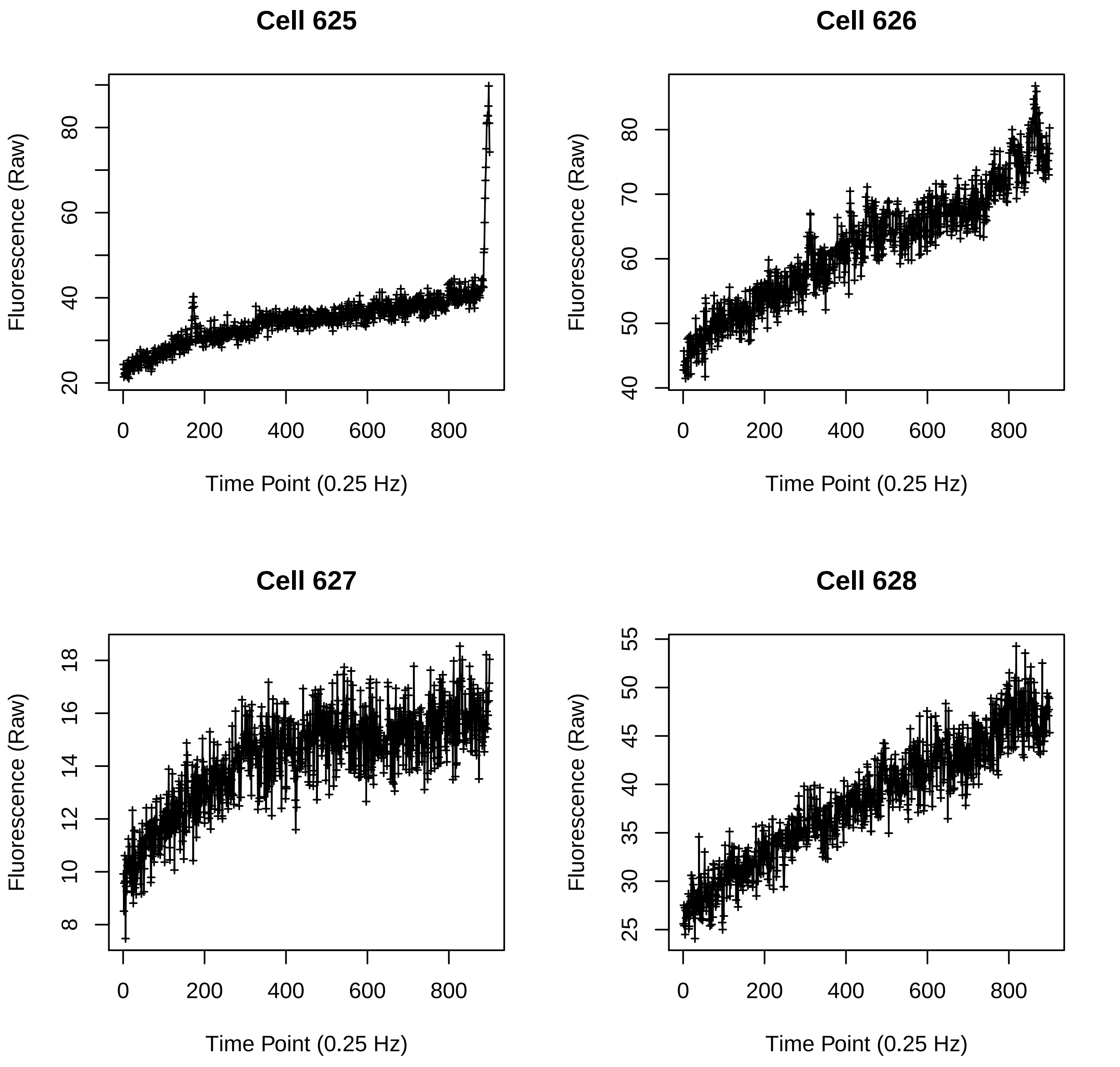

Cell 625
Cell 626
Cell 627
Cell 628
Fluorescence (Raw)
Time Point (0.25 Hz)
0
200
400
600
800

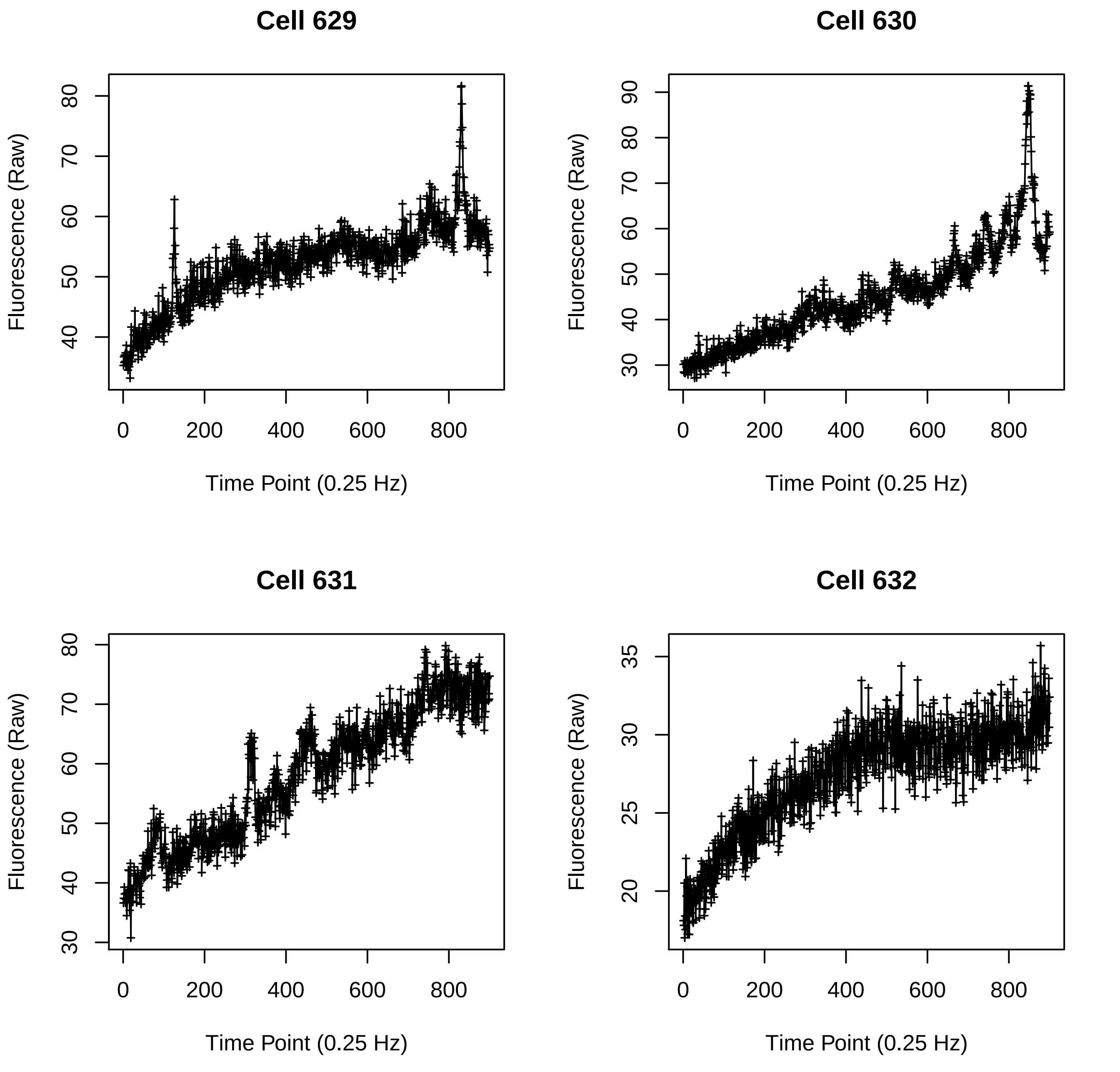
Cell 629
Cell 630
Cell 631
Cell 632
Fluorescence (Raw)
Time Point (0.25 Hz)
0
200
400
600
800

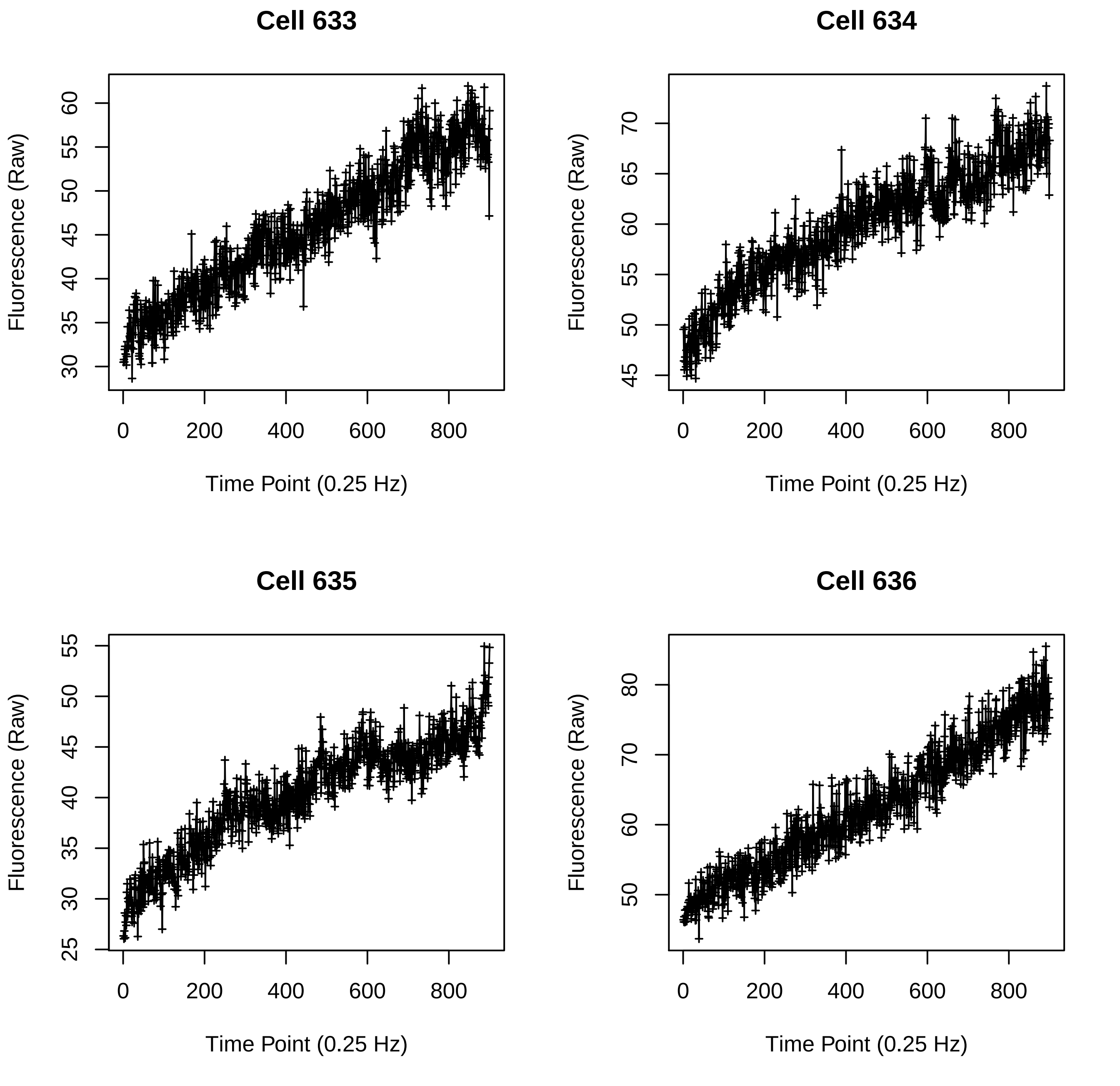
Cell 633
Cell 634
Cell 635
Cell 636
Fluorescence (Raw)
Time Point (0.25 Hz)
0
200
400
600
800
30
35
40
45
50
55
60
65
70
25
80

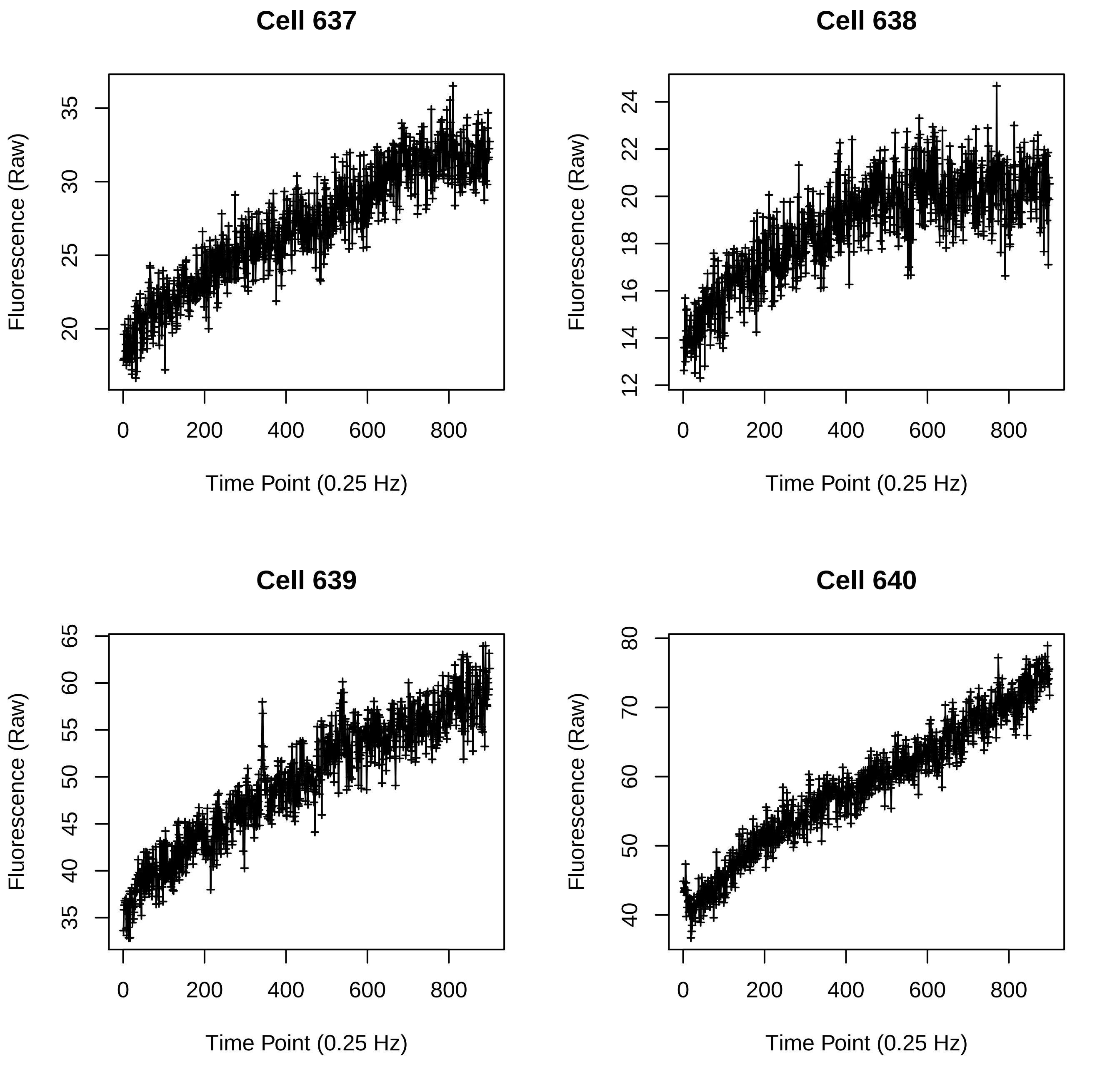
Cell 637
Fluorescence (Raw)
Time Point (0.25 Hz)
20
25
30
35
0
200
400
600
800
Cell 638
Fluorescence (Raw)
Time Point (0.25 Hz)
12
14
16
18
20
22
24
0
200
400
600
800
Cell 639
Fluorescence (Raw)
Time Point (0.25 Hz)
35
40
45
50
55
60
65
0
200
400
600
800
Cell 640
Fluorescence (Raw)
Time Point (0.25 Hz)
40
50
60
70
80
0
200
400
600
800

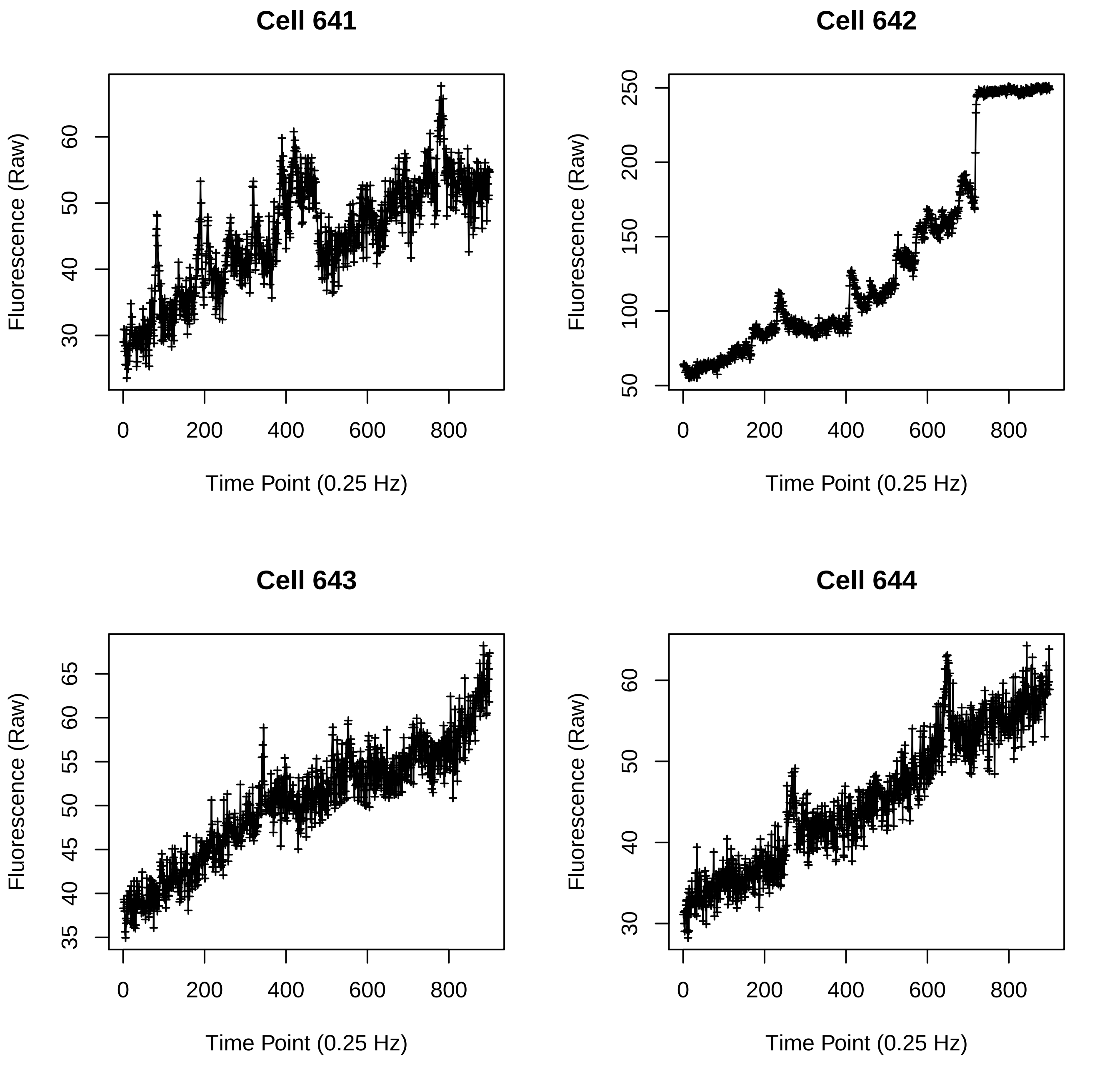
Cell 641
Fluorescence (Raw)
Time Point (0.25 Hz)
30
40
50
60
0
200
400
600
800
Cell 642
Fluorescence (Raw)
Time Point (0.25 Hz)
50
100
150
200
250
Cell 643
Fluorescence (Raw)
Time Point (0.25 Hz)
35
40
45
50
55
60
65
Cell 644
Fluorescence (Raw)
Time Point (0.25 Hz)

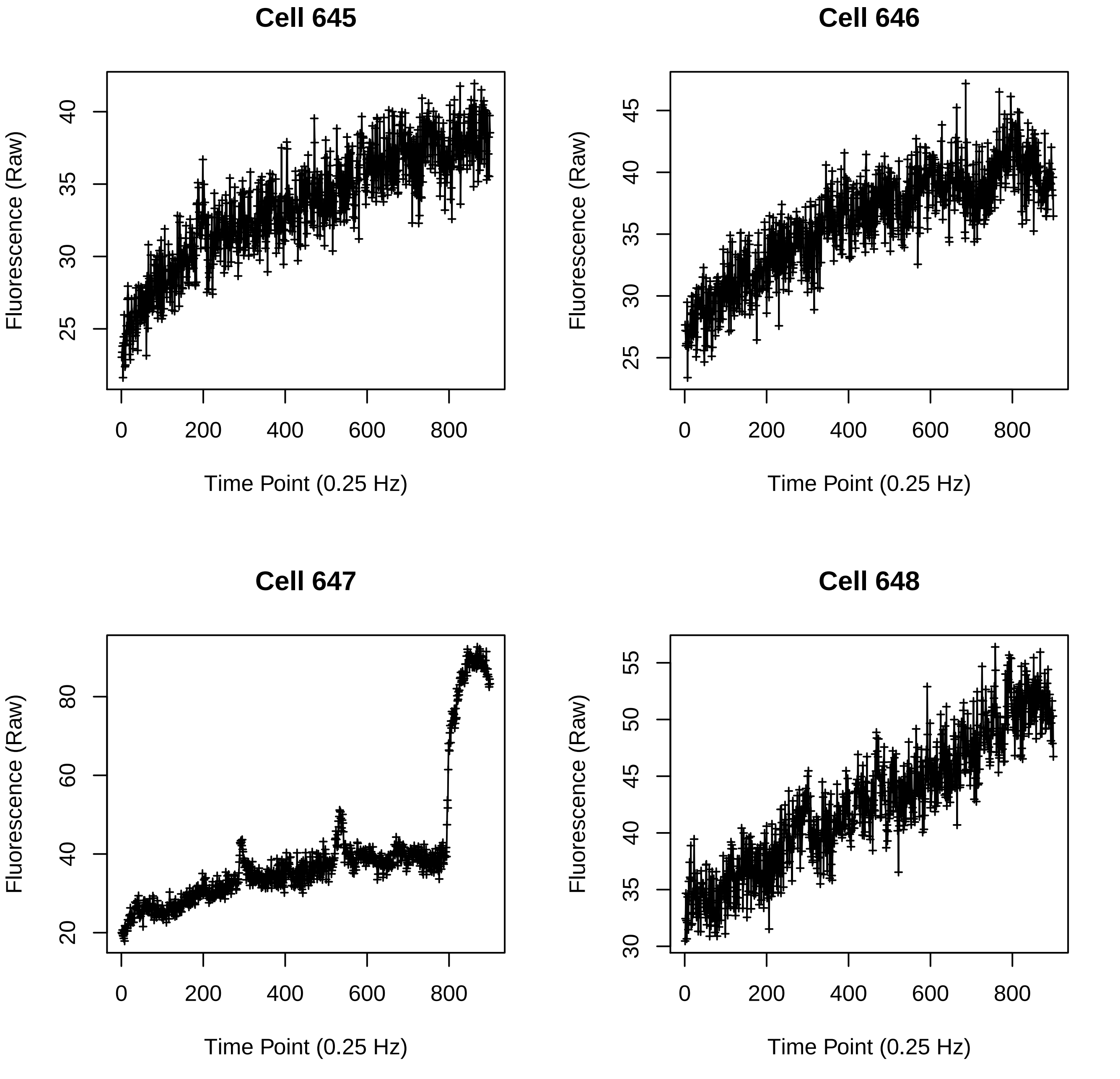
Cell 645
Cell 646
Cell 647
Cell 648
Fluorescence (Raw)
Time Point (0.25 Hz)
0
200
400
600
800
25
30
35
40
45
20
60
80
50
55

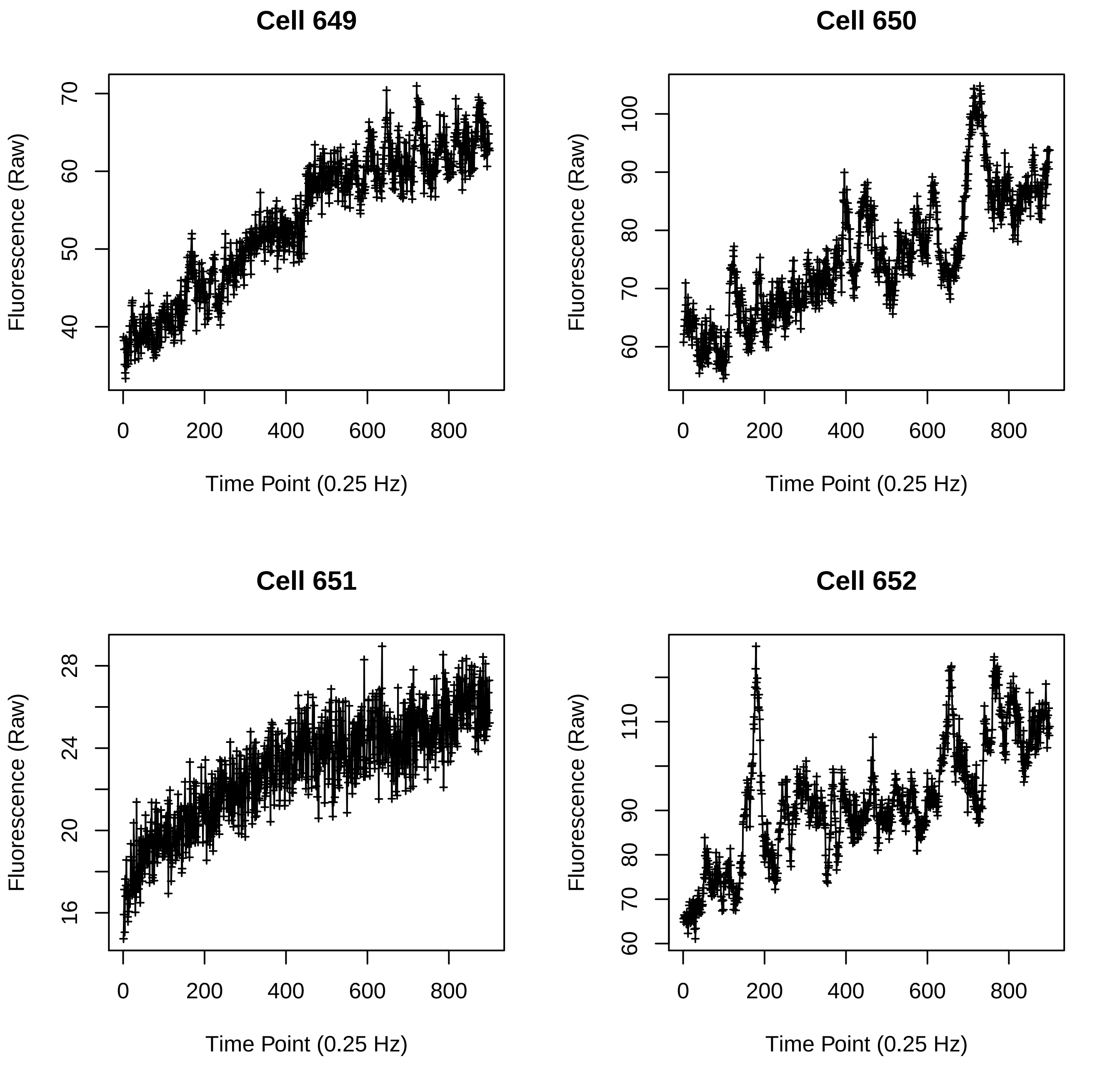
Cell 649
Cell 650
Cell 651
Cell 652
Fluorescence (Raw)
Time Point (0.25 Hz)
0
200
400
600
800
40
50
60
70
60
70
80
90
100
16
20
24
28
60
70
80
90
110

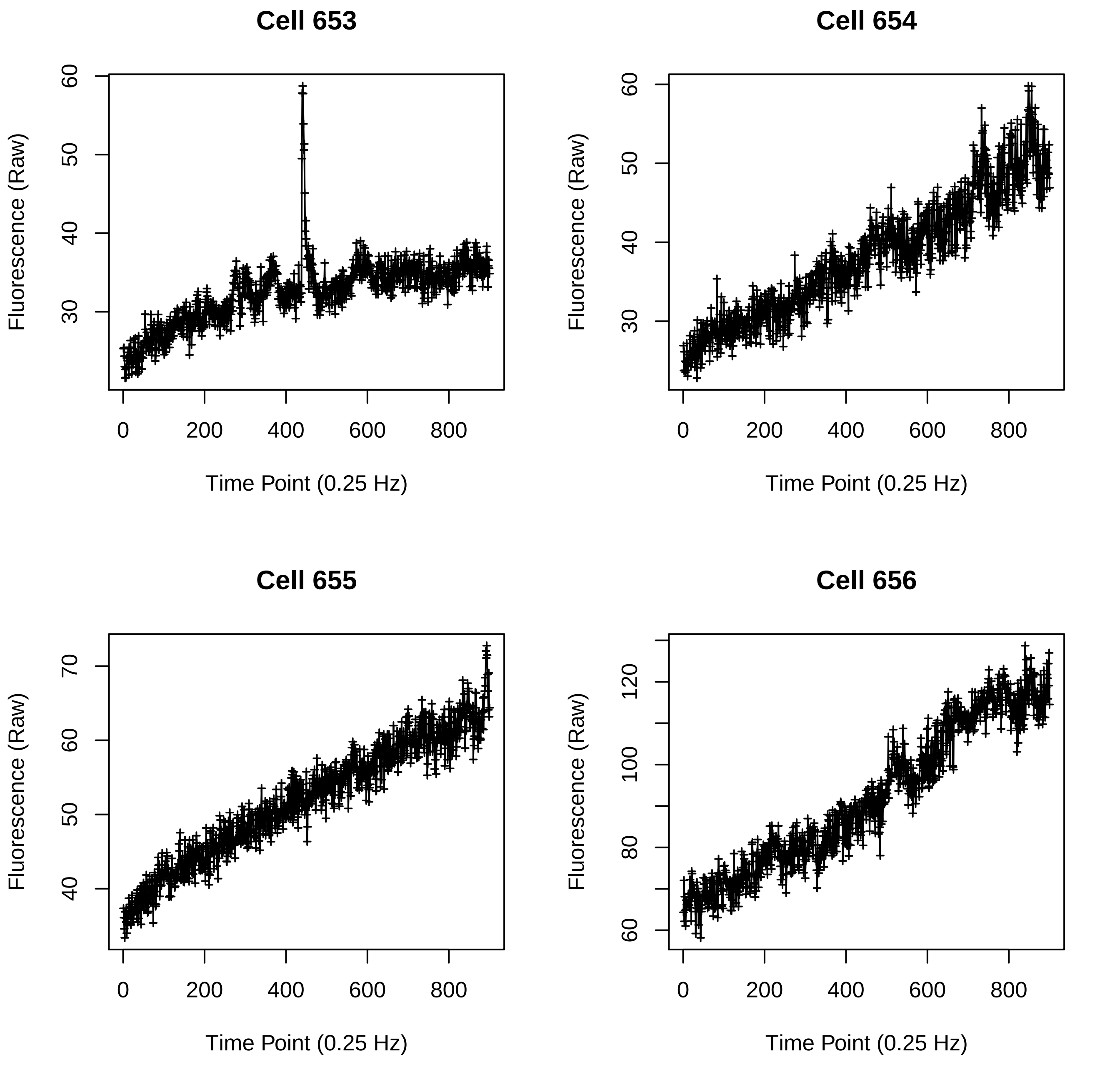
Cell 653
Cell 654
Cell 655
Cell 656
Fluorescence (Raw)
Time Point (0.25 Hz)
0
200
400
600
800
30
40
50
60
70
80
100
120

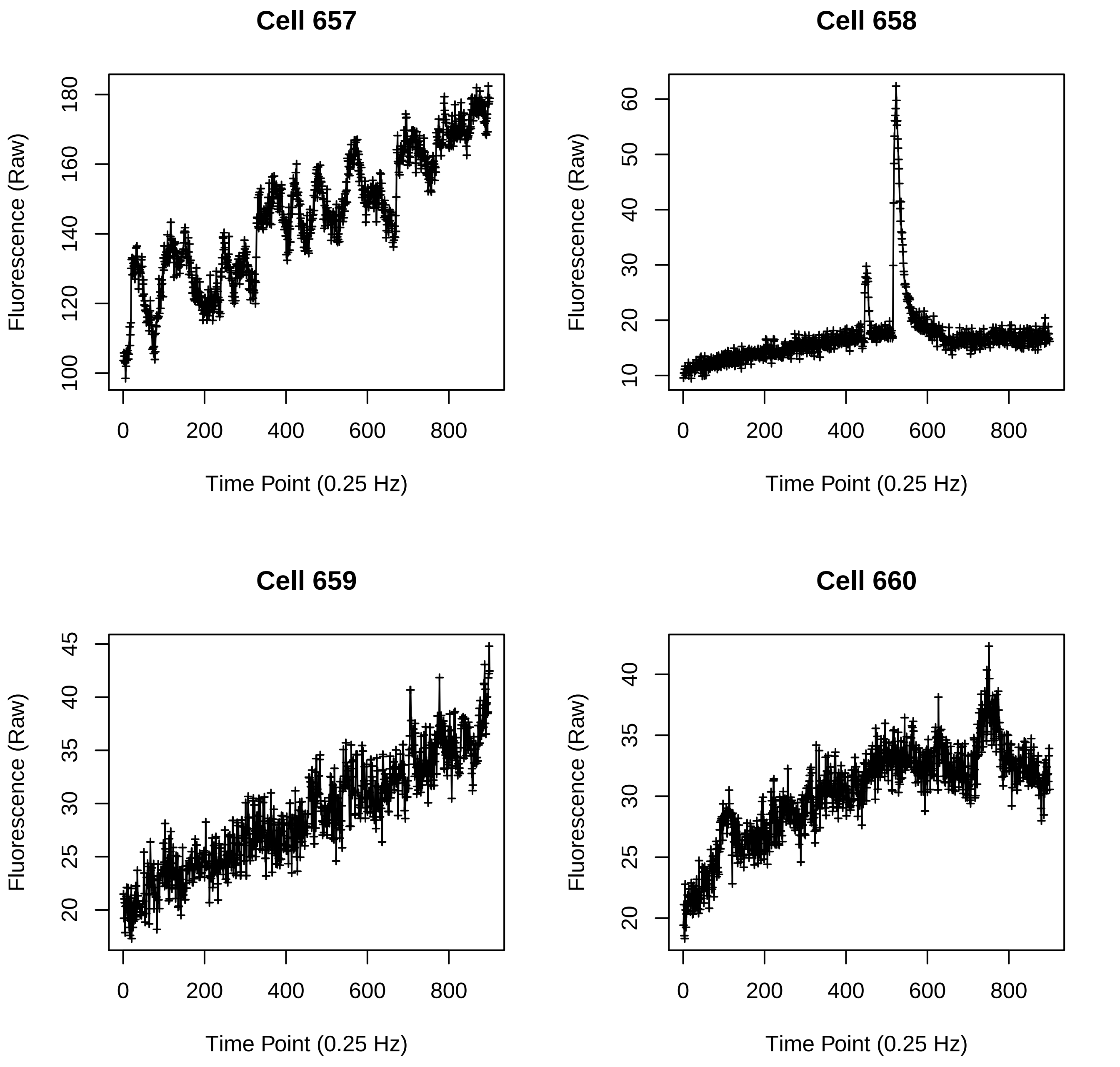
Cell 657
Fluorescence (Raw)
100
120
140
160
180
0
200
400
600
800
Time Point (0.25 Hz)
Cell 658
Fluorescence (Raw)
10
20
30
40
50
60
0
200
400
600
800
Time Point (0.25 Hz)
Cell 659
Fluorescence (Raw)
20
25
30
35
40
45
0
200
400
600
800
Time Point (0.25 Hz)
Cell 660
Fluorescence (Raw)
20
25
30
35
40
0
200
400
600
800
Time Point (0.25 Hz)

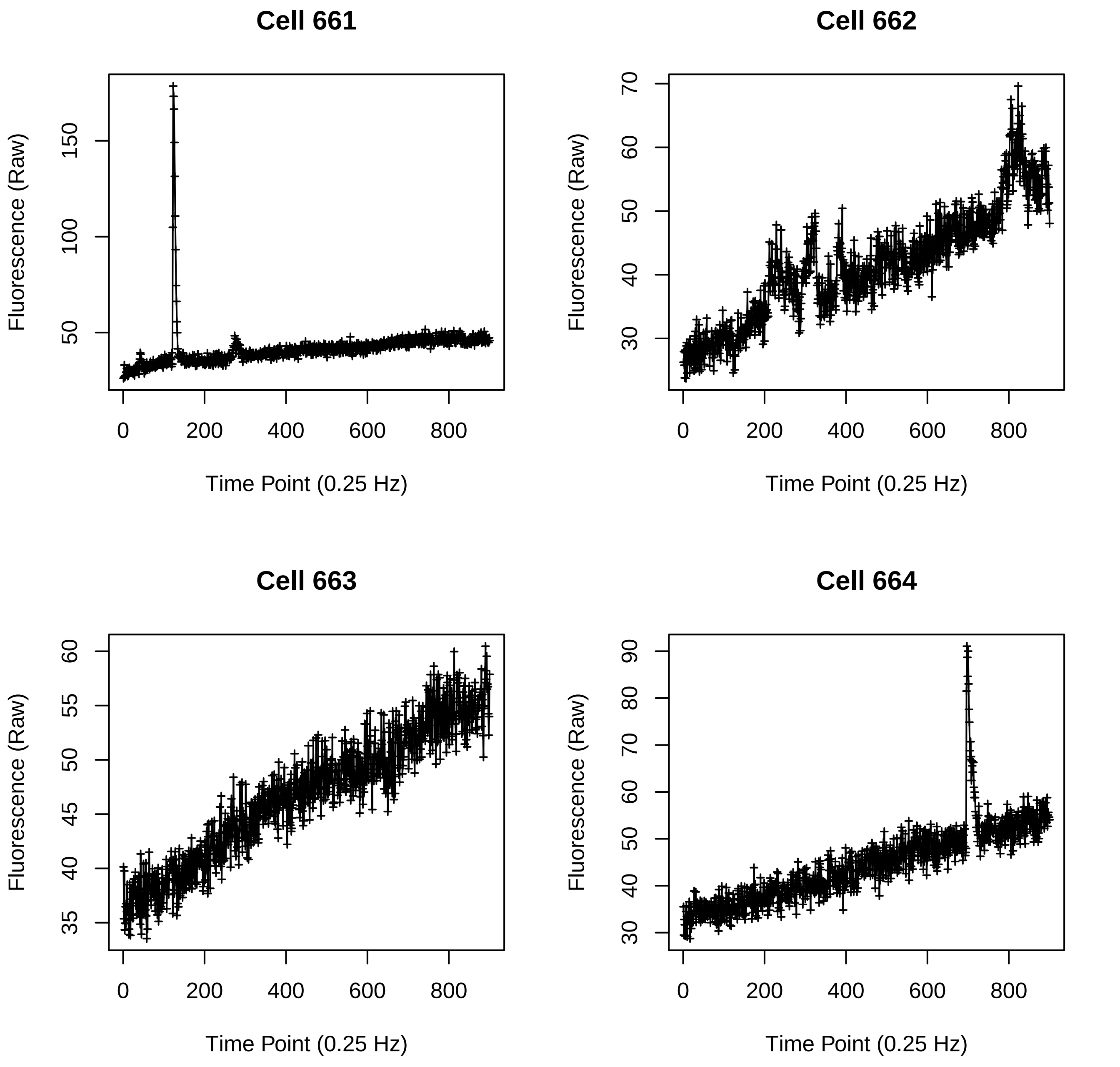
Cell 661
Fluorescence (Raw)
50
100
150
0
200
400
600
800
Time Point (0.25 Hz)
Cell 662
Fluorescence (Raw)
30
40
50
60
70
0
200
400
600
800
Time Point (0.25 Hz)
Cell 663
Fluorescence (Raw)
35
40
45
50
55
60
0
200
400
600
800
Time Point (0.25 Hz)
Cell 664
Fluorescence (Raw)
30
40
50
60
70
80
90
0
200
400
600
800
Time Point (0.25 Hz)

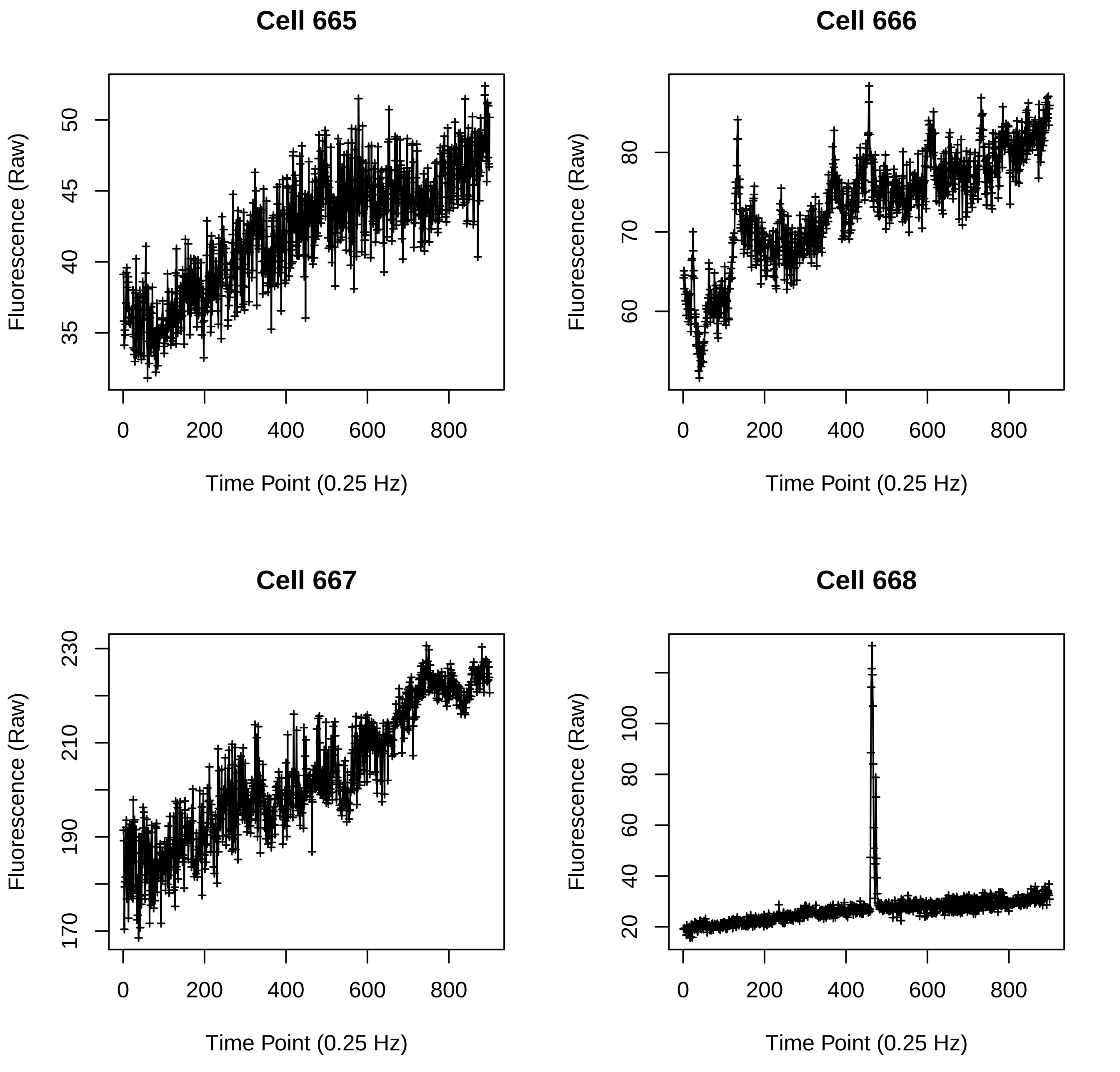
Cell 665
Cell 666
Cell 667
Cell 668
Fluorescence (Raw)
Time Point (0.25 Hz)
35
40
45
50
60
70
80
170
190
210
230
20
40
60
80
100
0
200
400
600
800

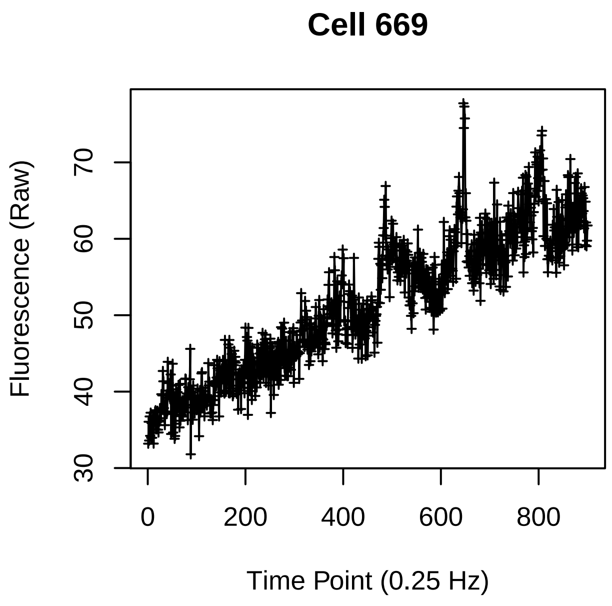
Cell 669
Fluorescence (Raw)
30
40
50
60
70
0
200
400
600
800
Time Point (0.25 Hz)

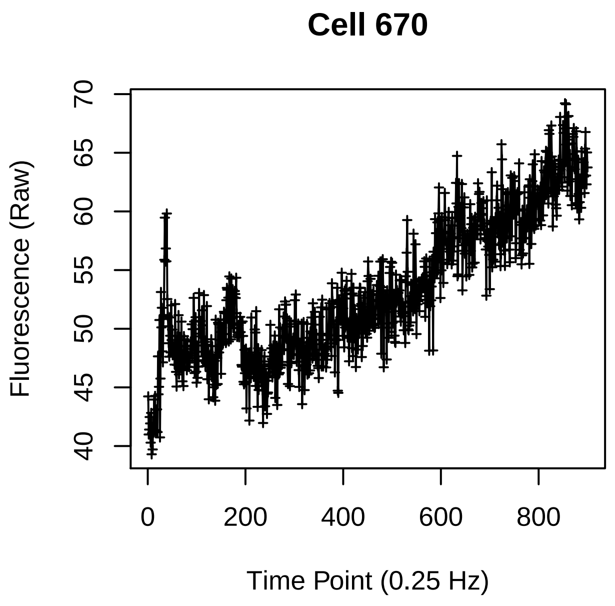
Cell 670
Fluorescence (Raw)
40
45
50
55
60
65
70
0
200
400
600
800
Time Point (0.25 Hz)

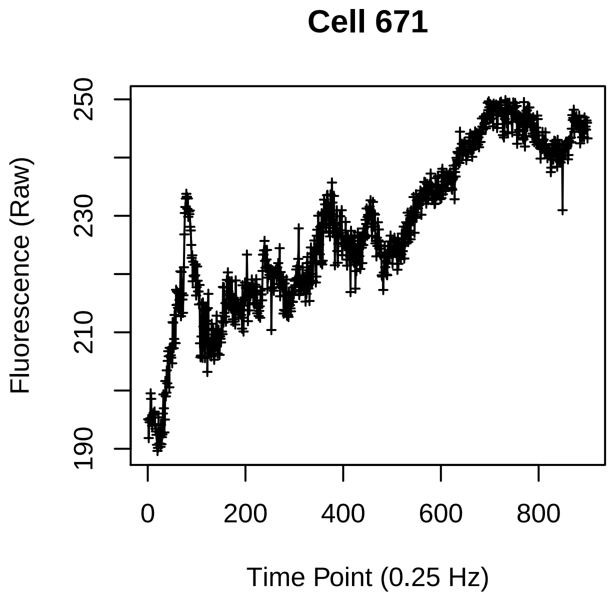
Cell 671
Fluorescence (Raw)
190
210
230
250
0
200
400
600
800
Time Point (0.25 Hz)

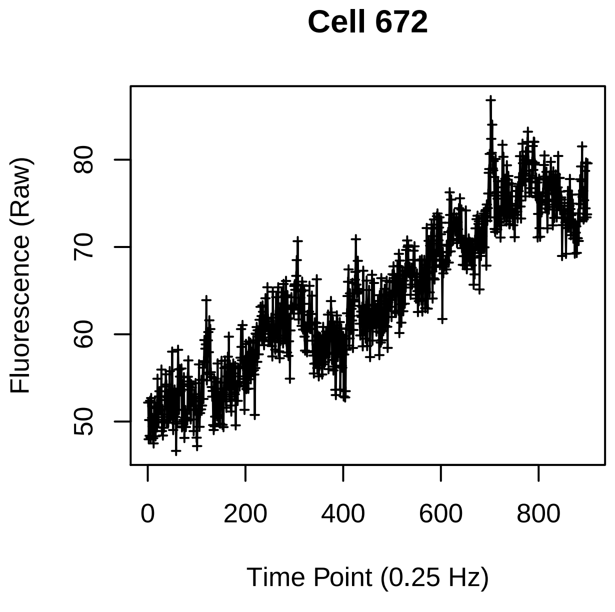
Cell 672
Fluorescence (Raw)
50
60
70
80
0
200
400
600
800
Time Point (0.25 Hz)

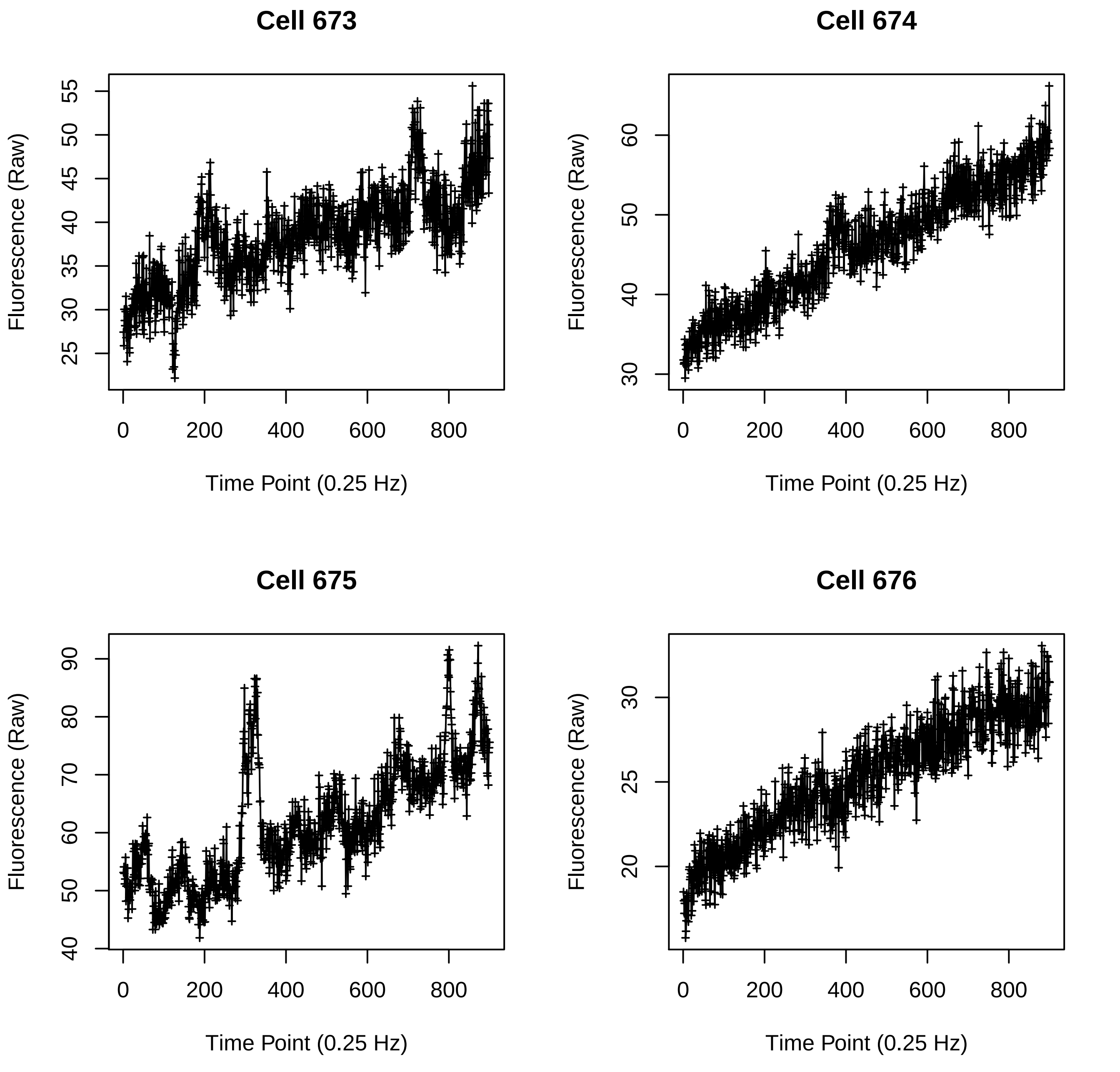
Cell 673
Cell 674
Cell 675
Cell 676
Fluorescence (Raw)
Time Point (0.25 Hz)
0
200
400
600
800
25
30
35
40
45
50
55
60
70
80
90
20

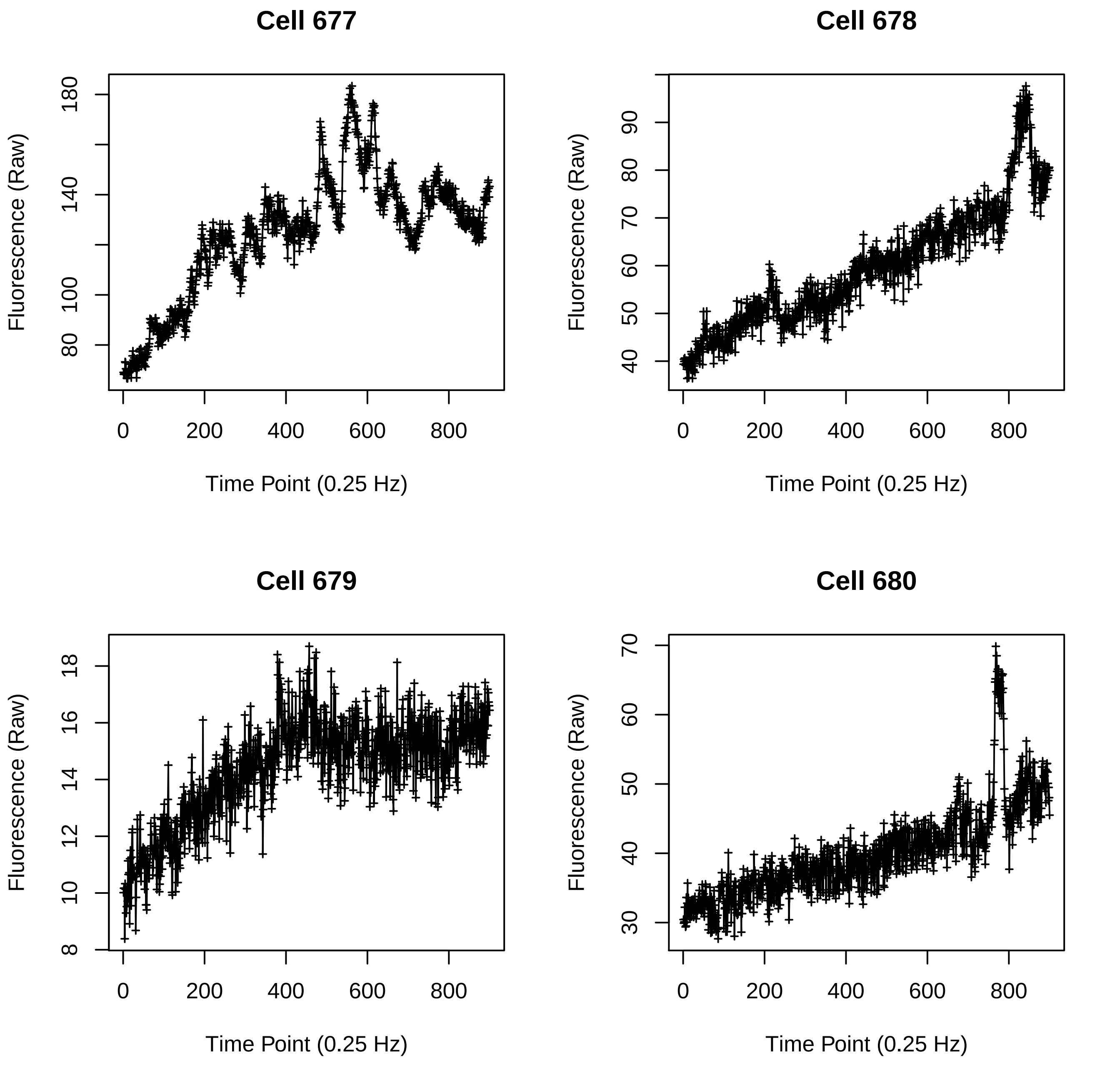

Cell 677
Cell 678
Cell 679
Cell 680
Fluorescence (Raw)
Time Point (0.25 Hz)
180
140
100
80
90
80
70
60
50
40
18
16
14
12
10
8
70
60
50
40
30
0
200
400
600
800

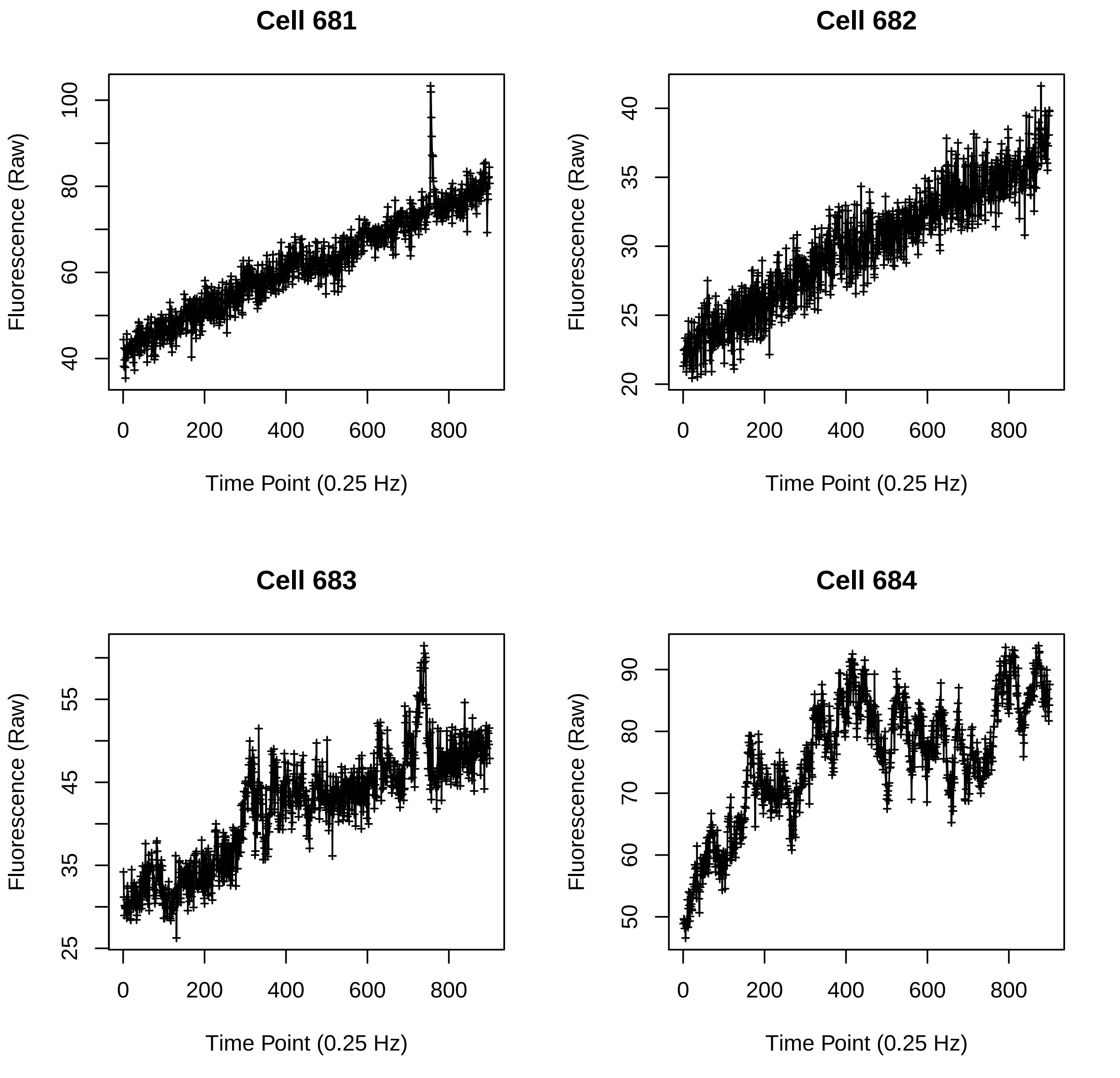
Cell 681
Fluorescence (Raw)
40
60
80
100
0
200
400
600
800
Time Point (0.25 Hz)
Cell 682
Fluorescence (Raw)
20
25
30
35
40
0
200
400
600
800
Time Point (0.25 Hz)
Cell 683
Fluorescence (Raw)
25
35
45
55
0
200
400
600
800
Time Point (0.25 Hz)
Cell 684
Fluorescence (Raw)
50
60
70
80
90
0
200
400
600
800
Time Point (0.25 Hz)

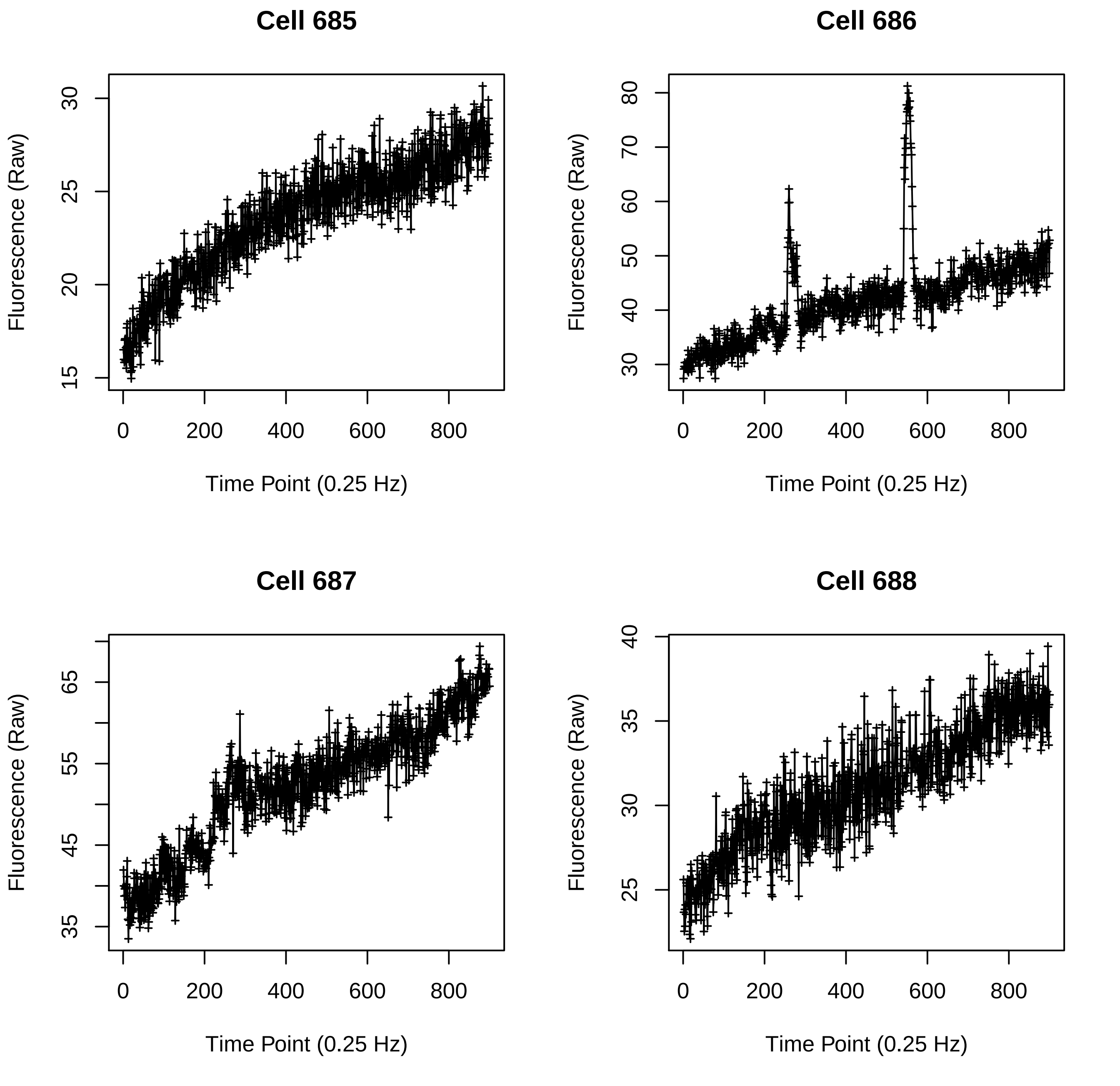

Cell 685
Fluorescence (Raw)
15
20
25
30
0
200
400
600
800
Time Point (0.25 Hz)
Cell 686
Fluorescence (Raw)
30
40
50
60
70
80
0
200
400
600
800
Time Point (0.25 Hz)
Cell 687
Fluorescence (Raw)
35
45
55
65
0
200
400
600
800
Time Point (0.25 Hz)
Cell 688
Fluorescence (Raw)
25
30
35
40
0
200
400
600
800
Time Point (0.25 Hz)

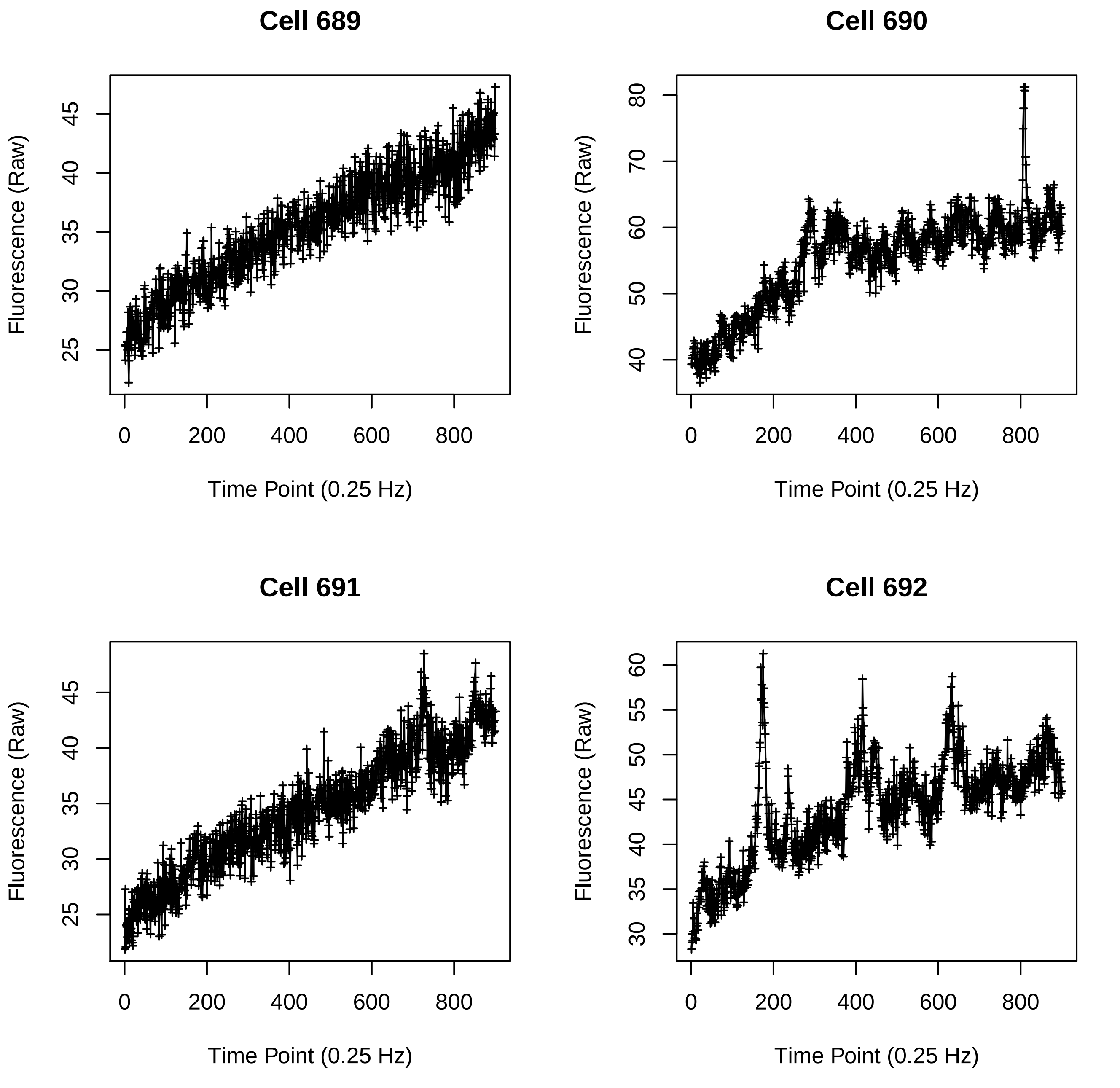
Cell 689
Fluorescence (Raw)
25
30
35
40
45
0
200
400
600
800
Time Point (0.25 Hz)
Cell 690
Fluorescence (Raw)
40
50
60
70
80
0
200
400
600
800
Time Point (0.25 Hz)
Cell 691
Fluorescence (Raw)
25
30
35
40
45
0
200
400
600
800
Time Point (0.25 Hz)
Cell 692
Fluorescence (Raw)
30
35
40
45
50
55
60
0
200
400
600
800
Time Point (0.25 Hz)

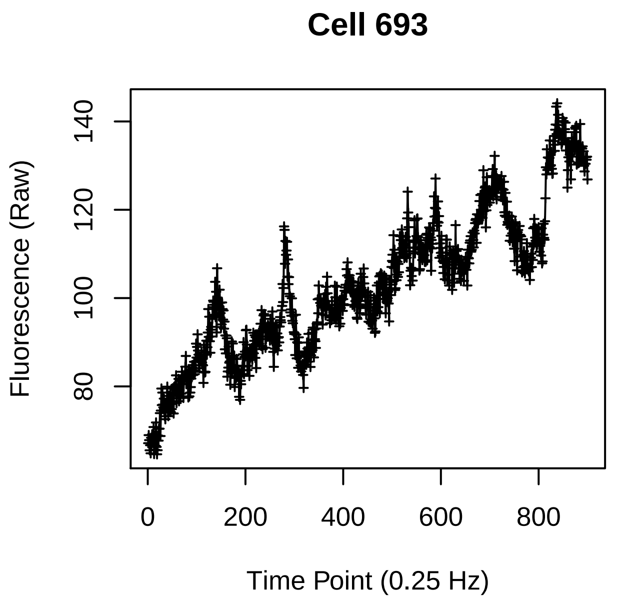
Cell 693
Fluorescence (Raw)
Time Point (0.25 Hz)
80
100
120
140
0
200
400
600
800

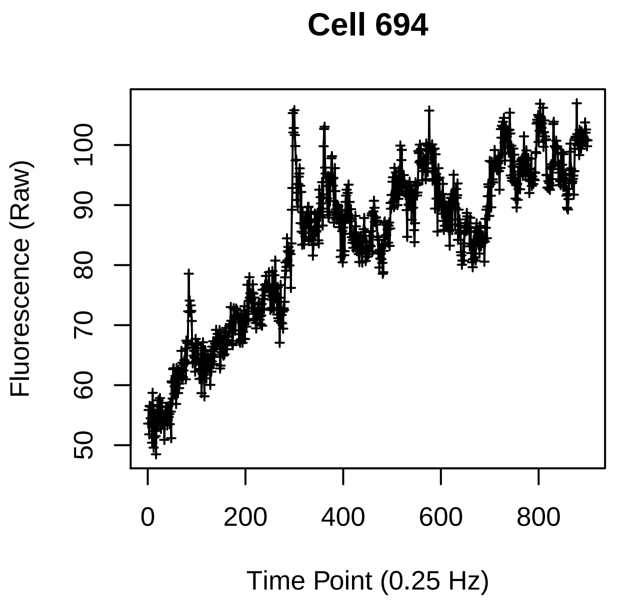
Cell 694
Fluorescence (Raw)
Time Point (0.25 Hz)
50
60
70
80
90
100
0
200
400
600
800

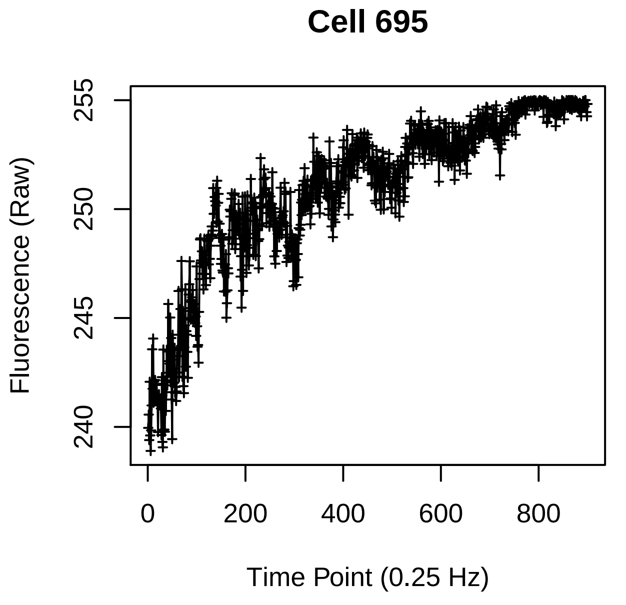
Cell 695
Fluorescence (Raw)
Time Point (0.25 Hz)
240
245
250
255
0
200
400
600
800

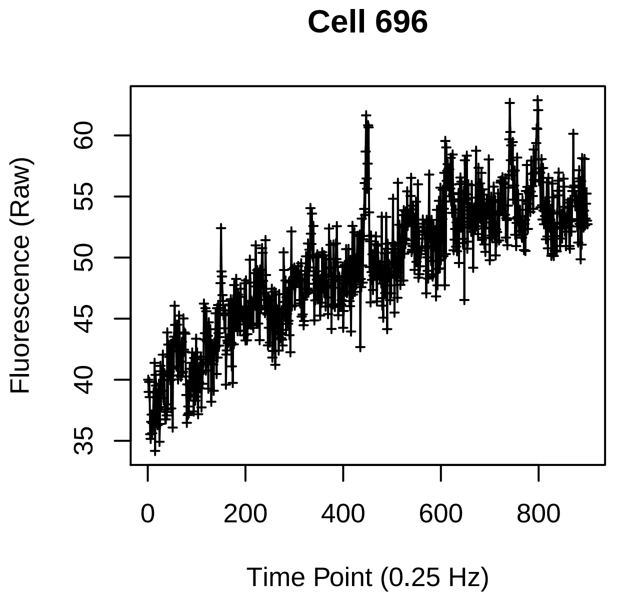
Cell 696
Fluorescence (Raw)
Time Point (0.25 Hz)
35
40
45
50
55
60
0
200
400
600
800

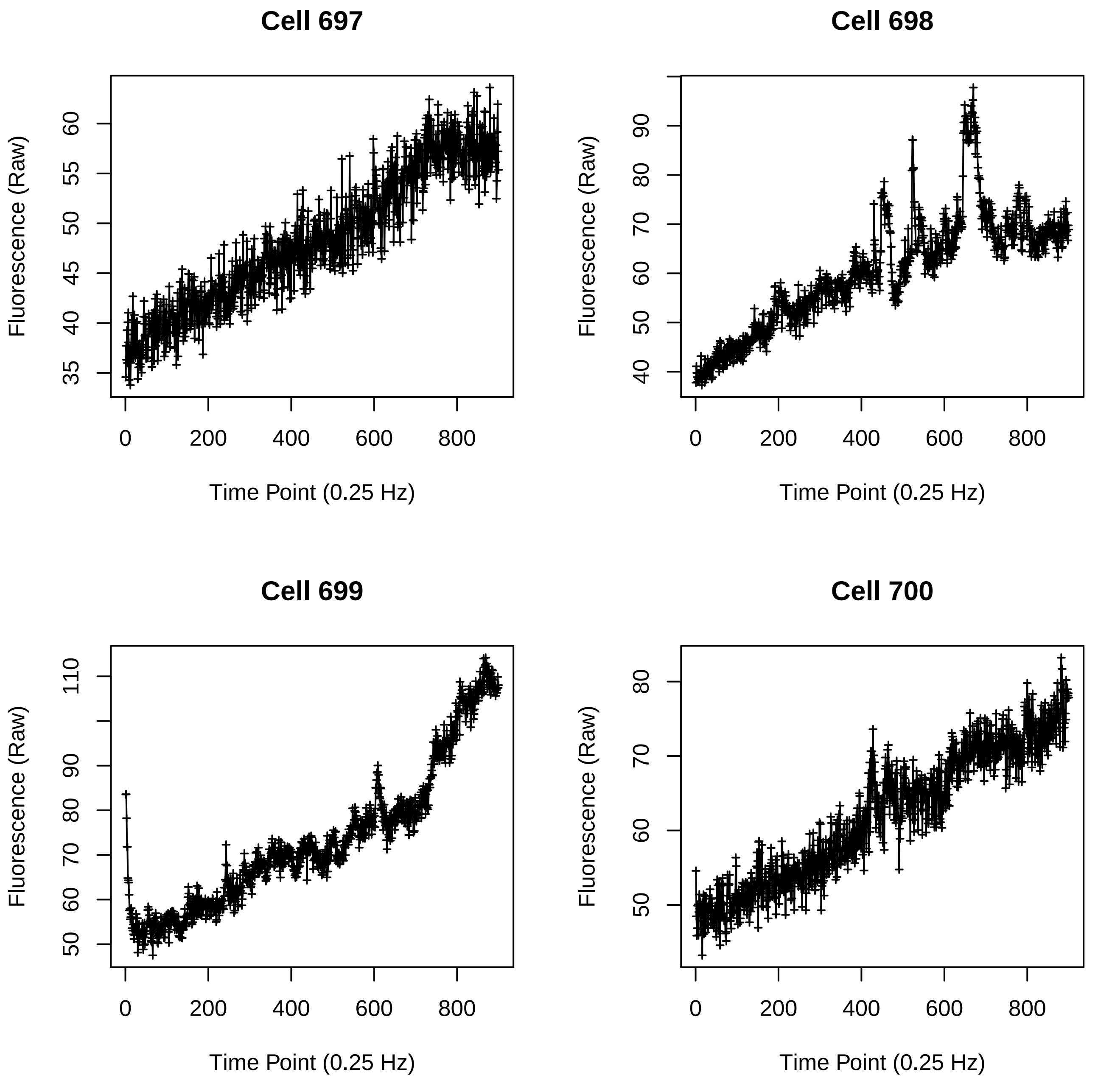
Cell 697
Fluorescence (Raw)
Time Point (0.25 Hz)
35
40
45
50
55
60
0
200
400
600
800
Cell 698
Fluorescence (Raw)
Time Point (0.25 Hz)
40
50
60
70
80
90
0
200
400
600
800
Cell 699
Fluorescence (Raw)
Time Point (0.25 Hz)
50
60
70
80
90
110
0
200
400
600
800
Cell 700
Fluorescence (Raw)
Time Point (0.25 Hz)
50
60
70
80
0
200
400
600
800

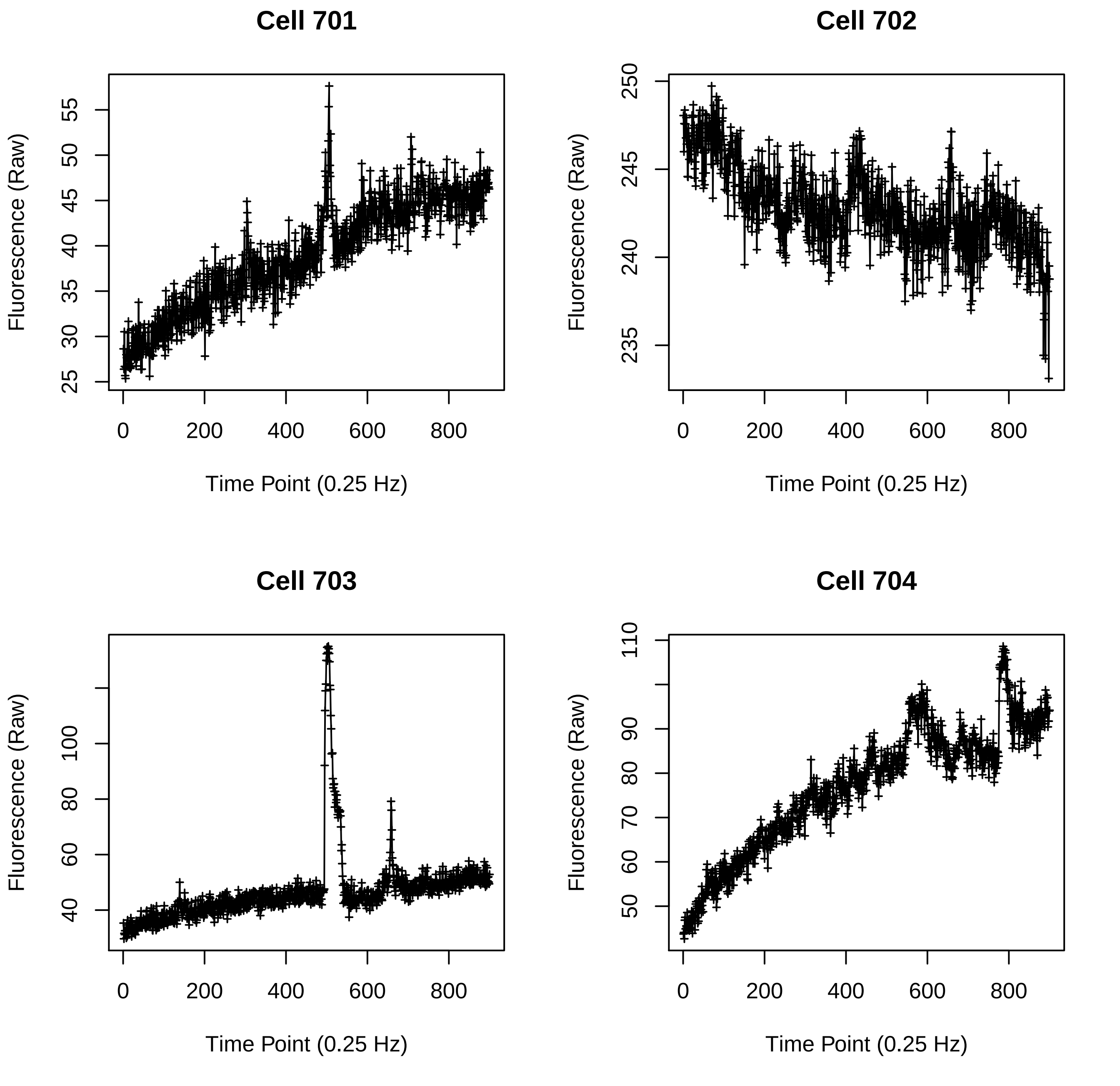
Cell 701
Fluorescence (Raw)
25
30
35
40
45
50
55
0
200
400
600
800
Time Point (0.25 Hz)
Cell 702
Fluorescence (Raw)
235
240
245
250
0
200
400
600
800
Time Point (0.25 Hz)
Cell 703
Fluorescence (Raw)
40
60
80
100
0
200
400
600
800
Time Point (0.25 Hz)
Cell 704
Fluorescence (Raw)
50
60
70
80
90
110
0
200
400
600
800
Time Point (0.25 Hz)

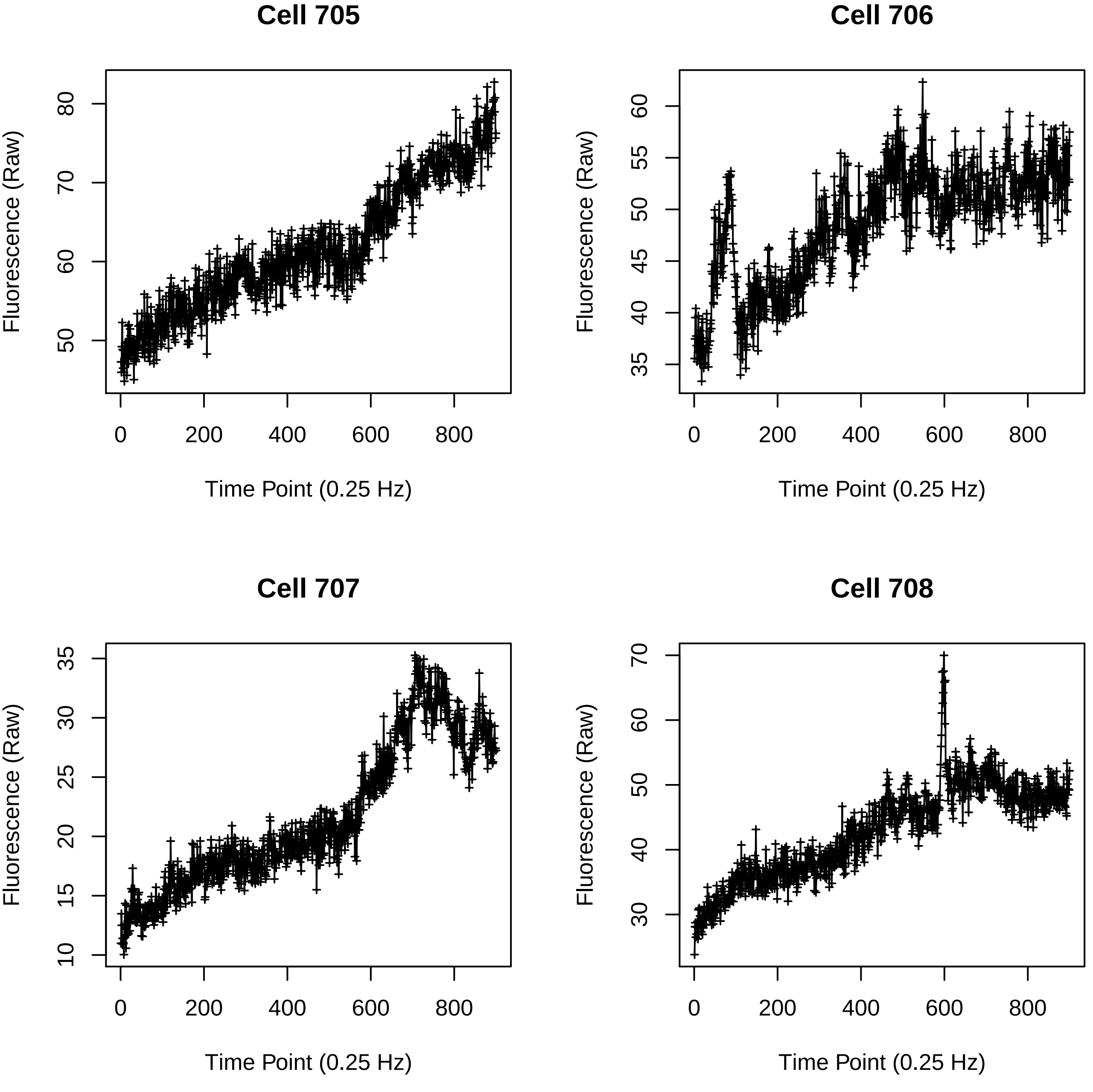
Cell 705
Cell 706
Cell 707
Cell 708
Fluorescence (Raw)
Time Point (0.25 Hz)
0
200
400
600
800
50
60
70
80
35
40
45
50
55
60
10
15
20
25
30
35
30
40
50
60
70

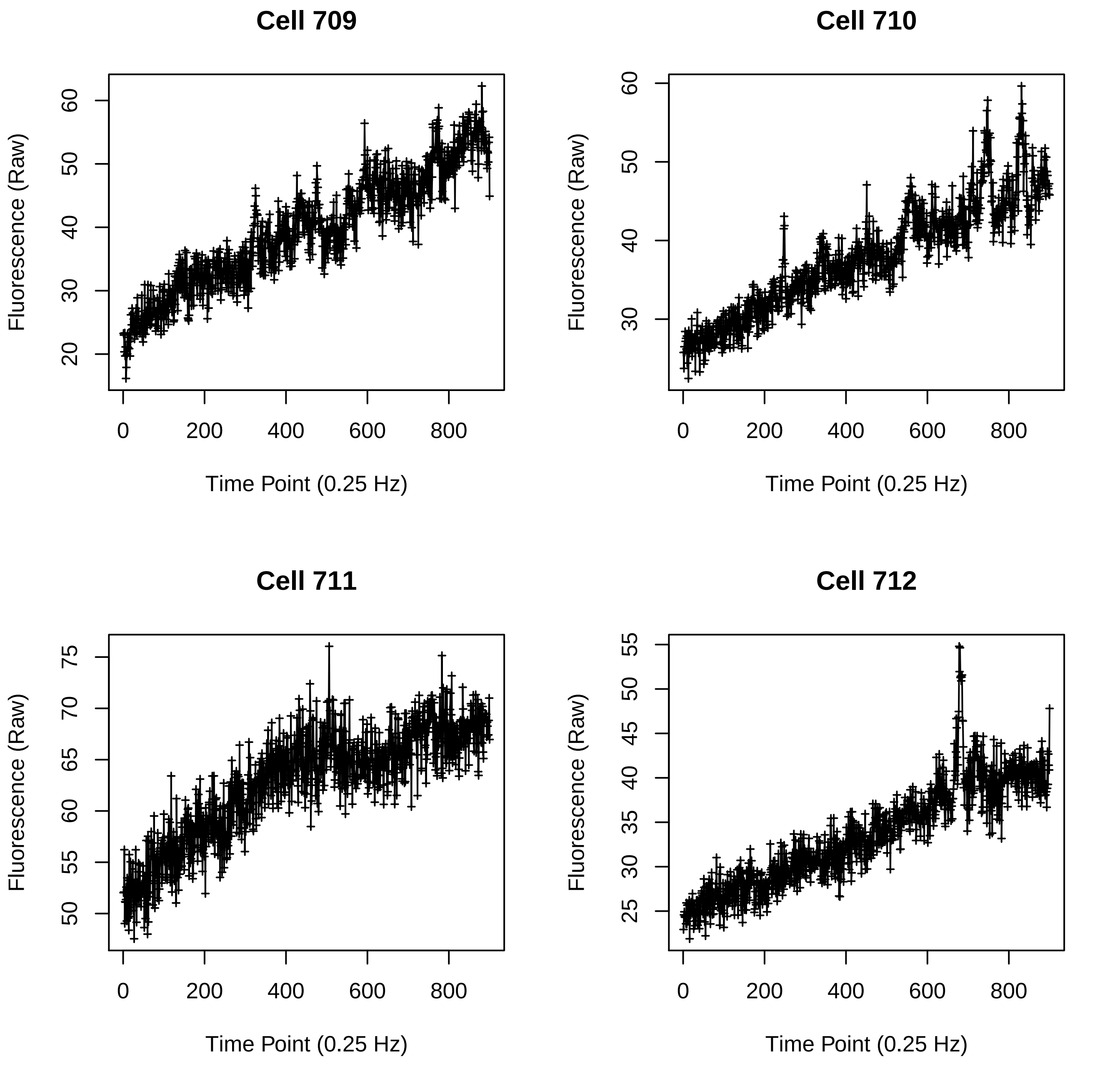

Cell 709
Cell 710
Cell 711
Cell 712
Fluorescence (Raw)
Time Point (0.25 Hz)
0
200
400
600
800
20
30
40
50
60
70
75
65
55
25
35
45

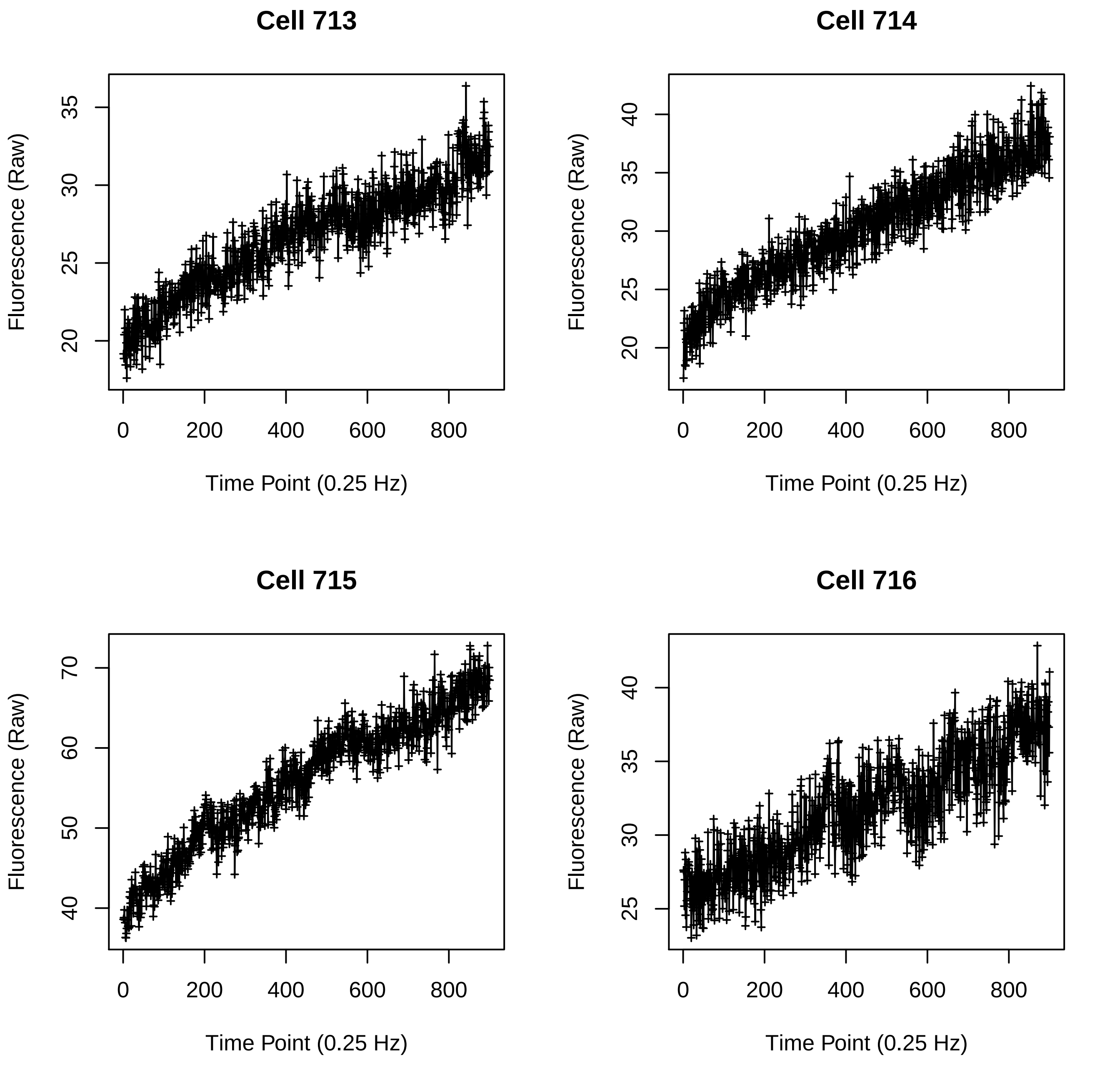
Cell 713
Fluorescence (Raw)
Time Point (0.25 Hz)
Cell 714
Fluorescence (Raw)
Time Point (0.25 Hz)
Cell 715
Fluorescence (Raw)
Time Point (0.25 Hz)
Cell 716
Fluorescence (Raw)
Time Point (0.25 Hz)

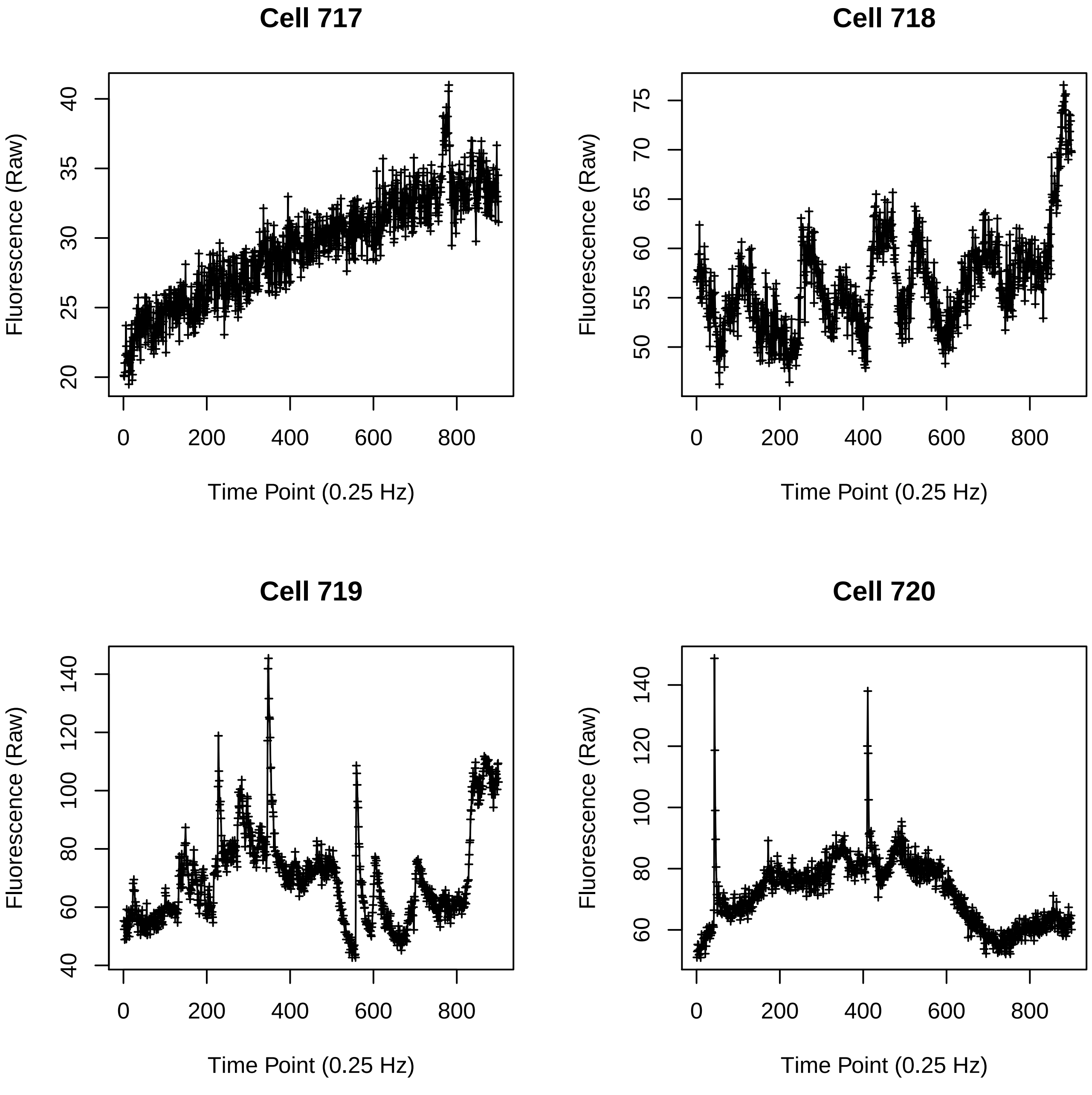
Cell 717
Cell 718
Cell 719
Cell 720
Fluorescence (Raw)
Time Point (0.25 Hz)
20
25
30
35
40
50
55
60
65
70
75
40
60
80
100
120
140
0
200
400
600
800

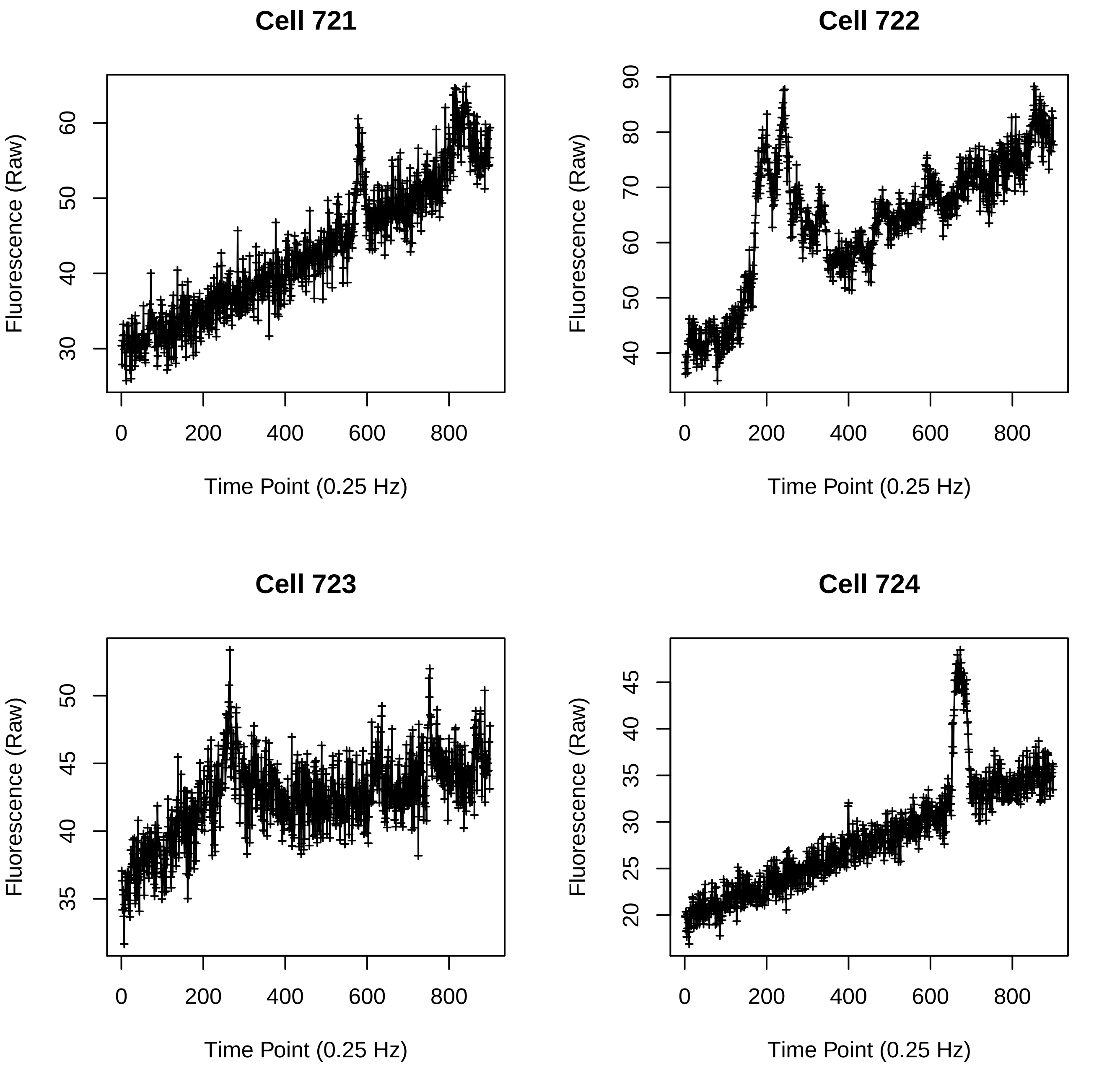
Cell 721
Cell 722
Cell 723
Cell 724
Fluorescence (Raw)
Time Point (0.25 Hz)
0
200
400
600
800
30
40
50
60
70
80
90
35
45
20
25

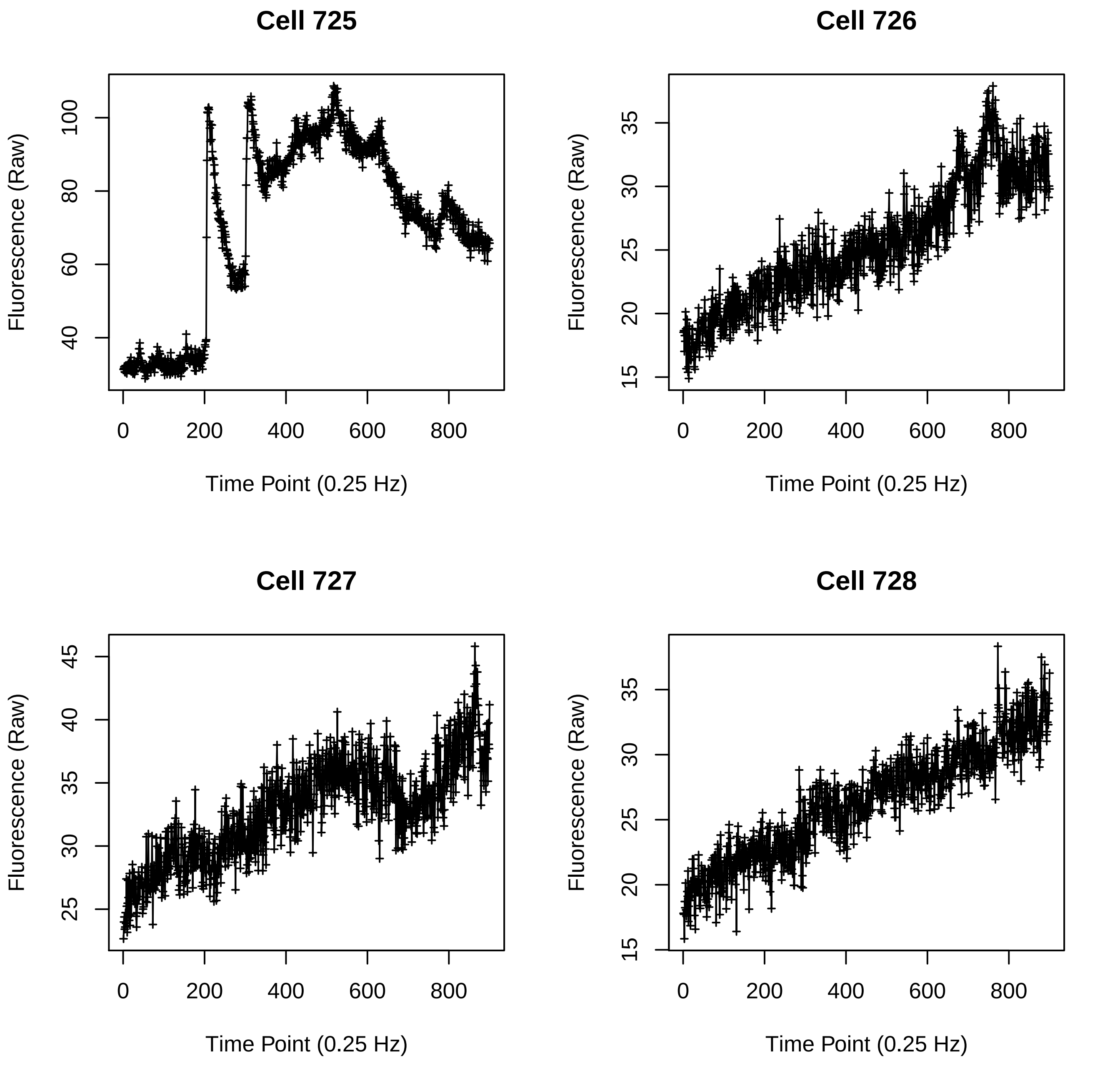

Cell 725
Cell 726
Cell 727
Cell 728
Fluorescence (Raw)
Time Point (0.25 Hz)
0
200
400
600
800
40
60
80
100
15
20
25
30
35
45

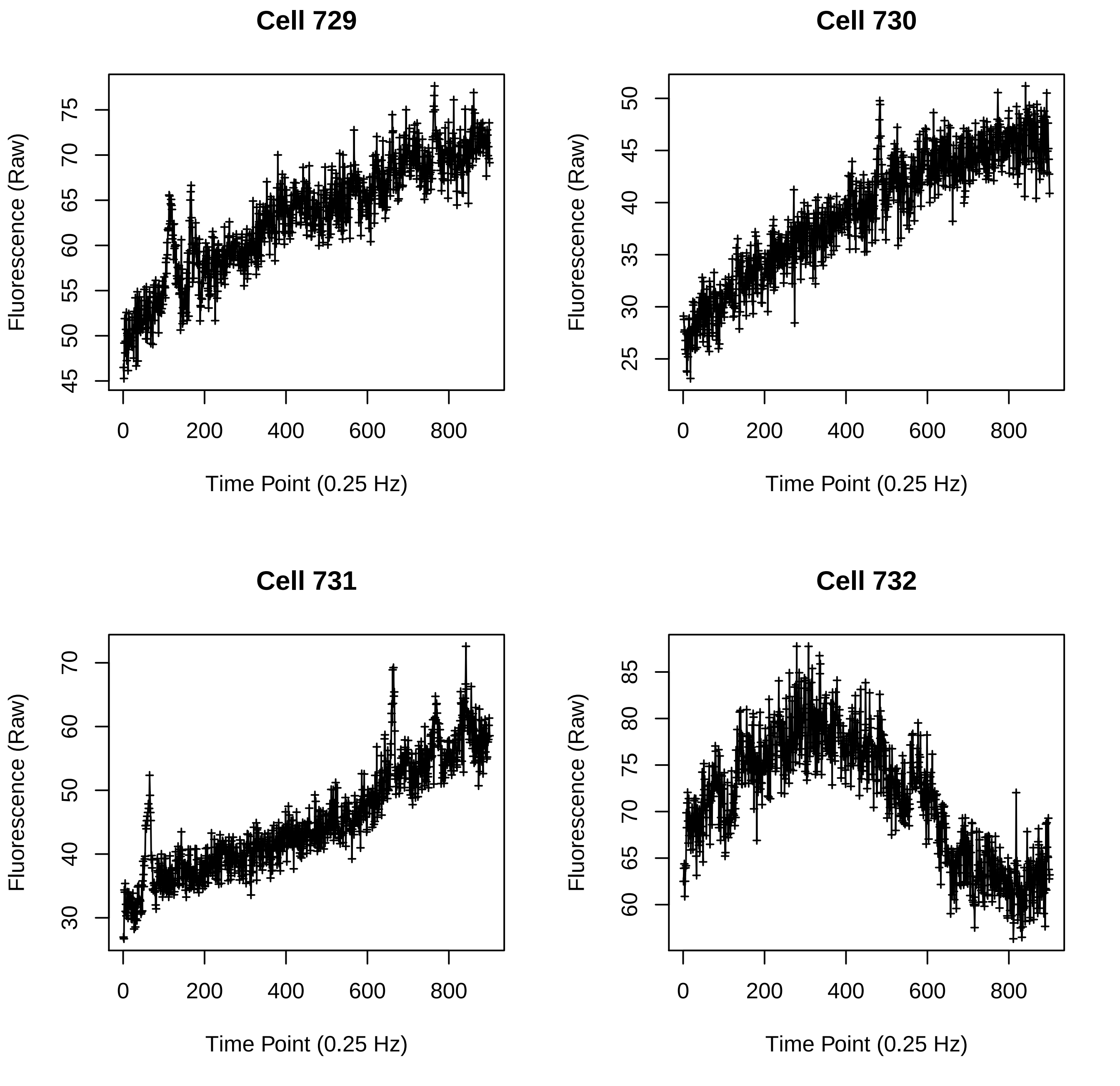

Cell 729
Cell 730
Cell 731
Cell 732
Fluorescence (Raw)
Time Point (0.25 Hz)
45
50
55
60
65
70
75
25
30
35
40
45
50
30
40
50
60
70
60
65
70
75
80
85
0
200
400
600
800

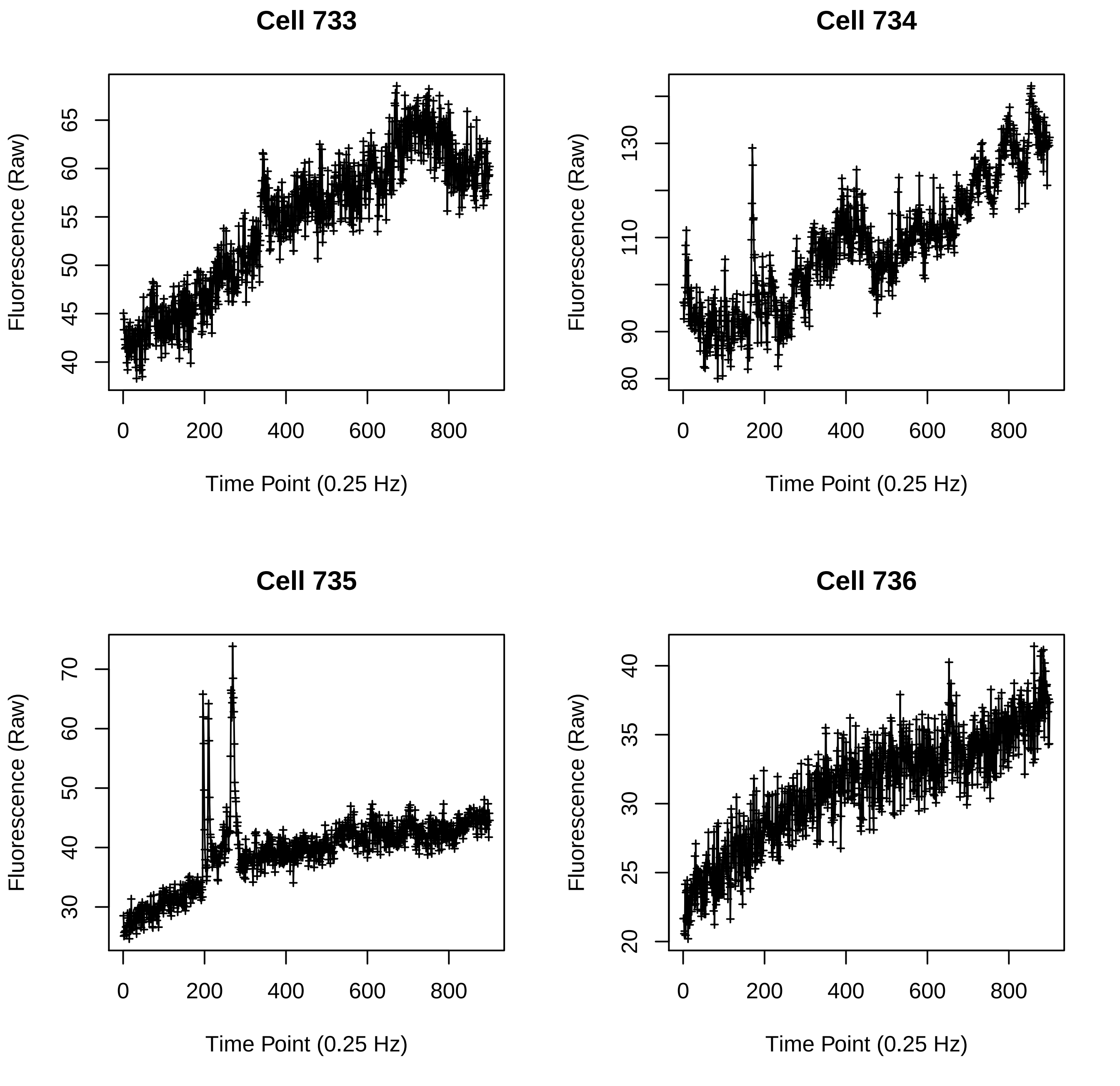

Cell 733
Cell 734
Cell 735
Cell 736
Fluorescence (Raw)
Time Point (0.25 Hz)
0
200
400
600
800
40
45
50
55
60
65
80
90
110
130
30
40
50
60
70
20
25
30
35
40

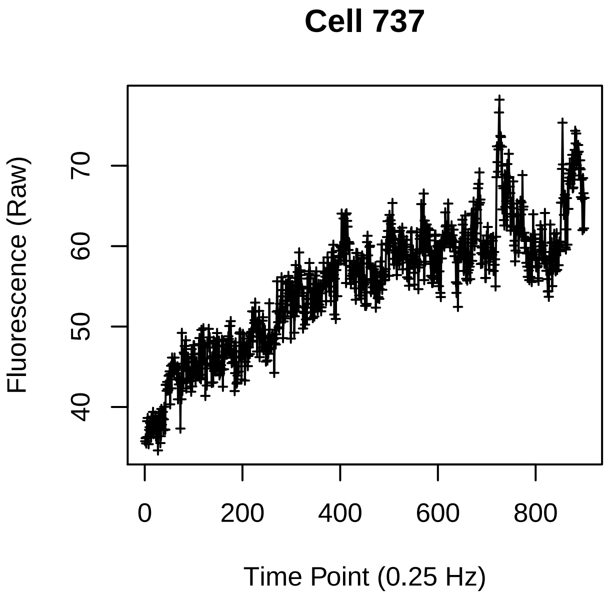
Cell 737
Fluorescence (Raw)
40
50
60
70
0
200
400
600
800
Time Point (0.25 Hz)

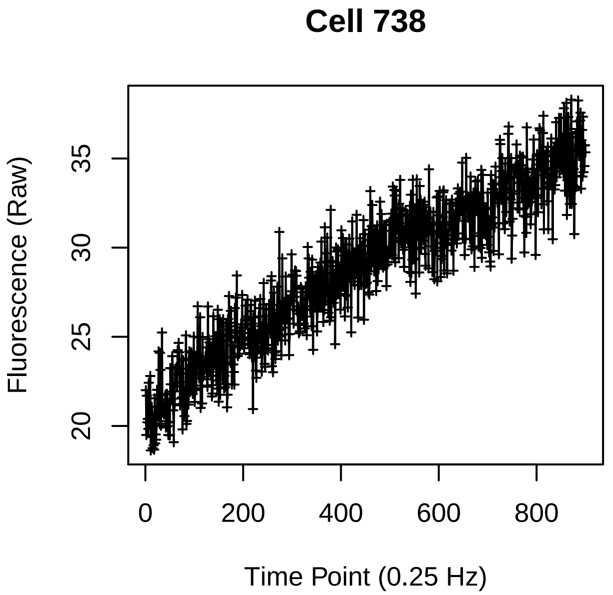
Cell 738
Fluorescence (Raw)
20
25
30
35
0
200
400
600
800
Time Point (0.25 Hz)

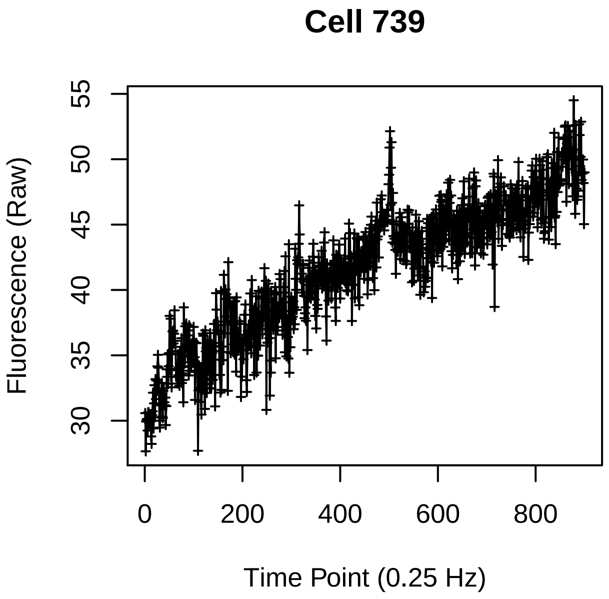
Cell 739
Fluorescence (Raw)
30
35
40
45
50
55
0
200
400
600
800
Time Point (0.25 Hz)

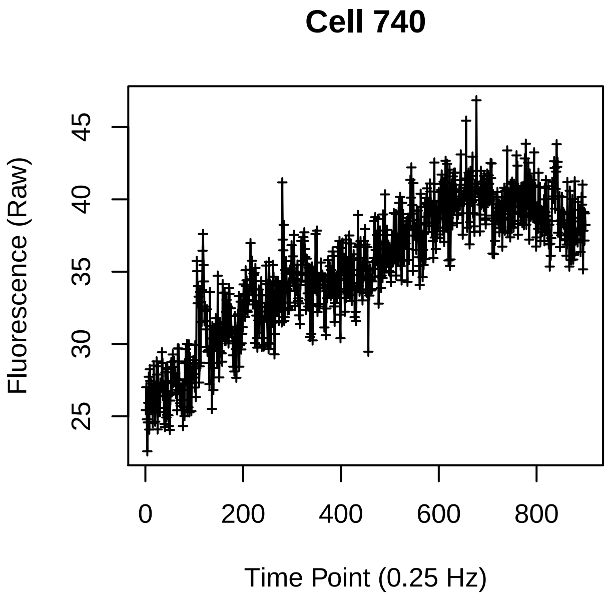
Cell 740
Fluorescence (Raw)
25
30
35
40
45
0
200
400
600
800
Time Point (0.25 Hz)

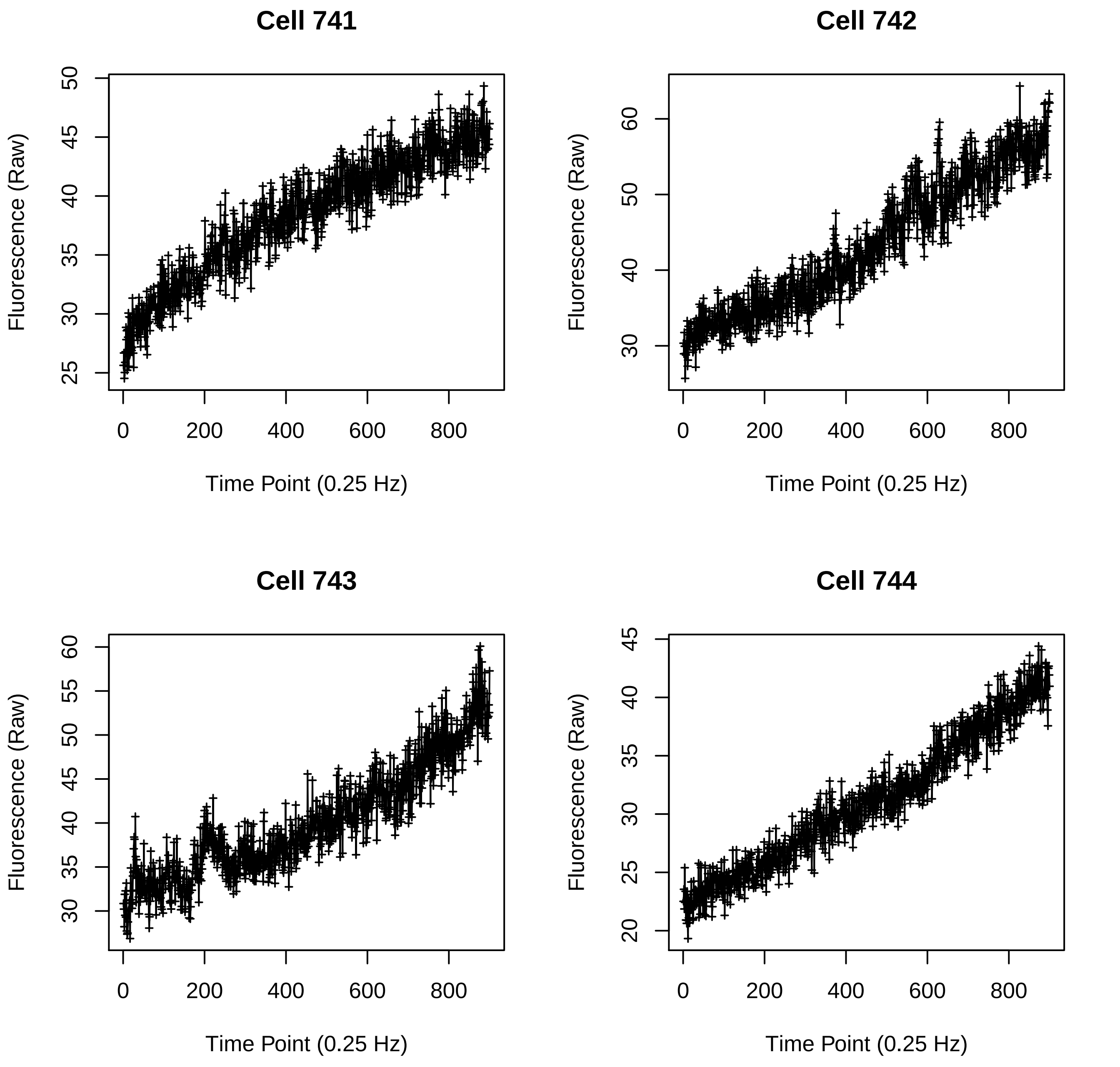
Cell 741
Cell 742
Cell 743
Cell 744
Fluorescence (Raw)
Time Point (0.25 Hz)
0
200
400
600
800
25
30
35
40
45
50
60
20

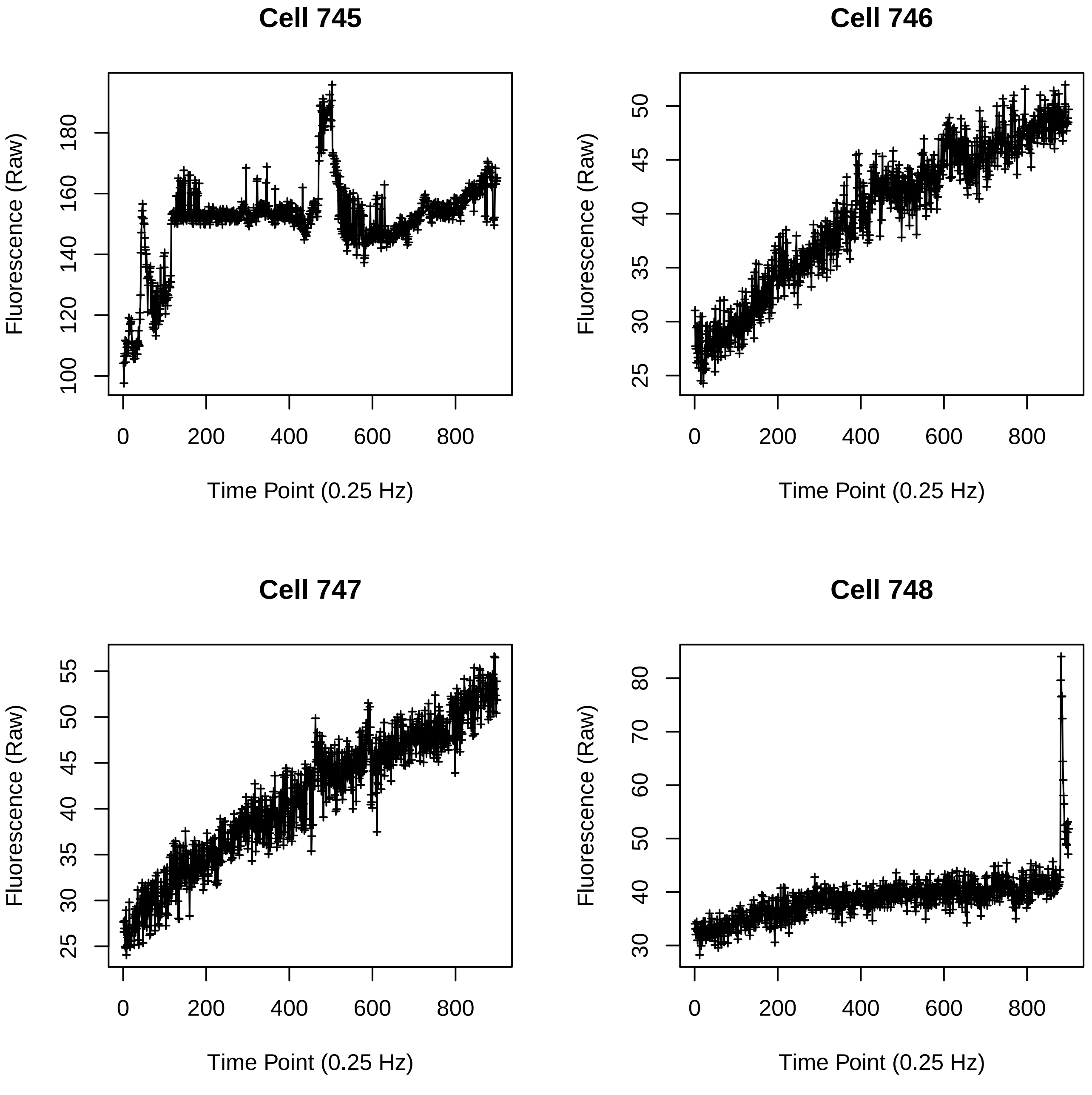
Cell 745
Fluorescence (Raw)
100
120
140
160
180
0
200
400
600
800
Time Point (0.25 Hz)
Cell 746
Fluorescence (Raw)
25
30
35
40
45
50
0
200
400
600
800
Time Point (0.25 Hz)
Cell 747
Fluorescence (Raw)
25
30
35
40
45
50
55
0
200
400
600
800
Time Point (0.25 Hz)
Cell 748
Fluorescence (Raw)
30
40
50
60
70
80
0
200
400
600
800
Time Point (0.25 Hz)

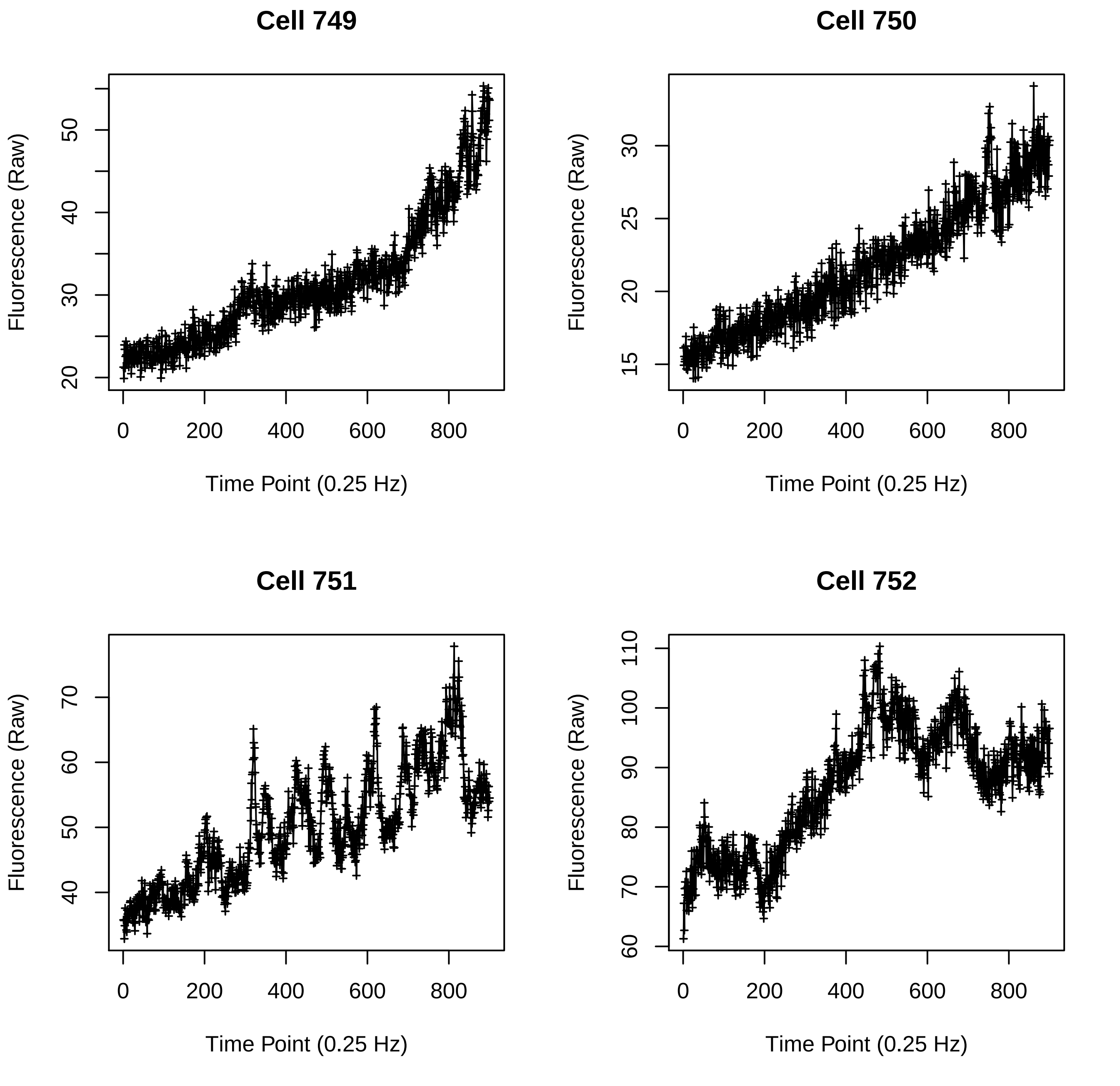
Cell 749
Fluorescence (Raw)
20
30
40
50
0
200
400
600
800
Time Point (0.25 Hz)
Cell 750
Fluorescence (Raw)
15
20
25
30
0
200
400
600
800
Time Point (0.25 Hz)
Cell 751
Fluorescence (Raw)
40
50
60
70
0
200
400
600
800
Time Point (0.25 Hz)
Cell 752
Fluorescence (Raw)
60
70
80
90
100
110
0
200
400
600
800
Time Point (0.25 Hz)

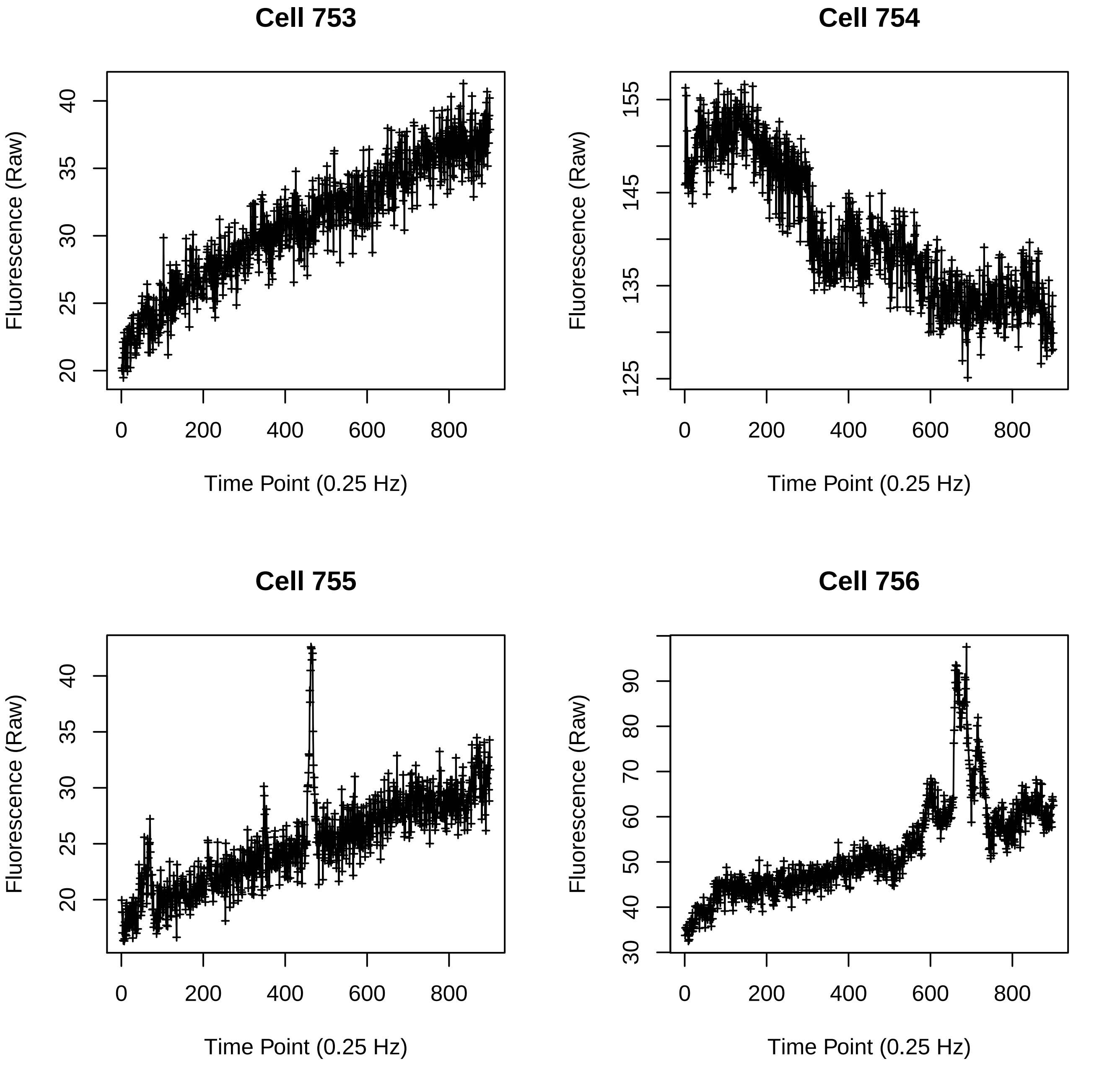
Cell 753
Cell 754
Cell 755
Cell 756
Fluorescence (Raw)
Time Point (0.25 Hz)
0
200
400
600
800
20
25
30
35
40
125
135
145
155
50
60
70
80
90

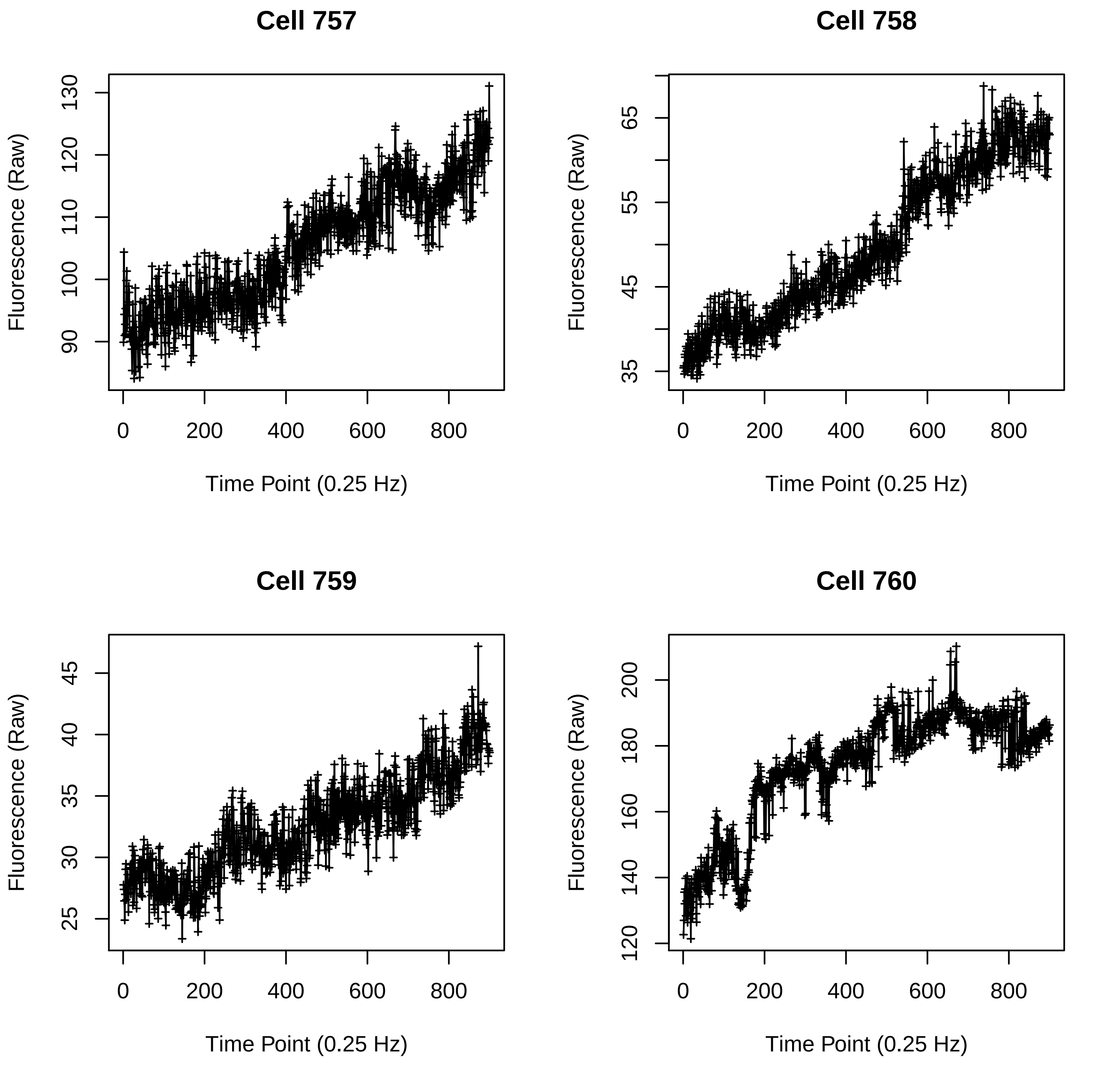
Cell 757
Fluorescence (Raw)
90
100
110
120
130
0
200
400
600
800
Time Point (0.25 Hz)
Cell 758
Fluorescence (Raw)
35
45
55
65
0
200
400
600
800
Time Point (0.25 Hz)
Cell 759
Fluorescence (Raw)
25
30
35
40
45
0
200
400
600
800
Time Point (0.25 Hz)
Cell 760
Fluorescence (Raw)
120
140
160
180
200
0
200
400
600
800
Time Point (0.25 Hz)

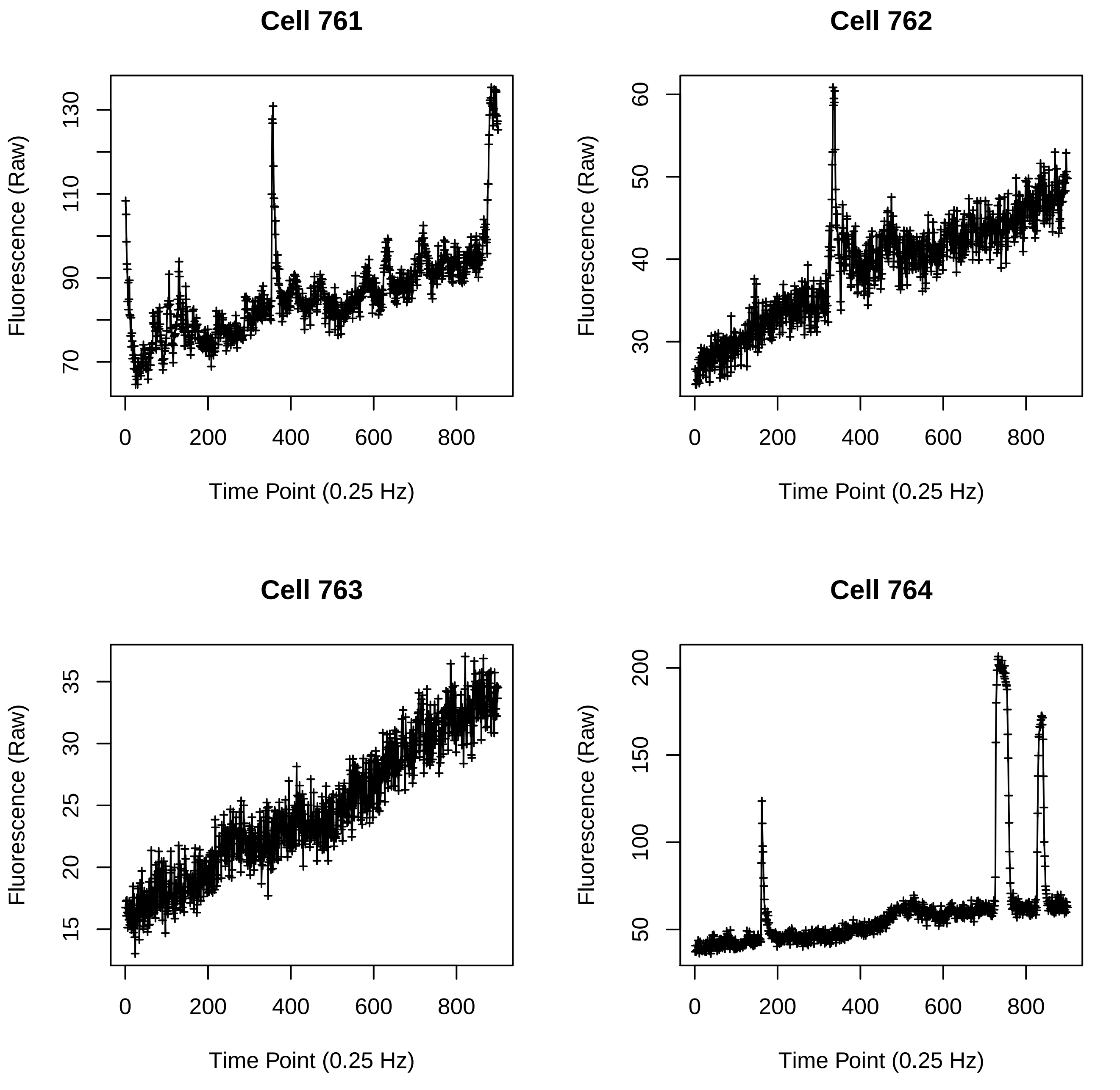
Cell 761
Cell 762
Cell 763
Cell 764
Fluorescence (Raw)
Time Point (0.25 Hz)
0
200
400
600
800
70
90
110
130
30
40
50
60
15
20
25
30
35
50
100
150
200

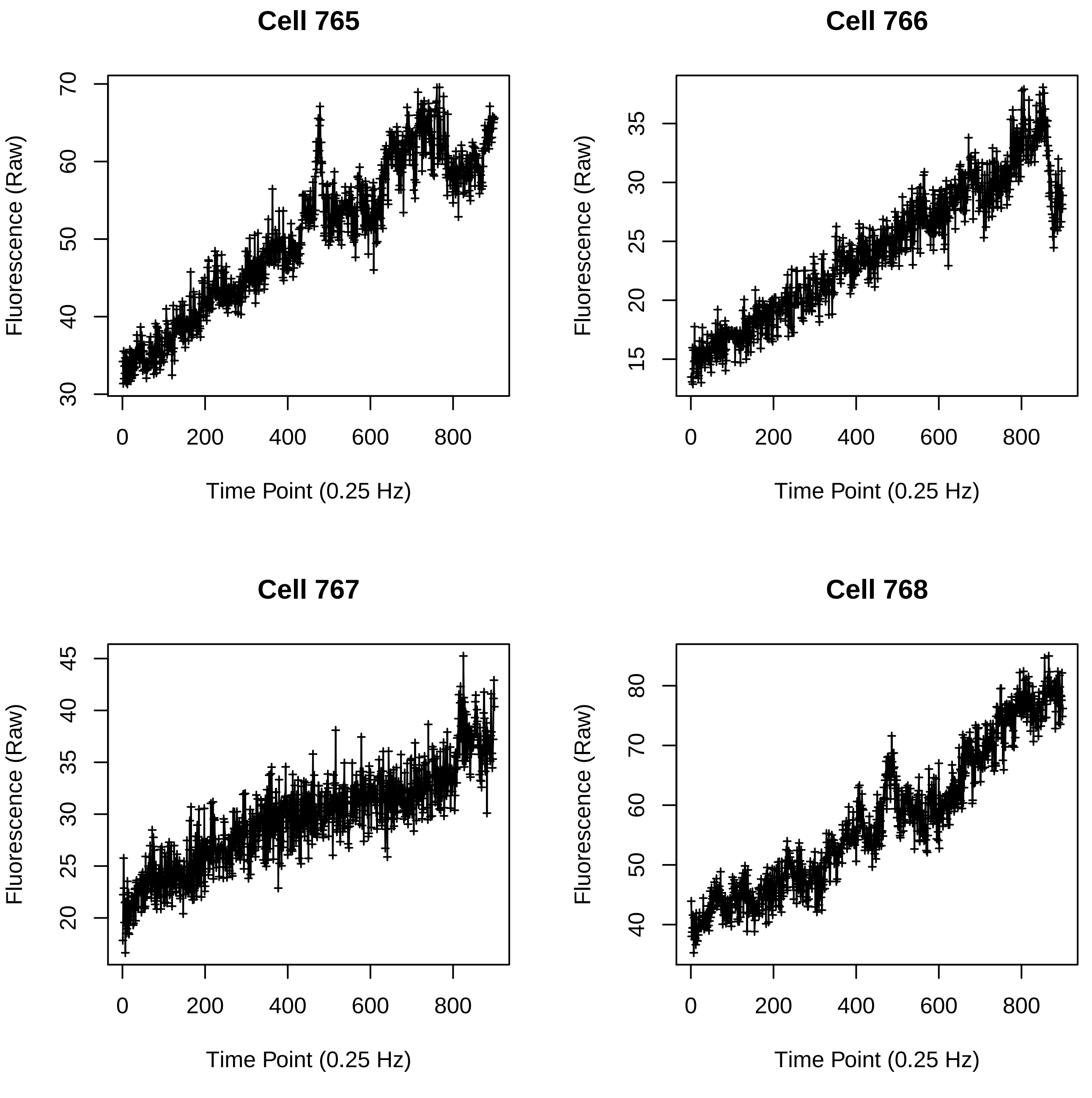
Cell 765
Fluorescence (Raw)
30
40
50
60
70
0
200
400
600
800
Time Point (0.25 Hz)
Cell 766
Fluorescence (Raw)
15
20
25
30
35
0
200
400
600
800
Time Point (0.25 Hz)
Cell 767
Fluorescence (Raw)
20
25
30
35
40
45
0
200
400
600
800
Time Point (0.25 Hz)
Cell 768
Fluorescence (Raw)
40
50
60
70
80
0
200
400
600
800
Time Point (0.25 Hz)

Cell 769

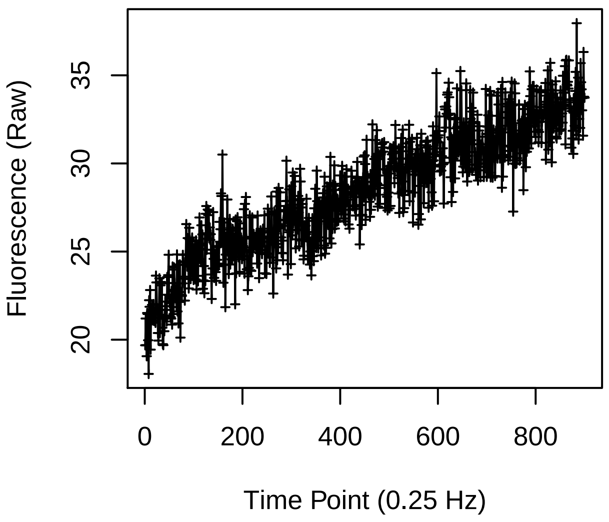

Cell 770

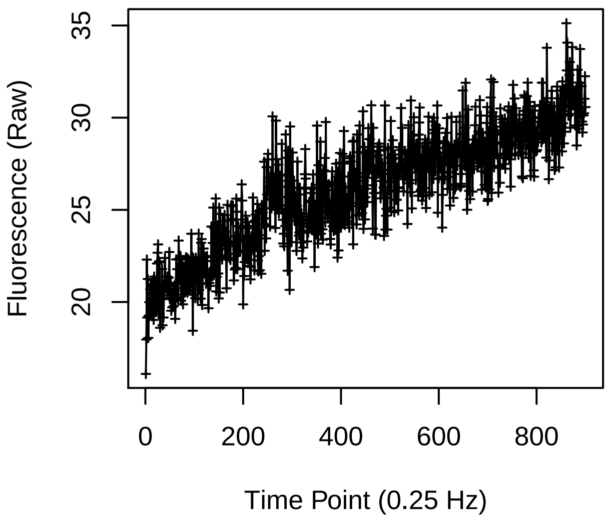

Cell 771

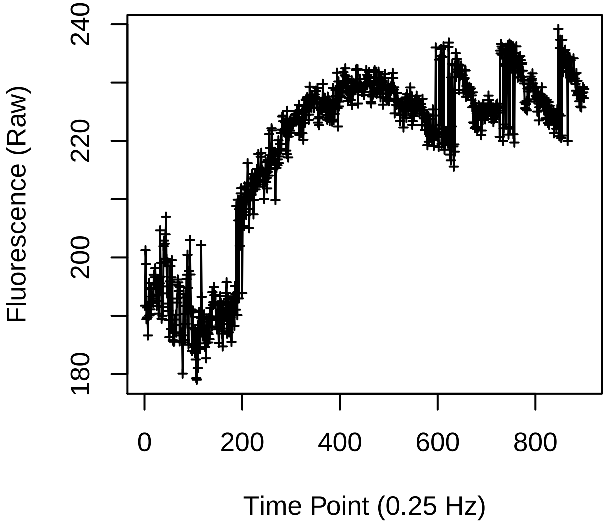

Cell 772

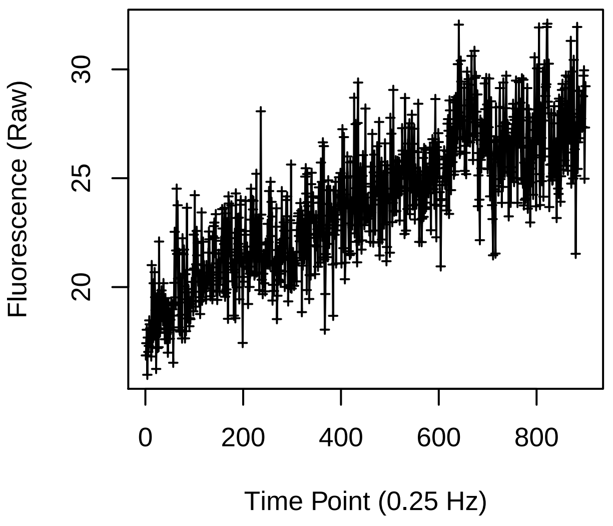

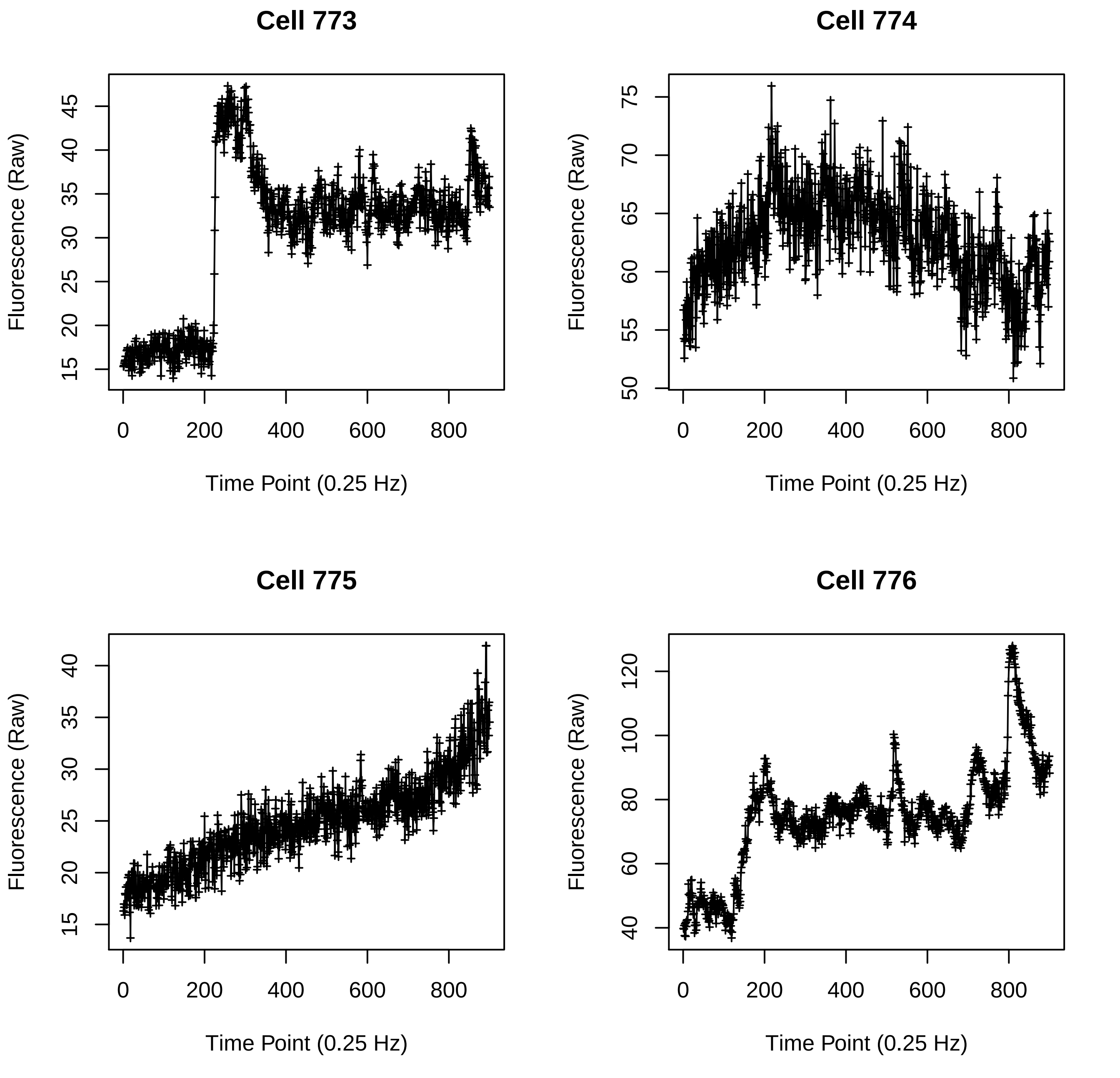
Cell 773
Fluorescence (Raw)
15
20
25
30
35
40
45
0
200
400
600
800
Time Point (0.25 Hz)
Cell 774
Fluorescence (Raw)
50
55
60
65
70
75
0
200
400
600
800
Time Point (0.25 Hz)
Cell 775
Fluorescence (Raw)
15
20
25
30
35
40
0
200
400
600
800
Time Point (0.25 Hz)
Cell 776
Fluorescence (Raw)
40
60
80
100
120
0
200
400
600
800
Time Point (0.25 Hz)

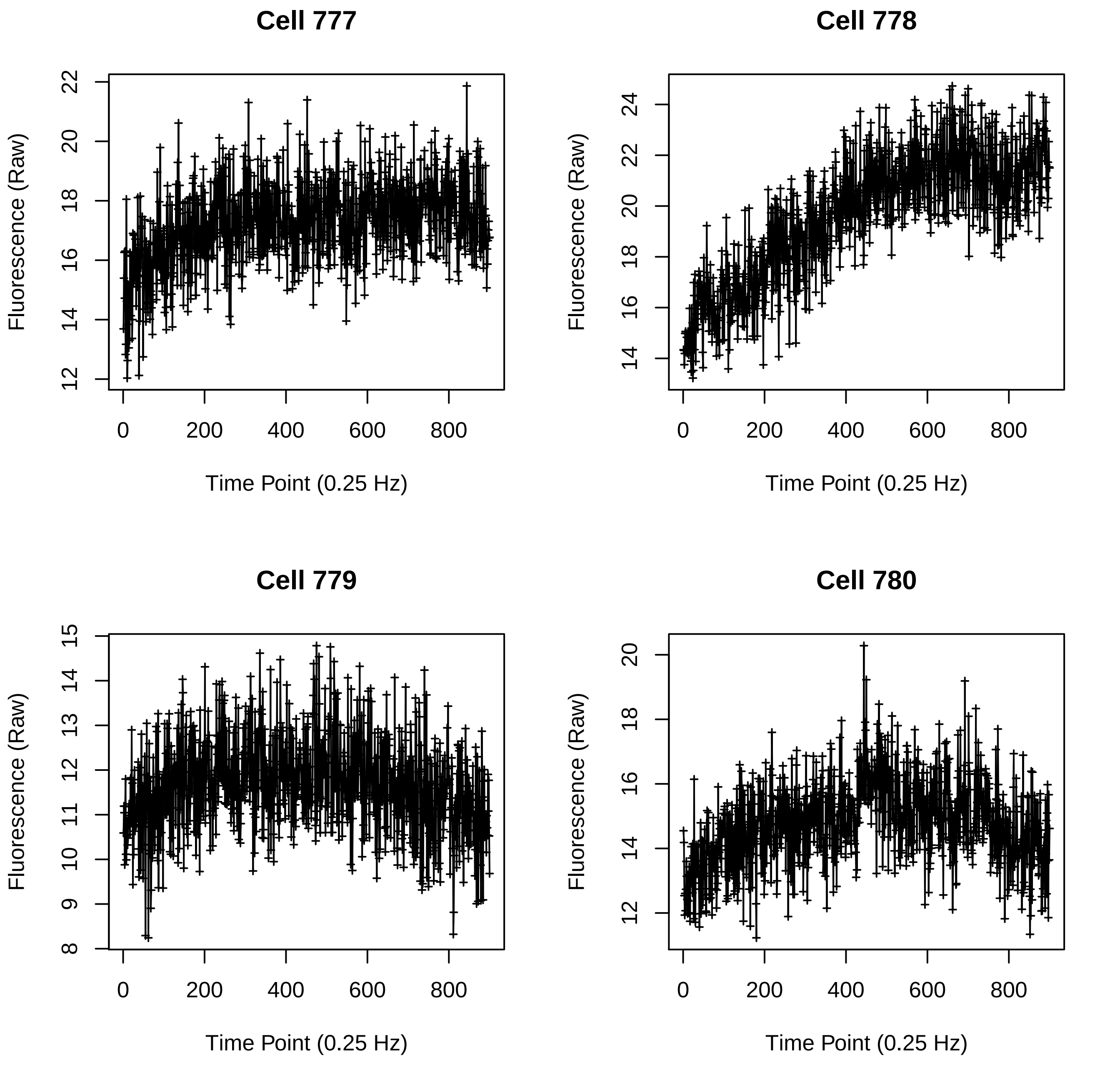

Cell 777
Cell 778
Cell 779
Cell 780
Fluorescence (Raw)
Time Point (0.25 Hz)
0
200
400
600
800
12
14
16
18
20
22
24
8
9
10
11
12
13
14
15

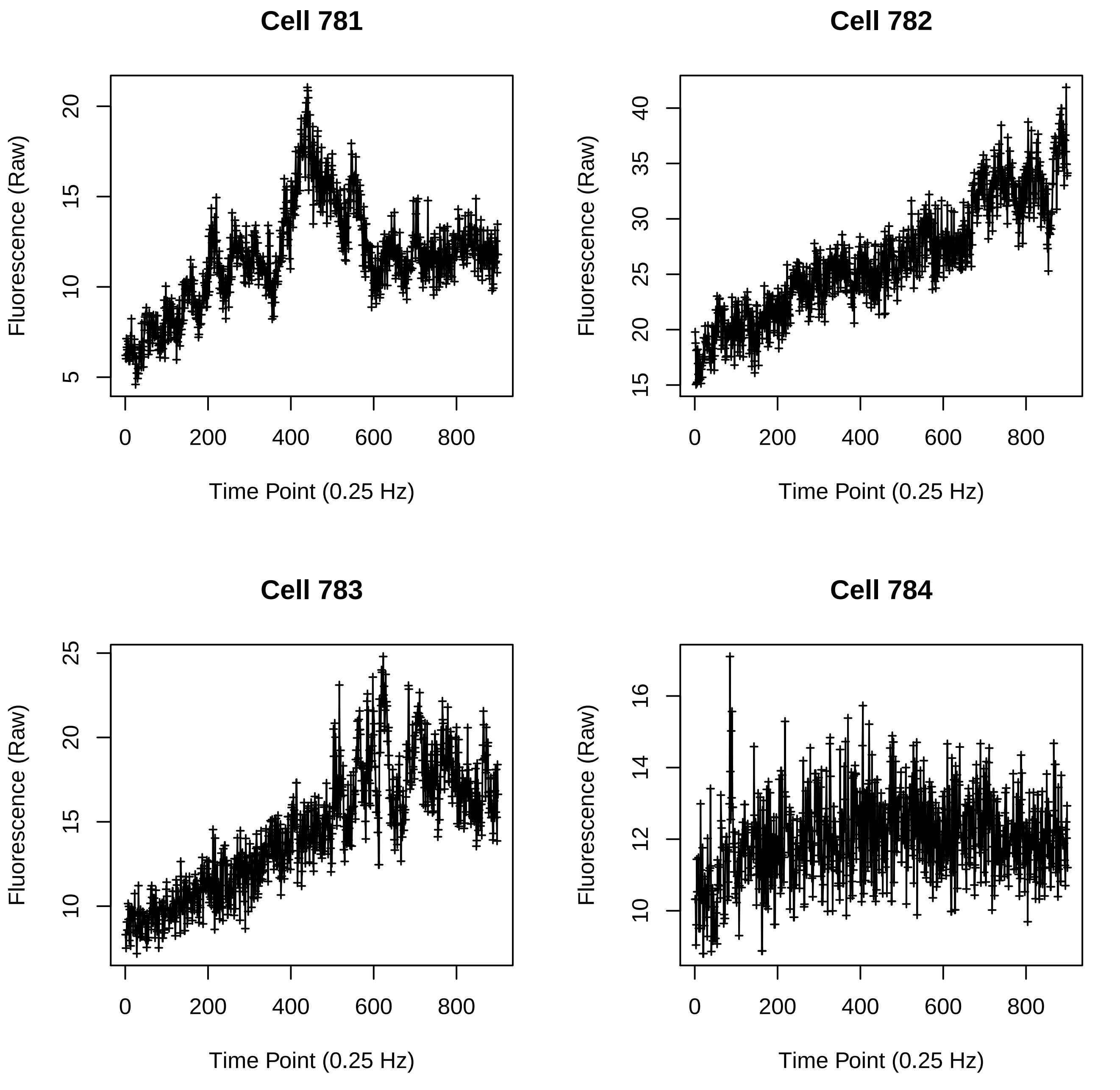
Cell 781
Fluorescence (Raw)
5
10
15
20
0
200
400
600
800
Time Point (0.25 Hz)
Cell 782
Fluorescence (Raw)
15
20
25
30
35
40
0
200
400
600
800
Time Point (0.25 Hz)
Cell 783
Fluorescence (Raw)
10
15
20
25
0
200
400
600
800
Time Point (0.25 Hz)
Cell 784
Fluorescence (Raw)
10
12
14
16
0
200
400
600
800
Time Point (0.25 Hz)

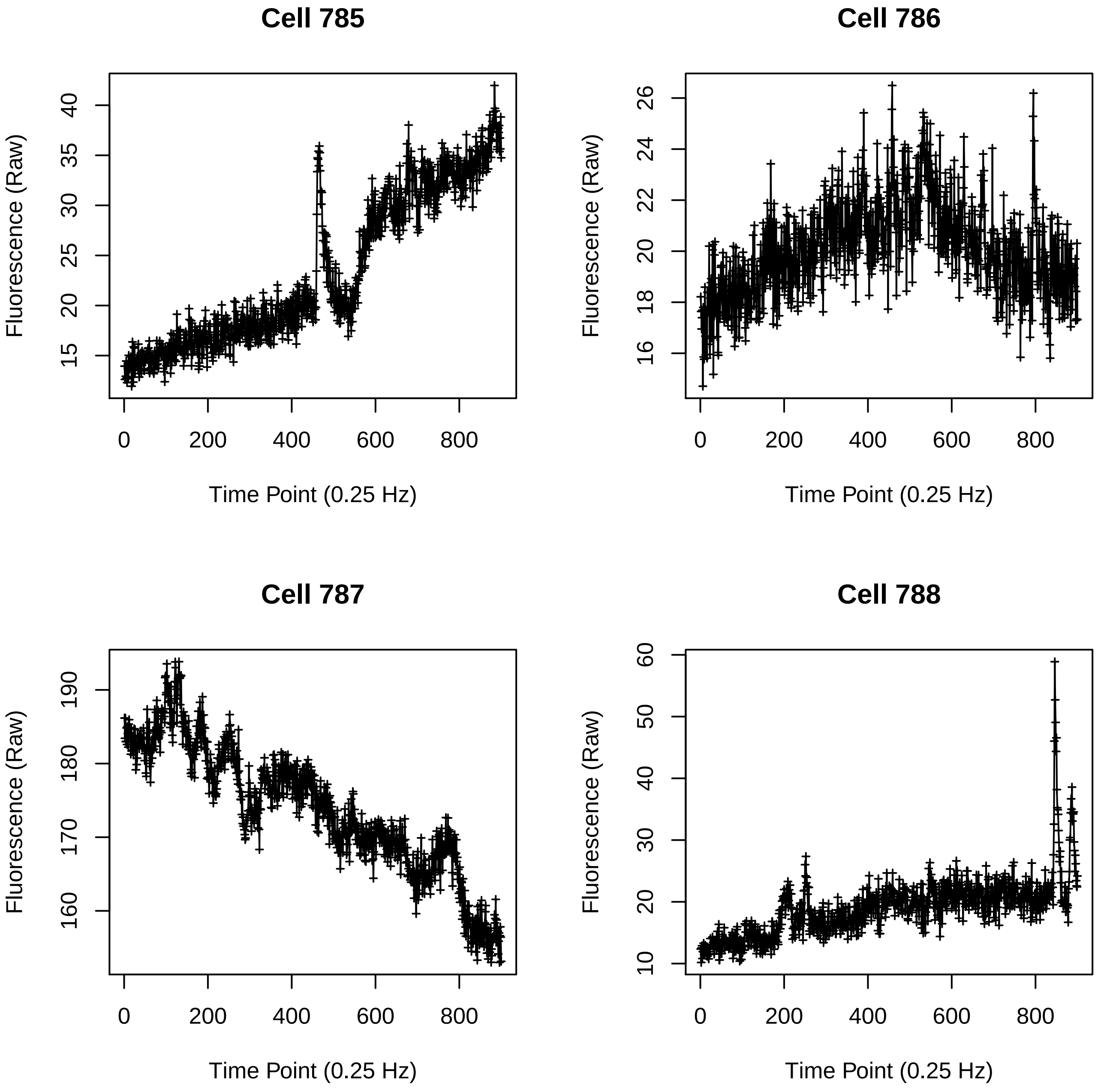
Cell 785
Fluorescence (Raw)
15
20
25
30
35
40
0
200
400
600
800
Time Point (0.25 Hz)
Cell 786
Fluorescence (Raw)
16
18
20
22
24
26
0
200
400
600
800
Time Point (0.25 Hz)
Cell 787
Fluorescence (Raw)
160
170
180
190
0
200
400
600
800
Time Point (0.25 Hz)
Cell 788
Fluorescence (Raw)
10
20
30
40
50
60
0
200
400
600
800
Time Point (0.25 Hz)

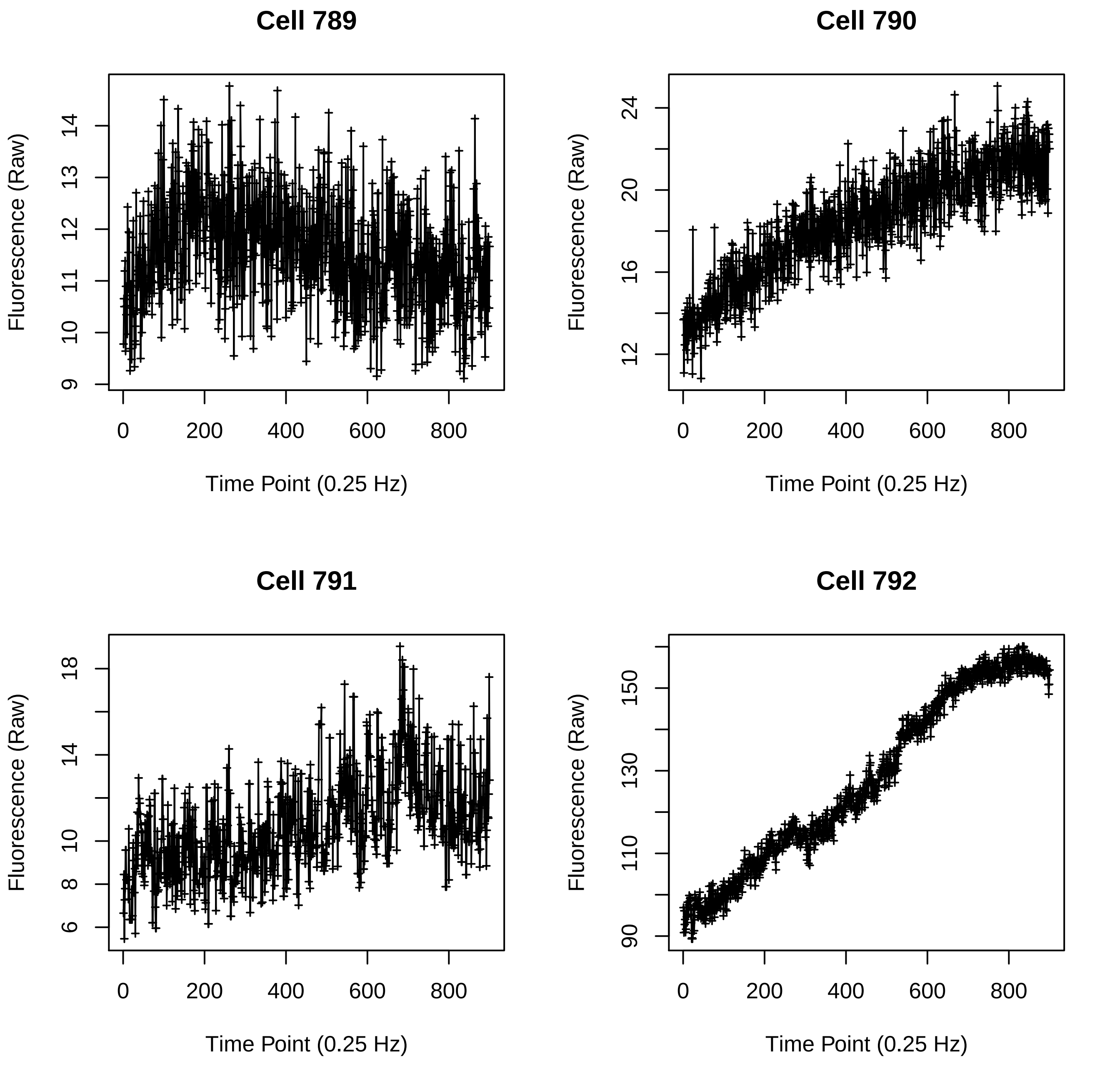
Cell 789
Cell 790
Cell 791
Cell 792
Fluorescence (Raw)
Time Point (0.25 Hz)
0
200
400
600
800
9
10
11
12
13
14
12
16
20
24
6
8
10
14
18
90
110
130
150

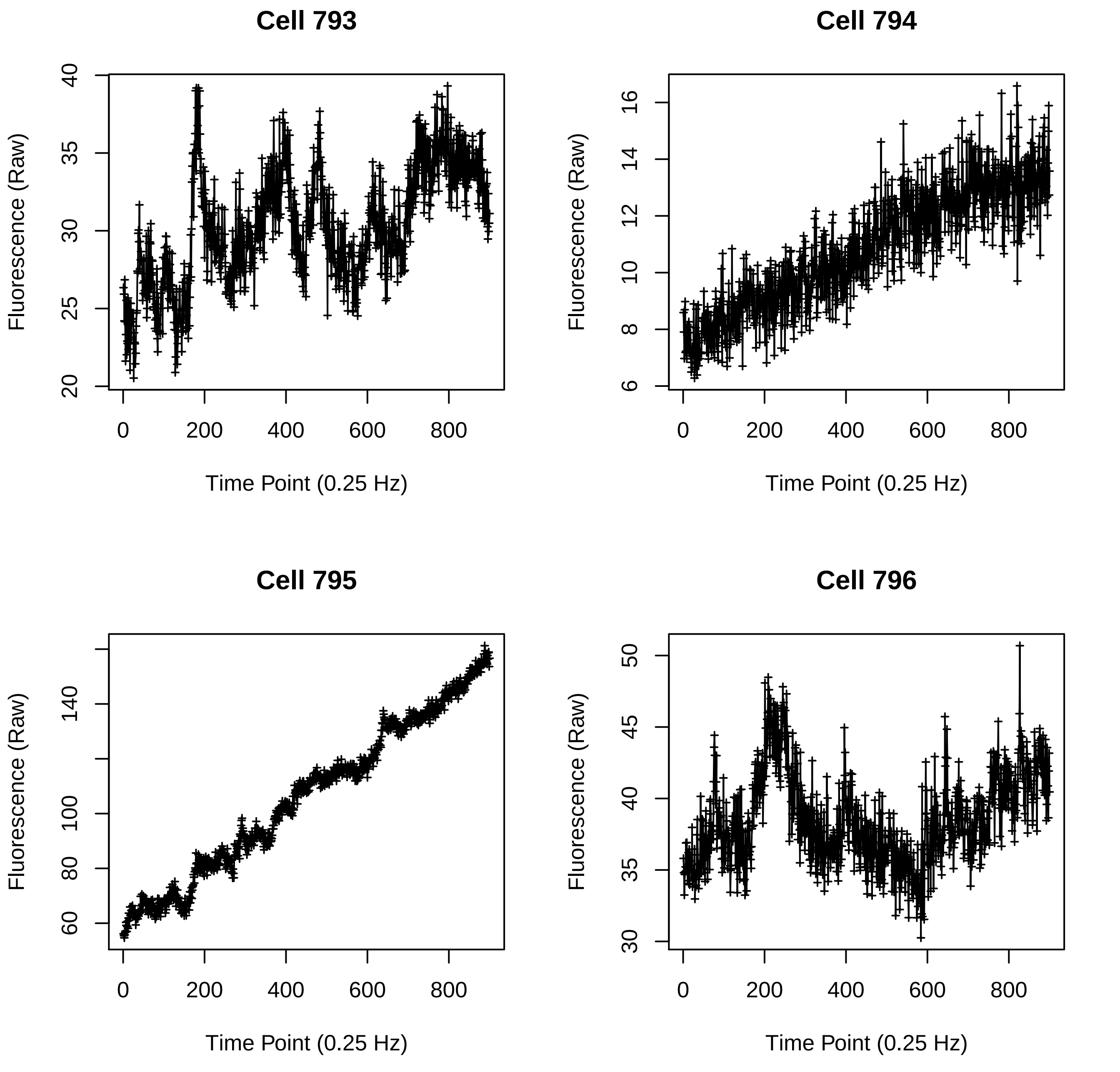

Cell 793
Fluorescence (Raw)
20
25
30
35
40
0
200
400
600
800
Time Point (0.25 Hz)
Cell 794
Fluorescence (Raw)
6
8
10
12
14
16
0
200
400
600
800
Time Point (0.25 Hz)
Cell 795
Fluorescence (Raw)
60
80
100
140
0
200
400
600
800
Time Point (0.25 Hz)
Cell 796
Fluorescence (Raw)
30
35
40
45
50
0
200
400
600
800
Time Point (0.25 Hz)

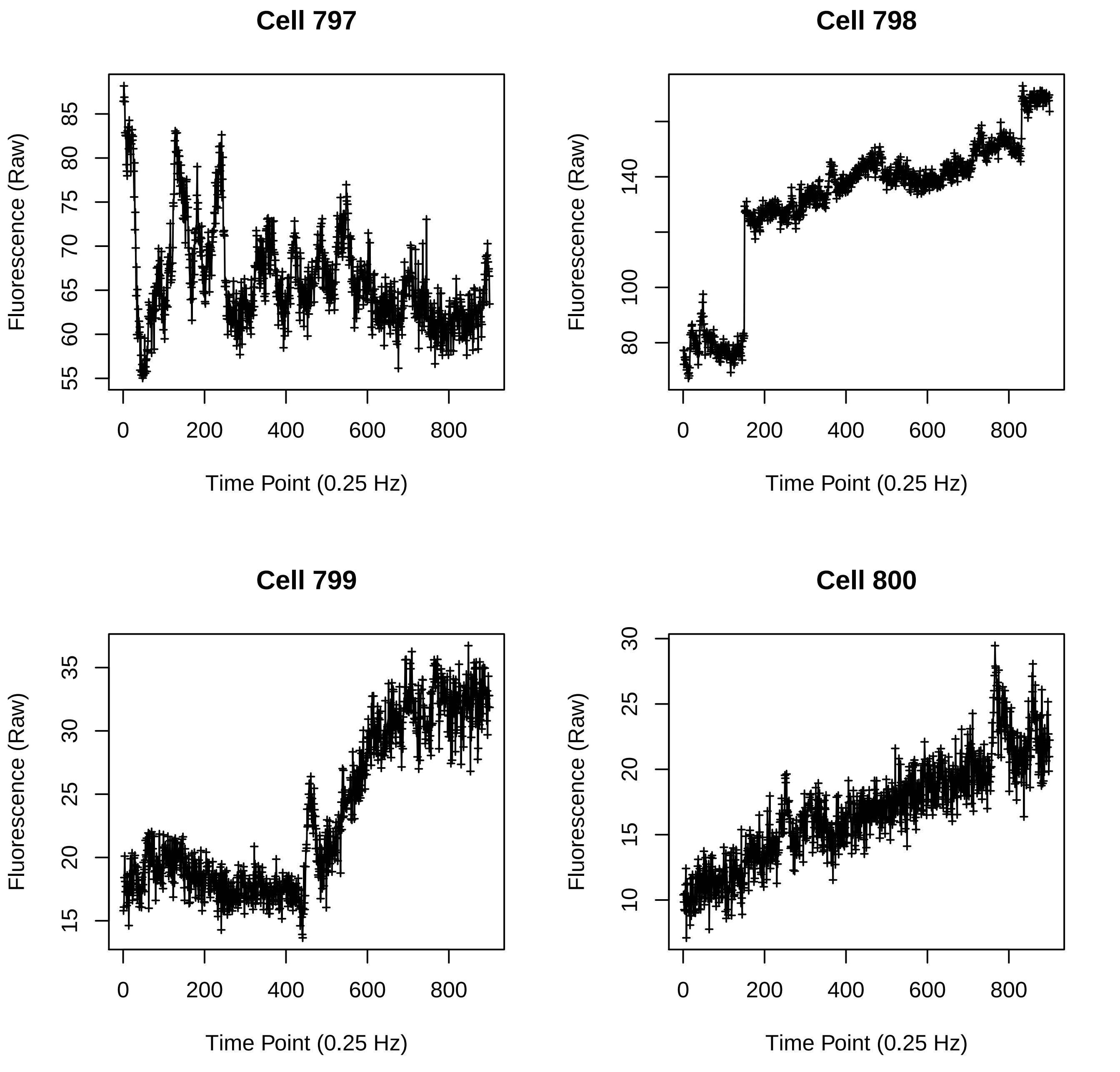
Cell 797
Fluorescence (Raw)
55
60
65
70
75
80
85
0
200
400
600
800
Time Point (0.25 Hz)
Cell 798
Fluorescence (Raw)
80
100
140
0
200
400
600
800
Time Point (0.25 Hz)
Cell 799
Fluorescence (Raw)
15
20
25
30
35
0
200
400
600
800
Time Point (0.25 Hz)
Cell 800
Fluorescence (Raw)
10
15
20
25
30
0
200
400
600
800
Time Point (0.25 Hz)

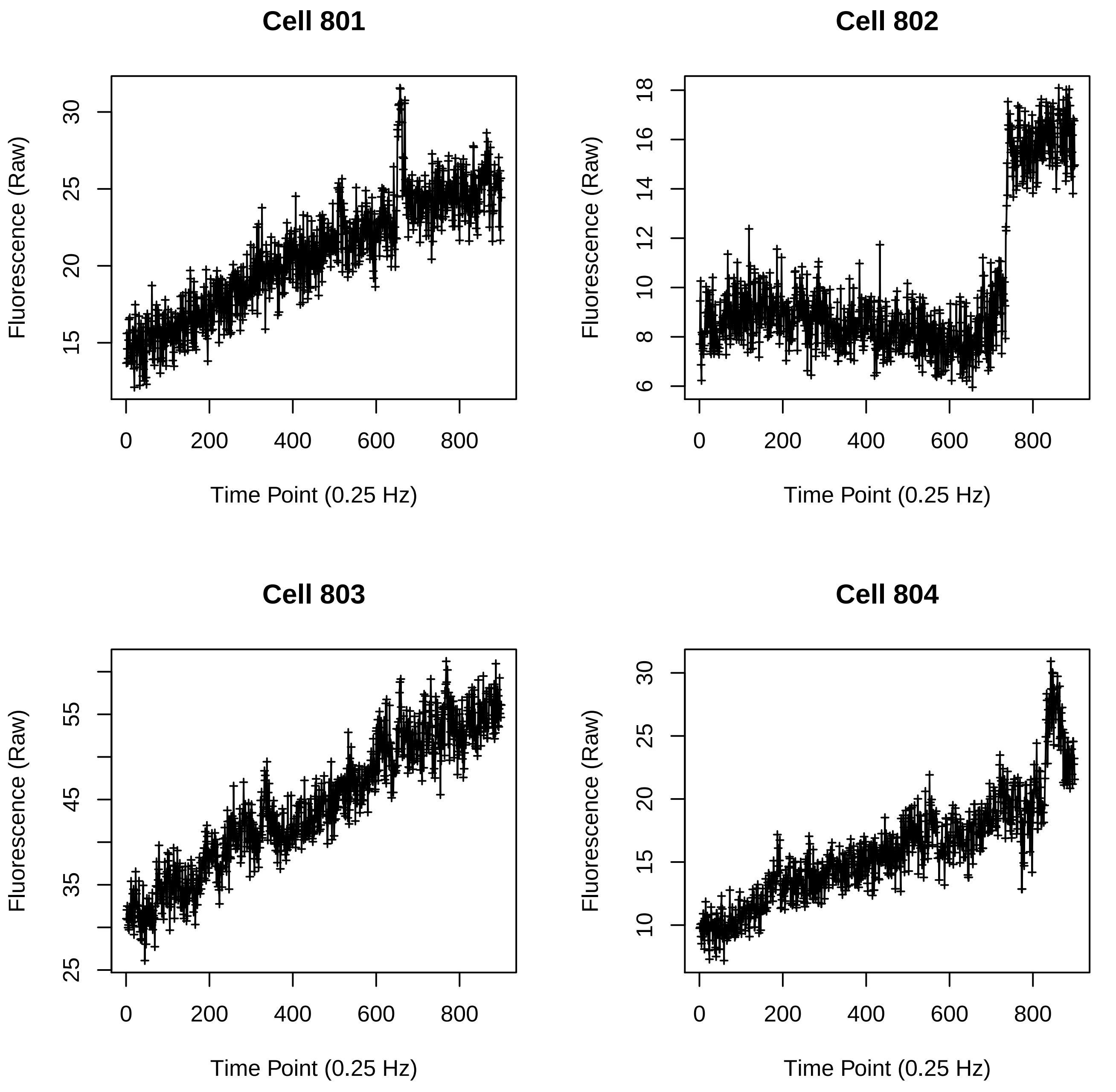
Cell 801
Fluorescence (Raw)
15
20
25
30
0
200
400
600
800
Time Point (0.25 Hz)
Cell 802
Fluorescence (Raw)
6
8
10
12
14
16
18
0
200
400
600
800
Time Point (0.25 Hz)
Cell 803
Fluorescence (Raw)
25
35
45
55
0
200
400
600
800
Time Point (0.25 Hz)
Cell 804
Fluorescence (Raw)
10
15
20
25
30
0
200
400
600
800
Time Point (0.25 Hz)

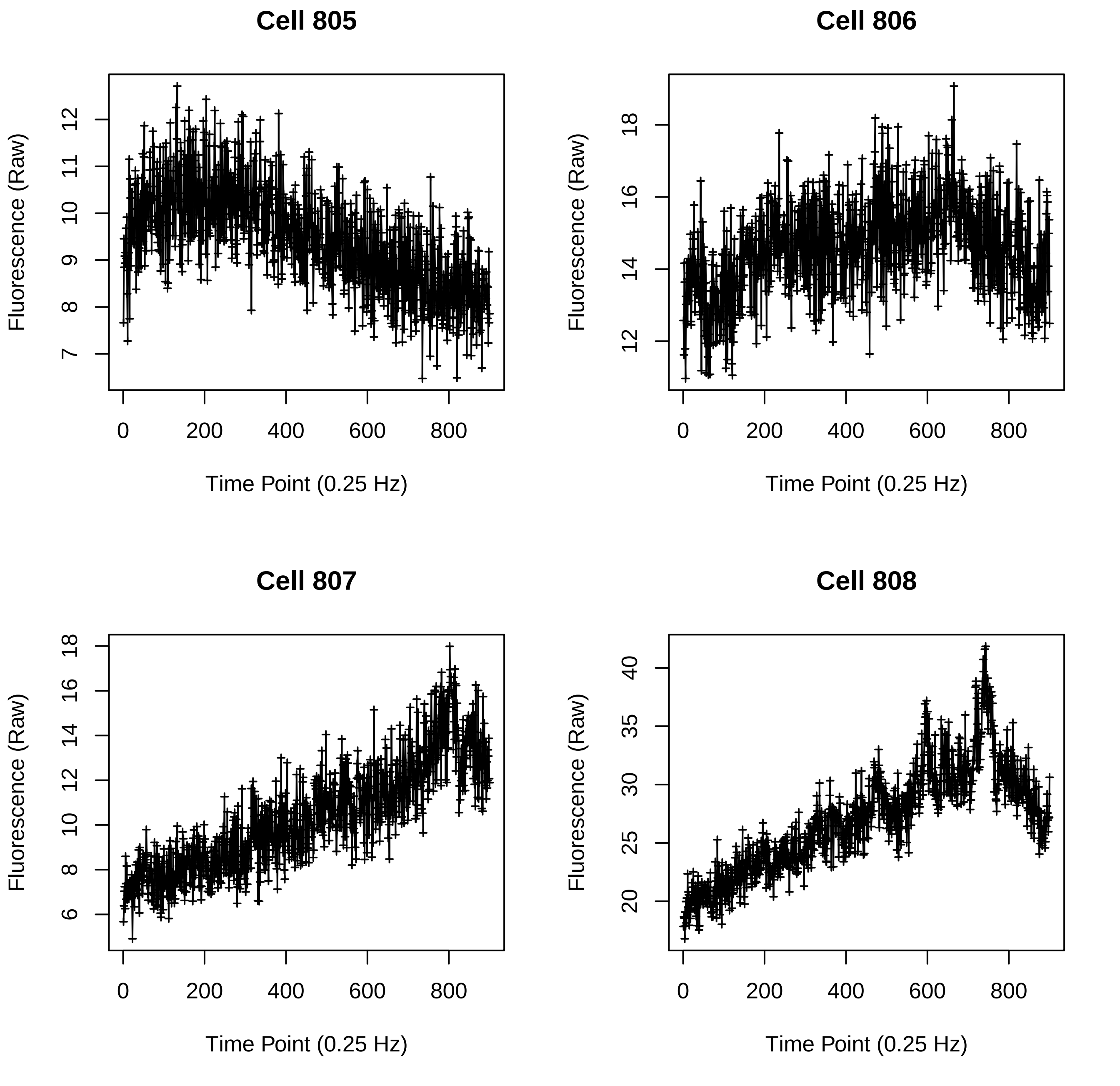
Cell 805
Cell 806
Cell 807
Cell 808
Fluorescence (Raw)
Time Point (0.25 Hz)
0
200
400
600
800

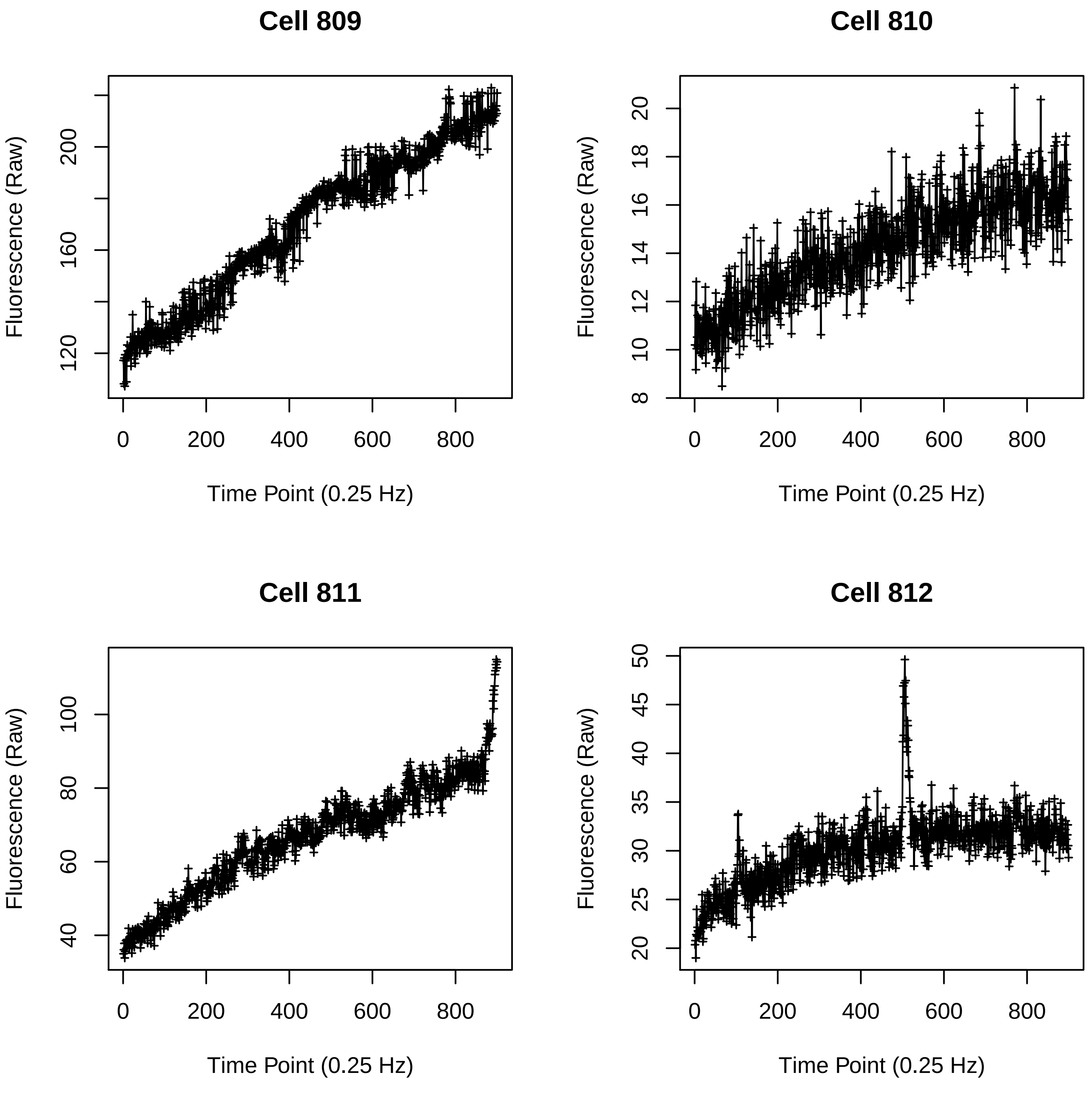
Cell 809
Fluorescence (Raw)
120
160
200
0
200
400
600
800
Time Point (0.25 Hz)
Cell 810
Fluorescence (Raw)
8
10
12
14
16
18
20
0
200
400
600
800
Time Point (0.25 Hz)
Cell 811
Fluorescence (Raw)
40
60
80
100
0
200
400
600
800
Time Point (0.25 Hz)
Cell 812
Fluorescence (Raw)
20
25
30
35
40
45
50
0
200
400
600
800
Time Point (0.25 Hz)

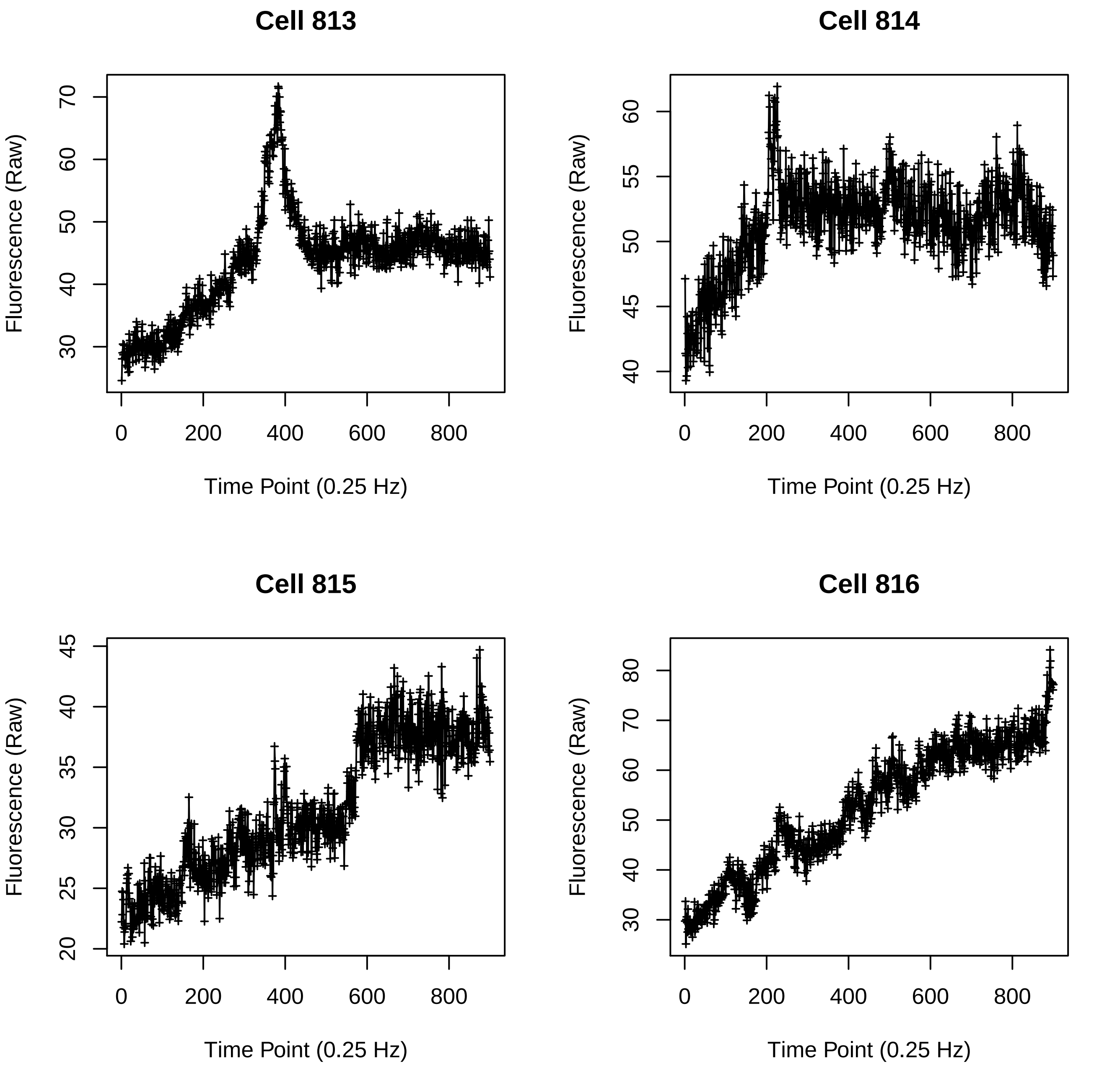

Cell 813
Fluorescence (Raw)
30
40
50
60
70
0
200
400
600
800
Time Point (0.25 Hz)
Cell 814
Fluorescence (Raw)
40
45
50
55
60
0
200
400
600
800
Time Point (0.25 Hz)
Cell 815
Fluorescence (Raw)
20
25
30
35
40
45
0
200
400
600
800
Time Point (0.25 Hz)
Cell 816
Fluorescence (Raw)
30
40
50
60
70
80
0
200
400
600
800
Time Point (0.25 Hz)

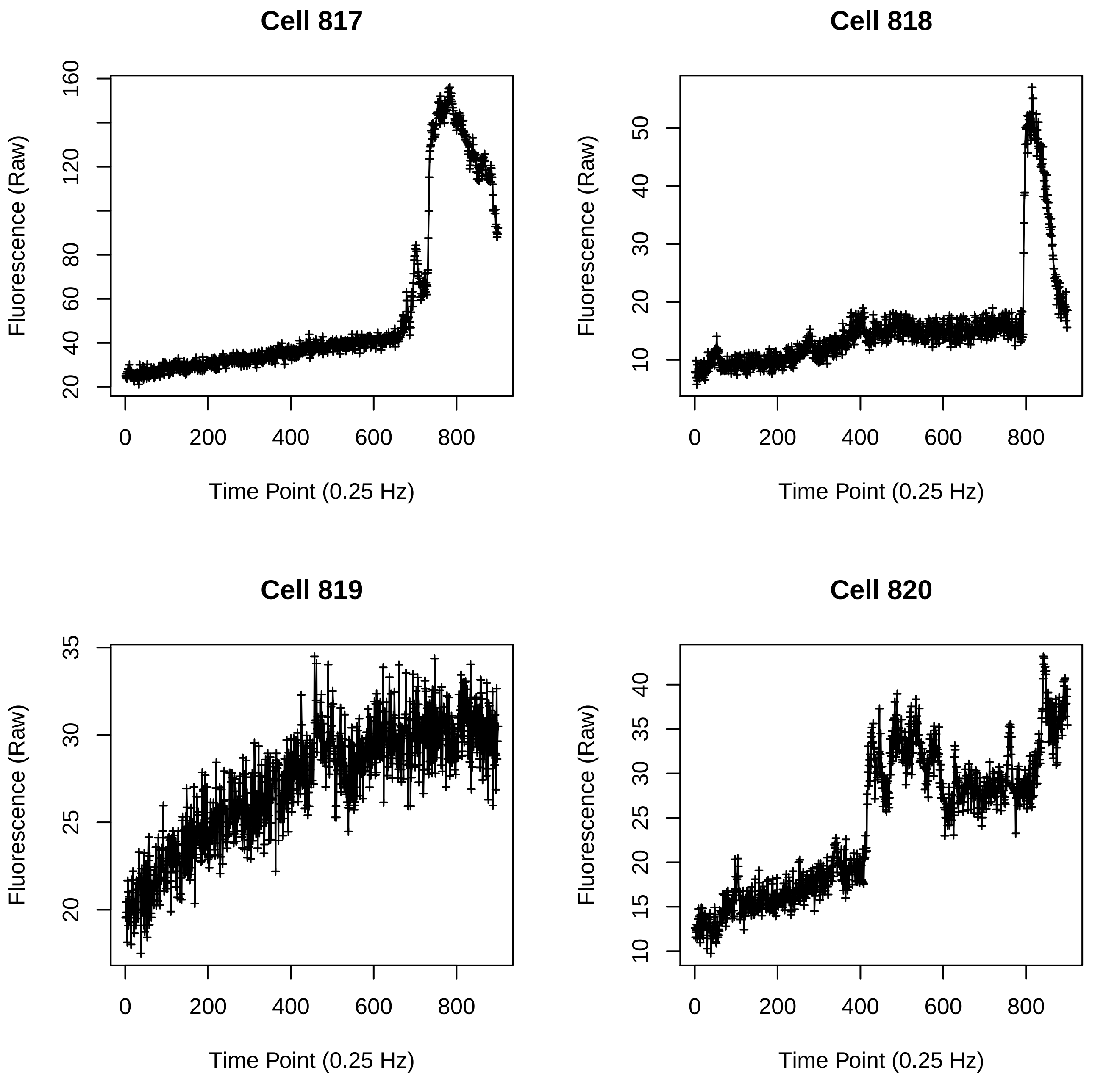

Cell 817
Cell 818
Cell 819
Cell 820
Fluorescence (Raw)
Time Point (0.25 Hz)
0
200
400
600
800
20
40
60
80
120
160
10
20
30
40
50
25
30
35
15

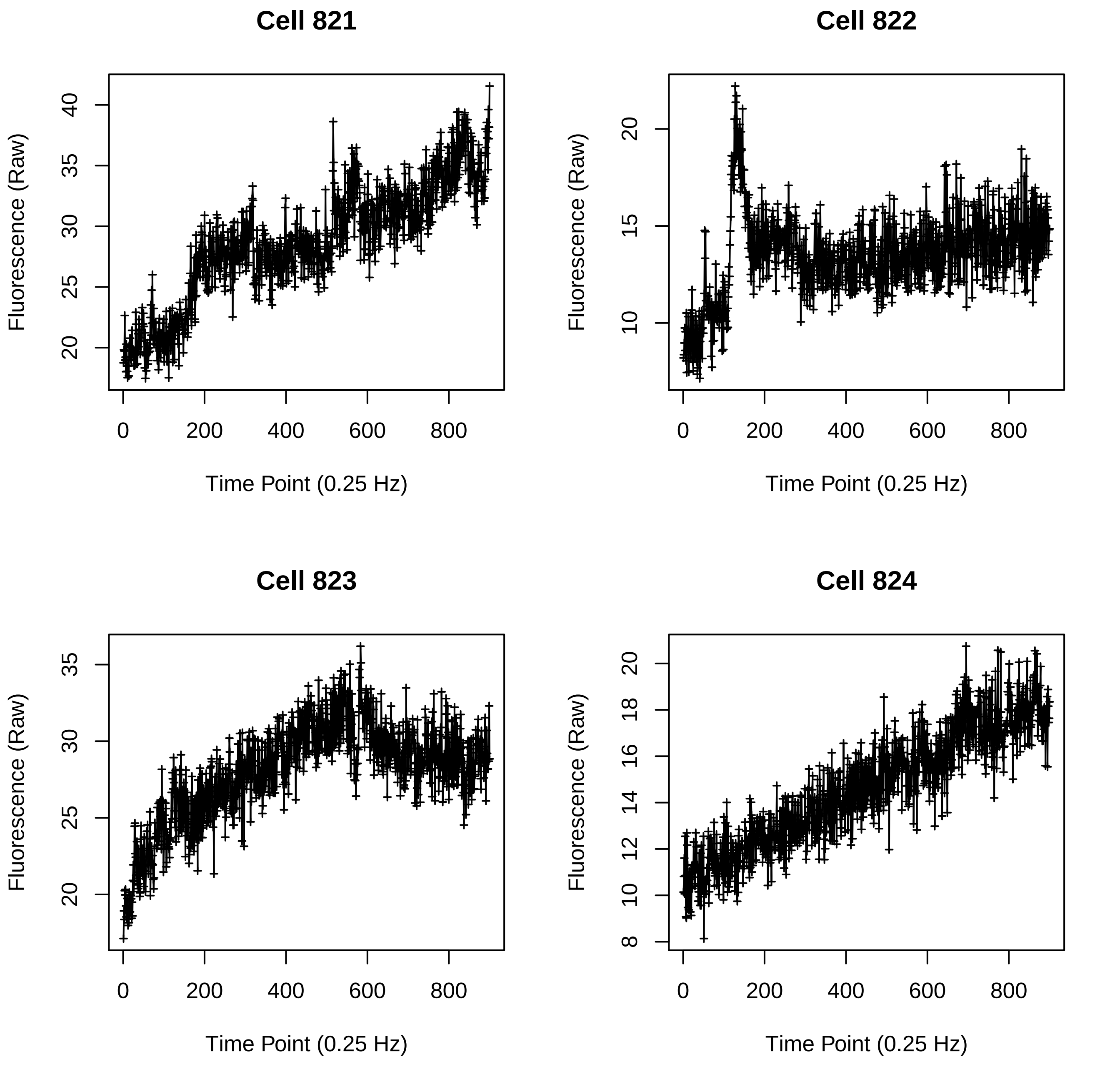

Cell 821
Cell 822
Cell 823
Cell 824
Fluorescence (Raw)
Time Point (0.25 Hz)
0
200
400
600
800
20
25
30
35
40
10
15
20
8
10
12
14
16
18

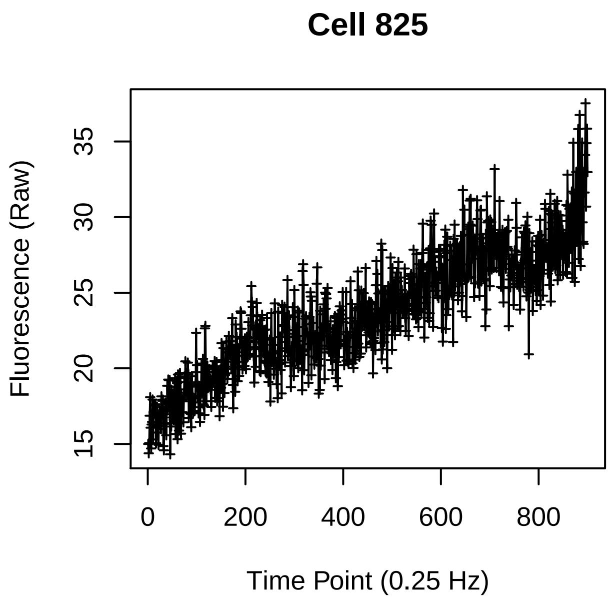
Cell 825
Fluorescence (Raw)
15
20
25
30
35
0
200
400
600
800
Time Point (0.25 Hz)

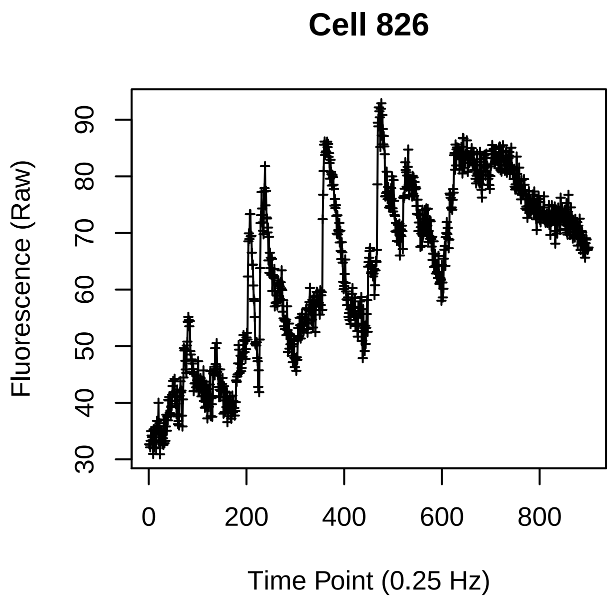
Cell 826
Fluorescence (Raw)
30
40
50
60
70
80
90
0
200
400
600
800
Time Point (0.25 Hz)

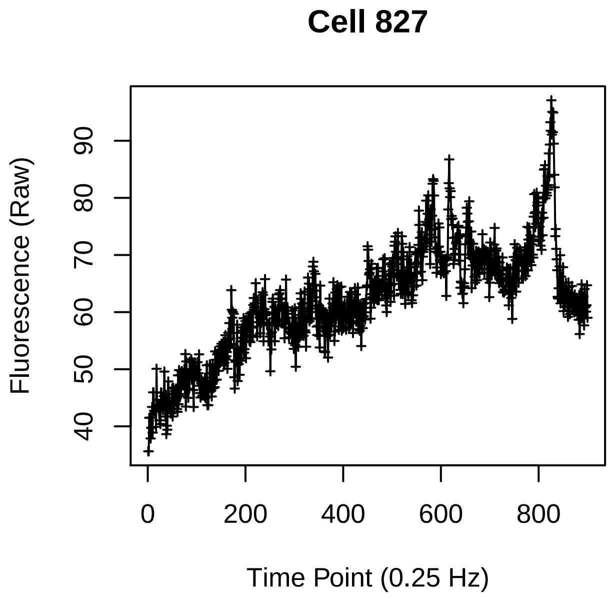
Cell 827
Fluorescence (Raw)
40
50
60
70
80
90
0
200
400
600
800
Time Point (0.25 Hz)

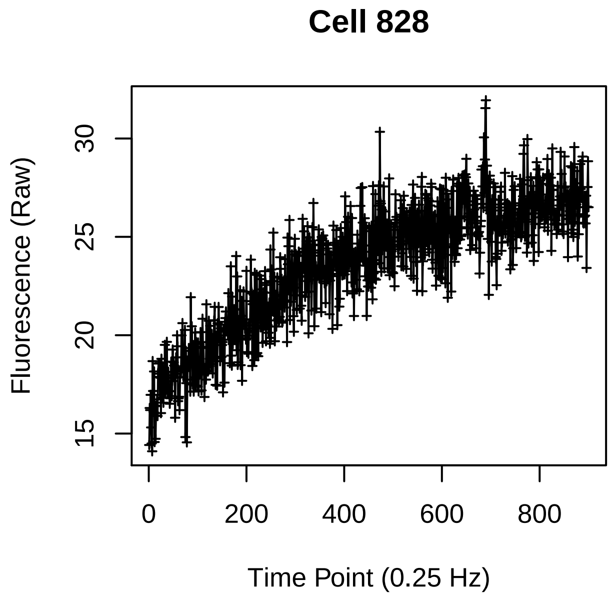
Cell 828
Fluorescence (Raw)
15
20
25
30
0
200
400
600
800
Time Point (0.25 Hz)

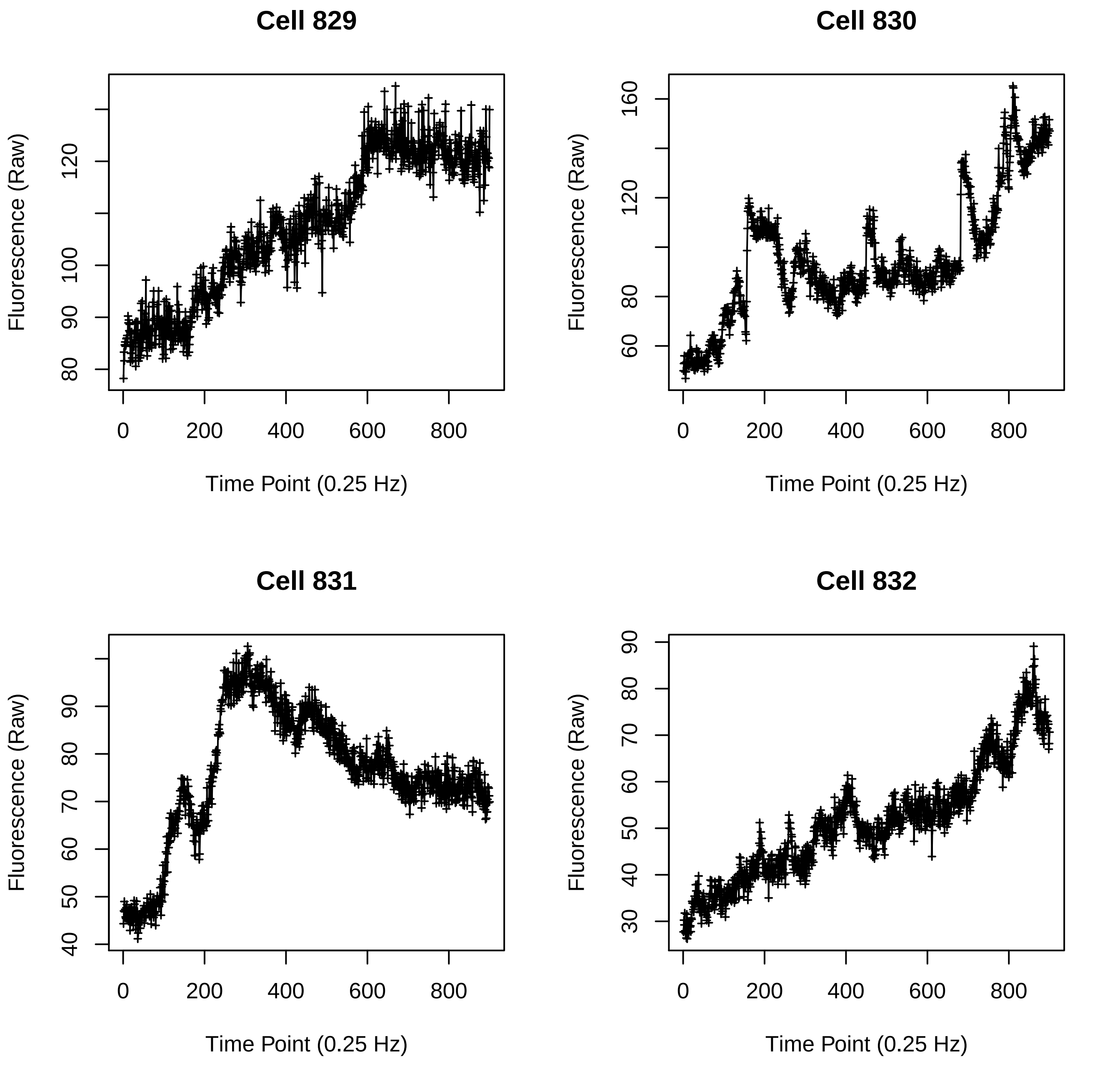
Cell 829
Cell 830
Cell 831
Cell 832
Fluorescence (Raw)
Time Point (0.25 Hz)
0
200
400
600
800
80
90
100
120
60
160
140
40
50
70
30

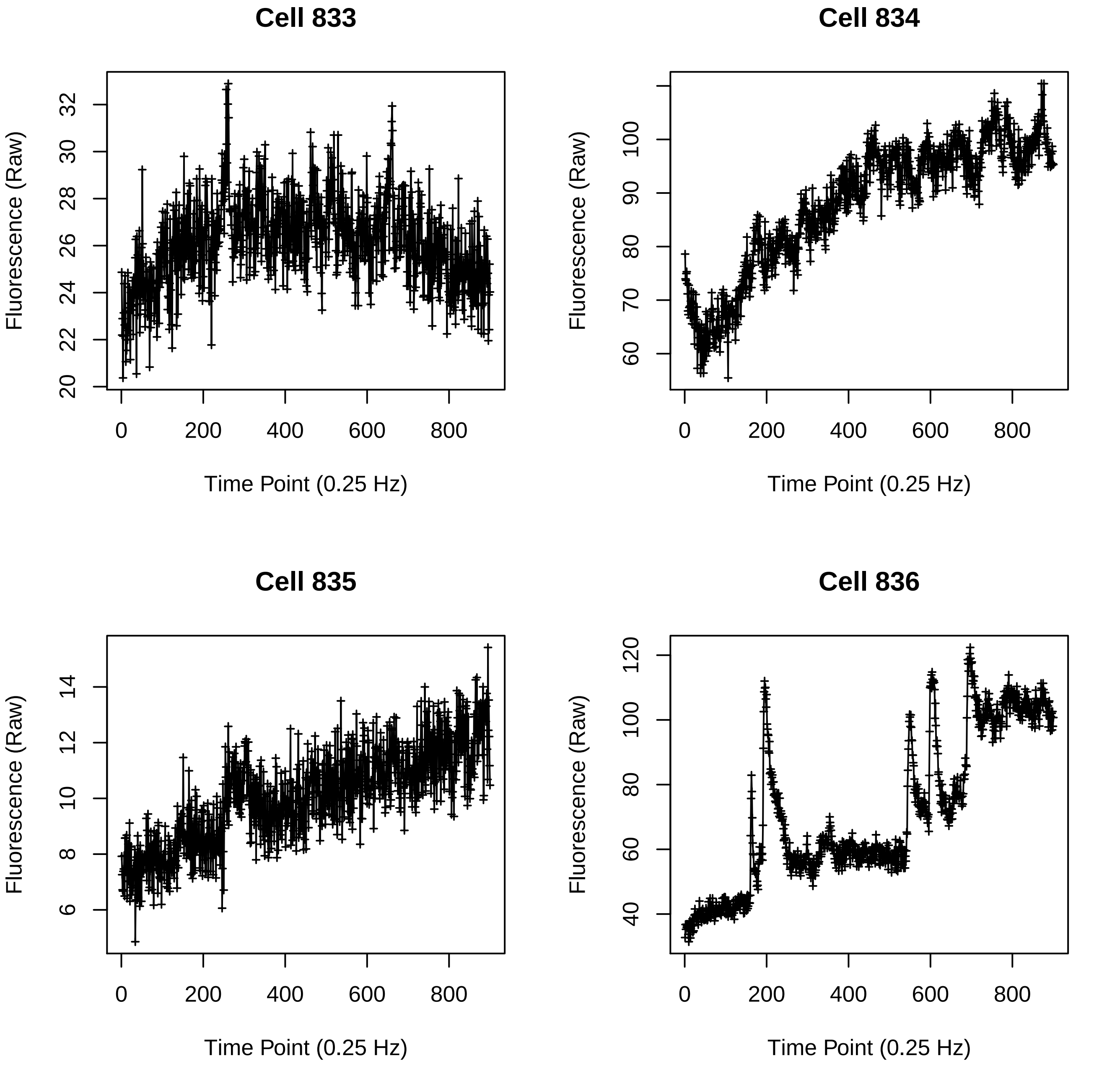
Cell 833
Fluorescence (Raw)
Time Point (0.25 Hz)
20
22
24
26
28
30
32
0
200
400
600
800
Cell 834
Fluorescence (Raw)
Time Point (0.25 Hz)
60
70
80
90
100
0
200
400
600
800
Cell 835
Fluorescence (Raw)
Time Point (0.25 Hz)
6
8
10
12
14
0
200
400
600
800
Cell 836
Fluorescence (Raw)
Time Point (0.25 Hz)
40
60
80
100
120
0
200
400
600
800

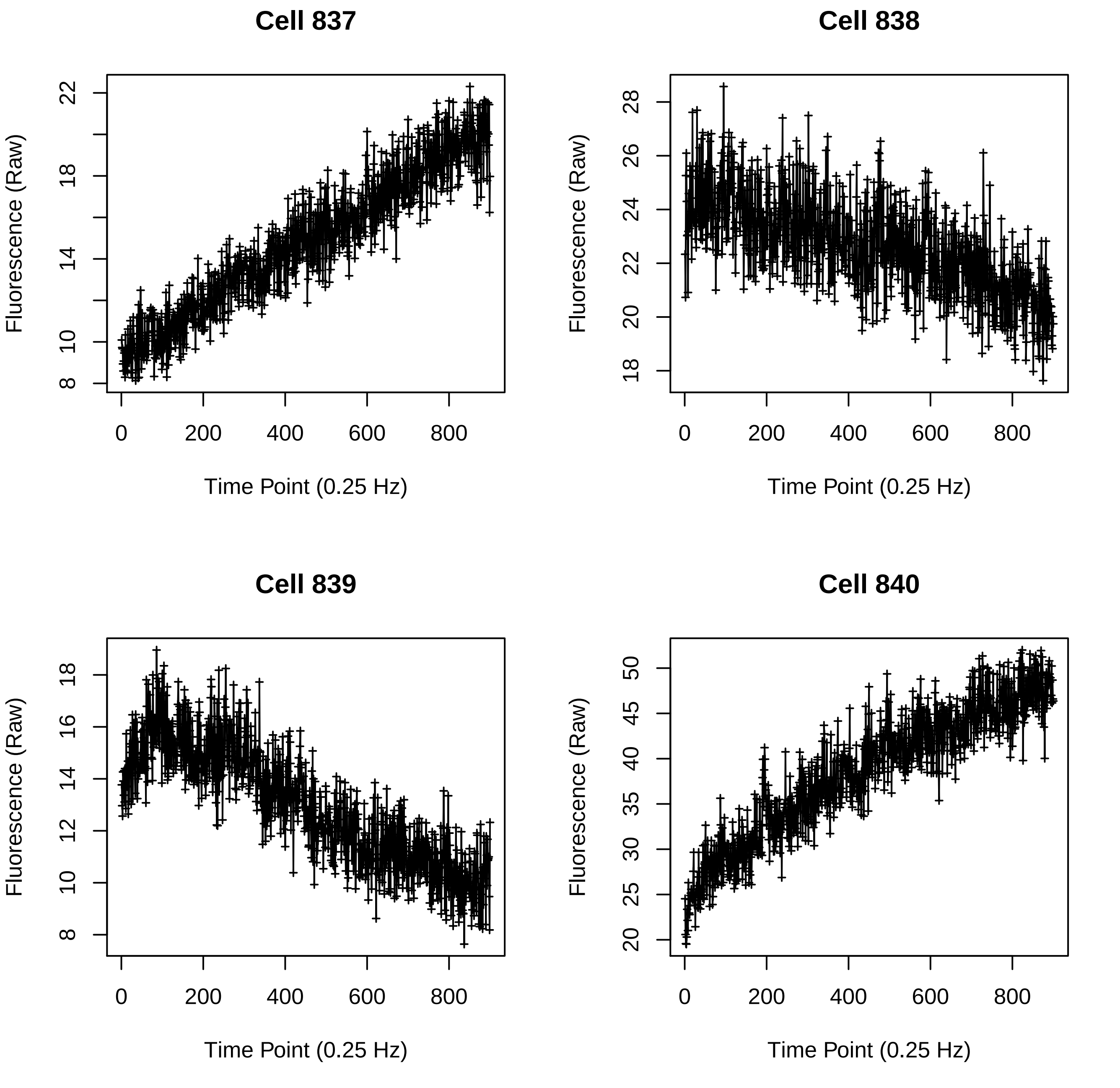

Cell 837
Cell 838
Cell 839
Cell 840
Fluorescence (Raw)
Time Point (0.25 Hz)
0
200
400
600
800
8
10
14
18
22
18
20
22
24
26
28
8
10
12
14
16
18
20
25
30
35
40
45
50

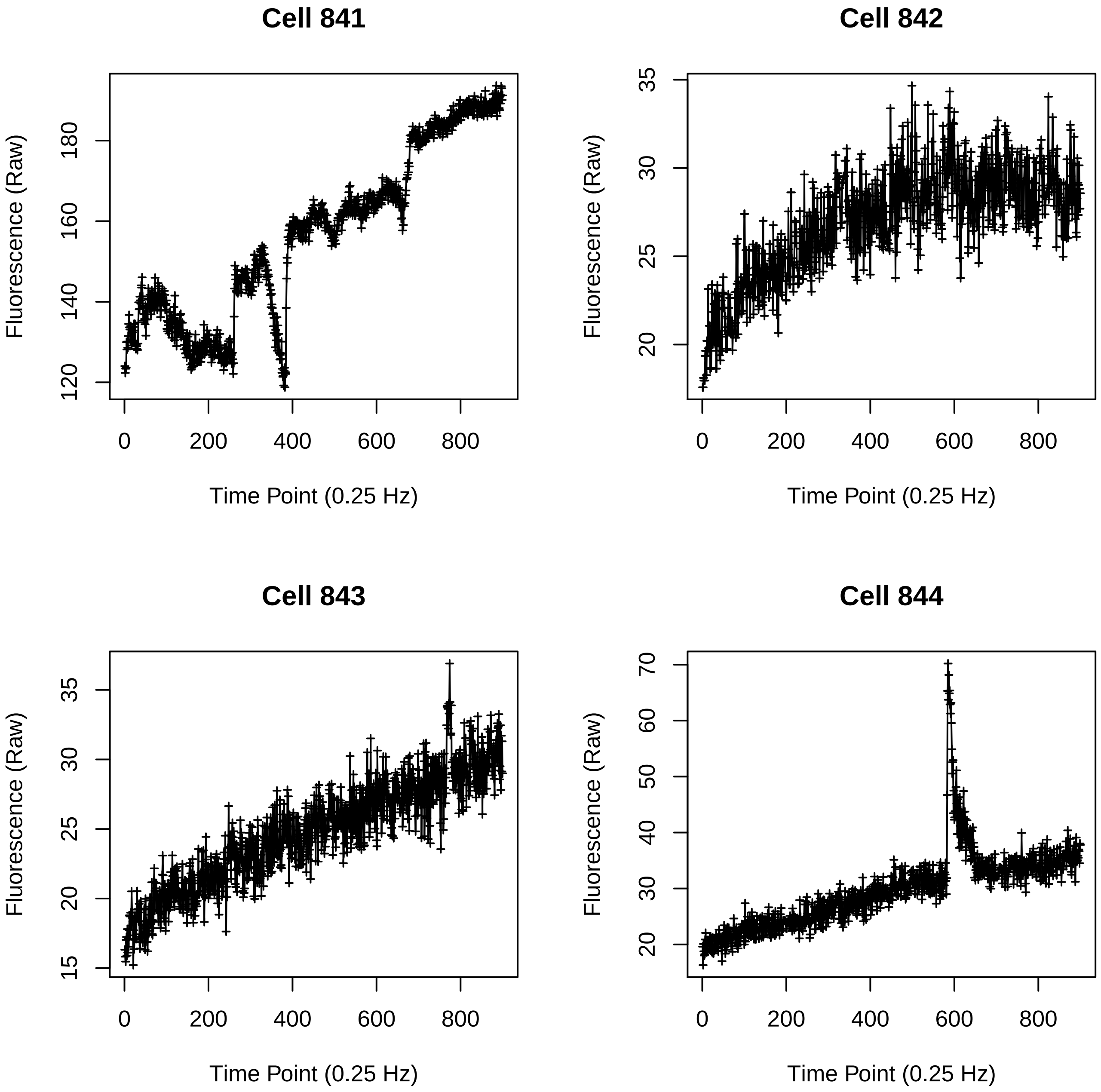
Cell 841
Fluorescence (Raw)
120
140
160
180
0
200
400
600
800
Time Point (0.25 Hz)
Cell 842
Fluorescence (Raw)
20
25
30
35
0
200
400
600
800
Time Point (0.25 Hz)
Cell 843
Fluorescence (Raw)
15
20
25
30
35
0
200
400
600
800
Time Point (0.25 Hz)
Cell 844
Fluorescence (Raw)
20
30
40
50
60
70
0
200
400
600
800
Time Point (0.25 Hz)

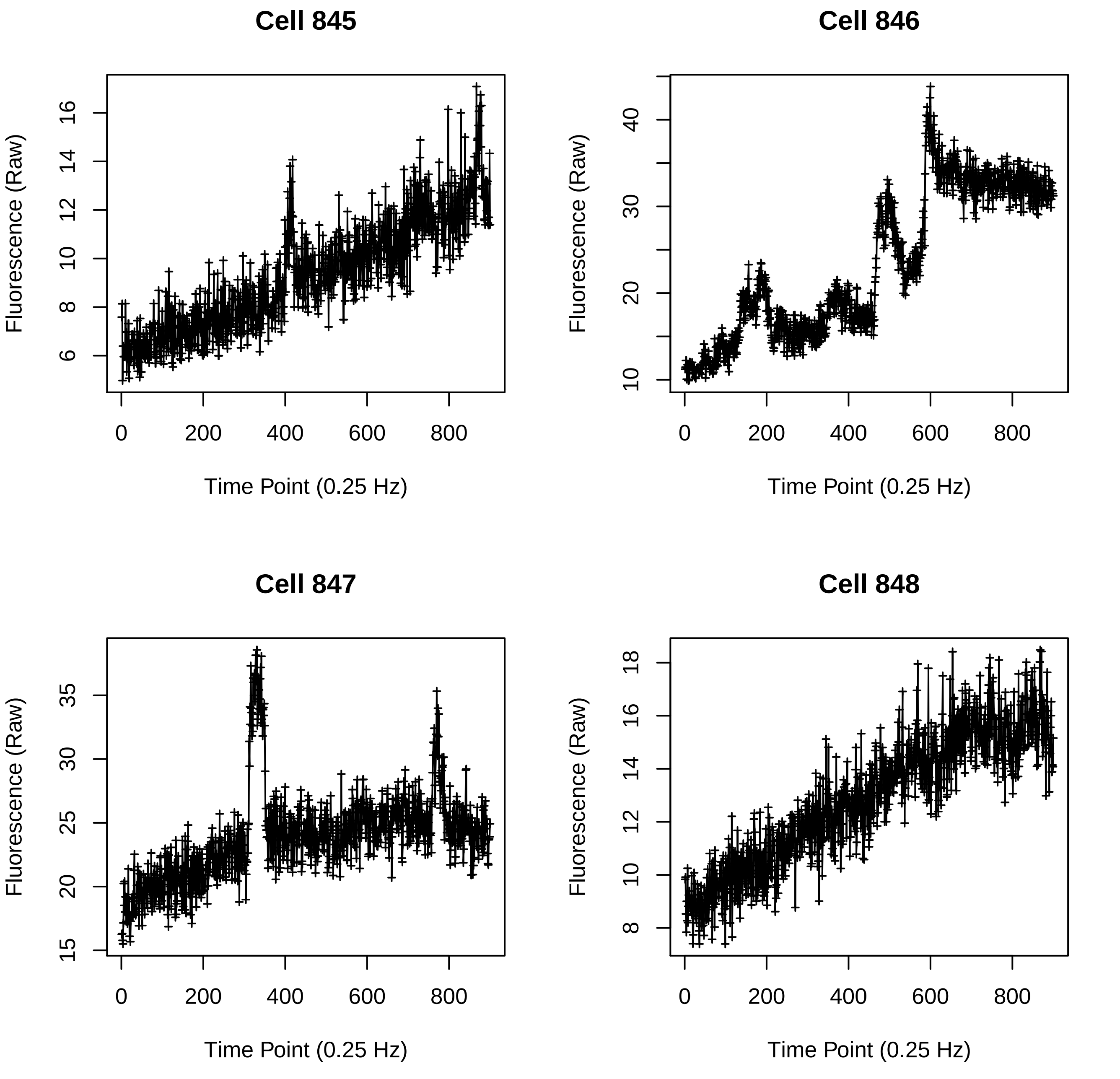

Cell 845
Fluorescence (Raw)
6
8
10
12
14
16
0
200
400
600
800
Time Point (0.25 Hz)
Cell 846
Fluorescence (Raw)
10
20
30
40
0
200
400
600
800
Time Point (0.25 Hz)
Cell 847
Fluorescence (Raw)
15
20
25
30
35
0
200
400
600
800
Time Point (0.25 Hz)
Cell 848
Fluorescence (Raw)
8
10
12
14
16
18
0
200
400
600
800
Time Point (0.25 Hz)

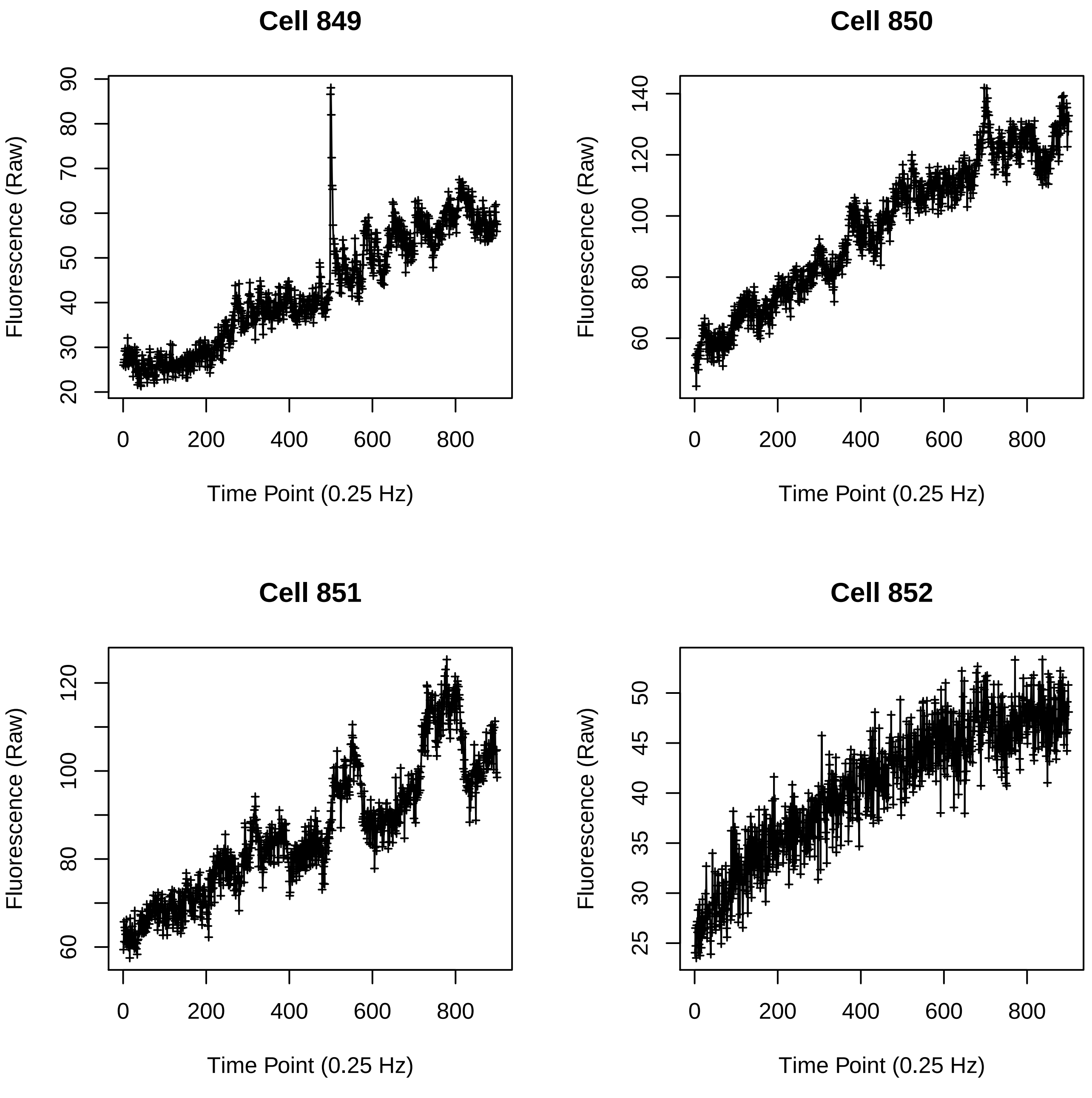

Cell 849
Fluorescence (Raw)
20
30
40
50
60
70
80
90
0
200
400
600
800
Time Point (0.25 Hz)
Cell 850
Fluorescence (Raw)
60
80
100
120
140
0
200
400
600
800
Time Point (0.25 Hz)
Cell 851
Fluorescence (Raw)
60
80
100
120
0
200
400
600
800
Time Point (0.25 Hz)
Cell 852
Fluorescence (Raw)
25
30
35
40
45
50
0
200
400
600
800
Time Point (0.25 Hz)

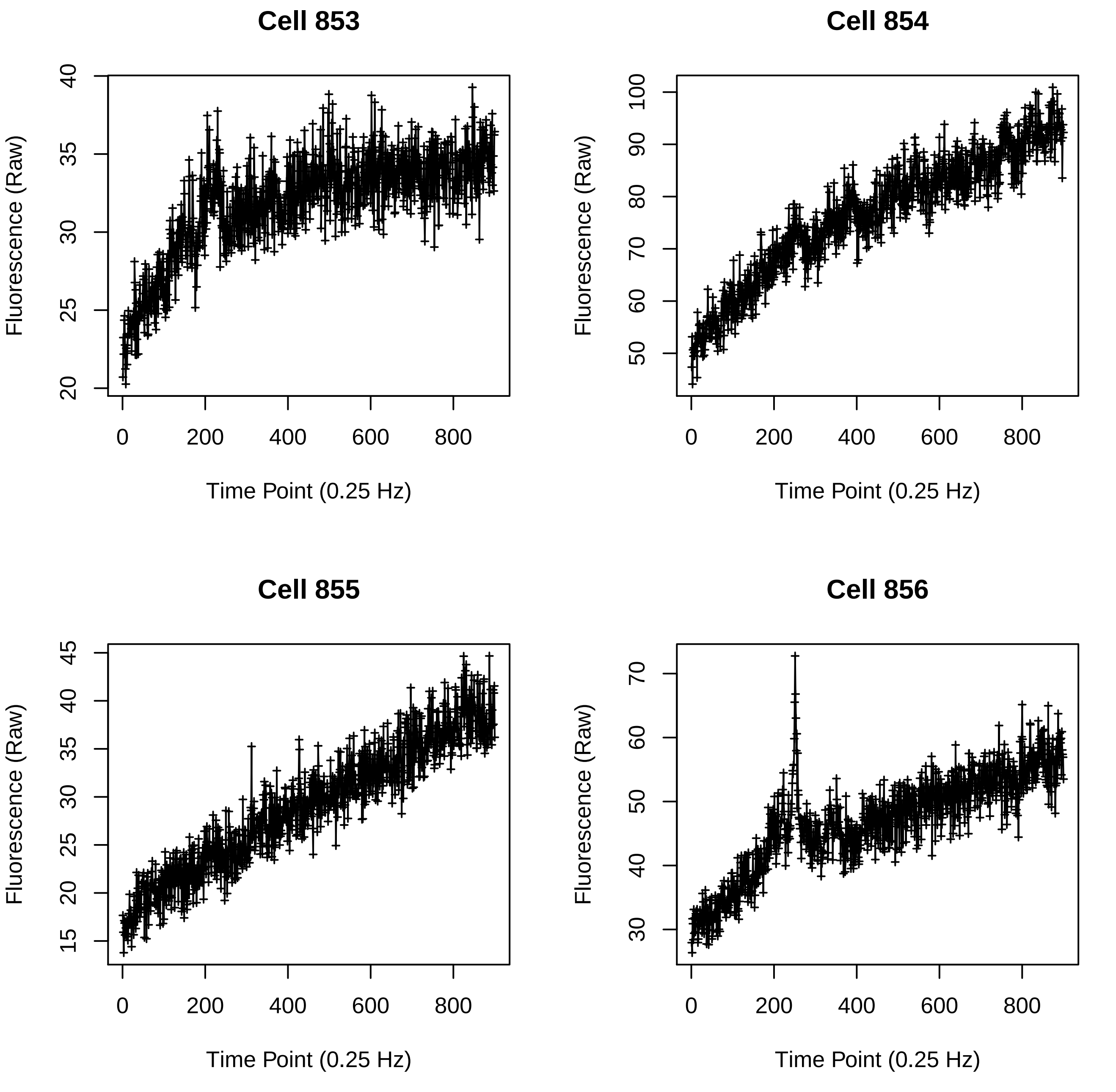
Cell 853
Fluorescence (Raw)
Time Point (0.25 Hz)
Cell 854
Fluorescence (Raw)
Time Point (0.25 Hz)
Cell 855
Fluorescence (Raw)
Time Point (0.25 Hz)
Cell 856
Fluorescence (Raw)
Time Point (0.25 Hz)

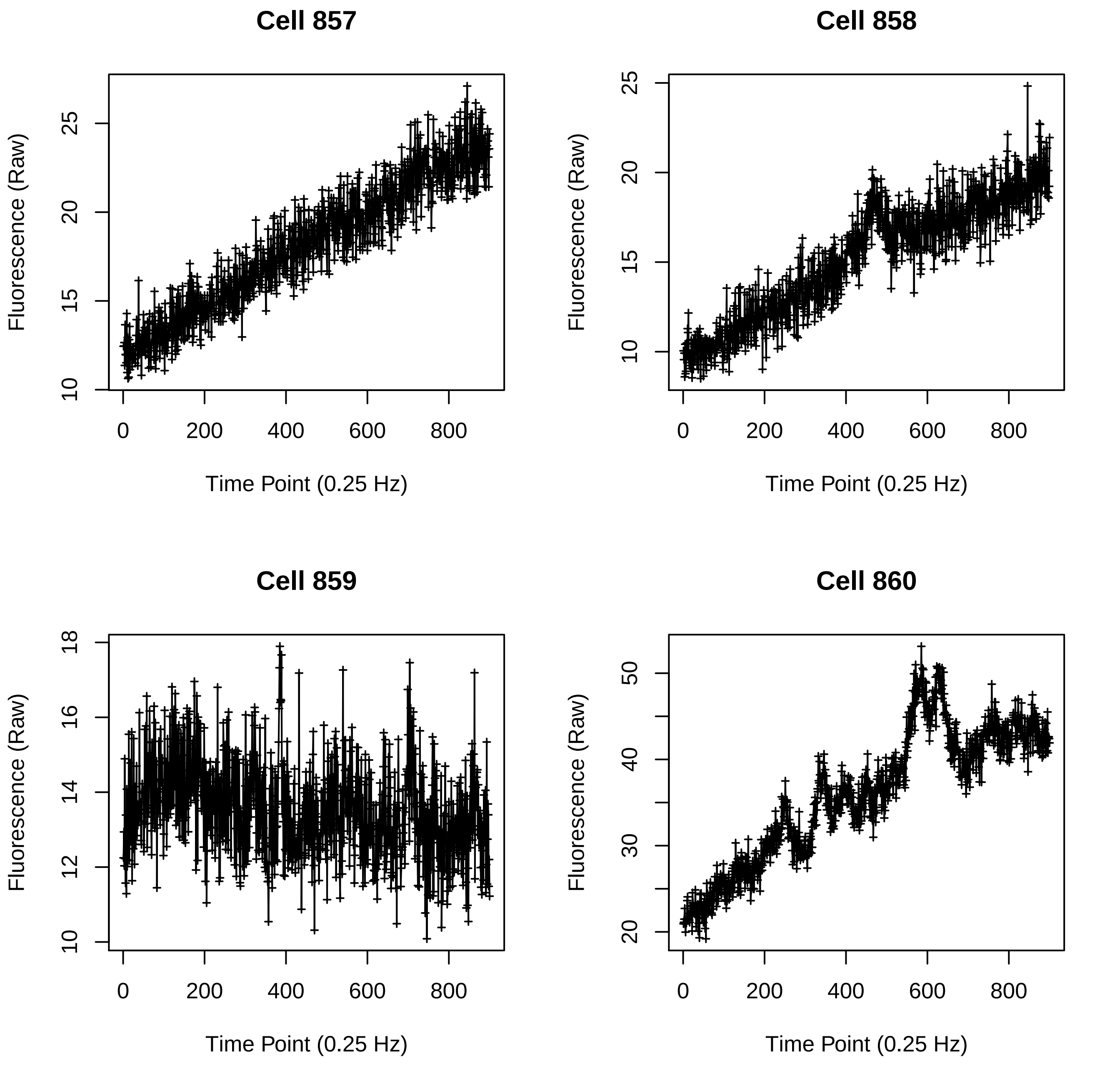

Cell 857
Cell 858
Cell 859
Cell 860
Fluorescence (Raw)
Time Point (0.25 Hz)
0
200
400
600
800
10
15
20
25
12
14
16
18
30
40
50

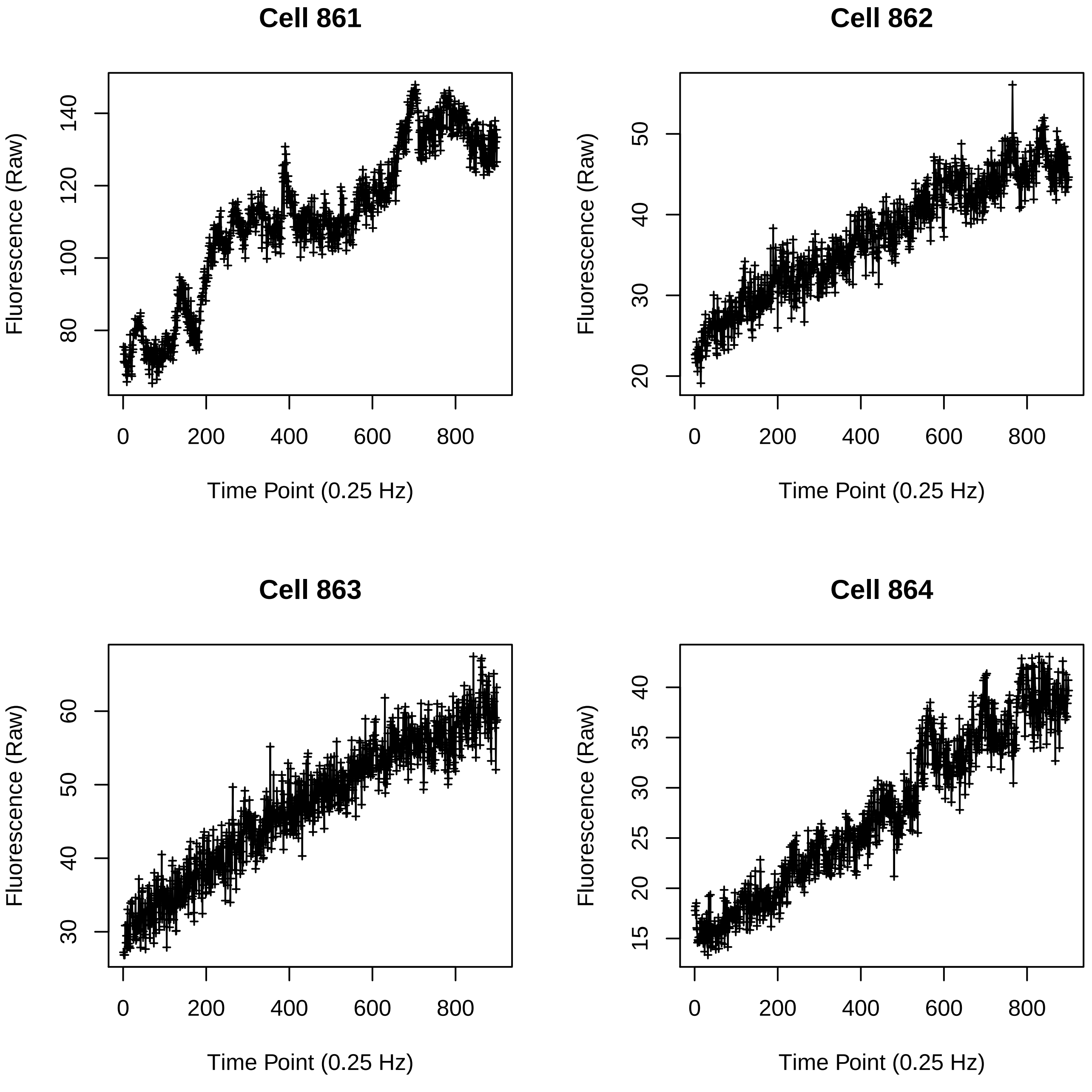
Cell 861
Cell 862
Cell 863
Cell 864
Fluorescence (Raw)
Time Point (0.25 Hz)
80
100
120
140
20
30
40
50
60
15
25
35
0
200
400
600
800

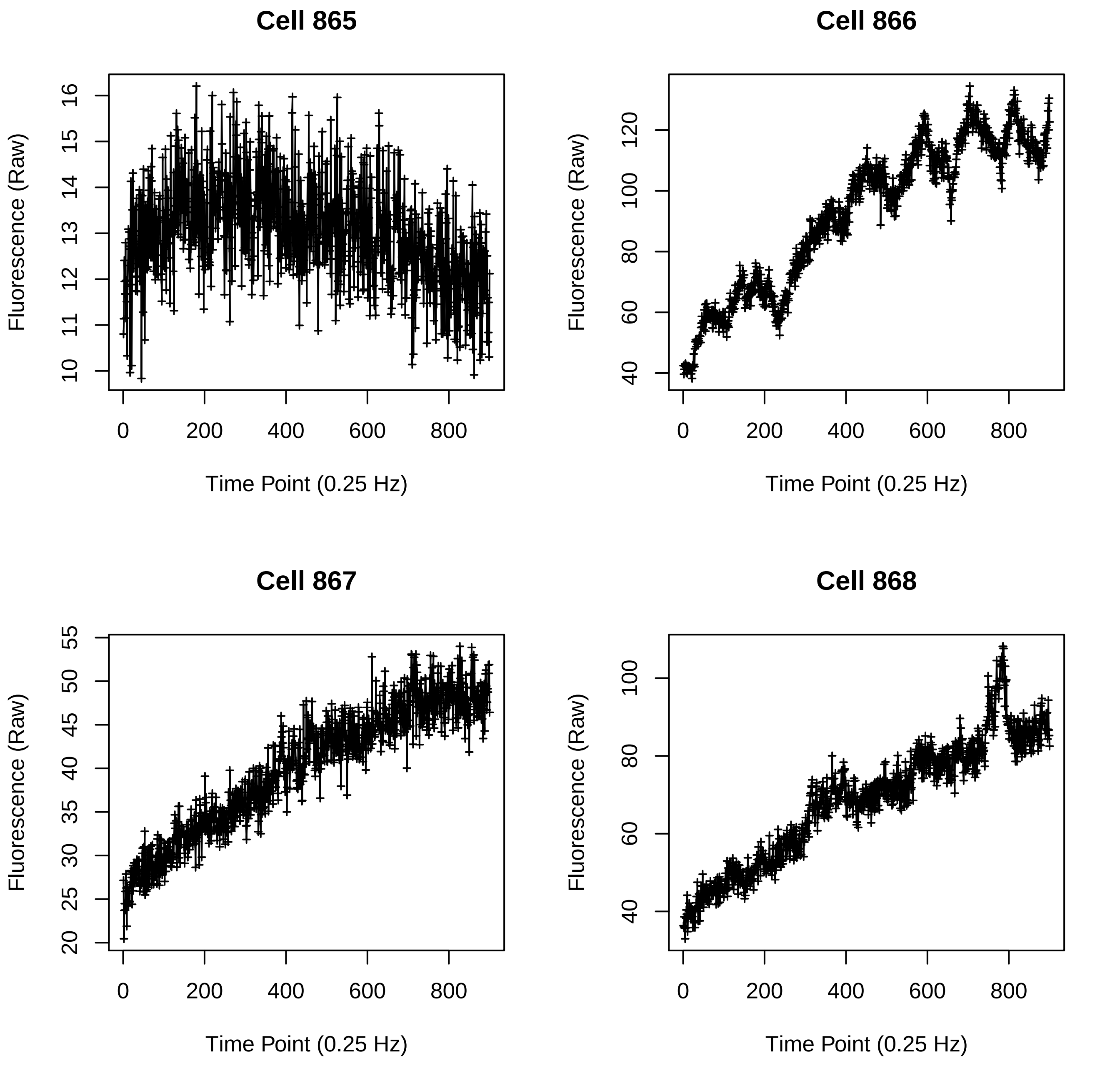

Cell 865
Cell 866
Cell 867
Cell 868
Fluorescence (Raw)
Time Point (0.25 Hz)
0
200
400
600
800
10
11
12
13
14
15
16
40
60
80
100
120
20
25
30
35
40
45
50
55

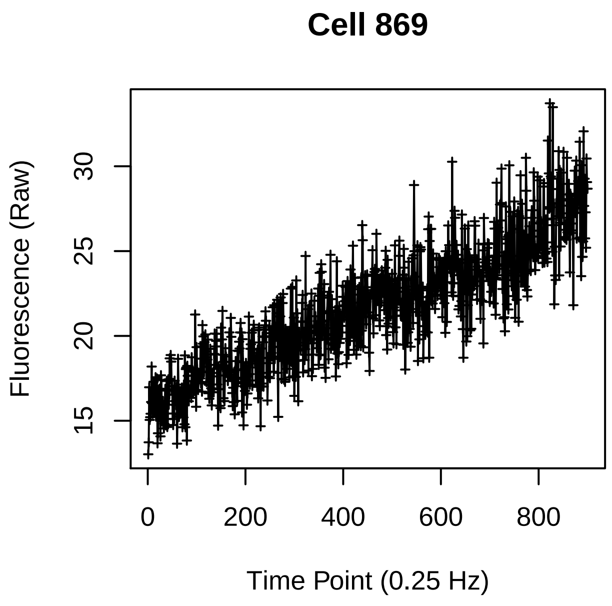
Cell 869
Fluorescence (Raw)
15
20
25
30
0
200
400
600
800
Time Point (0.25 Hz)

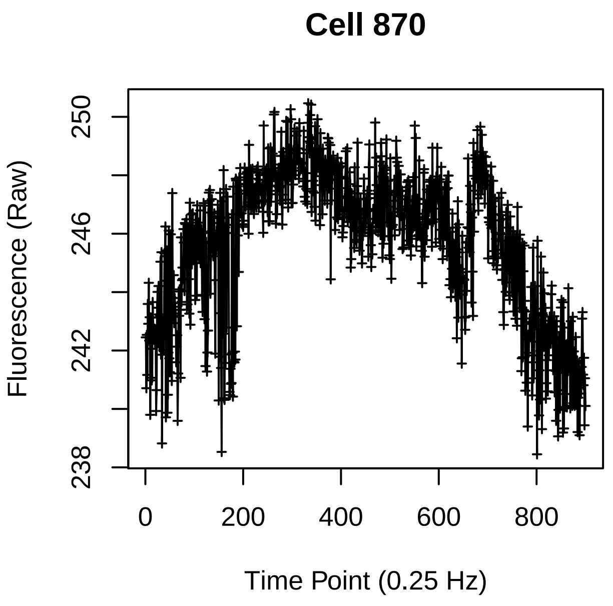
Cell 870
Fluorescence (Raw)
238
242
246
250
0
200
400
600
800
Time Point (0.25 Hz)

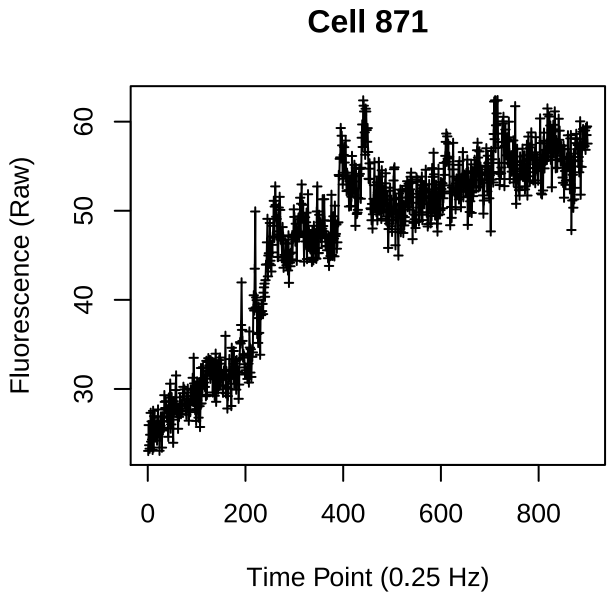
Cell 871
Fluorescence (Raw)
30
40
50
60
0
200
400
600
800
Time Point (0.25 Hz)

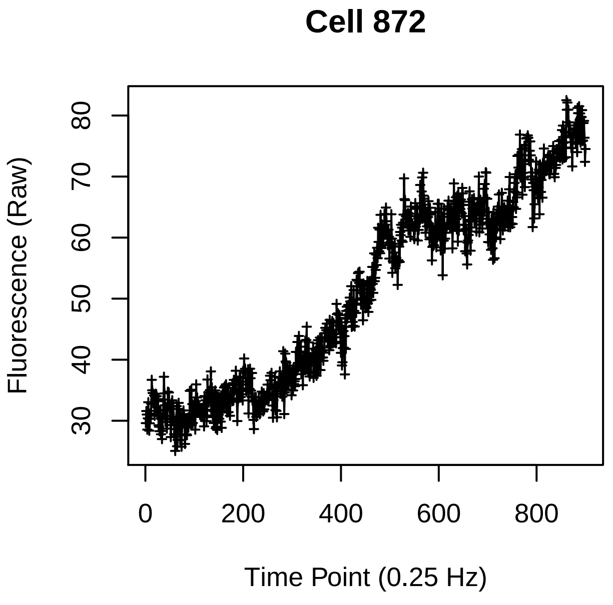
Cell 872
Fluorescence (Raw)
30
40
50
60
70
80
0
200
400
600
800
Time Point (0.25 Hz)

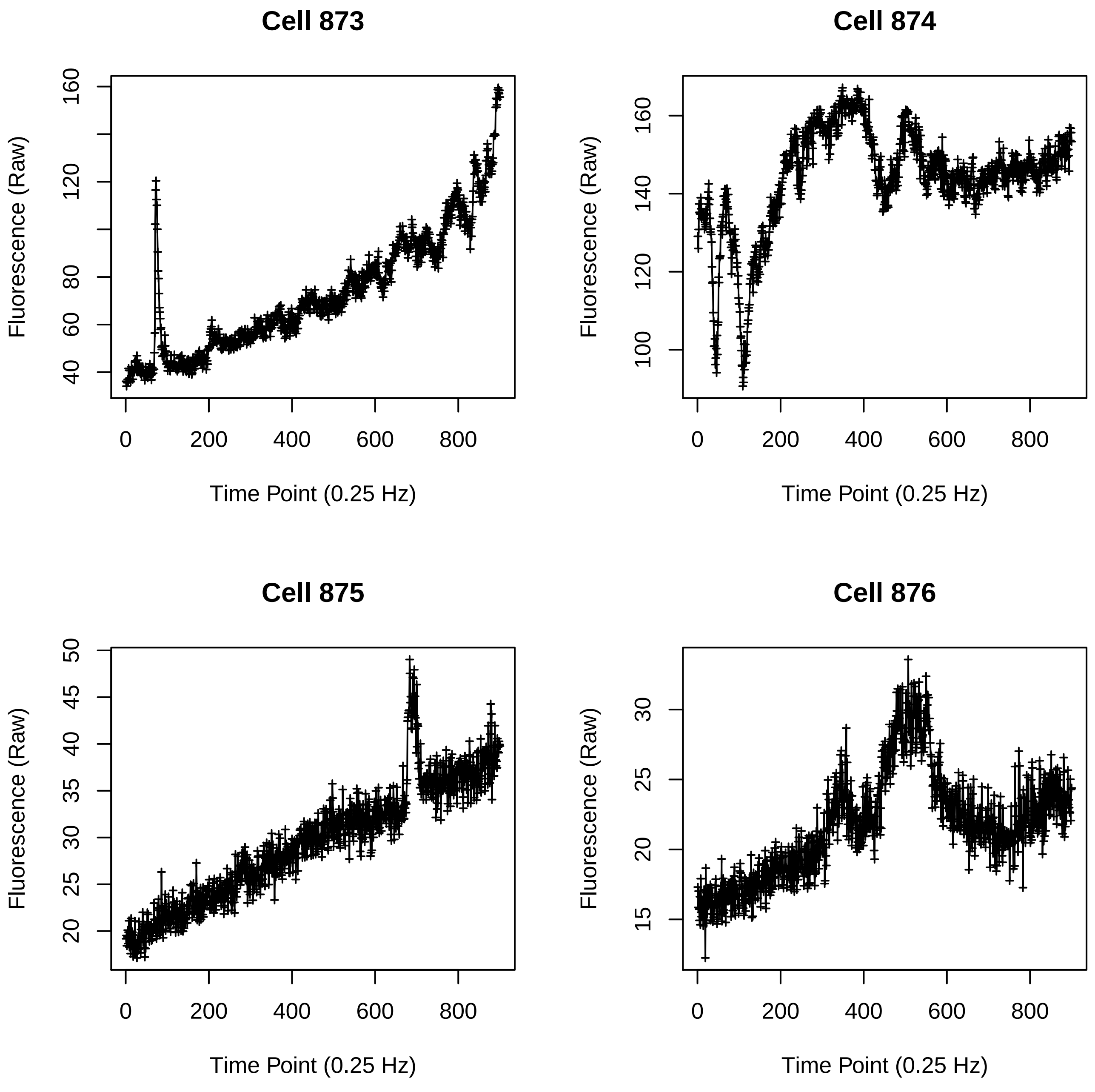

Cell 873
Cell 874
Cell 875
Cell 876
Fluorescence (Raw)
Time Point (0.25 Hz)
0
200
400
600
800
40
60
80
120
160
100
120
140
160
20
25
30
35
40
45
50
15
20
25
30

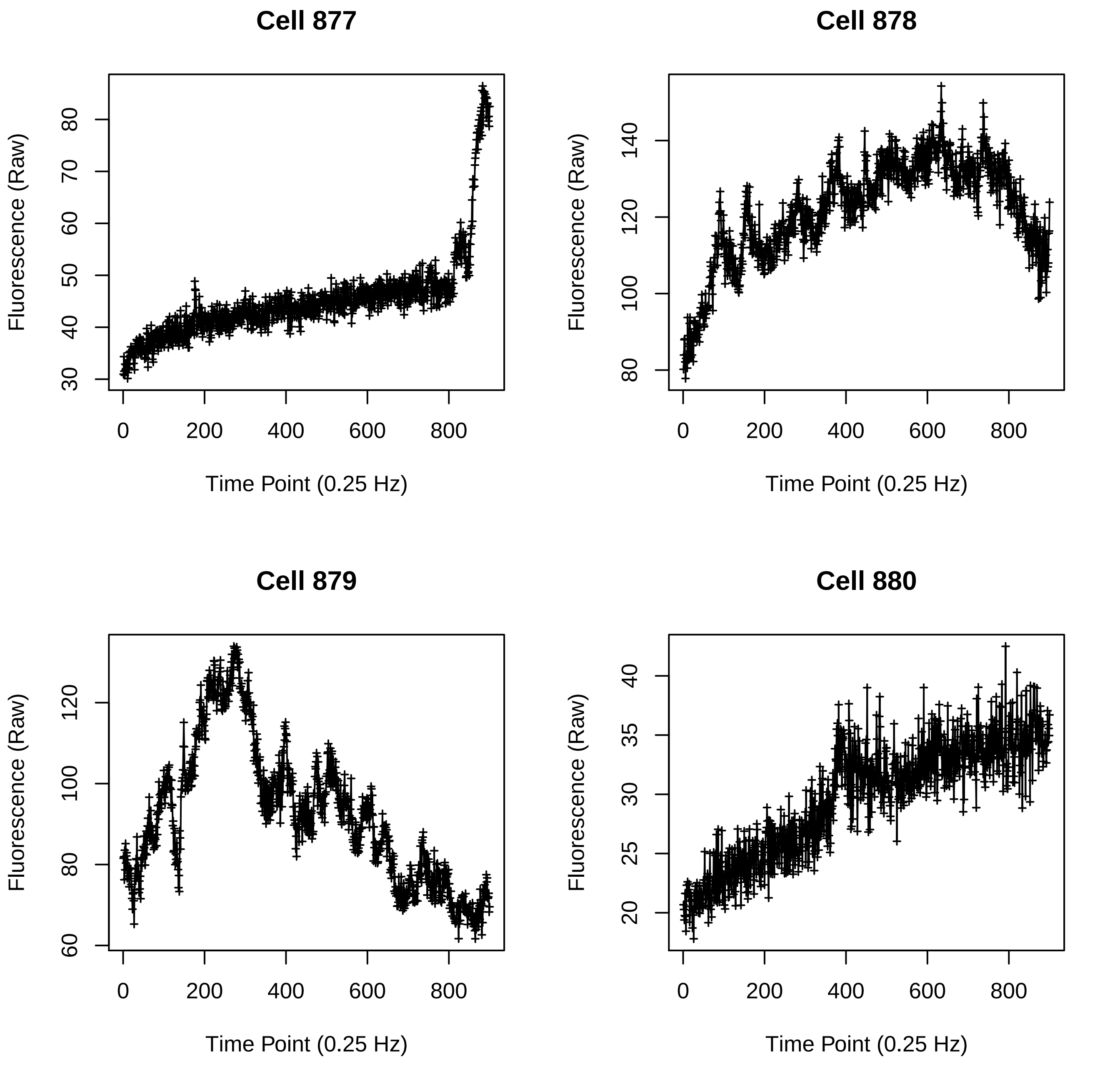

Cell 877
Fluorescence (Raw)
30
40
50
60
70
80
0
200
400
600
800
Time Point (0.25 Hz)
Cell 878
Fluorescence (Raw)
80
100
120
140
0
200
400
600
800
Time Point (0.25 Hz)
Cell 879
Fluorescence (Raw)
60
80
100
120
0
200
400
600
800
Time Point (0.25 Hz)
Cell 880
Fluorescence (Raw)
20
25
30
35
40
0
200
400
600
800
Time Point (0.25 Hz)

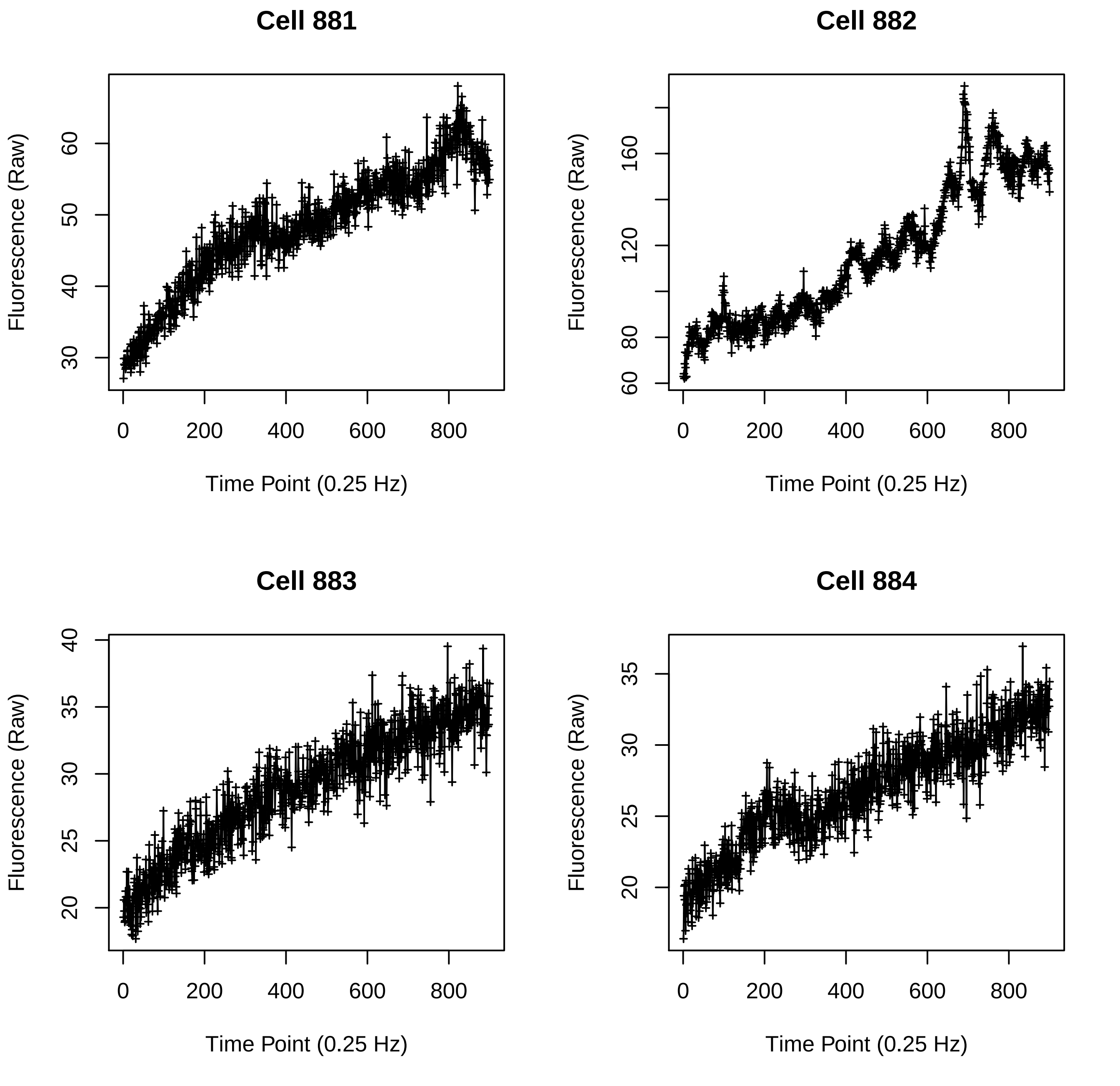
Cell 881
Fluorescence (Raw)
30
40
50
60
0
200
400
600
800
Time Point (0.25 Hz)
Cell 882
Fluorescence (Raw)
60
80
120
160
0
200
400
600
800
Time Point (0.25 Hz)
Cell 883
Fluorescence (Raw)
20
25
30
35
40
0
200
400
600
800
Time Point (0.25 Hz)
Cell 884
Fluorescence (Raw)
20
25
30
35
0
200
400
600
800
Time Point (0.25 Hz)

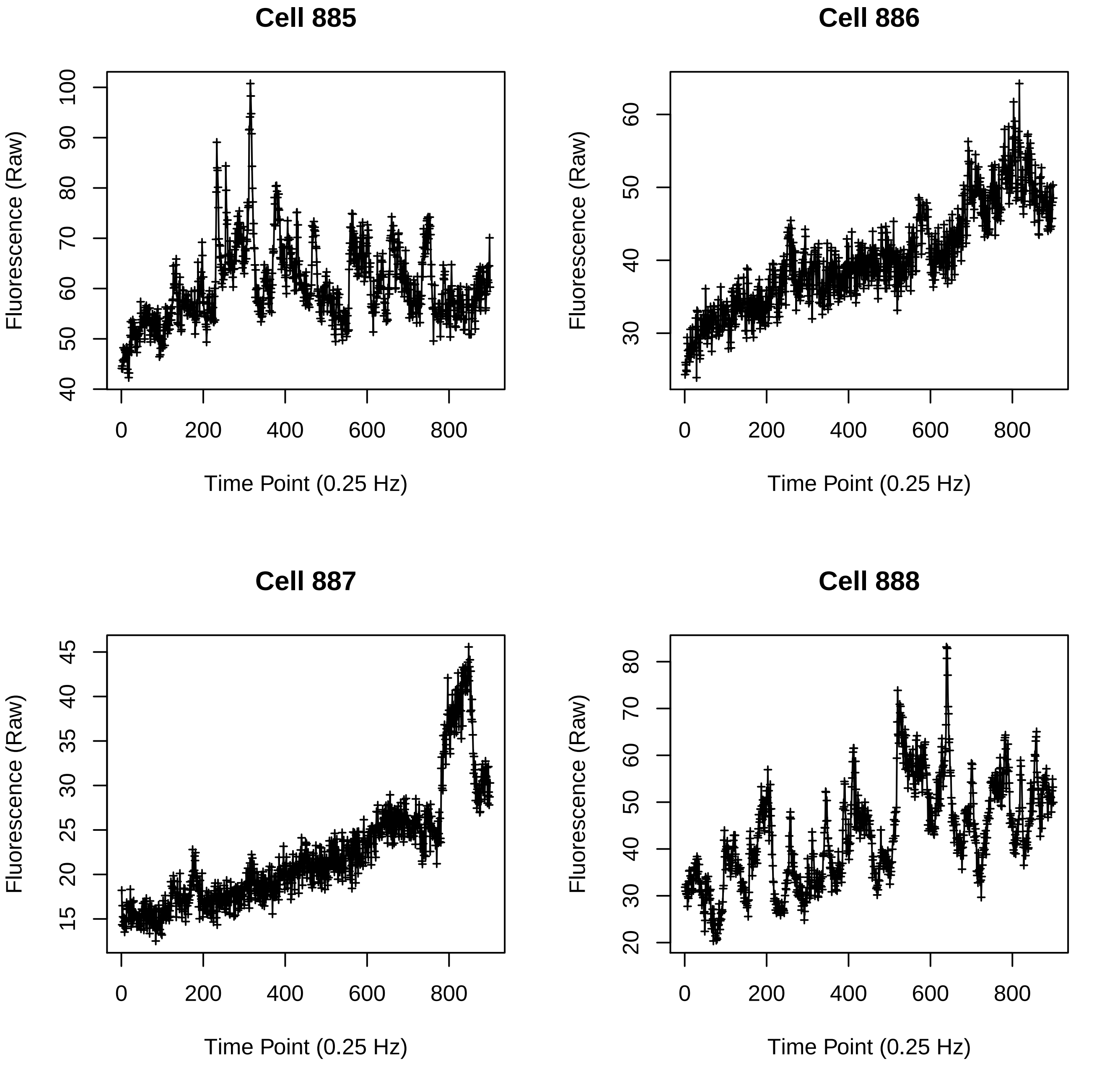

Cell 885
Fluorescence (Raw)
40
50
60
70
80
90
100
0
200
400
600
800
Time Point (0.25 Hz)
Cell 886
Fluorescence (Raw)
30
40
50
60
0
200
400
600
800
Time Point (0.25 Hz)
Cell 887
Fluorescence (Raw)
15
20
25
30
35
40
45
0
200
400
600
800
Time Point (0.25 Hz)
Cell 888
Fluorescence (Raw)
20
30
40
50
60
70
80
0
200
400
600
800
Time Point (0.25 Hz)

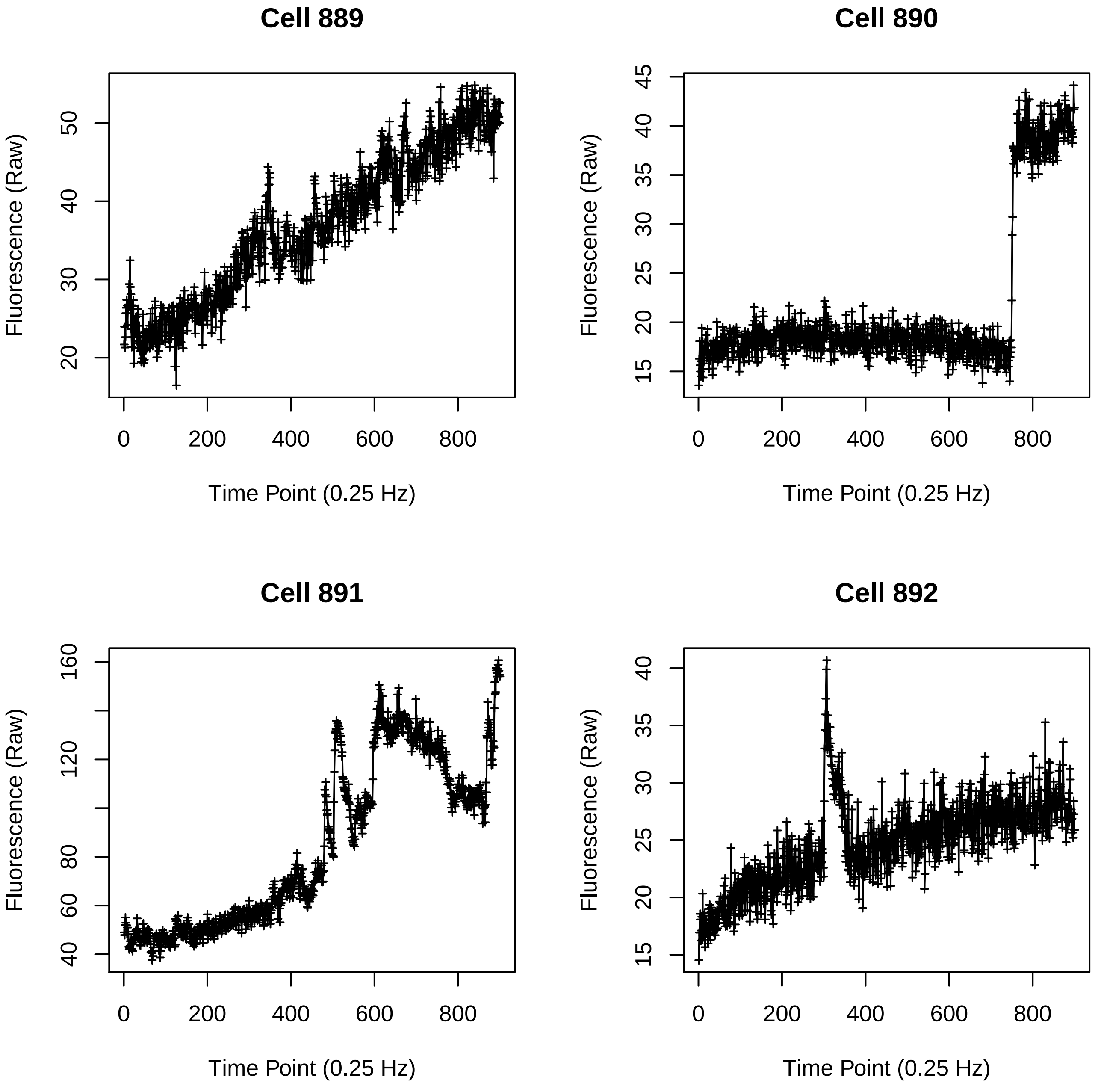
Cell 889
Cell 890
Cell 891
Cell 892
Fluorescence (Raw)
Time Point (0.25 Hz)
0
200
400
600
800
20
30
40
50
15
20
25
30
35
40
45
40
60
80
120
160

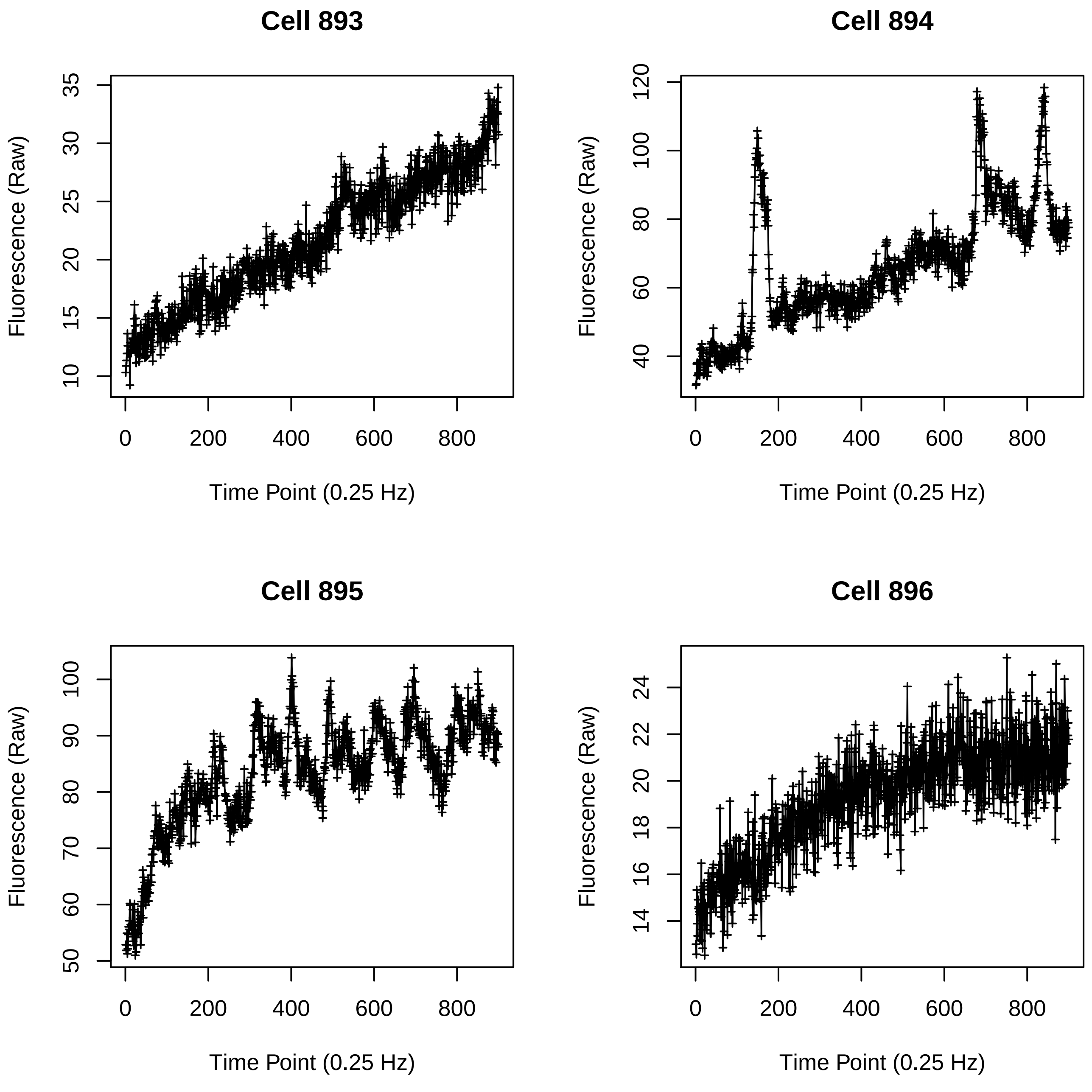
Cell 893
Fluorescence (Raw)
10
15
20
25
30
35
0
200
400
600
800
Time Point (0.25 Hz)
Cell 894
Fluorescence (Raw)
40
60
80
100
120
0
200
400
600
800
Time Point (0.25 Hz)
Cell 895
Fluorescence (Raw)
50
60
70
80
90
100
0
200
400
600
800
Time Point (0.25 Hz)
Cell 896
Fluorescence (Raw)
14
16
18
20
22
24
0
200
400
600
800
Time Point (0.25 Hz)

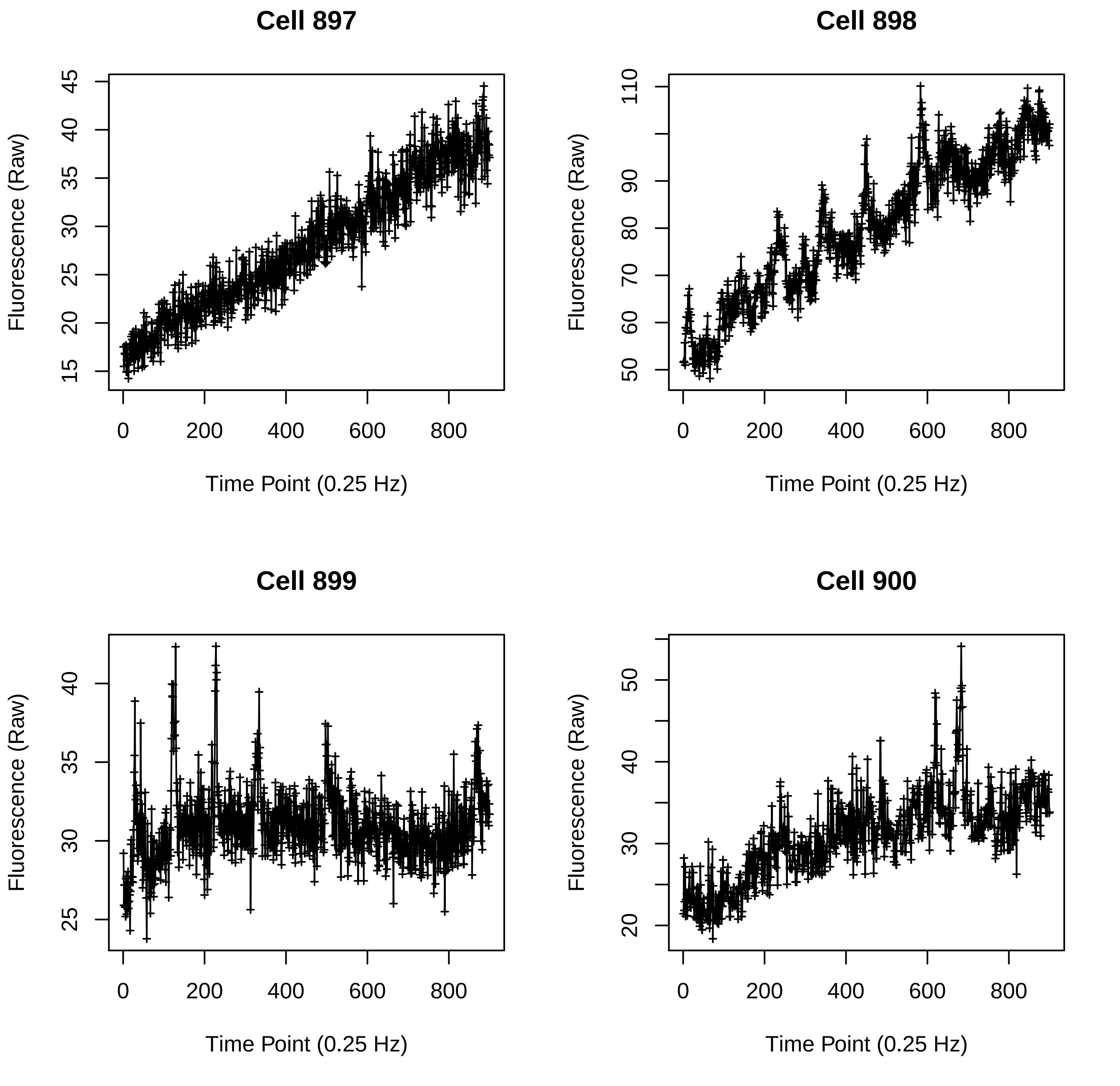

Cell 897
Cell 898
Cell 899
Cell 900
Fluorescence (Raw)
Time Point (0.25 Hz)
0
200
400
600
800
15
20
25
30
35
40
45
50
60
70
80
90
110

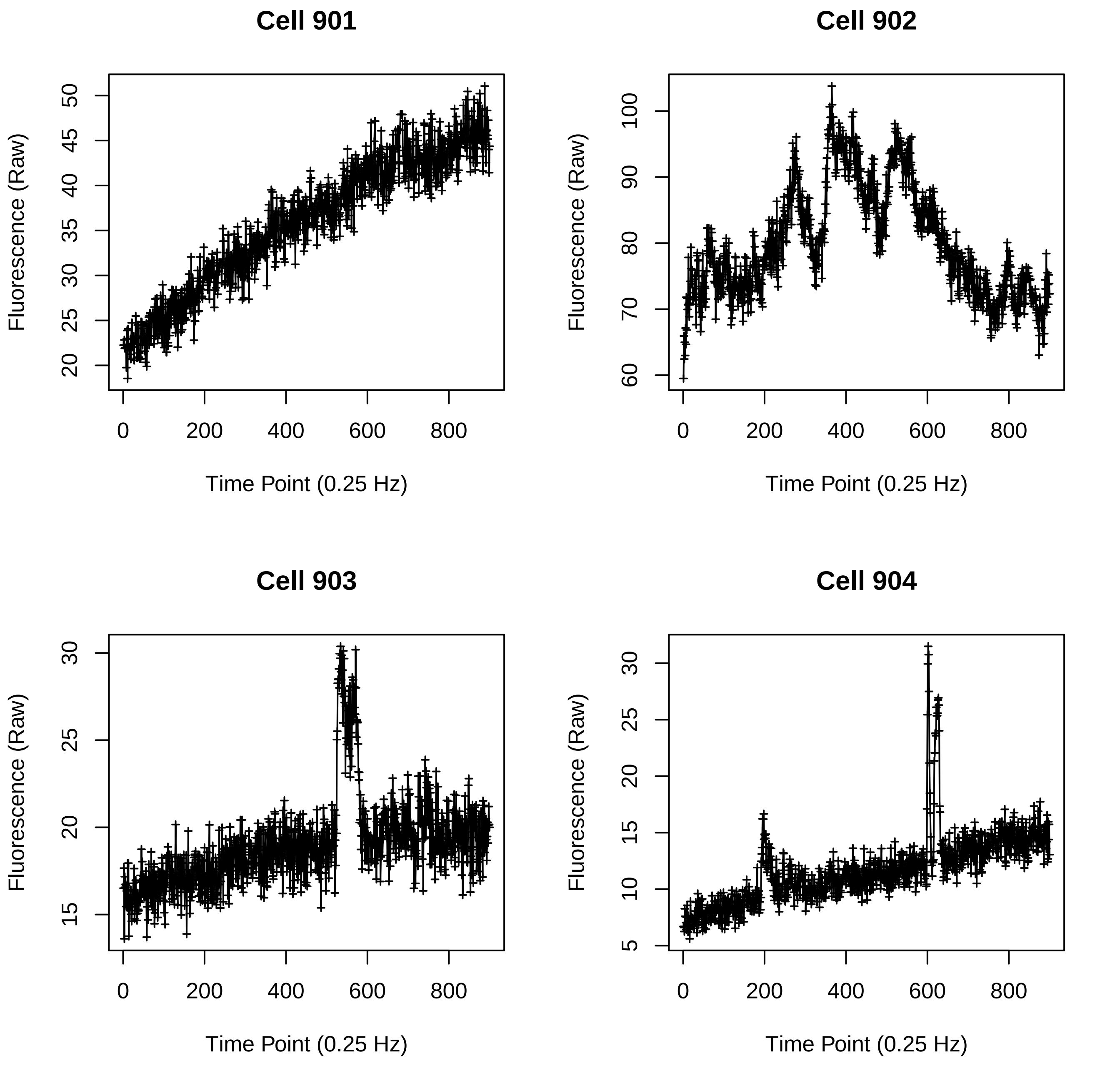

Cell 901
Fluorescence (Raw)
20
25
30
35
40
45
50
0
200
400
600
800
Time Point (0.25 Hz)
Cell 902
Fluorescence (Raw)
60
70
80
90
100
0
200
400
600
800
Time Point (0.25 Hz)
Cell 903
Fluorescence (Raw)
15
20
25
30
0
200
400
600
800
Time Point (0.25 Hz)
Cell 904
Fluorescence (Raw)
5
10
15
20
25
30
0
200
400
600
800
Time Point (0.25 Hz)

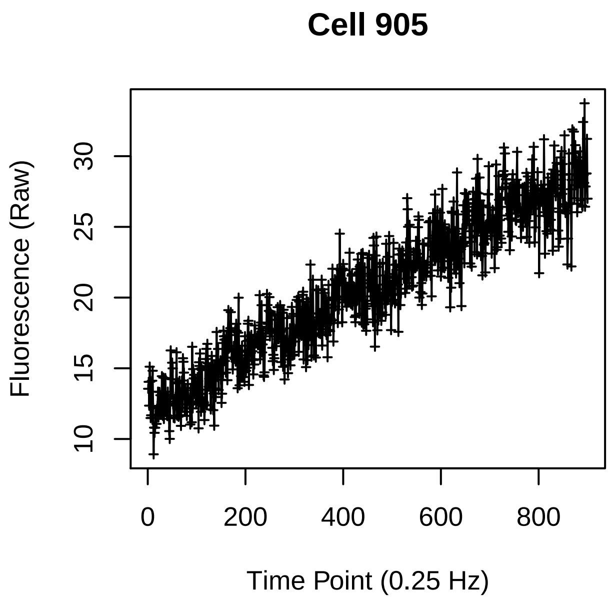
Cell 905
Fluorescence (Raw)
10
15
20
25
30
0
200
400
600
800
Time Point (0.25 Hz)

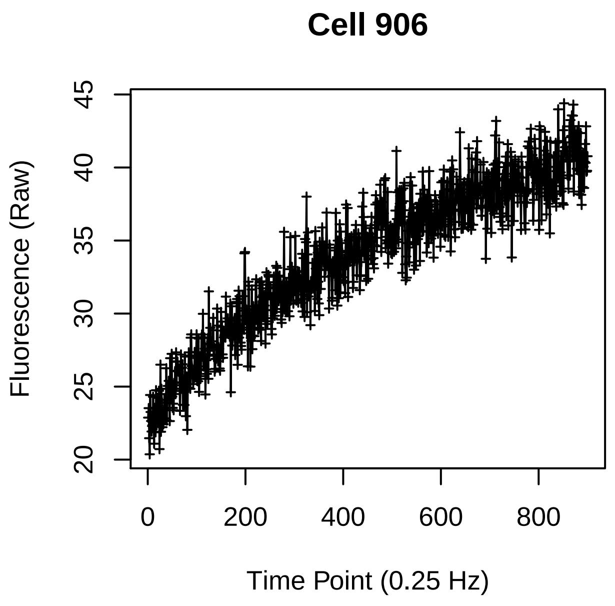
Cell 906
Fluorescence (Raw)
20
25
30
35
40
45
0
200
400
600
800
Time Point (0.25 Hz)

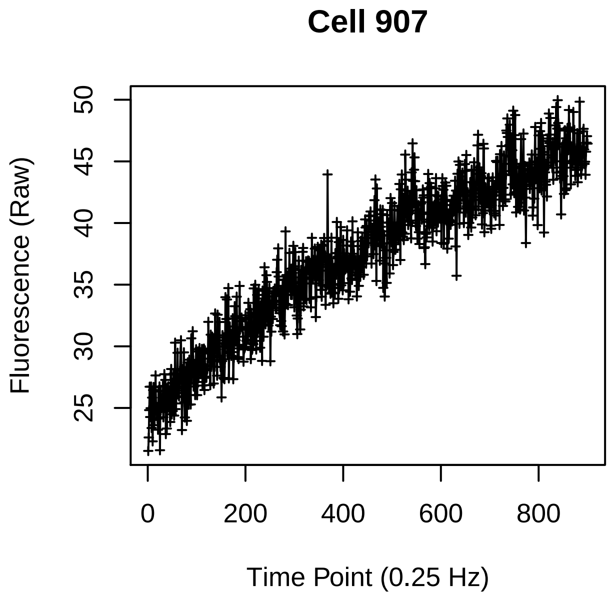
Cell 907
Fluorescence (Raw)
25
30
35
40
45
50
0
200
400
600
800
Time Point (0.25 Hz)

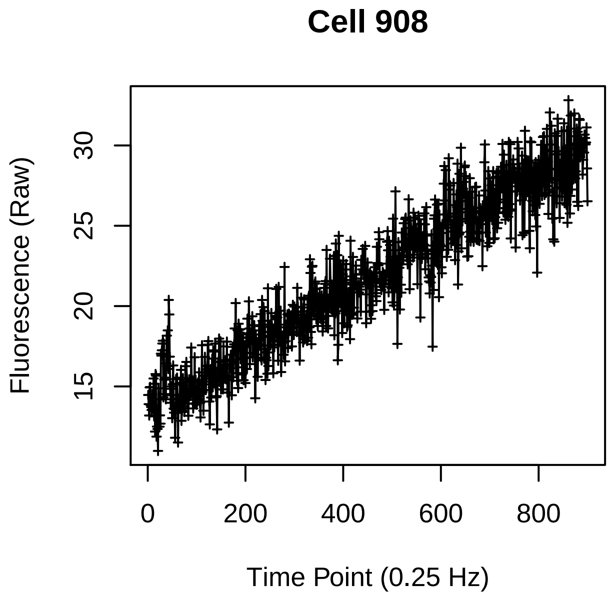
Cell 908
Fluorescence (Raw)
15
20
25
30
0
200
400
600
800
Time Point (0.25 Hz)

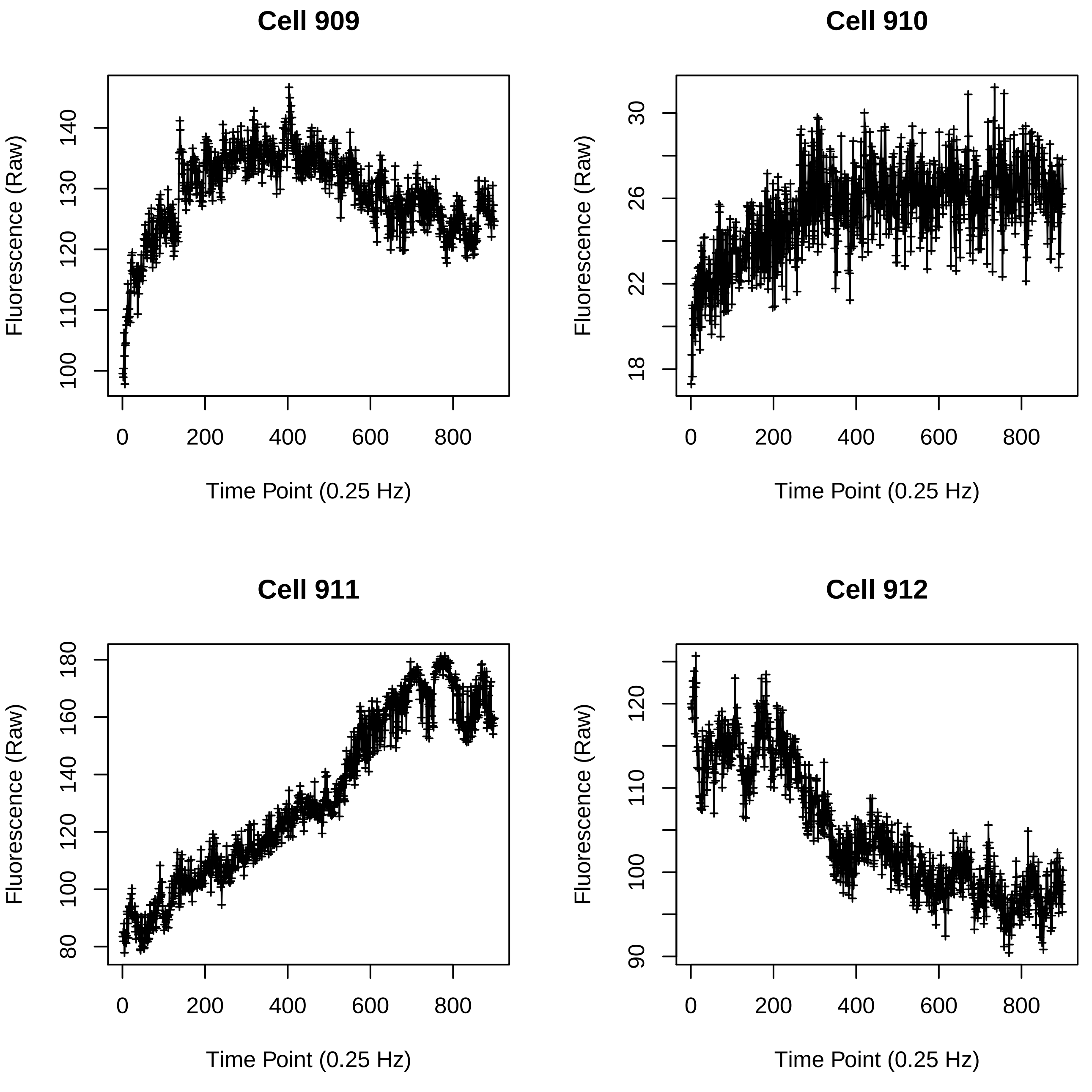
Cell 909
Fluorescence (Raw)
Time Point (0.25 Hz)
Cell 910
Fluorescence (Raw)
Time Point (0.25 Hz)
Cell 911
Fluorescence (Raw)
Time Point (0.25 Hz)
Cell 912
Fluorescence (Raw)
Time Point (0.25 Hz)

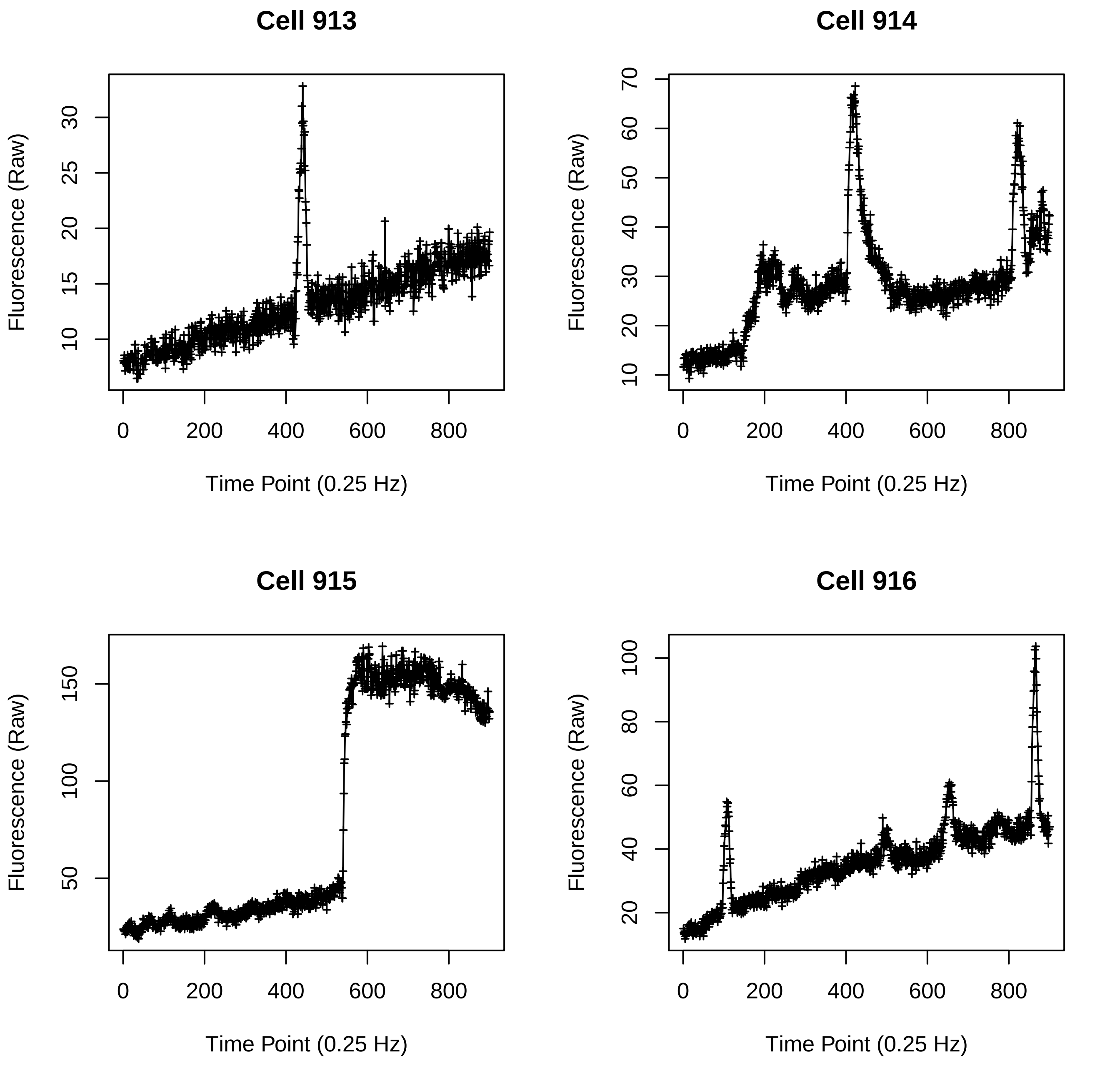
Cell 913
Fluorescence (Raw)
Time Point (0.25 Hz)
Cell 914
Fluorescence (Raw)
Time Point (0.25 Hz)
Cell 915
Fluorescence (Raw)
Time Point (0.25 Hz)
Cell 916
Fluorescence (Raw)
Time Point (0.25 Hz)

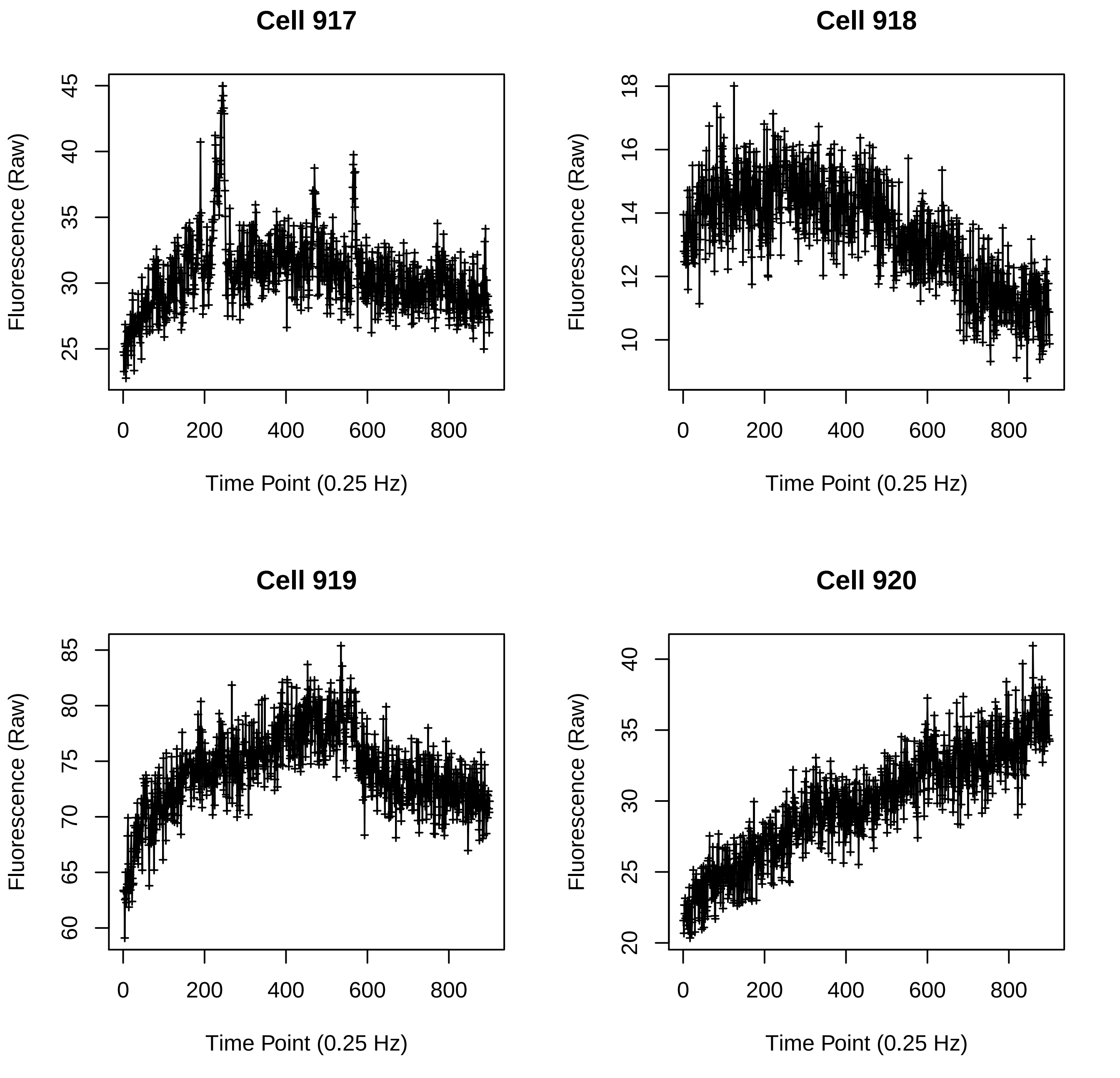
Cell 917
Fluorescence (Raw)
Time Point (0.25 Hz)
25
30
35
40
45
0
200
400
600
800
Cell 918
Fluorescence (Raw)
Time Point (0.25 Hz)
10
12
14
16
18
0
200
400
600
800
Cell 919
Fluorescence (Raw)
Time Point (0.25 Hz)
60
65
70
75
80
85
0
200
400
600
800
Cell 920
Fluorescence (Raw)
Time Point (0.25 Hz)
20
25
30
35
40
0
200
400
600
800

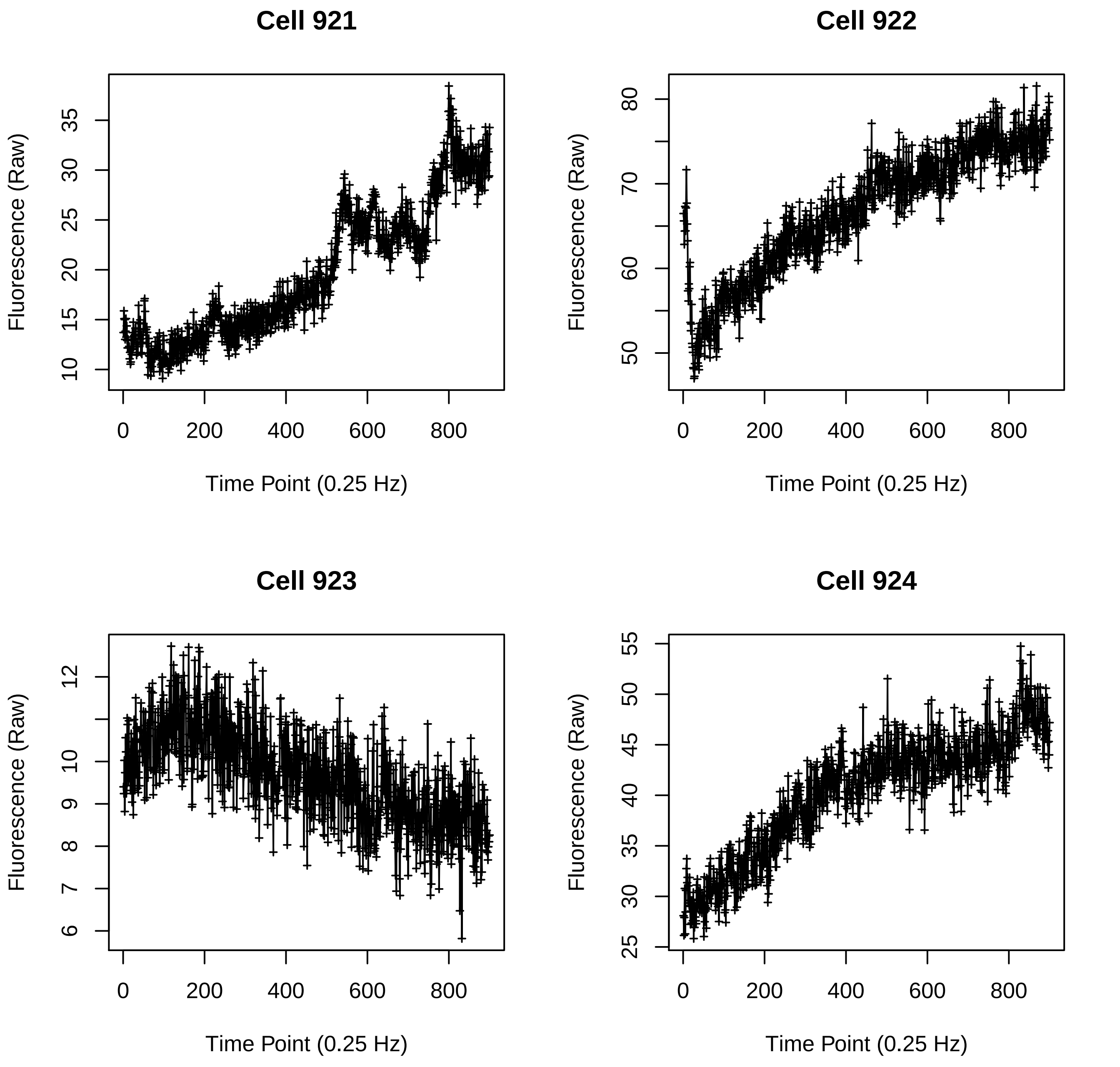

Cell 921
Cell 922
Cell 923
Cell 924
Fluorescence (Raw)
Time Point (0.25 Hz)
0
200
400
600
800
10
15
20
25
30
35
50
60
70
80
6
7
8
9
10
12
25
30
35
40
45
50
55

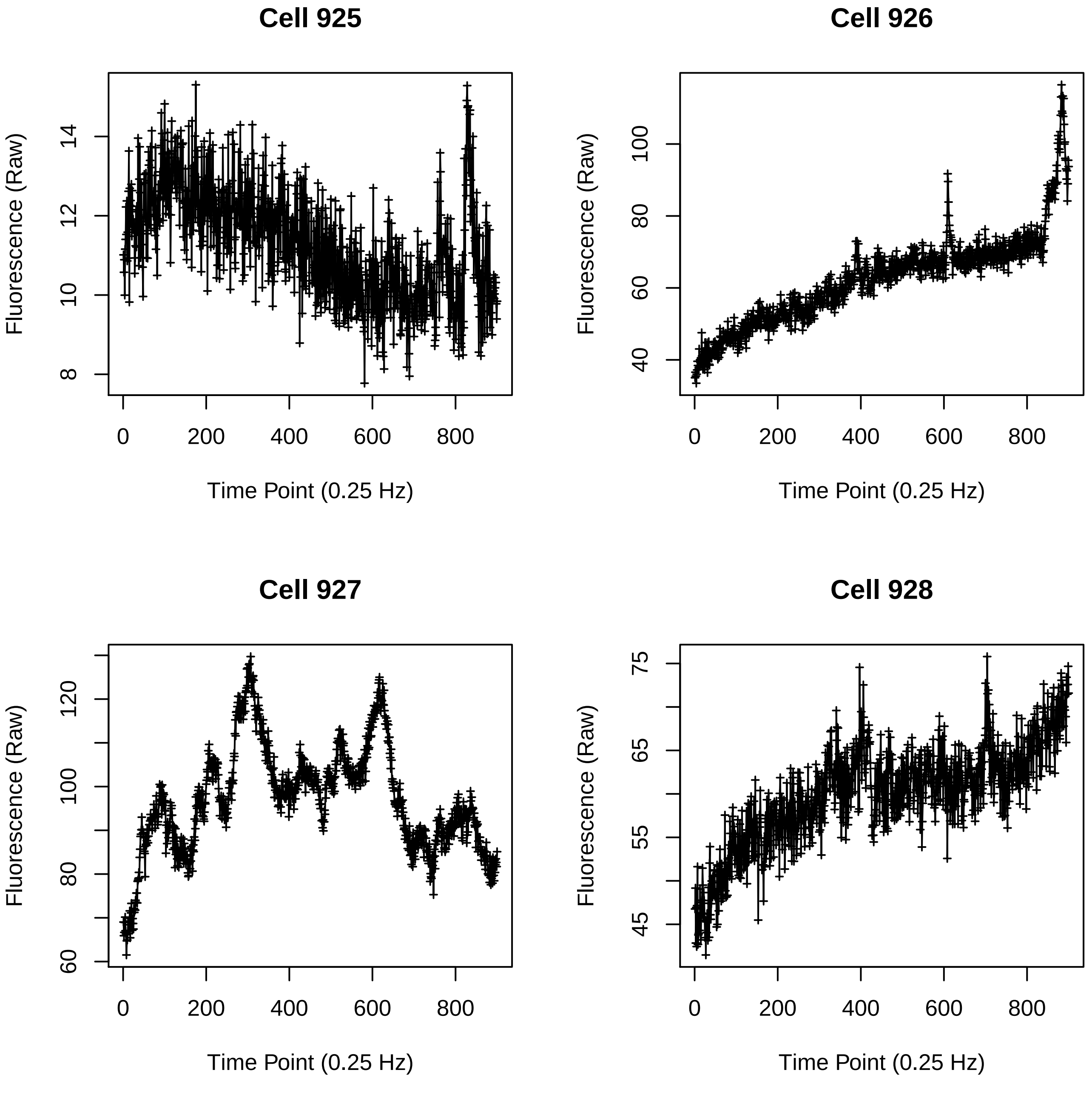
Cell 925
Fluorescence (Raw)
Time Point (0.25 Hz)
8
10
12
14
0
200
400
600
800
Cell 926
Fluorescence (Raw)
Time Point (0.25 Hz)
40
60
80
100
0
200
400
600
800
Cell 927
Fluorescence (Raw)
Time Point (0.25 Hz)
60
80
100
120
0
200
400
600
800
Cell 928
Fluorescence (Raw)
Time Point (0.25 Hz)
45
55
65
75
0
200
400
600
800

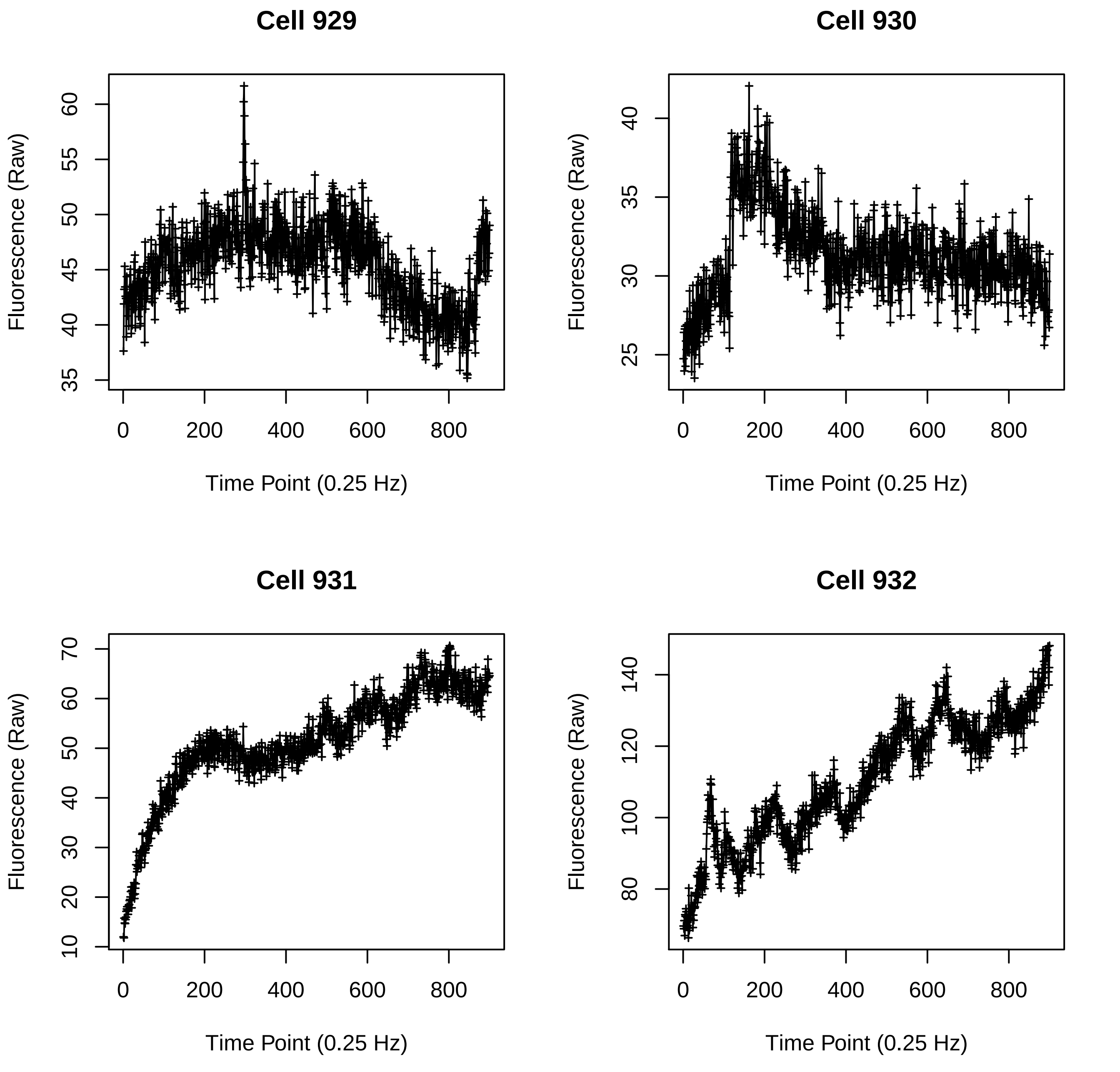
Cell 929
Cell 930
Cell 931
Cell 932
Fluorescence (Raw)
Time Point (0.25 Hz)
0
200
400
600
800
35
40
45
50
55
60
25
30
35
40
10
20
30
40
50
60
70
80
100
120
140

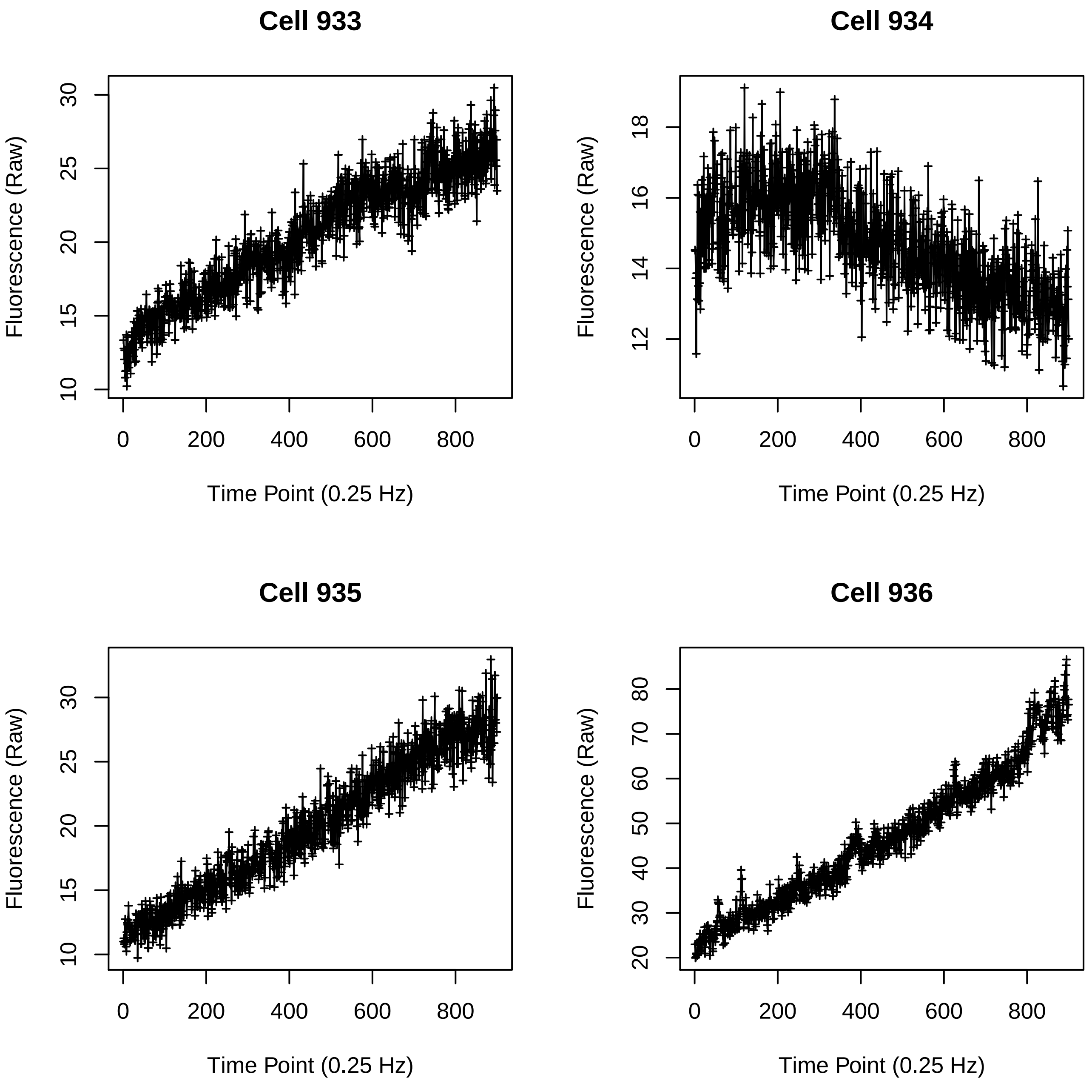
Cell 933
Fluorescence (Raw)
10
15
20
25
30
0
200
400
600
800
Time Point (0.25 Hz)
Cell 934
Fluorescence (Raw)
12
14
16
18
0
200
400
600
800
Time Point (0.25 Hz)
Cell 935
Fluorescence (Raw)
10
15
20
25
30
0
200
400
600
800
Time Point (0.25 Hz)
Cell 936
Fluorescence (Raw)
20
30
40
50
60
70
80
0
200
400
600
800
Time Point (0.25 Hz)

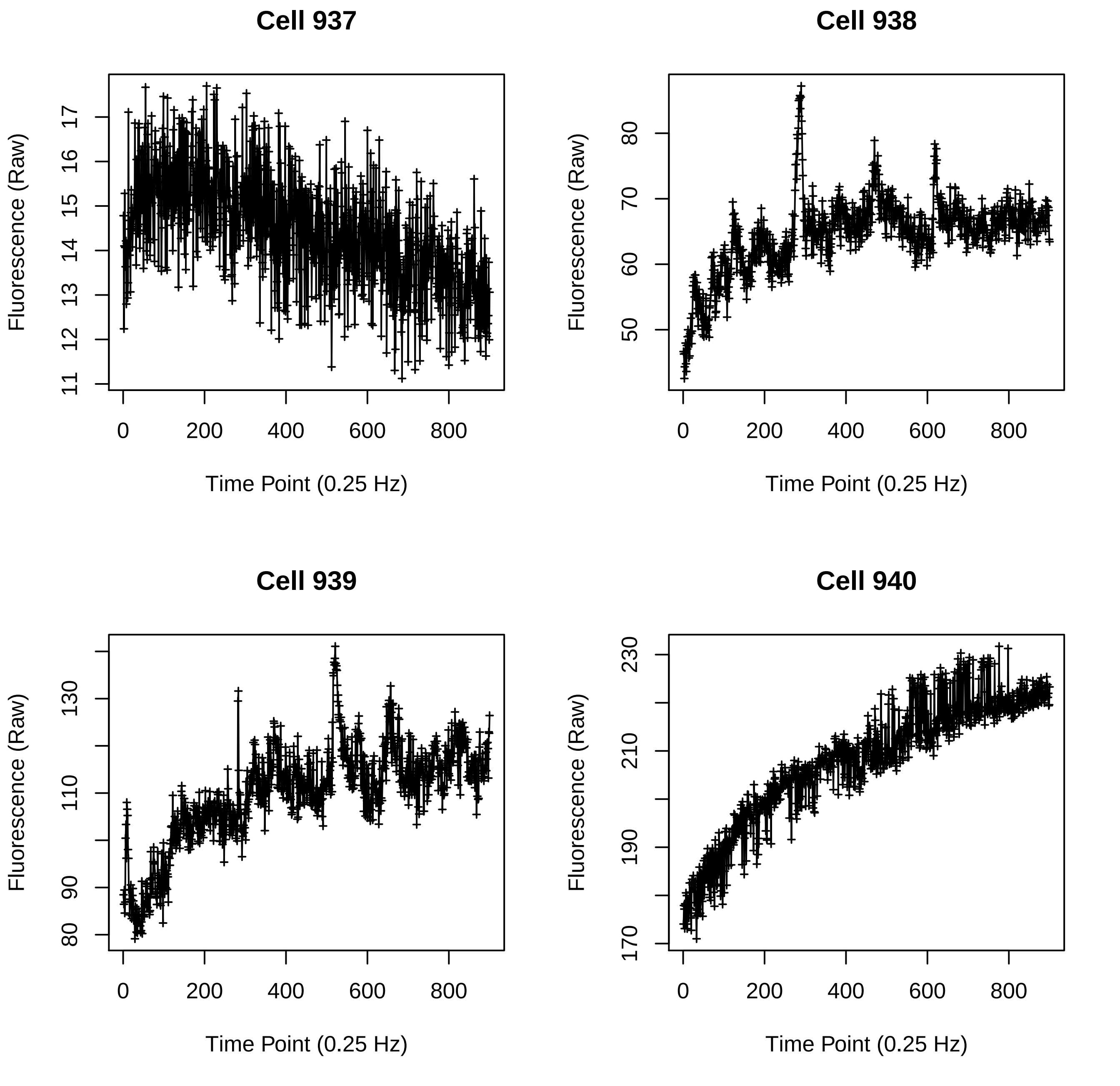
Cell 937
Fluorescence (Raw)
11
12
13
14
15
16
17
0
200
400
600
800
Time Point (0.25 Hz)
Cell 938
Fluorescence (Raw)
50
60
70
80
0
200
400
600
800
Time Point (0.25 Hz)
Cell 939
Fluorescence (Raw)
80
90
110
130
0
200
400
600
800
Time Point (0.25 Hz)
Cell 940
Fluorescence (Raw)
170
190
210
230
0
200
400
600
800
Time Point (0.25 Hz)

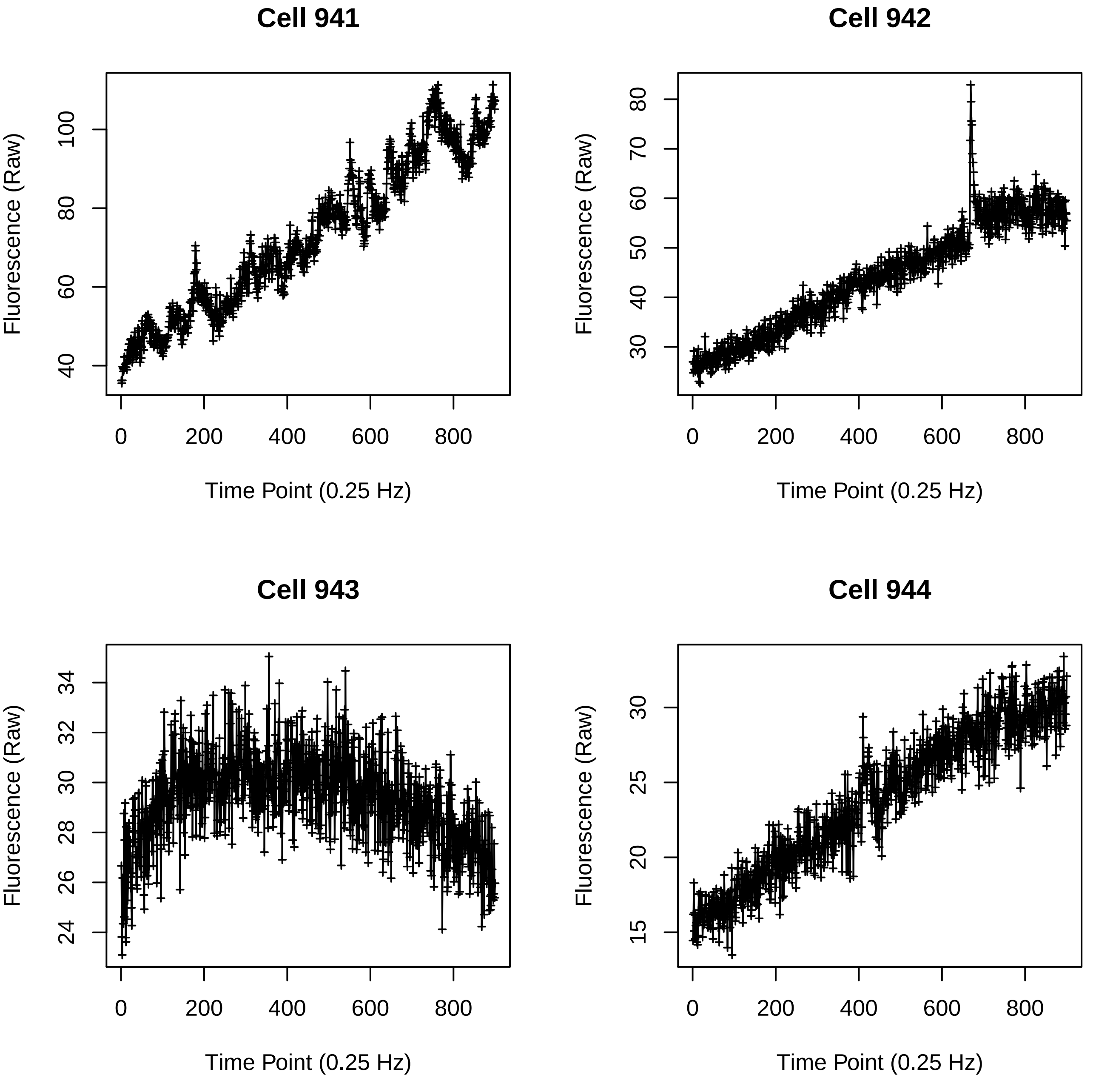
Cell 941
Fluorescence (Raw)
Time Point (0.25 Hz)
Cell 942
Fluorescence (Raw)
Time Point (0.25 Hz)
Cell 943
Fluorescence (Raw)
Time Point (0.25 Hz)
Cell 944
Fluorescence (Raw)
Time Point (0.25 Hz)

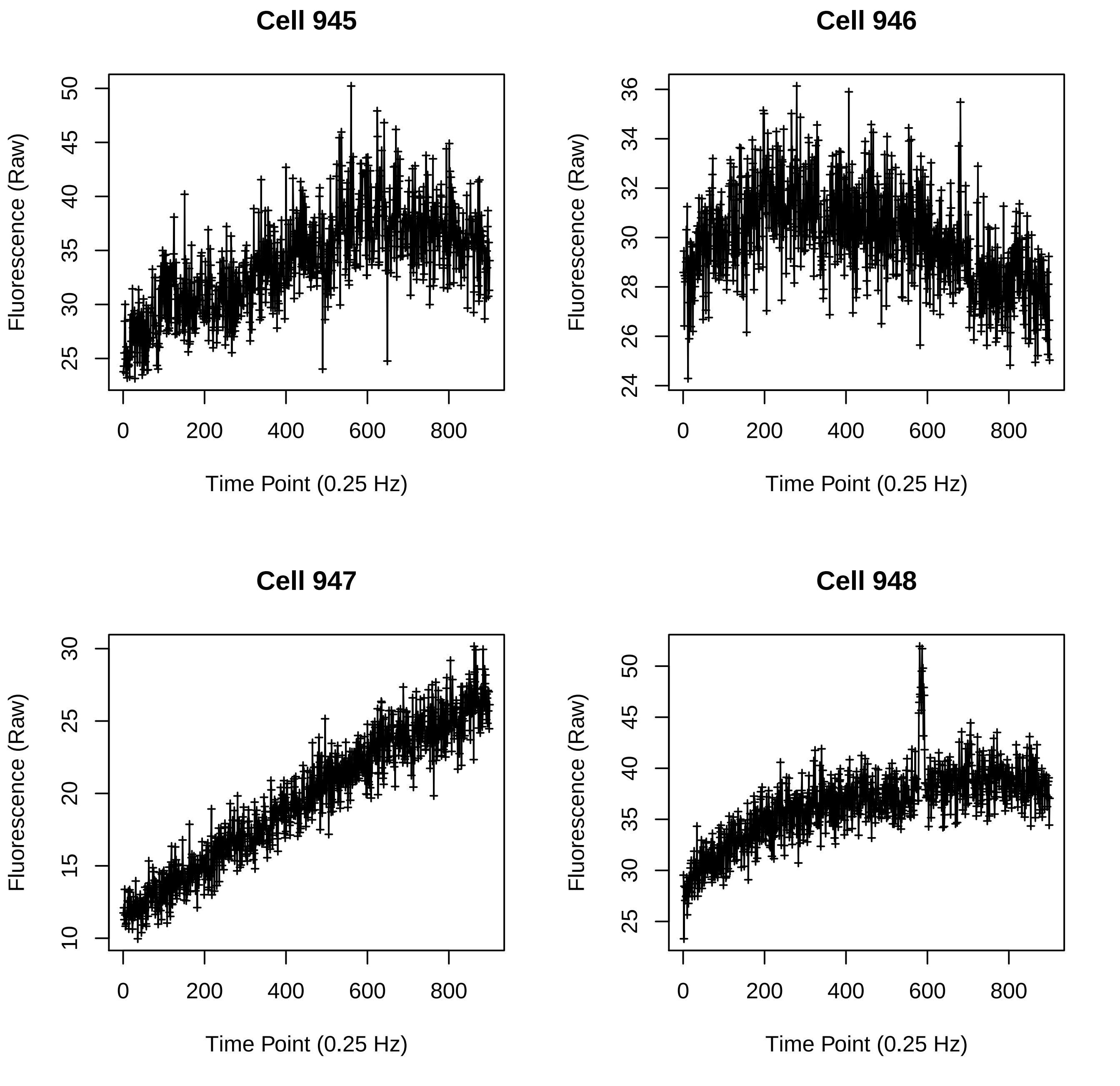

Cell 945
Cell 946
Cell 947
Cell 948
Fluorescence (Raw)
Time Point (0.25 Hz)
0
200
400
600
800
25
30
35
40
45
50
24
26
28
30
32
34
36
10
15
20
25
30

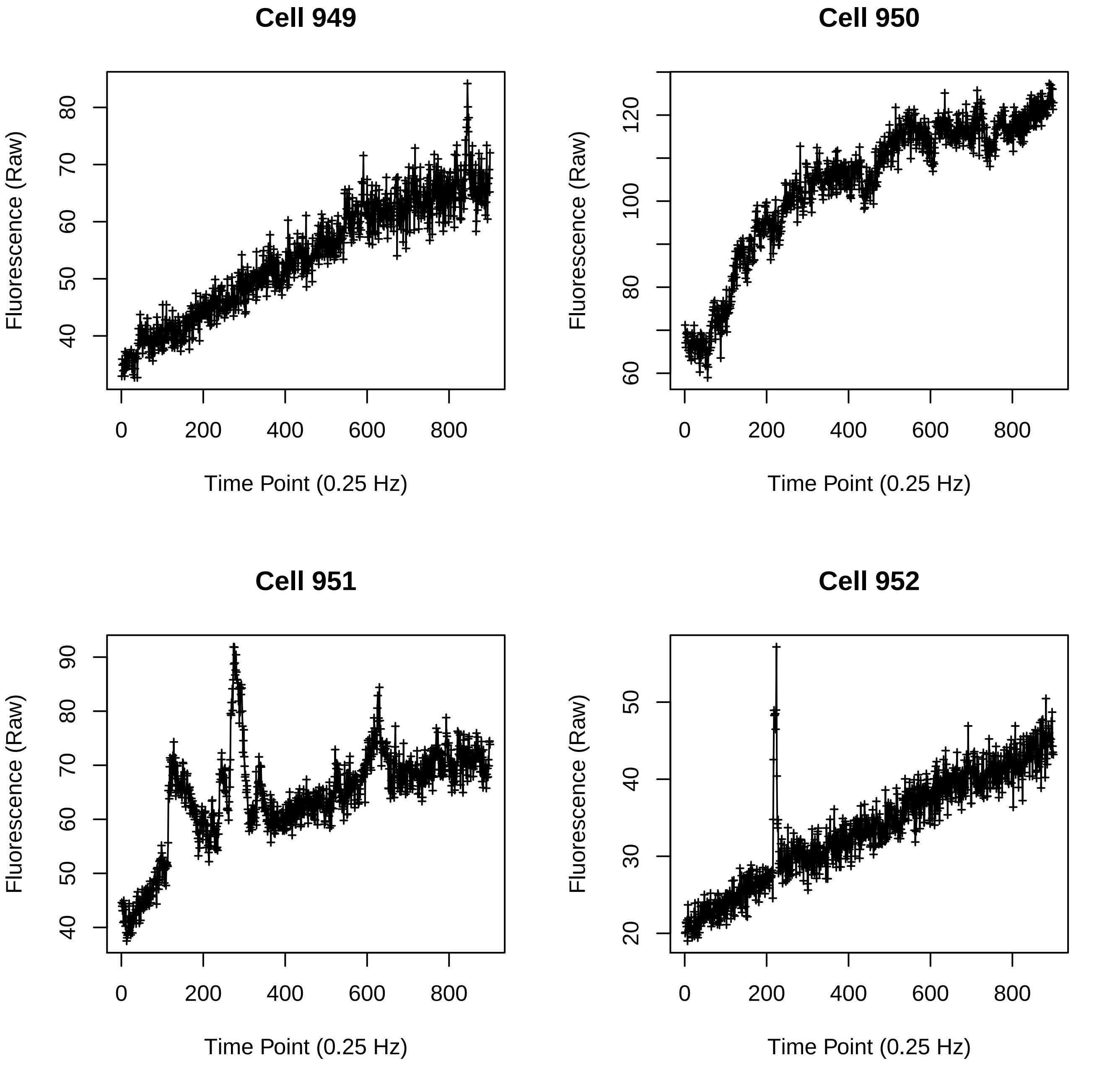

Cell 949
Fluorescence (Raw)
40
50
60
70
80
0
200
400
600
800
Time Point (0.25 Hz)
Cell 950
Fluorescence (Raw)
60
80
100
120
0
200
400
600
800
Time Point (0.25 Hz)
Cell 951
Fluorescence (Raw)
40
50
60
70
80
90
0
200
400
600
800
Time Point (0.25 Hz)
Cell 952
Fluorescence (Raw)
20
30
40
50
0
200
400
600
800
Time Point (0.25 Hz)

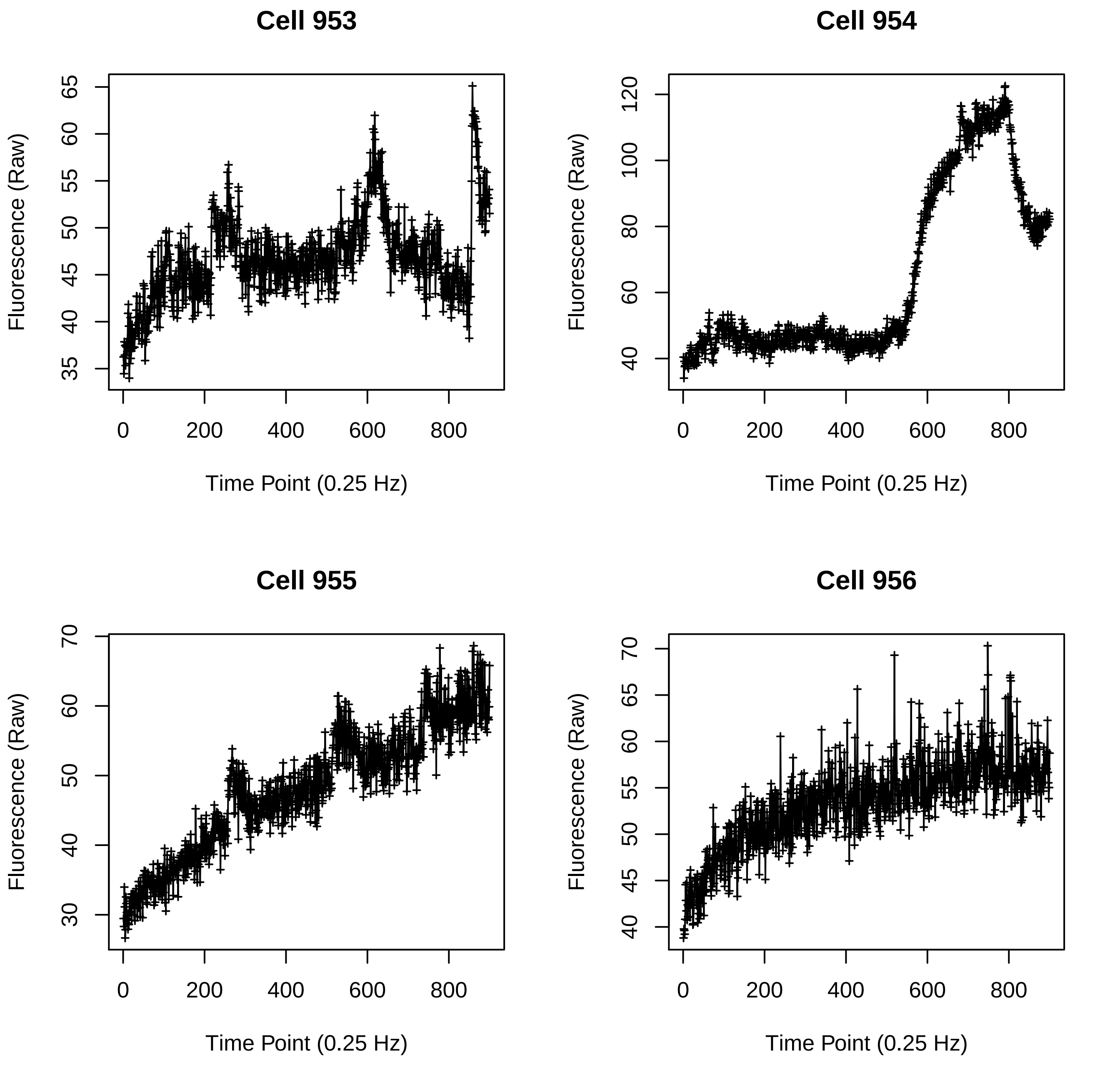

Cell 953
Fluorescence (Raw)
35
40
45
50
55
60
65
0
200
400
600
800
Time Point (0.25 Hz)
Cell 954
Fluorescence (Raw)
40
60
80
100
120
0
200
400
600
800
Time Point (0.25 Hz)
Cell 955
Fluorescence (Raw)
30
40
50
60
70
0
200
400
600
800
Time Point (0.25 Hz)
Cell 956
Fluorescence (Raw)
40
45
50
55
60
65
70
0
200
400
600
800
Time Point (0.25 Hz)

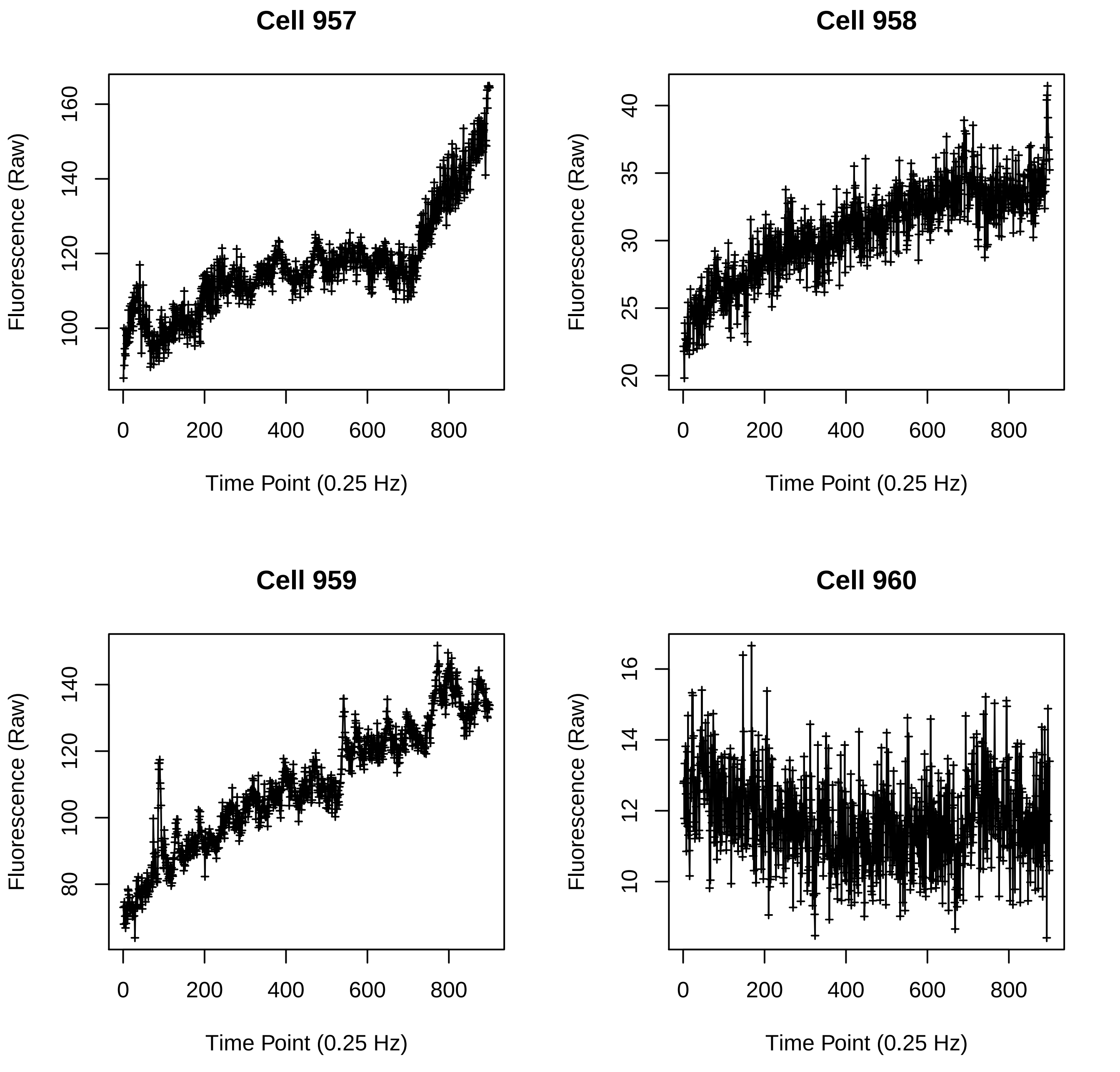
Cell 957
Fluorescence (Raw)
Time Point (0.25 Hz)
Cell 958
Fluorescence (Raw)
Time Point (0.25 Hz)
Cell 959
Fluorescence (Raw)
Time Point (0.25 Hz)
Cell 960
Fluorescence (Raw)
Time Point (0.25 Hz)

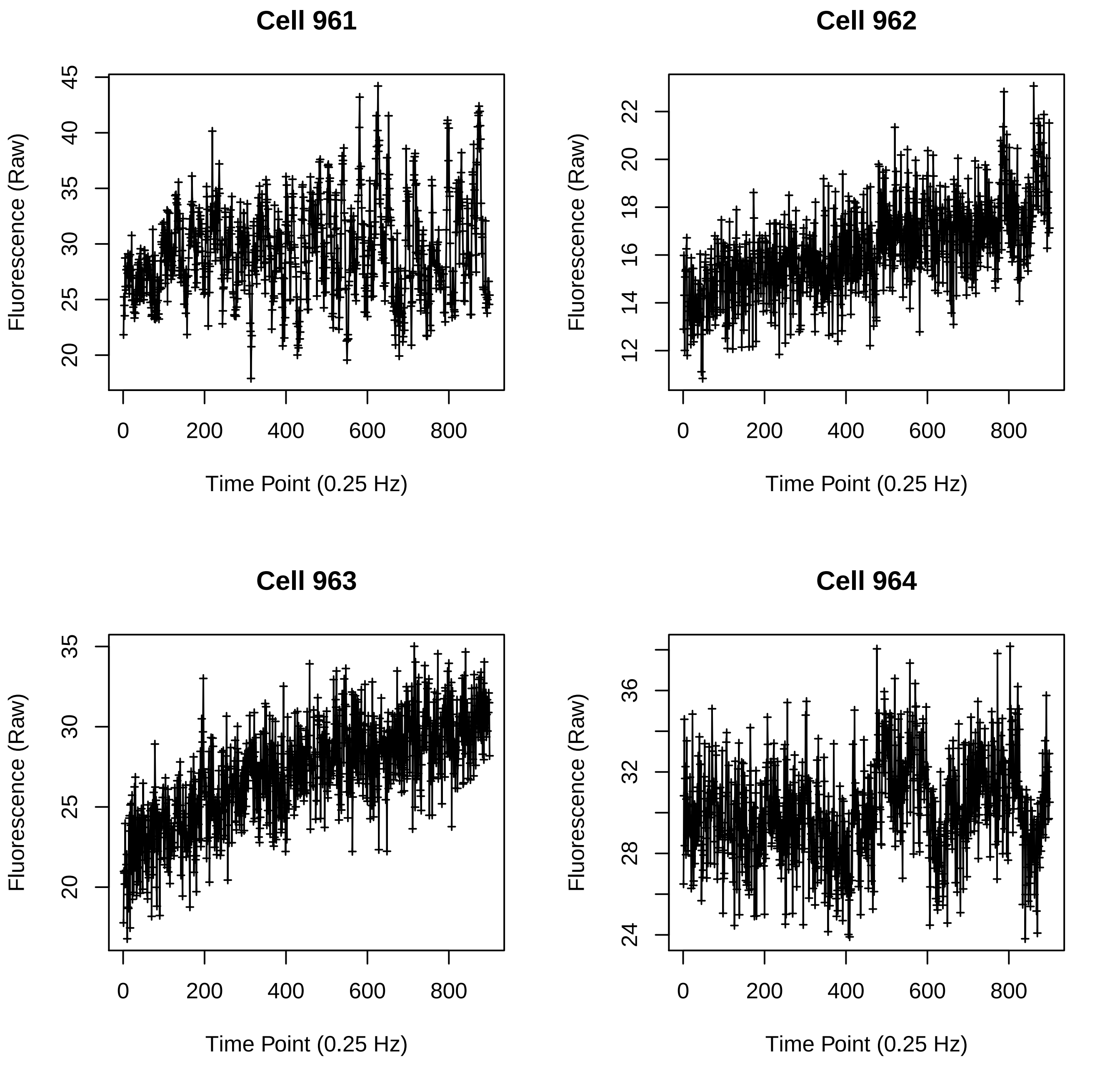

Cell 961
Cell 962
Cell 963
Cell 964
Fluorescence (Raw)
Time Point (0.25 Hz)
0
200
400
600
800
20
25
30
35
40
45
12
14
16
18
20
22
24
28
32
36

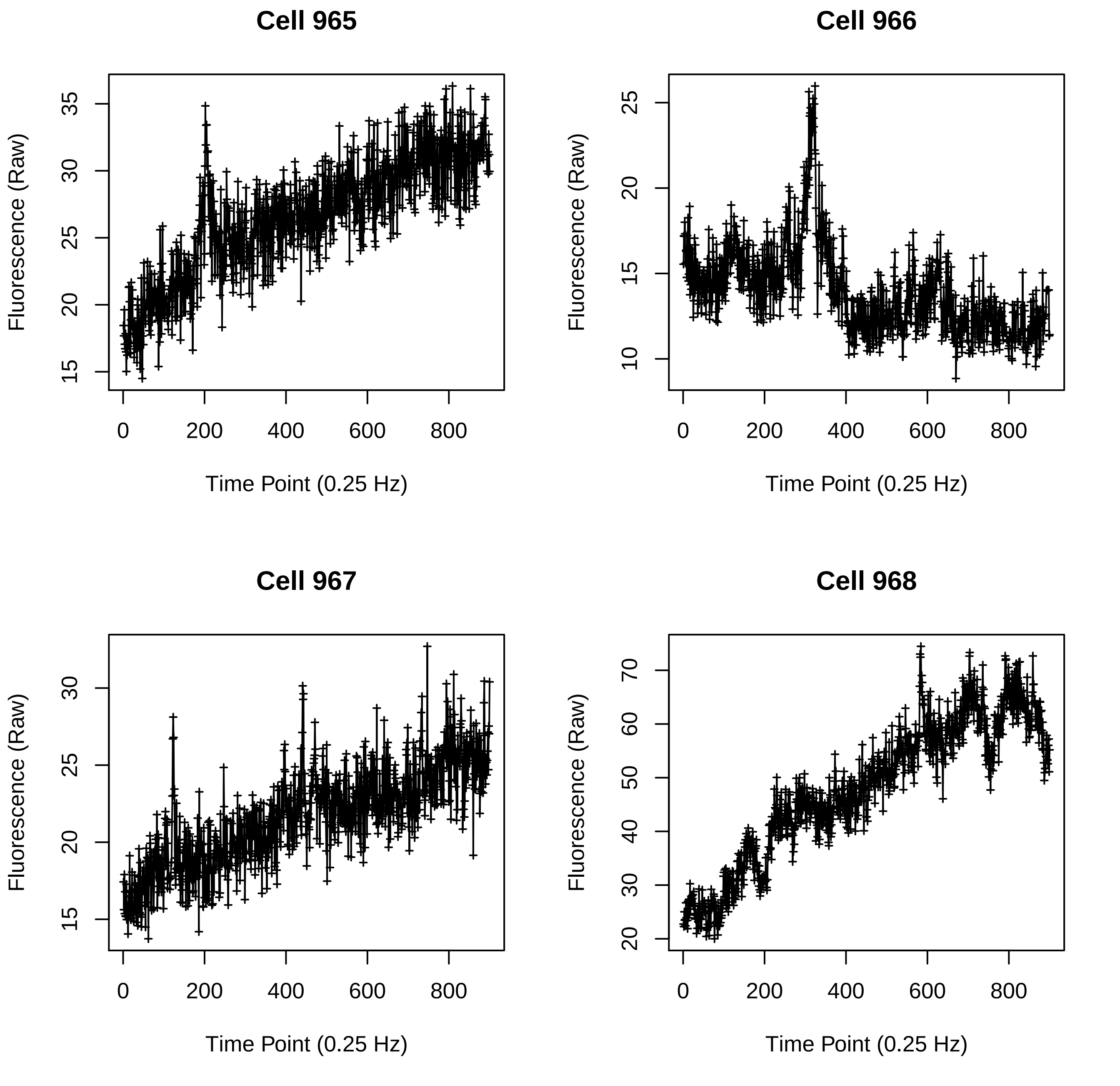

Cell 965
Cell 966
Cell 967
Cell 968
Fluorescence (Raw)
Time Point (0.25 Hz)
0
200
400
600
800
15
20
25
30
35
10
40
50
60
70

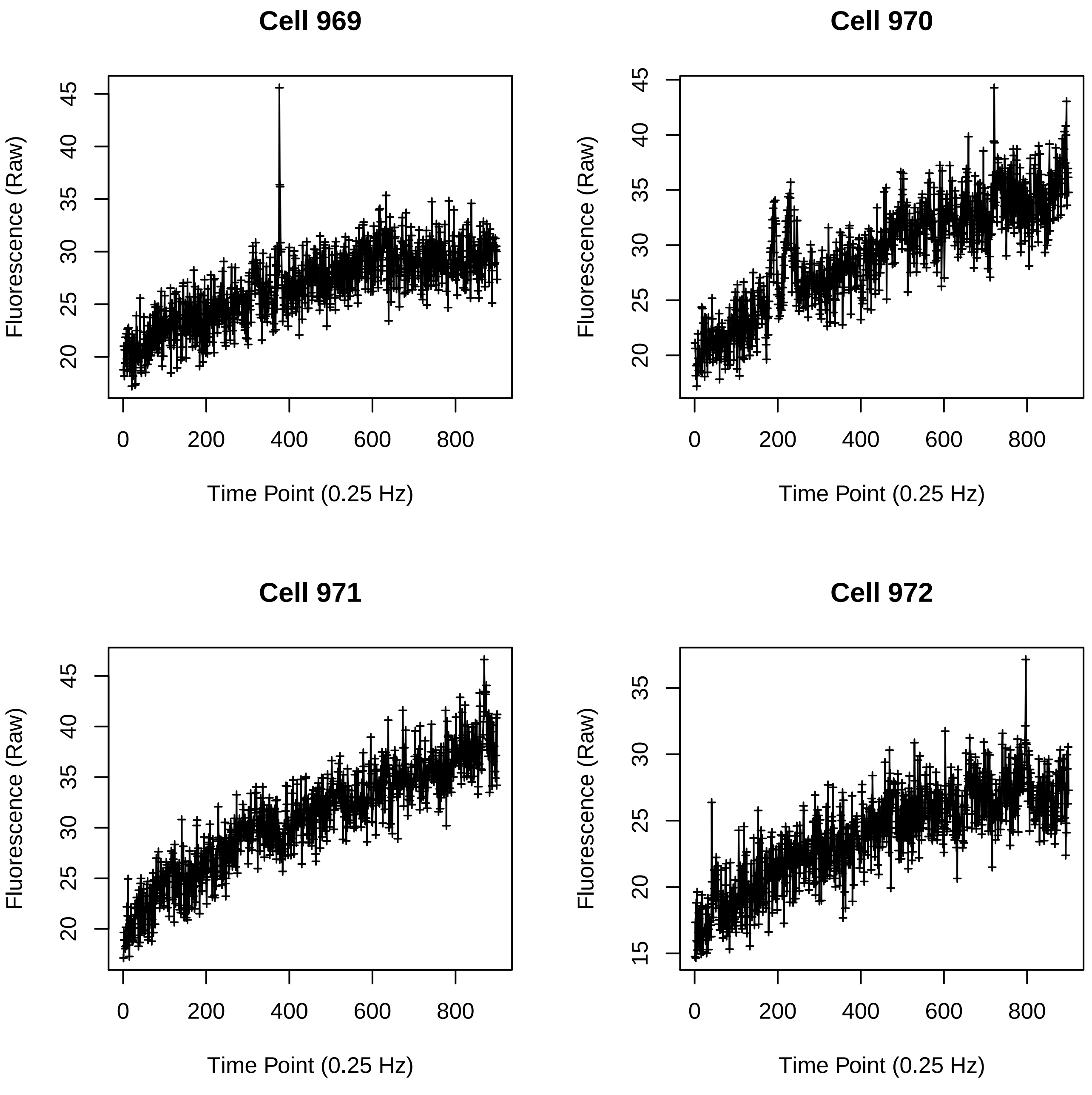

Cell 969
Cell 970
Cell 971
Cell 972
Fluorescence (Raw)
Time Point (0.25 Hz)
0
200
400
600
800
15
20
25
30
35
40
45

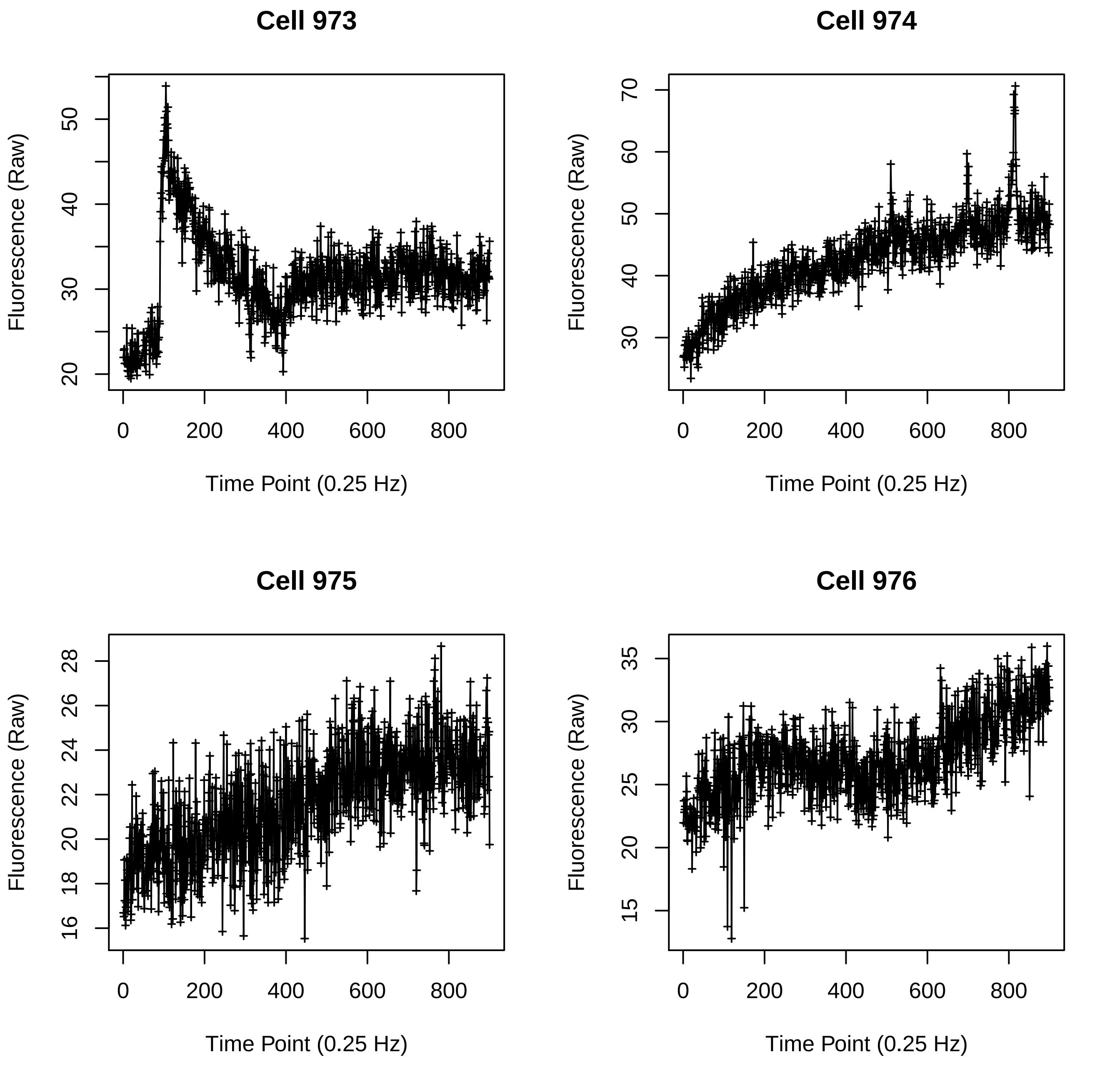
Cell 973
Cell 974
Cell 975
Cell 976
Fluorescence (Raw)
Time Point (0.25 Hz)
0
200
400
600
800
20
30
40
50
30
40
50
60
70
16
18
20
22
24
26
28
15
20
25
30
35

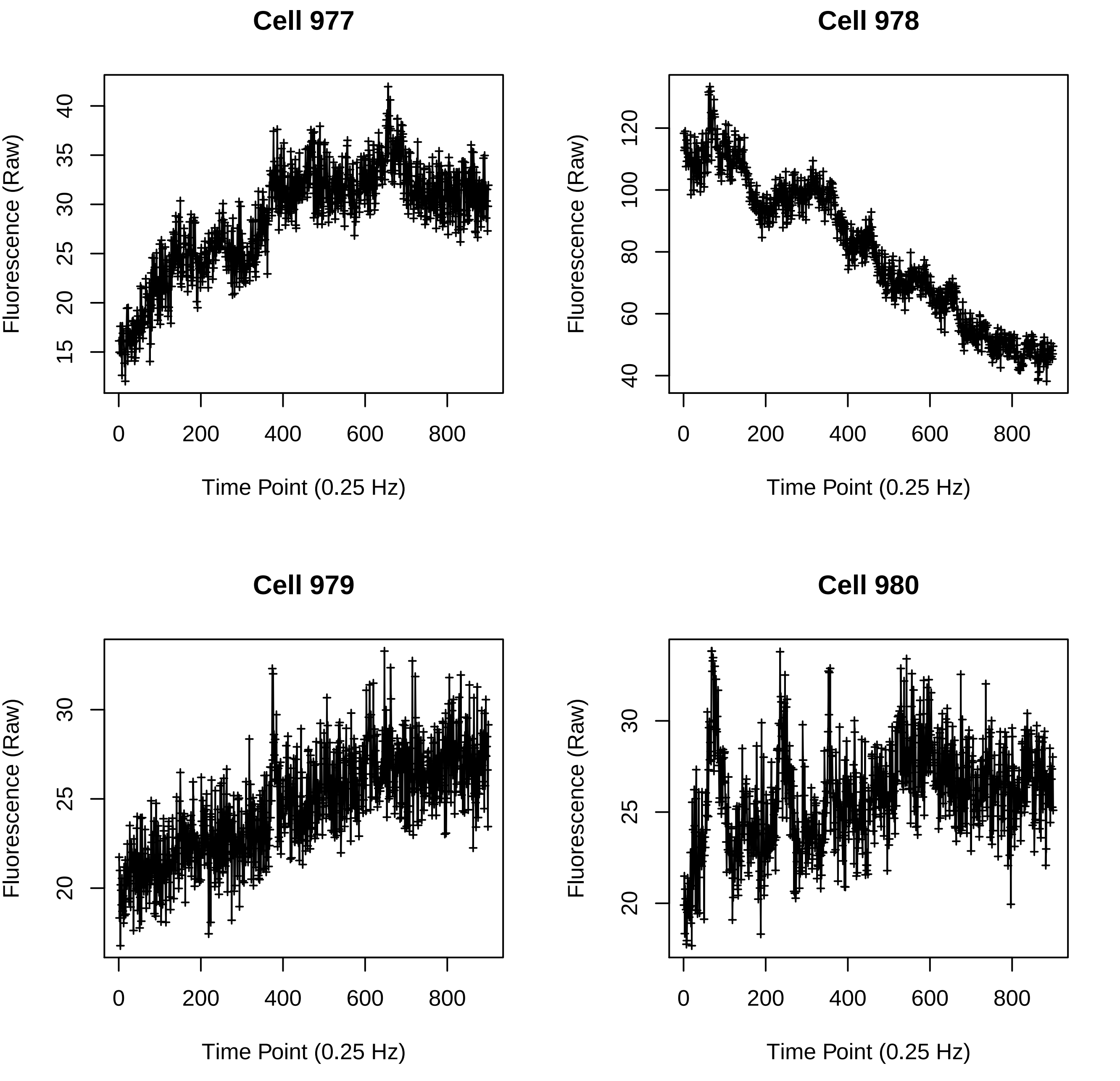

Cell 977
Fluorescence (Raw)
15
20
25
30
35
40
0
200
400
600
800
Time Point (0.25 Hz)
Cell 978
Fluorescence (Raw)
40
60
80
100
120
0
200
400
600
800
Time Point (0.25 Hz)
Cell 979
Fluorescence (Raw)
20
25
30
0
200
400
600
800
Time Point (0.25 Hz)
Cell 980
Fluorescence (Raw)
20
25
30
0
200
400
600
800
Time Point (0.25 Hz)

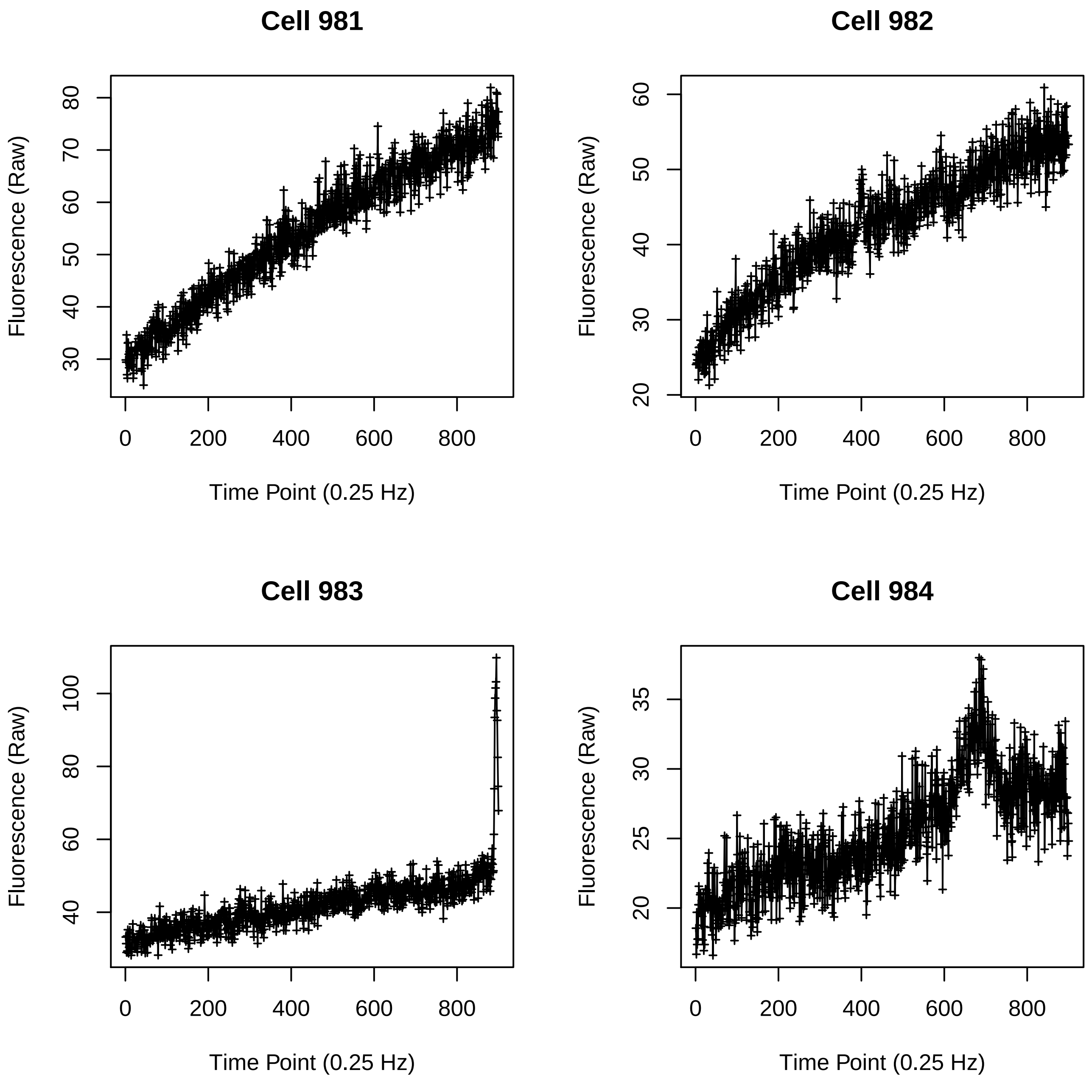
Cell 981
Cell 982
Cell 983
Cell 984
Fluorescence (Raw)
Time Point (0.25 Hz)
0
200
400
600
800
30
40
50
60
70
80
20
100
35
25

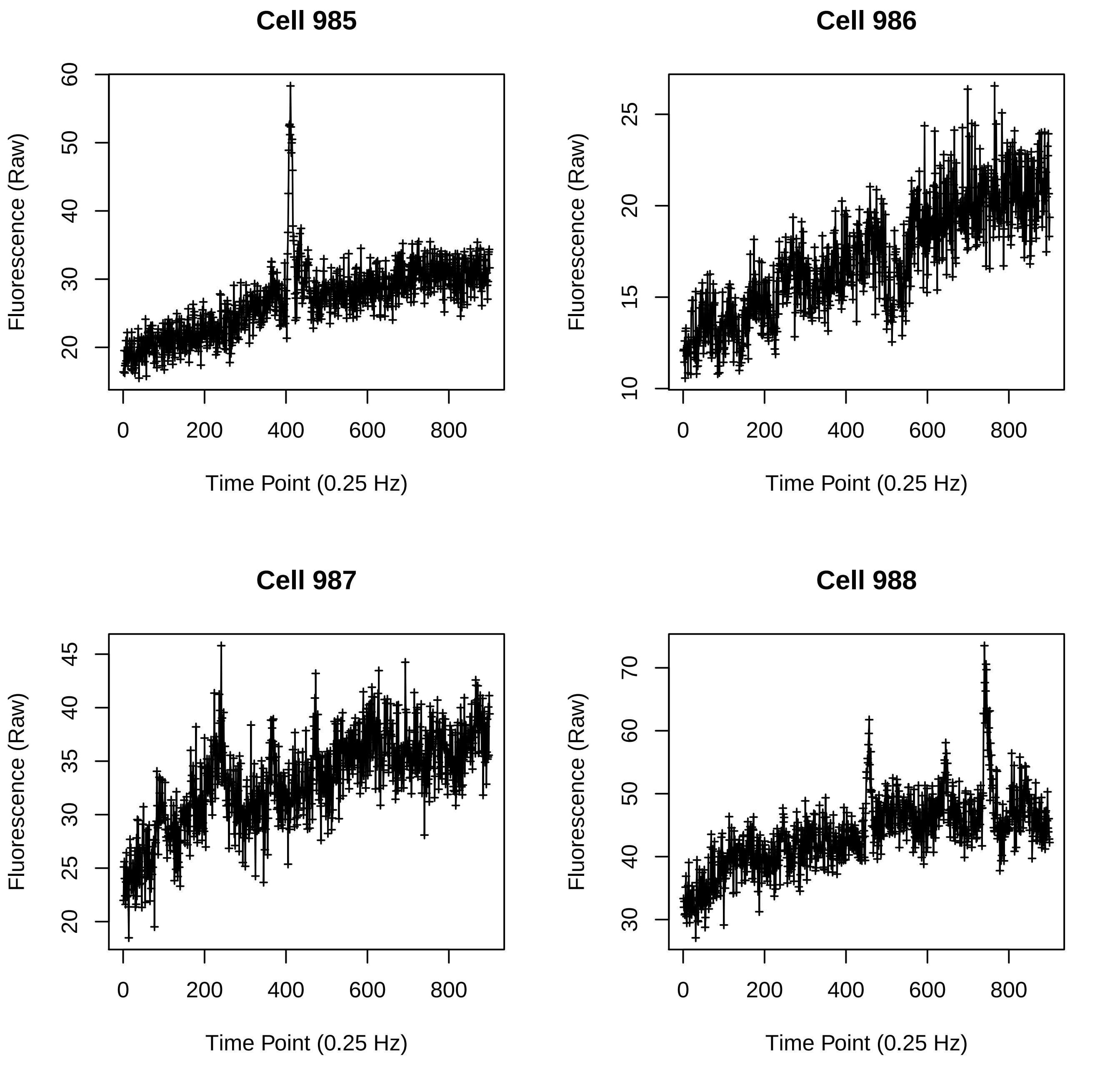

Cell 985
Cell 986
Cell 987
Cell 988
Fluorescence (Raw)
Time Point (0.25 Hz)
0
200
400
600
800
10
15
20
25
30
35
40
45
50
60
70

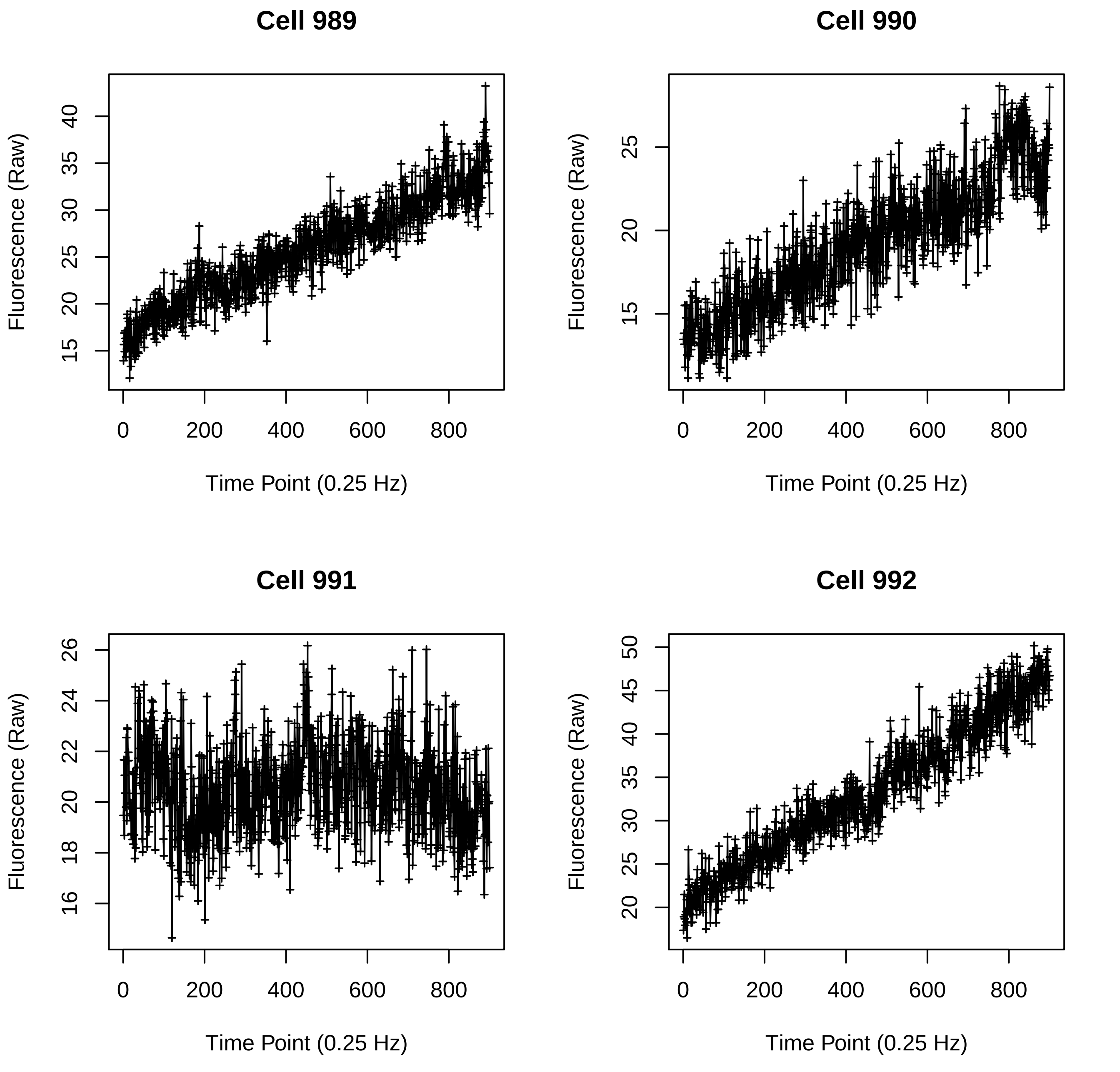
Cell 989
Cell 990
Cell 991
Cell 992
Fluorescence (Raw)
Time Point (0.25 Hz)
0
200
400
600
800
15
20
25
30
35
40
16
18
22
24
26
45
50

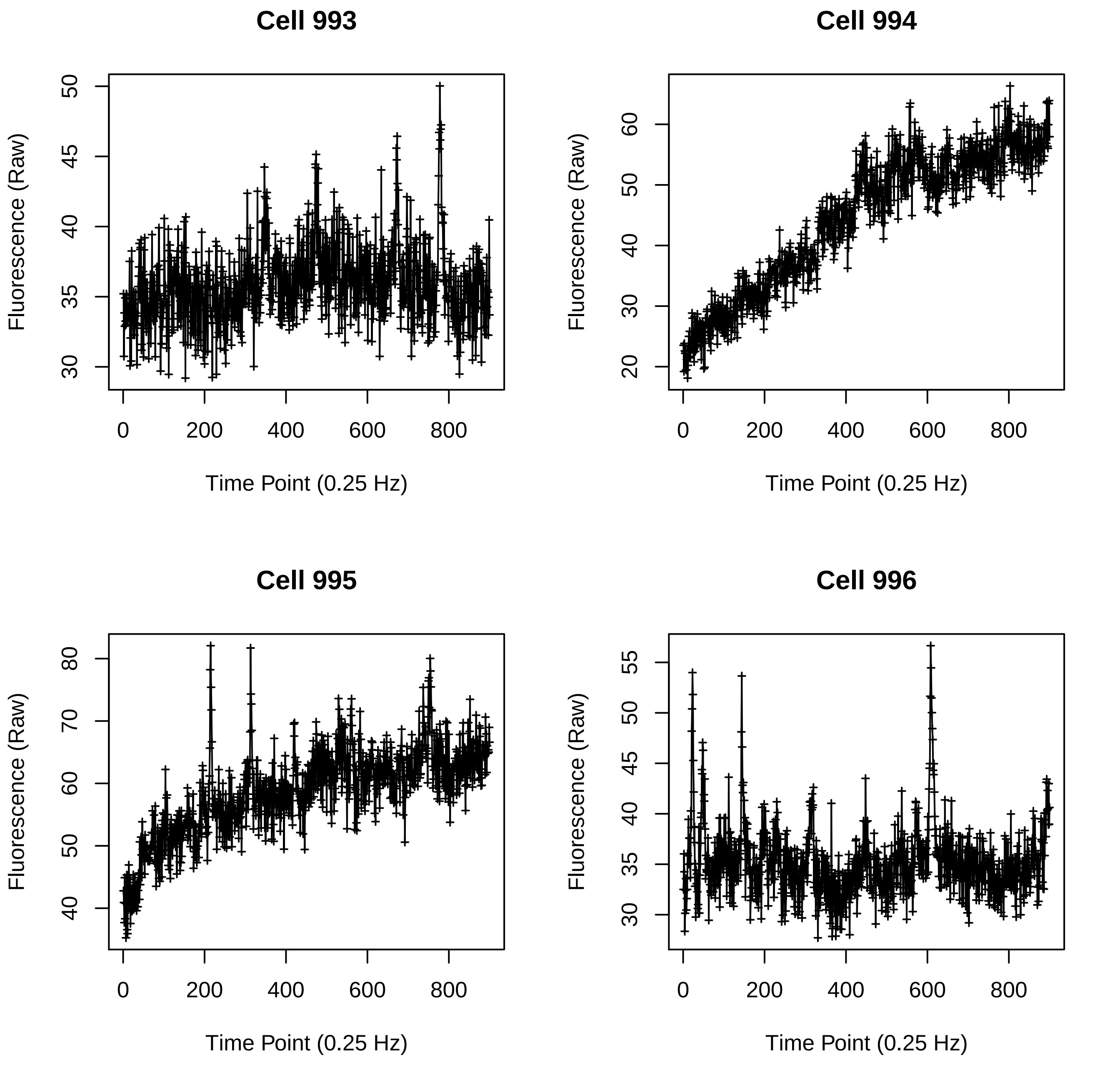
Cell 993
Cell 994
Cell 995
Cell 996
Fluorescence (Raw)
Time Point (0.25 Hz)
0
200
400
600
800
30
35
40
45
50
20
60
70
80
55

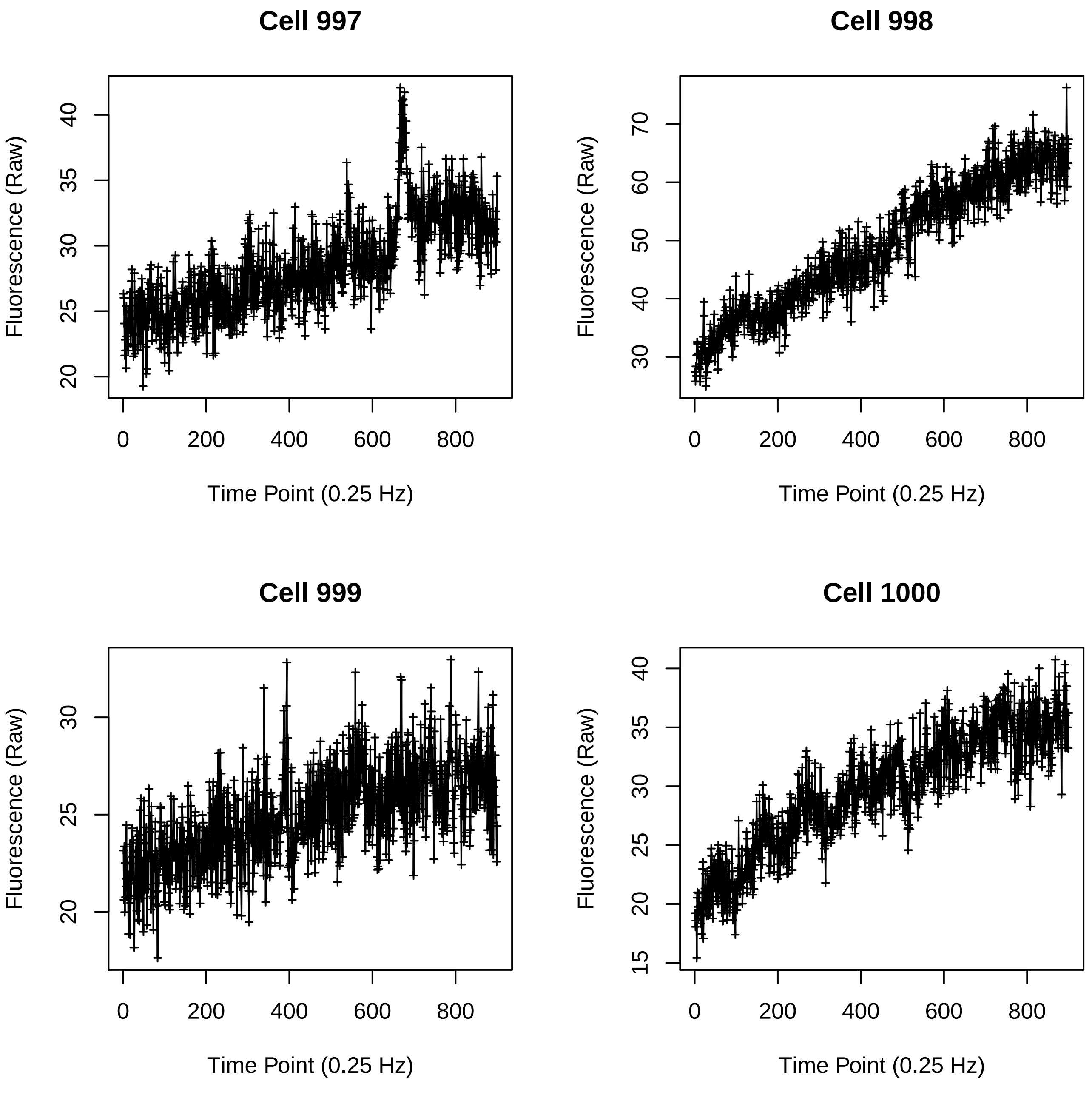

Cell 997
Cell 998
Cell 999
Cell 1000
Fluorescence (Raw)
Time Point (0.25 Hz)
0
200
400
600
800
20
25
30
35
40
50
60
70
15

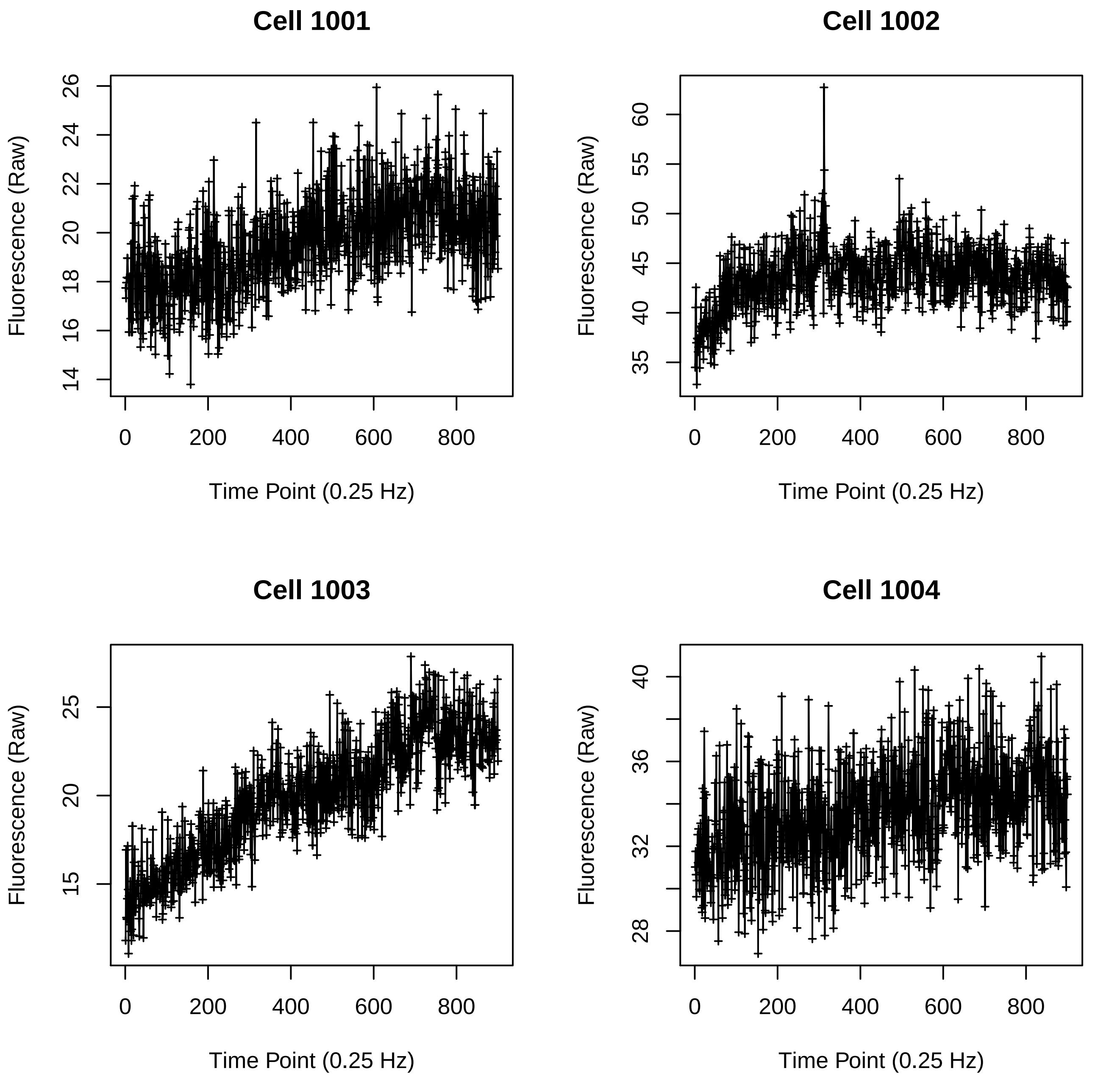

Cell 1001
Cell 1002
Cell 1003
Cell 1004
Fluorescence (Raw)
Time Point (0.25 Hz)
0
200
400
600
800
14
16
18
20
22
24
26
35
40
45
50
55
60
15
20
25
28
32
36
40

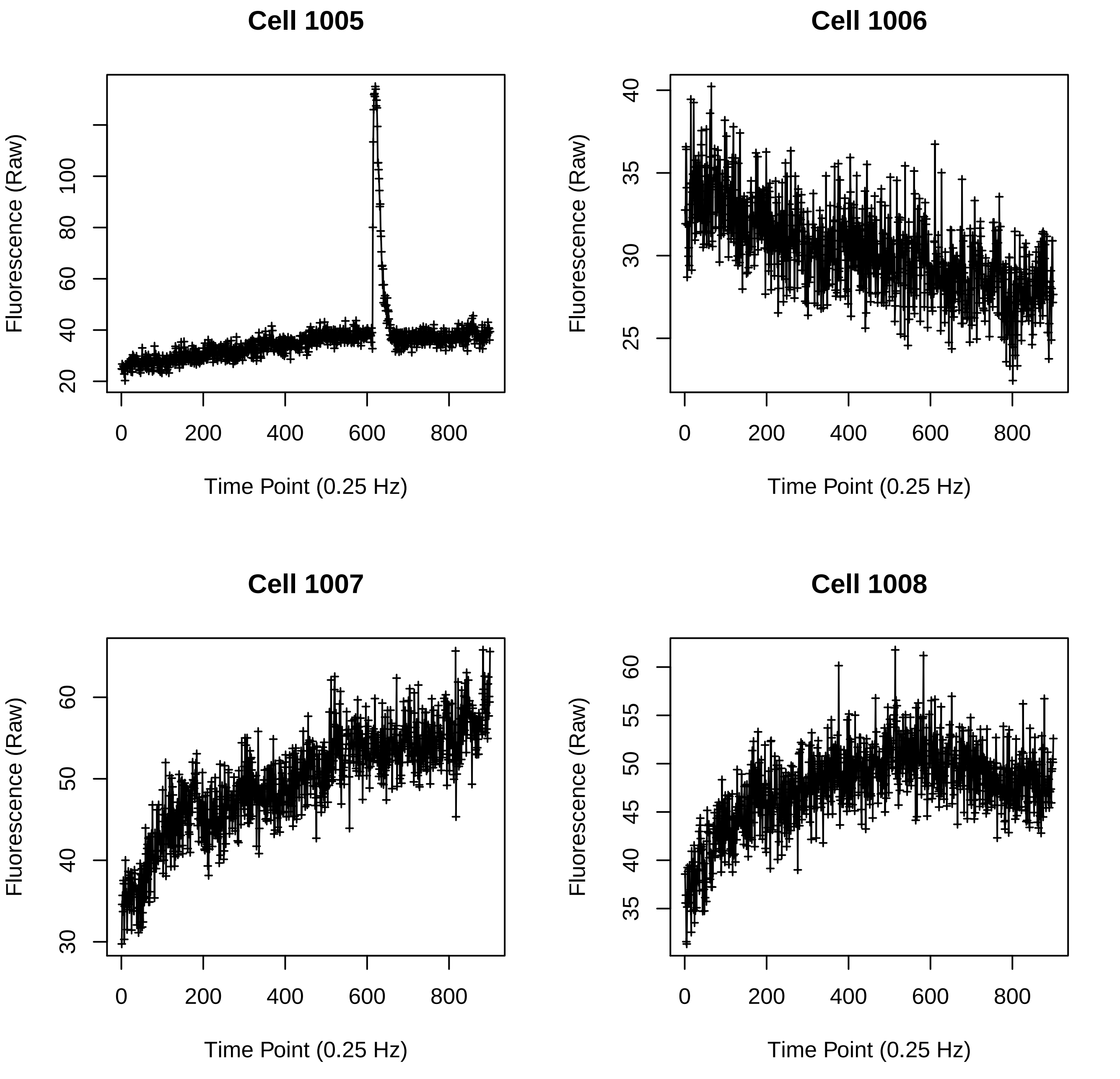
Cell 1005
Fluorescence (Raw)
20
40
60
80
100
0
200
400
600
800
Time Point (0.25 Hz)
Cell 1006
Fluorescence (Raw)
25
30
35
40
0
200
400
600
800
Time Point (0.25 Hz)
Cell 1007
Fluorescence (Raw)
30
40
50
60
0
200
400
600
800
Time Point (0.25 Hz)
Cell 1008
Fluorescence (Raw)
35
40
45
50
55
60
0
200
400
600
800
Time Point (0.25 Hz)

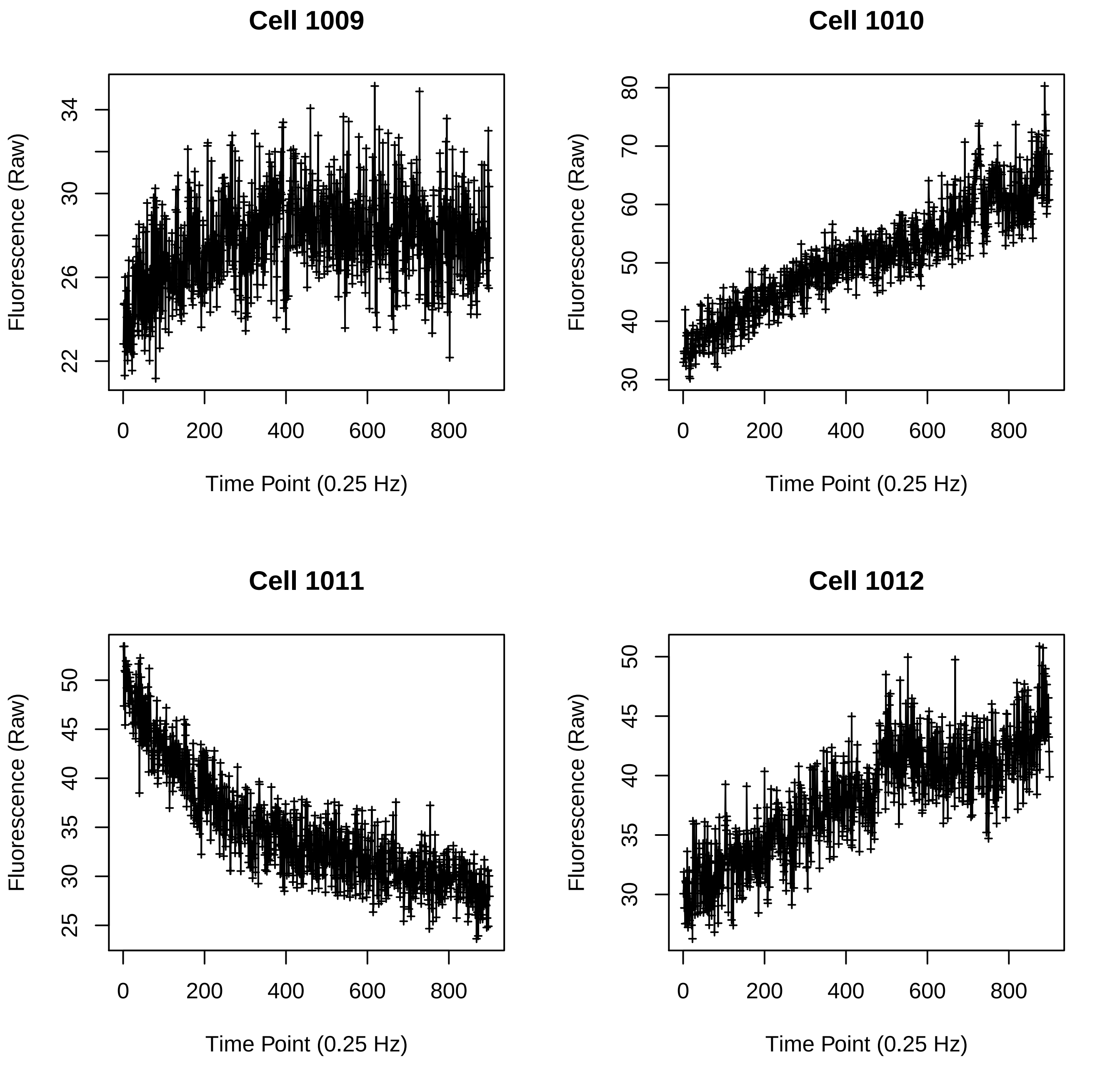
Cell 1009
Fluorescence (Raw)
22
26
30
34
0
200
400
600
800
Time Point (0.25 Hz)
Cell 1010
Fluorescence (Raw)
30
40
50
60
70
80
0
200
400
600
800
Time Point (0.25 Hz)
Cell 1011
Fluorescence (Raw)
25
30
35
40
45
50
0
200
400
600
800
Time Point (0.25 Hz)
Cell 1012
Fluorescence (Raw)
30
35
40
45
50
0
200
400
600
800
Time Point (0.25 Hz)

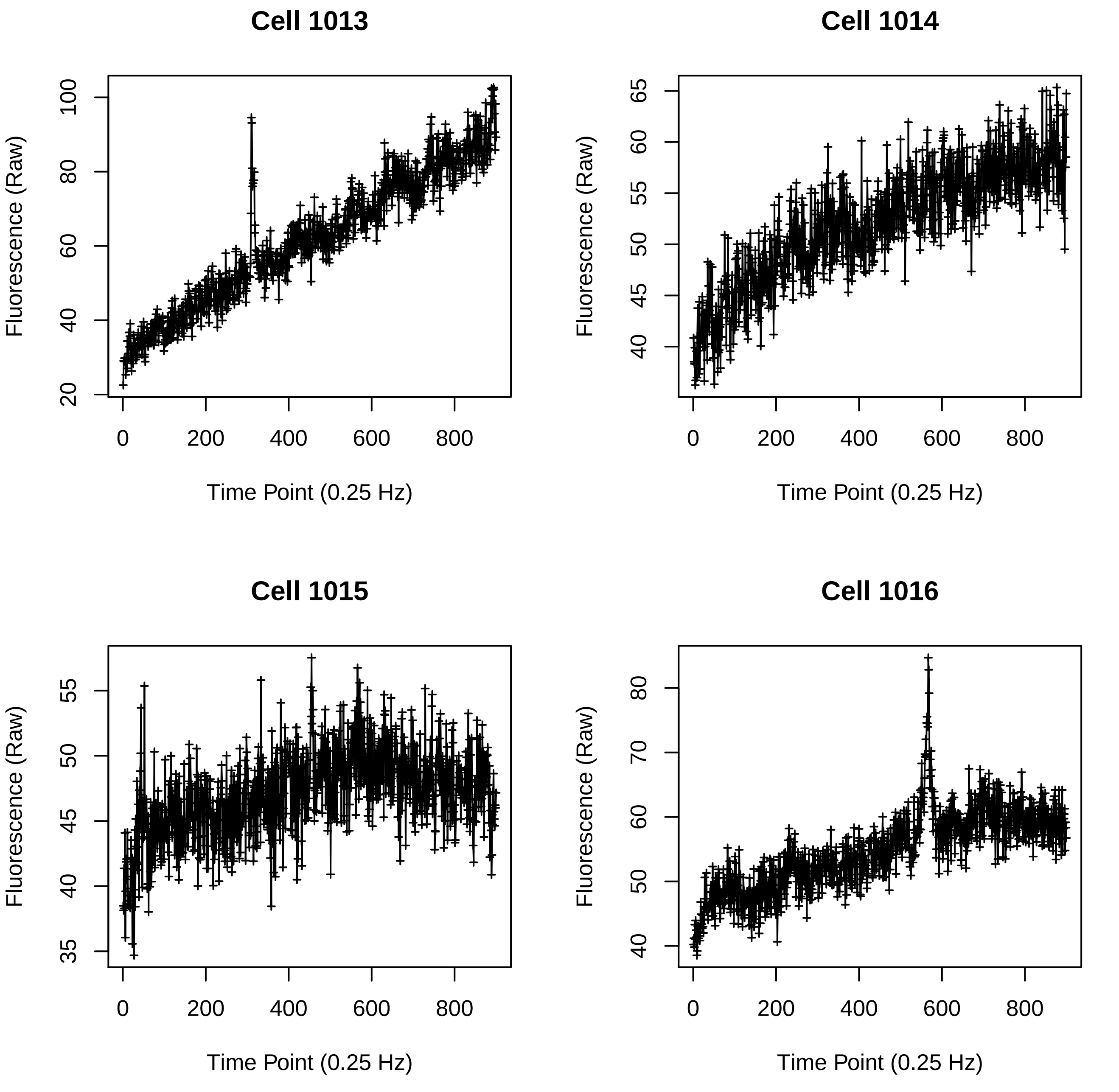
Cell 1013
Cell 1014
Cell 1015
Cell 1016
Fluorescence (Raw)
Time Point (0.25 Hz)
0
200
400
600
800
20
40
60
80
100
40
45
50
55
60
65
35
70

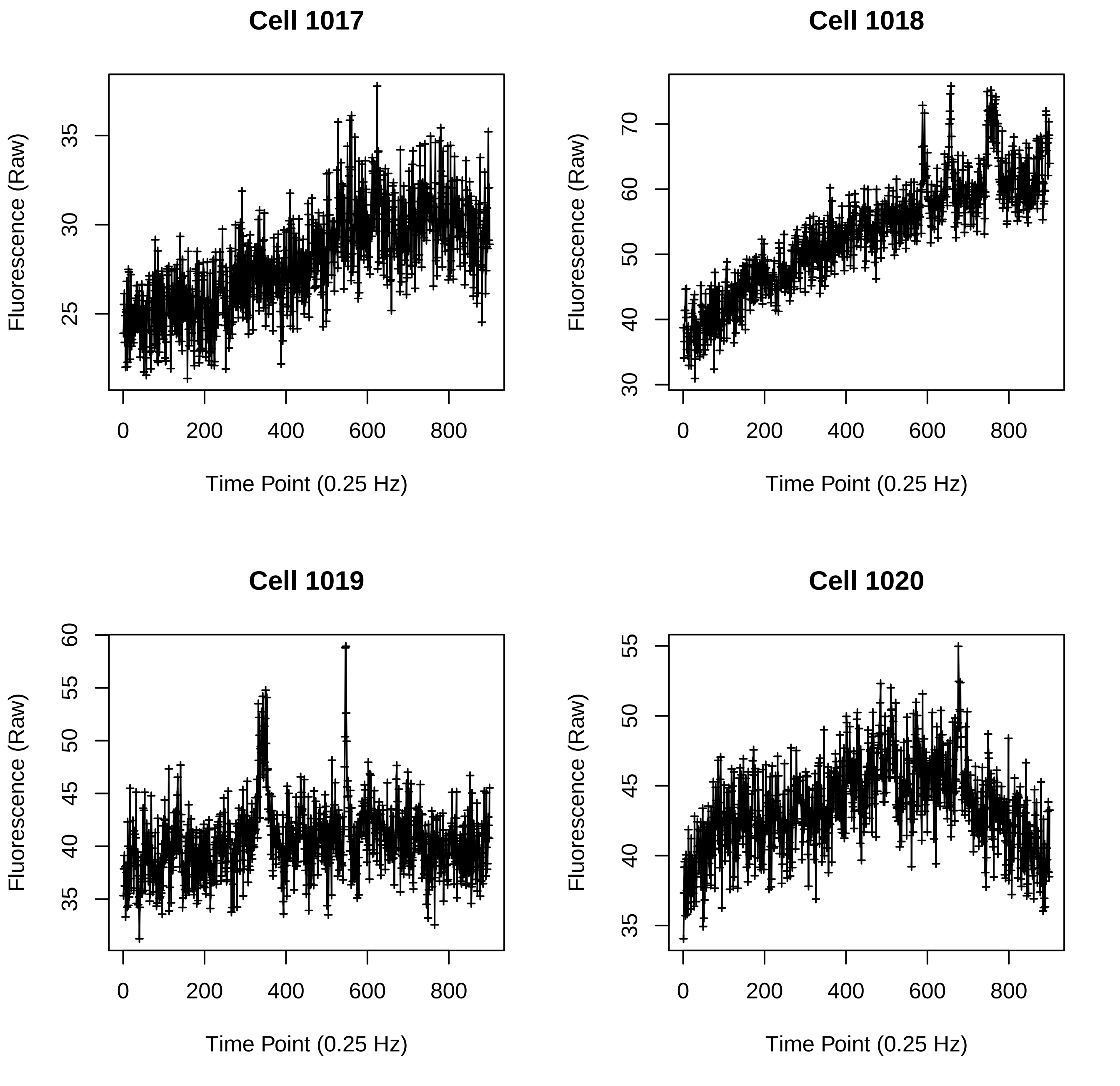

Cell 1017
Cell 1018
Cell 1019
Cell 1020
Fluorescence (Raw)
Time Point (0.25 Hz)
0
200
400
600
800
25
30
35
40
50
60
70
45
55

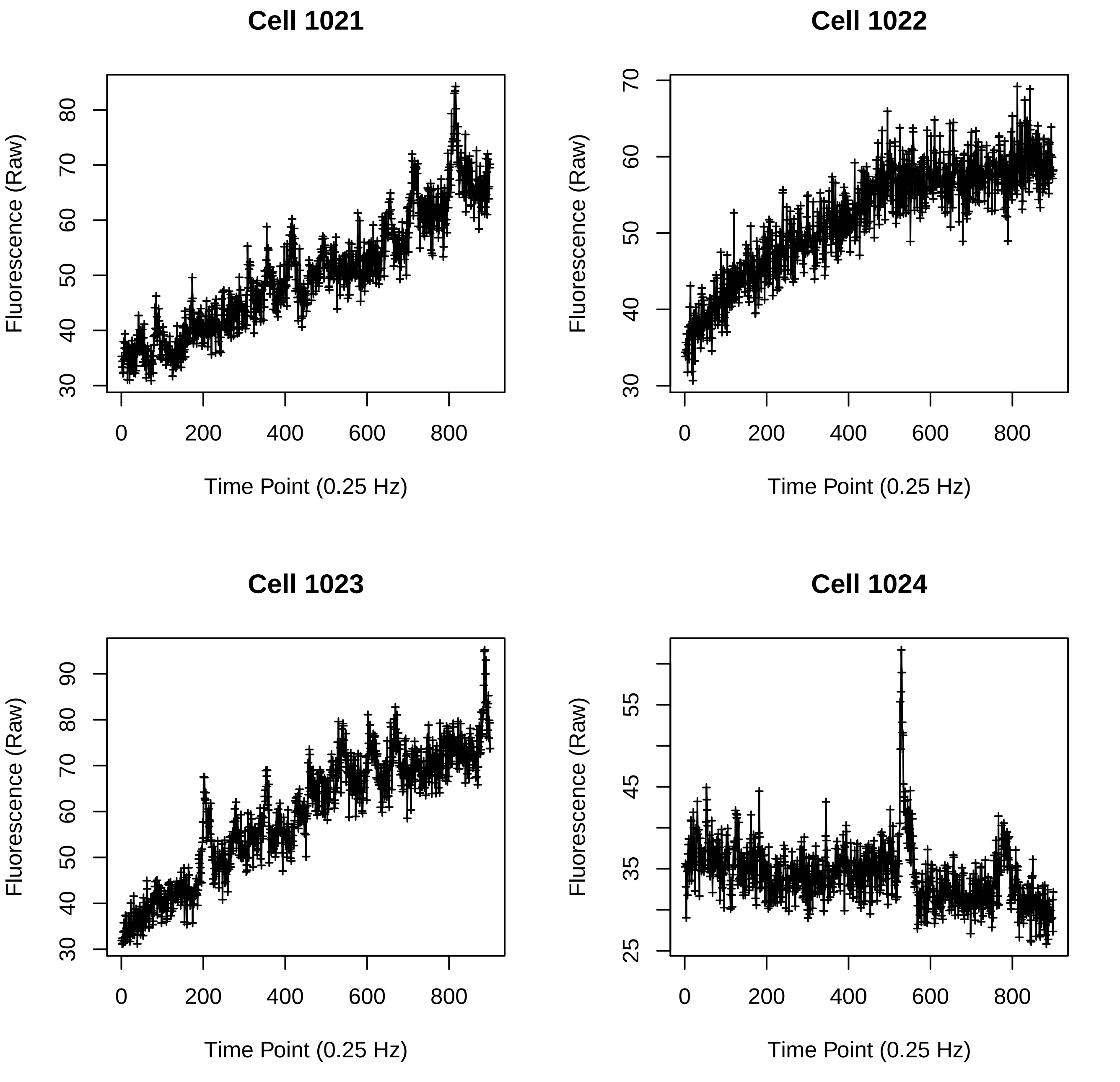
Cell 1021
Fluorescence (Raw)
30
40
50
60
70
80
0
200
400
600
800
Time Point (0.25 Hz)
Cell 1022
Fluorescence (Raw)
30
40
50
60
70
0
200
400
600
800
Time Point (0.25 Hz)
Cell 1023
Fluorescence (Raw)
30
40
50
60
70
80
90
0
200
400
600
800
Time Point (0.25 Hz)
Cell 1024
Fluorescence (Raw)
25
35
45
55
0
200
400
600
800
Time Point (0.25 Hz)

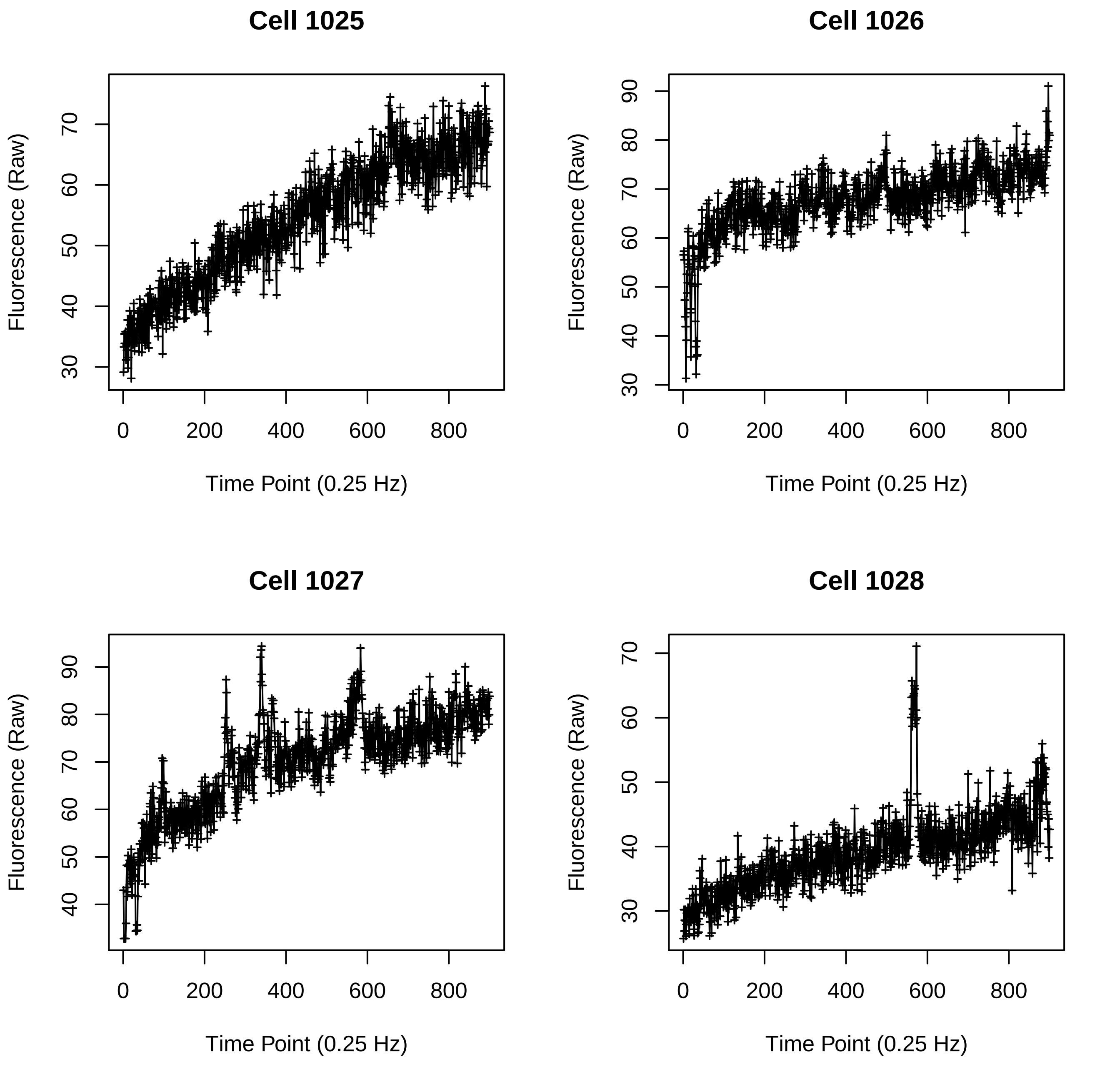

Cell 1025
Cell 1026
Cell 1027
Cell 1028
Fluorescence (Raw)
Time Point (0.25 Hz)
0
200
400
600
800
30
40
50
60
70
80
90

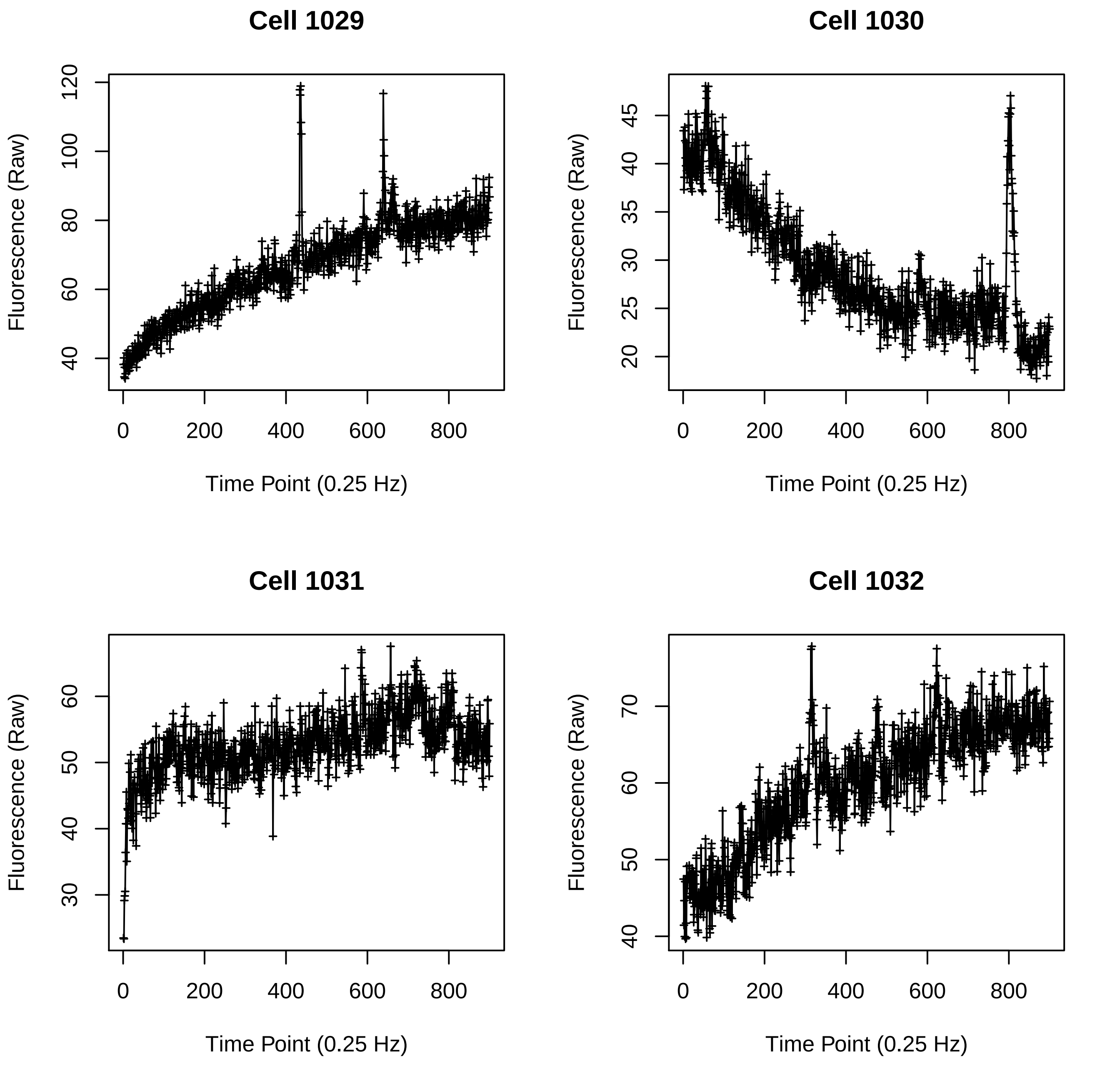
Cell 1029
Cell 1030
Cell 1031
Cell 1032
Fluorescence (Raw)
Time Point (0.25 Hz)
40
60
80
100
120
20
25
30
35
40
45
30
40
50
60
40
50
60
70
0
200
400
600
800

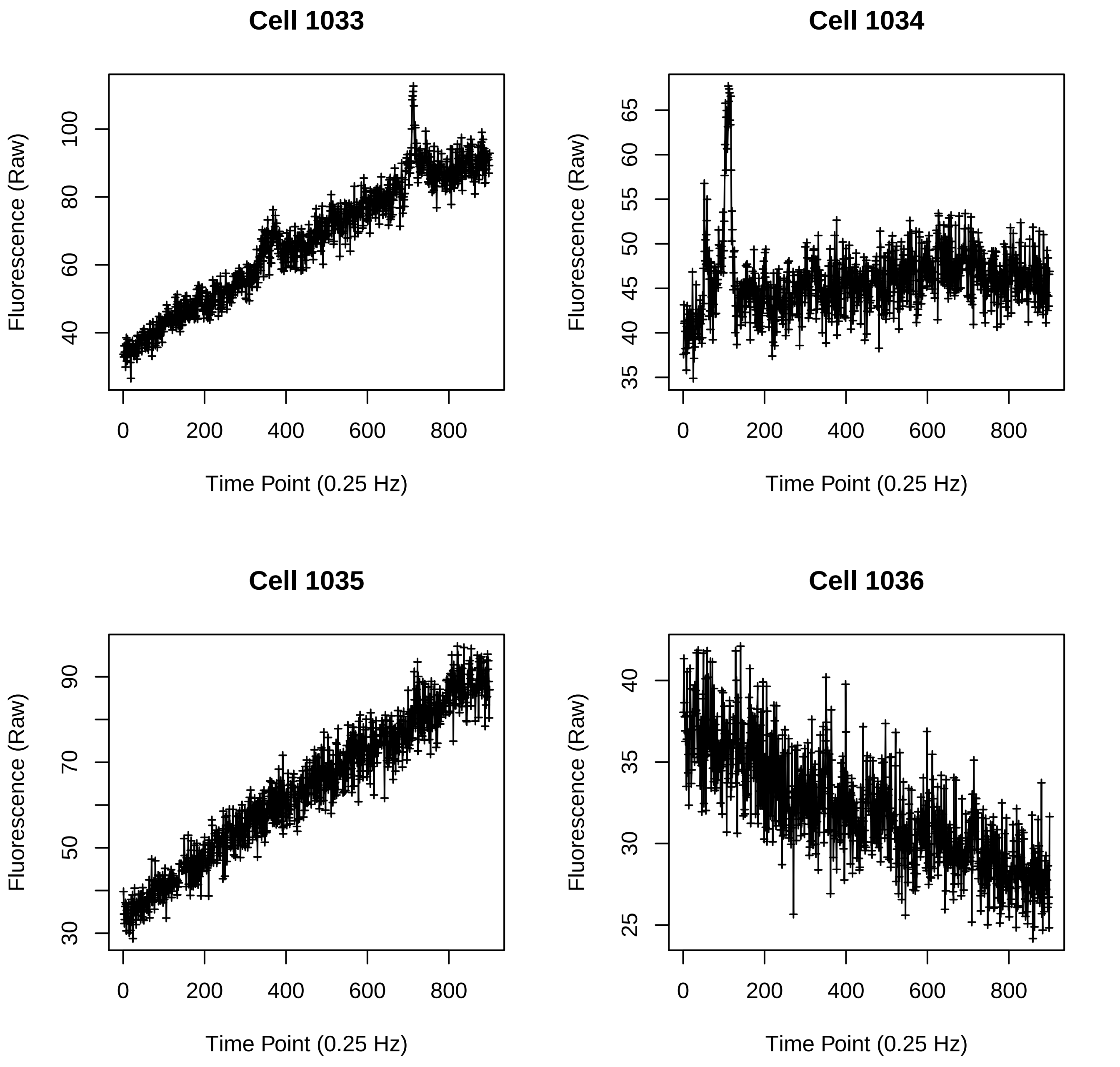
Cell 1033
Cell 1034
Cell 1035
Cell 1036
Fluorescence (Raw)
Time Point (0.25 Hz)
0
200
400
600
800
40
60
80
100
35
40
45
50
55
60
65
30
50
70
90
25
30
35
40

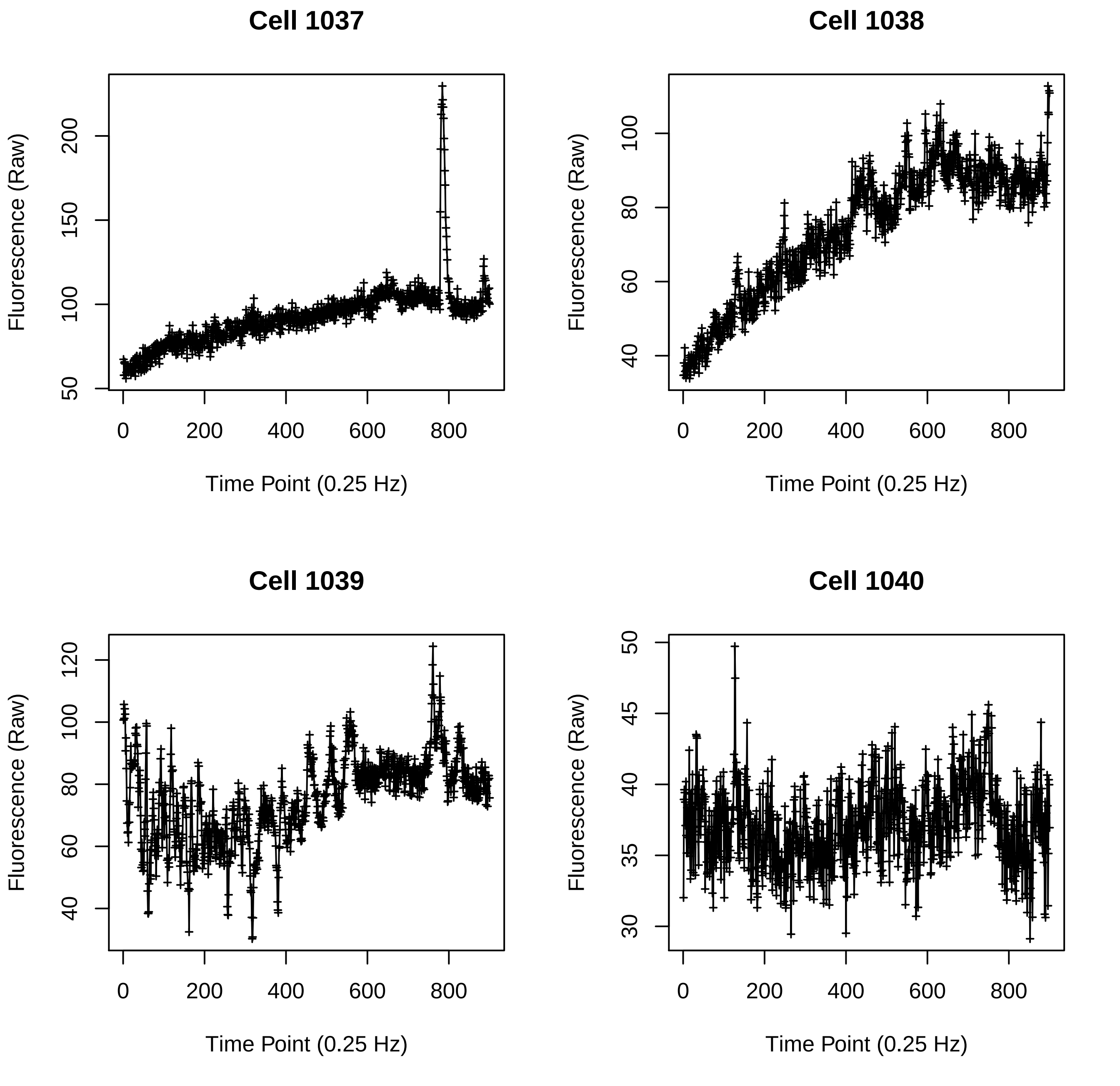

Cell 1037
Cell 1038
Cell 1039
Cell 1040
Fluorescence (Raw)
Time Point (0.25 Hz)
0
200
400
600
800
50
100
150
200
40
60
80
100
120
30
35
40
45
50

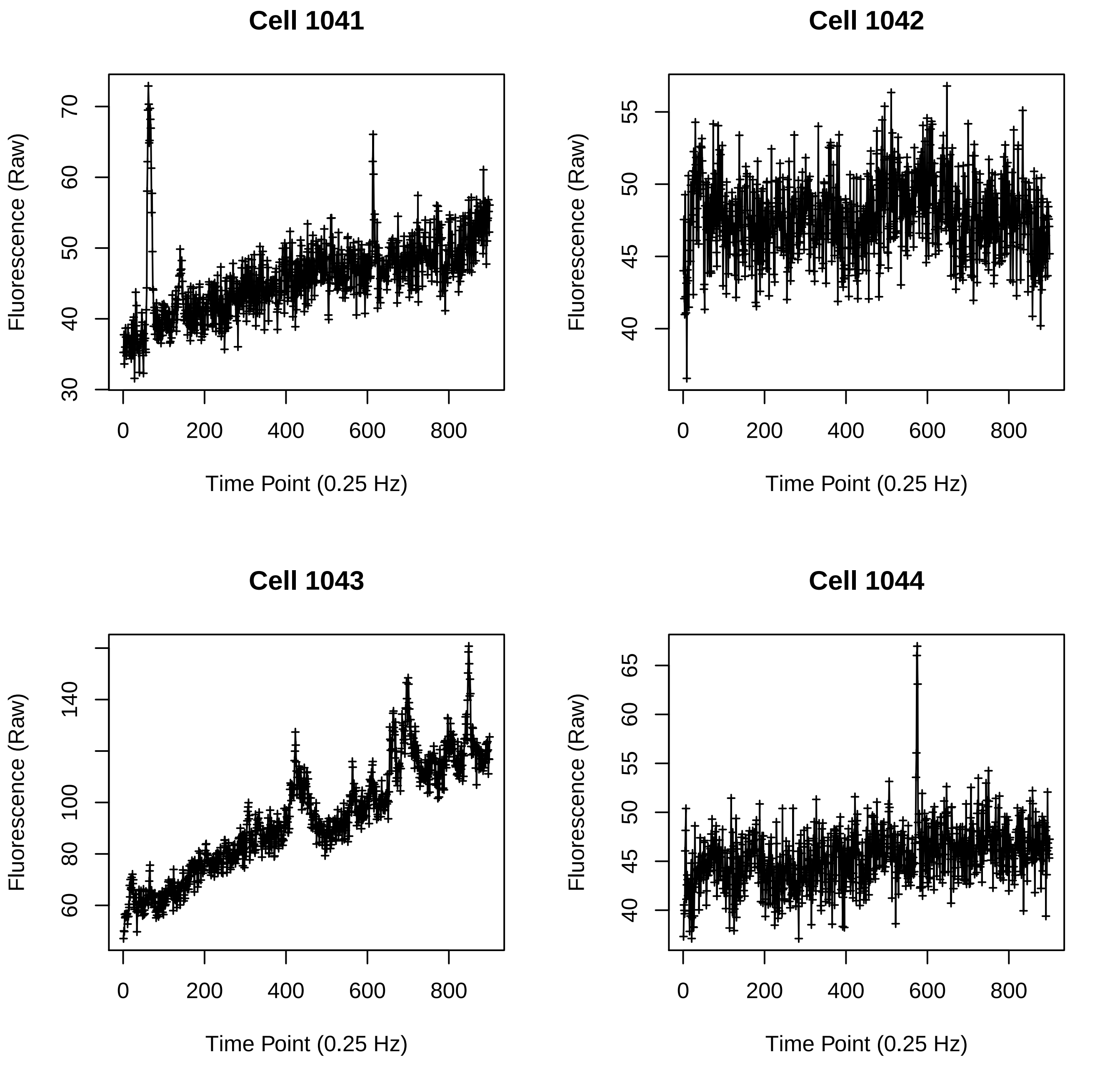
Cell 1041
Fluorescence (Raw)
30
40
50
60
70
0
200
400
600
800
Time Point (0.25 Hz)
Cell 1042
Fluorescence (Raw)
40
45
50
55
0
200
400
600
800
Time Point (0.25 Hz)
Cell 1043
Fluorescence (Raw)
60
80
100
140
0
200
400
600
800
Time Point (0.25 Hz)
Cell 1044
Fluorescence (Raw)
40
45
50
55
60
65
0
200
400
600
800
Time Point (0.25 Hz)

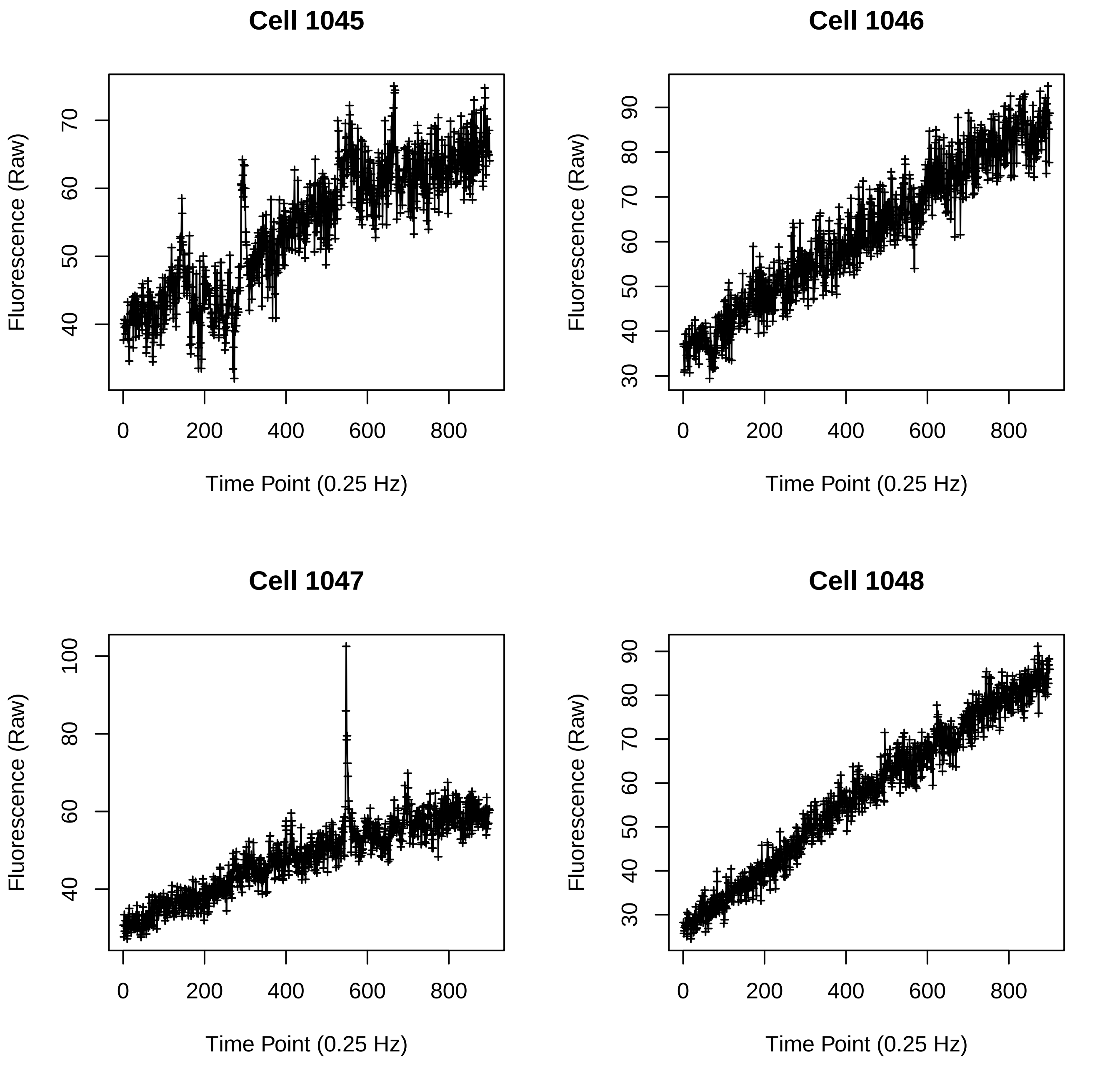
Cell 1045
Cell 1046
Cell 1047
Cell 1048
Fluorescence (Raw)
Time Point (0.25 Hz)
0
200
400
600
800
40
50
60
70
30
80
90
100

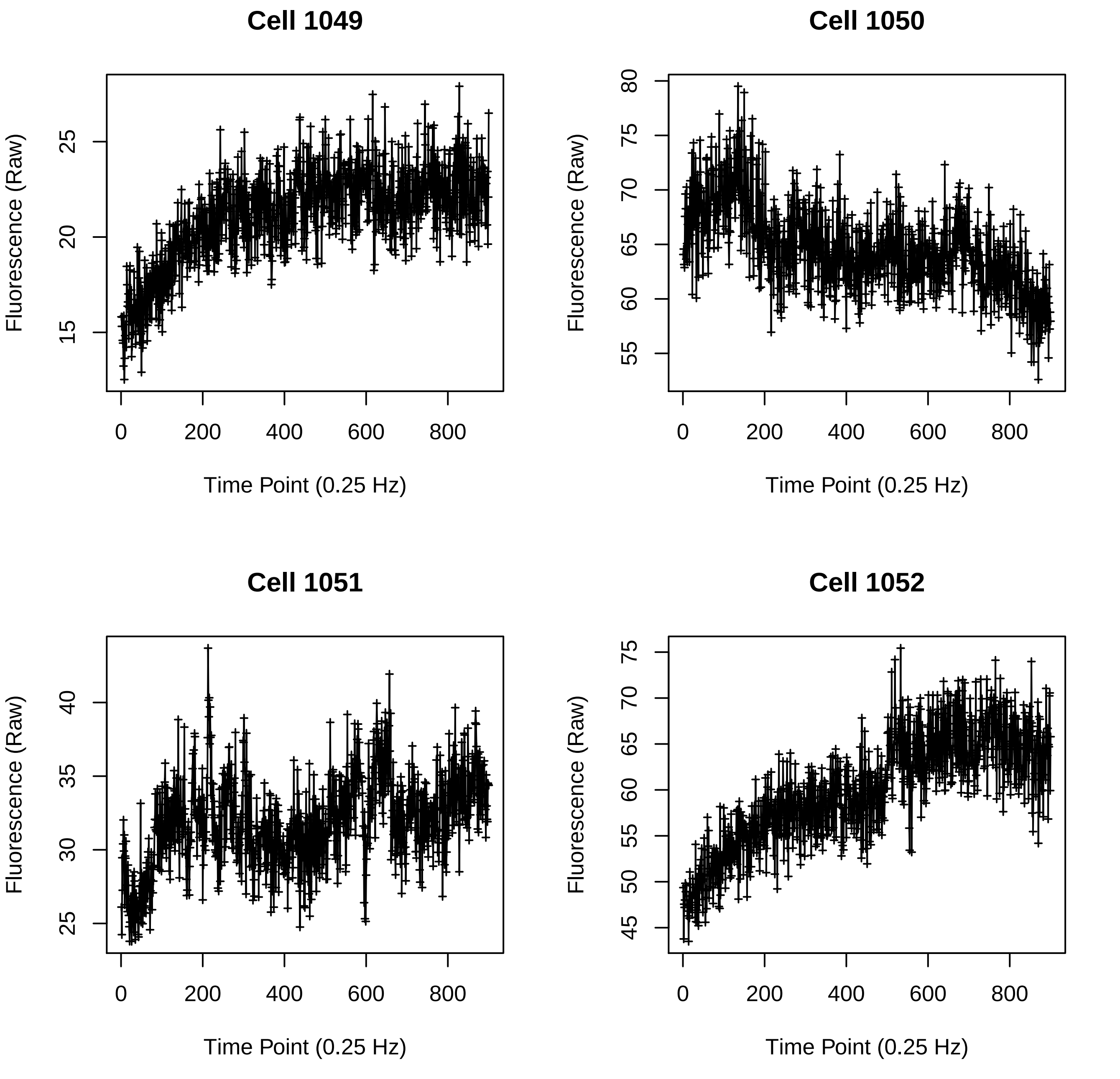
Cell 1049
Fluorescence (Raw)
15
20
25
0
200
400
600
800
Time Point (0.25 Hz)
Cell 1050
Fluorescence (Raw)
55
60
65
70
75
80
0
200
400
600
800
Time Point (0.25 Hz)
Cell 1051
Fluorescence (Raw)
25
30
35
40
0
200
400
600
800
Time Point (0.25 Hz)
Cell 1052
Fluorescence (Raw)
45
50
55
60
65
70
75
0
200
400
600
800
Time Point (0.25 Hz)

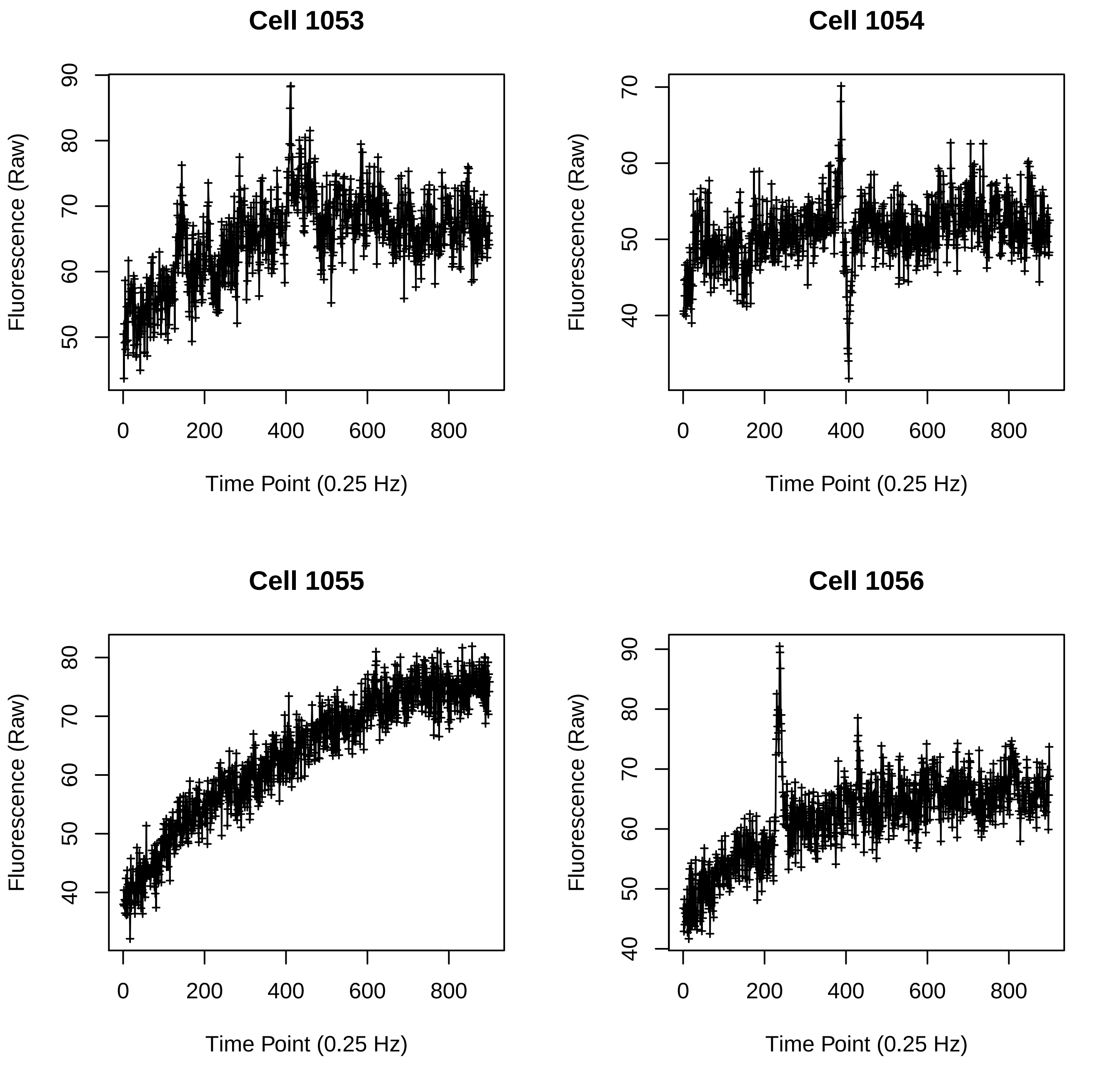
Cell 1053
Cell 1054
Cell 1055
Cell 1056
Fluorescence (Raw)
Time Point (0.25 Hz)
0
200
400
600
800
40
50
60
70
80
90

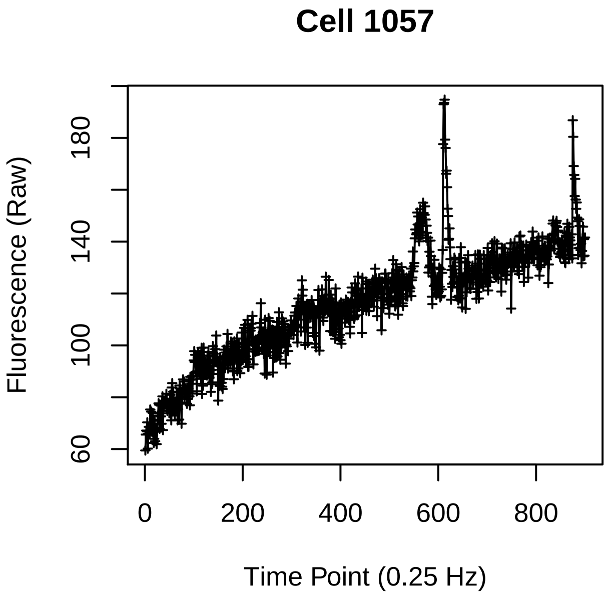
Cell 1057
Fluorescence (Raw)
60
100
140
180
0
200
400
600
800
Time Point (0.25 Hz)

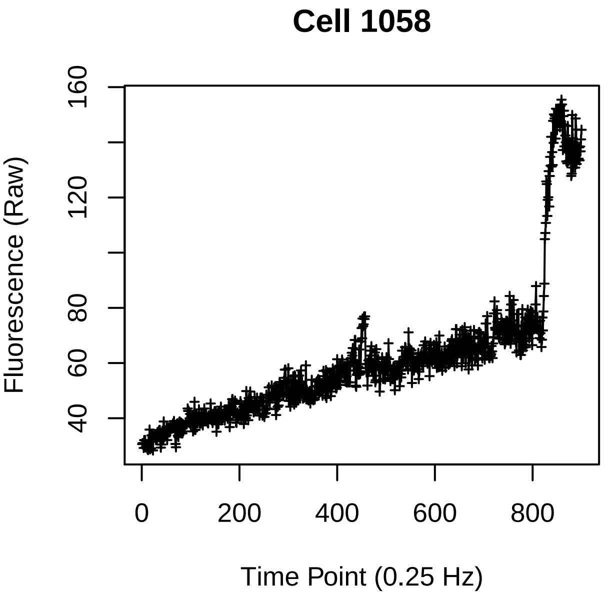
Cell 1058
Fluorescence (Raw)
40
60
80
120
160
0
200
400
600
800
Time Point (0.25 Hz)

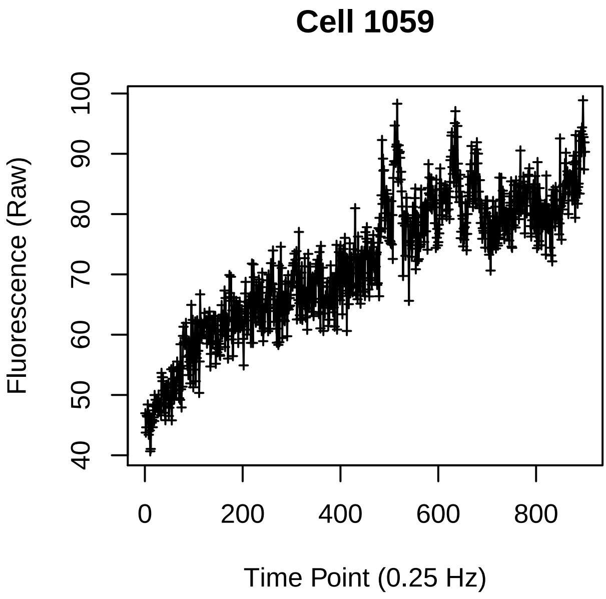
Cell 1059
Fluorescence (Raw)
40
50
60
70
80
90
100
0
200
400
600
800
Time Point (0.25 Hz)

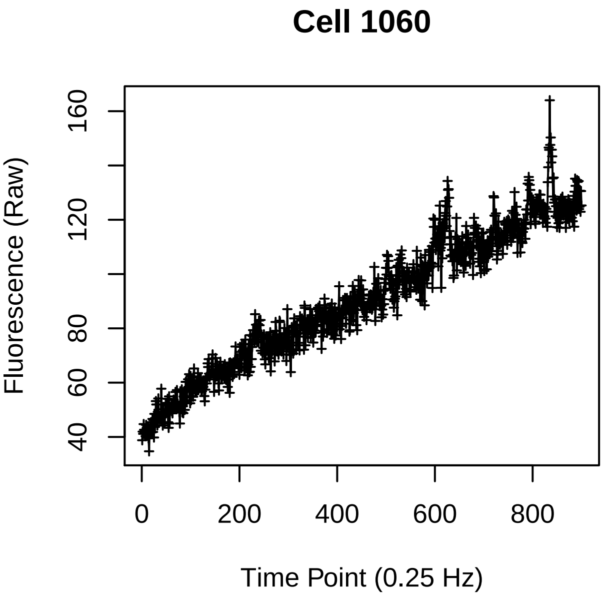
Cell 1060
Fluorescence (Raw)
40
60
80
120
160
0
200
400
600
800
Time Point (0.25 Hz)

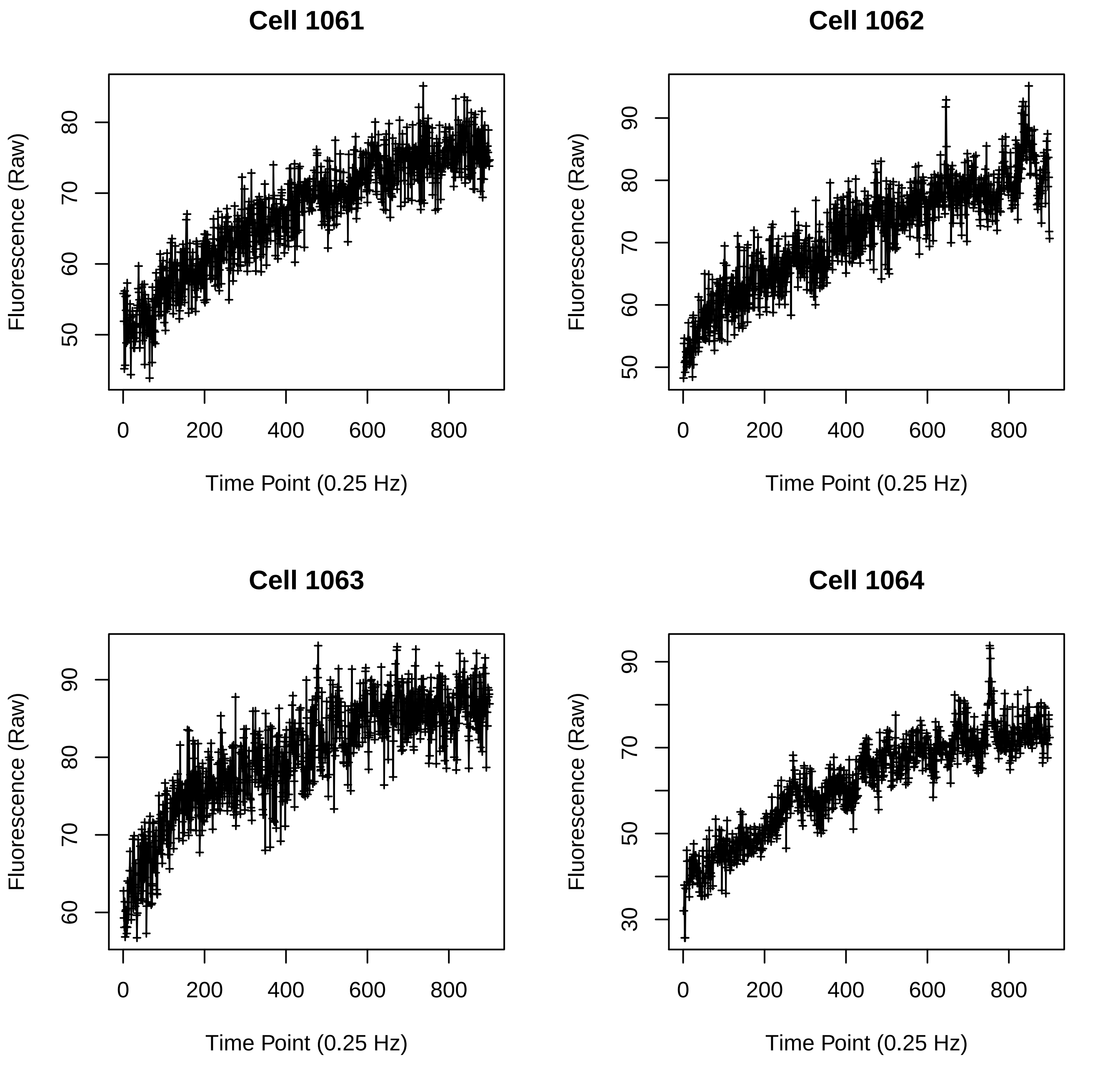
Cell 1061
Fluorescence (Raw)
50
60
70
80
0
200
400
600
800
Time Point (0.25 Hz)
Cell 1062
Fluorescence (Raw)
50
60
70
80
90
0
200
400
600
800
Time Point (0.25 Hz)
Cell 1063
Fluorescence (Raw)
60
70
80
90
0
200
400
600
800
Time Point (0.25 Hz)
Cell 1064
Fluorescence (Raw)
30
50
70
90
0
200
400
600
800
Time Point (0.25 Hz)

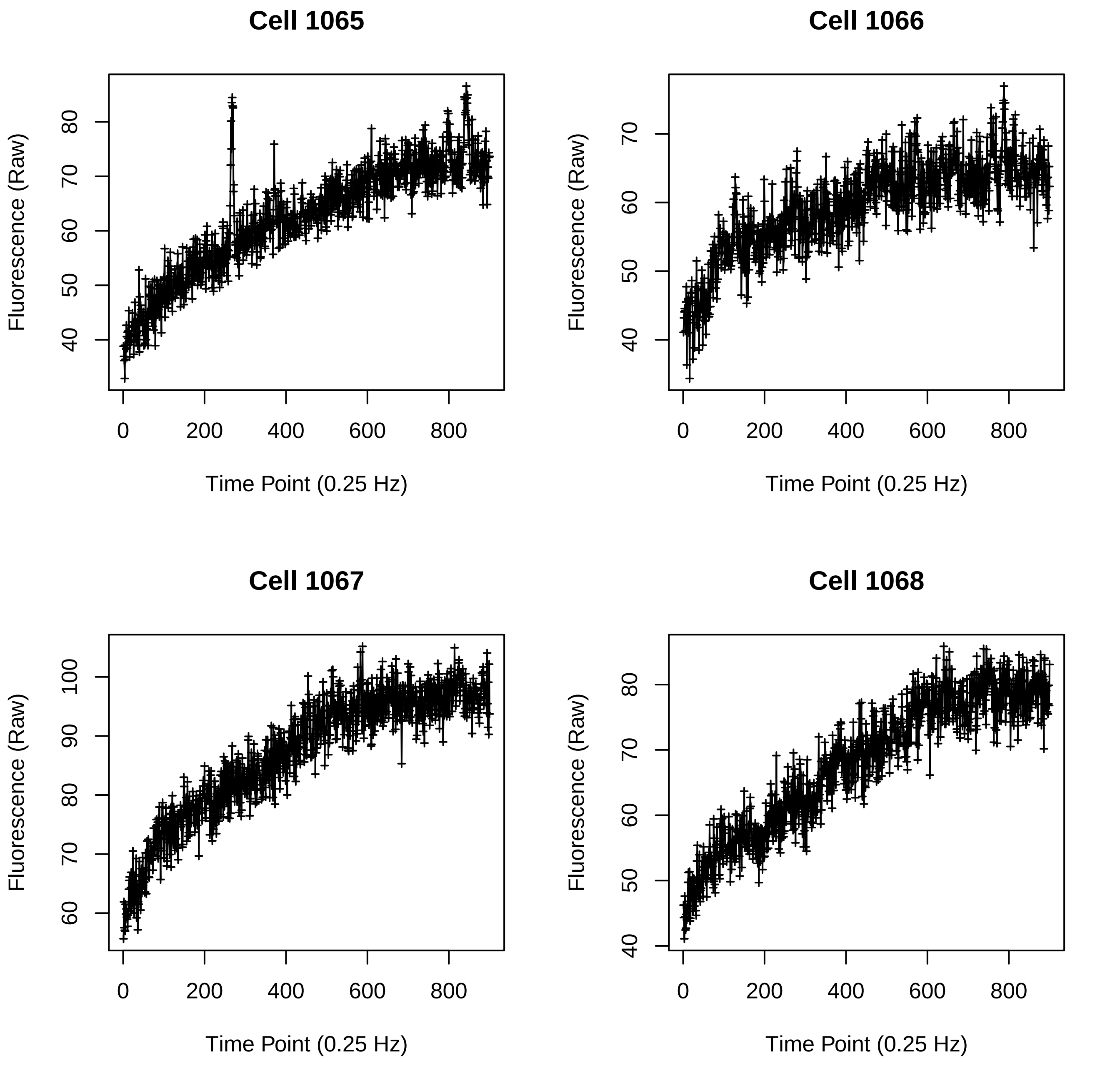

Cell 1065
Cell 1066
Cell 1067
Cell 1068
Fluorescence (Raw)
Time Point (0.25 Hz)
0
200
400
600
800
40
50
60
70
80
90
100

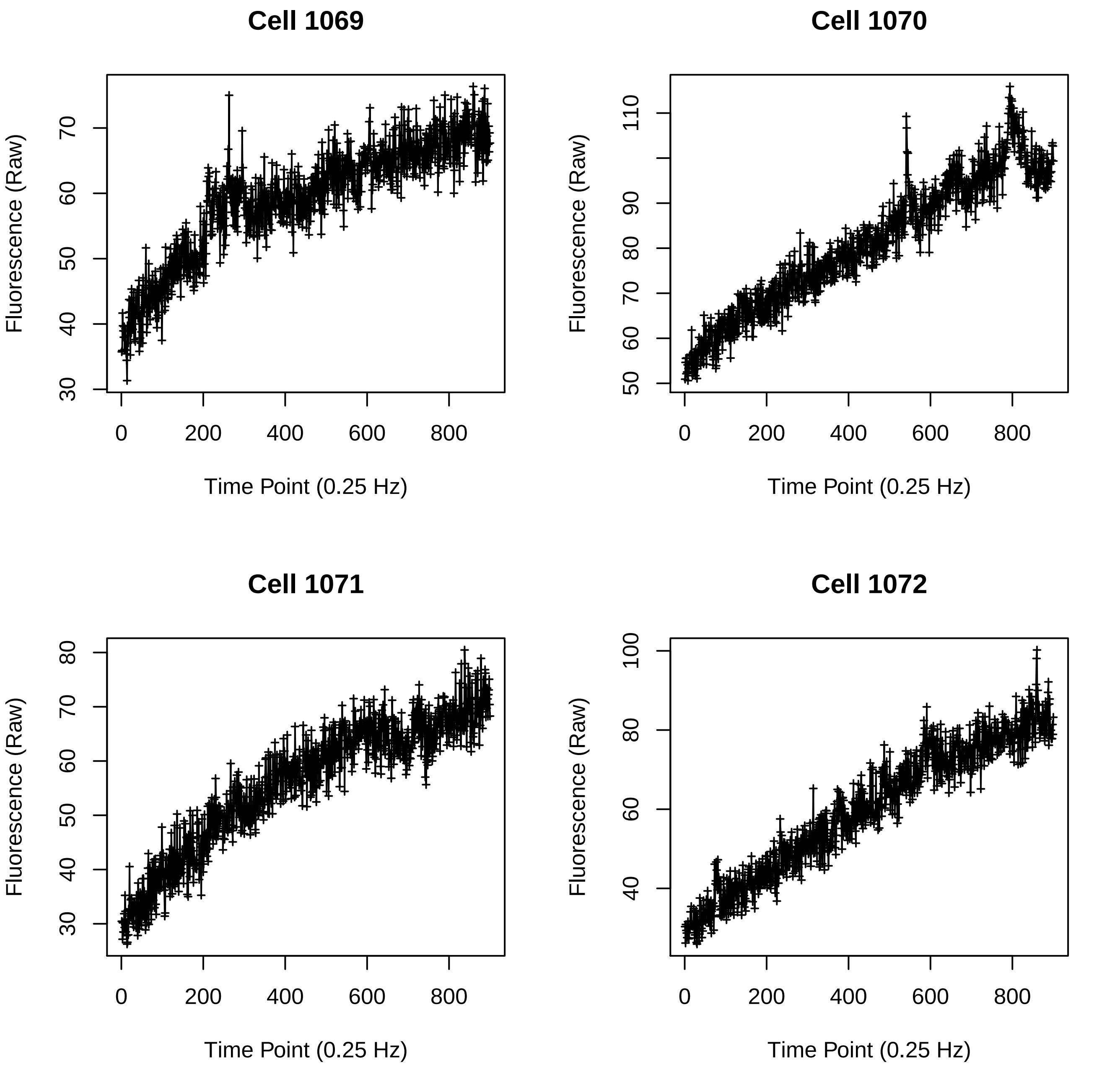
Cell 1069
Fluorescence (Raw)
30
40
50
60
70
0
200
400
600
800
Time Point (0.25 Hz)
Cell 1070
Fluorescence (Raw)
50
60
70
80
90
110
0
200
400
600
800
Time Point (0.25 Hz)
Cell 1071
Fluorescence (Raw)
30
40
50
60
70
80
0
200
400
600
800
Time Point (0.25 Hz)
Cell 1072
Fluorescence (Raw)
40
60
80
100
0
200
400
600
800
Time Point (0.25 Hz)

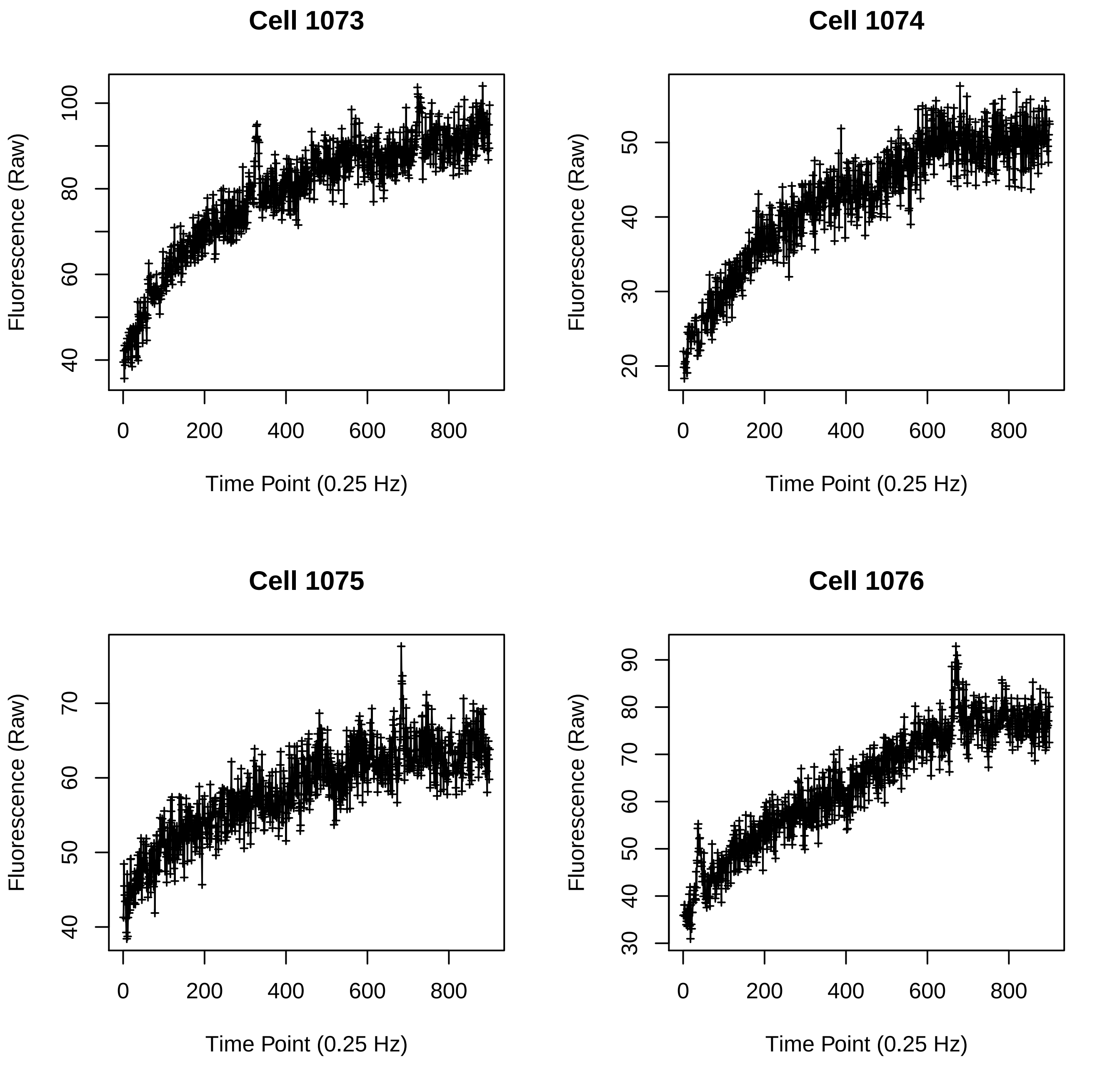
Cell 1073
Cell 1074
Cell 1075
Cell 1076
Fluorescence (Raw)
Time Point (0.25 Hz)
40
60
80
100
20
30
40
50
50
60
70
80
90
0
200
400
600
800

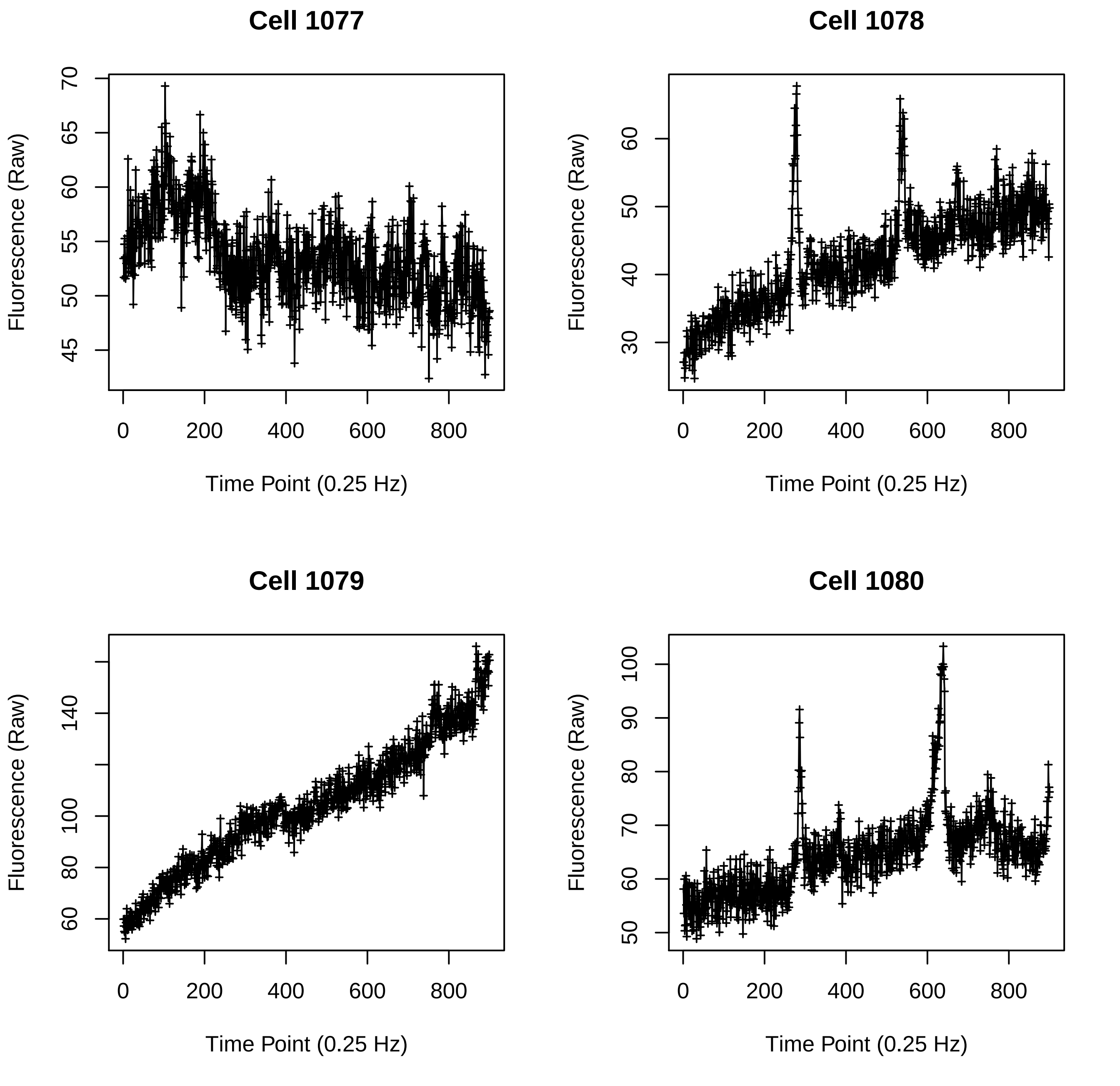

Cell 1077
Cell 1078
Cell 1079
Cell 1080
Fluorescence (Raw)
Time Point (0.25 Hz)
70
65
60
55
50
45
40
30
140
100
80
90
0
200
400
600
800

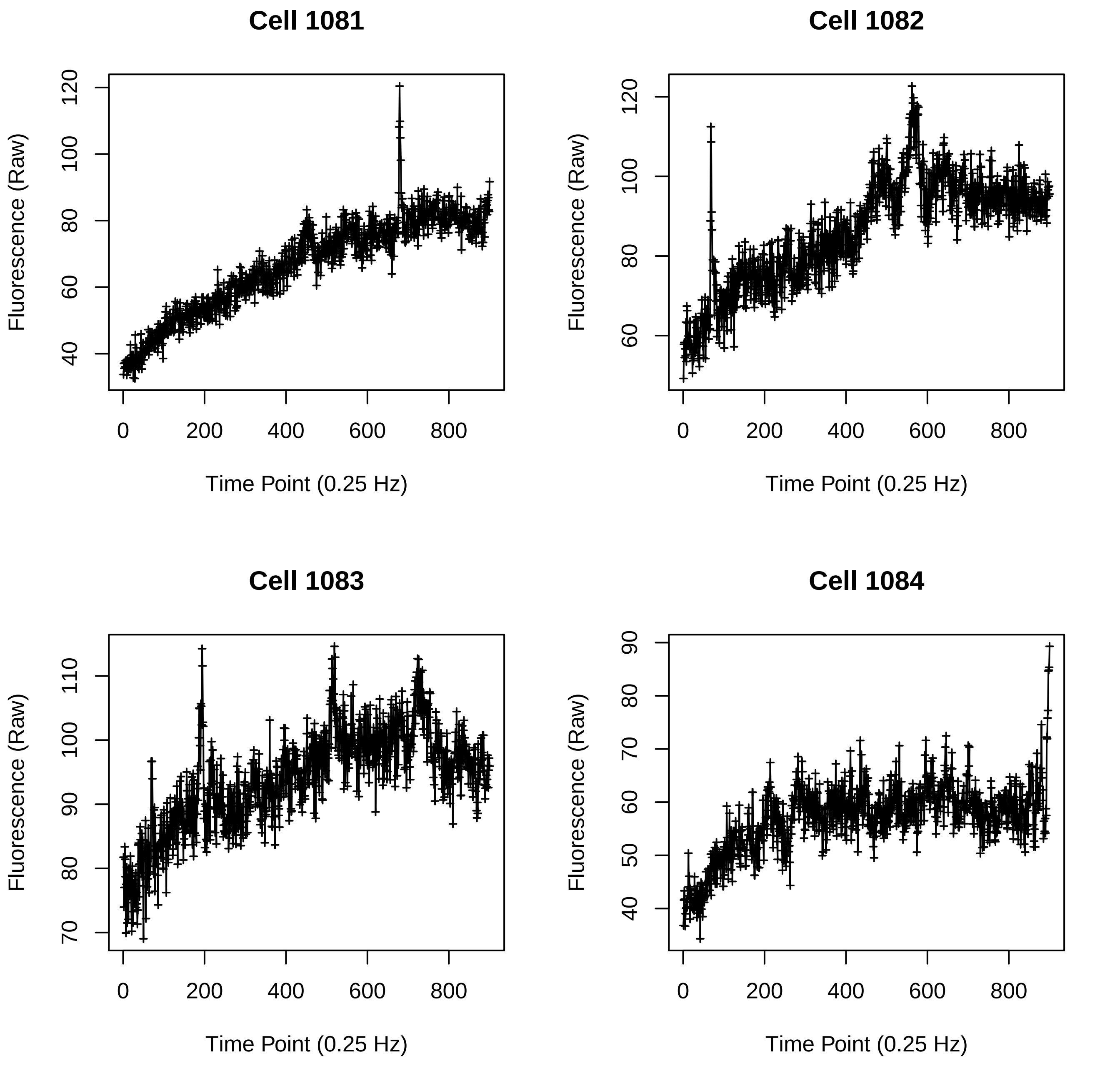
Cell 1081
Cell 1082
Cell 1083
Cell 1084
Fluorescence (Raw)
Time Point (0.25 Hz)
0
200
400
600
800
40
60
80
100
120
70
90
110
50

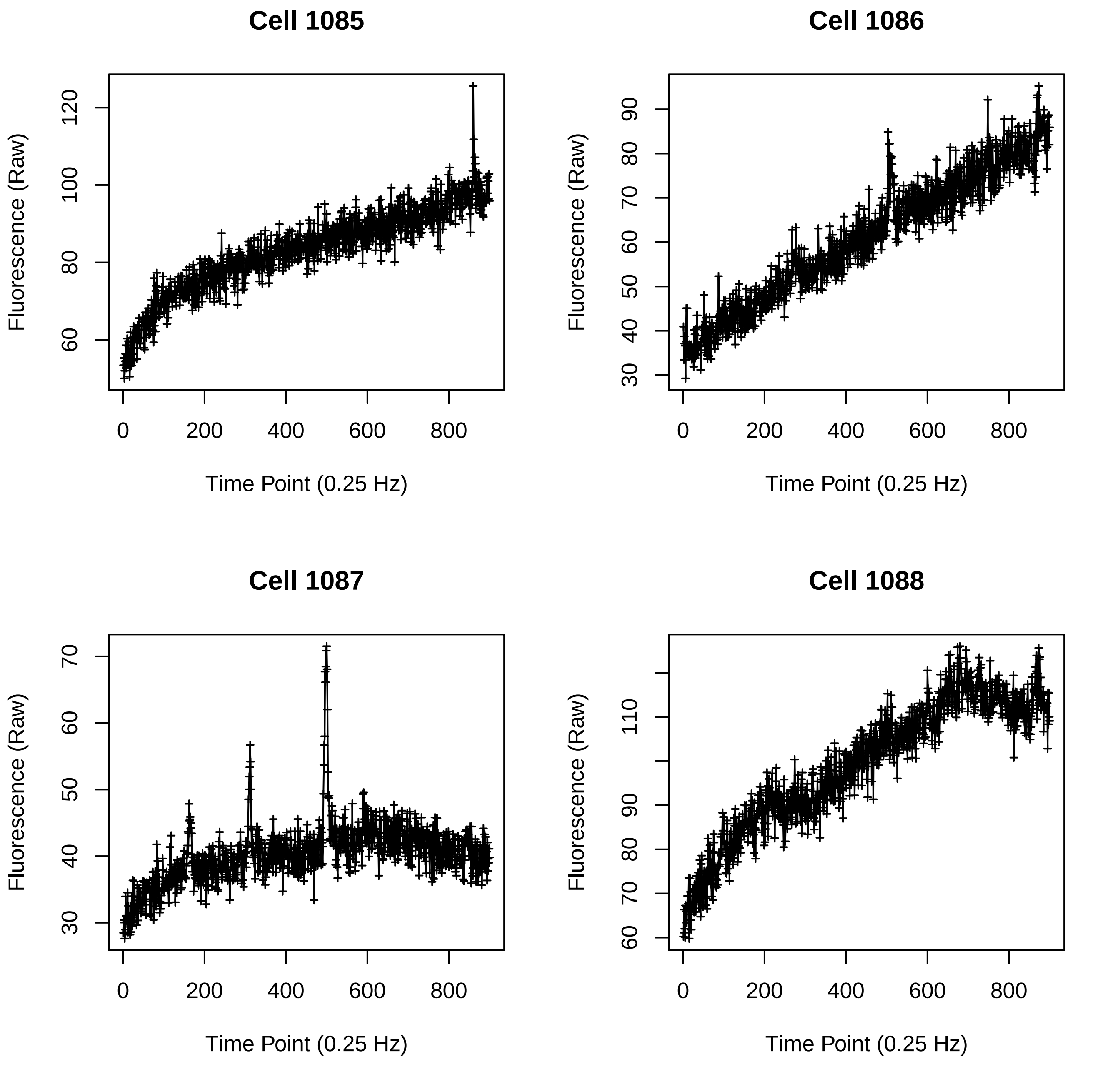
Cell 1085
Fluorescence (Raw)
60
80
100
120
0
200
400
600
800
Time Point (0.25 Hz)
Cell 1086
Fluorescence (Raw)
30
40
50
60
70
80
90
0
200
400
600
800
Time Point (0.25 Hz)
Cell 1087
Fluorescence (Raw)
30
40
50
60
70
0
200
400
600
800
Time Point (0.25 Hz)
Cell 1088
Fluorescence (Raw)
60
70
80
90
110
0
200
400
600
800
Time Point (0.25 Hz)

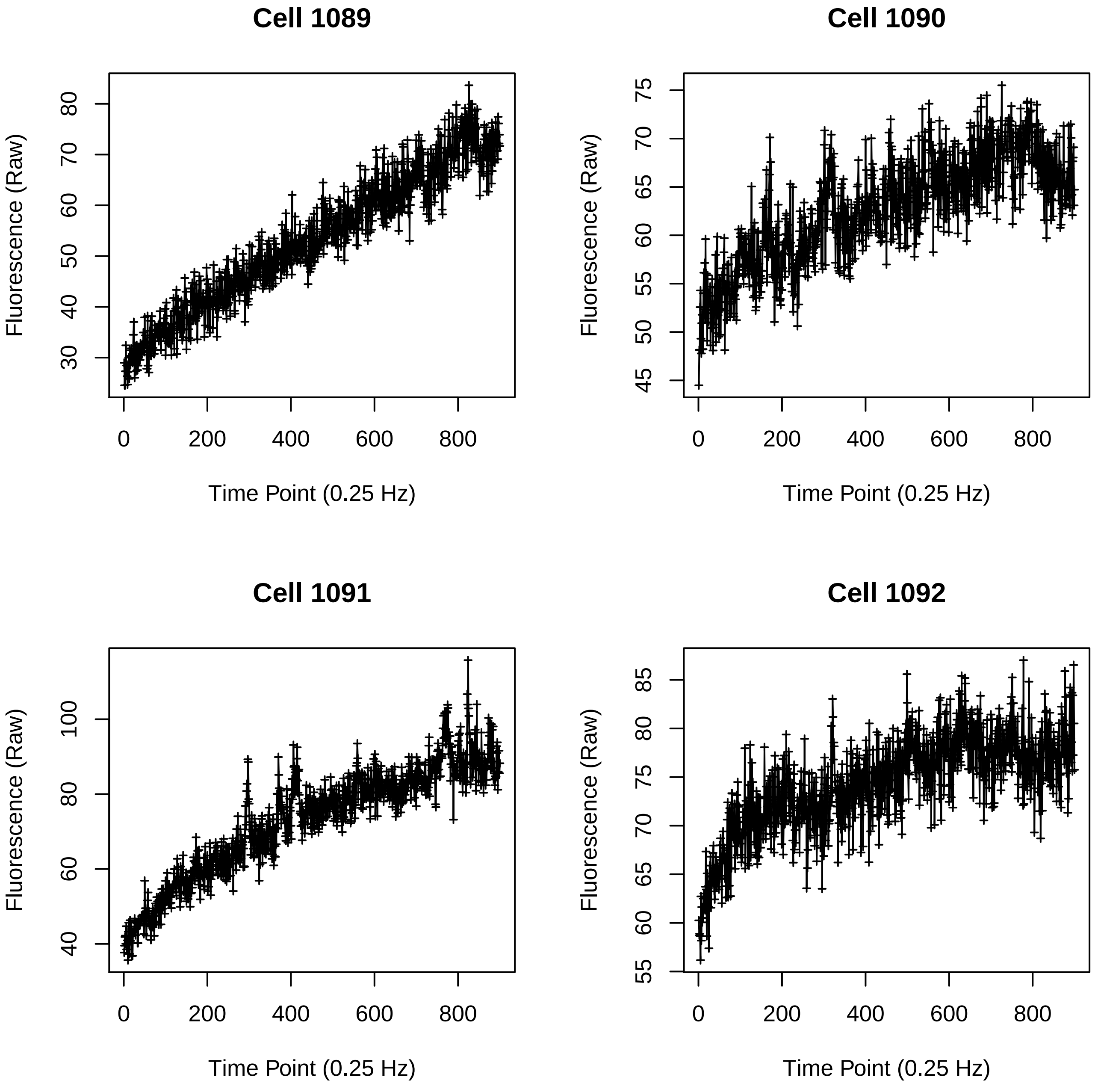

Cell 1089
Fluorescence (Raw)
30
40
50
60
70
80
0
200
400
600
800
Time Point (0.25 Hz)
Cell 1090
Fluorescence (Raw)
45
50
55
60
65
70
75
0
200
400
600
800
Time Point (0.25 Hz)
Cell 1091
Fluorescence (Raw)
40
60
80
100
0
200
400
600
800
Time Point (0.25 Hz)
Cell 1092
Fluorescence (Raw)
55
60
65
70
75
80
85
0
200
400
600
800
Time Point (0.25 Hz)

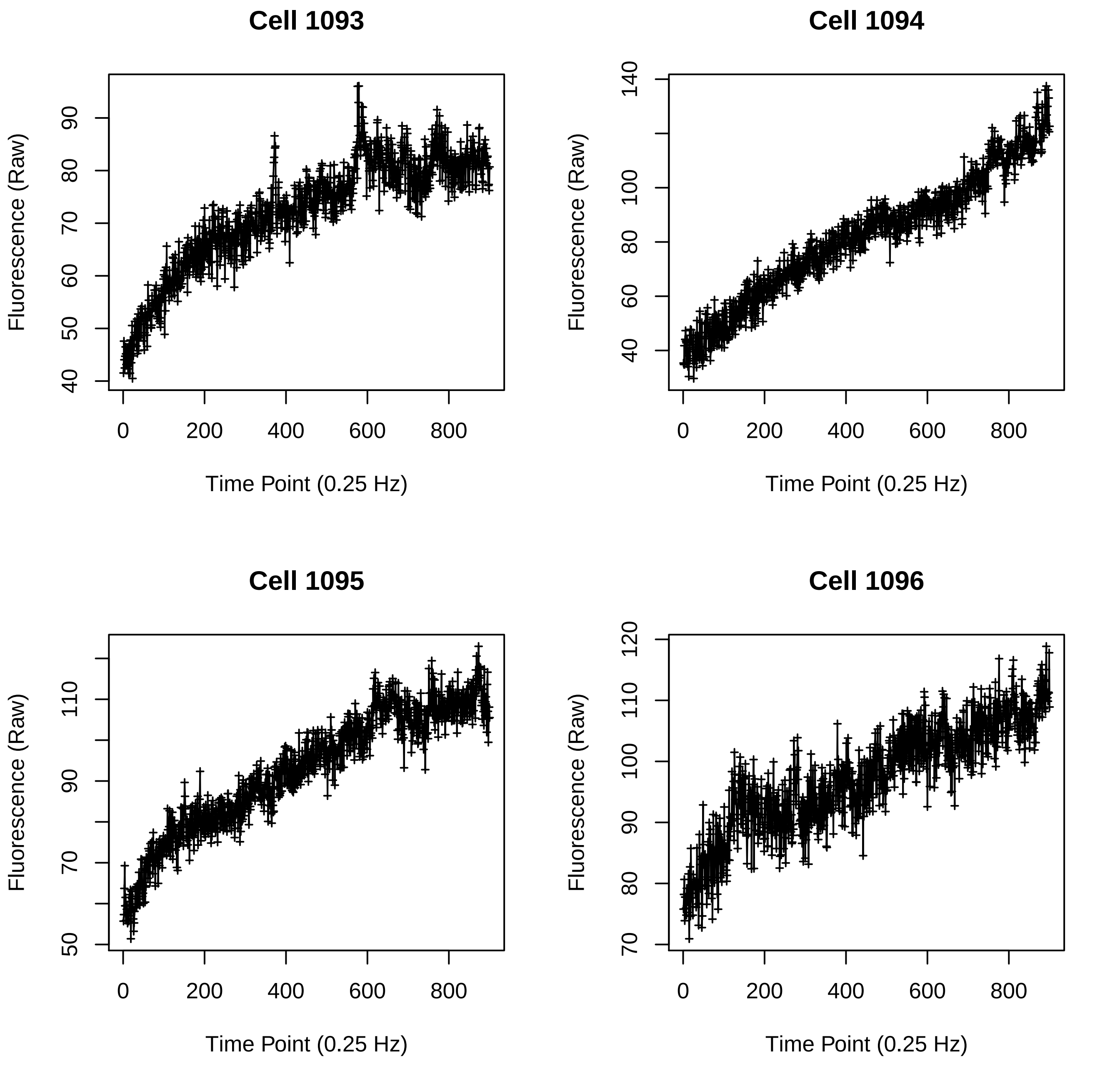
Cell 1093
Fluorescence (Raw)
40 50 60 70 80 90
0 200 400 600 800
Time Point (0.25 Hz)
Cell 1094
Fluorescence (Raw)
40 60 80 100 140
0 200 400 600 800
Time Point (0.25 Hz)
Cell 1095
Fluorescence (Raw)
50 70 90 110
0 200 400 600 800
Time Point (0.25 Hz)
Cell 1096
Fluorescence (Raw)
70 80 90 100 110 120
0 200 400 600 800
Time Point (0.25 Hz)

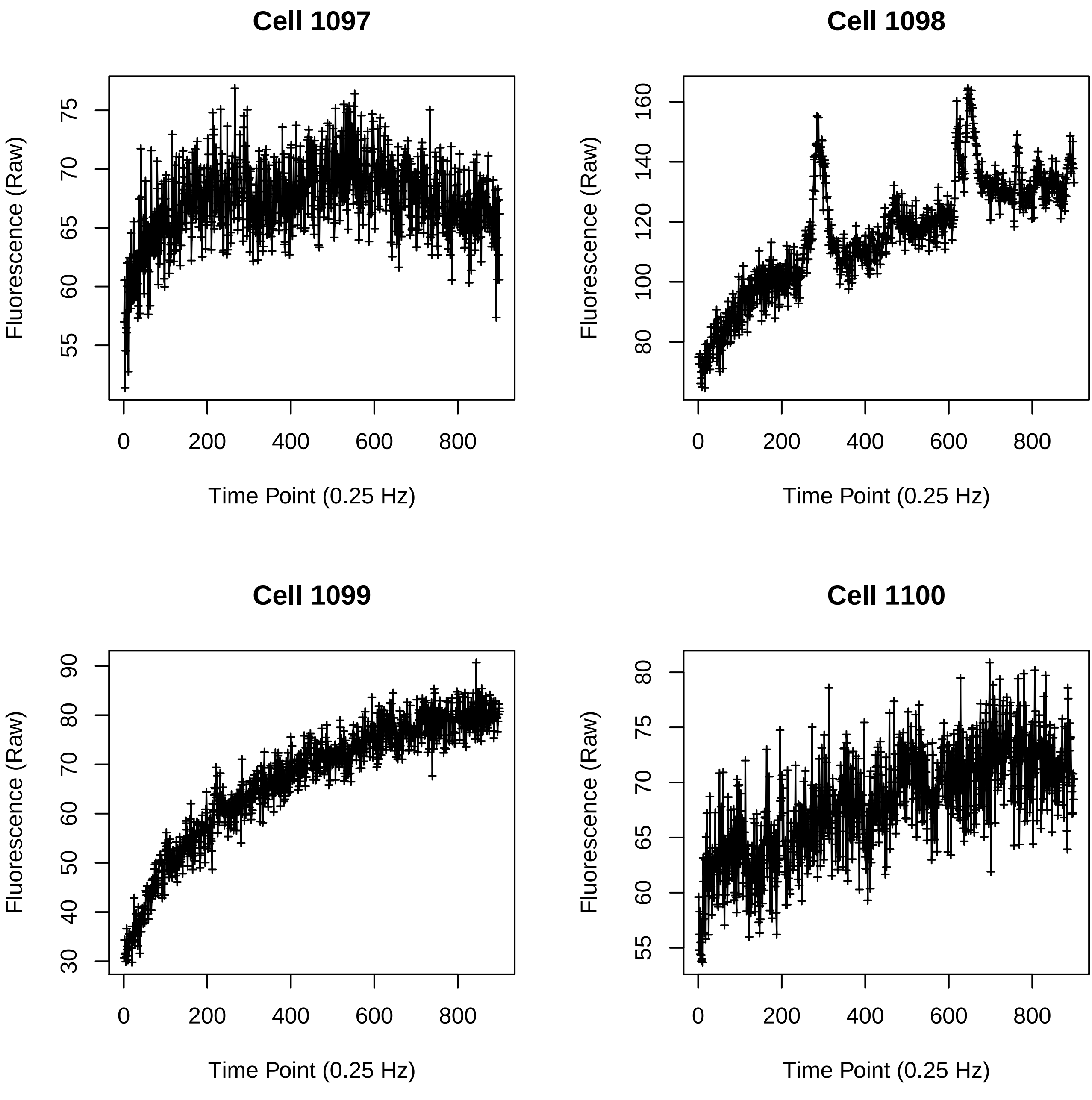

Cell 1097
Fluorescence (Raw)
55
60
65
70
75
0
200
400
600
800
Time Point (0.25 Hz)
Cell 1098
Fluorescence (Raw)
80
100
120
140
160
0
200
400
600
800
Time Point (0.25 Hz)
Cell 1099
Fluorescence (Raw)
30
40
50
60
70
80
90
0
200
400
600
800
Time Point (0.25 Hz)
Cell 1100
Fluorescence (Raw)
55
60
65
70
75
80
0
200
400
600
800
Time Point (0.25 Hz)

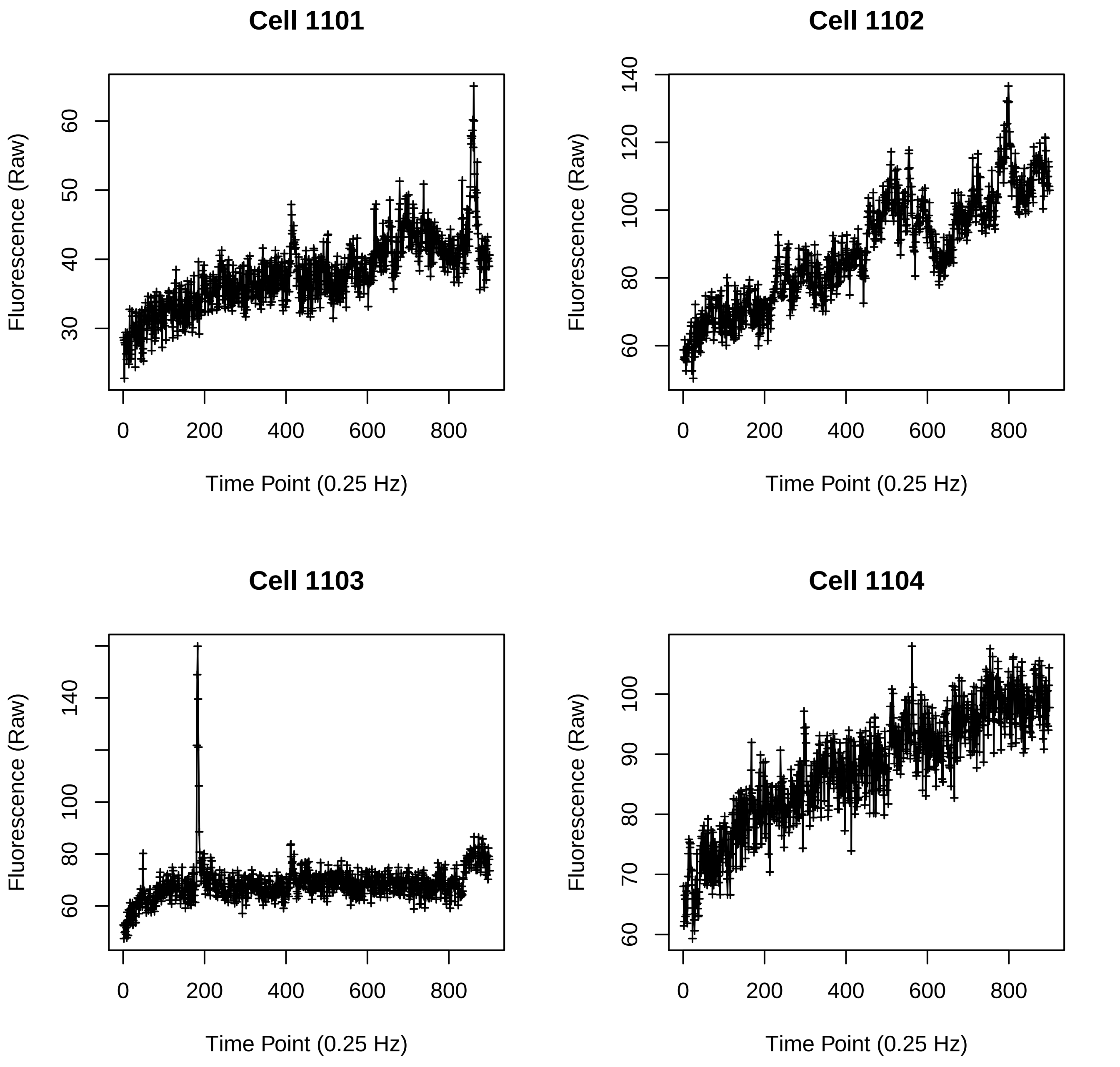
Cell 1101
Fluorescence (Raw)
30
40
50
60
0
200
400
600
800
Time Point (0.25 Hz)
Cell 1102
Fluorescence (Raw)
60
80
100
120
140
0
200
400
600
800
Time Point (0.25 Hz)
Cell 1103
Fluorescence (Raw)
60
80
100
140
0
200
400
600
800
Time Point (0.25 Hz)
Cell 1104
Fluorescence (Raw)
60
70
80
90
100
0
200
400
600
800
Time Point (0.25 Hz)

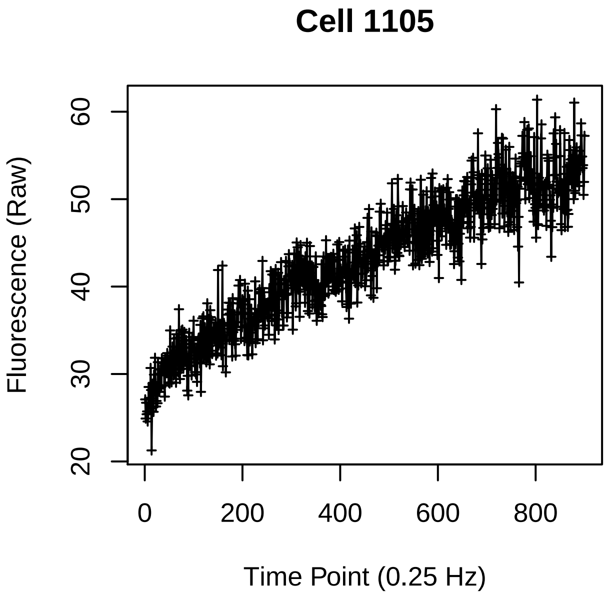
Cell 1105
Fluorescence (Raw)
20
30
40
50
60
0
200
400
600
800
Time Point (0.25 Hz)

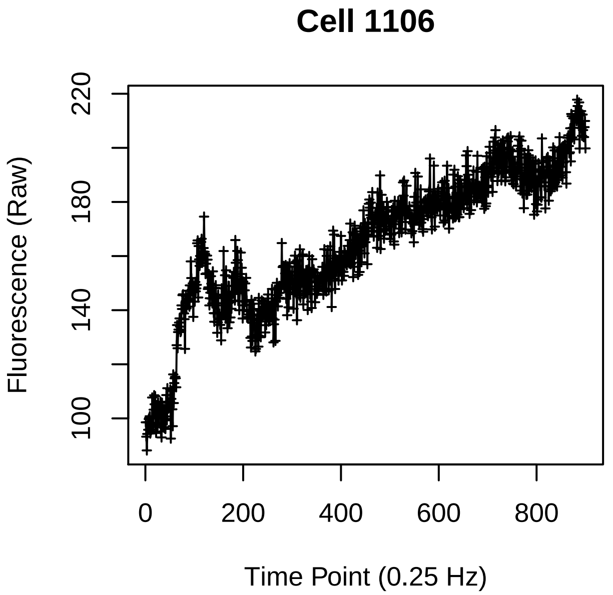
Cell 1106
Fluorescence (Raw)
100
140
180
220
0
200
400
600
800
Time Point (0.25 Hz)

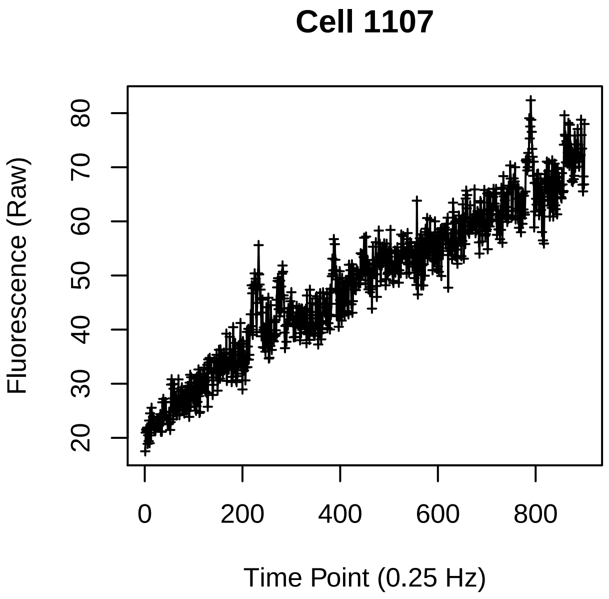
Cell 1107
Fluorescence (Raw)
20
30
40
50
60
70
80
0
200
400
600
800
Time Point (0.25 Hz)

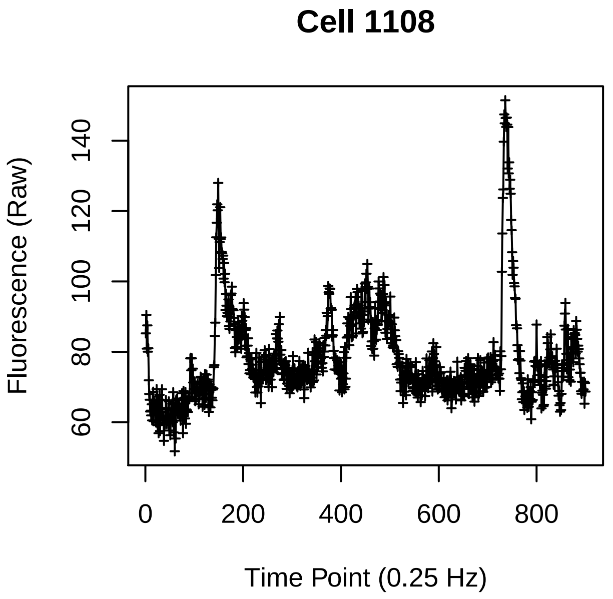
Cell 1108
Fluorescence (Raw)
60
80
100
120
140
0
200
400
600
800
Time Point (0.25 Hz)

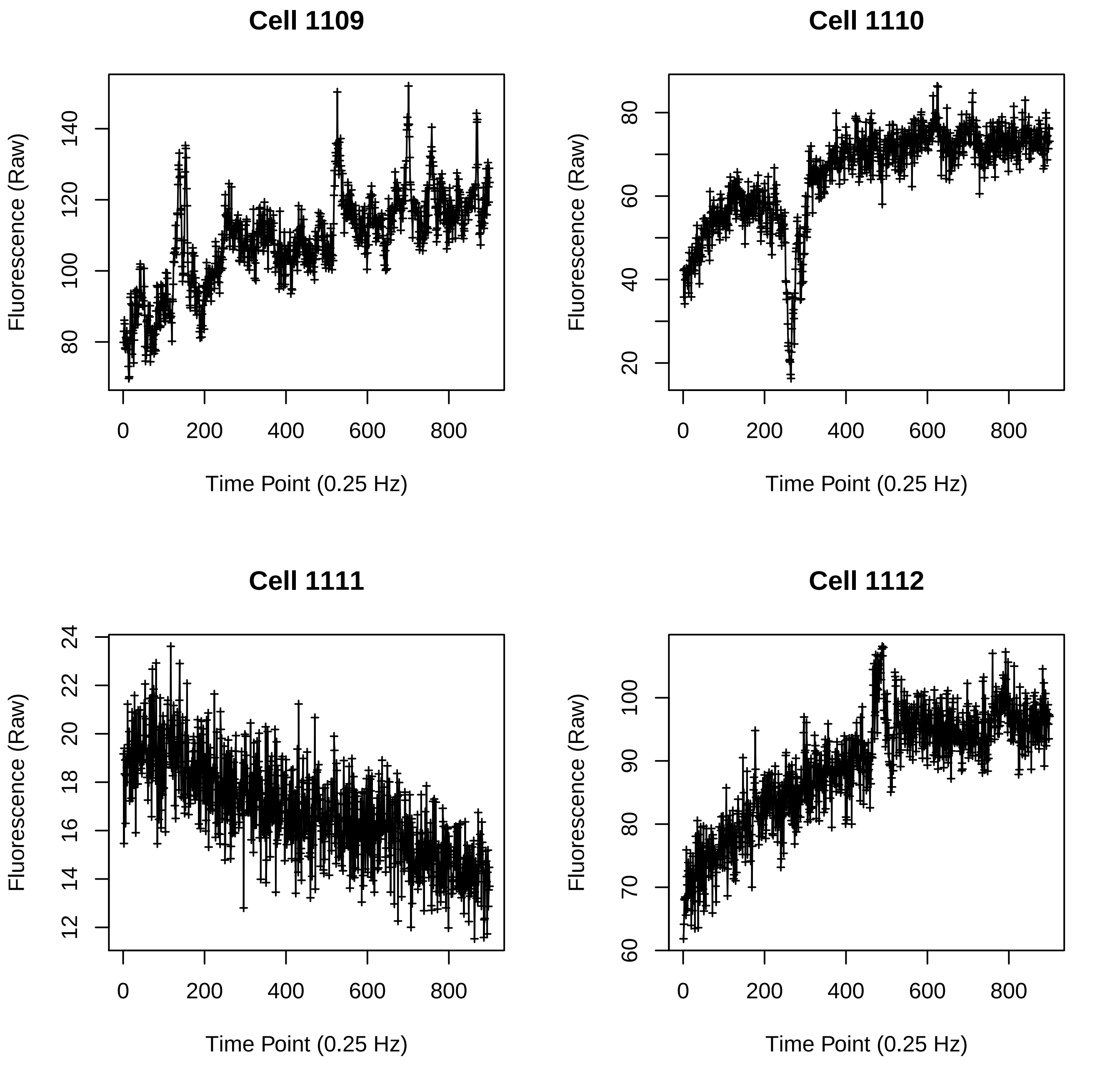
Cell 1109
Cell 1110
Cell 1111
Cell 1112
Fluorescence (Raw)
Time Point (0.25 Hz)
0
200
400
600
800
80
100
120
140
20
40
60
80
12
14
16
18
20
22
24
60
70
80
90
100

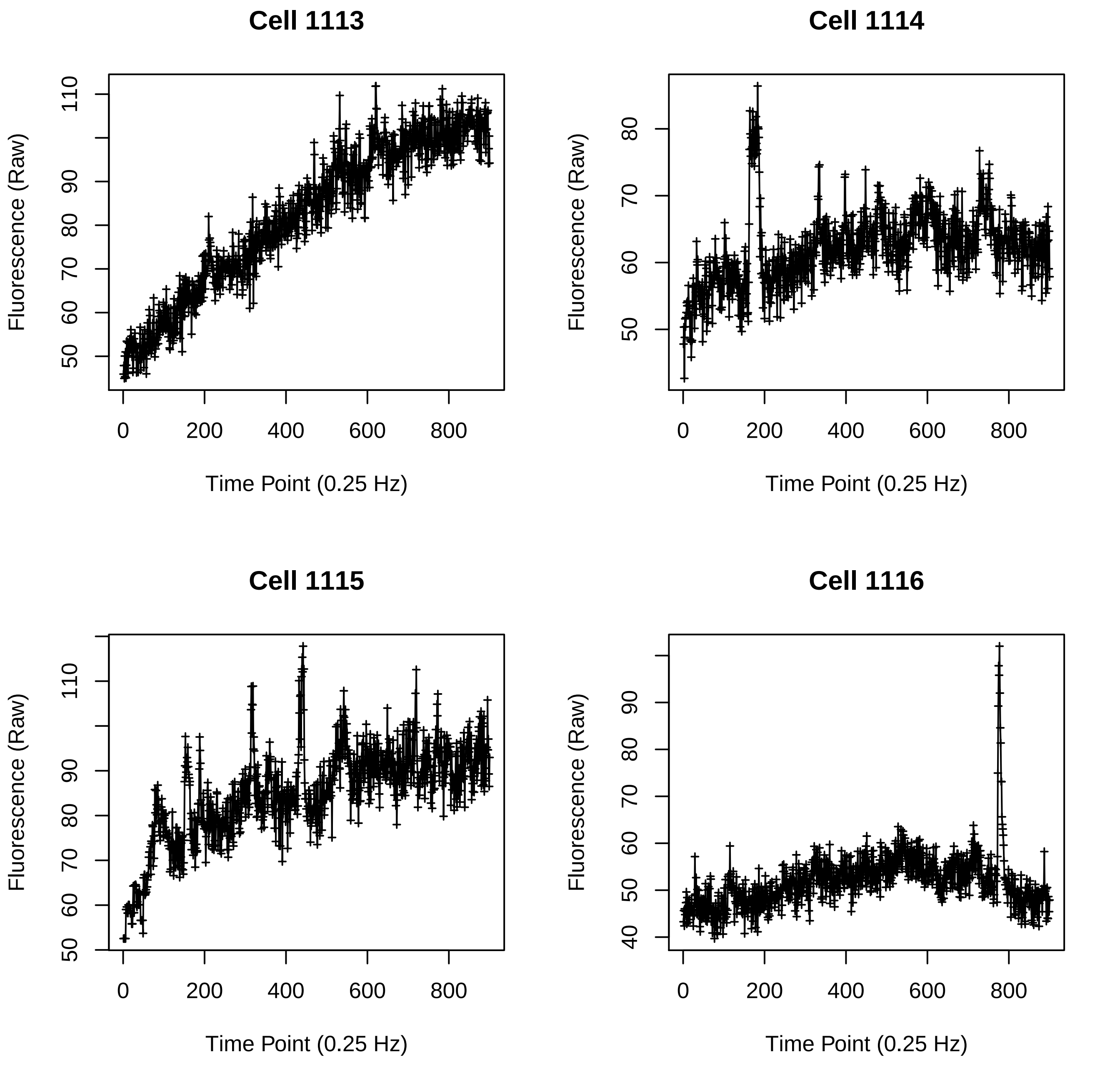
Cell 1113
Fluorescence (Raw)
50
60
70
80
90
110
0
200
400
600
800
Time Point (0.25 Hz)
Cell 1114
Fluorescence (Raw)
50
60
70
80
0
200
400
600
800
Time Point (0.25 Hz)
Cell 1115
Fluorescence (Raw)
50
60
70
80
90
110
0
200
400
600
800
Time Point (0.25 Hz)
Cell 1116
Fluorescence (Raw)
40
50
60
70
80
90
0
200
400
600
800
Time Point (0.25 Hz)

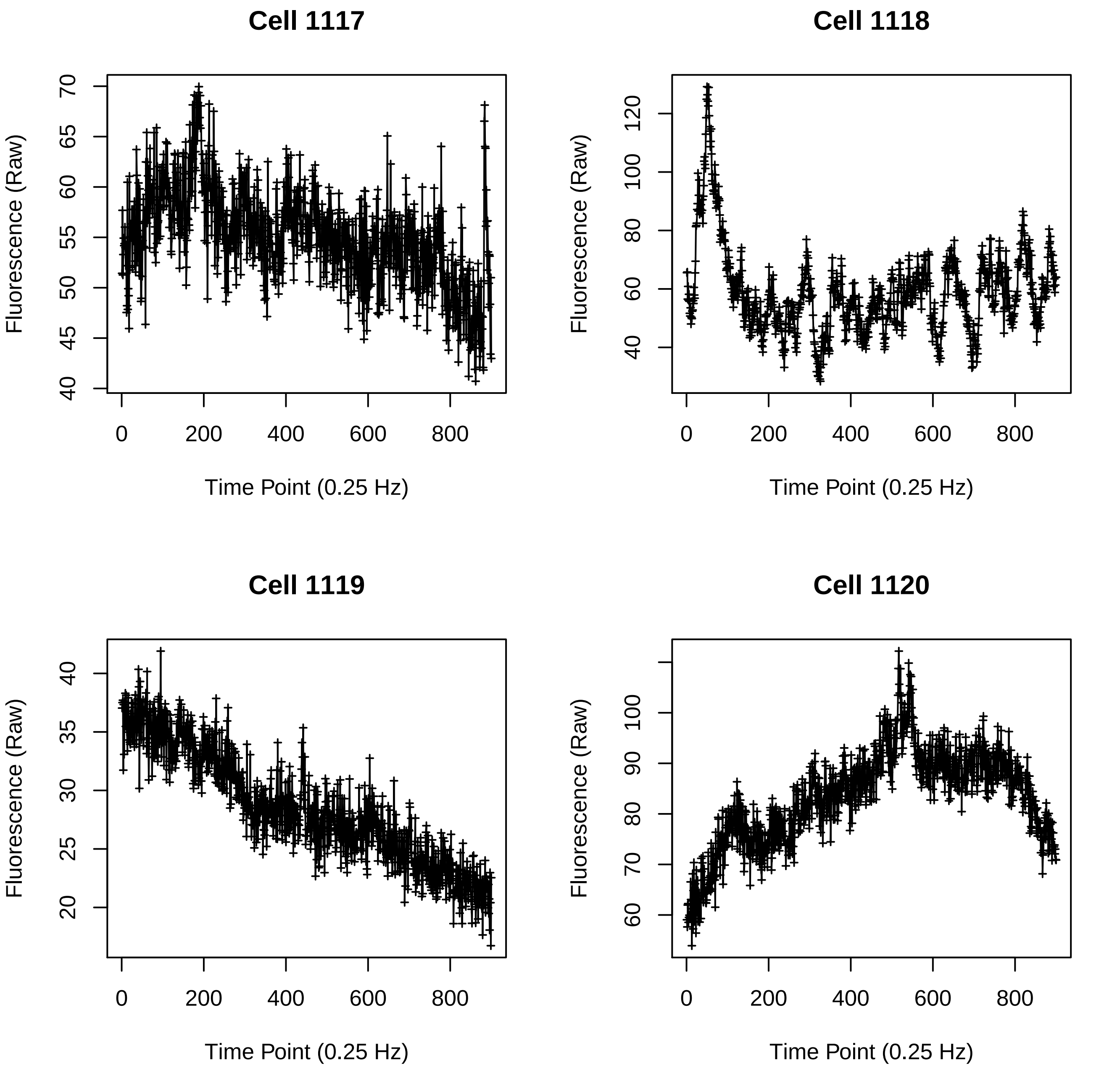
Cell 1117
Cell 1118
Cell 1119
Cell 1120
Fluorescence (Raw)
Time Point (0.25 Hz)
0
200
400
600
800
40
45
50
55
60
65
70
60
80
100
120
20
25
30
35
40
70
90

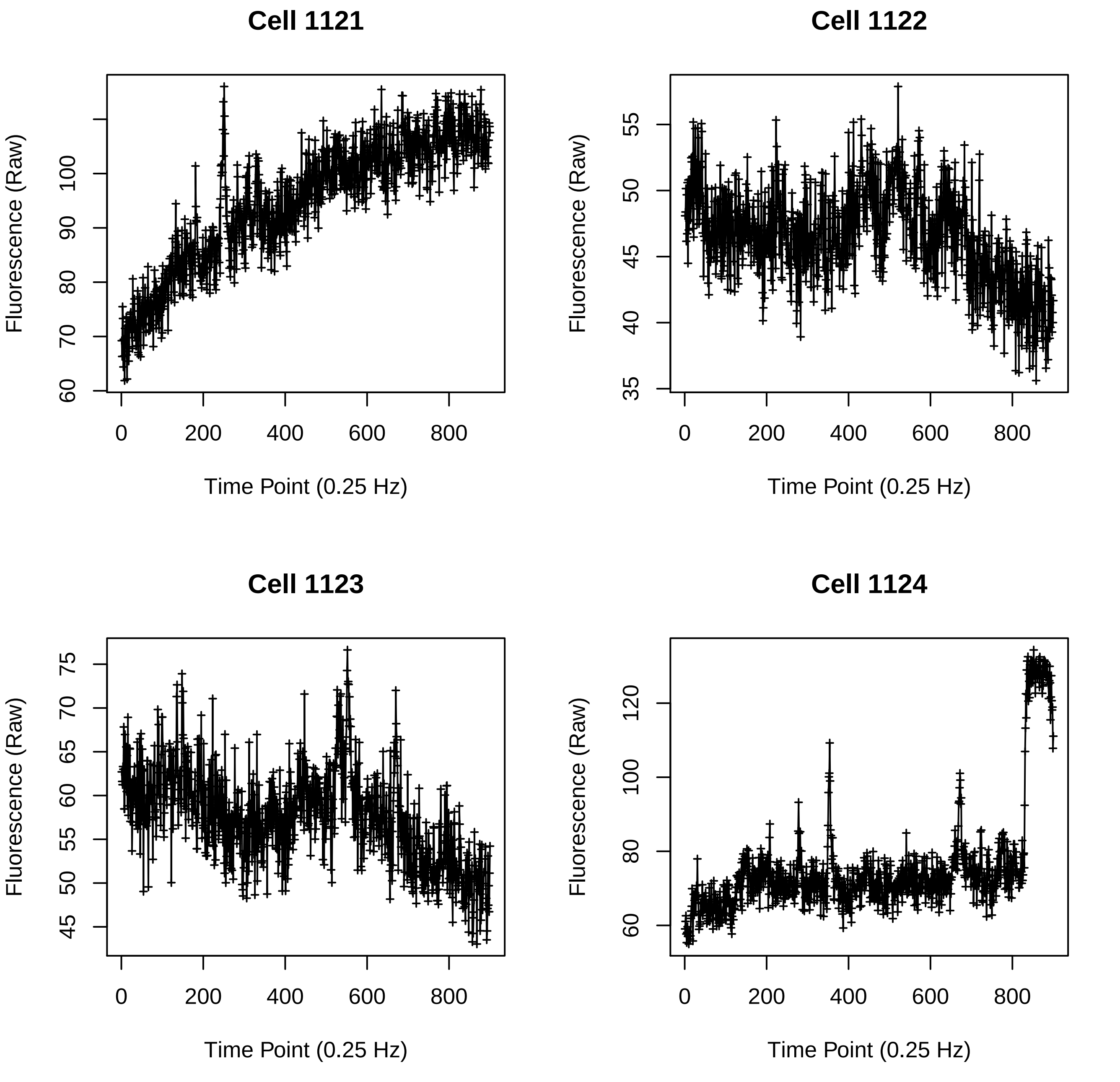
Cell 1121
Fluorescence (Raw)
Time Point (0.25 Hz)
60
70
80
90
100
0
200
400
600
800
Cell 1122
Fluorescence (Raw)
Time Point (0.25 Hz)
35
40
45
50
55
0
200
400
600
800
Cell 1123
Fluorescence (Raw)
Time Point (0.25 Hz)
45
50
55
60
65
70
75
0
200
400
600
800
Cell 1124
Fluorescence (Raw)
Time Point (0.25 Hz)
60
80
100
120
0
200
400
600
800

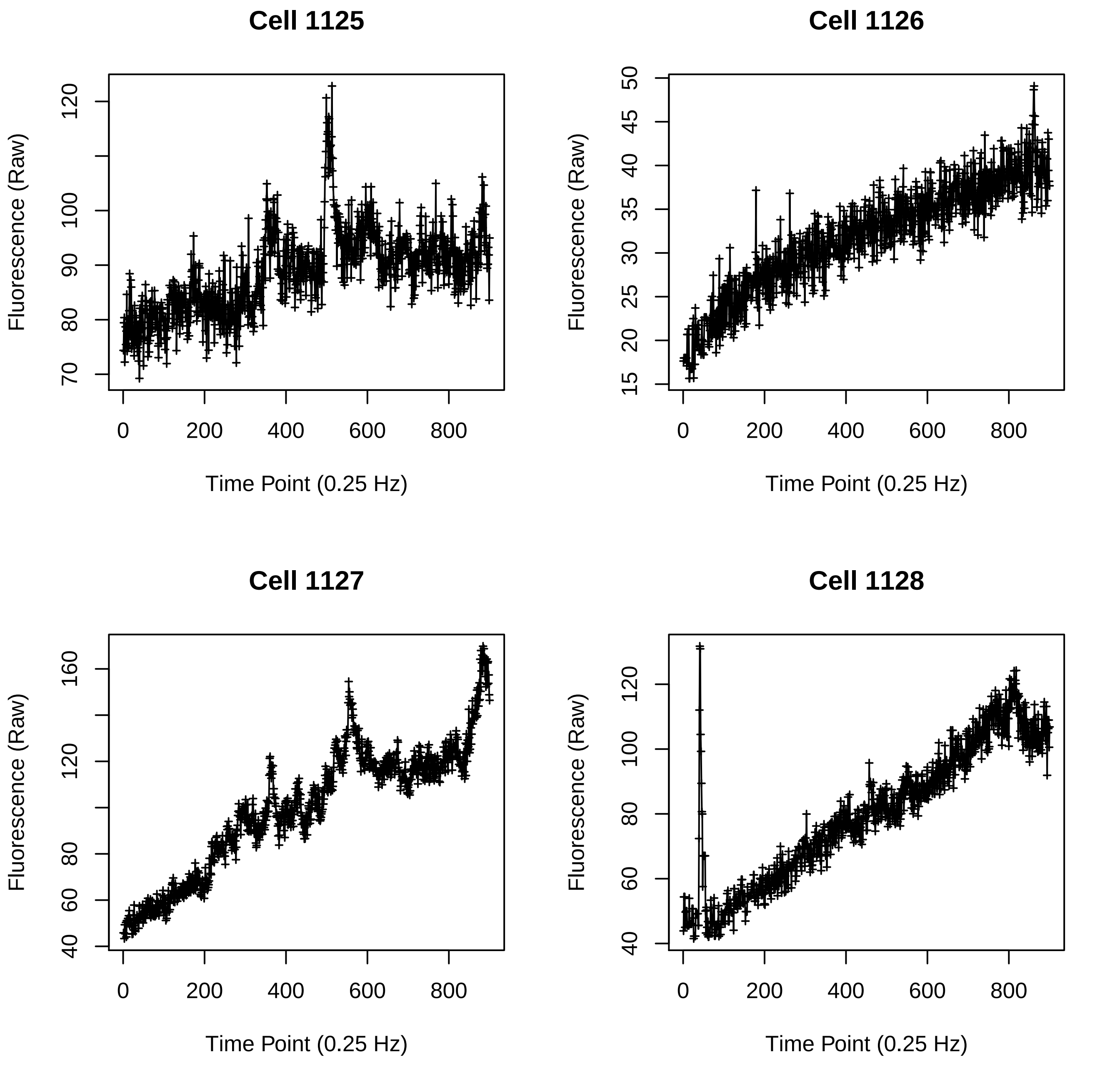
Cell 1125
Fluorescence (Raw)
70
80
90
100
120
0
200
400
600
800
Time Point (0.25 Hz)
Cell 1126
Fluorescence (Raw)
15
20
25
30
35
40
45
50
0
200
400
600
800
Time Point (0.25 Hz)
Cell 1127
Fluorescence (Raw)
40
60
80
120
160
0
200
400
600
800
Time Point (0.25 Hz)
Cell 1128
Fluorescence (Raw)
40
60
80
100
120
0
200
400
600
800
Time Point (0.25 Hz)

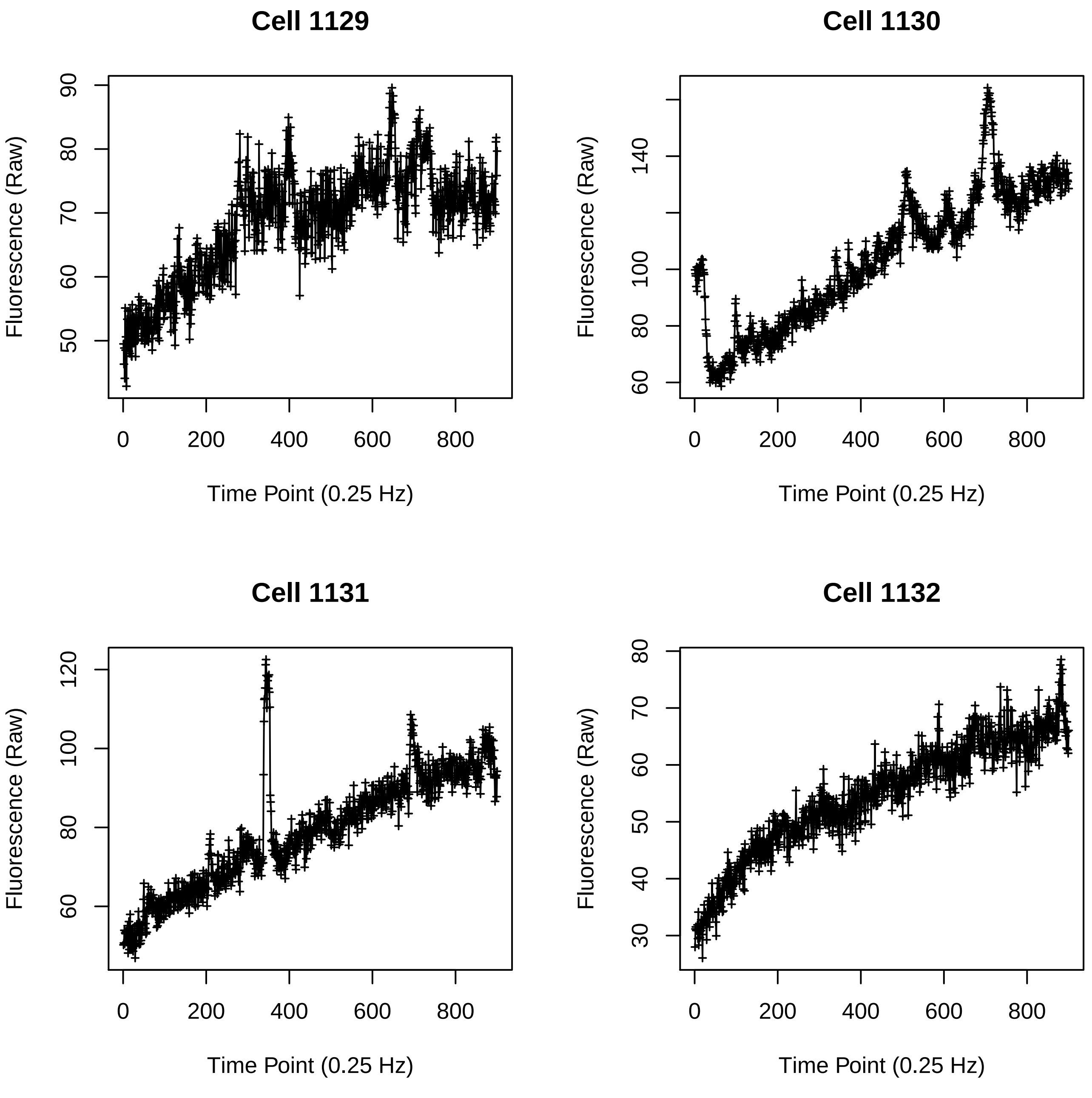
Cell 1129
Cell 1130
Cell 1131
Cell 1132
Fluorescence (Raw)
Time Point (0.25 Hz)
0
200
400
600
800
50
60
70
80
90
60
80
100
140
120
30
40

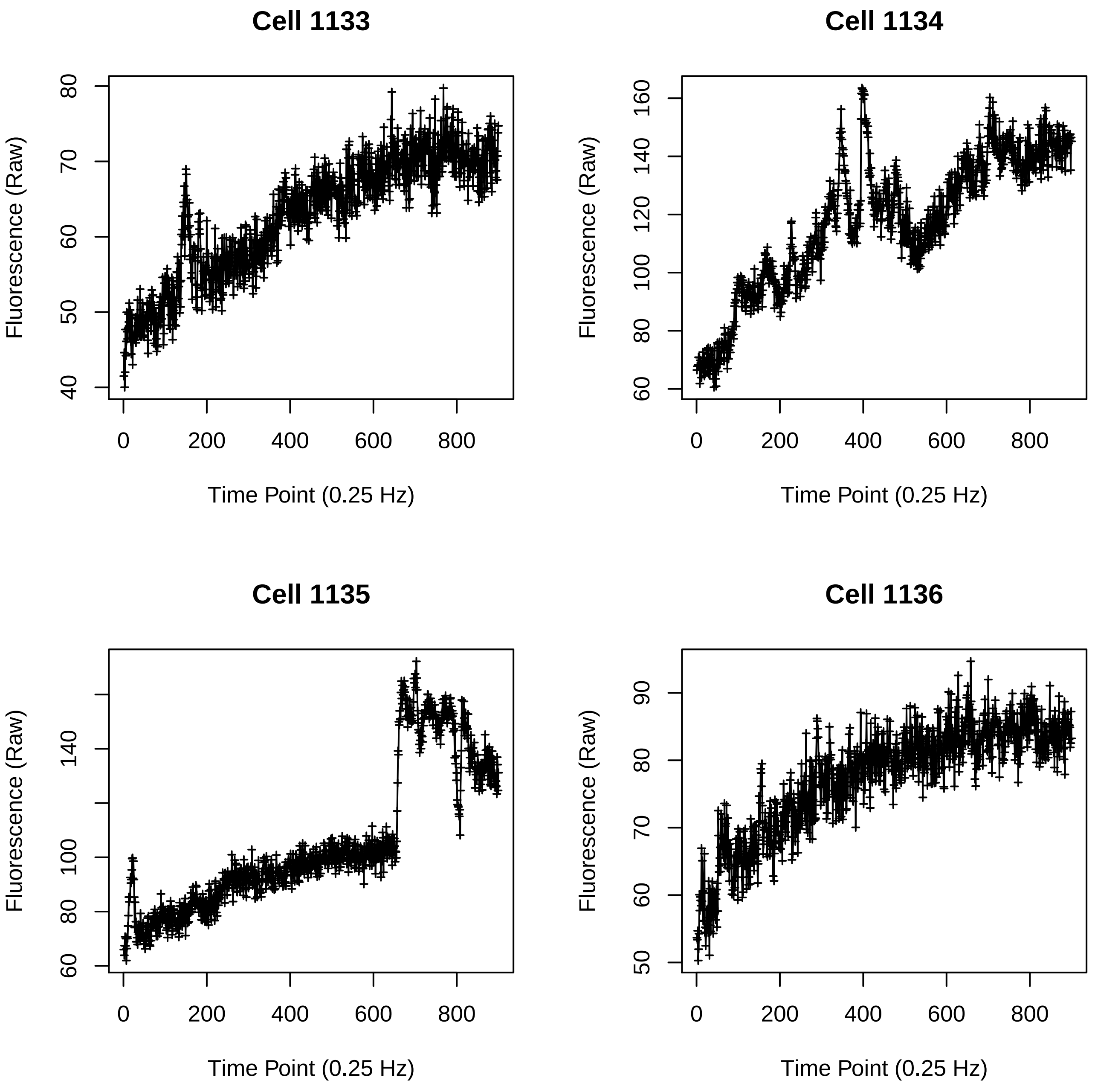
Cell 1133
Fluorescence (Raw)
40
50
60
70
80
0
200
400
600
800
Time Point (0.25 Hz)
Cell 1134
Fluorescence (Raw)
60
80
100
120
140
160
0
200
400
600
800
Time Point (0.25 Hz)
Cell 1135
Fluorescence (Raw)
60
80
100
140
0
200
400
600
800
Time Point (0.25 Hz)
Cell 1136
Fluorescence (Raw)
50
60
70
80
90
0
200
400
600
800
Time Point (0.25 Hz)

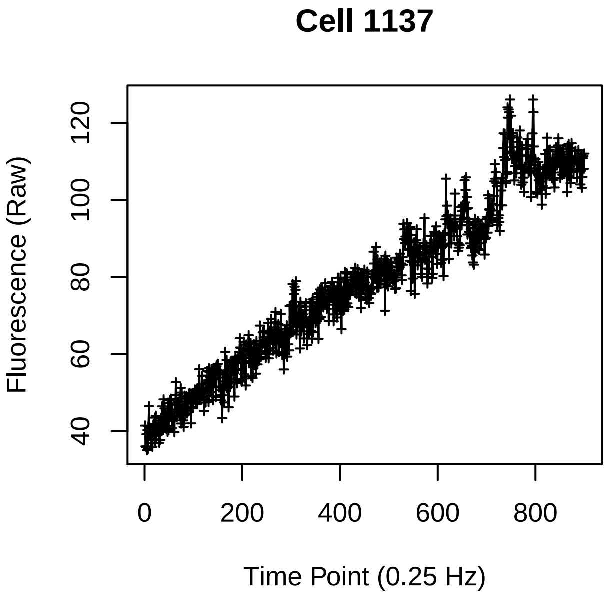
Cell 1137
Fluorescence (Raw)
40
60
80
100
120
0
200
400
600
800
Time Point (0.25 Hz)

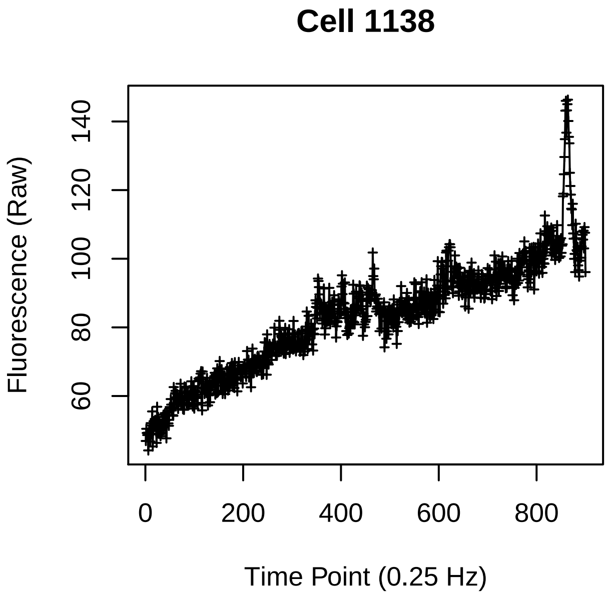
Cell 1138
Fluorescence (Raw)
60
80
100
120
140
0
200
400
600
800
Time Point (0.25 Hz)

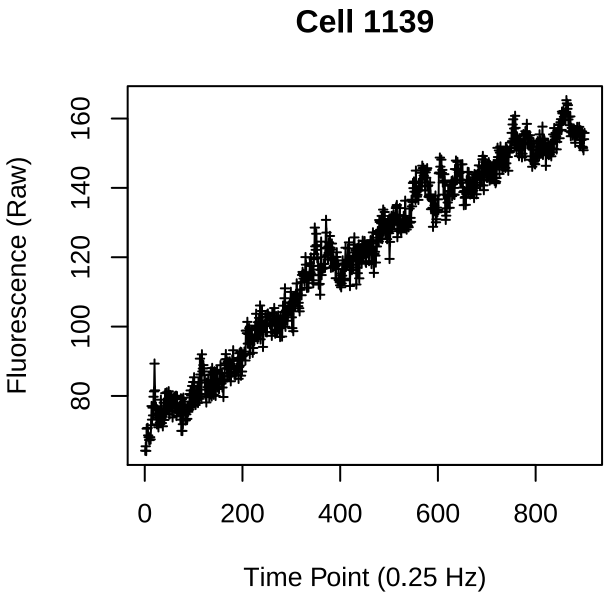
Cell 1139
Fluorescence (Raw)
80
100
120
140
160
0
200
400
600
800
Time Point (0.25 Hz)

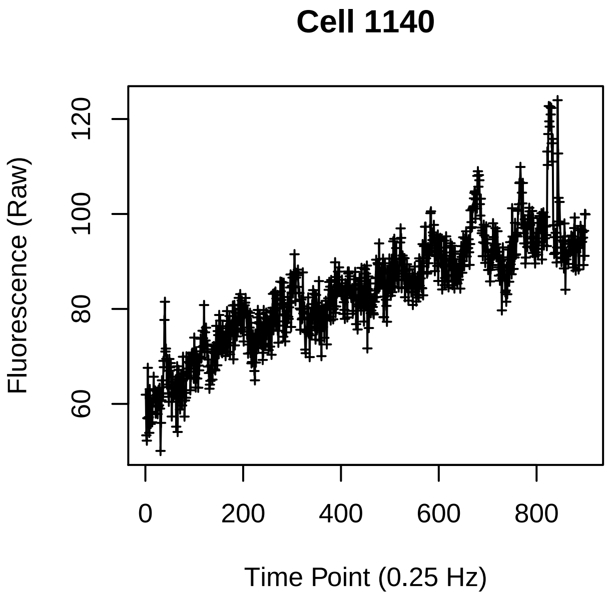
Cell 1140
Fluorescence (Raw)
60
80
100
120
0
200
400
600
800
Time Point (0.25 Hz)

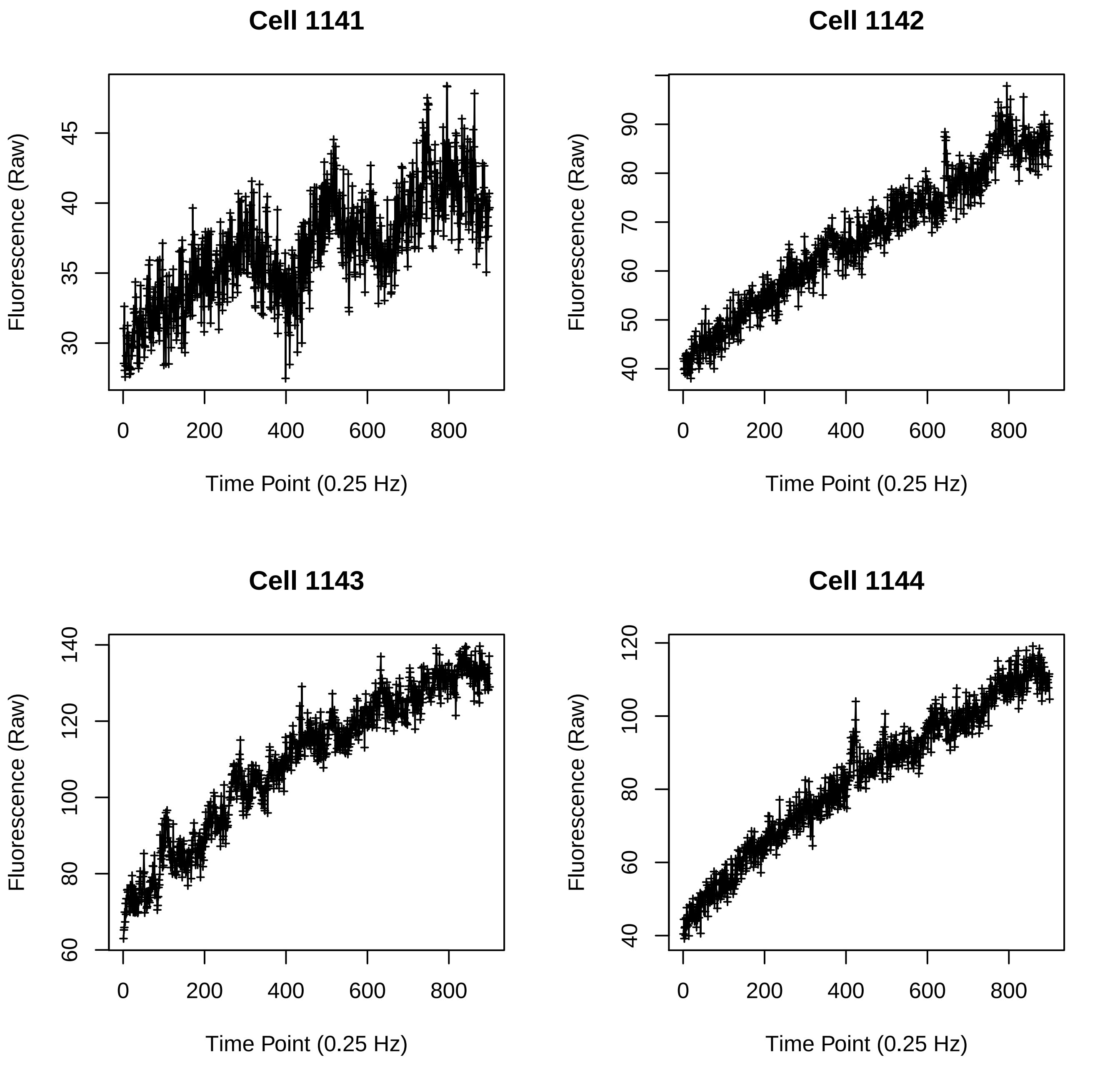
Cell 1141
Fluorescence (Raw)
30
35
40
45
0
200
400
600
800
Time Point (0.25 Hz)
Cell 1142
Fluorescence (Raw)
40
50
60
70
80
90
0
200
400
600
800
Time Point (0.25 Hz)
Cell 1143
Fluorescence (Raw)
60
80
100
120
140
0
200
400
600
800
Time Point (0.25 Hz)
Cell 1144
Fluorescence (Raw)
40
60
80
100
120
0
200
400
600
800
Time Point (0.25 Hz)

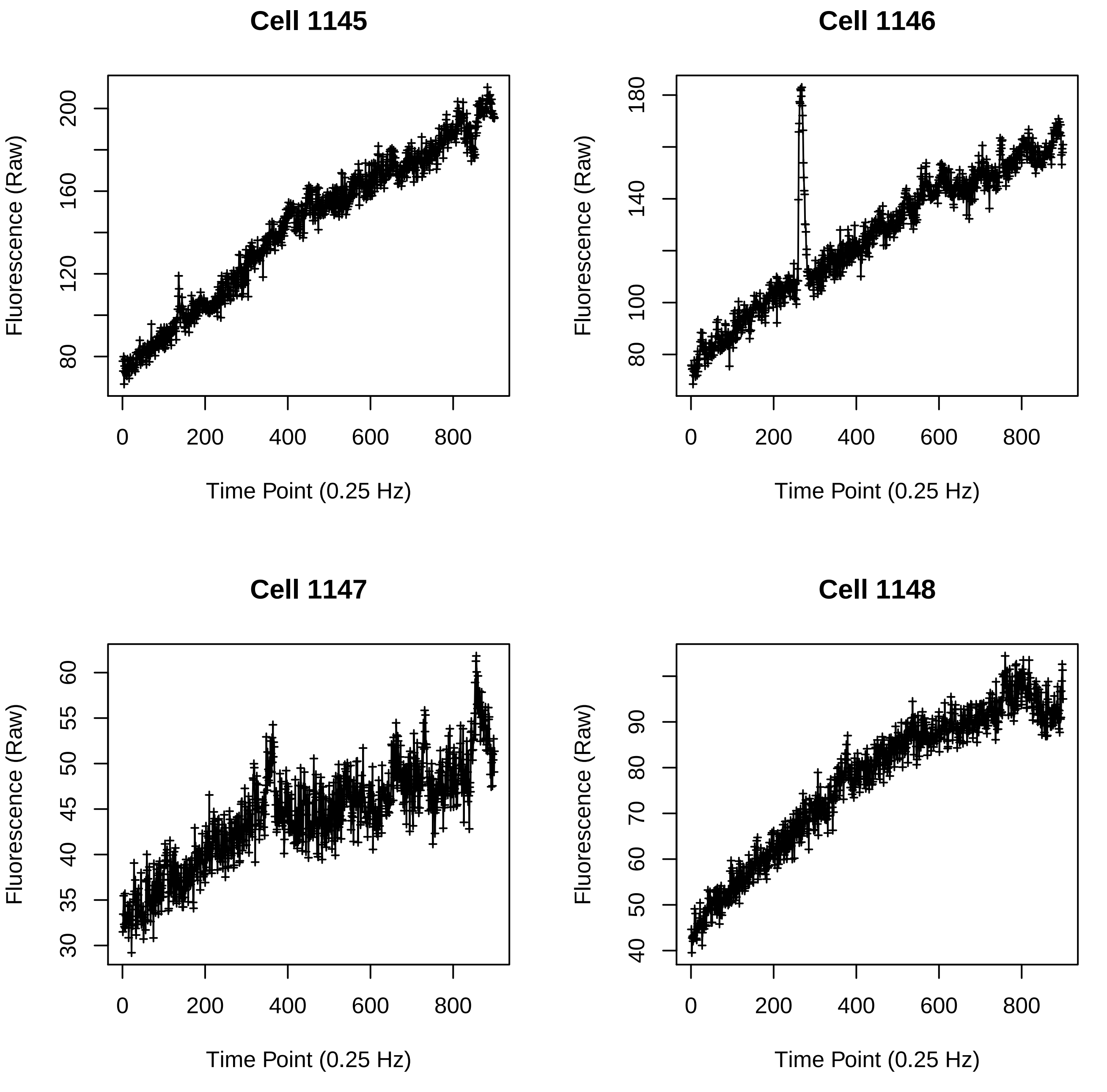
Cell 1145
Fluorescence (Raw)
80
120
160
200
0
200
400
600
800
Time Point (0.25 Hz)
Cell 1146
Fluorescence (Raw)
80
100
140
180
0
200
400
600
800
Time Point (0.25 Hz)
Cell 1147
Fluorescence (Raw)
30
35
40
45
50
55
60
0
200
400
600
800
Time Point (0.25 Hz)
Cell 1148
Fluorescence (Raw)
40
50
60
70
80
90
0
200
400
600
800
Time Point (0.25 Hz)

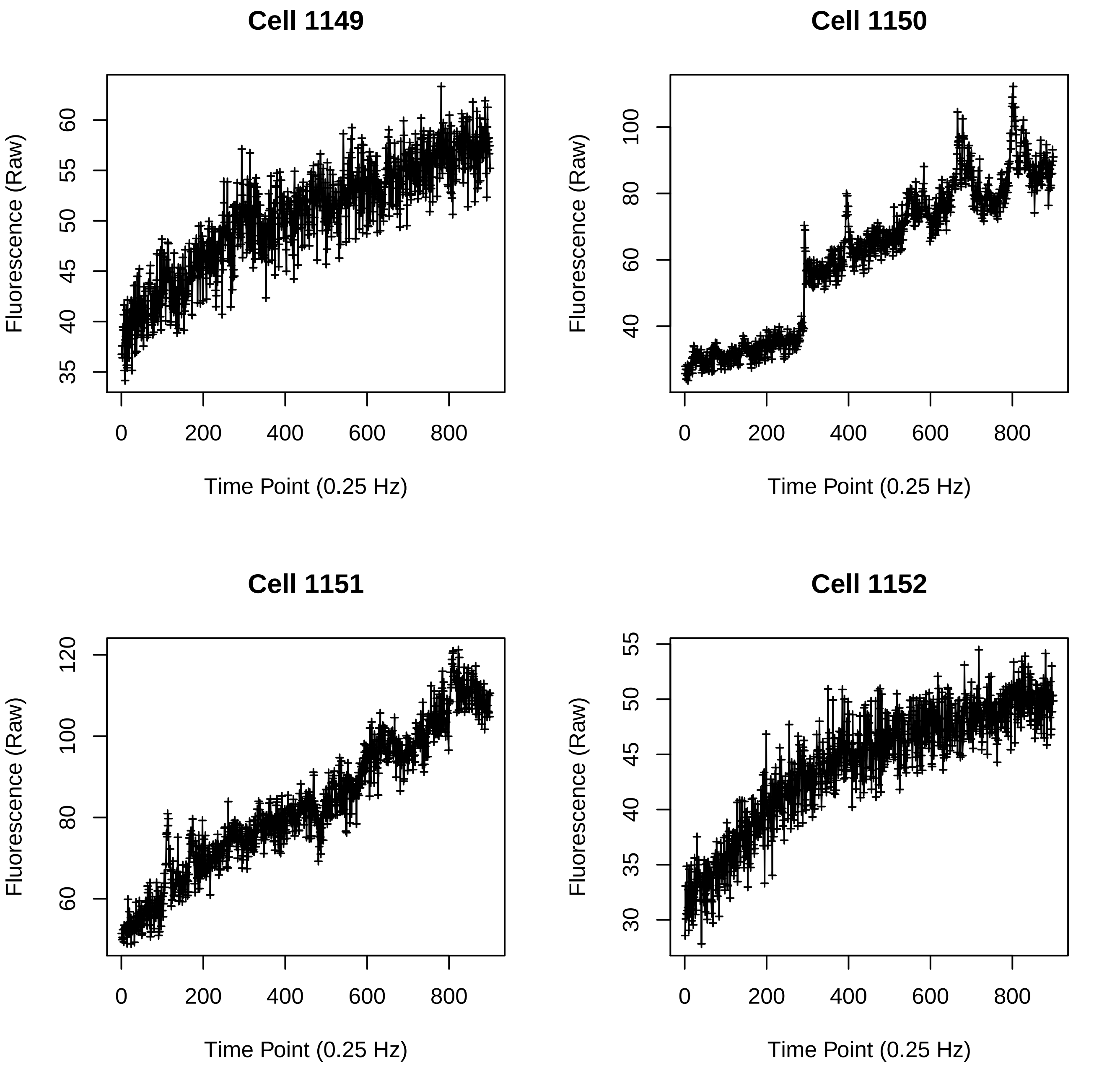
Cell 1149
Cell 1150
Cell 1151
Cell 1152
Fluorescence (Raw)
Time Point (0.25 Hz)
35
40
45
50
55
60
40
60
80
100
120
30
0
200
400
600
800

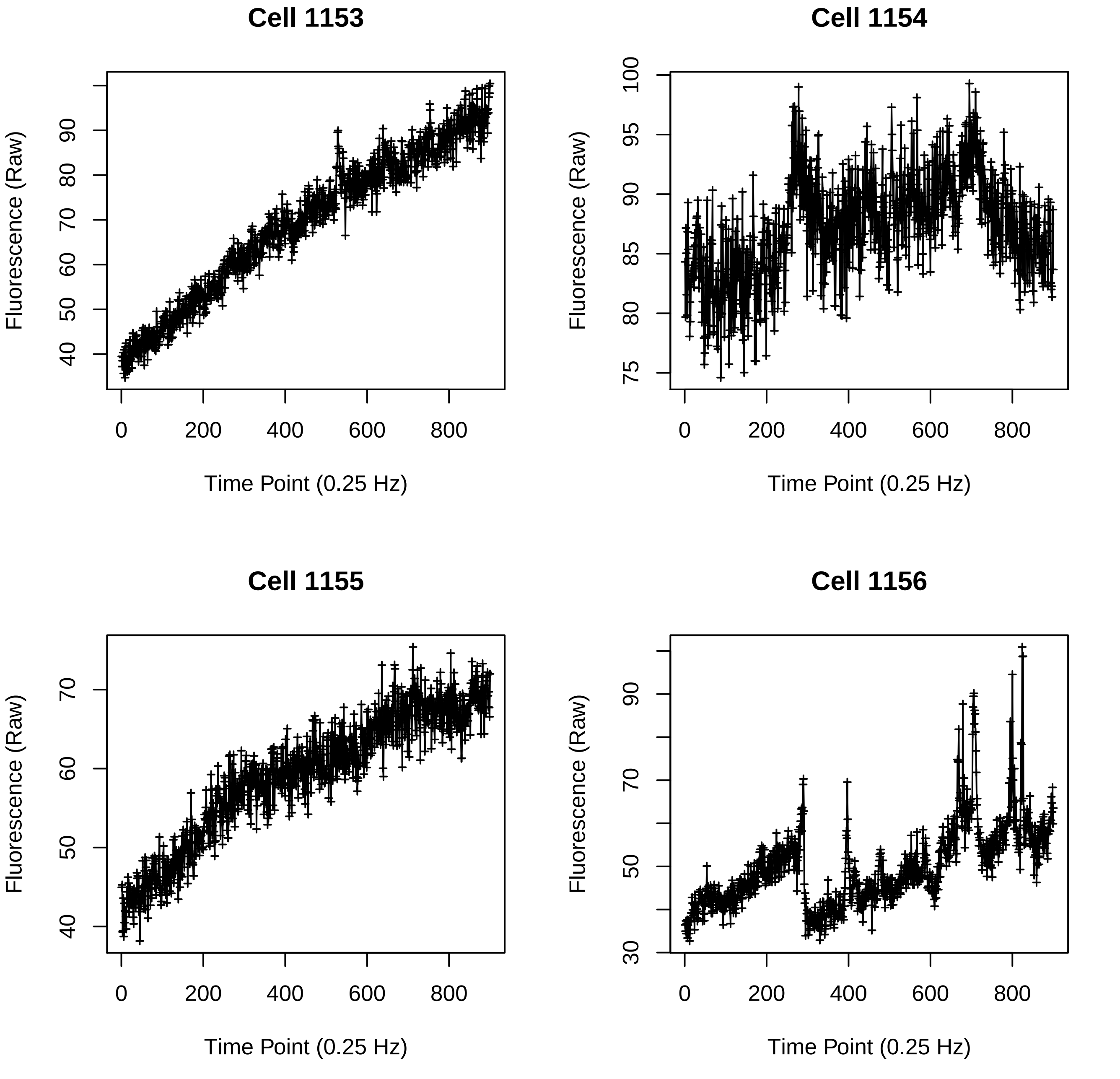
Cell 1153
Fluorescence (Raw)
40
50
60
70
80
90
0
200
400
600
800
Time Point (0.25 Hz)
Cell 1154
Fluorescence (Raw)
75
80
85
90
95
100
0
200
400
600
800
Time Point (0.25 Hz)
Cell 1155
Fluorescence (Raw)
40
50
60
70
0
200
400
600
800
Time Point (0.25 Hz)
Cell 1156
Fluorescence (Raw)
30
50
70
90
0
200
400
600
800
Time Point (0.25 Hz)

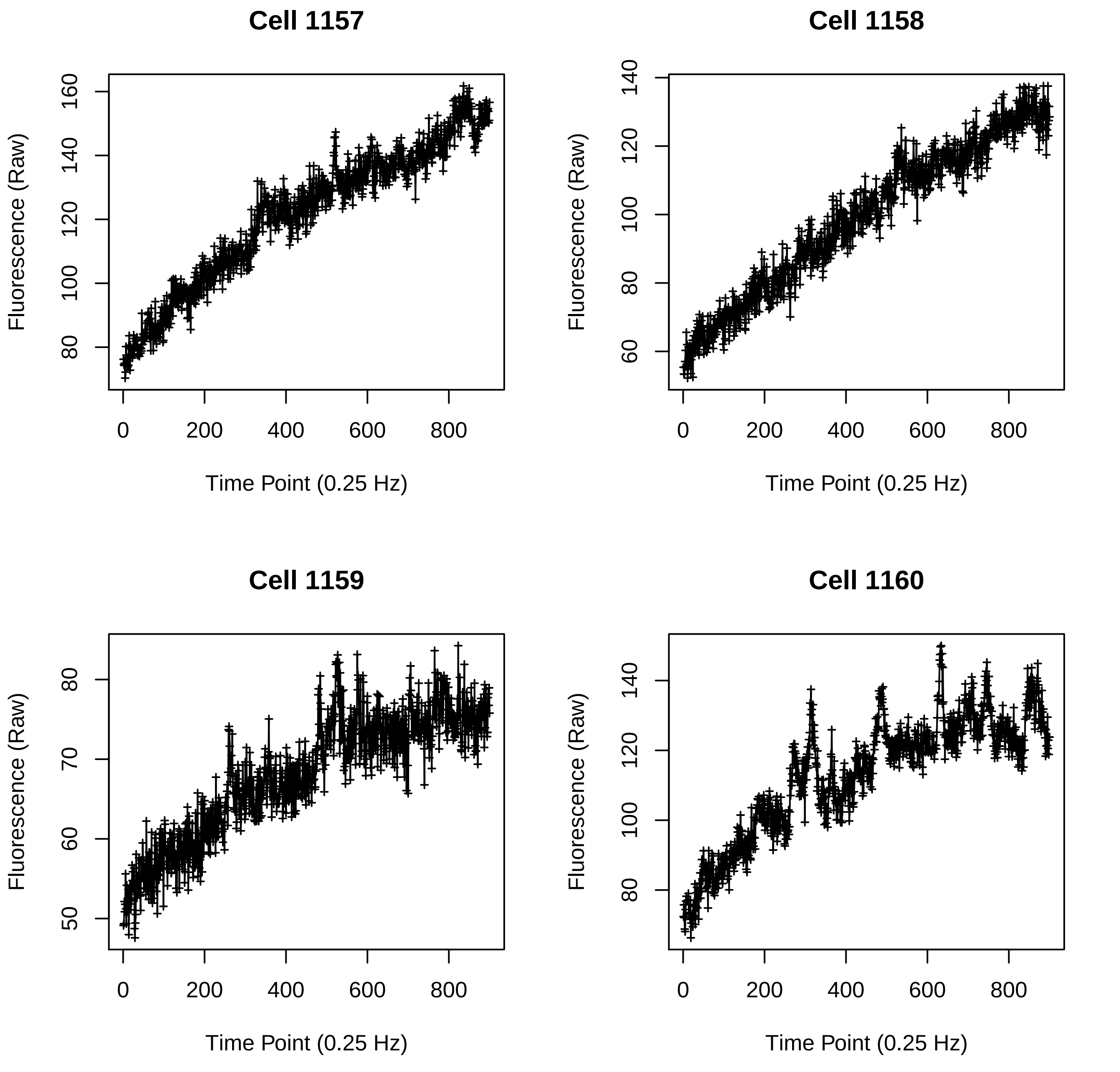
Cell 1157
Cell 1158
Cell 1159
Cell 1160
Fluorescence (Raw)
Time Point (0.25 Hz)
0
200
400
600
800
80
100
120
140
160
60
50
70

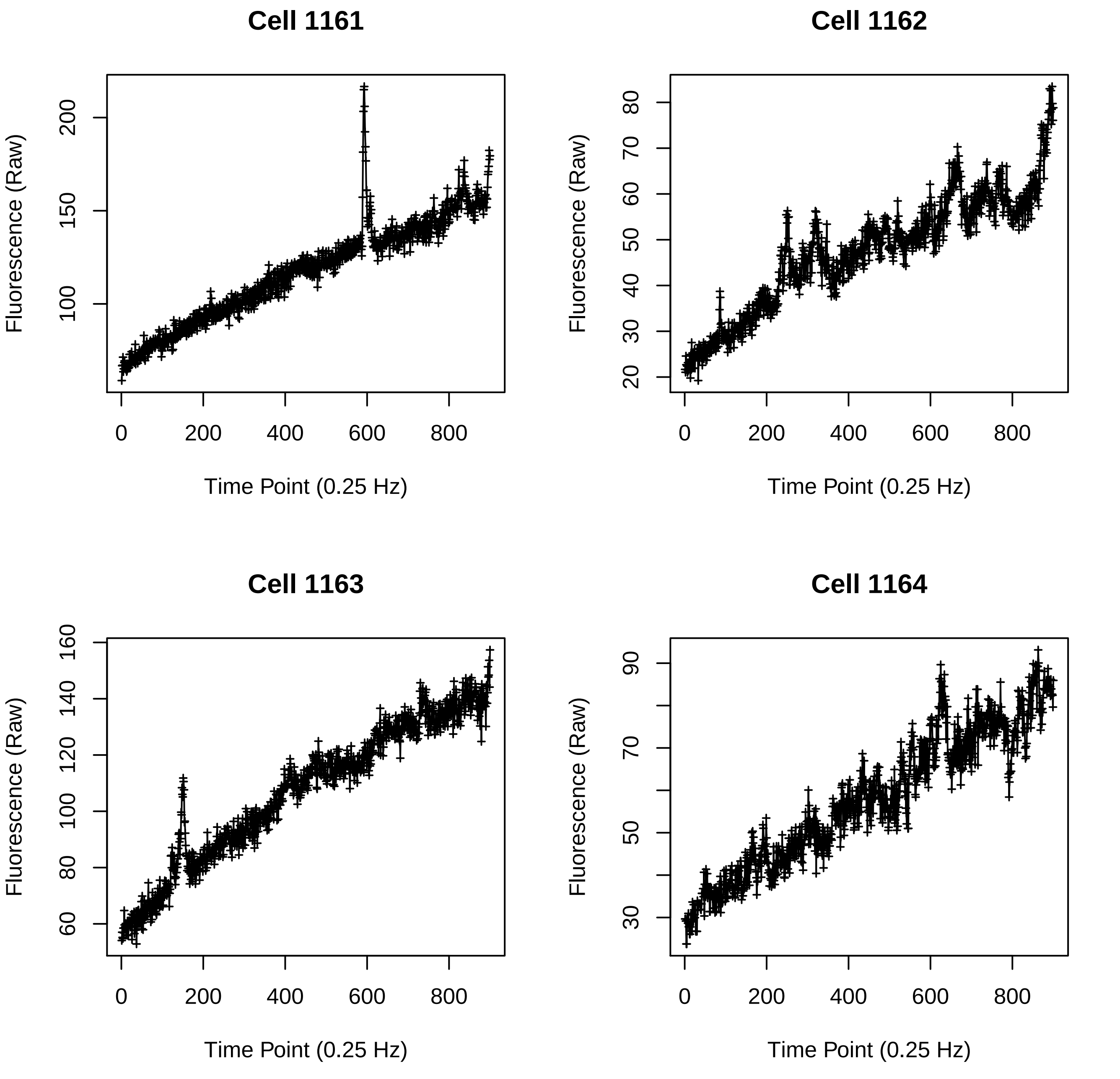
Cell 1161
Fluorescence (Raw)
100
150
200
0
200
400
600
800
Time Point (0.25 Hz)
Cell 1162
Fluorescence (Raw)
20
30
40
50
60
70
80
0
200
400
600
800
Time Point (0.25 Hz)
Cell 1163
Fluorescence (Raw)
60
80
100
120
140
160
0
200
400
600
800
Time Point (0.25 Hz)
Cell 1164
Fluorescence (Raw)
30
50
70
90
0
200
400
600
800
Time Point (0.25 Hz)

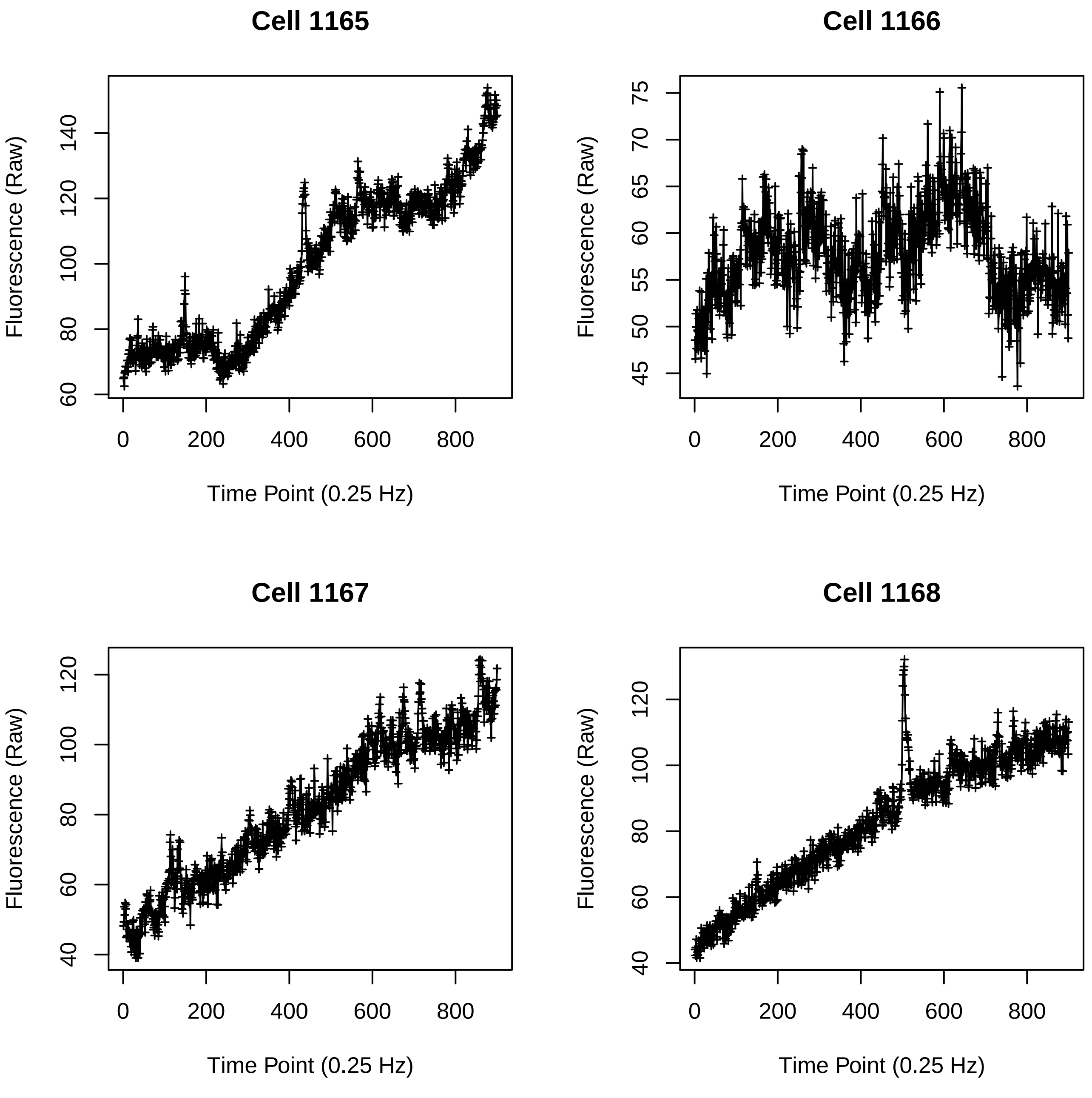
Cell 1165
Cell 1166
Cell 1167
Cell 1168
Fluorescence (Raw)
Time Point (0.25 Hz)
0
200
400
600
800
60
80
100
120
140
45
50
55
60
65
70
75
40

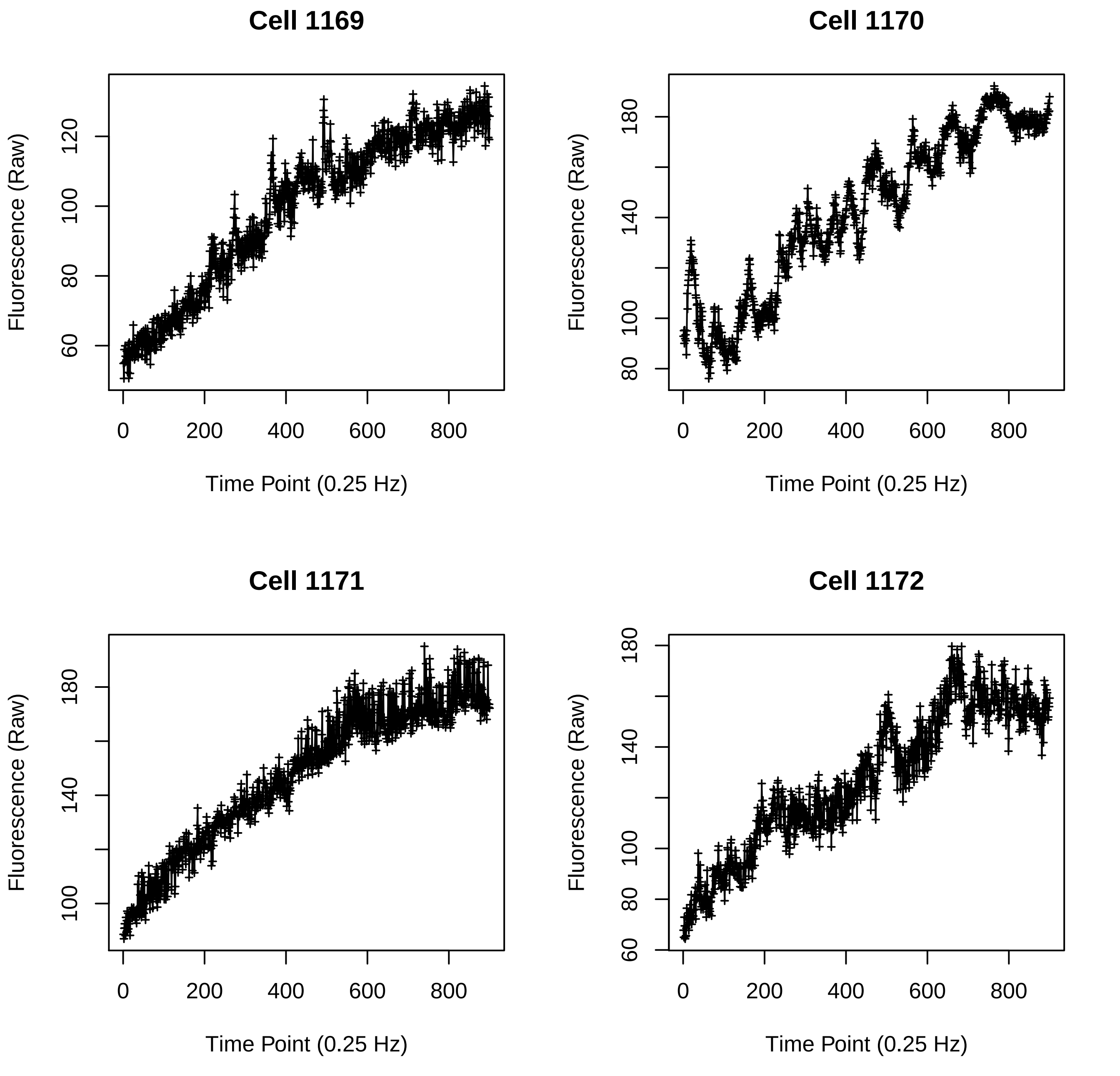

Cell 1169
Cell 1170
Cell 1171
Cell 1172
Fluorescence (Raw)
Time Point (0.25 Hz)
0
200
400
600
800
60
80
100
120
140
180

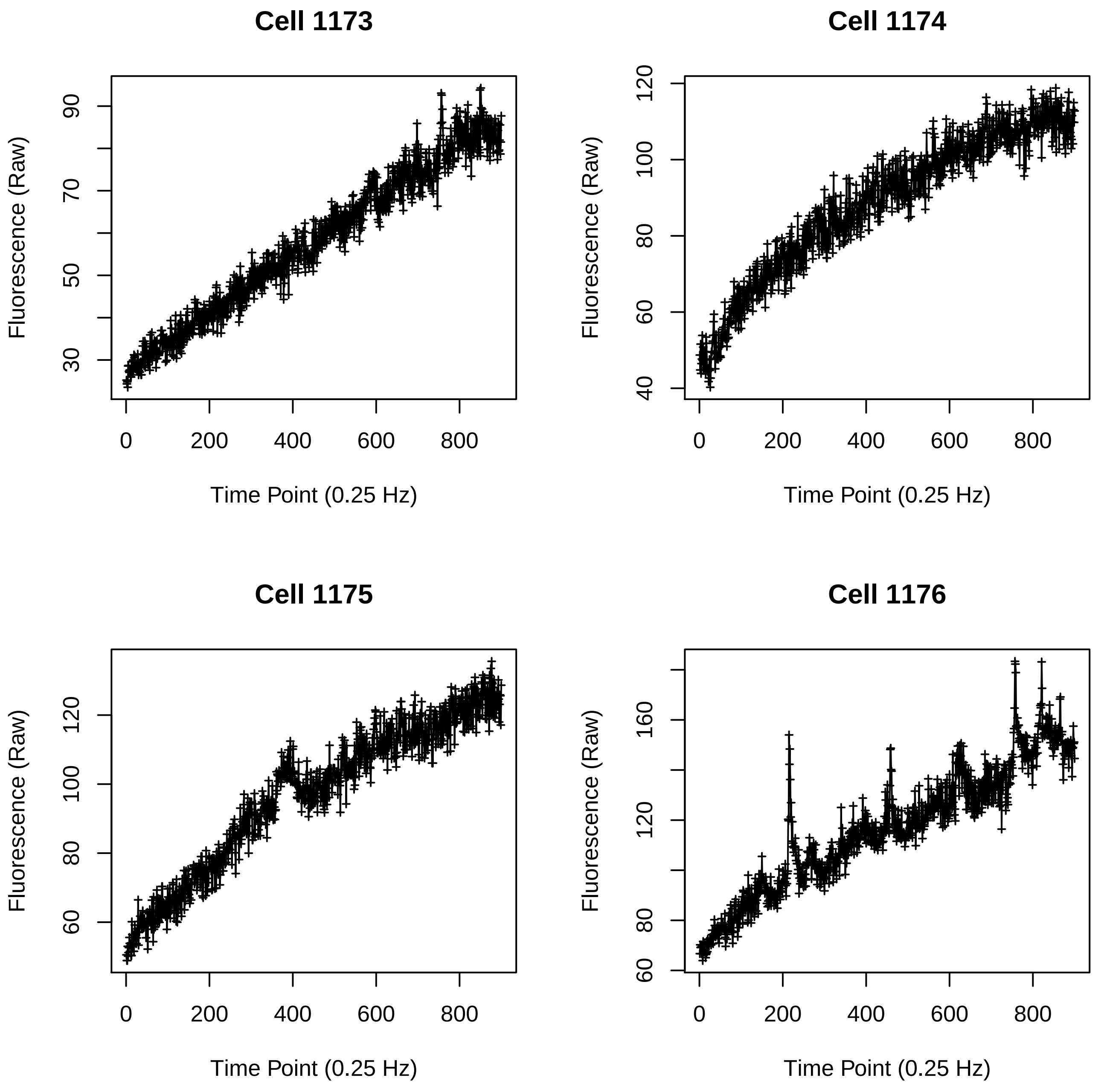

Cell 1173
Cell 1174
Cell 1175
Cell 1176
Fluorescence (Raw)
Time Point (0.25 Hz)
0
200
400
600
800
30
50
70
90
40
60
80
100
120
60
80
100
120
60
80
120
160

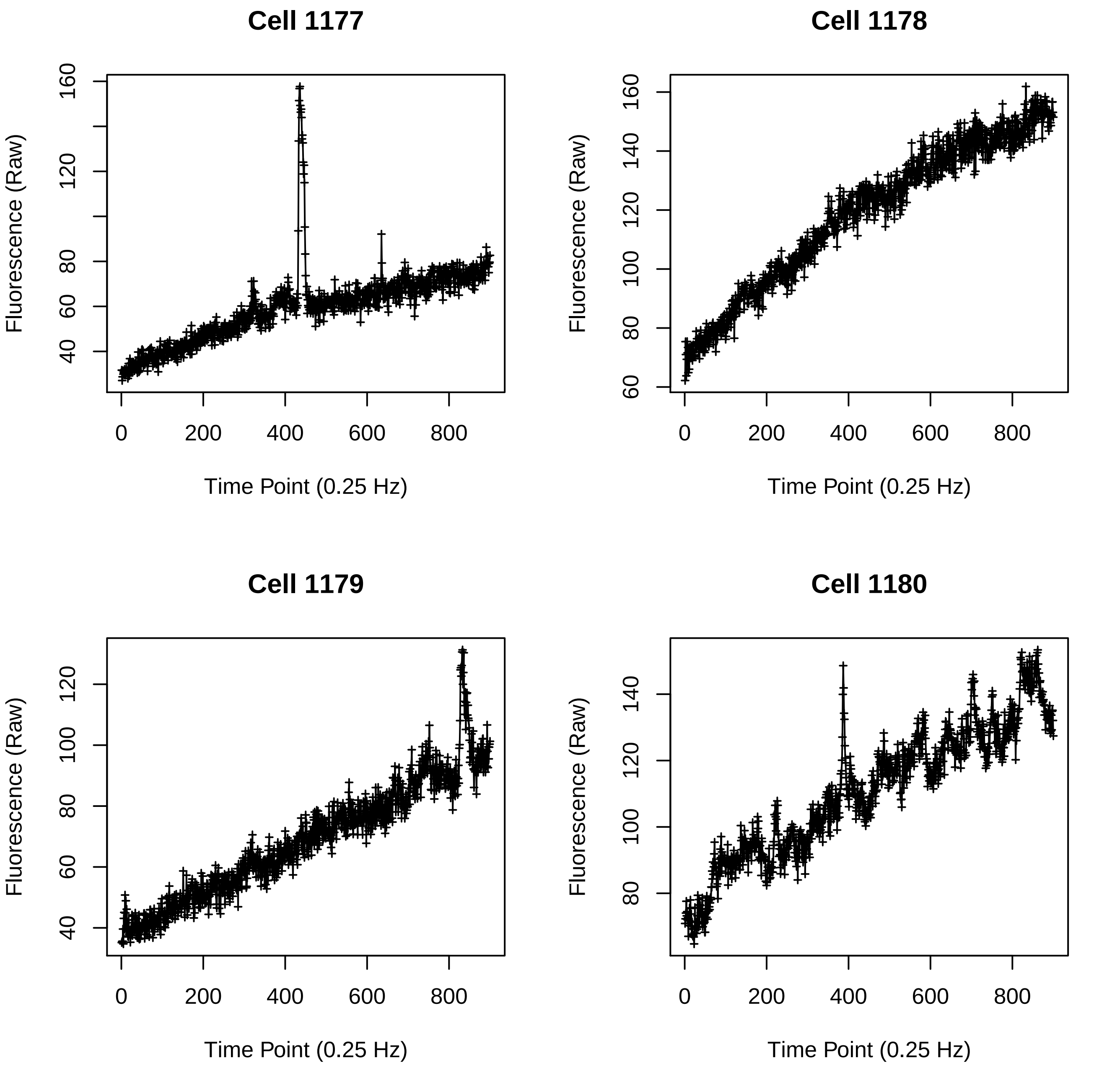
Cell 1177
Fluorescence (Raw)
40
60
80
120
160
0
200
400
600
800
Time Point (0.25 Hz)
Cell 1178
Fluorescence (Raw)
60
80
100
120
140
160
0
200
400
600
800
Time Point (0.25 Hz)
Cell 1179
Fluorescence (Raw)
40
60
80
100
120
0
200
400
600
800
Time Point (0.25 Hz)
Cell 1180
Fluorescence (Raw)
80
100
120
140
0
200
400
600
800
Time Point (0.25 Hz)

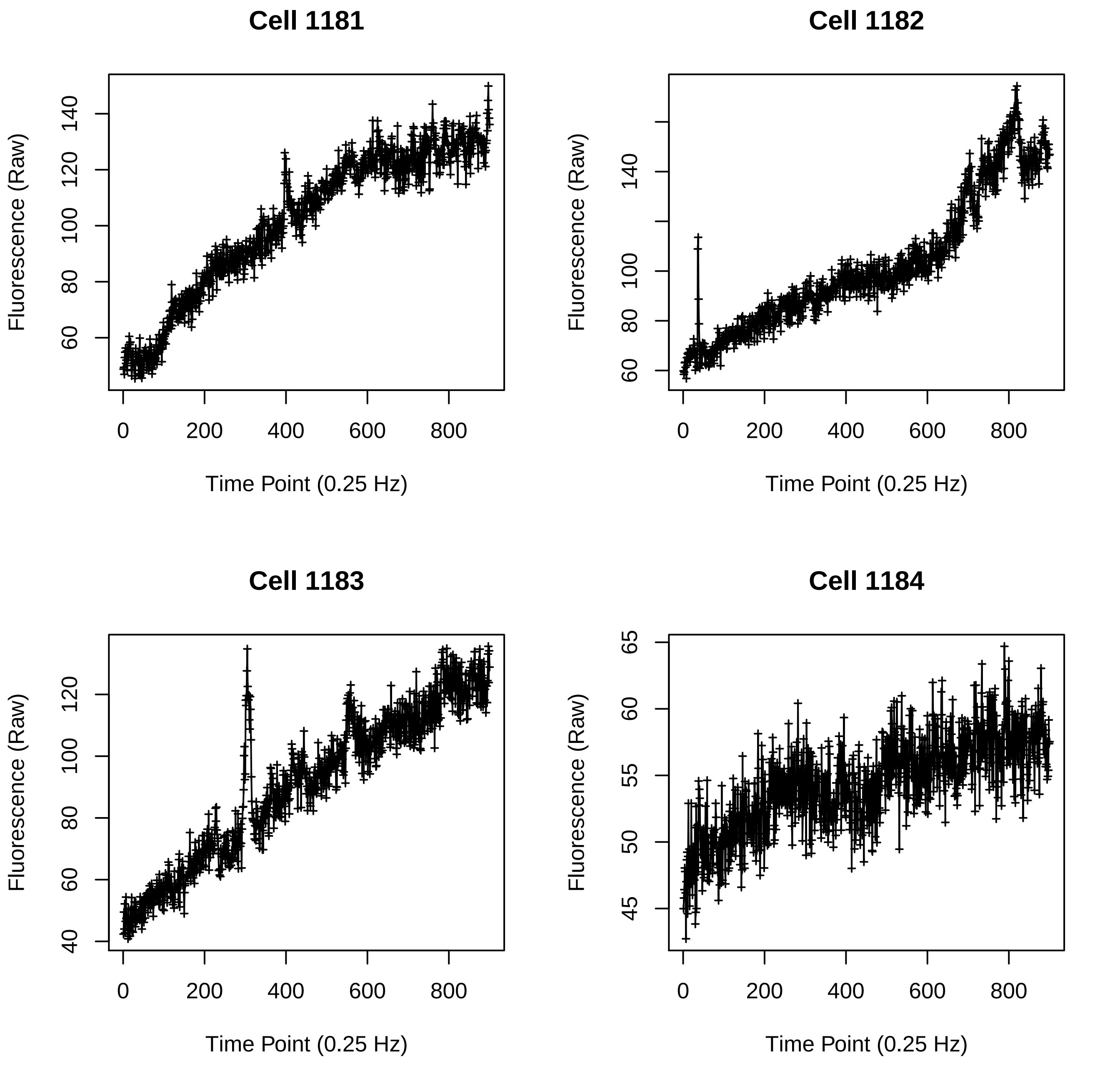
Cell 1181
Cell 1182
Cell 1183
Cell 1184
Fluorescence (Raw)
Time Point (0.25 Hz)
0
200
400
600
800
60
80
100
120
140
40
45
50
55
60
65

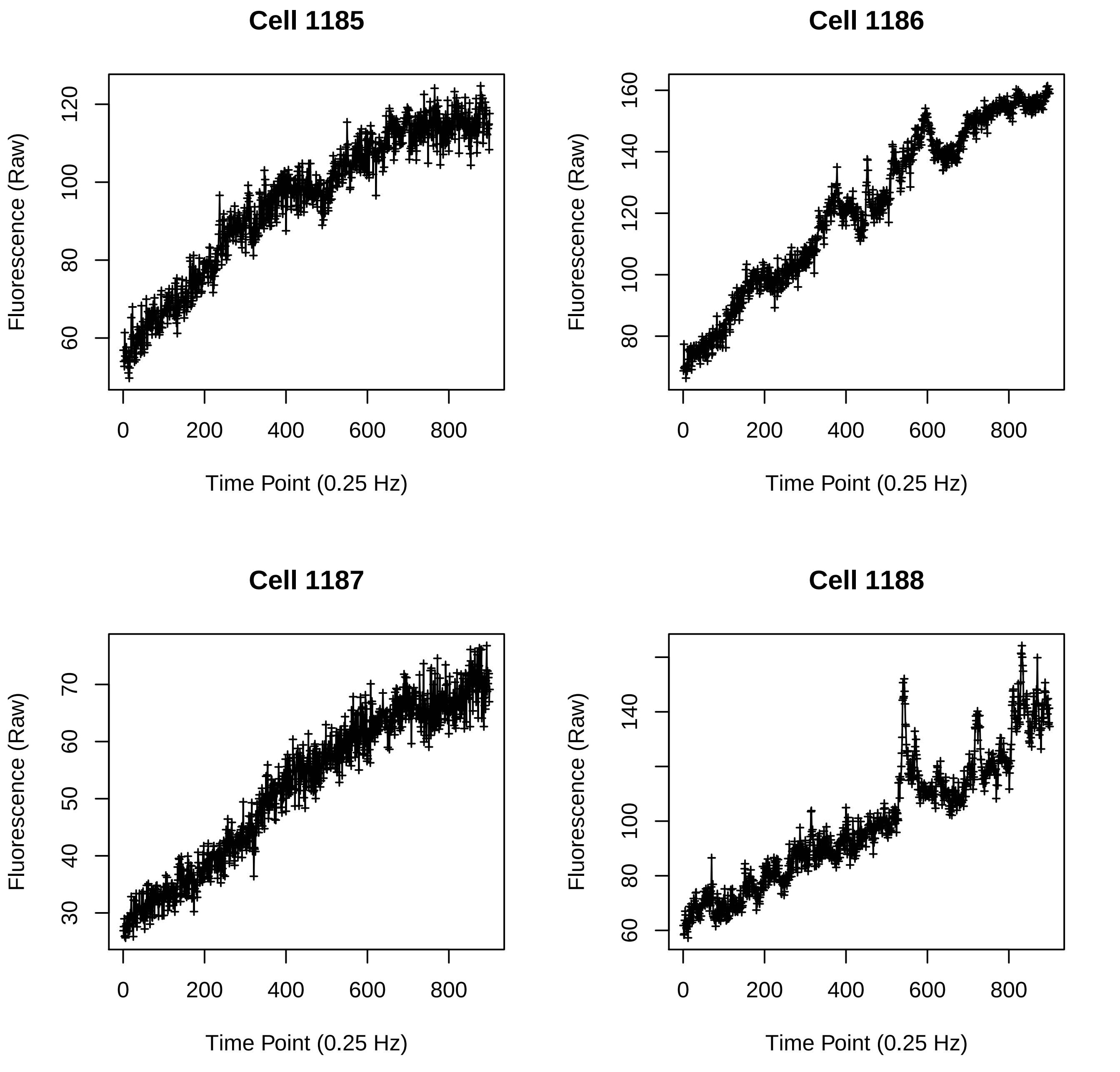

Cell 1185
Fluorescence (Raw)
60
80
100
120
0
200
400
600
800
Time Point (0.25 Hz)
Cell 1186
Fluorescence (Raw)
80
100
120
140
160
0
200
400
600
800
Time Point (0.25 Hz)
Cell 1187
Fluorescence (Raw)
30
40
50
60
70
0
200
400
600
800
Time Point (0.25 Hz)
Cell 1188
Fluorescence (Raw)
60
80
100
140
0
200
400
600
800
Time Point (0.25 Hz)

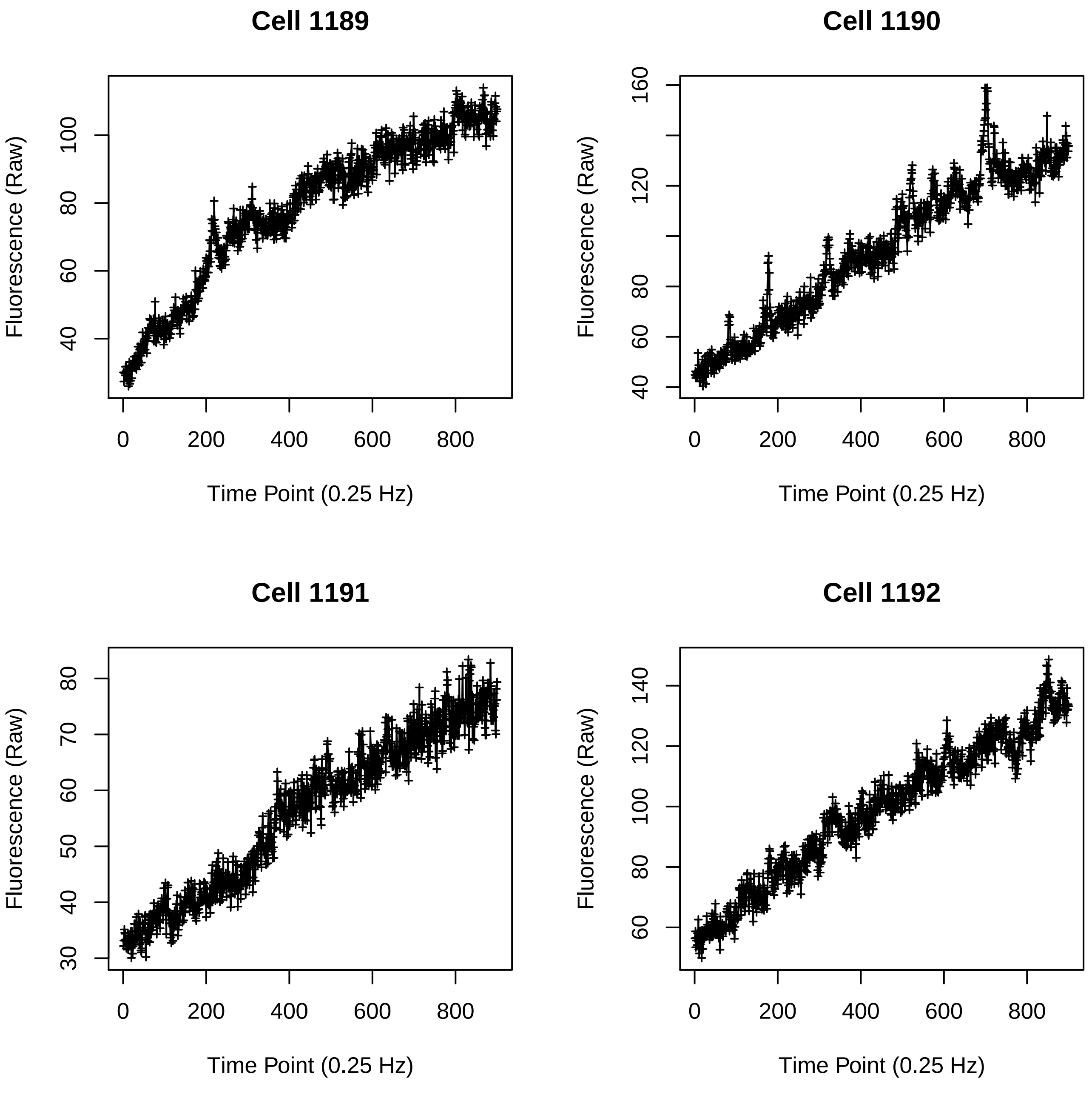
Cell 1189
Cell 1190
Cell 1191
Cell 1192
Fluorescence (Raw)
Time Point (0.25 Hz)
0
200
400
600
800
40
60
80
100
120
140
160
30
50
70

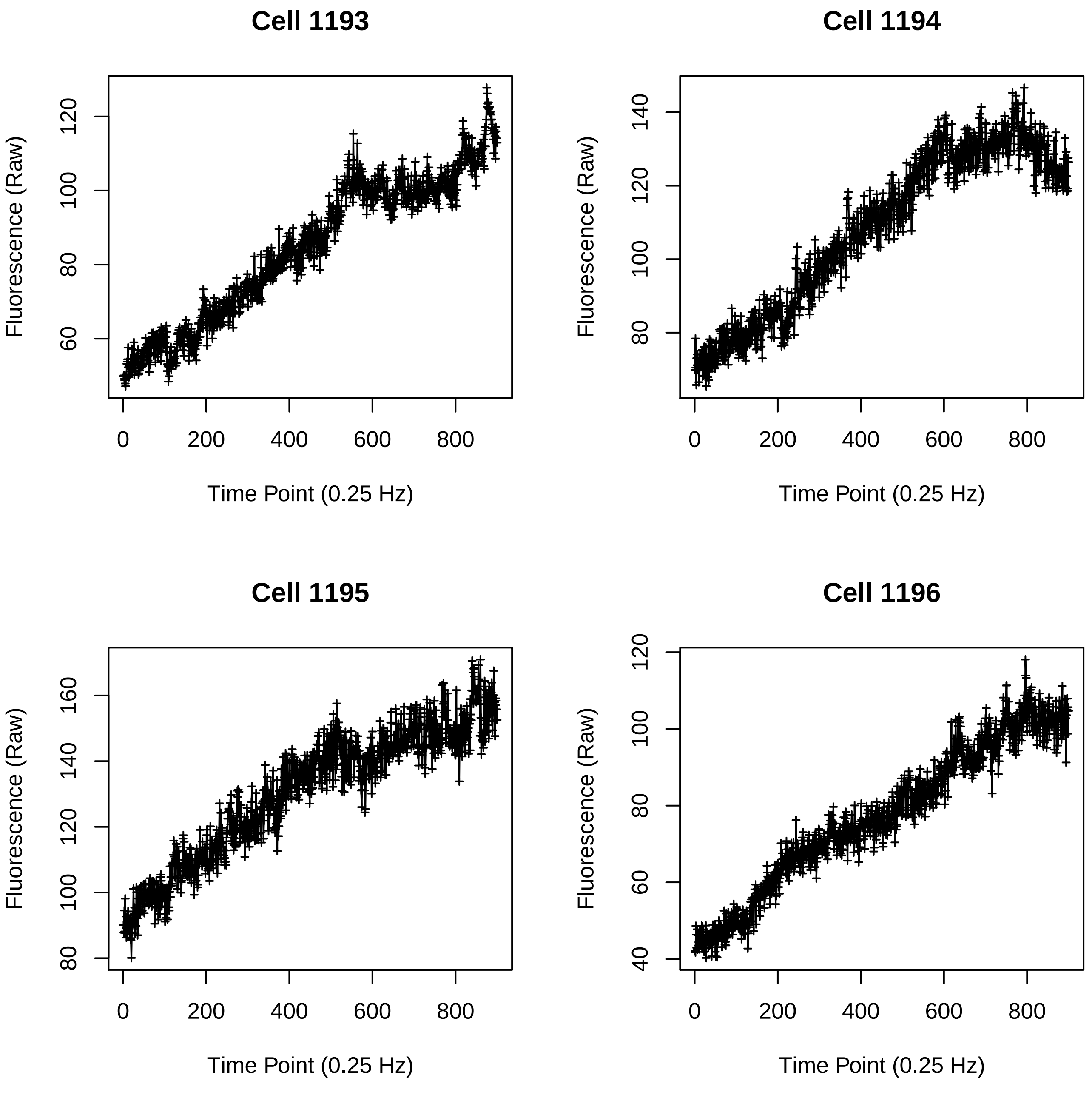
Cell 1193
Cell 1194
Cell 1195
Cell 1196
Fluorescence (Raw)
Time Point (0.25 Hz)
0
200
400
600
800
60
80
100
120
140
160
40

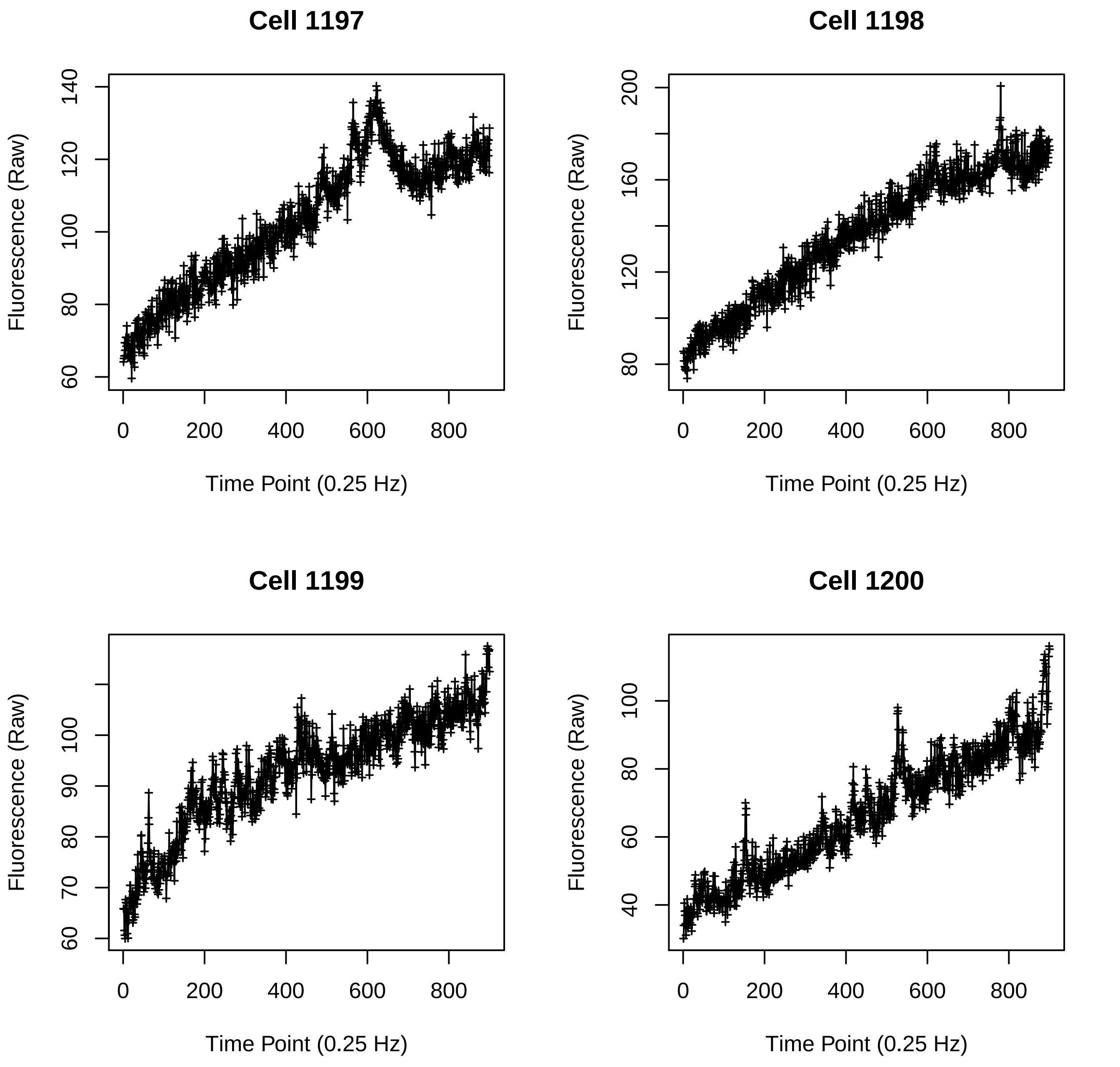

Cell 1197
Fluorescence (Raw)
60
80
100
120
140
0
200
400
600
800
Time Point (0.25 Hz)
Cell 1198
Fluorescence (Raw)
80
120
160
200
0
200
400
600
800
Time Point (0.25 Hz)
Cell 1199
Fluorescence (Raw)
60
70
80
90
100
0
200
400
600
800
Time Point (0.25 Hz)
Cell 1200
Fluorescence (Raw)
40
60
80
100
0
200
400
600
800
Time Point (0.25 Hz)

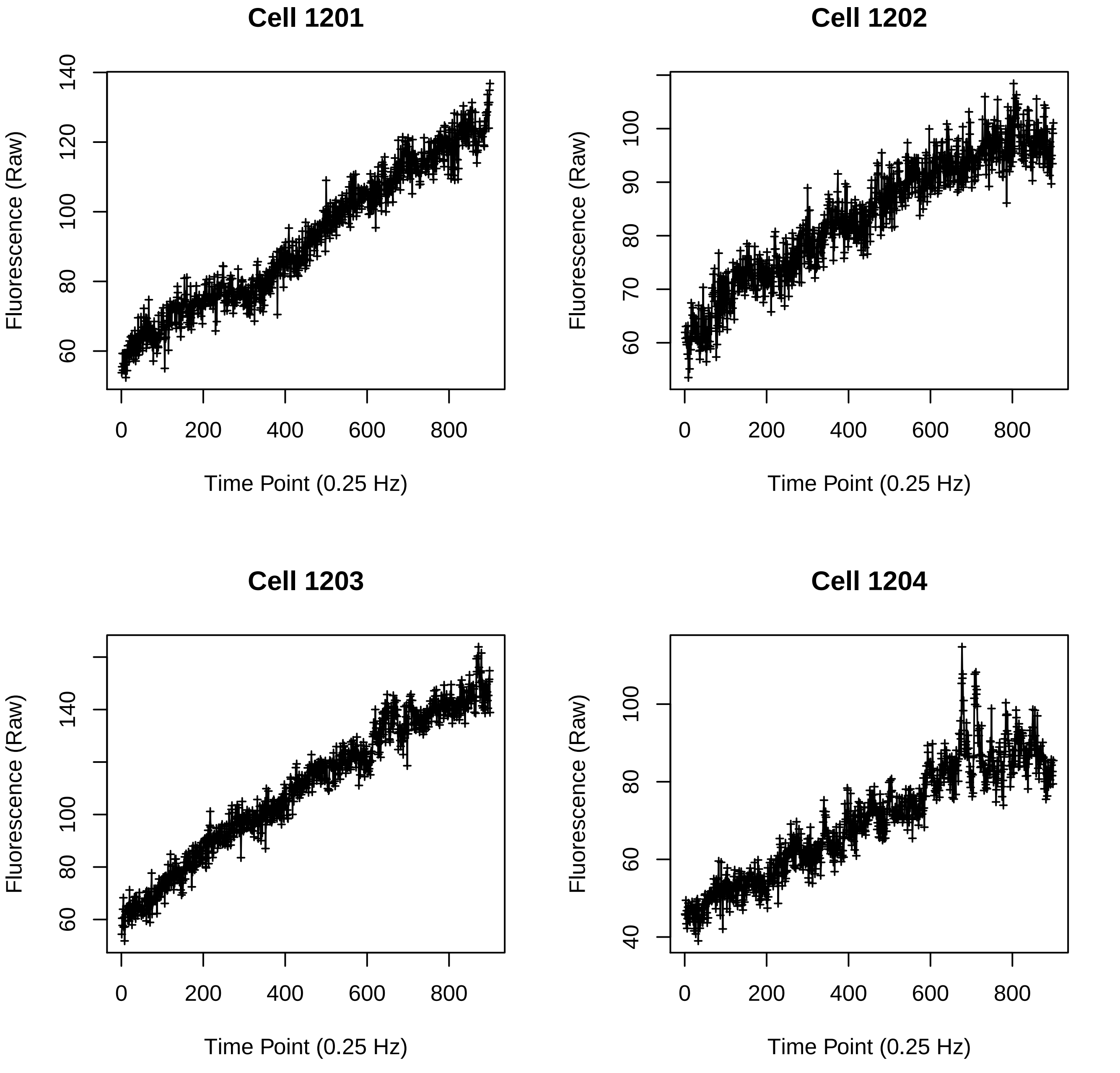
Cell 1201
Cell 1202
Cell 1203
Cell 1204
Fluorescence (Raw)
Time Point (0.25 Hz)
60
80
100
120
140
70
90
40
0
200
400
600
800

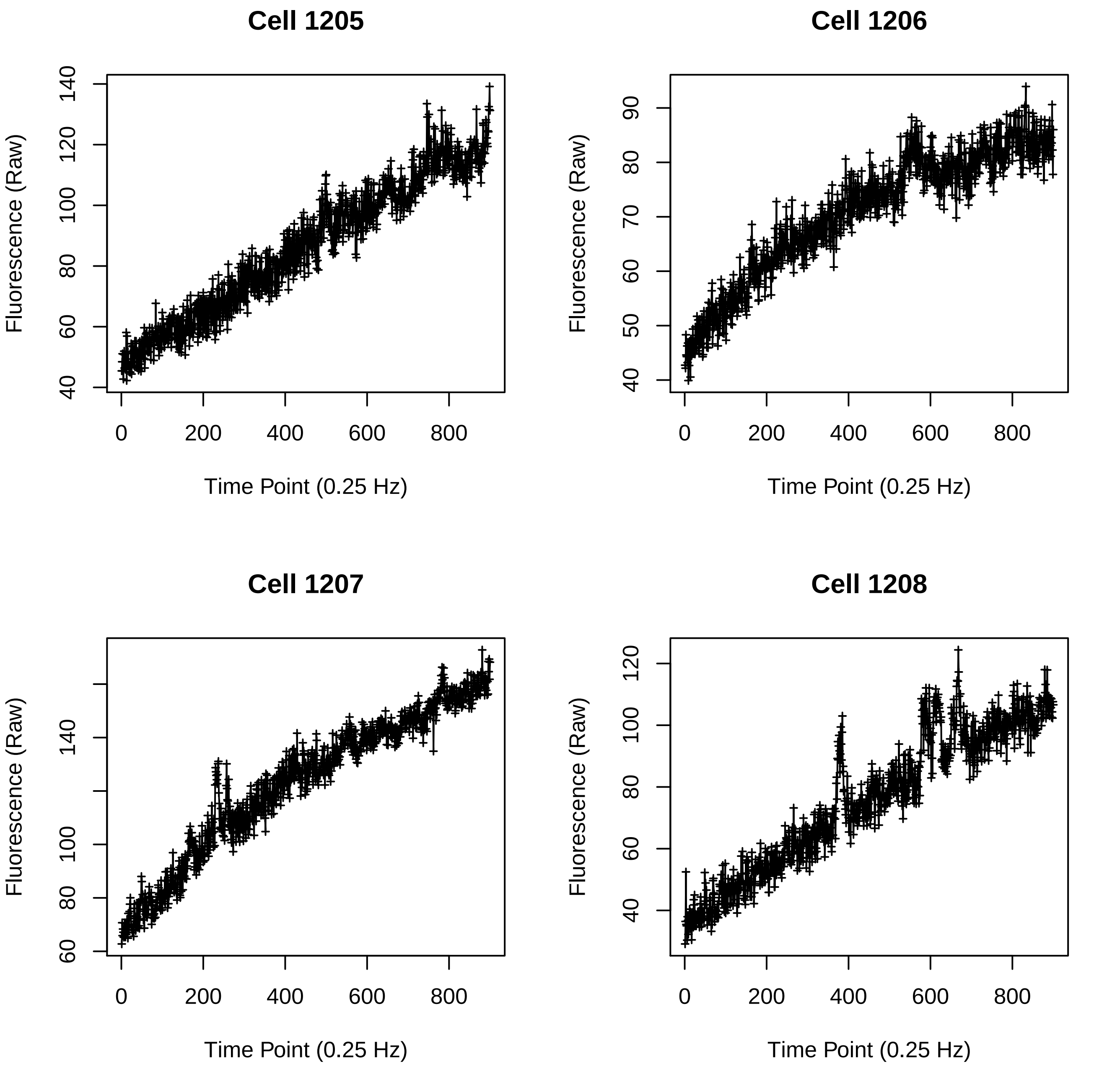
Cell 1205
Fluorescence (Raw)
40
60
80
100
120
140
0
200
400
600
800
Time Point (0.25 Hz)
Cell 1206
Fluorescence (Raw)
40
50
60
70
80
90
0
200
400
600
800
Time Point (0.25 Hz)
Cell 1207
Fluorescence (Raw)
60
80
100
140
0
200
400
600
800
Time Point (0.25 Hz)
Cell 1208
Fluorescence (Raw)
40
60
80
100
120
0
200
400
600
800
Time Point (0.25 Hz)

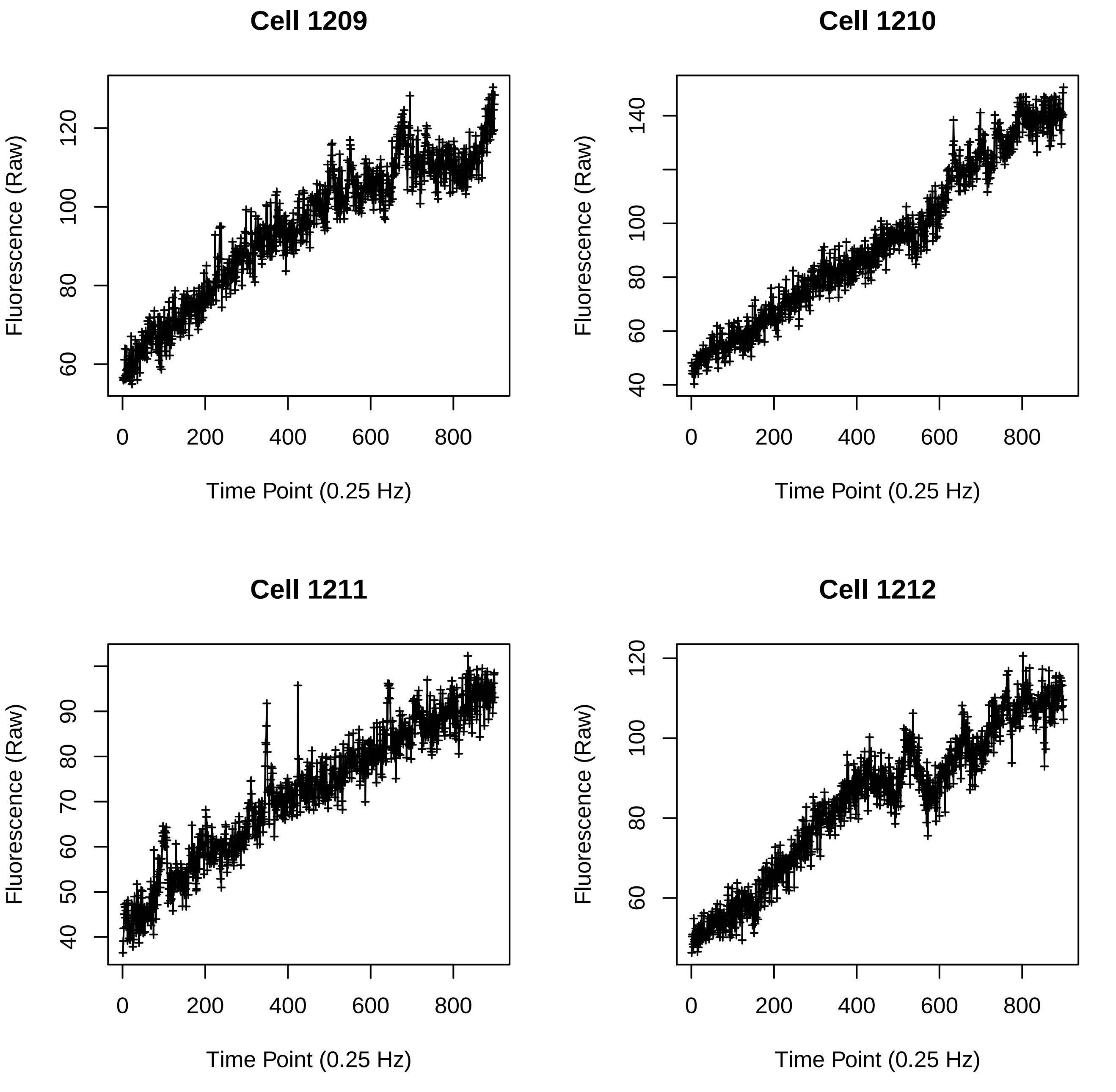

Cell 1209
Fluorescence (Raw)
60
80
100
120
0
200
400
600
800
Time Point (0.25 Hz)
Cell 1210
Fluorescence (Raw)
40
60
80
100
140
0
200
400
600
800
Time Point (0.25 Hz)
Cell 1211
Fluorescence (Raw)
40
50
60
70
80
90
0
200
400
600
800
Time Point (0.25 Hz)
Cell 1212
Fluorescence (Raw)
60
80
100
120
0
200
400
600
800
Time Point (0.25 Hz)

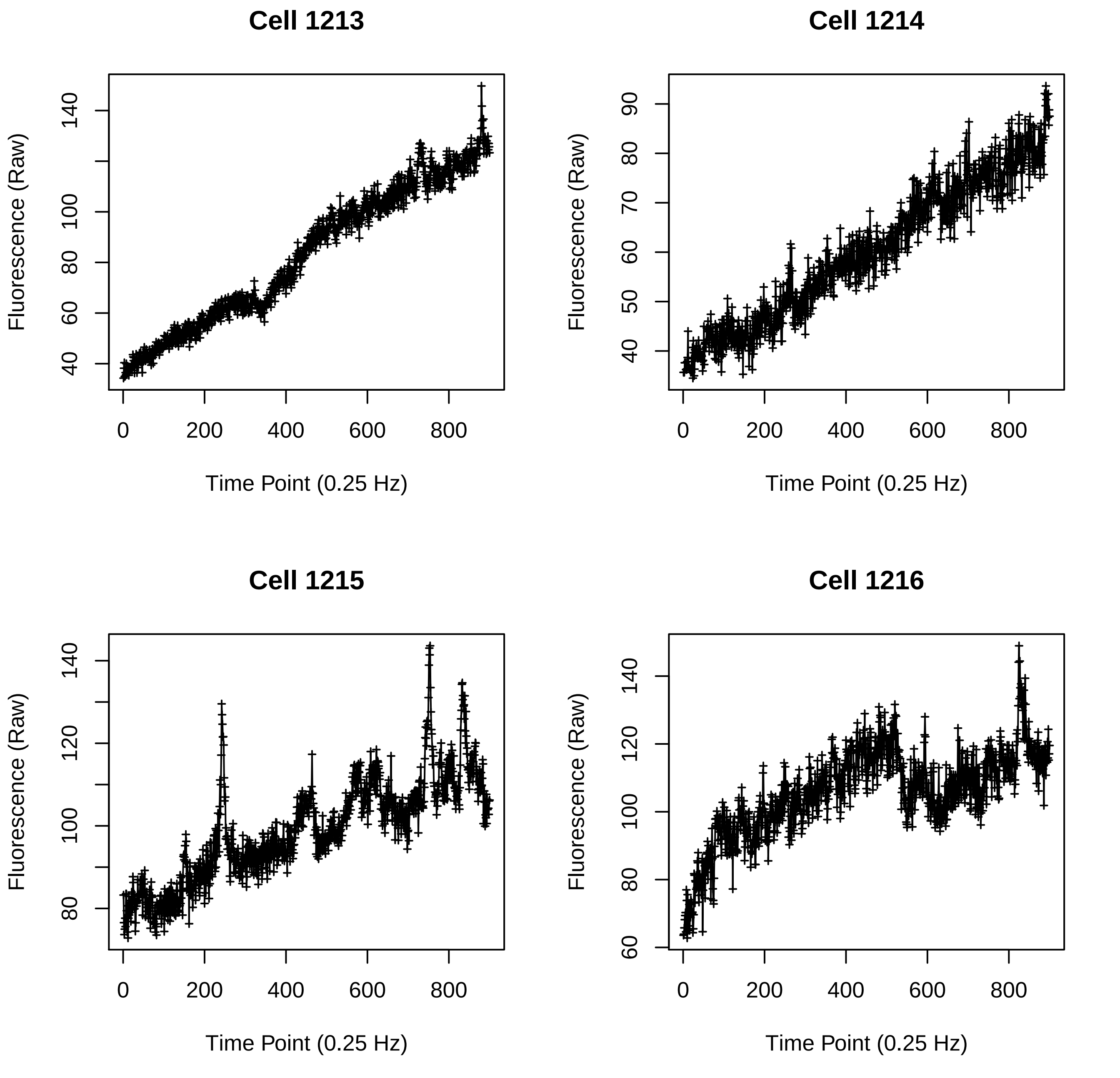

Cell 1213
Cell 1214
Cell 1215
Cell 1216
Fluorescence (Raw)
Time Point (0.25 Hz)
0
200
400
600
800
40
60
80
100
140
50
70
90
120

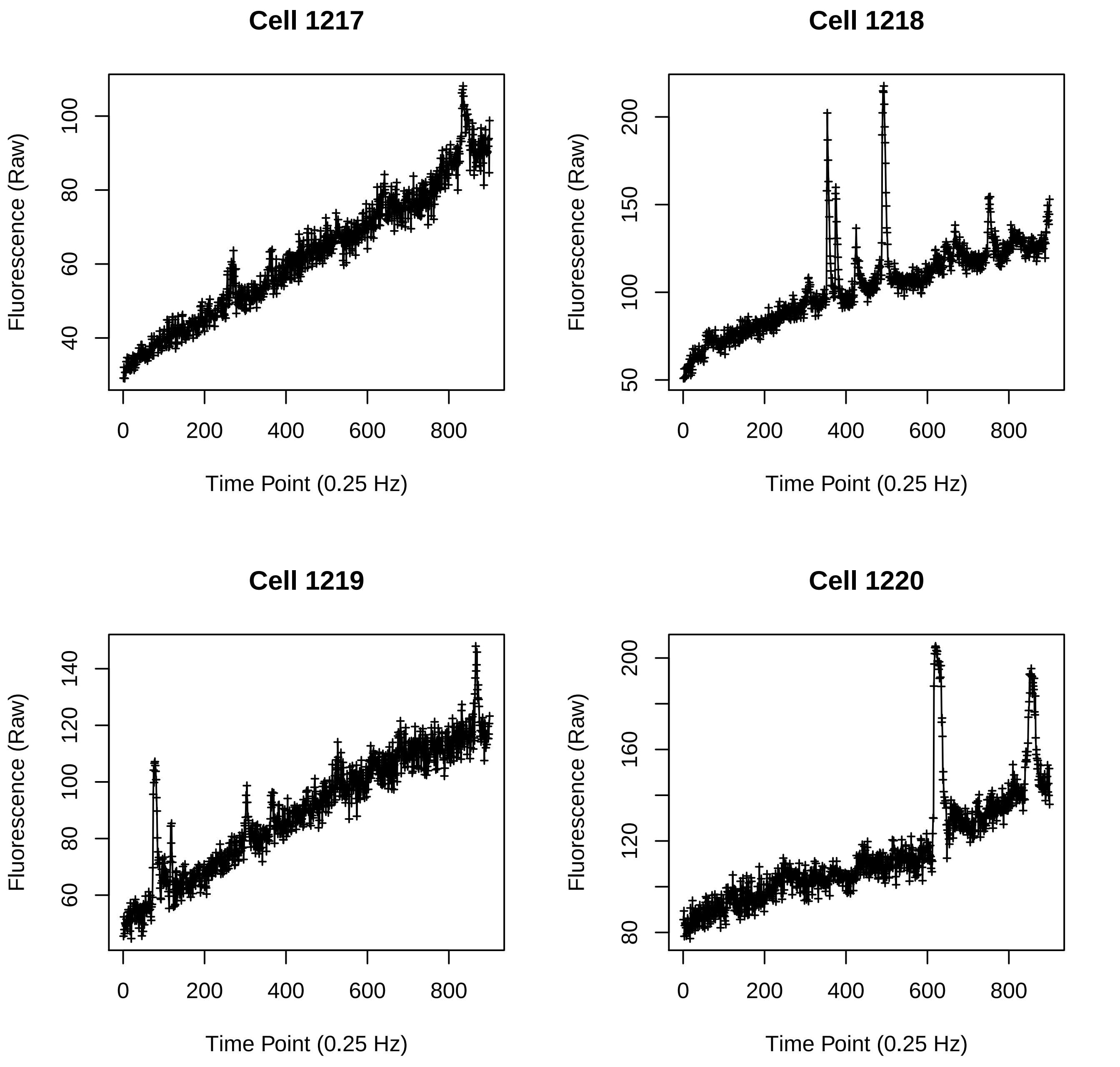
Cell 1217
Fluorescence (Raw)
40
60
80
100
0
200
400
600
800
Time Point (0.25 Hz)
Cell 1218
Fluorescence (Raw)
50
100
150
200
0
200
400
600
800
Time Point (0.25 Hz)
Cell 1219
Fluorescence (Raw)
60
80
100
120
140
0
200
400
600
800
Time Point (0.25 Hz)
Cell 1220
Fluorescence (Raw)
80
120
160
200
0
200
400
600
800
Time Point (0.25 Hz)

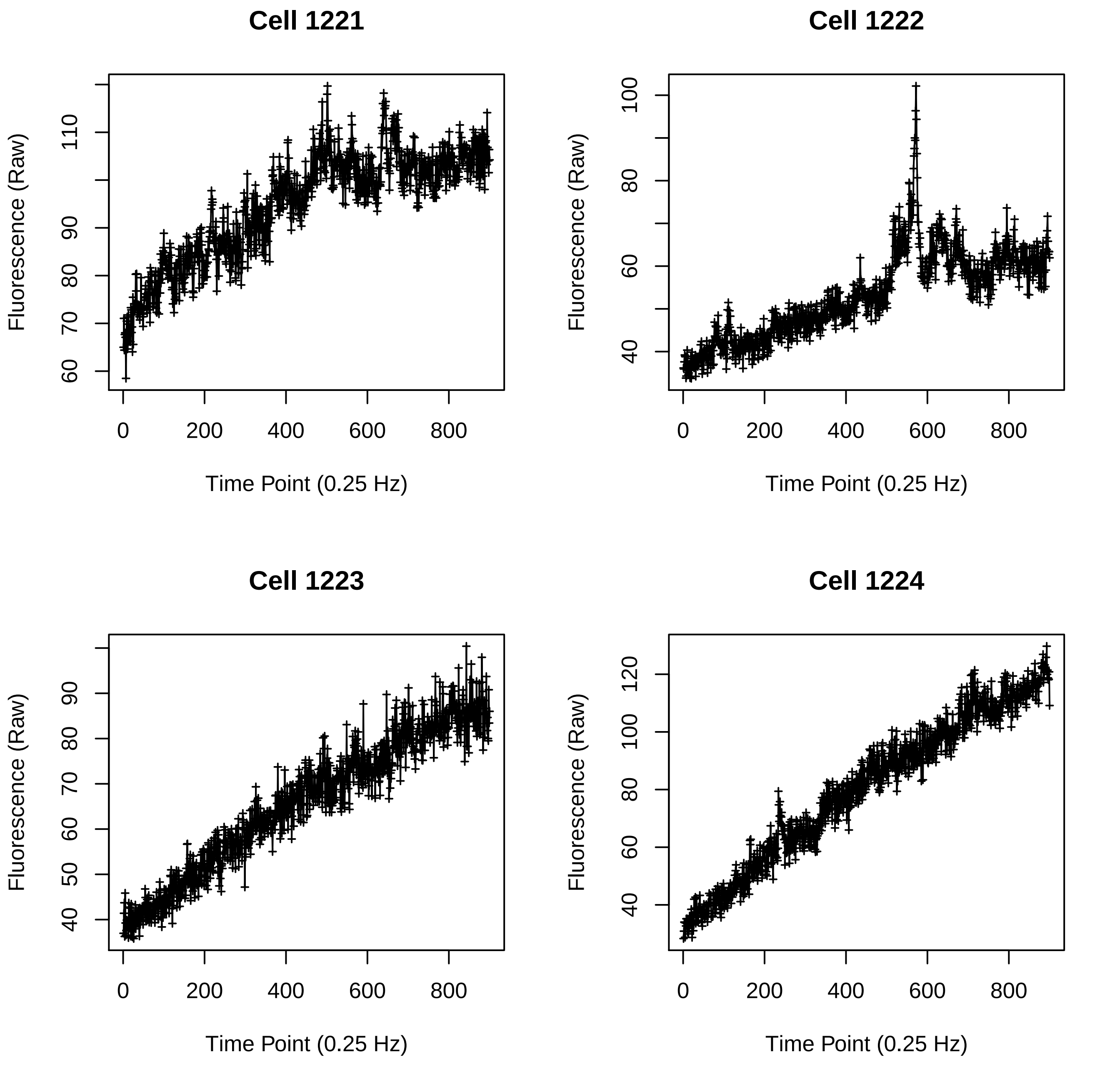
Cell 1221
Cell 1222
Cell 1223
Cell 1224
Fluorescence (Raw)
Time Point (0.25 Hz)
0
200
400
600
800
60
70
80
90
110
40
60
80
100
40
50
60
70
80
90
40
60
80
100
120

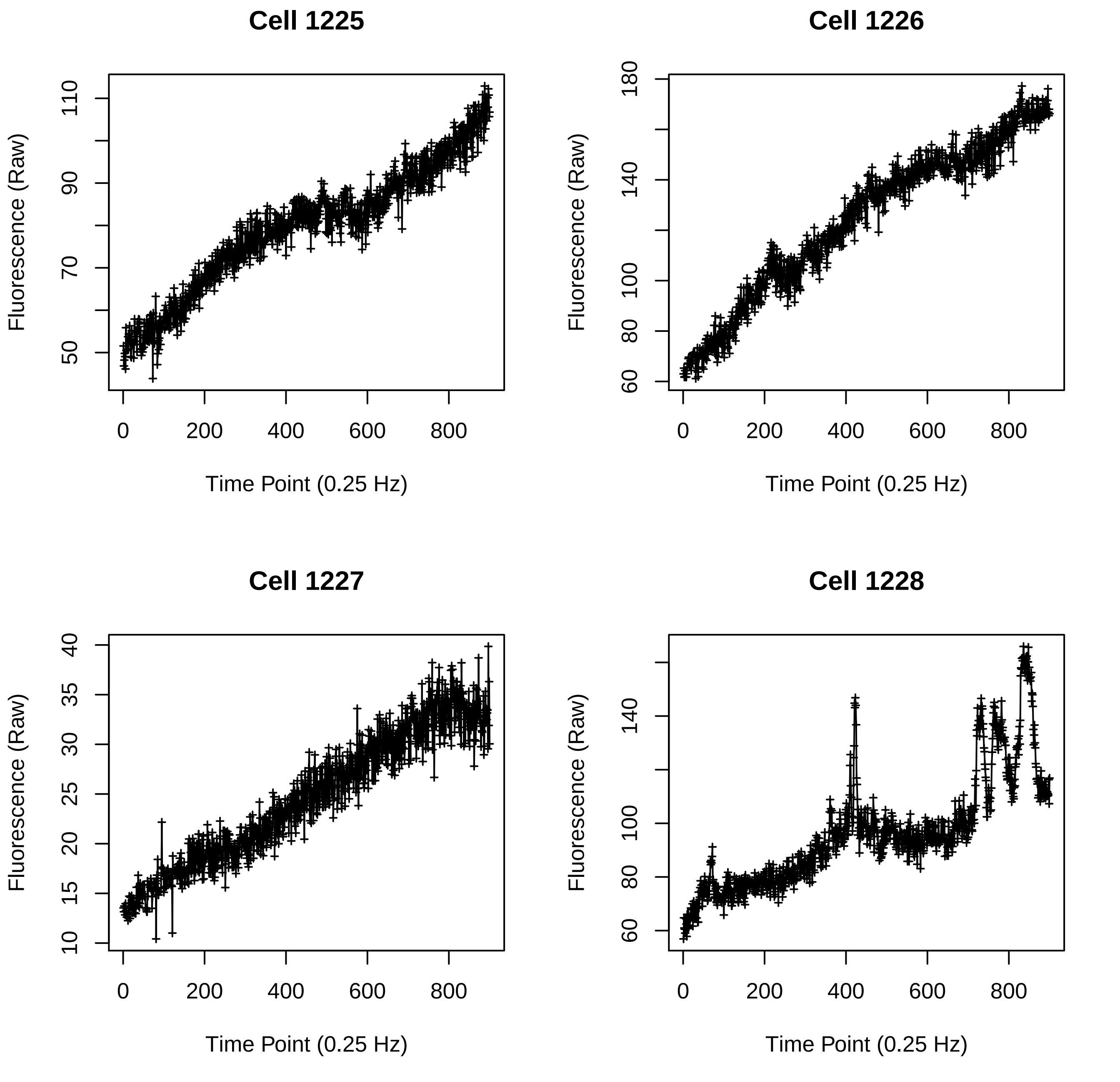
Cell 1225
Fluorescence (Raw)
50
70
90
110
0
200
400
600
800
Time Point (0.25 Hz)
Cell 1226
Fluorescence (Raw)
60
80
100
140
180
0
200
400
600
800
Time Point (0.25 Hz)
Cell 1227
Fluorescence (Raw)
10
15
20
25
30
35
40
0
200
400
600
800
Time Point (0.25 Hz)
Cell 1228
Fluorescence (Raw)
60
80
100
140
0
200
400
600
800
Time Point (0.25 Hz)

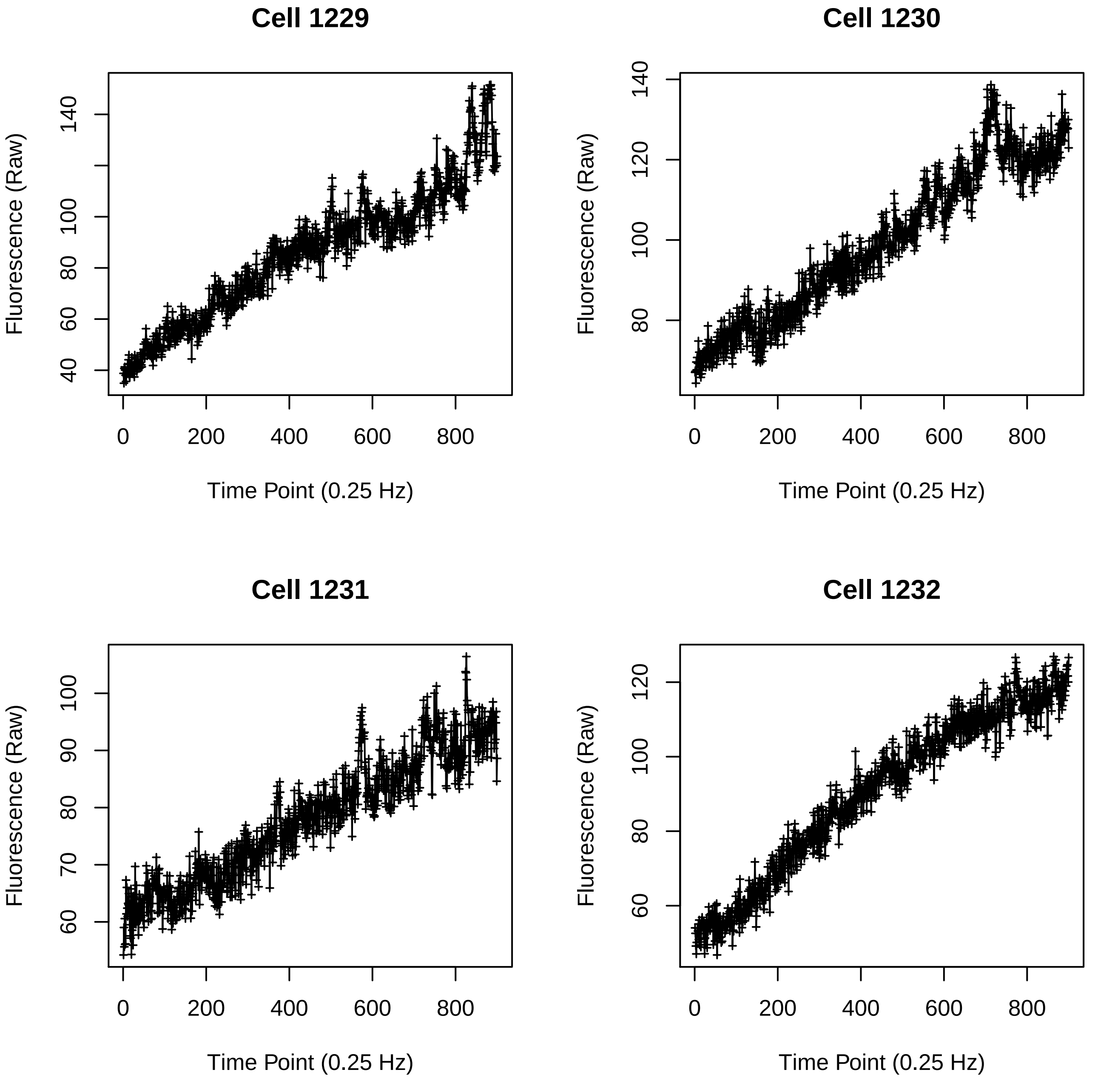

Cell 1229
Fluorescence (Raw)
40
60
80
100
140
0
200
400
600
800
Time Point (0.25 Hz)
Cell 1230
Fluorescence (Raw)
80
100
120
140
0
200
400
600
800
Time Point (0.25 Hz)
Cell 1231
Fluorescence (Raw)
60
70
80
90
100
0
200
400
600
800
Time Point (0.25 Hz)
Cell 1232
Fluorescence (Raw)
60
80
100
120
0
200
400
600
800
Time Point (0.25 Hz)

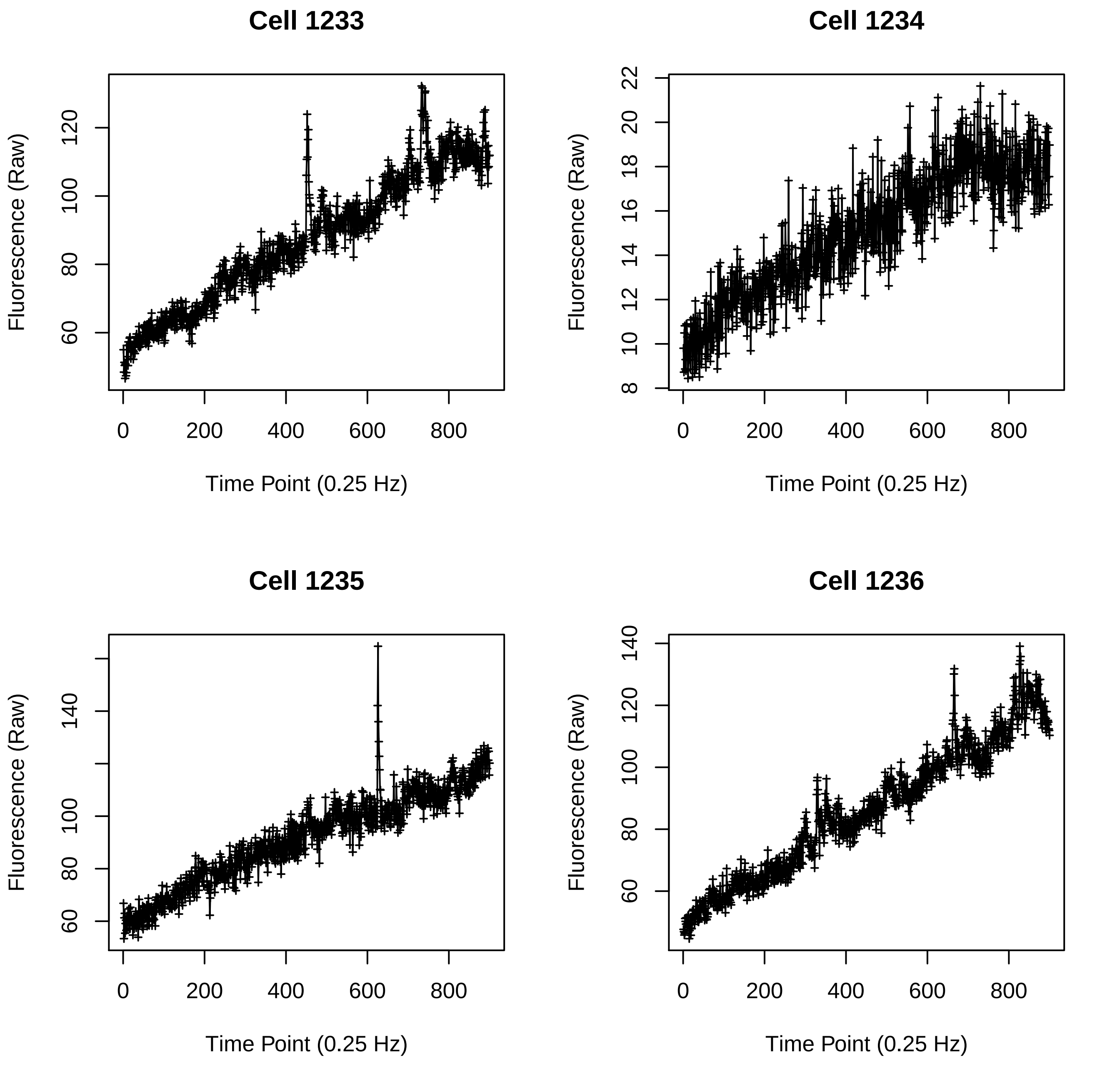
Cell 1233
Cell 1234
Cell 1235
Cell 1236
Fluorescence (Raw)
Time Point (0.25 Hz)
60
80
100
120
8
10
12
14
16
18
20
22
140
0
200
400
600
800

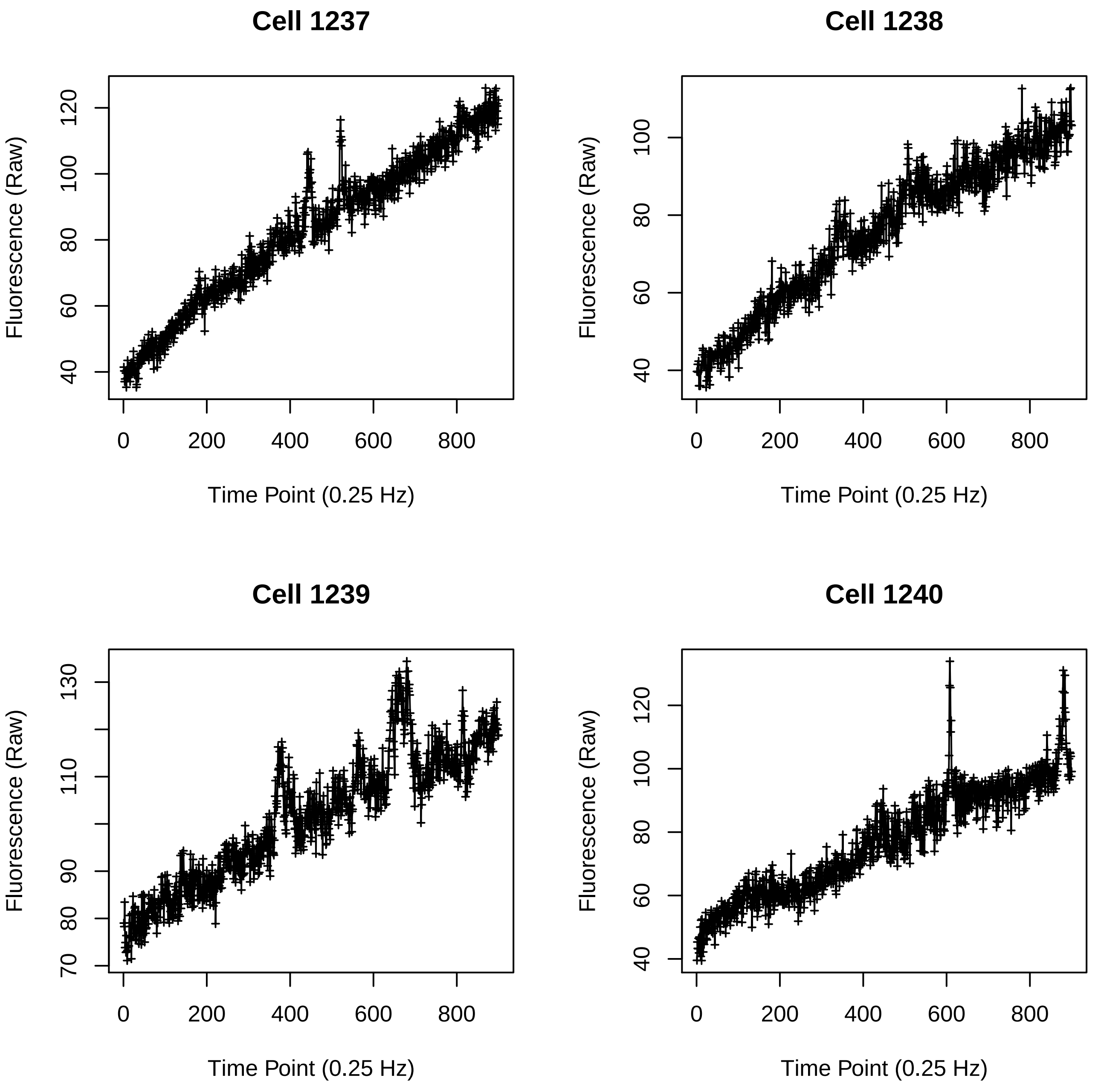
Cell 1237
Cell 1238
Cell 1239
Cell 1240
Fluorescence (Raw)
Time Point (0.25 Hz)
0
200
400
600
800
40
60
80
100
120
70
90
110
130

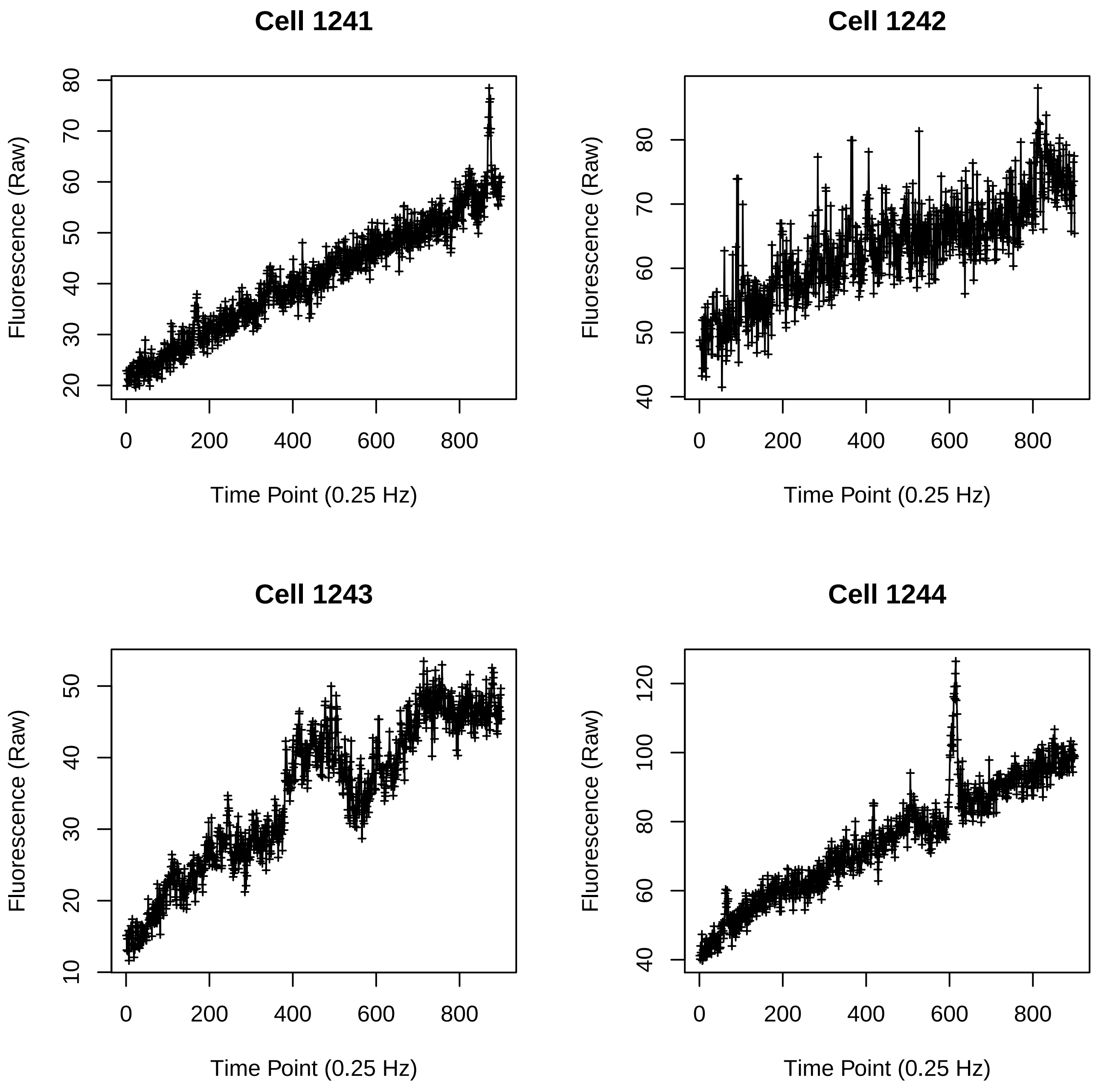
Cell 1241
Cell 1242
Cell 1243
Cell 1244
Fluorescence (Raw)
Time Point (0.25 Hz)
0
200
400
600
800
20
30
40
50
60
70
80
90
10
100
120

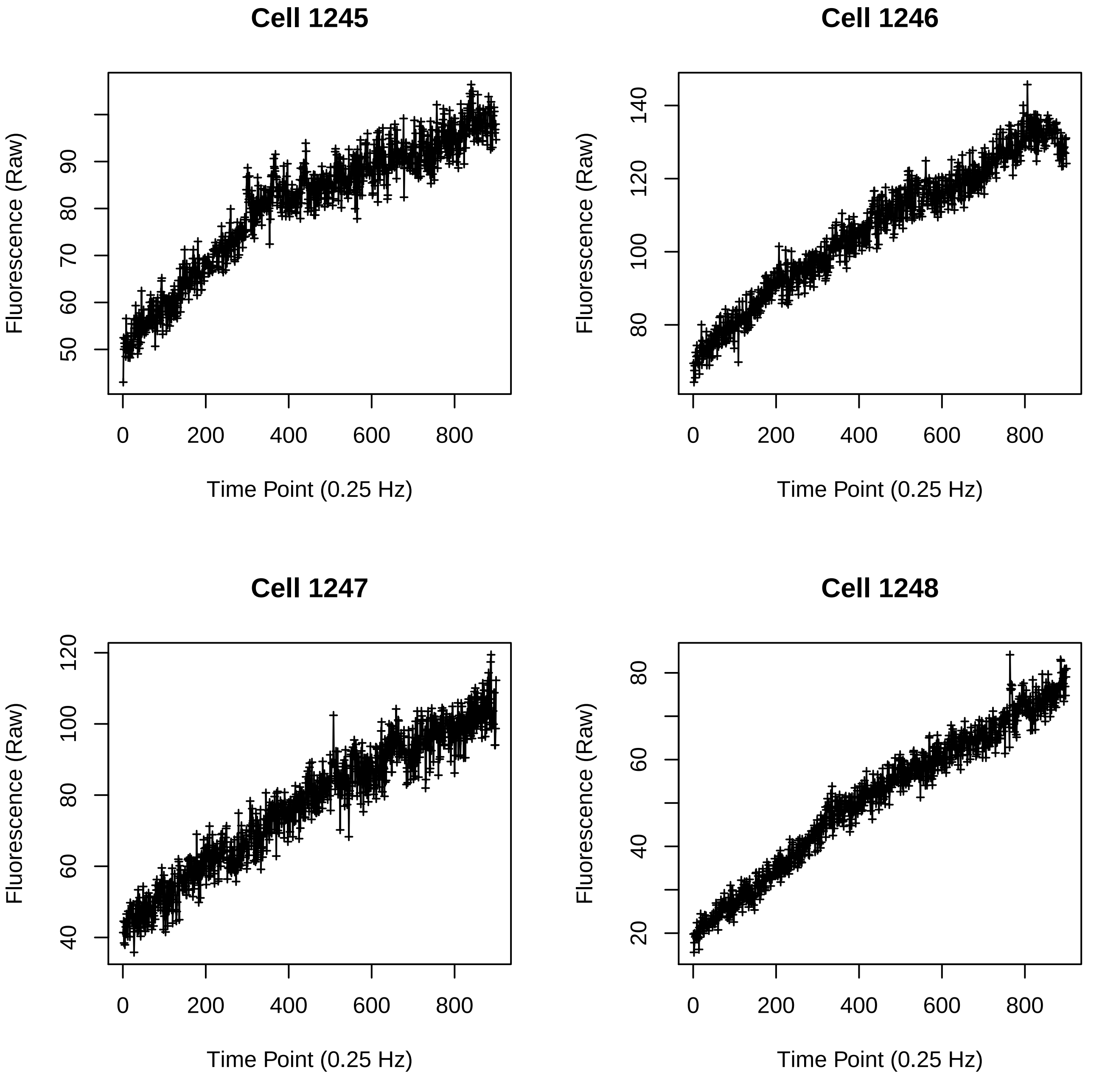
Cell 1245
Cell 1246
Cell 1247
Cell 1248
Fluorescence (Raw)
Time Point (0.25 Hz)
0
200
400
600
800
50
60
70
80
90
100
120
140
40
20

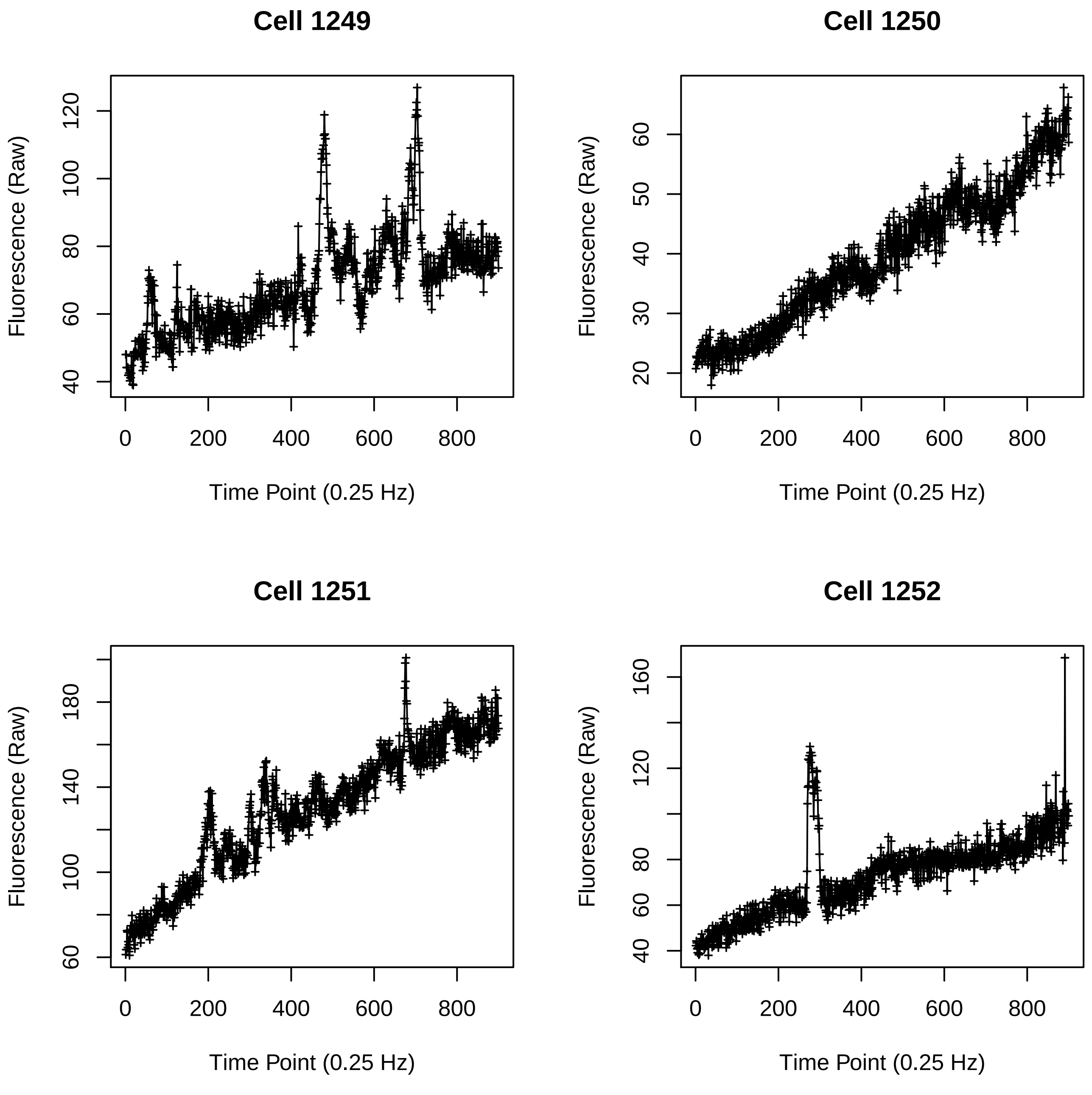

Cell 1249
Fluorescence (Raw)
40
60
80
100
120
0
200
400
600
800
Time Point (0.25 Hz)
Cell 1250
Fluorescence (Raw)
20
30
40
50
60
0
200
400
600
800
Time Point (0.25 Hz)
Cell 1251
Fluorescence (Raw)
60
100
140
180
0
200
400
600
800
Time Point (0.25 Hz)
Cell 1252
Fluorescence (Raw)
40
60
80
120
160
0
200
400
600
800
Time Point (0.25 Hz)

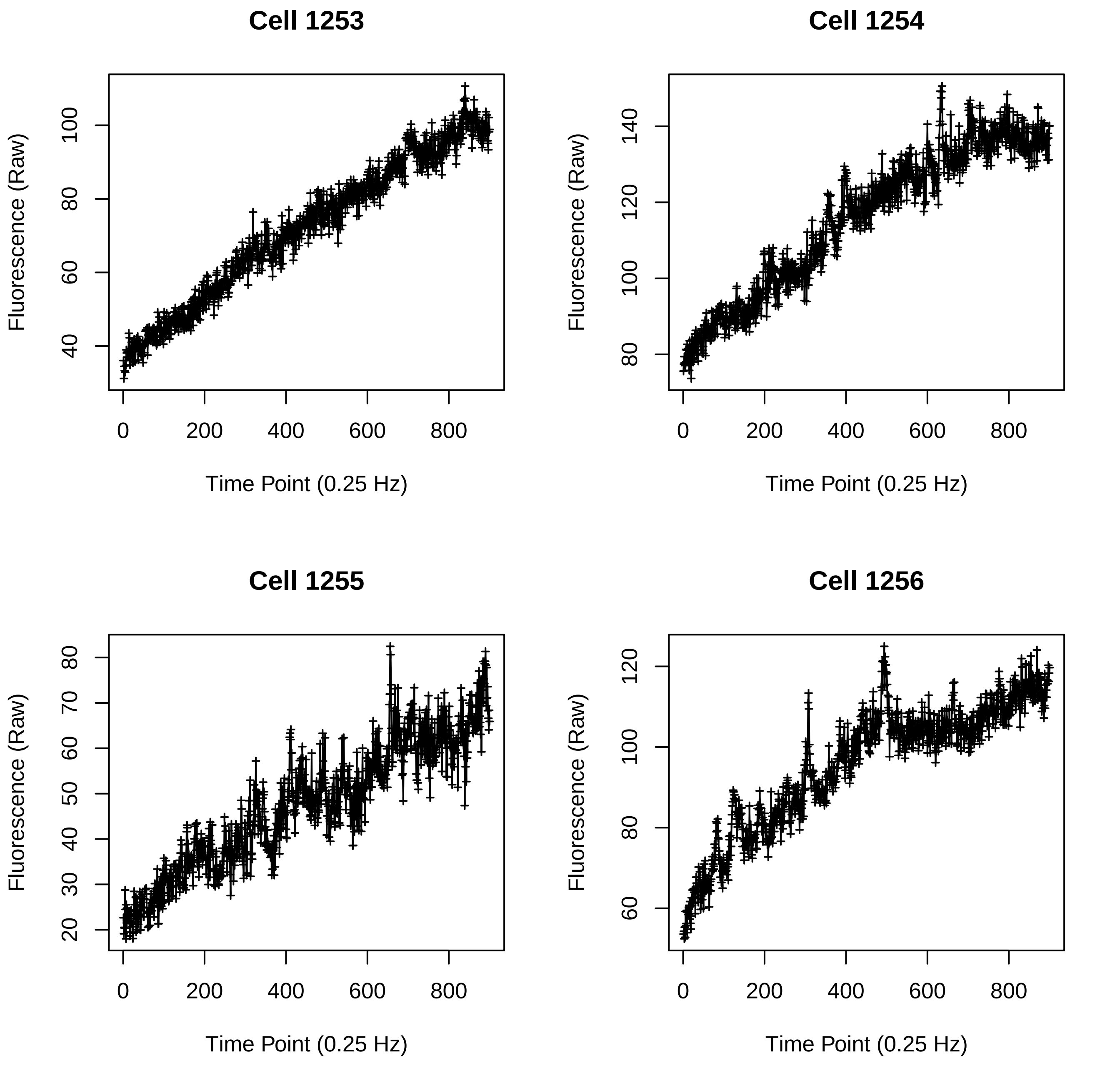
Cell 1253
Cell 1254
Cell 1255
Cell 1256
Fluorescence (Raw)
Time Point (0.25 Hz)
0
200
400
600
800
40
60
80
100
120
140
20
30
50
70

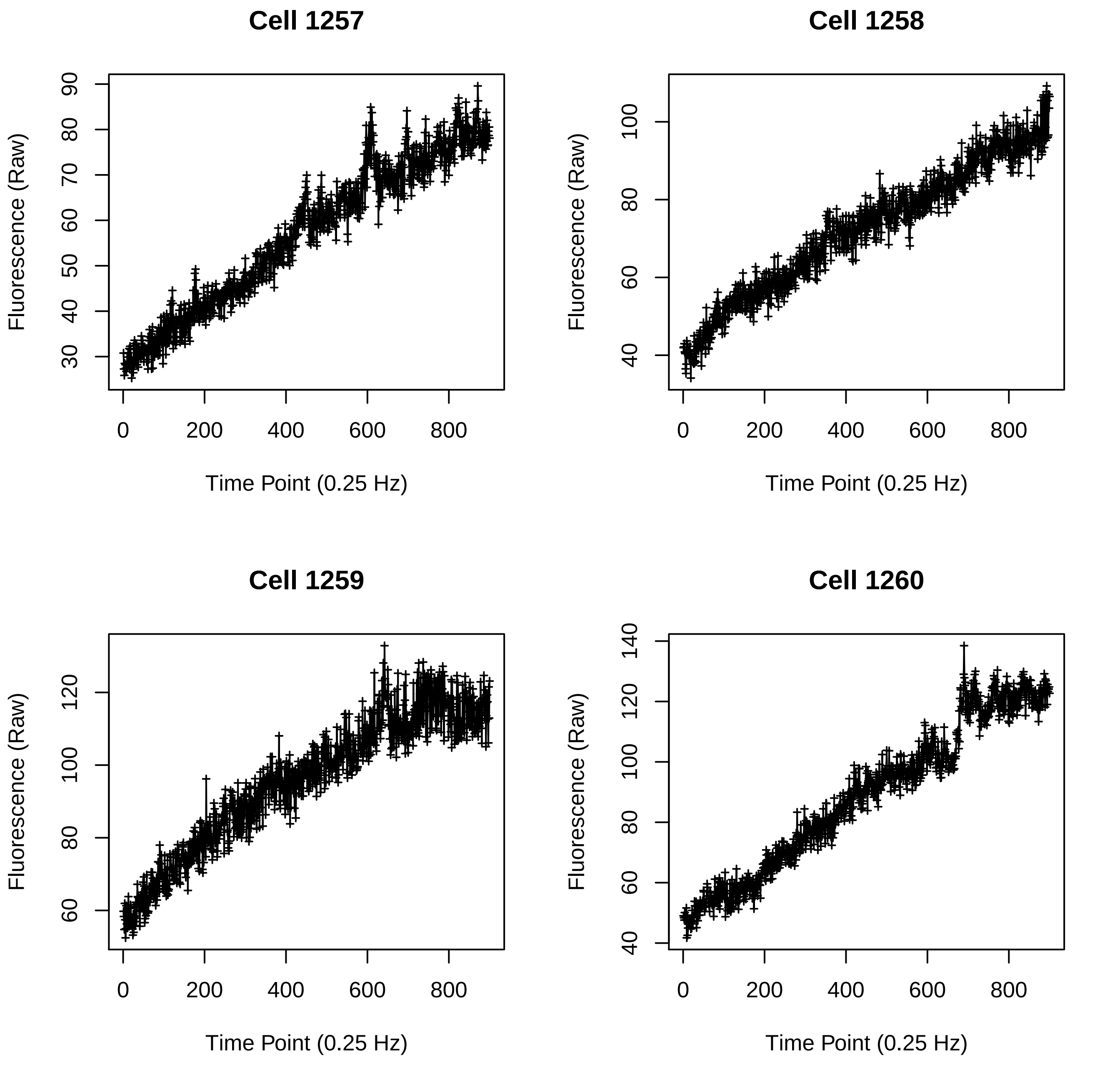
Cell 1257
Cell 1258
Cell 1259
Cell 1260
Fluorescence (Raw)
Time Point (0.25 Hz)
0
200
400
600
800
30
40
50
60
70
80
90
100
120
140

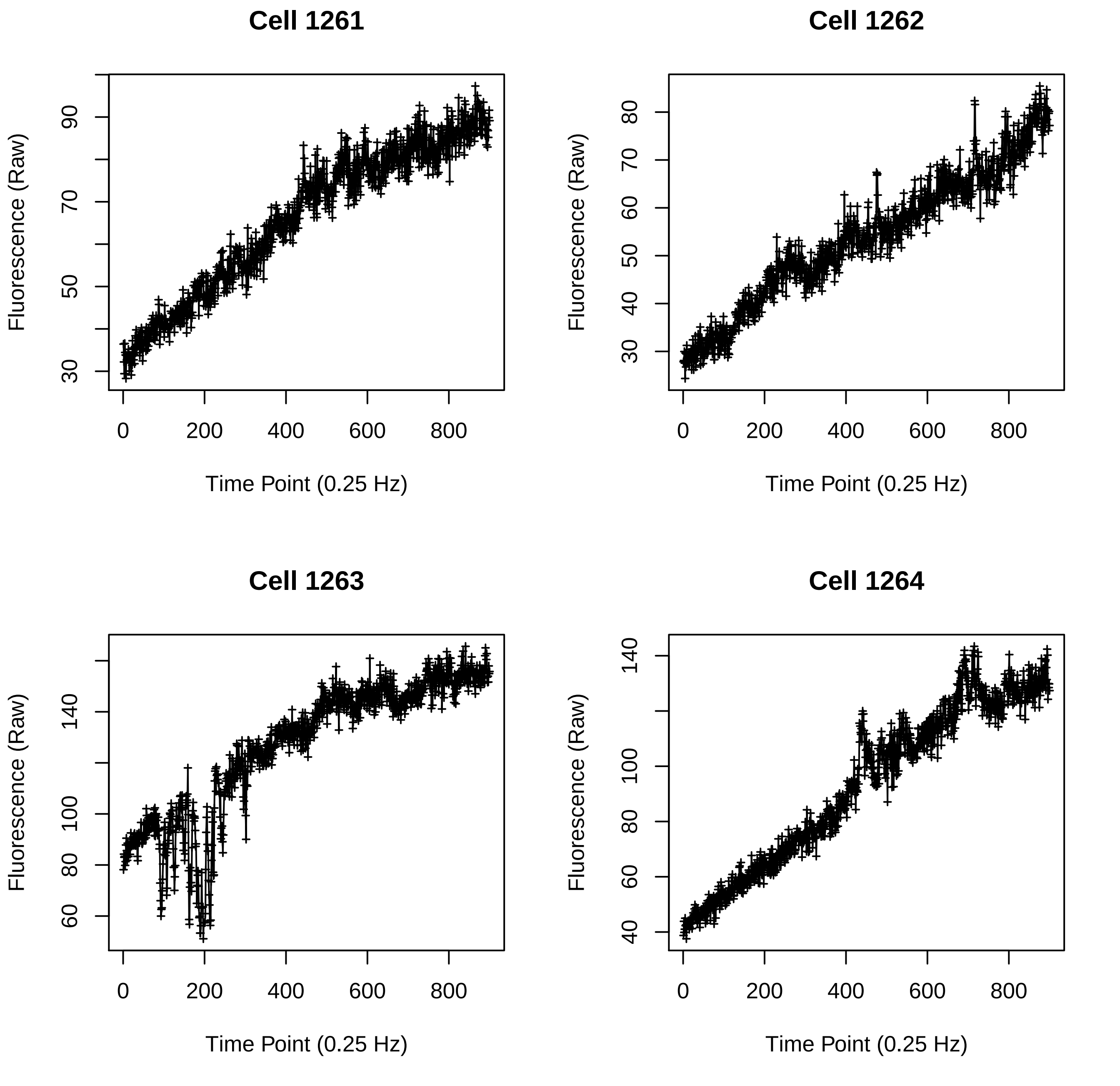

Cell 1261
Cell 1262
Cell 1263
Cell 1264
Fluorescence (Raw)
Time Point (0.25 Hz)
0
200
400
600
800
30
50
70
90
40
60
80
100
140

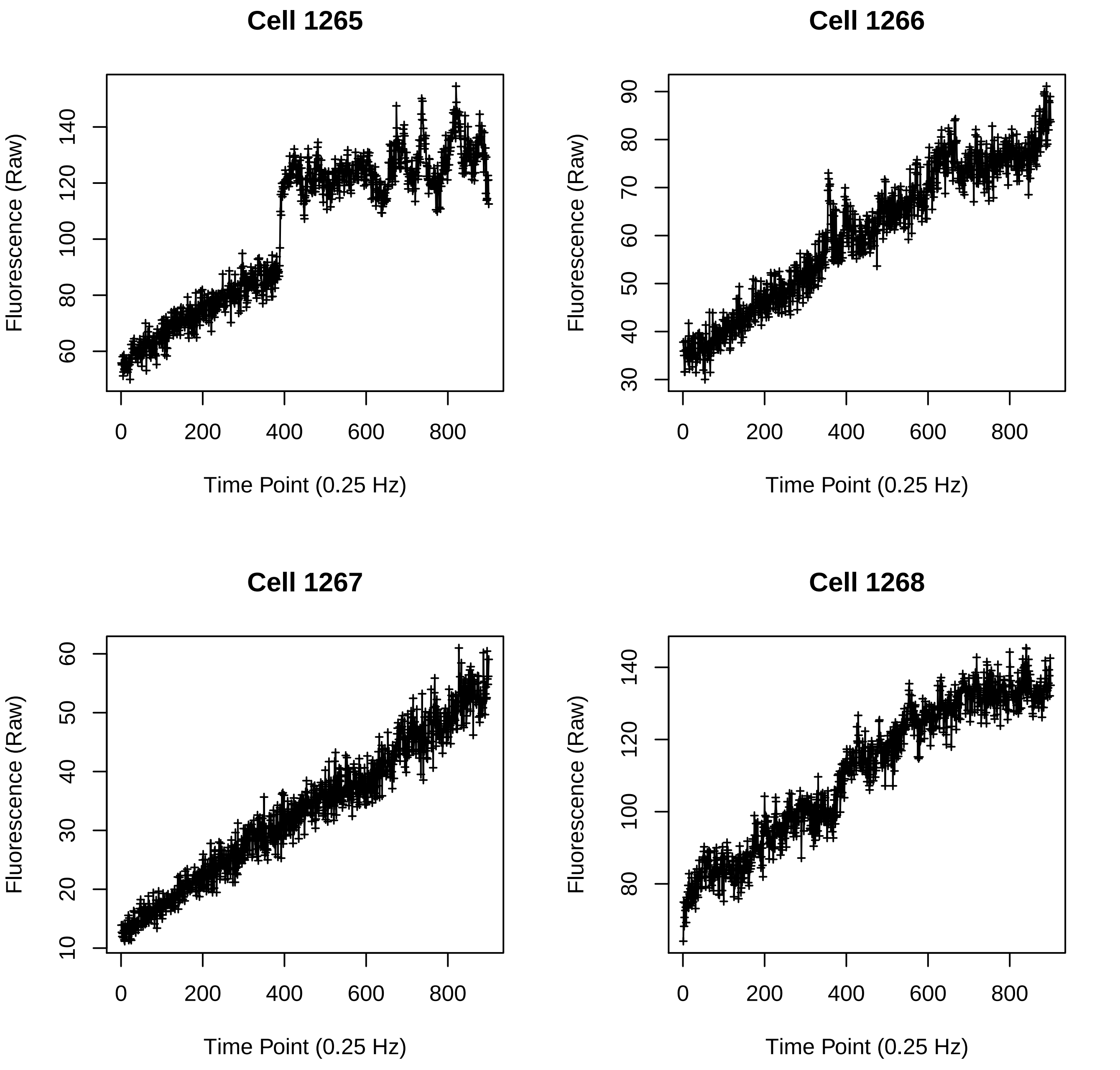

Cell 1265
Fluorescence (Raw)
60
80
100
120
140
0
200
400
600
800
Time Point (0.25 Hz)
Cell 1266
Fluorescence (Raw)
30
40
50
60
70
80
90
0
200
400
600
800
Time Point (0.25 Hz)
Cell 1267
Fluorescence (Raw)
10
20
30
40
50
60
0
200
400
600
800
Time Point (0.25 Hz)
Cell 1268
Fluorescence (Raw)
80
100
120
140
0
200
400
600
800
Time Point (0.25 Hz)

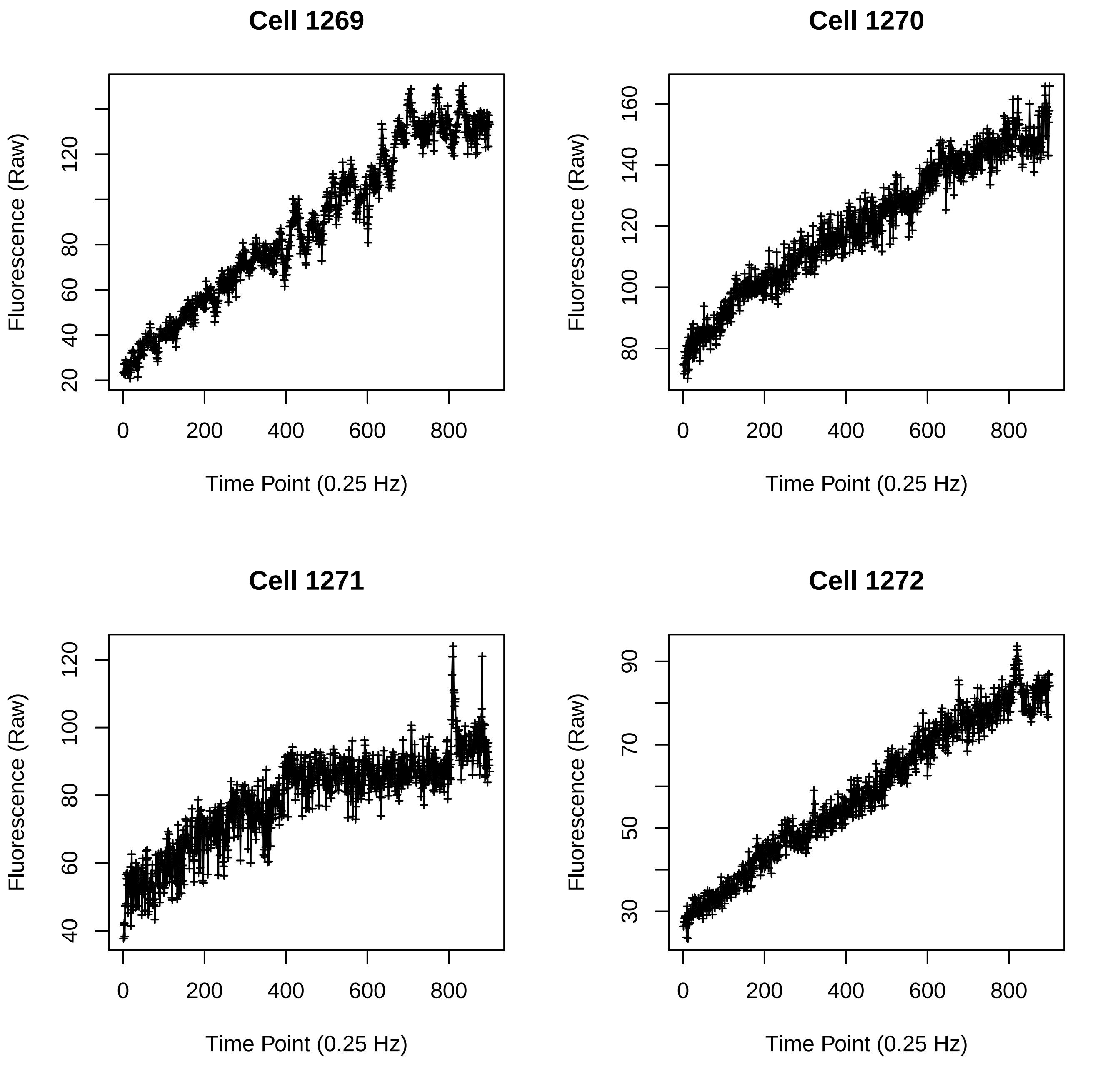
Cell 1269
Fluorescence (Raw)
20
40
60
80
120
0
200
400
600
800
Time Point (0.25 Hz)
Cell 1270
Fluorescence (Raw)
80
100
120
140
160
0
200
400
600
800
Time Point (0.25 Hz)
Cell 1271
Fluorescence (Raw)
40
60
80
100
120
0
200
400
600
800
Time Point (0.25 Hz)
Cell 1272
Fluorescence (Raw)
30
50
70
90
0
200
400
600
800
Time Point (0.25 Hz)

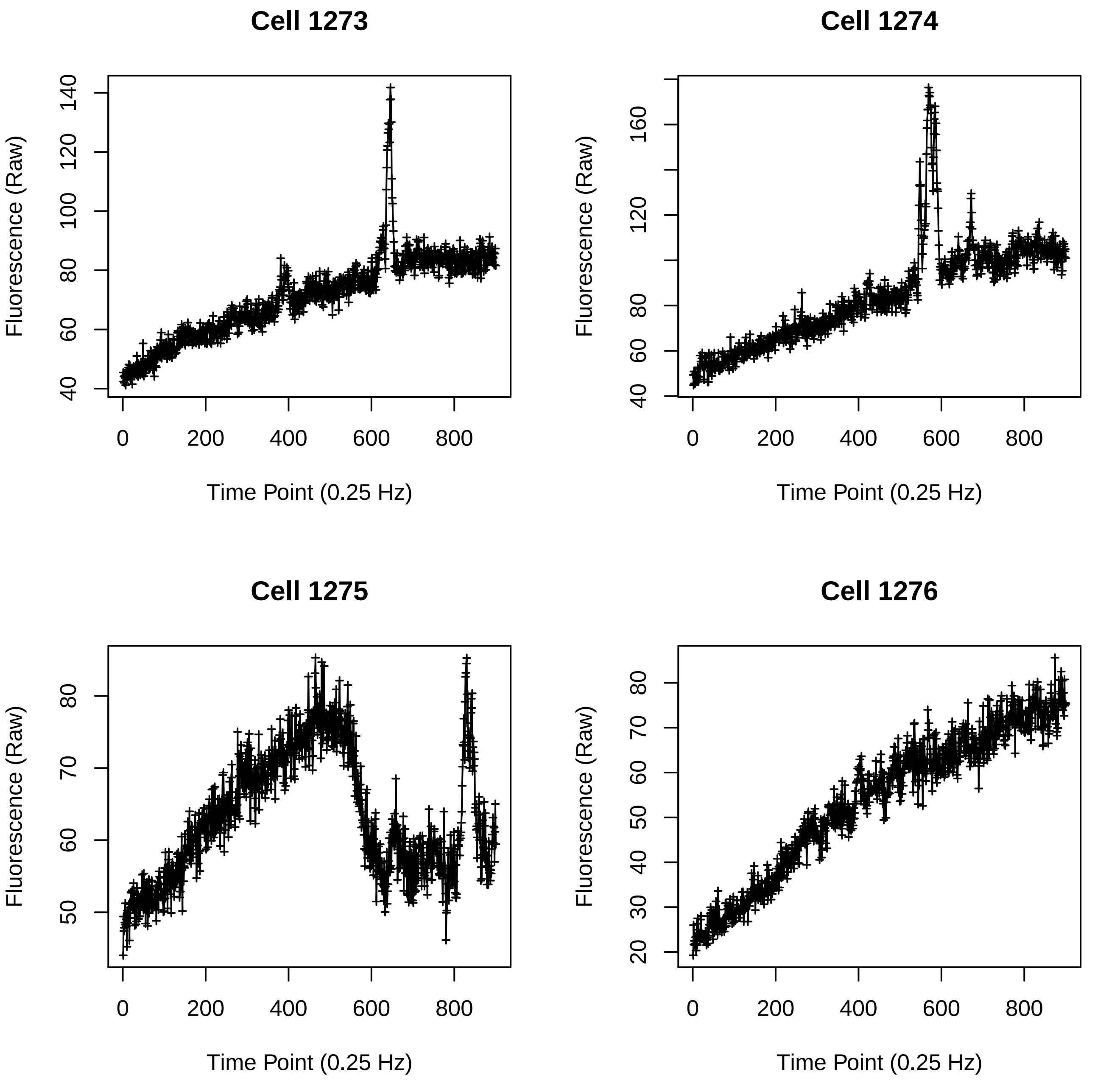
Cell 1273
Fluorescence (Raw)
40
60
80
100
120
140
0
200
400
600
800
Time Point (0.25 Hz)
Cell 1274
Fluorescence (Raw)
40
60
80
120
160
0
200
400
600
800
Time Point (0.25 Hz)
Cell 1275
Fluorescence (Raw)
50
60
70
80
0
200
400
600
800
Time Point (0.25 Hz)
Cell 1276
Fluorescence (Raw)
20
30
40
50
60
70
80
0
200
400
600
800
Time Point (0.25 Hz)

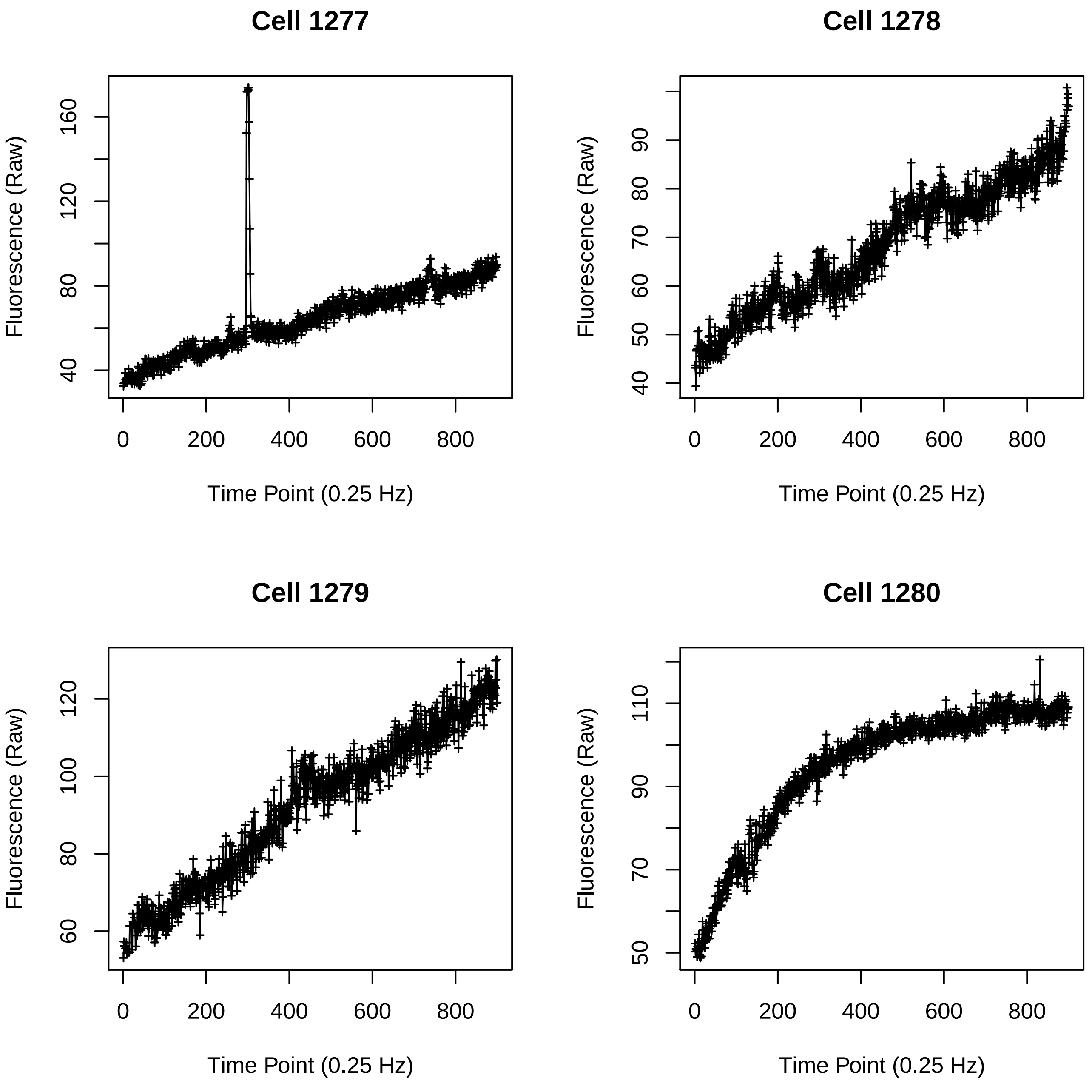
Cell 1277
Fluorescence (Raw)
40
80
120
160
0
200
400
600
800
Time Point (0.25 Hz)
Cell 1278
Fluorescence (Raw)
40
50
60
70
80
90
0
200
400
600
800
Time Point (0.25 Hz)
Cell 1279
Fluorescence (Raw)
60
80
100
120
0
200
400
600
800
Time Point (0.25 Hz)
Cell 1280
Fluorescence (Raw)
50
70
90
110
0
200
400
600
800
Time Point (0.25 Hz)

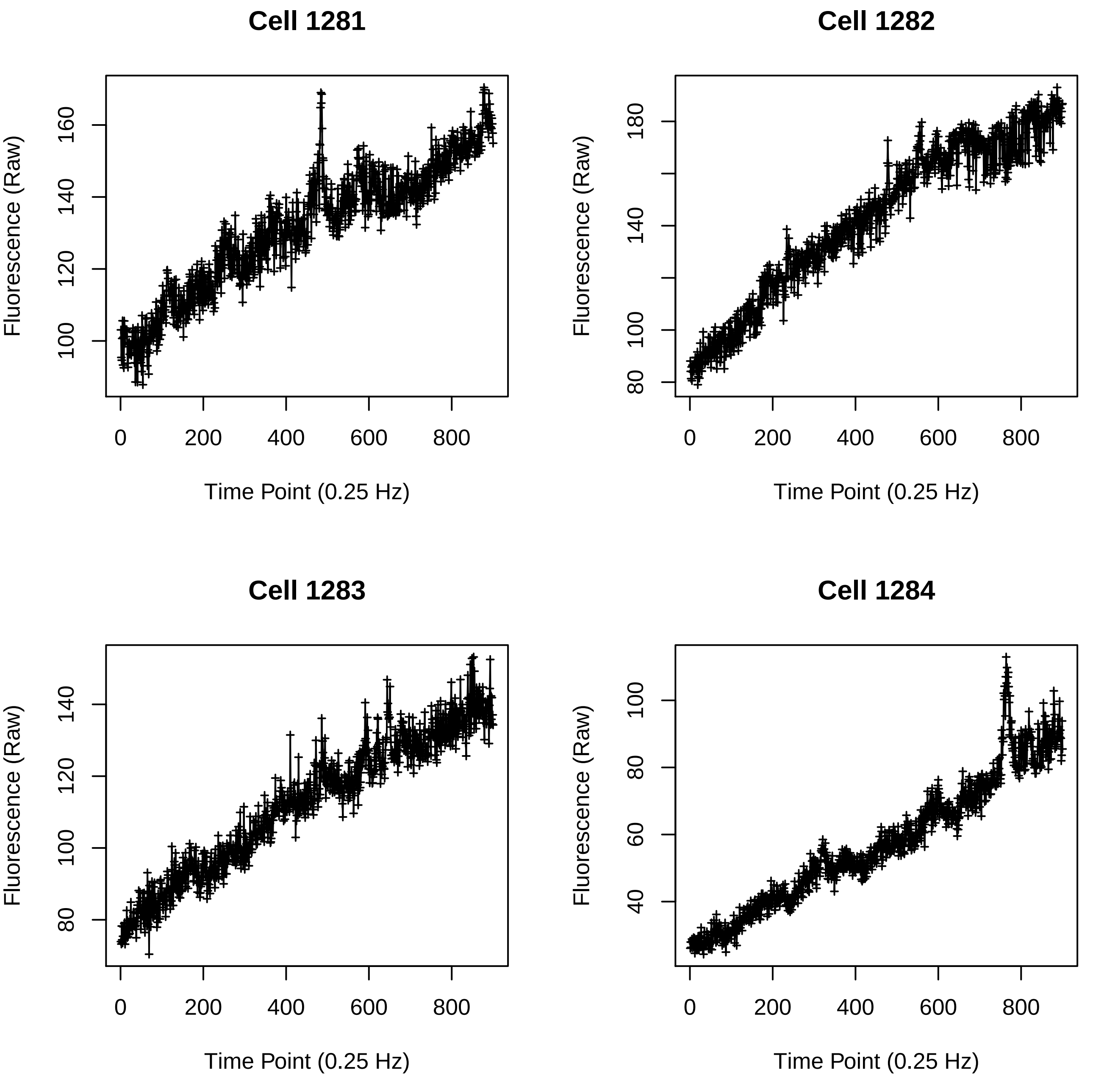
Cell 1281
Cell 1282
Cell 1283
Cell 1284
Fluorescence (Raw)
Time Point (0.25 Hz)
0
200
400
600
800
100
120
140
160
80
180
40
60

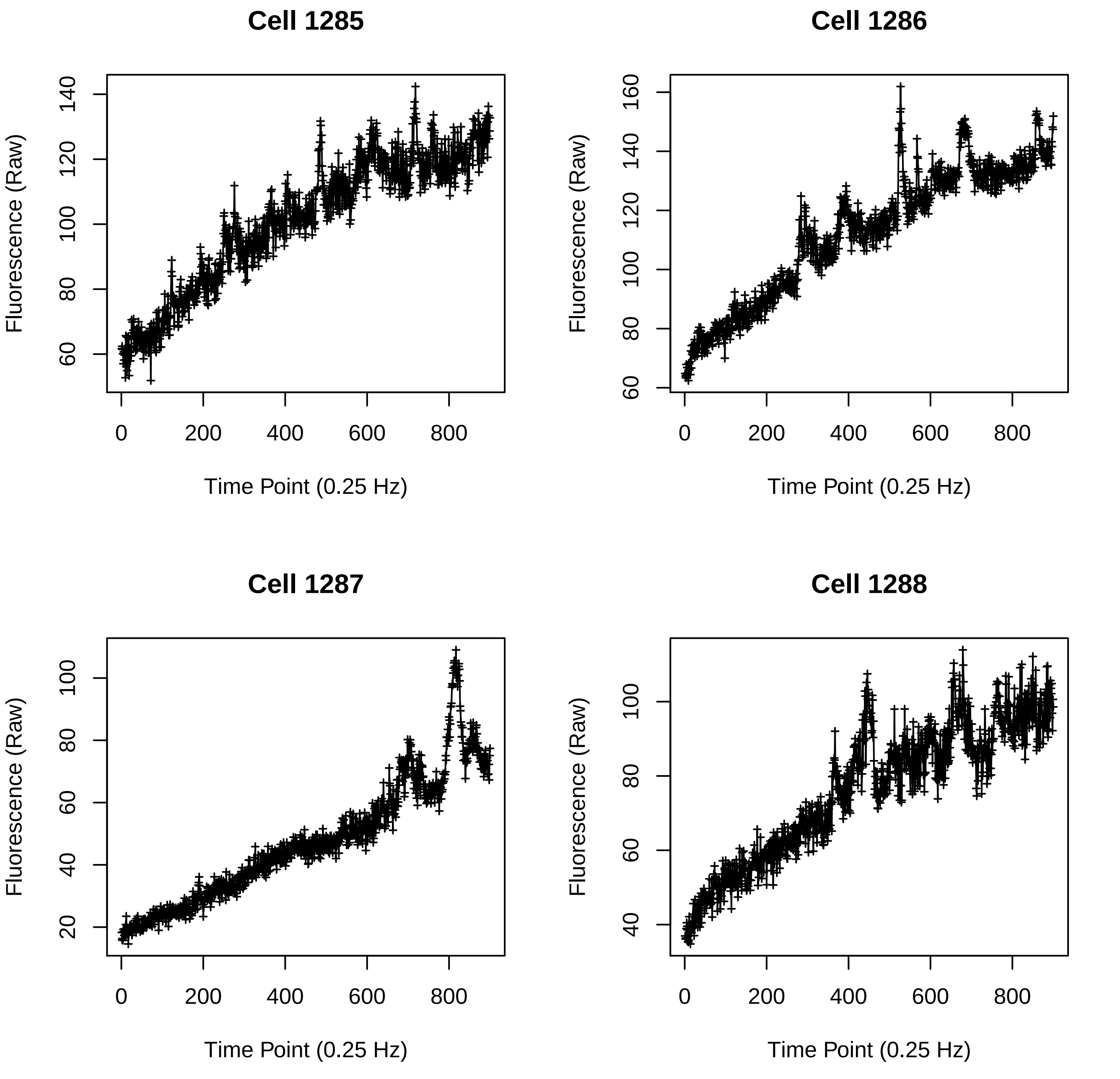
Cell 1285
Fluorescence (Raw)
60
80
100
120
140
0
200
400
600
800
Time Point (0.25 Hz)
Cell 1286
Fluorescence (Raw)
60
80
100
120
140
160
0
200
400
600
800
Time Point (0.25 Hz)
Cell 1287
Fluorescence (Raw)
20
40
60
80
100
0
200
400
600
800
Time Point (0.25 Hz)
Cell 1288
Fluorescence (Raw)
40
60
80
100
0
200
400
600
800
Time Point (0.25 Hz)

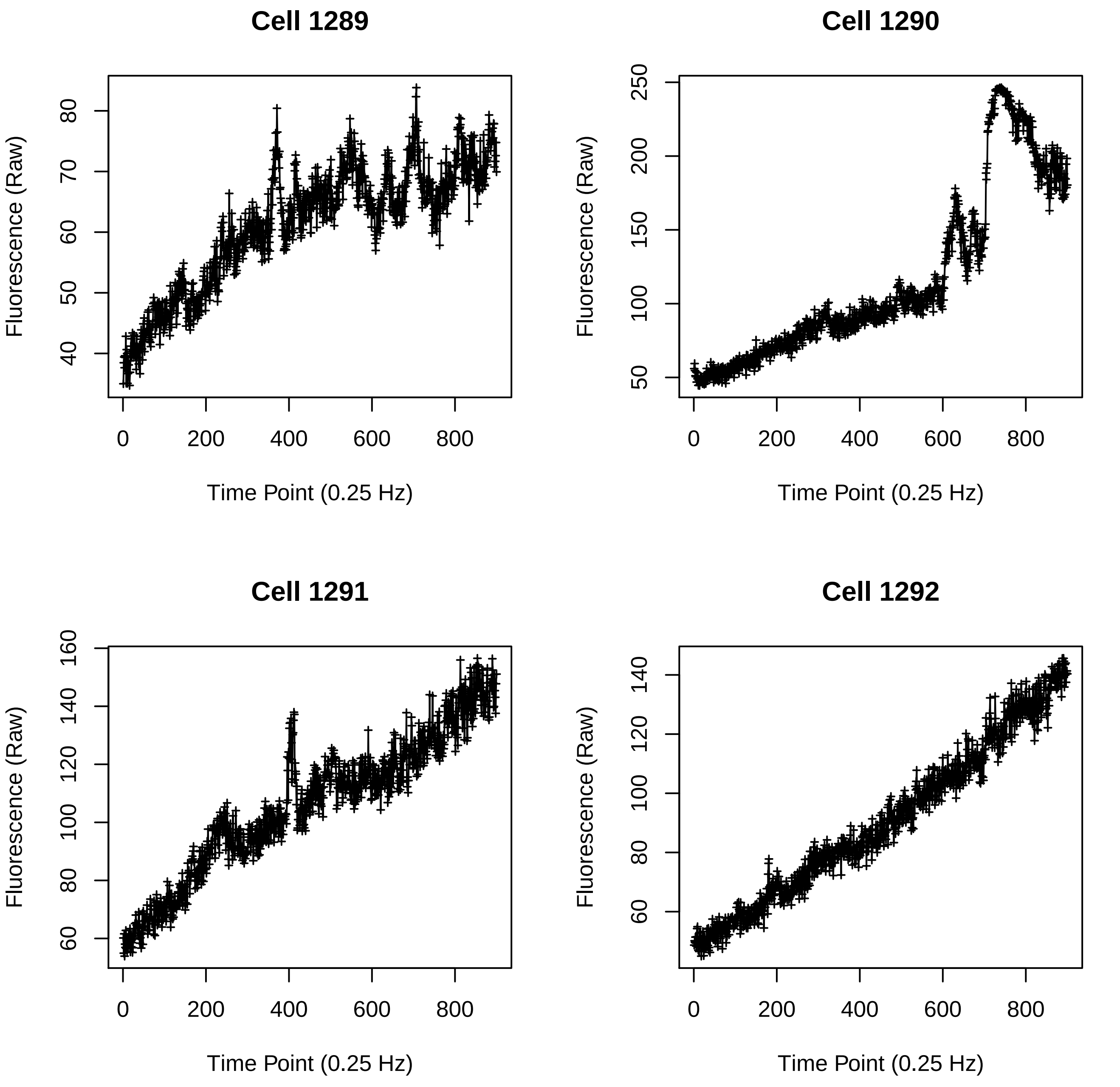
Cell 1289
Cell 1290
Cell 1291
Cell 1292
Fluorescence (Raw)
Time Point (0.25 Hz)
0
200
400
600
800
40
50
60
70
80
100
150
200
250
120
140
160

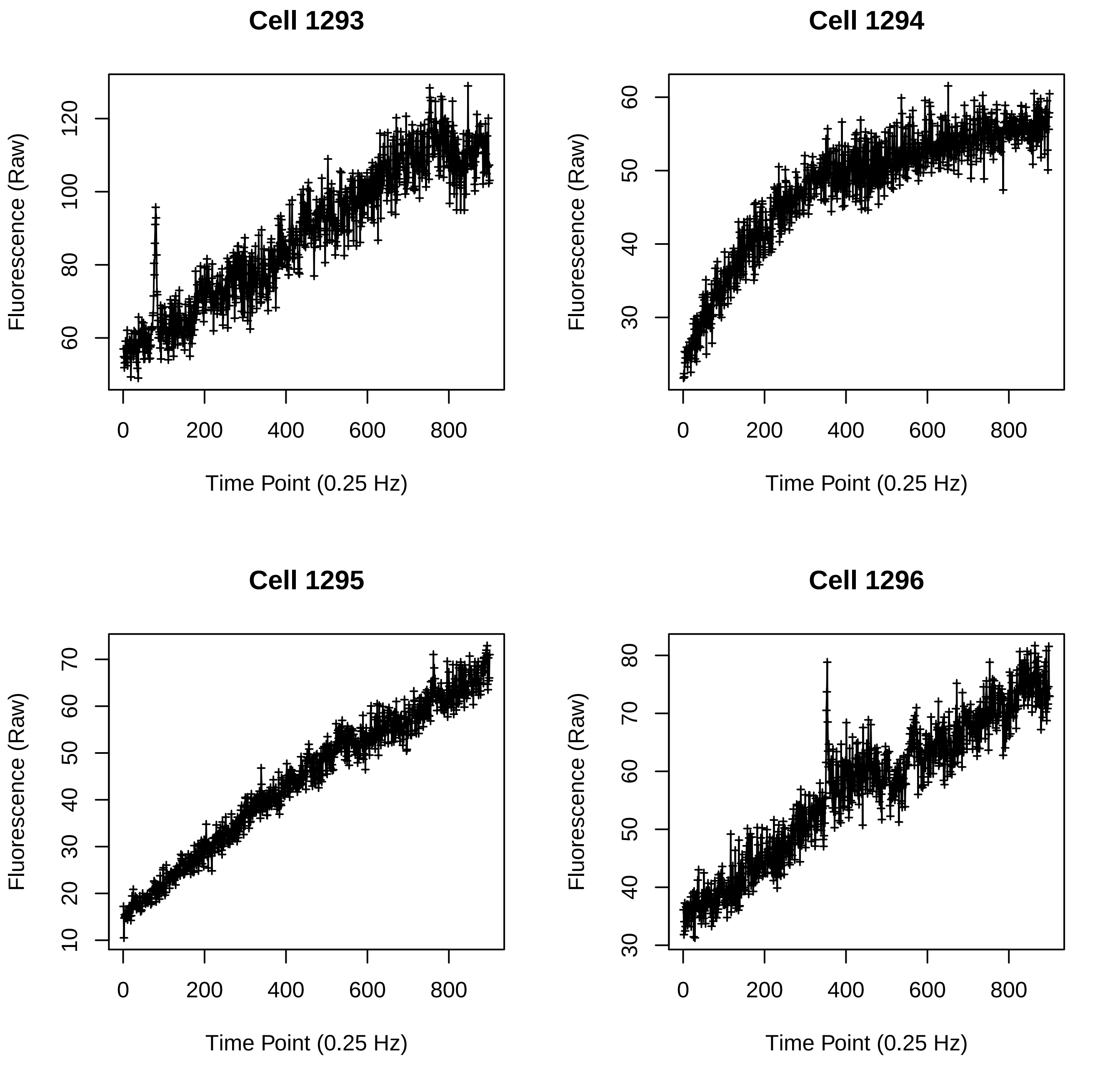

Cell 1293
Cell 1294
Cell 1295
Cell 1296
Fluorescence (Raw)
Time Point (0.25 Hz)
0
200
400
600
800
60
80
100
120
30
40
50
60
10
20
70

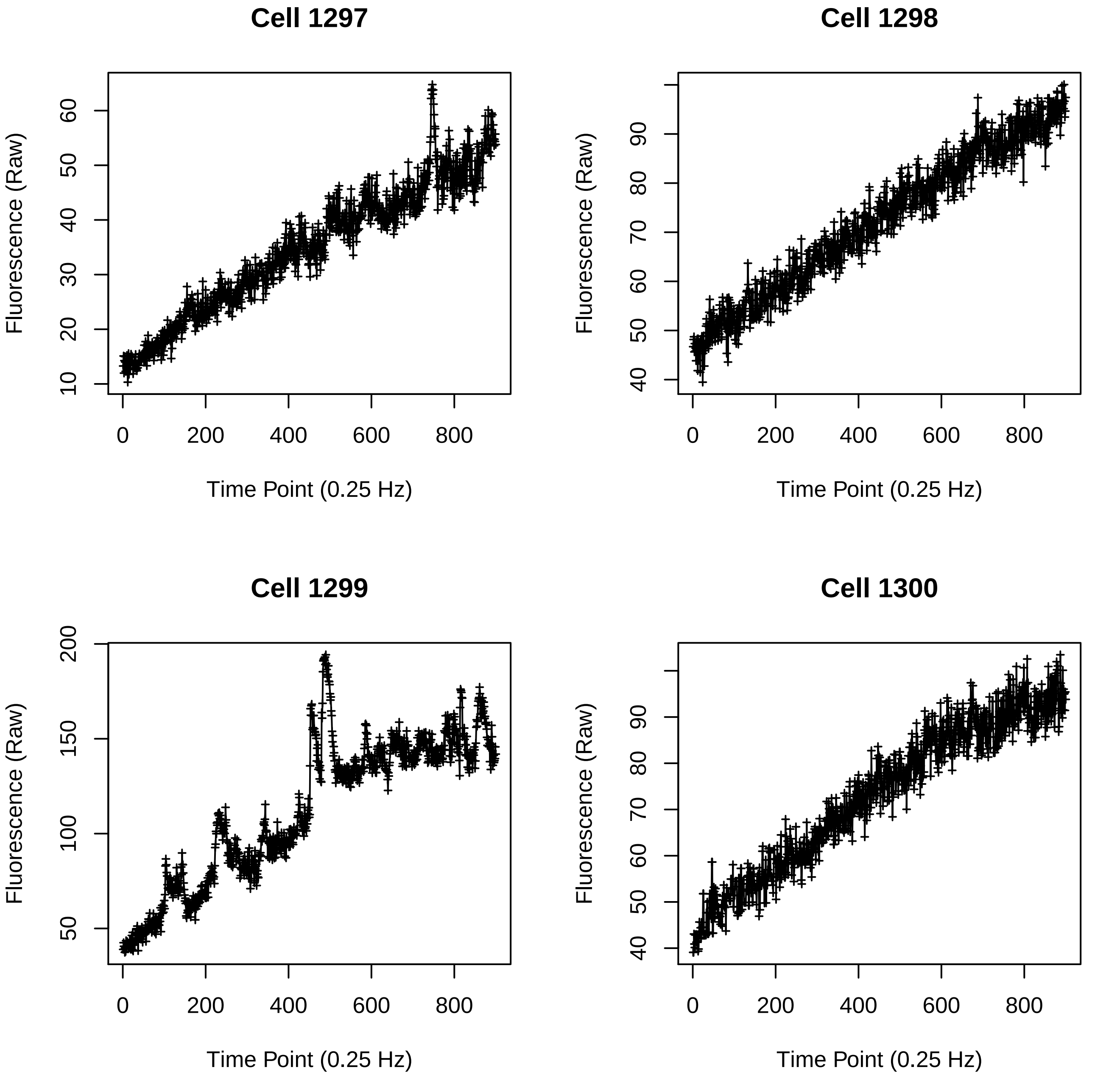
Cell 1297
Fluorescence (Raw)
10
20
30
40
50
60
0
200
400
600
800
Time Point (0.25 Hz)
Cell 1298
Fluorescence (Raw)
40
50
60
70
80
90
0
200
400
600
800
Time Point (0.25 Hz)
Cell 1299
Fluorescence (Raw)
50
100
150
200
0
200
400
600
800
Time Point (0.25 Hz)
Cell 1300
Fluorescence (Raw)
40
50
60
70
80
90
0
200
400
600
800
Time Point (0.25 Hz)

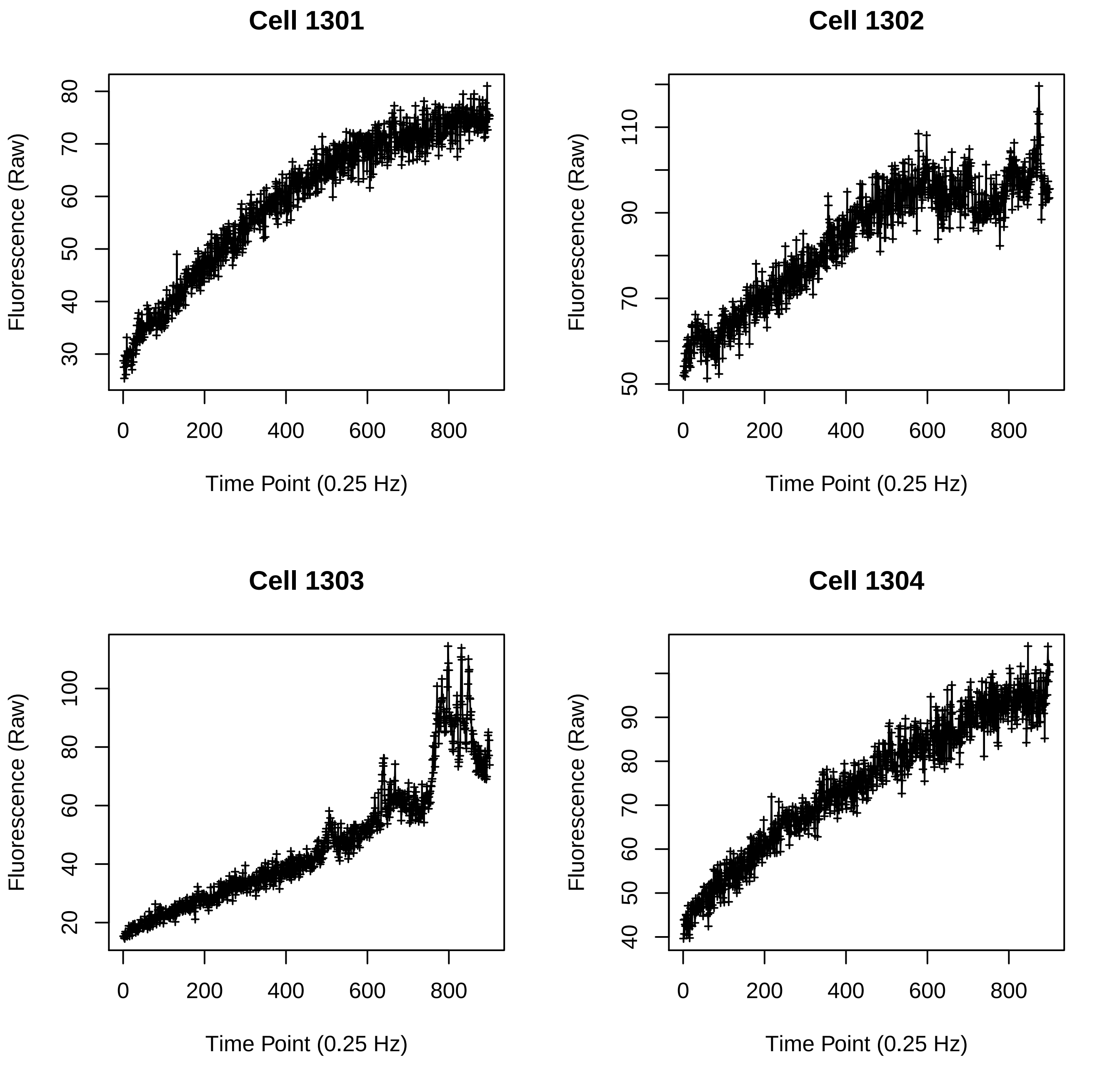
Cell 1301
Cell 1302
Cell 1303
Cell 1304
Fluorescence (Raw)
Time Point (0.25 Hz)
0
200
400
600
800
30
40
50
60
70
80
50
70
90
110
20
40
60
80
100
40
50
60
70
80
90

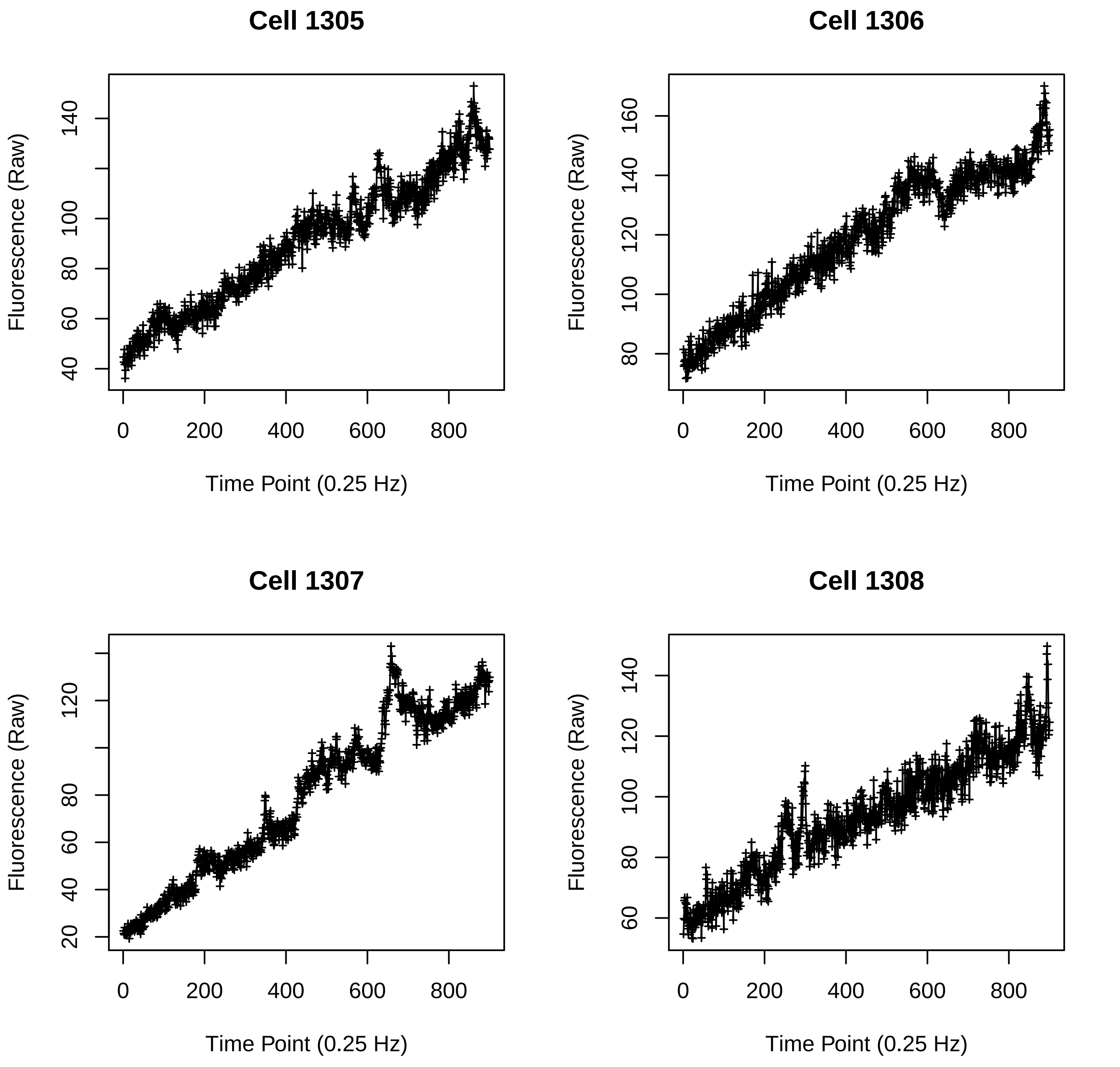

Cell 1305
Fluorescence (Raw)
Time Point (0.25 Hz)
Cell 1306
Fluorescence (Raw)
Time Point (0.25 Hz)
Cell 1307
Fluorescence (Raw)
Time Point (0.25 Hz)
Cell 1308
Fluorescence (Raw)
Time Point (0.25 Hz)

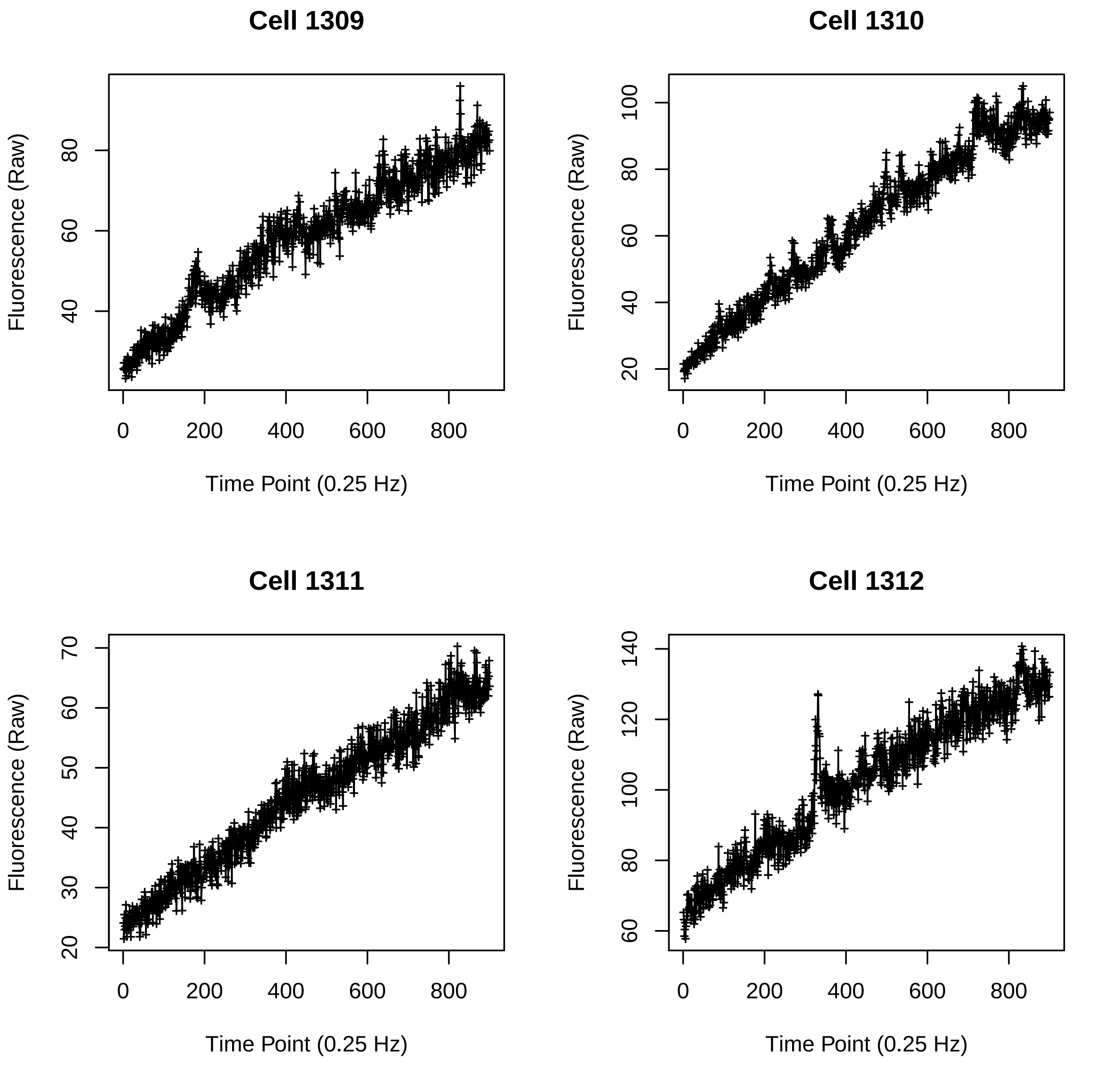

Cell 1309
Cell 1310
Cell 1311
Cell 1312
Fluorescence (Raw)
Time Point (0.25 Hz)
0
200
400
600
800
20
30
40
50
60
70
80
100
120
140

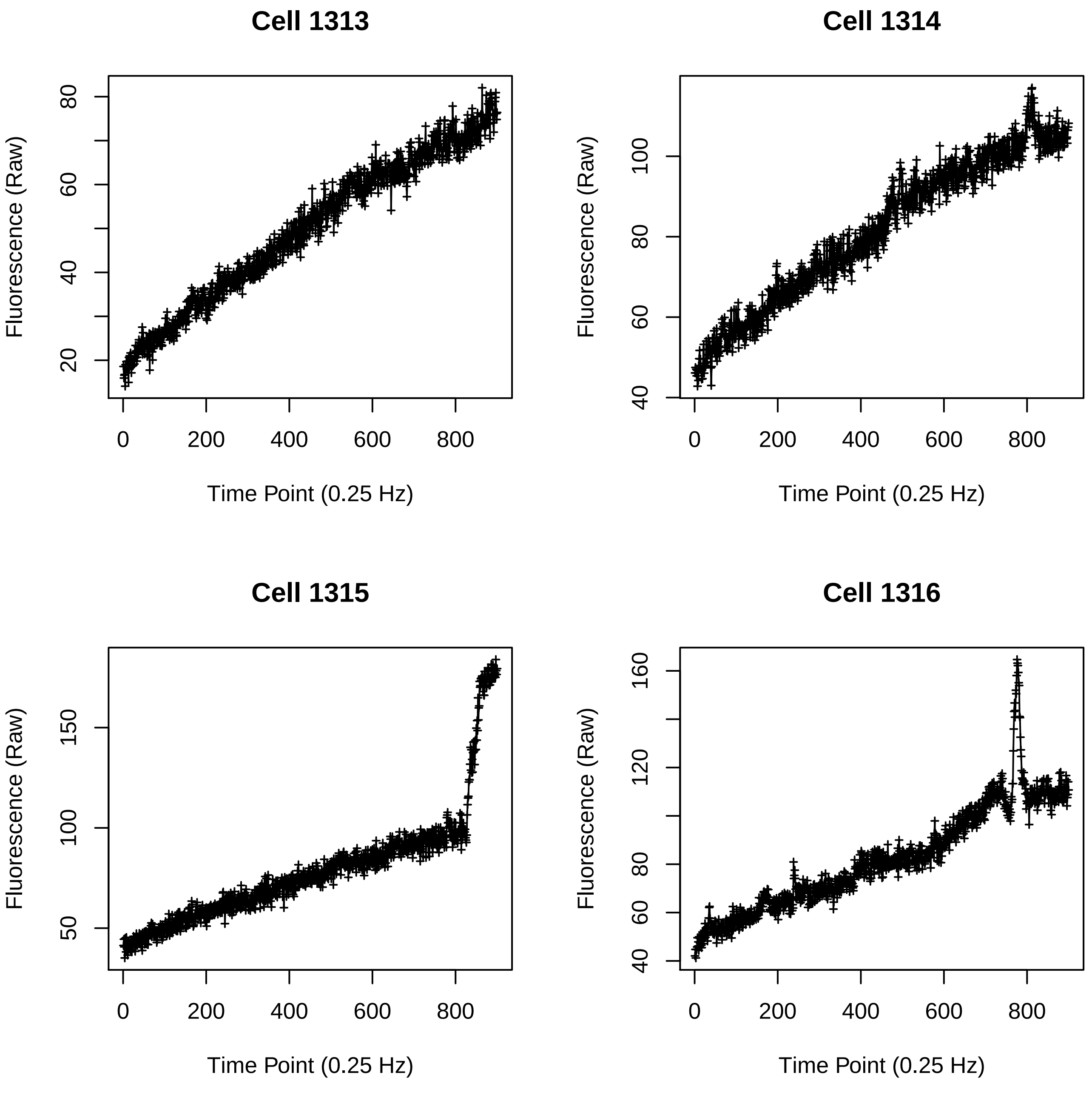

Cell 1313
Cell 1314
Cell 1315
Cell 1316
Fluorescence (Raw)
Time Point (0.25 Hz)
20
40
60
80
100
120
140
160
50
150
0
200
400
600
800

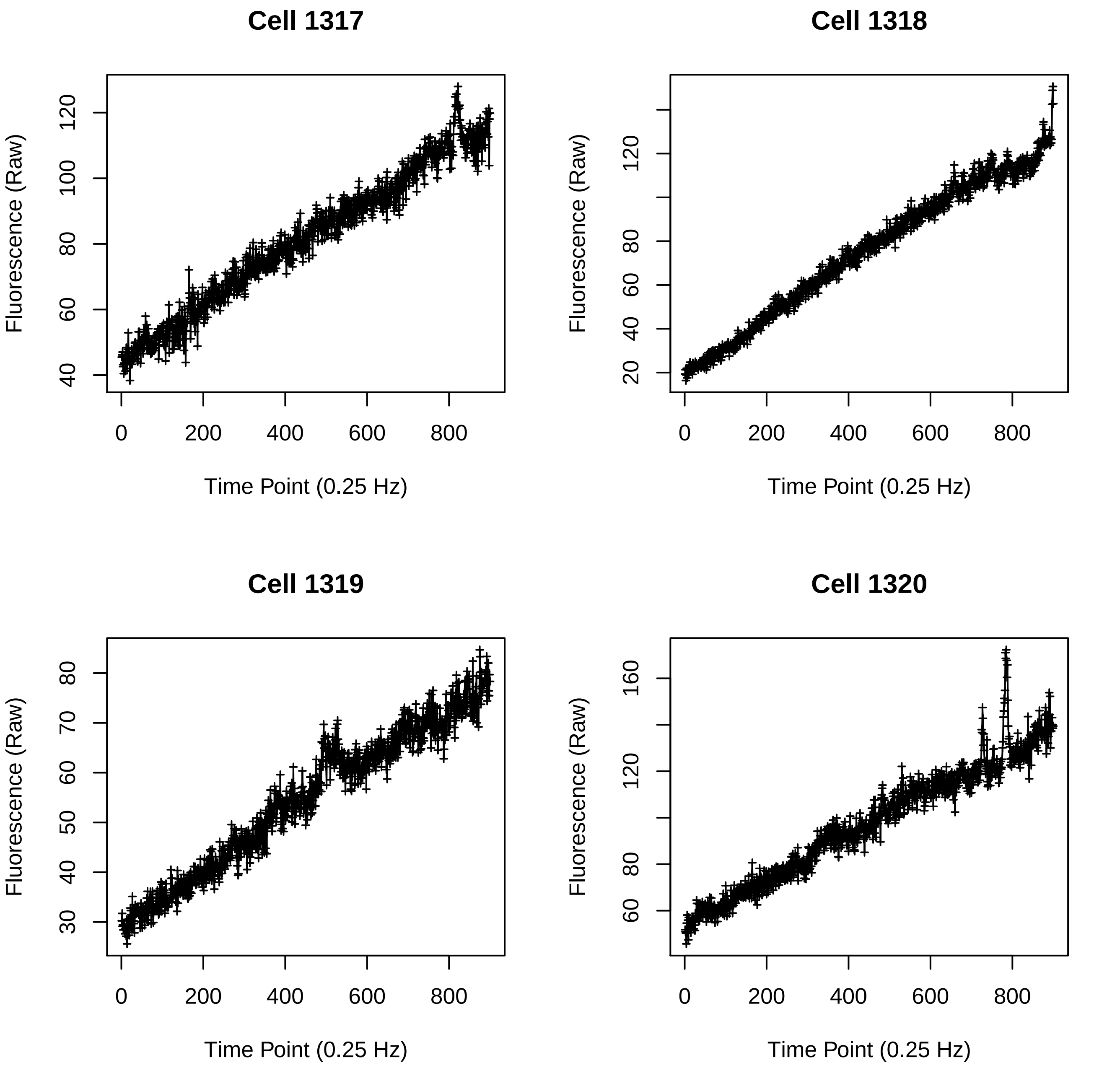
Cell 1317
Cell 1318
Cell 1319
Cell 1320
Fluorescence (Raw)
Time Point (0.25 Hz)
0
200
400
600
800
40
60
80
100
120
20
30
50
70
160

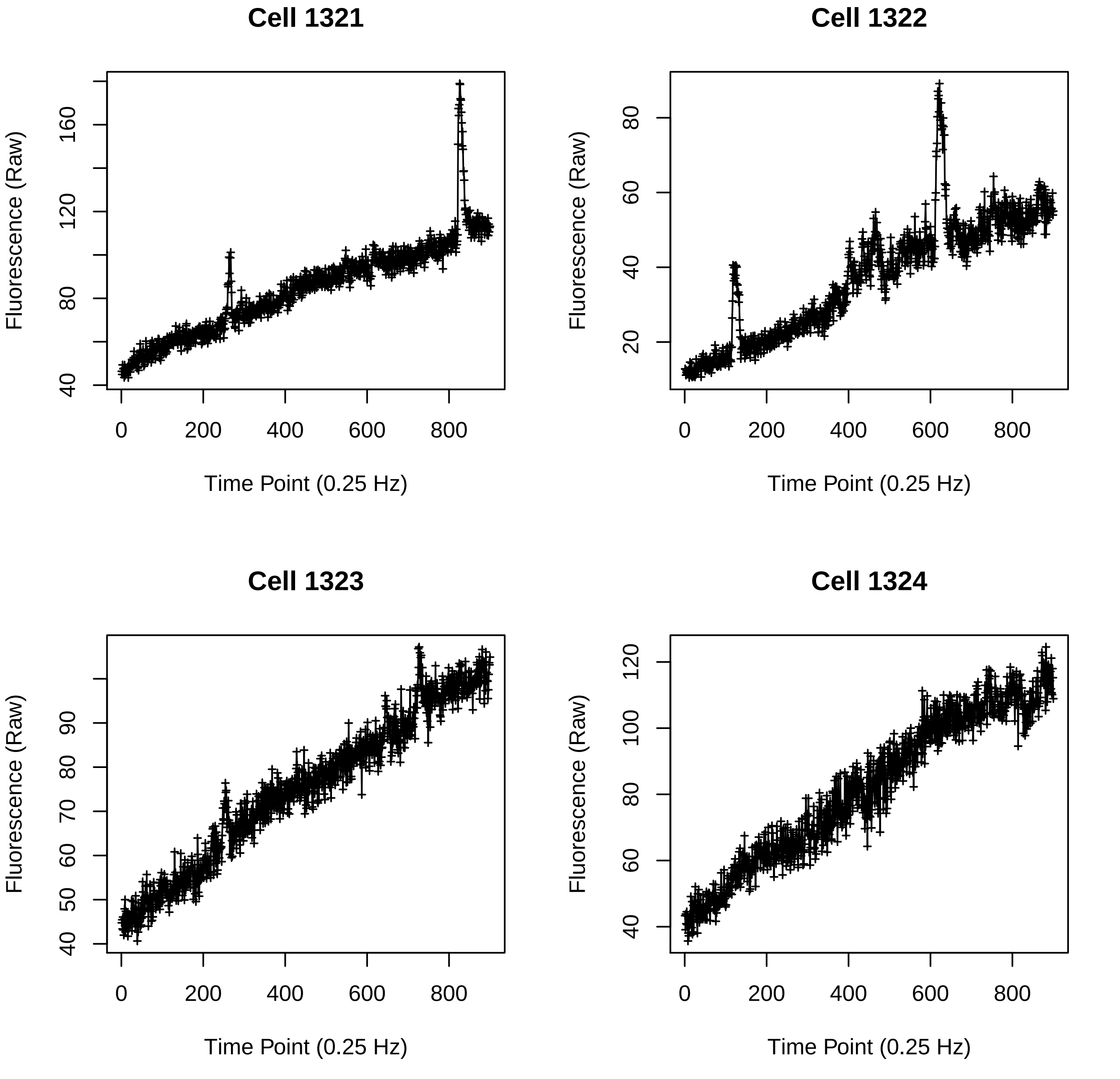
Cell 1321
Fluorescence (Raw)
40
80
120
160
0
200
400
600
800
Time Point (0.25 Hz)
Cell 1322
Fluorescence (Raw)
20
40
60
80
0
200
400
600
800
Time Point (0.25 Hz)
Cell 1323
Fluorescence (Raw)
40
50
60
70
80
90
0
200
400
600
800
Time Point (0.25 Hz)
Cell 1324
Fluorescence (Raw)
40
60
80
100
120
0
200
400
600
800
Time Point (0.25 Hz)

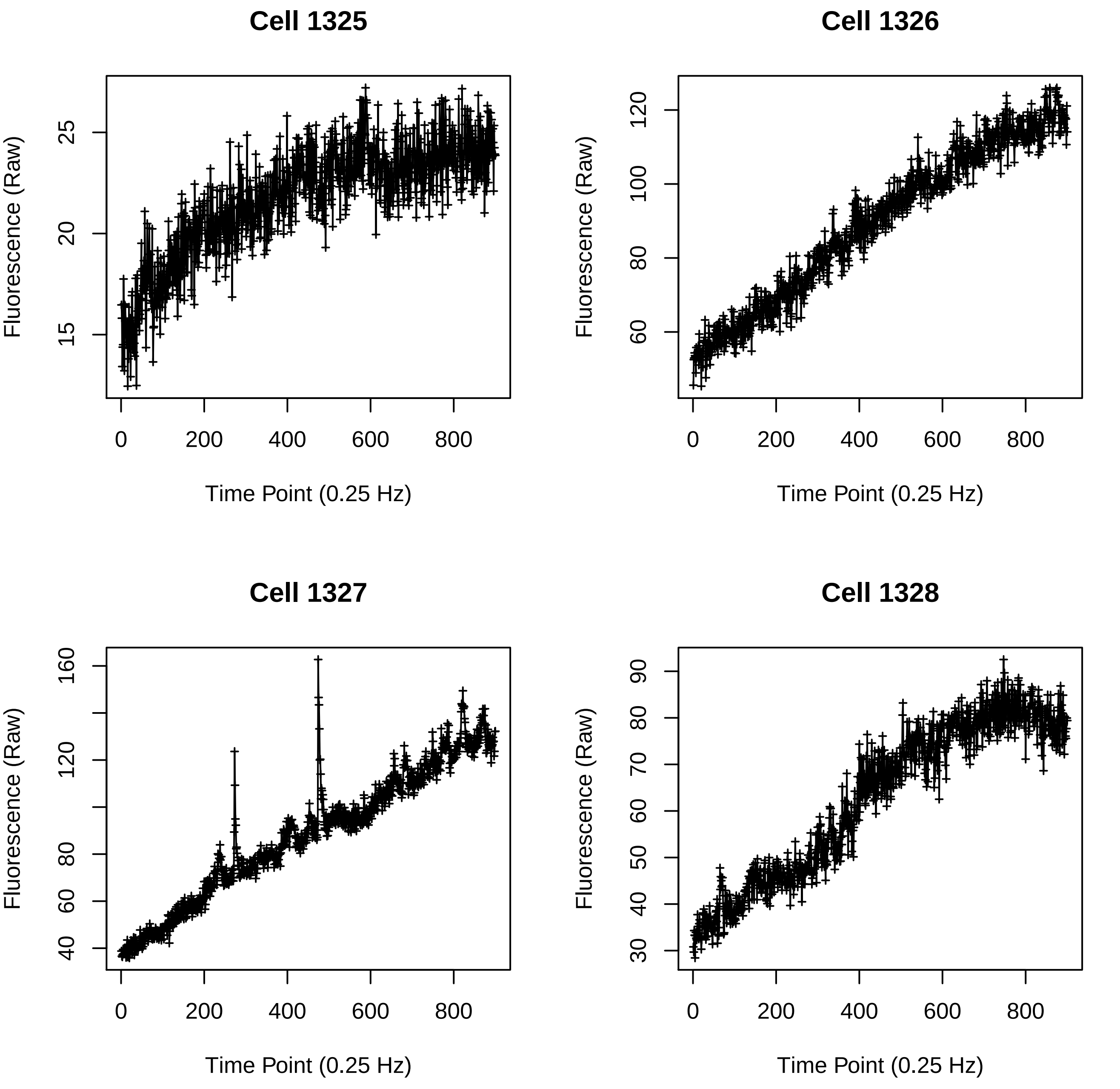
Cell 1325
Cell 1326
Cell 1327
Cell 1328
Fluorescence (Raw)
Time Point (0.25 Hz)
0
200
400
600
800
15
20
25
60
80
100
120
40
60
80
120
160
30
40
50
60
70
80
90

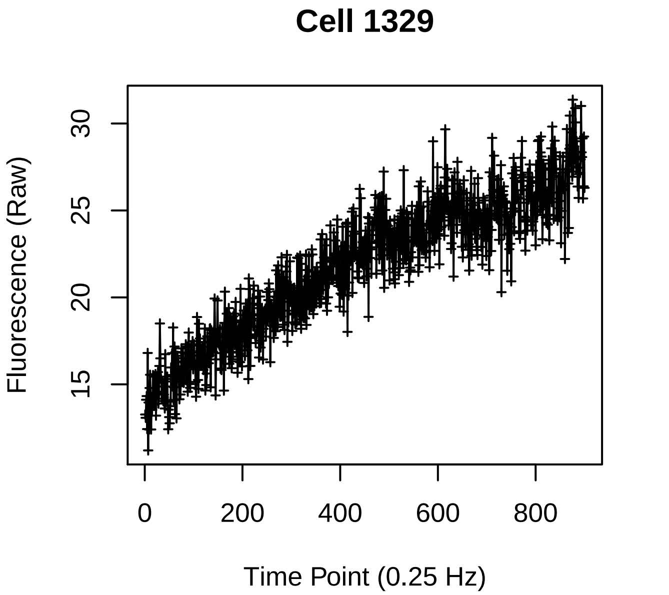
Cell 1329
Fluorescence (Raw)
15
20
25
30
0
200
400
600
800
Time Point (0.25 Hz)

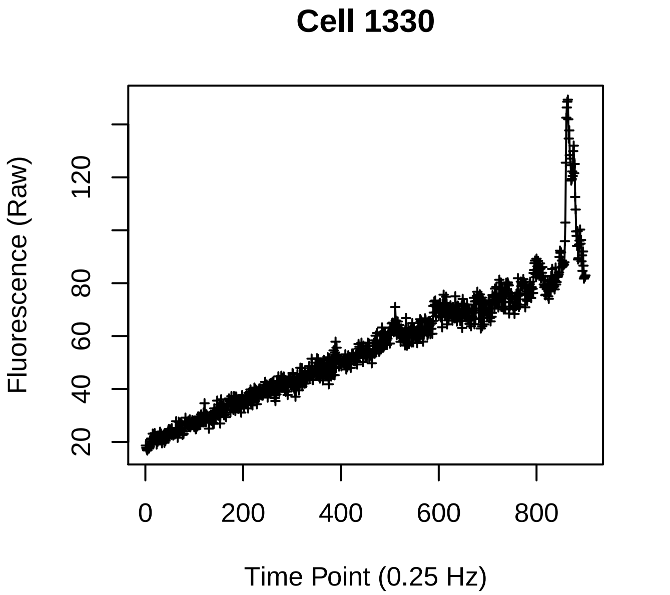
Cell 1330
Fluorescence (Raw)
20
40
60
80
120
0
200
400
600
800
Time Point (0.25 Hz)

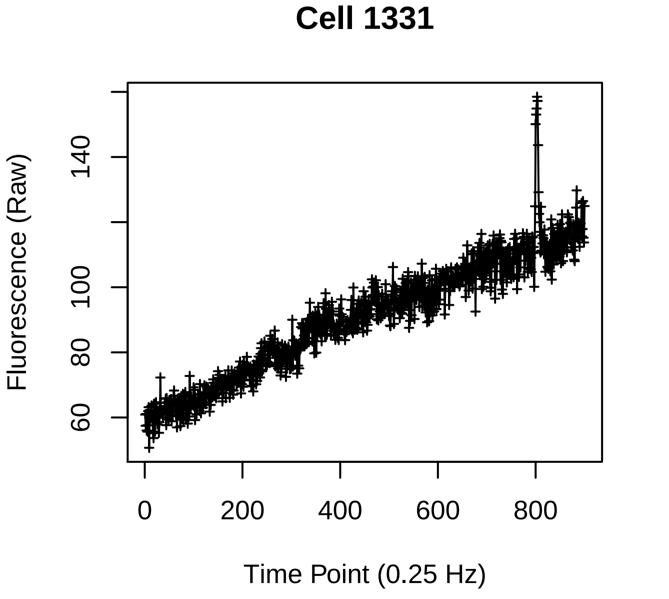
Cell 1331
Fluorescence (Raw)
60
80
100
140
0
200
400
600
800
Time Point (0.25 Hz)

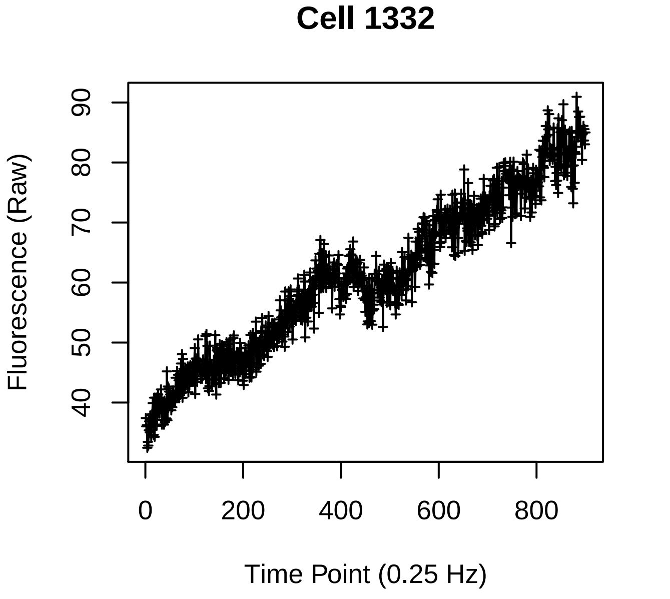
Cell 1332
Fluorescence (Raw)
40
50
60
70
80
90
0
200
400
600
800
Time Point (0.25 Hz)

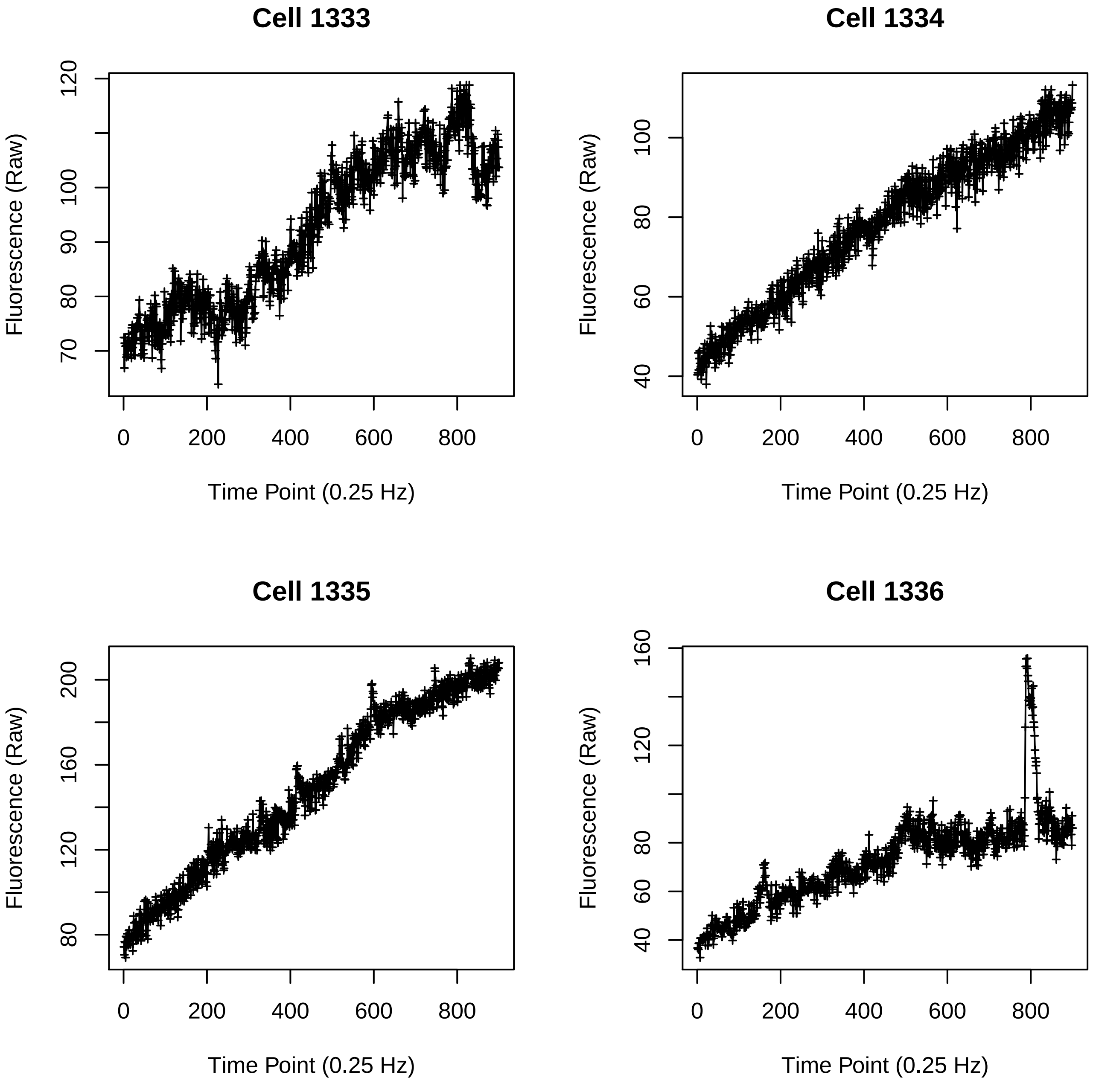
Cell 1333
Fluorescence (Raw)
70
80
90
100
120
0
200
400
600
800
Time Point (0.25 Hz)
Cell 1334
Fluorescence (Raw)
40
60
80
100
0
200
400
600
800
Time Point (0.25 Hz)
Cell 1335
Fluorescence (Raw)
80
120
160
200
0
200
400
600
800
Time Point (0.25 Hz)
Cell 1336
Fluorescence (Raw)
40
60
80
120
160
0
200
400
600
800
Time Point (0.25 Hz)

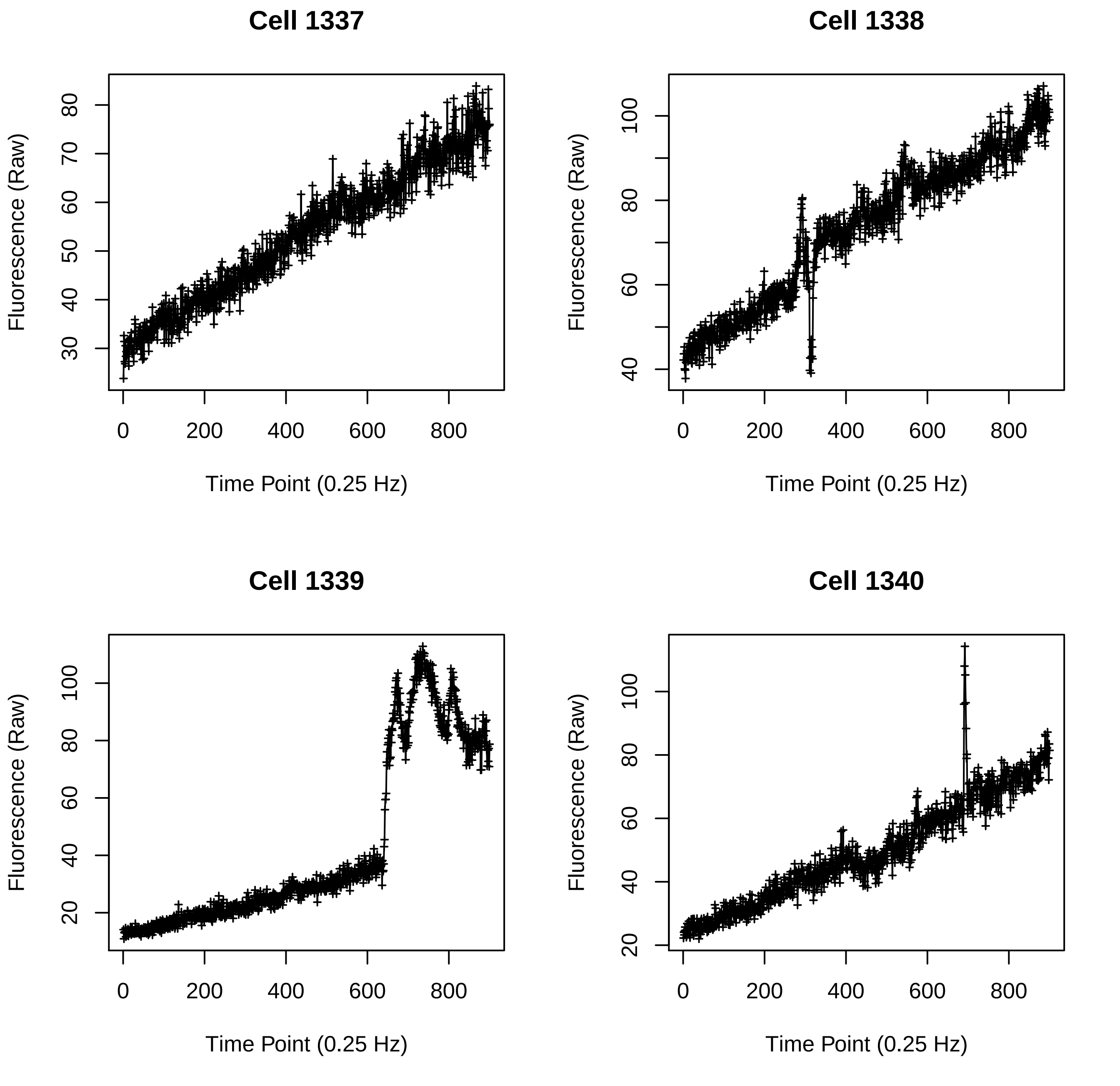

Cell 1337
Fluorescence (Raw)
Time Point (0.25 Hz)
Cell 1338
Fluorescence (Raw)
Time Point (0.25 Hz)
Cell 1339
Fluorescence (Raw)
Time Point (0.25 Hz)
Cell 1340
Fluorescence (Raw)
Time Point (0.25 Hz)

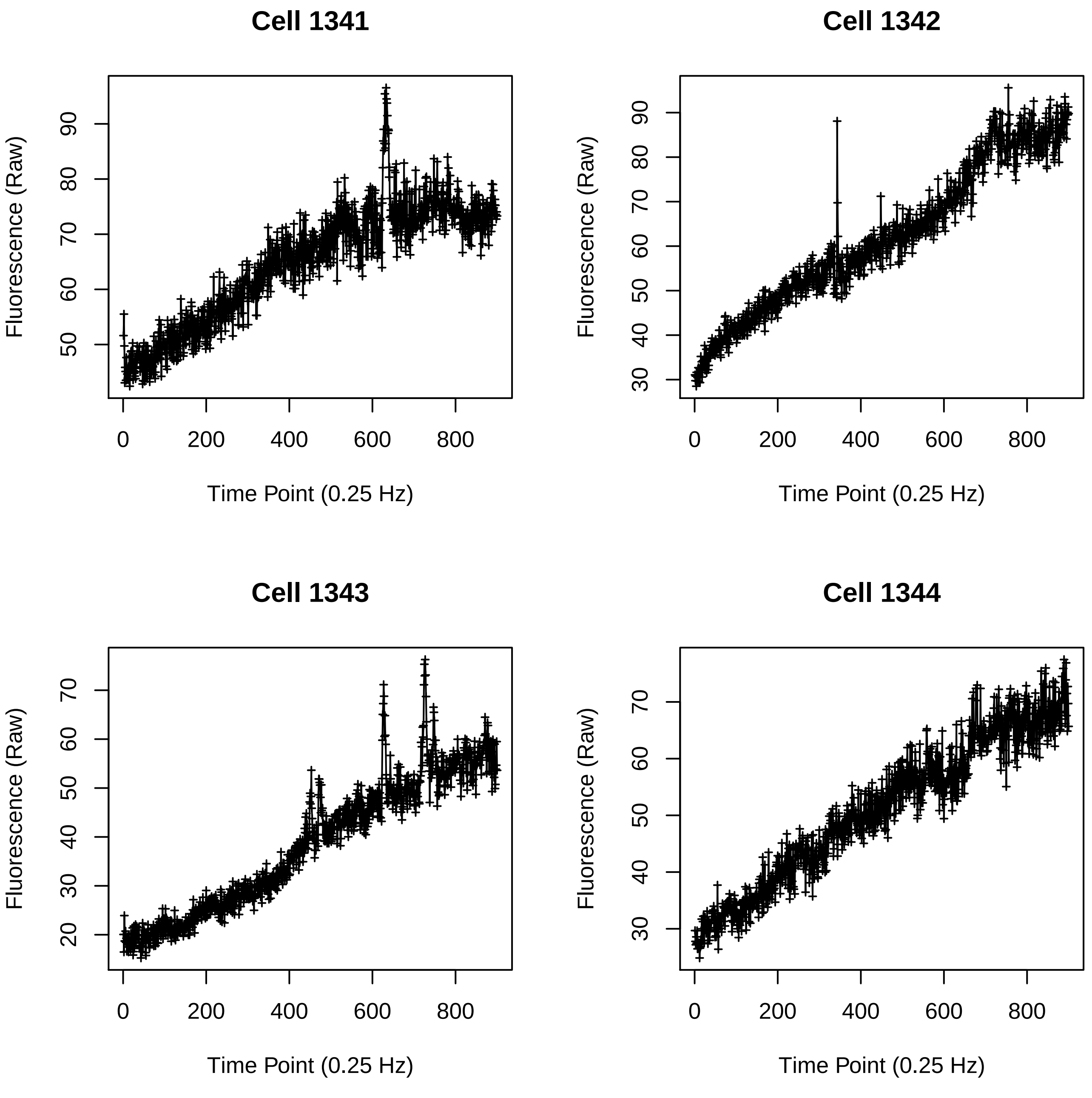

Cell 1341
Fluorescence (Raw)
Time Point (0.25 Hz)
50
60
70
80
90
0
200
400
600
800
Cell 1342
Fluorescence (Raw)
Time Point (0.25 Hz)
30
40
50
60
70
80
90
0
200
400
600
800
Cell 1343
Fluorescence (Raw)
Time Point (0.25 Hz)
20
30
40
50
60
70
0
200
400
600
800
Cell 1344
Fluorescence (Raw)
Time Point (0.25 Hz)
30
40
50
60
70
0
200
400
600
800

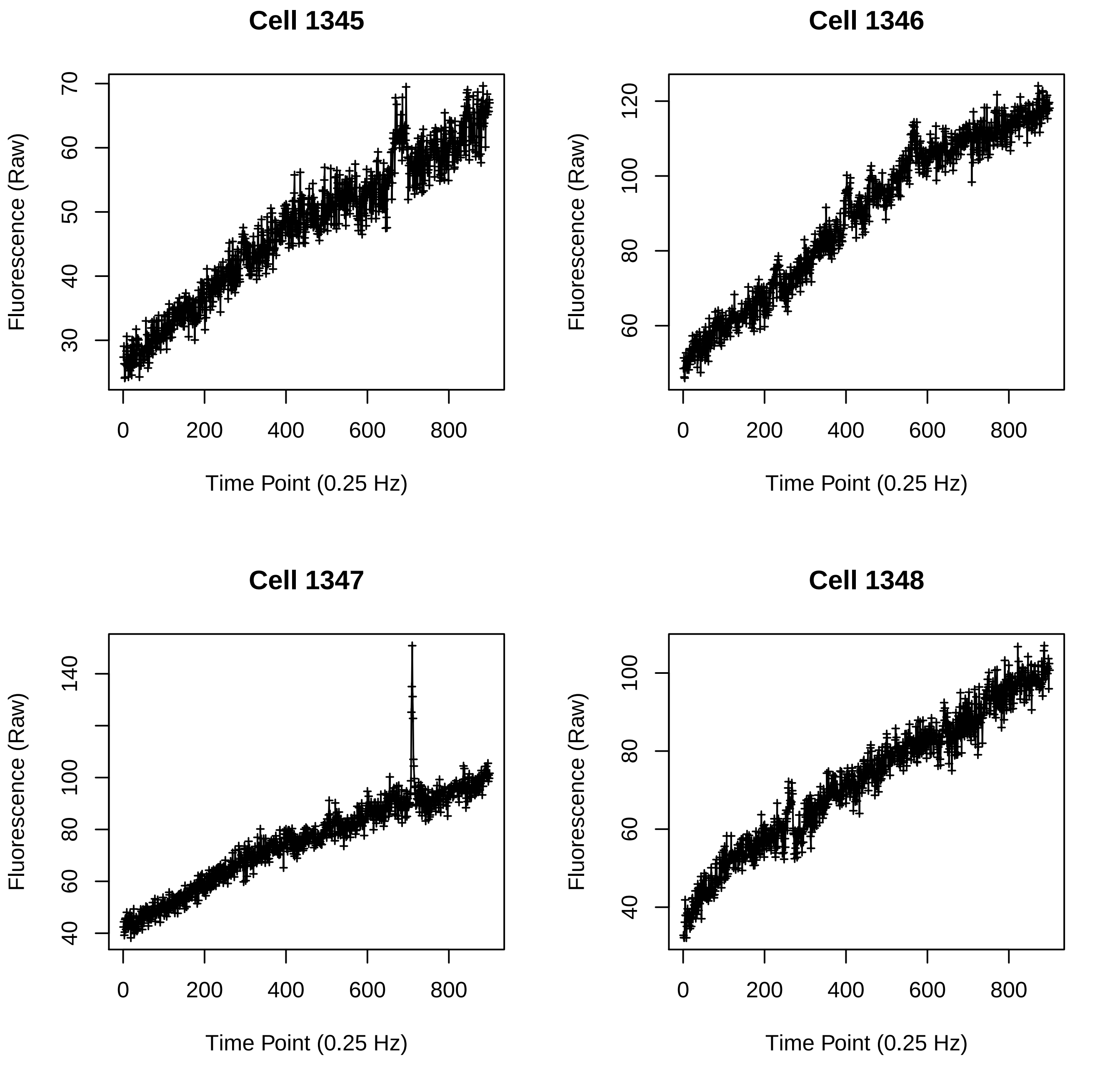
Cell 1345
Fluorescence (Raw)
30
40
50
60
70
0
200
400
600
800
Time Point (0.25 Hz)
Cell 1346
Fluorescence (Raw)
60
80
100
120
0
200
400
600
800
Time Point (0.25 Hz)
Cell 1347
Fluorescence (Raw)
40
60
80
100
140
0
200
400
600
800
Time Point (0.25 Hz)
Cell 1348
Fluorescence (Raw)
40
60
80
100
0
200
400
600
800
Time Point (0.25 Hz)

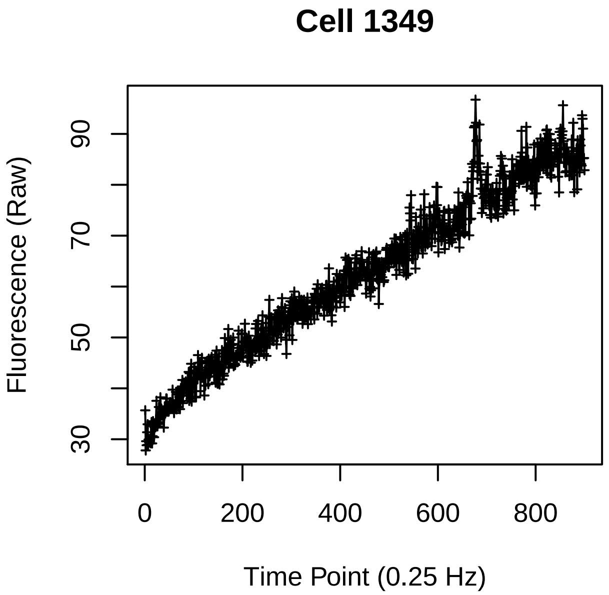
Cell 1349
Fluorescence (Raw)
Time Point (0.25 Hz)
30
50
70
90
0
200
400
600
800

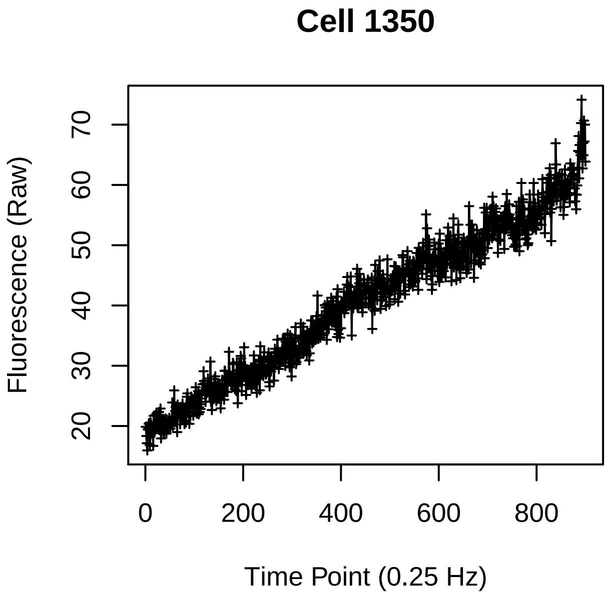
Cell 1350
Fluorescence (Raw)
Time Point (0.25 Hz)
20
30
40
50
60
70
0
200
400
600
800

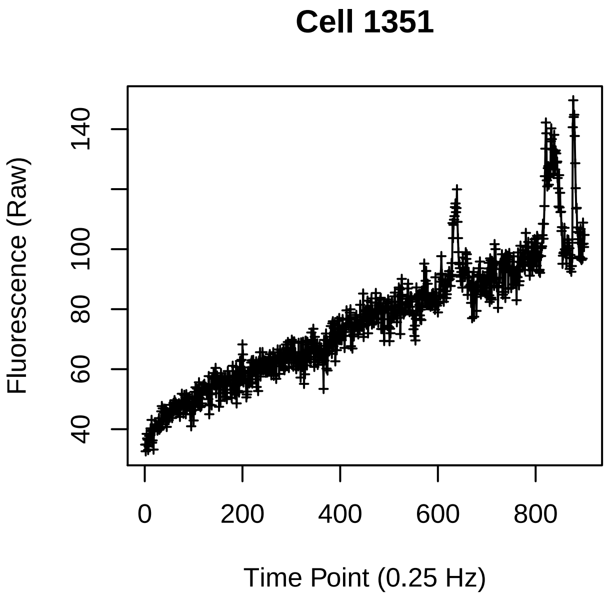
Cell 1351
Fluorescence (Raw)
Time Point (0.25 Hz)
40
60
80
100
140
0
200
400
600
800

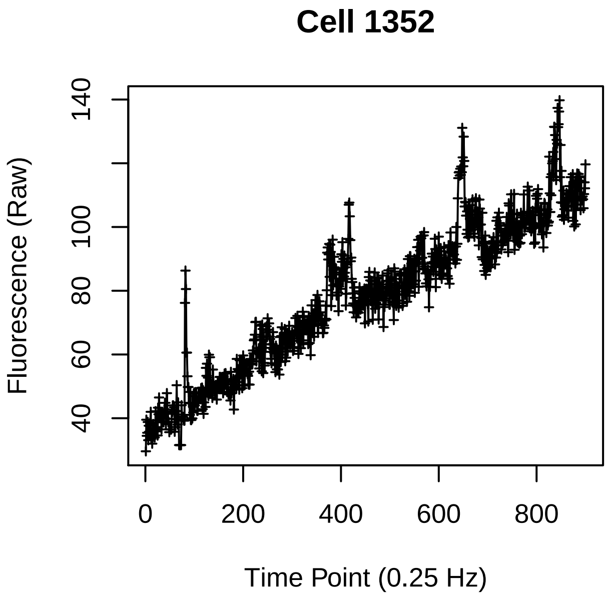
Cell 1352
Fluorescence (Raw)
Time Point (0.25 Hz)
40
60
80
100
140
0
200
400
600
800

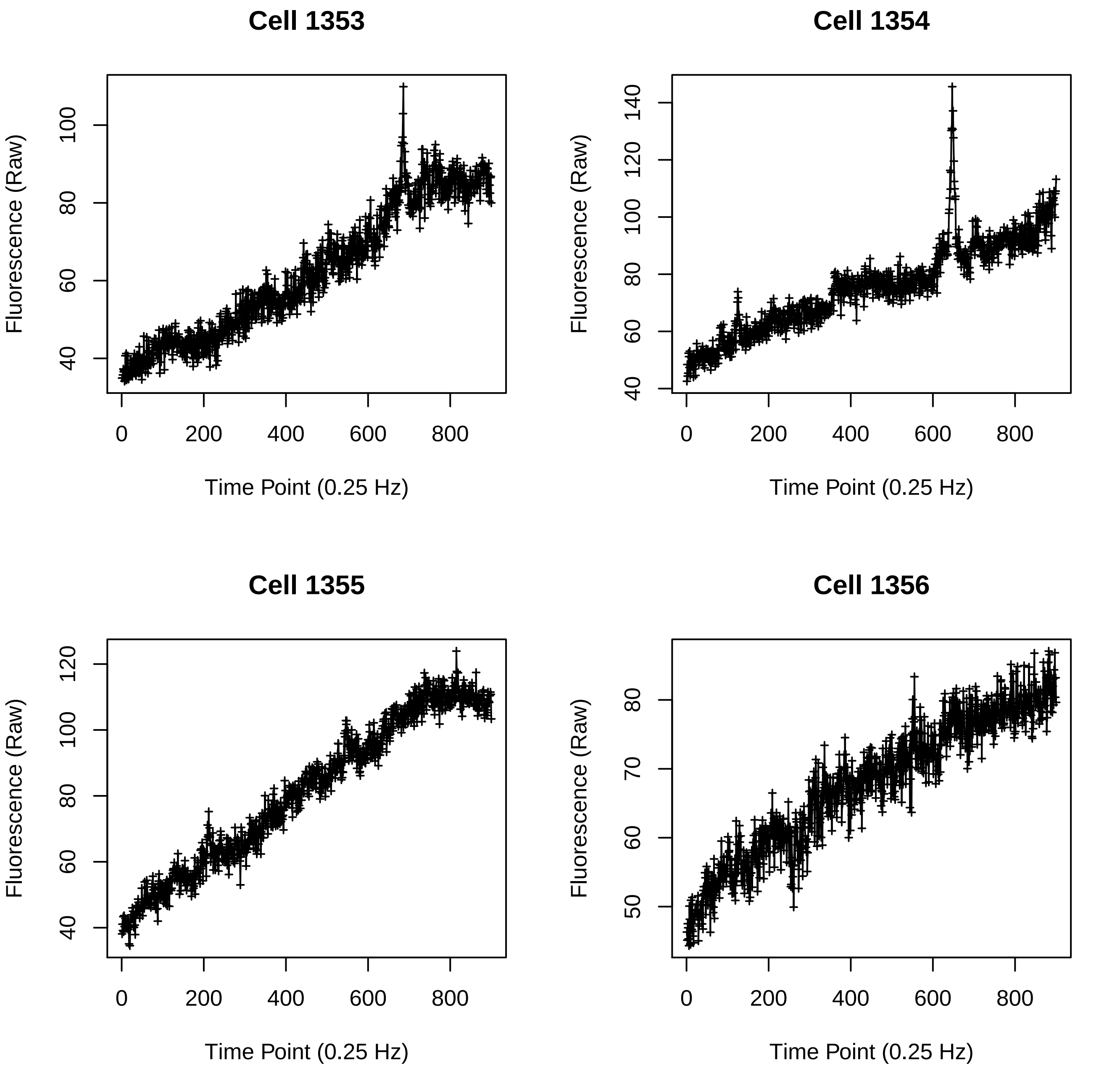

Cell 1353
Fluorescence (Raw)
Time Point (0.25 Hz)
Cell 1354
Fluorescence (Raw)
Time Point (0.25 Hz)
Cell 1355
Fluorescence (Raw)
Time Point (0.25 Hz)
Cell 1356
Fluorescence (Raw)
Time Point (0.25 Hz)

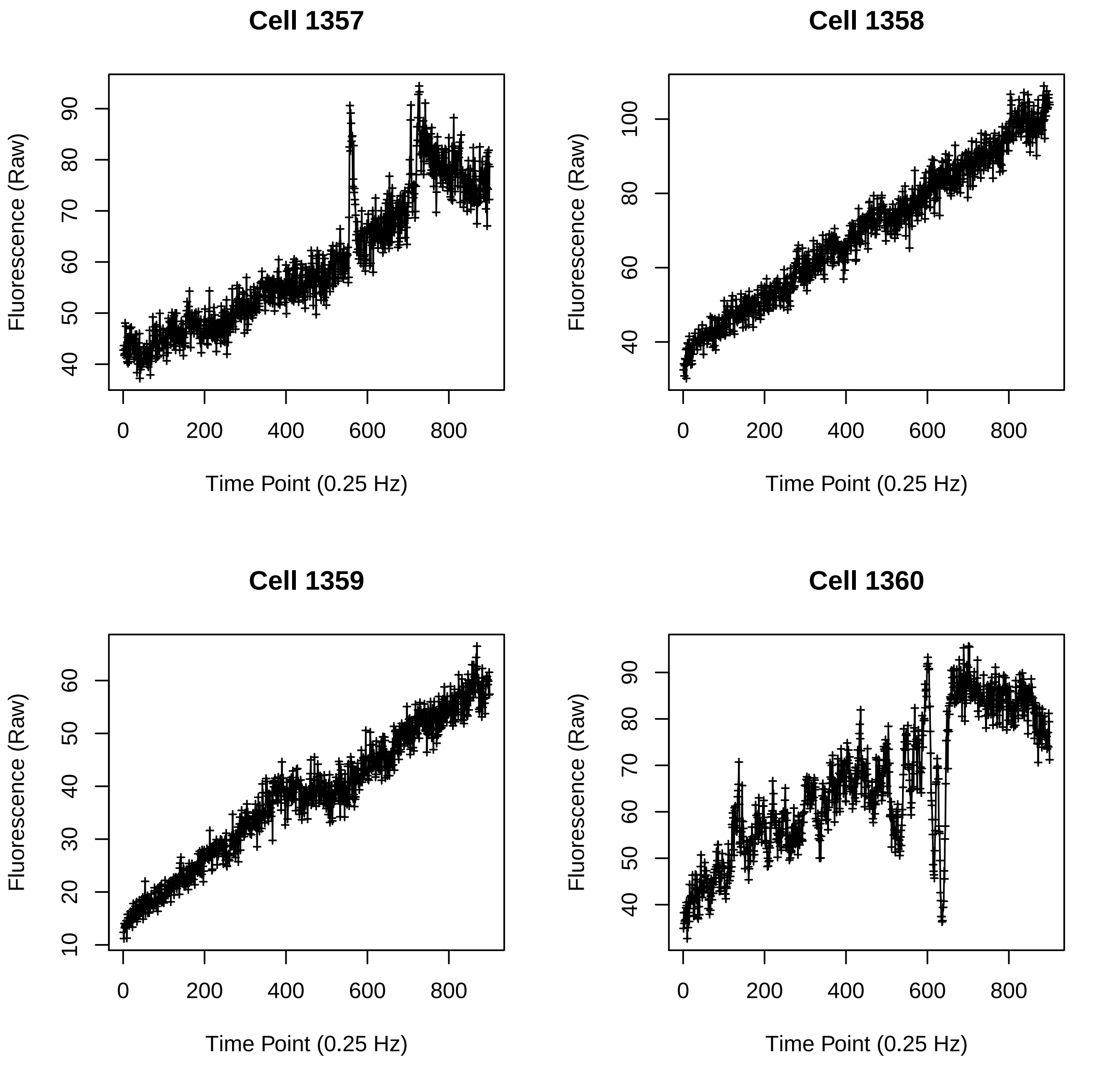
Cell 1357
Cell 1358
Cell 1359
Cell 1360
Fluorescence (Raw)
Time Point (0.25 Hz)
0
200
400
600
800
40
50
60
70
80
90
100
10
20
30

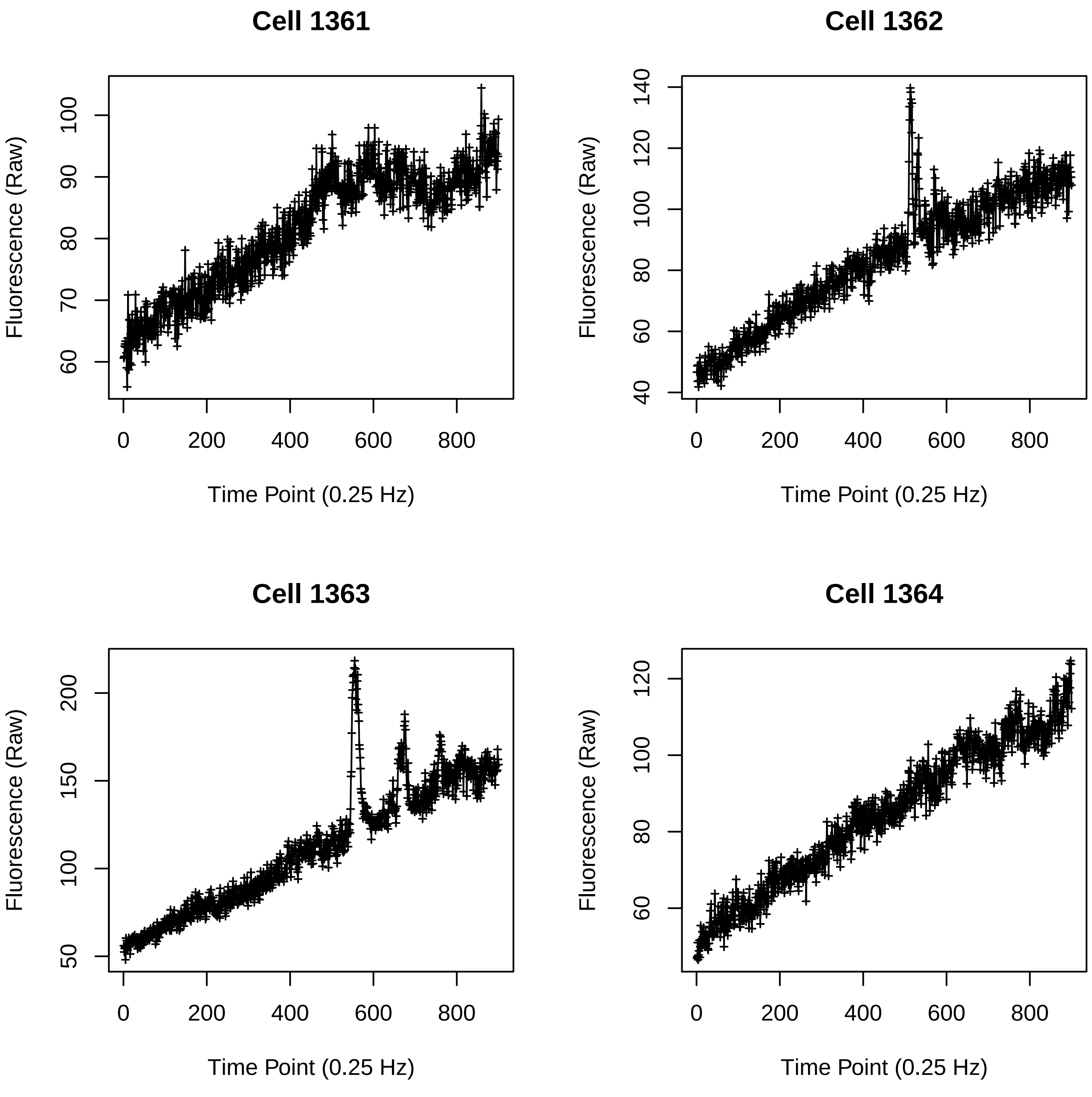

Cell 1361
Cell 1362
Cell 1363
Cell 1364
Fluorescence (Raw)
Time Point (0.25 Hz)
0
200
400
600
800
60
70
80
90
100
40
120
140
50
150
200

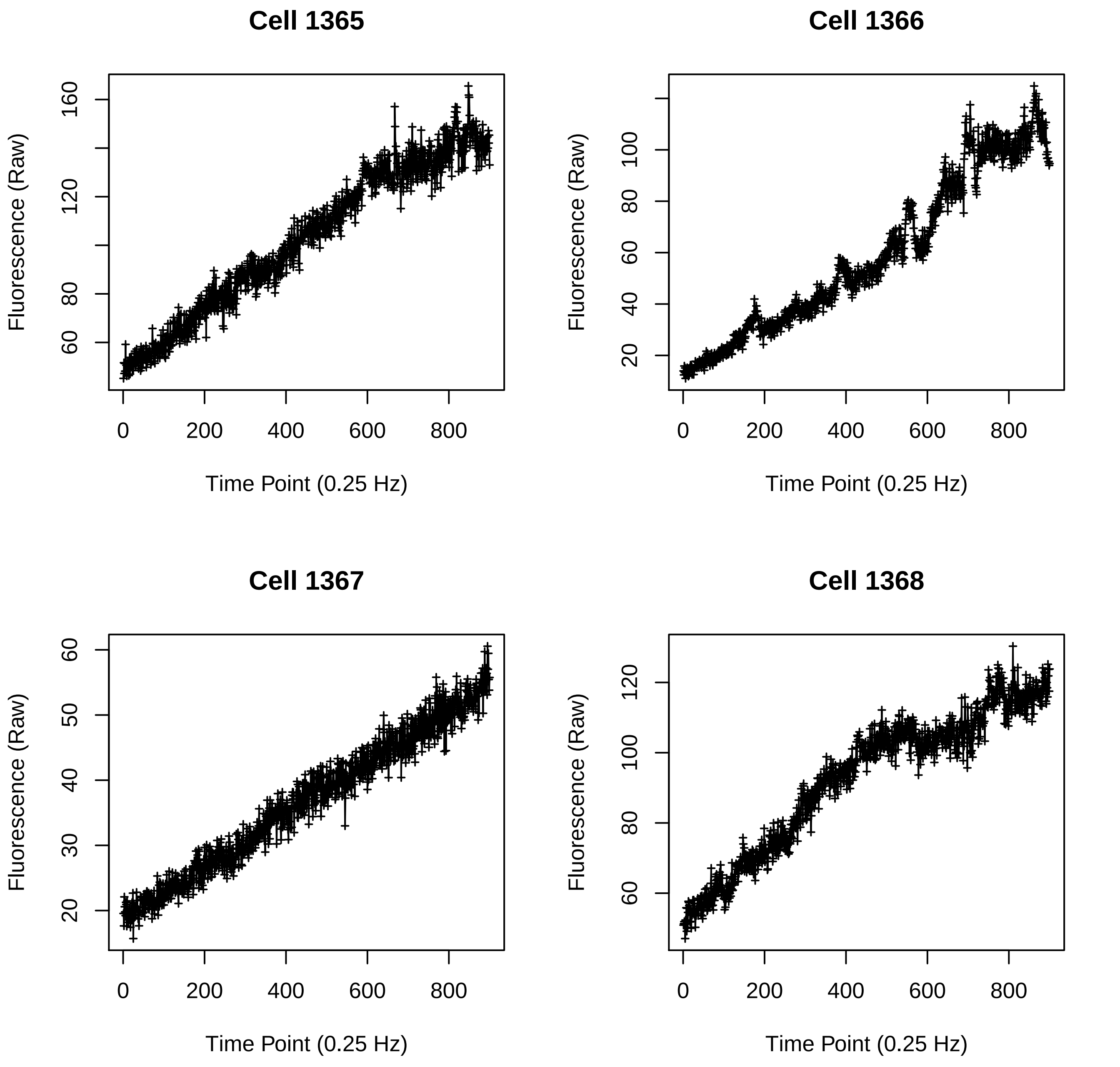
Cell 1365
Fluorescence (Raw)
60
80
120
160
0
200
400
600
800
Time Point (0.25 Hz)
Cell 1366
Fluorescence (Raw)
20
40
60
80
100
0
200
400
600
800
Time Point (0.25 Hz)
Cell 1367
Fluorescence (Raw)
20
30
40
50
60
0
200
400
600
800
Time Point (0.25 Hz)
Cell 1368
Fluorescence (Raw)
60
80
100
120
0
200
400
600
800
Time Point (0.25 Hz)

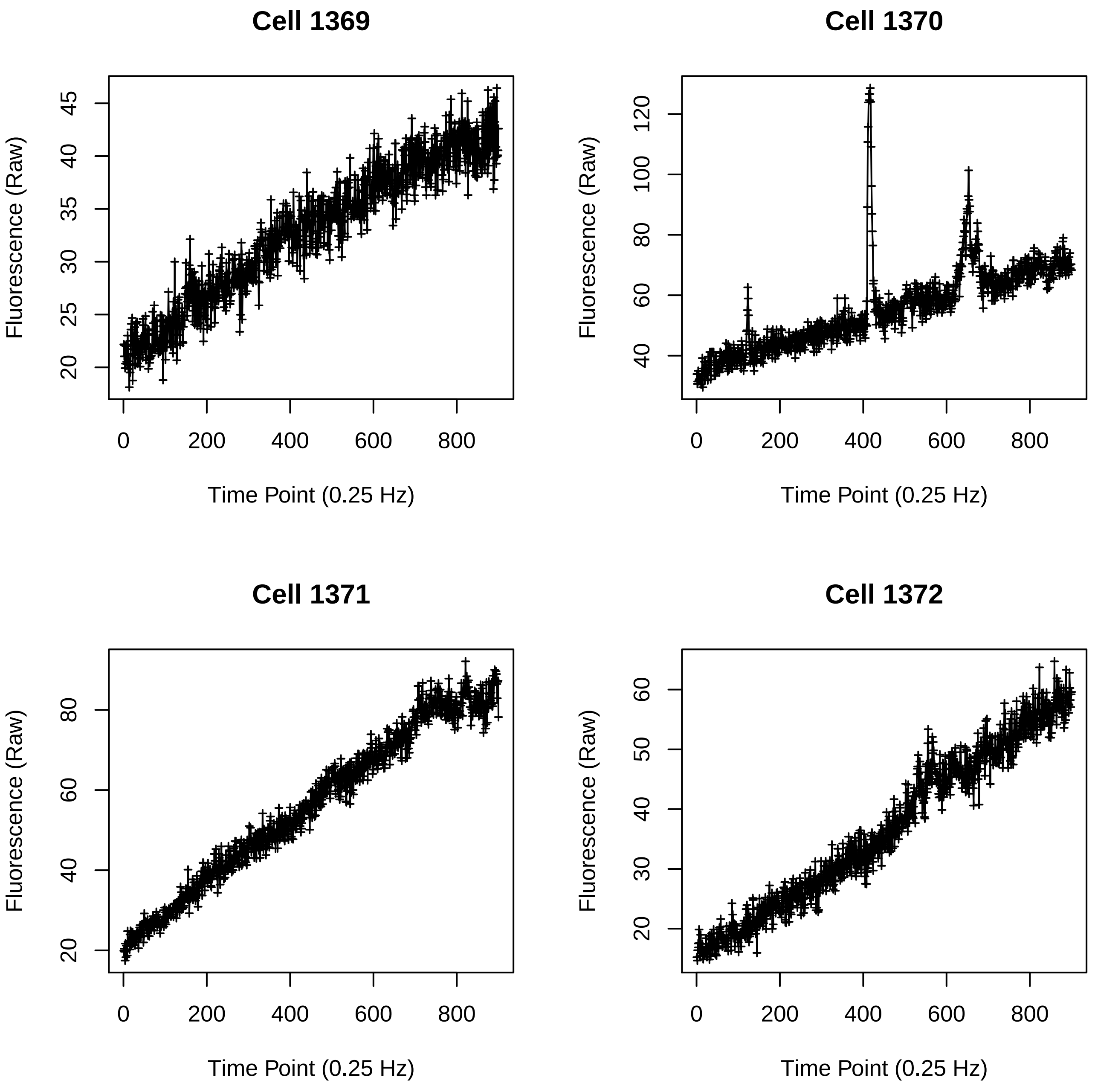
Cell 1369
Fluorescence (Raw)
20
25
30
35
40
45
0
200
400
600
800
Time Point (0.25 Hz)
Cell 1370
Fluorescence (Raw)
40
60
80
100
120
0
200
400
600
800
Time Point (0.25 Hz)
Cell 1371
Fluorescence (Raw)
20
40
60
80
0
200
400
600
800
Time Point (0.25 Hz)
Cell 1372
Fluorescence (Raw)
20
30
40
50
60
0
200
400
600
800
Time Point (0.25 Hz)

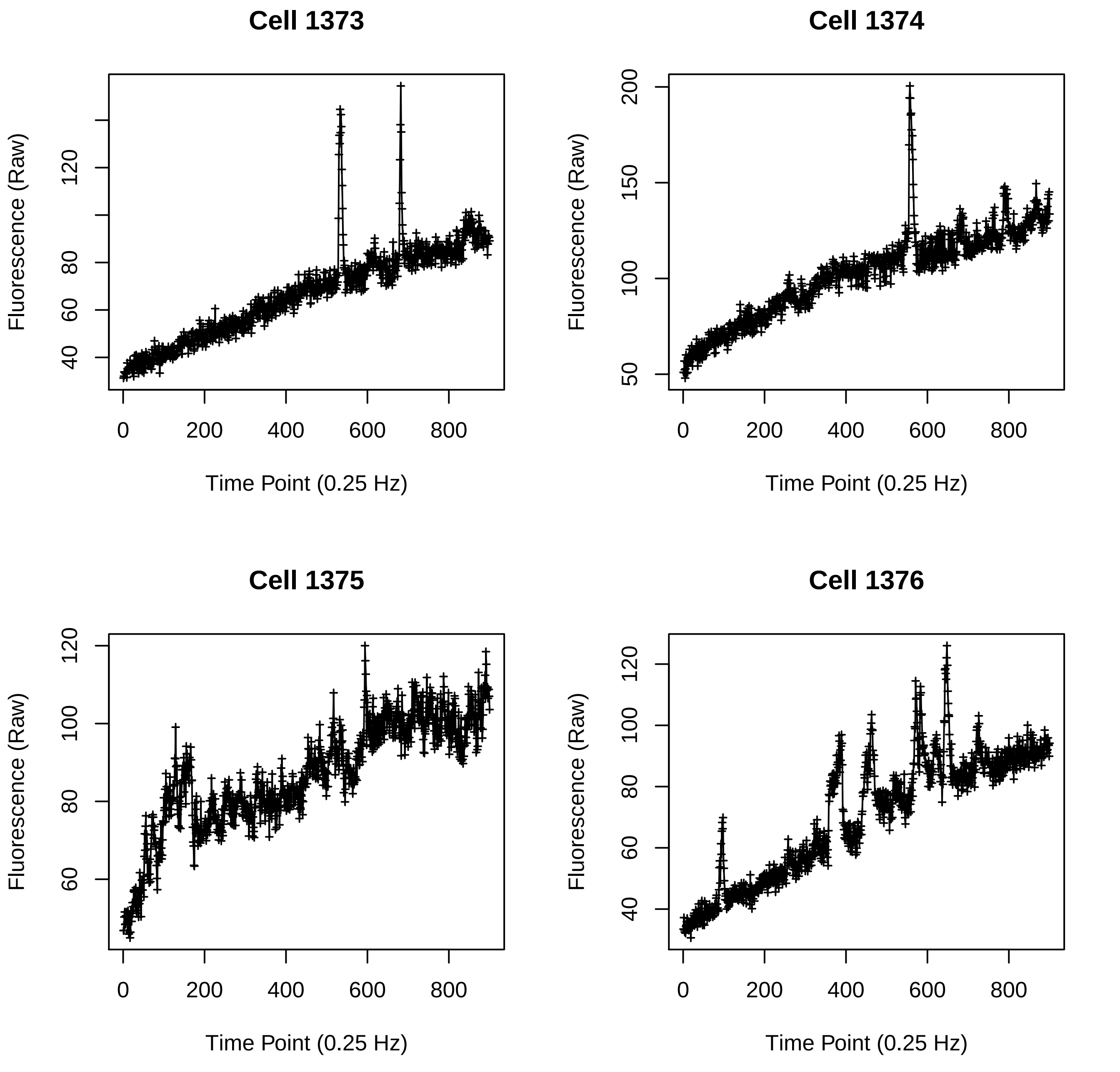
Cell 1373
Fluorescence (Raw)
40
60
80
120
0
200
400
600
800
Time Point (0.25 Hz)
Cell 1374
Fluorescence (Raw)
50
100
150
200
0
200
400
600
800
Time Point (0.25 Hz)
Cell 1375
Fluorescence (Raw)
60
80
100
120
0
200
400
600
800
Time Point (0.25 Hz)
Cell 1376
Fluorescence (Raw)
40
60
80
100
120
0
200
400
600
800
Time Point (0.25 Hz)

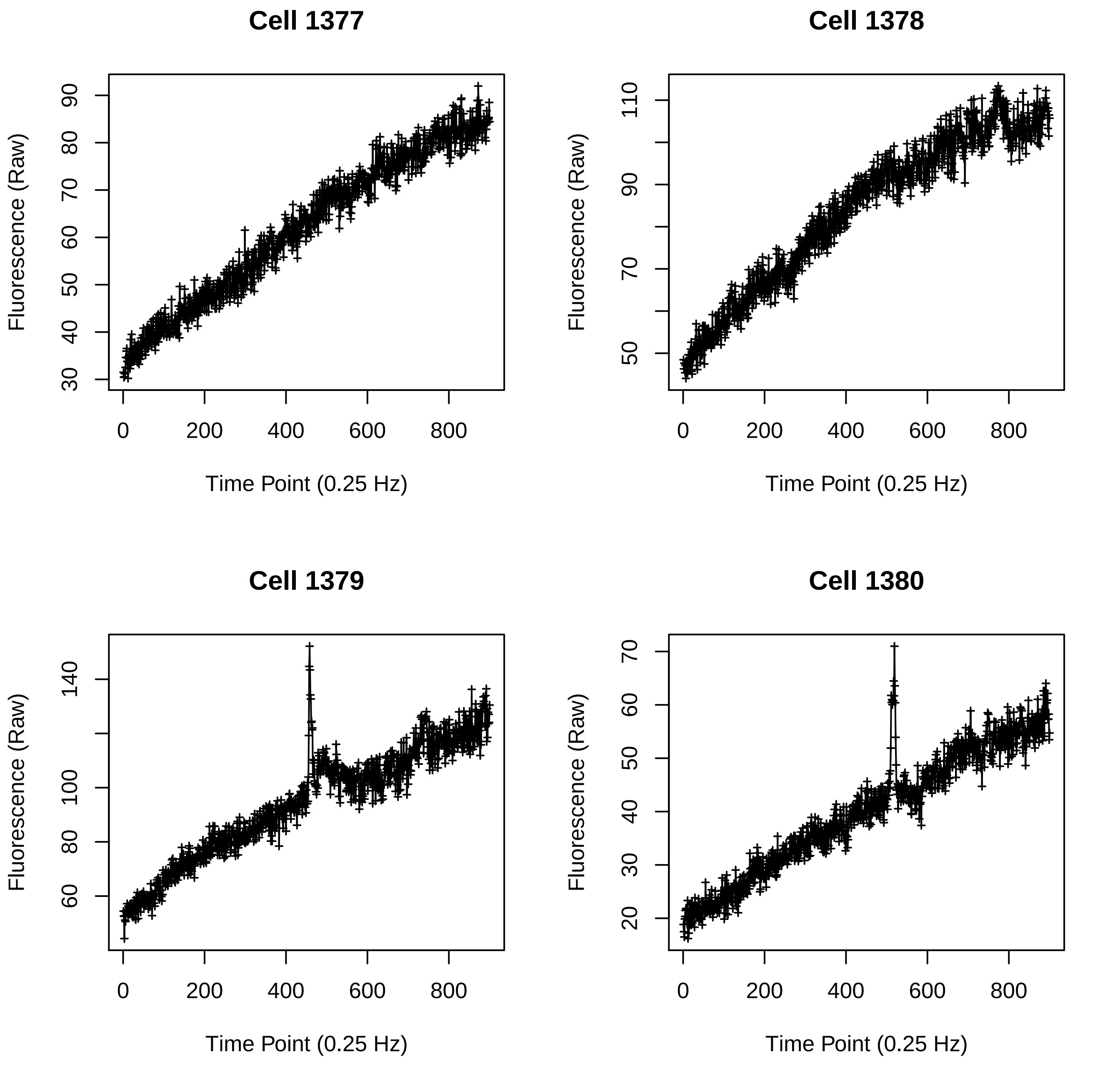
Cell 1377
Cell 1378
Cell 1379
Cell 1380
Fluorescence (Raw)
Time Point (0.25 Hz)
0
200
400
600
800
30
40
50
60
70
80
90
110
140
100
20

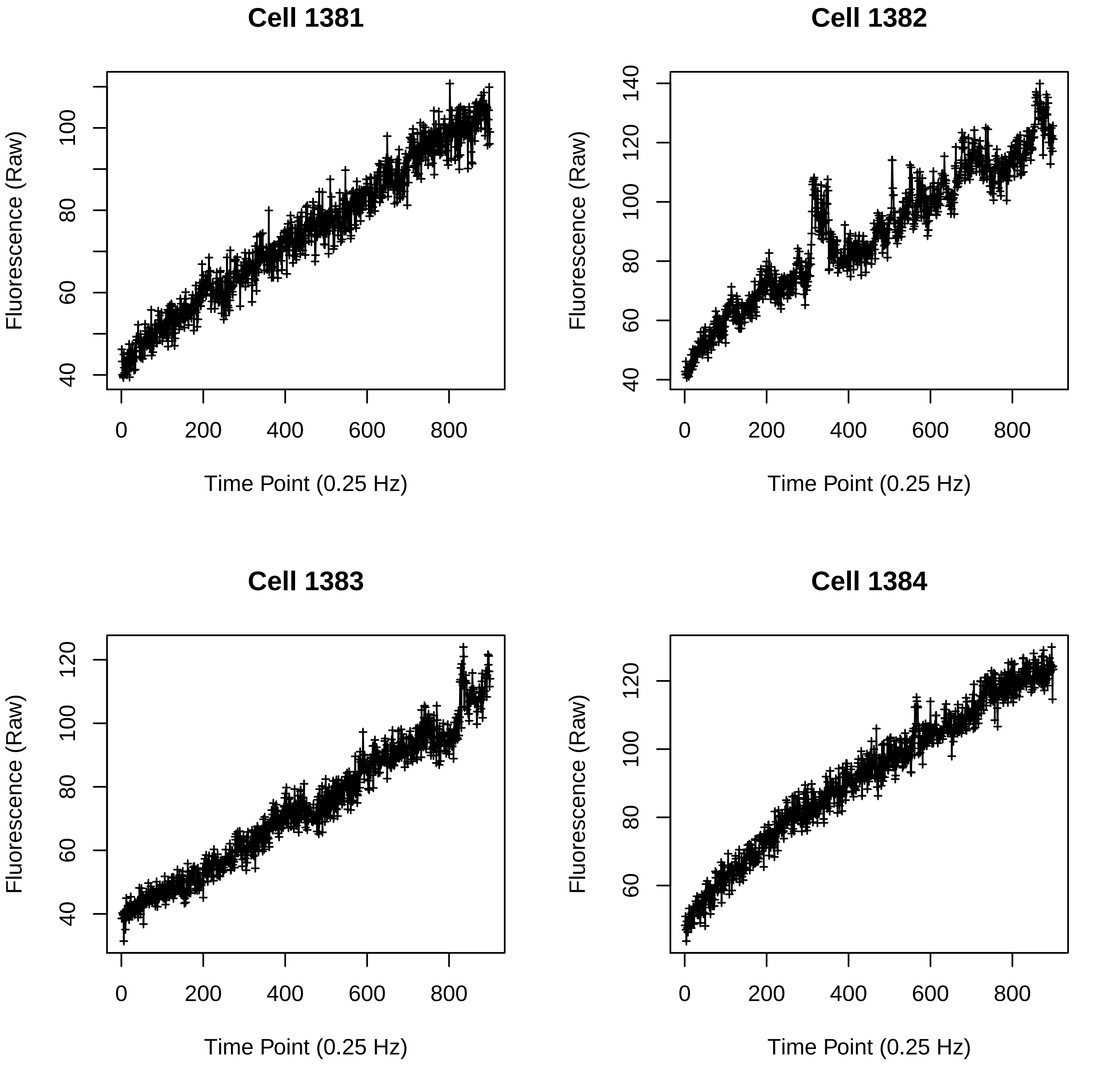

Cell 1381
Cell 1382
Cell 1383
Cell 1384
Fluorescence (Raw)
Time Point (0.25 Hz)
0
200
400
600
800
40
60
80
100
120
140

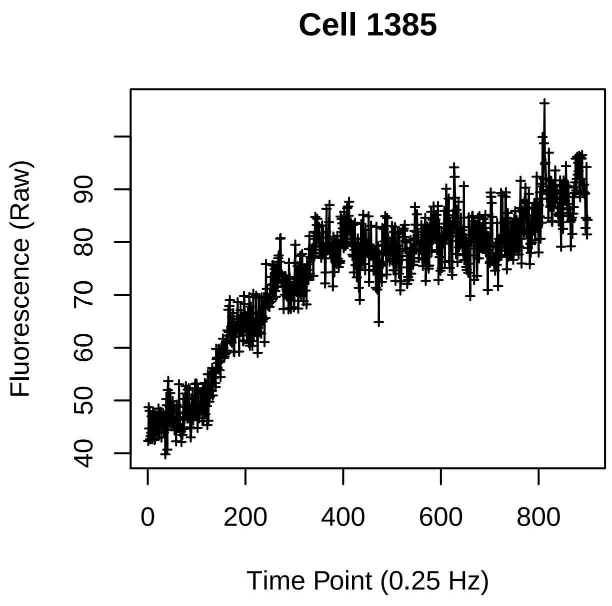
Cell 1385
Fluorescence (Raw)
40
50
60
70
80
90
0
200
400
600
800
Time Point (0.25 Hz)
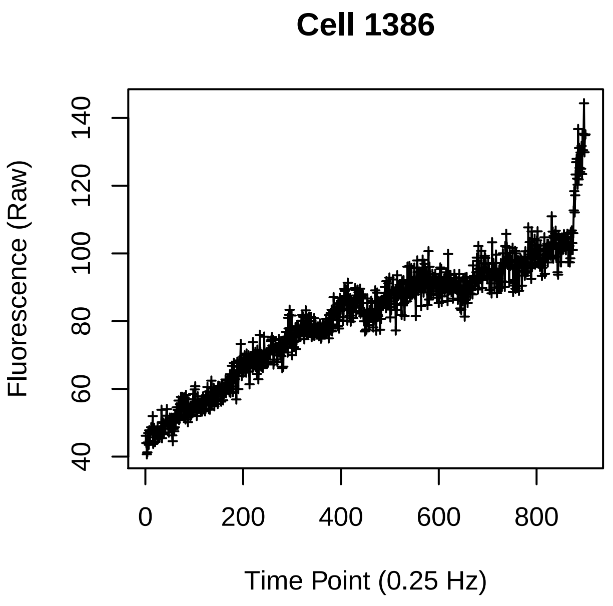
Cell 1386
Fluorescence (Raw)
40
60
80
100
120
140
0
200
400
600
800
Time Point (0.25 Hz)
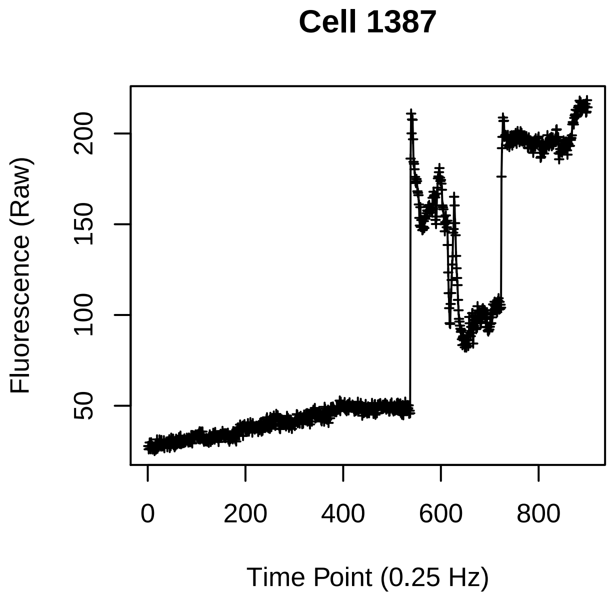
Cell 1387
Fluorescence (Raw)
50
100
150
200
0
200
400
600
800
Time Point (0.25 Hz)
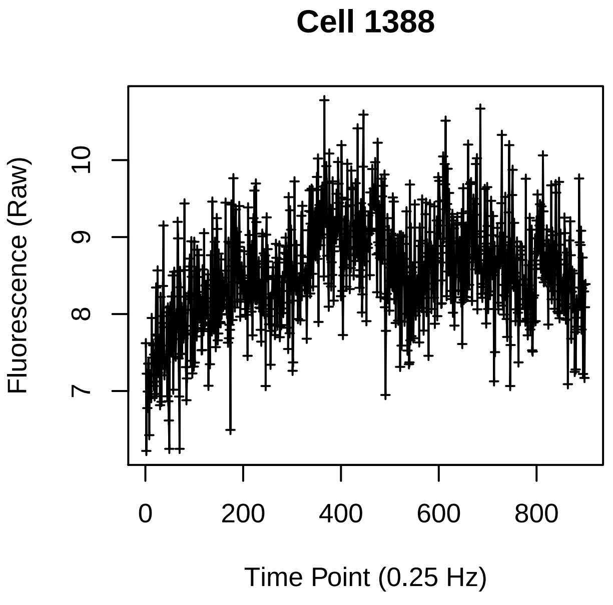
Cell 1388
Fluorescence (Raw)
7
8
9
10
0
200
400
600
800
Time Point (0.25 Hz)

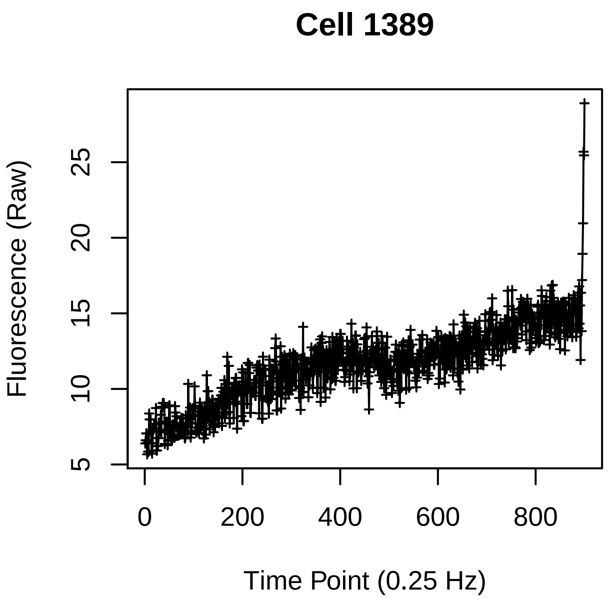
Cell 1389
Fluorescence (Raw)
Time Point (0.25 Hz)
5
10
15
20
25
0
200
400
600
800

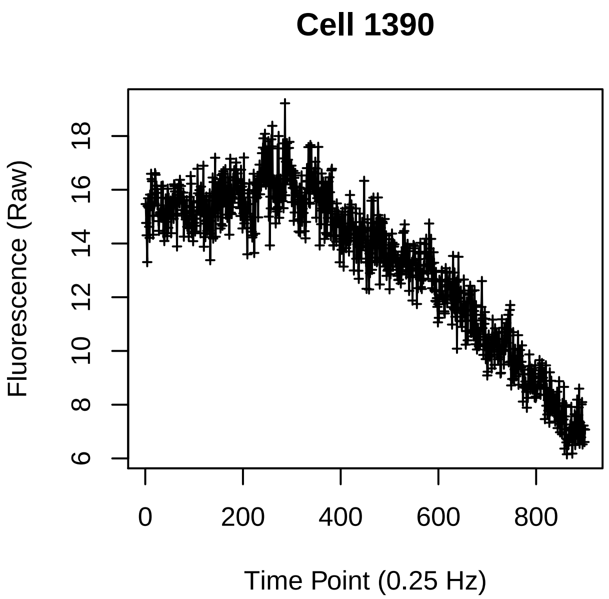
Cell 1390
Fluorescence (Raw)
Time Point (0.25 Hz)
6
8
10
12
14
16
18
0
200
400
600
800

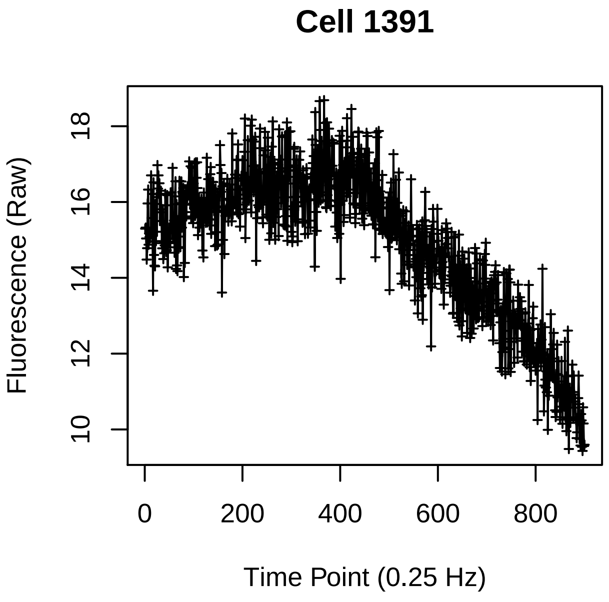
Cell 1391
Fluorescence (Raw)
Time Point (0.25 Hz)
10
12
14
16
18
0
200
400
600
800

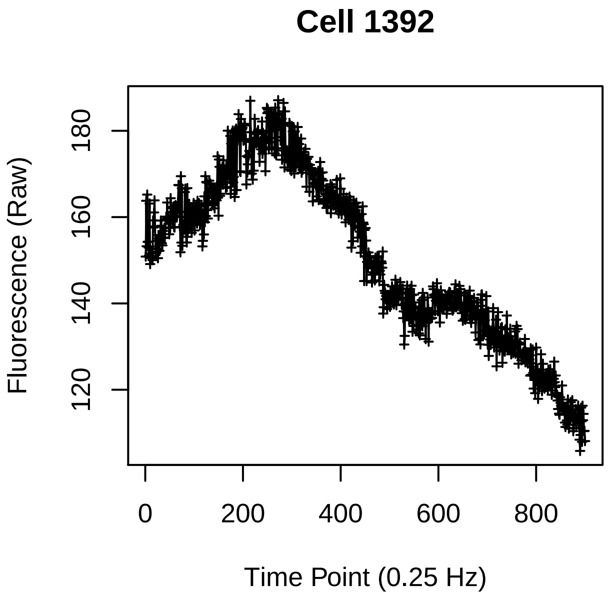
Cell 1392
Fluorescence (Raw)
Time Point (0.25 Hz)
120
140
160
180
0
200
400
600
800

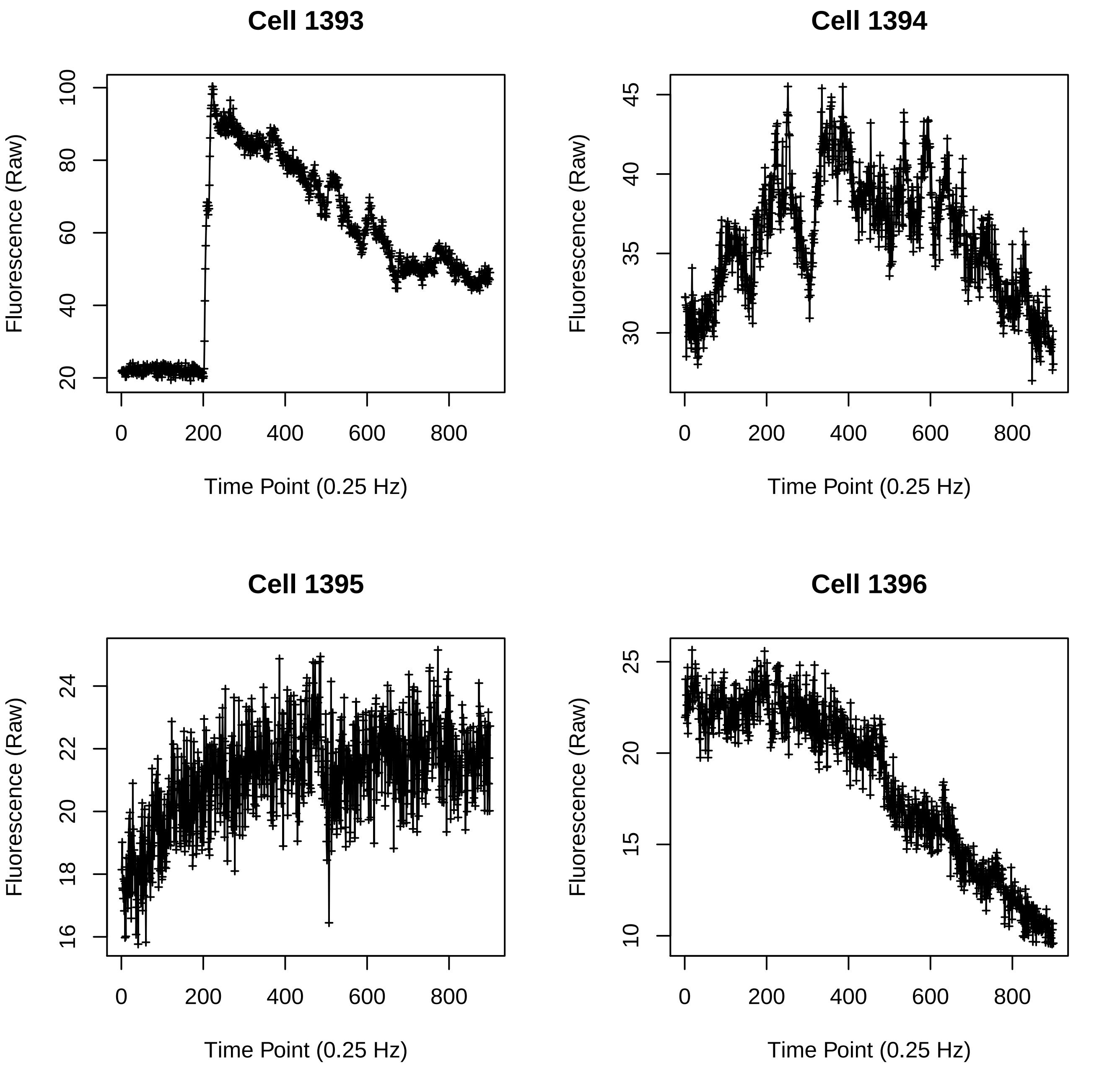
Cell 1393
Cell 1394
Cell 1395
Cell 1396
Fluorescence (Raw)
Time Point (0.25 Hz)
0
200
400
600
800
20
40
60
80
100
30
35
40
45
16
18
20
22
24
10
15
20
25

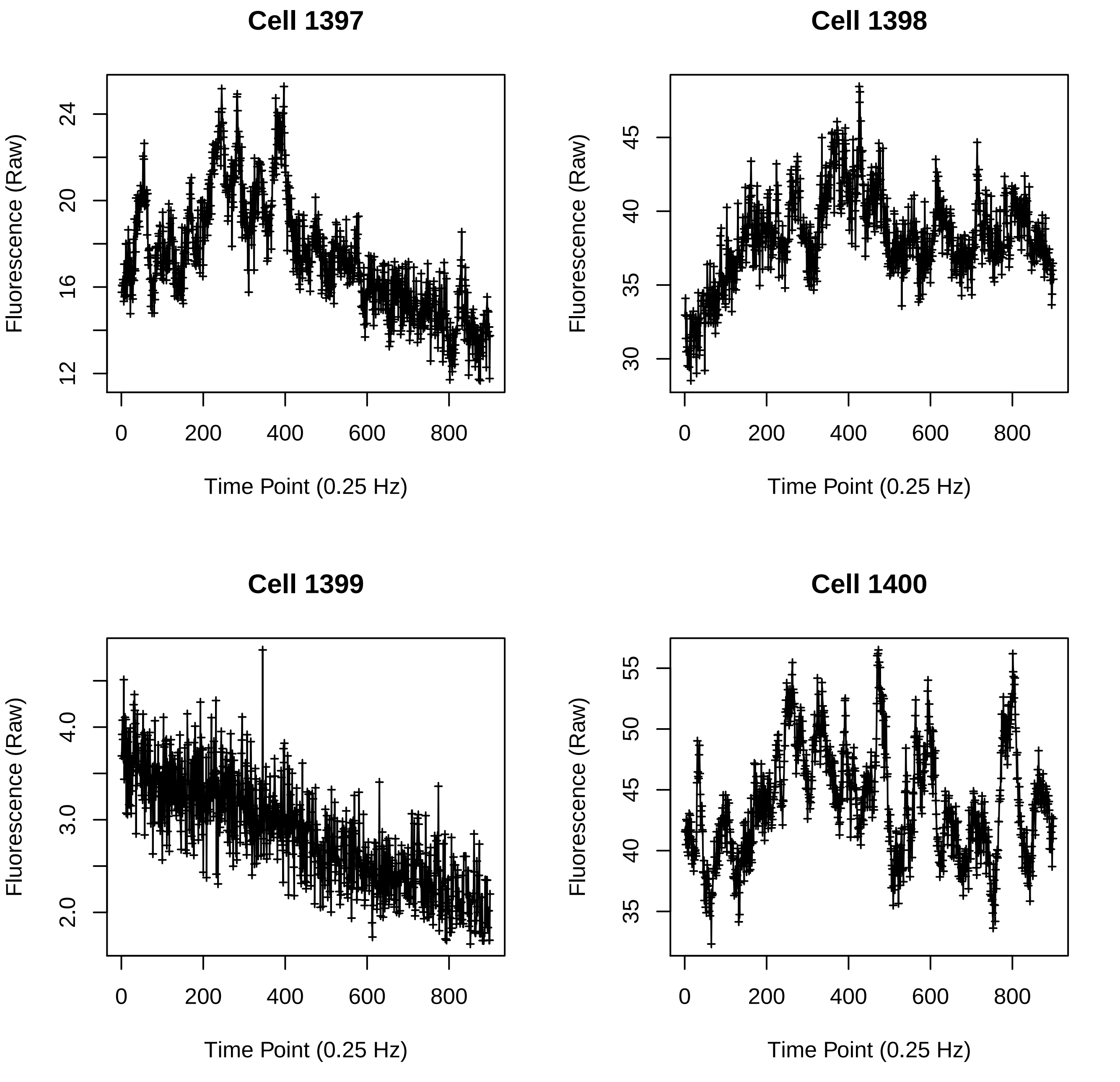
Cell 1397
Fluorescence (Raw)
12
16
20
24
Time Point (0.25 Hz)
0
200
400
600
800
Cell 1398
Fluorescence (Raw)
30
35
40
45
Time Point (0.25 Hz)
0
200
400
600
800
Cell 1399
Fluorescence (Raw)
2.0
3.0
4.0
Time Point (0.25 Hz)
0
200
400
600
800
Cell 1400
Fluorescence (Raw)
35
40
45
50
55
Time Point (0.25 Hz)
0
200
400
600
800

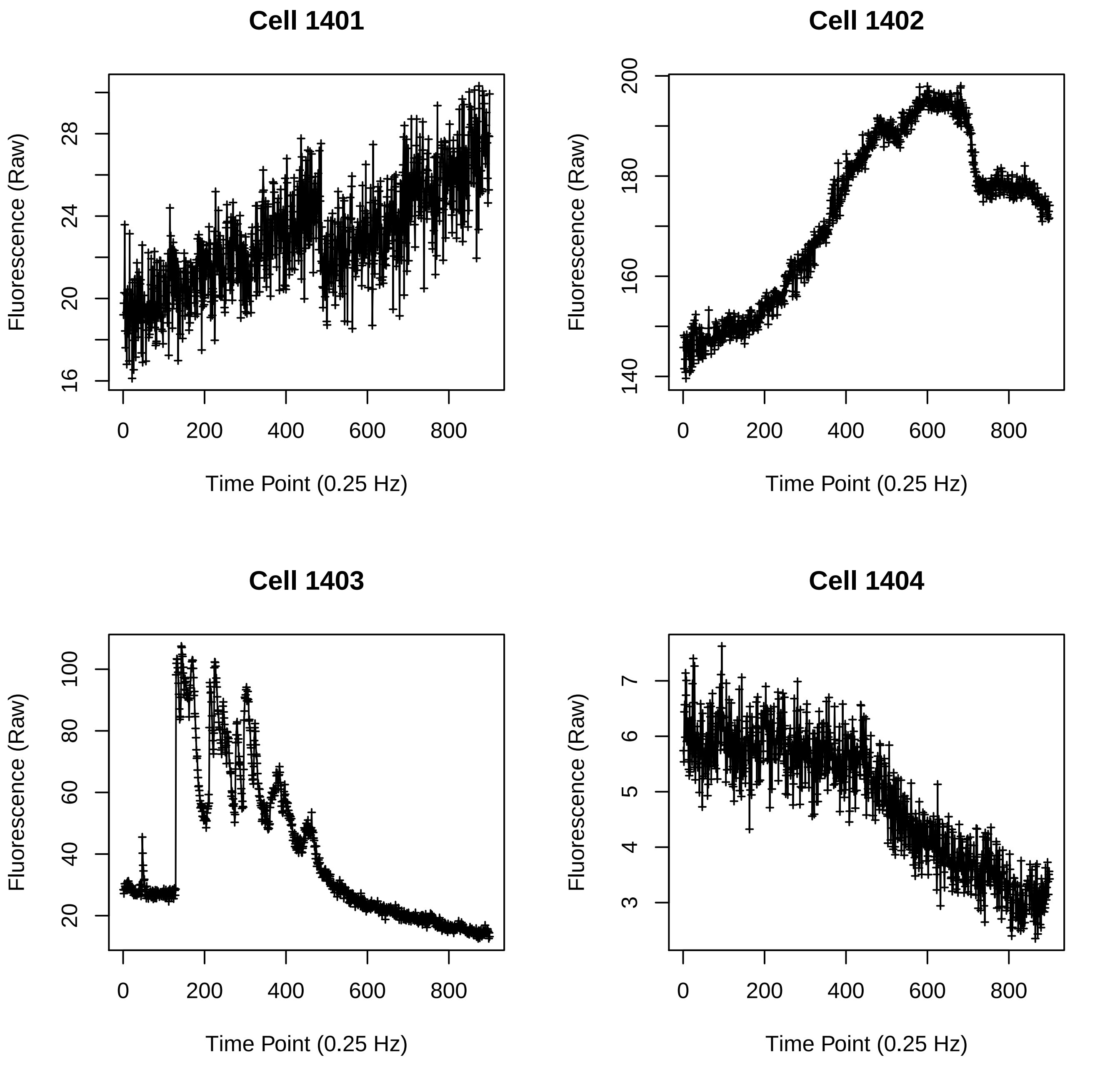
Cell 1401
Fluorescence (Raw)
16
20
24
28
0
200
400
600
800
Time Point (0.25 Hz)
Cell 1402
Fluorescence (Raw)
140
160
180
200
0
200
400
600
800
Time Point (0.25 Hz)
Cell 1403
Fluorescence (Raw)
20
40
60
80
100
0
200
400
600
800
Time Point (0.25 Hz)
Cell 1404
Fluorescence (Raw)
3
4
5
6
7
0
200
400
600
800
Time Point (0.25 Hz)

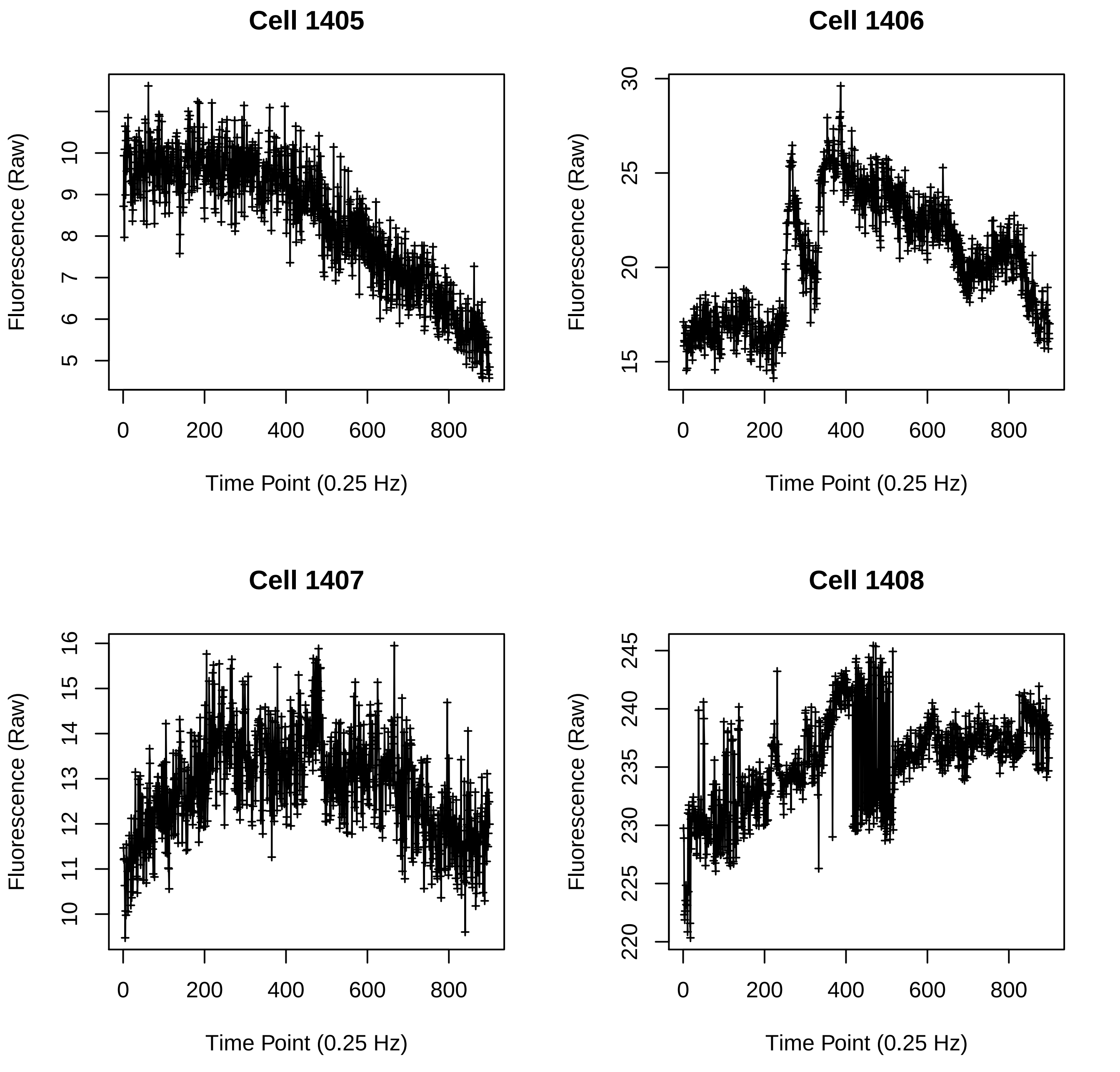
Cell 1405
Cell 1406
Cell 1407
Cell 1408
Fluorescence (Raw)
Time Point (0.25 Hz)
0
200
400
600
800
5
6
7
8
9
10
15
20
25
30
10
11
12
13
14
15
16
220
225
230
235
240
245

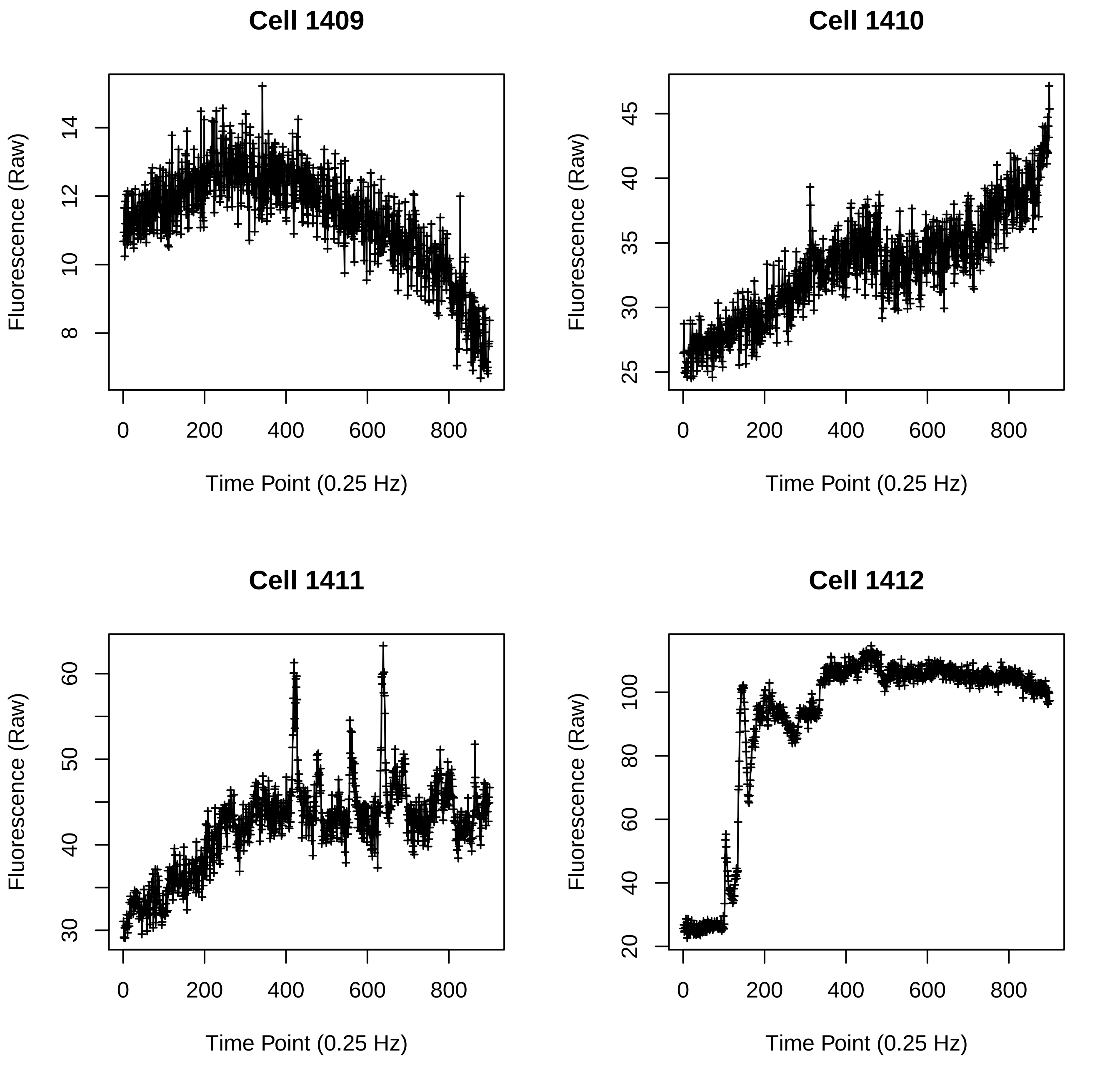

Cell 1409
Cell 1410
Cell 1411
Cell 1412
Fluorescence (Raw)
Time Point (0.25 Hz)
0
200
400
600
800
8
10
12
14
25
30
35
40
45
30
40
50
60
20
40
60
80
100

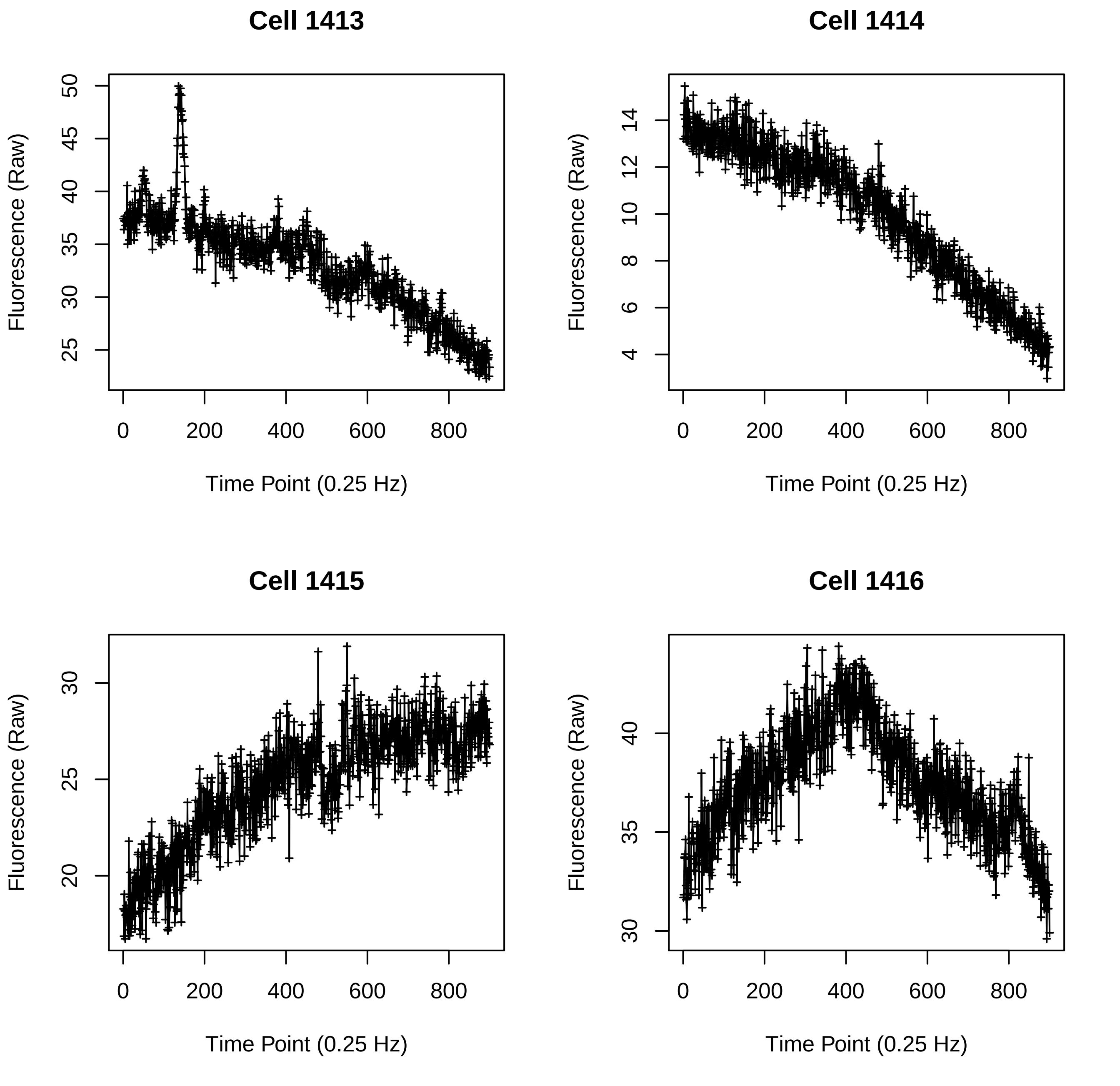

Cell 1413
Cell 1414
Cell 1415
Cell 1416
Fluorescence (Raw)
Time Point (0.25 Hz)
0
200
400
600
800
50
45
40
35
30
25
14
12
10
8
6
4
30
25
20
40
35
30

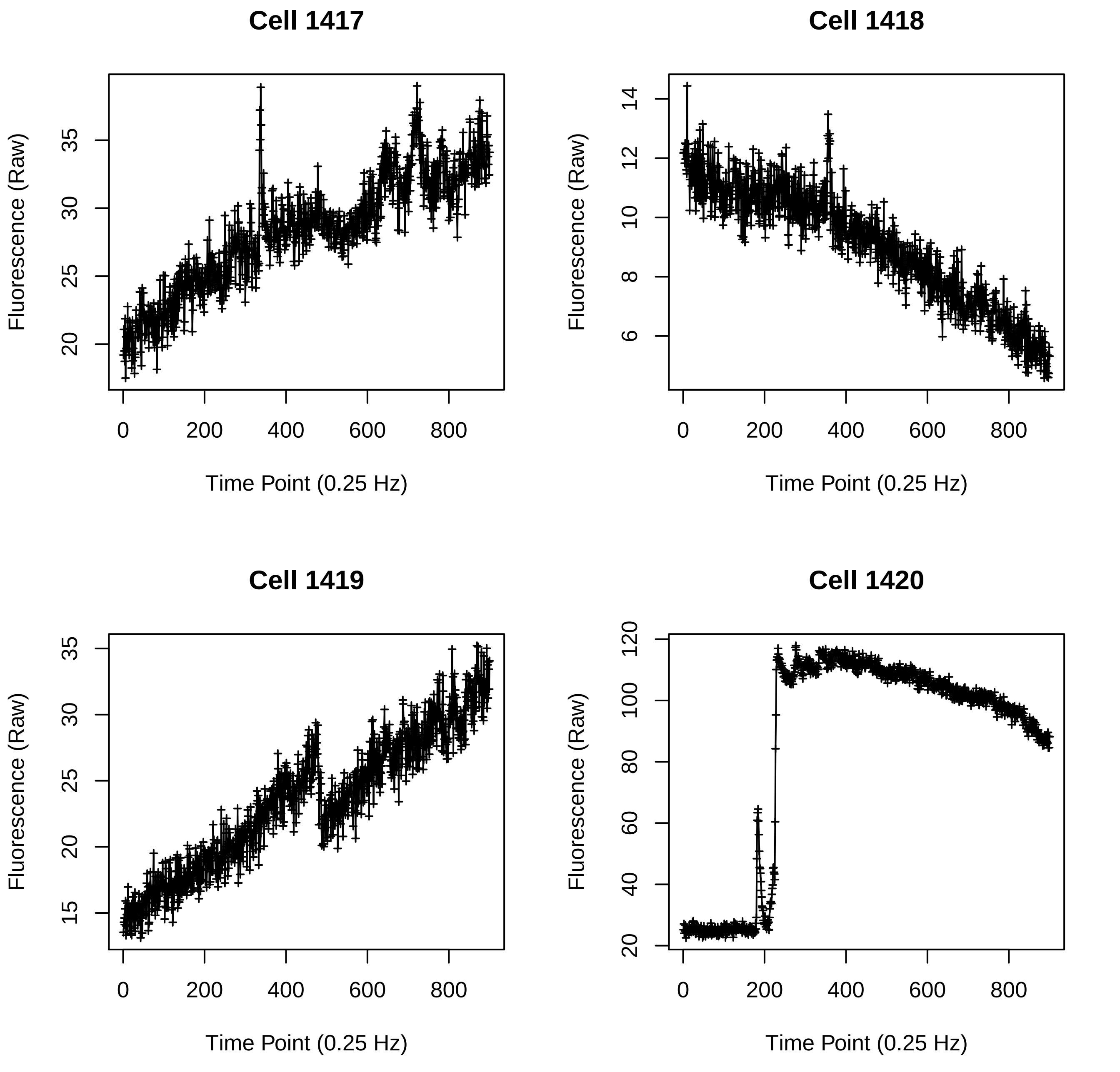
Cell 1417
Cell 1418
Cell 1419
Cell 1420
Fluorescence (Raw)
Time Point (0.25 Hz)
0
200
400
600
800
20
25
30
35
6
8
10
12
14
15
40
60
80
100
120

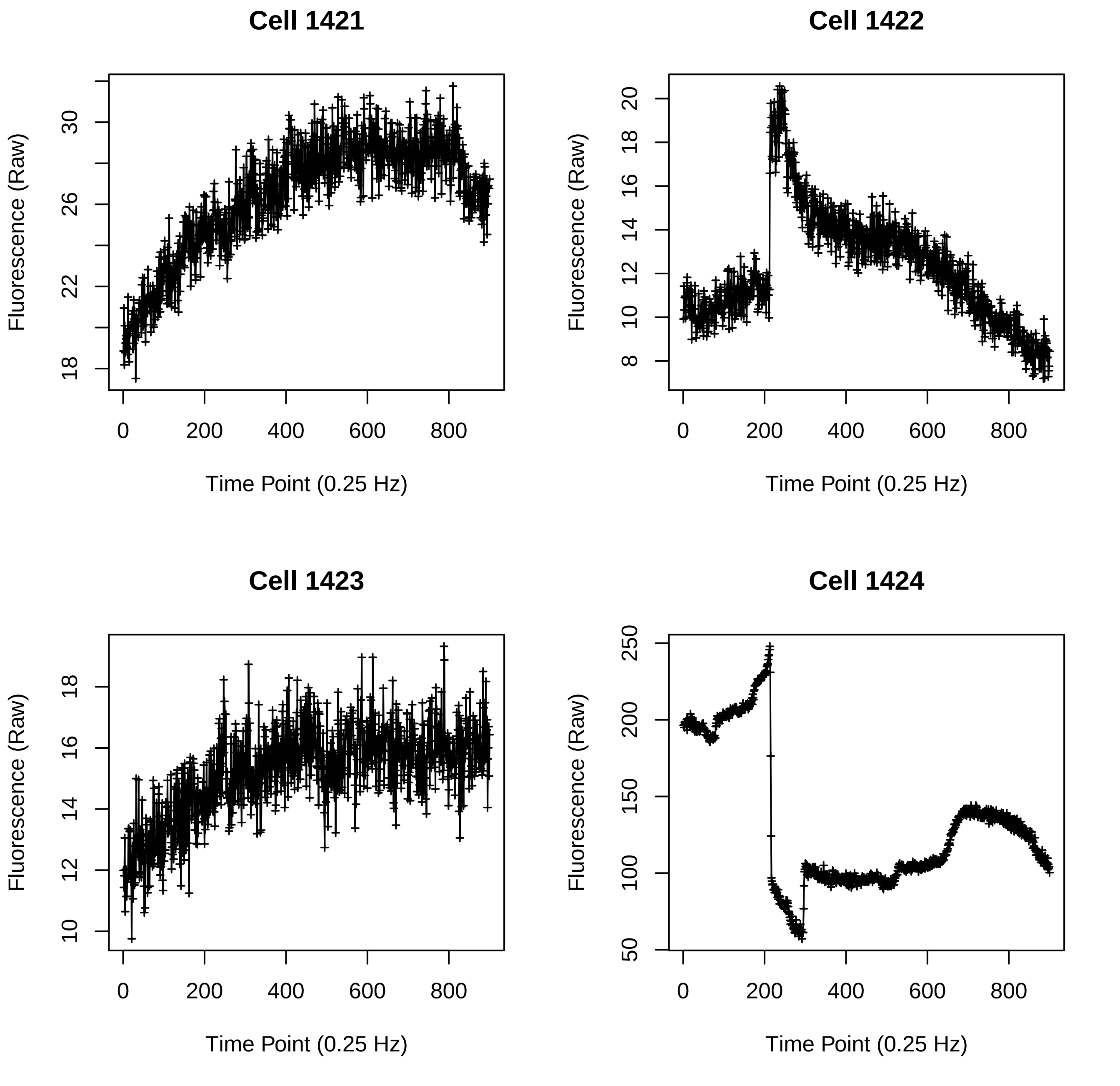

Cell 1421
Fluorescence (Raw)
18
22
26
30
0
200
400
600
800
Time Point (0.25 Hz)
Cell 1422
Fluorescence (Raw)
8
10
12
14
16
18
20
0
200
400
600
800
Time Point (0.25 Hz)
Cell 1423
Fluorescence (Raw)
10
12
14
16
18
0
200
400
600
800
Time Point (0.25 Hz)
Cell 1424
Fluorescence (Raw)
50
100
150
200
250
0
200
400
600
800
Time Point (0.25 Hz)

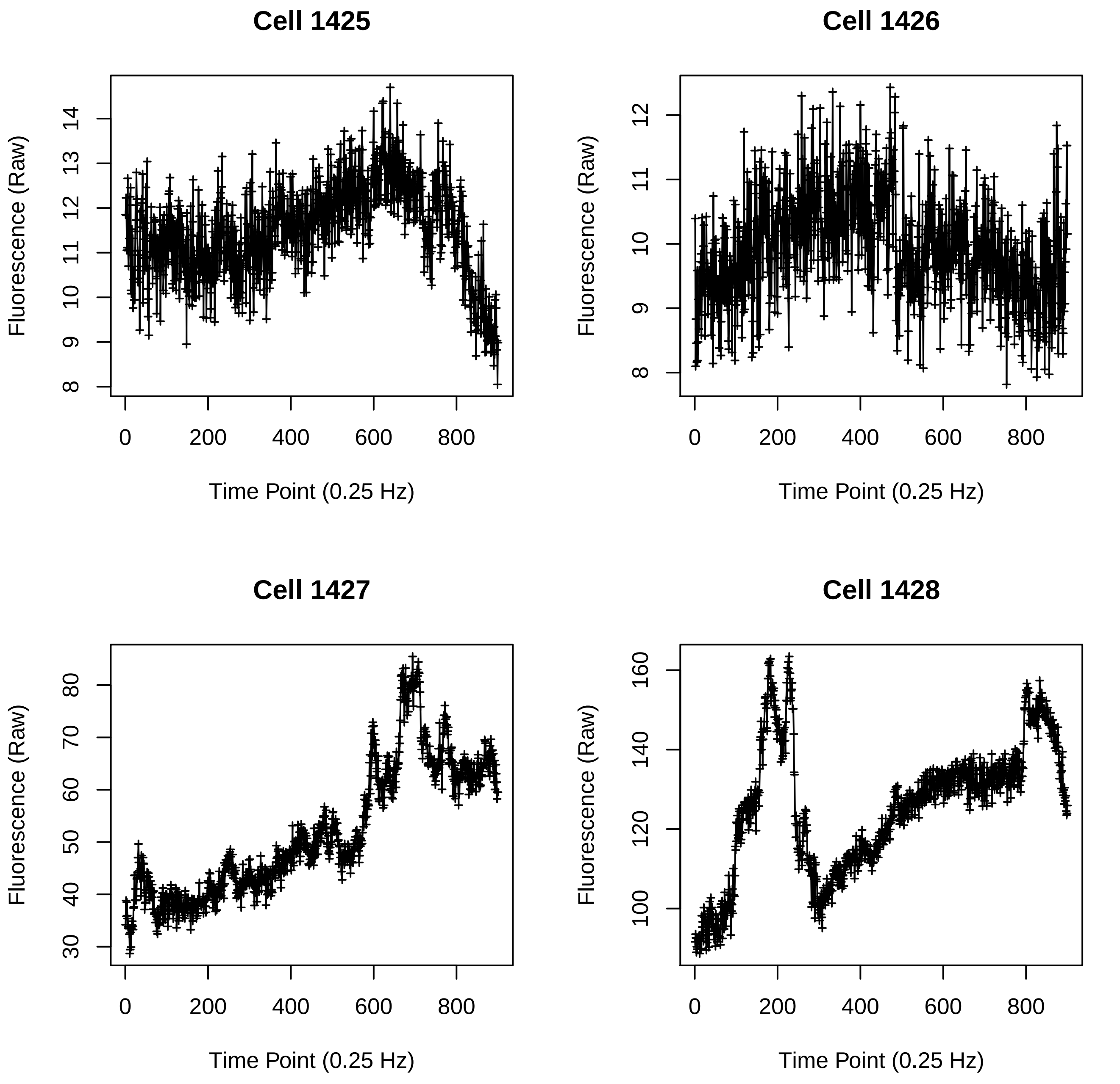
Cell 1425
Cell 1426
Cell 1427
Cell 1428
Fluorescence (Raw)
Time Point (0.25 Hz)
0
200
400
600
800

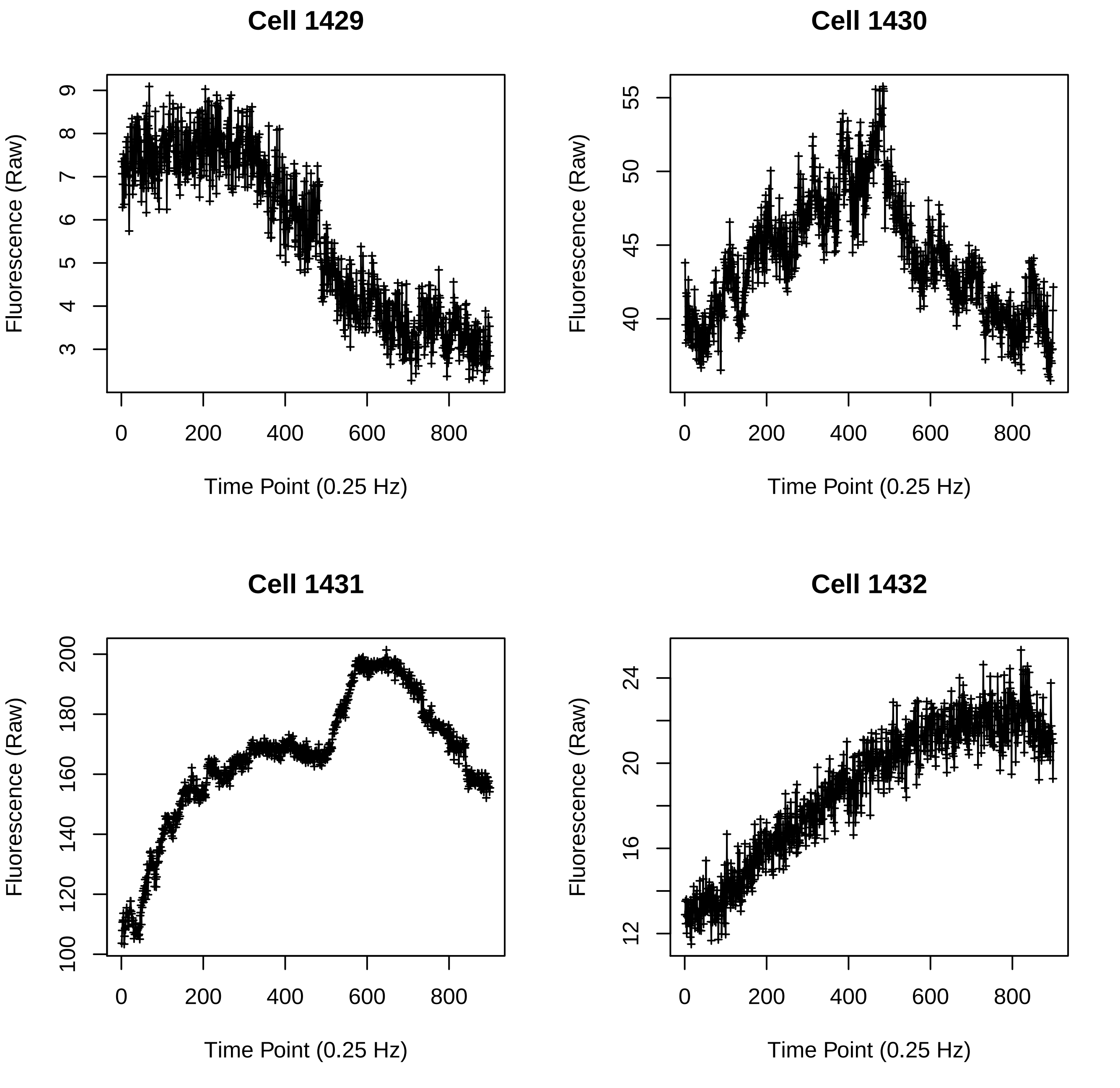

Cell 1429
Cell 1430
Cell 1431
Cell 1432
Fluorescence (Raw)
Time Point (0.25 Hz)
0
200
400
600
800
3
4
5
6
7
8
9
40
45
50
55
100
120
140
160
180
200
12
16
20
24

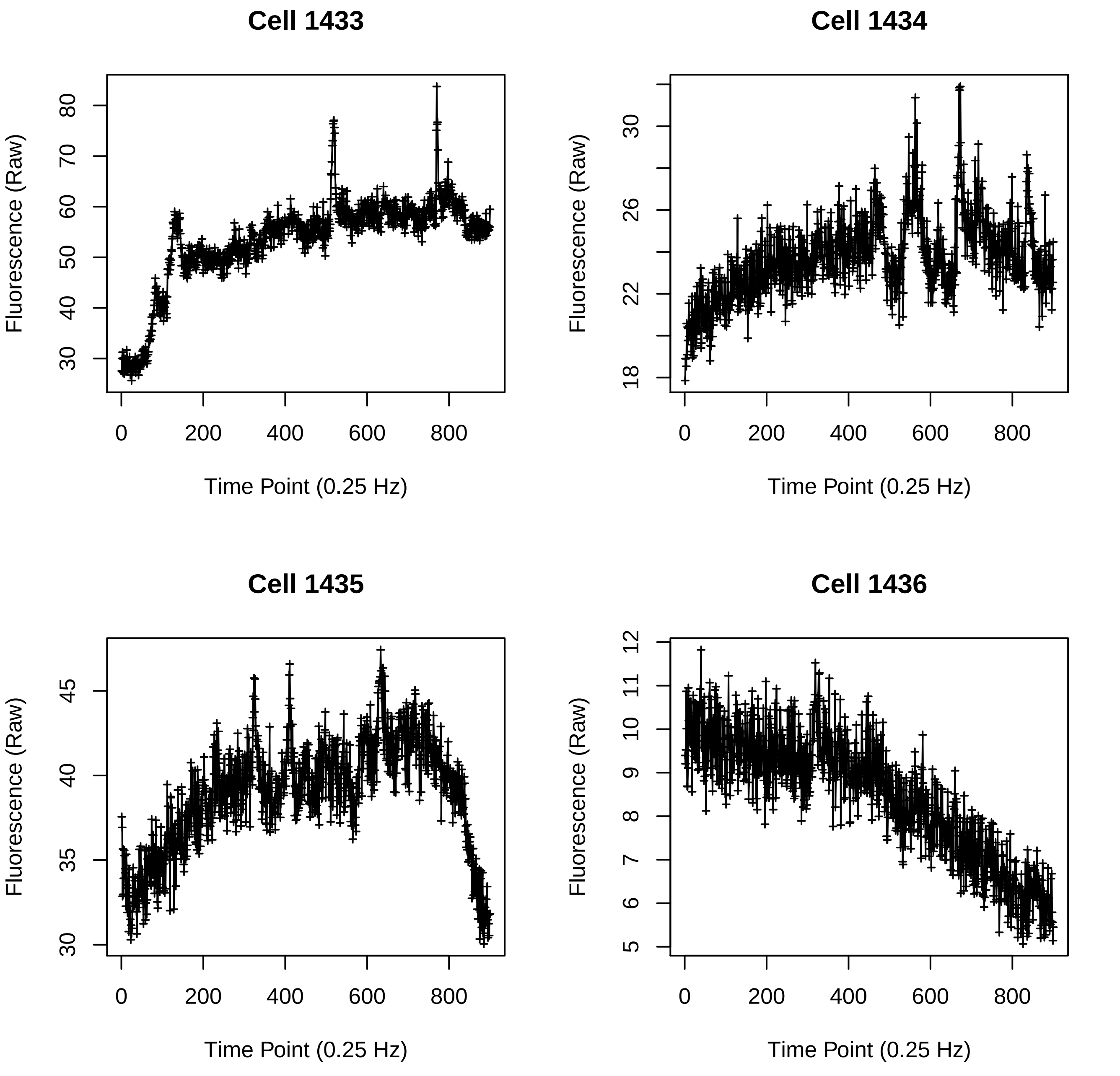
Cell 1433
Fluorescence (Raw)
30
40
50
60
70
80
0
200
400
600
800
Time Point (0.25 Hz)
Cell 1434
Fluorescence (Raw)
18
22
26
30
0
200
400
600
800
Time Point (0.25 Hz)
Cell 1435
Fluorescence (Raw)
30
35
40
45
0
200
400
600
800
Time Point (0.25 Hz)
Cell 1436
Fluorescence (Raw)
5
6
7
8
9
10
11
12
0
200
400
600
800
Time Point (0.25 Hz)

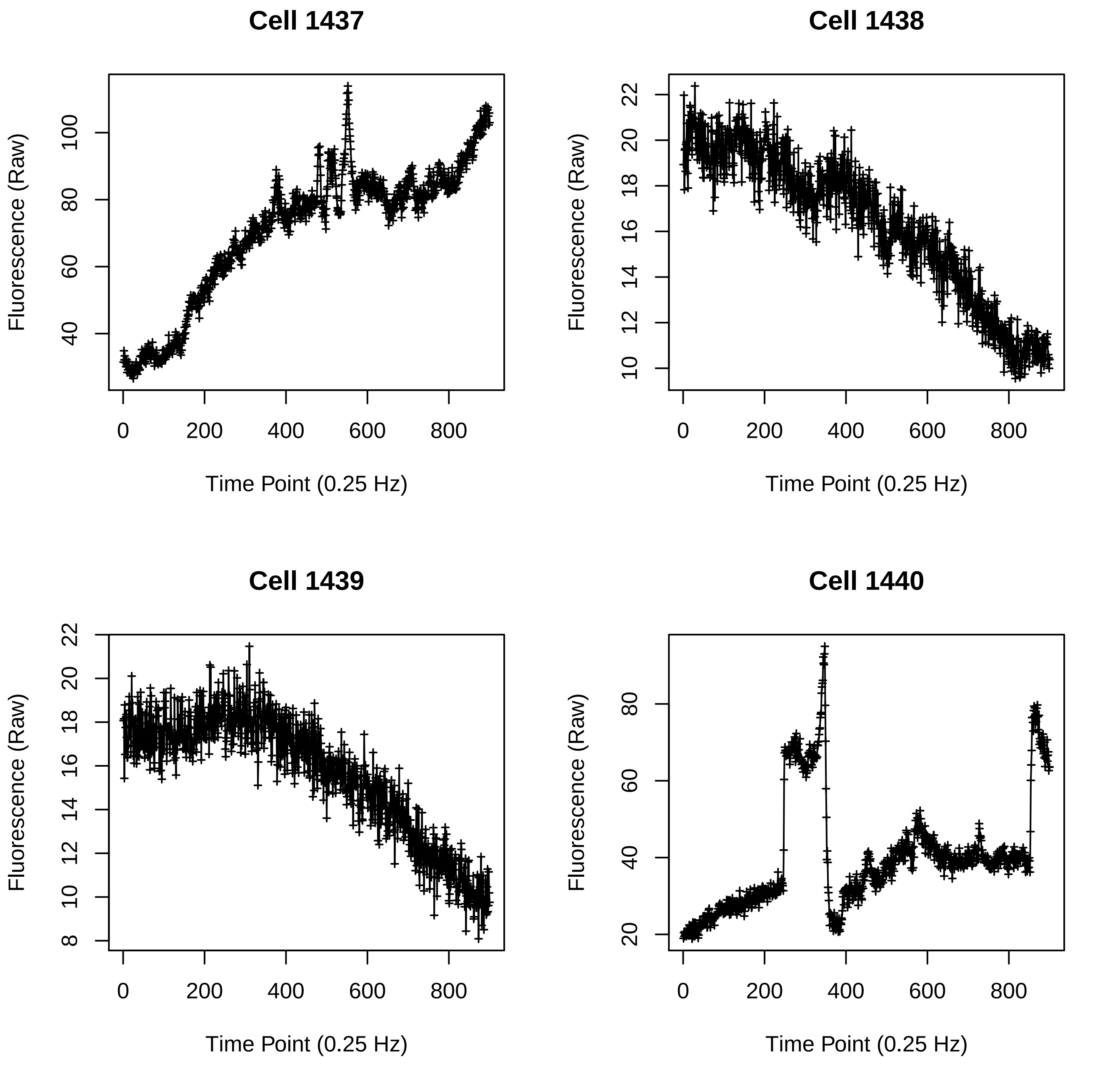

Cell 1437
Cell 1438
Cell 1439
Cell 1440
Fluorescence (Raw)
Time Point (0.25 Hz)
0
200
400
600
800
40
60
80
100
10
12
14
16
18
20
22
8

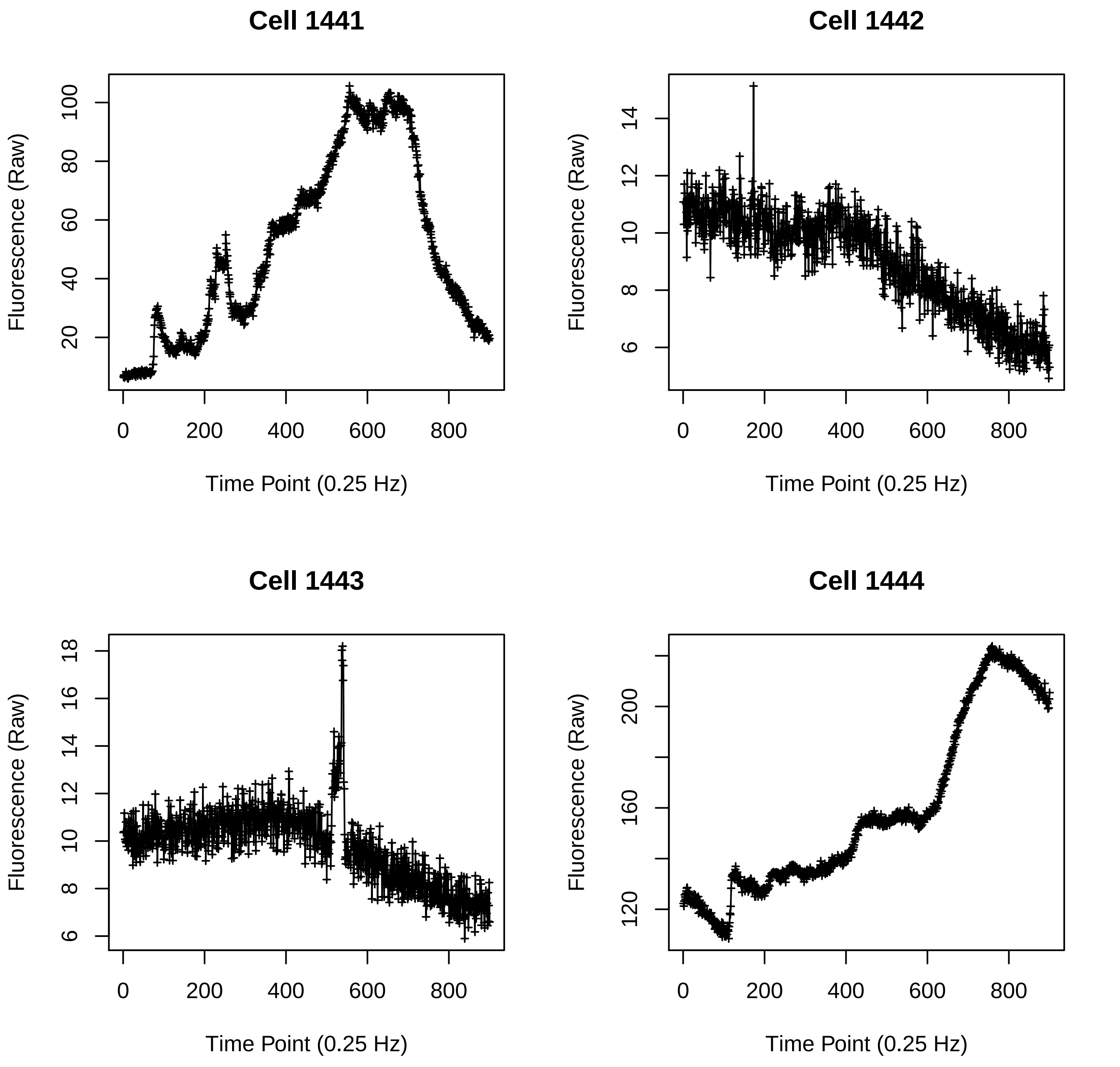
Cell 1441
Fluorescence (Raw)
20
40
60
80
100
0
200
400
600
800
Time Point (0.25 Hz)
Cell 1442
Fluorescence (Raw)
6
8
10
12
14
0
200
400
600
800
Time Point (0.25 Hz)
Cell 1443
Fluorescence (Raw)
6
8
10
12
14
16
18
0
200
400
600
800
Time Point (0.25 Hz)
Cell 1444
Fluorescence (Raw)
120
160
200
0
200
400
600
800
Time Point (0.25 Hz)

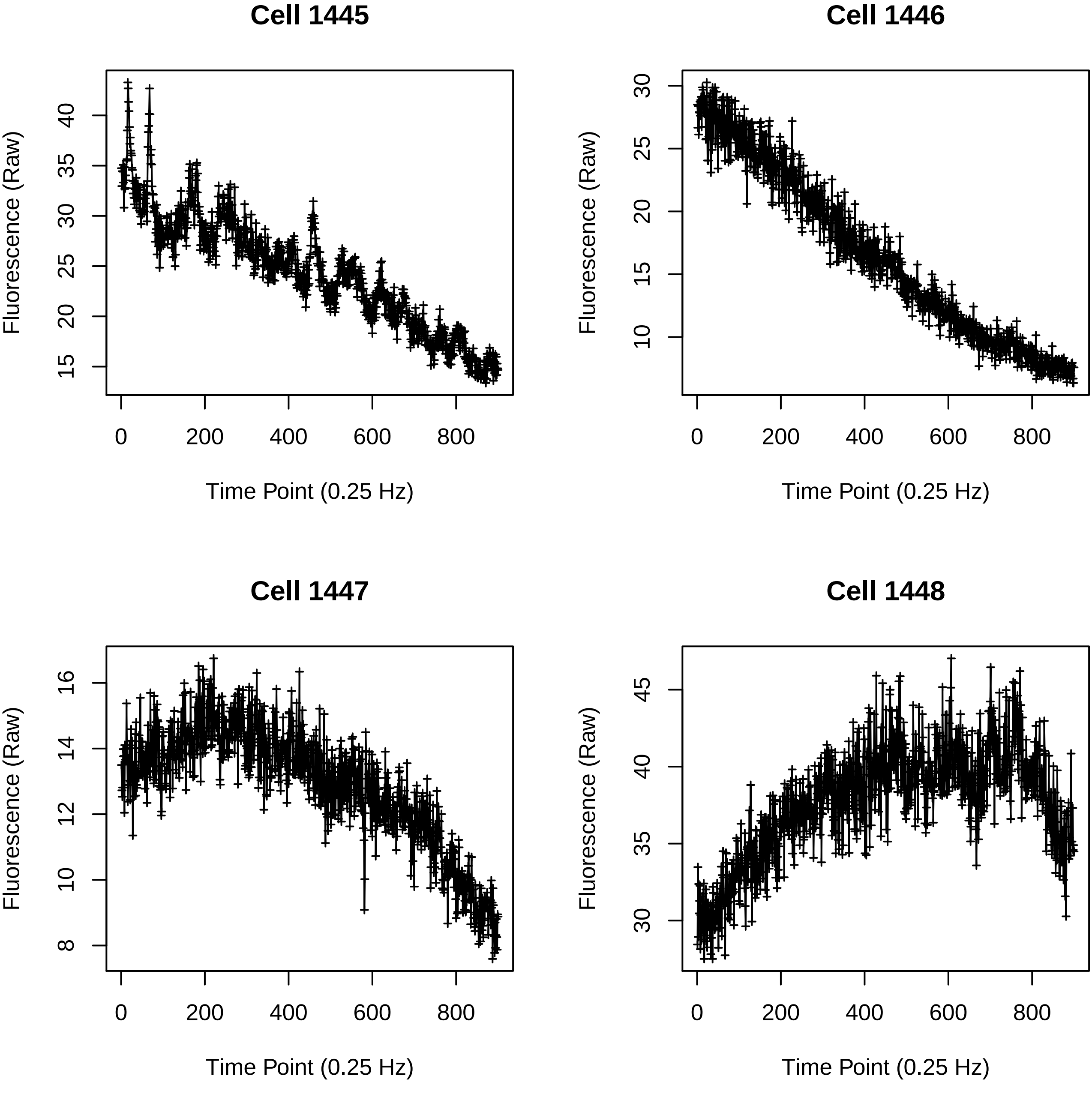
Cell 1445
Fluorescence (Raw)
15
20
25
30
35
40
0
200
400
600
800
Time Point (0.25 Hz)
Cell 1446
Fluorescence (Raw)
10
15
20
25
30
0
200
400
600
800
Time Point (0.25 Hz)
Cell 1447
Fluorescence (Raw)
8
10
12
14
16
0
200
400
600
800
Time Point (0.25 Hz)
Cell 1448
Fluorescence (Raw)
30
35
40
45
0
200
400
600
800
Time Point (0.25 Hz)

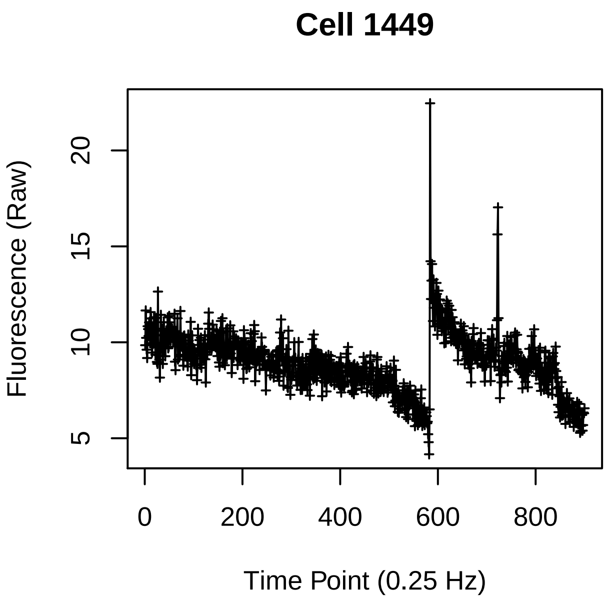
Cell 1449
Fluorescence (Raw)
5
10
15
20
0
200
400
600
800
Time Point (0.25 Hz)

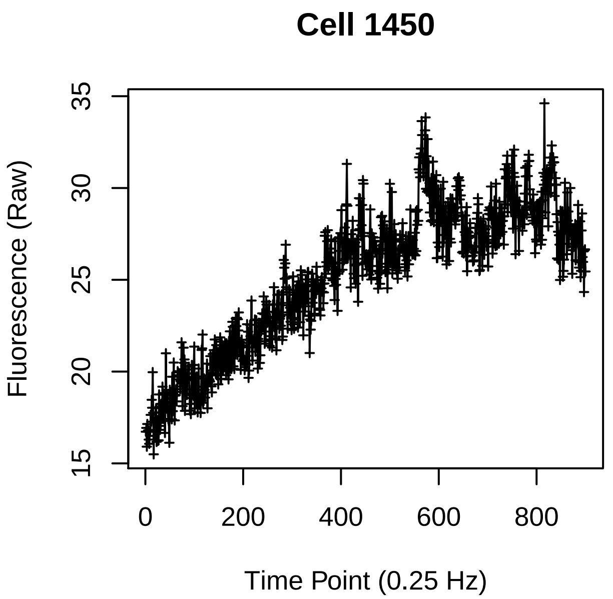
Cell 1450
Fluorescence (Raw)
15
20
25
30
35
0
200
400
600
800
Time Point (0.25 Hz)

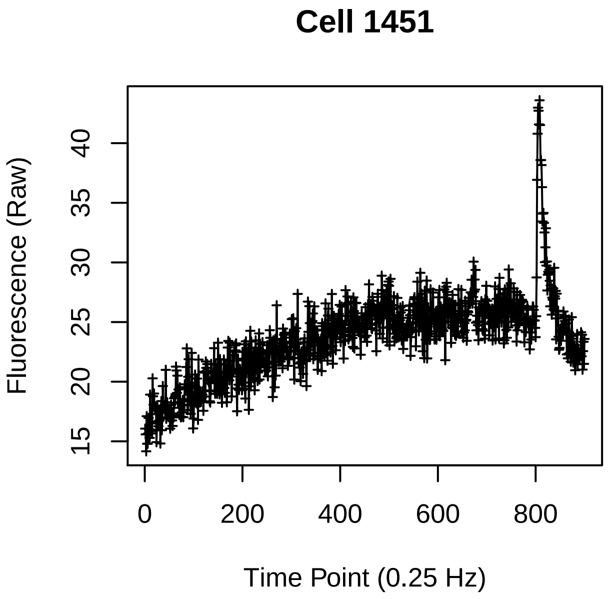
Cell 1451
Fluorescence (Raw)
15
20
25
30
35
40
0
200
400
600
800
Time Point (0.25 Hz)

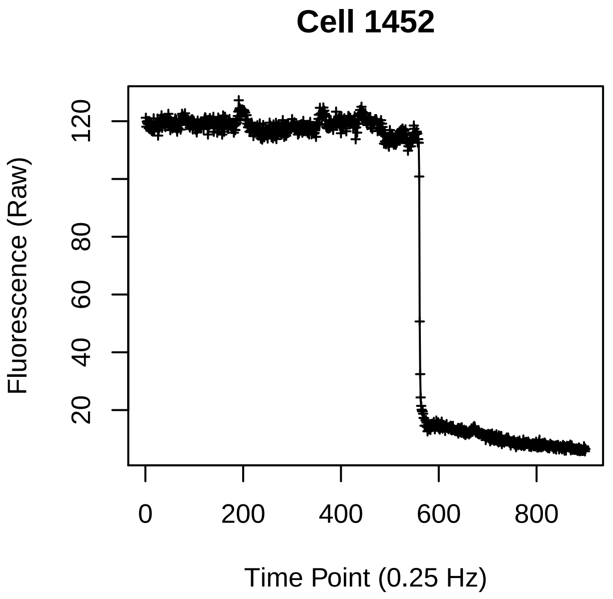
Cell 1452
Fluorescence (Raw)
20
40
60
80
120
0
200
400
600
800
Time Point (0.25 Hz)

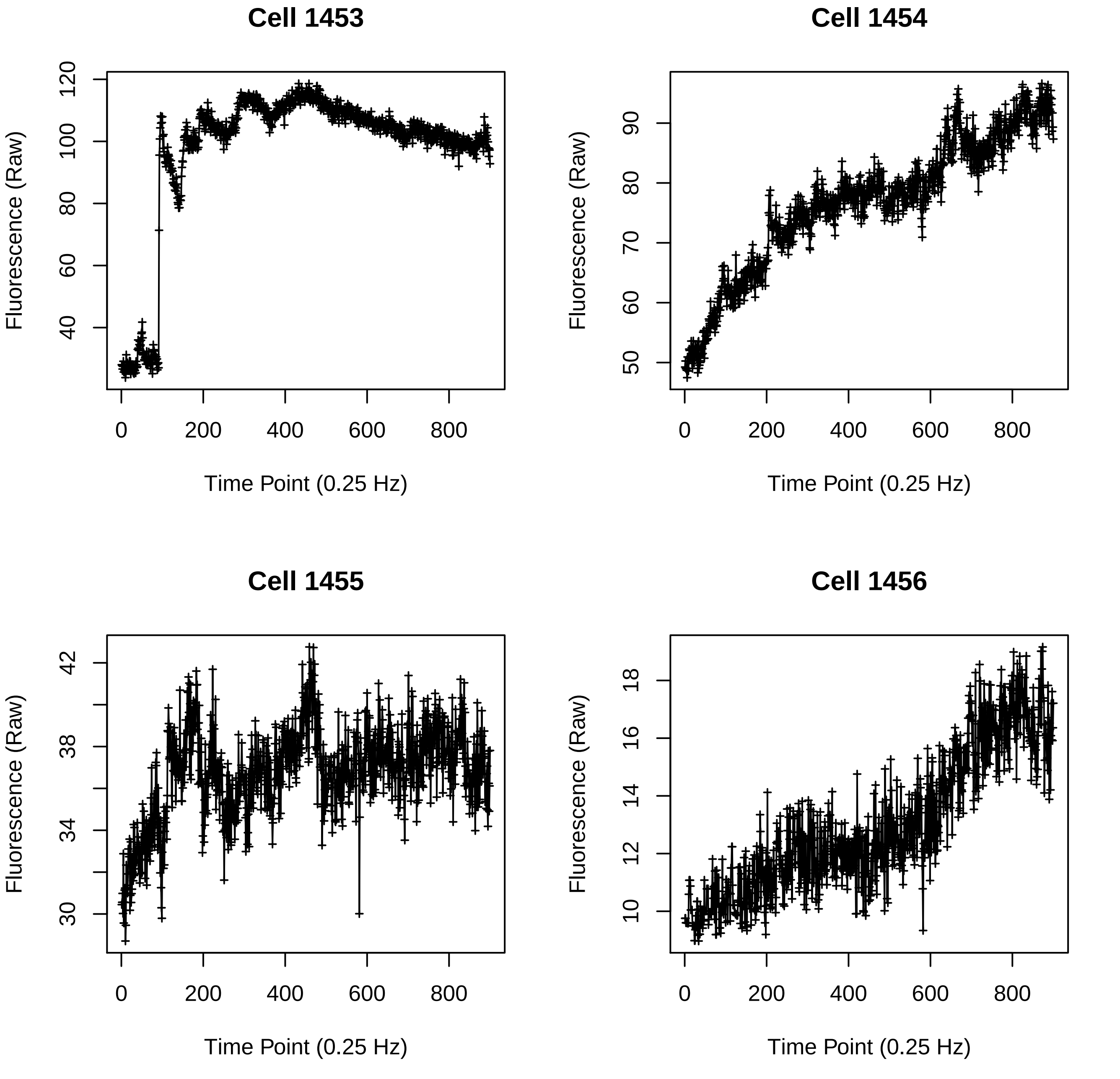
Cell 1453
Fluorescence (Raw)
Time Point (0.25 Hz)
Cell 1454
Fluorescence (Raw)
Time Point (0.25 Hz)
Cell 1455
Fluorescence (Raw)
Time Point (0.25 Hz)
Cell 1456
Fluorescence (Raw)
Time Point (0.25 Hz)

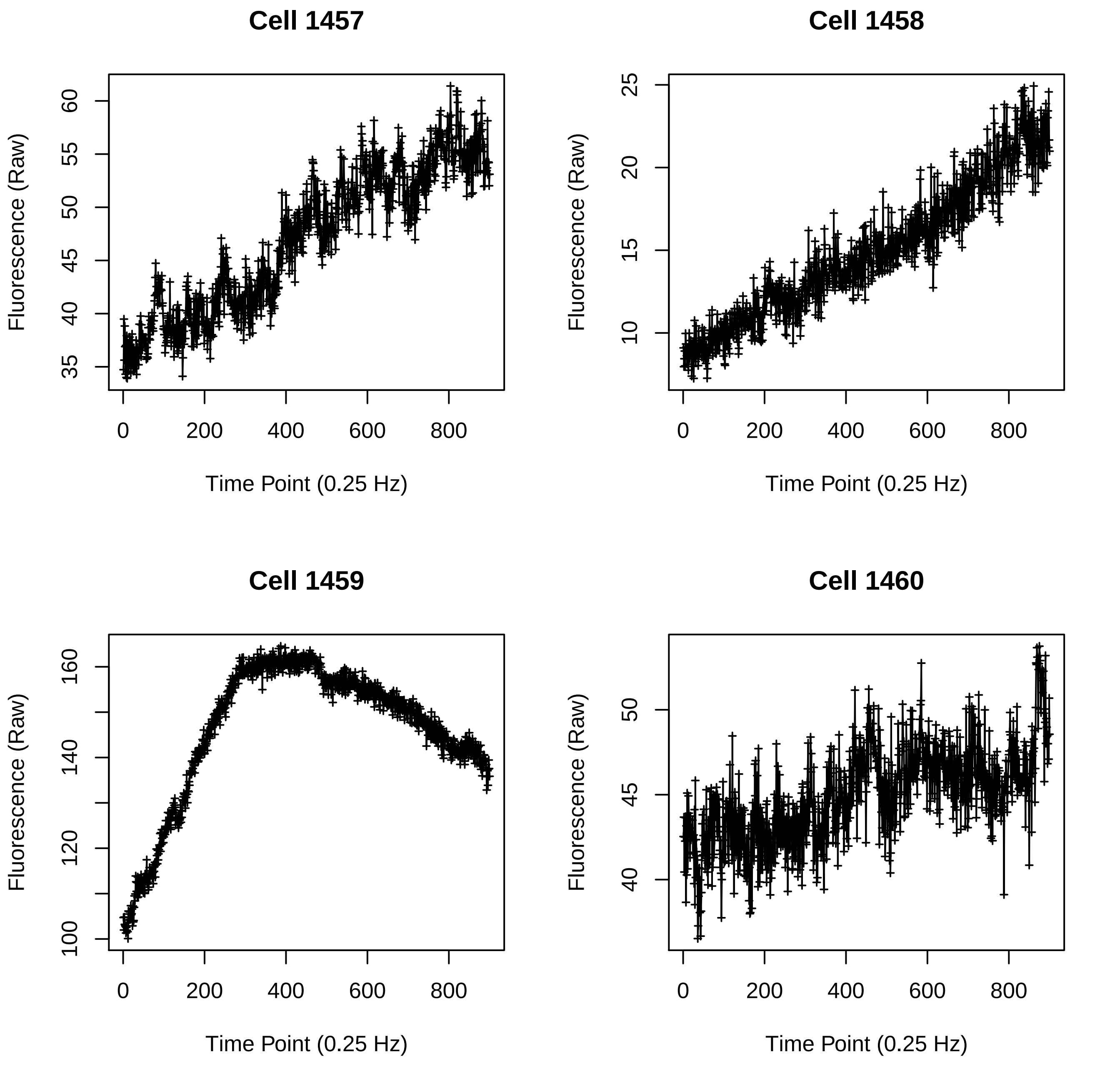

Cell 1457
Cell 1458
Cell 1459
Cell 1460
Fluorescence (Raw)
Time Point (0.25 Hz)
35
40
45
50
55
60
10
15
20
25
100
120
140
160
0
200
400
600
800

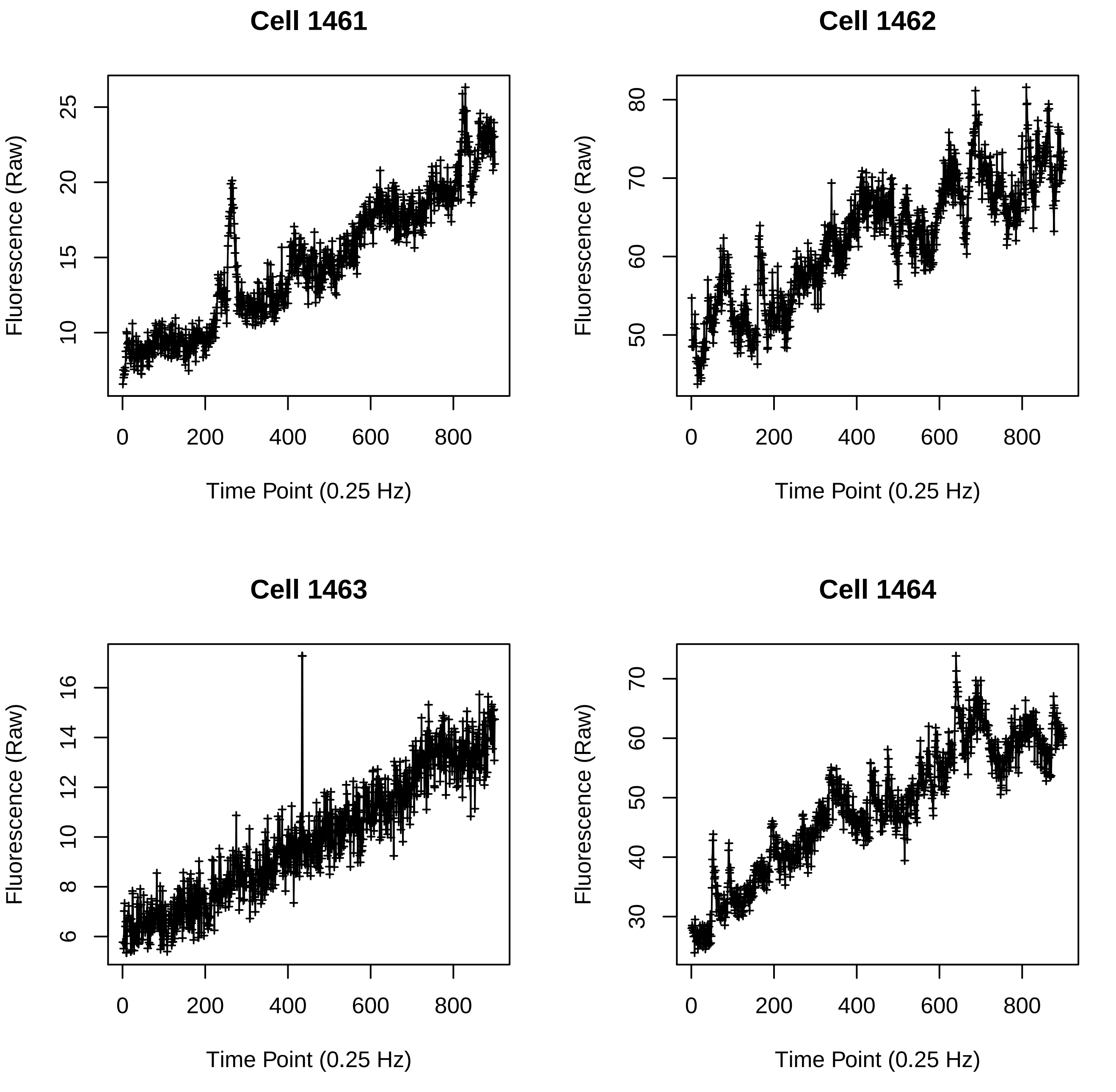
Cell 1461
Cell 1462
Cell 1463
Cell 1464
Fluorescence (Raw)
Time Point (0.25 Hz)
0
200
400
600
800
10
15
20
25
50
60
70
80
6
8
10
12
14
16
30
40
50
60
70

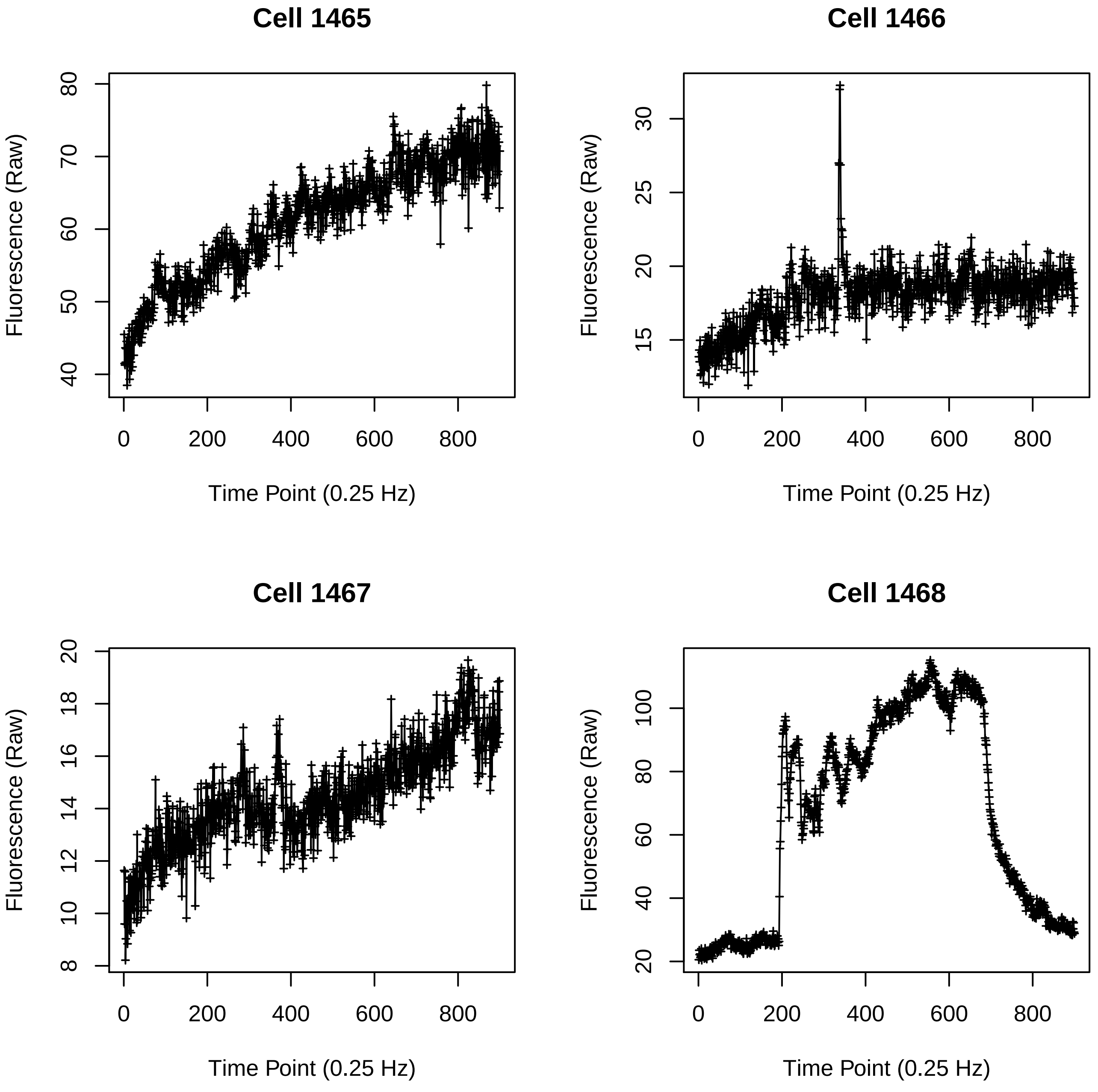
Cell 1465
Fluorescence (Raw)
40
50
60
70
80
0
200
400
600
800
Time Point (0.25 Hz)
Cell 1466
Fluorescence (Raw)
15
20
25
30
0
200
400
600
800
Time Point (0.25 Hz)
Cell 1467
Fluorescence (Raw)
8
10
12
14
16
18
20
0
200
400
600
800
Time Point (0.25 Hz)
Cell 1468
Fluorescence (Raw)
20
40
60
80
100
0
200
400
600
800
Time Point (0.25 Hz)

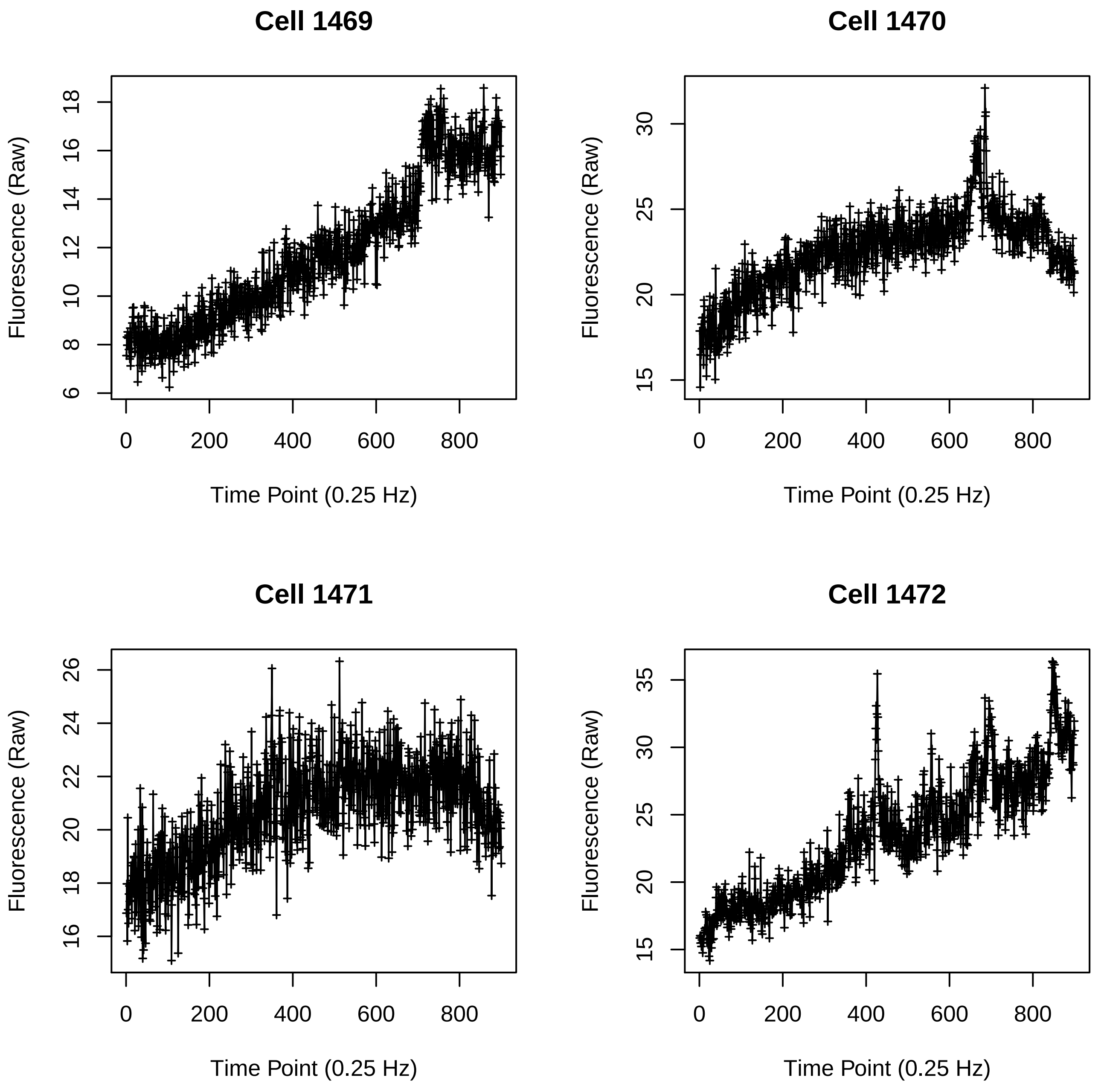
Cell 1469
Fluorescence (Raw)
6
8
10
12
14
16
18
0
200
400
600
800
Time Point (0.25 Hz)
Cell 1470
Fluorescence (Raw)
15
20
25
30
0
200
400
600
800
Time Point (0.25 Hz)
Cell 1471
Fluorescence (Raw)
16
18
20
22
24
26
0
200
400
600
800
Time Point (0.25 Hz)
Cell 1472
Fluorescence (Raw)
15
20
25
30
35
0
200
400
600
800
Time Point (0.25 Hz)

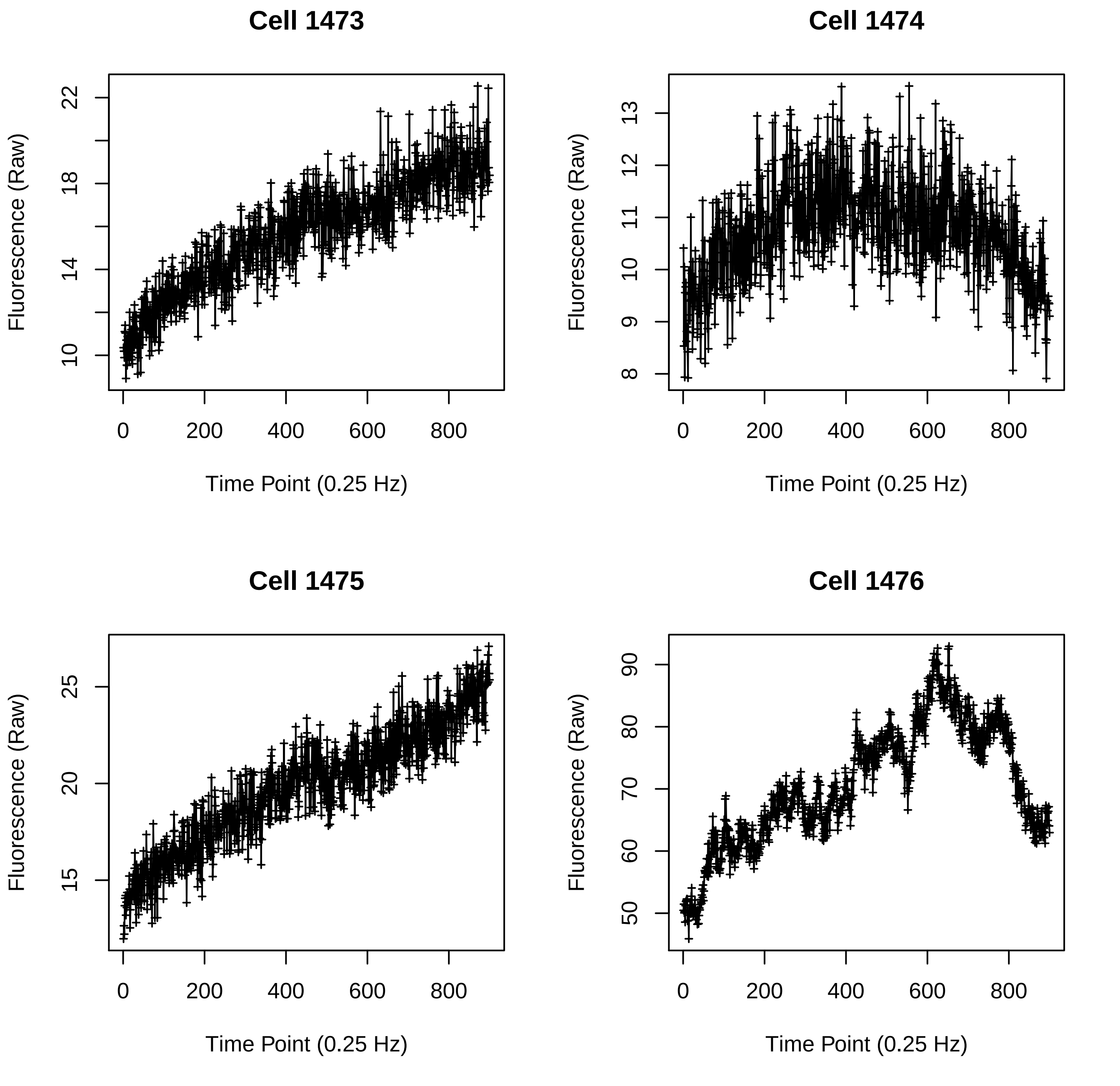

Cell 1473
Cell 1474
Cell 1475
Cell 1476
Fluorescence (Raw)
Time Point (0.25 Hz)
0
200
400
600
800
10
14
18
22
8
9
10
11
12
13
15
20
25
50
60
70
80
90

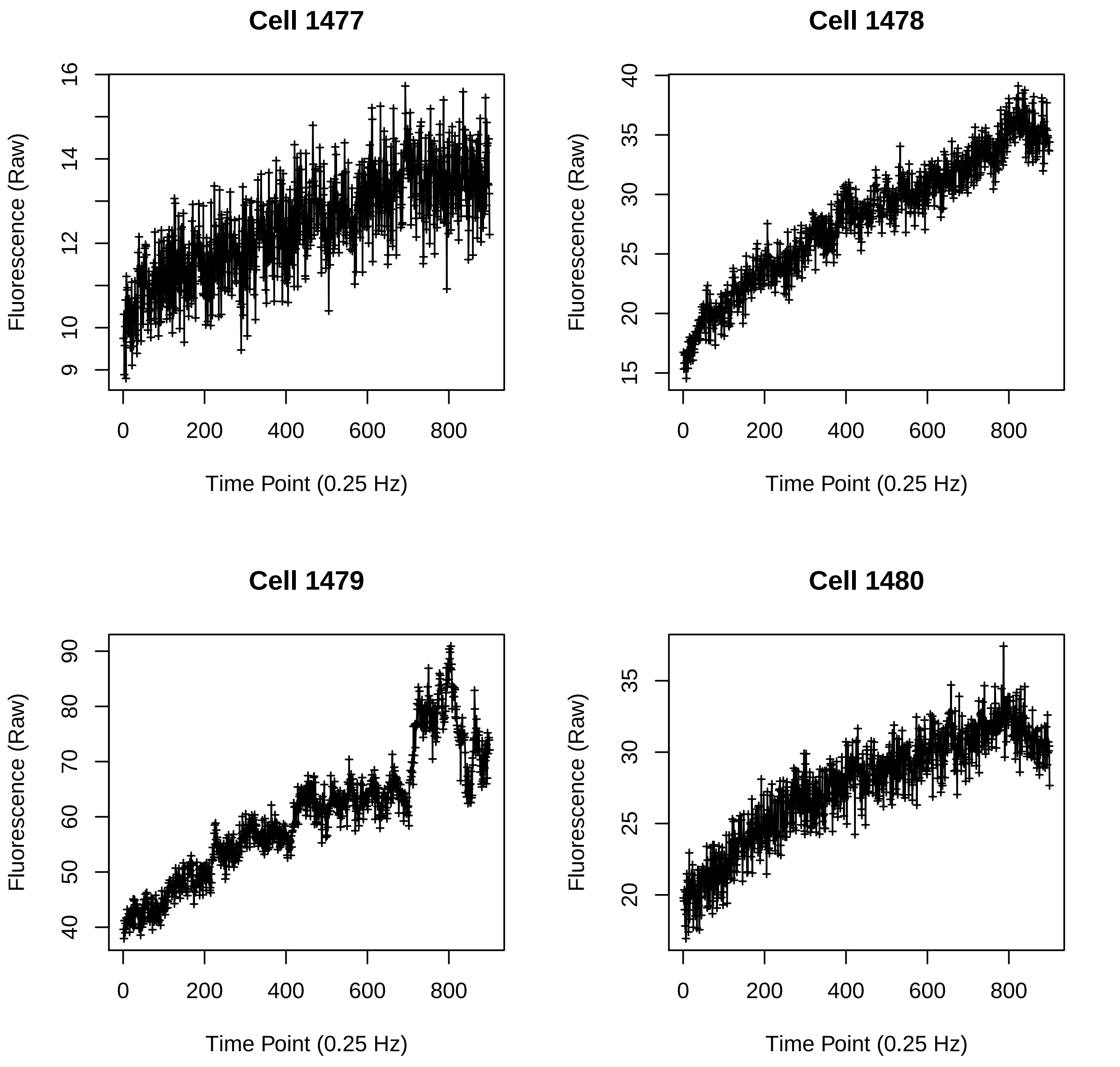

Cell 1477
Cell 1478
Cell 1479
Cell 1480
Fluorescence (Raw)
Time Point (0.25 Hz)
0
200
400
600
800
9
10
12
14
16
15
20
25
30
35
40
50
60
70
80
90

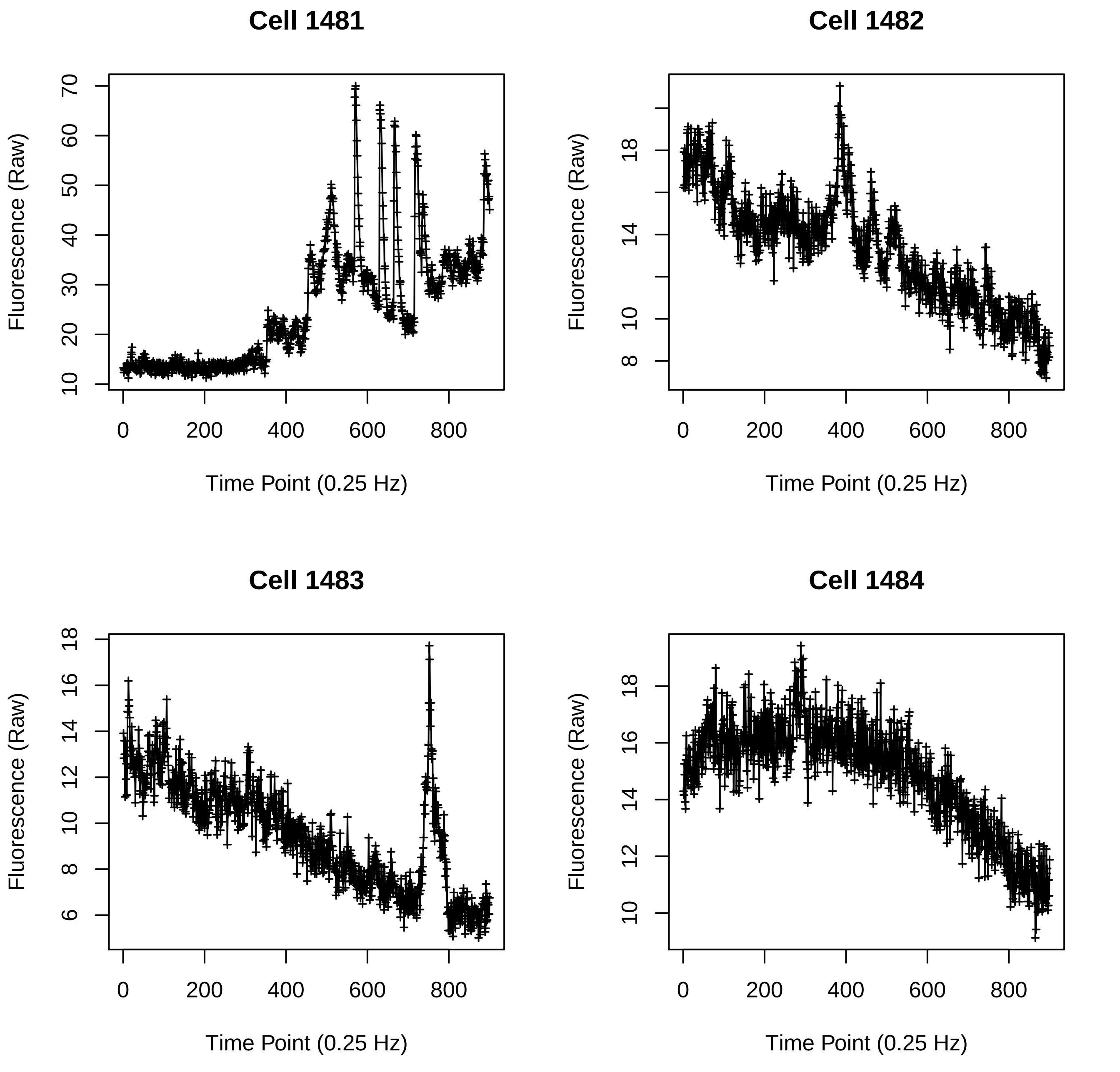
Cell 1481
Cell 1482
Cell 1483
Cell 1484
Fluorescence (Raw)
Time Point (0.25 Hz)
0
200
400
600
800
10
20
30
40
50
60
70
8
10
12
14
16
18

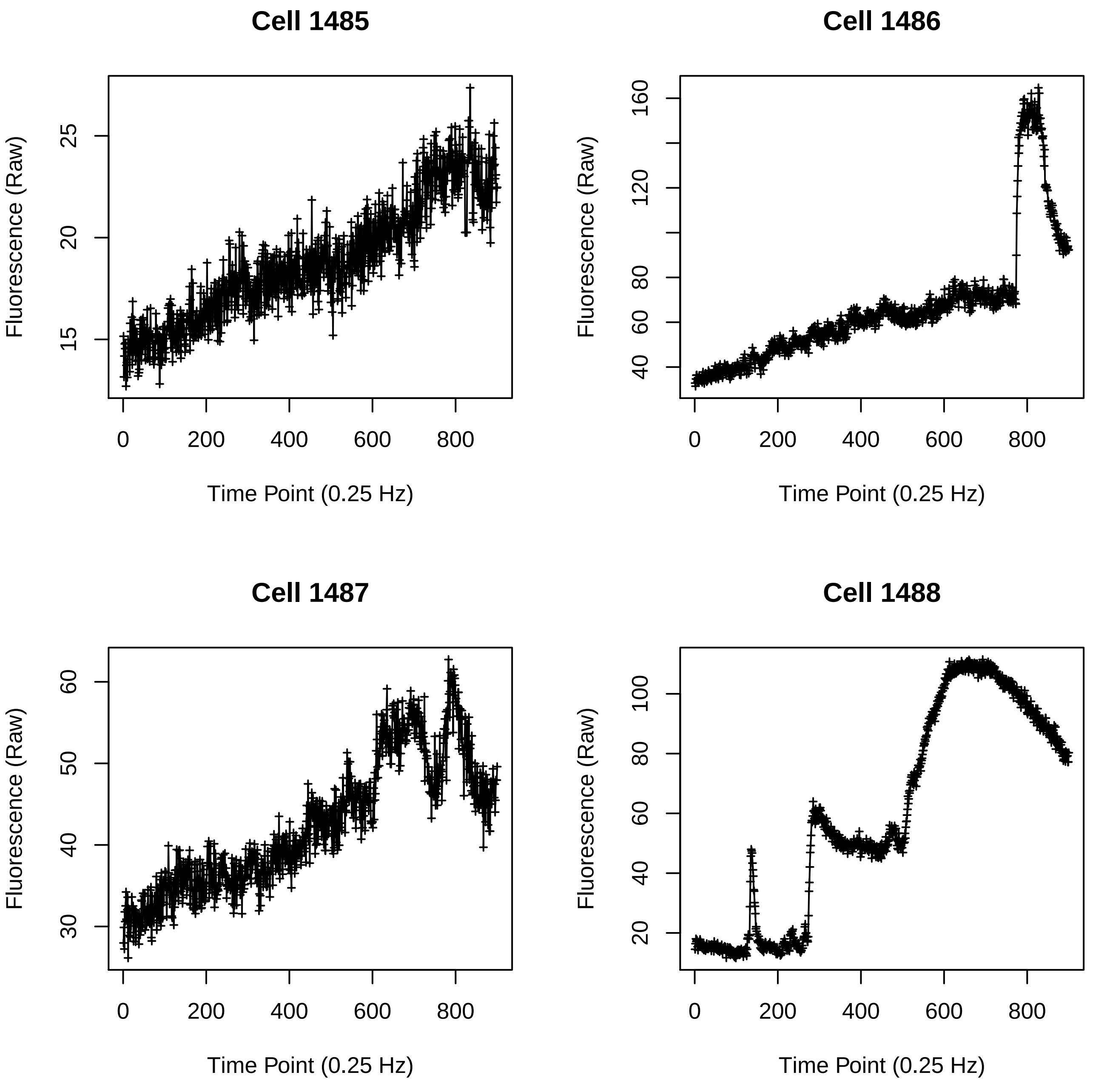
Cell 1485
Fluorescence (Raw)
15
20
25
0
200
400
600
800
Time Point (0.25 Hz)
Cell 1486
Fluorescence (Raw)
40
60
80
120
160
0
200
400
600
800
Time Point (0.25 Hz)
Cell 1487
Fluorescence (Raw)
30
40
50
60
0
200
400
600
800
Time Point (0.25 Hz)
Cell 1488
Fluorescence (Raw)
20
40
60
80
100
0
200
400
600
800
Time Point (0.25 Hz)

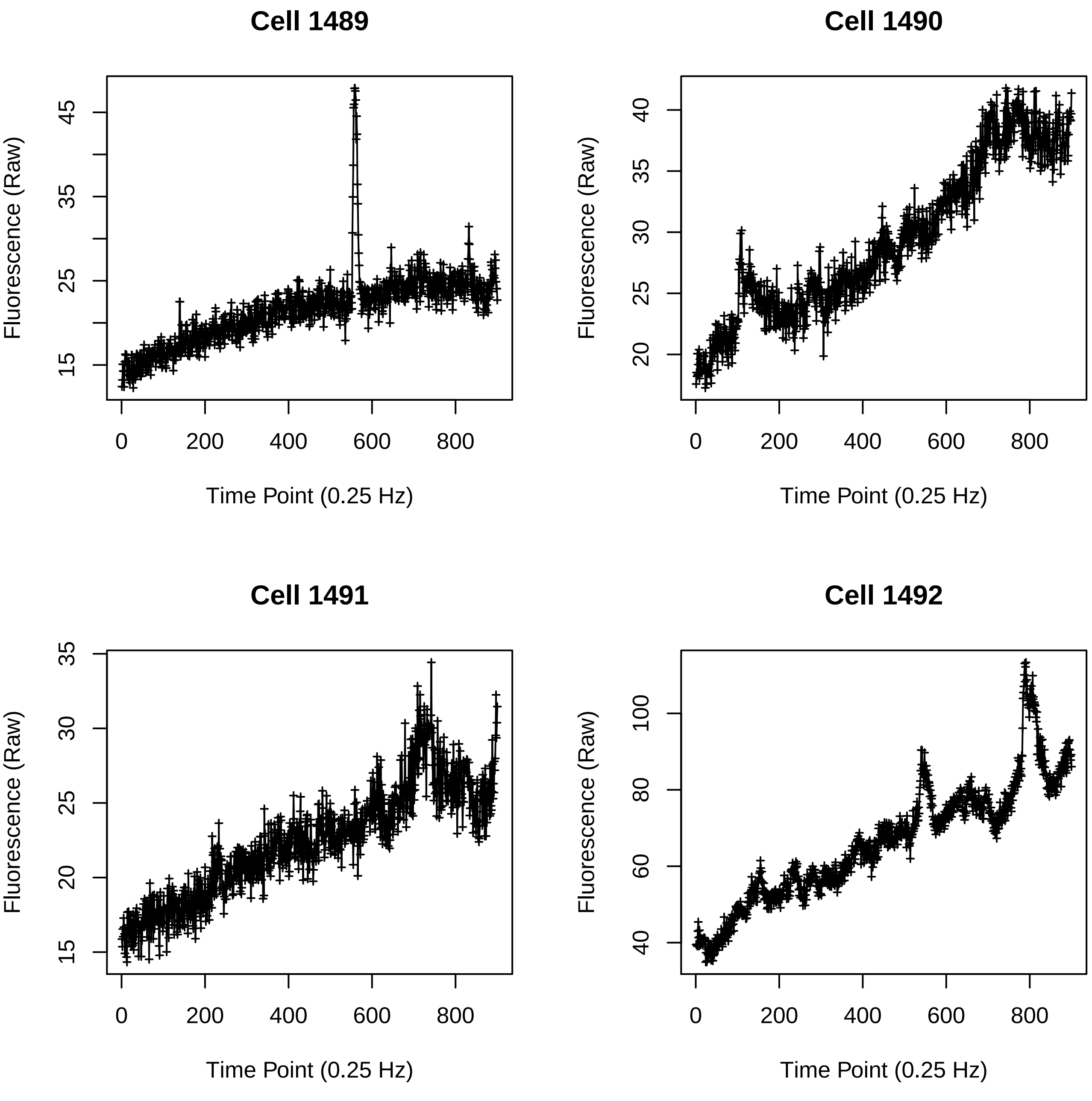

Cell 1489
Fluorescence (Raw)
15
25
35
45
0
200
400
600
800
Time Point (0.25 Hz)
Cell 1490
Fluorescence (Raw)
20
25
30
35
40
0
200
400
600
800
Time Point (0.25 Hz)
Cell 1491
Fluorescence (Raw)
15
20
25
30
35
0
200
400
600
800
Time Point (0.25 Hz)
Cell 1492
Fluorescence (Raw)
40
60
80
100
0
200
400
600
800
Time Point (0.25 Hz)

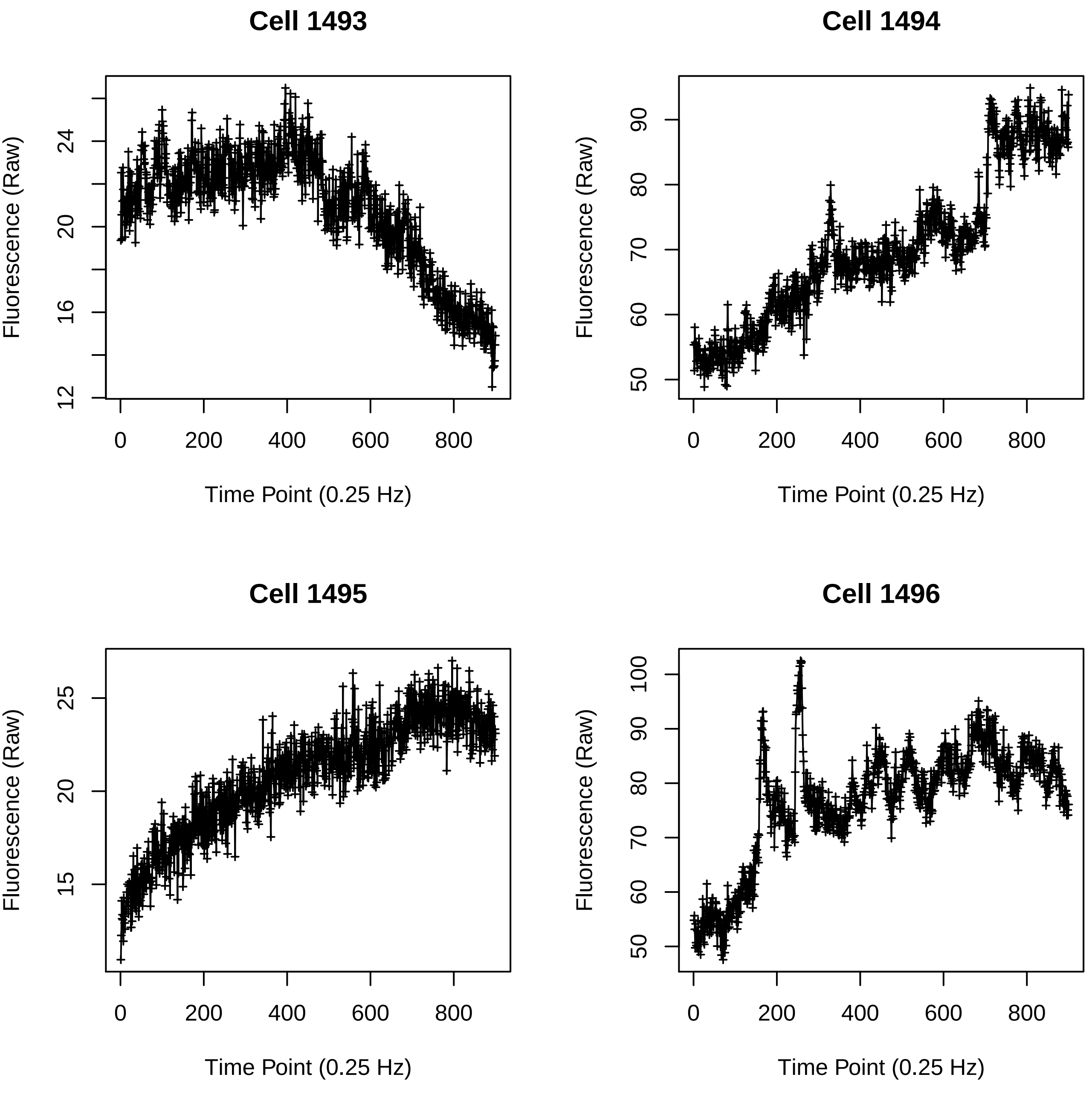
Cell 1493
Cell 1494
Cell 1495
Cell 1496
Fluorescence (Raw)
Time Point (0.25 Hz)
0
200
400
600
800
12
16
20
24
50
60
70
80
90
15
20
25
100

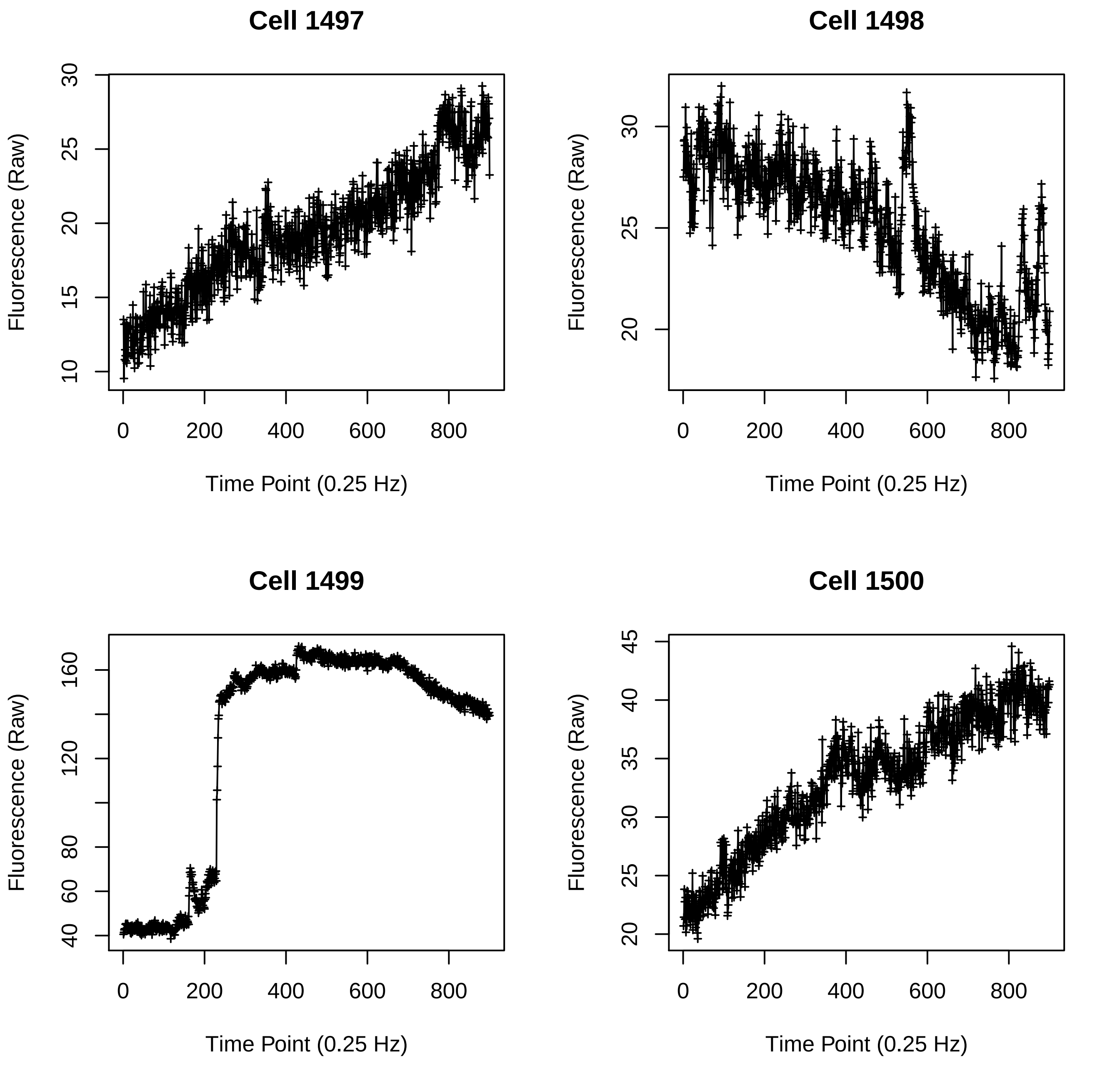
Cell 1497
Cell 1498
Cell 1499
Cell 1500
Fluorescence (Raw)
Time Point (0.25 Hz)
0
200
400
600
800
10
15
20
25
30
40
60
80
120
160
35
45

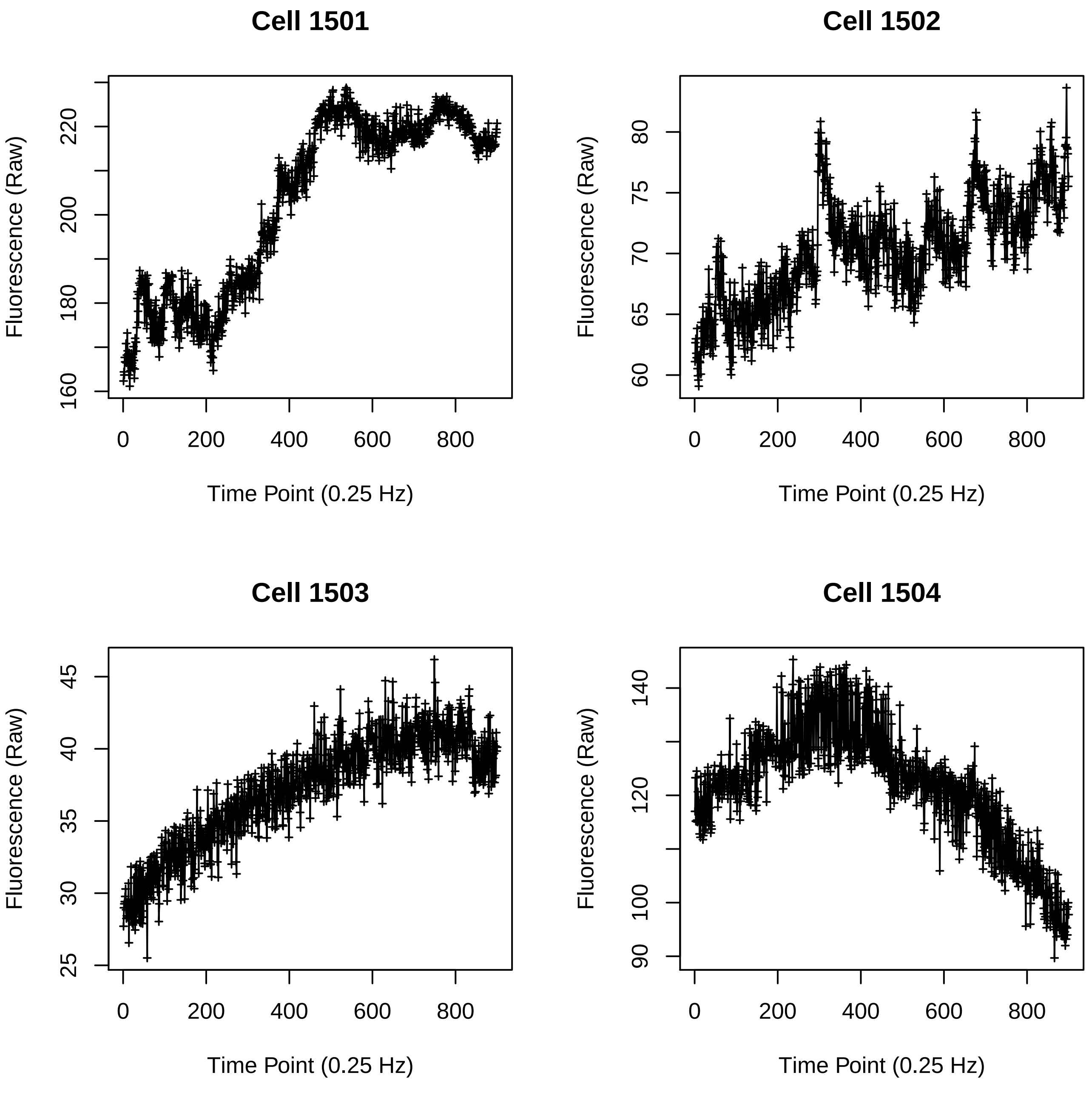

Cell 1501
Fluorescence (Raw)
160
180
200
220
0
200
400
600
800
Time Point (0.25 Hz)
Cell 1502
Fluorescence (Raw)
60
65
70
75
80
0
200
400
600
800
Time Point (0.25 Hz)
Cell 1503
Fluorescence (Raw)
25
30
35
40
45
0
200
400
600
800
Time Point (0.25 Hz)
Cell 1504
Fluorescence (Raw)
90
100
120
140
0
200
400
600
800
Time Point (0.25 Hz)

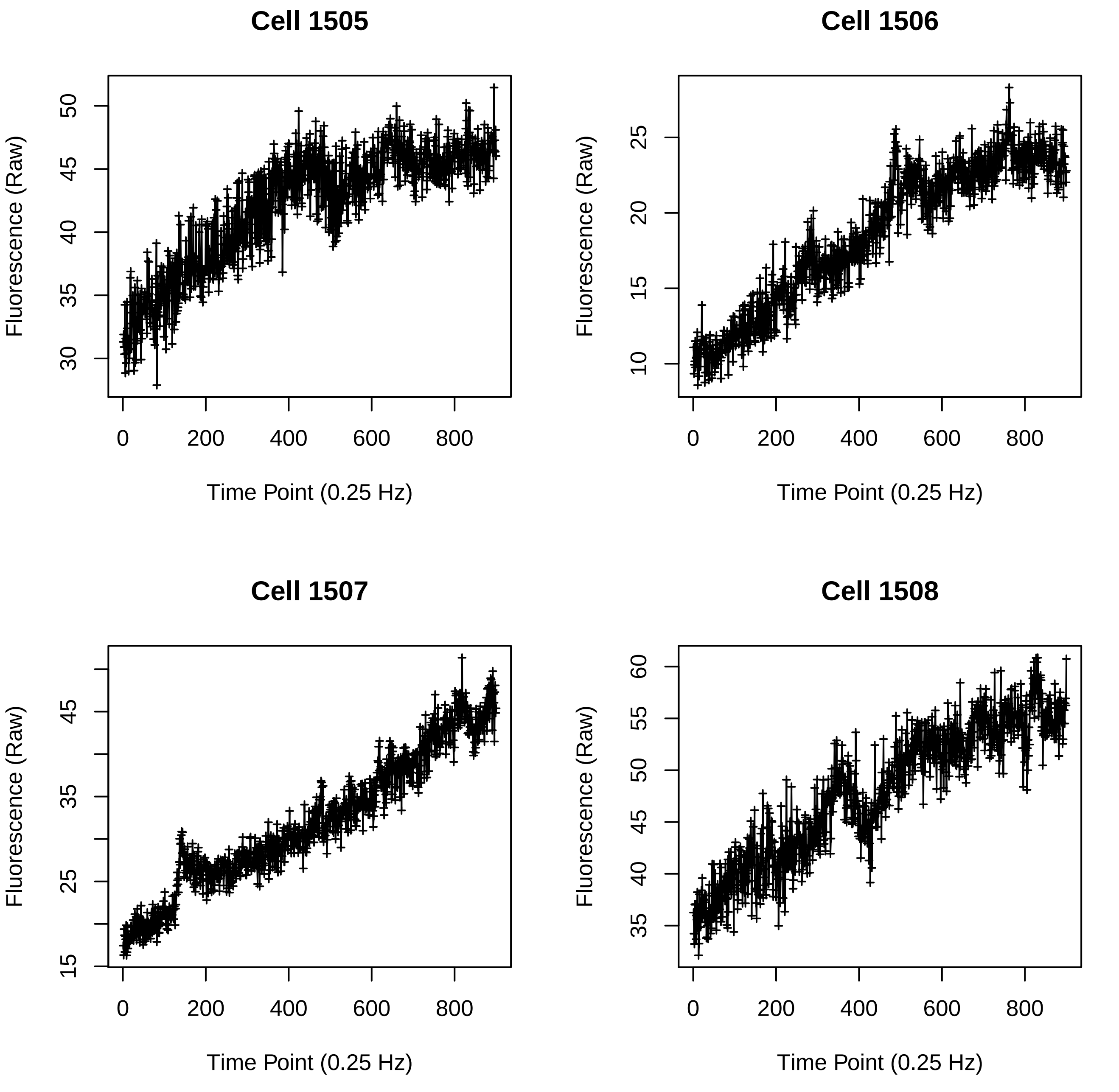

Cell 1505
Fluorescence (Raw)
Time Point (0.25 Hz)
30
35
40
45
50
0
200
400
600
800
Cell 1506
Fluorescence (Raw)
Time Point (0.25 Hz)
10
15
20
25
0
200
400
600
800
Cell 1507
Fluorescence (Raw)
Time Point (0.25 Hz)
15
25
35
45
0
200
400
600
800
Cell 1508
Fluorescence (Raw)
Time Point (0.25 Hz)
35
40
45
50
55
60
0
200
400
600
800

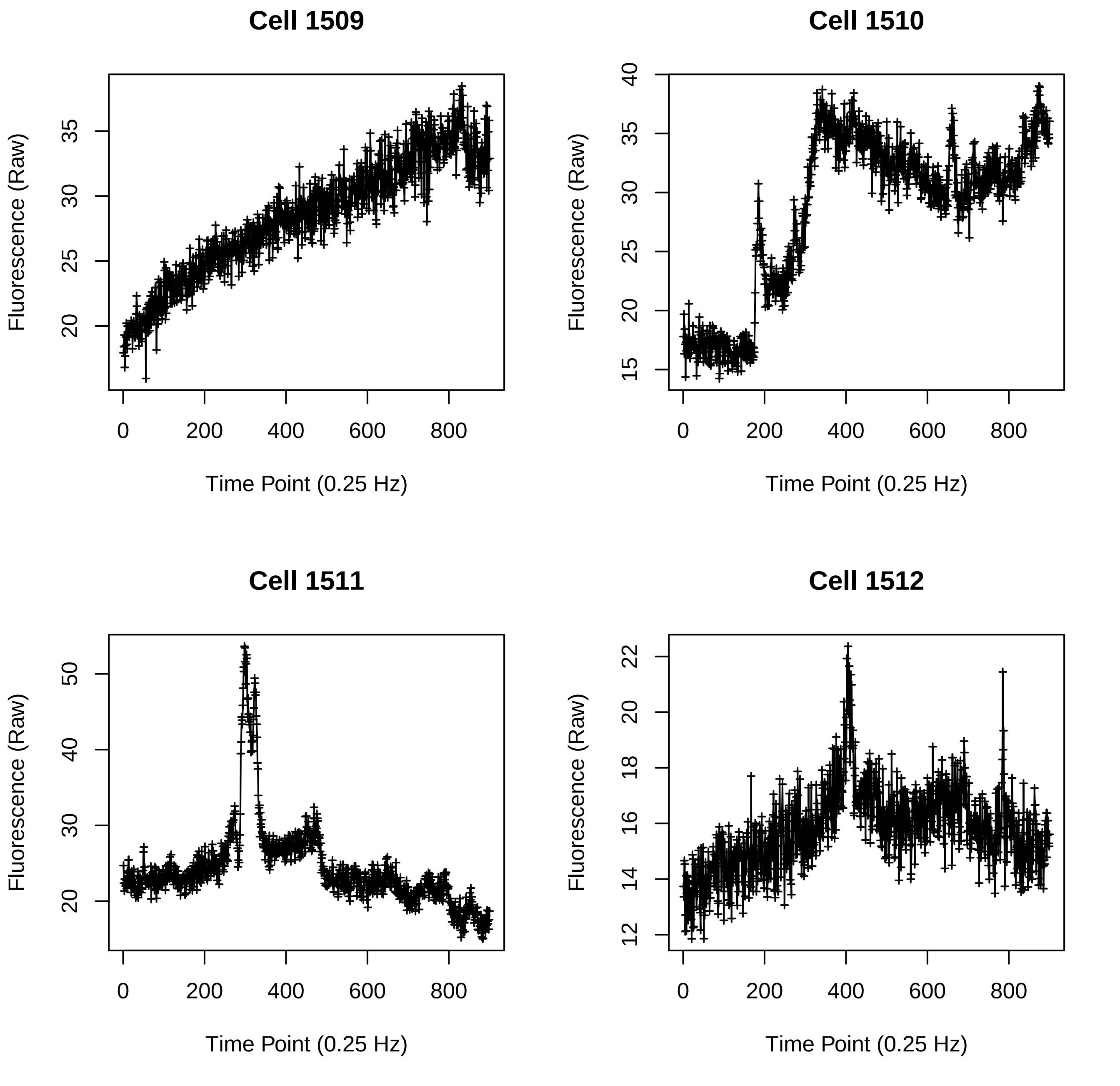
Cell 1509
Fluorescence (Raw)
20
25
30
35
0
200
400
600
800
Time Point (0.25 Hz)
Cell 1510
Fluorescence (Raw)
15
20
25
30
35
40
0
200
400
600
800
Time Point (0.25 Hz)
Cell 1511
Fluorescence (Raw)
20
30
40
50
0
200
400
600
800
Time Point (0.25 Hz)
Cell 1512
Fluorescence (Raw)
12
14
16
18
20
22
0
200
400
600
800
Time Point (0.25 Hz)

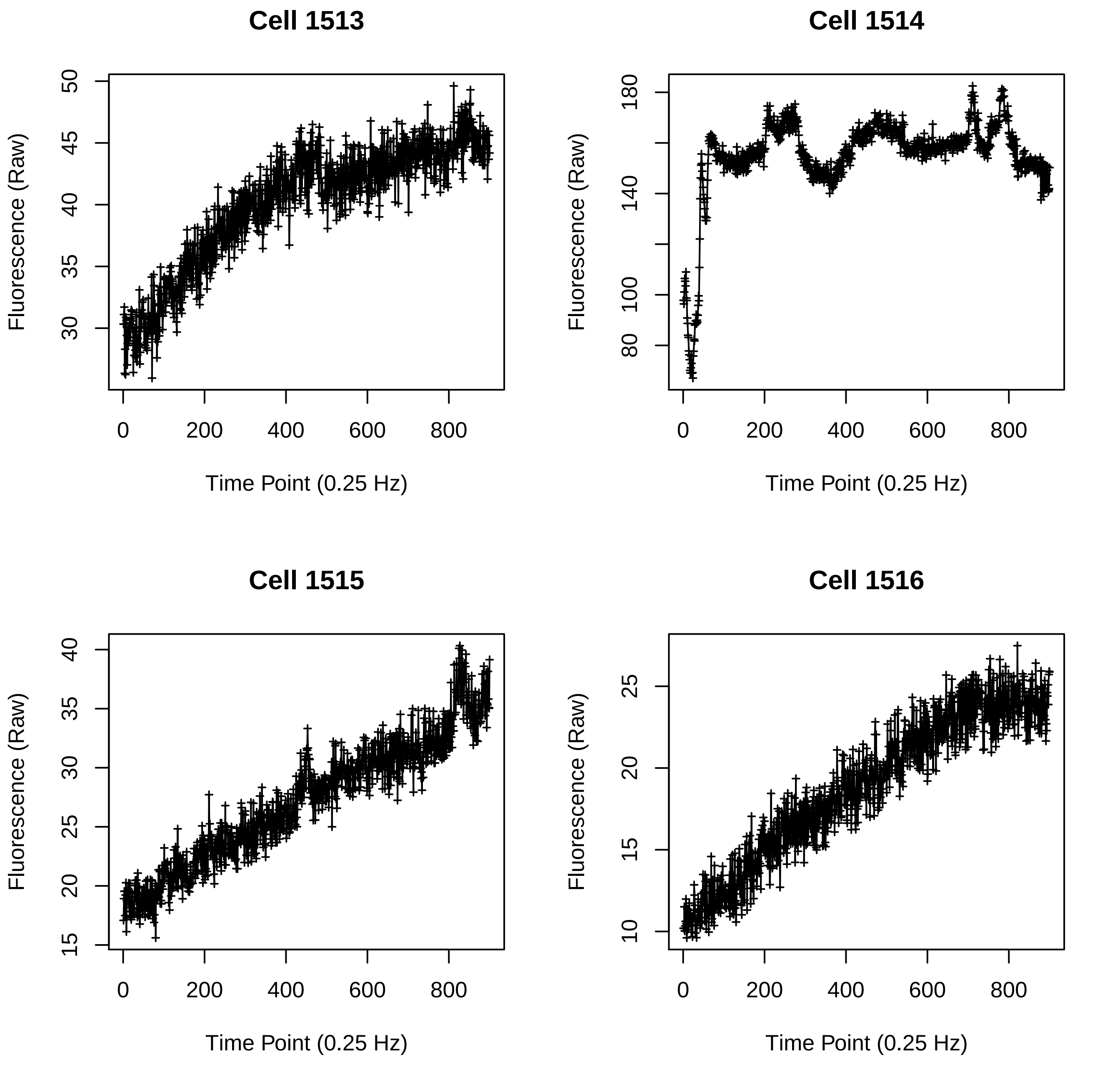
Cell 1513
Cell 1514
Cell 1515
Cell 1516
Fluorescence (Raw)
Time Point (0.25 Hz)
0
200
400
600
800
30
35
40
45
50
80
100
140
180
15
20
25
10

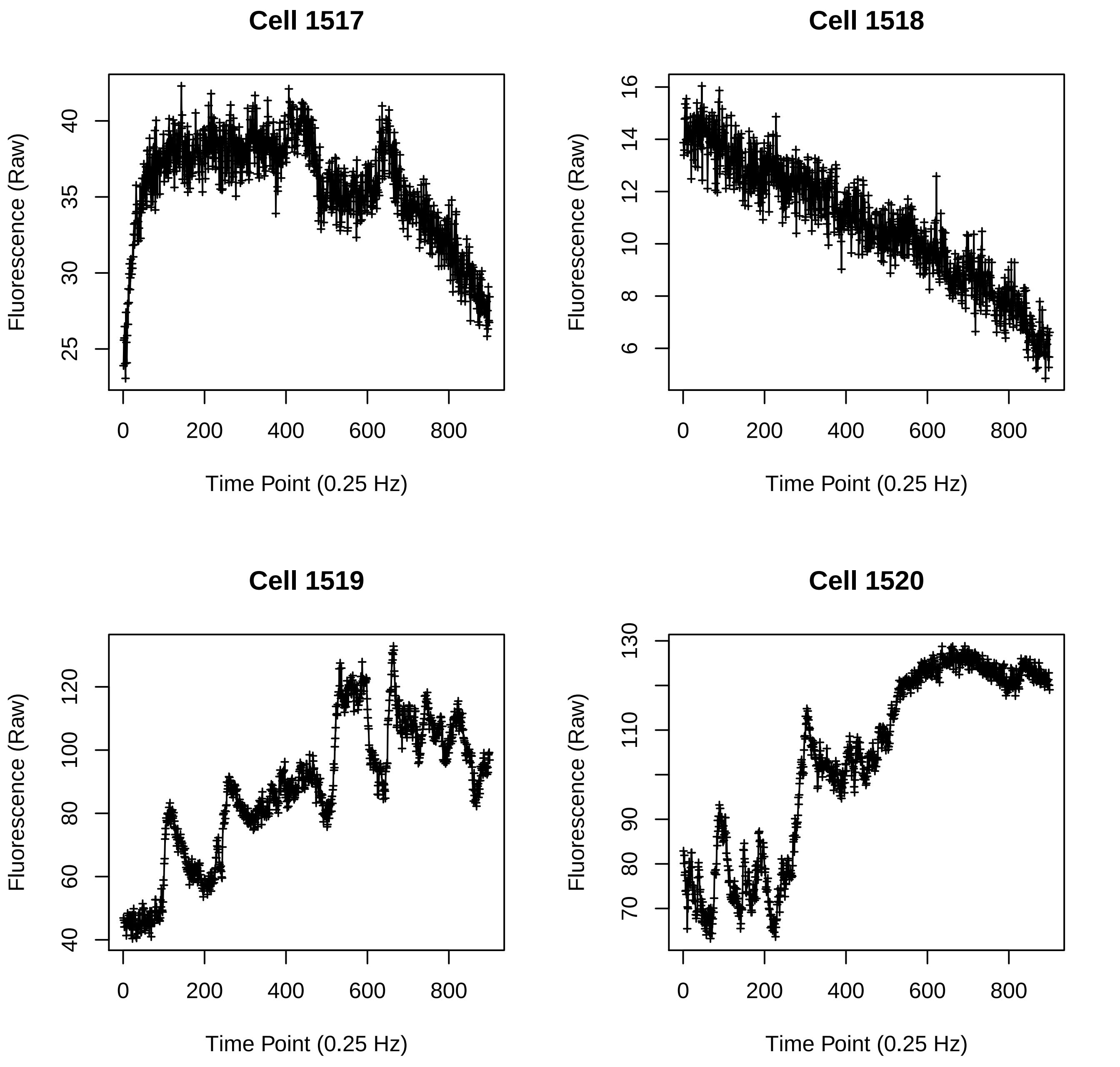
Cell 1517
Cell 1518
Cell 1519
Cell 1520
Fluorescence (Raw)
Time Point (0.25 Hz)
0
200
400
600
800
25
30
35
40
6
8
10
12
14
16
40
60
80
100
120
70
80
90
110
130

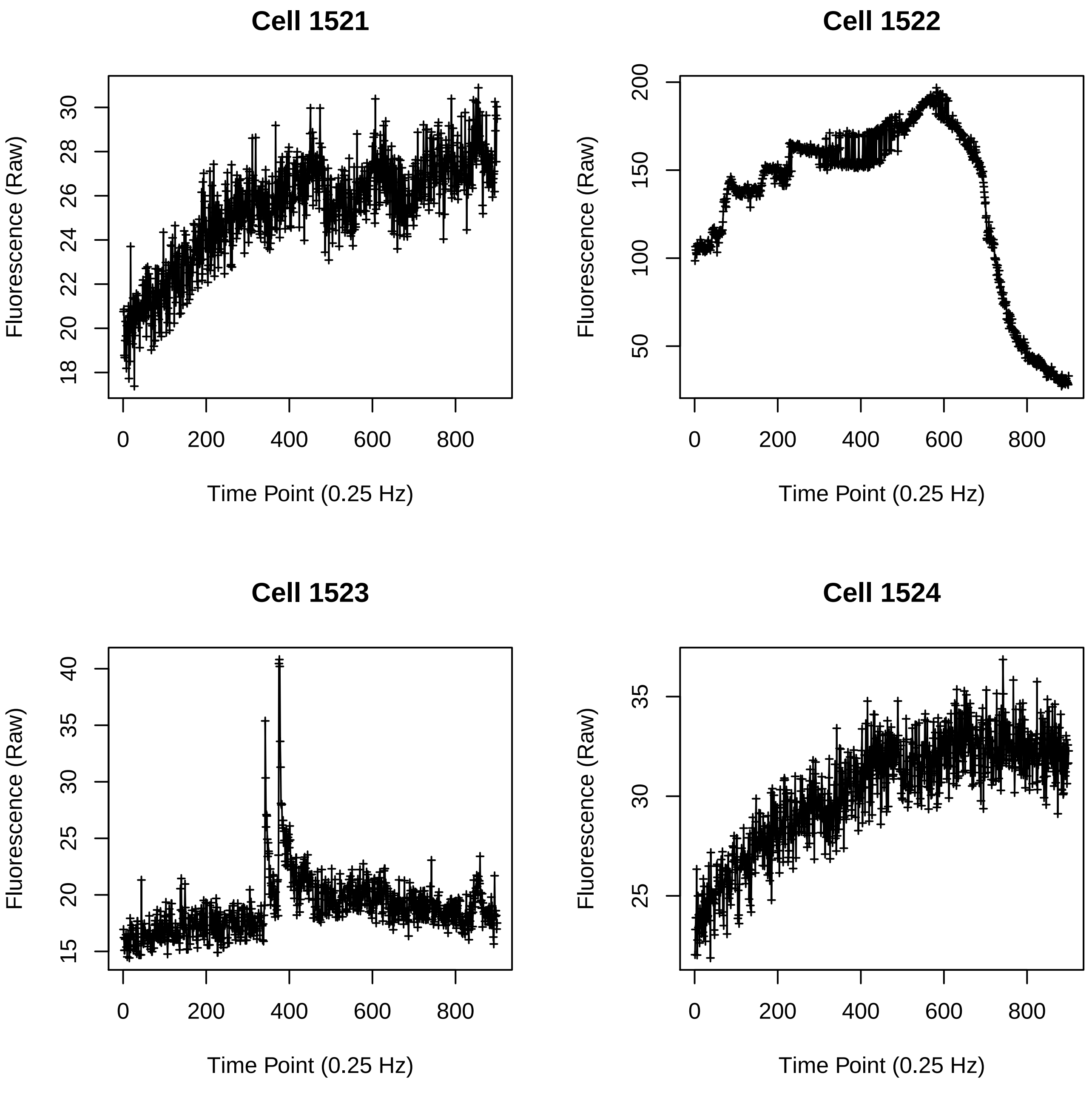
Cell 1521
Fluorescence (Raw)
18
20
22
24
26
28
30
0
200
400
600
800
Time Point (0.25 Hz)
Cell 1522
Fluorescence (Raw)
50
100
150
200
0
200
400
600
800
Time Point (0.25 Hz)
Cell 1523
Fluorescence (Raw)
15
20
25
30
35
40
0
200
400
600
800
Time Point (0.25 Hz)
Cell 1524
Fluorescence (Raw)
25
30
35
0
200
400
600
800
Time Point (0.25 Hz)

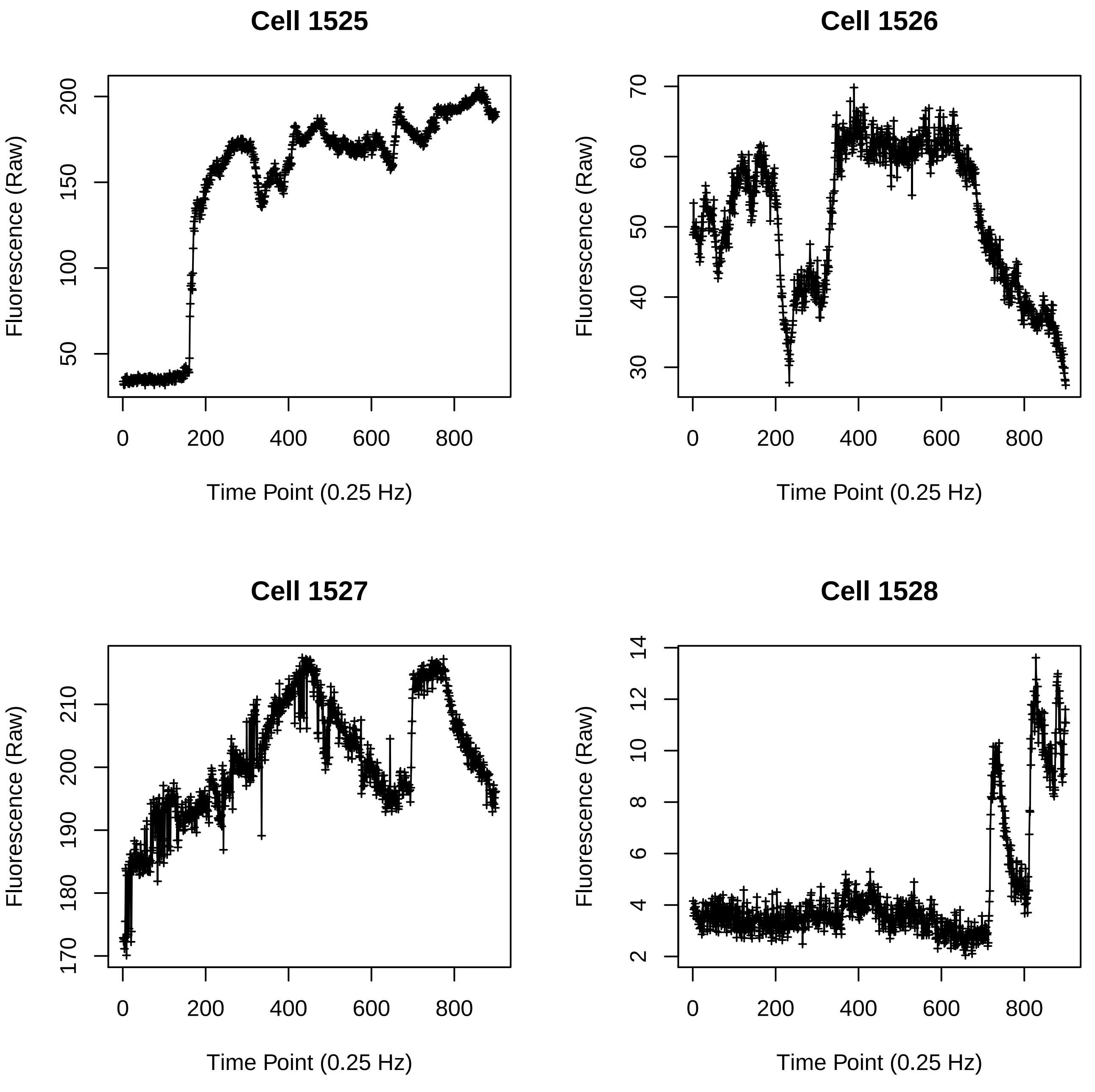
Cell 1525
Cell 1526
Cell 1527
Cell 1528
Fluorescence (Raw)
Time Point (0.25 Hz)
0
200
400
600
800
50
100
150
200
30
40
50
60
70
170
180
190
200
210
2
4
6
8
10
12
14

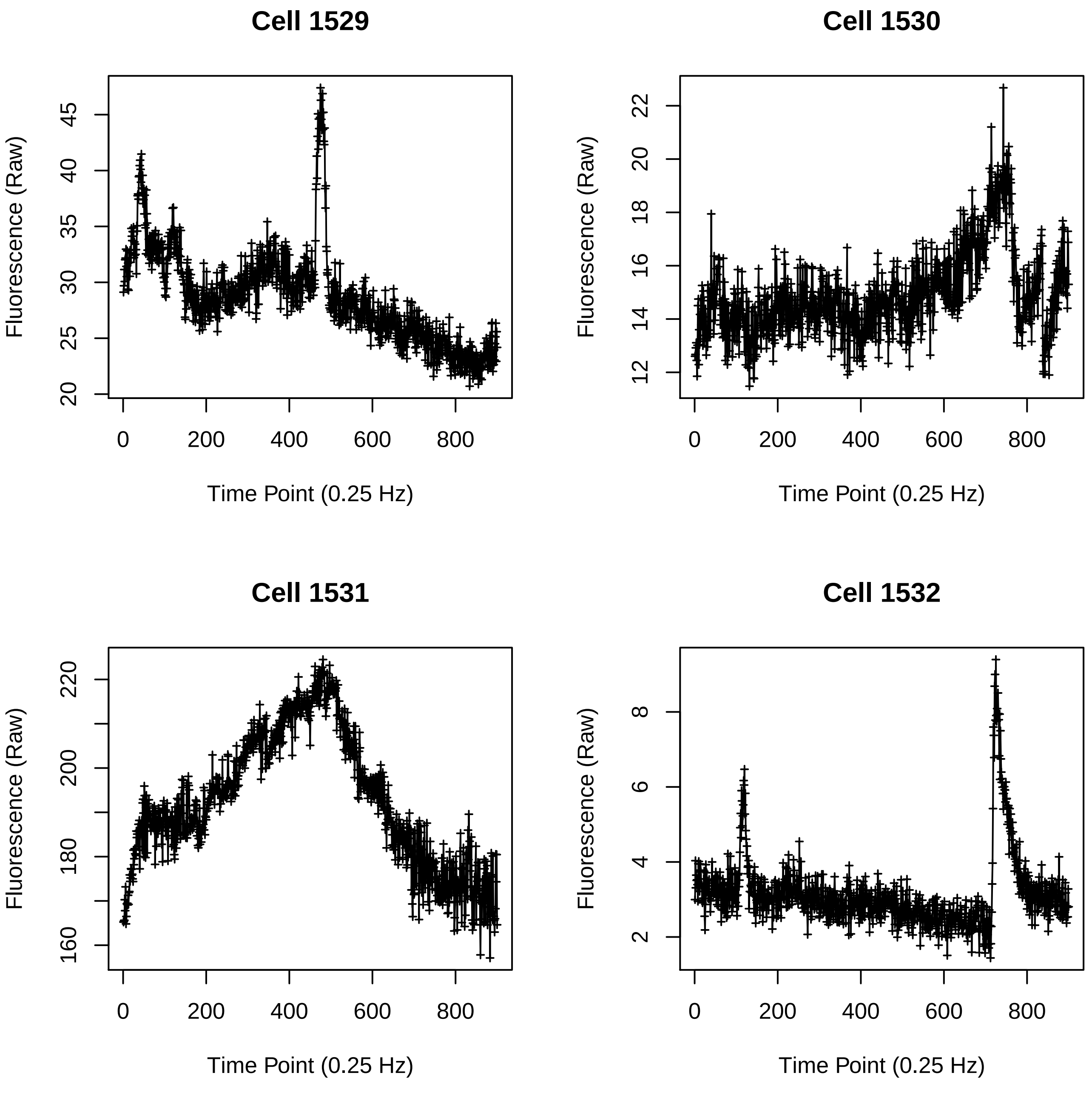
Cell 1529
Fluorescence (Raw)
20
25
30
35
40
45
0
200
400
600
800
Time Point (0.25 Hz)
Cell 1530
Fluorescence (Raw)
12
14
16
18
20
22
0
200
400
600
800
Time Point (0.25 Hz)
Cell 1531
Fluorescence (Raw)
160
180
200
220
0
200
400
600
800
Time Point (0.25 Hz)
Cell 1532
Fluorescence (Raw)
2
4
6
8
0
200
400
600
800
Time Point (0.25 Hz)

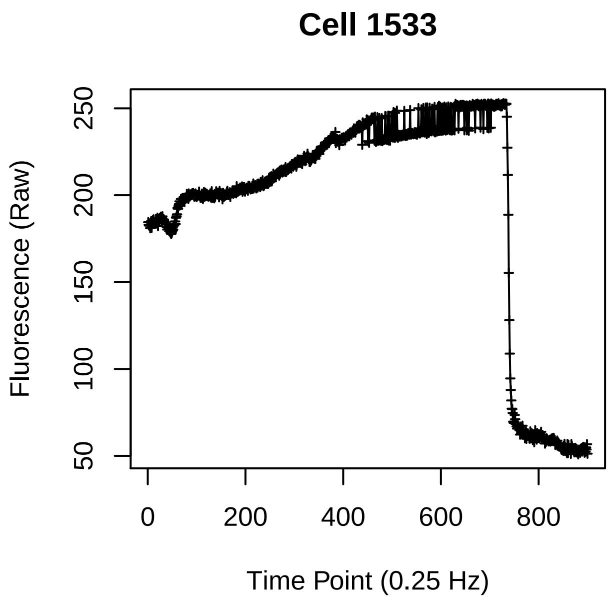
Cell 1533
Fluorescence (Raw)
50
100
150
200
250
0
200
400
600
800
Time Point (0.25 Hz)

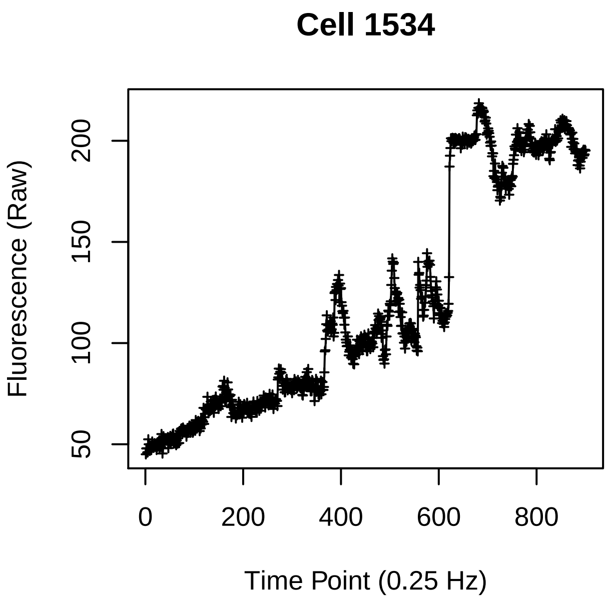
Cell 1534
Fluorescence (Raw)
50
100
150
200
0
200
400
600
800
Time Point (0.25 Hz)

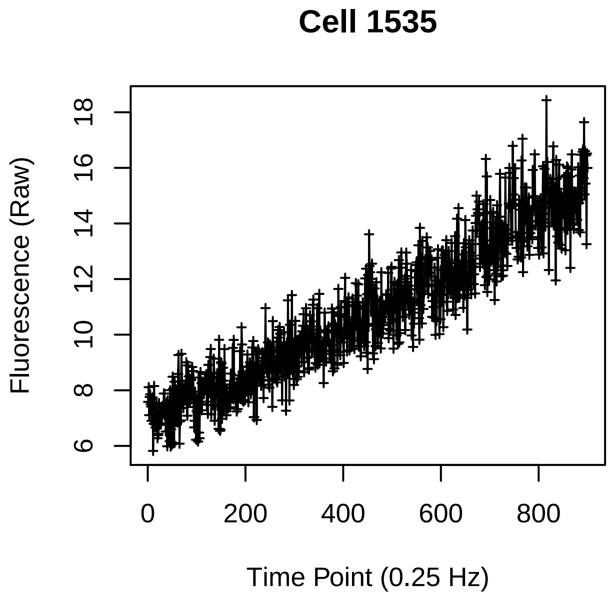
Cell 1535
Fluorescence (Raw)
6
8
10
12
14
16
18
0
200
400
600
800
Time Point (0.25 Hz)

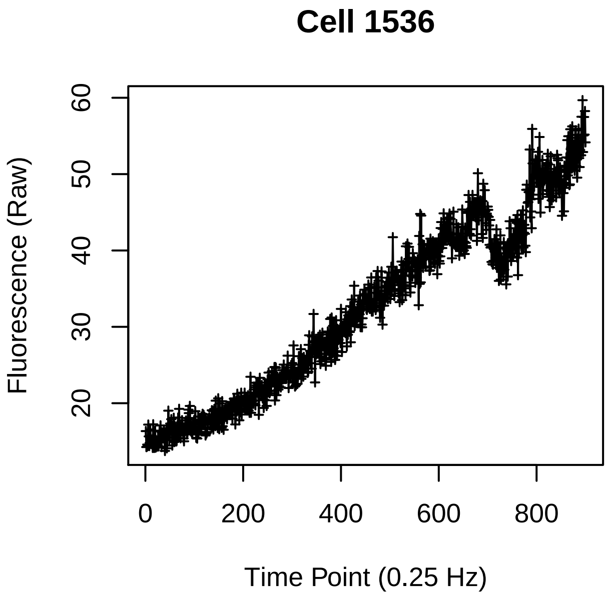
Cell 1536
Fluorescence (Raw)
20
30
40
50
60
0
200
400
600
800
Time Point (0.25 Hz)

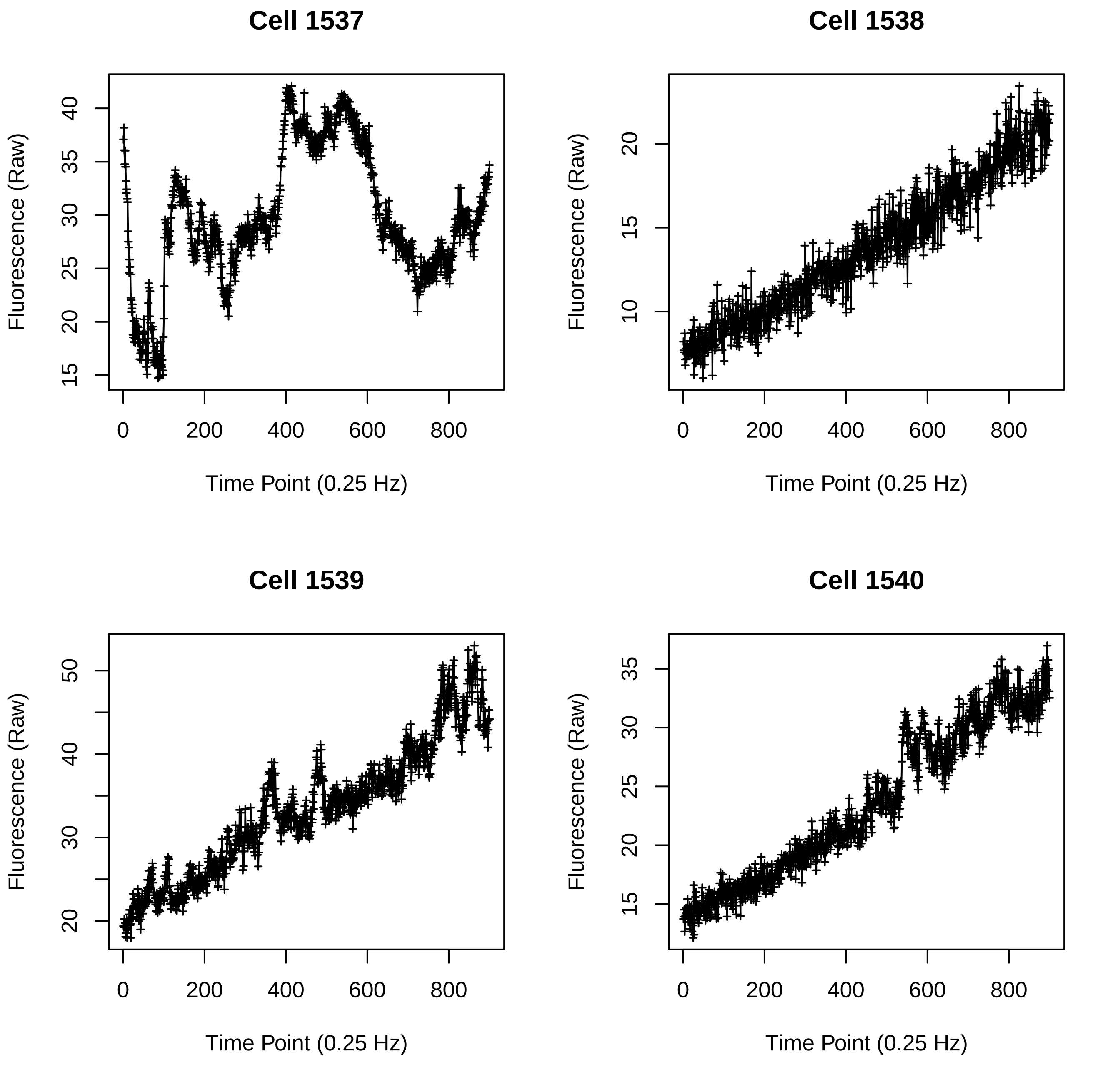
Cell 1537
Cell 1538
Cell 1539
Cell 1540
Fluorescence (Raw)
Time Point (0.25 Hz)
0
200
400
600
800
15
20
25
30
35
40
10
50

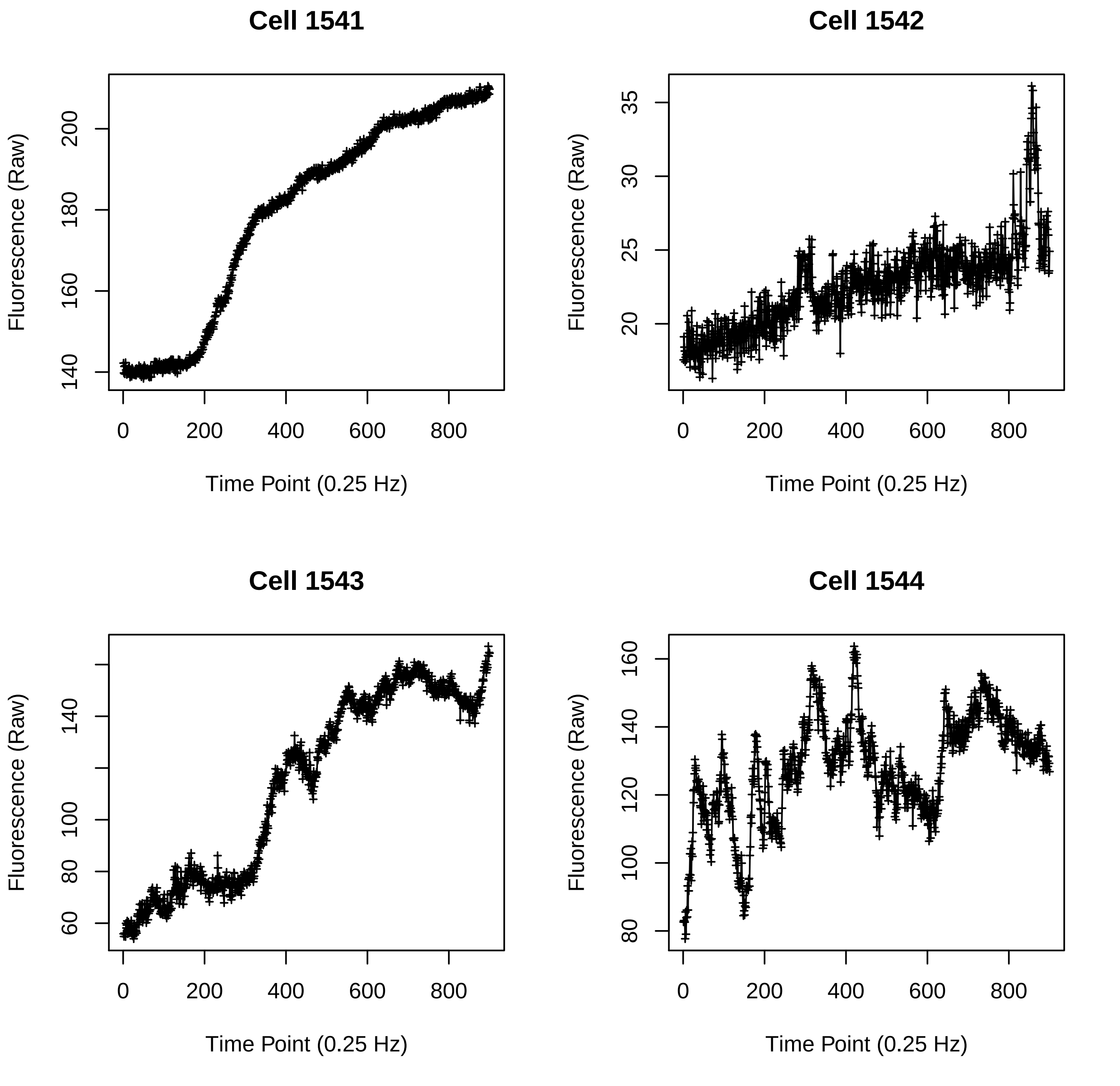

Cell 1541
Cell 1542
Cell 1543
Cell 1544
Fluorescence (Raw)
Time Point (0.25 Hz)
0
200
400
600
800
140
160
180
200
20
25
30
35
60
80
100
140
120

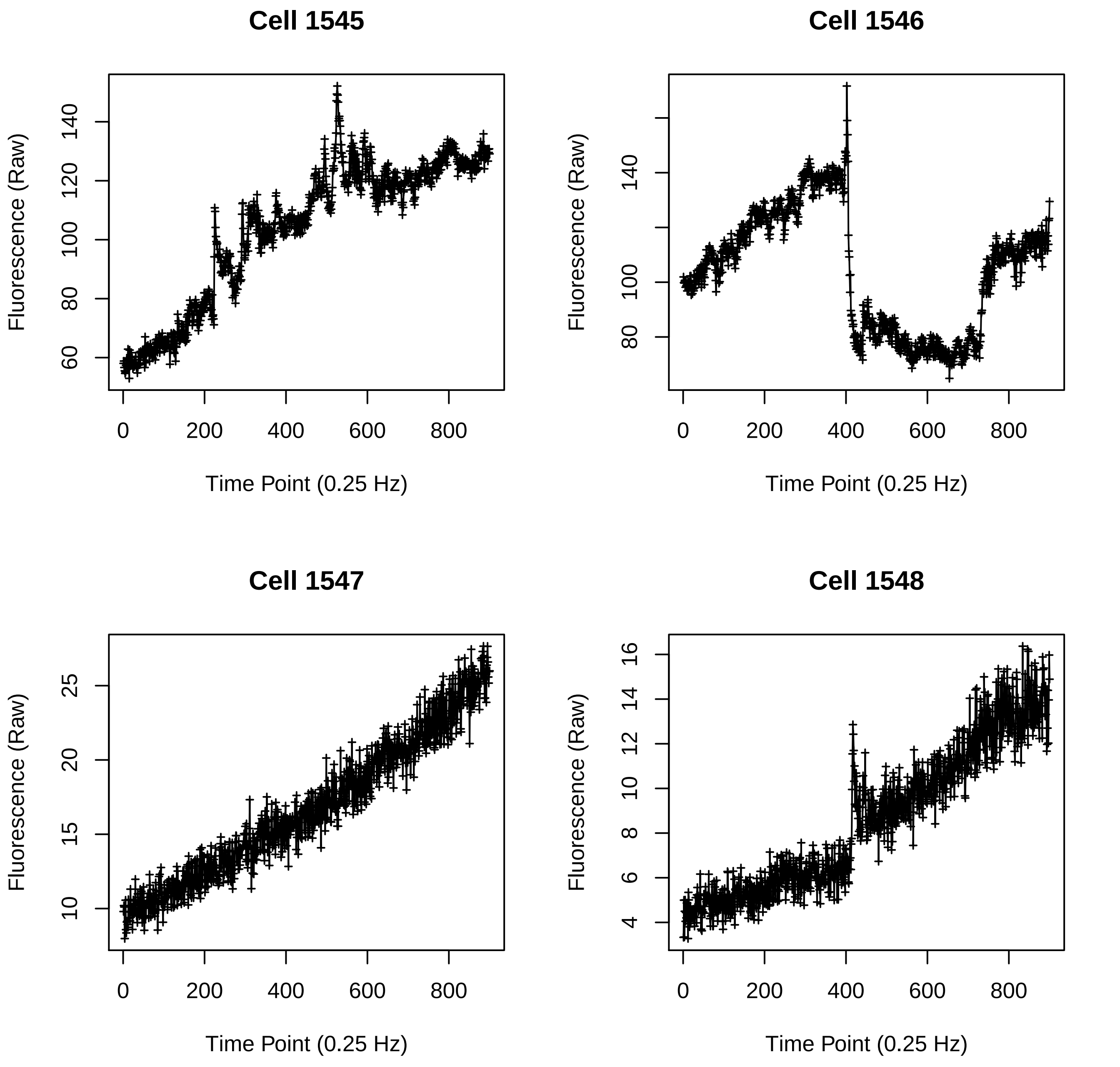
Cell 1545
Fluorescence (Raw)
60
80
100
120
140
0
200
400
600
800
Time Point (0.25 Hz)
Cell 1546
Fluorescence (Raw)
80
100
140
0
200
400
600
800
Time Point (0.25 Hz)
Cell 1547
Fluorescence (Raw)
10
15
20
25
0
200
400
600
800
Time Point (0.25 Hz)
Cell 1548
Fluorescence (Raw)
4
6
8
10
12
14
16
0
200
400
600
800
Time Point (0.25 Hz)

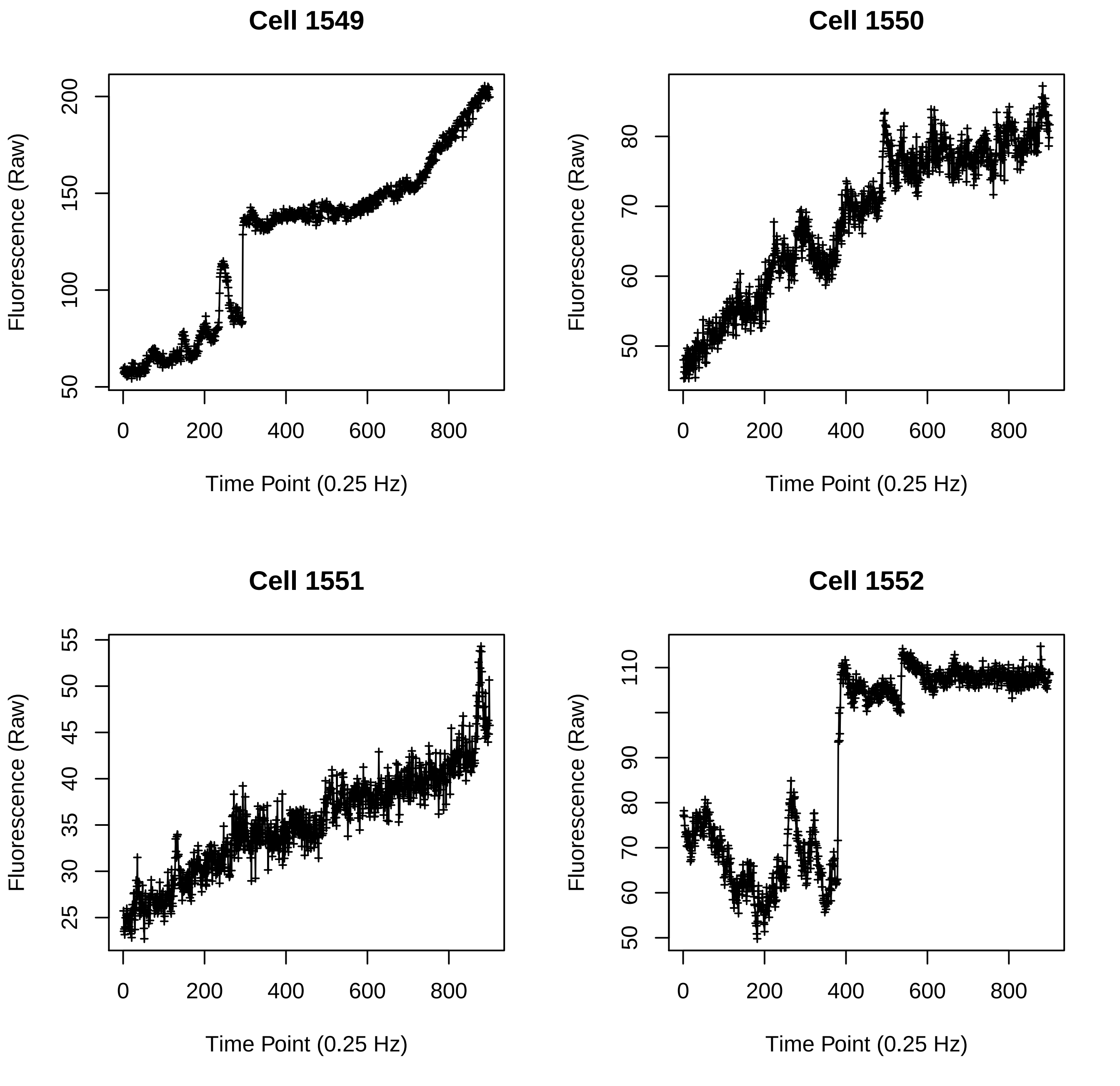

Cell 1549
Cell 1550
Cell 1551
Cell 1552
Fluorescence (Raw)
Time Point (0.25 Hz)
0
200
400
600
800
50
100
150
200
60
70
80
25
30
35
40
45
50
55
90
110

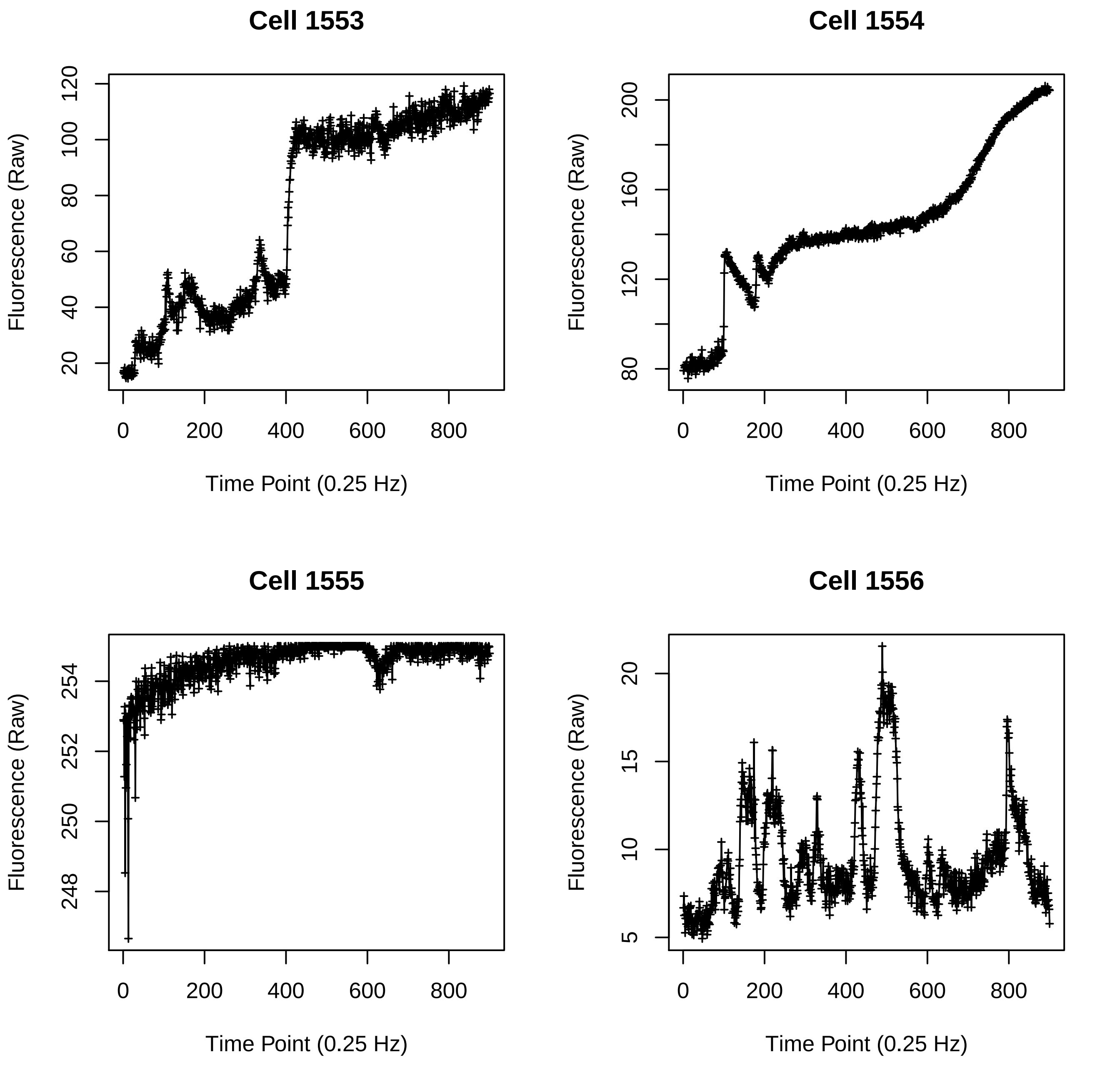

Cell 1553
Cell 1554
Cell 1555
Cell 1556
Fluorescence (Raw)
Time Point (0.25 Hz)
0
200
400
600
800
20
40
60
80
100
120
160
200
248
250
252
254
5
10
15
20

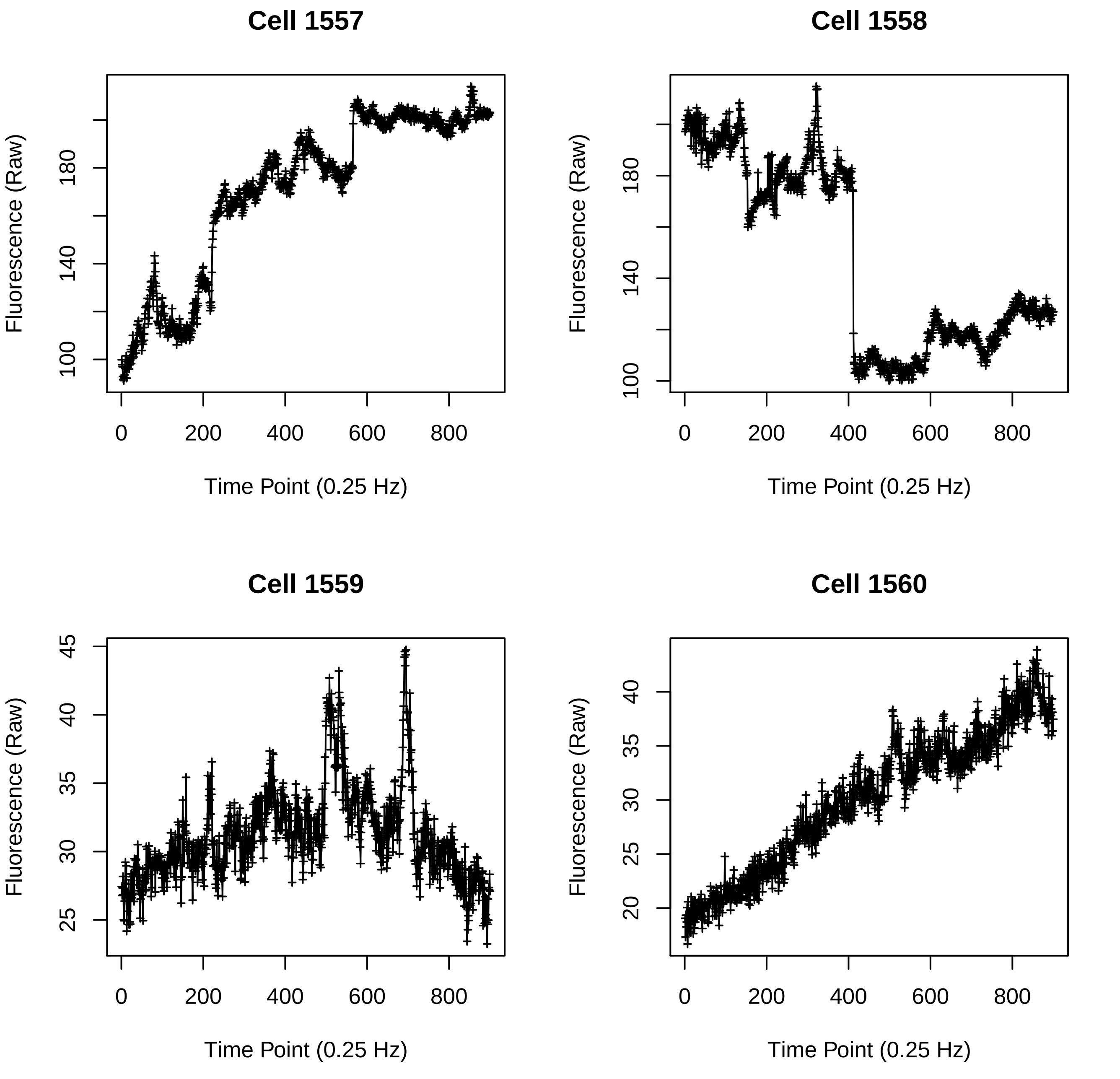

Cell 1557
Cell 1558
Cell 1559
Cell 1560
Fluorescence (Raw)
Time Point (0.25 Hz)
0
200
400
600
800
100
140
180
25
30
35
40
45
20

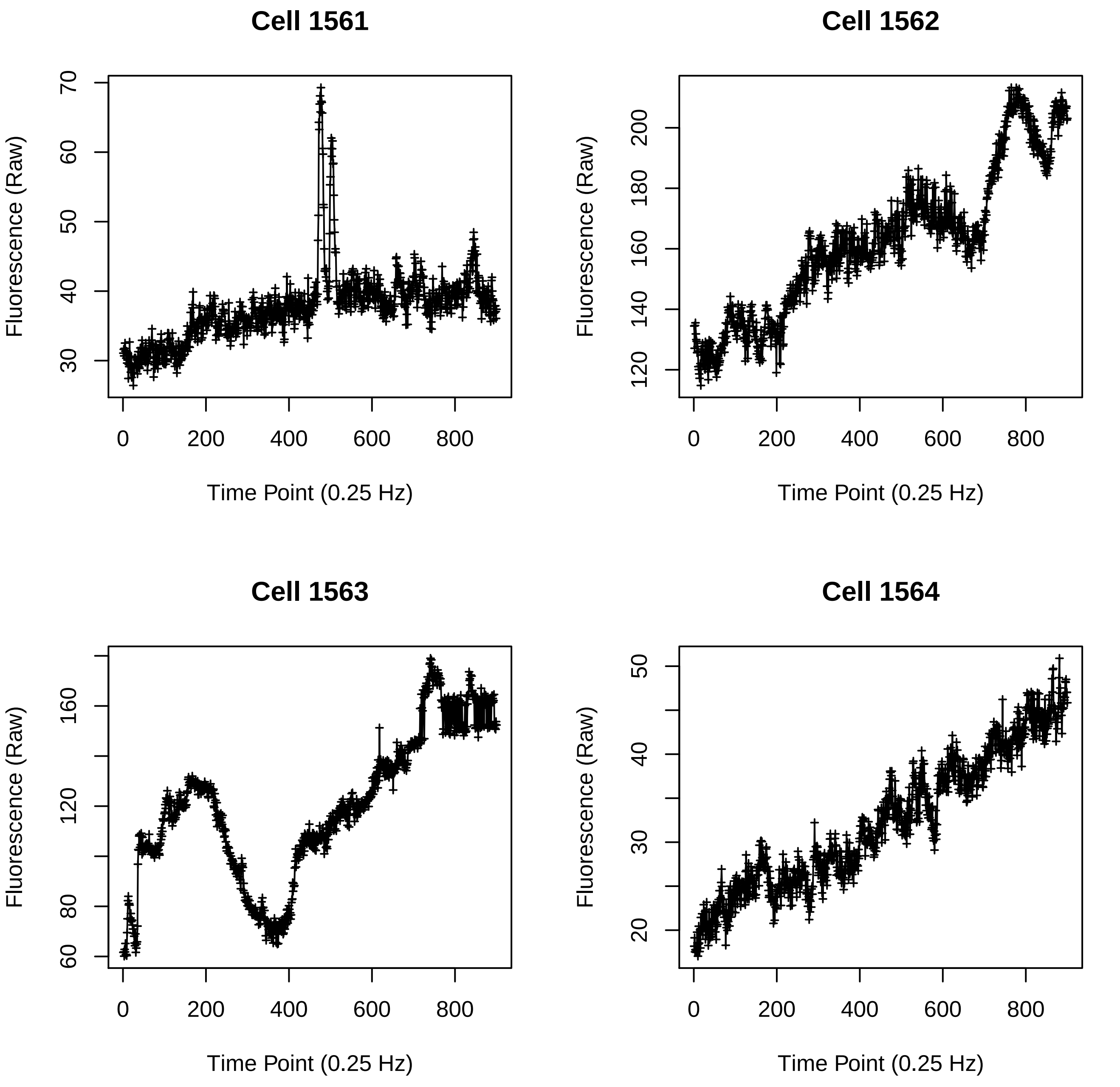

Cell 1561
Fluorescence (Raw)
30
40
50
60
70
0
200
400
600
800
Time Point (0.25 Hz)
Cell 1562
Fluorescence (Raw)
120
140
160
180
200
0
200
400
600
800
Time Point (0.25 Hz)
Cell 1563
Fluorescence (Raw)
60
80
120
160
0
200
400
600
800
Time Point (0.25 Hz)
Cell 1564
Fluorescence (Raw)
20
30
40
50
0
200
400
600
800
Time Point (0.25 Hz)

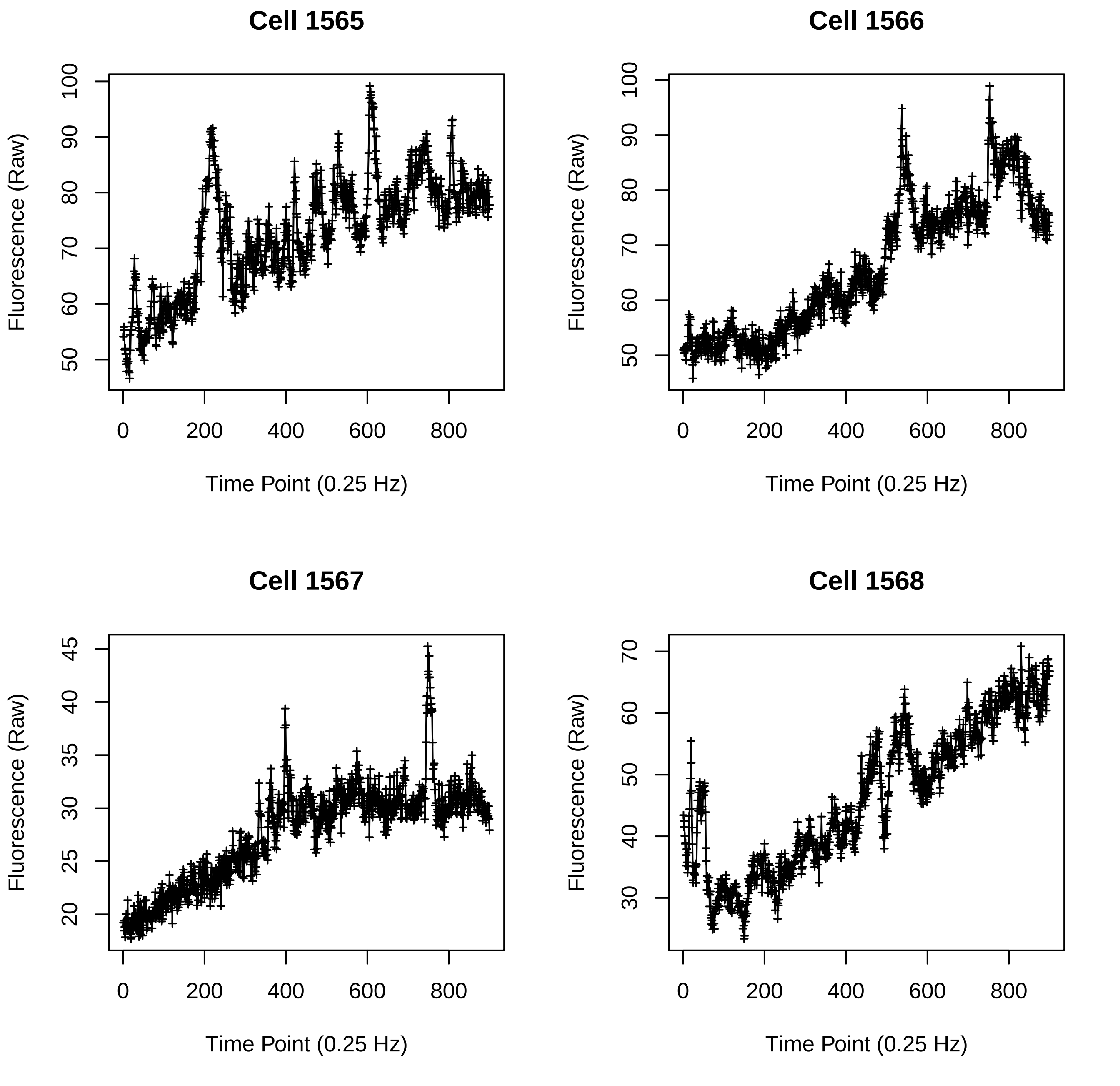

Cell 1565
Cell 1566
Cell 1567
Cell 1568
Fluorescence (Raw)
Time Point (0.25 Hz)
0
200
400
600
800
50
60
70
80
90
100
20
25
30
35
40
45
30
40
50
60
70

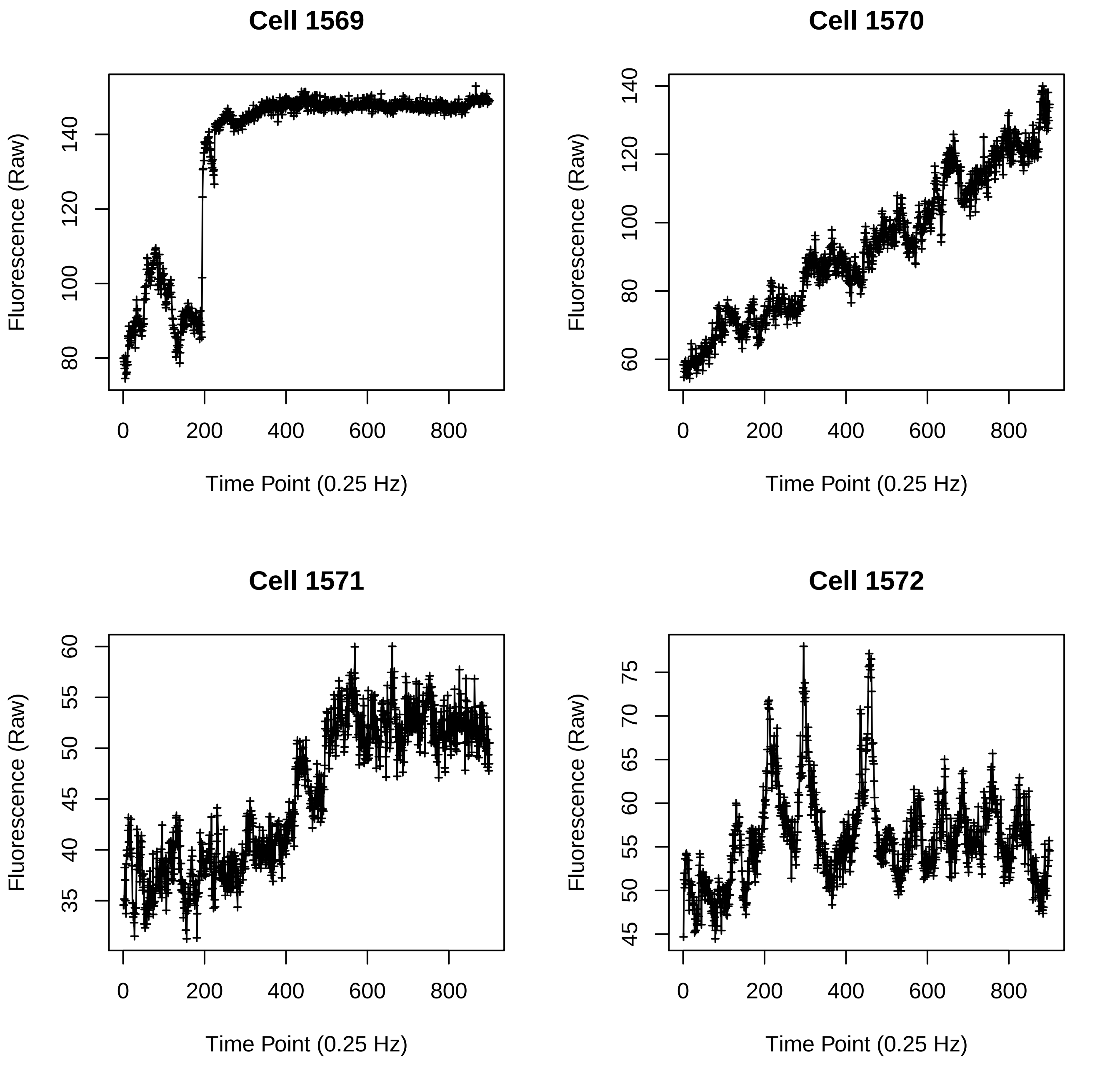
Cell 1569
Cell 1570
Cell 1571
Cell 1572
Fluorescence (Raw)
Time Point (0.25 Hz)
0
200
400
600
800
80
100
120
140
60
35
40
45
50
55
60
65
70
75

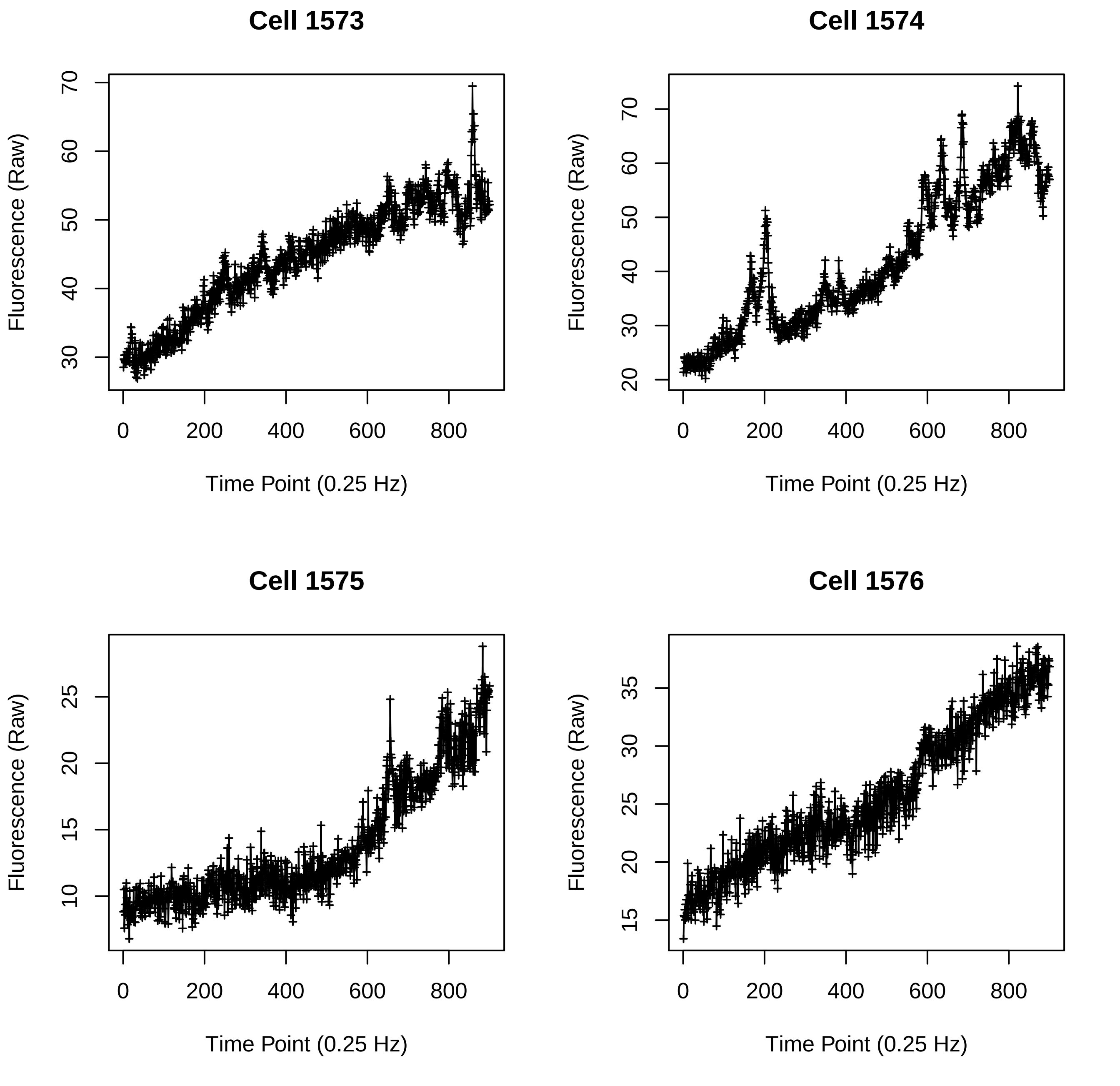

Cell 1573
Cell 1574
Cell 1575
Cell 1576
Fluorescence (Raw)
Time Point (0.25 Hz)
0
200
400
600
800
30
40
50
60
70
20
15
10
25
35

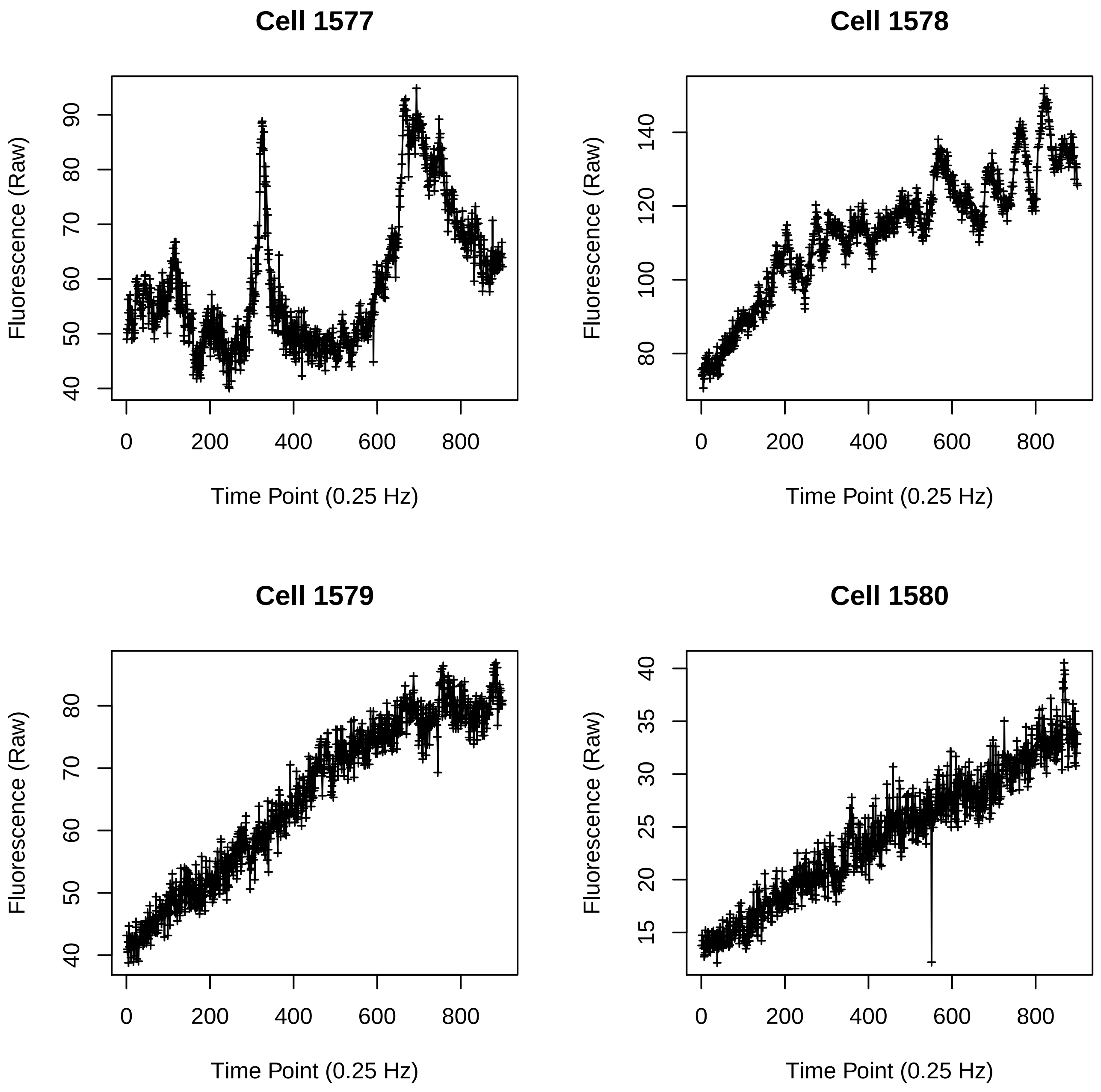
Cell 1577
Cell 1578
Cell 1579
Cell 1580
Fluorescence (Raw)
Time Point (0.25 Hz)
0
200
400
600
800
40
50
60
70
80
90
100
120
140
15
20
25
30
35

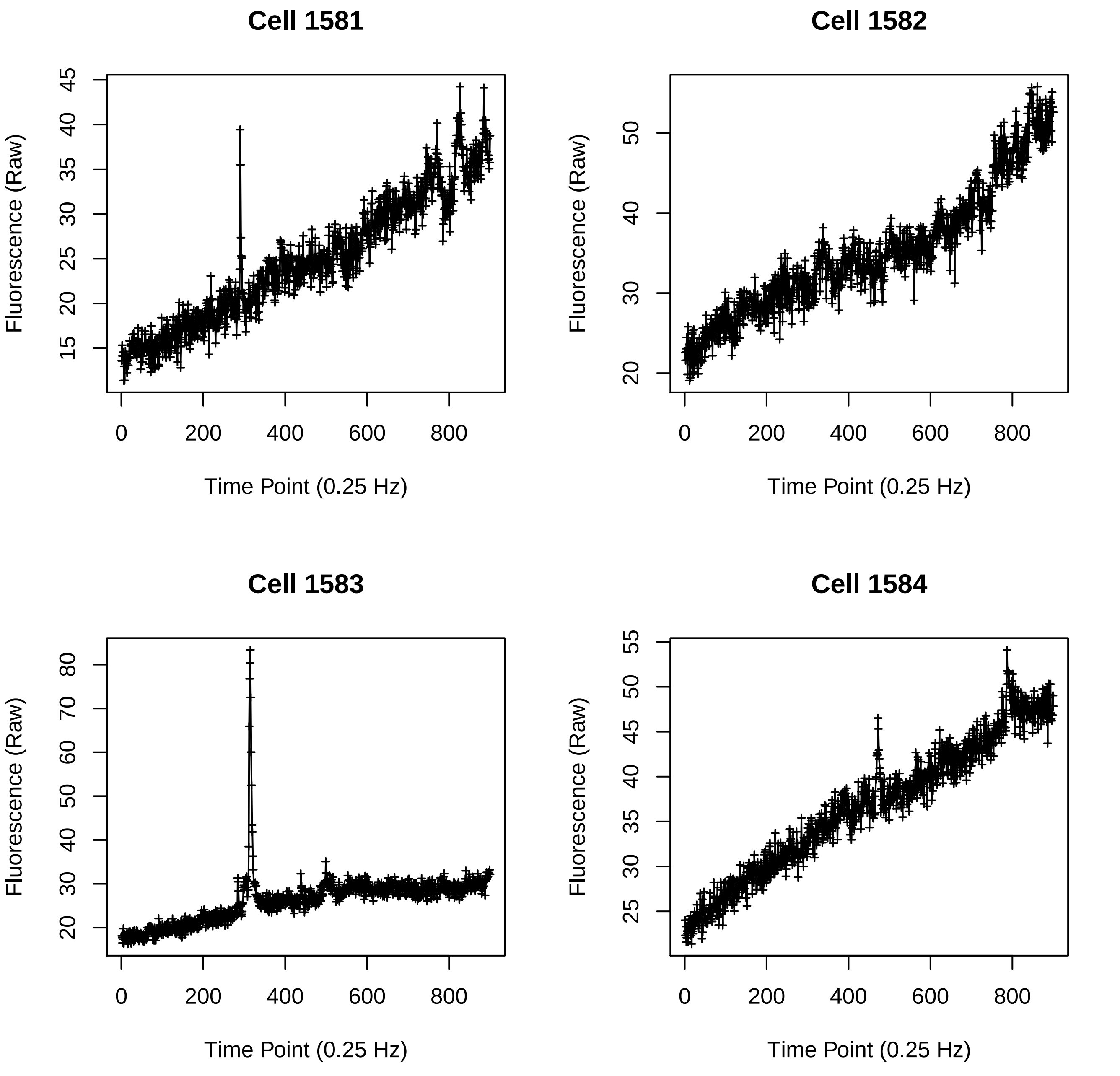
Cell 1581
Cell 1582
Cell 1583
Cell 1584
Fluorescence (Raw)
Time Point (0.25 Hz)
0
200
400
600
800
15
20
25
30
35
40
45
50
55
60
70
80

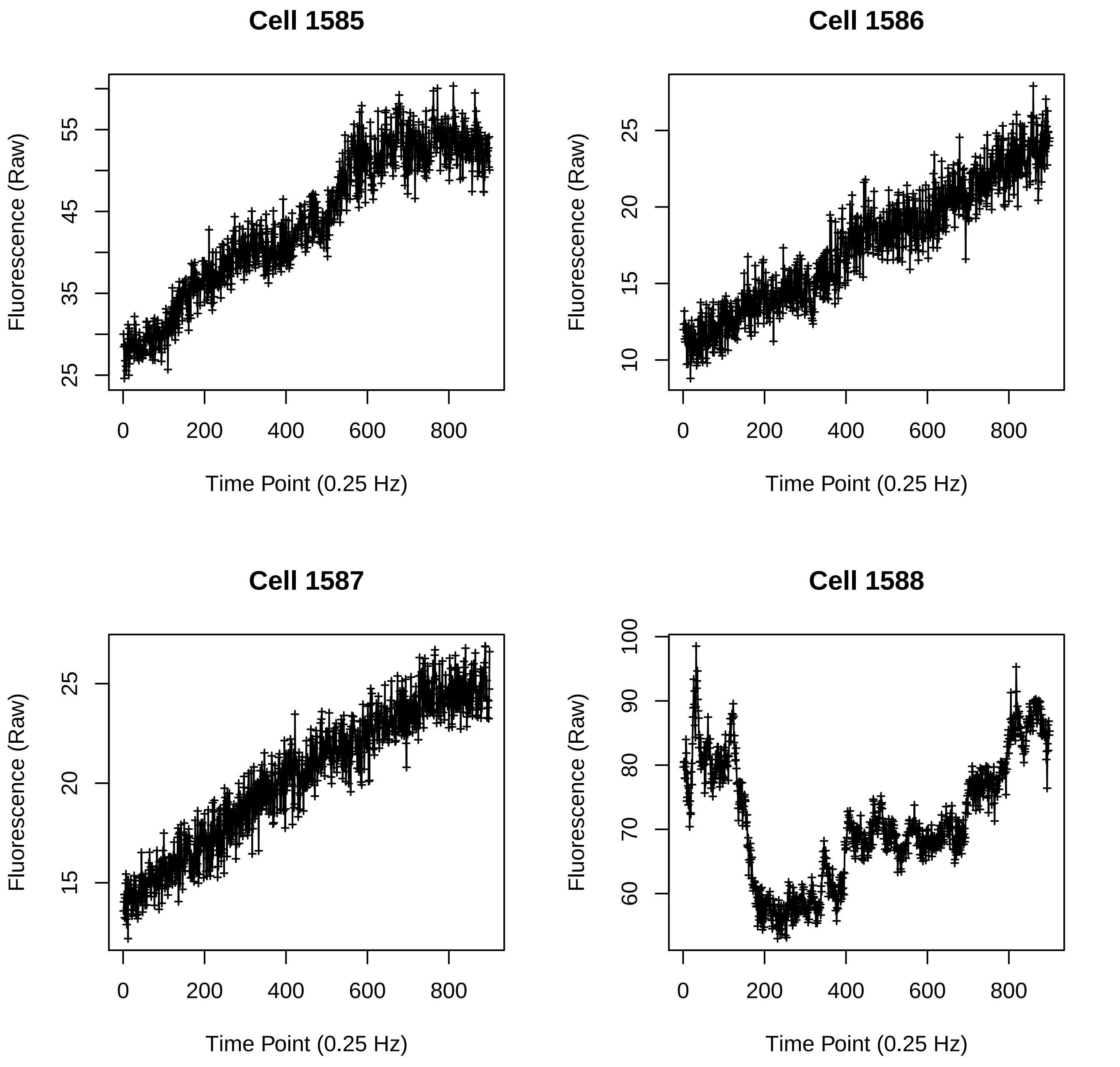

Cell 1585
Fluorescence (Raw)
25
35
45
55
0
200
400
600
800
Time Point (0.25 Hz)
Cell 1586
Fluorescence (Raw)
10
15
20
25
0
200
400
600
800
Time Point (0.25 Hz)
Cell 1587
Fluorescence (Raw)
15
20
25
0
200
400
600
800
Time Point (0.25 Hz)
Cell 1588
Fluorescence (Raw)
60
70
80
90
100
0
200
400
600
800
Time Point (0.25 Hz)

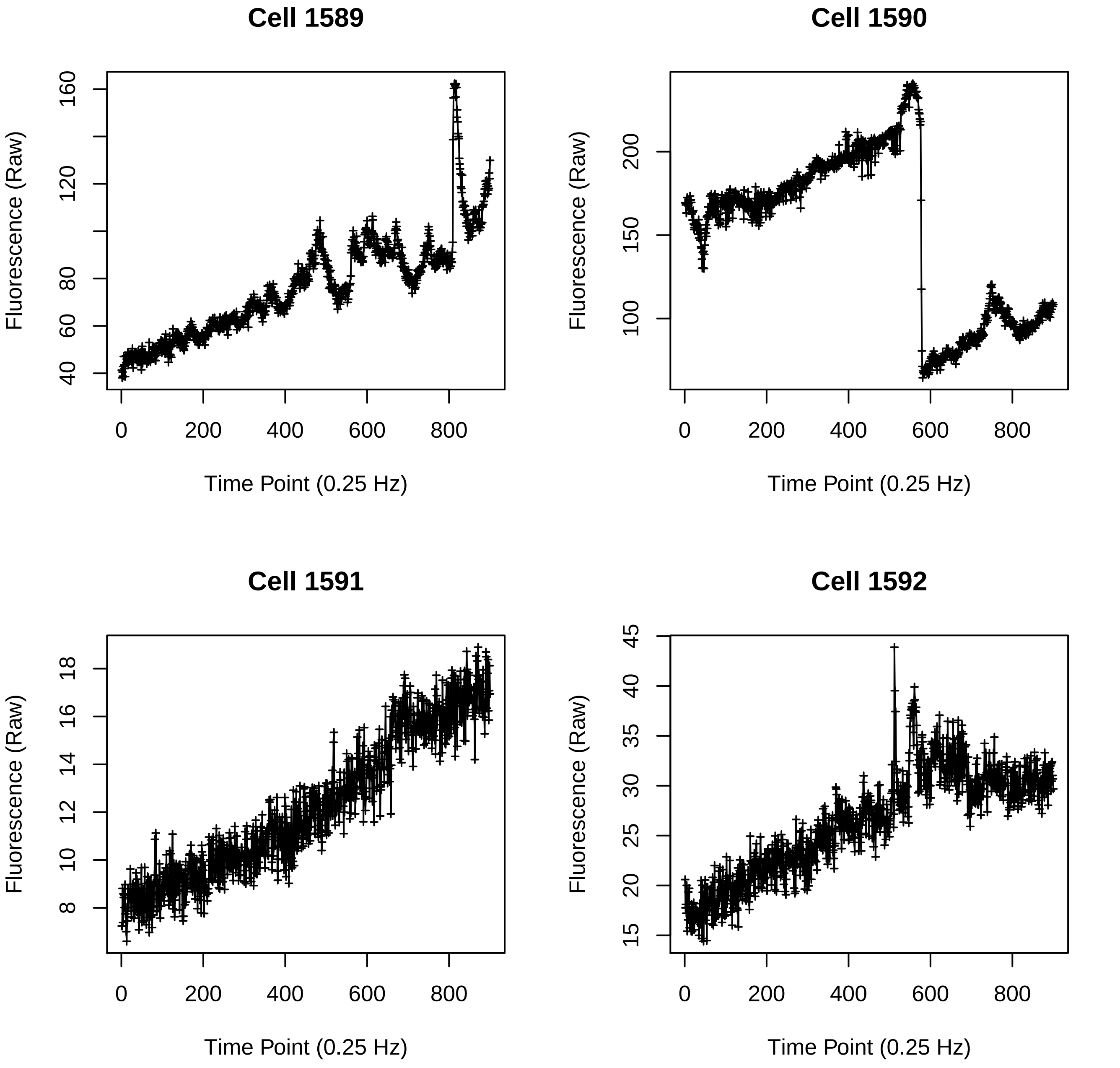
Cell 1589
Cell 1590
Cell 1591
Cell 1592
Fluorescence (Raw)
Time Point (0.25 Hz)
0
200
400
600
800
40
60
80
120
160
100
150
200
8
10
12
14
16
18
15
20
25
30
35
40
45

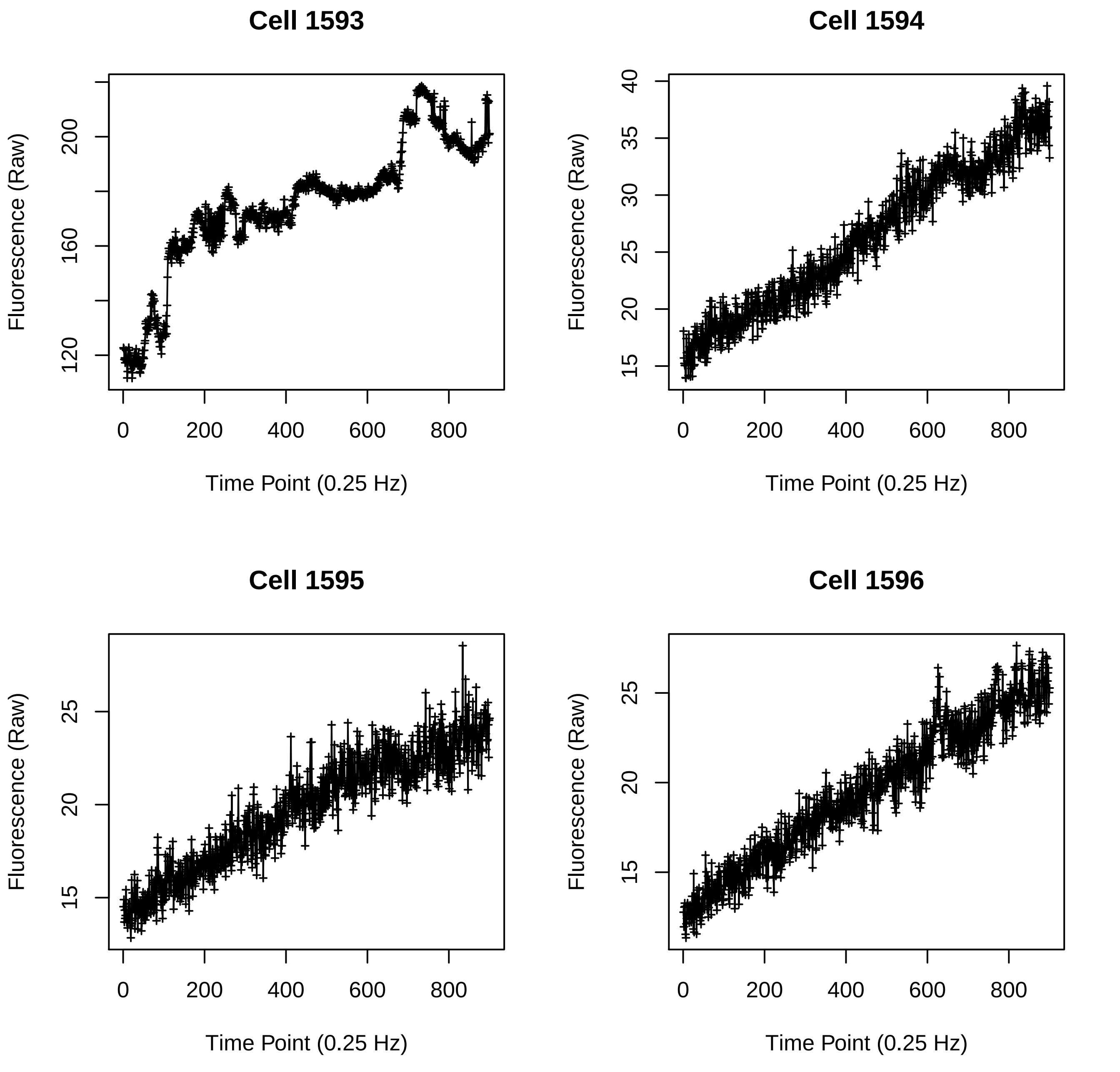
Cell 1593
Fluorescence (Raw)
120
160
200
0
200
400
600
800
Time Point (0.25 Hz)
Cell 1594
Fluorescence (Raw)
15
20
25
30
35
40
0
200
400
600
800
Time Point (0.25 Hz)
Cell 1595
Fluorescence (Raw)
15
20
25
0
200
400
600
800
Time Point (0.25 Hz)
Cell 1596
Fluorescence (Raw)
15
20
25
0
200
400
600
800
Time Point (0.25 Hz)

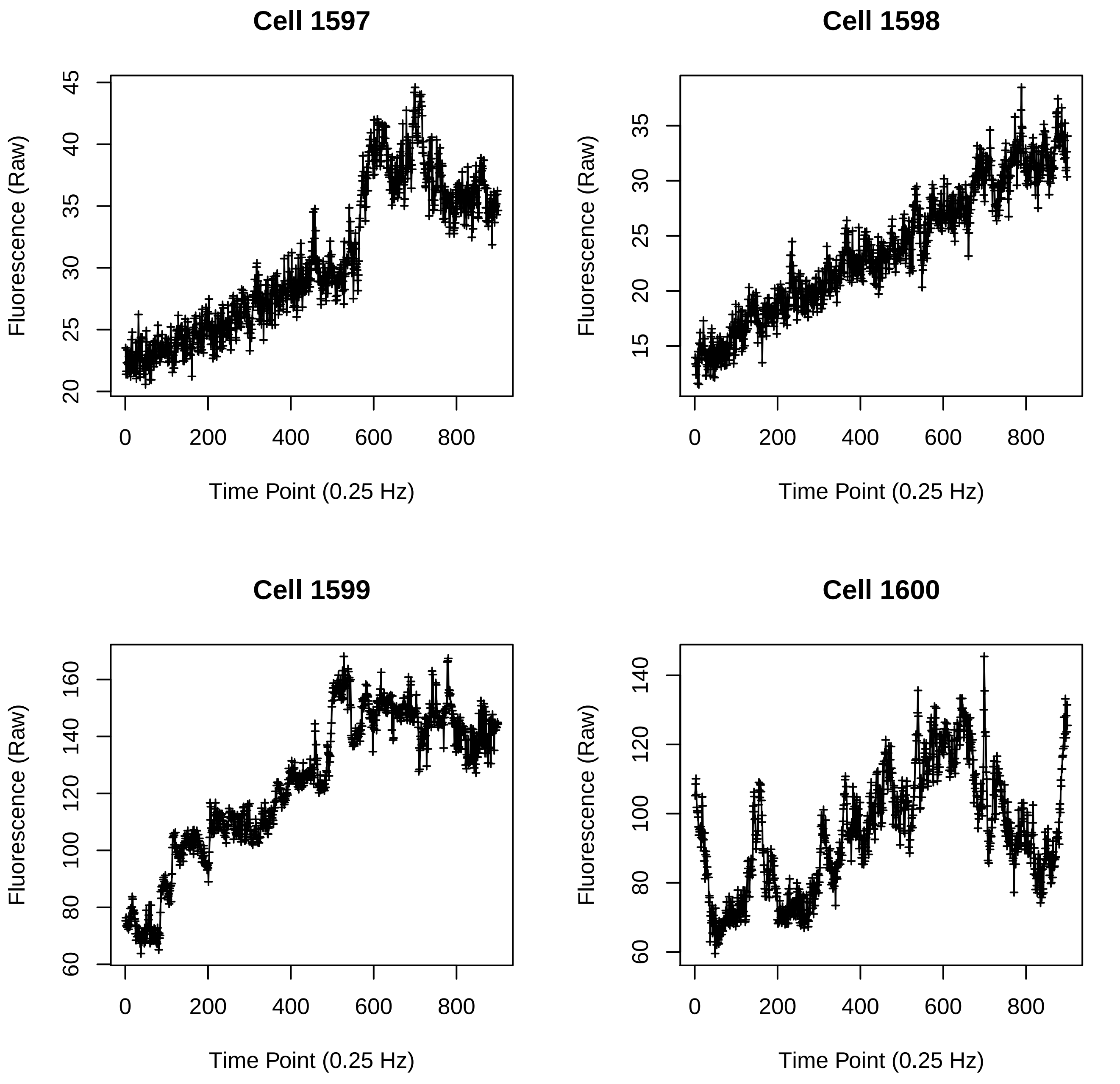

Cell 1597
Cell 1598
Cell 1599
Cell 1600
Fluorescence (Raw)
Time Point (0.25 Hz)
20
25
30
35
40
45
15
60
80
100
120
140
160
0
200
400
600
800

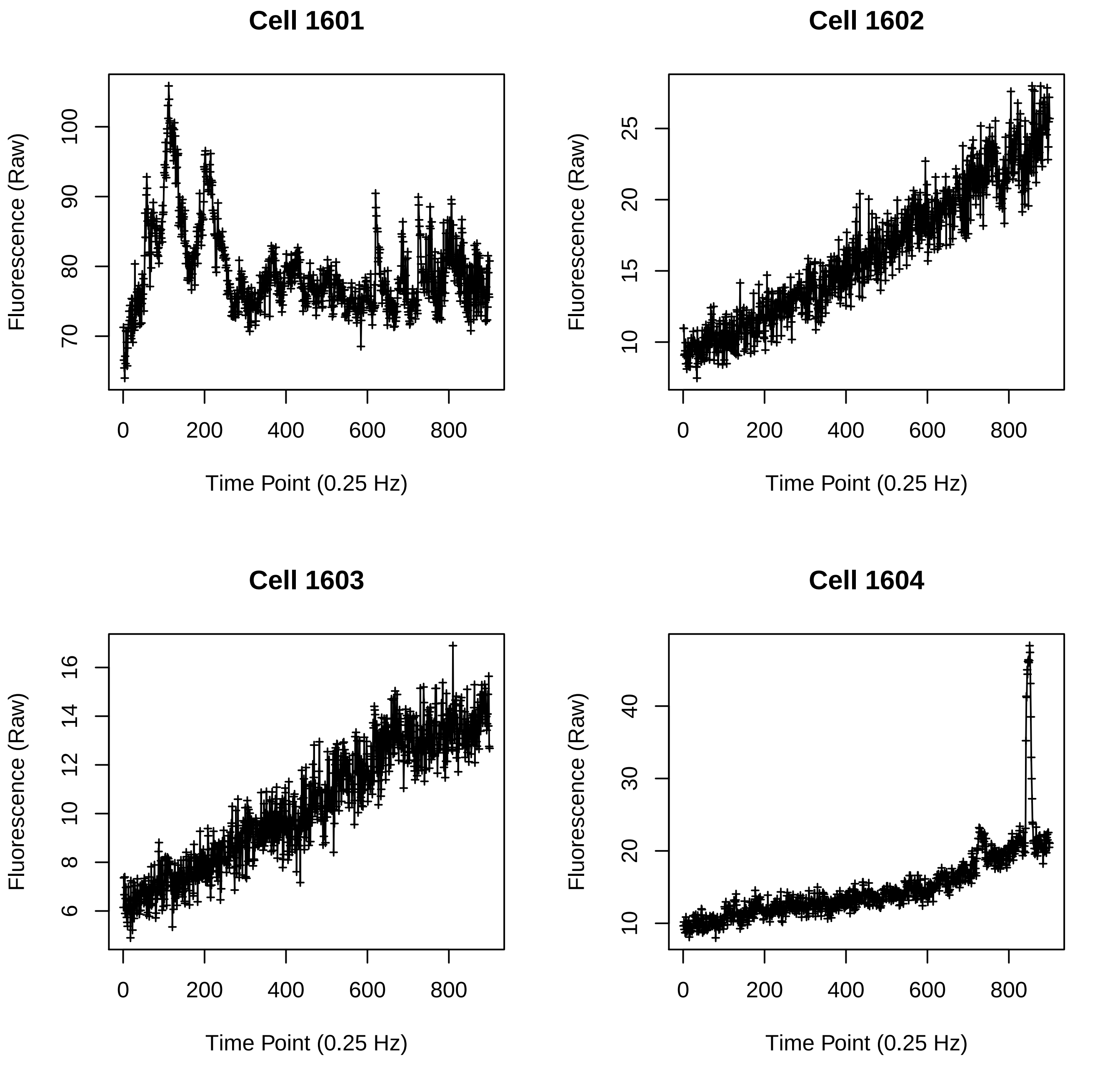
Cell 1601
Cell 1602
Cell 1603
Cell 1604
Fluorescence (Raw)
Time Point (0.25 Hz)
0
200
400
600
800
70
80
90
100
10
15
20
25
6
8
10
12
14
16
10
20
30
40

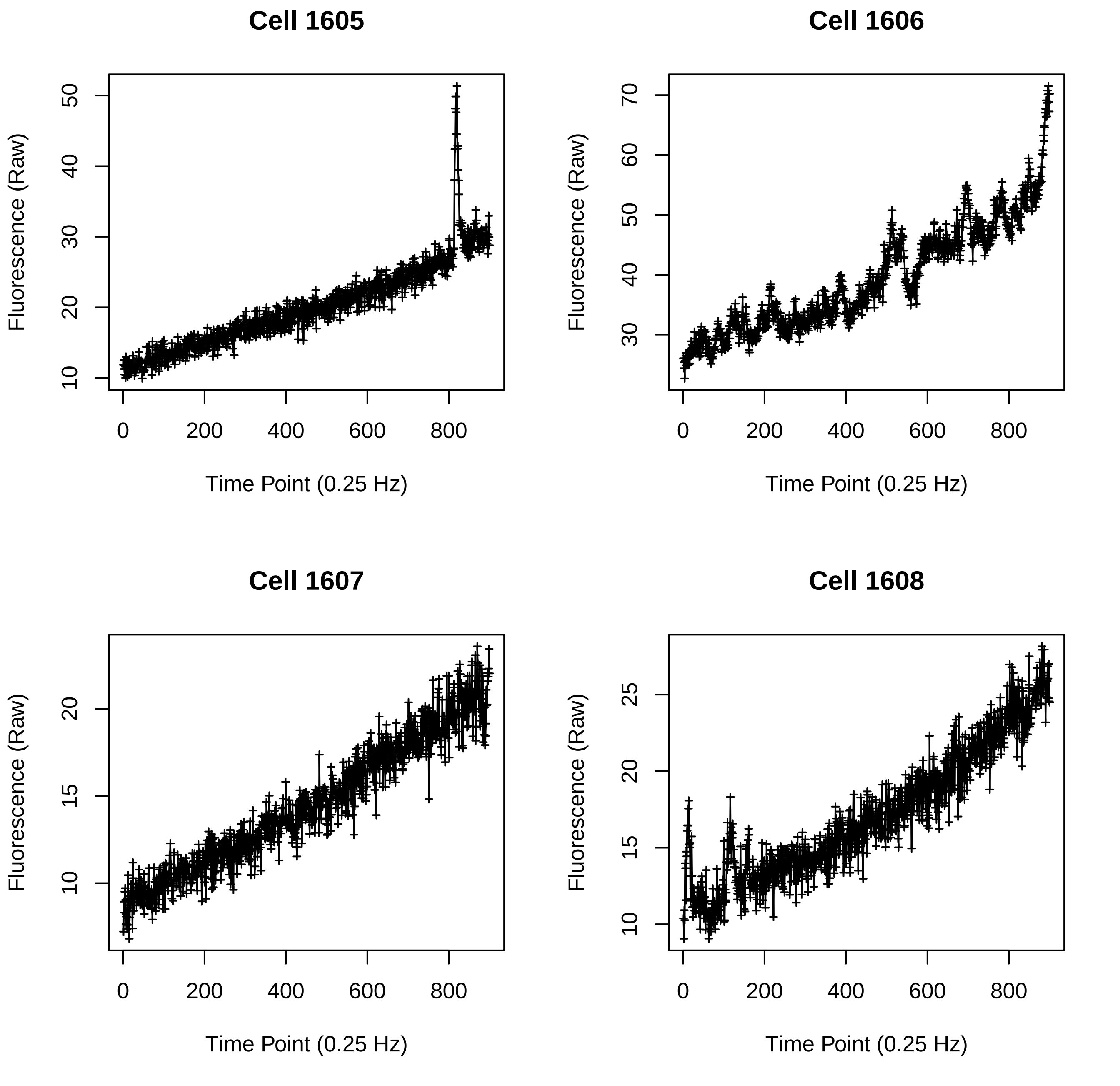

Cell 1605
Fluorescence (Raw)
10
20
30
40
50
0
200
400
600
800
Time Point (0.25 Hz)
Cell 1606
Fluorescence (Raw)
30
40
50
60
70
0
200
400
600
800
Time Point (0.25 Hz)
Cell 1607
Fluorescence (Raw)
10
15
20
0
200
400
600
800
Time Point (0.25 Hz)
Cell 1608
Fluorescence (Raw)
10
15
20
25
0
200
400
600
800
Time Point (0.25 Hz)

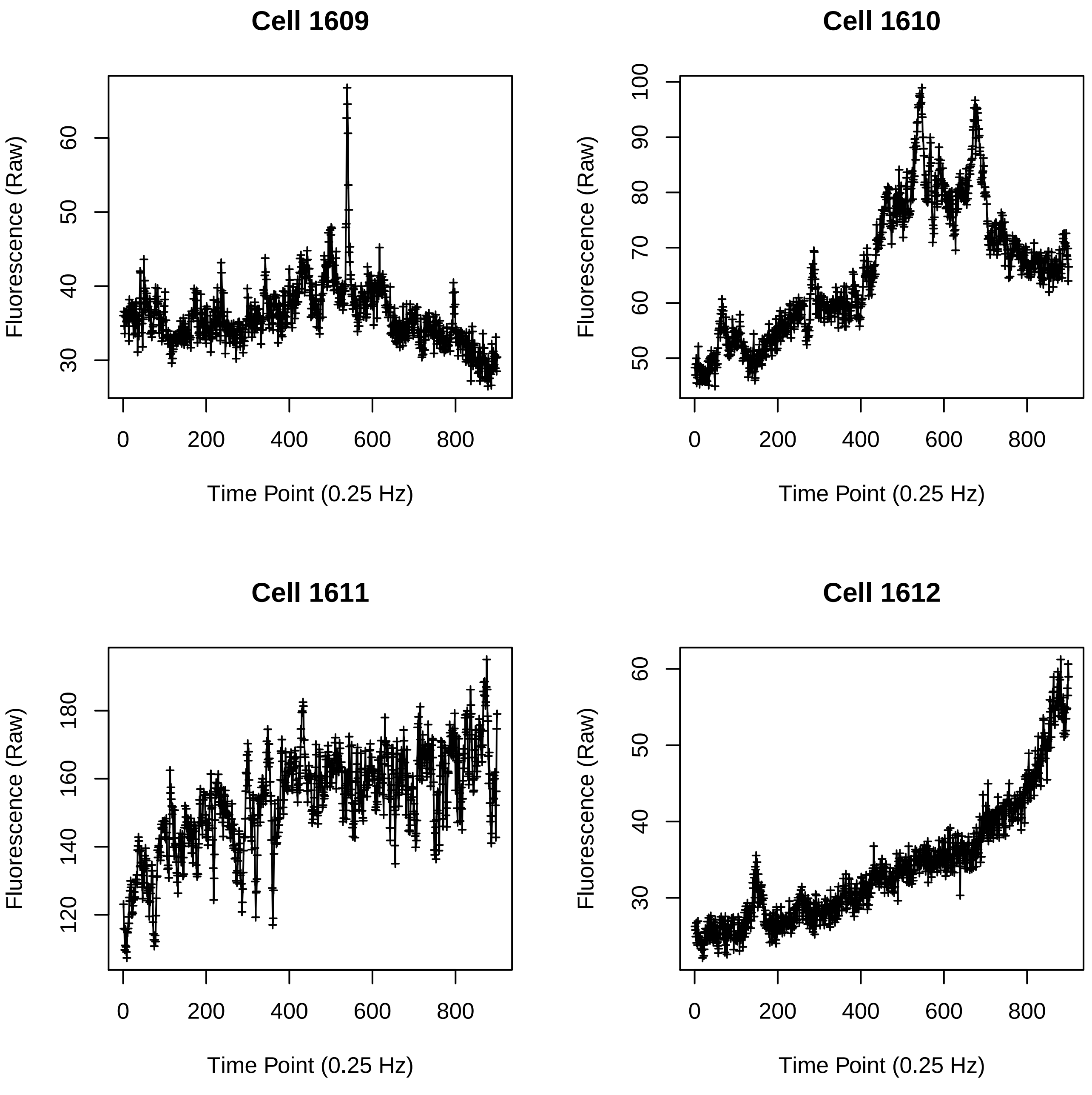

Cell 1609
Cell 1610
Cell 1611
Cell 1612
Fluorescence (Raw)
Time Point (0.25 Hz)
0
200
400
600
800
30
40
50
60
70
80
90
100
120
140
160
180

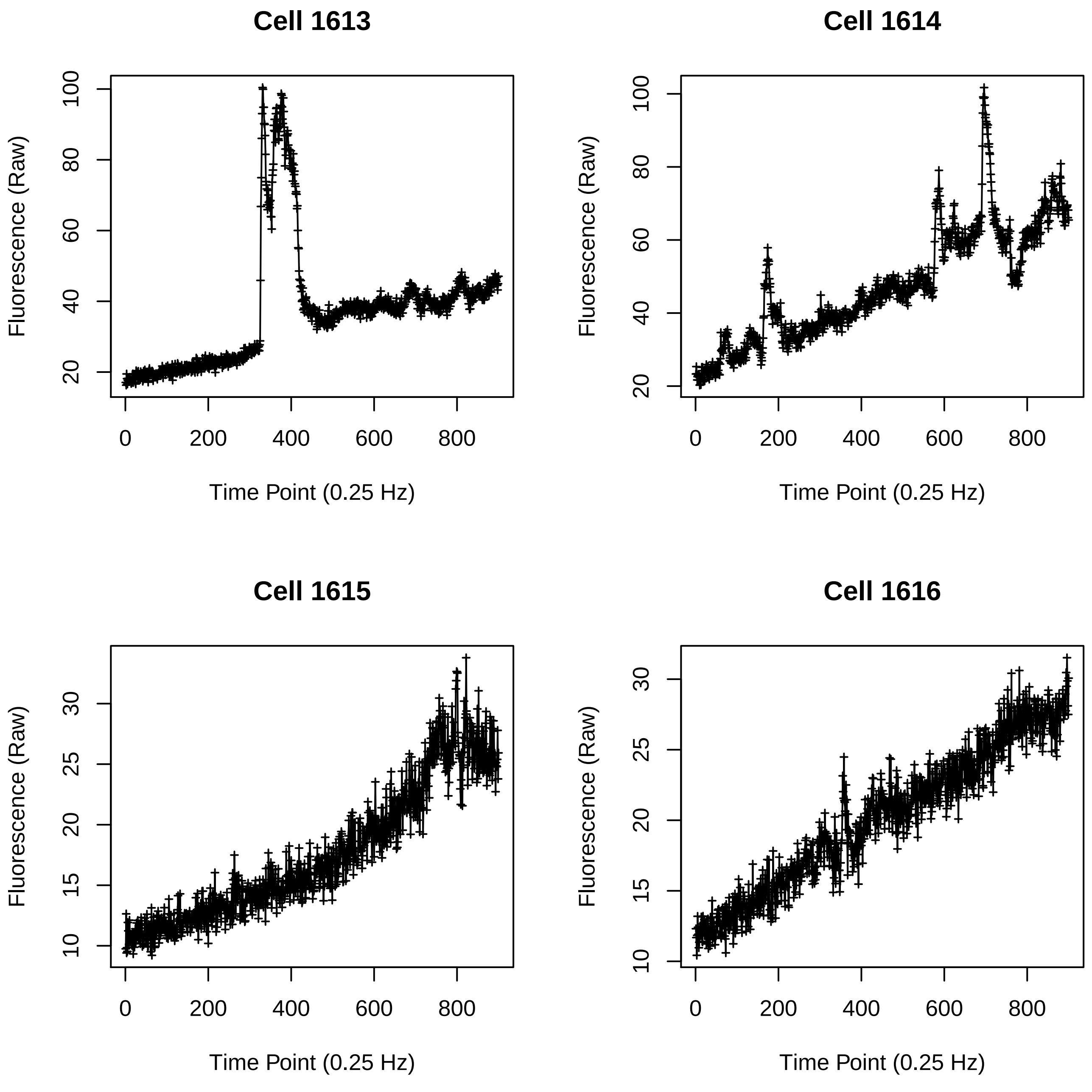

Cell 1613
Cell 1614
Cell 1615
Cell 1616
Fluorescence (Raw)
Time Point (0.25 Hz)
0
200
400
600
800
20
40
60
80
100
10
15
20
25
30

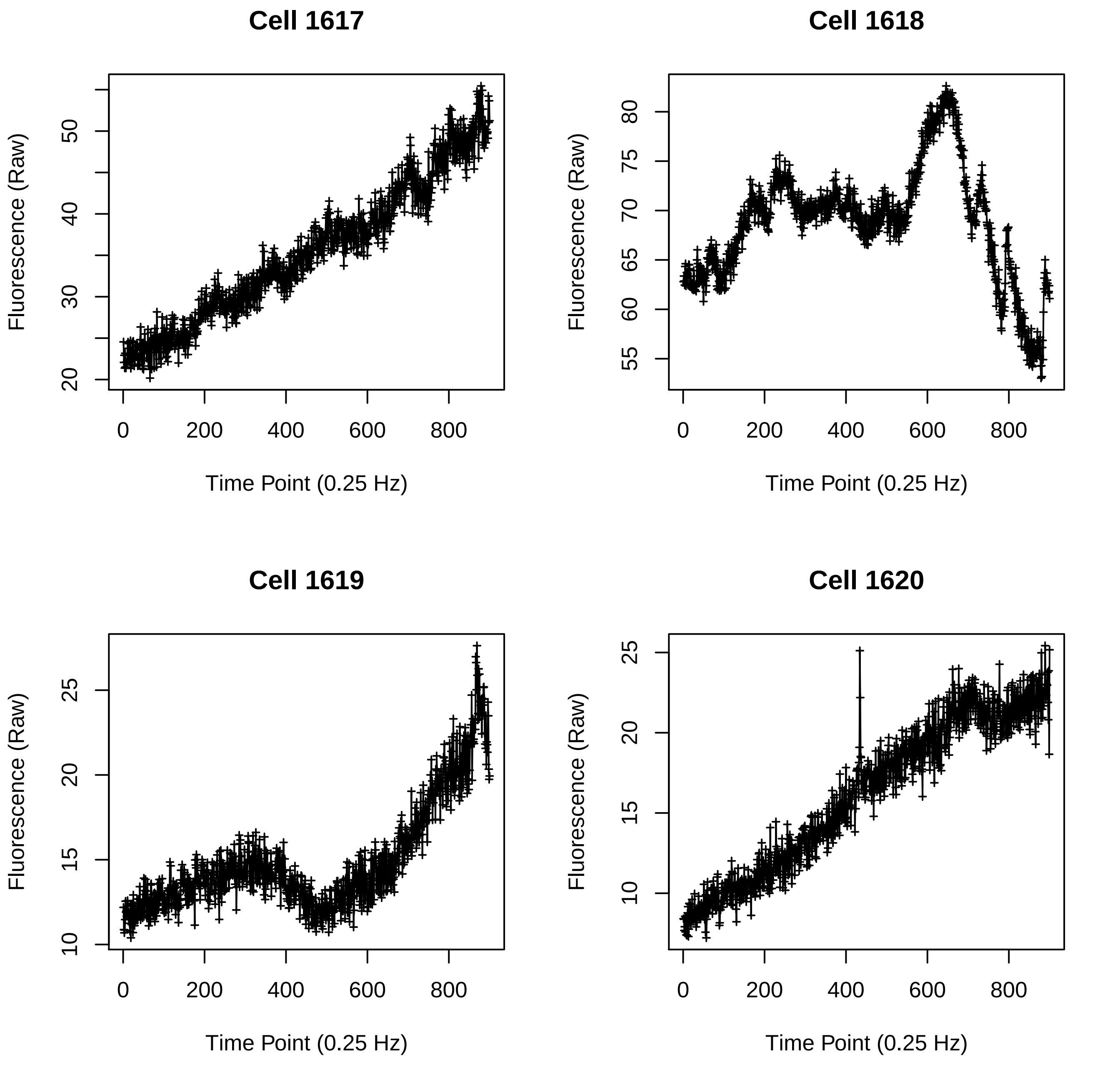
Cell 1617
Cell 1618
Cell 1619
Cell 1620
Fluorescence (Raw)
Time Point (0.25 Hz)
0
200
400
600
800
20
30
40
50
55
60
65
70
75
80
10
15
25

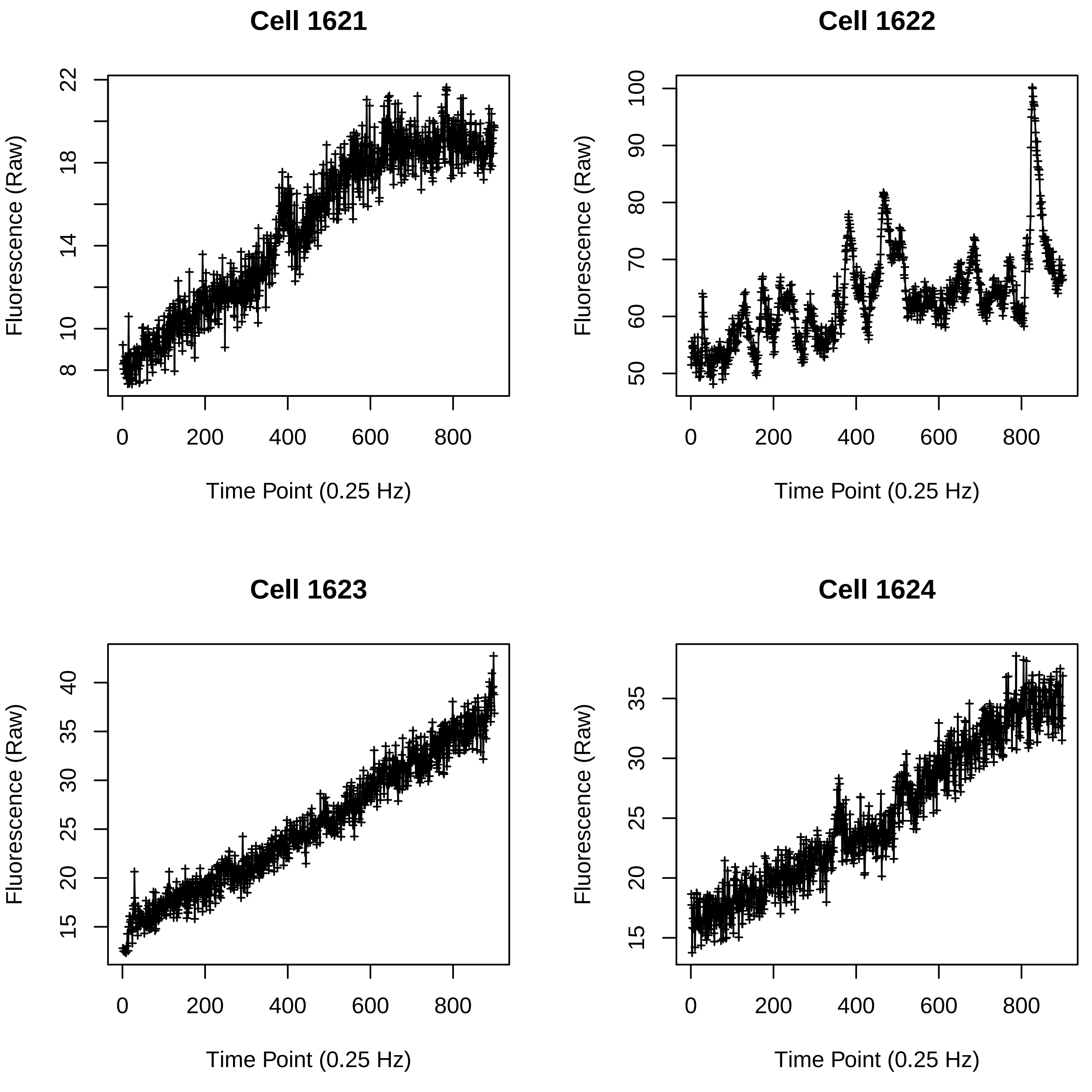

Cell 1621
Cell 1622
Cell 1623
Cell 1624
Fluorescence (Raw)
Time Point (0.25 Hz)
22
18
14
10
8
100
90
80
70
60
50
40
35
30
25
20
15
0
200
400
600
800

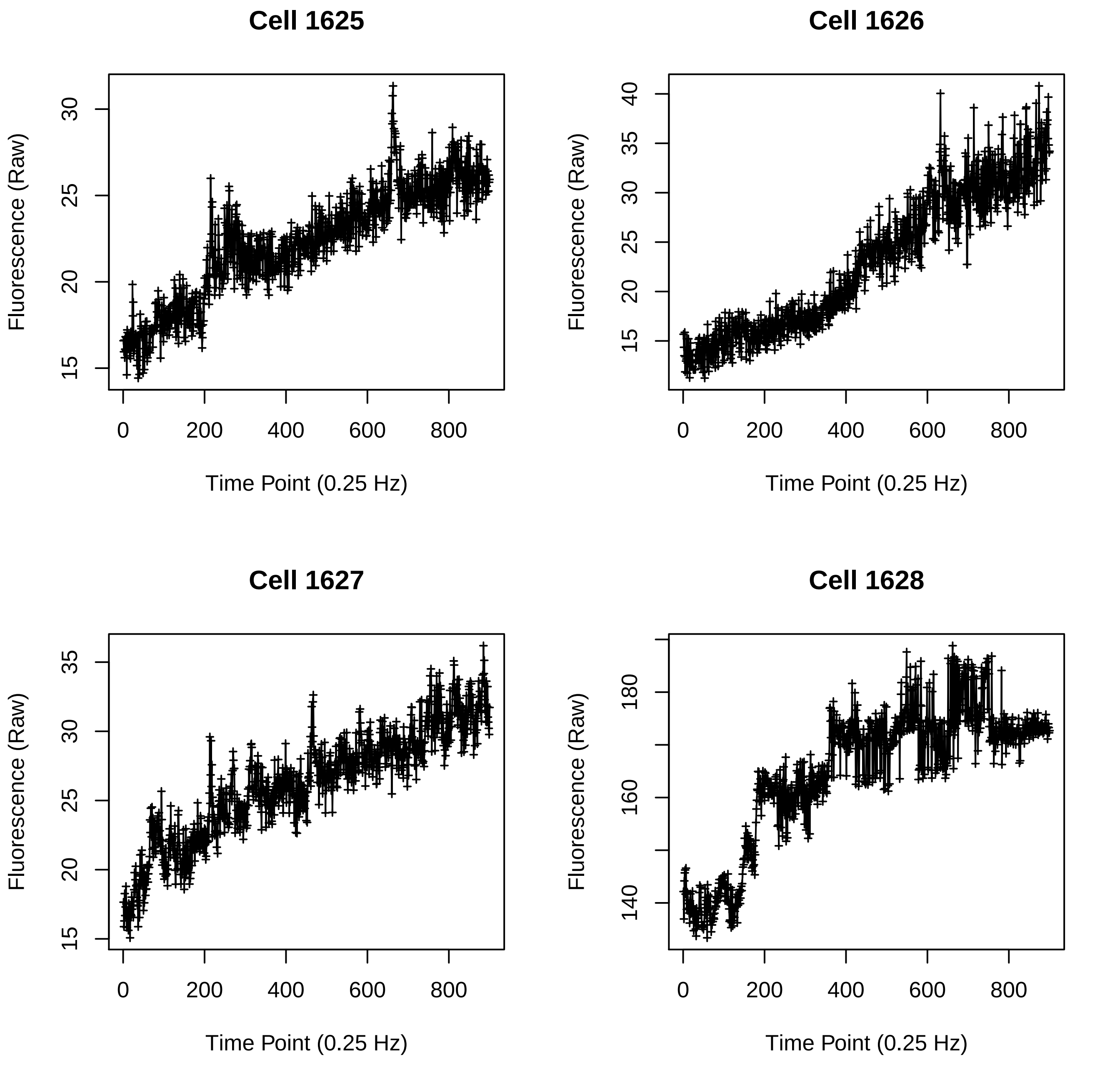
Cell 1625
Cell 1626
Cell 1627
Cell 1628
Fluorescence (Raw)
Time Point (0.25 Hz)
0
200
400
600
800
15
20
25
30
35
40
140
160
180

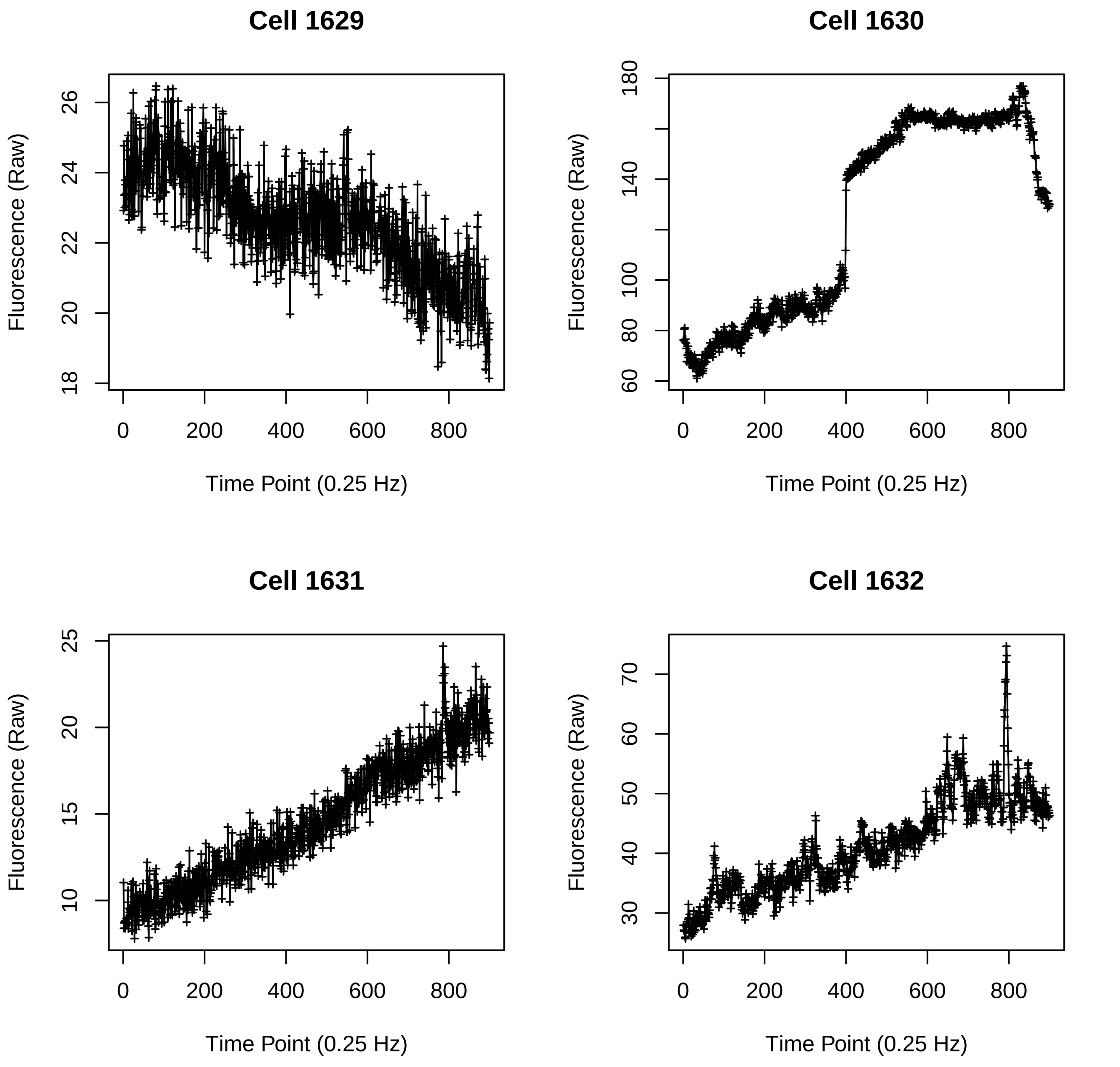

Cell 1629
Fluorescence (Raw)
Time Point (0.25 Hz)
18
20
22
24
26
0
200
400
600
800
Cell 1630
Fluorescence (Raw)
Time Point (0.25 Hz)
60
80
100
140
180
0
200
400
600
800
Cell 1631
Fluorescence (Raw)
Time Point (0.25 Hz)
10
15
20
25
0
200
400
600
800
Cell 1632
Fluorescence (Raw)
Time Point (0.25 Hz)
30
40
50
60
70
0
200
400
600
800

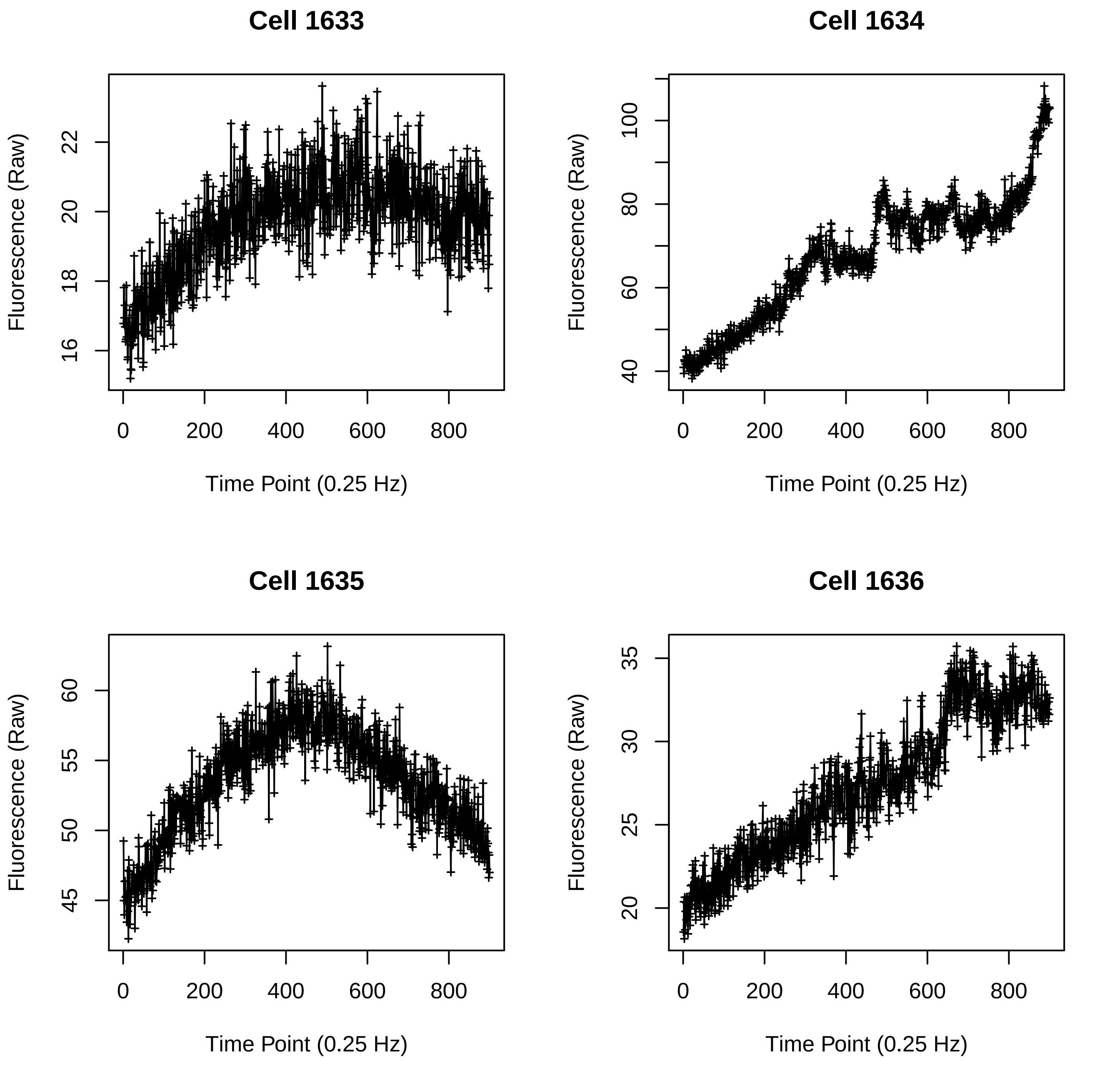

Cell 1633
Cell 1634
Cell 1635
Cell 1636
Fluorescence (Raw)
Time Point (0.25 Hz)
16
18
20
22
40
60
80
100
45
50
55
60
20
25
30
35
0
200
400
600
800

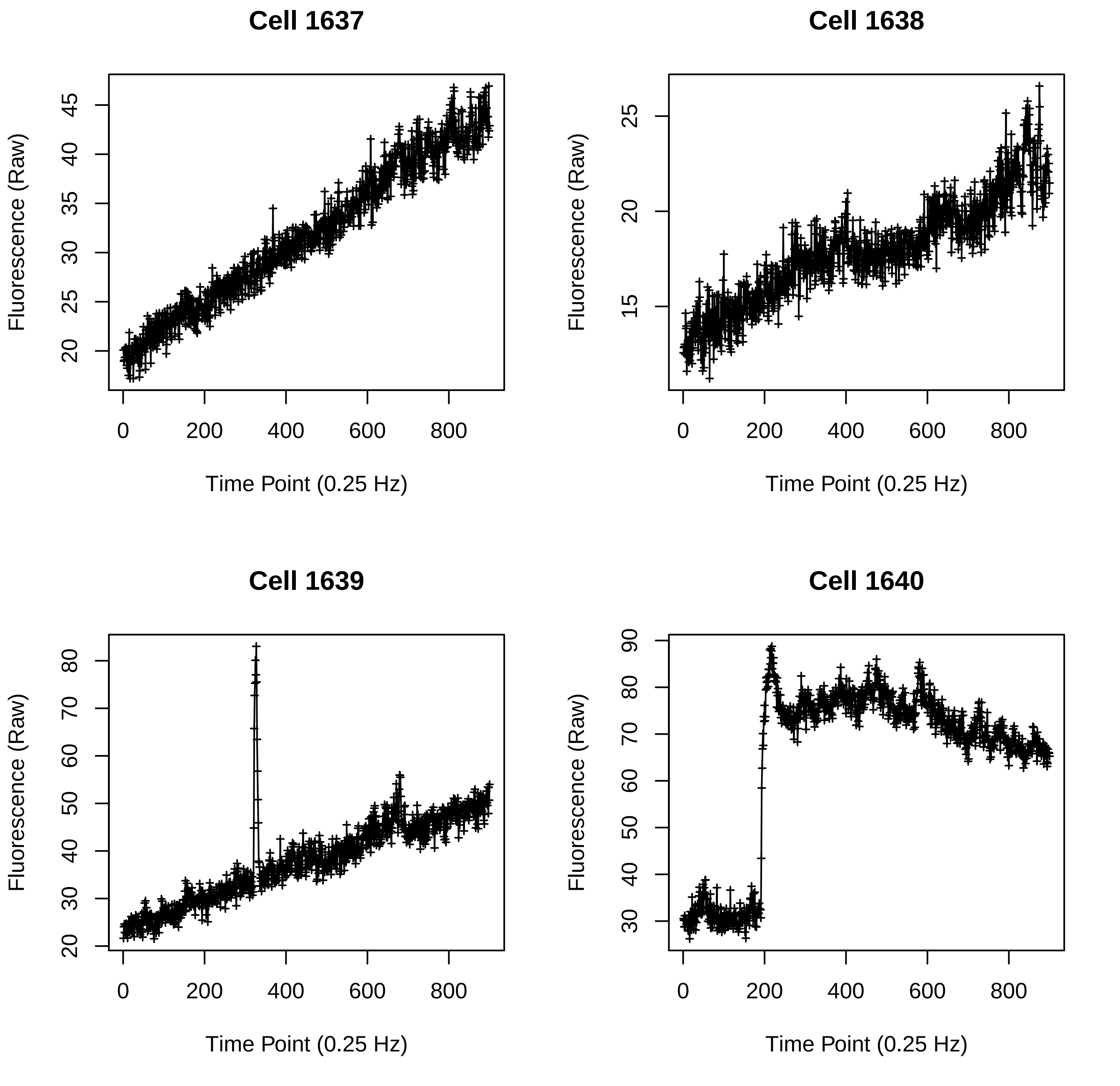
Cell 1637
Fluorescence (Raw)
20
25
30
35
40
45
0
200
400
600
800
Time Point (0.25 Hz)
Cell 1638
Fluorescence (Raw)
15
20
25
0
200
400
600
800
Time Point (0.25 Hz)
Cell 1639
Fluorescence (Raw)
20
30
40
50
60
70
80
0
200
400
600
800
Time Point (0.25 Hz)
Cell 1640
Fluorescence (Raw)
30
40
50
60
70
80
90
0
200
400
600
800
Time Point (0.25 Hz)

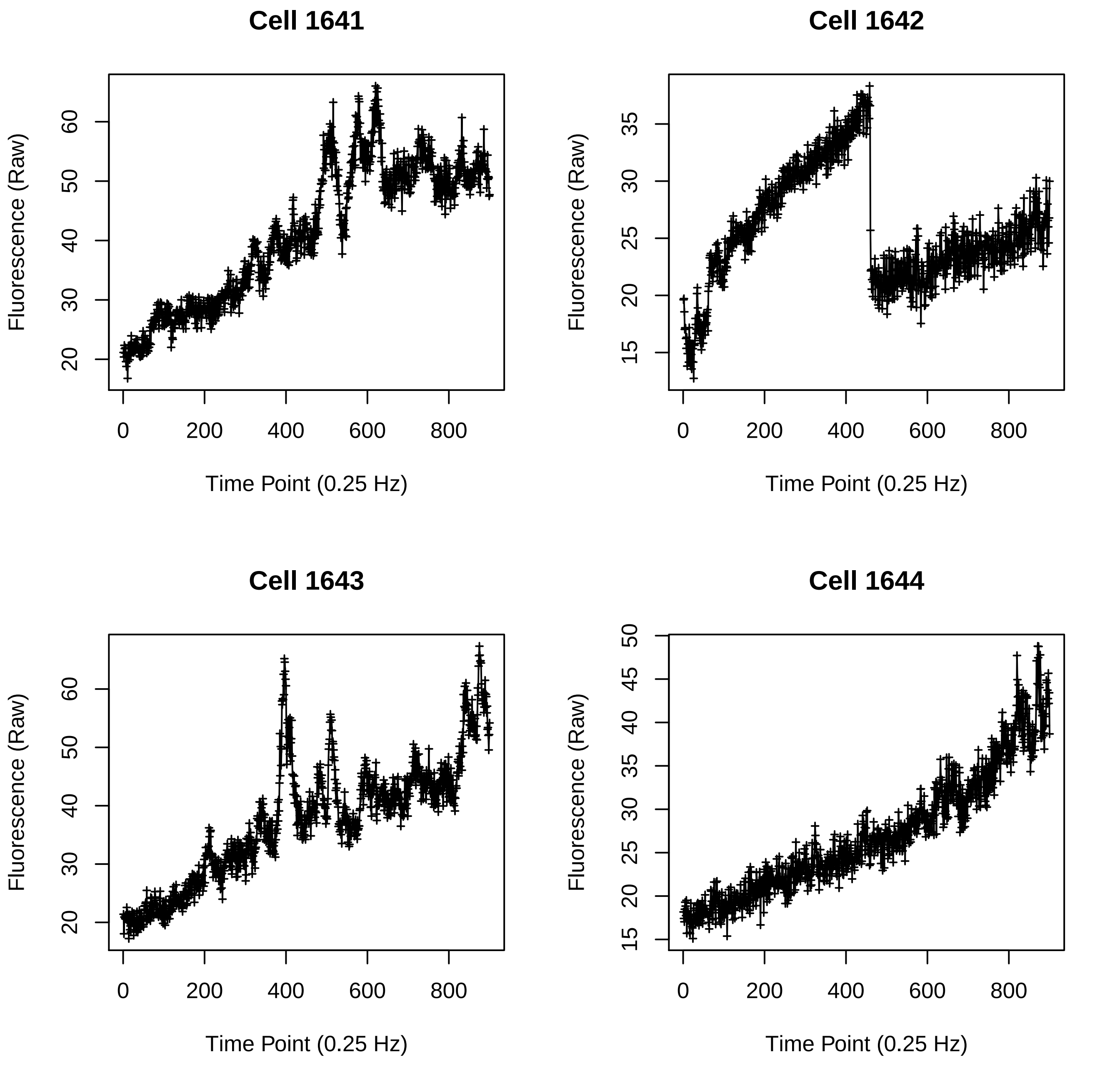
Cell 1641
Fluorescence (Raw)
20
30
40
50
60
0
200
400
600
800
Time Point (0.25 Hz)
Cell 1642
Fluorescence (Raw)
15
20
25
30
35
0
200
400
600
800
Time Point (0.25 Hz)
Cell 1643
Fluorescence (Raw)
20
30
40
50
60
0
200
400
600
800
Time Point (0.25 Hz)
Cell 1644
Fluorescence (Raw)
15
20
25
30
35
40
45
50
0
200
400
600
800
Time Point (0.25 Hz)

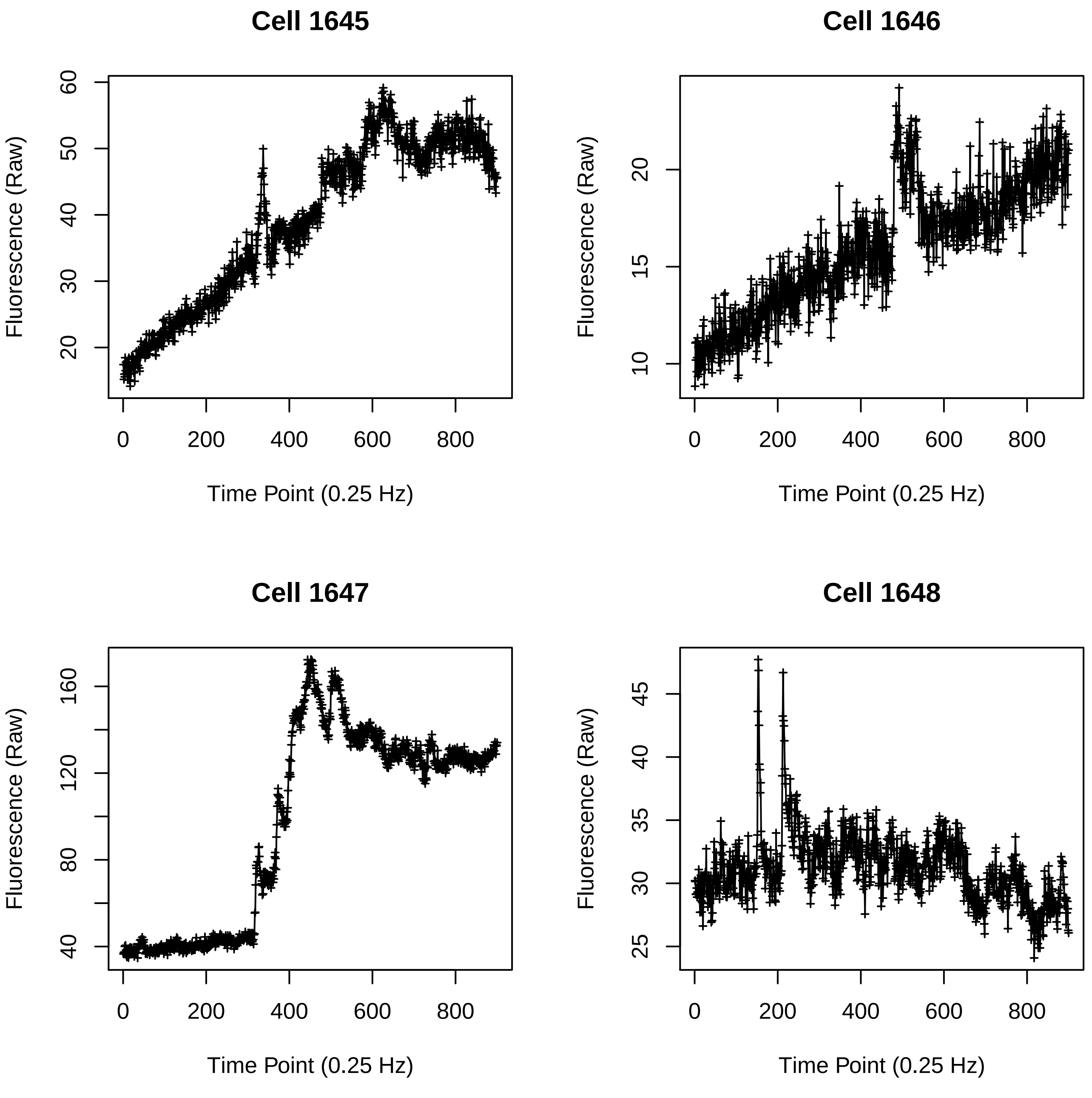
Cell 1645
Cell 1646
Cell 1647
Cell 1648
Fluorescence (Raw)
Time Point (0.25 Hz)
0
200
400
600
800
20
30
40
50
60
10
15
20
40
80
120
160
25
30
35
40
45

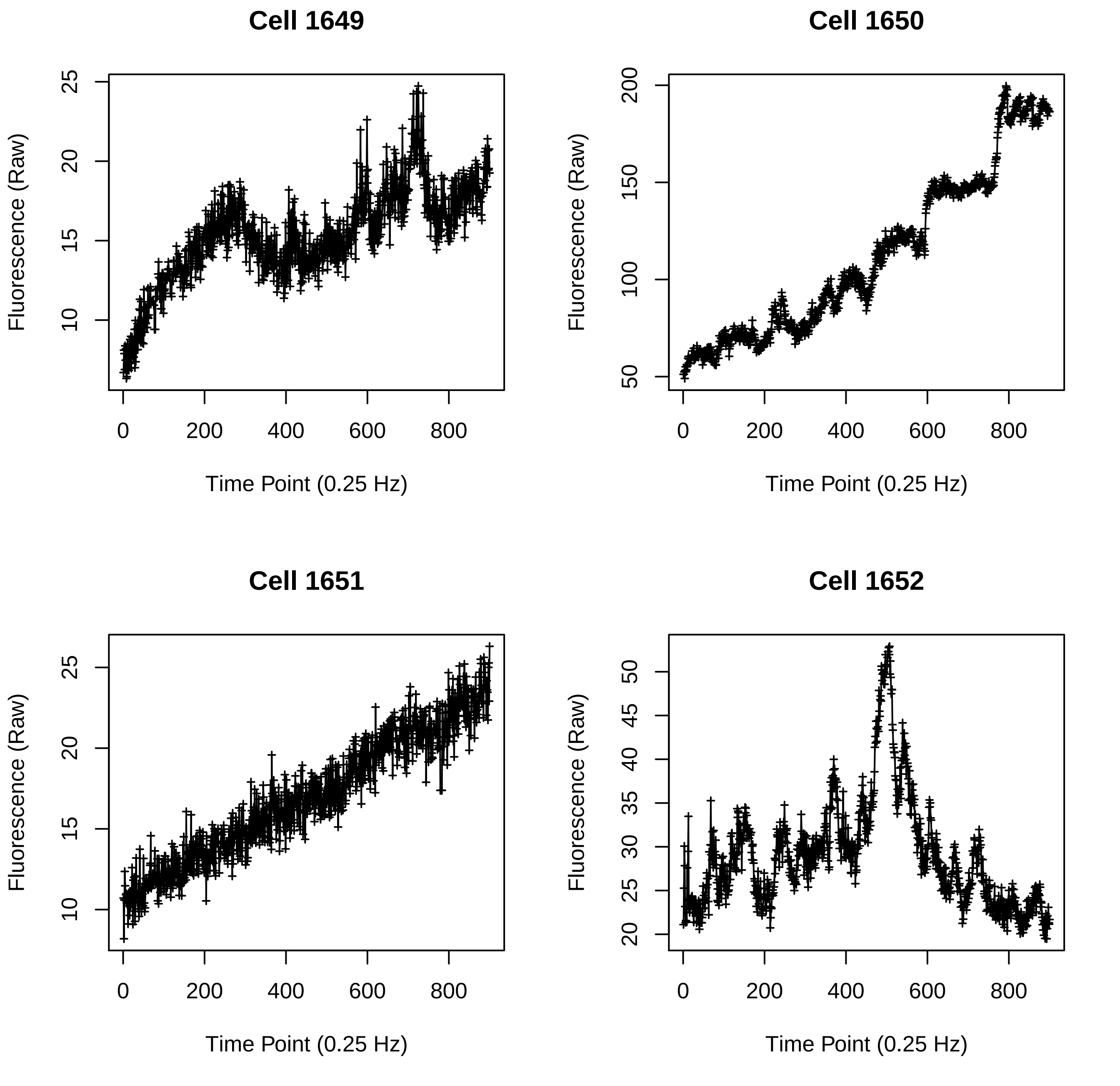
Cell 1649
Cell 1650
Cell 1651
Cell 1652
Fluorescence (Raw)
Time Point (0.25 Hz)
0
200
400
600
800
10
15
20
25
50
100
150
200
30
35
40
45

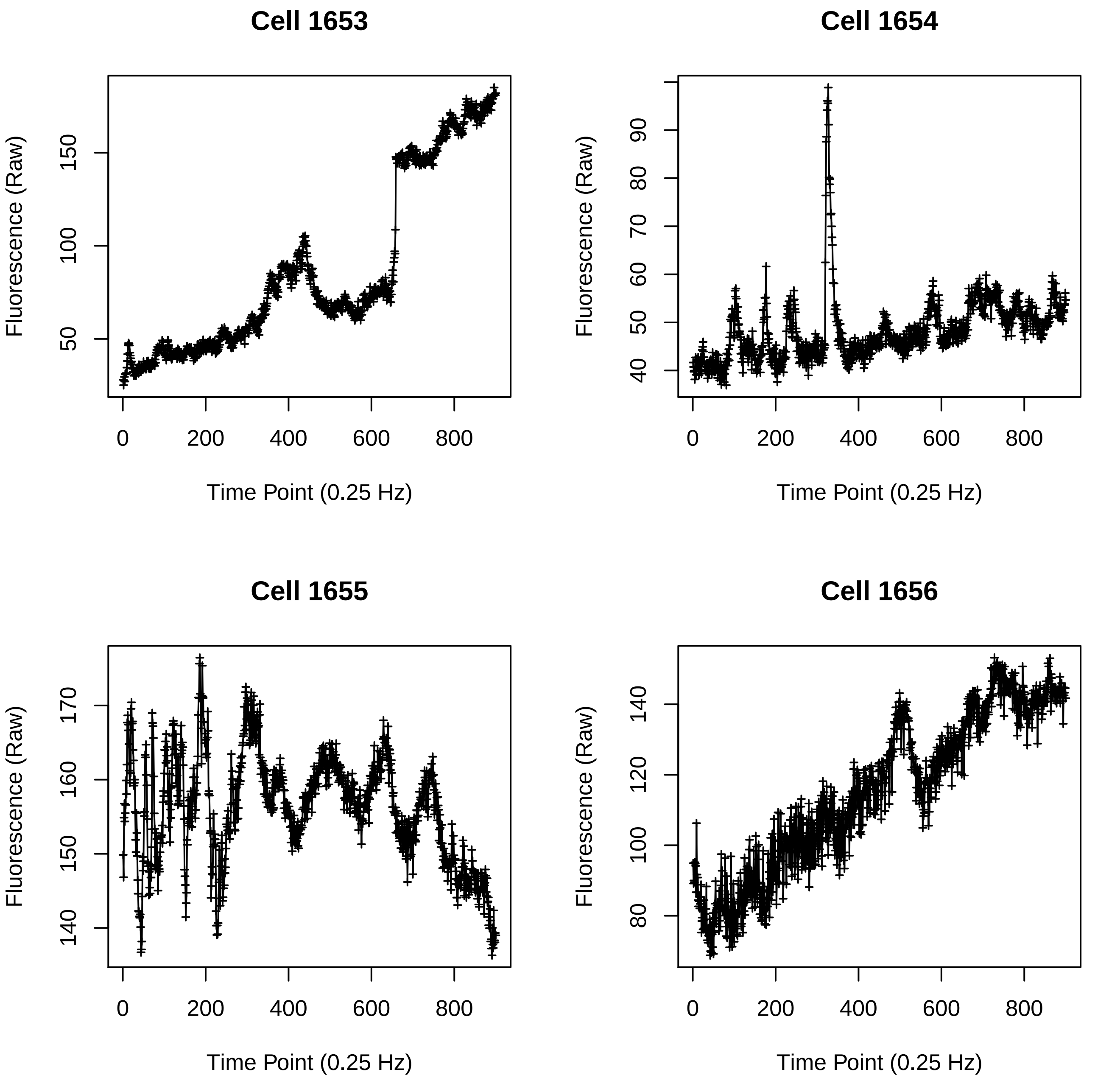
Cell 1653
Fluorescence (Raw)
50
100
150
0
200
400
600
800
Time Point (0.25 Hz)
Cell 1654
Fluorescence (Raw)
40
50
60
70
80
90
0
200
400
600
800
Time Point (0.25 Hz)
Cell 1655
Fluorescence (Raw)
140
150
160
170
0
200
400
600
800
Time Point (0.25 Hz)
Cell 1656
Fluorescence (Raw)
80
100
120
140
0
200
400
600
800
Time Point (0.25 Hz)

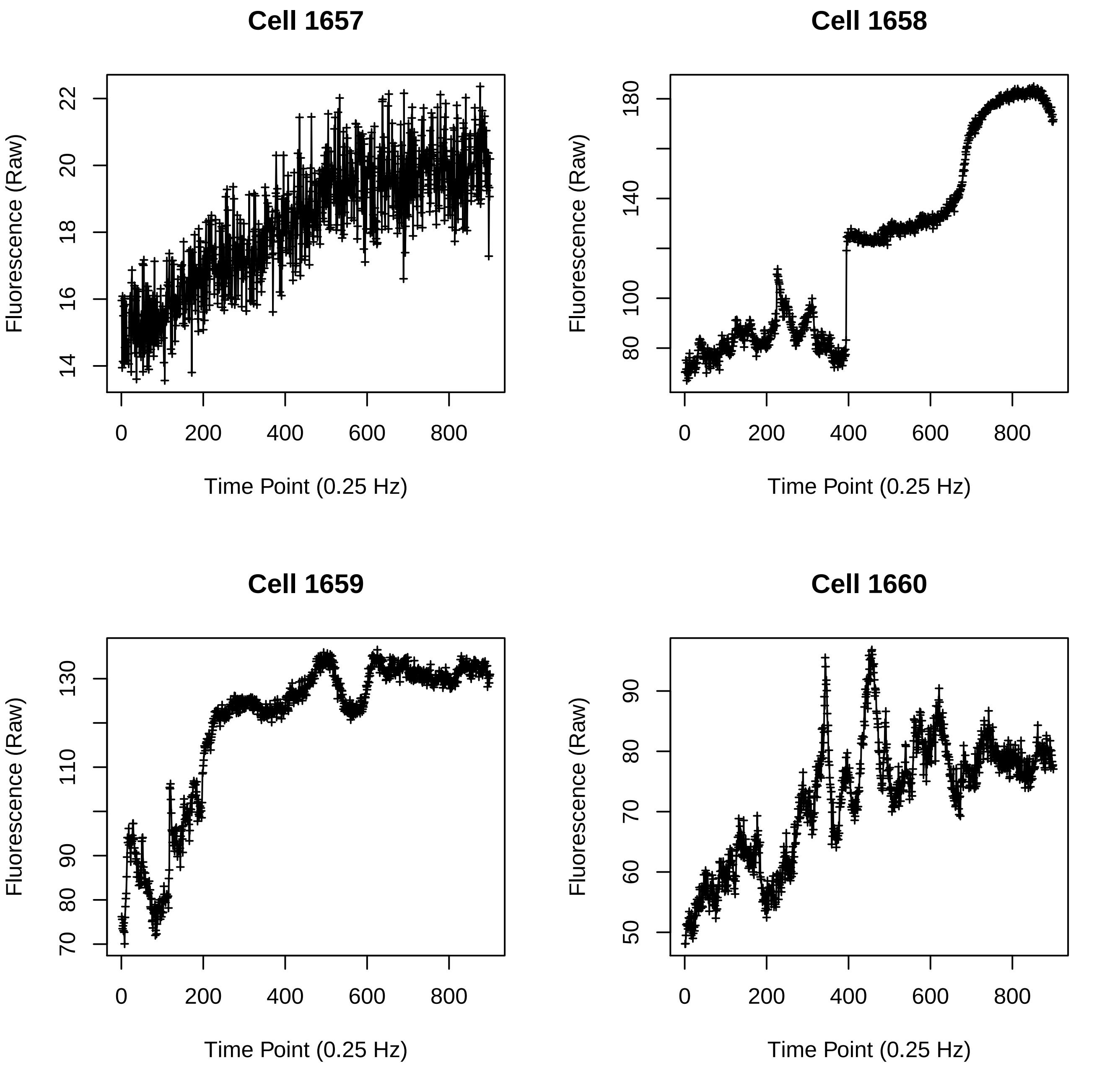

Cell 1657
Fluorescence (Raw)
14
16
18
20
22
0
200
400
600
800
Time Point (0.25 Hz)
Cell 1658
Fluorescence (Raw)
80
100
140
180
0
200
400
600
800
Time Point (0.25 Hz)
Cell 1659
Fluorescence (Raw)
70
80
90
110
130
0
200
400
600
800
Time Point (0.25 Hz)
Cell 1660
Fluorescence (Raw)
50
60
70
80
90
0
200
400
600
800
Time Point (0.25 Hz)

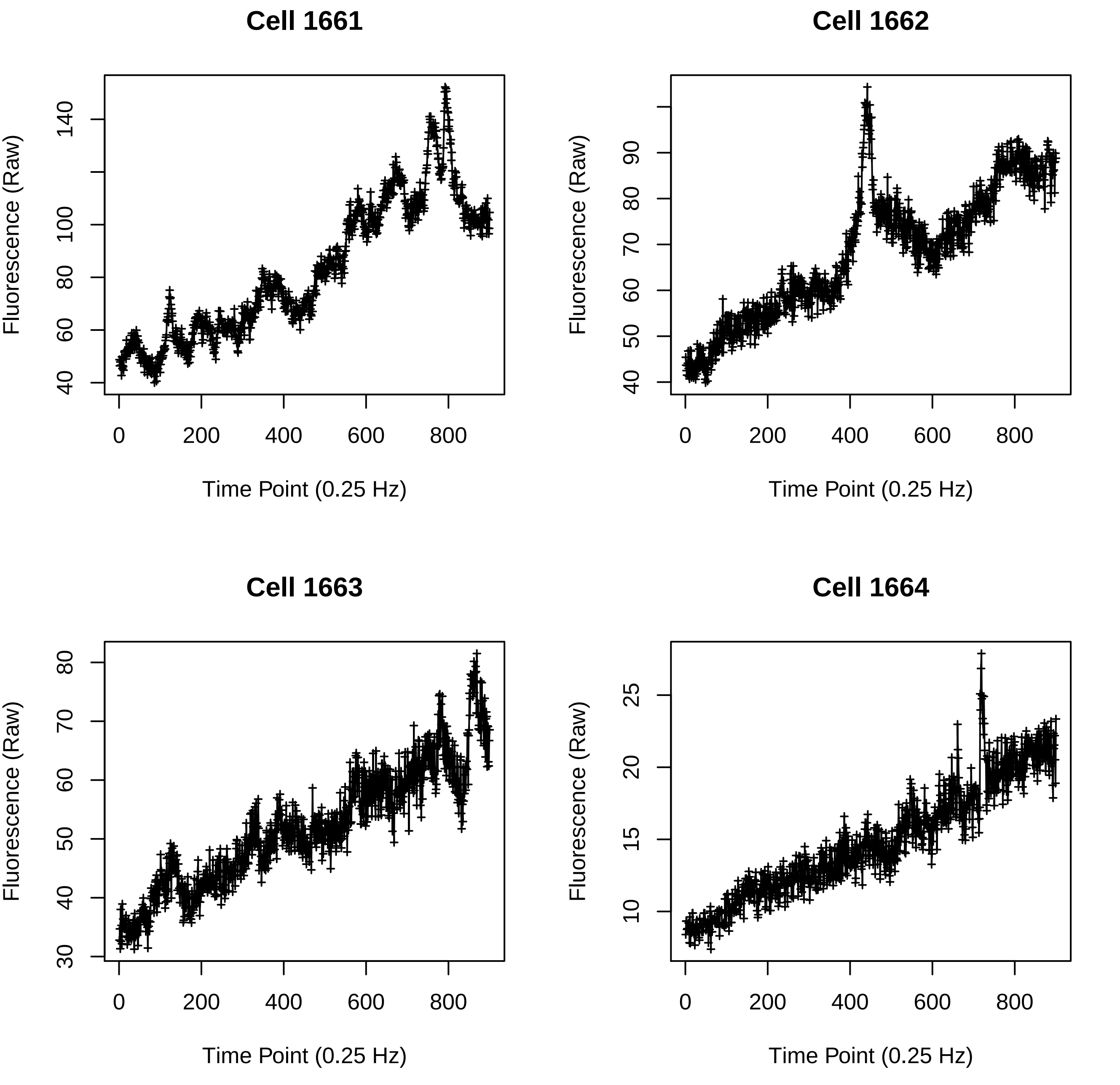
Cell 1661
Fluorescence (Raw)
40
60
80
100
140
0
200
400
600
800
Time Point (0.25 Hz)
Cell 1662
Fluorescence (Raw)
40
50
60
70
80
90
0
200
400
600
800
Time Point (0.25 Hz)
Cell 1663
Fluorescence (Raw)
30
40
50
60
70
80
0
200
400
600
800
Time Point (0.25 Hz)
Cell 1664
Fluorescence (Raw)
10
15
20
25
0
200
400
600
800
Time Point (0.25 Hz)

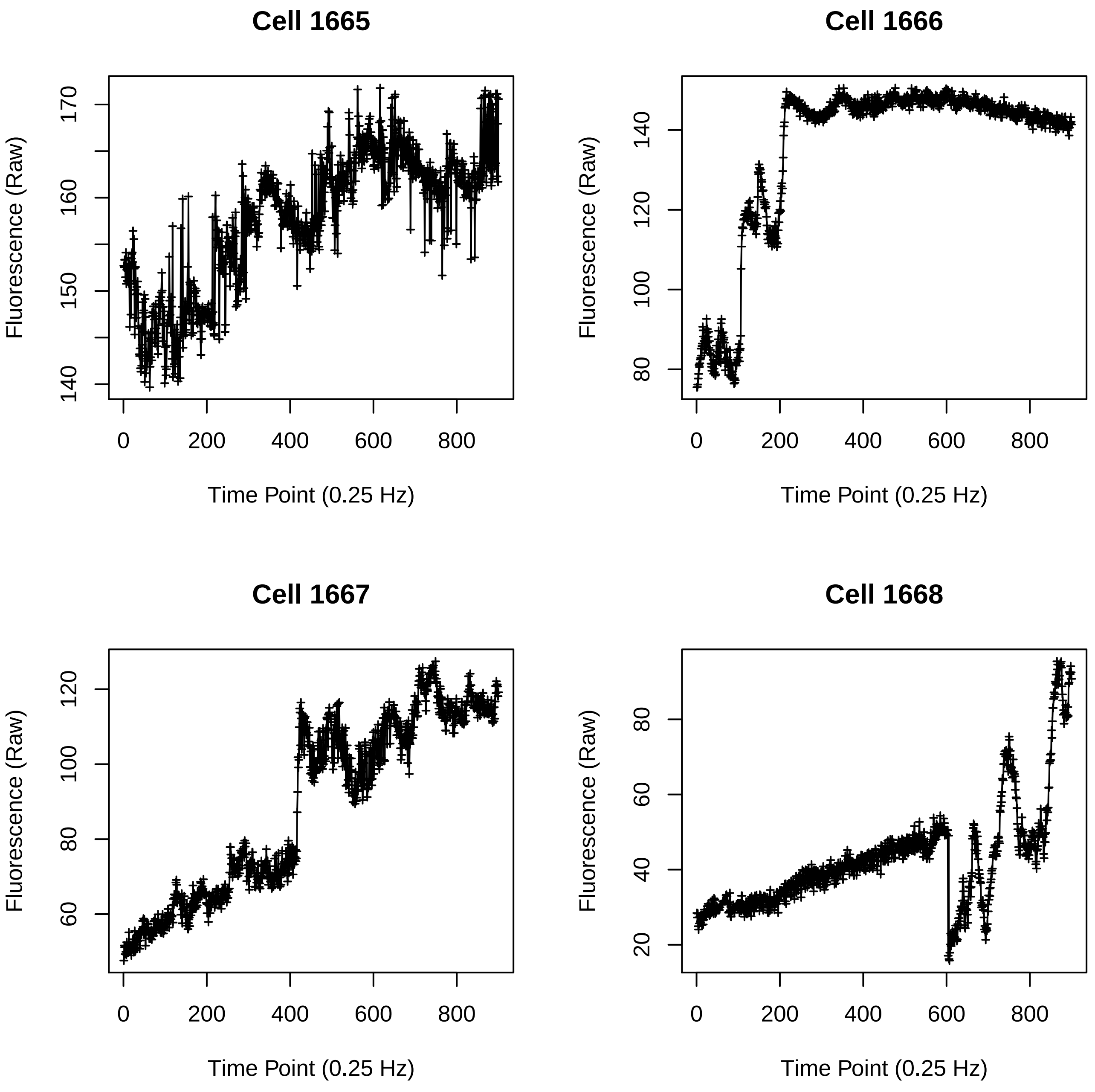

Cell 1665
Cell 1666
Cell 1667
Cell 1668
Fluorescence (Raw)
Time Point (0.25 Hz)
0
200
400
600
800
140
150
160
170
80
100
120
140
60
80
100
120
20
40
60
80

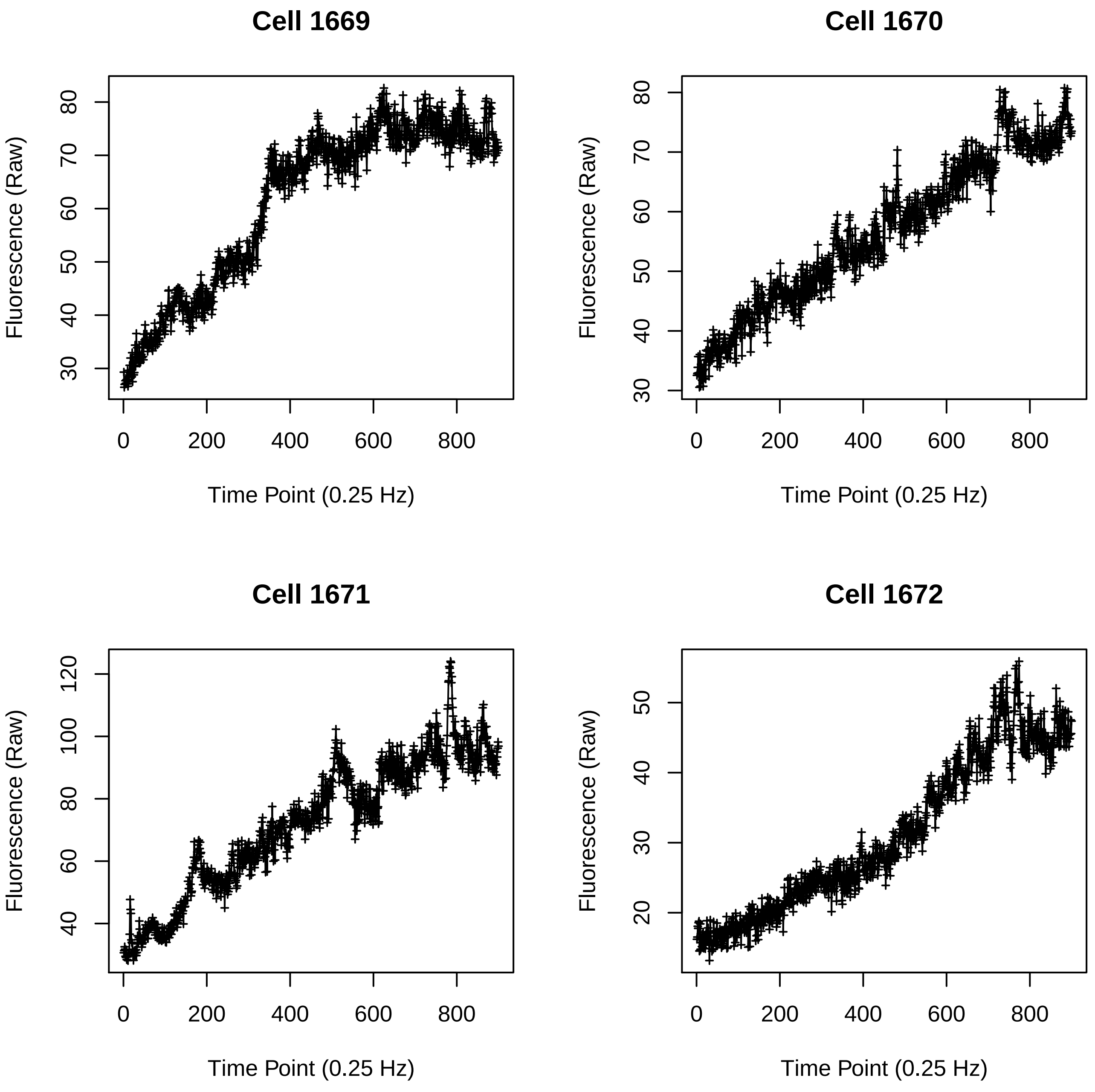
Cell 1669
Fluorescence (Raw)
Time Point (0.25 Hz)
30
40
50
60
70
80
0
200
400
600
800
Cell 1670
Fluorescence (Raw)
Time Point (0.25 Hz)
30
40
50
60
70
80
0
200
400
600
800
Cell 1671
Fluorescence (Raw)
Time Point (0.25 Hz)
40
60
80
100
120
0
200
400
600
800
Cell 1672
Fluorescence (Raw)
Time Point (0.25 Hz)
20
30
40
50
0
200
400
600
800

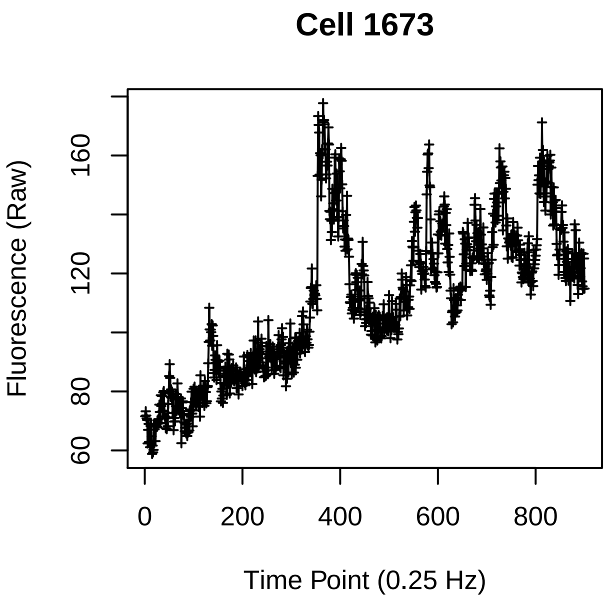
Cell 1673
Fluorescence (Raw)
60
80
120
160
0
200
400
600
800
Time Point (0.25 Hz)

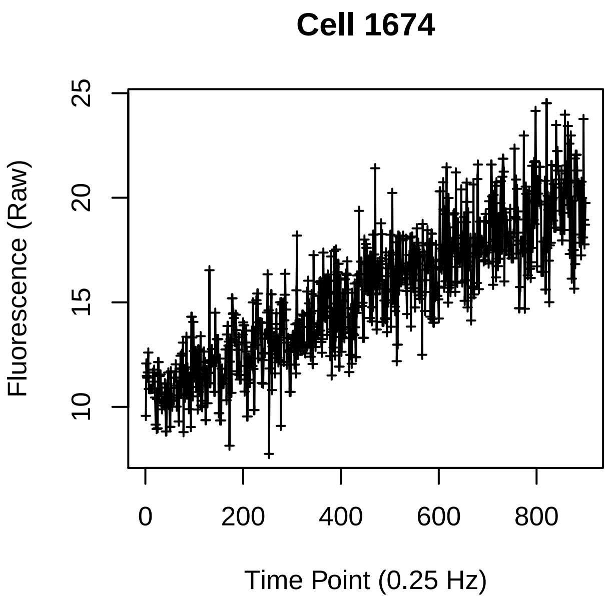
Cell 1674
Fluorescence (Raw)
10
15
20
25
0
200
400
600
800
Time Point (0.25 Hz)

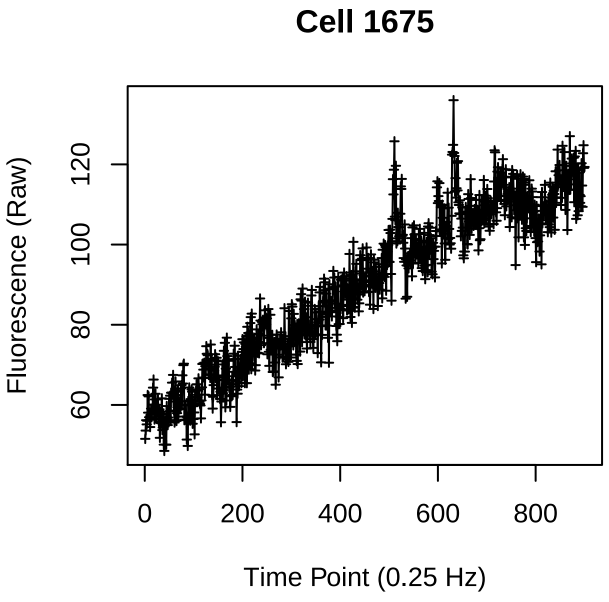
Cell 1675
Fluorescence (Raw)
60
80
100
120
0
200
400
600
800
Time Point (0.25 Hz)

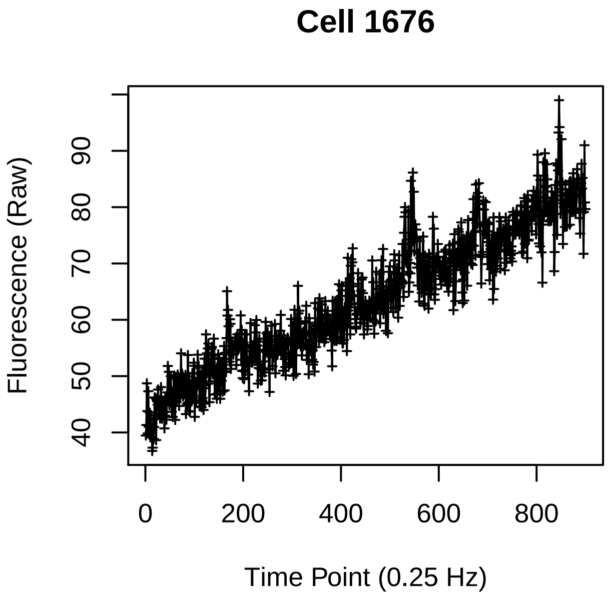
Cell 1676
Fluorescence (Raw)
40
50
60
70
80
90
0
200
400
600
800
Time Point (0.25 Hz)

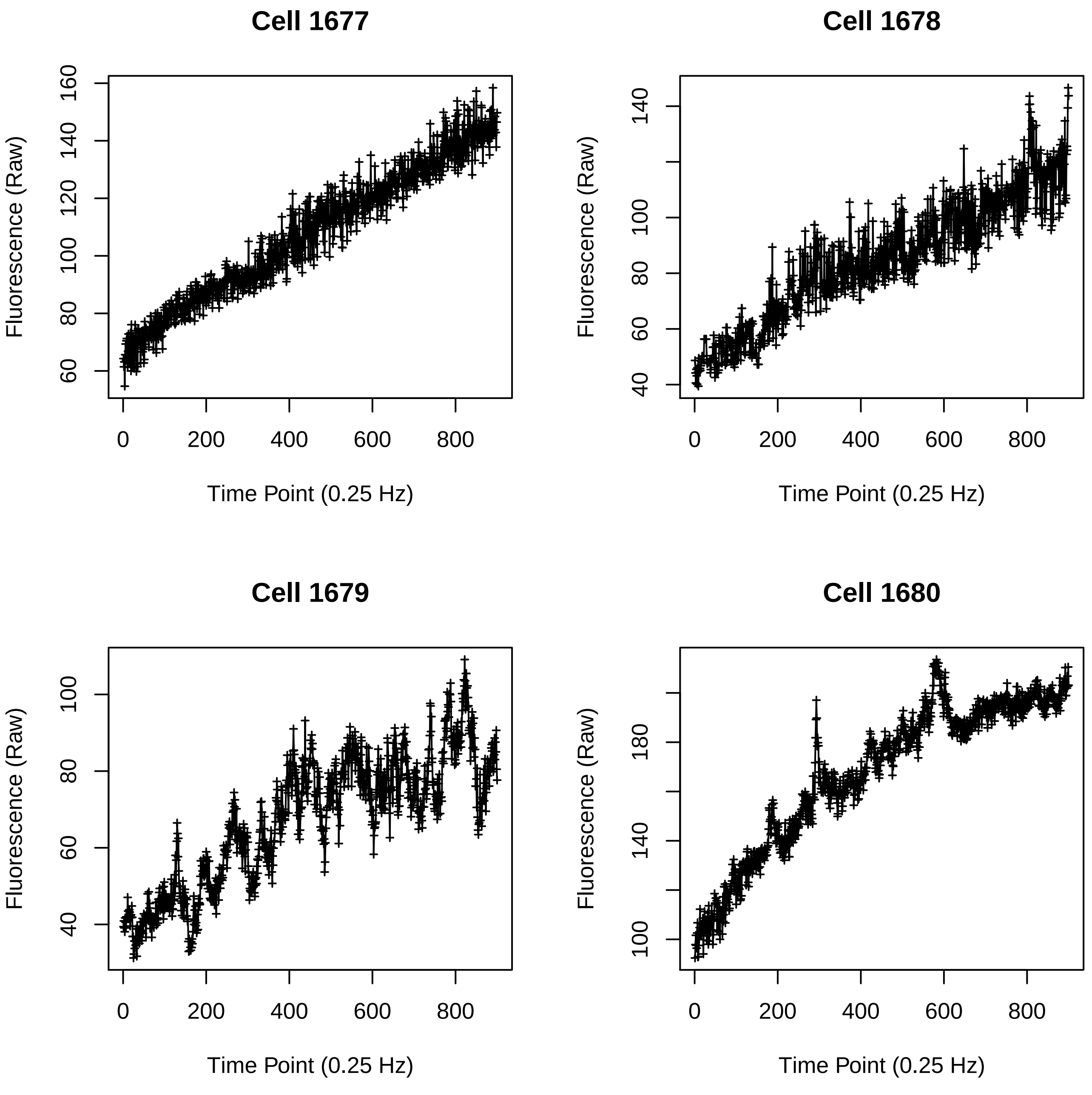

Cell 1677
Fluorescence (Raw)
60 80 100 120 140 160
Time Point (0.25 Hz)
0 200 400 600 800
Cell 1678
Fluorescence (Raw)
40 60 80 100 140
Time Point (0.25 Hz)
0 200 400 600 800
Cell 1679
Fluorescence (Raw)
40 60 80 100
Time Point (0.25 Hz)
0 200 400 600 800
Cell 1680
Fluorescence (Raw)
100 140 180
Time Point (0.25 Hz)
0 200 400 600 800

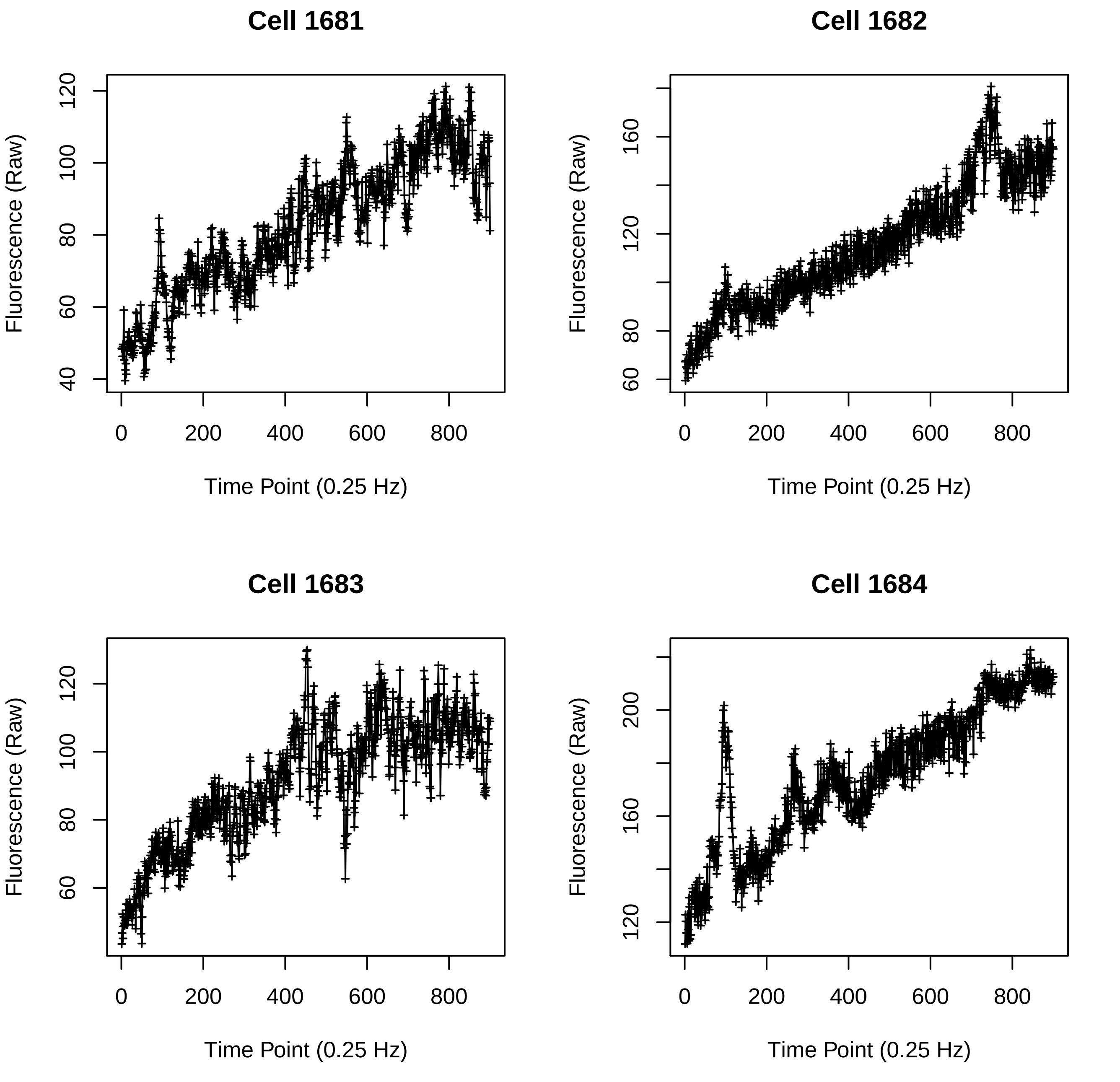
Cell 1681
Cell 1682
Cell 1683
Cell 1684
Fluorescence (Raw)
Time Point (0.25 Hz)
40
60
80
100
120
160
200
0
200
400
600
800

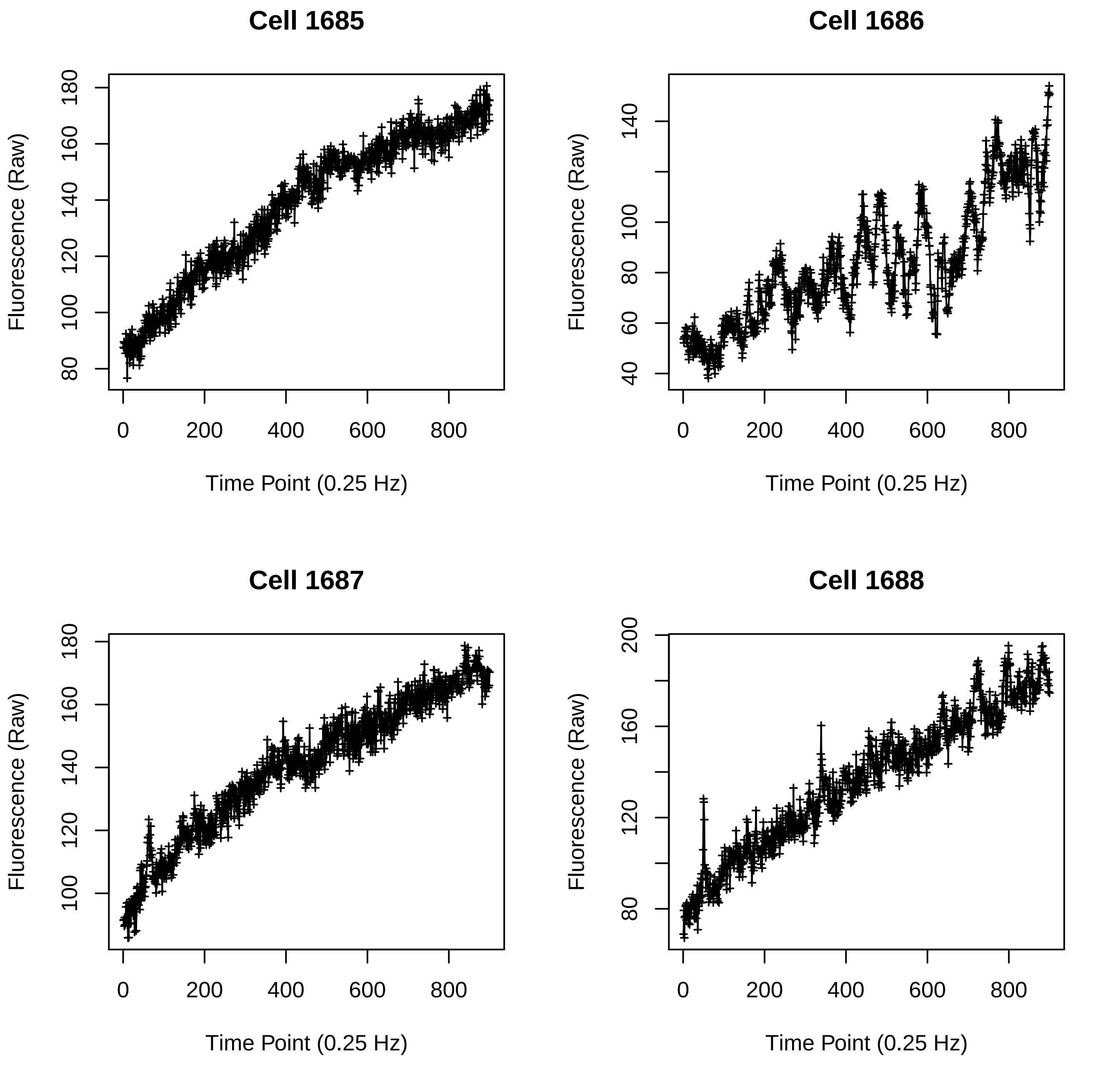
Cell 1685
Fluorescence (Raw)
80
100
120
140
160
180
0
200
400
600
800
Time Point (0.25 Hz)
Cell 1686
Fluorescence (Raw)
40
60
80
100
140
0
200
400
600
800
Time Point (0.25 Hz)
Cell 1687
Fluorescence (Raw)
100
120
140
160
180
0
200
400
600
800
Time Point (0.25 Hz)
Cell 1688
Fluorescence (Raw)
80
120
160
200
0
200
400
600
800
Time Point (0.25 Hz)

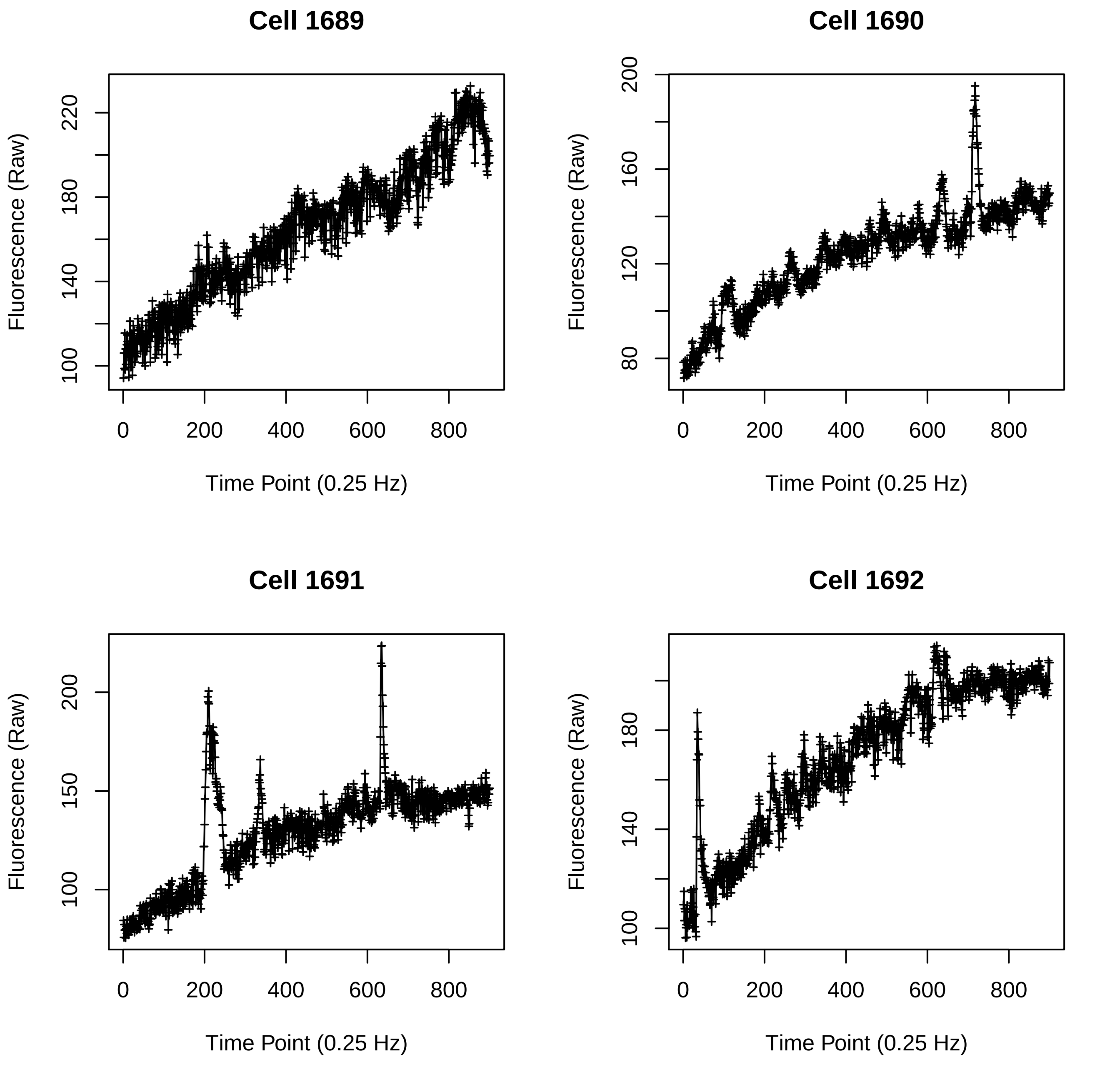

Cell 1689
Cell 1690
Cell 1691
Cell 1692
Fluorescence (Raw)
Time Point (0.25 Hz)
100
140
180
220
80
120
160
200
150
0
200
400
600
800

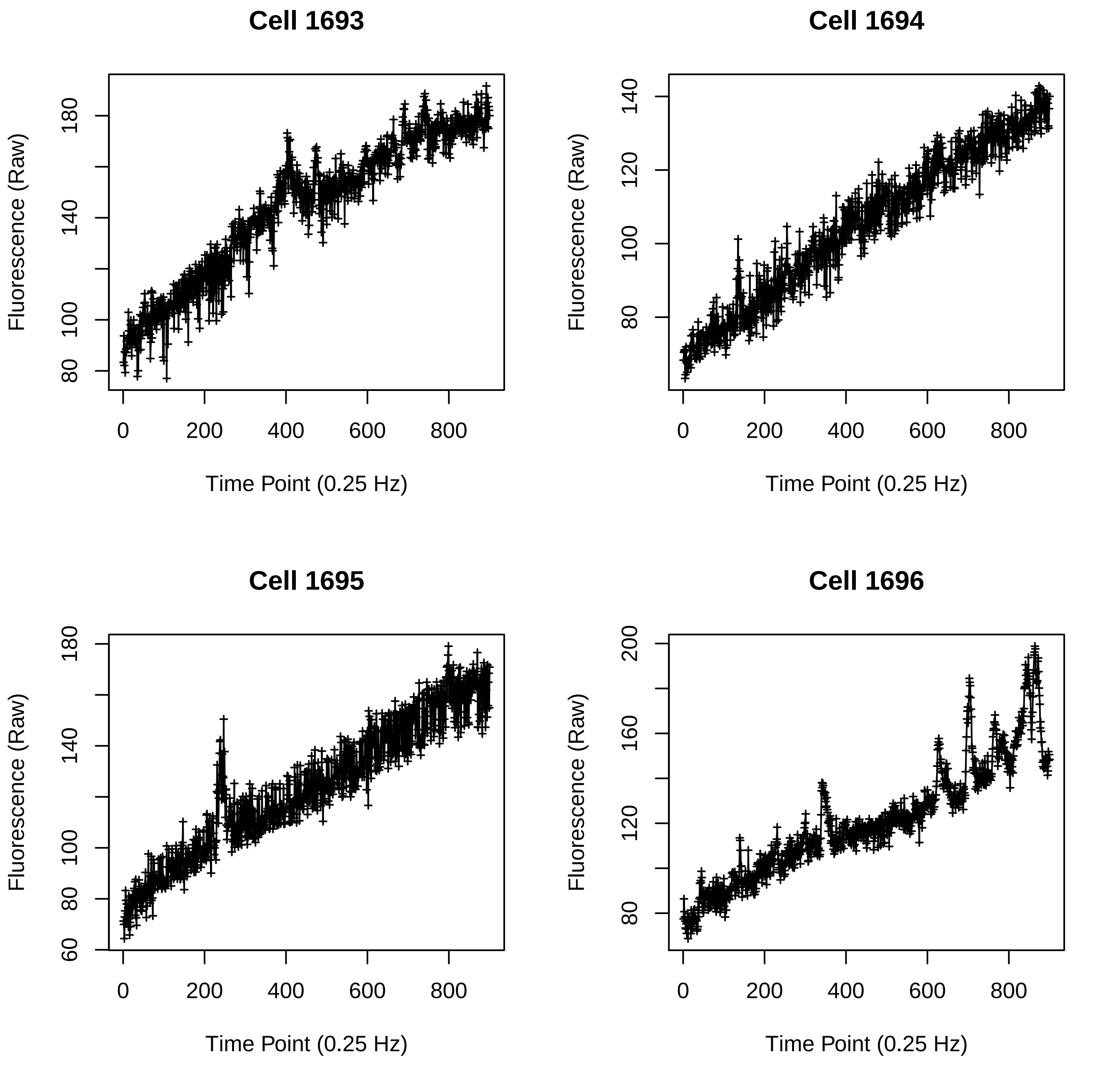

Cell 1693
Cell 1694
Cell 1695
Cell 1696
Fluorescence (Raw)
Time Point (0.25 Hz)

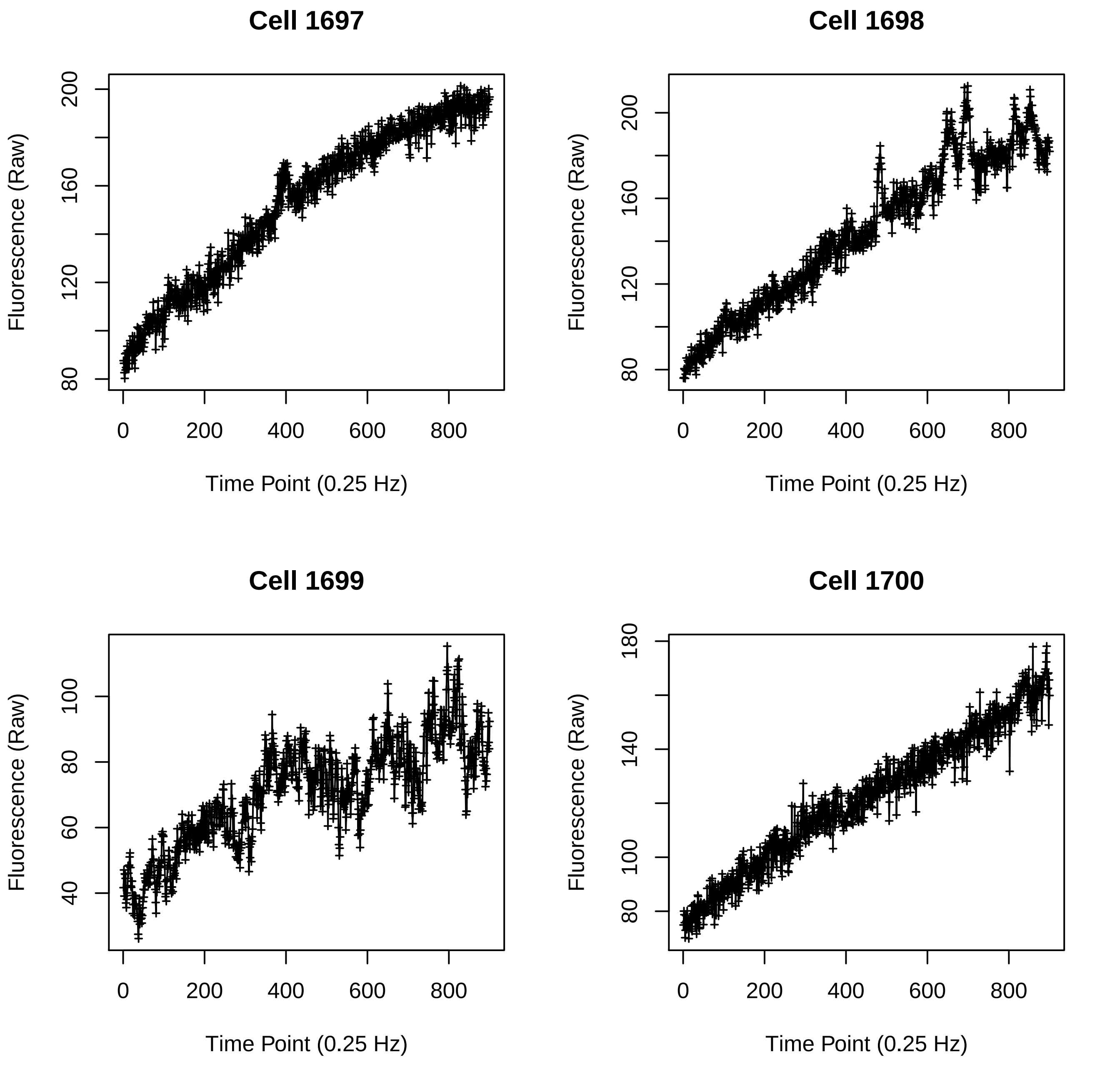

Cell 1697
Fluorescence (Raw)
80
120
160
200
0
200
400
600
800
Time Point (0.25 Hz)
Cell 1698
Fluorescence (Raw)
80
120
160
200
0
200
400
600
800
Time Point (0.25 Hz)
Cell 1699
Fluorescence (Raw)
40
60
80
100
0
200
400
600
800
Time Point (0.25 Hz)
Cell 1700
Fluorescence (Raw)
80
100
140
180
0
200
400
600
800
Time Point (0.25 Hz)

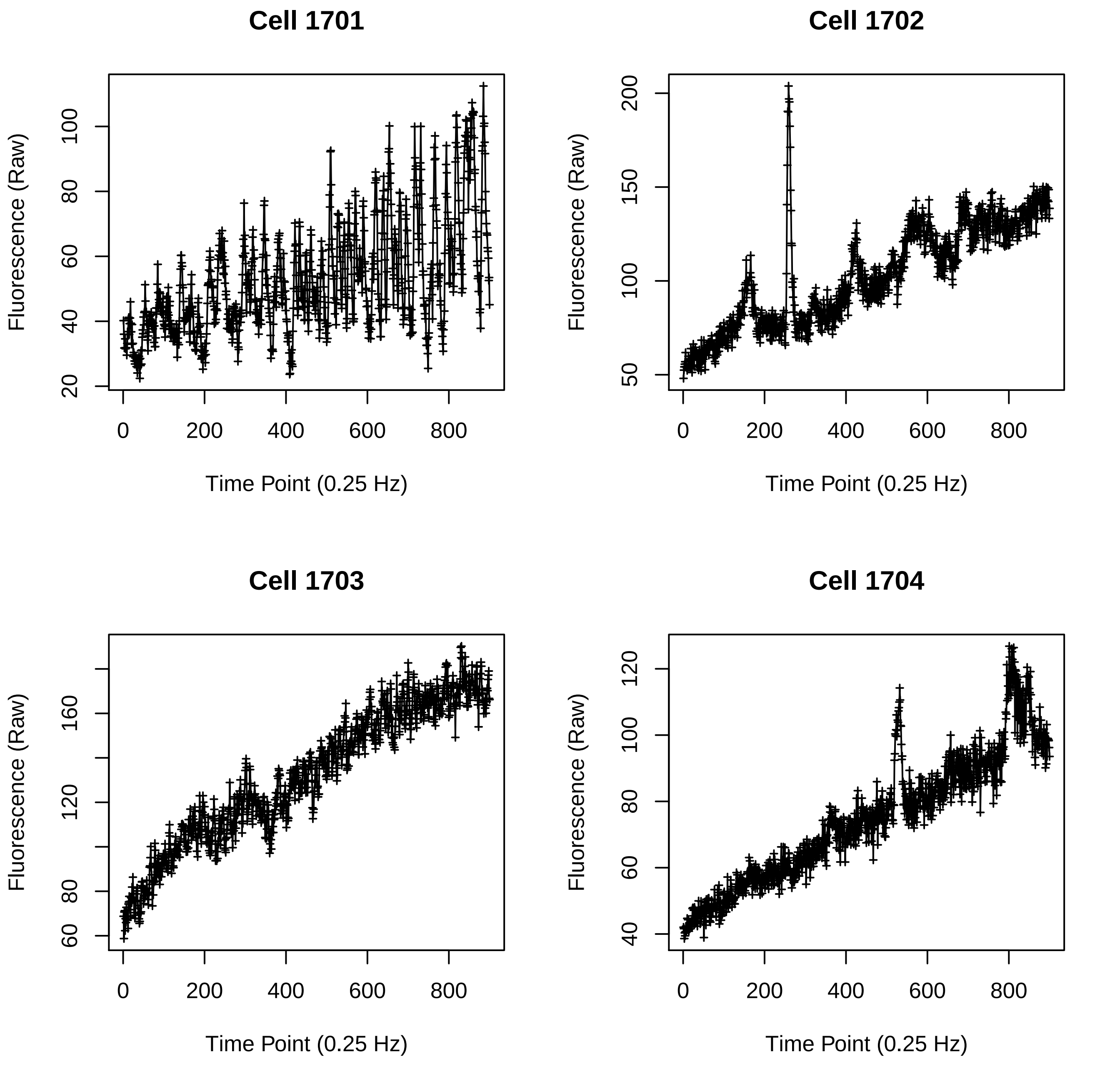
Cell 1701
Cell 1702
Cell 1703
Cell 1704
Fluorescence (Raw)
Time Point (0.25 Hz)
0
200
400
600
800
20
40
60
80
100
50
150
200
120
160

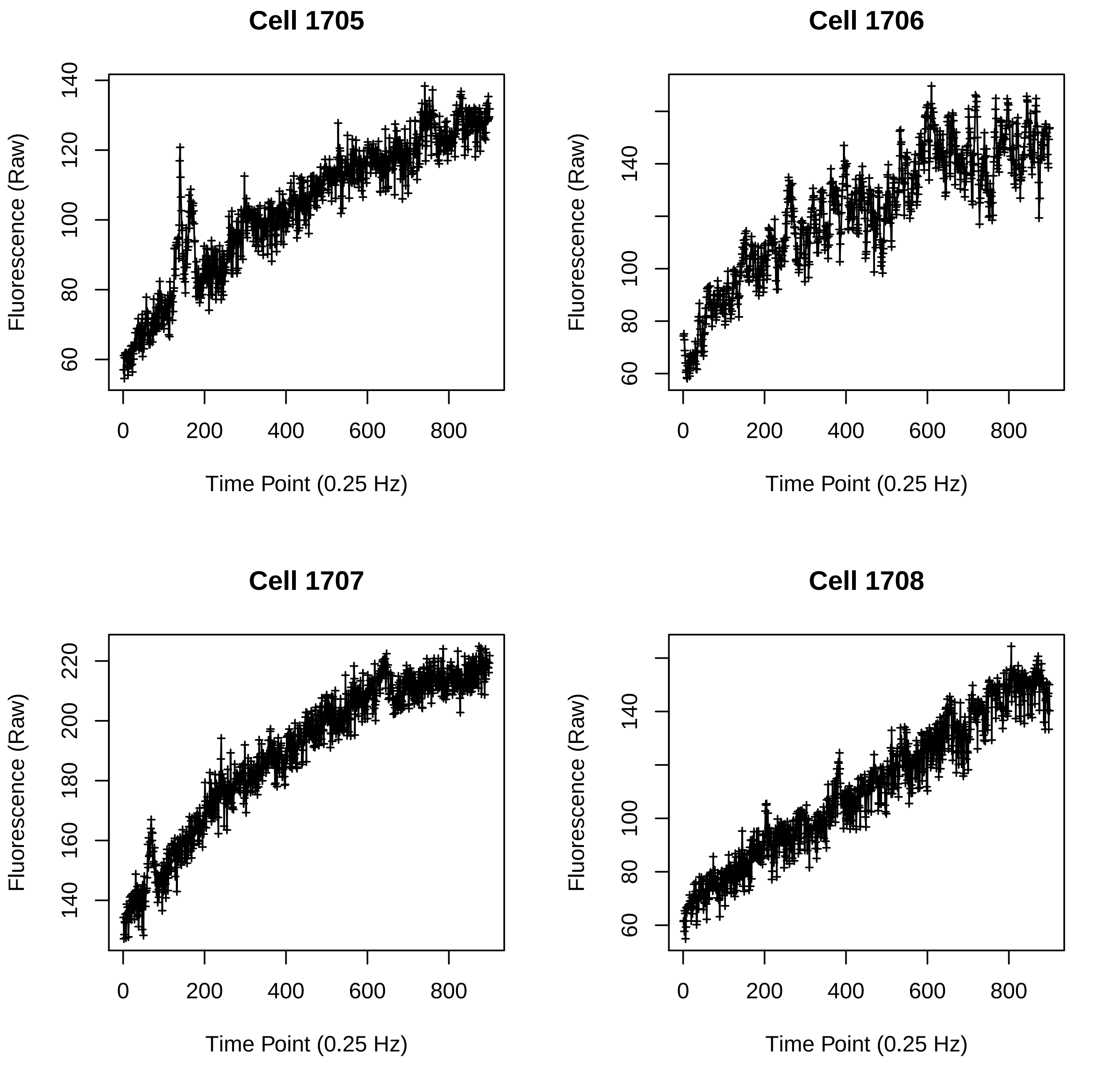

Cell 1705
Cell 1706
Cell 1707
Cell 1708
Fluorescence (Raw)
Time Point (0.25 Hz)
0
200
400
600
800
60
80
100
120
140
160
180
200
220

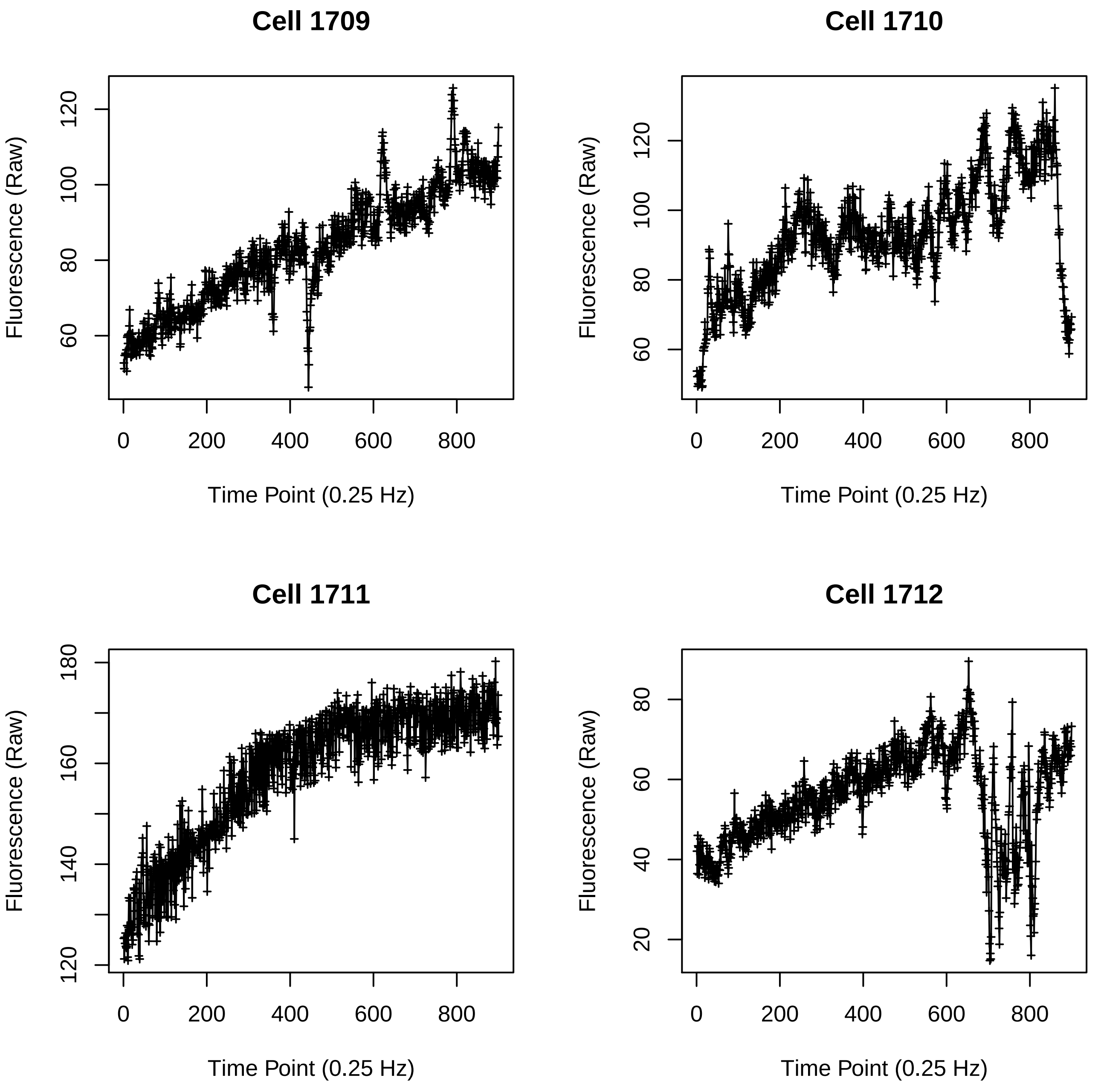
Cell 1709
Cell 1710
Cell 1711
Cell 1712
Fluorescence (Raw)
Time Point (0.25 Hz)
0
200
400
600
800
60
80
100
120
180
160
140
40
20

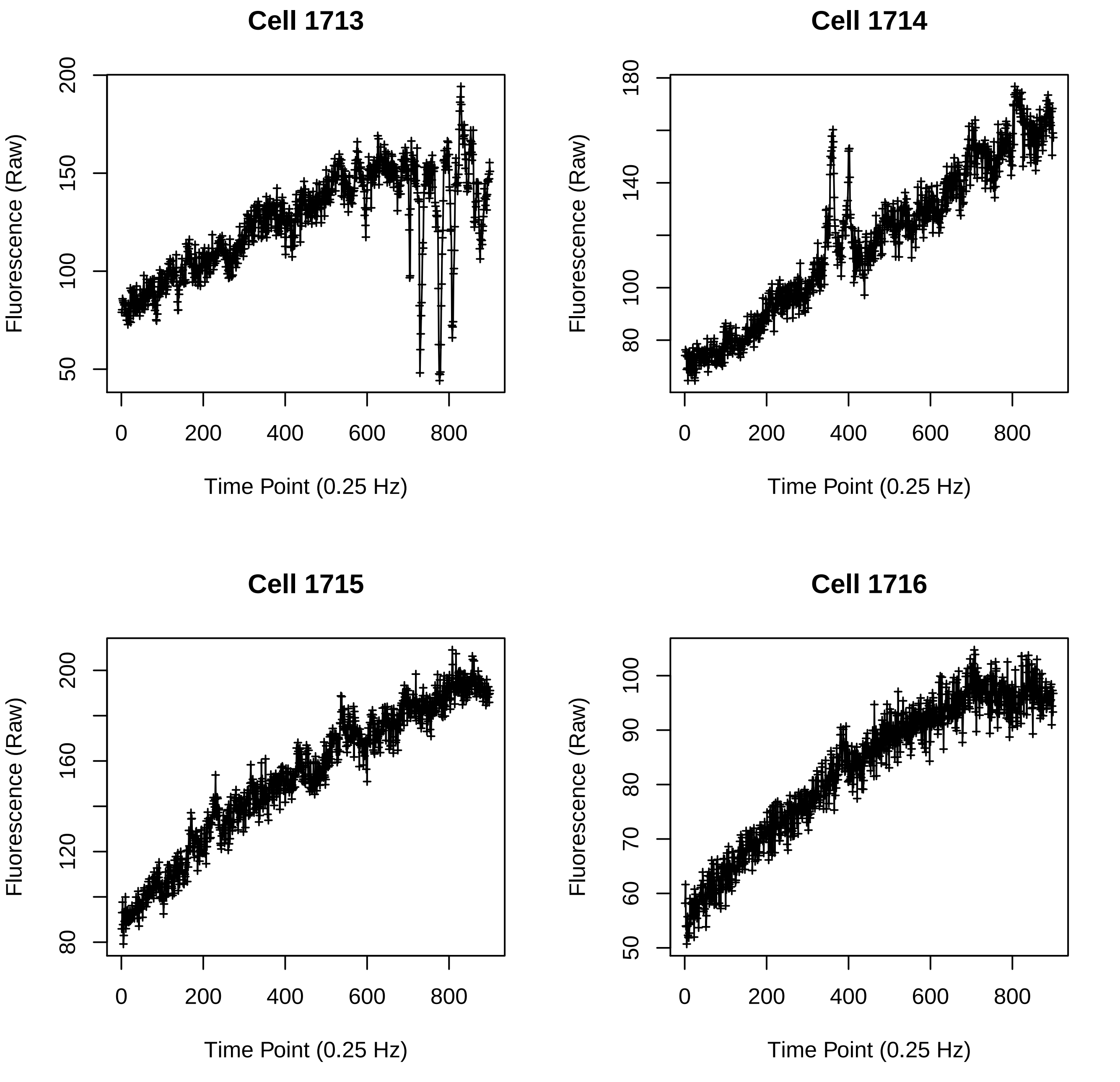
Cell 1713
Fluorescence (Raw)
200
150
100
50
0
200
400
600
800
Time Point (0.25 Hz)
Cell 1714
Fluorescence (Raw)
180
140
100
80
0
200
400
600
800
Time Point (0.25 Hz)
Cell 1715
Fluorescence (Raw)
200
160
120
80
0
200
400
600
800
Time Point (0.25 Hz)
Cell 1716
Fluorescence (Raw)
100
90
80
70
60
50
0
200
400
600
800
Time Point (0.25 Hz)

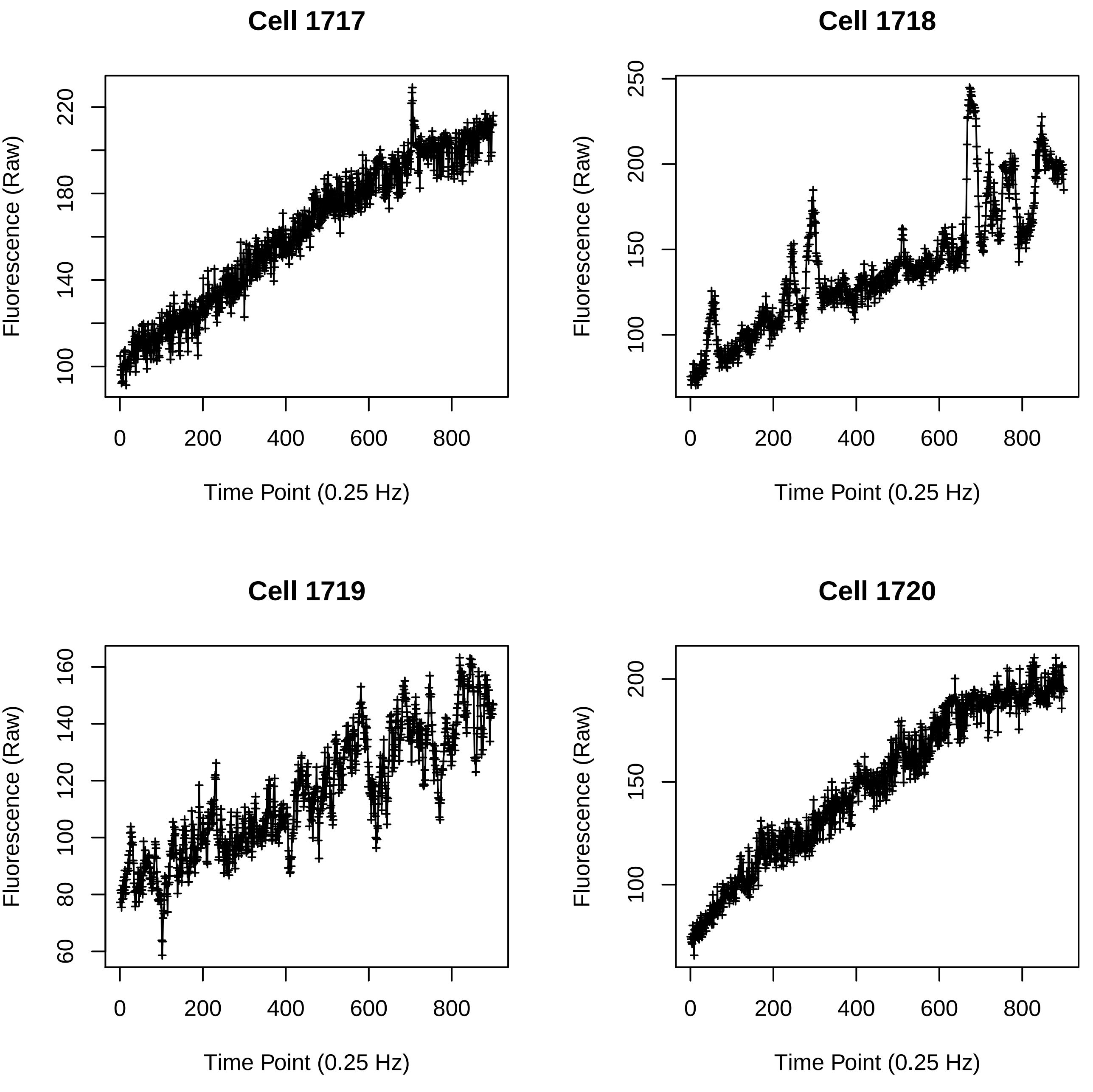

Cell 1717
Fluorescence (Raw)
100
140
180
220
0
200
400
600
800
Time Point (0.25 Hz)
Cell 1718
Fluorescence (Raw)
100
150
200
250
0
200
400
600
800
Time Point (0.25 Hz)
Cell 1719
Fluorescence (Raw)
60
80
100
120
140
160
0
200
400
600
800
Time Point (0.25 Hz)
Cell 1720
Fluorescence (Raw)
100
150
200
0
200
400
600
800
Time Point (0.25 Hz)

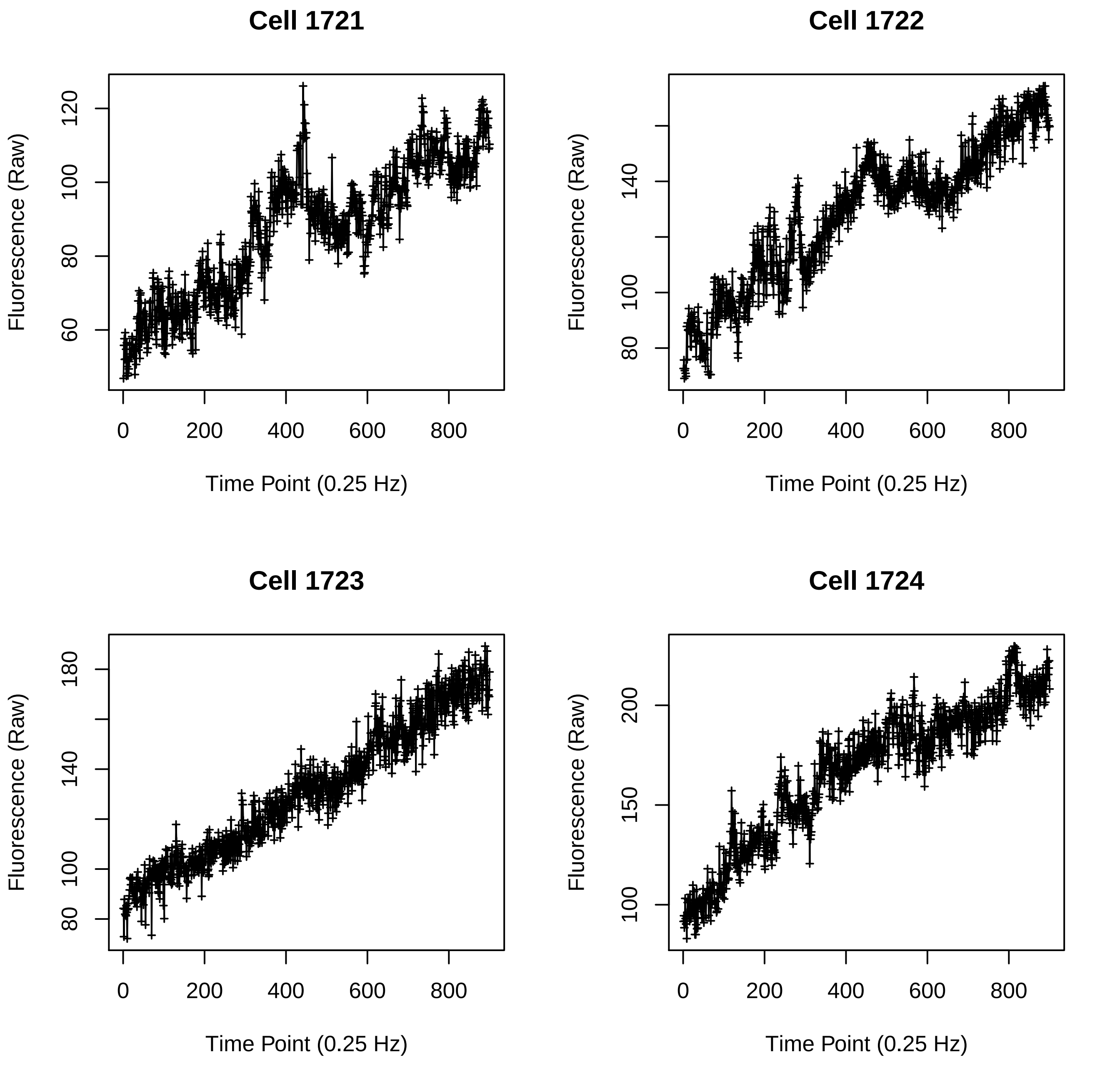

Cell 1721
Cell 1722
Cell 1723
Cell 1724
Fluorescence (Raw)
Time Point (0.25 Hz)
0
200
400
600
800
60
80
100
120
140
180
150
200

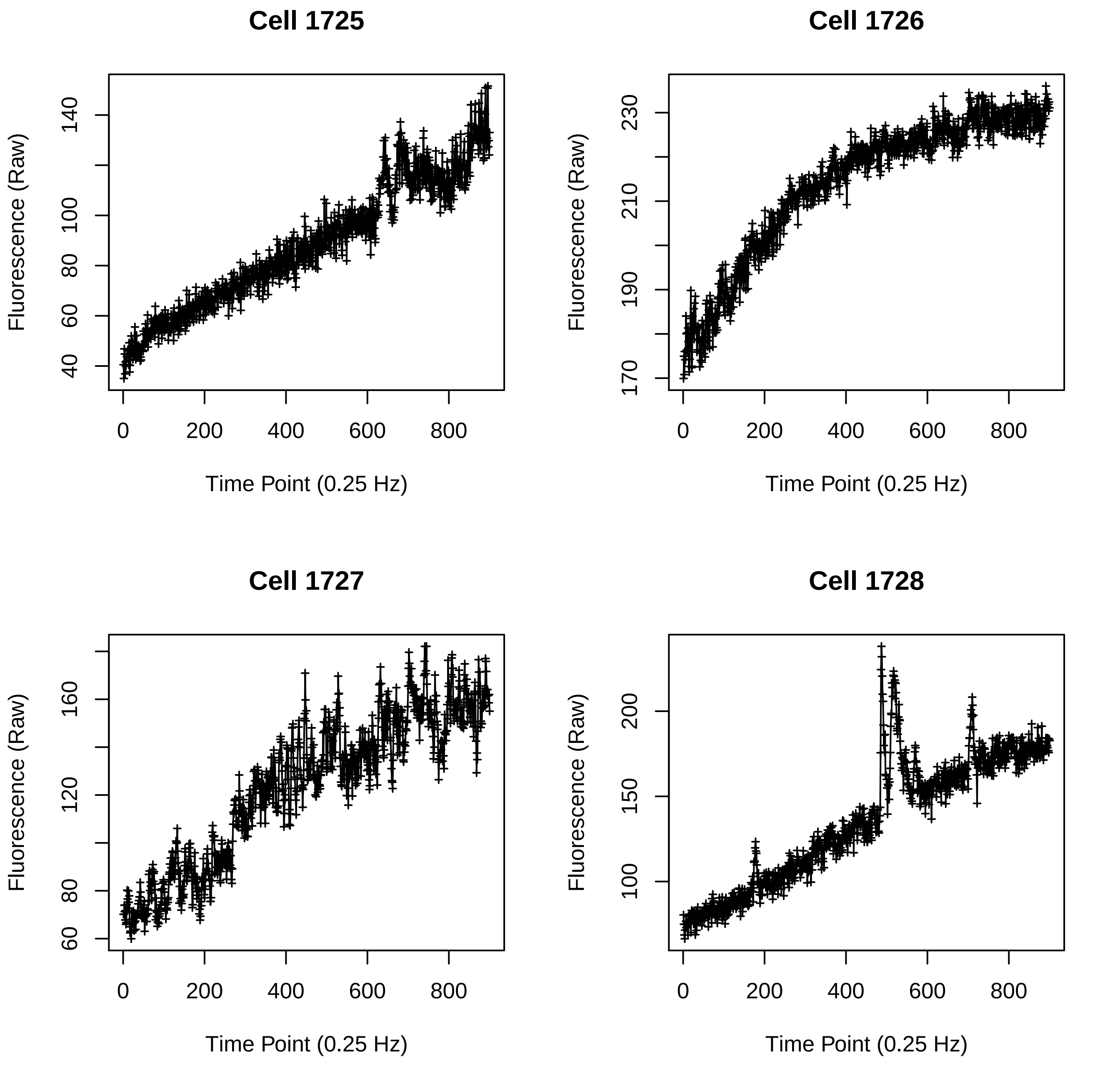

Cell 1725
Cell 1726
Cell 1727
Cell 1728
Fluorescence (Raw)
Time Point (0.25 Hz)
40
60
80
100
140
170
190
210
230
120
160
150
200
0
200
400
600
800

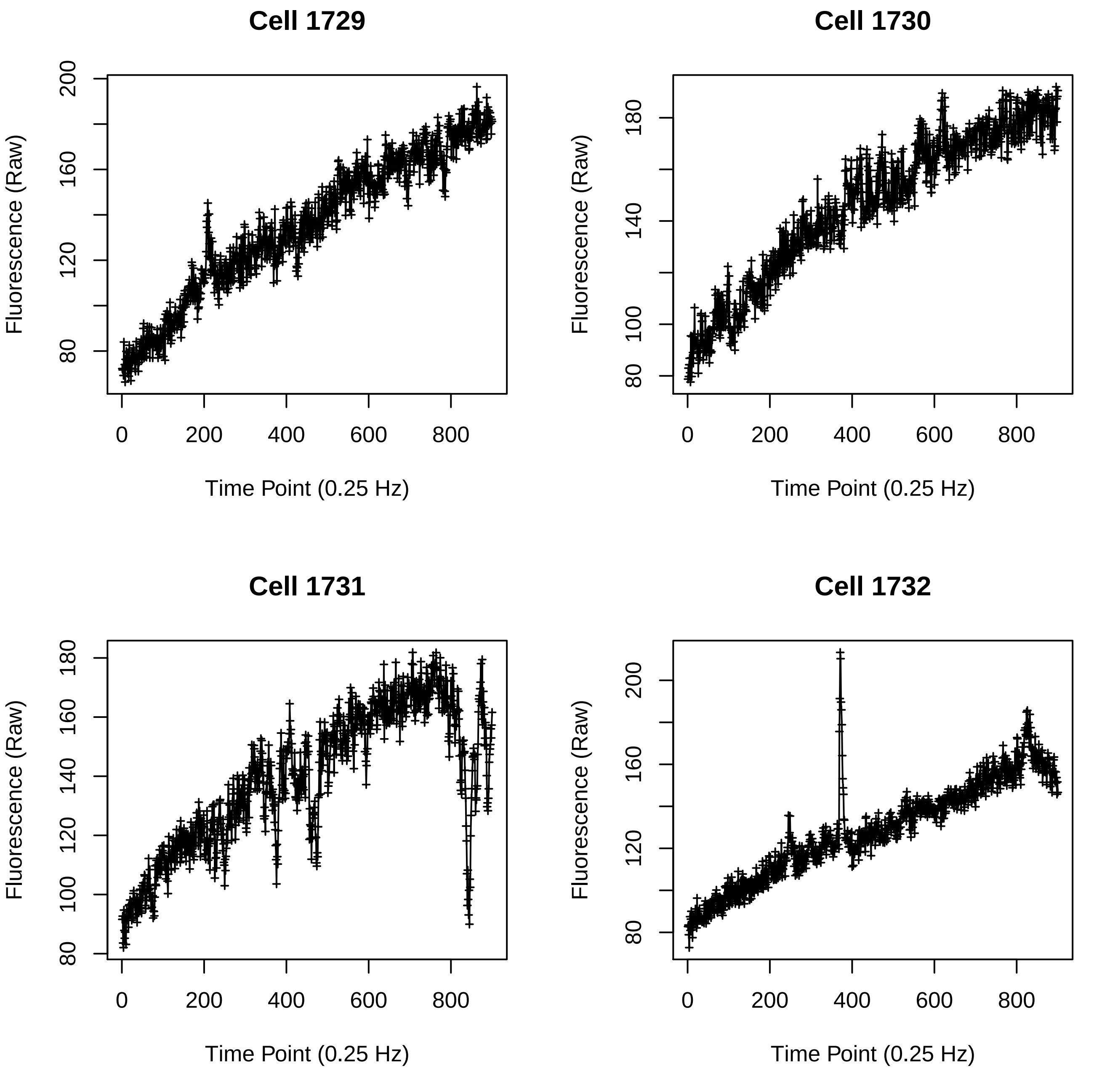

Cell 1729
Fluorescence (Raw)
80
120
160
200
0
200
400
600
800
Time Point (0.25 Hz)
Cell 1730
Fluorescence (Raw)
80
100
140
180
0
200
400
600
800
Time Point (0.25 Hz)
Cell 1731
Fluorescence (Raw)
80
100
120
140
160
180
0
200
400
600
800
Time Point (0.25 Hz)
Cell 1732
Fluorescence (Raw)
80
120
160
200
0
200
400
600
800
Time Point (0.25 Hz)

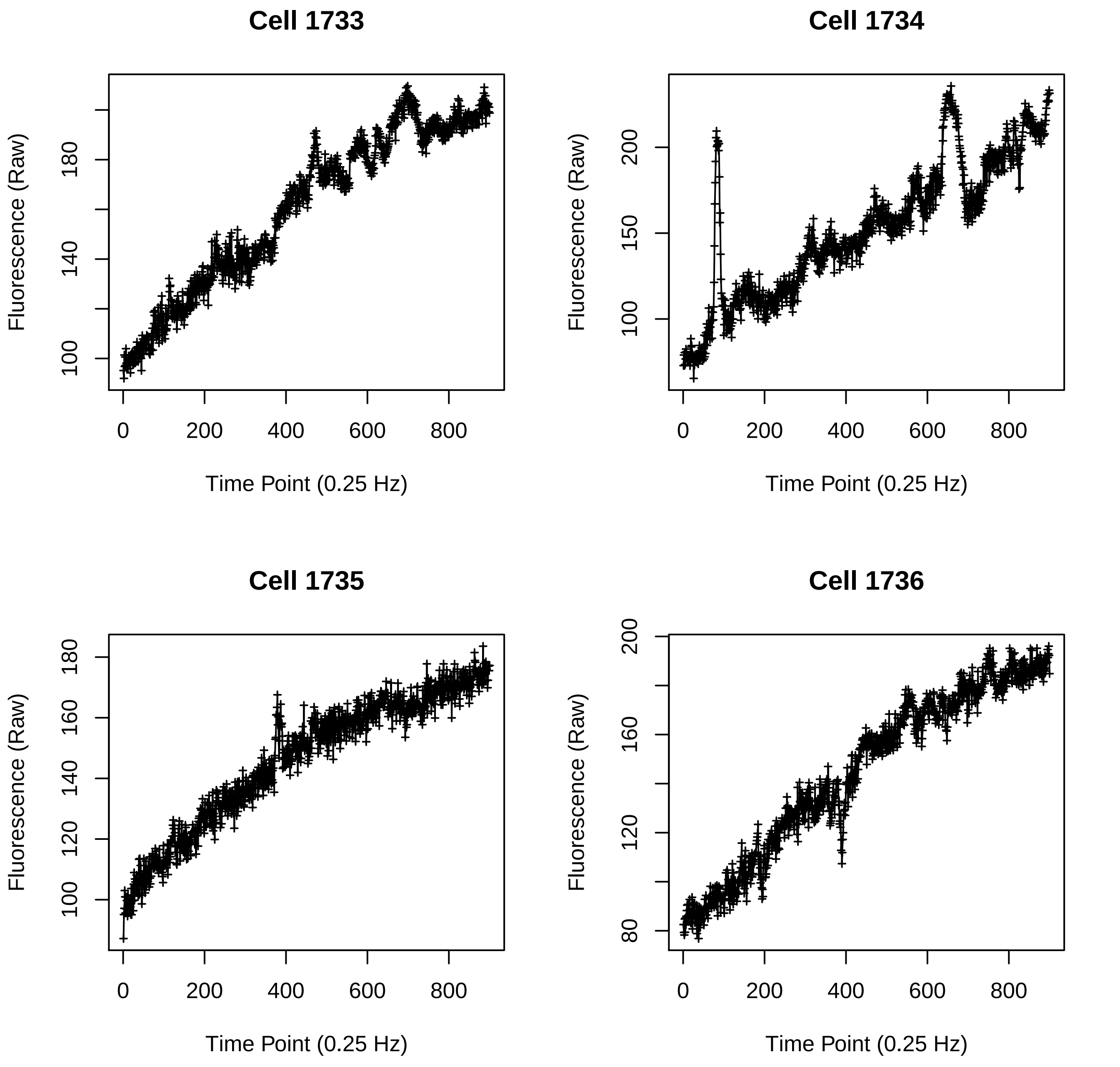
Cell 1733
Cell 1734
Cell 1735
Cell 1736
Fluorescence (Raw)
Time Point (0.25 Hz)
0
200
400
600
800
100
140
180
100
150
200
100
120
140
160
180
80
120
160
200

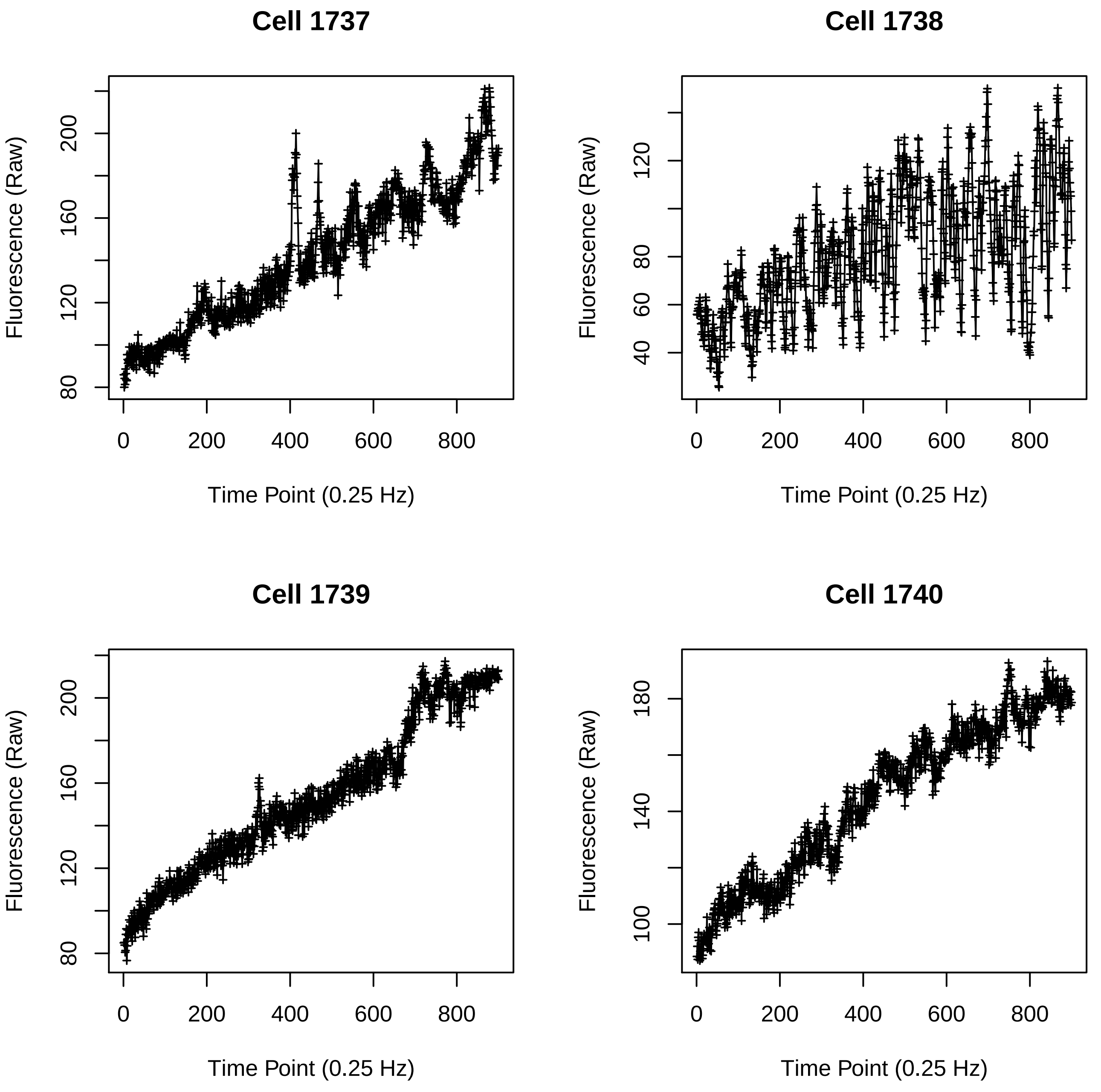
Cell 1737
Fluorescence (Raw)
80
120
160
200
0
200
400
600
800
Time Point (0.25 Hz)
Cell 1738
Fluorescence (Raw)
40
60
80
120
0
200
400
600
800
Time Point (0.25 Hz)
Cell 1739
Fluorescence (Raw)
80
120
160
200
0
200
400
600
800
Time Point (0.25 Hz)
Cell 1740
Fluorescence (Raw)
100
140
180
0
200
400
600
800
Time Point (0.25 Hz)

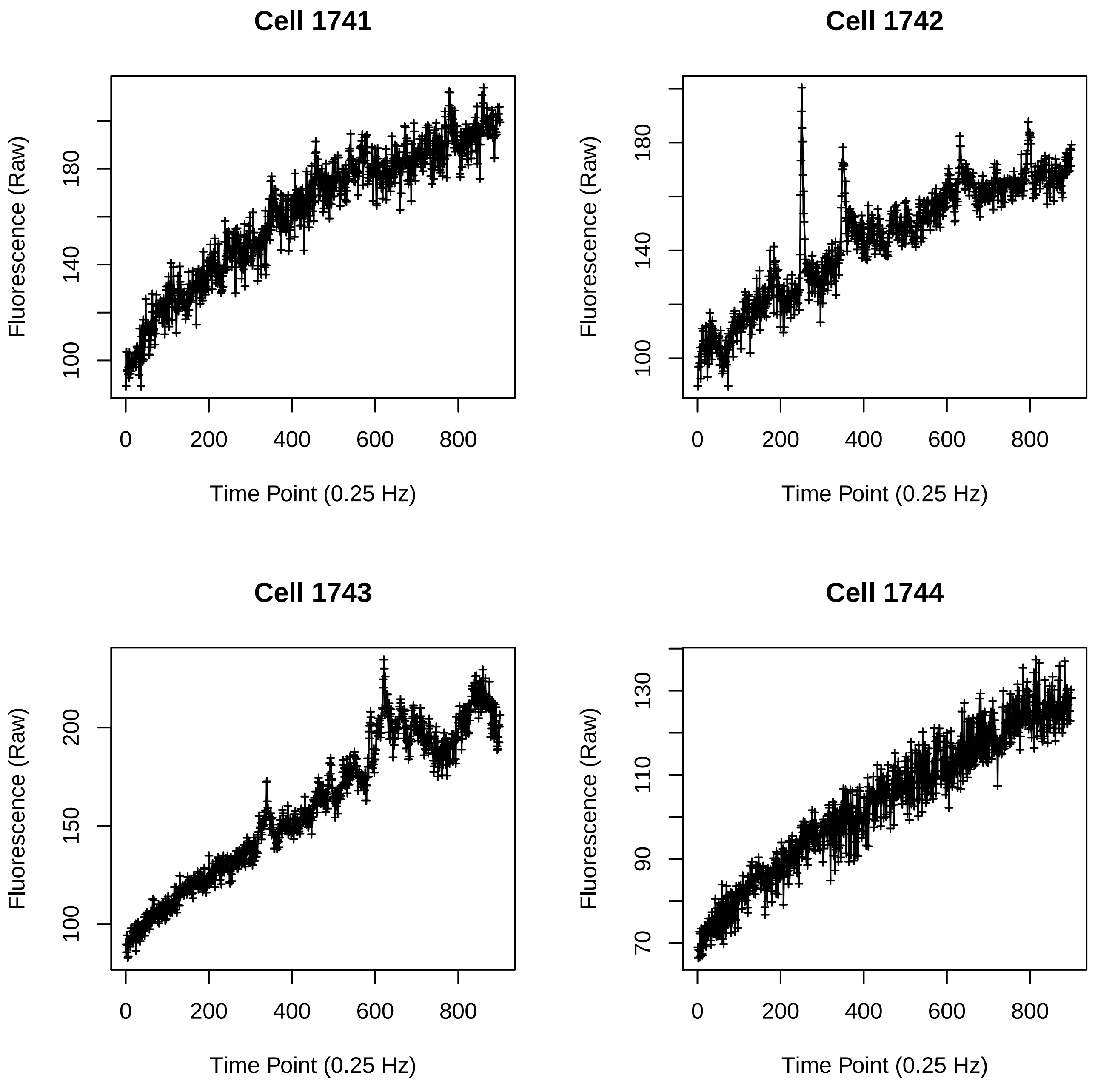

Cell 1741
Cell 1742
Cell 1743
Cell 1744
Fluorescence (Raw)
Time Point (0.25 Hz)
100
140
180
200
150
90
110
130
70
0
200
400
600
800

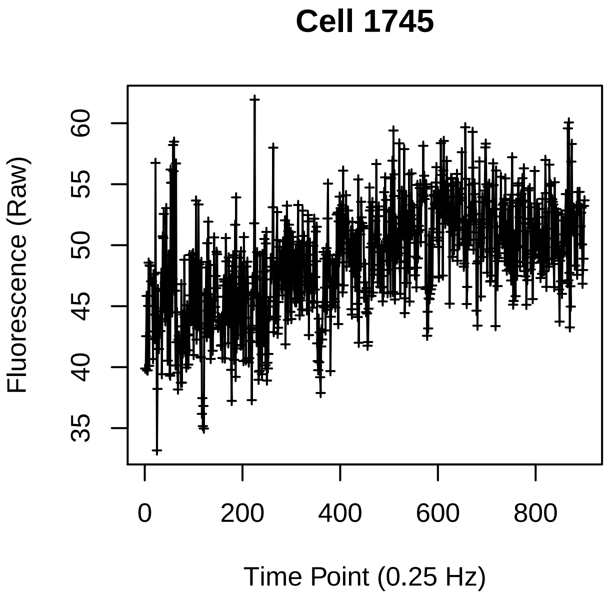
Cell 1745
Fluorescence (Raw)
35
40
45
50
55
60
0
200
400
600
800
Time Point (0.25 Hz)
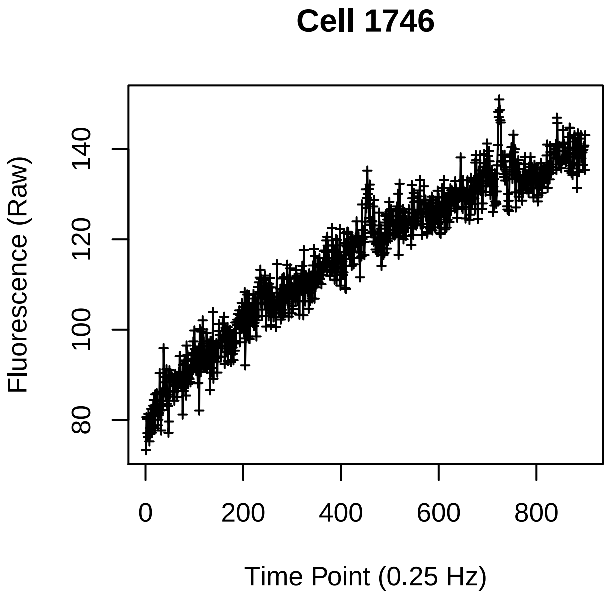
Cell 1746
Fluorescence (Raw)
80
100
120
140
0
200
400
600
800
Time Point (0.25 Hz)
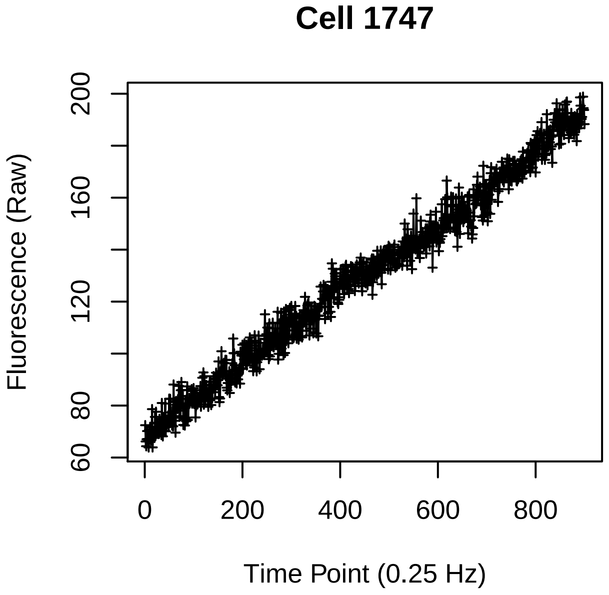
Cell 1747
Fluorescence (Raw)
60
80
120
160
200
0
200
400
600
800
Time Point (0.25 Hz)
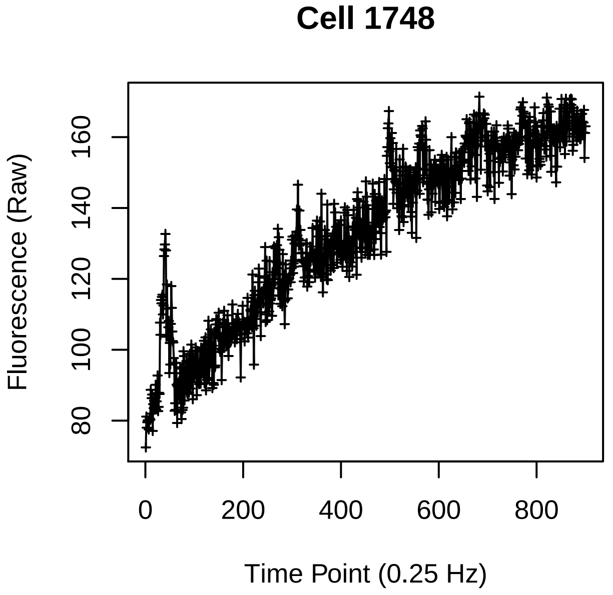
Cell 1748
Fluorescence (Raw)
80
100
120
140
160
0
200
400
600
800
Time Point (0.25 Hz)

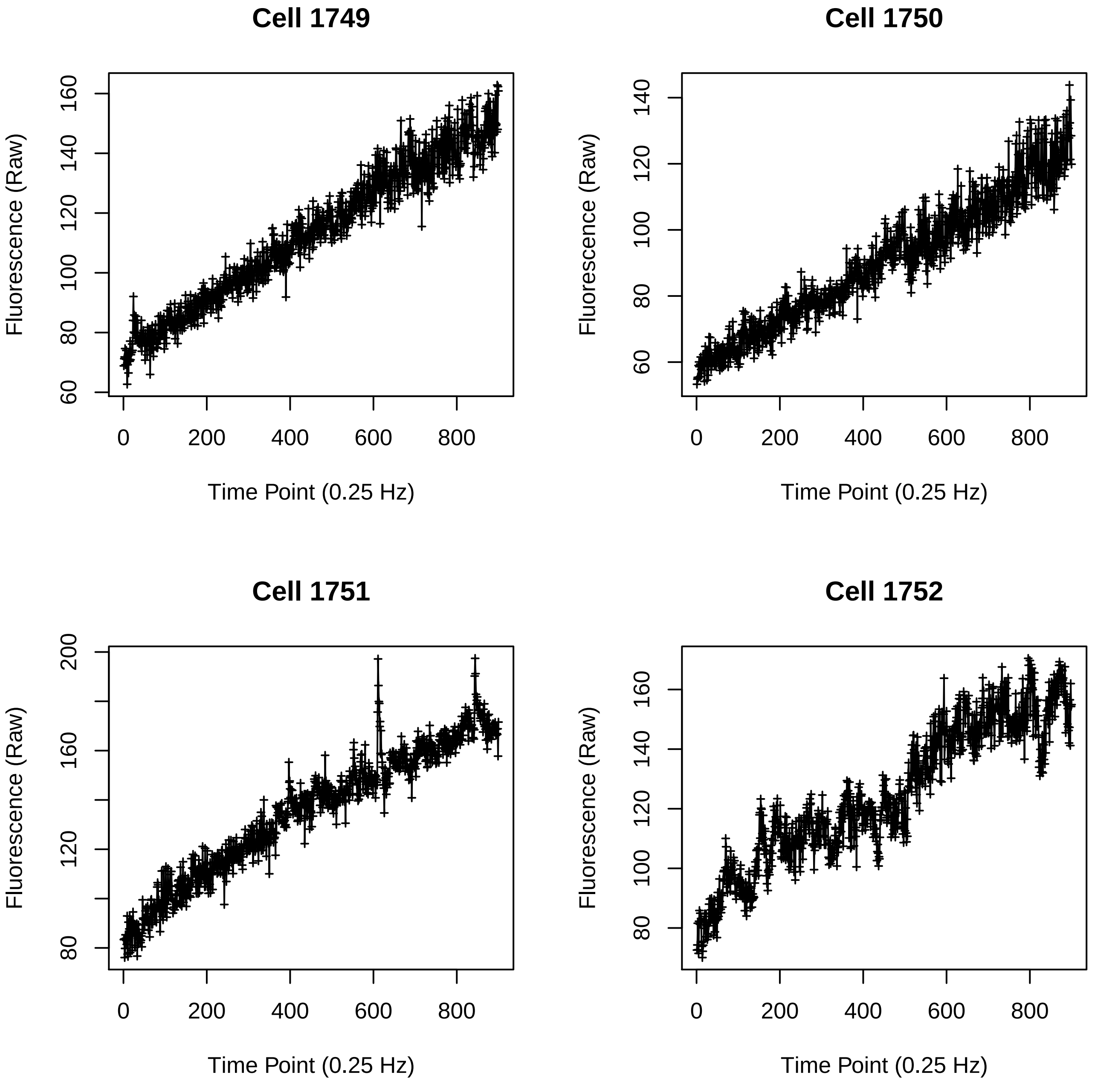

Cell 1749
Fluorescence (Raw)
60
80
100
120
140
160
0
200
400
600
800
Time Point (0.25 Hz)
Cell 1750
Fluorescence (Raw)
60
80
100
120
140
0
200
400
600
800
Time Point (0.25 Hz)
Cell 1751
Fluorescence (Raw)
80
120
160
200
0
200
400
600
800
Time Point (0.25 Hz)
Cell 1752
Fluorescence (Raw)
80
100
120
140
160
0
200
400
600
800
Time Point (0.25 Hz)

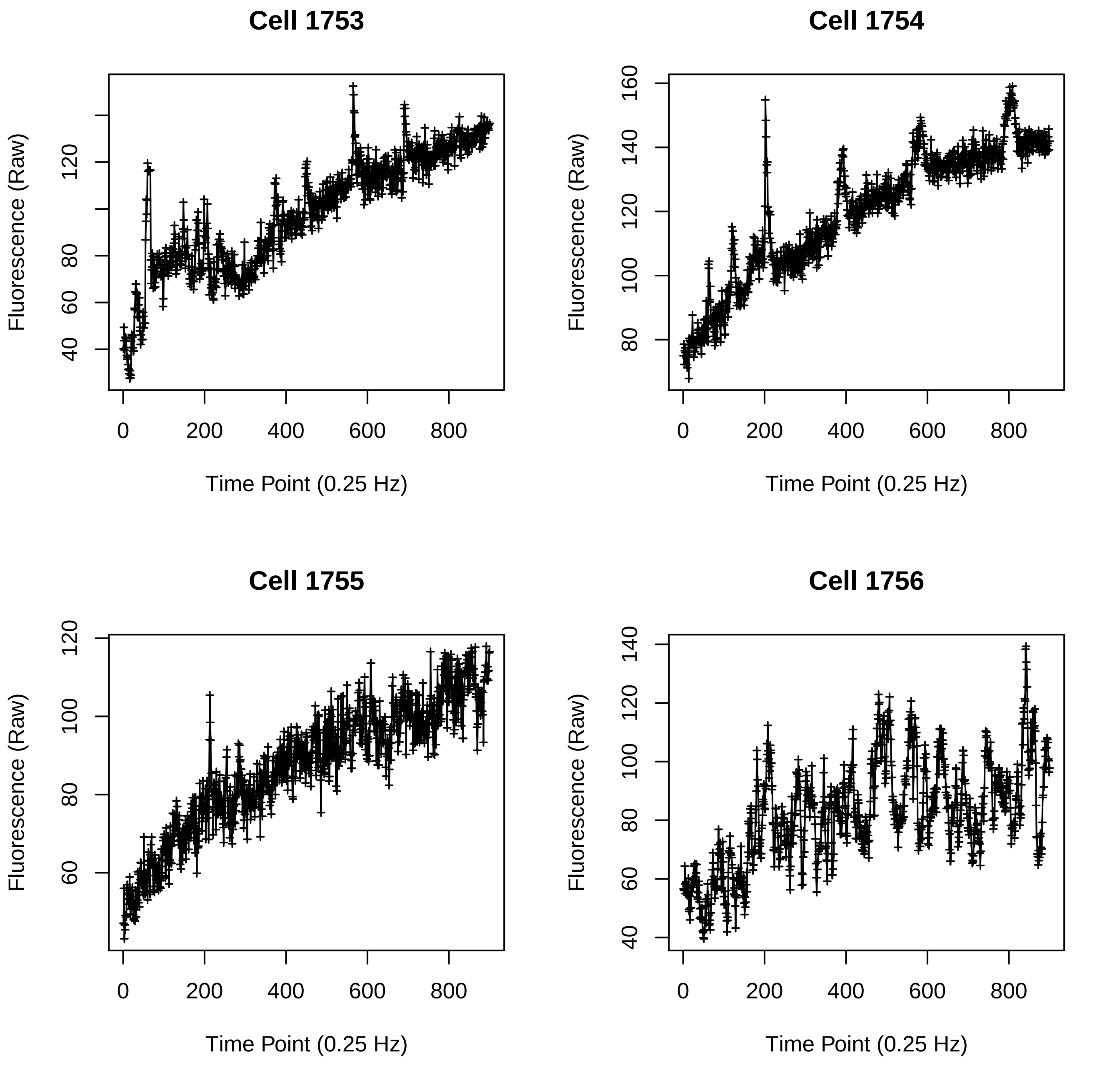
Cell 1753
Cell 1754
Cell 1755
Cell 1756
Fluorescence (Raw)
Time Point (0.25 Hz)
0
200
400
600
800
40
60
80
100
120
140
160

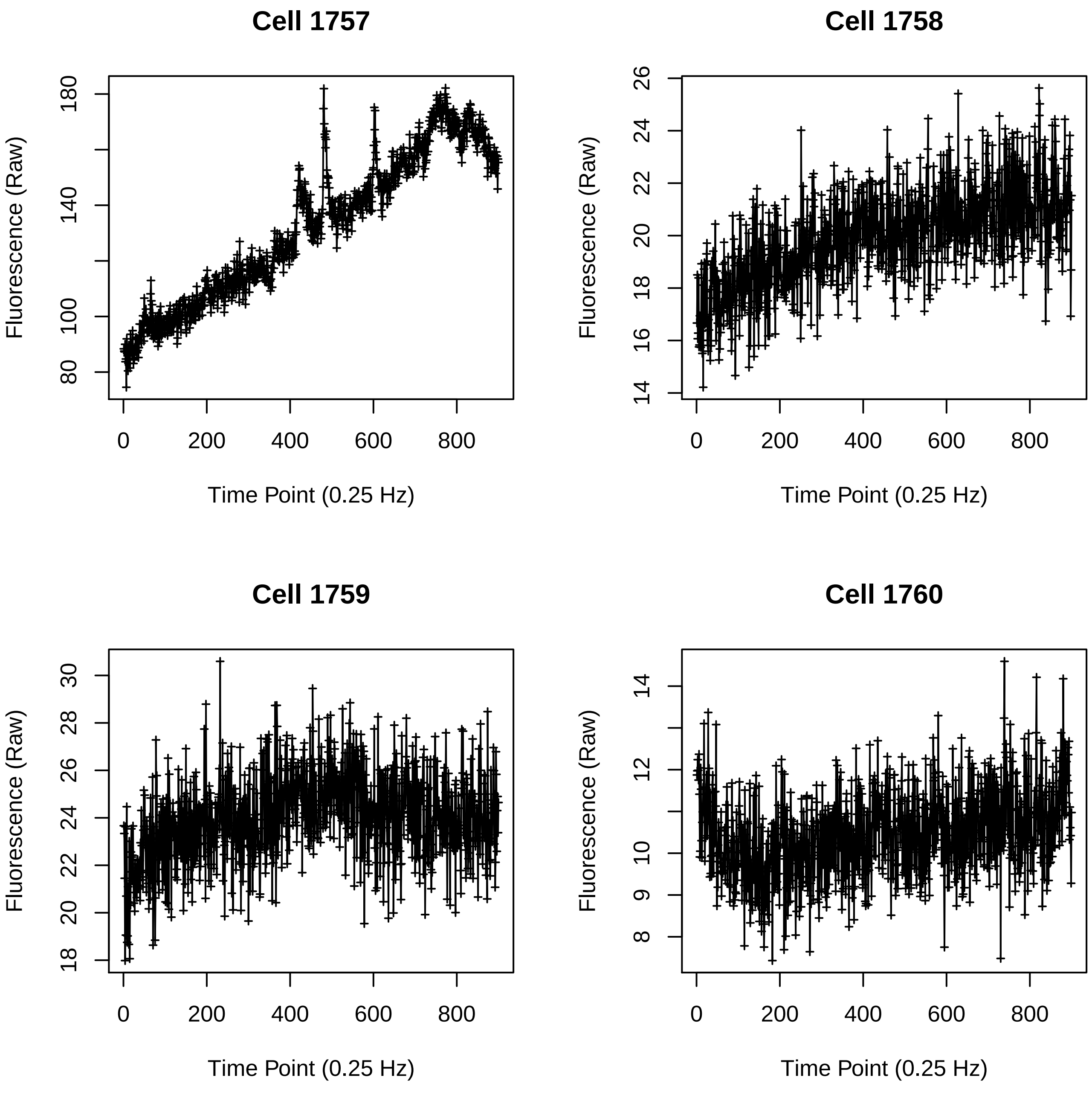
Cell 1757
Fluorescence (Raw)
80
100
140
180
0
200
400
600
800
Time Point (0.25 Hz)
Cell 1758
Fluorescence (Raw)
14
16
18
20
22
24
26
0
200
400
600
800
Time Point (0.25 Hz)
Cell 1759
Fluorescence (Raw)
18
20
22
24
26
28
30
0
200
400
600
800
Time Point (0.25 Hz)
Cell 1760
Fluorescence (Raw)
8
9
10
12
14
0
200
400
600
800
Time Point (0.25 Hz)

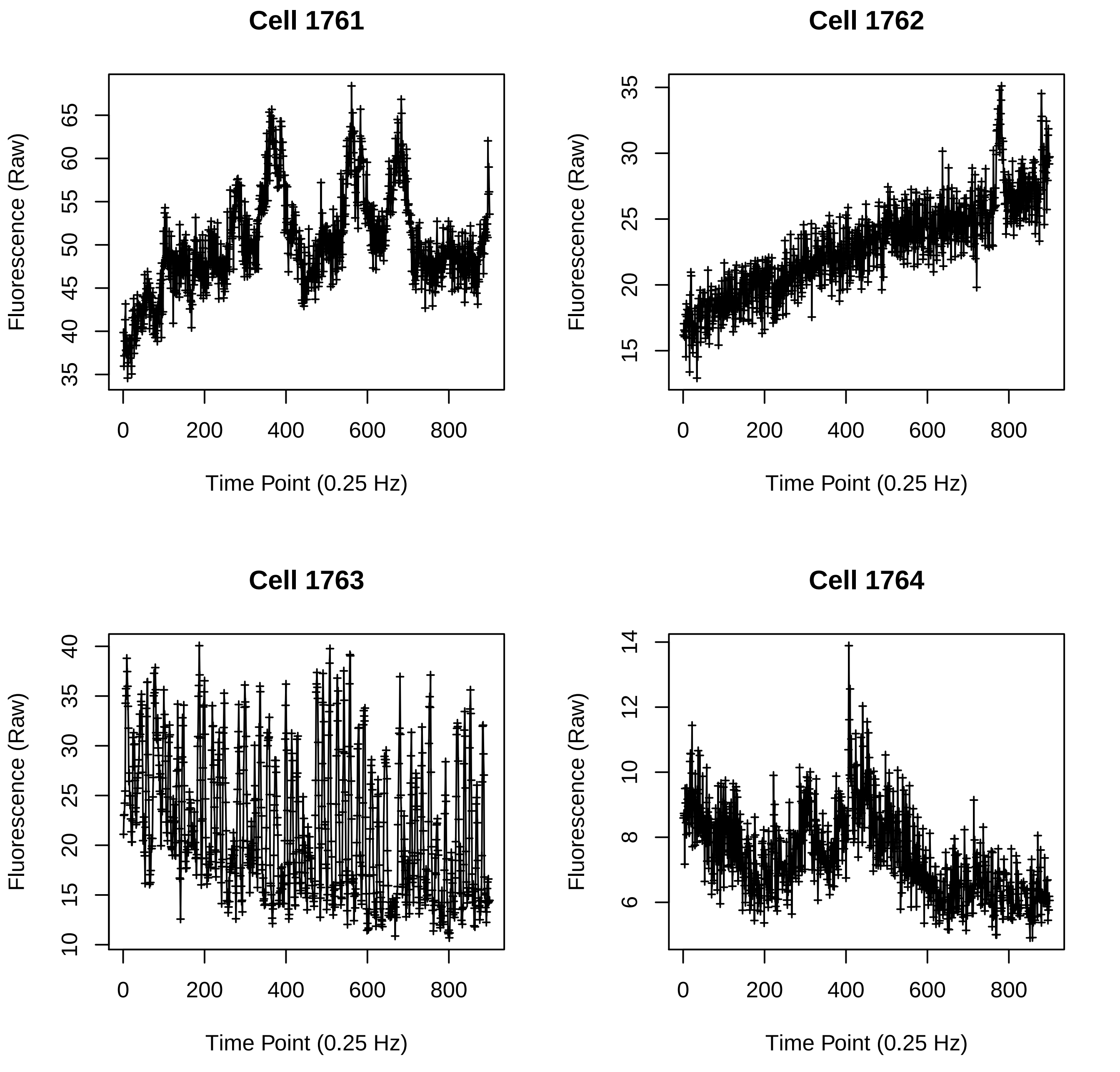
Cell 1761
Cell 1762
Cell 1763
Cell 1764
Fluorescence (Raw)
Time Point (0.25 Hz)
0
200
400
600
800
35
40
45
50
55
60
65
15
20
25
30
35
10
6
8
10
12
14

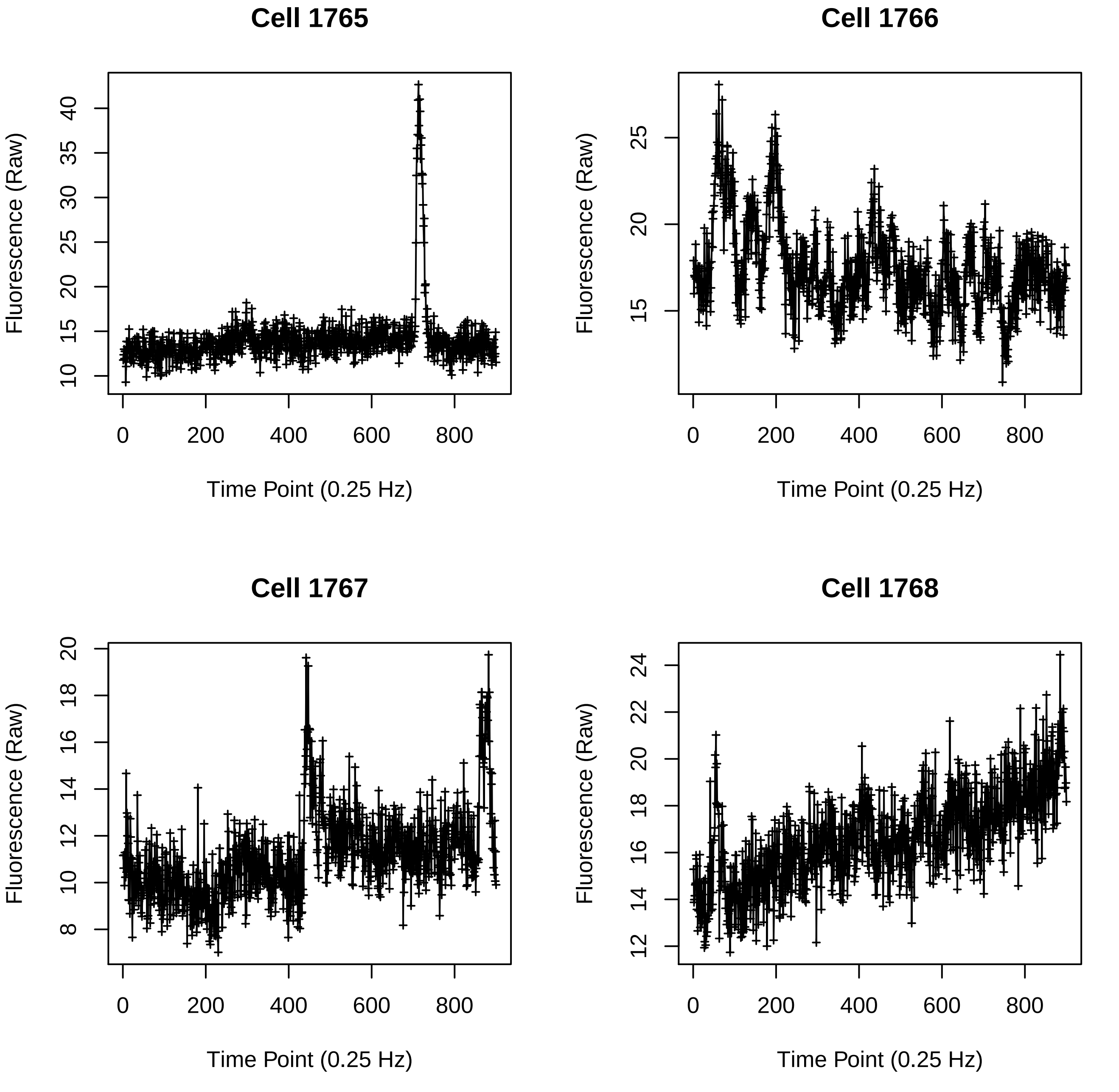
Cell 1765
Fluorescence (Raw)
10
15
20
25
30
35
40
0
200
400
600
800
Time Point (0.25 Hz)
Cell 1766
Fluorescence (Raw)
15
20
25
0
200
400
600
800
Time Point (0.25 Hz)
Cell 1767
Fluorescence (Raw)
8
10
12
14
16
18
20
0
200
400
600
800
Time Point (0.25 Hz)
Cell 1768
Fluorescence (Raw)
12
14
16
18
20
22
24
0
200
400
600
800
Time Point (0.25 Hz)

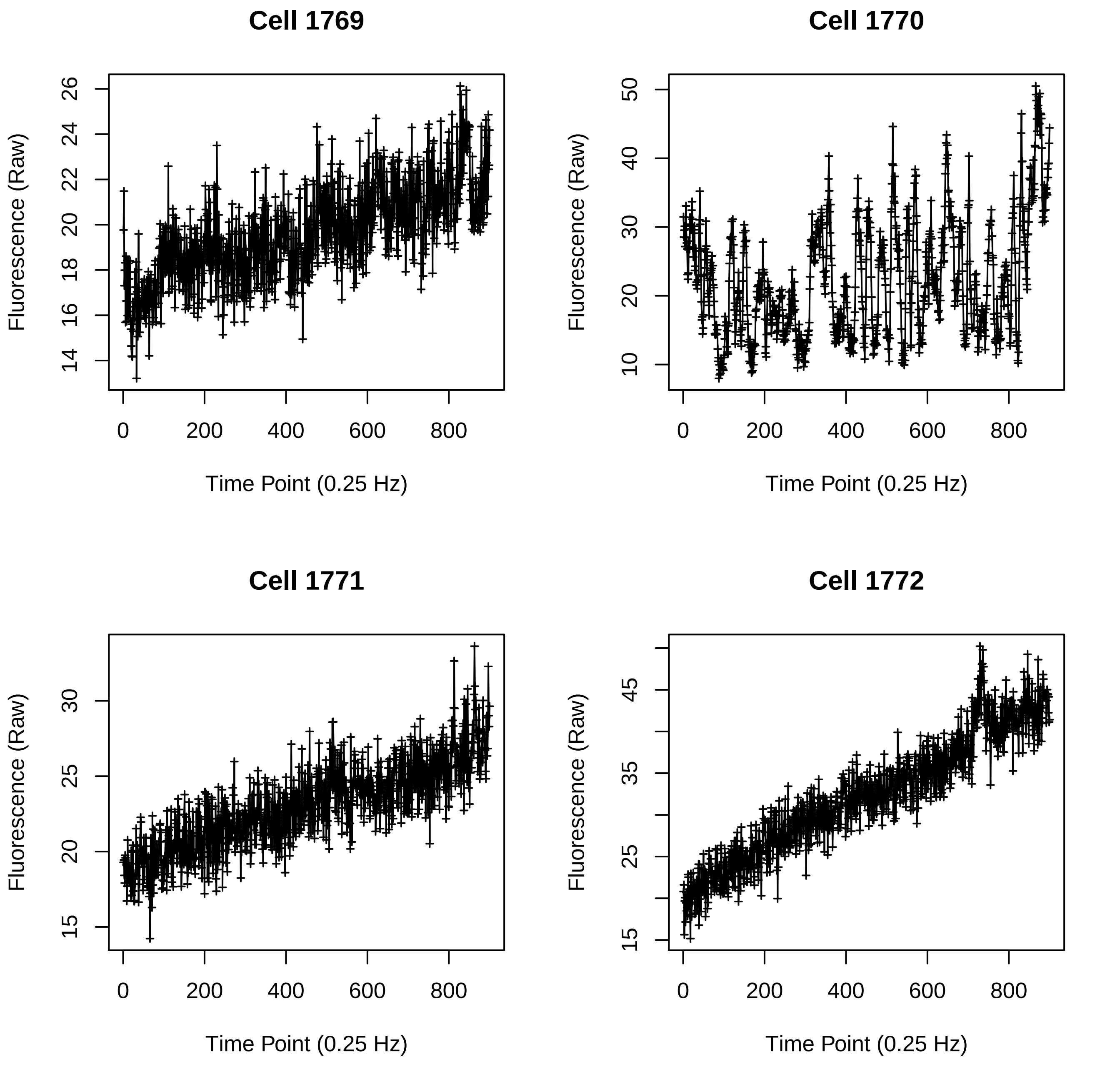

Cell 1769
Cell 1770
Cell 1771
Cell 1772
Fluorescence (Raw)
Time Point (0.25 Hz)
0
200
400
600
800
14
16
18
20
22
24
26
10
20
30
40
50
15
20
25
30
35
45

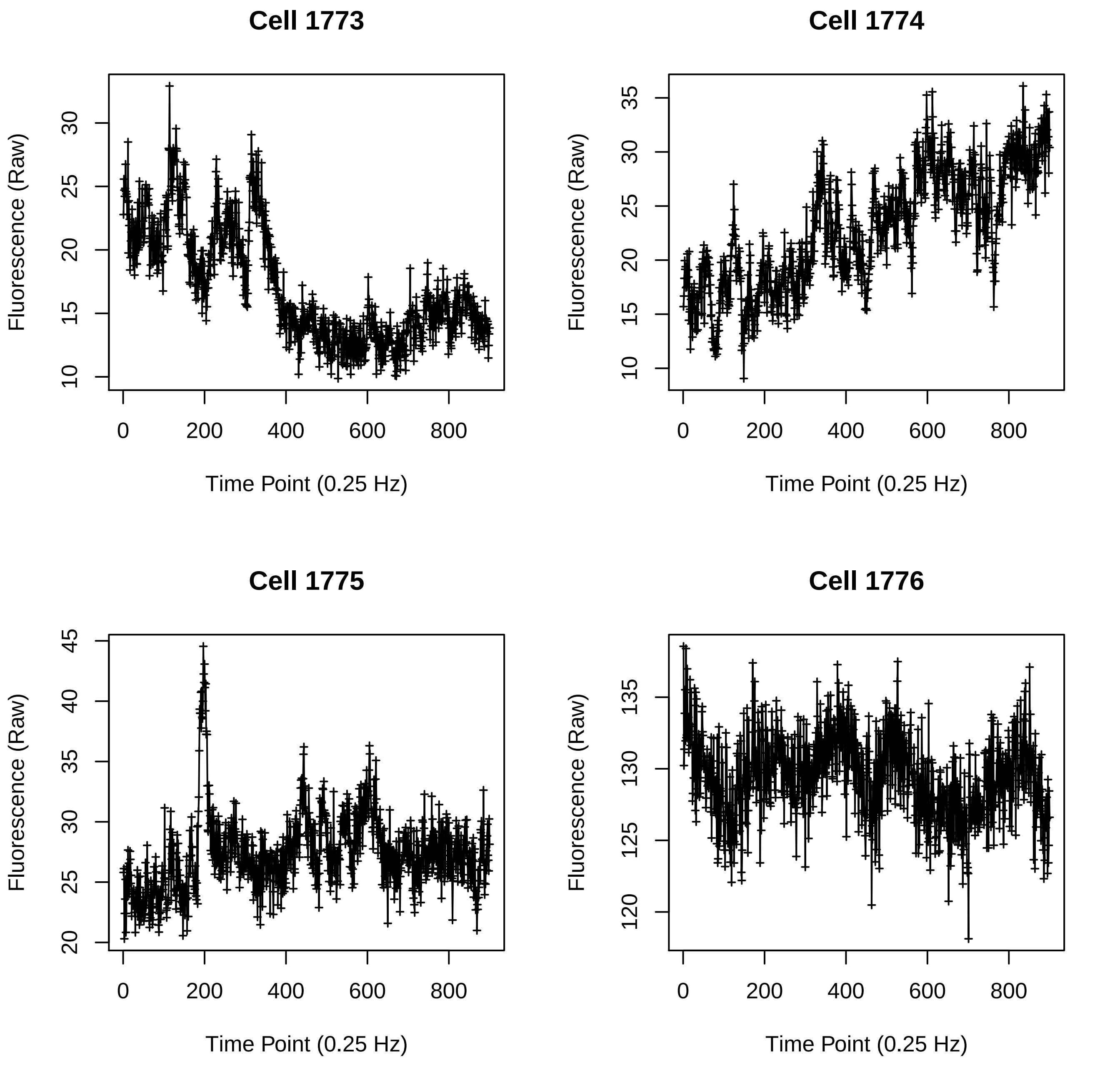
Cell 1773
Cell 1774
Cell 1775
Cell 1776
Fluorescence (Raw)
Time Point (0.25 Hz)
0
200
400
600
800
10
15
20
25
30
35
40
45
120
125
130
135

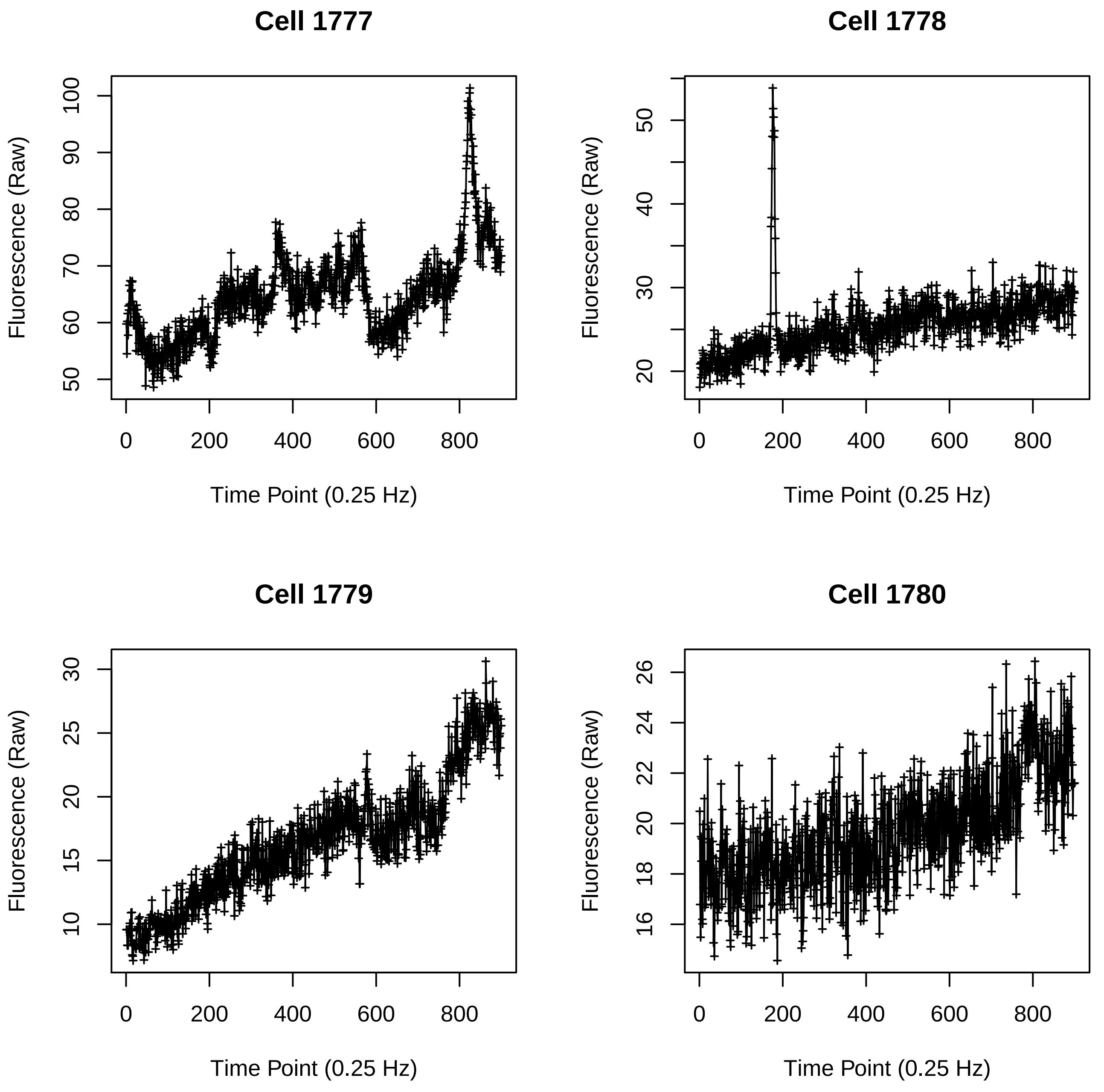

Cell 1777
Cell 1778
Cell 1779
Cell 1780
Fluorescence (Raw)
Time Point (0.25 Hz)
0
200
400
600
800
50
60
70
80
90
100
20
30
40
50
10
15
20
25
30
16
18
20
22
24
26

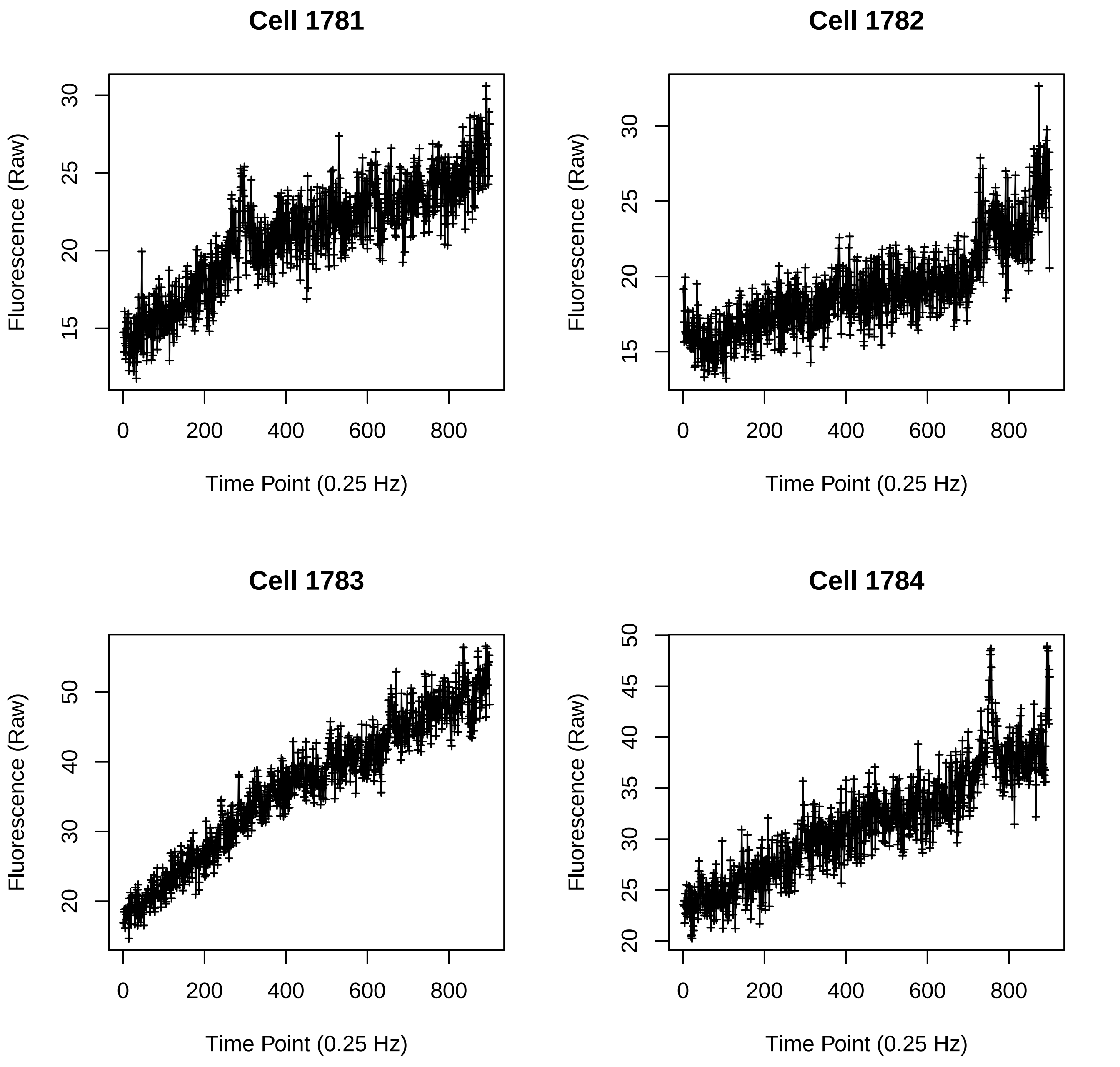
Cell 1781
Cell 1782
Cell 1783
Cell 1784
Fluorescence (Raw)
Time Point (0.25 Hz)
0
200
400
600
800
15
20
25
30
40
45
50

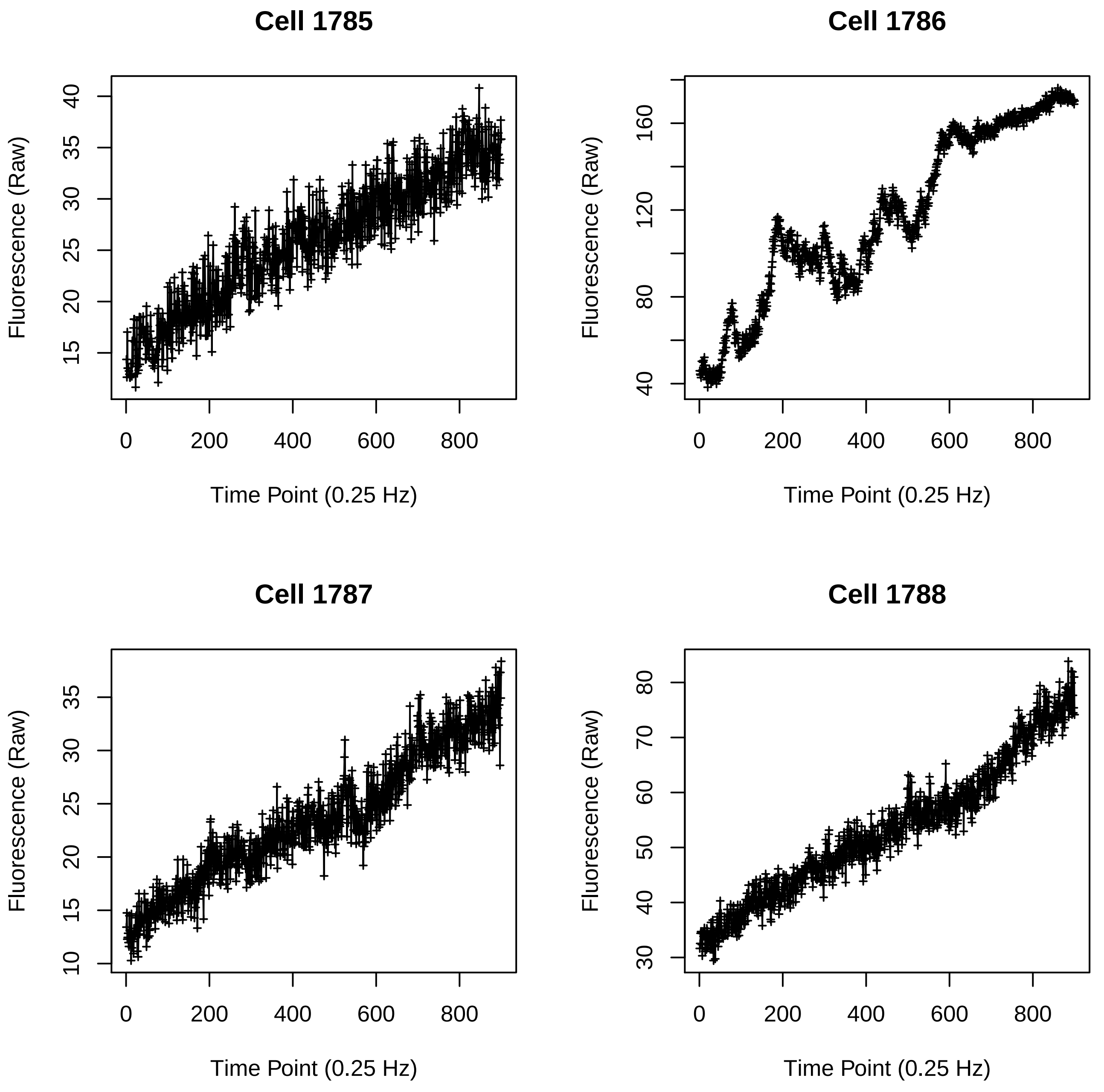
Cell 1785
Cell 1786
Cell 1787
Cell 1788
Fluorescence (Raw)
Time Point (0.25 Hz)
0
200
400
600
800
15
20
25
30
35
40
80
120
160
10
50
60
70

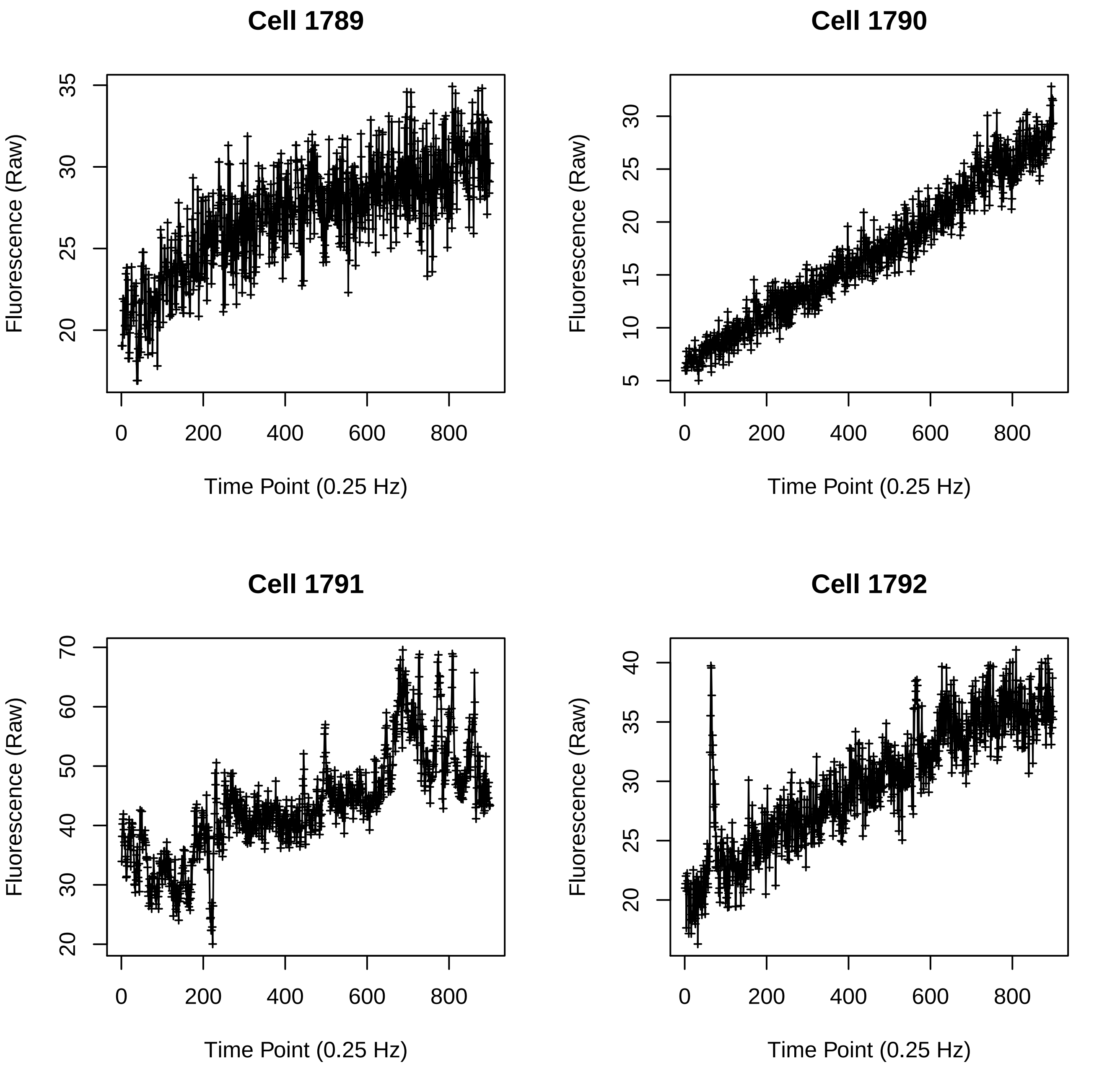

Cell 1789
Fluorescence (Raw)
20
25
30
35
0
200
400
600
800
Time Point (0.25 Hz)
Cell 1790
Fluorescence (Raw)
5
10
15
20
25
30
0
200
400
600
800
Time Point (0.25 Hz)
Cell 1791
Fluorescence (Raw)
20
30
40
50
60
70
0
200
400
600
800
Time Point (0.25 Hz)
Cell 1792
Fluorescence (Raw)
20
25
30
35
40
0
200
400
600
800
Time Point (0.25 Hz)

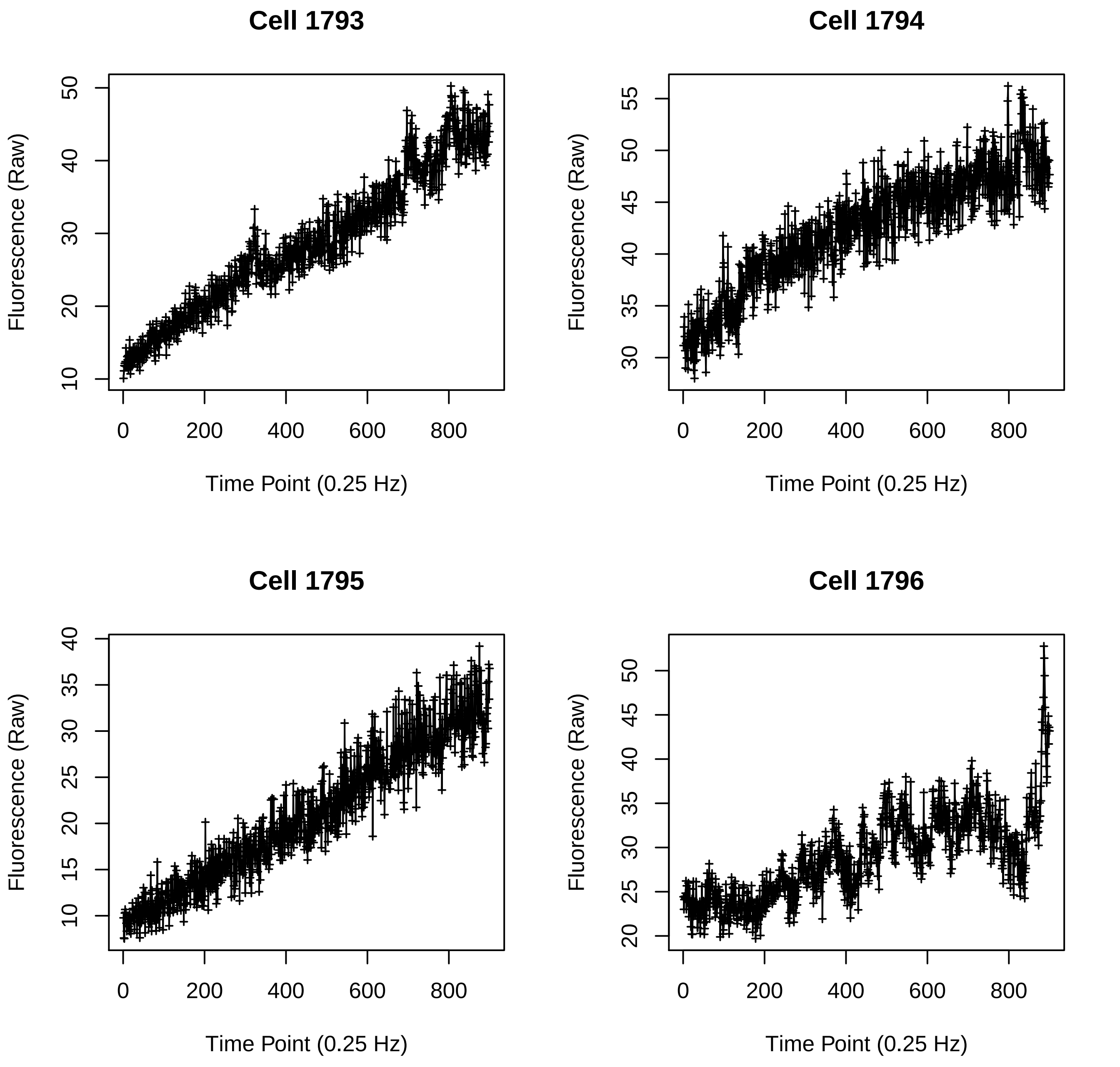

Cell 1793
Cell 1794
Cell 1795
Cell 1796
Fluorescence (Raw)
Time Point (0.25 Hz)
0
200
400
600
800
10
20
30
40
50
55
45
35
25
15

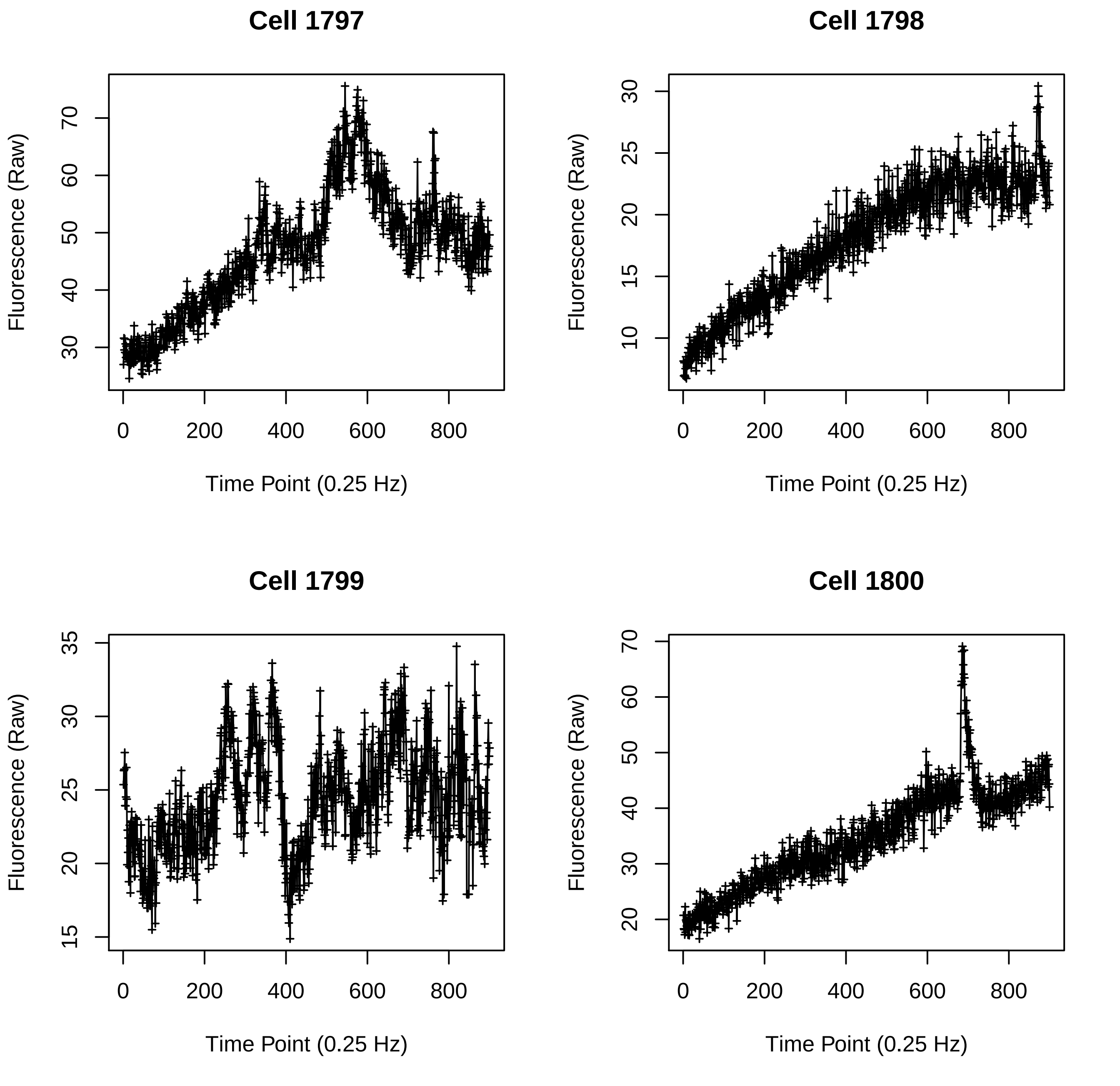

Cell 1797
Cell 1798
Cell 1799
Cell 1800
Fluorescence (Raw)
Time Point (0.25 Hz)
0
200
400
600
800
30
40
50
60
70
10
15
20
25
35

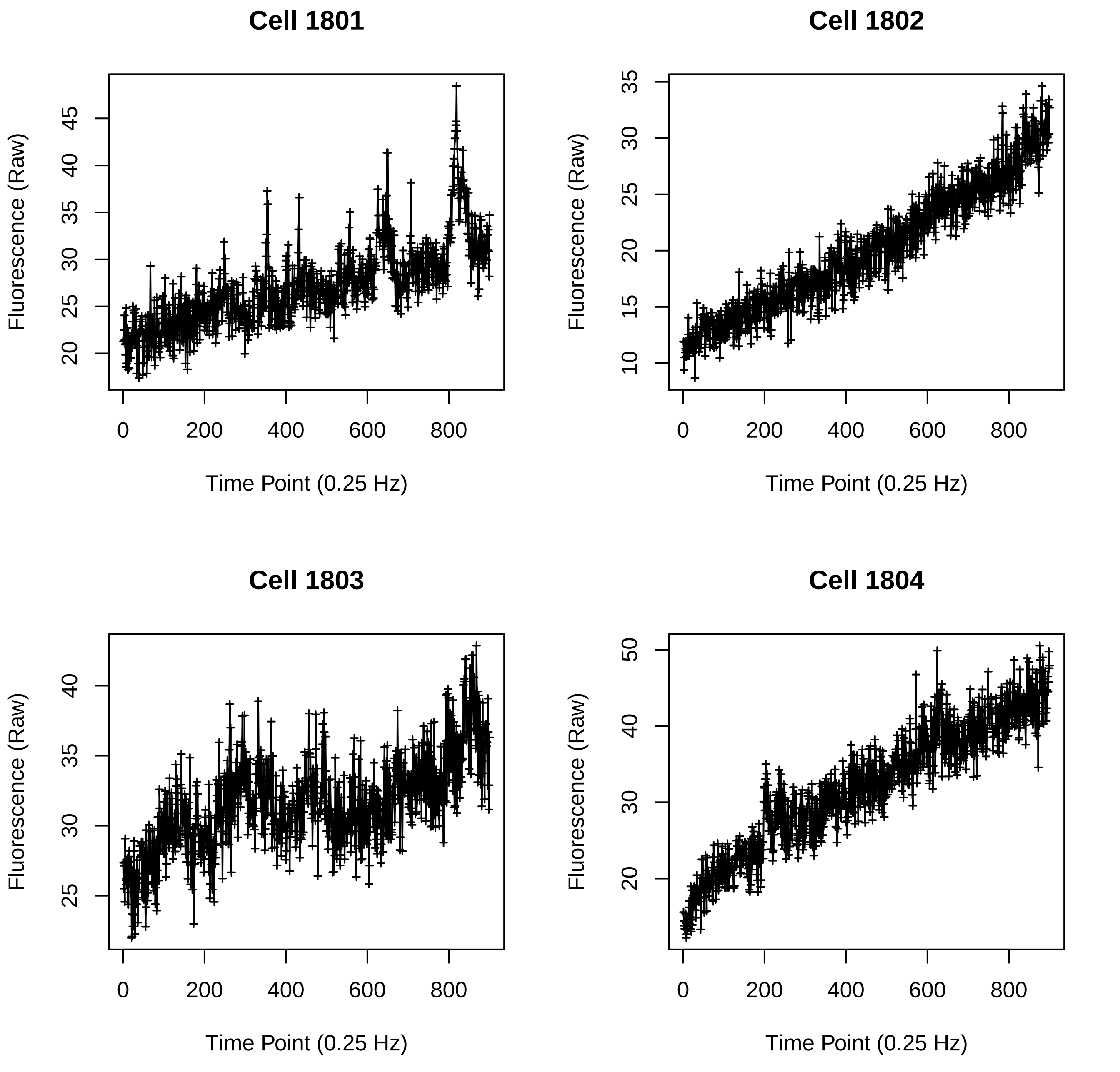
Cell 1801
Cell 1802
Cell 1803
Cell 1804
Fluorescence (Raw)
Time Point (0.25 Hz)
0
200
400
600
800
20
25
30
35
40
45
10
15
50

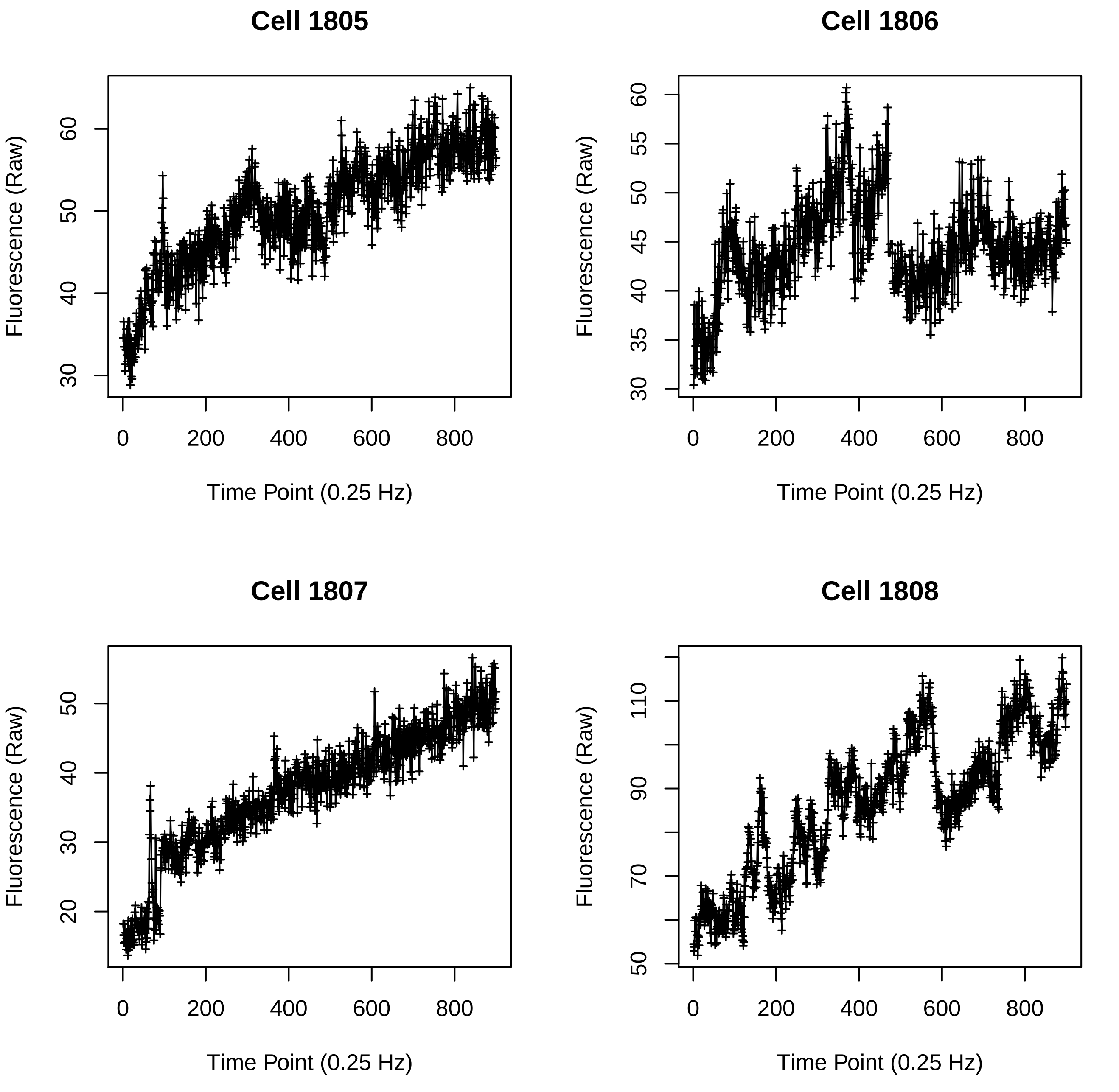
Cell 1805
Fluorescence (Raw)
30
40
50
60
0
200
400
600
800
Time Point (0.25 Hz)
Cell 1806
Fluorescence (Raw)
30
35
40
45
50
55
60
0
200
400
600
800
Time Point (0.25 Hz)
Cell 1807
Fluorescence (Raw)
20
30
40
50
0
200
400
600
800
Time Point (0.25 Hz)
Cell 1808
Fluorescence (Raw)
50
70
90
110
0
200
400
600
800
Time Point (0.25 Hz)

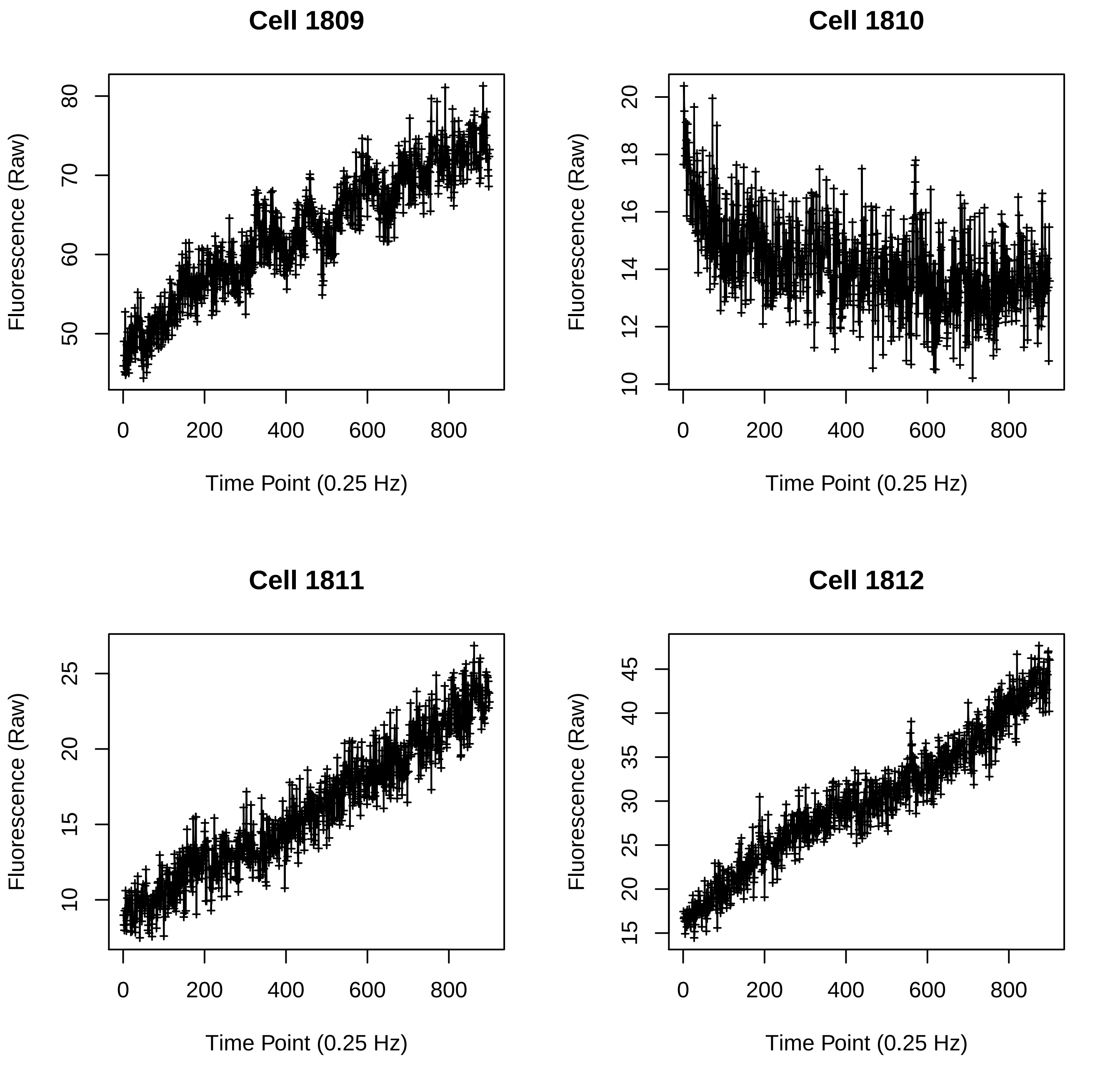

Cell 1809
Cell 1810
Cell 1811
Cell 1812
Fluorescence (Raw)
Time Point (0.25 Hz)
50
60
70
80
10
12
14
16
18
20
15
25
30
35
40
45
0
200
400
600
800

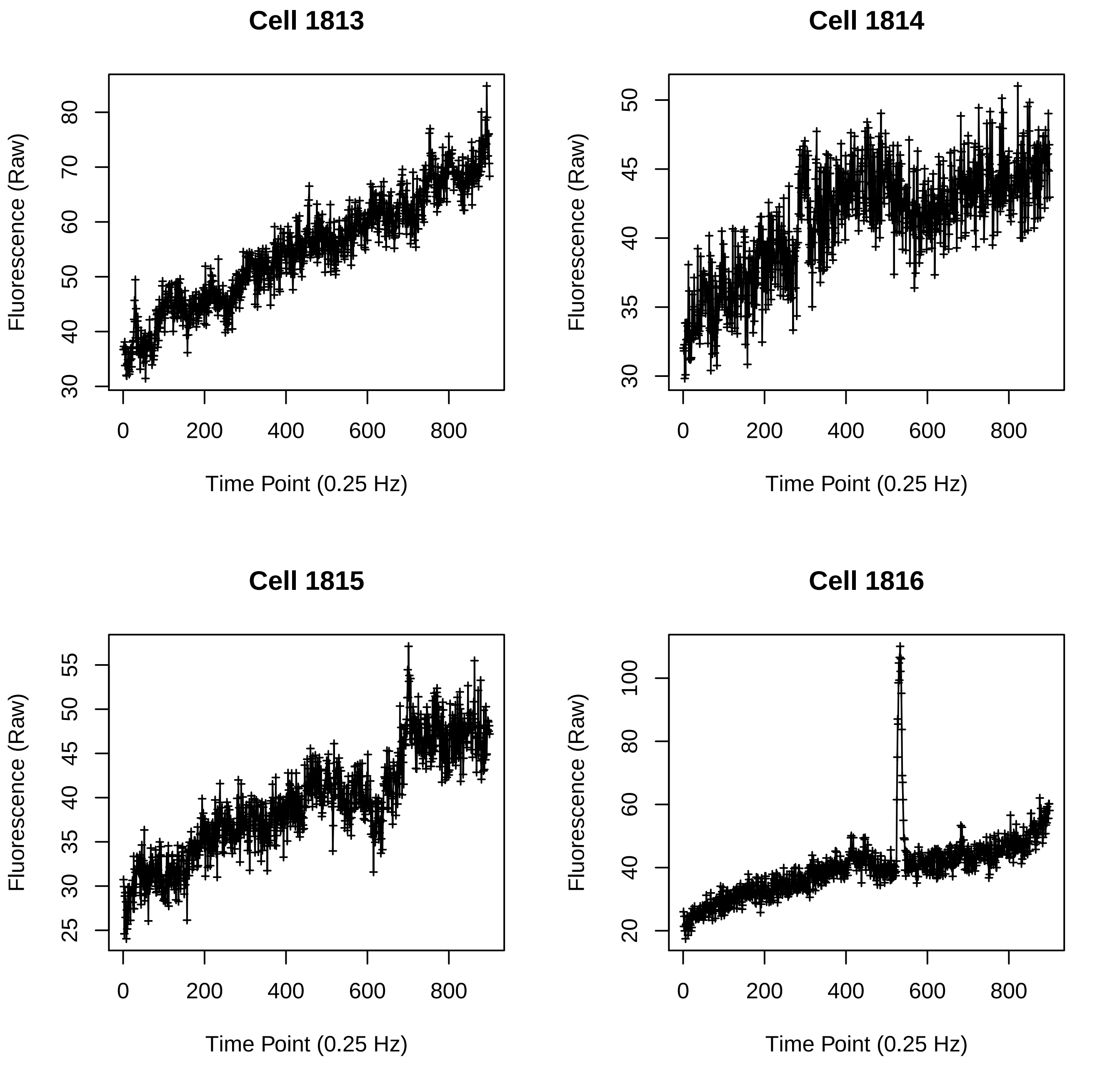
Cell 1813
Cell 1814
Cell 1815
Cell 1816
Fluorescence (Raw)
Time Point (0.25 Hz)
30
40
50
60
70
80
35
45
25
55
20
100
0
200
400
600
800

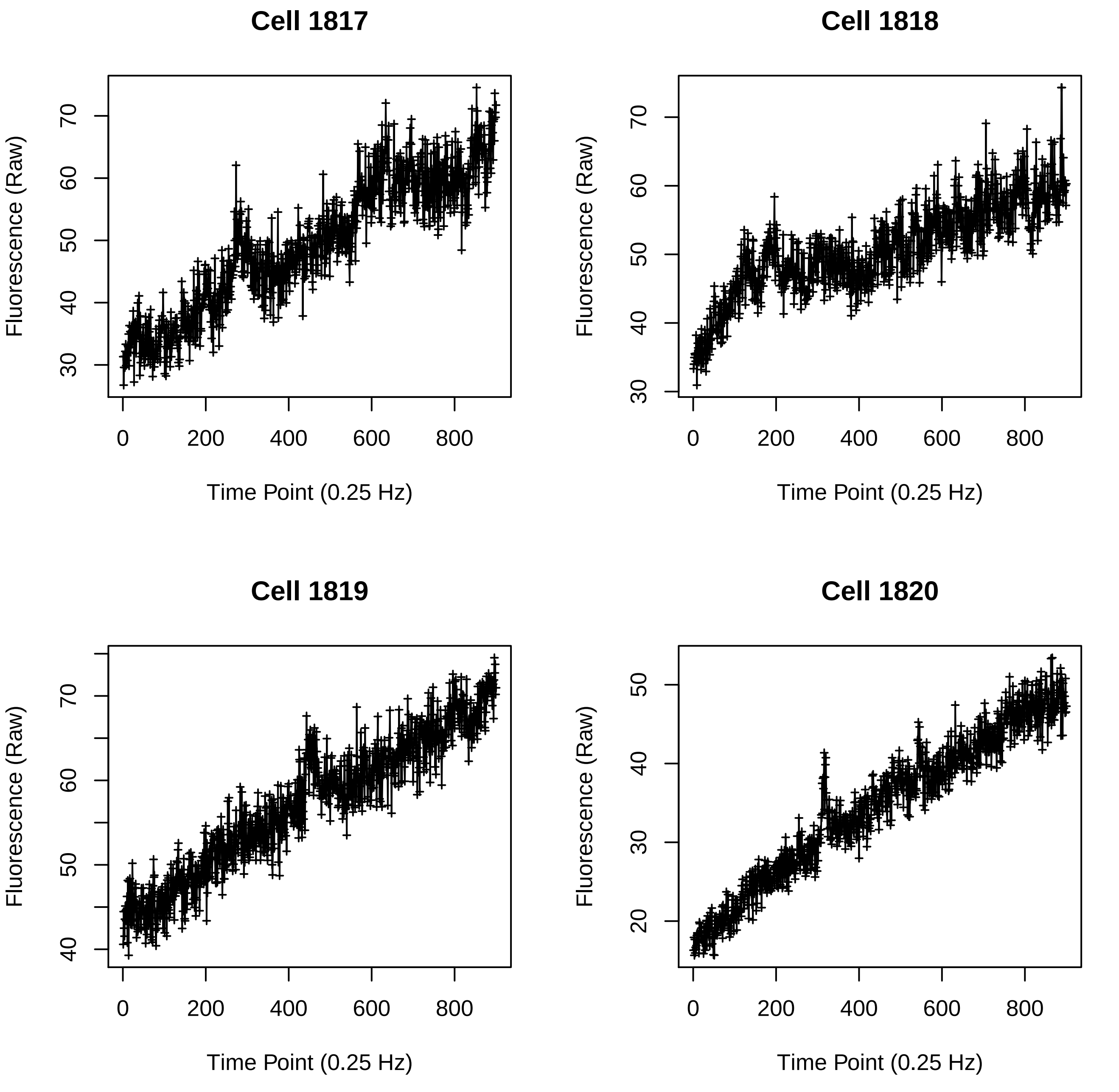

Cell 1817
Fluorescence (Raw)
Time Point (0.25 Hz)
Cell 1818
Fluorescence (Raw)
Time Point (0.25 Hz)
Cell 1819
Fluorescence (Raw)
Time Point (0.25 Hz)
Cell 1820
Fluorescence (Raw)
Time Point (0.25 Hz)

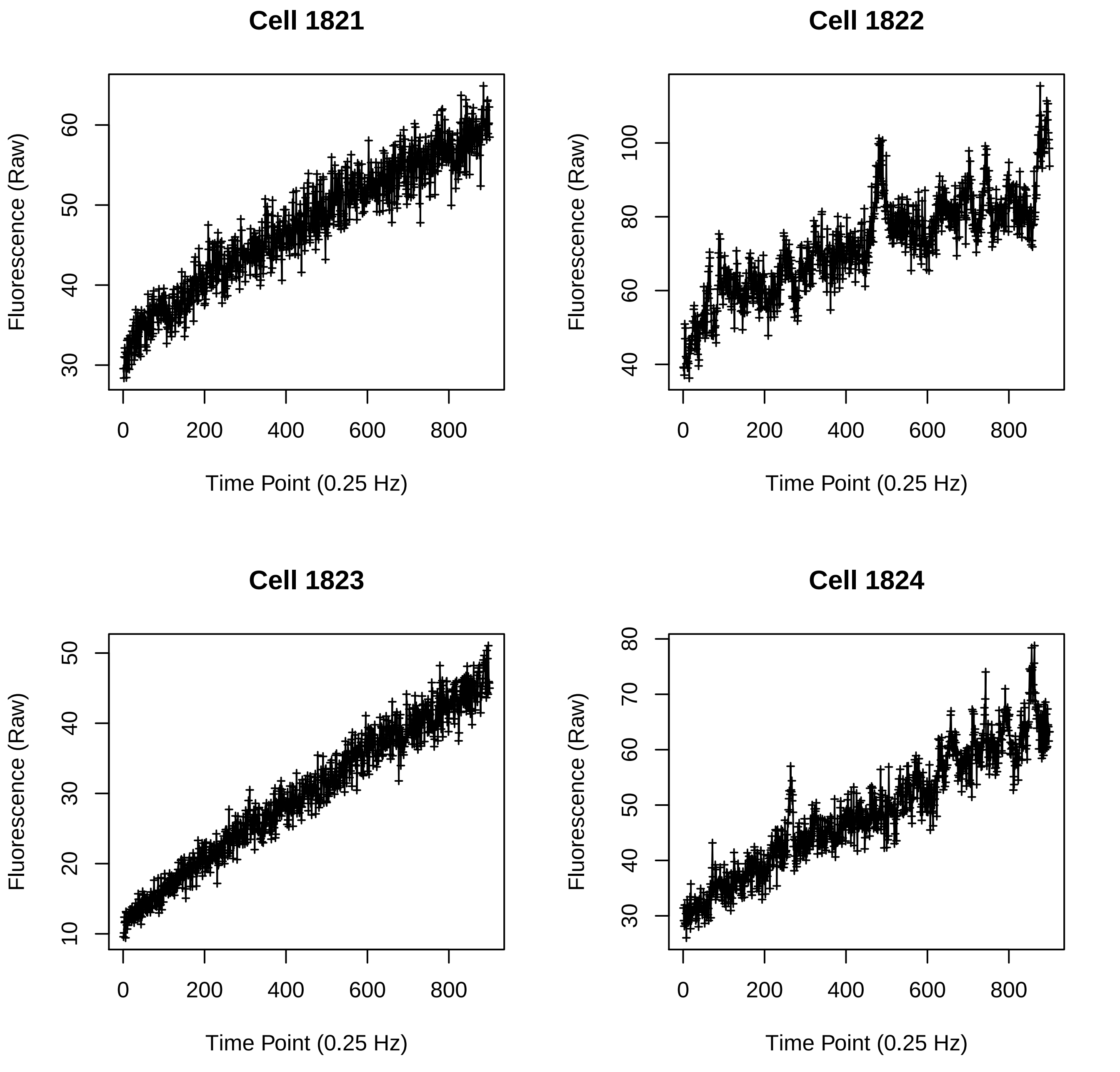

Cell 1821
Fluorescence (Raw)
30
40
50
60
0
200
400
600
800
Time Point (0.25 Hz)
Cell 1822
Fluorescence (Raw)
40
60
80
100
0
200
400
600
800
Time Point (0.25 Hz)
Cell 1823
Fluorescence (Raw)
10
20
30
40
50
0
200
400
600
800
Time Point (0.25 Hz)
Cell 1824
Fluorescence (Raw)
30
40
50
60
70
80
0
200
400
600
800
Time Point (0.25 Hz)

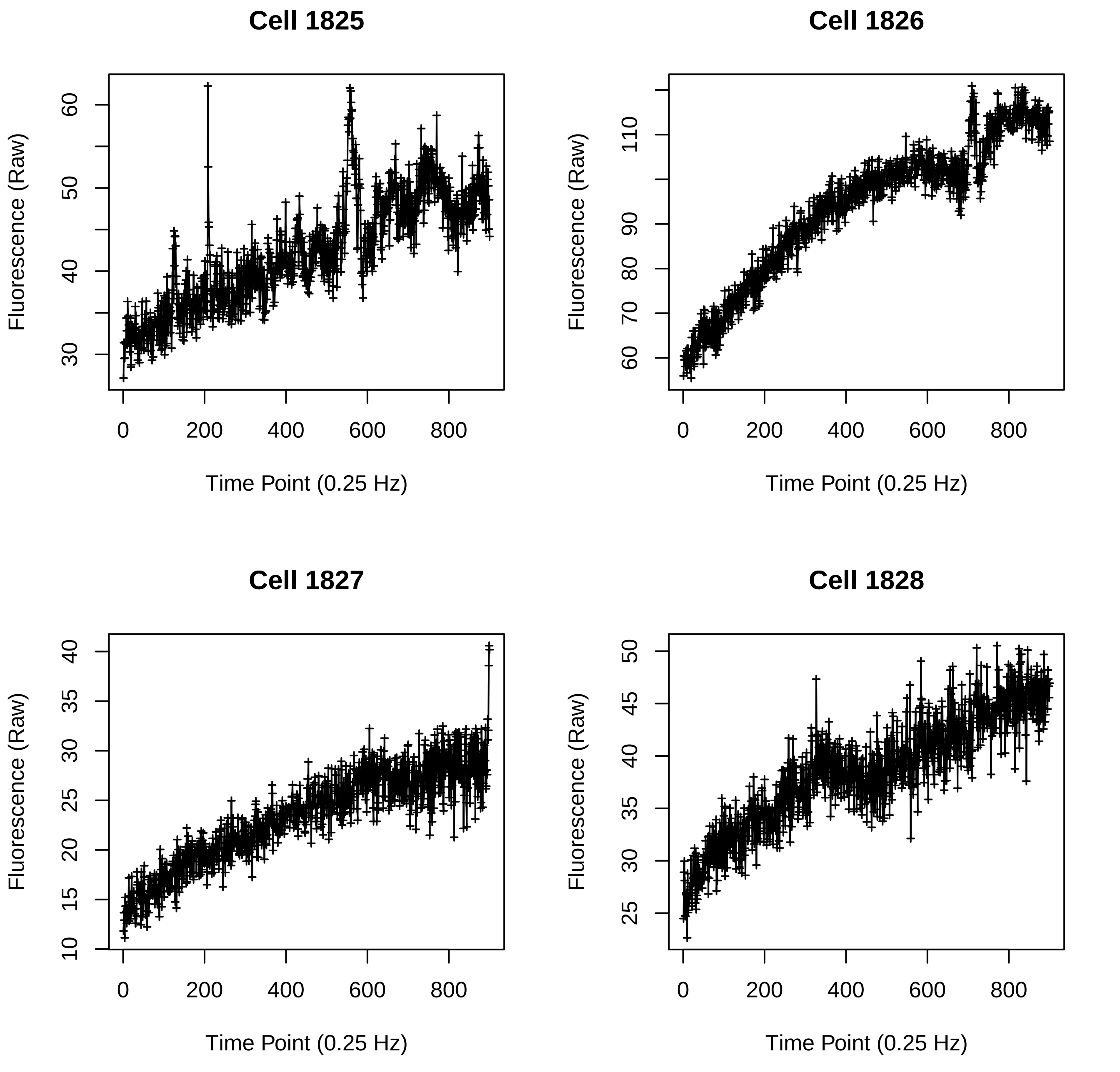

Cell 1825
Cell 1826
Cell 1827
Cell 1828
Fluorescence (Raw)
Time Point (0.25 Hz)
0
200
400
600
800
30
40
50
60
60
70
80
90
110
10
15
20
25
30
35
40
25
30
35
40
45
50

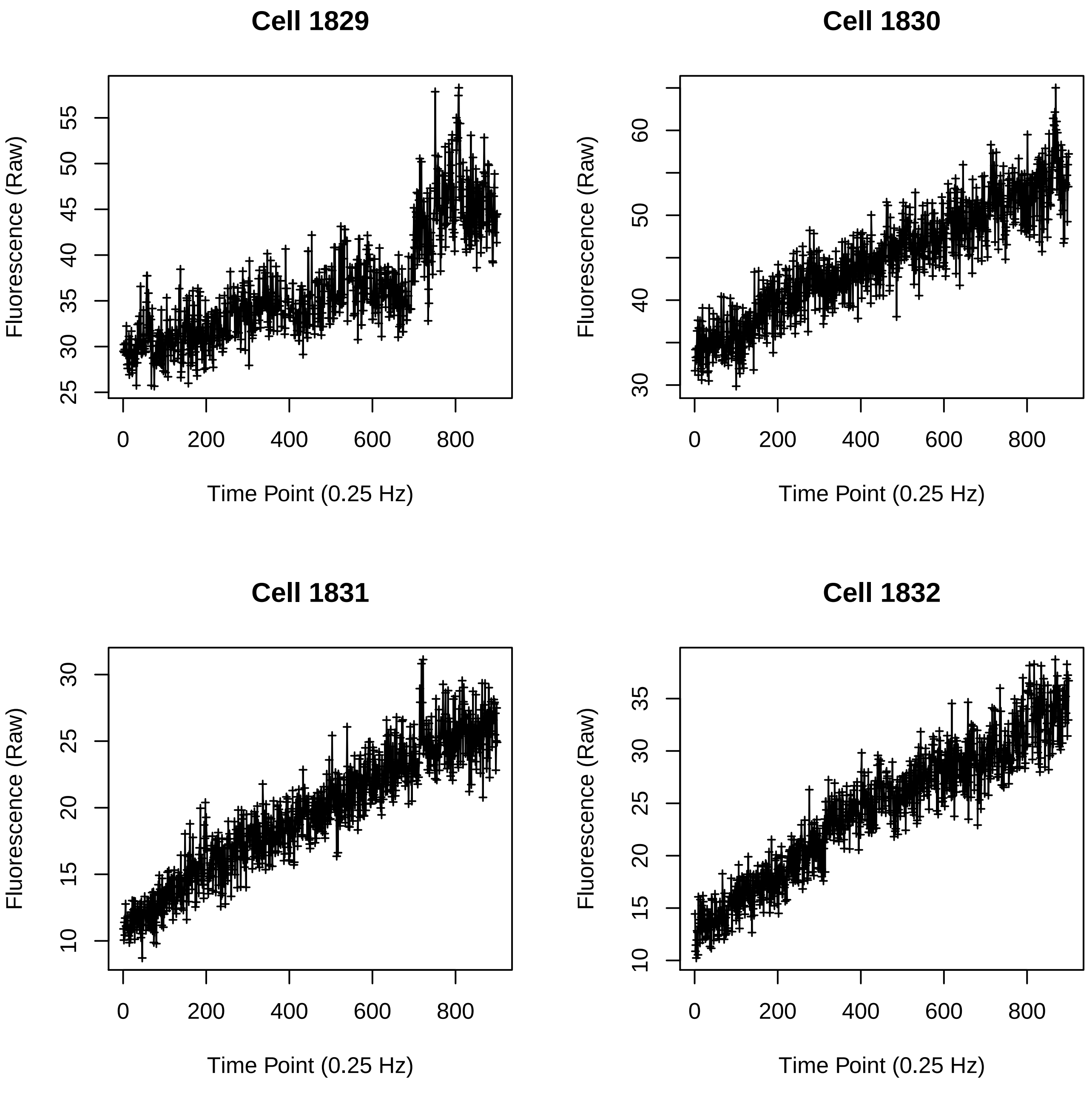
Cell 1829
Cell 1830
Cell 1831
Cell 1832
Fluorescence (Raw)
Time Point (0.25 Hz)
0
200
400
600
800
25
30
35
40
45
50
55
60
10
15
20

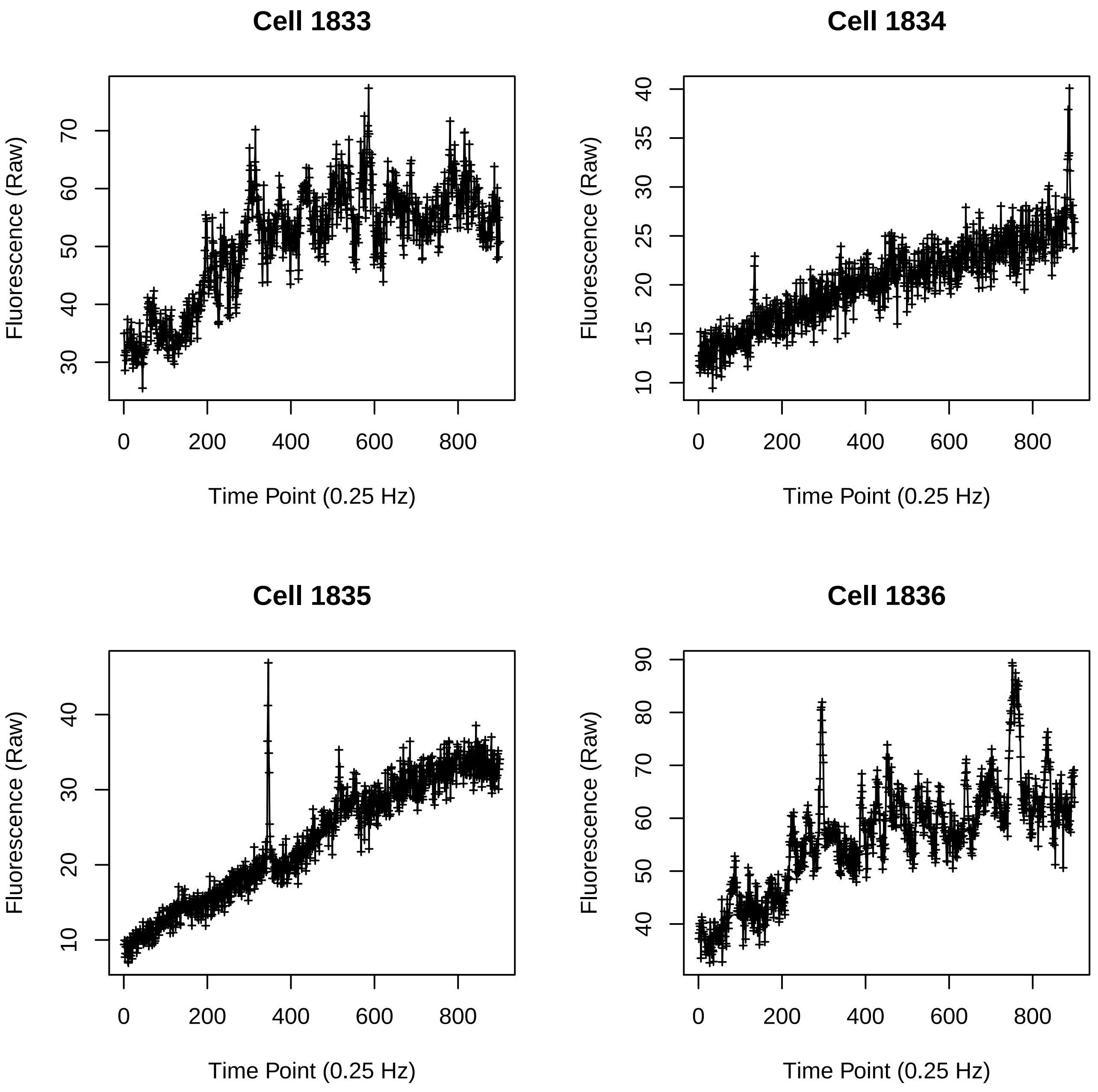

Cell 1833
Cell 1834
Cell 1835
Cell 1836
Fluorescence (Raw)
Time Point (0.25 Hz)
0
200
400
600
800
30
40
50
60
70
10
15
20
25
30
35
40
10
20
30
40
40
50
60
70
80
90

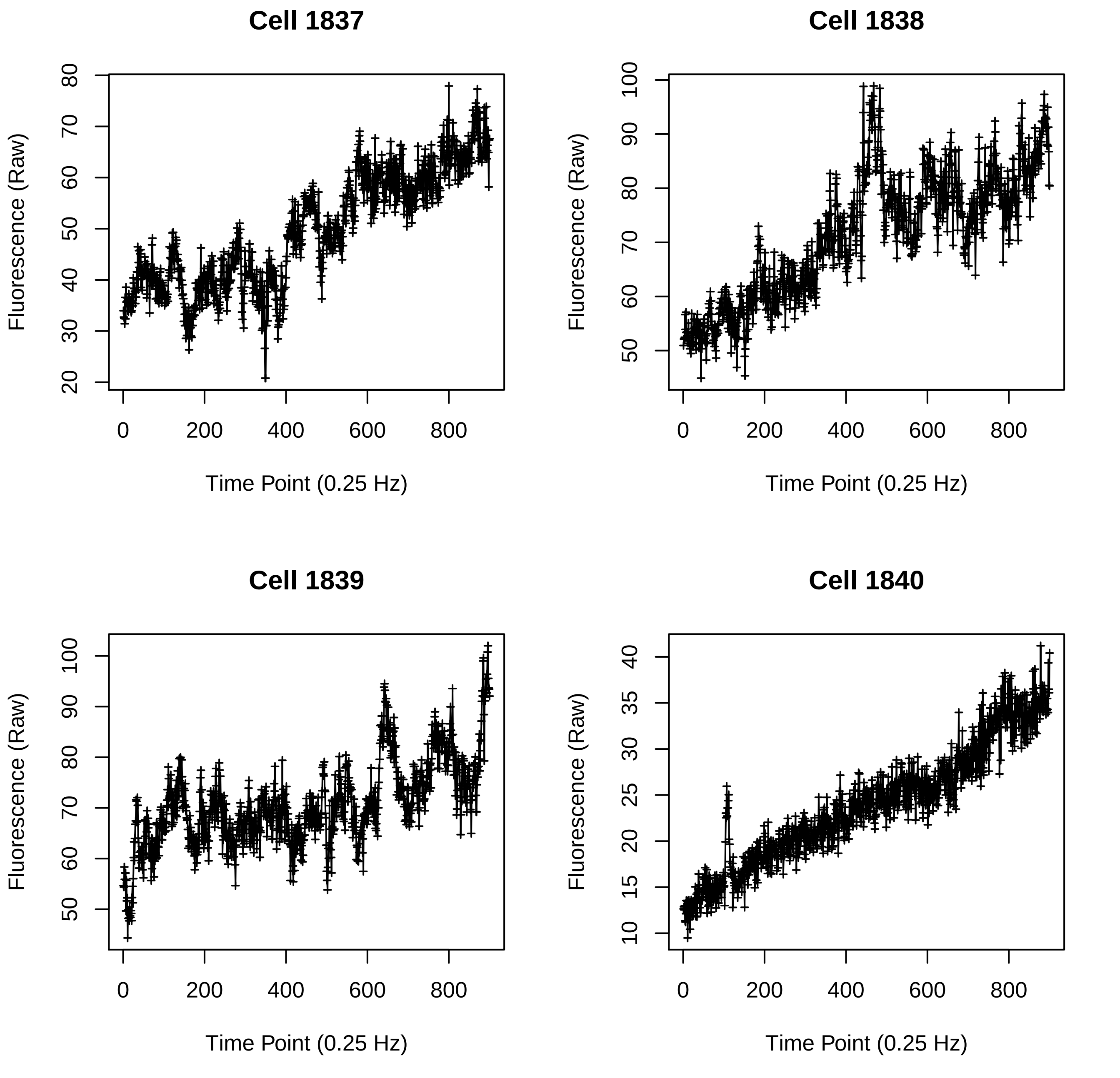

Cell 1837
Cell 1838
Cell 1839
Cell 1840
Fluorescence (Raw)
Time Point (0.25 Hz)
20
30
40
50
60
70
80
90
100
10
15
25
35
0
200
400
600
800

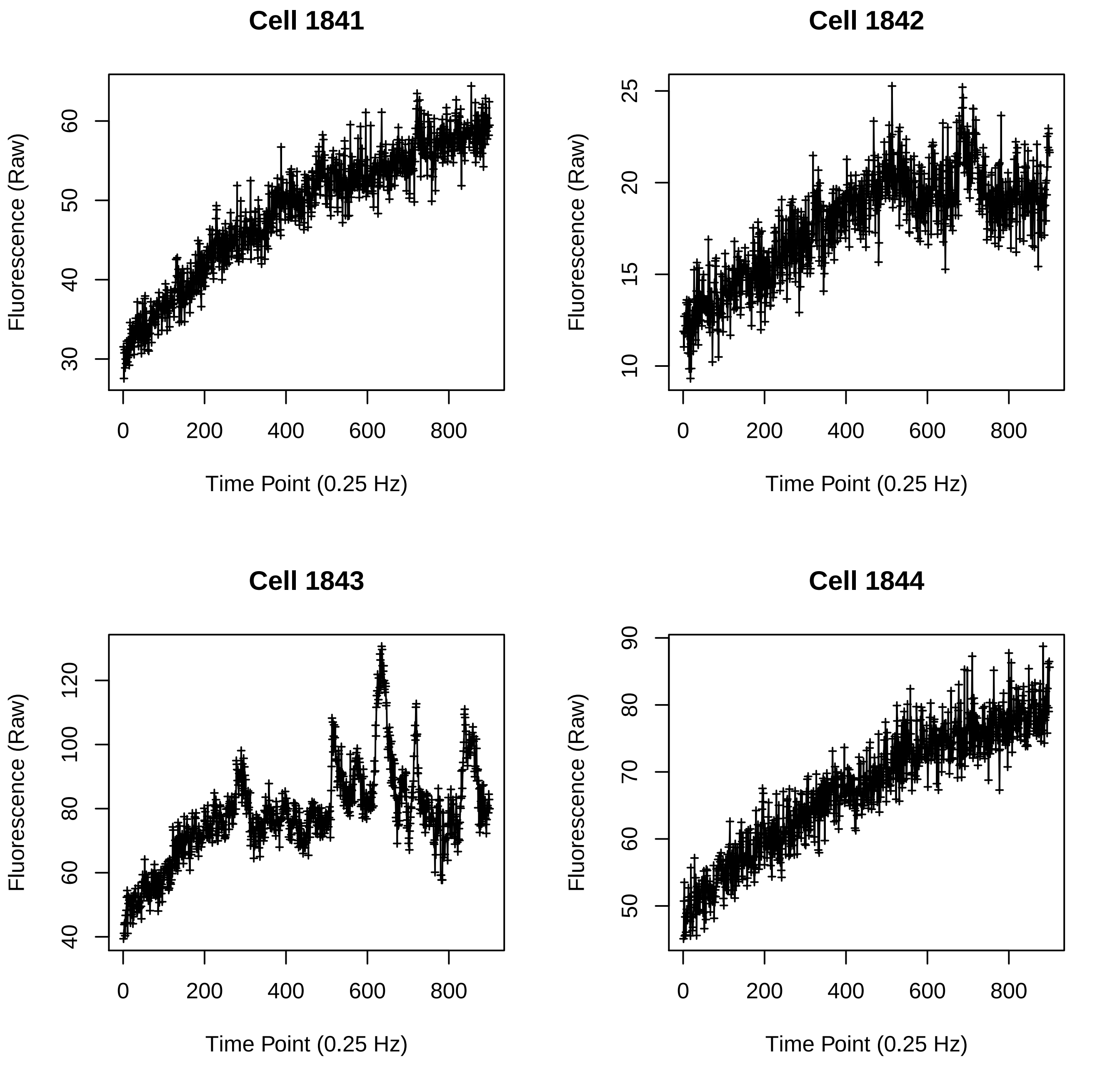

Cell 1841
Cell 1842
Cell 1843
Cell 1844
Fluorescence (Raw)
Time Point (0.25 Hz)
0
200
400
600
800
30
40
50
60
10
15
20
25
40
60
80
100
120
50
60
70
80
90

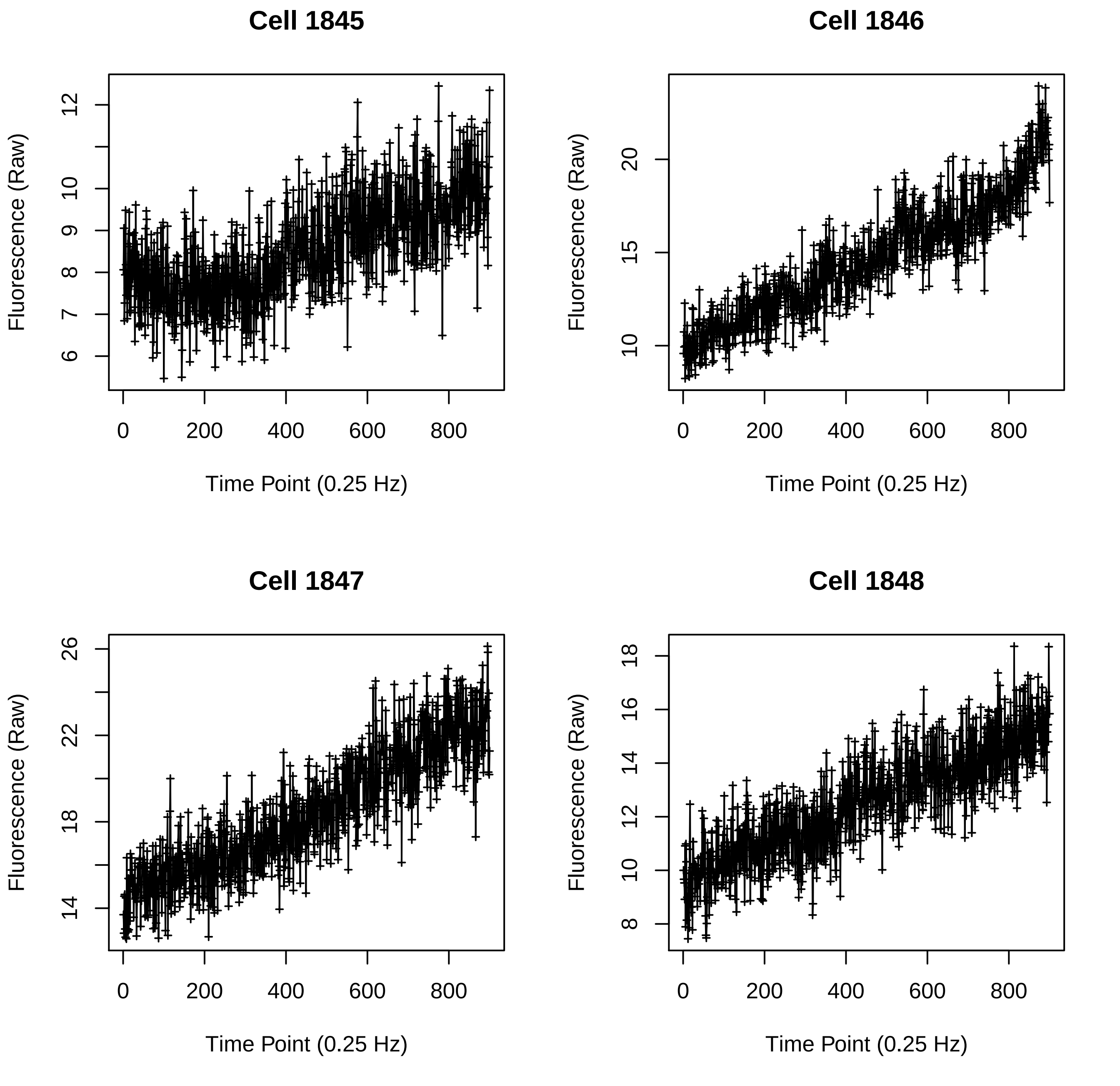

Cell 1845
Cell 1846
Cell 1847
Cell 1848
Fluorescence (Raw)
Time Point (0.25 Hz)
0
200
400
600
800
6
7
8
9
10
12
15
20
14
18
22
26
16

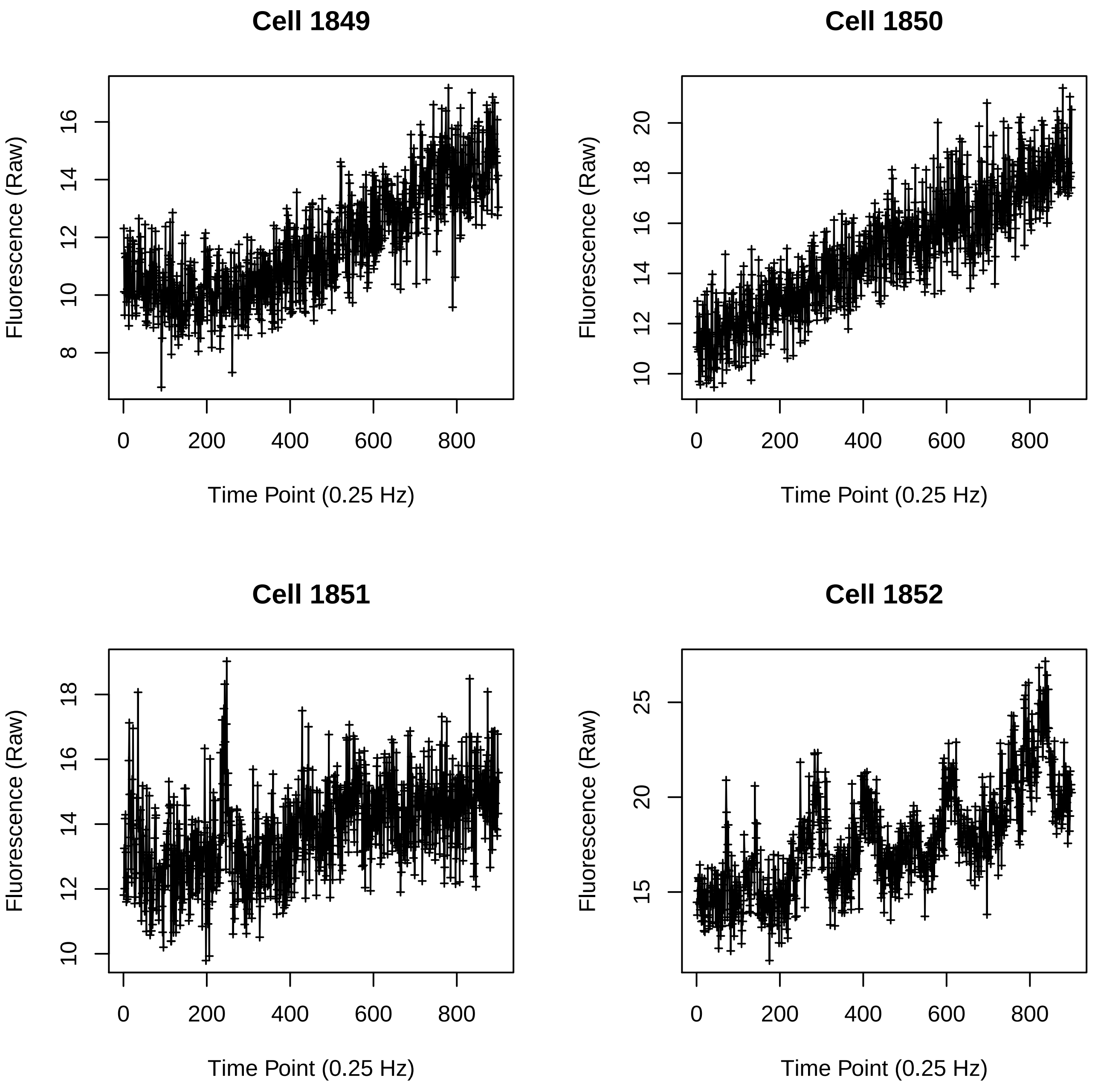
Cell 1849
Fluorescence (Raw)
8
10
12
14
16
0
200
400
600
800
Time Point (0.25 Hz)
Cell 1850
Fluorescence (Raw)
10
12
14
16
18
20
0
200
400
600
800
Time Point (0.25 Hz)
Cell 1851
Fluorescence (Raw)
10
12
14
16
18
0
200
400
600
800
Time Point (0.25 Hz)
Cell 1852
Fluorescence (Raw)
15
20
25
0
200
400
600
800
Time Point (0.25 Hz)

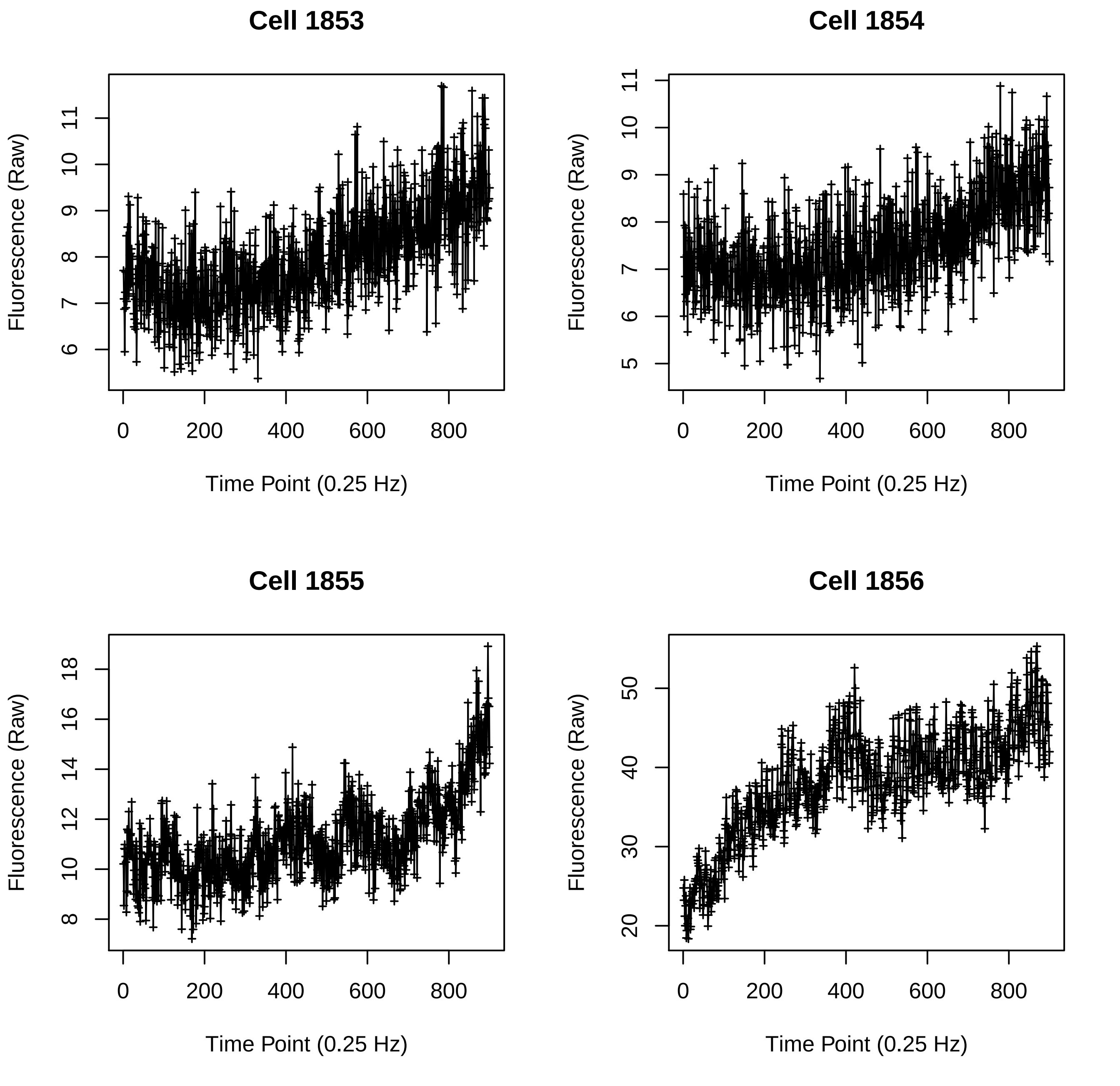

Cell 1853
Cell 1854
Cell 1855
Cell 1856
Fluorescence (Raw)
Time Point (0.25 Hz)
0
200
400
600
800
5
6
7
8
9
10
11
12
14
16
18
20
30
40
50

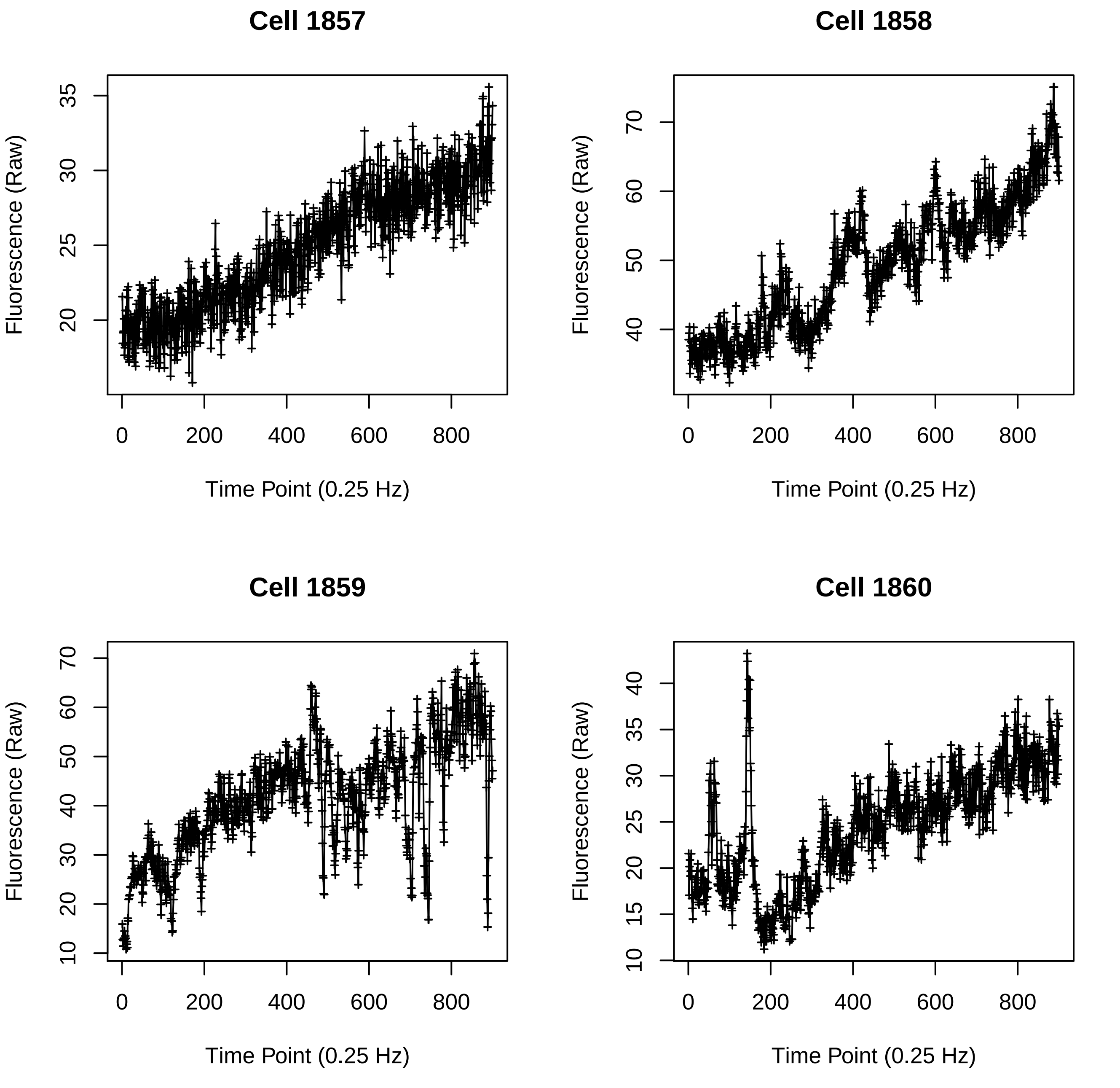
Cell 1857
Cell 1858
Cell 1859
Cell 1860
Fluorescence (Raw)
Time Point (0.25 Hz)
0
200
400
600
800
20
25
30
35
40
50
60
70
10
15

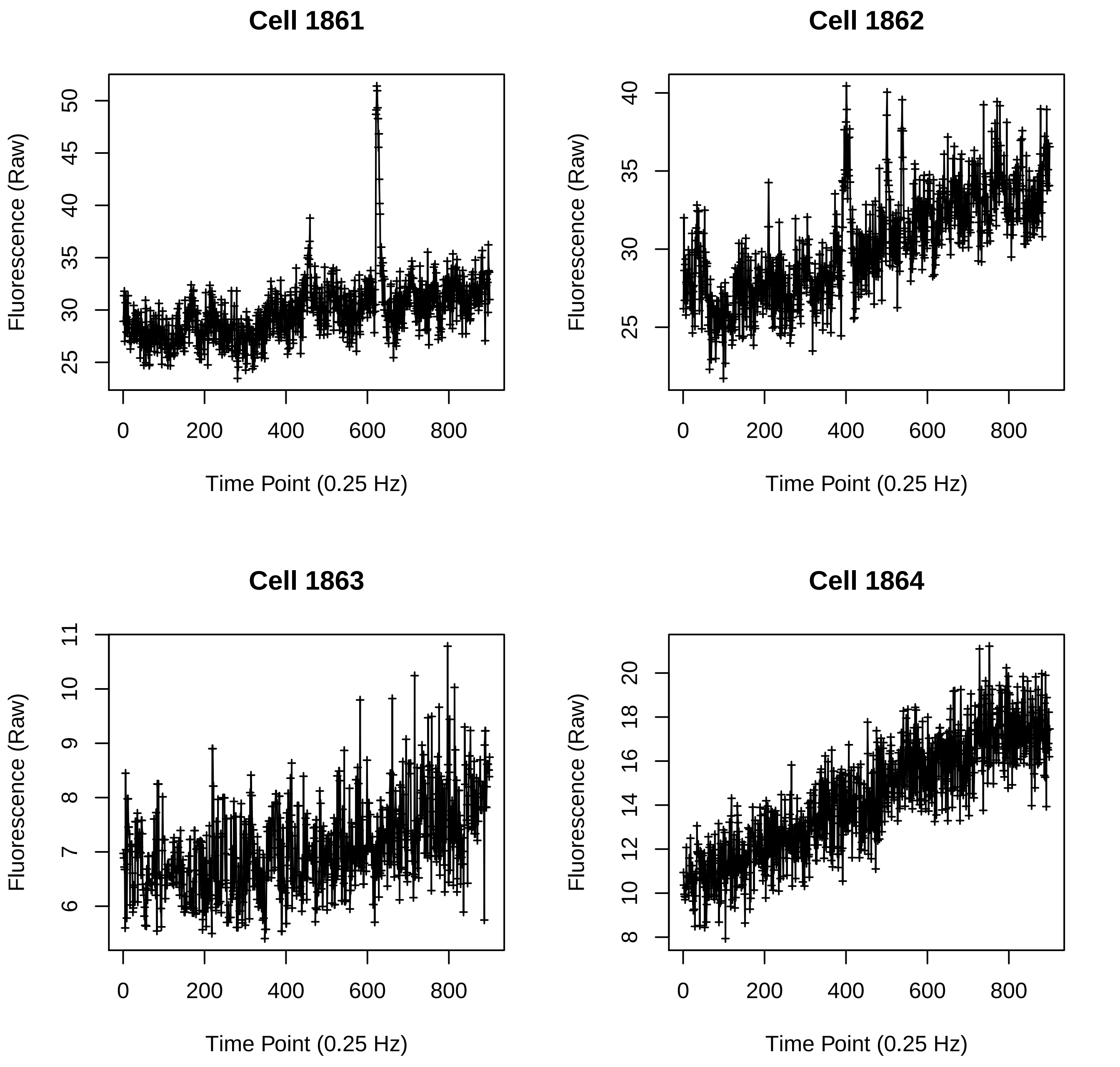

Cell 1861
Cell 1862
Cell 1863
Cell 1864
Fluorescence (Raw)
Time Point (0.25 Hz)
0
200
400
600
800

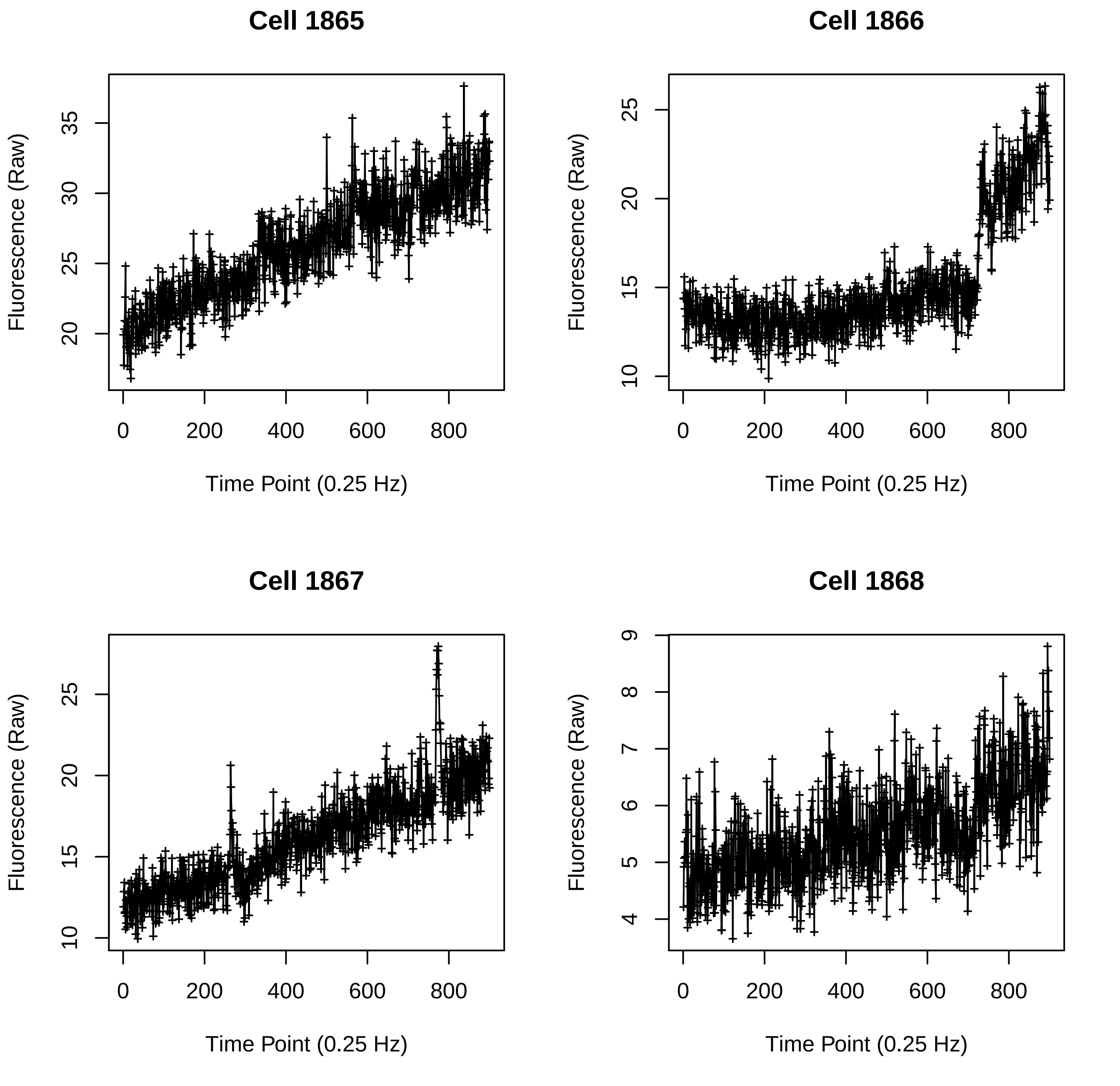

Cell 1865
Cell 1866
Cell 1867
Cell 1868
Fluorescence (Raw)
Time Point (0.25 Hz)

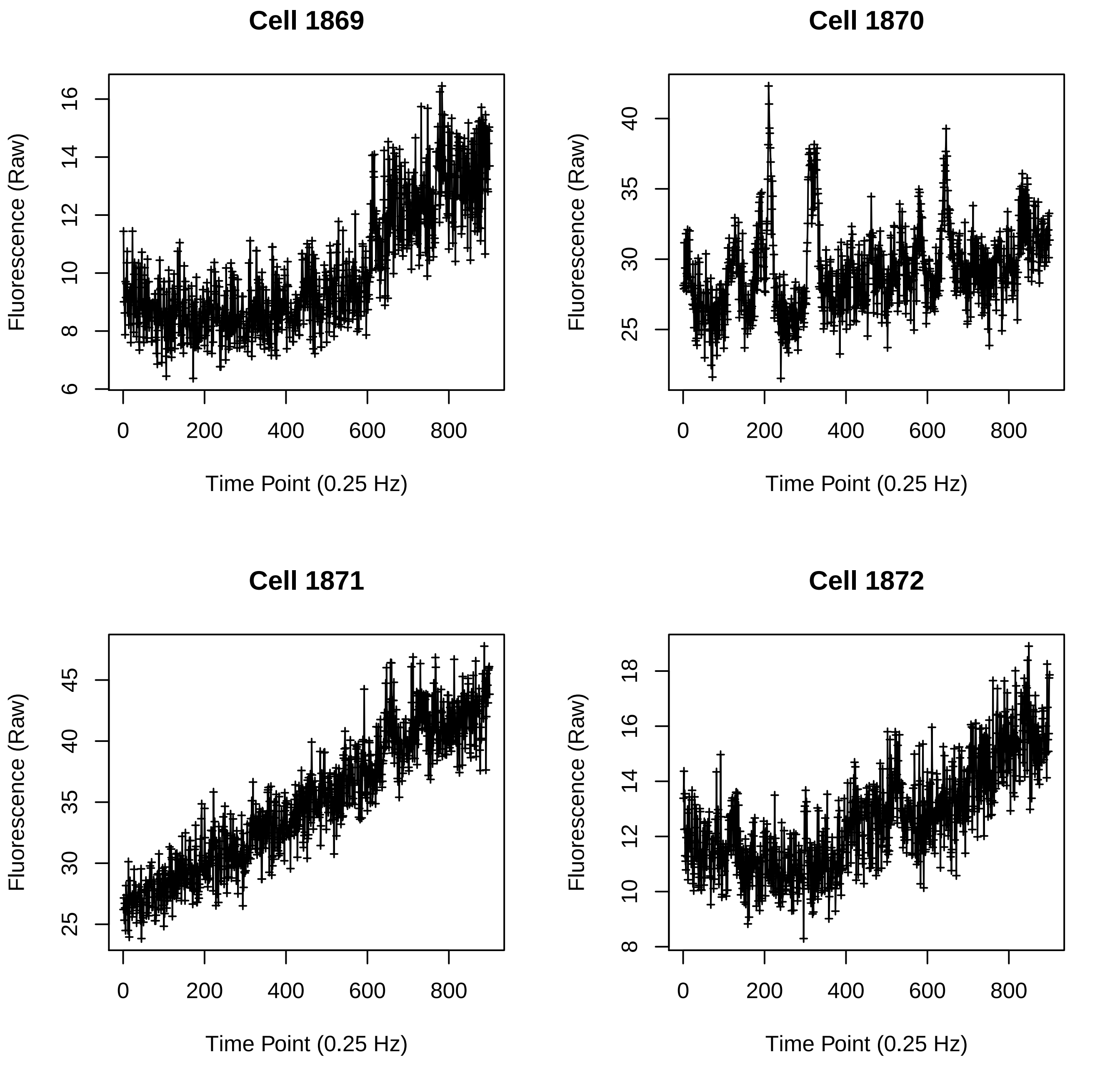
Cell 1869
Cell 1870
Cell 1871
Cell 1872
Fluorescence (Raw)
Time Point (0.25 Hz)
0
200
400
600
800
6
8
10
12
14
16
25
30
35
40
45
18

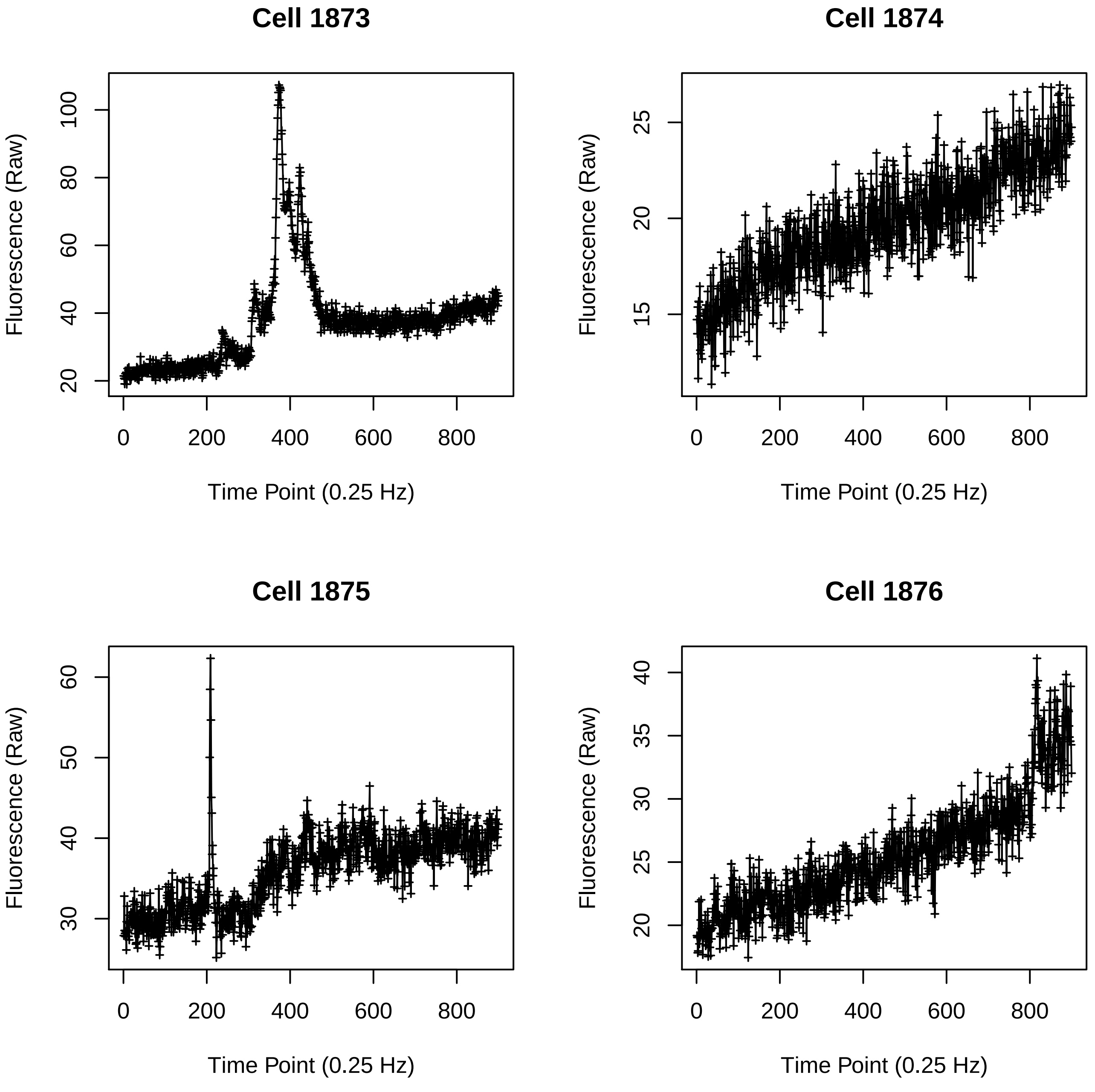

Cell 1873
Fluorescence (Raw)
20
40
60
80
100
0
200
400
600
800
Time Point (0.25 Hz)
Cell 1874
Fluorescence (Raw)
15
20
25
0
200
400
600
800
Time Point (0.25 Hz)
Cell 1875
Fluorescence (Raw)
30
40
50
60
0
200
400
600
800
Time Point (0.25 Hz)
Cell 1876
Fluorescence (Raw)
20
25
30
35
40
0
200
400
600
800
Time Point (0.25 Hz)

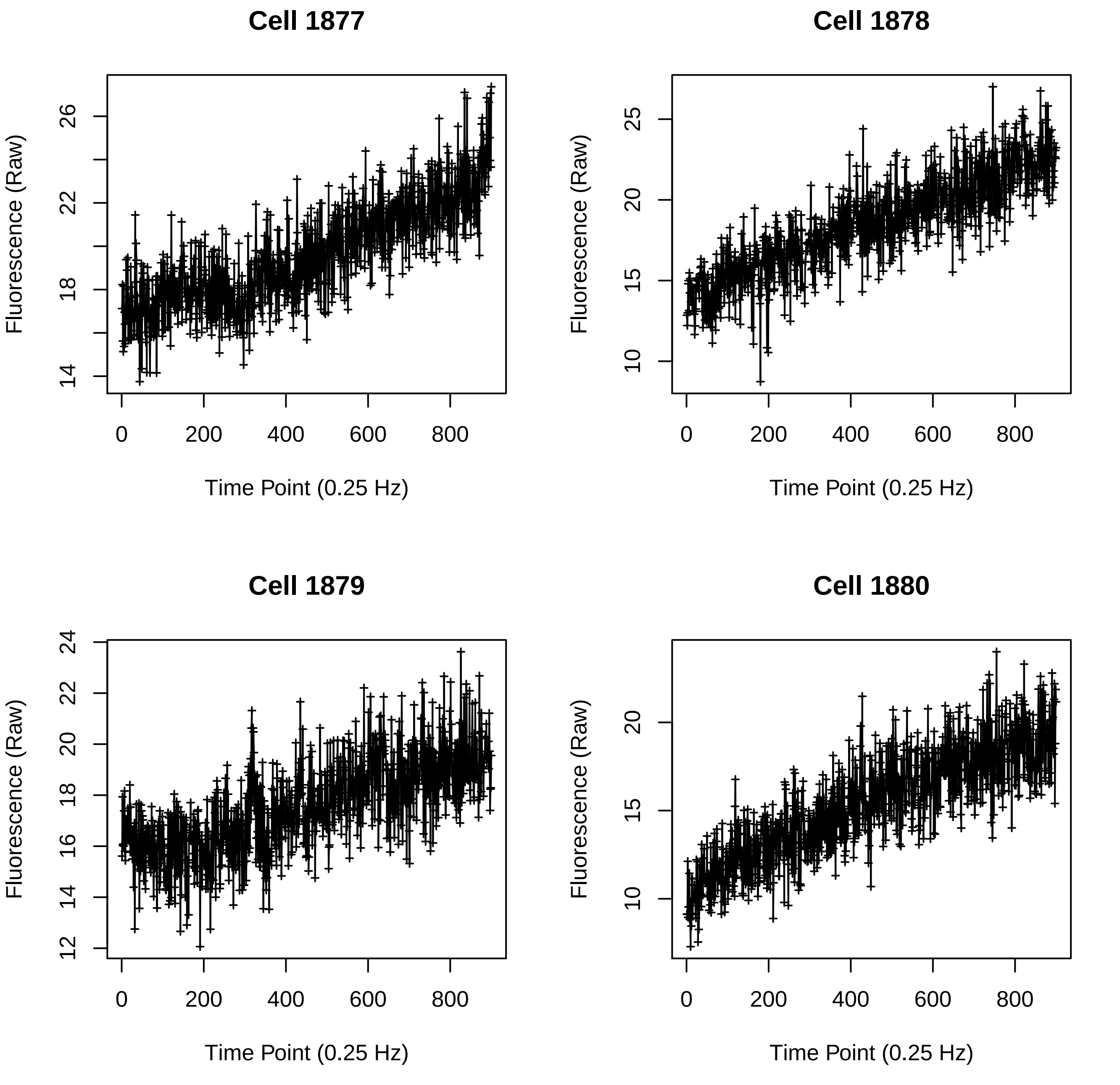
Cell 1877
Fluorescence (Raw)
14
18
22
26
0
200
400
600
800
Time Point (0.25 Hz)
Cell 1878
Fluorescence (Raw)
10
15
20
25
0
200
400
600
800
Time Point (0.25 Hz)
Cell 1879
Fluorescence (Raw)
12
14
16
18
20
22
24
0
200
400
600
800
Time Point (0.25 Hz)
Cell 1880
Fluorescence (Raw)
10
15
20
0
200
400
600
800
Time Point (0.25 Hz)

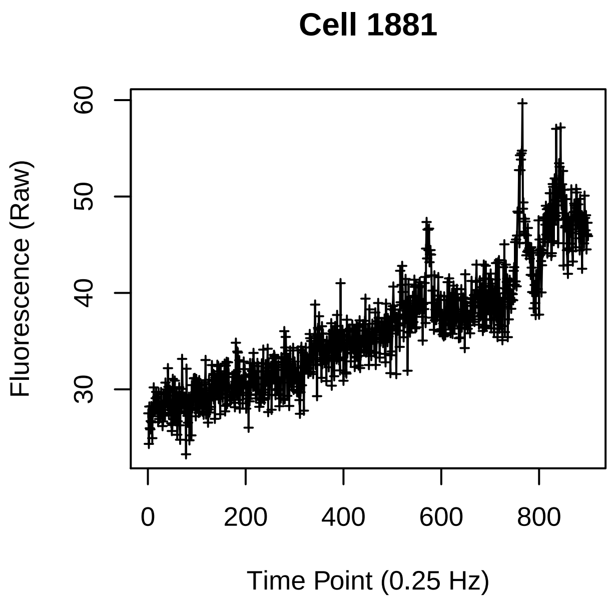
Cell 1881
Fluorescence (Raw)
30
40
50
60
0
200
400
600
800
Time Point (0.25 Hz)

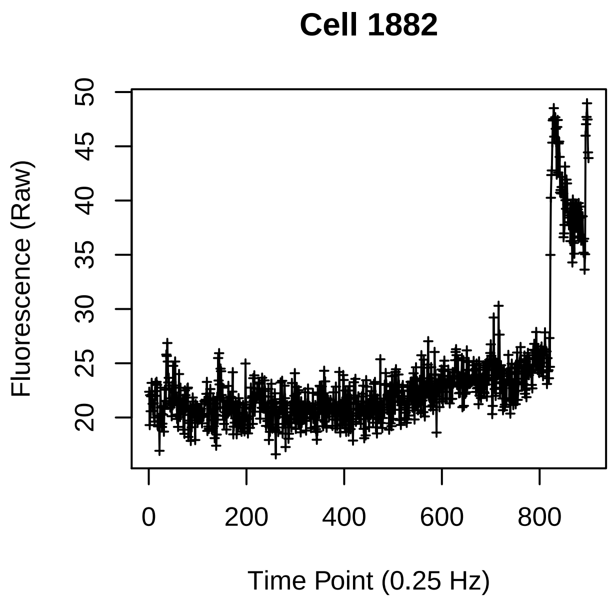
Cell 1882
Fluorescence (Raw)
20
25
30
35
40
45
50
0
200
400
600
800
Time Point (0.25 Hz)

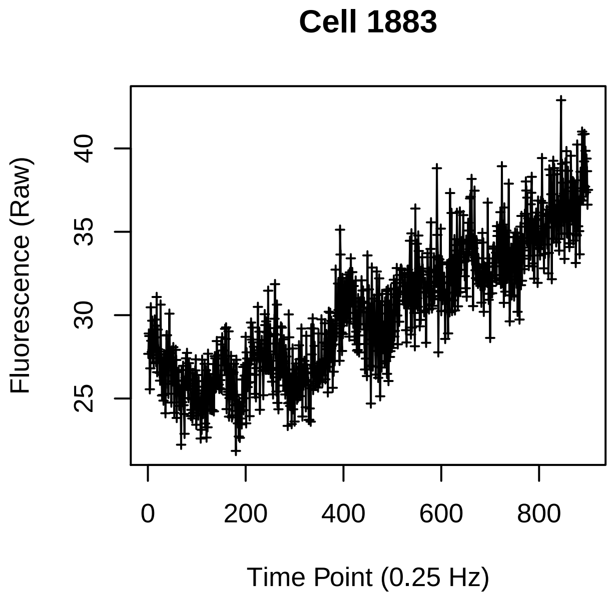
Cell 1883
Fluorescence (Raw)
25
30
35
40
0
200
400
600
800
Time Point (0.25 Hz)

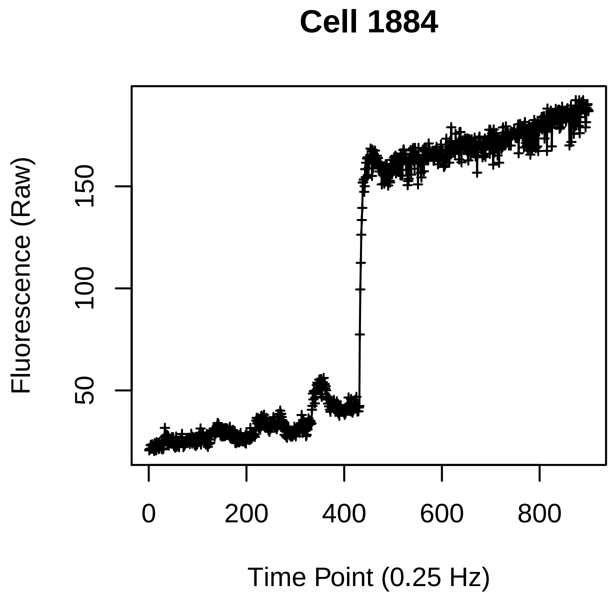
Cell 1884
Fluorescence (Raw)
50
100
150
0
200
400
600
800
Time Point (0.25 Hz)

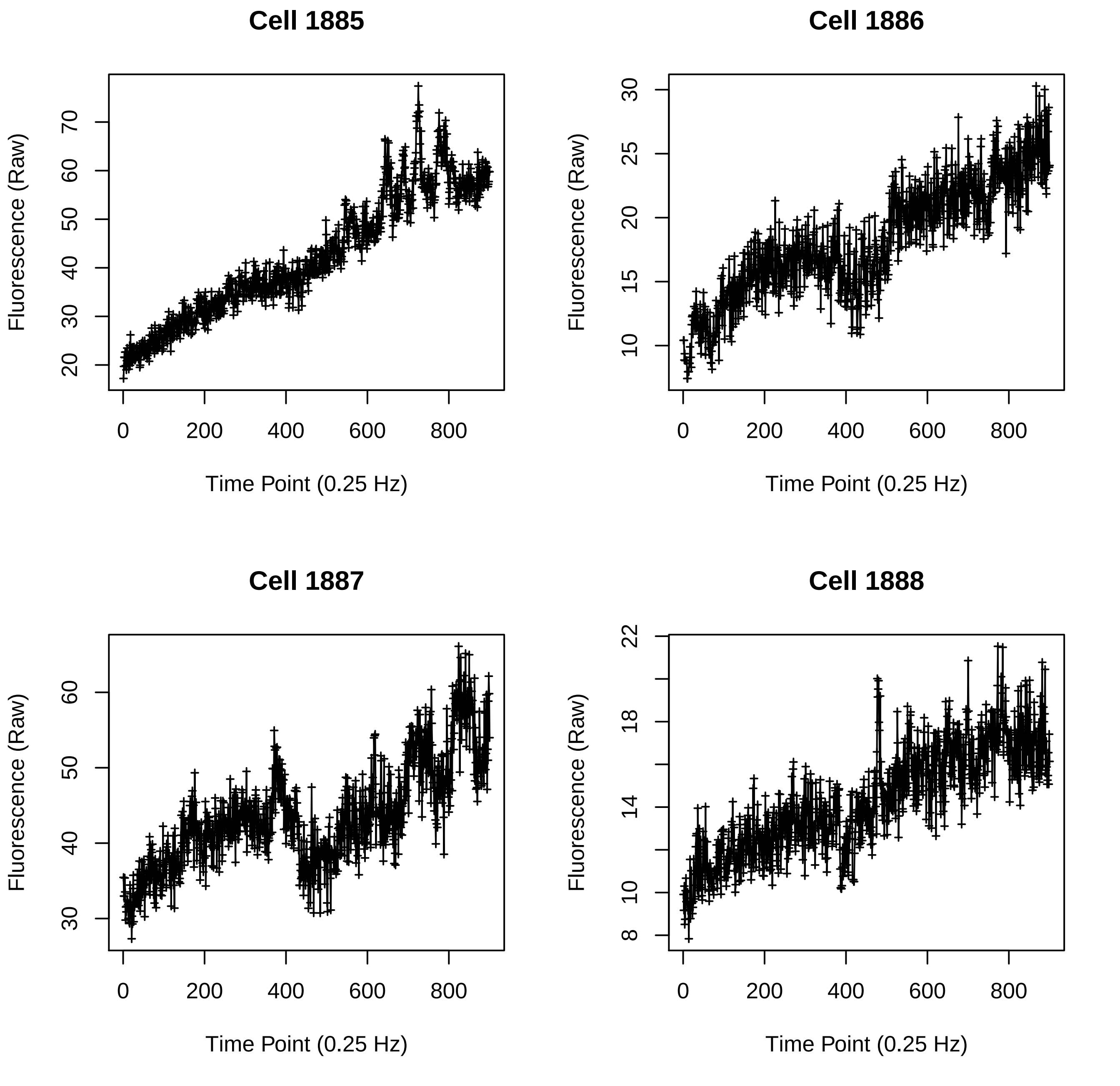

Cell 1885
Cell 1886
Cell 1887
Cell 1888
Fluorescence (Raw)
Time Point (0.25 Hz)
20
30
40
50
60
70
10
15
25
30
8
14
18
22
0
200
400
600
800

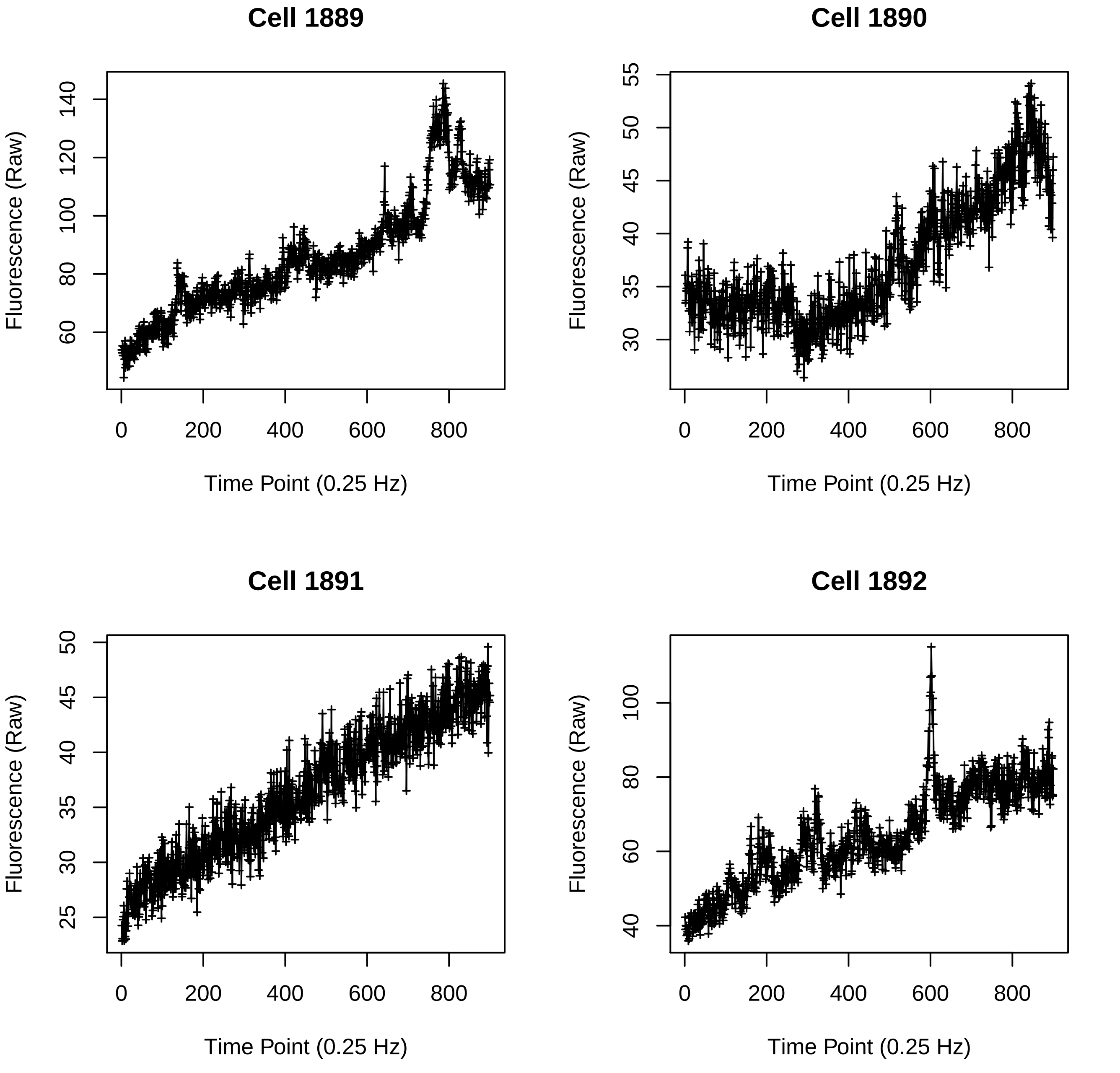

Cell 1889
Cell 1890
Cell 1891
Cell 1892
Fluorescence (Raw)
Time Point (0.25 Hz)
0
200
400
600
800
60
80
100
120
140
30
35
40
45
50
55
25
40

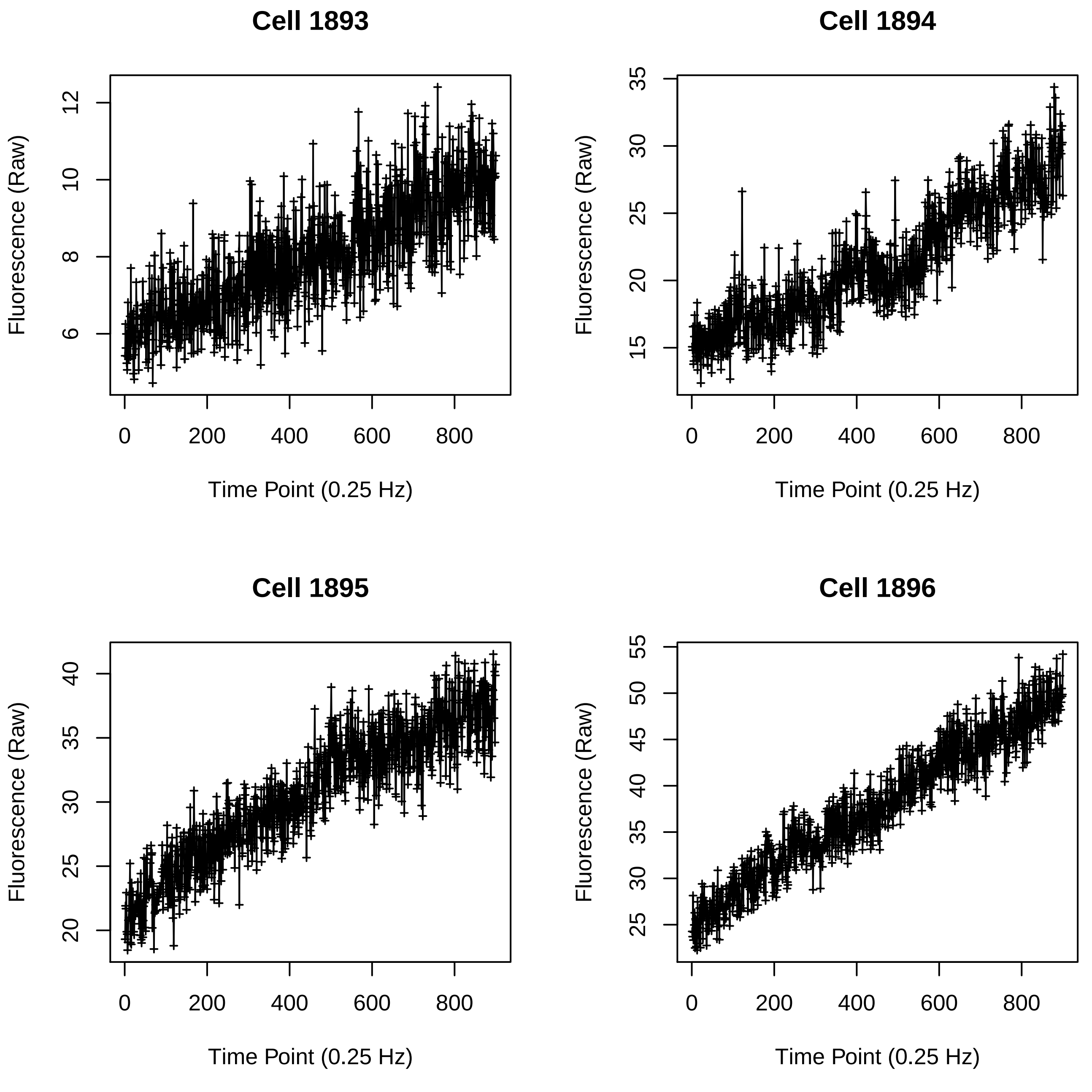

Cell 1893
Cell 1894
Cell 1895
Cell 1896
Fluorescence (Raw)
Time Point (0.25 Hz)
0
200
400
600
800
6
8
10
12
15
20
25
30
35
40
45
50
55

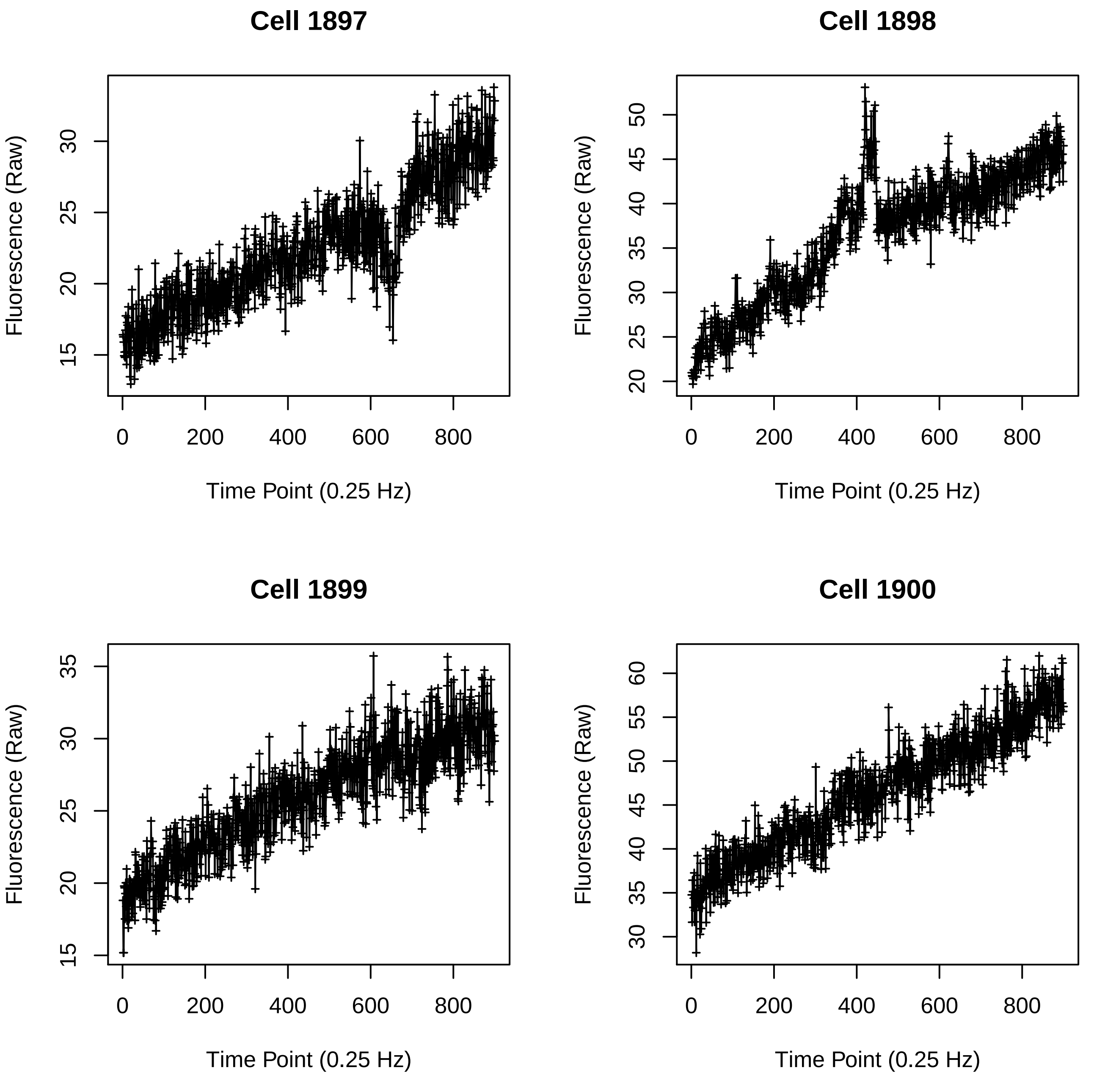

Cell 1897
Fluorescence (Raw)
15
20
25
30
0
200
400
600
800
Time Point (0.25 Hz)
Cell 1898
Fluorescence (Raw)
20
25
30
35
40
45
50
0
200
400
600
800
Time Point (0.25 Hz)
Cell 1899
Fluorescence (Raw)
15
20
25
30
35
0
200
400
600
800
Time Point (0.25 Hz)
Cell 1900
Fluorescence (Raw)
30
35
40
45
50
55
60
0
200
400
600
800
Time Point (0.25 Hz)

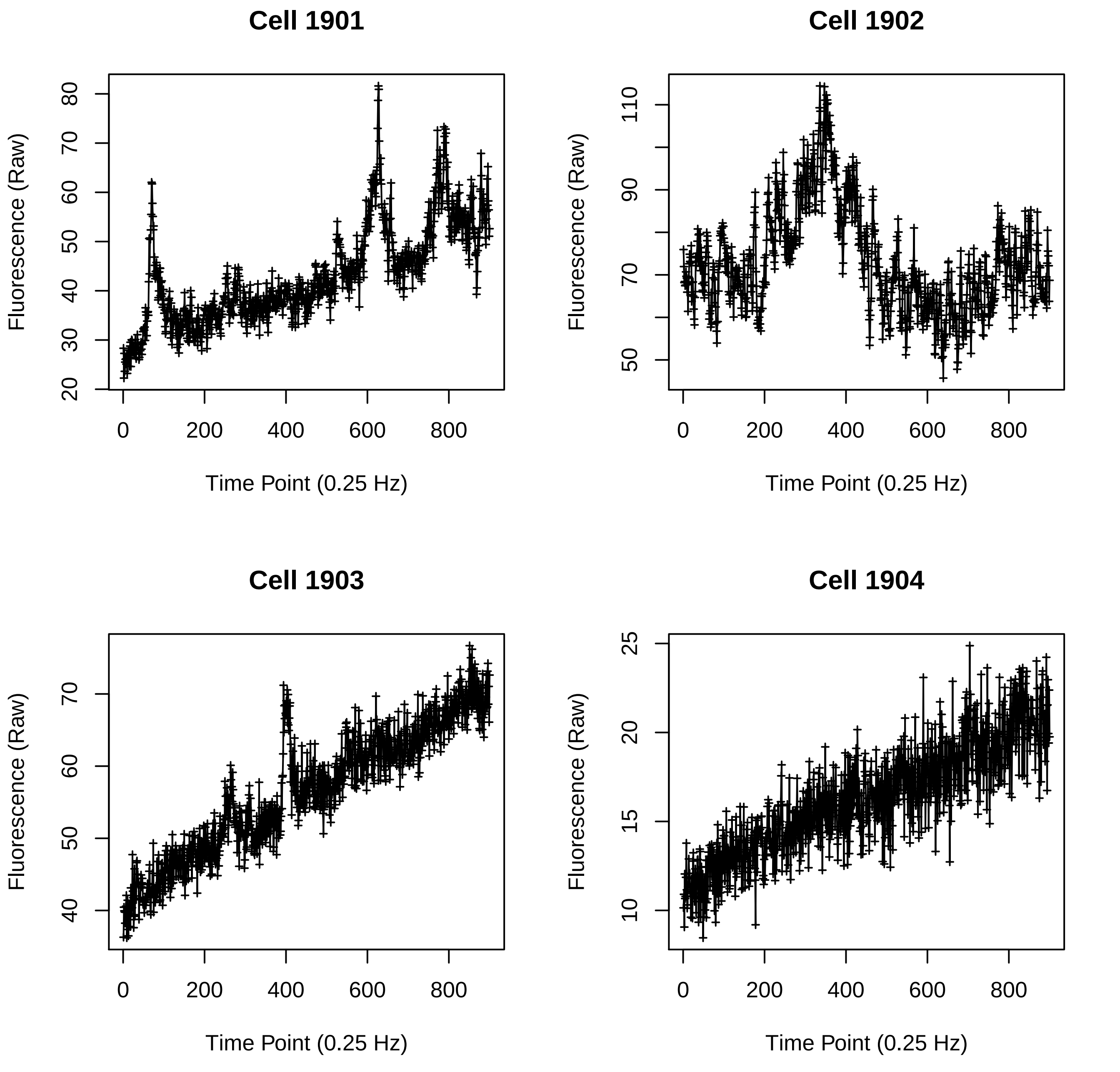
Cell 1901
Cell 1902
Cell 1903
Cell 1904
Fluorescence (Raw)
Time Point (0.25 Hz)
20
30
40
50
60
70
80
90
110
10
15
25
0
200
400
600
800

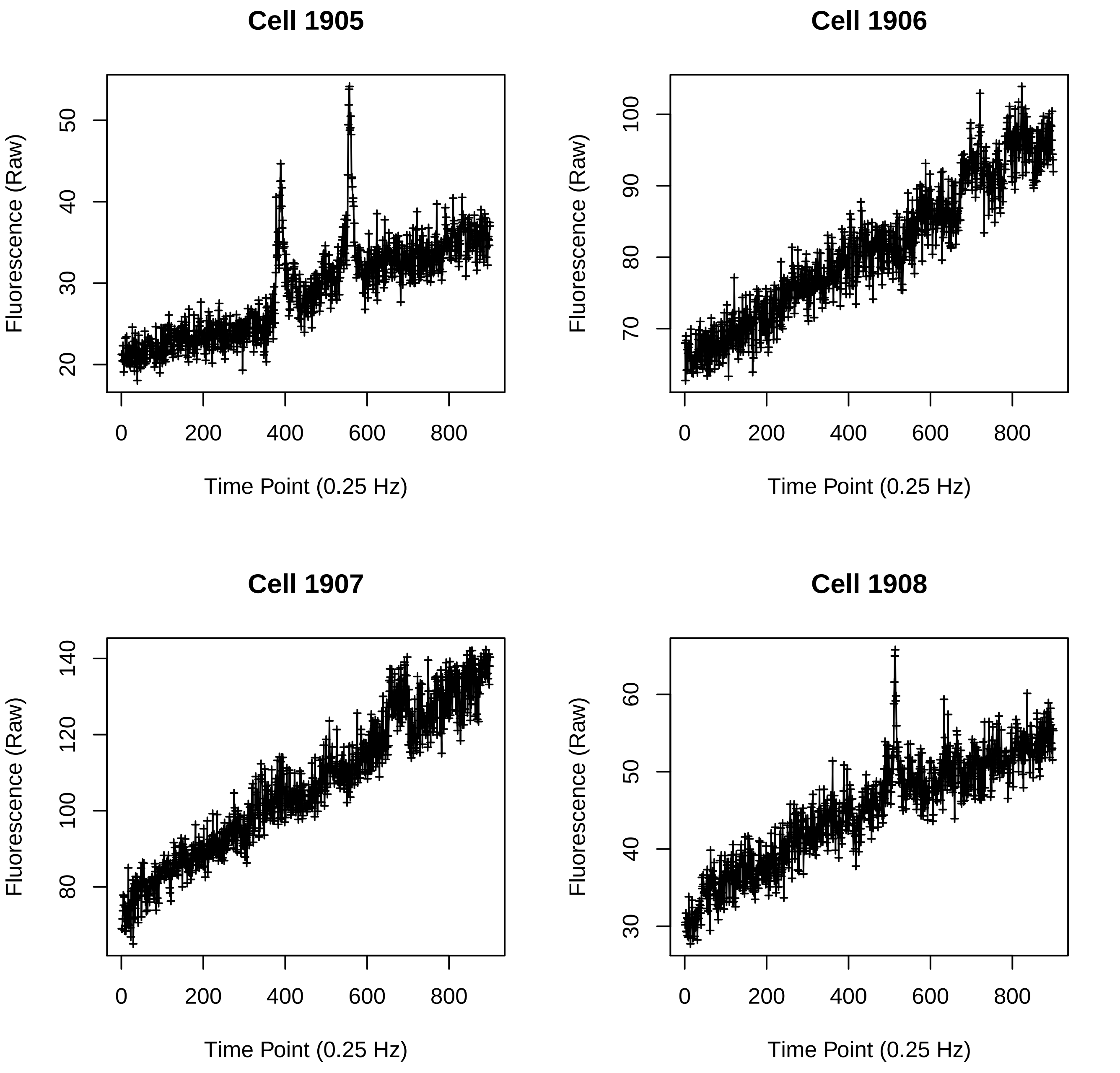

Cell 1905
Fluorescence (Raw)
20
30
40
50
0
200
400
600
800
Time Point (0.25 Hz)
Cell 1906
Fluorescence (Raw)
70
80
90
100
0
200
400
600
800
Time Point (0.25 Hz)
Cell 1907
Fluorescence (Raw)
80
100
120
140
0
200
400
600
800
Time Point (0.25 Hz)
Cell 1908
Fluorescence (Raw)
30
40
50
60
0
200
400
600
800
Time Point (0.25 Hz)

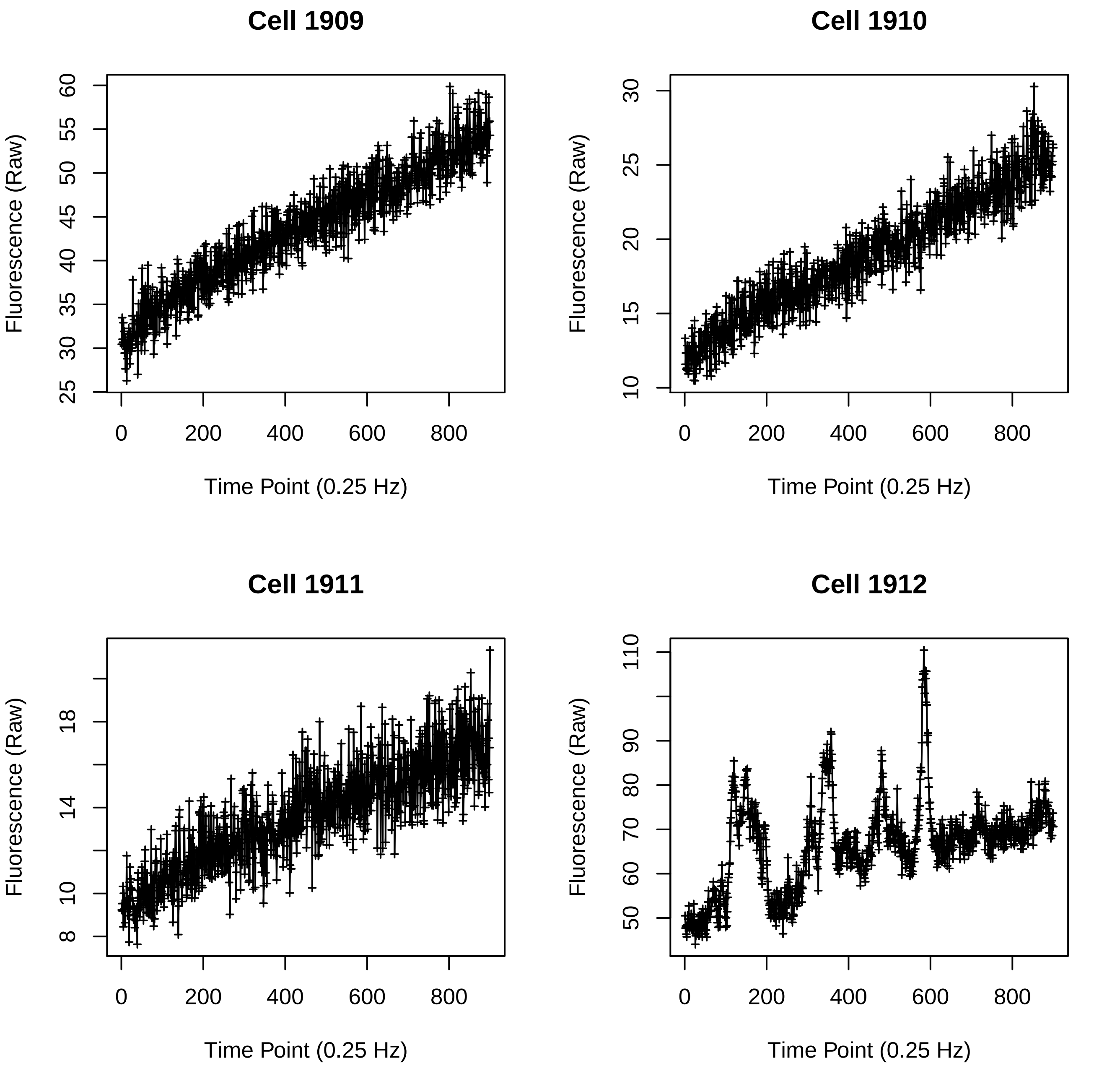
Cell 1909
Fluorescence (Raw)
25
30
35
40
45
50
55
60
0
200
400
600
800
Time Point (0.25 Hz)
Cell 1910
Fluorescence (Raw)
10
15
20
25
30
0
200
400
600
800
Time Point (0.25 Hz)
Cell 1911
Fluorescence (Raw)
8
10
14
18
0
200
400
600
800
Time Point (0.25 Hz)
Cell 1912
Fluorescence (Raw)
50
60
70
80
90
110
0
200
400
600
800
Time Point (0.25 Hz)

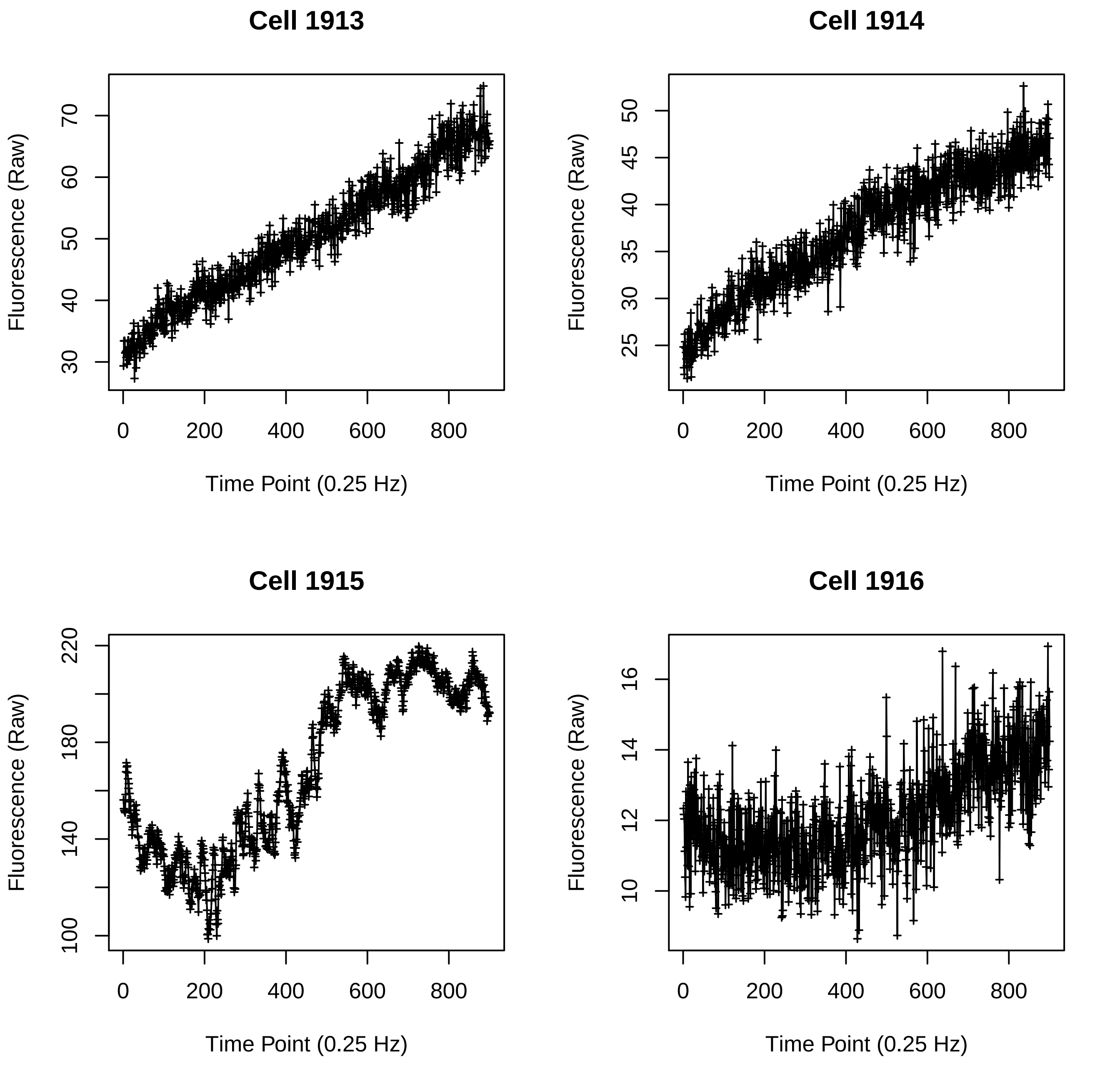
Cell 1913
Cell 1914
Cell 1915
Cell 1916
Fluorescence (Raw)
Time Point (0.25 Hz)
0
200
400
600
800
30
40
50
60
70
25
35
45
100
140
180
220
10
12
14
16

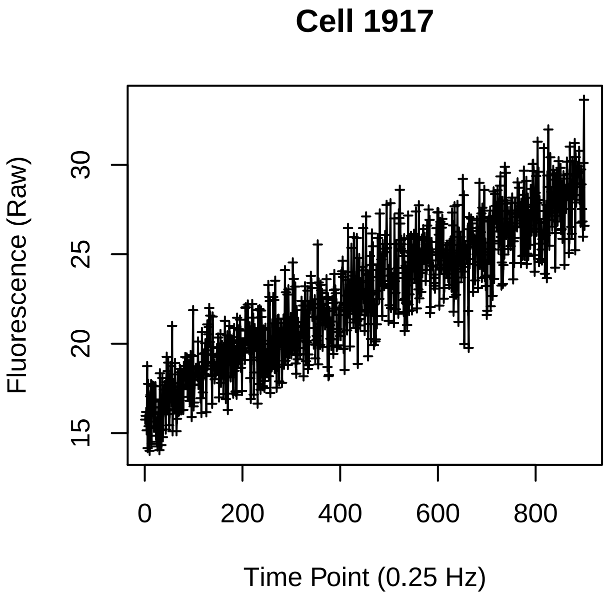
Cell 1917
Fluorescence (Raw)
15
20
25
30
0
200
400
600
800
Time Point (0.25 Hz)

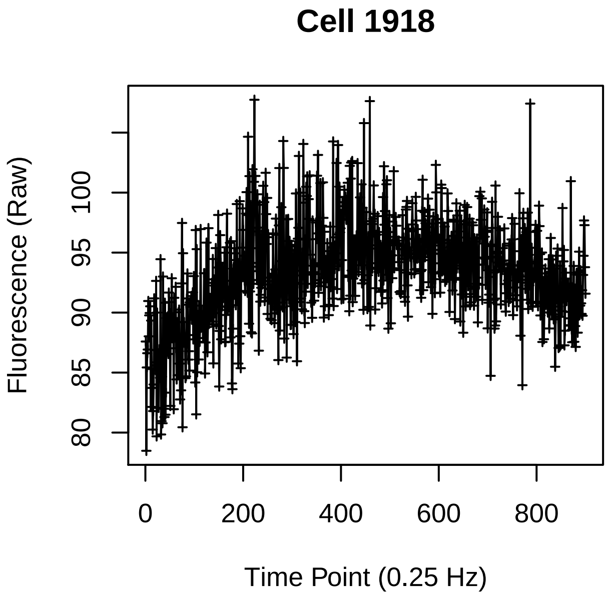
Cell 1918
Fluorescence (Raw)
80
85
90
95
100
0
200
400
600
800
Time Point (0.25 Hz)

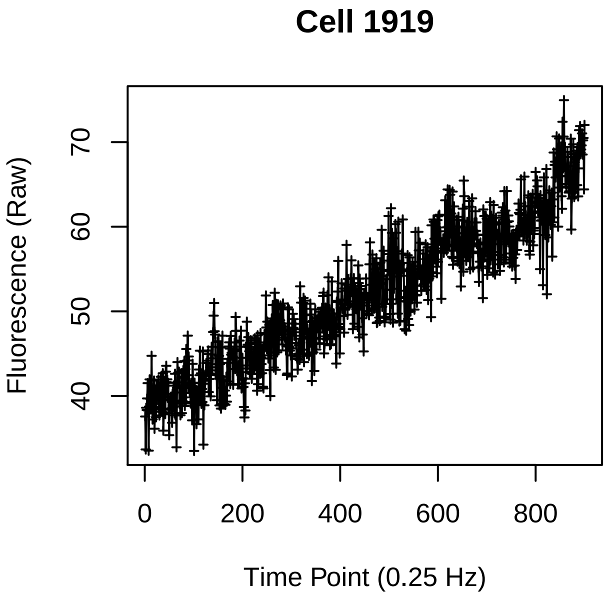
Cell 1919
Fluorescence (Raw)
40
50
60
70
0
200
400
600
800
Time Point (0.25 Hz)

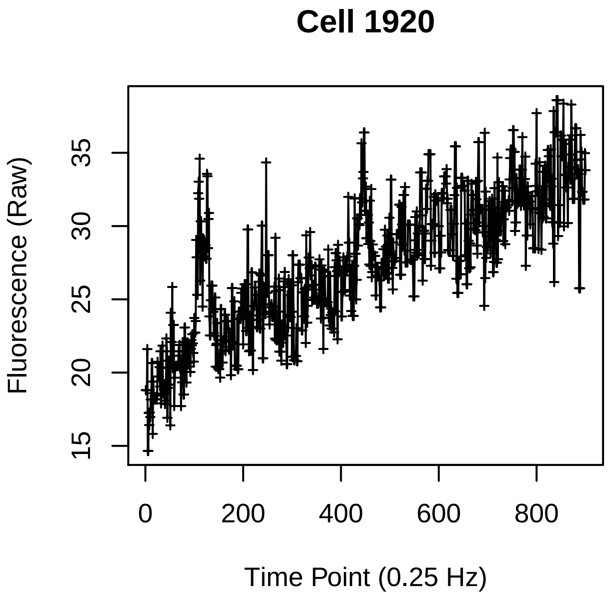
Cell 1920
Fluorescence (Raw)
15
20
25
30
35
0
200
400
600
800
Time Point (0.25 Hz)

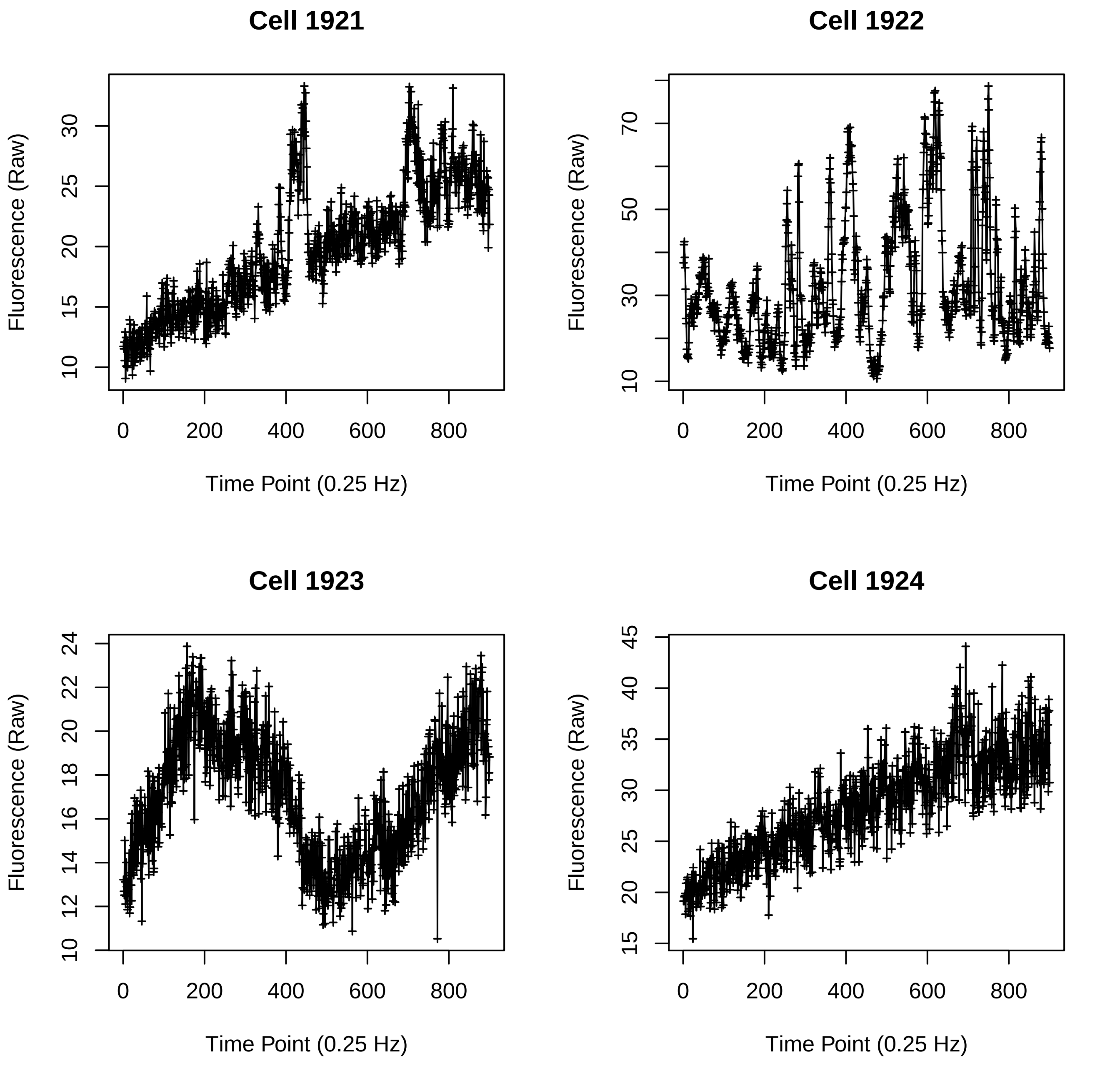
Cell 1921
Cell 1922
Cell 1923
Cell 1924
Fluorescence (Raw)
Time Point (0.25 Hz)
0
200
400
600
800
10
15
20
25
30
50
70
12
14
16
18
22
24
35
40
45

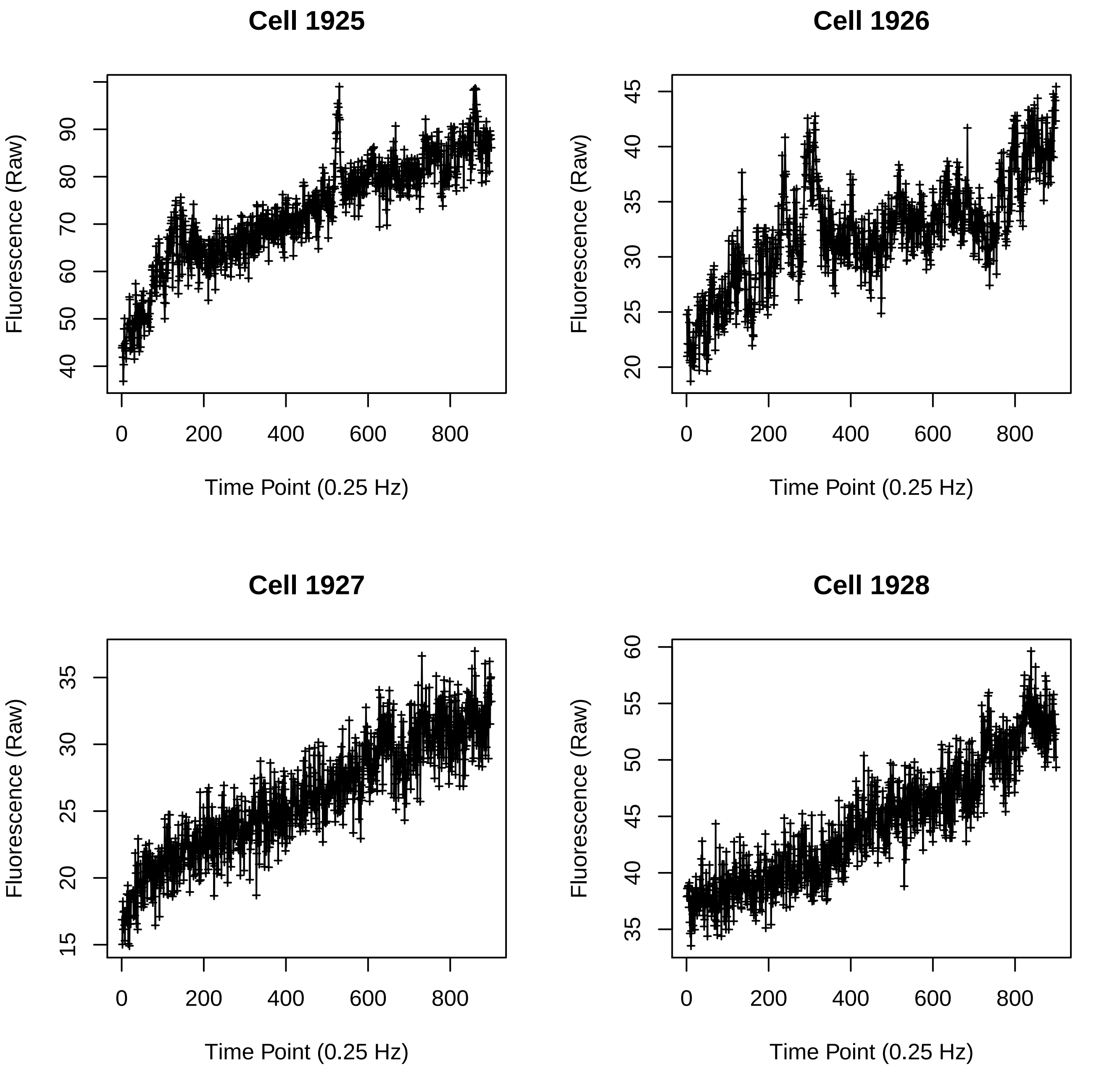
Cell 1925
Cell 1926
Cell 1927
Cell 1928
Fluorescence (Raw)
Time Point (0.25 Hz)
0
200
400
600
800
40
50
60
70
80
90
20
25
30
35
40
45
15
20
25
30
35
35
40
45
50
55
60

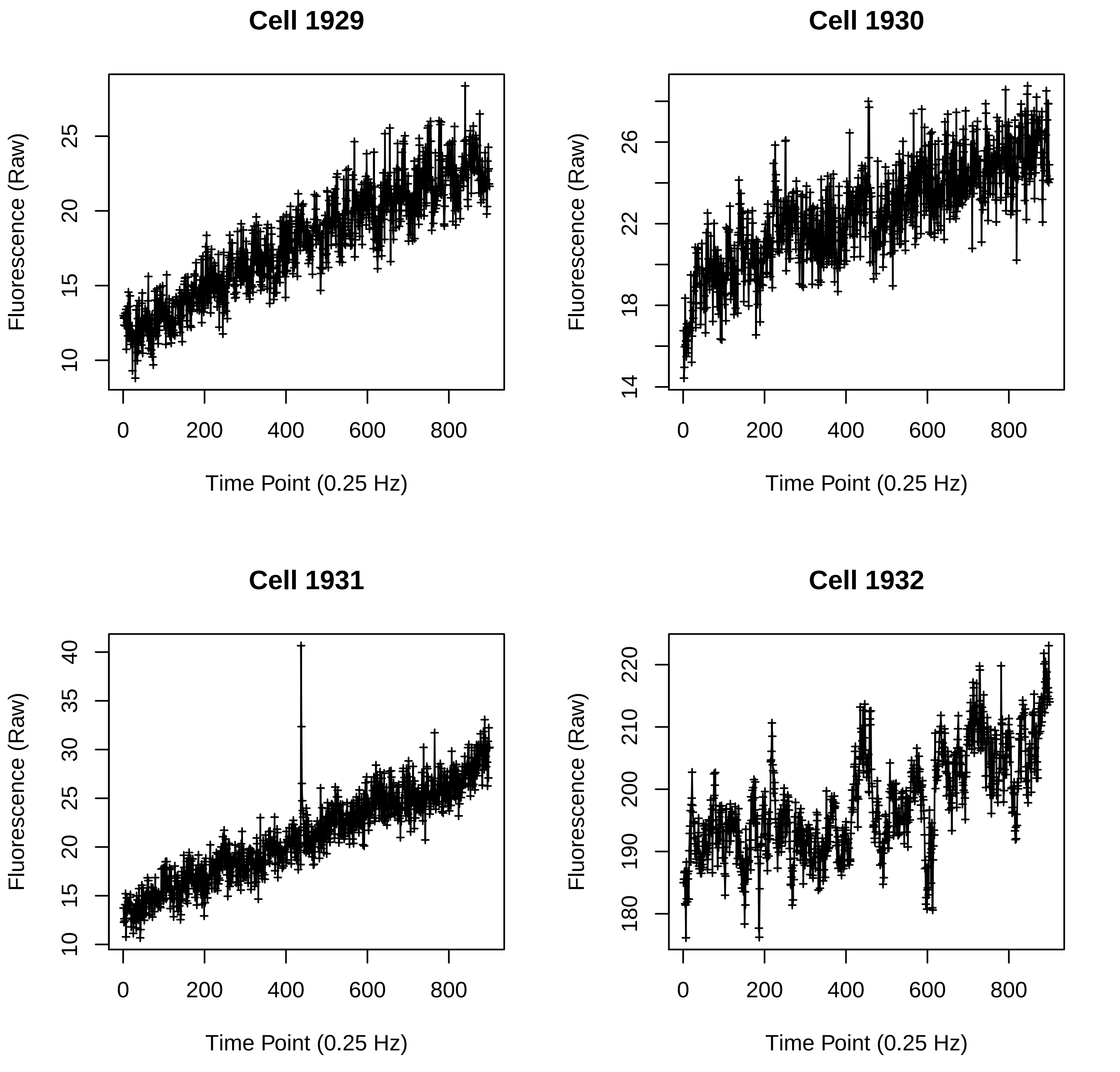

Cell 1929
Cell 1930
Cell 1931
Cell 1932
Fluorescence (Raw)
Time Point (0.25 Hz)
0
200
400
600
800
10
15
20
25
14
18
22
26
30
35
40
180
190
200
210
220

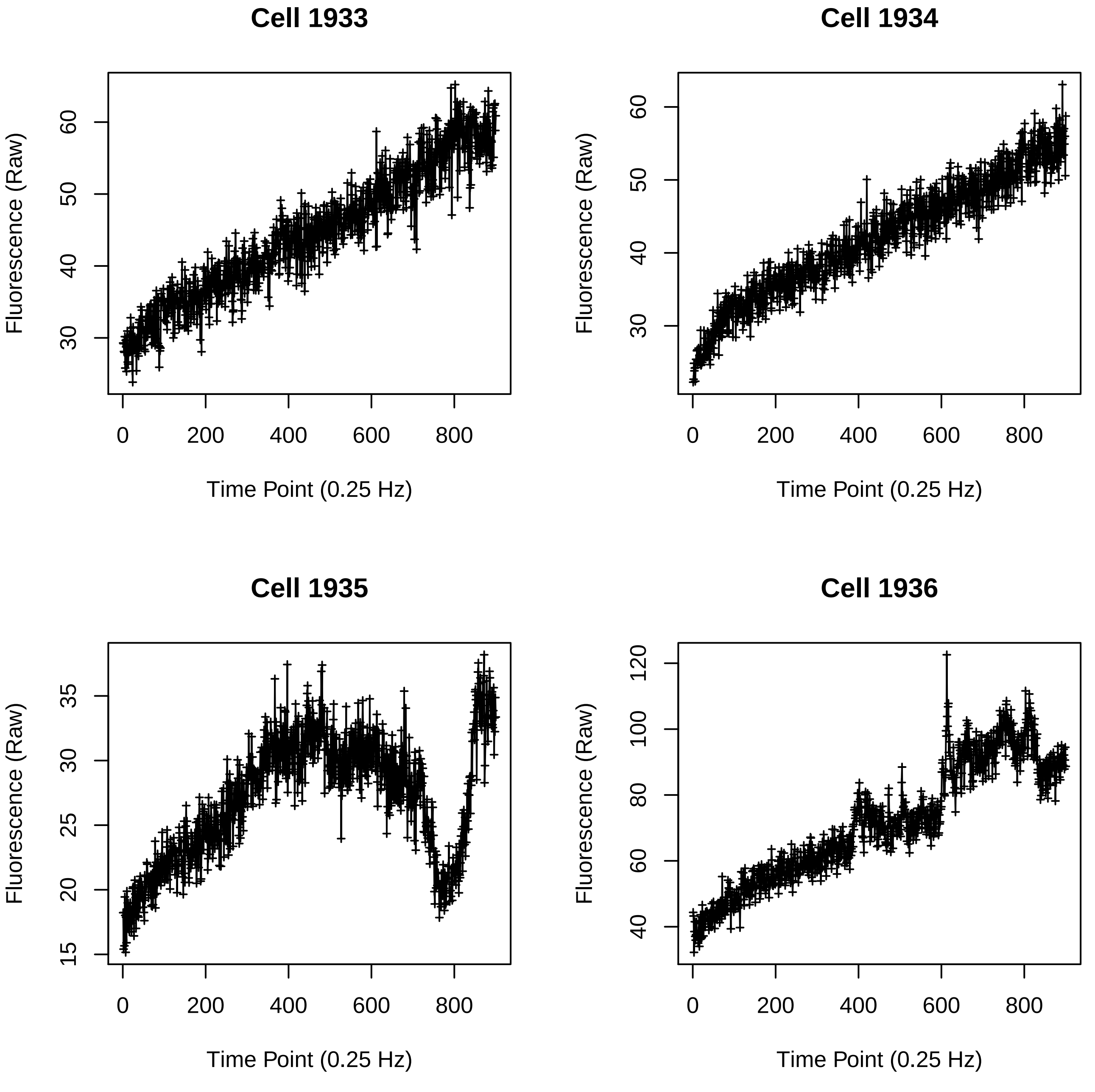
Cell 1933
Cell 1934
Cell 1935
Cell 1936
Fluorescence (Raw)
Time Point (0.25 Hz)
0
200
400
600
800
30
40
50
60
15
20
25
35
80
100
120

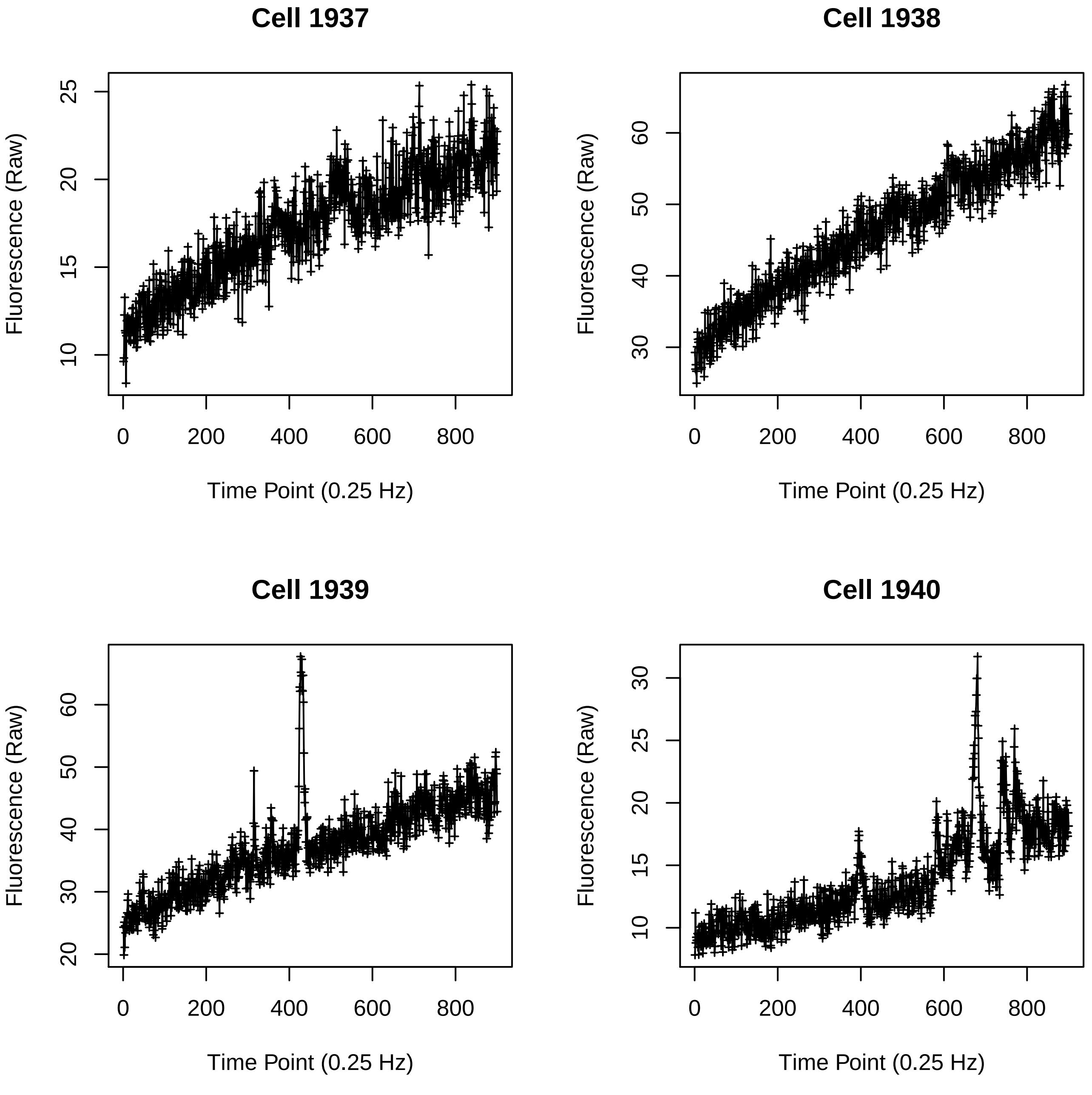
Cell 1937
Fluorescence (Raw)
10
15
20
25
0
200
400
600
800
Time Point (0.25 Hz)
Cell 1938
Fluorescence (Raw)
30
40
50
60
0
200
400
600
800
Time Point (0.25 Hz)
Cell 1939
Fluorescence (Raw)
20
30
40
50
60
0
200
400
600
800
Time Point (0.25 Hz)
Cell 1940
Fluorescence (Raw)
10
15
20
25
30
0
200
400
600
800
Time Point (0.25 Hz)

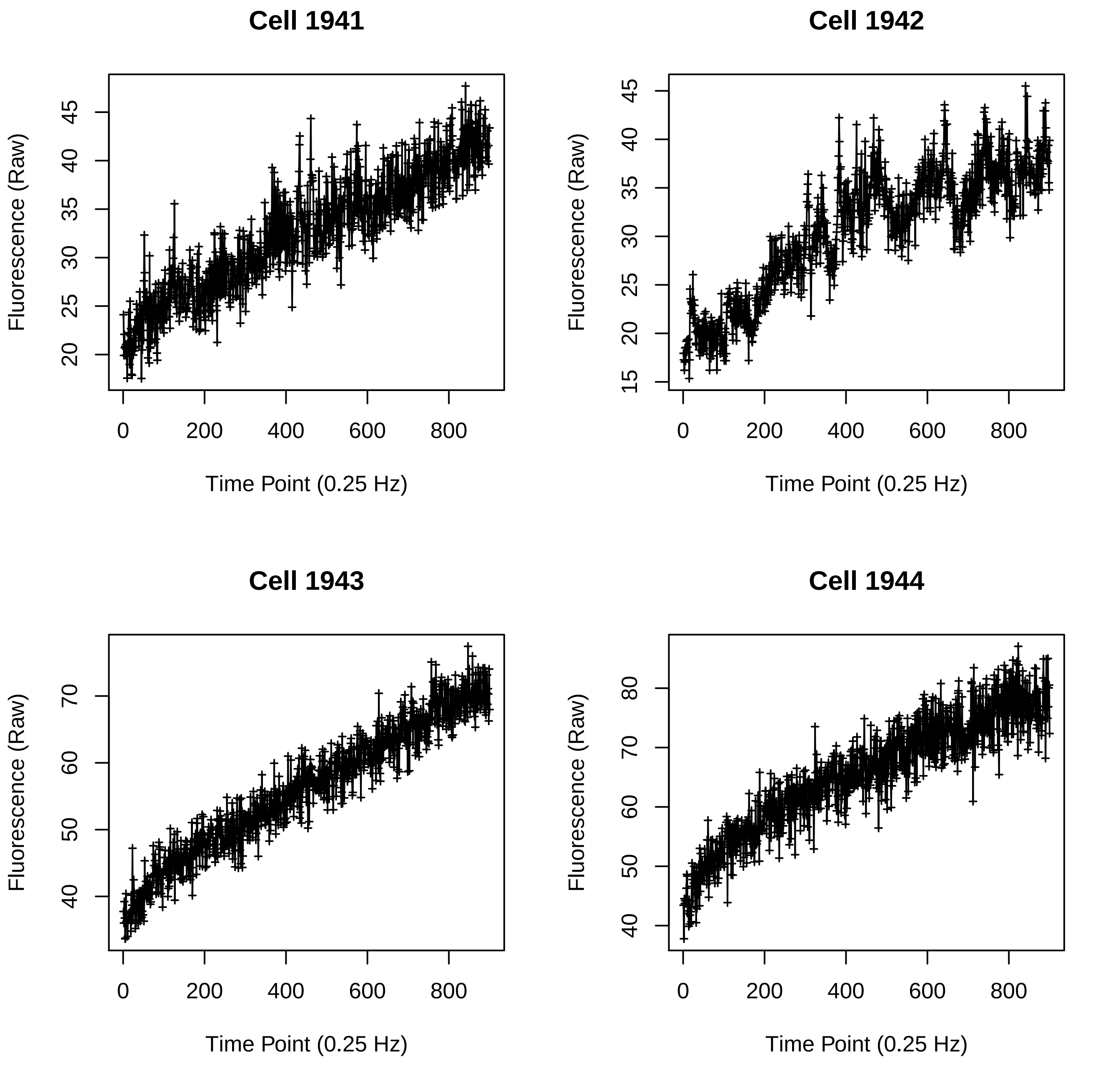

Cell 1941
Cell 1942
Cell 1943
Cell 1944
Fluorescence (Raw)
Time Point (0.25 Hz)
0
200
400
600
800
15
20
25
30
35
40
45
50
60
70
80

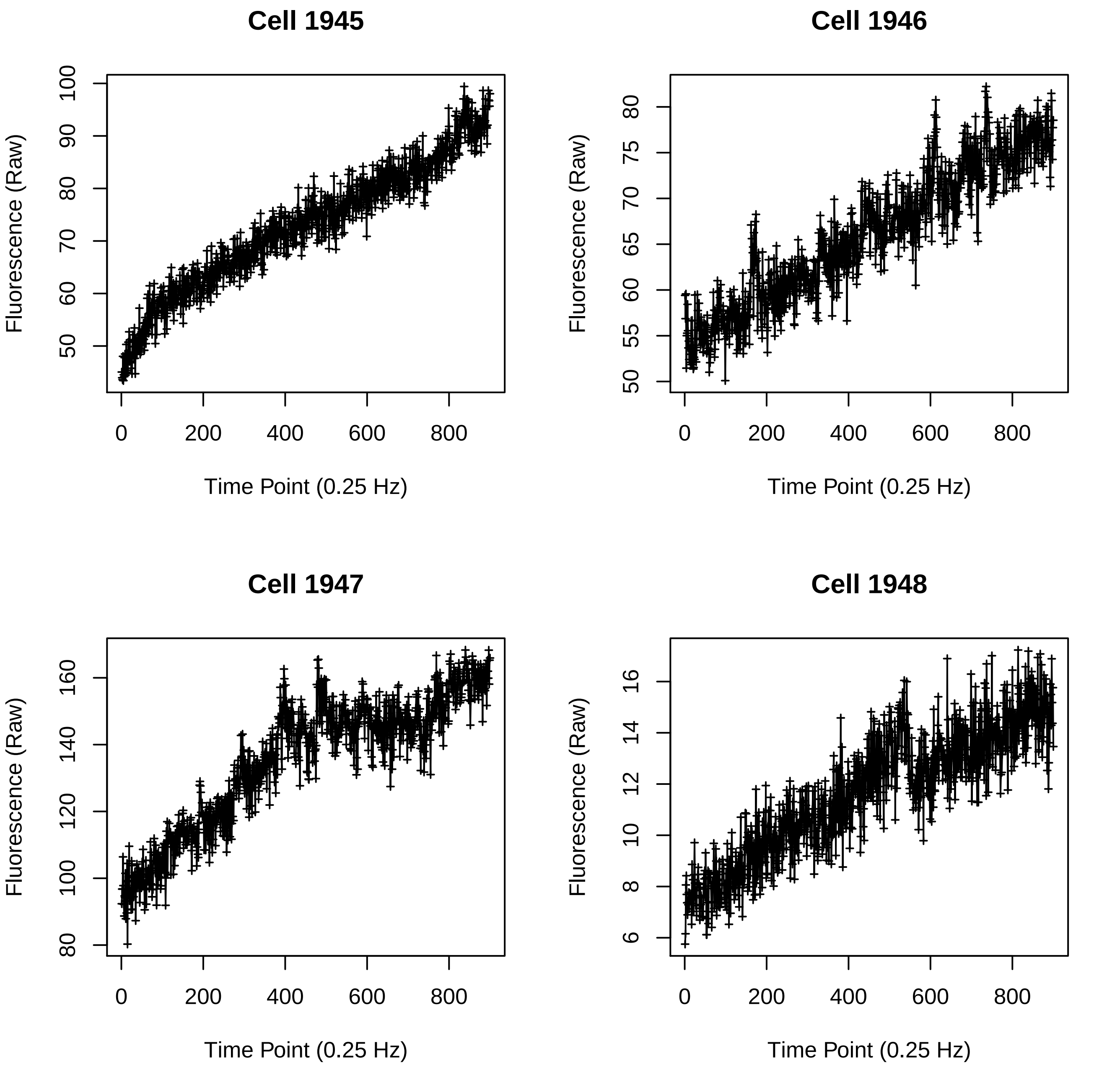
Cell 1945
Fluorescence (Raw)
50
60
70
80
90
100
0
200
400
600
800
Time Point (0.25 Hz)
Cell 1946
Fluorescence (Raw)
50
55
60
65
70
75
80
0
200
400
600
800
Time Point (0.25 Hz)
Cell 1947
Fluorescence (Raw)
80
100
120
140
160
0
200
400
600
800
Time Point (0.25 Hz)
Cell 1948
Fluorescence (Raw)
6
8
10
12
14
16
0
200
400
600
800
Time Point (0.25 Hz)

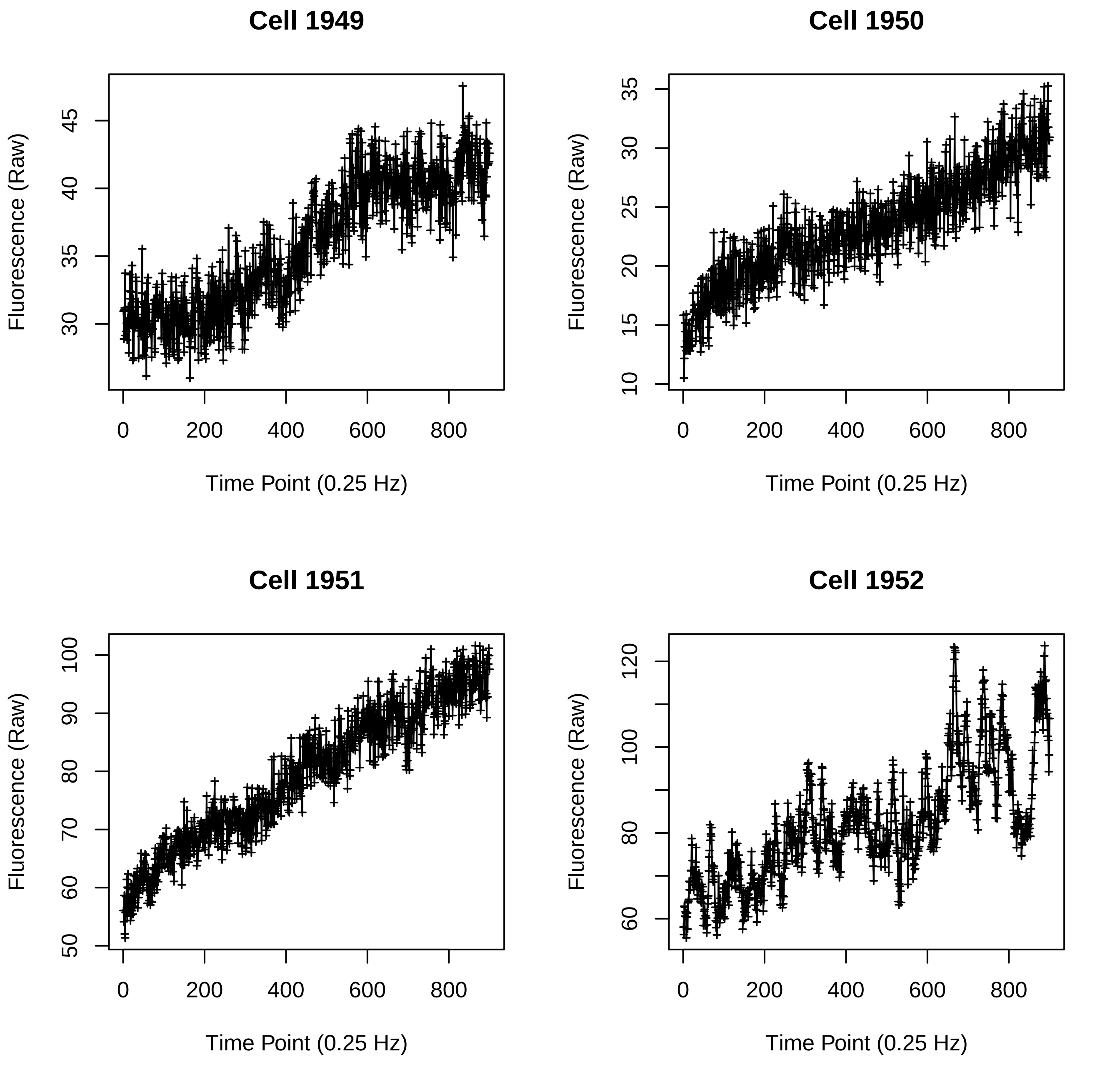
Cell 1949
Cell 1950
Cell 1951
Cell 1952
Fluorescence (Raw)
Time Point (0.25 Hz)
30
35
40
45
10
15
20
25
30
35
50
60
70
80
90
100
60
80
100
120
0
200
400
600
800

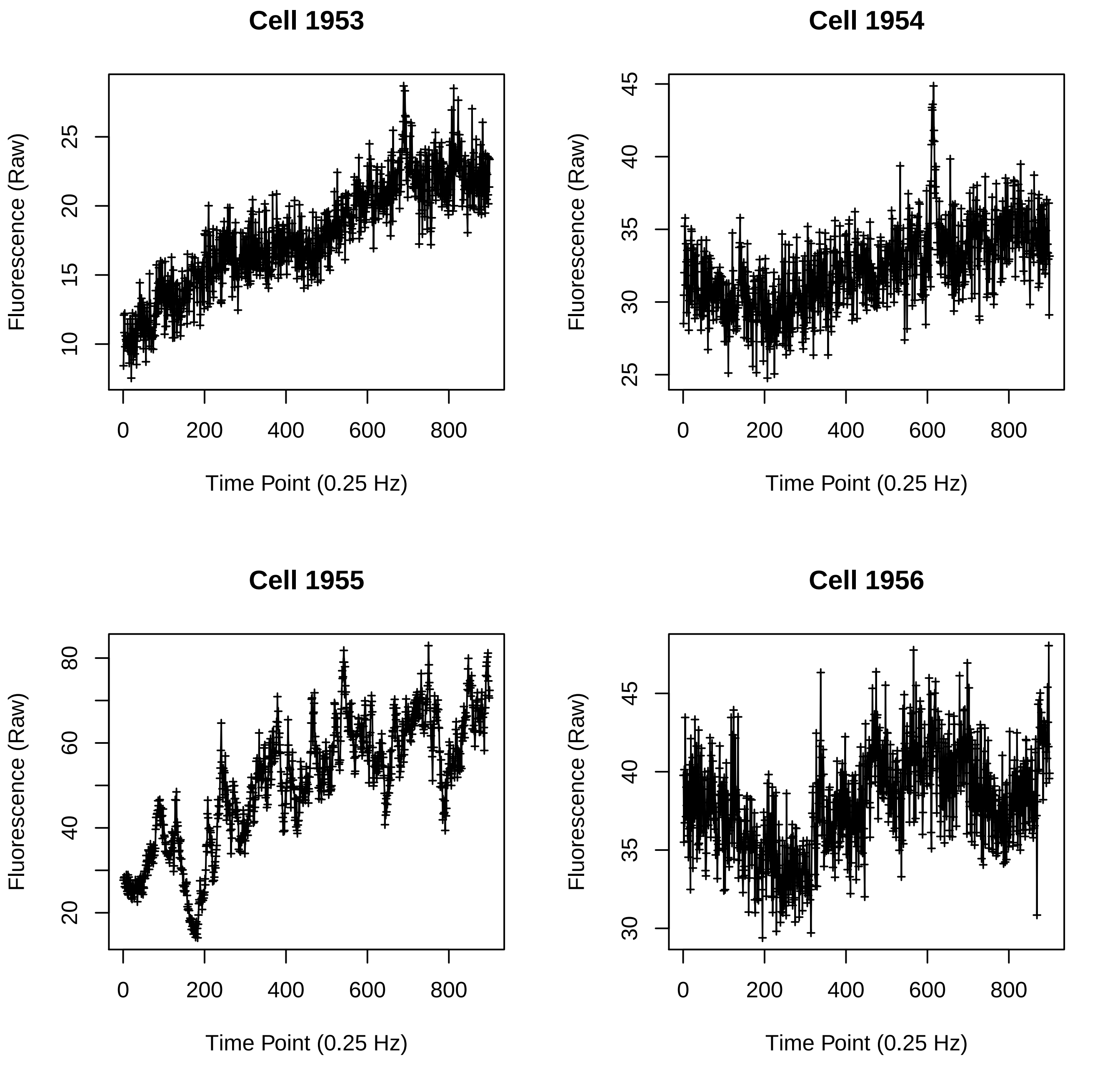
Cell 1953
Fluorescence (Raw)
10
15
20
25
0
200
400
600
800
Time Point (0.25 Hz)
Cell 1954
Fluorescence (Raw)
25
30
35
40
45
0
200
400
600
800
Time Point (0.25 Hz)
Cell 1955
Fluorescence (Raw)
20
40
60
80
0
200
400
600
800
Time Point (0.25 Hz)
Cell 1956
Fluorescence (Raw)
30
35
40
45
0
200
400
600
800
Time Point (0.25 Hz)

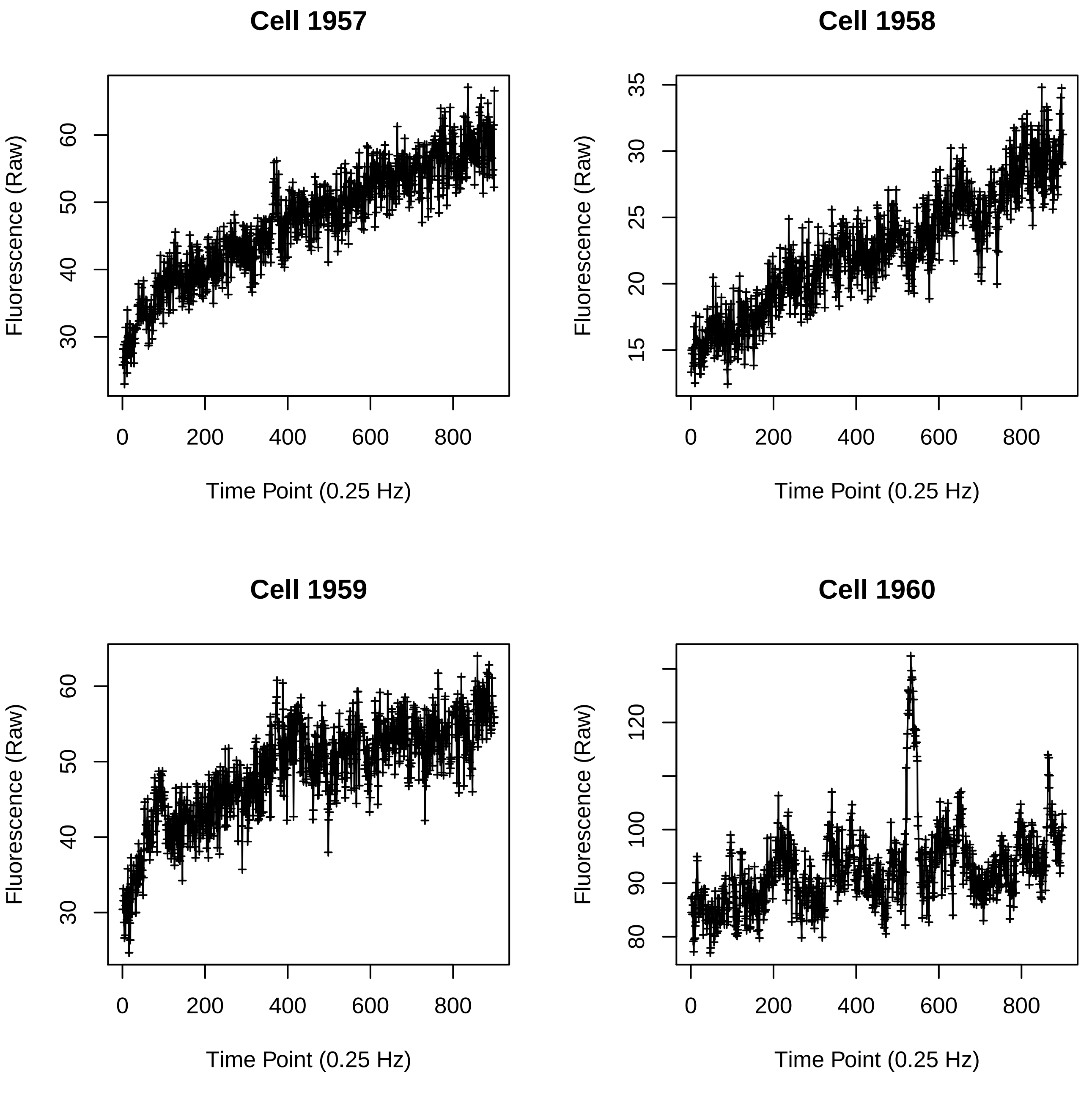
Cell 1957
Cell 1958
Cell 1959
Cell 1960
Fluorescence (Raw)
Time Point (0.25 Hz)
0
200
400
600
800
30
40
50
60
15
20
25
30
35
80
90
100
120

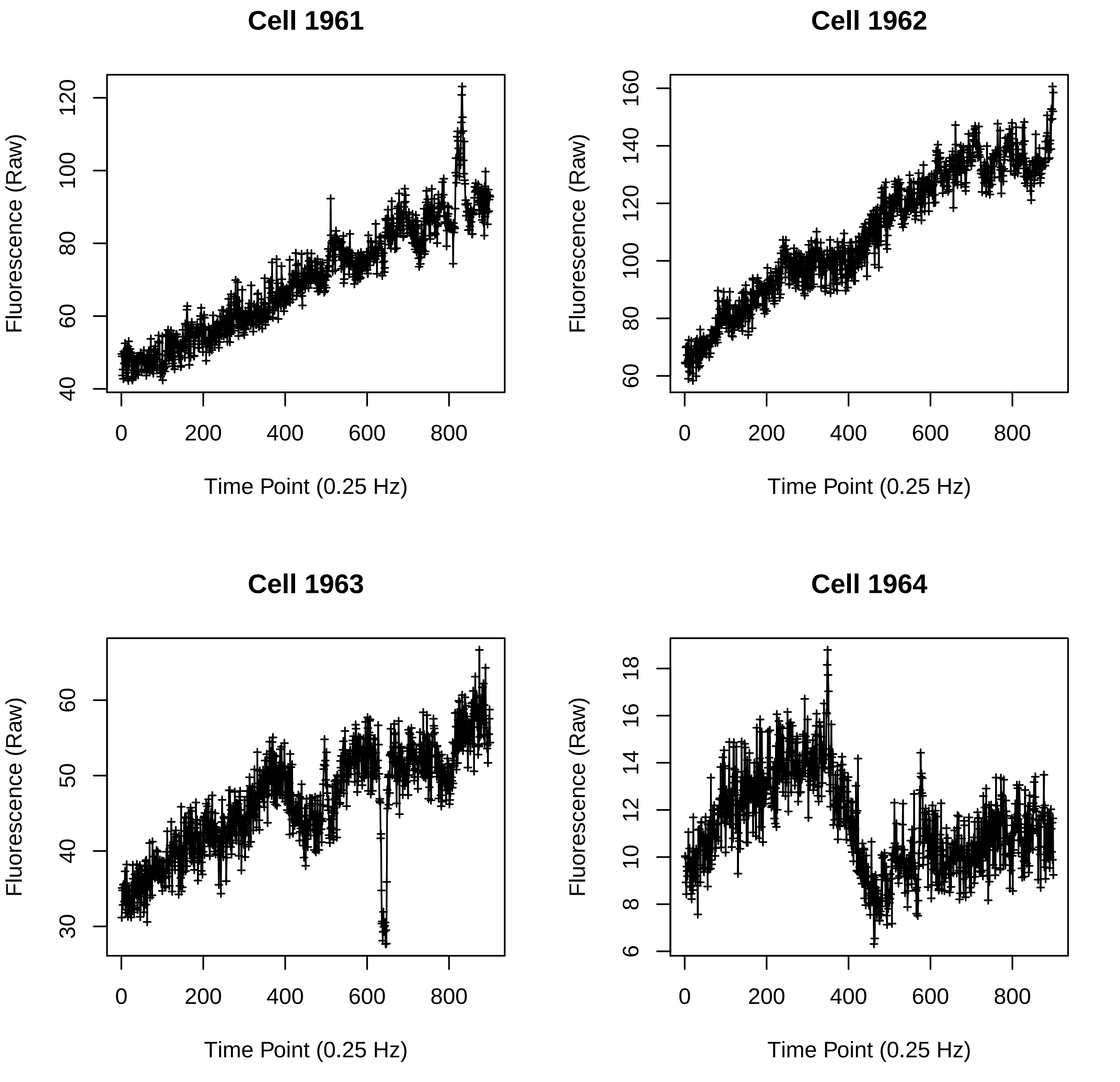

Cell 1961
Fluorescence (Raw)
40
60
80
100
120
0
200
400
600
800
Time Point (0.25 Hz)
Cell 1962
Fluorescence (Raw)
60
80
100
120
140
160
0
200
400
600
800
Time Point (0.25 Hz)
Cell 1963
Fluorescence (Raw)
30
40
50
60
0
200
400
600
800
Time Point (0.25 Hz)
Cell 1964
Fluorescence (Raw)
6
8
10
12
14
16
18
0
200
400
600
800
Time Point (0.25 Hz)

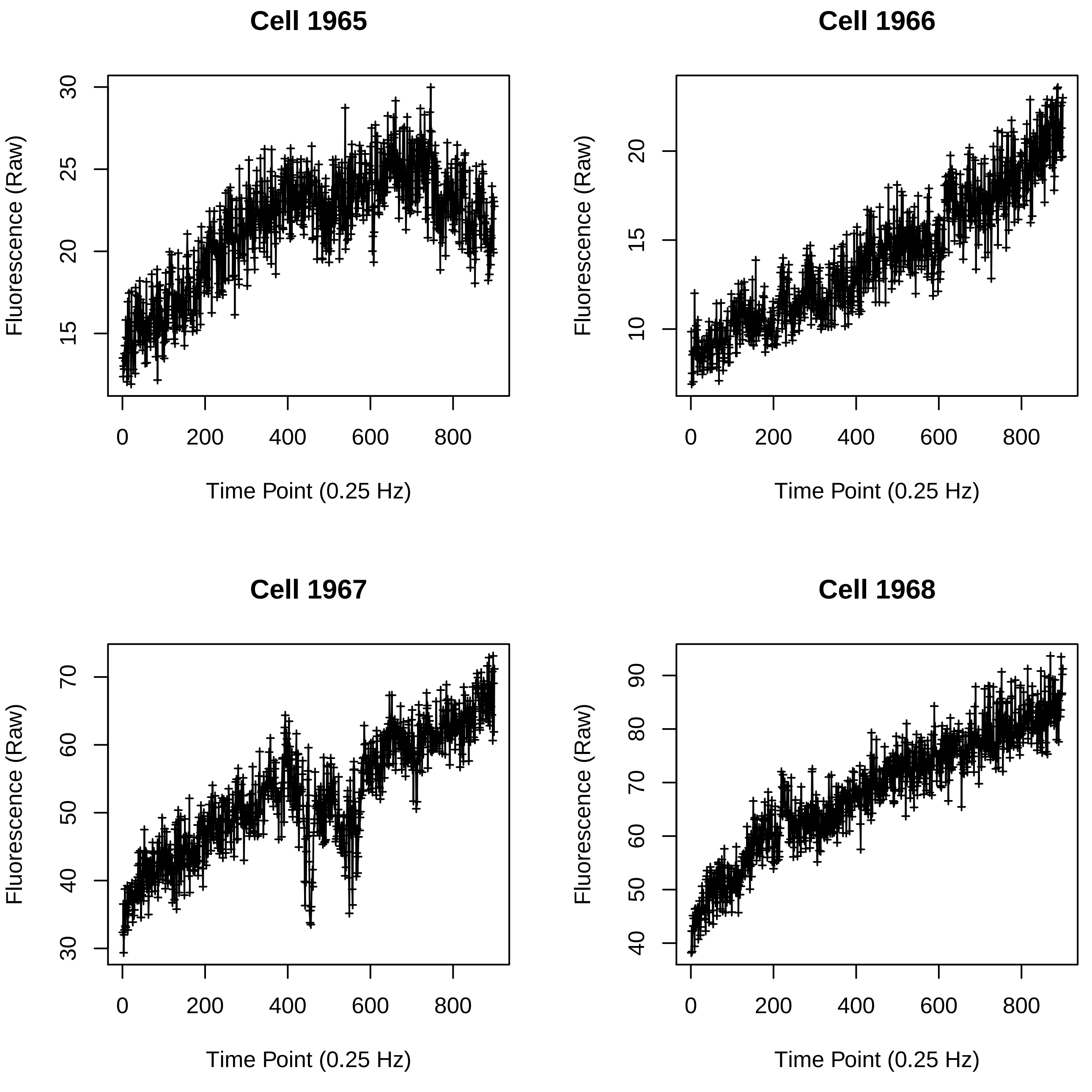

Cell 1965
Cell 1966
Cell 1967
Cell 1968
Fluorescence (Raw)
Time Point (0.25 Hz)
30
25
20
15
10
70
60
50
40
90
80
0
200
400
600
800

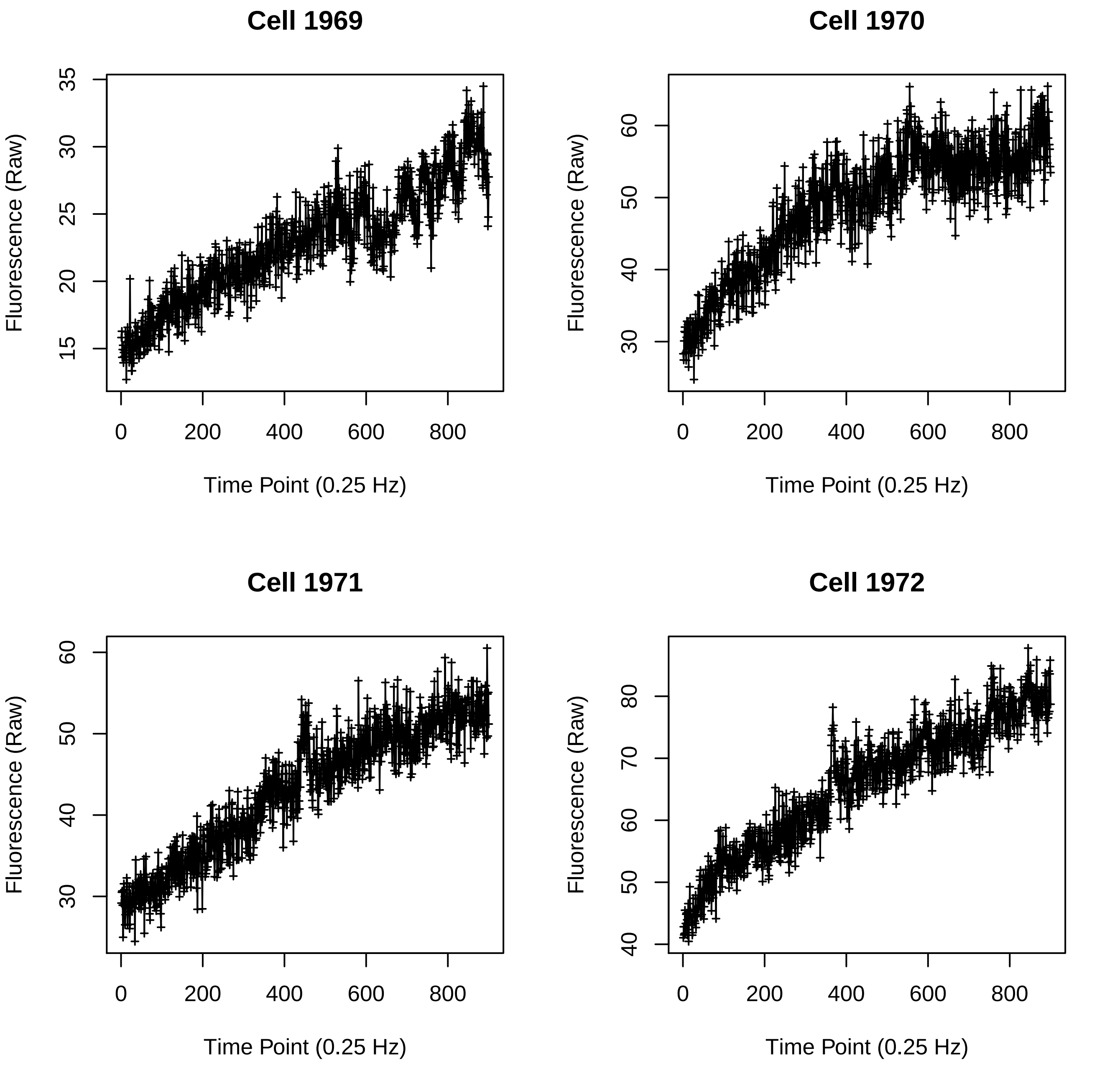
Cell 1969
Fluorescence (Raw)
Time Point (0.25 Hz)
15
20
25
30
35
0
200
400
600
800
Cell 1970
Fluorescence (Raw)
Time Point (0.25 Hz)
30
40
50
60
0
200
400
600
800
Cell 1971
Fluorescence (Raw)
Time Point (0.25 Hz)
30
40
50
60
0
200
400
600
800
Cell 1972
Fluorescence (Raw)
Time Point (0.25 Hz)
40
50
60
70
80
0
200
400
600
800

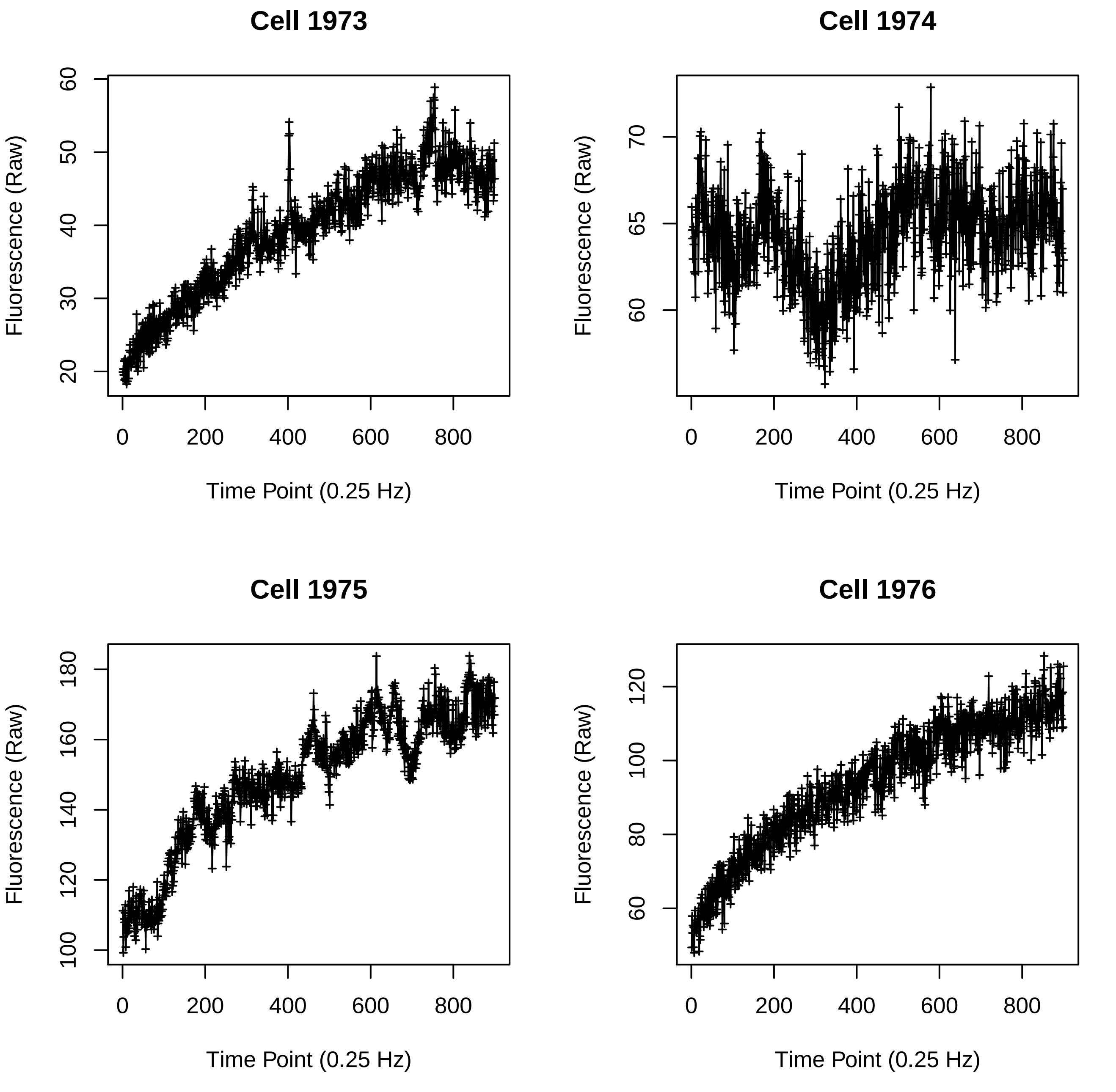
Cell 1973
Cell 1974
Cell 1975
Cell 1976
Fluorescence (Raw)
Time Point (0.25 Hz)
20
30
40
50
60
65
70
100
120
140
160
180
80
0
200
400
600
800

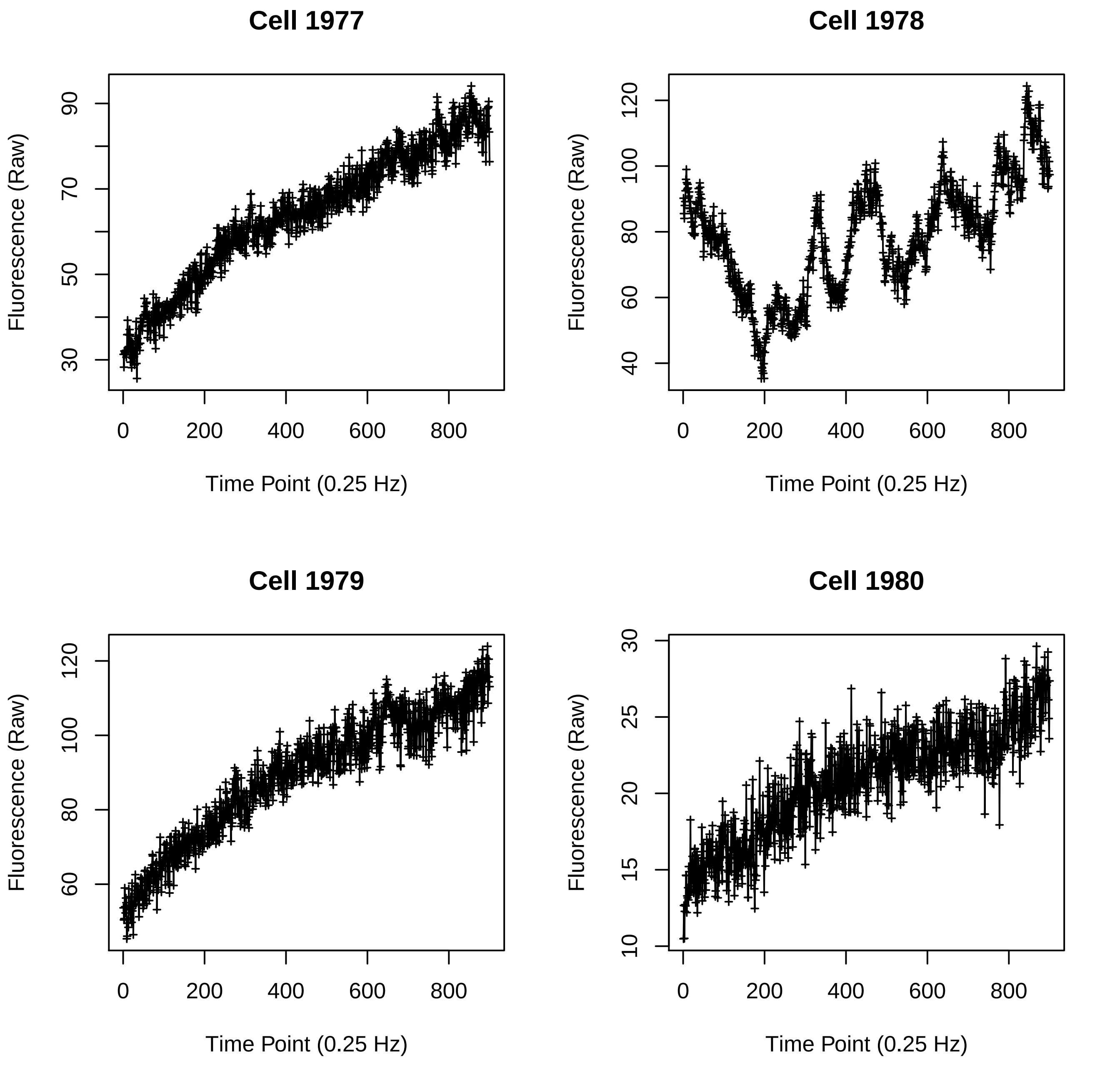

Cell 1977
Cell 1978
Cell 1979
Cell 1980
Fluorescence (Raw)
Time Point (0.25 Hz)
0
200
400
600
800
30
50
70
90
40
60
80
100
120
10
15
20
25
30

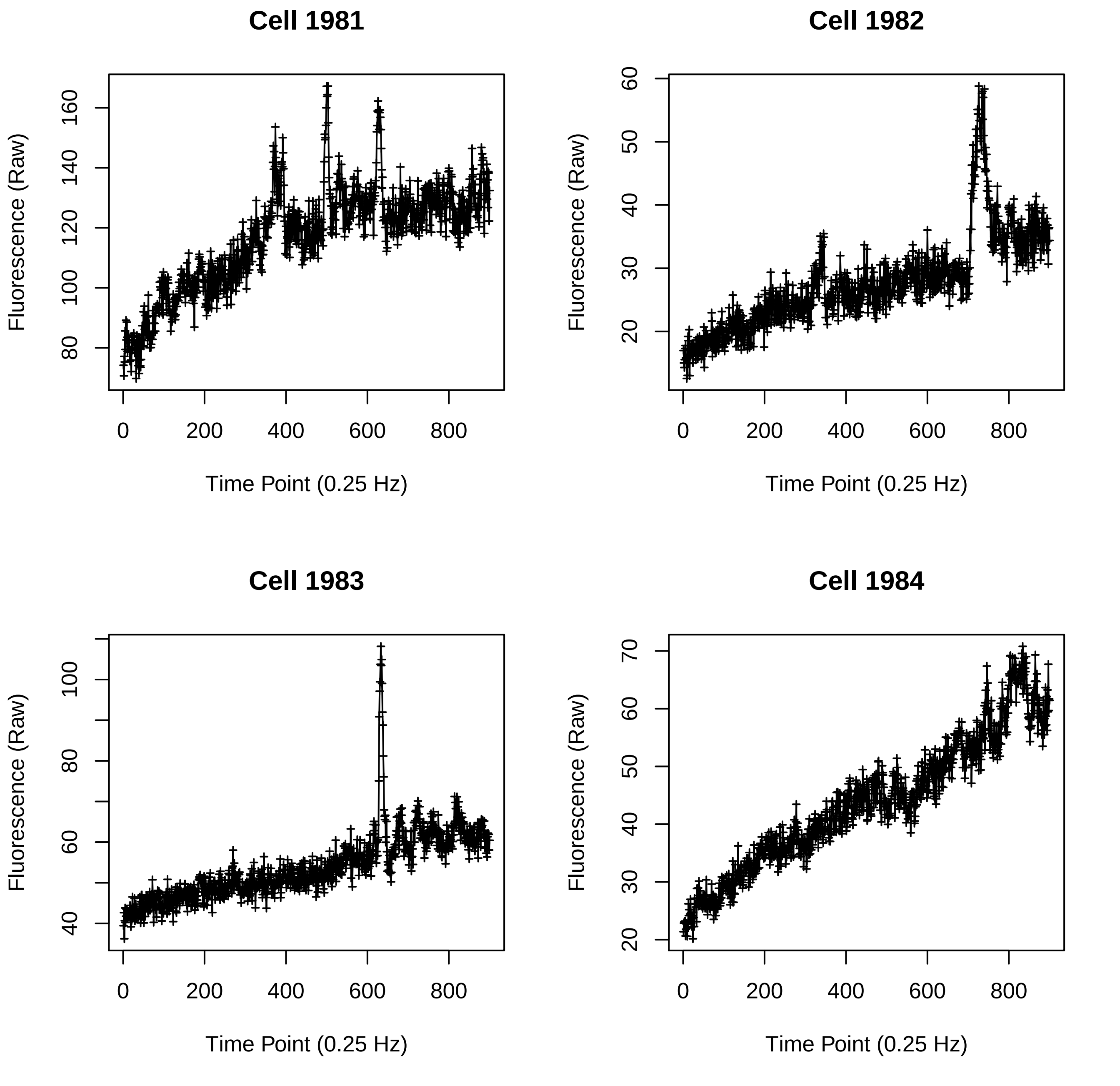
Cell 1981
Cell 1982
Cell 1983
Cell 1984
Fluorescence (Raw)
Time Point (0.25 Hz)
0
200
400
600
800
80
100
120
140
160
20
30
40
50
60
70

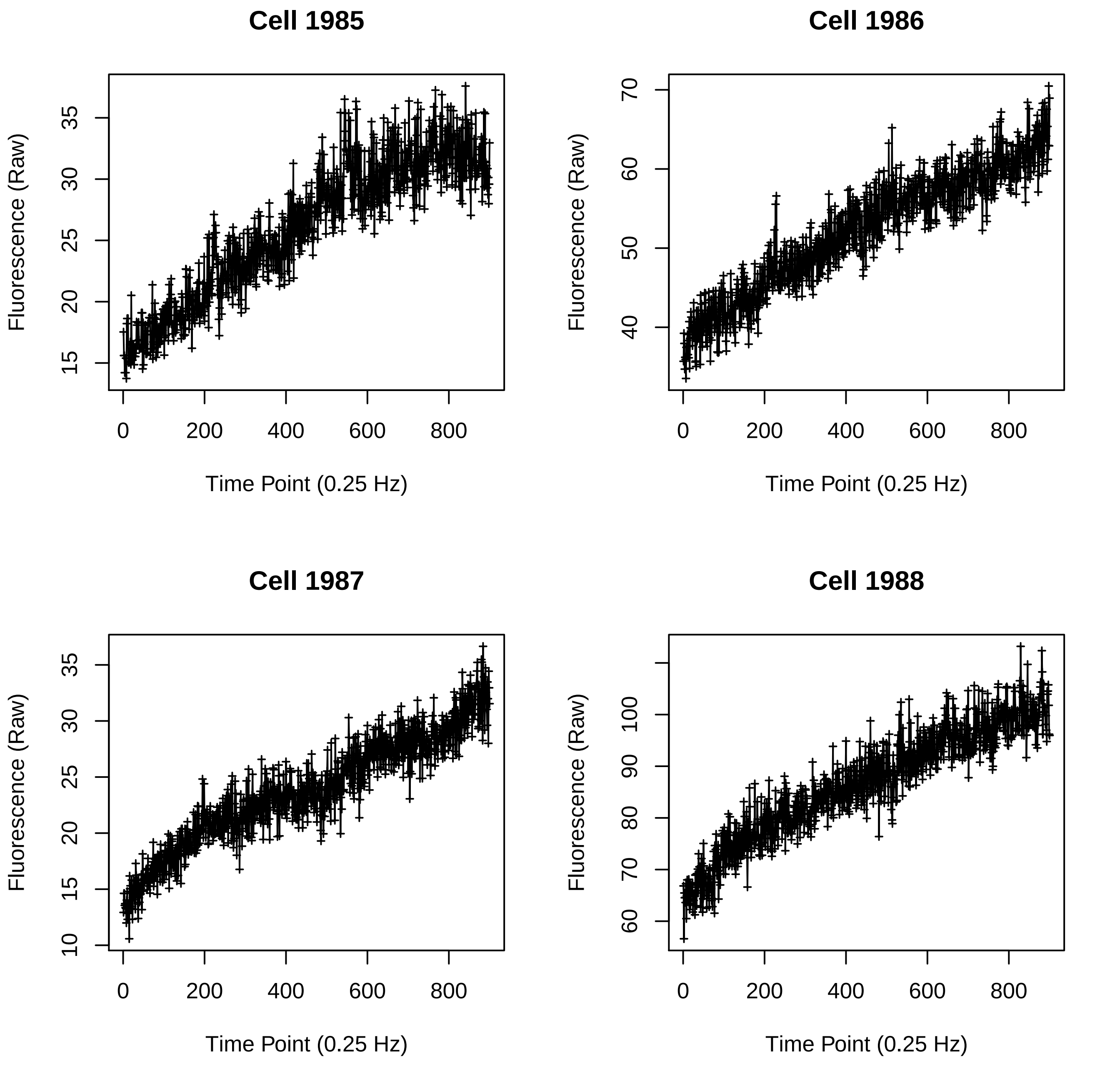

Cell 1985
Cell 1986
Cell 1987
Cell 1988
Fluorescence (Raw)
Time Point (0.25 Hz)
0
200
400
600
800
15
20
25
30
35
40
50
60
70
10
80
90
100

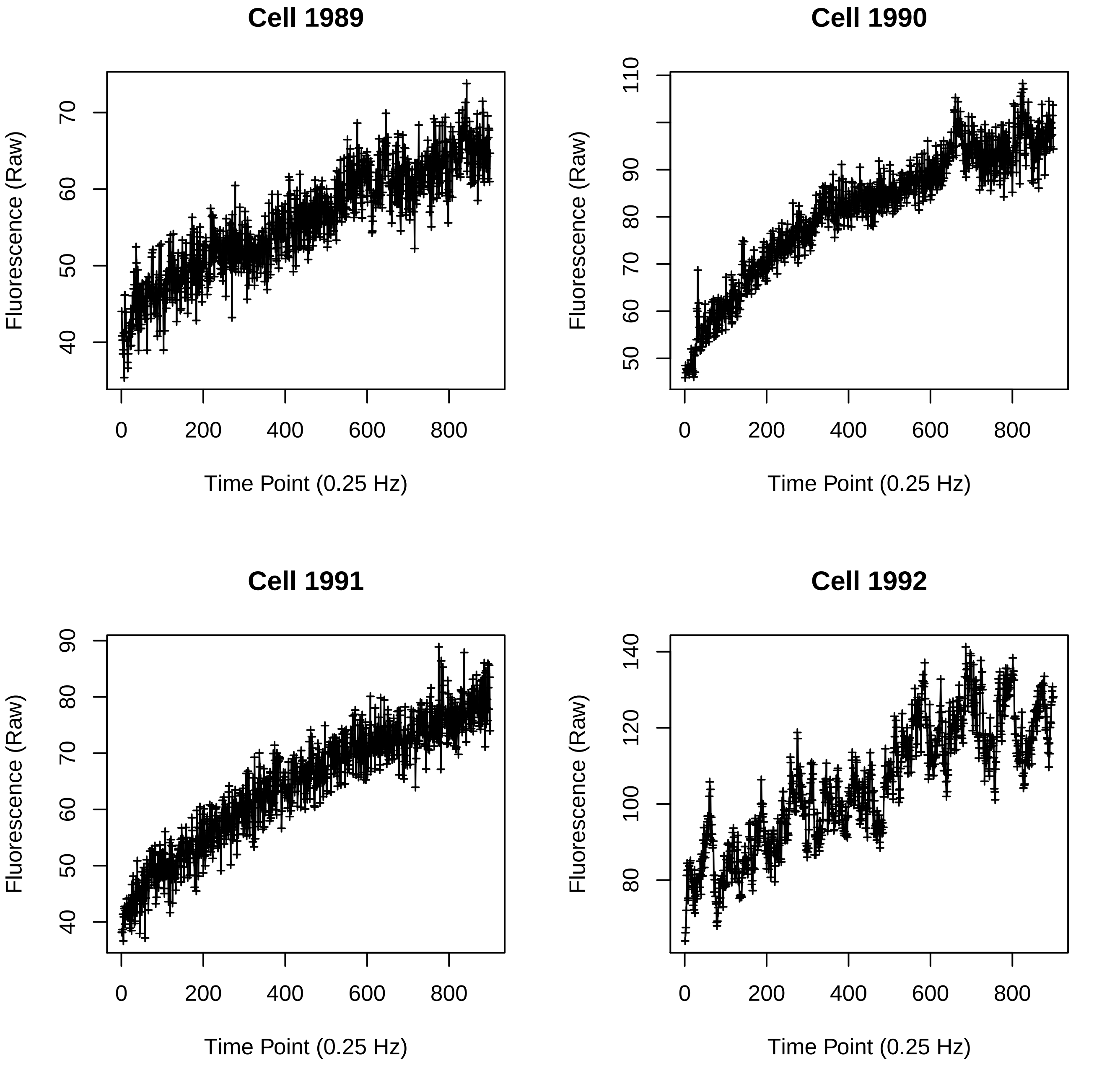

Cell 1989
Cell 1990
Cell 1991
Cell 1992
Fluorescence (Raw)
Time Point (0.25 Hz)
0
200
400
600
800
40
50
60
70
80
90
100
110
120
140

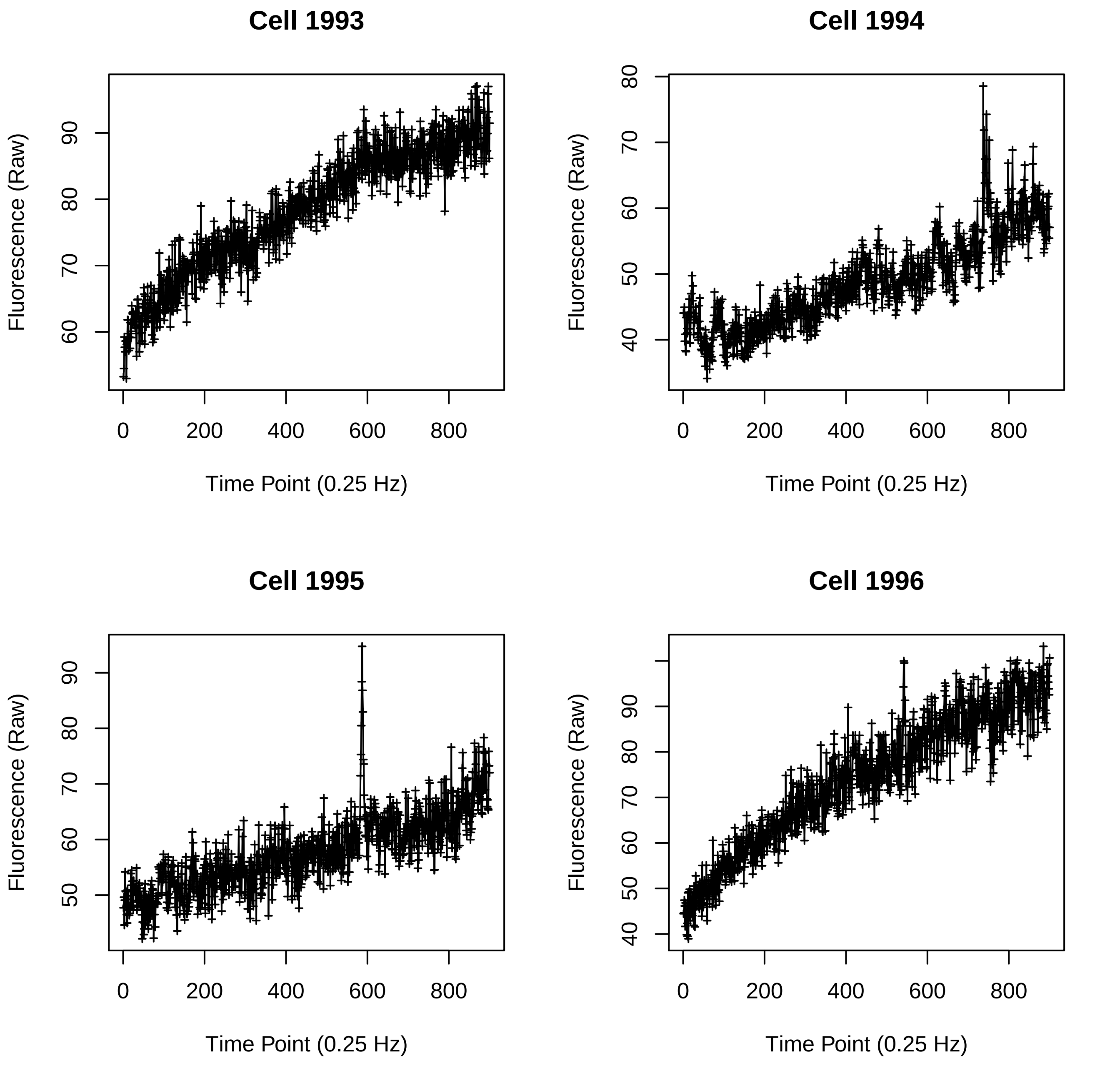
Cell 1993
Fluorescence (Raw)
60
70
80
90
0
200
400
600
800
Time Point (0.25 Hz)
Cell 1994
Fluorescence (Raw)
40
50
60
70
80
0
200
400
600
800
Time Point (0.25 Hz)
Cell 1995
Fluorescence (Raw)
50
60
70
80
90
0
200
400
600
800
Time Point (0.25 Hz)
Cell 1996
Fluorescence (Raw)
40
50
60
70
80
90
0
200
400
600
800
Time Point (0.25 Hz)

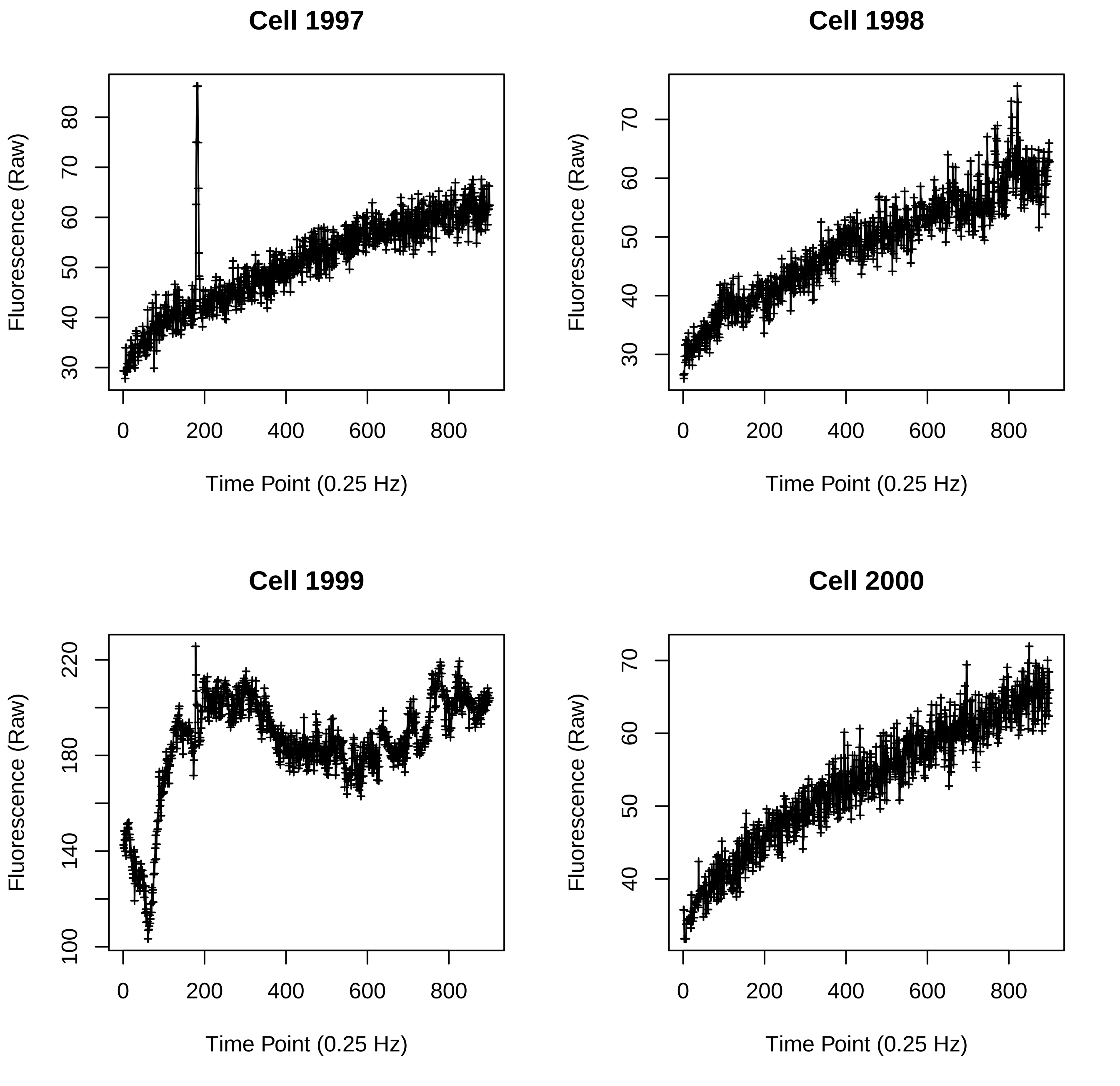

Cell 1997
Cell 1998
Cell 1999
Cell 2000
Fluorescence (Raw)
Time Point (0.25 Hz)
0
200
400
600
800
30
40
50
60
70
80
100
140
180
220

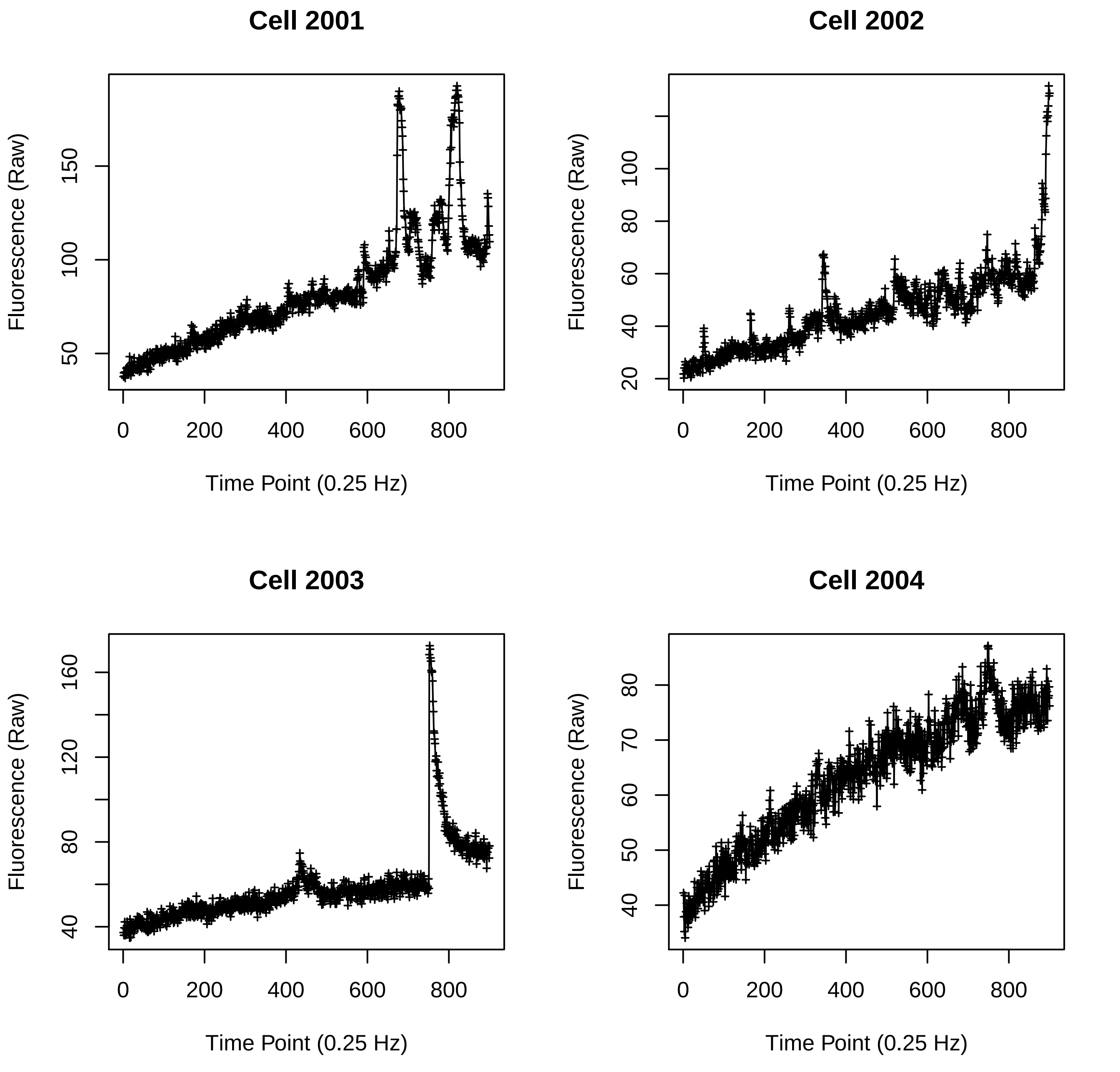
Cell 2001
Cell 2002
Cell 2003
Cell 2004
Fluorescence (Raw)
Time Point (0.25 Hz)
0
200
400
600
800
50
100
150
20
40
60
80
100
40
80
120
160
40
50
60
70
80

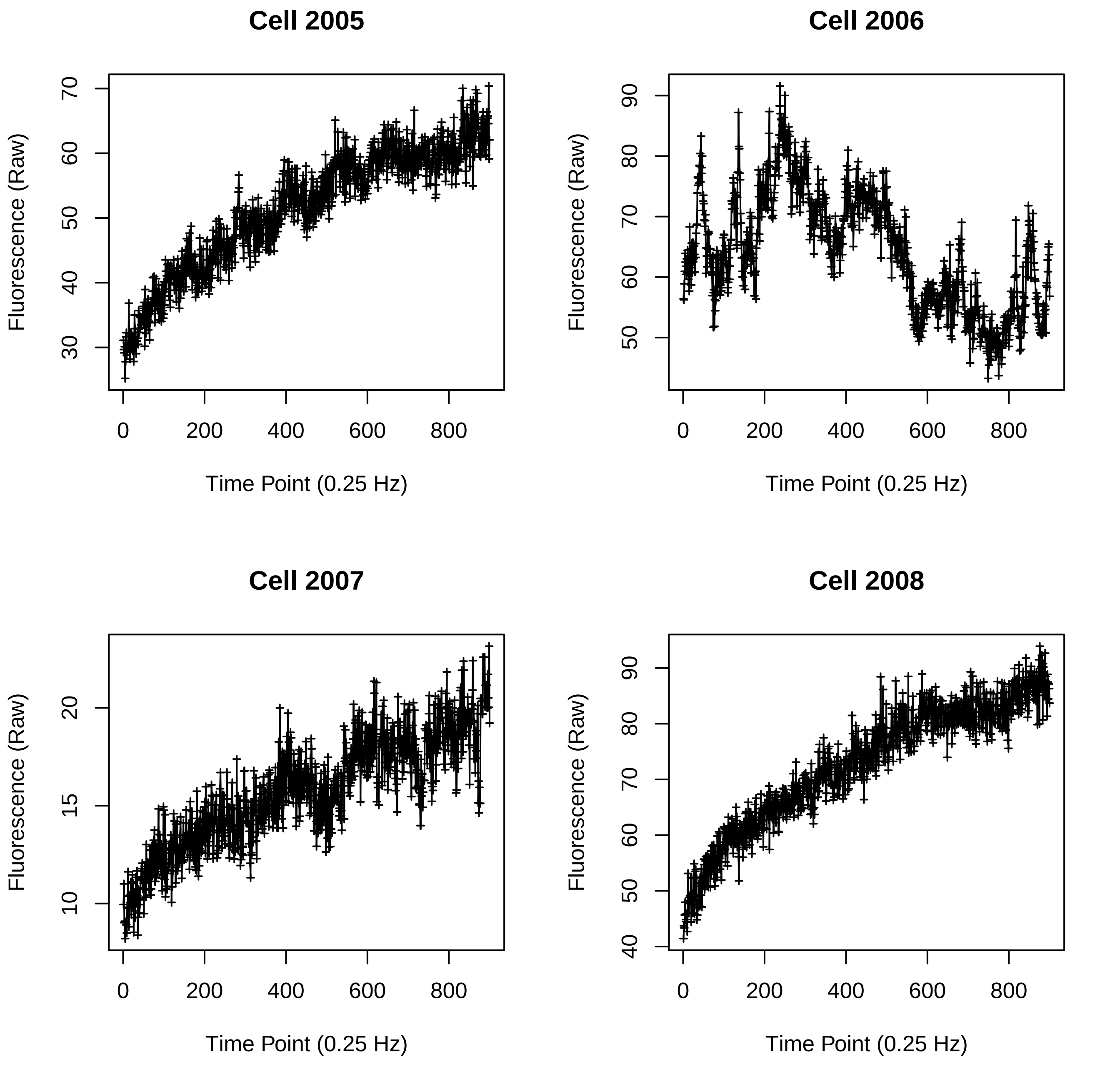
Cell 2005
Cell 2006
Cell 2007
Cell 2008
Fluorescence (Raw)
Time Point (0.25 Hz)
0
200
400
600
800
30
40
50
60
70
90
80
10
15
20

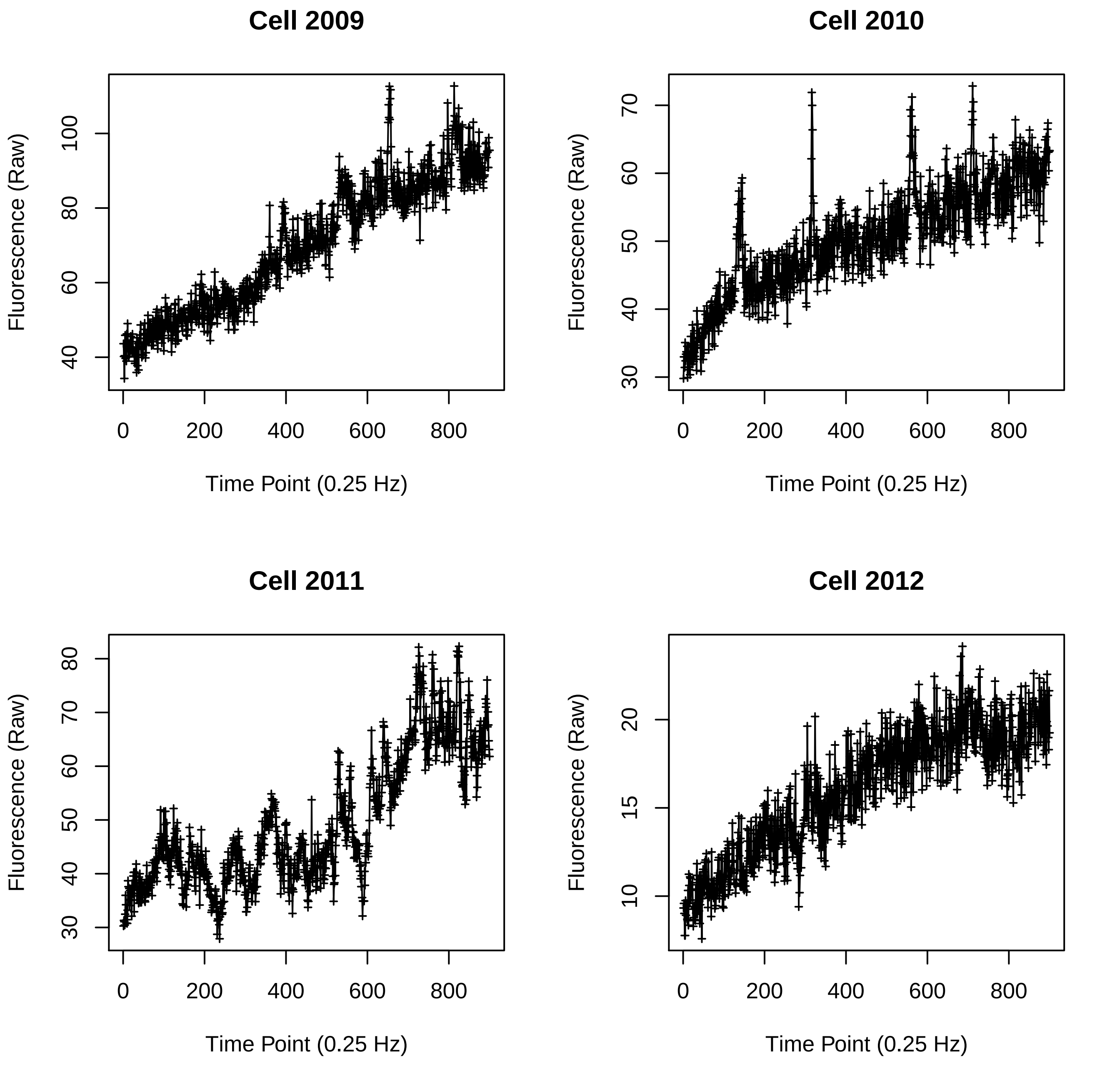
Cell 2009
Cell 2010
Cell 2011
Cell 2012
Fluorescence (Raw)
Time Point (0.25 Hz)
0
200
400
600
800
40
60
80
100
30
40
50
60
70
30
40
50
60
70
80
10
15
20

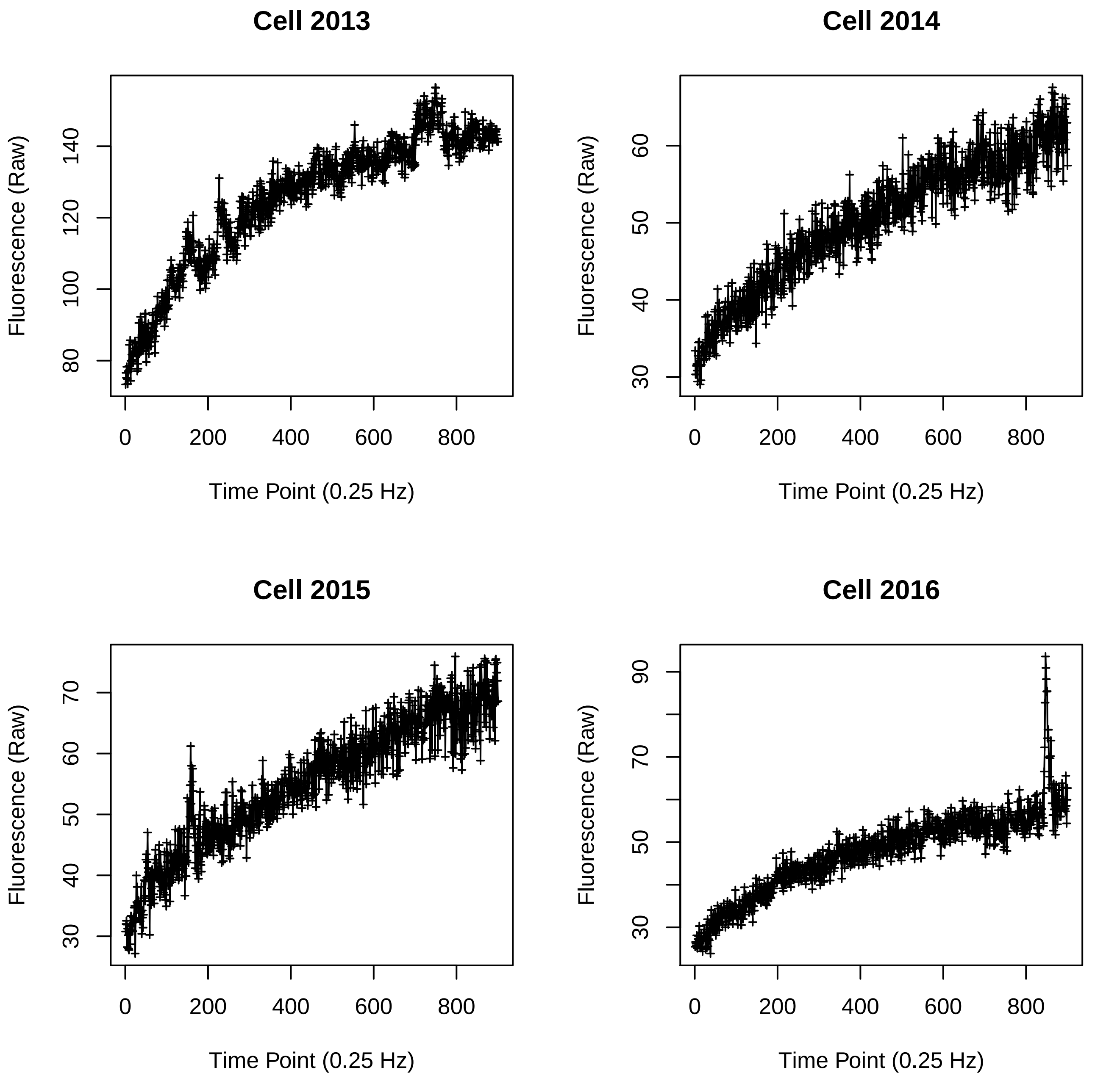
Cell 2013
Fluorescence (Raw)
Time Point (0.25 Hz)
80
100
120
140
0
200
400
600
800
Cell 2014
Fluorescence (Raw)
Time Point (0.25 Hz)
30
40
50
60
0
200
400
600
800
Cell 2015
Fluorescence (Raw)
Time Point (0.25 Hz)
30
40
50
60
70
0
200
400
600
800
Cell 2016
Fluorescence (Raw)
Time Point (0.25 Hz)
30
50
70
90
0
200
400
600
800

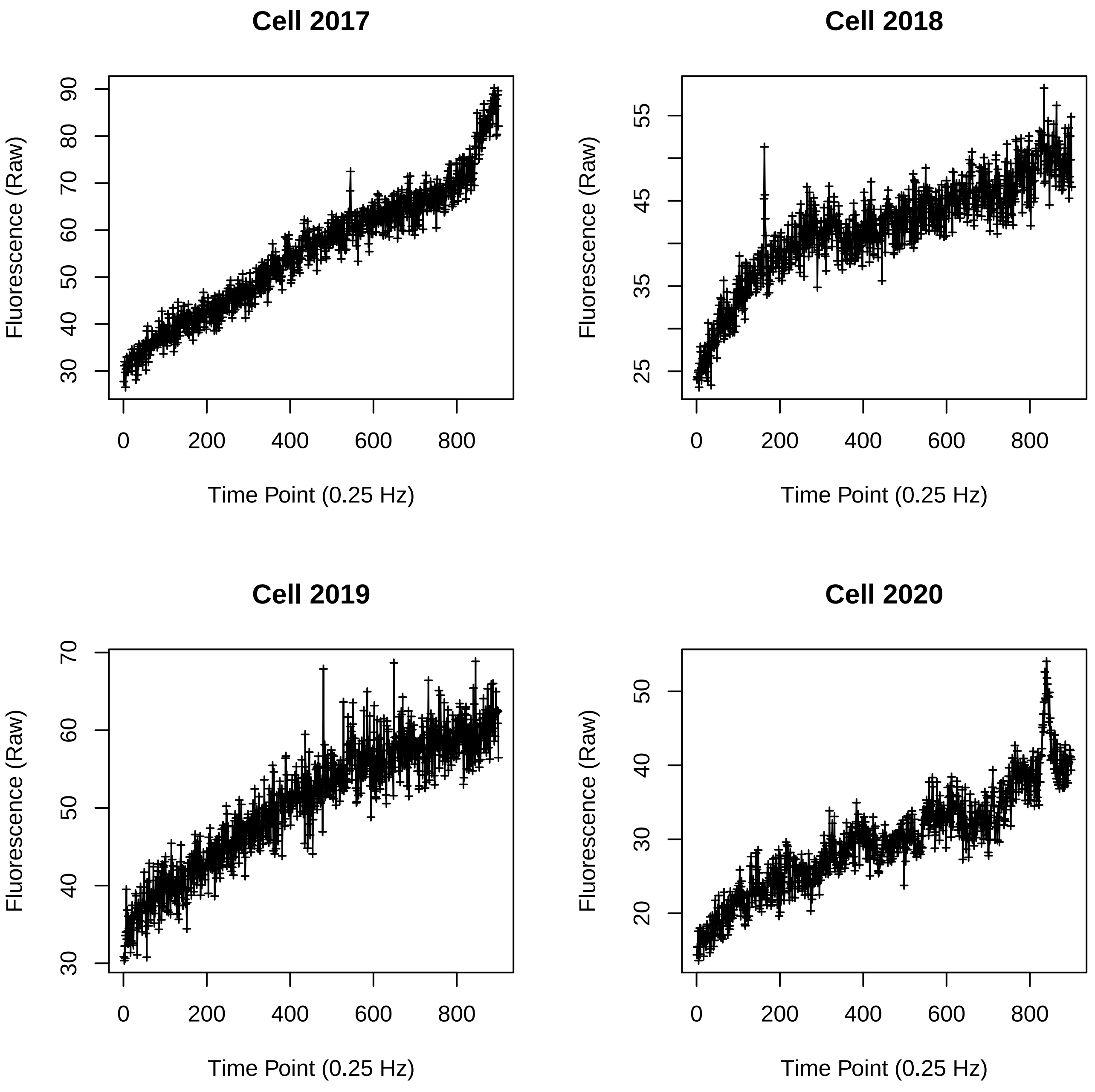
Cell 2017
Fluorescence (Raw)
30
40
50
60
70
80
90
0
200
400
600
800
Time Point (0.25 Hz)
Cell 2018
Fluorescence (Raw)
25
35
45
55
0
200
400
600
800
Time Point (0.25 Hz)
Cell 2019
Fluorescence (Raw)
30
40
50
60
70
0
200
400
600
800
Time Point (0.25 Hz)
Cell 2020
Fluorescence (Raw)
20
30
40
50
0
200
400
600
800
Time Point (0.25 Hz)

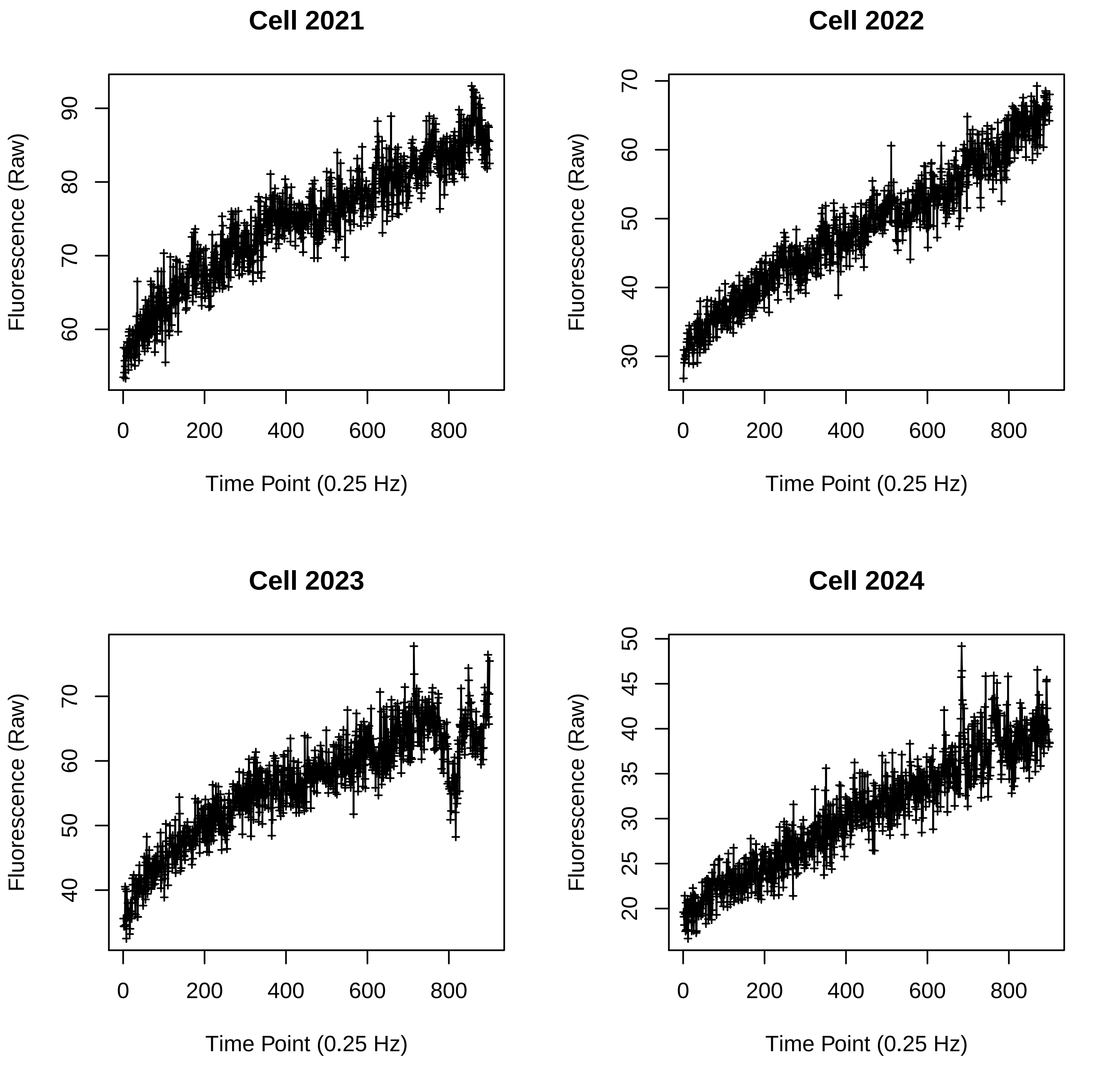

Cell 2021
Cell 2022
Cell 2023
Cell 2024
Fluorescence (Raw)
Time Point (0.25 Hz)
0
200
400
600
800
60
70
80
90
30
40
50
60
70
40
50
60
70
20
25
30
35
40
45
50

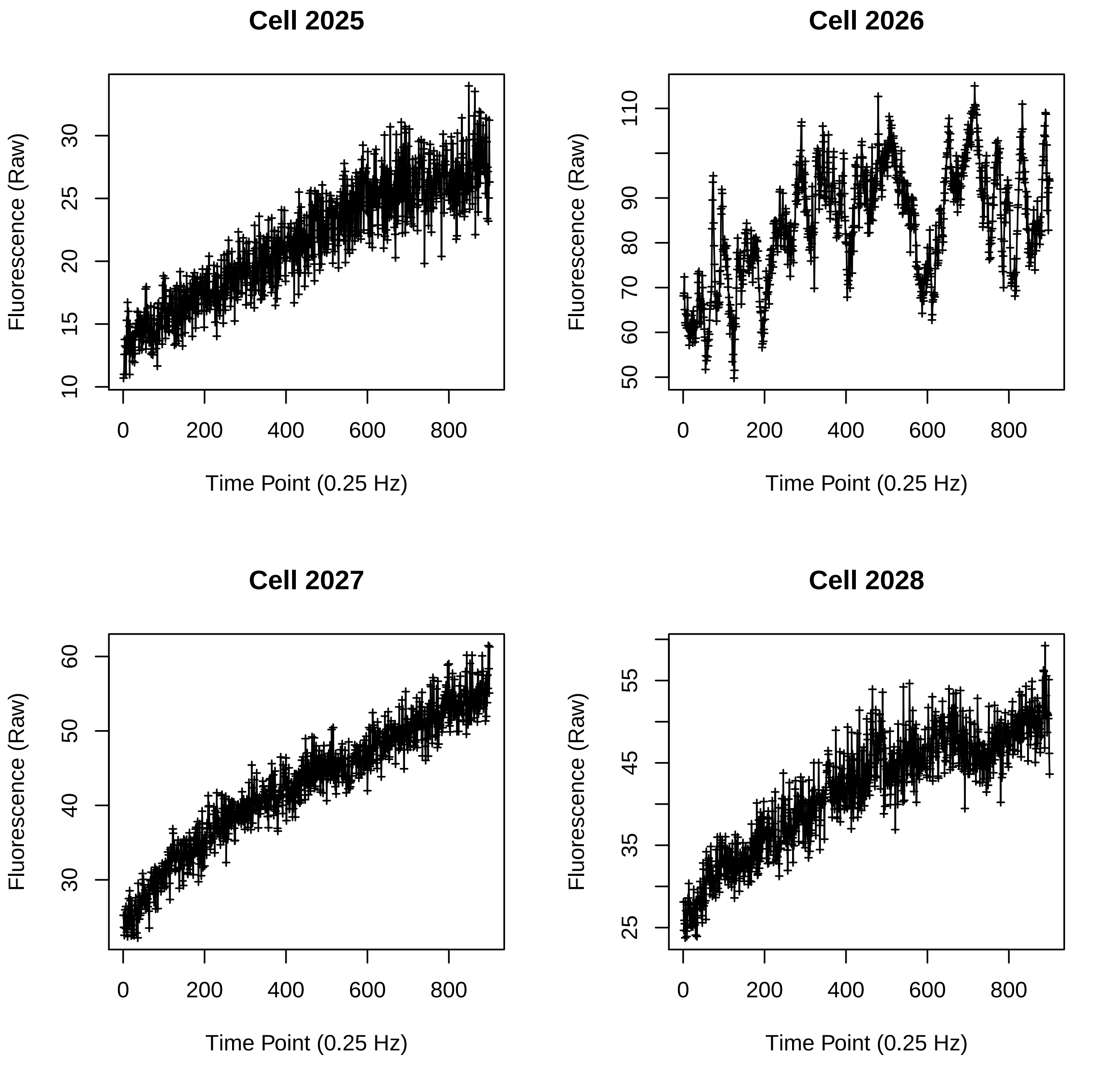

Cell 2025
Cell 2026
Cell 2027
Cell 2028
Fluorescence (Raw)
Time Point (0.25 Hz)
10
15
20
25
30
50
60
70
80
90
110
40
25
35
45
55
0
200
400
600
800

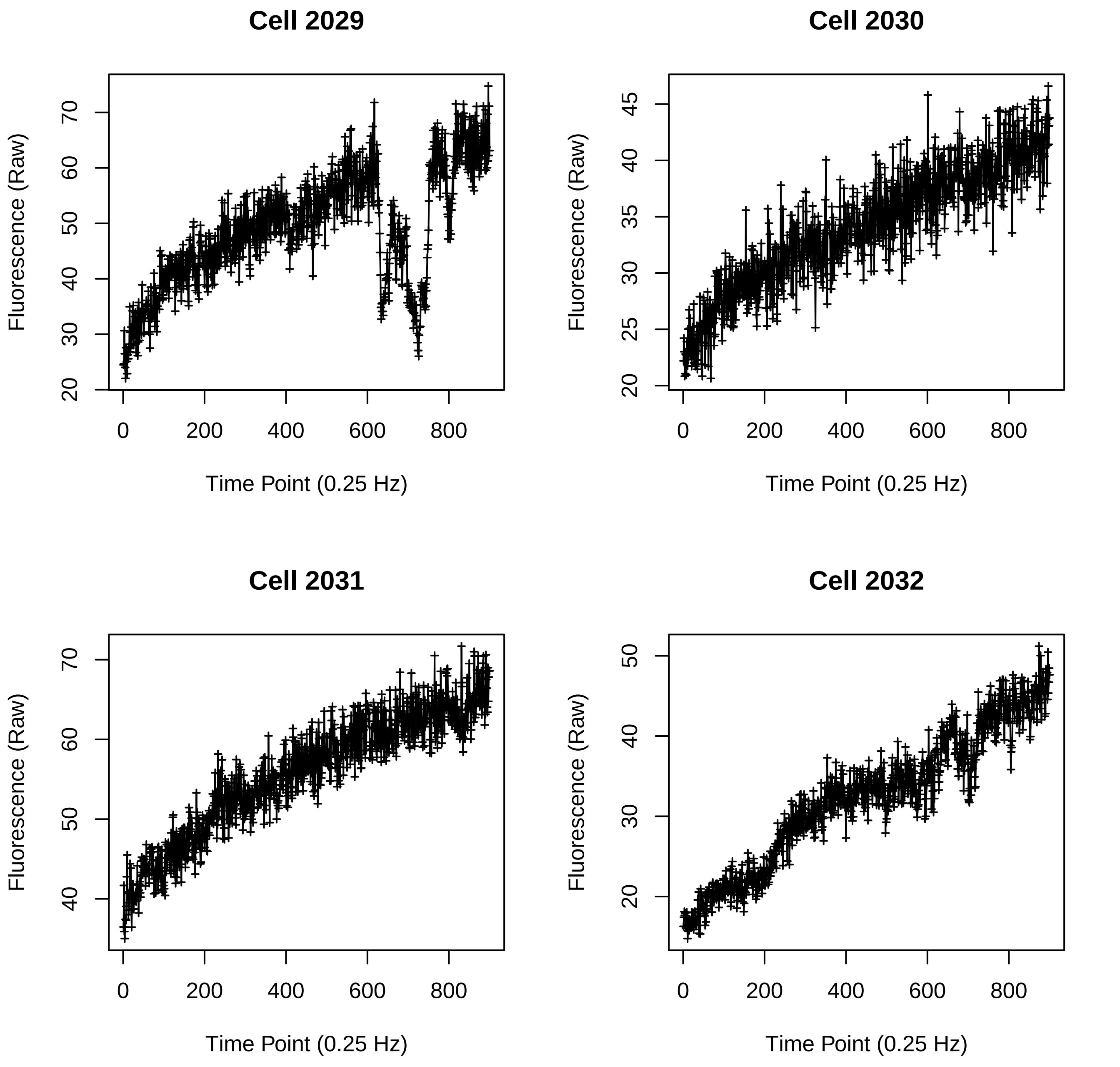
Cell 2029
Cell 2030
Cell 2031
Cell 2032
Fluorescence (Raw)
Time Point (0.25 Hz)
20
30
40
50
60
70
25
35
45
0
200
400
600
800

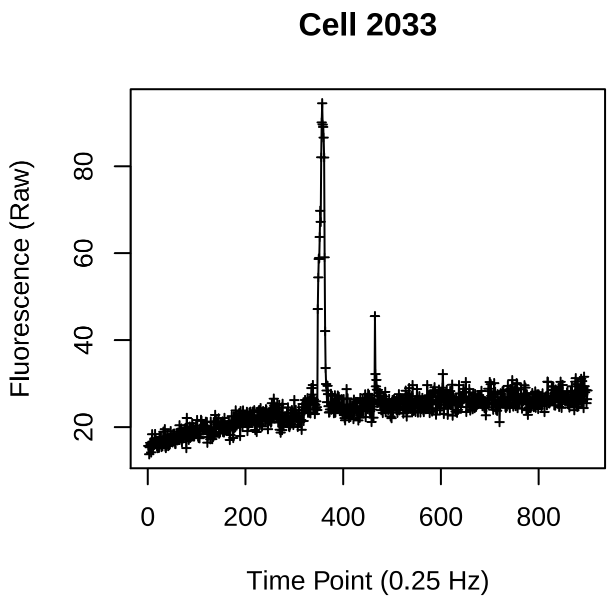

Cell 2033
Fluorescence (Raw)
Time Point (0.25 Hz)

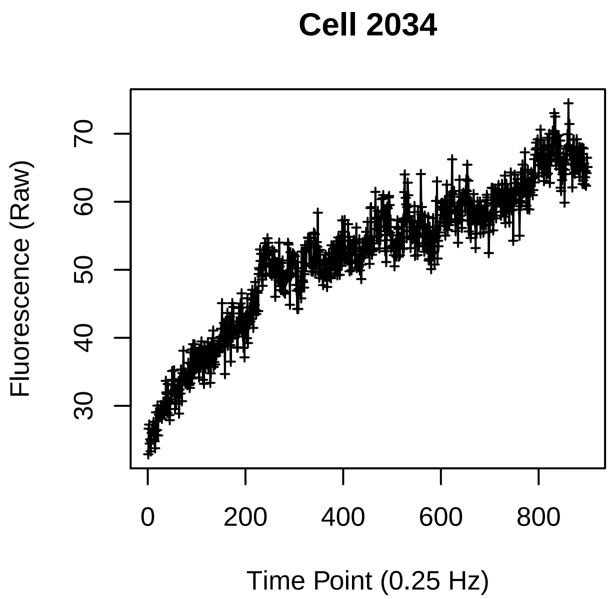

Cell 2034
Fluorescence (Raw)
Time Point (0.25 Hz)

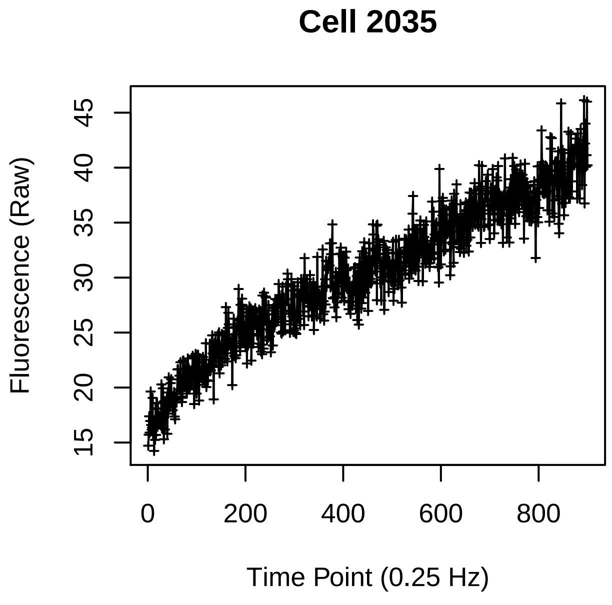

Cell 2035
Fluorescence (Raw)
Time Point (0.25 Hz)

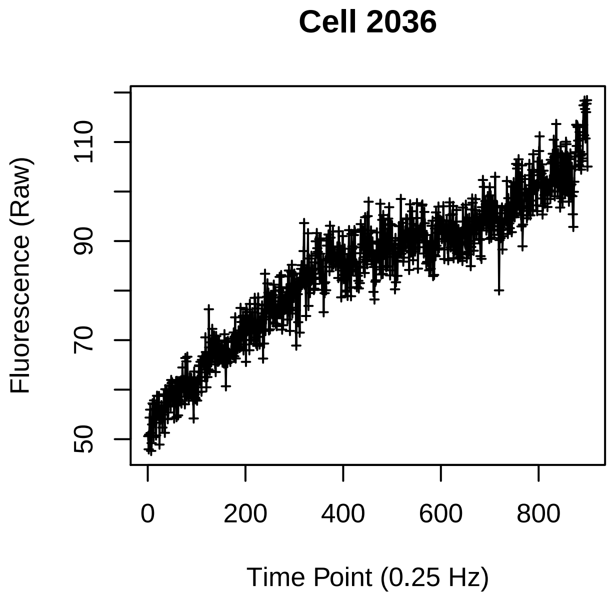

Cell 2036
Fluorescence (Raw)
Time Point (0.25 Hz)

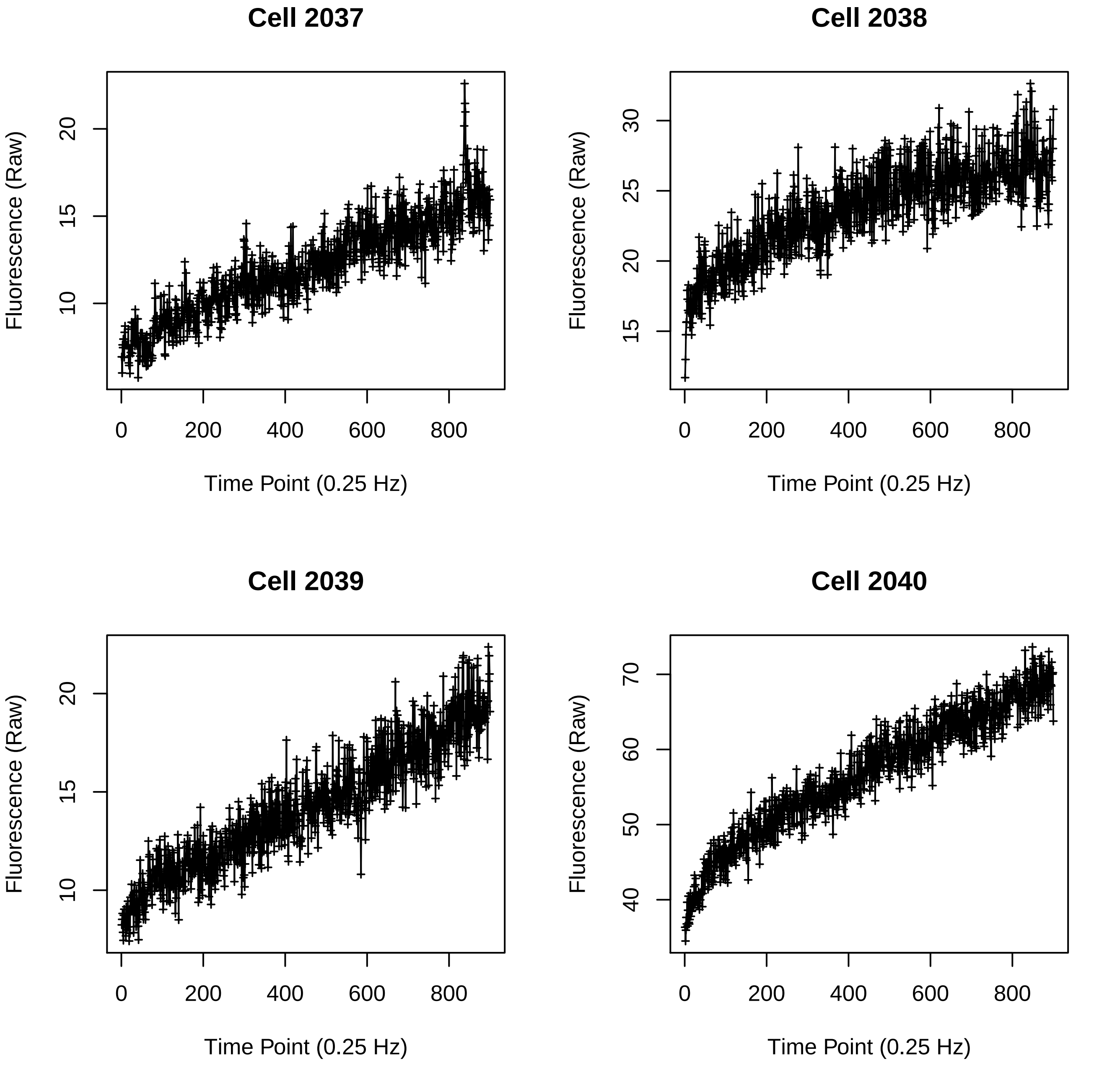
Cell 2037
Cell 2038
Cell 2039
Cell 2040
Fluorescence (Raw)
Time Point (0.25 Hz)
0
200
400
600
800
10
15
20
25
30
40
50
60
70

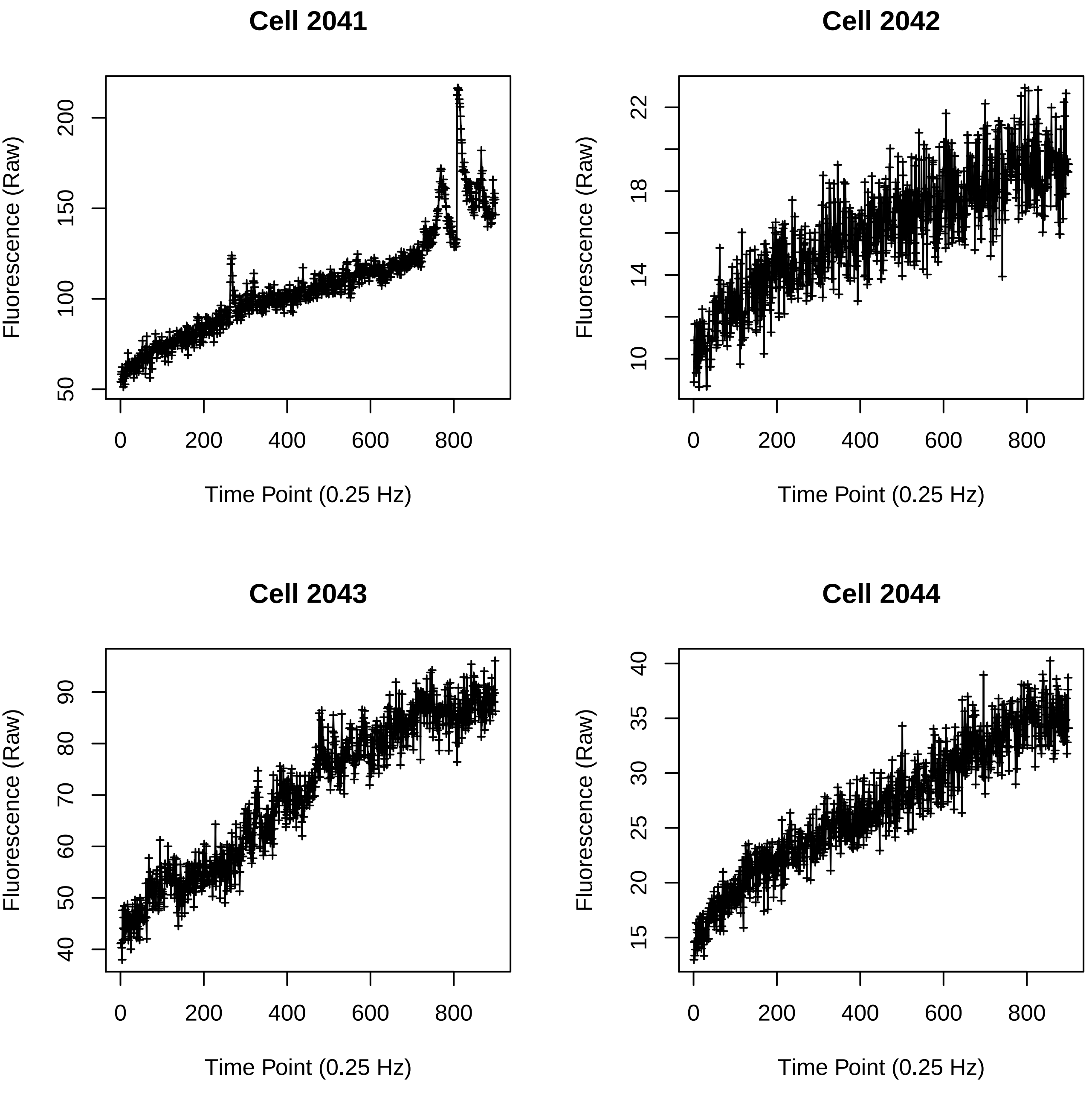

Cell 2041
Cell 2042
Cell 2043
Cell 2044
Fluorescence (Raw)
Time Point (0.25 Hz)
0
200
400
600
800
50
100
150
200
10
14
18
22
40
50
60
70
80
90
15
20
25
30
35
40

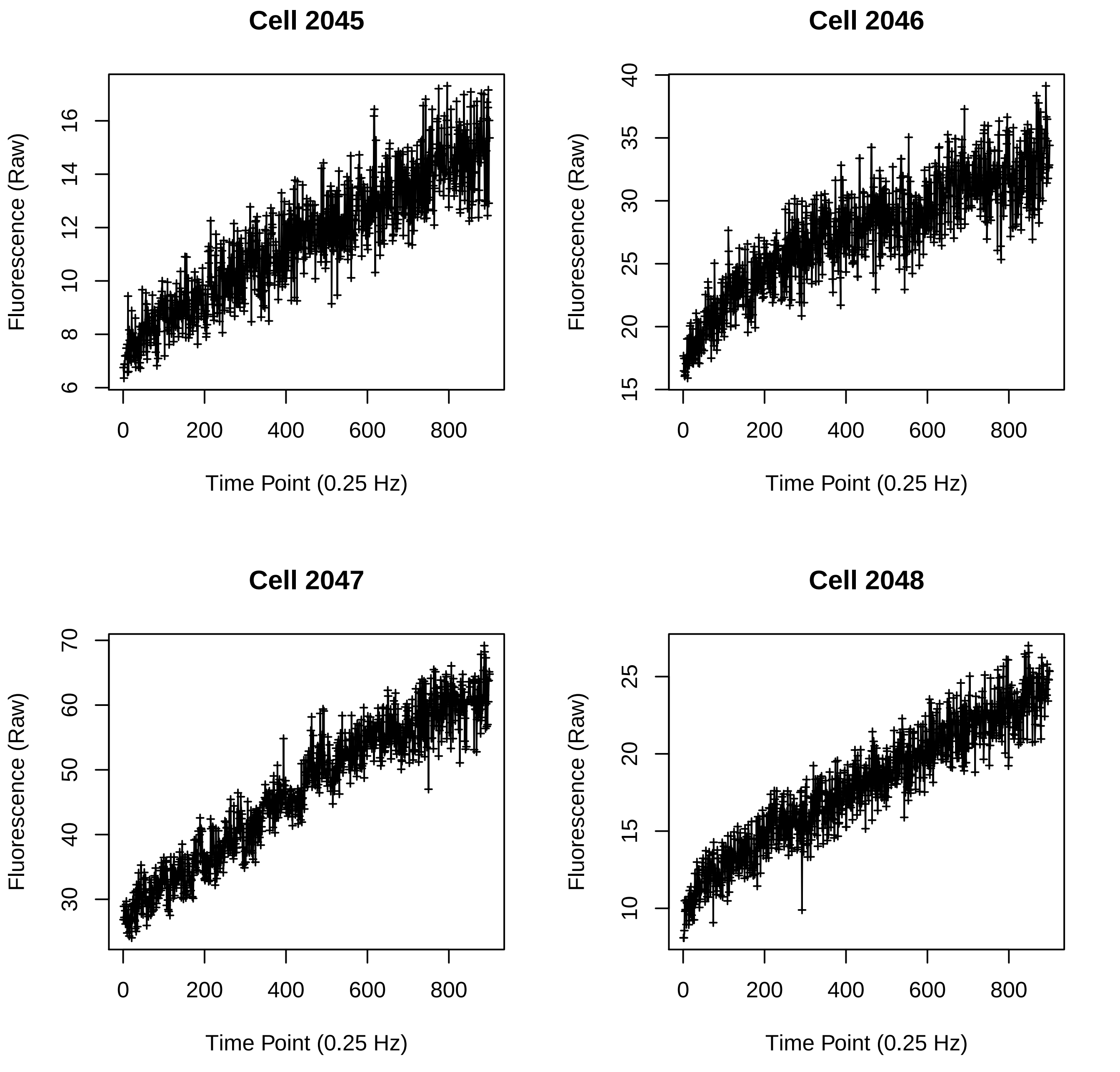
Cell 2045
Cell 2046
Cell 2047
Cell 2048
Fluorescence (Raw)
Time Point (0.25 Hz)
0
200
400
600
800
6
8
10
12
14
16
15
20
25
30
35
40
30
40
50
60
70
10
15
20
25

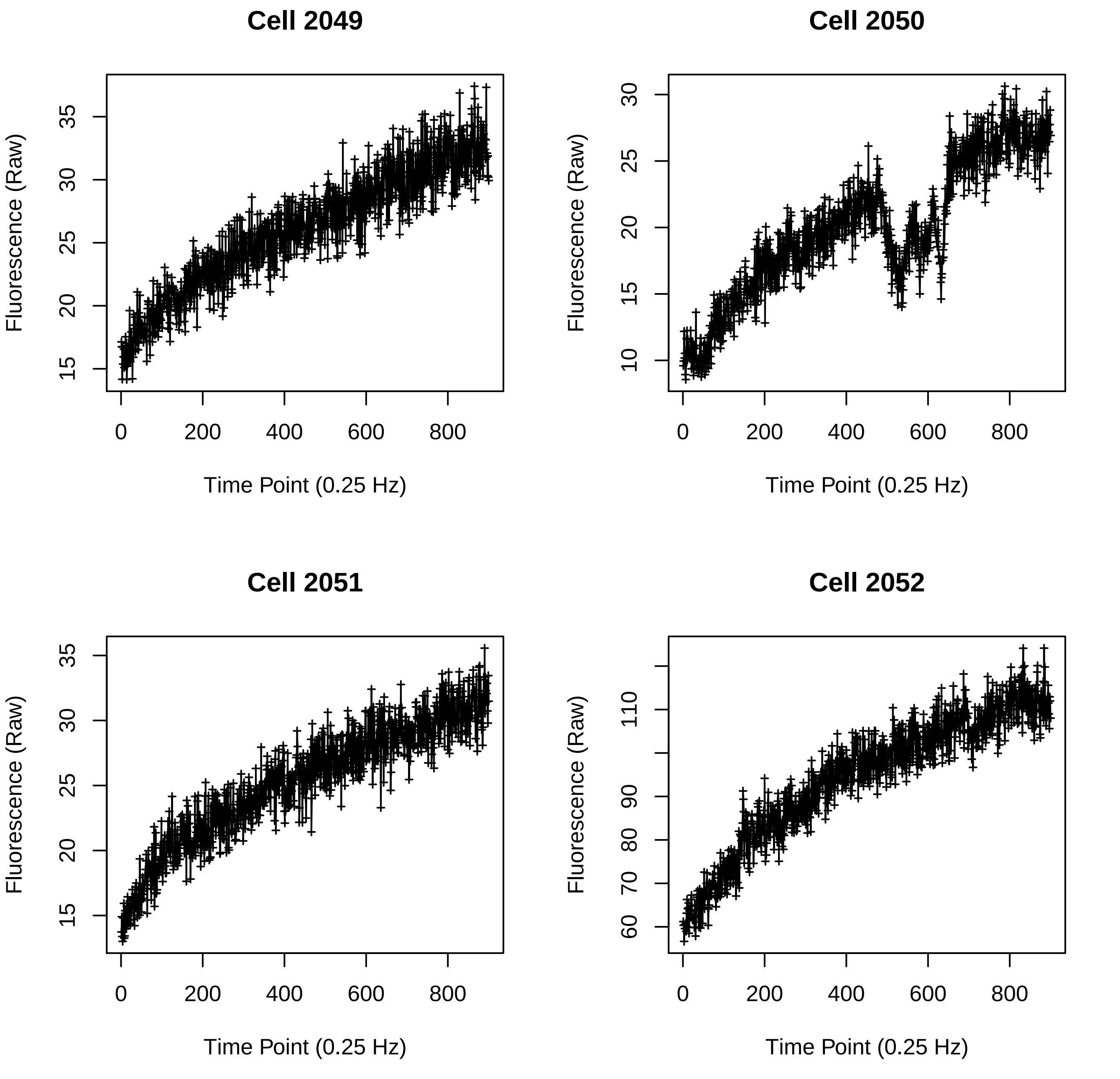
Cell 2049
Fluorescence (Raw)
15
20
25
30
35
0
200
400
600
800
Time Point (0.25 Hz)
Cell 2050
Fluorescence (Raw)
10
15
20
25
30
0
200
400
600
800
Time Point (0.25 Hz)
Cell 2051
Fluorescence (Raw)
15
20
25
30
35
0
200
400
600
800
Time Point (0.25 Hz)
Cell 2052
Fluorescence (Raw)
60
70
80
90
110
0
200
400
600
800
Time Point (0.25 Hz)

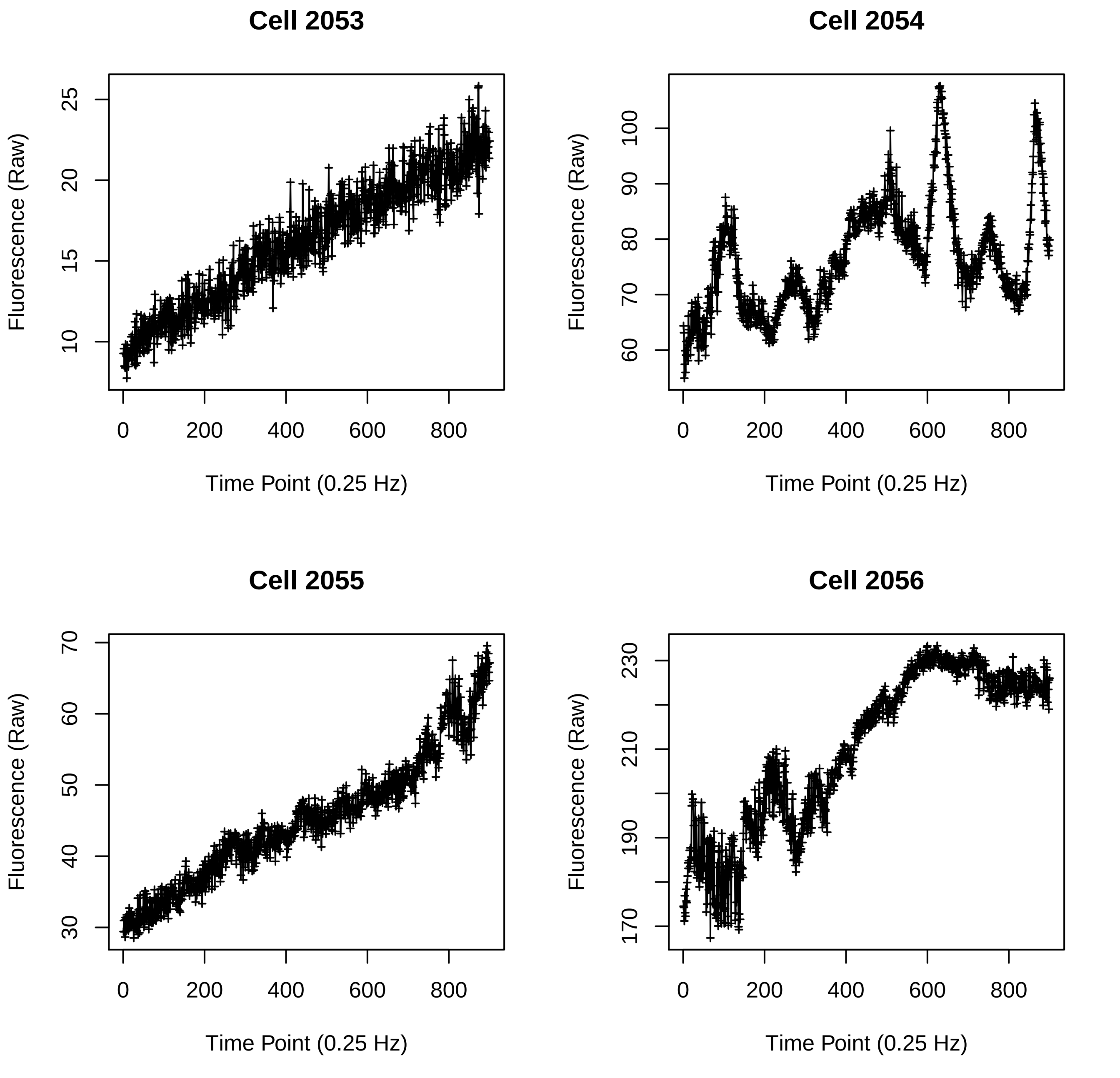

Cell 2053
Cell 2054
Cell 2055
Cell 2056
Fluorescence (Raw)
Time Point (0.25 Hz)
10
15
20
25
60
70
80
90
100
30
40
50
60
70
170
190
210
230
0
200
400
600
800

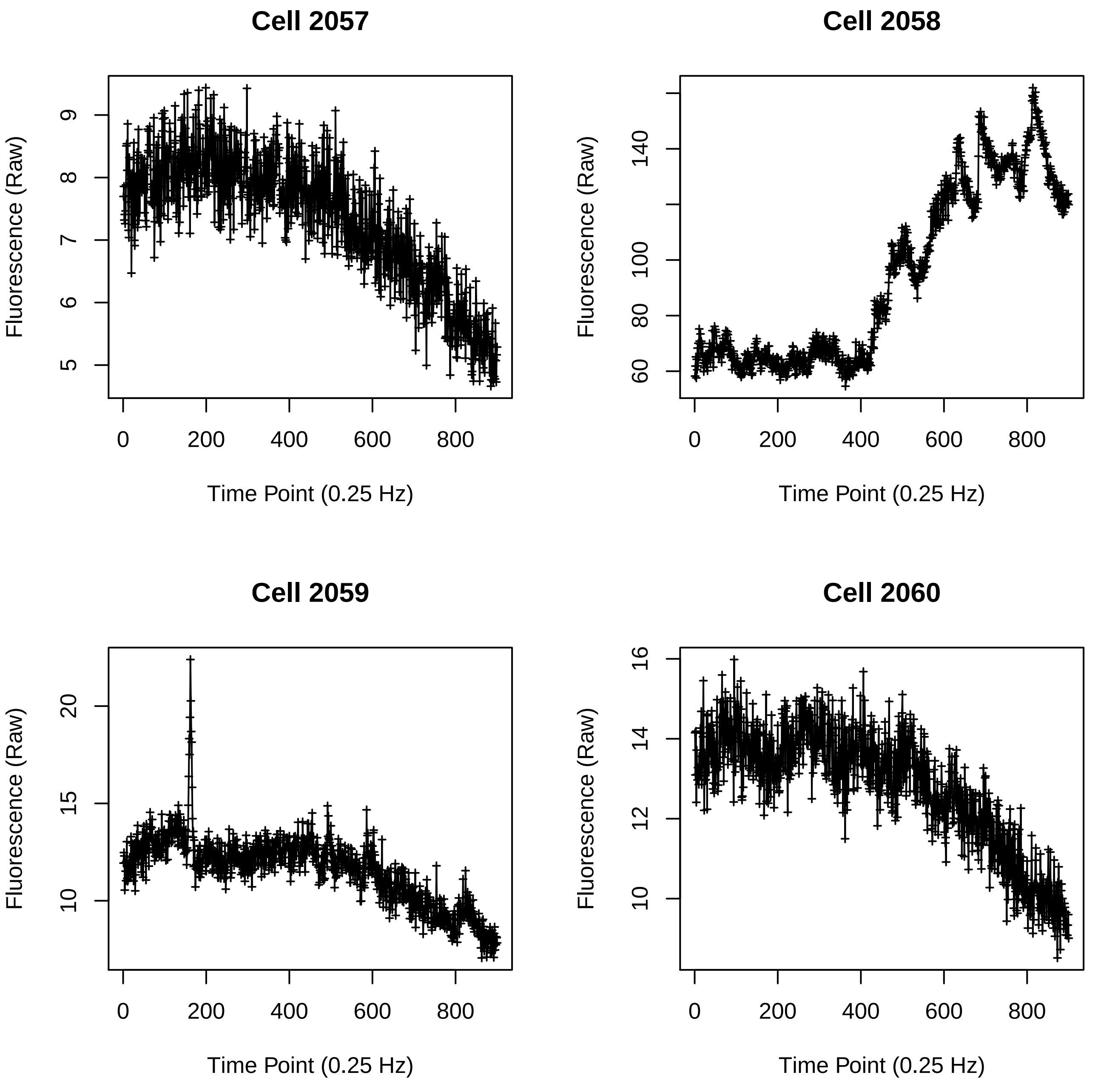
Cell 2057
Fluorescence (Raw)
9
8
7
6
5
0
200
400
600
800
Time Point (0.25 Hz)
Cell 2058
Fluorescence (Raw)
140
100
80
60
0
200
400
600
800
Time Point (0.25 Hz)
Cell 2059
Fluorescence (Raw)
20
15
10
0
200
400
600
800
Time Point (0.25 Hz)
Cell 2060
Fluorescence (Raw)
16
14
12
10
0
200
400
600
800
Time Point (0.25 Hz)

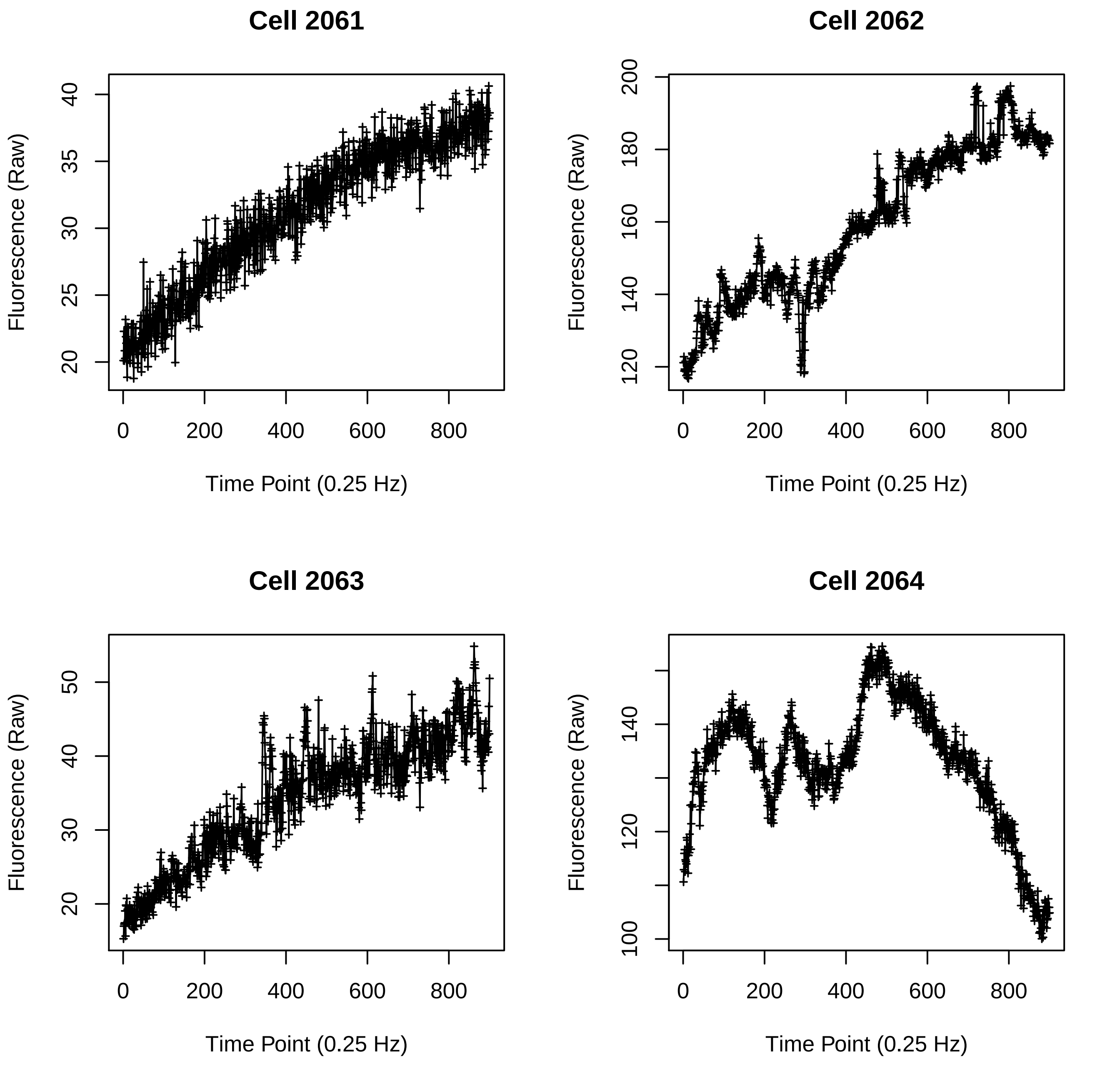

Cell 2061
Fluorescence (Raw)
20
25
30
35
40
0
200
400
600
800
Time Point (0.25 Hz)
Cell 2062
Fluorescence (Raw)
120
140
160
180
200
0
200
400
600
800
Time Point (0.25 Hz)
Cell 2063
Fluorescence (Raw)
20
30
40
50
0
200
400
600
800
Time Point (0.25 Hz)
Cell 2064
Fluorescence (Raw)
100
120
140
0
200
400
600
800
Time Point (0.25 Hz)

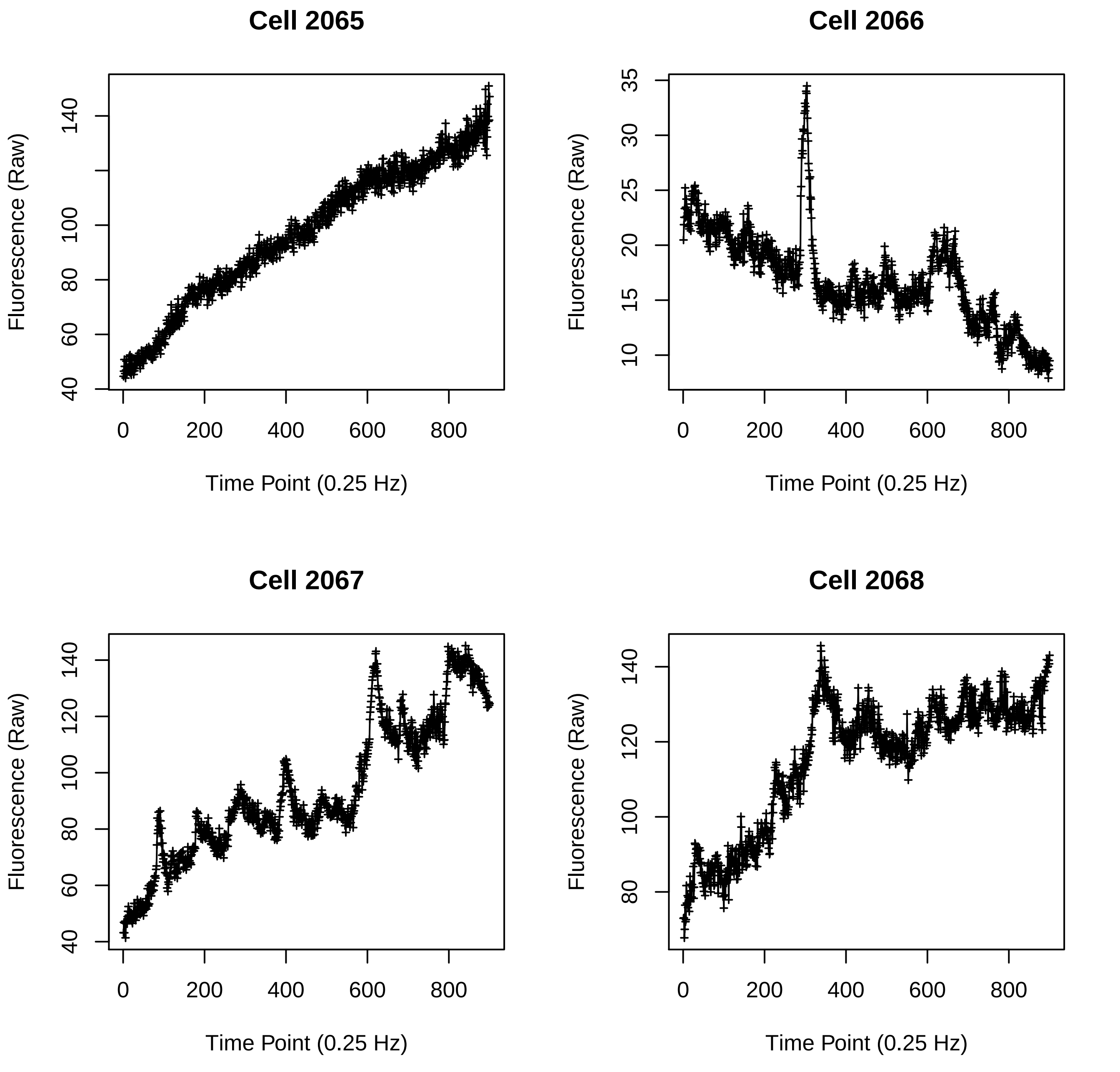
Cell 2065
Fluorescence (Raw)
40
60
80
100
140
0
200
400
600
800
Time Point (0.25 Hz)
Cell 2066
Fluorescence (Raw)
10
15
20
25
30
35
0
200
400
600
800
Time Point (0.25 Hz)
Cell 2067
Fluorescence (Raw)
40
60
80
100
120
140
0
200
400
600
800
Time Point (0.25 Hz)
Cell 2068
Fluorescence (Raw)
80
100
120
140
0
200
400
600
800
Time Point (0.25 Hz)

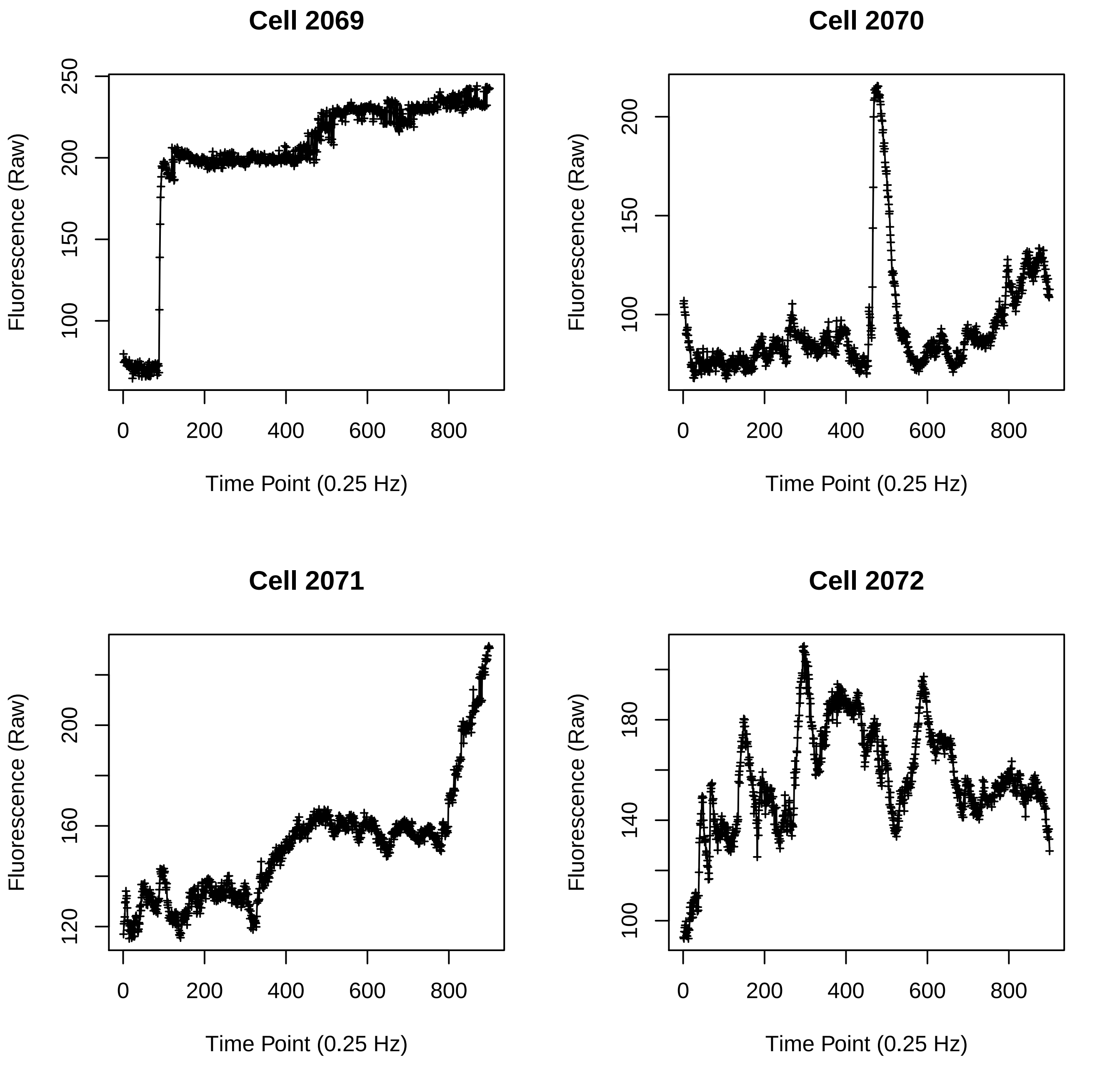
Cell 2069
Fluorescence (Raw)
100
150
200
250
0
200
400
600
800
Time Point (0.25 Hz)
Cell 2070
Fluorescence (Raw)
100
150
200
0
200
400
600
800
Time Point (0.25 Hz)
Cell 2071
Fluorescence (Raw)
120
160
200
0
200
400
600
800
Time Point (0.25 Hz)
Cell 2072
Fluorescence (Raw)
100
140
180
0
200
400
600
800
Time Point (0.25 Hz)

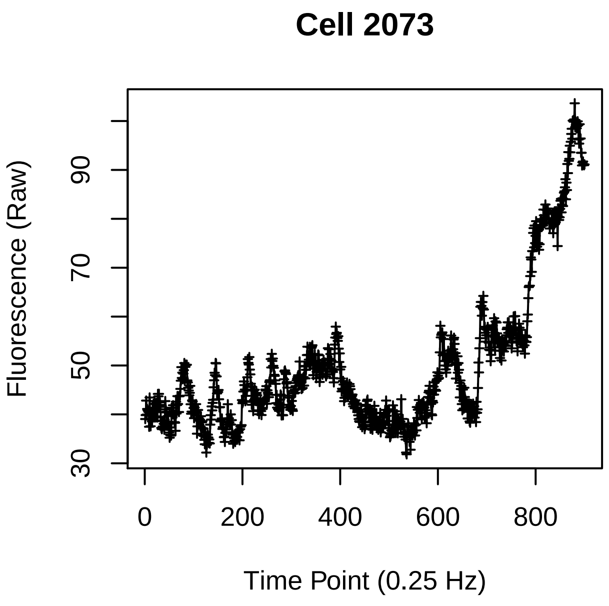
Cell 2073
Fluorescence (Raw)
30
50
70
90
0
200
400
600
800
Time Point (0.25 Hz)

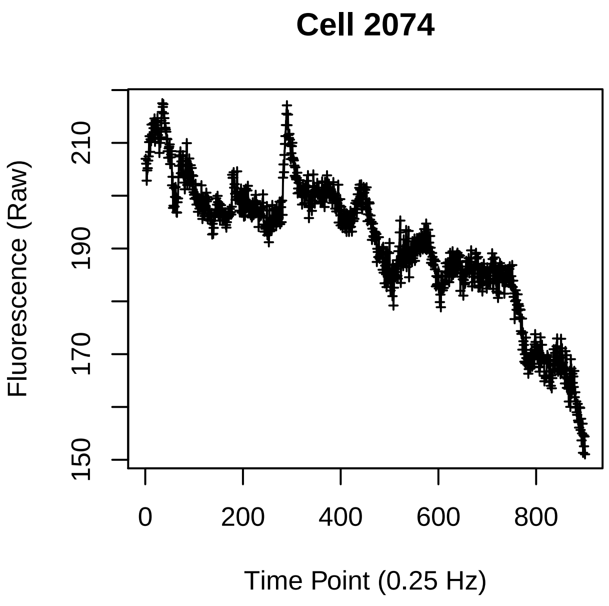
Cell 2074
Fluorescence (Raw)
150
170
190
210
0
200
400
600
800
Time Point (0.25 Hz)

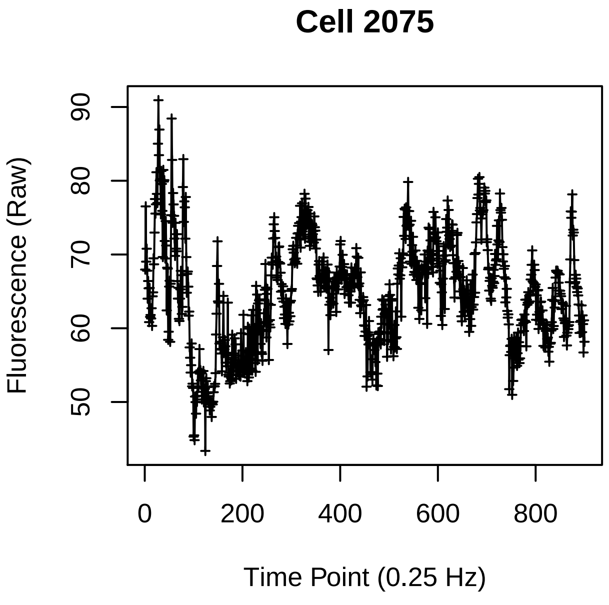
Cell 2075
Fluorescence (Raw)
50
60
70
80
90
0
200
400
600
800
Time Point (0.25 Hz)

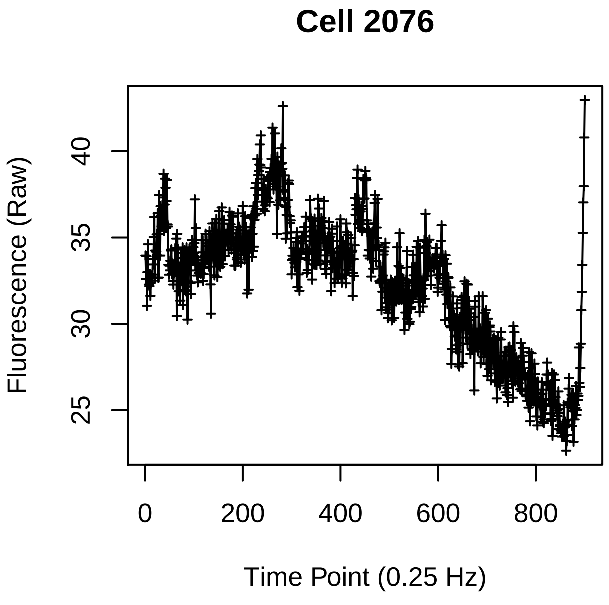
Cell 2076
Fluorescence (Raw)
25
30
35
40
0
200
400
600
800
Time Point (0.25 Hz)

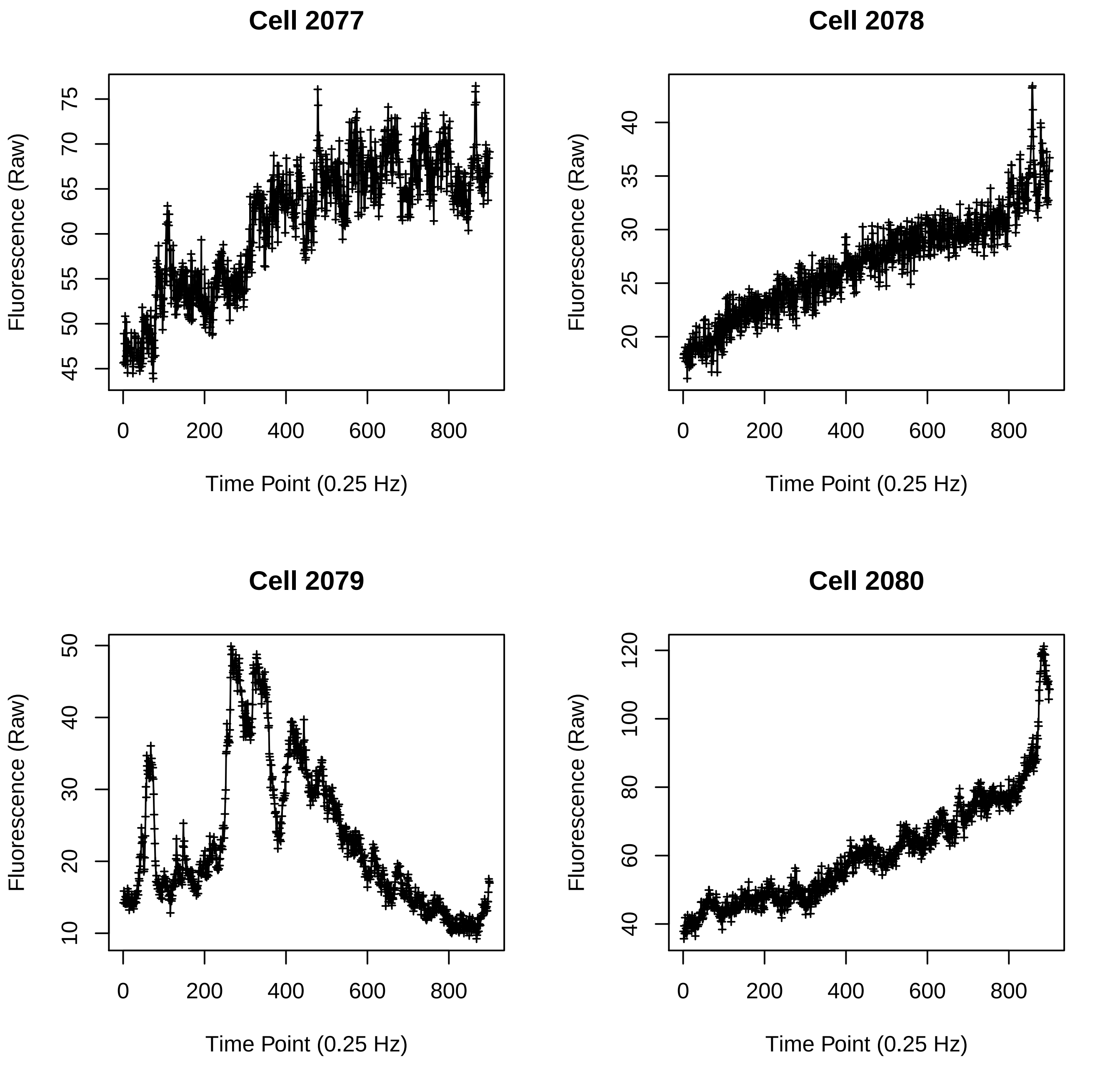

Cell 2077
Cell 2078
Cell 2079
Cell 2080
Fluorescence (Raw)
Time Point (0.25 Hz)
0
200
400
600
800
45
50
55
60
65
70
75
20
25
30
35
40
10
20
30
40
50
40
60
80
100
120

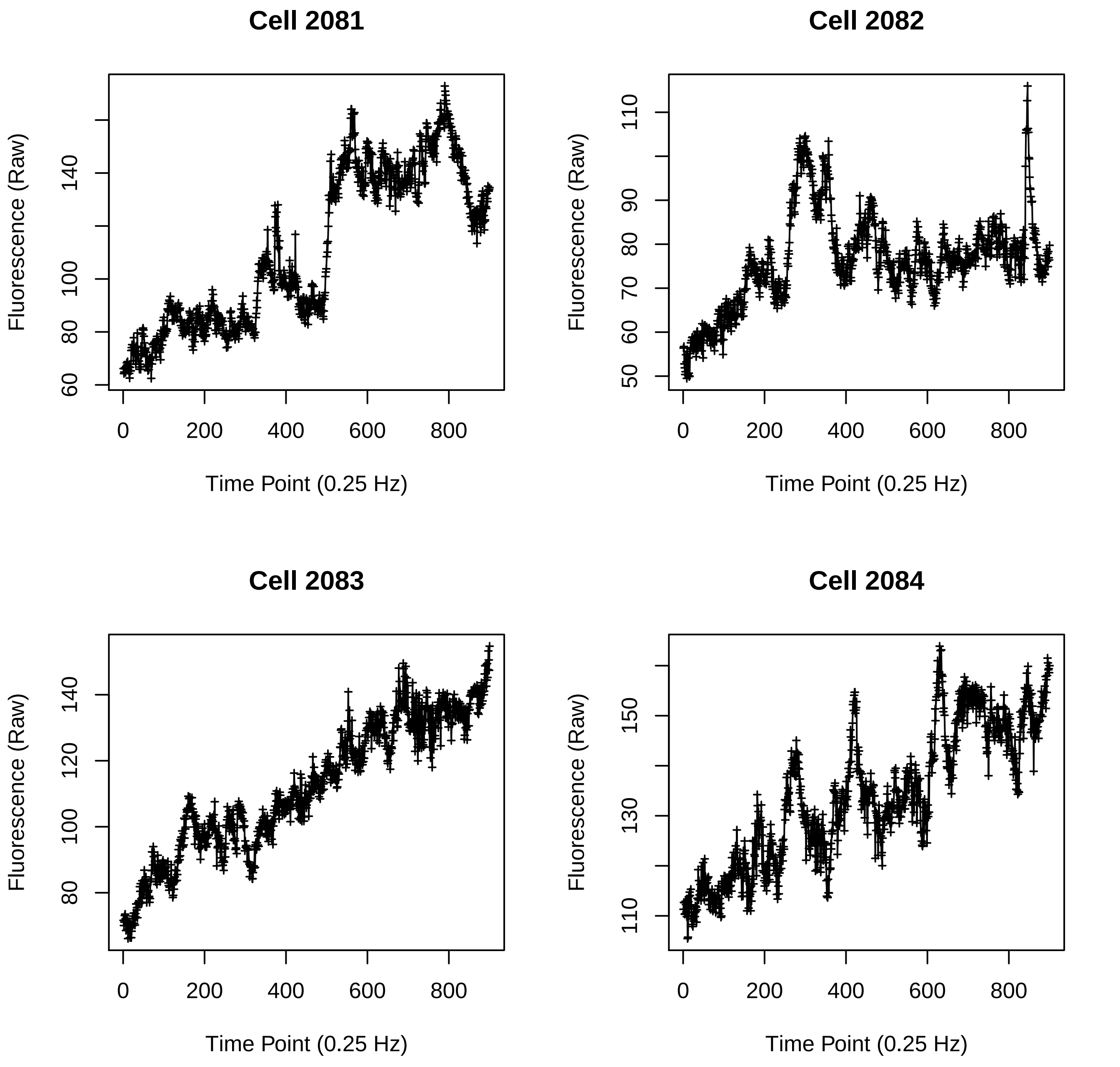
Cell 2081
Cell 2082
Cell 2083
Cell 2084
Fluorescence (Raw)
Time Point (0.25 Hz)
0
200
400
600
800
60
80
100
140
50
60
70
80
90
110
120
130
150

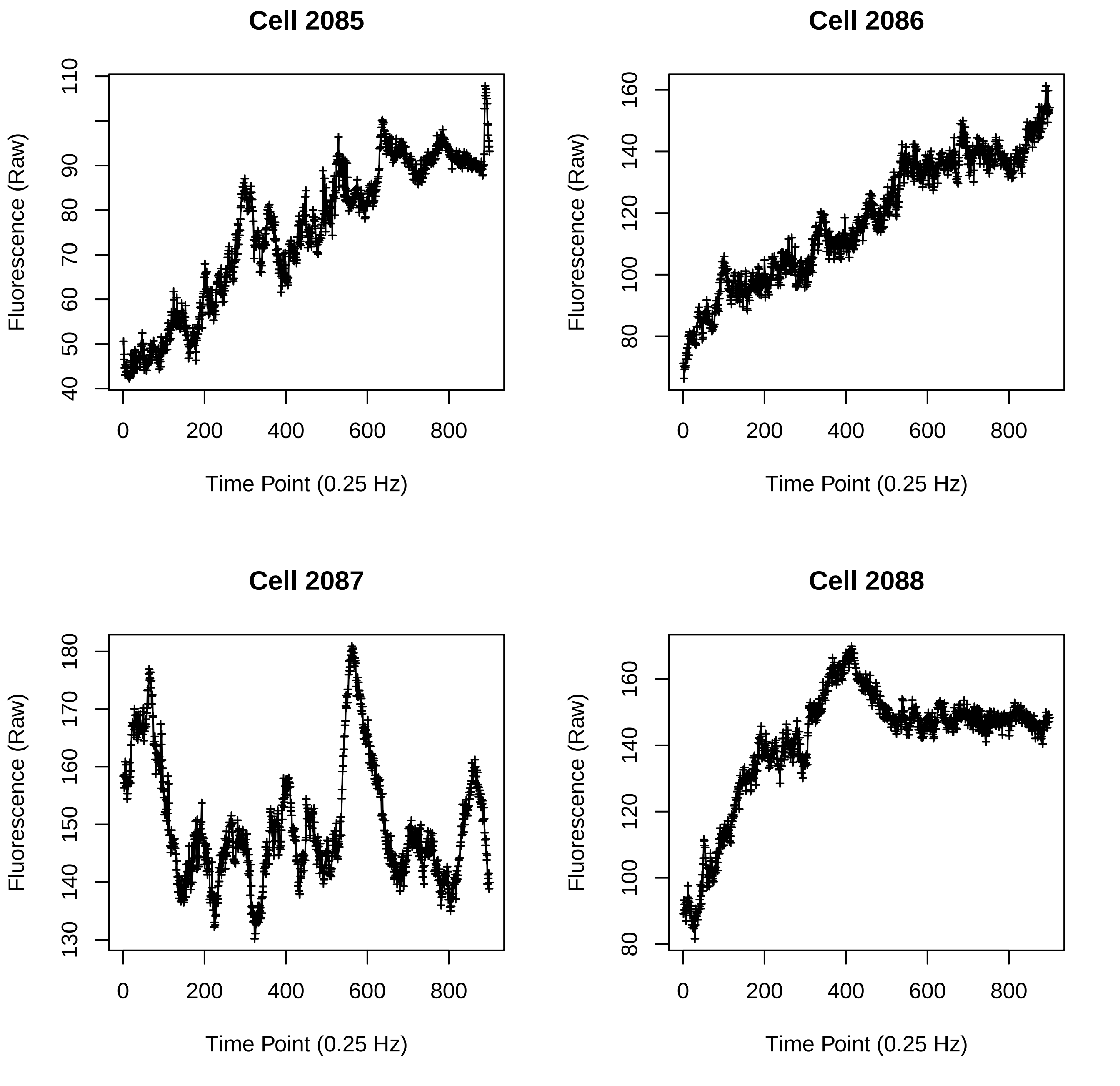
Cell 2085
Cell 2086
Cell 2087
Cell 2088
Fluorescence (Raw)
Time Point (0.25 Hz)
0
200
400
600
800
40
50
60
70
80
90
110
100
120
140
160
130
150
170
180

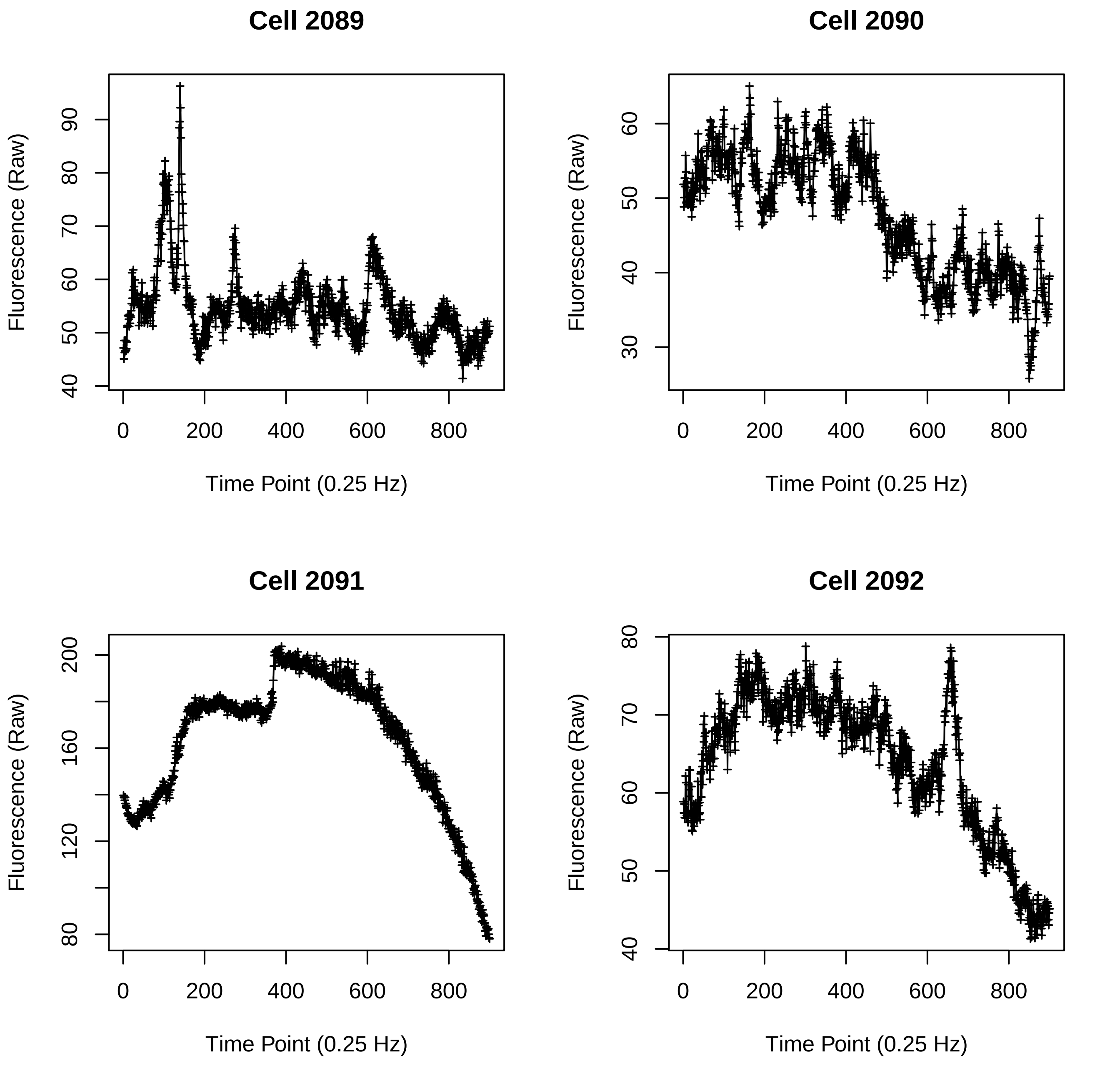

Cell 2089
Cell 2090
Cell 2091
Cell 2092
Fluorescence (Raw)
Time Point (0.25 Hz)
0
200
400
600
800
40
50
60
70
80
90
30
120
160
200

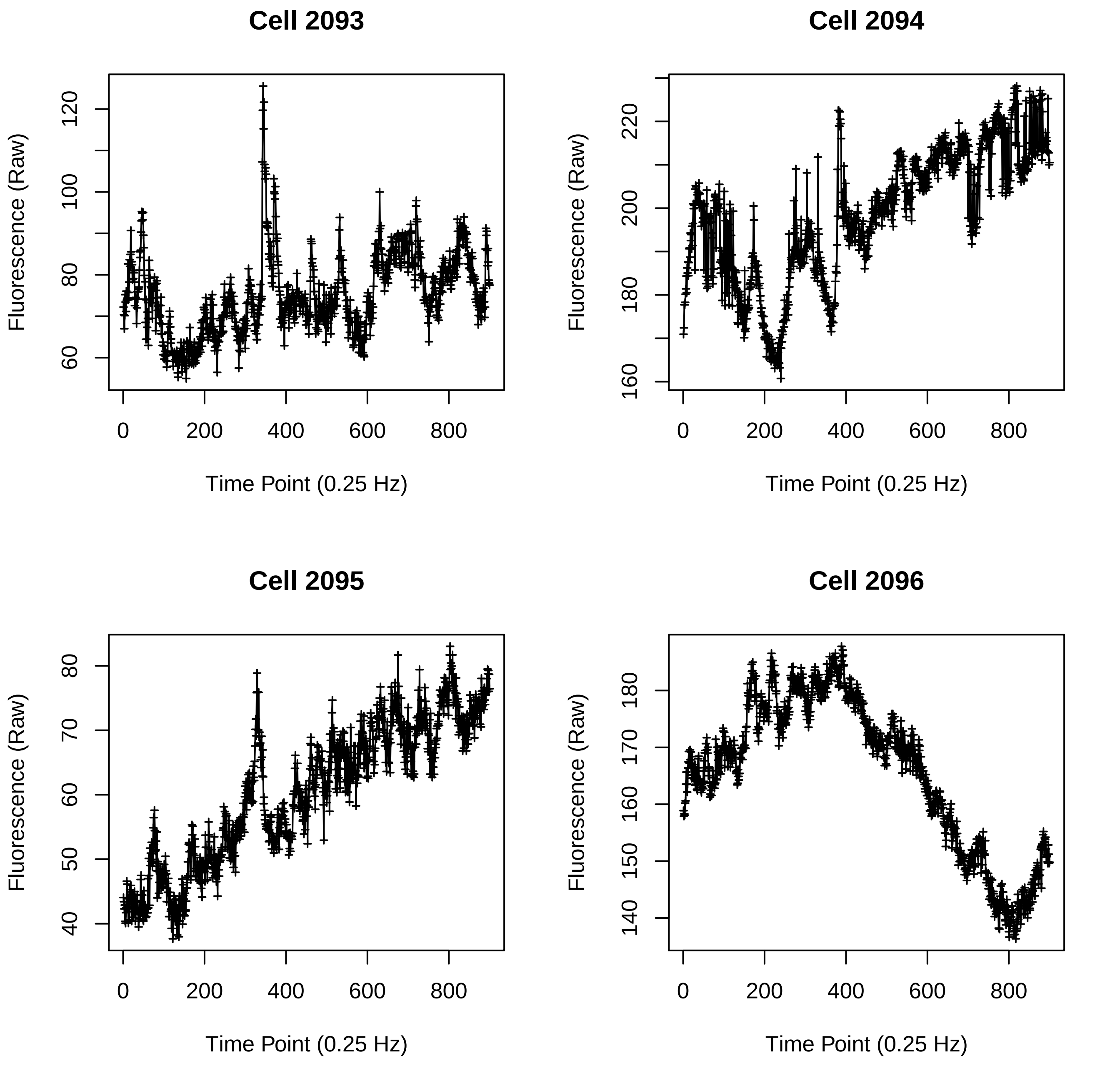
Cell 2093
Fluorescence (Raw)
60
80
100
120
0
200
400
600
800
Time Point (0.25 Hz)
Cell 2094
Fluorescence (Raw)
160
180
200
220
0
200
400
600
800
Time Point (0.25 Hz)
Cell 2095
Fluorescence (Raw)
40
50
60
70
80
0
200
400
600
800
Time Point (0.25 Hz)
Cell 2096
Fluorescence (Raw)
140
150
160
170
180
0
200
400
600
800
Time Point (0.25 Hz)

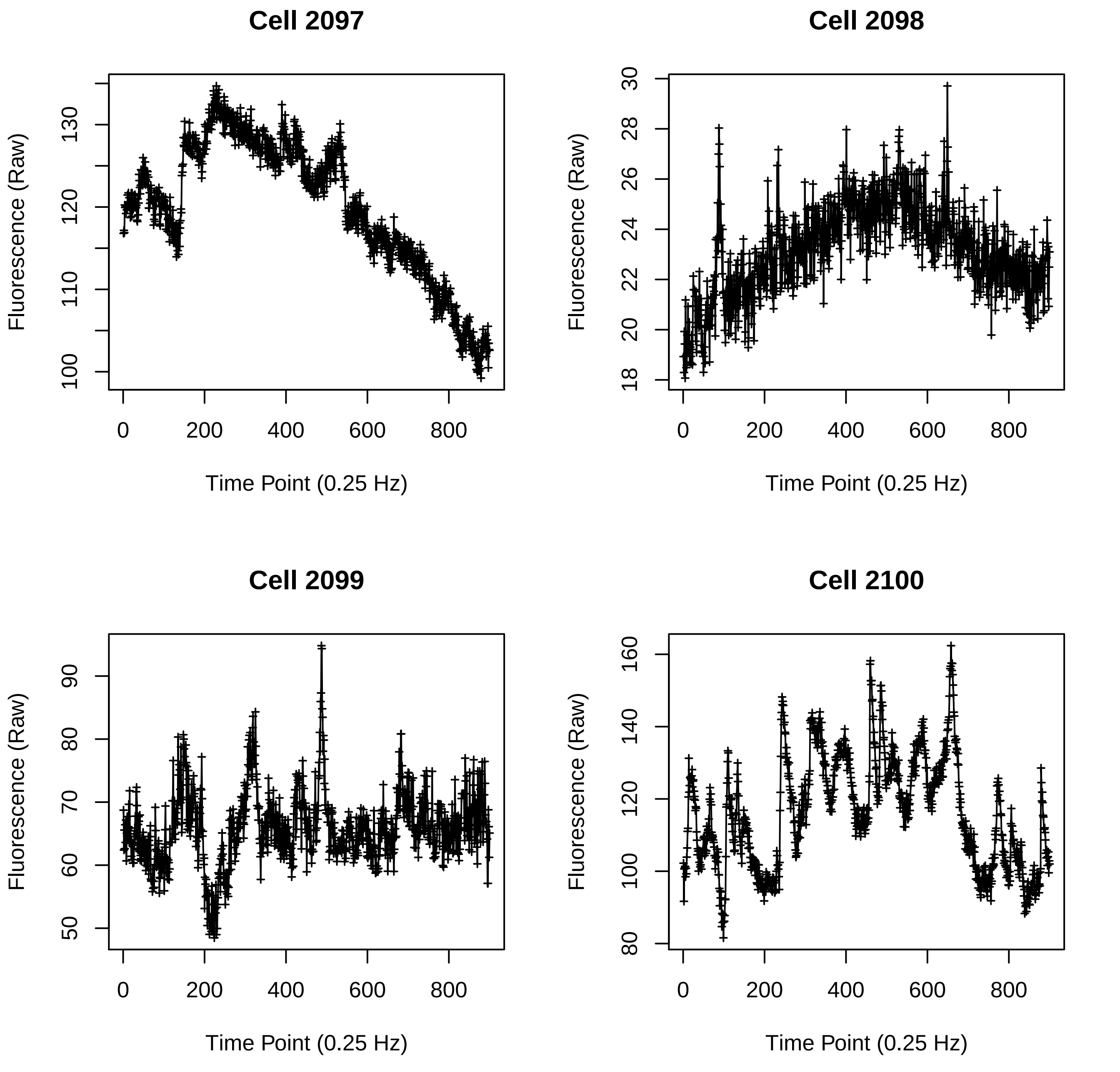
Cell 2097
Cell 2098
Cell 2099
Cell 2100
Fluorescence (Raw)
Time Point (0.25 Hz)
0
200
400
600
800
100
110
120
130
18
20
22
24
26
28
30
50
60
70
80
90
80
100
120
140
160

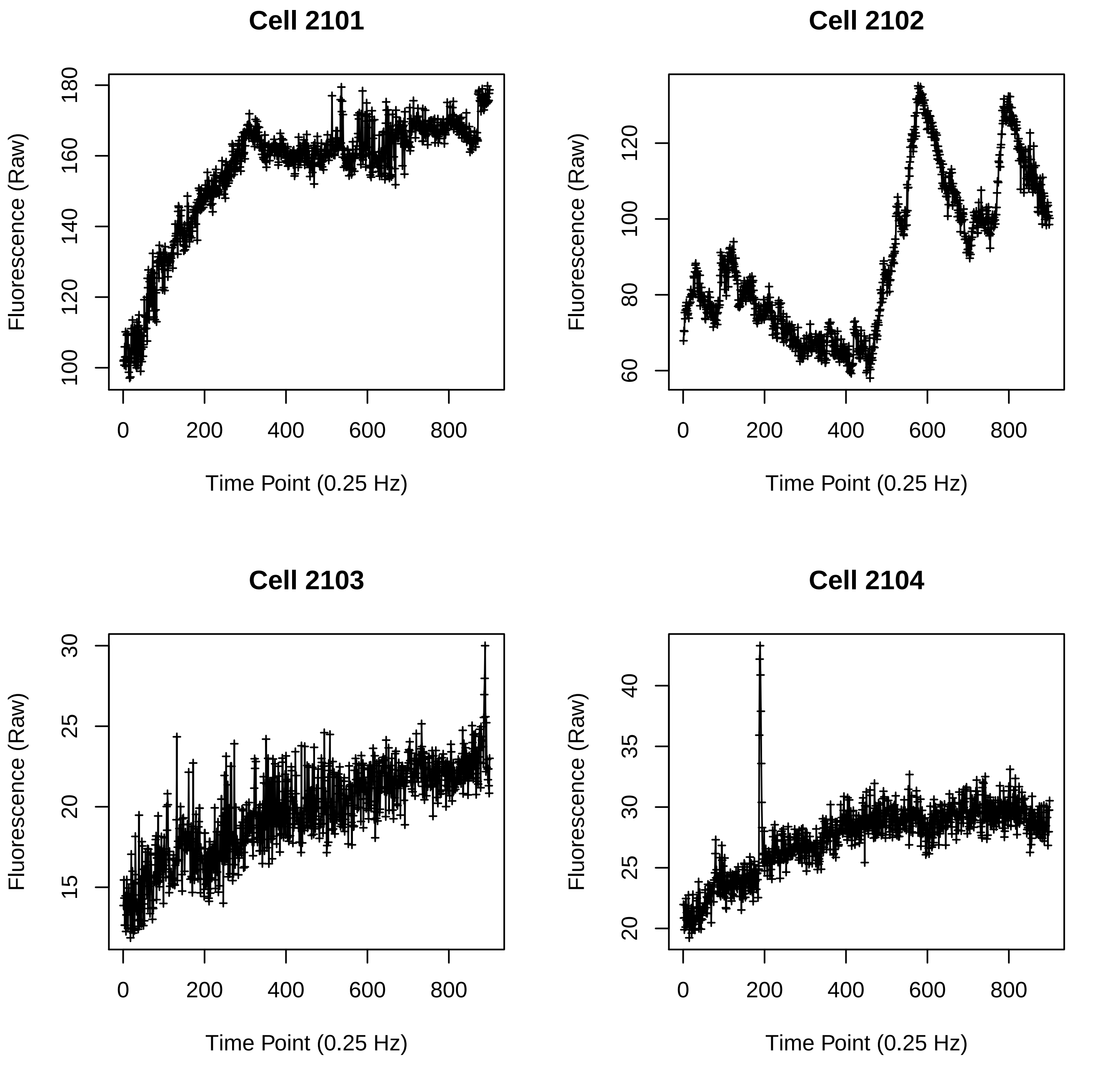
Cell 2101
Cell 2102
Cell 2103
Cell 2104
Fluorescence (Raw)
Time Point (0.25 Hz)
0
200
400
600
800
100
120
140
160
180
60
80
100
120
15
20
25
30
35
40

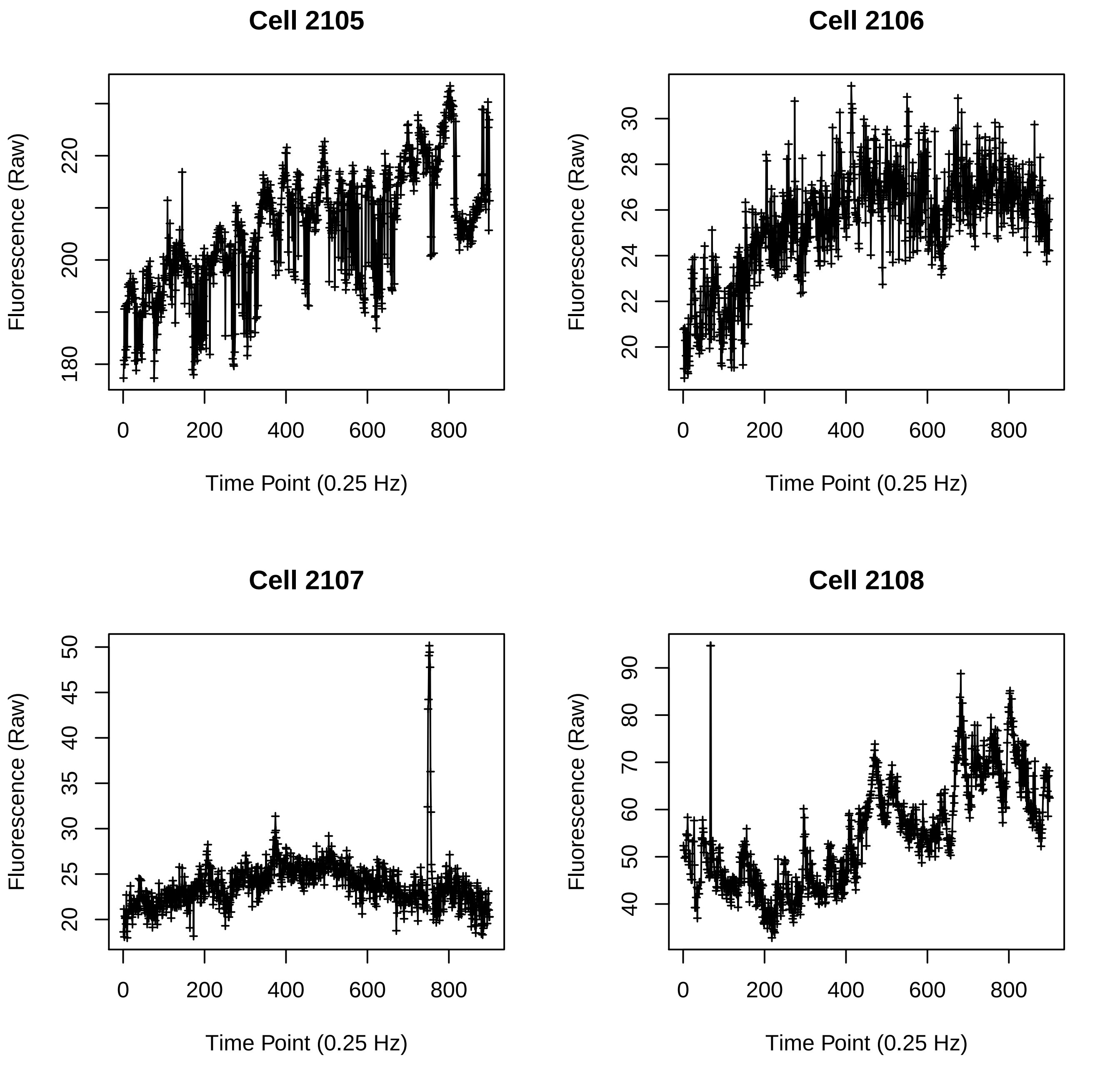
Cell 2105
Cell 2106
Cell 2107
Cell 2108
Fluorescence (Raw)
Time Point (0.25 Hz)
180
200
220
20
22
24
26
28
30
25
35
40
45
50
60
70
80
90
0
200
400
600
800

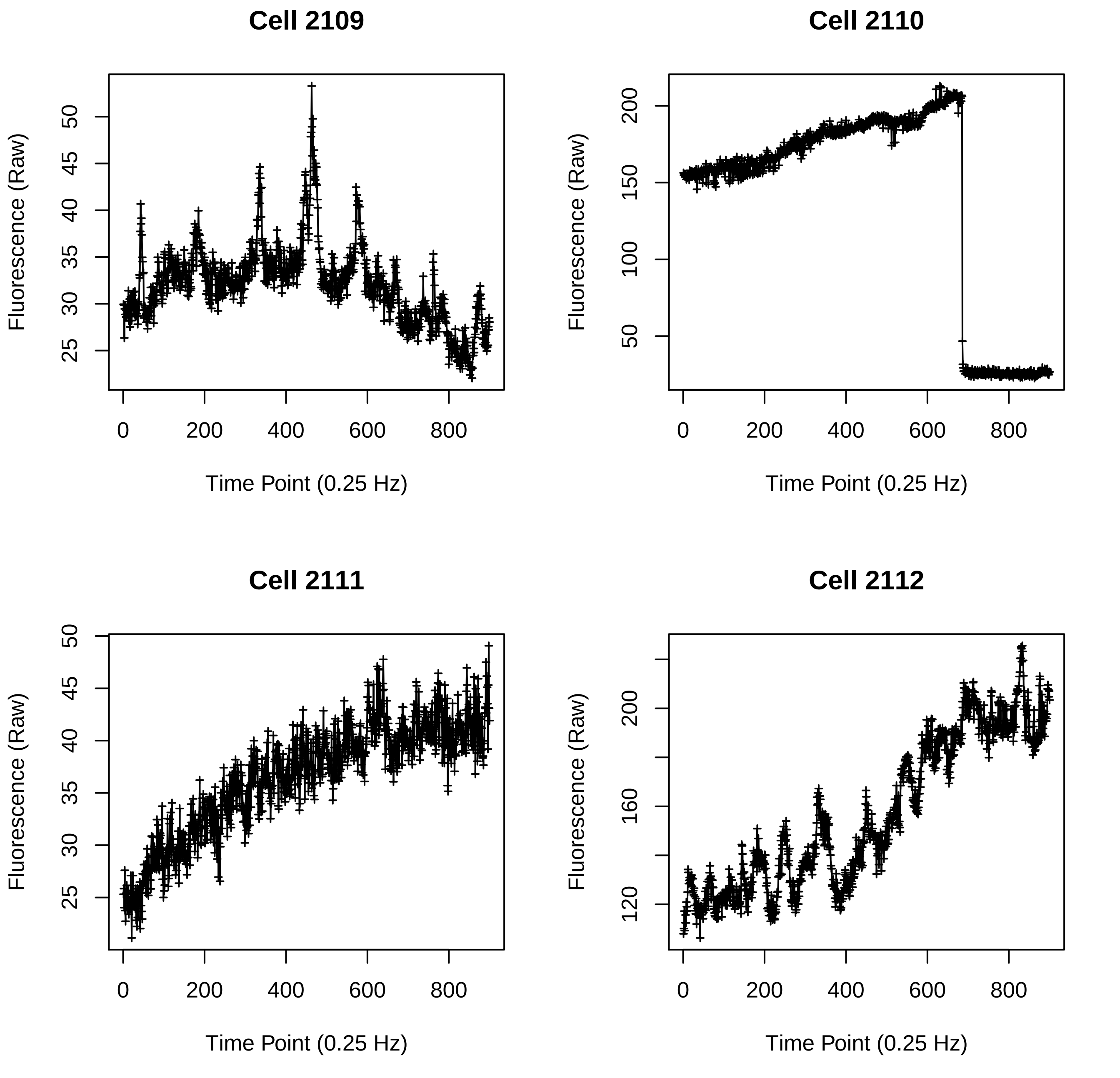
Cell 2109
Fluorescence (Raw)
25
30
35
40
45
50
0
200
400
600
800
Time Point (0.25 Hz)
Cell 2110
Fluorescence (Raw)
50
100
150
200
0
200
400
600
800
Time Point (0.25 Hz)
Cell 2111
Fluorescence (Raw)
25
30
35
40
45
50
0
200
400
600
800
Time Point (0.25 Hz)
Cell 2112
Fluorescence (Raw)
120
160
200
0
200
400
600
800
Time Point (0.25 Hz)

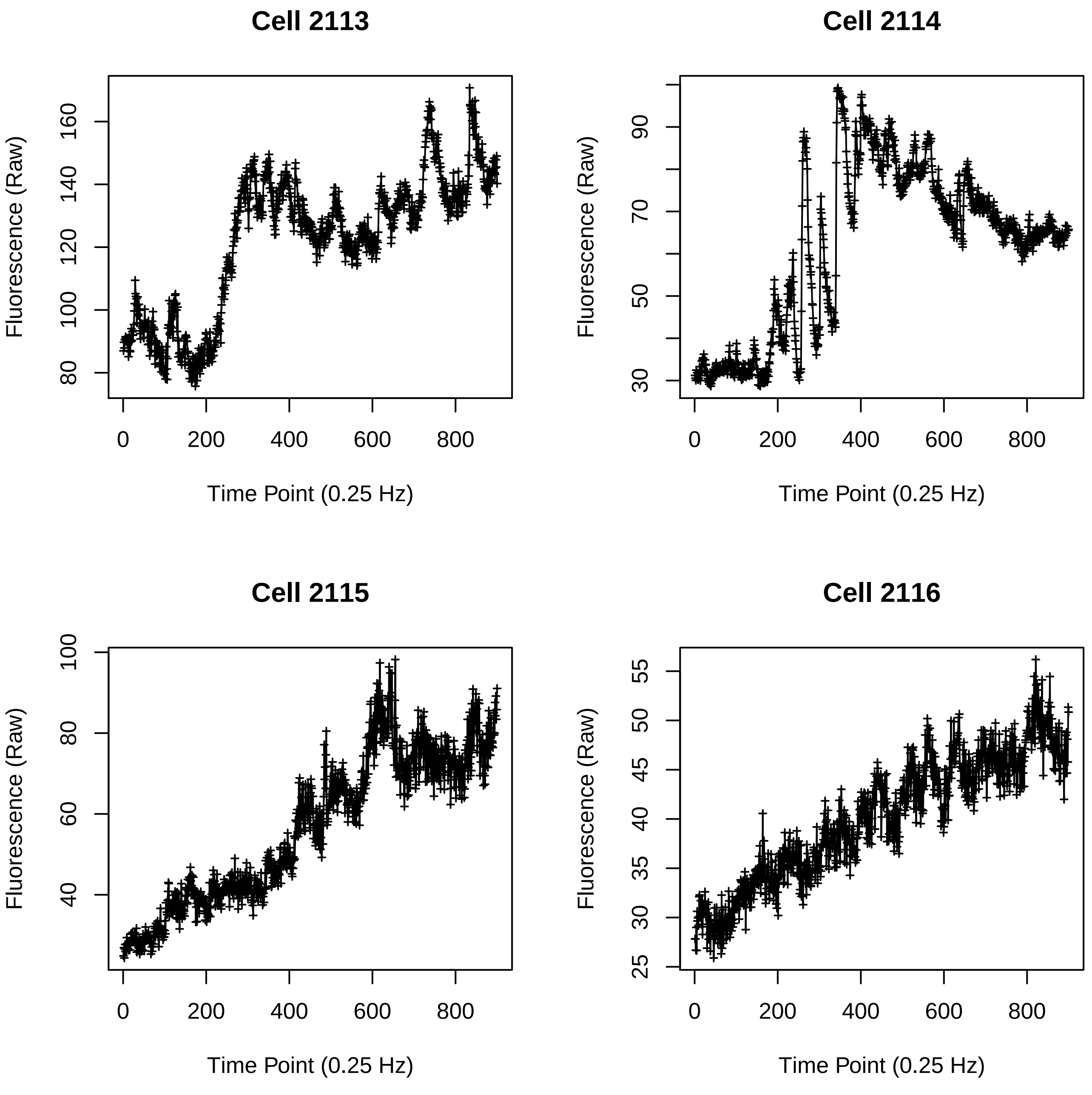
Cell 2113
Fluorescence (Raw)
80
100
120
140
160
0
200
400
600
800
Time Point (0.25 Hz)
Cell 2114
Fluorescence (Raw)
30
50
70
90
0
200
400
600
800
Time Point (0.25 Hz)
Cell 2115
Fluorescence (Raw)
40
60
80
100
0
200
400
600
800
Time Point (0.25 Hz)
Cell 2116
Fluorescence (Raw)
25
30
35
40
45
50
55
0
200
400
600
800
Time Point (0.25 Hz)

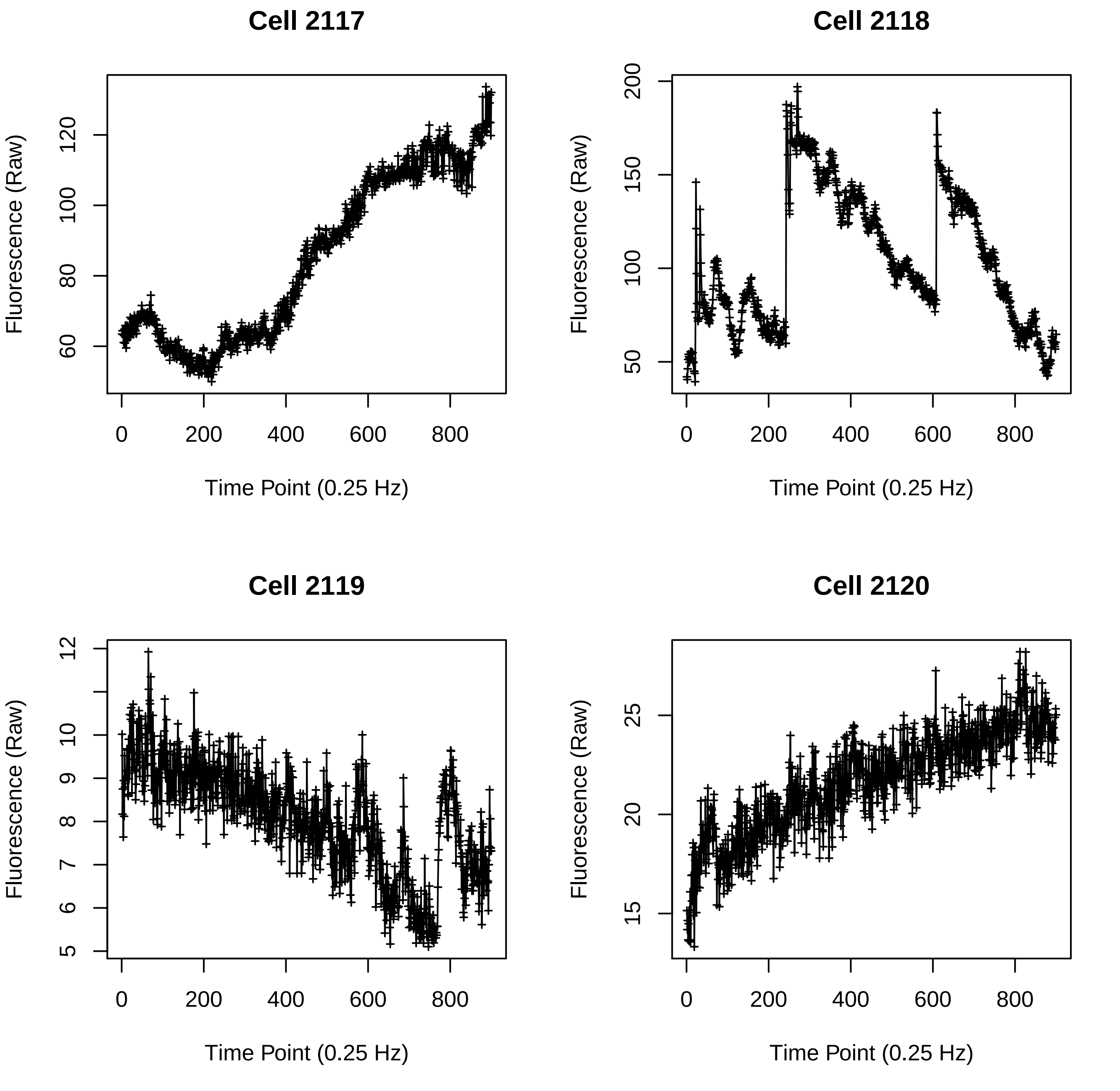

Cell 2117
Cell 2118
Cell 2119
Cell 2120
Fluorescence (Raw)
Time Point (0.25 Hz)
0
200
400
600
800
60
80
100
120
50
100
150
200
5
6
7
8
9
10
12
15
20
25

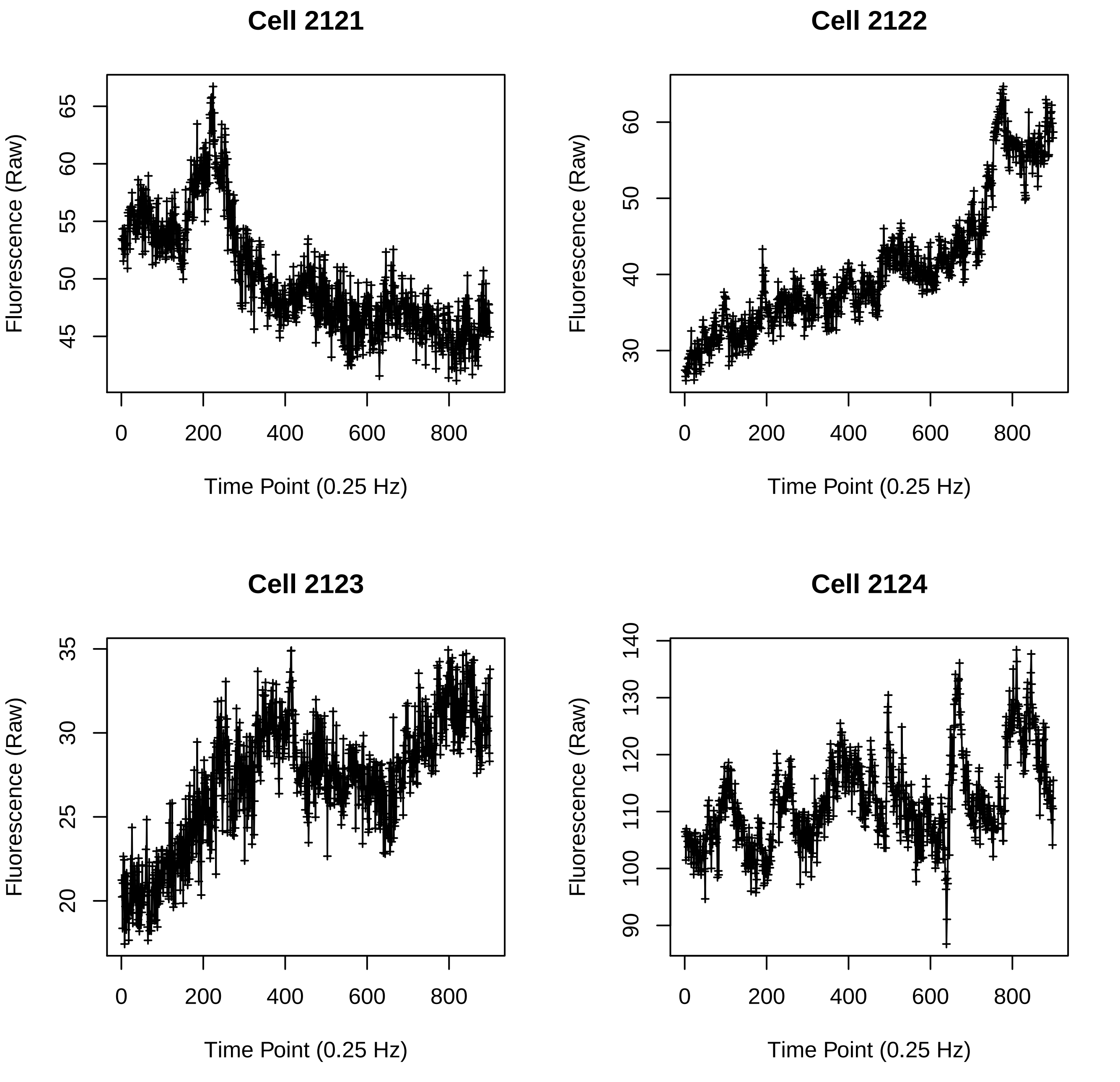
Cell 2121
Cell 2122
Cell 2123
Cell 2124
Fluorescence (Raw)
Time Point (0.25 Hz)
0
200
400
600
800
45
50
55
60
65
30
40
50
60
20
25
30
35
90
100
110
120
130
140

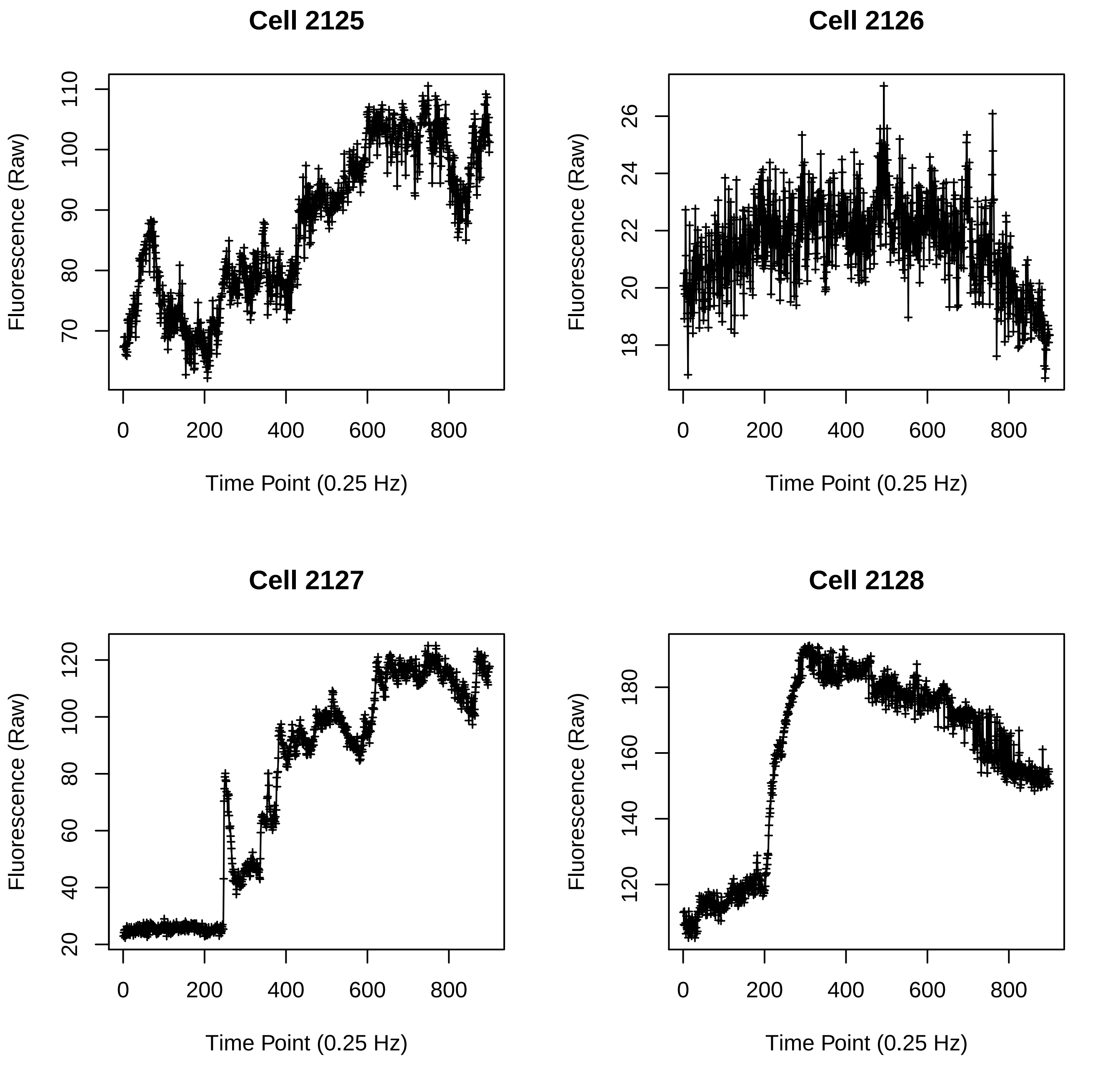
Cell 2125
Cell 2126
Cell 2127
Cell 2128
Fluorescence (Raw)
Time Point (0.25 Hz)
0
200
400
600
800
70
80
90
100
110
18
20
22
24
26
20
40
60
80
100
120
140
160
180

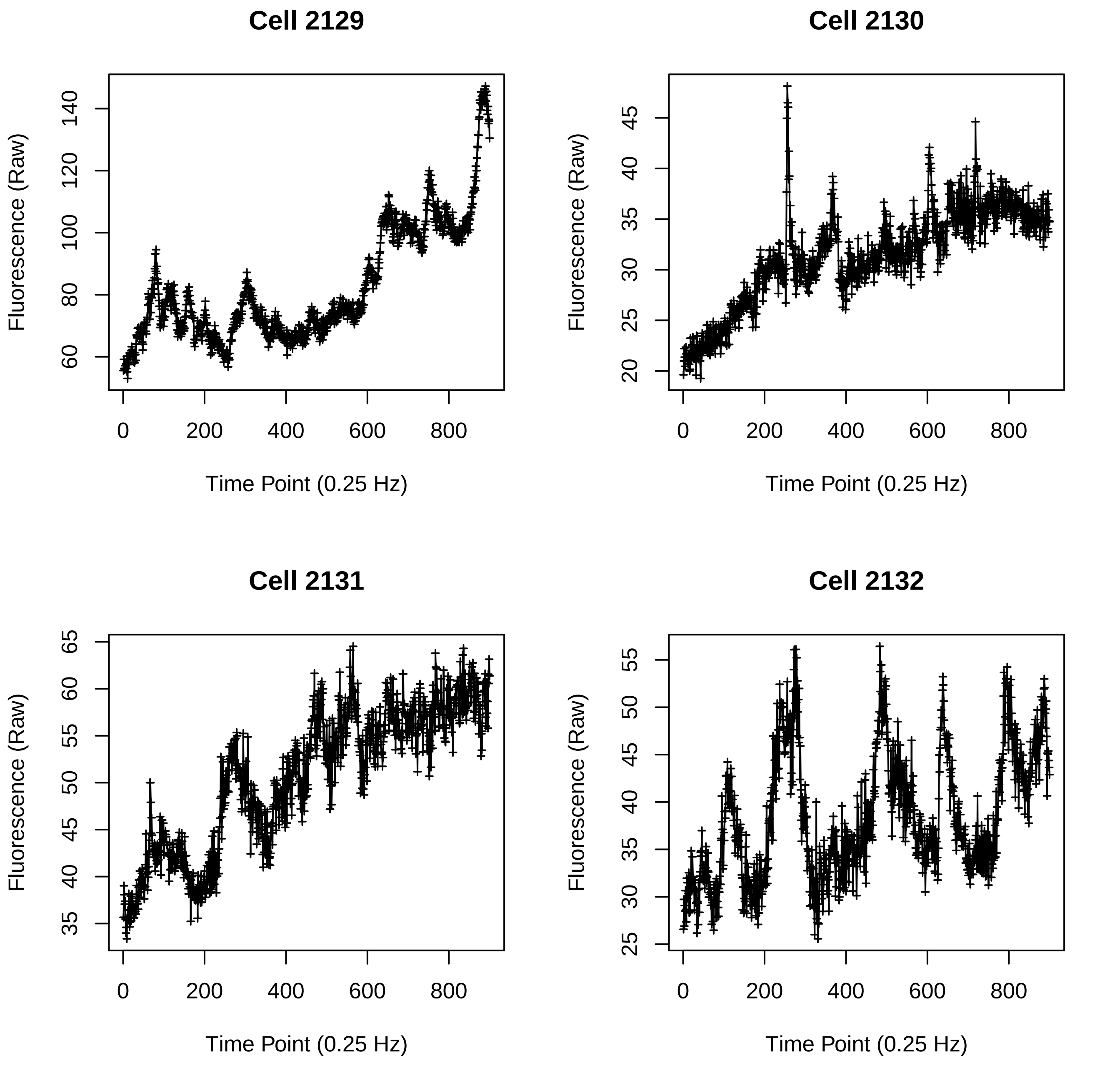
Cell 2129
Fluorescence (Raw)
Time Point (0.25 Hz)
Cell 2130
Fluorescence (Raw)
Time Point (0.25 Hz)
Cell 2131
Fluorescence (Raw)
Time Point (0.25 Hz)
Cell 2132
Fluorescence (Raw)
Time Point (0.25 Hz)

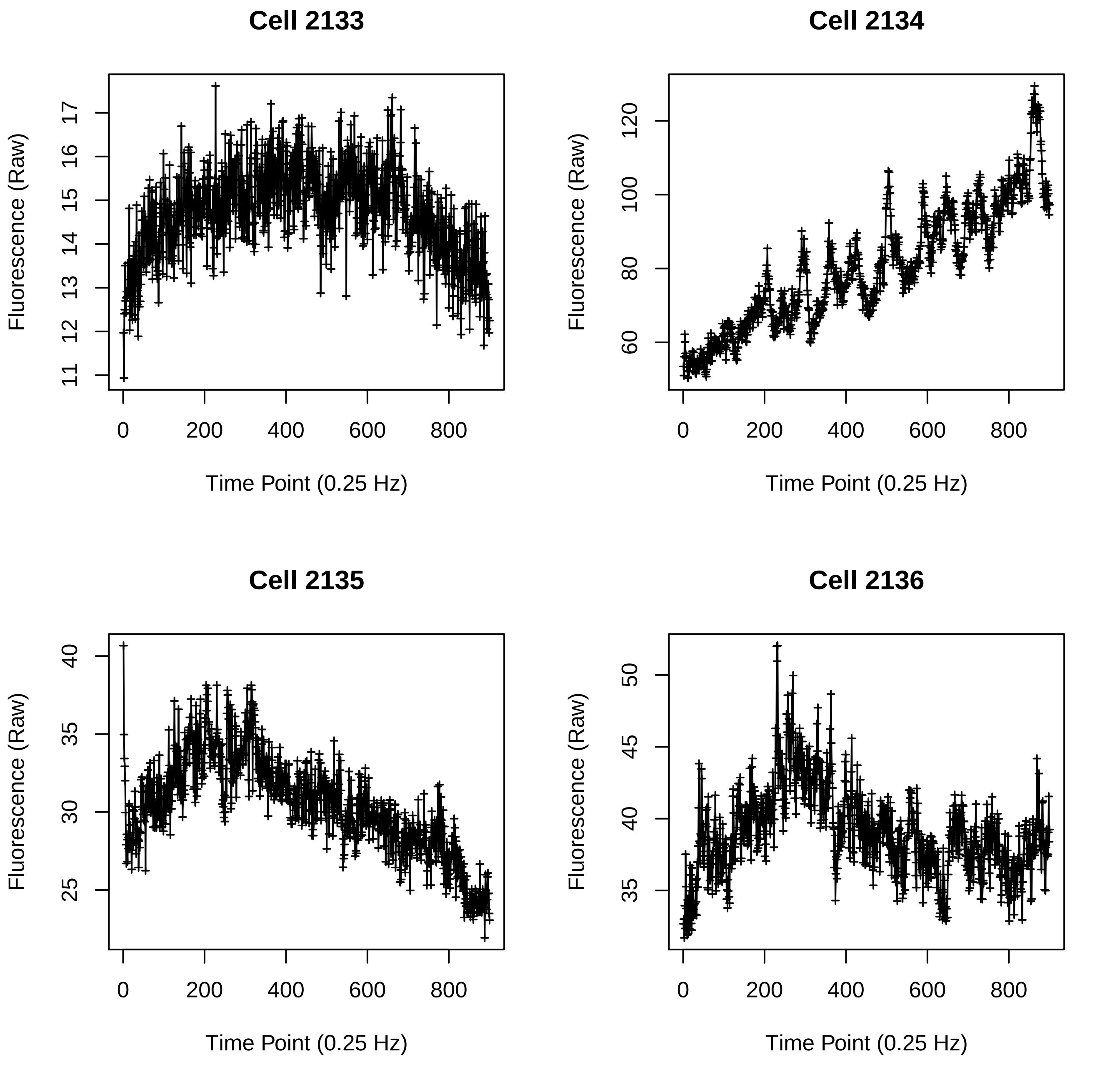

Cell 2133
Fluorescence (Raw)
Time Point (0.25 Hz)
Cell 2134
Fluorescence (Raw)
Time Point (0.25 Hz)
Cell 2135
Fluorescence (Raw)
Time Point (0.25 Hz)
Cell 2136
Fluorescence (Raw)
Time Point (0.25 Hz)

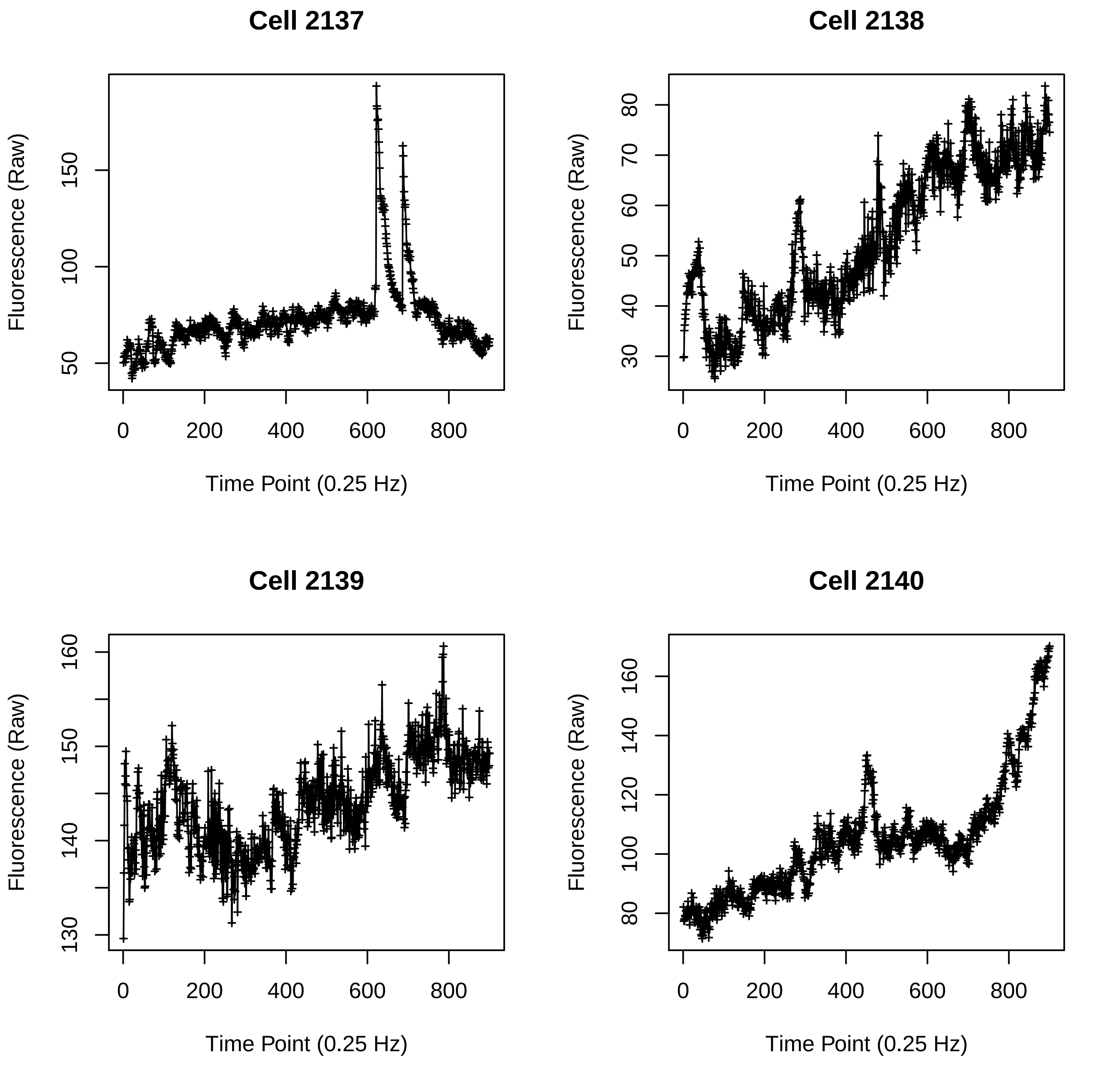
Cell 2137
Fluorescence (Raw)
50
100
150
0
200
400
600
800
Time Point (0.25 Hz)
Cell 2138
Fluorescence (Raw)
30
40
50
60
70
80
0
200
400
600
800
Time Point (0.25 Hz)
Cell 2139
Fluorescence (Raw)
130
140
150
160
0
200
400
600
800
Time Point (0.25 Hz)
Cell 2140
Fluorescence (Raw)
80
100
120
140
160
0
200
400
600
800
Time Point (0.25 Hz)

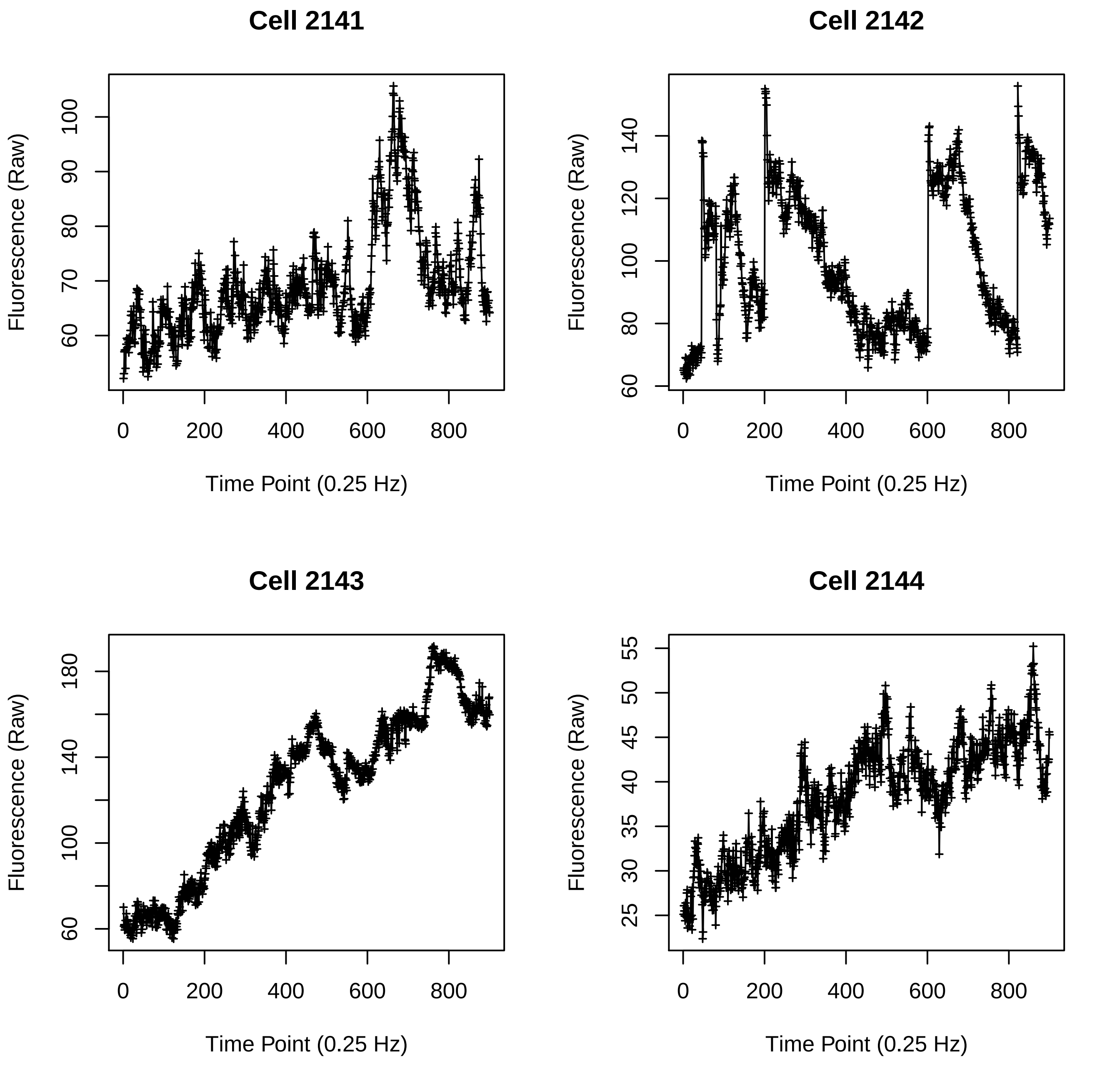

Cell 2141
Cell 2142
Cell 2143
Cell 2144
Fluorescence (Raw)
Time Point (0.25 Hz)
0
200
400
600
800

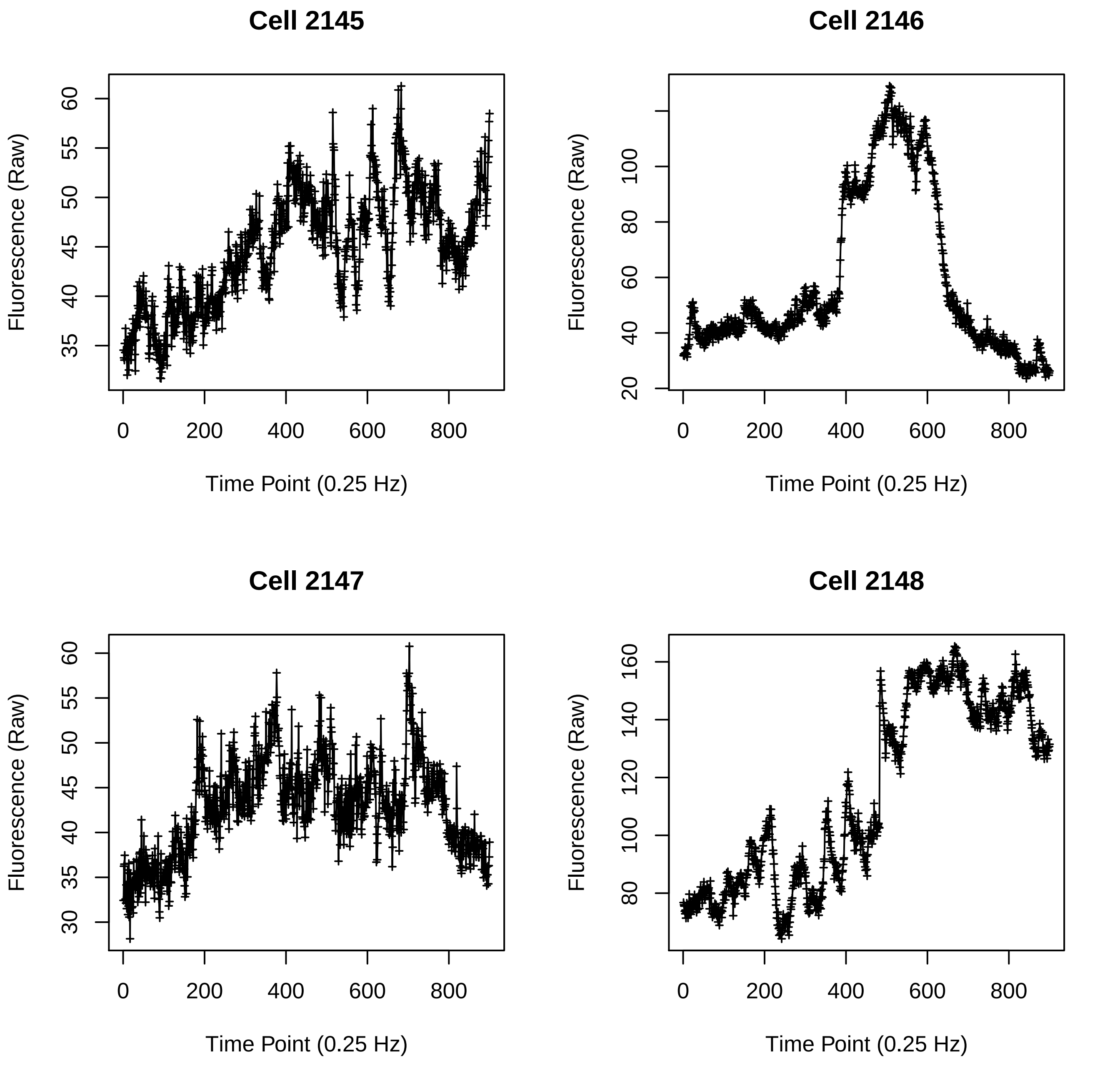
Cell 2145
Fluorescence (Raw)
Time Point (0.25 Hz)
Cell 2146
Fluorescence (Raw)
Time Point (0.25 Hz)
Cell 2147
Fluorescence (Raw)
Time Point (0.25 Hz)
Cell 2148
Fluorescence (Raw)
Time Point (0.25 Hz)

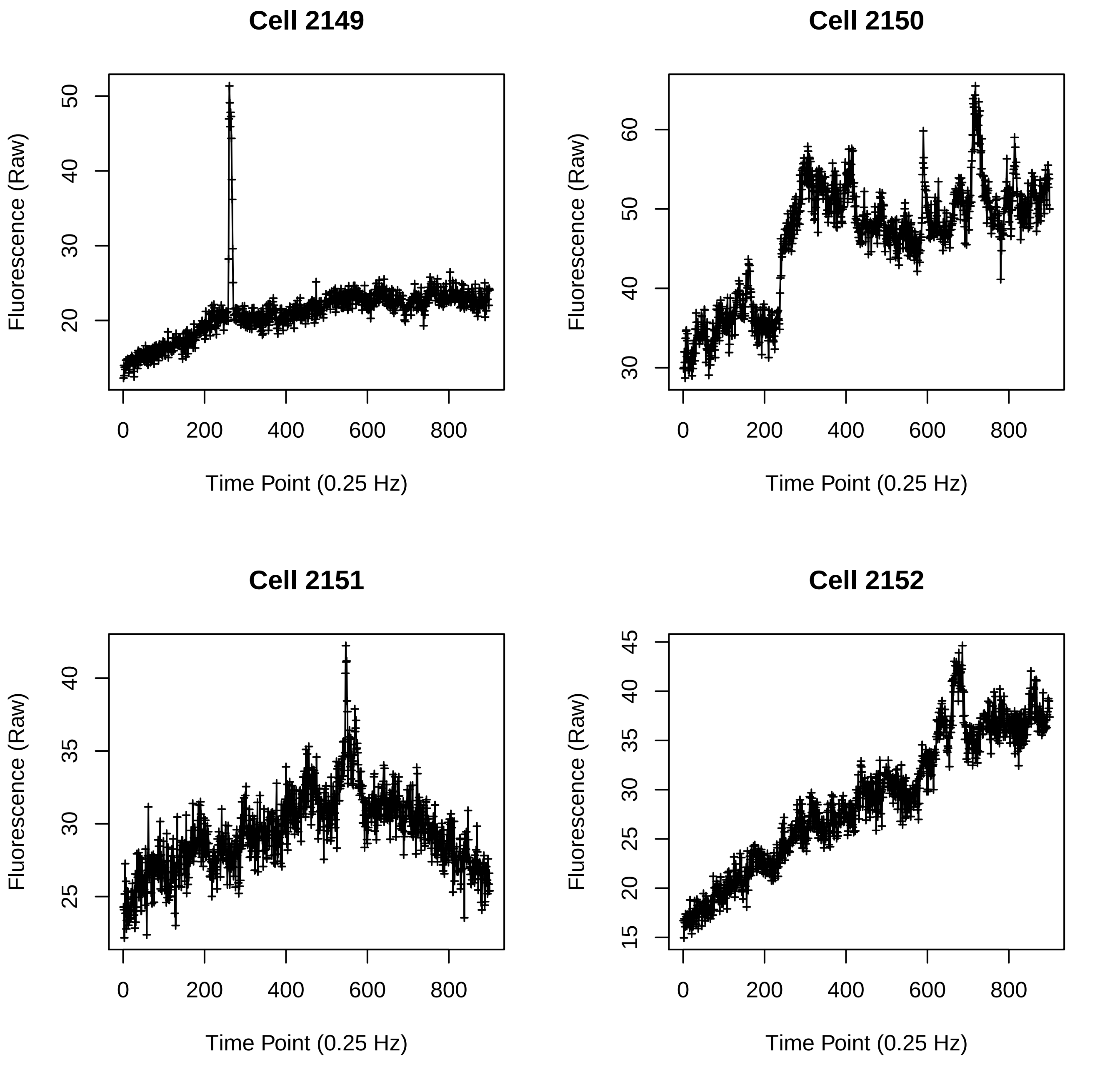
Cell 2149
Cell 2150
Cell 2151
Cell 2152
Fluorescence (Raw)
Time Point (0.25 Hz)
0
200
400
600
800
20
30
40
50
60
15
25
35
45

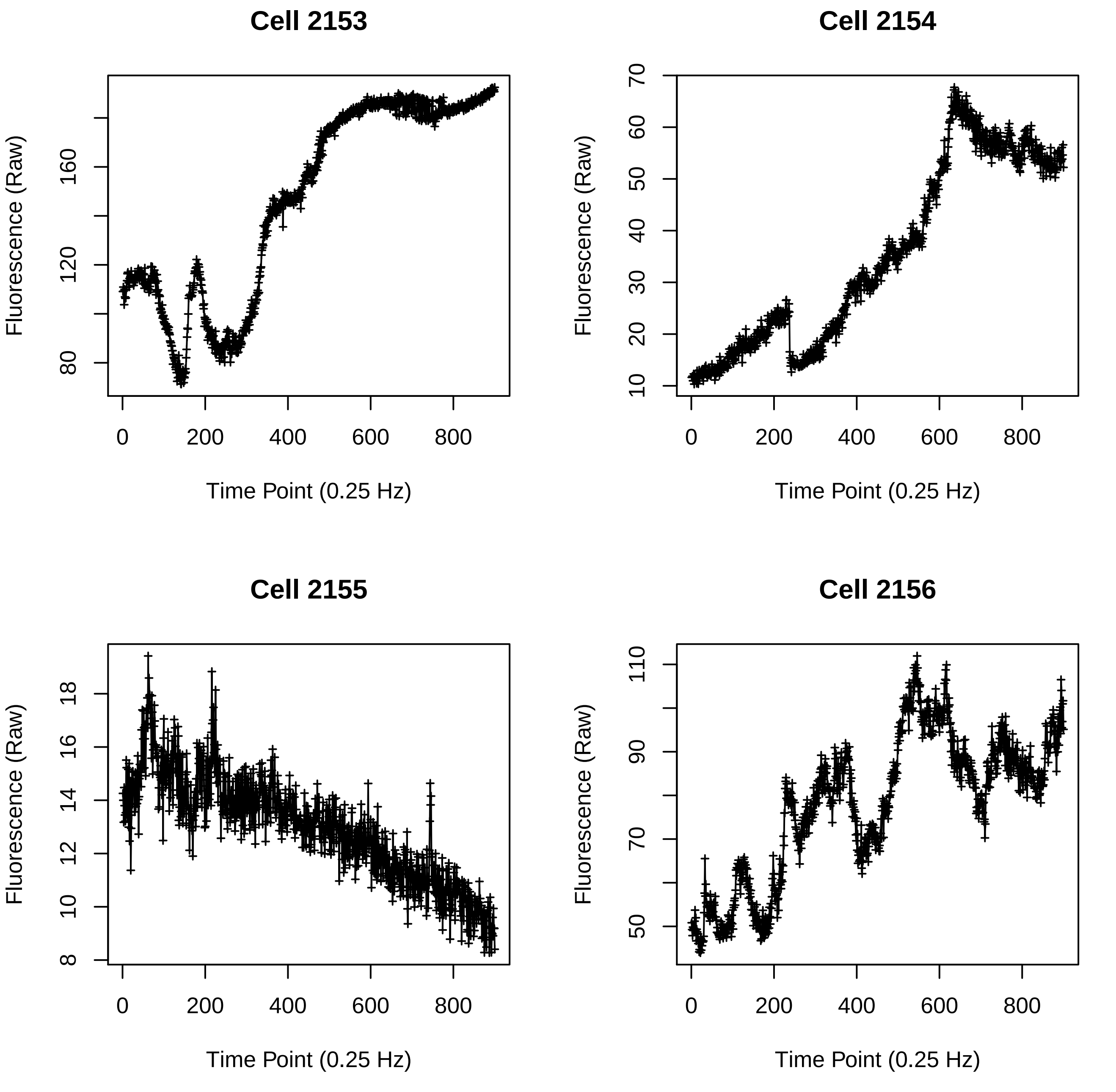

Cell 2153
Fluorescence (Raw)
80
120
160
0
200
400
600
800
Time Point (0.25 Hz)
Cell 2154
Fluorescence (Raw)
10
20
30
40
50
60
70
0
200
400
600
800
Time Point (0.25 Hz)
Cell 2155
Fluorescence (Raw)
8
10
12
14
16
18
0
200
400
600
800
Time Point (0.25 Hz)
Cell 2156
Fluorescence (Raw)
50
70
90
110
0
200
400
600
800
Time Point (0.25 Hz)

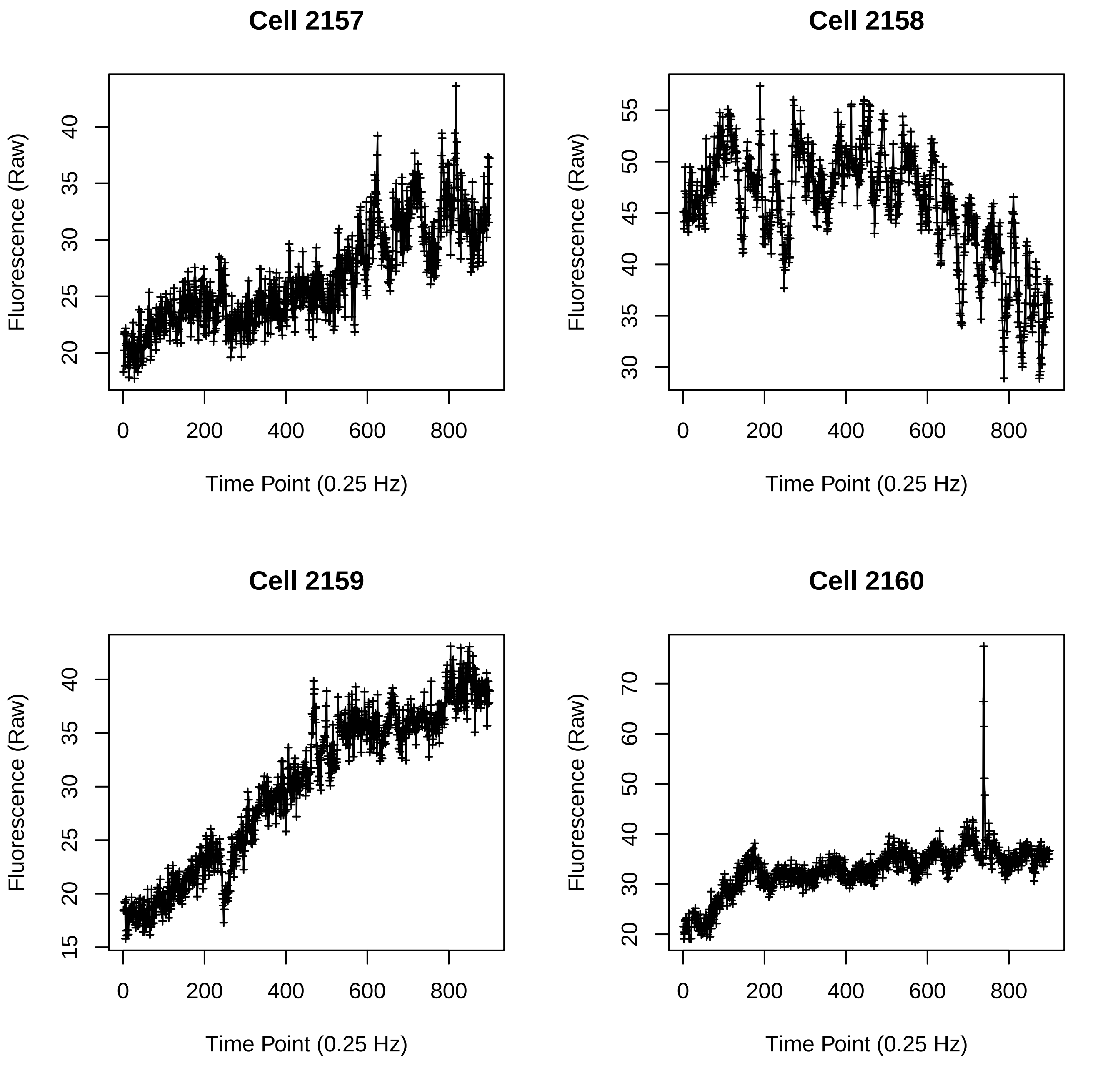
Cell 2157
Cell 2158
Cell 2159
Cell 2160
Fluorescence (Raw)
Time Point (0.25 Hz)
0
200
400
600
800
15
20
25
30
35
40
45
50
55
60
70

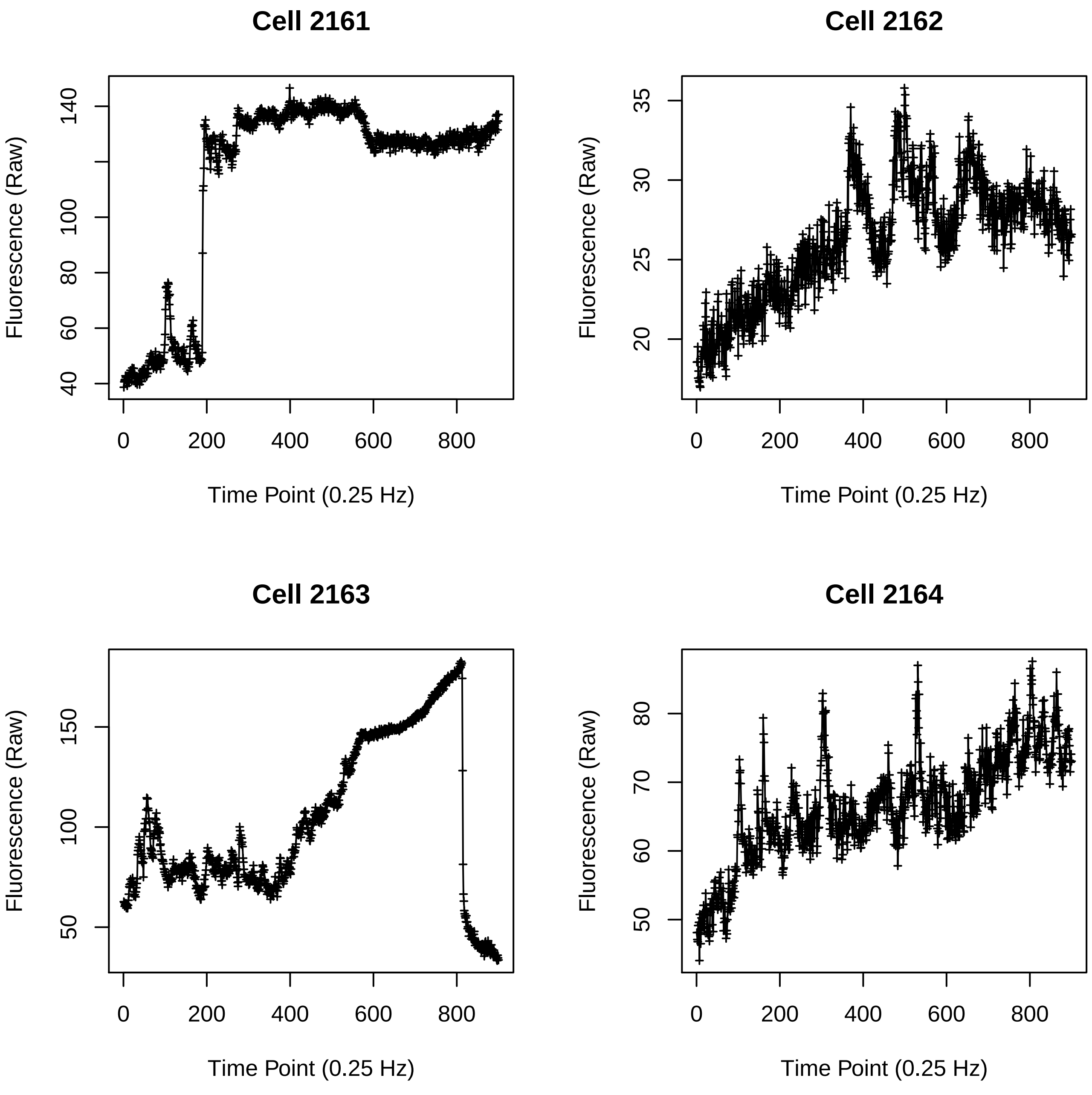
Cell 2161
Fluorescence (Raw)
40
60
80
100
140
0
200
400
600
800
Time Point (0.25 Hz)
Cell 2162
Fluorescence (Raw)
20
25
30
35
0
200
400
600
800
Time Point (0.25 Hz)
Cell 2163
Fluorescence (Raw)
50
100
150
0
200
400
600
800
Time Point (0.25 Hz)
Cell 2164
Fluorescence (Raw)
50
60
70
80
0
200
400
600
800
Time Point (0.25 Hz)

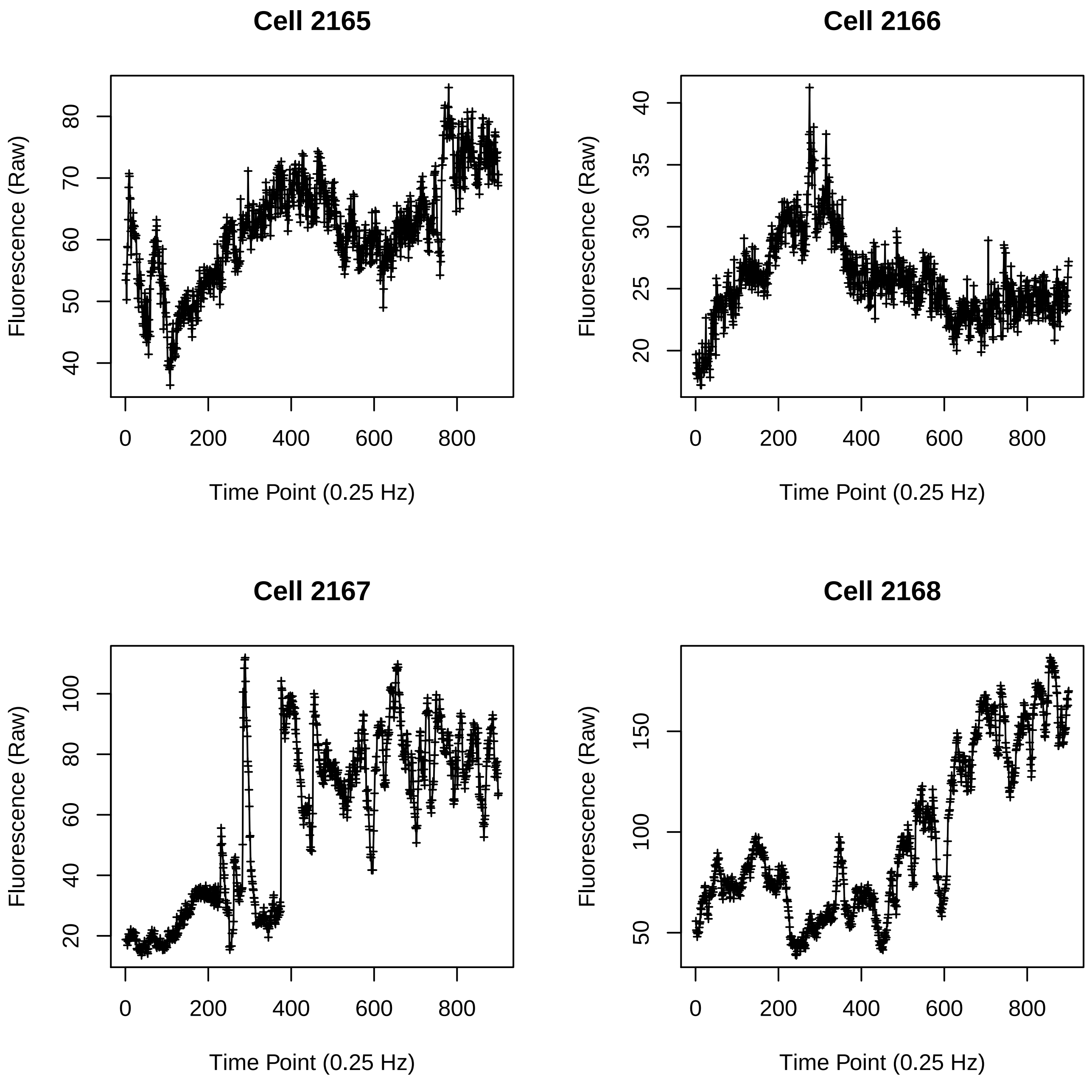

Cell 2165
Cell 2166
Cell 2167
Cell 2168
Fluorescence (Raw)
Time Point (0.25 Hz)
0
200
400
600
800
40
50
60
70
80
20
25
30
35
40
20
40
60
80
100
50
100
150

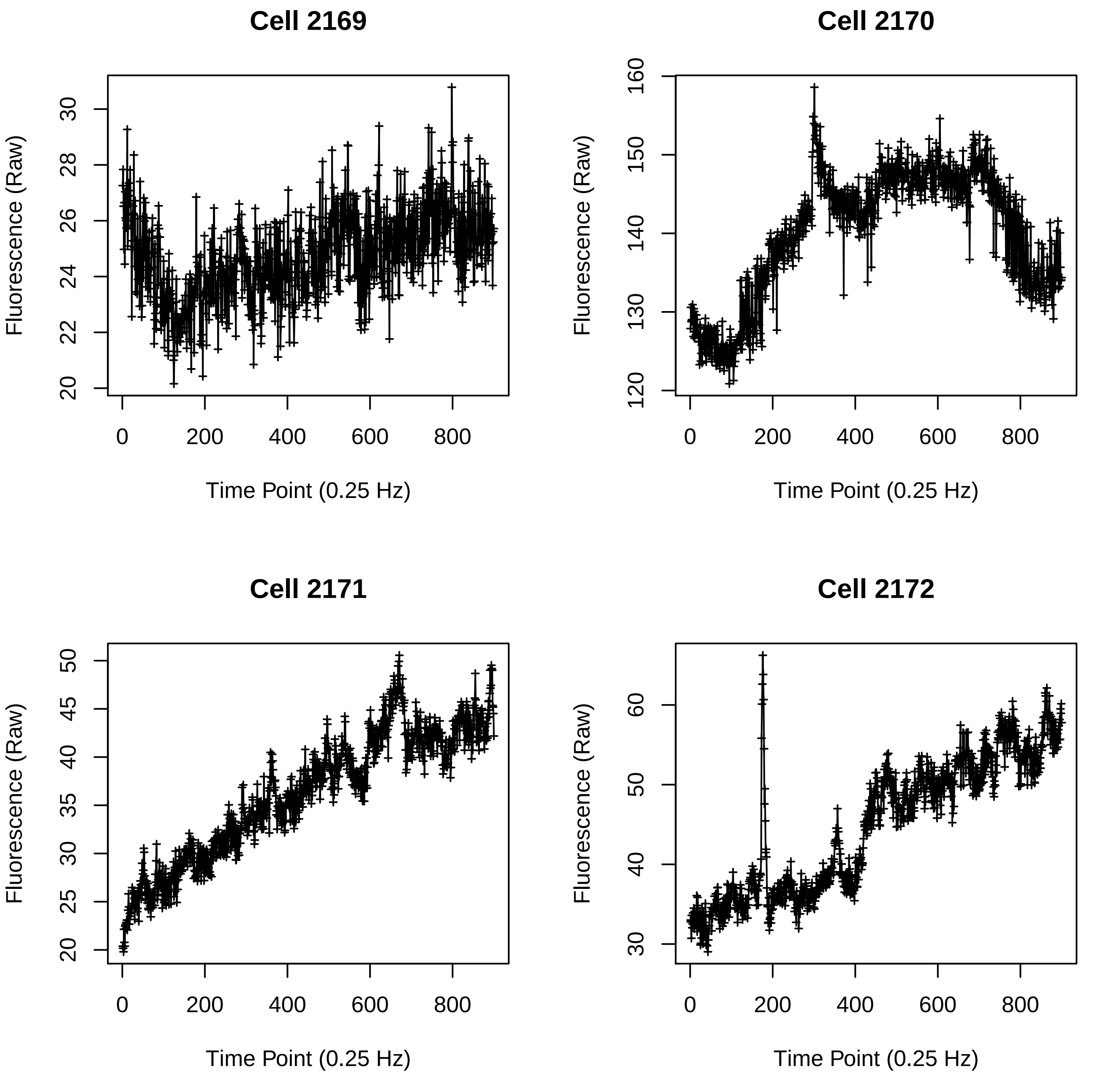
Cell 2169
Cell 2170
Cell 2171
Cell 2172
Fluorescence (Raw)
Time Point (0.25 Hz)
0
200
400
600
800
20
22
24
26
28
30
120
130
140
150
160
25
35
40
45
50
60

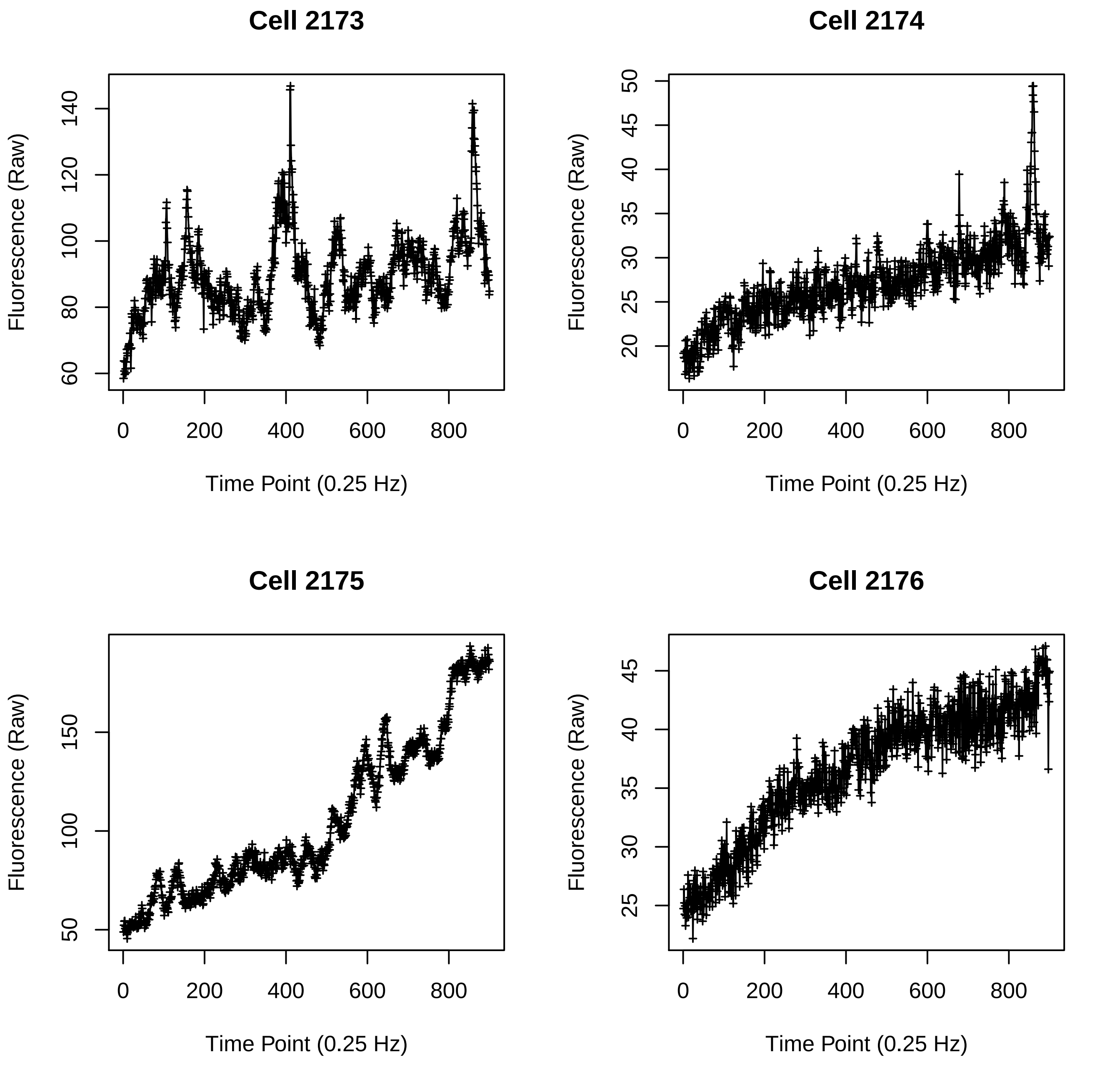
Cell 2173
Cell 2174
Cell 2175
Cell 2176
Fluorescence (Raw)
Time Point (0.25 Hz)
0
200
400
600
800
60
80
100
120
140
20
25
30
35
40
45
50
150

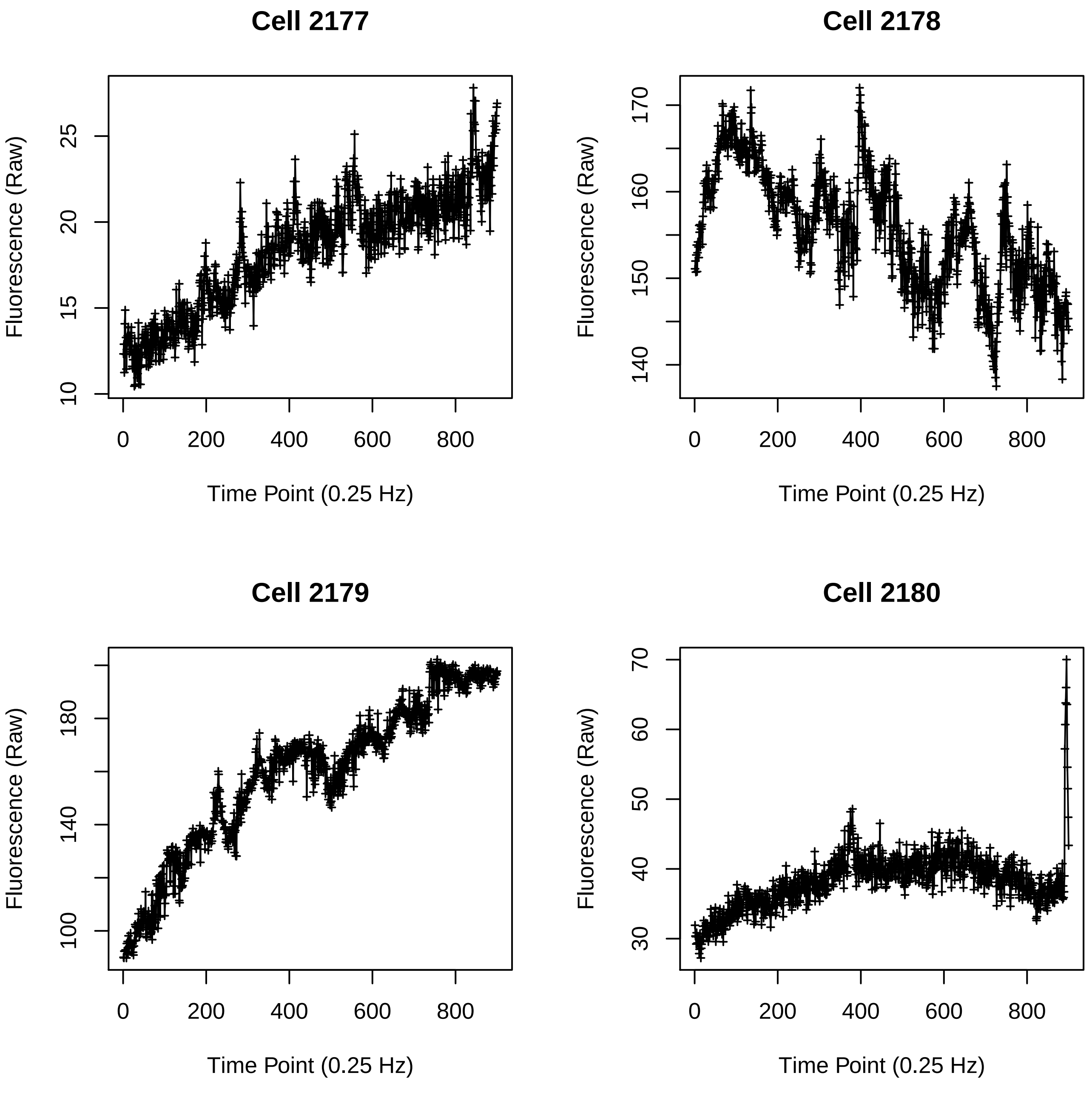
Cell 2177
Fluorescence (Raw)
10
15
20
25
0
200
400
600
800
Time Point (0.25 Hz)
Cell 2178
Fluorescence (Raw)
140
150
160
170
0
200
400
600
800
Time Point (0.25 Hz)
Cell 2179
Fluorescence (Raw)
100
140
180
0
200
400
600
800
Time Point (0.25 Hz)
Cell 2180
Fluorescence (Raw)
30
40
50
60
70
0
200
400
600
800
Time Point (0.25 Hz)

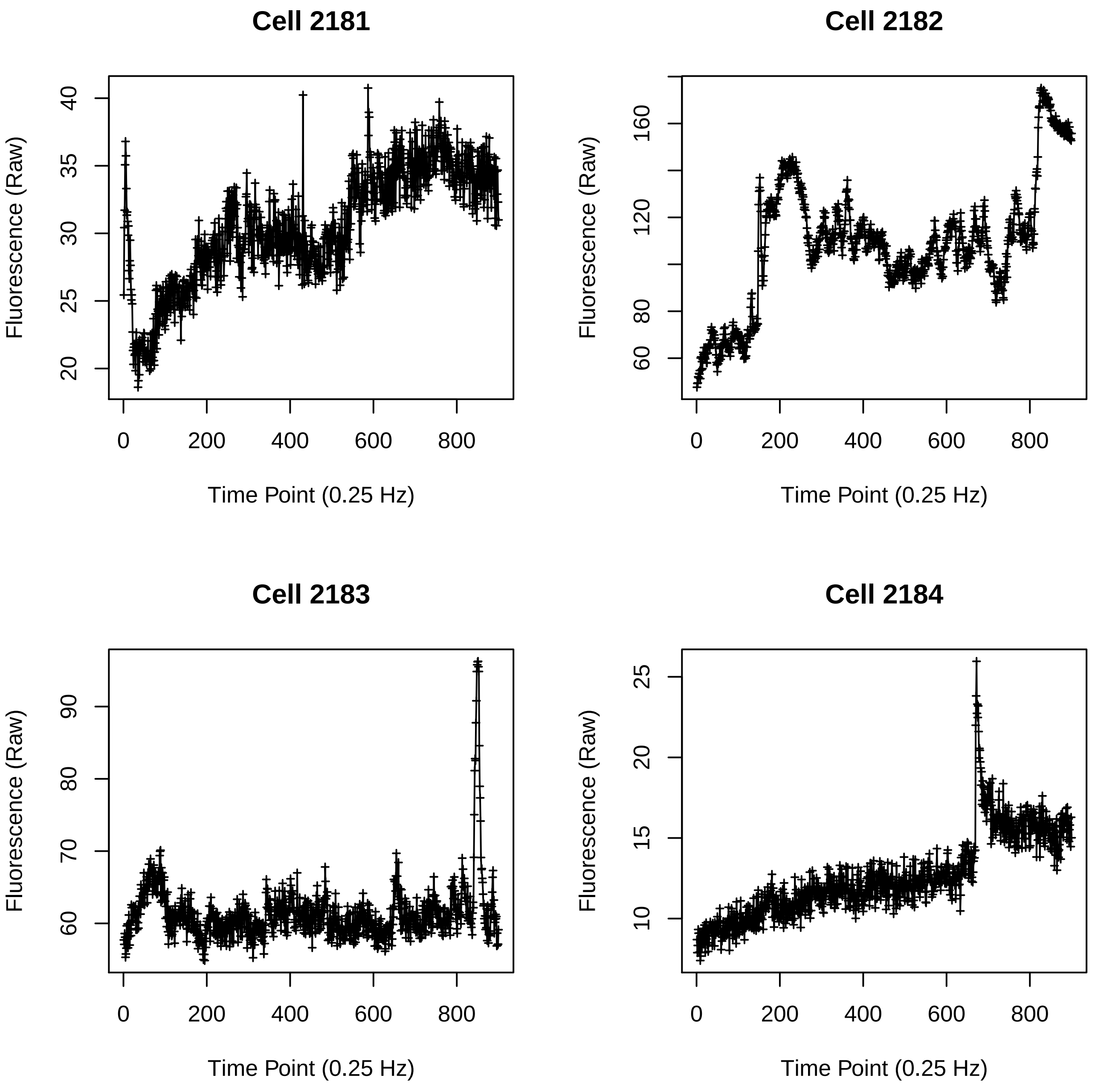
Cell 2181
Fluorescence (Raw)
20
25
30
35
40
0
200
400
600
800
Time Point (0.25 Hz)
Cell 2182
Fluorescence (Raw)
60
80
120
160
0
200
400
600
800
Time Point (0.25 Hz)
Cell 2183
Fluorescence (Raw)
60
70
80
90
0
200
400
600
800
Time Point (0.25 Hz)
Cell 2184
Fluorescence (Raw)
10
15
20
25
0
200
400
600
800
Time Point (0.25 Hz)

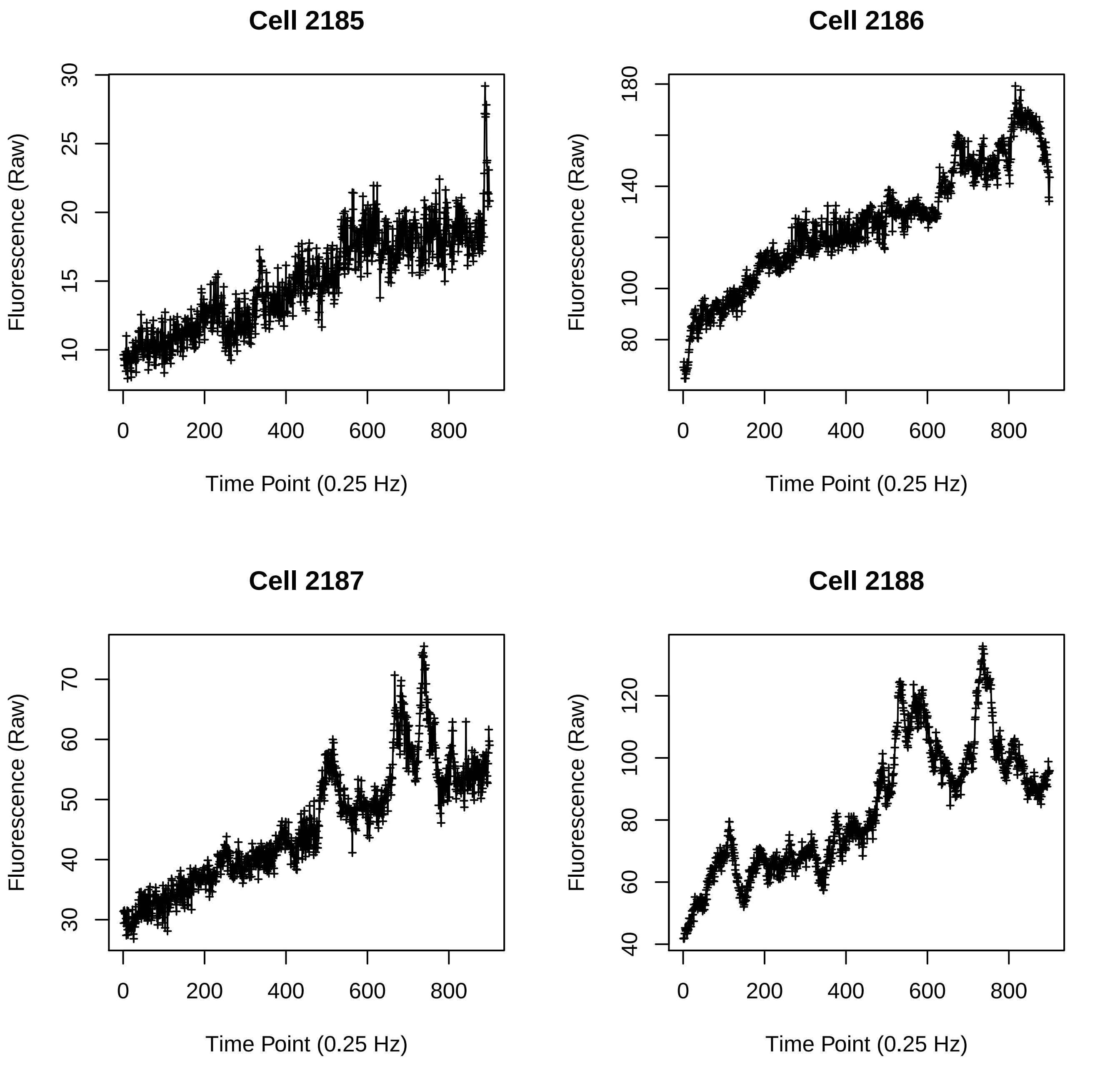
Cell 2185
Fluorescence (Raw)
10
15
20
25
30
Time Point (0.25 Hz)
0
200
400
600
800
Cell 2186
Fluorescence (Raw)
80
100
140
180
Time Point (0.25 Hz)
0
200
400
600
800
Cell 2187
Fluorescence (Raw)
30
40
50
60
70
Time Point (0.25 Hz)
0
200
400
600
800
Cell 2188
Fluorescence (Raw)
40
60
80
100
120
Time Point (0.25 Hz)
0
200
400
600
800

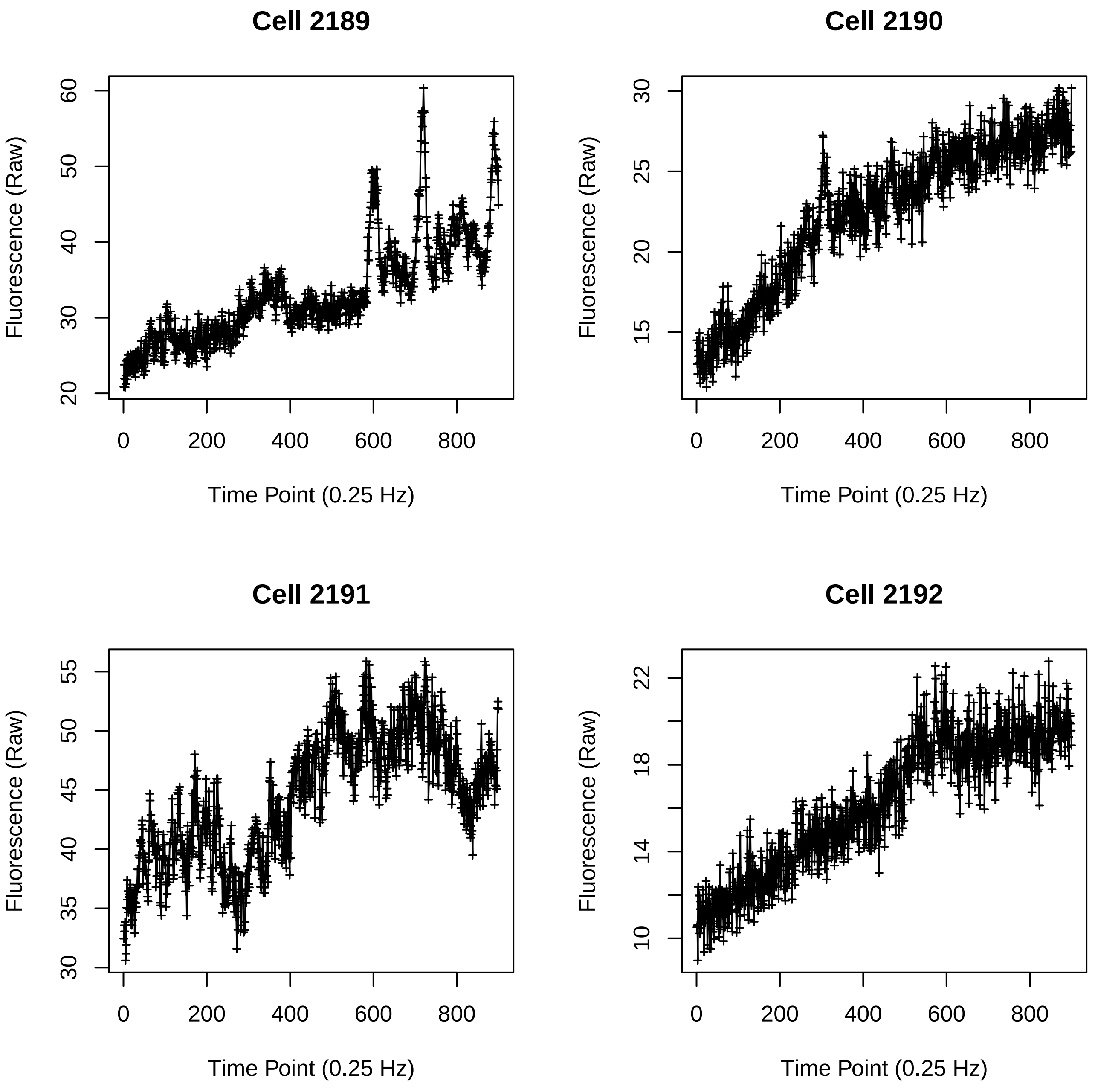

Cell 2189
Cell 2190
Cell 2191
Cell 2192
Fluorescence (Raw)
Time Point (0.25 Hz)
0
200
400
600
800
20
30
40
50
60
15
25
35
45
55
10
14
18
22

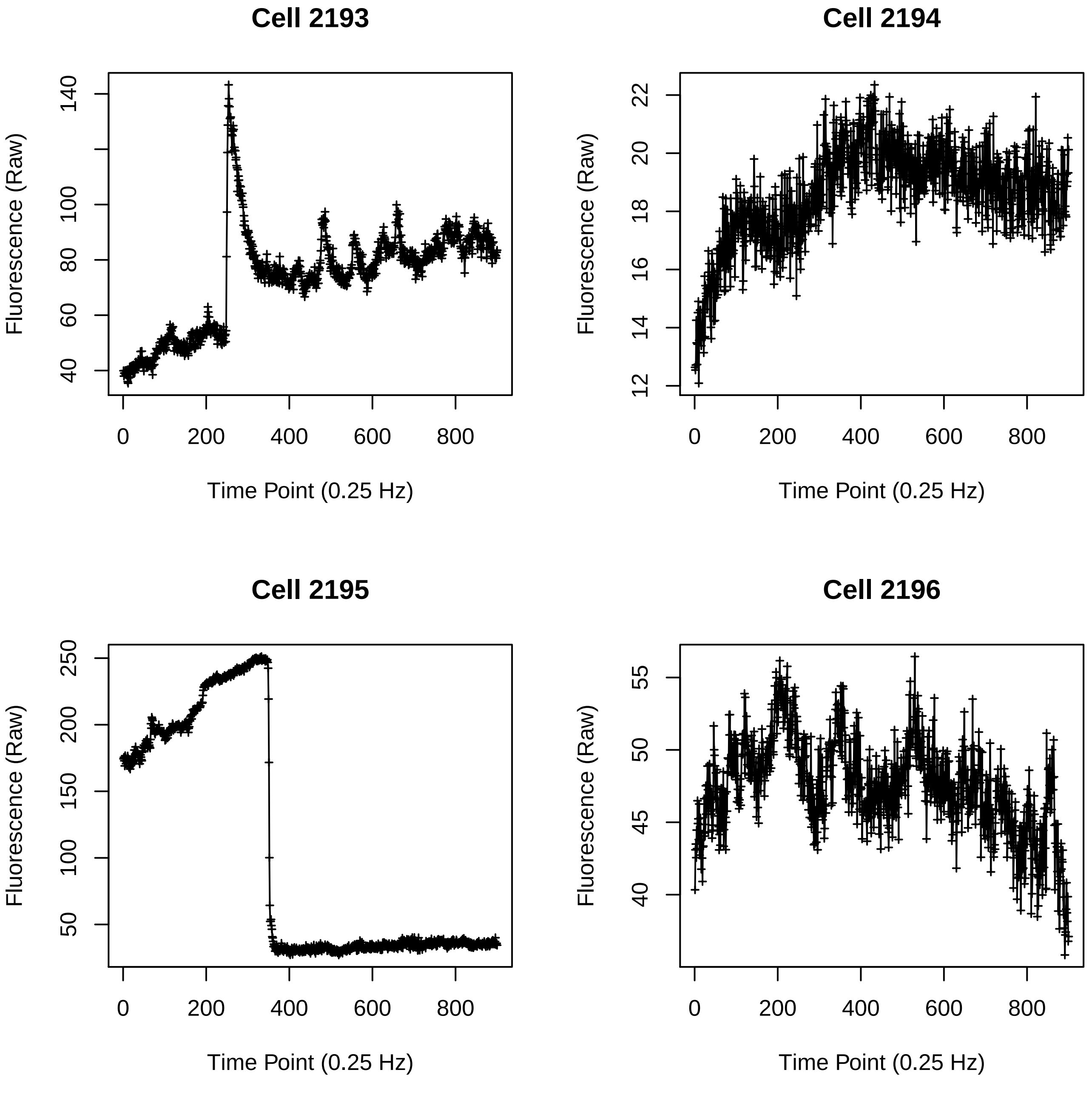
Cell 2193
Cell 2194
Cell 2195
Cell 2196
Fluorescence (Raw)
Time Point (0.25 Hz)
0
200
400
600
800
40
60
80
100
140
12
14
16
18
20
22
50
100
150
200
250
40
45
50
55

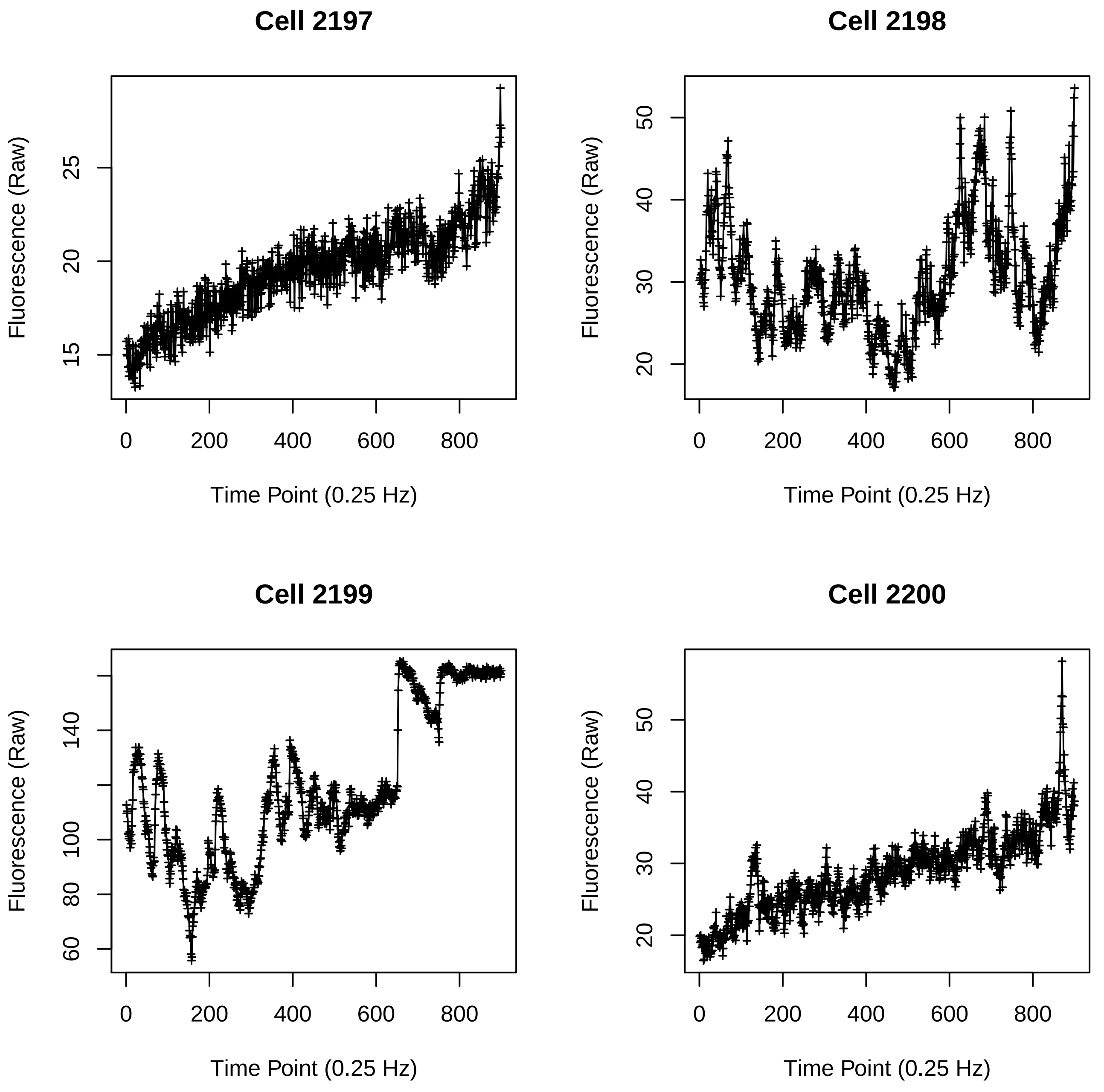
Cell 2197
Fluorescence (Raw)
15
20
25
0
200
400
600
800
Time Point (0.25 Hz)
Cell 2198
Fluorescence (Raw)
20
30
40
50
0
200
400
600
800
Time Point (0.25 Hz)
Cell 2199
Fluorescence (Raw)
60
80
100
140
0
200
400
600
800
Time Point (0.25 Hz)
Cell 2200
Fluorescence (Raw)
20
30
40
50
0
200
400
600
800
Time Point (0.25 Hz)

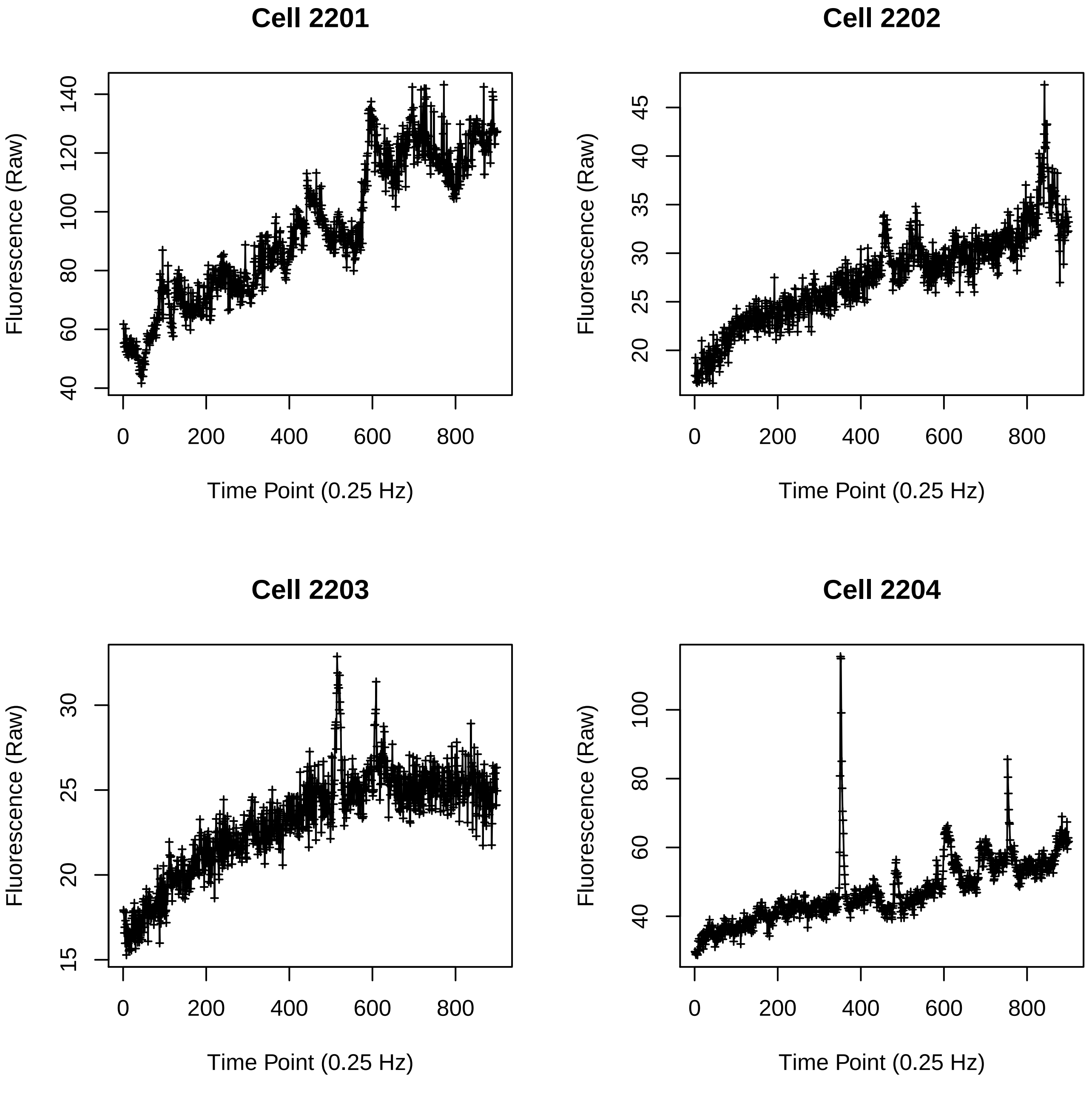

Cell 2201
Fluorescence (Raw)
40
60
80
100
120
140
0
200
400
600
800
Time Point (0.25 Hz)
Cell 2202
Fluorescence (Raw)
20
25
30
35
40
45
0
200
400
600
800
Time Point (0.25 Hz)
Cell 2203
Fluorescence (Raw)
15
20
25
30
0
200
400
600
800
Time Point (0.25 Hz)
Cell 2204
Fluorescence (Raw)
40
60
80
100
0
200
400
600
800
Time Point (0.25 Hz)

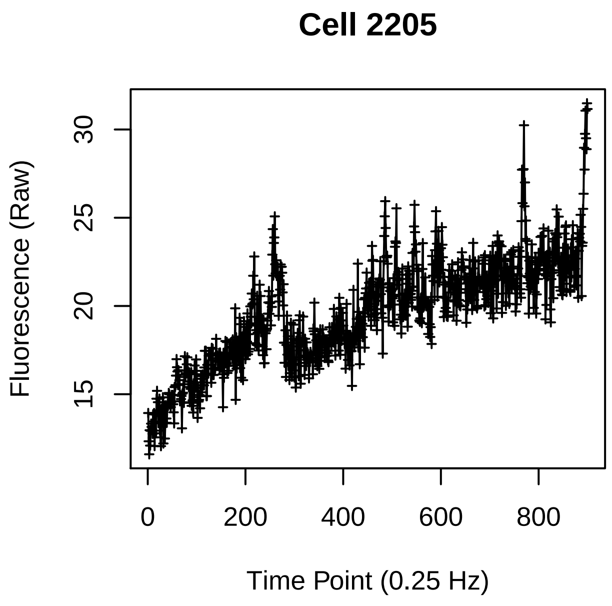
Cell 2205
Fluorescence (Raw)
15
20
25
30
0
200
400
600
800
Time Point (0.25 Hz)

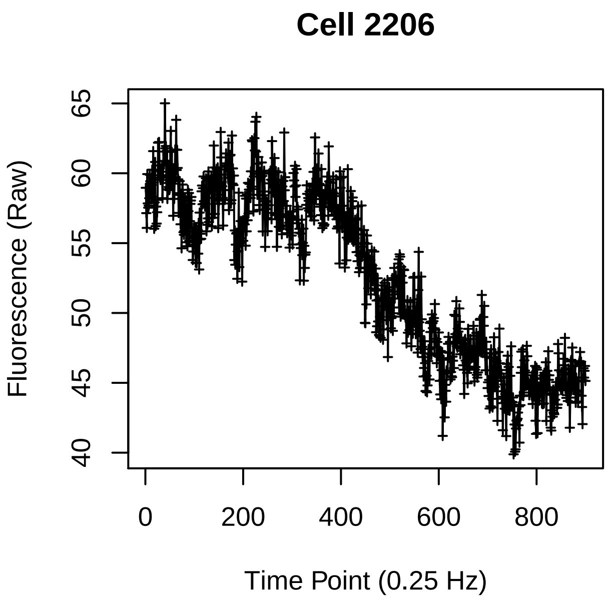
Cell 2206
Fluorescence (Raw)
40
45
50
55
60
65
0
200
400
600
800
Time Point (0.25 Hz)

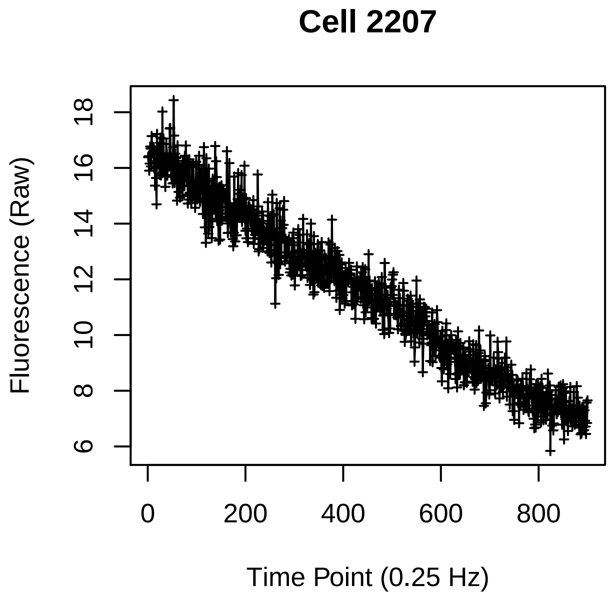
Cell 2207
Fluorescence (Raw)
6
8
10
12
14
16
18
0
200
400
600
800
Time Point (0.25 Hz)

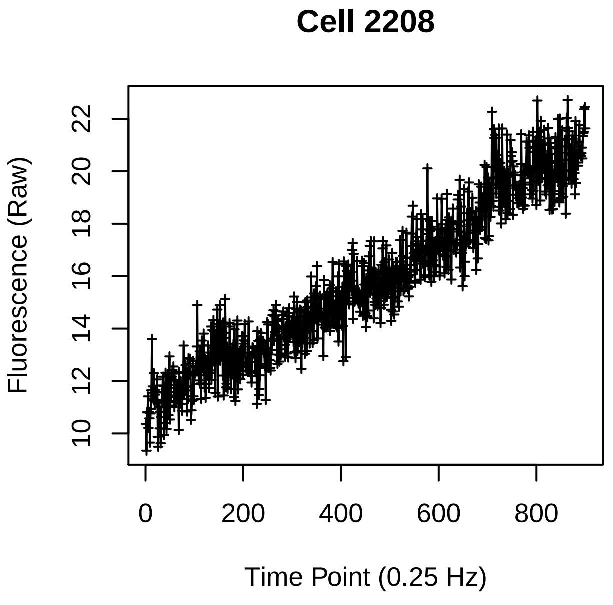
Cell 2208
Fluorescence (Raw)
10
12
14
16
18
20
22
0
200
400
600
800
Time Point (0.25 Hz)

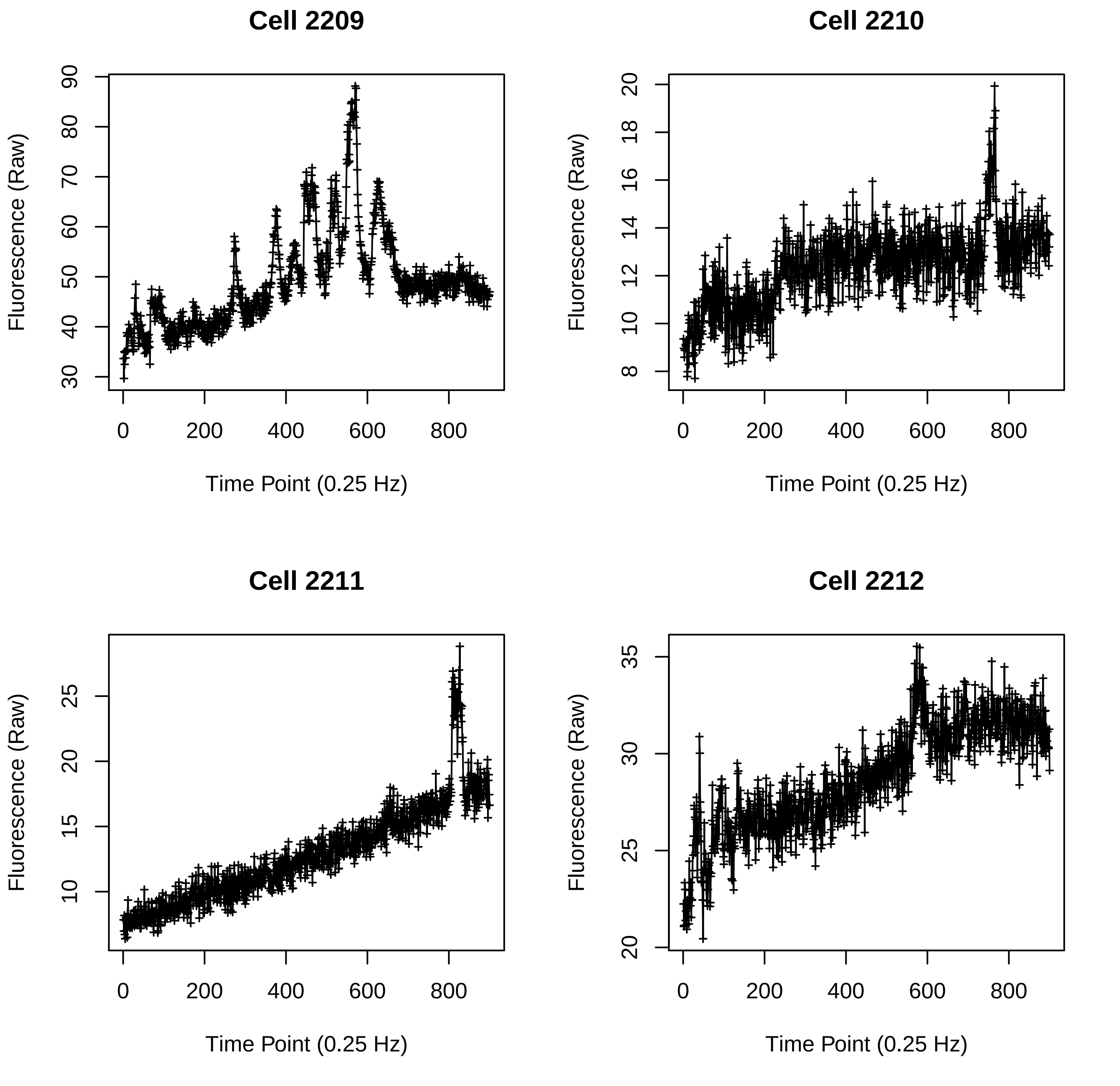
Cell 2209
Cell 2210
Cell 2211
Cell 2212
Fluorescence (Raw)
Time Point (0.25 Hz)
0
200
400
600
800
30
40
50
60
70
80
90
8
10
12
14
16
18
20
10
15
20
25
35

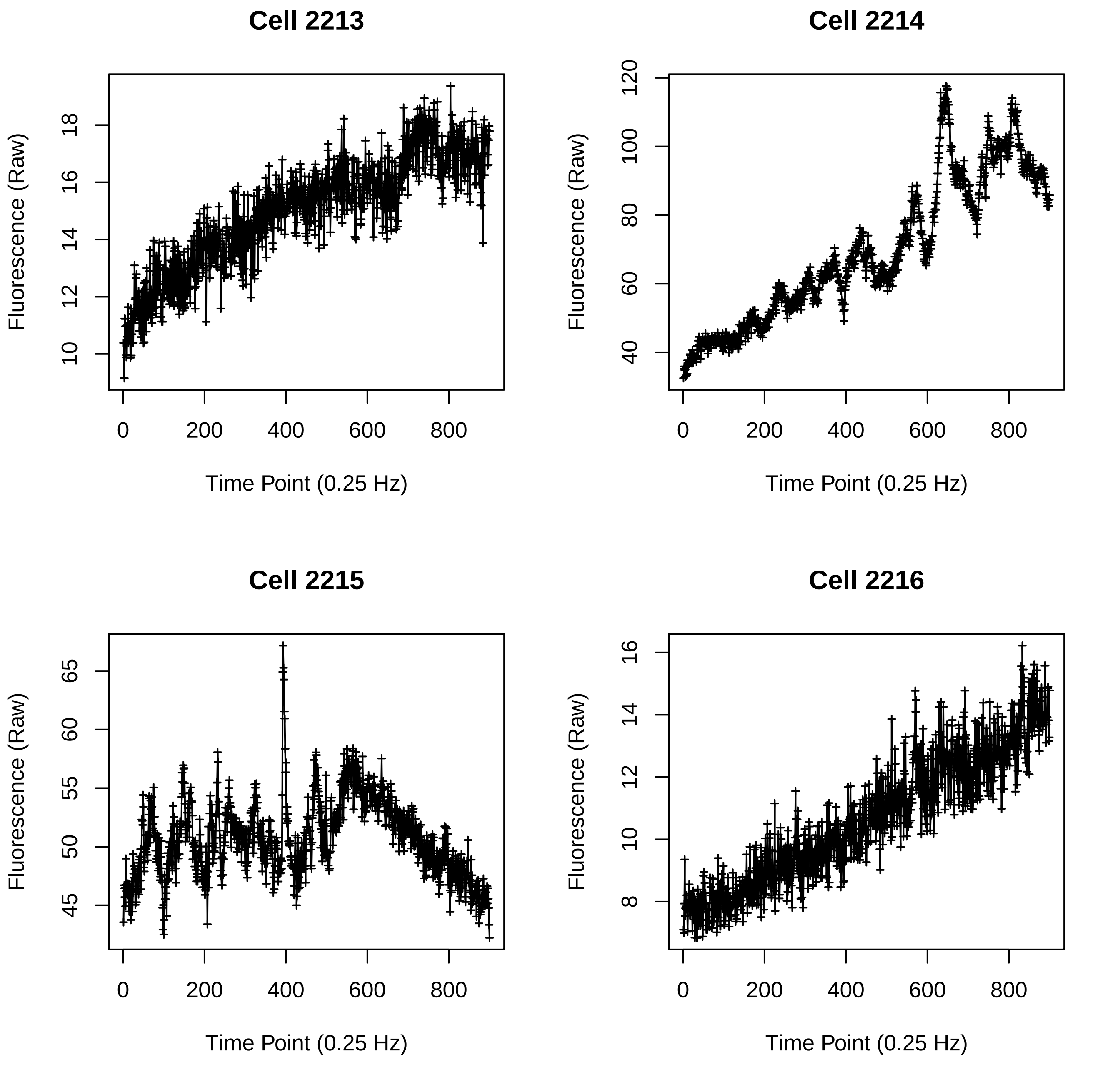
Cell 2213
Cell 2214
Cell 2215
Cell 2216
Fluorescence (Raw)
Time Point (0.25 Hz)
0
200
400
600
800
10
12
14
16
18
40
60
80
100
120
45
50
55
60
65
8
10
12
14
16

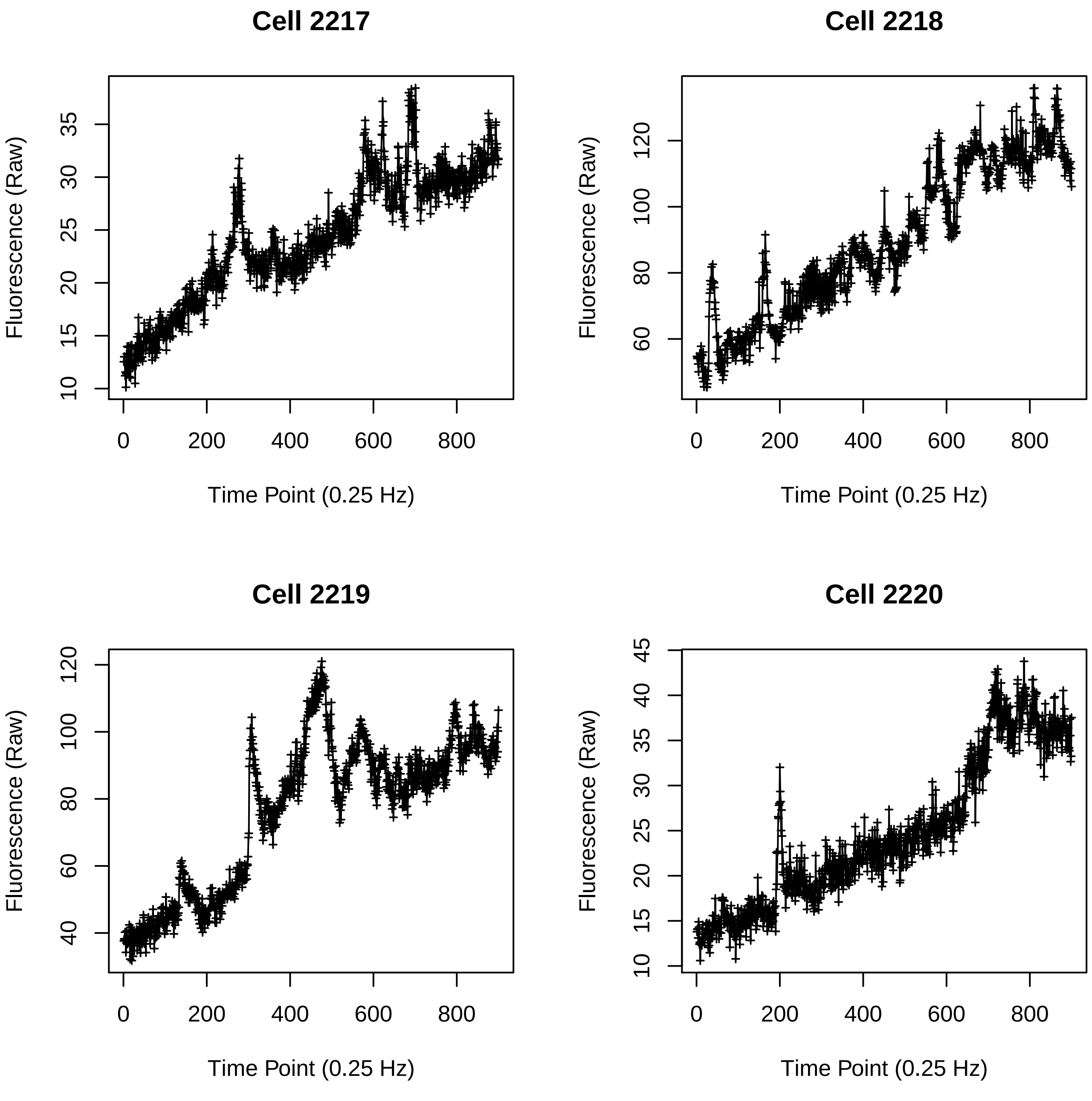
Cell 2217
Fluorescence (Raw)
10
15
20
25
30
35
Time Point (0.25 Hz)
0
200
400
600
800
Cell 2218
Fluorescence (Raw)
60
80
100
120
Time Point (0.25 Hz)
0
200
400
600
800
Cell 2219
Fluorescence (Raw)
40
60
80
100
120
Time Point (0.25 Hz)
0
200
400
600
800
Cell 2220
Fluorescence (Raw)
10
15
20
25
30
35
40
45
Time Point (0.25 Hz)
0
200
400
600
800

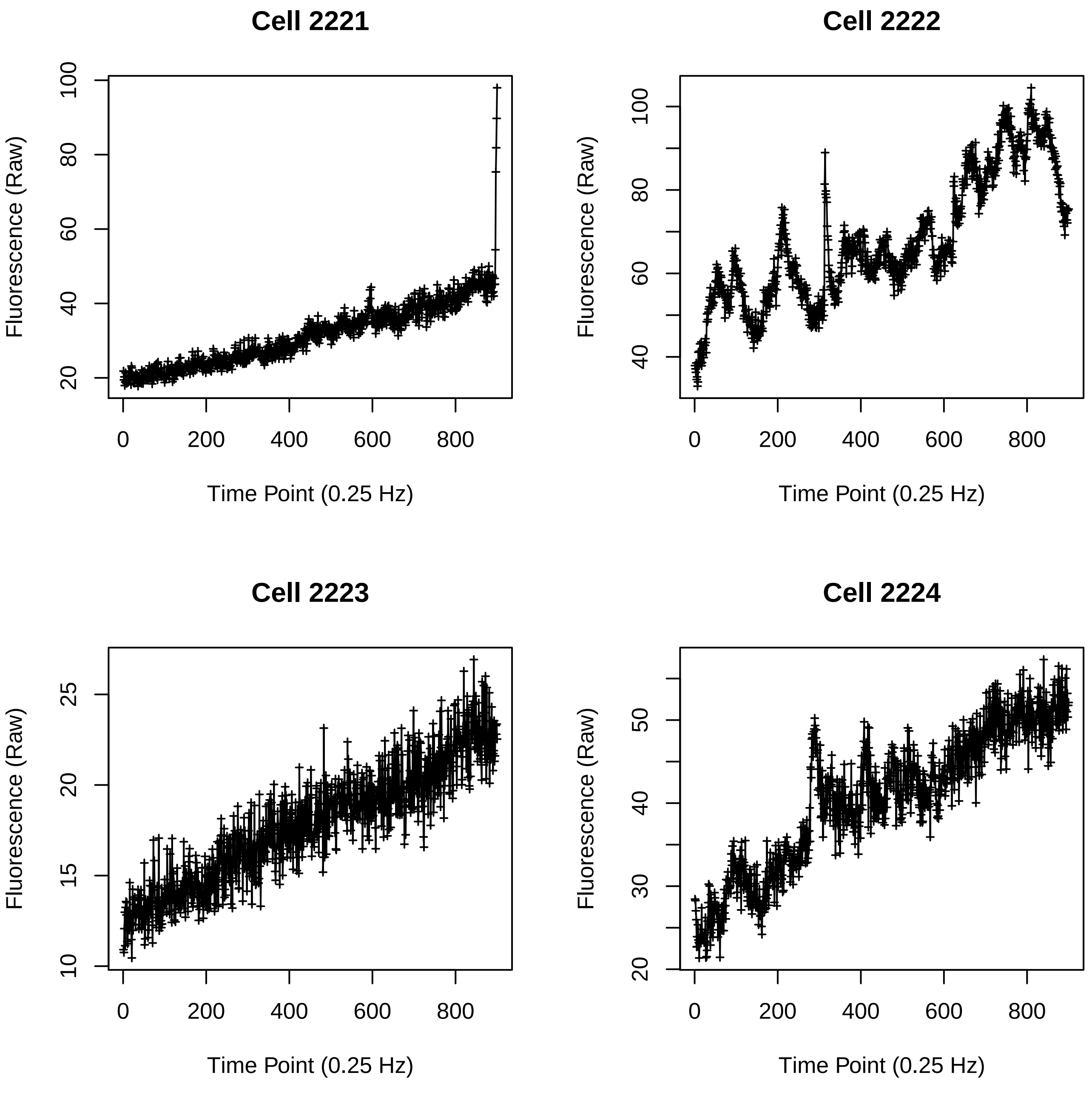
Cell 2221
Fluorescence (Raw)
20
40
60
80
100
0
200
400
600
800
Time Point (0.25 Hz)
Cell 2222
Fluorescence (Raw)
40
60
80
100
0
200
400
600
800
Time Point (0.25 Hz)
Cell 2223
Fluorescence (Raw)
10
15
20
25
0
200
400
600
800
Time Point (0.25 Hz)
Cell 2224
Fluorescence (Raw)
20
30
40
50
0
200
400
600
800
Time Point (0.25 Hz)

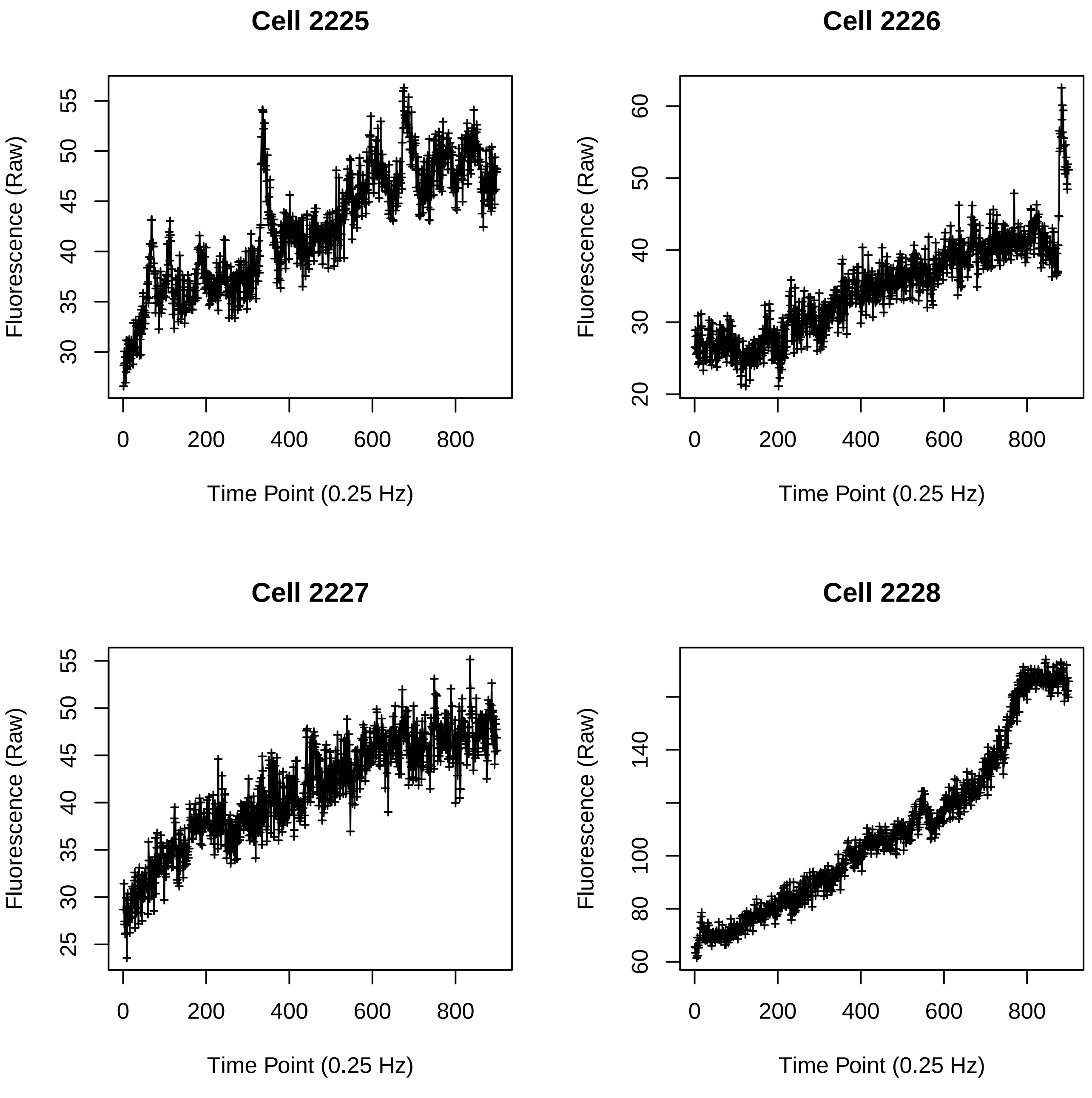

Cell 2225
55
50
45
40
35
30
Fluorescence (Raw)
0
200
400
600
800
Time Point (0.25 Hz)
Cell 2226
60
50
40
30
20
Fluorescence (Raw)
0
200
400
600
800
Time Point (0.25 Hz)
Cell 2227
55
50
45
40
35
30
25
Fluorescence (Raw)
0
200
400
600
800
Time Point (0.25 Hz)
Cell 2228
140
100
80
60
Fluorescence (Raw)
0
200
400
600
800
Time Point (0.25 Hz)

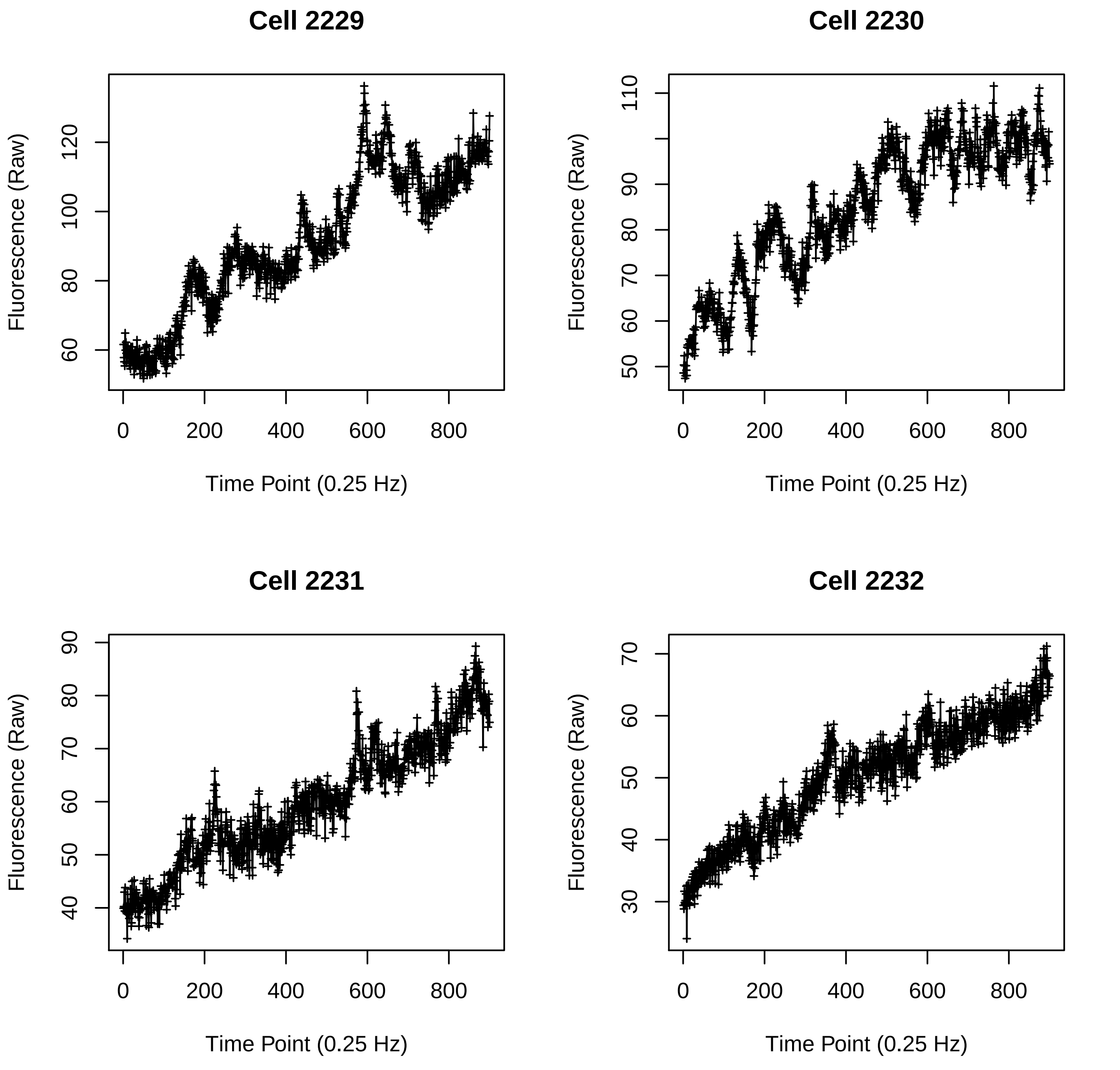

Cell 2229
Cell 2230
Cell 2231
Cell 2232
Fluorescence (Raw)
Time Point (0.25 Hz)
0
200
400
600
800
60
80
100
120
50
60
70
80
90
110
40
50
60
70
80
90
30
40
50
60
70

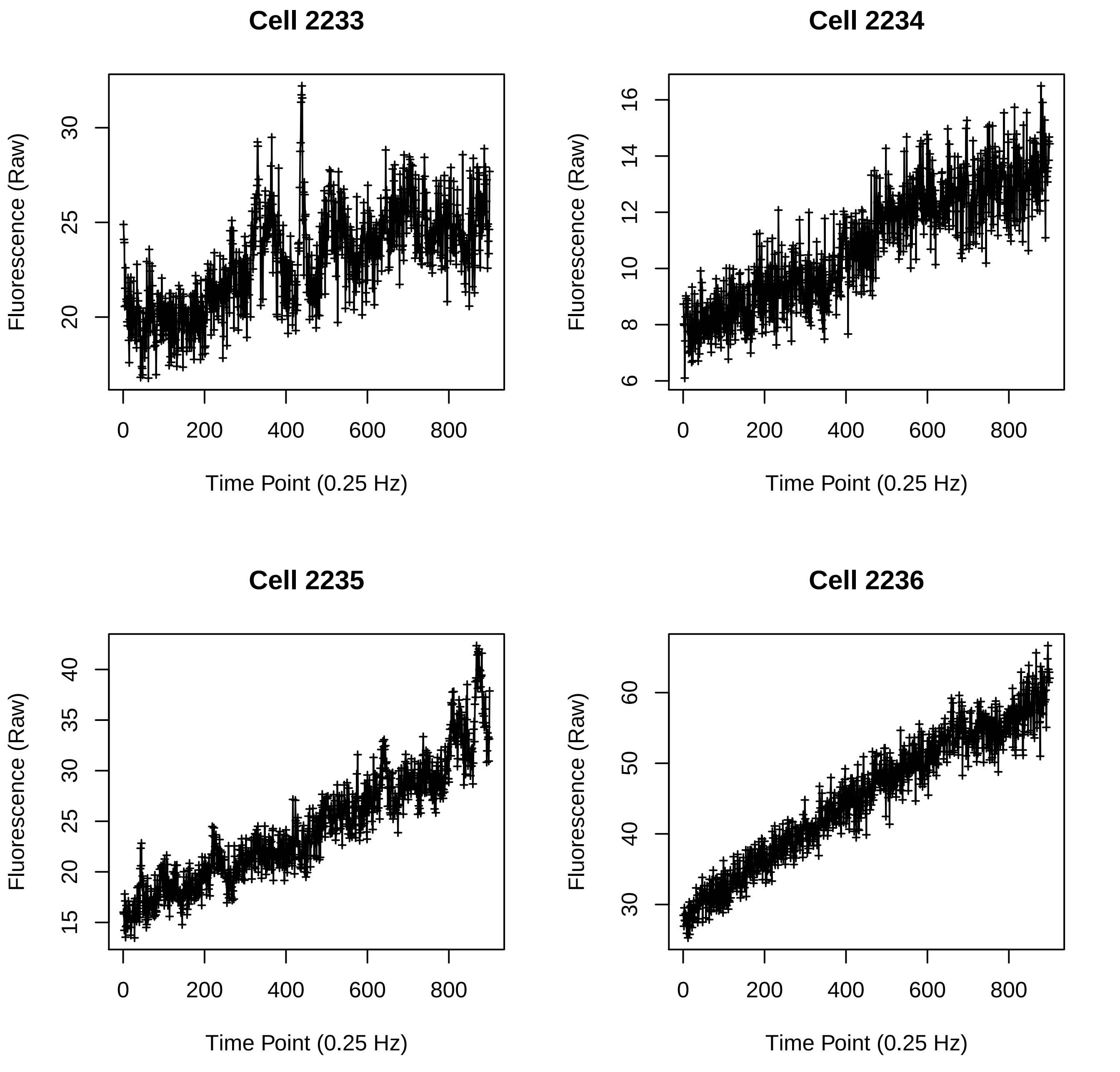
Cell 2233
Fluorescence (Raw)
20
25
30
0
200
400
600
800
Time Point (0.25 Hz)
Cell 2234
Fluorescence (Raw)
6
8
10
12
14
16
0
200
400
600
800
Time Point (0.25 Hz)
Cell 2235
Fluorescence (Raw)
15
20
25
30
35
40
0
200
400
600
800
Time Point (0.25 Hz)
Cell 2236
Fluorescence (Raw)
30
40
50
60
0
200
400
600
800
Time Point (0.25 Hz)

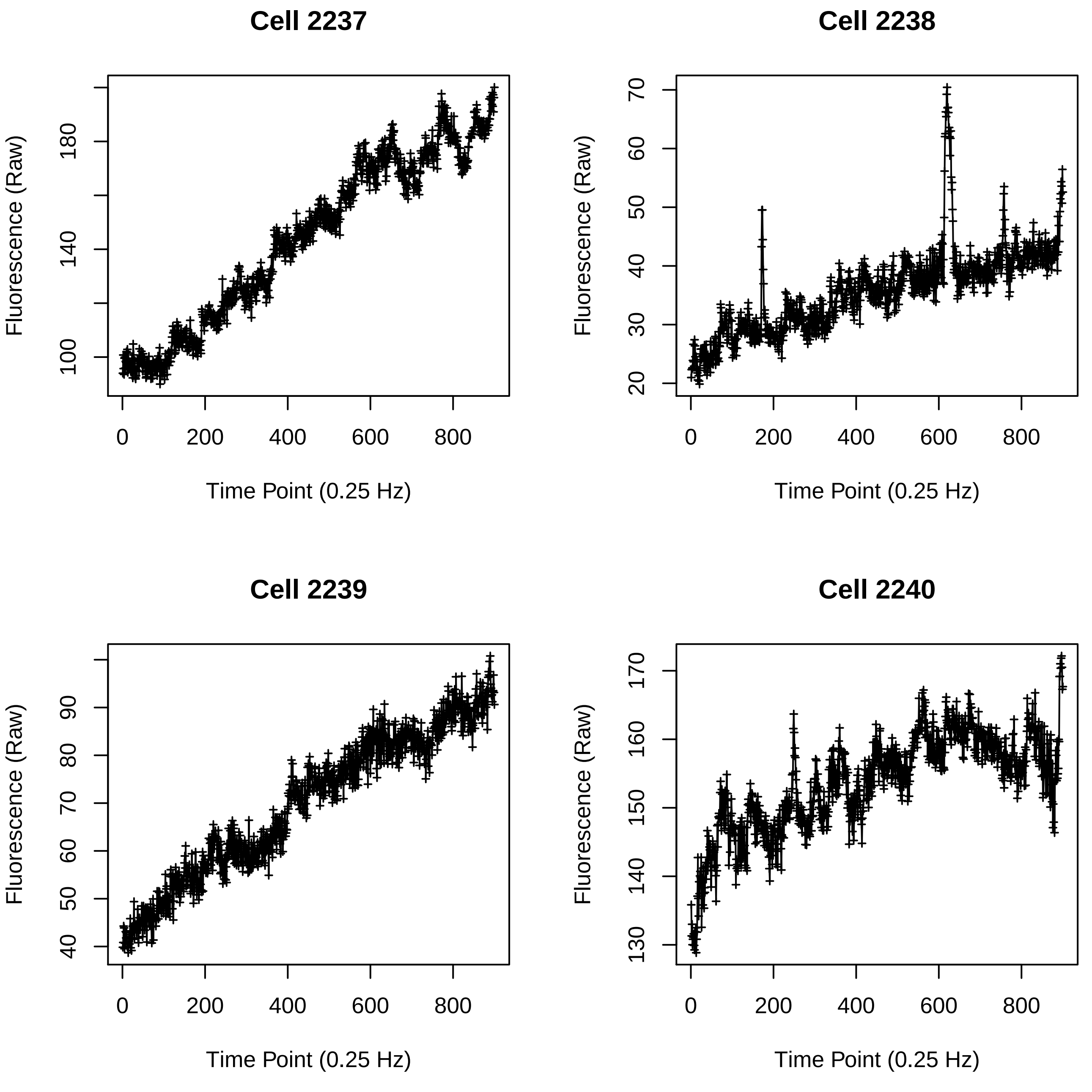
Cell 2237
Fluorescence (Raw)
100
140
180
0
200
400
600
800
Time Point (0.25 Hz)
Cell 2238
Fluorescence (Raw)
20
30
40
50
60
70
0
200
400
600
800
Time Point (0.25 Hz)
Cell 2239
Fluorescence (Raw)
40
50
60
70
80
90
0
200
400
600
800
Time Point (0.25 Hz)
Cell 2240
Fluorescence (Raw)
130
140
150
160
170
0
200
400
600
800
Time Point (0.25 Hz)

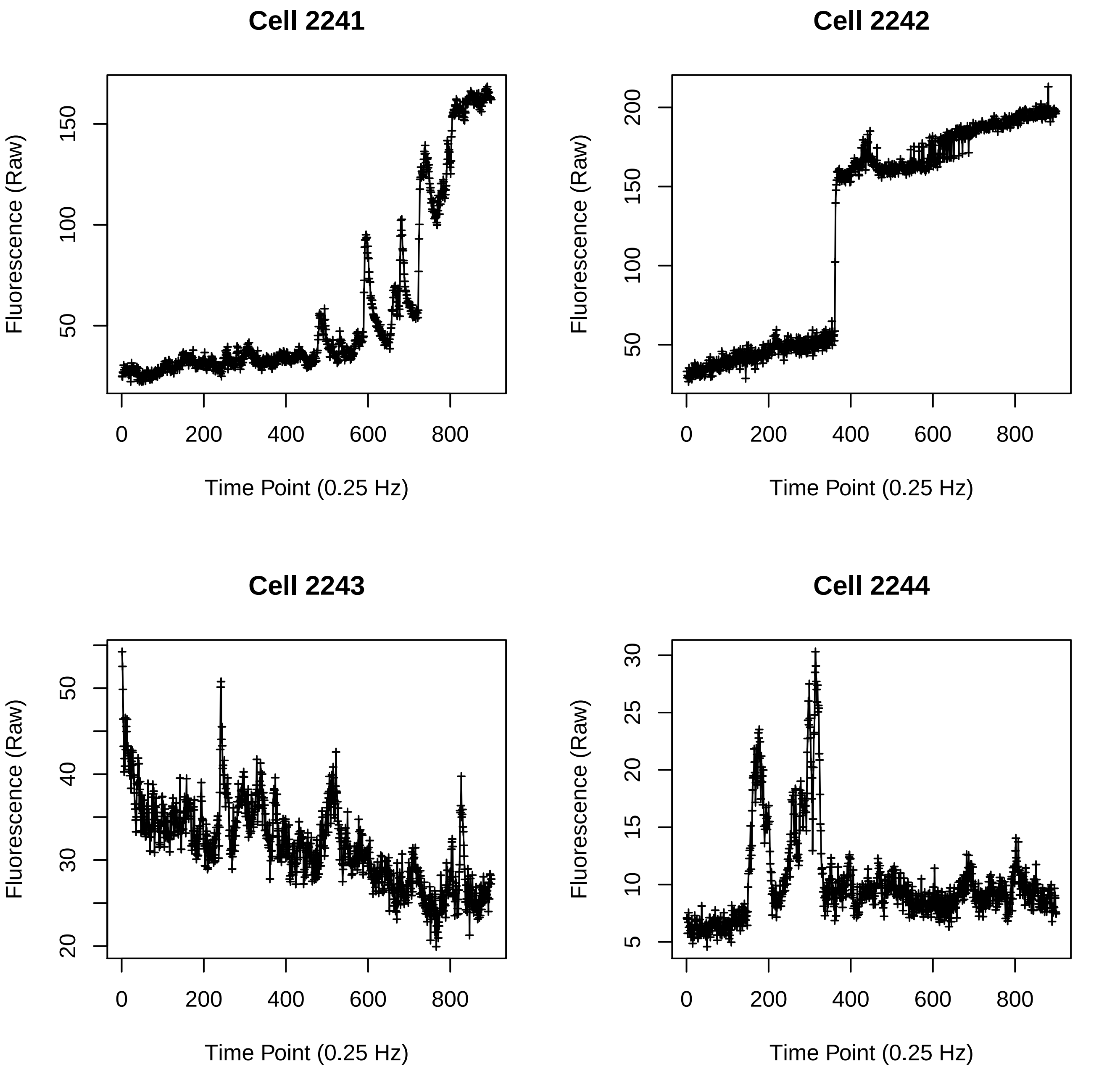
Cell 2241
Fluorescence (Raw)
50
100
150
0
200
400
600
800
Time Point (0.25 Hz)
Cell 2242
Fluorescence (Raw)
50
100
150
200
0
200
400
600
800
Time Point (0.25 Hz)
Cell 2243
Fluorescence (Raw)
20
30
40
50
0
200
400
600
800
Time Point (0.25 Hz)
Cell 2244
Fluorescence (Raw)
5
10
15
20
25
30
0
200
400
600
800
Time Point (0.25 Hz)

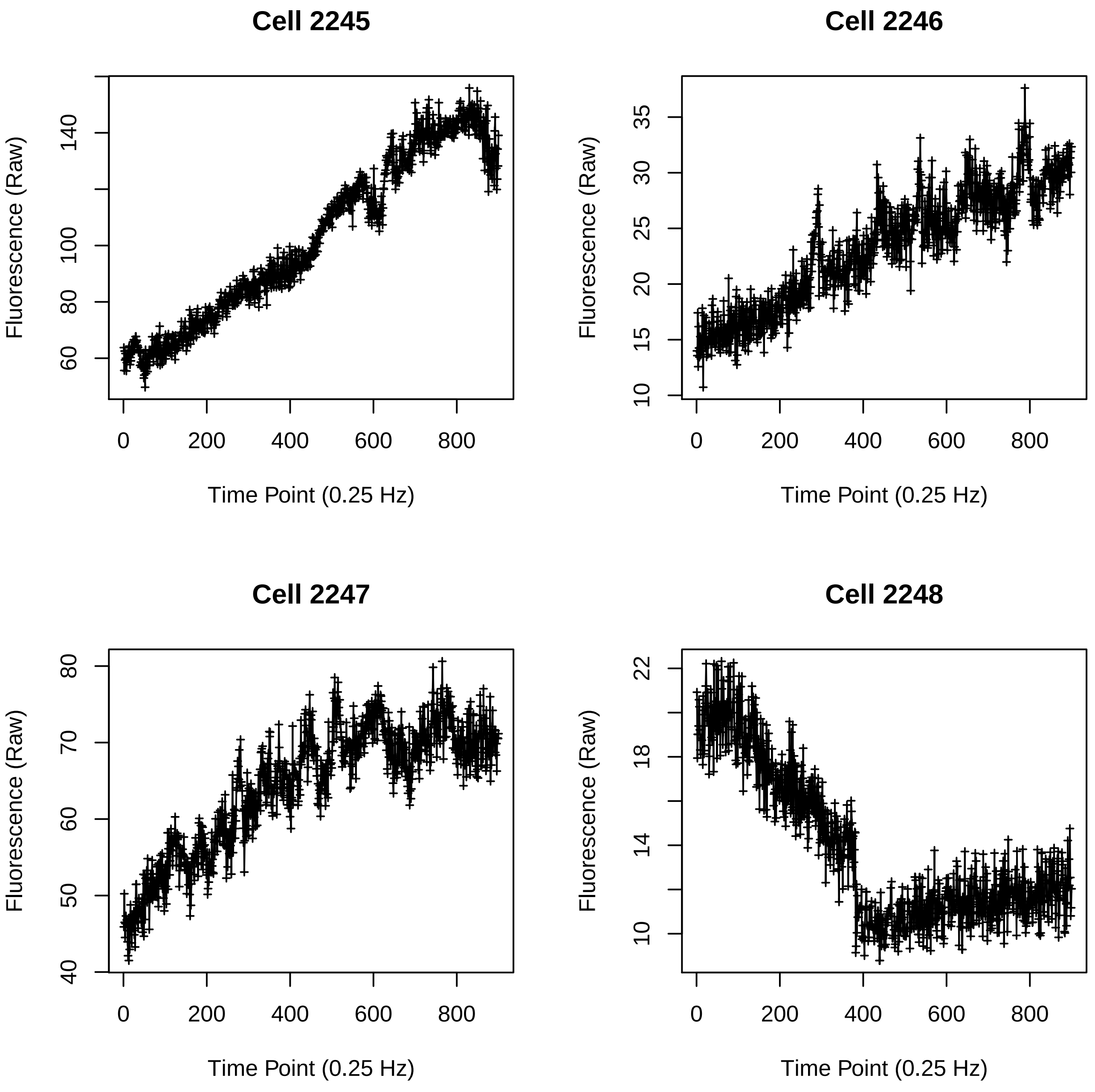
Cell 2245
Fluorescence (Raw)
60
80
100
140
0
200
400
600
800
Time Point (0.25 Hz)
Cell 2246
Fluorescence (Raw)
10
15
20
25
30
35
0
200
400
600
800
Time Point (0.25 Hz)
Cell 2247
Fluorescence (Raw)
40
50
60
70
80
0
200
400
600
800
Time Point (0.25 Hz)
Cell 2248
Fluorescence (Raw)
10
14
18
22
0
200
400
600
800
Time Point (0.25 Hz)

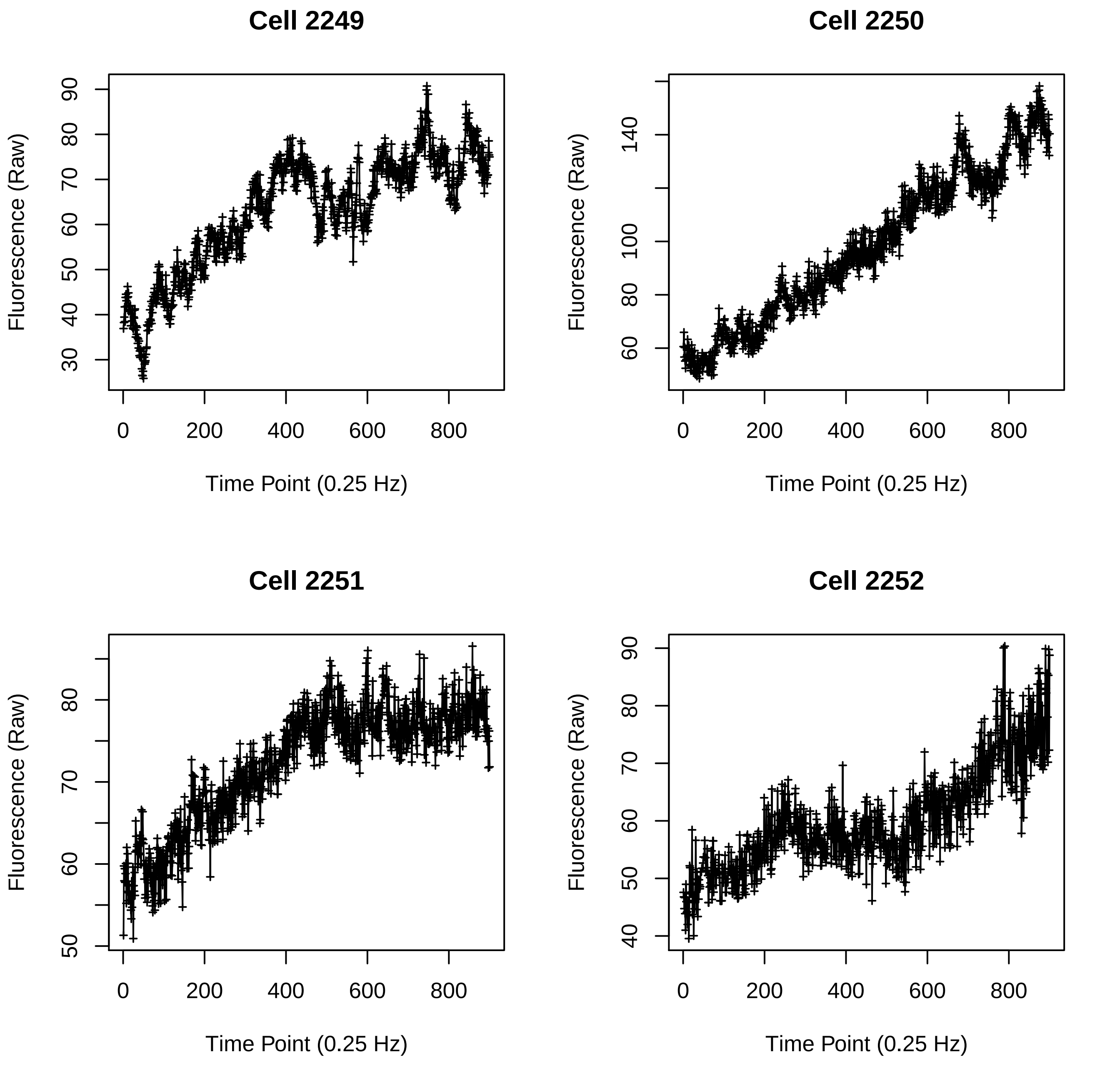
Cell 2249
Cell 2250
Cell 2251
Cell 2252
Fluorescence (Raw)
Time Point (0.25 Hz)
0
200
400
600
800
30
40
50
60
70
80
90
100
140

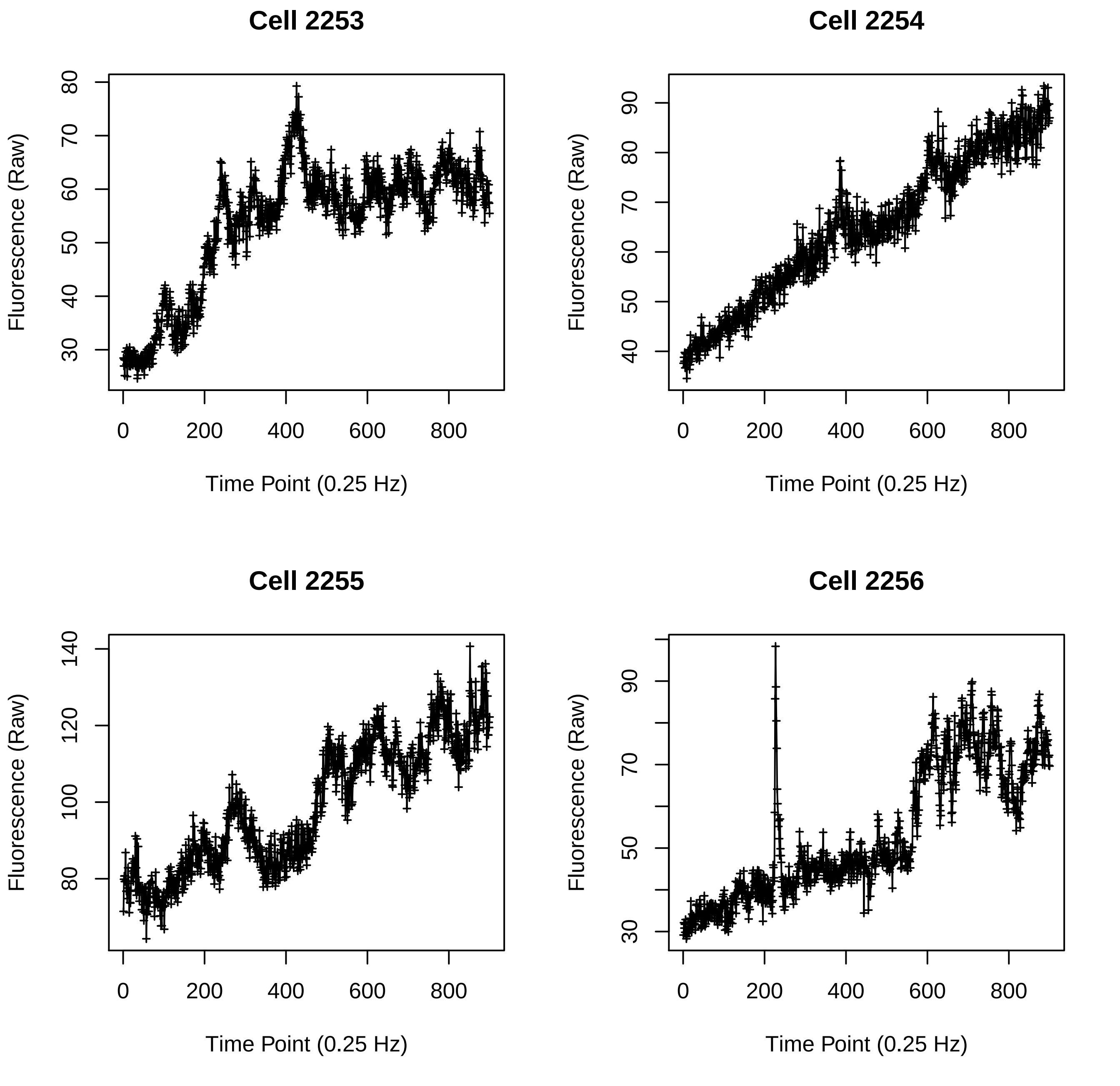
Cell 2253
Fluorescence (Raw)
30
40
50
60
70
80
0
200
400
600
800
Time Point (0.25 Hz)
Cell 2254
Fluorescence (Raw)
40
50
60
70
80
90
0
200
400
600
800
Time Point (0.25 Hz)
Cell 2255
Fluorescence (Raw)
80
100
120
140
0
200
400
600
800
Time Point (0.25 Hz)
Cell 2256
Fluorescence (Raw)
30
50
70
90
0
200
400
600
800
Time Point (0.25 Hz)

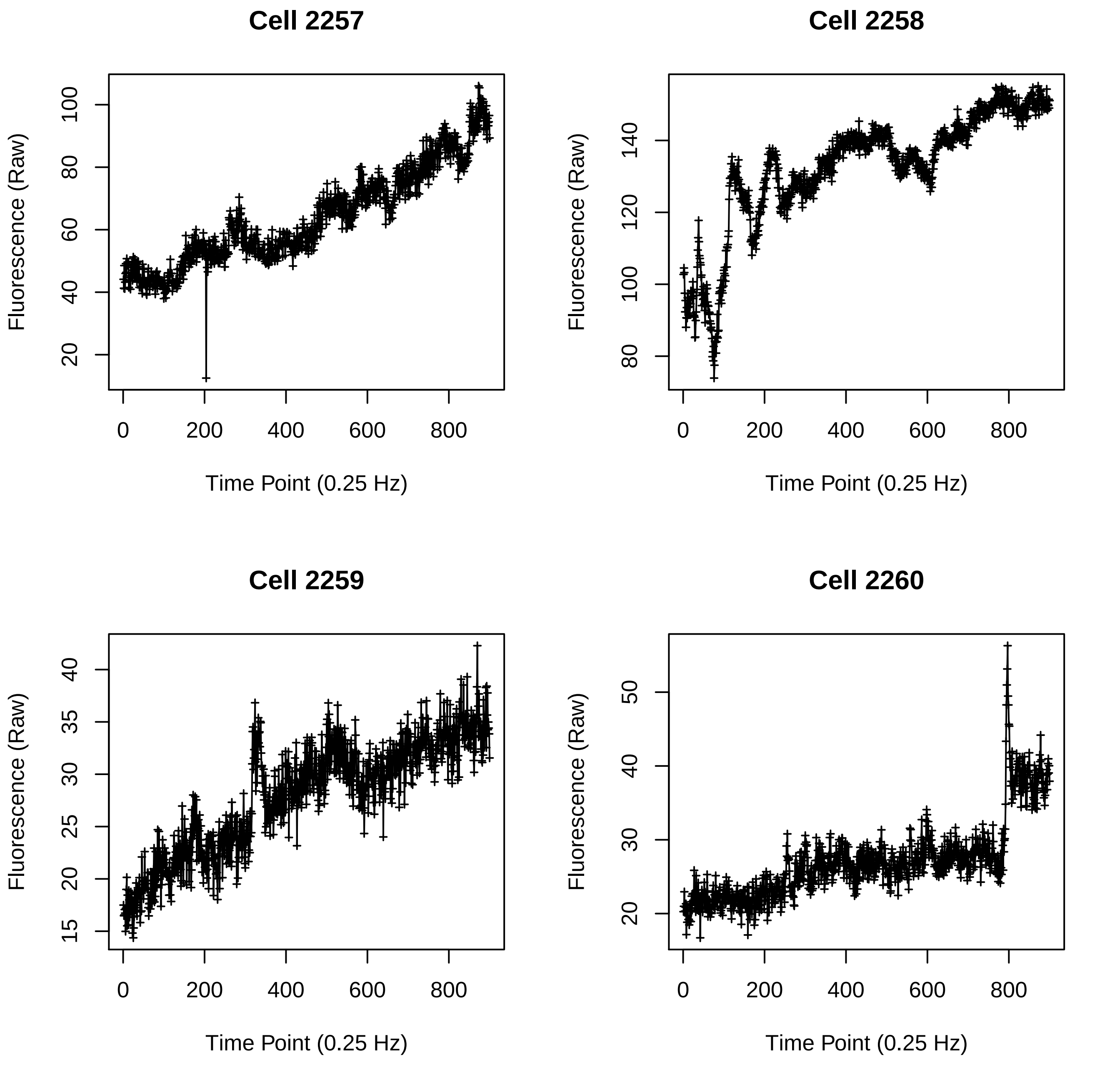
Cell 2257
Fluorescence (Raw)
Time Point (0.25 Hz)
Cell 2258
Fluorescence (Raw)
Time Point (0.25 Hz)
Cell 2259
Fluorescence (Raw)
Time Point (0.25 Hz)
Cell 2260
Fluorescence (Raw)
Time Point (0.25 Hz)

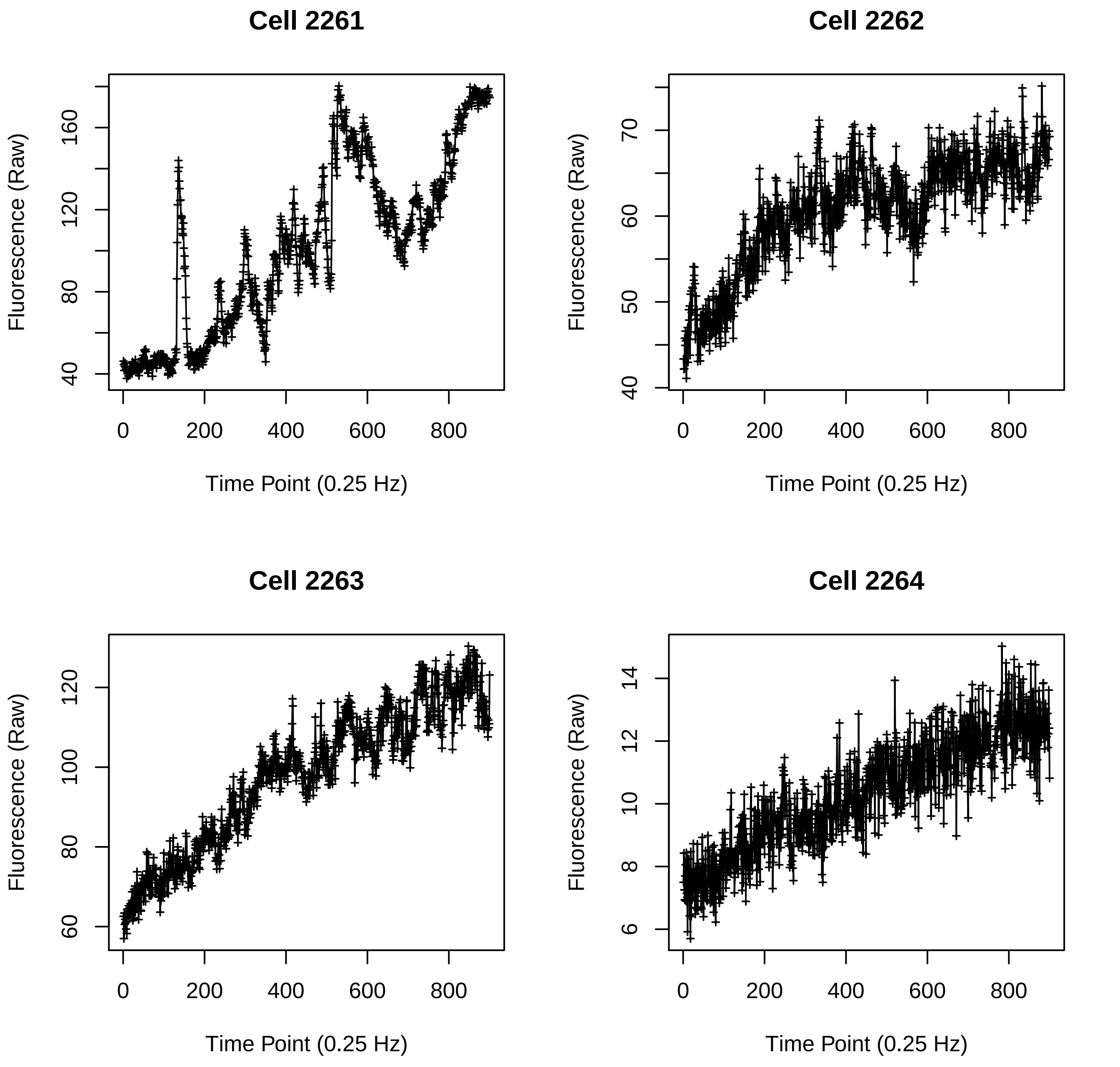
Cell 2261
Cell 2262
Cell 2263
Cell 2264
Fluorescence (Raw)
Time Point (0.25 Hz)
0
200
400
600
800
40
80
120
160
50
60
70
100
6
8
10
12
14

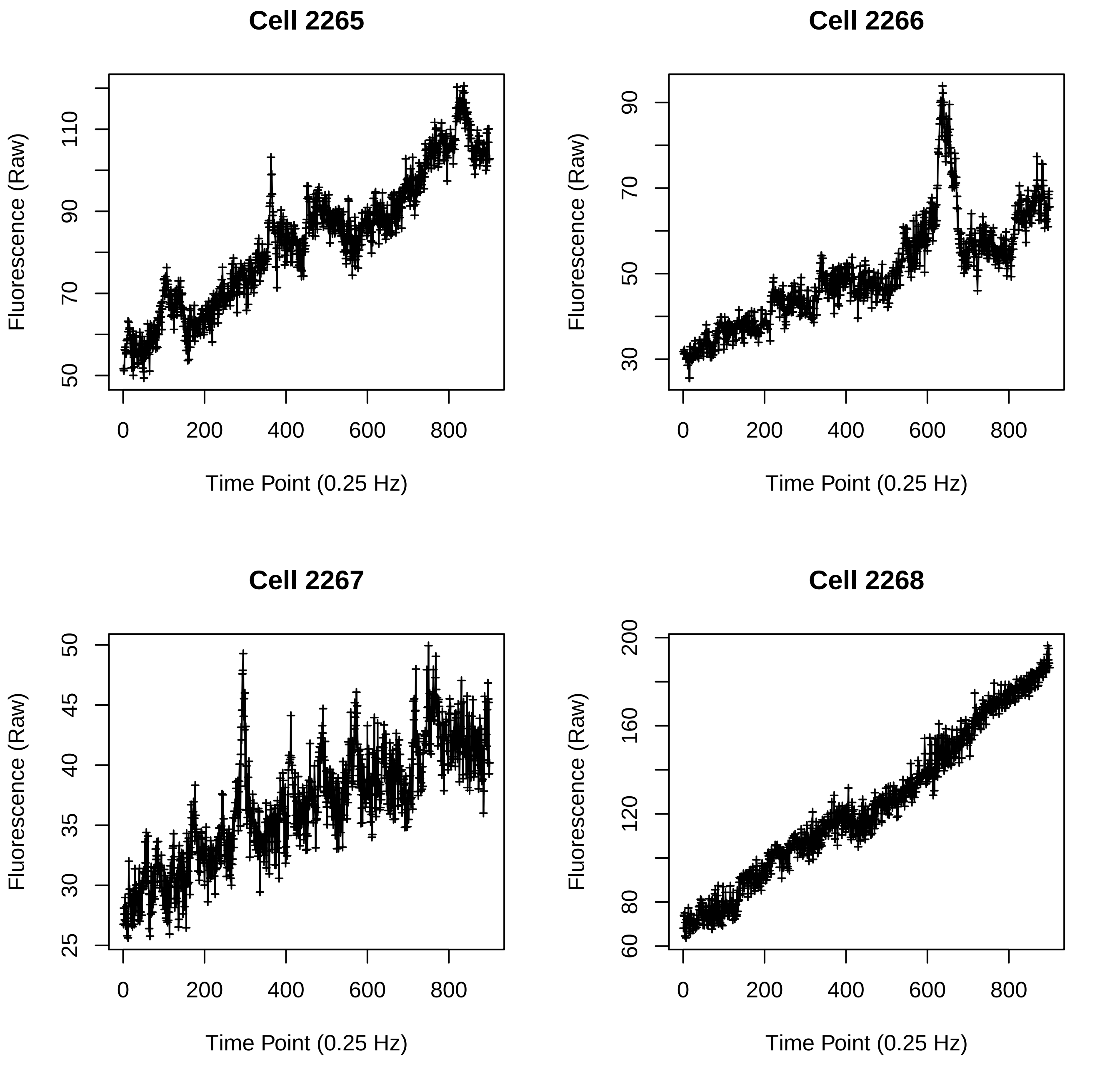
Cell 2265
Cell 2266
Cell 2267
Cell 2268
Fluorescence (Raw)
Time Point (0.25 Hz)
0
200
400
600
800
50
70
90
110
30
50
70
90
25
30
35
40
45
50
60
80
120
160
200

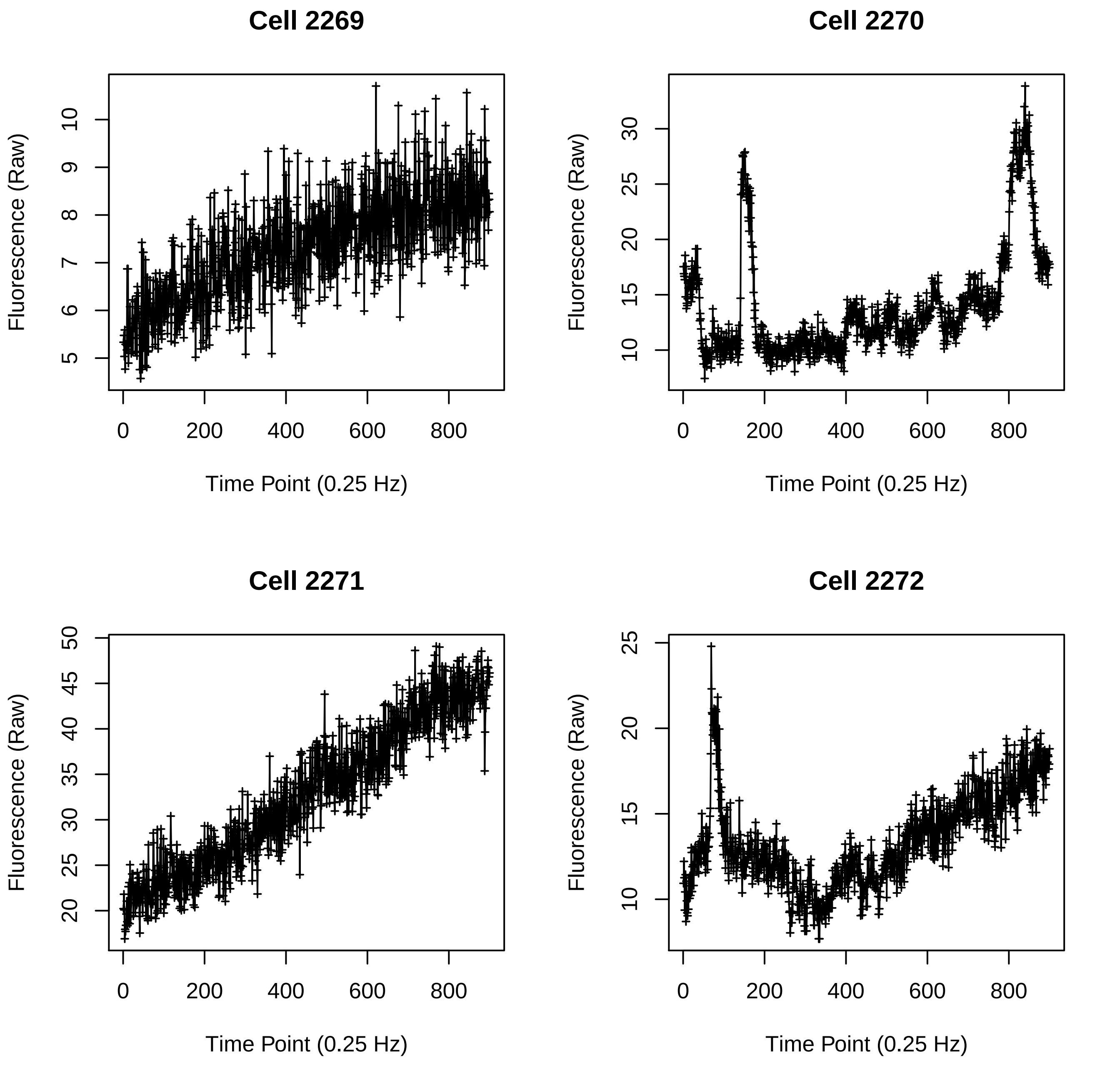
Cell 2269
Cell 2270
Cell 2271
Cell 2272
Fluorescence (Raw)
Time Point (0.25 Hz)
0
200
400
600
800
5
6
7
8
9
10
15
20
25
30
35
40
45
50

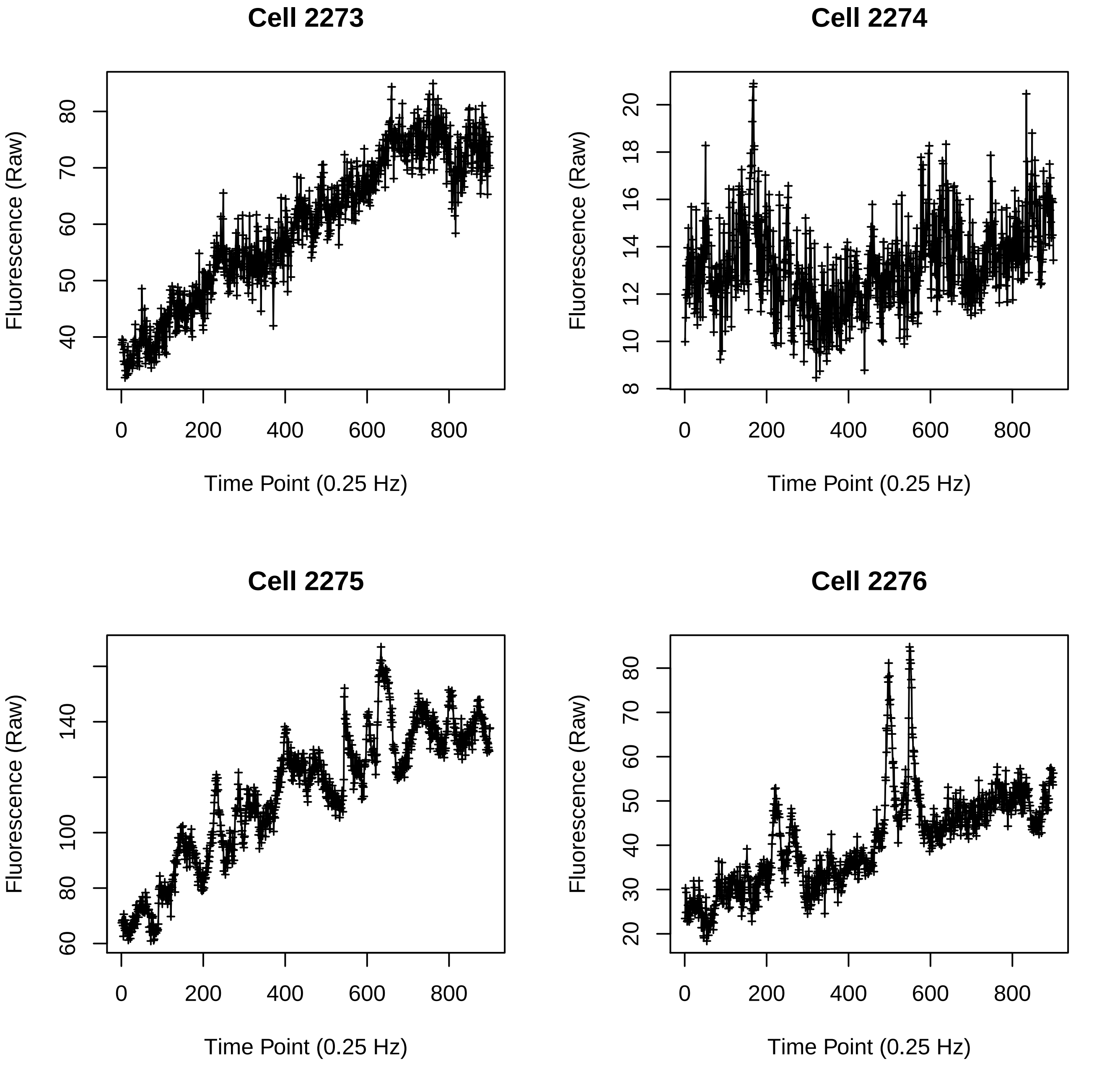

Cell 2273
Cell 2274
Cell 2275
Cell 2276
Fluorescence (Raw)
Time Point (0.25 Hz)
0
200
400
600
800
40
50
60
70
80
8
10
12
14
16
18
20
60
80
100
140
20
30
40
50
60
70
80

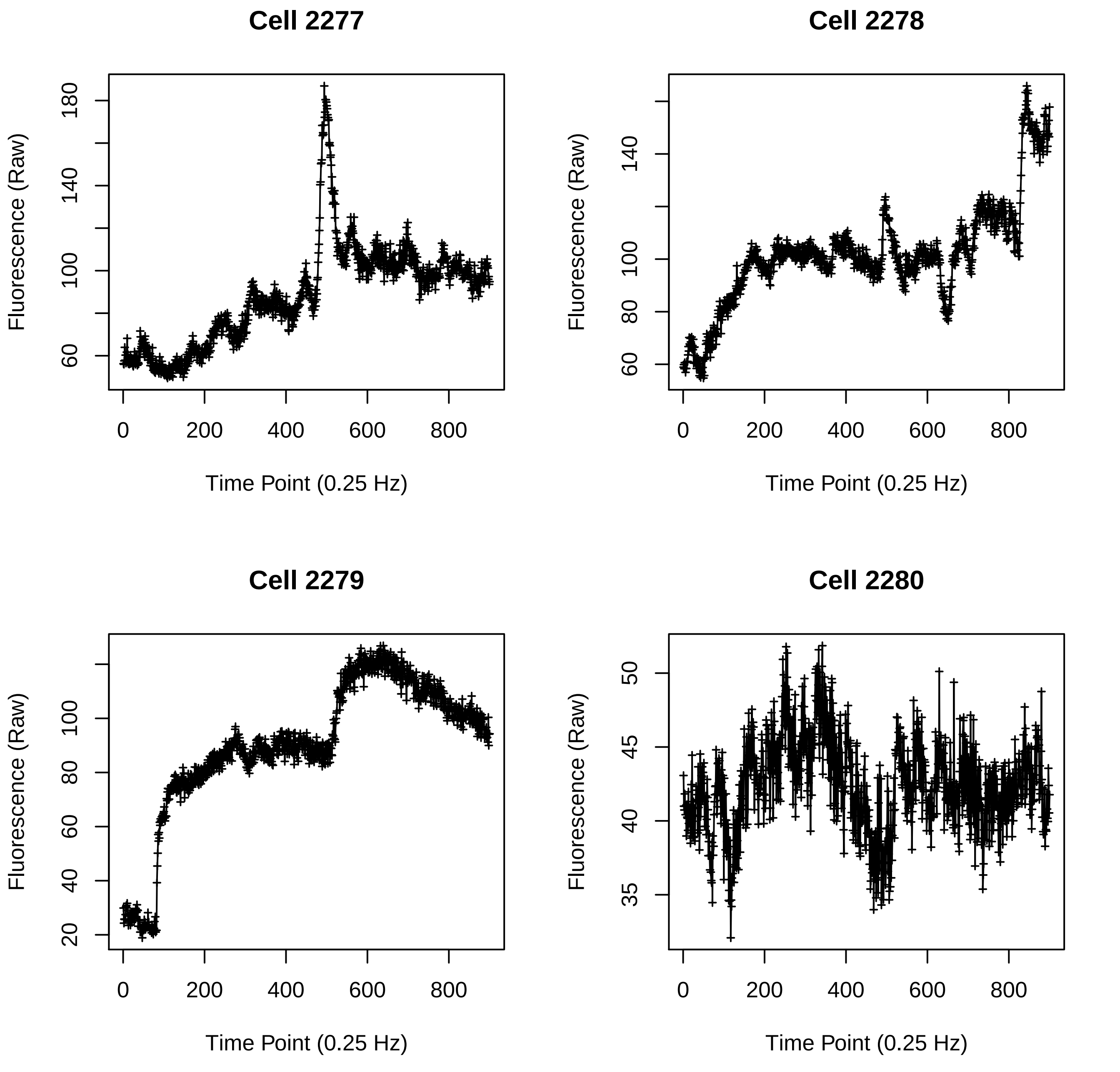

Cell 2277
Cell 2278
Cell 2279
Cell 2280
Fluorescence (Raw)
Time Point (0.25 Hz)
0
200
400
600
800
60
100
140
180
80
20
40
35
45
50

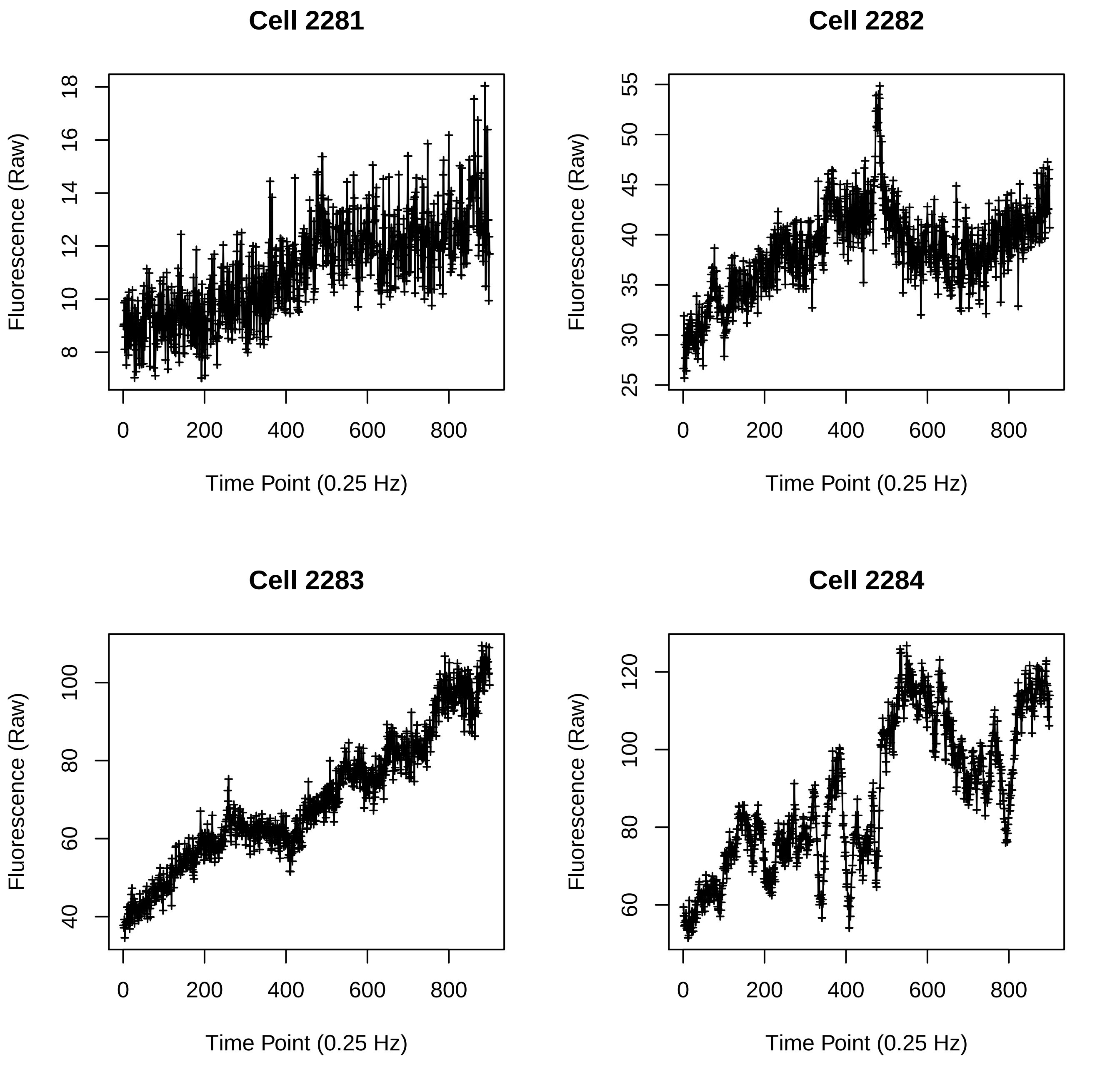
Cell 2281
Cell 2282
Cell 2283
Cell 2284
Fluorescence (Raw)
Time Point (0.25 Hz)
0
200
400
600
800
8
10
12
14
16
18
25
30
35
40
45
50
55
40
60
80
100
120

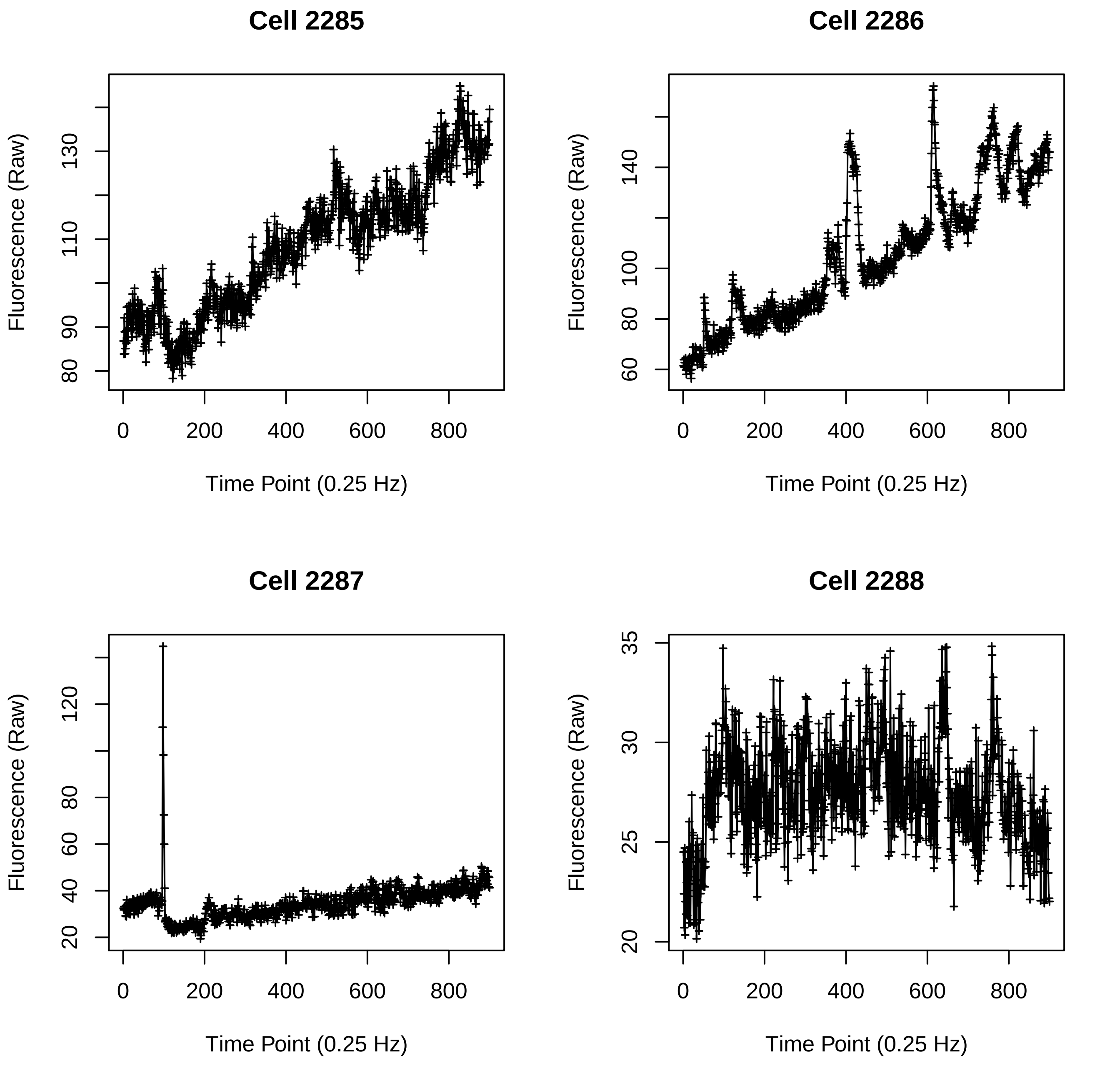

Cell 2285
Cell 2286
Cell 2287
Cell 2288
Fluorescence (Raw)
Time Point (0.25 Hz)
80
90
110
130
60
80
100
140
20
40
60
80
120
20
25
30
35
0
200
400
600
800

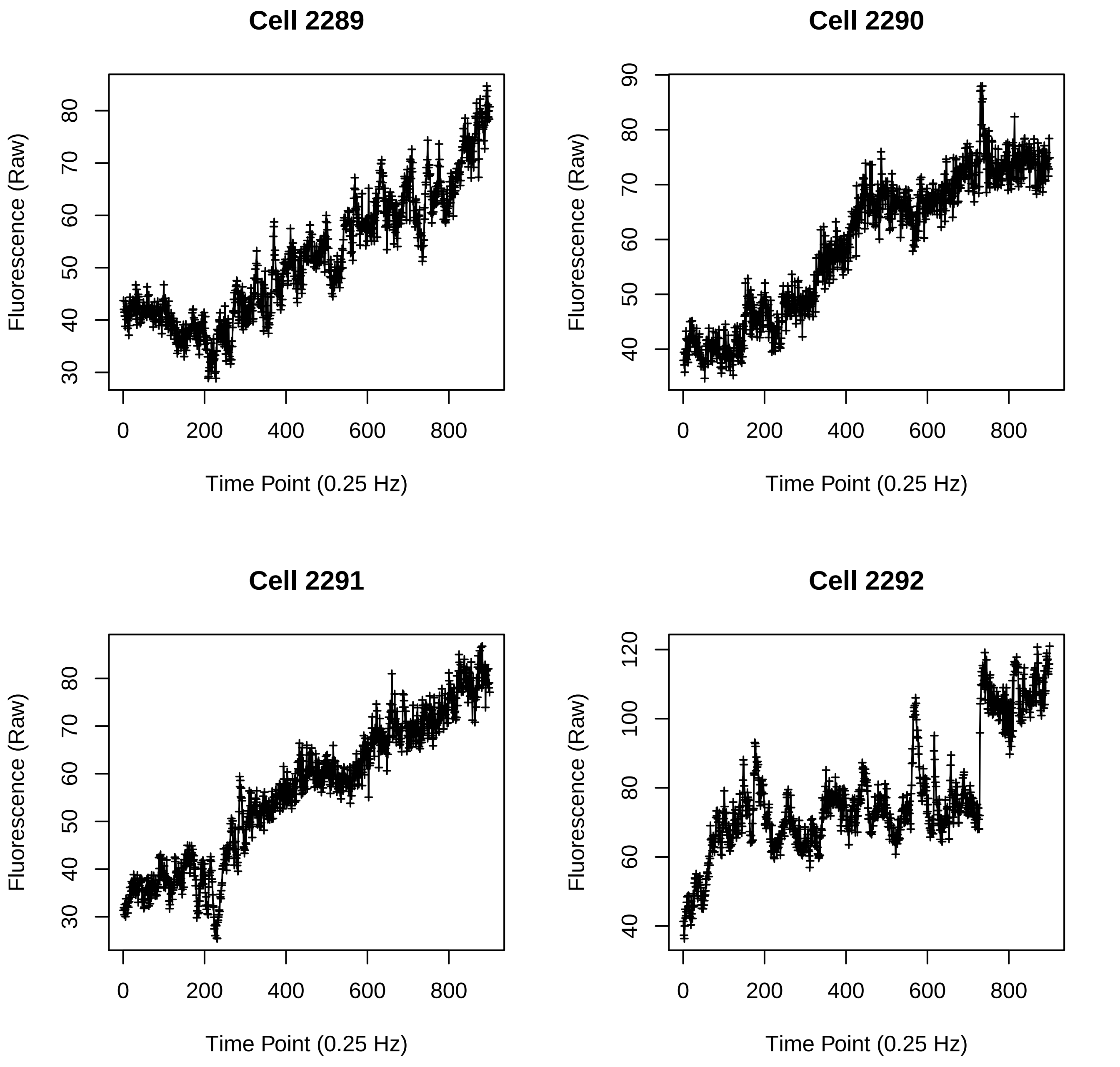
Cell 2289
Fluorescence (Raw)
30
40
50
60
70
80
0
200
400
600
800
Time Point (0.25 Hz)
Cell 2290
Fluorescence (Raw)
40
50
60
70
80
90
0
200
400
600
800
Time Point (0.25 Hz)
Cell 2291
Fluorescence (Raw)
30
40
50
60
70
80
0
200
400
600
800
Time Point (0.25 Hz)
Cell 2292
Fluorescence (Raw)
40
60
80
100
120
0
200
400
600
800
Time Point (0.25 Hz)

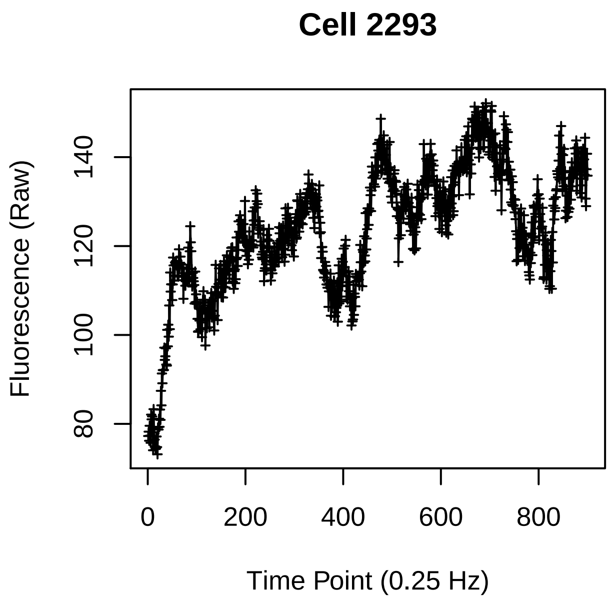
Cell 2293
Fluorescence (Raw)
80
100
120
140
0
200
400
600
800
Time Point (0.25 Hz)

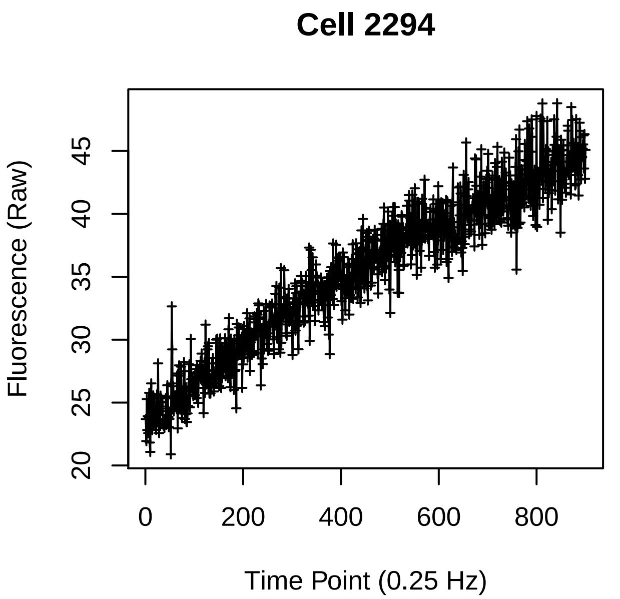
Cell 2294
Fluorescence (Raw)
20
25
30
35
40
45
0
200
400
600
800
Time Point (0.25 Hz)

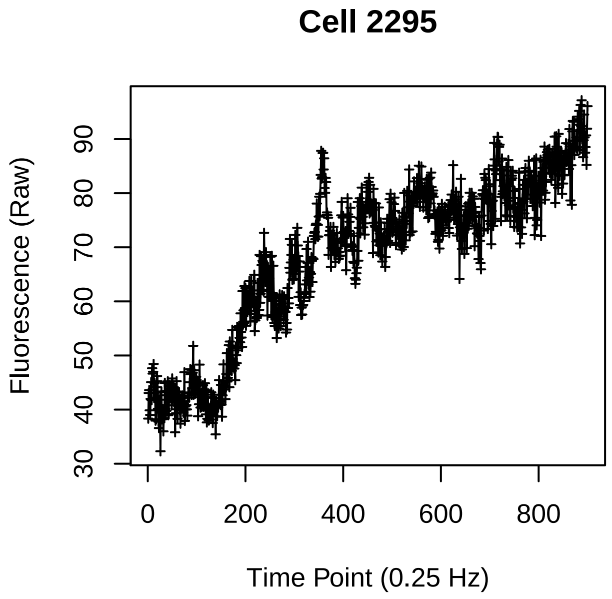
Cell 2295
Fluorescence (Raw)
30
40
50
60
70
80
90
0
200
400
600
800
Time Point (0.25 Hz)

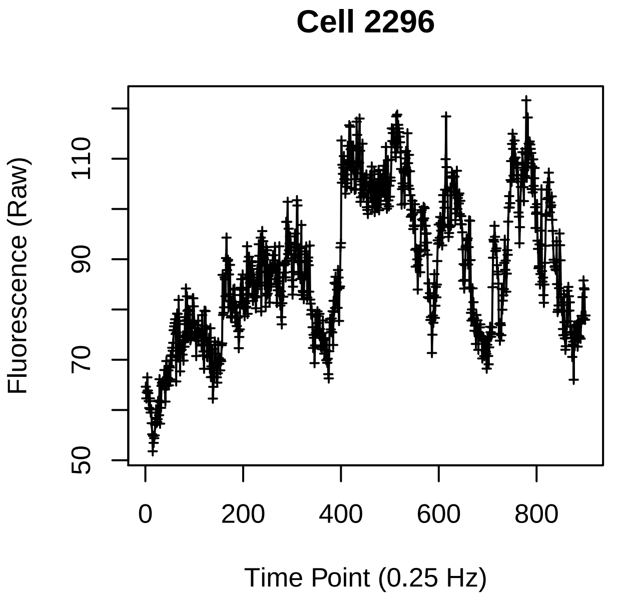
Cell 2296
Fluorescence (Raw)
50
70
90
110
0
200
400
600
800
Time Point (0.25 Hz)

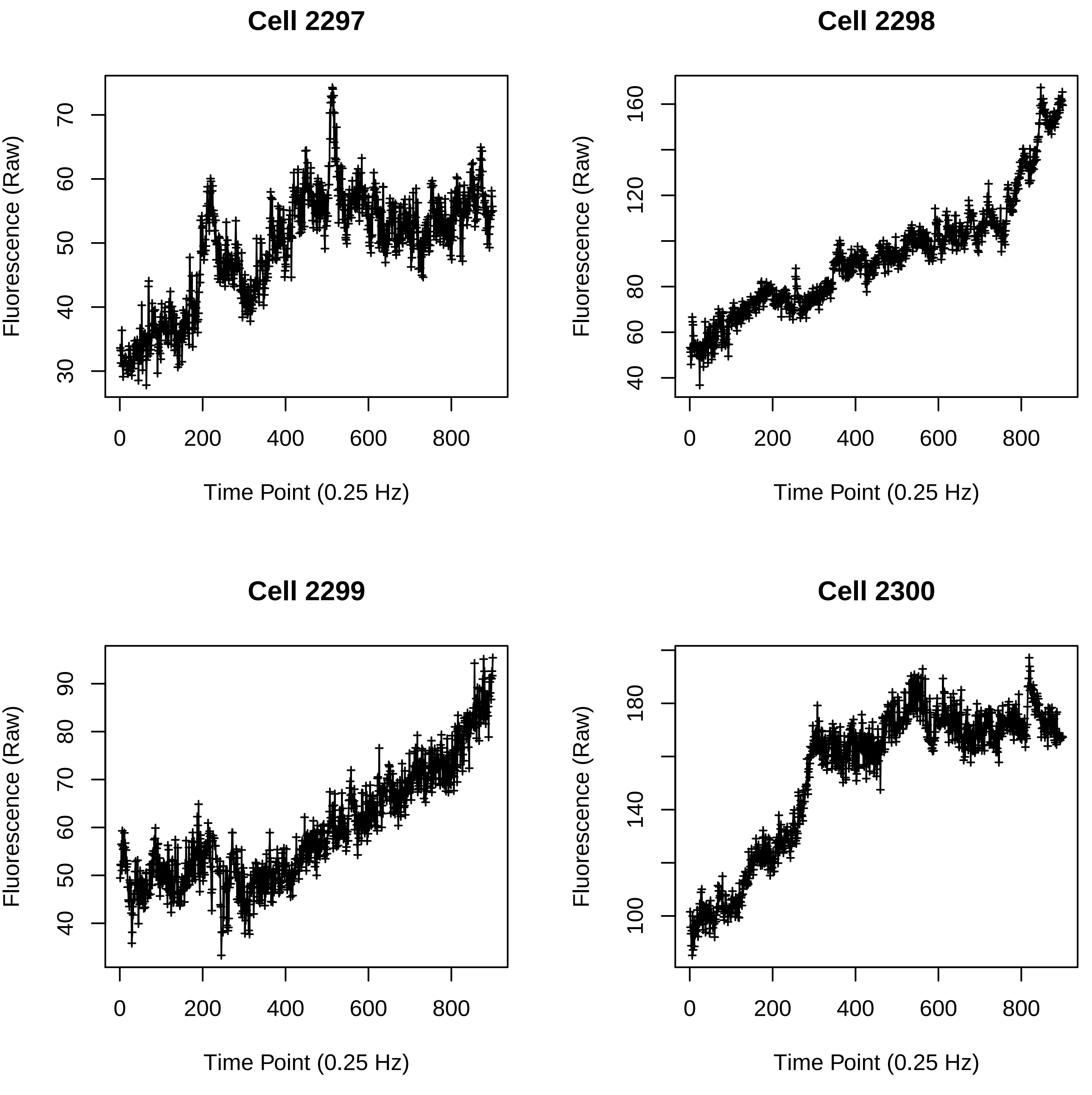
Cell 2297
Fluorescence (Raw)
30
40
50
60
70
0
200
400
600
800
Time Point (0.25 Hz)
Cell 2298
Fluorescence (Raw)
40
60
80
120
160
0
200
400
600
800
Time Point (0.25 Hz)
Cell 2299
Fluorescence (Raw)
40
50
60
70
80
90
0
200
400
600
800
Time Point (0.25 Hz)
Cell 2300
Fluorescence (Raw)
100
140
180
0
200
400
600
800
Time Point (0.25 Hz)

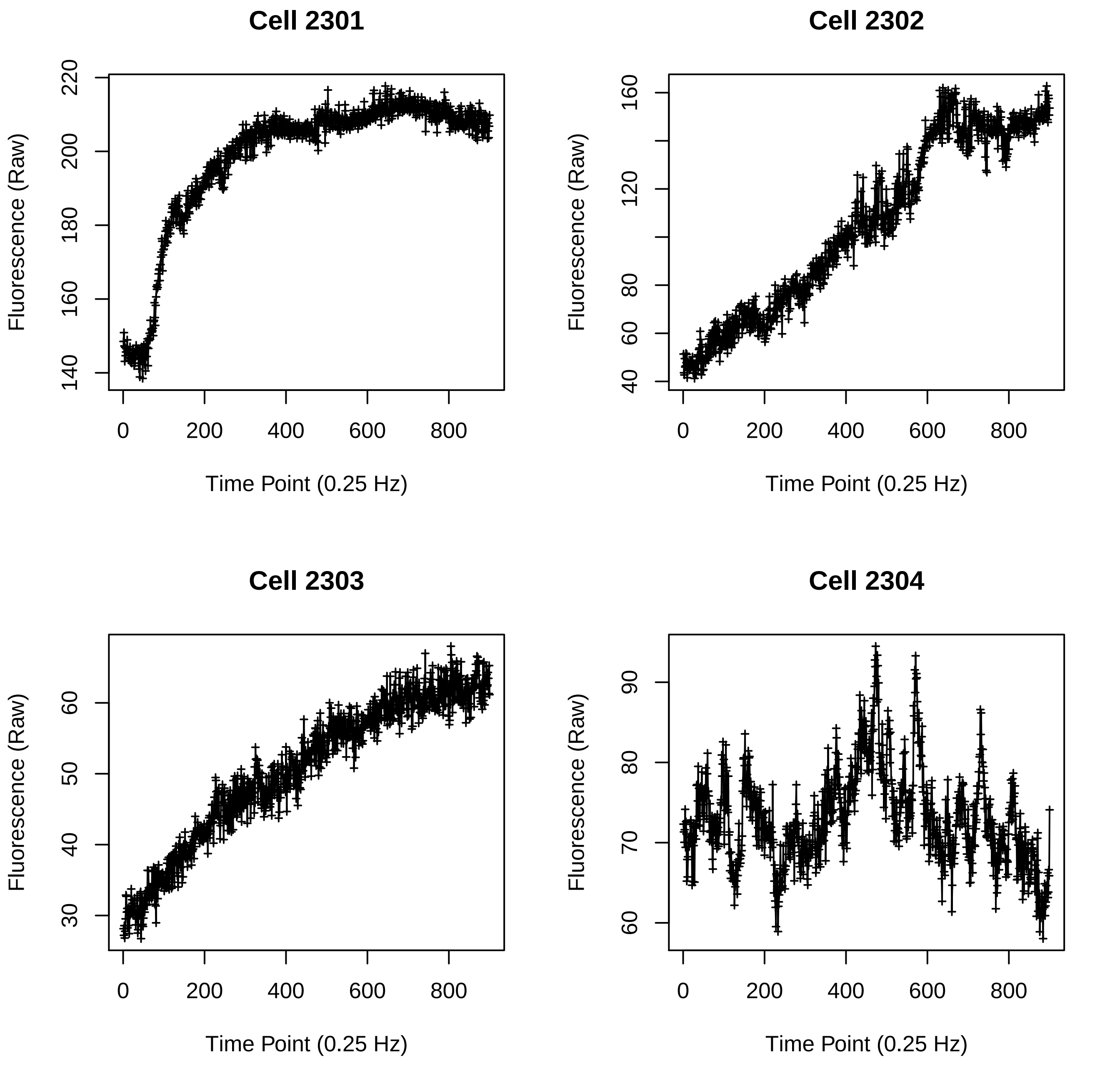
Cell 2301
Fluorescence (Raw)
140
160
180
200
220
Time Point (0.25 Hz)
0
200
400
600
800
Cell 2302
Fluorescence (Raw)
40
60
80
120
160
Time Point (0.25 Hz)
0
200
400
600
800
Cell 2303
Fluorescence (Raw)
30
40
50
60
Time Point (0.25 Hz)
0
200
400
600
800
Cell 2304
Fluorescence (Raw)
60
70
80
90
Time Point (0.25 Hz)
0
200
400
600
800

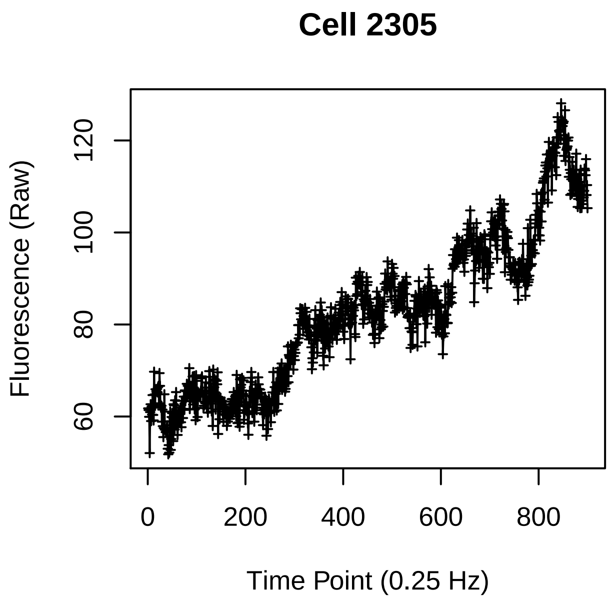
Cell 2305
Fluorescence (Raw)
60
80
100
120
0
200
400
600
800
Time Point (0.25 Hz)

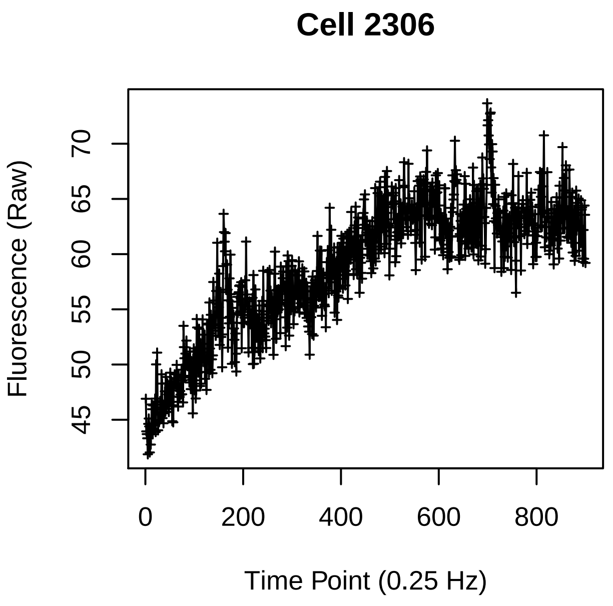
Cell 2306
Fluorescence (Raw)
45
50
55
60
65
70
0
200
400
600
800
Time Point (0.25 Hz)

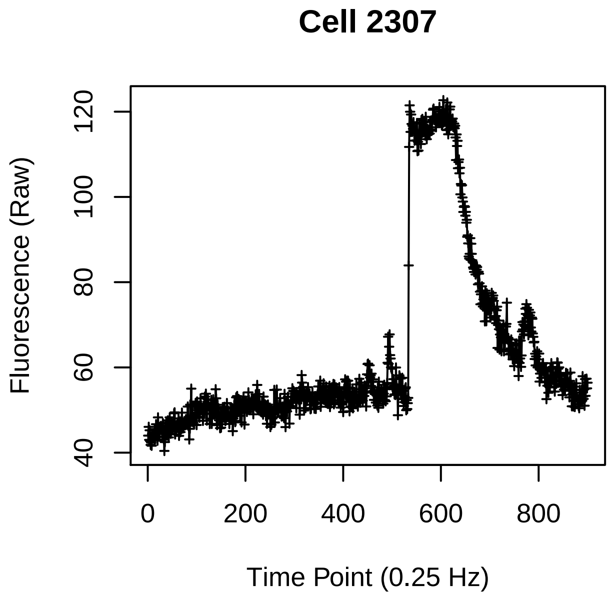
Cell 2307
Fluorescence (Raw)
40
60
80
100
120
0
200
400
600
800
Time Point (0.25 Hz)

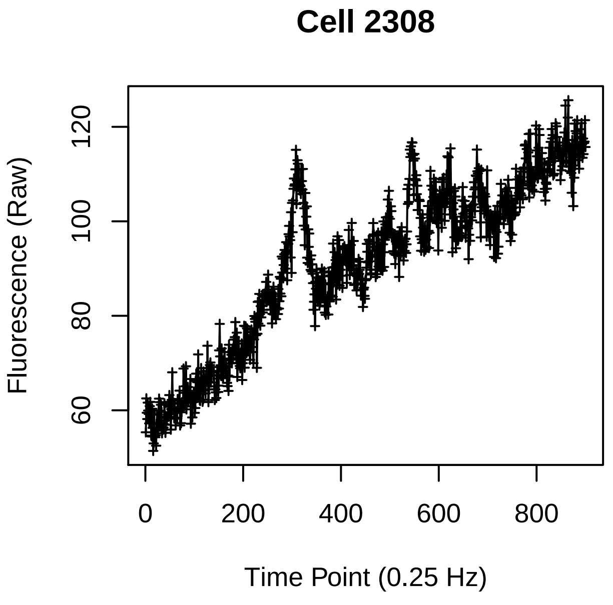
Cell 2308
Fluorescence (Raw)
60
80
100
120
0
200
400
600
800
Time Point (0.25 Hz)

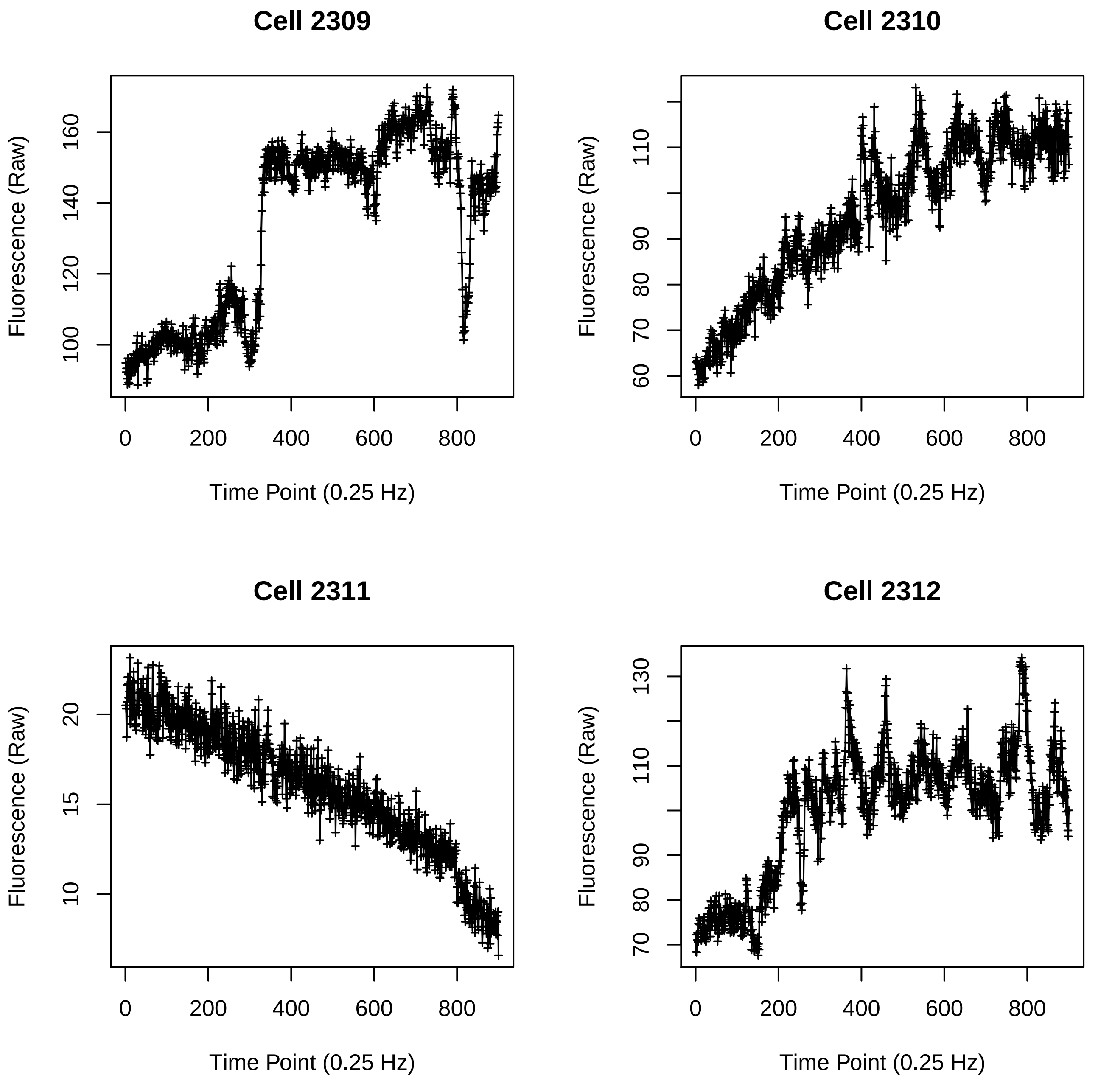
Cell 2309
Fluorescence (Raw)
Time Point (0.25 Hz)
Cell 2310
Fluorescence (Raw)
Time Point (0.25 Hz)
Cell 2311
Fluorescence (Raw)
Time Point (0.25 Hz)
Cell 2312
Fluorescence (Raw)
Time Point (0.25 Hz)

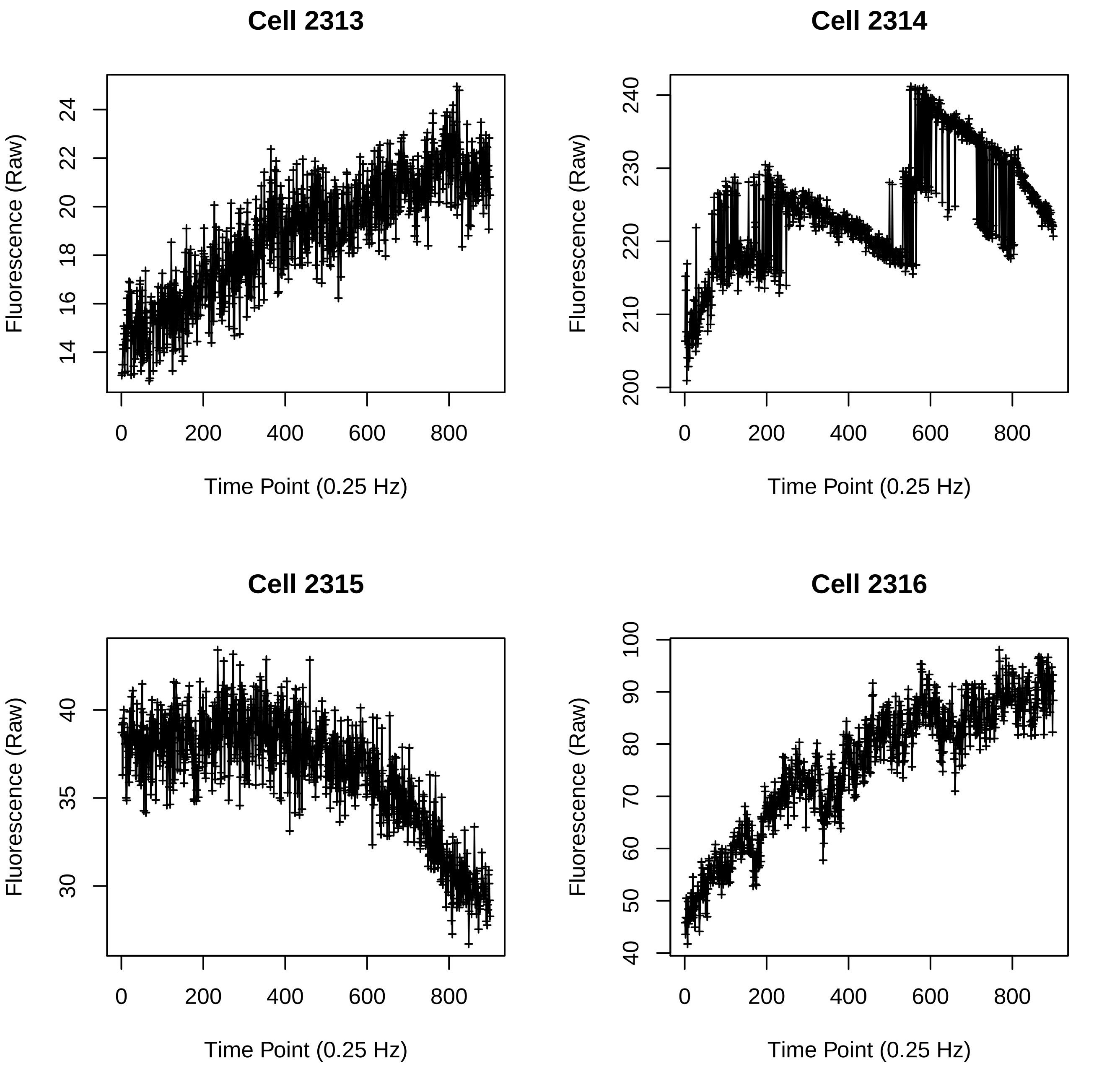
Cell 2313
Fluorescence (Raw)
14
16
18
20
22
24
0
200
400
600
800
Time Point (0.25 Hz)
Cell 2314
Fluorescence (Raw)
200
210
220
230
240
0
200
400
600
800
Time Point (0.25 Hz)
Cell 2315
Fluorescence (Raw)
30
35
40
0
200
400
600
800
Time Point (0.25 Hz)
Cell 2316
Fluorescence (Raw)
40
50
60
70
80
90
100
0
200
400
600
800
Time Point (0.25 Hz)

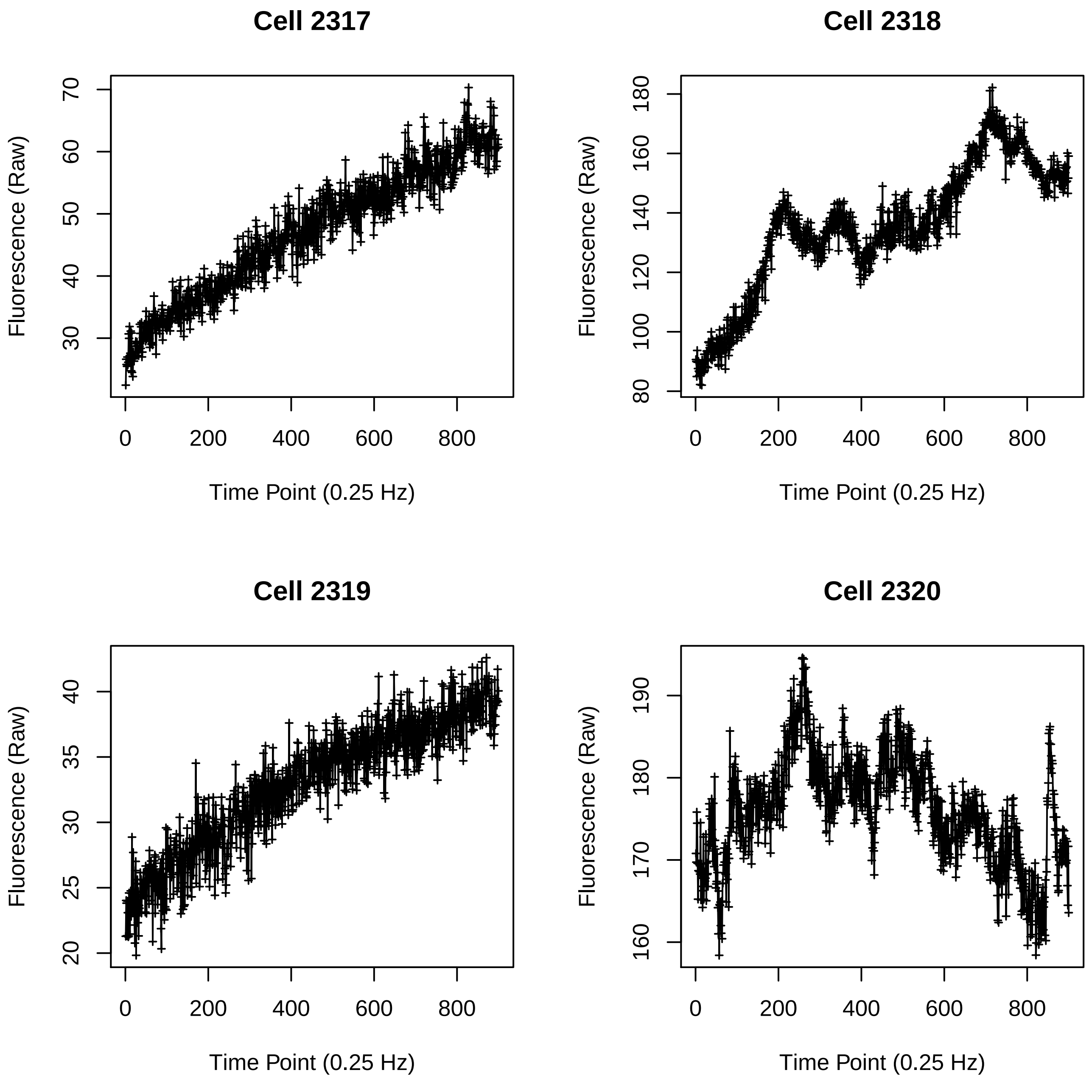
Cell 2317
Fluorescence (Raw)
Time Point (0.25 Hz)
Cell 2318
Fluorescence (Raw)
Time Point (0.25 Hz)
Cell 2319
Fluorescence (Raw)
Time Point (0.25 Hz)
Cell 2320
Fluorescence (Raw)
Time Point (0.25 Hz)

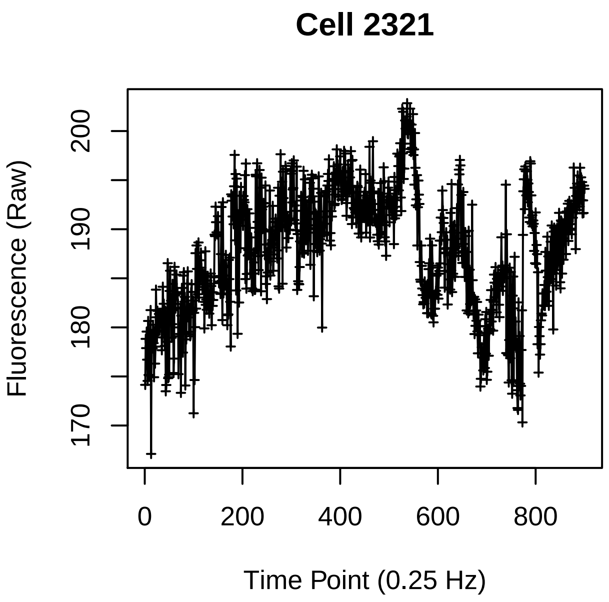
Cell 2321
Fluorescence (Raw)
170
180
190
200
0
200
400
600
800
Time Point (0.25 Hz)

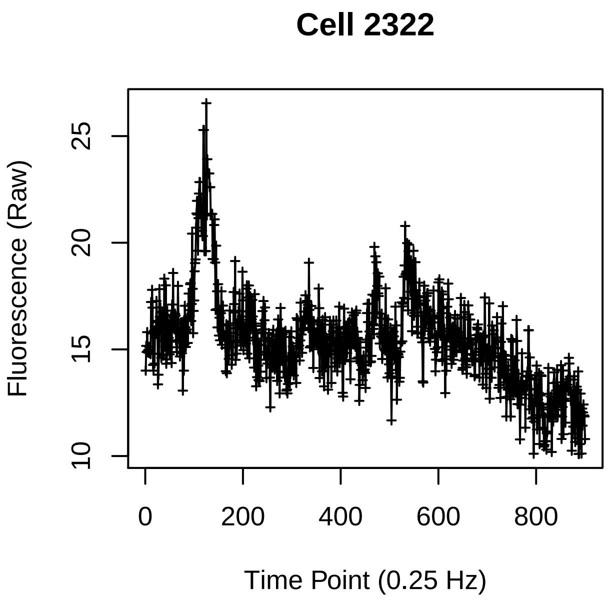
Cell 2322
Fluorescence (Raw)
10
15
20
25
0
200
400
600
800
Time Point (0.25 Hz)

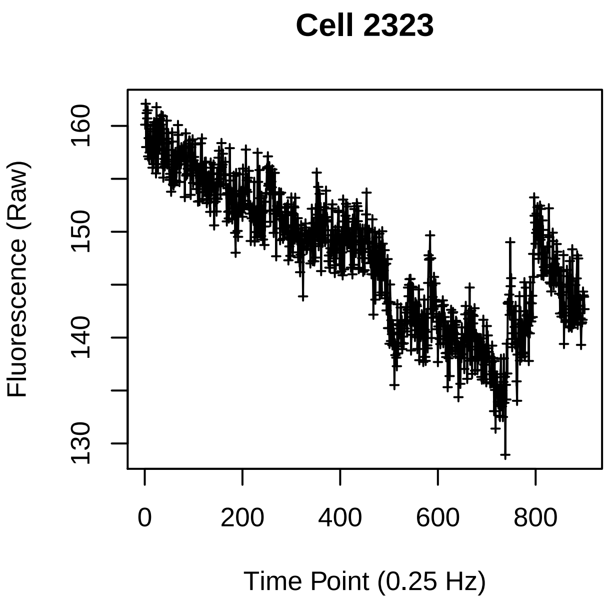
Cell 2323
Fluorescence (Raw)
130
140
150
160
0
200
400
600
800
Time Point (0.25 Hz)

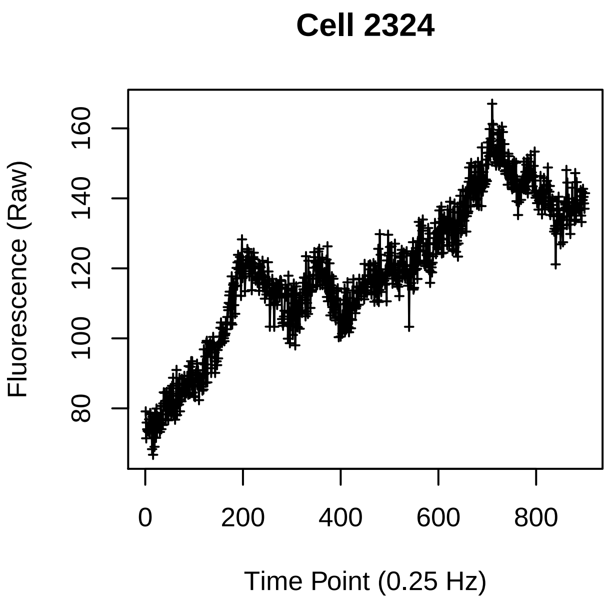
Cell 2324
Fluorescence (Raw)
80
100
120
140
160
0
200
400
600
800
Time Point (0.25 Hz)

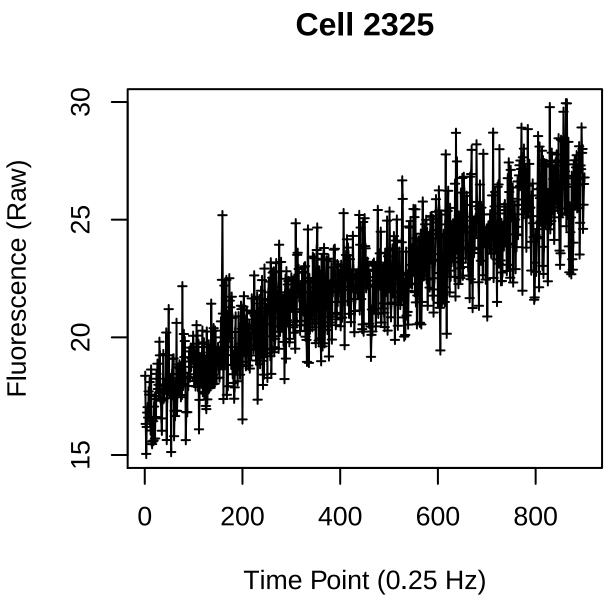
Cell 2325
Fluorescence (Raw)
15
20
25
30
0
200
400
600
800
Time Point (0.25 Hz)

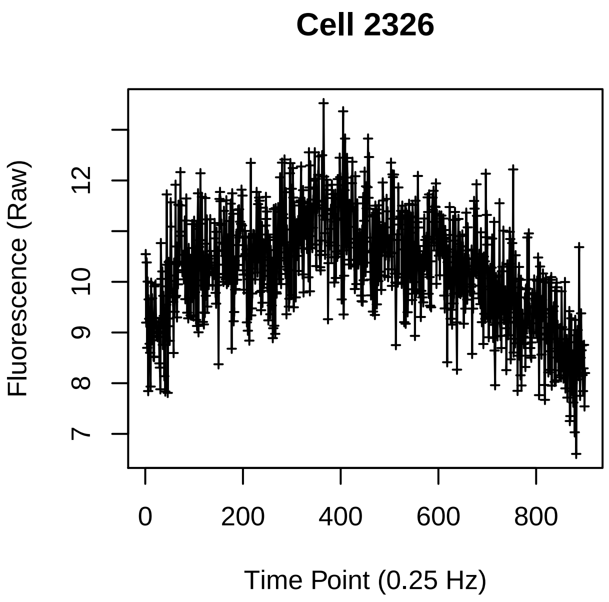
Cell 2326
Fluorescence (Raw)
7
8
9
10
12
0
200
400
600
800
Time Point (0.25 Hz)

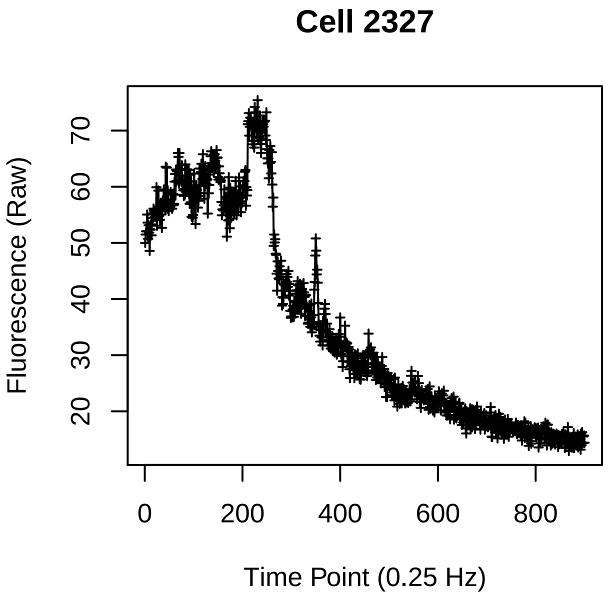
Cell 2327
Fluorescence (Raw)
20
30
40
50
60
70
0
200
400
600
800
Time Point (0.25 Hz)

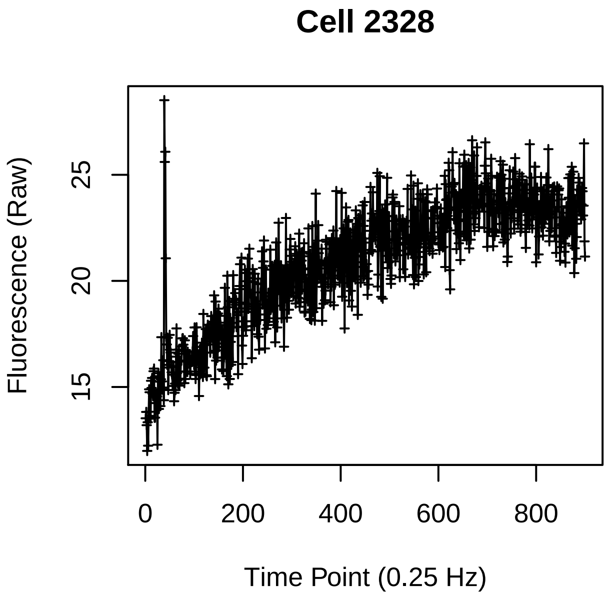
Cell 2328
Fluorescence (Raw)
15
20
25
0
200
400
600
800
Time Point (0.25 Hz)

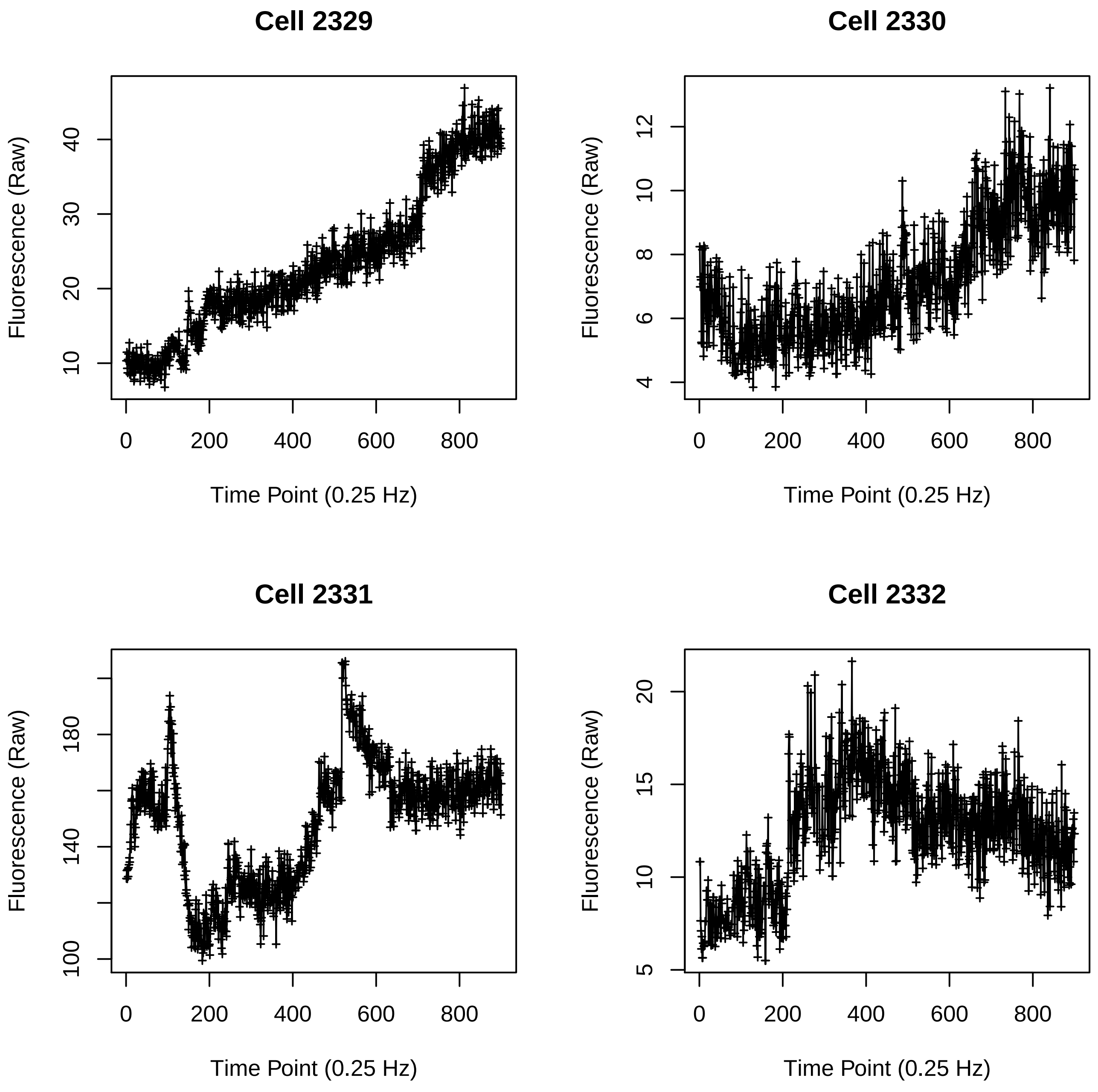
Cell 2329
Fluorescence (Raw)
10
20
30
40
Time Point (0.25 Hz)
0
200
400
600
800
Cell 2330
Fluorescence (Raw)
4
6
8
10
12
Time Point (0.25 Hz)
0
200
400
600
800
Cell 2331
Fluorescence (Raw)
100
140
180
Time Point (0.25 Hz)
0
200
400
600
800
Cell 2332
Fluorescence (Raw)
5
10
15
20
Time Point (0.25 Hz)
0
200
400
600
800

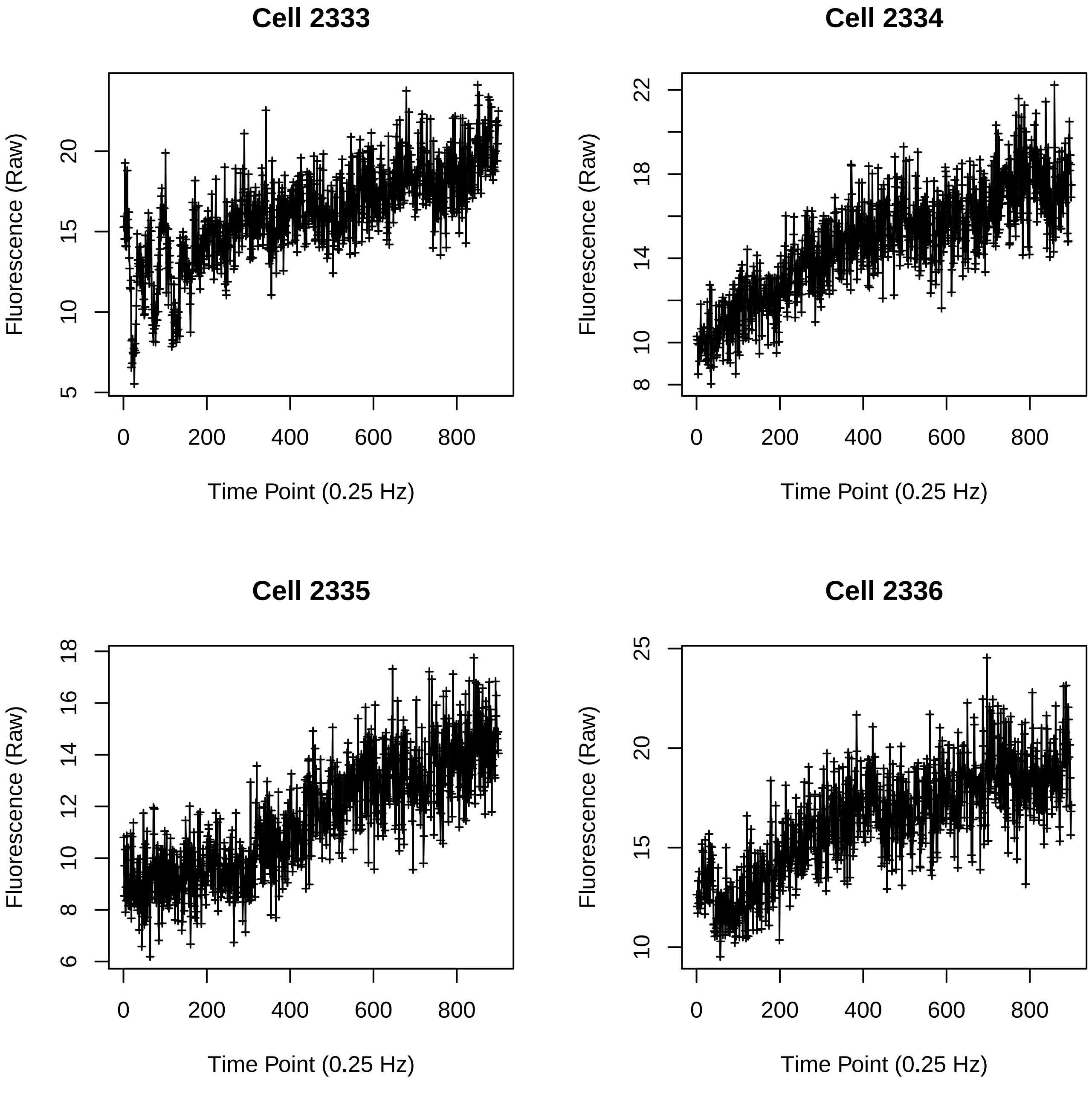
Cell 2333
Cell 2334
Cell 2335
Cell 2336
Fluorescence (Raw)
Time Point (0.25 Hz)
0
200
400
600
800
5
10
15
20
8
12
14
16
18
22
6
25

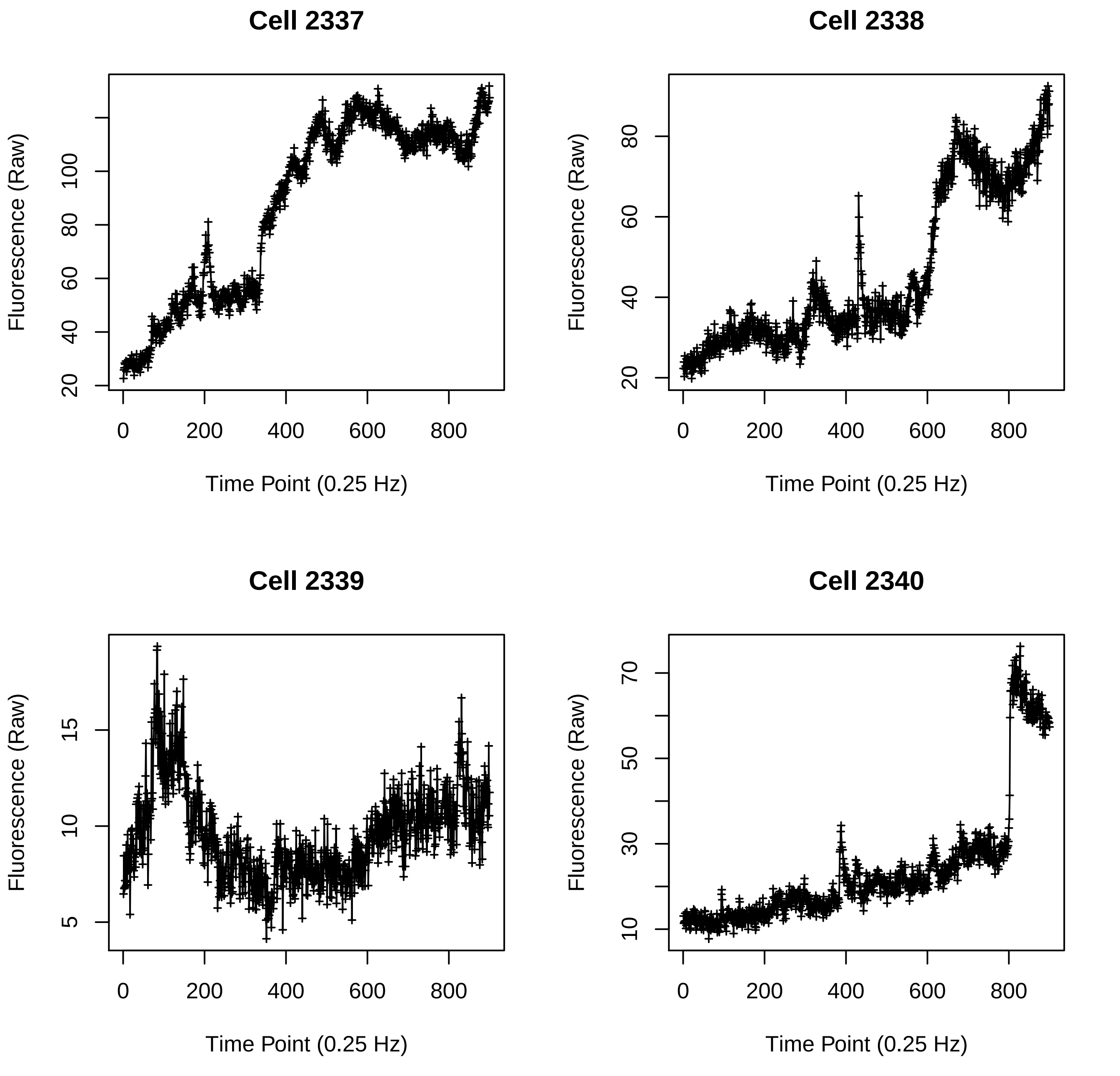
Cell 2337
Fluorescence (Raw)
20 40 60 80 100
0 200 400 600 800
Time Point (0.25 Hz)
Cell 2338
Fluorescence (Raw)
20 40 60 80
0 200 400 600 800
Time Point (0.25 Hz)
Cell 2339
Fluorescence (Raw)
5 10 15
0 200 400 600 800
Time Point (0.25 Hz)
Cell 2340
Fluorescence (Raw)
10 30 50 70
0 200 400 600 800
Time Point (0.25 Hz)

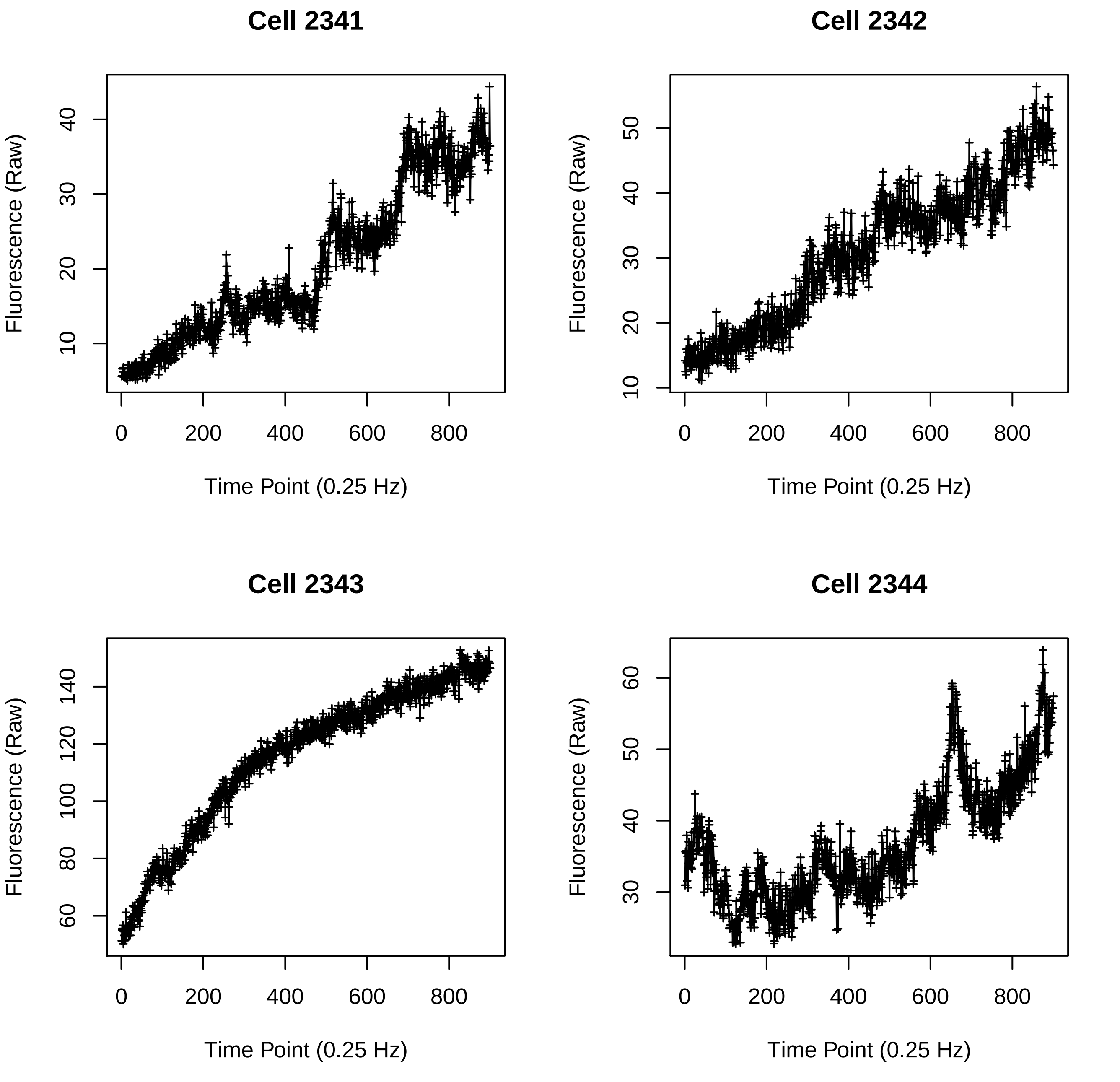
Cell 2341
Cell 2342
Cell 2343
Cell 2344
Fluorescence (Raw)
Time Point (0.25 Hz)
0
200
400
600
800
10
20
30
40
50
60
80
100
120
140

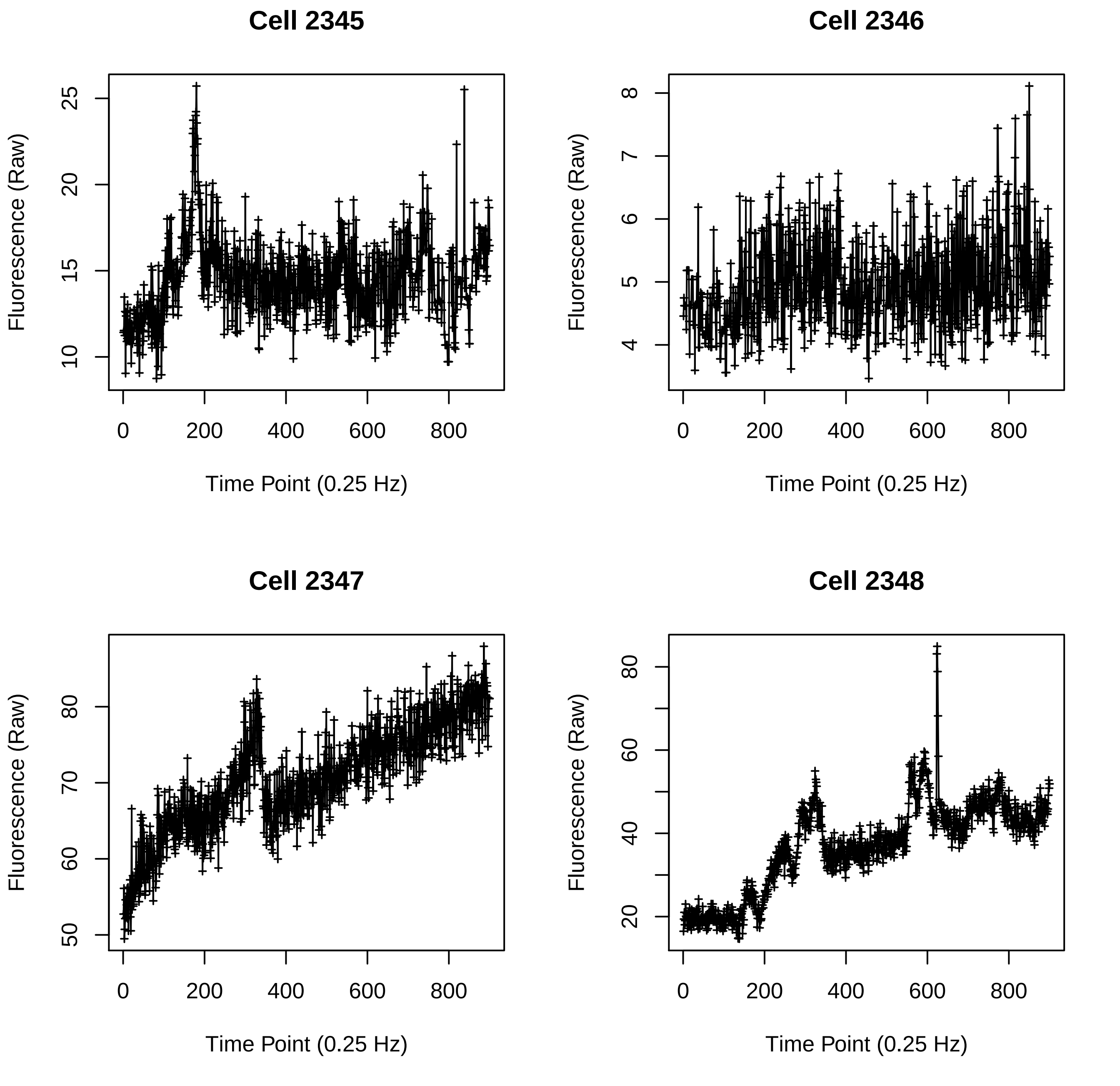
Cell 2345
Cell 2346
Cell 2347
Cell 2348
Fluorescence (Raw)
Time Point (0.25 Hz)
0
200
400
600
800
10
15
20
25
4
5
6
7
8
50
60
70
80
20
40
60
80

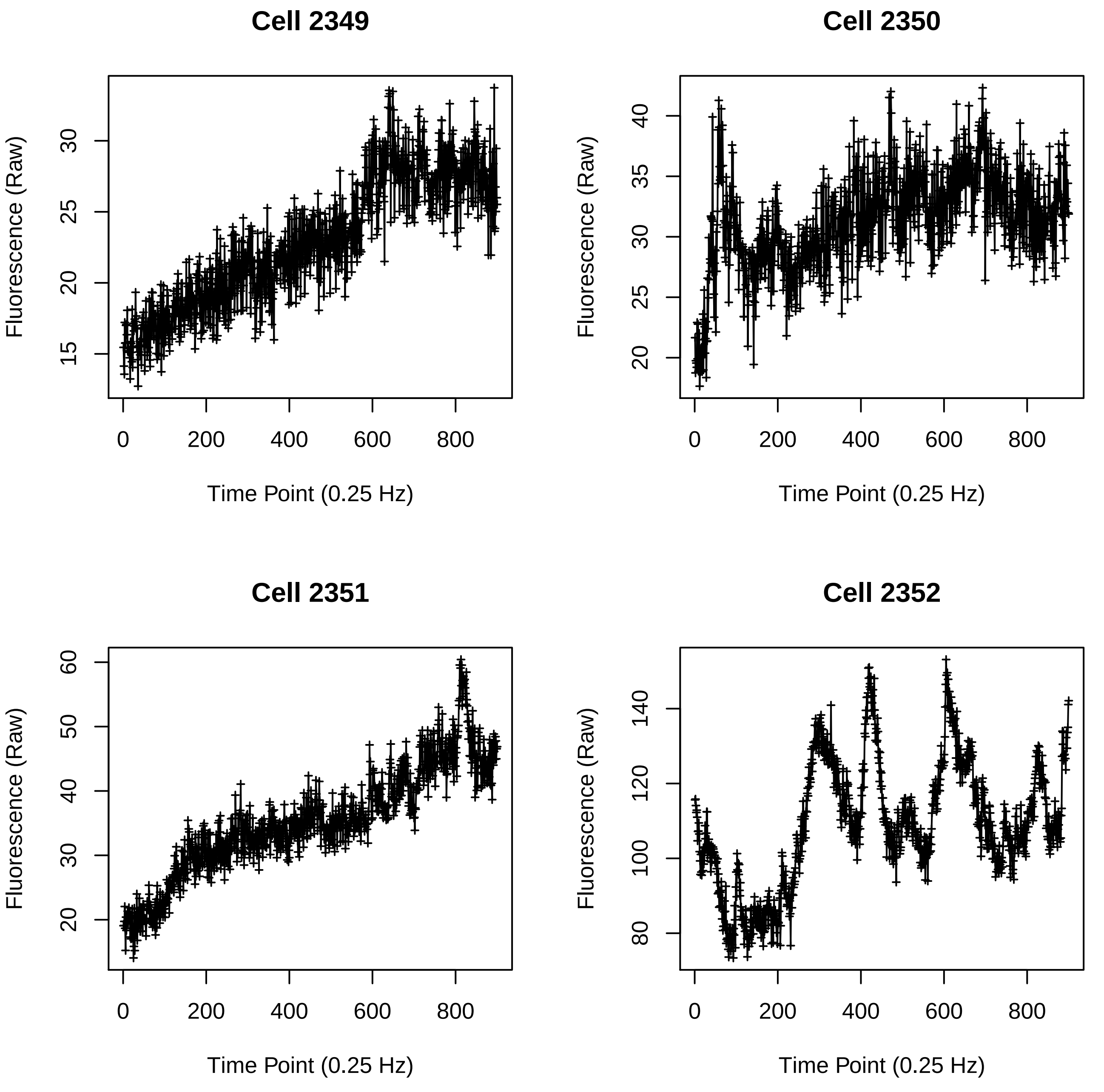

Cell 2349
Cell 2350
Cell 2351
Cell 2352
Fluorescence (Raw)
Time Point (0.25 Hz)
0
200
400
600
800
15
20
25
30
35
40
50
60
80
100
120
140

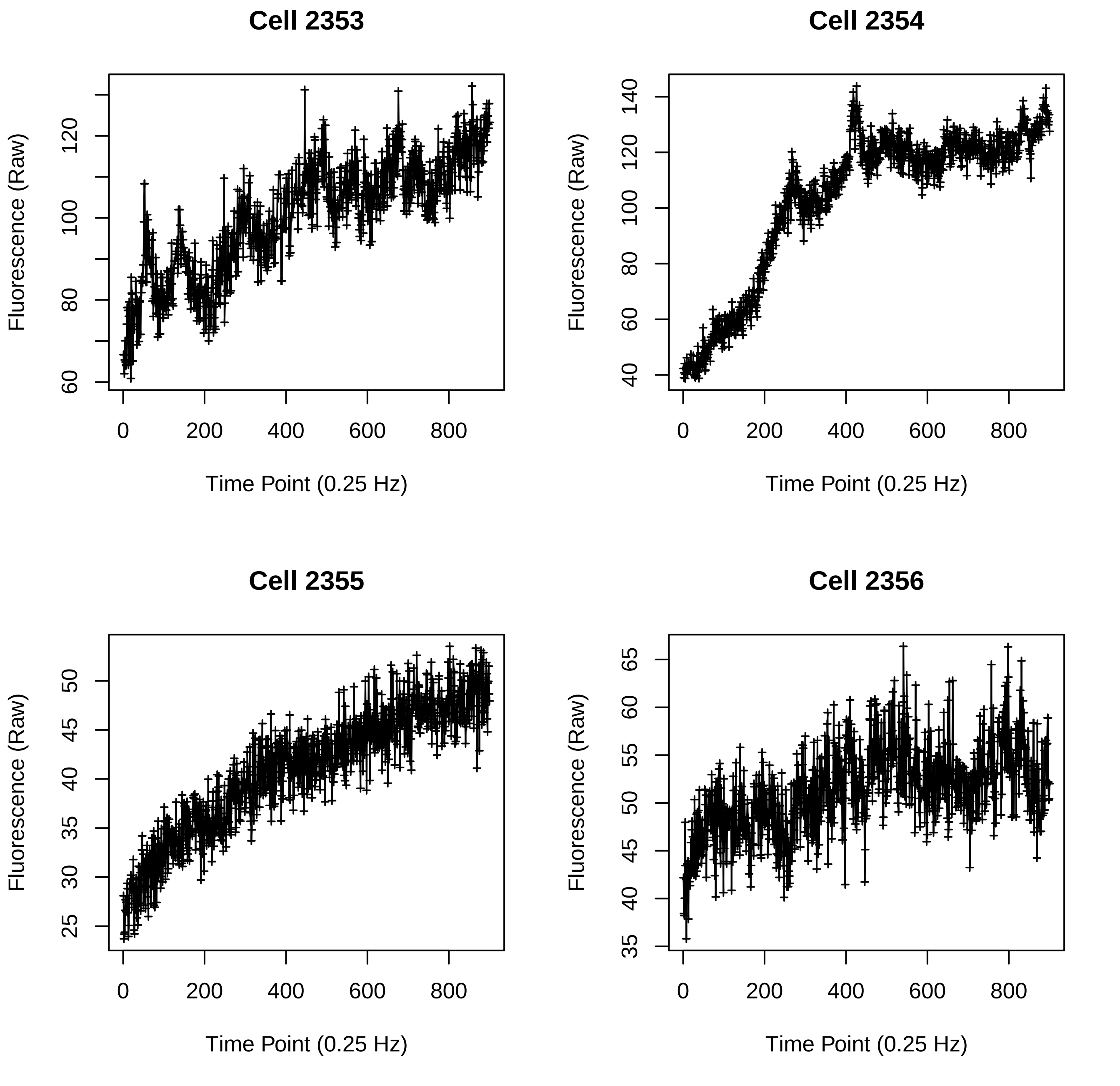
Cell 2353
Cell 2354
Cell 2355
Cell 2356
Fluorescence (Raw)
Time Point (0.25 Hz)
0
200
400
600
800

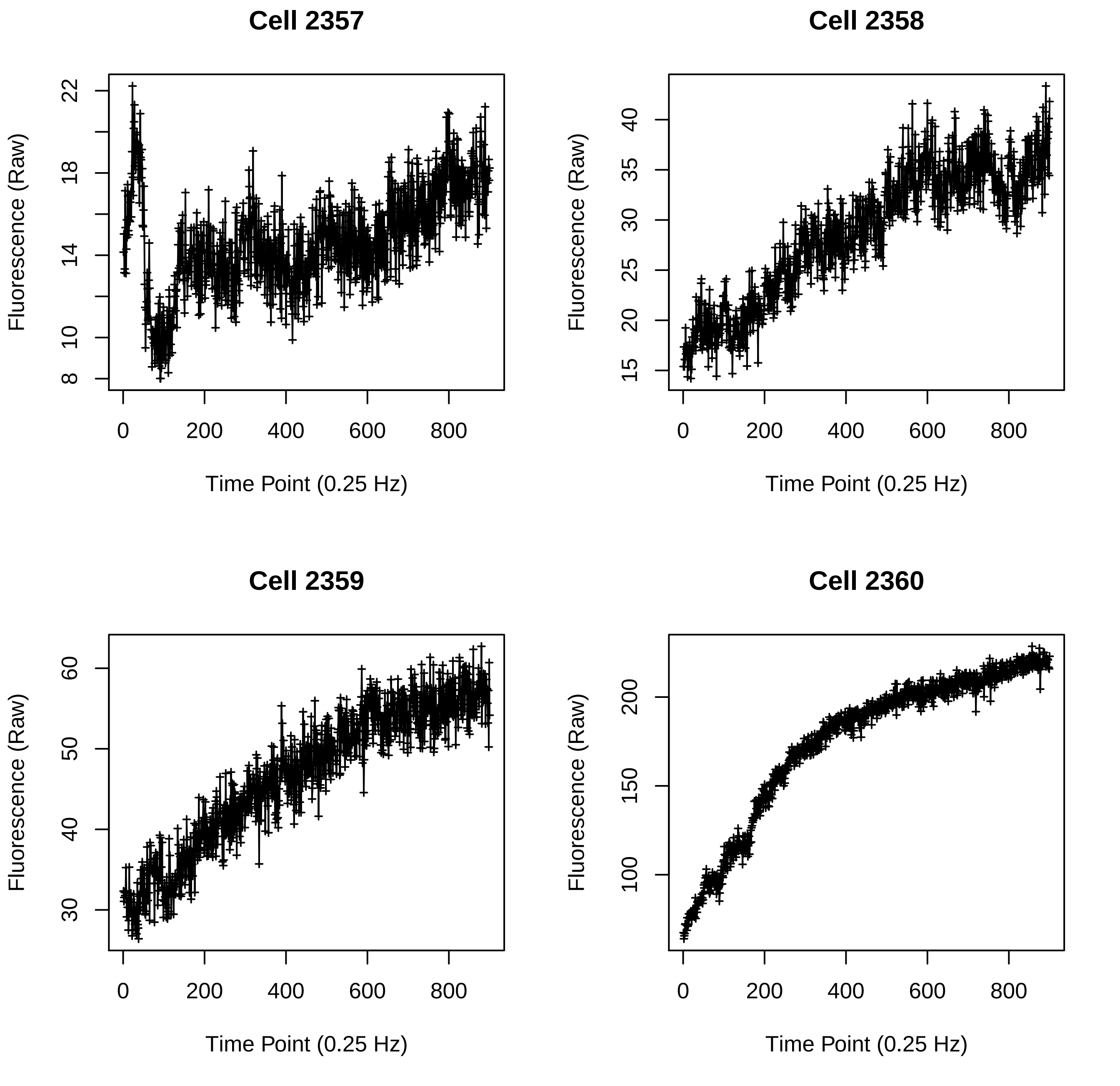
Cell 2357
Cell 2358
Cell 2359
Cell 2360
Fluorescence (Raw)
Time Point (0.25 Hz)
0
200
400
600
800
8
10
14
18
22
15
20
25
30
35
40
30
40
50
60
100
150
200

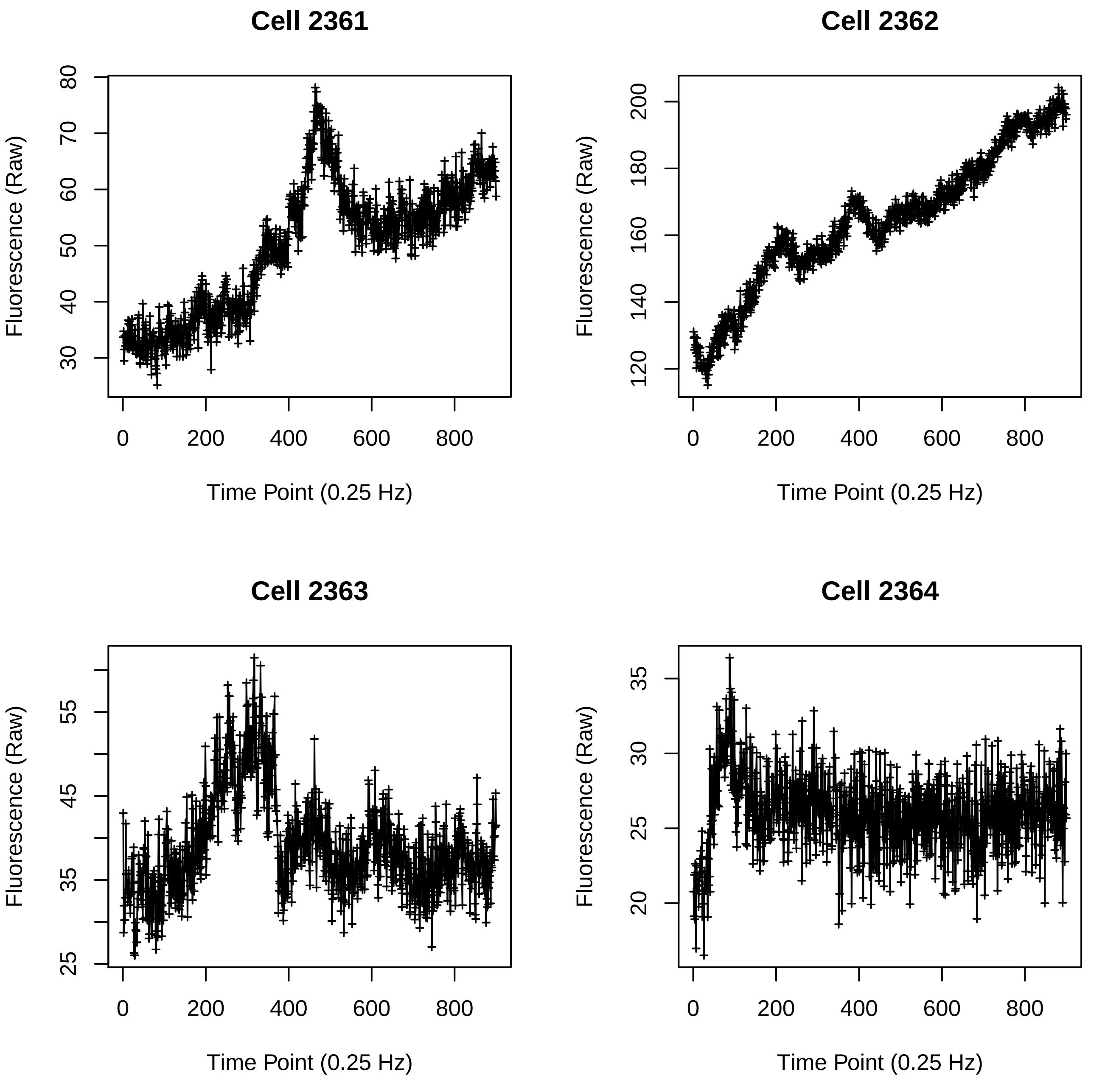
Cell 2361
Cell 2362
Cell 2363
Cell 2364
Fluorescence (Raw)
Time Point (0.25 Hz)
30
40
50
60
70
80
120
140
160
180
200
25
35
45
55
20
25
30
35
0
200
400
600
800

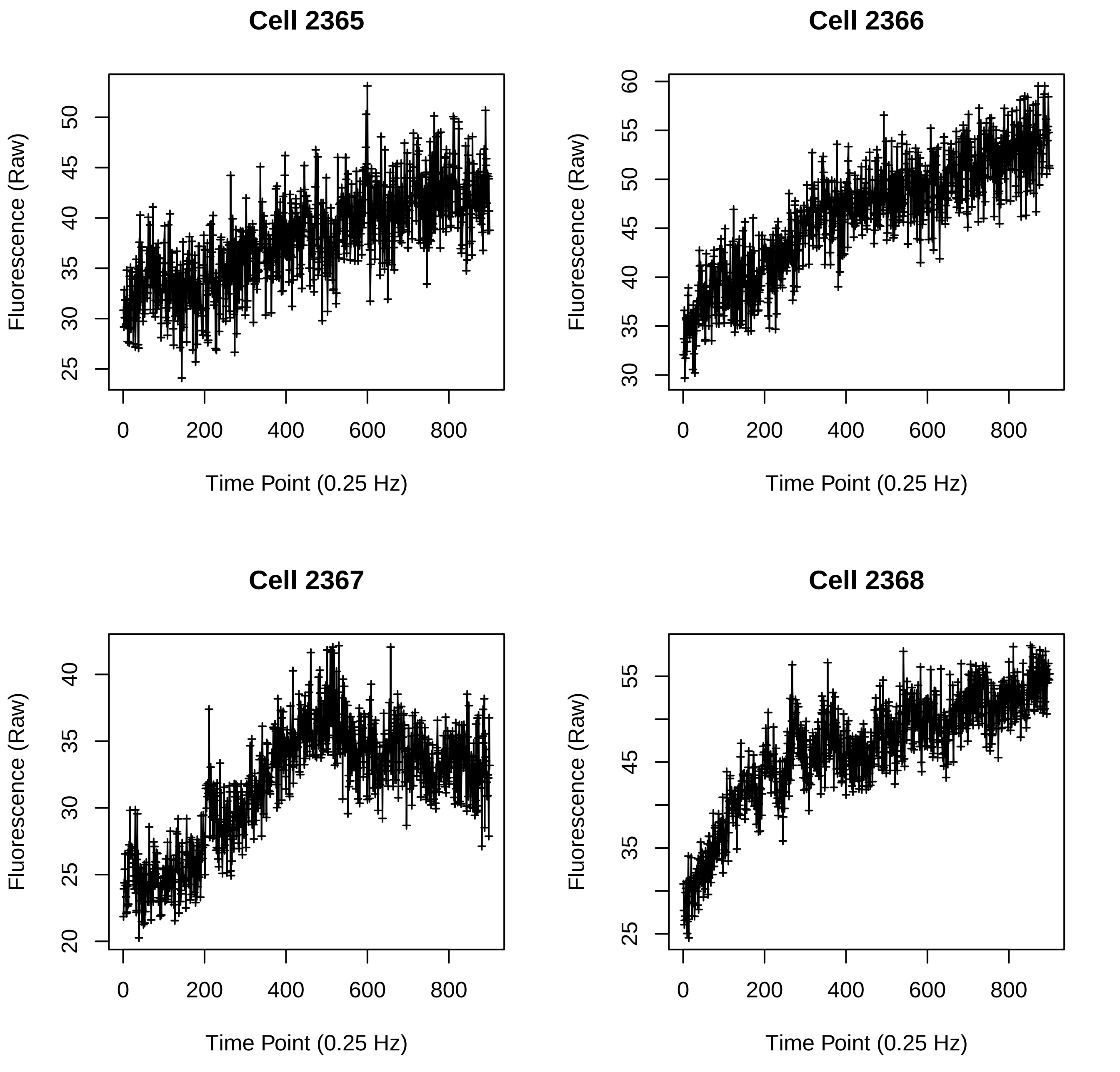

Cell 2365
Fluorescence (Raw)
25
30
35
40
45
50
0
200
400
600
800
Time Point (0.25 Hz)
Cell 2366
Fluorescence (Raw)
30
35
40
45
50
55
60
0
200
400
600
800
Time Point (0.25 Hz)
Cell 2367
Fluorescence (Raw)
20
25
30
35
40
0
200
400
600
800
Time Point (0.25 Hz)
Cell 2368
Fluorescence (Raw)
25
35
45
55
0
200
400
600
800
Time Point (0.25 Hz)

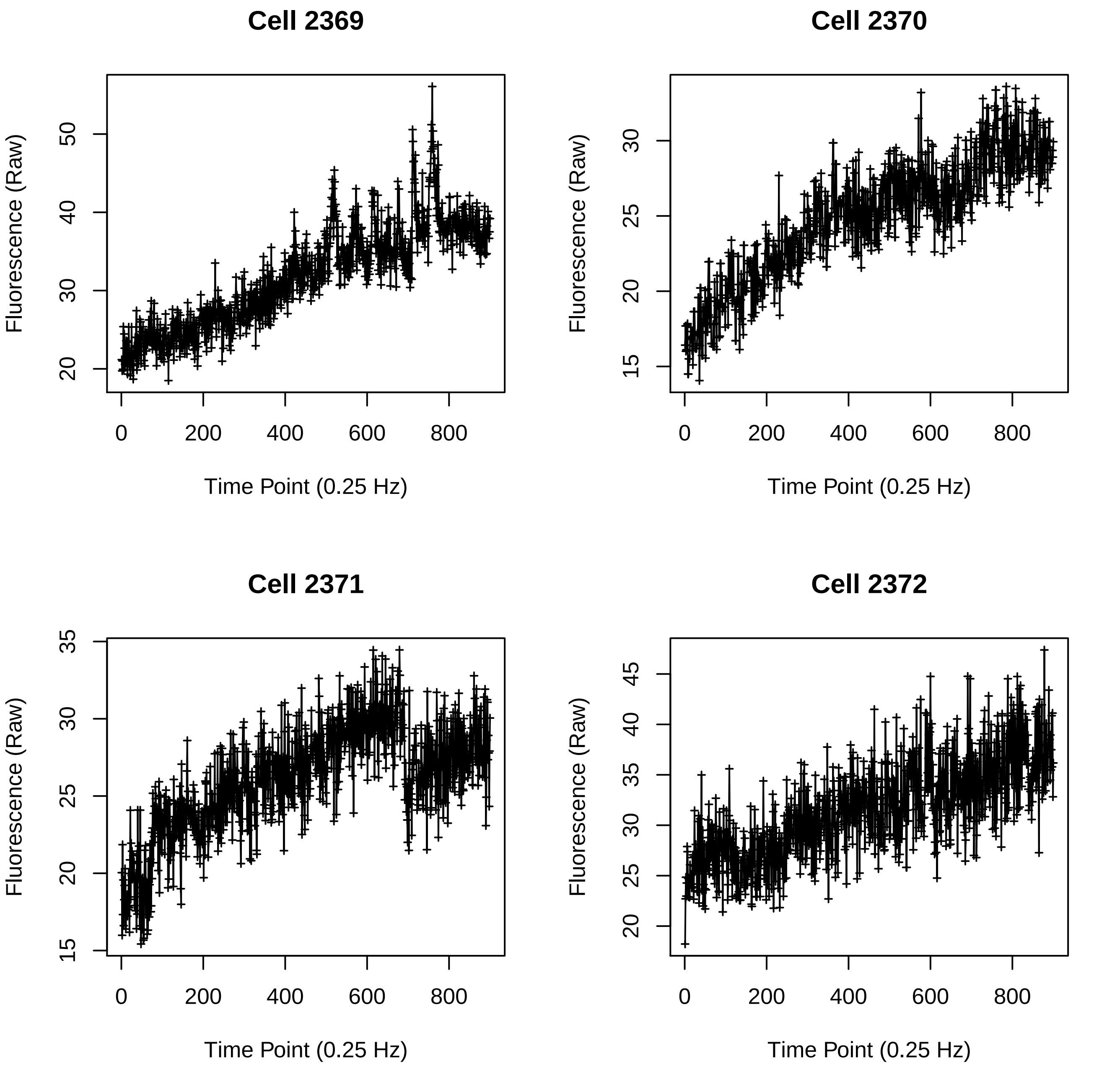
Cell 2369
Fluorescence (Raw)
20
30
40
50
0
200
400
600
800
Time Point (0.25 Hz)
Cell 2370
Fluorescence (Raw)
15
20
25
30
0
200
400
600
800
Time Point (0.25 Hz)
Cell 2371
Fluorescence (Raw)
15
20
25
30
35
0
200
400
600
800
Time Point (0.25 Hz)
Cell 2372
Fluorescence (Raw)
20
25
30
35
40
45
0
200
400
600
800
Time Point (0.25 Hz)

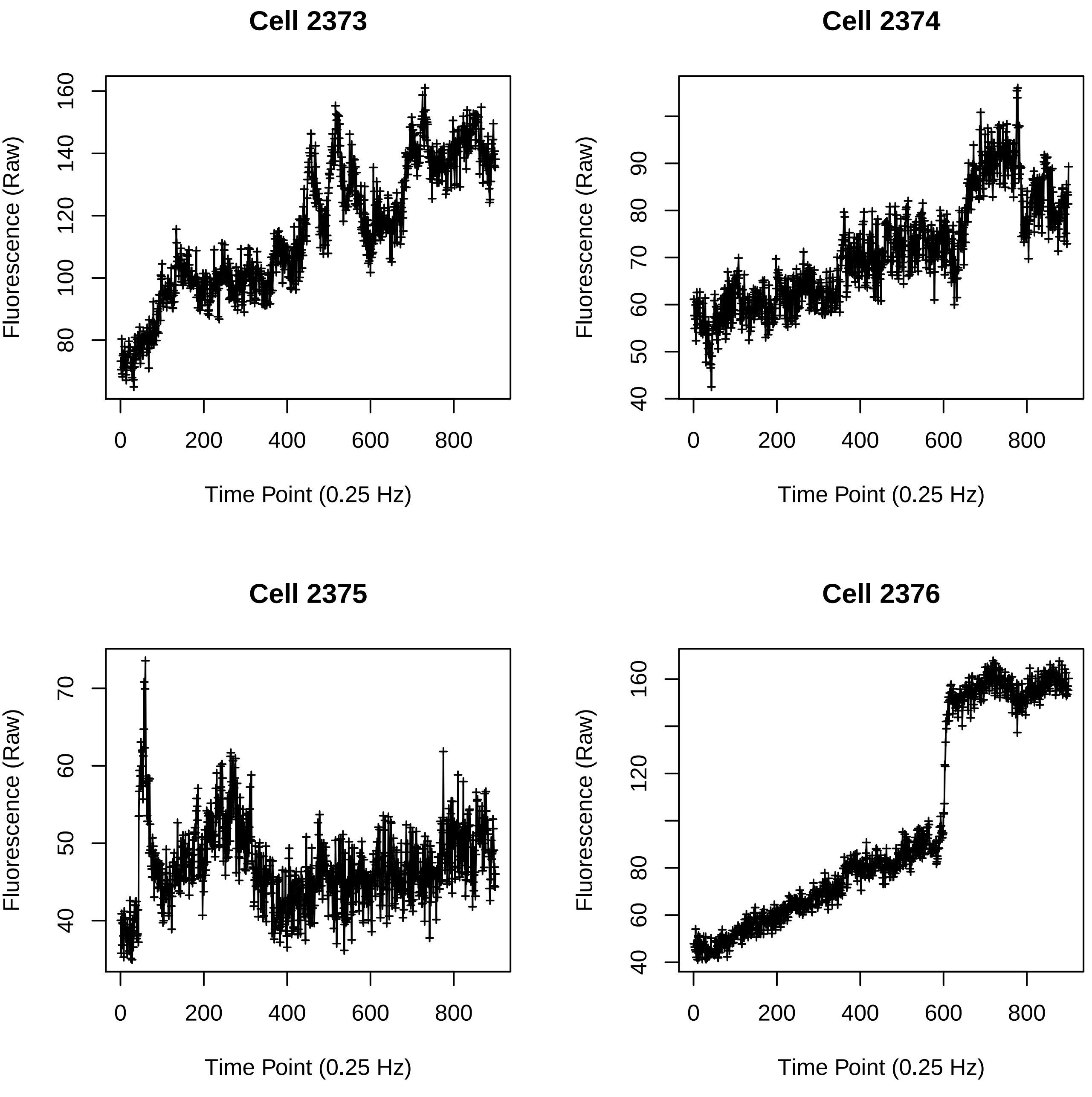
Cell 2373
Fluorescence (Raw)
80
100
120
140
160
0
200
400
600
800
Time Point (0.25 Hz)
Cell 2374
Fluorescence (Raw)
40
50
60
70
80
90
0
200
400
600
800
Time Point (0.25 Hz)
Cell 2375
Fluorescence (Raw)
40
50
60
70
0
200
400
600
800
Time Point (0.25 Hz)
Cell 2376
Fluorescence (Raw)
40
60
80
120
160
0
200
400
600
800
Time Point (0.25 Hz)

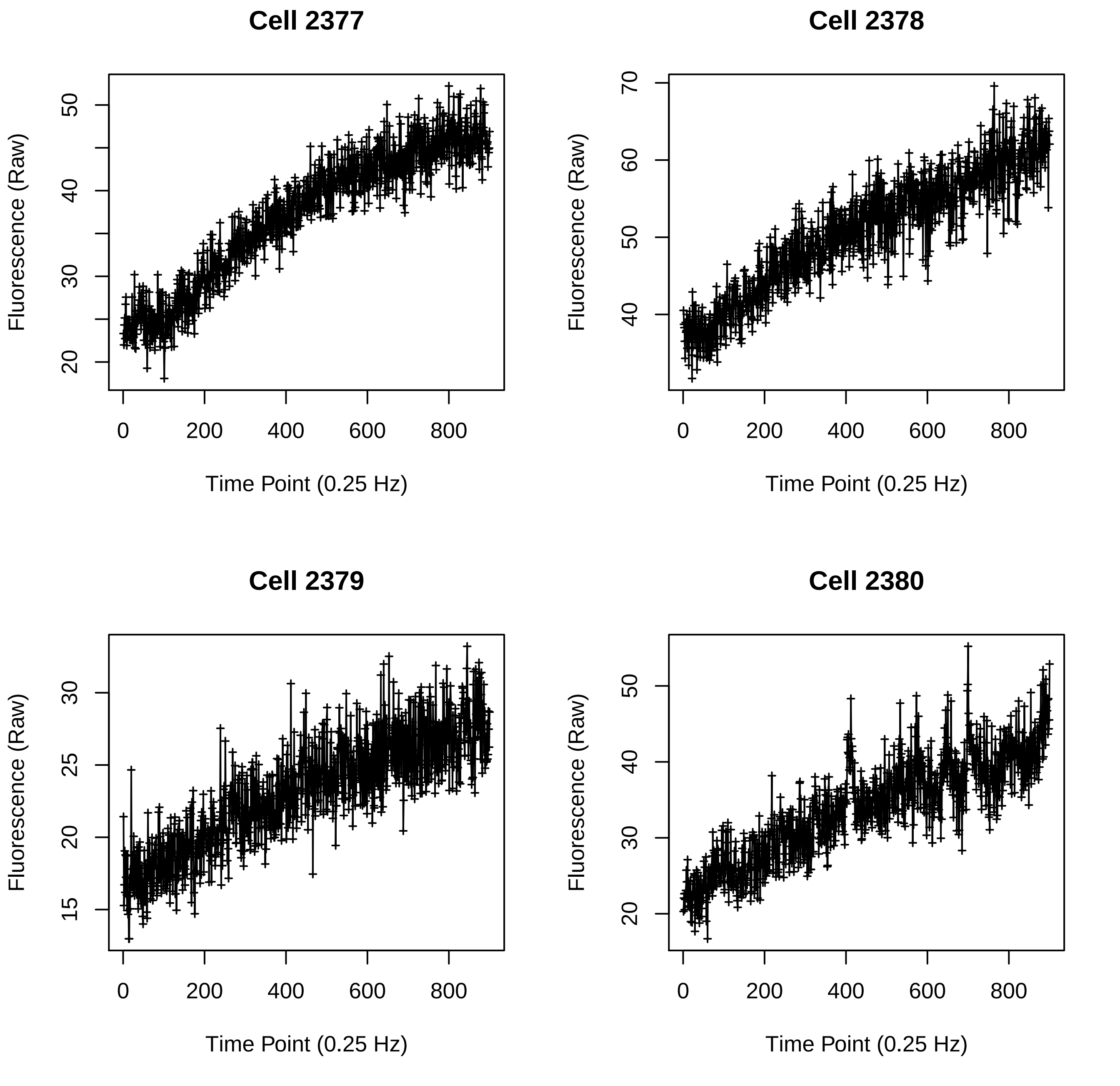
Cell 2377
Fluorescence (Raw)
Time Point (0.25 Hz)
Cell 2378
Fluorescence (Raw)
Time Point (0.25 Hz)
Cell 2379
Fluorescence (Raw)
Time Point (0.25 Hz)
Cell 2380
Fluorescence (Raw)
Time Point (0.25 Hz)

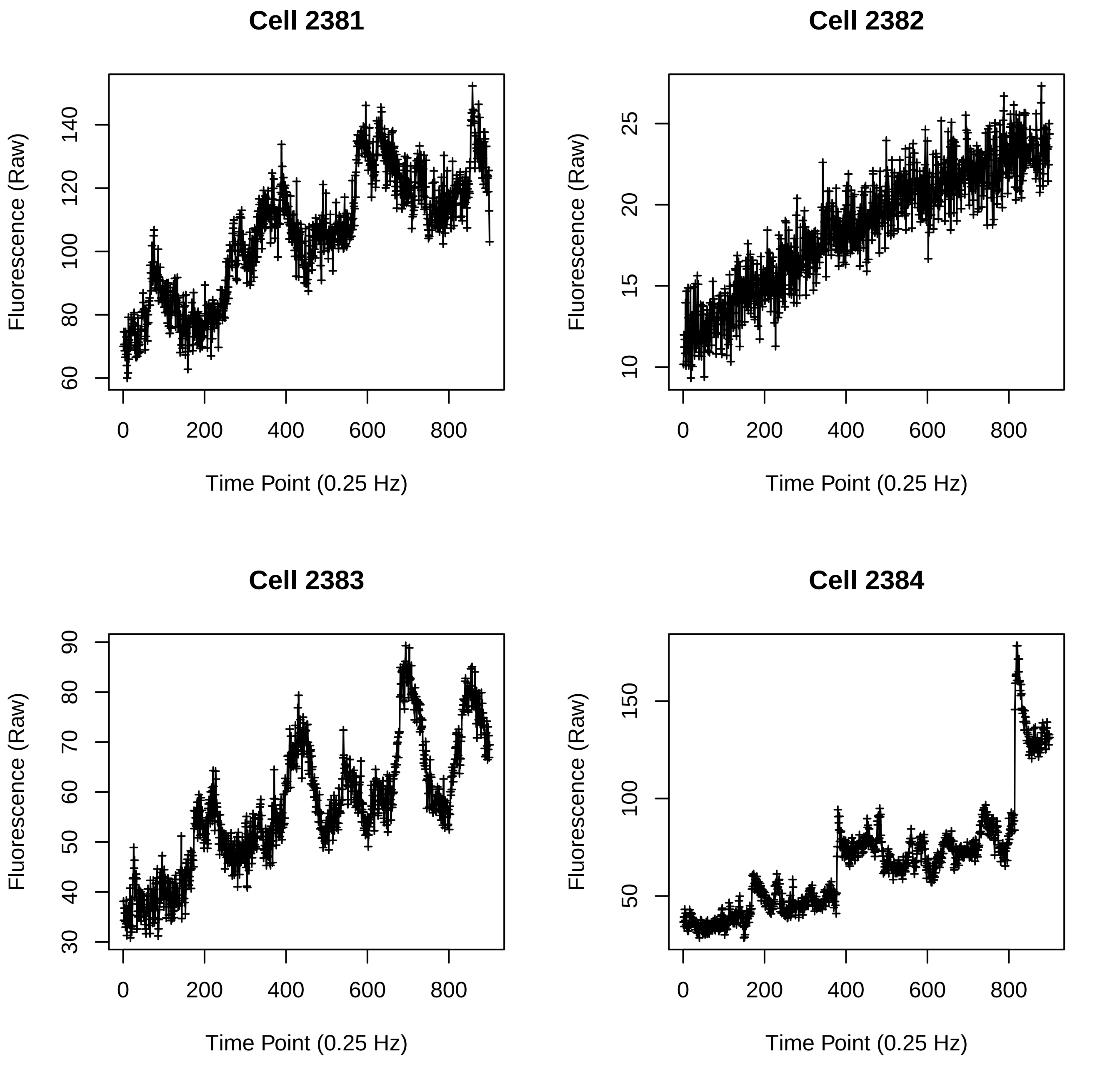
Cell 2381
Fluorescence (Raw)
60
80
100
120
140
0
200
400
600
800
Time Point (0.25 Hz)
Cell 2382
Fluorescence (Raw)
10
15
20
25
0
200
400
600
800
Time Point (0.25 Hz)
Cell 2383
Fluorescence (Raw)
30
40
50
60
70
80
90
0
200
400
600
800
Time Point (0.25 Hz)
Cell 2384
Fluorescence (Raw)
50
100
150
0
200
400
600
800
Time Point (0.25 Hz)

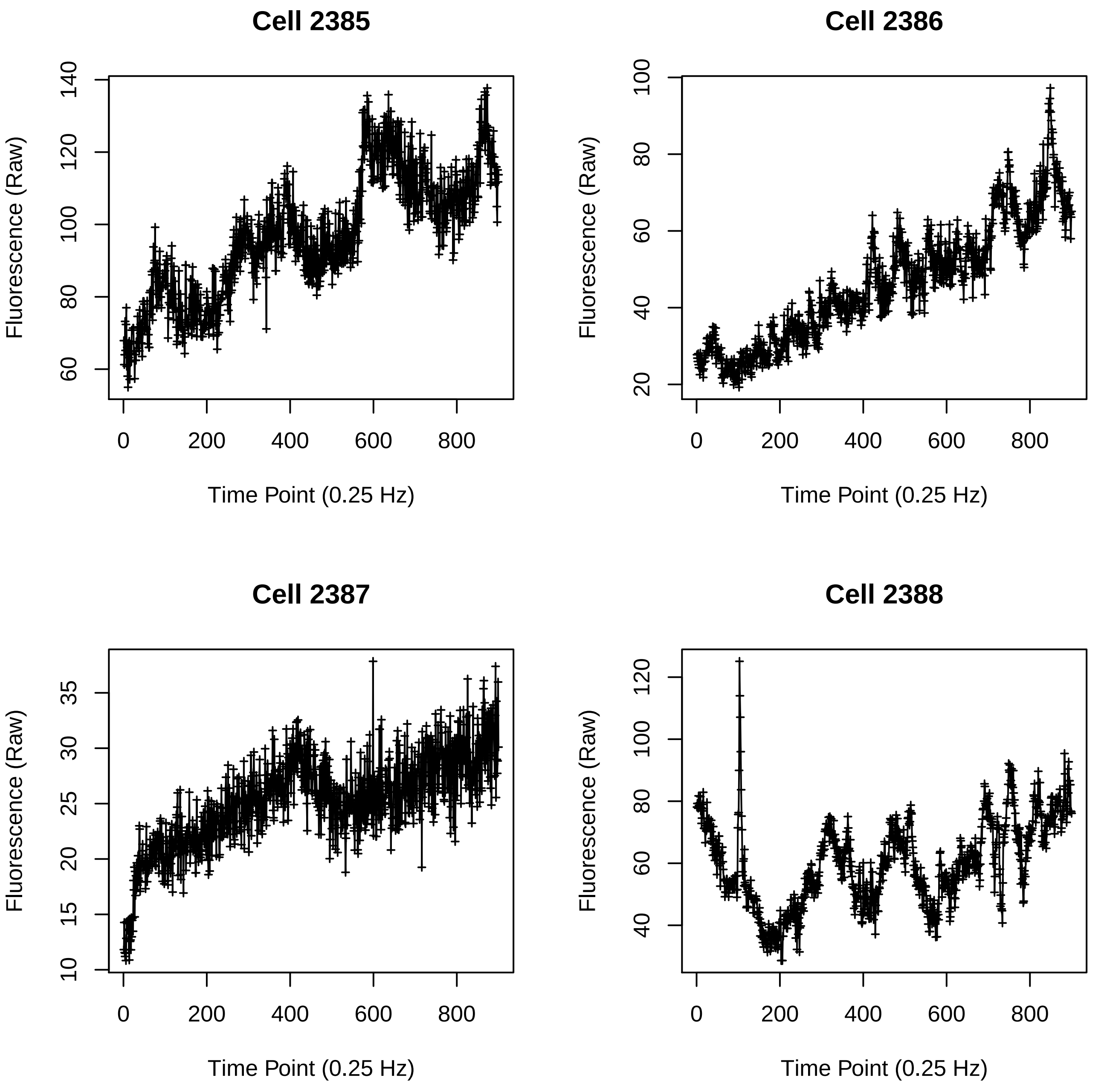
Cell 2385
Cell 2386
Cell 2387
Cell 2388
Fluorescence (Raw)
Time Point (0.25 Hz)
0
200
400
600
800
60
80
100
120
140
20
40
10
15
20
25
30
35

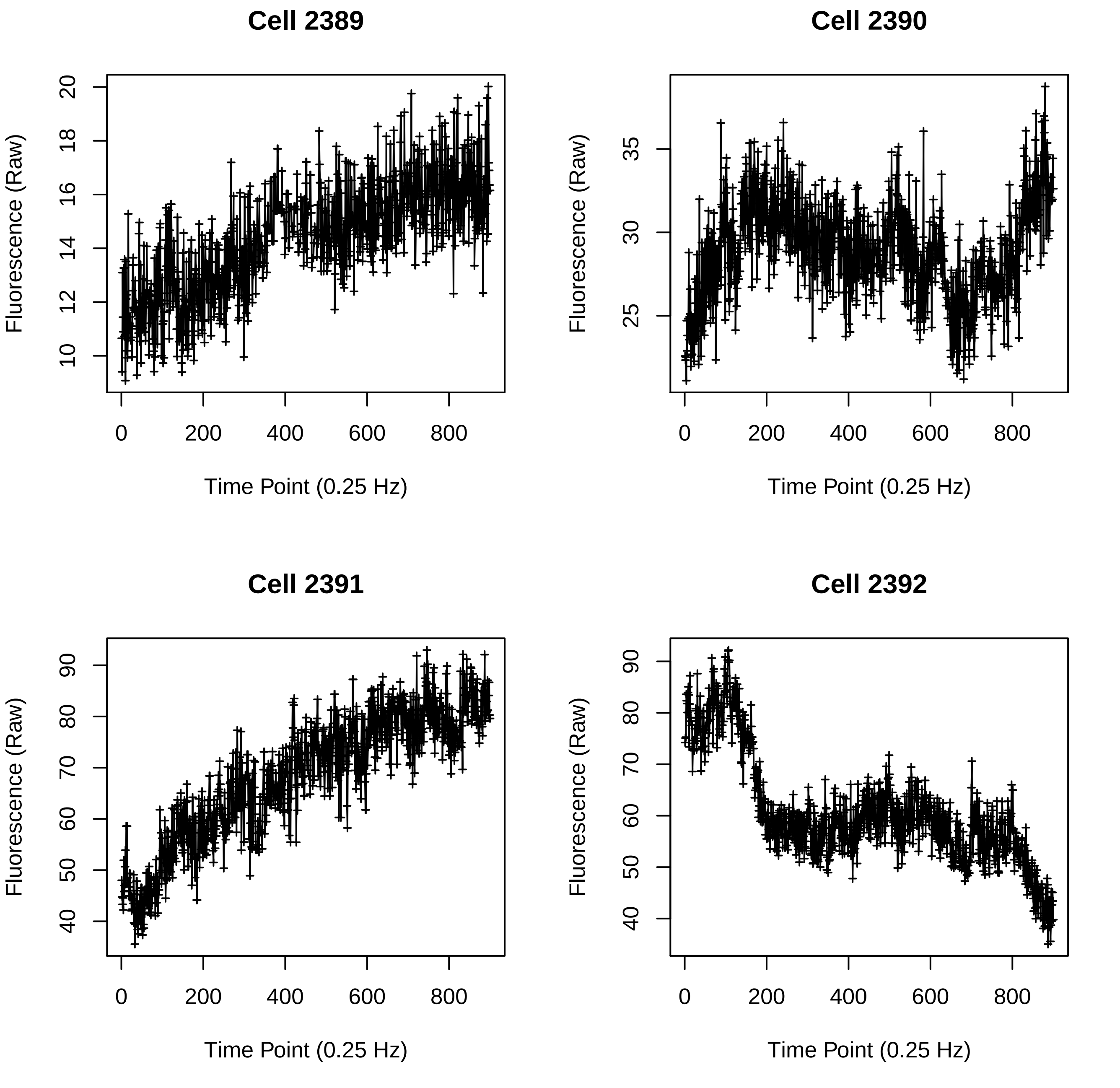
Cell 2389
Cell 2390
Cell 2391
Cell 2392
Fluorescence (Raw)
Time Point (0.25 Hz)
0
200
400
600
800
10
12
14
16
18
20
25
30
35
40
50
60
70
80
90

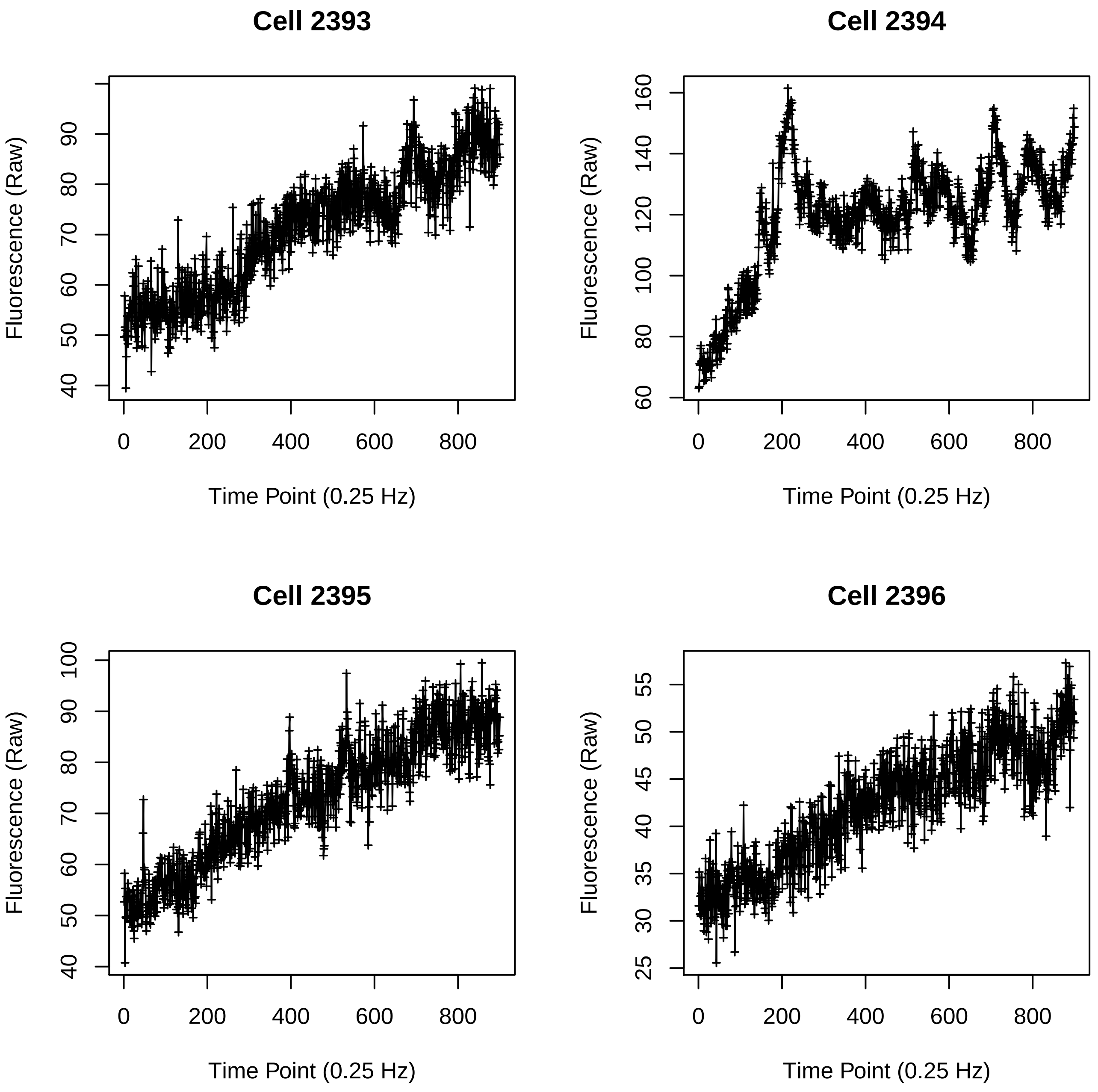
Cell 2393
Fluorescence (Raw)
40
50
60
70
80
90
0
200
400
600
800
Time Point (0.25 Hz)
Cell 2394
Fluorescence (Raw)
60
80
100
120
140
160
0
200
400
600
800
Time Point (0.25 Hz)
Cell 2395
Fluorescence (Raw)
40
50
60
70
80
90
100
0
200
400
600
800
Time Point (0.25 Hz)
Cell 2396
Fluorescence (Raw)
25
30
35
40
45
50
55
0
200
400
600
800
Time Point (0.25 Hz)

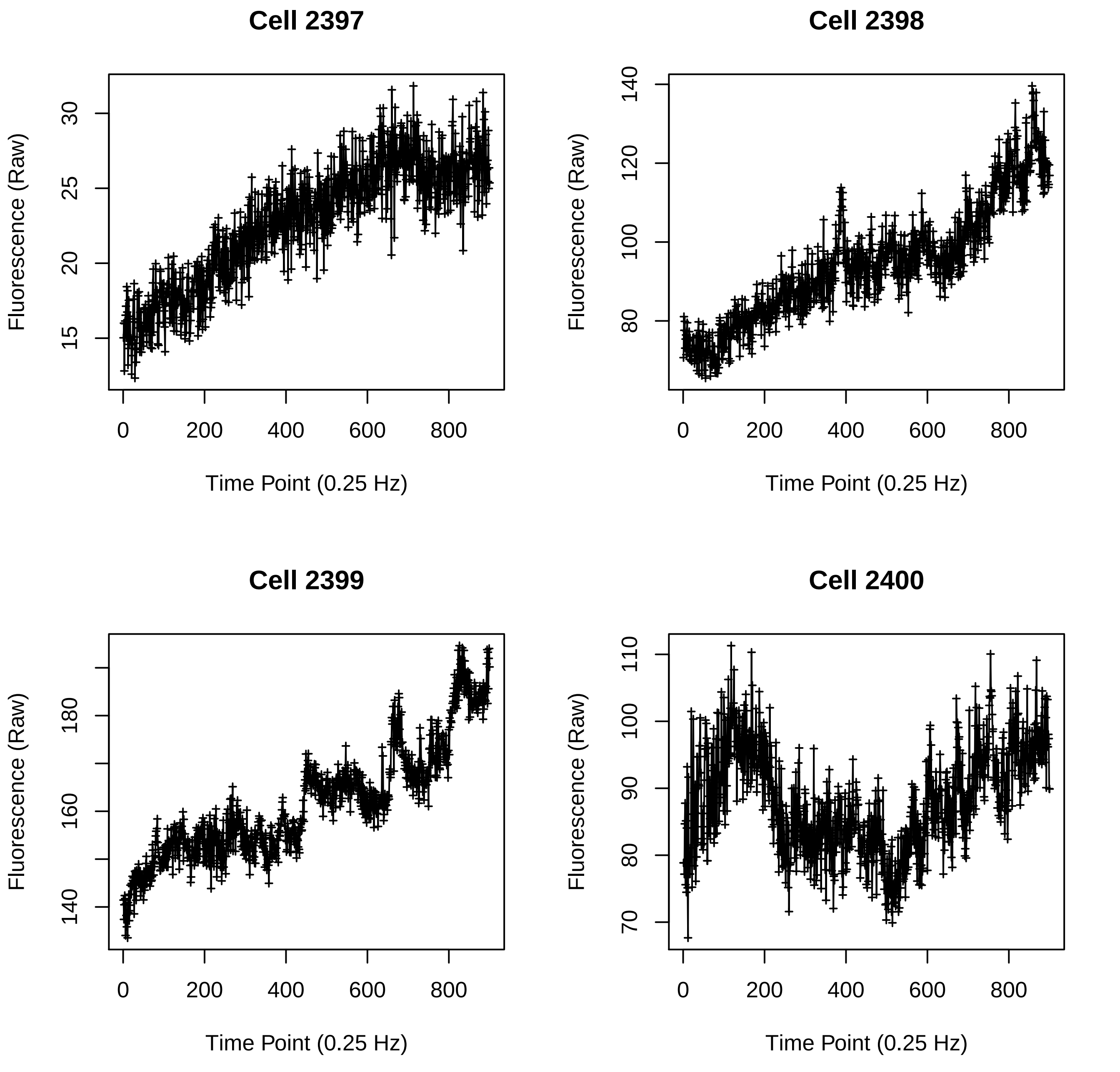

Cell 2397
Fluorescence (Raw)
15
20
25
30
0
200
400
600
800
Time Point (0.25 Hz)
Cell 2398
Fluorescence (Raw)
80
100
120
140
0
200
400
600
800
Time Point (0.25 Hz)
Cell 2399
Fluorescence (Raw)
140
160
180
0
200
400
600
800
Time Point (0.25 Hz)
Cell 2400
Fluorescence (Raw)
70
80
90
100
110
0
200
400
600
800
Time Point (0.25 Hz)

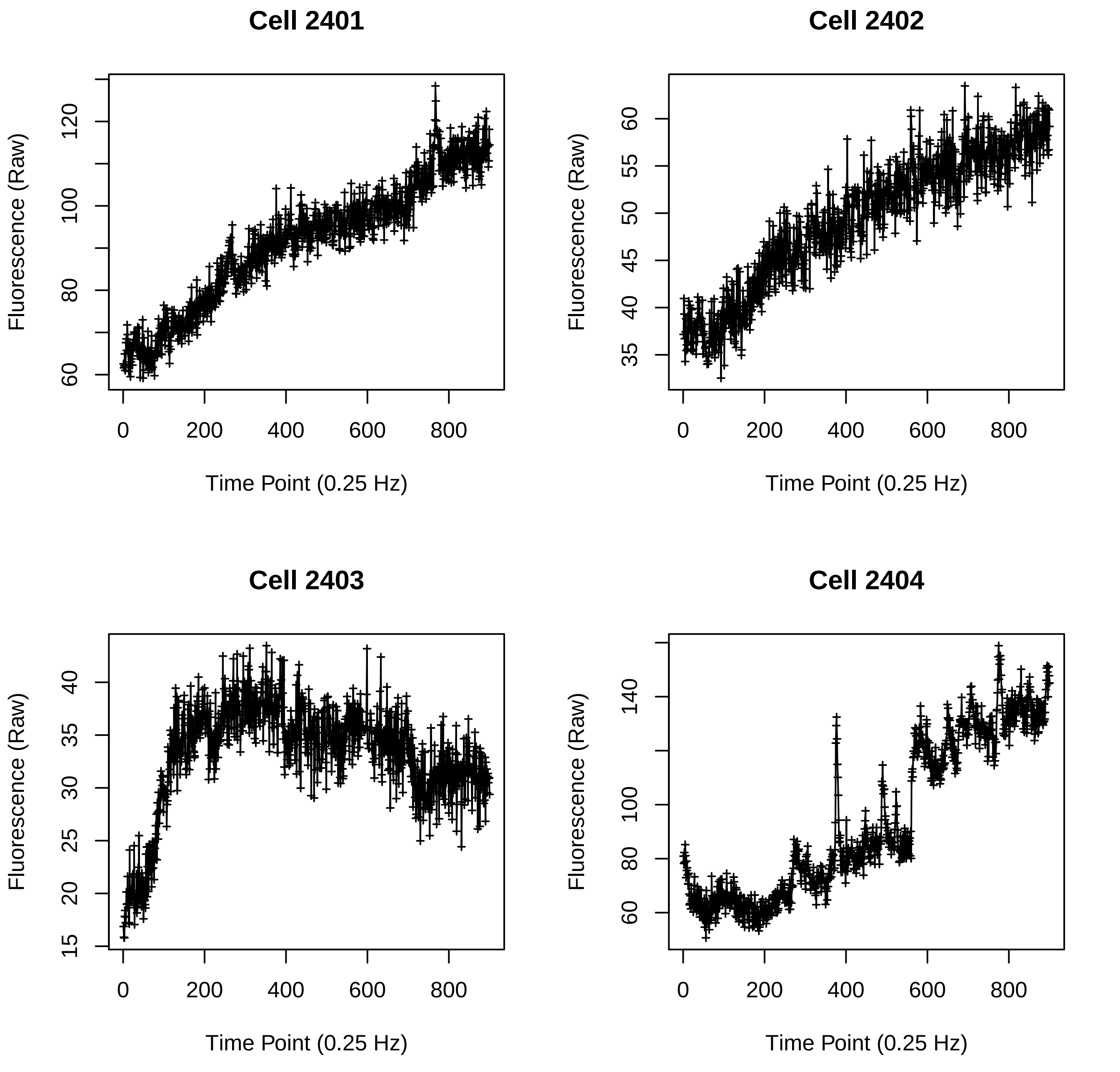

Cell 2401
Fluorescence (Raw)
60
80
100
120
0
200
400
600
800
Time Point (0.25 Hz)
Cell 2402
Fluorescence (Raw)
35
40
45
50
55
60
0
200
400
600
800
Time Point (0.25 Hz)
Cell 2403
Fluorescence (Raw)
15
20
25
30
35
40
0
200
400
600
800
Time Point (0.25 Hz)
Cell 2404
Fluorescence (Raw)
60
80
100
140
0
200
400
600
800
Time Point (0.25 Hz)

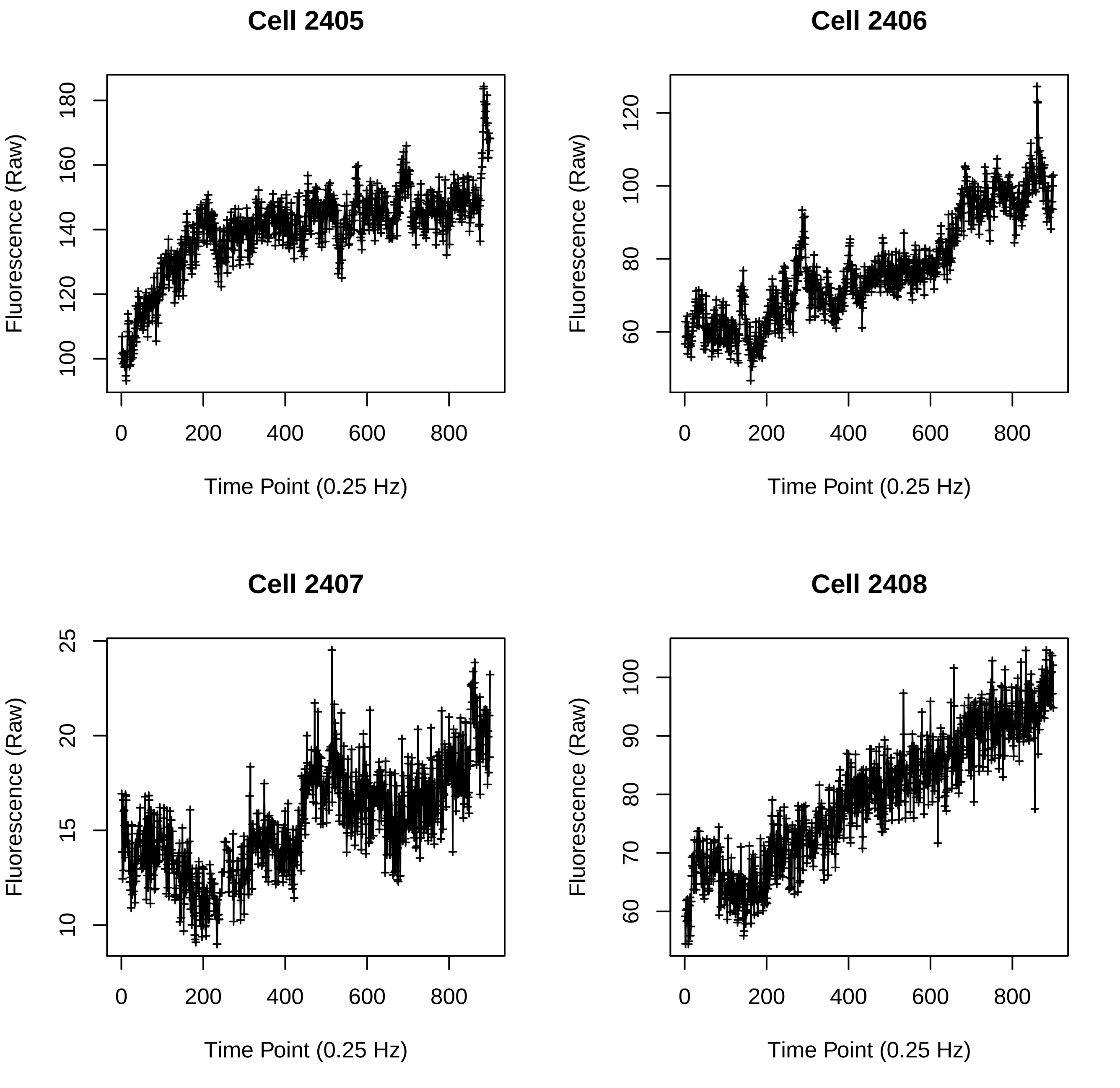

Cell 2405
Cell 2406
Cell 2407
Cell 2408
Fluorescence (Raw)
Time Point (0.25 Hz)
0
200
400
600
800
100
120
140
160
180
60
80
100
120
10
15
20
25
60
70
80
90
100

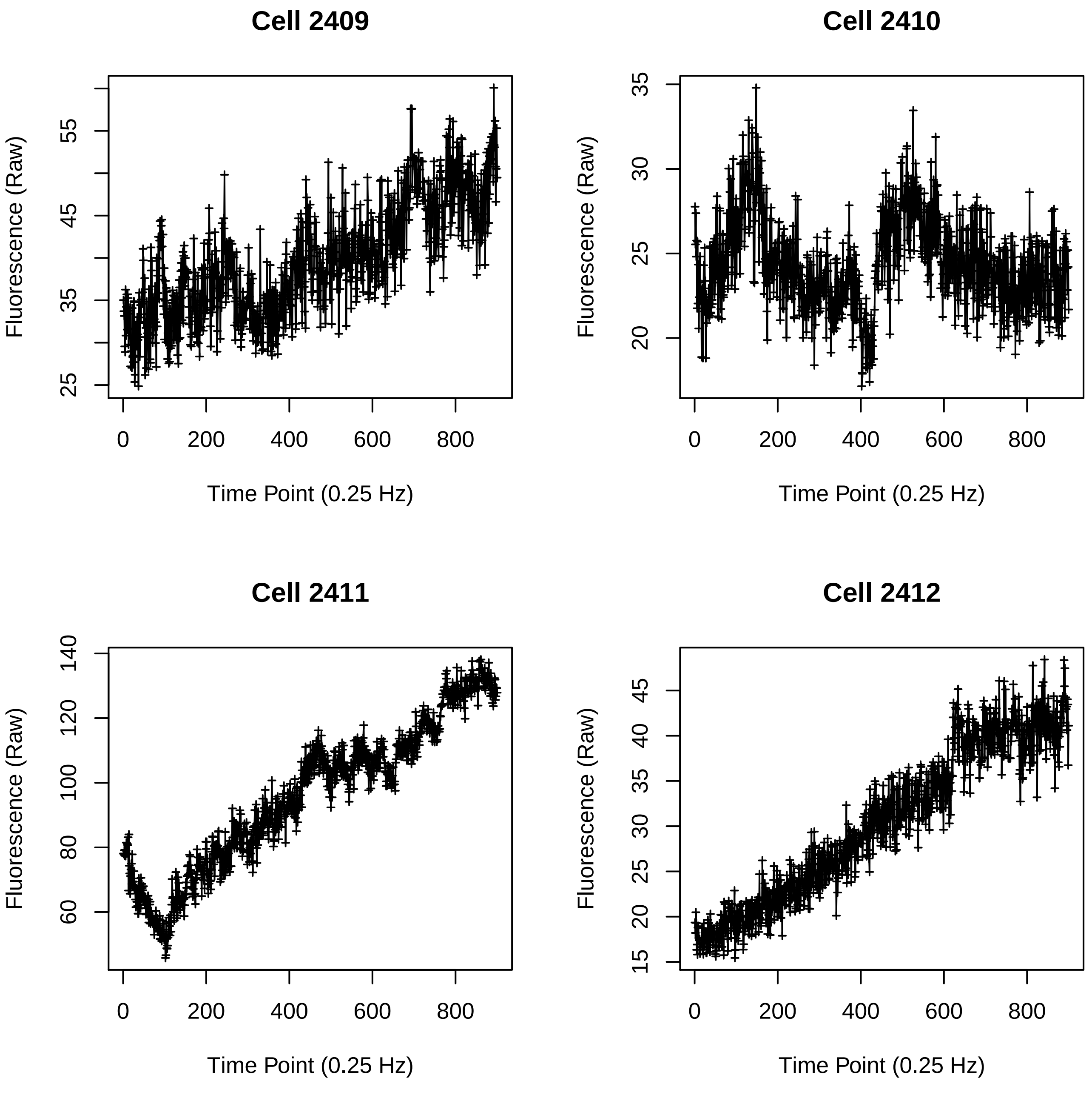

Cell 2409
Cell 2410
Cell 2411
Cell 2412
Fluorescence (Raw)
Time Point (0.25 Hz)
25
35
45
55
20
25
30
35
60
80
100
120
140
15
20
25
30
35
40
45
0
200
400
600
800

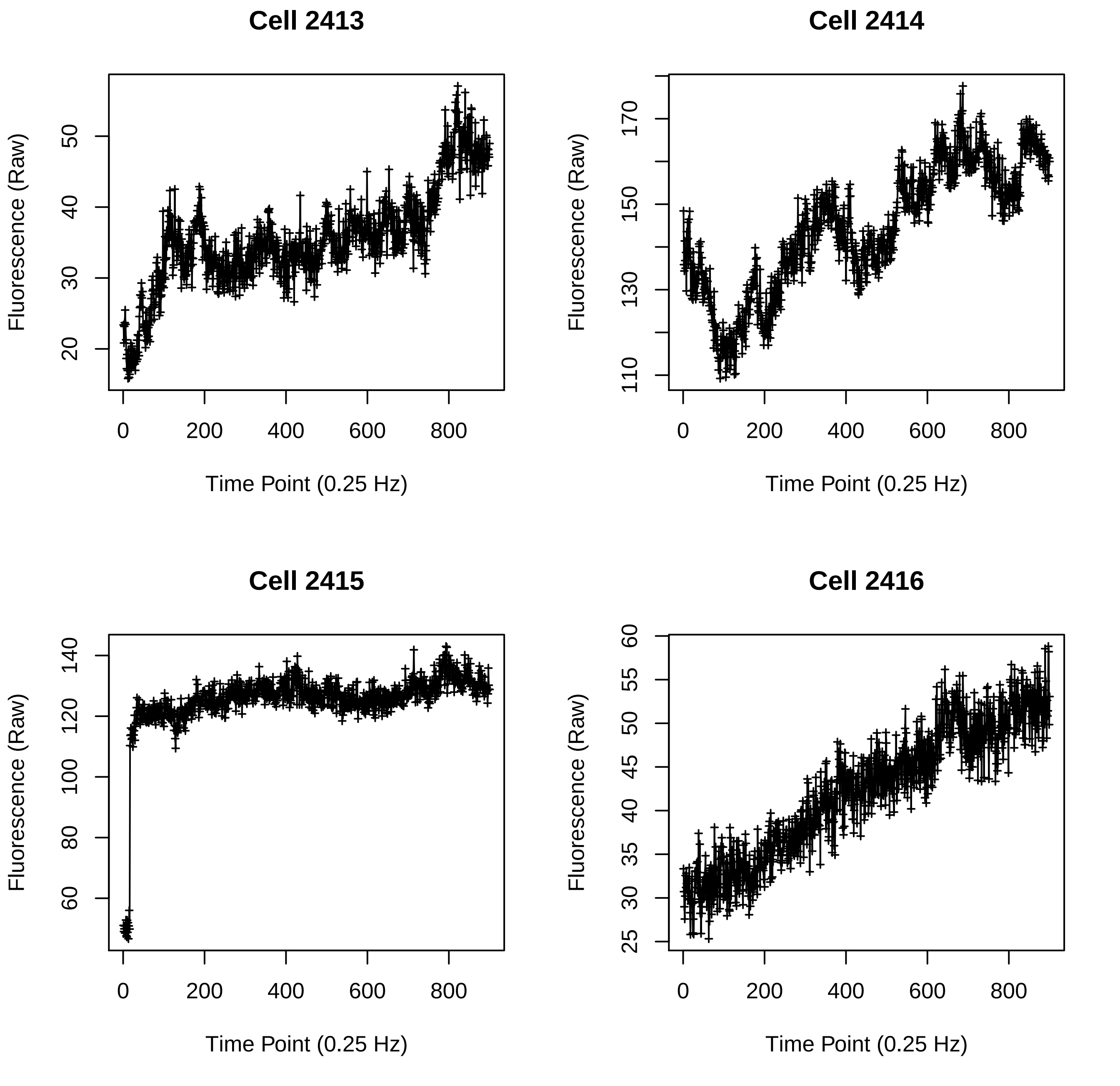

Cell 2413
Cell 2414
Cell 2415
Cell 2416
Fluorescence (Raw)
Time Point (0.25 Hz)
0
200
400
600
800
20
30
40
50
110
130
150
170
60
80
100
120
140
25
35
45
55

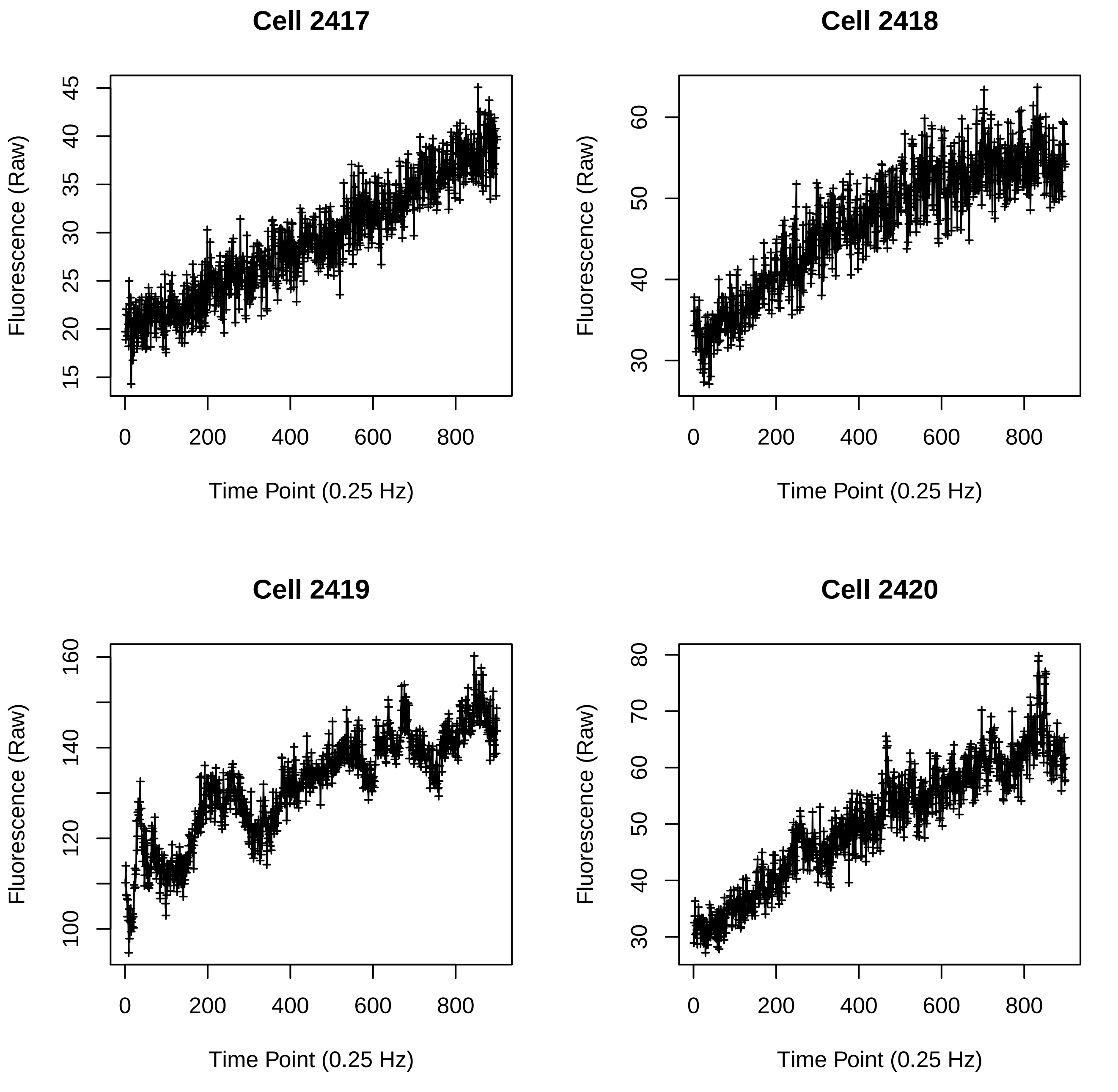

Cell 2417
Fluorescence (Raw)
15
20
25
30
35
40
45
0
200
400
600
800
Time Point (0.25 Hz)
Cell 2418
Fluorescence (Raw)
30
40
50
60
0
200
400
600
800
Time Point (0.25 Hz)
Cell 2419
Fluorescence (Raw)
100
120
140
160
0
200
400
600
800
Time Point (0.25 Hz)
Cell 2420
Fluorescence (Raw)
30
40
50
60
70
80
0
200
400
600
800
Time Point (0.25 Hz)

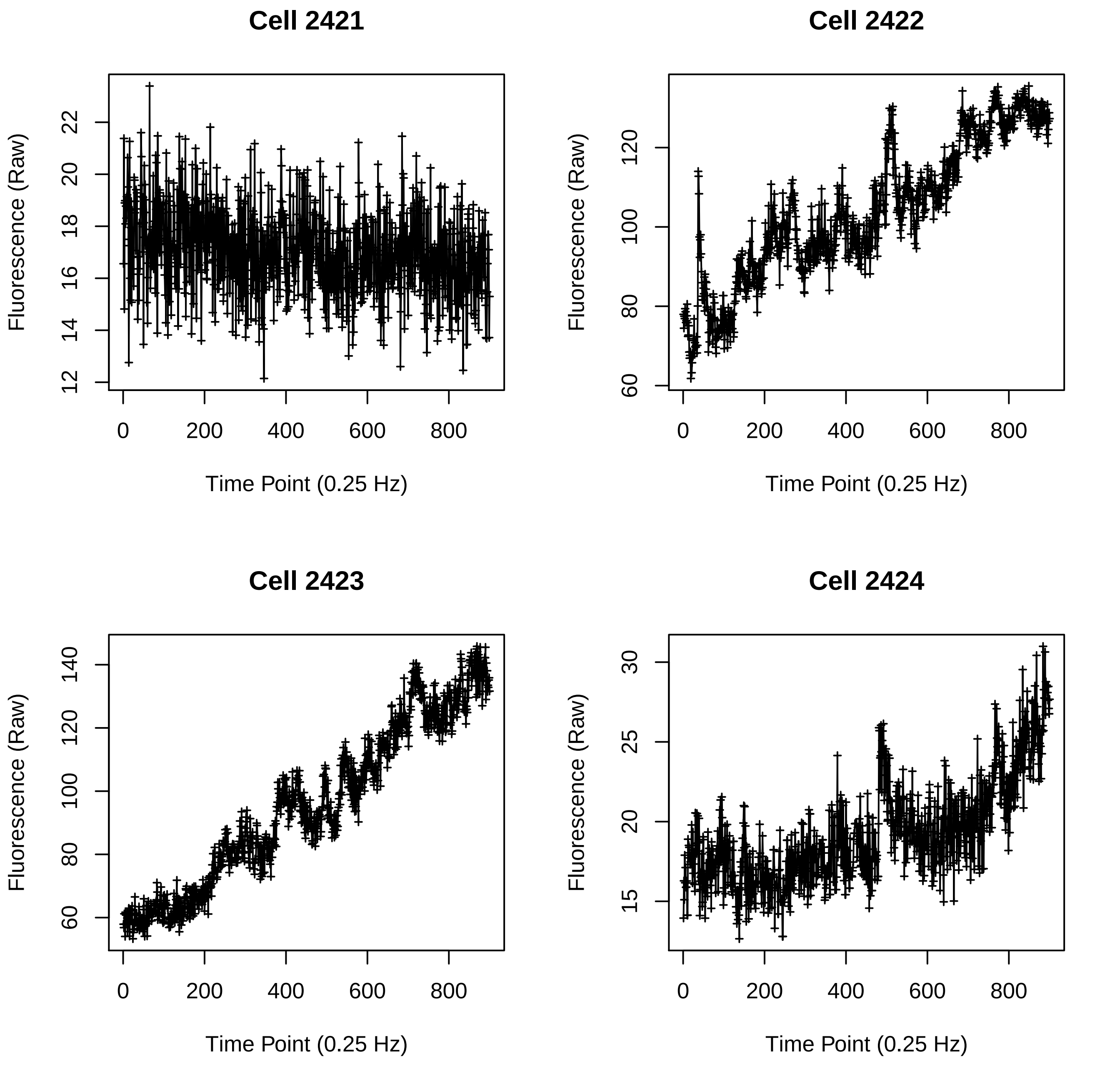
Cell 2421
Cell 2422
Cell 2423
Cell 2424
Fluorescence (Raw)
Time Point (0.25 Hz)
12
14
16
18
20
22
60
80
100
120
140
15
20
25
30
0
200
400
600
800

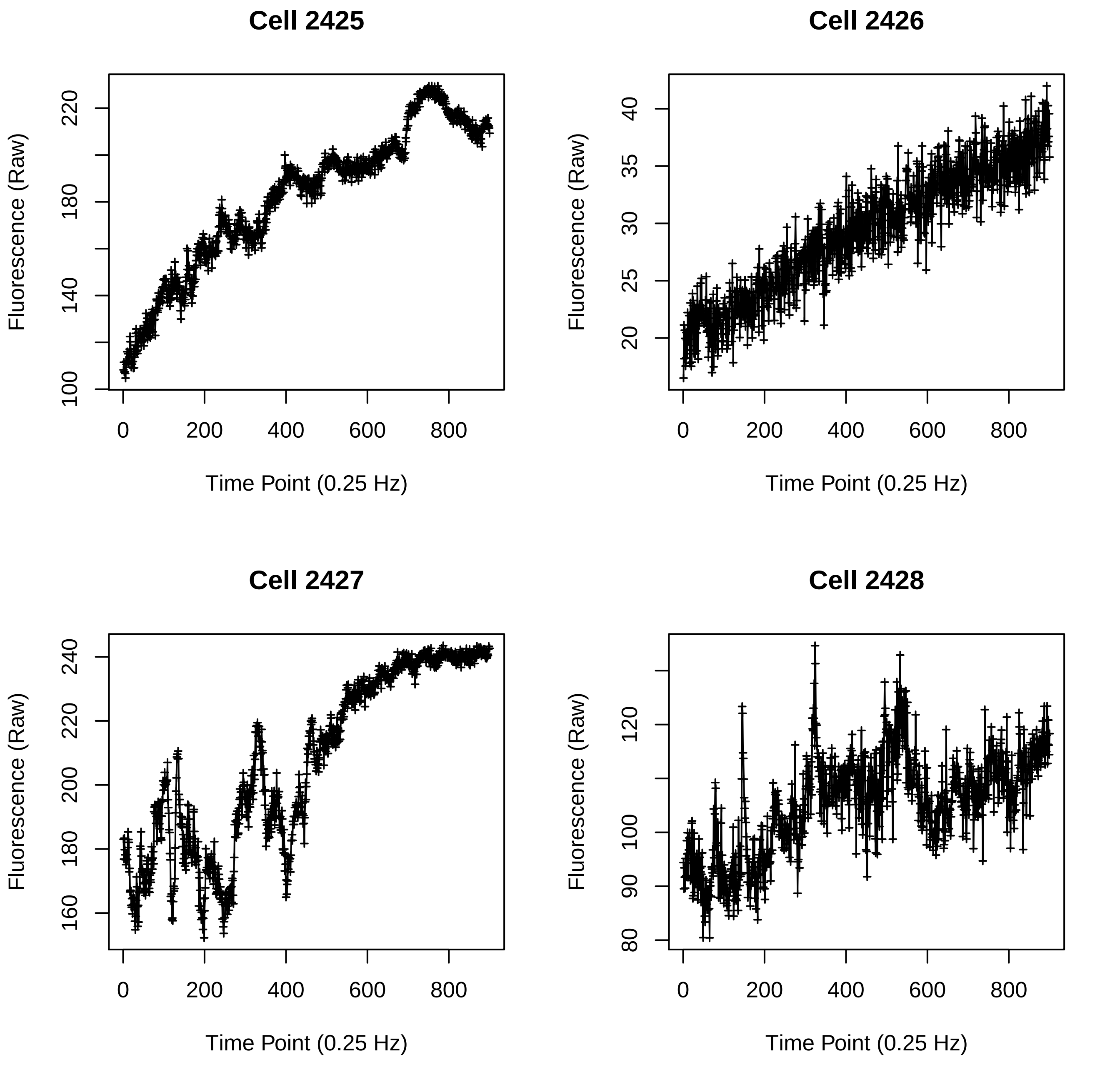
Cell 2425
Cell 2426
Cell 2427
Cell 2428
Fluorescence (Raw)
Time Point (0.25 Hz)
100
140
180
220
20
25
30
35
40
160
180
200
220
240
80
90
100
120
0
200
400
600
800

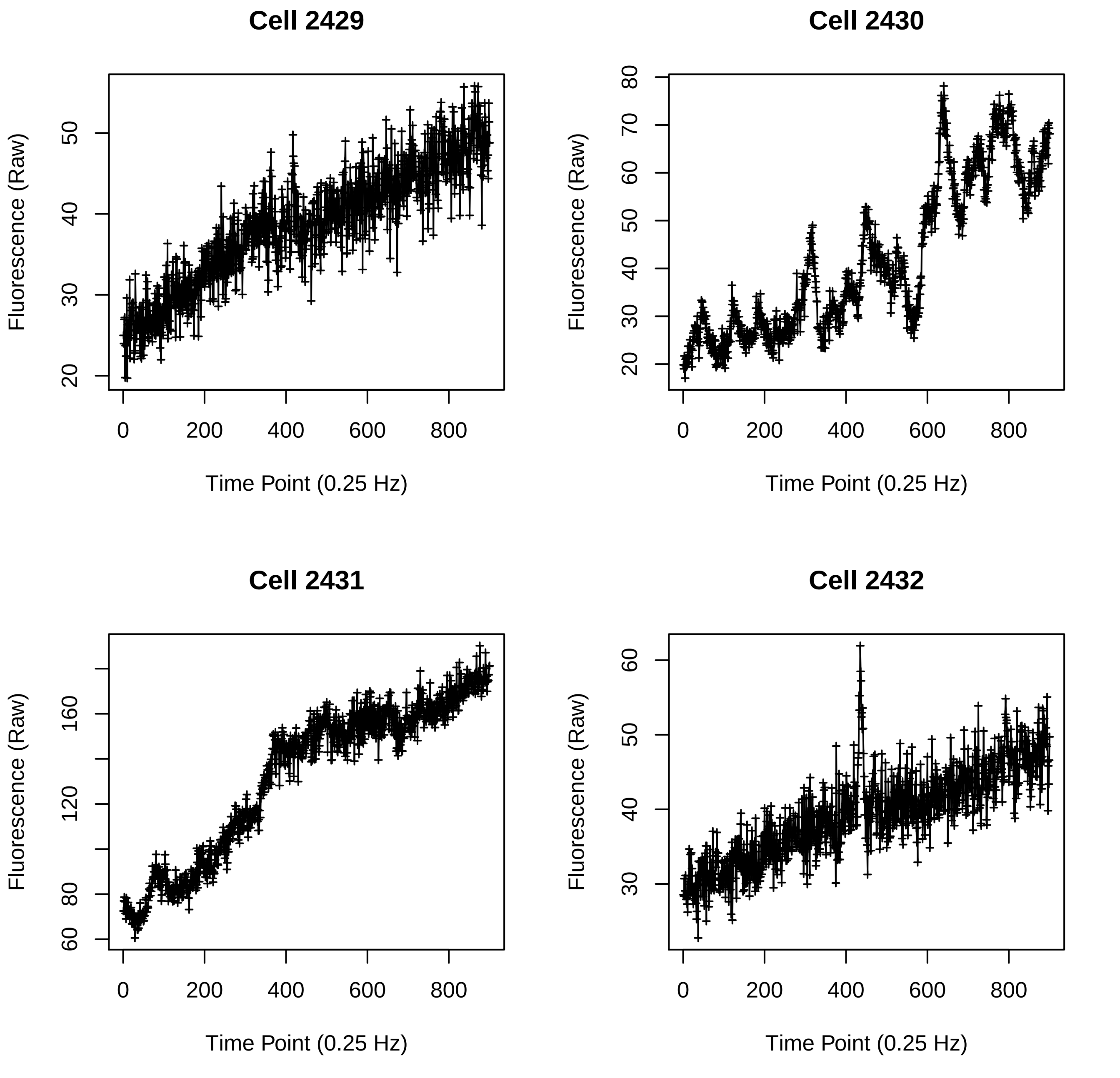

Cell 2429
Fluorescence (Raw)
20
30
40
50
0
200
400
600
800
Time Point (0.25 Hz)
Cell 2430
Fluorescence (Raw)
20
30
40
50
60
70
80
0
200
400
600
800
Time Point (0.25 Hz)
Cell 2431
Fluorescence (Raw)
60
80
120
160
0
200
400
600
800
Time Point (0.25 Hz)
Cell 2432
Fluorescence (Raw)
30
40
50
60
0
200
400
600
800
Time Point (0.25 Hz)

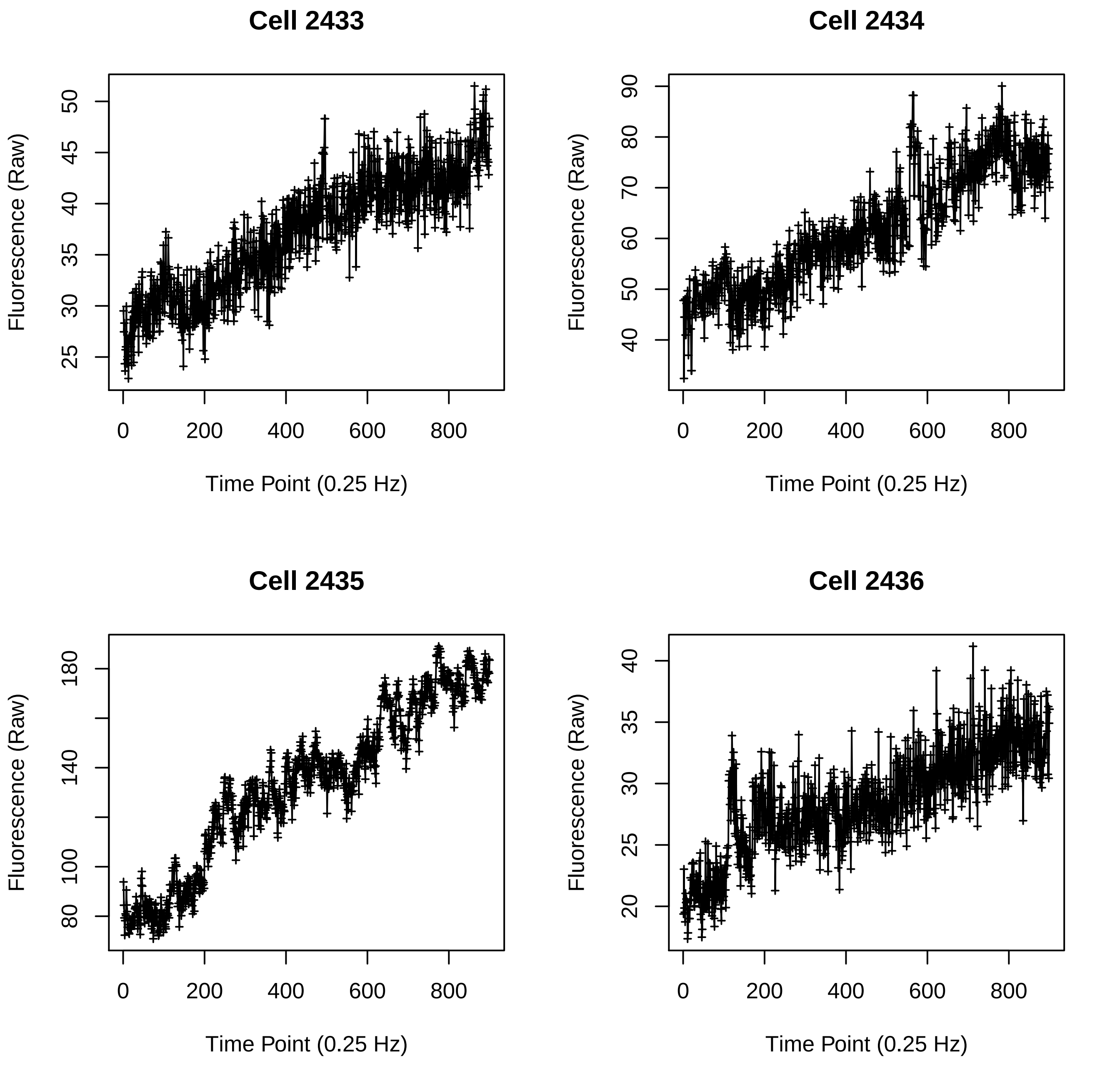
Cell 2433
Fluorescence (Raw)
25
30
35
40
45
50
0
200
400
600
800
Time Point (0.25 Hz)
Cell 2434
Fluorescence (Raw)
40
50
60
70
80
90
0
200
400
600
800
Time Point (0.25 Hz)
Cell 2435
Fluorescence (Raw)
80
100
140
180
0
200
400
600
800
Time Point (0.25 Hz)
Cell 2436
Fluorescence (Raw)
20
25
30
35
40
0
200
400
600
800
Time Point (0.25 Hz)

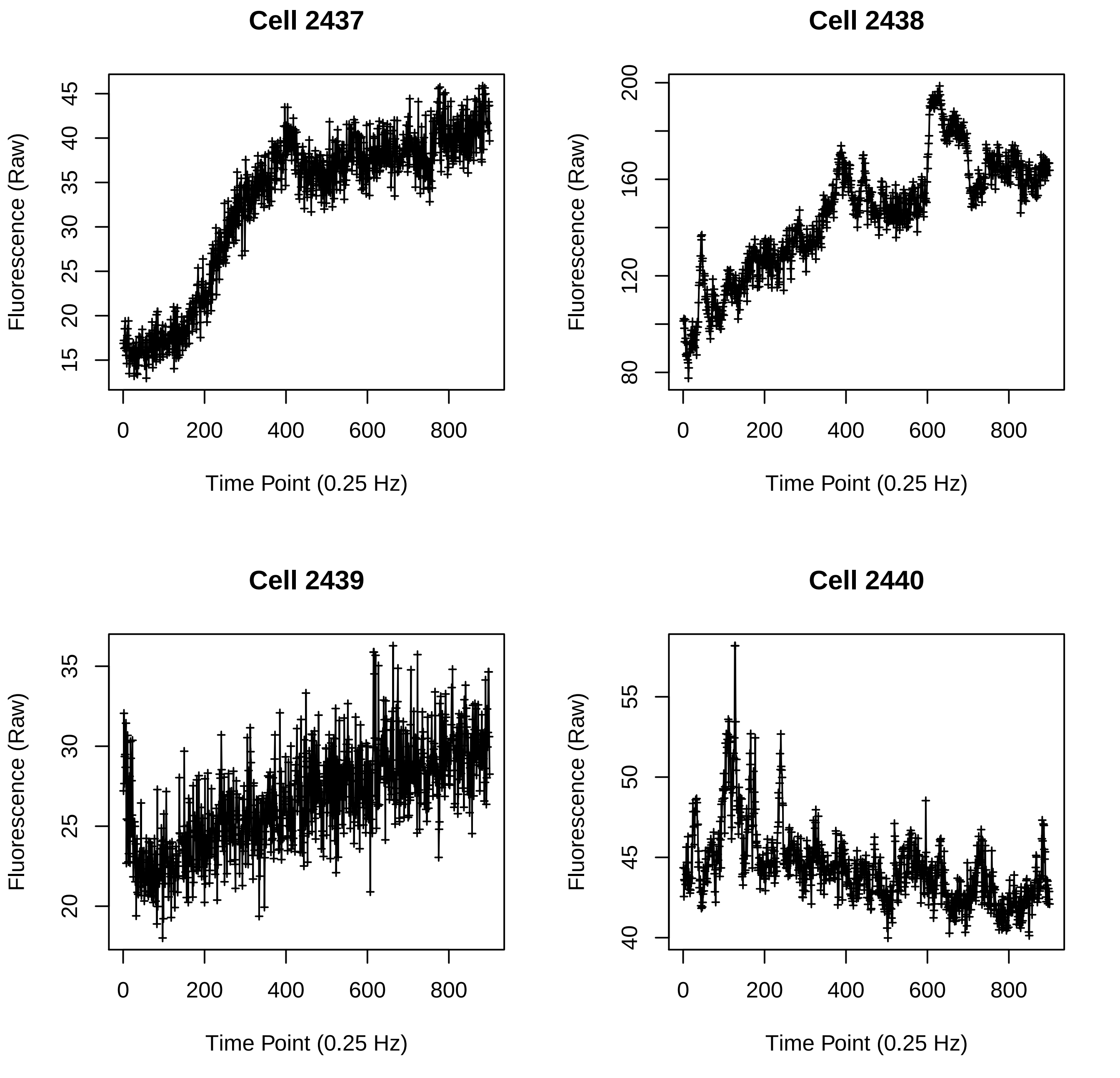
Cell 2437
Cell 2438
Cell 2439
Cell 2440
Fluorescence (Raw)
Time Point (0.25 Hz)
15
20
25
30
35
40
45
80
120
160
200
50
55
0
200
400
600
800

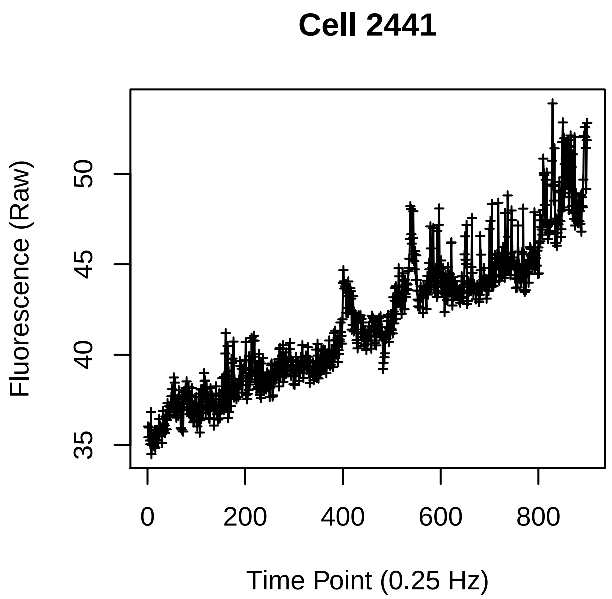
Cell 2441
Fluorescence (Raw)
35
40
45
50
0
200
400
600
800
Time Point (0.25 Hz)

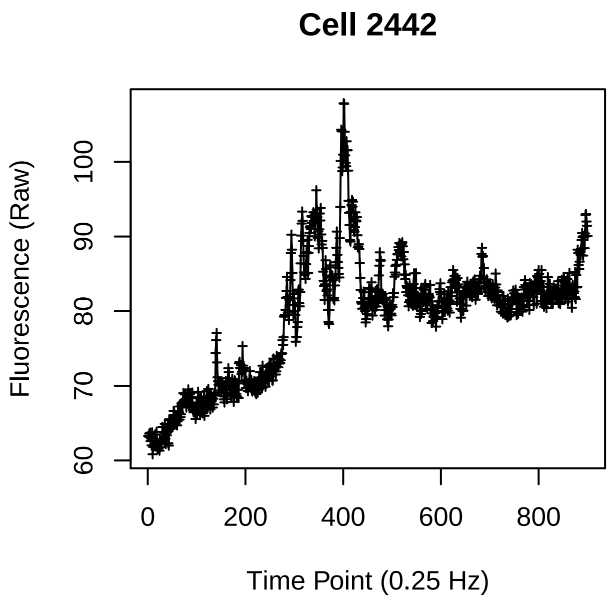
Cell 2442
Fluorescence (Raw)
60
70
80
90
100
0
200
400
600
800
Time Point (0.25 Hz)

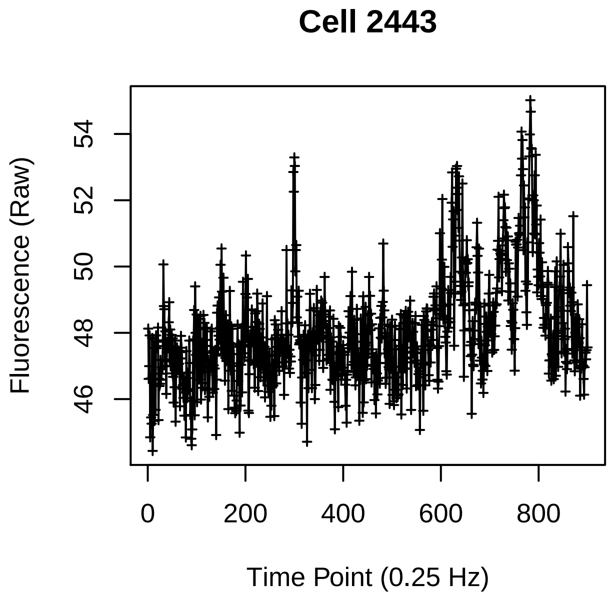
Cell 2443
Fluorescence (Raw)
46
48
50
52
54
0
200
400
600
800
Time Point (0.25 Hz)

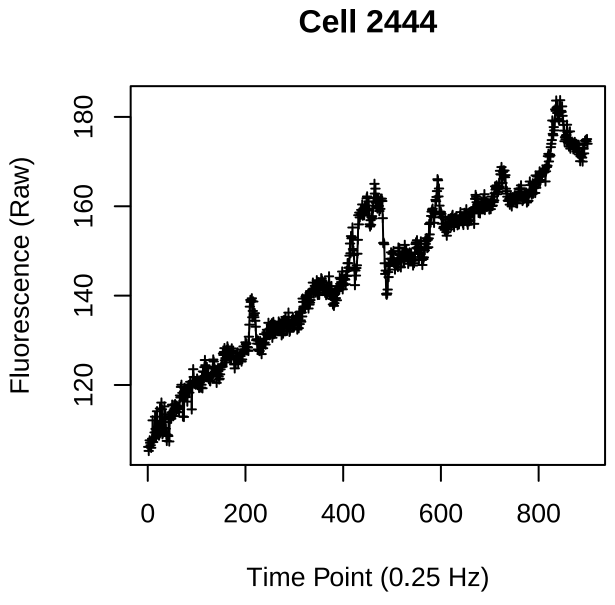
Cell 2444
Fluorescence (Raw)
120
140
160
180
0
200
400
600
800
Time Point (0.25 Hz)

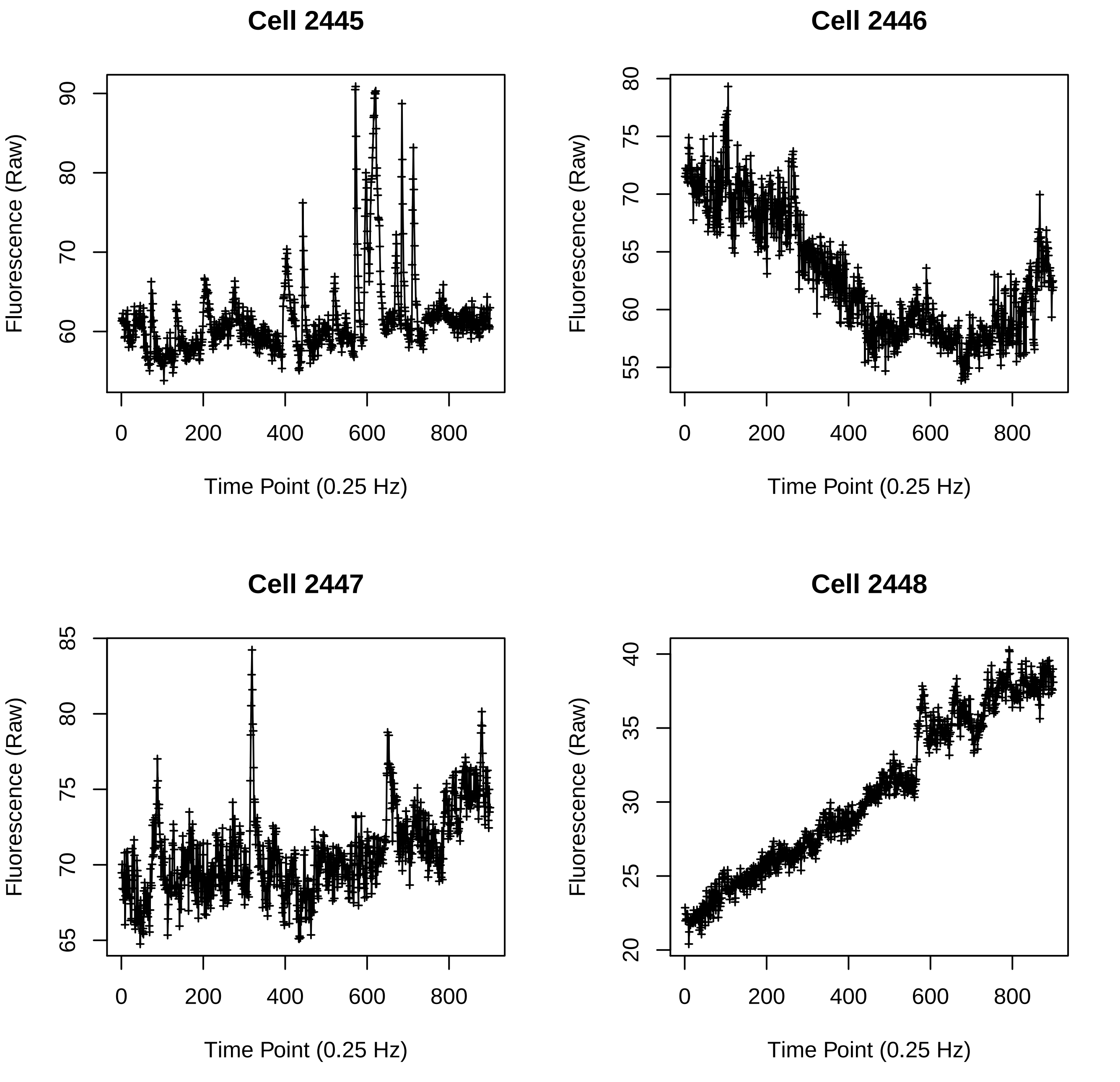

Cell 2445
Fluorescence (Raw)
60
70
80
90
0
200
400
600
800
Time Point (0.25 Hz)
Cell 2446
Fluorescence (Raw)
55
60
65
70
75
80
0
200
400
600
800
Time Point (0.25 Hz)
Cell 2447
Fluorescence (Raw)
65
70
75
80
85
0
200
400
600
800
Time Point (0.25 Hz)
Cell 2448
Fluorescence (Raw)
20
25
30
35
40
0
200
400
600
800
Time Point (0.25 Hz)

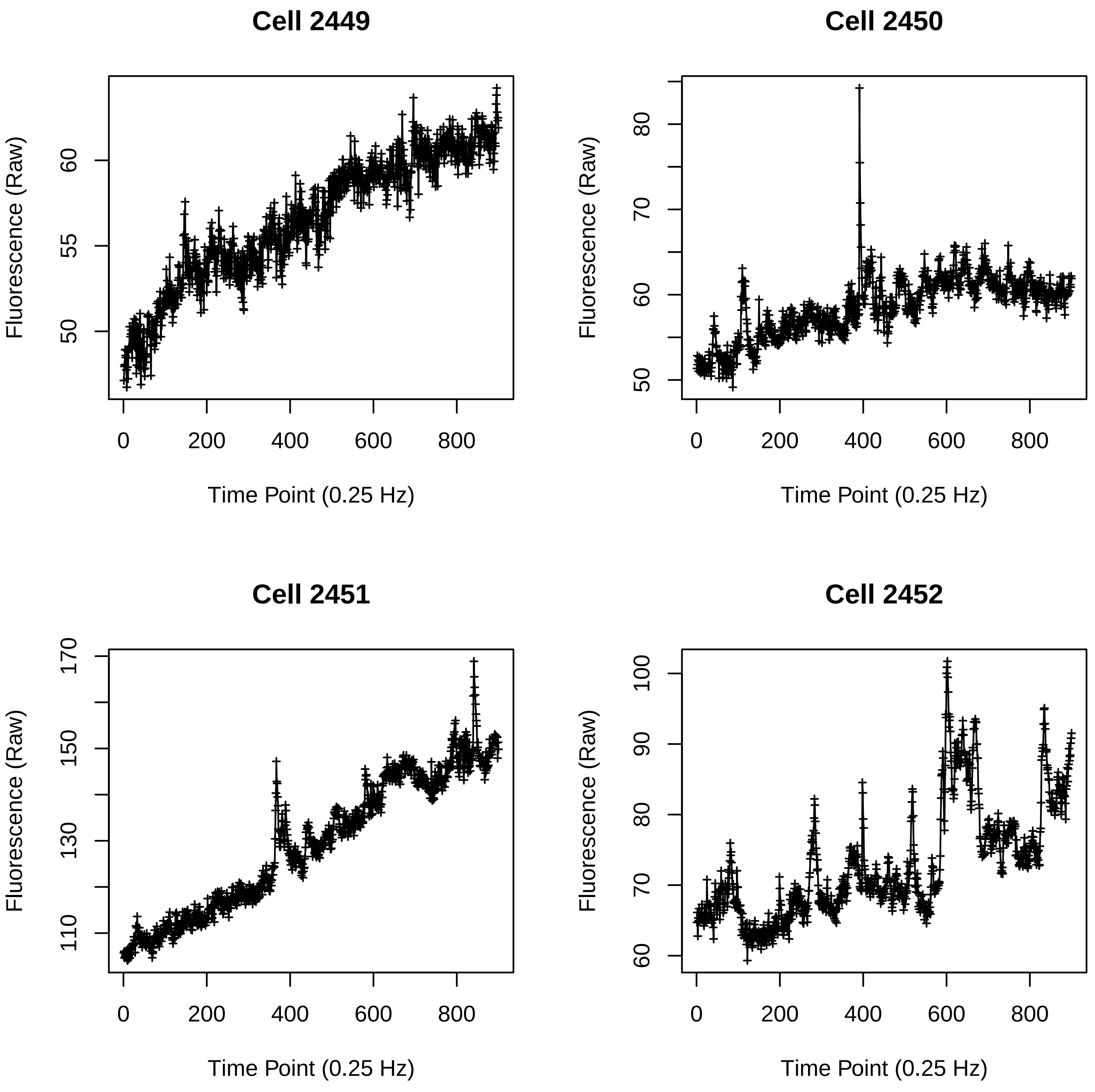
Cell 2449
Cell 2450
Cell 2451
Cell 2452
Fluorescence (Raw)
Time Point (0.25 Hz)
50
55
60
70
80
110
130
150
170
90
100
0
200
400
600
800

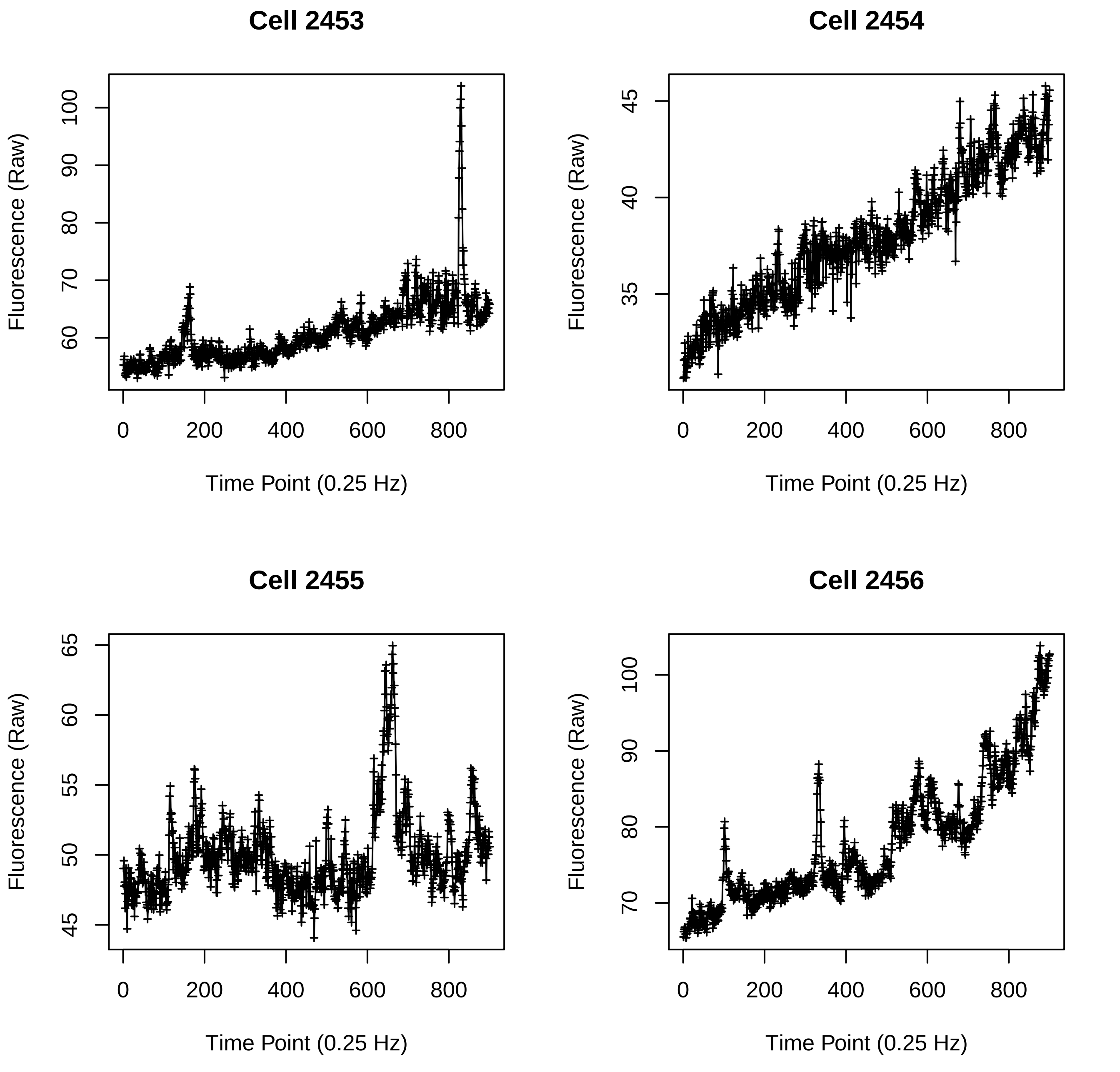
Cell 2453
Fluorescence (Raw)
60
70
80
90
100
0
200
400
600
800
Time Point (0.25 Hz)
Cell 2454
Fluorescence (Raw)
35
40
45
0
200
400
600
800
Time Point (0.25 Hz)
Cell 2455
Fluorescence (Raw)
45
50
55
60
65
0
200
400
600
800
Time Point (0.25 Hz)
Cell 2456
Fluorescence (Raw)
70
80
90
100
0
200
400
600
800
Time Point (0.25 Hz)

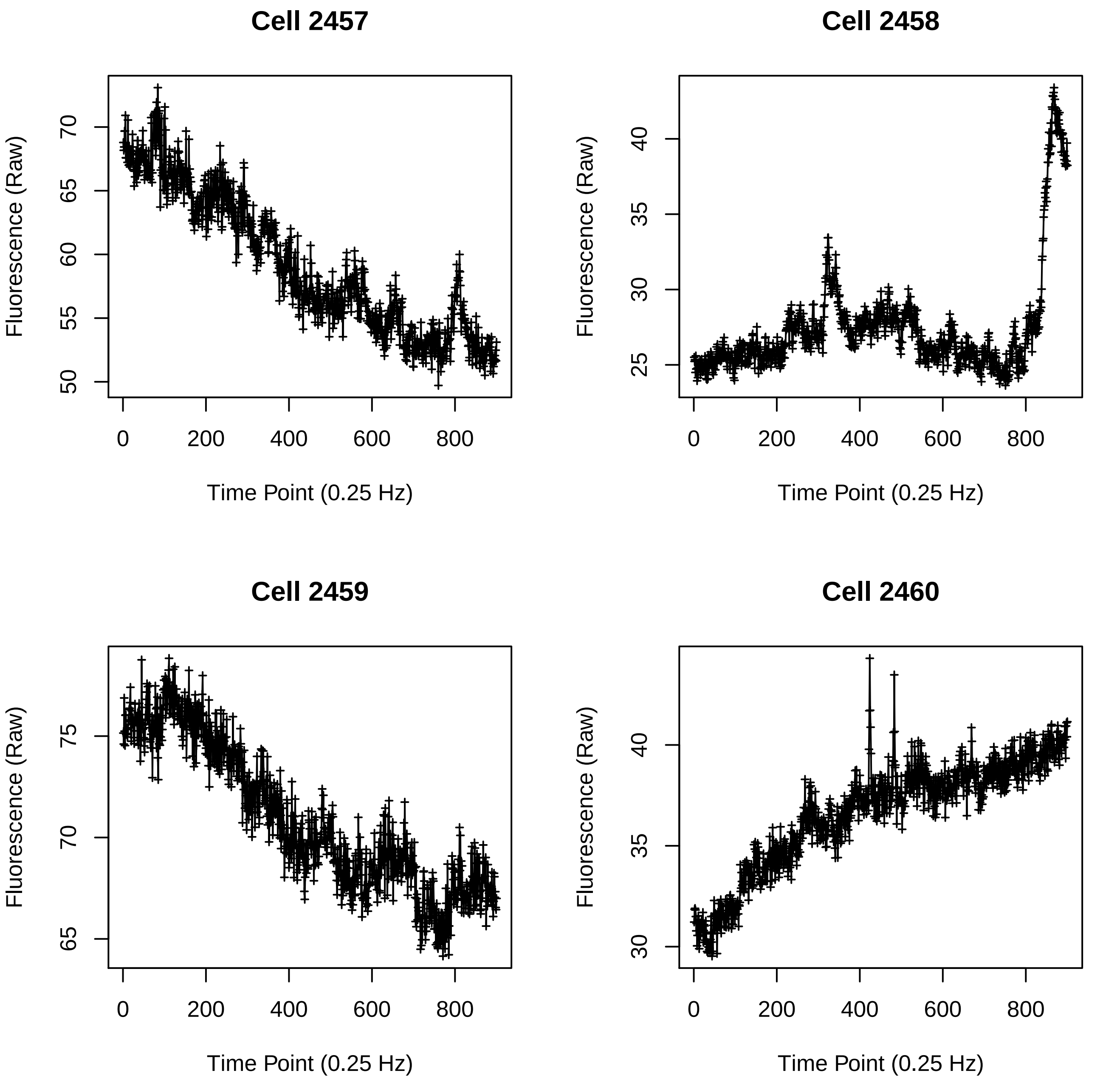
Cell 2457
Fluorescence (Raw)
50
55
60
65
70
Time Point (0.25 Hz)
0
200
400
600
800
Cell 2458
Fluorescence (Raw)
25
30
35
40
Time Point (0.25 Hz)
0
200
400
600
800
Cell 2459
Fluorescence (Raw)
65
70
75
Time Point (0.25 Hz)
0
200
400
600
800
Cell 2460
Fluorescence (Raw)
30
35
40
Time Point (0.25 Hz)
0
200
400
600
800

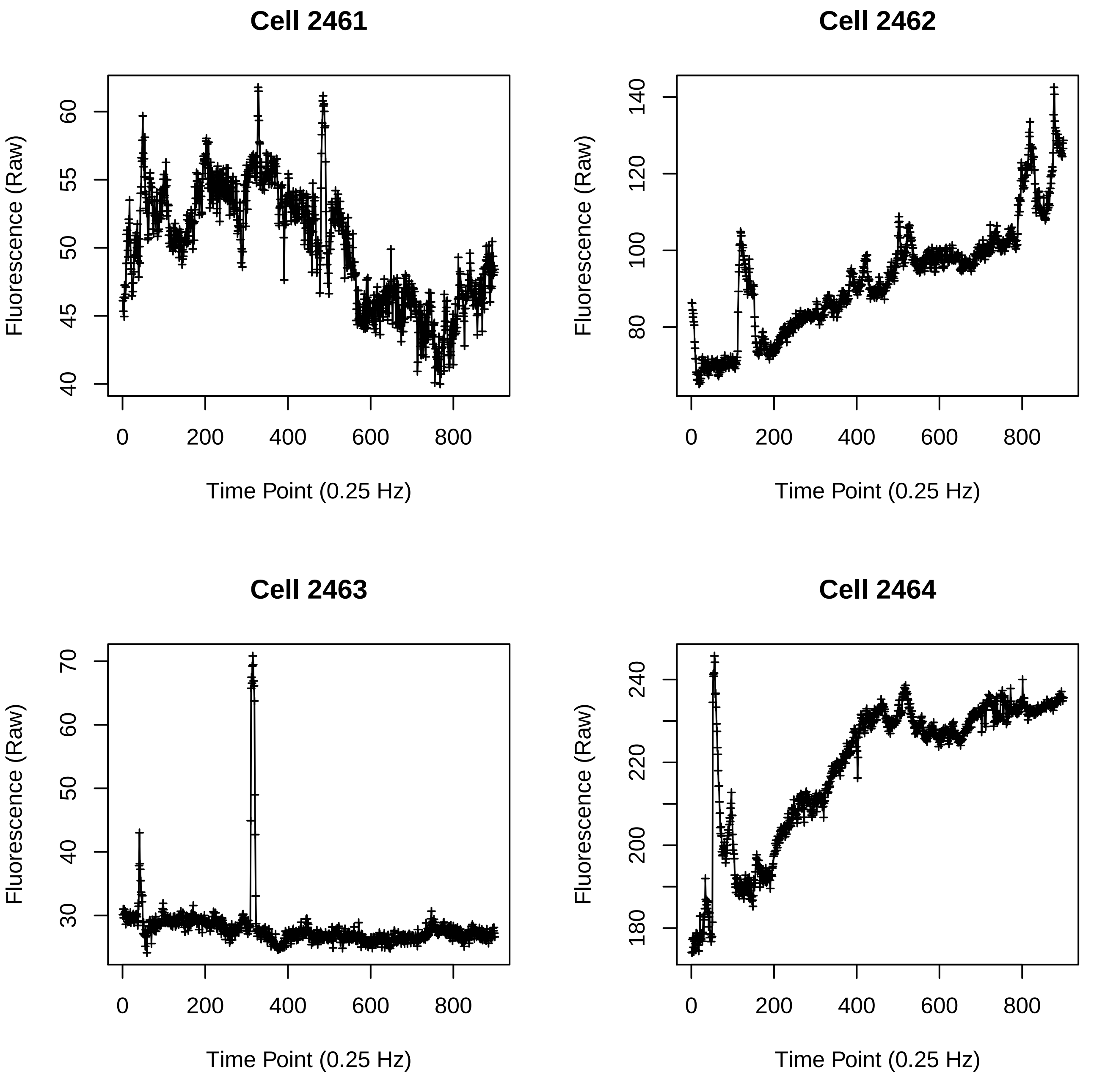
Cell 2461
Cell 2462
Cell 2463
Cell 2464
Fluorescence (Raw)
Time Point (0.25 Hz)
0
200
400
600
800
40
45
50
55
60
80
100
120
140
30
40
50
60
70
180
200
220
240

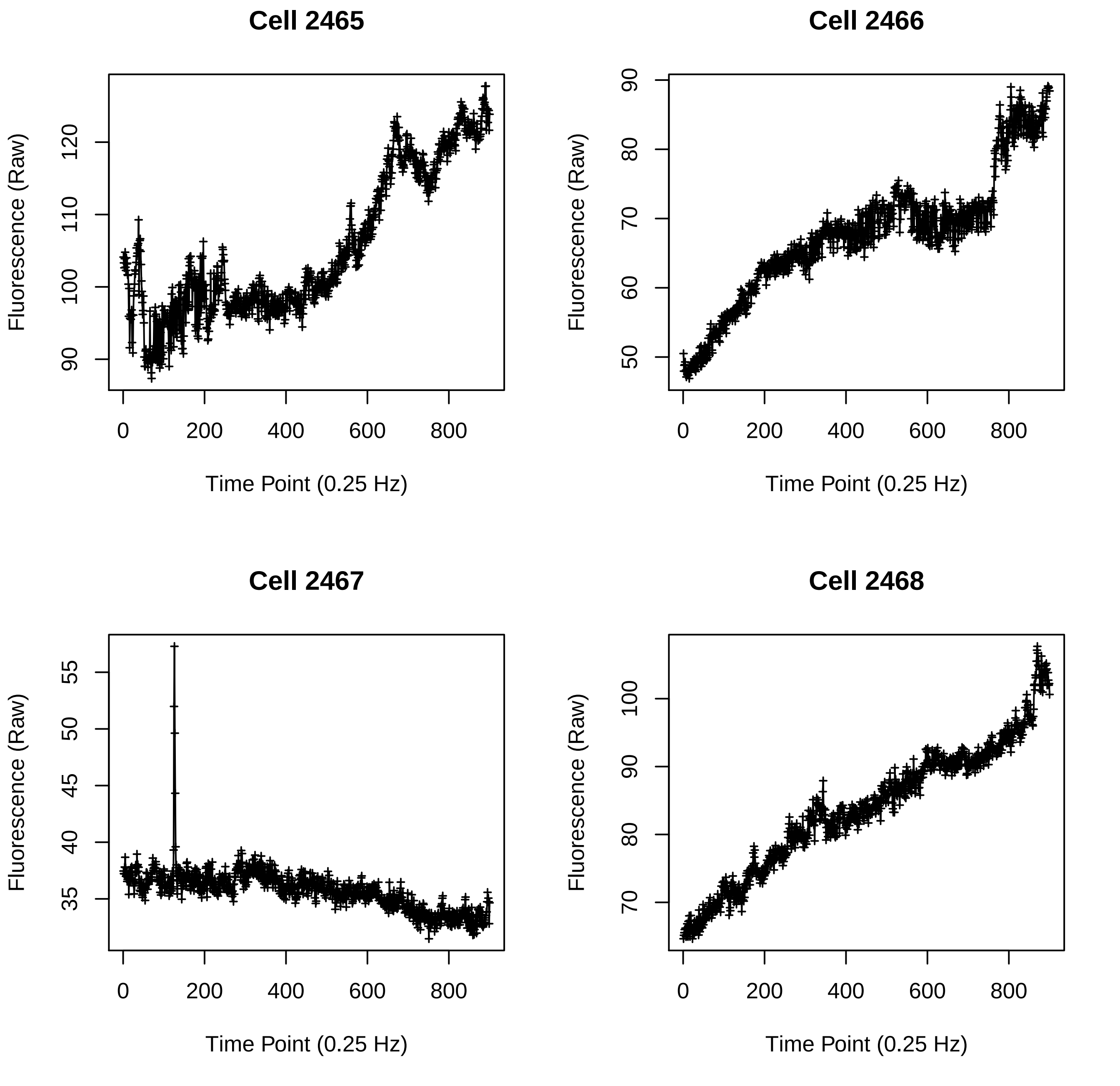
Cell 2465
Fluorescence (Raw)
90
100
110
120
0
200
400
600
800
Time Point (0.25 Hz)
Cell 2466
Fluorescence (Raw)
50
60
70
80
90
0
200
400
600
800
Time Point (0.25 Hz)
Cell 2467
Fluorescence (Raw)
35
40
45
50
55
0
200
400
600
800
Time Point (0.25 Hz)
Cell 2468
Fluorescence (Raw)
70
80
90
100
0
200
400
600
800
Time Point (0.25 Hz)

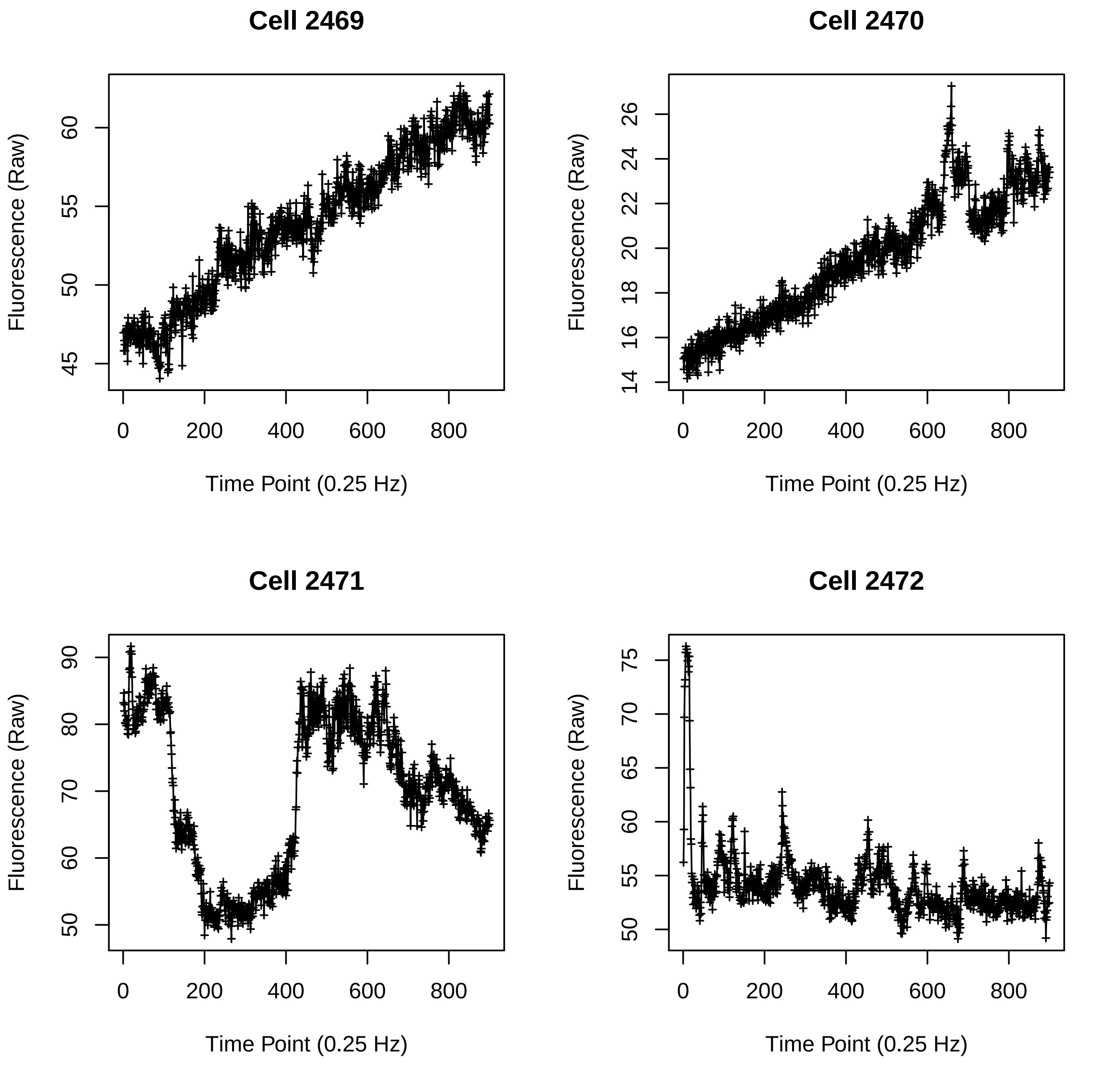

Cell 2469
Fluorescence (Raw)
45
50
55
60
0
200
400
600
800
Time Point (0.25 Hz)
Cell 2470
Fluorescence (Raw)
14
16
18
20
22
24
26
0
200
400
600
800
Time Point (0.25 Hz)
Cell 2471
Fluorescence (Raw)
50
60
70
80
90
0
200
400
600
800
Time Point (0.25 Hz)
Cell 2472
Fluorescence (Raw)
50
55
60
65
70
75
0
200
400
600
800
Time Point (0.25 Hz)

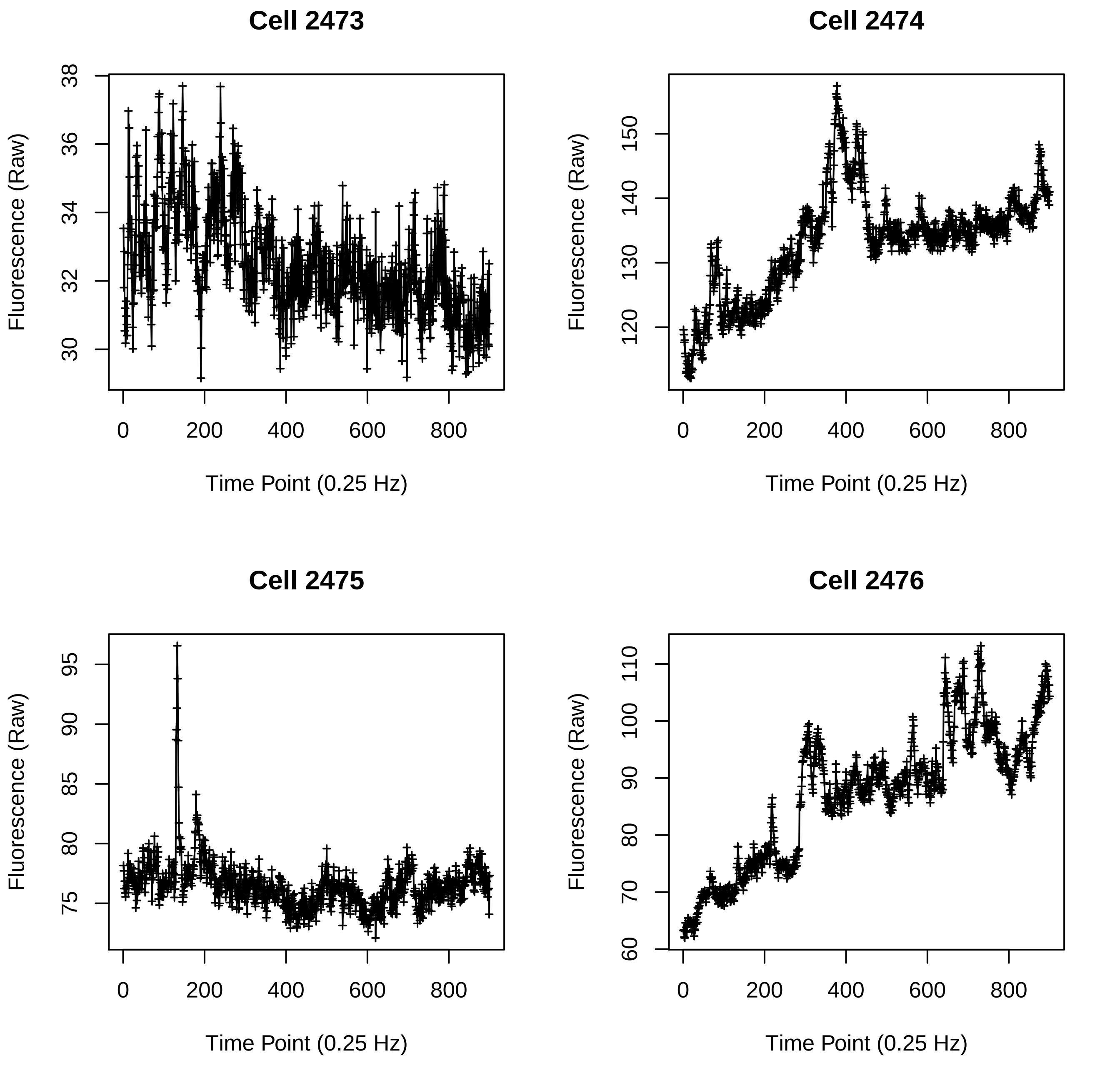
Cell 2473
Cell 2474
Cell 2475
Cell 2476
Fluorescence (Raw)
Time Point (0.25 Hz)
0
200
400
600
800
30
32
34
36
38
120
130
140
150
75
80
85
90
95
60
70
80
90
100
110

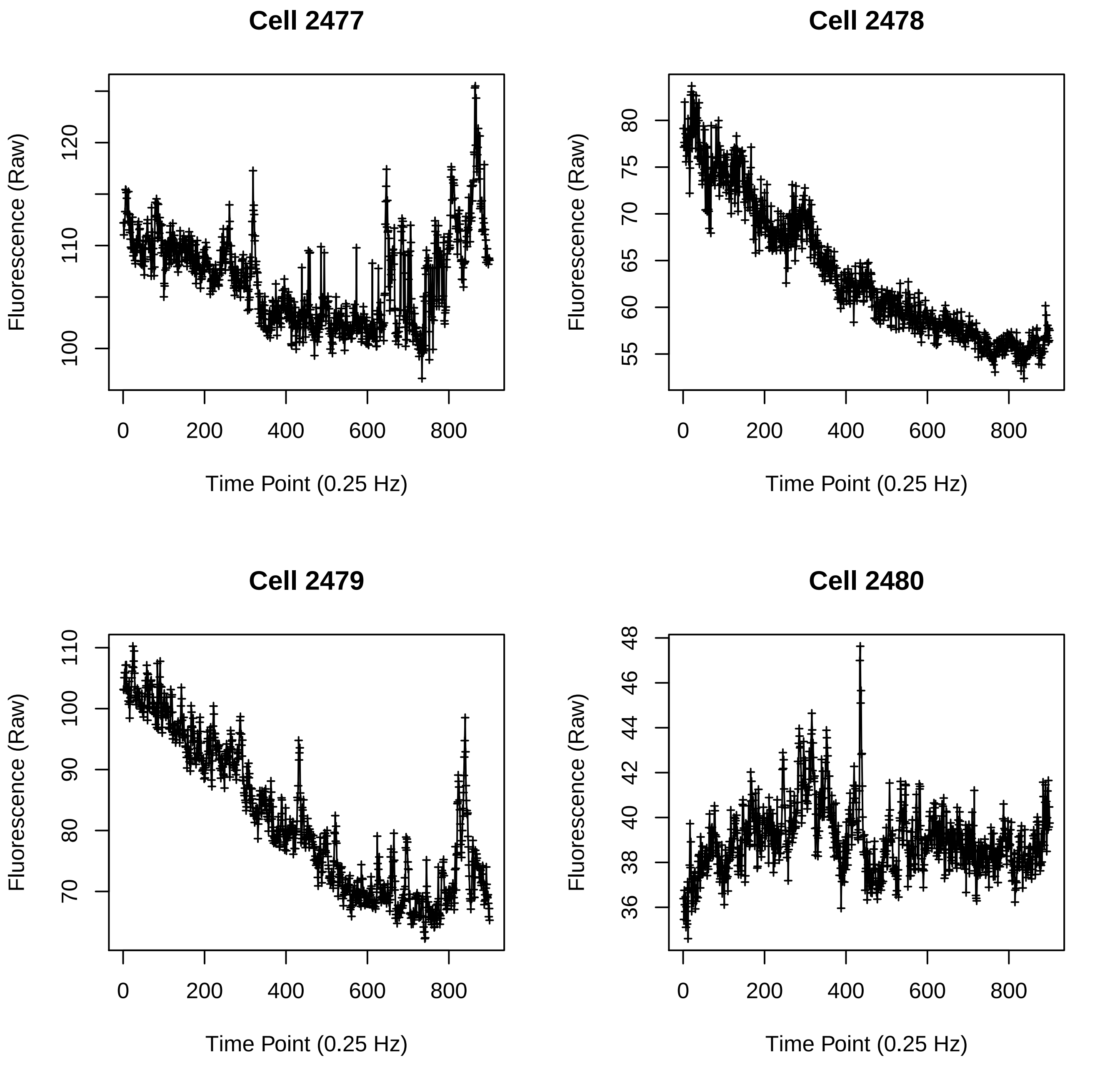
Cell 2477
Fluorescence (Raw)
100
110
120
0
200
400
600
800
Time Point (0.25 Hz)
Cell 2478
Fluorescence (Raw)
55
60
65
70
75
80
0
200
400
600
800
Time Point (0.25 Hz)
Cell 2479
Fluorescence (Raw)
70
80
90
100
110
0
200
400
600
800
Time Point (0.25 Hz)
Cell 2480
Fluorescence (Raw)
36
38
40
42
44
46
48
0
200
400
600
800
Time Point (0.25 Hz)

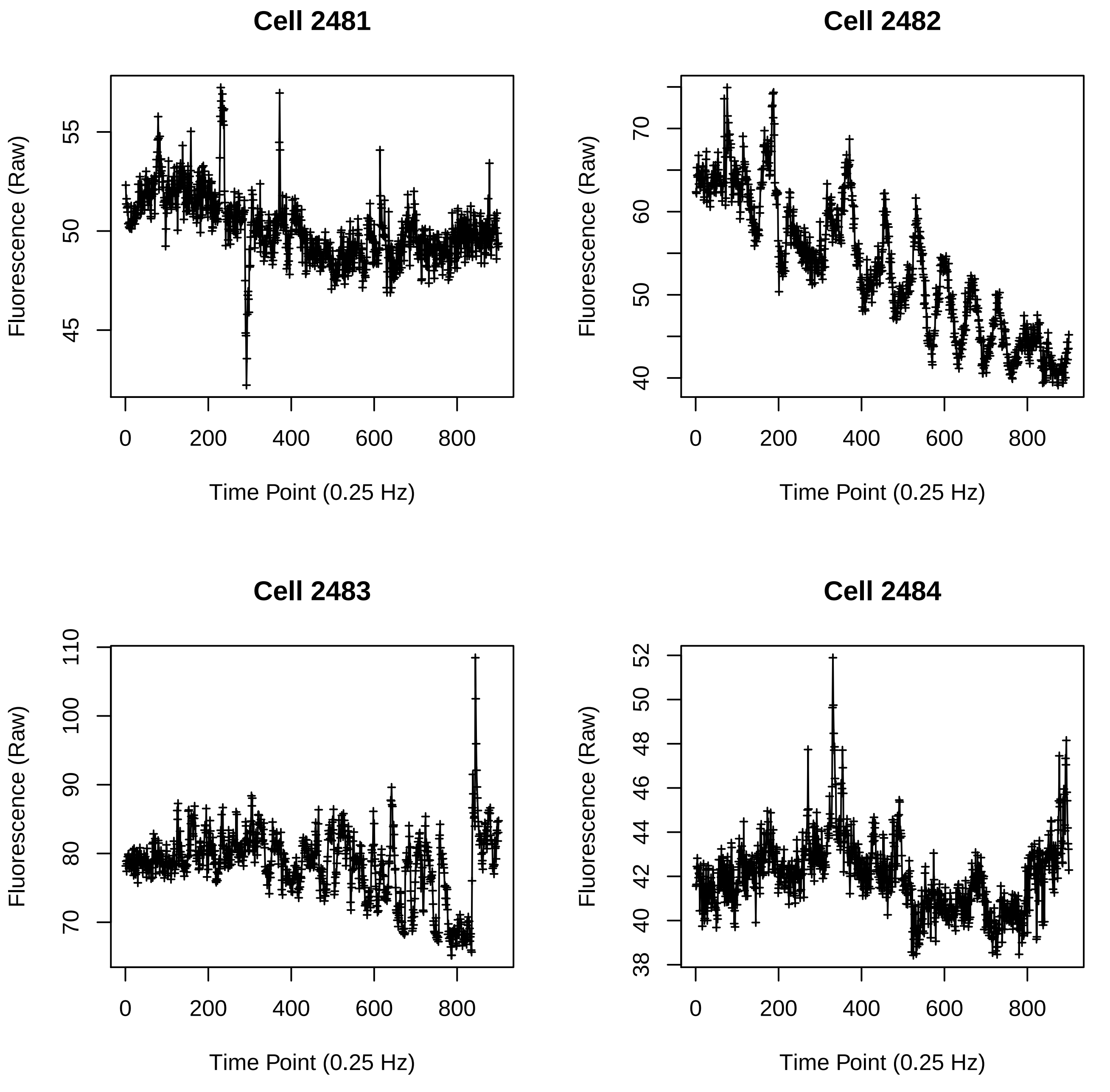
Cell 2481
Cell 2482
Cell 2483
Cell 2484
Fluorescence (Raw)
Time Point (0.25 Hz)
0
200
400
600
800
45
50
55
40
50
60
70
70
80
90
100
110
38
40
42
44
46
48
50
52

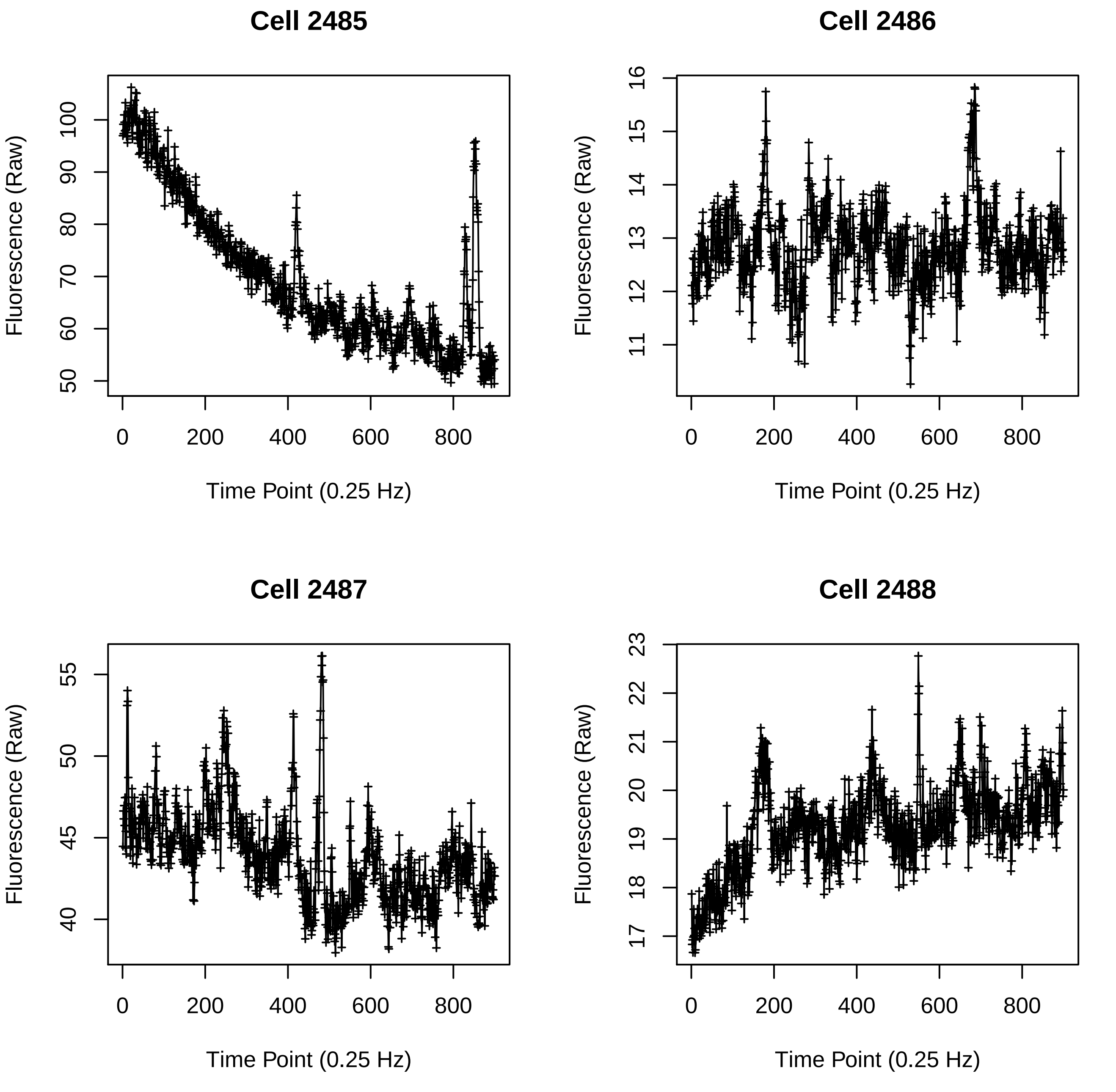
Cell 2485
Cell 2486
Cell 2487
Cell 2488
Fluorescence (Raw)
Time Point (0.25 Hz)
0
200
400
600
800
50
60
70
80
90
100
11
12
13
14
15
16
40
45
50
55
17
18
19
20
21
22
23

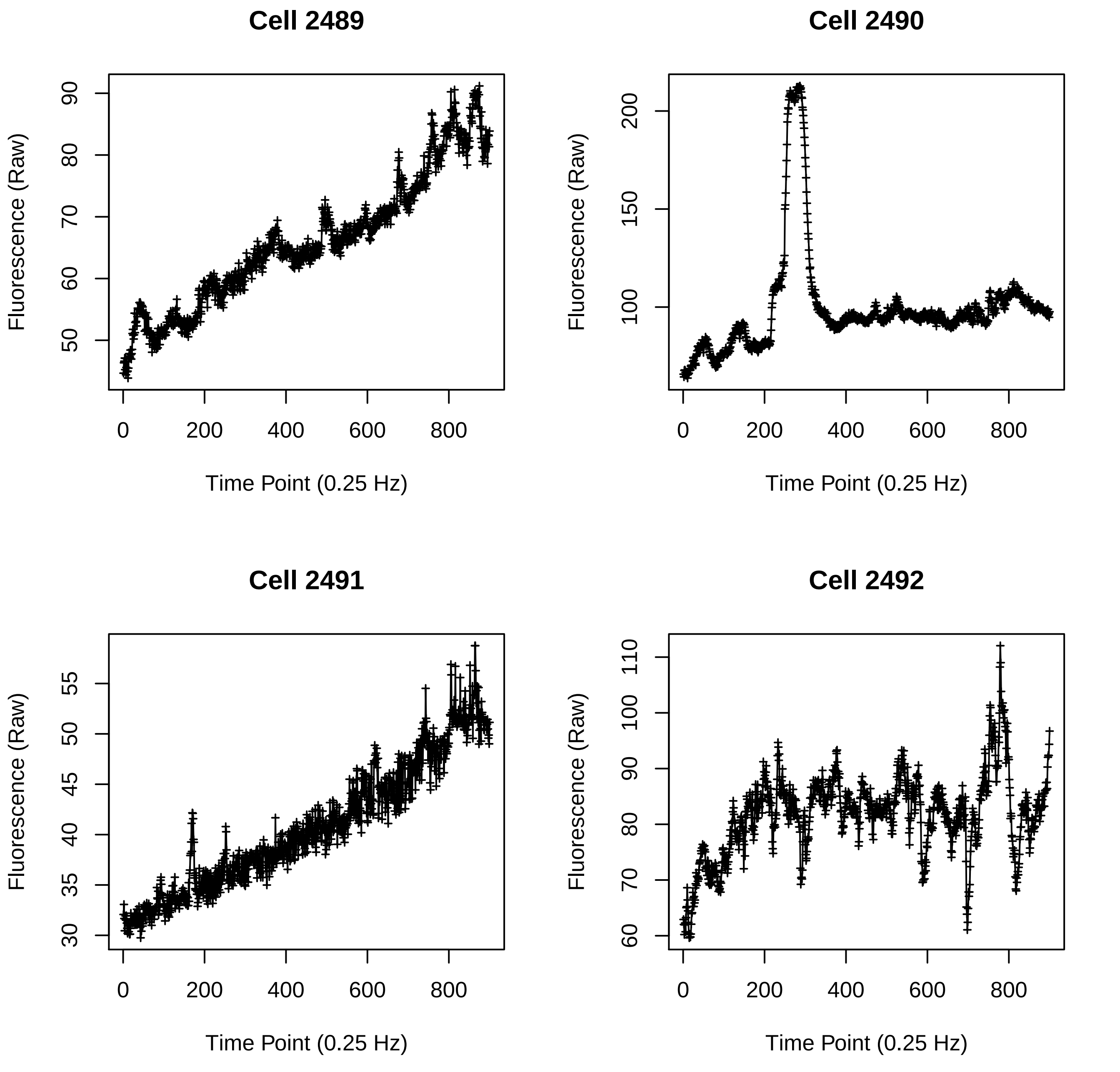
Cell 2489
Cell 2490
Cell 2491
Cell 2492
Fluorescence (Raw)
Time Point (0.25 Hz)
0
200
400
600
800
50
60
70
80
90
100
150
200
30
35
40
45
50
55
60
70
80
90
100
110

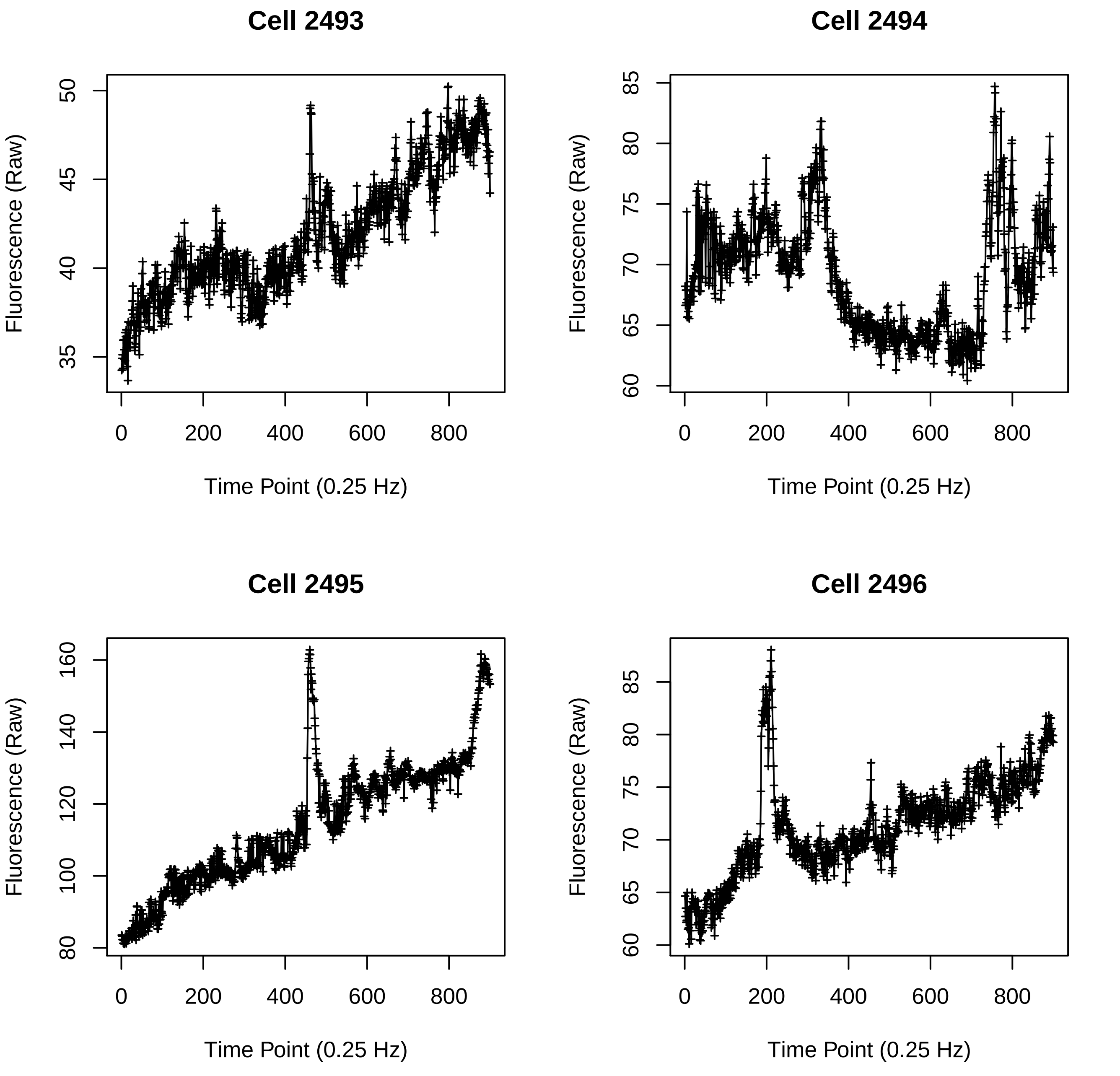

Cell 2493
Fluorescence (Raw)
35
40
45
50
0
200
400
600
800
Time Point (0.25 Hz)
Cell 2494
Fluorescence (Raw)
60
65
70
75
80
85
0
200
400
600
800
Time Point (0.25 Hz)
Cell 2495
Fluorescence (Raw)
80
100
120
140
160
0
200
400
600
800
Time Point (0.25 Hz)
Cell 2496
Fluorescence (Raw)
60
65
70
75
80
85
0
200
400
600
800
Time Point (0.25 Hz)

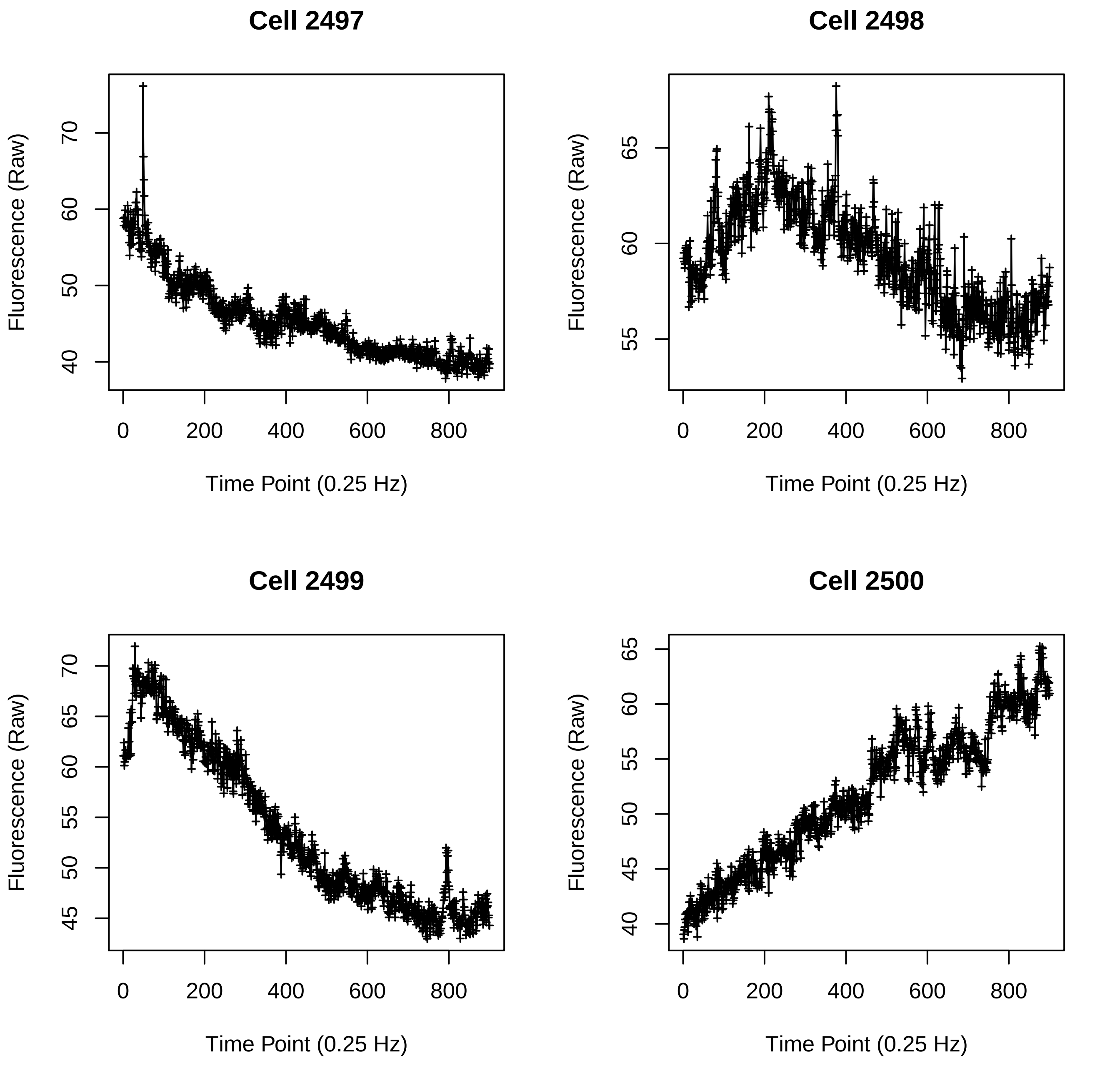

Cell 2497
Cell 2498
Cell 2499
Cell 2500
Fluorescence (Raw)
Time Point (0.25 Hz)
0
200
400
600
800
40
45
50
55
60
65
70

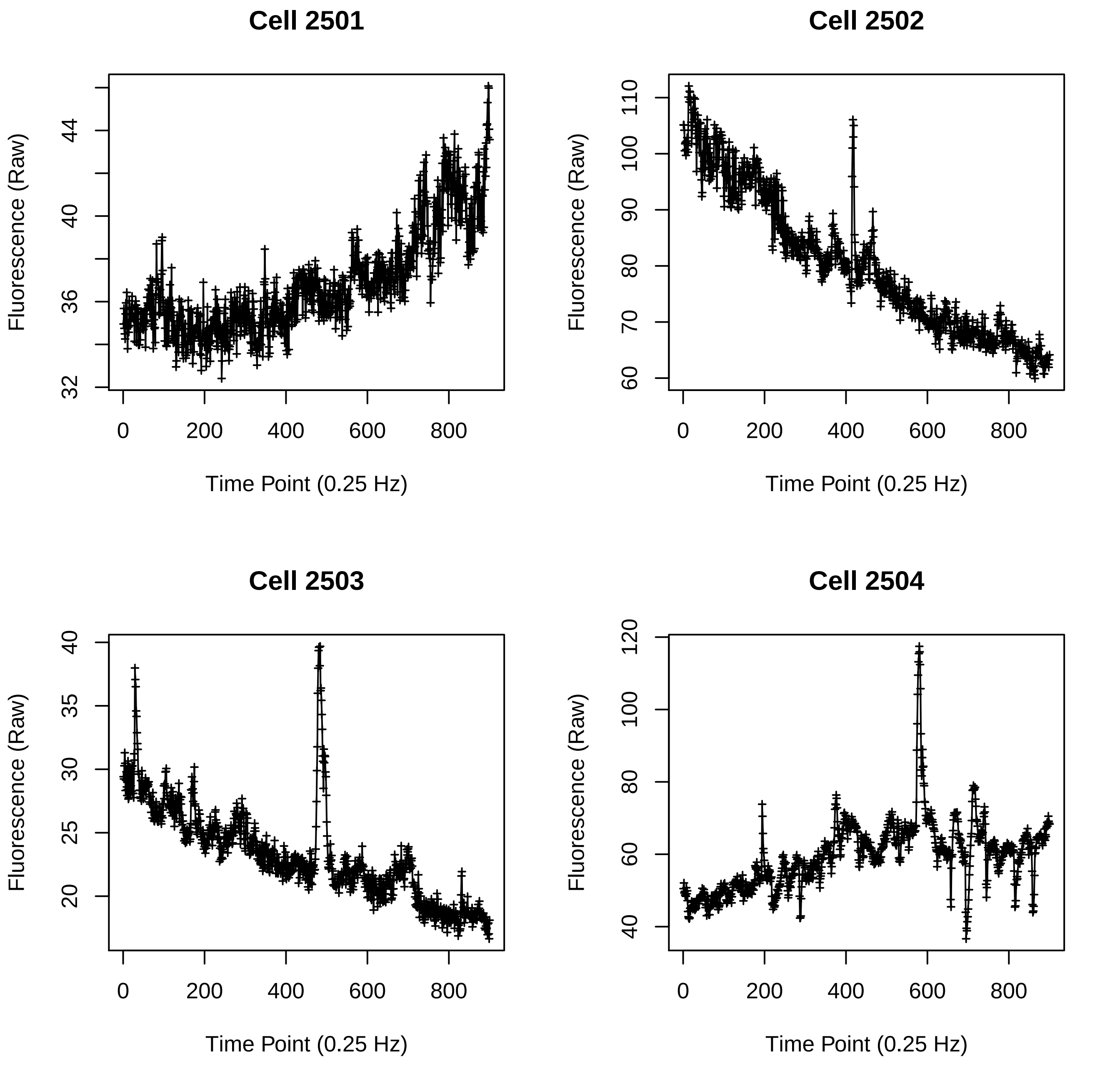
Cell 2501
Fluorescence (Raw)
32
36
40
44
0
200
400
600
800
Time Point (0.25 Hz)
Cell 2502
Fluorescence (Raw)
60
70
80
90
100
110
0
200
400
600
800
Time Point (0.25 Hz)
Cell 2503
Fluorescence (Raw)
20
25
30
35
40
0
200
400
600
800
Time Point (0.25 Hz)
Cell 2504
Fluorescence (Raw)
40
60
80
100
120
0
200
400
600
800
Time Point (0.25 Hz)

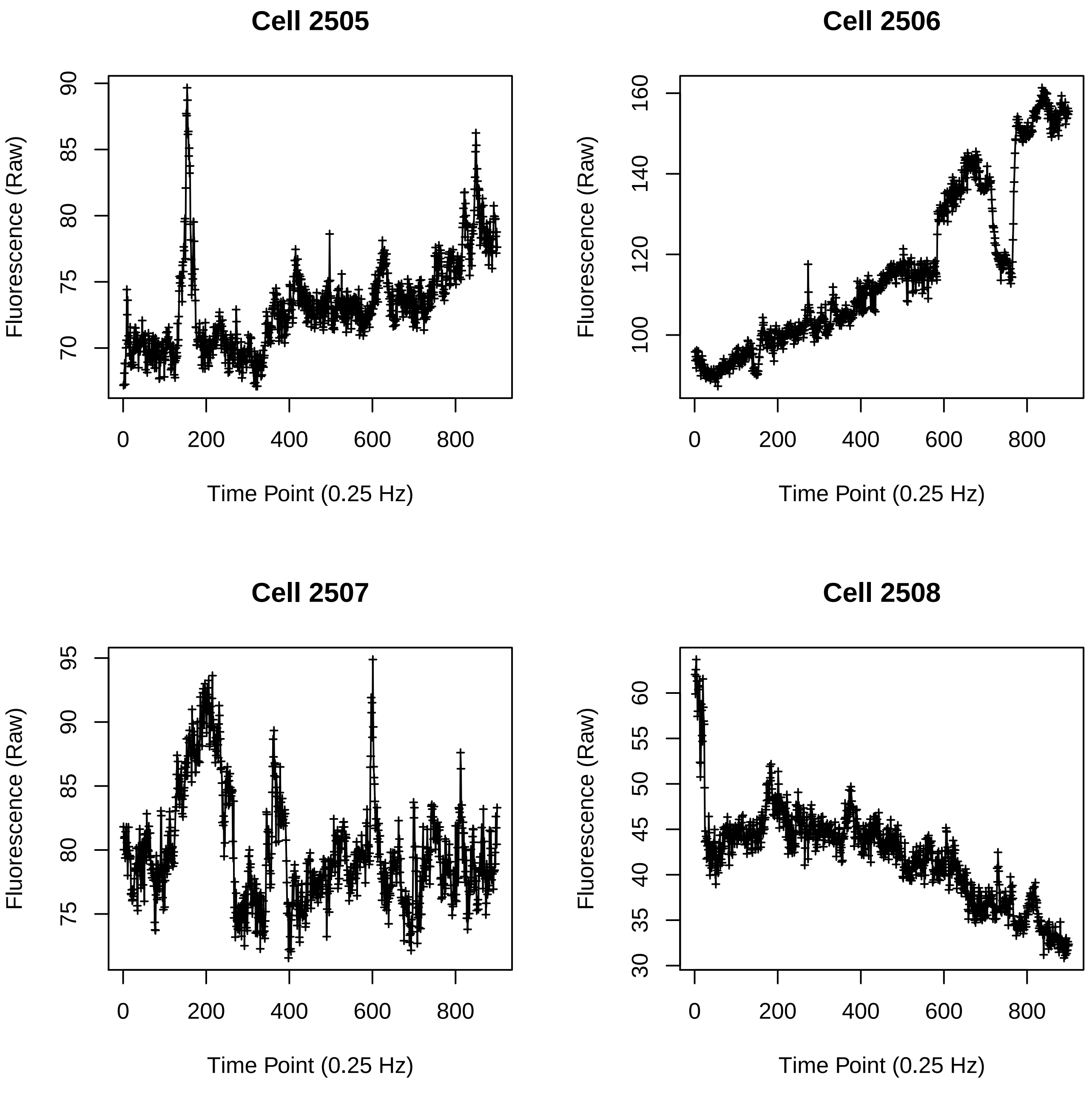
Cell 2505
Cell 2506
Cell 2507
Cell 2508
Fluorescence (Raw)
Time Point (0.25 Hz)
0
200
400
600
800
70
75
80
85
90
95
100
120
140
160
30
35
40
45
50
55
60

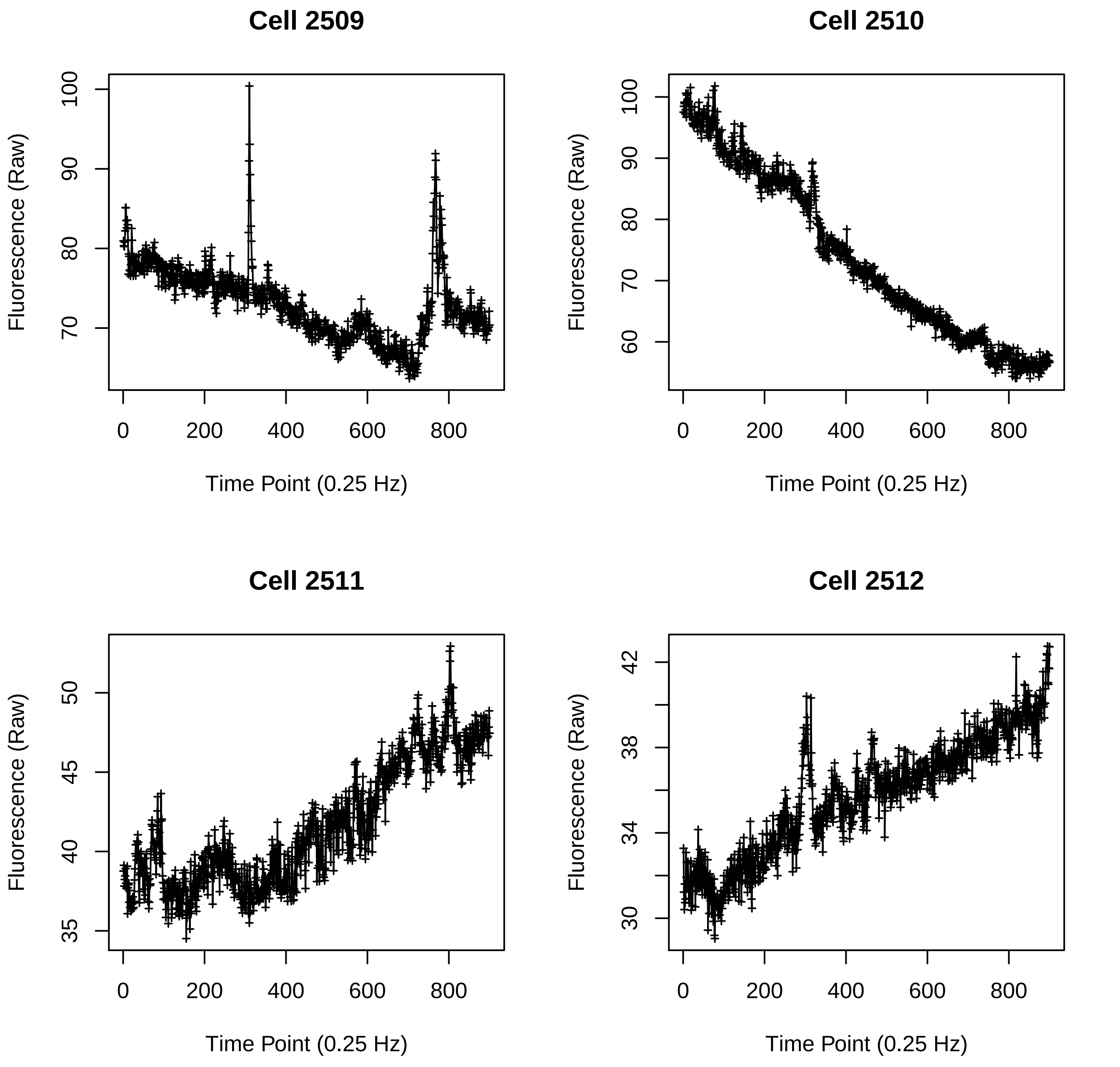
Cell 2509
Fluorescence (Raw)
70
80
90
100
0
200
400
600
800
Time Point (0.25 Hz)
Cell 2510
Fluorescence (Raw)
60
70
80
90
100
0
200
400
600
800
Time Point (0.25 Hz)
Cell 2511
Fluorescence (Raw)
35
40
45
50
0
200
400
600
800
Time Point (0.25 Hz)
Cell 2512
Fluorescence (Raw)
30
34
38
42
0
200
400
600
800
Time Point (0.25 Hz)

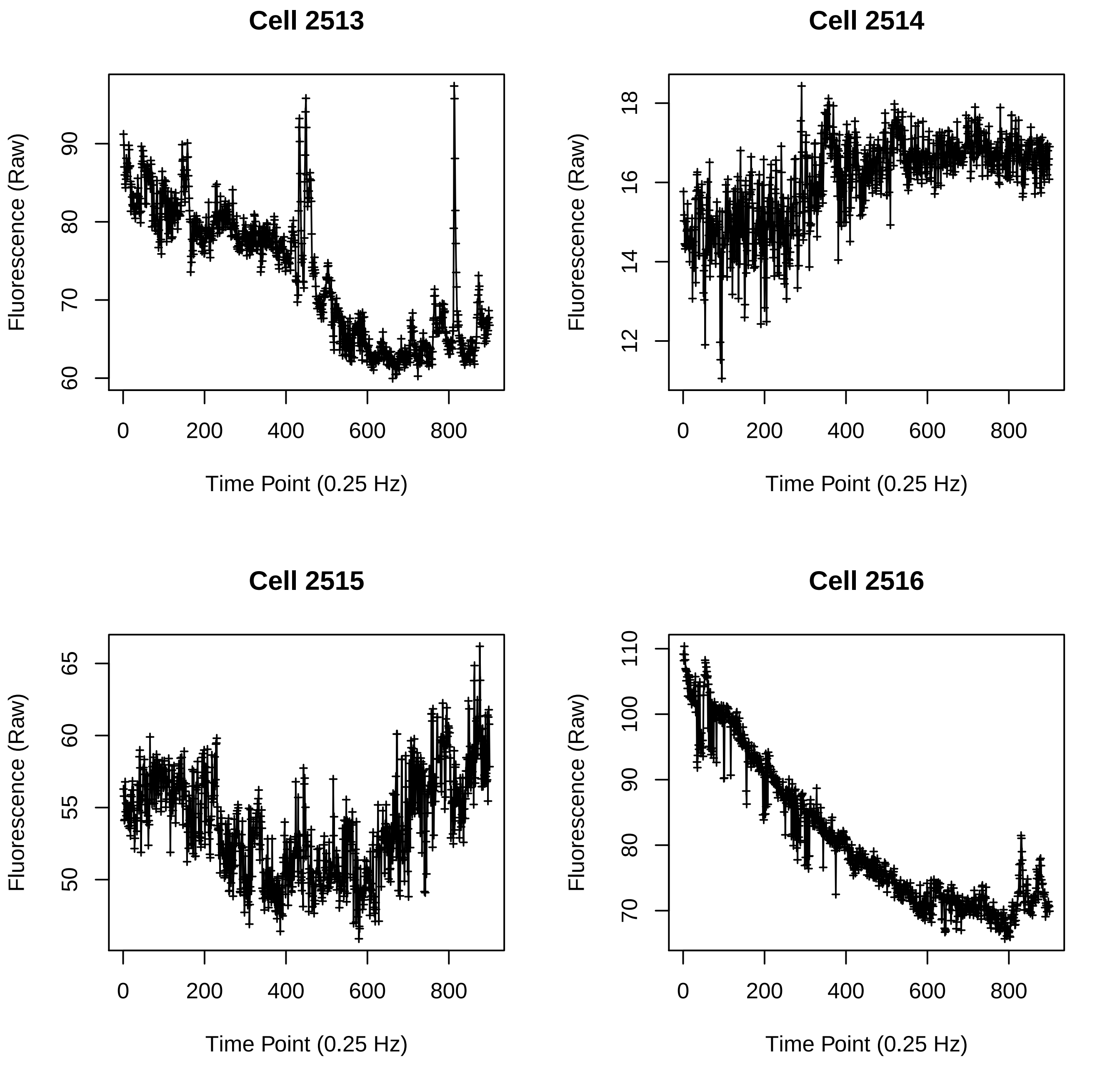
Cell 2513
Fluorescence (Raw)
60
70
80
90
0
200
400
600
800
Time Point (0.25 Hz)
Cell 2514
Fluorescence (Raw)
12
14
16
18
0
200
400
600
800
Time Point (0.25 Hz)
Cell 2515
Fluorescence (Raw)
50
55
60
65
0
200
400
600
800
Time Point (0.25 Hz)
Cell 2516
Fluorescence (Raw)
70
80
90
100
110
0
200
400
600
800
Time Point (0.25 Hz)

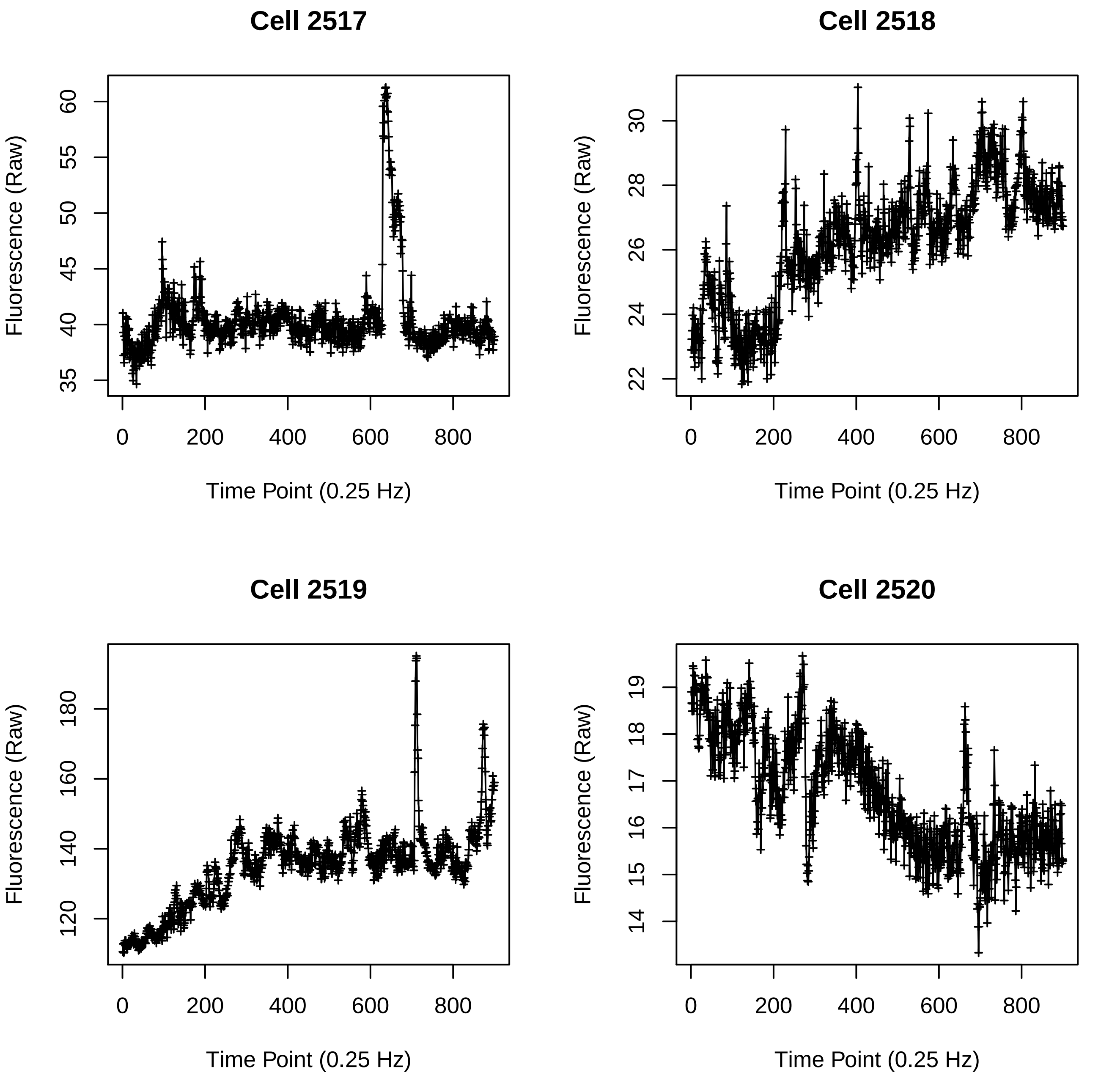

Cell 2517
Fluorescence (Raw)
35
40
45
50
55
60
0
200
400
600
800
Time Point (0.25 Hz)
Cell 2518
Fluorescence (Raw)
22
24
26
28
30
0
200
400
600
800
Time Point (0.25 Hz)
Cell 2519
Fluorescence (Raw)
120
140
160
180
0
200
400
600
800
Time Point (0.25 Hz)
Cell 2520
Fluorescence (Raw)
14
15
16
17
18
19
0
200
400
600
800
Time Point (0.25 Hz)

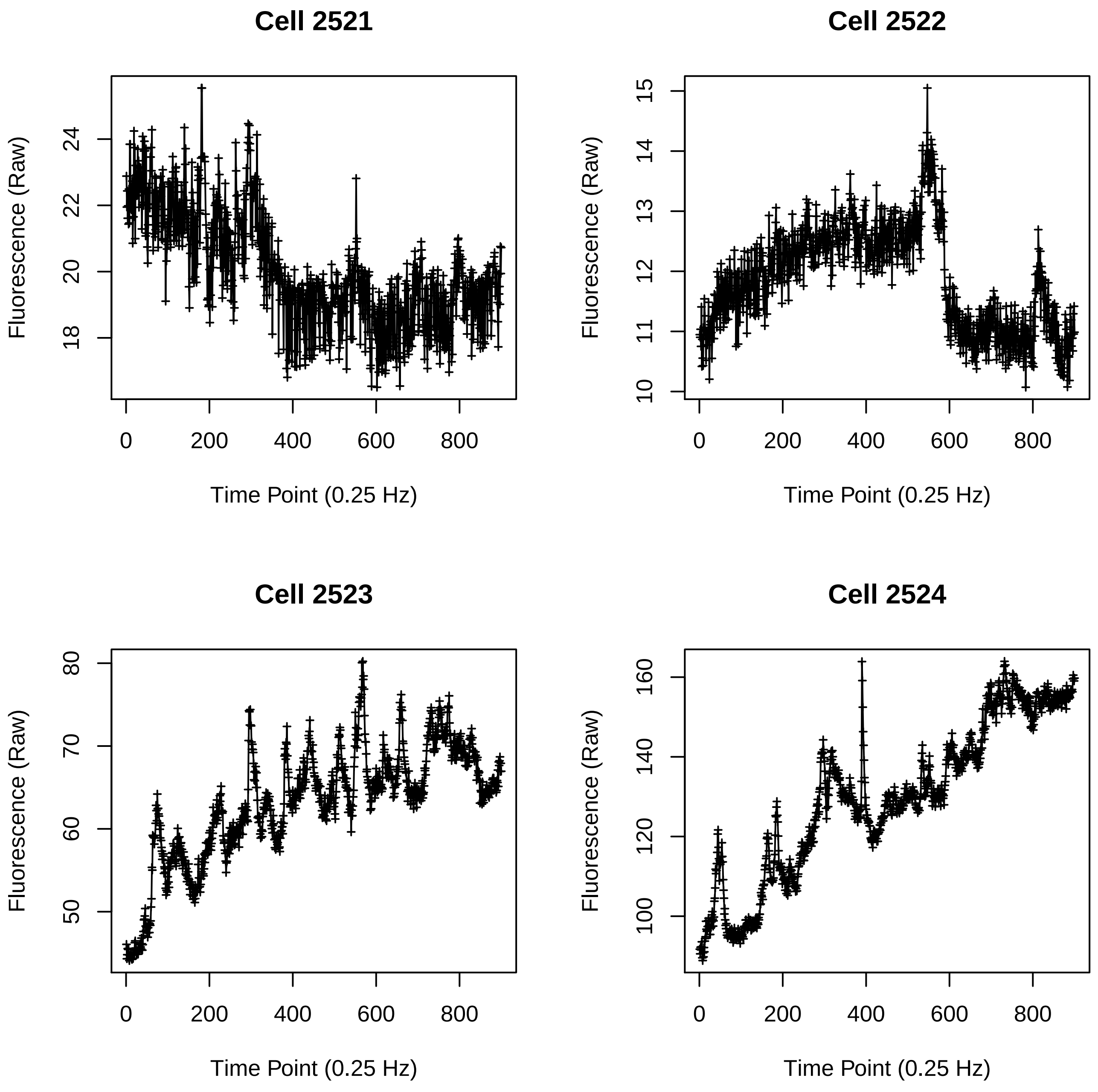
Cell 2521
Cell 2522
Cell 2523
Cell 2524
Fluorescence (Raw)
Time Point (0.25 Hz)
0
200
400
600
800
18
20
22
24
10
11
12
13
14
15
50
60
70
80
100
120
140
160

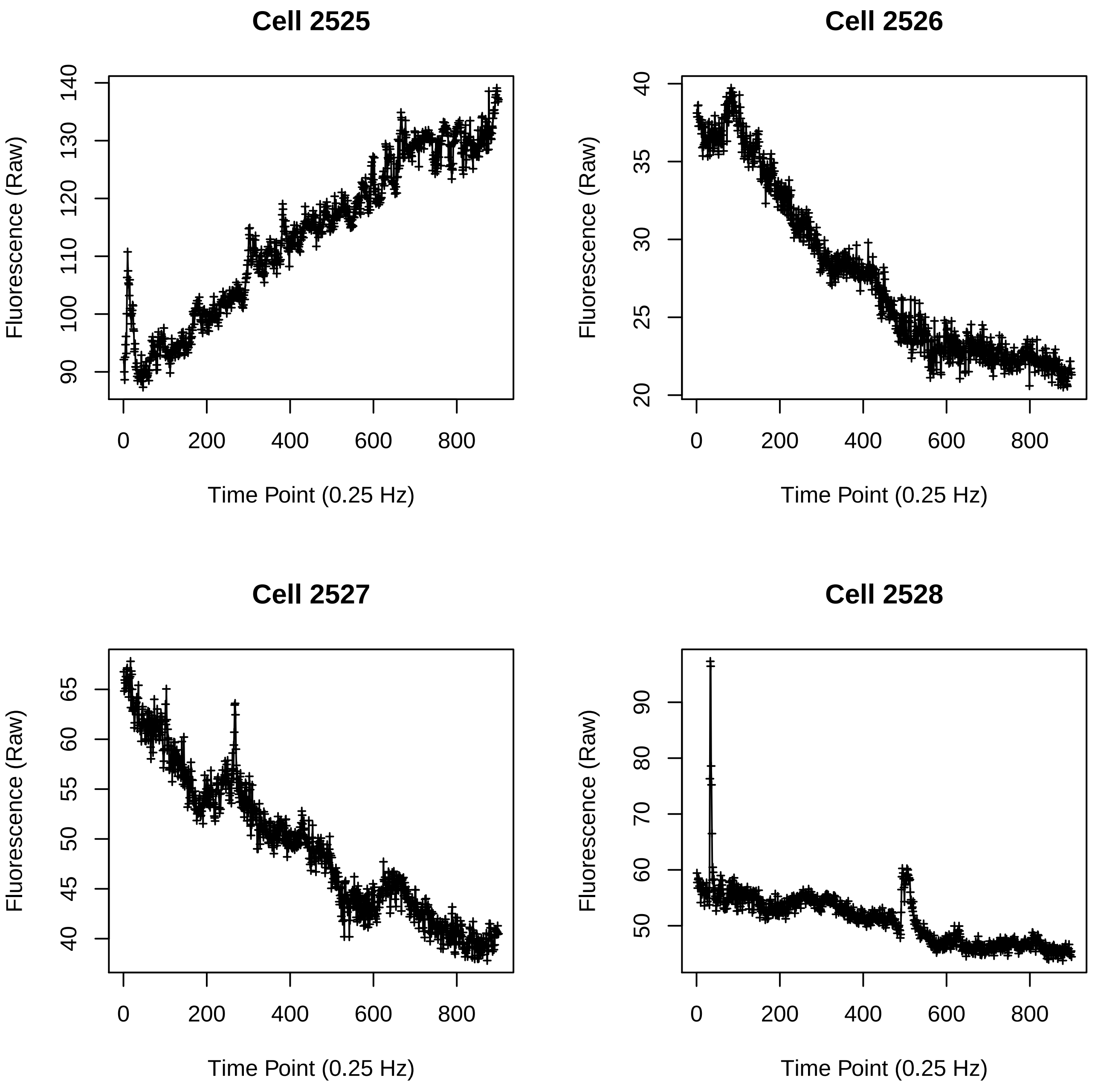
Cell 2525
Fluorescence (Raw)
90
100
110
120
130
140
0
200
400
600
800
Time Point (0.25 Hz)
Cell 2526
Fluorescence (Raw)
20
25
30
35
40
0
200
400
600
800
Time Point (0.25 Hz)
Cell 2527
Fluorescence (Raw)
40
45
50
55
60
65
0
200
400
600
800
Time Point (0.25 Hz)
Cell 2528
Fluorescence (Raw)
50
60
70
80
90
0
200
400
600
800
Time Point (0.25 Hz)

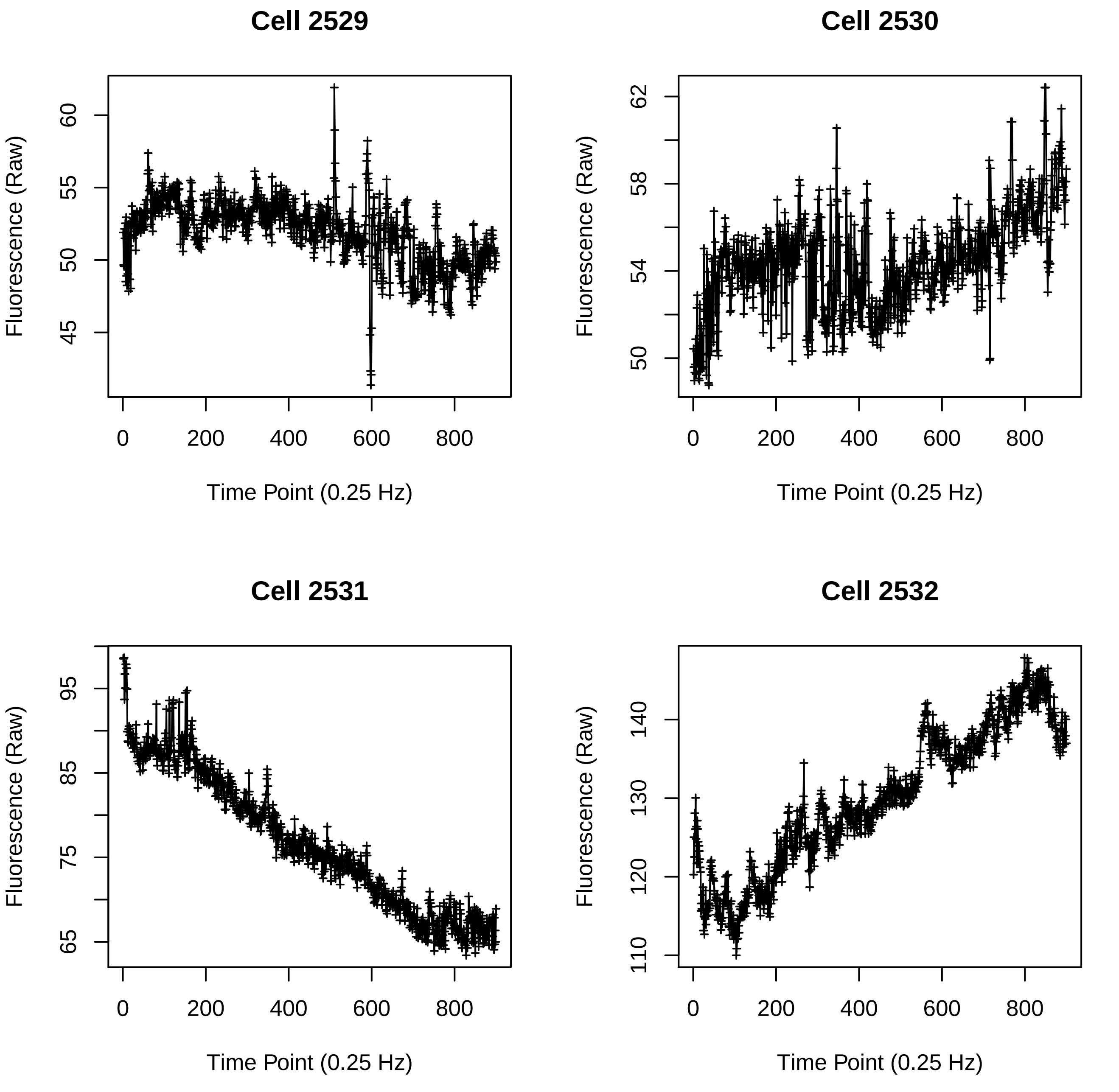
Cell 2529
Fluorescence (Raw)
45
50
55
60
0
200
400
600
800
Time Point (0.25 Hz)
Cell 2530
Fluorescence (Raw)
50
54
58
62
0
200
400
600
800
Time Point (0.25 Hz)
Cell 2531
Fluorescence (Raw)
65
75
85
95
0
200
400
600
800
Time Point (0.25 Hz)
Cell 2532
Fluorescence (Raw)
110
120
130
140
0
200
400
600
800
Time Point (0.25 Hz)

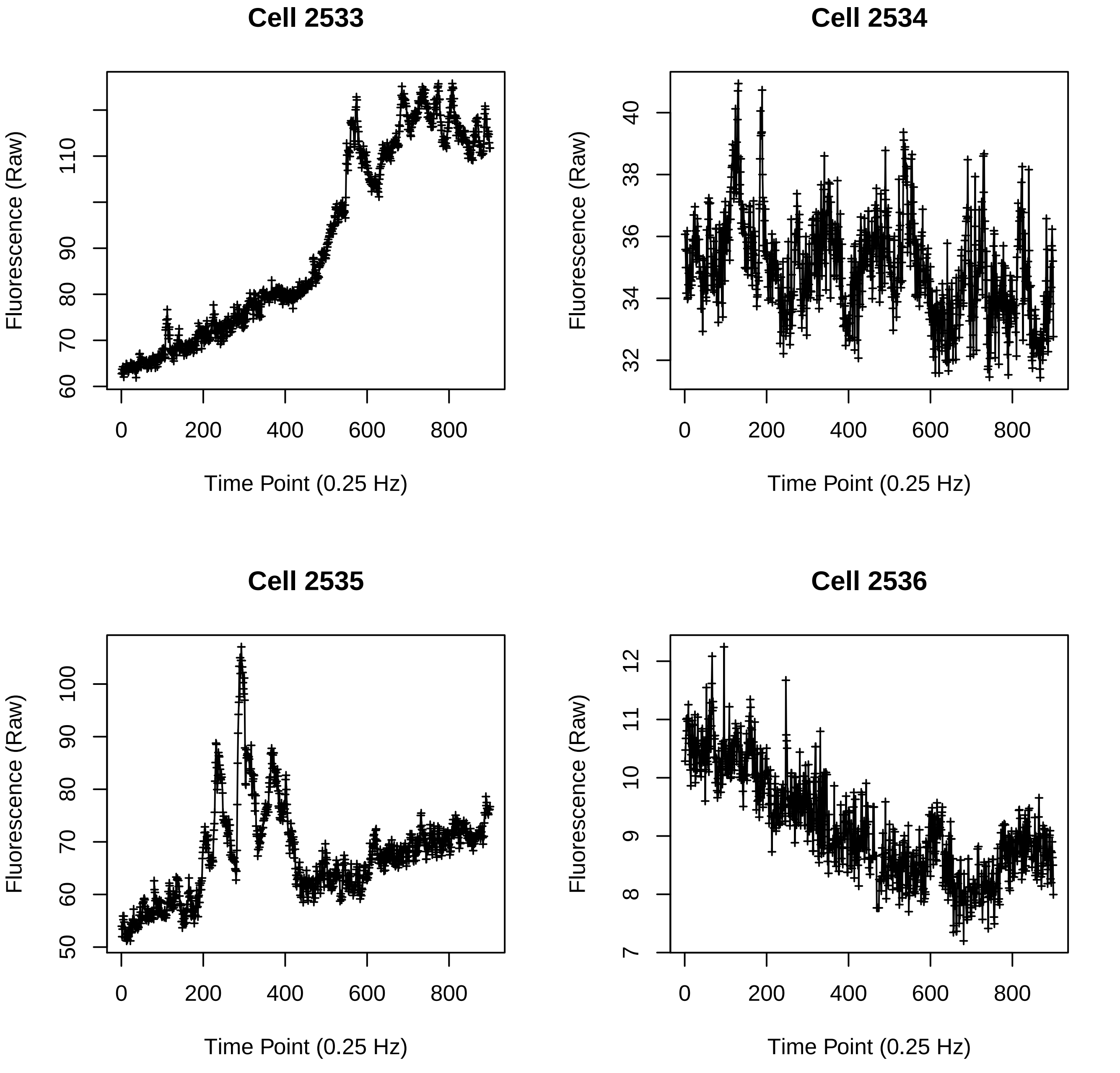

Cell 2533
Fluorescence (Raw)
60
70
80
90
110
0
200
400
600
800
Time Point (0.25 Hz)
Cell 2534
Fluorescence (Raw)
32
34
36
38
40
0
200
400
600
800
Time Point (0.25 Hz)
Cell 2535
Fluorescence (Raw)
50
60
70
80
90
100
0
200
400
600
800
Time Point (0.25 Hz)
Cell 2536
Fluorescence (Raw)
7
8
9
10
11
12
0
200
400
600
800
Time Point (0.25 Hz)

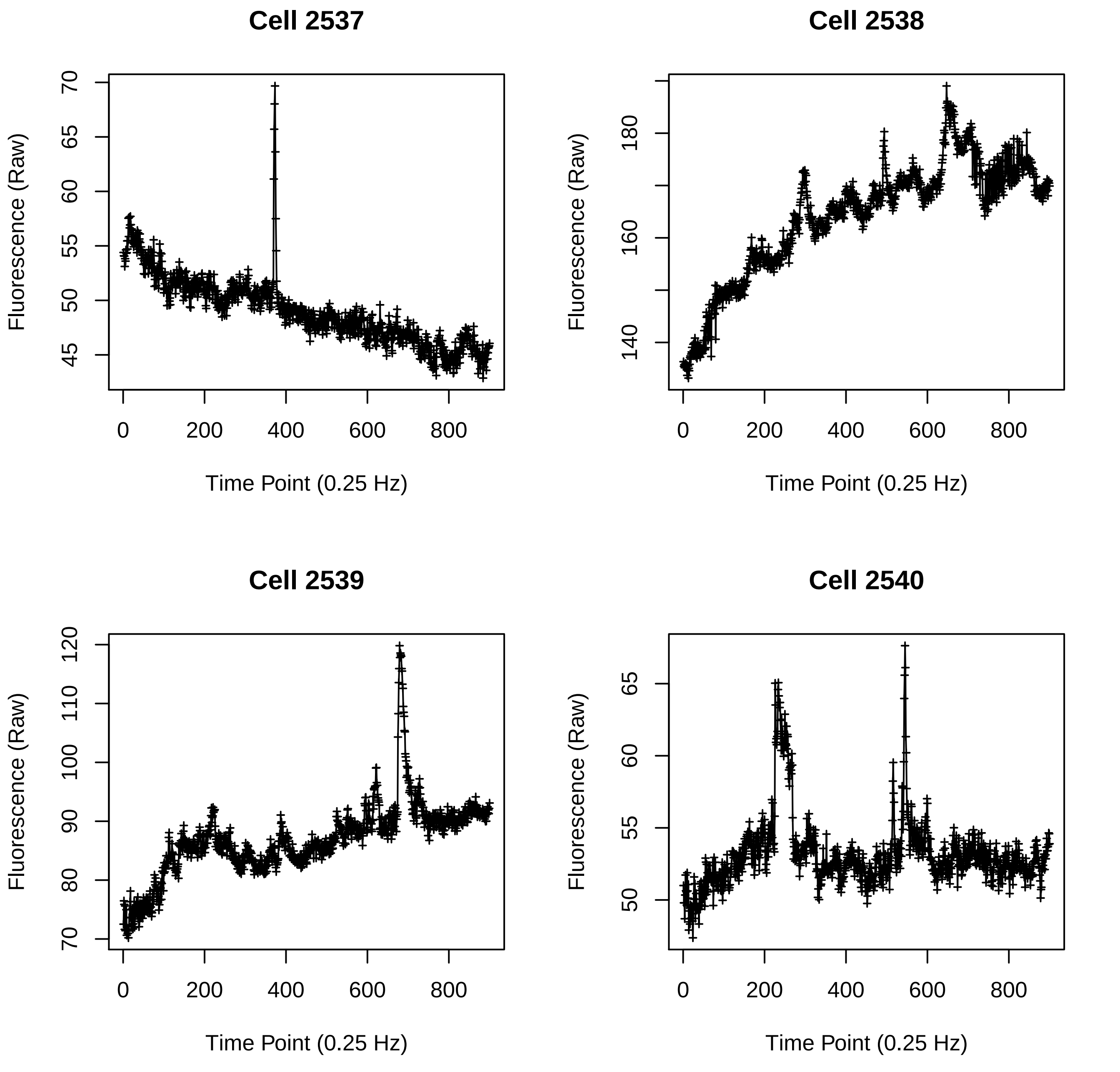

Cell 2537
Cell 2538
Cell 2539
Cell 2540
Fluorescence (Raw)
Time Point (0.25 Hz)
0
200
400
600
800
45
50
55
60
65
70
140
160
180
80
90
100
110
120

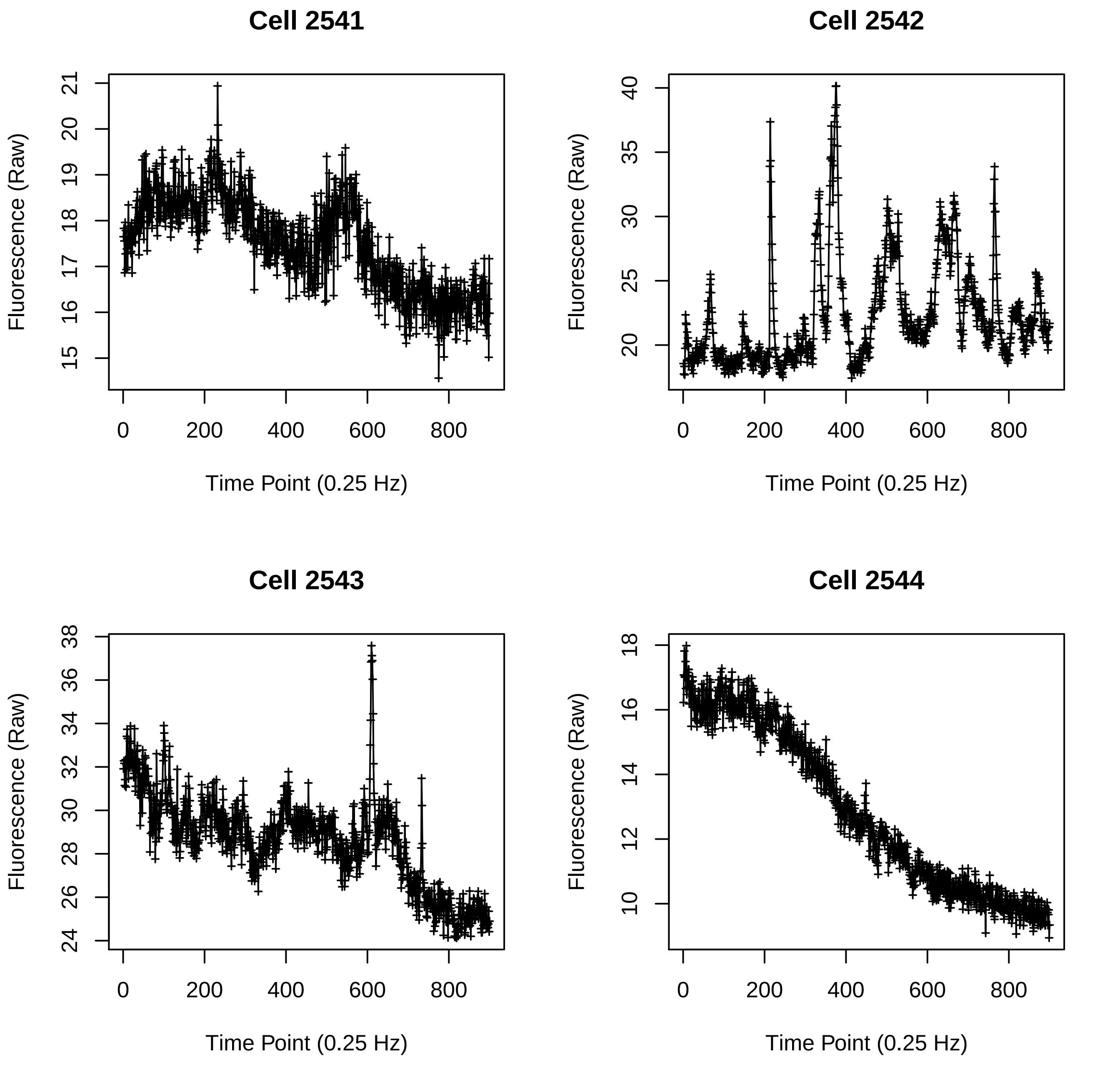
Cell 2541
Fluorescence (Raw)
15 16 17 18 19 20 21
0 200 400 600 800
Time Point (0.25 Hz)
Cell 2542
Fluorescence (Raw)
20 25 30 35 40
0 200 400 600 800
Time Point (0.25 Hz)
Cell 2543
Fluorescence (Raw)
24 26 28 30 32 34 36 38
0 200 400 600 800
Time Point (0.25 Hz)
Cell 2544
Fluorescence (Raw)
10 12 14 16 18
0 200 400 600 800
Time Point (0.25 Hz)

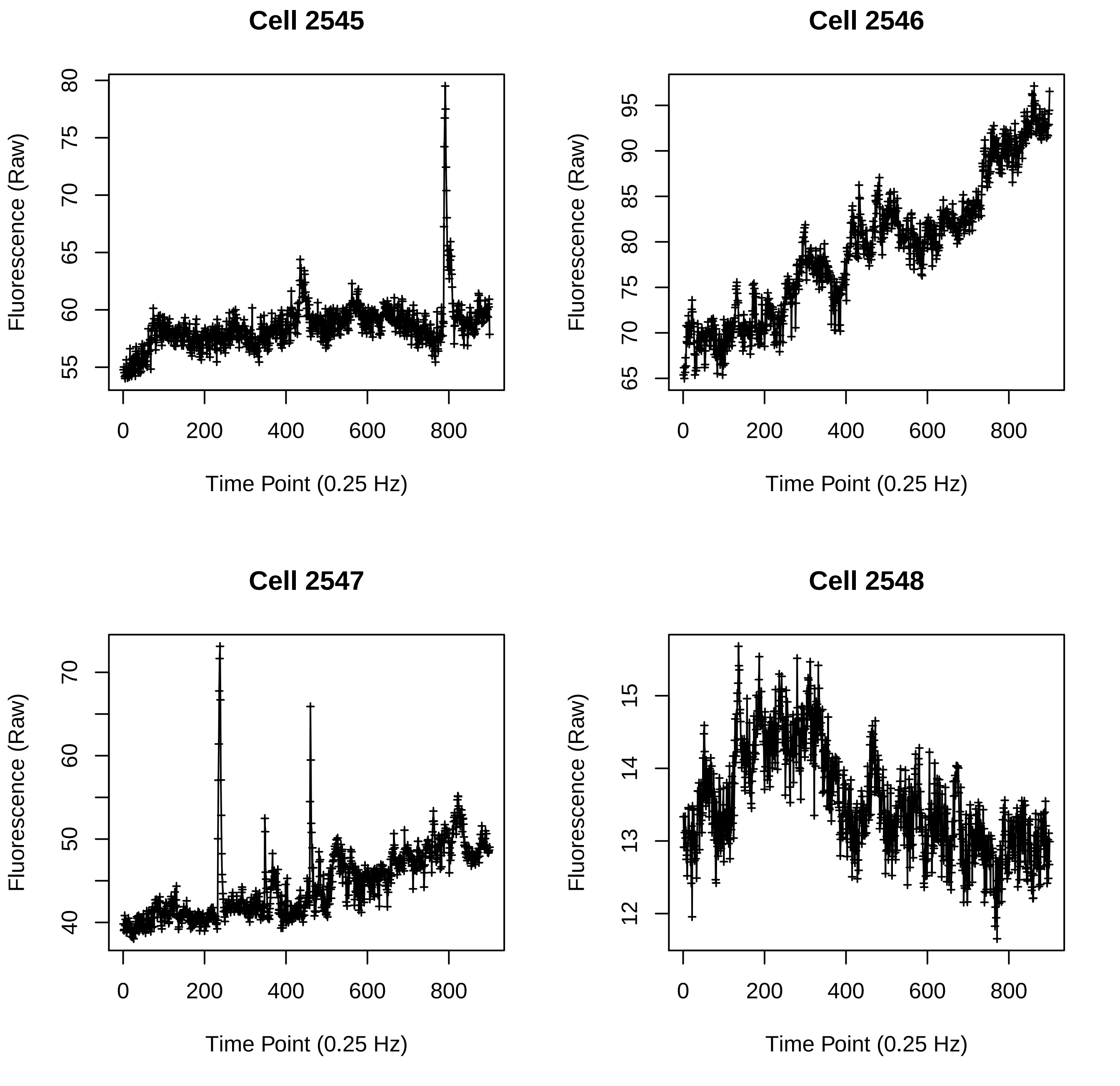

Cell 2545
Cell 2546
Cell 2547
Cell 2548
Fluorescence (Raw)
Time Point (0.25 Hz)
55
60
65
70
75
80
95
90
85
40
50
60
70
12
13
14
15
0
200
400
600
800

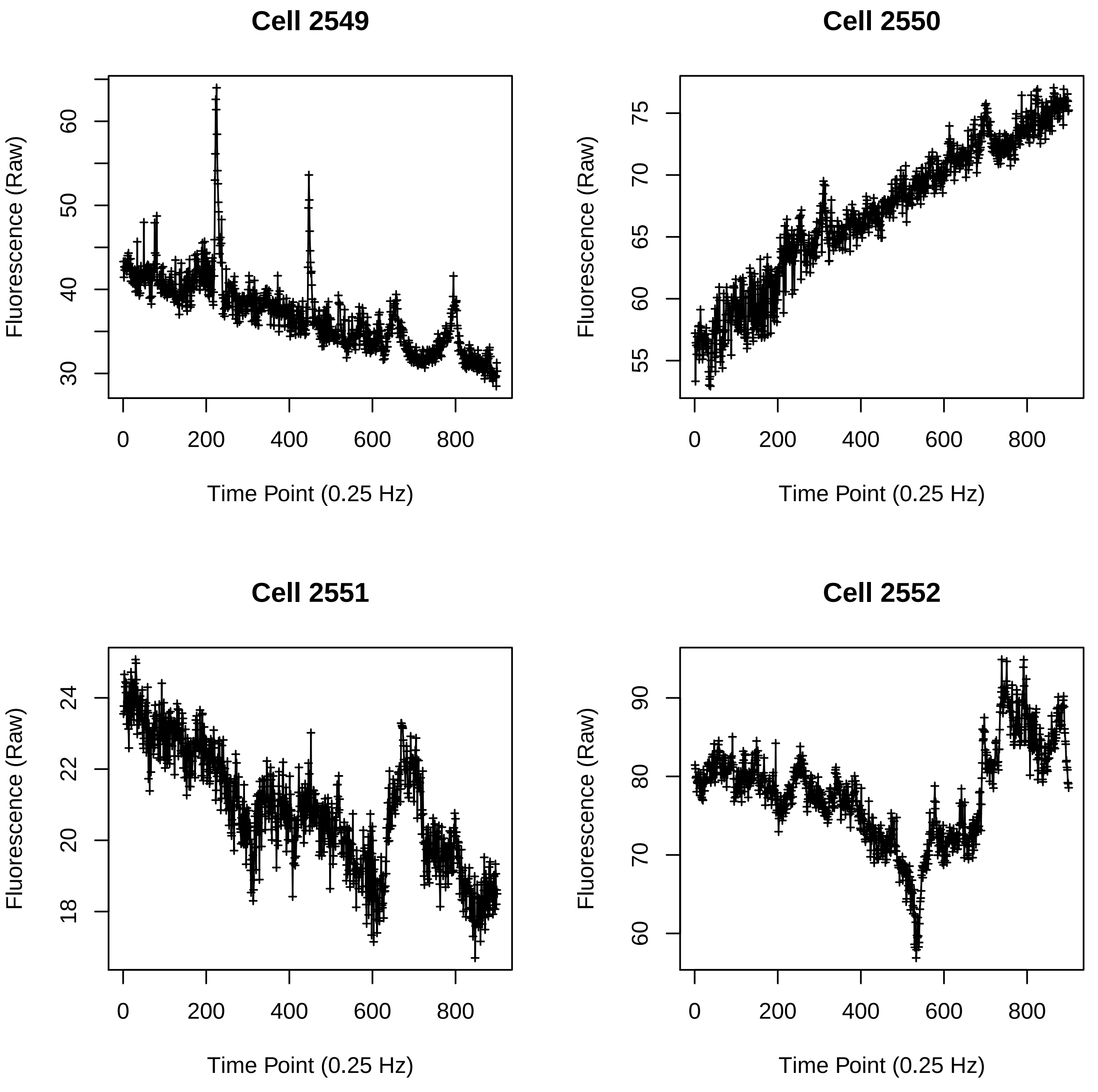
Cell 2549
Fluorescence (Raw)
Time Point (0.25 Hz)
Cell 2550
Fluorescence (Raw)
Time Point (0.25 Hz)
Cell 2551
Fluorescence (Raw)
Time Point (0.25 Hz)
Cell 2552
Fluorescence (Raw)
Time Point (0.25 Hz)

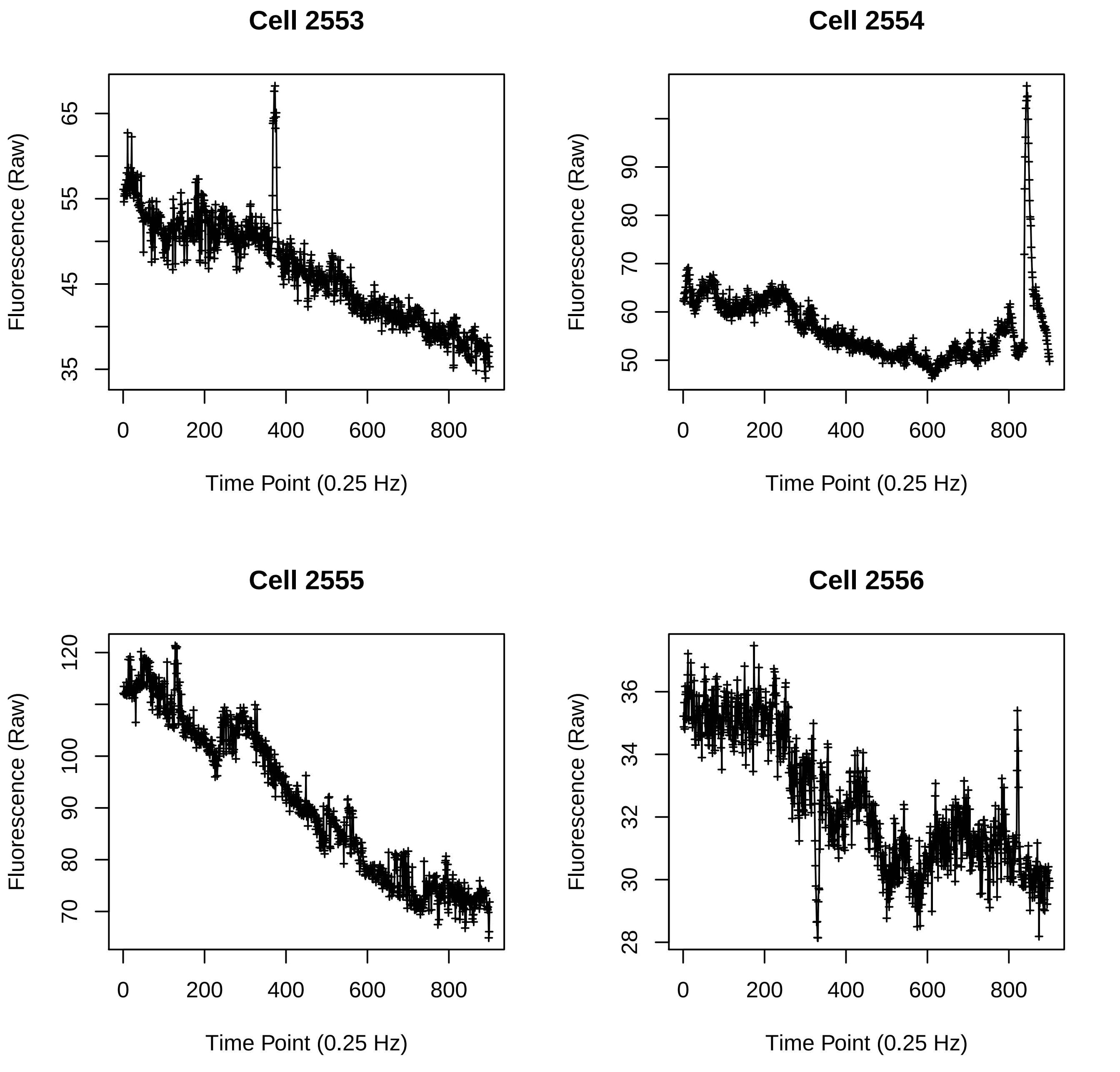

Cell 2553
Fluorescence (Raw)
35
45
55
65
Time Point (0.25 Hz)
0
200
400
600
800
Cell 2554
Fluorescence (Raw)
50
60
70
80
90
Time Point (0.25 Hz)
0
200
400
600
800
Cell 2555
Fluorescence (Raw)
70
80
90
100
120
Time Point (0.25 Hz)
0
200
400
600
800
Cell 2556
Fluorescence (Raw)
28
30
32
34
36
Time Point (0.25 Hz)
0
200
400
600
800

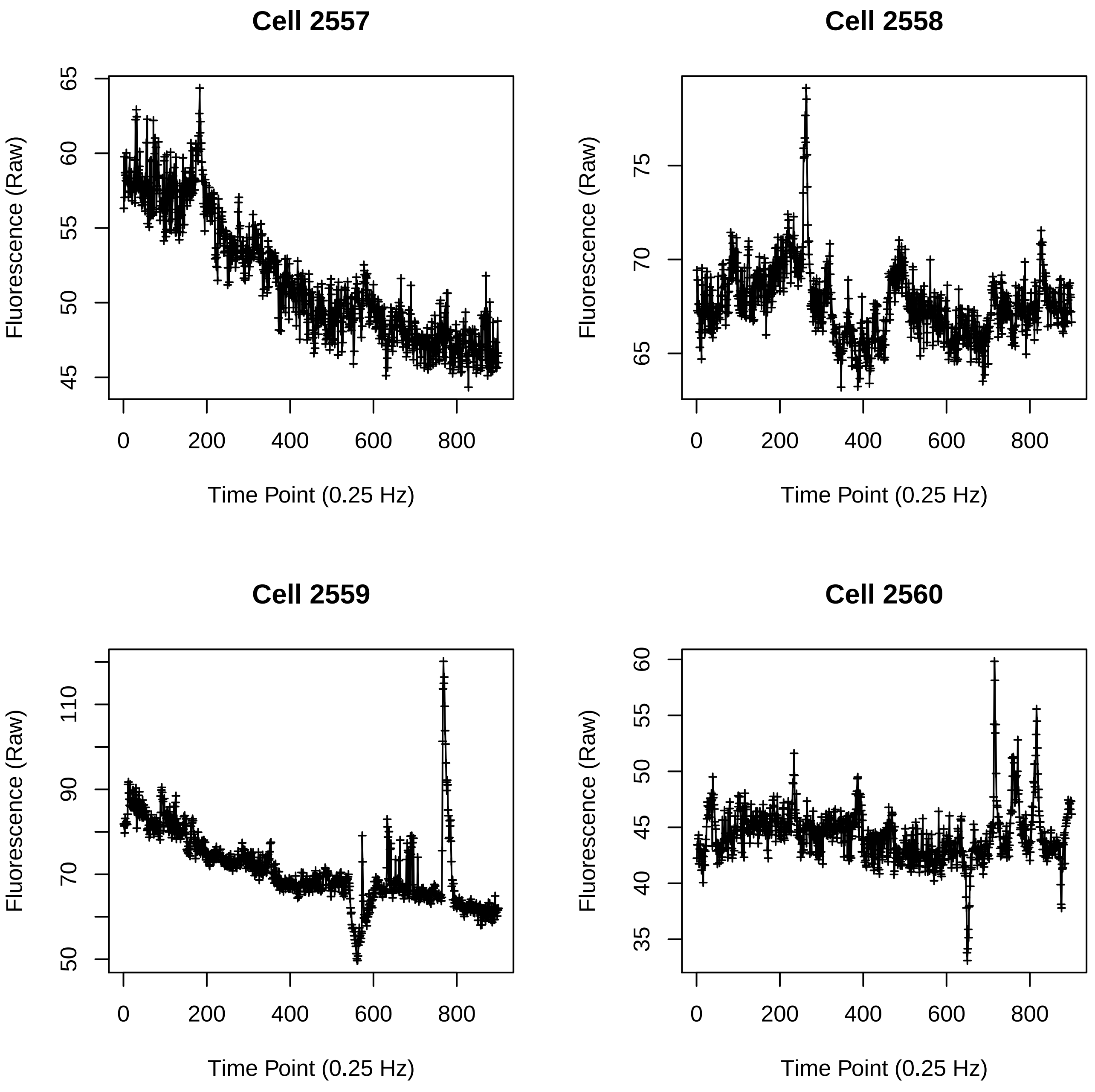
Cell 2557
Cell 2558
Cell 2559
Cell 2560
Fluorescence (Raw)
Time Point (0.25 Hz)
0
200
400
600
800
45
50
55
60
65
70
75
90
110
35
40

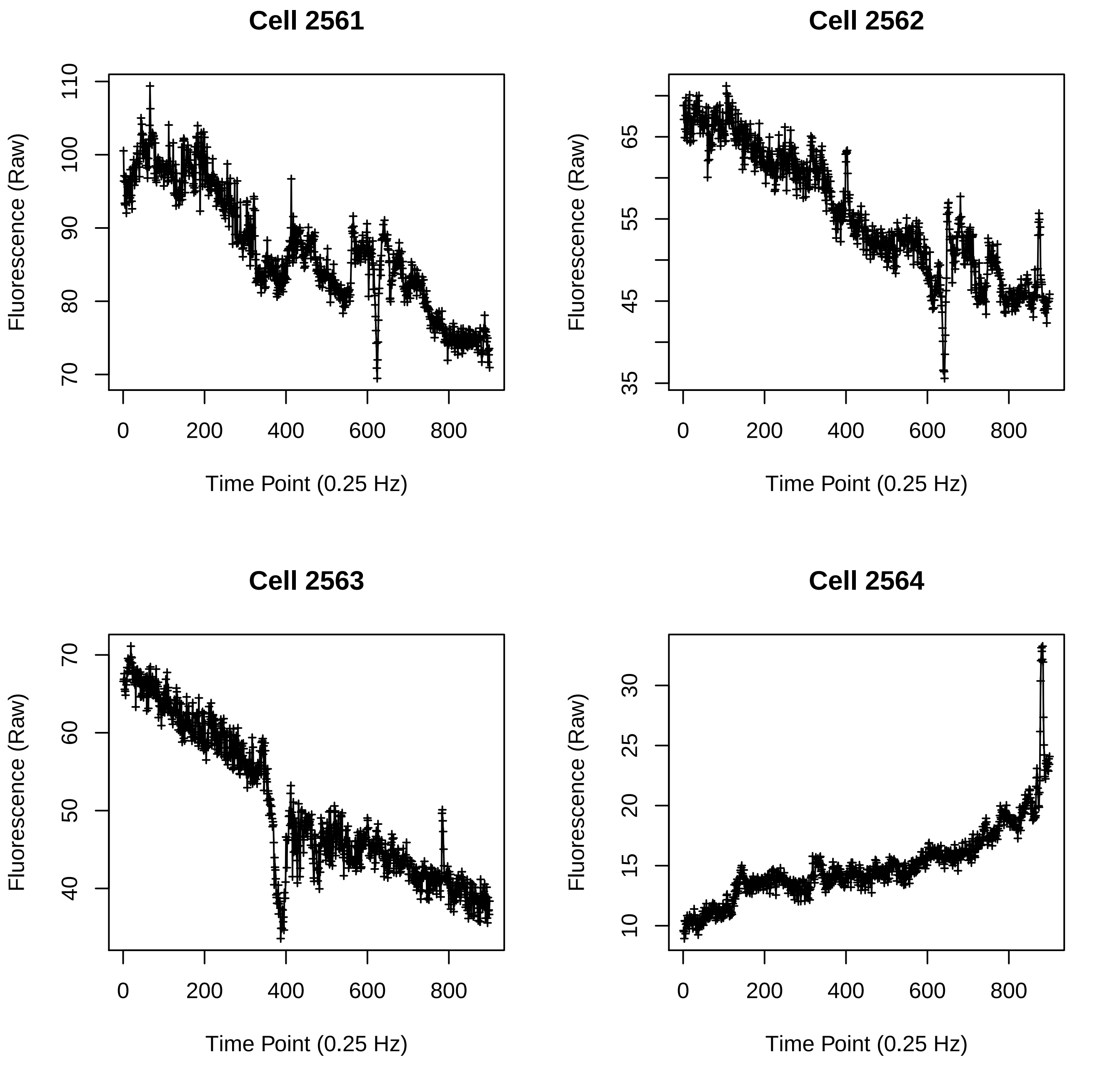

Cell 2561
Cell 2562
Cell 2563
Cell 2564
Fluorescence (Raw)
Time Point (0.25 Hz)
110
100
90
80
70
65
55
45
35
30
25
20
15
10
60
50
40
0
200
400
600
800

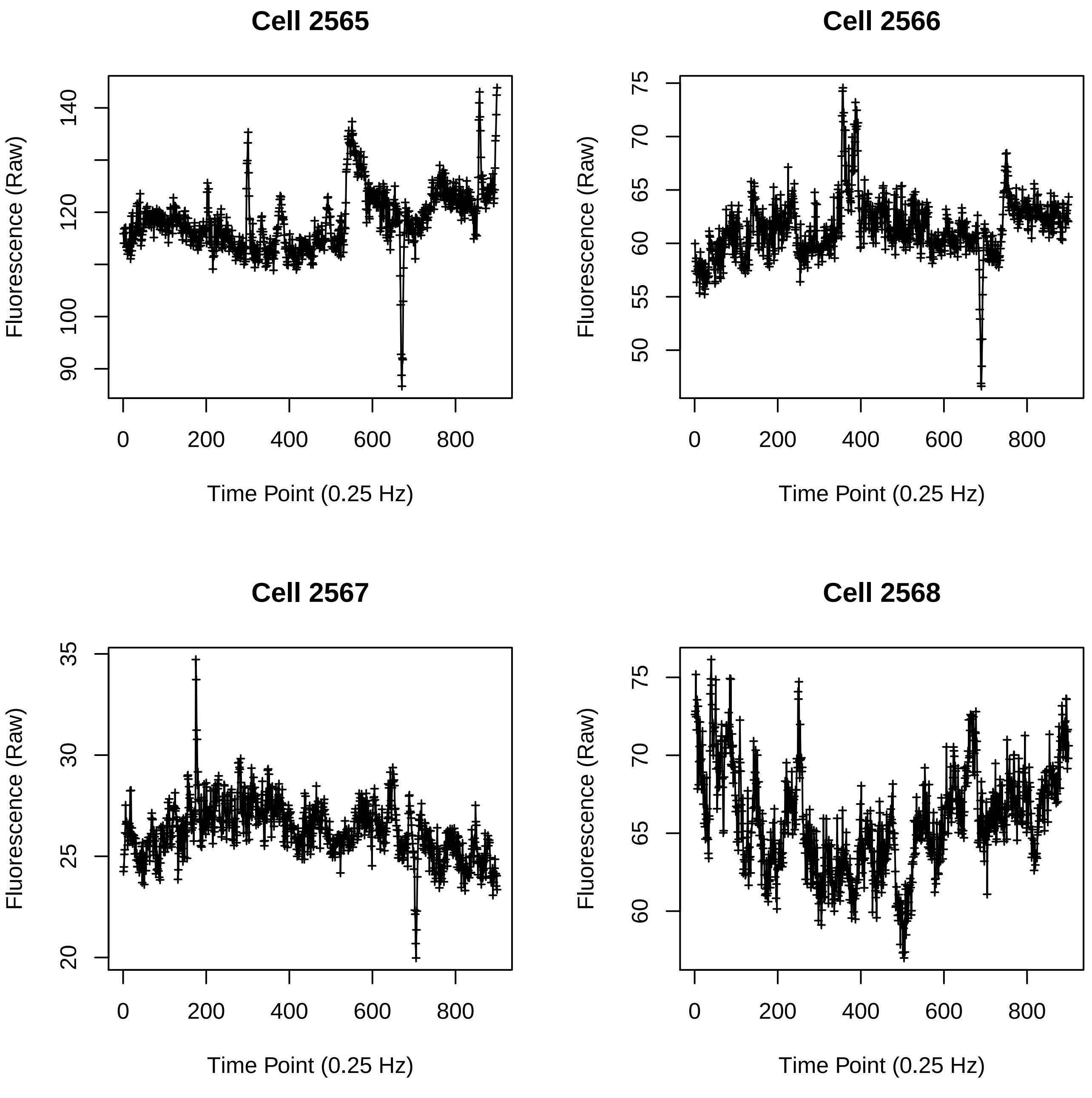

Cell 2565
Cell 2566
Cell 2567
Cell 2568
Fluorescence (Raw)
Time Point (0.25 Hz)
90
100
120
140
50
55
60
65
70
75
20
25
30
35
0
200
400
600
800

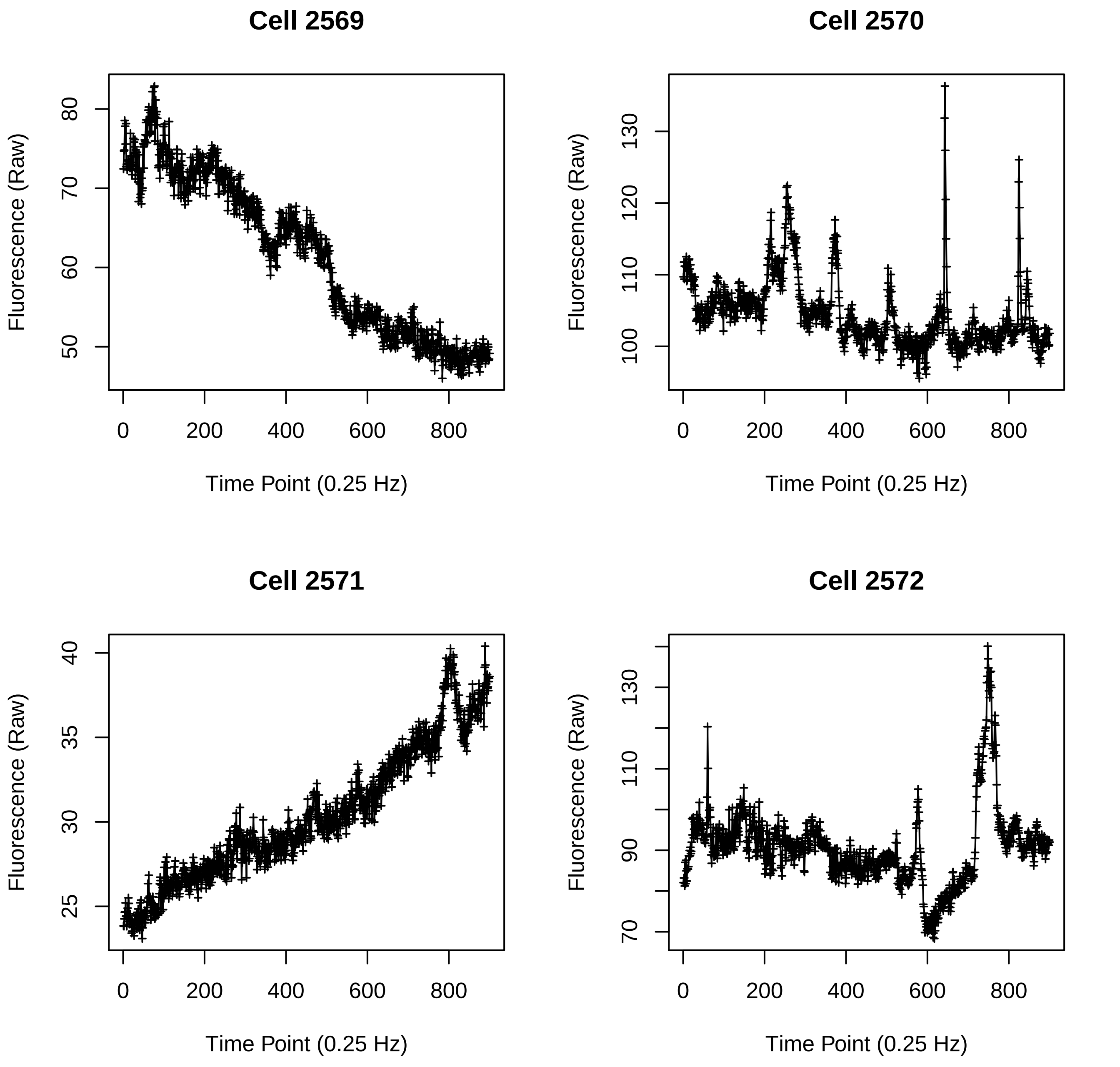

Cell 2569
Fluorescence (Raw)
50
60
70
80
0
200
400
600
800
Time Point (0.25 Hz)
Cell 2570
Fluorescence (Raw)
100
110
120
130
0
200
400
600
800
Time Point (0.25 Hz)
Cell 2571
Fluorescence (Raw)
25
30
35
40
0
200
400
600
800
Time Point (0.25 Hz)
Cell 2572
Fluorescence (Raw)
70
90
110
130
0
200
400
600
800
Time Point (0.25 Hz)

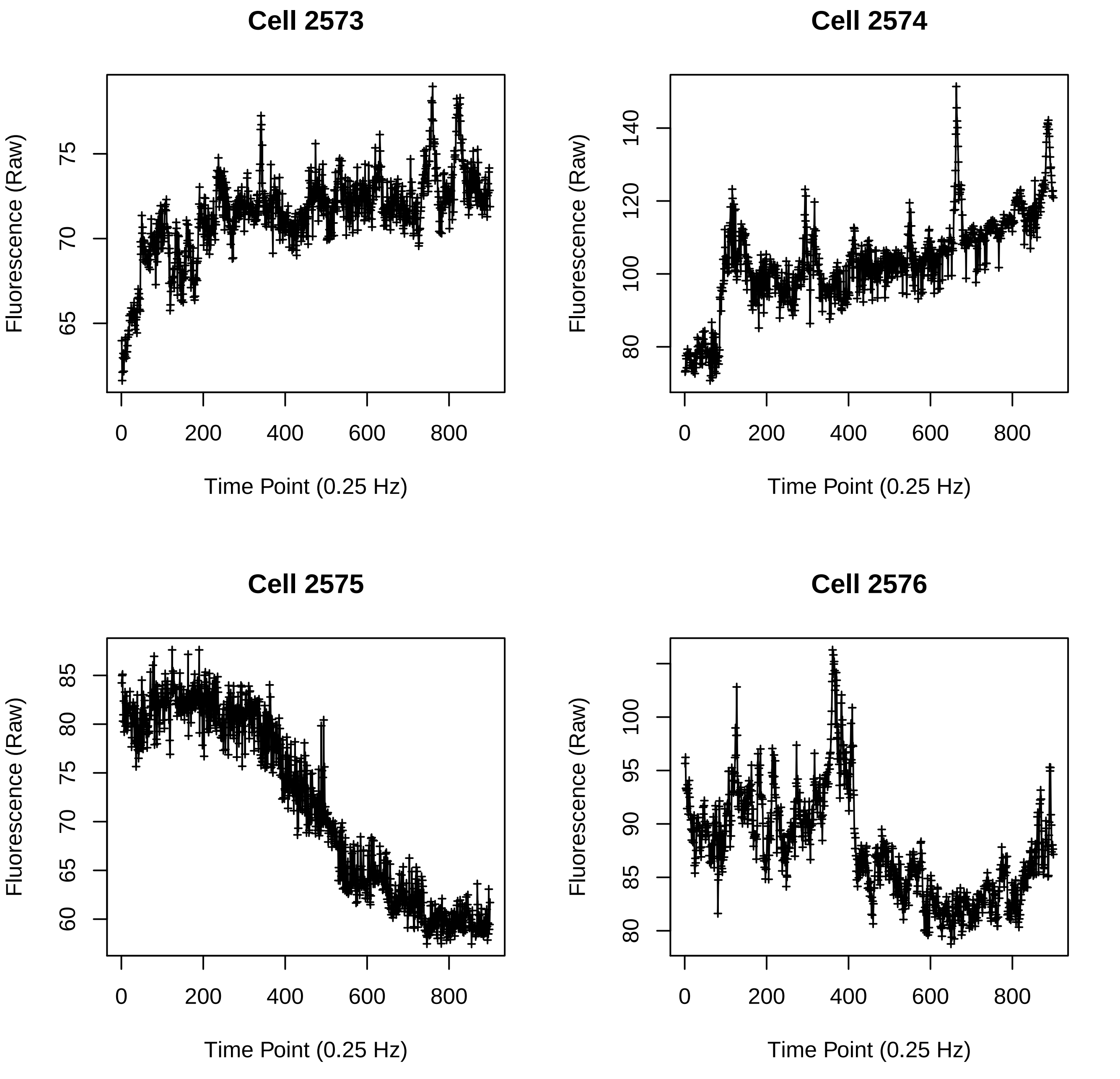
Cell 2573
Fluorescence (Raw)
65
70
75
0
200
400
600
800
Time Point (0.25 Hz)
Cell 2574
Fluorescence (Raw)
80
100
120
140
0
200
400
600
800
Time Point (0.25 Hz)
Cell 2575
Fluorescence (Raw)
60
65
70
75
80
85
0
200
400
600
800
Time Point (0.25 Hz)
Cell 2576
Fluorescence (Raw)
80
85
90
95
100
0
200
400
600
800
Time Point (0.25 Hz)

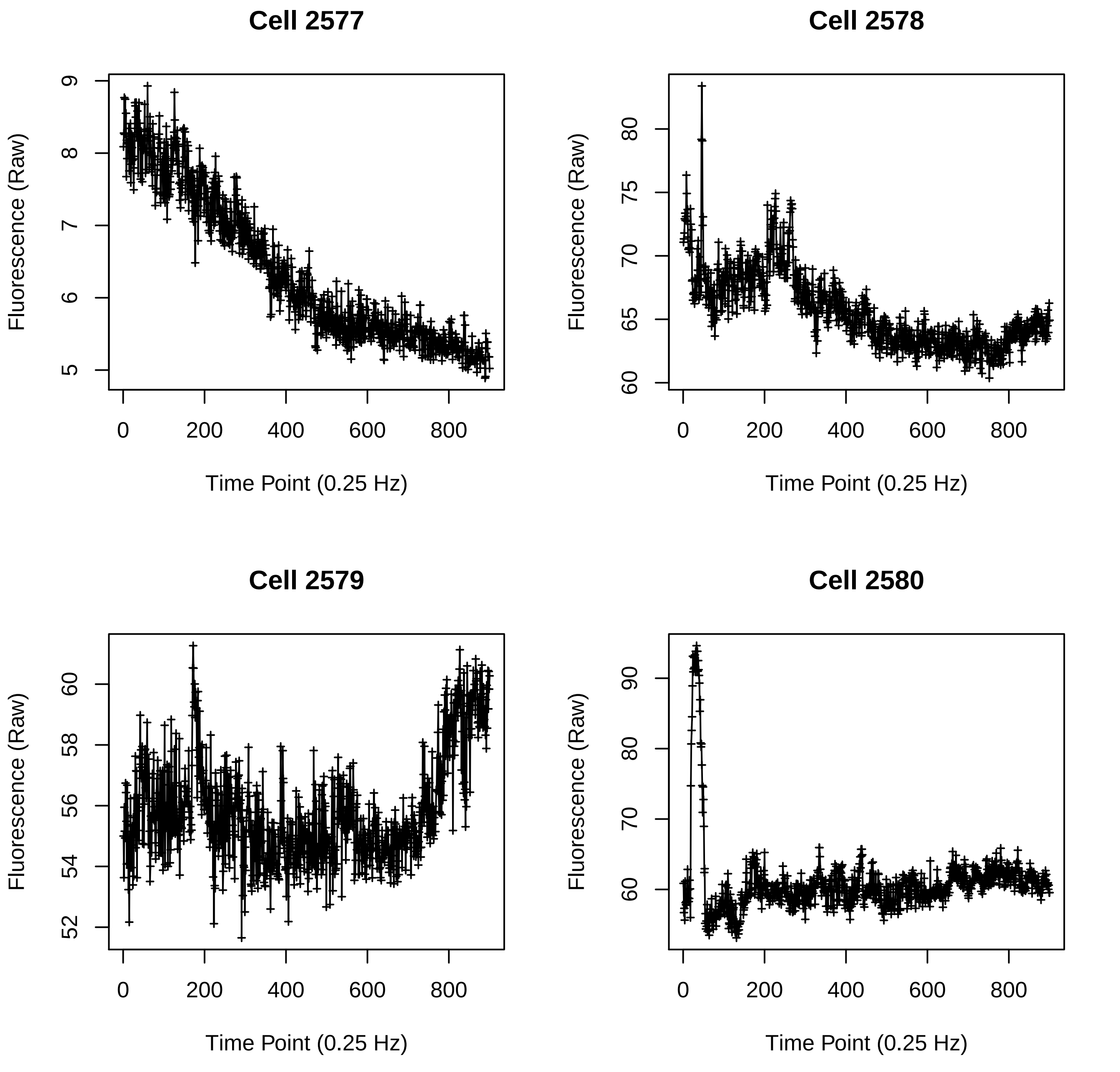
Cell 2577
Cell 2578
Cell 2579
Cell 2580
Fluorescence (Raw)
Time Point (0.25 Hz)
0
200
400
600
800
5
6
7
8
9
60
65
70
75
80
52
54
56
58
60
70
90

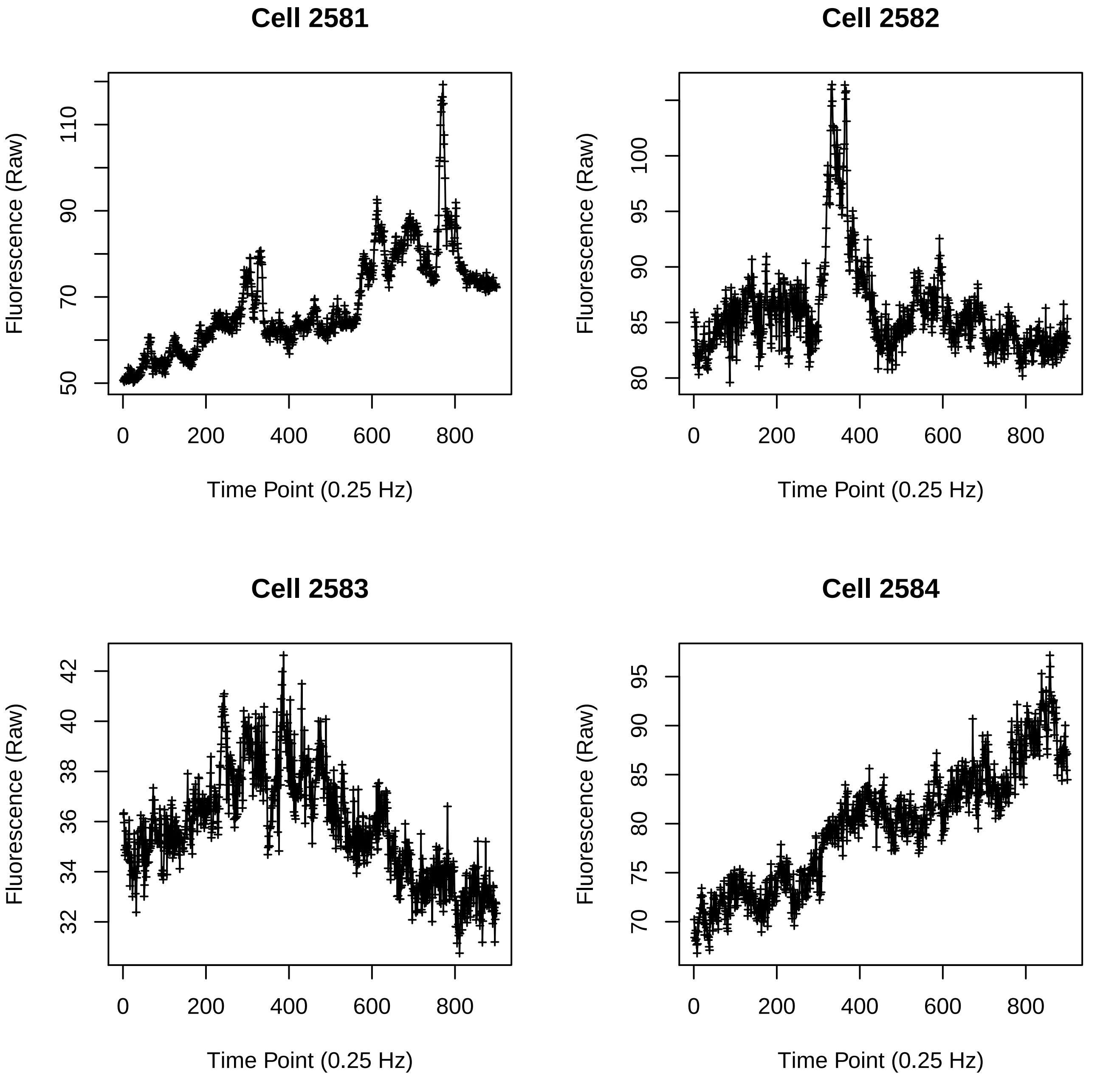

Cell 2581
Cell 2582
Cell 2583
Cell 2584
Fluorescence (Raw)
Time Point (0.25 Hz)
50
70
90
110
80
85
90
95
100
32
34
36
38
40
42
70
75
80
85
90
95
0
200
400
600
800

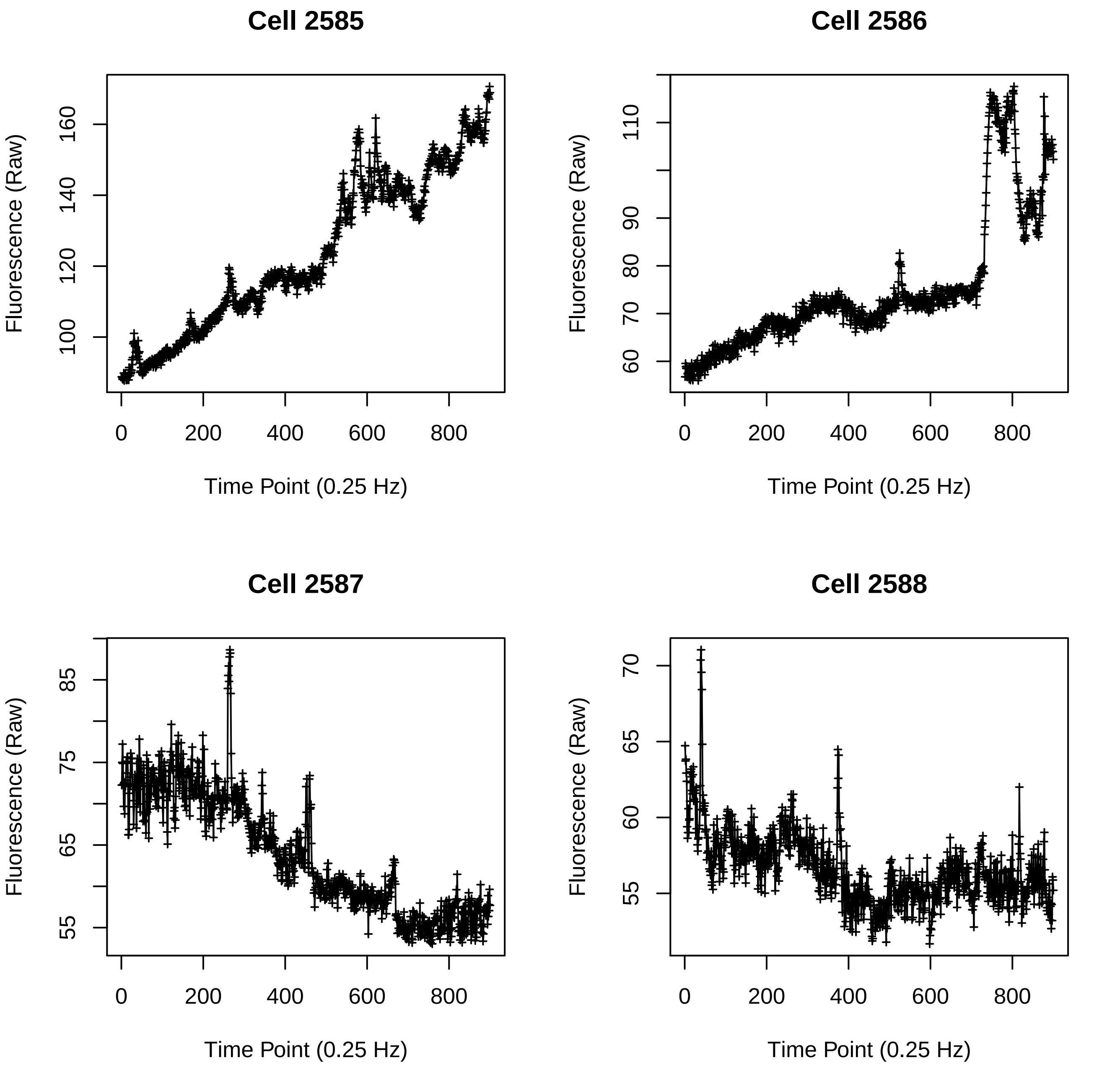
Cell 2585
Fluorescence (Raw)
100
120
140
160
0
200
400
600
800
Time Point (0.25 Hz)
Cell 2586
Fluorescence (Raw)
60
70
80
90
110
0
200
400
600
800
Time Point (0.25 Hz)
Cell 2587
Fluorescence (Raw)
55
65
75
85
0
200
400
600
800
Time Point (0.25 Hz)
Cell 2588
Fluorescence (Raw)
55
60
65
70
0
200
400
600
800
Time Point (0.25 Hz)

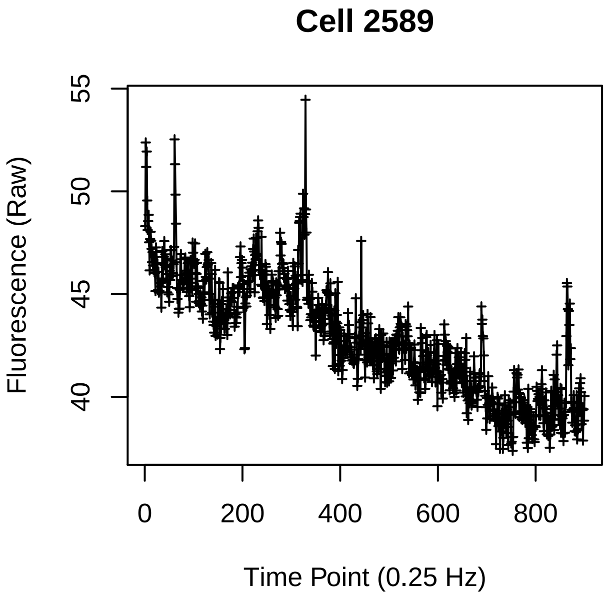
Cell 2589
Fluorescence (Raw)
40
45
50
55
0
200
400
600
800
Time Point (0.25 Hz)

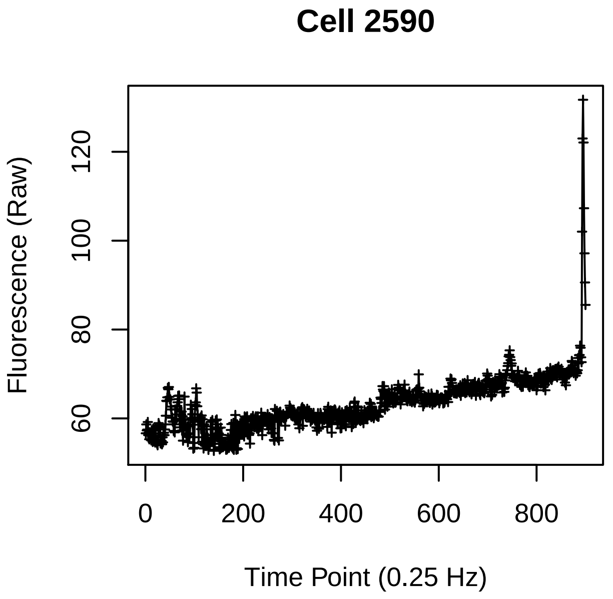
Cell 2590
Fluorescence (Raw)
60
80
100
120
0
200
400
600
800
Time Point (0.25 Hz)

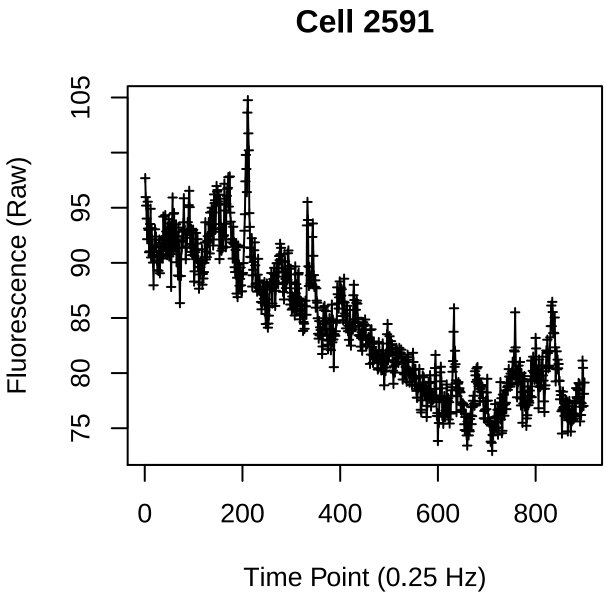
Cell 2591
Fluorescence (Raw)
75
80
85
90
95
105
0
200
400
600
800
Time Point (0.25 Hz)

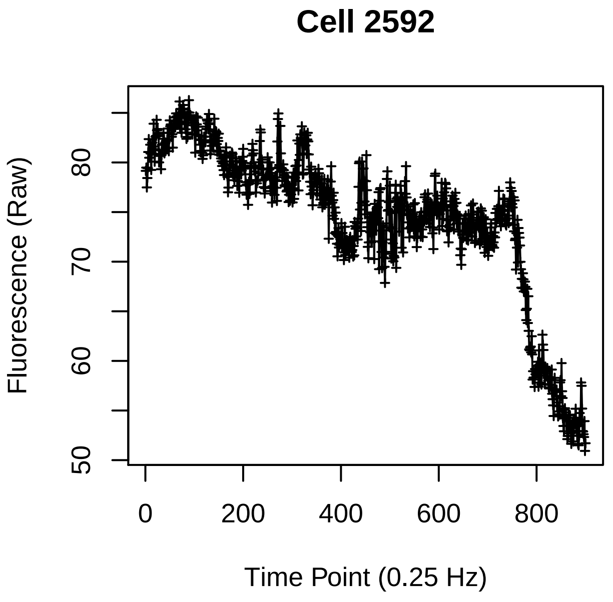
Cell 2592
Fluorescence (Raw)
50
60
70
80
0
200
400
600
800
Time Point (0.25 Hz)

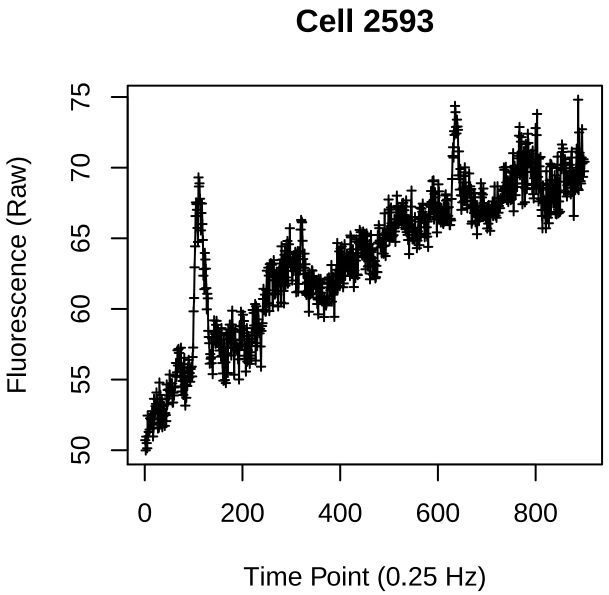
Cell 2593
Fluorescence (Raw)
50
55
60
65
70
75
0
200
400
600
800
Time Point (0.25 Hz)

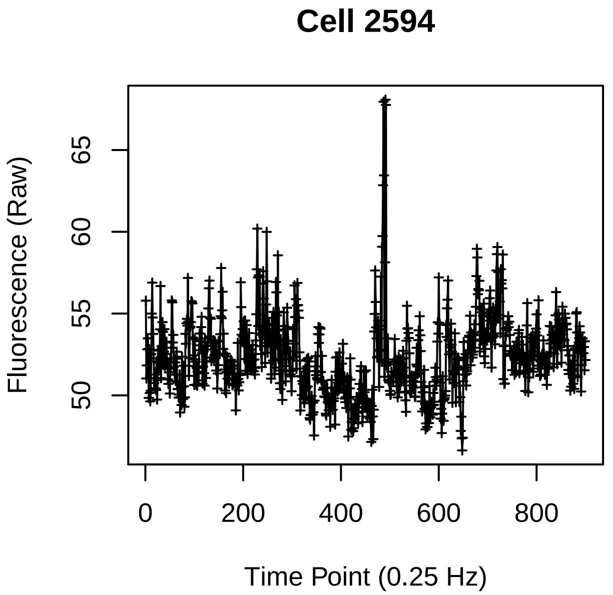
Cell 2594
Fluorescence (Raw)
50
55
60
65
0
200
400
600
800
Time Point (0.25 Hz)

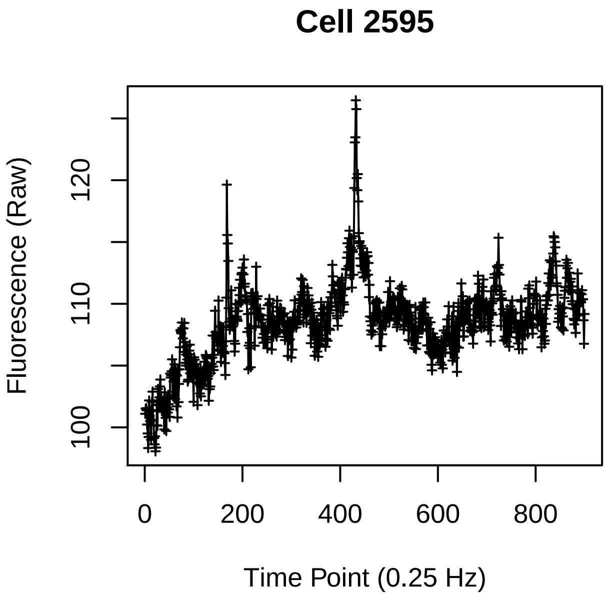
Cell 2595
Fluorescence (Raw)
100
110
120
0
200
400
600
800
Time Point (0.25 Hz)

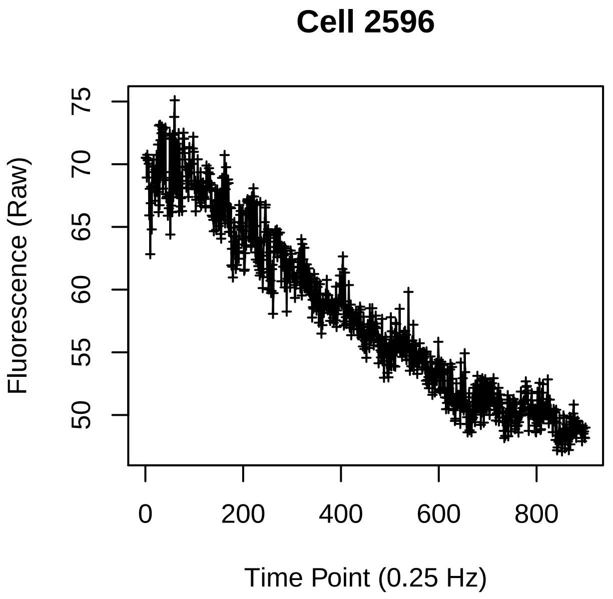
Cell 2596
Fluorescence (Raw)
50
55
60
65
70
75
0
200
400
600
800
Time Point (0.25 Hz)

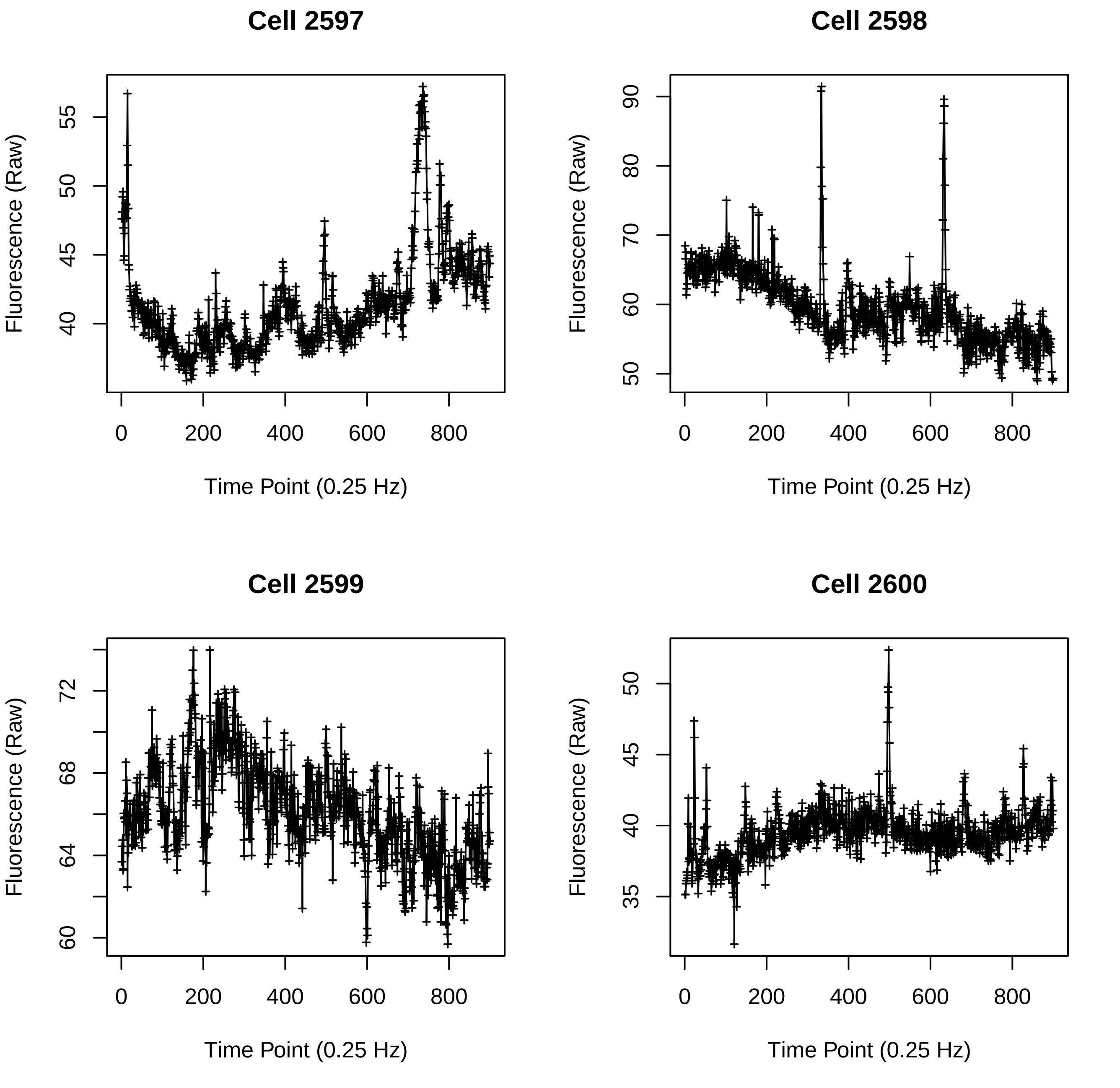
Cell 2597
Cell 2598
Cell 2599
Cell 2600
Fluorescence (Raw)
Time Point (0.25 Hz)
40
45
50
55
60
64
68
72
35
70
80
90
0
200
400
600
800

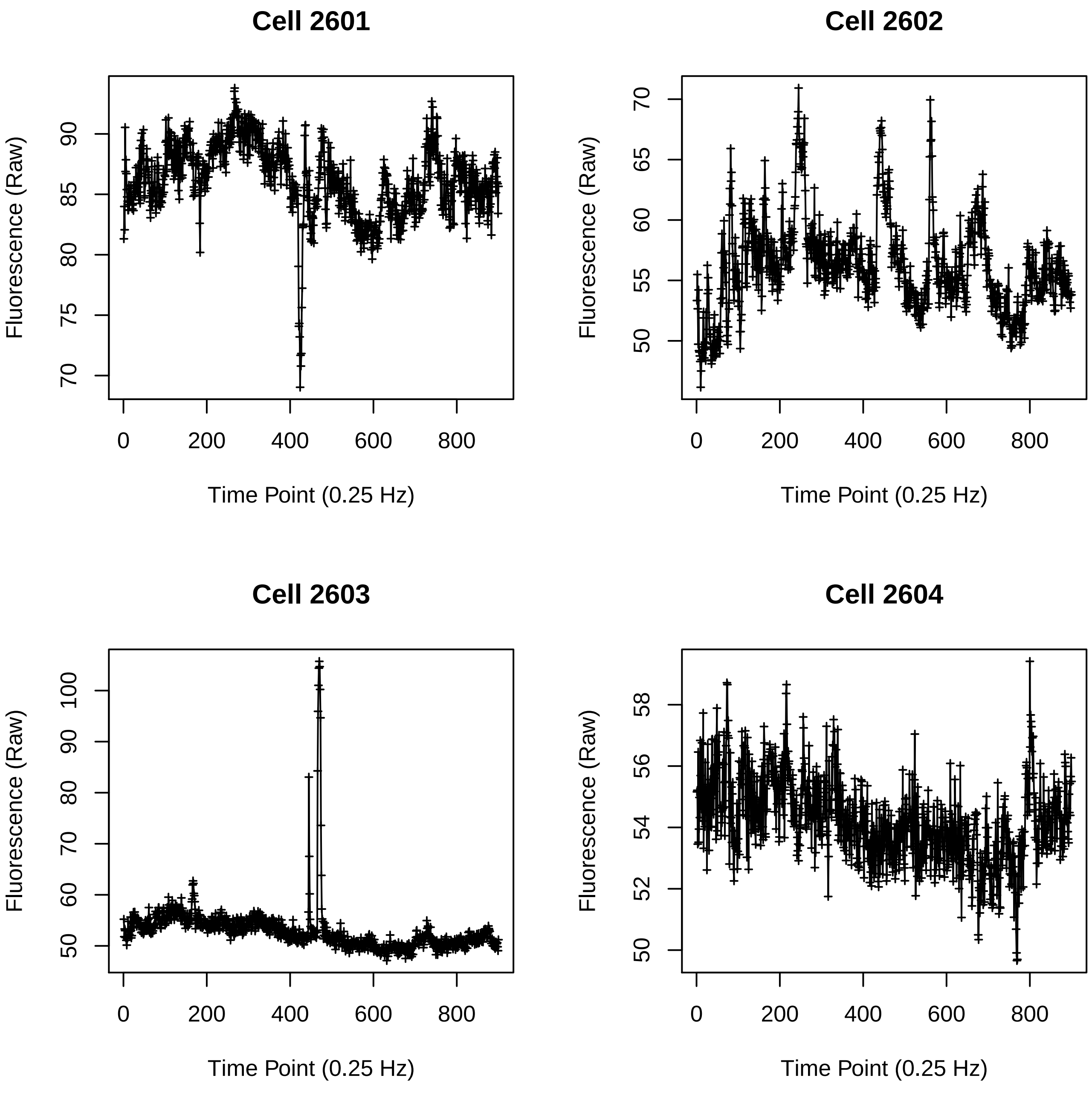
Cell 2601
Cell 2602
Cell 2603
Cell 2604
Fluorescence (Raw)
Time Point (0.25 Hz)
0
200
400
600
800
70
75
80
85
90
50
55
60
65
70
50
60
70
80
90
100
50
52
54
56
58

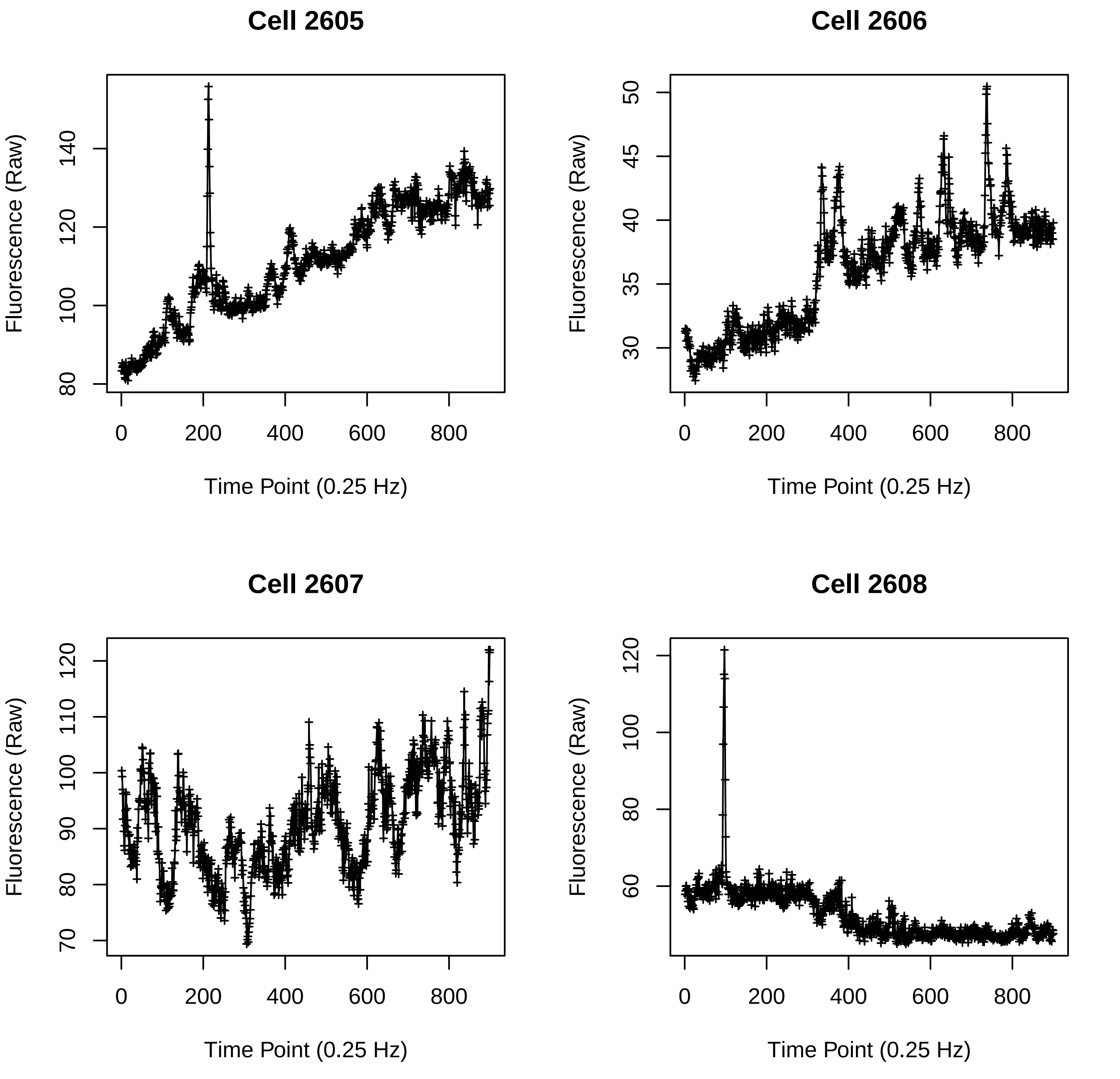
Cell 2605
Fluorescence (Raw)
80
100
120
140
0
200
400
600
800
Time Point (0.25 Hz)
Cell 2606
Fluorescence (Raw)
30
35
40
45
50
0
200
400
600
800
Time Point (0.25 Hz)
Cell 2607
Fluorescence (Raw)
70
80
90
100
110
120
0
200
400
600
800
Time Point (0.25 Hz)
Cell 2608
Fluorescence (Raw)
60
80
100
120
0
200
400
600
800
Time Point (0.25 Hz)

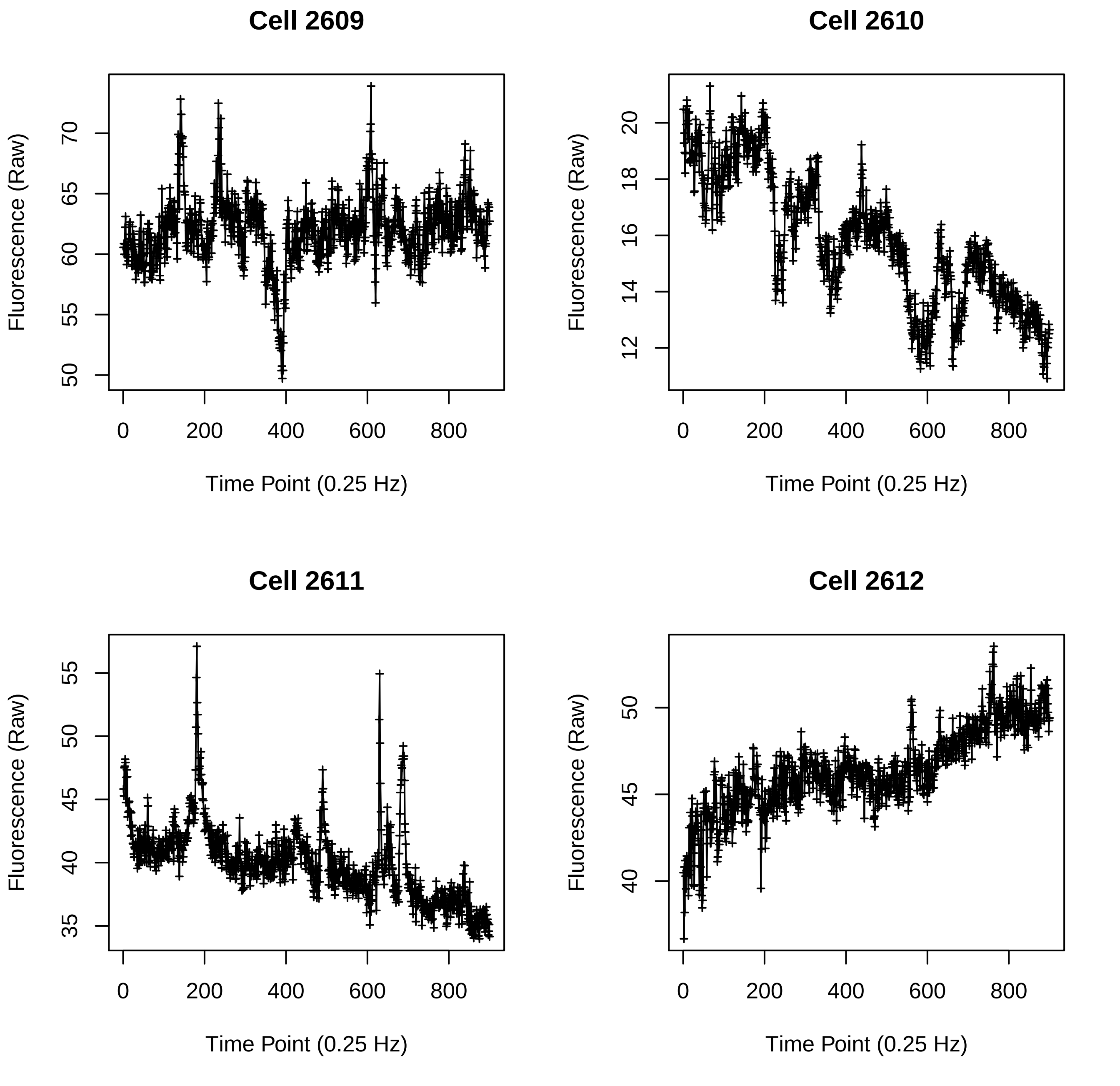
Cell 2609
Cell 2610
Cell 2611
Cell 2612
Fluorescence (Raw)
Time Point (0.25 Hz)
0
200
400
600
800
50
55
60
65
70
12
14
16
18
20
35
40
45
50
55
40
45
50

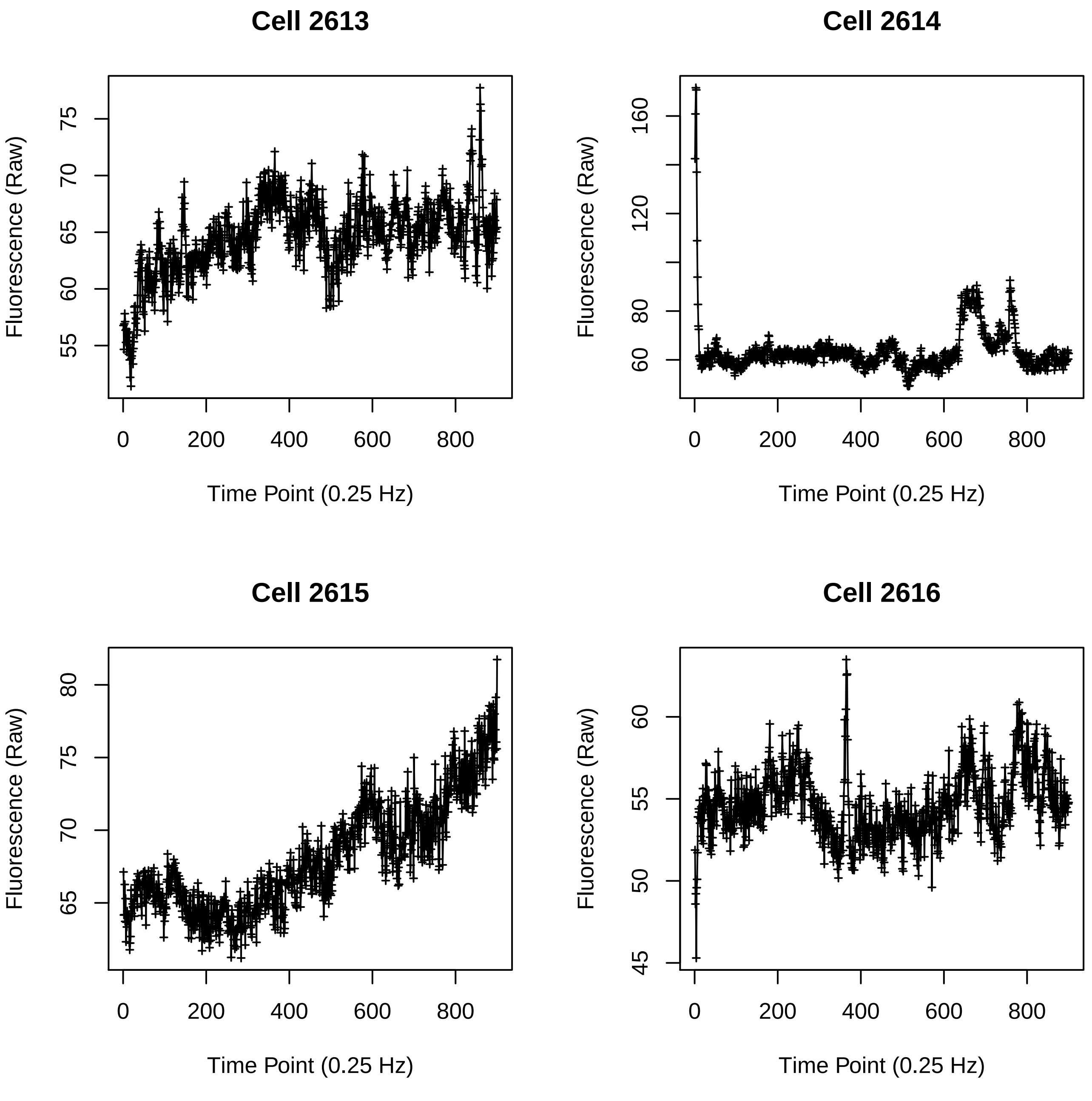
Cell 2613
Fluorescence (Raw)
Time Point (0.25 Hz)
Cell 2614
Fluorescence (Raw)
Time Point (0.25 Hz)
Cell 2615
Fluorescence (Raw)
Time Point (0.25 Hz)
Cell 2616
Fluorescence (Raw)
Time Point (0.25 Hz)

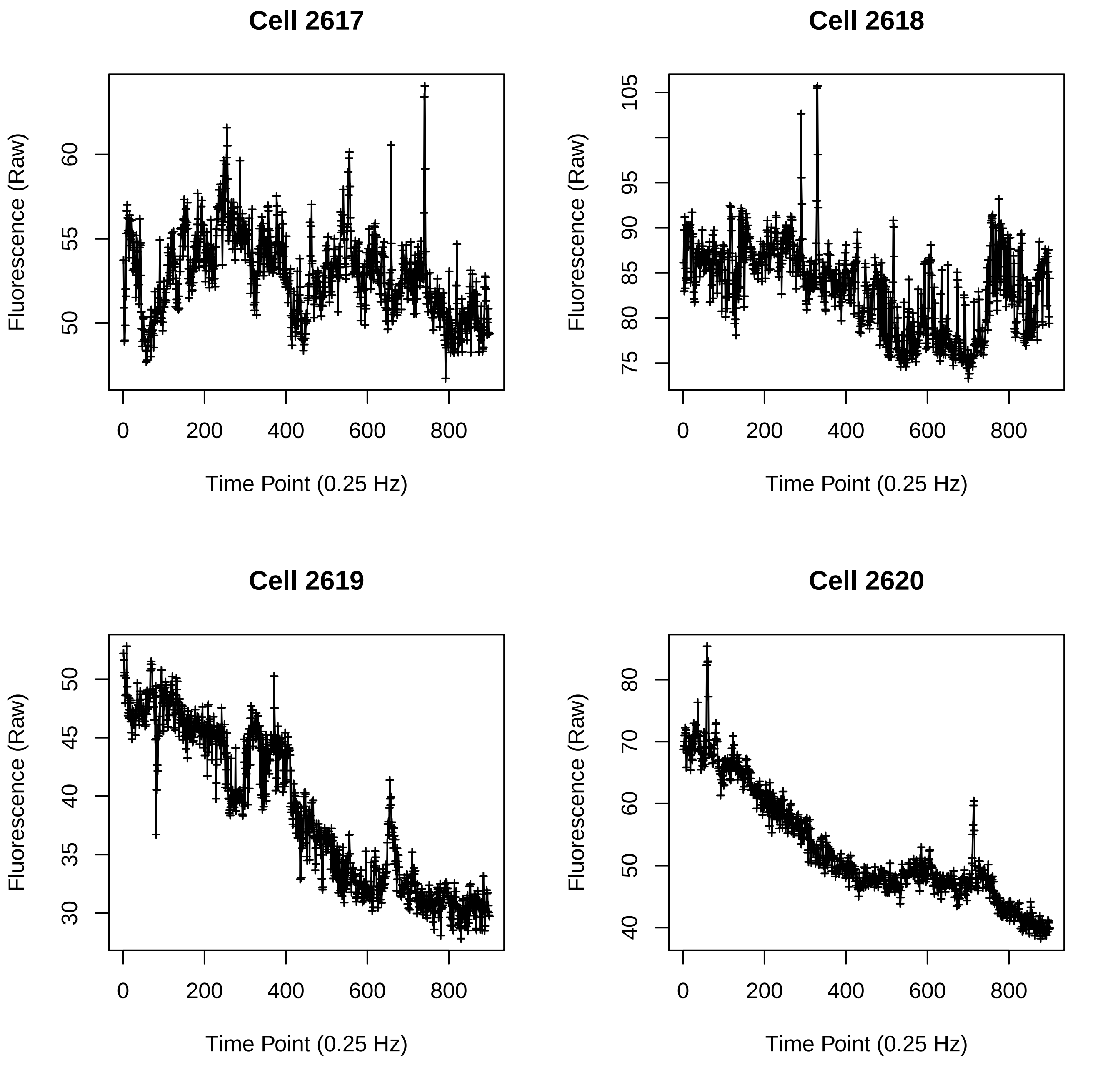
Cell 2617
Cell 2618
Cell 2619
Cell 2620
Fluorescence (Raw)
Time Point (0.25 Hz)
0
200
400
600
800
50
55
60
75
80
85
90
95
105
30
35
40
45
50
40
50
60
70
80

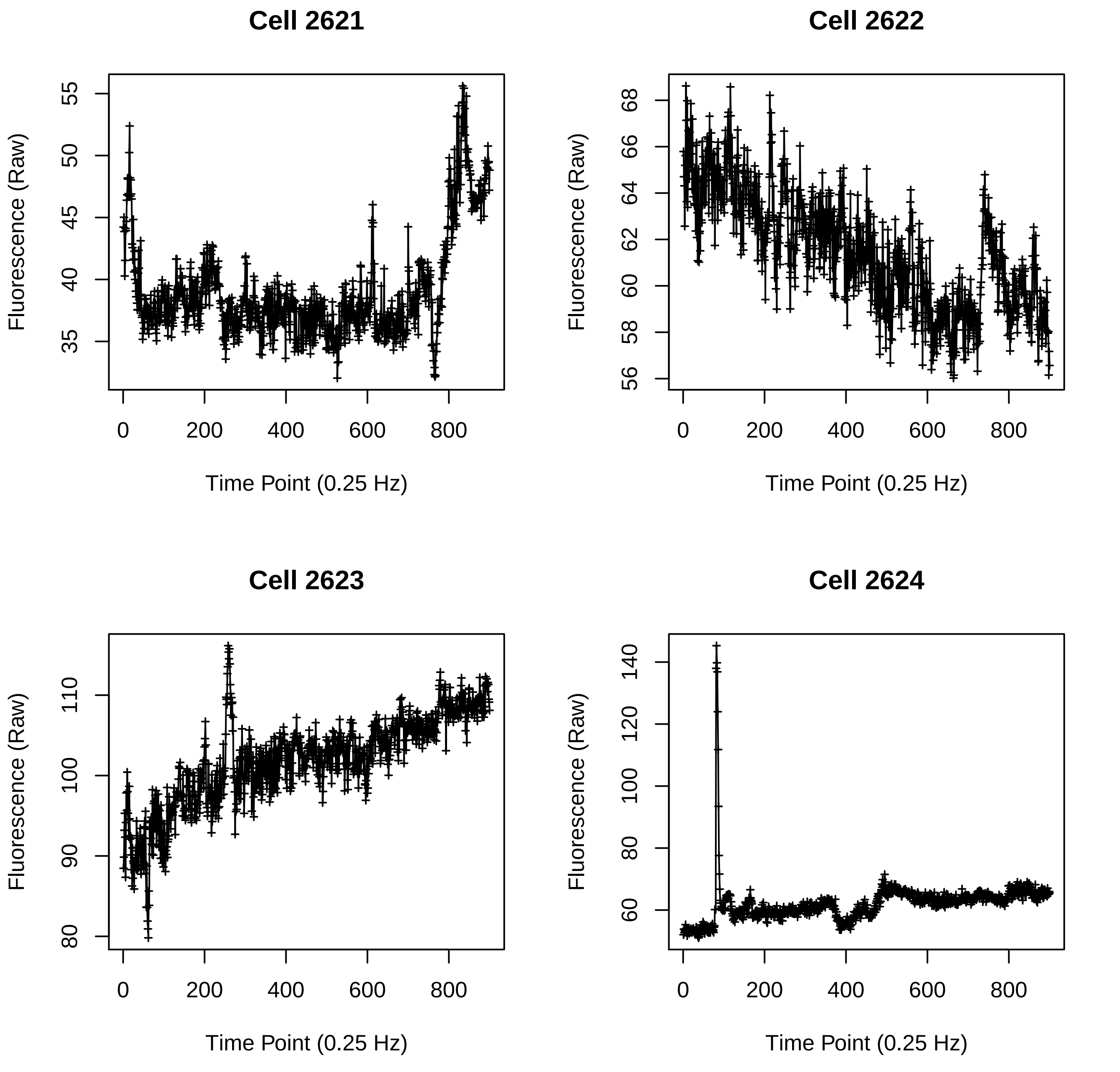

Cell 2621
Fluorescence (Raw)
Time Point (0.25 Hz)
Cell 2622
Fluorescence (Raw)
Time Point (0.25 Hz)
Cell 2623
Fluorescence (Raw)
Time Point (0.25 Hz)
Cell 2624
Fluorescence (Raw)
Time Point (0.25 Hz)

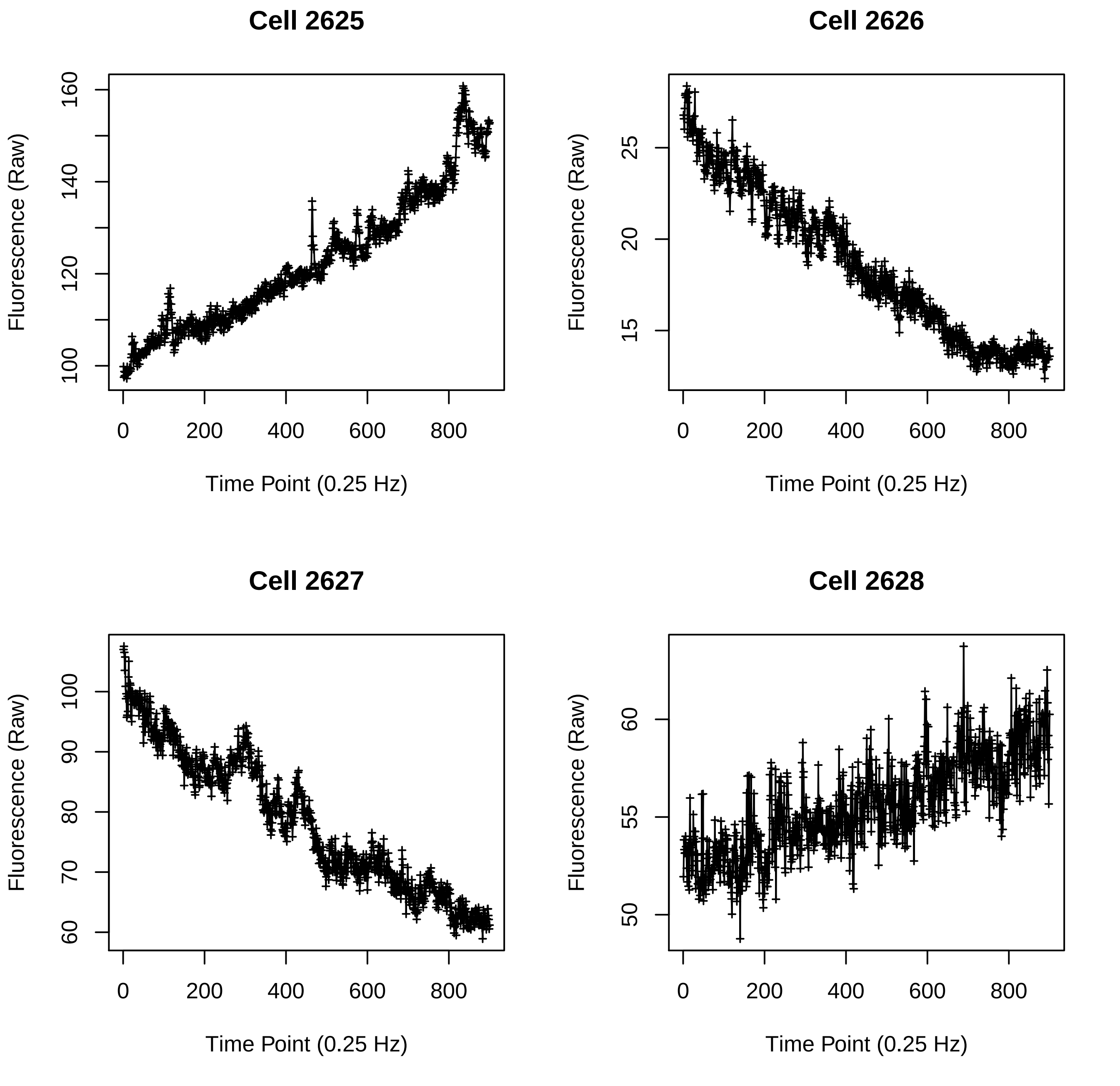
Cell 2625
Cell 2626
Cell 2627
Cell 2628
Fluorescence (Raw)
Time Point (0.25 Hz)
100
120
140
160
25
20
15
90
80
70
60
60
55
50
0
200
400
600
800

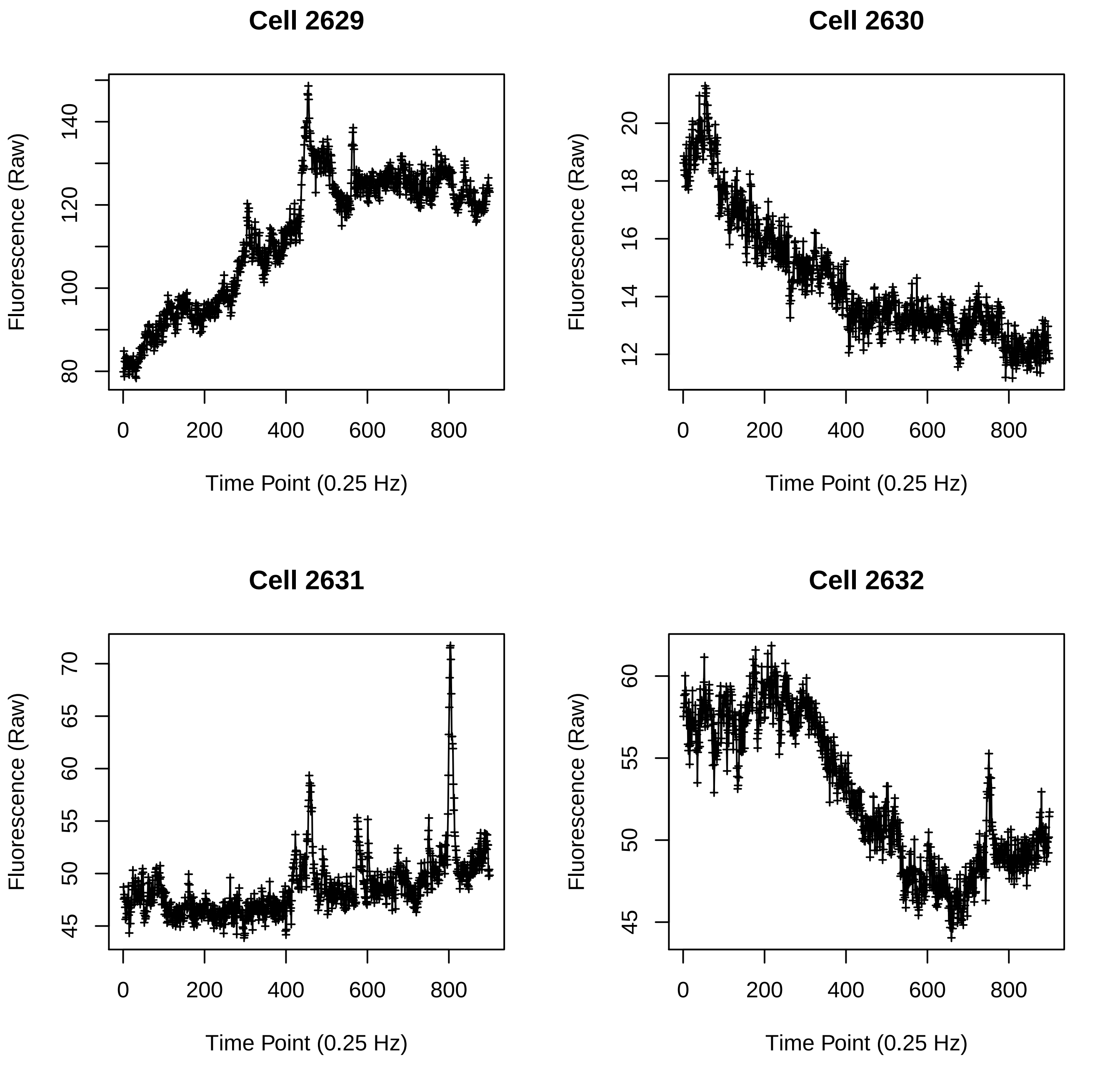

Cell 2629
Cell 2630
Cell 2631
Cell 2632
Fluorescence (Raw)
Time Point (0.25 Hz)
80
100
120
140
12
14
16
18
20
45
50
55
60
65
70
0
200
400
600
800

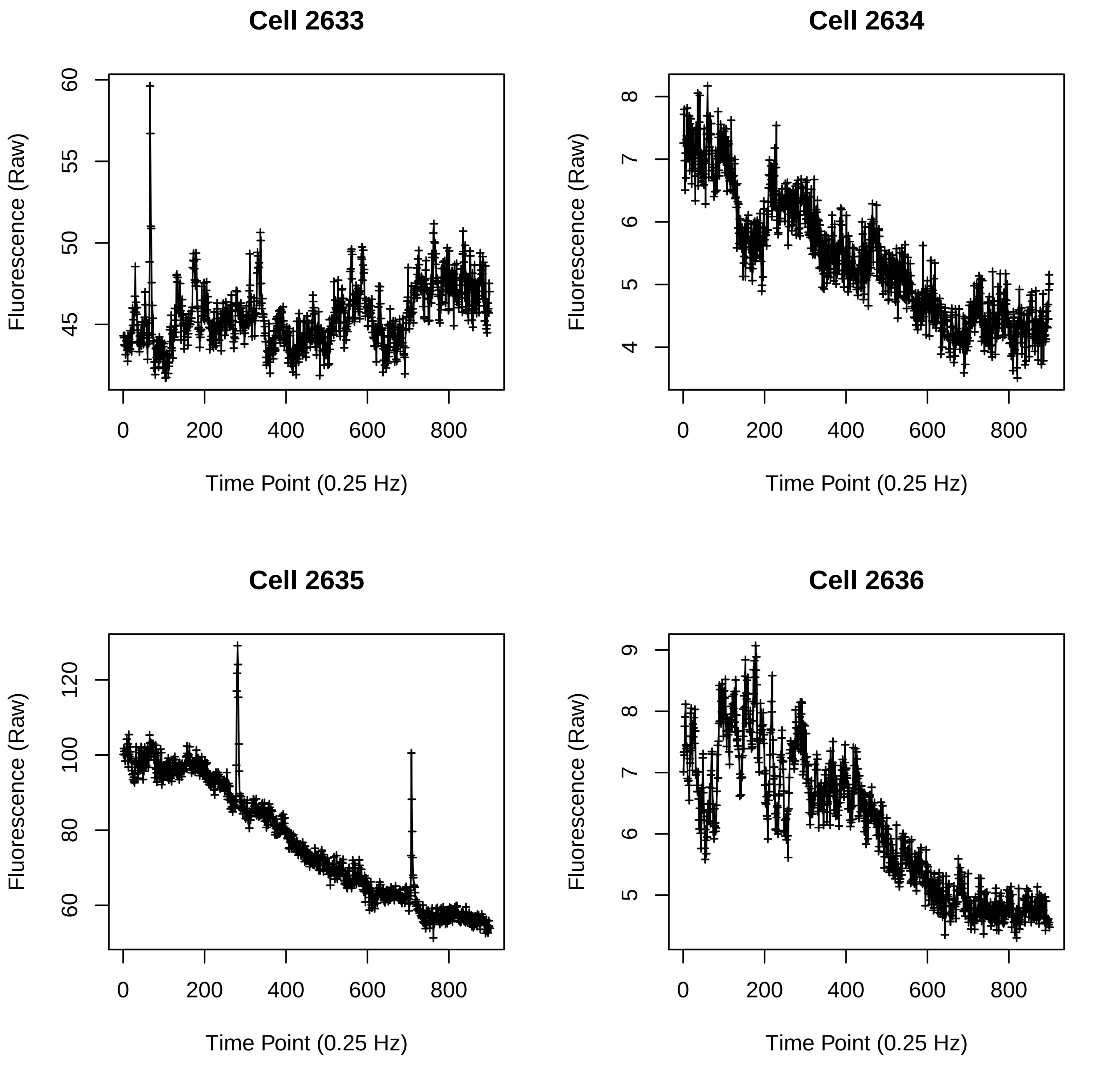
Cell 2633
Fluorescence (Raw)
45
50
55
60
0
200
400
600
800
Time Point (0.25 Hz)
Cell 2634
Fluorescence (Raw)
4
5
6
7
8
0
200
400
600
800
Time Point (0.25 Hz)
Cell 2635
Fluorescence (Raw)
60
80
100
120
0
200
400
600
800
Time Point (0.25 Hz)
Cell 2636
Fluorescence (Raw)
5
6
7
8
9
0
200
400
600
800
Time Point (0.25 Hz)

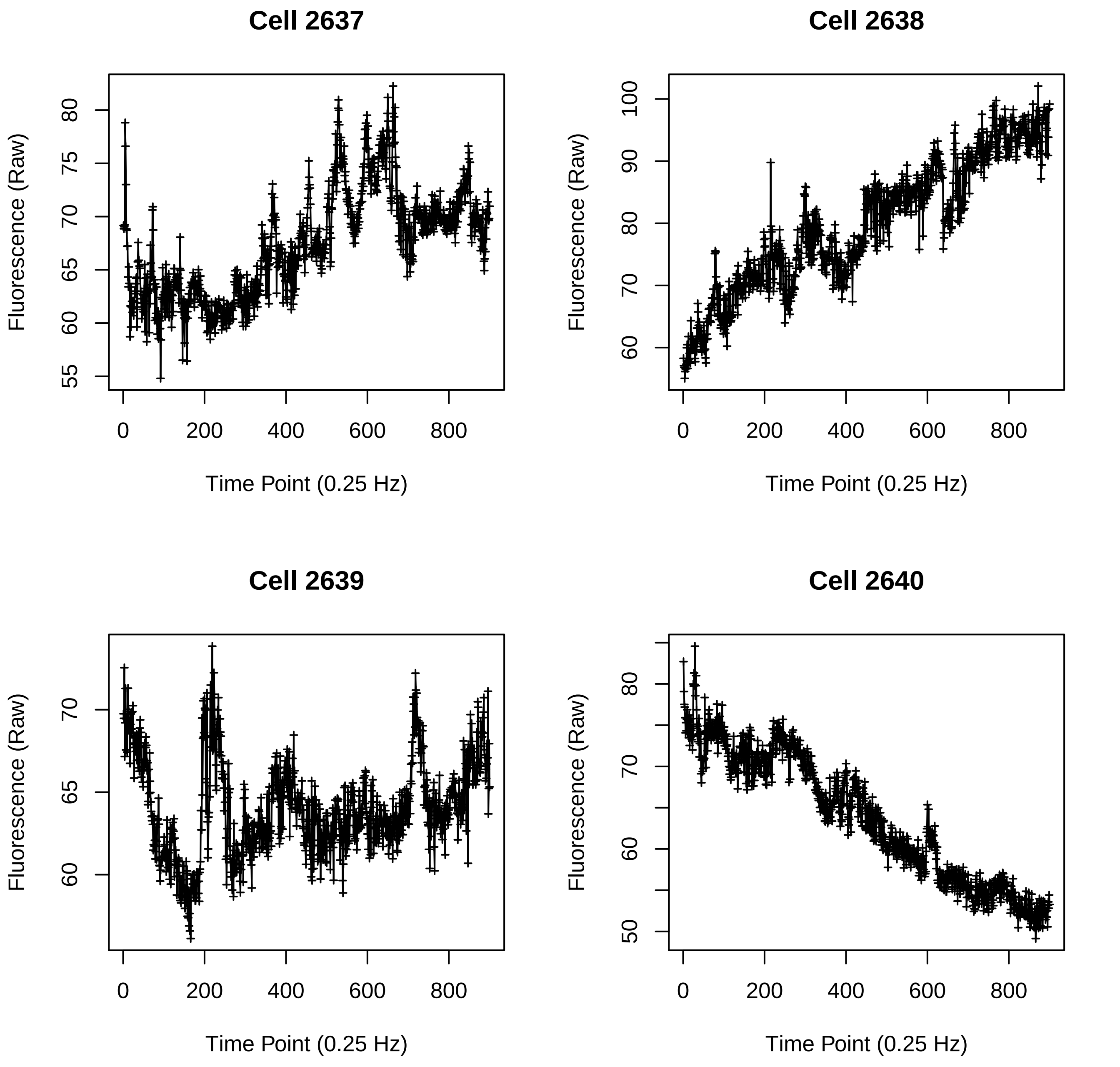
Cell 2637
Cell 2638
Cell 2639
Cell 2640
Fluorescence (Raw)
Time Point (0.25 Hz)
0
200
400
600
800
55
60
65
70
75
80
90
100
50

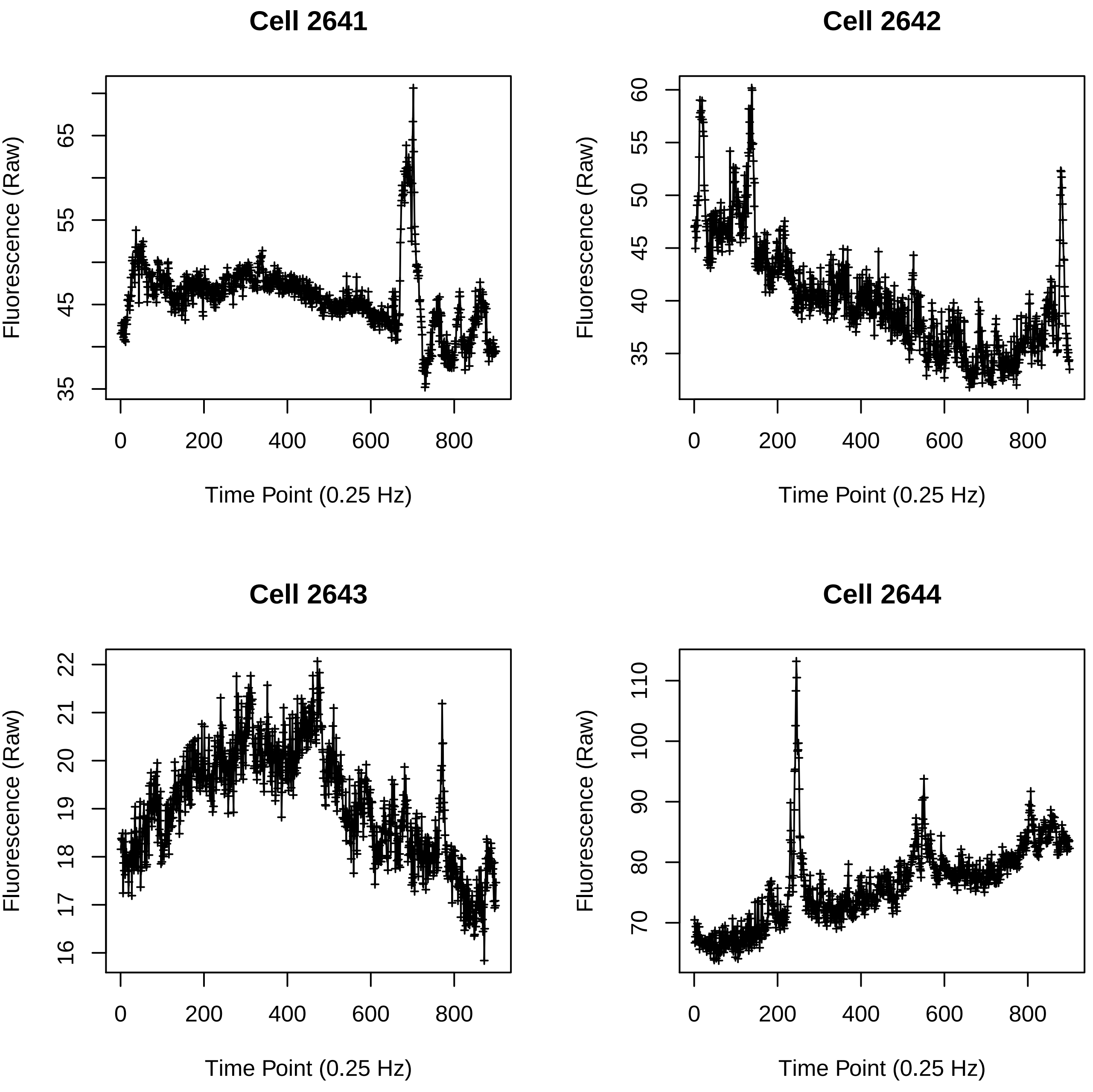
Cell 2641
Fluorescence (Raw)
35
45
55
65
0
200
400
600
800
Time Point (0.25 Hz)
Cell 2642
Fluorescence (Raw)
35
40
45
50
55
60
0
200
400
600
800
Time Point (0.25 Hz)
Cell 2643
Fluorescence (Raw)
16
17
18
19
20
21
22
0
200
400
600
800
Time Point (0.25 Hz)
Cell 2644
Fluorescence (Raw)
70
80
90
100
110
0
200
400
600
800
Time Point (0.25 Hz)

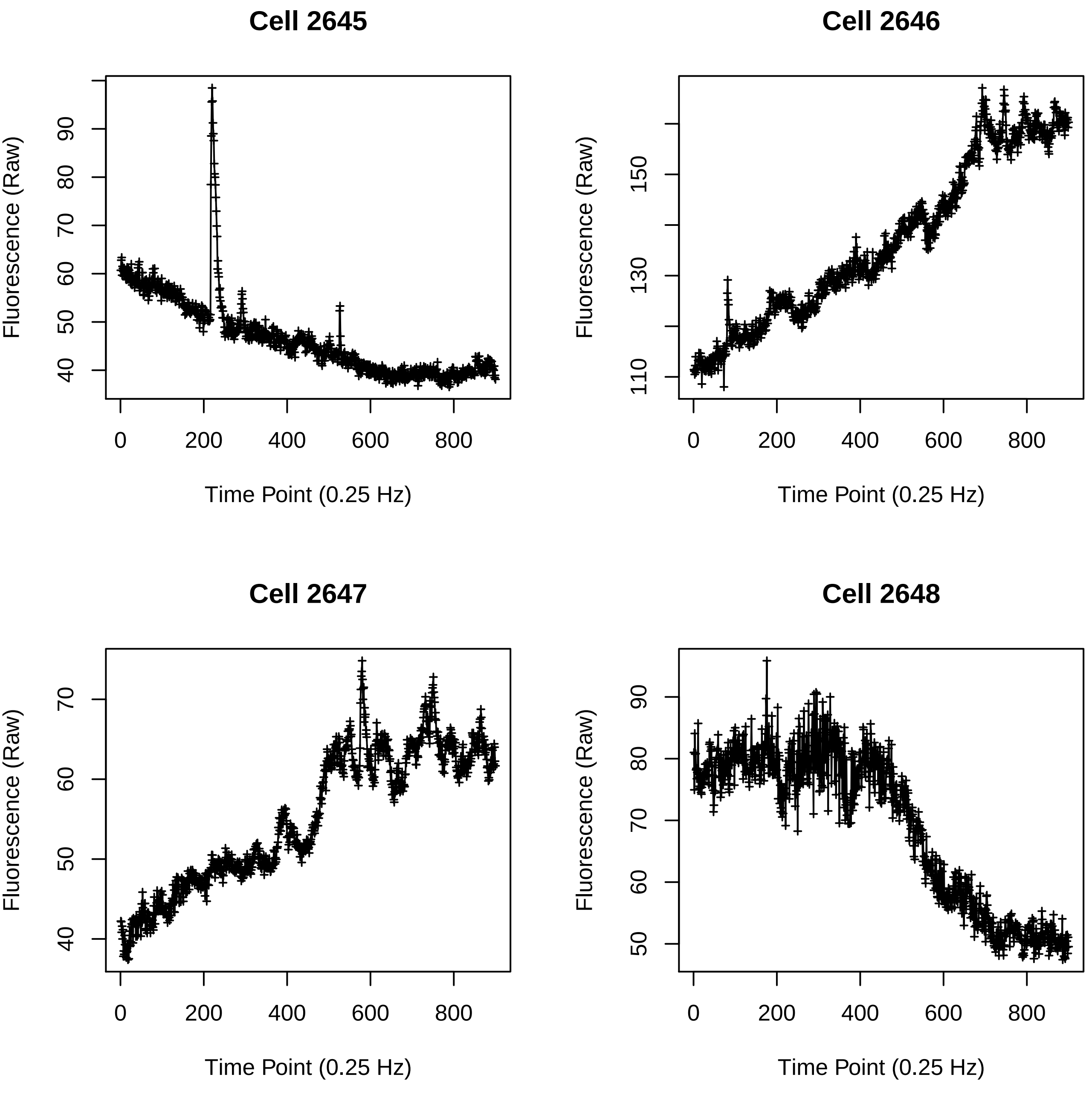
Cell 2645
Cell 2646
Cell 2647
Cell 2648
Fluorescence (Raw)
Time Point (0.25 Hz)
0
200
400
600
800
40
50
60
70
80
90
110
130
150

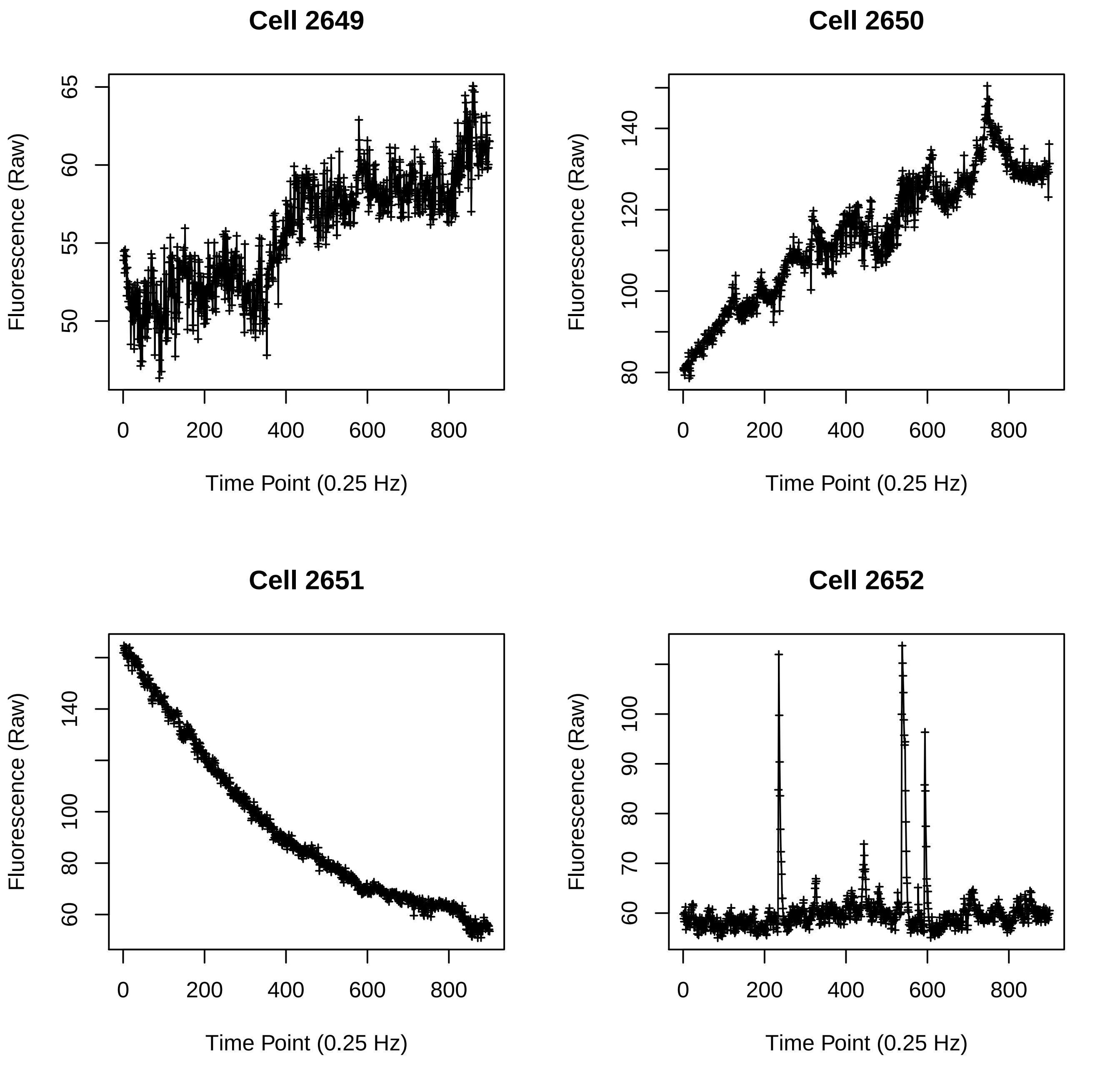
Cell 2649
Cell 2650
Cell 2651
Cell 2652
Fluorescence (Raw)
Time Point (0.25 Hz)
0
200
400
600
800
50
55
60
65
80
100
120
140
60
80
100
140
60
70
80
90
100

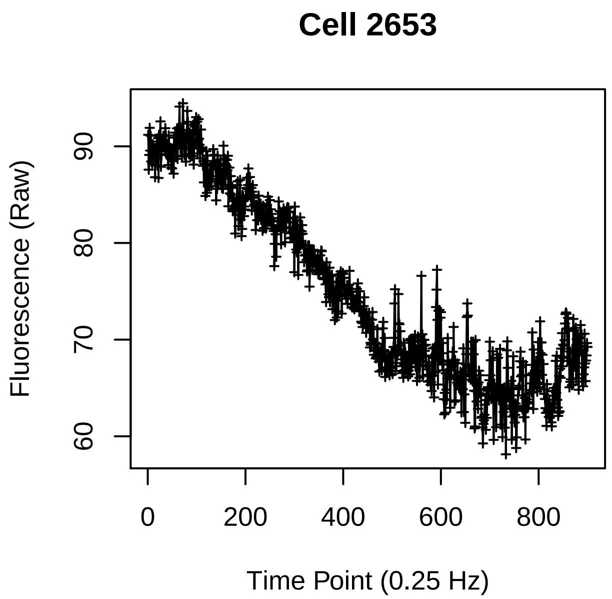
Cell 2653
Fluorescence (Raw)
60
70
80
90
0
200
400
600
800
Time Point (0.25 Hz)
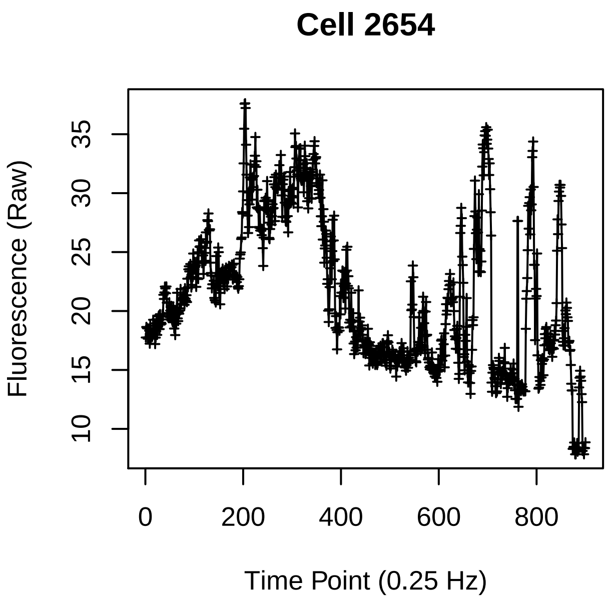
Cell 2654
Fluorescence (Raw)
10
15
20
25
30
35
0
200
400
600
800
Time Point (0.25 Hz)
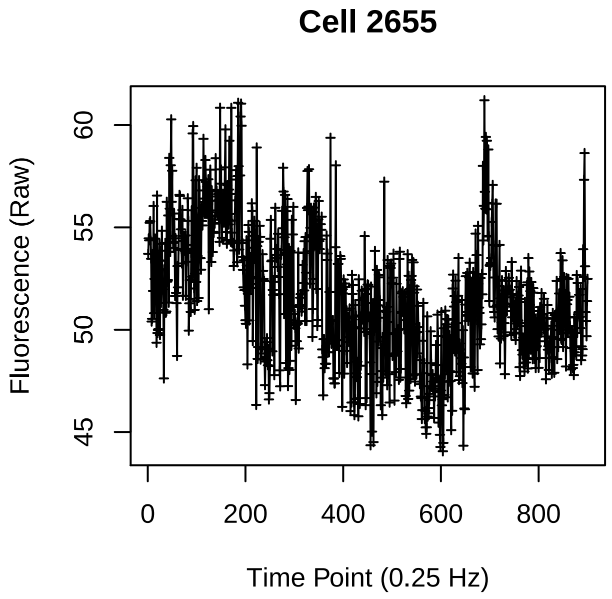
Cell 2655
Fluorescence (Raw)
45
50
55
60
0
200
400
600
800
Time Point (0.25 Hz)
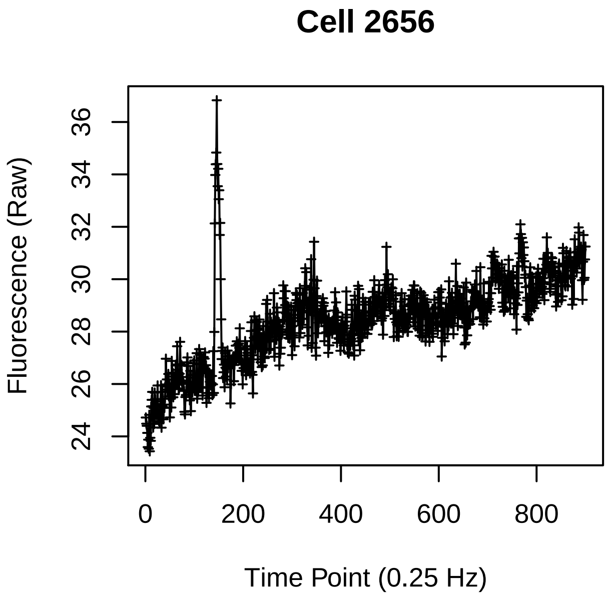
Cell 2656
Fluorescence (Raw)
24
26
28
30
32
34
36
0
200
400
600
800
Time Point (0.25 Hz)

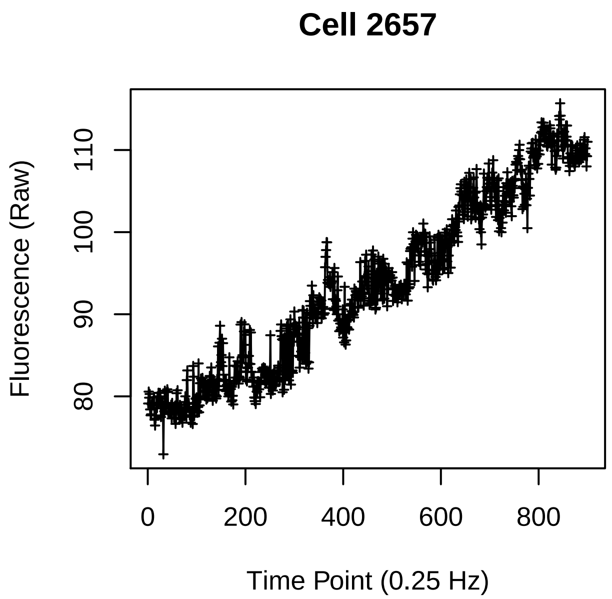
Cell 2657
Fluorescence (Raw)
80
90
100
110
0
200
400
600
800
Time Point (0.25 Hz)

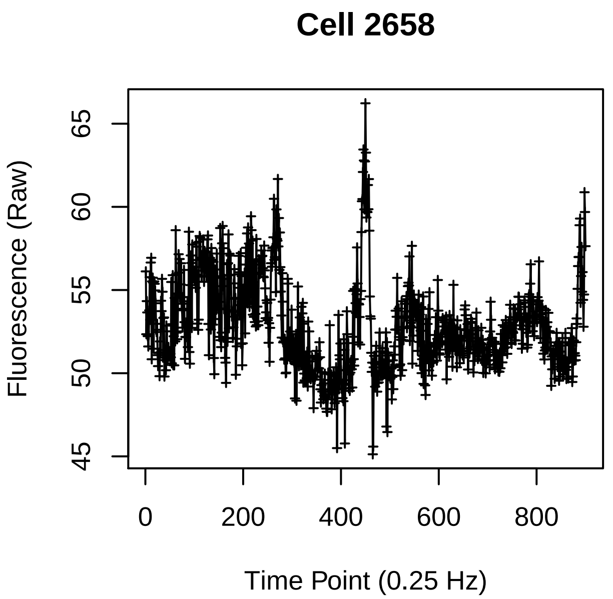
Cell 2658
Fluorescence (Raw)
45
50
55
60
65
0
200
400
600
800
Time Point (0.25 Hz)

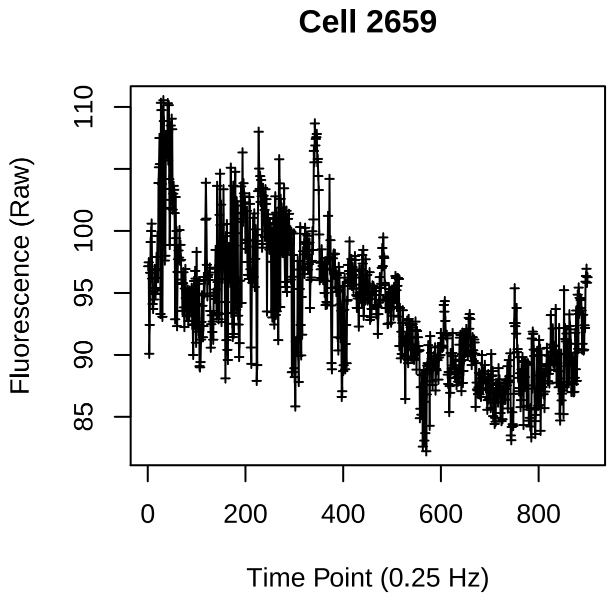
Cell 2659
Fluorescence (Raw)
85
90
95
100
110
0
200
400
600
800
Time Point (0.25 Hz)

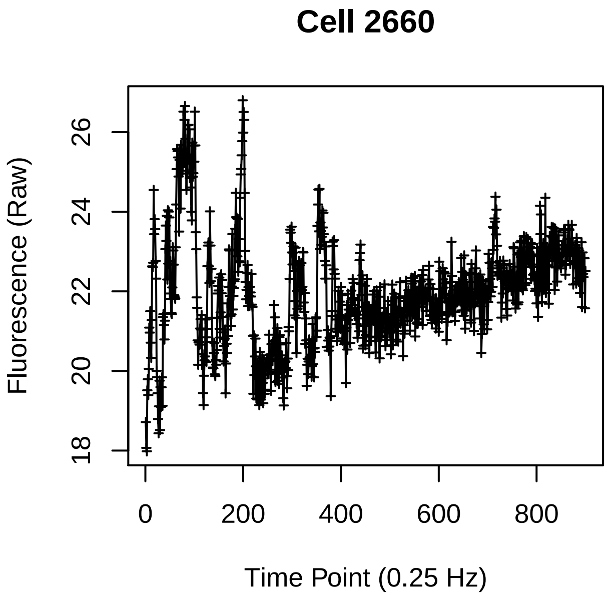
Cell 2660
Fluorescence (Raw)
18
20
22
24
26
0
200
400
600
800
Time Point (0.25 Hz)

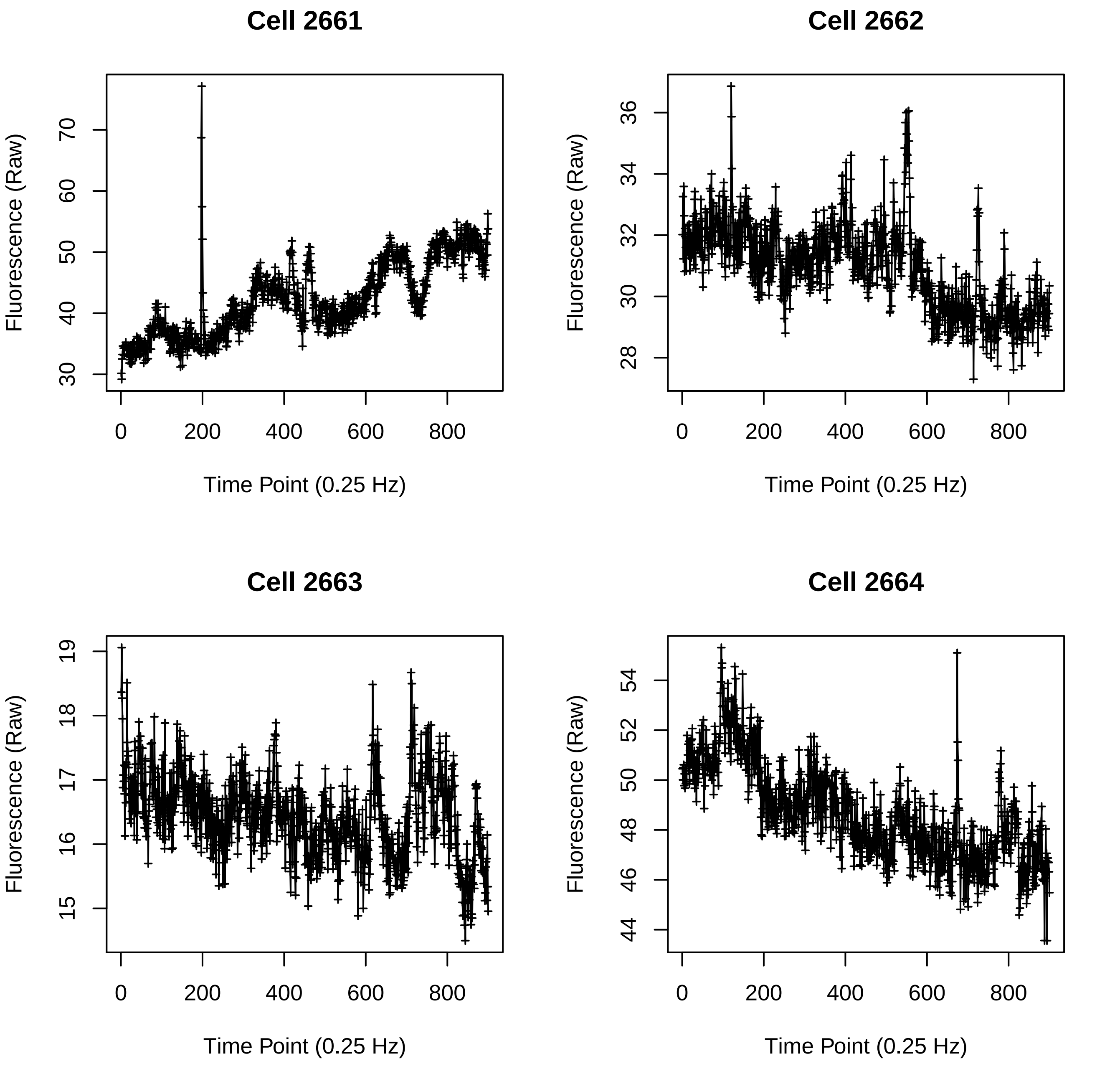
Cell 2661
Fluorescence (Raw)
30
40
50
60
70
0
200
400
600
800
Time Point (0.25 Hz)
Cell 2662
Fluorescence (Raw)
28
30
32
34
36
0
200
400
600
800
Time Point (0.25 Hz)
Cell 2663
Fluorescence (Raw)
15
16
17
18
19
0
200
400
600
800
Time Point (0.25 Hz)
Cell 2664
Fluorescence (Raw)
44
46
48
50
52
54
0
200
400
600
800
Time Point (0.25 Hz)
